# Venomics of the Enigmatic Andaman Cobra (*Naja sagittifera*) and the Preclinical Failure of Indian Antivenoms in Andaman and Nicobar Islands

**DOI:** 10.3389/fphar.2021.768210

**Published:** 2021-10-25

**Authors:** Saurabh Attarde, Suyog Khochare, Ashwin Iyer, Paulomi Dam, Gerard Martin, Kartik Sunagar

**Affiliations:** ^1^ Evolutionary Venomics Lab, Centre for Ecological Sciences, Indian Institute of Science, Bangalore, India; ^2^ The Liana Trust, Hunsur, India

**Keywords:** *Naja sagittifera*, *Naja naja*, venomics, Andaman and Nicobar Islands, antivenoms

## Abstract

The Andaman and Nicobar Islands are an abode to a diversity of flora and fauna, including the many endemic species of snakes, such as the elusive Andaman cobra (*Naja sagittifera*). However, the ecology and evolution of venomous snakes inhabiting these islands have remained entirely uninvestigated. This study aims to bridge this knowledge gap by investigating the evolutionary history of *N. sagittifera* and its venom proteomic, biochemical and toxicity profile. Phylogenetic reconstructions confirmed the close relationship between *N. sagittifera* and the Southeast Asian monocellate cobra (*N. kaouthia*). Overlooking this evolutionary history, a polyvalent antivenom manufactured using the venom of the spectacled cobra (*N. naja*) from mainland India is used for treating *N. sagittifera* envenomations. Comparative evaluation of venoms of these congeners revealed significant differences in their composition, functions and potencies. Given the close phylogenetic relatedness between *N. sagittifera* and *N. kaouthia*, we further assessed the cross-neutralising efficacy of Thai monovalent *N. kaouthia* antivenom against *N. sagittifera* venoms. Our findings revealed the inadequate preclinical performance of the Indian polyvalent and Thai monovalent antivenoms in neutralising *N. sagittifera* venoms. Moreover, the poor efficacy of the polyvalent antivenom against *N. naja* venom from southern India further revealed the critical need to manufacture region-specific Indian antivenoms.

## Introduction

Located 1,400 km off mainland India in the Bay of Bengal, the Andaman and Nicobar Islands are among India’s ten biogeographic zones. Over 85% of the total land area being covered by forests and only 38 out of the 572 islands are home to nearly 400,000 human inhabitants (Sawhney, 2019). Among these are the indigenous Andamanese people, the aboriginal residents belonging to many tribes, such as the Jarawa and Sentinelese. These islands are also an abode to many endemic plants and animals, including many species of enigmatic snakes. The Andaman cobra (*Naja sagittifera*), Andaman krait (*Bungarus andamanensis*), king cobra (*Ophiophagus hannah*) and several endemic pit vipers (*Trimeresurus* spp.) on the island are amongst the venomous snakes that are capable of inflicting severe clinical symptoms in humans. The Andaman cobra, in particular, is primarily distributed across the islands, but the ecology and evolution of venom of this enigmatic snake remain entirely uninvestigated.

Unlike in mainland India, the impact of snakebite to populations of Andaman and Nicobar Islands is unclear. While a study examining the Health Management Information System data from 2017 to 2019 estimated that between 23 and 28 people per 100,000 suffer snake envenoming on these islands (Salve et al., 2020), a rigorous snakebite epidemiological survey is warranted to precisely understand the burden of this socioeconomic disease. Moreover, completely neglecting the distinct biogeographic histories of these islands and the phylogenetic histories of snakes inhabiting them, a polyvalent antivenom manufactured against the “big four” Indian snakes [i.e., spectacled cobra (*N. naja*), common krait (*B. caeruleus*), Russell’s viper (*Daboia russelii*) and saw-scaled viper (*Echis carinatus*)] from Tamil Nadu is used for the treatment of snakebites. However, the effectiveness of this antivenom in treating bites from the phylogenetically distant snakes of these islands remains to be evaluated.

In this study, we unveil the venom composition, biochemistry, pharmacological activity, and potency of the Andaman cobra (*N. sagittifera*). We comparatively evaluate the venom of this enigmatic snake with its congener from mainland India: the spectacled cobra (*N. naja*). Phylogenetic reconstructions using mitochondrial markers revealed the distant evolutionary relationship between these *Naja* species. Consistent with previous report (Kazemi et al., 2021), *N. sagittifera* was found to have originated from the common ancestor of Southeast Asian monocled cobra (*N. kaouthia*). Furthermore, we assessed the effectiveness of the “big four” polyvalent Indian antivenom in cross-neutralising the venom of this phylogenetically distant elapid snake. Considering its close phylogenetic relationship with *N. kaouthia* from Southeast Asia, we also evaluated the potential use of Thai Red Cross Society’s *N. kaouthia* monovalent antivenom for the treatment of *N. sagittifera* bites. Our findings unravel the preclinical ineffectiveness of commercial antivenoms against *N. sagittifera* and underscore the necessity to manufacture a specific antivenom product for therapeutic use on these islands.

## Experimental Section

### Venoms and Antivenoms

Venom samples were sourced from an adult *N. naja* from Bannerghatta, Karnataka in mainland India, and a subadult *N. sagittifera* from Port Blair, Andaman Island. Since encountering *N. sagittifera* in the wild is extremely rare, we could not find full grown adult individuals of this species during our sampling trips. Following collection, *N. sagittifera* venom sample was immediately flash frozen and transported back to the lab in a vapor shipper. The snake was released back into the wild after sampling. Samples were collected with permissions from the Karnataka [PCCF(WL)/CR-06/2018-19)] and Andaman and Nicobar Islands [CWLW/WL/134(A)/253] Forest Departments. The venoms were flash-frozen immediately after extraction, lyophilised and stored at −80°C. Details of the commercial antivenoms and venoms tested in this study are provided in Table 1 and Supplementary Table S1, respectively.

**TABLE 1:** Details of antivenom samples investigated in this study.

Manufacturer	Batch	Manufacture (M) and expiry (E) dates	Protein content (mg/ml)	Marketed neutralising efficacy (mg/ml)
Bharat Serums and Vaccines Ltd.	A05318087	M: 10/2018	26.5 ± 0.77	*N. naja*: 0.60
		E: 09/2022		
Haffkine BioPharmaceutical Corporation Ltd.	AS180611	M: 06/2018	24.7 ± 0.5	*D. russelii*: 0.60
		E: 11/2022		
Premium Serums & Vaccines Pvt. Ltd.	ASVS(I)-Lyo013	M: 11/2018	26.2 ± 1.2	*E. carinatus*: 0.45
		E: 11/2022		
VINS Bioproducts Ltd.	01AS18067	M: 11/2018	31.4 ± 0.54	*B. caeruleus*: 0.45
		E: 10/2022		
Queen Saovabha Memorial Institute	NK00112	M: 03/2012	25.9 ± 0.44	*N. kaouthia*: 0.60
		E: 02/2017		

### Ethical Statements

Venom toxicity and neutralisation experiments were conducted in the murine model following WHO guidelines. Animal ethical approval was granted by the Institutional Animal Ethics Committee (IAEC), Indian Institute of Science (IISc), Bangalore (CAF/Ethics/770/2020 and CAF/Ethics/814/2021). Guidelines issued by the Committee for the Purpose of Control and Supervision of Experiments on Animals (CPCSEA) were followed while conducting these experiments. Collection of fresh blood for coagulation assay was obtained from healthy adult volunteers with written consent and approved by the Institutional Human Ethical Committee (IHEC No: 5-24072019 and 18/20201216).

### DNA Isolation and Sequencing

Scale samples were harvested from the ventral side of the snake using microscissors and preserved in molecular grade absolute ethanol. The genomic DNA was isolated using Xpress DNA Tissue kit following the manufacturer’s protocol (MagGenome, United Kingdom) and the purity of the isolate was determined by measuring the 260/280 nm wavelength absorbance ratio with an Epoch 2 microplate spectrophotometer (BioTek Instruments, Inc., United States). Mitochondrial markers (ND4 and cyt *b*) were amplified by performing polymerase chain reaction (PCR) on a ProFlex PCR System (Thermo Fisher Scientific, MA, United States) using universal primers (Arevalo et al., 1994; Palumbi, 1996; Harvey et al., 2000) (Supplementary Table S2). Components of PCR reaction mixtures for a total volume of 50 μl were: 2 μl of template DNA (∼50 ng), 25 μl of Taq DNA Polymerase Mastermix [Tris-HCl pH 8.5, (NH_4_)_2_SO_4_, 3 mM MgCl_2_, 0.2% Tween 20, 3 μl of 25 mM MgCl_2_, 0.4 mM dNTPs, Amplicon Taq polymerase], 2.5 μl each of forward and reverse primers and 18 μl nuclease-free water. The PCR program was set as follows: initial denaturation at 94°C for 5 min, followed by 35–40 cycles of denaturation (94°C for 30 s), annealing (T_A_ for 35 s) and extension (72°C for 2 min) and a final extension step for 10 min at 72°C. QIAquick PCR and Gel Cleanup Kit (Qiagen) were used for gel purification, and the amplicons were sequenced on an Applied Biosystems 3730xl platform (Thermo Fisher Scientific, MA, United States). The sequence data were acquired with Sequence Scanner Software v2.0. Datasets used for phylogenetic reconstructions, in addition to the cyt *b* and ND4 sequences generated in this study, have been provided in Supplementary Files S1, S2.

### Phylogenetics

Homologues of the sequenced mitochondrial markers were retrieved from NCBI’s GenBank repository using BLAST searches, and the nucleotide datasets were compiled and manually curated. Clustal-Omega algorithm was used for generating multiple sequence alignments (Sievers et al., 2011), and the phylogenetic histories of *Naja* spp. were reconstructed using a maximum likelihood approach implemented in the PhyML package (Guindon et al., 2010). Smart Model Selection (SMS) tool on the ATGC server (Lefort et al., 2017) was used to determine the best nucleotide substitution scheme and the phylogenetic trees were subsequently built using the Subtree-Pruning-Regrafting (SPR) method of tree topology search, and the node support was derived with 100 bootstrapping replicates. Additionally, Bayesian inference-based phylogenies were estimated using MrBayes 3.2.7 (Altekar et al., 2004; Ronquist et al., 2012). The analyses, which were parallelised on four independent runs, each executing eleven simultaneous Markov chain simulations, were programmed to terminate after 10 million generations or when the standard deviation of split frequencies converged to 0.01. At every 100th generation, trees and the corresponding parameter estimates were sampled, and the first 25% of these were discarded as burn-in. Finally, a majority-rule consensus tree was generated with the posterior probability for each node. The evolutionary divergence between sequences for each gene was determined by estimating *p*-distances using MEGA X (Kumar et al., 2018) with 100 bootstrap replicates.

### Protein Quantification

The total protein content of venoms and antivenoms were estimated using the Bradford method, wherein crude venom was incubated with 250 µl of Bradford reagent (Thermo Fisher Scientific, United States), and absorbance was recorded at 595 nm on an Epoch 2 microplate reader (BioTek Instruments, Inc., United States). Bovine Serum Albumin (BSA) and Bovine Gamma Globulin (BGG) served as standards for protein estimation of venoms and antivenoms, respectively (Bradford, 1976).

### Sodium Dodecyl Sulphate Polyacrylamide Gel Electrophoresis (SDS-PAGE)

*N. sagittifera* and *N. naja* venoms were subjected to reducing SDS-PAGE to evaluate the qualitative and quantitative differences in venoms. Briefly, venom samples (12 µg) were loaded into 12.5% gel, followed by an electrophoretic separation in Tris-Glycine-SDS buffer at 80 V (Smith, 1984). The Precision Plus Protein Dual Color Xtra Standard (Bio-Rad Laboratories, United States) was used as a marker, and the gel was stained with Coomassie Brilliant Blue R-250 (Sisco Research Laboratories Pvt. Ltd., India). iBright CL1000 gel documentation system (Thermo Fisher Scientific, United States) was then used for visualising the gel.

### Reversed Phase-High Performance Liquid Chromatography (RP-HPLC)

Snake venom samples (500 µg) were fractionated by RP-HPLC using a Shimadzu LC-20AD series system (Kyoto, Japan) equipped with Shimadzu C18 (25 cm × 4.6 mm, 5 µm particle size, 300 Å pore size) column equilibrated with solution A (0.1% trifluoroacetic acid in HPLC grade water) and a diode array detector (DAD). The elution was carried out at a flow rate of 1 ml/min with the following concentration gradients of solution B (0.1% trifluoroacetic acid in 100% acetonitrile): 5–15% for 10 min, 15–45% over 60 min, 45–70% over 10 min and 70% for 9 min (Lomonte and Calvete, 2017; Senji Laxme R. R. et al., 2021). The absorbance was monitored at 215 nm and venom fractions (1 ml/min) were collected semi-automatically using a fraction collector (Shimadzu FRC-10A, Japan).

### Liquid Chromatography-Tandem Mass Spectrometry (LC-MS/MS)

The RP-HPLC fractions of *N. sagittifera* and *N. naja* venom were subjected to in-solution trypsin digestion, followed by lyophilisation and storage at −80°C. The samples were then reconstituted moments before injection and characterisation with tandem mass spectrometry (Rashmi et al., 2021). Samples (40 µg) were first reduced with 10 mM dithiothreitol (DTT), alkylated using 30 mM iodoacetamide (IAA) and further digested with trypsin (0.2 µg/µl) overnight at 37°C. The digested samples were run through a C18 nano-LC column (50 cm × 75 µm, 3 µm particle size and 100 Å pore size) with a Thermo EASY nLC 1200 series system (Thermo Fisher Scientific, MA, United States). Elution was carried out at a constant flow rate of 300 nl/min for 120 min with buffer A (0.1% formic acid in HPLC grade water) and gradient buffer B (0.1% formic acid in 80% acetonitrile) as follows: 10–45% over 98 min, 45–95% over 4 min and 95% over 18 min. A Thermo Orbitrap Fusion Mass Spectrometer (Thermo Fisher Scientific, MA, United States) was used for mass spectrometric analyses of the samples. MS scans were performed using the following parameters: scan range (m/z) of 375–1700 with a resolution of 120,000 and maximum injection time of 50 ms. Fragment scans (MS/MS) were performed using an ion trap detector with high collision energy fragmentation (30%), scan range (m/z) of 100–2000, and maximum injection time of 35 ms.

For the identification of various toxin families in the proteomic profiles of venom fractions, the raw MS/MS spectra were searched against the NCBI-NR Serpentes databases (taxid: 8570) using PEAKS Studio X Plus (Bioinformatics Solutions Inc., ON, Canada) with the following parameters: parent and fragment mass error tolerance limits of 10 ppm and 0.6 Da, respectively; “monoisotopic” precursor ion search type; “semispecific” trypsin digestion; cysteine carbamidomethylation (+57.02) as a fixed modification; methionine oxidation (+15.99) as a variable modification and False Discovery Rate (FDR) of 0.1. Mass spectrometry data has been deposited to the ProteomeXchange Consortium *via* the PRIDE partner repository (Perez-Riverol et al., 2019) with data identifier: PXD025362. Protein families with at least one unique matching peptide were considered for downstream analyses. The redundant protein hits from each protein family were removed manually, and their relative abundance in a fraction was determined by calculating its area under the spectral intensity curve (AUC) relative to the overall AUC for all protein families in that fraction. AUC values obtained from PEAKS Studio analyses, representing the mean spectral intensities, were normalised across fractions using the percentage of peak areas for the respective RP-HPLC fractions (Tan et al., 2017; Rashmi et al., 2021). The relative abundance of a protein family hit (X) was estimated using the equation below, where “N” indicates the total number of fractions obtained from RP-HPLC and “n” corresponds to the fraction number.

$$Relative\;abundance\;of\;X\;\left(\%\right)\;=\overset{N}{\underset{n=1}{\sum }}\frac{AUC\;of\;X\;in\;Fraction\;F_{n\;}\times \;AUC\;of\;the\;chromatographic\;fraction\;F_{n}\;\left(\%\right)}{Total\;AUC\;of\;all\;protein\;families\;in\;Fraction\;F_{n}\;}$$

### Biochemical Characterisation

#### Phospholipase A_2_ Assay

PLA_2_ activity of *N. sagittifera* and *N. naja* venoms was evaluated using modifications to a previously described protocol (Marinetti, 1965; Joubert and Taljaard, 1980; Senji Laxme R. R. et al., 2021). In brief, a substrate solution containing chicken egg yolk dissolved in 0.9% NaCl was freshly prepared with an absorbance of 1 at 740 nm. Time-dependent kinetics of varying amounts of *Naja* venoms (0.01, 0.1, 0.5, and 1 µg) were assayed for their PLA_2_ activity in triplicates. 250 µl of the egg yolk substrate solution was added, and the absorbance was monitored at 1 min intervals for 60 min at 740 nm using an Epoch 2 microplate spectrophotometer (BioTek Instruments, Inc., United States). The unit PLA_2_ activity was estimated as the amount of crude venom that reduces the absorbance of the substrate by 0.01 OD unit per minute (Joubert and Taljaard, 1980).

#### Venom Protease Assay

*N. sagittifera* and *N. naja* venoms were analysed for their proteolytic activities using previously published protocols (Chowdhury et al., 1990). 10 µl of the crude venom was incubated with 80 µg azocasein (Sigma-Aldrich, United States) substrate for 90 min at 37°C. Following the incubation period, the enzymatic reaction was quenched by the addition of 200 µl of trichloroacetic acid. The supernatant of this mixture, obtained by centrifuging at 1,000 × g for 5 min, was mixed with equal volumes of 0.5 M NaOH and the absorbance was measured at 440 nm in an Epoch 2 microplate spectrophotometer (BioTek Instruments, Inc., United States). Purified bovine pancreatic protease (Sigma-Aldrich, United States) was used as a positive control to estimate the relative proteolytic activity of *Naja* venoms.

#### L-Amino Acid Oxidase Assay

The LAAO activity of *N. sagittifera* and *N. naja* venoms was evaluated with a previously described endpoint assay (Kishimoto and Takahashi, 2001; Senji Laxme R. R. et al., 2021). 90 µl of the L-leucine substrate solution (5 mM L-leucine, 50 nM Tris-HCl buffer, 5 IU/ml horseradish peroxidase, 2 mM o-phenylenediamine dihydrochloride) was incubated with the crude venom (10 µl) at 37°C for 60 min. Following incubation, 2 M H_2_SO_4_ solution was added to terminate the reaction, and the absorbance was recorded at 492 nm using an Epoch 2 microplate spectrophotometer.

#### DNase Assay

0.05 µg/µl of crude venom in 1X phosphate buffered saline (PBS) was mixed with purified calf thymus DNA (Sigma-Aldrich, United States) and incubated at 37°C for 60 min to assess the DNase activity of *N. sagittifera* and *N. naja* venoms (Gerceker et al., 2009; Senji Laxme R. R. et al., 2021). Post-incubation, these reaction mixtures were subjected to 0.8% agarose gel electrophoresis. GelRed (Sigma-Aldrich, United States) nucleic acid stain was used to visualise DNA in an iBright CL1000 gel documentation system (Thermo Fisher Scientific, United States). The percentage DNase activity of the venom samples, and that of the positive control, was calculated by densitometric analysis of the images using the ImageJ software (Schneider et al., 2012)

#### Fibrinogenolytic Assay

The fibrinogenolytic activity of *N. sagittifera* and *N. naja* venoms was elucidated using an electrophoresis-based protocol (Teng et al., 1985). A predefined concentration of venom (1.5 µg) based on protein content was incubated with human fibrinogen (15 µg) (Sigma-Aldrich, United States) in 1X PBS (pH 7.4) at 37°C for 60 min. An equal volume of the loading dye solution (1 M Tris-HCl, pH 6.8; 50% Glycerol; 0.5% Bromophenol blue; 10% SDS; and 20% β-mercaptoethanol) was added to the reaction mixture, and the resulting mixture was then heated at 70°C for 10 min. Samples were subjected to 15% SDS-PAGE, and the gel was subsequently stained with Coomassie Brilliant Blue R-250. An iBright CL1000 gel documentation system (Thermo Fisher Scientific, United States) was used to visualise the stained gels. A negative control containing human fibrinogen alone was used to compare and interpret the results.

### Plasma Coagulation Assays

The abilities of *N. sagittifera* and *N. naja* venoms in altering the two central blood coagulation cascades (i.e., the intrinsic and extrinsic pathway) was assessed by measuring the activated partial thromboplastin time (aPTT) and prothrombin time (PT). Whole blood from healthy human volunteers was collected in 2% Sodium citrate tubes (BD Vacutainer®) after obtaining written consent. Platelet-poor plasma (PPP) was obtained by centrifuging the whole blood at 3,000 × g for 10 min at 4°C. Reagents, specific for PT and aPTT tests [i.e., prewarmed calcium thromboplastin reagent (200 µl of Uniplastin; Tulip diagnostics, Mumbai) and activated cephaloplastin (100 µl of Liquicelin-E; Tulip diagnostics, Mumbai), along with 0.02 M calcium chloride (100 µl of CaCl_2_), respectively], was mixed with 50 µl PPP. This mixture was then treated with four distinct amounts of *Naja* venoms (5, 10, 20, and 40 µg), and the time taken for the formation of the first fibrin clot was measured on a Hemostar XF 2.0 coagulometer (Tulip Diagnostics).

### Haemolytic Assays

The extent of snake venom-induced haemolysis was measured by assessing the destruction of human red blood cells (RBCs) using a previously described method (Maisano et al., 2013; Senji Laxme et al., 2019). By centrifugation at 3,000 × g for 10 min at 4°C, RBCs were separated from the whole blood and resuspended in 1X PBS (pH 7.4). This step was repeated five times to remove undesired clotting factors and debris. An RBC solution (1%) was then prepared and mixed with varying concentrations of *N. naja* and *N. sagittifera* venoms (5, 10, 20, and 40 µg). These mixtures were then incubated at 37°C for 24 h and followed with centrifugation at 3,000 × g for 10 min. The absorbance of the supernatant was measured at 540 nm in an Epoch 2 microplate spectrophotometer (BioTek Instruments, Inc., United States). Triton X (0.5%) was used as the positive control, and the relative haemolytic activity of venoms was calculated with respect to this control.

### Determination of the Median Lethal Dose

Interspecific venom variation was assessed by estimating the median lethal dose or LD_50_ of the venom (the minimum amount of venom that can kill 50% of the test population) using the WHO-recommended murine model of envenoming (WHO, 2018). Five distinct concentrations of *N. naja* and *N. sagittifera* venoms, prepared in physiological saline (0.9% NaCl), were administered intravenously into the caudal vein of male CD-1 mice (200 µl/mouse). The death and survival patterns were recorded for each venom dose group (*n* = 5) 24 h post venom injection. Finally, using Probit analysis, the LD_50_ values were calculated with 95% confidence intervals (Finney, 1971).

### Indirect Enzyme-Linked Immunosorbent Assay

The *in vitro* venom recognition of the major Indian polyvalent and Thai *N. kaouthia* monovalent antivenoms were elucidated using a previously described ELISA protocol (Casewell et al., 2010; Senji Laxme et al., 2019). Venom samples (100 ng per well) were diluted in sodium carbonate-bicarbonate buffer (pH 9.6) and coated onto 96-well plates. Overnight incubation of these coated plates at 4°C was followed by washing with Tris-buffered saline and 1% Tween 20 (TBST). A blocking buffer (5% skimmed milk in TBST) was then added to the plates, and followed by incubation at room temperature (RT) for 3 hours. This was followed by another round of washing with TBST to remove the blocking solution. Various dilutions of antivenoms (Premium Serums, VINS, Bharat, Haffkine and QSMI) were added, and plates were incubated overnight at 4°C. Unbound antibodies were removed the next day by washing the plates with a TBST solution. Horseradish peroxidase (HRP)-conjugated rabbit anti-horse secondary antibody (Sigma-Aldrich, United States), diluted in PBS (1:1000), was then added to these plates, followed by an incubation period of 2 h at RT. The ABTS [2,2'-azino-bis(3-ethylbenzothiazoline-6-sulfonic acid)] substrate solution (Sigma-Aldrich, United States) was added to the plates post-incubation, and the absorbance was measured at a wavelength of 405 nm for 40 min in an Epoch 2 microplate reader. In addition, purified antibodies from naive horses (Bio-Rad Laboratories, United States) were used as a negative control.

### Western Blotting

Venom and antivenom immunoblotting experiments were performed by following a previously described protocol (Casewell et al., 2010). Firstly, crude venoms (20 µg) were subjected to SDS-PAGE in a 12.5% gel, followed by electrotransfer of the separated proteins onto a nitrocellulose membrane, as per the manufacturer’s instructions (Bio-Rad Laboratories, United States). Ponceau S reversible stain was used to verify the transfer efficacy, and the membrane was incubated overnight with a blocking buffer at 4°C. After washing with the TBST, the membrane was incubated overnight with a known concentration (1:200) of commercial antivenom at 4°C. On the following day, an HRP-conjugated rabbit anti-horse secondary antibody was added at a dilution of 1:2000, after six TBST washes to remove the unbound antivenom. Finally, following the manufacturer’s instructions (Thermo Fisher Scientific, United States), an enhanced chemiluminescence substrate was used to visualise the binding efficacy of commercial antivenoms to venom, and the membrane was imaged in an iBright CL1000 (Thermo Fisher Scientific, United States). Densitometric analyses of high- (>50 kDa), mid- (15–50 kDa) and low-molecular-weight bands (<15 kDa) in the immunoblots of all antivenoms against *N. naja* and *N. sagittifera* venoms were performed using ImageJ software (Schneider et al., 2012).

### Venom Neutralisation in the Mouse Model

A best binding antivenom (Bharat Serums) was selected for the *in vivo* venom neutralisation assays based on the results of the *in vitro* binding experiments (ELISA and western blotting). Furthermore, to evaluate whether the increased binding efficiency under *in vitro* conditions translates into effective *in vivo* neutralisation, we also performed these experiments with the antivenom that exhibited relatively poor binding (Premium Serums). The median effective dose (ED_50_), or the minimum amount of antivenom required to save 50% of the test population injected with the challenge dose of venom (five times the LD_50_), was determined for each of these antivenoms (WHO, 2018). Briefly, four dilutions of the antivenom were prepared in the physiological saline (0.9% NaCl) and mixed with the challenge dose. These mixtures were incubated for 30 min at 37°C, and followed with intravenous administration into their respective group male CD-1 mice (*n* = 5). The animals were kept under observation for 24 h, and the number of dead and surviving animals were documented. When the antivenom was found to be ineffective in neutralising 5X LD_50_ of the snake venom (*N. sagittifera*: 2.375 mg/kg; *N. naja*: 4.2 mg/kg), the experiment was repeated with a 3X LD_50_ challenge dose (*N. sagittifera*: 1.425 mg/kg; *N. naja*: 2.52 mg/kg). Probit analysis was used for calculating the ED_50_ values with 95% confidence intervals (Finney, 1971). Neutralisation potency of the antivenoms against *N. naja* and *N. sagittifera* venoms was calculated using the formula below, where ‘n’ indicates the number of LD_50_ used as the challenge dose (Mendonca-da-Silva et al., 2017; Senji Laxme et al., 2019).

$$Antivenom\;neutralisation\;potency\;\left(mg/ml\right)=\frac{\left(n-1\right)\;\times \;LD_{50}\;of\;venom\;\left(mg/mouse\right)}{ED_{50}\left(ml\right)}$$

Antivenom potencies were also described in terms of µl of antivenom per mg of the venom (Supplementary Table S8), following a previously described method (Ainsworth et al., 2020).

### Statistical Analyses

Statistical differences in the results of ELISA and biochemical assays were determined using two-way ANOVA and unpaired t-tests, respectively. These comparisons were made using GraphPad Prism (GraphPad Software 9.0, San Diego, California, United States, www.graphpad.com).

## Results

### Phylogenetic Reconstructions

**FIGURE 1:**
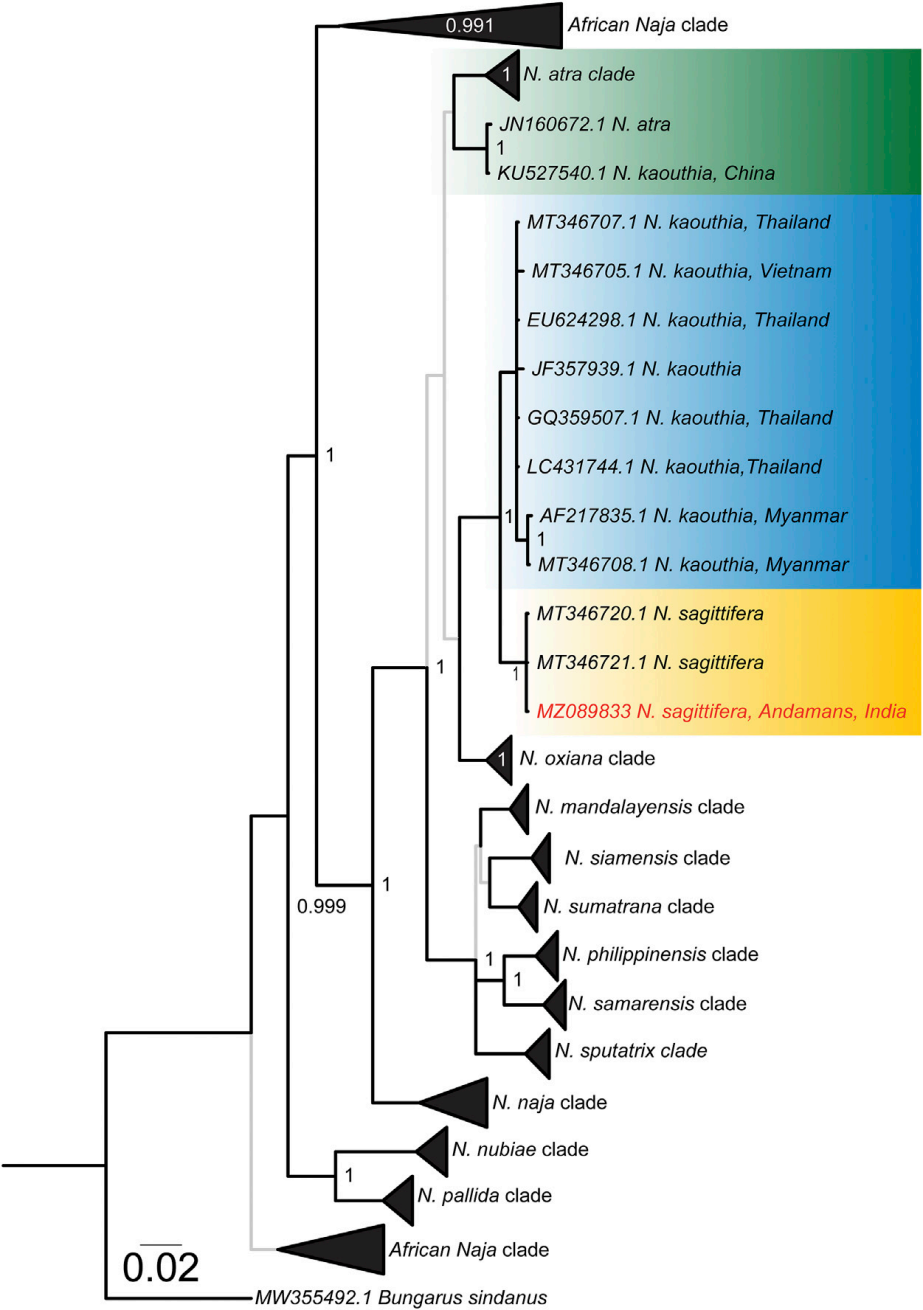
Bayesian cyt *b* phylogeny of *Naja* species. Lineages of interest have been shown in uniquely coloured boxes and the accession number of the individual sequenced in this study has been highlighted in red. Branches with superior (BPP ≥ 0.95) and relatively inferior (BPP ≤ 0.95) node support are shown in thick black and thin grey lines, respectively. Branch lengths are scaled by the number of nucleotide substitutions per site.

Phylogenetic reconstructions of the evolutionary histories of mitochondrial markers (cyt *b* and ND4) provided fascinating insights into the evolution of Asiatic cobras (Figure 1 and Supplementary Figures S1–S4). The overall topology of the *Naja* phylogeny was in agreement with the previously reported mitochondrial species tree (Kazemi et al., 2021). Consistent with the literature, *N. sagittifera* was recovered as a sister lineage to *N. kaouthia* (Figure 1 and Supplementary Figures S1–S4) with significant node support [Bayesian Posterior Probability (BPP): 1; bootstrap (BS): 100]. However, the *N. kaouthia* clade was found to be polyphyletic with two distinct lineages: one consisting of *N. kaouthia* and *N. atra* from China, and the other with the Southeast Asian *N. kaouthia* and *N. sagittifera* (Figure 1 and Supplementary Figures S1–S4). The estimation of evolutionary divergences further supported these findings (Supplementary Tables S3, S4). Limited pairwise differences were observed between cyt *b* (2.45–2.91%) and ND4 (1.51–1.85%) sequences of *N. sagittifera* and *N. kaouthia* from Southeast Asia, while significant differences were found when *N. sagittifera* sequences were compared to *N. kaouthia* from China (6.45 and 5.21%, respectively, for cyt *b* and ND4).

### Venom Proteomics

**FIGURE 2:**
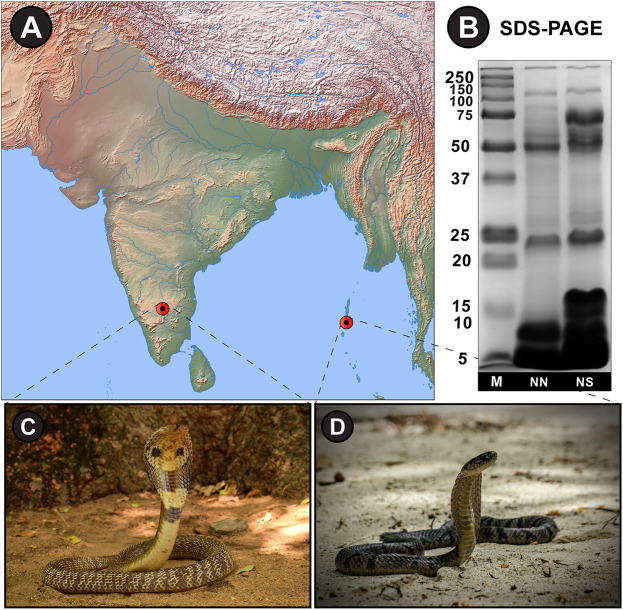
**(A)** Sampling locations from mainland India and Andaman and Nicobar Islands have been shown on a map of India prepared using (QGIS, 2019). **(B)** SDS-PAGE venom profiles and photographs of **(C)** *N. naja* and **(D)** *N. sagittifera*. M: Protein marker (units in kDa); NN: *N. naja*; NS: *N. sagittifera*.

The SDS-PAGE electrophoretic profiles of *N. naja* and *N. sagittifera* unveiled significant differences in band patterns and intensities, highlighting the considerable extent of venom variation in these congeners (Figure 2B).

**FIGURE 3:**
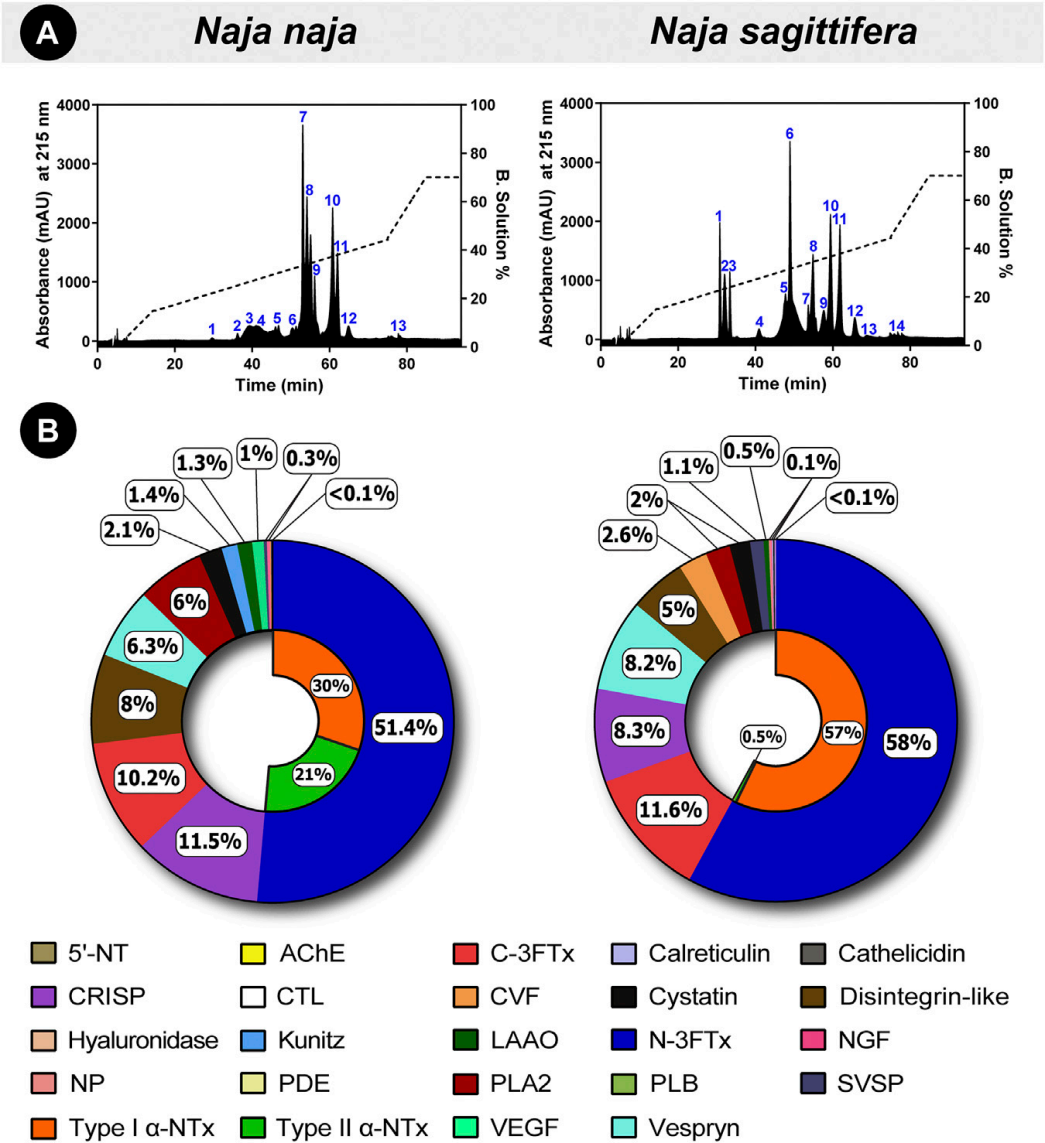
Comparative venom proteomics of *N. naja* and *N. sagittifera.* In this figure, **(A)** RP-HPLC profiles and **(B)** venom proteomes of *Naja* species are shown. Uniquely colour-coded individual fractions in RP-HPLC profiles are numbered from 1–14, whereas the relative abundance of each toxin family is indicated in the doughnut charts as percentages. The relative abundance of N-3FTx and C-3FTx in the two venoms has also been shown.

RP-HPLC profiles revealed remarkable differences in peak areas and intensities, particularly between the retention time of 30–60 min, highlighting the significant compositional differences in the venom proteomes of these snakes (Figure 3A). Furthermore, mass spectrometric analysis of individual RP-HPLC fractions of *N. naja* and *N. sagittifera* venoms against NCBI-NR Serpentes databases (taxid: 8570) identified 101 and 103 non-redundant protein families, respectively (Figure 3B; Supplementary Tables S5, S6; Supplementary File S3). The identified toxin proteins belonged to 20 toxin families, namely three-finger toxin [3FTx; neurotoxic-3FTx (N-3FTx) and cytotoxic-3FTx (C-3FTx)], cysteine-rich secretory proteins (CRISP), disintegrin-like, vespryn, phospholipase A_2_ (PLA_2_), cobra venom factor (CVF), cystatin, vascular endothelial growth factor (VEGF), L-amino-acid oxidase (LAAO), nerve growth factor (NGF), Kunitz-type serine protease inhibitor (Kunitz), calreticulin, 5’-nucleotidase (5’-NT), natriuretic peptide (NP), C-type lectin (CTL), phosphodiesterase (PDE), hyaluronidase, phospholipase B (PLB), acetylcholinesterase (AChE) and snake venom serine protease (SVSP) (Figure 3B; Supplementary Tables S5, S6). Interestingly, cathelicidin antimicrobial peptides (de Barros et al., 2019) were discovered in *N. naja* venom from Karnataka, emphasising the biodiscovery potential of Indian snake venoms. PLA_2_ inhibitors (PLI) were also detected in both *N. naja* and *N. sagittifera* venoms, which have been theorised to play a role in preventing self-envenomation in snakes (Kochva, 1978). These anti-toxins have also been previously reported from *N. naja* and *D. russelii* venoms from various parts of India (Senji Laxme RR. et al., 2021; Senji Laxme R. R. et al., 2021).

Further analysis of mass spectrometry data revealed that N-3FTx are the major toxic components in the venoms and they comprised 51% of *N. naja* and 58% of *N. sagittifera* protein content**.** However, considerable differences were noted in the relative abundance of α-neurotoxins (Figure 3B; Supplementary Tables S5, S6). Type I (or short) neurotoxins were found to be more prevalent in *N. sagittifera* venom (57%) than in the *N. naja* (30%) venom, whereas Type II (or long) neurotoxins were significantly more in *N. naja* venom (21%), in comparison to the trace amounts detected in *N. sagittifera* venom (0.5%). In contrast to the abundance of N-3FTx, C-3FTxs were nearly equally abundant in the *N. sagittifera* (12%) and *N. naja* (10%) venoms. Slight differences in the abundances of PLA_2_s were also observed. While 6% of the *N. naja* venom consisted of this toxin type, *N. sagittifera* venom contained only 2% (Figure 3B; Supplementary Tables S5, S6). Similarly, minor differences in the abundances of disintegrin, CRISP, vespryn, LAAO, Kunitz, SVSP and CVF were documented. Interestingly, certain toxin families, including NP, CTL, hyaluronidase, serpin, and calreticulin, were only detected in the venom of *N. sagittifera* (Figure 3B; Supplementary Tables S5, S6). Thus, our findings reveal that the major distinction between the proteomic composition of *N. naja* and *N. sagittifera* venom is in the relative abundance of α-neurotoxins.

### Venom Biochemistry

#### phospholipase A_2_ Assay

PLA_2_s are amongst the most important and widespread snake venom superfamilies and are known to be responsible for various pharmacological manifestations in bite victims (Kini, 2003; Tasoulis and Isbister, 2017). Taking the clinical significance of this toxin into account, we assessed the PLA_2_ activity of *N. naja* and *N. sagittifera* venoms. Despite the relatively lower abundance of PLA_2_s in the venom proteome of *N. sagittifera* (2%) in comparison to *N. naja* (6%), the former exhibited slightly increased PLA_2_ activity (*p* < 0.05; Table 2; Supplementary Figure S5A).

**TABLE 2:** Biochemical activities of *N. naja* and *N. sagittifera* venoms.

Enzyme activity	*N. naja*	*N. sagittifera*
Phospholipase A2 (Specific activity in U/mg)	89 ± 0.9	106 ± 1.1^a^
Protease (% Relative activity)	0.5 ± 0.4%	1.5 ± 0.8%
L-amino acid oxidase (Absorbance at 492 nm)	1.7 ± 0.05^a^	1.3 ± 0.03
DNase (% Relative activity)	93.1%	98.8%^a^

a Denotes comparisons that were statistically significant (*p* < 0.05).

#### Venom Protease Assay

Elapid venom proteases, such as snake venom serine proteases and disintegrin-like toxins, are known to inflict many coagulopathies, including the inhibition of platelet aggregation, and thrombin-, plasminogen-, and bradykinin-like effects (Kalogeropoulos et al., 2019; Senji Laxme R. R. et al., 2021). Therefore, we assayed the protease activities of *N. naja* and *N. sagittifera* venoms, in comparison to that of the bovine pancreatic protease (positive control). Both *Naja* venoms were found to exhibit limited ability in cleaving the azocasein substrate (*p* > 0.05; Table 2; Supplementary Figure S5B), which corresponds to the lower abundance of these proteases in the venoms of these snakes.

#### L-Amino Acid Oxidase Assay

LAAOs in snake venoms have been shown to exhibit cytotoxic effects and induce or inhibit platelet aggregation. In the presence of LAAO, L-amino acids undergo oxidative deamination to form α-keto acids and hydrogen peroxide (H_2_O_2_). H_2_O_2_ results in the accumulation of reactive oxygen species (ROS), leading to oxidative stress and cell death (Hiu and Yap, 2020). Upon assessing the LAAO activities of *Naja* venoms, it was found that the venom of *N. naja* has significantly higher LAAO activity (*p* < 0.05) compared to that of its island-counterpart (Table 2; Supplementary Figure S5C), which correlates with the relative abundance of LAAOs in the two venoms.

#### DNase Assay

DNases in elapid snake venoms are shown to work synergistically with other venom toxins and cause clinically severe manifestations in snakebite victims (Katkar et al., 2016). When the venoms of *N. naja* and *N. sagittifera* were tested for their ability to cleave the purified DNA from calf thymus, both venoms exhibited significant DNase activity (93.10 and 98.83%, respectively), which was comparable to that of the positive control: purified DNase I from bovine pancreas (94.11%; Table 2; Supplementary Figures S5D, S6).

#### Fibrinogenolytic Assay

Fibrinogen, also known as clotting factor I, is a soluble glycoprotein complex comprising of Aα, Bβ, and *γ* subunits. Upon infliction of a wound, fibrinogen undergoes enzymatic conversion in the presence of thrombin to form an insoluble fibrin clot, maintaining haemostasis (Weisel and Litvinov, 2017). Certain snake venom toxins, such as CTLs, SVSPs and snake venom metalloproteinases (SVMPs), have been documented to perturb haemostasis in bite victims by exhibiting fibrinogenolytic activity (Yamazaki and Morita, 2007). When the fibrinogenolytic activities of *N. naja* and *N. sagittifera* venoms were evaluated, both venoms were found to completely degrade the Aα subunit of the human fibrinogen, while both Bβ and γ subunits remained intact (Supplementary Figure S7).

### Coagulation Assays

**FIGURE 4:**
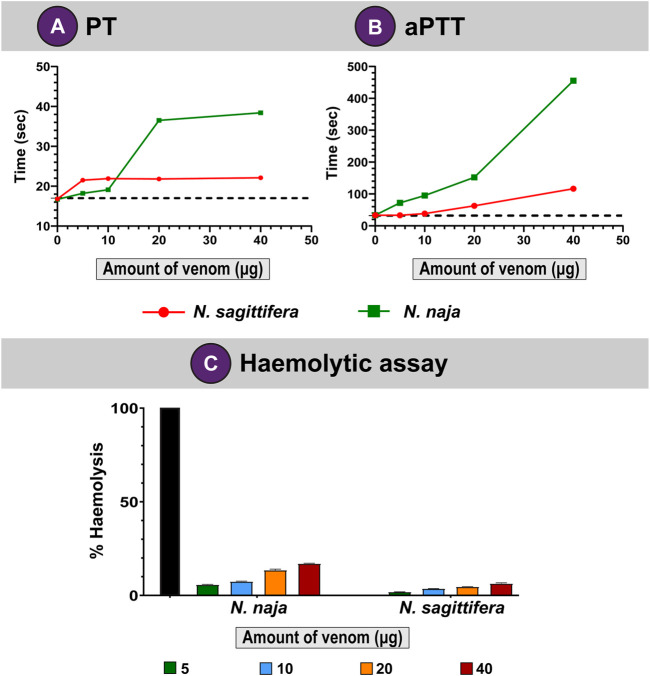
Coagulopathies induced by *N. naja* and *N. sagittifera* venoms. Effects of *N. naja and N. sagittifera* venoms on **(A)** extrinsic and **(B)** intrinsic pathways are depicted as line graphs. **(C)** Haemolytic abilities of *N. naja* and *N. sagittifera* venoms have also been shown. The horizontal dotted line in **(A,B)** indicate the plasma clotting time in seconds for the control sample.

Snake venoms are known to perturb the coagulation cascade of snakebite victims (Kumar et al., 2010; Gowtham et al., 2012; Das et al., 2013; Senji Laxme et al., 2019). Hence, we evaluated the abilities of *N. naja and N. sagittifera* venoms on the intrinsic and extrinsic coagulation cascades *via* PT and aPTT experiments, respectively. In these assays, the *N. naja* venom was found to affect the intrinsic coagulation pathway (aPTT) considerably and exhibited strong anticoagulatory properties (delayed coagulation by 422 s) under *in vitro* conditions at the highest tested concentration of the venom (40 µg; Figure 4B). Moreover, its congener from the island, *N. sagittifera* (40 µg)*,* altered the intrinsic pathway in a relatively limited manner (delayed coagulation by 83 s), highlighting the significant difference between the two venoms in inflicting these effects (Figure 4B). Similarly, *N. naja* venom (40 µg) affected the extrinsic (PT) coagulation cascade to a greater extent and more than doubled the time required for blood coagulation (a delay of coagulation by 20 s), when compared to *N. sagittifera* venom (a delay of coagulation by 4 s) which had limited effect on the extrinsic cascade (Figure 4A).

### Haemolytic Assay

Snake venom toxins such as PLA_2_s have been shown to hydrolyse the phospholipid membranes of red blood cells (RBCs) and disrupt haemostasis (Stoykova et al., 2013). When we evaluated the haemolytic potential of *N. naja* and *N. sagittifera* venoms on human RBCs and compared these with the Triton X-100 positive control, the *N. naja* venom (40 µg) showed appreciable haemolytic effects (17%). In contrast, *N. sagittifera* venom (40 µg) was found to exhibit limited haemolytic activity (7%) (Figure 4C). These results are consistent with the relative abundance of venom PLA_2_s in the two snake species (Figure 3B; Supplementary Tables S5, S6).

### *In Vitro* Venom Binding of Commercial Indian Antivenoms

**FIGURE 5:**
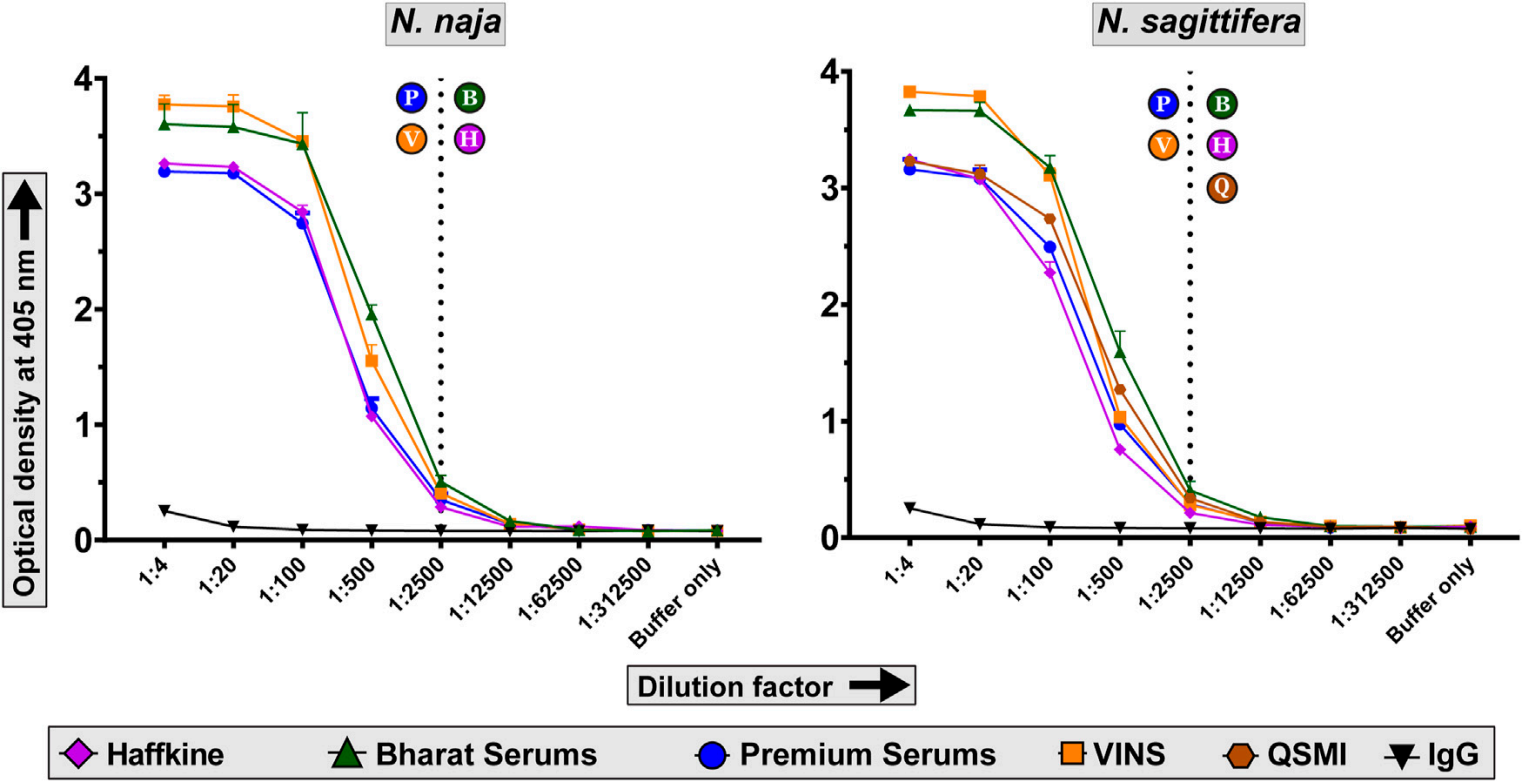
Venom recognition capabilities of commercial antivenoms. The line graphs in this figure indicate the *in vitro* venom binding of naive horse IgG and commercial Indian polyvalent and Thai monovalent antivenoms against *N. naja* and *N. sagittifera*. Multiple dilutions of antivenoms were evaluated in these indirect ELISA experiments. Dotted lines indicate the titre value of antivenoms against the two venoms. The alphabets in circles next to the dotted lines indicate the respective titres of antivenoms: P—Premium Serums and Vaccines Pvt. Ltd.; V—VINS Bioproducts Ltd.; B—Bharat Serums and Vaccines Ltd.; H—Haffkine Bio-Pharmaceutical Corporation Ltd. and Q—Queen Saovabha Memorial Institute monovalent *N*. *kaouthia* antivenom.

We evaluated the abilities of commercial Indian polyvalent antivenoms in recognising *N. naja* and *N. sagittifera* venoms using indirect ELISA assays. In these experiments, a fixed amount of the venom (100 ng) was incubated with varying concentrations of commercial antivenoms, and the absorbance, which corresponds to the venom binding capabilities of antivenoms, was measured at 405 nm. While all tested antivenoms exhibited a titre of 1:2500, Bharat Serums was found to be the best binding antivenom against both *N. naja* and *N. sagittifera* venoms (Figure 5). Bharat Serums exhibited between 1.4 to nearly two times better binding against the *N. sagittifera* venom and between 1.2 and 1.75 times better binding against the *N. naja* venom when compared to its Indian counterparts. Bharat Serums was closely followed by VINS and Premium Serums antivenoms, whereas the Haffkine antivenom exhibited the worst venom recognition capability. Considering their close phylogenetic relationship with *N. kaouthia* snakes, we also evaluated the cross-neutralising potential of Thai monovalent *N. kaouthia* antivenom against *N. sagittifera* venoms from the Andaman and Nicobar Islands. Similar to the Bharat Serums polyvalent antivenom, Thai monovalent *N. kaouthia* antivenom was very effective in recognising the *N. sagittifera* venom (Figure 5).

Moreover, when the binding efficacy of antivenoms to venom toxins was assessed using western blotting experiments, it was found that the Bharat Serums antivenom bound to many low-, mid-, and high-molecular-weight toxins in the venoms of *N. naja* and *N. sagittifera* (Supplementary Figures S8, S9). Further, Thai QSMI monovalent antivenom notably exhibited high binding to low-molecular-weight toxins (<15 kDa) in the *N. sagittifera* venom (Supplementary Figures S8, S9). In contrast, the antivenom manufactured by Premium Serums exhibited poor immunorecognition against both the *Naja* venoms, predominantly binding to high-molecular-weight toxins (>50 kDa), while poorly recognising their low-molecular-weight counterparts (Supplementary Figures S8, S9). Therefore, both *in vitro* binding experiments identified Bharat Serum and Premium Serums as the best- and worst-binding Indian antivenoms, respectively. Furthermore, similarly to the Bharat Serums antivenom, Thai QSMI monovalent antivenom exhibited increased *in vitro* binding to *N. sagittifera* venom (Supplementary Figures S8, S9).

### Toxicity Profiles

**FIGURE 6:**
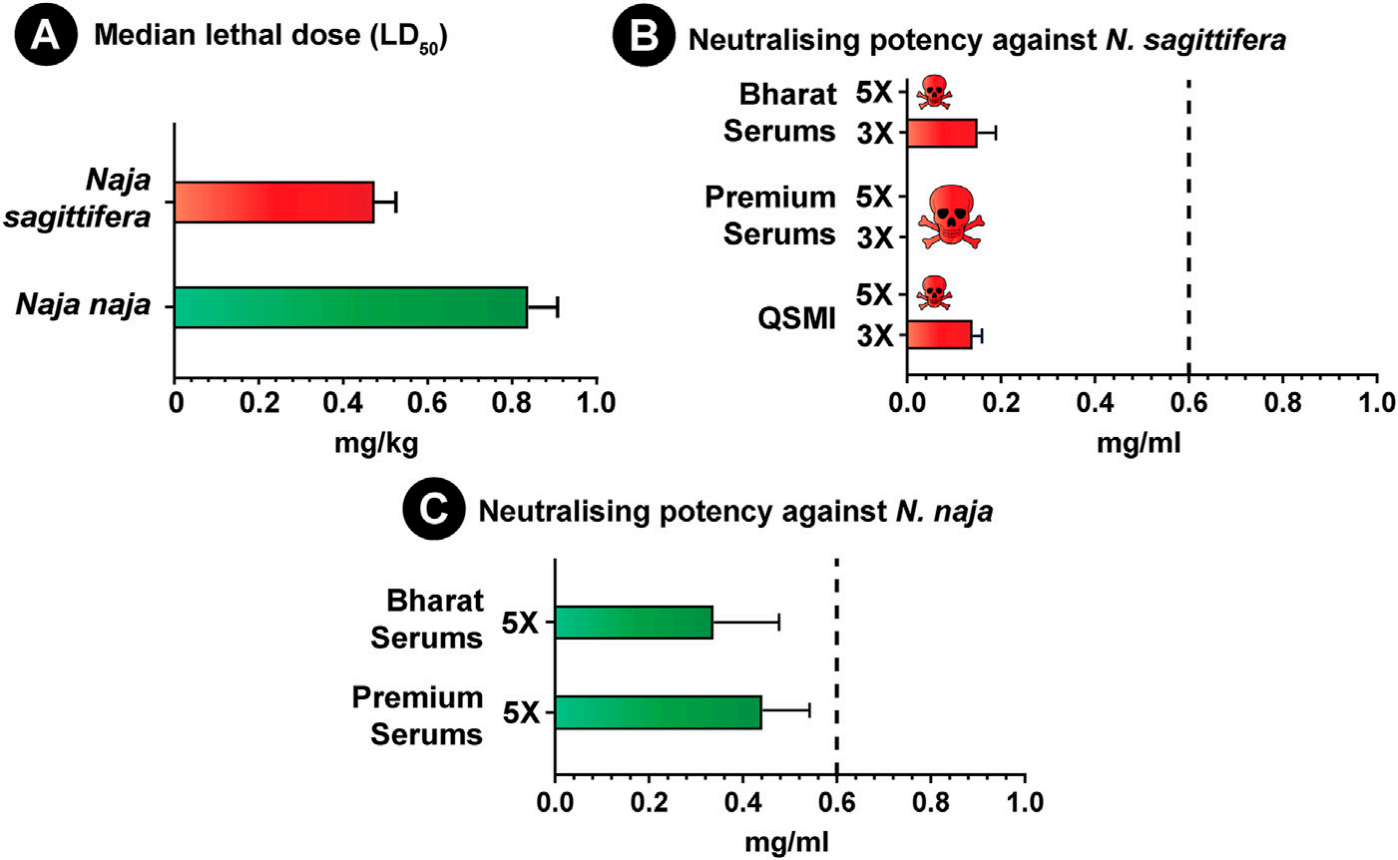
Venom toxicities of *N. naja* and *N. sagittifera* and the neutralisation potential of commercial antivenoms. **(A)** in this figure depicts the venom potencies (mg/kg) of *N. naja* and *N. sagittifera*, while the neutralising potencies (mg/ml) of Indian polyvalent (Premium Serums and Bharat Serums) and Thai monovalent antivenoms against the *Naja* venoms (3X and 5X LD50 challenge dose) are shown in **(B,C)**. The dotted vertical line in **(B,C)** represents the marketed claim of neutralisation (0.60 mg/ml) against *N. naja* and *N. kaouthia* for the Indian polyvalent and Thai monovalent antivenoms, whereas the error bars represent 95% confidence intervals.

The venom potencies of the endemic Andaman cobra and its congener from mainland India were evaluated in a murine model of envenoming using WHO-recommended protocols. The *N. sagittifera* venom (0.475 mg/kg) was found to be nearly two times as potent as the *N. naja* venom (0.84 mg/kg) from mainland India (Figure 6A; Supplementary Table S7). Although N-3FTxs dominated the venoms of both species (>50%), the significant difference in venom toxicities could be attributed to the differences in the abundance of Type I and Type II α-neurotoxins and variations in their sequence compositions. Type I α-neurotoxins were significantly more abundant in *N. sagittifera* venom (57%) than *N. naja* venom (30%).

### Preclinical Assessment of Antivenoms

Given the ethical considerations in animal usage, and based on the results of the *in vitro* binding studies (ELISA and western blotting), a single best binding polyvalent antivenom (Bharat Serums) was selected for the *in vivo* preclinical assessment. However, to evaluate whether the *in vitro* binding potential of antivenoms corresponds to their *in vivo* venom neutralisation efficacy, we also estimated the neutralisation potency of the relatively poor binding Premium Serums antivenom against the venoms of both *Naja* species. The outcomes of these experiments revealed the inadequacy of both the best and worst binding Indian polyvalent antivenoms in neutralising the *N. sagittifera* venom, indicating that the *in vitro* binding does not necessarily correlate with *in vivo* venom neutralisation (Figure 6B; Supplementary Table S8). Furthermore, as test animals rapidly (∼5 min) succumbed to the fatal effects of the venom in experiments with 5X LD_50_ of the challenge dose, neutralisation experiments were repeated by lowering the challenge dose to 3X LD_50_. In these experiments, Bharat Serums antivenom exhibited a meagre 0.151 mg/ml potency (Figure 6B; Supplementary Table S8), while Premium Serums was again completely ineffective in saving mice from the lethal effects of *N. sagittifera* envenoming. These results are suggestive of the preclinical ineffectiveness of Indian antivenoms in treating bites from the Andaman cobra.

As *N. sagittifera* is closely related to *N. kaouthia* from Southeast Asia, we further explored the cross-neutralisation effectiveness of Thai *N. kaouthia* monovalent antivenom manufactured by QSMI in treating *N. sagittifera* bites*.* Similar to its Indian polyvalent counterparts, Thai monovalent antivenom could not protect the experimental animals injected with 5X LD_50_ of venom. However, when the challenge dose was reduced to 3X LD_50_, this antivenom exhibited a very poor neutralisation potential (0.140 mg/ml; Figure 6B; Supplementary Table S8), highlighting the negative impact of geographical and phylogenetic divergence between the two species on antivenom’s effectiveness.

Both Indian antivenoms were also severely limited in their ability to neutralise the venom of *N. naja* from mainland India, as they were found to exhibit a neutralisation potential (Premium Serums: 0.442 mg/ml; Bharat Serums: 0.338 mg/ml; Figure 6C; Supplementary Table S8) that was significantly lower than the marketed value (0.6 mg/ml) when tested at 5X LD_50_ challenge dose. These results are congruent with a previous study, wherein the Indian polyvalent antivenom showed poor neutralising efficacy against *N. naja* venoms from various biogeographical zones of the Indian subcontinent (Senji Laxme R. R. et al., 2021).

## Discussion

### The Phylogenetic History of the Enigmatic Andaman Cobra

The Andaman and Nicobar Islands, which extend over an area of 800 km^2^, are separated from Southeast Asia and mainland India by the Andaman Sea and Bay of Bengal, respectively. The archipelago, comprising 572 islands, was once a part of the Asian mainland that later got detached due to geological events during the Upper Mesozoic and became submerged underwater [100 million years ago (MYA)] (Sivaperuman and Deepak, 2013). The archipelago finally re-emerged in the Late Miocene (10 MYA) (Lee and Lawver, 1995). Interestingly, the Andaman and Nicobar Islands were never a contiguous landmass and are geographically separated by a narrow water canal: the Ten-Degree (10°) channel (Ripley and Beehler, 1989). These islands are a part of two biodiversity hotspots: the Indo-Burma region consisting of the Andaman Islands, and Sundaland comprising the Nicobar Islands (Myers et al., 2000). It is believed that before its separation from the Asian mainland, the contiguous landmass of Andaman and Nicobar Islands may have had an influx of organisms from the mainland (Ripley and Beehler, 1989). This proposition is further corroborated by the phylogenetic affinity of Andaman and Nicobar herpetofauna with their counterparts in the Indo-China region (Das, 1999). However, as a consequence of geographic distance and saltwater barrier, species found on these islands experienced reduced gene flow that resulted in highly divergent populations/species (MacArthur and Wilson, 2001).

The Andaman and Nicobar Islands are an abode to a vast floral and faunal biodiversity due to the conducive tropical climatic conditions. In addition to mammalian and marine fauna, the archipelago harbours diverse herpetofauna with over 64 reptilian species, including 31 endemics (Tikader, 1992; Srivastava and Ambast, 2009). These include several species of snakes, such as Anderson’s pit viper (*T. andersonii*), Andaman krait (*B. andamanensis*) and Andaman cobra (*N. sagittifera*), that can potentially inflict medically significant envenomation in humans (Smith, 1941). Among these, the Andaman cobra probably causes a large number of snakebites, primarily due to its occurrence in the vicinity of human settlements and agricultural cultivations. Despite their clinical importance, *N. sagittifera* has received limited research attention. Though initially considered a conspecific of the monocled cobra (*N. kaouthia*), these snakes were elevated to species rank based on molecular phylogenetics (Wüster et al., 1995). Consistent with the literature (Kazemi et al., 2021), mitochondrial phylogeny of the genus *Naja* recovered *N. sagittifera* as a sister lineage to one of the branches of the polyphyletic *N. kaouthia* clades (Figure 1).

### Evolutionary Divergence has Profound Effects on Venom Composition, Activities and Toxicities

As a consequence of local adaptation to shifts in ecology and environment, stark inter and intraspecific differences in venom composition and toxicity profiles have been observed in snakes (Daltry et al., 1996; Sunagar et al., 2014; Rokyta et al., 2017; Casewell et al., 2020; Senji Laxme RR. et al., 2021; Senji Laxme R. R. et al., 2021), even within a restricted geographic locale (Rashmi et al., 2021). Consistently, the characterisation of venom proteomes in this study revealed considerable differences in the venoms of *N. naja* and *N. sagittifera*. While RP-HPLC profiling unravelled distinct differences in intensities and areas of peaks, tandem mass spectrometry of these fractions revealed the domination of N-3FTxs (>50%) in the venoms of both *Naja* species. However, a prominent difference in the relative abundance of Type I and Type II α-neurotoxins was noted. Perhaps, this variation in the relative abundance of α-neurotoxins, as well as sequence variations in venom toxins, underpins the increased toxicity of *N. sagittifera* venom to mice (0.475 mg/kg) when compared to *N. naja* (0.84 mg/kg)*.*

Moreover, as snake venoms are complex concoctions of various macromolecules, we also subjected these venoms to *in vitro* biochemical and pharmacological assays, including PLA_2_, protease, LAAO, fibrinogenolytic, blood coagulation, and haemolytic assays. Considerable differences in PLA_2_ (Supplementary Figure S5A), LAAO (Supplementary Figure S5C), PT and aPTT (Figure 4) activities of *N. naja* and *N. sagittifera* venoms were noted. Additional experiments are necessary to elucidate the biological and clinical relevance of these differences in activities. However, the significant variations documented in proteomic composition, toxicity profile and functional activity between the mainland *N. naja* and island-endemic *N. sagittifera* venoms are very likely to result in varied clinical manifestations and therapeutic implications in snakebite victims.

### The Preclinical Inefficacy of Indian Polyvalent Antivenoms in Andaman and Nicobar Islands

*In vitro* and *in vivo* experiments in this study revealed alarming preclinical deficiencies of the Indian polyvalent “big four” and Thai monovalent *N. kaouthia* antivenoms in neutralising the venoms of *N. naja* and *N. sagittifera.* Indirect ELISA and western blotting experiments identified Bharat Serums antivenom as the best binding Indian antivenom against both *N. naja* and *N. sagittifera* venoms, while VINS and Haffkine antivenoms showed intermediary *in vitro* venom recognition. In contrast, the Premium Serums antivenom exhibited the worst venom binding against both *Naja* venoms. In contrast, Thai *N. kaouthia* monovalent antivenom was also found to exhibit significant immunological cross-reactivity against the *N. sagittifera* venom.

Following the tenets of the three R’s in animal ethics (reduction, replacement and refinement), we utilised the results of the *in vitro* binding experiments to downselect the best binding antivenom for the *in vivo* preclinical assessment in mice. A statistically significant correlation between the *in vitro* binding effectiveness of antivenoms and their *in vivo* venom neutralising potency has been shown in the past (Theakston and Reid, 1979; Rungsiwongse and Ratanabanangkoon, 1991). To test whether this correlation holds in the case of the *Naja* species, we evaluated the neutralising efficacies of antivenoms that exhibited the best (Bharat Serum) and worst (Premium Serums) binding in ELISA and western blotting experiments. While Bharat Serum exhibited significantly better binding efficacy in our *in vitro* assays compared to its Indian antivenom counterparts, its preclinical neutralisation in the murine model was extremely poor against both *N. sagittifera* (0.151 mg/ml) and *N. naja* (0.338 mg/ml) and was on par with the neutralising potency of the worst binding Premium Serums antivenom (0.442 mg/ml against *N. naja*; 0 mg/ml against *N. sagittifera*). The plausible explanation for this discrepancy could be the presence of a higher abundance of antibodies against medically unimportant toxins or non-toxic proteins in snake venoms that do not inflict clinical manifestations in envenomed victims (Casewell et al., 2010). This could also be explained by the presence of antibodies against regions in toxins that are not involved in binding interactions with the receptor.

### Phylogenetically Relevant Thai Monovalent *N. kaouthia* Antivenoms are Inadequate for the Treatment of *N. sagittifera* Bites

Due to the presence of many shared toxin families across snakes, antivenoms may exhibit cross-neutralisation against non-target species (de Roodt et al., 1998; Tan et al., 2015; Whiteley et al., 2019; Casewell et al., 2020; Mora-Obando et al., 2021). This cross-neutralisation potential of antivenoms has been previously exploited for the treatment of snakebites. For example, Thai monovalent *N. kaouthia* antivenom*,* and Thai neuro polyvalent snake antivenom manufactured using the venoms of *N. kaouthia*, *O. hannah*, Malayan krait (*B. candidus*) and banded krait (*B. fasciatus*), have been shown to cross-neutralise the Malaysian sea snake (*Hydrophis spp.*) venoms (Tan et al., 2015). Similarly, commercial polyvalent antivenoms raised against the Indian “big four” snake species have been used to treat snakebites in regions where these snakes are absent. Until recently, antivenoms marketed by VINS BioProducts for Central Africa were formulated by hyperimmunising equines with the venoms of Indian Russell’s (*D. russelii*) and saw-scaled (*E. carinatus*) vipers. Other antivenom manufacturers have also adopted similar practices. For example, Premium Serums continues to market Indian “big four” antivenoms to treat hump-nosed pit viper (*Hypnale hypnale*) bites in Sri Lanka.

Considering the preclinical ineffectiveness of the “big four” Indian polyvalent antivenoms and the close phylogenetic relationship between *N. kaouthia* and *N. sagittifera*, we evaluated the potential use of *N. kaouthia* specific Thai monovalent antivenoms for the treatment of *N. sagittifera* envenomation in the Andaman and Nicobar Islands. Unfortunately, although *in vitro* experiments revealed that the QSMI monovalent antivenom binds effectively to *N. sagittifera* venom, our subsequent *in vivo* assessments revealed its limited neutralisation potential (0.14 mg/ml) against the *N. sagittifera* venom, falling well short of the marketed claim of neutralisation of 0.60 mg/ml against the Southeast Asian *N. kaouthia*. This disconcerting lack of neutralising efficacy exhibited by the Indian polyvalent and Thai monovalent antivenoms against *N. sagittifera* further highlights the pressing need to develop regional antivenoms tailored for the treatment of snakebites on the Andaman and Nicobar Islands.

## Conclusion

In conclusion, comparative proteomics, *in vitro* biochemical and pharmacological characterisation, and *in vivo* toxicity assessments revealed significant differences in the venoms of *N. naja* from mainland India (Karnataka) and *N. sagittifera* from Andaman and Nicobar Islands. When the Indian polyvalent antivenoms manufactured using the venoms of the “big four” snakes were tested for their *in vitro* immunorecognition capabilities, Bharat Serums and Premium Serums antivenoms were identified as the best- and worst-performing, respectively. Thai monovalent antivenom manufactured by QSMI using the phylogenetically relevant Southeast Asian *N. kaouthia* venoms too exhibited increased immunorecognition towards the *N. sagittifera* venom. Furthermore, *in vivo* venom neutralisation experiments in the murine model revealed that both the Indian polyvalent (Bharat Serums and Premium Serums) and Thai monovalent (QSMI) antivenoms are inefficacious in neutralising the lethal effects of *N. sagittifera* from Andaman and Nicobar Islands. Unfortunately, the Indian antivenoms were also found to lack desirable potency against the venoms of the Southern Indian population of *N. naja* from Karnataka. It should be noted that extensive sampling efforts across islands from individuals of various age groups of *N. sagittifera* are needed to reveal the extent of intraspecific and ontogenetic venom variation in this species, and its consequent impact on the effectiveness of snakebite therapy in Andaman and Nicobar Islands.

Overall, our results indicate the importance of preclinical assays in evaluating the efficacies of antivenoms and suggest the need to identify key toxins that are medically important. Immunisation of animals with the medically important toxins or toxin fractions, instead of the crude venom, is not only anticipated to reduce the abundance of therapeutically irrelevant antibodies in the finished product, but will also reduce the number of vials required to effect cure by enhancing antivenom’s dose effectiveness. Furthermore, in line with our previous findings (Senji Laxme RR. et al., 2021; Senji Laxme R. R. et al., 2021), the results of this study highlight the significance of phylogenetic and biogeographic considerations for the manufacture of commercial antivenoms in the Indian subcontinent. As our *in vivo* venom toxicity and neutralisation experiments revealed that *N. sagittifera* is more toxic than *N. naja* and tested antivenoms failed to reverse lethality of the venoms, it demonstrates the necessity to investigate the venoms of the medically important yet neglected species of Indian snakes [a.k.a. the “neglected many”; (Senji Laxme et al., 2019)]. Finally, while the preclinical effectiveness of the “big four” antivenom in treating bites of the other medically important snakes on the Andaman and Nicobar Islands is yet to be evaluated, our results demonstrate the need for a *N. sagittifera* specific antivenom product on these islands.

## Data Availability

The datasets presented in this study can be found in online repositories. The names of the repository/repositories and accession number(s) can be found in the article/Supplementary Material.
