# Supplementary figures and images for: Venomics of the Enigmatic Andaman Cobra (Naja sagittifera) and the Preclinical Failure of Indian Antivenoms in Andaman and Nicobar Islands (part 2 of 2)
Source: Front Pharmacol. 2021 Oct 25;12:768210. doi: 10.3389/fphar.2021.768210 (PMC8573199; doi:10.3389/fphar.2021.768210)

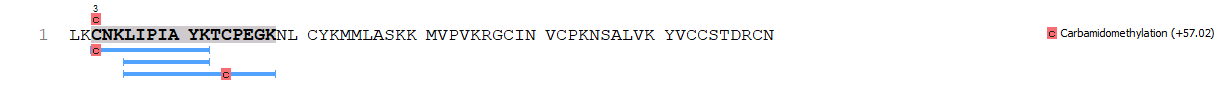

Supplement: Supplementary file 1 [file DataSheet3.ZIP › Naja naja/img/cov_1422.png]

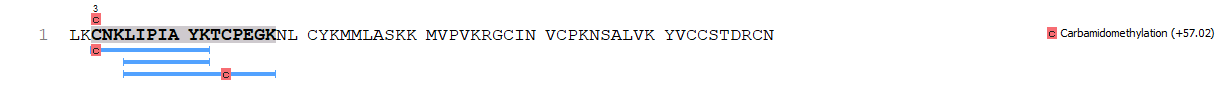

Supplement: Supplementary file 1 [file DataSheet3.ZIP › Naja naja/img/cov_1423.png]

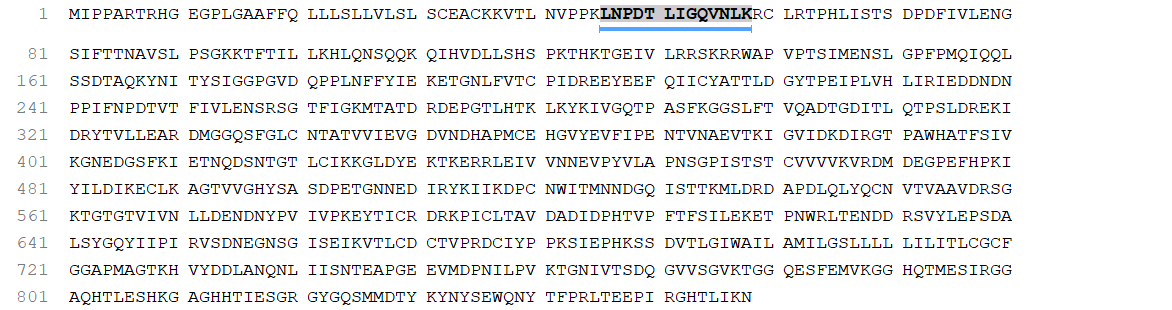

Supplement: Supplementary file 1 [file DataSheet3.ZIP › Naja naja/img/cov_1432.png]

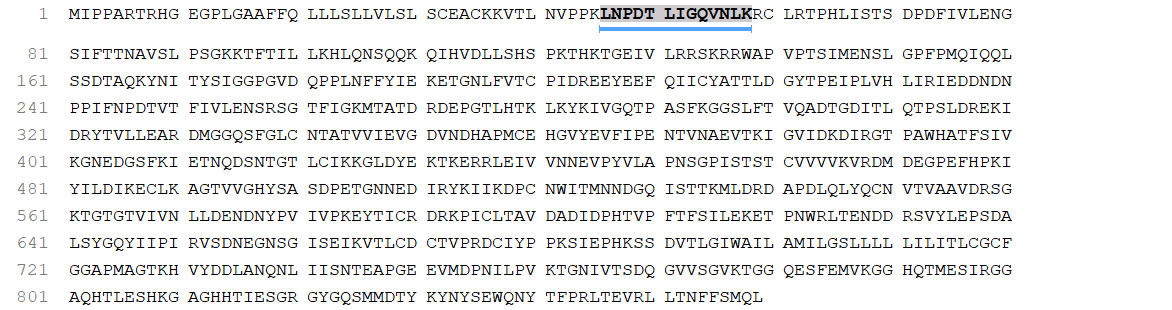

Supplement: Supplementary file 1 [file DataSheet3.ZIP › Naja naja/img/cov_1433.png]

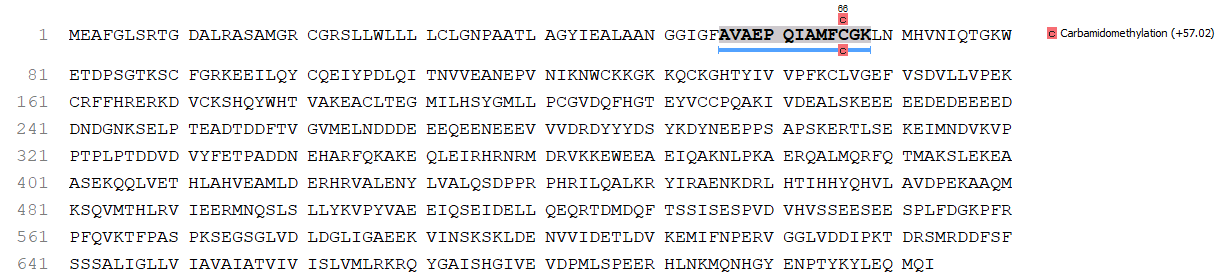

Supplement: Supplementary file 1 [file DataSheet3.ZIP › Naja naja/img/cov_1434.png]

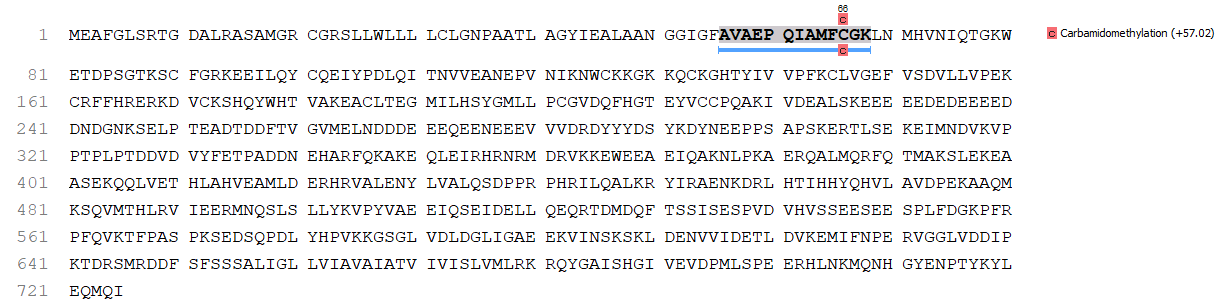

Supplement: Supplementary file 1 [file DataSheet3.ZIP › Naja naja/img/cov_1435.png]

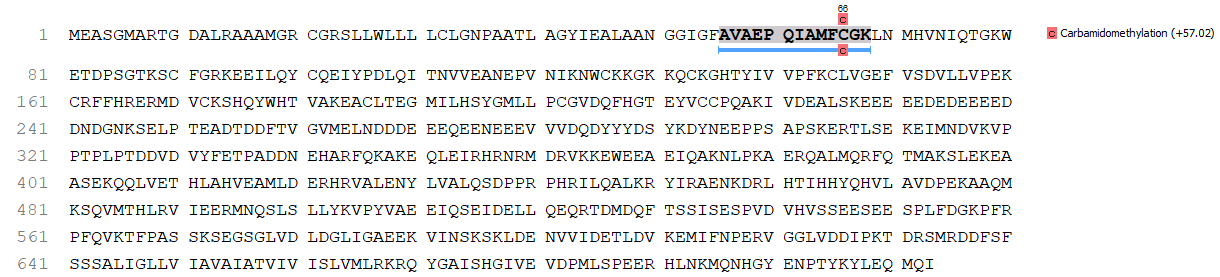

Supplement: Supplementary file 1 [file DataSheet3.ZIP › Naja naja/img/cov_1436.png]

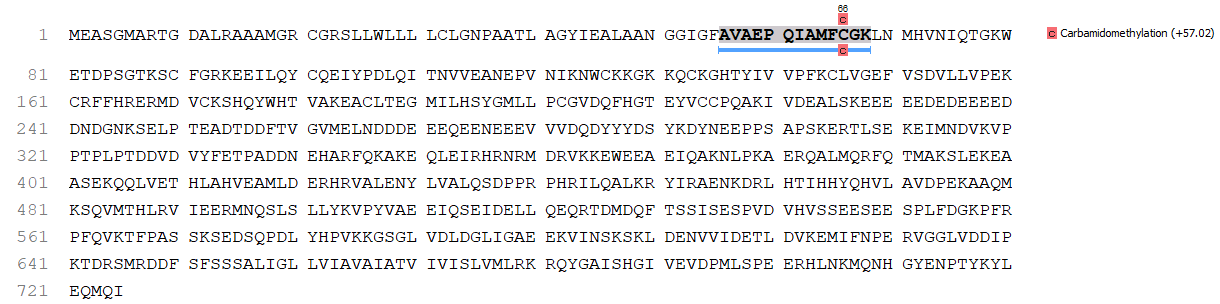

Supplement: Supplementary file 1 [file DataSheet3.ZIP › Naja naja/img/cov_1437.png]

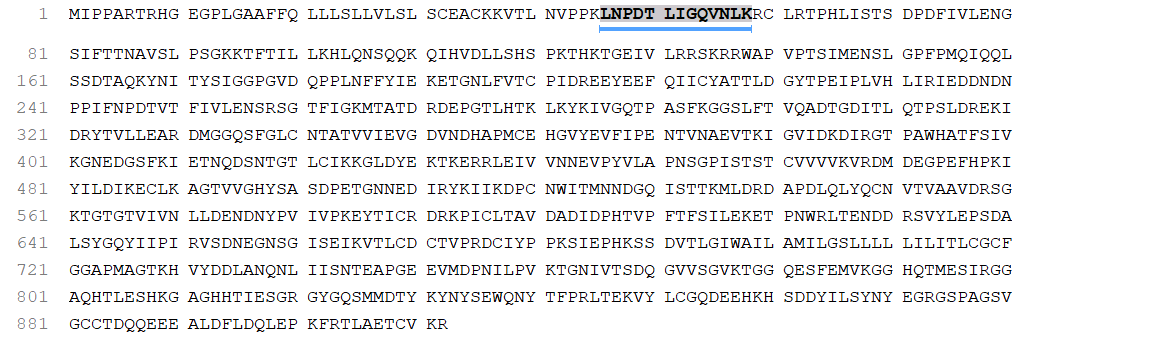

Supplement: Supplementary file 1 [file DataSheet3.ZIP › Naja naja/img/cov_1438.png]

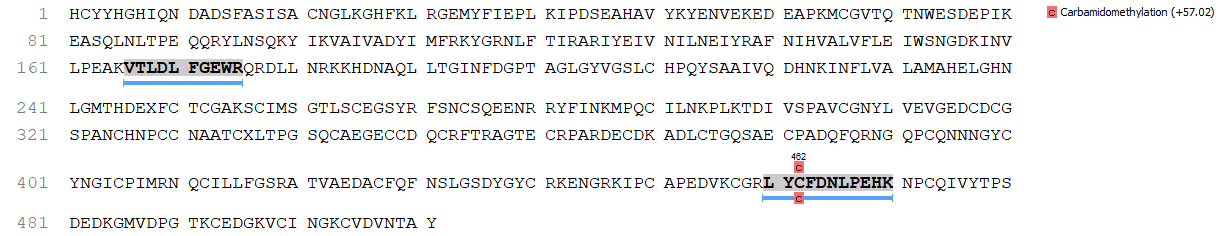

Supplement: Supplementary file 1 [file DataSheet3.ZIP › Naja naja/img/cov_1469.png]

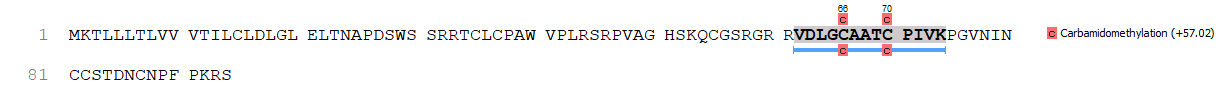

Supplement: Supplementary file 1 [file DataSheet3.ZIP › Naja naja/img/cov_1476.png]

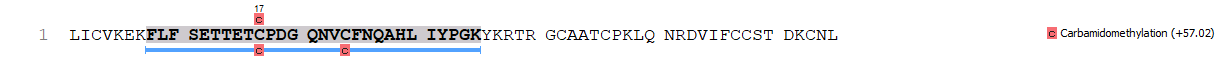

Supplement: Supplementary file 1 [file DataSheet3.ZIP › Naja naja/img/cov_1480.png]

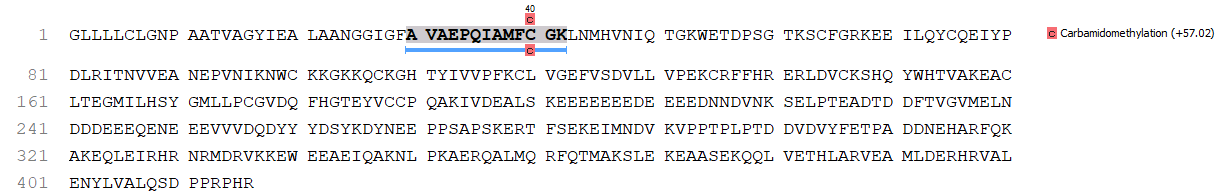

Supplement: Supplementary file 1 [file DataSheet3.ZIP › Naja naja/img/cov_1496.png]

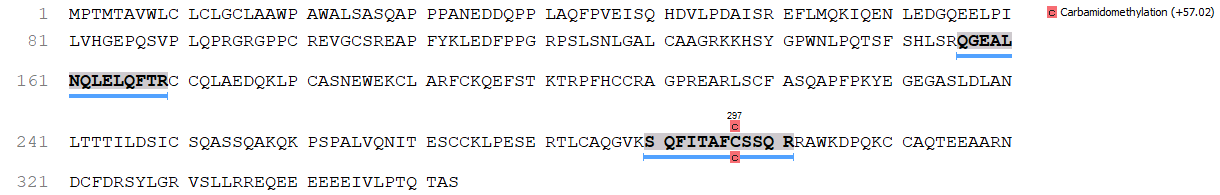

Supplement: Supplementary file 1 [file DataSheet3.ZIP › Naja naja/img/cov_1497.png]

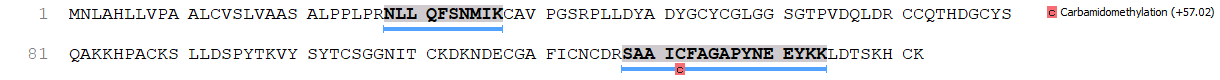

Supplement: Supplementary file 1 [file DataSheet3.ZIP › Naja naja/img/cov_1510.png]

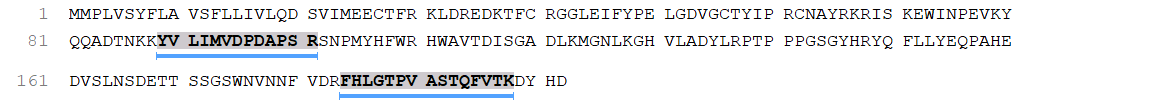

Supplement: Supplementary file 1 [file DataSheet3.ZIP › Naja naja/img/cov_1511.png]

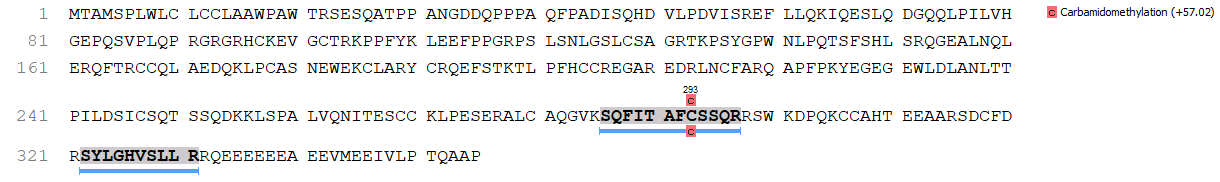

Supplement: Supplementary file 1 [file DataSheet3.ZIP › Naja naja/img/cov_1520.png]

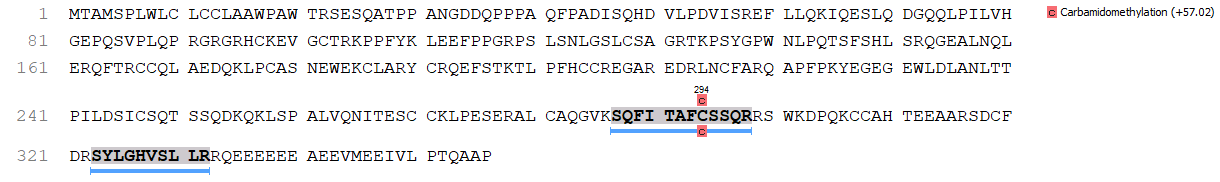

Supplement: Supplementary file 1 [file DataSheet3.ZIP › Naja naja/img/cov_1521.png]

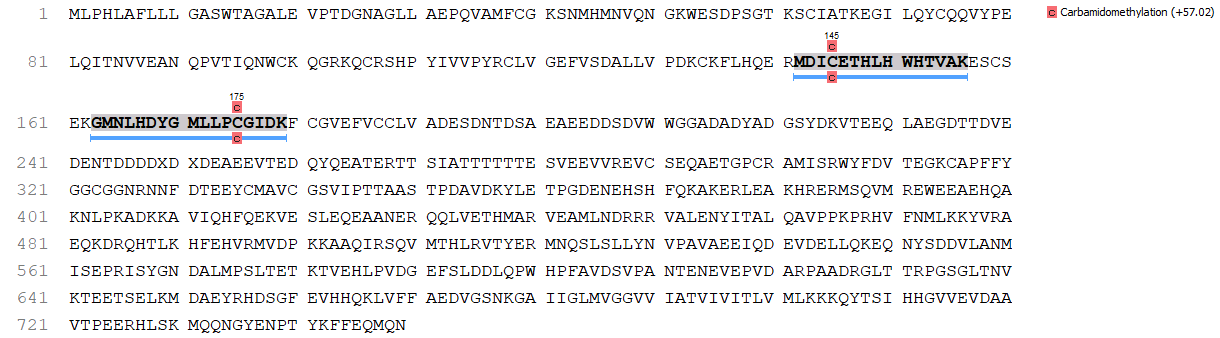

Supplement: Supplementary file 1 [file DataSheet3.ZIP › Naja naja/img/cov_1577.png]

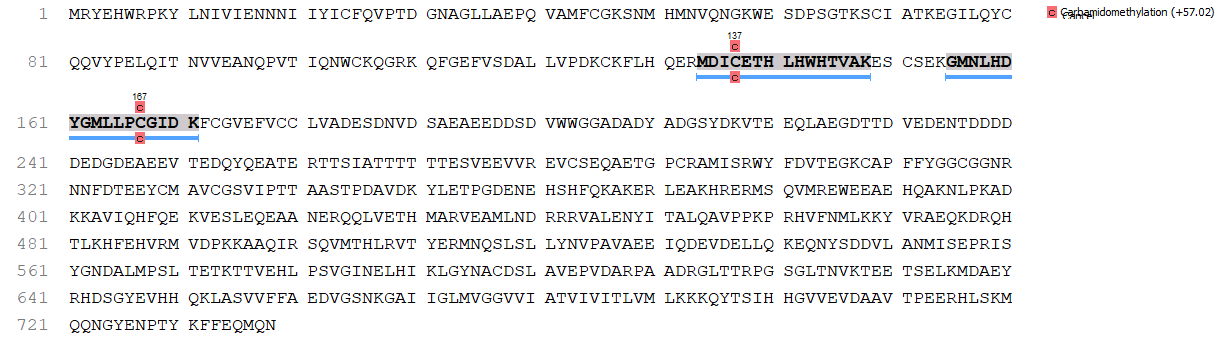

Supplement: Supplementary file 1 [file DataSheet3.ZIP › Naja naja/img/cov_1578.png]

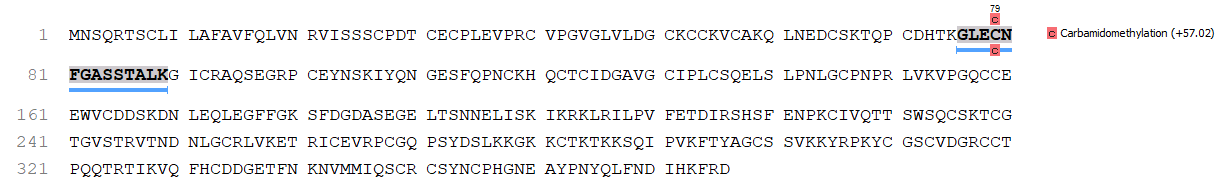

Supplement: Supplementary file 1 [file DataSheet3.ZIP › Naja naja/img/cov_1587.png]

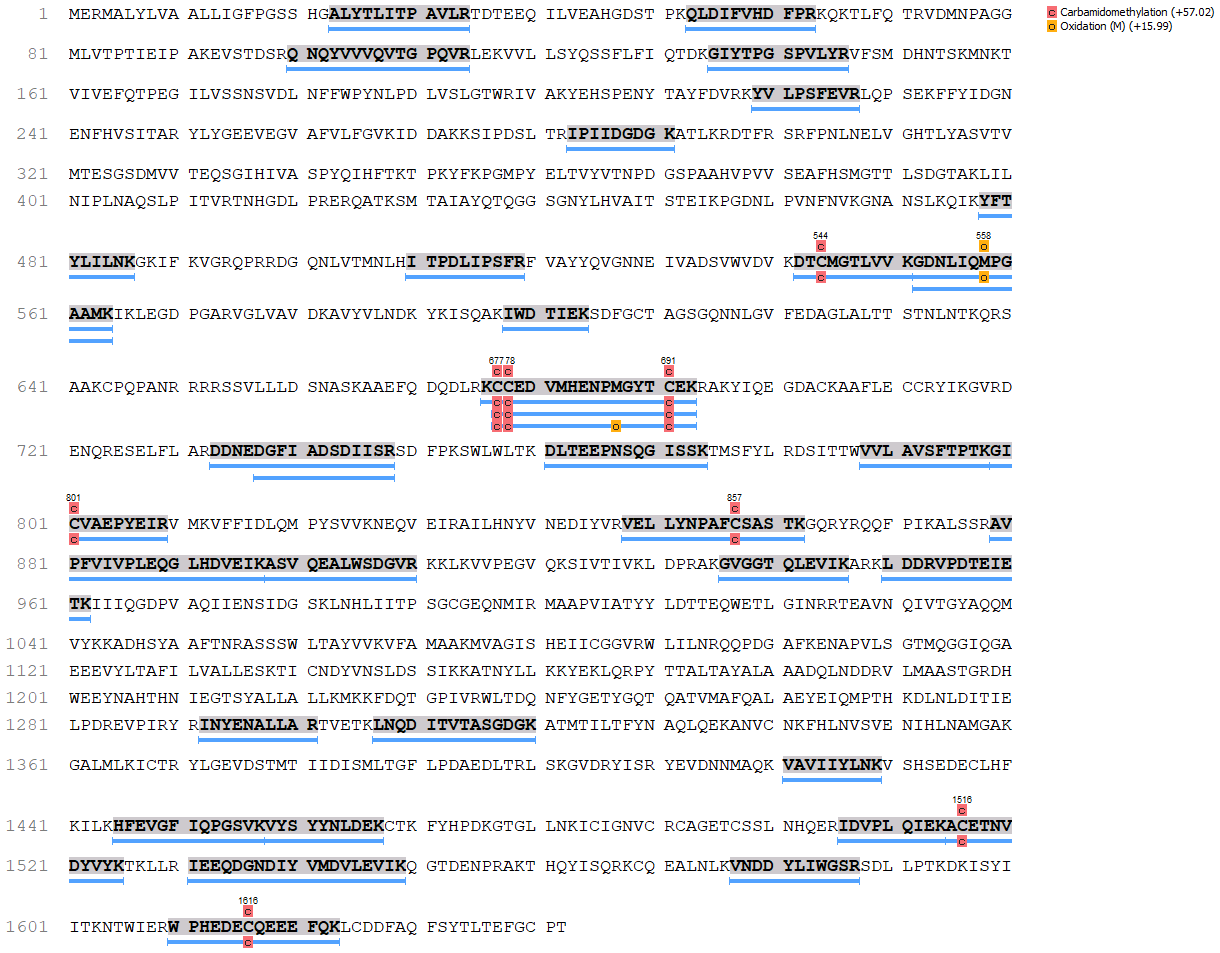

Supplement: Supplementary file 1 [file DataSheet3.ZIP › Naja naja/img/cov_16.png]

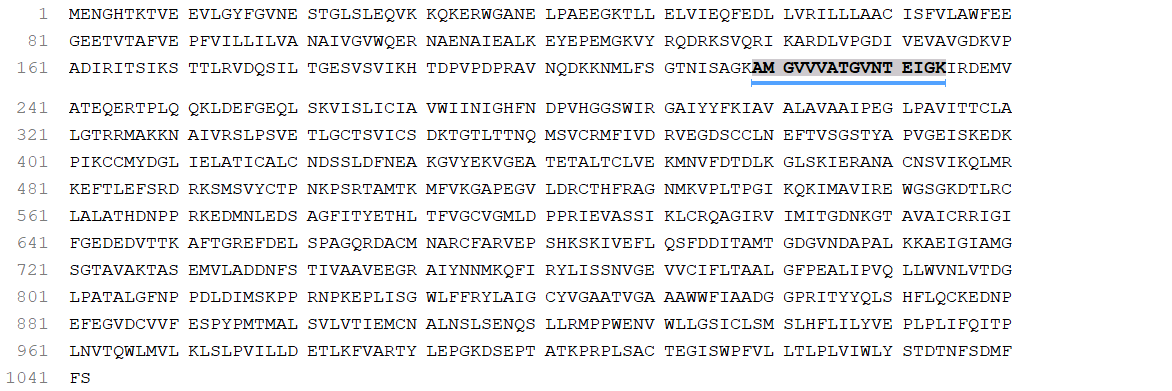

Supplement: Supplementary file 1 [file DataSheet3.ZIP › Naja naja/img/cov_1620.png]

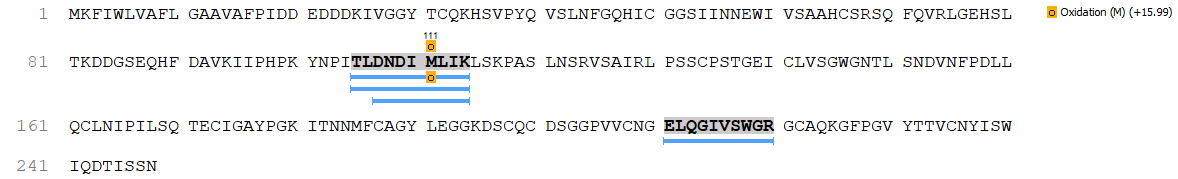

Supplement: Supplementary file 1 [file DataSheet3.ZIP › Naja naja/img/cov_1622.png]

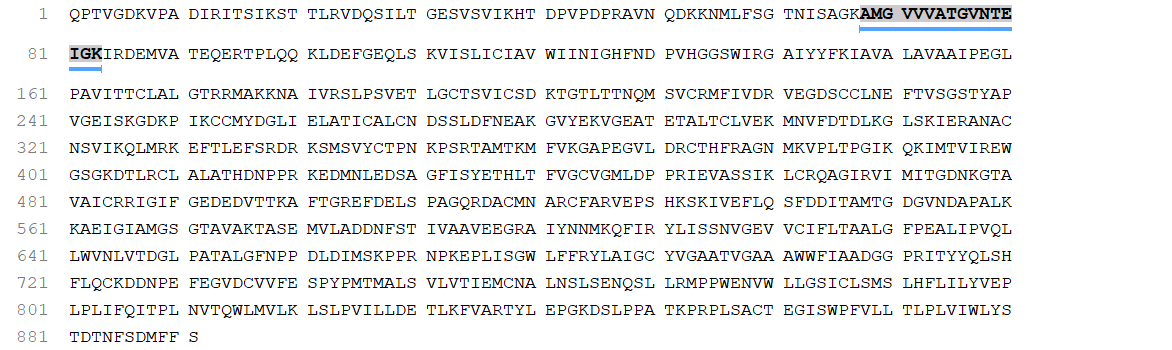

Supplement: Supplementary file 1 [file DataSheet3.ZIP › Naja naja/img/cov_1626.png]

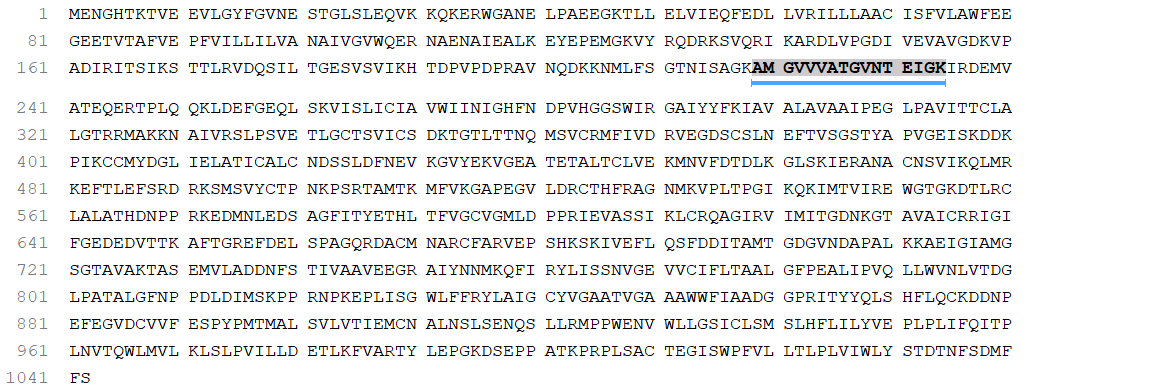

Supplement: Supplementary file 1 [file DataSheet3.ZIP › Naja naja/img/cov_1628.png]

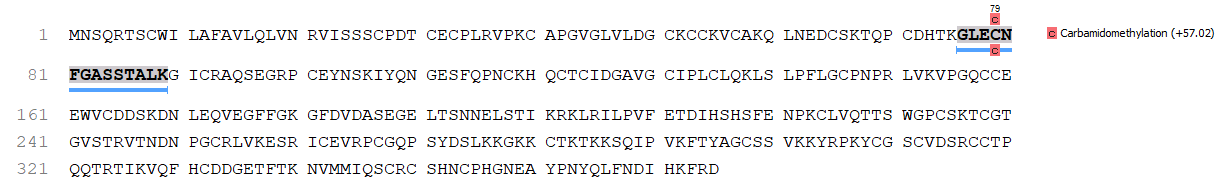

Supplement: Supplementary file 1 [file DataSheet3.ZIP › Naja naja/img/cov_1633.png]

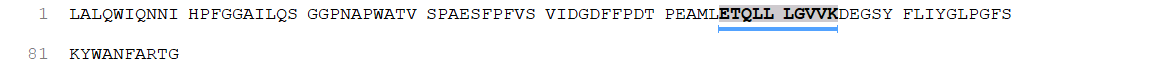

Supplement: Supplementary file 1 [file DataSheet3.ZIP › Naja naja/img/cov_1636.png]

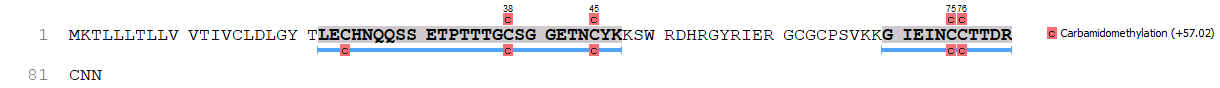

Supplement: Supplementary file 1 [file DataSheet3.ZIP › Naja naja/img/cov_1637.png]

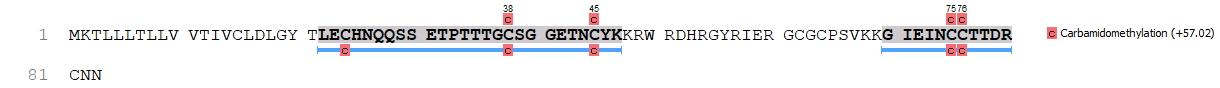

Supplement: Supplementary file 1 [file DataSheet3.ZIP › Naja naja/img/cov_1638.png]

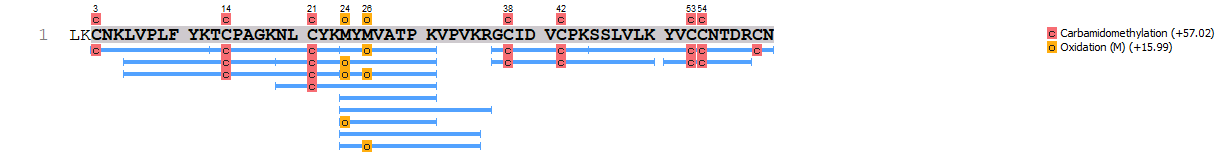

Supplement: Supplementary file 1 [file DataSheet3.ZIP › Naja naja/img/cov_164.png]

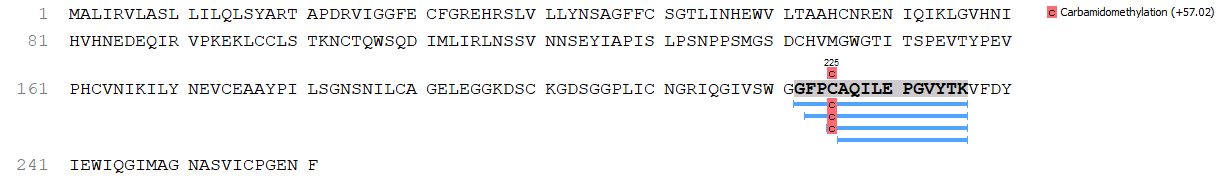

Supplement: Supplementary file 1 [file DataSheet3.ZIP › Naja naja/img/cov_1642.png]

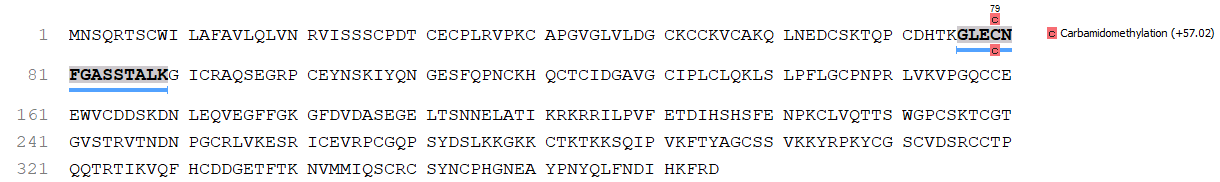

Supplement: Supplementary file 1 [file DataSheet3.ZIP › Naja naja/img/cov_1647.png]

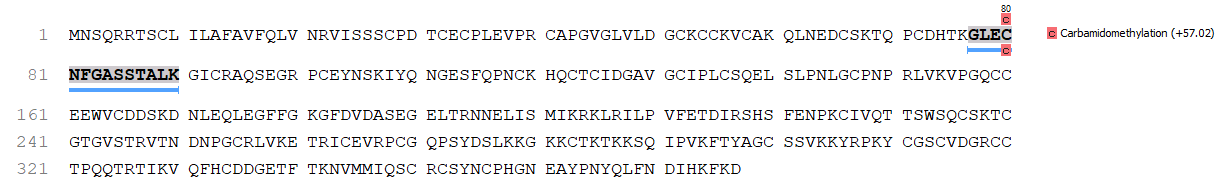

Supplement: Supplementary file 1 [file DataSheet3.ZIP › Naja naja/img/cov_1648.png]

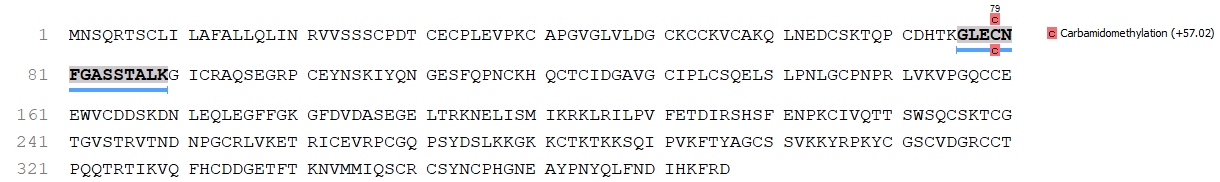

Supplement: Supplementary file 1 [file DataSheet3.ZIP › Naja naja/img/cov_1653.png]

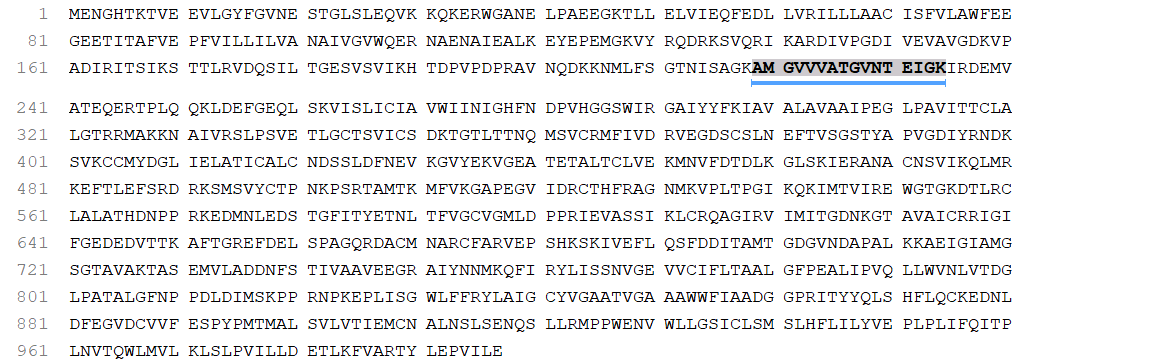

Supplement: Supplementary file 1 [file DataSheet3.ZIP › Naja naja/img/cov_1655.png]

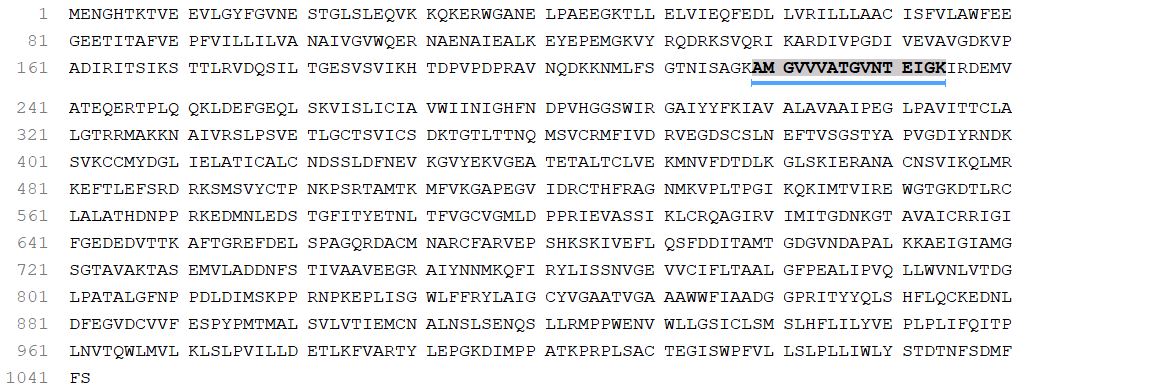

Supplement: Supplementary file 1 [file DataSheet3.ZIP › Naja naja/img/cov_1656.png]

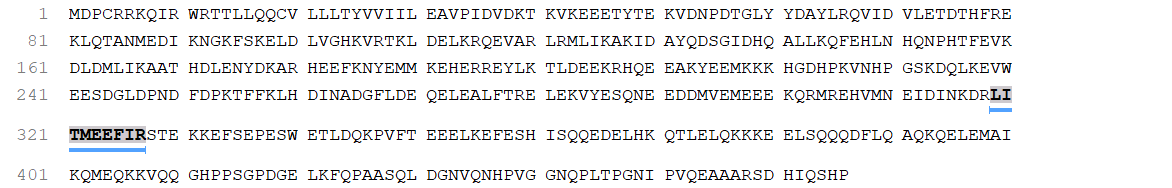

Supplement: Supplementary file 1 [file DataSheet3.ZIP › Naja naja/img/cov_1657.png]

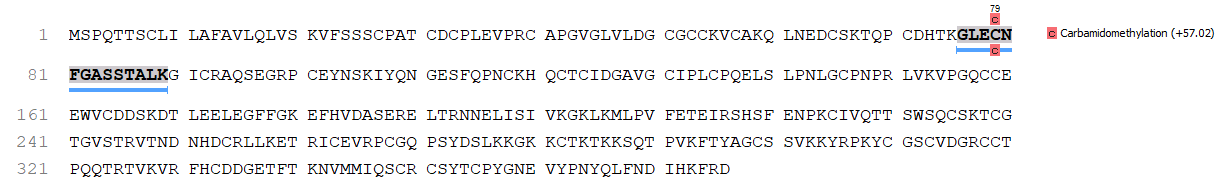

Supplement: Supplementary file 1 [file DataSheet3.ZIP › Naja naja/img/cov_1660.png]

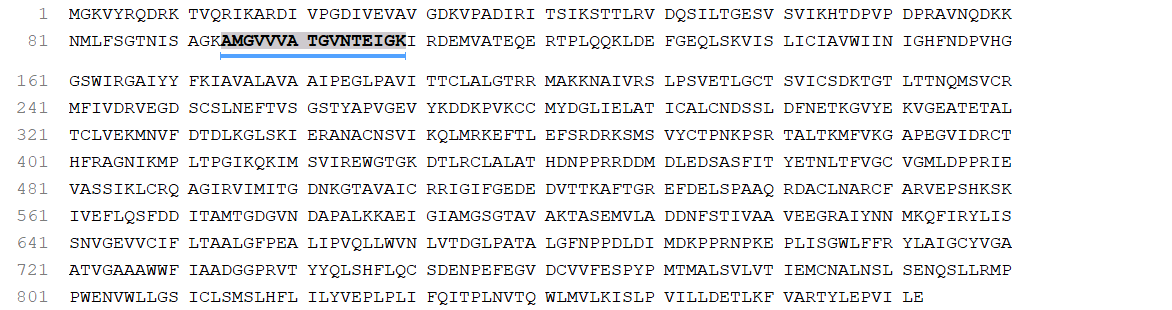

Supplement: Supplementary file 1 [file DataSheet3.ZIP › Naja naja/img/cov_1661.png]

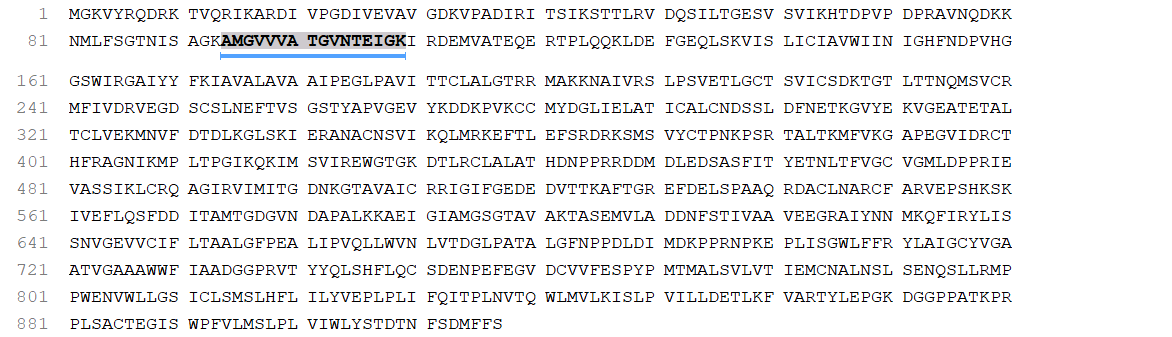

Supplement: Supplementary file 1 [file DataSheet3.ZIP › Naja naja/img/cov_1662.png]

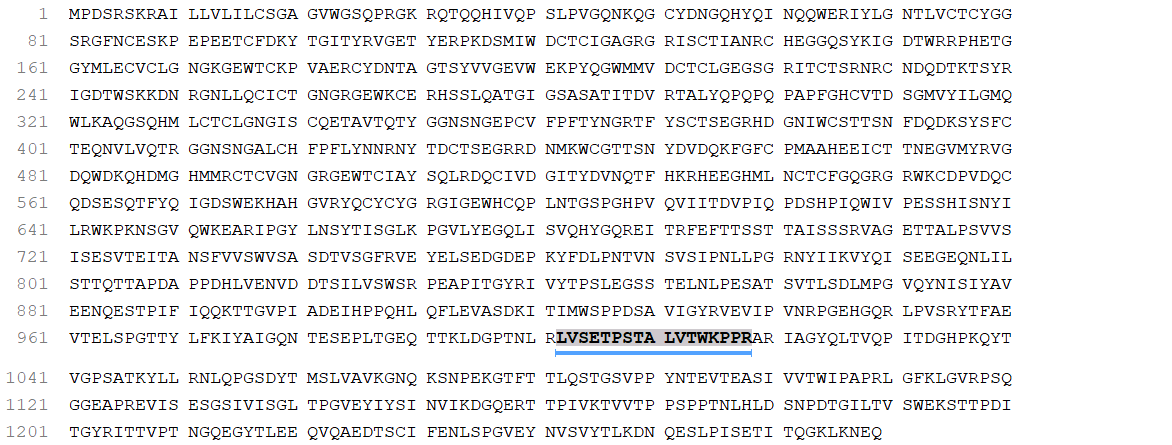

Supplement: Supplementary file 1 [file DataSheet3.ZIP › Naja naja/img/cov_1674.png]

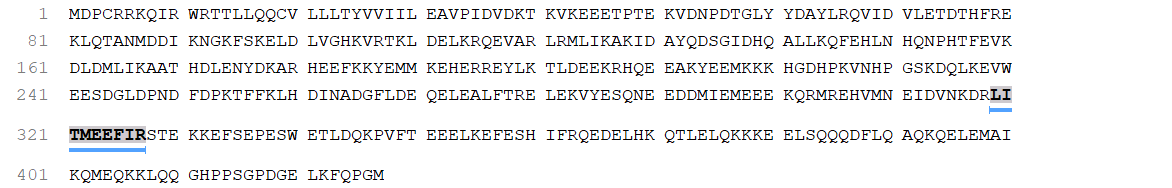

Supplement: Supplementary file 1 [file DataSheet3.ZIP › Naja naja/img/cov_1675.png]

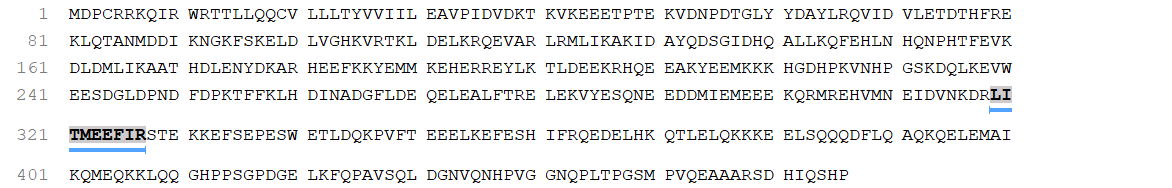

Supplement: Supplementary file 1 [file DataSheet3.ZIP › Naja naja/img/cov_1676.png]

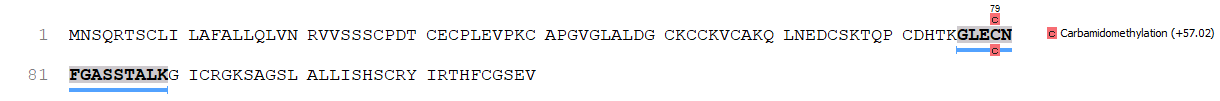

Supplement: Supplementary file 1 [file DataSheet3.ZIP › Naja naja/img/cov_1682.png]

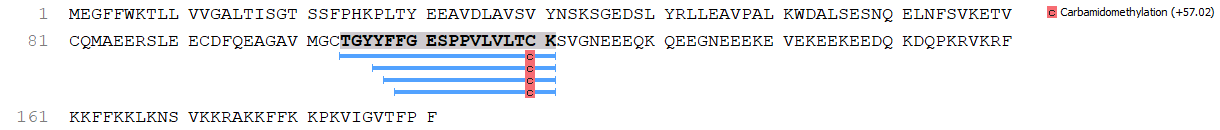

Supplement: Supplementary file 1 [file DataSheet3.ZIP › Naja naja/img/cov_1683.png]

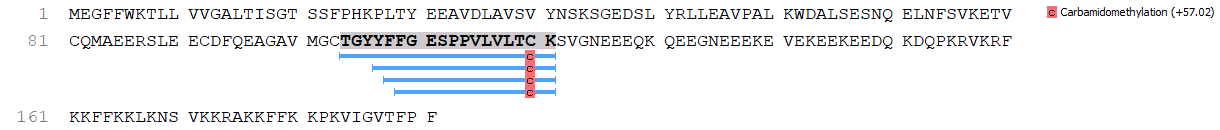

Supplement: Supplementary file 1 [file DataSheet3.ZIP › Naja naja/img/cov_1684.png]

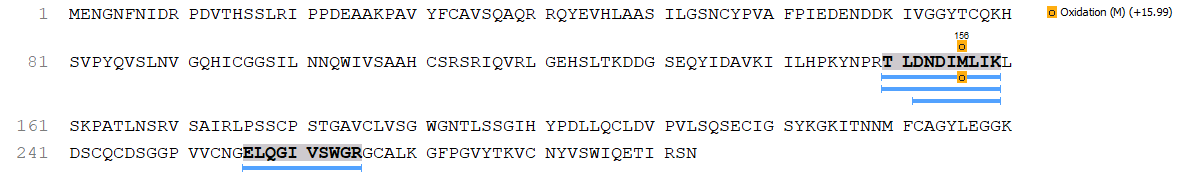

Supplement: Supplementary file 1 [file DataSheet3.ZIP › Naja naja/img/cov_1685.png]

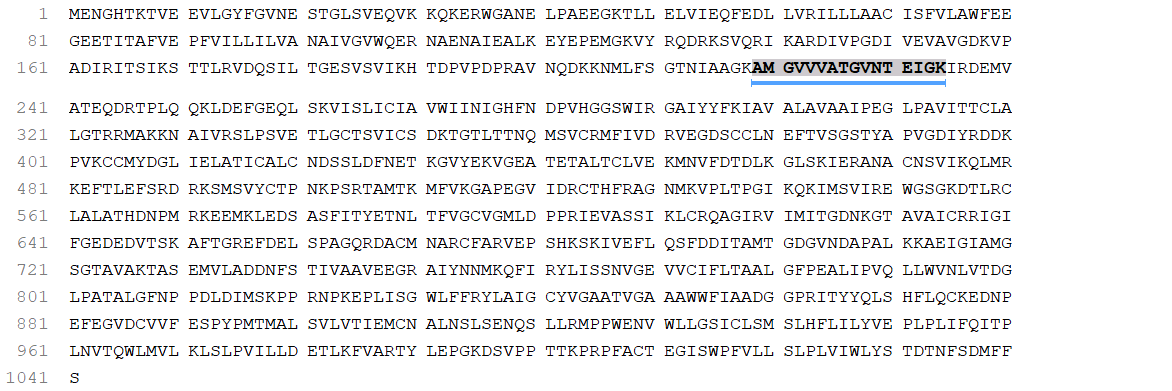

Supplement: Supplementary file 1 [file DataSheet3.ZIP › Naja naja/img/cov_1696.png]

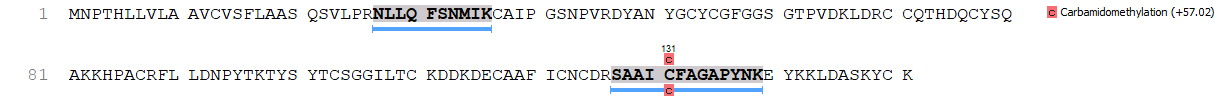

Supplement: Supplementary file 1 [file DataSheet3.ZIP › Naja naja/img/cov_1703.png]

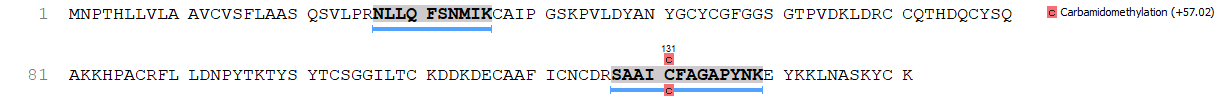

Supplement: Supplementary file 1 [file DataSheet3.ZIP › Naja naja/img/cov_1704.png]

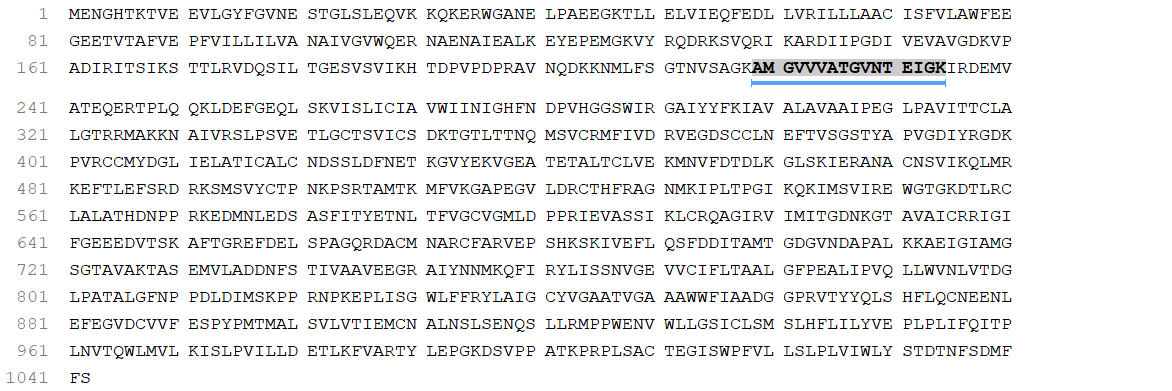

Supplement: Supplementary file 1 [file DataSheet3.ZIP › Naja naja/img/cov_1709.png]

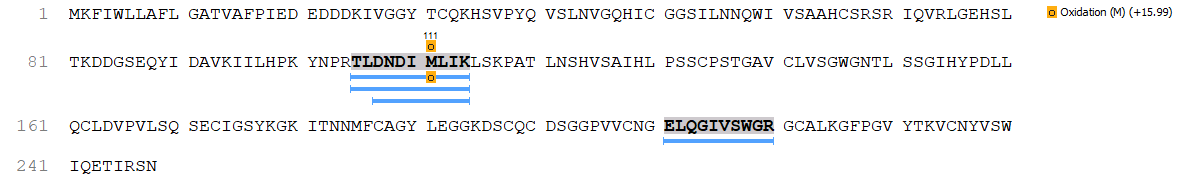

Supplement: Supplementary file 1 [file DataSheet3.ZIP › Naja naja/img/cov_1717.png]

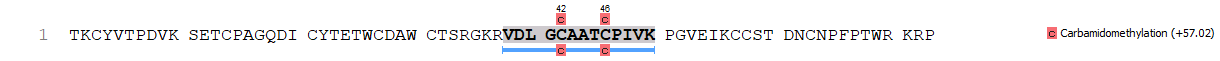

Supplement: Supplementary file 1 [file DataSheet3.ZIP › Naja naja/img/cov_1723.png]

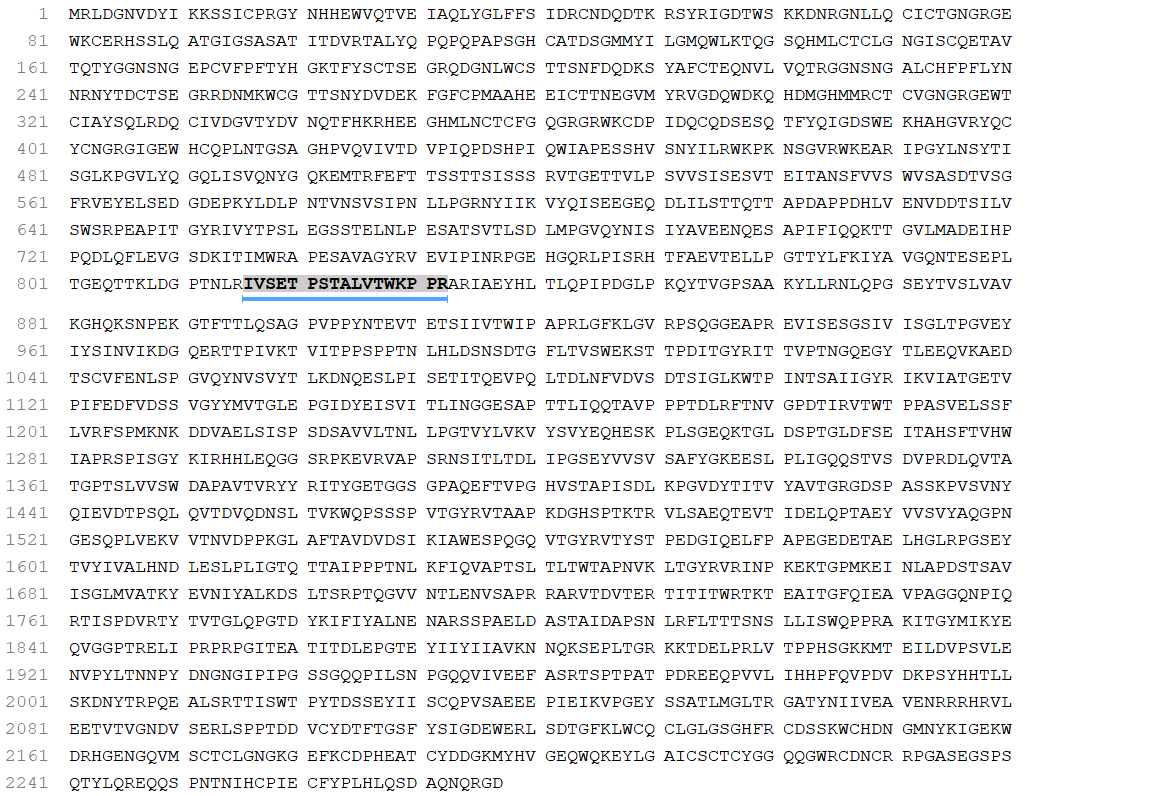

Supplement: Supplementary file 1 [file DataSheet3.ZIP › Naja naja/img/cov_1733.png]

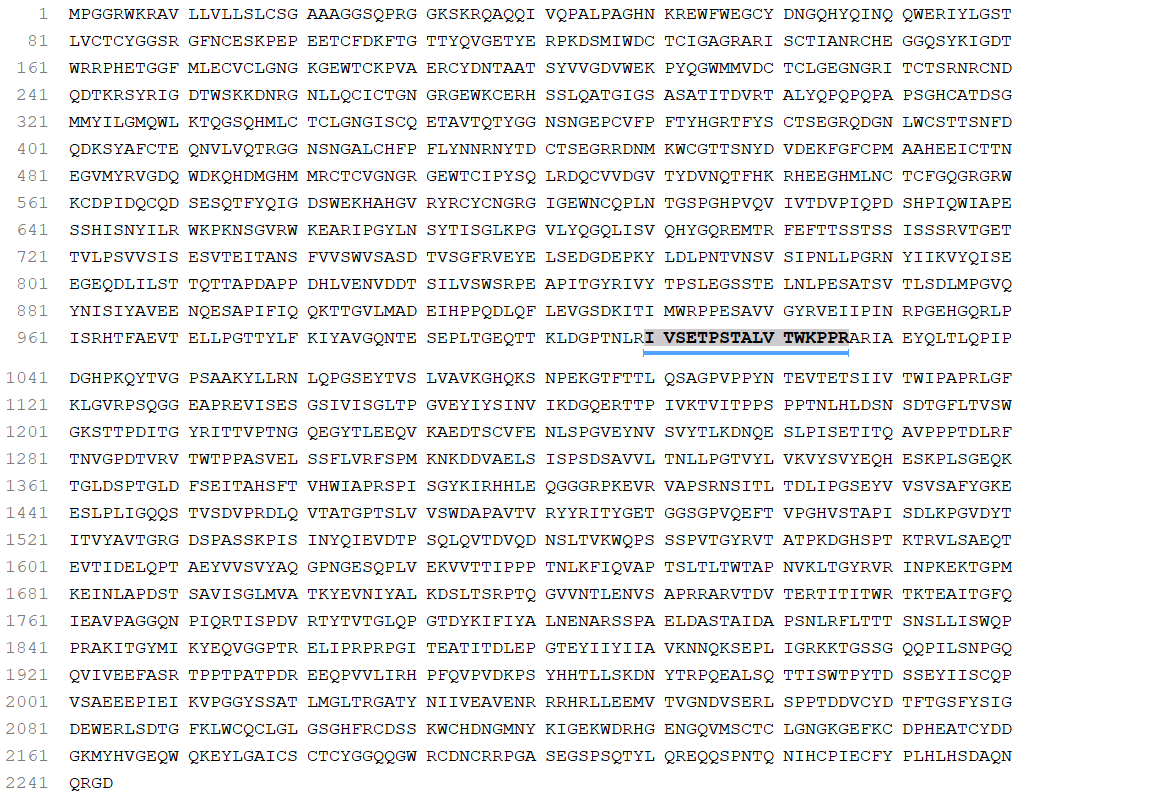

Supplement: Supplementary file 1 [file DataSheet3.ZIP › Naja naja/img/cov_1734.png]

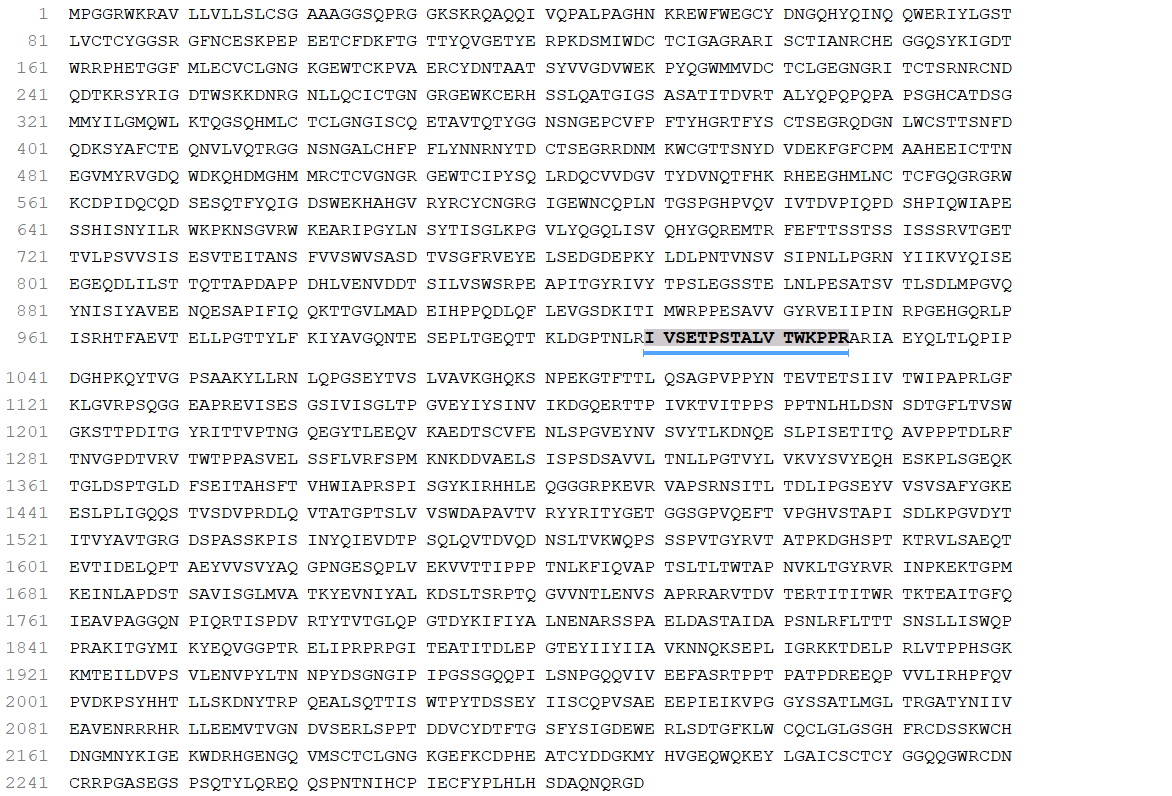

Supplement: Supplementary file 1 [file DataSheet3.ZIP › Naja naja/img/cov_1735.png]

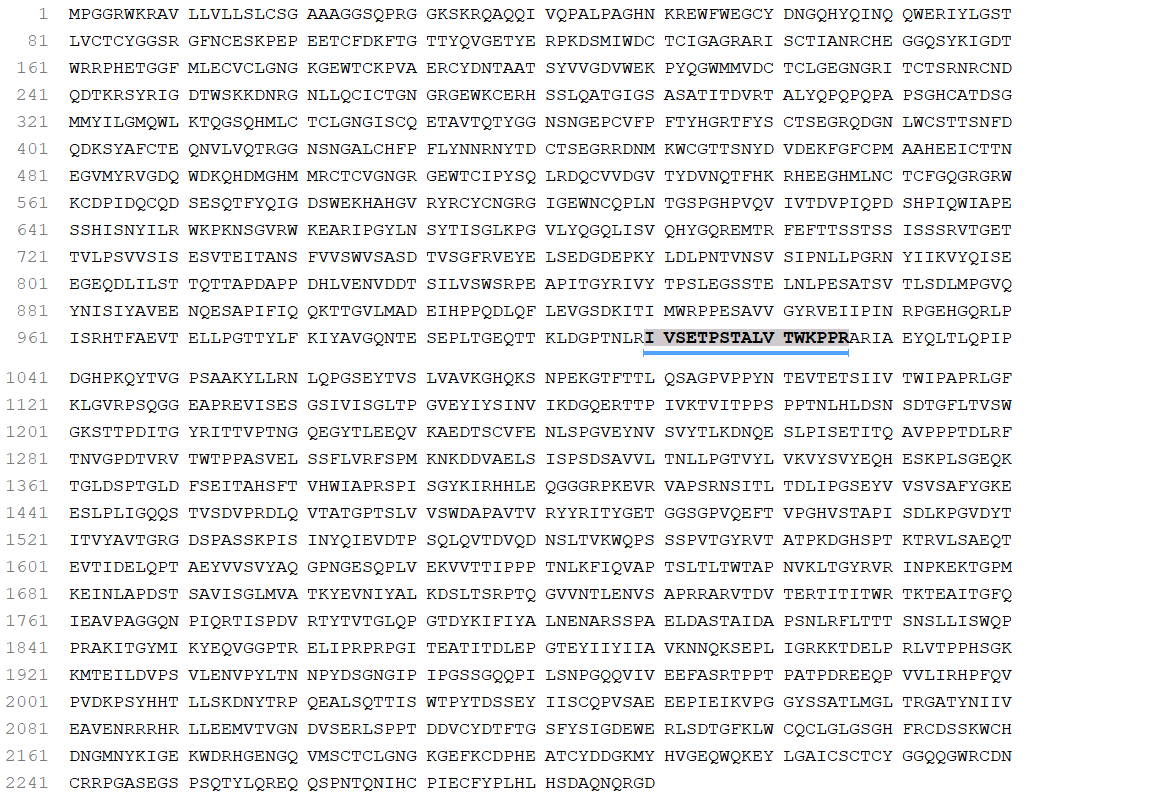

Supplement: Supplementary file 1 [file DataSheet3.ZIP › Naja naja/img/cov_1736.png]

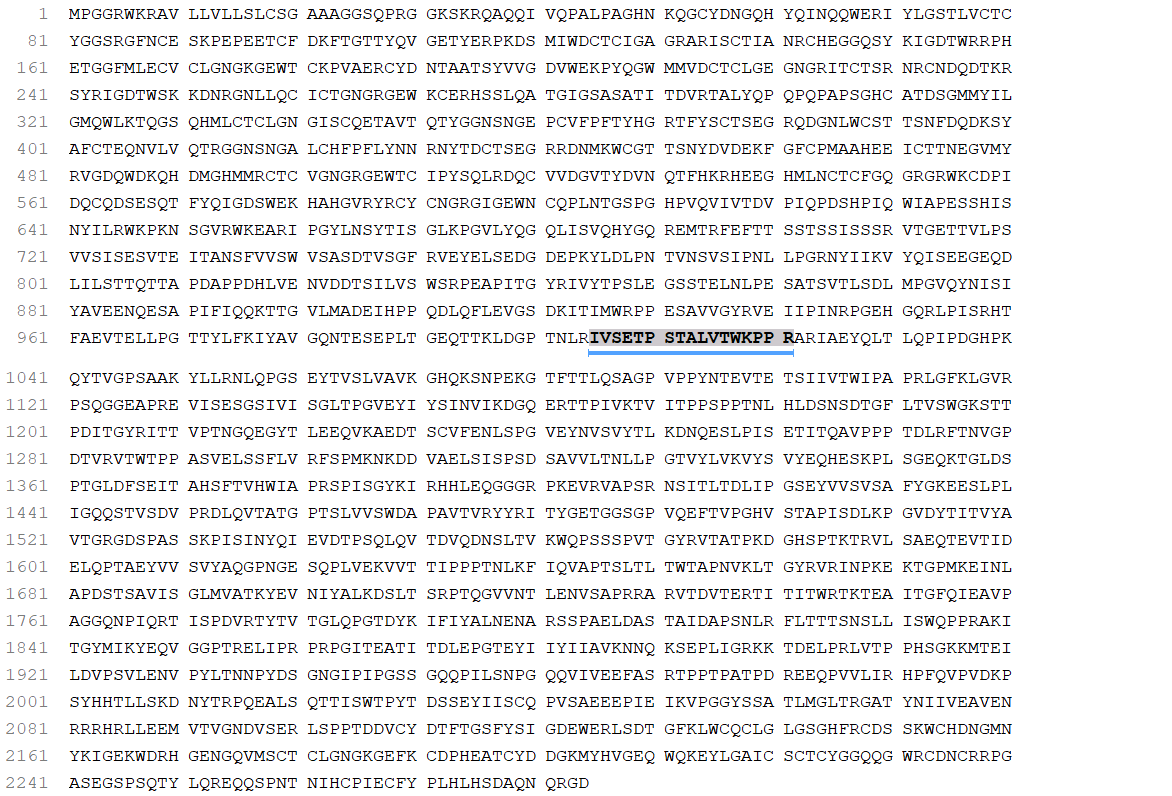

Supplement: Supplementary file 1 [file DataSheet3.ZIP › Naja naja/img/cov_1737.png]

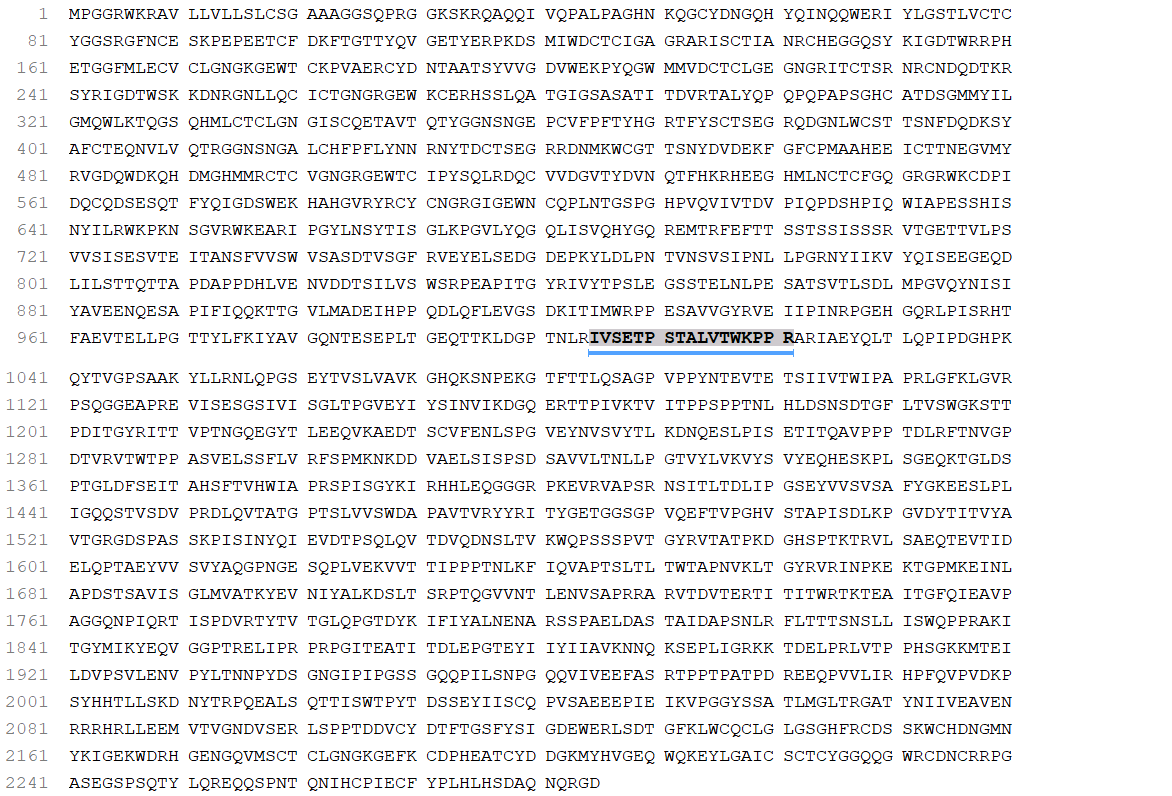

Supplement: Supplementary file 1 [file DataSheet3.ZIP › Naja naja/img/cov_1738.png]

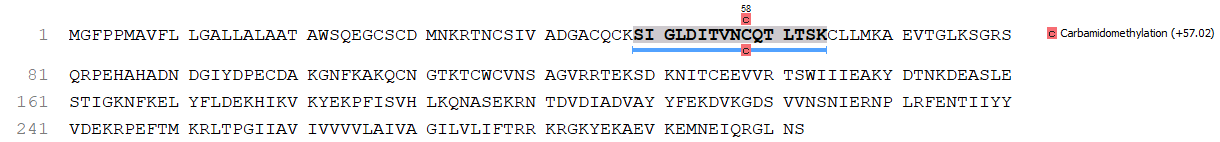

Supplement: Supplementary file 1 [file DataSheet3.ZIP › Naja naja/img/cov_1748.png]

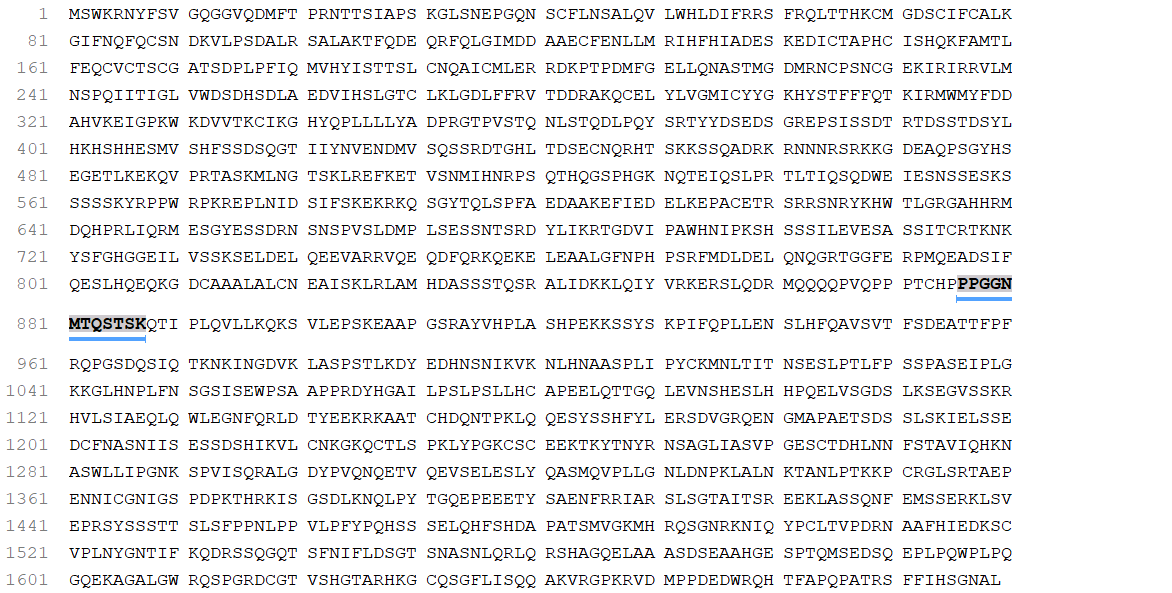

Supplement: Supplementary file 1 [file DataSheet3.ZIP › Naja naja/img/cov_1801.png]

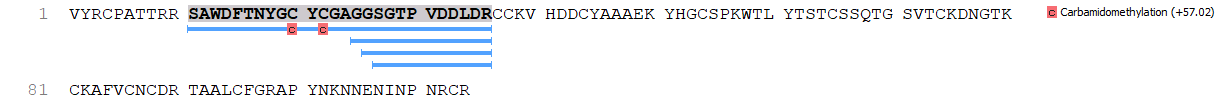

Supplement: Supplementary file 1 [file DataSheet3.ZIP › Naja naja/img/cov_1841.png]

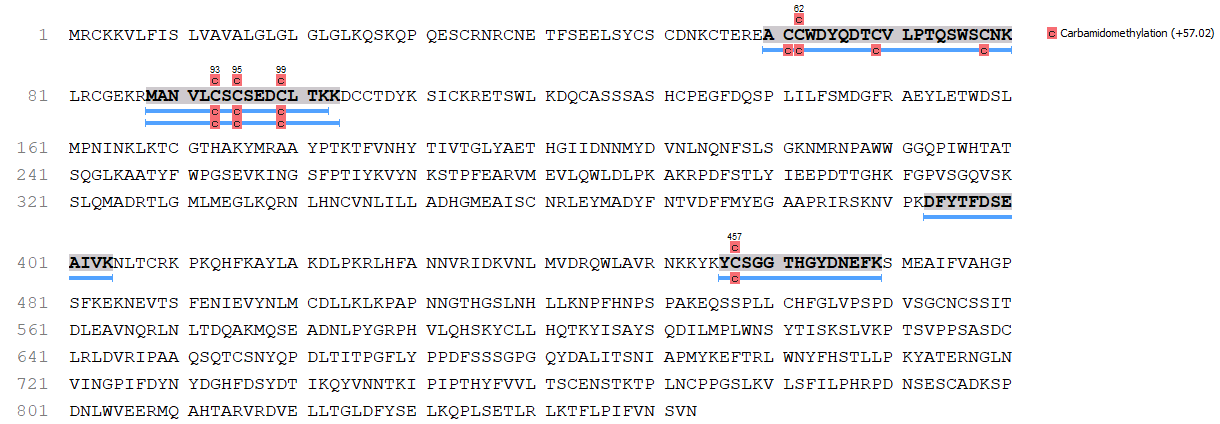

Supplement: Supplementary file 1 [file DataSheet3.ZIP › Naja naja/img/cov_187.png]

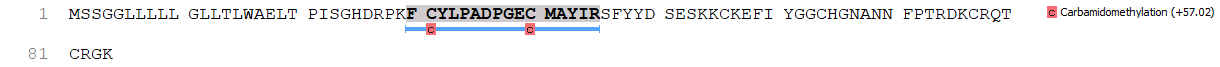

Supplement: Supplementary file 1 [file DataSheet3.ZIP › Naja naja/img/cov_1872.png]

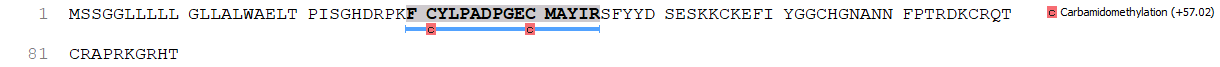

Supplement: Supplementary file 1 [file DataSheet3.ZIP › Naja naja/img/cov_1874.png]

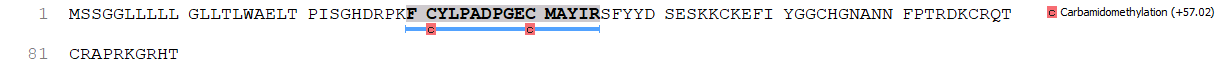

Supplement: Supplementary file 1 [file DataSheet3.ZIP › Naja naja/img/cov_1875.png]

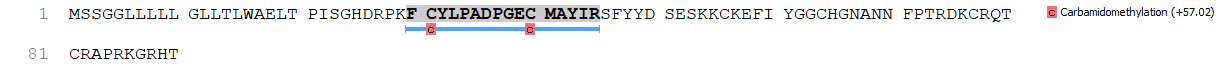

Supplement: Supplementary file 1 [file DataSheet3.ZIP › Naja naja/img/cov_1876.png]

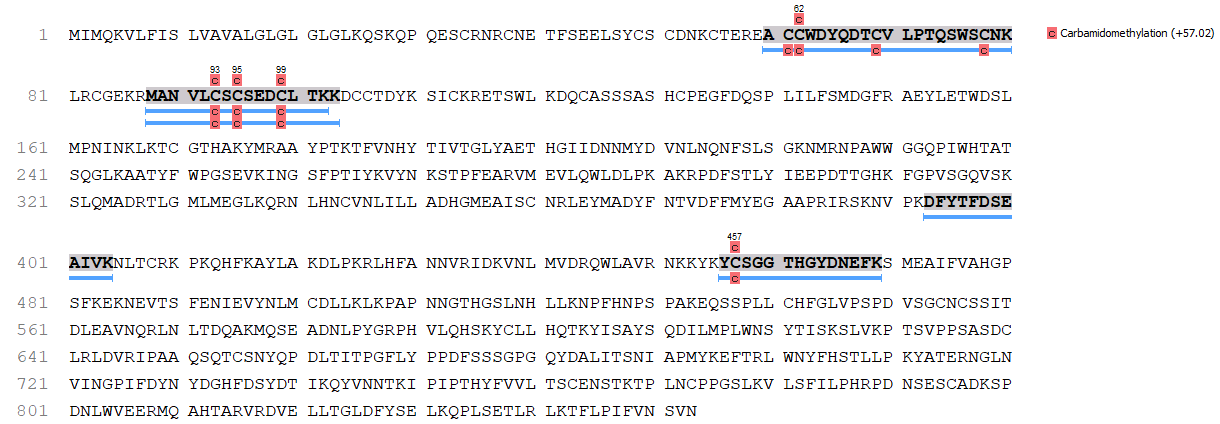

Supplement: Supplementary file 1 [file DataSheet3.ZIP › Naja naja/img/cov_188.png]

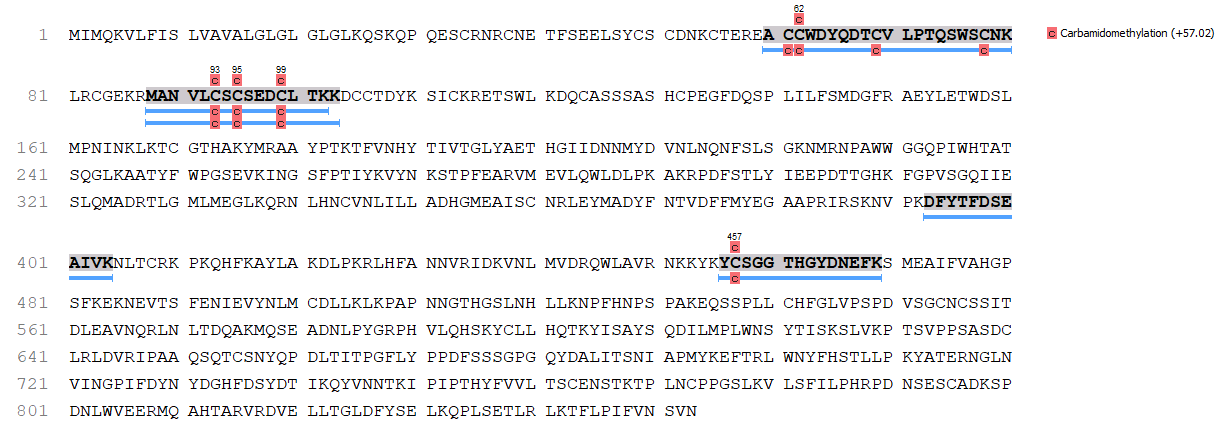

Supplement: Supplementary file 1 [file DataSheet3.ZIP › Naja naja/img/cov_189.png]
