# Supplementary figures and images for: Venomics of the Enigmatic Andaman Cobra (Naja sagittifera) and the Preclinical Failure of Indian Antivenoms in Andaman and Nicobar Islands (part 1 of 2)
Source: Front Pharmacol. 2021 Oct 25;12:768210. doi: 10.3389/fphar.2021.768210 (PMC8573199; doi:10.3389/fphar.2021.768210)

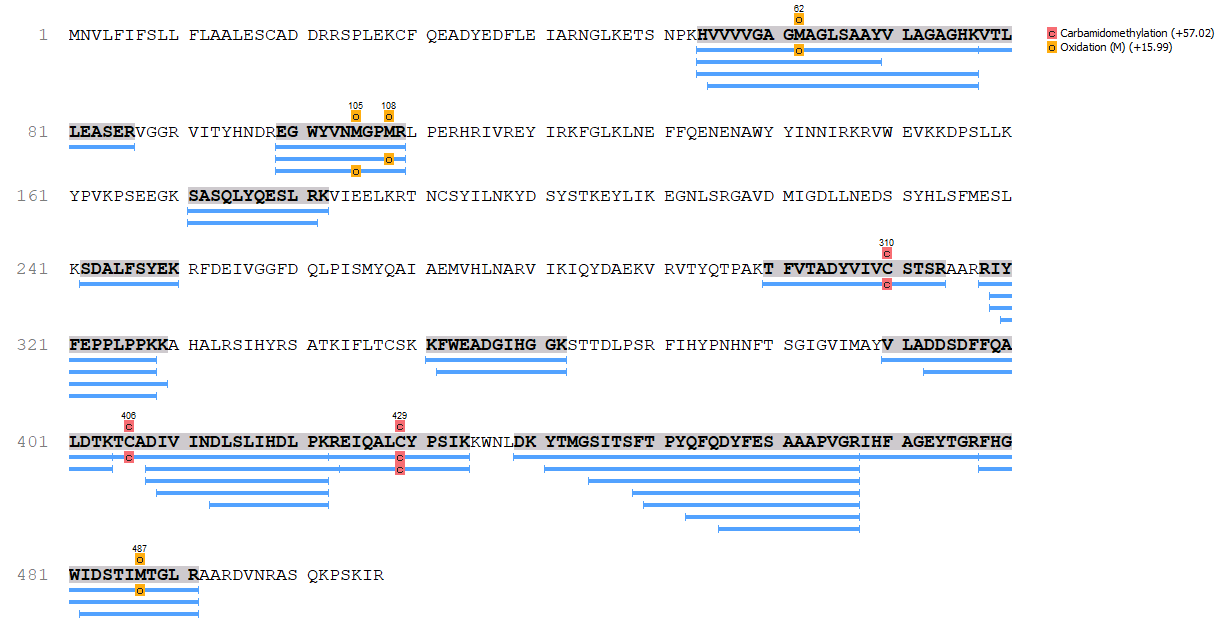

Supplement: Supplementary file 1 [file DataSheet3.ZIP › Naja naja/img/cov_1.png]

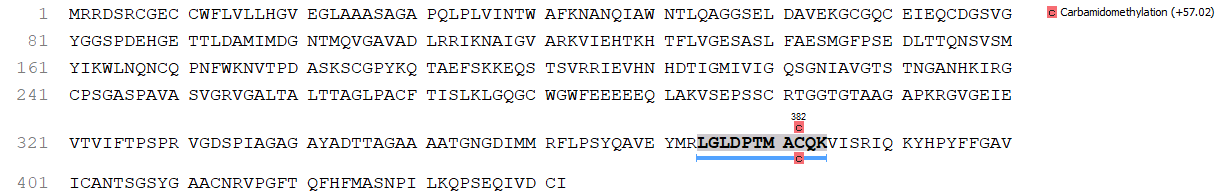

Supplement: Supplementary file 1 [file DataSheet3.ZIP › Naja naja/img/cov_1002.png]

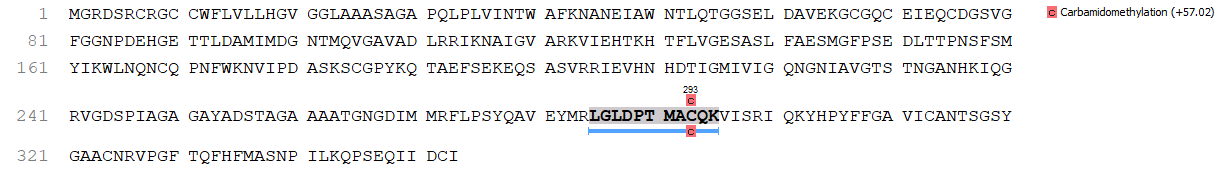

Supplement: Supplementary file 1 [file DataSheet3.ZIP › Naja naja/img/cov_1007.png]

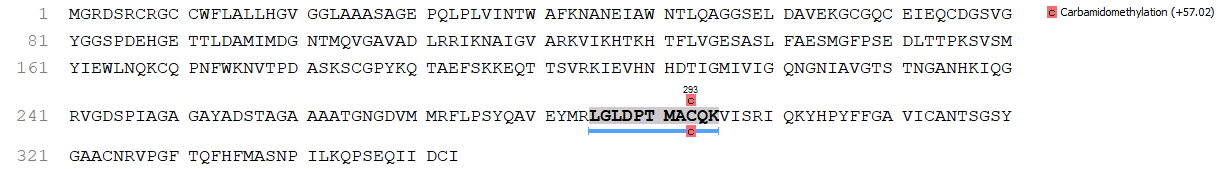

Supplement: Supplementary file 1 [file DataSheet3.ZIP › Naja naja/img/cov_1009.png]

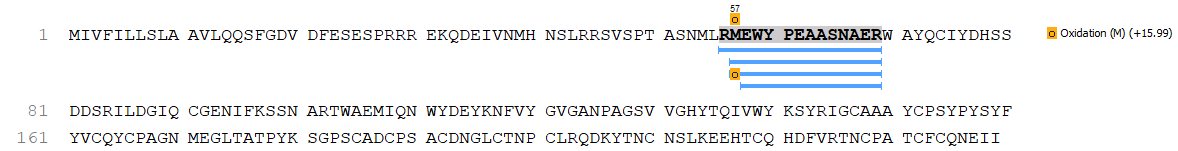

Supplement: Supplementary file 1 [file DataSheet3.ZIP › Naja naja/img/cov_1010.png]

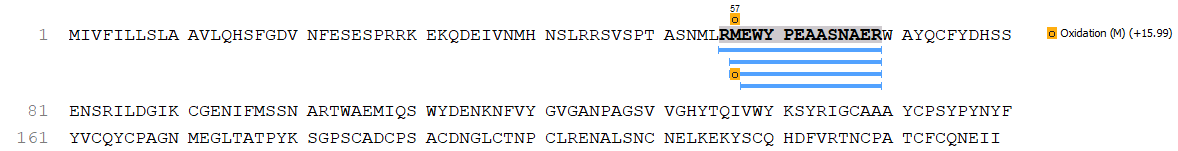

Supplement: Supplementary file 1 [file DataSheet3.ZIP › Naja naja/img/cov_1014.png]

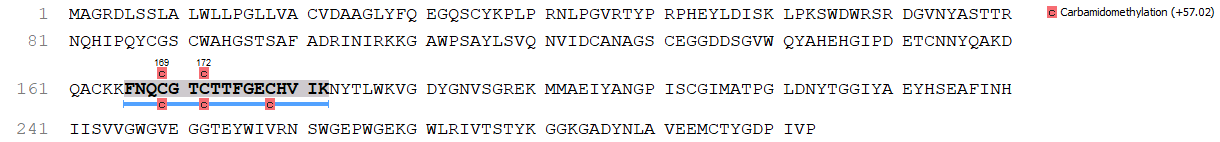

Supplement: Supplementary file 1 [file DataSheet3.ZIP › Naja naja/img/cov_1018.png]

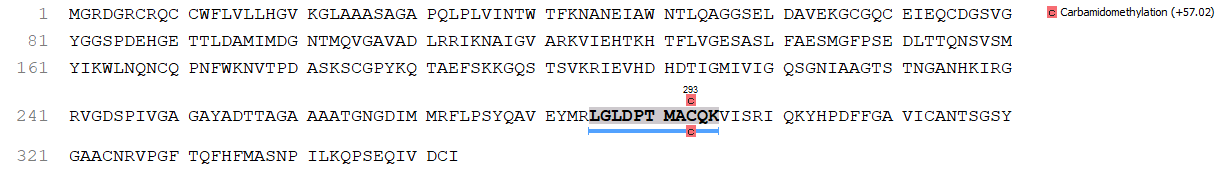

Supplement: Supplementary file 1 [file DataSheet3.ZIP › Naja naja/img/cov_1023.png]

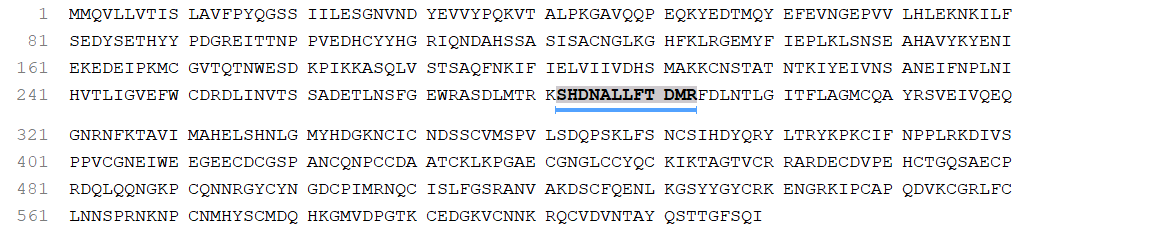

Supplement: Supplementary file 1 [file DataSheet3.ZIP › Naja naja/img/cov_1032.png]

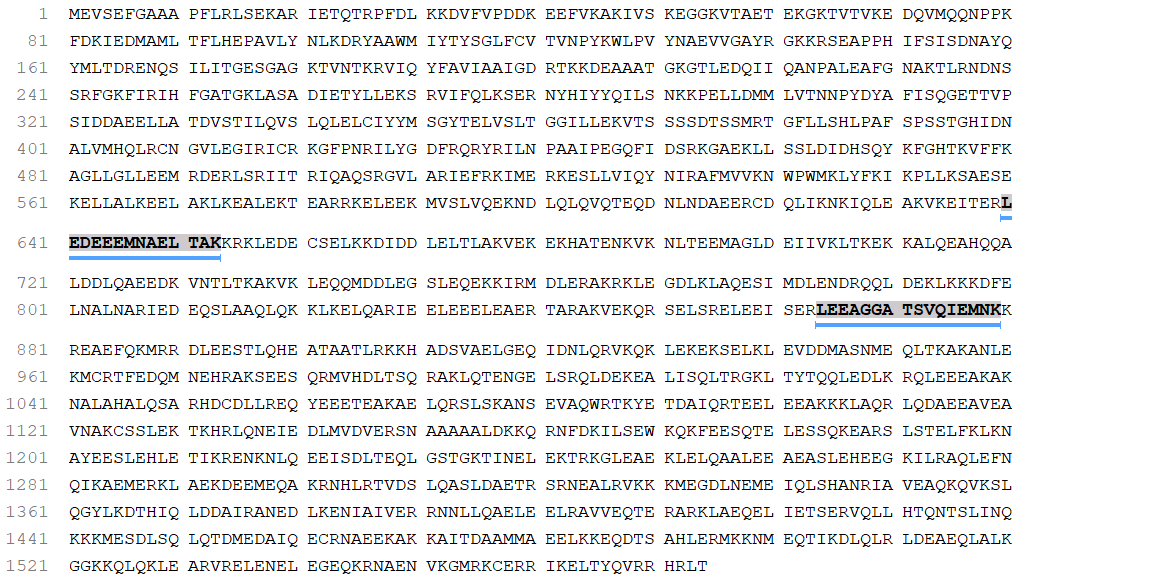

Supplement: Supplementary file 1 [file DataSheet3.ZIP › Naja naja/img/cov_1033.png]

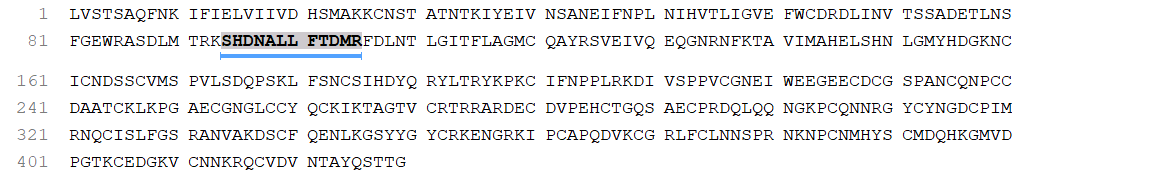

Supplement: Supplementary file 1 [file DataSheet3.ZIP › Naja naja/img/cov_1035.png]

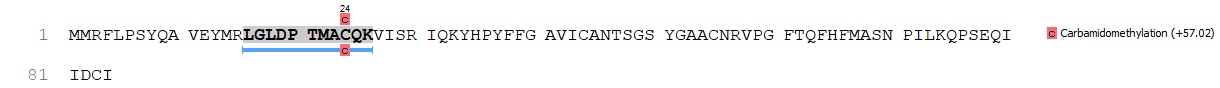

Supplement: Supplementary file 1 [file DataSheet3.ZIP › Naja naja/img/cov_1048.png]

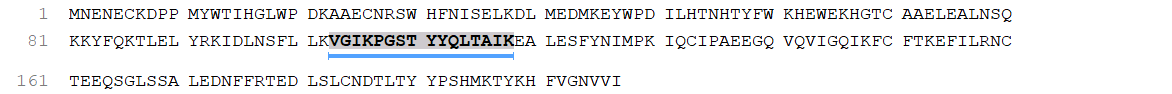

Supplement: Supplementary file 1 [file DataSheet3.ZIP › Naja naja/img/cov_1050.png]

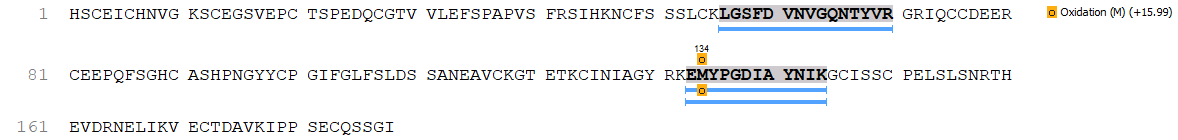

Supplement: Supplementary file 1 [file DataSheet3.ZIP › Naja naja/img/cov_1055.png]

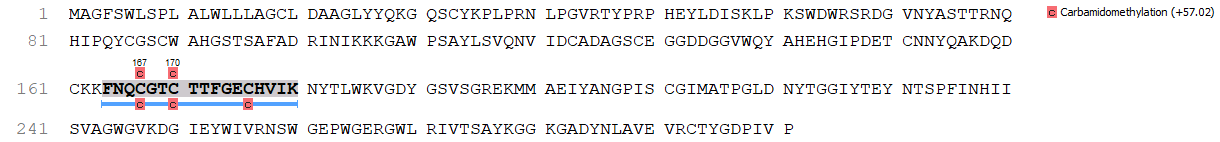

Supplement: Supplementary file 1 [file DataSheet3.ZIP › Naja naja/img/cov_1059.png]

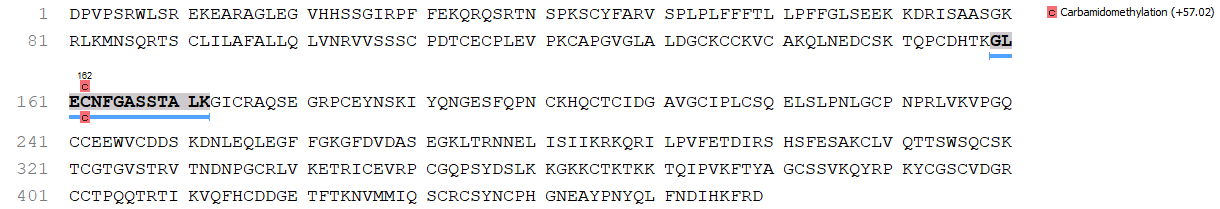

Supplement: Supplementary file 1 [file DataSheet3.ZIP › Naja naja/img/cov_1093.png]

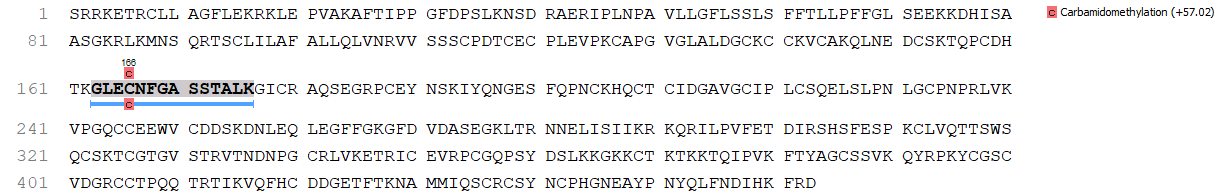

Supplement: Supplementary file 1 [file DataSheet3.ZIP › Naja naja/img/cov_1095.png]

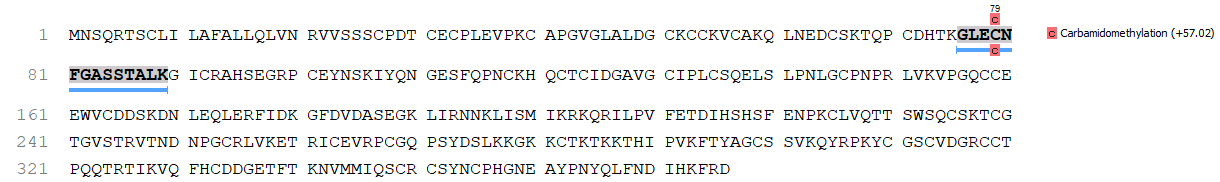

Supplement: Supplementary file 1 [file DataSheet3.ZIP › Naja naja/img/cov_1097.png]

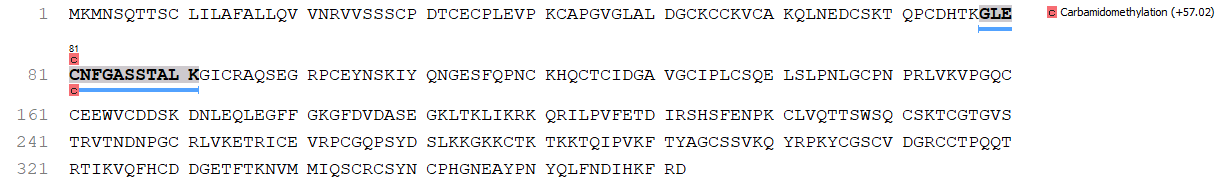

Supplement: Supplementary file 1 [file DataSheet3.ZIP › Naja naja/img/cov_1098.png]

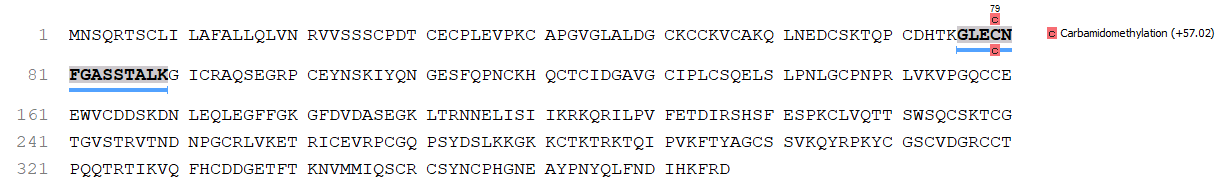

Supplement: Supplementary file 1 [file DataSheet3.ZIP › Naja naja/img/cov_1099.png]

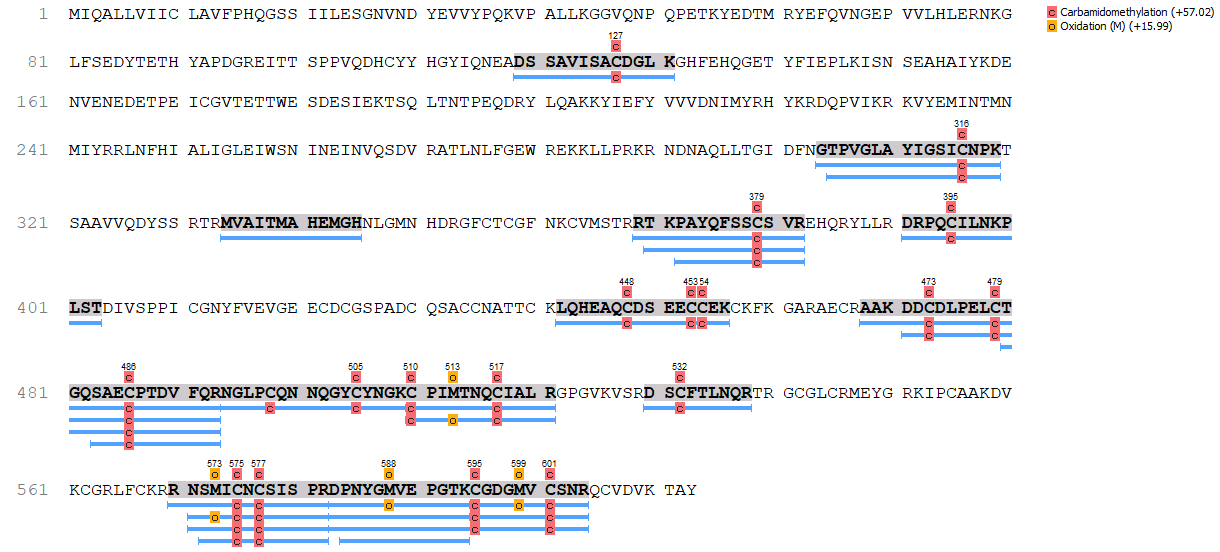

Supplement: Supplementary file 1 [file DataSheet3.ZIP › Naja naja/img/cov_11.png]

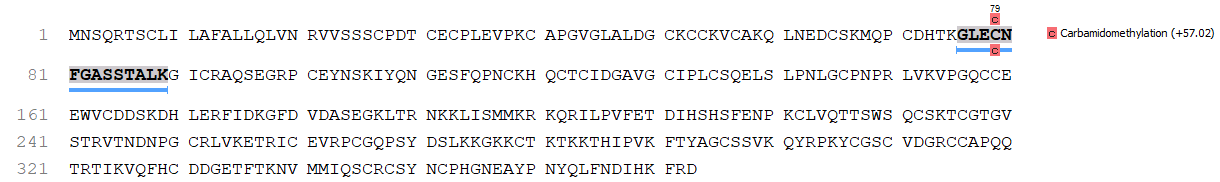

Supplement: Supplementary file 1 [file DataSheet3.ZIP › Naja naja/img/cov_1100.png]

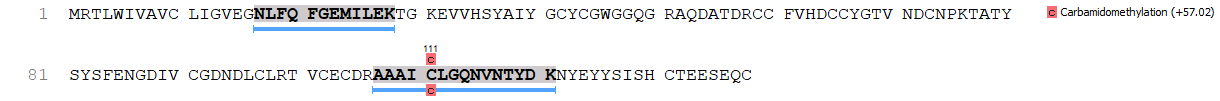

Supplement: Supplementary file 1 [file DataSheet3.ZIP › Naja naja/img/cov_1101.png]

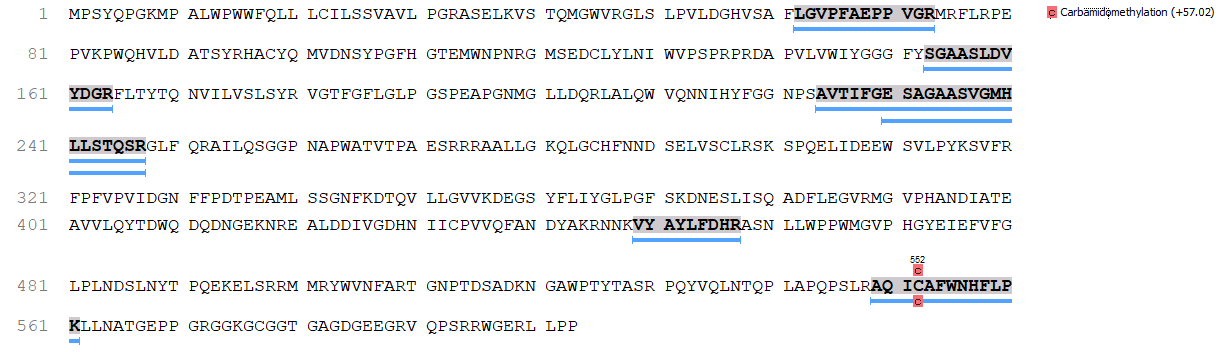

Supplement: Supplementary file 1 [file DataSheet3.ZIP › Naja naja/img/cov_111.png]

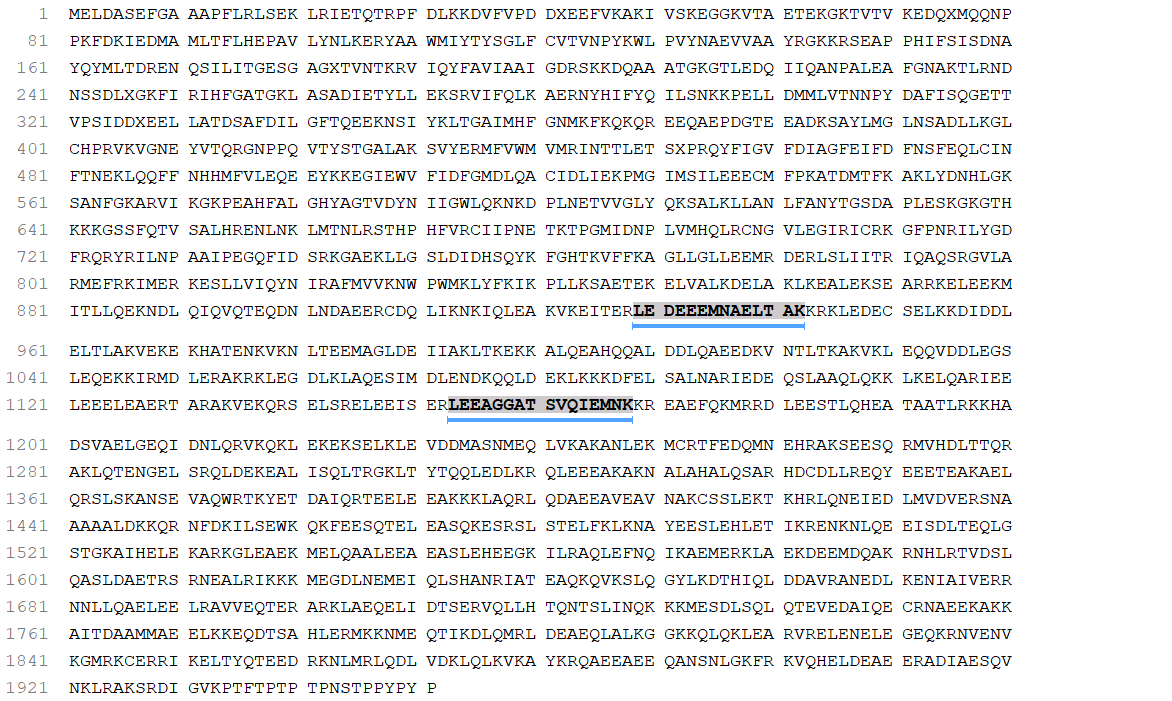

Supplement: Supplementary file 1 [file DataSheet3.ZIP › Naja naja/img/cov_1176.png]

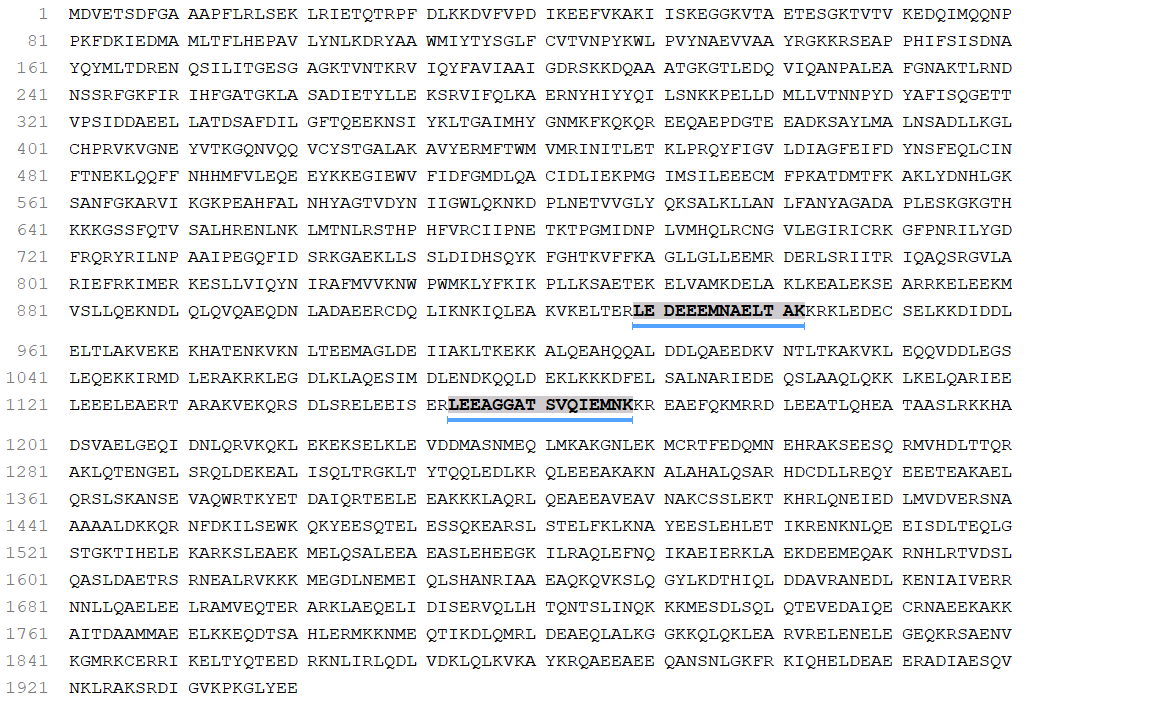

Supplement: Supplementary file 1 [file DataSheet3.ZIP › Naja naja/img/cov_1178.png]

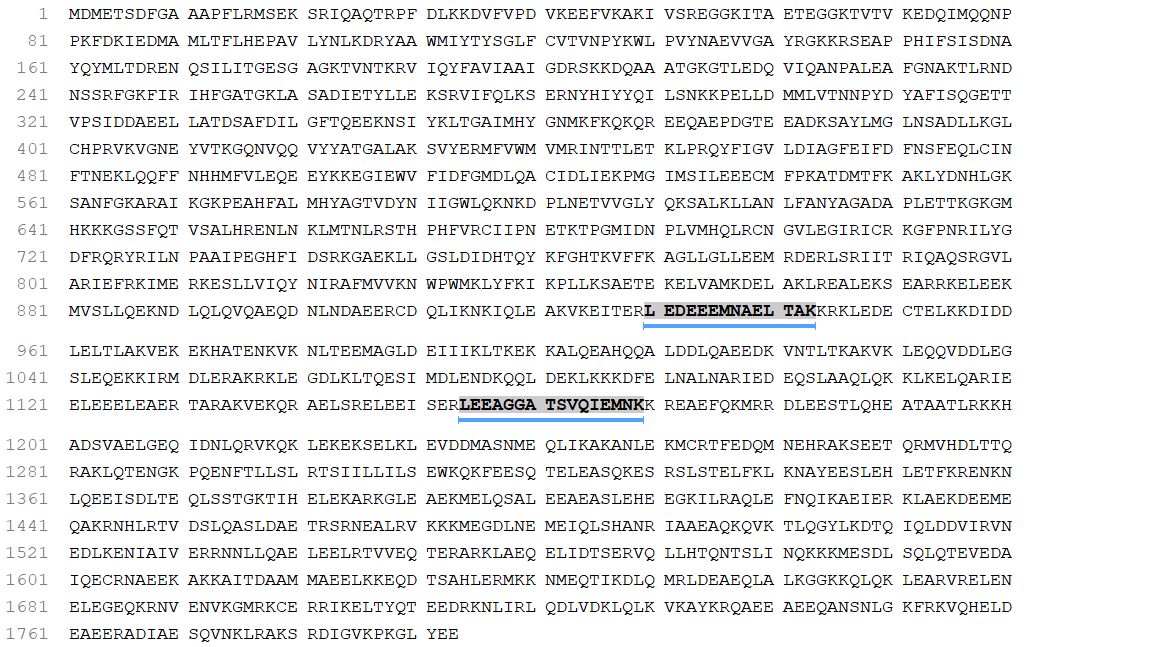

Supplement: Supplementary file 1 [file DataSheet3.ZIP › Naja naja/img/cov_1179.png]

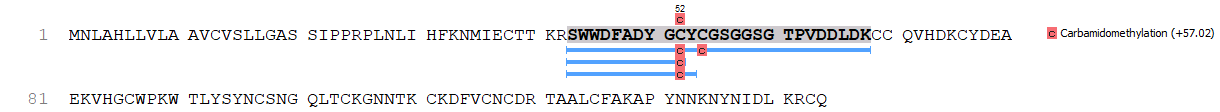

Supplement: Supplementary file 1 [file DataSheet3.ZIP › Naja naja/img/cov_1182.png]

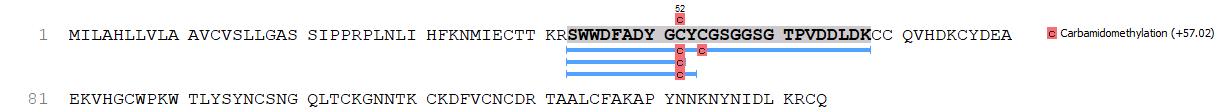

Supplement: Supplementary file 1 [file DataSheet3.ZIP › Naja naja/img/cov_1183.png]

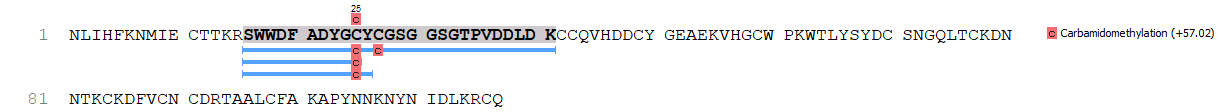

Supplement: Supplementary file 1 [file DataSheet3.ZIP › Naja naja/img/cov_1190.png]

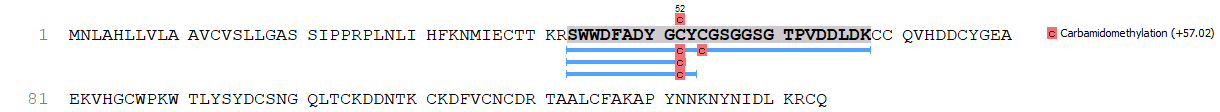

Supplement: Supplementary file 1 [file DataSheet3.ZIP › Naja naja/img/cov_1191.png]

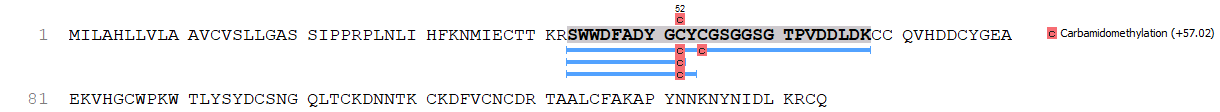

Supplement: Supplementary file 1 [file DataSheet3.ZIP › Naja naja/img/cov_1192.png]

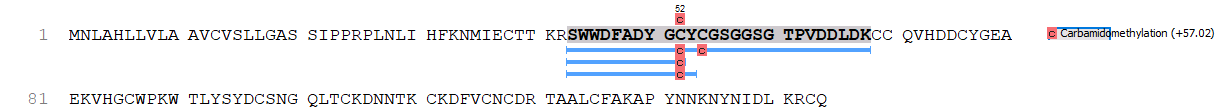

Supplement: Supplementary file 1 [file DataSheet3.ZIP › Naja naja/img/cov_1193.png]

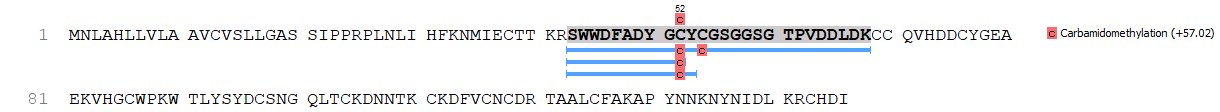

Supplement: Supplementary file 1 [file DataSheet3.ZIP › Naja naja/img/cov_1194.png]

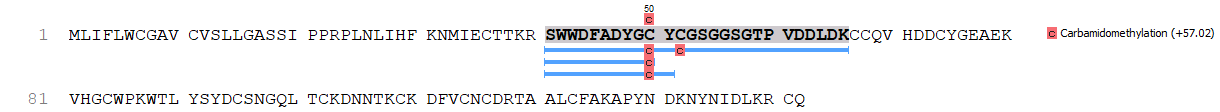

Supplement: Supplementary file 1 [file DataSheet3.ZIP › Naja naja/img/cov_1195.png]

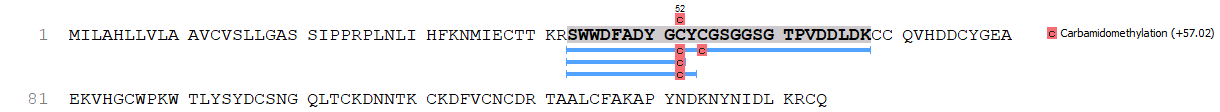

Supplement: Supplementary file 1 [file DataSheet3.ZIP › Naja naja/img/cov_1196.png]

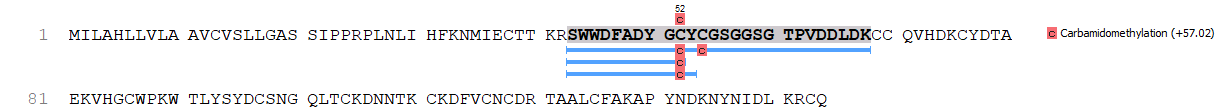

Supplement: Supplementary file 1 [file DataSheet3.ZIP › Naja naja/img/cov_1199.png]

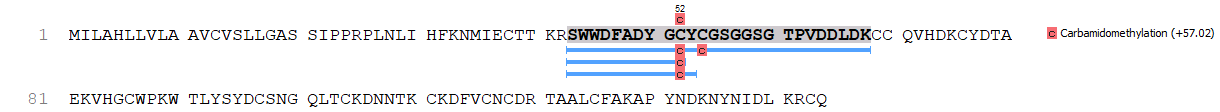

Supplement: Supplementary file 1 [file DataSheet3.ZIP › Naja naja/img/cov_1200.png]

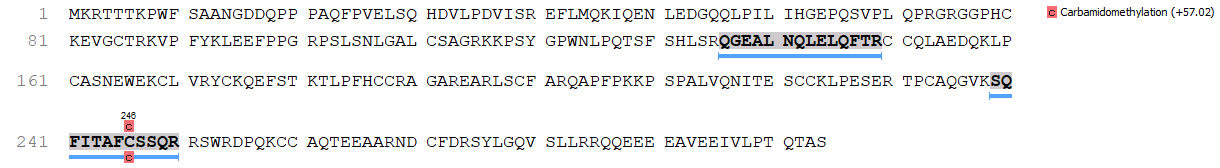

Supplement: Supplementary file 1 [file DataSheet3.ZIP › Naja naja/img/cov_1205.png]

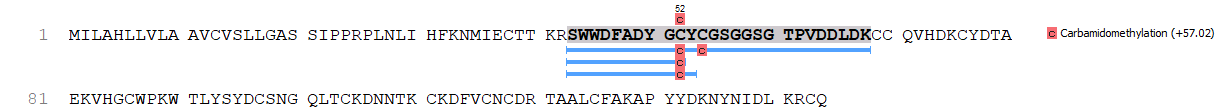

Supplement: Supplementary file 1 [file DataSheet3.ZIP › Naja naja/img/cov_1211.png]

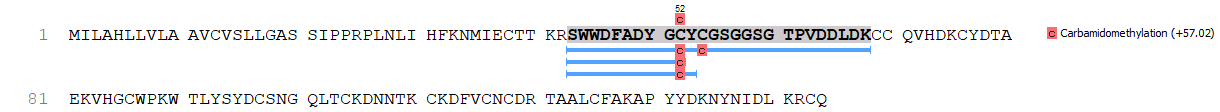

Supplement: Supplementary file 1 [file DataSheet3.ZIP › Naja naja/img/cov_1212.png]

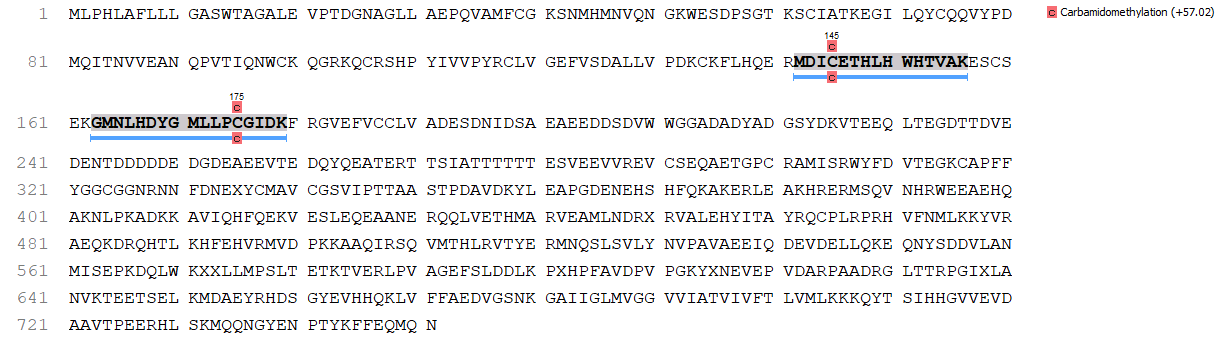

Supplement: Supplementary file 1 [file DataSheet3.ZIP › Naja naja/img/cov_1218.png]

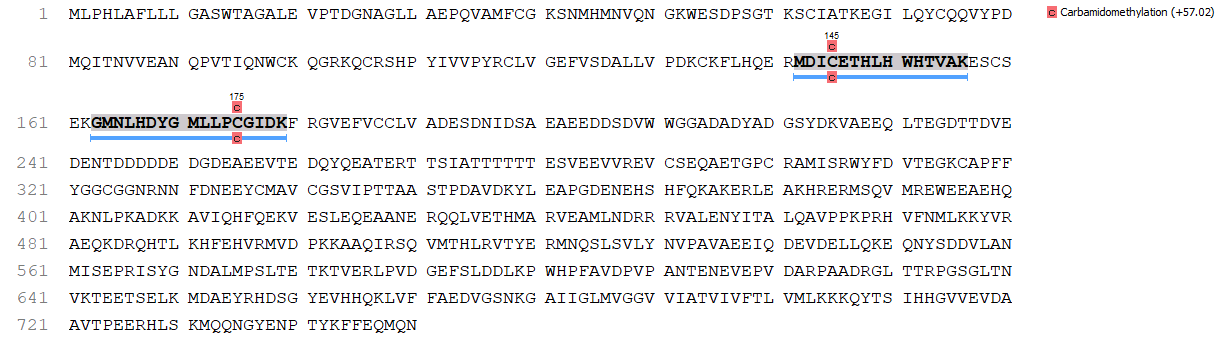

Supplement: Supplementary file 1 [file DataSheet3.ZIP › Naja naja/img/cov_1221.png]

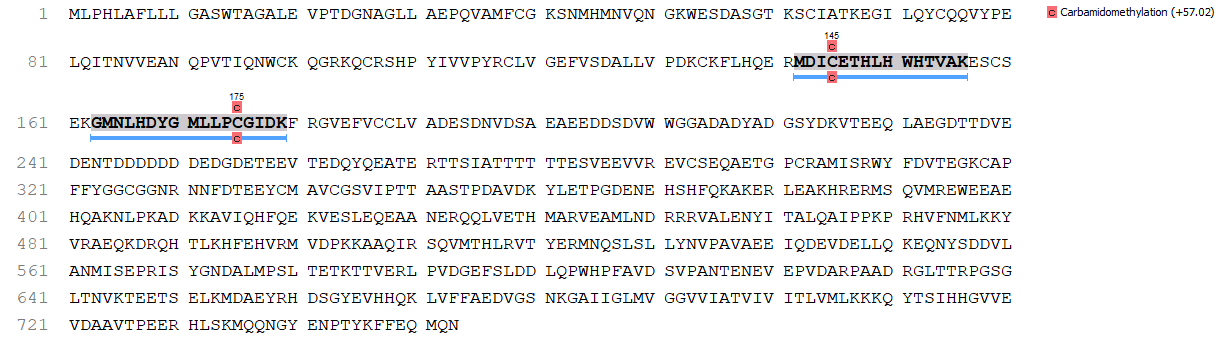

Supplement: Supplementary file 1 [file DataSheet3.ZIP › Naja naja/img/cov_1228.png]

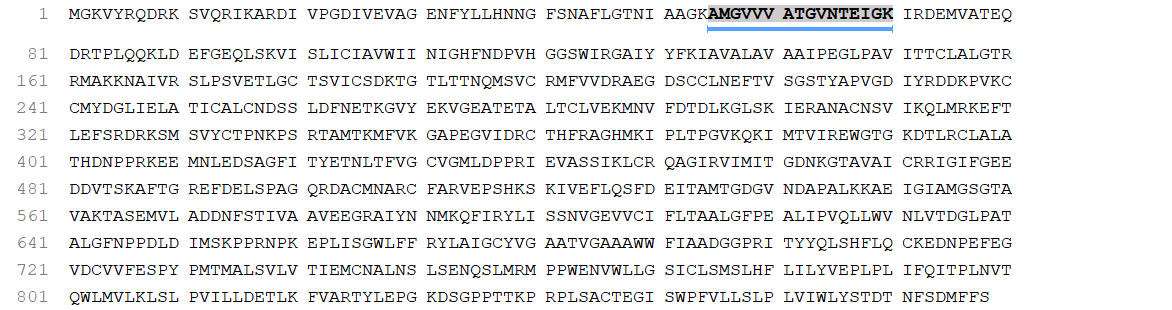

Supplement: Supplementary file 1 [file DataSheet3.ZIP › Naja naja/img/cov_1229.png]

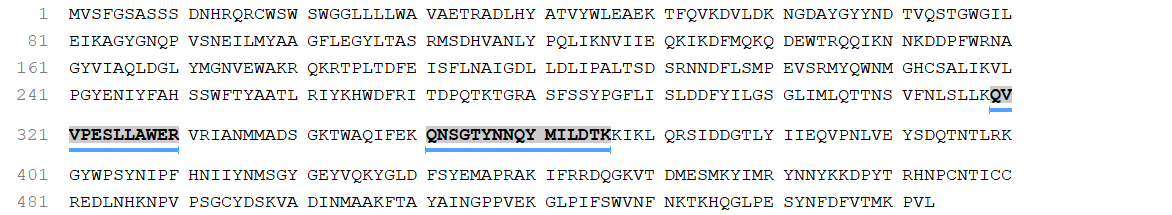

Supplement: Supplementary file 1 [file DataSheet3.ZIP › Naja naja/img/cov_1231.png]

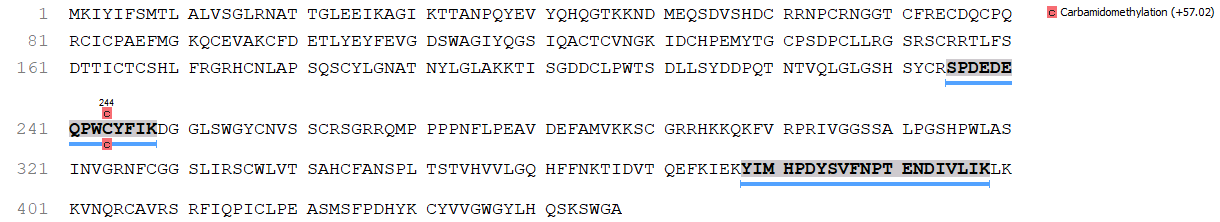

Supplement: Supplementary file 1 [file DataSheet3.ZIP › Naja naja/img/cov_1233.png]

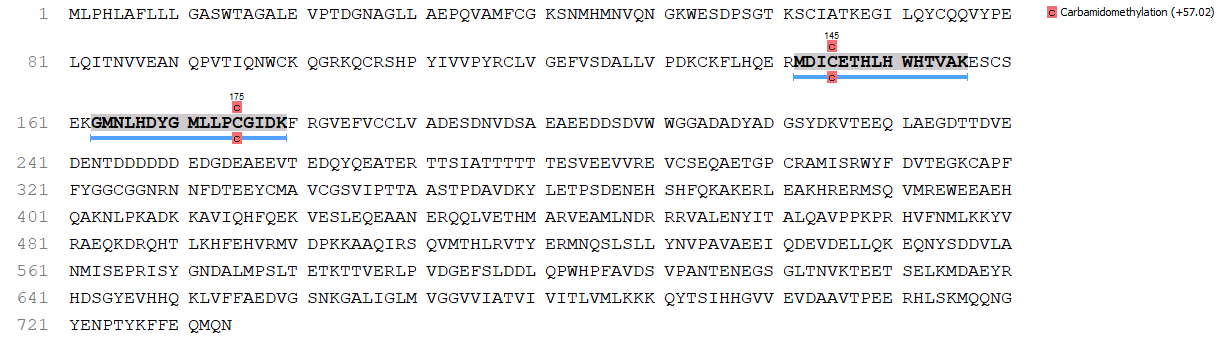

Supplement: Supplementary file 1 [file DataSheet3.ZIP › Naja naja/img/cov_1240.png]

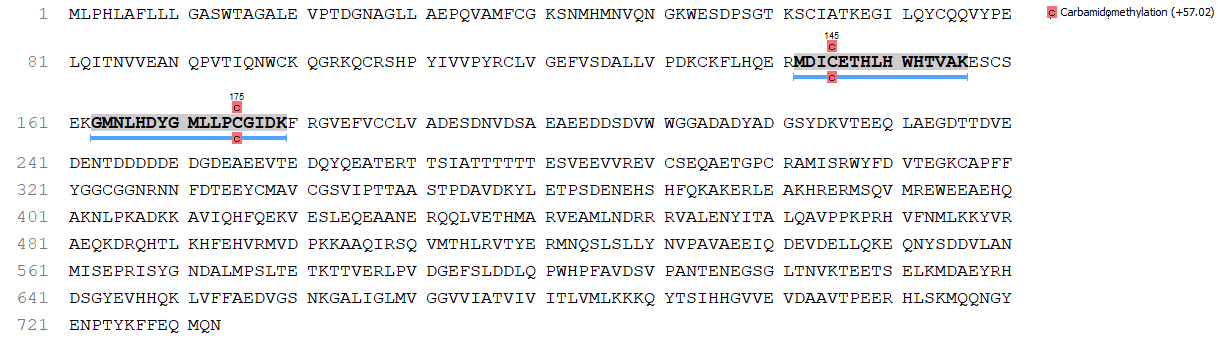

Supplement: Supplementary file 1 [file DataSheet3.ZIP › Naja naja/img/cov_1241.png]

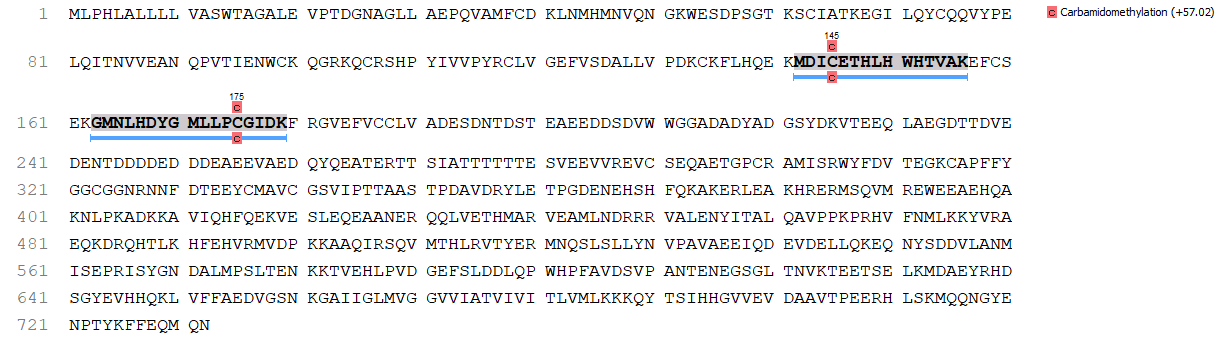

Supplement: Supplementary file 1 [file DataSheet3.ZIP › Naja naja/img/cov_1242.png]

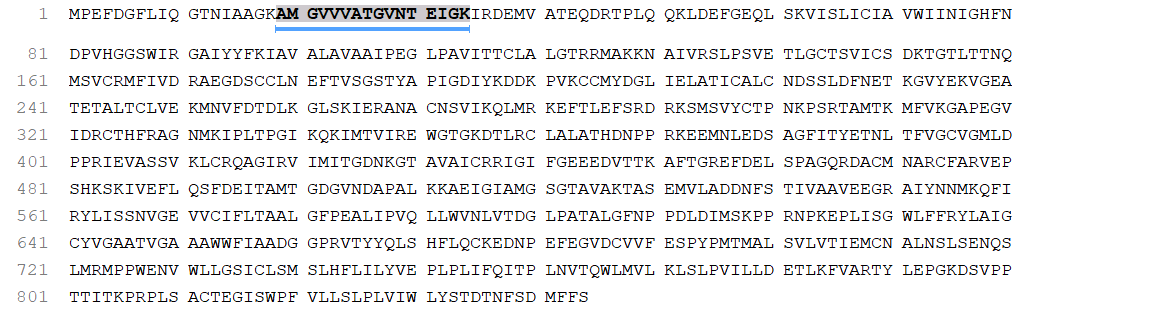

Supplement: Supplementary file 1 [file DataSheet3.ZIP › Naja naja/img/cov_1244.png]

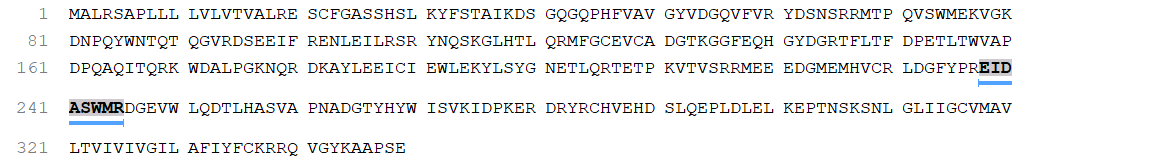

Supplement: Supplementary file 1 [file DataSheet3.ZIP › Naja naja/img/cov_1249.png]

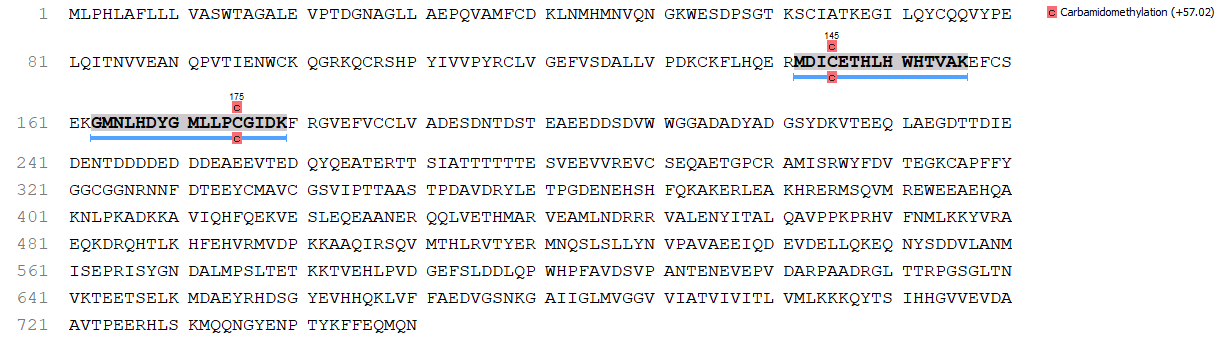

Supplement: Supplementary file 1 [file DataSheet3.ZIP › Naja naja/img/cov_1250.png]

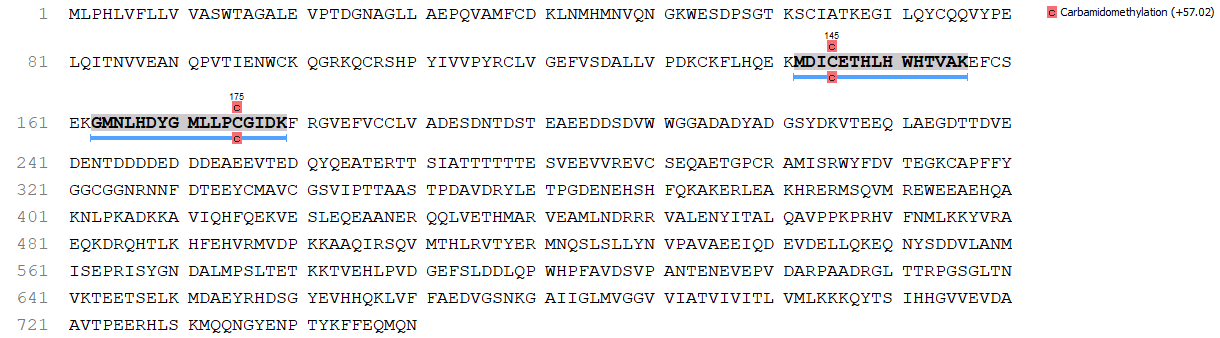

Supplement: Supplementary file 1 [file DataSheet3.ZIP › Naja naja/img/cov_1251.png]

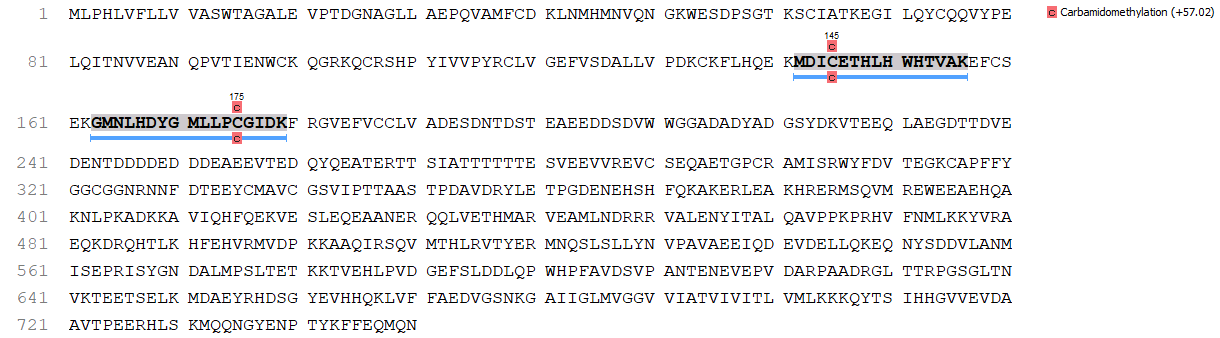

Supplement: Supplementary file 1 [file DataSheet3.ZIP › Naja naja/img/cov_1253.png]

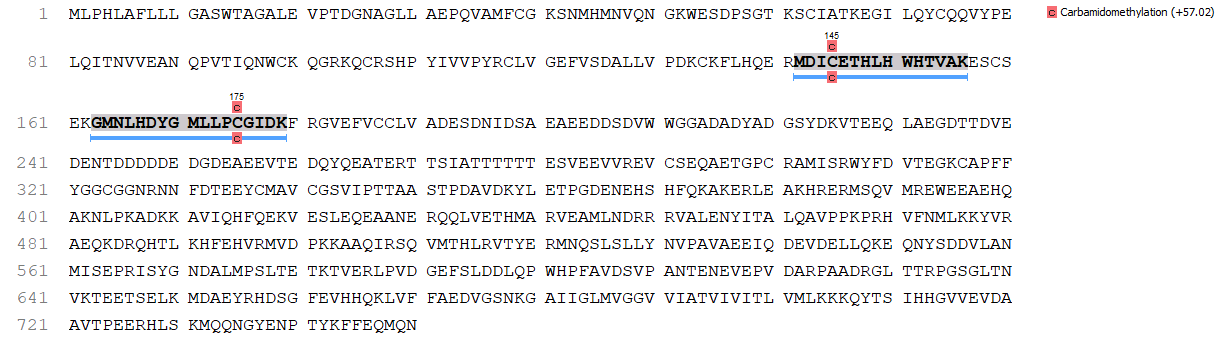

Supplement: Supplementary file 1 [file DataSheet3.ZIP › Naja naja/img/cov_1256.png]

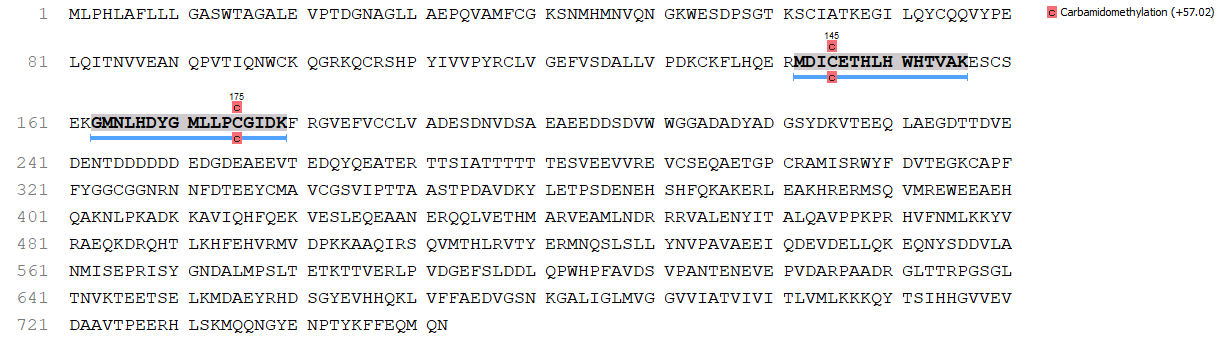

Supplement: Supplementary file 1 [file DataSheet3.ZIP › Naja naja/img/cov_1257.png]

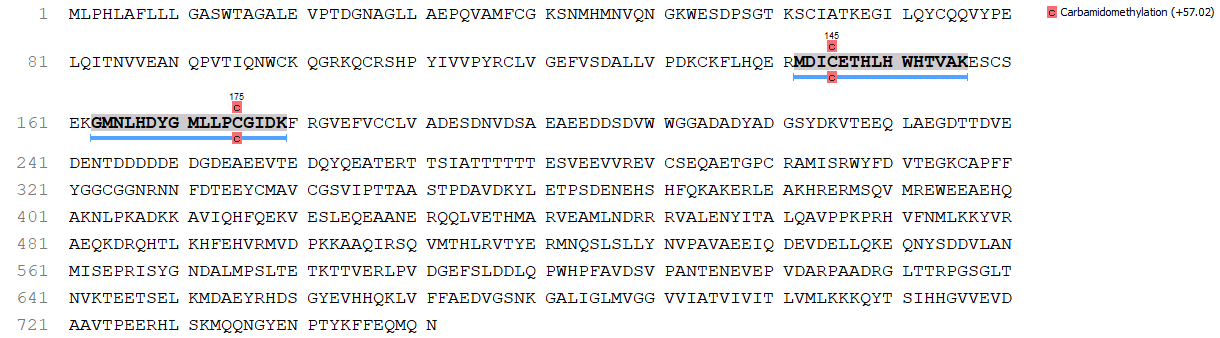

Supplement: Supplementary file 1 [file DataSheet3.ZIP › Naja naja/img/cov_1258.png]

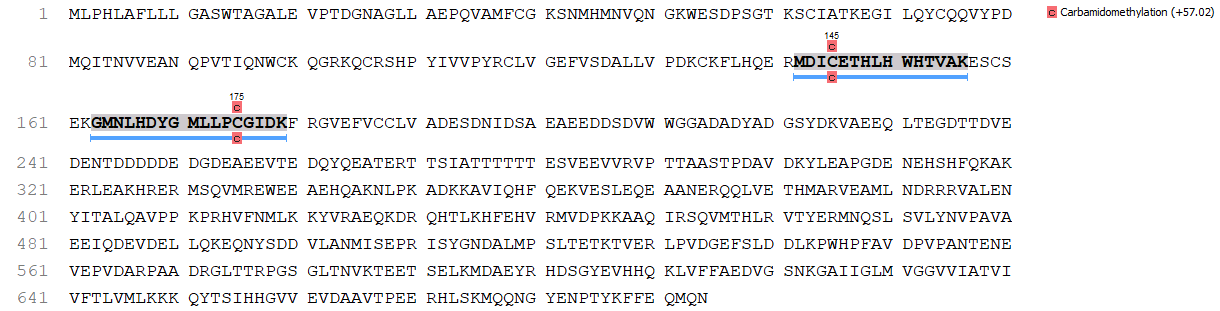

Supplement: Supplementary file 1 [file DataSheet3.ZIP › Naja naja/img/cov_1259.png]

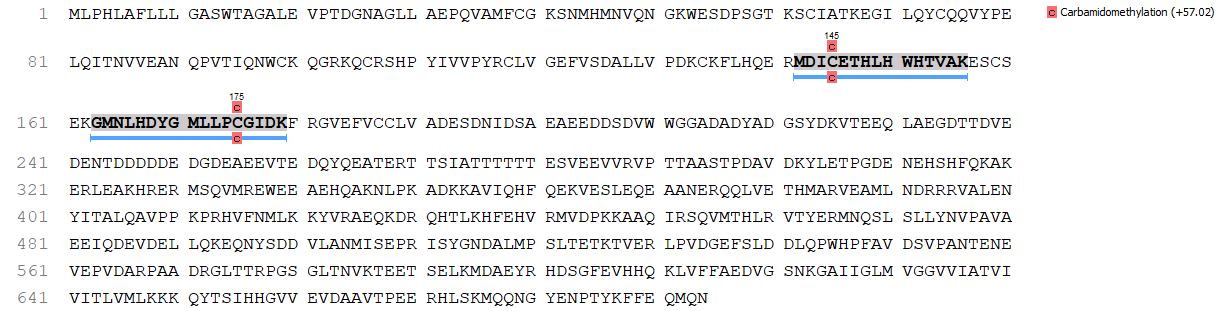

Supplement: Supplementary file 1 [file DataSheet3.ZIP › Naja naja/img/cov_1260.png]

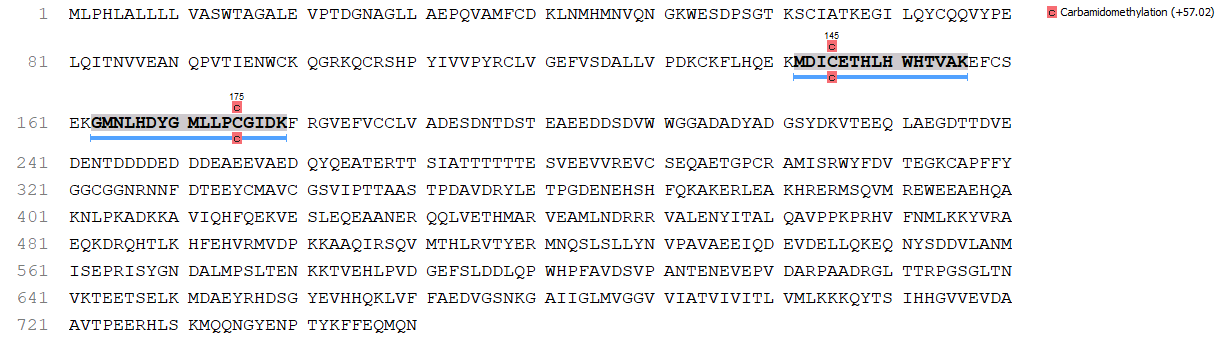

Supplement: Supplementary file 1 [file DataSheet3.ZIP › Naja naja/img/cov_1261.png]

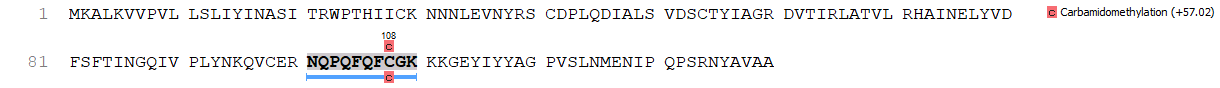

Supplement: Supplementary file 1 [file DataSheet3.ZIP › Naja naja/img/cov_1274.png]

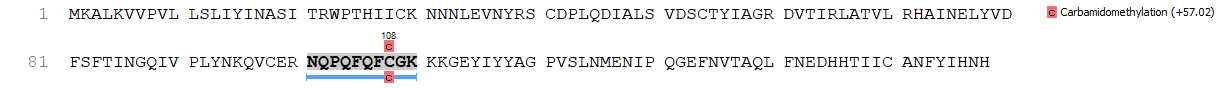

Supplement: Supplementary file 1 [file DataSheet3.ZIP › Naja naja/img/cov_1275.png]

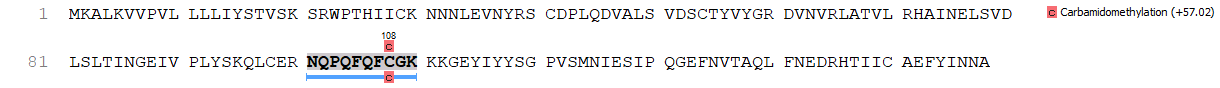

Supplement: Supplementary file 1 [file DataSheet3.ZIP › Naja naja/img/cov_1277.png]

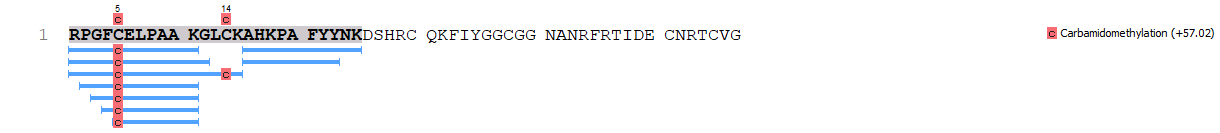

Supplement: Supplementary file 1 [file DataSheet3.ZIP › Naja naja/img/cov_1278.png]

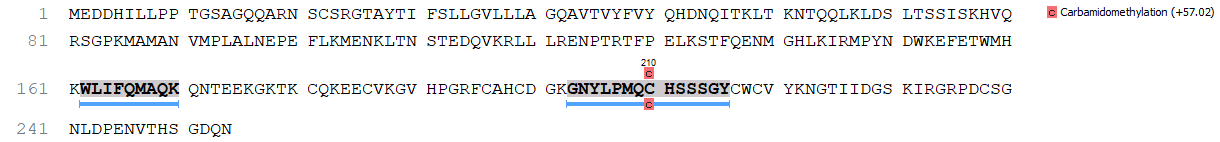

Supplement: Supplementary file 1 [file DataSheet3.ZIP › Naja naja/img/cov_1279.png]

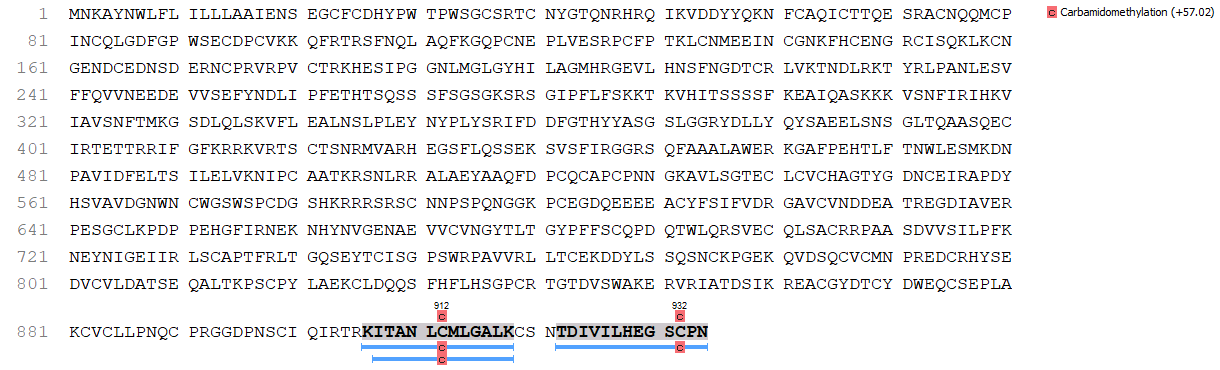

Supplement: Supplementary file 1 [file DataSheet3.ZIP › Naja naja/img/cov_1280.png]

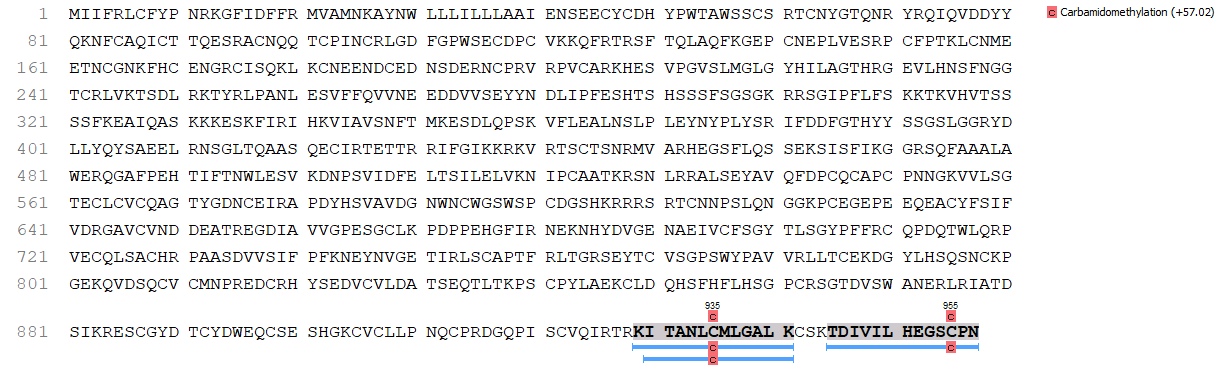

Supplement: Supplementary file 1 [file DataSheet3.ZIP › Naja naja/img/cov_1290.png]

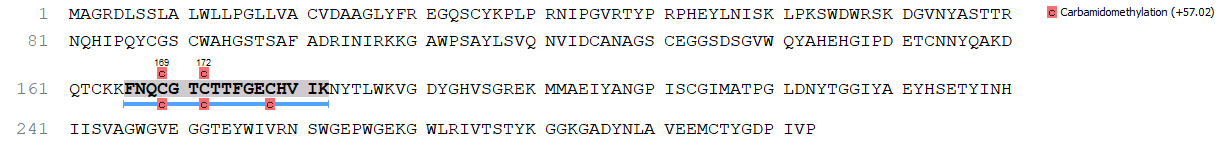

Supplement: Supplementary file 1 [file DataSheet3.ZIP › Naja naja/img/cov_1291.png]

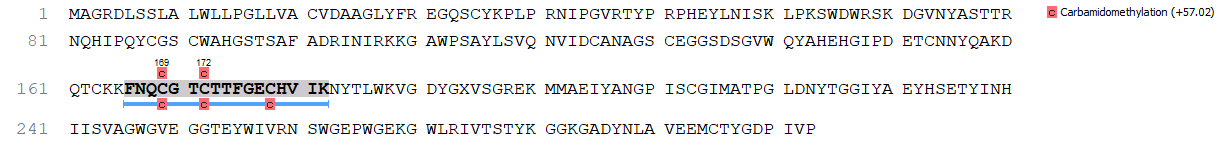

Supplement: Supplementary file 1 [file DataSheet3.ZIP › Naja naja/img/cov_1292.png]

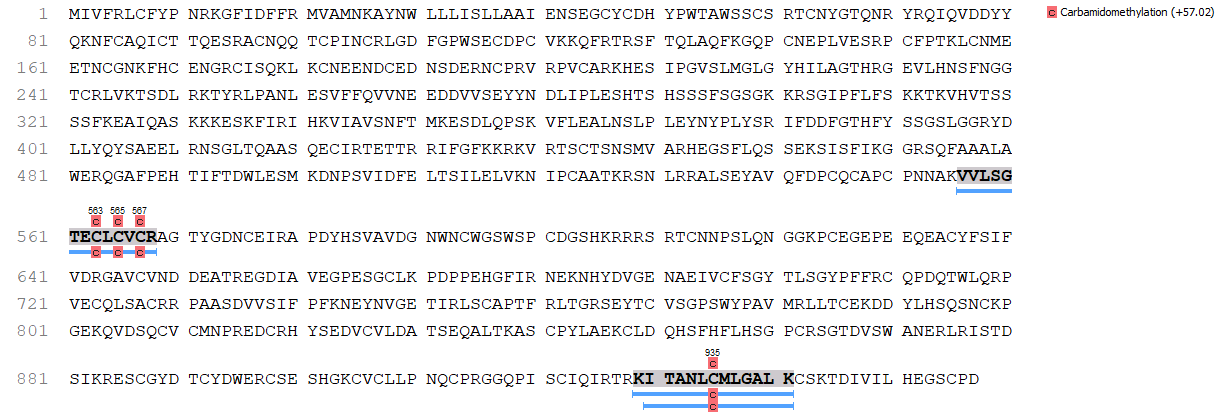

Supplement: Supplementary file 1 [file DataSheet3.ZIP › Naja naja/img/cov_1293.png]

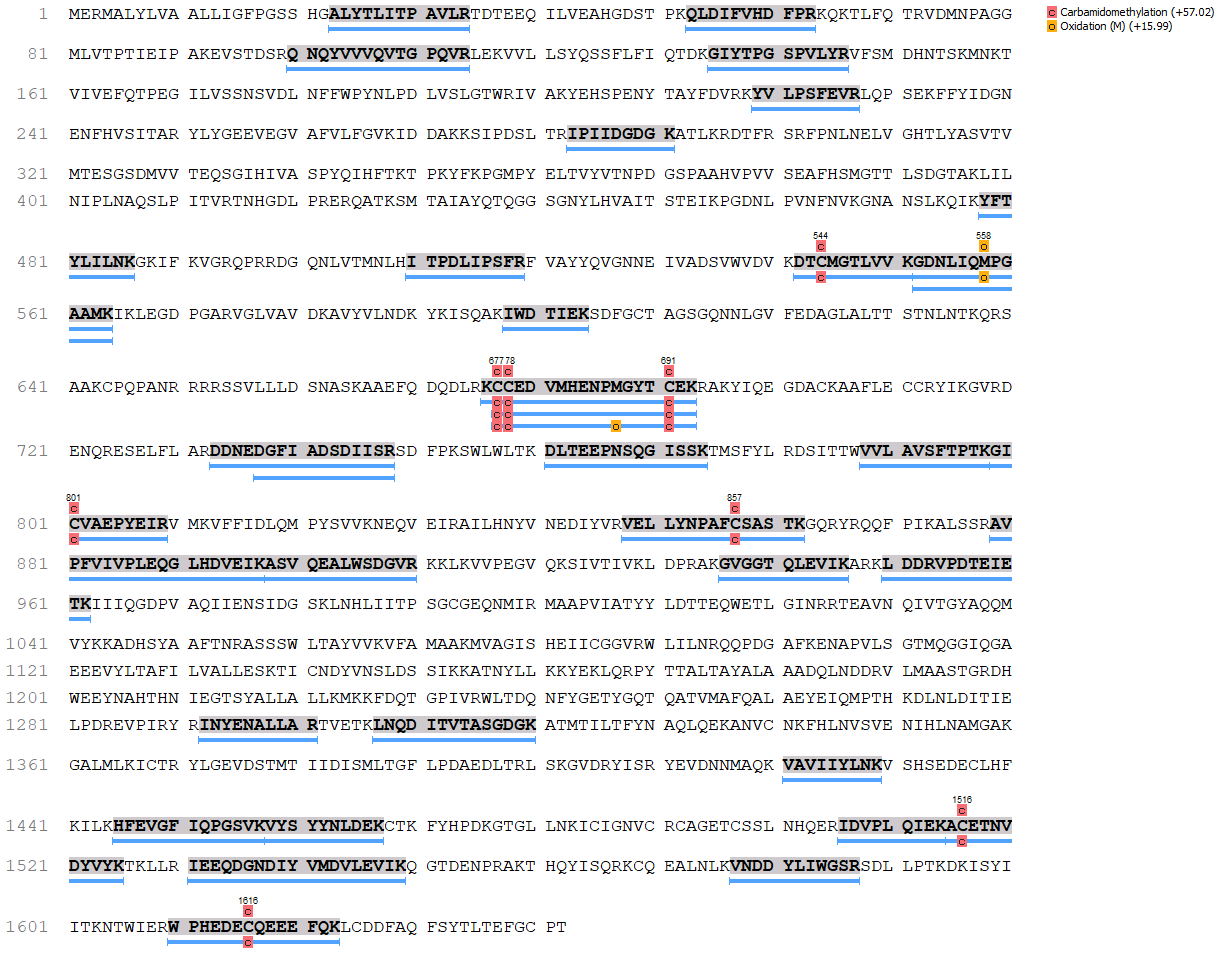

Supplement: Supplementary file 1 [file DataSheet3.ZIP › Naja naja/img/cov_13.png]

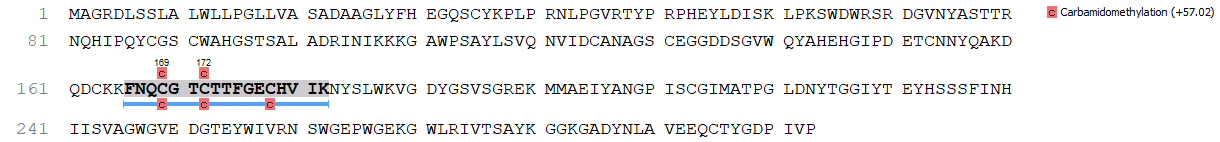

Supplement: Supplementary file 1 [file DataSheet3.ZIP › Naja naja/img/cov_1304.png]

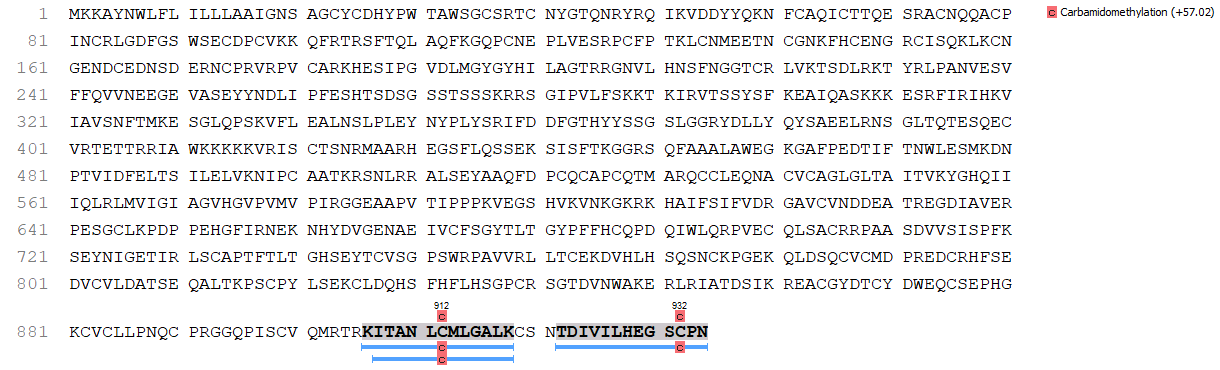

Supplement: Supplementary file 1 [file DataSheet3.ZIP › Naja naja/img/cov_1306.png]

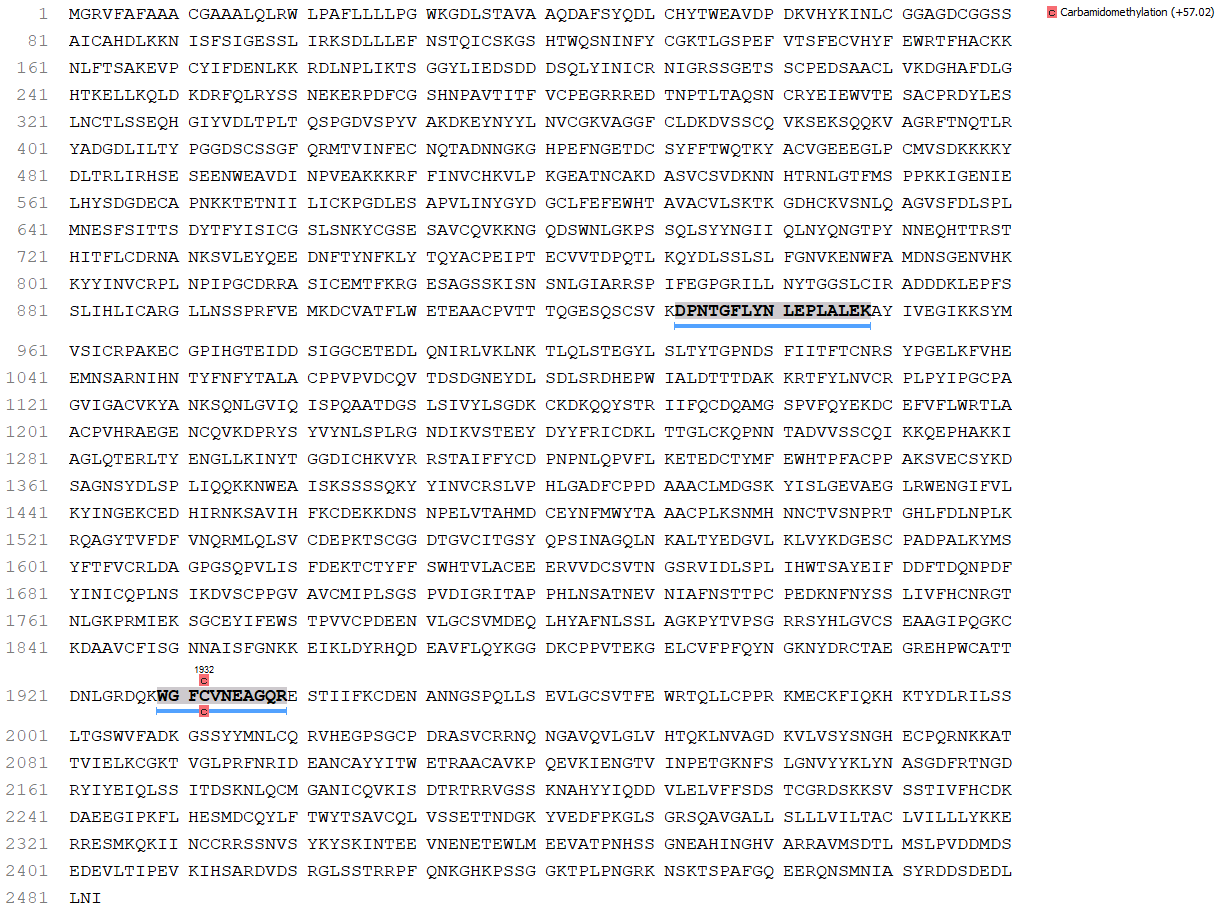

Supplement: Supplementary file 1 [file DataSheet3.ZIP › Naja naja/img/cov_1309.png]

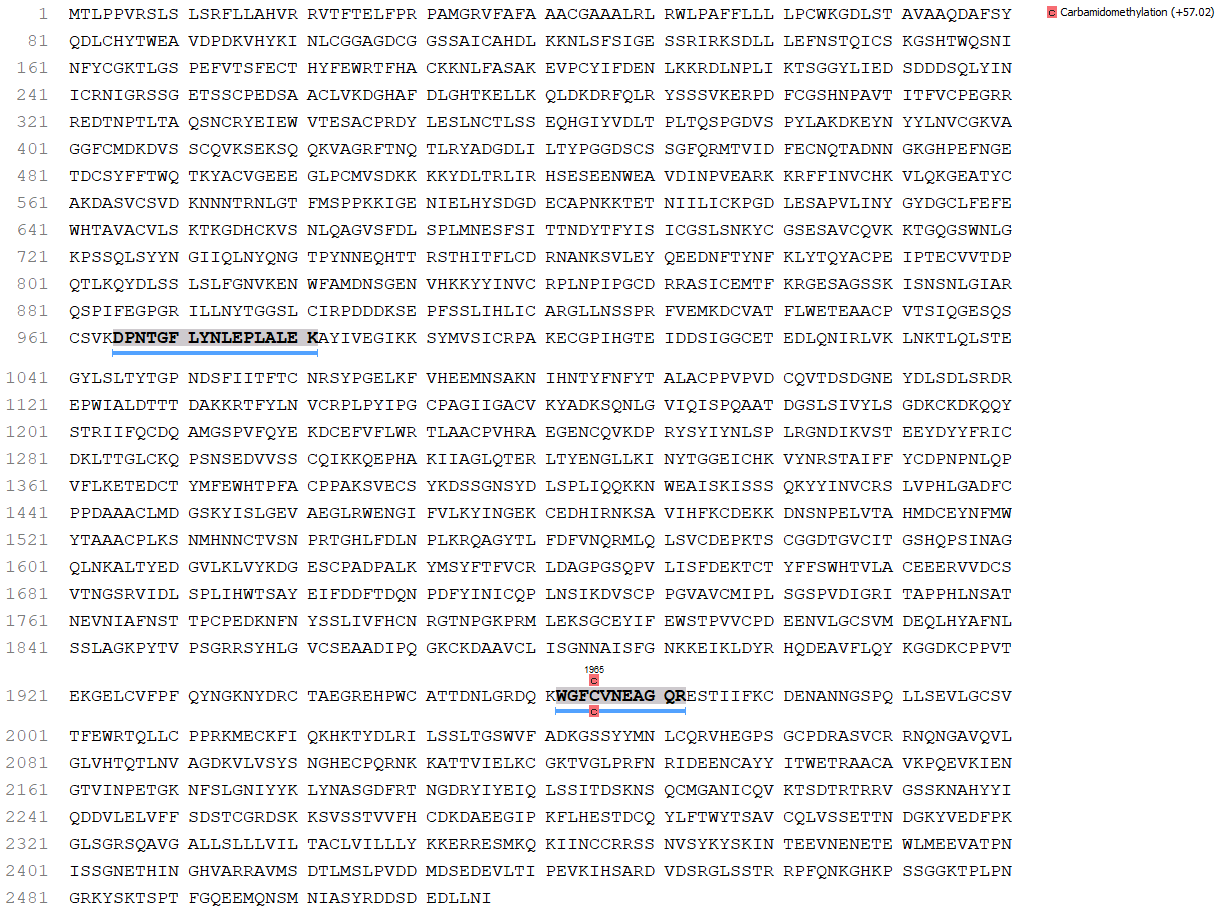

Supplement: Supplementary file 1 [file DataSheet3.ZIP › Naja naja/img/cov_1310.png]

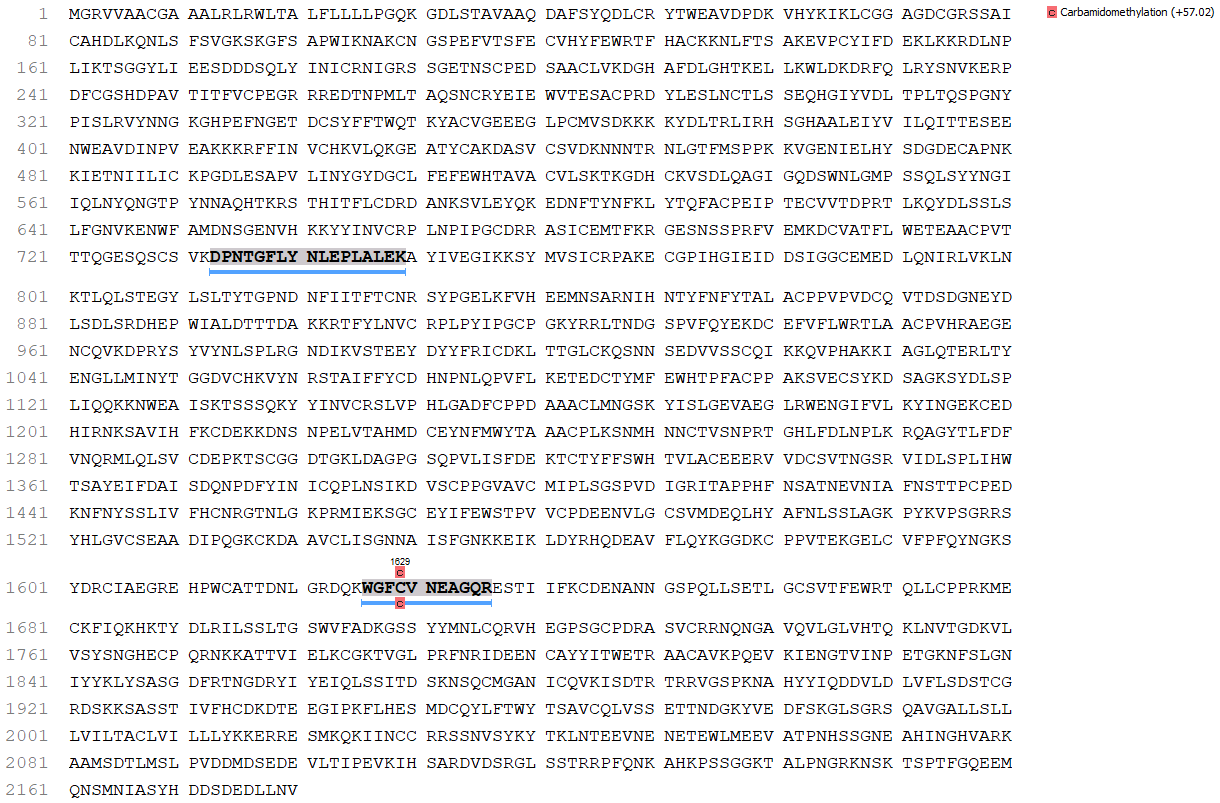

Supplement: Supplementary file 1 [file DataSheet3.ZIP › Naja naja/img/cov_1311.png]

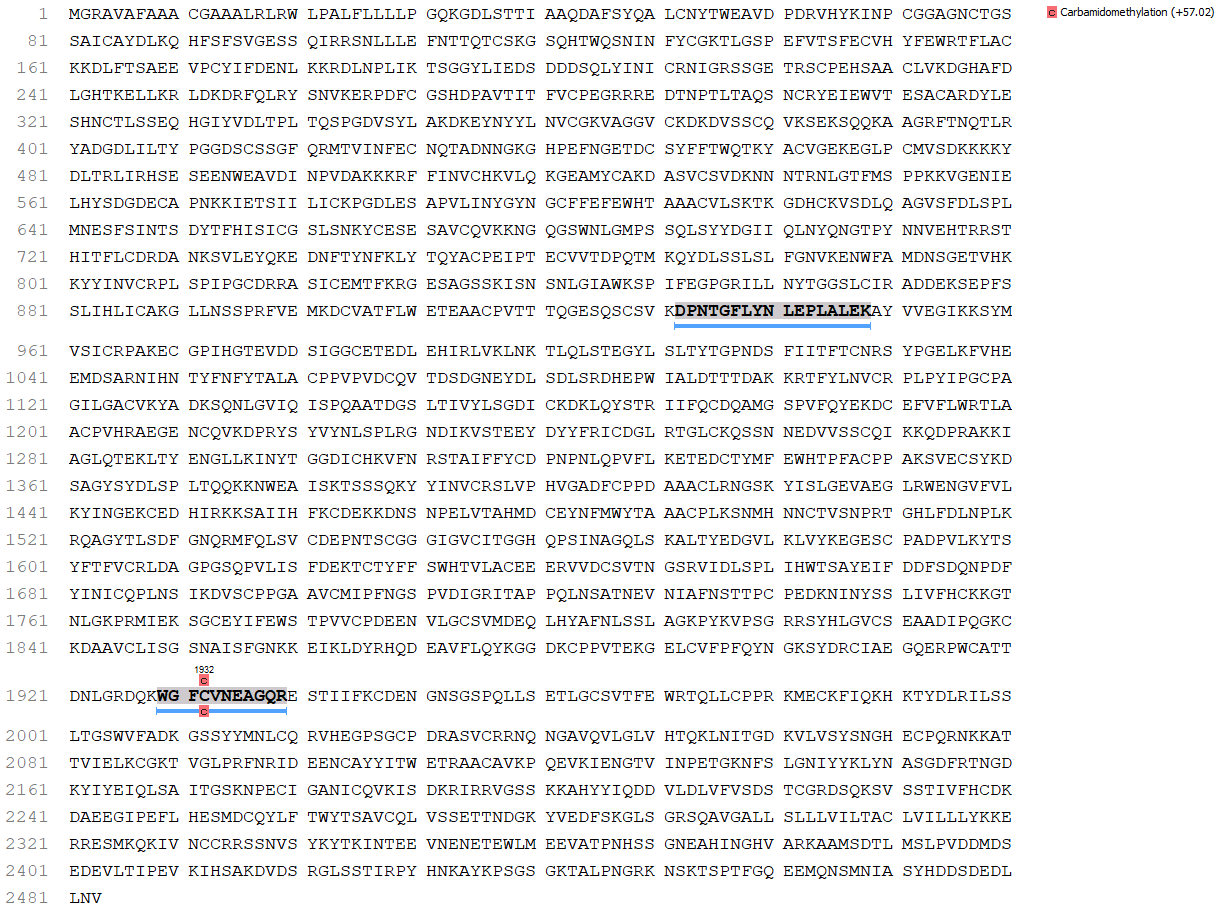

Supplement: Supplementary file 1 [file DataSheet3.ZIP › Naja naja/img/cov_1314.png]

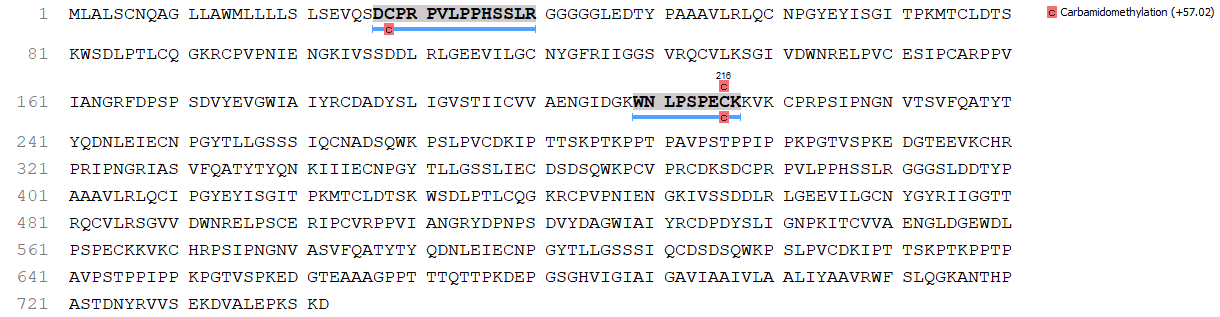

Supplement: Supplementary file 1 [file DataSheet3.ZIP › Naja naja/img/cov_1315.png]

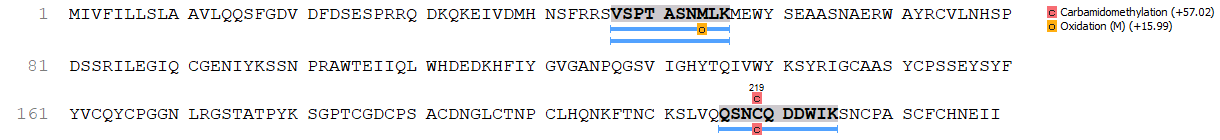

Supplement: Supplementary file 1 [file DataSheet3.ZIP › Naja naja/img/cov_1316.png]

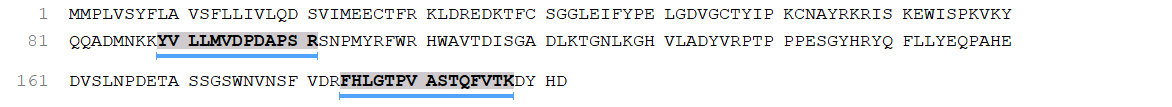

Supplement: Supplementary file 1 [file DataSheet3.ZIP › Naja naja/img/cov_1319.png]

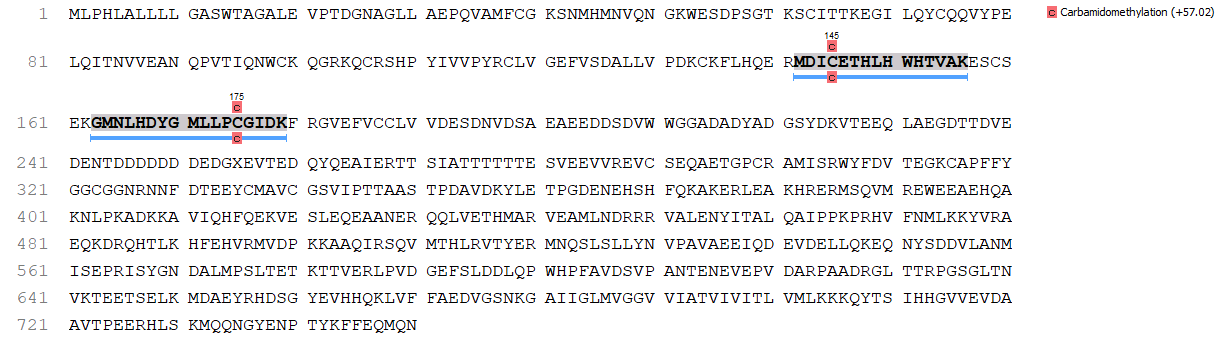

Supplement: Supplementary file 1 [file DataSheet3.ZIP › Naja naja/img/cov_1327.png]

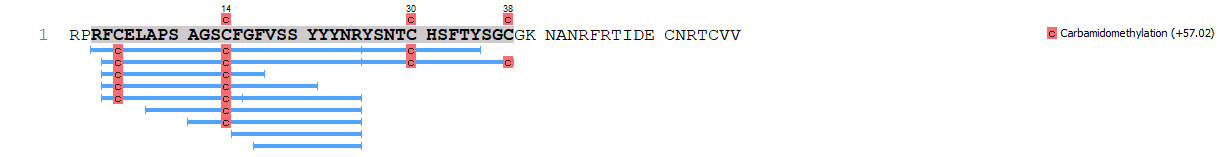

Supplement: Supplementary file 1 [file DataSheet3.ZIP › Naja naja/img/cov_1329.png]

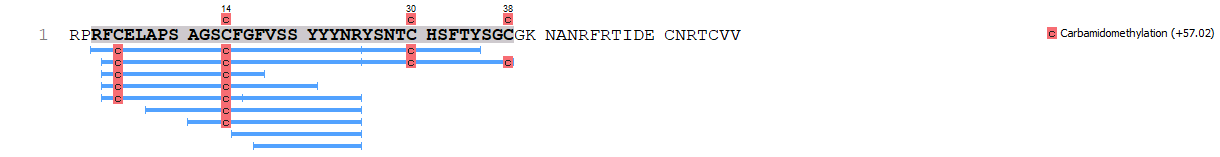

Supplement: Supplementary file 1 [file DataSheet3.ZIP › Naja naja/img/cov_1330.png]

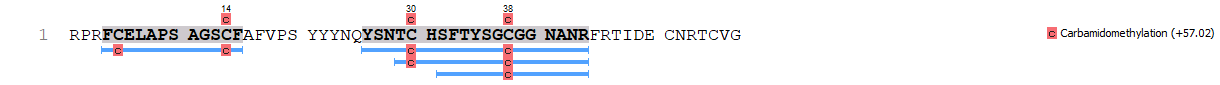

Supplement: Supplementary file 1 [file DataSheet3.ZIP › Naja naja/img/cov_1331.png]

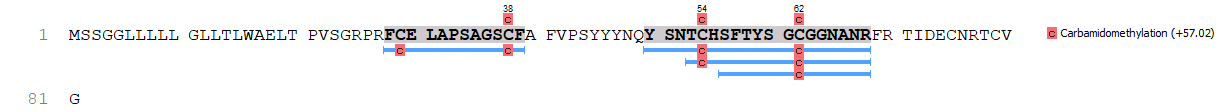

Supplement: Supplementary file 1 [file DataSheet3.ZIP › Naja naja/img/cov_1332.png]

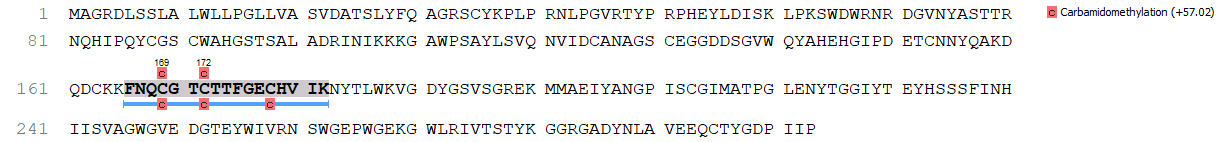

Supplement: Supplementary file 1 [file DataSheet3.ZIP › Naja naja/img/cov_1335.png]

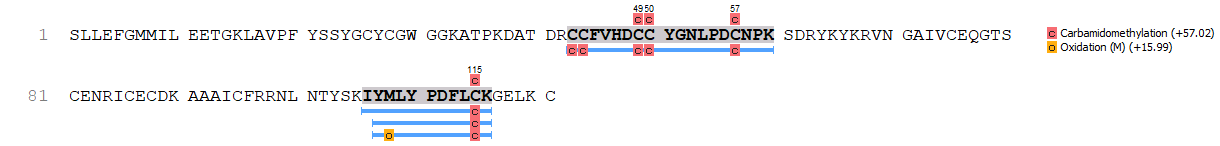

Supplement: Supplementary file 1 [file DataSheet3.ZIP › Naja naja/img/cov_1337.png]

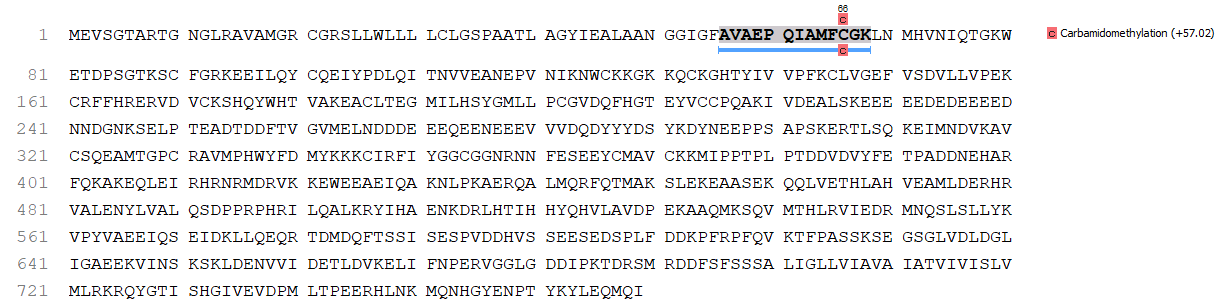

Supplement: Supplementary file 1 [file DataSheet3.ZIP › Naja naja/img/cov_1344.png]

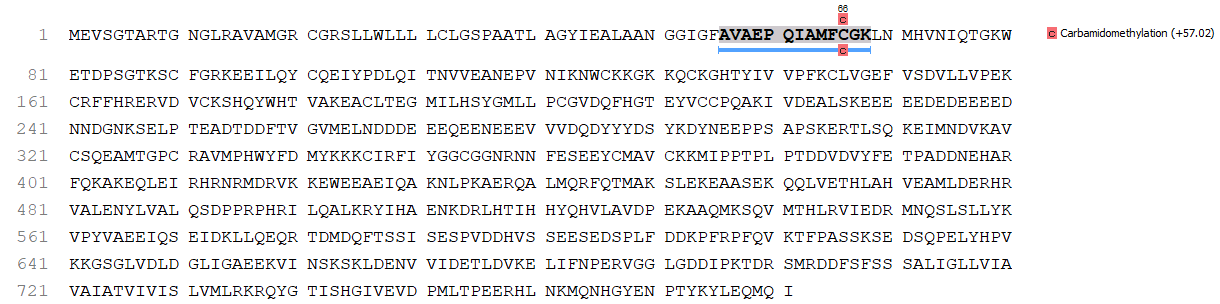

Supplement: Supplementary file 1 [file DataSheet3.ZIP › Naja naja/img/cov_1345.png]

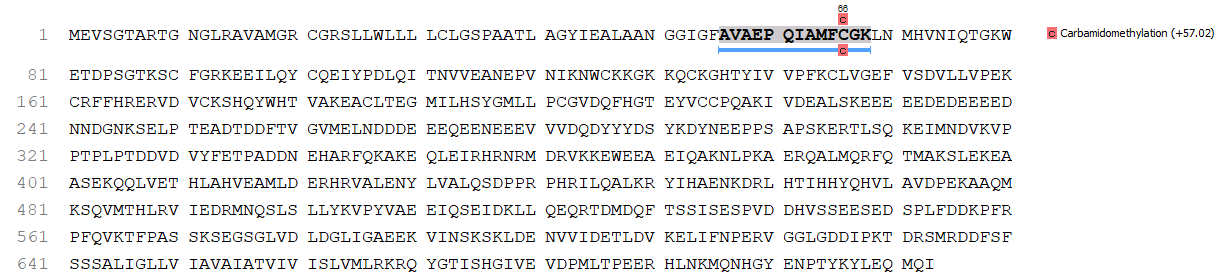

Supplement: Supplementary file 1 [file DataSheet3.ZIP › Naja naja/img/cov_1353.png]

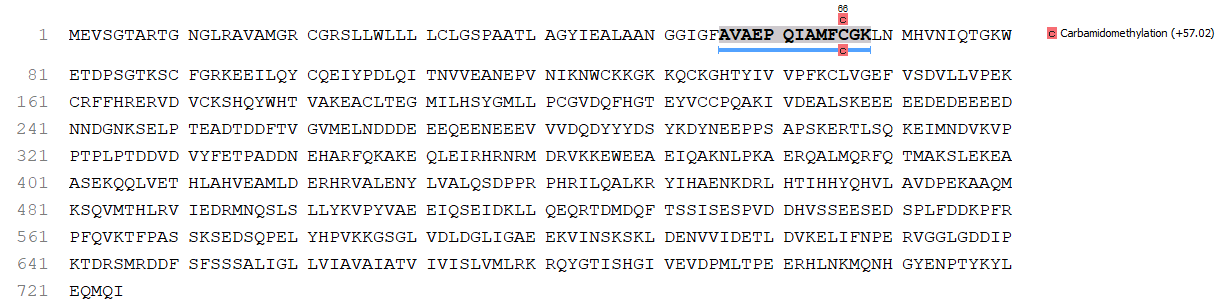

Supplement: Supplementary file 1 [file DataSheet3.ZIP › Naja naja/img/cov_1354.png]

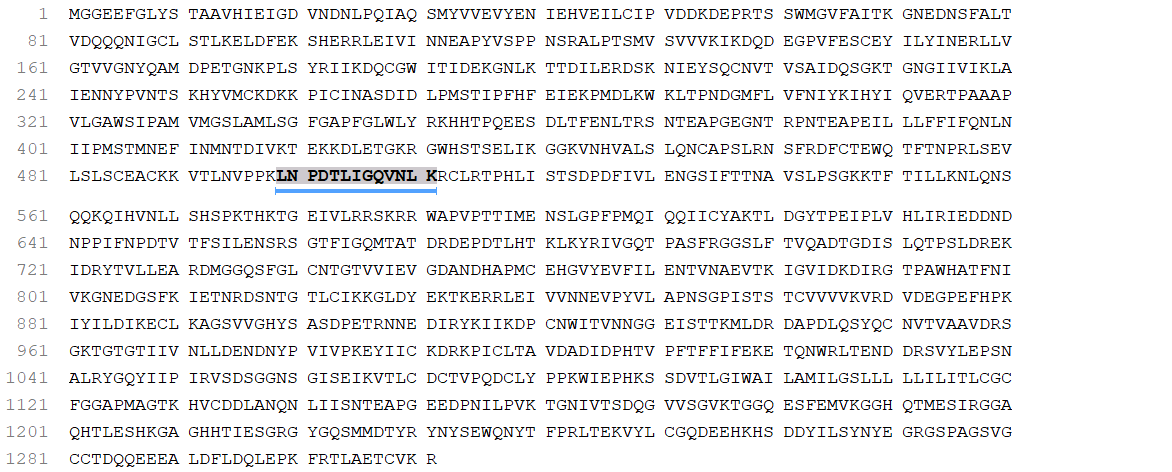

Supplement: Supplementary file 1 [file DataSheet3.ZIP › Naja naja/img/cov_1389.png]

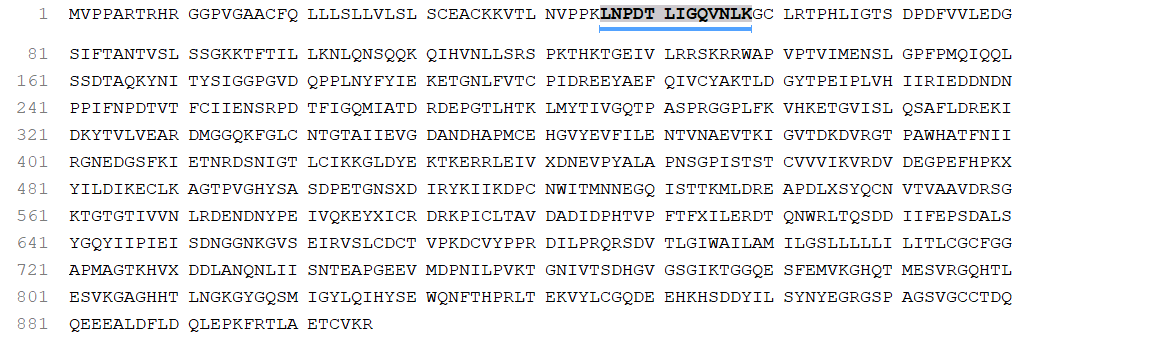

Supplement: Supplementary file 1 [file DataSheet3.ZIP › Naja naja/img/cov_1402.png]

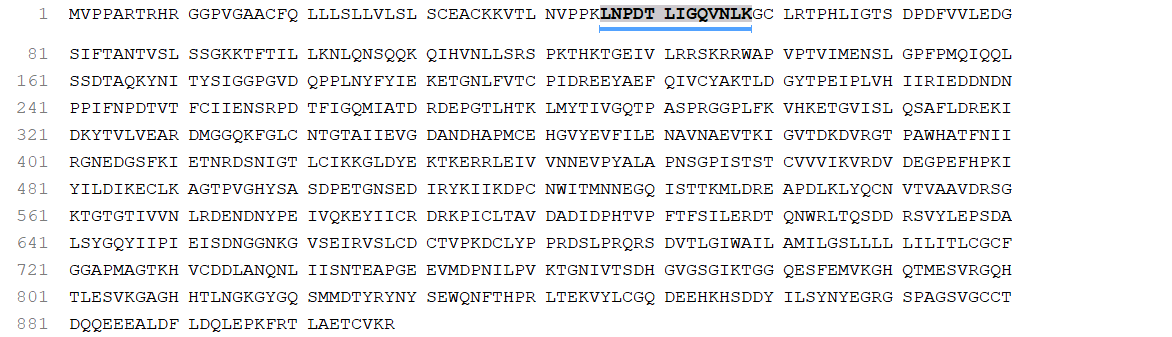

Supplement: Supplementary file 1 [file DataSheet3.ZIP › Naja naja/img/cov_1403.png]

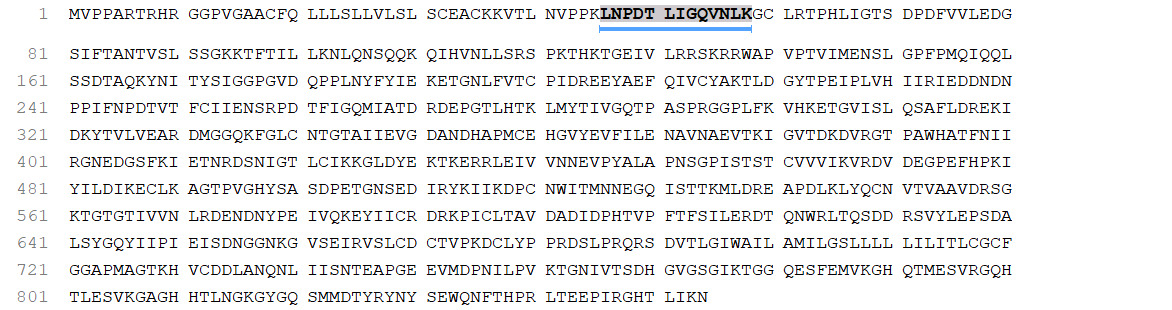

Supplement: Supplementary file 1 [file DataSheet3.ZIP › Naja naja/img/cov_1405.png]

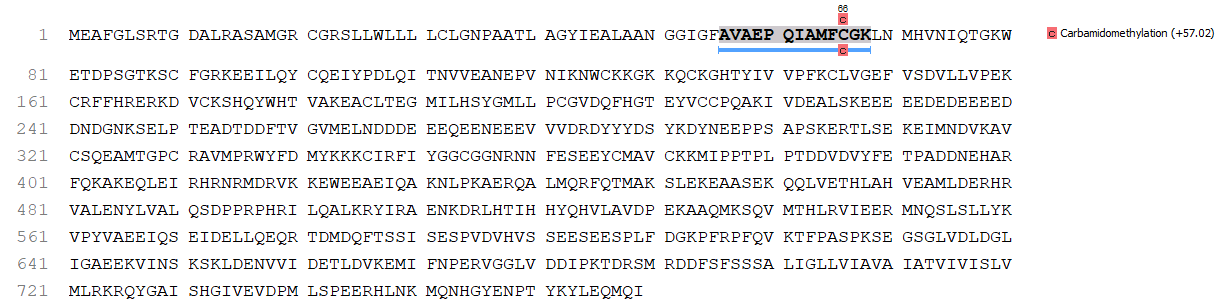

Supplement: Supplementary file 1 [file DataSheet3.ZIP › Naja naja/img/cov_1406.png]

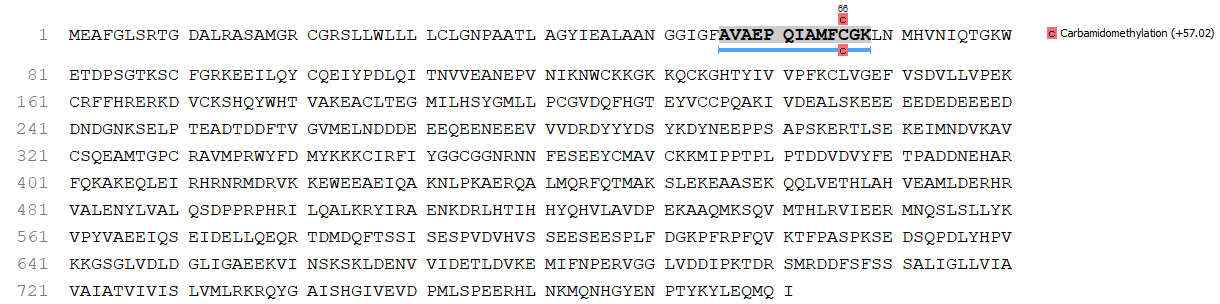

Supplement: Supplementary file 1 [file DataSheet3.ZIP › Naja naja/img/cov_1407.png]

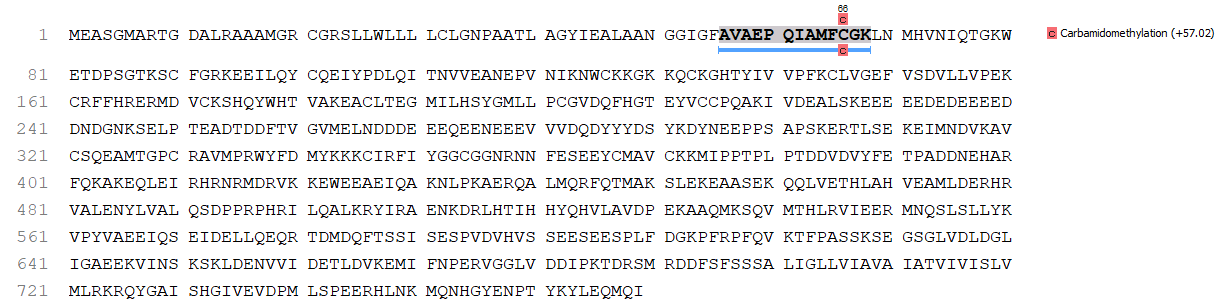

Supplement: Supplementary file 1 [file DataSheet3.ZIP › Naja naja/img/cov_1408.png]

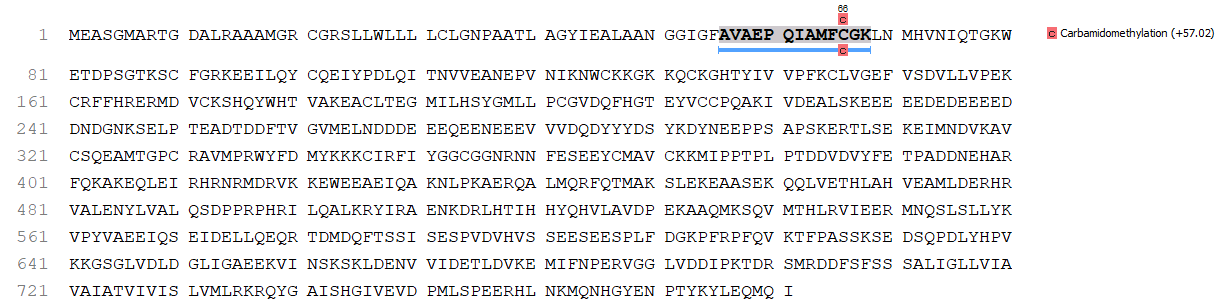

Supplement: Supplementary file 1 [file DataSheet3.ZIP › Naja naja/img/cov_1409.png]
