# Supplementary material for: Venomics of the Enigmatic Andaman Cobra (Naja sagittifera) and the Preclinical Failure of Indian Antivenoms in Andaman and Nicobar Islands
Source: Front Pharmacol. 2021 Oct 25;12:768210. doi: 10.3389/fphar.2021.768210 (PMC8573199; doi:10.3389/fphar.2021.768210)
Supplement: Supplementary file 2 [file DataSheet1.pdf]

Supplementary Material

Supplementary figures

Supplementary Figure 1. The Bayesian cytochrome *b* phylogeny of *Naja* species.

Supplementary Figure 2. The Bayesian ND4 phylogeny of *Naja* species.

Supplementary Figure 3. The maximum likelihood phylogeny (cyt *b* marker) for *Naja* species.

Supplementary Figure 4. The maximum likelihood phylogeny (ND4 marker) for *Naja* species.

Supplementary Figure 5. Biochemical activities of *N. naja* and *N. sagittifera* venoms.

Supplementary Figure 6. DNase activity of *N. naja* and *N. sagittifera* venoms.

Supplementary Figure 7. The fibrinogenolytic activity of *N. naja* and *N. sagittifera* venoms.

Supplementary Figure 8. Immunoblotting of Indian polyvalent and Thai monovalent antivenoms against *N. naja* and *N. sagittifera* venoms.

Supplementary Figure 9. Heatmap depicting the venom binding potential of commercial Indian polyvalent and Thai monovalent antivenoms against *N. naja* and *N. sagittifera* venoms.

Supplementary Tables

Supplementary Table 1. Details of venom samples investigated in this study.

Supplementary Table 2. The details of primers used in the amplification of mitochondrial markers.

Supplementary Table 3. Evolutionary divergence estimated as p-distance for cyt *b* marker.

Supplementary Table 4. Evolutionary divergence estimated as p-distance for ND4 marker.

Supplementary Table 5. The proteomic composition of *N. naja* venom from mainland India.

Supplementary Table 6. The proteomic composition of *N. sagittifera* venom.

Supplementary Table 7. Toxicity profiles of *N. naja* and *N. sagittifera* venoms.

Supplementary Table 8. Neutralisation potencies of commercial Indian polyvalent and Thai monovalent antivenoms.

Supplementary Files

Supplementary File 1. Multiple sequence alignment of ND4 sequences.

Supplementary File 2. Multiple sequence alignment of cyt *b* sequences.

Supplementary File 3. Mass spectrometric data.

Supplementary Materials

Supplementary Figures

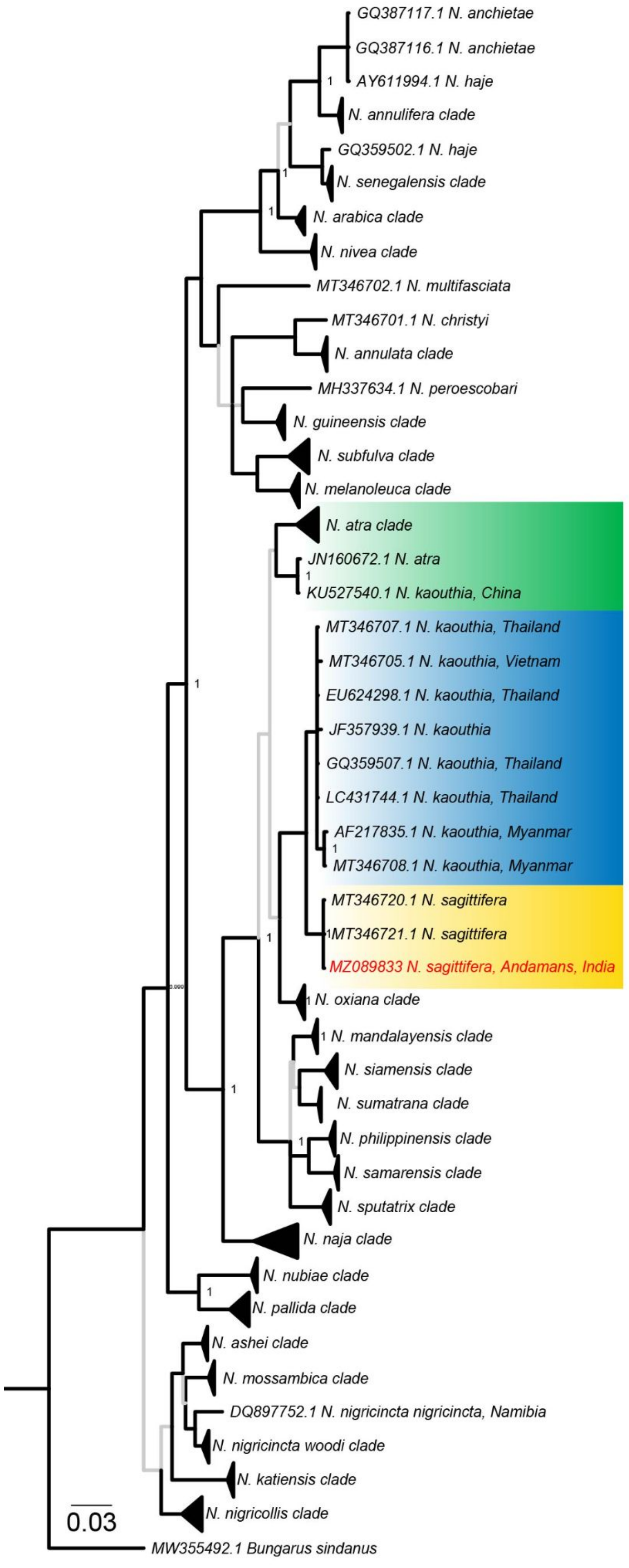

Supplementary Figure 1. The Bayesian cytochrome *b* phylogeny of *Naja* species. The Bayesian cytochrome b phylogeny of *Naja* species is depicted here. Lineages of interest have been shown in uniquely coloured boxes and the accession number of the individual sequenced in this study has been highlighted in red. Well supported branches (BPP ≥ 0.95) and branches with relatively inferior node support (BPP ≤ 0.95) are shown in thick black and thin grey lines, respectively. Branch lengths are scaled by the number of nucleotide substitutions per site.

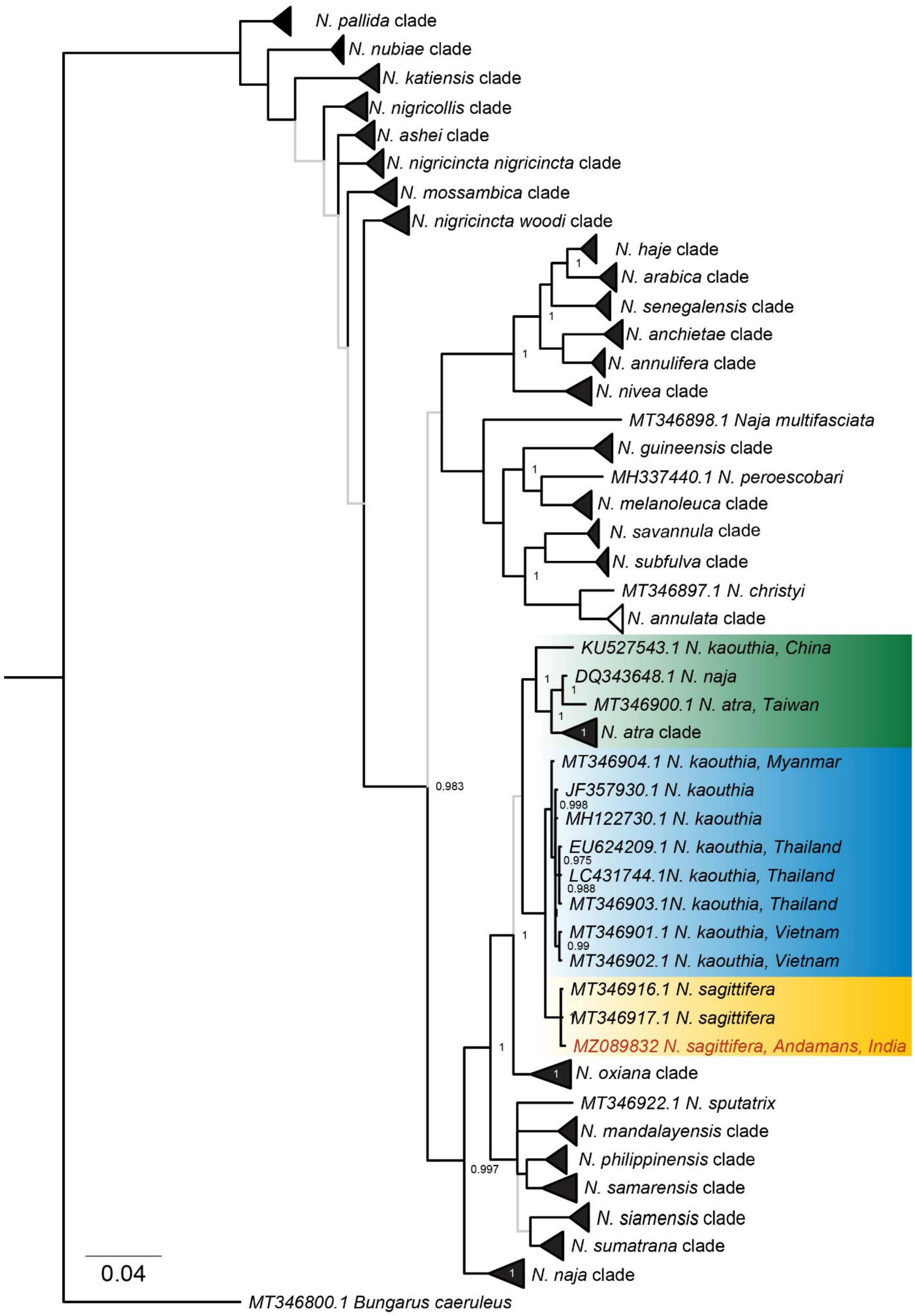

Supplementary Figure 2. The Bayesian ND4 phylogeny of *Naja* species. This figure depicts the phylogenetic relationships between various *Naja* species, built using ND4 sequences. Lineages of interest have been shown in uniquely coloured boxes and the accession number of the individual sequenced in this study has been highlighted in red.

Well supported branches (BPP ≥ 0.95) and branches with relatively inferior node support (BPP ≤ 0.95) are shown in thick black and thin grey lines, respectively. Branch lengths shown are scaled by the number of nucleotide substitutions per site.

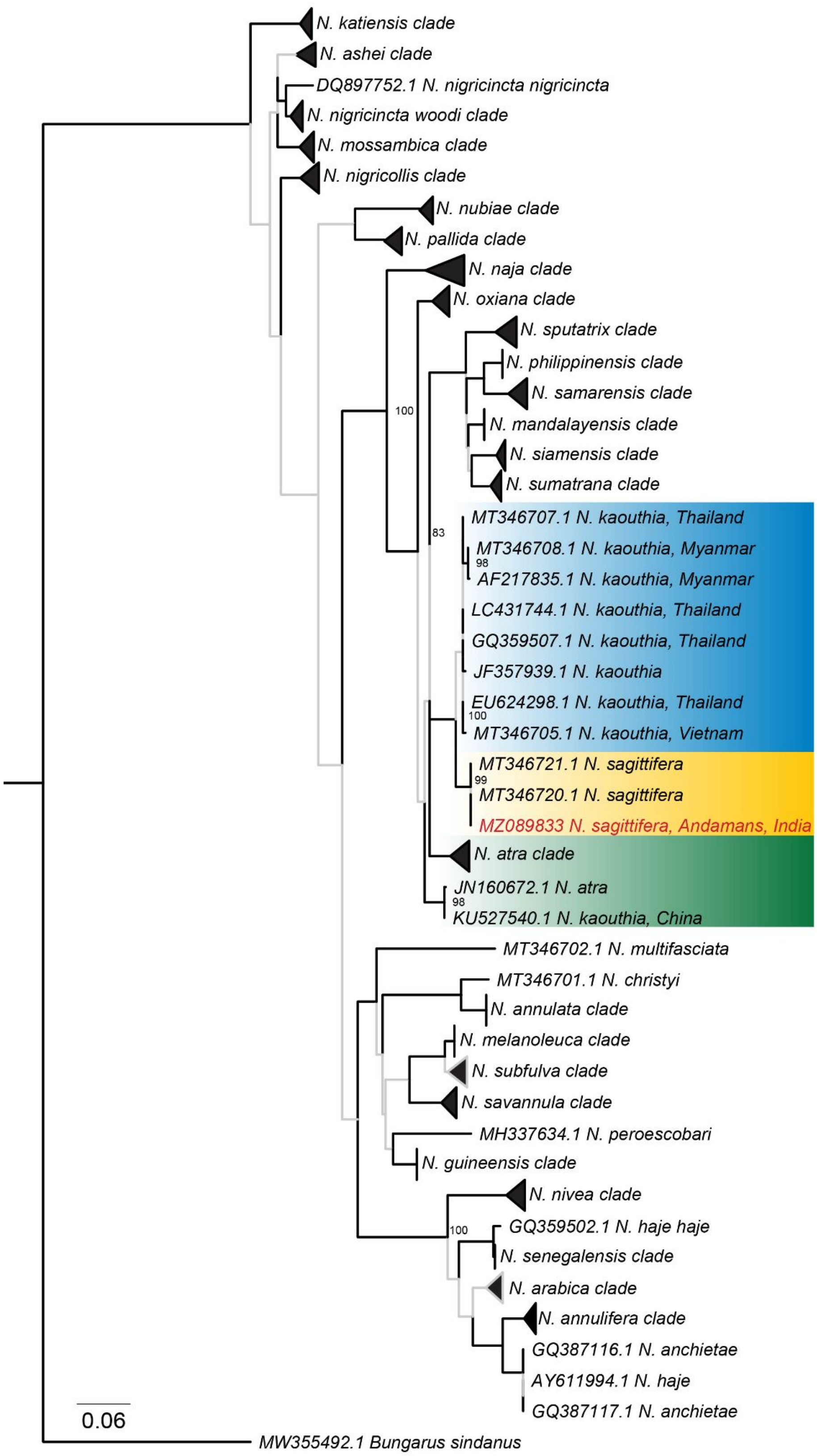

Supplementary Figure 3. The maximum likelihood phylogeny (cyt *b* marker) for *Naja* species. The ML tree depicting the phylogenetic relationships between various *Naja* species is shown here. Lineages of interest have been shown in uniquely coloured boxes

and the accession number of the sequence generated in this study has been highlighted in red. Well supported branches (BS ≥ 80) are shown in thick black while branches with inferior support (BS ≤ 80) are shown in thin grey lines, respectively. Branch lengths are scaled by the number of nucleotide substitutions per site.

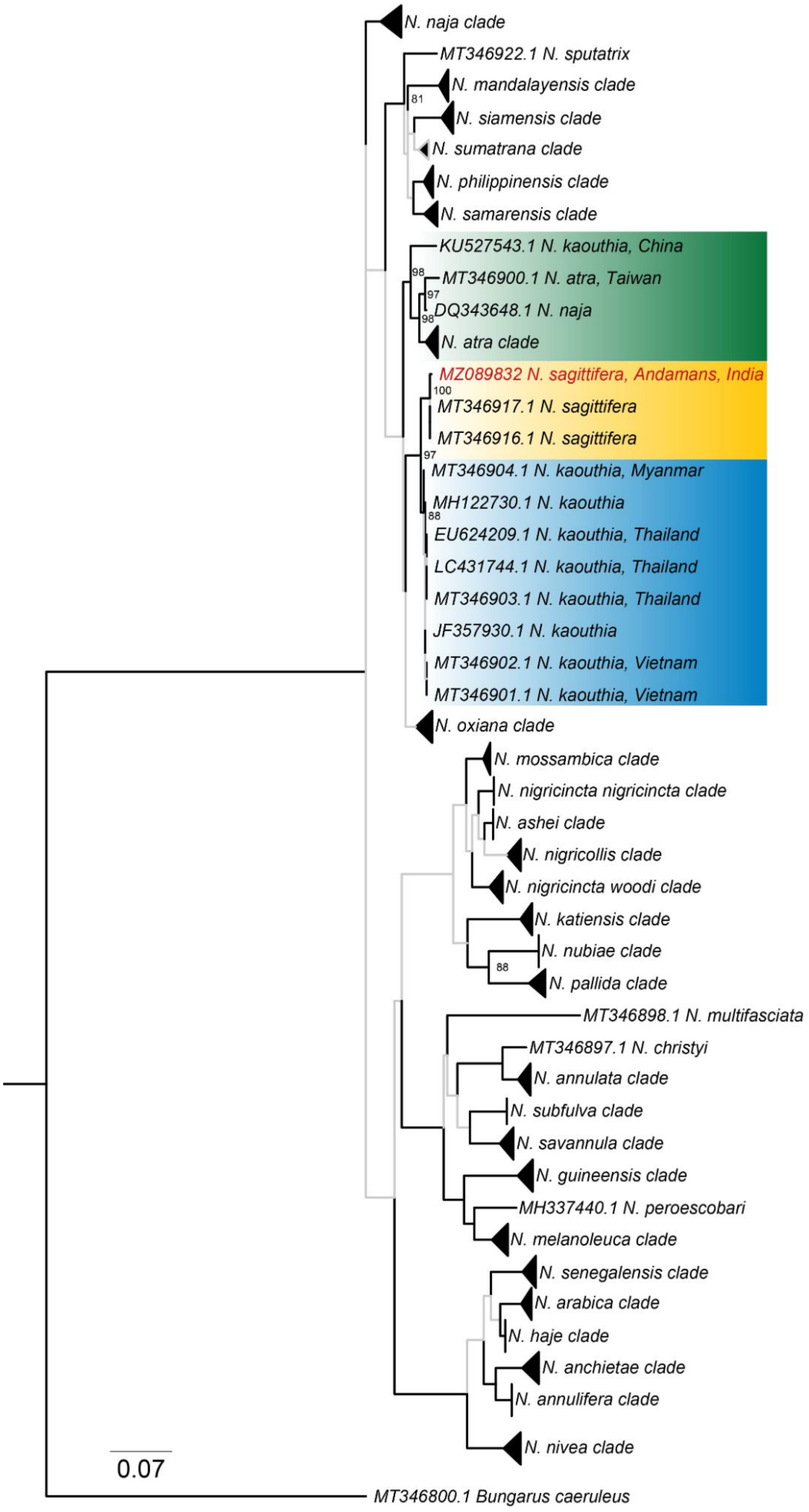

Supplementary Figure 4. The maximum likelihood phylogeny (ND4 marker) for *Naja* species. Built using ND4 sequences, this ML tree depicts the phylogenetic relationships between various *Naja* species. Lineages of interest have been shown in uniquely

coloured boxes. Well supported branches (BS ≥ 80) are shown in thick black while branches with inferior support (BS ≤ 80) are shown in thin grey lines, respectively. Branch lengths are scaled by the number of nucleotide substitutions per site. Branch lengths are scaled by the number of nucleotide substitutions per site.

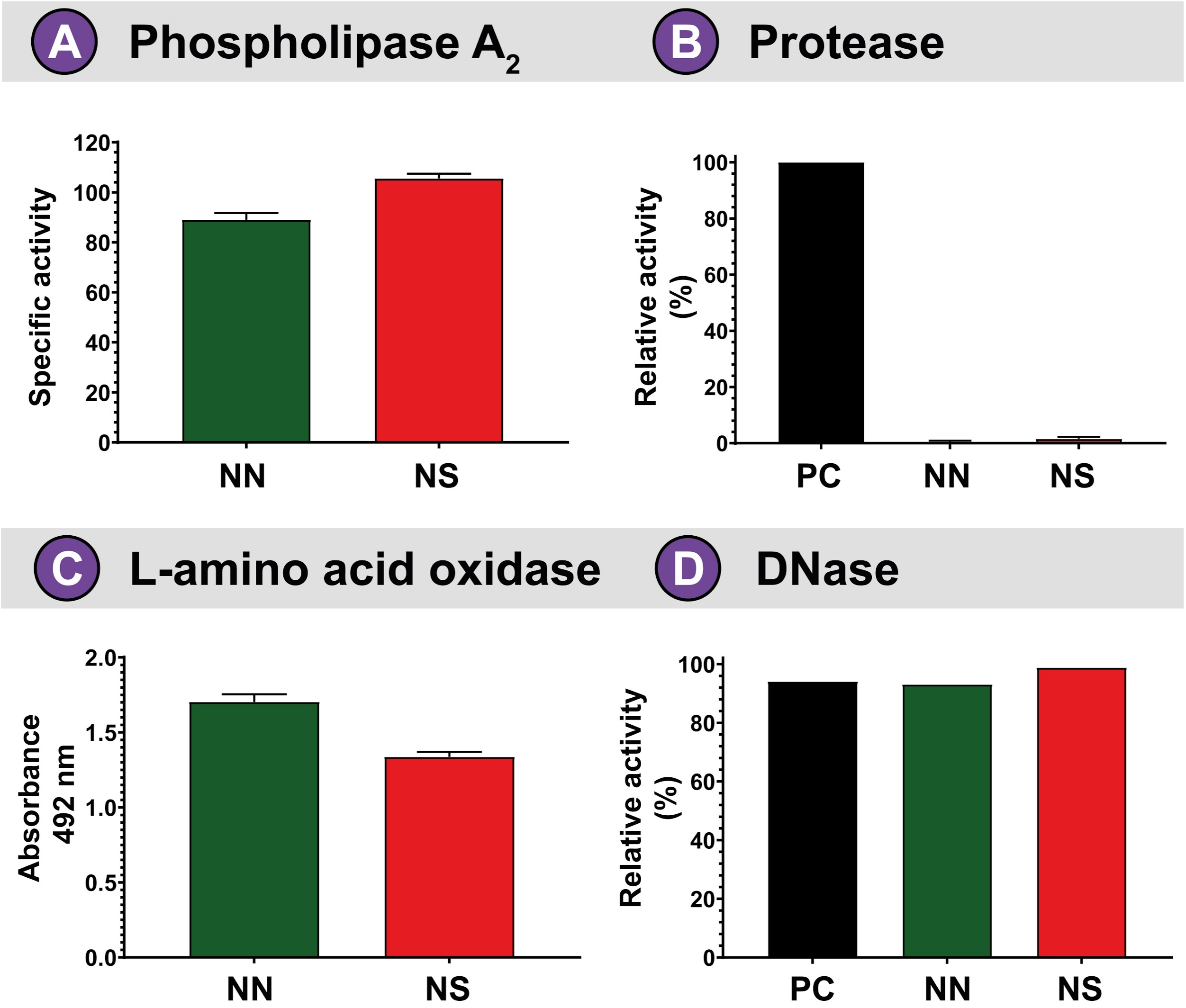

Supplementary Figure 5. Biochemical activities of *N. naja* and *N. sagittifera* venoms. The figure above represents the **(A)** phospholipase A_2, **(B)** protease, **(C)** L-amino acid oxidase, and **(D)** DNase activities of *N. naja* and *N. sagittifera*: venoms. In these graphs, the standard deviation is represented as error bars. Except for DNase, all other biochemical assays were performed in triplicates. Band intensities of agarose gel in DNase assay were measured using the ImageJ software (https://imagej.nih.gov/ij) (Schneider et al., 2012). **PC**: Positive control; **NN**: *N. naja*; **NS**: *N. sagittifera*.

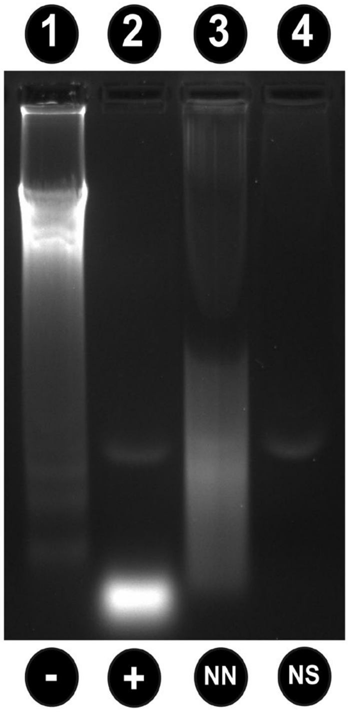

Supplementary Figure 6. DNase activity of *N. naja* and *N. sagittifera* venoms. The gel depicts the results of DNase assay, wherein, the DNA from calf thymus (500 ng) mixed with venoms or positive control, was subjected to electrophoresis. Lane **1**: purified DNA (negative control); **2**: DNA + bovine pancreatic DNase (positive control); **3**: DNA + *N. naja* (NN) venom; **4**: DNA + *N. sagittifera* (NS) venom.

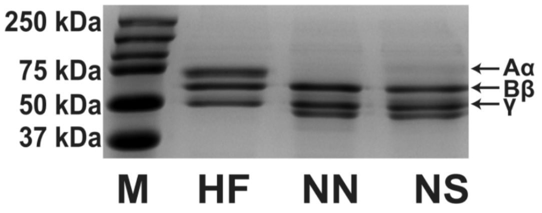

Supplementary Figure 7. The fibrinogenolytic activity of *N. naja* and *N. sagittifera* venoms. The gel above displays the results of enzymatic cleavage of human fibrinogen by the venoms of *N. naja and N. sagittifera* venoms. **M**: Pre-stained protein ladder; **HF**: Human fibrinogen only (negative control); **NN**: human fibrinogen mixed with *N. naja* venom; **NS**: human fibrinogen mixed with *N. sagittifera* venom

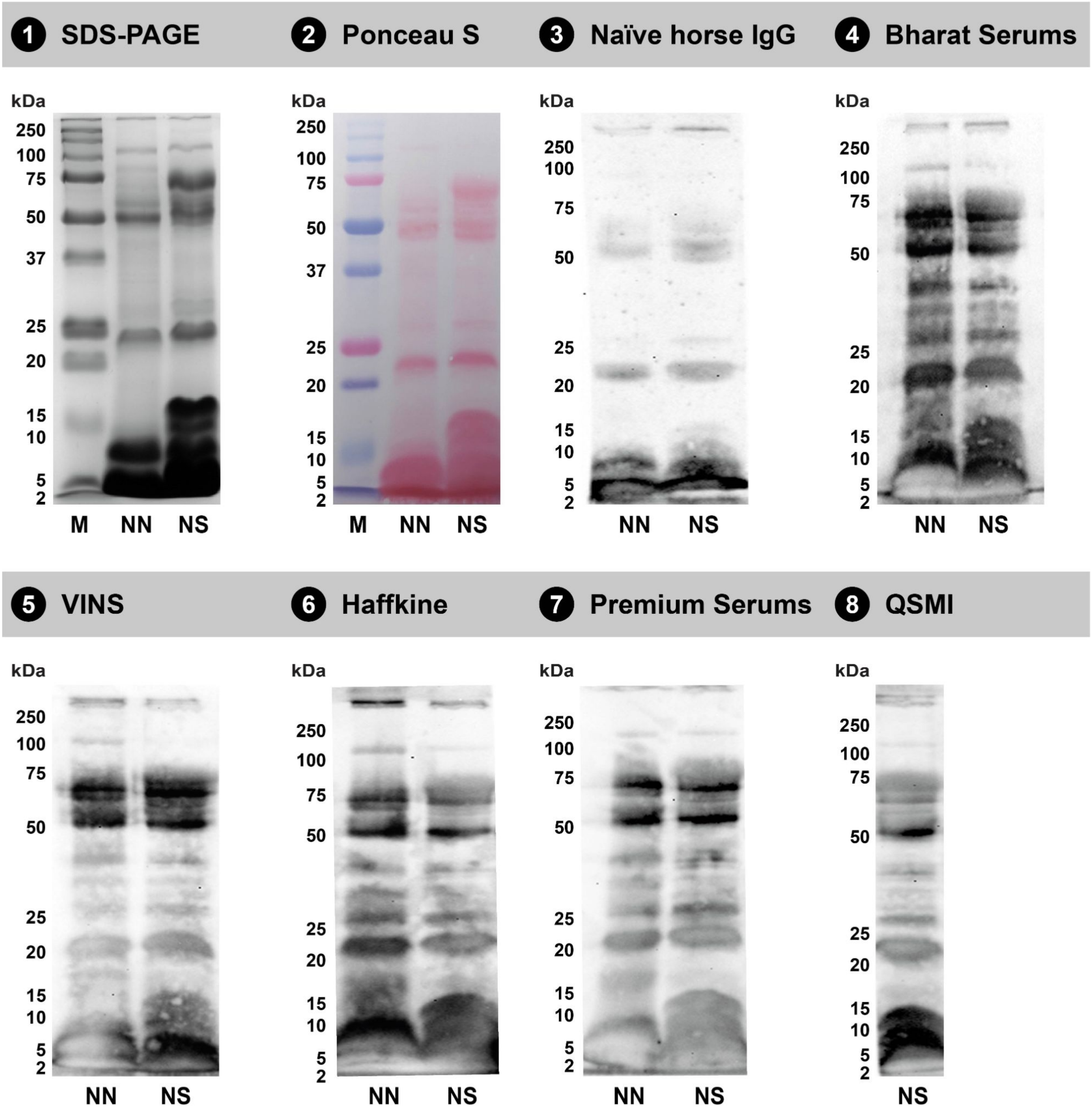

Supplementary Figure 8. Immunoblotting of Indian polyvalent and Thai monovalent antivenoms against *N. naja* and *N. sagittifera* venoms. The figure above shows the venom recognition potential of commercial Indian polyvalent 'big four' antivenoms (Bharat Serums, Haffkine, Premium Serums and VINS) and Thai monovalent *N. kaouthia* antivenom (QSMI) against *N. naja* and *N. sagittifera* venoms. **(1)** SDS-PAGE profiles; **(2)** Ponceau-S stained membrane; **(3)** Naive horse IgG; **(4)** Bharat Serums; **(5)** VINS; **(6)** Haffkine; **(7)** Premium Serums; and **(8)** QSMI antivenom. **NN**: *N. naja*; **NS**: *N. sagittifera*.

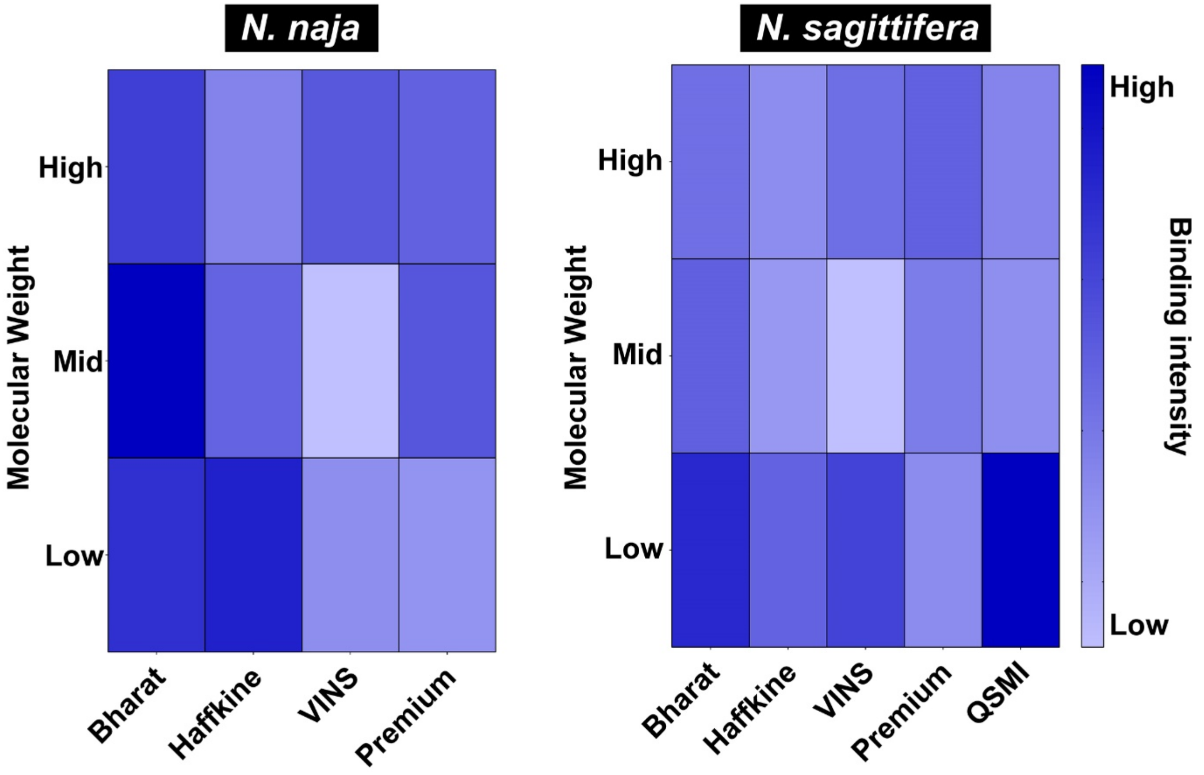

Supplementary Figure 9. Heatmap depicting the venom binding potential of commercial Indian polyvalent and Thai monovalent antivenoms against *N. naja* and *N. sagittifera* venoms. The venom recognition capabilities of commercial Indian polyvalent 'big four' antivenoms (Bharat, Haffkine, Premium and VINS) and Thai monovalent *N. kaouthia* antivenom (QSMI) against *N. naja* and *N. sagittifera* venoms are represented here. This heatmap, with a gradient of light blue (low binding) to dark blue (high binding), was generated using the values determined by densitometric analyses of high-(> 50kDa), mid- and low-molecular weight bands (<15 kDa) in the immunoblots of antivenoms, using ImageJ software (https://imagej.nih.gov/ij)(Schneider et al., 2012).

Supplementary Tables

Supplementary Table 1. Details of venom samples investigated in this study.

**Sample details**	**Number of individuals**	**Protein concentration** (mg/ml)	**Sampling location**
N. sagittifera NaSa01	1	0.100	Port Blair, Andaman & Nicobar Islands
N. naja NaNaKa16	1	0.160	Bannerghatta, Karnataka

Supplementary Table 2. The details of primers used in the amplification of mitochondrial markers.

Sr.no	Gene	Primer Name	Sense	Forward Primer Sequence (5'-3')	Amplicon Size	Annealing Temperature (T_A)	Cycles	Reference
1.	ND4	NADH4	Forward	CACCTATGACTACCAAAAGCTCATGTAGAAGC	919bp	57°C	39	Arevalo et al.,1994
2.		H12763V	Reverse	TTCTATCACTTGGATTTGCACCA				Arevalo et al.,1994
3.	Cytochrome *b*	Gludg	Forward	TGACTTGAARAACCAYCGTTG	785bp	47°C	35	Palumbi, 1996
4.		ATRCB3	Reverse	TGAGAAGTTTTCYGGGTCRTT				Harvey et al., 2000

Supplementary Table 3: Evolutionary divergence estimated as p-distance for cyt *b* marker.

	MT346720.1 *N. sagittifera*	MT346721.1 *N. sagittifera*	MT346705.1 *N. kaouthia*, Vietnam	MT346707.1 *N. kaouthia*, Thailand	MT346708.1 *N. kaouthia*, Myanmar	LC431744.1 *N. kaouthia*, Thailand	GQ359507.1 *N. kaouthia*, Thailand	EU624298.1 *N. kaouthia*, Thailand	JF357939.1 *N. kaouthia*	AF217835.1 *N. kaouthia*, Myanmar	KU527540.1 *N. kaouthia*, China	JN160672.1 *N. atra*	MZ089833 *N. sagittifera* _NaSaD01
MT346720.1 *N. sagittifera*	0	0	2.75	2.44	2.75	2.44	2.45	2.50	2.75	2.90	6.42	6.26	0
MT346721.1 *N. sagittifera*		0	2.73	2.43	2.73	2.43	2.44	2.49	2.73	2.89	6.39	6.24	0
MT346705.1 *N. kaouthia*, Vietnam			0	0.30	0.91	0.30	0.30	0.15	0.60	1.06	6.39	6.24	2.76
MT346707.1 *N. kaouthia*, Thailand				0	0.60	0	0	0	0.30	0.76	6.08	5.93	2.45
MT346708.1 *N. kaouthia*, Myanmar					0	0.60	0.61	0.62	0.91	0.15	6.39	6.24	2.76
LC431744.1 *N. kaouthia*, Thailand						0	0	0	0.30	0.76	6.08	5.93	2.45

GQ359507.1 *N. kaouthia,* Thailand							0	0	0.30	0.76	6.11	5.96	2.46
EU624298.1 *N. kaouthia,* Thailand								0	0.31	0.77	5.91	5.76	2.49
JF357939.1 *N. kaouthia*									0	1.06	6.39	6.24	2.76
AF217835.1 *N. kaouthia,* Myanmar										0	6.54	6.39	2.91
KU527540.1 *N. kaouthia,* China											0	0.15	6.45
JN160672.1 *N. atra*												0	6.29

This table shows the evolutionary distance between *Naja* species for cyt *b* marker calculated as p-distance. The accession number of the individual sequenced in this study has been highlighted in red.

Supplementary Table 4. Evolutionary divergence estimated as p-distance for ND4 marker.

	MT346916.1 *N. sagittifera*	MT346917.1 *N. sagittifera*	MT346904.1 *N. kaouthia*, Myanmar	MT346901.1 *N. kaouthia*, Vietnam	MT346902.1 *N. kaouthia*, Vietnam	JF357930.1 *N. kaouthia*	MH122730.1 *N. kaouthia*	EU624209.1 *N. kaouthia*, Thailand	LC431744.1 *N. kaouthia*, Thailand	MT346903.1 *N. kaouthia*, Thailand	MT346899.1 *N. atra*, China	KU527543.1 *N. kaouthia*, China	MZ089832 *N. sagittifera* _NaSaD01
MT346916.1 *N. sagittifera*	0	0	1.21	1.52	1.52	1.37	1.38	1.54	1.52	1.52	4.56	5.07	0.16
MT346917.1 *N. sagittifera*		0	1.21	1.52	1.52	1.37	1.38	1.54	1.52	1.52	4.56	5.07	0.16
MT346904.1*N. kaouthia*, Myanmar			0	0.30	0.30	0.15	0.15	0.30	0.30	0.30	3.95	4.12	1.51
MT346901.1 *N. kaouthia*, Vietnam				0	0	0.15	0.15	0.30	0.30	0.30	4.26	4.44	1.68
MT346902.1 *N. kaouthia*, Vietnam					0	0.15	0.15	0.30	0.30	0.30	4.26	4.44	1.68
JF357930.1 *N. kaouthia*						0	0	0.15	0.15	0.15	4.12	4.30	1.69

MH122730.1 *N. kaouthia*							0	0.15	0.15	0.15	3.99	4.30	1.69
EU624209.1 *N. kaouthia*, Thailand								0	0	0	4.16	4.44	1.85
LC431744.1 *N. kaouthia*, Thailand									0	0	4.26	4.44	1.85
MT346903.1 *N. kaouthia*, Thailand										0	4.26	4.44	1.85
MT346899.1 *N. atra*, China											0	4.12	4.04
KU527543.1 *N. kaouthia*, China												0	5.21

This table shows the evolutionary distance between *Naja* species for ND4 marker calculated as p-distance. The accession number of the individual sequenced in this study has been highlighted in red.

Raw MS/MS spectra were searched against the National Center for Biotechnology Information's (NCBI) non-redundant (nr) database (Serpentes: 8570) using Peaks Studio X+ for the identification of toxin classes present in the venom. The key results of these searches, including the accession numbers, species names, -10lgP values, number of high confidence peptides, unique peptides, percent abundance of each toxin hit, average molecular mass (kDa) and the toxin family of the matching NCBI entry and number of HPLC fraction in which the toxin was identified are listed here. The percentage indicated adjacent to the average mass column corresponds to its relative proportion in the venoms of *N. naja* **(Supplementary Table 5)** and *N. sagittifera* **(Supplementary Table 6)**.

Supplementary Table 5. The proteomic composition of *N. naja* venom from mainland India.

Sr. no.	Accession	Species	-10lgP	#Peptides	#Unique	Relative abundance of toxin hit (%)	Avg. Mass (kda)	Toxin Type	Fraction no.
**Neurotoxic three-finger toxins (N-3FTx): 51.380%**									
1	JAA74930.1	*Pseudonaja modesta*	87.54	1	1	0.0000	11.058	N-3FTx	5
2	P25679.2	*Naja kaouthia*	71.9	1	1	0.0026	7.438	N-3FTx	6
3	P82464.1	*Naja atra*	267.53	17	14	24.3360	7.624	Type I (short) muscarinic toxin	5-11
4	P82463.1	*Naja kaouthia*	234.15	8	8	1.0911	7.298	Type I (short) muscarinic toxin	4-12
5	CAB50691.1	*Bungarus multicinctus*	141.07	4	1	0.0002	9.934	Type I (short) muscarinic toxin	6
6	P82462.1	*Naja kaouthia*	107.17	1	1	2.6179	7.366	Type I (short) muscarinic toxin	7,8
7	P01427.1	*Naja oxiana*	200.03	8	8	0.0559	6.885	Type I (short) α-neurotoxin	1-4
8	APB88857.1	*Naja atra*	198.14	10	8	2.0013	9.695	Type I (short) α-neurotoxin	6,8,9
9	Q9W727.1	*Bungarus multicinctus*	141.07	4	1	0.0002	9.934	Type I (short) α-neurotoxin	6

10	1COD	*Naja atra*	134.23	3	2	0.0107	6.957	Type I (short) α-neurotoxin	1,2
11	Q9YGJ6.1	*Ophiophagus hannah*	75.68	2	1	0.1332	9.22	Type I (short) α-neurotoxin	1
12	1YI5	*Naja siamensis*	343.47	31	13	17.2824	7.831	Type II (long) α-neurotoxin	1-13
13	P25672.1	*Naja atra*	278.66	16	3	3.0548	7.889	Type II (long) α-neurotoxin	2-6
14	P25674.1	*Naja haje haje*	225.14	9	1	0.7302	7.821	Type II (long) α-neurotoxin	3
15	1NOR	*Naja sputatrix*	200.03	8	8	0.0004	6.885	Type II (long) α-neurotoxin	1-4
16	Q53B55.1	*Ophiophagus hannah*	75.55	1	1	0.0559	10.154	Type II (long) α-neurotoxin	5
17	JAA75006.1	*Vermicella annulata*	44.86	1	1	0.0001	10.504	Type II (long) α-neurotoxin	4
**Cysteine-rich secretory proteins (CRISP): 11.495%**									
18	2GIZ	*Naja atra*	363.58	50	35	2.4601	24.954	CRISP	5,7,10-13
19	ACH73168.1	*Naja kaouthia*	307.72	24	7	9.0249	26.216	CRISP	10-12
20	XP_013911763.1	*Thamnophis sirtalis*	152.33	3	1	0.0005	27.138	CRISP	12
21	AXL95289.1	*Spilotes sulphureus*	81.75	2	1	0.0097	26.943	CRISP	12
**Cytotoxic three-finger toxins (C-3FTx): 10.155%**									
22	P86540.2	*Naja naja*	220.16	10	2	0.0034	6.793	C-3FTx	6,7
23	P01440.1	*Naja naja*	219.97	12	2	0.0910	6.763	C-3FTx	8,10,12
24	Q9W6W9.1	*Naja atra*	212.61	10	1	0.0000	9.099	C-3FTx	6
25	AAB25732.1	*Naja atra*	192.05	9	3	6.2101	6.701	C-3FTx	6,8-10
26	1UG4	*Naja atra*	176.5	6	1	0.0136	6.689	C-3FTx	11
27	P49122.1	*Naja atra*	168.1	4	1	0.2911	9.086	C-3FTx	10,11
28	1CDT	*Naja mossambica*	114.67	3	1	3.5463	6.715	C-3FTx	5-10

**Disintegrin-like: 8.015%**									
29	Q9PVK7.1	*Naja kaouthia*	315.79	29	8	0.0001	67.662	Disintegrin-like	7-13
30	P82942.1	*SVMP*	287.12	18	12	5.8115	44.493	Disintegrin-like	5-7,9-13
31	ADG02948.1	*Naja atra*	252.64	13	3	0.7203	66.246	Disintegrin-like	2,3,5,6,10-12
32	JAS05092.1	*Micrurus tener*	169.48	6	1	0.0579	68.997	Disintegrin-like	6
33	AAM51550.1	*Naja mossambica*	143.55	4	2	0.0030	68.176	Disintegrin-like	13
34	AAZ39880.1	*Daboia russelii*	141.32	3	2	0.0053	69.555	Disintegrin-like	12
35	D5LMJ3.1	*Naja atra*	129.83	3	3	0.0003	68.254	Disintegrin-like	10-13
36	ADI47614.1	*Echis coloratus*	83.98	2	1	1.4116	57.183	Disintegrin-like	6
37	AAZ39881.1	*Daboia russelii*	74.52	1	1	0.0002	69.648	Disintegrin-like	12
38	XP_032084681.1	*Thamnophis elegans*	72.28	1	1	0.0000	69.288	Disintegrin-like	6,13
39	ADI47719.1	*Echis carinatus sochureki*	57.47	1	1	0.0039	37.367	Disintegrin-like	12
40	QGC85377.1	*Dispholidus typus*	42.88	1	1	0.0000	55.494	Disintegrin-like	13
**Vespryn: 6.321%**									
41	P82885.1	*Naja kaouthia*	202.6	6	6	6.3209	12.038	Vespryn	1,5,6,9-12
**Phospholipase A2 (PLA_2): 6.013%**									
42	CAA45372.1	*Naja naja*	346.63	45	1	0.0006	13.477	PLA2	6,7
43	PSNJ3K	*Naja kaouthia*	273.63	17	2	0.0442	13.271	PLA2	7,9
44	0508173A	*Naja naja*	272.58	20	4	0.4453	13.229	PLA2	7,9
45	1T37	*Naja sagittifera*	262.02	13	8	0.1675	13.162	PLA2	6,10,12
46	P60044.1	*Naja sagittifera*	242.58	16	1	0.9515	14.073	PLA2	9
47	JAA75025.1	*Suta fasciata*	240.27	9	6	0.0117	16.595	PLA2	5-12
48	4GFY	*Daboia russelii*	188.94	4	2	2.1837	13.611	PLA2	12
49	P00600.1	*Naja melanoleuca*	157.57	5	2	0.0043	13.427	PLA2	4,5,11-13
50	JAA75028.1	*Suta fasciata*	141.41	4	1	0.4203	16.452	PLA2	6
51	LAB46845.1	*Micrurus spixii*	133.22	4	1	0.0010	12.553	PLA2	9
52	P86368.1	*Daboia russelii*	132.62	3	1	0.3637	13.687	PLA2	7

53	AAB32582.1	*Naja kaouthia*	118.18	2	2	0.0005	20.452	PLA2	10-12
54	BAN08536.1	*Protobothrops flavoviridis*	105.21	2	1	0.4951	16.576	PLA2	6,9
55	JAS05124.1	*Micrurus tener*	92.82	3	1	0.0111	16.135	PLA2	7
56	JAA75029.1	*Suta fasciata*	84.58	2	1	0.1288	16.22	PLA2	9
57	P0DMT2.1	*Echis carinatus sochureki*	83.46	1	1	0.0138	13.865	PLA2	7,8
58	XP_032085287.1	*Thamnophis elegans*	82.09	2	1	0.6982	16.345	PLA2	10
59	ACD43466.1	*Daboia siamensis*	81.81	2	2	0.0242	15.421	PLA2	12
60	JAB52813.1	*Micrurus fulvius*	69	1	1	0.0001	16.274	PLA2	9,10
61	AAG17443.1	*Ophiophagus hannah*	45.43	1	1	0.0430	16.641	PLA2	5,6
**Cystatin: 2.068%**									
62	E3P6P4.1	*Naja kaouthia*	65.98	1	1	1.6102	15.772	Cystatin	9-11
63	XP_015680851.2	*Protobothrops mucrosquamatus*	61.93	1	1	0.2655	20.839	Cystatin	10
64	JAC94981.1	*Opheodrys aestivus*	47.55	1	1	0.1922	15.892	Cystatin	10
**Kunitz-type serine protease inhibitor (Kunitz): 1.412%**									
65	P19859.1	*Naja naja*	216.61	12	11	0.9865	6.508	Kunitz	3-9
66	P20229.1	*Naja naja*	183.03	9	9	0.3984	6.371	Kunitz	1-5
67	XP_026579406.1	*Pseudonaja textilis*	152.12	3	3	0.0182	22.428	Kunitz	5,6
68	CAE51866.1	*Naja atra*	151.33	4	3	0.0060	8.815	Kunitz	4,5,7
69	CAL69604.1	*Daboia siamensis*	74.51	1	1	0.0001	9.443	Kunitz	4
70	JAS03127.1	*Phalotris mertensi*	64.42	1	1	0.0025	20.534	Kunitz	6
**L-amino-acid oxidase (LAAO): 1.336%**									
71	AVX27607.1	*Naja atra*	357.18	34	12	1.2917	57.963	LAAO	2,4,5,8-13
72	P0DI91.1	*Naja oxiana*	221.4	11	4	0.0208	11.216	LAAO	5,6,9,10,12
73	P0DI84.1	*Vipera ammodytes ammodytes*	85.14	1	1	0.0239	54.748	LAAO	10

**Vascular endothelial growth factor (VEGF): 1.020%**									
74	JAB52939.1	*Micrurus fulvius*	148.24	5	5	0.0733	47.564	VEGF	2,3,8-10
75	LAB17920.1	*Micrurus spixii*	148.24	5	5	0.2328	32.584	VEGF	2,3,8-10
76	LAB42085.1	*Micrurus spixii*	100.91	3	3	0.7877	25.772	VEGF	6,8
**Snake venom serine protease (SVSP): 0.359%**									
77	XP_026575983.1	*Pseudonaja textilis*	190.28	8	7	0.0004	37.973	SVSP	7,8,10-13
78	XP_015680353.1	*Protobothrops mucrosquamatus*	54.64	1	1	0.1898	33.502	SVSP	12
79	XP_026544671.1	*Notechis scutatus*	312.39	21	2	0.0000	60.656	SVSP	7,12
80	XP_034291085.1	*Pantherophis guttatus*	303.83	21	2	0.0022	63.129	SVSP	11,12
81	XP_029140080.1	*Protobothrops mucrosquamatus*	253.06	12	1	0.0261	62.665	SVSP	6,7,9-12
82	P18964.1	*Daboia siamensis*	143.53	4	4	0.0573	26.182	SVSP	11,12
83	XP_026544110.1	*Notechis scutatus*	87.41	2	1	0.0025	50.196	SVSP	12
84	ABN72541.1	*Naja atra*	239.75	8	1	0.0006	31.137	SVSP	12
85	XP_026522175.1	*Notechis scutatus*	235.43	8	1	0.0002	31.53	SVSP	12
86	AJB84504.1	*Philodryas chamissonis*	164.86	4	1	0.0001	28.572	SVSP	12
87	2M99	*Naja atra*	151.33	4	3	0.0004	6.391	SVSP	4,5,7
88	E5L0E4.1	*Daboia siamensis*	71.71	1	1	0.0060	28.035	SVSP	10-12
**Nerve growth factor (NGF): 0.271%**									
89	AAS94269.1	*Naja sputatrix*	329.03	24	3	0.0074	27.03	NGF	5,6,9,12
**Cobra venom factor (CVF): 0.09%**									
90	3PRX	*Naja kaouthia*	299.72	33	22	0.0902	184.517	CVF	5,12,13
**Acetylcholinesterase (AChE): 0.054%**									
91	XP_026581281.1	*Pseudonaja textilis*	167.08	6	2	0.0375	67.027	AChE	10-13
92	4QWW	*Bungarus fasciatus*	160.76	6	2	0.0164	60.267	AChE	13
93	AXL96618.1	*Ahaetulla prasina*	122.29	3	1	0.0002	68.52	AChE	13

94	S68801	*Bungarus fasciatus*	64.29	1	1	0.0000	9.793	AChE	12
**5'-Nucleotidase (5'-NT): 0.006%**									
95	5H7W	*Naja atra*	218.28	11	3	0.0047	58.198	5'-NT	6,13
96	JAG67188.1	*Boiga irregularis*	198.32	9	1	0.0016	64.759	5'-NT	13
**Phosphodiesterase (PDE): 0.005%**									
97	5GZ4	*Naja atra*	188.79	6	2	0.2706	94.616	PDE	13
98	XP_026561288.1	*Pseudonaja textilis*	165.55	5	1	0.0042	96.749	PDE	4
**Cathelicidin: 0.0005%**									
99	ACF21000.1	*Naja atra*	129.81	4	4	0.0005	21.835	Cathelicidin	5,6,12
**Snaclec: 0.0001%**									
100	AAT91068.1	*Macrovipera lebetina*	51.75	1	1	0.0000	18.094	Snaclec	12
**Phospholipase B (PLB): 0.0001%**									
101	JAC94989.1	*Opheodrys aestivus*	69.37	2	1	0.0049	63.907	PLB	13

Supplementary Table 6. The proteomic composition of *N. sagittifera* venom.

Sr. no.	Accession	Species	-10lgP	#Peptides	#Unique	Relative abundance of toxin hit (%)	Avg. Mass (kda)	Toxin Type	Fraction no.
**Neurotoxic three-finger toxins (N-3FTx): 57.94%**									
1	P0DSM9.1	*Naja kaouthia*	153.62	6	1	0.1316	6.139	N-3FTx	4
2	Q9YGI4.1	*Naja atra*	149.05	4	1	0.0001	9.899	N-3FTx	5
3	P25679.2	*Naja atra*	146.47	4	1	0.0619	7.438	N-3FTx	6
4	P01415.1	*Naja haje haje*	96.84	3	3	0.0141	7.033	N-3FTx	5,7
5	P82462.1	*Naja kaouthia*	282.74	23	18	9.9084	7.366	Type I (short) muscarinic toxin	5-9,11,12
6	P82463.1	*Naja kaouthia*	219.84	11	9	8.1925	7.298	Type I (short) muscarinic toxin	5-12
7	Q9W727.1	*Bungarus multicinctus*	207.94	10	6	12.3866	9.934	Type I (short) muscarinic toxin	4-12
8	P82464.1	*Naja atra*	140.81	5	1	0.0002	7.624	Type I (short) muscarinic toxin	7
9	AAB28452.1	*Dendroaspis angusticeps*	65.36	2	1	0.0019	7.361	Type I (short) muscarinic toxin	5
10	1JE9	*Naja atra*	214.55	9	1	3.9880	6.859	Type I (short) α-neurotoxin	2,3
11	Q9DEQ3.1	*Naja atra*	207.94	10	6	12.3866	9.962	Type I (short) α-neurotoxin	4-12
12	CAB45156.1	*Naja atra*	164.05	7	6	0.2738	9.695	Type I (short) α-neurotoxin	4,8,9
13	ADN67584.1	*Naja atra*	155.73	4	2	8.4836	7.221	Type I (short) α-neurotoxin	1-5
14	P14613.1	*Naja kaouthia*	132.01	3	1	1.5817	6.983	Type I (short) α-neurotoxin	1,2

15	ADN67592.1	*Bungarus multicinctus*	58.07	2	2	0.0005	3.364	Type I (short) α-neurotoxin	7
16	2CTX	*Naja naja*	165.18	4	2	0.4798	7.831	Type II (long) α-neurotoxin	4,5,7-14
17	P25668.1	*Naja naja*	149.71	4	1	0.0487	7.847	Type II (long) α-neurotoxin	4,7
**Cytotoxic three-finger toxins (C-3FTx): 11.567%**									
18	P01446.1	*Naja kaouthia*	291.3	22	4	1.3631	6.717	C-3FTx	7,8
19	AAB25732.1	*Naja naja*	275.86	27	4	0.9479	6.701	C-3FTx	7,9,10
20	P14541.1	*Naja kaouthia*	269.9	18	1	2.2357	6.994	C-3FTx	8,9
21	P49122.1	*Naja atra*	243.68	18	12	6.5762	9.086	C-3FTx	3-12
22	Q98956.1	*Naja atra*	148.75	7	1	0.0043	8.894	C-3FTx	9
23	1CDT	*Naja mossambica*	138.81	4	1	0.0370	6.715	C-3FTx	7-9
24	P0DSN0.1	*Naja kaouthia*	89.79	3	3	0.4029	6.854	C-3FTx	5-9
**Cysteine-rich secretory proteins (CRISP): 8.334%**									
25	2GIZ	*Naja atra*	374.95	80	60	2.5229	24.954	CRISP	7,10-14
26	ACH73168.1	*Naja kaouthia*	319.84	36	33	5.8059	26.216	CRISP	9-14
27	XP_013911763.1	*Thamnophis sirtalis*	168.64	7	3	0.0049	27.138	CRISP	12
28	JAS04550.1	*Agkistrodon piscivorus conanti*	146.72	4	1	0.0001	26.681	CRISP	12
29	P60623.1	*Trimeresurus stejnegeri*	142.8	4	1	0.0001	26.294	CRISP	12
30	AAZ75607.1	*Trimorphodon biscutatus*	98.55	3	1	0.0001	26.648	CRISP	12
31	JAB52844.1	*Micrurus fulvius*	79.88	1	1	0.0000	39.958	CRISP	12
**Vespryn: 8.216%**									
32	P82885.1	*Python bivittatus*	283.09	19	18	8.2159	12.038	Vespryn	3,4,6-14
**Disintegrin-like: 5.063%**									
33	Q9PVK7.1	*Micrurus lemniscatus*	360.97	48	8	0.5210	67.662	Disintegrin-like	13,14

34	ACN50006.1	*Naja naja*	341.03	40	3	0.0048	69.181	Disintegrin-like	5,8,14
35	D5LMJ3.1	*Naja atra*	324.05	31	28	3.9863	68.254	Disintegrin-like	4,10-14
36	P82942.1	*Naja atra*	317.86	35	17	0.2269	44.493	Disintegrin-like	6-8,11-14
37	3K7N	*Naja atra*	272.2	27	1	0.0000	44.191	Disintegrin-like	13
38	AAM51550.1	*Naja mossambica*	214.69	12	9	0.1643	68.176	Disintegrin-like	13,14
39	AXL96651.1	*Ahaetulla prasina*	182.77	8	3	0.0200	68.041	Disintegrin-like	13,14
40	JAA74859.1	*Ahaetulla prasina*	182.48	8	2	0.0028	53.521	Disintegrin-like	11,14
41	JAS05092.1	*Hoplocephalus bungaroides*	165.32	6	2	0.0025	68.997	Disintegrin-like	13,14
42	ABQ01132.1	*Micrurus tener*	161.42	7	1	0.0874	68.09	Disintegrin-like	13,14
43	JAI08992.1	*Tropidechis carinatus*	158.06	6	1	0.0006	69.037	Disintegrin-like	14
44	B8K1W0.1	*Micrurus tener*	149.15	6	4	0.0002	69.555	Disintegrin-like	12-14
45	AJB84503.1	*Daboia russelii*	128.08	4	1	0.0020	68.466	Disintegrin-like	4
46	QGC85377.1	*Philodryas chamissonis*	83.76	2	1	0.0004	55.494	Disintegrin-like	13,14
47	ABN72547.1	*Dispholidus typus*	79.37	1	1	0.0001	69.403	Disintegrin-like	7
48	XP_032084681.1	*Thamnophis elegans*	73.27	1	1	0.0153	69.288	Disintegrin-like	13,14
49	JAS05411.1	*Thamnophis elegans*	67.25	2	1	0.0126	68.316	Disintegrin-like	14
50	ADJ51055.1	*Crotalus atrox*	58.56	1	1	0.0033	68.956	Disintegrin-like	11
51	AFJ49242.1	*Echis coloratus*	41.02	1	1	0.0126	67.993	Disintegrin-like	14
**Cobra venom factor (CVF): 2.578%**									
52	3PRX	*Naja kaouthia*	383.14	80	46	2.5780	184.517	CVF	3-5.7,11-14
53	C3NJ	*Naja kaouthia*	268.22	21	1	0.0003	184.926	CVF	14
**Phospholipase A2 (PLA_2): 2.179%**									
54	AAF82186.1	*Naja sputatrix*	262.8	19	1	0.0379	16.097	PLA2	8
55	1POB	*Naja atra*	255.48	16	1	0.0001	13.144	PLA2	12

56	1T37	*Naja sagittifera*	198.6	8	4	1.8813	13.162	PLA2	8,9
57	4GFY	*Daboia russelii*	180.96	6	3	0.0009	13.611	PLA2	12
58	P86368.1	*Daboia russelii*	167.63	5	2	0.0015	13.687	PLA2	12-14
59	S29299	*Daboia russelii*	114.93	3	3	0.0002	15.421	PLA2	12
60	P00601.1	*Daboia russelii*	110.09	2	1	0.2563	13.36	PLA2	5-9,12-14
61	AAB32582.1	*Naja kaouthia*	103.52	2	2	0.0009	20.452	PLA2	11-13
62	JAA75025.1	*Suta fasciata*	98.85	2	1	0.0001	16.595	PLA2	7
**Cystatin: 1.839%**									
63	ACR83850.1	*Naja kaouthia*	166.5	6	6	1.8387	15.772	Cystatin	9,10
**Snake venom serine protease (SVSP) 1.191%**									
64	XP_026575982.1	*Pseudonaja textilis*	125.13	3	1	0.0006	37.973	SVSP	12
65	ETE62885.1	*Ophiophagus hannah*	111.51	1	1	0.0140	38.69	SVSP	9
66	XP_015680353.1	*Protobothrops mucrosquamatus*	53.4	1	1	0.0000	33.502	SVSP	12
67	XP_034262198.1	*Pantherophis guttatus*	83.95	2	2	0.0000	140.811	SVSP	14
68	XP_026544671.1	*Notechis scutatus*	257.18	14	1	0.0005	60.656	SVSP	7
69	XP_034291087.1	*Pantherophis guttatus*	252.7	15	4	1.0885	63.129	SVSP	8-14
70	XP_029140080.1	*Protobothrops mucrosquamatus*	233.21	11	1	0.0707	62.665	SVSP	6-14
71	XP_026544110.1	*Notechis scutatus*	129.07	4	1	0.0125	50.196	SVSP	11,12
72	P18964.1	*Daboia siamensis*	95.59	3	3	0.0003	26.182	SVSP	11,12
73	AAB22477.1	*Daboia russelii*	95.56	2	2	0.0001	47.975	SVSP	12-14
74	JAS05177.1	*Micrurus tener*	87.92	2	2	0.0038	48.122	SVSP	5,8,9
75	JAC95044.1	*Pantherophis guttatus*	86.21	1	1	0.0001	86.011	SVSP	14
76	XP_026522175.1	*Agkistrodon piscivorus conanti*	206.93	7	1	0.0001	31.53	SVSP	12

77	ADP88560.1	*Pseudonaja textilis*	53.13	1	1	0.0000	28.035	SVSP	12
78	E5AJX2.1	*Vipera berus nikolskii*	44.9	1	1	0.0000	28.216	SVSP	12
**L-amino-acid oxidase (LAAO): 0.468%**									
79	5Z2G	*Naja atra*	417.14	85	38	0.4326	57.963	LAAO	7,8,10,12-14
80	AXL95287.1	*Spilotes sulphureus*	308.56	33	1	0.0081	58.594	LAAO	14
81	JAC95028.1	*Pantherophis guttatus*	283.56	21	1	0.0001	58.544	LAAO	14
82	P0DI91.1	*Naja oxiana*	260.03	15	5	0.0180	11.216	LAAO	11-14
83	P0DI84.1	*Vipera ammodytes ammodytes*	176.02	9	1	0.0079	54.748	LAAO	14
84	G8XQX1.1	*Daboia russelii*	143.15	6	2	0.0001	56.888	LAAO	14
85	XP_007444677.1	*Python bivittatus*	60.11	1	1	0.0014	20.584	LAAO	14
**Natriuretic peptides (NP): 0.153%**									
86	D9IX97.1	*Naja atra*	80.66	2	2	0.1529	17.345	NP	3,4
**Nerve growth factor (NGF): 0.131%**									
87	AAS94269.1	*Naja sputatrix*	167.52	5	5	0.1310	27.03	NGF	8-10,12
**Cathelicidin: 0.108%**									
88	ETE73213.1	*Ophiophagus hannah*	139.64	6	6	0.1078	48.455	Calreticulin	4,9-11
**Vascular endothelial growth factor (VEGF): 0.062%**									
89	LAA17506.1	*Vipera berus nikolskii*	74.18	1	1	0.0619	53.369	VEGF	7-9
**5'-Nucleotidase (5'-NT): 0.061%**									
90	A0A2I4HXH5.1	*Naja atra*	385.55	62	5	0.0610	58.198	5'-NT	13,14
91	JAS05143.1	*Micrurus tener*	364.2	53	1	0.0001	63.012	5'-NT	13
92	JAG67188.1	*Boiga irregularis*	315.97	26	1	0.0002	64.759	5'-NT	13,14
**Kunitz-type serine protease inhibitor (Kunitz): 0.05%**									
93	XP_026579406.1	*Pseudonaja textilis*	130.66	4	3	0.0498	22.428	Kunitz	5,6

**C-type lectin (CTL): 0.037%**									
94	Q90WI8.1	*Bungarus fasciatus*	125.65	3	2	0.0331	18.638	CTL	12
95	Q90WI7.1	*Bungarus fasciatus*	92.83	2	1	0.0034	18.254	CTL	9,12
96	LAA84028.1	*Micrurus lemniscatus*	60.42	1	1	0.0001	25.754	CTL	13,14
**Phosphodiesterase (PDE): 0.015%**									
97	5GZ4	*Naja atra*	280.62	23	14	0.0154	94.616	PDE	4,13,14
**Phospholipase B (PLB): 0.004%**									
98	JAC94989.1	*Opheodrys aestivus*	193.88	7	2	0.0029	63.907	PLB	12-14
99	LAA27322.1	*Opheodrys aestivus*	180.03	6	1	0.0012	63.374	PLB	14
**Hyaluronidases (HYL): 0.003%**									
100	XP_026524834.1	*Notechis scutatus*	200.49	11	11	0.0029	54.68	HYL	14
**Acetylcholinesterase (AChE): 0.0008%**									
101	XP_026581281.1	*Pseudonaja textilis*	181.47	9	2	0.0002	67.027	AChE	14
102	S68801	*Bungarus fasciatus*	94.99	2	1	0.0005	9.793	AChE	14
**Serpin: 0.0001%**									
103	ETE68149.1	*Micrurus lemniscatus*	68.11	1	1	0.0001	45.887	Serpin	13,14

Supplementary Table 7. Toxicity profiles of *N. naja* and *N. sagittifera* venoms.

**Name of sample**	**Venom Dose** (µg)					**Number of survivors**					**LD$_{50}$** (µg/mouse)	**LD$_{50}$** (mg/kg)
N. sagittifera	5.12	6.4	8	10	12.5	5	5	4	2	0	9.50<br>8.60- 10.50	0.475<br>0.43-0.52
N. naja	12	15	18.75	23.43	29.28	5	4	3	0	0	16.77<br>15.50-18.51	0.84<br>0.77-0.90

Supplementary Table 8. Neutralisation potencies of commercial Indian polyvalent and Thai monovalent antivenoms.

Sample details	**Antivenom used:** Indian polyvalent antivenom manufactured by Premium Serums and Vaccines Pvt. Ltd. (Batch No. ASVS-I Lyo.013)							
	**Challenge dose**	**Amount of antivenom injected in the venom-antivenom mixture** (µl)				**ED$_{50}$** (µl)	**ED$_{50}$** (µl antivenom/mg venom)	**Potency of antivenom** (mg/ml)
N. sagittifera	5X LD$_{50}$ 2.37 mg/kg	166.67	111.12	73.97	49.40	NIL	NIL	NIL
	3X LD$_{50}$ 1.42 mg/kg	166.67	111.12	73.97	49.40	NIL	NIL	NIL
N. naja	5X LD$_{50}$ 4.2 mg/kg	166.67	111.12	73.97	49.40	151.74<br>123.78-186	1809.66<br>1476.21-2218.49	0.442<br>0.361-0.542

Sample details	**Antivenom used**: Indian polyvalent antivenom manufactured by Bharat Serums and Vaccines Ltd. (Batch No. A05318087)							
	**Challenge dose**	**Amount of antivenom injected in the venom-antivenom mixture** (µl)				**ED$_{50}$** (µl)	**ED$_{50}$** (µl antivenom/ mg venom)	**Potency of antivenom** (mg/ml)
N. sagittifera	5X LD$_{50}$ 2.37 mg/kg	166.67	111.12	NE	NE	NIL	NIL	NIL
	3X LD$_{50}$ 1.42 mg/kg	166.67	111.12	73.97	49.40	126<br>100-158.32	4422.81<br>3508.77-5555.09	0.151<br>0.120-0.189
N. naja	5X LD$_{50}$ 4.2 mg/kg	166.67	111.12	73.97	49.40	198.46<br>140.51-280.32	23366.96<br>1675.73-3343.23	0.338<br>0.239-0.477

Sample details	**Antivenom used:** Thai monovalent antivenom manufactured by Queen Saovabha Memorial Institute (Batch No.- NK00112)							
	**Challenge dose**	**Amount of antivenom injected in the venom-antivenom mixture** (µl)				**ED$_{50}$** (µl)	**ED$_{50}$** (µl antivenom/ mg venom)	**Potency of antivenom** (mg/ml)
N. sagittifera	5X LD$_{50}$ 2.37 mg/kg	166.67	NE	NE	NE	NIL	NIL	NIL
	3X LD$_{50}$ 1.42 mg/kg	166.67	111.12	73.97	49.40	136<br>117.43-157.68	2704.83<br>2334.13-3134.35	0.140<br>0.120-0.162

The neutralisation potencies of Indian commercial polyvalent antivenoms and Thai monovalent antivenom against venoms of *N. naja* and *N. sagittifera* are shown in the table above.

Indian polyvalent antivenom produced by Premium Serums failed to neutralise *N. sagittifera* venom at 3X and 5X 'challenge dose'. Further, this antivenom showed limited neutralisation potency compared to marketed potency value (0.60 mg/ml) against *N. naja.* Another Indian polyvalent antivenom manufactured by Bharat Serums was unable to neutralise *N. sagittifera* venom at 5X 'challenge dose', however, it showed very poor neutralisation potency (0.151 mg/ml) at 3X 'challenge dose'. In addition, Thai monovalent *N. kaouthia* antivenom produced by QSMI

was found to be ineffective at 5X ‘challenge dose’ and showed potency value of 0.140 mg/ml at 3X ‘challenge dose’. (*NE - Not estimated)
