# Supplementary material for: Venomics of the Enigmatic Andaman Cobra (Naja sagittifera) and the Preclinical Failure of Indian Antivenoms in Andaman and Nicobar Islands
Source: Front Pharmacol. 2021 Oct 25;12:768210. doi: 10.3389/fphar.2021.768210 (PMC8573199; doi:10.3389/fphar.2021.768210)
Supplement: Supplementary file 1 [file DataSheet3.ZIP › Naja naja/Naja naja.html]

proteins


Summary

  

# 1. Notes

# 2. Result Statistics

**Figure 1.**
False discovery rate (FDR) curve. X axis is the number of peptide-spectrum matches (PSM) being kept. Y axis is the corresponding FDR.

  


  

**Figure 2.**
PSM score distribution.  **(a)**
Distribution of PEAKS peptide score;  **(b)**
Scatterplot of PEAKS peptide score versus precursor mass error.

**(a)**

**(b)**

**Table 1.**
Statistics of data.


|  |  |  |  |  |  |  |  |  |  |  |  |
| --- | --- | --- | --- | --- | --- | --- | --- | --- | --- | --- | --- |
|  | #Scans | | #Features | Identified | | | #Peptides | #Sequences | #Proteins^\*^ | | |
|  | MS1 | MS2 |  | #PSMs | #Scans | #Features |  |  | Groups | All | Top |
| Total | 185381 | 832887 | 1232000 | 15714 | 15117 | 121172 | 1004 | 920 | 179 | 1221 | 690 |
| F1 | 17747 | 25804 | 43172 | 151 | 145 | 1217 | 58 | 57 | 23 | 198 | 132 |
| F10 | 14450 | 66767 | 101661 | 1025 | 1000 | 10570 | 180 | 159 | 78 | 393 | 235 |
| F11 | 14901 | 60059 | 85015 | 702 | 688 | 8131 | 172 | 157 | 77 | 296 | 195 |
| F12 | 13572 | 65943 | 90270 | 2771 | 2719 | 7830 | 372 | 350 | 106 | 680 | 328 |
| F13 | 17804 | 25578 | 43974 | 851 | 840 | 2227 | 248 | 232 | 59 | 318 | 130 |
| F2 | 13957 | 63001 | 81914 | 746 | 739 | 6004 | 151 | 147 | 38 | 198 | 114 |
| F3 | 14375 | 66936 | 102262 | 835 | 824 | 10552 | 85 | 84 | 32 | 144 | 87 |
| F4 | 13555 | 75415 | 120379 | 1243 | 1194 | 14161 | 105 | 104 | 43 | 132 | 96 |
| F5 | 13188 | 79299 | 116370 | 1832 | 1731 | 14495 | 197 | 192 | 63 | 417 | 199 |
| F6 | 13394 | 73671 | 113396 | 1838 | 1749 | 11085 | 257 | 245 | 73 | 455 | 234 |
| F7 | 12386 | 78855 | 111633 | 1448 | 1370 | 10879 | 161 | 155 | 54 | 258 | 133 |
| F8 | 13868 | 73656 | 103980 | 910 | 834 | 12269 | 150 | 141 | 56 | 309 | 171 |
| F9 | 12184 | 77903 | 117974 | 1362 | 1284 | 11752 | 177 | 163 | 65 | 327 | 169 |

^\* proteins with significant peptides are used in counts.^

**Figure 3.**
Sample overlap for Proteins and Peptides (up to 8 samples).  **(a)**
All Proteins;  **(b)**
Top Proteins;  **(c)**
Peptides;

**(a)**

**Do not support more than 8 samples**

**(b)**

**Do not support more than 8 samples**

**(c)**

**Do not support more than 8 samples**

**Figure 4.**
Distribution of peptide feature detection.  **(a)**
Feature m/z distribution;  **(b)**
Feature RT distribution.

**(a)**

**(b)**

**Figure 5.**
Distribution of identified peptide features.  **(a)**
Feature abundance distribution;  **(b)**
 *De novo*
sequencing validation.

**(a)**

**(b)**

**Table 2.**
Result filtration parameters.

|  |  |
| --- | --- |
| Peptide -10lgP | ≥41.8 |
| PTM Ascore | ≥0 |
| Protein -10lgP | ≥20 |
| Proteins unique peptides | ≥1 |
| De novo score(%) | ≥50% |


**Table 3.**
Statistics of filtered result.

|  |  |
| --- | --- |
| FDR (Peptide-Spectrum Matches) | 0.1% |
| FDR (Peptide Sequences) | 1.3% |
| FDR (Protein Group) | 7.6% |
| De Novo Only Spectra | 248152 |

**Table 4.**
PTM profile.

| Name | ∆Mass | Position | #PSM | -10lgP | Abundance | AScore || Carbamidomethyl | 57.02 | C | 9818 | 103.21 | 6.19E7 | 1000.00 |
| Oxidation | 15.99 | M | 1088 | 91.90 | 6.56E8 | 1000.00 |

# 3. Experiment Control

**Figure 6.**
Precursor mass error of peptide-spectrum matches (PSM) in filtered result.  **(a)**
Distribution of precursor mass error in ppm;  **(b)**
Scatterplot of precursor m/z versus precursor mass error in ppm.

**(a)**

**(b)**

**Table 5.**
Number of identified peptides in each sample by the number of missed cleavages.

|  |  |  |  |  |  |
| --- | --- | --- | --- | --- | --- |
| Missed Cleavages | 0 | 1 | 2 | 3 | 4+ |
| F1 | 57 | 1 | 0 | 0 | 0 |
| F10 | 138 | 32 | 10 | 0 | 0 |
| F11 | 132 | 34 | 6 | 0 | 0 |
| F12 | 297 | 71 | 4 | 0 | 0 |
| F13 | 216 | 31 | 1 | 0 | 0 |
| F2 | 123 | 27 | 1 | 0 | 0 |
| F3 | 69 | 14 | 2 | 0 | 0 |
| F4 | 77 | 26 | 2 | 0 | 0 |
| F5 | 150 | 44 | 3 | 0 | 0 |
| F6 | 201 | 53 | 3 | 0 | 0 || ... |

# 4. Other Information

**Table 6.**
Search parameters.

Search Engine Name: PEAKS  
Parent Mass Error Tolerance: 10.0 ppm  
Fragment Mass Error Tolerance: 0.6 Da  
Precursor Mass Search Type: monoisotopic  
Enzyme: Trypsin  
Max Missed Cleavages: 2  
Digest Mode: Semispecific  
Fixed Modifications:   
  Carbamidomethylation: 57.02  
Variable Modifications:   
  Oxidation (M): 15.99  
Max Variable PTM Per Peptide: 3  
Database: NCBI\_Serpentes\_NR  
Taxon: All  
Contaminant Database: cRAP\_contaminants  
Searched Entry: 410049  
FDR Estimation: Enabled  
Merge Options: no merge  
Precursor Options: corrected  
Charge Options: no correction  
Filter Charge: 2 - 8  
Process: true  
Associate chimera: yes

**Table 7.**
Instrument parameters.

Fractions: NaNaKA16\_F1.raw, NaNaKA16\_F10.raw, NaNaKA16\_F11.raw,  
 NaNaKA16\_F12.raw, NaNaKA16\_F13.raw, NaNaKA16\_F2.raw, NaNaKA1  
6\_F3.raw, NaNaKA16\_F4.raw, NaNaKA16\_F5.raw, NaNaKA16\_F6.raw,   
NaNaKA16\_F7.raw, NaNaKA16\_F8.raw, NaNaKA16\_F9.raw  
Ion Source: ESI(nano-spray)  
Fragmentation Mode: CID, CAD(y and b ions)  
MS Scan Mode: FT-ICR/Orbitrap  
MS/MS Scan Mode: FT-ICR/Orbitrap

  

Protein List

  

Protein Accession Contains:

Protein Description Contains:

Protein Sample Area >=

Protein PTM Contains:

| Protein Group | Protein ID | Accession | -10lgP | Coverage (%) | Coverage (%) F1 | Coverage (%) F10 | Coverage (%) F11 | Coverage (%) F12 | Coverage (%) F13 | Coverage (%) F2 | Coverage (%) F3 | Coverage (%) F4 | Coverage (%) F5 | Coverage (%) F6 | Coverage (%) F7 | Coverage (%) F8 | Coverage (%) F9 | Area F1 | Area F10 | Area F11 | Area F12 | Area F13 | Area F2 | Area F3 | Area F4 | Area F5 | Area F6 | Area F7 | Area F8 | Area F9 | #Peptides | #Unique | #Spec F1 | #Spec F10 | #Spec F11 | #Spec F12 | #Spec F13 | #Spec F2 | #Spec F3 | #Spec F4 | #Spec F5 | #Spec F6 | #Spec F7 | #Spec F8 | #Spec F9 | PTM | Avg. Mass | Description |
| --- | --- | --- | --- | --- | --- | --- | --- | --- | --- | --- | --- | --- | --- | --- | --- | --- | --- | --- | --- | --- | --- | --- | --- | --- | --- | --- | --- | --- | --- | --- | --- | --- | --- | --- | --- | --- | --- | --- | --- | --- | --- | --- | --- | --- | --- | --- | --- | --- |
| 8 | 20 | 2GIZ | 363.58 | 74 | 0 | 8 | 19 | 74 | 36 | 0 | 0 | 0 | 6 | 0 | 7 | 0 | 8 |  | 5.4573E6 | 4.0163E7 | 4.8621E10 | 3.33E7 |  |  |  | 7.9009E6 |  | 1.8333E5 |  | 0 | 50 | 35 | 0 | 2 | 13 | 1663 | 37 | 0 | 0 | 0 | 4 | 0 | 1 | 0 | 1 | Y | 24954 | Chain B, Structural And Functional Analysis Of Natrin, A Member Of Crisp-3 Family Blocks A Variety Of Ion Channels |
| 8 | 21 | 1XX5 | 363.58 | 74 | 0 | 8 | 19 | 74 | 36 | 0 | 0 | 0 | 6 | 0 | 7 | 0 | 8 |  | 5.4573E6 | 4.0163E7 | 4.8621E10 | 3.33E7 |  |  |  | 7.9009E6 |  | 1.8333E5 |  | 0 | 50 | 35 | 0 | 2 | 13 | 1663 | 37 | 0 | 0 | 0 | 4 | 0 | 1 | 0 | 1 | Y | 24954 | Chain C, Natrin 1 |
| 8 | 22 | 3MZ8 | 363.58 | 74 | 0 | 8 | 19 | 74 | 36 | 0 | 0 | 0 | 6 | 0 | 7 | 0 | 8 |  | 5.4573E6 | 4.0163E7 | 4.8621E10 | 3.33E7 |  |  |  | 7.9009E6 |  | 1.8333E5 |  | 0 | 50 | 35 | 0 | 2 | 13 | 1663 | 37 | 0 | 0 | 0 | 4 | 0 | 1 | 0 | 1 | Y | 24954 | Chain B, Crystal Structure Of Zinc-Bound Natrin From Naja Atra |
| 8 | 23 | Q7T1K6.1 | 363.58 | 68 | 0 | 8 | 18 | 68 | 33 | 0 | 0 | 0 | 6 | 0 | 6 | 0 | 8 |  | 5.4573E6 | 4.0163E7 | 4.8621E10 | 3.33E7 |  |  |  | 7.9009E6 |  | 1.8333E5 |  | 0 | 50 | 35 | 0 | 2 | 13 | 1663 | 37 | 0 | 0 | 0 | 4 | 0 | 1 | 0 | 1 | Y | 26882 | RecName: Full=Cysteine-rich venom protein natrin-1; AltName: Full=Cysteine-rich venom protein 1; AltName: Full=NA-CRVP1; AltName: Full=Protein G2a; Flags: Precursor |
| 8 | 24 | AAP85301.1 | 363.58 | 68 | 0 | 8 | 18 | 68 | 33 | 0 | 0 | 0 | 6 | 0 | 6 | 0 | 8 |  | 5.4573E6 | 4.0163E7 | 4.8621E10 | 3.33E7 |  |  |  | 7.9009E6 |  | 1.8333E5 |  | 0 | 50 | 35 | 0 | 2 | 13 | 1663 | 37 | 0 | 0 | 0 | 4 | 0 | 1 | 0 | 1 | Y | 26882 | natrin [Naja atra] |
| 8 | 25 | ACH73167.1 | 363.58 | 68 | 0 | 8 | 18 | 68 | 33 | 0 | 0 | 0 | 6 | 0 | 6 | 0 | 8 |  | 5.4573E6 | 4.0163E7 | 4.8621E10 | 3.33E7 |  |  |  | 7.9009E6 |  | 1.8333E5 |  | 0 | 50 | 35 | 0 | 2 | 13 | 1663 | 37 | 0 | 0 | 0 | 4 | 0 | 1 | 0 | 1 | Y | 26846 | kaouthin-1 precursor [Naja kaouthia] |
| 8 | 26 | P84805.2 | 363.58 | 68 | 0 | 8 | 18 | 68 | 33 | 0 | 0 | 0 | 6 | 0 | 6 | 0 | 8 |  | 5.4573E6 | 4.0163E7 | 4.8621E10 | 3.33E7 |  |  |  | 7.9009E6 |  | 1.8333E5 |  | 0 | 50 | 35 | 0 | 2 | 13 | 1663 | 37 | 0 | 0 | 0 | 4 | 0 | 1 | 0 | 1 | Y | 26846 | RecName: Full=Cysteine-rich venom protein kaouthin-1; AltName: Full=Cysteine-rich venom protein 25; Short=CRVP-25k; Flags: Precursor |
| 42 | 1 | AVX27607.1 | 357.18 | 40 | 0 | 23 | 23 | 33 | 29 | 7 | 0 | 4 | 4 | 5 | 0 | 19 | 10 |  | 3.4205E7 | 1.3232E6 | 8.2977E7 | 6.7734E7 | 3.7271E7 |  | 8.2584E5 | 3.3503E6 |  |  | 9.9673E6 | 2.2165E7 | 34 | 12 | 0 | 59 | 35 | 112 | 82 | 15 | 0 | 7 | 3 | 2 | 0 | 14 | 28 | Y | 57963 | L-amino acid oxidase, partial [Naja atra] |
| 42 | 2 | 5Z2G | 357.18 | 40 | 0 | 23 | 23 | 33 | 29 | 7 | 0 | 4 | 4 | 5 | 0 | 19 | 10 |  | 3.4205E7 | 1.3232E6 | 8.2977E7 | 6.7734E7 | 3.7271E7 |  | 8.2584E5 | 3.3503E6 |  |  | 9.9673E6 | 2.2165E7 | 34 | 12 | 0 | 59 | 35 | 112 | 82 | 15 | 0 | 7 | 3 | 2 | 0 | 14 | 28 | Y | 57963 | Chain B, L-amino acid oxidase |
| 42 | 3 | A8QL58.2 | 357.18 | 40 | 0 | 23 | 23 | 33 | 29 | 7 | 0 | 4 | 4 | 5 | 0 | 19 | 10 |  | 3.4205E7 | 1.3232E6 | 8.2977E7 | 6.7734E7 | 3.7271E7 |  | 8.2584E5 | 3.3503E6 |  |  | 9.9673E6 | 2.2165E7 | 34 | 12 | 0 | 59 | 35 | 112 | 82 | 15 | 0 | 7 | 3 | 2 | 0 | 14 | 28 | Y | 57963 | RecName: Full=L-amino-acid oxidase; Short=LAO; Short=NA-LAAO; Flags: Precursor |
| 1 | 80 | CAA45372.1 | 346.63 | 88 | 0 | 48 | 37 | 48 | 48 | 12 | 0 | 19 | 48 | 83 | 88 | 88 | 59 |  |  |  |  |  |  |  |  |  | 6.9673E7 | 1.4844E7 |  |  | 45 | 1 | 0 | 36 | 22 | 38 | 10 | 2 | 0 | 3 | 29 | 391 | 953 | 388 | 448 | Y | 13477 | phospholipase a2 [Naja naja] |
| 1 | 82 | 1A3F | 346.63 | 89 | 0 | 49 | 37 | 49 | 49 | 12 | 0 | 19 | 49 | 84 | 89 | 89 | 60 |  |  |  |  |  |  |  |  |  | 6.9673E7 | 1.4844E7 |  |  | 45 | 1 | 0 | 36 | 22 | 38 | 10 | 2 | 0 | 3 | 29 | 391 | 953 | 388 | 448 | Y | 13346 | Chain C, Phospholipase A2 (Pla2) From Naja Naja Venom |
| 1 | 83 | 1A3D | 346.63 | 89 | 0 | 49 | 37 | 49 | 49 | 12 | 0 | 19 | 49 | 84 | 89 | 89 | 60 |  |  |  |  |  |  |  |  |  | 6.9673E7 | 1.4844E7 |  |  | 45 | 1 | 0 | 36 | 22 | 38 | 10 | 2 | 0 | 3 | 29 | 391 | 953 | 388 | 448 | Y | 13346 | Chain A, Phospholipase A2 |
| 1 | 84 | P15445.1 | 346.63 | 89 | 0 | 49 | 37 | 49 | 49 | 12 | 0 | 19 | 49 | 84 | 89 | 89 | 60 |  |  |  |  |  |  |  |  |  | 6.9673E7 | 1.4844E7 |  |  | 45 | 1 | 0 | 36 | 22 | 38 | 10 | 2 | 0 | 3 | 29 | 391 | 953 | 388 | 448 | Y | 13346 | RecName: Full=Acidic phospholipase A2 2; Short=PLA22; Short=svPLA2; AltName: Full=Phosphatidylcholine 2-acylhydrolase |
| 1 | 85 | 1PSH | 346.63 | 89 | 0 | 49 | 37 | 49 | 49 | 12 | 0 | 19 | 49 | 84 | 89 | 89 | 60 |  |  |  |  |  |  |  |  |  | 6.9673E7 | 1.4844E7 |  |  | 45 | 1 | 0 | 36 | 22 | 38 | 10 | 2 | 0 | 3 | 29 | 391 | 953 | 388 | 448 | Y | 13346 | Chain C, Phospholipase A2 |
| 2 | 221 | 1YI5 | 343.47 | 97 | 21 | 42 | 42 | 59 | 59 | 59 | 82 | 80 | 97 | 61 | 61 | 41 | 61 | 9.1527E6 | 1.686E8 | 7.9452E7 | 7.513E7 | 6.0306E6 | 5.5153E8 | 3.8575E10 | 6.2435E10 | 6.7316E10 | 3.819E9 | 8.6239E7 | 2.6489E7 | 3.5673E8 | 31 | 13 | 12 | 18 | 14 | 16 | 8 | 52 | 496 | 585 | 750 | 198 | 12 | 7 | 32 | Y | 7831 | Chain J, Long Neurotoxin 1 |
| 2 | 222 | 4AEA | 343.47 | 97 | 21 | 42 | 42 | 59 | 59 | 59 | 82 | 80 | 97 | 61 | 61 | 41 | 61 | 9.1527E6 | 1.686E8 | 7.9452E7 | 7.513E7 | 6.0306E6 | 5.5153E8 | 3.8575E10 | 6.2435E10 | 6.7316E10 | 3.819E9 | 8.6239E7 | 2.6489E7 | 3.5673E8 | 31 | 13 | 12 | 18 | 14 | 16 | 8 | 52 | 496 | 585 | 750 | 198 | 12 | 7 | 32 | Y | 7831 | Chain B, Long Neurotoxin 1 |
| 2 | 223 | 1CTX | 343.47 | 97 | 21 | 42 | 42 | 59 | 59 | 59 | 82 | 80 | 97 | 61 | 61 | 41 | 61 | 9.1527E6 | 1.686E8 | 7.9452E7 | 7.513E7 | 6.0306E6 | 5.5153E8 | 3.8575E10 | 6.2435E10 | 6.7316E10 | 3.819E9 | 8.6239E7 | 2.6489E7 | 3.5673E8 | 31 | 13 | 12 | 18 | 14 | 16 | 8 | 52 | 496 | 585 | 750 | 198 | 12 | 7 | 32 | Y | 7831 | Chain A, ALPHA-COBRATOXIN |
| 2 | 224 | 2CTX | 343.47 | 97 | 21 | 42 | 42 | 59 | 59 | 59 | 82 | 80 | 97 | 61 | 61 | 41 | 61 | 9.1527E6 | 1.686E8 | 7.9452E7 | 7.513E7 | 6.0306E6 | 5.5153E8 | 3.8575E10 | 6.2435E10 | 6.7316E10 | 3.819E9 | 8.6239E7 | 2.6489E7 | 3.5673E8 | 31 | 13 | 12 | 18 | 14 | 16 | 8 | 52 | 496 | 585 | 750 | 198 | 12 | 7 | 32 | Y | 7831 | Chain A, ALPHA-COBRATOXIN |
| 2 | 225 | P01391.1 | 343.47 | 97 | 21 | 42 | 42 | 59 | 59 | 59 | 82 | 80 | 97 | 61 | 61 | 41 | 61 | 9.1527E6 | 1.686E8 | 7.9452E7 | 7.513E7 | 6.0306E6 | 5.5153E8 | 3.8575E10 | 6.2435E10 | 6.7316E10 | 3.819E9 | 8.6239E7 | 2.6489E7 | 3.5673E8 | 31 | 13 | 12 | 18 | 14 | 16 | 8 | 52 | 496 | 585 | 750 | 198 | 12 | 7 | 32 | Y | 7831 | RecName: Full=Alpha-cobratoxin; Short=Alpha-CbT; Short=alpha-CT; Short=alpha-Cbtx; AltName: Full=Alpha-elapitoxin-Nk2a; Short=Alpha-EPTX-Nk2a; AltName: Full=Long neurotoxin 1; AltName: Full=Siamensis 3 |
| 2 | 226 | 1LXH | 343.47 | 97 | 21 | 42 | 42 | 59 | 59 | 59 | 82 | 80 | 97 | 61 | 61 | 41 | 61 | 9.1527E6 | 1.686E8 | 7.9452E7 | 7.513E7 | 6.0306E6 | 5.5153E8 | 3.8575E10 | 6.2435E10 | 6.7316E10 | 3.819E9 | 8.6239E7 | 2.6489E7 | 3.5673E8 | 31 | 13 | 12 | 18 | 14 | 16 | 8 | 52 | 496 | 585 | 750 | 198 | 12 | 7 | 32 | Y | 7831 | Chain A, LONG NEUROTOXIN 1 |
| 2 | 227 | 1LXG | 343.47 | 97 | 21 | 42 | 42 | 59 | 59 | 59 | 82 | 80 | 97 | 61 | 61 | 41 | 61 | 9.1527E6 | 1.686E8 | 7.9452E7 | 7.513E7 | 6.0306E6 | 5.5153E8 | 3.8575E10 | 6.2435E10 | 6.7316E10 | 3.819E9 | 8.6239E7 | 2.6489E7 | 3.5673E8 | 31 | 13 | 12 | 18 | 14 | 16 | 8 | 52 | 496 | 585 | 750 | 198 | 12 | 7 | 32 | Y | 7831 | Chain A, Long neurotoxin 1 |
| 37 | 297 | AAS94269.1 | 329.03 | 42 | 0 | 37 | 24 | 36 | 5 | 5 | 0 | 5 | 8 | 42 | 36 | 29 | 40 |  |  |  | 2.1402E6 |  |  |  |  | 2.8298E6 | 1.9319E9 |  |  | 7.0917E5 | 24 | 3 | 0 | 60 | 9 | 13 | 1 | 7 | 0 | 2 | 2 | 379 | 12 | 13 | 76 | Y | 27030 | nerve growth factor II [Naja sputatrix] |
| 37 | 298 | Q5YF89.1 | 329.03 | 42 | 0 | 37 | 24 | 36 | 5 | 5 | 0 | 5 | 8 | 42 | 36 | 29 | 40 |  |  |  | 2.1402E6 |  |  |  |  | 2.8298E6 | 1.9319E9 |  |  | 7.0917E5 | 24 | 3 | 0 | 60 | 9 | 13 | 1 | 7 | 0 | 2 | 2 | 379 | 12 | 13 | 76 | Y | 27030 | RecName: Full=Venom nerve growth factor 2; Short=v-NGF-2; Short=vNGF-2; AltName: Full=Nerve growth factor II; Flags: Precursor |
| 45 | 8 | Q9PVK7.1 | 315.79 | 40 | 0 | 4 | 4 | 8 | 30 | 0 | 0 | 2 | 8 | 16 | 8 | 12 | 6 |  | 7.7381E6 | 9.8751E5 | 1.0165E6 | 9.4121E7 |  |  |  |  |  | 1.432E7 | 9.6223E7 | 2.2471E7 | 29 | 8 | 0 | 4 | 2 | 6 | 193 | 0 | 0 | 1 | 24 | 51 | 5 | 16 | 6 | Y | 67662 | RecName: Full=Zinc metalloproteinase-disintegrin-like cobrin; AltName: Full=Snake venom metalloproteinase; Short=SVMP; Flags: Precursor |
| 45 | 9 | AAF00693.1 | 315.79 | 40 | 0 | 4 | 4 | 8 | 30 | 0 | 0 | 2 | 8 | 16 | 8 | 12 | 6 |  | 7.7381E6 | 9.8751E5 | 1.0165E6 | 9.4121E7 |  |  |  |  |  | 1.432E7 | 9.6223E7 | 2.2471E7 | 29 | 8 | 0 | 4 | 2 | 6 | 193 | 0 | 0 | 1 | 24 | 51 | 5 | 16 | 6 | Y | 67662 | cobrin precursor [Naja naja] |
| 59 | 31 | XP\_026544671.1 | 312.39 | 28 | 0 | 10 | 8 | 19 | 5 | 0 | 0 | 0 | 0 | 8 | 12 | 7 | 7 |  |  |  | 2.0337E6 |  |  |  |  |  |  | 8.9589E5 |  |  | 21 | 2 | 0 | 5 | 11 | 128 | 3 | 0 | 0 | 0 | 0 | 16 | 18 | 7 | 4 | Y | 60656 | tissue-type plasminogen activator, partial [Notechis scutatus] |
| 54 | 60 | ACH73168.1 | 307.72 | 61 | 0 | 38 | 56 | 56 | 0 | 0 | 0 | 0 | 0 | 0 | 0 | 0 | 0 |  | 1.217E8 | 1.2833E9 | 1.2554E8 |  |  |  |  |  |  |  |  |  | 24 | 7 | 0 | 17 | 174 | 41 | 0 | 0 | 0 | 0 | 0 | 0 | 0 | 0 | 0 | Y | 26216 | kaouthin-2 precursor [Naja kaouthia] |
| 54 | 61 | P84808.2 | 307.72 | 61 | 0 | 38 | 56 | 56 | 0 | 0 | 0 | 0 | 0 | 0 | 0 | 0 | 0 |  | 1.217E8 | 1.2833E9 | 1.2554E8 |  |  |  |  |  |  |  |  |  | 24 | 7 | 0 | 17 | 174 | 41 | 0 | 0 | 0 | 0 | 0 | 0 | 0 | 0 | 0 | Y | 26216 | RecName: Full=Cysteine-rich venom protein kaouthin-2; AltName: Full=Cysteine-rich venom protein 23; Short=CRVP-23k; Flags: Precursor |
| 54 | 62 | AHZ08822.1 | 307.72 | 61 | 0 | 38 | 56 | 56 | 0 | 0 | 0 | 0 | 0 | 0 | 0 | 0 | 0 |  | 1.217E8 | 1.2833E9 | 1.2554E8 |  |  |  |  |  |  |  |  |  | 24 | 7 | 0 | 17 | 174 | 41 | 0 | 0 | 0 | 0 | 0 | 0 | 0 | 0 | 0 | Y | 26216 | cysteine-rich secretory protein [Micropechis ikaheca] |
| 55 | 75 | XP\_034291085.1 | 303.83 | 23 | 0 | 11 | 10 | 18 | 2 | 0 | 0 | 0 | 0 | 5 | 5 | 9 | 9 |  | 0 | 1.4032E6 | 4.1585E8 |  |  |  |  |  |  |  |  |  | 21 | 2 | 0 | 8 | 15 | 153 | 2 | 0 | 0 | 0 | 0 | 13 | 14 | 8 | 5 | Y | 63129 | tissue-type plasminogen activator [Pantherophis guttatus] |
| 55 | 76 | XP\_034291087.1 | 303.83 | 23 | 0 | 11 | 10 | 18 | 2 | 0 | 0 | 0 | 0 | 5 | 5 | 9 | 9 |  | 0 | 1.4032E6 | 4.1585E8 |  |  |  |  |  |  |  |  |  | 21 | 2 | 0 | 8 | 15 | 153 | 2 | 0 | 0 | 0 | 0 | 13 | 14 | 8 | 5 | Y | 63129 | tissue-type plasminogen activator [Pantherophis guttatus] |
| 55 | 77 | XP\_034291086.1 | 303.83 | 23 | 0 | 11 | 10 | 18 | 2 | 0 | 0 | 0 | 0 | 5 | 5 | 9 | 9 |  | 0 | 1.4032E6 | 4.1585E8 |  |  |  |  |  |  |  |  |  | 21 | 2 | 0 | 8 | 15 | 153 | 2 | 0 | 0 | 0 | 0 | 13 | 14 | 8 | 5 | Y | 63129 | tissue-type plasminogen activator [Pantherophis guttatus] |
| 75 | 13 | 3PRX | 299.72 | 23 | 0 | 1 | 1 | 10 | 19 | 0 | 0 | 0 | 1 | 0 | 0 | 2 | 1 |  |  |  | 2.2798E7 | 6.2039E7 |  |  |  | 1.0651E8 |  |  |  |  | 33 | 22 | 0 | 6 | 1 | 26 | 62 | 0 | 0 | 0 | 7 | 0 | 0 | 2 | 1 | Y | 184517 | Chain D, Cobra Venom Factor |
| 75 | 14 | 3PVM | 299.72 | 23 | 0 | 1 | 1 | 10 | 19 | 0 | 0 | 0 | 1 | 0 | 0 | 2 | 1 |  |  |  | 2.2798E7 | 6.2039E7 |  |  |  | 1.0651E8 |  |  |  |  | 33 | 22 | 0 | 6 | 1 | 26 | 62 | 0 | 0 | 0 | 7 | 0 | 0 | 2 | 1 | Y | 184517 | Chain D, Cobra Venom Factor |
| 75 | 15 | Q91132.1 | 299.72 | 23 | 0 | 1 | 1 | 10 | 19 | 0 | 0 | 0 | 1 | 0 | 0 | 2 | 1 |  |  |  | 2.2798E7 | 6.2039E7 |  |  |  | 1.0651E8 |  |  |  |  | 33 | 22 | 0 | 6 | 1 | 26 | 62 | 0 | 0 | 0 | 7 | 0 | 0 | 2 | 1 | Y | 184517 | RecName: Full=Cobra venom factor; Short=CVF; Short=CVFk; AltName: Full=Complement C3 homolog; Contains: RecName: Full=Cobra venom factor alpha chain; Contains: RecName: Full=Cobra venom factor gamma chain; Contains: RecName: Full=C... |
| 75 | 16 | AAA68989.1 | 299.72 | 23 | 0 | 1 | 1 | 10 | 19 | 0 | 0 | 0 | 1 | 0 | 0 | 2 | 1 |  |  |  | 2.2798E7 | 6.2039E7 |  |  |  | 1.0651E8 |  |  |  |  | 33 | 22 | 0 | 6 | 1 | 26 | 62 | 0 | 0 | 0 | 7 | 0 | 0 | 2 | 1 | Y | 184517 | cobra venom factor precursor [Naja kaouthia] |
| 75 | 17 | CAI46845.1 | 299.72 | 23 | 0 | 1 | 1 | 10 | 19 | 0 | 0 | 0 | 1 | 0 | 0 | 2 | 1 |  |  |  | 2.2798E7 | 6.2039E7 |  |  |  | 1.0651E8 |  |  |  |  | 33 | 22 | 0 | 6 | 1 | 26 | 62 | 0 | 0 | 0 | 7 | 0 | 0 | 2 | 1 | Y | 184517 | unnamed protein product [Naja naja] |
| 75 | 18 | 6I2X | 299.72 | 23 | 0 | 1 | 1 | 10 | 19 | 0 | 0 | 0 | 1 | 0 | 0 | 2 | 1 |  |  |  | 2.2798E7 | 6.2039E7 |  |  |  | 1.0651E8 |  |  |  |  | 33 | 22 | 0 | 6 | 1 | 26 | 62 | 0 | 0 | 0 | 7 | 0 | 0 | 2 | 1 | Y | 184517 | Chain B, Cobra venom factor |
| 75 | 19 | I51018 | 299.72 | 23 | 0 | 1 | 1 | 10 | 19 | 0 | 0 | 0 | 1 | 0 | 0 | 2 | 1 |  |  |  | 2.2798E7 | 6.2039E7 |  |  |  | 1.0651E8 |  |  |  |  | 33 | 22 | 0 | 6 | 1 | 26 | 62 | 0 | 0 | 0 | 7 | 0 | 0 | 2 | 1 | Y | 184517 | cobra venom factor precursor - monocled cobra |
| 47 | 11 | ACN50006.1 | 298.91 | 30 | 0 | 0 | 2 | 4 | 25 | 0 | 0 | 2 | 13 | 16 | 5 | 5 | 2 |  |  |  |  | 0 |  |  |  |  |  |  |  |  | 23 | 1 | 0 | 0 | 1 | 2 | 236 | 0 | 0 | 1 | 29 | 20 | 3 | 8 | 1 | Y | 69181 | atragin precursor, partial [Naja atra] |
| 47 | 12 | D3TTC2.1 | 298.91 | 30 | 0 | 0 | 2 | 4 | 25 | 0 | 0 | 2 | 13 | 16 | 5 | 5 | 2 |  |  |  |  | 0 |  |  |  |  |  |  |  |  | 23 | 1 | 0 | 0 | 1 | 2 | 236 | 0 | 0 | 1 | 29 | 20 | 3 | 8 | 1 | Y | 69181 | RecName: Full=Zinc metalloproteinase-disintegrin-like atragin; AltName: Full=Snake venom metalloproteinase; Short=SVMP; Flags: Precursor |
| 72 | 35 | P82942.1 | 287.12 | 29 | 0 | 16 | 10 | 22 | 16 | 0 | 0 | 3 | 11 | 15 | 11 | 0 | 8 |  | 5.9195E6 | 8.1916E6 | 2.1966E7 | 5.7977E6 |  |  |  | 3.4085E8 | 2.8519E9 | 5.3659E7 |  | 7.1814E6 | 18 | 12 | 0 | 6 | 8 | 17 | 11 | 0 | 0 | 1 | 12 | 53 | 3 | 0 | 3 | Y | 44493 | RecName: Full=Hemorrhagic metalloproteinase-disintegrin-like kaouthiagin; AltName: Full=Snake venom metalloproteinase; Short=SVMP |
| 4 | 380 | 764177A | 280.64 | 93 | 0 | 14 | 33 | 47 | 49 | 49 | 93 | 67 | 67 | 49 | 34 | 33 | 34 |  |  |  |  |  |  | 0 |  |  |  |  |  |  | 17 | 1 | 0 | 7 | 9 | 16 | 14 | 67 | 434 | 701 | 691 | 211 | 11 | 7 | 19 | Y | 7706 | toxin B |
| 5 | 362 | P25668.1 | 279.47 | 93 | 0 | 28 | 32 | 32 | 34 | 48 | 93 | 93 | 93 | 75 | 34 | 32 | 48 |  | 5.247E6 |  |  |  | 5.4239E6 | 8.3364E9 | 2.3424E10 | 1.7299E10 | 5.9718E8 |  |  | 2.0334E7 | 16 | 2 | 0 | 9 | 9 | 15 | 9 | 52 | 433 | 648 | 701 | 201 | 11 | 7 | 20 | Y | 7847 | RecName: Full=Long neurotoxin 1; AltName: Full=Toxin A |
| 11 | 530 | P25672.1 | 278.66 | 93 | 0 | 14 | 14 | 32 | 32 | 44 | 89 | 93 | 93 | 73 | 32 | 14 | 32 |  |  |  |  |  | 1.1563E7 | 9.3448E7 | 4.4981E9 | 1.1598E10 | 2.9335E8 |  |  |  | 16 | 3 | 0 | 7 | 7 | 11 | 6 | 33 | 332 | 409 | 520 | 174 | 6 | 3 | 16 | Y | 7889 | RecName: Full=Long neurotoxin 4; AltName: Full=Toxin D |
| 18 | 241 | PSNJ3K | 273.63 | 55 | 0 | 29 | 18 | 19 | 19 | 12 | 0 | 19 | 29 | 51 | 54 | 42 | 50 |  |  |  |  |  |  |  |  |  |  | 5.6525E6 |  | 5.4823E7 | 17 | 2 | 0 | 16 | 12 | 26 | 6 | 2 | 0 | 3 | 20 | 283 | 369 | 159 | 177 | Y | 13271 | phospholipase A2 (EC 3.1.1.4) III - monocled cobra |
| 17 | 258 | 0508173A | 272.58 | 76 | 0 | 29 | 18 | 19 | 19 | 12 | 0 | 19 | 29 | 49 | 56 | 53 | 67 |  |  |  |  |  |  |  |  |  |  | 2.1553E7 |  | 1.4856E7 | 20 | 4 | 0 | 16 | 12 | 26 | 6 | 2 | 0 | 3 | 20 | 282 | 367 | 162 | 183 | Y | 13229 | phospholipase A2 E3 |
| 17 | 259 | P25498.1 | 272.58 | 76 | 0 | 29 | 18 | 19 | 19 | 12 | 0 | 19 | 29 | 49 | 56 | 53 | 67 |  |  |  |  |  |  |  |  |  |  | 2.1553E7 |  | 1.4856E7 | 20 | 4 | 0 | 16 | 12 | 26 | 6 | 2 | 0 | 3 | 20 | 282 | 367 | 162 | 183 | Y | 13229 | RecName: Full=Acidic phospholipase A2 E; Short=svPLA2; AltName: Full=Phosphatidylcholine 2-acylhydrolase |
| 70 | 532 | P82464.1 | 267.53 | 86 | 0 | 31 | 31 | 0 | 0 | 0 | 0 | 0 | 31 | 86 | 86 | 57 | 57 |  | 8.0424E6 | 3.6818E6 |  |  |  |  |  | 1.5657E7 | 4.073E8 | 7.6493E9 | 1.0533E8 | 5.0925E7 | 17 | 14 | 0 | 1 | 1 | 0 | 0 | 0 | 0 | 0 | 4 | 36 | 80 | 8 | 6 | Y | 7624 | RecName: Full=Muscarinic toxin-like protein 3; Short=MTLP-3 |
| 38 | 365 | 1T37 | 262.02 | 42 | 0 | 18 | 10 | 18 | 0 | 0 | 0 | 0 | 10 | 40 | 42 | 42 | 18 |  | 3.6037E7 |  | 3.3667E7 |  |  |  |  |  | 3.4246E9 | 2.7128E10 | 8.3689E9 | 1.506E10 | 13 | 8 | 0 | 13 | 3 | 7 | 0 | 0 | 0 | 0 | 2 | 127 | 151 | 88 | 120 | Y | 13162 | Chain A, Phospholipase A2 Isoform 3 |
| 38 | 366 | 1ZM6 | 262.02 | 42 | 0 | 18 | 10 | 18 | 0 | 0 | 0 | 0 | 10 | 40 | 42 | 42 | 18 |  | 3.6037E7 |  | 3.3667E7 |  |  |  |  |  | 3.4246E9 | 2.7128E10 | 8.3689E9 | 1.506E10 | 13 | 8 | 0 | 13 | 3 | 7 | 0 | 0 | 0 | 0 | 2 | 127 | 151 | 88 | 120 | Y | 13162 | Chain A, Phospholipase A2 Isoform 3 |
| 71 | 64 | XP\_029140080.1 | 253.06 | 19 | 0 | 8 | 6 | 12 | 3 | 0 | 0 | 0 | 0 | 10 | 12 | 3 | 6 |  | 1.2319E6 | 1.3702E6 | 9.6234E6 |  |  |  |  |  | 2.3912E7 | 7.3676E6 |  | 1.527E6 | 12 | 1 | 0 | 5 | 6 | 74 | 1 | 0 | 0 | 0 | 0 | 19 | 19 | 6 | 4 | Y | 62665 | tissue-type plasminogen activator [Protobothrops mucrosquamatus] |
| 71 | 70 | JAI11009.1 | 253.06 | 20 | 0 | 9 | 7 | 13 | 3 | 0 | 0 | 0 | 0 | 11 | 13 | 4 | 6 |  | 1.2319E6 | 1.3702E6 | 9.6234E6 |  |  |  |  |  | 2.3912E7 | 7.3676E6 |  | 1.527E6 | 12 | 1 | 0 | 5 | 6 | 74 | 1 | 0 | 0 | 0 | 0 | 19 | 19 | 6 | 4 | Y | 59716 | tissue-type plasminogen activator [Crotalus adamanteus] |
| 82 | 36 | ADG02948.1 | 252.64 | 18 | 0 | 4 | 4 | 12 | 4 | 4 | 4 | 2 | 4 | 11 | 0 | 0 | 2 |  | 1.7705E6 | 1.4324E6 | 6.6603E6 |  | 3.9675E6 | 5.5621E5 |  | 5.6803E7 | 1.7191E8 |  |  |  | 13 | 3 | 0 | 3 | 7 | 14 | 6 | 2 | 1 | 1 | 5 | 25 | 0 | 0 | 2 | Y | 66246 | metalloproteinase atrase B [Naja atra] |
| 82 | 37 | D6PXE8.1 | 252.64 | 18 | 0 | 4 | 4 | 12 | 4 | 4 | 4 | 2 | 4 | 11 | 0 | 0 | 2 |  | 1.7705E6 | 1.4324E6 | 6.6603E6 |  | 3.9675E6 | 5.5621E5 |  | 5.6803E7 | 1.7191E8 |  |  |  | 13 | 3 | 0 | 3 | 7 | 14 | 6 | 2 | 1 | 1 | 5 | 25 | 0 | 0 | 2 | Y | 66246 | RecName: Full=Zinc metalloproteinase-disintegrin-like atrase-B; AltName: Full=Snake venom metalloproteinase; Short=SVMP; Flags: Precursor |
| 82 | 39 | ACN50005.1 | 252.64 | 18 | 0 | 4 | 4 | 12 | 4 | 4 | 4 | 2 | 4 | 11 | 0 | 0 | 2 |  | 1.7705E6 | 1.4324E6 | 6.6603E6 |  | 3.9675E6 | 5.5621E5 |  | 5.6803E7 | 1.7191E8 |  |  |  | 13 | 3 | 0 | 3 | 7 | 14 | 6 | 2 | 1 | 1 | 5 | 25 | 0 | 0 | 2 | Y | 66292 | K-like metalloprotease precursor, partial [Naja atra] |
| 82 | 40 | D3TTC1.1 | 252.64 | 18 | 0 | 4 | 4 | 12 | 4 | 4 | 4 | 2 | 4 | 11 | 0 | 0 | 2 |  | 1.7705E6 | 1.4324E6 | 6.6603E6 |  | 3.9675E6 | 5.5621E5 |  | 5.6803E7 | 1.7191E8 |  |  |  | 13 | 3 | 0 | 3 | 7 | 14 | 6 | 2 | 1 | 1 | 5 | 25 | 0 | 0 | 2 | Y | 66292 | RecName: Full=Zinc metalloproteinase-disintegrin-like kaouthiagin-like; Short=K-like; AltName: Full=Snake venom metalloproteinase; Short=SVMP; Flags: Precursor |
| 35 | 590 | P60044.1 | 242.58 | 42 | 0 | 21 | 21 | 11 | 11 | 0 | 0 | 0 | 21 | 30 | 42 | 31 | 28 |  |  |  |  |  |  |  |  |  |  | 0 |  | 1.489E6 | 16 | 1 | 0 | 9 | 5 | 5 | 1 | 0 | 0 | 0 | 7 | 92 | 245 | 173 | 79 | Y | 14073 | RecName: Full=Acidic phospholipase A2 2; Short=svPLA2; AltName: Full=Phosphatidylcholine 2-acylhydrolase; Flags: Precursor |
| 35 | 591 | AAR00254.1 | 242.58 | 42 | 0 | 21 | 21 | 11 | 11 | 0 | 0 | 0 | 21 | 30 | 42 | 31 | 28 |  |  |  |  |  |  |  |  |  |  | 0 |  | 1.489E6 | 16 | 1 | 0 | 9 | 5 | 5 | 1 | 0 | 0 | 0 | 7 | 92 | 245 | 173 | 79 | Y | 14073 | phospholipase A2 isoform 2 precursor, partial [Naja sagittifera] |
| 35 | 619 | 1XXW | 242.58 | 45 | 0 | 22 | 22 | 12 | 12 | 0 | 0 | 0 | 22 | 32 | 45 | 33 | 29 |  |  |  |  |  |  |  |  |  |  | 0 |  | 1.489E6 | 16 | 1 | 0 | 9 | 5 | 5 | 1 | 0 | 0 | 0 | 7 | 92 | 245 | 173 | 79 | Y | 13277 | Chain B, Phospholipase A2 Isoform 2 |
| 35 | 620 | 1S6B | 242.58 | 45 | 0 | 22 | 22 | 12 | 12 | 0 | 0 | 0 | 22 | 32 | 45 | 33 | 29 |  |  |  |  |  |  |  |  |  |  | 0 |  | 1.489E6 | 16 | 1 | 0 | 9 | 5 | 5 | 1 | 0 | 0 | 0 | 7 | 92 | 245 | 173 | 79 | Y | 13277 | Chain B, Phospholipase A2 isoform 2 |
| 35 | 621 | 2RD4 | 242.58 | 45 | 0 | 22 | 22 | 12 | 12 | 0 | 0 | 0 | 22 | 32 | 45 | 33 | 29 |  |  |  |  |  |  |  |  |  |  | 0 |  | 1.489E6 | 16 | 1 | 0 | 9 | 5 | 5 | 1 | 0 | 0 | 0 | 7 | 92 | 245 | 173 | 79 | Y | 13277 | Chain B, Phospholipase A2 isoform 2 |
| 69 | 3267 | JAA75025.1 | 240.27 | 13 | 0 | 13 | 13 | 13 | 0 | 0 | 0 | 0 | 13 | 13 | 13 | 13 | 13 |  | 5.8775E6 | 2.4285E6 | 2.4039E6 |  |  |  |  | 1.2439E7 | 8.2398E9 | 2.1904E7 | 7.7818E6 | 6.0445E7 | 9 | 6 | 0 | 2 | 1 | 1 | 0 | 0 | 0 | 0 | 1 | 112 | 33 | 5 | 11 | Y | 16595 | PLA2-Sut-8 [Suta fasciata] |
| 67 | 635 | ABN72541.1 | 239.75 | 16 | 0 | 0 | 9 | 16 | 5 | 0 | 0 | 0 | 0 | 0 | 0 | 0 | 0 |  |  |  | 4.1304E6 |  |  |  |  |  |  |  |  |  | 8 | 1 | 0 | 0 | 3 | 143 | 2 | 0 | 0 | 0 | 0 | 0 | 0 | 0 | 0 | Y | 31137 | putative serine protease, partial [Naja atra] |
| 67 | 636 | ABN72545.1 | 239.75 | 16 | 0 | 0 | 9 | 16 | 5 | 0 | 0 | 0 | 0 | 0 | 0 | 0 | 0 |  |  |  | 4.1304E6 |  |  |  |  |  |  |  |  |  | 8 | 1 | 0 | 0 | 3 | 143 | 2 | 0 | 0 | 0 | 0 | 0 | 0 | 0 | 0 | Y | 31010 | putative serine protease, partial [Bungarus multicinctus] |
| 67 | 637 | A8QL57.1 | 239.75 | 16 | 0 | 0 | 9 | 16 | 5 | 0 | 0 | 0 | 0 | 0 | 0 | 0 | 0 |  |  |  | 4.1304E6 |  |  |  |  |  |  |  |  |  | 8 | 1 | 0 | 0 | 3 | 143 | 2 | 0 | 0 | 0 | 0 | 0 | 0 | 0 | 0 | Y | 31010 | RecName: Full=Snake venom serine protease BmSP; Short=SVSP; Flags: Precursor |
| 67 | 638 | A8QL53.1 | 239.75 | 16 | 0 | 0 | 9 | 16 | 5 | 0 | 0 | 0 | 0 | 0 | 0 | 0 | 0 |  |  |  | 4.1304E6 |  |  |  |  |  |  |  |  |  | 8 | 1 | 0 | 0 | 3 | 143 | 2 | 0 | 0 | 0 | 0 | 0 | 0 | 0 | 0 | Y | 31137 | RecName: Full=Snake venom serine protease NaSP; Short=SVSP; Flags: Precursor |
| 68 | 625 | XP\_026522175.1 | 235.43 | 21 | 0 | 0 | 8 | 21 | 5 | 0 | 0 | 0 | 0 | 0 | 0 | 0 | 0 |  |  |  | 1.7578E6 |  |  |  |  |  |  |  |  |  | 8 | 1 | 0 | 0 | 3 | 140 | 2 | 0 | 0 | 0 | 0 | 0 | 0 | 0 | 0 | Y | 31530 | snake venom serine protease NaSP isoform X1 [Notechis scutatus] |
| 68 | 650 | XP\_026522176.1 | 235.43 | 23 | 0 | 0 | 9 | 23 | 5 | 0 | 0 | 0 | 0 | 0 | 0 | 0 | 0 |  |  |  | 1.7578E6 |  |  |  |  |  |  |  |  |  | 8 | 1 | 0 | 0 | 3 | 140 | 2 | 0 | 0 | 0 | 0 | 0 | 0 | 0 | 0 | Y | 28988 | serine protease harobin isoform X2 [Notechis scutatus] |
| 68 | 679 | XP\_026522177.1 | 235.43 | 23 | 0 | 0 | 9 | 23 | 5 | 0 | 0 | 0 | 0 | 0 | 0 | 0 | 0 |  |  |  | 1.7578E6 |  |  |  |  |  |  |  |  |  | 8 | 1 | 0 | 0 | 3 | 140 | 2 | 0 | 0 | 0 | 0 | 0 | 0 | 0 | 0 | Y | 28056 | serine protease harobin isoform X3 [Notechis scutatus] |
| 58 | 889 | P82463.1 | 234.15 | 77 | 0 | 29 | 29 | 12 | 0 | 0 | 0 | 29 | 77 | 42 | 42 | 29 | 29 |  | 6.3064E6 | 2.4346E6 | 3.3475E5 |  |  |  | 2.938E6 | 1.9251E10 | 1.7838E8 | 6.0421E6 | 2.3887E6 | 1.6324E7 | 8 | 8 | 0 | 2 | 1 | 2 | 0 | 0 | 0 | 2 | 193 | 21 | 3 | 1 | 2 | Y | 7298 | RecName: Full=Muscarinic toxin-like protein 2; Short=MTLP-2 |
| 52 | 732 | P25674.1 | 225.14 | 44 | 18 | 0 | 0 | 18 | 18 | 37 | 42 | 38 | 38 | 37 | 18 | 0 | 18 |  |  |  |  |  |  | 4.6388E6 |  |  |  |  |  |  | 9 | 1 | 6 | 0 | 0 | 2 | 2 | 12 | 31 | 57 | 147 | 23 | 2 | 0 | 1 | Y | 7821 | RecName: Full=Long neurotoxin 1; AltName: Full=Toxin CM-5 |
| 90 | 242 | P0DI91.1 | 221.40 | 81 | 0 | 36 | 22 | 55 | 35 | 12 | 0 | 0 | 17 | 22 | 0 | 0 | 34 |  | 1.0135E6 |  | 2.4269E7 |  |  |  |  | 2.7322E6 | 1.8088E6 |  |  | 7.453E5 | 11 | 4 | 0 | 4 | 5 | 19 | 24 | 3 | 0 | 0 | 1 | 3 | 0 | 0 | 4 | Y | 11216 | RecName: Full=L-amino-acid oxidase; Short=LAAO; Short=LAO |
| 23 | 384 | P86540.2 | 220.16 | 88 | 0 | 35 | 13 | 23 | 0 | 12 | 13 | 12 | 62 | 83 | 88 | 67 | 35 |  |  |  |  |  |  |  |  |  | 5.2696E6 | 1.1409E6 |  |  | 10 | 2 | 0 | 2 | 1 | 1 | 0 | 1 | 1 | 2 | 24 | 29 | 35 | 16 | 3 | Y | 6793 | RecName: Full=Cytotoxin 8; Short=CTX8 |
| 34 | 164 | P01440.1 | 219.97 | 97 | 13 | 83 | 78 | 92 | 13 | 13 | 13 | 0 | 33 | 68 | 48 | 93 | 45 |  | 9.2923E5 |  | 3.3882E6 |  |  |  |  |  |  |  | 1.4076E6 |  | 12 | 2 | 2 | 167 | 179 | 21 | 4 | 1 | 1 | 0 | 3 | 22 | 9 | 20 | 19 | Y | 6763 | RecName: Full=Cytotoxin 2; AltName: Full=Cobramine-B; AltName: Full=Cytotoxin II |
| 118 | 29 | 5H7W | 218.28 | 26 | 0 | 0 | 2 | 7 | 22 | 0 | 0 | 0 | 0 | 2 | 0 | 4 | 4 |  |  |  |  | 2.7183E6 |  |  |  |  | 7.2729E6 |  |  |  | 11 | 3 | 0 | 0 | 1 | 4 | 23 | 0 | 0 | 0 | 0 | 2 | 0 | 1 | 5 | Y | 58198 | Chain B, venom 5'-nucleotidase |
| 118 | 30 | A0A2I4HXH5.1 | 218.28 | 26 | 0 | 0 | 2 | 7 | 22 | 0 | 0 | 0 | 0 | 2 | 0 | 4 | 4 |  |  |  |  | 2.7183E6 |  |  |  |  | 7.2729E6 |  |  |  | 11 | 3 | 0 | 0 | 1 | 4 | 23 | 0 | 0 | 0 | 0 | 2 | 0 | 1 | 5 | Y | 58198 | RecName: Full=Snake venom 5'-nucleotidase; Short=5'-NT; AltName: Full=Ecto-5'-nucleotidase; Flags: Precursor |
| 94 | 1329 | P19859.1 | 216.61 | 63 | 0 | 0 | 0 | 0 | 0 | 0 | 18 | 39 | 61 | 39 | 40 | 39 | 39 |  |  |  |  |  |  | 3.3057E7 | 4.0485E7 | 8.3572E8 | 7.8424E6 | 2.9747E7 | 1.4564E7 | 8.7373E6 | 12 | 11 | 0 | 0 | 0 | 0 | 0 | 0 | 3 | 5 | 35 | 1 | 4 | 2 | 3 | Y | 6508 | RecName: Full=Kunitz-type serine protease inhibitor; AltName: Full=Venom chymotrypsin inhibitor |
| 94 | 1330 | 1702215A | 216.61 | 63 | 0 | 0 | 0 | 0 | 0 | 0 | 18 | 39 | 61 | 39 | 40 | 39 | 39 |  |  |  |  |  |  | 3.3057E7 | 4.0485E7 | 8.3572E8 | 7.8424E6 | 2.9747E7 | 1.4564E7 | 8.7373E6 | 12 | 11 | 0 | 0 | 0 | 0 | 0 | 0 | 3 | 5 | 35 | 1 | 4 | 2 | 3 | Y | 6508 | chymotrypsin inhibitor |
| 93 | 198 | ETE68810.1 | 214.67 | 32 | 0 | 8 | 8 | 19 | 28 | 14 | 4 | 6 | 6 | 13 | 8 | 5 | 5 |  |  |  | 3.7961E6 | 2.8507E7 | 4.393E7 |  | 2.4421E6 | 3.064E6 | 4.1372E6 |  |  |  | 10 | 3 | 0 | 7 | 5 | 7 | 17 | 15 | 1 | 1 | 1 | 4 | 4 | 4 | 3 | Y | 29588 | Glutathione peroxidase 3, partial [Ophiophagus hannah] |
| 136 | 34 | XP\_026570202.1 | 213.47 | 10 | 0 | 3 | 3 | 8 | 0 | 0 | 0 | 0 | 1 | 1 | 0 | 0 | 0 |  | 2.6745E6 | 2.8881E6 | 7.1049E6 |  |  |  |  | 7.4916E6 | 5.7591E6 |  |  |  | 11 | 11 | 0 | 6 | 6 | 10 | 0 | 0 | 0 | 0 | 4 | 1 | 0 | 0 | 0 | Y | 133145 | Golgi apparatus protein 1 [Pseudonaja textilis] |
| 64 | 192 | Q9W6W9.1 | 212.61 | 72 | 0 | 46 | 52 | 37 | 0 | 0 | 10 | 0 | 10 | 52 | 26 | 53 | 30 |  |  |  |  |  |  |  |  |  | 3.5601E5 |  |  |  | 10 | 1 | 0 | 16 | 37 | 12 | 0 | 0 | 1 | 0 | 1 | 6 | 2 | 12 | 5 | Y | 9099 | RecName: Full=Cytotoxin 4N; AltName: Full=Cardiotoxin-4N; Short=CTX-4N; Flags: Precursor |
| 64 | 193 | CAB42057.1 | 212.61 | 72 | 0 | 46 | 52 | 37 | 0 | 0 | 10 | 0 | 10 | 52 | 26 | 53 | 30 |  |  |  |  |  |  |  |  |  | 3.5601E5 |  |  |  | 10 | 1 | 0 | 16 | 37 | 12 | 0 | 0 | 1 | 0 | 1 | 6 | 2 | 12 | 5 | Y | 9099 | cardiotoxin-4N [Naja atra] |
| 100 | 730 | P82885.1 | 202.60 | 56 | 16 | 48 | 32 | 56 | 0 | 0 | 0 | 0 | 37 | 32 | 0 | 0 | 13 | 4.4967E5 | 4.6854E8 | 1.1686E7 | 7.7052E6 |  |  |  |  | 7.2249E6 | 3.4117E6 |  |  | 5.3893E5 | 6 | 6 | 2 | 30 | 3 | 5 | 0 | 0 | 0 | 0 | 4 | 2 | 0 | 0 | 1 | Y | 12038 | RecName: Full=Thaicobrin |
| 91 | 533 | P01427.1 | 200.03 | 80 | 59 | 0 | 0 | 0 | 0 | 80 | 43 | 18 | 0 | 0 | 0 | 0 | 0 | 5.1027E7 |  |  |  |  | 6.0969E8 | 9.2136E6 | 8.5404E4 |  |  |  |  |  | 8 | 8 | 9 | 0 | 0 | 0 | 0 | 49 | 4 | 1 | 0 | 0 | 0 | 0 | 0 | Y | 6885 | RecName: Full=Short neurotoxin 1; AltName: Full=Neurotoxin II; Short=NT II; Short=NTII; Short=NTX II; AltName: Full=Neurotoxin alpha |
| 91 | 534 | 1NOR | 200.03 | 80 | 59 | 0 | 0 | 0 | 0 | 80 | 43 | 18 | 0 | 0 | 0 | 0 | 0 | 5.1027E7 |  |  |  |  | 6.0969E8 | 9.2136E6 | 8.5404E4 |  |  |  |  |  | 8 | 8 | 9 | 0 | 0 | 0 | 0 | 49 | 4 | 1 | 0 | 0 | 0 | 0 | 0 | Y | 6885 | Chain A, NEUROTOXIN II |
| 91 | 535 | 2MJ4 | 200.03 | 80 | 59 | 0 | 0 | 0 | 0 | 80 | 43 | 18 | 0 | 0 | 0 | 0 | 0 | 5.1027E7 |  |  |  |  | 6.0969E8 | 9.2136E6 | 8.5404E4 |  |  |  |  |  | 8 | 8 | 9 | 0 | 0 | 0 | 0 | 49 | 4 | 1 | 0 | 0 | 0 | 0 | 0 | Y | 6885 | Chain A, Short neurotoxin 1 |
| 124 | 63 | JAG67188.1 | 198.32 | 19 | 0 | 0 | 2 | 6 | 16 | 0 | 0 | 0 | 0 | 0 | 0 | 3 | 3 |  |  |  |  | 1.1341E6 |  |  |  |  |  |  |  |  | 9 | 1 | 0 | 0 | 1 | 4 | 19 | 0 | 0 | 0 | 0 | 0 | 0 | 1 | 5 | Y | 64759 | Snake venom 5'-nucleotidase [Boiga irregularis] |
| 108 | 995 | APB88857.1 | 198.14 | 42 | 0 | 0 | 0 | 0 | 0 | 0 | 0 | 0 | 0 | 19 | 0 | 36 | 42 |  |  |  |  |  |  |  |  |  | 6.8846E7 |  | 1.6894E7 | 1.3327E8 | 10 | 8 | 0 | 0 | 0 | 0 | 0 | 0 | 0 | 0 | 0 | 7 | 0 | 8 | 21 | Y | 9695 | neurotoxin-like protein [Naja naja] |
| 108 | 996 | CAB45156.1 | 198.14 | 42 | 0 | 0 | 0 | 0 | 0 | 0 | 0 | 0 | 0 | 19 | 0 | 36 | 42 |  |  |  |  |  |  |  |  |  | 6.8846E7 |  | 1.6894E7 | 1.3327E8 | 10 | 8 | 0 | 0 | 0 | 0 | 0 | 0 | 0 | 0 | 0 | 7 | 0 | 8 | 21 | Y | 9695 | neurotoxin-like protein [Naja atra] |
| 108 | 997 | Q9W717.1 | 198.14 | 42 | 0 | 0 | 0 | 0 | 0 | 0 | 0 | 0 | 0 | 19 | 0 | 36 | 42 |  |  |  |  |  |  |  |  |  | 6.8846E7 |  | 1.6894E7 | 1.3327E8 | 10 | 8 | 0 | 0 | 0 | 0 | 0 | 0 | 0 | 0 | 0 | 7 | 0 | 8 | 21 | Y | 9695 | RecName: Full=Neurotoxin-like protein NTL2; Flags: Precursor |
| 36 | 388 | AAB25732.1 | 192.05 | 80 | 0 | 48 | 48 | 35 | 22 | 0 | 13 | 0 | 13 | 58 | 35 | 77 | 45 |  | 6.926E6 |  |  |  |  |  |  |  | 1.1895E7 |  | 1.0889E8 | 6.0589E6 | 9 | 3 | 0 | 365 | 25 | 11 | 10 | 0 | 1 | 0 | 1 | 7 | 34 | 71 | 33 | Y | 6701 | cardiotoxin isoform 1, cytotoxin isoform 1, CTX-1 [Naja naja=Formosan cobra, ssp. atra, venom, Peptide, 60 aa] |
| 155 | 295 | XP\_026575983.1 | 190.28 | 19 | 0 | 5 | 4 | 10 | 3 | 0 | 0 | 0 | 0 | 0 | 3 | 3 | 0 |  | 6.3277E5 | 1.2185E5 | 4.4202E7 | 1.333E5 |  |  |  |  |  | 1.7428E6 | 3.131E6 |  | 8 | 7 | 0 | 1 | 1 | 11 | 1 | 0 | 0 | 0 | 0 | 0 | 1 | 1 | 0 | N | 37973 | cathepsin S [Pseudonaja textilis] |
| 155 | 296 | XP\_026575982.1 | 190.28 | 19 | 0 | 5 | 4 | 10 | 3 | 0 | 0 | 0 | 0 | 0 | 3 | 3 | 0 |  | 6.3277E5 | 1.2185E5 | 4.4202E7 | 1.333E5 |  |  |  |  |  | 1.7428E6 | 3.131E6 |  | 8 | 7 | 0 | 1 | 1 | 11 | 1 | 0 | 0 | 0 | 0 | 0 | 1 | 1 | 0 | N | 37973 | cathepsin S [Pseudonaja textilis] |
| 99 | 461 | 4GFY | 188.94 | 39 | 0 | 8 | 8 | 39 | 0 | 0 | 0 | 0 | 0 | 8 | 0 | 0 | 0 |  |  |  | 9.8858E7 |  |  |  |  |  |  |  |  |  | 4 | 2 | 0 | 2 | 1 | 34 | 0 | 0 | 0 | 0 | 0 | 1 | 0 | 0 | 0 | Y | 13611 | Chain A, Phospholipase A2 Vrv-pl-viiia |
| 99 | 462 | 1SV9 | 188.94 | 39 | 0 | 8 | 8 | 39 | 0 | 0 | 0 | 0 | 0 | 8 | 0 | 0 | 0 |  |  |  | 9.8858E7 |  |  |  |  |  |  |  |  |  | 4 | 2 | 0 | 2 | 1 | 34 | 0 | 0 | 0 | 0 | 0 | 1 | 0 | 0 | 0 | Y | 13611 | Chain A, Phospholipase A2 |
| 99 | 463 | 3G8F | 188.94 | 39 | 0 | 8 | 8 | 39 | 0 | 0 | 0 | 0 | 0 | 8 | 0 | 0 | 0 |  |  |  | 9.8858E7 |  |  |  |  |  |  |  |  |  | 4 | 2 | 0 | 2 | 1 | 34 | 0 | 0 | 0 | 0 | 0 | 1 | 0 | 0 | 0 | Y | 13611 | Chain A, Phospholipase A2 Vrv-pl-viiia |
| 99 | 464 | 1TK4 | 188.94 | 39 | 0 | 8 | 8 | 39 | 0 | 0 | 0 | 0 | 0 | 8 | 0 | 0 | 0 |  |  |  | 9.8858E7 |  |  |  |  |  |  |  |  |  | 4 | 2 | 0 | 2 | 1 | 34 | 0 | 0 | 0 | 0 | 0 | 1 | 0 | 0 | 0 | Y | 13611 | Chain A, Phospholipase A2 Vrv-pl-viiia |
| 99 | 465 | 2QVD | 188.94 | 39 | 0 | 8 | 8 | 39 | 0 | 0 | 0 | 0 | 0 | 8 | 0 | 0 | 0 |  |  |  | 9.8858E7 |  |  |  |  |  |  |  |  |  | 4 | 2 | 0 | 2 | 1 | 34 | 0 | 0 | 0 | 0 | 0 | 1 | 0 | 0 | 0 | Y | 13611 | Chain A, Phospholipase A2 Vrv-pl-viiia |
| 99 | 466 | 4EIX | 188.94 | 39 | 0 | 8 | 8 | 39 | 0 | 0 | 0 | 0 | 0 | 8 | 0 | 0 | 0 |  |  |  | 9.8858E7 |  |  |  |  |  |  |  |  |  | 4 | 2 | 0 | 2 | 1 | 34 | 0 | 0 | 0 | 0 | 0 | 1 | 0 | 0 | 0 | Y | 13611 | Chain A, Phospholipase A2 Vrv-pl-viiia |
| 99 | 467 | 3H1X | 188.94 | 39 | 0 | 8 | 8 | 39 | 0 | 0 | 0 | 0 | 0 | 8 | 0 | 0 | 0 |  |  |  | 9.8858E7 |  |  |  |  |  |  |  |  |  | 4 | 2 | 0 | 2 | 1 | 34 | 0 | 0 | 0 | 0 | 0 | 1 | 0 | 0 | 0 | Y | 13611 | Chain A, Simultaneous Inhibition Of Anti-Coagulation And Inflammation: Crystal Structure Of Phospholipase A2 Complexed With Indomethacin At 1.4 A Resolution Reveals The Presence Of The New Common Ligand Binding Site |
| 99 | 468 | 2B17 | 188.94 | 39 | 0 | 8 | 8 | 39 | 0 | 0 | 0 | 0 | 0 | 8 | 0 | 0 | 0 |  |  |  | 9.8858E7 |  |  |  |  |  |  |  |  |  | 4 | 2 | 0 | 2 | 1 | 34 | 0 | 0 | 0 | 0 | 0 | 1 | 0 | 0 | 0 | Y | 13611 | Chain A, Specific Binding Of Non-Steroidal Anti-Inflammatory Drugs (Nsaids) To Phospholipase A2: Crystal Structure Of The Complex Formed Between Phospholipase A2 And Diclofenac At 2.7 A Resolution: |
| 99 | 469 | 2ARM | 188.94 | 39 | 0 | 8 | 8 | 39 | 0 | 0 | 0 | 0 | 0 | 8 | 0 | 0 | 0 |  |  |  | 9.8858E7 |  |  |  |  |  |  |  |  |  | 4 | 2 | 0 | 2 | 1 | 34 | 0 | 0 | 0 | 0 | 0 | 1 | 0 | 0 | 0 | Y | 13611 | Chain A, Phospholipase A2 Vrv-pl-viiia |
| 99 | 470 | 1TG4 | 188.94 | 39 | 0 | 8 | 8 | 39 | 0 | 0 | 0 | 0 | 0 | 8 | 0 | 0 | 0 |  |  |  | 9.8858E7 |  |  |  |  |  |  |  |  |  | 4 | 2 | 0 | 2 | 1 | 34 | 0 | 0 | 0 | 0 | 0 | 1 | 0 | 0 | 0 | Y | 13611 | Chain A, Phospholipase A2 |
| 99 | 471 | 1SQZ | 188.94 | 39 | 0 | 8 | 8 | 39 | 0 | 0 | 0 | 0 | 0 | 8 | 0 | 0 | 0 |  |  |  | 9.8858E7 |  |  |  |  |  |  |  |  |  | 4 | 2 | 0 | 2 | 1 | 34 | 0 | 0 | 0 | 0 | 0 | 1 | 0 | 0 | 0 | Y | 13611 | Chain A, Phospholipase A2 |
| 99 | 472 | 3FG5 | 188.94 | 39 | 0 | 8 | 8 | 39 | 0 | 0 | 0 | 0 | 0 | 8 | 0 | 0 | 0 |  |  |  | 9.8858E7 |  |  |  |  |  |  |  |  |  | 4 | 2 | 0 | 2 | 1 | 34 | 0 | 0 | 0 | 0 | 0 | 1 | 0 | 0 | 0 | Y | 13611 | Chain A, Crystal Structure Determination Of A Ternary Complex Of Phospholipase A2 With A Pentapetide Flsyk And Ajmaline At 2.5 A Resolution |
| 99 | 473 | 1ZWP | 188.94 | 39 | 0 | 8 | 8 | 39 | 0 | 0 | 0 | 0 | 0 | 8 | 0 | 0 | 0 |  |  |  | 9.8858E7 |  |  |  |  |  |  |  |  |  | 4 | 2 | 0 | 2 | 1 | 34 | 0 | 0 | 0 | 0 | 0 | 1 | 0 | 0 | 0 | Y | 13611 | Chain A, Phospholipase A2 Vrv-pl-viiia |
| 99 | 474 | 2QU9 | 188.94 | 39 | 0 | 8 | 8 | 39 | 0 | 0 | 0 | 0 | 0 | 8 | 0 | 0 | 0 |  |  |  | 9.8858E7 |  |  |  |  |  |  |  |  |  | 4 | 2 | 0 | 2 | 1 | 34 | 0 | 0 | 0 | 0 | 0 | 1 | 0 | 0 | 0 | Y | 13611 | Chain A, Phospholipase A2 Vrv-pl-viiia |
| 99 | 475 | 1Y38 | 188.94 | 39 | 0 | 8 | 8 | 39 | 0 | 0 | 0 | 0 | 0 | 8 | 0 | 0 | 0 |  |  |  | 9.8858E7 |  |  |  |  |  |  |  |  |  | 4 | 2 | 0 | 2 | 1 | 34 | 0 | 0 | 0 | 0 | 0 | 1 | 0 | 0 | 0 | Y | 13611 | Chain B, Phospholipase A2 Vrv-pl-viiia |
| 99 | 476 | 4GLD | 188.94 | 39 | 0 | 8 | 8 | 39 | 0 | 0 | 0 | 0 | 0 | 8 | 0 | 0 | 0 |  |  |  | 9.8858E7 |  |  |  |  |  |  |  |  |  | 4 | 2 | 0 | 2 | 1 | 34 | 0 | 0 | 0 | 0 | 0 | 1 | 0 | 0 | 0 | Y | 13611 | Chain A, Phospholipase A2 Vrv-pl-viiia |
| 99 | 477 | 4FGA | 188.94 | 39 | 0 | 8 | 8 | 39 | 0 | 0 | 0 | 0 | 0 | 8 | 0 | 0 | 0 |  |  |  | 9.8858E7 |  |  |  |  |  |  |  |  |  | 4 | 2 | 0 | 2 | 1 | 34 | 0 | 0 | 0 | 0 | 0 | 1 | 0 | 0 | 0 | Y | 13611 | Chain A, Phospholipase A2 Vrv-pl-viiia |
| 99 | 478 | 1ZR8 | 188.94 | 39 | 0 | 8 | 8 | 39 | 0 | 0 | 0 | 0 | 0 | 8 | 0 | 0 | 0 |  |  |  | 9.8858E7 |  |  |  |  |  |  |  |  |  | 4 | 2 | 0 | 2 | 1 | 34 | 0 | 0 | 0 | 0 | 0 | 1 | 0 | 0 | 0 | Y | 13611 | Chain A, Phospholipase A2 Vrv-pl-viiia |
| 99 | 479 | 1TP2 | 188.94 | 39 | 0 | 8 | 8 | 39 | 0 | 0 | 0 | 0 | 0 | 8 | 0 | 0 | 0 |  |  |  | 9.8858E7 |  |  |  |  |  |  |  |  |  | 4 | 2 | 0 | 2 | 1 | 34 | 0 | 0 | 0 | 0 | 0 | 1 | 0 | 0 | 0 | Y | 13611 | Chain B, Phospholipase A2 Vrv-pl-viiia |
| 99 | 480 | 1Q7A | 188.94 | 39 | 0 | 8 | 8 | 39 | 0 | 0 | 0 | 0 | 0 | 8 | 0 | 0 | 0 |  |  |  | 9.8858E7 |  |  |  |  |  |  |  |  |  | 4 | 2 | 0 | 2 | 1 | 34 | 0 | 0 | 0 | 0 | 0 | 1 | 0 | 0 | 0 | Y | 13611 | Chain A, Phospholipase A2 Vrv-pl-viiia |
| 99 | 481 | 1TH6 | 188.94 | 39 | 0 | 8 | 8 | 39 | 0 | 0 | 0 | 0 | 0 | 8 | 0 | 0 | 0 |  |  |  | 9.8858E7 |  |  |  |  |  |  |  |  |  | 4 | 2 | 0 | 2 | 1 | 34 | 0 | 0 | 0 | 0 | 0 | 1 | 0 | 0 | 0 | Y | 13611 | Chain A, Phospholipase A2 |
| 99 | 482 | 4HMB | 188.94 | 39 | 0 | 8 | 8 | 39 | 0 | 0 | 0 | 0 | 0 | 8 | 0 | 0 | 0 |  |  |  | 9.8858E7 |  |  |  |  |  |  |  |  |  | 4 | 2 | 0 | 2 | 1 | 34 | 0 | 0 | 0 | 0 | 0 | 1 | 0 | 0 | 0 | Y | 13611 | Chain A, Phospholipase A2 Vrv-pl-viiia |
| 99 | 483 | 3CBI | 188.94 | 39 | 0 | 8 | 8 | 39 | 0 | 0 | 0 | 0 | 0 | 8 | 0 | 0 | 0 |  |  |  | 9.8858E7 |  |  |  |  |  |  |  |  |  | 4 | 2 | 0 | 2 | 1 | 34 | 0 | 0 | 0 | 0 | 0 | 1 | 0 | 0 | 0 | Y | 13611 | Chain D, Crystal Structure Of The Ternary Complex Of Phospholipase A2 With Ajmaline And Anisic Acid At 3.1 A Resolution |
| 99 | 484 | P59071.1 | 188.94 | 39 | 0 | 8 | 8 | 39 | 0 | 0 | 0 | 0 | 0 | 8 | 0 | 0 | 0 |  |  |  | 9.8858E7 |  |  |  |  |  |  |  |  |  | 4 | 2 | 0 | 2 | 1 | 34 | 0 | 0 | 0 | 0 | 0 | 1 | 0 | 0 | 0 | Y | 13611 | RecName: Full=Basic phospholipase A2 VRV-PL-VIIIa; Short=svPLA2; AltName: Full=DPLA2; AltName: Full=P1; AltName: Full=Phosphatidylcholine 2-acylhydrolase; AltName: Full=Phospholipase A2 4; Short=PLA24 |
| 99 | 485 | 2QUE | 188.94 | 39 | 0 | 8 | 8 | 39 | 0 | 0 | 0 | 0 | 0 | 8 | 0 | 0 | 0 |  |  |  | 9.8858E7 |  |  |  |  |  |  |  |  |  | 4 | 2 | 0 | 2 | 1 | 34 | 0 | 0 | 0 | 0 | 0 | 1 | 0 | 0 | 0 | Y | 13611 | Chain A, Phospholipase A2 Vrv-pl-viiia |
| 99 | 486 | 3FO7 | 188.94 | 39 | 0 | 8 | 8 | 39 | 0 | 0 | 0 | 0 | 0 | 8 | 0 | 0 | 0 |  |  |  | 9.8858E7 |  |  |  |  |  |  |  |  |  | 4 | 2 | 0 | 2 | 1 | 34 | 0 | 0 | 0 | 0 | 0 | 1 | 0 | 0 | 0 | Y | 13611 | Chain A, Simultaneous Inhibition Of Anti-Coagulation And Inflammation: Crystal Structure Of Phospholipase A2 Complexed With Indomethacin At 1.4 A Resolution Reveals The Presence Of The New Common Ligand Binding Site |
| 99 | 487 | AAZ53186.1 | 188.94 | 34 | 0 | 7 | 7 | 34 | 0 | 0 | 0 | 0 | 0 | 7 | 0 | 0 | 0 |  |  |  | 9.8858E7 |  |  |  |  |  |  |  |  |  | 4 | 2 | 0 | 2 | 1 | 34 | 0 | 0 | 0 | 0 | 0 | 1 | 0 | 0 | 0 | Y | 15353 | phospholipase A2 [Daboia russelii limitis] |
| 99 | 488 | AAZ53179.1 | 188.94 | 34 | 0 | 7 | 7 | 34 | 0 | 0 | 0 | 0 | 0 | 7 | 0 | 0 | 0 |  |  |  | 9.8858E7 |  |  |  |  |  |  |  |  |  | 4 | 2 | 0 | 2 | 1 | 34 | 0 | 0 | 0 | 0 | 0 | 1 | 0 | 0 | 0 | Y | 15381 | phospholipase A2 [Daboia siamensis] |
| 152 | 72 | 5GZ4 | 188.79 | 7 | 0 | 0 | 0 | 0 | 6 | 0 | 2 | 2 | 2 | 0 | 0 | 0 | 0 |  |  |  |  | 3.0397E6 |  |  |  |  |  |  |  |  | 6 | 2 | 0 | 0 | 0 | 0 | 9 | 0 | 2 | 4 | 1 | 0 | 0 | 0 | 0 | Y | 94616 | Chain A, Snake Venom Phosphodiesterase (pde) |
| 152 | 73 | A0A2D0TC04.1 | 188.79 | 7 | 0 | 0 | 0 | 0 | 6 | 0 | 2 | 2 | 2 | 0 | 0 | 0 | 0 |  |  |  |  | 3.0397E6 |  |  |  |  |  |  |  |  | 6 | 2 | 0 | 0 | 0 | 0 | 9 | 0 | 2 | 4 | 1 | 0 | 0 | 0 | 0 | Y | 94616 | RecName: Full=Venom phosphodiesterase; Short=PDE |
| 152 | 74 | 5GZ5 | 188.79 | 7 | 0 | 0 | 0 | 0 | 6 | 0 | 2 | 2 | 2 | 0 | 0 | 0 | 0 |  |  |  |  | 3.0397E6 |  |  |  |  |  |  |  |  | 6 | 2 | 0 | 0 | 0 | 0 | 9 | 0 | 2 | 4 | 1 | 0 | 0 | 0 | 0 | Y | 94616 | Chain A, Snake Venom Phosphodiesterase (pde) |
| 97 | 201 | XP\_026541908.1 | 188.16 | 33 | 0 | 8 | 8 | 19 | 29 | 9 | 4 | 0 | 6 | 18 | 8 | 5 | 5 |  |  |  | 1.2069E6 | 3.3174E6 |  |  |  | 1.1691E7 | 3.9836E6 |  |  |  | 8 | 1 | 0 | 7 | 5 | 7 | 13 | 5 | 1 | 0 | 2 | 4 | 4 | 4 | 3 | Y | 28574 | glutathione peroxidase 3 isoform X1 [Notechis scutatus] |
| 53 | 1278 | P20229.1 | 183.03 | 44 | 16 | 0 | 0 | 0 | 0 | 44 | 19 | 19 | 19 | 0 | 0 | 0 | 0 | 4.9737E5 |  |  |  |  | 2.0038E10 | 6.6671E6 | 1.2942E5 | 7.7812E4 |  |  |  |  | 9 | 9 | 3 | 0 | 0 | 0 | 0 | 237 | 5 | 1 | 1 | 0 | 0 | 0 | 0 | Y | 6371 | RecName: Full=Kunitz-type serine protease inhibitor; AltName: Full=Venom trypsin inhibitor |
| 120 | 674 | XP\_034258146.1 | 179.54 | 21 | 0 | 5 | 5 | 11 | 17 | 4 | 4 | 4 | 0 | 9 | 5 | 5 | 5 |  |  |  |  | 6.8309E5 |  |  | 1.2476E5 |  |  |  |  |  | 6 | 2 | 0 | 4 | 2 | 4 | 9 | 3 | 1 | 1 | 0 | 2 | 2 | 4 | 3 | Y | 28406 | glutathione peroxidase 3 [Pantherophis guttatus] |
| 87 | 567 | 1UG4 | 176.50 | 57 | 0 | 27 | 57 | 23 | 0 | 0 | 13 | 0 | 13 | 27 | 13 | 37 | 23 |  |  | 2.358E6 |  |  |  |  |  |  |  |  |  |  | 6 | 1 | 0 | 8 | 31 | 3 | 0 | 0 | 1 | 0 | 1 | 2 | 1 | 4 | 2 | Y | 6689 | Chain A, Crystal Structure Of Cardiotoxin Vi From Taiwan Cobra (Naja Atra) Venom |
| 87 | 568 | ADN67586.1 | 176.50 | 55 | 0 | 26 | 55 | 23 | 0 | 0 | 13 | 0 | 13 | 26 | 13 | 35 | 23 |  |  | 2.358E6 |  |  |  |  |  |  |  |  |  |  | 6 | 1 | 0 | 8 | 31 | 3 | 0 | 0 | 1 | 0 | 1 | 2 | 1 | 4 | 2 | Y | 6953 | three-finger toxin precursor, partial [Naja atra] |
| 87 | 569 | 2207174C | 176.50 | 42 | 0 | 20 | 42 | 17 | 0 | 0 | 10 | 0 | 10 | 20 | 10 | 27 | 17 |  |  | 2.358E6 |  |  |  |  |  |  |  |  |  |  | 6 | 1 | 0 | 8 | 31 | 3 | 0 | 0 | 1 | 0 | 1 | 2 | 1 | 4 | 2 | Y | 8952 | cardiotoxin:ISOTYPE=N |
| 87 | 570 | CAB42058.1 | 176.50 | 42 | 0 | 20 | 42 | 17 | 0 | 0 | 10 | 0 | 10 | 20 | 10 | 27 | 17 |  |  | 2.358E6 |  |  |  |  |  |  |  |  |  |  | 6 | 1 | 0 | 8 | 31 | 3 | 0 | 0 | 1 | 0 | 1 | 2 | 1 | 4 | 2 | Y | 8980 | cardiotoxin-N [Naja atra] |
| 87 | 571 | AAB86638.1 | 176.50 | 42 | 0 | 20 | 42 | 17 | 0 | 0 | 10 | 0 | 10 | 20 | 10 | 27 | 17 |  |  | 2.358E6 |  |  |  |  |  |  |  |  |  |  | 6 | 1 | 0 | 8 | 31 | 3 | 0 | 0 | 1 | 0 | 1 | 2 | 1 | 4 | 2 | Y | 8980 | cardiotoxin N [Naja atra] |
| 87 | 572 | CAA07687.1 | 176.50 | 42 | 0 | 20 | 42 | 17 | 0 | 0 | 10 | 0 | 10 | 20 | 10 | 27 | 17 |  |  | 2.358E6 |  |  |  |  |  |  |  |  |  |  | 6 | 1 | 0 | 8 | 31 | 3 | 0 | 0 | 1 | 0 | 1 | 2 | 1 | 4 | 2 | Y | 8980 | cardiotoxin 6 [Naja atra] |
| 87 | 573 | P80245.2 | 176.50 | 42 | 0 | 20 | 42 | 17 | 0 | 0 | 10 | 0 | 10 | 20 | 10 | 27 | 17 |  |  | 2.358E6 |  |  |  |  |  |  |  |  |  |  | 6 | 1 | 0 | 8 | 31 | 3 | 0 | 0 | 1 | 0 | 1 | 2 | 1 | 4 | 2 | Y | 8980 | RecName: Full=Cytotoxin 6; AltName: Full=Cardiotoxin 6; Short=CTX6; AltName: Full=Cardiotoxin A6; Short=CTX A6; AltName: Full=Cardiotoxin N; Short=CTXN; Short=Ctx-N; AltName: Full=Cytotoxin N; Flags: Precursor |
| 87 | 574 | CAA90966.1 | 176.50 | 42 | 0 | 20 | 42 | 17 | 0 | 0 | 10 | 0 | 10 | 20 | 10 | 27 | 17 |  |  | 2.358E6 |  |  |  |  |  |  |  |  |  |  | 6 | 1 | 0 | 8 | 31 | 3 | 0 | 0 | 1 | 0 | 1 | 2 | 1 | 4 | 2 | Y | 8952 | cardiotoxin N [Naja naja] |
| 87 | 575 | CAA69977.1 | 176.50 | 41 | 0 | 20 | 41 | 17 | 0 | 0 | 10 | 0 | 10 | 20 | 10 | 27 | 17 |  |  | 2.358E6 |  |  |  |  |  |  |  |  |  |  | 6 | 1 | 0 | 8 | 31 | 3 | 0 | 0 | 1 | 0 | 1 | 2 | 1 | 4 | 2 | Y | 9201 | cardiotoxin 6 [Naja atra] |
| 87 | 576 | Q98965.1 | 176.50 | 41 | 0 | 20 | 41 | 17 | 0 | 0 | 10 | 0 | 10 | 20 | 10 | 27 | 17 |  |  | 2.358E6 |  |  |  |  |  |  |  |  |  |  | 6 | 1 | 0 | 8 | 31 | 3 | 0 | 0 | 1 | 0 | 1 | 2 | 1 | 4 | 2 | Y | 9201 | RecName: Full=Cytotoxin 6; AltName: Full=Cardiotoxin-6; Short=CTX6; Flags: Precursor |
| 151 | 86 | CAA55333.1 | 173.37 | 14 | 0 | 1 | 0 | 6 | 8 | 0 | 0 | 0 | 0 | 0 | 0 | 0 | 0 |  |  |  | 3.4219E6 | 4.3738E6 |  |  |  |  |  |  |  |  | 8 | 6 | 0 | 1 | 0 | 10 | 7 | 0 | 0 | 0 | 0 | 0 | 0 | 0 | 0 | Y | 69799 | cobra serum albumin [Naja naja] |
| 151 | 87 | S59517 | 173.37 | 14 | 0 | 1 | 0 | 6 | 8 | 0 | 0 | 0 | 0 | 0 | 0 | 0 | 0 |  |  |  | 3.4219E6 | 4.3738E6 |  |  |  |  |  |  |  |  | 8 | 6 | 0 | 1 | 0 | 10 | 7 | 0 | 0 | 0 | 0 | 0 | 0 | 0 | 0 | Y | 69799 | serum albumin precursor - monocled cobra |
| 161 | 232 | JAS05092.1 | 169.48 | 13 | 0 | 0 | 0 | 2 | 6 | 0 | 0 | 2 | 0 | 5 | 0 | 0 | 0 |  |  |  |  |  |  |  |  |  | 2.1613E7 |  |  |  | 6 | 1 | 0 | 0 | 0 | 1 | 10 | 0 | 0 | 1 | 0 | 3 | 0 | 0 | 0 | Y | 68997 | Metalloproteinase type III 2 [Micrurus tener] |
| 85 | 911 | P49122.1 | 168.10 | 41 | 0 | 30 | 30 | 10 | 0 | 0 | 0 | 0 | 0 | 10 | 10 | 10 | 21 |  | 3.8675E6 | 4.1549E7 |  |  |  |  |  |  |  |  |  |  | 4 | 1 | 0 | 6 | 10 | 5 | 0 | 0 | 0 | 0 | 0 | 3 | 2 | 8 | 24 | Y | 9086 | RecName: Full=Cytotoxin 7; AltName: Full=Cardiotoxin-7; Short=CTX7; Short=Ctx-7; AltName: Full=Cardiotoxin-like basic protein 2; Short=CLBP2; Flags: Precursor |
| 85 | 912 | CAA77017.1 | 168.10 | 41 | 0 | 30 | 30 | 10 | 0 | 0 | 0 | 0 | 0 | 10 | 10 | 10 | 21 |  | 3.8675E6 | 4.1549E7 |  |  |  |  |  |  |  |  |  |  | 4 | 1 | 0 | 6 | 10 | 5 | 0 | 0 | 0 | 0 | 0 | 3 | 2 | 8 | 24 | Y | 9086 | cardiotoxin 7 precursor [Naja atra] |
| 85 | 913 | CAA90967.1 | 168.10 | 41 | 0 | 30 | 30 | 10 | 0 | 0 | 0 | 0 | 0 | 10 | 10 | 10 | 21 |  | 3.8675E6 | 4.1549E7 |  |  |  |  |  |  |  |  |  |  | 4 | 1 | 0 | 6 | 10 | 5 | 0 | 0 | 0 | 0 | 0 | 3 | 2 | 8 | 24 | Y | 9086 | cardiotoxin 7 [Naja naja] |
| 138 | 111 | XP\_026581281.1 | 167.08 | 12 | 0 | 4 | 6 | 6 | 5 | 0 | 0 | 0 | 0 | 0 | 0 | 0 | 3 |  | 1.5157E6 | 2.4175E6 | 2.4449E6 | 2.3712E6 |  |  |  |  |  |  |  |  | 6 | 2 | 0 | 4 | 2 | 3 | 11 | 0 | 0 | 0 | 0 | 0 | 0 | 0 | 1 | Y | 67027 | acetylcholinesterase, partial [Pseudonaja textilis] |
| 172 | 187 | XP\_026561288.1 | 165.55 | 7 | 0 | 0 | 0 | 0 | 3 | 0 | 2 | 4 | 2 | 0 | 0 | 0 | 0 |  |  |  |  |  |  |  | 6.225E6 |  |  |  |  |  | 5 | 1 | 0 | 0 | 0 | 0 | 2 | 0 | 2 | 5 | 1 | 0 | 0 | 0 | 0 | Y | 96749 | ectonucleotide pyrophosphatase/phosphodiesterase family member 3 isoform X4 [Pseudonaja textilis] |
| 172 | 188 | XP\_026561287.1 | 165.55 | 7 | 0 | 0 | 0 | 0 | 3 | 0 | 2 | 4 | 2 | 0 | 0 | 0 | 0 |  |  |  |  |  |  |  | 6.225E6 |  |  |  |  |  | 5 | 1 | 0 | 0 | 0 | 0 | 2 | 0 | 2 | 5 | 1 | 0 | 0 | 0 | 0 | Y | 96734 | ectonucleotide pyrophosphatase/phosphodiesterase family member 3 isoform X3 [Pseudonaja textilis] |
| 172 | 189 | XP\_026561289.1 | 165.55 | 7 | 0 | 0 | 0 | 0 | 3 | 0 | 2 | 4 | 2 | 0 | 0 | 0 | 0 |  |  |  |  |  |  |  | 6.225E6 |  |  |  |  |  | 5 | 1 | 0 | 0 | 0 | 0 | 2 | 0 | 2 | 5 | 1 | 0 | 0 | 0 | 0 | Y | 96775 | ectonucleotide pyrophosphatase/phosphodiesterase family member 3 isoform X5 [Pseudonaja textilis] |
| 172 | 190 | XP\_026561284.1 | 165.55 | 7 | 0 | 0 | 0 | 0 | 3 | 0 | 2 | 4 | 2 | 0 | 0 | 0 | 0 |  |  |  |  |  |  |  | 6.225E6 |  |  |  |  |  | 5 | 1 | 0 | 0 | 0 | 0 | 2 | 0 | 2 | 5 | 1 | 0 | 0 | 0 | 0 | Y | 100812 | ectonucleotide pyrophosphatase/phosphodiesterase family member 3 isoform X1 [Pseudonaja textilis] |
| 172 | 191 | XP\_026561286.1 | 165.55 | 7 | 0 | 0 | 0 | 0 | 3 | 0 | 2 | 4 | 2 | 0 | 0 | 0 | 0 |  |  |  |  |  |  |  | 6.225E6 |  |  |  |  |  | 5 | 1 | 0 | 0 | 0 | 0 | 2 | 0 | 2 | 5 | 1 | 0 | 0 | 0 | 0 | Y | 100853 | ectonucleotide pyrophosphatase/phosphodiesterase family member 3 isoform X2 [Pseudonaja textilis] |
| 96 | 1642 | AJB84504.1 | 164.86 | 6 | 0 | 0 | 5 | 6 | 5 | 0 | 0 | 0 | 0 | 0 | 0 | 0 | 0 |  |  |  | 8.0725E6 |  |  |  |  |  |  |  |  |  | 4 | 1 | 0 | 0 | 2 | 43 | 2 | 0 | 0 | 0 | 0 | 0 | 0 | 0 | 0 | Y | 28572 | serine protease precursor [Philodryas chamissonis] |
| 145 | 202 | 4QWW | 160.76 | 12 | 0 | 3 | 2 | 2 | 6 | 0 | 0 | 0 | 0 | 0 | 0 | 0 | 3 |  |  |  |  | 1.1881E7 |  |  |  |  |  |  |  |  | 6 | 2 | 0 | 2 | 1 | 2 | 15 | 0 | 0 | 0 | 0 | 0 | 0 | 0 | 1 | Y | 60267 | Chain B, Acetylcholinesterase |
| 145 | 203 | AAC59905.1 | 160.76 | 11 | 0 | 3 | 2 | 2 | 6 | 0 | 0 | 0 | 0 | 0 | 0 | 0 | 3 |  |  |  |  | 1.1881E7 |  |  |  |  |  |  |  |  | 6 | 2 | 0 | 2 | 1 | 2 | 15 | 0 | 0 | 0 | 0 | 0 | 0 | 0 | 1 | Y | 64723 | acetylcholinesterase [Bungarus fasciatus] |
| 40 | 947 | P00600.1 | 157.57 | 20 | 0 | 8 | 8 | 8 | 8 | 0 | 0 | 8 | 8 | 18 | 18 | 18 | 10 |  | 3.7449E8 | 7.0956E7 | 5.0959E7 | 2.0327E6 |  |  | 3.4961E5 | 1.7447E8 | 1.5647E10 | 3.8482E9 | 3.7982E9 | 1.0131E11 | 5 | 2 | 0 | 9 | 5 | 5 | 1 | 0 | 0 | 1 | 6 | 100 | 55 | 28 | 260 | Y | 13427 | RecName: Full=Acidic phospholipase A2 DE-II; Short=svPLA2; AltName: Full=Phosphatidylcholine 2-acylhydrolase |
| 40 | 950 | P00601.1 | 157.57 | 20 | 0 | 8 | 8 | 8 | 8 | 0 | 0 | 8 | 8 | 18 | 18 | 18 | 10 |  | 3.7449E8 | 7.0956E7 | 5.0959E7 | 2.0327E6 |  |  | 3.4961E5 | 1.7447E8 | 1.5647E10 | 3.8482E9 | 3.7982E9 | 1.0131E11 | 5 | 2 | 0 | 9 | 5 | 5 | 1 | 0 | 0 | 1 | 6 | 100 | 55 | 28 | 260 | Y | 13360 | RecName: Full=Acidic phospholipase A2 DE-III; Short=svPLA2; AltName: Full=Phosphatidylcholine 2-acylhydrolase |
| 46 | 1010 | XP\_013911763.1 | 152.33 | 6 | 0 | 0 | 5 | 6 | 5 | 0 | 0 | 0 | 0 | 0 | 0 | 0 | 0 |  |  |  | 1.1323E7 |  |  |  |  |  |  |  |  |  | 3 | 1 | 0 | 0 | 3 | 302 | 3 | 0 | 0 | 0 | 0 | 0 | 0 | 0 | 0 | Y | 27138 | PREDICTED: cysteine-rich venom protein TEL1-like [Thamnophis sirtalis] |
| 46 | 1014 | XP\_032069584.1 | 152.33 | 6 | 0 | 0 | 5 | 6 | 5 | 0 | 0 | 0 | 0 | 0 | 0 | 0 | 0 |  |  |  | 1.1323E7 |  |  |  |  |  |  |  |  |  | 3 | 1 | 0 | 0 | 3 | 302 | 3 | 0 | 0 | 0 | 0 | 0 | 0 | 0 | 0 | Y | 27051 | cysteine-rich venom protein TEL1-like [Thamnophis elegans] |
| 171 | 927 | XP\_026579406.1 | 152.12 | 15 | 0 | 0 | 0 | 0 | 0 | 0 | 0 | 0 | 14 | 15 | 0 | 0 | 0 |  |  |  |  |  |  |  |  | 5.9383E7 | 1.1749E8 |  |  |  | 3 | 3 | 0 | 0 | 0 | 0 | 0 | 0 | 0 | 0 | 3 | 10 | 0 | 0 | 0 | Y | 22428 | BPTI/Kunitz domain-containing protein-like isoform X3 [Pseudonaja textilis] |
| 171 | 928 | XP\_026579408.1 | 152.12 | 15 | 0 | 0 | 0 | 0 | 0 | 0 | 0 | 0 | 14 | 15 | 0 | 0 | 0 |  |  |  |  |  |  |  |  | 5.9383E7 | 1.1749E8 |  |  |  | 3 | 3 | 0 | 0 | 0 | 0 | 0 | 0 | 0 | 0 | 3 | 10 | 0 | 0 | 0 | Y | 22376 | BPTI/Kunitz domain-containing protein-like isoform X1 [Pseudonaja textilis] |
| 171 | 929 | XP\_026579405.1 | 152.12 | 15 | 0 | 0 | 0 | 0 | 0 | 0 | 0 | 0 | 14 | 15 | 0 | 0 | 0 |  |  |  |  |  |  |  |  | 5.9383E7 | 1.1749E8 |  |  |  | 3 | 3 | 0 | 0 | 0 | 0 | 0 | 0 | 0 | 0 | 3 | 10 | 0 | 0 | 0 | Y | 22372 | BPTI/Kunitz domain-containing protein-like isoform X2 [Pseudonaja textilis] |
| 171 | 930 | XP\_026579404.1 | 152.12 | 15 | 0 | 0 | 0 | 0 | 0 | 0 | 0 | 0 | 14 | 15 | 0 | 0 | 0 |  |  |  |  |  |  |  |  | 5.9383E7 | 1.1749E8 |  |  |  | 3 | 3 | 0 | 0 | 0 | 0 | 0 | 0 | 0 | 0 | 3 | 10 | 0 | 0 | 0 | Y | 22376 | BPTI/Kunitz domain-containing protein-like isoform X1 [Pseudonaja textilis] |
| 171 | 931 | XP\_026579409.1 | 152.12 | 15 | 0 | 0 | 0 | 0 | 0 | 0 | 0 | 0 | 14 | 15 | 0 | 0 | 0 |  |  |  |  |  |  |  |  | 5.9383E7 | 1.1749E8 |  |  |  | 3 | 3 | 0 | 0 | 0 | 0 | 0 | 0 | 0 | 0 | 3 | 10 | 0 | 0 | 0 | Y | 22372 | BPTI/Kunitz domain-containing protein-like isoform X2 [Pseudonaja textilis] |
| 185 | 1331 | 2M99 | 151.33 | 54 | 0 | 0 | 0 | 0 | 0 | 0 | 0 | 33 | 54 | 0 | 33 | 0 | 0 |  |  |  |  |  |  |  | 7.3152E5 | 1.395E8 |  | 4.2021E5 |  |  | 4 | 3 | 0 | 0 | 0 | 0 | 0 | 0 | 0 | 1 | 7 | 0 | 1 | 0 | 0 | Y | 6391 | Chain A, Protease inhibitor NACI |
| 185 | 1332 | CAE51866.1 | 151.33 | 38 | 0 | 0 | 0 | 0 | 0 | 0 | 0 | 23 | 38 | 0 | 23 | 0 | 0 |  |  |  |  |  |  |  | 7.3152E5 | 1.395E8 |  | 4.2021E5 |  |  | 4 | 3 | 0 | 0 | 0 | 0 | 0 | 0 | 0 | 1 | 7 | 0 | 1 | 0 | 0 | Y | 8815 | chymotrypsin inhibitor [Naja atra] |
| 185 | 1333 | Q5ZPJ7.1 | 151.33 | 38 | 0 | 0 | 0 | 0 | 0 | 0 | 0 | 23 | 38 | 0 | 23 | 0 | 0 |  |  |  |  |  |  |  | 7.3152E5 | 1.395E8 |  | 4.2021E5 |  |  | 4 | 3 | 0 | 0 | 0 | 0 | 0 | 0 | 0 | 1 | 7 | 0 | 1 | 0 | 0 | Y | 8815 | RecName: Full=Kunitz-type serine protease inhibitor NACI; AltName: Full=Naja atra chymotrypsin inhibitor; Short=ACI; Short=NA-CI; Flags: Precursor |
| 185 | 1334 | CAE51867.1 | 151.33 | 38 | 0 | 0 | 0 | 0 | 0 | 0 | 0 | 23 | 38 | 0 | 23 | 0 | 0 |  |  |  |  |  |  |  | 7.3152E5 | 1.395E8 |  | 4.2021E5 |  |  | 4 | 3 | 0 | 0 | 0 | 0 | 0 | 0 | 0 | 1 | 7 | 0 | 1 | 0 | 0 | Y | 8815 | chymotrypsin inhibitor precursor [Naja atra] |
| 162 | 398 | JAB52939.1 | 148.24 | 13 | 0 | 3 | 0 | 0 | 0 | 8 | 4 | 0 | 0 | 0 | 0 | 3 | 3 |  | 9.1753E6 |  |  |  | 2.7328E6 | 1.4164E5 |  |  |  |  | 1.5718E6 | 2.8923E6 | 5 | 5 | 0 | 5 | 0 | 0 | 0 | 5 | 1 | 0 | 0 | 0 | 0 | 2 | 1 | Y | 47564 | vascular endothelial growth factor C [Micrurus fulvius] |
| 162 | 399 | JAI08988.1 | 148.24 | 13 | 0 | 3 | 0 | 0 | 0 | 8 | 4 | 0 | 0 | 0 | 0 | 3 | 3 |  | 9.1753E6 |  |  |  | 2.7328E6 | 1.4164E5 |  |  |  |  | 1.5718E6 | 2.8923E6 | 5 | 5 | 0 | 5 | 0 | 0 | 0 | 5 | 1 | 0 | 0 | 0 | 0 | 2 | 1 | Y | 47564 | Vascular endothelial growth factor 2 [Micrurus fulvius] |
| 162 | 400 | JAI09156.1 | 148.24 | 13 | 0 | 3 | 0 | 0 | 0 | 8 | 4 | 0 | 0 | 0 | 0 | 3 | 3 |  | 9.1753E6 |  |  |  | 2.7328E6 | 1.4164E5 |  |  |  |  | 1.5718E6 | 2.8923E6 | 5 | 5 | 0 | 5 | 0 | 0 | 0 | 5 | 1 | 0 | 0 | 0 | 0 | 2 | 1 | Y | 47564 | Vascular endothelial growth factor C-like protein [Micrurus fulvius] |
| 162 | 401 | JAS04977.1 | 148.24 | 13 | 0 | 3 | 0 | 0 | 0 | 8 | 4 | 0 | 0 | 0 | 0 | 3 | 3 |  | 9.1753E6 |  |  |  | 2.7328E6 | 1.4164E5 |  |  |  |  | 1.5718E6 | 2.8923E6 | 5 | 5 | 0 | 5 | 0 | 0 | 0 | 5 | 1 | 0 | 0 | 0 | 0 | 2 | 1 | Y | 47564 | Vascular endothelial growth factor 2 [Micrurus fulvius] |
| 162 | 402 | JAC95037.1 | 148.24 | 13 | 0 | 3 | 0 | 0 | 0 | 8 | 4 | 0 | 0 | 0 | 0 | 3 | 3 |  | 9.1753E6 |  |  |  | 2.7328E6 | 1.4164E5 |  |  |  |  | 1.5718E6 | 2.8923E6 | 5 | 5 | 0 | 5 | 0 | 0 | 0 | 5 | 1 | 0 | 0 | 0 | 0 | 2 | 1 | Y | 47507 | Vascular endothelial growth factor C [Pantherophis guttatus] |
| 162 | 403 | XP\_034298588.1 | 148.24 | 13 | 0 | 3 | 0 | 0 | 0 | 8 | 4 | 0 | 0 | 0 | 0 | 3 | 3 |  | 9.1753E6 |  |  |  | 2.7328E6 | 1.4164E5 |  |  |  |  | 1.5718E6 | 2.8923E6 | 5 | 5 | 0 | 5 | 0 | 0 | 0 | 5 | 1 | 0 | 0 | 0 | 0 | 2 | 1 | Y | 47507 | vascular endothelial growth factor C isoform X1 [Pantherophis guttatus] |
| 162 | 404 | XP\_026522979.1 | 148.24 | 13 | 0 | 3 | 0 | 0 | 0 | 8 | 4 | 0 | 0 | 0 | 0 | 3 | 3 |  | 9.1753E6 |  |  |  | 2.7328E6 | 1.4164E5 |  |  |  |  | 1.5718E6 | 2.8923E6 | 5 | 5 | 0 | 5 | 0 | 0 | 0 | 5 | 1 | 0 | 0 | 0 | 0 | 2 | 1 | Y | 47528 | vascular endothelial growth factor C [Notechis scutatus] |
| 162 | 405 | ETE60014.1 | 148.24 | 13 | 0 | 3 | 0 | 0 | 0 | 8 | 4 | 0 | 0 | 0 | 0 | 3 | 3 |  | 9.1753E6 |  |  |  | 2.7328E6 | 1.4164E5 |  |  |  |  | 1.5718E6 | 2.8923E6 | 5 | 5 | 0 | 5 | 0 | 0 | 0 | 5 | 1 | 0 | 0 | 0 | 0 | 2 | 1 | Y | 47557 | Vascular endothelial growth factor C [Ophiophagus hannah] |
| 162 | 406 | XP\_026555459.1 | 148.24 | 13 | 0 | 3 | 0 | 0 | 0 | 8 | 4 | 0 | 0 | 0 | 0 | 3 | 3 |  | 9.1753E6 |  |  |  | 2.7328E6 | 1.4164E5 |  |  |  |  | 1.5718E6 | 2.8923E6 | 5 | 5 | 0 | 5 | 0 | 0 | 0 | 5 | 1 | 0 | 0 | 0 | 0 | 2 | 1 | Y | 47615 | vascular endothelial growth factor C [Pseudonaja textilis] |
| 162 | 407 | LAA17506.1 | 148.24 | 11 | 0 | 3 | 0 | 0 | 0 | 7 | 3 | 0 | 0 | 0 | 0 | 3 | 3 |  | 9.1753E6 |  |  |  | 2.7328E6 | 1.4164E5 |  |  |  |  | 1.5718E6 | 2.8923E6 | 5 | 5 | 0 | 5 | 0 | 0 | 0 | 5 | 1 | 0 | 0 | 0 | 0 | 2 | 1 | Y | 53369 | hypothetical protein, partial [Micrurus lemniscatus carvalhoi] |
| 162 | 408 | LAB17921.1 | 148.24 | 11 | 0 | 3 | 0 | 0 | 0 | 7 | 3 | 0 | 0 | 0 | 0 | 3 | 3 |  | 9.1753E6 |  |  |  | 2.7328E6 | 1.4164E5 |  |  |  |  | 1.5718E6 | 2.8923E6 | 5 | 5 | 0 | 5 | 0 | 0 | 0 | 5 | 1 | 0 | 0 | 0 | 0 | 2 | 1 | Y | 53456 | hypothetical protein, partial [Micrurus spixii] |
| 162 | 409 | XP\_034298590.1 | 148.24 | 15 | 0 | 4 | 0 | 0 | 0 | 9 | 5 | 0 | 0 | 0 | 0 | 4 | 4 |  | 9.1753E6 |  |  |  | 2.7328E6 | 1.4164E5 |  |  |  |  | 1.5718E6 | 2.8923E6 | 5 | 5 | 0 | 5 | 0 | 0 | 0 | 5 | 1 | 0 | 0 | 0 | 0 | 2 | 1 | Y | 40062 | vascular endothelial growth factor C isoform X3 [Pantherophis guttatus] |
| 162 | 410 | XP\_034298589.1 | 148.24 | 14 | 0 | 4 | 0 | 0 | 0 | 9 | 4 | 0 | 0 | 0 | 0 | 4 | 4 |  | 9.1753E6 |  |  |  | 2.7328E6 | 1.4164E5 |  |  |  |  | 1.5718E6 | 2.8923E6 | 5 | 5 | 0 | 5 | 0 | 0 | 0 | 5 | 1 | 0 | 0 | 0 | 0 | 2 | 1 | Y | 42840 | vascular endothelial growth factor C isoform X2 [Pantherophis guttatus] |
| 162 | 411 | JAC96561.1 | 148.24 | 13 | 0 | 3 | 0 | 0 | 0 | 8 | 4 | 0 | 0 | 0 | 0 | 3 | 3 |  | 9.1753E6 |  |  |  | 2.7328E6 | 1.4164E5 |  |  |  |  | 1.5718E6 | 2.8923E6 | 5 | 5 | 0 | 5 | 0 | 0 | 0 | 5 | 1 | 0 | 0 | 0 | 0 | 2 | 1 | Y | 47649 | Vascular endothelial growth factor C [Echis coloratus] |
| 162 | 412 | XP\_029139565.1 | 148.24 | 13 | 0 | 3 | 0 | 0 | 0 | 8 | 4 | 0 | 0 | 0 | 0 | 3 | 3 |  | 9.1753E6 |  |  |  | 2.7328E6 | 1.4164E5 |  |  |  |  | 1.5718E6 | 2.8923E6 | 5 | 5 | 0 | 5 | 0 | 0 | 0 | 5 | 1 | 0 | 0 | 0 | 0 | 2 | 1 | Y | 47709 | vascular endothelial growth factor C [Protobothrops mucrosquamatus] |
| 162 | 413 | JAI10614.1 | 148.24 | 13 | 0 | 3 | 0 | 0 | 0 | 8 | 4 | 0 | 0 | 0 | 0 | 3 | 3 |  | 9.1753E6 |  |  |  | 2.7328E6 | 1.4164E5 |  |  |  |  | 1.5718E6 | 2.8923E6 | 5 | 5 | 0 | 5 | 0 | 0 | 0 | 5 | 1 | 0 | 0 | 0 | 0 | 2 | 1 | Y | 47697 | Vascular endothelial growth factor C-like protein [Crotalus adamanteus] |
| 162 | 414 | JAV48556.1 | 148.24 | 13 | 0 | 3 | 0 | 0 | 0 | 8 | 4 | 0 | 0 | 0 | 0 | 3 | 3 |  | 9.1753E6 |  |  |  | 2.7328E6 | 1.4164E5 |  |  |  |  | 1.5718E6 | 2.8923E6 | 5 | 5 | 0 | 5 | 0 | 0 | 0 | 5 | 1 | 0 | 0 | 0 | 0 | 2 | 1 | Y | 47653 | Vascular endothelial growth factor C-like protein [Agkistrodon contortrix contortrix] |
| 162 | 415 | XP\_013912093.1 | 148.24 | 13 | 0 | 3 | 0 | 0 | 0 | 8 | 4 | 0 | 0 | 0 | 0 | 3 | 3 |  | 9.1753E6 |  |  |  | 2.7328E6 | 1.4164E5 |  |  |  |  | 1.5718E6 | 2.8923E6 | 5 | 5 | 0 | 5 | 0 | 0 | 0 | 5 | 1 | 0 | 0 | 0 | 0 | 2 | 1 | Y | 47484 | PREDICTED: vascular endothelial growth factor C [Thamnophis sirtalis] |
| 162 | 417 | JAC94973.1 | 148.24 | 15 | 0 | 4 | 0 | 0 | 0 | 9 | 5 | 0 | 0 | 0 | 0 | 4 | 4 |  | 9.1753E6 |  |  |  | 2.7328E6 | 1.4164E5 |  |  |  |  | 1.5718E6 | 2.8923E6 | 5 | 5 | 0 | 5 | 0 | 0 | 0 | 5 | 1 | 0 | 0 | 0 | 0 | 2 | 1 | Y | 40057 | Vascular endothelial growth factor C [Opheodrys aestivus] |
| 162 | 418 | SMD28268.1 | 148.24 | 13 | 0 | 3 | 0 | 0 | 0 | 8 | 4 | 0 | 0 | 0 | 0 | 3 | 3 |  | 9.1753E6 |  |  |  | 2.7328E6 | 1.4164E5 |  |  |  |  | 1.5718E6 | 2.8923E6 | 5 | 5 | 0 | 5 | 0 | 0 | 0 | 5 | 1 | 0 | 0 | 0 | 0 | 2 | 1 | Y | 47611 | vascular endothelial growth factor CdtVEGF3 [Crotalus durissus terrificus] |
| 162 | 419 | JAA95033.1 | 148.24 | 13 | 0 | 3 | 0 | 0 | 0 | 8 | 4 | 0 | 0 | 0 | 0 | 3 | 3 |  | 9.1753E6 |  |  |  | 2.7328E6 | 1.4164E5 |  |  |  |  | 1.5718E6 | 2.8923E6 | 5 | 5 | 0 | 5 | 0 | 0 | 0 | 5 | 1 | 0 | 0 | 0 | 0 | 2 | 1 | Y | 47639 | Vascular endothelial growth factor C-like protein [Crotalus horridus] |
| 162 | 443 | LAB17920.1 | 148.24 | 18 | 0 | 5 | 0 | 0 | 0 | 11 | 6 | 0 | 0 | 0 | 0 | 5 | 5 |  | 9.1753E6 |  |  |  | 2.7328E6 | 1.4164E5 |  |  |  |  | 1.5718E6 | 2.8923E6 | 5 | 5 | 0 | 5 | 0 | 0 | 0 | 5 | 1 | 0 | 0 | 0 | 0 | 2 | 1 | Y | 32584 | hypothetical protein [Micrurus spixii] |
| 189 | 639 | XP\_026575343.1 | 146.67 | 5 | 0 | 0 | 0 | 5 | 0 | 0 | 0 | 0 | 0 | 0 | 0 | 0 | 0 |  |  |  | 5.649E6 |  |  |  |  |  |  |  |  |  | 3 | 3 | 0 | 0 | 0 | 6 | 0 | 0 | 0 | 0 | 0 | 0 | 0 | 0 | 0 | N | 97316 | peptidyl-glycine alpha-amidating monooxygenase isoform X3 [Pseudonaja textilis] |
| 189 | 640 | XP\_026575334.1 | 146.67 | 5 | 0 | 0 | 0 | 5 | 0 | 0 | 0 | 0 | 0 | 0 | 0 | 0 | 0 |  |  |  | 5.649E6 |  |  |  |  |  |  |  |  |  | 3 | 3 | 0 | 0 | 0 | 6 | 0 | 0 | 0 | 0 | 0 | 0 | 0 | 0 | 0 | N | 109505 | peptidyl-glycine alpha-amidating monooxygenase isoform X2 [Pseudonaja textilis] |
| 189 | 641 | XP\_026575314.1 | 146.67 | 5 | 0 | 0 | 0 | 5 | 0 | 0 | 0 | 0 | 0 | 0 | 0 | 0 | 0 |  |  |  | 5.649E6 |  |  |  |  |  |  |  |  |  | 3 | 3 | 0 | 0 | 0 | 6 | 0 | 0 | 0 | 0 | 0 | 0 | 0 | 0 | 0 | N | 109576 | peptidyl-glycine alpha-amidating monooxygenase isoform X1 [Pseudonaja textilis] |
| 189 | 642 | XP\_026575325.1 | 146.67 | 5 | 0 | 0 | 0 | 5 | 0 | 0 | 0 | 0 | 0 | 0 | 0 | 0 | 0 |  |  |  | 5.649E6 |  |  |  |  |  |  |  |  |  | 3 | 3 | 0 | 0 | 0 | 6 | 0 | 0 | 0 | 0 | 0 | 0 | 0 | 0 | 0 | N | 109576 | peptidyl-glycine alpha-amidating monooxygenase isoform X1 [Pseudonaja textilis] |
| 189 | 643 | XP\_026575319.1 | 146.67 | 5 | 0 | 0 | 0 | 5 | 0 | 0 | 0 | 0 | 0 | 0 | 0 | 0 | 0 |  |  |  | 5.649E6 |  |  |  |  |  |  |  |  |  | 3 | 3 | 0 | 0 | 0 | 6 | 0 | 0 | 0 | 0 | 0 | 0 | 0 | 0 | 0 | N | 109576 | peptidyl-glycine alpha-amidating monooxygenase isoform X1 [Pseudonaja textilis] |
| 122 | 437 | XP\_026570451.1 | 145.89 | 7 | 0 | 0 | 0 | 2 | 5 | 3 | 0 | 2 | 3 | 3 | 2 | 2 | 2 |  |  |  |  | 1.4999E6 |  |  |  |  |  |  |  |  | 4 | 2 | 0 | 0 | 0 | 3 | 6 | 4 | 0 | 1 | 3 | 6 | 1 | 2 | 1 | N | 62685 | keratin, type II cytoskeletal 5-like [Pseudonaja textilis] |
| 150 | 357 | AAM51550.1 | 143.55 | 8 | 0 | 2 | 0 | 2 | 6 | 0 | 0 | 0 | 0 | 4 | 2 | 2 | 2 |  |  |  |  | 3.8745E6 |  |  |  |  |  |  |  |  | 4 | 2 | 0 | 1 | 0 | 2 | 9 | 0 | 0 | 0 | 0 | 4 | 1 | 2 | 2 | Y | 68176 | mocarhagin 1 [Naja mossambica] |
| 150 | 358 | Q10749.3 | 143.55 | 8 | 0 | 2 | 0 | 2 | 6 | 0 | 0 | 0 | 0 | 4 | 2 | 2 | 2 |  |  |  |  | 3.8745E6 |  |  |  |  |  |  |  |  | 4 | 2 | 0 | 1 | 0 | 2 | 9 | 0 | 0 | 0 | 0 | 4 | 1 | 2 | 2 | Y | 68176 | RecName: Full=Snake venom metalloproteinase-disintegrin-like mocarhagin; Short=MOC; Short=Mocarhagin-1; Short=SVMP; AltName: Full=Zinc metalloproteinase mocarhagin; Flags: Precursor |
| 193 | 624 | P18964.1 | 143.53 | 28 | 0 | 0 | 6 | 28 | 0 | 0 | 0 | 0 | 0 | 0 | 0 | 0 | 0 |  |  | 3.2408E5 | 1.5397E7 |  |  |  |  |  |  |  |  |  | 4 | 4 | 0 | 0 | 1 | 9 | 0 | 0 | 0 | 0 | 0 | 0 | 0 | 0 | 0 | Y | 26182 | RecName: Full=Factor V activator RVV-V alpha; AltName: Full=Russel's viper venom FV activator alpha; Short=RVV-V alpha; AltName: Full=Snake venom serine protease; Short=SVSP |
| 193 | 654 | P18965.2 | 143.53 | 25 | 0 | 0 | 5 | 25 | 0 | 0 | 0 | 0 | 0 | 0 | 0 | 0 | 0 |  |  | 3.2408E5 | 1.5397E7 |  |  |  |  |  |  |  |  |  | 4 | 4 | 0 | 0 | 1 | 9 | 0 | 0 | 0 | 0 | 0 | 0 | 0 | 0 | 0 | Y | 28823 | RecName: Full=Factor V activator RVV-V gamma; AltName: Full=Russel's viper venom FV activator gamma; Short=RVV-V gamma; AltName: Full=Snake venom serine protease; Short=SVSP; Flags: Precursor |
| 193 | 673 | ADP88558.1 | 143.53 | 25 | 0 | 0 | 5 | 25 | 0 | 0 | 0 | 0 | 0 | 0 | 0 | 0 | 0 |  |  | 3.2408E5 | 1.5397E7 |  |  |  |  |  |  |  |  |  | 4 | 4 | 0 | 0 | 1 | 9 | 0 | 0 | 0 | 0 | 0 | 0 | 0 | 0 | 0 | Y | 28814 | RVV-V gamma-like protein precursor [Daboia siamensis] |
| 148 | 390 | LAA86163.1 | 141.65 | 8 | 0 | 0 | 0 | 0 | 0 | 2 | 2 | 2 | 2 | 6 | 0 | 0 | 0 |  |  |  |  |  | 1.6308E7 | 1.6776E6 | 2.4729E5 | 9.5826E6 | 1.3887E7 |  |  |  | 4 | 4 | 0 | 0 | 0 | 0 | 0 | 5 | 5 | 2 | 4 | 4 | 0 | 0 | 0 | Y | 67294 | hypothetical protein [Micrurus lemniscatus lemniscatus] |
| 111 | 2144 | JAA75028.1 | 141.41 | 14 | 0 | 0 | 0 | 0 | 0 | 0 | 0 | 0 | 0 | 14 | 8 | 8 | 0 |  |  |  |  |  |  |  |  |  | 7.4633E6 |  |  |  | 4 | 1 | 0 | 0 | 0 | 0 | 0 | 0 | 0 | 0 | 0 | 22 | 26 | 1 | 0 | Y | 16452 | PLA2-Sut-1 [Suta fasciata] |
| 191 | 439 | AAZ39880.1 | 141.32 | 7 | 0 | 0 | 0 | 5 | 0 | 0 | 0 | 0 | 0 | 0 | 2 | 2 | 0 |  |  |  | 6.8373E6 |  |  |  |  |  |  |  |  |  | 3 | 2 | 0 | 0 | 0 | 5 | 0 | 0 | 0 | 0 | 0 | 0 | 1 | 1 | 0 | Y | 69555 | hemorrhagic metalloproteinase russelysin [Daboia russelii] |
| 191 | 440 | B8K1W0.1 | 141.32 | 7 | 0 | 0 | 0 | 5 | 0 | 0 | 0 | 0 | 0 | 0 | 2 | 2 | 0 |  |  |  | 6.8373E6 |  |  |  |  |  |  |  |  |  | 3 | 2 | 0 | 0 | 0 | 5 | 0 | 0 | 0 | 0 | 0 | 0 | 1 | 1 | 0 | Y | 69555 | RecName: Full=Zinc metalloproteinase-disintegrin-like daborhagin-K; AltName: Full=Haemorrhagic metalloproteinase russelysin; AltName: Full=Snake venom metalloproteinase; Short=SVMP; Flags: Precursor |
| 146 | 982 | Q9W727.1 | 141.07 | 43 | 0 | 0 | 0 | 0 | 0 | 0 | 0 | 0 | 0 | 43 | 12 | 0 | 0 |  |  |  |  |  |  |  |  |  | 1.6607E6 |  |  |  | 4 | 1 | 0 | 0 | 0 | 0 | 0 | 0 | 0 | 0 | 0 | 8 | 7 | 0 | 0 | Y | 9934 | RecName: Full=Muscarinic toxin-like protein; Flags: Precursor |
| 146 | 983 | Q9DEQ3.1 | 141.07 | 43 | 0 | 0 | 0 | 0 | 0 | 0 | 0 | 0 | 0 | 43 | 12 | 0 | 0 |  |  |  |  |  |  |  |  |  | 1.6607E6 |  |  |  | 4 | 1 | 0 | 0 | 0 | 0 | 0 | 0 | 0 | 0 | 0 | 8 | 7 | 0 | 0 | Y | 9962 | RecName: Full=Neurotoxin homolog NL1; Flags: Precursor |
| 146 | 984 | CAB50691.1 | 141.07 | 43 | 0 | 0 | 0 | 0 | 0 | 0 | 0 | 0 | 0 | 43 | 12 | 0 | 0 |  |  |  |  |  |  |  |  |  | 1.6607E6 |  |  |  | 4 | 1 | 0 | 0 | 0 | 0 | 0 | 0 | 0 | 0 | 0 | 8 | 7 | 0 | 0 | Y | 9934 | snake venom [Bungarus multicinctus multicinctus] |
| 146 | 985 | CAC08183.1 | 141.07 | 43 | 0 | 0 | 0 | 0 | 0 | 0 | 0 | 0 | 0 | 43 | 12 | 0 | 0 |  |  |  |  |  |  |  |  |  | 1.6607E6 |  |  |  | 4 | 1 | 0 | 0 | 0 | 0 | 0 | 0 | 0 | 0 | 0 | 8 | 7 | 0 | 0 | Y | 9962 | unnamed protein product (plasmid) [Naja atra] |
| 175 | 1218 | XP\_032075406.1 | 137.06 | 4 | 0 | 0 | 0 | 0 | 0 | 0 | 0 | 0 | 4 | 0 | 0 | 0 | 0 |  |  |  |  |  |  |  |  | 4.9768E7 |  |  |  |  | 2 | 2 | 0 | 0 | 0 | 0 | 0 | 0 | 0 | 0 | 12 | 0 | 0 | 0 | 0 | Y | 85050 | LOW QUALITY PROTEIN: amyloid-beta precursor protein [Thamnophis elegans] |
| 175 | 1221 | XP\_013912685.1 | 137.06 | 4 | 0 | 0 | 0 | 0 | 0 | 0 | 0 | 0 | 4 | 0 | 0 | 0 | 0 |  |  |  |  |  |  |  |  | 4.9768E7 |  |  |  |  | 2 | 2 | 0 | 0 | 0 | 0 | 0 | 0 | 0 | 0 | 12 | 0 | 0 | 0 | 0 | Y | 84599 | PREDICTED: amyloid beta A4 protein isoform X1 [Thamnophis sirtalis] |
| 175 | 1228 | LAB26479.1 | 137.06 | 4 | 0 | 0 | 0 | 0 | 0 | 0 | 0 | 0 | 4 | 0 | 0 | 0 | 0 |  |  |  |  |  |  |  |  | 4.9768E7 |  |  |  |  | 2 | 2 | 0 | 0 | 0 | 0 | 0 | 0 | 0 | 0 | 12 | 0 | 0 | 0 | 0 | Y | 84918 | hypothetical protein [Micrurus spixii] |
| 175 | 1240 | XP\_026519907.1 | 137.06 | 4 | 0 | 0 | 0 | 0 | 0 | 0 | 0 | 0 | 4 | 0 | 0 | 0 | 0 |  |  |  |  |  |  |  |  | 4.9768E7 |  |  |  |  | 2 | 2 | 0 | 0 | 0 | 0 | 0 | 0 | 0 | 0 | 12 | 0 | 0 | 0 | 0 | Y | 82911 | amyloid-beta A4 protein isoform X2 [Notechis scutatus] |
| 175 | 1241 | XP\_026556422.1 | 137.06 | 4 | 0 | 0 | 0 | 0 | 0 | 0 | 0 | 0 | 4 | 0 | 0 | 0 | 0 |  |  |  |  |  |  |  |  | 4.9768E7 |  |  |  |  | 2 | 2 | 0 | 0 | 0 | 0 | 0 | 0 | 0 | 0 | 12 | 0 | 0 | 0 | 0 | Y | 82796 | amyloid-beta A4 protein isoform X2 [Pseudonaja textilis] |
| 175 | 1242 | XP\_015676850.1 | 137.06 | 4 | 0 | 0 | 0 | 0 | 0 | 0 | 0 | 0 | 4 | 0 | 0 | 0 | 0 |  |  |  |  |  |  |  |  | 4.9768E7 |  |  |  |  | 2 | 2 | 0 | 0 | 0 | 0 | 0 | 0 | 0 | 0 | 12 | 0 | 0 | 0 | 0 | Y | 82886 | amyloid-beta precursor protein isoform X2 [Protobothrops mucrosquamatus] |
| 175 | 1250 | JAV51273.1 | 137.06 | 4 | 0 | 0 | 0 | 0 | 0 | 0 | 0 | 0 | 4 | 0 | 0 | 0 | 0 |  |  |  |  |  |  |  |  | 4.9768E7 |  |  |  |  | 2 | 2 | 0 | 0 | 0 | 0 | 0 | 0 | 0 | 0 | 12 | 0 | 0 | 0 | 0 | Y | 84882 | amyloid beta A4 protein-like protein [Agkistrodon contortrix contortrix] |
| 175 | 1251 | AFJ49397.1 | 137.06 | 4 | 0 | 0 | 0 | 0 | 0 | 0 | 0 | 0 | 4 | 0 | 0 | 0 | 0 |  |  |  |  |  |  |  |  | 4.9768E7 |  |  |  |  | 2 | 2 | 0 | 0 | 0 | 0 | 0 | 0 | 0 | 0 | 12 | 0 | 0 | 0 | 0 | Y | 84854 | Amyloid beta A4 protein-like [Crotalus adamanteus] |
| 175 | 1252 | JAG47493.1 | 137.06 | 4 | 0 | 0 | 0 | 0 | 0 | 0 | 0 | 0 | 4 | 0 | 0 | 0 | 0 |  |  |  |  |  |  |  |  | 4.9768E7 |  |  |  |  | 2 | 2 | 0 | 0 | 0 | 0 | 0 | 0 | 0 | 0 | 12 | 0 | 0 | 0 | 0 | Y | 84854 | amyloid beta A4 protein-like protein [Crotalus horridus] |
| 175 | 1253 | JAG45861.1 | 137.06 | 4 | 0 | 0 | 0 | 0 | 0 | 0 | 0 | 0 | 4 | 0 | 0 | 0 | 0 |  |  |  |  |  |  |  |  | 4.9768E7 |  |  |  |  | 2 | 2 | 0 | 0 | 0 | 0 | 0 | 0 | 0 | 0 | 12 | 0 | 0 | 0 | 0 | Y | 84854 | amyloid beta A4 protein-like protein [Crotalus horridus] |
| 175 | 1254 | JAI14367.1 | 137.06 | 4 | 0 | 0 | 0 | 0 | 0 | 0 | 0 | 0 | 4 | 0 | 0 | 0 | 0 |  |  |  |  |  |  |  |  | 4.9768E7 |  |  |  |  | 2 | 2 | 0 | 0 | 0 | 0 | 0 | 0 | 0 | 0 | 12 | 0 | 0 | 0 | 0 | Y | 84854 | amyloid beta A4 protein-like protein [Crotalus adamanteus] |
| 175 | 1255 | JAA97793.1 | 137.06 | 4 | 0 | 0 | 0 | 0 | 0 | 0 | 0 | 0 | 4 | 0 | 0 | 0 | 0 |  |  |  |  |  |  |  |  | 4.9768E7 |  |  |  |  | 2 | 2 | 0 | 0 | 0 | 0 | 0 | 0 | 0 | 0 | 12 | 0 | 0 | 0 | 0 | Y | 84854 | amyloid beta A4 protein-like protein [Crotalus horridus] |
| 175 | 1256 | XP\_034283168.1 | 137.06 | 4 | 0 | 0 | 0 | 0 | 0 | 0 | 0 | 0 | 4 | 0 | 0 | 0 | 0 |  |  |  |  |  |  |  |  | 4.9768E7 |  |  |  |  | 2 | 2 | 0 | 0 | 0 | 0 | 0 | 0 | 0 | 0 | 12 | 0 | 0 | 0 | 0 | Y | 84566 | amyloid-beta precursor protein isoform X1 [Pantherophis guttatus] |
| 175 | 1257 | XP\_026519906.1 | 137.06 | 4 | 0 | 0 | 0 | 0 | 0 | 0 | 0 | 0 | 4 | 0 | 0 | 0 | 0 |  |  |  |  |  |  |  |  | 4.9768E7 |  |  |  |  | 2 | 2 | 0 | 0 | 0 | 0 | 0 | 0 | 0 | 0 | 12 | 0 | 0 | 0 | 0 | Y | 84815 | amyloid-beta A4 protein isoform X1 [Notechis scutatus] |
| 175 | 1258 | XP\_026556420.1 | 137.06 | 4 | 0 | 0 | 0 | 0 | 0 | 0 | 0 | 0 | 4 | 0 | 0 | 0 | 0 |  |  |  |  |  |  |  |  | 4.9768E7 |  |  |  |  | 2 | 2 | 0 | 0 | 0 | 0 | 0 | 0 | 0 | 0 | 12 | 0 | 0 | 0 | 0 | Y | 84700 | amyloid-beta A4 protein isoform X1 [Pseudonaja textilis] |
| 175 | 1259 | XP\_013912686.1 | 137.06 | 5 | 0 | 0 | 0 | 0 | 0 | 0 | 0 | 0 | 5 | 0 | 0 | 0 | 0 |  |  |  |  |  |  |  |  | 4.9768E7 |  |  |  |  | 2 | 2 | 0 | 0 | 0 | 0 | 0 | 0 | 0 | 0 | 12 | 0 | 0 | 0 | 0 | Y | 78403 | PREDICTED: amyloid beta A4 protein isoform X2 [Thamnophis sirtalis] |
| 175 | 1260 | XP\_034283217.1 | 137.06 | 5 | 0 | 0 | 0 | 0 | 0 | 0 | 0 | 0 | 5 | 0 | 0 | 0 | 0 |  |  |  |  |  |  |  |  | 4.9768E7 |  |  |  |  | 2 | 2 | 0 | 0 | 0 | 0 | 0 | 0 | 0 | 0 | 12 | 0 | 0 | 0 | 0 | Y | 78383 | amyloid-beta precursor protein isoform X2 [Pantherophis guttatus] |
| 175 | 1261 | XP\_015676849.1 | 137.06 | 4 | 0 | 0 | 0 | 0 | 0 | 0 | 0 | 0 | 4 | 0 | 0 | 0 | 0 |  |  |  |  |  |  |  |  | 4.9768E7 |  |  |  |  | 2 | 2 | 0 | 0 | 0 | 0 | 0 | 0 | 0 | 0 | 12 | 0 | 0 | 0 | 0 | Y | 84789 | amyloid-beta precursor protein isoform X1 [Protobothrops mucrosquamatus] |
| 175 | 1327 | JAI10297.1 | 137.06 | 4 | 0 | 0 | 0 | 0 | 0 | 0 | 0 | 0 | 4 | 0 | 0 | 0 | 0 |  |  |  |  |  |  |  |  | 4.9768E7 |  |  |  |  | 2 | 2 | 0 | 0 | 0 | 0 | 0 | 0 | 0 | 0 | 12 | 0 | 0 | 0 | 0 | Y | 84624 | amyloid beta A4 protein-like protein [Micrurus fulvius] |
| 175 | 1577 | JAG68744.1 | 137.06 | 4 | 0 | 0 | 0 | 0 | 0 | 0 | 0 | 0 | 4 | 0 | 0 | 0 | 0 |  |  |  |  |  |  |  |  | 4.9768E7 |  |  |  |  | 2 | 2 | 0 | 0 | 0 | 0 | 0 | 0 | 0 | 0 | 12 | 0 | 0 | 0 | 0 | Y | 84419 | amyloid beta A4 protein-like protein [Boiga irregularis] |
| 175 | 1578 | ETE71713.1 | 137.06 | 4 | 0 | 0 | 0 | 0 | 0 | 0 | 0 | 0 | 4 | 0 | 0 | 0 | 0 |  |  |  |  |  |  |  |  | 4.9768E7 |  |  |  |  | 2 | 2 | 0 | 0 | 0 | 0 | 0 | 0 | 0 | 0 | 12 | 0 | 0 | 0 | 0 | Y | 83462 | Amyloid beta A4 protein, partial [Ophiophagus hannah] |
| 179 | 360 | XP\_026556851.1 | 135.58 | 10 | 0 | 1 | 0 | 2 | 0 | 0 | 0 | 0 | 0 | 7 | 0 | 1 | 1 |  | 1.8048E6 |  | 1.5704E5 |  |  |  |  |  | 1.672E7 |  | 0 | 0 | 5 | 5 | 0 | 1 | 0 | 1 | 0 | 0 | 0 | 0 | 0 | 7 | 0 | 1 | 2 | Y | 81535 | hepatocyte growth factor isoform X2 [Pseudonaja textilis] |
| 179 | 361 | XP\_026556843.1 | 135.58 | 10 | 0 | 1 | 0 | 2 | 0 | 0 | 0 | 0 | 0 | 7 | 0 | 1 | 1 |  | 1.8048E6 |  | 1.5704E5 |  |  |  |  |  | 1.672E7 |  | 0 | 0 | 5 | 5 | 0 | 1 | 0 | 1 | 0 | 0 | 0 | 0 | 0 | 7 | 0 | 1 | 2 | Y | 82067 | hepatocyte growth factor isoform X1 [Pseudonaja textilis] |
| 158 | 2050 | 1COD | 134.23 | 61 | 61 | 0 | 0 | 0 | 0 | 61 | 0 | 0 | 0 | 0 | 0 | 0 | 0 | 9.9654E6 |  |  |  |  | 1.1582E8 |  |  |  |  |  |  |  | 3 | 2 | 9 | 0 | 0 | 0 | 0 | 8 | 0 | 0 | 0 | 0 | 0 | 0 | 0 | Y | 6957 | Chain A, COBROTOXIN |
| 158 | 2051 | 1V6P | 134.23 | 61 | 61 | 0 | 0 | 0 | 0 | 61 | 0 | 0 | 0 | 0 | 0 | 0 | 0 | 9.9654E6 |  |  |  |  | 1.1582E8 |  |  |  |  |  |  |  | 3 | 2 | 9 | 0 | 0 | 0 | 0 | 8 | 0 | 0 | 0 | 0 | 0 | 0 | 0 | Y | 6957 | Chain B, Cobrotoxin |
| 158 | 2052 | 1COE | 134.23 | 61 | 61 | 0 | 0 | 0 | 0 | 61 | 0 | 0 | 0 | 0 | 0 | 0 | 0 | 9.9654E6 |  |  |  |  | 1.1582E8 |  |  |  |  |  |  |  | 3 | 2 | 9 | 0 | 0 | 0 | 0 | 8 | 0 | 0 | 0 | 0 | 0 | 0 | 0 | Y | 6957 | Chain A, COBROTOXIN |
| 158 | 2053 | AAB25735.1 | 134.23 | 61 | 61 | 0 | 0 | 0 | 0 | 61 | 0 | 0 | 0 | 0 | 0 | 0 | 0 | 9.9654E6 |  |  |  |  | 1.1582E8 |  |  |  |  |  |  |  | 3 | 2 | 9 | 0 | 0 | 0 | 0 | 8 | 0 | 0 | 0 | 0 | 0 | 0 | 0 | Y | 6957 | neurotoxin, NTX [Naja naja=Formosan cobra, ssp. atra, venom, Peptide, 62 aa] |
| 158 | 2054 | ADN67584.1 | 134.23 | 59 | 59 | 0 | 0 | 0 | 0 | 59 | 0 | 0 | 0 | 0 | 0 | 0 | 0 | 9.9654E6 |  |  |  |  | 1.1582E8 |  |  |  |  |  |  |  | 3 | 2 | 9 | 0 | 0 | 0 | 0 | 8 | 0 | 0 | 0 | 0 | 0 | 0 | 0 | Y | 7221 | three-finger toxin precursor, partial [Naja atra] |
| 158 | 2055 | AAB36932.1 | 134.23 | 46 | 46 | 0 | 0 | 0 | 0 | 46 | 0 | 0 | 0 | 0 | 0 | 0 | 0 | 9.9654E6 |  |  |  |  | 1.1582E8 |  |  |  |  |  |  |  | 3 | 2 | 9 | 0 | 0 | 0 | 0 | 8 | 0 | 0 | 0 | 0 | 0 | 0 | 0 | Y | 9262 | cobrotoxin''' [Naja atra] |
| 158 | 2056 | Q9PTT0.1 | 134.23 | 46 | 46 | 0 | 0 | 0 | 0 | 46 | 0 | 0 | 0 | 0 | 0 | 0 | 0 | 9.9654E6 |  |  |  |  | 1.1582E8 |  |  |  |  |  |  |  | 3 | 2 | 9 | 0 | 0 | 0 | 0 | 8 | 0 | 0 | 0 | 0 | 0 | 0 | 0 | Y | 9262 | RecName: Full=Cobrotoxin homolog; AltName: Full=Short neurotoxin; Flags: Precursor |
| 158 | 2057 | P60770.1 | 134.23 | 46 | 46 | 0 | 0 | 0 | 0 | 46 | 0 | 0 | 0 | 0 | 0 | 0 | 0 | 9.9654E6 |  |  |  |  | 1.1582E8 |  |  |  |  |  |  |  | 3 | 2 | 9 | 0 | 0 | 0 | 0 | 8 | 0 | 0 | 0 | 0 | 0 | 0 | 0 | Y | 9262 | RecName: Full=Cobrotoxin; Short=CBT; AltName: Full=Atratoxin; AltName: Full=Short neurotoxin 1; Flags: Precursor |
| 158 | 2058 | P60771.2 | 134.23 | 46 | 46 | 0 | 0 | 0 | 0 | 46 | 0 | 0 | 0 | 0 | 0 | 0 | 0 | 9.9654E6 |  |  |  |  | 1.1582E8 |  |  |  |  |  |  |  | 3 | 2 | 9 | 0 | 0 | 0 | 0 | 8 | 0 | 0 | 0 | 0 | 0 | 0 | 0 | Y | 9262 | RecName: Full=Cobrotoxin; Short=CBT; AltName: Full=Short neurotoxin I; Short=NT1; Flags: Precursor |
| 158 | 2059 | AAY63884.1 | 134.23 | 46 | 46 | 0 | 0 | 0 | 0 | 46 | 0 | 0 | 0 | 0 | 0 | 0 | 0 | 9.9654E6 |  |  |  |  | 1.1582E8 |  |  |  |  |  |  |  | 3 | 2 | 9 | 0 | 0 | 0 | 0 | 8 | 0 | 0 | 0 | 0 | 0 | 0 | 0 | Y | 9262 | neurotoxin [Naja kaouthia] |
| 158 | 2060 | AAB36930.1 | 134.23 | 46 | 46 | 0 | 0 | 0 | 0 | 46 | 0 | 0 | 0 | 0 | 0 | 0 | 0 | 9.9654E6 |  |  |  |  | 1.1582E8 |  |  |  |  |  |  |  | 3 | 2 | 9 | 0 | 0 | 0 | 0 | 8 | 0 | 0 | 0 | 0 | 0 | 0 | 0 | Y | 9262 | cobrotoxin' [Naja atra] |
| 158 | 2061 | AAB03222.1 | 134.23 | 46 | 46 | 0 | 0 | 0 | 0 | 46 | 0 | 0 | 0 | 0 | 0 | 0 | 0 | 9.9654E6 |  |  |  |  | 1.1582E8 |  |  |  |  |  |  |  | 3 | 2 | 9 | 0 | 0 | 0 | 0 | 8 | 0 | 0 | 0 | 0 | 0 | 0 | 0 | Y | 9262 | cobrotoxin'' [Naja atra] |
| 158 | 2062 | CAA73097.2 | 134.23 | 46 | 46 | 0 | 0 | 0 | 0 | 46 | 0 | 0 | 0 | 0 | 0 | 0 | 0 | 9.9654E6 |  |  |  |  | 1.1582E8 |  |  |  |  |  |  |  | 3 | 2 | 9 | 0 | 0 | 0 | 0 | 8 | 0 | 0 | 0 | 0 | 0 | 0 | 0 | Y | 9262 | cobrotoxin [Naja atra] |
| 158 | 2063 | AAB03221.1 | 134.23 | 46 | 46 | 0 | 0 | 0 | 0 | 46 | 0 | 0 | 0 | 0 | 0 | 0 | 0 | 9.9654E6 |  |  |  |  | 1.1582E8 |  |  |  |  |  |  |  | 3 | 2 | 9 | 0 | 0 | 0 | 0 | 8 | 0 | 0 | 0 | 0 | 0 | 0 | 0 | Y | 9262 | cobrotoxin' [Naja atra] |
| 158 | 2064 | ADN67574.1 | 134.23 | 46 | 46 | 0 | 0 | 0 | 0 | 46 | 0 | 0 | 0 | 0 | 0 | 0 | 0 | 9.9654E6 |  |  |  |  | 1.1582E8 |  |  |  |  |  |  |  | 3 | 2 | 9 | 0 | 0 | 0 | 0 | 8 | 0 | 0 | 0 | 0 | 0 | 0 | 0 | Y | 9262 | three-finger toxin precursor [Naja atra] |
| 158 | 2065 | AAB03223.1 | 134.23 | 46 | 46 | 0 | 0 | 0 | 0 | 46 | 0 | 0 | 0 | 0 | 0 | 0 | 0 | 9.9654E6 |  |  |  |  | 1.1582E8 |  |  |  |  |  |  |  | 3 | 2 | 9 | 0 | 0 | 0 | 0 | 8 | 0 | 0 | 0 | 0 | 0 | 0 | 0 | Y | 9262 | cobrotoxin''' [Naja atra] |
| 158 | 2066 | AAB36931.1 | 134.23 | 46 | 46 | 0 | 0 | 0 | 0 | 46 | 0 | 0 | 0 | 0 | 0 | 0 | 0 | 9.9654E6 |  |  |  |  | 1.1582E8 |  |  |  |  |  |  |  | 3 | 2 | 9 | 0 | 0 | 0 | 0 | 8 | 0 | 0 | 0 | 0 | 0 | 0 | 0 | Y | 9262 | cobrotoxin'' [Naja atra] |
| 158 | 2067 | AAB01538.1 | 134.23 | 46 | 46 | 0 | 0 | 0 | 0 | 46 | 0 | 0 | 0 | 0 | 0 | 0 | 0 | 9.9654E6 |  |  |  |  | 1.1582E8 |  |  |  |  |  |  |  | 3 | 2 | 9 | 0 | 0 | 0 | 0 | 8 | 0 | 0 | 0 | 0 | 0 | 0 | 0 | Y | 9262 | cobrotoxin [Naja atra] |
| 158 | 2068 | AAF21774.1 | 134.23 | 46 | 46 | 0 | 0 | 0 | 0 | 46 | 0 | 0 | 0 | 0 | 0 | 0 | 0 | 9.9654E6 |  |  |  |  | 1.1582E8 |  |  |  |  |  |  |  | 3 | 2 | 9 | 0 | 0 | 0 | 0 | 8 | 0 | 0 | 0 | 0 | 0 | 0 | 0 | Y | 9262 | neurotoxin preprotein precursor [Naja naja] |
| 158 | 2069 | AAR33035.1 | 134.23 | 46 | 46 | 0 | 0 | 0 | 0 | 46 | 0 | 0 | 0 | 0 | 0 | 0 | 0 | 9.9654E6 |  |  |  |  | 1.1582E8 |  |  |  |  |  |  |  | 3 | 2 | 9 | 0 | 0 | 0 | 0 | 8 | 0 | 0 | 0 | 0 | 0 | 0 | 0 | Y | 9262 | atratoxin [Naja atra] |
| 113 | 1841 | LAB46845.1 | 133.22 | 23 | 0 | 0 | 0 | 0 | 0 | 0 | 0 | 0 | 0 | 11 | 11 | 11 | 23 |  |  |  |  |  |  |  |  |  |  |  |  | 4.6144E7 | 4 | 1 | 0 | 0 | 0 | 0 | 0 | 0 | 0 | 0 | 0 | 20 | 26 | 1 | 1 | Y | 12553 | hypothetical protein, partial [Micrurus spixii] |
| 113 | 1978 | LAA90412.1 | 133.22 | 23 | 0 | 0 | 0 | 0 | 0 | 0 | 0 | 0 | 0 | 11 | 11 | 11 | 23 |  |  |  |  |  |  |  |  |  |  |  |  | 4.6144E7 | 4 | 1 | 0 | 0 | 0 | 0 | 0 | 0 | 0 | 0 | 0 | 20 | 26 | 1 | 1 | Y | 12188 | hypothetical protein, partial [Micrurus lemniscatus lemniscatus] |
| 113 | 1979 | LAA90421.1 | 133.22 | 23 | 0 | 0 | 0 | 0 | 0 | 0 | 0 | 0 | 0 | 11 | 11 | 11 | 23 |  |  |  |  |  |  |  |  |  |  |  |  | 4.6144E7 | 4 | 1 | 0 | 0 | 0 | 0 | 0 | 0 | 0 | 0 | 0 | 20 | 26 | 1 | 1 | Y | 12261 | hypothetical protein, partial [Micrurus lemniscatus lemniscatus] |
| 113 | 2175 | LAB46837.1 | 133.22 | 23 | 0 | 0 | 0 | 0 | 0 | 0 | 0 | 0 | 0 | 11 | 11 | 11 | 23 |  |  |  |  |  |  |  |  |  |  |  |  | 4.6144E7 | 4 | 1 | 0 | 0 | 0 | 0 | 0 | 0 | 0 | 0 | 0 | 20 | 26 | 1 | 1 | Y | 12466 | hypothetical protein, partial [Micrurus spixii] |
| 113 | 2176 | LAB46855.1 | 133.22 | 23 | 0 | 0 | 0 | 0 | 0 | 0 | 0 | 0 | 0 | 11 | 11 | 11 | 23 |  |  |  |  |  |  |  |  |  |  |  |  | 4.6144E7 | 4 | 1 | 0 | 0 | 0 | 0 | 0 | 0 | 0 | 0 | 0 | 20 | 26 | 1 | 1 | Y | 12539 | hypothetical protein, partial [Micrurus spixii] |
| 131 | 622 | XP\_032067281.1 | 133.02 | 5 | 0 | 0 | 0 | 2 | 2 | 4 | 2 | 2 | 4 | 4 | 2 | 2 | 2 |  |  |  |  |  |  | 3.5293E6 |  |  |  |  |  |  | 3 | 1 | 0 | 0 | 0 | 3 | 2 | 4 | 1 | 1 | 3 | 6 | 1 | 2 | 1 | N | 59706 | keratin, type II cytoskeletal 6A-like [Thamnophis elegans] |
| 131 | 623 | XP\_013915209.1 | 133.02 | 5 | 0 | 0 | 0 | 2 | 2 | 4 | 2 | 2 | 4 | 4 | 2 | 2 | 2 |  |  |  |  |  |  | 3.5293E6 |  |  |  |  |  |  | 3 | 1 | 0 | 0 | 0 | 3 | 2 | 4 | 1 | 1 | 3 | 6 | 1 | 2 | 1 | N | 59764 | PREDICTED: keratin, type II cytoskeletal 6A-like [Thamnophis sirtalis] |
| 137 | 1337 | P86368.1 | 132.62 | 24 | 0 | 8 | 8 | 23 | 0 | 0 | 0 | 0 | 0 | 8 | 9 | 0 | 0 |  |  |  |  |  |  |  |  |  |  | 2.1087E5 |  |  | 3 | 1 | 0 | 2 | 1 | 15 | 0 | 0 | 0 | 0 | 0 | 1 | 1 | 0 | 0 | Y | 13687 | RecName: Full=Basic phospholipase A2 3; Short=PLA23; Short=svPLA2; AltName: Full=Phosphatidylcholine 2-acylhydrolase |
| 163 | 594 | JAG66642.1 | 132.14 | 30 | 24 | 0 | 0 | 10 | 0 | 0 | 0 | 0 | 16 | 0 | 0 | 0 | 0 | 2.1518E7 |  |  | 8.563E5 |  |  |  |  | 1.0437E6 |  |  |  |  | 4 | 4 | 11 | 0 | 0 | 1 | 0 | 0 | 0 | 0 | 2 | 0 | 0 | 0 | 0 | N | 17979 | ubiquitin-40S ribosomal protein S27a [Boiga irregularis] |
| 163 | 595 | JAB53407.1 | 132.14 | 30 | 24 | 0 | 0 | 10 | 0 | 0 | 0 | 0 | 16 | 0 | 0 | 0 | 0 | 2.1518E7 |  |  | 8.563E5 |  |  |  |  | 1.0437E6 |  |  |  |  | 4 | 4 | 11 | 0 | 0 | 1 | 0 | 0 | 0 | 0 | 2 | 0 | 0 | 0 | 0 | N | 17979 | Ubiquitin-40S ribosomal protein S27a [Micrurus fulvius] |
| 163 | 596 | JAI11580.1 | 132.14 | 30 | 24 | 0 | 0 | 10 | 0 | 0 | 0 | 0 | 16 | 0 | 0 | 0 | 0 | 2.1518E7 |  |  | 8.563E5 |  |  |  |  | 1.0437E6 |  |  |  |  | 4 | 4 | 11 | 0 | 0 | 1 | 0 | 0 | 0 | 0 | 2 | 0 | 0 | 0 | 0 | N | 17979 | ubiquitin-40S ribosomal protein S27a [Crotalus adamanteus] |
| 163 | 597 | XP\_015686894.1 | 132.14 | 30 | 24 | 0 | 0 | 10 | 0 | 0 | 0 | 0 | 16 | 0 | 0 | 0 | 0 | 2.1518E7 |  |  | 8.563E5 |  |  |  |  | 1.0437E6 |  |  |  |  | 4 | 4 | 11 | 0 | 0 | 1 | 0 | 0 | 0 | 0 | 2 | 0 | 0 | 0 | 0 | N | 17979 | ubiquitin-40S ribosomal protein S27a [Protobothrops mucrosquamatus] |
| 163 | 598 | XP\_007432078.1 | 132.14 | 30 | 24 | 0 | 0 | 10 | 0 | 0 | 0 | 0 | 16 | 0 | 0 | 0 | 0 | 2.1518E7 |  |  | 8.563E5 |  |  |  |  | 1.0437E6 |  |  |  |  | 4 | 4 | 11 | 0 | 0 | 1 | 0 | 0 | 0 | 0 | 2 | 0 | 0 | 0 | 0 | N | 17979 | ubiquitin-40S ribosomal protein S27a [Python bivittatus] |
| 163 | 599 | JAV49286.1 | 132.14 | 30 | 24 | 0 | 0 | 10 | 0 | 0 | 0 | 0 | 16 | 0 | 0 | 0 | 0 | 2.1518E7 |  |  | 8.563E5 |  |  |  |  | 1.0437E6 |  |  |  |  | 4 | 4 | 11 | 0 | 0 | 1 | 0 | 0 | 0 | 0 | 2 | 0 | 0 | 0 | 0 | N | 17979 | ubiquitin-40S ribosomal protein S27a [Agkistrodon contortrix contortrix] |
| 163 | 600 | LAA53283.1 | 132.14 | 30 | 24 | 0 | 0 | 10 | 0 | 0 | 0 | 0 | 16 | 0 | 0 | 0 | 0 | 2.1518E7 |  |  | 8.563E5 |  |  |  |  | 1.0437E6 |  |  |  |  | 4 | 4 | 11 | 0 | 0 | 1 | 0 | 0 | 0 | 0 | 2 | 0 | 0 | 0 | 0 | N | 17979 | hypothetical protein [Micrurus corallinus] |
| 163 | 601 | XP\_015686900.1 | 132.14 | 30 | 24 | 0 | 0 | 10 | 0 | 0 | 0 | 0 | 16 | 0 | 0 | 0 | 0 | 2.1518E7 |  |  | 8.563E5 |  |  |  |  | 1.0437E6 |  |  |  |  | 4 | 4 | 11 | 0 | 0 | 1 | 0 | 0 | 0 | 0 | 2 | 0 | 0 | 0 | 0 | N | 17979 | ubiquitin-40S ribosomal protein S27a [Protobothrops mucrosquamatus] |
| 163 | 602 | XP\_007432077.1 | 132.14 | 30 | 24 | 0 | 0 | 10 | 0 | 0 | 0 | 0 | 16 | 0 | 0 | 0 | 0 | 2.1518E7 |  |  | 8.563E5 |  |  |  |  | 1.0437E6 |  |  |  |  | 4 | 4 | 11 | 0 | 0 | 1 | 0 | 0 | 0 | 0 | 2 | 0 | 0 | 0 | 0 | N | 17979 | ubiquitin-40S ribosomal protein S27a [Python bivittatus] |
| 163 | 603 | XP\_026551822.1 | 132.14 | 30 | 24 | 0 | 0 | 10 | 0 | 0 | 0 | 0 | 16 | 0 | 0 | 0 | 0 | 2.1518E7 |  |  | 8.563E5 |  |  |  |  | 1.0437E6 |  |  |  |  | 4 | 4 | 11 | 0 | 0 | 1 | 0 | 0 | 0 | 0 | 2 | 0 | 0 | 0 | 0 | N | 17979 | ubiquitin-40S ribosomal protein S27a [Pseudonaja textilis] |
| 163 | 604 | AFJ51304.1 | 132.14 | 30 | 24 | 0 | 0 | 10 | 0 | 0 | 0 | 0 | 16 | 0 | 0 | 0 | 0 | 2.1518E7 |  |  | 8.563E5 |  |  |  |  | 1.0437E6 |  |  |  |  | 4 | 4 | 11 | 0 | 0 | 1 | 0 | 0 | 0 | 0 | 2 | 0 | 0 | 0 | 0 | N | 17979 | Ubiquitin-40S ribosomal protein S27a-like [Crotalus adamanteus] |
| 163 | 605 | XP\_034283249.1 | 132.14 | 30 | 24 | 0 | 0 | 10 | 0 | 0 | 0 | 0 | 16 | 0 | 0 | 0 | 0 | 2.1518E7 |  |  | 8.563E5 |  |  |  |  | 1.0437E6 |  |  |  |  | 4 | 4 | 11 | 0 | 0 | 1 | 0 | 0 | 0 | 0 | 2 | 0 | 0 | 0 | 0 | N | 17979 | ubiquitin-40S ribosomal protein S27a [Pantherophis guttatus] |
| 163 | 606 | XP\_026551821.1 | 132.14 | 30 | 24 | 0 | 0 | 10 | 0 | 0 | 0 | 0 | 16 | 0 | 0 | 0 | 0 | 2.1518E7 |  |  | 8.563E5 |  |  |  |  | 1.0437E6 |  |  |  |  | 4 | 4 | 11 | 0 | 0 | 1 | 0 | 0 | 0 | 0 | 2 | 0 | 0 | 0 | 0 | N | 17979 | ubiquitin-40S ribosomal protein S27a [Pseudonaja textilis] |
| 163 | 607 | JAA95751.1 | 132.14 | 30 | 24 | 0 | 0 | 10 | 0 | 0 | 0 | 0 | 16 | 0 | 0 | 0 | 0 | 2.1518E7 |  |  | 8.563E5 |  |  |  |  | 1.0437E6 |  |  |  |  | 4 | 4 | 11 | 0 | 0 | 1 | 0 | 0 | 0 | 0 | 2 | 0 | 0 | 0 | 0 | N | 17979 | ubiquitin-40S ribosomal protein S27a [Crotalus horridus] |
| 163 | 608 | XP\_032072309.1 | 132.14 | 30 | 24 | 0 | 0 | 10 | 0 | 0 | 0 | 0 | 16 | 0 | 0 | 0 | 0 | 2.1518E7 |  |  | 8.563E5 |  |  |  |  | 1.0437E6 |  |  |  |  | 4 | 4 | 11 | 0 | 0 | 1 | 0 | 0 | 0 | 0 | 2 | 0 | 0 | 0 | 0 | N | 17979 | ubiquitin-40S ribosomal protein S27a [Thamnophis elegans] |
| 163 | 609 | XP\_026532495.1 | 132.14 | 30 | 24 | 0 | 0 | 10 | 0 | 0 | 0 | 0 | 16 | 0 | 0 | 0 | 0 | 2.1518E7 |  |  | 8.563E5 |  |  |  |  | 1.0437E6 |  |  |  |  | 4 | 4 | 11 | 0 | 0 | 1 | 0 | 0 | 0 | 0 | 2 | 0 | 0 | 0 | 0 | N | 17979 | ubiquitin-40S ribosomal protein S27a [Notechis scutatus] |
| 163 | 610 | XP\_026532494.1 | 132.14 | 30 | 24 | 0 | 0 | 10 | 0 | 0 | 0 | 0 | 16 | 0 | 0 | 0 | 0 | 2.1518E7 |  |  | 8.563E5 |  |  |  |  | 1.0437E6 |  |  |  |  | 4 | 4 | 11 | 0 | 0 | 1 | 0 | 0 | 0 | 0 | 2 | 0 | 0 | 0 | 0 | N | 17979 | ubiquitin-40S ribosomal protein S27a [Notechis scutatus] |
| 163 | 611 | JAI09470.1 | 132.14 | 30 | 24 | 0 | 0 | 10 | 0 | 0 | 0 | 0 | 16 | 0 | 0 | 0 | 0 | 2.1518E7 |  |  | 8.563E5 |  |  |  |  | 1.0437E6 |  |  |  |  | 4 | 4 | 11 | 0 | 0 | 1 | 0 | 0 | 0 | 0 | 2 | 0 | 0 | 0 | 0 | N | 17979 | ubiquitin-40S ribosomal protein S27a [Micrurus fulvius] |
| 163 | 612 | XP\_032072311.1 | 132.14 | 30 | 24 | 0 | 0 | 10 | 0 | 0 | 0 | 0 | 16 | 0 | 0 | 0 | 0 | 2.1518E7 |  |  | 8.563E5 |  |  |  |  | 1.0437E6 |  |  |  |  | 4 | 4 | 11 | 0 | 0 | 1 | 0 | 0 | 0 | 0 | 2 | 0 | 0 | 0 | 0 | N | 17979 | ubiquitin-40S ribosomal protein S27a [Thamnophis elegans] |
| 163 | 613 | XP\_034283248.1 | 132.14 | 30 | 24 | 0 | 0 | 10 | 0 | 0 | 0 | 0 | 16 | 0 | 0 | 0 | 0 | 2.1518E7 |  |  | 8.563E5 |  |  |  |  | 1.0437E6 |  |  |  |  | 4 | 4 | 11 | 0 | 0 | 1 | 0 | 0 | 0 | 0 | 2 | 0 | 0 | 0 | 0 | N | 17979 | ubiquitin-40S ribosomal protein S27a [Pantherophis guttatus] |
| 163 | 614 | LAB39333.1 | 132.14 | 30 | 24 | 0 | 0 | 10 | 0 | 0 | 0 | 0 | 16 | 0 | 0 | 0 | 0 | 2.1518E7 |  |  | 8.563E5 |  |  |  |  | 1.0437E6 |  |  |  |  | 4 | 4 | 11 | 0 | 0 | 1 | 0 | 0 | 0 | 0 | 2 | 0 | 0 | 0 | 0 | N | 17979 | hypothetical protein [Micrurus spixii] |
| 163 | 615 | LAB39331.1 | 132.14 | 28 | 22 | 0 | 0 | 9 | 0 | 0 | 0 | 0 | 15 | 0 | 0 | 0 | 0 | 2.1518E7 |  |  | 8.563E5 |  |  |  |  | 1.0437E6 |  |  |  |  | 4 | 4 | 11 | 0 | 0 | 1 | 0 | 0 | 0 | 0 | 2 | 0 | 0 | 0 | 0 | N | 19424 | hypothetical protein [Micrurus spixii] |
| 163 | 616 | LAA53282.1 | 132.14 | 26 | 21 | 0 | 0 | 9 | 0 | 0 | 0 | 0 | 14 | 0 | 0 | 0 | 0 | 2.1518E7 |  |  | 8.563E5 |  |  |  |  | 1.0437E6 |  |  |  |  | 4 | 4 | 11 | 0 | 0 | 1 | 0 | 0 | 0 | 0 | 2 | 0 | 0 | 0 | 0 | N | 20590 | hypothetical protein, partial [Micrurus corallinus] |
| 163 | 685 | XP\_013929631.1 | 132.14 | 37 | 30 | 0 | 0 | 12 | 0 | 0 | 0 | 0 | 20 | 0 | 0 | 0 | 0 | 2.1518E7 |  |  | 8.563E5 |  |  |  |  | 1.0437E6 |  |  |  |  | 4 | 4 | 11 | 0 | 0 | 1 | 0 | 0 | 0 | 0 | 2 | 0 | 0 | 0 | 0 | N | 14674 | PREDICTED: ubiquitin-60S ribosomal protein L40 [Thamnophis sirtalis] |
| 163 | 686 | XP\_013929630.1 | 132.14 | 37 | 30 | 0 | 0 | 12 | 0 | 0 | 0 | 0 | 20 | 0 | 0 | 0 | 0 | 2.1518E7 |  |  | 8.563E5 |  |  |  |  | 1.0437E6 |  |  |  |  | 4 | 4 | 11 | 0 | 0 | 1 | 0 | 0 | 0 | 0 | 2 | 0 | 0 | 0 | 0 | N | 14674 | PREDICTED: ubiquitin-60S ribosomal protein L40 [Thamnophis sirtalis] |
| 163 | 720 | JAI09497.1 | 132.14 | 37 | 30 | 0 | 0 | 12 | 0 | 0 | 0 | 0 | 20 | 0 | 0 | 0 | 0 | 2.1518E7 |  |  | 8.563E5 |  |  |  |  | 1.0437E6 |  |  |  |  | 4 | 4 | 11 | 0 | 0 | 1 | 0 | 0 | 0 | 0 | 2 | 0 | 0 | 0 | 0 | N | 14728 | 60S ribosomal protein L40 [Micrurus fulvius] |
| 163 | 721 | JAB53439.1 | 132.14 | 37 | 30 | 0 | 0 | 12 | 0 | 0 | 0 | 0 | 20 | 0 | 0 | 0 | 0 | 2.1518E7 |  |  | 8.563E5 |  |  |  |  | 1.0437E6 |  |  |  |  | 4 | 4 | 11 | 0 | 0 | 1 | 0 | 0 | 0 | 0 | 2 | 0 | 0 | 0 | 0 | N | 14728 | Ubiquitin-60S ribosomal protein L40 [Micrurus fulvius] |
| 163 | 722 | JAI11613.1 | 132.14 | 37 | 30 | 0 | 0 | 12 | 0 | 0 | 0 | 0 | 20 | 0 | 0 | 0 | 0 | 2.1518E7 |  |  | 8.563E5 |  |  |  |  | 1.0437E6 |  |  |  |  | 4 | 4 | 11 | 0 | 0 | 1 | 0 | 0 | 0 | 0 | 2 | 0 | 0 | 0 | 0 | N | 14728 | 60S ribosomal protein L40 [Crotalus adamanteus] |
| 163 | 723 | AFJ51273.1 | 132.14 | 37 | 30 | 0 | 0 | 12 | 0 | 0 | 0 | 0 | 20 | 0 | 0 | 0 | 0 | 2.1518E7 |  |  | 8.563E5 |  |  |  |  | 1.0437E6 |  |  |  |  | 4 | 4 | 11 | 0 | 0 | 1 | 0 | 0 | 0 | 0 | 2 | 0 | 0 | 0 | 0 | N | 14728 | Ubiquitin-60S ribosomal protein L40 [Crotalus adamanteus] |
| 163 | 724 | JAG66673.1 | 132.14 | 37 | 30 | 0 | 0 | 12 | 0 | 0 | 0 | 0 | 20 | 0 | 0 | 0 | 0 | 2.1518E7 |  |  | 8.563E5 |  |  |  |  | 1.0437E6 |  |  |  |  | 4 | 4 | 11 | 0 | 0 | 1 | 0 | 0 | 0 | 0 | 2 | 0 | 0 | 0 | 0 | N | 14728 | 60S ribosomal protein L40 [Boiga irregularis] |
| 163 | 725 | JAA95781.1 | 132.14 | 37 | 30 | 0 | 0 | 12 | 0 | 0 | 0 | 0 | 20 | 0 | 0 | 0 | 0 | 2.1518E7 |  |  | 8.563E5 |  |  |  |  | 1.0437E6 |  |  |  |  | 4 | 4 | 11 | 0 | 0 | 1 | 0 | 0 | 0 | 0 | 2 | 0 | 0 | 0 | 0 | N | 14728 | 60S ribosomal protein L40 [Crotalus horridus] |
| 163 | 726 | JAB53440.1 | 132.14 | 37 | 30 | 0 | 0 | 12 | 0 | 0 | 0 | 0 | 20 | 0 | 0 | 0 | 0 | 2.1518E7 |  |  | 8.563E5 |  |  |  |  | 1.0437E6 |  |  |  |  | 4 | 4 | 11 | 0 | 0 | 1 | 0 | 0 | 0 | 0 | 2 | 0 | 0 | 0 | 0 | N | 14716 | ubiquitin-60S ribosomal protein L40 [Micrurus fulvius] |
| 163 | 727 | AAG17445.1 | 132.14 | 37 | 30 | 0 | 0 | 12 | 0 | 0 | 0 | 0 | 20 | 0 | 0 | 0 | 0 | 2.1518E7 |  |  | 8.563E5 |  |  |  |  | 1.0437E6 |  |  |  |  | 4 | 4 | 11 | 0 | 0 | 1 | 0 | 0 | 0 | 0 | 2 | 0 | 0 | 0 | 0 | N | 14728 | ubiquitin fusion protein [Ophiophagus hannah] |
| 163 | 740 | JAI10738.1 | 132.14 | 48 | 39 | 0 | 0 | 16 | 0 | 0 | 0 | 0 | 26 | 0 | 0 | 0 | 0 | 2.1518E7 |  |  | 8.563E5 |  |  |  |  | 1.0437E6 |  |  |  |  | 4 | 4 | 11 | 0 | 0 | 1 | 0 | 0 | 0 | 0 | 2 | 0 | 0 | 0 | 0 | N | 10986 | ubiquitin C [Crotalus adamanteus] |
| 163 | 741 | XP\_013928637.1 | 132.14 | 21 | 17 | 0 | 0 | 7 | 0 | 0 | 0 | 0 | 11 | 0 | 0 | 0 | 0 | 2.1518E7 |  |  | 8.563E5 |  |  |  |  | 1.0437E6 |  |  |  |  | 4 | 4 | 11 | 0 | 0 | 1 | 0 | 0 | 0 | 0 | 2 | 0 | 0 | 0 | 0 | N | 25822 | PREDICTED: polyubiquitin-B [Thamnophis sirtalis] |
| 163 | 742 | AFJ51941.1 | 132.14 | 21 | 17 | 0 | 0 | 7 | 0 | 0 | 0 | 0 | 11 | 0 | 0 | 0 | 0 | 2.1518E7 |  |  | 8.563E5 |  |  |  |  | 1.0437E6 |  |  |  |  | 4 | 4 | 11 | 0 | 0 | 1 | 0 | 0 | 0 | 0 | 2 | 0 | 0 | 0 | 0 | N | 25806 | putative ubiquitin B [Crotalus adamanteus] |
| 163 | 745 | JAB53023.1 | 132.14 | 12 | 10 | 0 | 0 | 4 | 0 | 0 | 0 | 0 | 7 | 0 | 0 | 0 | 0 | 2.1518E7 |  |  | 8.563E5 |  |  |  |  | 1.0437E6 |  |  |  |  | 4 | 4 | 11 | 0 | 0 | 1 | 0 | 0 | 0 | 0 | 2 | 0 | 0 | 0 | 0 | N | 42865 | polyubiquitin-C isoform 2 [Micrurus fulvius] |
| 123 | 648 | LAA22167.1 | 131.33 | 7 | 7 | 0 | 0 | 0 | 0 | 0 | 0 | 0 | 0 | 0 | 0 | 0 | 0 | 8.7118E6 |  |  |  |  |  |  |  |  |  |  |  |  | 3 | 3 | 26 | 0 | 0 | 0 | 0 | 0 | 0 | 0 | 0 | 0 | 0 | 0 | 0 | N | 61958 | hypothetical protein, partial [Micrurus lemniscatus carvalhoi] |
| 123 | 657 | XP\_007444287.1 | 131.33 | 8 | 8 | 0 | 0 | 0 | 0 | 0 | 0 | 0 | 0 | 0 | 0 | 0 | 0 | 8.7118E6 |  |  |  |  |  |  |  |  |  |  |  |  | 3 | 3 | 26 | 0 | 0 | 0 | 0 | 0 | 0 | 0 | 0 | 0 | 0 | 0 | 0 | N | 57067 | ATP synthase subunit alpha, mitochondrial [Python bivittatus] |
| 123 | 658 | XP\_026569669.1 | 131.33 | 8 | 8 | 0 | 0 | 0 | 0 | 0 | 0 | 0 | 0 | 0 | 0 | 0 | 0 | 8.7118E6 |  |  |  |  |  |  |  |  |  |  |  |  | 3 | 3 | 26 | 0 | 0 | 0 | 0 | 0 | 0 | 0 | 0 | 0 | 0 | 0 | 0 | N | 60057 | ATP synthase subunit alpha, mitochondrial [Pseudonaja textilis] |
| 123 | 659 | JAB53920.1 | 131.33 | 8 | 8 | 0 | 0 | 0 | 0 | 0 | 0 | 0 | 0 | 0 | 0 | 0 | 0 | 8.7118E6 |  |  |  |  |  |  |  |  |  |  |  |  | 3 | 3 | 26 | 0 | 0 | 0 | 0 | 0 | 0 | 0 | 0 | 0 | 0 | 0 | 0 | N | 59947 | ATP synthase subunit alpha [Micrurus fulvius] |
| 123 | 660 | XP\_026544199.1 | 131.33 | 8 | 8 | 0 | 0 | 0 | 0 | 0 | 0 | 0 | 0 | 0 | 0 | 0 | 0 | 8.7118E6 |  |  |  |  |  |  |  |  |  |  |  |  | 3 | 3 | 26 | 0 | 0 | 0 | 0 | 0 | 0 | 0 | 0 | 0 | 0 | 0 | 0 | N | 60073 | ATP synthase subunit alpha, mitochondrial [Notechis scutatus] |
| 123 | 681 | JAG47406.1 | 131.33 | 8 | 8 | 0 | 0 | 0 | 0 | 0 | 0 | 0 | 0 | 0 | 0 | 0 | 0 | 8.7118E6 |  |  |  |  |  |  |  |  |  |  |  |  | 3 | 3 | 26 | 0 | 0 | 0 | 0 | 0 | 0 | 0 | 0 | 0 | 0 | 0 | 0 | N | 59979 | ATP synthase subunit alpha, mitochondrial-like protein [Crotalus horridus] |
| 123 | 682 | JAI14245.1 | 131.33 | 8 | 8 | 0 | 0 | 0 | 0 | 0 | 0 | 0 | 0 | 0 | 0 | 0 | 0 | 8.7118E6 |  |  |  |  |  |  |  |  |  |  |  |  | 3 | 3 | 26 | 0 | 0 | 0 | 0 | 0 | 0 | 0 | 0 | 0 | 0 | 0 | 0 | N | 59936 | ATP synthase subunit alpha, mitochondrial-like protein [Crotalus adamanteus] |
| 123 | 683 | JAG45747.1 | 131.33 | 8 | 8 | 0 | 0 | 0 | 0 | 0 | 0 | 0 | 0 | 0 | 0 | 0 | 0 | 8.7118E6 |  |  |  |  |  |  |  |  |  |  |  |  | 3 | 3 | 26 | 0 | 0 | 0 | 0 | 0 | 0 | 0 | 0 | 0 | 0 | 0 | 0 | N | 59979 | ATP synthase subunit alpha, mitochondrial-like protein [Crotalus horridus] |
| 123 | 684 | JAA97708.1 | 131.33 | 8 | 8 | 0 | 0 | 0 | 0 | 0 | 0 | 0 | 0 | 0 | 0 | 0 | 0 | 8.7118E6 |  |  |  |  |  |  |  |  |  |  |  |  | 3 | 3 | 26 | 0 | 0 | 0 | 0 | 0 | 0 | 0 | 0 | 0 | 0 | 0 | 0 | N | 59979 | ATP synthase subunit alpha, mitochondrial-like protein [Crotalus horridus] |
| 123 | 690 | XP\_013922009.1 | 131.33 | 8 | 8 | 0 | 0 | 0 | 0 | 0 | 0 | 0 | 0 | 0 | 0 | 0 | 0 | 8.7118E6 |  |  |  |  |  |  |  |  |  |  |  |  | 3 | 3 | 26 | 0 | 0 | 0 | 0 | 0 | 0 | 0 | 0 | 0 | 0 | 0 | 0 | N | 58891 | PREDICTED: ATP synthase subunit alpha, mitochondrial [Thamnophis sirtalis] |
| 123 | 691 | XP\_032069128.1 | 131.33 | 8 | 8 | 0 | 0 | 0 | 0 | 0 | 0 | 0 | 0 | 0 | 0 | 0 | 0 | 8.7118E6 |  |  |  |  |  |  |  |  |  |  |  |  | 3 | 3 | 26 | 0 | 0 | 0 | 0 | 0 | 0 | 0 | 0 | 0 | 0 | 0 | 0 | N | 59972 | ATP synthase subunit alpha, mitochondrial [Thamnophis elegans] |
| 147 | 528 | D5LMJ3.1 | 129.83 | 4 | 0 | 2 | 2 | 2 | 4 | 0 | 0 | 0 | 0 | 0 | 0 | 0 | 0 |  | 5.6566E7 | 1.1334E8 | 8.156E7 | 3.8861E5 |  |  |  |  |  |  |  |  | 3 | 3 | 0 | 3 | 4 | 13 | 2 | 0 | 0 | 0 | 0 | 0 | 0 | 0 | 0 | Y | 68254 | RecName: Full=Zinc metalloproteinase-disintegrin-like atrase-A; AltName: Full=Snake venom metalloproteinase; Short=SVMP; Flags: Precursor |
| 147 | 529 | ADF43026.1 | 129.83 | 4 | 0 | 2 | 2 | 2 | 4 | 0 | 0 | 0 | 0 | 0 | 0 | 0 | 0 |  | 5.6566E7 | 1.1334E8 | 8.156E7 | 3.8861E5 |  |  |  |  |  |  |  |  | 3 | 3 | 0 | 3 | 4 | 13 | 2 | 0 | 0 | 0 | 0 | 0 | 0 | 0 | 0 | Y | 68254 | metalloproteinase atrase A [Naja atra] |
| 201 | 1683 | ACF21000.1 | 129.81 | 9 | 0 | 0 | 0 | 9 | 0 | 0 | 0 | 0 | 7 | 7 | 0 | 0 | 0 |  |  |  | 5.7554E6 |  |  |  |  | 3.4892E6 | 1.1315E6 |  |  |  | 4 | 4 | 0 | 0 | 0 | 4 | 0 | 0 | 0 | 0 | 2 | 1 | 0 | 0 | 0 | Y | 21835 | cathelicidin-related protein precursor [Naja atra] |
| 201 | 1684 | B6S2X0.1 | 129.81 | 9 | 0 | 0 | 0 | 9 | 0 | 0 | 0 | 0 | 7 | 7 | 0 | 0 | 0 |  |  |  | 5.7554E6 |  |  |  |  | 3.4892E6 | 1.1315E6 |  |  |  | 4 | 4 | 0 | 0 | 0 | 4 | 0 | 0 | 0 | 0 | 2 | 1 | 0 | 0 | 0 | Y | 21835 | RecName: Full=Cathelicidin-related antimicrobial peptide Na\_CRAMP; AltName: Full=Cathelicidin-NA antimicrobial peptide; AltName: Full=Vipericidin; Flags: Precursor |
| 126 | 312 | JAG47405.1 | 129.27 | 7 | 7 | 0 | 0 | 0 | 0 | 0 | 0 | 0 | 0 | 0 | 0 | 0 | 0 | 3.6919E6 |  |  |  |  |  |  |  |  |  |  |  |  | 3 | 3 | 15 | 0 | 0 | 0 | 0 | 0 | 0 | 0 | 0 | 0 | 0 | 0 | 0 | N | 56548 | ATP synthase subunit beta, mitochondrial-like protein [Crotalus horridus] |
| 126 | 313 | JAA97707.1 | 129.27 | 7 | 7 | 0 | 0 | 0 | 0 | 0 | 0 | 0 | 0 | 0 | 0 | 0 | 0 | 3.6919E6 |  |  |  |  |  |  |  |  |  |  |  |  | 3 | 3 | 15 | 0 | 0 | 0 | 0 | 0 | 0 | 0 | 0 | 0 | 0 | 0 | 0 | N | 56562 | ATP synthase subunit beta, mitochondrial-like protein [Crotalus horridus] |
| 126 | 314 | JAG45746.1 | 129.27 | 7 | 7 | 0 | 0 | 0 | 0 | 0 | 0 | 0 | 0 | 0 | 0 | 0 | 0 | 3.6919E6 |  |  |  |  |  |  |  |  |  |  |  |  | 3 | 3 | 15 | 0 | 0 | 0 | 0 | 0 | 0 | 0 | 0 | 0 | 0 | 0 | 0 | N | 56580 | ATP synthase subunit beta, mitochondrial-like protein [Crotalus horridus] |
| 126 | 315 | JAV51185.1 | 129.27 | 7 | 7 | 0 | 0 | 0 | 0 | 0 | 0 | 0 | 0 | 0 | 0 | 0 | 0 | 3.6919E6 |  |  |  |  |  |  |  |  |  |  |  |  | 3 | 3 | 15 | 0 | 0 | 0 | 0 | 0 | 0 | 0 | 0 | 0 | 0 | 0 | 0 | N | 56592 | ATP synthase subunit beta, mitochondrial-like protein [Agkistrodon contortrix contortrix] |
| 126 | 316 | AFJ49478.1 | 129.27 | 7 | 7 | 0 | 0 | 0 | 0 | 0 | 0 | 0 | 0 | 0 | 0 | 0 | 0 | 3.6919E6 |  |  |  |  |  |  |  |  |  |  |  |  | 3 | 3 | 15 | 0 | 0 | 0 | 0 | 0 | 0 | 0 | 0 | 0 | 0 | 0 | 0 | N | 56574 | ATP synthase subunit beta, mitochondrial [Crotalus adamanteus] |
| 126 | 317 | JAI14244.1 | 129.27 | 7 | 7 | 0 | 0 | 0 | 0 | 0 | 0 | 0 | 0 | 0 | 0 | 0 | 0 | 3.6919E6 |  |  |  |  |  |  |  |  |  |  |  |  | 3 | 3 | 15 | 0 | 0 | 0 | 0 | 0 | 0 | 0 | 0 | 0 | 0 | 0 | 0 | N | 56574 | ATP synthase subunit beta, mitochondrial-like protein [Crotalus adamanteus] |
| 180 | 584 | XP\_025032248.1 | 127.98 | 11 | 0 | 0 | 0 | 0 | 0 | 0 | 0 | 0 | 9 | 2 | 0 | 0 | 0 |  |  |  |  |  |  |  |  | 5.9367E7 |  |  |  |  | 5 | 2 | 0 | 0 | 0 | 0 | 0 | 0 | 0 | 0 | 9 | 1 | 0 | 0 | 0 | Y | 41355 | insulin-like growth factor-binding protein 3, partial [Python bivittatus] |
| 180 | 766 | XP\_013925299.1 | 127.98 | 14 | 0 | 0 | 0 | 0 | 0 | 0 | 0 | 0 | 11 | 3 | 0 | 0 | 0 |  |  |  |  |  |  |  |  | 5.9367E7 |  |  |  |  | 5 | 2 | 0 | 0 | 0 | 0 | 0 | 0 | 0 | 0 | 9 | 1 | 0 | 0 | 0 | Y | 32409 | PREDICTED: insulin-like growth factor-binding protein 3 [Thamnophis sirtalis] |
| 180 | 772 | XP\_032093683.1 | 127.98 | 14 | 0 | 0 | 0 | 0 | 0 | 0 | 0 | 0 | 11 | 3 | 0 | 0 | 0 |  |  |  |  |  |  |  |  | 5.9367E7 |  |  |  |  | 5 | 2 | 0 | 0 | 0 | 0 | 0 | 0 | 0 | 0 | 9 | 1 | 0 | 0 | 0 | Y | 32147 | insulin-like growth factor-binding protein 3 [Thamnophis elegans] |
| 180 | 871 | XP\_015680080.1 | 127.98 | 14 | 0 | 0 | 0 | 0 | 0 | 0 | 0 | 0 | 11 | 3 | 0 | 0 | 0 |  |  |  |  |  |  |  |  | 5.9367E7 |  |  |  |  | 5 | 2 | 0 | 0 | 0 | 0 | 0 | 0 | 0 | 0 | 9 | 1 | 0 | 0 | 0 | Y | 31982 | LOW QUALITY PROTEIN: insulin-like growth factor-binding protein 3 [Protobothrops mucrosquamatus] |
| 221 | 1293 | XP\_026571876.1 | 123.92 | 3 | 0 | 0 | 0 | 0 | 0 | 0 | 0 | 1 | 1 | 0 | 0 | 0 | 0 |  |  |  |  |  |  |  | 2.542E5 |  |  |  |  |  | 3 | 1 | 0 | 0 | 0 | 0 | 0 | 0 | 0 | 1 | 4 | 0 | 0 | 0 | 0 | Y | 108071 | complement component C6-like [Pseudonaja textilis] |
| 184 | 435 | XP\_026558105.1 | 123.87 | 16 | 0 | 0 | 0 | 0 | 0 | 0 | 0 | 0 | 14 | 3 | 0 | 0 | 0 |  |  |  |  |  |  |  |  | 2.241E8 |  |  |  |  | 4 | 1 | 0 | 0 | 0 | 0 | 0 | 0 | 0 | 0 | 8 | 1 | 0 | 0 | 0 | Y | 32240 | insulin-like growth factor-binding protein 3 isoform X1 [Pseudonaja textilis] |
| 170 | 490 | AXL96618.1 | 122.29 | 6 | 0 | 0 | 2 | 2 | 4 | 0 | 0 | 0 | 0 | 0 | 0 | 0 | 0 |  |  |  |  | 1.3261E5 |  |  |  |  |  |  |  |  | 3 | 1 | 0 | 0 | 1 | 2 | 8 | 0 | 0 | 0 | 0 | 0 | 0 | 0 | 0 | Y | 68520 | acetycholinesterase 2, partial [Ahaetulla prasina] |
| 170 | 491 | JAC96629.1 | 122.29 | 6 | 0 | 0 | 2 | 2 | 4 | 0 | 0 | 0 | 0 | 0 | 0 | 0 | 0 |  |  |  |  | 1.3261E5 |  |  |  |  |  |  |  |  | 3 | 1 | 0 | 0 | 1 | 2 | 8 | 0 | 0 | 0 | 0 | 0 | 0 | 0 | 0 | Y | 69005 | Acetylcholinesterase transcript 1 [Echis coloratus] |
| 170 | 492 | AHY20009.1 | 122.29 | 6 | 0 | 0 | 2 | 2 | 4 | 0 | 0 | 0 | 0 | 0 | 0 | 0 | 0 |  |  |  |  | 1.3261E5 |  |  |  |  |  |  |  |  | 3 | 1 | 0 | 0 | 1 | 2 | 8 | 0 | 0 | 0 | 0 | 0 | 0 | 0 | 0 | Y | 68986 | acetylcholinesterase [Echis coloratus] |
| 170 | 493 | ASU45022.1 | 122.29 | 7 | 0 | 0 | 2 | 2 | 5 | 0 | 0 | 0 | 0 | 0 | 0 | 0 | 0 |  |  |  |  | 1.3261E5 |  |  |  |  |  |  |  |  | 3 | 1 | 0 | 0 | 1 | 2 | 8 | 0 | 0 | 0 | 0 | 0 | 0 | 0 | 0 | Y | 62791 | acetylcholinesterase, partial [Daboia russelii] |
| 220 | 1280 | XP\_029140716.1 | 122.26 | 3 | 0 | 0 | 0 | 0 | 0 | 0 | 0 | 0 | 3 | 0 | 0 | 0 | 0 |  |  |  |  |  |  |  |  | 2.402E6 |  |  |  |  | 3 | 1 | 0 | 0 | 0 | 0 | 0 | 0 | 0 | 0 | 6 | 0 | 0 | 0 | 0 | Y | 104965 | complement component C6 [Protobothrops mucrosquamatus] |
| 220 | 1290 | XP\_026522231.1 | 122.26 | 3 | 0 | 0 | 0 | 0 | 0 | 0 | 0 | 0 | 3 | 0 | 0 | 0 | 0 |  |  |  |  |  |  |  |  | 2.402E6 |  |  |  |  | 3 | 1 | 0 | 0 | 0 | 0 | 0 | 0 | 0 | 0 | 6 | 0 | 0 | 0 | 0 | Y | 108124 | complement component C6-like [Notechis scutatus] |
| 220 | 1306 | XP\_032070501.1 | 122.26 | 3 | 0 | 0 | 0 | 0 | 0 | 0 | 0 | 0 | 3 | 0 | 0 | 0 | 0 |  |  |  |  |  |  |  |  | 2.402E6 |  |  |  |  | 3 | 1 | 0 | 0 | 0 | 0 | 0 | 0 | 0 | 0 | 6 | 0 | 0 | 0 | 0 | Y | 104608 | complement component C6-like [Thamnophis elegans] |
| 167 | 1055 | AAB32582.1 | 118.18 | 14 | 0 | 14 | 14 | 14 | 0 | 0 | 0 | 0 | 0 | 0 | 0 | 0 | 8 |  | 3.0482E7 | 1.5311E7 | 7.9103E6 |  |  |  |  |  |  |  |  | 0 | 2 | 2 | 0 | 3 | 5 | 3 | 0 | 0 | 0 | 0 | 0 | 0 | 0 | 0 | 1 | Y | 20452 | phospholipase A2 31 kda subunit, PLA2 31 kda subunit=urokinase-type plasminogen activator receptor homolog [Naja naja kaouthia=Thailand cobras, blood, Peptide, 188 aa] |
| 167 | 1056 | Q7LZI1.1 | 118.18 | 14 | 0 | 14 | 14 | 14 | 0 | 0 | 0 | 0 | 0 | 0 | 0 | 0 | 8 |  | 3.0482E7 | 1.5311E7 | 7.9103E6 |  |  |  |  |  |  |  |  | 0 | 2 | 2 | 0 | 3 | 5 | 3 | 0 | 0 | 0 | 0 | 0 | 0 | 0 | 0 | 1 | Y | 20452 | RecName: Full=Phospholipase A2 inhibitor 31 kDa subunit; AltName: Full=gamma-PLI |
| 183 | 527 | ETE59846.1 | 117.37 | 5 | 0 | 1 | 0 | 3 | 2 | 0 | 0 | 0 | 0 | 0 | 0 | 0 | 0 |  |  |  | 8.1726E6 |  |  |  |  |  |  |  |  |  | 3 | 1 | 0 | 1 | 0 | 6 | 2 | 0 | 0 | 0 | 0 | 0 | 0 | 0 | 0 | Y | 72383 | Alpha-fetoprotein, partial [Ophiophagus hannah] |
| 182 | 827 | XP\_026555558.1 | 115.66 | 8 | 0 | 5 | 3 | 5 | 0 | 0 | 0 | 0 | 0 | 0 | 0 | 0 | 0 |  |  |  | 1.2567E6 |  |  |  |  |  |  |  |  |  | 3 | 1 | 0 | 2 | 2 | 6 | 0 | 0 | 0 | 0 | 0 | 0 | 0 | 0 | 0 | Y | 61349 | hepatocyte growth factor activator [Pseudonaja textilis] |
| 182 | 841 | XP\_034282171.1 | 115.66 | 10 | 0 | 7 | 3 | 7 | 0 | 0 | 0 | 0 | 0 | 0 | 0 | 0 | 0 |  |  |  | 1.2567E6 |  |  |  |  |  |  |  |  |  | 3 | 1 | 0 | 2 | 2 | 6 | 0 | 0 | 0 | 0 | 0 | 0 | 0 | 0 | 0 | Y | 46138 | hepatocyte growth factor activator [Pantherophis guttatus] |
| 24 | 1422 | 1CDT | 114.67 | 27 | 0 | 22 | 0 | 0 | 0 | 12 | 0 | 12 | 22 | 27 | 27 | 27 | 22 |  | 2.4904E8 |  |  |  |  |  |  | 2.7013E6 | 5.7501E7 | 1.6839E6 | 3.5494E6 | 1.9896E6 | 3 | 1 | 0 | 21 | 0 | 0 | 0 | 1 | 0 | 2 | 14 | 8 | 7 | 5 | 2 | Y | 6715 | Chain B, CARDIOTOXIN VII4 |
| 24 | 1423 | P01452.1 | 114.67 | 27 | 0 | 22 | 0 | 0 | 0 | 12 | 0 | 12 | 22 | 27 | 27 | 27 | 22 |  | 2.4904E8 |  |  |  |  |  |  | 2.7013E6 | 5.7501E7 | 1.6839E6 | 3.5494E6 | 1.9896E6 | 3 | 1 | 0 | 21 | 0 | 0 | 0 | 1 | 0 | 2 | 14 | 8 | 7 | 5 | 2 | Y | 6715 | RecName: Full=Cytotoxin 4; AltName: Full=CTX M3; AltName: Full=Cardiotoxin V(II)4 |
| 128 | 1622 | XP\_013925940.1 | 113.49 | 8 | 4 | 0 | 0 | 8 | 4 | 4 | 4 | 4 | 4 | 4 | 0 | 0 | 0 |  |  |  | 3.8043E5 |  |  |  |  |  |  |  |  |  | 3 | 1 | 2 | 0 | 0 | 2 | 1 | 10 | 2 | 2 | 2 | 1 | 0 | 0 | 0 | Y | 26870 | PREDICTED: cationic trypsin-3-like [Thamnophis sirtalis] |
| 128 | 1685 | XP\_029139862.1 | 113.49 | 7 | 3 | 0 | 0 | 7 | 3 | 3 | 3 | 3 | 3 | 3 | 0 | 0 | 0 |  |  |  | 3.8043E5 |  |  |  |  |  |  |  |  |  | 3 | 1 | 2 | 0 | 0 | 2 | 1 | 10 | 2 | 2 | 2 | 1 | 0 | 0 | 0 | Y | 31859 | cationic trypsin-3 [Protobothrops mucrosquamatus] |
| 128 | 1717 | XP\_015670838.1 | 113.49 | 8 | 4 | 0 | 0 | 8 | 4 | 4 | 4 | 4 | 4 | 4 | 0 | 0 | 0 |  |  |  | 3.8043E5 |  |  |  |  |  |  |  |  |  | 3 | 1 | 2 | 0 | 0 | 2 | 1 | 10 | 2 | 2 | 2 | 1 | 0 | 0 | 0 | Y | 26884 | cationic trypsin-3-like [Protobothrops mucrosquamatus] |
| 160 | 1205 | ETE63009.1 | 111.48 | 9 | 0 | 9 | 9 | 5 | 0 | 0 | 0 | 0 | 0 | 0 | 0 | 0 | 0 |  | 4.1542E7 | 1.6447E7 | 4.5596E6 |  |  |  |  |  |  |  |  |  | 2 | 1 | 0 | 7 | 7 | 4 | 0 | 0 | 0 | 0 | 0 | 0 | 0 | 0 | 0 | Y | 34271 | Extracellular matrix protein 1, partial [Ophiophagus hannah] |
| 160 | 1497 | XP\_034280930.1 | 111.48 | 7 | 0 | 7 | 7 | 4 | 0 | 0 | 0 | 0 | 0 | 0 | 0 | 0 | 0 |  | 4.1542E7 | 1.6447E7 | 4.5596E6 |  |  |  |  |  |  |  |  |  | 2 | 1 | 0 | 7 | 7 | 4 | 0 | 0 | 0 | 0 | 0 | 0 | 0 | 0 | 0 | Y | 39235 | extracellular matrix protein 1 [Pantherophis guttatus] |
| 139 | 809 | ETE67452.1 | 111.12 | 9 | 0 | 0 | 4 | 4 | 0 | 0 | 0 | 0 | 0 | 6 | 0 | 0 | 0 |  |  | 1.6125E6 | 9.1581E5 |  |  |  |  |  | 8.0495E7 |  |  |  | 3 | 3 | 0 | 0 | 1 | 2 | 0 | 0 | 0 | 0 | 0 | 18 | 0 | 0 | 0 | Y | 39348 | Tumor necrosis factor receptor superfamily member 11B [Ophiophagus hannah] |
| 181 | 1520 | XP\_026576072.1 | 108.26 | 6 | 0 | 6 | 3 | 3 | 0 | 0 | 0 | 0 | 0 | 0 | 0 | 0 | 0 |  | 1.3801E5 |  | 5.5659E6 |  |  |  |  |  |  |  |  |  | 2 | 1 | 0 | 3 | 2 | 6 | 0 | 0 | 0 | 0 | 0 | 0 | 0 | 0 | 0 | Y | 39960 | extracellular matrix protein 1 isoform X2 [Pseudonaja textilis] |
| 181 | 1521 | XP\_026576070.1 | 108.26 | 6 | 0 | 6 | 3 | 3 | 0 | 0 | 0 | 0 | 0 | 0 | 0 | 0 | 0 |  | 1.3801E5 |  | 5.5659E6 |  |  |  |  |  |  |  |  |  | 2 | 1 | 0 | 3 | 2 | 6 | 0 | 0 | 0 | 0 | 0 | 0 | 0 | 0 | 0 | Y | 40088 | extracellular matrix protein 1 isoform X1 [Pseudonaja textilis] |
| 224 | 542 | JAG67228.1 | 107.84 | 12 | 0 | 0 | 0 | 0 | 12 | 0 | 0 | 0 | 0 | 0 | 0 | 0 | 0 |  |  |  |  | 5.1493E5 |  |  |  |  |  |  |  |  | 4 | 1 | 0 | 0 | 0 | 0 | 5 | 0 | 0 | 0 | 0 | 0 | 0 | 0 | 0 | Y | 44109 | Glia-derived nexin-like protein [Boiga irregularis] |
| 224 | 555 | XP\_034295297.1 | 107.84 | 12 | 0 | 0 | 0 | 0 | 12 | 0 | 0 | 0 | 0 | 0 | 0 | 0 | 0 |  |  |  |  | 5.1493E5 |  |  |  |  |  |  |  |  | 4 | 1 | 0 | 0 | 0 | 0 | 5 | 0 | 0 | 0 | 0 | 0 | 0 | 0 | 0 | Y | 44043 | glia-derived nexin [Pantherophis guttatus] |
| 214 | 1480 | P82462.1 | 107.17 | 43 | 0 | 0 | 0 | 0 | 0 | 0 | 0 | 0 | 0 | 0 | 43 | 43 | 0 |  |  |  |  |  |  |  |  |  |  | 4.7405E7 | 4.4988E7 |  | 1 | 1 | 0 | 0 | 0 | 0 | 0 | 0 | 0 | 0 | 0 | 0 | 1 | 2 | 0 | Y | 7366 | RecName: Full=Muscarinic toxin-like protein 1; Short=MTLP-1 |
| 202 | 1703 | BAN08536.1 | 105.21 | 15 | 0 | 7 | 7 | 0 | 0 | 0 | 0 | 0 | 0 | 9 | 0 | 0 | 9 |  |  |  |  |  |  |  |  |  | 7.9588E6 |  |  | 1.268E6 | 2 | 1 | 0 | 2 | 1 | 0 | 0 | 0 | 0 | 0 | 0 | 2 | 0 | 0 | 2 | Y | 16576 | phospholipase A2 [Protobothrops flavoviridis] |
| 202 | 1704 | BAM76245.1 | 105.21 | 15 | 0 | 7 | 7 | 0 | 0 | 0 | 0 | 0 | 0 | 9 | 0 | 0 | 9 |  |  |  |  |  |  |  |  |  | 7.9588E6 |  |  | 1.268E6 | 2 | 1 | 0 | 2 | 1 | 0 | 0 | 0 | 0 | 0 | 0 | 2 | 0 | 0 | 2 | Y | 16546 | pancreatic phospholipase A2 [Protobothrops elegans] |
| 168 | 2029 | XP\_034292109.1 | 103.54 | 7 | 0 | 0 | 0 | 0 | 0 | 7 | 6 | 6 | 6 | 0 | 0 | 0 | 0 |  |  |  |  |  | 1.3046E7 | 3.9264E7 | 4.6158E7 | 4.2897E6 |  |  |  |  | 2 | 2 | 0 | 0 | 0 | 0 | 0 | 4 | 3 | 3 | 1 | 0 | 0 | 0 | 0 | Y | 33533 | neural proliferation differentiation and control protein 1 [Pantherophis guttatus] |
| 199 | 876 | JAB53674.1 | 100.91 | 16 | 0 | 0 | 0 | 0 | 0 | 0 | 0 | 0 | 0 | 8 | 0 | 8 | 0 |  |  |  |  |  |  |  |  |  | 4.8944E6 |  | 1.4123E7 |  | 3 | 3 | 0 | 0 | 0 | 0 | 0 | 0 | 0 | 0 | 0 | 3 | 0 | 3 | 0 | Y | 27667 | platelet-derived growth factor A chain long form [Micrurus fulvius] |
| 199 | 888 | LAB42085.1 | 100.91 | 17 | 0 | 0 | 0 | 0 | 0 | 0 | 0 | 0 | 0 | 8 | 0 | 9 | 0 |  |  |  |  |  |  |  |  |  | 4.8944E6 |  | 1.4123E7 |  | 3 | 3 | 0 | 0 | 0 | 0 | 0 | 0 | 0 | 0 | 0 | 3 | 0 | 3 | 0 | Y | 25772 | hypothetical protein, partial [Micrurus spixii] |
| 199 | 899 | LAB42082.1 | 100.91 | 14 | 0 | 0 | 0 | 0 | 0 | 0 | 0 | 0 | 0 | 7 | 0 | 7 | 0 |  |  |  |  |  |  |  |  |  | 4.8944E6 |  | 1.4123E7 |  | 3 | 3 | 0 | 0 | 0 | 0 | 0 | 0 | 0 | 0 | 0 | 3 | 0 | 3 | 0 | Y | 31036 | hypothetical protein, partial [Micrurus spixii] |
| 199 | 906 | XP\_026560089.1 | 100.91 | 20 | 0 | 0 | 0 | 0 | 0 | 0 | 0 | 0 | 0 | 10 | 0 | 10 | 0 |  |  |  |  |  |  |  |  |  | 4.8944E6 |  | 1.4123E7 |  | 3 | 3 | 0 | 0 | 0 | 0 | 0 | 0 | 0 | 0 | 0 | 3 | 0 | 3 | 0 | Y | 22535 | platelet-derived growth factor subunit A isoform X3 [Pseudonaja textilis] |
| 199 | 917 | XP\_026560087.1 | 100.91 | 18 | 0 | 0 | 0 | 0 | 0 | 0 | 0 | 0 | 0 | 9 | 0 | 9 | 0 |  |  |  |  |  |  |  |  |  | 4.8944E6 |  | 1.4123E7 |  | 3 | 3 | 0 | 0 | 0 | 0 | 0 | 0 | 0 | 0 | 0 | 3 | 0 | 3 | 0 | Y | 24924 | platelet-derived growth factor subunit A isoform X1 [Pseudonaja textilis] |
| 199 | 918 | LAA51478.1 | 100.91 | 33 | 0 | 0 | 0 | 0 | 0 | 0 | 0 | 0 | 0 | 16 | 0 | 17 | 0 |  |  |  |  |  |  |  |  |  | 4.8944E6 |  | 1.4123E7 |  | 3 | 3 | 0 | 0 | 0 | 0 | 0 | 0 | 0 | 0 | 0 | 3 | 0 | 3 | 0 | Y | 13713 | hypothetical protein, partial [Micrurus corallinus] |
| 199 | 919 | XP\_034286468.1 | 100.91 | 25 | 0 | 0 | 0 | 0 | 0 | 0 | 0 | 0 | 0 | 12 | 0 | 13 | 0 |  |  |  |  |  |  |  |  |  | 4.8944E6 |  | 1.4123E7 |  | 3 | 3 | 0 | 0 | 0 | 0 | 0 | 0 | 0 | 0 | 0 | 3 | 0 | 3 | 0 | Y | 18298 | platelet-derived growth factor subunit A, partial [Pantherophis guttatus] |
| 199 | 921 | XP\_026560088.1 | 100.91 | 18 | 0 | 0 | 0 | 0 | 0 | 0 | 0 | 0 | 0 | 9 | 0 | 9 | 0 |  |  |  |  |  |  |  |  |  | 4.8944E6 |  | 1.4123E7 |  | 3 | 3 | 0 | 0 | 0 | 0 | 0 | 0 | 0 | 0 | 0 | 3 | 0 | 3 | 0 | Y | 24698 | platelet-derived growth factor subunit A isoform X2 [Pseudonaja textilis] |
| 199 | 940 | AFJ50955.1 | 100.91 | 20 | 0 | 0 | 0 | 0 | 0 | 0 | 0 | 0 | 0 | 10 | 0 | 10 | 0 |  |  |  |  |  |  |  |  |  | 4.8944E6 |  | 1.4123E7 |  | 3 | 3 | 0 | 0 | 0 | 0 | 0 | 0 | 0 | 0 | 0 | 3 | 0 | 3 | 0 | Y | 22576 | Platelet-derived growth factor subunit A preproprotein [Crotalus adamanteus] |
| 199 | 941 | JAI12034.1 | 100.91 | 20 | 0 | 0 | 0 | 0 | 0 | 0 | 0 | 0 | 0 | 10 | 0 | 10 | 0 |  |  |  |  |  |  |  |  |  | 4.8944E6 |  | 1.4123E7 |  | 3 | 3 | 0 | 0 | 0 | 0 | 0 | 0 | 0 | 0 | 0 | 3 | 0 | 3 | 0 | Y | 22576 | platelet-derived growth factor subunit A [Crotalus adamanteus] |
| 199 | 942 | XP\_015676418.1 | 100.91 | 20 | 0 | 0 | 0 | 0 | 0 | 0 | 0 | 0 | 0 | 10 | 0 | 10 | 0 |  |  |  |  |  |  |  |  |  | 4.8944E6 |  | 1.4123E7 |  | 3 | 3 | 0 | 0 | 0 | 0 | 0 | 0 | 0 | 0 | 0 | 3 | 0 | 3 | 0 | Y | 22562 | platelet-derived growth factor subunit A [Protobothrops mucrosquamatus] |
| 199 | 943 | LAA51474.1 | 100.91 | 33 | 0 | 0 | 0 | 0 | 0 | 0 | 0 | 0 | 0 | 16 | 0 | 17 | 0 |  |  |  |  |  |  |  |  |  | 4.8944E6 |  | 1.4123E7 |  | 3 | 3 | 0 | 0 | 0 | 0 | 0 | 0 | 0 | 0 | 0 | 3 | 0 | 3 | 0 | Y | 13798 | hypothetical protein, partial [Micrurus corallinus] |
| 199 | 944 | XP\_007426296.1 | 100.91 | 20 | 0 | 0 | 0 | 0 | 0 | 0 | 0 | 0 | 0 | 10 | 0 | 10 | 0 |  |  |  |  |  |  |  |  |  | 4.8944E6 |  | 1.4123E7 |  | 3 | 3 | 0 | 0 | 0 | 0 | 0 | 0 | 0 | 0 | 0 | 3 | 0 | 3 | 0 | Y | 22561 | platelet-derived growth factor subunit A isoform X3 [Python bivittatus] |
| 199 | 945 | XP\_025021193.1 | 100.91 | 19 | 0 | 0 | 0 | 0 | 0 | 0 | 0 | 0 | 0 | 9 | 0 | 10 | 0 |  |  |  |  |  |  |  |  |  | 4.8944E6 |  | 1.4123E7 |  | 3 | 3 | 0 | 0 | 0 | 0 | 0 | 0 | 0 | 0 | 0 | 3 | 0 | 3 | 0 | Y | 23303 | platelet-derived growth factor subunit A isoform X2 [Python bivittatus] |
| 199 | 946 | XP\_015743230.1 | 100.91 | 18 | 0 | 0 | 0 | 0 | 0 | 0 | 0 | 0 | 0 | 9 | 0 | 9 | 0 |  |  |  |  |  |  |  |  |  | 4.8944E6 |  | 1.4123E7 |  | 3 | 3 | 0 | 0 | 0 | 0 | 0 | 0 | 0 | 0 | 0 | 3 | 0 | 3 | 0 | Y | 24934 | platelet-derived growth factor subunit A isoform X1 [Python bivittatus] |
| 199 | 949 | XP\_026540879.1 | 100.91 | 18 | 0 | 0 | 0 | 0 | 0 | 0 | 0 | 0 | 0 | 9 | 0 | 9 | 0 |  |  |  |  |  |  |  |  |  | 4.8944E6 |  | 1.4123E7 |  | 3 | 3 | 0 | 0 | 0 | 0 | 0 | 0 | 0 | 0 | 0 | 3 | 0 | 3 | 0 | Y | 25552 | platelet-derived growth factor subunit A [Notechis scutatus] |
| 245 | 933 | ETE63349.1 | 99.67 | 4 | 0 | 0 | 0 | 4 | 0 | 0 | 0 | 0 | 0 | 0 | 0 | 0 | 0 |  |  |  | 1.9395E6 |  |  |  |  |  |  |  |  |  | 2 | 2 | 0 | 0 | 0 | 2 | 0 | 0 | 0 | 0 | 0 | 0 | 0 | 0 | 0 | Y | 74315 | Polymeric immunoglobulin receptor [Ophiophagus hannah] |
| 194 | 2409 | ETE61477.1 | 98.50 | 35 | 0 | 15 | 15 | 35 | 0 | 0 | 0 | 0 | 0 | 0 | 0 | 0 | 0 |  |  |  | 6.87E5 |  |  |  |  |  |  |  |  |  | 2 | 1 | 0 | 1 | 2 | 6 | 0 | 0 | 0 | 0 | 0 | 0 | 0 | 0 | 0 | Y | 10789 | Hepatocyte growth factor activator, partial [Ophiophagus hannah] |
| 159 | 1182 | JAS05124.1 | 92.82 | 18 | 0 | 0 | 0 | 0 | 0 | 0 | 0 | 0 | 0 | 8 | 18 | 8 | 0 |  |  |  |  |  |  |  |  |  |  | 5.5123E7 |  |  | 3 | 1 | 0 | 0 | 0 | 0 | 0 | 0 | 0 | 0 | 0 | 1 | 3 | 1 | 0 | Y | 16135 | Phospholipase A2 5a [Micrurus tener] |
| 159 | 1183 | JAS05123.1 | 92.82 | 18 | 0 | 0 | 0 | 0 | 0 | 0 | 0 | 0 | 0 | 8 | 18 | 8 | 0 |  |  |  |  |  |  |  |  |  |  | 5.5123E7 |  |  | 3 | 1 | 0 | 0 | 0 | 0 | 0 | 0 | 0 | 0 | 0 | 1 | 3 | 1 | 0 | Y | 16135 | Phospholipase A2 5b [Micrurus tener] |
| 159 | 1190 | AET85561.1 | 92.82 | 22 | 0 | 0 | 0 | 0 | 0 | 0 | 0 | 0 | 0 | 9 | 22 | 9 | 0 |  |  |  |  |  |  |  |  |  |  | 5.5123E7 |  |  | 3 | 1 | 0 | 0 | 0 | 0 | 0 | 0 | 0 | 0 | 0 | 1 | 3 | 1 | 0 | Y | 13385 | phospholipase A2, partial [Micrurus tener tener] |
| 159 | 1191 | JAS05125.1 | 92.82 | 18 | 0 | 0 | 0 | 0 | 0 | 0 | 0 | 0 | 0 | 8 | 18 | 8 | 0 |  |  |  |  |  |  |  |  |  |  | 5.5123E7 |  |  | 3 | 1 | 0 | 0 | 0 | 0 | 0 | 0 | 0 | 0 | 0 | 1 | 3 | 1 | 0 | Y | 16124 | Phospholipase A2 4d [Micrurus tener] |
| 159 | 1192 | JAS05128.1 | 92.82 | 18 | 0 | 0 | 0 | 0 | 0 | 0 | 0 | 0 | 0 | 8 | 18 | 8 | 0 |  |  |  |  |  |  |  |  |  |  | 5.5123E7 |  |  | 3 | 1 | 0 | 0 | 0 | 0 | 0 | 0 | 0 | 0 | 0 | 1 | 3 | 1 | 0 | Y | 16122 | Phospholipase A2 4a [Micrurus tener] |
| 159 | 1193 | JAS05127.1 | 92.82 | 18 | 0 | 0 | 0 | 0 | 0 | 0 | 0 | 0 | 0 | 8 | 18 | 8 | 0 |  |  |  |  |  |  |  |  |  |  | 5.5123E7 |  |  | 3 | 1 | 0 | 0 | 0 | 0 | 0 | 0 | 0 | 0 | 0 | 1 | 3 | 1 | 0 | Y | 16123 | Phospholipase A2 4b [Micrurus tener] |
| 159 | 1194 | JAS05126.1 | 92.82 | 18 | 0 | 0 | 0 | 0 | 0 | 0 | 0 | 0 | 0 | 8 | 18 | 8 | 0 |  |  |  |  |  |  |  |  |  |  | 5.5123E7 |  |  | 3 | 1 | 0 | 0 | 0 | 0 | 0 | 0 | 0 | 0 | 0 | 1 | 3 | 1 | 0 | Y | 16361 | Phospholipase A2 4c [Micrurus tener] |
| 159 | 1195 | JAB52812.1 | 92.82 | 18 | 0 | 0 | 0 | 0 | 0 | 0 | 0 | 0 | 0 | 8 | 18 | 8 | 0 |  |  |  |  |  |  |  |  |  |  | 5.5123E7 |  |  | 3 | 1 | 0 | 0 | 0 | 0 | 0 | 0 | 0 | 0 | 0 | 1 | 3 | 1 | 0 | Y | 16012 | phospholipase A2 11 [Micrurus fulvius] |
| 159 | 1196 | JAB52803.1 | 92.82 | 18 | 0 | 0 | 0 | 0 | 0 | 0 | 0 | 0 | 0 | 8 | 18 | 8 | 0 |  |  |  |  |  |  |  |  |  |  | 5.5123E7 |  |  | 3 | 1 | 0 | 0 | 0 | 0 | 0 | 0 | 0 | 0 | 0 | 1 | 3 | 1 | 0 | Y | 16123 | phospholipase A2 1a [Micrurus fulvius] |
| 159 | 1197 | JAS05033.1 | 92.82 | 18 | 0 | 0 | 0 | 0 | 0 | 0 | 0 | 0 | 0 | 8 | 18 | 8 | 0 |  |  |  |  |  |  |  |  |  |  | 5.5123E7 |  |  | 3 | 1 | 0 | 0 | 0 | 0 | 0 | 0 | 0 | 0 | 0 | 1 | 3 | 1 | 0 | Y | 16123 | Phospholipase A2 2a [Micrurus fulvius] |
| 159 | 1198 | JAI09044.1 | 92.82 | 18 | 0 | 0 | 0 | 0 | 0 | 0 | 0 | 0 | 0 | 8 | 18 | 8 | 0 |  |  |  |  |  |  |  |  |  |  | 5.5123E7 |  |  | 3 | 1 | 0 | 0 | 0 | 0 | 0 | 0 | 0 | 0 | 0 | 1 | 3 | 1 | 0 | Y | 16123 | Phospholipase A2 2a [Micrurus fulvius] |
| 159 | 1199 | JAS05025.1 | 92.82 | 18 | 0 | 0 | 0 | 0 | 0 | 0 | 0 | 0 | 0 | 8 | 18 | 8 | 0 |  |  |  |  |  |  |  |  |  |  | 5.5123E7 |  |  | 3 | 1 | 0 | 0 | 0 | 0 | 0 | 0 | 0 | 0 | 0 | 1 | 3 | 1 | 0 | Y | 16167 | Phospholipase A2 2c [Micrurus fulvius] |
| 159 | 1200 | JAS05019.1 | 92.82 | 18 | 0 | 0 | 0 | 0 | 0 | 0 | 0 | 0 | 0 | 8 | 18 | 8 | 0 |  |  |  |  |  |  |  |  |  |  | 5.5123E7 |  |  | 3 | 1 | 0 | 0 | 0 | 0 | 0 | 0 | 0 | 0 | 0 | 1 | 3 | 1 | 0 | Y | 16167 | Phospholipase A2 2i [Micrurus fulvius] |
| 159 | 1201 | JAI09030.1 | 92.82 | 18 | 0 | 0 | 0 | 0 | 0 | 0 | 0 | 0 | 0 | 8 | 18 | 8 | 0 |  |  |  |  |  |  |  |  |  |  | 5.5123E7 |  |  | 3 | 1 | 0 | 0 | 0 | 0 | 0 | 0 | 0 | 0 | 0 | 1 | 3 | 1 | 0 | Y | 16167 | Phospholipase A2 2i [Micrurus fulvius] |
| 159 | 1202 | JAI09036.1 | 92.82 | 18 | 0 | 0 | 0 | 0 | 0 | 0 | 0 | 0 | 0 | 8 | 18 | 8 | 0 |  |  |  |  |  |  |  |  |  |  | 5.5123E7 |  |  | 3 | 1 | 0 | 0 | 0 | 0 | 0 | 0 | 0 | 0 | 0 | 1 | 3 | 1 | 0 | Y | 16167 | Phospholipase A2 2c [Micrurus fulvius] |
| 159 | 1203 | JAB52789.1 | 92.82 | 18 | 0 | 0 | 0 | 0 | 0 | 0 | 0 | 0 | 0 | 8 | 18 | 8 | 0 |  |  |  |  |  |  |  |  |  |  | 5.5123E7 |  |  | 3 | 1 | 0 | 0 | 0 | 0 | 0 | 0 | 0 | 0 | 0 | 1 | 3 | 1 | 0 | Y | 16167 | phospholipase A2 2a [Micrurus fulvius] |
| 159 | 1211 | JAS05020.1 | 92.82 | 18 | 0 | 0 | 0 | 0 | 0 | 0 | 0 | 0 | 0 | 8 | 18 | 8 | 0 |  |  |  |  |  |  |  |  |  |  | 5.5123E7 |  |  | 3 | 1 | 0 | 0 | 0 | 0 | 0 | 0 | 0 | 0 | 0 | 1 | 3 | 1 | 0 | Y | 16216 | Phospholipase A2 2h [Micrurus fulvius] |
| 159 | 1212 | JAI09031.1 | 92.82 | 18 | 0 | 0 | 0 | 0 | 0 | 0 | 0 | 0 | 0 | 8 | 18 | 8 | 0 |  |  |  |  |  |  |  |  |  |  | 5.5123E7 |  |  | 3 | 1 | 0 | 0 | 0 | 0 | 0 | 0 | 0 | 0 | 0 | 1 | 3 | 1 | 0 | Y | 16216 | Phospholipase A2 2h [Micrurus fulvius] |
| 159 | 1213 | JAB52781.1 | 92.82 | 18 | 0 | 0 | 0 | 0 | 0 | 0 | 0 | 0 | 0 | 8 | 18 | 8 | 0 |  |  |  |  |  |  |  |  |  |  | 5.5123E7 |  |  | 3 | 1 | 0 | 0 | 0 | 0 | 0 | 0 | 0 | 0 | 0 | 1 | 3 | 1 | 0 | Y | 16216 | phospholipase A2 2i [Micrurus fulvius] |
| 239 | 2427 | ETE59007.1 | 92.64 | 32 | 0 | 0 | 0 | 32 | 0 | 0 | 0 | 0 | 0 | 0 | 0 | 0 | 0 |  |  |  | 9.9997E6 |  |  |  |  |  |  |  |  |  | 2 | 2 | 0 | 0 | 0 | 3 | 0 | 0 | 0 | 0 | 0 | 0 | 0 | 0 | 0 | N | 9348 | hypothetical protein L345\_15265, partial [Ophiophagus hannah] |
| 176 | 1279 | ETE65528.1 | 92.44 | 9 | 0 | 4 | 4 | 4 | 0 | 6 | 0 | 0 | 0 | 0 | 4 | 4 | 4 |  |  |  |  |  | 1.6385E6 |  |  |  |  |  |  |  | 2 | 1 | 0 | 1 | 2 | 2 | 0 | 1 | 0 | 0 | 0 | 0 | 1 | 2 | 1 | Y | 28804 | H-2 class II histocompatibility antigen gamma chain [Ophiophagus hannah] |
| 257 | 1309 | XP\_026560868.1 | 91.01 | 1 | 0 | 1 | 0 | 0 | 0 | 0 | 0 | 0 | 0 | 0 | 0 | 0 | 0 |  | 1.0243E6 |  |  |  |  |  |  | 0 |  |  |  |  | 2 | 2 | 0 | 1 | 0 | 0 | 0 | 0 | 0 | 0 | 1 | 0 | 0 | 0 | 0 | Y | 277539 | cation-independent mannose-6-phosphate receptor [Pseudonaja textilis] |
| 257 | 1310 | XP\_026531033.1 | 91.01 | 1 | 0 | 1 | 0 | 0 | 0 | 0 | 0 | 0 | 0 | 0 | 0 | 0 | 0 |  | 1.0243E6 |  |  |  |  |  |  | 0 |  |  |  |  | 2 | 2 | 0 | 1 | 0 | 0 | 0 | 0 | 0 | 0 | 1 | 0 | 0 | 0 | 0 | Y | 281509 | cation-independent mannose-6-phosphate receptor [Notechis scutatus] |
| 257 | 1311 | ETE64374.1 | 91.01 | 1 | 0 | 1 | 0 | 0 | 0 | 0 | 0 | 0 | 1 | 0 | 0 | 0 | 0 |  | 1.0243E6 |  |  |  |  |  |  | 0 |  |  |  |  | 2 | 2 | 0 | 1 | 0 | 0 | 0 | 0 | 0 | 0 | 1 | 0 | 0 | 0 | 0 | Y | 244985 | Cation-independent mannose-6-phosphate receptor [Ophiophagus hannah] |
| 257 | 1314 | XP\_034284419.1 | 91.01 | 1 | 0 | 1 | 0 | 0 | 0 | 0 | 0 | 0 | 0 | 0 | 0 | 0 | 0 |  | 1.0243E6 |  |  |  |  |  |  | 0 |  |  |  |  | 2 | 2 | 0 | 1 | 0 | 0 | 0 | 0 | 0 | 0 | 1 | 0 | 0 | 0 | 0 | Y | 277267 | cation-independent mannose-6-phosphate receptor [Pantherophis guttatus] |
| 209 | 986 | XP\_032090066.1 | 87.95 | 9 | 0 | 3 | 3 | 5 | 0 | 0 | 0 | 0 | 0 | 5 | 0 | 0 | 0 |  |  |  | 1.5737E6 |  |  |  |  |  | 0 |  |  |  | 2 | 1 | 0 | 2 | 2 | 1 | 0 | 0 | 0 | 0 | 0 | 1 | 0 | 0 | 0 | Y | 39123 | extracellular matrix protein 1 isoform X2 [Thamnophis elegans] |
| 209 | 987 | XP\_032090065.1 | 87.95 | 9 | 0 | 3 | 3 | 5 | 0 | 0 | 0 | 0 | 0 | 5 | 0 | 0 | 0 |  |  |  | 1.5737E6 |  |  |  |  |  | 0 |  |  |  | 2 | 1 | 0 | 2 | 2 | 1 | 0 | 0 | 0 | 0 | 0 | 1 | 0 | 0 | 0 | Y | 40343 | extracellular matrix protein 1 isoform X1 [Thamnophis elegans] |
| 240 | 4386 | JAA74930.1 | 87.54 | 14 | 0 | 0 | 0 | 0 | 0 | 0 | 0 | 0 | 14 | 0 | 0 | 0 | 0 |  |  |  |  |  |  |  |  | 8.4235E5 |  |  |  |  | 1 | 1 | 0 | 0 | 0 | 0 | 0 | 0 | 0 | 0 | 1 | 0 | 0 | 0 | 0 | Y | 11058 | 3FTx-Pse-99 [Pseudonaja modesta] |
| 222 | 1233 | XP\_026544110.1 | 87.41 | 8 | 0 | 3 | 0 | 5 | 0 | 0 | 0 | 0 | 0 | 0 | 0 | 0 | 0 |  |  |  | 1.4523E7 |  |  |  |  |  |  |  |  |  | 2 | 1 | 0 | 1 | 0 | 2 | 0 | 0 | 0 | 0 | 0 | 0 | 0 | 0 | 0 | Y | 50196 | hepatocyte growth factor activator [Notechis scutatus] |
| 264 | 835 | P0DI84.1 | 85.14 | 5 | 0 | 5 | 0 | 0 | 0 | 0 | 0 | 0 | 0 | 0 | 0 | 0 | 0 |  | 1.7954E6 |  |  |  |  |  |  |  |  |  |  |  | 1 | 1 | 0 | 2 | 0 | 0 | 0 | 0 | 0 | 0 | 0 | 0 | 0 | 0 | 0 | N | 54748 | RecName: Full=L-amino-acid oxidase; Short=LAO; Short=VAA-LAAO I |
| 264 | 836 | 3KVE | 85.14 | 5 | 0 | 5 | 0 | 0 | 0 | 0 | 0 | 0 | 0 | 0 | 0 | 0 | 0 |  | 1.7954E6 |  |  |  |  |  |  |  |  |  |  |  | 1 | 1 | 0 | 2 | 0 | 0 | 0 | 0 | 0 | 0 | 0 | 0 | 0 | 0 | 0 | N | 54933 | Chain D, Structure Of Native L-Amino Acid Oxidase From Vipera Ammodytes Ammodytes: Stabilization Of The Quaternary Structure By Divalent Ions And Structural Changes In The Dynamic Active Site |
| 226 | 2271 | JAA75029.1 | 84.58 | 18 | 0 | 0 | 0 | 0 | 0 | 0 | 0 | 0 | 0 | 0 | 7 | 0 | 18 |  |  |  |  |  |  |  |  |  |  |  |  | 1.751E6 | 2 | 1 | 0 | 0 | 0 | 0 | 0 | 0 | 0 | 0 | 0 | 0 | 1 | 0 | 1 | Y | 16220 | PLA2-Sut-25 [Suta fasciata] |
| 232 | 1469 | ADI47614.1 | 83.98 | 4 | 0 | 0 | 0 | 0 | 0 | 0 | 0 | 0 | 0 | 2 | 2 | 2 | 0 |  |  |  |  |  |  |  |  |  | 1.1234E6 |  |  |  | 2 | 1 | 0 | 0 | 0 | 0 | 0 | 0 | 0 | 0 | 0 | 1 | 1 | 1 | 0 | Y | 57183 | metalloproteinase, partial [Echis coloratus] |
| 266 | 2288 | P0DMT2.1 | 83.46 | 15 | 0 | 0 | 0 | 0 | 0 | 0 | 0 | 0 | 0 | 0 | 15 | 15 | 0 |  |  |  |  |  |  |  |  |  |  | 1.7369E6 | 1.2456E7 |  | 1 | 1 | 0 | 0 | 0 | 0 | 0 | 0 | 0 | 0 | 0 | 0 | 1 | 2 | 0 | Y | 13865 | RecName: Full=Phospholipase A2 homolog; Short=svPLA2 homolog; AltName: Full=ECS\_00014 |
| 266 | 2379 | 3BJW | 83.46 | 15 | 0 | 0 | 0 | 0 | 0 | 0 | 0 | 0 | 0 | 0 | 15 | 15 | 0 |  |  |  |  |  |  |  |  |  |  | 1.7369E6 | 1.2456E7 |  | 1 | 1 | 0 | 0 | 0 | 0 | 0 | 0 | 0 | 0 | 0 | 0 | 1 | 2 | 0 | Y | 13819 | Chain H, Phospholipase A2 |
| 266 | 2380 | 2QHD | 83.46 | 15 | 0 | 0 | 0 | 0 | 0 | 0 | 0 | 0 | 0 | 0 | 15 | 15 | 0 |  |  |  |  |  |  |  |  |  |  | 1.7369E6 | 1.2456E7 |  | 1 | 1 | 0 | 0 | 0 | 0 | 0 | 0 | 0 | 0 | 0 | 0 | 1 | 2 | 0 | Y | 13819 | Chain B, Phospholipase A2 |
| 266 | 2381 | P48650.1 | 83.46 | 15 | 0 | 0 | 0 | 0 | 0 | 0 | 0 | 0 | 0 | 0 | 15 | 15 | 0 |  |  |  |  |  |  |  |  |  |  | 1.7369E6 | 1.2456E7 |  | 1 | 1 | 0 | 0 | 0 | 0 | 0 | 0 | 0 | 0 | 0 | 0 | 1 | 2 | 0 | Y | 13819 | RecName: Full=Basic phospholipase A2 homolog ecarpholin S; Short=Ecs-S49; Short=svPLA2 homolog |
| 266 | 2382 | 2QHE | 83.46 | 15 | 0 | 0 | 0 | 0 | 0 | 0 | 0 | 0 | 0 | 0 | 15 | 15 | 0 |  |  |  |  |  |  |  |  |  |  | 1.7369E6 | 1.2456E7 |  | 1 | 1 | 0 | 0 | 0 | 0 | 0 | 0 | 0 | 0 | 0 | 0 | 1 | 2 | 0 | Y | 13819 | Chain A, Phospholipase A2 |
| 200 | 1315 | XP\_026571379.1 | 83.09 | 3 | 0 | 1 | 3 | 1 | 0 | 0 | 0 | 0 | 0 | 0 | 0 | 0 | 1 |  | 1.8284E6 | 1.5489E7 | 7.0028E6 |  |  |  |  |  |  |  |  | 4.4868E6 | 2 | 2 | 0 | 1 | 2 | 3 | 0 | 0 | 0 | 0 | 0 | 0 | 0 | 0 | 2 | Y | 80026 | C4b-binding protein alpha chain-like isoform X1 [Pseudonaja textilis] |
| 215 | 1510 | XP\_032085287.1 | 82.09 | 18 | 0 | 18 | 7 | 0 | 0 | 0 | 0 | 0 | 0 | 0 | 0 | 0 | 0 |  | 1.8111E6 |  |  |  |  |  |  |  |  |  |  |  | 2 | 1 | 0 | 3 | 1 | 0 | 0 | 0 | 0 | 0 | 0 | 0 | 0 | 0 | 0 | Y | 16345 | phospholipase A2 [Thamnophis elegans] |
| 292 | 988 | JAG46496.1 | 82.04 | 5 | 0 | 0 | 0 | 5 | 0 | 0 | 0 | 0 | 0 | 0 | 0 | 0 | 0 |  |  |  | 2.3013E6 |  |  |  |  |  |  |  |  |  | 2 | 1 | 0 | 0 | 0 | 2 | 0 | 0 | 0 | 0 | 0 | 0 | 0 | 0 | 0 | N | 49179 | legumain preproprotein [Crotalus horridus] |
| 249 | 1101 | ACD43466.1 | 81.81 | 20 | 0 | 0 | 0 | 20 | 0 | 0 | 0 | 0 | 0 | 0 | 0 | 0 | 0 |  |  |  | 2.7298E6 |  |  |  |  |  |  |  |  |  | 2 | 2 | 0 | 0 | 0 | 4 | 0 | 0 | 0 | 0 | 0 | 0 | 0 | 0 | 0 | Y | 15421 | acidic phospholipase A2 [Daboia siamensis] |
| 249 | 1102 | P31100.1 | 81.81 | 20 | 0 | 0 | 0 | 20 | 0 | 0 | 0 | 0 | 0 | 0 | 0 | 0 | 0 |  |  |  | 2.7298E6 |  |  |  |  |  |  |  |  |  | 2 | 2 | 0 | 0 | 0 | 4 | 0 | 0 | 0 | 0 | 0 | 0 | 0 | 0 | 0 | Y | 15421 | RecName: Full=Acidic phospholipase A2 RV-7; Short=svPLA2; AltName: Full=F7; AltName: Full=Phosphatidylcholine 2-acylhydrolase; AltName: Full=Phospholipase A2 inhibitor; AltName: Full=S4; AltName: Full=Viperotoxin F; AltName: Full=... |
| 249 | 1103 | CAA48457.1 | 81.81 | 20 | 0 | 0 | 0 | 20 | 0 | 0 | 0 | 0 | 0 | 0 | 0 | 0 | 0 |  |  |  | 2.7298E6 |  |  |  |  |  |  |  |  |  | 2 | 2 | 0 | 0 | 0 | 4 | 0 | 0 | 0 | 0 | 0 | 0 | 0 | 0 | 0 | Y | 15421 | phospholipase a2 [Daboia russelii] |
| 249 | 1104 | S29299 | 81.81 | 20 | 0 | 0 | 0 | 20 | 0 | 0 | 0 | 0 | 0 | 0 | 0 | 0 | 0 |  |  |  | 2.7298E6 |  |  |  |  |  |  |  |  |  | 2 | 2 | 0 | 0 | 0 | 4 | 0 | 0 | 0 | 0 | 0 | 0 | 0 | 0 | 0 | Y | 15421 | phospholipase A2 (EC 3.1.1.4) - Russell's viper |
| 249 | 1105 | AAZ53184.1 | 81.81 | 20 | 0 | 0 | 0 | 20 | 0 | 0 | 0 | 0 | 0 | 0 | 0 | 0 | 0 |  |  |  | 2.7298E6 |  |  |  |  |  |  |  |  |  | 2 | 2 | 0 | 0 | 0 | 4 | 0 | 0 | 0 | 0 | 0 | 0 | 0 | 0 | 0 | Y | 15421 | acidic phospholipase A2 [Daboia russelii limitis] |
| 249 | 1106 | ACD43468.1 | 81.81 | 20 | 0 | 0 | 0 | 20 | 0 | 0 | 0 | 0 | 0 | 0 | 0 | 0 | 0 |  |  |  | 2.7298E6 |  |  |  |  |  |  |  |  |  | 2 | 2 | 0 | 0 | 0 | 4 | 0 | 0 | 0 | 0 | 0 | 0 | 0 | 0 | 0 | Y | 15421 | acidic phospholipase A2 [Daboia siamensis] |
| 141 | 1316 | AXL95289.1 | 81.75 | 8 | 0 | 0 | 0 | 8 | 0 | 0 | 0 | 0 | 0 | 0 | 0 | 0 | 0 |  |  |  | 2.2338E8 |  |  |  |  |  |  |  |  |  | 2 | 1 | 0 | 0 | 0 | 13 | 0 | 0 | 0 | 0 | 0 | 0 | 0 | 0 | 0 | Y | 26943 | cysteine-rich secretory protein, partial [Spilotes sulphureus] |
| 242 | 1033 | XP\_034262239.1 | 77.68 | 2 | 2 | 0 | 0 | 0 | 0 | 0 | 0 | 0 | 0 | 0 | 0 | 0 | 0 | 1.6814E6 |  |  |  |  |  |  |  |  |  |  |  |  | 2 | 2 | 3 | 0 | 0 | 0 | 0 | 0 | 0 | 0 | 0 | 0 | 0 | 0 | 0 | N | 181278 | myosin-7 [Pantherophis guttatus] |
| 242 | 1176 | XP\_032092515.1 | 77.68 | 2 | 2 | 0 | 0 | 0 | 0 | 0 | 0 | 0 | 0 | 0 | 0 | 0 | 0 | 1.6814E6 |  |  |  |  |  |  |  |  |  |  |  |  | 2 | 2 | 3 | 0 | 0 | 0 | 0 | 0 | 0 | 0 | 0 | 0 | 0 | 0 | 0 | N | 224393 | LOW QUALITY PROTEIN: myosin-7-like [Thamnophis elegans] |
| 242 | 1178 | XP\_026538839.1 | 77.68 | 2 | 2 | 0 | 0 | 0 | 0 | 0 | 0 | 0 | 0 | 0 | 0 | 0 | 0 | 1.6814E6 |  |  |  |  |  |  |  |  |  |  |  |  | 2 | 2 | 3 | 0 | 0 | 0 | 0 | 0 | 0 | 0 | 0 | 0 | 0 | 0 | 0 | N | 223333 | myosin-7 [Notechis scutatus] |
| 242 | 1179 | XP\_015677713.1 | 77.68 | 2 | 2 | 0 | 0 | 0 | 0 | 0 | 0 | 0 | 0 | 0 | 0 | 0 | 0 | 1.6814E6 |  |  |  |  |  |  |  |  |  |  |  |  | 2 | 2 | 3 | 0 | 0 | 0 | 0 | 0 | 0 | 0 | 0 | 0 | 0 | 0 | 0 | N | 206763 | myosin-7-like [Protobothrops mucrosquamatus] |
| 253 | 1002 | ETE67243.1 | 76.56 | 2 | 0 | 0 | 0 | 2 | 0 | 0 | 0 | 0 | 0 | 0 | 0 | 0 | 0 |  |  |  | 1.6499E5 |  |  |  |  |  |  |  |  |  | 1 | 1 | 0 | 0 | 0 | 1 | 0 | 0 | 0 | 0 | 0 | 0 | 0 | 0 | 0 | Y | 46824 | N(4)-(beta-N-acetylglucosaminyl)-L-asparaginase, partial [Ophiophagus hannah] |
| 253 | 1007 | XP\_032079951.1 | 76.56 | 3 | 0 | 0 | 0 | 3 | 0 | 0 | 0 | 0 | 0 | 0 | 0 | 0 | 0 |  |  |  | 1.6499E5 |  |  |  |  |  |  |  |  |  | 1 | 1 | 0 | 0 | 0 | 1 | 0 | 0 | 0 | 0 | 0 | 0 | 0 | 0 | 0 | Y | 37830 | N(4)-(beta-N-acetylglucosaminyl)-L-asparaginase [Thamnophis elegans] |
| 253 | 1009 | XP\_034298591.1 | 76.56 | 3 | 0 | 0 | 0 | 3 | 0 | 0 | 0 | 0 | 0 | 0 | 0 | 0 | 0 |  |  |  | 1.6499E5 |  |  |  |  |  |  |  |  |  | 1 | 1 | 0 | 0 | 0 | 1 | 0 | 0 | 0 | 0 | 0 | 0 | 0 | 0 | 0 | Y | 37788 | N(4)-(beta-N-acetylglucosaminyl)-L-asparaginase [Pantherophis guttatus] |
| 253 | 1023 | XP\_026555457.1 | 76.56 | 3 | 0 | 0 | 0 | 3 | 0 | 0 | 0 | 0 | 0 | 0 | 0 | 0 | 0 |  |  |  | 1.6499E5 |  |  |  |  |  |  |  |  |  | 1 | 1 | 0 | 0 | 0 | 1 | 0 | 0 | 0 | 0 | 0 | 0 | 0 | 0 | 0 | Y | 37785 | N(4)-(beta-N-acetylglucosaminyl)-L-asparaginase [Pseudonaja textilis] |
| 253 | 1048 | XP\_013912092.1 | 76.56 | 13 | 0 | 0 | 0 | 13 | 0 | 0 | 0 | 0 | 0 | 0 | 0 | 0 | 0 |  |  |  | 1.6499E5 |  |  |  |  |  |  |  |  |  | 1 | 1 | 0 | 0 | 0 | 1 | 0 | 0 | 0 | 0 | 0 | 0 | 0 | 0 | 0 | Y | 9501 | PREDICTED: N(4)-(beta-N-acetylglucosaminyl)-L-asparaginase-like [Thamnophis sirtalis] |
| 254 | 1637 | Q9YGJ6.1 | 75.68 | 45 | 31 | 0 | 0 | 0 | 0 | 13 | 0 | 0 | 0 | 0 | 0 | 0 | 0 | 1.582E8 |  |  |  |  |  |  |  |  |  |  |  |  | 2 | 1 | 2 | 0 | 0 | 0 | 0 | 2 | 0 | 0 | 0 | 0 | 0 | 0 | 0 | Y | 9220 | RecName: Full=Alpha-neurotoxin NTX-1; Short=NTX1; Flags: Precursor |
| 254 | 1638 | O57326.1 | 75.68 | 45 | 31 | 0 | 0 | 0 | 0 | 13 | 0 | 0 | 0 | 0 | 0 | 0 | 0 | 1.582E8 |  |  |  |  |  |  |  |  |  |  |  |  | 2 | 1 | 2 | 0 | 0 | 0 | 0 | 2 | 0 | 0 | 0 | 0 | 0 | 0 | 0 | Y | 9289 | RecName: Full=Alpha-neurotoxin NTX-3; Short=NTX3; Flags: Precursor |
| 254 | 1639 | AAC69915.1 | 75.68 | 45 | 31 | 0 | 0 | 0 | 0 | 13 | 0 | 0 | 0 | 0 | 0 | 0 | 0 | 1.582E8 |  |  |  |  |  |  |  |  |  |  |  |  | 2 | 1 | 2 | 0 | 0 | 0 | 0 | 2 | 0 | 0 | 0 | 0 | 0 | 0 | 0 | Y | 9289 | post synaptic alpha neurotoxin precursor [Naja sputatrix] |
| 254 | 1640 | AAD08812.1 | 75.68 | 45 | 31 | 0 | 0 | 0 | 0 | 13 | 0 | 0 | 0 | 0 | 0 | 0 | 0 | 1.582E8 |  |  |  |  |  |  |  |  |  |  |  |  | 2 | 1 | 2 | 0 | 0 | 0 | 0 | 2 | 0 | 0 | 0 | 0 | 0 | 0 | 0 | Y | 9220 | post synaptic alpha neurotoxin precursor [Naja sputatrix] |
| 235 | 1476 | Q53B55.1 | 75.55 | 14 | 0 | 0 | 0 | 0 | 0 | 0 | 0 | 0 | 14 | 0 | 0 | 0 | 0 |  |  |  |  |  |  |  |  | 2.7231E6 |  |  |  |  | 1 | 1 | 0 | 0 | 0 | 0 | 0 | 0 | 0 | 0 | 1 | 0 | 0 | 0 | 0 | Y | 10154 | RecName: Full=Long neurotoxin-like OH-31; Flags: Precursor |
| 235 | 1477 | AAT97253.1 | 75.55 | 14 | 0 | 0 | 0 | 0 | 0 | 0 | 0 | 0 | 14 | 0 | 0 | 0 | 0 |  |  |  |  |  |  |  |  | 2.7231E6 |  |  |  |  | 1 | 1 | 0 | 0 | 0 | 0 | 0 | 0 | 0 | 0 | 1 | 0 | 0 | 0 | 0 | Y | 10154 | long chain neurotoxin-like protein [Ophiophagus hannah] |
| 235 | 1723 | P01387.1 | 75.55 | 18 | 0 | 0 | 0 | 0 | 0 | 0 | 0 | 0 | 18 | 0 | 0 | 0 | 0 |  |  |  |  |  |  |  |  | 2.7231E6 |  |  |  |  | 1 | 1 | 0 | 0 | 0 | 0 | 0 | 0 | 0 | 0 | 1 | 0 | 0 | 0 | 0 | Y | 8106 | RecName: Full=Long neurotoxin 1; AltName: Full=Neurotoxin A |
| 235 | 1945 | Q53B53.1 | 75.55 | 14 | 0 | 0 | 0 | 0 | 0 | 0 | 0 | 0 | 14 | 0 | 0 | 0 | 0 |  |  |  |  |  |  |  |  | 2.7231E6 |  |  |  |  | 1 | 1 | 0 | 0 | 0 | 0 | 0 | 0 | 0 | 0 | 1 | 0 | 0 | 0 | 0 | Y | 9867 | RecName: Full=Long neurotoxin OH-34; Flags: Precursor |
| 235 | 1946 | AAT97255.1 | 75.55 | 14 | 0 | 0 | 0 | 0 | 0 | 0 | 0 | 0 | 14 | 0 | 0 | 0 | 0 |  |  |  |  |  |  |  |  | 2.7231E6 |  |  |  |  | 1 | 1 | 0 | 0 | 0 | 0 | 0 | 0 | 0 | 0 | 1 | 0 | 0 | 0 | 0 | Y | 9867 | long chain neurotoxin precursor, partial [Ophiophagus hannah] |
| 229 | 1032 | AAZ39881.1 | 74.52 | 2 | 0 | 0 | 0 | 2 | 0 | 0 | 0 | 0 | 0 | 0 | 0 | 0 | 0 |  |  |  | 7.5878E4 |  |  |  |  |  |  |  |  |  | 1 | 1 | 0 | 0 | 0 | 1 | 0 | 0 | 0 | 0 | 0 | 0 | 0 | 0 | 0 | N | 69648 | RVV-X-heavy chain [Daboia russelii] |
| 229 | 1035 | AAB22477.1 | 74.52 | 3 | 0 | 0 | 0 | 3 | 0 | 0 | 0 | 0 | 0 | 0 | 0 | 0 | 0 |  |  |  | 7.5878E4 |  |  |  |  |  |  |  |  |  | 1 | 1 | 0 | 0 | 0 | 1 | 0 | 0 | 0 | 0 | 0 | 0 | 0 | 0 | 0 | N | 47975 | coagulation factor X activating enzyme heavy chain, RVV-X-heavy chain=metalloproteinase with disintegrin (platelet aggregation inhibitor)-like and C-type lectin-like domains [Vipera russelli=Russell's viper, Siamensis, venom, Peptide, 429 aa] |
| 311 | 1872 | CAL69604.1 | 74.51 | 19 | 0 | 0 | 0 | 0 | 0 | 0 | 0 | 19 | 0 | 0 | 0 | 0 | 0 |  |  |  |  |  |  |  | 1.1621E6 |  |  |  |  |  | 1 | 1 | 0 | 0 | 0 | 0 | 0 | 0 | 0 | 1 | 0 | 0 | 0 | 0 | 0 | Y | 9443 | trypsin inhibitor-3 precursor [Daboia siamensis] |
| 311 | 1873 | A8Y7N6.1 | 74.51 | 19 | 0 | 0 | 0 | 0 | 0 | 0 | 0 | 19 | 0 | 0 | 0 | 0 | 0 |  |  |  |  |  |  |  | 1.1621E6 |  |  |  |  |  | 1 | 1 | 0 | 0 | 0 | 0 | 0 | 0 | 0 | 1 | 0 | 0 | 0 | 0 | 0 | Y | 9443 | RecName: Full=Kunitz-type serine protease inhibitor C3; AltName: Full=BPTI-3; AltName: Full=Trypsin inhibitor 3; AltName: Full=Trypsin inhibitor C3; Flags: Precursor |
| 311 | 1874 | AFD04724.1 | 74.51 | 18 | 0 | 0 | 0 | 0 | 0 | 0 | 0 | 18 | 0 | 0 | 0 | 0 | 0 |  |  |  |  |  |  |  | 1.1621E6 |  |  |  |  |  | 1 | 1 | 0 | 0 | 0 | 0 | 0 | 0 | 0 | 1 | 0 | 0 | 0 | 0 | 0 | Y | 10132 | Kunitz-type protease inhibitor [Daboia russelii] |
| 311 | 1875 | CAL69605.1 | 74.51 | 18 | 0 | 0 | 0 | 0 | 0 | 0 | 0 | 18 | 0 | 0 | 0 | 0 | 0 |  |  |  |  |  |  |  | 1.1621E6 |  |  |  |  |  | 1 | 1 | 0 | 0 | 0 | 0 | 0 | 0 | 0 | 1 | 0 | 0 | 0 | 0 | 0 | Y | 10162 | trypsin inhibitor-4 precursor [Daboia siamensis] |
| 311 | 1876 | A8Y7N7.1 | 74.51 | 18 | 0 | 0 | 0 | 0 | 0 | 0 | 0 | 18 | 0 | 0 | 0 | 0 | 0 |  |  |  |  |  |  |  | 1.1621E6 |  |  |  |  |  | 1 | 1 | 0 | 0 | 0 | 0 | 0 | 0 | 0 | 1 | 0 | 0 | 0 | 0 | 0 | Y | 10162 | RecName: Full=Kunitz-type serine protease inhibitor C4; AltName: Full=BPTI-4; AltName: Full=Trypsin inhibitor 4; AltName: Full=Trypsin inhibitor C4; Flags: Precursor |
| 311 | 2824 | ABD24042.1 | 74.51 | 19 | 0 | 0 | 0 | 0 | 0 | 0 | 0 | 19 | 0 | 0 | 0 | 0 | 0 |  |  |  |  |  |  |  | 1.1621E6 |  |  |  |  |  | 1 | 1 | 0 | 0 | 0 | 0 | 0 | 0 | 0 | 1 | 0 | 0 | 0 | 0 | 0 | Y | 9417 | Kunitz protease inhibitor-III [Daboia russelii russelii] |
| 311 | 2825 | Q2ES48.1 | 74.51 | 19 | 0 | 0 | 0 | 0 | 0 | 0 | 0 | 19 | 0 | 0 | 0 | 0 | 0 |  |  |  |  |  |  |  | 1.1621E6 |  |  |  |  |  | 1 | 1 | 0 | 0 | 0 | 0 | 0 | 0 | 0 | 1 | 0 | 0 | 0 | 0 | 0 | Y | 9417 | RecName: Full=Kunitz-type serine protease inhibitor 3; AltName: Full=Kunitz protease inhibitor 3; AltName: Full=Kunitz protease inhibitor III; Flags: Precursor |
| 213 | 2019 | XP\_032084681.1 | 72.28 | 2 | 0 | 0 | 0 | 0 | 2 | 0 | 0 | 0 | 0 | 2 | 0 | 0 | 0 |  |  |  |  | 6.4023E5 |  |  |  |  | 2.2329E7 |  |  |  | 1 | 1 | 0 | 0 | 0 | 0 | 1 | 0 | 0 | 0 | 0 | 3 | 0 | 0 | 0 | Y | 69288 | zinc metalloproteinase-disintegrin-like MTP9 [Thamnophis elegans] |
| 213 | 2715 | XP\_013922279.1 | 72.28 | 7 | 0 | 0 | 0 | 0 | 7 | 0 | 0 | 0 | 0 | 7 | 0 | 0 | 0 |  |  |  |  | 6.4023E5 |  |  |  |  | 2.2329E7 |  |  |  | 1 | 1 | 0 | 0 | 0 | 0 | 1 | 0 | 0 | 0 | 0 | 3 | 0 | 0 | 0 | Y | 18221 | PREDICTED: hemorrhagic metalloproteinase-disintegrin-like kaouthiagin, partial [Thamnophis sirtalis] |
| 297 | 1929 | P25679.2 | 71.90 | 20 | 0 | 0 | 0 | 0 | 0 | 0 | 0 | 0 | 0 | 20 | 0 | 0 | 0 |  |  |  |  |  |  |  |  |  | 1.8887E7 |  |  |  | 1 | 1 | 0 | 0 | 0 | 0 | 0 | 0 | 0 | 0 | 0 | 2 | 0 | 0 | 0 | Y | 7438 | RecName: Full=Weak toxin CM-9a |
| 297 | 1960 | P60814.1 | 71.90 | 15 | 0 | 0 | 0 | 0 | 0 | 0 | 0 | 0 | 0 | 15 | 0 | 0 | 0 |  |  |  |  |  |  |  |  |  | 1.8887E7 |  |  |  | 1 | 1 | 0 | 0 | 0 | 0 | 0 | 0 | 0 | 0 | 0 | 2 | 0 | 0 | 0 | Y | 9811 | RecName: Full=Probable weak neurotoxin NNAM2I; Flags: Precursor |
| 297 | 1961 | CAA06888.1 | 71.90 | 15 | 0 | 0 | 0 | 0 | 0 | 0 | 0 | 0 | 0 | 15 | 0 | 0 | 0 |  |  |  |  |  |  |  |  |  | 1.8887E7 |  |  |  | 1 | 1 | 0 | 0 | 0 | 0 | 0 | 0 | 0 | 0 | 0 | 2 | 0 | 0 | 0 | Y | 9811 | neurotoxin [Naja atra] |
| 297 | 2168 | 2MJ0 | 71.90 | 20 | 0 | 0 | 0 | 0 | 0 | 0 | 0 | 0 | 0 | 20 | 0 | 0 | 0 |  |  |  |  |  |  |  |  |  | 1.8887E7 |  |  |  | 1 | 1 | 0 | 0 | 0 | 0 | 0 | 0 | 0 | 0 | 0 | 2 | 0 | 0 | 0 | Y | 7729 | Chain A, Weak tryptophan-containing neurotoxin |
| 297 | 2259 | P82935.2 | 71.90 | 15 | 0 | 0 | 0 | 0 | 0 | 0 | 0 | 0 | 0 | 15 | 0 | 0 | 0 |  |  |  |  |  |  |  |  |  | 1.8887E7 |  |  |  | 1 | 1 | 0 | 0 | 0 | 0 | 0 | 0 | 0 | 0 | 0 | 2 | 0 | 0 | 0 | Y | 9915 | RecName: Full=Tryptophan-containing weak neurotoxin; Short=WTX; Flags: Precursor |
| 297 | 2315 | CAA04578.1 | 71.90 | 15 | 0 | 0 | 0 | 0 | 0 | 0 | 0 | 0 | 0 | 15 | 0 | 0 | 0 |  |  |  |  |  |  |  |  |  | 1.8887E7 |  |  |  | 1 | 1 | 0 | 0 | 0 | 0 | 0 | 0 | 0 | 0 | 0 | 2 | 0 | 0 | 0 | Y | 9885 | unnamed protein product (plasmid) [Naja atra] |
| 297 | 2363 | AAL87467.1 | 71.90 | 15 | 0 | 0 | 0 | 0 | 0 | 0 | 0 | 0 | 0 | 15 | 0 | 0 | 0 |  |  |  |  |  |  |  |  |  | 1.8887E7 |  |  |  | 1 | 1 | 0 | 0 | 0 | 0 | 0 | 0 | 0 | 0 | 0 | 2 | 0 | 0 | 0 | Y | 9806 | weak neurotoxin 5 precursor [Naja sputatrix] |
| 297 | 2364 | O42255.1 | 71.90 | 15 | 0 | 0 | 0 | 0 | 0 | 0 | 0 | 0 | 0 | 15 | 0 | 0 | 0 |  |  |  |  |  |  |  |  |  | 1.8887E7 |  |  |  | 1 | 1 | 0 | 0 | 0 | 0 | 0 | 0 | 0 | 0 | 0 | 2 | 0 | 0 | 0 | Y | 9806 | RecName: Full=Weak neurotoxin 5; Short=Wntx-5; Flags: Precursor |
| 297 | 2365 | AAB87415.1 | 71.90 | 15 | 0 | 0 | 0 | 0 | 0 | 0 | 0 | 0 | 0 | 15 | 0 | 0 | 0 |  |  |  |  |  |  |  |  |  | 1.8887E7 |  |  |  | 1 | 1 | 0 | 0 | 0 | 0 | 0 | 0 | 0 | 0 | 0 | 2 | 0 | 0 | 0 | Y | 9806 | weak neurotoxin isoform precursor [Naja sputatrix] |
| 297 | 2449 | AAB87416.1 | 71.90 | 15 | 0 | 0 | 0 | 0 | 0 | 0 | 0 | 0 | 0 | 15 | 0 | 0 | 0 |  |  |  |  |  |  |  |  |  | 1.8887E7 |  |  |  | 1 | 1 | 0 | 0 | 0 | 0 | 0 | 0 | 0 | 0 | 0 | 2 | 0 | 0 | 0 | Y | 9807 | weak neurotoxin isoform precursor [Naja sputatrix] |
| 297 | 2450 | O42256.1 | 71.90 | 15 | 0 | 0 | 0 | 0 | 0 | 0 | 0 | 0 | 0 | 15 | 0 | 0 | 0 |  |  |  |  |  |  |  |  |  | 1.8887E7 |  |  |  | 1 | 1 | 0 | 0 | 0 | 0 | 0 | 0 | 0 | 0 | 0 | 2 | 0 | 0 | 0 | Y | 9807 | RecName: Full=Weak neurotoxin 6; Short=Wntx-6; Flags: Precursor |
| 297 | 2480 | CAA10530.1 | 71.90 | 15 | 0 | 0 | 0 | 0 | 0 | 0 | 0 | 0 | 0 | 15 | 0 | 0 | 0 |  |  |  |  |  |  |  |  |  | 1.8887E7 |  |  |  | 1 | 1 | 0 | 0 | 0 | 0 | 0 | 0 | 0 | 0 | 0 | 2 | 0 | 0 | 0 | Y | 9899 | neurotoxin homolog [Naja atra] |
| 297 | 2481 | Q9YGI4.1 | 71.90 | 15 | 0 | 0 | 0 | 0 | 0 | 0 | 0 | 0 | 0 | 15 | 0 | 0 | 0 |  |  |  |  |  |  |  |  |  | 1.8887E7 |  |  |  | 1 | 1 | 0 | 0 | 0 | 0 | 0 | 0 | 0 | 0 | 0 | 2 | 0 | 0 | 0 | Y | 9899 | RecName: Full=Probable weak neurotoxin NNAM2; AltName: Full=TA-N9; Flags: Precursor |
| 297 | 2622 | AAL87466.1 | 71.90 | 15 | 0 | 0 | 0 | 0 | 0 | 0 | 0 | 0 | 0 | 15 | 0 | 0 | 0 |  |  |  |  |  |  |  |  |  | 1.8887E7 |  |  |  | 1 | 1 | 0 | 0 | 0 | 0 | 0 | 0 | 0 | 0 | 0 | 2 | 0 | 0 | 0 | Y | 9823 | weak neurotoxin 8 precursor [Naja sputatrix] |
| 297 | 2623 | AAD39353.1 | 71.90 | 15 | 0 | 0 | 0 | 0 | 0 | 0 | 0 | 0 | 0 | 15 | 0 | 0 | 0 |  |  |  |  |  |  |  |  |  | 1.8887E7 |  |  |  | 1 | 1 | 0 | 0 | 0 | 0 | 0 | 0 | 0 | 0 | 0 | 2 | 0 | 0 | 0 | Y | 9809 | weak neurotoxin precursor [Naja sputatrix] |
| 297 | 2624 | Q802B3.2 | 71.90 | 15 | 0 | 0 | 0 | 0 | 0 | 0 | 0 | 0 | 0 | 15 | 0 | 0 | 0 |  |  |  |  |  |  |  |  |  | 1.8887E7 |  |  |  | 1 | 1 | 0 | 0 | 0 | 0 | 0 | 0 | 0 | 0 | 0 | 2 | 0 | 0 | 0 | Y | 9809 | RecName: Full=Weak neurotoxin 8; Short=Wntx-8; Flags: Precursor |
| 297 | 2832 | AAD39354.1 | 71.90 | 15 | 0 | 0 | 0 | 0 | 0 | 0 | 0 | 0 | 0 | 15 | 0 | 0 | 0 |  |  |  |  |  |  |  |  |  | 1.8887E7 |  |  |  | 1 | 1 | 0 | 0 | 0 | 0 | 0 | 0 | 0 | 0 | 0 | 2 | 0 | 0 | 0 | Y | 9836 | weak neurotoxin precursor [Naja sputatrix] |
| 297 | 2833 | Q9W7I3.1 | 71.90 | 15 | 0 | 0 | 0 | 0 | 0 | 0 | 0 | 0 | 0 | 15 | 0 | 0 | 0 |  |  |  |  |  |  |  |  |  | 1.8887E7 |  |  |  | 1 | 1 | 0 | 0 | 0 | 0 | 0 | 0 | 0 | 0 | 0 | 2 | 0 | 0 | 0 | Y | 9836 | RecName: Full=Weak neurotoxin 9; Short=Wntx-9; Flags: Precursor |
| 297 | 2892 | AAL87465.1 | 71.90 | 15 | 0 | 0 | 0 | 0 | 0 | 0 | 0 | 0 | 0 | 15 | 0 | 0 | 0 |  |  |  |  |  |  |  |  |  | 1.8887E7 |  |  |  | 1 | 1 | 0 | 0 | 0 | 0 | 0 | 0 | 0 | 0 | 0 | 2 | 0 | 0 | 0 | Y | 9824 | weak neurotoxin 6 precursor [Naja sputatrix] |
| 297 | 3761 | C0HJW9.2 | 71.90 | 39 | 0 | 0 | 0 | 0 | 0 | 0 | 0 | 0 | 0 | 39 | 0 | 0 | 0 |  |  |  |  |  |  |  |  |  | 1.8887E7 |  |  |  | 1 | 1 | 0 | 0 | 0 | 0 | 0 | 0 | 0 | 0 | 0 | 2 | 0 | 0 | 0 | Y | 3943 | RecName: Full=Neurotoxin Nk-3FTx |
| 225 | 1914 | E5L0E4.1 | 71.71 | 6 | 0 | 6 | 6 | 6 | 0 | 0 | 0 | 0 | 0 | 0 | 0 | 0 | 0 |  | 3.9103E6 | 3.6573E6 | 1.9316E6 |  |  |  |  |  |  |  |  |  | 1 | 1 | 0 | 1 | 3 | 1 | 0 | 0 | 0 | 0 | 0 | 0 | 0 | 0 | 0 | N | 28035 | RecName: Full=Beta-fibrinogenase-like; Short=RVBF; AltName: Full=Snake venom serine protease; Short=SVSP; Flags: Precursor |
| 225 | 1915 | ADP88560.1 | 71.71 | 6 | 0 | 6 | 6 | 6 | 0 | 0 | 0 | 0 | 0 | 0 | 0 | 0 | 0 |  | 3.9103E6 | 3.6573E6 | 1.9316E6 |  |  |  |  |  |  |  |  |  | 1 | 1 | 0 | 1 | 3 | 1 | 0 | 0 | 0 | 0 | 0 | 0 | 0 | 0 | 0 | N | 28035 | serine beta-fibrinogenase-like protein precursor [Daboia siamensis] |
| 228 | 1389 | ETE73990.1 | 71.44 | 1 | 0 | 1 | 0 | 0 | 0 | 0 | 0 | 0 | 0 | 0 | 0 | 0 | 1 |  | 7.3137E6 |  |  |  |  |  |  |  |  |  |  | 4.6581E6 | 1 | 1 | 0 | 2 | 0 | 0 | 0 | 0 | 0 | 0 | 0 | 0 | 0 | 0 | 2 | N | 146075 | Desmocollin-2 [Ophiophagus hannah] |
| 228 | 1402 | XP\_032078632.1 | 71.44 | 1 | 0 | 1 | 0 | 0 | 0 | 0 | 0 | 0 | 0 | 0 | 0 | 0 | 1 |  | 7.3137E6 |  |  |  |  |  |  |  |  |  |  | 4.6581E6 | 1 | 1 | 0 | 2 | 0 | 0 | 0 | 0 | 0 | 0 | 0 | 0 | 0 | 0 | 2 | N | 100258 | LOW QUALITY PROTEIN: desmocollin-2-like [Thamnophis elegans] |
| 228 | 1403 | XP\_013911097.1 | 71.44 | 1 | 0 | 1 | 0 | 0 | 0 | 0 | 0 | 0 | 0 | 0 | 0 | 0 | 1 |  | 7.3137E6 |  |  |  |  |  |  |  |  |  |  | 4.6581E6 | 1 | 1 | 0 | 2 | 0 | 0 | 0 | 0 | 0 | 0 | 0 | 0 | 0 | 0 | 2 | N | 100546 | PREDICTED: desmocollin-2-like isoform X1 [Thamnophis sirtalis] |
| 228 | 1404 | XP\_013911096.1 | 71.44 | 1 | 0 | 1 | 0 | 0 | 0 | 0 | 0 | 0 | 0 | 0 | 0 | 0 | 1 |  | 7.3137E6 |  |  |  |  |  |  |  |  |  |  | 4.6581E6 | 1 | 1 | 0 | 2 | 0 | 0 | 0 | 0 | 0 | 0 | 0 | 0 | 0 | 0 | 2 | N | 100546 | PREDICTED: desmocollin-2-like isoform X1 [Thamnophis sirtalis] |
| 228 | 1405 | XP\_013911098.1 | 71.44 | 2 | 0 | 2 | 0 | 0 | 0 | 0 | 0 | 0 | 0 | 0 | 0 | 0 | 2 |  | 7.3137E6 |  |  |  |  |  |  |  |  |  |  | 4.6581E6 | 1 | 1 | 0 | 2 | 0 | 0 | 0 | 0 | 0 | 0 | 0 | 0 | 0 | 0 | 2 | N | 94407 | PREDICTED: desmocollin-2-like isoform X2 [Thamnophis sirtalis] |
| 228 | 1432 | XP\_026526159.1 | 71.44 | 2 | 0 | 2 | 0 | 0 | 0 | 0 | 0 | 0 | 0 | 0 | 0 | 0 | 2 |  | 7.3137E6 |  |  |  |  |  |  |  |  |  |  | 4.6581E6 | 1 | 1 | 0 | 2 | 0 | 0 | 0 | 0 | 0 | 0 | 0 | 0 | 0 | 0 | 2 | N | 94797 | desmocollin-2-like isoform X3 [Notechis scutatus] |
| 228 | 1433 | XP\_026526158.1 | 71.44 | 2 | 0 | 2 | 0 | 0 | 0 | 0 | 0 | 0 | 0 | 0 | 0 | 0 | 2 |  | 7.3137E6 |  |  |  |  |  |  |  |  |  |  | 4.6581E6 | 1 | 1 | 0 | 2 | 0 | 0 | 0 | 0 | 0 | 0 | 0 | 0 | 0 | 0 | 2 | N | 94988 | desmocollin-2-like isoform X2 [Notechis scutatus] |
| 228 | 1438 | XP\_026526157.1 | 71.44 | 1 | 0 | 1 | 0 | 0 | 0 | 0 | 0 | 0 | 0 | 0 | 0 | 0 | 1 |  | 7.3137E6 |  |  |  |  |  |  |  |  |  |  | 4.6581E6 | 1 | 1 | 0 | 2 | 0 | 0 | 0 | 0 | 0 | 0 | 0 | 0 | 0 | 0 | 2 | N | 100936 | desmocollin-2-like isoform X1 [Notechis scutatus] |
| 218 | 3715 | ETE67110.1 | 70.63 | 8 | 0 | 8 | 8 | 0 | 0 | 0 | 0 | 0 | 0 | 0 | 0 | 0 | 0 |  | 1.0165E6 | 1.4658E6 |  |  |  |  |  |  |  |  |  |  | 1 | 1 | 0 | 2 | 3 | 0 | 0 | 0 | 0 | 0 | 0 | 0 | 0 | 0 | 0 | N | 16724 | Insulin-like growth factor-binding protein 6 [Ophiophagus hannah] |
| 271 | 1319 | JAB53705.1 | 70.54 | 14 | 0 | 0 | 0 | 14 | 0 | 0 | 0 | 0 | 0 | 0 | 0 | 0 | 0 |  |  |  | 1.596E6 |  |  |  |  |  |  |  |  |  | 2 | 2 | 0 | 0 | 0 | 3 | 0 | 0 | 0 | 0 | 0 | 0 | 0 | 0 | 0 | N | 23247 | phosphatidylethanolamine-binding protein 4 [Micrurus fulvius] |
| 271 | 1511 | XP\_026539772.1 | 70.54 | 14 | 0 | 0 | 0 | 14 | 0 | 0 | 0 | 0 | 0 | 0 | 0 | 0 | 0 |  |  |  | 1.596E6 |  |  |  |  |  |  |  |  |  | 2 | 2 | 0 | 0 | 0 | 3 | 0 | 0 | 0 | 0 | 0 | 0 | 0 | 0 | 0 | N | 23342 | phosphatidylethanolamine-binding protein 4 [Notechis scutatus] |
| 271 | 1512 | XP\_026539773.1 | 70.54 | 14 | 0 | 0 | 0 | 14 | 0 | 0 | 0 | 0 | 0 | 0 | 0 | 0 | 0 |  |  |  | 1.596E6 |  |  |  |  |  |  |  |  |  | 2 | 2 | 0 | 0 | 0 | 3 | 0 | 0 | 0 | 0 | 0 | 0 | 0 | 0 | 0 | N | 23342 | phosphatidylethanolamine-binding protein 4 [Notechis scutatus] |
| 271 | 2455 | XP\_026570986.1 | 70.54 | 14 | 0 | 0 | 0 | 14 | 0 | 0 | 0 | 0 | 0 | 0 | 0 | 0 | 0 |  |  |  | 1.596E6 |  |  |  |  |  |  |  |  |  | 2 | 2 | 0 | 0 | 0 | 3 | 0 | 0 | 0 | 0 | 0 | 0 | 0 | 0 | 0 | N | 23371 | phosphatidylethanolamine-binding protein 4 [Pseudonaja textilis] |
| 271 | 2456 | XP\_026570987.1 | 70.54 | 14 | 0 | 0 | 0 | 14 | 0 | 0 | 0 | 0 | 0 | 0 | 0 | 0 | 0 |  |  |  | 1.596E6 |  |  |  |  |  |  |  |  |  | 2 | 2 | 0 | 0 | 0 | 3 | 0 | 0 | 0 | 0 | 0 | 0 | 0 | 0 | 0 | N | 23371 | phosphatidylethanolamine-binding protein 4 [Pseudonaja textilis] |
| 270 | 1231 | JAC94989.1 | 69.37 | 5 | 0 | 0 | 0 | 0 | 5 | 0 | 0 | 0 | 0 | 0 | 0 | 0 | 0 |  |  |  |  | 1.7683E4 |  |  |  |  |  |  |  |  | 2 | 1 | 0 | 0 | 0 | 0 | 2 | 0 | 0 | 0 | 0 | 0 | 0 | 0 | 0 | N | 63907 | Phospholipase B [Opheodrys aestivus] |
| 304 | 2520 | JAB52813.1 | 69.00 | 13 | 0 | 13 | 0 | 0 | 0 | 0 | 0 | 0 | 0 | 0 | 0 | 0 | 13 |  | 2.1439E6 |  |  |  |  |  |  |  |  |  |  | 1.8341E6 | 1 | 1 | 0 | 1 | 0 | 0 | 0 | 0 | 0 | 0 | 0 | 0 | 0 | 0 | 1 | Y | 16274 | Phospholipase A2 10 [Micrurus fulvius] |
| 304 | 2521 | JAI09045.1 | 69.00 | 13 | 0 | 13 | 0 | 0 | 0 | 0 | 0 | 0 | 0 | 0 | 0 | 0 | 13 |  | 2.1439E6 |  |  |  |  |  |  |  |  |  |  | 1.8341E6 | 1 | 1 | 0 | 1 | 0 | 0 | 0 | 0 | 0 | 0 | 0 | 0 | 0 | 0 | 1 | Y | 16274 | Phospholipase A2 1 [Micrurus fulvius] |
| 304 | 2522 | JAS05034.1 | 69.00 | 13 | 0 | 13 | 0 | 0 | 0 | 0 | 0 | 0 | 0 | 0 | 0 | 0 | 13 |  | 2.1439E6 |  |  |  |  |  |  |  |  |  |  | 1.8341E6 | 1 | 1 | 0 | 1 | 0 | 0 | 0 | 0 | 0 | 0 | 0 | 0 | 0 | 0 | 1 | Y | 16274 | Phospholipase A2 1 [Micrurus fulvius] |
| 304 | 2523 | JAS05141.1 | 69.00 | 13 | 0 | 13 | 0 | 0 | 0 | 0 | 0 | 0 | 0 | 0 | 0 | 0 | 13 |  | 2.1439E6 |  |  |  |  |  |  |  |  |  |  | 1.8341E6 | 1 | 1 | 0 | 1 | 0 | 0 | 0 | 0 | 0 | 0 | 0 | 0 | 0 | 0 | 1 | Y | 16274 | Phospholipase A2 1 [Micrurus tener] |
| 494 | 2519 | XP\_026558006.1 | 68.99 | 8 | 0 | 8 | 0 | 0 | 0 | 0 | 0 | 0 | 0 | 0 | 0 | 0 | 0 |  | 9.5542E6 |  |  |  |  |  |  |  |  |  |  |  | 1 | 1 | 0 | 1 | 0 | 0 | 0 | 0 | 0 | 0 | 0 | 0 | 0 | 0 | 0 | Y | 40576 | MANSC domain-containing protein 1 [Pseudonaja textilis] |
| 289 | 1344 | XP\_032084613.1 | 68.76 | 2 | 0 | 2 | 0 | 0 | 0 | 0 | 0 | 0 | 0 | 0 | 0 | 0 | 0 |  | 0 |  |  |  |  |  |  |  |  |  |  |  | 1 | 1 | 0 | 1 | 0 | 0 | 0 | 0 | 0 | 0 | 0 | 0 | 0 | 0 | 0 | Y | 87537 | amyloid-like protein 2 isoform X2 [Thamnophis elegans] |
| 289 | 1345 | XP\_032084612.1 | 68.76 | 2 | 0 | 2 | 0 | 0 | 0 | 0 | 0 | 0 | 0 | 0 | 0 | 0 | 0 |  | 0 |  |  |  |  |  |  |  |  |  |  |  | 1 | 1 | 0 | 1 | 0 | 0 | 0 | 0 | 0 | 0 | 0 | 0 | 0 | 0 | 0 | Y | 88960 | amyloid-like protein 2 isoform X1 [Thamnophis elegans] |
| 289 | 1353 | XP\_032084615.1 | 68.76 | 2 | 0 | 2 | 0 | 0 | 0 | 0 | 0 | 0 | 0 | 0 | 0 | 0 | 0 |  | 0 |  |  |  |  |  |  |  |  |  |  |  | 1 | 1 | 0 | 1 | 0 | 0 | 0 | 0 | 0 | 0 | 0 | 0 | 0 | 0 | 0 | Y | 81056 | amyloid-like protein 2 isoform X4 [Thamnophis elegans] |
| 289 | 1354 | XP\_032084614.1 | 68.76 | 2 | 0 | 2 | 0 | 0 | 0 | 0 | 0 | 0 | 0 | 0 | 0 | 0 | 0 |  | 0 |  |  |  |  |  |  |  |  |  |  |  | 1 | 1 | 0 | 1 | 0 | 0 | 0 | 0 | 0 | 0 | 0 | 0 | 0 | 0 | 0 | Y | 82479 | amyloid-like protein 2 isoform X3 [Thamnophis elegans] |
| 289 | 1406 | XP\_026534403.1 | 68.76 | 2 | 0 | 2 | 0 | 0 | 0 | 0 | 0 | 0 | 0 | 0 | 0 | 0 | 0 |  | 0 |  |  |  |  |  |  |  |  |  |  |  | 1 | 1 | 0 | 1 | 0 | 0 | 0 | 0 | 0 | 0 | 0 | 0 | 0 | 0 | 0 | Y | 87705 | amyloid-like protein 2 isoform X2 [Notechis scutatus] |
| 289 | 1407 | XP\_026534402.1 | 68.76 | 2 | 0 | 2 | 0 | 0 | 0 | 0 | 0 | 0 | 0 | 0 | 0 | 0 | 0 |  | 0 |  |  |  |  |  |  |  |  |  |  |  | 1 | 1 | 0 | 1 | 0 | 0 | 0 | 0 | 0 | 0 | 0 | 0 | 0 | 0 | 0 | Y | 89114 | amyloid-like protein 2 isoform X1 [Notechis scutatus] |
| 289 | 1408 | XP\_026564759.1 | 68.76 | 2 | 0 | 2 | 0 | 0 | 0 | 0 | 0 | 0 | 0 | 0 | 0 | 0 | 0 |  | 0 |  |  |  |  |  |  |  |  |  |  |  | 1 | 1 | 0 | 1 | 0 | 0 | 0 | 0 | 0 | 0 | 0 | 0 | 0 | 0 | 0 | Y | 87596 | amyloid-like protein 2 isoform X2 [Pseudonaja textilis] |
| 289 | 1409 | XP\_026564758.1 | 68.76 | 2 | 0 | 2 | 0 | 0 | 0 | 0 | 0 | 0 | 0 | 0 | 0 | 0 | 0 |  | 0 |  |  |  |  |  |  |  |  |  |  |  | 1 | 1 | 0 | 1 | 0 | 0 | 0 | 0 | 0 | 0 | 0 | 0 | 0 | 0 | 0 | Y | 89005 | amyloid-like protein 2 isoform X1 [Pseudonaja textilis] |
| 289 | 1434 | XP\_026534405.1 | 68.76 | 2 | 0 | 2 | 0 | 0 | 0 | 0 | 0 | 0 | 0 | 0 | 0 | 0 | 0 |  | 0 |  |  |  |  |  |  |  |  |  |  |  | 1 | 1 | 0 | 1 | 0 | 0 | 0 | 0 | 0 | 0 | 0 | 0 | 0 | 0 | 0 | Y | 81206 | amyloid-like protein 2 isoform X4 [Notechis scutatus] |
| 289 | 1435 | XP\_026534404.1 | 68.76 | 2 | 0 | 2 | 0 | 0 | 0 | 0 | 0 | 0 | 0 | 0 | 0 | 0 | 0 |  | 0 |  |  |  |  |  |  |  |  |  |  |  | 1 | 1 | 0 | 1 | 0 | 0 | 0 | 0 | 0 | 0 | 0 | 0 | 0 | 0 | 0 | Y | 82614 | amyloid-like protein 2 isoform X3 [Notechis scutatus] |
| 289 | 1436 | XP\_026564761.1 | 68.76 | 2 | 0 | 2 | 0 | 0 | 0 | 0 | 0 | 0 | 0 | 0 | 0 | 0 | 0 |  | 0 |  |  |  |  |  |  |  |  |  |  |  | 1 | 1 | 0 | 1 | 0 | 0 | 0 | 0 | 0 | 0 | 0 | 0 | 0 | 0 | 0 | Y | 81096 | amyloid-like protein 2 isoform X4 [Pseudonaja textilis] |
| 289 | 1437 | XP\_026564760.1 | 68.76 | 2 | 0 | 2 | 0 | 0 | 0 | 0 | 0 | 0 | 0 | 0 | 0 | 0 | 0 |  | 0 |  |  |  |  |  |  |  |  |  |  |  | 1 | 1 | 0 | 1 | 0 | 0 | 0 | 0 | 0 | 0 | 0 | 0 | 0 | 0 | 0 | Y | 82505 | amyloid-like protein 2 isoform X3 [Pseudonaja textilis] |
| 289 | 1496 | LAA59850.1 | 68.76 | 3 | 0 | 3 | 0 | 0 | 0 | 0 | 0 | 0 | 0 | 0 | 0 | 0 | 0 |  | 0 |  |  |  |  |  |  |  |  |  |  |  | 1 | 1 | 0 | 1 | 0 | 0 | 0 | 0 | 0 | 0 | 0 | 0 | 0 | 0 | 0 | Y | 47699 | hypothetical protein, partial [Micrurus corallinus] |
| 319 | 4096 | ETE57873.1 | 67.63 | 12 | 0 | 0 | 0 | 12 | 0 | 0 | 0 | 0 | 0 | 0 | 0 | 0 | 0 |  |  |  | 8.0605E5 |  |  |  |  |  |  |  |  |  | 1 | 1 | 0 | 0 | 0 | 2 | 0 | 0 | 0 | 0 | 0 | 0 | 0 | 0 | 0 | N | 13885 | hypothetical protein L345\_16412, partial [Ophiophagus hannah] |
| 149 | 2588 | E3P6P4.1 | 65.98 | 6 | 0 | 6 | 6 | 0 | 0 | 0 | 0 | 0 | 0 | 0 | 0 | 0 | 6 |  | 1.1945E8 | 5.9073E5 |  |  |  |  |  |  |  |  |  | 1.7714E6 | 1 | 1 | 0 | 15 | 1 | 0 | 0 | 0 | 0 | 0 | 0 | 0 | 0 | 0 | 2 | Y | 15772 | RecName: Full=Cystatin; Flags: Precursor |
| 149 | 2589 | ACR83850.1 | 65.98 | 6 | 0 | 6 | 6 | 0 | 0 | 0 | 0 | 0 | 0 | 0 | 0 | 0 | 6 |  | 1.1945E8 | 5.9073E5 |  |  |  |  |  |  |  |  |  | 1.7714E6 | 1 | 1 | 0 | 15 | 1 | 0 | 0 | 0 | 0 | 0 | 0 | 0 | 0 | 0 | 2 | Y | 15772 | cystatin precursor [Naja kaouthia] |
| 306 | 2490 | JAS03127.1 | 64.42 | 8 | 0 | 0 | 0 | 0 | 0 | 0 | 0 | 0 | 0 | 8 | 0 | 0 | 0 |  |  |  |  |  |  |  |  |  | 1.7968E7 |  |  |  | 1 | 1 | 0 | 0 | 0 | 0 | 0 | 0 | 0 | 0 | 0 | 2 | 0 | 0 | 0 | Y | 20534 | Papilin, partial [Phalotris mertensi] |
| 237 | 1636 | S68801 | 64.29 | 11 | 0 | 0 | 0 | 11 | 0 | 0 | 0 | 0 | 0 | 0 | 0 | 0 | 0 |  |  |  | 2.7542E5 |  |  |  |  |  |  |  |  |  | 1 | 1 | 0 | 0 | 0 | 1 | 0 | 0 | 0 | 0 | 0 | 0 | 0 | 0 | 0 | N | 9793 | acetylcholinesterase (EC 3.1.1.7) - banded krait (fragments) |
| 251 | 1657 | XP\_026524106.1 | 63.65 | 2 | 0 | 0 | 2 | 2 | 0 | 0 | 0 | 0 | 0 | 0 | 0 | 0 | 0 |  |  | 6.5993E5 | 6.3396E5 |  |  |  |  |  |  |  |  |  | 1 | 1 | 0 | 0 | 1 | 1 | 0 | 0 | 0 | 0 | 0 | 0 | 0 | 0 | 0 | N | 55065 | nucleobindin-2 [Notechis scutatus] |
| 251 | 1675 | XP\_026562300.1 | 63.65 | 2 | 0 | 0 | 2 | 2 | 0 | 0 | 0 | 0 | 0 | 0 | 0 | 0 | 0 |  |  | 6.5993E5 | 6.3396E5 |  |  |  |  |  |  |  |  |  | 1 | 1 | 0 | 0 | 1 | 1 | 0 | 0 | 0 | 0 | 0 | 0 | 0 | 0 | 0 | N | 51084 | nucleobindin-2 isoform X2 [Pseudonaja textilis] |
| 251 | 1676 | XP\_026562299.1 | 63.65 | 2 | 0 | 0 | 2 | 2 | 0 | 0 | 0 | 0 | 0 | 0 | 0 | 0 | 0 |  |  | 6.5993E5 | 6.3396E5 |  |  |  |  |  |  |  |  |  | 1 | 1 | 0 | 0 | 1 | 1 | 0 | 0 | 0 | 0 | 0 | 0 | 0 | 0 | 0 | N | 55120 | nucleobindin-2 isoform X1 [Pseudonaja textilis] |
| 255 | 2184 | XP\_034262406.1 | 62.29 | 4 | 0 | 0 | 0 | 4 | 0 | 0 | 0 | 0 | 0 | 0 | 0 | 0 | 0 |  |  |  | 1.462E7 |  |  |  |  |  |  |  |  |  | 1 | 1 | 0 | 0 | 0 | 3 | 0 | 0 | 0 | 0 | 0 | 0 | 0 | 0 | 0 | N | 25308 | serum amyloid P-component-like, partial [Pantherophis guttatus] |
| 255 | 3229 | XP\_015678953.1 | 62.29 | 4 | 0 | 0 | 0 | 4 | 0 | 0 | 0 | 0 | 0 | 0 | 0 | 0 | 0 |  |  |  | 1.462E7 |  |  |  |  |  |  |  |  |  | 1 | 1 | 0 | 0 | 0 | 3 | 0 | 0 | 0 | 0 | 0 | 0 | 0 | 0 | 0 | N | 25637 | C-reactive protein [Protobothrops mucrosquamatus] |
| 255 | 3626 | JAG66558.1 | 62.29 | 4 | 0 | 0 | 0 | 4 | 0 | 0 | 0 | 0 | 0 | 0 | 0 | 0 | 0 |  |  |  | 1.462E7 |  |  |  |  |  |  |  |  |  | 1 | 1 | 0 | 0 | 0 | 3 | 0 | 0 | 0 | 0 | 0 | 0 | 0 | 0 | 0 | N | 25320 | serum amyloid P-component-like [Boiga irregularis] |
| 255 | 3683 | XP\_026545516.1 | 62.29 | 4 | 0 | 0 | 0 | 4 | 0 | 0 | 0 | 0 | 0 | 0 | 0 | 0 | 0 |  |  |  | 1.462E7 |  |  |  |  |  |  |  |  |  | 1 | 1 | 0 | 0 | 0 | 3 | 0 | 0 | 0 | 0 | 0 | 0 | 0 | 0 | 0 | N | 25122 | C-reactive protein-like [Notechis scutatus] |
| 255 | 3772 | ETE56526.1 | 62.29 | 5 | 0 | 0 | 0 | 5 | 0 | 0 | 0 | 0 | 0 | 0 | 0 | 0 | 0 |  |  |  | 1.462E7 |  |  |  |  |  |  |  |  |  | 1 | 1 | 0 | 0 | 0 | 3 | 0 | 0 | 0 | 0 | 0 | 0 | 0 | 0 | 0 | N | 22732 | Serum amyloid P-component, partial [Ophiophagus hannah] |
| 255 | 3879 | XP\_034261801.1 | 62.29 | 4 | 0 | 0 | 0 | 4 | 0 | 0 | 0 | 0 | 0 | 0 | 0 | 0 | 0 |  |  |  | 1.462E7 |  |  |  |  |  |  |  |  |  | 1 | 1 | 0 | 0 | 0 | 3 | 0 | 0 | 0 | 0 | 0 | 0 | 0 | 0 | 0 | N | 25330 | C-reactive protein-like [Pantherophis guttatus] |
| 255 | 4298 | XP\_026572193.1 | 62.29 | 4 | 0 | 0 | 0 | 4 | 0 | 0 | 0 | 0 | 0 | 0 | 0 | 0 | 0 |  |  |  | 1.462E7 |  |  |  |  |  |  |  |  |  | 1 | 1 | 0 | 0 | 0 | 3 | 0 | 0 | 0 | 0 | 0 | 0 | 0 | 0 | 0 | N | 25165 | serum amyloid P-component-like [Pseudonaja textilis] |
| 255 | 4299 | XP\_015678948.1 | 62.29 | 4 | 0 | 0 | 0 | 4 | 0 | 0 | 0 | 0 | 0 | 0 | 0 | 0 | 0 |  |  |  | 1.462E7 |  |  |  |  |  |  |  |  |  | 1 | 1 | 0 | 0 | 0 | 3 | 0 | 0 | 0 | 0 | 0 | 0 | 0 | 0 | 0 | N | 25142 | serum amyloid P-component-like [Protobothrops mucrosquamatus] |
| 310 | 2625 | XP\_015680851.2 | 61.93 | 5 | 0 | 5 | 0 | 0 | 0 | 0 | 0 | 0 | 0 | 0 | 0 | 0 | 0 |  | 1.9911E7 |  |  |  |  |  |  |  |  |  |  |  | 1 | 1 | 0 | 2 | 0 | 0 | 0 | 0 | 0 | 0 | 0 | 0 | 0 | 0 | 0 | Y | 20839 | cystatin-1-like [Protobothrops mucrosquamatus] |
| 318 | 4411 | XP\_026553567.1 | 61.47 | 5 | 0 | 0 | 0 | 0 | 0 | 0 | 0 | 0 | 5 | 0 | 0 | 0 | 0 |  |  |  |  |  |  |  |  | 5.4621E5 |  |  |  |  | 1 | 1 | 0 | 0 | 0 | 0 | 0 | 0 | 0 | 0 | 1 | 0 | 0 | 0 | 0 | N | 29143 | somatomedin-B and thrombospondin type-1 domain-containing protein-like [Pseudonaja textilis] |
| 449 | 1748 | XP\_013928420.1 | 60.77 | 5 | 0 | 0 | 0 | 0 | 0 | 5 | 0 | 0 | 0 | 0 | 0 | 0 | 0 |  |  |  |  |  | 7.5948E5 |  |  |  |  |  |  |  | 1 | 1 | 0 | 0 | 0 | 0 | 0 | 1 | 0 | 0 | 0 | 0 | 0 | 0 | 0 | Y | 33841 | PREDICTED: epithelial cell adhesion molecule [Thamnophis sirtalis] |
| 449 | 2118 | ETE70163.1 | 60.77 | 5 | 0 | 0 | 0 | 0 | 0 | 5 | 0 | 0 | 0 | 0 | 0 | 0 | 0 |  |  |  |  |  | 7.5948E5 |  |  |  |  |  |  |  | 1 | 1 | 0 | 0 | 0 | 0 | 0 | 1 | 0 | 0 | 0 | 0 | 0 | 0 | 0 | Y | 33908 | Epithelial cell adhesion molecule [Ophiophagus hannah] |
| 178 | 2667 | XP\_032073624.1 | 60.71 | 3 | 0 | 3 | 3 | 3 | 0 | 0 | 0 | 0 | 0 | 0 | 0 | 0 | 0 |  | 1.2432E7 | 8.5152E7 | 3.2948E6 |  |  |  |  |  |  |  |  |  | 1 | 1 | 0 | 3 | 6 | 2 | 0 | 0 | 0 | 0 | 0 | 0 | 0 | 0 | 0 | Y | 41736 | membrane cofactor protein-like isoform X1 [Thamnophis elegans] |
| 178 | 3625 | XP\_032073625.1 | 60.71 | 3 | 0 | 3 | 3 | 3 | 0 | 0 | 0 | 0 | 0 | 0 | 0 | 0 | 0 |  | 1.2432E7 | 8.5152E7 | 3.2948E6 |  |  |  |  |  |  |  |  |  | 1 | 1 | 0 | 3 | 6 | 2 | 0 | 0 | 0 | 0 | 0 | 0 | 0 | 0 | 0 | Y | 39347 | membrane cofactor protein-like isoform X2 [Thamnophis elegans] |
| 178 | 3847 | XP\_032073626.1 | 60.71 | 3 | 0 | 3 | 3 | 3 | 0 | 0 | 0 | 0 | 0 | 0 | 0 | 0 | 0 |  | 1.2432E7 | 8.5152E7 | 3.2948E6 |  |  |  |  |  |  |  |  |  | 1 | 1 | 0 | 3 | 6 | 2 | 0 | 0 | 0 | 0 | 0 | 0 | 0 | 0 | 0 | Y | 38075 | zona pellucida sperm-binding protein 3 receptor-like isoform X3 [Thamnophis elegans] |
| 317 | 1050 | ETE72142.1 | 60.64 | 8 | 0 | 0 | 0 | 8 | 0 | 0 | 0 | 0 | 0 | 0 | 0 | 0 | 0 |  |  |  | 0 |  |  |  |  |  |  |  |  |  | 1 | 1 | 0 | 0 | 0 | 1 | 0 | 0 | 0 | 0 | 0 | 0 | 0 | 0 | 0 | N | 24461 | Ribonuclease T2 [Ophiophagus hannah] |
| 248 | 1938 | LAB36464.1 | 59.68 | 2 | 0 | 0 | 0 | 0 | 0 | 0 | 0 | 0 | 0 | 0 | 0 | 2 | 0 |  |  |  |  |  |  |  |  |  |  |  | 2.7719E6 |  | 1 | 1 | 0 | 0 | 0 | 0 | 0 | 0 | 0 | 0 | 0 | 0 | 0 | 3 | 0 | N | 97420 | hypothetical protein [Micrurus spixii] |
| 256 | 4301 | XP\_015686076.1 | 59.64 | 5 | 0 | 0 | 0 | 5 | 0 | 0 | 0 | 0 | 0 | 0 | 0 | 0 | 0 |  |  |  | 1.2509E7 |  |  |  |  |  |  |  |  |  | 1 | 1 | 0 | 0 | 0 | 3 | 0 | 0 | 0 | 0 | 0 | 0 | 0 | 0 | 0 | N | 23560 | serum amyloid P-component-like, partial [Protobothrops mucrosquamatus] |
| 238 | 2072 | XP\_034262785.1 | 58.50 | 1 | 0 | 1 | 1 | 0 | 0 | 0 | 0 | 0 | 0 | 0 | 0 | 0 | 0 |  | 1.4943E6 | 8.1117E5 |  |  |  |  |  |  |  |  |  |  | 1 | 1 | 0 | 3 | 2 | 0 | 0 | 0 | 0 | 0 | 0 | 0 | 0 | 0 | 0 | N | 113116 | lysosomal alpha-mannosidase [Pantherophis guttatus] |
| 238 | 2782 | LAB27223.1 | 58.50 | 1 | 0 | 1 | 1 | 0 | 0 | 0 | 0 | 0 | 0 | 0 | 0 | 0 | 0 |  | 1.4943E6 | 8.1117E5 |  |  |  |  |  |  |  |  |  |  | 1 | 1 | 0 | 3 | 2 | 0 | 0 | 0 | 0 | 0 | 0 | 0 | 0 | 0 | 0 | N | 113239 | hypothetical protein, partial [Micrurus spixii] |
| 238 | 2982 | XP\_026537394.1 | 58.50 | 1 | 0 | 1 | 1 | 0 | 0 | 0 | 0 | 0 | 0 | 0 | 0 | 0 | 0 |  | 1.4943E6 | 8.1117E5 |  |  |  |  |  |  |  |  |  |  | 1 | 1 | 0 | 3 | 2 | 0 | 0 | 0 | 0 | 0 | 0 | 0 | 0 | 0 | 0 | N | 111874 | lysosomal alpha-mannosidase-like [Notechis scutatus] |
| 238 | 2983 | XP\_026537257.1 | 58.50 | 1 | 0 | 1 | 1 | 0 | 0 | 0 | 0 | 0 | 0 | 0 | 0 | 0 | 0 |  | 1.4943E6 | 8.1117E5 |  |  |  |  |  |  |  |  |  |  | 1 | 1 | 0 | 3 | 2 | 0 | 0 | 0 | 0 | 0 | 0 | 0 | 0 | 0 | 0 | N | 111925 | lysosomal alpha-mannosidase-like [Notechis scutatus] |
| 238 | 2984 | XP\_026552070.1 | 58.50 | 1 | 0 | 1 | 1 | 0 | 0 | 0 | 0 | 0 | 0 | 0 | 0 | 0 | 0 |  | 1.4943E6 | 8.1117E5 |  |  |  |  |  |  |  |  |  |  | 1 | 1 | 0 | 3 | 2 | 0 | 0 | 0 | 0 | 0 | 0 | 0 | 0 | 0 | 0 | N | 112008 | lysosomal alpha-mannosidase [Pseudonaja textilis] |
| 238 | 3485 | LAB59085.1 | 58.50 | 4 | 0 | 4 | 4 | 0 | 0 | 0 | 0 | 0 | 0 | 0 | 0 | 0 | 0 |  | 1.4943E6 | 8.1117E5 |  |  |  |  |  |  |  |  |  |  | 1 | 1 | 0 | 3 | 2 | 0 | 0 | 0 | 0 | 0 | 0 | 0 | 0 | 0 | 0 | N | 37828 | hypothetical protein, partial [Micrurus surinamensis] |
| 238 | 3486 | LAB59082.1 | 58.50 | 3 | 0 | 3 | 3 | 0 | 0 | 0 | 0 | 0 | 0 | 0 | 0 | 0 | 0 |  | 1.4943E6 | 8.1117E5 |  |  |  |  |  |  |  |  |  |  | 1 | 1 | 0 | 3 | 2 | 0 | 0 | 0 | 0 | 0 | 0 | 0 | 0 | 0 | 0 | N | 53569 | hypothetical protein, partial [Micrurus surinamensis] |
| 238 | 3542 | LAA55510.1 | 58.50 | 3 | 0 | 3 | 3 | 0 | 0 | 0 | 0 | 0 | 0 | 0 | 0 | 0 | 0 |  | 1.4943E6 | 8.1117E5 |  |  |  |  |  |  |  |  |  |  | 1 | 1 | 0 | 3 | 2 | 0 | 0 | 0 | 0 | 0 | 0 | 0 | 0 | 0 | 0 | N | 55498 | hypothetical protein, partial [Micrurus corallinus] |
| 238 | 3942 | LAB59086.1 | 58.50 | 4 | 0 | 4 | 4 | 0 | 0 | 0 | 0 | 0 | 0 | 0 | 0 | 0 | 0 |  | 1.4943E6 | 8.1117E5 |  |  |  |  |  |  |  |  |  |  | 1 | 1 | 0 | 3 | 2 | 0 | 0 | 0 | 0 | 0 | 0 | 0 | 0 | 0 | 0 | N | 37555 | hypothetical protein, partial [Micrurus surinamensis] |
| 259 | 2188 | ADI47719.1 | 57.47 | 4 | 0 | 0 | 0 | 4 | 0 | 0 | 0 | 0 | 0 | 0 | 0 | 0 | 0 |  |  |  | 8.4323E5 |  |  |  |  |  |  |  |  |  | 1 | 1 | 0 | 0 | 0 | 2 | 0 | 0 | 0 | 0 | 0 | 0 | 0 | 0 | 0 | N | 37367 | metalloproteinase, partial [Echis carinatus sochureki] |
| 259 | 2205 | ADI47718.1 | 57.47 | 6 | 0 | 0 | 0 | 6 | 0 | 0 | 0 | 0 | 0 | 0 | 0 | 0 | 0 |  |  |  | 8.4323E5 |  |  |  |  |  |  |  |  |  | 1 | 1 | 0 | 0 | 0 | 2 | 0 | 0 | 0 | 0 | 0 | 0 | 0 | 0 | 0 | N | 27679 | metalloproteinase, partial [Echis carinatus sochureki] |
| 267 | 2372 | JAA95034.1 | 57.46 | 8 | 8 | 0 | 0 | 0 | 0 | 0 | 0 | 0 | 0 | 0 | 0 | 0 | 0 | 3.2728E6 |  |  |  |  |  |  |  |  |  |  |  |  | 1 | 1 | 1 | 0 | 0 | 0 | 0 | 0 | 0 | 0 | 0 | 0 | 0 | 0 | 0 | N | 30806 | Voltage-dependent anion-selective channel protein 1-like protein [Crotalus horridus] |
| 267 | 2642 | XP\_032066440.1 | 57.46 | 7 | 7 | 0 | 0 | 0 | 0 | 0 | 0 | 0 | 0 | 0 | 0 | 0 | 0 | 3.2728E6 |  |  |  |  |  |  |  |  |  |  |  |  | 1 | 1 | 1 | 0 | 0 | 0 | 0 | 0 | 0 | 0 | 0 | 0 | 0 | 0 | 0 | N | 34999 | voltage-dependent anion-selective channel protein 1 [Thamnophis elegans] |
| 458 | 1018 | XP\_015680353.1 | 54.64 | 6 | 0 | 0 | 0 | 6 | 0 | 0 | 0 | 0 | 0 | 0 | 0 | 0 | 0 |  |  |  | 5.9741E5 |  |  |  |  |  |  |  |  |  | 1 | 1 | 0 | 0 | 0 | 1 | 0 | 0 | 0 | 0 | 0 | 0 | 0 | 0 | 0 | Y | 33502 | cathepsin Z [Protobothrops mucrosquamatus] |
| 458 | 1059 | XP\_032073085.1 | 54.64 | 6 | 0 | 0 | 0 | 6 | 0 | 0 | 0 | 0 | 0 | 0 | 0 | 0 | 0 |  |  |  | 5.9741E5 |  |  |  |  |  |  |  |  |  | 1 | 1 | 0 | 0 | 0 | 1 | 0 | 0 | 0 | 0 | 0 | 0 | 0 | 0 | 0 | Y | 33380 | cathepsin Z-like [Thamnophis elegans] |
| 458 | 1291 | JAA97490.1 | 54.64 | 6 | 0 | 0 | 0 | 6 | 0 | 0 | 0 | 0 | 0 | 0 | 0 | 0 | 0 |  |  |  | 5.9741E5 |  |  |  |  |  |  |  |  |  | 1 | 1 | 0 | 0 | 0 | 1 | 0 | 0 | 0 | 0 | 0 | 0 | 0 | 0 | 0 | Y | 33544 | Cathepsin Z-like protein [Crotalus horridus] |
| 458 | 1292 | JAG45445.1 | 54.64 | 6 | 0 | 0 | 0 | 6 | 0 | 0 | 0 | 0 | 0 | 0 | 0 | 0 | 0 |  |  |  | 5.9741E5 |  |  |  |  |  |  |  |  |  | 1 | 1 | 0 | 0 | 0 | 1 | 0 | 0 | 0 | 0 | 0 | 0 | 0 | 0 | 0 | Y | 33525 | Cathepsin Z-like protein [Crotalus horridus] |
| 458 | 1304 | XP\_034289001.1 | 54.64 | 6 | 0 | 0 | 0 | 6 | 0 | 0 | 0 | 0 | 0 | 0 | 0 | 0 | 0 |  |  |  | 5.9741E5 |  |  |  |  |  |  |  |  |  | 1 | 1 | 0 | 0 | 0 | 1 | 0 | 0 | 0 | 0 | 0 | 0 | 0 | 0 | 0 | Y | 33408 | cathepsin Z-like [Pantherophis guttatus] |
| 458 | 1335 | XP\_026520698.1 | 54.64 | 6 | 0 | 0 | 0 | 6 | 0 | 0 | 0 | 0 | 0 | 0 | 0 | 0 | 0 |  |  |  | 5.9741E5 |  |  |  |  |  |  |  |  |  | 1 | 1 | 0 | 0 | 0 | 1 | 0 | 0 | 0 | 0 | 0 | 0 | 0 | 0 | 0 | Y | 33585 | cathepsin Z [Notechis scutatus] |
| 458 | 2669 | LAA68286.1 | 54.64 | 5 | 0 | 0 | 0 | 5 | 0 | 0 | 0 | 0 | 0 | 0 | 0 | 0 | 0 |  |  |  | 5.9741E5 |  |  |  |  |  |  |  |  |  | 1 | 1 | 0 | 0 | 0 | 1 | 0 | 0 | 0 | 0 | 0 | 0 | 0 | 0 | 0 | Y | 36128 | hypothetical protein, partial [Micrurus lemniscatus lemniscatus] |
| 458 | 2946 | XP\_026553211.1 | 54.64 | 6 | 0 | 0 | 0 | 6 | 0 | 0 | 0 | 0 | 0 | 0 | 0 | 0 | 0 |  |  |  | 5.9741E5 |  |  |  |  |  |  |  |  |  | 1 | 1 | 0 | 0 | 0 | 1 | 0 | 0 | 0 | 0 | 0 | 0 | 0 | 0 | 0 | Y | 34145 | cathepsin Z isoform X1 [Pseudonaja textilis] |
| 458 | 3056 | XP\_032074969.1 | 54.64 | 5 | 0 | 0 | 0 | 5 | 0 | 0 | 0 | 0 | 0 | 0 | 0 | 0 | 0 |  |  |  | 5.9741E5 |  |  |  |  |  |  |  |  |  | 1 | 1 | 0 | 0 | 0 | 1 | 0 | 0 | 0 | 0 | 0 | 0 | 0 | 0 | 0 | Y | 34490 | cathepsin Z-like [Thamnophis elegans] |
| 458 | 3355 | XP\_026553213.1 | 54.64 | 6 | 0 | 0 | 0 | 6 | 0 | 0 | 0 | 0 | 0 | 0 | 0 | 0 | 0 |  |  |  | 5.9741E5 |  |  |  |  |  |  |  |  |  | 1 | 1 | 0 | 0 | 0 | 1 | 0 | 0 | 0 | 0 | 0 | 0 | 0 | 0 | 0 | Y | 33561 | cathepsin Z isoform X2 [Pseudonaja textilis] |
| 458 | 3762 | XP\_034289002.1 | 54.64 | 6 | 0 | 0 | 0 | 6 | 0 | 0 | 0 | 0 | 0 | 0 | 0 | 0 | 0 |  |  |  | 5.9741E5 |  |  |  |  |  |  |  |  |  | 1 | 1 | 0 | 0 | 0 | 1 | 0 | 0 | 0 | 0 | 0 | 0 | 0 | 0 | 0 | Y | 32467 | cathepsin Z-like [Pantherophis guttatus] |
| 458 | 4305 | LAB21704.1 | 54.64 | 13 | 0 | 0 | 0 | 13 | 0 | 0 | 0 | 0 | 0 | 0 | 0 | 0 | 0 |  |  |  | 5.9741E5 |  |  |  |  |  |  |  |  |  | 1 | 1 | 0 | 0 | 0 | 1 | 0 | 0 | 0 | 0 | 0 | 0 | 0 | 0 | 0 | Y | 14536 | hypothetical protein, partial [Micrurus spixii] |
| 458 | 4306 | XP\_015685975.1 | 54.64 | 12 | 0 | 0 | 0 | 12 | 0 | 0 | 0 | 0 | 0 | 0 | 0 | 0 | 0 |  |  |  | 5.9741E5 |  |  |  |  |  |  |  |  |  | 1 | 1 | 0 | 0 | 0 | 1 | 0 | 0 | 0 | 0 | 0 | 0 | 0 | 0 | 0 | Y | 15743 | cathepsin Z-like, partial [Protobothrops mucrosquamatus] |
| 458 | 4307 | LAA25473.1 | 54.64 | 10 | 0 | 0 | 0 | 10 | 0 | 0 | 0 | 0 | 0 | 0 | 0 | 0 | 0 |  |  |  | 5.9741E5 |  |  |  |  |  |  |  |  |  | 1 | 1 | 0 | 0 | 0 | 1 | 0 | 0 | 0 | 0 | 0 | 0 | 0 | 0 | 0 | Y | 19121 | hypothetical protein, partial [Micrurus lemniscatus carvalhoi] |
| 458 | 4308 | XP\_015679450.1 | 54.64 | 8 | 0 | 0 | 0 | 8 | 0 | 0 | 0 | 0 | 0 | 0 | 0 | 0 | 0 |  |  |  | 5.9741E5 |  |  |  |  |  |  |  |  |  | 1 | 1 | 0 | 0 | 0 | 1 | 0 | 0 | 0 | 0 | 0 | 0 | 0 | 0 | 0 | Y | 24752 | cathepsin Z, partial [Protobothrops mucrosquamatus] |
| 458 | 4309 | XP\_026520697.1 | 54.64 | 6 | 0 | 0 | 0 | 6 | 0 | 0 | 0 | 0 | 0 | 0 | 0 | 0 | 0 |  |  |  | 5.9741E5 |  |  |  |  |  |  |  |  |  | 1 | 1 | 0 | 0 | 0 | 1 | 0 | 0 | 0 | 0 | 0 | 0 | 0 | 0 | 0 | Y | 34179 | cathepsin Z-like [Notechis scutatus] |
| 203 | 3138 | LAA93895.1 | 53.72 | 5 | 0 | 0 | 0 | 5 | 5 | 5 | 0 | 0 | 0 | 5 | 0 | 0 | 0 |  |  |  | 1.1234E6 | 9.9306E5 | 3.8362E5 |  |  |  | 5.7822E5 |  |  |  | 1 | 1 | 0 | 0 | 0 | 2 | 3 | 1 | 0 | 0 | 0 | 1 | 0 | 0 | 0 | N | 19716 | hypothetical protein, partial [Micrurus lemniscatus lemniscatus] |
| 474 | 2633 | AFJ50537.1 | 53.67 | 6 | 6 | 0 | 0 | 0 | 0 | 0 | 0 | 0 | 0 | 0 | 0 | 0 | 0 | 3.364E5 |  |  |  |  |  |  |  |  |  |  |  |  | 1 | 1 | 1 | 0 | 0 | 0 | 0 | 0 | 0 | 0 | 0 | 0 | 0 | 0 | 0 | N | 36788 | Malate dehydrogenase, cytoplasmic-like [Crotalus adamanteus] |
| 474 | 2634 | JAI12652.1 | 53.67 | 6 | 6 | 0 | 0 | 0 | 0 | 0 | 0 | 0 | 0 | 0 | 0 | 0 | 0 | 3.364E5 |  |  |  |  |  |  |  |  |  |  |  |  | 1 | 1 | 1 | 0 | 0 | 0 | 0 | 0 | 0 | 0 | 0 | 0 | 0 | 0 | 0 | N | 36788 | Malate dehydrogenase, cytoplasmic-like protein [Crotalus adamanteus] |
| 474 | 2635 | JAV50078.1 | 53.67 | 6 | 6 | 0 | 0 | 0 | 0 | 0 | 0 | 0 | 0 | 0 | 0 | 0 | 0 | 3.364E5 |  |  |  |  |  |  |  |  |  |  |  |  | 1 | 1 | 1 | 0 | 0 | 0 | 0 | 0 | 0 | 0 | 0 | 0 | 0 | 0 | 0 | N | 36728 | Malate dehydrogenase, cytoplasmic-like protein [Agkistrodon contortrix contortrix] |
| 474 | 2636 | JAA96599.1 | 53.67 | 6 | 6 | 0 | 0 | 0 | 0 | 0 | 0 | 0 | 0 | 0 | 0 | 0 | 0 | 3.364E5 |  |  |  |  |  |  |  |  |  |  |  |  | 1 | 1 | 1 | 0 | 0 | 0 | 0 | 0 | 0 | 0 | 0 | 0 | 0 | 0 | 0 | N | 36816 | Malate dehydrogenase, cytoplasmic-like protein [Crotalus horridus] |
| 474 | 2637 | JAG44071.1 | 53.67 | 6 | 6 | 0 | 0 | 0 | 0 | 0 | 0 | 0 | 0 | 0 | 0 | 0 | 0 | 3.364E5 |  |  |  |  |  |  |  |  |  |  |  |  | 1 | 1 | 1 | 0 | 0 | 0 | 0 | 0 | 0 | 0 | 0 | 0 | 0 | 0 | 0 | N | 36816 | Malate dehydrogenase, cytoplasmic-like protein [Crotalus horridus] |
| 474 | 2638 | JAG46413.1 | 53.67 | 6 | 6 | 0 | 0 | 0 | 0 | 0 | 0 | 0 | 0 | 0 | 0 | 0 | 0 | 3.364E5 |  |  |  |  |  |  |  |  |  |  |  |  | 1 | 1 | 1 | 0 | 0 | 0 | 0 | 0 | 0 | 0 | 0 | 0 | 0 | 0 | 0 | N | 36816 | Malate dehydrogenase, cytoplasmic-like protein [Crotalus horridus] |
| 344 | 1093 | LAB38707.1 | 53.63 | 3 | 0 | 0 | 0 | 0 | 0 | 0 | 0 | 0 | 0 | 3 | 0 | 0 | 0 |  |  |  |  |  |  |  |  |  | 2.8031E6 |  |  |  | 1 | 1 | 0 | 0 | 0 | 0 | 0 | 0 | 0 | 0 | 0 | 1 | 0 | 0 | 0 | Y | 51122 | hypothetical protein, partial [Micrurus spixii] |
| 344 | 1095 | LAB61292.1 | 53.63 | 3 | 0 | 0 | 0 | 0 | 0 | 0 | 0 | 0 | 0 | 3 | 0 | 0 | 0 |  |  |  |  |  |  |  |  |  | 2.8031E6 |  |  |  | 1 | 1 | 0 | 0 | 0 | 0 | 0 | 0 | 0 | 0 | 0 | 1 | 0 | 0 | 0 | Y | 51312 | hypothetical protein, partial [Micrurus surinamensis] |
| 344 | 1097 | XP\_026529938.1 | 53.63 | 4 | 0 | 0 | 0 | 0 | 0 | 0 | 0 | 0 | 0 | 4 | 0 | 0 | 0 |  |  |  |  |  |  |  |  |  | 2.8031E6 |  |  |  | 1 | 1 | 0 | 0 | 0 | 0 | 0 | 0 | 0 | 0 | 0 | 1 | 0 | 0 | 0 | Y | 41834 | protein CYR61 [Notechis scutatus] |
| 344 | 1098 | ETE65500.1 | 53.63 | 4 | 0 | 0 | 0 | 0 | 0 | 0 | 0 | 0 | 0 | 4 | 0 | 0 | 0 |  |  |  |  |  |  |  |  |  | 2.8031E6 |  |  |  | 1 | 1 | 0 | 0 | 0 | 0 | 0 | 0 | 0 | 0 | 0 | 1 | 0 | 0 | 0 | Y | 41174 | Protein CYR61 [Ophiophagus hannah] |
| 344 | 1099 | LAA35956.1 | 53.63 | 4 | 0 | 0 | 0 | 0 | 0 | 0 | 0 | 0 | 0 | 4 | 0 | 0 | 0 |  |  |  |  |  |  |  |  |  | 2.8031E6 |  |  |  | 1 | 1 | 0 | 0 | 0 | 0 | 0 | 0 | 0 | 0 | 0 | 1 | 0 | 0 | 0 | Y | 41684 | hypothetical protein [Micrurus lemniscatus carvalhoi] |
| 344 | 1100 | XP\_026556047.1 | 53.63 | 4 | 0 | 0 | 0 | 0 | 0 | 0 | 0 | 0 | 0 | 4 | 0 | 0 | 0 |  |  |  |  |  |  |  |  |  | 2.8031E6 |  |  |  | 1 | 1 | 0 | 0 | 0 | 0 | 0 | 0 | 0 | 0 | 0 | 1 | 0 | 0 | 0 | Y | 41498 | protein CYR61 [Pseudonaja textilis] |
| 344 | 1587 | JAG68264.1 | 53.63 | 4 | 0 | 0 | 0 | 0 | 0 | 0 | 0 | 0 | 0 | 4 | 0 | 0 | 0 |  |  |  |  |  |  |  |  |  | 2.8031E6 |  |  |  | 1 | 1 | 0 | 0 | 0 | 0 | 0 | 0 | 0 | 0 | 0 | 1 | 0 | 0 | 0 | Y | 41736 | Cyr61 protein [Boiga irregularis] |
| 344 | 1633 | XP\_032074982.1 | 53.63 | 4 | 0 | 0 | 0 | 0 | 0 | 0 | 0 | 0 | 0 | 4 | 0 | 0 | 0 |  |  |  |  |  |  |  |  |  | 2.8031E6 |  |  |  | 1 | 1 | 0 | 0 | 0 | 0 | 0 | 0 | 0 | 0 | 0 | 1 | 0 | 0 | 0 | Y | 41543 | CCN family member 1 [Thamnophis elegans] |
| 344 | 1647 | XP\_013913807.1 | 53.63 | 4 | 0 | 0 | 0 | 0 | 0 | 0 | 0 | 0 | 0 | 4 | 0 | 0 | 0 |  |  |  |  |  |  |  |  |  | 2.8031E6 |  |  |  | 1 | 1 | 0 | 0 | 0 | 0 | 0 | 0 | 0 | 0 | 0 | 1 | 0 | 0 | 0 | Y | 41596 | PREDICTED: protein CYR61 [Thamnophis sirtalis] |
| 344 | 1648 | XP\_034293020.1 | 53.63 | 4 | 0 | 0 | 0 | 0 | 0 | 0 | 0 | 0 | 0 | 4 | 0 | 0 | 0 |  |  |  |  |  |  |  |  |  | 2.8031E6 |  |  |  | 1 | 1 | 0 | 0 | 0 | 0 | 0 | 0 | 0 | 0 | 0 | 1 | 0 | 0 | 0 | Y | 41891 | CCN family member 1 [Pantherophis guttatus] |
| 344 | 1653 | JAC95858.1 | 53.63 | 4 | 0 | 0 | 0 | 0 | 0 | 0 | 0 | 0 | 0 | 4 | 0 | 0 | 0 |  |  |  |  |  |  |  |  |  | 2.8031E6 |  |  |  | 1 | 1 | 0 | 0 | 0 | 0 | 0 | 0 | 0 | 0 | 0 | 1 | 0 | 0 | 0 | Y | 41729 | protein CYR61 [Hypsiglena sp. JMG-2014] |
| 344 | 1660 | XP\_007444773.1 | 53.63 | 4 | 0 | 0 | 0 | 0 | 0 | 0 | 0 | 0 | 0 | 4 | 0 | 0 | 0 |  |  |  |  |  |  |  |  |  | 2.8031E6 |  |  |  | 1 | 1 | 0 | 0 | 0 | 0 | 0 | 0 | 0 | 0 | 0 | 1 | 0 | 0 | 0 | Y | 41726 | protein CYR61 [Python bivittatus] |
| 344 | 1682 | LAA35964.1 | 53.63 | 12 | 0 | 0 | 0 | 0 | 0 | 0 | 0 | 0 | 0 | 12 | 0 | 0 | 0 |  |  |  |  |  |  |  |  |  | 2.8031E6 |  |  |  | 1 | 1 | 0 | 0 | 0 | 0 | 0 | 0 | 0 | 0 | 0 | 1 | 0 | 0 | 0 | Y | 12702 | hypothetical protein [Micrurus lemniscatus carvalhoi] |
| 344 | 2204 | XP\_015674483.1 | 53.63 | 4 | 0 | 0 | 0 | 0 | 0 | 0 | 0 | 0 | 0 | 4 | 0 | 0 | 0 |  |  |  |  |  |  |  |  |  | 2.8031E6 |  |  |  | 1 | 1 | 0 | 0 | 0 | 0 | 0 | 0 | 0 | 0 | 0 | 1 | 0 | 0 | 0 | Y | 41672 | protein CYR61 [Protobothrops mucrosquamatus] |
| 252 | 1985 | ETE57293.1 | 53.44 | 7 | 0 | 7 | 0 | 0 | 0 | 0 | 0 | 0 | 0 | 0 | 0 | 0 | 7 |  | 1.4409E6 |  |  |  |  |  |  |  |  |  |  | 4.0652E6 | 1 | 1 | 0 | 1 | 0 | 0 | 0 | 0 | 0 | 0 | 0 | 0 | 0 | 0 | 2 | N | 17619 | Major histocompatibility complex class I-related protein, partial [Ophiophagus hannah] |
| 252 | 2283 | XP\_015683643.1 | 53.44 | 3 | 0 | 3 | 0 | 0 | 0 | 0 | 0 | 0 | 0 | 0 | 0 | 0 | 3 |  | 1.4409E6 |  |  |  |  |  |  |  |  |  |  | 4.0652E6 | 1 | 1 | 0 | 1 | 0 | 0 | 0 | 0 | 0 | 0 | 0 | 0 | 0 | 0 | 2 | N | 40693 | major histocompatibility complex class I-related gene protein-like [Protobothrops mucrosquamatus] |
| 252 | 2323 | XP\_032065690.1 | 53.44 | 3 | 0 | 3 | 0 | 0 | 0 | 0 | 0 | 0 | 0 | 0 | 0 | 0 | 3 |  | 1.4409E6 |  |  |  |  |  |  |  |  |  |  | 4.0652E6 | 1 | 1 | 0 | 1 | 0 | 0 | 0 | 0 | 0 | 0 | 0 | 0 | 0 | 0 | 2 | N | 41122 | major histocompatibility complex class I-related gene protein-like [Thamnophis elegans] |
| 252 | 2327 | XP\_032065704.1 | 53.44 | 3 | 0 | 3 | 0 | 0 | 0 | 0 | 0 | 0 | 0 | 0 | 0 | 0 | 3 |  | 1.4409E6 |  |  |  |  |  |  |  |  |  |  | 4.0652E6 | 1 | 1 | 0 | 1 | 0 | 0 | 0 | 0 | 0 | 0 | 0 | 0 | 0 | 0 | 2 | N | 38503 | major histocompatibility complex class I-related gene protein-like isoform X1 [Thamnophis elegans] |
| 252 | 2336 | XP\_032065705.1 | 53.44 | 3 | 0 | 3 | 0 | 0 | 0 | 0 | 0 | 0 | 0 | 0 | 0 | 0 | 3 |  | 1.4409E6 |  |  |  |  |  |  |  |  |  |  | 4.0652E6 | 1 | 1 | 0 | 1 | 0 | 0 | 0 | 0 | 0 | 0 | 0 | 0 | 0 | 0 | 2 | N | 38501 | major histocompatibility complex class I-related gene protein-like isoform X2 [Thamnophis elegans] |
| 252 | 2337 | XP\_032065706.1 | 53.44 | 3 | 0 | 3 | 0 | 0 | 0 | 0 | 0 | 0 | 0 | 0 | 0 | 0 | 3 |  | 1.4409E6 |  |  |  |  |  |  |  |  |  |  | 4.0652E6 | 1 | 1 | 0 | 1 | 0 | 0 | 0 | 0 | 0 | 0 | 0 | 0 | 0 | 0 | 2 | N | 38529 | major histocompatibility complex class I-related gene protein-like isoform X3 [Thamnophis elegans] |
| 247 | 1801 | XP\_034291155.1 | 53.27 | 1 | 0 | 0 | 0 | 0 | 0 | 0 | 1 | 1 | 1 | 0 | 0 | 0 | 0 |  |  |  |  |  |  | 8.9276E6 | 4.6145E6 | 8.6948E6 |  |  |  |  | 1 | 1 | 0 | 0 | 0 | 0 | 0 | 0 | 1 | 1 | 1 | 0 | 0 | 0 | 0 | N | 188004 | inactive ubiquitin carboxyl-terminal hydrolase 54 isoform X1 [Pantherophis guttatus] |
| 473 | 2351 | XP\_026531535.1 | 52.58 | 2 | 0 | 0 | 0 | 2 | 0 | 0 | 0 | 0 | 0 | 0 | 0 | 0 | 0 |  |  |  | 8.0879E4 |  |  |  |  |  |  |  |  |  | 1 | 1 | 0 | 0 | 0 | 1 | 0 | 0 | 0 | 0 | 0 | 0 | 0 | 0 | 0 | N | 58696 | brain-specific angiogenesis inhibitor 1-associated protein 2 isoform X2 [Notechis scutatus] |
| 473 | 2352 | XP\_026531534.1 | 52.58 | 2 | 0 | 0 | 0 | 2 | 0 | 0 | 0 | 0 | 0 | 0 | 0 | 0 | 0 |  |  |  | 8.0879E4 |  |  |  |  |  |  |  |  |  | 1 | 1 | 0 | 0 | 0 | 1 | 0 | 0 | 0 | 0 | 0 | 0 | 0 | 0 | 0 | N | 60215 | brain-specific angiogenesis inhibitor 1-associated protein 2 isoform X1 [Notechis scutatus] |
| 473 | 2841 | ETE74005.1 | 52.58 | 3 | 0 | 0 | 0 | 3 | 0 | 0 | 0 | 0 | 0 | 0 | 0 | 0 | 0 |  |  |  | 8.0879E4 |  |  |  |  |  |  |  |  |  | 1 | 1 | 0 | 0 | 0 | 1 | 0 | 0 | 0 | 0 | 0 | 0 | 0 | 0 | 0 | N | 53391 | Brain-specific angiogenesis inhibitor 1-associated protein 2, partial [Ophiophagus hannah] |
| 473 | 2842 | JAB54701.1 | 52.58 | 3 | 0 | 0 | 0 | 3 | 0 | 0 | 0 | 0 | 0 | 0 | 0 | 0 | 0 |  |  |  | 8.0879E4 |  |  |  |  |  |  |  |  |  | 1 | 1 | 0 | 0 | 0 | 1 | 0 | 0 | 0 | 0 | 0 | 0 | 0 | 0 | 0 | N | 57090 | Brain-specific angiogenesis inhibitor 1-associated protein 2 [Micrurus fulvius] |
| 473 | 2843 | XP\_026557565.1 | 52.58 | 2 | 0 | 0 | 0 | 2 | 0 | 0 | 0 | 0 | 0 | 0 | 0 | 0 | 0 |  |  |  | 8.0879E4 |  |  |  |  |  |  |  |  |  | 1 | 1 | 0 | 0 | 0 | 1 | 0 | 0 | 0 | 0 | 0 | 0 | 0 | 0 | 0 | N | 58744 | brain-specific angiogenesis inhibitor 1-associated protein 2 isoform X2 [Pseudonaja textilis] |
| 473 | 2844 | LAB18462.1 | 52.58 | 2 | 0 | 0 | 0 | 2 | 0 | 0 | 0 | 0 | 0 | 0 | 0 | 0 | 0 |  |  |  | 8.0879E4 |  |  |  |  |  |  |  |  |  | 1 | 1 | 0 | 0 | 0 | 1 | 0 | 0 | 0 | 0 | 0 | 0 | 0 | 0 | 0 | N | 59460 | hypothetical protein, partial [Micrurus spixii] |
| 473 | 2845 | XP\_026557564.1 | 52.58 | 2 | 0 | 0 | 0 | 2 | 0 | 0 | 0 | 0 | 0 | 0 | 0 | 0 | 0 |  |  |  | 8.0879E4 |  |  |  |  |  |  |  |  |  | 1 | 1 | 0 | 0 | 0 | 1 | 0 | 0 | 0 | 0 | 0 | 0 | 0 | 0 | 0 | N | 60263 | brain-specific angiogenesis inhibitor 1-associated protein 2 isoform X1 [Pseudonaja textilis] |
| 473 | 2846 | LAA60085.1 | 52.58 | 2 | 0 | 0 | 0 | 2 | 0 | 0 | 0 | 0 | 0 | 0 | 0 | 0 | 0 |  |  |  | 8.0879E4 |  |  |  |  |  |  |  |  |  | 1 | 1 | 0 | 0 | 0 | 1 | 0 | 0 | 0 | 0 | 0 | 0 | 0 | 0 | 0 | N | 61049 | hypothetical protein [Micrurus corallinus] |
| 473 | 2847 | LAB18463.1 | 52.58 | 2 | 0 | 0 | 0 | 2 | 0 | 0 | 0 | 0 | 0 | 0 | 0 | 0 | 0 |  |  |  | 8.0879E4 |  |  |  |  |  |  |  |  |  | 1 | 1 | 0 | 0 | 0 | 1 | 0 | 0 | 0 | 0 | 0 | 0 | 0 | 0 | 0 | N | 60994 | hypothetical protein, partial [Micrurus spixii] |
| 473 | 2848 | LAA60082.1 | 52.58 | 2 | 0 | 0 | 0 | 2 | 0 | 0 | 0 | 0 | 0 | 0 | 0 | 0 | 0 |  |  |  | 8.0879E4 |  |  |  |  |  |  |  |  |  | 1 | 1 | 0 | 0 | 0 | 1 | 0 | 0 | 0 | 0 | 0 | 0 | 0 | 0 | 0 | N | 62569 | hypothetical protein [Micrurus corallinus] |
| 473 | 4048 | LAA19315.1 | 52.58 | 7 | 0 | 0 | 0 | 7 | 0 | 0 | 0 | 0 | 0 | 0 | 0 | 0 | 0 |  |  |  | 8.0879E4 |  |  |  |  |  |  |  |  |  | 1 | 1 | 0 | 0 | 0 | 1 | 0 | 0 | 0 | 0 | 0 | 0 | 0 | 0 | 0 | N | 19568 | hypothetical protein, partial [Micrurus lemniscatus carvalhoi] |
| 473 | 4049 | LAA19316.1 | 52.58 | 6 | 0 | 0 | 0 | 6 | 0 | 0 | 0 | 0 | 0 | 0 | 0 | 0 | 0 |  |  |  | 8.0879E4 |  |  |  |  |  |  |  |  |  | 1 | 1 | 0 | 0 | 0 | 1 | 0 | 0 | 0 | 0 | 0 | 0 | 0 | 0 | 0 | N | 23658 | hypothetical protein, partial [Micrurus lemniscatus carvalhoi] |
| 315 | 3746 | AAT91068.1 | 51.75 | 6 | 0 | 0 | 0 | 6 | 0 | 0 | 0 | 0 | 0 | 0 | 0 | 0 | 0 |  |  |  | 1.2405E6 |  |  |  |  |  |  |  |  |  | 1 | 1 | 0 | 0 | 0 | 2 | 0 | 0 | 0 | 0 | 0 | 0 | 0 | 0 | 0 | Y | 18094 | factor X activator light chain 2 [Macrovipera lebetina] |
| 315 | 3747 | Q696W1.1 | 51.75 | 6 | 0 | 0 | 0 | 6 | 0 | 0 | 0 | 0 | 0 | 0 | 0 | 0 | 0 |  |  |  | 1.2405E6 |  |  |  |  |  |  |  |  |  | 1 | 1 | 0 | 0 | 0 | 2 | 0 | 0 | 0 | 0 | 0 | 0 | 0 | 0 | 0 | Y | 18094 | RecName: Full=Snaclec coagulation factor X-activating enzyme light chain 2; AltName: Full=VL factor X activator light chain 2; Short=VLFXA light chain 2; Flags: Precursor |
| 315 | 4316 | ADJ67473.1 | 51.75 | 6 | 0 | 0 | 0 | 6 | 0 | 0 | 0 | 0 | 0 | 0 | 0 | 0 | 0 |  |  |  | 1.2405E6 |  |  |  |  |  |  |  |  |  | 1 | 1 | 0 | 0 | 0 | 2 | 0 | 0 | 0 | 0 | 0 | 0 | 0 | 0 | 0 | Y | 18273 | factor X activator light chain 2 [Daboia russelii russelii] |
| 499 | 3048 | XP\_026572109.1 | 49.86 | 2 | 2 | 0 | 0 | 0 | 0 | 0 | 0 | 0 | 0 | 0 | 0 | 0 | 0 | 2.9152E5 |  |  |  |  |  |  |  |  |  |  |  |  | 1 | 1 | 1 | 0 | 0 | 0 | 0 | 0 | 0 | 0 | 0 | 0 | 0 | 0 | 0 | N | 113983 | NAD(P) transhydrogenase, mitochondrial [Pseudonaja textilis] |
| 499 | 3280 | XP\_032070506.1 | 49.86 | 2 | 2 | 0 | 0 | 0 | 0 | 0 | 0 | 0 | 0 | 0 | 0 | 0 | 0 | 2.9152E5 |  |  |  |  |  |  |  |  |  |  |  |  | 1 | 1 | 1 | 0 | 0 | 0 | 0 | 0 | 0 | 0 | 0 | 0 | 0 | 0 | 0 | N | 114140 | NAD(P) transhydrogenase, mitochondrial [Thamnophis elegans] |
| 265 | 1249 | XP\_034260774.1 | 49.84 | 2 | 0 | 0 | 0 | 2 | 0 | 0 | 0 | 0 | 0 | 0 | 0 | 0 | 0 |  |  |  | 3.2782E6 |  |  |  |  |  |  |  |  |  | 1 | 1 | 0 | 0 | 0 | 2 | 0 | 0 | 0 | 0 | 0 | 0 | 0 | 0 | 0 | N | 40023 | H-2 class I histocompatibility antigen, Q9 alpha chain-like [Pantherophis guttatus] |
| 262 | 1674 | XP\_034289344.1 | 48.34 | 1 | 0 | 0 | 0 | 1 | 0 | 0 | 0 | 0 | 0 | 0 | 0 | 0 | 0 |  |  |  | 1.1177E5 |  |  |  |  |  |  |  |  |  | 1 | 1 | 0 | 0 | 0 | 1 | 0 | 0 | 0 | 0 | 0 | 0 | 0 | 0 | 0 | N | 140635 | fibronectin-like [Pantherophis guttatus] |
| 262 | 1733 | XP\_013916282.1 | 48.34 | 1 | 0 | 0 | 0 | 1 | 0 | 0 | 0 | 0 | 0 | 0 | 0 | 0 | 0 |  |  |  | 1.1177E5 |  |  |  |  |  |  |  |  |  | 1 | 1 | 0 | 0 | 0 | 1 | 0 | 0 | 0 | 0 | 0 | 0 | 0 | 0 | 0 | N | 250820 | PREDICTED: fibronectin [Thamnophis sirtalis] |
| 262 | 1734 | XP\_032094866.1 | 48.34 | 1 | 0 | 0 | 0 | 1 | 0 | 0 | 0 | 0 | 0 | 0 | 0 | 0 | 0 |  |  |  | 1.1177E5 |  |  |  |  |  |  |  |  |  | 1 | 1 | 0 | 0 | 0 | 1 | 0 | 0 | 0 | 0 | 0 | 0 | 0 | 0 | 0 | N | 247750 | fibronectin isoform X4 [Thamnophis elegans] |
| 262 | 1735 | XP\_032094851.1 | 48.34 | 1 | 0 | 0 | 0 | 1 | 0 | 0 | 0 | 0 | 0 | 0 | 0 | 0 | 0 |  |  |  | 1.1177E5 |  |  |  |  |  |  |  |  |  | 1 | 1 | 0 | 0 | 0 | 1 | 0 | 0 | 0 | 0 | 0 | 0 | 0 | 0 | 0 | N | 252633 | fibronectin isoform X2 [Thamnophis elegans] |
| 262 | 1736 | XP\_032094842.1 | 48.34 | 1 | 0 | 0 | 0 | 1 | 0 | 0 | 0 | 0 | 0 | 0 | 0 | 0 | 0 |  |  |  | 1.1177E5 |  |  |  |  |  |  |  |  |  | 1 | 1 | 0 | 0 | 0 | 1 | 0 | 0 | 0 | 0 | 0 | 0 | 0 | 0 | 0 | N | 252761 | fibronectin isoform X1 [Thamnophis elegans] |
| 262 | 1737 | XP\_032094874.1 | 48.34 | 1 | 0 | 0 | 0 | 1 | 0 | 0 | 0 | 0 | 0 | 0 | 0 | 0 | 0 |  |  |  | 1.1177E5 |  |  |  |  |  |  |  |  |  | 1 | 1 | 0 | 0 | 0 | 1 | 0 | 0 | 0 | 0 | 0 | 0 | 0 | 0 | 0 | N | 251827 | fibronectin isoform X5 [Thamnophis elegans] |
| 262 | 1738 | XP\_032094858.1 | 48.34 | 1 | 0 | 0 | 0 | 1 | 0 | 0 | 0 | 0 | 0 | 0 | 0 | 0 | 0 |  |  |  | 1.1177E5 |  |  |  |  |  |  |  |  |  | 1 | 1 | 0 | 0 | 0 | 1 | 0 | 0 | 0 | 0 | 0 | 0 | 0 | 0 | 0 | N | 251955 | fibronectin isoform X3 [Thamnophis elegans] |
| 261 | 1229 | XP\_026542806.1 | 47.72 | 2 | 2 | 0 | 0 | 0 | 0 | 0 | 0 | 0 | 0 | 0 | 0 | 0 | 0 | 1.7359E6 |  |  |  |  |  |  |  |  |  |  |  |  | 1 | 1 | 2 | 0 | 0 | 0 | 0 | 0 | 0 | 0 | 0 | 0 | 0 | 0 | 0 | N | 96457 | sarcoplasmic/endoplasmic reticulum calcium ATPase 2 [Notechis scutatus] |
| 261 | 1244 | XP\_026580482.1 | 47.72 | 2 | 2 | 0 | 0 | 0 | 0 | 0 | 0 | 0 | 0 | 0 | 0 | 0 | 0 | 1.7359E6 |  |  |  |  |  |  |  |  |  |  |  |  | 1 | 1 | 2 | 0 | 0 | 0 | 0 | 0 | 0 | 0 | 0 | 0 | 0 | 0 | 0 | N | 92634 | sarcoplasmic/endoplasmic reticulum calcium ATPase 2 [Pseudonaja textilis] |
| 261 | 1245 | XP\_026580485.1 | 47.72 | 2 | 2 | 0 | 0 | 0 | 0 | 0 | 0 | 0 | 0 | 0 | 0 | 0 | 0 | 1.7359E6 |  |  |  |  |  |  |  |  |  |  |  |  | 1 | 1 | 2 | 0 | 0 | 0 | 0 | 0 | 0 | 0 | 0 | 0 | 0 | 0 | 0 | N | 92634 | sarcoplasmic/endoplasmic reticulum calcium ATPase 2 [Pseudonaja textilis] |
| 261 | 1620 | JAV49206.1 | 47.72 | 2 | 2 | 0 | 0 | 0 | 0 | 0 | 0 | 0 | 0 | 0 | 0 | 0 | 0 | 1.7359E6 |  |  |  |  |  |  |  |  |  |  |  |  | 1 | 1 | 2 | 0 | 0 | 0 | 0 | 0 | 0 | 0 | 0 | 0 | 0 | 0 | 0 | N | 114644 | sarcoplasmic/endoplasmic reticulum calcium ATPase 2 isoform a [Agkistrodon contortrix contortrix] |
| 261 | 1626 | XP\_015684492.1 | 47.72 | 2 | 2 | 0 | 0 | 0 | 0 | 0 | 0 | 0 | 0 | 0 | 0 | 0 | 0 | 1.7359E6 |  |  |  |  |  |  |  |  |  |  |  |  | 1 | 1 | 2 | 0 | 0 | 0 | 0 | 0 | 0 | 0 | 0 | 0 | 0 | 0 | 0 | N | 97509 | sarcoplasmic/endoplasmic reticulum calcium ATPase 2, partial [Protobothrops mucrosquamatus] |
| 261 | 1628 | JAI11473.1 | 47.72 | 2 | 2 | 0 | 0 | 0 | 0 | 0 | 0 | 0 | 0 | 0 | 0 | 0 | 0 | 1.7359E6 |  |  |  |  |  |  |  |  |  |  |  |  | 1 | 1 | 2 | 0 | 0 | 0 | 0 | 0 | 0 | 0 | 0 | 0 | 0 | 0 | 0 | N | 114668 | sarcoplasmic/endoplasmic reticulum calcium ATPase 2 [Crotalus adamanteus] |
| 261 | 1655 | XP\_034284638.1 | 47.72 | 2 | 2 | 0 | 0 | 0 | 0 | 0 | 0 | 0 | 0 | 0 | 0 | 0 | 0 | 1.7359E6 |  |  |  |  |  |  |  |  |  |  |  |  | 1 | 1 | 2 | 0 | 0 | 0 | 0 | 0 | 0 | 0 | 0 | 0 | 0 | 0 | 0 | N | 109765 | sarcoplasmic/endoplasmic reticulum calcium ATPase 2 isoform X2 [Pantherophis guttatus] |
| 261 | 1656 | XP\_034284637.1 | 47.72 | 2 | 2 | 0 | 0 | 0 | 0 | 0 | 0 | 0 | 0 | 0 | 0 | 0 | 0 | 1.7359E6 |  |  |  |  |  |  |  |  |  |  |  |  | 1 | 1 | 2 | 0 | 0 | 0 | 0 | 0 | 0 | 0 | 0 | 0 | 0 | 0 | 0 | N | 114798 | sarcoplasmic/endoplasmic reticulum calcium ATPase 2 isoform X1 [Pantherophis guttatus] |
| 261 | 1661 | XP\_025025635.1 | 47.72 | 2 | 2 | 0 | 0 | 0 | 0 | 0 | 0 | 0 | 0 | 0 | 0 | 0 | 0 | 1.7359E6 |  |  |  |  |  |  |  |  |  |  |  |  | 1 | 1 | 2 | 0 | 0 | 0 | 0 | 0 | 0 | 0 | 0 | 0 | 0 | 0 | 0 | N | 95653 | sarcoplasmic/endoplasmic reticulum calcium ATPase 2 isoform X1 [Python bivittatus] |
| 261 | 1662 | XP\_007433335.1 | 47.72 | 2 | 2 | 0 | 0 | 0 | 0 | 0 | 0 | 0 | 0 | 0 | 0 | 0 | 0 | 1.7359E6 |  |  |  |  |  |  |  |  |  |  |  |  | 1 | 1 | 2 | 0 | 0 | 0 | 0 | 0 | 0 | 0 | 0 | 0 | 0 | 0 | 0 | N | 100560 | sarcoplasmic/endoplasmic reticulum calcium ATPase 2 isoform X2 [Python bivittatus] |
| 261 | 1696 | JAB54287.1 | 47.72 | 2 | 2 | 0 | 0 | 0 | 0 | 0 | 0 | 0 | 0 | 0 | 0 | 0 | 0 | 1.7359E6 |  |  |  |  |  |  |  |  |  |  |  |  | 1 | 1 | 2 | 0 | 0 | 0 | 0 | 0 | 0 | 0 | 0 | 0 | 0 | 0 | 0 | N | 114706 | sarcoplasmic/endoplasmic reticulum calcium ATPase 2a [Micrurus fulvius] |
| 261 | 1709 | JAG66604.1 | 47.72 | 2 | 2 | 0 | 0 | 0 | 0 | 0 | 0 | 0 | 0 | 0 | 0 | 0 | 0 | 1.7359E6 |  |  |  |  |  |  |  |  |  |  |  |  | 1 | 1 | 2 | 0 | 0 | 0 | 0 | 0 | 0 | 0 | 0 | 0 | 0 | 0 | 0 | N | 114711 | sarcoplasmic/endoplasmic reticulum calcium ATPase 2 [Boiga irregularis] |
| 261 | 1954 | XP\_032085178.1 | 47.72 | 2 | 2 | 0 | 0 | 0 | 0 | 0 | 0 | 0 | 0 | 0 | 0 | 0 | 0 | 1.7359E6 |  |  |  |  |  |  |  |  |  |  |  |  | 1 | 1 | 2 | 0 | 0 | 0 | 0 | 0 | 0 | 0 | 0 | 0 | 0 | 0 | 0 | N | 114797 | sarcoplasmic/endoplasmic reticulum calcium ATPase 2 [Thamnophis elegans] |
| 261 | 3661 | LAB57412.1 | 47.72 | 10 | 10 | 0 | 0 | 0 | 0 | 0 | 0 | 0 | 0 | 0 | 0 | 0 | 0 | 1.7359E6 |  |  |  |  |  |  |  |  |  |  |  |  | 1 | 1 | 2 | 0 | 0 | 0 | 0 | 0 | 0 | 0 | 0 | 0 | 0 | 0 | 0 | N | 17527 | hypothetical protein, partial [Micrurus surinamensis] |
| 261 | 3662 | LAB57416.1 | 47.72 | 10 | 10 | 0 | 0 | 0 | 0 | 0 | 0 | 0 | 0 | 0 | 0 | 0 | 0 | 1.7359E6 |  |  |  |  |  |  |  |  |  |  |  |  | 1 | 1 | 2 | 0 | 0 | 0 | 0 | 0 | 0 | 0 | 0 | 0 | 0 | 0 | 0 | N | 17527 | hypothetical protein, partial [Micrurus surinamensis] |
| 261 | 3663 | LAA36529.1 | 47.72 | 5 | 5 | 0 | 0 | 0 | 0 | 0 | 0 | 0 | 0 | 0 | 0 | 0 | 0 | 1.7359E6 |  |  |  |  |  |  |  |  |  |  |  |  | 1 | 1 | 2 | 0 | 0 | 0 | 0 | 0 | 0 | 0 | 0 | 0 | 0 | 0 | 0 | N | 39001 | hypothetical protein, partial [Micrurus corallinus] |
| 241 | 2823 | JAC94981.1 | 47.55 | 7 | 0 | 7 | 0 | 0 | 0 | 0 | 0 | 0 | 0 | 0 | 0 | 0 | 0 |  | 1.441E7 |  |  |  |  |  |  |  |  |  |  |  | 1 | 1 | 0 | 3 | 0 | 0 | 0 | 0 | 0 | 0 | 0 | 0 | 0 | 0 | 0 | N | 15892 | Cystatin E/M [Opheodrys aestivus] |
| 313 | 4415 | XP\_015678946.1 | 46.46 | 3 | 0 | 0 | 0 | 3 | 0 | 0 | 0 | 0 | 0 | 0 | 0 | 0 | 0 |  |  |  | 4.1362E6 |  |  |  |  |  |  |  |  |  | 1 | 1 | 0 | 0 | 0 | 2 | 0 | 0 | 0 | 0 | 0 | 0 | 0 | 0 | 0 | N | 26257 | serum amyloid P-component-like [Protobothrops mucrosquamatus] |
| 314 | 4371 | XP\_032064447.1 | 46.08 | 6 | 0 | 0 | 0 | 0 | 0 | 6 | 0 | 0 | 0 | 0 | 0 | 0 | 0 |  |  |  |  |  | 1.2045E5 |  |  |  |  |  |  |  | 1 | 1 | 0 | 0 | 0 | 0 | 0 | 1 | 0 | 0 | 0 | 0 | 0 | 0 | 0 | N | 21987 | peroxiredoxin-2 [Thamnophis elegans] |
| 314 | 4372 | XP\_026537276.1 | 46.08 | 6 | 0 | 0 | 0 | 0 | 0 | 6 | 0 | 0 | 0 | 0 | 0 | 0 | 0 |  |  |  |  |  | 1.2045E5 |  |  |  |  |  |  |  | 1 | 1 | 0 | 0 | 0 | 0 | 0 | 1 | 0 | 0 | 0 | 0 | 0 | 0 | 0 | N | 22065 | peroxiredoxin-2-like [Notechis scutatus] |
| 314 | 4373 | XP\_015670807.1 | 46.08 | 6 | 0 | 0 | 0 | 0 | 0 | 6 | 0 | 0 | 0 | 0 | 0 | 0 | 0 |  |  |  |  |  | 1.2045E5 |  |  |  |  |  |  |  | 1 | 1 | 0 | 0 | 0 | 0 | 0 | 1 | 0 | 0 | 0 | 0 | 0 | 0 | 0 | N | 21997 | peroxiredoxin-2 isoform X2 [Protobothrops mucrosquamatus] |
| 314 | 4374 | XP\_007421035.1 | 46.08 | 6 | 0 | 0 | 0 | 0 | 0 | 6 | 0 | 0 | 0 | 0 | 0 | 0 | 0 |  |  |  |  |  | 1.2045E5 |  |  |  |  |  |  |  | 1 | 1 | 0 | 0 | 0 | 0 | 0 | 1 | 0 | 0 | 0 | 0 | 0 | 0 | 0 | N | 22036 | peroxiredoxin-2 [Python bivittatus] |
| 314 | 4375 | XP\_026552097.1 | 46.08 | 6 | 0 | 0 | 0 | 0 | 0 | 6 | 0 | 0 | 0 | 0 | 0 | 0 | 0 |  |  |  |  |  | 1.2045E5 |  |  |  |  |  |  |  | 1 | 1 | 0 | 0 | 0 | 0 | 0 | 1 | 0 | 0 | 0 | 0 | 0 | 0 | 0 | N | 22051 | peroxiredoxin-2 [Pseudonaja textilis] |
| 314 | 4376 | XP\_032064446.1 | 46.08 | 6 | 0 | 0 | 0 | 0 | 0 | 6 | 0 | 0 | 0 | 0 | 0 | 0 | 0 |  |  |  |  |  | 1.2045E5 |  |  |  |  |  |  |  | 1 | 1 | 0 | 0 | 0 | 0 | 0 | 1 | 0 | 0 | 0 | 0 | 0 | 0 | 0 | N | 21987 | peroxiredoxin-2 [Thamnophis elegans] |
| 314 | 4377 | XP\_013928980.1 | 46.08 | 6 | 0 | 0 | 0 | 0 | 0 | 6 | 0 | 0 | 0 | 0 | 0 | 0 | 0 |  |  |  |  |  | 1.2045E5 |  |  |  |  |  |  |  | 1 | 1 | 0 | 0 | 0 | 0 | 0 | 1 | 0 | 0 | 0 | 0 | 0 | 0 | 0 | N | 21987 | PREDICTED: peroxiredoxin-2 [Thamnophis sirtalis] |
| 314 | 4378 | XP\_026537409.1 | 46.08 | 6 | 0 | 0 | 0 | 0 | 0 | 6 | 0 | 0 | 0 | 0 | 0 | 0 | 0 |  |  |  |  |  | 1.2045E5 |  |  |  |  |  |  |  | 1 | 1 | 0 | 0 | 0 | 0 | 0 | 1 | 0 | 0 | 0 | 0 | 0 | 0 | 0 | N | 22065 | peroxiredoxin-2-like [Notechis scutatus] |
| 314 | 4379 | XP\_015670805.1 | 46.08 | 6 | 0 | 0 | 0 | 0 | 0 | 6 | 0 | 0 | 0 | 0 | 0 | 0 | 0 |  |  |  |  |  | 1.2045E5 |  |  |  |  |  |  |  | 1 | 1 | 0 | 0 | 0 | 0 | 0 | 1 | 0 | 0 | 0 | 0 | 0 | 0 | 0 | N | 21997 | peroxiredoxin-2 isoform X1 [Protobothrops mucrosquamatus] |
| 234 | 2136 | AAG17443.1 | 45.43 | 6 | 0 | 0 | 0 | 0 | 0 | 0 | 0 | 0 | 6 | 6 | 0 | 0 | 0 |  |  |  |  |  |  |  |  | 2.7171E6 | 3.4765E7 |  |  |  | 1 | 1 | 0 | 0 | 0 | 0 | 0 | 0 | 0 | 0 | 1 | 1 | 0 | 0 | 0 | Y | 16641 | phospholipase A2 [Ophiophagus hannah] |
| 234 | 2137 | Q9DF56.1 | 45.43 | 6 | 0 | 0 | 0 | 0 | 0 | 0 | 0 | 0 | 6 | 6 | 0 | 0 | 0 |  |  |  |  |  |  |  |  | 2.7171E6 | 3.4765E7 |  |  |  | 1 | 1 | 0 | 0 | 0 | 0 | 0 | 0 | 0 | 0 | 1 | 1 | 0 | 0 | 0 | Y | 16641 | RecName: Full=Acidic phospholipase A2; Short=svPLA2; AltName: Full=Phosphatidylcholine 2-acylhydrolase; Flags: Precursor |
| 485 | 2732 | XP\_026558429.1 | 45.05 | 1 | 0 | 0 | 1 | 0 | 0 | 0 | 0 | 0 | 0 | 0 | 0 | 0 | 0 |  |  | 1.8019E5 |  |  |  |  |  |  |  |  |  |  | 1 | 1 | 0 | 0 | 1 | 0 | 0 | 0 | 0 | 0 | 0 | 0 | 0 | 0 | 0 | N | 121887 | dyslexia-associated protein KIAA0319-like protein homolog isoform X1 [Pseudonaja textilis] |
| 485 | 2970 | JAB54031.1 | 45.05 | 1 | 0 | 0 | 1 | 0 | 0 | 0 | 0 | 0 | 0 | 0 | 0 | 0 | 0 |  |  | 1.8019E5 |  |  |  |  |  |  |  |  |  |  | 1 | 1 | 0 | 0 | 1 | 0 | 0 | 0 | 0 | 0 | 0 | 0 | 0 | 0 | 0 | N | 122551 | KIAA0319-like protein [Micrurus fulvius] |
| 498 | 2807 | XP\_026544668.1 | 44.91 | 6 | 0 | 0 | 0 | 6 | 0 | 0 | 0 | 0 | 0 | 0 | 0 | 0 | 0 |  |  |  | 1.1359E6 |  |  |  |  |  |  |  |  |  | 1 | 1 | 0 | 0 | 0 | 1 | 0 | 0 | 0 | 0 | 0 | 0 | 0 | 0 | 0 | N | 30609 | serum amyloid P-component-like, partial [Notechis scutatus] |
| 210 | 4471 | JAA75006.1 | 44.86 | 10 | 0 | 0 | 0 | 0 | 0 | 0 | 0 | 10 | 0 | 0 | 0 | 0 | 0 |  |  |  |  |  |  |  | 1.2644E8 |  |  |  |  |  | 1 | 1 | 0 | 0 | 0 | 0 | 0 | 0 | 0 | 2 | 0 | 0 | 0 | 0 | 0 | Y | 10504 | 3FTx-Ver-5 [Vermicella annulata] |
| 495 | 2619 | XP\_015666657.1 | 44.69 | 6 | 0 | 0 | 6 | 0 | 0 | 0 | 0 | 0 | 0 | 0 | 0 | 0 | 0 |  |  | 2.6513E5 |  |  |  |  |  |  |  |  |  |  | 1 | 1 | 0 | 0 | 1 | 0 | 0 | 0 | 0 | 0 | 0 | 0 | 0 | 0 | 0 | N | 25001 | mammalian ependymin-related protein 1 [Protobothrops mucrosquamatus] |
| 495 | 3871 | ETE58174.1 | 44.69 | 6 | 0 | 0 | 6 | 0 | 0 | 0 | 0 | 0 | 0 | 0 | 0 | 0 | 0 |  |  | 2.6513E5 |  |  |  |  |  |  |  |  |  |  | 1 | 1 | 0 | 0 | 1 | 0 | 0 | 0 | 0 | 0 | 0 | 0 | 0 | 0 | 0 | N | 24063 | Mammalian ependymin-related protein 1, partial [Ophiophagus hannah] |
| 433 | 2701 | XP\_026544778.1 | 44.50 | 2 | 0 | 0 | 0 | 2 | 0 | 0 | 0 | 0 | 0 | 0 | 0 | 0 | 0 |  |  |  | 7.2941E5 |  |  |  |  |  |  |  |  |  | 1 | 1 | 0 | 0 | 0 | 1 | 0 | 0 | 0 | 0 | 0 | 0 | 0 | 0 | 0 | N | 62803 | liver carboxylesterase 1 isoform X2 [Notechis scutatus] |
| 433 | 2736 | XP\_013920173.1 | 44.50 | 2 | 0 | 0 | 0 | 2 | 0 | 0 | 0 | 0 | 0 | 0 | 0 | 0 | 0 |  |  |  | 7.2941E5 |  |  |  |  |  |  |  |  |  | 1 | 1 | 0 | 0 | 0 | 1 | 0 | 0 | 0 | 0 | 0 | 0 | 0 | 0 | 0 | N | 62622 | PREDICTED: liver carboxylesterase 1 [Thamnophis sirtalis] |
| 433 | 2749 | XP\_032086793.1 | 44.50 | 1 | 0 | 0 | 0 | 1 | 0 | 0 | 0 | 0 | 0 | 0 | 0 | 0 | 0 |  |  |  | 7.2941E5 |  |  |  |  |  |  |  |  |  | 1 | 1 | 0 | 0 | 0 | 1 | 0 | 0 | 0 | 0 | 0 | 0 | 0 | 0 | 0 | N | 84309 | LOW QUALITY PROTEIN: fatty acyl-CoA hydrolase precursor, medium chain-like [Thamnophis elegans] |
| 433 | 2928 | XP\_034281500.1 | 44.50 | 2 | 0 | 0 | 0 | 2 | 0 | 0 | 0 | 0 | 0 | 0 | 0 | 0 | 0 |  |  |  | 7.2941E5 |  |  |  |  |  |  |  |  |  | 1 | 1 | 0 | 0 | 0 | 1 | 0 | 0 | 0 | 0 | 0 | 0 | 0 | 0 | 0 | N | 62740 | fatty acyl-CoA hydrolase precursor, medium chain-like [Pantherophis guttatus] |
| 433 | 2929 | XP\_034281501.1 | 44.50 | 2 | 0 | 0 | 0 | 2 | 0 | 0 | 0 | 0 | 0 | 0 | 0 | 0 | 0 |  |  |  | 7.2941E5 |  |  |  |  |  |  |  |  |  | 1 | 1 | 0 | 0 | 0 | 1 | 0 | 0 | 0 | 0 | 0 | 0 | 0 | 0 | 0 | N | 62740 | fatty acyl-CoA hydrolase precursor, medium chain-like [Pantherophis guttatus] |
| 433 | 3316 | XP\_026544777.1 | 44.50 | 2 | 0 | 0 | 0 | 2 | 0 | 0 | 0 | 0 | 0 | 0 | 0 | 0 | 0 |  |  |  | 7.2941E5 |  |  |  |  |  |  |  |  |  | 1 | 1 | 0 | 0 | 0 | 1 | 0 | 0 | 0 | 0 | 0 | 0 | 0 | 0 | 0 | N | 62773 | liver carboxylesterase 1 isoform X1 [Notechis scutatus] |
| 433 | 3619 | XP\_026570249.1 | 44.50 | 2 | 0 | 0 | 0 | 2 | 0 | 0 | 0 | 0 | 0 | 0 | 0 | 0 | 0 |  |  |  | 7.2941E5 |  |  |  |  |  |  |  |  |  | 1 | 1 | 0 | 0 | 0 | 1 | 0 | 0 | 0 | 0 | 0 | 0 | 0 | 0 | 0 | N | 52765 | fatty acyl-CoA hydrolase precursor, medium chain-like isoform X1 [Pseudonaja textilis] |
| 433 | 3620 | XP\_026570250.1 | 44.50 | 2 | 0 | 0 | 0 | 2 | 0 | 0 | 0 | 0 | 0 | 0 | 0 | 0 | 0 |  |  |  | 7.2941E5 |  |  |  |  |  |  |  |  |  | 1 | 1 | 0 | 0 | 0 | 1 | 0 | 0 | 0 | 0 | 0 | 0 | 0 | 0 | 0 | N | 52795 | fatty acyl-CoA hydrolase precursor, medium chain-like isoform X2 [Pseudonaja textilis] |
| 433 | 3862 | LAA68381.1 | 44.50 | 5 | 0 | 0 | 0 | 5 | 0 | 0 | 0 | 0 | 0 | 0 | 0 | 0 | 0 |  |  |  | 7.2941E5 |  |  |  |  |  |  |  |  |  | 1 | 1 | 0 | 0 | 0 | 1 | 0 | 0 | 0 | 0 | 0 | 0 | 0 | 0 | 0 | N | 27008 | hypothetical protein, partial [Micrurus lemniscatus lemniscatus] |
| 433 | 3914 | XP\_029139218.1 | 44.50 | 2 | 0 | 0 | 0 | 2 | 0 | 0 | 0 | 0 | 0 | 0 | 0 | 0 | 0 |  |  |  | 7.2941E5 |  |  |  |  |  |  |  |  |  | 1 | 1 | 0 | 0 | 0 | 1 | 0 | 0 | 0 | 0 | 0 | 0 | 0 | 0 | 0 | N | 65042 | LOW QUALITY PROTEIN: fatty acyl-CoA hydrolase precursor, medium chain-like [Protobothrops mucrosquamatus] |
| 433 | 4133 | XP\_025028950.1 | 44.50 | 3 | 0 | 0 | 0 | 3 | 0 | 0 | 0 | 0 | 0 | 0 | 0 | 0 | 0 |  |  |  | 7.2941E5 |  |  |  |  |  |  |  |  |  | 1 | 1 | 0 | 0 | 0 | 1 | 0 | 0 | 0 | 0 | 0 | 0 | 0 | 0 | 0 | N | 37660 | putative inactive carboxylesterase 4 isoform X2 [Python bivittatus] |
| 433 | 4369 | XP\_007438289.2 | 44.50 | 2 | 0 | 0 | 0 | 2 | 0 | 0 | 0 | 0 | 0 | 0 | 0 | 0 | 0 |  |  |  | 7.2941E5 |  |  |  |  |  |  |  |  |  | 1 | 1 | 0 | 0 | 0 | 1 | 0 | 0 | 0 | 0 | 0 | 0 | 0 | 0 | 0 | N | 53443 | fatty acyl-CoA hydrolase precursor, medium chain-like isoform X1 [Python bivittatus] |
| 437 | 1958 | XP\_034279548.1 | 44.11 | 2 | 0 | 0 | 0 | 0 | 0 | 0 | 0 | 2 | 0 | 0 | 0 | 0 | 0 |  |  |  |  |  |  |  | 0 |  |  |  |  |  | 1 | 1 | 0 | 0 | 0 | 0 | 0 | 0 | 0 | 1 | 0 | 0 | 0 | 0 | 0 | Y | 75325 | amyloid-like protein 1 isoform X1 [Pantherophis guttatus] |
| 437 | 1968 | XP\_034279549.1 | 44.11 | 2 | 0 | 0 | 0 | 0 | 0 | 0 | 0 | 2 | 0 | 0 | 0 | 0 | 0 |  |  |  |  |  |  |  | 0 |  |  |  |  |  | 1 | 1 | 0 | 0 | 0 | 0 | 0 | 0 | 0 | 1 | 0 | 0 | 0 | 0 | 0 | Y | 76186 | amyloid-like protein 1 isoform X2 [Pantherophis guttatus] |
| 437 | 2105 | XP\_029140167.1 | 44.11 | 2 | 0 | 0 | 0 | 0 | 0 | 0 | 0 | 2 | 0 | 0 | 0 | 0 | 0 |  |  |  |  |  |  |  | 0 |  |  |  |  |  | 1 | 1 | 0 | 0 | 0 | 0 | 0 | 0 | 0 | 1 | 0 | 0 | 0 | 0 | 0 | Y | 76188 | amyloid-like protein 1 [Protobothrops mucrosquamatus] |
| 437 | 2123 | XP\_026558172.1 | 44.11 | 2 | 0 | 0 | 0 | 0 | 0 | 0 | 0 | 2 | 0 | 0 | 0 | 0 | 0 |  |  |  |  |  |  |  | 0 |  |  |  |  |  | 1 | 1 | 0 | 0 | 0 | 0 | 0 | 0 | 0 | 1 | 0 | 0 | 0 | 0 | 0 | Y | 80173 | amyloid-like protein 1 [Pseudonaja textilis] |
| 437 | 2127 | XP\_007441538.2 | 44.11 | 2 | 0 | 0 | 0 | 0 | 0 | 0 | 0 | 2 | 0 | 0 | 0 | 0 | 0 |  |  |  |  |  |  |  | 0 |  |  |  |  |  | 1 | 1 | 0 | 0 | 0 | 0 | 0 | 0 | 0 | 1 | 0 | 0 | 0 | 0 | 0 | Y | 77187 | amyloid-like protein 1 isoform X1 [Python bivittatus] |
| 437 | 2146 | ETE66347.1 | 44.11 | 3 | 0 | 0 | 0 | 0 | 0 | 0 | 0 | 3 | 0 | 0 | 0 | 0 | 0 |  |  |  |  |  |  |  | 0 |  |  |  |  |  | 1 | 1 | 0 | 0 | 0 | 0 | 0 | 0 | 0 | 1 | 0 | 0 | 0 | 0 | 0 | Y | 57192 | Amyloid-like protein 1 [Ophiophagus hannah] |
| 437 | 2147 | XP\_026535207.1 | 44.11 | 3 | 0 | 0 | 0 | 0 | 0 | 0 | 0 | 3 | 0 | 0 | 0 | 0 | 0 |  |  |  |  |  |  |  | 0 |  |  |  |  |  | 1 | 1 | 0 | 0 | 0 | 0 | 0 | 0 | 0 | 1 | 0 | 0 | 0 | 0 | 0 | Y | 65333 | amyloid-like protein 1 [Notechis scutatus] |
| 437 | 2148 | XP\_007441537.2 | 44.11 | 2 | 0 | 0 | 0 | 0 | 0 | 0 | 0 | 2 | 0 | 0 | 0 | 0 | 0 |  |  |  |  |  |  |  | 0 |  |  |  |  |  | 1 | 1 | 0 | 0 | 0 | 0 | 0 | 0 | 0 | 1 | 0 | 0 | 0 | 0 | 0 | Y | 76326 | amyloid-like protein 2 isoform X2 [Python bivittatus] |
| 437 | 2369 | XP\_032084106.1 | 44.11 | 2 | 0 | 0 | 0 | 0 | 0 | 0 | 0 | 2 | 0 | 0 | 0 | 0 | 0 |  |  |  |  |  |  |  | 0 |  |  |  |  |  | 1 | 1 | 0 | 0 | 0 | 0 | 0 | 0 | 0 | 1 | 0 | 0 | 0 | 0 | 0 | Y | 79520 | amyloid-like protein 1 isoform X2 [Thamnophis elegans] |
| 437 | 2370 | XP\_032084105.1 | 44.11 | 2 | 0 | 0 | 0 | 0 | 0 | 0 | 0 | 2 | 0 | 0 | 0 | 0 | 0 |  |  |  |  |  |  |  | 0 |  |  |  |  |  | 1 | 1 | 0 | 0 | 0 | 0 | 0 | 0 | 0 | 1 | 0 | 0 | 0 | 0 | 0 | Y | 80365 | amyloid-like protein 1 isoform X1 [Thamnophis elegans] |
| 437 | 2420 | XP\_013924423.1 | 44.11 | 3 | 0 | 0 | 0 | 0 | 0 | 0 | 0 | 3 | 0 | 0 | 0 | 0 | 0 |  |  |  |  |  |  |  | 0 |  |  |  |  |  | 1 | 1 | 0 | 0 | 0 | 0 | 0 | 0 | 0 | 1 | 0 | 0 | 0 | 0 | 0 | Y | 66589 | PREDICTED: amyloid-like protein 1 [Thamnophis sirtalis] |
| 437 | 2514 | XP\_015685842.1 | 44.11 | 7 | 0 | 0 | 0 | 0 | 0 | 0 | 0 | 7 | 0 | 0 | 0 | 0 | 0 |  |  |  |  |  |  |  | 0 |  |  |  |  |  | 1 | 1 | 0 | 0 | 0 | 0 | 0 | 0 | 0 | 1 | 0 | 0 | 0 | 0 | 0 | Y | 25758 | amyloid-beta A4 protein-like, partial [Protobothrops mucrosquamatus] |
| 359 | 2177 | XP\_026519977.1 | 42.97 | 0 | 0 | 0 | 0 | 0 | 0 | 0 | 0 | 0 | 0 | 0 | 0 | 0 | 0 |  |  |  |  |  |  | 3.9466E5 |  |  |  |  |  |  | 1 | 1 | 0 | 0 | 0 | 0 | 0 | 0 | 1 | 0 | 0 | 0 | 0 | 0 | 0 | N | 291780 | protein SON isoform X4 [Notechis scutatus] |
| 359 | 2178 | XP\_026519976.1 | 42.97 | 0 | 0 | 0 | 0 | 0 | 0 | 0 | 0 | 0 | 0 | 0 | 0 | 0 | 0 |  |  |  |  |  |  | 3.9466E5 |  |  |  |  |  |  | 1 | 1 | 0 | 0 | 0 | 0 | 0 | 0 | 1 | 0 | 0 | 0 | 0 | 0 | 0 | N | 313728 | protein SON isoform X3 [Notechis scutatus] |
| 359 | 2179 | XP\_026519975.1 | 42.97 | 0 | 0 | 0 | 0 | 0 | 0 | 0 | 0 | 0 | 0 | 0 | 0 | 0 | 0 |  |  |  |  |  |  | 3.9466E5 |  |  |  |  |  |  | 1 | 1 | 0 | 0 | 0 | 0 | 0 | 0 | 1 | 0 | 0 | 0 | 0 | 0 | 0 | N | 315606 | protein SON isoform X2 [Notechis scutatus] |
| 359 | 2181 | XP\_026519974.1 | 42.97 | 0 | 0 | 0 | 0 | 0 | 0 | 0 | 0 | 0 | 0 | 0 | 0 | 0 | 0 |  |  |  |  |  |  | 3.9466E5 |  |  |  |  |  |  | 1 | 1 | 0 | 0 | 0 | 0 | 0 | 0 | 1 | 0 | 0 | 0 | 0 | 0 | 0 | N | 330544 | protein SON isoform X1 [Notechis scutatus] |
| 388 | 2264 | QGC85377.1 | 42.88 | 3 | 0 | 0 | 0 | 0 | 3 | 0 | 0 | 0 | 0 | 0 | 0 | 0 | 0 |  |  |  |  | 4.1894E5 |  |  |  |  |  |  |  |  | 1 | 1 | 0 | 0 | 0 | 0 | 1 | 0 | 0 | 0 | 0 | 0 | 0 | 0 | 0 | N | 55494 | metalloproteinase, partial [Dispholidus typus] |
| 273 | 1274 | XP\_032078280.1 | 42.65 | 7 | 0 | 0 | 0 | 0 | 7 | 0 | 0 | 0 | 0 | 0 | 0 | 0 | 0 |  |  |  |  | 1.5541E6 |  |  |  |  |  |  |  |  | 1 | 1 | 0 | 0 | 0 | 0 | 1 | 0 | 0 | 0 | 0 | 0 | 0 | 0 | 0 | Y | 15886 | lymphocyte antigen 86 isoform X2 [Thamnophis elegans] |
| 273 | 1275 | XP\_032078278.1 | 42.65 | 6 | 0 | 0 | 0 | 0 | 6 | 0 | 0 | 0 | 0 | 0 | 0 | 0 | 0 |  |  |  |  | 1.5541E6 |  |  |  |  |  |  |  |  | 1 | 1 | 0 | 0 | 0 | 0 | 1 | 0 | 0 | 0 | 0 | 0 | 0 | 0 | 0 | Y | 18124 | lymphocyte antigen 86 isoform X1 [Thamnophis elegans] |
| 273 | 1276 | XP\_032078279.1 | 42.65 | 6 | 0 | 0 | 0 | 0 | 6 | 0 | 0 | 0 | 0 | 0 | 0 | 0 | 0 |  |  |  |  | 1.5541E6 |  |  |  |  |  |  |  |  | 1 | 1 | 0 | 0 | 0 | 0 | 1 | 0 | 0 | 0 | 0 | 0 | 0 | 0 | 0 | Y | 18124 | lymphocyte antigen 86 isoform X1 [Thamnophis elegans] |
| 273 | 1277 | LAB44273.1 | 42.65 | 6 | 0 | 0 | 0 | 0 | 6 | 0 | 0 | 0 | 0 | 0 | 0 | 0 | 0 |  |  |  |  | 1.5541E6 |  |  |  |  |  |  |  |  | 1 | 1 | 0 | 0 | 0 | 0 | 1 | 0 | 0 | 0 | 0 | 0 | 0 | 0 | 0 | Y | 17981 | hypothetical protein [Micrurus spixii] |
| 273 | 3247 | LAA56518.1 | 42.65 | 6 | 0 | 0 | 0 | 0 | 6 | 0 | 0 | 0 | 0 | 0 | 0 | 0 | 0 |  |  |  |  | 1.5541E6 |  |  |  |  |  |  |  |  | 1 | 1 | 0 | 0 | 0 | 0 | 1 | 0 | 0 | 0 | 0 | 0 | 0 | 0 | 0 | Y | 17452 | hypothetical protein, partial [Micrurus corallinus] |
| 273 | 3248 | XP\_026570835.1 | 42.65 | 6 | 0 | 0 | 0 | 0 | 6 | 0 | 0 | 0 | 0 | 0 | 0 | 0 | 0 |  |  |  |  | 1.5541E6 |  |  |  |  |  |  |  |  | 1 | 1 | 0 | 0 | 0 | 0 | 1 | 0 | 0 | 0 | 0 | 0 | 0 | 0 | 0 | Y | 19799 | lymphocyte antigen 86 [Pseudonaja textilis] |
| 273 | 3249 | XP\_026532440.1 | 42.65 | 6 | 0 | 0 | 0 | 0 | 6 | 0 | 0 | 0 | 0 | 0 | 0 | 0 | 0 |  |  |  |  | 1.5541E6 |  |  |  |  |  |  |  |  | 1 | 1 | 0 | 0 | 0 | 0 | 1 | 0 | 0 | 0 | 0 | 0 | 0 | 0 | 0 | Y | 19701 | lymphocyte antigen 86 [Notechis scutatus] |
| 288 | 4170 | XP\_015673175.1 | 42.36 | 6 | 0 | 0 | 6 | 0 | 0 | 0 | 0 | 0 | 0 | 0 | 0 | 0 | 0 |  |  | 2.6866E6 |  |  |  |  |  |  |  |  |  |  | 1 | 1 | 0 | 0 | 1 | 0 | 0 | 0 | 0 | 0 | 0 | 0 | 0 | 0 | 0 | Y | 22501 | testis-expressed protein 36 [Protobothrops mucrosquamatus] |
| total 681 proteins |  |  |  |  |  |  |  |  |  |  |  |  |  |  |  |  |  |  |  |  |  |  |  |  |  |  |  |  |  |  |  |  |  |  |  |  |  |  |  |  |  |  |  |  |  |  |  |  |
| --- | --- | --- | --- | --- | --- | --- | --- | --- | --- | --- | --- | --- | --- | --- | --- | --- | --- | --- | --- | --- | --- | --- | --- | --- | --- | --- | --- | --- | --- | --- | --- | --- | --- | --- | --- | --- | --- | --- | --- | --- | --- | --- | --- | --- | --- | --- | --- | --- |

  

2GIZ

back to list

  

| Protein Coverage
| Supporting Peptides
|

Protein Coverage:

Supporting Peptides:

| Peptide | Uniq | -10lgP | Mass | Length | ppm | m/z | z | RT | Fraction | Scan | Source File | Area F1 | Area F10 | Area F11 | Area F12 | Area F13 | Area F2 | Area F3 | Area F4 | Area F5 | Area F6 | Area F7 | Area F8 | Area F9 | #Feature | #Feature F1 | #Feature F10 | #Feature F11 | #Feature F12 | #Feature F13 | #Feature F2 | #Feature F3 | #Feature F4 | #Feature F5 | #Feature F6 | #Feature F7 | #Feature F8 | #Feature F9 | Start | End | PTM | AScore | Found By |
| --- | --- | --- | --- | --- | --- | --- | --- | --- | --- | --- | --- | --- | --- | --- | --- | --- | --- | --- | --- | --- | --- | --- | --- | --- | --- | --- | --- | --- | --- | --- | --- | --- | --- | --- | --- | --- | --- | --- | --- | --- | --- | --- | --- |
| R.TWTEIIHLWHDEYK.N | Y | 96.42 | 1869.9049 | 14 | 0.2 | 935.9600 | 2 | 67.88 | 4 | F4:47092 | NaNaKA16\_F12.raw |  |  |  | 1.192E10 | 1.3265E7 |  |  |  |  |  |  |  |  | 7 | 0 | 0 | 0 | 5 | 2 | 0 | 0 | 0 | 0 | 0 | 0 | 0 | 0 | 85 | 98 |  |  | PEAKS DB |
| R.WANTC(+57.02)SLNHSPDNLR.V | Y | 95.37 | 1783.8060 | 15 | -0.2 | 892.9102 | 2 | 20.57 | 4 | F4:6149 | NaNaKA16\_F12.raw |  |  |  | 2.5809E10 | 7.535E6 |  |  |  |  |  | 1.8333E5 |  |  | 20 | 0 | 0 | 0 | 16 | 3 | 0 | 0 | 0 | 0 | 0 | 1 | 0 | 0 | 52 | 66 | Carbamidomethylation | C5:Carbamidomethylation:1000.00 | PEAKS DB |
| R.VLEGIQC(+57.02)GESIYMSSNAR.T | Y | 94.89 | 2012.9296 | 18 | 0.0 | 1007.4721 | 2 | 61.49 | 4 | F4:39775 | NaNaKA16\_F12.raw |  | 5.2943E6 | 3.3626E7 | 7.5365E9 | 1.1193E7 |  |  |  |  |  |  |  | 0 | 16 | 0 | 1 | 2 | 9 | 4 | 0 | 0 | 0 | 0 | 0 | 0 | 0 | 0 | 67 | 84 | Carbamidomethylation | C7:Carbamidomethylation:1000.00 | PEAKS DB |
| R.VLEGIQC(+57.02)GESIYM(+15.99)SSNAR.T | Y | 91.90 | 2028.9244 | 18 | -0.3 | 1015.4692 | 2 | 47.15 | 4 | F4:28194 | NaNaKA16\_F12.raw |  | 1.6305E5 | 4.9762E6 | 1.1167E9 | 1.3064E6 |  |  |  |  |  |  |  |  | 10 | 0 | 1 | 1 | 7 | 1 | 0 | 0 | 0 | 0 | 0 | 0 | 0 | 0 | 67 | 84 | Carbamidomethylation; Oxidation (M) | C7:Carbamidomethylation:1000.00;M13:Oxidation (M):1000.00 | PEAKS DB |
| V.TGHYTQIVWYQTYR.A | Y | 79.15 | 1814.8740 | 14 | 0.5 | 908.4447 | 2 | 44.15 | 4 | F4:25452 | NaNaKA16\_F12.raw |  |  |  | 1.3608E8 |  |  |  |  |  |  |  |  |  | 2 | 0 | 0 | 0 | 2 | 0 | 0 | 0 | 0 | 0 | 0 | 0 | 0 | 0 | 113 | 126 |  |  | PEAKS DB |
| R.VSPTASNMLKMEWYPEAASNAER.W | N | 79.04 | 2581.1941 | 23 | 2.4 | 861.4074 | 3 | 60.50 | 4 | F4:38986 | NaNaKA16\_F12.raw |  |  |  | 7.3936E7 |  |  |  |  |  |  |  |  |  | 2 | 0 | 0 | 0 | 2 | 0 | 0 | 0 | 0 | 0 | 0 | 0 | 0 | 0 | 29 | 51 |  |  | PEAKS DB |
| Y.FYVC(+57.02)QYC(+57.02)PSGNFQGK.T | Y | 77.96 | 1853.7865 | 15 | -0.4 | 927.9001 | 2 | 45.62 | 4 | F4:26578 | NaNaKA16\_F12.raw |  |  |  | 2.3019E7 |  |  |  |  |  |  |  |  |  | 1 | 0 | 0 | 0 | 1 | 0 | 0 | 0 | 0 | 0 | 0 | 0 | 0 | 0 | 142 | 156 | Carbamidomethylation | C4:Carbamidomethylation:1000.00;C7:Carbamidomethylation:1000.00 | PEAKS DB |
| K.MEWYPEAASNAER.W | N | 77.76 | 1552.6616 | 13 | 0.7 | 518.5615 | 3 | 44.66 | 4 | F4:25052 | NaNaKA16\_F12.raw |  |  | 1.7754E7 | 3.6E10 | 1.0543E7 |  |  |  |  |  |  |  |  | 15 | 0 | 0 | 1 | 12 | 2 | 0 | 0 | 0 | 0 | 0 | 0 | 0 | 0 | 39 | 51 |  |  | PEAKS DB |
| W.SYFYVC(+57.02)QYC(+57.02)PSGNFQGK.T | Y | 76.97 | 2103.8818 | 17 | 0.0 | 1052.9482 | 2 | 56.99 | 4 | F4:35993 | NaNaKA16\_F12.raw |  |  |  | 2.0613E7 |  |  |  |  |  |  |  |  |  | 1 | 0 | 0 | 0 | 1 | 0 | 0 | 0 | 0 | 0 | 0 | 0 | 0 | 0 | 140 | 156 | Carbamidomethylation | C6:Carbamidomethylation:1000.00;C9:Carbamidomethylation:1000.00 | PEAKS DB |
| K.QKEIVDLHNSLR.R | N | 76.76 | 1450.7892 | 12 | -0.1 | 726.4018 | 2 | 11.75 | 4 | F4:2056 | NaNaKA16\_F12.raw |  |  |  | 8.0169E7 |  |  |  |  |  |  |  |  |  | 7 | 0 | 0 | 0 | 7 | 0 | 0 | 0 | 0 | 0 | 0 | 0 | 0 | 0 | 15 | 26 |  |  | PEAKS DB |
| K.M(+15.99)EWYPEAASNAER.W | N | 75.60 | 1568.6565 | 13 | 1.2 | 785.3364 | 2 | 31.92 | 4 | F4:18885 | NaNaKA16\_F12.raw |  |  |  | 4.1615E9 |  |  |  |  |  |  |  |  |  | 21 | 0 | 0 | 0 | 21 | 0 | 0 | 0 | 0 | 0 | 0 | 0 | 0 | 0 | 39 | 51 | Oxidation (M) | M1:Oxidation (M):1000.00 | PEAKS DB |
| L.RVLEGIQC(+57.02)GESIYMSSNAR.T | Y | 75.04 | 2169.0308 | 19 | -0.9 | 724.0169 | 3 | 42.93 | 4 | F4:24321 | NaNaKA16\_F12.raw |  |  | 1.5612E6 | 3.8584E6 |  |  |  |  |  |  |  |  |  | 2 | 0 | 0 | 1 | 1 | 0 | 0 | 0 | 0 | 0 | 0 | 0 | 0 | 0 | 66 | 84 | Carbamidomethylation | C8:Carbamidomethylation:1000.00 | PEAKS DB |
| N.GLC(+57.02)TNPC(+57.02)TIYNK.L | Y | 73.43 | 1439.6537 | 12 | 0.2 | 720.8342 | 2 | 27.20 | 4 | F4:10879 | NaNaKA16\_F12.raw |  |  |  | 3.9014E7 |  |  |  |  |  |  |  |  |  | 1 | 0 | 0 | 0 | 1 | 0 | 0 | 0 | 0 | 0 | 0 | 0 | 0 | 0 | 177 | 188 | Carbamidomethylation | C3:Carbamidomethylation:1000.00;C7:Carbamidomethylation:1000.00 | PEAKS DB |
| F.YVC(+57.02)QYC(+57.02)PSGNFQGK.T | Y | 71.81 | 1706.7181 | 14 | 0.0 | 854.3663 | 2 | 25.92 | 4 | F4:10821 | NaNaKA16\_F12.raw |  |  |  | 4.1439E8 |  |  |  |  |  |  |  |  |  | 1 | 0 | 0 | 0 | 1 | 0 | 0 | 0 | 0 | 0 | 0 | 0 | 0 | 0 | 143 | 156 | Carbamidomethylation | C3:Carbamidomethylation:1000.00;C6:Carbamidomethylation:1000.00 | PEAKS DB |
| E.GIQC(+57.02)GESIYMSSNAR.T | Y | 70.11 | 1671.7345 | 15 | 0.6 | 836.8750 | 2 | 29.85 | 4 | F4:13323 | NaNaKA16\_F12.raw |  |  |  | 1.3244E6 |  |  |  |  |  |  |  |  |  | 1 | 0 | 0 | 0 | 1 | 0 | 0 | 0 | 0 | 0 | 0 | 0 | 0 | 0 | 70 | 84 | Carbamidomethylation | C4:Carbamidomethylation:1000.00 | PEAKS DB |
| R.RVSPTASNMLK.M | Y | 69.72 | 1202.6442 | 11 | 0.3 | 602.3295 | 2 | 11.45 | 4 | F4:1801 | NaNaKA16\_F12.raw |  |  |  | 1.7511E7 |  |  |  |  |  |  |  |  |  | 2 | 0 | 0 | 0 | 2 | 0 | 0 | 0 | 0 | 0 | 0 | 0 | 0 | 0 | 28 | 38 |  |  | PEAKS DB |
| L.KMEWYPEAASNAER.W | N | 69.69 | 1680.7566 | 14 | -0.9 | 561.2590 | 3 | 29.03 | 4 | F4:12760 | NaNaKA16\_F12.raw |  |  |  | 3.1822E7 |  |  |  |  |  |  |  |  |  | 2 | 0 | 0 | 0 | 2 | 0 | 0 | 0 | 0 | 0 | 0 | 0 | 0 | 0 | 38 | 51 |  |  | PEAKS DB |
| NVDFNSESTR.R | N | 69.40 | 1167.5156 | 10 | 0.6 | 584.7654 | 2 | 12.15 | 4 | F4:2376 | NaNaKA16\_F12.raw |  |  |  | 8.6869E7 |  |  |  |  |  |  |  |  |  | 1 | 0 | 0 | 0 | 1 | 0 | 0 | 0 | 0 | 0 | 0 | 0 | 0 | 0 | 1 | 10 |  |  | PEAKS DB |
| W.TEIIHLWHDEYK.N | Y | 68.88 | 1582.7780 | 12 | 0.3 | 528.6001 | 3 | 38.63 | 4 | F4:20823 | NaNaKA16\_F12.raw |  |  |  | 1.545E7 |  |  |  |  |  |  |  |  |  | 4 | 0 | 0 | 0 | 4 | 0 | 0 | 0 | 0 | 0 | 0 | 0 | 0 | 0 | 87 | 98 |  |  | PEAKS DB |
| C.DNGLC(+57.02)TNPC(+57.02)TIYNK.L | Y | 68.73 | 1668.7236 | 14 | 0.5 | 835.3695 | 2 | 36.61 | 4 | F4:19046 | NaNaKA16\_F12.raw |  |  |  | 6.9202E6 |  |  |  |  | 7.9009E6 |  |  |  |  | 3 | 0 | 0 | 0 | 1 | 0 | 0 | 0 | 0 | 2 | 0 | 0 | 0 | 0 | 175 | 188 | Carbamidomethylation | C5:Carbamidomethylation:1000.00;C9:Carbamidomethylation:1000.00 | PEAKS DB |
| K.EIVDLHNSLR.R | N | 66.53 | 1194.6356 | 10 | -0.4 | 598.3248 | 2 | 26.22 | 4 | F4:11038 | NaNaKA16\_F12.raw |  |  | 2.0613E5 | 9.1517E8 | 1.6717E6 |  |  |  |  |  |  |  |  | 7 | 0 | 0 | 1 | 5 | 1 | 0 | 0 | 0 | 0 | 0 | 0 | 0 | 0 | 17 | 26 |  |  | PEAKS DB |
| A.RTWTEIIHLWHDEYK.N | Y | 65.27 | 2026.0061 | 15 | -0.1 | 507.5088 | 4 | 59.52 | 4 | F4:38212 | NaNaKA16\_F12.raw |  |  |  | 2.6065E6 |  |  |  |  |  |  |  |  |  | 1 | 0 | 0 | 0 | 1 | 0 | 0 | 0 | 0 | 0 | 0 | 0 | 0 | 0 | 84 | 98 |  |  | PEAKS DB |
| NVDFNSESTRR.K | N | 65.26 | 1323.6167 | 11 | -0.1 | 662.8156 | 2 | 11.44 | 4 | F4:1806 | NaNaKA16\_F12.raw |  |  |  | 6.0739E6 |  |  |  |  |  |  |  |  |  | 2 | 0 | 0 | 0 | 2 | 0 | 0 | 0 | 0 | 0 | 0 | 0 | 0 | 0 | 1 | 11 |  |  | PEAKS DB |
| K.SNC(+57.02)PASC(+57.02)FC(+57.02)R.N | N | 62.02 | 1257.4689 | 10 | 0.2 | 629.7418 | 2 | 12.25 | 4 | F4:2426 | NaNaKA16\_F12.raw |  |  |  | 6.4924E7 | 9.521E3 |  |  |  |  |  |  |  |  | 2 | 0 | 0 | 0 | 1 | 1 | 0 | 0 | 0 | 0 | 0 | 0 | 0 | 0 | 208 | 217 | Carbamidomethylation | C3:Carbamidomethylation:1000.00;C7:Carbamidomethylation:1000.00;C9:Carbamidomethylation:1000.00 | PEAKS DB |
| H.YTQIVWYQTYR.A | Y | 61.61 | 1519.7460 | 11 | 0.1 | 760.8804 | 2 | 55.75 | 4 | F4:34986 | NaNaKA16\_F12.raw |  |  |  | 1.6068E8 |  |  |  |  |  |  |  |  |  | 2 | 0 | 0 | 0 | 2 | 0 | 0 | 0 | 0 | 0 | 0 | 0 | 0 | 0 | 116 | 126 |  |  | PEAKS DB |
| R.TWTEIIHLWH.D | Y | 60.78 | 1334.6771 | 10 | 0.3 | 668.3461 | 2 | 73.98 | 4 | F4:49966 | NaNaKA16\_F12.raw |  |  |  | 4.783E6 |  |  |  |  |  |  |  |  |  | 1 | 0 | 0 | 0 | 1 | 0 | 0 | 0 | 0 | 0 | 0 | 0 | 0 | 0 | 85 | 94 |  |  | PEAKS DB |
| R.VLEGIQC(+57.02)GESIYMSSN.A | Y | 58.92 | 1785.7914 | 16 | -0.4 | 893.9026 | 2 | 68.70 | 4 | F4:45706 | NaNaKA16\_F12.raw |  |  |  | 1.3545E8 |  |  |  |  |  |  |  |  |  | 1 | 0 | 0 | 0 | 1 | 0 | 0 | 0 | 0 | 0 | 0 | 0 | 0 | 0 | 67 | 82 | Carbamidomethylation | C7:Carbamidomethylation:1000.00 | PEAKS DB |
| N.TC(+57.02)SLNHSPDNLR.V | Y | 56.85 | 1412.6466 | 12 | 0.5 | 707.3309 | 2 | 11.48 | 4 | F4:1809 | NaNaKA16\_F12.raw |  |  |  | 4.5467E6 |  |  |  |  |  |  |  |  |  | 2 | 0 | 0 | 0 | 2 | 0 | 0 | 0 | 0 | 0 | 0 | 0 | 0 | 0 | 55 | 66 | Carbamidomethylation | C2:Carbamidomethylation:1000.00 | PEAKS DB |
| R.VSPTASNM(+15.99)LK.M | N | 56.73 | 1062.5380 | 10 | 0.7 | 532.2766 | 2 | 11.45 | 4 | F4:1800 | NaNaKA16\_F12.raw |  |  |  | 1.2798E7 |  |  |  |  |  |  |  |  |  | 1 | 0 | 0 | 0 | 1 | 0 | 0 | 0 | 0 | 0 | 0 | 0 | 0 | 0 | 29 | 38 | Oxidation (M) | M8:Oxidation (M):1000.00 | PEAKS DB |
| K.LGPPC(+57.02)GDC(+57.02)PSAC(+57.02)DNGLC(+57.02)TNPC(+57.02)TIYNK.L | Y | 56.61 | 2940.1970 | 26 | 1.2 | 981.0742 | 3 | 49.74 | 4 | F4:29707 | NaNaKA16\_F12.raw |  |  |  | 9.9063E8 |  |  |  |  |  |  |  |  |  | 2 | 0 | 0 | 0 | 2 | 0 | 0 | 0 | 0 | 0 | 0 | 0 | 0 | 0 | 163 | 188 | Carbamidomethylation | C5:Carbamidomethylation:1000.00;C8:Carbamidomethylation:1000.00;C12:Carbamidomethylation:1000.00;C17:Carbamidomethylation:1000.00;C21:Carbamidomethylation:1000.00 | PEAKS DB |
| Y.TQIVWYQTYR.A | Y | 56.03 | 1356.6826 | 10 | 0.6 | 679.3490 | 2 | 49.37 | 4 | F4:29565 | NaNaKA16\_F12.raw |  |  |  | 3.2787E7 |  |  |  |  |  |  |  |  |  | 1 | 0 | 0 | 0 | 1 | 0 | 0 | 0 | 0 | 0 | 0 | 0 | 0 | 0 | 117 | 126 |  |  | PEAKS DB |
| Y.VC(+57.02)QYC(+57.02)PSGNFQGK.T | Y | 54.51 | 1543.6548 | 13 | -0.3 | 772.8344 | 2 | 11.76 | 4 | F4:2117 | NaNaKA16\_F12.raw |  |  |  | 1.3951E6 |  |  |  |  |  |  |  |  |  | 1 | 0 | 0 | 0 | 1 | 0 | 0 | 0 | 0 | 0 | 0 | 0 | 0 | 0 | 144 | 156 | Carbamidomethylation | C2:Carbamidomethylation:1000.00;C5:Carbamidomethylation:1000.00 | PEAKS DB |
| R.RVSPTASNM(+15.99)LK.M | Y | 54.31 | 1218.6390 | 11 | 0.7 | 407.2206 | 3 | 11.48 | 4 | F4:1808 | NaNaKA16\_F12.raw |  |  |  | 4.4814E5 |  |  |  |  |  |  |  |  |  | 1 | 0 | 0 | 0 | 1 | 0 | 0 | 0 | 0 | 0 | 0 | 0 | 0 | 0 | 28 | 38 | Oxidation (M) | M9:Oxidation (M):1000.00 | PEAKS DB |
| R.VSPTASNMLK.M | N | 54.02 | 1046.5430 | 10 | -0.7 | 524.2784 | 2 | 11.74 | 4 | F4:2287 | NaNaKA16\_F12.raw |  |  |  | 1.7067E8 |  |  |  |  |  |  |  |  |  | 2 | 0 | 0 | 0 | 2 | 0 | 0 | 0 | 0 | 0 | 0 | 0 | 0 | 0 | 29 | 38 |  |  | PEAKS DB |
| I.VDLHNSLR.R | N | 53.27 | 952.5090 | 8 | 0.1 | 477.2618 | 2 | 26.22 | 4 | F4:10364 | NaNaKA16\_F12.raw |  |  |  | 3.6616E7 |  |  |  |  |  |  |  |  |  | 1 | 0 | 0 | 0 | 1 | 0 | 0 | 0 | 0 | 0 | 0 | 0 | 0 | 0 | 19 | 26 |  |  | PEAKS DB |
| M.EWYPEAASNAER.W | N | 53.15 | 1421.6211 | 12 | 0.6 | 711.8182 | 2 | 30.28 | 4 | F4:13787 | NaNaKA16\_F12.raw |  |  |  | 1.7579E7 |  |  |  |  |  |  |  |  |  | 1 | 0 | 0 | 0 | 1 | 0 | 0 | 0 | 0 | 0 | 0 | 0 | 0 | 0 | 40 | 51 |  |  | PEAKS DB |
| N.VDFNSESTR.R | N | 52.01 | 1053.4727 | 9 | 0.6 | 527.7439 | 2 | 11.66 | 4 | F4:1931 | NaNaKA16\_F12.raw |  |  |  | 9.3775E6 |  |  |  |  |  |  |  |  |  | 1 | 0 | 0 | 0 | 1 | 0 | 0 | 0 | 0 | 0 | 0 | 0 | 0 | 0 | 2 | 10 |  |  | PEAKS DB |
| S.AC(+57.02)DNGLC(+57.02)TNPC(+57.02)TIYNK.L | Y | 51.74 | 1899.7914 | 16 | -0.3 | 950.9027 | 2 | 33.35 | 4 | F4:16304 | NaNaKA16\_F12.raw |  |  |  | 3.0888E6 |  |  |  |  |  |  |  |  |  | 1 | 0 | 0 | 0 | 1 | 0 | 0 | 0 | 0 | 0 | 0 | 0 | 0 | 0 | 173 | 188 | Carbamidomethylation | C2:Carbamidomethylation:1000.00;C7:Carbamidomethylation:1000.00;C11:Carbamidomethylation:1000.00 | PEAKS DB |
| C.SLNHSPDNLR.V | Y | 50.57 | 1151.5684 | 10 | -0.3 | 384.8633 | 3 | 11.45 | 4 | F4:1803 | NaNaKA16\_F12.raw |  |  |  | 3.3986E6 |  |  |  |  |  |  |  |  |  | 2 | 0 | 0 | 0 | 2 | 0 | 0 | 0 | 0 | 0 | 0 | 0 | 0 | 0 | 57 | 66 |  |  | PEAKS DB |
| R.TWTEIIHLWHDEYKN.F | Y | 49.28 | 1983.9479 | 15 | -0.2 | 496.9941 | 4 | 67.43 | 4 | F4:44938 | NaNaKA16\_F12.raw |  |  |  | 1.7614E7 |  |  |  |  |  |  |  |  |  | 2 | 0 | 0 | 0 | 2 | 0 | 0 | 0 | 0 | 0 | 0 | 0 | 0 | 0 | 85 | 99 |  |  | PEAKS DB |
| L.EGIQC(+57.02)GESIYMSSNAR.T | Y | 48.30 | 1800.7771 | 16 | 0.9 | 901.3966 | 2 | 35.87 | 4 | F4:18384 | NaNaKA16\_F12.raw |  |  |  | 0 |  |  |  |  |  |  |  |  |  | 0 | 0 | 0 | 0 | 0 | 0 | 0 | 0 | 0 | 0 | 0 | 0 | 0 | 0 | 69 | 84 | Carbamidomethylation | C5:Carbamidomethylation:1000.00 | PEAKS DB |
| R.VLEGIQC(+57.02)GESIYM(+15.99)SSN.A | Y | 48.10 | 1801.7863 | 16 | 0.0 | 901.9004 | 2 | 59.91 | 4 | F4:38428 | NaNaKA16\_F12.raw |  |  |  | 1.9592E7 |  |  |  |  |  |  |  |  |  | 1 | 0 | 0 | 0 | 1 | 0 | 0 | 0 | 0 | 0 | 0 | 0 | 0 | 0 | 67 | 82 | Carbamidomethylation; Oxidation (M) | C7:Carbamidomethylation:1000.00;M13:Oxidation (M):1000.00 | PEAKS DB |
| Y.KLGPPC(+57.02)GDC(+57.02)PSAC(+57.02)DNGLC(+57.02)TNPC(+57.02)TIYNK.L | Y | 48.02 | 3068.2917 | 27 | 1.2 | 1023.7724 | 3 | 40.46 | 4 | F4:22361 | NaNaKA16\_F12.raw |  |  |  | 7.6949E6 |  |  |  |  |  |  |  |  |  | 1 | 0 | 0 | 0 | 1 | 0 | 0 | 0 | 0 | 0 | 0 | 0 | 0 | 0 | 162 | 188 | Carbamidomethylation | C6:Carbamidomethylation:1000.00;C9:Carbamidomethylation:1000.00;C13:Carbamidomethylation:1000.00;C18:Carbamidomethylation:1000.00;C22:Carbamidomethylation:1000.00 | PEAKS DB |
| Q.IVWYQTYR.A | Y | 47.45 | 1127.5764 | 8 | -5.9 | 564.7922 | 2 | 43.62 | 4 | F4:24799 | NaNaKA16\_F12.raw |  |  |  | 3.1475E7 |  |  |  |  |  |  |  |  |  | 2 | 0 | 0 | 0 | 2 | 0 | 0 | 0 | 0 | 0 | 0 | 0 | 0 | 0 | 119 | 126 |  |  | PEAKS DB |
| R.TWTEIIHLWHD.E | Y | 47.44 | 1449.7041 | 11 | 0.4 | 484.2422 | 3 | 76.70 | 4 | F4:52262 | NaNaKA16\_F12.raw |  |  |  | 2.7409E6 |  |  |  |  |  |  |  |  |  | 1 | 0 | 0 | 0 | 1 | 0 | 0 | 0 | 0 | 0 | 0 | 0 | 0 | 0 | 85 | 95 |  |  | PEAKS DB |
| T.QIVWYQTYR.A | Y | 46.03 | 1255.6349 | 9 | 0.0 | 628.8247 | 2 | 47.28 | 4 | F4:27952 | NaNaKA16\_F12.raw |  |  |  | 5.8793E6 |  |  |  |  |  |  |  |  |  | 1 | 0 | 0 | 0 | 1 | 0 | 0 | 0 | 0 | 0 | 0 | 0 | 0 | 0 | 118 | 126 |  |  | PEAKS DB |
| E.IIHLWHDEYK.N | N | 45.95 | 1352.6877 | 10 | -0.5 | 451.9030 | 3 | 26.85 | 4 | F4:10987 | NaNaKA16\_F12.raw |  |  |  | 8.808E5 |  |  |  |  |  |  |  |  |  | 1 | 0 | 0 | 0 | 1 | 0 | 0 | 0 | 0 | 0 | 0 | 0 | 0 | 0 | 89 | 98 |  |  | PEAKS DB |
| T.EIIHLWHDEYK.N | Y | 44.66 | 1481.7302 | 11 | 0.1 | 494.9174 | 3 | 34.79 | 4 | F4:17343 | NaNaKA16\_F12.raw |  |  |  | 4.613E5 |  |  |  |  |  |  |  |  |  | 1 | 0 | 0 | 0 | 1 | 0 | 0 | 0 | 0 | 0 | 0 | 0 | 0 | 0 | 88 | 98 |  |  | PEAKS DB |
| R.VLEGIQC(+57.02)GESIY.M | Y | 44.56 | 1366.6438 | 12 | 0.4 | 684.3295 | 2 | 64.32 | 4 | F4:42048 | NaNaKA16\_F12.raw |  |  |  | 1.267E8 |  |  |  |  |  |  |  |  |  | 1 | 0 | 0 | 0 | 1 | 0 | 0 | 0 | 0 | 0 | 0 | 0 | 0 | 0 | 67 | 78 | Carbamidomethylation | C7:Carbamidomethylation:1000.00 | PEAKS DB |
| D.C(+57.02)PSAC(+57.02)DNGLC(+57.02)TNPC(+57.02)TIYNK.L | Y | 44.19 | 2243.9067 | 19 | 0.6 | 1122.9613 | 2 | 36.03 | 4 | F4:18539 | NaNaKA16\_F12.raw |  |  |  | 1.1206E6 |  |  |  |  |  |  |  |  |  | 1 | 0 | 0 | 0 | 1 | 0 | 0 | 0 | 0 | 0 | 0 | 0 | 0 | 0 | 170 | 188 | Carbamidomethylation | C1:Carbamidomethylation:1000.00;C5:Carbamidomethylation:1000.00;C10:Carbamidomethylation:1000.00;C14:Carbamidomethylation:1000.00 | PEAKS DB |
| L.RVLEGIQC(+57.02)GESIYM(+15.99)SSNAR.T | Y | 43.71 | 2185.0256 | 19 | 0.8 | 729.3497 | 3 | 36.11 | 4 | F4:18487 | NaNaKA16\_F12.raw |  |  |  | 6.1752E5 |  |  |  |  |  |  |  |  |  | 1 | 0 | 0 | 0 | 1 | 0 | 0 | 0 | 0 | 0 | 0 | 0 | 0 | 0 | 66 | 84 | Carbamidomethylation; Oxidation (M) | C8:Carbamidomethylation:1000.00;M14:Oxidation (M):1000.00 | PEAKS DB |
| R.RVSPTASNMLKMEWYPEAASNAER.W | Y | 43.34 | 2737.2952 | 24 | 1.4 | 685.3320 | 4 | 51.07 | 4 | F4:31143 | NaNaKA16\_F12.raw |  |  |  | 1.6942E6 |  |  |  |  |  |  |  |  |  | 1 | 0 | 0 | 0 | 1 | 0 | 0 | 0 | 0 | 0 | 0 | 0 | 0 | 0 | 28 | 51 |  |  | PEAKS DB |
| K.QKEIVDLHN.S | N | 43.18 | 1094.5720 | 9 | -0.5 | 548.2930 | 2 | 11.57 | 4 | F4:1902 | NaNaKA16\_F12.raw |  |  |  | 1.3713E5 |  |  |  |  |  |  |  |  |  | 1 | 0 | 0 | 0 | 1 | 0 | 0 | 0 | 0 | 0 | 0 | 0 | 0 | 0 | 15 | 23 |  |  | PEAKS DB |
| R.TWTEIIHLW.H | Y | 42.97 | 1197.6183 | 9 | 0.1 | 599.8165 | 2 | 92.08 | 4 | F4:64706 | NaNaKA16\_F12.raw |  |  |  | 2.1391E6 |  |  |  |  |  |  |  |  |  | 1 | 0 | 0 | 0 | 1 | 0 | 0 | 0 | 0 | 0 | 0 | 0 | 0 | 0 | 85 | 93 |  |  | PEAKS DB |
| N.MLKMEWYPEAASNAER.W | N | 42.97 | 1924.8811 | 16 | 0.5 | 642.6346 | 3 | 48.79 | 4 | F4:29214 | NaNaKA16\_F12.raw |  |  |  | 1.0448E6 |  |  |  |  |  |  |  |  |  | 1 | 0 | 0 | 0 | 1 | 0 | 0 | 0 | 0 | 0 | 0 | 0 | 0 | 0 | 36 | 51 |  |  | PEAKS DB |
| T.GHYTQIVWYQTYR.A | Y | 42.91 | 1713.8263 | 13 | -0.2 | 572.2826 | 3 | 43.93 | 4 | F4:25228 | NaNaKA16\_F12.raw |  |  |  | 2.5056E6 |  |  |  |  |  |  |  |  |  | 1 | 0 | 0 | 0 | 1 | 0 | 0 | 0 | 0 | 0 | 0 | 0 | 0 | 0 | 114 | 126 |  |  | PEAKS DB |
| R.VSPTASNMLKM(+15.99)EWYPEAASNAER.W | N | 41.81 | 2597.1890 | 23 | -0.1 | 866.7368 | 3 | 60.59 | 4 | F4:38922 | NaNaKA16\_F12.raw |  |  |  | 1.7447E6 |  |  |  |  |  |  |  |  |  | 1 | 0 | 0 | 0 | 1 | 0 | 0 | 0 | 0 | 0 | 0 | 0 | 0 | 0 | 29 | 51 | Oxidation (M) | M11:Oxidation (M):22.34 | PEAKS DB |
| total 57 peptides |  |  |  |  |  |  |  |  |  |  |  |  |  |  |  |  |  |  |  |  |  |  |  |  |  |  |  |  |  |  |  |  |  |  |  |  |  |  |  |  |  |  |  |
| --- | --- | --- | --- | --- | --- | --- | --- | --- | --- | --- | --- | --- | --- | --- | --- | --- | --- | --- | --- | --- | --- | --- | --- | --- | --- | --- | --- | --- | --- | --- | --- | --- | --- | --- | --- | --- | --- | --- | --- | --- | --- | --- | --- |

1XX5

back to list

  

| Protein Coverage
| Supporting Peptides
|

Protein Coverage:

Supporting Peptides:

| Peptide | Uniq | -10lgP | Mass | Length | ppm | m/z | z | RT | Fraction | Scan | Source File | Area F1 | Area F10 | Area F11 | Area F12 | Area F13 | Area F2 | Area F3 | Area F4 | Area F5 | Area F6 | Area F7 | Area F8 | Area F9 | #Feature | #Feature F1 | #Feature F10 | #Feature F11 | #Feature F12 | #Feature F13 | #Feature F2 | #Feature F3 | #Feature F4 | #Feature F5 | #Feature F6 | #Feature F7 | #Feature F8 | #Feature F9 | Start | End | PTM | AScore | Found By |
| --- | --- | --- | --- | --- | --- | --- | --- | --- | --- | --- | --- | --- | --- | --- | --- | --- | --- | --- | --- | --- | --- | --- | --- | --- | --- | --- | --- | --- | --- | --- | --- | --- | --- | --- | --- | --- | --- | --- | --- | --- | --- | --- | --- |
| R.TWTEIIHLWHDEYK.N | Y | 96.42 | 1869.9049 | 14 | 0.2 | 935.9600 | 2 | 67.88 | 4 | F4:47092 | NaNaKA16\_F12.raw |  |  |  | 1.192E10 | 1.3265E7 |  |  |  |  |  |  |  |  | 7 | 0 | 0 | 0 | 5 | 2 | 0 | 0 | 0 | 0 | 0 | 0 | 0 | 0 | 85 | 98 |  |  | PEAKS DB |
| R.WANTC(+57.02)SLNHSPDNLR.V | Y | 95.37 | 1783.8060 | 15 | -0.2 | 892.9102 | 2 | 20.57 | 4 | F4:6149 | NaNaKA16\_F12.raw |  |  |  | 2.5809E10 | 7.535E6 |  |  |  |  |  | 1.8333E5 |  |  | 20 | 0 | 0 | 0 | 16 | 3 | 0 | 0 | 0 | 0 | 0 | 1 | 0 | 0 | 52 | 66 | Carbamidomethylation | C5:Carbamidomethylation:1000.00 | PEAKS DB |
| R.VLEGIQC(+57.02)GESIYMSSNAR.T | Y | 94.89 | 2012.9296 | 18 | 0.0 | 1007.4721 | 2 | 61.49 | 4 | F4:39775 | NaNaKA16\_F12.raw |  | 5.2943E6 | 3.3626E7 | 7.5365E9 | 1.1193E7 |  |  |  |  |  |  |  | 0 | 16 | 0 | 1 | 2 | 9 | 4 | 0 | 0 | 0 | 0 | 0 | 0 | 0 | 0 | 67 | 84 | Carbamidomethylation | C7:Carbamidomethylation:1000.00 | PEAKS DB |
| R.VLEGIQC(+57.02)GESIYM(+15.99)SSNAR.T | Y | 91.90 | 2028.9244 | 18 | -0.3 | 1015.4692 | 2 | 47.15 | 4 | F4:28194 | NaNaKA16\_F12.raw |  | 1.6305E5 | 4.9762E6 | 1.1167E9 | 1.3064E6 |  |  |  |  |  |  |  |  | 10 | 0 | 1 | 1 | 7 | 1 | 0 | 0 | 0 | 0 | 0 | 0 | 0 | 0 | 67 | 84 | Carbamidomethylation; Oxidation (M) | C7:Carbamidomethylation:1000.00;M13:Oxidation (M):1000.00 | PEAKS DB |
| V.TGHYTQIVWYQTYR.A | Y | 79.15 | 1814.8740 | 14 | 0.5 | 908.4447 | 2 | 44.15 | 4 | F4:25452 | NaNaKA16\_F12.raw |  |  |  | 1.3608E8 |  |  |  |  |  |  |  |  |  | 2 | 0 | 0 | 0 | 2 | 0 | 0 | 0 | 0 | 0 | 0 | 0 | 0 | 0 | 113 | 126 |  |  | PEAKS DB |
| R.VSPTASNMLKMEWYPEAASNAER.W | N | 79.04 | 2581.1941 | 23 | 2.4 | 861.4074 | 3 | 60.50 | 4 | F4:38986 | NaNaKA16\_F12.raw |  |  |  | 7.3936E7 |  |  |  |  |  |  |  |  |  | 2 | 0 | 0 | 0 | 2 | 0 | 0 | 0 | 0 | 0 | 0 | 0 | 0 | 0 | 29 | 51 |  |  | PEAKS DB |
| Y.FYVC(+57.02)QYC(+57.02)PSGNFQGK.T | Y | 77.96 | 1853.7865 | 15 | -0.4 | 927.9001 | 2 | 45.62 | 4 | F4:26578 | NaNaKA16\_F12.raw |  |  |  | 2.3019E7 |  |  |  |  |  |  |  |  |  | 1 | 0 | 0 | 0 | 1 | 0 | 0 | 0 | 0 | 0 | 0 | 0 | 0 | 0 | 142 | 156 | Carbamidomethylation | C4:Carbamidomethylation:1000.00;C7:Carbamidomethylation:1000.00 | PEAKS DB |
| K.MEWYPEAASNAER.W | N | 77.76 | 1552.6616 | 13 | 0.7 | 518.5615 | 3 | 44.66 | 4 | F4:25052 | NaNaKA16\_F12.raw |  |  | 1.7754E7 | 3.6E10 | 1.0543E7 |  |  |  |  |  |  |  |  | 15 | 0 | 0 | 1 | 12 | 2 | 0 | 0 | 0 | 0 | 0 | 0 | 0 | 0 | 39 | 51 |  |  | PEAKS DB |
| W.SYFYVC(+57.02)QYC(+57.02)PSGNFQGK.T | Y | 76.97 | 2103.8818 | 17 | 0.0 | 1052.9482 | 2 | 56.99 | 4 | F4:35993 | NaNaKA16\_F12.raw |  |  |  | 2.0613E7 |  |  |  |  |  |  |  |  |  | 1 | 0 | 0 | 0 | 1 | 0 | 0 | 0 | 0 | 0 | 0 | 0 | 0 | 0 | 140 | 156 | Carbamidomethylation | C6:Carbamidomethylation:1000.00;C9:Carbamidomethylation:1000.00 | PEAKS DB |
| K.QKEIVDLHNSLR.R | N | 76.76 | 1450.7892 | 12 | -0.1 | 726.4018 | 2 | 11.75 | 4 | F4:2056 | NaNaKA16\_F12.raw |  |  |  | 8.0169E7 |  |  |  |  |  |  |  |  |  | 7 | 0 | 0 | 0 | 7 | 0 | 0 | 0 | 0 | 0 | 0 | 0 | 0 | 0 | 15 | 26 |  |  | PEAKS DB |
| K.M(+15.99)EWYPEAASNAER.W | N | 75.60 | 1568.6565 | 13 | 1.2 | 785.3364 | 2 | 31.92 | 4 | F4:18885 | NaNaKA16\_F12.raw |  |  |  | 4.1615E9 |  |  |  |  |  |  |  |  |  | 21 | 0 | 0 | 0 | 21 | 0 | 0 | 0 | 0 | 0 | 0 | 0 | 0 | 0 | 39 | 51 | Oxidation (M) | M1:Oxidation (M):1000.00 | PEAKS DB |
| L.RVLEGIQC(+57.02)GESIYMSSNAR.T | Y | 75.04 | 2169.0308 | 19 | -0.9 | 724.0169 | 3 | 42.93 | 4 | F4:24321 | NaNaKA16\_F12.raw |  |  | 1.5612E6 | 3.8584E6 |  |  |  |  |  |  |  |  |  | 2 | 0 | 0 | 1 | 1 | 0 | 0 | 0 | 0 | 0 | 0 | 0 | 0 | 0 | 66 | 84 | Carbamidomethylation | C8:Carbamidomethylation:1000.00 | PEAKS DB |
| N.GLC(+57.02)TNPC(+57.02)TIYNK.L | Y | 73.43 | 1439.6537 | 12 | 0.2 | 720.8342 | 2 | 27.20 | 4 | F4:10879 | NaNaKA16\_F12.raw |  |  |  | 3.9014E7 |  |  |  |  |  |  |  |  |  | 1 | 0 | 0 | 0 | 1 | 0 | 0 | 0 | 0 | 0 | 0 | 0 | 0 | 0 | 177 | 188 | Carbamidomethylation | C3:Carbamidomethylation:1000.00;C7:Carbamidomethylation:1000.00 | PEAKS DB |
| F.YVC(+57.02)QYC(+57.02)PSGNFQGK.T | Y | 71.81 | 1706.7181 | 14 | 0.0 | 854.3663 | 2 | 25.92 | 4 | F4:10821 | NaNaKA16\_F12.raw |  |  |  | 4.1439E8 |  |  |  |  |  |  |  |  |  | 1 | 0 | 0 | 0 | 1 | 0 | 0 | 0 | 0 | 0 | 0 | 0 | 0 | 0 | 143 | 156 | Carbamidomethylation | C3:Carbamidomethylation:1000.00;C6:Carbamidomethylation:1000.00 | PEAKS DB |
| E.GIQC(+57.02)GESIYMSSNAR.T | Y | 70.11 | 1671.7345 | 15 | 0.6 | 836.8750 | 2 | 29.85 | 4 | F4:13323 | NaNaKA16\_F12.raw |  |  |  | 1.3244E6 |  |  |  |  |  |  |  |  |  | 1 | 0 | 0 | 0 | 1 | 0 | 0 | 0 | 0 | 0 | 0 | 0 | 0 | 0 | 70 | 84 | Carbamidomethylation | C4:Carbamidomethylation:1000.00 | PEAKS DB |
| R.RVSPTASNMLK.M | Y | 69.72 | 1202.6442 | 11 | 0.3 | 602.3295 | 2 | 11.45 | 4 | F4:1801 | NaNaKA16\_F12.raw |  |  |  | 1.7511E7 |  |  |  |  |  |  |  |  |  | 2 | 0 | 0 | 0 | 2 | 0 | 0 | 0 | 0 | 0 | 0 | 0 | 0 | 0 | 28 | 38 |  |  | PEAKS DB |
| L.KMEWYPEAASNAER.W | N | 69.69 | 1680.7566 | 14 | -0.9 | 561.2590 | 3 | 29.03 | 4 | F4:12760 | NaNaKA16\_F12.raw |  |  |  | 3.1822E7 |  |  |  |  |  |  |  |  |  | 2 | 0 | 0 | 0 | 2 | 0 | 0 | 0 | 0 | 0 | 0 | 0 | 0 | 0 | 38 | 51 |  |  | PEAKS DB |
| NVDFNSESTR.R | N | 69.40 | 1167.5156 | 10 | 0.6 | 584.7654 | 2 | 12.15 | 4 | F4:2376 | NaNaKA16\_F12.raw |  |  |  | 8.6869E7 |  |  |  |  |  |  |  |  |  | 1 | 0 | 0 | 0 | 1 | 0 | 0 | 0 | 0 | 0 | 0 | 0 | 0 | 0 | 1 | 10 |  |  | PEAKS DB |
| W.TEIIHLWHDEYK.N | Y | 68.88 | 1582.7780 | 12 | 0.3 | 528.6001 | 3 | 38.63 | 4 | F4:20823 | NaNaKA16\_F12.raw |  |  |  | 1.545E7 |  |  |  |  |  |  |  |  |  | 4 | 0 | 0 | 0 | 4 | 0 | 0 | 0 | 0 | 0 | 0 | 0 | 0 | 0 | 87 | 98 |  |  | PEAKS DB |
| C.DNGLC(+57.02)TNPC(+57.02)TIYNK.L | Y | 68.73 | 1668.7236 | 14 | 0.5 | 835.3695 | 2 | 36.61 | 4 | F4:19046 | NaNaKA16\_F12.raw |  |  |  | 6.9202E6 |  |  |  |  | 7.9009E6 |  |  |  |  | 3 | 0 | 0 | 0 | 1 | 0 | 0 | 0 | 0 | 2 | 0 | 0 | 0 | 0 | 175 | 188 | Carbamidomethylation | C5:Carbamidomethylation:1000.00;C9:Carbamidomethylation:1000.00 | PEAKS DB |
| K.EIVDLHNSLR.R | N | 66.53 | 1194.6356 | 10 | -0.4 | 598.3248 | 2 | 26.22 | 4 | F4:11038 | NaNaKA16\_F12.raw |  |  | 2.0613E5 | 9.1517E8 | 1.6717E6 |  |  |  |  |  |  |  |  | 7 | 0 | 0 | 1 | 5 | 1 | 0 | 0 | 0 | 0 | 0 | 0 | 0 | 0 | 17 | 26 |  |  | PEAKS DB |
| A.RTWTEIIHLWHDEYK.N | Y | 65.27 | 2026.0061 | 15 | -0.1 | 507.5088 | 4 | 59.52 | 4 | F4:38212 | NaNaKA16\_F12.raw |  |  |  | 2.6065E6 |  |  |  |  |  |  |  |  |  | 1 | 0 | 0 | 0 | 1 | 0 | 0 | 0 | 0 | 0 | 0 | 0 | 0 | 0 | 84 | 98 |  |  | PEAKS DB |
| NVDFNSESTRR.K | N | 65.26 | 1323.6167 | 11 | -0.1 | 662.8156 | 2 | 11.44 | 4 | F4:1806 | NaNaKA16\_F12.raw |  |  |  | 6.0739E6 |  |  |  |  |  |  |  |  |  | 2 | 0 | 0 | 0 | 2 | 0 | 0 | 0 | 0 | 0 | 0 | 0 | 0 | 0 | 1 | 11 |  |  | PEAKS DB |
| K.SNC(+57.02)PASC(+57.02)FC(+57.02)R.N | N | 62.02 | 1257.4689 | 10 | 0.2 | 629.7418 | 2 | 12.25 | 4 | F4:2426 | NaNaKA16\_F12.raw |  |  |  | 6.4924E7 | 9.521E3 |  |  |  |  |  |  |  |  | 2 | 0 | 0 | 0 | 1 | 1 | 0 | 0 | 0 | 0 | 0 | 0 | 0 | 0 | 208 | 217 | Carbamidomethylation | C3:Carbamidomethylation:1000.00;C7:Carbamidomethylation:1000.00;C9:Carbamidomethylation:1000.00 | PEAKS DB |
| H.YTQIVWYQTYR.A | Y | 61.61 | 1519.7460 | 11 | 0.1 | 760.8804 | 2 | 55.75 | 4 | F4:34986 | NaNaKA16\_F12.raw |  |  |  | 1.6068E8 |  |  |  |  |  |  |  |  |  | 2 | 0 | 0 | 0 | 2 | 0 | 0 | 0 | 0 | 0 | 0 | 0 | 0 | 0 | 116 | 126 |  |  | PEAKS DB |
| R.TWTEIIHLWH.D | Y | 60.78 | 1334.6771 | 10 | 0.3 | 668.3461 | 2 | 73.98 | 4 | F4:49966 | NaNaKA16\_F12.raw |  |  |  | 4.783E6 |  |  |  |  |  |  |  |  |  | 1 | 0 | 0 | 0 | 1 | 0 | 0 | 0 | 0 | 0 | 0 | 0 | 0 | 0 | 85 | 94 |  |  | PEAKS DB |
| R.VLEGIQC(+57.02)GESIYMSSN.A | Y | 58.92 | 1785.7914 | 16 | -0.4 | 893.9026 | 2 | 68.70 | 4 | F4:45706 | NaNaKA16\_F12.raw |  |  |  | 1.3545E8 |  |  |  |  |  |  |  |  |  | 1 | 0 | 0 | 0 | 1 | 0 | 0 | 0 | 0 | 0 | 0 | 0 | 0 | 0 | 67 | 82 | Carbamidomethylation | C7:Carbamidomethylation:1000.00 | PEAKS DB |
| N.TC(+57.02)SLNHSPDNLR.V | Y | 56.85 | 1412.6466 | 12 | 0.5 | 707.3309 | 2 | 11.48 | 4 | F4:1809 | NaNaKA16\_F12.raw |  |  |  | 4.5467E6 |  |  |  |  |  |  |  |  |  | 2 | 0 | 0 | 0 | 2 | 0 | 0 | 0 | 0 | 0 | 0 | 0 | 0 | 0 | 55 | 66 | Carbamidomethylation | C2:Carbamidomethylation:1000.00 | PEAKS DB |
| R.VSPTASNM(+15.99)LK.M | N | 56.73 | 1062.5380 | 10 | 0.7 | 532.2766 | 2 | 11.45 | 4 | F4:1800 | NaNaKA16\_F12.raw |  |  |  | 1.2798E7 |  |  |  |  |  |  |  |  |  | 1 | 0 | 0 | 0 | 1 | 0 | 0 | 0 | 0 | 0 | 0 | 0 | 0 | 0 | 29 | 38 | Oxidation (M) | M8:Oxidation (M):1000.00 | PEAKS DB |
| K.LGPPC(+57.02)GDC(+57.02)PSAC(+57.02)DNGLC(+57.02)TNPC(+57.02)TIYNK.L | Y | 56.61 | 2940.1970 | 26 | 1.2 | 981.0742 | 3 | 49.74 | 4 | F4:29707 | NaNaKA16\_F12.raw |  |  |  | 9.9063E8 |  |  |  |  |  |  |  |  |  | 2 | 0 | 0 | 0 | 2 | 0 | 0 | 0 | 0 | 0 | 0 | 0 | 0 | 0 | 163 | 188 | Carbamidomethylation | C5:Carbamidomethylation:1000.00;C8:Carbamidomethylation:1000.00;C12:Carbamidomethylation:1000.00;C17:Carbamidomethylation:1000.00;C21:Carbamidomethylation:1000.00 | PEAKS DB |
| Y.TQIVWYQTYR.A | Y | 56.03 | 1356.6826 | 10 | 0.6 | 679.3490 | 2 | 49.37 | 4 | F4:29565 | NaNaKA16\_F12.raw |  |  |  | 3.2787E7 |  |  |  |  |  |  |  |  |  | 1 | 0 | 0 | 0 | 1 | 0 | 0 | 0 | 0 | 0 | 0 | 0 | 0 | 0 | 117 | 126 |  |  | PEAKS DB |
| Y.VC(+57.02)QYC(+57.02)PSGNFQGK.T | Y | 54.51 | 1543.6548 | 13 | -0.3 | 772.8344 | 2 | 11.76 | 4 | F4:2117 | NaNaKA16\_F12.raw |  |  |  | 1.3951E6 |  |  |  |  |  |  |  |  |  | 1 | 0 | 0 | 0 | 1 | 0 | 0 | 0 | 0 | 0 | 0 | 0 | 0 | 0 | 144 | 156 | Carbamidomethylation | C2:Carbamidomethylation:1000.00;C5:Carbamidomethylation:1000.00 | PEAKS DB |
| R.RVSPTASNM(+15.99)LK.M | Y | 54.31 | 1218.6390 | 11 | 0.7 | 407.2206 | 3 | 11.48 | 4 | F4:1808 | NaNaKA16\_F12.raw |  |  |  | 4.4814E5 |  |  |  |  |  |  |  |  |  | 1 | 0 | 0 | 0 | 1 | 0 | 0 | 0 | 0 | 0 | 0 | 0 | 0 | 0 | 28 | 38 | Oxidation (M) | M9:Oxidation (M):1000.00 | PEAKS DB |
| R.VSPTASNMLK.M | N | 54.02 | 1046.5430 | 10 | -0.7 | 524.2784 | 2 | 11.74 | 4 | F4:2287 | NaNaKA16\_F12.raw |  |  |  | 1.7067E8 |  |  |  |  |  |  |  |  |  | 2 | 0 | 0 | 0 | 2 | 0 | 0 | 0 | 0 | 0 | 0 | 0 | 0 | 0 | 29 | 38 |  |  | PEAKS DB |
| I.VDLHNSLR.R | N | 53.27 | 952.5090 | 8 | 0.1 | 477.2618 | 2 | 26.22 | 4 | F4:10364 | NaNaKA16\_F12.raw |  |  |  | 3.6616E7 |  |  |  |  |  |  |  |  |  | 1 | 0 | 0 | 0 | 1 | 0 | 0 | 0 | 0 | 0 | 0 | 0 | 0 | 0 | 19 | 26 |  |  | PEAKS DB |
| M.EWYPEAASNAER.W | N | 53.15 | 1421.6211 | 12 | 0.6 | 711.8182 | 2 | 30.28 | 4 | F4:13787 | NaNaKA16\_F12.raw |  |  |  | 1.7579E7 |  |  |  |  |  |  |  |  |  | 1 | 0 | 0 | 0 | 1 | 0 | 0 | 0 | 0 | 0 | 0 | 0 | 0 | 0 | 40 | 51 |  |  | PEAKS DB |
| N.VDFNSESTR.R | N | 52.01 | 1053.4727 | 9 | 0.6 | 527.7439 | 2 | 11.66 | 4 | F4:1931 | NaNaKA16\_F12.raw |  |  |  | 9.3775E6 |  |  |  |  |  |  |  |  |  | 1 | 0 | 0 | 0 | 1 | 0 | 0 | 0 | 0 | 0 | 0 | 0 | 0 | 0 | 2 | 10 |  |  | PEAKS DB |
| S.AC(+57.02)DNGLC(+57.02)TNPC(+57.02)TIYNK.L | Y | 51.74 | 1899.7914 | 16 | -0.3 | 950.9027 | 2 | 33.35 | 4 | F4:16304 | NaNaKA16\_F12.raw |  |  |  | 3.0888E6 |  |  |  |  |  |  |  |  |  | 1 | 0 | 0 | 0 | 1 | 0 | 0 | 0 | 0 | 0 | 0 | 0 | 0 | 0 | 173 | 188 | Carbamidomethylation | C2:Carbamidomethylation:1000.00;C7:Carbamidomethylation:1000.00;C11:Carbamidomethylation:1000.00 | PEAKS DB |
| C.SLNHSPDNLR.V | Y | 50.57 | 1151.5684 | 10 | -0.3 | 384.8633 | 3 | 11.45 | 4 | F4:1803 | NaNaKA16\_F12.raw |  |  |  | 3.3986E6 |  |  |  |  |  |  |  |  |  | 2 | 0 | 0 | 0 | 2 | 0 | 0 | 0 | 0 | 0 | 0 | 0 | 0 | 0 | 57 | 66 |  |  | PEAKS DB |
| R.TWTEIIHLWHDEYKN.F | Y | 49.28 | 1983.9479 | 15 | -0.2 | 496.9941 | 4 | 67.43 | 4 | F4:44938 | NaNaKA16\_F12.raw |  |  |  | 1.7614E7 |  |  |  |  |  |  |  |  |  | 2 | 0 | 0 | 0 | 2 | 0 | 0 | 0 | 0 | 0 | 0 | 0 | 0 | 0 | 85 | 99 |  |  | PEAKS DB |
| L.EGIQC(+57.02)GESIYMSSNAR.T | Y | 48.30 | 1800.7771 | 16 | 0.9 | 901.3966 | 2 | 35.87 | 4 | F4:18384 | NaNaKA16\_F12.raw |  |  |  | 0 |  |  |  |  |  |  |  |  |  | 0 | 0 | 0 | 0 | 0 | 0 | 0 | 0 | 0 | 0 | 0 | 0 | 0 | 0 | 69 | 84 | Carbamidomethylation | C5:Carbamidomethylation:1000.00 | PEAKS DB |
| R.VLEGIQC(+57.02)GESIYM(+15.99)SSN.A | Y | 48.10 | 1801.7863 | 16 | 0.0 | 901.9004 | 2 | 59.91 | 4 | F4:38428 | NaNaKA16\_F12.raw |  |  |  | 1.9592E7 |  |  |  |  |  |  |  |  |  | 1 | 0 | 0 | 0 | 1 | 0 | 0 | 0 | 0 | 0 | 0 | 0 | 0 | 0 | 67 | 82 | Carbamidomethylation; Oxidation (M) | C7:Carbamidomethylation:1000.00;M13:Oxidation (M):1000.00 | PEAKS DB |
| Y.KLGPPC(+57.02)GDC(+57.02)PSAC(+57.02)DNGLC(+57.02)TNPC(+57.02)TIYNK.L | Y | 48.02 | 3068.2917 | 27 | 1.2 | 1023.7724 | 3 | 40.46 | 4 | F4:22361 | NaNaKA16\_F12.raw |  |  |  | 7.6949E6 |  |  |  |  |  |  |  |  |  | 1 | 0 | 0 | 0 | 1 | 0 | 0 | 0 | 0 | 0 | 0 | 0 | 0 | 0 | 162 | 188 | Carbamidomethylation | C6:Carbamidomethylation:1000.00;C9:Carbamidomethylation:1000.00;C13:Carbamidomethylation:1000.00;C18:Carbamidomethylation:1000.00;C22:Carbamidomethylation:1000.00 | PEAKS DB |
| Q.IVWYQTYR.A | Y | 47.45 | 1127.5764 | 8 | -5.9 | 564.7922 | 2 | 43.62 | 4 | F4:24799 | NaNaKA16\_F12.raw |  |  |  | 3.1475E7 |  |  |  |  |  |  |  |  |  | 2 | 0 | 0 | 0 | 2 | 0 | 0 | 0 | 0 | 0 | 0 | 0 | 0 | 0 | 119 | 126 |  |  | PEAKS DB |
| R.TWTEIIHLWHD.E | Y | 47.44 | 1449.7041 | 11 | 0.4 | 484.2422 | 3 | 76.70 | 4 | F4:52262 | NaNaKA16\_F12.raw |  |  |  | 2.7409E6 |  |  |  |  |  |  |  |  |  | 1 | 0 | 0 | 0 | 1 | 0 | 0 | 0 | 0 | 0 | 0 | 0 | 0 | 0 | 85 | 95 |  |  | PEAKS DB |
| T.QIVWYQTYR.A | Y | 46.03 | 1255.6349 | 9 | 0.0 | 628.8247 | 2 | 47.28 | 4 | F4:27952 | NaNaKA16\_F12.raw |  |  |  | 5.8793E6 |  |  |  |  |  |  |  |  |  | 1 | 0 | 0 | 0 | 1 | 0 | 0 | 0 | 0 | 0 | 0 | 0 | 0 | 0 | 118 | 126 |  |  | PEAKS DB |
| E.IIHLWHDEYK.N | N | 45.95 | 1352.6877 | 10 | -0.5 | 451.9030 | 3 | 26.85 | 4 | F4:10987 | NaNaKA16\_F12.raw |  |  |  | 8.808E5 |  |  |  |  |  |  |  |  |  | 1 | 0 | 0 | 0 | 1 | 0 | 0 | 0 | 0 | 0 | 0 | 0 | 0 | 0 | 89 | 98 |  |  | PEAKS DB |
| T.EIIHLWHDEYK.N | Y | 44.66 | 1481.7302 | 11 | 0.1 | 494.9174 | 3 | 34.79 | 4 | F4:17343 | NaNaKA16\_F12.raw |  |  |  | 4.613E5 |  |  |  |  |  |  |  |  |  | 1 | 0 | 0 | 0 | 1 | 0 | 0 | 0 | 0 | 0 | 0 | 0 | 0 | 0 | 88 | 98 |  |  | PEAKS DB |
| R.VLEGIQC(+57.02)GESIY.M | Y | 44.56 | 1366.6438 | 12 | 0.4 | 684.3295 | 2 | 64.32 | 4 | F4:42048 | NaNaKA16\_F12.raw |  |  |  | 1.267E8 |  |  |  |  |  |  |  |  |  | 1 | 0 | 0 | 0 | 1 | 0 | 0 | 0 | 0 | 0 | 0 | 0 | 0 | 0 | 67 | 78 | Carbamidomethylation | C7:Carbamidomethylation:1000.00 | PEAKS DB |
| D.C(+57.02)PSAC(+57.02)DNGLC(+57.02)TNPC(+57.02)TIYNK.L | Y | 44.19 | 2243.9067 | 19 | 0.6 | 1122.9613 | 2 | 36.03 | 4 | F4:18539 | NaNaKA16\_F12.raw |  |  |  | 1.1206E6 |  |  |  |  |  |  |  |  |  | 1 | 0 | 0 | 0 | 1 | 0 | 0 | 0 | 0 | 0 | 0 | 0 | 0 | 0 | 170 | 188 | Carbamidomethylation | C1:Carbamidomethylation:1000.00;C5:Carbamidomethylation:1000.00;C10:Carbamidomethylation:1000.00;C14:Carbamidomethylation:1000.00 | PEAKS DB |
| L.RVLEGIQC(+57.02)GESIYM(+15.99)SSNAR.T | Y | 43.71 | 2185.0256 | 19 | 0.8 | 729.3497 | 3 | 36.11 | 4 | F4:18487 | NaNaKA16\_F12.raw |  |  |  | 6.1752E5 |  |  |  |  |  |  |  |  |  | 1 | 0 | 0 | 0 | 1 | 0 | 0 | 0 | 0 | 0 | 0 | 0 | 0 | 0 | 66 | 84 | Carbamidomethylation; Oxidation (M) | C8:Carbamidomethylation:1000.00;M14:Oxidation (M):1000.00 | PEAKS DB |
| R.RVSPTASNMLKMEWYPEAASNAER.W | Y | 43.34 | 2737.2952 | 24 | 1.4 | 685.3320 | 4 | 51.07 | 4 | F4:31143 | NaNaKA16\_F12.raw |  |  |  | 1.6942E6 |  |  |  |  |  |  |  |  |  | 1 | 0 | 0 | 0 | 1 | 0 | 0 | 0 | 0 | 0 | 0 | 0 | 0 | 0 | 28 | 51 |  |  | PEAKS DB |
| K.QKEIVDLHN.S | N | 43.18 | 1094.5720 | 9 | -0.5 | 548.2930 | 2 | 11.57 | 4 | F4:1902 | NaNaKA16\_F12.raw |  |  |  | 1.3713E5 |  |  |  |  |  |  |  |  |  | 1 | 0 | 0 | 0 | 1 | 0 | 0 | 0 | 0 | 0 | 0 | 0 | 0 | 0 | 15 | 23 |  |  | PEAKS DB |
| R.TWTEIIHLW.H | Y | 42.97 | 1197.6183 | 9 | 0.1 | 599.8165 | 2 | 92.08 | 4 | F4:64706 | NaNaKA16\_F12.raw |  |  |  | 2.1391E6 |  |  |  |  |  |  |  |  |  | 1 | 0 | 0 | 0 | 1 | 0 | 0 | 0 | 0 | 0 | 0 | 0 | 0 | 0 | 85 | 93 |  |  | PEAKS DB |
| N.MLKMEWYPEAASNAER.W | N | 42.97 | 1924.8811 | 16 | 0.5 | 642.6346 | 3 | 48.79 | 4 | F4:29214 | NaNaKA16\_F12.raw |  |  |  | 1.0448E6 |  |  |  |  |  |  |  |  |  | 1 | 0 | 0 | 0 | 1 | 0 | 0 | 0 | 0 | 0 | 0 | 0 | 0 | 0 | 36 | 51 |  |  | PEAKS DB |
| T.GHYTQIVWYQTYR.A | Y | 42.91 | 1713.8263 | 13 | -0.2 | 572.2826 | 3 | 43.93 | 4 | F4:25228 | NaNaKA16\_F12.raw |  |  |  | 2.5056E6 |  |  |  |  |  |  |  |  |  | 1 | 0 | 0 | 0 | 1 | 0 | 0 | 0 | 0 | 0 | 0 | 0 | 0 | 0 | 114 | 126 |  |  | PEAKS DB |
| R.VSPTASNMLKM(+15.99)EWYPEAASNAER.W | N | 41.81 | 2597.1890 | 23 | -0.1 | 866.7368 | 3 | 60.59 | 4 | F4:38922 | NaNaKA16\_F12.raw |  |  |  | 1.7447E6 |  |  |  |  |  |  |  |  |  | 1 | 0 | 0 | 0 | 1 | 0 | 0 | 0 | 0 | 0 | 0 | 0 | 0 | 0 | 29 | 51 | Oxidation (M) | M11:Oxidation (M):22.34 | PEAKS DB |
| total 57 peptides |  |  |  |  |  |  |  |  |  |  |  |  |  |  |  |  |  |  |  |  |  |  |  |  |  |  |  |  |  |  |  |  |  |  |  |  |  |  |  |  |  |  |  |
| --- | --- | --- | --- | --- | --- | --- | --- | --- | --- | --- | --- | --- | --- | --- | --- | --- | --- | --- | --- | --- | --- | --- | --- | --- | --- | --- | --- | --- | --- | --- | --- | --- | --- | --- | --- | --- | --- | --- | --- | --- | --- | --- | --- |

3MZ8

back to list

  

| Protein Coverage
| Supporting Peptides
|

Protein Coverage:

Supporting Peptides:

| Peptide | Uniq | -10lgP | Mass | Length | ppm | m/z | z | RT | Fraction | Scan | Source File | Area F1 | Area F10 | Area F11 | Area F12 | Area F13 | Area F2 | Area F3 | Area F4 | Area F5 | Area F6 | Area F7 | Area F8 | Area F9 | #Feature | #Feature F1 | #Feature F10 | #Feature F11 | #Feature F12 | #Feature F13 | #Feature F2 | #Feature F3 | #Feature F4 | #Feature F5 | #Feature F6 | #Feature F7 | #Feature F8 | #Feature F9 | Start | End | PTM | AScore | Found By |
| --- | --- | --- | --- | --- | --- | --- | --- | --- | --- | --- | --- | --- | --- | --- | --- | --- | --- | --- | --- | --- | --- | --- | --- | --- | --- | --- | --- | --- | --- | --- | --- | --- | --- | --- | --- | --- | --- | --- | --- | --- | --- | --- | --- |
| R.TWTEIIHLWHDEYK.N | Y | 96.42 | 1869.9049 | 14 | 0.2 | 935.9600 | 2 | 67.88 | 4 | F4:47092 | NaNaKA16\_F12.raw |  |  |  | 1.192E10 | 1.3265E7 |  |  |  |  |  |  |  |  | 7 | 0 | 0 | 0 | 5 | 2 | 0 | 0 | 0 | 0 | 0 | 0 | 0 | 0 | 85 | 98 |  |  | PEAKS DB |
| R.WANTC(+57.02)SLNHSPDNLR.V | Y | 95.37 | 1783.8060 | 15 | -0.2 | 892.9102 | 2 | 20.57 | 4 | F4:6149 | NaNaKA16\_F12.raw |  |  |  | 2.5809E10 | 7.535E6 |  |  |  |  |  | 1.8333E5 |  |  | 20 | 0 | 0 | 0 | 16 | 3 | 0 | 0 | 0 | 0 | 0 | 1 | 0 | 0 | 52 | 66 | Carbamidomethylation | C5:Carbamidomethylation:1000.00 | PEAKS DB |
| R.VLEGIQC(+57.02)GESIYMSSNAR.T | Y | 94.89 | 2012.9296 | 18 | 0.0 | 1007.4721 | 2 | 61.49 | 4 | F4:39775 | NaNaKA16\_F12.raw |  | 5.2943E6 | 3.3626E7 | 7.5365E9 | 1.1193E7 |  |  |  |  |  |  |  | 0 | 16 | 0 | 1 | 2 | 9 | 4 | 0 | 0 | 0 | 0 | 0 | 0 | 0 | 0 | 67 | 84 | Carbamidomethylation | C7:Carbamidomethylation:1000.00 | PEAKS DB |
| R.VLEGIQC(+57.02)GESIYM(+15.99)SSNAR.T | Y | 91.90 | 2028.9244 | 18 | -0.3 | 1015.4692 | 2 | 47.15 | 4 | F4:28194 | NaNaKA16\_F12.raw |  | 1.6305E5 | 4.9762E6 | 1.1167E9 | 1.3064E6 |  |  |  |  |  |  |  |  | 10 | 0 | 1 | 1 | 7 | 1 | 0 | 0 | 0 | 0 | 0 | 0 | 0 | 0 | 67 | 84 | Carbamidomethylation; Oxidation (M) | C7:Carbamidomethylation:1000.00;M13:Oxidation (M):1000.00 | PEAKS DB |
| V.TGHYTQIVWYQTYR.A | Y | 79.15 | 1814.8740 | 14 | 0.5 | 908.4447 | 2 | 44.15 | 4 | F4:25452 | NaNaKA16\_F12.raw |  |  |  | 1.3608E8 |  |  |  |  |  |  |  |  |  | 2 | 0 | 0 | 0 | 2 | 0 | 0 | 0 | 0 | 0 | 0 | 0 | 0 | 0 | 113 | 126 |  |  | PEAKS DB |
| R.VSPTASNMLKMEWYPEAASNAER.W | N | 79.04 | 2581.1941 | 23 | 2.4 | 861.4074 | 3 | 60.50 | 4 | F4:38986 | NaNaKA16\_F12.raw |  |  |  | 7.3936E7 |  |  |  |  |  |  |  |  |  | 2 | 0 | 0 | 0 | 2 | 0 | 0 | 0 | 0 | 0 | 0 | 0 | 0 | 0 | 29 | 51 |  |  | PEAKS DB |
| Y.FYVC(+57.02)QYC(+57.02)PSGNFQGK.T | Y | 77.96 | 1853.7865 | 15 | -0.4 | 927.9001 | 2 | 45.62 | 4 | F4:26578 | NaNaKA16\_F12.raw |  |  |  | 2.3019E7 |  |  |  |  |  |  |  |  |  | 1 | 0 | 0 | 0 | 1 | 0 | 0 | 0 | 0 | 0 | 0 | 0 | 0 | 0 | 142 | 156 | Carbamidomethylation | C4:Carbamidomethylation:1000.00;C7:Carbamidomethylation:1000.00 | PEAKS DB |
| K.MEWYPEAASNAER.W | N | 77.76 | 1552.6616 | 13 | 0.7 | 518.5615 | 3 | 44.66 | 4 | F4:25052 | NaNaKA16\_F12.raw |  |  | 1.7754E7 | 3.6E10 | 1.0543E7 |  |  |  |  |  |  |  |  | 15 | 0 | 0 | 1 | 12 | 2 | 0 | 0 | 0 | 0 | 0 | 0 | 0 | 0 | 39 | 51 |  |  | PEAKS DB |
| W.SYFYVC(+57.02)QYC(+57.02)PSGNFQGK.T | Y | 76.97 | 2103.8818 | 17 | 0.0 | 1052.9482 | 2 | 56.99 | 4 | F4:35993 | NaNaKA16\_F12.raw |  |  |  | 2.0613E7 |  |  |  |  |  |  |  |  |  | 1 | 0 | 0 | 0 | 1 | 0 | 0 | 0 | 0 | 0 | 0 | 0 | 0 | 0 | 140 | 156 | Carbamidomethylation | C6:Carbamidomethylation:1000.00;C9:Carbamidomethylation:1000.00 | PEAKS DB |
| K.QKEIVDLHNSLR.R | N | 76.76 | 1450.7892 | 12 | -0.1 | 726.4018 | 2 | 11.75 | 4 | F4:2056 | NaNaKA16\_F12.raw |  |  |  | 8.0169E7 |  |  |  |  |  |  |  |  |  | 7 | 0 | 0 | 0 | 7 | 0 | 0 | 0 | 0 | 0 | 0 | 0 | 0 | 0 | 15 | 26 |  |  | PEAKS DB |
| K.M(+15.99)EWYPEAASNAER.W | N | 75.60 | 1568.6565 | 13 | 1.2 | 785.3364 | 2 | 31.92 | 4 | F4:18885 | NaNaKA16\_F12.raw |  |  |  | 4.1615E9 |  |  |  |  |  |  |  |  |  | 21 | 0 | 0 | 0 | 21 | 0 | 0 | 0 | 0 | 0 | 0 | 0 | 0 | 0 | 39 | 51 | Oxidation (M) | M1:Oxidation (M):1000.00 | PEAKS DB |
| L.RVLEGIQC(+57.02)GESIYMSSNAR.T | Y | 75.04 | 2169.0308 | 19 | -0.9 | 724.0169 | 3 | 42.93 | 4 | F4:24321 | NaNaKA16\_F12.raw |  |  | 1.5612E6 | 3.8584E6 |  |  |  |  |  |  |  |  |  | 2 | 0 | 0 | 1 | 1 | 0 | 0 | 0 | 0 | 0 | 0 | 0 | 0 | 0 | 66 | 84 | Carbamidomethylation | C8:Carbamidomethylation:1000.00 | PEAKS DB |
| N.GLC(+57.02)TNPC(+57.02)TIYNK.L | Y | 73.43 | 1439.6537 | 12 | 0.2 | 720.8342 | 2 | 27.20 | 4 | F4:10879 | NaNaKA16\_F12.raw |  |  |  | 3.9014E7 |  |  |  |  |  |  |  |  |  | 1 | 0 | 0 | 0 | 1 | 0 | 0 | 0 | 0 | 0 | 0 | 0 | 0 | 0 | 177 | 188 | Carbamidomethylation | C3:Carbamidomethylation:1000.00;C7:Carbamidomethylation:1000.00 | PEAKS DB |
| F.YVC(+57.02)QYC(+57.02)PSGNFQGK.T | Y | 71.81 | 1706.7181 | 14 | 0.0 | 854.3663 | 2 | 25.92 | 4 | F4:10821 | NaNaKA16\_F12.raw |  |  |  | 4.1439E8 |  |  |  |  |  |  |  |  |  | 1 | 0 | 0 | 0 | 1 | 0 | 0 | 0 | 0 | 0 | 0 | 0 | 0 | 0 | 143 | 156 | Carbamidomethylation | C3:Carbamidomethylation:1000.00;C6:Carbamidomethylation:1000.00 | PEAKS DB |
| E.GIQC(+57.02)GESIYMSSNAR.T | Y | 70.11 | 1671.7345 | 15 | 0.6 | 836.8750 | 2 | 29.85 | 4 | F4:13323 | NaNaKA16\_F12.raw |  |  |  | 1.3244E6 |  |  |  |  |  |  |  |  |  | 1 | 0 | 0 | 0 | 1 | 0 | 0 | 0 | 0 | 0 | 0 | 0 | 0 | 0 | 70 | 84 | Carbamidomethylation | C4:Carbamidomethylation:1000.00 | PEAKS DB |
| R.RVSPTASNMLK.M | Y | 69.72 | 1202.6442 | 11 | 0.3 | 602.3295 | 2 | 11.45 | 4 | F4:1801 | NaNaKA16\_F12.raw |  |  |  | 1.7511E7 |  |  |  |  |  |  |  |  |  | 2 | 0 | 0 | 0 | 2 | 0 | 0 | 0 | 0 | 0 | 0 | 0 | 0 | 0 | 28 | 38 |  |  | PEAKS DB |
| L.KMEWYPEAASNAER.W | N | 69.69 | 1680.7566 | 14 | -0.9 | 561.2590 | 3 | 29.03 | 4 | F4:12760 | NaNaKA16\_F12.raw |  |  |  | 3.1822E7 |  |  |  |  |  |  |  |  |  | 2 | 0 | 0 | 0 | 2 | 0 | 0 | 0 | 0 | 0 | 0 | 0 | 0 | 0 | 38 | 51 |  |  | PEAKS DB |
| NVDFNSESTR.R | N | 69.40 | 1167.5156 | 10 | 0.6 | 584.7654 | 2 | 12.15 | 4 | F4:2376 | NaNaKA16\_F12.raw |  |  |  | 8.6869E7 |  |  |  |  |  |  |  |  |  | 1 | 0 | 0 | 0 | 1 | 0 | 0 | 0 | 0 | 0 | 0 | 0 | 0 | 0 | 1 | 10 |  |  | PEAKS DB |
| W.TEIIHLWHDEYK.N | Y | 68.88 | 1582.7780 | 12 | 0.3 | 528.6001 | 3 | 38.63 | 4 | F4:20823 | NaNaKA16\_F12.raw |  |  |  | 1.545E7 |  |  |  |  |  |  |  |  |  | 4 | 0 | 0 | 0 | 4 | 0 | 0 | 0 | 0 | 0 | 0 | 0 | 0 | 0 | 87 | 98 |  |  | PEAKS DB |
| C.DNGLC(+57.02)TNPC(+57.02)TIYNK.L | Y | 68.73 | 1668.7236 | 14 | 0.5 | 835.3695 | 2 | 36.61 | 4 | F4:19046 | NaNaKA16\_F12.raw |  |  |  | 6.9202E6 |  |  |  |  | 7.9009E6 |  |  |  |  | 3 | 0 | 0 | 0 | 1 | 0 | 0 | 0 | 0 | 2 | 0 | 0 | 0 | 0 | 175 | 188 | Carbamidomethylation | C5:Carbamidomethylation:1000.00;C9:Carbamidomethylation:1000.00 | PEAKS DB |
| K.EIVDLHNSLR.R | N | 66.53 | 1194.6356 | 10 | -0.4 | 598.3248 | 2 | 26.22 | 4 | F4:11038 | NaNaKA16\_F12.raw |  |  | 2.0613E5 | 9.1517E8 | 1.6717E6 |  |  |  |  |  |  |  |  | 7 | 0 | 0 | 1 | 5 | 1 | 0 | 0 | 0 | 0 | 0 | 0 | 0 | 0 | 17 | 26 |  |  | PEAKS DB |
| A.RTWTEIIHLWHDEYK.N | Y | 65.27 | 2026.0061 | 15 | -0.1 | 507.5088 | 4 | 59.52 | 4 | F4:38212 | NaNaKA16\_F12.raw |  |  |  | 2.6065E6 |  |  |  |  |  |  |  |  |  | 1 | 0 | 0 | 0 | 1 | 0 | 0 | 0 | 0 | 0 | 0 | 0 | 0 | 0 | 84 | 98 |  |  | PEAKS DB |
| NVDFNSESTRR.K | N | 65.26 | 1323.6167 | 11 | -0.1 | 662.8156 | 2 | 11.44 | 4 | F4:1806 | NaNaKA16\_F12.raw |  |  |  | 6.0739E6 |  |  |  |  |  |  |  |  |  | 2 | 0 | 0 | 0 | 2 | 0 | 0 | 0 | 0 | 0 | 0 | 0 | 0 | 0 | 1 | 11 |  |  | PEAKS DB |
| K.SNC(+57.02)PASC(+57.02)FC(+57.02)R.N | N | 62.02 | 1257.4689 | 10 | 0.2 | 629.7418 | 2 | 12.25 | 4 | F4:2426 | NaNaKA16\_F12.raw |  |  |  | 6.4924E7 | 9.521E3 |  |  |  |  |  |  |  |  | 2 | 0 | 0 | 0 | 1 | 1 | 0 | 0 | 0 | 0 | 0 | 0 | 0 | 0 | 208 | 217 | Carbamidomethylation | C3:Carbamidomethylation:1000.00;C7:Carbamidomethylation:1000.00;C9:Carbamidomethylation:1000.00 | PEAKS DB |
| H.YTQIVWYQTYR.A | Y | 61.61 | 1519.7460 | 11 | 0.1 | 760.8804 | 2 | 55.75 | 4 | F4:34986 | NaNaKA16\_F12.raw |  |  |  | 1.6068E8 |  |  |  |  |  |  |  |  |  | 2 | 0 | 0 | 0 | 2 | 0 | 0 | 0 | 0 | 0 | 0 | 0 | 0 | 0 | 116 | 126 |  |  | PEAKS DB |
| R.TWTEIIHLWH.D | Y | 60.78 | 1334.6771 | 10 | 0.3 | 668.3461 | 2 | 73.98 | 4 | F4:49966 | NaNaKA16\_F12.raw |  |  |  | 4.783E6 |  |  |  |  |  |  |  |  |  | 1 | 0 | 0 | 0 | 1 | 0 | 0 | 0 | 0 | 0 | 0 | 0 | 0 | 0 | 85 | 94 |  |  | PEAKS DB |
| R.VLEGIQC(+57.02)GESIYMSSN.A | Y | 58.92 | 1785.7914 | 16 | -0.4 | 893.9026 | 2 | 68.70 | 4 | F4:45706 | NaNaKA16\_F12.raw |  |  |  | 1.3545E8 |  |  |  |  |  |  |  |  |  | 1 | 0 | 0 | 0 | 1 | 0 | 0 | 0 | 0 | 0 | 0 | 0 | 0 | 0 | 67 | 82 | Carbamidomethylation | C7:Carbamidomethylation:1000.00 | PEAKS DB |
| N.TC(+57.02)SLNHSPDNLR.V | Y | 56.85 | 1412.6466 | 12 | 0.5 | 707.3309 | 2 | 11.48 | 4 | F4:1809 | NaNaKA16\_F12.raw |  |  |  | 4.5467E6 |  |  |  |  |  |  |  |  |  | 2 | 0 | 0 | 0 | 2 | 0 | 0 | 0 | 0 | 0 | 0 | 0 | 0 | 0 | 55 | 66 | Carbamidomethylation | C2:Carbamidomethylation:1000.00 | PEAKS DB |
| R.VSPTASNM(+15.99)LK.M | N | 56.73 | 1062.5380 | 10 | 0.7 | 532.2766 | 2 | 11.45 | 4 | F4:1800 | NaNaKA16\_F12.raw |  |  |  | 1.2798E7 |  |  |  |  |  |  |  |  |  | 1 | 0 | 0 | 0 | 1 | 0 | 0 | 0 | 0 | 0 | 0 | 0 | 0 | 0 | 29 | 38 | Oxidation (M) | M8:Oxidation (M):1000.00 | PEAKS DB |
| K.LGPPC(+57.02)GDC(+57.02)PSAC(+57.02)DNGLC(+57.02)TNPC(+57.02)TIYNK.L | Y | 56.61 | 2940.1970 | 26 | 1.2 | 981.0742 | 3 | 49.74 | 4 | F4:29707 | NaNaKA16\_F12.raw |  |  |  | 9.9063E8 |  |  |  |  |  |  |  |  |  | 2 | 0 | 0 | 0 | 2 | 0 | 0 | 0 | 0 | 0 | 0 | 0 | 0 | 0 | 163 | 188 | Carbamidomethylation | C5:Carbamidomethylation:1000.00;C8:Carbamidomethylation:1000.00;C12:Carbamidomethylation:1000.00;C17:Carbamidomethylation:1000.00;C21:Carbamidomethylation:1000.00 | PEAKS DB |
| Y.TQIVWYQTYR.A | Y | 56.03 | 1356.6826 | 10 | 0.6 | 679.3490 | 2 | 49.37 | 4 | F4:29565 | NaNaKA16\_F12.raw |  |  |  | 3.2787E7 |  |  |  |  |  |  |  |  |  | 1 | 0 | 0 | 0 | 1 | 0 | 0 | 0 | 0 | 0 | 0 | 0 | 0 | 0 | 117 | 126 |  |  | PEAKS DB |
| Y.VC(+57.02)QYC(+57.02)PSGNFQGK.T | Y | 54.51 | 1543.6548 | 13 | -0.3 | 772.8344 | 2 | 11.76 | 4 | F4:2117 | NaNaKA16\_F12.raw |  |  |  | 1.3951E6 |  |  |  |  |  |  |  |  |  | 1 | 0 | 0 | 0 | 1 | 0 | 0 | 0 | 0 | 0 | 0 | 0 | 0 | 0 | 144 | 156 | Carbamidomethylation | C2:Carbamidomethylation:1000.00;C5:Carbamidomethylation:1000.00 | PEAKS DB |
| R.RVSPTASNM(+15.99)LK.M | Y | 54.31 | 1218.6390 | 11 | 0.7 | 407.2206 | 3 | 11.48 | 4 | F4:1808 | NaNaKA16\_F12.raw |  |  |  | 4.4814E5 |  |  |  |  |  |  |  |  |  | 1 | 0 | 0 | 0 | 1 | 0 | 0 | 0 | 0 | 0 | 0 | 0 | 0 | 0 | 28 | 38 | Oxidation (M) | M9:Oxidation (M):1000.00 | PEAKS DB |
| R.VSPTASNMLK.M | N | 54.02 | 1046.5430 | 10 | -0.7 | 524.2784 | 2 | 11.74 | 4 | F4:2287 | NaNaKA16\_F12.raw |  |  |  | 1.7067E8 |  |  |  |  |  |  |  |  |  | 2 | 0 | 0 | 0 | 2 | 0 | 0 | 0 | 0 | 0 | 0 | 0 | 0 | 0 | 29 | 38 |  |  | PEAKS DB |
| I.VDLHNSLR.R | N | 53.27 | 952.5090 | 8 | 0.1 | 477.2618 | 2 | 26.22 | 4 | F4:10364 | NaNaKA16\_F12.raw |  |  |  | 3.6616E7 |  |  |  |  |  |  |  |  |  | 1 | 0 | 0 | 0 | 1 | 0 | 0 | 0 | 0 | 0 | 0 | 0 | 0 | 0 | 19 | 26 |  |  | PEAKS DB |
| M.EWYPEAASNAER.W | N | 53.15 | 1421.6211 | 12 | 0.6 | 711.8182 | 2 | 30.28 | 4 | F4:13787 | NaNaKA16\_F12.raw |  |  |  | 1.7579E7 |  |  |  |  |  |  |  |  |  | 1 | 0 | 0 | 0 | 1 | 0 | 0 | 0 | 0 | 0 | 0 | 0 | 0 | 0 | 40 | 51 |  |  | PEAKS DB |
| N.VDFNSESTR.R | N | 52.01 | 1053.4727 | 9 | 0.6 | 527.7439 | 2 | 11.66 | 4 | F4:1931 | NaNaKA16\_F12.raw |  |  |  | 9.3775E6 |  |  |  |  |  |  |  |  |  | 1 | 0 | 0 | 0 | 1 | 0 | 0 | 0 | 0 | 0 | 0 | 0 | 0 | 0 | 2 | 10 |  |  | PEAKS DB |
| S.AC(+57.02)DNGLC(+57.02)TNPC(+57.02)TIYNK.L | Y | 51.74 | 1899.7914 | 16 | -0.3 | 950.9027 | 2 | 33.35 | 4 | F4:16304 | NaNaKA16\_F12.raw |  |  |  | 3.0888E6 |  |  |  |  |  |  |  |  |  | 1 | 0 | 0 | 0 | 1 | 0 | 0 | 0 | 0 | 0 | 0 | 0 | 0 | 0 | 173 | 188 | Carbamidomethylation | C2:Carbamidomethylation:1000.00;C7:Carbamidomethylation:1000.00;C11:Carbamidomethylation:1000.00 | PEAKS DB |
| C.SLNHSPDNLR.V | Y | 50.57 | 1151.5684 | 10 | -0.3 | 384.8633 | 3 | 11.45 | 4 | F4:1803 | NaNaKA16\_F12.raw |  |  |  | 3.3986E6 |  |  |  |  |  |  |  |  |  | 2 | 0 | 0 | 0 | 2 | 0 | 0 | 0 | 0 | 0 | 0 | 0 | 0 | 0 | 57 | 66 |  |  | PEAKS DB |
| R.TWTEIIHLWHDEYKN.F | Y | 49.28 | 1983.9479 | 15 | -0.2 | 496.9941 | 4 | 67.43 | 4 | F4:44938 | NaNaKA16\_F12.raw |  |  |  | 1.7614E7 |  |  |  |  |  |  |  |  |  | 2 | 0 | 0 | 0 | 2 | 0 | 0 | 0 | 0 | 0 | 0 | 0 | 0 | 0 | 85 | 99 |  |  | PEAKS DB |
| L.EGIQC(+57.02)GESIYMSSNAR.T | Y | 48.30 | 1800.7771 | 16 | 0.9 | 901.3966 | 2 | 35.87 | 4 | F4:18384 | NaNaKA16\_F12.raw |  |  |  | 0 |  |  |  |  |  |  |  |  |  | 0 | 0 | 0 | 0 | 0 | 0 | 0 | 0 | 0 | 0 | 0 | 0 | 0 | 0 | 69 | 84 | Carbamidomethylation | C5:Carbamidomethylation:1000.00 | PEAKS DB |
| R.VLEGIQC(+57.02)GESIYM(+15.99)SSN.A | Y | 48.10 | 1801.7863 | 16 | 0.0 | 901.9004 | 2 | 59.91 | 4 | F4:38428 | NaNaKA16\_F12.raw |  |  |  | 1.9592E7 |  |  |  |  |  |  |  |  |  | 1 | 0 | 0 | 0 | 1 | 0 | 0 | 0 | 0 | 0 | 0 | 0 | 0 | 0 | 67 | 82 | Carbamidomethylation; Oxidation (M) | C7:Carbamidomethylation:1000.00;M13:Oxidation (M):1000.00 | PEAKS DB |
| Y.KLGPPC(+57.02)GDC(+57.02)PSAC(+57.02)DNGLC(+57.02)TNPC(+57.02)TIYNK.L | Y | 48.02 | 3068.2917 | 27 | 1.2 | 1023.7724 | 3 | 40.46 | 4 | F4:22361 | NaNaKA16\_F12.raw |  |  |  | 7.6949E6 |  |  |  |  |  |  |  |  |  | 1 | 0 | 0 | 0 | 1 | 0 | 0 | 0 | 0 | 0 | 0 | 0 | 0 | 0 | 162 | 188 | Carbamidomethylation | C6:Carbamidomethylation:1000.00;C9:Carbamidomethylation:1000.00;C13:Carbamidomethylation:1000.00;C18:Carbamidomethylation:1000.00;C22:Carbamidomethylation:1000.00 | PEAKS DB |
| Q.IVWYQTYR.A | Y | 47.45 | 1127.5764 | 8 | -5.9 | 564.7922 | 2 | 43.62 | 4 | F4:24799 | NaNaKA16\_F12.raw |  |  |  | 3.1475E7 |  |  |  |  |  |  |  |  |  | 2 | 0 | 0 | 0 | 2 | 0 | 0 | 0 | 0 | 0 | 0 | 0 | 0 | 0 | 119 | 126 |  |  | PEAKS DB |
| R.TWTEIIHLWHD.E | Y | 47.44 | 1449.7041 | 11 | 0.4 | 484.2422 | 3 | 76.70 | 4 | F4:52262 | NaNaKA16\_F12.raw |  |  |  | 2.7409E6 |  |  |  |  |  |  |  |  |  | 1 | 0 | 0 | 0 | 1 | 0 | 0 | 0 | 0 | 0 | 0 | 0 | 0 | 0 | 85 | 95 |  |  | PEAKS DB |
| T.QIVWYQTYR.A | Y | 46.03 | 1255.6349 | 9 | 0.0 | 628.8247 | 2 | 47.28 | 4 | F4:27952 | NaNaKA16\_F12.raw |  |  |  | 5.8793E6 |  |  |  |  |  |  |  |  |  | 1 | 0 | 0 | 0 | 1 | 0 | 0 | 0 | 0 | 0 | 0 | 0 | 0 | 0 | 118 | 126 |  |  | PEAKS DB |
| E.IIHLWHDEYK.N | N | 45.95 | 1352.6877 | 10 | -0.5 | 451.9030 | 3 | 26.85 | 4 | F4:10987 | NaNaKA16\_F12.raw |  |  |  | 8.808E5 |  |  |  |  |  |  |  |  |  | 1 | 0 | 0 | 0 | 1 | 0 | 0 | 0 | 0 | 0 | 0 | 0 | 0 | 0 | 89 | 98 |  |  | PEAKS DB |
| T.EIIHLWHDEYK.N | Y | 44.66 | 1481.7302 | 11 | 0.1 | 494.9174 | 3 | 34.79 | 4 | F4:17343 | NaNaKA16\_F12.raw |  |  |  | 4.613E5 |  |  |  |  |  |  |  |  |  | 1 | 0 | 0 | 0 | 1 | 0 | 0 | 0 | 0 | 0 | 0 | 0 | 0 | 0 | 88 | 98 |  |  | PEAKS DB |
| R.VLEGIQC(+57.02)GESIY.M | Y | 44.56 | 1366.6438 | 12 | 0.4 | 684.3295 | 2 | 64.32 | 4 | F4:42048 | NaNaKA16\_F12.raw |  |  |  | 1.267E8 |  |  |  |  |  |  |  |  |  | 1 | 0 | 0 | 0 | 1 | 0 | 0 | 0 | 0 | 0 | 0 | 0 | 0 | 0 | 67 | 78 | Carbamidomethylation | C7:Carbamidomethylation:1000.00 | PEAKS DB |
| D.C(+57.02)PSAC(+57.02)DNGLC(+57.02)TNPC(+57.02)TIYNK.L | Y | 44.19 | 2243.9067 | 19 | 0.6 | 1122.9613 | 2 | 36.03 | 4 | F4:18539 | NaNaKA16\_F12.raw |  |  |  | 1.1206E6 |  |  |  |  |  |  |  |  |  | 1 | 0 | 0 | 0 | 1 | 0 | 0 | 0 | 0 | 0 | 0 | 0 | 0 | 0 | 170 | 188 | Carbamidomethylation | C1:Carbamidomethylation:1000.00;C5:Carbamidomethylation:1000.00;C10:Carbamidomethylation:1000.00;C14:Carbamidomethylation:1000.00 | PEAKS DB |
| L.RVLEGIQC(+57.02)GESIYM(+15.99)SSNAR.T | Y | 43.71 | 2185.0256 | 19 | 0.8 | 729.3497 | 3 | 36.11 | 4 | F4:18487 | NaNaKA16\_F12.raw |  |  |  | 6.1752E5 |  |  |  |  |  |  |  |  |  | 1 | 0 | 0 | 0 | 1 | 0 | 0 | 0 | 0 | 0 | 0 | 0 | 0 | 0 | 66 | 84 | Carbamidomethylation; Oxidation (M) | C8:Carbamidomethylation:1000.00;M14:Oxidation (M):1000.00 | PEAKS DB |
| R.RVSPTASNMLKMEWYPEAASNAER.W | Y | 43.34 | 2737.2952 | 24 | 1.4 | 685.3320 | 4 | 51.07 | 4 | F4:31143 | NaNaKA16\_F12.raw |  |  |  | 1.6942E6 |  |  |  |  |  |  |  |  |  | 1 | 0 | 0 | 0 | 1 | 0 | 0 | 0 | 0 | 0 | 0 | 0 | 0 | 0 | 28 | 51 |  |  | PEAKS DB |
| K.QKEIVDLHN.S | N | 43.18 | 1094.5720 | 9 | -0.5 | 548.2930 | 2 | 11.57 | 4 | F4:1902 | NaNaKA16\_F12.raw |  |  |  | 1.3713E5 |  |  |  |  |  |  |  |  |  | 1 | 0 | 0 | 0 | 1 | 0 | 0 | 0 | 0 | 0 | 0 | 0 | 0 | 0 | 15 | 23 |  |  | PEAKS DB |
| R.TWTEIIHLW.H | Y | 42.97 | 1197.6183 | 9 | 0.1 | 599.8165 | 2 | 92.08 | 4 | F4:64706 | NaNaKA16\_F12.raw |  |  |  | 2.1391E6 |  |  |  |  |  |  |  |  |  | 1 | 0 | 0 | 0 | 1 | 0 | 0 | 0 | 0 | 0 | 0 | 0 | 0 | 0 | 85 | 93 |  |  | PEAKS DB |
| N.MLKMEWYPEAASNAER.W | N | 42.97 | 1924.8811 | 16 | 0.5 | 642.6346 | 3 | 48.79 | 4 | F4:29214 | NaNaKA16\_F12.raw |  |  |  | 1.0448E6 |  |  |  |  |  |  |  |  |  | 1 | 0 | 0 | 0 | 1 | 0 | 0 | 0 | 0 | 0 | 0 | 0 | 0 | 0 | 36 | 51 |  |  | PEAKS DB |
| T.GHYTQIVWYQTYR.A | Y | 42.91 | 1713.8263 | 13 | -0.2 | 572.2826 | 3 | 43.93 | 4 | F4:25228 | NaNaKA16\_F12.raw |  |  |  | 2.5056E6 |  |  |  |  |  |  |  |  |  | 1 | 0 | 0 | 0 | 1 | 0 | 0 | 0 | 0 | 0 | 0 | 0 | 0 | 0 | 114 | 126 |  |  | PEAKS DB |
| R.VSPTASNMLKM(+15.99)EWYPEAASNAER.W | N | 41.81 | 2597.1890 | 23 | -0.1 | 866.7368 | 3 | 60.59 | 4 | F4:38922 | NaNaKA16\_F12.raw |  |  |  | 1.7447E6 |  |  |  |  |  |  |  |  |  | 1 | 0 | 0 | 0 | 1 | 0 | 0 | 0 | 0 | 0 | 0 | 0 | 0 | 0 | 29 | 51 | Oxidation (M) | M11:Oxidation (M):22.34 | PEAKS DB |
| total 57 peptides |  |  |  |  |  |  |  |  |  |  |  |  |  |  |  |  |  |  |  |  |  |  |  |  |  |  |  |  |  |  |  |  |  |  |  |  |  |  |  |  |  |  |  |
| --- | --- | --- | --- | --- | --- | --- | --- | --- | --- | --- | --- | --- | --- | --- | --- | --- | --- | --- | --- | --- | --- | --- | --- | --- | --- | --- | --- | --- | --- | --- | --- | --- | --- | --- | --- | --- | --- | --- | --- | --- | --- | --- | --- |

Q7T1K6.1

back to list

  

| Protein Coverage
| Supporting Peptides
|

Protein Coverage:

Supporting Peptides:

| Peptide | Uniq | -10lgP | Mass | Length | ppm | m/z | z | RT | Fraction | Scan | Source File | Area F1 | Area F10 | Area F11 | Area F12 | Area F13 | Area F2 | Area F3 | Area F4 | Area F5 | Area F6 | Area F7 | Area F8 | Area F9 | #Feature | #Feature F1 | #Feature F10 | #Feature F11 | #Feature F12 | #Feature F13 | #Feature F2 | #Feature F3 | #Feature F4 | #Feature F5 | #Feature F6 | #Feature F7 | #Feature F8 | #Feature F9 | Start | End | PTM | AScore | Found By |
| --- | --- | --- | --- | --- | --- | --- | --- | --- | --- | --- | --- | --- | --- | --- | --- | --- | --- | --- | --- | --- | --- | --- | --- | --- | --- | --- | --- | --- | --- | --- | --- | --- | --- | --- | --- | --- | --- | --- | --- | --- | --- | --- | --- |
| R.TWTEIIHLWHDEYK.N | Y | 96.42 | 1869.9049 | 14 | 0.2 | 935.9600 | 2 | 67.88 | 4 | F4:47092 | NaNaKA16\_F12.raw |  |  |  | 1.192E10 | 1.3265E7 |  |  |  |  |  |  |  |  | 7 | 0 | 0 | 0 | 5 | 2 | 0 | 0 | 0 | 0 | 0 | 0 | 0 | 0 | 103 | 116 |  |  | PEAKS DB |
| R.WANTC(+57.02)SLNHSPDNLR.V | Y | 95.37 | 1783.8060 | 15 | -0.2 | 892.9102 | 2 | 20.57 | 4 | F4:6149 | NaNaKA16\_F12.raw |  |  |  | 2.5809E10 | 7.535E6 |  |  |  |  |  | 1.8333E5 |  |  | 20 | 0 | 0 | 0 | 16 | 3 | 0 | 0 | 0 | 0 | 0 | 1 | 0 | 0 | 70 | 84 | Carbamidomethylation | C5:Carbamidomethylation:1000.00 | PEAKS DB |
| R.VLEGIQC(+57.02)GESIYMSSNAR.T | Y | 94.89 | 2012.9296 | 18 | 0.0 | 1007.4721 | 2 | 61.49 | 4 | F4:39775 | NaNaKA16\_F12.raw |  | 5.2943E6 | 3.3626E7 | 7.5365E9 | 1.1193E7 |  |  |  |  |  |  |  | 0 | 16 | 0 | 1 | 2 | 9 | 4 | 0 | 0 | 0 | 0 | 0 | 0 | 0 | 0 | 85 | 102 | Carbamidomethylation | C7:Carbamidomethylation:1000.00 | PEAKS DB |
| R.VLEGIQC(+57.02)GESIYM(+15.99)SSNAR.T | Y | 91.90 | 2028.9244 | 18 | -0.3 | 1015.4692 | 2 | 47.15 | 4 | F4:28194 | NaNaKA16\_F12.raw |  | 1.6305E5 | 4.9762E6 | 1.1167E9 | 1.3064E6 |  |  |  |  |  |  |  |  | 10 | 0 | 1 | 1 | 7 | 1 | 0 | 0 | 0 | 0 | 0 | 0 | 0 | 0 | 85 | 102 | Carbamidomethylation; Oxidation (M) | C7:Carbamidomethylation:1000.00;M13:Oxidation (M):1000.00 | PEAKS DB |
| V.TGHYTQIVWYQTYR.A | Y | 79.15 | 1814.8740 | 14 | 0.5 | 908.4447 | 2 | 44.15 | 4 | F4:25452 | NaNaKA16\_F12.raw |  |  |  | 1.3608E8 |  |  |  |  |  |  |  |  |  | 2 | 0 | 0 | 0 | 2 | 0 | 0 | 0 | 0 | 0 | 0 | 0 | 0 | 0 | 131 | 144 |  |  | PEAKS DB |
| R.VSPTASNMLKMEWYPEAASNAER.W | N | 79.04 | 2581.1941 | 23 | 2.4 | 861.4074 | 3 | 60.50 | 4 | F4:38986 | NaNaKA16\_F12.raw |  |  |  | 7.3936E7 |  |  |  |  |  |  |  |  |  | 2 | 0 | 0 | 0 | 2 | 0 | 0 | 0 | 0 | 0 | 0 | 0 | 0 | 0 | 47 | 69 |  |  | PEAKS DB |
| Y.FYVC(+57.02)QYC(+57.02)PSGNFQGK.T | Y | 77.96 | 1853.7865 | 15 | -0.4 | 927.9001 | 2 | 45.62 | 4 | F4:26578 | NaNaKA16\_F12.raw |  |  |  | 2.3019E7 |  |  |  |  |  |  |  |  |  | 1 | 0 | 0 | 0 | 1 | 0 | 0 | 0 | 0 | 0 | 0 | 0 | 0 | 0 | 160 | 174 | Carbamidomethylation | C4:Carbamidomethylation:1000.00;C7:Carbamidomethylation:1000.00 | PEAKS DB |
| K.MEWYPEAASNAER.W | N | 77.76 | 1552.6616 | 13 | 0.7 | 518.5615 | 3 | 44.66 | 4 | F4:25052 | NaNaKA16\_F12.raw |  |  | 1.7754E7 | 3.6E10 | 1.0543E7 |  |  |  |  |  |  |  |  | 15 | 0 | 0 | 1 | 12 | 2 | 0 | 0 | 0 | 0 | 0 | 0 | 0 | 0 | 57 | 69 |  |  | PEAKS DB |
| W.SYFYVC(+57.02)QYC(+57.02)PSGNFQGK.T | Y | 76.97 | 2103.8818 | 17 | 0.0 | 1052.9482 | 2 | 56.99 | 4 | F4:35993 | NaNaKA16\_F12.raw |  |  |  | 2.0613E7 |  |  |  |  |  |  |  |  |  | 1 | 0 | 0 | 0 | 1 | 0 | 0 | 0 | 0 | 0 | 0 | 0 | 0 | 0 | 158 | 174 | Carbamidomethylation | C6:Carbamidomethylation:1000.00;C9:Carbamidomethylation:1000.00 | PEAKS DB |
| K.QKEIVDLHNSLR.R | N | 76.76 | 1450.7892 | 12 | -0.1 | 726.4018 | 2 | 11.75 | 4 | F4:2056 | NaNaKA16\_F12.raw |  |  |  | 8.0169E7 |  |  |  |  |  |  |  |  |  | 7 | 0 | 0 | 0 | 7 | 0 | 0 | 0 | 0 | 0 | 0 | 0 | 0 | 0 | 33 | 44 |  |  | PEAKS DB |
| K.M(+15.99)EWYPEAASNAER.W | N | 75.60 | 1568.6565 | 13 | 1.2 | 785.3364 | 2 | 31.92 | 4 | F4:18885 | NaNaKA16\_F12.raw |  |  |  | 4.1615E9 |  |  |  |  |  |  |  |  |  | 21 | 0 | 0 | 0 | 21 | 0 | 0 | 0 | 0 | 0 | 0 | 0 | 0 | 0 | 57 | 69 | Oxidation (M) | M1:Oxidation (M):1000.00 | PEAKS DB |
| L.RVLEGIQC(+57.02)GESIYMSSNAR.T | Y | 75.04 | 2169.0308 | 19 | -0.9 | 724.0169 | 3 | 42.93 | 4 | F4:24321 | NaNaKA16\_F12.raw |  |  | 1.5612E6 | 3.8584E6 |  |  |  |  |  |  |  |  |  | 2 | 0 | 0 | 1 | 1 | 0 | 0 | 0 | 0 | 0 | 0 | 0 | 0 | 0 | 84 | 102 | Carbamidomethylation | C8:Carbamidomethylation:1000.00 | PEAKS DB |
| N.GLC(+57.02)TNPC(+57.02)TIYNK.L | Y | 73.43 | 1439.6537 | 12 | 0.2 | 720.8342 | 2 | 27.20 | 4 | F4:10879 | NaNaKA16\_F12.raw |  |  |  | 3.9014E7 |  |  |  |  |  |  |  |  |  | 1 | 0 | 0 | 0 | 1 | 0 | 0 | 0 | 0 | 0 | 0 | 0 | 0 | 0 | 195 | 206 | Carbamidomethylation | C3:Carbamidomethylation:1000.00;C7:Carbamidomethylation:1000.00 | PEAKS DB |
| F.YVC(+57.02)QYC(+57.02)PSGNFQGK.T | Y | 71.81 | 1706.7181 | 14 | 0.0 | 854.3663 | 2 | 25.92 | 4 | F4:10821 | NaNaKA16\_F12.raw |  |  |  | 4.1439E8 |  |  |  |  |  |  |  |  |  | 1 | 0 | 0 | 0 | 1 | 0 | 0 | 0 | 0 | 0 | 0 | 0 | 0 | 0 | 161 | 174 | Carbamidomethylation | C3:Carbamidomethylation:1000.00;C6:Carbamidomethylation:1000.00 | PEAKS DB |
| E.GIQC(+57.02)GESIYMSSNAR.T | Y | 70.11 | 1671.7345 | 15 | 0.6 | 836.8750 | 2 | 29.85 | 4 | F4:13323 | NaNaKA16\_F12.raw |  |  |  | 1.3244E6 |  |  |  |  |  |  |  |  |  | 1 | 0 | 0 | 0 | 1 | 0 | 0 | 0 | 0 | 0 | 0 | 0 | 0 | 0 | 88 | 102 | Carbamidomethylation | C4:Carbamidomethylation:1000.00 | PEAKS DB |
| R.RVSPTASNMLK.M | Y | 69.72 | 1202.6442 | 11 | 0.3 | 602.3295 | 2 | 11.45 | 4 | F4:1801 | NaNaKA16\_F12.raw |  |  |  | 1.7511E7 |  |  |  |  |  |  |  |  |  | 2 | 0 | 0 | 0 | 2 | 0 | 0 | 0 | 0 | 0 | 0 | 0 | 0 | 0 | 46 | 56 |  |  | PEAKS DB |
| L.KMEWYPEAASNAER.W | N | 69.69 | 1680.7566 | 14 | -0.9 | 561.2590 | 3 | 29.03 | 4 | F4:12760 | NaNaKA16\_F12.raw |  |  |  | 3.1822E7 |  |  |  |  |  |  |  |  |  | 2 | 0 | 0 | 0 | 2 | 0 | 0 | 0 | 0 | 0 | 0 | 0 | 0 | 0 | 56 | 69 |  |  | PEAKS DB |
| G.NVDFNSESTR.R | N | 69.40 | 1167.5156 | 10 | 0.6 | 584.7654 | 2 | 12.15 | 4 | F4:2376 | NaNaKA16\_F12.raw |  |  |  | 8.6869E7 |  |  |  |  |  |  |  |  |  | 1 | 0 | 0 | 0 | 1 | 0 | 0 | 0 | 0 | 0 | 0 | 0 | 0 | 0 | 19 | 28 |  |  | PEAKS DB |
| W.TEIIHLWHDEYK.N | Y | 68.88 | 1582.7780 | 12 | 0.3 | 528.6001 | 3 | 38.63 | 4 | F4:20823 | NaNaKA16\_F12.raw |  |  |  | 1.545E7 |  |  |  |  |  |  |  |  |  | 4 | 0 | 0 | 0 | 4 | 0 | 0 | 0 | 0 | 0 | 0 | 0 | 0 | 0 | 105 | 116 |  |  | PEAKS DB |
| C.DNGLC(+57.02)TNPC(+57.02)TIYNK.L | Y | 68.73 | 1668.7236 | 14 | 0.5 | 835.3695 | 2 | 36.61 | 4 | F4:19046 | NaNaKA16\_F12.raw |  |  |  | 6.9202E6 |  |  |  |  | 7.9009E6 |  |  |  |  | 3 | 0 | 0 | 0 | 1 | 0 | 0 | 0 | 0 | 2 | 0 | 0 | 0 | 0 | 193 | 206 | Carbamidomethylation | C5:Carbamidomethylation:1000.00;C9:Carbamidomethylation:1000.00 | PEAKS DB |
| K.EIVDLHNSLR.R | N | 66.53 | 1194.6356 | 10 | -0.4 | 598.3248 | 2 | 26.22 | 4 | F4:11038 | NaNaKA16\_F12.raw |  |  | 2.0613E5 | 9.1517E8 | 1.6717E6 |  |  |  |  |  |  |  |  | 7 | 0 | 0 | 1 | 5 | 1 | 0 | 0 | 0 | 0 | 0 | 0 | 0 | 0 | 35 | 44 |  |  | PEAKS DB |
| A.RTWTEIIHLWHDEYK.N | Y | 65.27 | 2026.0061 | 15 | -0.1 | 507.5088 | 4 | 59.52 | 4 | F4:38212 | NaNaKA16\_F12.raw |  |  |  | 2.6065E6 |  |  |  |  |  |  |  |  |  | 1 | 0 | 0 | 0 | 1 | 0 | 0 | 0 | 0 | 0 | 0 | 0 | 0 | 0 | 102 | 116 |  |  | PEAKS DB |
| G.NVDFNSESTRR.K | N | 65.26 | 1323.6167 | 11 | -0.1 | 662.8156 | 2 | 11.44 | 4 | F4:1806 | NaNaKA16\_F12.raw |  |  |  | 6.0739E6 |  |  |  |  |  |  |  |  |  | 2 | 0 | 0 | 0 | 2 | 0 | 0 | 0 | 0 | 0 | 0 | 0 | 0 | 0 | 19 | 29 |  |  | PEAKS DB |
| K.SNC(+57.02)PASC(+57.02)FC(+57.02)R.N | N | 62.02 | 1257.4689 | 10 | 0.2 | 629.7418 | 2 | 12.25 | 4 | F4:2426 | NaNaKA16\_F12.raw |  |  |  | 6.4924E7 | 9.521E3 |  |  |  |  |  |  |  |  | 2 | 0 | 0 | 0 | 1 | 1 | 0 | 0 | 0 | 0 | 0 | 0 | 0 | 0 | 226 | 235 | Carbamidomethylation | C3:Carbamidomethylation:1000.00;C7:Carbamidomethylation:1000.00;C9:Carbamidomethylation:1000.00 | PEAKS DB |
| H.YTQIVWYQTYR.A | Y | 61.61 | 1519.7460 | 11 | 0.1 | 760.8804 | 2 | 55.75 | 4 | F4:34986 | NaNaKA16\_F12.raw |  |  |  | 1.6068E8 |  |  |  |  |  |  |  |  |  | 2 | 0 | 0 | 0 | 2 | 0 | 0 | 0 | 0 | 0 | 0 | 0 | 0 | 0 | 134 | 144 |  |  | PEAKS DB |
| R.TWTEIIHLWH.D | Y | 60.78 | 1334.6771 | 10 | 0.3 | 668.3461 | 2 | 73.98 | 4 | F4:49966 | NaNaKA16\_F12.raw |  |  |  | 4.783E6 |  |  |  |  |  |  |  |  |  | 1 | 0 | 0 | 0 | 1 | 0 | 0 | 0 | 0 | 0 | 0 | 0 | 0 | 0 | 103 | 112 |  |  | PEAKS DB |
| R.VLEGIQC(+57.02)GESIYMSSN.A | Y | 58.92 | 1785.7914 | 16 | -0.4 | 893.9026 | 2 | 68.70 | 4 | F4:45706 | NaNaKA16\_F12.raw |  |  |  | 1.3545E8 |  |  |  |  |  |  |  |  |  | 1 | 0 | 0 | 0 | 1 | 0 | 0 | 0 | 0 | 0 | 0 | 0 | 0 | 0 | 85 | 100 | Carbamidomethylation | C7:Carbamidomethylation:1000.00 | PEAKS DB |
| N.TC(+57.02)SLNHSPDNLR.V | Y | 56.85 | 1412.6466 | 12 | 0.5 | 707.3309 | 2 | 11.48 | 4 | F4:1809 | NaNaKA16\_F12.raw |  |  |  | 4.5467E6 |  |  |  |  |  |  |  |  |  | 2 | 0 | 0 | 0 | 2 | 0 | 0 | 0 | 0 | 0 | 0 | 0 | 0 | 0 | 73 | 84 | Carbamidomethylation | C2:Carbamidomethylation:1000.00 | PEAKS DB |
| R.VSPTASNM(+15.99)LK.M | N | 56.73 | 1062.5380 | 10 | 0.7 | 532.2766 | 2 | 11.45 | 4 | F4:1800 | NaNaKA16\_F12.raw |  |  |  | 1.2798E7 |  |  |  |  |  |  |  |  |  | 1 | 0 | 0 | 0 | 1 | 0 | 0 | 0 | 0 | 0 | 0 | 0 | 0 | 0 | 47 | 56 | Oxidation (M) | M8:Oxidation (M):1000.00 | PEAKS DB |
| K.LGPPC(+57.02)GDC(+57.02)PSAC(+57.02)DNGLC(+57.02)TNPC(+57.02)TIYNK.L | Y | 56.61 | 2940.1970 | 26 | 1.2 | 981.0742 | 3 | 49.74 | 4 | F4:29707 | NaNaKA16\_F12.raw |  |  |  | 9.9063E8 |  |  |  |  |  |  |  |  |  | 2 | 0 | 0 | 0 | 2 | 0 | 0 | 0 | 0 | 0 | 0 | 0 | 0 | 0 | 181 | 206 | Carbamidomethylation | C5:Carbamidomethylation:1000.00;C8:Carbamidomethylation:1000.00;C12:Carbamidomethylation:1000.00;C17:Carbamidomethylation:1000.00;C21:Carbamidomethylation:1000.00 | PEAKS DB |
| Y.TQIVWYQTYR.A | Y | 56.03 | 1356.6826 | 10 | 0.6 | 679.3490 | 2 | 49.37 | 4 | F4:29565 | NaNaKA16\_F12.raw |  |  |  | 3.2787E7 |  |  |  |  |  |  |  |  |  | 1 | 0 | 0 | 0 | 1 | 0 | 0 | 0 | 0 | 0 | 0 | 0 | 0 | 0 | 135 | 144 |  |  | PEAKS DB |
| Y.VC(+57.02)QYC(+57.02)PSGNFQGK.T | Y | 54.51 | 1543.6548 | 13 | -0.3 | 772.8344 | 2 | 11.76 | 4 | F4:2117 | NaNaKA16\_F12.raw |  |  |  | 1.3951E6 |  |  |  |  |  |  |  |  |  | 1 | 0 | 0 | 0 | 1 | 0 | 0 | 0 | 0 | 0 | 0 | 0 | 0 | 0 | 162 | 174 | Carbamidomethylation | C2:Carbamidomethylation:1000.00;C5:Carbamidomethylation:1000.00 | PEAKS DB |
| R.RVSPTASNM(+15.99)LK.M | Y | 54.31 | 1218.6390 | 11 | 0.7 | 407.2206 | 3 | 11.48 | 4 | F4:1808 | NaNaKA16\_F12.raw |  |  |  | 4.4814E5 |  |  |  |  |  |  |  |  |  | 1 | 0 | 0 | 0 | 1 | 0 | 0 | 0 | 0 | 0 | 0 | 0 | 0 | 0 | 46 | 56 | Oxidation (M) | M9:Oxidation (M):1000.00 | PEAKS DB |
| R.VSPTASNMLK.M | N | 54.02 | 1046.5430 | 10 | -0.7 | 524.2784 | 2 | 11.74 | 4 | F4:2287 | NaNaKA16\_F12.raw |  |  |  | 1.7067E8 |  |  |  |  |  |  |  |  |  | 2 | 0 | 0 | 0 | 2 | 0 | 0 | 0 | 0 | 0 | 0 | 0 | 0 | 0 | 47 | 56 |  |  | PEAKS DB |
| I.VDLHNSLR.R | N | 53.27 | 952.5090 | 8 | 0.1 | 477.2618 | 2 | 26.22 | 4 | F4:10364 | NaNaKA16\_F12.raw |  |  |  | 3.6616E7 |  |  |  |  |  |  |  |  |  | 1 | 0 | 0 | 0 | 1 | 0 | 0 | 0 | 0 | 0 | 0 | 0 | 0 | 0 | 37 | 44 |  |  | PEAKS DB |
| M.EWYPEAASNAER.W | N | 53.15 | 1421.6211 | 12 | 0.6 | 711.8182 | 2 | 30.28 | 4 | F4:13787 | NaNaKA16\_F12.raw |  |  |  | 1.7579E7 |  |  |  |  |  |  |  |  |  | 1 | 0 | 0 | 0 | 1 | 0 | 0 | 0 | 0 | 0 | 0 | 0 | 0 | 0 | 58 | 69 |  |  | PEAKS DB |
| N.VDFNSESTR.R | N | 52.01 | 1053.4727 | 9 | 0.6 | 527.7439 | 2 | 11.66 | 4 | F4:1931 | NaNaKA16\_F12.raw |  |  |  | 9.3775E6 |  |  |  |  |  |  |  |  |  | 1 | 0 | 0 | 0 | 1 | 0 | 0 | 0 | 0 | 0 | 0 | 0 | 0 | 0 | 20 | 28 |  |  | PEAKS DB |
| S.AC(+57.02)DNGLC(+57.02)TNPC(+57.02)TIYNK.L | Y | 51.74 | 1899.7914 | 16 | -0.3 | 950.9027 | 2 | 33.35 | 4 | F4:16304 | NaNaKA16\_F12.raw |  |  |  | 3.0888E6 |  |  |  |  |  |  |  |  |  | 1 | 0 | 0 | 0 | 1 | 0 | 0 | 0 | 0 | 0 | 0 | 0 | 0 | 0 | 191 | 206 | Carbamidomethylation | C2:Carbamidomethylation:1000.00;C7:Carbamidomethylation:1000.00;C11:Carbamidomethylation:1000.00 | PEAKS DB |
| C.SLNHSPDNLR.V | Y | 50.57 | 1151.5684 | 10 | -0.3 | 384.8633 | 3 | 11.45 | 4 | F4:1803 | NaNaKA16\_F12.raw |  |  |  | 3.3986E6 |  |  |  |  |  |  |  |  |  | 2 | 0 | 0 | 0 | 2 | 0 | 0 | 0 | 0 | 0 | 0 | 0 | 0 | 0 | 75 | 84 |  |  | PEAKS DB |
| R.TWTEIIHLWHDEYKN.F | Y | 49.28 | 1983.9479 | 15 | -0.2 | 496.9941 | 4 | 67.43 | 4 | F4:44938 | NaNaKA16\_F12.raw |  |  |  | 1.7614E7 |  |  |  |  |  |  |  |  |  | 2 | 0 | 0 | 0 | 2 | 0 | 0 | 0 | 0 | 0 | 0 | 0 | 0 | 0 | 103 | 117 |  |  | PEAKS DB |
| L.EGIQC(+57.02)GESIYMSSNAR.T | Y | 48.30 | 1800.7771 | 16 | 0.9 | 901.3966 | 2 | 35.87 | 4 | F4:18384 | NaNaKA16\_F12.raw |  |  |  | 0 |  |  |  |  |  |  |  |  |  | 0 | 0 | 0 | 0 | 0 | 0 | 0 | 0 | 0 | 0 | 0 | 0 | 0 | 0 | 87 | 102 | Carbamidomethylation | C5:Carbamidomethylation:1000.00 | PEAKS DB |
| R.VLEGIQC(+57.02)GESIYM(+15.99)SSN.A | Y | 48.10 | 1801.7863 | 16 | 0.0 | 901.9004 | 2 | 59.91 | 4 | F4:38428 | NaNaKA16\_F12.raw |  |  |  | 1.9592E7 |  |  |  |  |  |  |  |  |  | 1 | 0 | 0 | 0 | 1 | 0 | 0 | 0 | 0 | 0 | 0 | 0 | 0 | 0 | 85 | 100 | Carbamidomethylation; Oxidation (M) | C7:Carbamidomethylation:1000.00;M13:Oxidation (M):1000.00 | PEAKS DB |
| Y.KLGPPC(+57.02)GDC(+57.02)PSAC(+57.02)DNGLC(+57.02)TNPC(+57.02)TIYNK.L | Y | 48.02 | 3068.2917 | 27 | 1.2 | 1023.7724 | 3 | 40.46 | 4 | F4:22361 | NaNaKA16\_F12.raw |  |  |  | 7.6949E6 |  |  |  |  |  |  |  |  |  | 1 | 0 | 0 | 0 | 1 | 0 | 0 | 0 | 0 | 0 | 0 | 0 | 0 | 0 | 180 | 206 | Carbamidomethylation | C6:Carbamidomethylation:1000.00;C9:Carbamidomethylation:1000.00;C13:Carbamidomethylation:1000.00;C18:Carbamidomethylation:1000.00;C22:Carbamidomethylation:1000.00 | PEAKS DB |
| Q.IVWYQTYR.A | Y | 47.45 | 1127.5764 | 8 | -5.9 | 564.7922 | 2 | 43.62 | 4 | F4:24799 | NaNaKA16\_F12.raw |  |  |  | 3.1475E7 |  |  |  |  |  |  |  |  |  | 2 | 0 | 0 | 0 | 2 | 0 | 0 | 0 | 0 | 0 | 0 | 0 | 0 | 0 | 137 | 144 |  |  | PEAKS DB |
| R.TWTEIIHLWHD.E | Y | 47.44 | 1449.7041 | 11 | 0.4 | 484.2422 | 3 | 76.70 | 4 | F4:52262 | NaNaKA16\_F12.raw |  |  |  | 2.7409E6 |  |  |  |  |  |  |  |  |  | 1 | 0 | 0 | 0 | 1 | 0 | 0 | 0 | 0 | 0 | 0 | 0 | 0 | 0 | 103 | 113 |  |  | PEAKS DB |
| T.QIVWYQTYR.A | Y | 46.03 | 1255.6349 | 9 | 0.0 | 628.8247 | 2 | 47.28 | 4 | F4:27952 | NaNaKA16\_F12.raw |  |  |  | 5.8793E6 |  |  |  |  |  |  |  |  |  | 1 | 0 | 0 | 0 | 1 | 0 | 0 | 0 | 0 | 0 | 0 | 0 | 0 | 0 | 136 | 144 |  |  | PEAKS DB |
| E.IIHLWHDEYK.N | N | 45.95 | 1352.6877 | 10 | -0.5 | 451.9030 | 3 | 26.85 | 4 | F4:10987 | NaNaKA16\_F12.raw |  |  |  | 8.808E5 |  |  |  |  |  |  |  |  |  | 1 | 0 | 0 | 0 | 1 | 0 | 0 | 0 | 0 | 0 | 0 | 0 | 0 | 0 | 107 | 116 |  |  | PEAKS DB |
| T.EIIHLWHDEYK.N | Y | 44.66 | 1481.7302 | 11 | 0.1 | 494.9174 | 3 | 34.79 | 4 | F4:17343 | NaNaKA16\_F12.raw |  |  |  | 4.613E5 |  |  |  |  |  |  |  |  |  | 1 | 0 | 0 | 0 | 1 | 0 | 0 | 0 | 0 | 0 | 0 | 0 | 0 | 0 | 106 | 116 |  |  | PEAKS DB |
| R.VLEGIQC(+57.02)GESIY.M | Y | 44.56 | 1366.6438 | 12 | 0.4 | 684.3295 | 2 | 64.32 | 4 | F4:42048 | NaNaKA16\_F12.raw |  |  |  | 1.267E8 |  |  |  |  |  |  |  |  |  | 1 | 0 | 0 | 0 | 1 | 0 | 0 | 0 | 0 | 0 | 0 | 0 | 0 | 0 | 85 | 96 | Carbamidomethylation | C7:Carbamidomethylation:1000.00 | PEAKS DB |
| D.C(+57.02)PSAC(+57.02)DNGLC(+57.02)TNPC(+57.02)TIYNK.L | Y | 44.19 | 2243.9067 | 19 | 0.6 | 1122.9613 | 2 | 36.03 | 4 | F4:18539 | NaNaKA16\_F12.raw |  |  |  | 1.1206E6 |  |  |  |  |  |  |  |  |  | 1 | 0 | 0 | 0 | 1 | 0 | 0 | 0 | 0 | 0 | 0 | 0 | 0 | 0 | 188 | 206 | Carbamidomethylation | C1:Carbamidomethylation:1000.00;C5:Carbamidomethylation:1000.00;C10:Carbamidomethylation:1000.00;C14:Carbamidomethylation:1000.00 | PEAKS DB |
| L.RVLEGIQC(+57.02)GESIYM(+15.99)SSNAR.T | Y | 43.71 | 2185.0256 | 19 | 0.8 | 729.3497 | 3 | 36.11 | 4 | F4:18487 | NaNaKA16\_F12.raw |  |  |  | 6.1752E5 |  |  |  |  |  |  |  |  |  | 1 | 0 | 0 | 0 | 1 | 0 | 0 | 0 | 0 | 0 | 0 | 0 | 0 | 0 | 84 | 102 | Carbamidomethylation; Oxidation (M) | C8:Carbamidomethylation:1000.00;M14:Oxidation (M):1000.00 | PEAKS DB |
| R.RVSPTASNMLKMEWYPEAASNAER.W | Y | 43.34 | 2737.2952 | 24 | 1.4 | 685.3320 | 4 | 51.07 | 4 | F4:31143 | NaNaKA16\_F12.raw |  |  |  | 1.6942E6 |  |  |  |  |  |  |  |  |  | 1 | 0 | 0 | 0 | 1 | 0 | 0 | 0 | 0 | 0 | 0 | 0 | 0 | 0 | 46 | 69 |  |  | PEAKS DB |
| K.QKEIVDLHN.S | N | 43.18 | 1094.5720 | 9 | -0.5 | 548.2930 | 2 | 11.57 | 4 | F4:1902 | NaNaKA16\_F12.raw |  |  |  | 1.3713E5 |  |  |  |  |  |  |  |  |  | 1 | 0 | 0 | 0 | 1 | 0 | 0 | 0 | 0 | 0 | 0 | 0 | 0 | 0 | 33 | 41 |  |  | PEAKS DB |
| R.TWTEIIHLW.H | Y | 42.97 | 1197.6183 | 9 | 0.1 | 599.8165 | 2 | 92.08 | 4 | F4:64706 | NaNaKA16\_F12.raw |  |  |  | 2.1391E6 |  |  |  |  |  |  |  |  |  | 1 | 0 | 0 | 0 | 1 | 0 | 0 | 0 | 0 | 0 | 0 | 0 | 0 | 0 | 103 | 111 |  |  | PEAKS DB |
| N.MLKMEWYPEAASNAER.W | N | 42.97 | 1924.8811 | 16 | 0.5 | 642.6346 | 3 | 48.79 | 4 | F4:29214 | NaNaKA16\_F12.raw |  |  |  | 1.0448E6 |  |  |  |  |  |  |  |  |  | 1 | 0 | 0 | 0 | 1 | 0 | 0 | 0 | 0 | 0 | 0 | 0 | 0 | 0 | 54 | 69 |  |  | PEAKS DB |
| T.GHYTQIVWYQTYR.A | Y | 42.91 | 1713.8263 | 13 | -0.2 | 572.2826 | 3 | 43.93 | 4 | F4:25228 | NaNaKA16\_F12.raw |  |  |  | 2.5056E6 |  |  |  |  |  |  |  |  |  | 1 | 0 | 0 | 0 | 1 | 0 | 0 | 0 | 0 | 0 | 0 | 0 | 0 | 0 | 132 | 144 |  |  | PEAKS DB |
| R.VSPTASNMLKM(+15.99)EWYPEAASNAER.W | N | 41.81 | 2597.1890 | 23 | -0.1 | 866.7368 | 3 | 60.59 | 4 | F4:38922 | NaNaKA16\_F12.raw |  |  |  | 1.7447E6 |  |  |  |  |  |  |  |  |  | 1 | 0 | 0 | 0 | 1 | 0 | 0 | 0 | 0 | 0 | 0 | 0 | 0 | 0 | 47 | 69 | Oxidation (M) | M11:Oxidation (M):22.34 | PEAKS DB |
| total 57 peptides |  |  |  |  |  |  |  |  |  |  |  |  |  |  |  |  |  |  |  |  |  |  |  |  |  |  |  |  |  |  |  |  |  |  |  |  |  |  |  |  |  |  |  |
| --- | --- | --- | --- | --- | --- | --- | --- | --- | --- | --- | --- | --- | --- | --- | --- | --- | --- | --- | --- | --- | --- | --- | --- | --- | --- | --- | --- | --- | --- | --- | --- | --- | --- | --- | --- | --- | --- | --- | --- | --- | --- | --- | --- |

AAP85301.1

back to list

  

| Protein Coverage
| Supporting Peptides
|

Protein Coverage:

Supporting Peptides:

| Peptide | Uniq | -10lgP | Mass | Length | ppm | m/z | z | RT | Fraction | Scan | Source File | Area F1 | Area F10 | Area F11 | Area F12 | Area F13 | Area F2 | Area F3 | Area F4 | Area F5 | Area F6 | Area F7 | Area F8 | Area F9 | #Feature | #Feature F1 | #Feature F10 | #Feature F11 | #Feature F12 | #Feature F13 | #Feature F2 | #Feature F3 | #Feature F4 | #Feature F5 | #Feature F6 | #Feature F7 | #Feature F8 | #Feature F9 | Start | End | PTM | AScore | Found By |
| --- | --- | --- | --- | --- | --- | --- | --- | --- | --- | --- | --- | --- | --- | --- | --- | --- | --- | --- | --- | --- | --- | --- | --- | --- | --- | --- | --- | --- | --- | --- | --- | --- | --- | --- | --- | --- | --- | --- | --- | --- | --- | --- | --- |
| R.TWTEIIHLWHDEYK.N | Y | 96.42 | 1869.9049 | 14 | 0.2 | 935.9600 | 2 | 67.88 | 4 | F4:47092 | NaNaKA16\_F12.raw |  |  |  | 1.192E10 | 1.3265E7 |  |  |  |  |  |  |  |  | 7 | 0 | 0 | 0 | 5 | 2 | 0 | 0 | 0 | 0 | 0 | 0 | 0 | 0 | 103 | 116 |  |  | PEAKS DB |
| R.WANTC(+57.02)SLNHSPDNLR.V | Y | 95.37 | 1783.8060 | 15 | -0.2 | 892.9102 | 2 | 20.57 | 4 | F4:6149 | NaNaKA16\_F12.raw |  |  |  | 2.5809E10 | 7.535E6 |  |  |  |  |  | 1.8333E5 |  |  | 20 | 0 | 0 | 0 | 16 | 3 | 0 | 0 | 0 | 0 | 0 | 1 | 0 | 0 | 70 | 84 | Carbamidomethylation | C5:Carbamidomethylation:1000.00 | PEAKS DB |
| R.VLEGIQC(+57.02)GESIYMSSNAR.T | Y | 94.89 | 2012.9296 | 18 | 0.0 | 1007.4721 | 2 | 61.49 | 4 | F4:39775 | NaNaKA16\_F12.raw |  | 5.2943E6 | 3.3626E7 | 7.5365E9 | 1.1193E7 |  |  |  |  |  |  |  | 0 | 16 | 0 | 1 | 2 | 9 | 4 | 0 | 0 | 0 | 0 | 0 | 0 | 0 | 0 | 85 | 102 | Carbamidomethylation | C7:Carbamidomethylation:1000.00 | PEAKS DB |
| R.VLEGIQC(+57.02)GESIYM(+15.99)SSNAR.T | Y | 91.90 | 2028.9244 | 18 | -0.3 | 1015.4692 | 2 | 47.15 | 4 | F4:28194 | NaNaKA16\_F12.raw |  | 1.6305E5 | 4.9762E6 | 1.1167E9 | 1.3064E6 |  |  |  |  |  |  |  |  | 10 | 0 | 1 | 1 | 7 | 1 | 0 | 0 | 0 | 0 | 0 | 0 | 0 | 0 | 85 | 102 | Carbamidomethylation; Oxidation (M) | C7:Carbamidomethylation:1000.00;M13:Oxidation (M):1000.00 | PEAKS DB |
| V.TGHYTQIVWYQTYR.A | Y | 79.15 | 1814.8740 | 14 | 0.5 | 908.4447 | 2 | 44.15 | 4 | F4:25452 | NaNaKA16\_F12.raw |  |  |  | 1.3608E8 |  |  |  |  |  |  |  |  |  | 2 | 0 | 0 | 0 | 2 | 0 | 0 | 0 | 0 | 0 | 0 | 0 | 0 | 0 | 131 | 144 |  |  | PEAKS DB |
| R.VSPTASNMLKMEWYPEAASNAER.W | N | 79.04 | 2581.1941 | 23 | 2.4 | 861.4074 | 3 | 60.50 | 4 | F4:38986 | NaNaKA16\_F12.raw |  |  |  | 7.3936E7 |  |  |  |  |  |  |  |  |  | 2 | 0 | 0 | 0 | 2 | 0 | 0 | 0 | 0 | 0 | 0 | 0 | 0 | 0 | 47 | 69 |  |  | PEAKS DB |
| Y.FYVC(+57.02)QYC(+57.02)PSGNFQGK.T | Y | 77.96 | 1853.7865 | 15 | -0.4 | 927.9001 | 2 | 45.62 | 4 | F4:26578 | NaNaKA16\_F12.raw |  |  |  | 2.3019E7 |  |  |  |  |  |  |  |  |  | 1 | 0 | 0 | 0 | 1 | 0 | 0 | 0 | 0 | 0 | 0 | 0 | 0 | 0 | 160 | 174 | Carbamidomethylation | C4:Carbamidomethylation:1000.00;C7:Carbamidomethylation:1000.00 | PEAKS DB |
| K.MEWYPEAASNAER.W | N | 77.76 | 1552.6616 | 13 | 0.7 | 518.5615 | 3 | 44.66 | 4 | F4:25052 | NaNaKA16\_F12.raw |  |  | 1.7754E7 | 3.6E10 | 1.0543E7 |  |  |  |  |  |  |  |  | 15 | 0 | 0 | 1 | 12 | 2 | 0 | 0 | 0 | 0 | 0 | 0 | 0 | 0 | 57 | 69 |  |  | PEAKS DB |
| W.SYFYVC(+57.02)QYC(+57.02)PSGNFQGK.T | Y | 76.97 | 2103.8818 | 17 | 0.0 | 1052.9482 | 2 | 56.99 | 4 | F4:35993 | NaNaKA16\_F12.raw |  |  |  | 2.0613E7 |  |  |  |  |  |  |  |  |  | 1 | 0 | 0 | 0 | 1 | 0 | 0 | 0 | 0 | 0 | 0 | 0 | 0 | 0 | 158 | 174 | Carbamidomethylation | C6:Carbamidomethylation:1000.00;C9:Carbamidomethylation:1000.00 | PEAKS DB |
| K.QKEIVDLHNSLR.R | N | 76.76 | 1450.7892 | 12 | -0.1 | 726.4018 | 2 | 11.75 | 4 | F4:2056 | NaNaKA16\_F12.raw |  |  |  | 8.0169E7 |  |  |  |  |  |  |  |  |  | 7 | 0 | 0 | 0 | 7 | 0 | 0 | 0 | 0 | 0 | 0 | 0 | 0 | 0 | 33 | 44 |  |  | PEAKS DB |
| K.M(+15.99)EWYPEAASNAER.W | N | 75.60 | 1568.6565 | 13 | 1.2 | 785.3364 | 2 | 31.92 | 4 | F4:18885 | NaNaKA16\_F12.raw |  |  |  | 4.1615E9 |  |  |  |  |  |  |  |  |  | 21 | 0 | 0 | 0 | 21 | 0 | 0 | 0 | 0 | 0 | 0 | 0 | 0 | 0 | 57 | 69 | Oxidation (M) | M1:Oxidation (M):1000.00 | PEAKS DB |
| L.RVLEGIQC(+57.02)GESIYMSSNAR.T | Y | 75.04 | 2169.0308 | 19 | -0.9 | 724.0169 | 3 | 42.93 | 4 | F4:24321 | NaNaKA16\_F12.raw |  |  | 1.5612E6 | 3.8584E6 |  |  |  |  |  |  |  |  |  | 2 | 0 | 0 | 1 | 1 | 0 | 0 | 0 | 0 | 0 | 0 | 0 | 0 | 0 | 84 | 102 | Carbamidomethylation | C8:Carbamidomethylation:1000.00 | PEAKS DB |
| N.GLC(+57.02)TNPC(+57.02)TIYNK.L | Y | 73.43 | 1439.6537 | 12 | 0.2 | 720.8342 | 2 | 27.20 | 4 | F4:10879 | NaNaKA16\_F12.raw |  |  |  | 3.9014E7 |  |  |  |  |  |  |  |  |  | 1 | 0 | 0 | 0 | 1 | 0 | 0 | 0 | 0 | 0 | 0 | 0 | 0 | 0 | 195 | 206 | Carbamidomethylation | C3:Carbamidomethylation:1000.00;C7:Carbamidomethylation:1000.00 | PEAKS DB |
| F.YVC(+57.02)QYC(+57.02)PSGNFQGK.T | Y | 71.81 | 1706.7181 | 14 | 0.0 | 854.3663 | 2 | 25.92 | 4 | F4:10821 | NaNaKA16\_F12.raw |  |  |  | 4.1439E8 |  |  |  |  |  |  |  |  |  | 1 | 0 | 0 | 0 | 1 | 0 | 0 | 0 | 0 | 0 | 0 | 0 | 0 | 0 | 161 | 174 | Carbamidomethylation | C3:Carbamidomethylation:1000.00;C6:Carbamidomethylation:1000.00 | PEAKS DB |
| E.GIQC(+57.02)GESIYMSSNAR.T | Y | 70.11 | 1671.7345 | 15 | 0.6 | 836.8750 | 2 | 29.85 | 4 | F4:13323 | NaNaKA16\_F12.raw |  |  |  | 1.3244E6 |  |  |  |  |  |  |  |  |  | 1 | 0 | 0 | 0 | 1 | 0 | 0 | 0 | 0 | 0 | 0 | 0 | 0 | 0 | 88 | 102 | Carbamidomethylation | C4:Carbamidomethylation:1000.00 | PEAKS DB |
| R.RVSPTASNMLK.M | Y | 69.72 | 1202.6442 | 11 | 0.3 | 602.3295 | 2 | 11.45 | 4 | F4:1801 | NaNaKA16\_F12.raw |  |  |  | 1.7511E7 |  |  |  |  |  |  |  |  |  | 2 | 0 | 0 | 0 | 2 | 0 | 0 | 0 | 0 | 0 | 0 | 0 | 0 | 0 | 46 | 56 |  |  | PEAKS DB |
| L.KMEWYPEAASNAER.W | N | 69.69 | 1680.7566 | 14 | -0.9 | 561.2590 | 3 | 29.03 | 4 | F4:12760 | NaNaKA16\_F12.raw |  |  |  | 3.1822E7 |  |  |  |  |  |  |  |  |  | 2 | 0 | 0 | 0 | 2 | 0 | 0 | 0 | 0 | 0 | 0 | 0 | 0 | 0 | 56 | 69 |  |  | PEAKS DB |
| G.NVDFNSESTR.R | N | 69.40 | 1167.5156 | 10 | 0.6 | 584.7654 | 2 | 12.15 | 4 | F4:2376 | NaNaKA16\_F12.raw |  |  |  | 8.6869E7 |  |  |  |  |  |  |  |  |  | 1 | 0 | 0 | 0 | 1 | 0 | 0 | 0 | 0 | 0 | 0 | 0 | 0 | 0 | 19 | 28 |  |  | PEAKS DB |
| W.TEIIHLWHDEYK.N | Y | 68.88 | 1582.7780 | 12 | 0.3 | 528.6001 | 3 | 38.63 | 4 | F4:20823 | NaNaKA16\_F12.raw |  |  |  | 1.545E7 |  |  |  |  |  |  |  |  |  | 4 | 0 | 0 | 0 | 4 | 0 | 0 | 0 | 0 | 0 | 0 | 0 | 0 | 0 | 105 | 116 |  |  | PEAKS DB |
| C.DNGLC(+57.02)TNPC(+57.02)TIYNK.L | Y | 68.73 | 1668.7236 | 14 | 0.5 | 835.3695 | 2 | 36.61 | 4 | F4:19046 | NaNaKA16\_F12.raw |  |  |  | 6.9202E6 |  |  |  |  | 7.9009E6 |  |  |  |  | 3 | 0 | 0 | 0 | 1 | 0 | 0 | 0 | 0 | 2 | 0 | 0 | 0 | 0 | 193 | 206 | Carbamidomethylation | C5:Carbamidomethylation:1000.00;C9:Carbamidomethylation:1000.00 | PEAKS DB |
| K.EIVDLHNSLR.R | N | 66.53 | 1194.6356 | 10 | -0.4 | 598.3248 | 2 | 26.22 | 4 | F4:11038 | NaNaKA16\_F12.raw |  |  | 2.0613E5 | 9.1517E8 | 1.6717E6 |  |  |  |  |  |  |  |  | 7 | 0 | 0 | 1 | 5 | 1 | 0 | 0 | 0 | 0 | 0 | 0 | 0 | 0 | 35 | 44 |  |  | PEAKS DB |
| A.RTWTEIIHLWHDEYK.N | Y | 65.27 | 2026.0061 | 15 | -0.1 | 507.5088 | 4 | 59.52 | 4 | F4:38212 | NaNaKA16\_F12.raw |  |  |  | 2.6065E6 |  |  |  |  |  |  |  |  |  | 1 | 0 | 0 | 0 | 1 | 0 | 0 | 0 | 0 | 0 | 0 | 0 | 0 | 0 | 102 | 116 |  |  | PEAKS DB |
| G.NVDFNSESTRR.K | N | 65.26 | 1323.6167 | 11 | -0.1 | 662.8156 | 2 | 11.44 | 4 | F4:1806 | NaNaKA16\_F12.raw |  |  |  | 6.0739E6 |  |  |  |  |  |  |  |  |  | 2 | 0 | 0 | 0 | 2 | 0 | 0 | 0 | 0 | 0 | 0 | 0 | 0 | 0 | 19 | 29 |  |  | PEAKS DB |
| K.SNC(+57.02)PASC(+57.02)FC(+57.02)R.N | N | 62.02 | 1257.4689 | 10 | 0.2 | 629.7418 | 2 | 12.25 | 4 | F4:2426 | NaNaKA16\_F12.raw |  |  |  | 6.4924E7 | 9.521E3 |  |  |  |  |  |  |  |  | 2 | 0 | 0 | 0 | 1 | 1 | 0 | 0 | 0 | 0 | 0 | 0 | 0 | 0 | 226 | 235 | Carbamidomethylation | C3:Carbamidomethylation:1000.00;C7:Carbamidomethylation:1000.00;C9:Carbamidomethylation:1000.00 | PEAKS DB |
| H.YTQIVWYQTYR.A | Y | 61.61 | 1519.7460 | 11 | 0.1 | 760.8804 | 2 | 55.75 | 4 | F4:34986 | NaNaKA16\_F12.raw |  |  |  | 1.6068E8 |  |  |  |  |  |  |  |  |  | 2 | 0 | 0 | 0 | 2 | 0 | 0 | 0 | 0 | 0 | 0 | 0 | 0 | 0 | 134 | 144 |  |  | PEAKS DB |
| R.TWTEIIHLWH.D | Y | 60.78 | 1334.6771 | 10 | 0.3 | 668.3461 | 2 | 73.98 | 4 | F4:49966 | NaNaKA16\_F12.raw |  |  |  | 4.783E6 |  |  |  |  |  |  |  |  |  | 1 | 0 | 0 | 0 | 1 | 0 | 0 | 0 | 0 | 0 | 0 | 0 | 0 | 0 | 103 | 112 |  |  | PEAKS DB |
| R.VLEGIQC(+57.02)GESIYMSSN.A | Y | 58.92 | 1785.7914 | 16 | -0.4 | 893.9026 | 2 | 68.70 | 4 | F4:45706 | NaNaKA16\_F12.raw |  |  |  | 1.3545E8 |  |  |  |  |  |  |  |  |  | 1 | 0 | 0 | 0 | 1 | 0 | 0 | 0 | 0 | 0 | 0 | 0 | 0 | 0 | 85 | 100 | Carbamidomethylation | C7:Carbamidomethylation:1000.00 | PEAKS DB |
| N.TC(+57.02)SLNHSPDNLR.V | Y | 56.85 | 1412.6466 | 12 | 0.5 | 707.3309 | 2 | 11.48 | 4 | F4:1809 | NaNaKA16\_F12.raw |  |  |  | 4.5467E6 |  |  |  |  |  |  |  |  |  | 2 | 0 | 0 | 0 | 2 | 0 | 0 | 0 | 0 | 0 | 0 | 0 | 0 | 0 | 73 | 84 | Carbamidomethylation | C2:Carbamidomethylation:1000.00 | PEAKS DB |
| R.VSPTASNM(+15.99)LK.M | N | 56.73 | 1062.5380 | 10 | 0.7 | 532.2766 | 2 | 11.45 | 4 | F4:1800 | NaNaKA16\_F12.raw |  |  |  | 1.2798E7 |  |  |  |  |  |  |  |  |  | 1 | 0 | 0 | 0 | 1 | 0 | 0 | 0 | 0 | 0 | 0 | 0 | 0 | 0 | 47 | 56 | Oxidation (M) | M8:Oxidation (M):1000.00 | PEAKS DB |
| K.LGPPC(+57.02)GDC(+57.02)PSAC(+57.02)DNGLC(+57.02)TNPC(+57.02)TIYNK.L | Y | 56.61 | 2940.1970 | 26 | 1.2 | 981.0742 | 3 | 49.74 | 4 | F4:29707 | NaNaKA16\_F12.raw |  |  |  | 9.9063E8 |  |  |  |  |  |  |  |  |  | 2 | 0 | 0 | 0 | 2 | 0 | 0 | 0 | 0 | 0 | 0 | 0 | 0 | 0 | 181 | 206 | Carbamidomethylation | C5:Carbamidomethylation:1000.00;C8:Carbamidomethylation:1000.00;C12:Carbamidomethylation:1000.00;C17:Carbamidomethylation:1000.00;C21:Carbamidomethylation:1000.00 | PEAKS DB |
| Y.TQIVWYQTYR.A | Y | 56.03 | 1356.6826 | 10 | 0.6 | 679.3490 | 2 | 49.37 | 4 | F4:29565 | NaNaKA16\_F12.raw |  |  |  | 3.2787E7 |  |  |  |  |  |  |  |  |  | 1 | 0 | 0 | 0 | 1 | 0 | 0 | 0 | 0 | 0 | 0 | 0 | 0 | 0 | 135 | 144 |  |  | PEAKS DB |
| Y.VC(+57.02)QYC(+57.02)PSGNFQGK.T | Y | 54.51 | 1543.6548 | 13 | -0.3 | 772.8344 | 2 | 11.76 | 4 | F4:2117 | NaNaKA16\_F12.raw |  |  |  | 1.3951E6 |  |  |  |  |  |  |  |  |  | 1 | 0 | 0 | 0 | 1 | 0 | 0 | 0 | 0 | 0 | 0 | 0 | 0 | 0 | 162 | 174 | Carbamidomethylation | C2:Carbamidomethylation:1000.00;C5:Carbamidomethylation:1000.00 | PEAKS DB |
| R.RVSPTASNM(+15.99)LK.M | Y | 54.31 | 1218.6390 | 11 | 0.7 | 407.2206 | 3 | 11.48 | 4 | F4:1808 | NaNaKA16\_F12.raw |  |  |  | 4.4814E5 |  |  |  |  |  |  |  |  |  | 1 | 0 | 0 | 0 | 1 | 0 | 0 | 0 | 0 | 0 | 0 | 0 | 0 | 0 | 46 | 56 | Oxidation (M) | M9:Oxidation (M):1000.00 | PEAKS DB |
| R.VSPTASNMLK.M | N | 54.02 | 1046.5430 | 10 | -0.7 | 524.2784 | 2 | 11.74 | 4 | F4:2287 | NaNaKA16\_F12.raw |  |  |  | 1.7067E8 |  |  |  |  |  |  |  |  |  | 2 | 0 | 0 | 0 | 2 | 0 | 0 | 0 | 0 | 0 | 0 | 0 | 0 | 0 | 47 | 56 |  |  | PEAKS DB |
| I.VDLHNSLR.R | N | 53.27 | 952.5090 | 8 | 0.1 | 477.2618 | 2 | 26.22 | 4 | F4:10364 | NaNaKA16\_F12.raw |  |  |  | 3.6616E7 |  |  |  |  |  |  |  |  |  | 1 | 0 | 0 | 0 | 1 | 0 | 0 | 0 | 0 | 0 | 0 | 0 | 0 | 0 | 37 | 44 |  |  | PEAKS DB |
| M.EWYPEAASNAER.W | N | 53.15 | 1421.6211 | 12 | 0.6 | 711.8182 | 2 | 30.28 | 4 | F4:13787 | NaNaKA16\_F12.raw |  |  |  | 1.7579E7 |  |  |  |  |  |  |  |  |  | 1 | 0 | 0 | 0 | 1 | 0 | 0 | 0 | 0 | 0 | 0 | 0 | 0 | 0 | 58 | 69 |  |  | PEAKS DB |
| N.VDFNSESTR.R | N | 52.01 | 1053.4727 | 9 | 0.6 | 527.7439 | 2 | 11.66 | 4 | F4:1931 | NaNaKA16\_F12.raw |  |  |  | 9.3775E6 |  |  |  |  |  |  |  |  |  | 1 | 0 | 0 | 0 | 1 | 0 | 0 | 0 | 0 | 0 | 0 | 0 | 0 | 0 | 20 | 28 |  |  | PEAKS DB |
| S.AC(+57.02)DNGLC(+57.02)TNPC(+57.02)TIYNK.L | Y | 51.74 | 1899.7914 | 16 | -0.3 | 950.9027 | 2 | 33.35 | 4 | F4:16304 | NaNaKA16\_F12.raw |  |  |  | 3.0888E6 |  |  |  |  |  |  |  |  |  | 1 | 0 | 0 | 0 | 1 | 0 | 0 | 0 | 0 | 0 | 0 | 0 | 0 | 0 | 191 | 206 | Carbamidomethylation | C2:Carbamidomethylation:1000.00;C7:Carbamidomethylation:1000.00;C11:Carbamidomethylation:1000.00 | PEAKS DB |
| C.SLNHSPDNLR.V | Y | 50.57 | 1151.5684 | 10 | -0.3 | 384.8633 | 3 | 11.45 | 4 | F4:1803 | NaNaKA16\_F12.raw |  |  |  | 3.3986E6 |  |  |  |  |  |  |  |  |  | 2 | 0 | 0 | 0 | 2 | 0 | 0 | 0 | 0 | 0 | 0 | 0 | 0 | 0 | 75 | 84 |  |  | PEAKS DB |
| R.TWTEIIHLWHDEYKN.F | Y | 49.28 | 1983.9479 | 15 | -0.2 | 496.9941 | 4 | 67.43 | 4 | F4:44938 | NaNaKA16\_F12.raw |  |  |  | 1.7614E7 |  |  |  |  |  |  |  |  |  | 2 | 0 | 0 | 0 | 2 | 0 | 0 | 0 | 0 | 0 | 0 | 0 | 0 | 0 | 103 | 117 |  |  | PEAKS DB |
| L.EGIQC(+57.02)GESIYMSSNAR.T | Y | 48.30 | 1800.7771 | 16 | 0.9 | 901.3966 | 2 | 35.87 | 4 | F4:18384 | NaNaKA16\_F12.raw |  |  |  | 0 |  |  |  |  |  |  |  |  |  | 0 | 0 | 0 | 0 | 0 | 0 | 0 | 0 | 0 | 0 | 0 | 0 | 0 | 0 | 87 | 102 | Carbamidomethylation | C5:Carbamidomethylation:1000.00 | PEAKS DB |
| R.VLEGIQC(+57.02)GESIYM(+15.99)SSN.A | Y | 48.10 | 1801.7863 | 16 | 0.0 | 901.9004 | 2 | 59.91 | 4 | F4:38428 | NaNaKA16\_F12.raw |  |  |  | 1.9592E7 |  |  |  |  |  |  |  |  |  | 1 | 0 | 0 | 0 | 1 | 0 | 0 | 0 | 0 | 0 | 0 | 0 | 0 | 0 | 85 | 100 | Carbamidomethylation; Oxidation (M) | C7:Carbamidomethylation:1000.00;M13:Oxidation (M):1000.00 | PEAKS DB |
| Y.KLGPPC(+57.02)GDC(+57.02)PSAC(+57.02)DNGLC(+57.02)TNPC(+57.02)TIYNK.L | Y | 48.02 | 3068.2917 | 27 | 1.2 | 1023.7724 | 3 | 40.46 | 4 | F4:22361 | NaNaKA16\_F12.raw |  |  |  | 7.6949E6 |  |  |  |  |  |  |  |  |  | 1 | 0 | 0 | 0 | 1 | 0 | 0 | 0 | 0 | 0 | 0 | 0 | 0 | 0 | 180 | 206 | Carbamidomethylation | C6:Carbamidomethylation:1000.00;C9:Carbamidomethylation:1000.00;C13:Carbamidomethylation:1000.00;C18:Carbamidomethylation:1000.00;C22:Carbamidomethylation:1000.00 | PEAKS DB |
| Q.IVWYQTYR.A | Y | 47.45 | 1127.5764 | 8 | -5.9 | 564.7922 | 2 | 43.62 | 4 | F4:24799 | NaNaKA16\_F12.raw |  |  |  | 3.1475E7 |  |  |  |  |  |  |  |  |  | 2 | 0 | 0 | 0 | 2 | 0 | 0 | 0 | 0 | 0 | 0 | 0 | 0 | 0 | 137 | 144 |  |  | PEAKS DB |
| R.TWTEIIHLWHD.E | Y | 47.44 | 1449.7041 | 11 | 0.4 | 484.2422 | 3 | 76.70 | 4 | F4:52262 | NaNaKA16\_F12.raw |  |  |  | 2.7409E6 |  |  |  |  |  |  |  |  |  | 1 | 0 | 0 | 0 | 1 | 0 | 0 | 0 | 0 | 0 | 0 | 0 | 0 | 0 | 103 | 113 |  |  | PEAKS DB |
| T.QIVWYQTYR.A | Y | 46.03 | 1255.6349 | 9 | 0.0 | 628.8247 | 2 | 47.28 | 4 | F4:27952 | NaNaKA16\_F12.raw |  |  |  | 5.8793E6 |  |  |  |  |  |  |  |  |  | 1 | 0 | 0 | 0 | 1 | 0 | 0 | 0 | 0 | 0 | 0 | 0 | 0 | 0 | 136 | 144 |  |  | PEAKS DB |
| E.IIHLWHDEYK.N | N | 45.95 | 1352.6877 | 10 | -0.5 | 451.9030 | 3 | 26.85 | 4 | F4:10987 | NaNaKA16\_F12.raw |  |  |  | 8.808E5 |  |  |  |  |  |  |  |  |  | 1 | 0 | 0 | 0 | 1 | 0 | 0 | 0 | 0 | 0 | 0 | 0 | 0 | 0 | 107 | 116 |  |  | PEAKS DB |
| T.EIIHLWHDEYK.N | Y | 44.66 | 1481.7302 | 11 | 0.1 | 494.9174 | 3 | 34.79 | 4 | F4:17343 | NaNaKA16\_F12.raw |  |  |  | 4.613E5 |  |  |  |  |  |  |  |  |  | 1 | 0 | 0 | 0 | 1 | 0 | 0 | 0 | 0 | 0 | 0 | 0 | 0 | 0 | 106 | 116 |  |  | PEAKS DB |
| R.VLEGIQC(+57.02)GESIY.M | Y | 44.56 | 1366.6438 | 12 | 0.4 | 684.3295 | 2 | 64.32 | 4 | F4:42048 | NaNaKA16\_F12.raw |  |  |  | 1.267E8 |  |  |  |  |  |  |  |  |  | 1 | 0 | 0 | 0 | 1 | 0 | 0 | 0 | 0 | 0 | 0 | 0 | 0 | 0 | 85 | 96 | Carbamidomethylation | C7:Carbamidomethylation:1000.00 | PEAKS DB |
| D.C(+57.02)PSAC(+57.02)DNGLC(+57.02)TNPC(+57.02)TIYNK.L | Y | 44.19 | 2243.9067 | 19 | 0.6 | 1122.9613 | 2 | 36.03 | 4 | F4:18539 | NaNaKA16\_F12.raw |  |  |  | 1.1206E6 |  |  |  |  |  |  |  |  |  | 1 | 0 | 0 | 0 | 1 | 0 | 0 | 0 | 0 | 0 | 0 | 0 | 0 | 0 | 188 | 206 | Carbamidomethylation | C1:Carbamidomethylation:1000.00;C5:Carbamidomethylation:1000.00;C10:Carbamidomethylation:1000.00;C14:Carbamidomethylation:1000.00 | PEAKS DB |
| L.RVLEGIQC(+57.02)GESIYM(+15.99)SSNAR.T | Y | 43.71 | 2185.0256 | 19 | 0.8 | 729.3497 | 3 | 36.11 | 4 | F4:18487 | NaNaKA16\_F12.raw |  |  |  | 6.1752E5 |  |  |  |  |  |  |  |  |  | 1 | 0 | 0 | 0 | 1 | 0 | 0 | 0 | 0 | 0 | 0 | 0 | 0 | 0 | 84 | 102 | Carbamidomethylation; Oxidation (M) | C8:Carbamidomethylation:1000.00;M14:Oxidation (M):1000.00 | PEAKS DB |
| R.RVSPTASNMLKMEWYPEAASNAER.W | Y | 43.34 | 2737.2952 | 24 | 1.4 | 685.3320 | 4 | 51.07 | 4 | F4:31143 | NaNaKA16\_F12.raw |  |  |  | 1.6942E6 |  |  |  |  |  |  |  |  |  | 1 | 0 | 0 | 0 | 1 | 0 | 0 | 0 | 0 | 0 | 0 | 0 | 0 | 0 | 46 | 69 |  |  | PEAKS DB |
| K.QKEIVDLHN.S | N | 43.18 | 1094.5720 | 9 | -0.5 | 548.2930 | 2 | 11.57 | 4 | F4:1902 | NaNaKA16\_F12.raw |  |  |  | 1.3713E5 |  |  |  |  |  |  |  |  |  | 1 | 0 | 0 | 0 | 1 | 0 | 0 | 0 | 0 | 0 | 0 | 0 | 0 | 0 | 33 | 41 |  |  | PEAKS DB |
| R.TWTEIIHLW.H | Y | 42.97 | 1197.6183 | 9 | 0.1 | 599.8165 | 2 | 92.08 | 4 | F4:64706 | NaNaKA16\_F12.raw |  |  |  | 2.1391E6 |  |  |  |  |  |  |  |  |  | 1 | 0 | 0 | 0 | 1 | 0 | 0 | 0 | 0 | 0 | 0 | 0 | 0 | 0 | 103 | 111 |  |  | PEAKS DB |
| N.MLKMEWYPEAASNAER.W | N | 42.97 | 1924.8811 | 16 | 0.5 | 642.6346 | 3 | 48.79 | 4 | F4:29214 | NaNaKA16\_F12.raw |  |  |  | 1.0448E6 |  |  |  |  |  |  |  |  |  | 1 | 0 | 0 | 0 | 1 | 0 | 0 | 0 | 0 | 0 | 0 | 0 | 0 | 0 | 54 | 69 |  |  | PEAKS DB |
| T.GHYTQIVWYQTYR.A | Y | 42.91 | 1713.8263 | 13 | -0.2 | 572.2826 | 3 | 43.93 | 4 | F4:25228 | NaNaKA16\_F12.raw |  |  |  | 2.5056E6 |  |  |  |  |  |  |  |  |  | 1 | 0 | 0 | 0 | 1 | 0 | 0 | 0 | 0 | 0 | 0 | 0 | 0 | 0 | 132 | 144 |  |  | PEAKS DB |
| R.VSPTASNMLKM(+15.99)EWYPEAASNAER.W | N | 41.81 | 2597.1890 | 23 | -0.1 | 866.7368 | 3 | 60.59 | 4 | F4:38922 | NaNaKA16\_F12.raw |  |  |  | 1.7447E6 |  |  |  |  |  |  |  |  |  | 1 | 0 | 0 | 0 | 1 | 0 | 0 | 0 | 0 | 0 | 0 | 0 | 0 | 0 | 47 | 69 | Oxidation (M) | M11:Oxidation (M):22.34 | PEAKS DB |
| total 57 peptides |  |  |  |  |  |  |  |  |  |  |  |  |  |  |  |  |  |  |  |  |  |  |  |  |  |  |  |  |  |  |  |  |  |  |  |  |  |  |  |  |  |  |  |
| --- | --- | --- | --- | --- | --- | --- | --- | --- | --- | --- | --- | --- | --- | --- | --- | --- | --- | --- | --- | --- | --- | --- | --- | --- | --- | --- | --- | --- | --- | --- | --- | --- | --- | --- | --- | --- | --- | --- | --- | --- | --- | --- | --- |

ACH73167.1

back to list

  

| Protein Coverage
| Supporting Peptides
|

Protein Coverage:

Supporting Peptides:

| Peptide | Uniq | -10lgP | Mass | Length | ppm | m/z | z | RT | Fraction | Scan | Source File | Area F1 | Area F10 | Area F11 | Area F12 | Area F13 | Area F2 | Area F3 | Area F4 | Area F5 | Area F6 | Area F7 | Area F8 | Area F9 | #Feature | #Feature F1 | #Feature F10 | #Feature F11 | #Feature F12 | #Feature F13 | #Feature F2 | #Feature F3 | #Feature F4 | #Feature F5 | #Feature F6 | #Feature F7 | #Feature F8 | #Feature F9 | Start | End | PTM | AScore | Found By |
| --- | --- | --- | --- | --- | --- | --- | --- | --- | --- | --- | --- | --- | --- | --- | --- | --- | --- | --- | --- | --- | --- | --- | --- | --- | --- | --- | --- | --- | --- | --- | --- | --- | --- | --- | --- | --- | --- | --- | --- | --- | --- | --- | --- |
| R.TWTEIIHLWHDEYK.N | Y | 96.42 | 1869.9049 | 14 | 0.2 | 935.9600 | 2 | 67.88 | 4 | F4:47092 | NaNaKA16\_F12.raw |  |  |  | 1.192E10 | 1.3265E7 |  |  |  |  |  |  |  |  | 7 | 0 | 0 | 0 | 5 | 2 | 0 | 0 | 0 | 0 | 0 | 0 | 0 | 0 | 103 | 116 |  |  | PEAKS DB |
| R.WANTC(+57.02)SLNHSPDNLR.V | Y | 95.37 | 1783.8060 | 15 | -0.2 | 892.9102 | 2 | 20.57 | 4 | F4:6149 | NaNaKA16\_F12.raw |  |  |  | 2.5809E10 | 7.535E6 |  |  |  |  |  | 1.8333E5 |  |  | 20 | 0 | 0 | 0 | 16 | 3 | 0 | 0 | 0 | 0 | 0 | 1 | 0 | 0 | 70 | 84 | Carbamidomethylation | C5:Carbamidomethylation:1000.00 | PEAKS DB |
| R.VLEGIQC(+57.02)GESIYMSSNAR.T | Y | 94.89 | 2012.9296 | 18 | 0.0 | 1007.4721 | 2 | 61.49 | 4 | F4:39775 | NaNaKA16\_F12.raw |  | 5.2943E6 | 3.3626E7 | 7.5365E9 | 1.1193E7 |  |  |  |  |  |  |  | 0 | 16 | 0 | 1 | 2 | 9 | 4 | 0 | 0 | 0 | 0 | 0 | 0 | 0 | 0 | 85 | 102 | Carbamidomethylation | C7:Carbamidomethylation:1000.00 | PEAKS DB |
| R.VLEGIQC(+57.02)GESIYM(+15.99)SSNAR.T | Y | 91.90 | 2028.9244 | 18 | -0.3 | 1015.4692 | 2 | 47.15 | 4 | F4:28194 | NaNaKA16\_F12.raw |  | 1.6305E5 | 4.9762E6 | 1.1167E9 | 1.3064E6 |  |  |  |  |  |  |  |  | 10 | 0 | 1 | 1 | 7 | 1 | 0 | 0 | 0 | 0 | 0 | 0 | 0 | 0 | 85 | 102 | Carbamidomethylation; Oxidation (M) | C7:Carbamidomethylation:1000.00;M13:Oxidation (M):1000.00 | PEAKS DB |
| V.TGHYTQIVWYQTYR.A | Y | 79.15 | 1814.8740 | 14 | 0.5 | 908.4447 | 2 | 44.15 | 4 | F4:25452 | NaNaKA16\_F12.raw |  |  |  | 1.3608E8 |  |  |  |  |  |  |  |  |  | 2 | 0 | 0 | 0 | 2 | 0 | 0 | 0 | 0 | 0 | 0 | 0 | 0 | 0 | 131 | 144 |  |  | PEAKS DB |
| R.VSPTASNMLKMEWYPEAASNAER.W | N | 79.04 | 2581.1941 | 23 | 2.4 | 861.4074 | 3 | 60.50 | 4 | F4:38986 | NaNaKA16\_F12.raw |  |  |  | 7.3936E7 |  |  |  |  |  |  |  |  |  | 2 | 0 | 0 | 0 | 2 | 0 | 0 | 0 | 0 | 0 | 0 | 0 | 0 | 0 | 47 | 69 |  |  | PEAKS DB |
| Y.FYVC(+57.02)QYC(+57.02)PSGNFQGK.T | Y | 77.96 | 1853.7865 | 15 | -0.4 | 927.9001 | 2 | 45.62 | 4 | F4:26578 | NaNaKA16\_F12.raw |  |  |  | 2.3019E7 |  |  |  |  |  |  |  |  |  | 1 | 0 | 0 | 0 | 1 | 0 | 0 | 0 | 0 | 0 | 0 | 0 | 0 | 0 | 160 | 174 | Carbamidomethylation | C4:Carbamidomethylation:1000.00;C7:Carbamidomethylation:1000.00 | PEAKS DB |
| K.MEWYPEAASNAER.W | N | 77.76 | 1552.6616 | 13 | 0.7 | 518.5615 | 3 | 44.66 | 4 | F4:25052 | NaNaKA16\_F12.raw |  |  | 1.7754E7 | 3.6E10 | 1.0543E7 |  |  |  |  |  |  |  |  | 15 | 0 | 0 | 1 | 12 | 2 | 0 | 0 | 0 | 0 | 0 | 0 | 0 | 0 | 57 | 69 |  |  | PEAKS DB |
| W.SYFYVC(+57.02)QYC(+57.02)PSGNFQGK.T | Y | 76.97 | 2103.8818 | 17 | 0.0 | 1052.9482 | 2 | 56.99 | 4 | F4:35993 | NaNaKA16\_F12.raw |  |  |  | 2.0613E7 |  |  |  |  |  |  |  |  |  | 1 | 0 | 0 | 0 | 1 | 0 | 0 | 0 | 0 | 0 | 0 | 0 | 0 | 0 | 158 | 174 | Carbamidomethylation | C6:Carbamidomethylation:1000.00;C9:Carbamidomethylation:1000.00 | PEAKS DB |
| K.QKEIVDLHNSLR.R | N | 76.76 | 1450.7892 | 12 | -0.1 | 726.4018 | 2 | 11.75 | 4 | F4:2056 | NaNaKA16\_F12.raw |  |  |  | 8.0169E7 |  |  |  |  |  |  |  |  |  | 7 | 0 | 0 | 0 | 7 | 0 | 0 | 0 | 0 | 0 | 0 | 0 | 0 | 0 | 33 | 44 |  |  | PEAKS DB |
| K.M(+15.99)EWYPEAASNAER.W | N | 75.60 | 1568.6565 | 13 | 1.2 | 785.3364 | 2 | 31.92 | 4 | F4:18885 | NaNaKA16\_F12.raw |  |  |  | 4.1615E9 |  |  |  |  |  |  |  |  |  | 21 | 0 | 0 | 0 | 21 | 0 | 0 | 0 | 0 | 0 | 0 | 0 | 0 | 0 | 57 | 69 | Oxidation (M) | M1:Oxidation (M):1000.00 | PEAKS DB |
| L.RVLEGIQC(+57.02)GESIYMSSNAR.T | Y | 75.04 | 2169.0308 | 19 | -0.9 | 724.0169 | 3 | 42.93 | 4 | F4:24321 | NaNaKA16\_F12.raw |  |  | 1.5612E6 | 3.8584E6 |  |  |  |  |  |  |  |  |  | 2 | 0 | 0 | 1 | 1 | 0 | 0 | 0 | 0 | 0 | 0 | 0 | 0 | 0 | 84 | 102 | Carbamidomethylation | C8:Carbamidomethylation:1000.00 | PEAKS DB |
| N.GLC(+57.02)TNPC(+57.02)TIYNK.L | Y | 73.43 | 1439.6537 | 12 | 0.2 | 720.8342 | 2 | 27.20 | 4 | F4:10879 | NaNaKA16\_F12.raw |  |  |  | 3.9014E7 |  |  |  |  |  |  |  |  |  | 1 | 0 | 0 | 0 | 1 | 0 | 0 | 0 | 0 | 0 | 0 | 0 | 0 | 0 | 195 | 206 | Carbamidomethylation | C3:Carbamidomethylation:1000.00;C7:Carbamidomethylation:1000.00 | PEAKS DB |
| F.YVC(+57.02)QYC(+57.02)PSGNFQGK.T | Y | 71.81 | 1706.7181 | 14 | 0.0 | 854.3663 | 2 | 25.92 | 4 | F4:10821 | NaNaKA16\_F12.raw |  |  |  | 4.1439E8 |  |  |  |  |  |  |  |  |  | 1 | 0 | 0 | 0 | 1 | 0 | 0 | 0 | 0 | 0 | 0 | 0 | 0 | 0 | 161 | 174 | Carbamidomethylation | C3:Carbamidomethylation:1000.00;C6:Carbamidomethylation:1000.00 | PEAKS DB |
| E.GIQC(+57.02)GESIYMSSNAR.T | Y | 70.11 | 1671.7345 | 15 | 0.6 | 836.8750 | 2 | 29.85 | 4 | F4:13323 | NaNaKA16\_F12.raw |  |  |  | 1.3244E6 |  |  |  |  |  |  |  |  |  | 1 | 0 | 0 | 0 | 1 | 0 | 0 | 0 | 0 | 0 | 0 | 0 | 0 | 0 | 88 | 102 | Carbamidomethylation | C4:Carbamidomethylation:1000.00 | PEAKS DB |
| R.RVSPTASNMLK.M | Y | 69.72 | 1202.6442 | 11 | 0.3 | 602.3295 | 2 | 11.45 | 4 | F4:1801 | NaNaKA16\_F12.raw |  |  |  | 1.7511E7 |  |  |  |  |  |  |  |  |  | 2 | 0 | 0 | 0 | 2 | 0 | 0 | 0 | 0 | 0 | 0 | 0 | 0 | 0 | 46 | 56 |  |  | PEAKS DB |
| L.KMEWYPEAASNAER.W | N | 69.69 | 1680.7566 | 14 | -0.9 | 561.2590 | 3 | 29.03 | 4 | F4:12760 | NaNaKA16\_F12.raw |  |  |  | 3.1822E7 |  |  |  |  |  |  |  |  |  | 2 | 0 | 0 | 0 | 2 | 0 | 0 | 0 | 0 | 0 | 0 | 0 | 0 | 0 | 56 | 69 |  |  | PEAKS DB |
| G.NVDFNSESTR.R | N | 69.40 | 1167.5156 | 10 | 0.6 | 584.7654 | 2 | 12.15 | 4 | F4:2376 | NaNaKA16\_F12.raw |  |  |  | 8.6869E7 |  |  |  |  |  |  |  |  |  | 1 | 0 | 0 | 0 | 1 | 0 | 0 | 0 | 0 | 0 | 0 | 0 | 0 | 0 | 19 | 28 |  |  | PEAKS DB |
| W.TEIIHLWHDEYK.N | Y | 68.88 | 1582.7780 | 12 | 0.3 | 528.6001 | 3 | 38.63 | 4 | F4:20823 | NaNaKA16\_F12.raw |  |  |  | 1.545E7 |  |  |  |  |  |  |  |  |  | 4 | 0 | 0 | 0 | 4 | 0 | 0 | 0 | 0 | 0 | 0 | 0 | 0 | 0 | 105 | 116 |  |  | PEAKS DB |
| C.DNGLC(+57.02)TNPC(+57.02)TIYNK.L | Y | 68.73 | 1668.7236 | 14 | 0.5 | 835.3695 | 2 | 36.61 | 4 | F4:19046 | NaNaKA16\_F12.raw |  |  |  | 6.9202E6 |  |  |  |  | 7.9009E6 |  |  |  |  | 3 | 0 | 0 | 0 | 1 | 0 | 0 | 0 | 0 | 2 | 0 | 0 | 0 | 0 | 193 | 206 | Carbamidomethylation | C5:Carbamidomethylation:1000.00;C9:Carbamidomethylation:1000.00 | PEAKS DB |
| K.EIVDLHNSLR.R | N | 66.53 | 1194.6356 | 10 | -0.4 | 598.3248 | 2 | 26.22 | 4 | F4:11038 | NaNaKA16\_F12.raw |  |  | 2.0613E5 | 9.1517E8 | 1.6717E6 |  |  |  |  |  |  |  |  | 7 | 0 | 0 | 1 | 5 | 1 | 0 | 0 | 0 | 0 | 0 | 0 | 0 | 0 | 35 | 44 |  |  | PEAKS DB |
| A.RTWTEIIHLWHDEYK.N | Y | 65.27 | 2026.0061 | 15 | -0.1 | 507.5088 | 4 | 59.52 | 4 | F4:38212 | NaNaKA16\_F12.raw |  |  |  | 2.6065E6 |  |  |  |  |  |  |  |  |  | 1 | 0 | 0 | 0 | 1 | 0 | 0 | 0 | 0 | 0 | 0 | 0 | 0 | 0 | 102 | 116 |  |  | PEAKS DB |
| G.NVDFNSESTRR.K | N | 65.26 | 1323.6167 | 11 | -0.1 | 662.8156 | 2 | 11.44 | 4 | F4:1806 | NaNaKA16\_F12.raw |  |  |  | 6.0739E6 |  |  |  |  |  |  |  |  |  | 2 | 0 | 0 | 0 | 2 | 0 | 0 | 0 | 0 | 0 | 0 | 0 | 0 | 0 | 19 | 29 |  |  | PEAKS DB |
| K.SNC(+57.02)PASC(+57.02)FC(+57.02)R.N | N | 62.02 | 1257.4689 | 10 | 0.2 | 629.7418 | 2 | 12.25 | 4 | F4:2426 | NaNaKA16\_F12.raw |  |  |  | 6.4924E7 | 9.521E3 |  |  |  |  |  |  |  |  | 2 | 0 | 0 | 0 | 1 | 1 | 0 | 0 | 0 | 0 | 0 | 0 | 0 | 0 | 226 | 235 | Carbamidomethylation | C3:Carbamidomethylation:1000.00;C7:Carbamidomethylation:1000.00;C9:Carbamidomethylation:1000.00 | PEAKS DB |
| H.YTQIVWYQTYR.A | Y | 61.61 | 1519.7460 | 11 | 0.1 | 760.8804 | 2 | 55.75 | 4 | F4:34986 | NaNaKA16\_F12.raw |  |  |  | 1.6068E8 |  |  |  |  |  |  |  |  |  | 2 | 0 | 0 | 0 | 2 | 0 | 0 | 0 | 0 | 0 | 0 | 0 | 0 | 0 | 134 | 144 |  |  | PEAKS DB |
| R.TWTEIIHLWH.D | Y | 60.78 | 1334.6771 | 10 | 0.3 | 668.3461 | 2 | 73.98 | 4 | F4:49966 | NaNaKA16\_F12.raw |  |  |  | 4.783E6 |  |  |  |  |  |  |  |  |  | 1 | 0 | 0 | 0 | 1 | 0 | 0 | 0 | 0 | 0 | 0 | 0 | 0 | 0 | 103 | 112 |  |  | PEAKS DB |
| R.VLEGIQC(+57.02)GESIYMSSN.A | Y | 58.92 | 1785.7914 | 16 | -0.4 | 893.9026 | 2 | 68.70 | 4 | F4:45706 | NaNaKA16\_F12.raw |  |  |  | 1.3545E8 |  |  |  |  |  |  |  |  |  | 1 | 0 | 0 | 0 | 1 | 0 | 0 | 0 | 0 | 0 | 0 | 0 | 0 | 0 | 85 | 100 | Carbamidomethylation | C7:Carbamidomethylation:1000.00 | PEAKS DB |
| N.TC(+57.02)SLNHSPDNLR.V | Y | 56.85 | 1412.6466 | 12 | 0.5 | 707.3309 | 2 | 11.48 | 4 | F4:1809 | NaNaKA16\_F12.raw |  |  |  | 4.5467E6 |  |  |  |  |  |  |  |  |  | 2 | 0 | 0 | 0 | 2 | 0 | 0 | 0 | 0 | 0 | 0 | 0 | 0 | 0 | 73 | 84 | Carbamidomethylation | C2:Carbamidomethylation:1000.00 | PEAKS DB |
| R.VSPTASNM(+15.99)LK.M | N | 56.73 | 1062.5380 | 10 | 0.7 | 532.2766 | 2 | 11.45 | 4 | F4:1800 | NaNaKA16\_F12.raw |  |  |  | 1.2798E7 |  |  |  |  |  |  |  |  |  | 1 | 0 | 0 | 0 | 1 | 0 | 0 | 0 | 0 | 0 | 0 | 0 | 0 | 0 | 47 | 56 | Oxidation (M) | M8:Oxidation (M):1000.00 | PEAKS DB |
| K.LGPPC(+57.02)GDC(+57.02)PSAC(+57.02)DNGLC(+57.02)TNPC(+57.02)TIYNK.L | Y | 56.61 | 2940.1970 | 26 | 1.2 | 981.0742 | 3 | 49.74 | 4 | F4:29707 | NaNaKA16\_F12.raw |  |  |  | 9.9063E8 |  |  |  |  |  |  |  |  |  | 2 | 0 | 0 | 0 | 2 | 0 | 0 | 0 | 0 | 0 | 0 | 0 | 0 | 0 | 181 | 206 | Carbamidomethylation | C5:Carbamidomethylation:1000.00;C8:Carbamidomethylation:1000.00;C12:Carbamidomethylation:1000.00;C17:Carbamidomethylation:1000.00;C21:Carbamidomethylation:1000.00 | PEAKS DB |
| Y.TQIVWYQTYR.A | Y | 56.03 | 1356.6826 | 10 | 0.6 | 679.3490 | 2 | 49.37 | 4 | F4:29565 | NaNaKA16\_F12.raw |  |  |  | 3.2787E7 |  |  |  |  |  |  |  |  |  | 1 | 0 | 0 | 0 | 1 | 0 | 0 | 0 | 0 | 0 | 0 | 0 | 0 | 0 | 135 | 144 |  |  | PEAKS DB |
| Y.VC(+57.02)QYC(+57.02)PSGNFQGK.T | Y | 54.51 | 1543.6548 | 13 | -0.3 | 772.8344 | 2 | 11.76 | 4 | F4:2117 | NaNaKA16\_F12.raw |  |  |  | 1.3951E6 |  |  |  |  |  |  |  |  |  | 1 | 0 | 0 | 0 | 1 | 0 | 0 | 0 | 0 | 0 | 0 | 0 | 0 | 0 | 162 | 174 | Carbamidomethylation | C2:Carbamidomethylation:1000.00;C5:Carbamidomethylation:1000.00 | PEAKS DB |
| R.RVSPTASNM(+15.99)LK.M | Y | 54.31 | 1218.6390 | 11 | 0.7 | 407.2206 | 3 | 11.48 | 4 | F4:1808 | NaNaKA16\_F12.raw |  |  |  | 4.4814E5 |  |  |  |  |  |  |  |  |  | 1 | 0 | 0 | 0 | 1 | 0 | 0 | 0 | 0 | 0 | 0 | 0 | 0 | 0 | 46 | 56 | Oxidation (M) | M9:Oxidation (M):1000.00 | PEAKS DB |
| R.VSPTASNMLK.M | N | 54.02 | 1046.5430 | 10 | -0.7 | 524.2784 | 2 | 11.74 | 4 | F4:2287 | NaNaKA16\_F12.raw |  |  |  | 1.7067E8 |  |  |  |  |  |  |  |  |  | 2 | 0 | 0 | 0 | 2 | 0 | 0 | 0 | 0 | 0 | 0 | 0 | 0 | 0 | 47 | 56 |  |  | PEAKS DB |
| I.VDLHNSLR.R | N | 53.27 | 952.5090 | 8 | 0.1 | 477.2618 | 2 | 26.22 | 4 | F4:10364 | NaNaKA16\_F12.raw |  |  |  | 3.6616E7 |  |  |  |  |  |  |  |  |  | 1 | 0 | 0 | 0 | 1 | 0 | 0 | 0 | 0 | 0 | 0 | 0 | 0 | 0 | 37 | 44 |  |  | PEAKS DB |
| M.EWYPEAASNAER.W | N | 53.15 | 1421.6211 | 12 | 0.6 | 711.8182 | 2 | 30.28 | 4 | F4:13787 | NaNaKA16\_F12.raw |  |  |  | 1.7579E7 |  |  |  |  |  |  |  |  |  | 1 | 0 | 0 | 0 | 1 | 0 | 0 | 0 | 0 | 0 | 0 | 0 | 0 | 0 | 58 | 69 |  |  | PEAKS DB |
| N.VDFNSESTR.R | N | 52.01 | 1053.4727 | 9 | 0.6 | 527.7439 | 2 | 11.66 | 4 | F4:1931 | NaNaKA16\_F12.raw |  |  |  | 9.3775E6 |  |  |  |  |  |  |  |  |  | 1 | 0 | 0 | 0 | 1 | 0 | 0 | 0 | 0 | 0 | 0 | 0 | 0 | 0 | 20 | 28 |  |  | PEAKS DB |
| S.AC(+57.02)DNGLC(+57.02)TNPC(+57.02)TIYNK.L | Y | 51.74 | 1899.7914 | 16 | -0.3 | 950.9027 | 2 | 33.35 | 4 | F4:16304 | NaNaKA16\_F12.raw |  |  |  | 3.0888E6 |  |  |  |  |  |  |  |  |  | 1 | 0 | 0 | 0 | 1 | 0 | 0 | 0 | 0 | 0 | 0 | 0 | 0 | 0 | 191 | 206 | Carbamidomethylation | C2:Carbamidomethylation:1000.00;C7:Carbamidomethylation:1000.00;C11:Carbamidomethylation:1000.00 | PEAKS DB |
| C.SLNHSPDNLR.V | Y | 50.57 | 1151.5684 | 10 | -0.3 | 384.8633 | 3 | 11.45 | 4 | F4:1803 | NaNaKA16\_F12.raw |  |  |  | 3.3986E6 |  |  |  |  |  |  |  |  |  | 2 | 0 | 0 | 0 | 2 | 0 | 0 | 0 | 0 | 0 | 0 | 0 | 0 | 0 | 75 | 84 |  |  | PEAKS DB |
| R.TWTEIIHLWHDEYKN.F | Y | 49.28 | 1983.9479 | 15 | -0.2 | 496.9941 | 4 | 67.43 | 4 | F4:44938 | NaNaKA16\_F12.raw |  |  |  | 1.7614E7 |  |  |  |  |  |  |  |  |  | 2 | 0 | 0 | 0 | 2 | 0 | 0 | 0 | 0 | 0 | 0 | 0 | 0 | 0 | 103 | 117 |  |  | PEAKS DB |
| L.EGIQC(+57.02)GESIYMSSNAR.T | Y | 48.30 | 1800.7771 | 16 | 0.9 | 901.3966 | 2 | 35.87 | 4 | F4:18384 | NaNaKA16\_F12.raw |  |  |  | 0 |  |  |  |  |  |  |  |  |  | 0 | 0 | 0 | 0 | 0 | 0 | 0 | 0 | 0 | 0 | 0 | 0 | 0 | 0 | 87 | 102 | Carbamidomethylation | C5:Carbamidomethylation:1000.00 | PEAKS DB |
| R.VLEGIQC(+57.02)GESIYM(+15.99)SSN.A | Y | 48.10 | 1801.7863 | 16 | 0.0 | 901.9004 | 2 | 59.91 | 4 | F4:38428 | NaNaKA16\_F12.raw |  |  |  | 1.9592E7 |  |  |  |  |  |  |  |  |  | 1 | 0 | 0 | 0 | 1 | 0 | 0 | 0 | 0 | 0 | 0 | 0 | 0 | 0 | 85 | 100 | Carbamidomethylation; Oxidation (M) | C7:Carbamidomethylation:1000.00;M13:Oxidation (M):1000.00 | PEAKS DB |
| Y.KLGPPC(+57.02)GDC(+57.02)PSAC(+57.02)DNGLC(+57.02)TNPC(+57.02)TIYNK.L | Y | 48.02 | 3068.2917 | 27 | 1.2 | 1023.7724 | 3 | 40.46 | 4 | F4:22361 | NaNaKA16\_F12.raw |  |  |  | 7.6949E6 |  |  |  |  |  |  |  |  |  | 1 | 0 | 0 | 0 | 1 | 0 | 0 | 0 | 0 | 0 | 0 | 0 | 0 | 0 | 180 | 206 | Carbamidomethylation | C6:Carbamidomethylation:1000.00;C9:Carbamidomethylation:1000.00;C13:Carbamidomethylation:1000.00;C18:Carbamidomethylation:1000.00;C22:Carbamidomethylation:1000.00 | PEAKS DB |
| Q.IVWYQTYR.A | Y | 47.45 | 1127.5764 | 8 | -5.9 | 564.7922 | 2 | 43.62 | 4 | F4:24799 | NaNaKA16\_F12.raw |  |  |  | 3.1475E7 |  |  |  |  |  |  |  |  |  | 2 | 0 | 0 | 0 | 2 | 0 | 0 | 0 | 0 | 0 | 0 | 0 | 0 | 0 | 137 | 144 |  |  | PEAKS DB |
| R.TWTEIIHLWHD.E | Y | 47.44 | 1449.7041 | 11 | 0.4 | 484.2422 | 3 | 76.70 | 4 | F4:52262 | NaNaKA16\_F12.raw |  |  |  | 2.7409E6 |  |  |  |  |  |  |  |  |  | 1 | 0 | 0 | 0 | 1 | 0 | 0 | 0 | 0 | 0 | 0 | 0 | 0 | 0 | 103 | 113 |  |  | PEAKS DB |
| T.QIVWYQTYR.A | Y | 46.03 | 1255.6349 | 9 | 0.0 | 628.8247 | 2 | 47.28 | 4 | F4:27952 | NaNaKA16\_F12.raw |  |  |  | 5.8793E6 |  |  |  |  |  |  |  |  |  | 1 | 0 | 0 | 0 | 1 | 0 | 0 | 0 | 0 | 0 | 0 | 0 | 0 | 0 | 136 | 144 |  |  | PEAKS DB |
| E.IIHLWHDEYK.N | N | 45.95 | 1352.6877 | 10 | -0.5 | 451.9030 | 3 | 26.85 | 4 | F4:10987 | NaNaKA16\_F12.raw |  |  |  | 8.808E5 |  |  |  |  |  |  |  |  |  | 1 | 0 | 0 | 0 | 1 | 0 | 0 | 0 | 0 | 0 | 0 | 0 | 0 | 0 | 107 | 116 |  |  | PEAKS DB |
| T.EIIHLWHDEYK.N | Y | 44.66 | 1481.7302 | 11 | 0.1 | 494.9174 | 3 | 34.79 | 4 | F4:17343 | NaNaKA16\_F12.raw |  |  |  | 4.613E5 |  |  |  |  |  |  |  |  |  | 1 | 0 | 0 | 0 | 1 | 0 | 0 | 0 | 0 | 0 | 0 | 0 | 0 | 0 | 106 | 116 |  |  | PEAKS DB |
| R.VLEGIQC(+57.02)GESIY.M | Y | 44.56 | 1366.6438 | 12 | 0.4 | 684.3295 | 2 | 64.32 | 4 | F4:42048 | NaNaKA16\_F12.raw |  |  |  | 1.267E8 |  |  |  |  |  |  |  |  |  | 1 | 0 | 0 | 0 | 1 | 0 | 0 | 0 | 0 | 0 | 0 | 0 | 0 | 0 | 85 | 96 | Carbamidomethylation | C7:Carbamidomethylation:1000.00 | PEAKS DB |
| D.C(+57.02)PSAC(+57.02)DNGLC(+57.02)TNPC(+57.02)TIYNK.L | Y | 44.19 | 2243.9067 | 19 | 0.6 | 1122.9613 | 2 | 36.03 | 4 | F4:18539 | NaNaKA16\_F12.raw |  |  |  | 1.1206E6 |  |  |  |  |  |  |  |  |  | 1 | 0 | 0 | 0 | 1 | 0 | 0 | 0 | 0 | 0 | 0 | 0 | 0 | 0 | 188 | 206 | Carbamidomethylation | C1:Carbamidomethylation:1000.00;C5:Carbamidomethylation:1000.00;C10:Carbamidomethylation:1000.00;C14:Carbamidomethylation:1000.00 | PEAKS DB |
| L.RVLEGIQC(+57.02)GESIYM(+15.99)SSNAR.T | Y | 43.71 | 2185.0256 | 19 | 0.8 | 729.3497 | 3 | 36.11 | 4 | F4:18487 | NaNaKA16\_F12.raw |  |  |  | 6.1752E5 |  |  |  |  |  |  |  |  |  | 1 | 0 | 0 | 0 | 1 | 0 | 0 | 0 | 0 | 0 | 0 | 0 | 0 | 0 | 84 | 102 | Carbamidomethylation; Oxidation (M) | C8:Carbamidomethylation:1000.00;M14:Oxidation (M):1000.00 | PEAKS DB |
| R.RVSPTASNMLKMEWYPEAASNAER.W | Y | 43.34 | 2737.2952 | 24 | 1.4 | 685.3320 | 4 | 51.07 | 4 | F4:31143 | NaNaKA16\_F12.raw |  |  |  | 1.6942E6 |  |  |  |  |  |  |  |  |  | 1 | 0 | 0 | 0 | 1 | 0 | 0 | 0 | 0 | 0 | 0 | 0 | 0 | 0 | 46 | 69 |  |  | PEAKS DB |
| K.QKEIVDLHN.S | N | 43.18 | 1094.5720 | 9 | -0.5 | 548.2930 | 2 | 11.57 | 4 | F4:1902 | NaNaKA16\_F12.raw |  |  |  | 1.3713E5 |  |  |  |  |  |  |  |  |  | 1 | 0 | 0 | 0 | 1 | 0 | 0 | 0 | 0 | 0 | 0 | 0 | 0 | 0 | 33 | 41 |  |  | PEAKS DB |
| R.TWTEIIHLW.H | Y | 42.97 | 1197.6183 | 9 | 0.1 | 599.8165 | 2 | 92.08 | 4 | F4:64706 | NaNaKA16\_F12.raw |  |  |  | 2.1391E6 |  |  |  |  |  |  |  |  |  | 1 | 0 | 0 | 0 | 1 | 0 | 0 | 0 | 0 | 0 | 0 | 0 | 0 | 0 | 103 | 111 |  |  | PEAKS DB |
| N.MLKMEWYPEAASNAER.W | N | 42.97 | 1924.8811 | 16 | 0.5 | 642.6346 | 3 | 48.79 | 4 | F4:29214 | NaNaKA16\_F12.raw |  |  |  | 1.0448E6 |  |  |  |  |  |  |  |  |  | 1 | 0 | 0 | 0 | 1 | 0 | 0 | 0 | 0 | 0 | 0 | 0 | 0 | 0 | 54 | 69 |  |  | PEAKS DB |
| T.GHYTQIVWYQTYR.A | Y | 42.91 | 1713.8263 | 13 | -0.2 | 572.2826 | 3 | 43.93 | 4 | F4:25228 | NaNaKA16\_F12.raw |  |  |  | 2.5056E6 |  |  |  |  |  |  |  |  |  | 1 | 0 | 0 | 0 | 1 | 0 | 0 | 0 | 0 | 0 | 0 | 0 | 0 | 0 | 132 | 144 |  |  | PEAKS DB |
| R.VSPTASNMLKM(+15.99)EWYPEAASNAER.W | N | 41.81 | 2597.1890 | 23 | -0.1 | 866.7368 | 3 | 60.59 | 4 | F4:38922 | NaNaKA16\_F12.raw |  |  |  | 1.7447E6 |  |  |  |  |  |  |  |  |  | 1 | 0 | 0 | 0 | 1 | 0 | 0 | 0 | 0 | 0 | 0 | 0 | 0 | 0 | 47 | 69 | Oxidation (M) | M11:Oxidation (M):22.34 | PEAKS DB |
| total 57 peptides |  |  |  |  |  |  |  |  |  |  |  |  |  |  |  |  |  |  |  |  |  |  |  |  |  |  |  |  |  |  |  |  |  |  |  |  |  |  |  |  |  |  |  |
| --- | --- | --- | --- | --- | --- | --- | --- | --- | --- | --- | --- | --- | --- | --- | --- | --- | --- | --- | --- | --- | --- | --- | --- | --- | --- | --- | --- | --- | --- | --- | --- | --- | --- | --- | --- | --- | --- | --- | --- | --- | --- | --- | --- |

P84805.2

back to list

  

| Protein Coverage
| Supporting Peptides
|

Protein Coverage:

Supporting Peptides:

| Peptide | Uniq | -10lgP | Mass | Length | ppm | m/z | z | RT | Fraction | Scan | Source File | Area F1 | Area F10 | Area F11 | Area F12 | Area F13 | Area F2 | Area F3 | Area F4 | Area F5 | Area F6 | Area F7 | Area F8 | Area F9 | #Feature | #Feature F1 | #Feature F10 | #Feature F11 | #Feature F12 | #Feature F13 | #Feature F2 | #Feature F3 | #Feature F4 | #Feature F5 | #Feature F6 | #Feature F7 | #Feature F8 | #Feature F9 | Start | End | PTM | AScore | Found By |
| --- | --- | --- | --- | --- | --- | --- | --- | --- | --- | --- | --- | --- | --- | --- | --- | --- | --- | --- | --- | --- | --- | --- | --- | --- | --- | --- | --- | --- | --- | --- | --- | --- | --- | --- | --- | --- | --- | --- | --- | --- | --- | --- | --- |
| R.TWTEIIHLWHDEYK.N | Y | 96.42 | 1869.9049 | 14 | 0.2 | 935.9600 | 2 | 67.88 | 4 | F4:47092 | NaNaKA16\_F12.raw |  |  |  | 1.192E10 | 1.3265E7 |  |  |  |  |  |  |  |  | 7 | 0 | 0 | 0 | 5 | 2 | 0 | 0 | 0 | 0 | 0 | 0 | 0 | 0 | 103 | 116 |  |  | PEAKS DB |
| R.WANTC(+57.02)SLNHSPDNLR.V | Y | 95.37 | 1783.8060 | 15 | -0.2 | 892.9102 | 2 | 20.57 | 4 | F4:6149 | NaNaKA16\_F12.raw |  |  |  | 2.5809E10 | 7.535E6 |  |  |  |  |  | 1.8333E5 |  |  | 20 | 0 | 0 | 0 | 16 | 3 | 0 | 0 | 0 | 0 | 0 | 1 | 0 | 0 | 70 | 84 | Carbamidomethylation | C5:Carbamidomethylation:1000.00 | PEAKS DB |
| R.VLEGIQC(+57.02)GESIYMSSNAR.T | Y | 94.89 | 2012.9296 | 18 | 0.0 | 1007.4721 | 2 | 61.49 | 4 | F4:39775 | NaNaKA16\_F12.raw |  | 5.2943E6 | 3.3626E7 | 7.5365E9 | 1.1193E7 |  |  |  |  |  |  |  | 0 | 16 | 0 | 1 | 2 | 9 | 4 | 0 | 0 | 0 | 0 | 0 | 0 | 0 | 0 | 85 | 102 | Carbamidomethylation | C7:Carbamidomethylation:1000.00 | PEAKS DB |
| R.VLEGIQC(+57.02)GESIYM(+15.99)SSNAR.T | Y | 91.90 | 2028.9244 | 18 | -0.3 | 1015.4692 | 2 | 47.15 | 4 | F4:28194 | NaNaKA16\_F12.raw |  | 1.6305E5 | 4.9762E6 | 1.1167E9 | 1.3064E6 |  |  |  |  |  |  |  |  | 10 | 0 | 1 | 1 | 7 | 1 | 0 | 0 | 0 | 0 | 0 | 0 | 0 | 0 | 85 | 102 | Carbamidomethylation; Oxidation (M) | C7:Carbamidomethylation:1000.00;M13:Oxidation (M):1000.00 | PEAKS DB |
| V.TGHYTQIVWYQTYR.A | Y | 79.15 | 1814.8740 | 14 | 0.5 | 908.4447 | 2 | 44.15 | 4 | F4:25452 | NaNaKA16\_F12.raw |  |  |  | 1.3608E8 |  |  |  |  |  |  |  |  |  | 2 | 0 | 0 | 0 | 2 | 0 | 0 | 0 | 0 | 0 | 0 | 0 | 0 | 0 | 131 | 144 |  |  | PEAKS DB |
| R.VSPTASNMLKMEWYPEAASNAER.W | N | 79.04 | 2581.1941 | 23 | 2.4 | 861.4074 | 3 | 60.50 | 4 | F4:38986 | NaNaKA16\_F12.raw |  |  |  | 7.3936E7 |  |  |  |  |  |  |  |  |  | 2 | 0 | 0 | 0 | 2 | 0 | 0 | 0 | 0 | 0 | 0 | 0 | 0 | 0 | 47 | 69 |  |  | PEAKS DB |
| Y.FYVC(+57.02)QYC(+57.02)PSGNFQGK.T | Y | 77.96 | 1853.7865 | 15 | -0.4 | 927.9001 | 2 | 45.62 | 4 | F4:26578 | NaNaKA16\_F12.raw |  |  |  | 2.3019E7 |  |  |  |  |  |  |  |  |  | 1 | 0 | 0 | 0 | 1 | 0 | 0 | 0 | 0 | 0 | 0 | 0 | 0 | 0 | 160 | 174 | Carbamidomethylation | C4:Carbamidomethylation:1000.00;C7:Carbamidomethylation:1000.00 | PEAKS DB |
| K.MEWYPEAASNAER.W | N | 77.76 | 1552.6616 | 13 | 0.7 | 518.5615 | 3 | 44.66 | 4 | F4:25052 | NaNaKA16\_F12.raw |  |  | 1.7754E7 | 3.6E10 | 1.0543E7 |  |  |  |  |  |  |  |  | 15 | 0 | 0 | 1 | 12 | 2 | 0 | 0 | 0 | 0 | 0 | 0 | 0 | 0 | 57 | 69 |  |  | PEAKS DB |
| W.SYFYVC(+57.02)QYC(+57.02)PSGNFQGK.T | Y | 76.97 | 2103.8818 | 17 | 0.0 | 1052.9482 | 2 | 56.99 | 4 | F4:35993 | NaNaKA16\_F12.raw |  |  |  | 2.0613E7 |  |  |  |  |  |  |  |  |  | 1 | 0 | 0 | 0 | 1 | 0 | 0 | 0 | 0 | 0 | 0 | 0 | 0 | 0 | 158 | 174 | Carbamidomethylation | C6:Carbamidomethylation:1000.00;C9:Carbamidomethylation:1000.00 | PEAKS DB |
| K.QKEIVDLHNSLR.R | N | 76.76 | 1450.7892 | 12 | -0.1 | 726.4018 | 2 | 11.75 | 4 | F4:2056 | NaNaKA16\_F12.raw |  |  |  | 8.0169E7 |  |  |  |  |  |  |  |  |  | 7 | 0 | 0 | 0 | 7 | 0 | 0 | 0 | 0 | 0 | 0 | 0 | 0 | 0 | 33 | 44 |  |  | PEAKS DB |
| K.M(+15.99)EWYPEAASNAER.W | N | 75.60 | 1568.6565 | 13 | 1.2 | 785.3364 | 2 | 31.92 | 4 | F4:18885 | NaNaKA16\_F12.raw |  |  |  | 4.1615E9 |  |  |  |  |  |  |  |  |  | 21 | 0 | 0 | 0 | 21 | 0 | 0 | 0 | 0 | 0 | 0 | 0 | 0 | 0 | 57 | 69 | Oxidation (M) | M1:Oxidation (M):1000.00 | PEAKS DB |
| L.RVLEGIQC(+57.02)GESIYMSSNAR.T | Y | 75.04 | 2169.0308 | 19 | -0.9 | 724.0169 | 3 | 42.93 | 4 | F4:24321 | NaNaKA16\_F12.raw |  |  | 1.5612E6 | 3.8584E6 |  |  |  |  |  |  |  |  |  | 2 | 0 | 0 | 1 | 1 | 0 | 0 | 0 | 0 | 0 | 0 | 0 | 0 | 0 | 84 | 102 | Carbamidomethylation | C8:Carbamidomethylation:1000.00 | PEAKS DB |
| N.GLC(+57.02)TNPC(+57.02)TIYNK.L | Y | 73.43 | 1439.6537 | 12 | 0.2 | 720.8342 | 2 | 27.20 | 4 | F4:10879 | NaNaKA16\_F12.raw |  |  |  | 3.9014E7 |  |  |  |  |  |  |  |  |  | 1 | 0 | 0 | 0 | 1 | 0 | 0 | 0 | 0 | 0 | 0 | 0 | 0 | 0 | 195 | 206 | Carbamidomethylation | C3:Carbamidomethylation:1000.00;C7:Carbamidomethylation:1000.00 | PEAKS DB |
| F.YVC(+57.02)QYC(+57.02)PSGNFQGK.T | Y | 71.81 | 1706.7181 | 14 | 0.0 | 854.3663 | 2 | 25.92 | 4 | F4:10821 | NaNaKA16\_F12.raw |  |  |  | 4.1439E8 |  |  |  |  |  |  |  |  |  | 1 | 0 | 0 | 0 | 1 | 0 | 0 | 0 | 0 | 0 | 0 | 0 | 0 | 0 | 161 | 174 | Carbamidomethylation | C3:Carbamidomethylation:1000.00;C6:Carbamidomethylation:1000.00 | PEAKS DB |
| E.GIQC(+57.02)GESIYMSSNAR.T | Y | 70.11 | 1671.7345 | 15 | 0.6 | 836.8750 | 2 | 29.85 | 4 | F4:13323 | NaNaKA16\_F12.raw |  |  |  | 1.3244E6 |  |  |  |  |  |  |  |  |  | 1 | 0 | 0 | 0 | 1 | 0 | 0 | 0 | 0 | 0 | 0 | 0 | 0 | 0 | 88 | 102 | Carbamidomethylation | C4:Carbamidomethylation:1000.00 | PEAKS DB |
| R.RVSPTASNMLK.M | Y | 69.72 | 1202.6442 | 11 | 0.3 | 602.3295 | 2 | 11.45 | 4 | F4:1801 | NaNaKA16\_F12.raw |  |  |  | 1.7511E7 |  |  |  |  |  |  |  |  |  | 2 | 0 | 0 | 0 | 2 | 0 | 0 | 0 | 0 | 0 | 0 | 0 | 0 | 0 | 46 | 56 |  |  | PEAKS DB |
| L.KMEWYPEAASNAER.W | N | 69.69 | 1680.7566 | 14 | -0.9 | 561.2590 | 3 | 29.03 | 4 | F4:12760 | NaNaKA16\_F12.raw |  |  |  | 3.1822E7 |  |  |  |  |  |  |  |  |  | 2 | 0 | 0 | 0 | 2 | 0 | 0 | 0 | 0 | 0 | 0 | 0 | 0 | 0 | 56 | 69 |  |  | PEAKS DB |
| G.NVDFNSESTR.R | N | 69.40 | 1167.5156 | 10 | 0.6 | 584.7654 | 2 | 12.15 | 4 | F4:2376 | NaNaKA16\_F12.raw |  |  |  | 8.6869E7 |  |  |  |  |  |  |  |  |  | 1 | 0 | 0 | 0 | 1 | 0 | 0 | 0 | 0 | 0 | 0 | 0 | 0 | 0 | 19 | 28 |  |  | PEAKS DB |
| W.TEIIHLWHDEYK.N | Y | 68.88 | 1582.7780 | 12 | 0.3 | 528.6001 | 3 | 38.63 | 4 | F4:20823 | NaNaKA16\_F12.raw |  |  |  | 1.545E7 |  |  |  |  |  |  |  |  |  | 4 | 0 | 0 | 0 | 4 | 0 | 0 | 0 | 0 | 0 | 0 | 0 | 0 | 0 | 105 | 116 |  |  | PEAKS DB |
| C.DNGLC(+57.02)TNPC(+57.02)TIYNK.L | Y | 68.73 | 1668.7236 | 14 | 0.5 | 835.3695 | 2 | 36.61 | 4 | F4:19046 | NaNaKA16\_F12.raw |  |  |  | 6.9202E6 |  |  |  |  | 7.9009E6 |  |  |  |  | 3 | 0 | 0 | 0 | 1 | 0 | 0 | 0 | 0 | 2 | 0 | 0 | 0 | 0 | 193 | 206 | Carbamidomethylation | C5:Carbamidomethylation:1000.00;C9:Carbamidomethylation:1000.00 | PEAKS DB |
| K.EIVDLHNSLR.R | N | 66.53 | 1194.6356 | 10 | -0.4 | 598.3248 | 2 | 26.22 | 4 | F4:11038 | NaNaKA16\_F12.raw |  |  | 2.0613E5 | 9.1517E8 | 1.6717E6 |  |  |  |  |  |  |  |  | 7 | 0 | 0 | 1 | 5 | 1 | 0 | 0 | 0 | 0 | 0 | 0 | 0 | 0 | 35 | 44 |  |  | PEAKS DB |
| A.RTWTEIIHLWHDEYK.N | Y | 65.27 | 2026.0061 | 15 | -0.1 | 507.5088 | 4 | 59.52 | 4 | F4:38212 | NaNaKA16\_F12.raw |  |  |  | 2.6065E6 |  |  |  |  |  |  |  |  |  | 1 | 0 | 0 | 0 | 1 | 0 | 0 | 0 | 0 | 0 | 0 | 0 | 0 | 0 | 102 | 116 |  |  | PEAKS DB |
| G.NVDFNSESTRR.K | N | 65.26 | 1323.6167 | 11 | -0.1 | 662.8156 | 2 | 11.44 | 4 | F4:1806 | NaNaKA16\_F12.raw |  |  |  | 6.0739E6 |  |  |  |  |  |  |  |  |  | 2 | 0 | 0 | 0 | 2 | 0 | 0 | 0 | 0 | 0 | 0 | 0 | 0 | 0 | 19 | 29 |  |  | PEAKS DB |
| K.SNC(+57.02)PASC(+57.02)FC(+57.02)R.N | N | 62.02 | 1257.4689 | 10 | 0.2 | 629.7418 | 2 | 12.25 | 4 | F4:2426 | NaNaKA16\_F12.raw |  |  |  | 6.4924E7 | 9.521E3 |  |  |  |  |  |  |  |  | 2 | 0 | 0 | 0 | 1 | 1 | 0 | 0 | 0 | 0 | 0 | 0 | 0 | 0 | 226 | 235 | Carbamidomethylation | C3:Carbamidomethylation:1000.00;C7:Carbamidomethylation:1000.00;C9:Carbamidomethylation:1000.00 | PEAKS DB |
| H.YTQIVWYQTYR.A | Y | 61.61 | 1519.7460 | 11 | 0.1 | 760.8804 | 2 | 55.75 | 4 | F4:34986 | NaNaKA16\_F12.raw |  |  |  | 1.6068E8 |  |  |  |  |  |  |  |  |  | 2 | 0 | 0 | 0 | 2 | 0 | 0 | 0 | 0 | 0 | 0 | 0 | 0 | 0 | 134 | 144 |  |  | PEAKS DB |
| R.TWTEIIHLWH.D | Y | 60.78 | 1334.6771 | 10 | 0.3 | 668.3461 | 2 | 73.98 | 4 | F4:49966 | NaNaKA16\_F12.raw |  |  |  | 4.783E6 |  |  |  |  |  |  |  |  |  | 1 | 0 | 0 | 0 | 1 | 0 | 0 | 0 | 0 | 0 | 0 | 0 | 0 | 0 | 103 | 112 |  |  | PEAKS DB |
| R.VLEGIQC(+57.02)GESIYMSSN.A | Y | 58.92 | 1785.7914 | 16 | -0.4 | 893.9026 | 2 | 68.70 | 4 | F4:45706 | NaNaKA16\_F12.raw |  |  |  | 1.3545E8 |  |  |  |  |  |  |  |  |  | 1 | 0 | 0 | 0 | 1 | 0 | 0 | 0 | 0 | 0 | 0 | 0 | 0 | 0 | 85 | 100 | Carbamidomethylation | C7:Carbamidomethylation:1000.00 | PEAKS DB |
| N.TC(+57.02)SLNHSPDNLR.V | Y | 56.85 | 1412.6466 | 12 | 0.5 | 707.3309 | 2 | 11.48 | 4 | F4:1809 | NaNaKA16\_F12.raw |  |  |  | 4.5467E6 |  |  |  |  |  |  |  |  |  | 2 | 0 | 0 | 0 | 2 | 0 | 0 | 0 | 0 | 0 | 0 | 0 | 0 | 0 | 73 | 84 | Carbamidomethylation | C2:Carbamidomethylation:1000.00 | PEAKS DB |
| R.VSPTASNM(+15.99)LK.M | N | 56.73 | 1062.5380 | 10 | 0.7 | 532.2766 | 2 | 11.45 | 4 | F4:1800 | NaNaKA16\_F12.raw |  |  |  | 1.2798E7 |  |  |  |  |  |  |  |  |  | 1 | 0 | 0 | 0 | 1 | 0 | 0 | 0 | 0 | 0 | 0 | 0 | 0 | 0 | 47 | 56 | Oxidation (M) | M8:Oxidation (M):1000.00 | PEAKS DB |
| K.LGPPC(+57.02)GDC(+57.02)PSAC(+57.02)DNGLC(+57.02)TNPC(+57.02)TIYNK.L | Y | 56.61 | 2940.1970 | 26 | 1.2 | 981.0742 | 3 | 49.74 | 4 | F4:29707 | NaNaKA16\_F12.raw |  |  |  | 9.9063E8 |  |  |  |  |  |  |  |  |  | 2 | 0 | 0 | 0 | 2 | 0 | 0 | 0 | 0 | 0 | 0 | 0 | 0 | 0 | 181 | 206 | Carbamidomethylation | C5:Carbamidomethylation:1000.00;C8:Carbamidomethylation:1000.00;C12:Carbamidomethylation:1000.00;C17:Carbamidomethylation:1000.00;C21:Carbamidomethylation:1000.00 | PEAKS DB |
| Y.TQIVWYQTYR.A | Y | 56.03 | 1356.6826 | 10 | 0.6 | 679.3490 | 2 | 49.37 | 4 | F4:29565 | NaNaKA16\_F12.raw |  |  |  | 3.2787E7 |  |  |  |  |  |  |  |  |  | 1 | 0 | 0 | 0 | 1 | 0 | 0 | 0 | 0 | 0 | 0 | 0 | 0 | 0 | 135 | 144 |  |  | PEAKS DB |
| Y.VC(+57.02)QYC(+57.02)PSGNFQGK.T | Y | 54.51 | 1543.6548 | 13 | -0.3 | 772.8344 | 2 | 11.76 | 4 | F4:2117 | NaNaKA16\_F12.raw |  |  |  | 1.3951E6 |  |  |  |  |  |  |  |  |  | 1 | 0 | 0 | 0 | 1 | 0 | 0 | 0 | 0 | 0 | 0 | 0 | 0 | 0 | 162 | 174 | Carbamidomethylation | C2:Carbamidomethylation:1000.00;C5:Carbamidomethylation:1000.00 | PEAKS DB |
| R.RVSPTASNM(+15.99)LK.M | Y | 54.31 | 1218.6390 | 11 | 0.7 | 407.2206 | 3 | 11.48 | 4 | F4:1808 | NaNaKA16\_F12.raw |  |  |  | 4.4814E5 |  |  |  |  |  |  |  |  |  | 1 | 0 | 0 | 0 | 1 | 0 | 0 | 0 | 0 | 0 | 0 | 0 | 0 | 0 | 46 | 56 | Oxidation (M) | M9:Oxidation (M):1000.00 | PEAKS DB |
| R.VSPTASNMLK.M | N | 54.02 | 1046.5430 | 10 | -0.7 | 524.2784 | 2 | 11.74 | 4 | F4:2287 | NaNaKA16\_F12.raw |  |  |  | 1.7067E8 |  |  |  |  |  |  |  |  |  | 2 | 0 | 0 | 0 | 2 | 0 | 0 | 0 | 0 | 0 | 0 | 0 | 0 | 0 | 47 | 56 |  |  | PEAKS DB |
| I.VDLHNSLR.R | N | 53.27 | 952.5090 | 8 | 0.1 | 477.2618 | 2 | 26.22 | 4 | F4:10364 | NaNaKA16\_F12.raw |  |  |  | 3.6616E7 |  |  |  |  |  |  |  |  |  | 1 | 0 | 0 | 0 | 1 | 0 | 0 | 0 | 0 | 0 | 0 | 0 | 0 | 0 | 37 | 44 |  |  | PEAKS DB |
| M.EWYPEAASNAER.W | N | 53.15 | 1421.6211 | 12 | 0.6 | 711.8182 | 2 | 30.28 | 4 | F4:13787 | NaNaKA16\_F12.raw |  |  |  | 1.7579E7 |  |  |  |  |  |  |  |  |  | 1 | 0 | 0 | 0 | 1 | 0 | 0 | 0 | 0 | 0 | 0 | 0 | 0 | 0 | 58 | 69 |  |  | PEAKS DB |
| N.VDFNSESTR.R | N | 52.01 | 1053.4727 | 9 | 0.6 | 527.7439 | 2 | 11.66 | 4 | F4:1931 | NaNaKA16\_F12.raw |  |  |  | 9.3775E6 |  |  |  |  |  |  |  |  |  | 1 | 0 | 0 | 0 | 1 | 0 | 0 | 0 | 0 | 0 | 0 | 0 | 0 | 0 | 20 | 28 |  |  | PEAKS DB |
| S.AC(+57.02)DNGLC(+57.02)TNPC(+57.02)TIYNK.L | Y | 51.74 | 1899.7914 | 16 | -0.3 | 950.9027 | 2 | 33.35 | 4 | F4:16304 | NaNaKA16\_F12.raw |  |  |  | 3.0888E6 |  |  |  |  |  |  |  |  |  | 1 | 0 | 0 | 0 | 1 | 0 | 0 | 0 | 0 | 0 | 0 | 0 | 0 | 0 | 191 | 206 | Carbamidomethylation | C2:Carbamidomethylation:1000.00;C7:Carbamidomethylation:1000.00;C11:Carbamidomethylation:1000.00 | PEAKS DB |
| C.SLNHSPDNLR.V | Y | 50.57 | 1151.5684 | 10 | -0.3 | 384.8633 | 3 | 11.45 | 4 | F4:1803 | NaNaKA16\_F12.raw |  |  |  | 3.3986E6 |  |  |  |  |  |  |  |  |  | 2 | 0 | 0 | 0 | 2 | 0 | 0 | 0 | 0 | 0 | 0 | 0 | 0 | 0 | 75 | 84 |  |  | PEAKS DB |
| R.TWTEIIHLWHDEYKN.F | Y | 49.28 | 1983.9479 | 15 | -0.2 | 496.9941 | 4 | 67.43 | 4 | F4:44938 | NaNaKA16\_F12.raw |  |  |  | 1.7614E7 |  |  |  |  |  |  |  |  |  | 2 | 0 | 0 | 0 | 2 | 0 | 0 | 0 | 0 | 0 | 0 | 0 | 0 | 0 | 103 | 117 |  |  | PEAKS DB |
| L.EGIQC(+57.02)GESIYMSSNAR.T | Y | 48.30 | 1800.7771 | 16 | 0.9 | 901.3966 | 2 | 35.87 | 4 | F4:18384 | NaNaKA16\_F12.raw |  |  |  | 0 |  |  |  |  |  |  |  |  |  | 0 | 0 | 0 | 0 | 0 | 0 | 0 | 0 | 0 | 0 | 0 | 0 | 0 | 0 | 87 | 102 | Carbamidomethylation | C5:Carbamidomethylation:1000.00 | PEAKS DB |
| R.VLEGIQC(+57.02)GESIYM(+15.99)SSN.A | Y | 48.10 | 1801.7863 | 16 | 0.0 | 901.9004 | 2 | 59.91 | 4 | F4:38428 | NaNaKA16\_F12.raw |  |  |  | 1.9592E7 |  |  |  |  |  |  |  |  |  | 1 | 0 | 0 | 0 | 1 | 0 | 0 | 0 | 0 | 0 | 0 | 0 | 0 | 0 | 85 | 100 | Carbamidomethylation; Oxidation (M) | C7:Carbamidomethylation:1000.00;M13:Oxidation (M):1000.00 | PEAKS DB |
| Y.KLGPPC(+57.02)GDC(+57.02)PSAC(+57.02)DNGLC(+57.02)TNPC(+57.02)TIYNK.L | Y | 48.02 | 3068.2917 | 27 | 1.2 | 1023.7724 | 3 | 40.46 | 4 | F4:22361 | NaNaKA16\_F12.raw |  |  |  | 7.6949E6 |  |  |  |  |  |  |  |  |  | 1 | 0 | 0 | 0 | 1 | 0 | 0 | 0 | 0 | 0 | 0 | 0 | 0 | 0 | 180 | 206 | Carbamidomethylation | C6:Carbamidomethylation:1000.00;C9:Carbamidomethylation:1000.00;C13:Carbamidomethylation:1000.00;C18:Carbamidomethylation:1000.00;C22:Carbamidomethylation:1000.00 | PEAKS DB |
| Q.IVWYQTYR.A | Y | 47.45 | 1127.5764 | 8 | -5.9 | 564.7922 | 2 | 43.62 | 4 | F4:24799 | NaNaKA16\_F12.raw |  |  |  | 3.1475E7 |  |  |  |  |  |  |  |  |  | 2 | 0 | 0 | 0 | 2 | 0 | 0 | 0 | 0 | 0 | 0 | 0 | 0 | 0 | 137 | 144 |  |  | PEAKS DB |
| R.TWTEIIHLWHD.E | Y | 47.44 | 1449.7041 | 11 | 0.4 | 484.2422 | 3 | 76.70 | 4 | F4:52262 | NaNaKA16\_F12.raw |  |  |  | 2.7409E6 |  |  |  |  |  |  |  |  |  | 1 | 0 | 0 | 0 | 1 | 0 | 0 | 0 | 0 | 0 | 0 | 0 | 0 | 0 | 103 | 113 |  |  | PEAKS DB |
| T.QIVWYQTYR.A | Y | 46.03 | 1255.6349 | 9 | 0.0 | 628.8247 | 2 | 47.28 | 4 | F4:27952 | NaNaKA16\_F12.raw |  |  |  | 5.8793E6 |  |  |  |  |  |  |  |  |  | 1 | 0 | 0 | 0 | 1 | 0 | 0 | 0 | 0 | 0 | 0 | 0 | 0 | 0 | 136 | 144 |  |  | PEAKS DB |
| E.IIHLWHDEYK.N | N | 45.95 | 1352.6877 | 10 | -0.5 | 451.9030 | 3 | 26.85 | 4 | F4:10987 | NaNaKA16\_F12.raw |  |  |  | 8.808E5 |  |  |  |  |  |  |  |  |  | 1 | 0 | 0 | 0 | 1 | 0 | 0 | 0 | 0 | 0 | 0 | 0 | 0 | 0 | 107 | 116 |  |  | PEAKS DB |
| T.EIIHLWHDEYK.N | Y | 44.66 | 1481.7302 | 11 | 0.1 | 494.9174 | 3 | 34.79 | 4 | F4:17343 | NaNaKA16\_F12.raw |  |  |  | 4.613E5 |  |  |  |  |  |  |  |  |  | 1 | 0 | 0 | 0 | 1 | 0 | 0 | 0 | 0 | 0 | 0 | 0 | 0 | 0 | 106 | 116 |  |  | PEAKS DB |
| R.VLEGIQC(+57.02)GESIY.M | Y | 44.56 | 1366.6438 | 12 | 0.4 | 684.3295 | 2 | 64.32 | 4 | F4:42048 | NaNaKA16\_F12.raw |  |  |  | 1.267E8 |  |  |  |  |  |  |  |  |  | 1 | 0 | 0 | 0 | 1 | 0 | 0 | 0 | 0 | 0 | 0 | 0 | 0 | 0 | 85 | 96 | Carbamidomethylation | C7:Carbamidomethylation:1000.00 | PEAKS DB |
| D.C(+57.02)PSAC(+57.02)DNGLC(+57.02)TNPC(+57.02)TIYNK.L | Y | 44.19 | 2243.9067 | 19 | 0.6 | 1122.9613 | 2 | 36.03 | 4 | F4:18539 | NaNaKA16\_F12.raw |  |  |  | 1.1206E6 |  |  |  |  |  |  |  |  |  | 1 | 0 | 0 | 0 | 1 | 0 | 0 | 0 | 0 | 0 | 0 | 0 | 0 | 0 | 188 | 206 | Carbamidomethylation | C1:Carbamidomethylation:1000.00;C5:Carbamidomethylation:1000.00;C10:Carbamidomethylation:1000.00;C14:Carbamidomethylation:1000.00 | PEAKS DB |
| L.RVLEGIQC(+57.02)GESIYM(+15.99)SSNAR.T | Y | 43.71 | 2185.0256 | 19 | 0.8 | 729.3497 | 3 | 36.11 | 4 | F4:18487 | NaNaKA16\_F12.raw |  |  |  | 6.1752E5 |  |  |  |  |  |  |  |  |  | 1 | 0 | 0 | 0 | 1 | 0 | 0 | 0 | 0 | 0 | 0 | 0 | 0 | 0 | 84 | 102 | Carbamidomethylation; Oxidation (M) | C8:Carbamidomethylation:1000.00;M14:Oxidation (M):1000.00 | PEAKS DB |
| R.RVSPTASNMLKMEWYPEAASNAER.W | Y | 43.34 | 2737.2952 | 24 | 1.4 | 685.3320 | 4 | 51.07 | 4 | F4:31143 | NaNaKA16\_F12.raw |  |  |  | 1.6942E6 |  |  |  |  |  |  |  |  |  | 1 | 0 | 0 | 0 | 1 | 0 | 0 | 0 | 0 | 0 | 0 | 0 | 0 | 0 | 46 | 69 |  |  | PEAKS DB |
| K.QKEIVDLHN.S | N | 43.18 | 1094.5720 | 9 | -0.5 | 548.2930 | 2 | 11.57 | 4 | F4:1902 | NaNaKA16\_F12.raw |  |  |  | 1.3713E5 |  |  |  |  |  |  |  |  |  | 1 | 0 | 0 | 0 | 1 | 0 | 0 | 0 | 0 | 0 | 0 | 0 | 0 | 0 | 33 | 41 |  |  | PEAKS DB |
| R.TWTEIIHLW.H | Y | 42.97 | 1197.6183 | 9 | 0.1 | 599.8165 | 2 | 92.08 | 4 | F4:64706 | NaNaKA16\_F12.raw |  |  |  | 2.1391E6 |  |  |  |  |  |  |  |  |  | 1 | 0 | 0 | 0 | 1 | 0 | 0 | 0 | 0 | 0 | 0 | 0 | 0 | 0 | 103 | 111 |  |  | PEAKS DB |
| N.MLKMEWYPEAASNAER.W | N | 42.97 | 1924.8811 | 16 | 0.5 | 642.6346 | 3 | 48.79 | 4 | F4:29214 | NaNaKA16\_F12.raw |  |  |  | 1.0448E6 |  |  |  |  |  |  |  |  |  | 1 | 0 | 0 | 0 | 1 | 0 | 0 | 0 | 0 | 0 | 0 | 0 | 0 | 0 | 54 | 69 |  |  | PEAKS DB |
| T.GHYTQIVWYQTYR.A | Y | 42.91 | 1713.8263 | 13 | -0.2 | 572.2826 | 3 | 43.93 | 4 | F4:25228 | NaNaKA16\_F12.raw |  |  |  | 2.5056E6 |  |  |  |  |  |  |  |  |  | 1 | 0 | 0 | 0 | 1 | 0 | 0 | 0 | 0 | 0 | 0 | 0 | 0 | 0 | 132 | 144 |  |  | PEAKS DB |
| R.VSPTASNMLKM(+15.99)EWYPEAASNAER.W | N | 41.81 | 2597.1890 | 23 | -0.1 | 866.7368 | 3 | 60.59 | 4 | F4:38922 | NaNaKA16\_F12.raw |  |  |  | 1.7447E6 |  |  |  |  |  |  |  |  |  | 1 | 0 | 0 | 0 | 1 | 0 | 0 | 0 | 0 | 0 | 0 | 0 | 0 | 0 | 47 | 69 | Oxidation (M) | M11:Oxidation (M):22.34 | PEAKS DB |
| total 57 peptides |  |  |  |  |  |  |  |  |  |  |  |  |  |  |  |  |  |  |  |  |  |  |  |  |  |  |  |  |  |  |  |  |  |  |  |  |  |  |  |  |  |  |  |
| --- | --- | --- | --- | --- | --- | --- | --- | --- | --- | --- | --- | --- | --- | --- | --- | --- | --- | --- | --- | --- | --- | --- | --- | --- | --- | --- | --- | --- | --- | --- | --- | --- | --- | --- | --- | --- | --- | --- | --- | --- | --- | --- | --- |

AVX27607.1

back to list

  

| Protein Coverage
| Supporting Peptides
|

Protein Coverage:

Supporting Peptides:

| Peptide | Uniq | -10lgP | Mass | Length | ppm | m/z | z | RT | Fraction | Scan | Source File | Area F1 | Area F10 | Area F11 | Area F12 | Area F13 | Area F2 | Area F3 | Area F4 | Area F5 | Area F6 | Area F7 | Area F8 | Area F9 | #Feature | #Feature F1 | #Feature F10 | #Feature F11 | #Feature F12 | #Feature F13 | #Feature F2 | #Feature F3 | #Feature F4 | #Feature F5 | #Feature F6 | #Feature F7 | #Feature F8 | #Feature F9 | Start | End | PTM | AScore | Found By |
| --- | --- | --- | --- | --- | --- | --- | --- | --- | --- | --- | --- | --- | --- | --- | --- | --- | --- | --- | --- | --- | --- | --- | --- | --- | --- | --- | --- | --- | --- | --- | --- | --- | --- | --- | --- | --- | --- | --- | --- | --- | --- | --- | --- |
| K.HVVVVGAGMAGLSAAYVLAGAGHK.V | N | 116.89 | 2234.1992 | 24 | 0.4 | 745.7407 | 3 | 62.90 | 13 | F13:39164 | NaNaKA16\_F9.raw |  | 1.1179E8 | 1.0168E7 | 7.8612E6 |  |  |  |  |  |  |  | 4.7423E6 | 2.5143E7 | 12 | 0 | 3 | 2 | 2 | 0 | 0 | 0 | 0 | 0 | 0 | 0 | 2 | 3 | 54 | 77 |  |  | PEAKS DB |
| K.TC(+57.02)ADIVINDLSLIHDLPK.R | N | 85.41 | 2036.0612 | 18 | 1.1 | 679.6951 | 3 | 82.21 | 3 | F3:55460 | NaNaKA16\_F11.raw |  | 6.8273E6 | 2.6259E6 | 1.2527E7 |  |  |  |  |  |  |  |  |  | 5 | 0 | 2 | 1 | 2 | 0 | 0 | 0 | 0 | 0 | 0 | 0 | 0 | 0 | 405 | 422 | Carbamidomethylation | C2:Carbamidomethylation:1000.00 | PEAKS DB |
| K.TFVTADYVIVC(+57.02)STSR.A | N | 84.19 | 1717.8345 | 15 | 0.1 | 859.9246 | 2 | 61.23 | 4 | F4:39837 | NaNaKA16\_F12.raw |  | 1.1036E6 | 1.0862E6 | 1.2599E7 | 8.2211E5 |  |  |  |  |  |  | 5.3578E5 |  | 6 | 0 | 1 | 1 | 2 | 1 | 0 | 0 | 0 | 0 | 0 | 0 | 1 | 0 | 300 | 314 | Carbamidomethylation | C11:Carbamidomethylation:1000.00 | PEAKS DB |
| R.FHGWIDSTIMTGLR.A | Y | 83.58 | 1632.8082 | 14 | 0.3 | 817.4116 | 2 | 67.68 | 4 | F4:45214 | NaNaKA16\_F12.raw |  | 3.2243E7 | 8.0897E5 | 6.2437E7 |  |  |  |  |  |  |  | 8.2725E6 | 2.2165E7 | 17 | 0 | 2 | 1 | 10 | 0 | 0 | 0 | 0 | 0 | 0 | 0 | 2 | 2 | 478 | 491 |  |  | PEAKS DB |
| H.VVVVGAGMAGLSAAYVLAGAGHK.V | N | 76.18 | 2097.1404 | 23 | 0.9 | 700.0547 | 3 | 74.20 | 2 | F2:49558 | NaNaKA16\_F10.raw |  | 1.7277E6 |  |  |  |  |  |  |  |  |  |  |  | 1 | 0 | 1 | 0 | 0 | 0 | 0 | 0 | 0 | 0 | 0 | 0 | 0 | 0 | 55 | 77 |  |  | PEAKS DB |
| F.TPYQFQDYFESAAAPVGR.I | Y | 75.29 | 2045.9482 | 18 | 0.0 | 1023.9814 | 2 | 72.61 | 4 | F4:48939 | NaNaKA16\_F12.raw |  |  |  | 1.9418E6 |  |  |  |  |  |  |  |  |  | 1 | 0 | 0 | 0 | 1 | 0 | 0 | 0 | 0 | 0 | 0 | 0 | 0 | 0 | 450 | 467 |  |  | PEAKS DB |
| R.IYFEPPLPPK.K | N | 75.25 | 1199.6589 | 10 | 0.1 | 600.8368 | 2 | 57.78 | 4 | F4:36626 | NaNaKA16\_F12.raw |  | 3.3521E6 | 4.7257E6 | 2.469E7 | 2.9029E7 |  |  | 9.7178E6 |  |  |  |  |  | 7 | 0 | 1 | 1 | 1 | 1 | 0 | 0 | 3 | 0 | 0 | 0 | 0 | 0 | 319 | 328 |  |  | PEAKS DB |
| Y.VLADDSDFFQALDTK.T | N | 75.15 | 1683.7991 | 15 | 0.9 | 842.9075 | 2 | 76.95 | 5 | F5:31672 | NaNaKA16\_F13.raw |  |  |  |  | 1.2528E7 |  |  |  |  |  |  |  |  | 1 | 0 | 0 | 0 | 0 | 1 | 0 | 0 | 0 | 0 | 0 | 0 | 0 | 0 | 390 | 404 |  |  | PEAKS DB |
| K.REIQALC(+57.02)YPSIK.K | N | 74.57 | 1476.7759 | 12 | -0.3 | 493.2658 | 3 | 32.85 | 4 | F4:15922 | NaNaKA16\_F12.raw |  |  |  | 1.3566E7 | 2.8894E6 |  |  |  |  |  |  |  | 2.7998E5 | 4 | 0 | 0 | 0 | 2 | 1 | 0 | 0 | 0 | 0 | 0 | 0 | 0 | 1 | 423 | 434 | Carbamidomethylation | C7:Carbamidomethylation:1000.00 | PEAKS DB |
| A.DIVINDLSLIHDLPK.R | N | 73.95 | 1703.9458 | 15 | 0.6 | 852.9807 | 2 | 80.37 | 4 | F4:55290 | NaNaKA16\_F12.raw |  | 2.9714E7 | 9.3836E6 | 3.0023E7 |  |  |  |  |  |  |  | 6.9266E5 |  | 7 | 0 | 2 | 2 | 2 | 0 | 0 | 0 | 0 | 0 | 0 | 0 | 1 | 0 | 408 | 422 |  |  | PEAKS DB |
| Y.QFQDYFESAAAPVGR.I | Y | 73.75 | 1684.7844 | 15 | 1.1 | 843.4004 | 2 | 59.42 | 4 | F4:38157 | NaNaKA16\_F12.raw |  |  |  | 1.1155E7 | 7.003E6 |  |  |  |  |  |  |  |  | 6 | 0 | 0 | 0 | 3 | 3 | 0 | 0 | 0 | 0 | 0 | 0 | 0 | 0 | 453 | 467 |  |  | PEAKS DB |
| R.EIQALC(+57.02)YPSIK.K | N | 73.42 | 1320.6747 | 11 | -0.2 | 661.3445 | 2 | 47.89 | 3 | F3:27602 | NaNaKA16\_F11.raw |  | 0 | 1.5106E7 | 3.3878E7 | 2.1775E7 |  |  |  |  |  |  |  | 3.2256E6 | 4 | 0 | 0 | 1 | 1 | 1 | 0 | 0 | 0 | 0 | 0 | 0 | 0 | 1 | 424 | 434 | Carbamidomethylation | C6:Carbamidomethylation:1000.00 | PEAKS DB |
| K.HVVVVGAGM(+15.99)AGLSAAYVLAGAGHK.V | N | 72.68 | 2250.1943 | 24 | 0.0 | 563.5558 | 4 | 52.57 | 2 | F2:30632 | NaNaKA16\_F10.raw |  | 4.3956E6 |  | 2.4715E5 |  |  |  |  |  |  |  |  | 2.5443E6 | 4 | 0 | 2 | 0 | 1 | 0 | 0 | 0 | 0 | 0 | 0 | 0 | 0 | 1 | 54 | 77 | Oxidation (M) | M9:Oxidation (M):1000.00 | PEAKS DB |
| A.DDSDFFQALDTK.T | N | 70.63 | 1400.6095 | 12 | 0.5 | 701.3124 | 2 | 73.59 | 4 | F4:49585 | NaNaKA16\_F12.raw |  |  |  | 6.7842E6 |  |  |  |  |  | 7.1261E5 |  |  |  | 2 | 0 | 0 | 0 | 1 | 0 | 0 | 0 | 0 | 0 | 1 | 0 | 0 | 0 | 393 | 404 |  |  | PEAKS DB |
| K.SASQLYQESLR.K | Y | 67.47 | 1280.6360 | 11 | -2.1 | 641.3239 | 2 | 26.70 | 6 | F6:12667 | NaNaKA16\_F2.raw |  |  |  |  | 4.263E7 | 2.3745E7 |  |  | 3.3503E6 |  |  |  |  | 3 | 0 | 0 | 0 | 0 | 1 | 1 | 0 | 0 | 1 | 0 | 0 | 0 | 0 | 171 | 181 |  |  | PEAKS DB |
| Q.DYFESAAAPVGR.I | Y | 66.83 | 1281.5989 | 12 | 0.8 | 641.8073 | 2 | 43.00 | 6 | F6:26794 | NaNaKA16\_F2.raw |  | 1.3876E6 | 5.1422E5 |  |  | 1.2058E7 |  |  |  |  |  |  |  | 3 | 0 | 1 | 1 | 0 | 0 | 1 | 0 | 0 | 0 | 0 | 0 | 0 | 0 | 456 | 467 |  |  | PEAKS DB |
| R.RIYFEPPLPPK.K | N | 66.53 | 1355.7601 | 11 | 0.2 | 452.9274 | 3 | 43.83 | 4 | F4:25641 | NaNaKA16\_F12.raw |  | 5.7564E5 | 1.1183E6 | 4.1789E6 | 3.0828E6 |  |  |  |  |  |  |  |  | 6 | 0 | 3 | 1 | 1 | 1 | 0 | 0 | 0 | 0 | 0 | 0 | 0 | 0 | 318 | 328 |  |  | PEAKS DB |
| K.KFWEADGIHGGK.S | N | 66.46 | 1343.6622 | 12 | -0.8 | 448.8943 | 3 | 17.58 | 5 | F5:3842 | NaNaKA16\_F13.raw |  |  |  |  | 2.6107E6 |  |  |  |  |  |  |  |  | 2 | 0 | 0 | 0 | 0 | 2 | 0 | 0 | 0 | 0 | 0 | 0 | 0 | 0 | 351 | 362 |  |  | PEAKS DB |
| R.EGWYVNMGPMR.L | N | 63.13 | 1338.5850 | 11 | 0.2 | 670.2999 | 2 | 58.22 | 9 | F9:38419 | NaNaKA16\_F5.raw |  | 2.3017E6 | 3.4741E6 | 8.3473E6 | 1.0741E7 |  |  |  | 1.8901E6 | 4.3579E5 |  | 2.2555E6 |  | 7 | 0 | 1 | 1 | 1 | 1 | 0 | 0 | 0 | 1 | 1 | 0 | 1 | 0 | 99 | 109 |  |  | PEAKS DB |
| N.DLSLIHDLPK.R | N | 62.72 | 1149.6393 | 10 | 1.4 | 575.8277 | 2 | 52.52 | 3 | F3:29824 | NaNaKA16\_F11.raw |  | 1.6899E6 | 7.7612E6 | 4.7179E6 |  |  |  |  |  |  |  |  |  | 4 | 0 | 1 | 1 | 2 | 0 | 0 | 0 | 0 | 0 | 0 | 0 | 0 | 0 | 413 | 422 |  |  | PEAKS DB |
| R.FHGWIDSTIM(+15.99)TGLR.A | Y | 60.71 | 1648.8031 | 14 | -0.3 | 550.6082 | 3 | 55.70 | 4 | F4:35031 | NaNaKA16\_F12.raw |  | 5.7355E5 |  | 2.9168E6 |  |  |  |  |  |  |  |  |  | 2 | 0 | 1 | 0 | 1 | 0 | 0 | 0 | 0 | 0 | 0 | 0 | 0 | 0 | 478 | 491 | Oxidation (M) | M10:Oxidation (M):1000.00 | PEAKS DB |
| K.HVVVVGAGMAGLSAAY.V | N | 59.94 | 1500.7759 | 16 | 0.4 | 751.3955 | 2 | 61.71 | 5 | F5:25523 | NaNaKA16\_F13.raw |  |  |  |  | 6.9379E5 |  |  |  |  |  |  |  |  | 1 | 0 | 0 | 0 | 0 | 1 | 0 | 0 | 0 | 0 | 0 | 0 | 0 | 0 | 54 | 69 |  |  | PEAKS DB |
| R.IHFAGEYTGR.F | Y | 59.47 | 1149.5566 | 10 | 1.4 | 384.1934 | 3 | 17.69 | 5 | F5:3877 | NaNaKA16\_F13.raw |  |  |  | 0 | 1.8101E7 |  |  |  |  |  |  |  |  | 2 | 0 | 0 | 0 | 0 | 2 | 0 | 0 | 0 | 0 | 0 | 0 | 0 | 0 | 468 | 477 |  |  | PEAKS DB |
| K.FWEADGIHGGK.S | N | 57.27 | 1215.5673 | 11 | -0.2 | 608.7908 | 2 | 22.40 | 5 | F5:5921 | NaNaKA16\_F13.raw |  |  |  |  | 1.8542E7 |  |  |  |  |  |  |  |  | 2 | 0 | 0 | 0 | 0 | 2 | 0 | 0 | 0 | 0 | 0 | 0 | 0 | 0 | 352 | 362 |  |  | PEAKS DB |
| G.SITSFTPYQFQDYFESAAAPVGR.I | Y | 55.78 | 2581.2124 | 23 | 0.6 | 861.4119 | 3 | 92.74 | 4 | F4:65226 | NaNaKA16\_F12.raw |  |  |  | 6.4315E5 |  |  |  |  |  |  |  |  |  | 1 | 0 | 0 | 0 | 1 | 0 | 0 | 0 | 0 | 0 | 0 | 0 | 0 | 0 | 445 | 467 |  |  | PEAKS DB |
| K.SDALFSYEK.R | N | 53.51 | 1058.4919 | 9 | 1.2 | 530.2539 | 2 | 36.12 | 5 | F5:13813 | NaNaKA16\_F13.raw |  |  |  | 1.7934E6 | 2.9321E7 |  |  |  |  |  |  |  |  | 2 | 0 | 0 | 0 | 1 | 1 | 0 | 0 | 0 | 0 | 0 | 0 | 0 | 0 | 242 | 250 |  |  | PEAKS DB |
| R.EGWYVNM(+15.99)GPMR.L | N | 53.18 | 1354.5798 | 11 | 0.0 | 678.2972 | 2 | 41.87 | 4 | F4:23544 | NaNaKA16\_F12.raw |  |  |  | 1.2884E6 | 1.7705E6 |  |  |  |  |  |  |  |  | 2 | 0 | 0 | 0 | 1 | 1 | 0 | 0 | 0 | 0 | 0 | 0 | 0 | 0 | 99 | 109 | Oxidation (M) | M7:Oxidation (M):38.16 | PEAKS DB |
| R.IYFEPPLPPKK.A | N | 51.41 | 1327.7539 | 11 | 0.3 | 664.8844 | 2 | 40.66 | 5 | F5:16431 | NaNaKA16\_F13.raw |  |  |  | 7.8644E5 | 7.4644E5 |  |  |  |  |  |  |  |  | 2 | 0 | 0 | 0 | 1 | 1 | 0 | 0 | 0 | 0 | 0 | 0 | 0 | 0 | 319 | 329 |  |  | PEAKS DB |
| L.DKYTMGSITSFTPYQFQDYFESAAAPVGR.I | Y | 50.86 | 3276.5073 | 29 | 1.2 | 1093.1777 | 3 | 94.70 | 4 | F4:66626 | NaNaKA16\_F12.raw |  |  |  | 3.1812E6 |  |  |  |  |  |  |  |  |  | 1 | 0 | 0 | 0 | 1 | 0 | 0 | 0 | 0 | 0 | 0 | 0 | 0 | 0 | 439 | 467 |  |  | PEAKS DB |
| I.YFEPPLPPK.K | N | 50.02 | 1086.5750 | 9 | -0.2 | 544.2946 | 2 | 44.20 | 6 | F6:28085 | NaNaKA16\_F2.raw |  |  |  |  |  | 8.7131E6 |  |  |  |  |  |  |  | 1 | 0 | 0 | 0 | 0 | 0 | 1 | 0 | 0 | 0 | 0 | 0 | 0 | 0 | 320 | 328 |  |  | PEAKS DB |
| A.DIVINDLSLIHDLPKR.E | N | 48.00 | 1860.0469 | 16 | 0.2 | 621.0230 | 3 | 65.80 | 2 | F2:42289 | NaNaKA16\_F10.raw |  | 7.1576E5 |  |  |  |  |  |  |  |  |  |  |  | 1 | 0 | 1 | 0 | 0 | 0 | 0 | 0 | 0 | 0 | 0 | 0 | 0 | 0 | 408 | 423 |  |  | PEAKS DB |
| R.EGWYVNMGPM(+15.99)R.L | N | 47.08 | 1354.5798 | 11 | 0.3 | 678.2974 | 2 | 42.61 | 5 | F5:17152 | NaNaKA16\_F13.raw |  |  |  |  | 1.7705E6 |  |  |  |  |  |  |  |  | 1 | 0 | 0 | 0 | 0 | 1 | 0 | 0 | 0 | 0 | 0 | 0 | 0 | 0 | 99 | 109 | Oxidation (M) | M10:Oxidation (M):20.70 | PEAKS DB |
| K.SASQLYQESLRK.V | Y | 45.54 | 1408.7310 | 12 | 0.0 | 470.5843 | 3 | 12.22 | 6 | F6:2235 | NaNaKA16\_F2.raw |  |  |  |  |  | 1.4677E6 |  |  |  |  |  |  |  | 1 | 0 | 0 | 0 | 0 | 0 | 1 | 0 | 0 | 0 | 0 | 0 | 0 | 0 | 171 | 182 |  |  | PEAKS DB |
| S.FTPYQFQDYFESAAAPVGR.I | Y | 45.47 | 2193.0166 | 19 | 0.0 | 1097.5156 | 2 | 83.82 | 12 | F12:59508 | NaNaKA16\_F8.raw |  |  |  |  |  |  |  |  |  |  |  | 1.6948E6 |  | 1 | 0 | 0 | 0 | 0 | 0 | 0 | 0 | 0 | 0 | 0 | 0 | 1 | 0 | 449 | 467 |  |  | PEAKS DB |
| K.VTLLEASER.V | N | 44.72 | 1016.5502 | 9 | 0.2 | 509.2825 | 2 | 26.63 | 5 | F5:8576 | NaNaKA16\_F13.raw |  |  |  |  | 4.1415E7 |  |  |  |  |  |  |  |  | 1 | 0 | 0 | 0 | 0 | 1 | 0 | 0 | 0 | 0 | 0 | 0 | 0 | 0 | 78 | 86 |  |  | PEAKS DB |
| W.IDSTIMTGLR.A | Y | 43.99 | 1105.5801 | 10 | 6.1 | 553.7977 | 2 | 42.74 | 8 | F8:23703 | NaNaKA16\_F4.raw |  |  |  |  |  |  |  | 8.2584E5 |  |  |  |  |  | 1 | 0 | 0 | 0 | 0 | 0 | 0 | 0 | 1 | 0 | 0 | 0 | 0 | 0 | 482 | 491 |  |  | PEAKS DB |
| K.YTMGSITSFTPYQFQDYFESAAAPVGR.I | Y | 43.77 | 3033.3855 | 27 | 0.6 | 1012.1364 | 3 | 100.71 | 4 | F4:70262 | NaNaKA16\_F12.raw |  |  |  | 7.0268E5 |  |  |  |  |  |  |  |  |  | 1 | 0 | 0 | 0 | 1 | 0 | 0 | 0 | 0 | 0 | 0 | 0 | 0 | 0 | 441 | 467 |  |  | PEAKS DB |
| D.IVINDLSLIHDLPK.R | N | 42.85 | 1588.9188 | 14 | -0.1 | 530.6469 | 3 | 68.04 | 4 | F4:45149 | NaNaKA16\_F12.raw |  |  |  | 0 |  |  |  |  |  |  |  |  |  | 0 | 0 | 0 | 0 | 0 | 0 | 0 | 0 | 0 | 0 | 0 | 0 | 0 | 0 | 409 | 422 |  |  | PEAKS DB |
| total 38 peptides |  |  |  |  |  |  |  |  |  |  |  |  |  |  |  |  |  |  |  |  |  |  |  |  |  |  |  |  |  |  |  |  |  |  |  |  |  |  |  |  |  |  |  |
| --- | --- | --- | --- | --- | --- | --- | --- | --- | --- | --- | --- | --- | --- | --- | --- | --- | --- | --- | --- | --- | --- | --- | --- | --- | --- | --- | --- | --- | --- | --- | --- | --- | --- | --- | --- | --- | --- | --- | --- | --- | --- | --- | --- |

5Z2G

back to list

  

| Protein Coverage
| Supporting Peptides
|

Protein Coverage:

Supporting Peptides:

| Peptide | Uniq | -10lgP | Mass | Length | ppm | m/z | z | RT | Fraction | Scan | Source File | Area F1 | Area F10 | Area F11 | Area F12 | Area F13 | Area F2 | Area F3 | Area F4 | Area F5 | Area F6 | Area F7 | Area F8 | Area F9 | #Feature | #Feature F1 | #Feature F10 | #Feature F11 | #Feature F12 | #Feature F13 | #Feature F2 | #Feature F3 | #Feature F4 | #Feature F5 | #Feature F6 | #Feature F7 | #Feature F8 | #Feature F9 | Start | End | PTM | AScore | Found By |
| --- | --- | --- | --- | --- | --- | --- | --- | --- | --- | --- | --- | --- | --- | --- | --- | --- | --- | --- | --- | --- | --- | --- | --- | --- | --- | --- | --- | --- | --- | --- | --- | --- | --- | --- | --- | --- | --- | --- | --- | --- | --- | --- | --- |
| K.HVVVVGAGMAGLSAAYVLAGAGHK.V | N | 116.89 | 2234.1992 | 24 | 0.4 | 745.7407 | 3 | 62.90 | 13 | F13:39164 | NaNaKA16\_F9.raw |  | 1.1179E8 | 1.0168E7 | 7.8612E6 |  |  |  |  |  |  |  | 4.7423E6 | 2.5143E7 | 12 | 0 | 3 | 2 | 2 | 0 | 0 | 0 | 0 | 0 | 0 | 0 | 2 | 3 | 54 | 77 |  |  | PEAKS DB |
| K.TC(+57.02)ADIVINDLSLIHDLPK.R | N | 85.41 | 2036.0612 | 18 | 1.1 | 679.6951 | 3 | 82.21 | 3 | F3:55460 | NaNaKA16\_F11.raw |  | 6.8273E6 | 2.6259E6 | 1.2527E7 |  |  |  |  |  |  |  |  |  | 5 | 0 | 2 | 1 | 2 | 0 | 0 | 0 | 0 | 0 | 0 | 0 | 0 | 0 | 405 | 422 | Carbamidomethylation | C2:Carbamidomethylation:1000.00 | PEAKS DB |
| K.TFVTADYVIVC(+57.02)STSR.A | N | 84.19 | 1717.8345 | 15 | 0.1 | 859.9246 | 2 | 61.23 | 4 | F4:39837 | NaNaKA16\_F12.raw |  | 1.1036E6 | 1.0862E6 | 1.2599E7 | 8.2211E5 |  |  |  |  |  |  | 5.3578E5 |  | 6 | 0 | 1 | 1 | 2 | 1 | 0 | 0 | 0 | 0 | 0 | 0 | 1 | 0 | 300 | 314 | Carbamidomethylation | C11:Carbamidomethylation:1000.00 | PEAKS DB |
| R.FHGWIDSTIMTGLR.A | Y | 83.58 | 1632.8082 | 14 | 0.3 | 817.4116 | 2 | 67.68 | 4 | F4:45214 | NaNaKA16\_F12.raw |  | 3.2243E7 | 8.0897E5 | 6.2437E7 |  |  |  |  |  |  |  | 8.2725E6 | 2.2165E7 | 17 | 0 | 2 | 1 | 10 | 0 | 0 | 0 | 0 | 0 | 0 | 0 | 2 | 2 | 478 | 491 |  |  | PEAKS DB |
| H.VVVVGAGMAGLSAAYVLAGAGHK.V | N | 76.18 | 2097.1404 | 23 | 0.9 | 700.0547 | 3 | 74.20 | 2 | F2:49558 | NaNaKA16\_F10.raw |  | 1.7277E6 |  |  |  |  |  |  |  |  |  |  |  | 1 | 0 | 1 | 0 | 0 | 0 | 0 | 0 | 0 | 0 | 0 | 0 | 0 | 0 | 55 | 77 |  |  | PEAKS DB |
| F.TPYQFQDYFESAAAPVGR.I | Y | 75.29 | 2045.9482 | 18 | 0.0 | 1023.9814 | 2 | 72.61 | 4 | F4:48939 | NaNaKA16\_F12.raw |  |  |  | 1.9418E6 |  |  |  |  |  |  |  |  |  | 1 | 0 | 0 | 0 | 1 | 0 | 0 | 0 | 0 | 0 | 0 | 0 | 0 | 0 | 450 | 467 |  |  | PEAKS DB |
| R.IYFEPPLPPK.K | N | 75.25 | 1199.6589 | 10 | 0.1 | 600.8368 | 2 | 57.78 | 4 | F4:36626 | NaNaKA16\_F12.raw |  | 3.3521E6 | 4.7257E6 | 2.469E7 | 2.9029E7 |  |  | 9.7178E6 |  |  |  |  |  | 7 | 0 | 1 | 1 | 1 | 1 | 0 | 0 | 3 | 0 | 0 | 0 | 0 | 0 | 319 | 328 |  |  | PEAKS DB |
| Y.VLADDSDFFQALDTK.T | N | 75.15 | 1683.7991 | 15 | 0.9 | 842.9075 | 2 | 76.95 | 5 | F5:31672 | NaNaKA16\_F13.raw |  |  |  |  | 1.2528E7 |  |  |  |  |  |  |  |  | 1 | 0 | 0 | 0 | 0 | 1 | 0 | 0 | 0 | 0 | 0 | 0 | 0 | 0 | 390 | 404 |  |  | PEAKS DB |
| K.REIQALC(+57.02)YPSIK.K | N | 74.57 | 1476.7759 | 12 | -0.3 | 493.2658 | 3 | 32.85 | 4 | F4:15922 | NaNaKA16\_F12.raw |  |  |  | 1.3566E7 | 2.8894E6 |  |  |  |  |  |  |  | 2.7998E5 | 4 | 0 | 0 | 0 | 2 | 1 | 0 | 0 | 0 | 0 | 0 | 0 | 0 | 1 | 423 | 434 | Carbamidomethylation | C7:Carbamidomethylation:1000.00 | PEAKS DB |
| A.DIVINDLSLIHDLPK.R | N | 73.95 | 1703.9458 | 15 | 0.6 | 852.9807 | 2 | 80.37 | 4 | F4:55290 | NaNaKA16\_F12.raw |  | 2.9714E7 | 9.3836E6 | 3.0023E7 |  |  |  |  |  |  |  | 6.9266E5 |  | 7 | 0 | 2 | 2 | 2 | 0 | 0 | 0 | 0 | 0 | 0 | 0 | 1 | 0 | 408 | 422 |  |  | PEAKS DB |
| Y.QFQDYFESAAAPVGR.I | Y | 73.75 | 1684.7844 | 15 | 1.1 | 843.4004 | 2 | 59.42 | 4 | F4:38157 | NaNaKA16\_F12.raw |  |  |  | 1.1155E7 | 7.003E6 |  |  |  |  |  |  |  |  | 6 | 0 | 0 | 0 | 3 | 3 | 0 | 0 | 0 | 0 | 0 | 0 | 0 | 0 | 453 | 467 |  |  | PEAKS DB |
| R.EIQALC(+57.02)YPSIK.K | N | 73.42 | 1320.6747 | 11 | -0.2 | 661.3445 | 2 | 47.89 | 3 | F3:27602 | NaNaKA16\_F11.raw |  | 0 | 1.5106E7 | 3.3878E7 | 2.1775E7 |  |  |  |  |  |  |  | 3.2256E6 | 4 | 0 | 0 | 1 | 1 | 1 | 0 | 0 | 0 | 0 | 0 | 0 | 0 | 1 | 424 | 434 | Carbamidomethylation | C6:Carbamidomethylation:1000.00 | PEAKS DB |
| K.HVVVVGAGM(+15.99)AGLSAAYVLAGAGHK.V | N | 72.68 | 2250.1943 | 24 | 0.0 | 563.5558 | 4 | 52.57 | 2 | F2:30632 | NaNaKA16\_F10.raw |  | 4.3956E6 |  | 2.4715E5 |  |  |  |  |  |  |  |  | 2.5443E6 | 4 | 0 | 2 | 0 | 1 | 0 | 0 | 0 | 0 | 0 | 0 | 0 | 0 | 1 | 54 | 77 | Oxidation (M) | M9:Oxidation (M):1000.00 | PEAKS DB |
| A.DDSDFFQALDTK.T | N | 70.63 | 1400.6095 | 12 | 0.5 | 701.3124 | 2 | 73.59 | 4 | F4:49585 | NaNaKA16\_F12.raw |  |  |  | 6.7842E6 |  |  |  |  |  | 7.1261E5 |  |  |  | 2 | 0 | 0 | 0 | 1 | 0 | 0 | 0 | 0 | 0 | 1 | 0 | 0 | 0 | 393 | 404 |  |  | PEAKS DB |
| K.SASQLYQESLR.K | Y | 67.47 | 1280.6360 | 11 | -2.1 | 641.3239 | 2 | 26.70 | 6 | F6:12667 | NaNaKA16\_F2.raw |  |  |  |  | 4.263E7 | 2.3745E7 |  |  | 3.3503E6 |  |  |  |  | 3 | 0 | 0 | 0 | 0 | 1 | 1 | 0 | 0 | 1 | 0 | 0 | 0 | 0 | 171 | 181 |  |  | PEAKS DB |
| Q.DYFESAAAPVGR.I | Y | 66.83 | 1281.5989 | 12 | 0.8 | 641.8073 | 2 | 43.00 | 6 | F6:26794 | NaNaKA16\_F2.raw |  | 1.3876E6 | 5.1422E5 |  |  | 1.2058E7 |  |  |  |  |  |  |  | 3 | 0 | 1 | 1 | 0 | 0 | 1 | 0 | 0 | 0 | 0 | 0 | 0 | 0 | 456 | 467 |  |  | PEAKS DB |
| R.RIYFEPPLPPK.K | N | 66.53 | 1355.7601 | 11 | 0.2 | 452.9274 | 3 | 43.83 | 4 | F4:25641 | NaNaKA16\_F12.raw |  | 5.7564E5 | 1.1183E6 | 4.1789E6 | 3.0828E6 |  |  |  |  |  |  |  |  | 6 | 0 | 3 | 1 | 1 | 1 | 0 | 0 | 0 | 0 | 0 | 0 | 0 | 0 | 318 | 328 |  |  | PEAKS DB |
| K.KFWEADGIHGGK.S | N | 66.46 | 1343.6622 | 12 | -0.8 | 448.8943 | 3 | 17.58 | 5 | F5:3842 | NaNaKA16\_F13.raw |  |  |  |  | 2.6107E6 |  |  |  |  |  |  |  |  | 2 | 0 | 0 | 0 | 0 | 2 | 0 | 0 | 0 | 0 | 0 | 0 | 0 | 0 | 351 | 362 |  |  | PEAKS DB |
| R.EGWYVNMGPMR.L | N | 63.13 | 1338.5850 | 11 | 0.2 | 670.2999 | 2 | 58.22 | 9 | F9:38419 | NaNaKA16\_F5.raw |  | 2.3017E6 | 3.4741E6 | 8.3473E6 | 1.0741E7 |  |  |  | 1.8901E6 | 4.3579E5 |  | 2.2555E6 |  | 7 | 0 | 1 | 1 | 1 | 1 | 0 | 0 | 0 | 1 | 1 | 0 | 1 | 0 | 99 | 109 |  |  | PEAKS DB |
| N.DLSLIHDLPK.R | N | 62.72 | 1149.6393 | 10 | 1.4 | 575.8277 | 2 | 52.52 | 3 | F3:29824 | NaNaKA16\_F11.raw |  | 1.6899E6 | 7.7612E6 | 4.7179E6 |  |  |  |  |  |  |  |  |  | 4 | 0 | 1 | 1 | 2 | 0 | 0 | 0 | 0 | 0 | 0 | 0 | 0 | 0 | 413 | 422 |  |  | PEAKS DB |
| R.FHGWIDSTIM(+15.99)TGLR.A | Y | 60.71 | 1648.8031 | 14 | -0.3 | 550.6082 | 3 | 55.70 | 4 | F4:35031 | NaNaKA16\_F12.raw |  | 5.7355E5 |  | 2.9168E6 |  |  |  |  |  |  |  |  |  | 2 | 0 | 1 | 0 | 1 | 0 | 0 | 0 | 0 | 0 | 0 | 0 | 0 | 0 | 478 | 491 | Oxidation (M) | M10:Oxidation (M):1000.00 | PEAKS DB |
| K.HVVVVGAGMAGLSAAY.V | N | 59.94 | 1500.7759 | 16 | 0.4 | 751.3955 | 2 | 61.71 | 5 | F5:25523 | NaNaKA16\_F13.raw |  |  |  |  | 6.9379E5 |  |  |  |  |  |  |  |  | 1 | 0 | 0 | 0 | 0 | 1 | 0 | 0 | 0 | 0 | 0 | 0 | 0 | 0 | 54 | 69 |  |  | PEAKS DB |
| R.IHFAGEYTGR.F | Y | 59.47 | 1149.5566 | 10 | 1.4 | 384.1934 | 3 | 17.69 | 5 | F5:3877 | NaNaKA16\_F13.raw |  |  |  | 0 | 1.8101E7 |  |  |  |  |  |  |  |  | 2 | 0 | 0 | 0 | 0 | 2 | 0 | 0 | 0 | 0 | 0 | 0 | 0 | 0 | 468 | 477 |  |  | PEAKS DB |
| K.FWEADGIHGGK.S | N | 57.27 | 1215.5673 | 11 | -0.2 | 608.7908 | 2 | 22.40 | 5 | F5:5921 | NaNaKA16\_F13.raw |  |  |  |  | 1.8542E7 |  |  |  |  |  |  |  |  | 2 | 0 | 0 | 0 | 0 | 2 | 0 | 0 | 0 | 0 | 0 | 0 | 0 | 0 | 352 | 362 |  |  | PEAKS DB |
| G.SITSFTPYQFQDYFESAAAPVGR.I | Y | 55.78 | 2581.2124 | 23 | 0.6 | 861.4119 | 3 | 92.74 | 4 | F4:65226 | NaNaKA16\_F12.raw |  |  |  | 6.4315E5 |  |  |  |  |  |  |  |  |  | 1 | 0 | 0 | 0 | 1 | 0 | 0 | 0 | 0 | 0 | 0 | 0 | 0 | 0 | 445 | 467 |  |  | PEAKS DB |
| K.SDALFSYEK.R | N | 53.51 | 1058.4919 | 9 | 1.2 | 530.2539 | 2 | 36.12 | 5 | F5:13813 | NaNaKA16\_F13.raw |  |  |  | 1.7934E6 | 2.9321E7 |  |  |  |  |  |  |  |  | 2 | 0 | 0 | 0 | 1 | 1 | 0 | 0 | 0 | 0 | 0 | 0 | 0 | 0 | 242 | 250 |  |  | PEAKS DB |
| R.EGWYVNM(+15.99)GPMR.L | N | 53.18 | 1354.5798 | 11 | 0.0 | 678.2972 | 2 | 41.87 | 4 | F4:23544 | NaNaKA16\_F12.raw |  |  |  | 1.2884E6 | 1.7705E6 |  |  |  |  |  |  |  |  | 2 | 0 | 0 | 0 | 1 | 1 | 0 | 0 | 0 | 0 | 0 | 0 | 0 | 0 | 99 | 109 | Oxidation (M) | M7:Oxidation (M):38.16 | PEAKS DB |
| R.IYFEPPLPPKK.A | N | 51.41 | 1327.7539 | 11 | 0.3 | 664.8844 | 2 | 40.66 | 5 | F5:16431 | NaNaKA16\_F13.raw |  |  |  | 7.8644E5 | 7.4644E5 |  |  |  |  |  |  |  |  | 2 | 0 | 0 | 0 | 1 | 1 | 0 | 0 | 0 | 0 | 0 | 0 | 0 | 0 | 319 | 329 |  |  | PEAKS DB |
| L.DKYTMGSITSFTPYQFQDYFESAAAPVGR.I | Y | 50.86 | 3276.5073 | 29 | 1.2 | 1093.1777 | 3 | 94.70 | 4 | F4:66626 | NaNaKA16\_F12.raw |  |  |  | 3.1812E6 |  |  |  |  |  |  |  |  |  | 1 | 0 | 0 | 0 | 1 | 0 | 0 | 0 | 0 | 0 | 0 | 0 | 0 | 0 | 439 | 467 |  |  | PEAKS DB |
| I.YFEPPLPPK.K | N | 50.02 | 1086.5750 | 9 | -0.2 | 544.2946 | 2 | 44.20 | 6 | F6:28085 | NaNaKA16\_F2.raw |  |  |  |  |  | 8.7131E6 |  |  |  |  |  |  |  | 1 | 0 | 0 | 0 | 0 | 0 | 1 | 0 | 0 | 0 | 0 | 0 | 0 | 0 | 320 | 328 |  |  | PEAKS DB |
| A.DIVINDLSLIHDLPKR.E | N | 48.00 | 1860.0469 | 16 | 0.2 | 621.0230 | 3 | 65.80 | 2 | F2:42289 | NaNaKA16\_F10.raw |  | 7.1576E5 |  |  |  |  |  |  |  |  |  |  |  | 1 | 0 | 1 | 0 | 0 | 0 | 0 | 0 | 0 | 0 | 0 | 0 | 0 | 0 | 408 | 423 |  |  | PEAKS DB |
| R.EGWYVNMGPM(+15.99)R.L | N | 47.08 | 1354.5798 | 11 | 0.3 | 678.2974 | 2 | 42.61 | 5 | F5:17152 | NaNaKA16\_F13.raw |  |  |  |  | 1.7705E6 |  |  |  |  |  |  |  |  | 1 | 0 | 0 | 0 | 0 | 1 | 0 | 0 | 0 | 0 | 0 | 0 | 0 | 0 | 99 | 109 | Oxidation (M) | M10:Oxidation (M):20.70 | PEAKS DB |
| K.SASQLYQESLRK.V | Y | 45.54 | 1408.7310 | 12 | 0.0 | 470.5843 | 3 | 12.22 | 6 | F6:2235 | NaNaKA16\_F2.raw |  |  |  |  |  | 1.4677E6 |  |  |  |  |  |  |  | 1 | 0 | 0 | 0 | 0 | 0 | 1 | 0 | 0 | 0 | 0 | 0 | 0 | 0 | 171 | 182 |  |  | PEAKS DB |
| S.FTPYQFQDYFESAAAPVGR.I | Y | 45.47 | 2193.0166 | 19 | 0.0 | 1097.5156 | 2 | 83.82 | 12 | F12:59508 | NaNaKA16\_F8.raw |  |  |  |  |  |  |  |  |  |  |  | 1.6948E6 |  | 1 | 0 | 0 | 0 | 0 | 0 | 0 | 0 | 0 | 0 | 0 | 0 | 1 | 0 | 449 | 467 |  |  | PEAKS DB |
| K.VTLLEASER.V | N | 44.72 | 1016.5502 | 9 | 0.2 | 509.2825 | 2 | 26.63 | 5 | F5:8576 | NaNaKA16\_F13.raw |  |  |  |  | 4.1415E7 |  |  |  |  |  |  |  |  | 1 | 0 | 0 | 0 | 0 | 1 | 0 | 0 | 0 | 0 | 0 | 0 | 0 | 0 | 78 | 86 |  |  | PEAKS DB |
| W.IDSTIMTGLR.A | Y | 43.99 | 1105.5801 | 10 | 6.1 | 553.7977 | 2 | 42.74 | 8 | F8:23703 | NaNaKA16\_F4.raw |  |  |  |  |  |  |  | 8.2584E5 |  |  |  |  |  | 1 | 0 | 0 | 0 | 0 | 0 | 0 | 0 | 1 | 0 | 0 | 0 | 0 | 0 | 482 | 491 |  |  | PEAKS DB |
| K.YTMGSITSFTPYQFQDYFESAAAPVGR.I | Y | 43.77 | 3033.3855 | 27 | 0.6 | 1012.1364 | 3 | 100.71 | 4 | F4:70262 | NaNaKA16\_F12.raw |  |  |  | 7.0268E5 |  |  |  |  |  |  |  |  |  | 1 | 0 | 0 | 0 | 1 | 0 | 0 | 0 | 0 | 0 | 0 | 0 | 0 | 0 | 441 | 467 |  |  | PEAKS DB |
| D.IVINDLSLIHDLPK.R | N | 42.85 | 1588.9188 | 14 | -0.1 | 530.6469 | 3 | 68.04 | 4 | F4:45149 | NaNaKA16\_F12.raw |  |  |  | 0 |  |  |  |  |  |  |  |  |  | 0 | 0 | 0 | 0 | 0 | 0 | 0 | 0 | 0 | 0 | 0 | 0 | 0 | 0 | 409 | 422 |  |  | PEAKS DB |
| total 38 peptides |  |  |  |  |  |  |  |  |  |  |  |  |  |  |  |  |  |  |  |  |  |  |  |  |  |  |  |  |  |  |  |  |  |  |  |  |  |  |  |  |  |  |  |
| --- | --- | --- | --- | --- | --- | --- | --- | --- | --- | --- | --- | --- | --- | --- | --- | --- | --- | --- | --- | --- | --- | --- | --- | --- | --- | --- | --- | --- | --- | --- | --- | --- | --- | --- | --- | --- | --- | --- | --- | --- | --- | --- | --- |

A8QL58.2

back to list

  

| Protein Coverage
| Supporting Peptides
|

Protein Coverage:

Supporting Peptides:

| Peptide | Uniq | -10lgP | Mass | Length | ppm | m/z | z | RT | Fraction | Scan | Source File | Area F1 | Area F10 | Area F11 | Area F12 | Area F13 | Area F2 | Area F3 | Area F4 | Area F5 | Area F6 | Area F7 | Area F8 | Area F9 | #Feature | #Feature F1 | #Feature F10 | #Feature F11 | #Feature F12 | #Feature F13 | #Feature F2 | #Feature F3 | #Feature F4 | #Feature F5 | #Feature F6 | #Feature F7 | #Feature F8 | #Feature F9 | Start | End | PTM | AScore | Found By |
| --- | --- | --- | --- | --- | --- | --- | --- | --- | --- | --- | --- | --- | --- | --- | --- | --- | --- | --- | --- | --- | --- | --- | --- | --- | --- | --- | --- | --- | --- | --- | --- | --- | --- | --- | --- | --- | --- | --- | --- | --- | --- | --- | --- |
| K.HVVVVGAGMAGLSAAYVLAGAGHK.V | N | 116.89 | 2234.1992 | 24 | 0.4 | 745.7407 | 3 | 62.90 | 13 | F13:39164 | NaNaKA16\_F9.raw |  | 1.1179E8 | 1.0168E7 | 7.8612E6 |  |  |  |  |  |  |  | 4.7423E6 | 2.5143E7 | 12 | 0 | 3 | 2 | 2 | 0 | 0 | 0 | 0 | 0 | 0 | 0 | 2 | 3 | 54 | 77 |  |  | PEAKS DB |
| K.TC(+57.02)ADIVINDLSLIHDLPK.R | N | 85.41 | 2036.0612 | 18 | 1.1 | 679.6951 | 3 | 82.21 | 3 | F3:55460 | NaNaKA16\_F11.raw |  | 6.8273E6 | 2.6259E6 | 1.2527E7 |  |  |  |  |  |  |  |  |  | 5 | 0 | 2 | 1 | 2 | 0 | 0 | 0 | 0 | 0 | 0 | 0 | 0 | 0 | 405 | 422 | Carbamidomethylation | C2:Carbamidomethylation:1000.00 | PEAKS DB |
| K.TFVTADYVIVC(+57.02)STSR.A | N | 84.19 | 1717.8345 | 15 | 0.1 | 859.9246 | 2 | 61.23 | 4 | F4:39837 | NaNaKA16\_F12.raw |  | 1.1036E6 | 1.0862E6 | 1.2599E7 | 8.2211E5 |  |  |  |  |  |  | 5.3578E5 |  | 6 | 0 | 1 | 1 | 2 | 1 | 0 | 0 | 0 | 0 | 0 | 0 | 1 | 0 | 300 | 314 | Carbamidomethylation | C11:Carbamidomethylation:1000.00 | PEAKS DB |
| R.FHGWIDSTIMTGLR.A | Y | 83.58 | 1632.8082 | 14 | 0.3 | 817.4116 | 2 | 67.68 | 4 | F4:45214 | NaNaKA16\_F12.raw |  | 3.2243E7 | 8.0897E5 | 6.2437E7 |  |  |  |  |  |  |  | 8.2725E6 | 2.2165E7 | 17 | 0 | 2 | 1 | 10 | 0 | 0 | 0 | 0 | 0 | 0 | 0 | 2 | 2 | 478 | 491 |  |  | PEAKS DB |
| H.VVVVGAGMAGLSAAYVLAGAGHK.V | N | 76.18 | 2097.1404 | 23 | 0.9 | 700.0547 | 3 | 74.20 | 2 | F2:49558 | NaNaKA16\_F10.raw |  | 1.7277E6 |  |  |  |  |  |  |  |  |  |  |  | 1 | 0 | 1 | 0 | 0 | 0 | 0 | 0 | 0 | 0 | 0 | 0 | 0 | 0 | 55 | 77 |  |  | PEAKS DB |
| F.TPYQFQDYFESAAAPVGR.I | Y | 75.29 | 2045.9482 | 18 | 0.0 | 1023.9814 | 2 | 72.61 | 4 | F4:48939 | NaNaKA16\_F12.raw |  |  |  | 1.9418E6 |  |  |  |  |  |  |  |  |  | 1 | 0 | 0 | 0 | 1 | 0 | 0 | 0 | 0 | 0 | 0 | 0 | 0 | 0 | 450 | 467 |  |  | PEAKS DB |
| R.IYFEPPLPPK.K | N | 75.25 | 1199.6589 | 10 | 0.1 | 600.8368 | 2 | 57.78 | 4 | F4:36626 | NaNaKA16\_F12.raw |  | 3.3521E6 | 4.7257E6 | 2.469E7 | 2.9029E7 |  |  | 9.7178E6 |  |  |  |  |  | 7 | 0 | 1 | 1 | 1 | 1 | 0 | 0 | 3 | 0 | 0 | 0 | 0 | 0 | 319 | 328 |  |  | PEAKS DB |
| Y.VLADDSDFFQALDTK.T | N | 75.15 | 1683.7991 | 15 | 0.9 | 842.9075 | 2 | 76.95 | 5 | F5:31672 | NaNaKA16\_F13.raw |  |  |  |  | 1.2528E7 |  |  |  |  |  |  |  |  | 1 | 0 | 0 | 0 | 0 | 1 | 0 | 0 | 0 | 0 | 0 | 0 | 0 | 0 | 390 | 404 |  |  | PEAKS DB |
| K.REIQALC(+57.02)YPSIK.K | N | 74.57 | 1476.7759 | 12 | -0.3 | 493.2658 | 3 | 32.85 | 4 | F4:15922 | NaNaKA16\_F12.raw |  |  |  | 1.3566E7 | 2.8894E6 |  |  |  |  |  |  |  | 2.7998E5 | 4 | 0 | 0 | 0 | 2 | 1 | 0 | 0 | 0 | 0 | 0 | 0 | 0 | 1 | 423 | 434 | Carbamidomethylation | C7:Carbamidomethylation:1000.00 | PEAKS DB |
| A.DIVINDLSLIHDLPK.R | N | 73.95 | 1703.9458 | 15 | 0.6 | 852.9807 | 2 | 80.37 | 4 | F4:55290 | NaNaKA16\_F12.raw |  | 2.9714E7 | 9.3836E6 | 3.0023E7 |  |  |  |  |  |  |  | 6.9266E5 |  | 7 | 0 | 2 | 2 | 2 | 0 | 0 | 0 | 0 | 0 | 0 | 0 | 1 | 0 | 408 | 422 |  |  | PEAKS DB |
| Y.QFQDYFESAAAPVGR.I | Y | 73.75 | 1684.7844 | 15 | 1.1 | 843.4004 | 2 | 59.42 | 4 | F4:38157 | NaNaKA16\_F12.raw |  |  |  | 1.1155E7 | 7.003E6 |  |  |  |  |  |  |  |  | 6 | 0 | 0 | 0 | 3 | 3 | 0 | 0 | 0 | 0 | 0 | 0 | 0 | 0 | 453 | 467 |  |  | PEAKS DB |
| R.EIQALC(+57.02)YPSIK.K | N | 73.42 | 1320.6747 | 11 | -0.2 | 661.3445 | 2 | 47.89 | 3 | F3:27602 | NaNaKA16\_F11.raw |  | 0 | 1.5106E7 | 3.3878E7 | 2.1775E7 |  |  |  |  |  |  |  | 3.2256E6 | 4 | 0 | 0 | 1 | 1 | 1 | 0 | 0 | 0 | 0 | 0 | 0 | 0 | 1 | 424 | 434 | Carbamidomethylation | C6:Carbamidomethylation:1000.00 | PEAKS DB |
| K.HVVVVGAGM(+15.99)AGLSAAYVLAGAGHK.V | N | 72.68 | 2250.1943 | 24 | 0.0 | 563.5558 | 4 | 52.57 | 2 | F2:30632 | NaNaKA16\_F10.raw |  | 4.3956E6 |  | 2.4715E5 |  |  |  |  |  |  |  |  | 2.5443E6 | 4 | 0 | 2 | 0 | 1 | 0 | 0 | 0 | 0 | 0 | 0 | 0 | 0 | 1 | 54 | 77 | Oxidation (M) | M9:Oxidation (M):1000.00 | PEAKS DB |
| A.DDSDFFQALDTK.T | N | 70.63 | 1400.6095 | 12 | 0.5 | 701.3124 | 2 | 73.59 | 4 | F4:49585 | NaNaKA16\_F12.raw |  |  |  | 6.7842E6 |  |  |  |  |  | 7.1261E5 |  |  |  | 2 | 0 | 0 | 0 | 1 | 0 | 0 | 0 | 0 | 0 | 1 | 0 | 0 | 0 | 393 | 404 |  |  | PEAKS DB |
| K.SASQLYQESLR.K | Y | 67.47 | 1280.6360 | 11 | -2.1 | 641.3239 | 2 | 26.70 | 6 | F6:12667 | NaNaKA16\_F2.raw |  |  |  |  | 4.263E7 | 2.3745E7 |  |  | 3.3503E6 |  |  |  |  | 3 | 0 | 0 | 0 | 0 | 1 | 1 | 0 | 0 | 1 | 0 | 0 | 0 | 0 | 171 | 181 |  |  | PEAKS DB |
| Q.DYFESAAAPVGR.I | Y | 66.83 | 1281.5989 | 12 | 0.8 | 641.8073 | 2 | 43.00 | 6 | F6:26794 | NaNaKA16\_F2.raw |  | 1.3876E6 | 5.1422E5 |  |  | 1.2058E7 |  |  |  |  |  |  |  | 3 | 0 | 1 | 1 | 0 | 0 | 1 | 0 | 0 | 0 | 0 | 0 | 0 | 0 | 456 | 467 |  |  | PEAKS DB |
| R.RIYFEPPLPPK.K | N | 66.53 | 1355.7601 | 11 | 0.2 | 452.9274 | 3 | 43.83 | 4 | F4:25641 | NaNaKA16\_F12.raw |  | 5.7564E5 | 1.1183E6 | 4.1789E6 | 3.0828E6 |  |  |  |  |  |  |  |  | 6 | 0 | 3 | 1 | 1 | 1 | 0 | 0 | 0 | 0 | 0 | 0 | 0 | 0 | 318 | 328 |  |  | PEAKS DB |
| K.KFWEADGIHGGK.S | N | 66.46 | 1343.6622 | 12 | -0.8 | 448.8943 | 3 | 17.58 | 5 | F5:3842 | NaNaKA16\_F13.raw |  |  |  |  | 2.6107E6 |  |  |  |  |  |  |  |  | 2 | 0 | 0 | 0 | 0 | 2 | 0 | 0 | 0 | 0 | 0 | 0 | 0 | 0 | 351 | 362 |  |  | PEAKS DB |
| R.EGWYVNMGPMR.L | N | 63.13 | 1338.5850 | 11 | 0.2 | 670.2999 | 2 | 58.22 | 9 | F9:38419 | NaNaKA16\_F5.raw |  | 2.3017E6 | 3.4741E6 | 8.3473E6 | 1.0741E7 |  |  |  | 1.8901E6 | 4.3579E5 |  | 2.2555E6 |  | 7 | 0 | 1 | 1 | 1 | 1 | 0 | 0 | 0 | 1 | 1 | 0 | 1 | 0 | 99 | 109 |  |  | PEAKS DB |
| N.DLSLIHDLPK.R | N | 62.72 | 1149.6393 | 10 | 1.4 | 575.8277 | 2 | 52.52 | 3 | F3:29824 | NaNaKA16\_F11.raw |  | 1.6899E6 | 7.7612E6 | 4.7179E6 |  |  |  |  |  |  |  |  |  | 4 | 0 | 1 | 1 | 2 | 0 | 0 | 0 | 0 | 0 | 0 | 0 | 0 | 0 | 413 | 422 |  |  | PEAKS DB |
| R.FHGWIDSTIM(+15.99)TGLR.A | Y | 60.71 | 1648.8031 | 14 | -0.3 | 550.6082 | 3 | 55.70 | 4 | F4:35031 | NaNaKA16\_F12.raw |  | 5.7355E5 |  | 2.9168E6 |  |  |  |  |  |  |  |  |  | 2 | 0 | 1 | 0 | 1 | 0 | 0 | 0 | 0 | 0 | 0 | 0 | 0 | 0 | 478 | 491 | Oxidation (M) | M10:Oxidation (M):1000.00 | PEAKS DB |
| K.HVVVVGAGMAGLSAAY.V | N | 59.94 | 1500.7759 | 16 | 0.4 | 751.3955 | 2 | 61.71 | 5 | F5:25523 | NaNaKA16\_F13.raw |  |  |  |  | 6.9379E5 |  |  |  |  |  |  |  |  | 1 | 0 | 0 | 0 | 0 | 1 | 0 | 0 | 0 | 0 | 0 | 0 | 0 | 0 | 54 | 69 |  |  | PEAKS DB |
| R.IHFAGEYTGR.F | Y | 59.47 | 1149.5566 | 10 | 1.4 | 384.1934 | 3 | 17.69 | 5 | F5:3877 | NaNaKA16\_F13.raw |  |  |  | 0 | 1.8101E7 |  |  |  |  |  |  |  |  | 2 | 0 | 0 | 0 | 0 | 2 | 0 | 0 | 0 | 0 | 0 | 0 | 0 | 0 | 468 | 477 |  |  | PEAKS DB |
| K.FWEADGIHGGK.S | N | 57.27 | 1215.5673 | 11 | -0.2 | 608.7908 | 2 | 22.40 | 5 | F5:5921 | NaNaKA16\_F13.raw |  |  |  |  | 1.8542E7 |  |  |  |  |  |  |  |  | 2 | 0 | 0 | 0 | 0 | 2 | 0 | 0 | 0 | 0 | 0 | 0 | 0 | 0 | 352 | 362 |  |  | PEAKS DB |
| G.SITSFTPYQFQDYFESAAAPVGR.I | Y | 55.78 | 2581.2124 | 23 | 0.6 | 861.4119 | 3 | 92.74 | 4 | F4:65226 | NaNaKA16\_F12.raw |  |  |  | 6.4315E5 |  |  |  |  |  |  |  |  |  | 1 | 0 | 0 | 0 | 1 | 0 | 0 | 0 | 0 | 0 | 0 | 0 | 0 | 0 | 445 | 467 |  |  | PEAKS DB |
| K.SDALFSYEK.R | N | 53.51 | 1058.4919 | 9 | 1.2 | 530.2539 | 2 | 36.12 | 5 | F5:13813 | NaNaKA16\_F13.raw |  |  |  | 1.7934E6 | 2.9321E7 |  |  |  |  |  |  |  |  | 2 | 0 | 0 | 0 | 1 | 1 | 0 | 0 | 0 | 0 | 0 | 0 | 0 | 0 | 242 | 250 |  |  | PEAKS DB |
| R.EGWYVNM(+15.99)GPMR.L | N | 53.18 | 1354.5798 | 11 | 0.0 | 678.2972 | 2 | 41.87 | 4 | F4:23544 | NaNaKA16\_F12.raw |  |  |  | 1.2884E6 | 1.7705E6 |  |  |  |  |  |  |  |  | 2 | 0 | 0 | 0 | 1 | 1 | 0 | 0 | 0 | 0 | 0 | 0 | 0 | 0 | 99 | 109 | Oxidation (M) | M7:Oxidation (M):38.16 | PEAKS DB |
| R.IYFEPPLPPKK.A | N | 51.41 | 1327.7539 | 11 | 0.3 | 664.8844 | 2 | 40.66 | 5 | F5:16431 | NaNaKA16\_F13.raw |  |  |  | 7.8644E5 | 7.4644E5 |  |  |  |  |  |  |  |  | 2 | 0 | 0 | 0 | 1 | 1 | 0 | 0 | 0 | 0 | 0 | 0 | 0 | 0 | 319 | 329 |  |  | PEAKS DB |
| L.DKYTMGSITSFTPYQFQDYFESAAAPVGR.I | Y | 50.86 | 3276.5073 | 29 | 1.2 | 1093.1777 | 3 | 94.70 | 4 | F4:66626 | NaNaKA16\_F12.raw |  |  |  | 3.1812E6 |  |  |  |  |  |  |  |  |  | 1 | 0 | 0 | 0 | 1 | 0 | 0 | 0 | 0 | 0 | 0 | 0 | 0 | 0 | 439 | 467 |  |  | PEAKS DB |
| I.YFEPPLPPK.K | N | 50.02 | 1086.5750 | 9 | -0.2 | 544.2946 | 2 | 44.20 | 6 | F6:28085 | NaNaKA16\_F2.raw |  |  |  |  |  | 8.7131E6 |  |  |  |  |  |  |  | 1 | 0 | 0 | 0 | 0 | 0 | 1 | 0 | 0 | 0 | 0 | 0 | 0 | 0 | 320 | 328 |  |  | PEAKS DB |
| A.DIVINDLSLIHDLPKR.E | N | 48.00 | 1860.0469 | 16 | 0.2 | 621.0230 | 3 | 65.80 | 2 | F2:42289 | NaNaKA16\_F10.raw |  | 7.1576E5 |  |  |  |  |  |  |  |  |  |  |  | 1 | 0 | 1 | 0 | 0 | 0 | 0 | 0 | 0 | 0 | 0 | 0 | 0 | 0 | 408 | 423 |  |  | PEAKS DB |
| R.EGWYVNMGPM(+15.99)R.L | N | 47.08 | 1354.5798 | 11 | 0.3 | 678.2974 | 2 | 42.61 | 5 | F5:17152 | NaNaKA16\_F13.raw |  |  |  |  | 1.7705E6 |  |  |  |  |  |  |  |  | 1 | 0 | 0 | 0 | 0 | 1 | 0 | 0 | 0 | 0 | 0 | 0 | 0 | 0 | 99 | 109 | Oxidation (M) | M10:Oxidation (M):20.70 | PEAKS DB |
| K.SASQLYQESLRK.V | Y | 45.54 | 1408.7310 | 12 | 0.0 | 470.5843 | 3 | 12.22 | 6 | F6:2235 | NaNaKA16\_F2.raw |  |  |  |  |  | 1.4677E6 |  |  |  |  |  |  |  | 1 | 0 | 0 | 0 | 0 | 0 | 1 | 0 | 0 | 0 | 0 | 0 | 0 | 0 | 171 | 182 |  |  | PEAKS DB |
| S.FTPYQFQDYFESAAAPVGR.I | Y | 45.47 | 2193.0166 | 19 | 0.0 | 1097.5156 | 2 | 83.82 | 12 | F12:59508 | NaNaKA16\_F8.raw |  |  |  |  |  |  |  |  |  |  |  | 1.6948E6 |  | 1 | 0 | 0 | 0 | 0 | 0 | 0 | 0 | 0 | 0 | 0 | 0 | 1 | 0 | 449 | 467 |  |  | PEAKS DB |
| K.VTLLEASER.V | N | 44.72 | 1016.5502 | 9 | 0.2 | 509.2825 | 2 | 26.63 | 5 | F5:8576 | NaNaKA16\_F13.raw |  |  |  |  | 4.1415E7 |  |  |  |  |  |  |  |  | 1 | 0 | 0 | 0 | 0 | 1 | 0 | 0 | 0 | 0 | 0 | 0 | 0 | 0 | 78 | 86 |  |  | PEAKS DB |
| W.IDSTIMTGLR.A | Y | 43.99 | 1105.5801 | 10 | 6.1 | 553.7977 | 2 | 42.74 | 8 | F8:23703 | NaNaKA16\_F4.raw |  |  |  |  |  |  |  | 8.2584E5 |  |  |  |  |  | 1 | 0 | 0 | 0 | 0 | 0 | 0 | 0 | 1 | 0 | 0 | 0 | 0 | 0 | 482 | 491 |  |  | PEAKS DB |
| K.YTMGSITSFTPYQFQDYFESAAAPVGR.I | Y | 43.77 | 3033.3855 | 27 | 0.6 | 1012.1364 | 3 | 100.71 | 4 | F4:70262 | NaNaKA16\_F12.raw |  |  |  | 7.0268E5 |  |  |  |  |  |  |  |  |  | 1 | 0 | 0 | 0 | 1 | 0 | 0 | 0 | 0 | 0 | 0 | 0 | 0 | 0 | 441 | 467 |  |  | PEAKS DB |
| D.IVINDLSLIHDLPK.R | N | 42.85 | 1588.9188 | 14 | -0.1 | 530.6469 | 3 | 68.04 | 4 | F4:45149 | NaNaKA16\_F12.raw |  |  |  | 0 |  |  |  |  |  |  |  |  |  | 0 | 0 | 0 | 0 | 0 | 0 | 0 | 0 | 0 | 0 | 0 | 0 | 0 | 0 | 409 | 422 |  |  | PEAKS DB |
| total 38 peptides |  |  |  |  |  |  |  |  |  |  |  |  |  |  |  |  |  |  |  |  |  |  |  |  |  |  |  |  |  |  |  |  |  |  |  |  |  |  |  |  |  |  |  |
| --- | --- | --- | --- | --- | --- | --- | --- | --- | --- | --- | --- | --- | --- | --- | --- | --- | --- | --- | --- | --- | --- | --- | --- | --- | --- | --- | --- | --- | --- | --- | --- | --- | --- | --- | --- | --- | --- | --- | --- | --- | --- | --- | --- |

CAA45372.1

back to list

  

| Protein Coverage
| Supporting Peptides
|

Protein Coverage:

Supporting Peptides:

| Peptide | Uniq | -10lgP | Mass | Length | ppm | m/z | z | RT | Fraction | Scan | Source File | Area F1 | Area F10 | Area F11 | Area F12 | Area F13 | Area F2 | Area F3 | Area F4 | Area F5 | Area F6 | Area F7 | Area F8 | Area F9 | #Feature | #Feature F1 | #Feature F10 | #Feature F11 | #Feature F12 | #Feature F13 | #Feature F2 | #Feature F3 | #Feature F4 | #Feature F5 | #Feature F6 | #Feature F7 | #Feature F8 | #Feature F9 | Start | End | PTM | AScore | Found By |
| --- | --- | --- | --- | --- | --- | --- | --- | --- | --- | --- | --- | --- | --- | --- | --- | --- | --- | --- | --- | --- | --- | --- | --- | --- | --- | --- | --- | --- | --- | --- | --- | --- | --- | --- | --- | --- | --- | --- | --- | --- | --- | --- | --- |
| K.ISGC(+57.02)WPYFKTYSYEC(+57.02)SQGTLTC(+57.02)K.G | N | 89.48 | 2835.2341 | 23 | -1.5 | 946.0839 | 3 | 57.58 | 11 | F11:37368 | NaNaKA16\_F7.raw |  |  |  |  |  |  |  |  |  | 1.3653E7 | 7.0731E7 | 1.9929E7 | 6.9253E6 | 5 | 0 | 0 | 0 | 0 | 0 | 0 | 0 | 0 | 0 | 1 | 2 | 1 | 1 | 58 | 80 | Carbamidomethylation | C4:Carbamidomethylation:1000.00;C15:Carbamidomethylation:1000.00;C22:Carbamidomethylation:1000.00 | PEAKS DB |
| K.TYSYEC(+57.02)SQGTLTC(+57.02)K.G | N | 89.30 | 1696.7073 | 14 | 0.3 | 849.3611 | 2 | 21.20 | 9 | F9:9021 | NaNaKA16\_F5.raw |  | 7.5988E5 |  | 1.6097E7 | 6.3725E6 | 1.1263E6 |  | 2.9128E6 | 6.1482E8 | 3.4541E10 | 2.2713E9 | 9.1534E6 | 4.1844E8 | 46 | 0 | 1 | 0 | 3 | 1 | 1 | 0 | 1 | 4 | 18 | 3 | 2 | 12 | 67 | 80 | Carbamidomethylation | C6:Carbamidomethylation:1000.00;C13:Carbamidomethylation:1000.00 | PEAKS DB |
| R.LAAIC(+57.02)FAGAPYNDNNYNIDLKAR.C | N | 86.87 | 2583.2539 | 23 | 0.0 | 862.0919 | 3 | 62.98 | 12 | F12:41772 | NaNaKA16\_F8.raw |  |  |  |  |  |  |  |  |  | 4.515E6 | 2.8262E7 | 1.4923E7 | 1.4692E7 | 5 | 0 | 0 | 0 | 0 | 0 | 0 | 0 | 0 | 0 | 1 | 2 | 1 | 1 | 96 | 118 | Carbamidomethylation | C5:Carbamidomethylation:1000.00 | PEAKS DB |
| R.LAAIC(+57.02)FAGAPYNDNNYNIDLK.A | N | 82.29 | 2356.1157 | 21 | -2.1 | 1179.0626 | 2 | 73.79 | 11 | F11:49962 | NaNaKA16\_F7.raw |  | 2.7376E8 | 2.6984E7 | 7.0178E7 | 6.5633E6 |  |  |  | 3.1038E7 | 2.7883E9 | 4.7656E10 | 2.4584E9 | 2.4535E10 | 74 | 0 | 4 | 4 | 2 | 2 | 0 | 0 | 0 | 1 | 2 | 23 | 11 | 25 | 96 | 116 | Carbamidomethylation | C5:Carbamidomethylation:1000.00 | PEAKS DB |
| S.RSWWDFADYGC(+57.02)YC(+57.02)GR.G | N | 77.58 | 1997.7937 | 15 | 1.2 | 666.9393 | 3 | 70.64 | 11 | F11:48142 | NaNaKA16\_F7.raw |  |  |  |  |  |  |  |  |  |  | 3.4823E7 | 9.6445E6 |  | 4 | 0 | 0 | 0 | 0 | 0 | 0 | 0 | 0 | 0 | 0 | 2 | 2 | 0 | 17 | 31 | Carbamidomethylation | C11:Carbamidomethylation:1000.00;C13:Carbamidomethylation:1000.00 | PEAKS DB |
| R.SWWDFADYGC(+57.02)YC(+57.02)GR.G | N | 77.10 | 1841.6926 | 14 | 2.4 | 921.8558 | 2 | 86.60 | 11 | F11:61115 | NaNaKA16\_F7.raw |  | 2.3849E7 | 3.1182E6 | 1.2353E7 | 9.152E5 |  |  |  | 6.0399E6 | 1.4952E9 | 5.4032E10 | 1.5192E10 | 2.1735E9 | 46 | 0 | 2 | 1 | 2 | 1 | 0 | 0 | 0 | 1 | 3 | 17 | 6 | 13 | 18 | 31 | Carbamidomethylation | C10:Carbamidomethylation:1000.00;C12:Carbamidomethylation:1000.00 | PEAKS DB |
| G.APYNDNNYNIDLK.A | N | 74.44 | 1552.7157 | 13 | 0.1 | 777.3652 | 2 | 38.38 | 4 | F4:20647 | NaNaKA16\_F12.raw |  | 1.0864E7 | 7.8409E6 | 1.7533E7 | 6.5956E5 |  |  |  | 1.5969E7 | 3.4996E9 | 2.5425E9 | 1.4323E8 | 4.1292E8 | 10 | 0 | 1 | 1 | 1 | 1 | 0 | 0 | 0 | 1 | 1 | 1 | 1 | 2 | 104 | 116 |  |  | PEAKS DB |
| F.AGAPYNDNNYNIDLK.A | N | 74.15 | 1680.7743 | 15 | 0.8 | 841.3951 | 2 | 39.58 | 12 | F12:22604 | NaNaKA16\_F8.raw |  | 1.9233E6 | 9.618E5 | 3.9059E6 |  |  |  |  |  | 4.7877E8 | 2.5646E8 | 4.8059E7 | 1.8544E9 | 9 | 0 | 1 | 1 | 1 | 0 | 0 | 0 | 0 | 0 | 1 | 3 | 1 | 1 | 102 | 116 |  |  | PEAKS DB |
| D.RLAAIC(+57.02)FAGAPYNDNNYNIDLK.A | N | 71.67 | 2512.2168 | 22 | 0.3 | 838.4131 | 3 | 61.74 | 11 | F11:40640 | NaNaKA16\_F7.raw |  |  |  |  |  |  |  |  |  |  | 5.8961E7 | 7.0057E7 | 3.3382E7 | 4 | 0 | 0 | 0 | 0 | 0 | 0 | 0 | 0 | 0 | 0 | 2 | 1 | 1 | 95 | 116 | Carbamidomethylation | C6:Carbamidomethylation:1000.00 | PEAKS DB |
| C.FAGAPYNDNNYNIDLK.A | N | 70.87 | 1827.8428 | 16 | 0.3 | 914.9290 | 2 | 51.38 | 13 | F13:29549 | NaNaKA16\_F9.raw |  |  |  |  |  |  |  |  |  | 3.3389E7 | 6.5117E7 | 8.1033E6 | 2.2994E8 | 5 | 0 | 0 | 0 | 0 | 0 | 0 | 0 | 0 | 0 | 1 | 1 | 1 | 2 | 101 | 116 |  |  | PEAKS DB |
| K.ISGC(+57.02)WPYFK.T | N | 70.32 | 1156.5375 | 9 | 0.1 | 579.2761 | 2 | 68.82 | 11 | F11:46583 | NaNaKA16\_F7.raw |  | 3.6915E8 | 1.4139E8 | 6.9856E7 | 2.3823E6 |  |  | 3.6353E6 | 3.5126E8 | 2.1014E10 | 1.2727E11 | 1.9979E10 | 1.2073E10 | 71 | 0 | 1 | 1 | 3 | 1 | 0 | 0 | 1 | 1 | 15 | 17 | 10 | 21 | 58 | 66 | Carbamidomethylation | C4:Carbamidomethylation:1000.00 | PEAKS DB |
| K.GDNNAC(+57.02)AASVC(+57.02)DC(+57.02)DR.L | N | 70.22 | 1683.6035 | 15 | 0.2 | 842.8092 | 2 | 12.08 | 11 | F11:1839 | NaNaKA16\_F7.raw |  |  |  |  |  |  |  |  |  | 6.3052E6 | 1.0666E6 | 0 |  | 2 | 0 | 0 | 0 | 0 | 0 | 0 | 0 | 0 | 0 | 1 | 1 | 0 | 0 | 81 | 95 | Carbamidomethylation | C6:Carbamidomethylation:1000.00;C11:Carbamidomethylation:1000.00;C13:Carbamidomethylation:1000.00 | PEAKS DB |
| G.APYNDNNYNIDLKAR.C | N | 70.03 | 1779.8540 | 15 | 0.1 | 594.2920 | 3 | 28.22 | 11 | F11:14195 | NaNaKA16\_F7.raw |  |  |  |  |  |  |  |  |  | 3.8065E6 | 1.1348E7 |  |  | 3 | 0 | 0 | 0 | 0 | 0 | 0 | 0 | 0 | 0 | 1 | 2 | 0 | 0 | 104 | 118 |  |  | PEAKS DB |
| E.AEKISGC(+57.02)WPYFK.T | N | 69.57 | 1484.7122 | 12 | 0.0 | 743.3633 | 2 | 40.74 | 11 | F11:24212 | NaNaKA16\_F7.raw |  |  |  |  |  |  |  |  |  | 9.0796E6 | 5.2934E7 | 1.0921E7 |  | 9 | 0 | 0 | 0 | 0 | 0 | 0 | 0 | 0 | 0 | 2 | 5 | 2 | 0 | 55 | 66 | Carbamidomethylation | C7:Carbamidomethylation:1000.00 | PEAKS DB |
| R.GGSGTPVDDLDR.C | N | 67.81 | 1187.5417 | 12 | 0.8 | 594.7786 | 2 | 19.63 | 10 | F10:6988 | NaNaKA16\_F6.raw |  |  |  |  |  |  |  |  |  | 5.7224E7 | 2.6509E9 | 3.9395E6 |  | 4 | 0 | 0 | 0 | 0 | 0 | 0 | 0 | 0 | 0 | 2 | 1 | 1 | 0 | 32 | 43 |  |  | PEAKS DB |
| M.NLYQFKNMIKC(+57.02)TVPSR.S | N | 66.48 | 1998.0179 | 16 | 0.3 | 667.0134 | 3 | 40.68 | 12 | F12:23498 | NaNaKA16\_F8.raw |  |  |  |  |  |  |  |  |  |  |  | 3.4886E6 |  | 1 | 0 | 0 | 0 | 0 | 0 | 0 | 0 | 0 | 0 | 0 | 0 | 1 | 0 | 2 | 17 | Carbamidomethylation | C11:Carbamidomethylation:1000.00 | PEAKS DB |
| T.YSYEC(+57.02)SQGTLTC(+57.02)K.G | N | 65.61 | 1595.6595 | 13 | -0.2 | 798.8369 | 2 | 19.78 | 10 | F10:7072 | NaNaKA16\_F6.raw |  |  |  |  |  |  |  |  |  | 9.5485E6 |  |  |  | 1 | 0 | 0 | 0 | 0 | 0 | 0 | 0 | 0 | 0 | 1 | 0 | 0 | 0 | 68 | 80 | Carbamidomethylation | C5:Carbamidomethylation:1000.00;C12:Carbamidomethylation:1000.00 | PEAKS DB |
| Y.SYEC(+57.02)SQGTLTC(+57.02)K.G | N | 65.06 | 1432.5963 | 12 | 0.7 | 717.3060 | 2 | 11.79 | 10 | F10:1902 | NaNaKA16\_F6.raw |  |  |  |  |  |  |  |  |  | 7.186E5 |  |  |  | 1 | 0 | 0 | 0 | 0 | 0 | 0 | 0 | 0 | 0 | 1 | 0 | 0 | 0 | 69 | 80 | Carbamidomethylation | C4:Carbamidomethylation:1000.00;C11:Carbamidomethylation:1000.00 | PEAKS DB |
| A.IC(+57.02)FAGAPYNDNNYNIDLK.A | N | 61.89 | 2100.9575 | 18 | 0.5 | 1051.4866 | 2 | 63.78 | 13 | F13:39698 | NaNaKA16\_F9.raw |  |  |  |  |  |  |  |  |  | 9.6858E6 | 1.8845E7 | 6.9038E6 | 3.858E7 | 4 | 0 | 0 | 0 | 0 | 0 | 0 | 0 | 0 | 0 | 1 | 1 | 1 | 1 | 99 | 116 | Carbamidomethylation | C2:Carbamidomethylation:1000.00 | PEAKS DB |
| R.SWWDFADYGC(+57.02)YC(+57.02)GRG.G | N | 61.68 | 1898.7141 | 15 | 1.5 | 950.3657 | 2 | 84.85 | 11 | F11:59757 | NaNaKA16\_F7.raw |  |  |  |  |  |  |  |  |  |  | 1.1046E7 |  |  | 1 | 0 | 0 | 0 | 0 | 0 | 0 | 0 | 0 | 0 | 0 | 1 | 0 | 0 | 18 | 32 | Carbamidomethylation | C10:Carbamidomethylation:1000.00;C12:Carbamidomethylation:1000.00 | PEAKS DB |
| A.AIC(+57.02)FAGAPYNDNNYNIDLK.A | N | 61.56 | 2171.9946 | 19 | 1.2 | 1087.0059 | 2 | 65.58 | 13 | F13:41436 | NaNaKA16\_F9.raw |  |  |  |  |  |  |  |  |  |  | 1.0912E7 |  | 1.9483E7 | 2 | 0 | 0 | 0 | 0 | 0 | 0 | 0 | 0 | 0 | 0 | 1 | 0 | 1 | 98 | 116 | Carbamidomethylation | C3:Carbamidomethylation:1000.00 | PEAKS DB |
| G.GSGTPVDDLDR.C | N | 60.74 | 1130.5204 | 11 | -0.3 | 566.2673 | 2 | 21.08 | 11 | F11:8172 | NaNaKA16\_F7.raw |  |  |  |  |  |  |  |  |  | 1.4052E5 | 1.2671E7 |  |  | 2 | 0 | 0 | 0 | 0 | 0 | 0 | 0 | 0 | 0 | 1 | 1 | 0 | 0 | 33 | 43 |  |  | PEAKS DB |
| A.GAPYNDNNYNIDLK.A | N | 58.77 | 1609.7372 | 14 | -0.1 | 805.8758 | 2 | 38.63 | 10 | F10:22911 | NaNaKA16\_F6.raw |  |  |  |  |  |  |  |  |  | 4.2863E6 | 2.6962E6 |  | 1.5111E7 | 3 | 0 | 0 | 0 | 0 | 0 | 0 | 0 | 0 | 0 | 1 | 1 | 0 | 1 | 103 | 116 |  |  | PEAKS DB |
| K.TYSYEC(+57.02)SQGTLTC(+57.02)KGDNNAC(+57.02)AASVC(+57.02)DC(+57.02)DR.L | Y | 58.08 | 3362.3003 | 29 | 0.4 | 1121.7745 | 3 | 27.28 | 11 | F11:13291 | NaNaKA16\_F7.raw |  |  |  |  |  |  |  |  |  | 6.9673E7 | 1.4844E7 |  |  | 2 | 0 | 0 | 0 | 0 | 0 | 0 | 0 | 0 | 0 | 1 | 1 | 0 | 0 | 67 | 95 | Carbamidomethylation | C6:Carbamidomethylation:1000.00;C13:Carbamidomethylation:1000.00;C20:Carbamidomethylation:1000.00;C25:Carbamidomethylation:1000.00;C27:Carbamidomethylation:1000.00 | PEAKS DB |
| D.FADYGC(+57.02)YC(+57.02)GR.G | N | 57.15 | 1267.4750 | 10 | 0.8 | 634.7452 | 2 | 25.42 | 11 | F11:11882 | NaNaKA16\_F7.raw |  |  |  |  |  |  |  |  |  | 2.1506E6 | 1.834E7 |  |  | 2 | 0 | 0 | 0 | 0 | 0 | 0 | 0 | 0 | 0 | 1 | 1 | 0 | 0 | 22 | 31 | Carbamidomethylation | C6:Carbamidomethylation:1000.00;C8:Carbamidomethylation:1000.00 | PEAKS DB |
| R.SWWDFADYGC(+57.02)YC(+57.02)GRGGSGTPVDDLDR.C | N | 56.86 | 3011.2239 | 26 | 0.6 | 1004.7491 | 3 | 81.63 | 11 | F11:57027 | NaNaKA16\_F7.raw |  |  |  |  |  |  |  |  |  |  | 4.9169E6 |  |  | 1 | 0 | 0 | 0 | 0 | 0 | 0 | 0 | 0 | 0 | 0 | 1 | 0 | 0 | 18 | 43 | Carbamidomethylation | C10:Carbamidomethylation:1000.00;C12:Carbamidomethylation:1000.00 | PEAKS DB |
| S.WWDFADYGC(+57.02)YC(+57.02)GR.G | N | 56.83 | 1754.6605 | 13 | 0.8 | 878.3382 | 2 | 78.92 | 12 | F12:55446 | NaNaKA16\_F8.raw |  |  |  |  |  |  |  |  |  |  | 1.3475E7 | 3.6699E6 |  | 2 | 0 | 0 | 0 | 0 | 0 | 0 | 0 | 0 | 0 | 0 | 1 | 1 | 0 | 19 | 31 | Carbamidomethylation | C9:Carbamidomethylation:1000.00;C11:Carbamidomethylation:1000.00 | PEAKS DB |
| L.AAIC(+57.02)FAGAPYNDNNYNIDLK.A | N | 56.55 | 2243.0317 | 20 | -0.4 | 1122.5227 | 2 | 66.82 | 13 | F13:42492 | NaNaKA16\_F9.raw |  |  |  |  |  |  |  |  |  |  |  |  | 1.837E7 | 1 | 0 | 0 | 0 | 0 | 0 | 0 | 0 | 0 | 0 | 0 | 0 | 0 | 1 | 97 | 116 | Carbamidomethylation | C4:Carbamidomethylation:1000.00 | PEAKS DB |
| M.NLYQFKNMIK.C | N | 55.84 | 1297.6853 | 10 | 0.6 | 649.8503 | 2 | 34.48 | 11 | F11:19260 | NaNaKA16\_F7.raw |  |  |  |  |  |  |  |  |  | 2.422E7 | 1.1905E8 | 3.5531E7 | 5.6701E7 | 7 | 0 | 0 | 0 | 0 | 0 | 0 | 0 | 0 | 0 | 2 | 2 | 1 | 2 | 2 | 11 |  |  | PEAKS DB |
| P.YNDNNYNIDLK.A | N | 54.97 | 1384.6259 | 11 | -5.4 | 693.3165 | 2 | 32.55 | 10 | F10:17533 | NaNaKA16\_F6.raw |  |  |  |  |  |  |  |  |  | 8.5654E7 |  |  |  | 1 | 0 | 0 | 0 | 0 | 0 | 0 | 0 | 0 | 0 | 1 | 0 | 0 | 0 | 106 | 116 |  |  | PEAKS DB |
| Y.FKTYSYEC(+57.02)SQGTLTC(+57.02)K.G | N | 54.26 | 1971.8706 | 16 | 0.6 | 658.2979 | 3 | 23.41 | 11 | F11:10060 | NaNaKA16\_F7.raw |  |  |  |  |  |  |  |  |  | 3.532E6 | 1.7096E6 |  |  | 2 | 0 | 0 | 0 | 0 | 0 | 0 | 0 | 0 | 0 | 1 | 1 | 0 | 0 | 65 | 80 | Carbamidomethylation | C8:Carbamidomethylation:1000.00;C15:Carbamidomethylation:1000.00 | PEAKS DB |
| R.LAAIC(+57.02)FAGAPYN.D | N | 53.90 | 1266.6067 | 12 | 0.8 | 634.3111 | 2 | 70.83 | 11 | F11:49747 | NaNaKA16\_F7.raw |  | 2.6932E8 | 6.3964E7 |  |  |  |  |  | 3.6922E8 | 7.2309E9 | 3.8533E9 | 2.4096E9 | 1.5572E9 | 11 | 0 | 1 | 1 | 0 | 0 | 0 | 0 | 0 | 1 | 2 | 3 | 2 | 1 | 96 | 107 | Carbamidomethylation | C5:Carbamidomethylation:1000.00 | PEAKS DB |
| I.SGC(+57.02)WPYFK.T | N | 53.87 | 1043.4535 | 8 | -1.4 | 522.7333 | 2 | 44.38 | 11 | F11:27176 | NaNaKA16\_F7.raw |  |  |  |  |  |  |  |  |  | 8.1441E6 | 5.2634E6 |  |  | 2 | 0 | 0 | 0 | 0 | 0 | 0 | 0 | 0 | 0 | 1 | 1 | 0 | 0 | 59 | 66 | Carbamidomethylation | C3:Carbamidomethylation:1000.00 | PEAKS DB |
| E.KISGC(+57.02)WPYFK.T | N | 51.04 | 1284.6324 | 10 | 0.2 | 643.3236 | 2 | 38.26 | 11 | F11:22081 | NaNaKA16\_F7.raw |  |  |  |  |  |  |  |  |  |  | 3.8432E6 |  |  | 2 | 0 | 0 | 0 | 0 | 0 | 0 | 0 | 0 | 0 | 0 | 2 | 0 | 0 | 57 | 66 | Carbamidomethylation | C5:Carbamidomethylation:1000.00 | PEAKS DB |
| W.WDFADYGC(+57.02)YC(+57.02)GR.G | N | 50.34 | 1568.5813 | 12 | 0.5 | 785.2983 | 2 | 61.41 | 11 | F11:40293 | NaNaKA16\_F7.raw |  |  |  |  |  |  |  |  |  |  | 5.6691E6 |  |  | 1 | 0 | 0 | 0 | 0 | 0 | 0 | 0 | 0 | 0 | 0 | 1 | 0 | 0 | 20 | 31 | Carbamidomethylation | C8:Carbamidomethylation:1000.00;C10:Carbamidomethylation:1000.00 | PEAKS DB |
| W.DFADYGC(+57.02)YC(+57.02)GR.G | N | 49.85 | 1382.5020 | 11 | 0.3 | 692.2585 | 2 | 40.50 | 10 | F10:24767 | NaNaKA16\_F6.raw |  |  |  |  |  |  |  |  | 5.0674E6 | 1.1493E7 | 1.4257E6 |  |  | 3 | 0 | 0 | 0 | 0 | 0 | 0 | 0 | 0 | 1 | 1 | 1 | 0 | 0 | 21 | 31 | Carbamidomethylation | C7:Carbamidomethylation:1000.00;C9:Carbamidomethylation:1000.00 | PEAKS DB |
| K.NMIKC(+57.02)TVPSR.S | N | 48.35 | 1204.6056 | 10 | 0.1 | 603.3101 | 2 | 12.09 | 11 | F11:1822 | NaNaKA16\_F7.raw |  |  |  |  |  |  |  |  |  |  | 1.7697E7 |  |  | 2 | 0 | 0 | 0 | 0 | 0 | 0 | 0 | 0 | 0 | 0 | 2 | 0 | 0 | 8 | 17 | Carbamidomethylation | C5:Carbamidomethylation:1000.00 | PEAKS DB |
| A.PYNDNNYNIDLK.A | N | 48.26 | 1481.6786 | 12 | 0.7 | 741.8471 | 2 | 34.63 | 13 | F13:15900 | NaNaKA16\_F9.raw |  |  |  |  |  |  |  |  |  |  | 1.9835E6 |  | 2.598E6 | 2 | 0 | 0 | 0 | 0 | 0 | 0 | 0 | 0 | 0 | 0 | 1 | 0 | 1 | 105 | 116 |  |  | PEAKS DB |
| K.C(+57.02)TVPSRSWWDFADYGC(+57.02)YC(+57.02)GR.G | N | 48.21 | 2542.0251 | 20 | -0.3 | 848.3488 | 3 | 69.09 | 11 | F11:46749 | NaNaKA16\_F7.raw |  |  |  |  |  |  |  |  |  |  | 6.2414E6 |  |  | 1 | 0 | 0 | 0 | 0 | 0 | 0 | 0 | 0 | 0 | 0 | 1 | 0 | 0 | 12 | 31 | Carbamidomethylation | C1:Carbamidomethylation:1000.00;C16:Carbamidomethylation:1000.00;C18:Carbamidomethylation:1000.00 | PEAKS DB |
| M.NLYQFKNM(+15.99)IK.C | N | 46.70 | 1313.6802 | 10 | 0.4 | 657.8476 | 2 | 22.54 | 11 | F11:9423 | NaNaKA16\_F7.raw |  |  |  |  |  |  |  |  |  |  | 2.8165E6 |  |  | 1 | 0 | 0 | 0 | 0 | 0 | 0 | 0 | 0 | 0 | 0 | 1 | 0 | 0 | 2 | 11 | Oxidation (M) | M8:Oxidation (M):1000.00 | PEAKS DB |
| R.SWWDFADYGC(+57.02).Y | N | 45.71 | 1305.4761 | 10 | 0.8 | 653.7458 | 2 | 109.86 | 11 | F11:80150 | NaNaKA16\_F7.raw |  |  |  |  |  |  |  |  |  |  | 6.5059E6 |  |  | 1 | 0 | 0 | 0 | 0 | 0 | 0 | 0 | 0 | 0 | 0 | 1 | 0 | 0 | 18 | 27 | Carbamidomethylation | C10:Carbamidomethylation:1000.00 | PEAKS DB |
| G.SGTPVDDLDR.C | N | 45.52 | 1073.4989 | 10 | 0.9 | 537.7572 | 2 | 20.23 | 11 | F11:7497 | NaNaKA16\_F7.raw |  |  |  |  |  |  |  |  |  |  | 1.1072E7 |  |  | 1 | 0 | 0 | 0 | 0 | 0 | 0 | 0 | 0 | 0 | 0 | 1 | 0 | 0 | 34 | 43 |  |  | PEAKS DB |
| K.TYSYEC(+57.02)SQGTLTC(+57.02)KG.D | N | 45.40 | 1753.7288 | 15 | 1.4 | 877.8729 | 2 | 21.92 | 10 | F10:8643 | NaNaKA16\_F6.raw |  |  |  |  |  |  |  |  |  | 0 |  |  |  | 0 | 0 | 0 | 0 | 0 | 0 | 0 | 0 | 0 | 0 | 0 | 0 | 0 | 0 | 67 | 81 | Carbamidomethylation | C6:Carbamidomethylation:1000.00;C13:Carbamidomethylation:1000.00 | PEAKS DB |
| R.SWWDFADYGC(+57.02)Y.C | N | 44.81 | 1468.5394 | 11 | 0.1 | 735.2771 | 2 | 111.77 | 10 | F10:82113 | NaNaKA16\_F6.raw |  |  |  |  |  |  |  |  |  | 1.9586E7 |  | 1.4496E7 |  | 2 | 0 | 0 | 0 | 0 | 0 | 0 | 0 | 0 | 0 | 1 | 0 | 1 | 0 | 18 | 28 | Carbamidomethylation | C10:Carbamidomethylation:1000.00 | PEAKS DB |
| N.DNNYNIDLK.A | N | 44.51 | 1107.5197 | 9 | 1.1 | 554.7677 | 2 | 32.05 | 10 | F10:17657 | NaNaKA16\_F6.raw |  |  |  |  |  |  |  |  |  | 4.8851E7 | 9.5595E6 |  |  | 3 | 0 | 0 | 0 | 0 | 0 | 0 | 0 | 0 | 0 | 2 | 1 | 0 | 0 | 108 | 116 |  |  | PEAKS DB |
| F.AGAPYNDNNYNIDLKAR.C | N | 42.93 | 1907.9126 | 17 | 0.4 | 636.9784 | 3 | 29.66 | 13 | F13:11769 | NaNaKA16\_F9.raw |  |  |  |  |  |  |  |  |  |  |  |  | 8.9691E5 | 1 | 0 | 0 | 0 | 0 | 0 | 0 | 0 | 0 | 0 | 0 | 0 | 0 | 1 | 102 | 118 |  |  | PEAKS DB |
| total 46 peptides |  |  |  |  |  |  |  |  |  |  |  |  |  |  |  |  |  |  |  |  |  |  |  |  |  |  |  |  |  |  |  |  |  |  |  |  |  |  |  |  |  |  |  |
| --- | --- | --- | --- | --- | --- | --- | --- | --- | --- | --- | --- | --- | --- | --- | --- | --- | --- | --- | --- | --- | --- | --- | --- | --- | --- | --- | --- | --- | --- | --- | --- | --- | --- | --- | --- | --- | --- | --- | --- | --- | --- | --- | --- |

1A3F

back to list

  

| Protein Coverage
| Supporting Peptides
|

Protein Coverage:

Supporting Peptides:

| Peptide | Uniq | -10lgP | Mass | Length | ppm | m/z | z | RT | Fraction | Scan | Source File | Area F1 | Area F10 | Area F11 | Area F12 | Area F13 | Area F2 | Area F3 | Area F4 | Area F5 | Area F6 | Area F7 | Area F8 | Area F9 | #Feature | #Feature F1 | #Feature F10 | #Feature F11 | #Feature F12 | #Feature F13 | #Feature F2 | #Feature F3 | #Feature F4 | #Feature F5 | #Feature F6 | #Feature F7 | #Feature F8 | #Feature F9 | Start | End | PTM | AScore | Found By |
| --- | --- | --- | --- | --- | --- | --- | --- | --- | --- | --- | --- | --- | --- | --- | --- | --- | --- | --- | --- | --- | --- | --- | --- | --- | --- | --- | --- | --- | --- | --- | --- | --- | --- | --- | --- | --- | --- | --- | --- | --- | --- | --- | --- |
| K.ISGC(+57.02)WPYFKTYSYEC(+57.02)SQGTLTC(+57.02)K.G | N | 89.48 | 2835.2341 | 23 | -1.5 | 946.0839 | 3 | 57.58 | 11 | F11:37368 | NaNaKA16\_F7.raw |  |  |  |  |  |  |  |  |  | 1.3653E7 | 7.0731E7 | 1.9929E7 | 6.9253E6 | 5 | 0 | 0 | 0 | 0 | 0 | 0 | 0 | 0 | 0 | 1 | 2 | 1 | 1 | 57 | 79 | Carbamidomethylation | C4:Carbamidomethylation:1000.00;C15:Carbamidomethylation:1000.00;C22:Carbamidomethylation:1000.00 | PEAKS DB |
| K.TYSYEC(+57.02)SQGTLTC(+57.02)K.G | N | 89.30 | 1696.7073 | 14 | 0.3 | 849.3611 | 2 | 21.20 | 9 | F9:9021 | NaNaKA16\_F5.raw |  | 7.5988E5 |  | 1.6097E7 | 6.3725E6 | 1.1263E6 |  | 2.9128E6 | 6.1482E8 | 3.4541E10 | 2.2713E9 | 9.1534E6 | 4.1844E8 | 46 | 0 | 1 | 0 | 3 | 1 | 1 | 0 | 1 | 4 | 18 | 3 | 2 | 12 | 66 | 79 | Carbamidomethylation | C6:Carbamidomethylation:1000.00;C13:Carbamidomethylation:1000.00 | PEAKS DB |
| R.LAAIC(+57.02)FAGAPYNDNNYNIDLKAR.C | N | 86.87 | 2583.2539 | 23 | 0.0 | 862.0919 | 3 | 62.98 | 12 | F12:41772 | NaNaKA16\_F8.raw |  |  |  |  |  |  |  |  |  | 4.515E6 | 2.8262E7 | 1.4923E7 | 1.4692E7 | 5 | 0 | 0 | 0 | 0 | 0 | 0 | 0 | 0 | 0 | 1 | 2 | 1 | 1 | 95 | 117 | Carbamidomethylation | C5:Carbamidomethylation:1000.00 | PEAKS DB |
| R.LAAIC(+57.02)FAGAPYNDNNYNIDLK.A | N | 82.29 | 2356.1157 | 21 | -2.1 | 1179.0626 | 2 | 73.79 | 11 | F11:49962 | NaNaKA16\_F7.raw |  | 2.7376E8 | 2.6984E7 | 7.0178E7 | 6.5633E6 |  |  |  | 3.1038E7 | 2.7883E9 | 4.7656E10 | 2.4584E9 | 2.4535E10 | 74 | 0 | 4 | 4 | 2 | 2 | 0 | 0 | 0 | 1 | 2 | 23 | 11 | 25 | 95 | 115 | Carbamidomethylation | C5:Carbamidomethylation:1000.00 | PEAKS DB |
| S.RSWWDFADYGC(+57.02)YC(+57.02)GR.G | N | 77.58 | 1997.7937 | 15 | 1.2 | 666.9393 | 3 | 70.64 | 11 | F11:48142 | NaNaKA16\_F7.raw |  |  |  |  |  |  |  |  |  |  | 3.4823E7 | 9.6445E6 |  | 4 | 0 | 0 | 0 | 0 | 0 | 0 | 0 | 0 | 0 | 0 | 2 | 2 | 0 | 16 | 30 | Carbamidomethylation | C11:Carbamidomethylation:1000.00;C13:Carbamidomethylation:1000.00 | PEAKS DB |
| R.SWWDFADYGC(+57.02)YC(+57.02)GR.G | N | 77.10 | 1841.6926 | 14 | 2.4 | 921.8558 | 2 | 86.60 | 11 | F11:61115 | NaNaKA16\_F7.raw |  | 2.3849E7 | 3.1182E6 | 1.2353E7 | 9.152E5 |  |  |  | 6.0399E6 | 1.4952E9 | 5.4032E10 | 1.5192E10 | 2.1735E9 | 46 | 0 | 2 | 1 | 2 | 1 | 0 | 0 | 0 | 1 | 3 | 17 | 6 | 13 | 17 | 30 | Carbamidomethylation | C10:Carbamidomethylation:1000.00;C12:Carbamidomethylation:1000.00 | PEAKS DB |
| G.APYNDNNYNIDLK.A | N | 74.44 | 1552.7157 | 13 | 0.1 | 777.3652 | 2 | 38.38 | 4 | F4:20647 | NaNaKA16\_F12.raw |  | 1.0864E7 | 7.8409E6 | 1.7533E7 | 6.5956E5 |  |  |  | 1.5969E7 | 3.4996E9 | 2.5425E9 | 1.4323E8 | 4.1292E8 | 10 | 0 | 1 | 1 | 1 | 1 | 0 | 0 | 0 | 1 | 1 | 1 | 1 | 2 | 103 | 115 |  |  | PEAKS DB |
| F.AGAPYNDNNYNIDLK.A | N | 74.15 | 1680.7743 | 15 | 0.8 | 841.3951 | 2 | 39.58 | 12 | F12:22604 | NaNaKA16\_F8.raw |  | 1.9233E6 | 9.618E5 | 3.9059E6 |  |  |  |  |  | 4.7877E8 | 2.5646E8 | 4.8059E7 | 1.8544E9 | 9 | 0 | 1 | 1 | 1 | 0 | 0 | 0 | 0 | 0 | 1 | 3 | 1 | 1 | 101 | 115 |  |  | PEAKS DB |
| D.RLAAIC(+57.02)FAGAPYNDNNYNIDLK.A | N | 71.67 | 2512.2168 | 22 | 0.3 | 838.4131 | 3 | 61.74 | 11 | F11:40640 | NaNaKA16\_F7.raw |  |  |  |  |  |  |  |  |  |  | 5.8961E7 | 7.0057E7 | 3.3382E7 | 4 | 0 | 0 | 0 | 0 | 0 | 0 | 0 | 0 | 0 | 0 | 2 | 1 | 1 | 94 | 115 | Carbamidomethylation | C6:Carbamidomethylation:1000.00 | PEAKS DB |
| C.FAGAPYNDNNYNIDLK.A | N | 70.87 | 1827.8428 | 16 | 0.3 | 914.9290 | 2 | 51.38 | 13 | F13:29549 | NaNaKA16\_F9.raw |  |  |  |  |  |  |  |  |  | 3.3389E7 | 6.5117E7 | 8.1033E6 | 2.2994E8 | 5 | 0 | 0 | 0 | 0 | 0 | 0 | 0 | 0 | 0 | 1 | 1 | 1 | 2 | 100 | 115 |  |  | PEAKS DB |
| K.ISGC(+57.02)WPYFK.T | N | 70.32 | 1156.5375 | 9 | 0.1 | 579.2761 | 2 | 68.82 | 11 | F11:46583 | NaNaKA16\_F7.raw |  | 3.6915E8 | 1.4139E8 | 6.9856E7 | 2.3823E6 |  |  | 3.6353E6 | 3.5126E8 | 2.1014E10 | 1.2727E11 | 1.9979E10 | 1.2073E10 | 71 | 0 | 1 | 1 | 3 | 1 | 0 | 0 | 1 | 1 | 15 | 17 | 10 | 21 | 57 | 65 | Carbamidomethylation | C4:Carbamidomethylation:1000.00 | PEAKS DB |
| K.GDNNAC(+57.02)AASVC(+57.02)DC(+57.02)DR.L | N | 70.22 | 1683.6035 | 15 | 0.2 | 842.8092 | 2 | 12.08 | 11 | F11:1839 | NaNaKA16\_F7.raw |  |  |  |  |  |  |  |  |  | 6.3052E6 | 1.0666E6 | 0 |  | 2 | 0 | 0 | 0 | 0 | 0 | 0 | 0 | 0 | 0 | 1 | 1 | 0 | 0 | 80 | 94 | Carbamidomethylation | C6:Carbamidomethylation:1000.00;C11:Carbamidomethylation:1000.00;C13:Carbamidomethylation:1000.00 | PEAKS DB |
| G.APYNDNNYNIDLKAR.C | N | 70.03 | 1779.8540 | 15 | 0.1 | 594.2920 | 3 | 28.22 | 11 | F11:14195 | NaNaKA16\_F7.raw |  |  |  |  |  |  |  |  |  | 3.8065E6 | 1.1348E7 |  |  | 3 | 0 | 0 | 0 | 0 | 0 | 0 | 0 | 0 | 0 | 1 | 2 | 0 | 0 | 103 | 117 |  |  | PEAKS DB |
| E.AEKISGC(+57.02)WPYFK.T | N | 69.57 | 1484.7122 | 12 | 0.0 | 743.3633 | 2 | 40.74 | 11 | F11:24212 | NaNaKA16\_F7.raw |  |  |  |  |  |  |  |  |  | 9.0796E6 | 5.2934E7 | 1.0921E7 |  | 9 | 0 | 0 | 0 | 0 | 0 | 0 | 0 | 0 | 0 | 2 | 5 | 2 | 0 | 54 | 65 | Carbamidomethylation | C7:Carbamidomethylation:1000.00 | PEAKS DB |
| R.GGSGTPVDDLDR.C | N | 67.81 | 1187.5417 | 12 | 0.8 | 594.7786 | 2 | 19.63 | 10 | F10:6988 | NaNaKA16\_F6.raw |  |  |  |  |  |  |  |  |  | 5.7224E7 | 2.6509E9 | 3.9395E6 |  | 4 | 0 | 0 | 0 | 0 | 0 | 0 | 0 | 0 | 0 | 2 | 1 | 1 | 0 | 31 | 42 |  |  | PEAKS DB |
| NLYQFKNMIKC(+57.02)TVPSR.S | N | 66.48 | 1998.0179 | 16 | 0.3 | 667.0134 | 3 | 40.68 | 12 | F12:23498 | NaNaKA16\_F8.raw |  |  |  |  |  |  |  |  |  |  |  | 3.4886E6 |  | 1 | 0 | 0 | 0 | 0 | 0 | 0 | 0 | 0 | 0 | 0 | 0 | 1 | 0 | 1 | 16 | Carbamidomethylation | C11:Carbamidomethylation:1000.00 | PEAKS DB |
| T.YSYEC(+57.02)SQGTLTC(+57.02)K.G | N | 65.61 | 1595.6595 | 13 | -0.2 | 798.8369 | 2 | 19.78 | 10 | F10:7072 | NaNaKA16\_F6.raw |  |  |  |  |  |  |  |  |  | 9.5485E6 |  |  |  | 1 | 0 | 0 | 0 | 0 | 0 | 0 | 0 | 0 | 0 | 1 | 0 | 0 | 0 | 67 | 79 | Carbamidomethylation | C5:Carbamidomethylation:1000.00;C12:Carbamidomethylation:1000.00 | PEAKS DB |
| Y.SYEC(+57.02)SQGTLTC(+57.02)K.G | N | 65.06 | 1432.5963 | 12 | 0.7 | 717.3060 | 2 | 11.79 | 10 | F10:1902 | NaNaKA16\_F6.raw |  |  |  |  |  |  |  |  |  | 7.186E5 |  |  |  | 1 | 0 | 0 | 0 | 0 | 0 | 0 | 0 | 0 | 0 | 1 | 0 | 0 | 0 | 68 | 79 | Carbamidomethylation | C4:Carbamidomethylation:1000.00;C11:Carbamidomethylation:1000.00 | PEAKS DB |
| A.IC(+57.02)FAGAPYNDNNYNIDLK.A | N | 61.89 | 2100.9575 | 18 | 0.5 | 1051.4866 | 2 | 63.78 | 13 | F13:39698 | NaNaKA16\_F9.raw |  |  |  |  |  |  |  |  |  | 9.6858E6 | 1.8845E7 | 6.9038E6 | 3.858E7 | 4 | 0 | 0 | 0 | 0 | 0 | 0 | 0 | 0 | 0 | 1 | 1 | 1 | 1 | 98 | 115 | Carbamidomethylation | C2:Carbamidomethylation:1000.00 | PEAKS DB |
| R.SWWDFADYGC(+57.02)YC(+57.02)GRG.G | N | 61.68 | 1898.7141 | 15 | 1.5 | 950.3657 | 2 | 84.85 | 11 | F11:59757 | NaNaKA16\_F7.raw |  |  |  |  |  |  |  |  |  |  | 1.1046E7 |  |  | 1 | 0 | 0 | 0 | 0 | 0 | 0 | 0 | 0 | 0 | 0 | 1 | 0 | 0 | 17 | 31 | Carbamidomethylation | C10:Carbamidomethylation:1000.00;C12:Carbamidomethylation:1000.00 | PEAKS DB |
| A.AIC(+57.02)FAGAPYNDNNYNIDLK.A | N | 61.56 | 2171.9946 | 19 | 1.2 | 1087.0059 | 2 | 65.58 | 13 | F13:41436 | NaNaKA16\_F9.raw |  |  |  |  |  |  |  |  |  |  | 1.0912E7 |  | 1.9483E7 | 2 | 0 | 0 | 0 | 0 | 0 | 0 | 0 | 0 | 0 | 0 | 1 | 0 | 1 | 97 | 115 | Carbamidomethylation | C3:Carbamidomethylation:1000.00 | PEAKS DB |
| G.GSGTPVDDLDR.C | N | 60.74 | 1130.5204 | 11 | -0.3 | 566.2673 | 2 | 21.08 | 11 | F11:8172 | NaNaKA16\_F7.raw |  |  |  |  |  |  |  |  |  | 1.4052E5 | 1.2671E7 |  |  | 2 | 0 | 0 | 0 | 0 | 0 | 0 | 0 | 0 | 0 | 1 | 1 | 0 | 0 | 32 | 42 |  |  | PEAKS DB |
| A.GAPYNDNNYNIDLK.A | N | 58.77 | 1609.7372 | 14 | -0.1 | 805.8758 | 2 | 38.63 | 10 | F10:22911 | NaNaKA16\_F6.raw |  |  |  |  |  |  |  |  |  | 4.2863E6 | 2.6962E6 |  | 1.5111E7 | 3 | 0 | 0 | 0 | 0 | 0 | 0 | 0 | 0 | 0 | 1 | 1 | 0 | 1 | 102 | 115 |  |  | PEAKS DB |
| K.TYSYEC(+57.02)SQGTLTC(+57.02)KGDNNAC(+57.02)AASVC(+57.02)DC(+57.02)DR.L | Y | 58.08 | 3362.3003 | 29 | 0.4 | 1121.7745 | 3 | 27.28 | 11 | F11:13291 | NaNaKA16\_F7.raw |  |  |  |  |  |  |  |  |  | 6.9673E7 | 1.4844E7 |  |  | 2 | 0 | 0 | 0 | 0 | 0 | 0 | 0 | 0 | 0 | 1 | 1 | 0 | 0 | 66 | 94 | Carbamidomethylation | C6:Carbamidomethylation:1000.00;C13:Carbamidomethylation:1000.00;C20:Carbamidomethylation:1000.00;C25:Carbamidomethylation:1000.00;C27:Carbamidomethylation:1000.00 | PEAKS DB |
| D.FADYGC(+57.02)YC(+57.02)GR.G | N | 57.15 | 1267.4750 | 10 | 0.8 | 634.7452 | 2 | 25.42 | 11 | F11:11882 | NaNaKA16\_F7.raw |  |  |  |  |  |  |  |  |  | 2.1506E6 | 1.834E7 |  |  | 2 | 0 | 0 | 0 | 0 | 0 | 0 | 0 | 0 | 0 | 1 | 1 | 0 | 0 | 21 | 30 | Carbamidomethylation | C6:Carbamidomethylation:1000.00;C8:Carbamidomethylation:1000.00 | PEAKS DB |
| R.SWWDFADYGC(+57.02)YC(+57.02)GRGGSGTPVDDLDR.C | N | 56.86 | 3011.2239 | 26 | 0.6 | 1004.7491 | 3 | 81.63 | 11 | F11:57027 | NaNaKA16\_F7.raw |  |  |  |  |  |  |  |  |  |  | 4.9169E6 |  |  | 1 | 0 | 0 | 0 | 0 | 0 | 0 | 0 | 0 | 0 | 0 | 1 | 0 | 0 | 17 | 42 | Carbamidomethylation | C10:Carbamidomethylation:1000.00;C12:Carbamidomethylation:1000.00 | PEAKS DB |
| S.WWDFADYGC(+57.02)YC(+57.02)GR.G | N | 56.83 | 1754.6605 | 13 | 0.8 | 878.3382 | 2 | 78.92 | 12 | F12:55446 | NaNaKA16\_F8.raw |  |  |  |  |  |  |  |  |  |  | 1.3475E7 | 3.6699E6 |  | 2 | 0 | 0 | 0 | 0 | 0 | 0 | 0 | 0 | 0 | 0 | 1 | 1 | 0 | 18 | 30 | Carbamidomethylation | C9:Carbamidomethylation:1000.00;C11:Carbamidomethylation:1000.00 | PEAKS DB |
| L.AAIC(+57.02)FAGAPYNDNNYNIDLK.A | N | 56.55 | 2243.0317 | 20 | -0.4 | 1122.5227 | 2 | 66.82 | 13 | F13:42492 | NaNaKA16\_F9.raw |  |  |  |  |  |  |  |  |  |  |  |  | 1.837E7 | 1 | 0 | 0 | 0 | 0 | 0 | 0 | 0 | 0 | 0 | 0 | 0 | 0 | 1 | 96 | 115 | Carbamidomethylation | C4:Carbamidomethylation:1000.00 | PEAKS DB |
| NLYQFKNMIK.C | N | 55.84 | 1297.6853 | 10 | 0.6 | 649.8503 | 2 | 34.48 | 11 | F11:19260 | NaNaKA16\_F7.raw |  |  |  |  |  |  |  |  |  | 2.422E7 | 1.1905E8 | 3.5531E7 | 5.6701E7 | 7 | 0 | 0 | 0 | 0 | 0 | 0 | 0 | 0 | 0 | 2 | 2 | 1 | 2 | 1 | 10 |  |  | PEAKS DB |
| P.YNDNNYNIDLK.A | N | 54.97 | 1384.6259 | 11 | -5.4 | 693.3165 | 2 | 32.55 | 10 | F10:17533 | NaNaKA16\_F6.raw |  |  |  |  |  |  |  |  |  | 8.5654E7 |  |  |  | 1 | 0 | 0 | 0 | 0 | 0 | 0 | 0 | 0 | 0 | 1 | 0 | 0 | 0 | 105 | 115 |  |  | PEAKS DB |
| Y.FKTYSYEC(+57.02)SQGTLTC(+57.02)K.G | N | 54.26 | 1971.8706 | 16 | 0.6 | 658.2979 | 3 | 23.41 | 11 | F11:10060 | NaNaKA16\_F7.raw |  |  |  |  |  |  |  |  |  | 3.532E6 | 1.7096E6 |  |  | 2 | 0 | 0 | 0 | 0 | 0 | 0 | 0 | 0 | 0 | 1 | 1 | 0 | 0 | 64 | 79 | Carbamidomethylation | C8:Carbamidomethylation:1000.00;C15:Carbamidomethylation:1000.00 | PEAKS DB |
| R.LAAIC(+57.02)FAGAPYN.D | N | 53.90 | 1266.6067 | 12 | 0.8 | 634.3111 | 2 | 70.83 | 11 | F11:49747 | NaNaKA16\_F7.raw |  | 2.6932E8 | 6.3964E7 |  |  |  |  |  | 3.6922E8 | 7.2309E9 | 3.8533E9 | 2.4096E9 | 1.5572E9 | 11 | 0 | 1 | 1 | 0 | 0 | 0 | 0 | 0 | 1 | 2 | 3 | 2 | 1 | 95 | 106 | Carbamidomethylation | C5:Carbamidomethylation:1000.00 | PEAKS DB |
| I.SGC(+57.02)WPYFK.T | N | 53.87 | 1043.4535 | 8 | -1.4 | 522.7333 | 2 | 44.38 | 11 | F11:27176 | NaNaKA16\_F7.raw |  |  |  |  |  |  |  |  |  | 8.1441E6 | 5.2634E6 |  |  | 2 | 0 | 0 | 0 | 0 | 0 | 0 | 0 | 0 | 0 | 1 | 1 | 0 | 0 | 58 | 65 | Carbamidomethylation | C3:Carbamidomethylation:1000.00 | PEAKS DB |
| E.KISGC(+57.02)WPYFK.T | N | 51.04 | 1284.6324 | 10 | 0.2 | 643.3236 | 2 | 38.26 | 11 | F11:22081 | NaNaKA16\_F7.raw |  |  |  |  |  |  |  |  |  |  | 3.8432E6 |  |  | 2 | 0 | 0 | 0 | 0 | 0 | 0 | 0 | 0 | 0 | 0 | 2 | 0 | 0 | 56 | 65 | Carbamidomethylation | C5:Carbamidomethylation:1000.00 | PEAKS DB |
| W.WDFADYGC(+57.02)YC(+57.02)GR.G | N | 50.34 | 1568.5813 | 12 | 0.5 | 785.2983 | 2 | 61.41 | 11 | F11:40293 | NaNaKA16\_F7.raw |  |  |  |  |  |  |  |  |  |  | 5.6691E6 |  |  | 1 | 0 | 0 | 0 | 0 | 0 | 0 | 0 | 0 | 0 | 0 | 1 | 0 | 0 | 19 | 30 | Carbamidomethylation | C8:Carbamidomethylation:1000.00;C10:Carbamidomethylation:1000.00 | PEAKS DB |
| W.DFADYGC(+57.02)YC(+57.02)GR.G | N | 49.85 | 1382.5020 | 11 | 0.3 | 692.2585 | 2 | 40.50 | 10 | F10:24767 | NaNaKA16\_F6.raw |  |  |  |  |  |  |  |  | 5.0674E6 | 1.1493E7 | 1.4257E6 |  |  | 3 | 0 | 0 | 0 | 0 | 0 | 0 | 0 | 0 | 1 | 1 | 1 | 0 | 0 | 20 | 30 | Carbamidomethylation | C7:Carbamidomethylation:1000.00;C9:Carbamidomethylation:1000.00 | PEAKS DB |
| K.NMIKC(+57.02)TVPSR.S | N | 48.35 | 1204.6056 | 10 | 0.1 | 603.3101 | 2 | 12.09 | 11 | F11:1822 | NaNaKA16\_F7.raw |  |  |  |  |  |  |  |  |  |  | 1.7697E7 |  |  | 2 | 0 | 0 | 0 | 0 | 0 | 0 | 0 | 0 | 0 | 0 | 2 | 0 | 0 | 7 | 16 | Carbamidomethylation | C5:Carbamidomethylation:1000.00 | PEAKS DB |
| A.PYNDNNYNIDLK.A | N | 48.26 | 1481.6786 | 12 | 0.7 | 741.8471 | 2 | 34.63 | 13 | F13:15900 | NaNaKA16\_F9.raw |  |  |  |  |  |  |  |  |  |  | 1.9835E6 |  | 2.598E6 | 2 | 0 | 0 | 0 | 0 | 0 | 0 | 0 | 0 | 0 | 0 | 1 | 0 | 1 | 104 | 115 |  |  | PEAKS DB |
| K.C(+57.02)TVPSRSWWDFADYGC(+57.02)YC(+57.02)GR.G | N | 48.21 | 2542.0251 | 20 | -0.3 | 848.3488 | 3 | 69.09 | 11 | F11:46749 | NaNaKA16\_F7.raw |  |  |  |  |  |  |  |  |  |  | 6.2414E6 |  |  | 1 | 0 | 0 | 0 | 0 | 0 | 0 | 0 | 0 | 0 | 0 | 1 | 0 | 0 | 11 | 30 | Carbamidomethylation | C1:Carbamidomethylation:1000.00;C16:Carbamidomethylation:1000.00;C18:Carbamidomethylation:1000.00 | PEAKS DB |
| NLYQFKNM(+15.99)IK.C | N | 46.70 | 1313.6802 | 10 | 0.4 | 657.8476 | 2 | 22.54 | 11 | F11:9423 | NaNaKA16\_F7.raw |  |  |  |  |  |  |  |  |  |  | 2.8165E6 |  |  | 1 | 0 | 0 | 0 | 0 | 0 | 0 | 0 | 0 | 0 | 0 | 1 | 0 | 0 | 1 | 10 | Oxidation (M) | M8:Oxidation (M):1000.00 | PEAKS DB |
| R.SWWDFADYGC(+57.02).Y | N | 45.71 | 1305.4761 | 10 | 0.8 | 653.7458 | 2 | 109.86 | 11 | F11:80150 | NaNaKA16\_F7.raw |  |  |  |  |  |  |  |  |  |  | 6.5059E6 |  |  | 1 | 0 | 0 | 0 | 0 | 0 | 0 | 0 | 0 | 0 | 0 | 1 | 0 | 0 | 17 | 26 | Carbamidomethylation | C10:Carbamidomethylation:1000.00 | PEAKS DB |
| G.SGTPVDDLDR.C | N | 45.52 | 1073.4989 | 10 | 0.9 | 537.7572 | 2 | 20.23 | 11 | F11:7497 | NaNaKA16\_F7.raw |  |  |  |  |  |  |  |  |  |  | 1.1072E7 |  |  | 1 | 0 | 0 | 0 | 0 | 0 | 0 | 0 | 0 | 0 | 0 | 1 | 0 | 0 | 33 | 42 |  |  | PEAKS DB |
| K.TYSYEC(+57.02)SQGTLTC(+57.02)KG.D | N | 45.40 | 1753.7288 | 15 | 1.4 | 877.8729 | 2 | 21.92 | 10 | F10:8643 | NaNaKA16\_F6.raw |  |  |  |  |  |  |  |  |  | 0 |  |  |  | 0 | 0 | 0 | 0 | 0 | 0 | 0 | 0 | 0 | 0 | 0 | 0 | 0 | 0 | 66 | 80 | Carbamidomethylation | C6:Carbamidomethylation:1000.00;C13:Carbamidomethylation:1000.00 | PEAKS DB |
| R.SWWDFADYGC(+57.02)Y.C | N | 44.81 | 1468.5394 | 11 | 0.1 | 735.2771 | 2 | 111.77 | 10 | F10:82113 | NaNaKA16\_F6.raw |  |  |  |  |  |  |  |  |  | 1.9586E7 |  | 1.4496E7 |  | 2 | 0 | 0 | 0 | 0 | 0 | 0 | 0 | 0 | 0 | 1 | 0 | 1 | 0 | 17 | 27 | Carbamidomethylation | C10:Carbamidomethylation:1000.00 | PEAKS DB |
| N.DNNYNIDLK.A | N | 44.51 | 1107.5197 | 9 | 1.1 | 554.7677 | 2 | 32.05 | 10 | F10:17657 | NaNaKA16\_F6.raw |  |  |  |  |  |  |  |  |  | 4.8851E7 | 9.5595E6 |  |  | 3 | 0 | 0 | 0 | 0 | 0 | 0 | 0 | 0 | 0 | 2 | 1 | 0 | 0 | 107 | 115 |  |  | PEAKS DB |
| F.AGAPYNDNNYNIDLKAR.C | N | 42.93 | 1907.9126 | 17 | 0.4 | 636.9784 | 3 | 29.66 | 13 | F13:11769 | NaNaKA16\_F9.raw |  |  |  |  |  |  |  |  |  |  |  |  | 8.9691E5 | 1 | 0 | 0 | 0 | 0 | 0 | 0 | 0 | 0 | 0 | 0 | 0 | 0 | 1 | 101 | 117 |  |  | PEAKS DB |
| total 46 peptides |  |  |  |  |  |  |  |  |  |  |  |  |  |  |  |  |  |  |  |  |  |  |  |  |  |  |  |  |  |  |  |  |  |  |  |  |  |  |  |  |  |  |  |
| --- | --- | --- | --- | --- | --- | --- | --- | --- | --- | --- | --- | --- | --- | --- | --- | --- | --- | --- | --- | --- | --- | --- | --- | --- | --- | --- | --- | --- | --- | --- | --- | --- | --- | --- | --- | --- | --- | --- | --- | --- | --- | --- | --- |

1A3D

back to list

  

| Protein Coverage
| Supporting Peptides
|

Protein Coverage:

Supporting Peptides:

| Peptide | Uniq | -10lgP | Mass | Length | ppm | m/z | z | RT | Fraction | Scan | Source File | Area F1 | Area F10 | Area F11 | Area F12 | Area F13 | Area F2 | Area F3 | Area F4 | Area F5 | Area F6 | Area F7 | Area F8 | Area F9 | #Feature | #Feature F1 | #Feature F10 | #Feature F11 | #Feature F12 | #Feature F13 | #Feature F2 | #Feature F3 | #Feature F4 | #Feature F5 | #Feature F6 | #Feature F7 | #Feature F8 | #Feature F9 | Start | End | PTM | AScore | Found By |
| --- | --- | --- | --- | --- | --- | --- | --- | --- | --- | --- | --- | --- | --- | --- | --- | --- | --- | --- | --- | --- | --- | --- | --- | --- | --- | --- | --- | --- | --- | --- | --- | --- | --- | --- | --- | --- | --- | --- | --- | --- | --- | --- | --- |
| K.ISGC(+57.02)WPYFKTYSYEC(+57.02)SQGTLTC(+57.02)K.G | N | 89.48 | 2835.2341 | 23 | -1.5 | 946.0839 | 3 | 57.58 | 11 | F11:37368 | NaNaKA16\_F7.raw |  |  |  |  |  |  |  |  |  | 1.3653E7 | 7.0731E7 | 1.9929E7 | 6.9253E6 | 5 | 0 | 0 | 0 | 0 | 0 | 0 | 0 | 0 | 0 | 1 | 2 | 1 | 1 | 57 | 79 | Carbamidomethylation | C4:Carbamidomethylation:1000.00;C15:Carbamidomethylation:1000.00;C22:Carbamidomethylation:1000.00 | PEAKS DB |
| K.TYSYEC(+57.02)SQGTLTC(+57.02)K.G | N | 89.30 | 1696.7073 | 14 | 0.3 | 849.3611 | 2 | 21.20 | 9 | F9:9021 | NaNaKA16\_F5.raw |  | 7.5988E5 |  | 1.6097E7 | 6.3725E6 | 1.1263E6 |  | 2.9128E6 | 6.1482E8 | 3.4541E10 | 2.2713E9 | 9.1534E6 | 4.1844E8 | 46 | 0 | 1 | 0 | 3 | 1 | 1 | 0 | 1 | 4 | 18 | 3 | 2 | 12 | 66 | 79 | Carbamidomethylation | C6:Carbamidomethylation:1000.00;C13:Carbamidomethylation:1000.00 | PEAKS DB |
| R.LAAIC(+57.02)FAGAPYNDNNYNIDLKAR.C | N | 86.87 | 2583.2539 | 23 | 0.0 | 862.0919 | 3 | 62.98 | 12 | F12:41772 | NaNaKA16\_F8.raw |  |  |  |  |  |  |  |  |  | 4.515E6 | 2.8262E7 | 1.4923E7 | 1.4692E7 | 5 | 0 | 0 | 0 | 0 | 0 | 0 | 0 | 0 | 0 | 1 | 2 | 1 | 1 | 95 | 117 | Carbamidomethylation | C5:Carbamidomethylation:1000.00 | PEAKS DB |
| R.LAAIC(+57.02)FAGAPYNDNNYNIDLK.A | N | 82.29 | 2356.1157 | 21 | -2.1 | 1179.0626 | 2 | 73.79 | 11 | F11:49962 | NaNaKA16\_F7.raw |  | 2.7376E8 | 2.6984E7 | 7.0178E7 | 6.5633E6 |  |  |  | 3.1038E7 | 2.7883E9 | 4.7656E10 | 2.4584E9 | 2.4535E10 | 74 | 0 | 4 | 4 | 2 | 2 | 0 | 0 | 0 | 1 | 2 | 23 | 11 | 25 | 95 | 115 | Carbamidomethylation | C5:Carbamidomethylation:1000.00 | PEAKS DB |
| S.RSWWDFADYGC(+57.02)YC(+57.02)GR.G | N | 77.58 | 1997.7937 | 15 | 1.2 | 666.9393 | 3 | 70.64 | 11 | F11:48142 | NaNaKA16\_F7.raw |  |  |  |  |  |  |  |  |  |  | 3.4823E7 | 9.6445E6 |  | 4 | 0 | 0 | 0 | 0 | 0 | 0 | 0 | 0 | 0 | 0 | 2 | 2 | 0 | 16 | 30 | Carbamidomethylation | C11:Carbamidomethylation:1000.00;C13:Carbamidomethylation:1000.00 | PEAKS DB |
| R.SWWDFADYGC(+57.02)YC(+57.02)GR.G | N | 77.10 | 1841.6926 | 14 | 2.4 | 921.8558 | 2 | 86.60 | 11 | F11:61115 | NaNaKA16\_F7.raw |  | 2.3849E7 | 3.1182E6 | 1.2353E7 | 9.152E5 |  |  |  | 6.0399E6 | 1.4952E9 | 5.4032E10 | 1.5192E10 | 2.1735E9 | 46 | 0 | 2 | 1 | 2 | 1 | 0 | 0 | 0 | 1 | 3 | 17 | 6 | 13 | 17 | 30 | Carbamidomethylation | C10:Carbamidomethylation:1000.00;C12:Carbamidomethylation:1000.00 | PEAKS DB |
| G.APYNDNNYNIDLK.A | N | 74.44 | 1552.7157 | 13 | 0.1 | 777.3652 | 2 | 38.38 | 4 | F4:20647 | NaNaKA16\_F12.raw |  | 1.0864E7 | 7.8409E6 | 1.7533E7 | 6.5956E5 |  |  |  | 1.5969E7 | 3.4996E9 | 2.5425E9 | 1.4323E8 | 4.1292E8 | 10 | 0 | 1 | 1 | 1 | 1 | 0 | 0 | 0 | 1 | 1 | 1 | 1 | 2 | 103 | 115 |  |  | PEAKS DB |
| F.AGAPYNDNNYNIDLK.A | N | 74.15 | 1680.7743 | 15 | 0.8 | 841.3951 | 2 | 39.58 | 12 | F12:22604 | NaNaKA16\_F8.raw |  | 1.9233E6 | 9.618E5 | 3.9059E6 |  |  |  |  |  | 4.7877E8 | 2.5646E8 | 4.8059E7 | 1.8544E9 | 9 | 0 | 1 | 1 | 1 | 0 | 0 | 0 | 0 | 0 | 1 | 3 | 1 | 1 | 101 | 115 |  |  | PEAKS DB |
| D.RLAAIC(+57.02)FAGAPYNDNNYNIDLK.A | N | 71.67 | 2512.2168 | 22 | 0.3 | 838.4131 | 3 | 61.74 | 11 | F11:40640 | NaNaKA16\_F7.raw |  |  |  |  |  |  |  |  |  |  | 5.8961E7 | 7.0057E7 | 3.3382E7 | 4 | 0 | 0 | 0 | 0 | 0 | 0 | 0 | 0 | 0 | 0 | 2 | 1 | 1 | 94 | 115 | Carbamidomethylation | C6:Carbamidomethylation:1000.00 | PEAKS DB |
| C.FAGAPYNDNNYNIDLK.A | N | 70.87 | 1827.8428 | 16 | 0.3 | 914.9290 | 2 | 51.38 | 13 | F13:29549 | NaNaKA16\_F9.raw |  |  |  |  |  |  |  |  |  | 3.3389E7 | 6.5117E7 | 8.1033E6 | 2.2994E8 | 5 | 0 | 0 | 0 | 0 | 0 | 0 | 0 | 0 | 0 | 1 | 1 | 1 | 2 | 100 | 115 |  |  | PEAKS DB |
| K.ISGC(+57.02)WPYFK.T | N | 70.32 | 1156.5375 | 9 | 0.1 | 579.2761 | 2 | 68.82 | 11 | F11:46583 | NaNaKA16\_F7.raw |  | 3.6915E8 | 1.4139E8 | 6.9856E7 | 2.3823E6 |  |  | 3.6353E6 | 3.5126E8 | 2.1014E10 | 1.2727E11 | 1.9979E10 | 1.2073E10 | 71 | 0 | 1 | 1 | 3 | 1 | 0 | 0 | 1 | 1 | 15 | 17 | 10 | 21 | 57 | 65 | Carbamidomethylation | C4:Carbamidomethylation:1000.00 | PEAKS DB |
| K.GDNNAC(+57.02)AASVC(+57.02)DC(+57.02)DR.L | N | 70.22 | 1683.6035 | 15 | 0.2 | 842.8092 | 2 | 12.08 | 11 | F11:1839 | NaNaKA16\_F7.raw |  |  |  |  |  |  |  |  |  | 6.3052E6 | 1.0666E6 | 0 |  | 2 | 0 | 0 | 0 | 0 | 0 | 0 | 0 | 0 | 0 | 1 | 1 | 0 | 0 | 80 | 94 | Carbamidomethylation | C6:Carbamidomethylation:1000.00;C11:Carbamidomethylation:1000.00;C13:Carbamidomethylation:1000.00 | PEAKS DB |
| G.APYNDNNYNIDLKAR.C | N | 70.03 | 1779.8540 | 15 | 0.1 | 594.2920 | 3 | 28.22 | 11 | F11:14195 | NaNaKA16\_F7.raw |  |  |  |  |  |  |  |  |  | 3.8065E6 | 1.1348E7 |  |  | 3 | 0 | 0 | 0 | 0 | 0 | 0 | 0 | 0 | 0 | 1 | 2 | 0 | 0 | 103 | 117 |  |  | PEAKS DB |
| E.AEKISGC(+57.02)WPYFK.T | N | 69.57 | 1484.7122 | 12 | 0.0 | 743.3633 | 2 | 40.74 | 11 | F11:24212 | NaNaKA16\_F7.raw |  |  |  |  |  |  |  |  |  | 9.0796E6 | 5.2934E7 | 1.0921E7 |  | 9 | 0 | 0 | 0 | 0 | 0 | 0 | 0 | 0 | 0 | 2 | 5 | 2 | 0 | 54 | 65 | Carbamidomethylation | C7:Carbamidomethylation:1000.00 | PEAKS DB |
| R.GGSGTPVDDLDR.C | N | 67.81 | 1187.5417 | 12 | 0.8 | 594.7786 | 2 | 19.63 | 10 | F10:6988 | NaNaKA16\_F6.raw |  |  |  |  |  |  |  |  |  | 5.7224E7 | 2.6509E9 | 3.9395E6 |  | 4 | 0 | 0 | 0 | 0 | 0 | 0 | 0 | 0 | 0 | 2 | 1 | 1 | 0 | 31 | 42 |  |  | PEAKS DB |
| NLYQFKNMIKC(+57.02)TVPSR.S | N | 66.48 | 1998.0179 | 16 | 0.3 | 667.0134 | 3 | 40.68 | 12 | F12:23498 | NaNaKA16\_F8.raw |  |  |  |  |  |  |  |  |  |  |  | 3.4886E6 |  | 1 | 0 | 0 | 0 | 0 | 0 | 0 | 0 | 0 | 0 | 0 | 0 | 1 | 0 | 1 | 16 | Carbamidomethylation | C11:Carbamidomethylation:1000.00 | PEAKS DB |
| T.YSYEC(+57.02)SQGTLTC(+57.02)K.G | N | 65.61 | 1595.6595 | 13 | -0.2 | 798.8369 | 2 | 19.78 | 10 | F10:7072 | NaNaKA16\_F6.raw |  |  |  |  |  |  |  |  |  | 9.5485E6 |  |  |  | 1 | 0 | 0 | 0 | 0 | 0 | 0 | 0 | 0 | 0 | 1 | 0 | 0 | 0 | 67 | 79 | Carbamidomethylation | C5:Carbamidomethylation:1000.00;C12:Carbamidomethylation:1000.00 | PEAKS DB |
| Y.SYEC(+57.02)SQGTLTC(+57.02)K.G | N | 65.06 | 1432.5963 | 12 | 0.7 | 717.3060 | 2 | 11.79 | 10 | F10:1902 | NaNaKA16\_F6.raw |  |  |  |  |  |  |  |  |  | 7.186E5 |  |  |  | 1 | 0 | 0 | 0 | 0 | 0 | 0 | 0 | 0 | 0 | 1 | 0 | 0 | 0 | 68 | 79 | Carbamidomethylation | C4:Carbamidomethylation:1000.00;C11:Carbamidomethylation:1000.00 | PEAKS DB |
| A.IC(+57.02)FAGAPYNDNNYNIDLK.A | N | 61.89 | 2100.9575 | 18 | 0.5 | 1051.4866 | 2 | 63.78 | 13 | F13:39698 | NaNaKA16\_F9.raw |  |  |  |  |  |  |  |  |  | 9.6858E6 | 1.8845E7 | 6.9038E6 | 3.858E7 | 4 | 0 | 0 | 0 | 0 | 0 | 0 | 0 | 0 | 0 | 1 | 1 | 1 | 1 | 98 | 115 | Carbamidomethylation | C2:Carbamidomethylation:1000.00 | PEAKS DB |
| R.SWWDFADYGC(+57.02)YC(+57.02)GRG.G | N | 61.68 | 1898.7141 | 15 | 1.5 | 950.3657 | 2 | 84.85 | 11 | F11:59757 | NaNaKA16\_F7.raw |  |  |  |  |  |  |  |  |  |  | 1.1046E7 |  |  | 1 | 0 | 0 | 0 | 0 | 0 | 0 | 0 | 0 | 0 | 0 | 1 | 0 | 0 | 17 | 31 | Carbamidomethylation | C10:Carbamidomethylation:1000.00;C12:Carbamidomethylation:1000.00 | PEAKS DB |
| A.AIC(+57.02)FAGAPYNDNNYNIDLK.A | N | 61.56 | 2171.9946 | 19 | 1.2 | 1087.0059 | 2 | 65.58 | 13 | F13:41436 | NaNaKA16\_F9.raw |  |  |  |  |  |  |  |  |  |  | 1.0912E7 |  | 1.9483E7 | 2 | 0 | 0 | 0 | 0 | 0 | 0 | 0 | 0 | 0 | 0 | 1 | 0 | 1 | 97 | 115 | Carbamidomethylation | C3:Carbamidomethylation:1000.00 | PEAKS DB |
| G.GSGTPVDDLDR.C | N | 60.74 | 1130.5204 | 11 | -0.3 | 566.2673 | 2 | 21.08 | 11 | F11:8172 | NaNaKA16\_F7.raw |  |  |  |  |  |  |  |  |  | 1.4052E5 | 1.2671E7 |  |  | 2 | 0 | 0 | 0 | 0 | 0 | 0 | 0 | 0 | 0 | 1 | 1 | 0 | 0 | 32 | 42 |  |  | PEAKS DB |
| A.GAPYNDNNYNIDLK.A | N | 58.77 | 1609.7372 | 14 | -0.1 | 805.8758 | 2 | 38.63 | 10 | F10:22911 | NaNaKA16\_F6.raw |  |  |  |  |  |  |  |  |  | 4.2863E6 | 2.6962E6 |  | 1.5111E7 | 3 | 0 | 0 | 0 | 0 | 0 | 0 | 0 | 0 | 0 | 1 | 1 | 0 | 1 | 102 | 115 |  |  | PEAKS DB |
| K.TYSYEC(+57.02)SQGTLTC(+57.02)KGDNNAC(+57.02)AASVC(+57.02)DC(+57.02)DR.L | Y | 58.08 | 3362.3003 | 29 | 0.4 | 1121.7745 | 3 | 27.28 | 11 | F11:13291 | NaNaKA16\_F7.raw |  |  |  |  |  |  |  |  |  | 6.9673E7 | 1.4844E7 |  |  | 2 | 0 | 0 | 0 | 0 | 0 | 0 | 0 | 0 | 0 | 1 | 1 | 0 | 0 | 66 | 94 | Carbamidomethylation | C6:Carbamidomethylation:1000.00;C13:Carbamidomethylation:1000.00;C20:Carbamidomethylation:1000.00;C25:Carbamidomethylation:1000.00;C27:Carbamidomethylation:1000.00 | PEAKS DB |
| D.FADYGC(+57.02)YC(+57.02)GR.G | N | 57.15 | 1267.4750 | 10 | 0.8 | 634.7452 | 2 | 25.42 | 11 | F11:11882 | NaNaKA16\_F7.raw |  |  |  |  |  |  |  |  |  | 2.1506E6 | 1.834E7 |  |  | 2 | 0 | 0 | 0 | 0 | 0 | 0 | 0 | 0 | 0 | 1 | 1 | 0 | 0 | 21 | 30 | Carbamidomethylation | C6:Carbamidomethylation:1000.00;C8:Carbamidomethylation:1000.00 | PEAKS DB |
| R.SWWDFADYGC(+57.02)YC(+57.02)GRGGSGTPVDDLDR.C | N | 56.86 | 3011.2239 | 26 | 0.6 | 1004.7491 | 3 | 81.63 | 11 | F11:57027 | NaNaKA16\_F7.raw |  |  |  |  |  |  |  |  |  |  | 4.9169E6 |  |  | 1 | 0 | 0 | 0 | 0 | 0 | 0 | 0 | 0 | 0 | 0 | 1 | 0 | 0 | 17 | 42 | Carbamidomethylation | C10:Carbamidomethylation:1000.00;C12:Carbamidomethylation:1000.00 | PEAKS DB |
| S.WWDFADYGC(+57.02)YC(+57.02)GR.G | N | 56.83 | 1754.6605 | 13 | 0.8 | 878.3382 | 2 | 78.92 | 12 | F12:55446 | NaNaKA16\_F8.raw |  |  |  |  |  |  |  |  |  |  | 1.3475E7 | 3.6699E6 |  | 2 | 0 | 0 | 0 | 0 | 0 | 0 | 0 | 0 | 0 | 0 | 1 | 1 | 0 | 18 | 30 | Carbamidomethylation | C9:Carbamidomethylation:1000.00;C11:Carbamidomethylation:1000.00 | PEAKS DB |
| L.AAIC(+57.02)FAGAPYNDNNYNIDLK.A | N | 56.55 | 2243.0317 | 20 | -0.4 | 1122.5227 | 2 | 66.82 | 13 | F13:42492 | NaNaKA16\_F9.raw |  |  |  |  |  |  |  |  |  |  |  |  | 1.837E7 | 1 | 0 | 0 | 0 | 0 | 0 | 0 | 0 | 0 | 0 | 0 | 0 | 0 | 1 | 96 | 115 | Carbamidomethylation | C4:Carbamidomethylation:1000.00 | PEAKS DB |
| NLYQFKNMIK.C | N | 55.84 | 1297.6853 | 10 | 0.6 | 649.8503 | 2 | 34.48 | 11 | F11:19260 | NaNaKA16\_F7.raw |  |  |  |  |  |  |  |  |  | 2.422E7 | 1.1905E8 | 3.5531E7 | 5.6701E7 | 7 | 0 | 0 | 0 | 0 | 0 | 0 | 0 | 0 | 0 | 2 | 2 | 1 | 2 | 1 | 10 |  |  | PEAKS DB |
| P.YNDNNYNIDLK.A | N | 54.97 | 1384.6259 | 11 | -5.4 | 693.3165 | 2 | 32.55 | 10 | F10:17533 | NaNaKA16\_F6.raw |  |  |  |  |  |  |  |  |  | 8.5654E7 |  |  |  | 1 | 0 | 0 | 0 | 0 | 0 | 0 | 0 | 0 | 0 | 1 | 0 | 0 | 0 | 105 | 115 |  |  | PEAKS DB |
| Y.FKTYSYEC(+57.02)SQGTLTC(+57.02)K.G | N | 54.26 | 1971.8706 | 16 | 0.6 | 658.2979 | 3 | 23.41 | 11 | F11:10060 | NaNaKA16\_F7.raw |  |  |  |  |  |  |  |  |  | 3.532E6 | 1.7096E6 |  |  | 2 | 0 | 0 | 0 | 0 | 0 | 0 | 0 | 0 | 0 | 1 | 1 | 0 | 0 | 64 | 79 | Carbamidomethylation | C8:Carbamidomethylation:1000.00;C15:Carbamidomethylation:1000.00 | PEAKS DB |
| R.LAAIC(+57.02)FAGAPYN.D | N | 53.90 | 1266.6067 | 12 | 0.8 | 634.3111 | 2 | 70.83 | 11 | F11:49747 | NaNaKA16\_F7.raw |  | 2.6932E8 | 6.3964E7 |  |  |  |  |  | 3.6922E8 | 7.2309E9 | 3.8533E9 | 2.4096E9 | 1.5572E9 | 11 | 0 | 1 | 1 | 0 | 0 | 0 | 0 | 0 | 1 | 2 | 3 | 2 | 1 | 95 | 106 | Carbamidomethylation | C5:Carbamidomethylation:1000.00 | PEAKS DB |
| I.SGC(+57.02)WPYFK.T | N | 53.87 | 1043.4535 | 8 | -1.4 | 522.7333 | 2 | 44.38 | 11 | F11:27176 | NaNaKA16\_F7.raw |  |  |  |  |  |  |  |  |  | 8.1441E6 | 5.2634E6 |  |  | 2 | 0 | 0 | 0 | 0 | 0 | 0 | 0 | 0 | 0 | 1 | 1 | 0 | 0 | 58 | 65 | Carbamidomethylation | C3:Carbamidomethylation:1000.00 | PEAKS DB |
| E.KISGC(+57.02)WPYFK.T | N | 51.04 | 1284.6324 | 10 | 0.2 | 643.3236 | 2 | 38.26 | 11 | F11:22081 | NaNaKA16\_F7.raw |  |  |  |  |  |  |  |  |  |  | 3.8432E6 |  |  | 2 | 0 | 0 | 0 | 0 | 0 | 0 | 0 | 0 | 0 | 0 | 2 | 0 | 0 | 56 | 65 | Carbamidomethylation | C5:Carbamidomethylation:1000.00 | PEAKS DB |
| W.WDFADYGC(+57.02)YC(+57.02)GR.G | N | 50.34 | 1568.5813 | 12 | 0.5 | 785.2983 | 2 | 61.41 | 11 | F11:40293 | NaNaKA16\_F7.raw |  |  |  |  |  |  |  |  |  |  | 5.6691E6 |  |  | 1 | 0 | 0 | 0 | 0 | 0 | 0 | 0 | 0 | 0 | 0 | 1 | 0 | 0 | 19 | 30 | Carbamidomethylation | C8:Carbamidomethylation:1000.00;C10:Carbamidomethylation:1000.00 | PEAKS DB |
| W.DFADYGC(+57.02)YC(+57.02)GR.G | N | 49.85 | 1382.5020 | 11 | 0.3 | 692.2585 | 2 | 40.50 | 10 | F10:24767 | NaNaKA16\_F6.raw |  |  |  |  |  |  |  |  | 5.0674E6 | 1.1493E7 | 1.4257E6 |  |  | 3 | 0 | 0 | 0 | 0 | 0 | 0 | 0 | 0 | 1 | 1 | 1 | 0 | 0 | 20 | 30 | Carbamidomethylation | C7:Carbamidomethylation:1000.00;C9:Carbamidomethylation:1000.00 | PEAKS DB |
| K.NMIKC(+57.02)TVPSR.S | N | 48.35 | 1204.6056 | 10 | 0.1 | 603.3101 | 2 | 12.09 | 11 | F11:1822 | NaNaKA16\_F7.raw |  |  |  |  |  |  |  |  |  |  | 1.7697E7 |  |  | 2 | 0 | 0 | 0 | 0 | 0 | 0 | 0 | 0 | 0 | 0 | 2 | 0 | 0 | 7 | 16 | Carbamidomethylation | C5:Carbamidomethylation:1000.00 | PEAKS DB |
| A.PYNDNNYNIDLK.A | N | 48.26 | 1481.6786 | 12 | 0.7 | 741.8471 | 2 | 34.63 | 13 | F13:15900 | NaNaKA16\_F9.raw |  |  |  |  |  |  |  |  |  |  | 1.9835E6 |  | 2.598E6 | 2 | 0 | 0 | 0 | 0 | 0 | 0 | 0 | 0 | 0 | 0 | 1 | 0 | 1 | 104 | 115 |  |  | PEAKS DB |
| K.C(+57.02)TVPSRSWWDFADYGC(+57.02)YC(+57.02)GR.G | N | 48.21 | 2542.0251 | 20 | -0.3 | 848.3488 | 3 | 69.09 | 11 | F11:46749 | NaNaKA16\_F7.raw |  |  |  |  |  |  |  |  |  |  | 6.2414E6 |  |  | 1 | 0 | 0 | 0 | 0 | 0 | 0 | 0 | 0 | 0 | 0 | 1 | 0 | 0 | 11 | 30 | Carbamidomethylation | C1:Carbamidomethylation:1000.00;C16:Carbamidomethylation:1000.00;C18:Carbamidomethylation:1000.00 | PEAKS DB |
| NLYQFKNM(+15.99)IK.C | N | 46.70 | 1313.6802 | 10 | 0.4 | 657.8476 | 2 | 22.54 | 11 | F11:9423 | NaNaKA16\_F7.raw |  |  |  |  |  |  |  |  |  |  | 2.8165E6 |  |  | 1 | 0 | 0 | 0 | 0 | 0 | 0 | 0 | 0 | 0 | 0 | 1 | 0 | 0 | 1 | 10 | Oxidation (M) | M8:Oxidation (M):1000.00 | PEAKS DB |
| R.SWWDFADYGC(+57.02).Y | N | 45.71 | 1305.4761 | 10 | 0.8 | 653.7458 | 2 | 109.86 | 11 | F11:80150 | NaNaKA16\_F7.raw |  |  |  |  |  |  |  |  |  |  | 6.5059E6 |  |  | 1 | 0 | 0 | 0 | 0 | 0 | 0 | 0 | 0 | 0 | 0 | 1 | 0 | 0 | 17 | 26 | Carbamidomethylation | C10:Carbamidomethylation:1000.00 | PEAKS DB |
| G.SGTPVDDLDR.C | N | 45.52 | 1073.4989 | 10 | 0.9 | 537.7572 | 2 | 20.23 | 11 | F11:7497 | NaNaKA16\_F7.raw |  |  |  |  |  |  |  |  |  |  | 1.1072E7 |  |  | 1 | 0 | 0 | 0 | 0 | 0 | 0 | 0 | 0 | 0 | 0 | 1 | 0 | 0 | 33 | 42 |  |  | PEAKS DB |
| K.TYSYEC(+57.02)SQGTLTC(+57.02)KG.D | N | 45.40 | 1753.7288 | 15 | 1.4 | 877.8729 | 2 | 21.92 | 10 | F10:8643 | NaNaKA16\_F6.raw |  |  |  |  |  |  |  |  |  | 0 |  |  |  | 0 | 0 | 0 | 0 | 0 | 0 | 0 | 0 | 0 | 0 | 0 | 0 | 0 | 0 | 66 | 80 | Carbamidomethylation | C6:Carbamidomethylation:1000.00;C13:Carbamidomethylation:1000.00 | PEAKS DB |
| R.SWWDFADYGC(+57.02)Y.C | N | 44.81 | 1468.5394 | 11 | 0.1 | 735.2771 | 2 | 111.77 | 10 | F10:82113 | NaNaKA16\_F6.raw |  |  |  |  |  |  |  |  |  | 1.9586E7 |  | 1.4496E7 |  | 2 | 0 | 0 | 0 | 0 | 0 | 0 | 0 | 0 | 0 | 1 | 0 | 1 | 0 | 17 | 27 | Carbamidomethylation | C10:Carbamidomethylation:1000.00 | PEAKS DB |
| N.DNNYNIDLK.A | N | 44.51 | 1107.5197 | 9 | 1.1 | 554.7677 | 2 | 32.05 | 10 | F10:17657 | NaNaKA16\_F6.raw |  |  |  |  |  |  |  |  |  | 4.8851E7 | 9.5595E6 |  |  | 3 | 0 | 0 | 0 | 0 | 0 | 0 | 0 | 0 | 0 | 2 | 1 | 0 | 0 | 107 | 115 |  |  | PEAKS DB |
| F.AGAPYNDNNYNIDLKAR.C | N | 42.93 | 1907.9126 | 17 | 0.4 | 636.9784 | 3 | 29.66 | 13 | F13:11769 | NaNaKA16\_F9.raw |  |  |  |  |  |  |  |  |  |  |  |  | 8.9691E5 | 1 | 0 | 0 | 0 | 0 | 0 | 0 | 0 | 0 | 0 | 0 | 0 | 0 | 1 | 101 | 117 |  |  | PEAKS DB |
| total 46 peptides |  |  |  |  |  |  |  |  |  |  |  |  |  |  |  |  |  |  |  |  |  |  |  |  |  |  |  |  |  |  |  |  |  |  |  |  |  |  |  |  |  |  |  |
| --- | --- | --- | --- | --- | --- | --- | --- | --- | --- | --- | --- | --- | --- | --- | --- | --- | --- | --- | --- | --- | --- | --- | --- | --- | --- | --- | --- | --- | --- | --- | --- | --- | --- | --- | --- | --- | --- | --- | --- | --- | --- | --- | --- |

P15445.1

back to list

  

| Protein Coverage
| Supporting Peptides
|

Protein Coverage:

Supporting Peptides:

| Peptide | Uniq | -10lgP | Mass | Length | ppm | m/z | z | RT | Fraction | Scan | Source File | Area F1 | Area F10 | Area F11 | Area F12 | Area F13 | Area F2 | Area F3 | Area F4 | Area F5 | Area F6 | Area F7 | Area F8 | Area F9 | #Feature | #Feature F1 | #Feature F10 | #Feature F11 | #Feature F12 | #Feature F13 | #Feature F2 | #Feature F3 | #Feature F4 | #Feature F5 | #Feature F6 | #Feature F7 | #Feature F8 | #Feature F9 | Start | End | PTM | AScore | Found By |
| --- | --- | --- | --- | --- | --- | --- | --- | --- | --- | --- | --- | --- | --- | --- | --- | --- | --- | --- | --- | --- | --- | --- | --- | --- | --- | --- | --- | --- | --- | --- | --- | --- | --- | --- | --- | --- | --- | --- | --- | --- | --- | --- | --- |
| K.ISGC(+57.02)WPYFKTYSYEC(+57.02)SQGTLTC(+57.02)K.G | N | 89.48 | 2835.2341 | 23 | -1.5 | 946.0839 | 3 | 57.58 | 11 | F11:37368 | NaNaKA16\_F7.raw |  |  |  |  |  |  |  |  |  | 1.3653E7 | 7.0731E7 | 1.9929E7 | 6.9253E6 | 5 | 0 | 0 | 0 | 0 | 0 | 0 | 0 | 0 | 0 | 1 | 2 | 1 | 1 | 57 | 79 | Carbamidomethylation | C4:Carbamidomethylation:1000.00;C15:Carbamidomethylation:1000.00;C22:Carbamidomethylation:1000.00 | PEAKS DB |
| K.TYSYEC(+57.02)SQGTLTC(+57.02)K.G | N | 89.30 | 1696.7073 | 14 | 0.3 | 849.3611 | 2 | 21.20 | 9 | F9:9021 | NaNaKA16\_F5.raw |  | 7.5988E5 |  | 1.6097E7 | 6.3725E6 | 1.1263E6 |  | 2.9128E6 | 6.1482E8 | 3.4541E10 | 2.2713E9 | 9.1534E6 | 4.1844E8 | 46 | 0 | 1 | 0 | 3 | 1 | 1 | 0 | 1 | 4 | 18 | 3 | 2 | 12 | 66 | 79 | Carbamidomethylation | C6:Carbamidomethylation:1000.00;C13:Carbamidomethylation:1000.00 | PEAKS DB |
| R.LAAIC(+57.02)FAGAPYNDNNYNIDLKAR.C | N | 86.87 | 2583.2539 | 23 | 0.0 | 862.0919 | 3 | 62.98 | 12 | F12:41772 | NaNaKA16\_F8.raw |  |  |  |  |  |  |  |  |  | 4.515E6 | 2.8262E7 | 1.4923E7 | 1.4692E7 | 5 | 0 | 0 | 0 | 0 | 0 | 0 | 0 | 0 | 0 | 1 | 2 | 1 | 1 | 95 | 117 | Carbamidomethylation | C5:Carbamidomethylation:1000.00 | PEAKS DB |
| R.LAAIC(+57.02)FAGAPYNDNNYNIDLK.A | N | 82.29 | 2356.1157 | 21 | -2.1 | 1179.0626 | 2 | 73.79 | 11 | F11:49962 | NaNaKA16\_F7.raw |  | 2.7376E8 | 2.6984E7 | 7.0178E7 | 6.5633E6 |  |  |  | 3.1038E7 | 2.7883E9 | 4.7656E10 | 2.4584E9 | 2.4535E10 | 74 | 0 | 4 | 4 | 2 | 2 | 0 | 0 | 0 | 1 | 2 | 23 | 11 | 25 | 95 | 115 | Carbamidomethylation | C5:Carbamidomethylation:1000.00 | PEAKS DB |
| S.RSWWDFADYGC(+57.02)YC(+57.02)GR.G | N | 77.58 | 1997.7937 | 15 | 1.2 | 666.9393 | 3 | 70.64 | 11 | F11:48142 | NaNaKA16\_F7.raw |  |  |  |  |  |  |  |  |  |  | 3.4823E7 | 9.6445E6 |  | 4 | 0 | 0 | 0 | 0 | 0 | 0 | 0 | 0 | 0 | 0 | 2 | 2 | 0 | 16 | 30 | Carbamidomethylation | C11:Carbamidomethylation:1000.00;C13:Carbamidomethylation:1000.00 | PEAKS DB |
| R.SWWDFADYGC(+57.02)YC(+57.02)GR.G | N | 77.10 | 1841.6926 | 14 | 2.4 | 921.8558 | 2 | 86.60 | 11 | F11:61115 | NaNaKA16\_F7.raw |  | 2.3849E7 | 3.1182E6 | 1.2353E7 | 9.152E5 |  |  |  | 6.0399E6 | 1.4952E9 | 5.4032E10 | 1.5192E10 | 2.1735E9 | 46 | 0 | 2 | 1 | 2 | 1 | 0 | 0 | 0 | 1 | 3 | 17 | 6 | 13 | 17 | 30 | Carbamidomethylation | C10:Carbamidomethylation:1000.00;C12:Carbamidomethylation:1000.00 | PEAKS DB |
| G.APYNDNNYNIDLK.A | N | 74.44 | 1552.7157 | 13 | 0.1 | 777.3652 | 2 | 38.38 | 4 | F4:20647 | NaNaKA16\_F12.raw |  | 1.0864E7 | 7.8409E6 | 1.7533E7 | 6.5956E5 |  |  |  | 1.5969E7 | 3.4996E9 | 2.5425E9 | 1.4323E8 | 4.1292E8 | 10 | 0 | 1 | 1 | 1 | 1 | 0 | 0 | 0 | 1 | 1 | 1 | 1 | 2 | 103 | 115 |  |  | PEAKS DB |
| F.AGAPYNDNNYNIDLK.A | N | 74.15 | 1680.7743 | 15 | 0.8 | 841.3951 | 2 | 39.58 | 12 | F12:22604 | NaNaKA16\_F8.raw |  | 1.9233E6 | 9.618E5 | 3.9059E6 |  |  |  |  |  | 4.7877E8 | 2.5646E8 | 4.8059E7 | 1.8544E9 | 9 | 0 | 1 | 1 | 1 | 0 | 0 | 0 | 0 | 0 | 1 | 3 | 1 | 1 | 101 | 115 |  |  | PEAKS DB |
| D.RLAAIC(+57.02)FAGAPYNDNNYNIDLK.A | N | 71.67 | 2512.2168 | 22 | 0.3 | 838.4131 | 3 | 61.74 | 11 | F11:40640 | NaNaKA16\_F7.raw |  |  |  |  |  |  |  |  |  |  | 5.8961E7 | 7.0057E7 | 3.3382E7 | 4 | 0 | 0 | 0 | 0 | 0 | 0 | 0 | 0 | 0 | 0 | 2 | 1 | 1 | 94 | 115 | Carbamidomethylation | C6:Carbamidomethylation:1000.00 | PEAKS DB |
| C.FAGAPYNDNNYNIDLK.A | N | 70.87 | 1827.8428 | 16 | 0.3 | 914.9290 | 2 | 51.38 | 13 | F13:29549 | NaNaKA16\_F9.raw |  |  |  |  |  |  |  |  |  | 3.3389E7 | 6.5117E7 | 8.1033E6 | 2.2994E8 | 5 | 0 | 0 | 0 | 0 | 0 | 0 | 0 | 0 | 0 | 1 | 1 | 1 | 2 | 100 | 115 |  |  | PEAKS DB |
| K.ISGC(+57.02)WPYFK.T | N | 70.32 | 1156.5375 | 9 | 0.1 | 579.2761 | 2 | 68.82 | 11 | F11:46583 | NaNaKA16\_F7.raw |  | 3.6915E8 | 1.4139E8 | 6.9856E7 | 2.3823E6 |  |  | 3.6353E6 | 3.5126E8 | 2.1014E10 | 1.2727E11 | 1.9979E10 | 1.2073E10 | 71 | 0 | 1 | 1 | 3 | 1 | 0 | 0 | 1 | 1 | 15 | 17 | 10 | 21 | 57 | 65 | Carbamidomethylation | C4:Carbamidomethylation:1000.00 | PEAKS DB |
| K.GDNNAC(+57.02)AASVC(+57.02)DC(+57.02)DR.L | N | 70.22 | 1683.6035 | 15 | 0.2 | 842.8092 | 2 | 12.08 | 11 | F11:1839 | NaNaKA16\_F7.raw |  |  |  |  |  |  |  |  |  | 6.3052E6 | 1.0666E6 | 0 |  | 2 | 0 | 0 | 0 | 0 | 0 | 0 | 0 | 0 | 0 | 1 | 1 | 0 | 0 | 80 | 94 | Carbamidomethylation | C6:Carbamidomethylation:1000.00;C11:Carbamidomethylation:1000.00;C13:Carbamidomethylation:1000.00 | PEAKS DB |
| G.APYNDNNYNIDLKAR.C | N | 70.03 | 1779.8540 | 15 | 0.1 | 594.2920 | 3 | 28.22 | 11 | F11:14195 | NaNaKA16\_F7.raw |  |  |  |  |  |  |  |  |  | 3.8065E6 | 1.1348E7 |  |  | 3 | 0 | 0 | 0 | 0 | 0 | 0 | 0 | 0 | 0 | 1 | 2 | 0 | 0 | 103 | 117 |  |  | PEAKS DB |
| E.AEKISGC(+57.02)WPYFK.T | N | 69.57 | 1484.7122 | 12 | 0.0 | 743.3633 | 2 | 40.74 | 11 | F11:24212 | NaNaKA16\_F7.raw |  |  |  |  |  |  |  |  |  | 9.0796E6 | 5.2934E7 | 1.0921E7 |  | 9 | 0 | 0 | 0 | 0 | 0 | 0 | 0 | 0 | 0 | 2 | 5 | 2 | 0 | 54 | 65 | Carbamidomethylation | C7:Carbamidomethylation:1000.00 | PEAKS DB |
| R.GGSGTPVDDLDR.C | N | 67.81 | 1187.5417 | 12 | 0.8 | 594.7786 | 2 | 19.63 | 10 | F10:6988 | NaNaKA16\_F6.raw |  |  |  |  |  |  |  |  |  | 5.7224E7 | 2.6509E9 | 3.9395E6 |  | 4 | 0 | 0 | 0 | 0 | 0 | 0 | 0 | 0 | 0 | 2 | 1 | 1 | 0 | 31 | 42 |  |  | PEAKS DB |
| NLYQFKNMIKC(+57.02)TVPSR.S | N | 66.48 | 1998.0179 | 16 | 0.3 | 667.0134 | 3 | 40.68 | 12 | F12:23498 | NaNaKA16\_F8.raw |  |  |  |  |  |  |  |  |  |  |  | 3.4886E6 |  | 1 | 0 | 0 | 0 | 0 | 0 | 0 | 0 | 0 | 0 | 0 | 0 | 1 | 0 | 1 | 16 | Carbamidomethylation | C11:Carbamidomethylation:1000.00 | PEAKS DB |
| T.YSYEC(+57.02)SQGTLTC(+57.02)K.G | N | 65.61 | 1595.6595 | 13 | -0.2 | 798.8369 | 2 | 19.78 | 10 | F10:7072 | NaNaKA16\_F6.raw |  |  |  |  |  |  |  |  |  | 9.5485E6 |  |  |  | 1 | 0 | 0 | 0 | 0 | 0 | 0 | 0 | 0 | 0 | 1 | 0 | 0 | 0 | 67 | 79 | Carbamidomethylation | C5:Carbamidomethylation:1000.00;C12:Carbamidomethylation:1000.00 | PEAKS DB |
| Y.SYEC(+57.02)SQGTLTC(+57.02)K.G | N | 65.06 | 1432.5963 | 12 | 0.7 | 717.3060 | 2 | 11.79 | 10 | F10:1902 | NaNaKA16\_F6.raw |  |  |  |  |  |  |  |  |  | 7.186E5 |  |  |  | 1 | 0 | 0 | 0 | 0 | 0 | 0 | 0 | 0 | 0 | 1 | 0 | 0 | 0 | 68 | 79 | Carbamidomethylation | C4:Carbamidomethylation:1000.00;C11:Carbamidomethylation:1000.00 | PEAKS DB |
| A.IC(+57.02)FAGAPYNDNNYNIDLK.A | N | 61.89 | 2100.9575 | 18 | 0.5 | 1051.4866 | 2 | 63.78 | 13 | F13:39698 | NaNaKA16\_F9.raw |  |  |  |  |  |  |  |  |  | 9.6858E6 | 1.8845E7 | 6.9038E6 | 3.858E7 | 4 | 0 | 0 | 0 | 0 | 0 | 0 | 0 | 0 | 0 | 1 | 1 | 1 | 1 | 98 | 115 | Carbamidomethylation | C2:Carbamidomethylation:1000.00 | PEAKS DB |
| R.SWWDFADYGC(+57.02)YC(+57.02)GRG.G | N | 61.68 | 1898.7141 | 15 | 1.5 | 950.3657 | 2 | 84.85 | 11 | F11:59757 | NaNaKA16\_F7.raw |  |  |  |  |  |  |  |  |  |  | 1.1046E7 |  |  | 1 | 0 | 0 | 0 | 0 | 0 | 0 | 0 | 0 | 0 | 0 | 1 | 0 | 0 | 17 | 31 | Carbamidomethylation | C10:Carbamidomethylation:1000.00;C12:Carbamidomethylation:1000.00 | PEAKS DB |
| A.AIC(+57.02)FAGAPYNDNNYNIDLK.A | N | 61.56 | 2171.9946 | 19 | 1.2 | 1087.0059 | 2 | 65.58 | 13 | F13:41436 | NaNaKA16\_F9.raw |  |  |  |  |  |  |  |  |  |  | 1.0912E7 |  | 1.9483E7 | 2 | 0 | 0 | 0 | 0 | 0 | 0 | 0 | 0 | 0 | 0 | 1 | 0 | 1 | 97 | 115 | Carbamidomethylation | C3:Carbamidomethylation:1000.00 | PEAKS DB |
| G.GSGTPVDDLDR.C | N | 60.74 | 1130.5204 | 11 | -0.3 | 566.2673 | 2 | 21.08 | 11 | F11:8172 | NaNaKA16\_F7.raw |  |  |  |  |  |  |  |  |  | 1.4052E5 | 1.2671E7 |  |  | 2 | 0 | 0 | 0 | 0 | 0 | 0 | 0 | 0 | 0 | 1 | 1 | 0 | 0 | 32 | 42 |  |  | PEAKS DB |
| A.GAPYNDNNYNIDLK.A | N | 58.77 | 1609.7372 | 14 | -0.1 | 805.8758 | 2 | 38.63 | 10 | F10:22911 | NaNaKA16\_F6.raw |  |  |  |  |  |  |  |  |  | 4.2863E6 | 2.6962E6 |  | 1.5111E7 | 3 | 0 | 0 | 0 | 0 | 0 | 0 | 0 | 0 | 0 | 1 | 1 | 0 | 1 | 102 | 115 |  |  | PEAKS DB |
| K.TYSYEC(+57.02)SQGTLTC(+57.02)KGDNNAC(+57.02)AASVC(+57.02)DC(+57.02)DR.L | Y | 58.08 | 3362.3003 | 29 | 0.4 | 1121.7745 | 3 | 27.28 | 11 | F11:13291 | NaNaKA16\_F7.raw |  |  |  |  |  |  |  |  |  | 6.9673E7 | 1.4844E7 |  |  | 2 | 0 | 0 | 0 | 0 | 0 | 0 | 0 | 0 | 0 | 1 | 1 | 0 | 0 | 66 | 94 | Carbamidomethylation | C6:Carbamidomethylation:1000.00;C13:Carbamidomethylation:1000.00;C20:Carbamidomethylation:1000.00;C25:Carbamidomethylation:1000.00;C27:Carbamidomethylation:1000.00 | PEAKS DB |
| D.FADYGC(+57.02)YC(+57.02)GR.G | N | 57.15 | 1267.4750 | 10 | 0.8 | 634.7452 | 2 | 25.42 | 11 | F11:11882 | NaNaKA16\_F7.raw |  |  |  |  |  |  |  |  |  | 2.1506E6 | 1.834E7 |  |  | 2 | 0 | 0 | 0 | 0 | 0 | 0 | 0 | 0 | 0 | 1 | 1 | 0 | 0 | 21 | 30 | Carbamidomethylation | C6:Carbamidomethylation:1000.00;C8:Carbamidomethylation:1000.00 | PEAKS DB |
| R.SWWDFADYGC(+57.02)YC(+57.02)GRGGSGTPVDDLDR.C | N | 56.86 | 3011.2239 | 26 | 0.6 | 1004.7491 | 3 | 81.63 | 11 | F11:57027 | NaNaKA16\_F7.raw |  |  |  |  |  |  |  |  |  |  | 4.9169E6 |  |  | 1 | 0 | 0 | 0 | 0 | 0 | 0 | 0 | 0 | 0 | 0 | 1 | 0 | 0 | 17 | 42 | Carbamidomethylation | C10:Carbamidomethylation:1000.00;C12:Carbamidomethylation:1000.00 | PEAKS DB |
| S.WWDFADYGC(+57.02)YC(+57.02)GR.G | N | 56.83 | 1754.6605 | 13 | 0.8 | 878.3382 | 2 | 78.92 | 12 | F12:55446 | NaNaKA16\_F8.raw |  |  |  |  |  |  |  |  |  |  | 1.3475E7 | 3.6699E6 |  | 2 | 0 | 0 | 0 | 0 | 0 | 0 | 0 | 0 | 0 | 0 | 1 | 1 | 0 | 18 | 30 | Carbamidomethylation | C9:Carbamidomethylation:1000.00;C11:Carbamidomethylation:1000.00 | PEAKS DB |
| L.AAIC(+57.02)FAGAPYNDNNYNIDLK.A | N | 56.55 | 2243.0317 | 20 | -0.4 | 1122.5227 | 2 | 66.82 | 13 | F13:42492 | NaNaKA16\_F9.raw |  |  |  |  |  |  |  |  |  |  |  |  | 1.837E7 | 1 | 0 | 0 | 0 | 0 | 0 | 0 | 0 | 0 | 0 | 0 | 0 | 0 | 1 | 96 | 115 | Carbamidomethylation | C4:Carbamidomethylation:1000.00 | PEAKS DB |
| NLYQFKNMIK.C | N | 55.84 | 1297.6853 | 10 | 0.6 | 649.8503 | 2 | 34.48 | 11 | F11:19260 | NaNaKA16\_F7.raw |  |  |  |  |  |  |  |  |  | 2.422E7 | 1.1905E8 | 3.5531E7 | 5.6701E7 | 7 | 0 | 0 | 0 | 0 | 0 | 0 | 0 | 0 | 0 | 2 | 2 | 1 | 2 | 1 | 10 |  |  | PEAKS DB |
| P.YNDNNYNIDLK.A | N | 54.97 | 1384.6259 | 11 | -5.4 | 693.3165 | 2 | 32.55 | 10 | F10:17533 | NaNaKA16\_F6.raw |  |  |  |  |  |  |  |  |  | 8.5654E7 |  |  |  | 1 | 0 | 0 | 0 | 0 | 0 | 0 | 0 | 0 | 0 | 1 | 0 | 0 | 0 | 105 | 115 |  |  | PEAKS DB |
| Y.FKTYSYEC(+57.02)SQGTLTC(+57.02)K.G | N | 54.26 | 1971.8706 | 16 | 0.6 | 658.2979 | 3 | 23.41 | 11 | F11:10060 | NaNaKA16\_F7.raw |  |  |  |  |  |  |  |  |  | 3.532E6 | 1.7096E6 |  |  | 2 | 0 | 0 | 0 | 0 | 0 | 0 | 0 | 0 | 0 | 1 | 1 | 0 | 0 | 64 | 79 | Carbamidomethylation | C8:Carbamidomethylation:1000.00;C15:Carbamidomethylation:1000.00 | PEAKS DB |
| R.LAAIC(+57.02)FAGAPYN.D | N | 53.90 | 1266.6067 | 12 | 0.8 | 634.3111 | 2 | 70.83 | 11 | F11:49747 | NaNaKA16\_F7.raw |  | 2.6932E8 | 6.3964E7 |  |  |  |  |  | 3.6922E8 | 7.2309E9 | 3.8533E9 | 2.4096E9 | 1.5572E9 | 11 | 0 | 1 | 1 | 0 | 0 | 0 | 0 | 0 | 1 | 2 | 3 | 2 | 1 | 95 | 106 | Carbamidomethylation | C5:Carbamidomethylation:1000.00 | PEAKS DB |
| I.SGC(+57.02)WPYFK.T | N | 53.87 | 1043.4535 | 8 | -1.4 | 522.7333 | 2 | 44.38 | 11 | F11:27176 | NaNaKA16\_F7.raw |  |  |  |  |  |  |  |  |  | 8.1441E6 | 5.2634E6 |  |  | 2 | 0 | 0 | 0 | 0 | 0 | 0 | 0 | 0 | 0 | 1 | 1 | 0 | 0 | 58 | 65 | Carbamidomethylation | C3:Carbamidomethylation:1000.00 | PEAKS DB |
| E.KISGC(+57.02)WPYFK.T | N | 51.04 | 1284.6324 | 10 | 0.2 | 643.3236 | 2 | 38.26 | 11 | F11:22081 | NaNaKA16\_F7.raw |  |  |  |  |  |  |  |  |  |  | 3.8432E6 |  |  | 2 | 0 | 0 | 0 | 0 | 0 | 0 | 0 | 0 | 0 | 0 | 2 | 0 | 0 | 56 | 65 | Carbamidomethylation | C5:Carbamidomethylation:1000.00 | PEAKS DB |
| W.WDFADYGC(+57.02)YC(+57.02)GR.G | N | 50.34 | 1568.5813 | 12 | 0.5 | 785.2983 | 2 | 61.41 | 11 | F11:40293 | NaNaKA16\_F7.raw |  |  |  |  |  |  |  |  |  |  | 5.6691E6 |  |  | 1 | 0 | 0 | 0 | 0 | 0 | 0 | 0 | 0 | 0 | 0 | 1 | 0 | 0 | 19 | 30 | Carbamidomethylation | C8:Carbamidomethylation:1000.00;C10:Carbamidomethylation:1000.00 | PEAKS DB |
| W.DFADYGC(+57.02)YC(+57.02)GR.G | N | 49.85 | 1382.5020 | 11 | 0.3 | 692.2585 | 2 | 40.50 | 10 | F10:24767 | NaNaKA16\_F6.raw |  |  |  |  |  |  |  |  | 5.0674E6 | 1.1493E7 | 1.4257E6 |  |  | 3 | 0 | 0 | 0 | 0 | 0 | 0 | 0 | 0 | 1 | 1 | 1 | 0 | 0 | 20 | 30 | Carbamidomethylation | C7:Carbamidomethylation:1000.00;C9:Carbamidomethylation:1000.00 | PEAKS DB |
| K.NMIKC(+57.02)TVPSR.S | N | 48.35 | 1204.6056 | 10 | 0.1 | 603.3101 | 2 | 12.09 | 11 | F11:1822 | NaNaKA16\_F7.raw |  |  |  |  |  |  |  |  |  |  | 1.7697E7 |  |  | 2 | 0 | 0 | 0 | 0 | 0 | 0 | 0 | 0 | 0 | 0 | 2 | 0 | 0 | 7 | 16 | Carbamidomethylation | C5:Carbamidomethylation:1000.00 | PEAKS DB |
| A.PYNDNNYNIDLK.A | N | 48.26 | 1481.6786 | 12 | 0.7 | 741.8471 | 2 | 34.63 | 13 | F13:15900 | NaNaKA16\_F9.raw |  |  |  |  |  |  |  |  |  |  | 1.9835E6 |  | 2.598E6 | 2 | 0 | 0 | 0 | 0 | 0 | 0 | 0 | 0 | 0 | 0 | 1 | 0 | 1 | 104 | 115 |  |  | PEAKS DB |
| K.C(+57.02)TVPSRSWWDFADYGC(+57.02)YC(+57.02)GR.G | N | 48.21 | 2542.0251 | 20 | -0.3 | 848.3488 | 3 | 69.09 | 11 | F11:46749 | NaNaKA16\_F7.raw |  |  |  |  |  |  |  |  |  |  | 6.2414E6 |  |  | 1 | 0 | 0 | 0 | 0 | 0 | 0 | 0 | 0 | 0 | 0 | 1 | 0 | 0 | 11 | 30 | Carbamidomethylation | C1:Carbamidomethylation:1000.00;C16:Carbamidomethylation:1000.00;C18:Carbamidomethylation:1000.00 | PEAKS DB |
| NLYQFKNM(+15.99)IK.C | N | 46.70 | 1313.6802 | 10 | 0.4 | 657.8476 | 2 | 22.54 | 11 | F11:9423 | NaNaKA16\_F7.raw |  |  |  |  |  |  |  |  |  |  | 2.8165E6 |  |  | 1 | 0 | 0 | 0 | 0 | 0 | 0 | 0 | 0 | 0 | 0 | 1 | 0 | 0 | 1 | 10 | Oxidation (M) | M8:Oxidation (M):1000.00 | PEAKS DB |
| R.SWWDFADYGC(+57.02).Y | N | 45.71 | 1305.4761 | 10 | 0.8 | 653.7458 | 2 | 109.86 | 11 | F11:80150 | NaNaKA16\_F7.raw |  |  |  |  |  |  |  |  |  |  | 6.5059E6 |  |  | 1 | 0 | 0 | 0 | 0 | 0 | 0 | 0 | 0 | 0 | 0 | 1 | 0 | 0 | 17 | 26 | Carbamidomethylation | C10:Carbamidomethylation:1000.00 | PEAKS DB |
| G.SGTPVDDLDR.C | N | 45.52 | 1073.4989 | 10 | 0.9 | 537.7572 | 2 | 20.23 | 11 | F11:7497 | NaNaKA16\_F7.raw |  |  |  |  |  |  |  |  |  |  | 1.1072E7 |  |  | 1 | 0 | 0 | 0 | 0 | 0 | 0 | 0 | 0 | 0 | 0 | 1 | 0 | 0 | 33 | 42 |  |  | PEAKS DB |
| K.TYSYEC(+57.02)SQGTLTC(+57.02)KG.D | N | 45.40 | 1753.7288 | 15 | 1.4 | 877.8729 | 2 | 21.92 | 10 | F10:8643 | NaNaKA16\_F6.raw |  |  |  |  |  |  |  |  |  | 0 |  |  |  | 0 | 0 | 0 | 0 | 0 | 0 | 0 | 0 | 0 | 0 | 0 | 0 | 0 | 0 | 66 | 80 | Carbamidomethylation | C6:Carbamidomethylation:1000.00;C13:Carbamidomethylation:1000.00 | PEAKS DB |
| R.SWWDFADYGC(+57.02)Y.C | N | 44.81 | 1468.5394 | 11 | 0.1 | 735.2771 | 2 | 111.77 | 10 | F10:82113 | NaNaKA16\_F6.raw |  |  |  |  |  |  |  |  |  | 1.9586E7 |  | 1.4496E7 |  | 2 | 0 | 0 | 0 | 0 | 0 | 0 | 0 | 0 | 0 | 1 | 0 | 1 | 0 | 17 | 27 | Carbamidomethylation | C10:Carbamidomethylation:1000.00 | PEAKS DB |
| N.DNNYNIDLK.A | N | 44.51 | 1107.5197 | 9 | 1.1 | 554.7677 | 2 | 32.05 | 10 | F10:17657 | NaNaKA16\_F6.raw |  |  |  |  |  |  |  |  |  | 4.8851E7 | 9.5595E6 |  |  | 3 | 0 | 0 | 0 | 0 | 0 | 0 | 0 | 0 | 0 | 2 | 1 | 0 | 0 | 107 | 115 |  |  | PEAKS DB |
| F.AGAPYNDNNYNIDLKAR.C | N | 42.93 | 1907.9126 | 17 | 0.4 | 636.9784 | 3 | 29.66 | 13 | F13:11769 | NaNaKA16\_F9.raw |  |  |  |  |  |  |  |  |  |  |  |  | 8.9691E5 | 1 | 0 | 0 | 0 | 0 | 0 | 0 | 0 | 0 | 0 | 0 | 0 | 0 | 1 | 101 | 117 |  |  | PEAKS DB |
| total 46 peptides |  |  |  |  |  |  |  |  |  |  |  |  |  |  |  |  |  |  |  |  |  |  |  |  |  |  |  |  |  |  |  |  |  |  |  |  |  |  |  |  |  |  |  |
| --- | --- | --- | --- | --- | --- | --- | --- | --- | --- | --- | --- | --- | --- | --- | --- | --- | --- | --- | --- | --- | --- | --- | --- | --- | --- | --- | --- | --- | --- | --- | --- | --- | --- | --- | --- | --- | --- | --- | --- | --- | --- | --- | --- |

1PSH

back to list

  

| Protein Coverage
| Supporting Peptides
|

Protein Coverage:

Supporting Peptides:

| Peptide | Uniq | -10lgP | Mass | Length | ppm | m/z | z | RT | Fraction | Scan | Source File | Area F1 | Area F10 | Area F11 | Area F12 | Area F13 | Area F2 | Area F3 | Area F4 | Area F5 | Area F6 | Area F7 | Area F8 | Area F9 | #Feature | #Feature F1 | #Feature F10 | #Feature F11 | #Feature F12 | #Feature F13 | #Feature F2 | #Feature F3 | #Feature F4 | #Feature F5 | #Feature F6 | #Feature F7 | #Feature F8 | #Feature F9 | Start | End | PTM | AScore | Found By |
| --- | --- | --- | --- | --- | --- | --- | --- | --- | --- | --- | --- | --- | --- | --- | --- | --- | --- | --- | --- | --- | --- | --- | --- | --- | --- | --- | --- | --- | --- | --- | --- | --- | --- | --- | --- | --- | --- | --- | --- | --- | --- | --- | --- |
| K.ISGC(+57.02)WPYFKTYSYEC(+57.02)SQGTLTC(+57.02)K.G | N | 89.48 | 2835.2341 | 23 | -1.5 | 946.0839 | 3 | 57.58 | 11 | F11:37368 | NaNaKA16\_F7.raw |  |  |  |  |  |  |  |  |  | 1.3653E7 | 7.0731E7 | 1.9929E7 | 6.9253E6 | 5 | 0 | 0 | 0 | 0 | 0 | 0 | 0 | 0 | 0 | 1 | 2 | 1 | 1 | 57 | 79 | Carbamidomethylation | C4:Carbamidomethylation:1000.00;C15:Carbamidomethylation:1000.00;C22:Carbamidomethylation:1000.00 | PEAKS DB |
| K.TYSYEC(+57.02)SQGTLTC(+57.02)K.G | N | 89.30 | 1696.7073 | 14 | 0.3 | 849.3611 | 2 | 21.20 | 9 | F9:9021 | NaNaKA16\_F5.raw |  | 7.5988E5 |  | 1.6097E7 | 6.3725E6 | 1.1263E6 |  | 2.9128E6 | 6.1482E8 | 3.4541E10 | 2.2713E9 | 9.1534E6 | 4.1844E8 | 46 | 0 | 1 | 0 | 3 | 1 | 1 | 0 | 1 | 4 | 18 | 3 | 2 | 12 | 66 | 79 | Carbamidomethylation | C6:Carbamidomethylation:1000.00;C13:Carbamidomethylation:1000.00 | PEAKS DB |
| R.LAAIC(+57.02)FAGAPYNDNNYNIDLKAR.C | N | 86.87 | 2583.2539 | 23 | 0.0 | 862.0919 | 3 | 62.98 | 12 | F12:41772 | NaNaKA16\_F8.raw |  |  |  |  |  |  |  |  |  | 4.515E6 | 2.8262E7 | 1.4923E7 | 1.4692E7 | 5 | 0 | 0 | 0 | 0 | 0 | 0 | 0 | 0 | 0 | 1 | 2 | 1 | 1 | 95 | 117 | Carbamidomethylation | C5:Carbamidomethylation:1000.00 | PEAKS DB |
| R.LAAIC(+57.02)FAGAPYNDNNYNIDLK.A | N | 82.29 | 2356.1157 | 21 | -2.1 | 1179.0626 | 2 | 73.79 | 11 | F11:49962 | NaNaKA16\_F7.raw |  | 2.7376E8 | 2.6984E7 | 7.0178E7 | 6.5633E6 |  |  |  | 3.1038E7 | 2.7883E9 | 4.7656E10 | 2.4584E9 | 2.4535E10 | 74 | 0 | 4 | 4 | 2 | 2 | 0 | 0 | 0 | 1 | 2 | 23 | 11 | 25 | 95 | 115 | Carbamidomethylation | C5:Carbamidomethylation:1000.00 | PEAKS DB |
| S.RSWWDFADYGC(+57.02)YC(+57.02)GR.G | N | 77.58 | 1997.7937 | 15 | 1.2 | 666.9393 | 3 | 70.64 | 11 | F11:48142 | NaNaKA16\_F7.raw |  |  |  |  |  |  |  |  |  |  | 3.4823E7 | 9.6445E6 |  | 4 | 0 | 0 | 0 | 0 | 0 | 0 | 0 | 0 | 0 | 0 | 2 | 2 | 0 | 16 | 30 | Carbamidomethylation | C11:Carbamidomethylation:1000.00;C13:Carbamidomethylation:1000.00 | PEAKS DB |
| R.SWWDFADYGC(+57.02)YC(+57.02)GR.G | N | 77.10 | 1841.6926 | 14 | 2.4 | 921.8558 | 2 | 86.60 | 11 | F11:61115 | NaNaKA16\_F7.raw |  | 2.3849E7 | 3.1182E6 | 1.2353E7 | 9.152E5 |  |  |  | 6.0399E6 | 1.4952E9 | 5.4032E10 | 1.5192E10 | 2.1735E9 | 46 | 0 | 2 | 1 | 2 | 1 | 0 | 0 | 0 | 1 | 3 | 17 | 6 | 13 | 17 | 30 | Carbamidomethylation | C10:Carbamidomethylation:1000.00;C12:Carbamidomethylation:1000.00 | PEAKS DB |
| G.APYNDNNYNIDLK.A | N | 74.44 | 1552.7157 | 13 | 0.1 | 777.3652 | 2 | 38.38 | 4 | F4:20647 | NaNaKA16\_F12.raw |  | 1.0864E7 | 7.8409E6 | 1.7533E7 | 6.5956E5 |  |  |  | 1.5969E7 | 3.4996E9 | 2.5425E9 | 1.4323E8 | 4.1292E8 | 10 | 0 | 1 | 1 | 1 | 1 | 0 | 0 | 0 | 1 | 1 | 1 | 1 | 2 | 103 | 115 |  |  | PEAKS DB |
| F.AGAPYNDNNYNIDLK.A | N | 74.15 | 1680.7743 | 15 | 0.8 | 841.3951 | 2 | 39.58 | 12 | F12:22604 | NaNaKA16\_F8.raw |  | 1.9233E6 | 9.618E5 | 3.9059E6 |  |  |  |  |  | 4.7877E8 | 2.5646E8 | 4.8059E7 | 1.8544E9 | 9 | 0 | 1 | 1 | 1 | 0 | 0 | 0 | 0 | 0 | 1 | 3 | 1 | 1 | 101 | 115 |  |  | PEAKS DB |
| D.RLAAIC(+57.02)FAGAPYNDNNYNIDLK.A | N | 71.67 | 2512.2168 | 22 | 0.3 | 838.4131 | 3 | 61.74 | 11 | F11:40640 | NaNaKA16\_F7.raw |  |  |  |  |  |  |  |  |  |  | 5.8961E7 | 7.0057E7 | 3.3382E7 | 4 | 0 | 0 | 0 | 0 | 0 | 0 | 0 | 0 | 0 | 0 | 2 | 1 | 1 | 94 | 115 | Carbamidomethylation | C6:Carbamidomethylation:1000.00 | PEAKS DB |
| C.FAGAPYNDNNYNIDLK.A | N | 70.87 | 1827.8428 | 16 | 0.3 | 914.9290 | 2 | 51.38 | 13 | F13:29549 | NaNaKA16\_F9.raw |  |  |  |  |  |  |  |  |  | 3.3389E7 | 6.5117E7 | 8.1033E6 | 2.2994E8 | 5 | 0 | 0 | 0 | 0 | 0 | 0 | 0 | 0 | 0 | 1 | 1 | 1 | 2 | 100 | 115 |  |  | PEAKS DB |
| K.ISGC(+57.02)WPYFK.T | N | 70.32 | 1156.5375 | 9 | 0.1 | 579.2761 | 2 | 68.82 | 11 | F11:46583 | NaNaKA16\_F7.raw |  | 3.6915E8 | 1.4139E8 | 6.9856E7 | 2.3823E6 |  |  | 3.6353E6 | 3.5126E8 | 2.1014E10 | 1.2727E11 | 1.9979E10 | 1.2073E10 | 71 | 0 | 1 | 1 | 3 | 1 | 0 | 0 | 1 | 1 | 15 | 17 | 10 | 21 | 57 | 65 | Carbamidomethylation | C4:Carbamidomethylation:1000.00 | PEAKS DB |
| K.GDNNAC(+57.02)AASVC(+57.02)DC(+57.02)DR.L | N | 70.22 | 1683.6035 | 15 | 0.2 | 842.8092 | 2 | 12.08 | 11 | F11:1839 | NaNaKA16\_F7.raw |  |  |  |  |  |  |  |  |  | 6.3052E6 | 1.0666E6 | 0 |  | 2 | 0 | 0 | 0 | 0 | 0 | 0 | 0 | 0 | 0 | 1 | 1 | 0 | 0 | 80 | 94 | Carbamidomethylation | C6:Carbamidomethylation:1000.00;C11:Carbamidomethylation:1000.00;C13:Carbamidomethylation:1000.00 | PEAKS DB |
| G.APYNDNNYNIDLKAR.C | N | 70.03 | 1779.8540 | 15 | 0.1 | 594.2920 | 3 | 28.22 | 11 | F11:14195 | NaNaKA16\_F7.raw |  |  |  |  |  |  |  |  |  | 3.8065E6 | 1.1348E7 |  |  | 3 | 0 | 0 | 0 | 0 | 0 | 0 | 0 | 0 | 0 | 1 | 2 | 0 | 0 | 103 | 117 |  |  | PEAKS DB |
| E.AEKISGC(+57.02)WPYFK.T | N | 69.57 | 1484.7122 | 12 | 0.0 | 743.3633 | 2 | 40.74 | 11 | F11:24212 | NaNaKA16\_F7.raw |  |  |  |  |  |  |  |  |  | 9.0796E6 | 5.2934E7 | 1.0921E7 |  | 9 | 0 | 0 | 0 | 0 | 0 | 0 | 0 | 0 | 0 | 2 | 5 | 2 | 0 | 54 | 65 | Carbamidomethylation | C7:Carbamidomethylation:1000.00 | PEAKS DB |
| R.GGSGTPVDDLDR.C | N | 67.81 | 1187.5417 | 12 | 0.8 | 594.7786 | 2 | 19.63 | 10 | F10:6988 | NaNaKA16\_F6.raw |  |  |  |  |  |  |  |  |  | 5.7224E7 | 2.6509E9 | 3.9395E6 |  | 4 | 0 | 0 | 0 | 0 | 0 | 0 | 0 | 0 | 0 | 2 | 1 | 1 | 0 | 31 | 42 |  |  | PEAKS DB |
| NLYQFKNMIKC(+57.02)TVPSR.S | N | 66.48 | 1998.0179 | 16 | 0.3 | 667.0134 | 3 | 40.68 | 12 | F12:23498 | NaNaKA16\_F8.raw |  |  |  |  |  |  |  |  |  |  |  | 3.4886E6 |  | 1 | 0 | 0 | 0 | 0 | 0 | 0 | 0 | 0 | 0 | 0 | 0 | 1 | 0 | 1 | 16 | Carbamidomethylation | C11:Carbamidomethylation:1000.00 | PEAKS DB |
| T.YSYEC(+57.02)SQGTLTC(+57.02)K.G | N | 65.61 | 1595.6595 | 13 | -0.2 | 798.8369 | 2 | 19.78 | 10 | F10:7072 | NaNaKA16\_F6.raw |  |  |  |  |  |  |  |  |  | 9.5485E6 |  |  |  | 1 | 0 | 0 | 0 | 0 | 0 | 0 | 0 | 0 | 0 | 1 | 0 | 0 | 0 | 67 | 79 | Carbamidomethylation | C5:Carbamidomethylation:1000.00;C12:Carbamidomethylation:1000.00 | PEAKS DB |
| Y.SYEC(+57.02)SQGTLTC(+57.02)K.G | N | 65.06 | 1432.5963 | 12 | 0.7 | 717.3060 | 2 | 11.79 | 10 | F10:1902 | NaNaKA16\_F6.raw |  |  |  |  |  |  |  |  |  | 7.186E5 |  |  |  | 1 | 0 | 0 | 0 | 0 | 0 | 0 | 0 | 0 | 0 | 1 | 0 | 0 | 0 | 68 | 79 | Carbamidomethylation | C4:Carbamidomethylation:1000.00;C11:Carbamidomethylation:1000.00 | PEAKS DB |
| A.IC(+57.02)FAGAPYNDNNYNIDLK.A | N | 61.89 | 2100.9575 | 18 | 0.5 | 1051.4866 | 2 | 63.78 | 13 | F13:39698 | NaNaKA16\_F9.raw |  |  |  |  |  |  |  |  |  | 9.6858E6 | 1.8845E7 | 6.9038E6 | 3.858E7 | 4 | 0 | 0 | 0 | 0 | 0 | 0 | 0 | 0 | 0 | 1 | 1 | 1 | 1 | 98 | 115 | Carbamidomethylation | C2:Carbamidomethylation:1000.00 | PEAKS DB |
| R.SWWDFADYGC(+57.02)YC(+57.02)GRG.G | N | 61.68 | 1898.7141 | 15 | 1.5 | 950.3657 | 2 | 84.85 | 11 | F11:59757 | NaNaKA16\_F7.raw |  |  |  |  |  |  |  |  |  |  | 1.1046E7 |  |  | 1 | 0 | 0 | 0 | 0 | 0 | 0 | 0 | 0 | 0 | 0 | 1 | 0 | 0 | 17 | 31 | Carbamidomethylation | C10:Carbamidomethylation:1000.00;C12:Carbamidomethylation:1000.00 | PEAKS DB |
| A.AIC(+57.02)FAGAPYNDNNYNIDLK.A | N | 61.56 | 2171.9946 | 19 | 1.2 | 1087.0059 | 2 | 65.58 | 13 | F13:41436 | NaNaKA16\_F9.raw |  |  |  |  |  |  |  |  |  |  | 1.0912E7 |  | 1.9483E7 | 2 | 0 | 0 | 0 | 0 | 0 | 0 | 0 | 0 | 0 | 0 | 1 | 0 | 1 | 97 | 115 | Carbamidomethylation | C3:Carbamidomethylation:1000.00 | PEAKS DB |
| G.GSGTPVDDLDR.C | N | 60.74 | 1130.5204 | 11 | -0.3 | 566.2673 | 2 | 21.08 | 11 | F11:8172 | NaNaKA16\_F7.raw |  |  |  |  |  |  |  |  |  | 1.4052E5 | 1.2671E7 |  |  | 2 | 0 | 0 | 0 | 0 | 0 | 0 | 0 | 0 | 0 | 1 | 1 | 0 | 0 | 32 | 42 |  |  | PEAKS DB |
| A.GAPYNDNNYNIDLK.A | N | 58.77 | 1609.7372 | 14 | -0.1 | 805.8758 | 2 | 38.63 | 10 | F10:22911 | NaNaKA16\_F6.raw |  |  |  |  |  |  |  |  |  | 4.2863E6 | 2.6962E6 |  | 1.5111E7 | 3 | 0 | 0 | 0 | 0 | 0 | 0 | 0 | 0 | 0 | 1 | 1 | 0 | 1 | 102 | 115 |  |  | PEAKS DB |
| K.TYSYEC(+57.02)SQGTLTC(+57.02)KGDNNAC(+57.02)AASVC(+57.02)DC(+57.02)DR.L | Y | 58.08 | 3362.3003 | 29 | 0.4 | 1121.7745 | 3 | 27.28 | 11 | F11:13291 | NaNaKA16\_F7.raw |  |  |  |  |  |  |  |  |  | 6.9673E7 | 1.4844E7 |  |  | 2 | 0 | 0 | 0 | 0 | 0 | 0 | 0 | 0 | 0 | 1 | 1 | 0 | 0 | 66 | 94 | Carbamidomethylation | C6:Carbamidomethylation:1000.00;C13:Carbamidomethylation:1000.00;C20:Carbamidomethylation:1000.00;C25:Carbamidomethylation:1000.00;C27:Carbamidomethylation:1000.00 | PEAKS DB |
| D.FADYGC(+57.02)YC(+57.02)GR.G | N | 57.15 | 1267.4750 | 10 | 0.8 | 634.7452 | 2 | 25.42 | 11 | F11:11882 | NaNaKA16\_F7.raw |  |  |  |  |  |  |  |  |  | 2.1506E6 | 1.834E7 |  |  | 2 | 0 | 0 | 0 | 0 | 0 | 0 | 0 | 0 | 0 | 1 | 1 | 0 | 0 | 21 | 30 | Carbamidomethylation | C6:Carbamidomethylation:1000.00;C8:Carbamidomethylation:1000.00 | PEAKS DB |
| R.SWWDFADYGC(+57.02)YC(+57.02)GRGGSGTPVDDLDR.C | N | 56.86 | 3011.2239 | 26 | 0.6 | 1004.7491 | 3 | 81.63 | 11 | F11:57027 | NaNaKA16\_F7.raw |  |  |  |  |  |  |  |  |  |  | 4.9169E6 |  |  | 1 | 0 | 0 | 0 | 0 | 0 | 0 | 0 | 0 | 0 | 0 | 1 | 0 | 0 | 17 | 42 | Carbamidomethylation | C10:Carbamidomethylation:1000.00;C12:Carbamidomethylation:1000.00 | PEAKS DB |
| S.WWDFADYGC(+57.02)YC(+57.02)GR.G | N | 56.83 | 1754.6605 | 13 | 0.8 | 878.3382 | 2 | 78.92 | 12 | F12:55446 | NaNaKA16\_F8.raw |  |  |  |  |  |  |  |  |  |  | 1.3475E7 | 3.6699E6 |  | 2 | 0 | 0 | 0 | 0 | 0 | 0 | 0 | 0 | 0 | 0 | 1 | 1 | 0 | 18 | 30 | Carbamidomethylation | C9:Carbamidomethylation:1000.00;C11:Carbamidomethylation:1000.00 | PEAKS DB |
| L.AAIC(+57.02)FAGAPYNDNNYNIDLK.A | N | 56.55 | 2243.0317 | 20 | -0.4 | 1122.5227 | 2 | 66.82 | 13 | F13:42492 | NaNaKA16\_F9.raw |  |  |  |  |  |  |  |  |  |  |  |  | 1.837E7 | 1 | 0 | 0 | 0 | 0 | 0 | 0 | 0 | 0 | 0 | 0 | 0 | 0 | 1 | 96 | 115 | Carbamidomethylation | C4:Carbamidomethylation:1000.00 | PEAKS DB |
| NLYQFKNMIK.C | N | 55.84 | 1297.6853 | 10 | 0.6 | 649.8503 | 2 | 34.48 | 11 | F11:19260 | NaNaKA16\_F7.raw |  |  |  |  |  |  |  |  |  | 2.422E7 | 1.1905E8 | 3.5531E7 | 5.6701E7 | 7 | 0 | 0 | 0 | 0 | 0 | 0 | 0 | 0 | 0 | 2 | 2 | 1 | 2 | 1 | 10 |  |  | PEAKS DB |
| P.YNDNNYNIDLK.A | N | 54.97 | 1384.6259 | 11 | -5.4 | 693.3165 | 2 | 32.55 | 10 | F10:17533 | NaNaKA16\_F6.raw |  |  |  |  |  |  |  |  |  | 8.5654E7 |  |  |  | 1 | 0 | 0 | 0 | 0 | 0 | 0 | 0 | 0 | 0 | 1 | 0 | 0 | 0 | 105 | 115 |  |  | PEAKS DB |
| Y.FKTYSYEC(+57.02)SQGTLTC(+57.02)K.G | N | 54.26 | 1971.8706 | 16 | 0.6 | 658.2979 | 3 | 23.41 | 11 | F11:10060 | NaNaKA16\_F7.raw |  |  |  |  |  |  |  |  |  | 3.532E6 | 1.7096E6 |  |  | 2 | 0 | 0 | 0 | 0 | 0 | 0 | 0 | 0 | 0 | 1 | 1 | 0 | 0 | 64 | 79 | Carbamidomethylation | C8:Carbamidomethylation:1000.00;C15:Carbamidomethylation:1000.00 | PEAKS DB |
| R.LAAIC(+57.02)FAGAPYN.D | N | 53.90 | 1266.6067 | 12 | 0.8 | 634.3111 | 2 | 70.83 | 11 | F11:49747 | NaNaKA16\_F7.raw |  | 2.6932E8 | 6.3964E7 |  |  |  |  |  | 3.6922E8 | 7.2309E9 | 3.8533E9 | 2.4096E9 | 1.5572E9 | 11 | 0 | 1 | 1 | 0 | 0 | 0 | 0 | 0 | 1 | 2 | 3 | 2 | 1 | 95 | 106 | Carbamidomethylation | C5:Carbamidomethylation:1000.00 | PEAKS DB |
| I.SGC(+57.02)WPYFK.T | N | 53.87 | 1043.4535 | 8 | -1.4 | 522.7333 | 2 | 44.38 | 11 | F11:27176 | NaNaKA16\_F7.raw |  |  |  |  |  |  |  |  |  | 8.1441E6 | 5.2634E6 |  |  | 2 | 0 | 0 | 0 | 0 | 0 | 0 | 0 | 0 | 0 | 1 | 1 | 0 | 0 | 58 | 65 | Carbamidomethylation | C3:Carbamidomethylation:1000.00 | PEAKS DB |
| E.KISGC(+57.02)WPYFK.T | N | 51.04 | 1284.6324 | 10 | 0.2 | 643.3236 | 2 | 38.26 | 11 | F11:22081 | NaNaKA16\_F7.raw |  |  |  |  |  |  |  |  |  |  | 3.8432E6 |  |  | 2 | 0 | 0 | 0 | 0 | 0 | 0 | 0 | 0 | 0 | 0 | 2 | 0 | 0 | 56 | 65 | Carbamidomethylation | C5:Carbamidomethylation:1000.00 | PEAKS DB |
| W.WDFADYGC(+57.02)YC(+57.02)GR.G | N | 50.34 | 1568.5813 | 12 | 0.5 | 785.2983 | 2 | 61.41 | 11 | F11:40293 | NaNaKA16\_F7.raw |  |  |  |  |  |  |  |  |  |  | 5.6691E6 |  |  | 1 | 0 | 0 | 0 | 0 | 0 | 0 | 0 | 0 | 0 | 0 | 1 | 0 | 0 | 19 | 30 | Carbamidomethylation | C8:Carbamidomethylation:1000.00;C10:Carbamidomethylation:1000.00 | PEAKS DB |
| W.DFADYGC(+57.02)YC(+57.02)GR.G | N | 49.85 | 1382.5020 | 11 | 0.3 | 692.2585 | 2 | 40.50 | 10 | F10:24767 | NaNaKA16\_F6.raw |  |  |  |  |  |  |  |  | 5.0674E6 | 1.1493E7 | 1.4257E6 |  |  | 3 | 0 | 0 | 0 | 0 | 0 | 0 | 0 | 0 | 1 | 1 | 1 | 0 | 0 | 20 | 30 | Carbamidomethylation | C7:Carbamidomethylation:1000.00;C9:Carbamidomethylation:1000.00 | PEAKS DB |
| K.NMIKC(+57.02)TVPSR.S | N | 48.35 | 1204.6056 | 10 | 0.1 | 603.3101 | 2 | 12.09 | 11 | F11:1822 | NaNaKA16\_F7.raw |  |  |  |  |  |  |  |  |  |  | 1.7697E7 |  |  | 2 | 0 | 0 | 0 | 0 | 0 | 0 | 0 | 0 | 0 | 0 | 2 | 0 | 0 | 7 | 16 | Carbamidomethylation | C5:Carbamidomethylation:1000.00 | PEAKS DB |
| A.PYNDNNYNIDLK.A | N | 48.26 | 1481.6786 | 12 | 0.7 | 741.8471 | 2 | 34.63 | 13 | F13:15900 | NaNaKA16\_F9.raw |  |  |  |  |  |  |  |  |  |  | 1.9835E6 |  | 2.598E6 | 2 | 0 | 0 | 0 | 0 | 0 | 0 | 0 | 0 | 0 | 0 | 1 | 0 | 1 | 104 | 115 |  |  | PEAKS DB |
| K.C(+57.02)TVPSRSWWDFADYGC(+57.02)YC(+57.02)GR.G | N | 48.21 | 2542.0251 | 20 | -0.3 | 848.3488 | 3 | 69.09 | 11 | F11:46749 | NaNaKA16\_F7.raw |  |  |  |  |  |  |  |  |  |  | 6.2414E6 |  |  | 1 | 0 | 0 | 0 | 0 | 0 | 0 | 0 | 0 | 0 | 0 | 1 | 0 | 0 | 11 | 30 | Carbamidomethylation | C1:Carbamidomethylation:1000.00;C16:Carbamidomethylation:1000.00;C18:Carbamidomethylation:1000.00 | PEAKS DB |
| NLYQFKNM(+15.99)IK.C | N | 46.70 | 1313.6802 | 10 | 0.4 | 657.8476 | 2 | 22.54 | 11 | F11:9423 | NaNaKA16\_F7.raw |  |  |  |  |  |  |  |  |  |  | 2.8165E6 |  |  | 1 | 0 | 0 | 0 | 0 | 0 | 0 | 0 | 0 | 0 | 0 | 1 | 0 | 0 | 1 | 10 | Oxidation (M) | M8:Oxidation (M):1000.00 | PEAKS DB |
| R.SWWDFADYGC(+57.02).Y | N | 45.71 | 1305.4761 | 10 | 0.8 | 653.7458 | 2 | 109.86 | 11 | F11:80150 | NaNaKA16\_F7.raw |  |  |  |  |  |  |  |  |  |  | 6.5059E6 |  |  | 1 | 0 | 0 | 0 | 0 | 0 | 0 | 0 | 0 | 0 | 0 | 1 | 0 | 0 | 17 | 26 | Carbamidomethylation | C10:Carbamidomethylation:1000.00 | PEAKS DB |
| G.SGTPVDDLDR.C | N | 45.52 | 1073.4989 | 10 | 0.9 | 537.7572 | 2 | 20.23 | 11 | F11:7497 | NaNaKA16\_F7.raw |  |  |  |  |  |  |  |  |  |  | 1.1072E7 |  |  | 1 | 0 | 0 | 0 | 0 | 0 | 0 | 0 | 0 | 0 | 0 | 1 | 0 | 0 | 33 | 42 |  |  | PEAKS DB |
| K.TYSYEC(+57.02)SQGTLTC(+57.02)KG.D | N | 45.40 | 1753.7288 | 15 | 1.4 | 877.8729 | 2 | 21.92 | 10 | F10:8643 | NaNaKA16\_F6.raw |  |  |  |  |  |  |  |  |  | 0 |  |  |  | 0 | 0 | 0 | 0 | 0 | 0 | 0 | 0 | 0 | 0 | 0 | 0 | 0 | 0 | 66 | 80 | Carbamidomethylation | C6:Carbamidomethylation:1000.00;C13:Carbamidomethylation:1000.00 | PEAKS DB |
| R.SWWDFADYGC(+57.02)Y.C | N | 44.81 | 1468.5394 | 11 | 0.1 | 735.2771 | 2 | 111.77 | 10 | F10:82113 | NaNaKA16\_F6.raw |  |  |  |  |  |  |  |  |  | 1.9586E7 |  | 1.4496E7 |  | 2 | 0 | 0 | 0 | 0 | 0 | 0 | 0 | 0 | 0 | 1 | 0 | 1 | 0 | 17 | 27 | Carbamidomethylation | C10:Carbamidomethylation:1000.00 | PEAKS DB |
| N.DNNYNIDLK.A | N | 44.51 | 1107.5197 | 9 | 1.1 | 554.7677 | 2 | 32.05 | 10 | F10:17657 | NaNaKA16\_F6.raw |  |  |  |  |  |  |  |  |  | 4.8851E7 | 9.5595E6 |  |  | 3 | 0 | 0 | 0 | 0 | 0 | 0 | 0 | 0 | 0 | 2 | 1 | 0 | 0 | 107 | 115 |  |  | PEAKS DB |
| F.AGAPYNDNNYNIDLKAR.C | N | 42.93 | 1907.9126 | 17 | 0.4 | 636.9784 | 3 | 29.66 | 13 | F13:11769 | NaNaKA16\_F9.raw |  |  |  |  |  |  |  |  |  |  |  |  | 8.9691E5 | 1 | 0 | 0 | 0 | 0 | 0 | 0 | 0 | 0 | 0 | 0 | 0 | 0 | 1 | 101 | 117 |  |  | PEAKS DB |
| total 46 peptides |  |  |  |  |  |  |  |  |  |  |  |  |  |  |  |  |  |  |  |  |  |  |  |  |  |  |  |  |  |  |  |  |  |  |  |  |  |  |  |  |  |  |  |
| --- | --- | --- | --- | --- | --- | --- | --- | --- | --- | --- | --- | --- | --- | --- | --- | --- | --- | --- | --- | --- | --- | --- | --- | --- | --- | --- | --- | --- | --- | --- | --- | --- | --- | --- | --- | --- | --- | --- | --- | --- | --- | --- | --- |

1YI5

back to list

  

| Protein Coverage
| Supporting Peptides
|

Protein Coverage:

Supporting Peptides:

| Peptide | Uniq | -10lgP | Mass | Length | ppm | m/z | z | RT | Fraction | Scan | Source File | Area F1 | Area F10 | Area F11 | Area F12 | Area F13 | Area F2 | Area F3 | Area F4 | Area F5 | Area F6 | Area F7 | Area F8 | Area F9 | #Feature | #Feature F1 | #Feature F10 | #Feature F11 | #Feature F12 | #Feature F13 | #Feature F2 | #Feature F3 | #Feature F4 | #Feature F5 | #Feature F6 | #Feature F7 | #Feature F8 | #Feature F9 | Start | End | PTM | AScore | Found By |
| --- | --- | --- | --- | --- | --- | --- | --- | --- | --- | --- | --- | --- | --- | --- | --- | --- | --- | --- | --- | --- | --- | --- | --- | --- | --- | --- | --- | --- | --- | --- | --- | --- | --- | --- | --- | --- | --- | --- | --- | --- | --- | --- | --- |
| R.C(+57.02)FITPDITSKDC(+57.02)PNGHVC(+57.02)YTK.T | N | 97.70 | 2512.1184 | 21 | -0.2 | 838.3799 | 3 | 32.91 | 9 | F9:17528 | NaNaKA16\_F5.raw |  |  |  |  |  |  | 1.3652E7 | 5.3048E7 | 1.1015E8 |  |  |  |  | 10 | 0 | 0 | 0 | 0 | 0 | 0 | 3 | 4 | 3 | 0 | 0 | 0 | 0 | 3 | 23 | Carbamidomethylation | C1:Carbamidomethylation:1000.00;C12:Carbamidomethylation:1000.00;C18:Carbamidomethylation:1000.00 | PEAKS DB |
| K.TGVDIQC(+57.02)C(+57.02)STDNC(+57.02)NPFPTRK.R | Y | 97.32 | 2369.0198 | 20 | 5.0 | 1185.5167 | 2 | 30.34 | 8 | F8:13936 | NaNaKA16\_F4.raw |  | 5.2751E6 | 1.4938E6 |  |  |  | 4.2829E8 | 1.4347E9 | 1.578E9 | 3.3235E7 | 3.1822E6 |  | 6.4482E6 | 17 | 0 | 1 | 1 | 0 | 0 | 0 | 3 | 5 | 4 | 1 | 1 | 0 | 1 | 50 | 69 | Carbamidomethylation | C7:Carbamidomethylation:1000.00;C8:Carbamidomethylation:1000.00;C13:Carbamidomethylation:1000.00 | PEAKS DB |
| K.TGVDIQC(+57.02)C(+57.02)STDNC(+57.02)NPFPTR.K | Y | 94.74 | 2240.9248 | 19 | 1.1 | 1121.4709 | 2 | 44.69 | 3 | F3:24754 | NaNaKA16\_F11.raw |  | 1.6333E8 | 7.7958E7 | 7.513E7 | 6.0306E6 | 5.3603E8 | 3.7554E10 | 5.9156E10 | 6.2024E10 | 3.6074E9 | 8.3057E7 | 2.6489E7 | 3.5028E8 | 108 | 0 | 2 | 2 | 2 | 2 | 2 | 19 | 35 | 30 | 3 | 2 | 2 | 7 | 50 | 68 | Carbamidomethylation | C7:Carbamidomethylation:1000.00;C8:Carbamidomethylation:1000.00;C13:Carbamidomethylation:1000.00 | PEAKS DB |
| R.VDLGC(+57.02)AATC(+57.02)PTVK.T | N | 93.61 | 1390.6584 | 13 | 6.1 | 696.3372 | 2 | 26.78 | 7 | F7:8603 | NaNaKA16\_F3.raw |  |  |  | 1.5246E7 | 1.5932E6 | 5.0659E7 | 1.9121E8 | 2.2473E10 | 6.1192E10 | 1.9939E9 | 5.3549E6 |  | 0 | 56 | 0 | 0 | 0 | 1 | 1 | 1 | 2 | 13 | 32 | 5 | 1 | 0 | 0 | 37 | 49 | Carbamidomethylation | C5:Carbamidomethylation:1000.00;C9:Carbamidomethylation:1000.00 | PEAKS DB |
| T.GVDIQC(+57.02)C(+57.02)STDNC(+57.02)NPFPTR.K | Y | 89.23 | 2139.8772 | 18 | 4.9 | 1070.9458 | 2 | 42.08 | 7 | F7:20869 | NaNaKA16\_F3.raw |  |  |  |  |  |  | 1.8625E7 | 1.2871E7 | 2.0018E7 |  |  |  |  | 5 | 0 | 0 | 0 | 0 | 0 | 0 | 1 | 1 | 3 | 0 | 0 | 0 | 0 | 51 | 68 | Carbamidomethylation | C6:Carbamidomethylation:1000.00;C7:Carbamidomethylation:1000.00;C12:Carbamidomethylation:1000.00 | PEAKS DB |
| G.VDIQC(+57.02)C(+57.02)STDNC(+57.02)NPFPTR.K | Y | 86.06 | 2082.8557 | 17 | 5.7 | 1042.4355 | 2 | 40.37 | 8 | F8:21741 | NaNaKA16\_F4.raw |  |  |  |  |  |  | 9.4539E6 | 2.0548E7 | 4.7809E7 |  |  |  |  | 4 | 0 | 0 | 0 | 0 | 0 | 0 | 1 | 1 | 2 | 0 | 0 | 0 | 0 | 52 | 68 | Carbamidomethylation | C5:Carbamidomethylation:1000.00;C6:Carbamidomethylation:1000.00;C11:Carbamidomethylation:1000.00 | PEAKS DB |
| D.IQC(+57.02)C(+57.02)STDNC(+57.02)NPFPTR.K | Y | 84.44 | 1868.7604 | 15 | 0.1 | 935.3876 | 2 | 26.32 | 9 | F9:12175 | NaNaKA16\_F5.raw | 8.3412E6 |  |  |  |  | 6.6128E6 | 1.3076E6 | 2.197E8 | 3.1221E8 |  |  |  |  | 9 | 1 | 0 | 0 | 0 | 0 | 1 | 1 | 3 | 3 | 0 | 0 | 0 | 0 | 54 | 68 | Carbamidomethylation | C3:Carbamidomethylation:1000.00;C4:Carbamidomethylation:1000.00;C9:Carbamidomethylation:1000.00 | PEAKS DB |
| V.DIQC(+57.02)C(+57.02)STDNC(+57.02)NPFPTR.K | Y | 83.87 | 1983.7874 | 16 | 6.2 | 992.9019 | 2 | 37.25 | 8 | F8:19226 | NaNaKA16\_F4.raw |  |  |  |  |  | 8.8806E6 | 1.0298E7 | 1.017E7 | 5.0018E8 | 3.4519E7 |  |  |  | 9 | 0 | 0 | 0 | 0 | 0 | 1 | 1 | 1 | 4 | 2 | 0 | 0 | 0 | 53 | 68 | Carbamidomethylation | C4:Carbamidomethylation:1000.00;C5:Carbamidomethylation:1000.00;C10:Carbamidomethylation:1000.00 | PEAKS DB |
| Q.C(+57.02)C(+57.02)STDNC(+57.02)NPFPTR.K | N | 81.03 | 1627.6178 | 13 | 6.1 | 814.8168 | 2 | 20.86 | 8 | F8:6556 | NaNaKA16\_F4.raw | 4.0032E5 |  |  |  |  | 2.1346E6 | 1.8581E7 | 2.2172E6 | 1.8648E7 | 7.3726E5 |  |  |  | 12 | 1 | 0 | 0 | 0 | 0 | 1 | 4 | 2 | 3 | 1 | 0 | 0 | 0 | 56 | 68 | Carbamidomethylation | C1:Carbamidomethylation:1000.00;C2:Carbamidomethylation:1000.00;C7:Carbamidomethylation:1000.00 | PEAKS DB |
| R.C(+57.02)FITPDITSK.D | N | 75.82 | 1180.5798 | 10 | 7.7 | 591.2988 | 2 | 43.13 | 7 | F7:21737 | NaNaKA16\_F3.raw |  | 2.2482E7 | 8.6587E7 | 7.912E7 | 7.0808E6 | 5.0807E8 | 6.1225E10 | 8.5353E10 | 1.2307E11 | 5.4499E9 | 1.0861E8 | 6.3864E7 | 2.9206E8 | 82 | 0 | 4 | 2 | 4 | 2 | 4 | 9 | 21 | 14 | 13 | 2 | 2 | 5 | 3 | 12 | Carbamidomethylation | C1:Carbamidomethylation:1000.00 | PEAKS DB |
| V.KTGVDIQC(+57.02)C(+57.02)STDNC(+57.02)NPFPTR.K | Y | 73.92 | 2369.0198 | 20 | -0.3 | 1185.5168 | 2 | 30.58 | 9 | F9:15182 | NaNaKA16\_F5.raw |  |  |  |  |  |  | 4.0817E8 | 1.4066E9 | 1.5575E9 |  |  |  |  | 5 | 0 | 0 | 0 | 0 | 0 | 0 | 1 | 2 | 2 | 0 | 0 | 0 | 0 | 49 | 68 | Carbamidomethylation | C8:Carbamidomethylation:1000.00;C9:Carbamidomethylation:1000.00;C14:Carbamidomethylation:1000.00 | PEAKS DB |
| C.STDNC(+57.02)NPFPTR.K | N | 69.23 | 1307.5564 | 11 | 4.8 | 654.7853 | 2 | 11.47 | 7 | F7:1955 | NaNaKA16\_F3.raw |  |  |  |  |  | 3.2217E6 | 1.3046E7 | 2.4922E6 | 1.0115E7 |  |  |  |  | 5 | 0 | 0 | 0 | 0 | 0 | 1 | 1 | 1 | 2 | 0 | 0 | 0 | 0 | 58 | 68 | Carbamidomethylation | C5:Carbamidomethylation:1000.00 | PEAKS DB |
| IRC(+57.02)FITPDITSK.D | N | 67.07 | 1449.7650 | 12 | 0.1 | 484.2623 | 3 | 34.24 | 9 | F9:18401 | NaNaKA16\_F5.raw |  |  |  |  |  |  | 2.4051E7 | 7.2194E7 | 8.0035E7 |  |  |  |  | 10 | 0 | 0 | 0 | 0 | 0 | 0 | 3 | 4 | 3 | 0 | 0 | 0 | 0 | 1 | 12 | Carbamidomethylation | C3:Carbamidomethylation:1000.00 | PEAKS DB |
| C.C(+57.02)STDNC(+57.02)NPFPTR.K | N | 66.15 | 1467.5872 | 12 | 0.1 | 734.8009 | 2 | 20.98 | 6 | F6:8194 | NaNaKA16\_F2.raw | 3.5509E6 |  |  |  |  | 1.6348E6 | 1.0498E7 |  | 1.4874E7 |  |  |  |  | 5 | 1 | 0 | 0 | 0 | 0 | 1 | 2 | 0 | 1 | 0 | 0 | 0 | 0 | 57 | 68 | Carbamidomethylation | C1:Carbamidomethylation:1000.00;C6:Carbamidomethylation:1000.00 | PEAKS DB |
| K.TGVDIQC(+57.02)C(+57.02)STDNC(+57.02)NPFPTRKRP | Y | 58.58 | 2622.1738 | 22 | 5.5 | 656.5510 | 4 | 25.48 | 7 | F7:7710 | NaNaKA16\_F3.raw |  |  |  |  |  |  | 0 |  | 1.7072E7 |  |  |  |  | 2 | 0 | 0 | 0 | 0 | 0 | 0 | 0 | 0 | 2 | 0 | 0 | 0 | 0 | 50 | 71 | Carbamidomethylation | C7:Carbamidomethylation:1000.00;C8:Carbamidomethylation:1000.00;C13:Carbamidomethylation:1000.00 | PEAKS DB |
| R.C(+57.02)FITPDITSKD.C | N | 53.03 | 1295.6067 | 11 | 0.2 | 648.8107 | 2 | 44.47 | 9 | F9:26660 | NaNaKA16\_F5.raw |  |  |  |  |  |  | 7.9006E5 | 3.8625E6 | 1.7287E7 |  |  |  |  | 6 | 0 | 0 | 0 | 0 | 0 | 0 | 1 | 2 | 3 | 0 | 0 | 0 | 0 | 3 | 13 | Carbamidomethylation | C1:Carbamidomethylation:1000.00 | PEAKS DB |
| V.DIQC(+57.02)C(+57.02)STDNC(+57.02)NPFPTRK.R | Y | 52.18 | 2111.8823 | 17 | 0.2 | 704.9682 | 3 | 22.55 | 9 | F9:9549 | NaNaKA16\_F5.raw |  |  |  |  |  |  |  |  | 3.3337E6 |  |  |  |  | 1 | 0 | 0 | 0 | 0 | 0 | 0 | 0 | 0 | 1 | 0 | 0 | 0 | 0 | 53 | 69 | Carbamidomethylation | C4:Carbamidomethylation:1000.00;C5:Carbamidomethylation:1000.00;C10:Carbamidomethylation:1000.00 | PEAKS DB |
| K.TGVDIQC(+57.02)C(+57.02)STDNC(+57.02)NPFPT.R | Y | 50.82 | 2084.8237 | 18 | 7.4 | 1043.4213 | 2 | 61.82 | 8 | F8:39511 | NaNaKA16\_F4.raw |  |  |  |  |  |  | 1.4461E8 | 1.6598E8 | 1.2556E9 | 1.4388E8 |  |  |  | 4 | 0 | 0 | 0 | 0 | 0 | 0 | 1 | 1 | 1 | 1 | 0 | 0 | 0 | 50 | 67 | Carbamidomethylation | C7:Carbamidomethylation:1000.00;C8:Carbamidomethylation:1000.00;C13:Carbamidomethylation:1000.00 | PEAKS DB |
| K.TWC(+57.02)DAFC(+57.02)SIR.G | N | 50.20 | 1314.5486 | 10 | 0.4 | 658.2819 | 2 | 55.05 | 9 | F9:35981 | NaNaKA16\_F5.raw |  |  |  |  |  |  |  |  | 1.2559E7 |  |  |  |  | 1 | 0 | 0 | 0 | 0 | 0 | 0 | 0 | 0 | 1 | 0 | 0 | 0 | 0 | 24 | 33 | Carbamidomethylation | C3:Carbamidomethylation:1000.00;C7:Carbamidomethylation:1000.00 | PEAKS DB |
| K.DC(+57.02)PNGHVC(+57.02)YTK.T | N | 50.09 | 1349.5493 | 11 | -0.1 | 450.8570 | 3 | 15.59 | 9 | F9:3959 | NaNaKA16\_F5.raw |  |  |  |  |  |  |  |  | 0 |  |  |  |  | 0 | 0 | 0 | 0 | 0 | 0 | 0 | 0 | 0 | 0 | 0 | 0 | 0 | 0 | 13 | 23 | Carbamidomethylation | C2:Carbamidomethylation:1000.00;C8:Carbamidomethylation:1000.00 | PEAKS DB |
| D.LGC(+57.02)AATC(+57.02)PTVK.T | N | 49.50 | 1176.5631 | 11 | 4.9 | 589.2886 | 2 | 11.80 | 8 | F8:1936 | NaNaKA16\_F4.raw |  |  |  |  |  |  |  | 1.7287E5 |  |  |  |  |  | 1 | 0 | 0 | 0 | 0 | 0 | 0 | 0 | 1 | 0 | 0 | 0 | 0 | 0 | 39 | 49 | Carbamidomethylation | C3:Carbamidomethylation:1000.00;C7:Carbamidomethylation:1000.00 | PEAKS DB |
| I.RC(+57.02)FITPDITSK.D | N | 48.93 | 1336.6809 | 11 | 0.7 | 446.5679 | 3 | 25.75 | 9 | F9:12007 | NaNaKA16\_F5.raw |  |  |  |  |  |  |  |  | 8.2828E6 |  |  |  |  | 1 | 0 | 0 | 0 | 0 | 0 | 0 | 0 | 0 | 1 | 0 | 0 | 0 | 0 | 2 | 12 | Carbamidomethylation | C2:Carbamidomethylation:1000.00 | PEAKS DB |
| I.QC(+57.02)C(+57.02)STDNC(+57.02)NPFPTR.K | Y | 47.30 | 1755.6764 | 14 | 1.0 | 878.8463 | 2 | 22.00 | 1 | F1:7265 | NaNaKA16\_F1.raw | 8.1149E5 |  |  |  |  |  |  |  |  |  |  |  |  | 1 | 1 | 0 | 0 | 0 | 0 | 0 | 0 | 0 | 0 | 0 | 0 | 0 | 0 | 55 | 68 | Carbamidomethylation | C2:Carbamidomethylation:1000.00;C3:Carbamidomethylation:1000.00;C8:Carbamidomethylation:1000.00 | PEAKS DB |
| C.FITPDITSK.D | N | 45.76 | 1020.5491 | 9 | -2.0 | 511.2808 | 2 | 35.97 | 9 | F9:20225 | NaNaKA16\_F5.raw |  |  |  |  |  |  |  | 2.3378E8 | 1.8E8 |  |  |  |  | 3 | 0 | 0 | 0 | 0 | 0 | 0 | 0 | 2 | 1 | 0 | 0 | 0 | 0 | 4 | 12 |  |  | PEAKS DB |
| R.VDLGC(+57.02)AATC(+57.02)PTV.K | N | 45.13 | 1262.5635 | 12 | -0.9 | 632.2885 | 2 | 50.91 | 10 | F10:33445 | NaNaKA16\_F6.raw |  |  |  |  |  |  | 1.1303E7 |  |  | 5.2132E7 |  |  |  | 2 | 0 | 0 | 0 | 0 | 0 | 0 | 1 | 0 | 0 | 1 | 0 | 0 | 0 | 37 | 48 | Carbamidomethylation | C5:Carbamidomethylation:1000.00;C9:Carbamidomethylation:1000.00 | PEAKS DB |
| T.DNC(+57.02)NPFPTR.K | N | 44.94 | 1119.4767 | 9 | 0.7 | 560.7460 | 2 | 23.18 | 6 | F6:10002 | NaNaKA16\_F2.raw |  |  |  |  |  | 3.0548E5 |  |  |  |  |  |  |  | 1 | 0 | 0 | 0 | 0 | 0 | 1 | 0 | 0 | 0 | 0 | 0 | 0 | 0 | 60 | 68 | Carbamidomethylation | C3:Carbamidomethylation:1000.00 | PEAKS DB |
| A.TC(+57.02)PTVKTGVDIQC(+57.02)C(+57.02)STDNC(+57.02)NPFPTR.K | Y | 44.77 | 2927.2671 | 25 | -7.1 | 976.7509 | 3 | 63.76 | 8 | F8:41290 | NaNaKA16\_F4.raw |  |  |  |  |  |  |  | 7.0141E6 |  |  |  |  |  | 1 | 0 | 0 | 0 | 0 | 0 | 0 | 0 | 1 | 0 | 0 | 0 | 0 | 0 | 44 | 68 | Carbamidomethylation | C2:Carbamidomethylation:1000.00;C13:Carbamidomethylation:1000.00;C14:Carbamidomethylation:1000.00;C19:Carbamidomethylation:1000.00 | PEAKS DB |
| K.RVDLGC(+57.02)AATC(+57.02)PTVK.T | N | 44.70 | 1546.7595 | 14 | -0.8 | 516.5934 | 3 | 18.27 | 9 | F9:5871 | NaNaKA16\_F5.raw |  |  |  |  |  |  |  | 1.5478E5 | 2.2798E6 |  |  |  |  | 2 | 0 | 0 | 0 | 0 | 0 | 0 | 0 | 1 | 1 | 0 | 0 | 0 | 0 | 36 | 49 | Carbamidomethylation | C6:Carbamidomethylation:1000.00;C10:Carbamidomethylation:1000.00 | PEAKS DB |
| F.ITPDITSK.D | N | 42.90 | 873.4807 | 8 | 4.9 | 437.7476 | 2 | 11.46 | 7 | F7:1934 | NaNaKA16\_F3.raw |  |  |  |  |  |  | 1.0681E6 |  |  |  |  |  |  | 1 | 0 | 0 | 0 | 0 | 0 | 0 | 1 | 0 | 0 | 0 | 0 | 0 | 0 | 5 | 12 |  |  | PEAKS DB |
| V.KTGVDIQC(+57.02)C(+57.02)STDNC(+57.02)NPFPTRK.R | Y | 42.67 | 2497.1147 | 21 | 5.2 | 625.2859 | 4 | 20.10 | 8 | F8:6040 | NaNaKA16\_F4.raw |  |  |  |  |  |  |  | 1.4274E6 |  |  |  |  |  | 1 | 0 | 0 | 0 | 0 | 0 | 0 | 0 | 1 | 0 | 0 | 0 | 0 | 0 | 49 | 69 | Carbamidomethylation | C8:Carbamidomethylation:1000.00;C9:Carbamidomethylation:1000.00;C14:Carbamidomethylation:1000.00 | PEAKS DB |
| D.NC(+57.02)NPFPTR.K | N | 41.96 | 1004.4498 | 8 | -0.2 | 503.2321 | 2 | 43.27 | 9 | F9:25860 | NaNaKA16\_F5.raw |  |  |  |  |  |  |  |  | 0 |  |  |  |  | 0 | 0 | 0 | 0 | 0 | 0 | 0 | 0 | 0 | 0 | 0 | 0 | 0 | 0 | 61 | 68 | Carbamidomethylation | C2:Carbamidomethylation:1000.00 | PEAKS DB |
| total 31 peptides |  |  |  |  |  |  |  |  |  |  |  |  |  |  |  |  |  |  |  |  |  |  |  |  |  |  |  |  |  |  |  |  |  |  |  |  |  |  |  |  |  |  |  |
| --- | --- | --- | --- | --- | --- | --- | --- | --- | --- | --- | --- | --- | --- | --- | --- | --- | --- | --- | --- | --- | --- | --- | --- | --- | --- | --- | --- | --- | --- | --- | --- | --- | --- | --- | --- | --- | --- | --- | --- | --- | --- | --- | --- |

4AEA

back to list

  

| Protein Coverage
| Supporting Peptides
|

Protein Coverage:

Supporting Peptides:

| Peptide | Uniq | -10lgP | Mass | Length | ppm | m/z | z | RT | Fraction | Scan | Source File | Area F1 | Area F10 | Area F11 | Area F12 | Area F13 | Area F2 | Area F3 | Area F4 | Area F5 | Area F6 | Area F7 | Area F8 | Area F9 | #Feature | #Feature F1 | #Feature F10 | #Feature F11 | #Feature F12 | #Feature F13 | #Feature F2 | #Feature F3 | #Feature F4 | #Feature F5 | #Feature F6 | #Feature F7 | #Feature F8 | #Feature F9 | Start | End | PTM | AScore | Found By |
| --- | --- | --- | --- | --- | --- | --- | --- | --- | --- | --- | --- | --- | --- | --- | --- | --- | --- | --- | --- | --- | --- | --- | --- | --- | --- | --- | --- | --- | --- | --- | --- | --- | --- | --- | --- | --- | --- | --- | --- | --- | --- | --- | --- |
| R.C(+57.02)FITPDITSKDC(+57.02)PNGHVC(+57.02)YTK.T | N | 97.70 | 2512.1184 | 21 | -0.2 | 838.3799 | 3 | 32.91 | 9 | F9:17528 | NaNaKA16\_F5.raw |  |  |  |  |  |  | 1.3652E7 | 5.3048E7 | 1.1015E8 |  |  |  |  | 10 | 0 | 0 | 0 | 0 | 0 | 0 | 3 | 4 | 3 | 0 | 0 | 0 | 0 | 3 | 23 | Carbamidomethylation | C1:Carbamidomethylation:1000.00;C12:Carbamidomethylation:1000.00;C18:Carbamidomethylation:1000.00 | PEAKS DB |
| K.TGVDIQC(+57.02)C(+57.02)STDNC(+57.02)NPFPTRK.R | Y | 97.32 | 2369.0198 | 20 | 5.0 | 1185.5167 | 2 | 30.34 | 8 | F8:13936 | NaNaKA16\_F4.raw |  | 5.2751E6 | 1.4938E6 |  |  |  | 4.2829E8 | 1.4347E9 | 1.578E9 | 3.3235E7 | 3.1822E6 |  | 6.4482E6 | 17 | 0 | 1 | 1 | 0 | 0 | 0 | 3 | 5 | 4 | 1 | 1 | 0 | 1 | 50 | 69 | Carbamidomethylation | C7:Carbamidomethylation:1000.00;C8:Carbamidomethylation:1000.00;C13:Carbamidomethylation:1000.00 | PEAKS DB |
| K.TGVDIQC(+57.02)C(+57.02)STDNC(+57.02)NPFPTR.K | Y | 94.74 | 2240.9248 | 19 | 1.1 | 1121.4709 | 2 | 44.69 | 3 | F3:24754 | NaNaKA16\_F11.raw |  | 1.6333E8 | 7.7958E7 | 7.513E7 | 6.0306E6 | 5.3603E8 | 3.7554E10 | 5.9156E10 | 6.2024E10 | 3.6074E9 | 8.3057E7 | 2.6489E7 | 3.5028E8 | 108 | 0 | 2 | 2 | 2 | 2 | 2 | 19 | 35 | 30 | 3 | 2 | 2 | 7 | 50 | 68 | Carbamidomethylation | C7:Carbamidomethylation:1000.00;C8:Carbamidomethylation:1000.00;C13:Carbamidomethylation:1000.00 | PEAKS DB |
| R.VDLGC(+57.02)AATC(+57.02)PTVK.T | N | 93.61 | 1390.6584 | 13 | 6.1 | 696.3372 | 2 | 26.78 | 7 | F7:8603 | NaNaKA16\_F3.raw |  |  |  | 1.5246E7 | 1.5932E6 | 5.0659E7 | 1.9121E8 | 2.2473E10 | 6.1192E10 | 1.9939E9 | 5.3549E6 |  | 0 | 56 | 0 | 0 | 0 | 1 | 1 | 1 | 2 | 13 | 32 | 5 | 1 | 0 | 0 | 37 | 49 | Carbamidomethylation | C5:Carbamidomethylation:1000.00;C9:Carbamidomethylation:1000.00 | PEAKS DB |
| T.GVDIQC(+57.02)C(+57.02)STDNC(+57.02)NPFPTR.K | Y | 89.23 | 2139.8772 | 18 | 4.9 | 1070.9458 | 2 | 42.08 | 7 | F7:20869 | NaNaKA16\_F3.raw |  |  |  |  |  |  | 1.8625E7 | 1.2871E7 | 2.0018E7 |  |  |  |  | 5 | 0 | 0 | 0 | 0 | 0 | 0 | 1 | 1 | 3 | 0 | 0 | 0 | 0 | 51 | 68 | Carbamidomethylation | C6:Carbamidomethylation:1000.00;C7:Carbamidomethylation:1000.00;C12:Carbamidomethylation:1000.00 | PEAKS DB |
| G.VDIQC(+57.02)C(+57.02)STDNC(+57.02)NPFPTR.K | Y | 86.06 | 2082.8557 | 17 | 5.7 | 1042.4355 | 2 | 40.37 | 8 | F8:21741 | NaNaKA16\_F4.raw |  |  |  |  |  |  | 9.4539E6 | 2.0548E7 | 4.7809E7 |  |  |  |  | 4 | 0 | 0 | 0 | 0 | 0 | 0 | 1 | 1 | 2 | 0 | 0 | 0 | 0 | 52 | 68 | Carbamidomethylation | C5:Carbamidomethylation:1000.00;C6:Carbamidomethylation:1000.00;C11:Carbamidomethylation:1000.00 | PEAKS DB |
| D.IQC(+57.02)C(+57.02)STDNC(+57.02)NPFPTR.K | Y | 84.44 | 1868.7604 | 15 | 0.1 | 935.3876 | 2 | 26.32 | 9 | F9:12175 | NaNaKA16\_F5.raw | 8.3412E6 |  |  |  |  | 6.6128E6 | 1.3076E6 | 2.197E8 | 3.1221E8 |  |  |  |  | 9 | 1 | 0 | 0 | 0 | 0 | 1 | 1 | 3 | 3 | 0 | 0 | 0 | 0 | 54 | 68 | Carbamidomethylation | C3:Carbamidomethylation:1000.00;C4:Carbamidomethylation:1000.00;C9:Carbamidomethylation:1000.00 | PEAKS DB |
| V.DIQC(+57.02)C(+57.02)STDNC(+57.02)NPFPTR.K | Y | 83.87 | 1983.7874 | 16 | 6.2 | 992.9019 | 2 | 37.25 | 8 | F8:19226 | NaNaKA16\_F4.raw |  |  |  |  |  | 8.8806E6 | 1.0298E7 | 1.017E7 | 5.0018E8 | 3.4519E7 |  |  |  | 9 | 0 | 0 | 0 | 0 | 0 | 1 | 1 | 1 | 4 | 2 | 0 | 0 | 0 | 53 | 68 | Carbamidomethylation | C4:Carbamidomethylation:1000.00;C5:Carbamidomethylation:1000.00;C10:Carbamidomethylation:1000.00 | PEAKS DB |
| Q.C(+57.02)C(+57.02)STDNC(+57.02)NPFPTR.K | N | 81.03 | 1627.6178 | 13 | 6.1 | 814.8168 | 2 | 20.86 | 8 | F8:6556 | NaNaKA16\_F4.raw | 4.0032E5 |  |  |  |  | 2.1346E6 | 1.8581E7 | 2.2172E6 | 1.8648E7 | 7.3726E5 |  |  |  | 12 | 1 | 0 | 0 | 0 | 0 | 1 | 4 | 2 | 3 | 1 | 0 | 0 | 0 | 56 | 68 | Carbamidomethylation | C1:Carbamidomethylation:1000.00;C2:Carbamidomethylation:1000.00;C7:Carbamidomethylation:1000.00 | PEAKS DB |
| R.C(+57.02)FITPDITSK.D | N | 75.82 | 1180.5798 | 10 | 7.7 | 591.2988 | 2 | 43.13 | 7 | F7:21737 | NaNaKA16\_F3.raw |  | 2.2482E7 | 8.6587E7 | 7.912E7 | 7.0808E6 | 5.0807E8 | 6.1225E10 | 8.5353E10 | 1.2307E11 | 5.4499E9 | 1.0861E8 | 6.3864E7 | 2.9206E8 | 82 | 0 | 4 | 2 | 4 | 2 | 4 | 9 | 21 | 14 | 13 | 2 | 2 | 5 | 3 | 12 | Carbamidomethylation | C1:Carbamidomethylation:1000.00 | PEAKS DB |
| V.KTGVDIQC(+57.02)C(+57.02)STDNC(+57.02)NPFPTR.K | Y | 73.92 | 2369.0198 | 20 | -0.3 | 1185.5168 | 2 | 30.58 | 9 | F9:15182 | NaNaKA16\_F5.raw |  |  |  |  |  |  | 4.0817E8 | 1.4066E9 | 1.5575E9 |  |  |  |  | 5 | 0 | 0 | 0 | 0 | 0 | 0 | 1 | 2 | 2 | 0 | 0 | 0 | 0 | 49 | 68 | Carbamidomethylation | C8:Carbamidomethylation:1000.00;C9:Carbamidomethylation:1000.00;C14:Carbamidomethylation:1000.00 | PEAKS DB |
| C.STDNC(+57.02)NPFPTR.K | N | 69.23 | 1307.5564 | 11 | 4.8 | 654.7853 | 2 | 11.47 | 7 | F7:1955 | NaNaKA16\_F3.raw |  |  |  |  |  | 3.2217E6 | 1.3046E7 | 2.4922E6 | 1.0115E7 |  |  |  |  | 5 | 0 | 0 | 0 | 0 | 0 | 1 | 1 | 1 | 2 | 0 | 0 | 0 | 0 | 58 | 68 | Carbamidomethylation | C5:Carbamidomethylation:1000.00 | PEAKS DB |
| IRC(+57.02)FITPDITSK.D | N | 67.07 | 1449.7650 | 12 | 0.1 | 484.2623 | 3 | 34.24 | 9 | F9:18401 | NaNaKA16\_F5.raw |  |  |  |  |  |  | 2.4051E7 | 7.2194E7 | 8.0035E7 |  |  |  |  | 10 | 0 | 0 | 0 | 0 | 0 | 0 | 3 | 4 | 3 | 0 | 0 | 0 | 0 | 1 | 12 | Carbamidomethylation | C3:Carbamidomethylation:1000.00 | PEAKS DB |
| C.C(+57.02)STDNC(+57.02)NPFPTR.K | N | 66.15 | 1467.5872 | 12 | 0.1 | 734.8009 | 2 | 20.98 | 6 | F6:8194 | NaNaKA16\_F2.raw | 3.5509E6 |  |  |  |  | 1.6348E6 | 1.0498E7 |  | 1.4874E7 |  |  |  |  | 5 | 1 | 0 | 0 | 0 | 0 | 1 | 2 | 0 | 1 | 0 | 0 | 0 | 0 | 57 | 68 | Carbamidomethylation | C1:Carbamidomethylation:1000.00;C6:Carbamidomethylation:1000.00 | PEAKS DB |
| K.TGVDIQC(+57.02)C(+57.02)STDNC(+57.02)NPFPTRKRP | Y | 58.58 | 2622.1738 | 22 | 5.5 | 656.5510 | 4 | 25.48 | 7 | F7:7710 | NaNaKA16\_F3.raw |  |  |  |  |  |  | 0 |  | 1.7072E7 |  |  |  |  | 2 | 0 | 0 | 0 | 0 | 0 | 0 | 0 | 0 | 2 | 0 | 0 | 0 | 0 | 50 | 71 | Carbamidomethylation | C7:Carbamidomethylation:1000.00;C8:Carbamidomethylation:1000.00;C13:Carbamidomethylation:1000.00 | PEAKS DB |
| R.C(+57.02)FITPDITSKD.C | N | 53.03 | 1295.6067 | 11 | 0.2 | 648.8107 | 2 | 44.47 | 9 | F9:26660 | NaNaKA16\_F5.raw |  |  |  |  |  |  | 7.9006E5 | 3.8625E6 | 1.7287E7 |  |  |  |  | 6 | 0 | 0 | 0 | 0 | 0 | 0 | 1 | 2 | 3 | 0 | 0 | 0 | 0 | 3 | 13 | Carbamidomethylation | C1:Carbamidomethylation:1000.00 | PEAKS DB |
| V.DIQC(+57.02)C(+57.02)STDNC(+57.02)NPFPTRK.R | Y | 52.18 | 2111.8823 | 17 | 0.2 | 704.9682 | 3 | 22.55 | 9 | F9:9549 | NaNaKA16\_F5.raw |  |  |  |  |  |  |  |  | 3.3337E6 |  |  |  |  | 1 | 0 | 0 | 0 | 0 | 0 | 0 | 0 | 0 | 1 | 0 | 0 | 0 | 0 | 53 | 69 | Carbamidomethylation | C4:Carbamidomethylation:1000.00;C5:Carbamidomethylation:1000.00;C10:Carbamidomethylation:1000.00 | PEAKS DB |
| K.TGVDIQC(+57.02)C(+57.02)STDNC(+57.02)NPFPT.R | Y | 50.82 | 2084.8237 | 18 | 7.4 | 1043.4213 | 2 | 61.82 | 8 | F8:39511 | NaNaKA16\_F4.raw |  |  |  |  |  |  | 1.4461E8 | 1.6598E8 | 1.2556E9 | 1.4388E8 |  |  |  | 4 | 0 | 0 | 0 | 0 | 0 | 0 | 1 | 1 | 1 | 1 | 0 | 0 | 0 | 50 | 67 | Carbamidomethylation | C7:Carbamidomethylation:1000.00;C8:Carbamidomethylation:1000.00;C13:Carbamidomethylation:1000.00 | PEAKS DB |
| K.TWC(+57.02)DAFC(+57.02)SIR.G | N | 50.20 | 1314.5486 | 10 | 0.4 | 658.2819 | 2 | 55.05 | 9 | F9:35981 | NaNaKA16\_F5.raw |  |  |  |  |  |  |  |  | 1.2559E7 |  |  |  |  | 1 | 0 | 0 | 0 | 0 | 0 | 0 | 0 | 0 | 1 | 0 | 0 | 0 | 0 | 24 | 33 | Carbamidomethylation | C3:Carbamidomethylation:1000.00;C7:Carbamidomethylation:1000.00 | PEAKS DB |
| K.DC(+57.02)PNGHVC(+57.02)YTK.T | N | 50.09 | 1349.5493 | 11 | -0.1 | 450.8570 | 3 | 15.59 | 9 | F9:3959 | NaNaKA16\_F5.raw |  |  |  |  |  |  |  |  | 0 |  |  |  |  | 0 | 0 | 0 | 0 | 0 | 0 | 0 | 0 | 0 | 0 | 0 | 0 | 0 | 0 | 13 | 23 | Carbamidomethylation | C2:Carbamidomethylation:1000.00;C8:Carbamidomethylation:1000.00 | PEAKS DB |
| D.LGC(+57.02)AATC(+57.02)PTVK.T | N | 49.50 | 1176.5631 | 11 | 4.9 | 589.2886 | 2 | 11.80 | 8 | F8:1936 | NaNaKA16\_F4.raw |  |  |  |  |  |  |  | 1.7287E5 |  |  |  |  |  | 1 | 0 | 0 | 0 | 0 | 0 | 0 | 0 | 1 | 0 | 0 | 0 | 0 | 0 | 39 | 49 | Carbamidomethylation | C3:Carbamidomethylation:1000.00;C7:Carbamidomethylation:1000.00 | PEAKS DB |
| I.RC(+57.02)FITPDITSK.D | N | 48.93 | 1336.6809 | 11 | 0.7 | 446.5679 | 3 | 25.75 | 9 | F9:12007 | NaNaKA16\_F5.raw |  |  |  |  |  |  |  |  | 8.2828E6 |  |  |  |  | 1 | 0 | 0 | 0 | 0 | 0 | 0 | 0 | 0 | 1 | 0 | 0 | 0 | 0 | 2 | 12 | Carbamidomethylation | C2:Carbamidomethylation:1000.00 | PEAKS DB |
| I.QC(+57.02)C(+57.02)STDNC(+57.02)NPFPTR.K | Y | 47.30 | 1755.6764 | 14 | 1.0 | 878.8463 | 2 | 22.00 | 1 | F1:7265 | NaNaKA16\_F1.raw | 8.1149E5 |  |  |  |  |  |  |  |  |  |  |  |  | 1 | 1 | 0 | 0 | 0 | 0 | 0 | 0 | 0 | 0 | 0 | 0 | 0 | 0 | 55 | 68 | Carbamidomethylation | C2:Carbamidomethylation:1000.00;C3:Carbamidomethylation:1000.00;C8:Carbamidomethylation:1000.00 | PEAKS DB |
| C.FITPDITSK.D | N | 45.76 | 1020.5491 | 9 | -2.0 | 511.2808 | 2 | 35.97 | 9 | F9:20225 | NaNaKA16\_F5.raw |  |  |  |  |  |  |  | 2.3378E8 | 1.8E8 |  |  |  |  | 3 | 0 | 0 | 0 | 0 | 0 | 0 | 0 | 2 | 1 | 0 | 0 | 0 | 0 | 4 | 12 |  |  | PEAKS DB |
| R.VDLGC(+57.02)AATC(+57.02)PTV.K | N | 45.13 | 1262.5635 | 12 | -0.9 | 632.2885 | 2 | 50.91 | 10 | F10:33445 | NaNaKA16\_F6.raw |  |  |  |  |  |  | 1.1303E7 |  |  | 5.2132E7 |  |  |  | 2 | 0 | 0 | 0 | 0 | 0 | 0 | 1 | 0 | 0 | 1 | 0 | 0 | 0 | 37 | 48 | Carbamidomethylation | C5:Carbamidomethylation:1000.00;C9:Carbamidomethylation:1000.00 | PEAKS DB |
| T.DNC(+57.02)NPFPTR.K | N | 44.94 | 1119.4767 | 9 | 0.7 | 560.7460 | 2 | 23.18 | 6 | F6:10002 | NaNaKA16\_F2.raw |  |  |  |  |  | 3.0548E5 |  |  |  |  |  |  |  | 1 | 0 | 0 | 0 | 0 | 0 | 1 | 0 | 0 | 0 | 0 | 0 | 0 | 0 | 60 | 68 | Carbamidomethylation | C3:Carbamidomethylation:1000.00 | PEAKS DB |
| A.TC(+57.02)PTVKTGVDIQC(+57.02)C(+57.02)STDNC(+57.02)NPFPTR.K | Y | 44.77 | 2927.2671 | 25 | -7.1 | 976.7509 | 3 | 63.76 | 8 | F8:41290 | NaNaKA16\_F4.raw |  |  |  |  |  |  |  | 7.0141E6 |  |  |  |  |  | 1 | 0 | 0 | 0 | 0 | 0 | 0 | 0 | 1 | 0 | 0 | 0 | 0 | 0 | 44 | 68 | Carbamidomethylation | C2:Carbamidomethylation:1000.00;C13:Carbamidomethylation:1000.00;C14:Carbamidomethylation:1000.00;C19:Carbamidomethylation:1000.00 | PEAKS DB |
| K.RVDLGC(+57.02)AATC(+57.02)PTVK.T | N | 44.70 | 1546.7595 | 14 | -0.8 | 516.5934 | 3 | 18.27 | 9 | F9:5871 | NaNaKA16\_F5.raw |  |  |  |  |  |  |  | 1.5478E5 | 2.2798E6 |  |  |  |  | 2 | 0 | 0 | 0 | 0 | 0 | 0 | 0 | 1 | 1 | 0 | 0 | 0 | 0 | 36 | 49 | Carbamidomethylation | C6:Carbamidomethylation:1000.00;C10:Carbamidomethylation:1000.00 | PEAKS DB |
| F.ITPDITSK.D | N | 42.90 | 873.4807 | 8 | 4.9 | 437.7476 | 2 | 11.46 | 7 | F7:1934 | NaNaKA16\_F3.raw |  |  |  |  |  |  | 1.0681E6 |  |  |  |  |  |  | 1 | 0 | 0 | 0 | 0 | 0 | 0 | 1 | 0 | 0 | 0 | 0 | 0 | 0 | 5 | 12 |  |  | PEAKS DB |
| V.KTGVDIQC(+57.02)C(+57.02)STDNC(+57.02)NPFPTRK.R | Y | 42.67 | 2497.1147 | 21 | 5.2 | 625.2859 | 4 | 20.10 | 8 | F8:6040 | NaNaKA16\_F4.raw |  |  |  |  |  |  |  | 1.4274E6 |  |  |  |  |  | 1 | 0 | 0 | 0 | 0 | 0 | 0 | 0 | 1 | 0 | 0 | 0 | 0 | 0 | 49 | 69 | Carbamidomethylation | C8:Carbamidomethylation:1000.00;C9:Carbamidomethylation:1000.00;C14:Carbamidomethylation:1000.00 | PEAKS DB |
| D.NC(+57.02)NPFPTR.K | N | 41.96 | 1004.4498 | 8 | -0.2 | 503.2321 | 2 | 43.27 | 9 | F9:25860 | NaNaKA16\_F5.raw |  |  |  |  |  |  |  |  | 0 |  |  |  |  | 0 | 0 | 0 | 0 | 0 | 0 | 0 | 0 | 0 | 0 | 0 | 0 | 0 | 0 | 61 | 68 | Carbamidomethylation | C2:Carbamidomethylation:1000.00 | PEAKS DB |
| total 31 peptides |  |  |  |  |  |  |  |  |  |  |  |  |  |  |  |  |  |  |  |  |  |  |  |  |  |  |  |  |  |  |  |  |  |  |  |  |  |  |  |  |  |  |  |
| --- | --- | --- | --- | --- | --- | --- | --- | --- | --- | --- | --- | --- | --- | --- | --- | --- | --- | --- | --- | --- | --- | --- | --- | --- | --- | --- | --- | --- | --- | --- | --- | --- | --- | --- | --- | --- | --- | --- | --- | --- | --- | --- | --- |

1CTX

back to list

  

| Protein Coverage
| Supporting Peptides
|

Protein Coverage:

Supporting Peptides:

| Peptide | Uniq | -10lgP | Mass | Length | ppm | m/z | z | RT | Fraction | Scan | Source File | Area F1 | Area F10 | Area F11 | Area F12 | Area F13 | Area F2 | Area F3 | Area F4 | Area F5 | Area F6 | Area F7 | Area F8 | Area F9 | #Feature | #Feature F1 | #Feature F10 | #Feature F11 | #Feature F12 | #Feature F13 | #Feature F2 | #Feature F3 | #Feature F4 | #Feature F5 | #Feature F6 | #Feature F7 | #Feature F8 | #Feature F9 | Start | End | PTM | AScore | Found By |
| --- | --- | --- | --- | --- | --- | --- | --- | --- | --- | --- | --- | --- | --- | --- | --- | --- | --- | --- | --- | --- | --- | --- | --- | --- | --- | --- | --- | --- | --- | --- | --- | --- | --- | --- | --- | --- | --- | --- | --- | --- | --- | --- | --- |
| R.C(+57.02)FITPDITSKDC(+57.02)PNGHVC(+57.02)YTK.T | N | 97.70 | 2512.1184 | 21 | -0.2 | 838.3799 | 3 | 32.91 | 9 | F9:17528 | NaNaKA16\_F5.raw |  |  |  |  |  |  | 1.3652E7 | 5.3048E7 | 1.1015E8 |  |  |  |  | 10 | 0 | 0 | 0 | 0 | 0 | 0 | 3 | 4 | 3 | 0 | 0 | 0 | 0 | 3 | 23 | Carbamidomethylation | C1:Carbamidomethylation:1000.00;C12:Carbamidomethylation:1000.00;C18:Carbamidomethylation:1000.00 | PEAKS DB |
| K.TGVDIQC(+57.02)C(+57.02)STDNC(+57.02)NPFPTRK.R | Y | 97.32 | 2369.0198 | 20 | 5.0 | 1185.5167 | 2 | 30.34 | 8 | F8:13936 | NaNaKA16\_F4.raw |  | 5.2751E6 | 1.4938E6 |  |  |  | 4.2829E8 | 1.4347E9 | 1.578E9 | 3.3235E7 | 3.1822E6 |  | 6.4482E6 | 17 | 0 | 1 | 1 | 0 | 0 | 0 | 3 | 5 | 4 | 1 | 1 | 0 | 1 | 50 | 69 | Carbamidomethylation | C7:Carbamidomethylation:1000.00;C8:Carbamidomethylation:1000.00;C13:Carbamidomethylation:1000.00 | PEAKS DB |
| K.TGVDIQC(+57.02)C(+57.02)STDNC(+57.02)NPFPTR.K | Y | 94.74 | 2240.9248 | 19 | 1.1 | 1121.4709 | 2 | 44.69 | 3 | F3:24754 | NaNaKA16\_F11.raw |  | 1.6333E8 | 7.7958E7 | 7.513E7 | 6.0306E6 | 5.3603E8 | 3.7554E10 | 5.9156E10 | 6.2024E10 | 3.6074E9 | 8.3057E7 | 2.6489E7 | 3.5028E8 | 108 | 0 | 2 | 2 | 2 | 2 | 2 | 19 | 35 | 30 | 3 | 2 | 2 | 7 | 50 | 68 | Carbamidomethylation | C7:Carbamidomethylation:1000.00;C8:Carbamidomethylation:1000.00;C13:Carbamidomethylation:1000.00 | PEAKS DB |
| R.VDLGC(+57.02)AATC(+57.02)PTVK.T | N | 93.61 | 1390.6584 | 13 | 6.1 | 696.3372 | 2 | 26.78 | 7 | F7:8603 | NaNaKA16\_F3.raw |  |  |  | 1.5246E7 | 1.5932E6 | 5.0659E7 | 1.9121E8 | 2.2473E10 | 6.1192E10 | 1.9939E9 | 5.3549E6 |  | 0 | 56 | 0 | 0 | 0 | 1 | 1 | 1 | 2 | 13 | 32 | 5 | 1 | 0 | 0 | 37 | 49 | Carbamidomethylation | C5:Carbamidomethylation:1000.00;C9:Carbamidomethylation:1000.00 | PEAKS DB |
| T.GVDIQC(+57.02)C(+57.02)STDNC(+57.02)NPFPTR.K | Y | 89.23 | 2139.8772 | 18 | 4.9 | 1070.9458 | 2 | 42.08 | 7 | F7:20869 | NaNaKA16\_F3.raw |  |  |  |  |  |  | 1.8625E7 | 1.2871E7 | 2.0018E7 |  |  |  |  | 5 | 0 | 0 | 0 | 0 | 0 | 0 | 1 | 1 | 3 | 0 | 0 | 0 | 0 | 51 | 68 | Carbamidomethylation | C6:Carbamidomethylation:1000.00;C7:Carbamidomethylation:1000.00;C12:Carbamidomethylation:1000.00 | PEAKS DB |
| G.VDIQC(+57.02)C(+57.02)STDNC(+57.02)NPFPTR.K | Y | 86.06 | 2082.8557 | 17 | 5.7 | 1042.4355 | 2 | 40.37 | 8 | F8:21741 | NaNaKA16\_F4.raw |  |  |  |  |  |  | 9.4539E6 | 2.0548E7 | 4.7809E7 |  |  |  |  | 4 | 0 | 0 | 0 | 0 | 0 | 0 | 1 | 1 | 2 | 0 | 0 | 0 | 0 | 52 | 68 | Carbamidomethylation | C5:Carbamidomethylation:1000.00;C6:Carbamidomethylation:1000.00;C11:Carbamidomethylation:1000.00 | PEAKS DB |
| D.IQC(+57.02)C(+57.02)STDNC(+57.02)NPFPTR.K | Y | 84.44 | 1868.7604 | 15 | 0.1 | 935.3876 | 2 | 26.32 | 9 | F9:12175 | NaNaKA16\_F5.raw | 8.3412E6 |  |  |  |  | 6.6128E6 | 1.3076E6 | 2.197E8 | 3.1221E8 |  |  |  |  | 9 | 1 | 0 | 0 | 0 | 0 | 1 | 1 | 3 | 3 | 0 | 0 | 0 | 0 | 54 | 68 | Carbamidomethylation | C3:Carbamidomethylation:1000.00;C4:Carbamidomethylation:1000.00;C9:Carbamidomethylation:1000.00 | PEAKS DB |
| V.DIQC(+57.02)C(+57.02)STDNC(+57.02)NPFPTR.K | Y | 83.87 | 1983.7874 | 16 | 6.2 | 992.9019 | 2 | 37.25 | 8 | F8:19226 | NaNaKA16\_F4.raw |  |  |  |  |  | 8.8806E6 | 1.0298E7 | 1.017E7 | 5.0018E8 | 3.4519E7 |  |  |  | 9 | 0 | 0 | 0 | 0 | 0 | 1 | 1 | 1 | 4 | 2 | 0 | 0 | 0 | 53 | 68 | Carbamidomethylation | C4:Carbamidomethylation:1000.00;C5:Carbamidomethylation:1000.00;C10:Carbamidomethylation:1000.00 | PEAKS DB |
| Q.C(+57.02)C(+57.02)STDNC(+57.02)NPFPTR.K | N | 81.03 | 1627.6178 | 13 | 6.1 | 814.8168 | 2 | 20.86 | 8 | F8:6556 | NaNaKA16\_F4.raw | 4.0032E5 |  |  |  |  | 2.1346E6 | 1.8581E7 | 2.2172E6 | 1.8648E7 | 7.3726E5 |  |  |  | 12 | 1 | 0 | 0 | 0 | 0 | 1 | 4 | 2 | 3 | 1 | 0 | 0 | 0 | 56 | 68 | Carbamidomethylation | C1:Carbamidomethylation:1000.00;C2:Carbamidomethylation:1000.00;C7:Carbamidomethylation:1000.00 | PEAKS DB |
| R.C(+57.02)FITPDITSK.D | N | 75.82 | 1180.5798 | 10 | 7.7 | 591.2988 | 2 | 43.13 | 7 | F7:21737 | NaNaKA16\_F3.raw |  | 2.2482E7 | 8.6587E7 | 7.912E7 | 7.0808E6 | 5.0807E8 | 6.1225E10 | 8.5353E10 | 1.2307E11 | 5.4499E9 | 1.0861E8 | 6.3864E7 | 2.9206E8 | 82 | 0 | 4 | 2 | 4 | 2 | 4 | 9 | 21 | 14 | 13 | 2 | 2 | 5 | 3 | 12 | Carbamidomethylation | C1:Carbamidomethylation:1000.00 | PEAKS DB |
| V.KTGVDIQC(+57.02)C(+57.02)STDNC(+57.02)NPFPTR.K | Y | 73.92 | 2369.0198 | 20 | -0.3 | 1185.5168 | 2 | 30.58 | 9 | F9:15182 | NaNaKA16\_F5.raw |  |  |  |  |  |  | 4.0817E8 | 1.4066E9 | 1.5575E9 |  |  |  |  | 5 | 0 | 0 | 0 | 0 | 0 | 0 | 1 | 2 | 2 | 0 | 0 | 0 | 0 | 49 | 68 | Carbamidomethylation | C8:Carbamidomethylation:1000.00;C9:Carbamidomethylation:1000.00;C14:Carbamidomethylation:1000.00 | PEAKS DB |
| C.STDNC(+57.02)NPFPTR.K | N | 69.23 | 1307.5564 | 11 | 4.8 | 654.7853 | 2 | 11.47 | 7 | F7:1955 | NaNaKA16\_F3.raw |  |  |  |  |  | 3.2217E6 | 1.3046E7 | 2.4922E6 | 1.0115E7 |  |  |  |  | 5 | 0 | 0 | 0 | 0 | 0 | 1 | 1 | 1 | 2 | 0 | 0 | 0 | 0 | 58 | 68 | Carbamidomethylation | C5:Carbamidomethylation:1000.00 | PEAKS DB |
| IRC(+57.02)FITPDITSK.D | N | 67.07 | 1449.7650 | 12 | 0.1 | 484.2623 | 3 | 34.24 | 9 | F9:18401 | NaNaKA16\_F5.raw |  |  |  |  |  |  | 2.4051E7 | 7.2194E7 | 8.0035E7 |  |  |  |  | 10 | 0 | 0 | 0 | 0 | 0 | 0 | 3 | 4 | 3 | 0 | 0 | 0 | 0 | 1 | 12 | Carbamidomethylation | C3:Carbamidomethylation:1000.00 | PEAKS DB |
| C.C(+57.02)STDNC(+57.02)NPFPTR.K | N | 66.15 | 1467.5872 | 12 | 0.1 | 734.8009 | 2 | 20.98 | 6 | F6:8194 | NaNaKA16\_F2.raw | 3.5509E6 |  |  |  |  | 1.6348E6 | 1.0498E7 |  | 1.4874E7 |  |  |  |  | 5 | 1 | 0 | 0 | 0 | 0 | 1 | 2 | 0 | 1 | 0 | 0 | 0 | 0 | 57 | 68 | Carbamidomethylation | C1:Carbamidomethylation:1000.00;C6:Carbamidomethylation:1000.00 | PEAKS DB |
| K.TGVDIQC(+57.02)C(+57.02)STDNC(+57.02)NPFPTRKRP | Y | 58.58 | 2622.1738 | 22 | 5.5 | 656.5510 | 4 | 25.48 | 7 | F7:7710 | NaNaKA16\_F3.raw |  |  |  |  |  |  | 0 |  | 1.7072E7 |  |  |  |  | 2 | 0 | 0 | 0 | 0 | 0 | 0 | 0 | 0 | 2 | 0 | 0 | 0 | 0 | 50 | 71 | Carbamidomethylation | C7:Carbamidomethylation:1000.00;C8:Carbamidomethylation:1000.00;C13:Carbamidomethylation:1000.00 | PEAKS DB |
| R.C(+57.02)FITPDITSKD.C | N | 53.03 | 1295.6067 | 11 | 0.2 | 648.8107 | 2 | 44.47 | 9 | F9:26660 | NaNaKA16\_F5.raw |  |  |  |  |  |  | 7.9006E5 | 3.8625E6 | 1.7287E7 |  |  |  |  | 6 | 0 | 0 | 0 | 0 | 0 | 0 | 1 | 2 | 3 | 0 | 0 | 0 | 0 | 3 | 13 | Carbamidomethylation | C1:Carbamidomethylation:1000.00 | PEAKS DB |
| V.DIQC(+57.02)C(+57.02)STDNC(+57.02)NPFPTRK.R | Y | 52.18 | 2111.8823 | 17 | 0.2 | 704.9682 | 3 | 22.55 | 9 | F9:9549 | NaNaKA16\_F5.raw |  |  |  |  |  |  |  |  | 3.3337E6 |  |  |  |  | 1 | 0 | 0 | 0 | 0 | 0 | 0 | 0 | 0 | 1 | 0 | 0 | 0 | 0 | 53 | 69 | Carbamidomethylation | C4:Carbamidomethylation:1000.00;C5:Carbamidomethylation:1000.00;C10:Carbamidomethylation:1000.00 | PEAKS DB |
| K.TGVDIQC(+57.02)C(+57.02)STDNC(+57.02)NPFPT.R | Y | 50.82 | 2084.8237 | 18 | 7.4 | 1043.4213 | 2 | 61.82 | 8 | F8:39511 | NaNaKA16\_F4.raw |  |  |  |  |  |  | 1.4461E8 | 1.6598E8 | 1.2556E9 | 1.4388E8 |  |  |  | 4 | 0 | 0 | 0 | 0 | 0 | 0 | 1 | 1 | 1 | 1 | 0 | 0 | 0 | 50 | 67 | Carbamidomethylation | C7:Carbamidomethylation:1000.00;C8:Carbamidomethylation:1000.00;C13:Carbamidomethylation:1000.00 | PEAKS DB |
| K.TWC(+57.02)DAFC(+57.02)SIR.G | N | 50.20 | 1314.5486 | 10 | 0.4 | 658.2819 | 2 | 55.05 | 9 | F9:35981 | NaNaKA16\_F5.raw |  |  |  |  |  |  |  |  | 1.2559E7 |  |  |  |  | 1 | 0 | 0 | 0 | 0 | 0 | 0 | 0 | 0 | 1 | 0 | 0 | 0 | 0 | 24 | 33 | Carbamidomethylation | C3:Carbamidomethylation:1000.00;C7:Carbamidomethylation:1000.00 | PEAKS DB |
| K.DC(+57.02)PNGHVC(+57.02)YTK.T | N | 50.09 | 1349.5493 | 11 | -0.1 | 450.8570 | 3 | 15.59 | 9 | F9:3959 | NaNaKA16\_F5.raw |  |  |  |  |  |  |  |  | 0 |  |  |  |  | 0 | 0 | 0 | 0 | 0 | 0 | 0 | 0 | 0 | 0 | 0 | 0 | 0 | 0 | 13 | 23 | Carbamidomethylation | C2:Carbamidomethylation:1000.00;C8:Carbamidomethylation:1000.00 | PEAKS DB |
| D.LGC(+57.02)AATC(+57.02)PTVK.T | N | 49.50 | 1176.5631 | 11 | 4.9 | 589.2886 | 2 | 11.80 | 8 | F8:1936 | NaNaKA16\_F4.raw |  |  |  |  |  |  |  | 1.7287E5 |  |  |  |  |  | 1 | 0 | 0 | 0 | 0 | 0 | 0 | 0 | 1 | 0 | 0 | 0 | 0 | 0 | 39 | 49 | Carbamidomethylation | C3:Carbamidomethylation:1000.00;C7:Carbamidomethylation:1000.00 | PEAKS DB |
| I.RC(+57.02)FITPDITSK.D | N | 48.93 | 1336.6809 | 11 | 0.7 | 446.5679 | 3 | 25.75 | 9 | F9:12007 | NaNaKA16\_F5.raw |  |  |  |  |  |  |  |  | 8.2828E6 |  |  |  |  | 1 | 0 | 0 | 0 | 0 | 0 | 0 | 0 | 0 | 1 | 0 | 0 | 0 | 0 | 2 | 12 | Carbamidomethylation | C2:Carbamidomethylation:1000.00 | PEAKS DB |
| I.QC(+57.02)C(+57.02)STDNC(+57.02)NPFPTR.K | Y | 47.30 | 1755.6764 | 14 | 1.0 | 878.8463 | 2 | 22.00 | 1 | F1:7265 | NaNaKA16\_F1.raw | 8.1149E5 |  |  |  |  |  |  |  |  |  |  |  |  | 1 | 1 | 0 | 0 | 0 | 0 | 0 | 0 | 0 | 0 | 0 | 0 | 0 | 0 | 55 | 68 | Carbamidomethylation | C2:Carbamidomethylation:1000.00;C3:Carbamidomethylation:1000.00;C8:Carbamidomethylation:1000.00 | PEAKS DB |
| C.FITPDITSK.D | N | 45.76 | 1020.5491 | 9 | -2.0 | 511.2808 | 2 | 35.97 | 9 | F9:20225 | NaNaKA16\_F5.raw |  |  |  |  |  |  |  | 2.3378E8 | 1.8E8 |  |  |  |  | 3 | 0 | 0 | 0 | 0 | 0 | 0 | 0 | 2 | 1 | 0 | 0 | 0 | 0 | 4 | 12 |  |  | PEAKS DB |
| R.VDLGC(+57.02)AATC(+57.02)PTV.K | N | 45.13 | 1262.5635 | 12 | -0.9 | 632.2885 | 2 | 50.91 | 10 | F10:33445 | NaNaKA16\_F6.raw |  |  |  |  |  |  | 1.1303E7 |  |  | 5.2132E7 |  |  |  | 2 | 0 | 0 | 0 | 0 | 0 | 0 | 1 | 0 | 0 | 1 | 0 | 0 | 0 | 37 | 48 | Carbamidomethylation | C5:Carbamidomethylation:1000.00;C9:Carbamidomethylation:1000.00 | PEAKS DB |
| T.DNC(+57.02)NPFPTR.K | N | 44.94 | 1119.4767 | 9 | 0.7 | 560.7460 | 2 | 23.18 | 6 | F6:10002 | NaNaKA16\_F2.raw |  |  |  |  |  | 3.0548E5 |  |  |  |  |  |  |  | 1 | 0 | 0 | 0 | 0 | 0 | 1 | 0 | 0 | 0 | 0 | 0 | 0 | 0 | 60 | 68 | Carbamidomethylation | C3:Carbamidomethylation:1000.00 | PEAKS DB |
| A.TC(+57.02)PTVKTGVDIQC(+57.02)C(+57.02)STDNC(+57.02)NPFPTR.K | Y | 44.77 | 2927.2671 | 25 | -7.1 | 976.7509 | 3 | 63.76 | 8 | F8:41290 | NaNaKA16\_F4.raw |  |  |  |  |  |  |  | 7.0141E6 |  |  |  |  |  | 1 | 0 | 0 | 0 | 0 | 0 | 0 | 0 | 1 | 0 | 0 | 0 | 0 | 0 | 44 | 68 | Carbamidomethylation | C2:Carbamidomethylation:1000.00;C13:Carbamidomethylation:1000.00;C14:Carbamidomethylation:1000.00;C19:Carbamidomethylation:1000.00 | PEAKS DB |
| K.RVDLGC(+57.02)AATC(+57.02)PTVK.T | N | 44.70 | 1546.7595 | 14 | -0.8 | 516.5934 | 3 | 18.27 | 9 | F9:5871 | NaNaKA16\_F5.raw |  |  |  |  |  |  |  | 1.5478E5 | 2.2798E6 |  |  |  |  | 2 | 0 | 0 | 0 | 0 | 0 | 0 | 0 | 1 | 1 | 0 | 0 | 0 | 0 | 36 | 49 | Carbamidomethylation | C6:Carbamidomethylation:1000.00;C10:Carbamidomethylation:1000.00 | PEAKS DB |
| F.ITPDITSK.D | N | 42.90 | 873.4807 | 8 | 4.9 | 437.7476 | 2 | 11.46 | 7 | F7:1934 | NaNaKA16\_F3.raw |  |  |  |  |  |  | 1.0681E6 |  |  |  |  |  |  | 1 | 0 | 0 | 0 | 0 | 0 | 0 | 1 | 0 | 0 | 0 | 0 | 0 | 0 | 5 | 12 |  |  | PEAKS DB |
| V.KTGVDIQC(+57.02)C(+57.02)STDNC(+57.02)NPFPTRK.R | Y | 42.67 | 2497.1147 | 21 | 5.2 | 625.2859 | 4 | 20.10 | 8 | F8:6040 | NaNaKA16\_F4.raw |  |  |  |  |  |  |  | 1.4274E6 |  |  |  |  |  | 1 | 0 | 0 | 0 | 0 | 0 | 0 | 0 | 1 | 0 | 0 | 0 | 0 | 0 | 49 | 69 | Carbamidomethylation | C8:Carbamidomethylation:1000.00;C9:Carbamidomethylation:1000.00;C14:Carbamidomethylation:1000.00 | PEAKS DB |
| D.NC(+57.02)NPFPTR.K | N | 41.96 | 1004.4498 | 8 | -0.2 | 503.2321 | 2 | 43.27 | 9 | F9:25860 | NaNaKA16\_F5.raw |  |  |  |  |  |  |  |  | 0 |  |  |  |  | 0 | 0 | 0 | 0 | 0 | 0 | 0 | 0 | 0 | 0 | 0 | 0 | 0 | 0 | 61 | 68 | Carbamidomethylation | C2:Carbamidomethylation:1000.00 | PEAKS DB |
| total 31 peptides |  |  |  |  |  |  |  |  |  |  |  |  |  |  |  |  |  |  |  |  |  |  |  |  |  |  |  |  |  |  |  |  |  |  |  |  |  |  |  |  |  |  |  |
| --- | --- | --- | --- | --- | --- | --- | --- | --- | --- | --- | --- | --- | --- | --- | --- | --- | --- | --- | --- | --- | --- | --- | --- | --- | --- | --- | --- | --- | --- | --- | --- | --- | --- | --- | --- | --- | --- | --- | --- | --- | --- | --- | --- |

2CTX

back to list

  

| Protein Coverage
| Supporting Peptides
|

Protein Coverage:

Supporting Peptides:

| Peptide | Uniq | -10lgP | Mass | Length | ppm | m/z | z | RT | Fraction | Scan | Source File | Area F1 | Area F10 | Area F11 | Area F12 | Area F13 | Area F2 | Area F3 | Area F4 | Area F5 | Area F6 | Area F7 | Area F8 | Area F9 | #Feature | #Feature F1 | #Feature F10 | #Feature F11 | #Feature F12 | #Feature F13 | #Feature F2 | #Feature F3 | #Feature F4 | #Feature F5 | #Feature F6 | #Feature F7 | #Feature F8 | #Feature F9 | Start | End | PTM | AScore | Found By |
| --- | --- | --- | --- | --- | --- | --- | --- | --- | --- | --- | --- | --- | --- | --- | --- | --- | --- | --- | --- | --- | --- | --- | --- | --- | --- | --- | --- | --- | --- | --- | --- | --- | --- | --- | --- | --- | --- | --- | --- | --- | --- | --- | --- |
| R.C(+57.02)FITPDITSKDC(+57.02)PNGHVC(+57.02)YTK.T | N | 97.70 | 2512.1184 | 21 | -0.2 | 838.3799 | 3 | 32.91 | 9 | F9:17528 | NaNaKA16\_F5.raw |  |  |  |  |  |  | 1.3652E7 | 5.3048E7 | 1.1015E8 |  |  |  |  | 10 | 0 | 0 | 0 | 0 | 0 | 0 | 3 | 4 | 3 | 0 | 0 | 0 | 0 | 3 | 23 | Carbamidomethylation | C1:Carbamidomethylation:1000.00;C12:Carbamidomethylation:1000.00;C18:Carbamidomethylation:1000.00 | PEAKS DB |
| K.TGVDIQC(+57.02)C(+57.02)STDNC(+57.02)NPFPTRK.R | Y | 97.32 | 2369.0198 | 20 | 5.0 | 1185.5167 | 2 | 30.34 | 8 | F8:13936 | NaNaKA16\_F4.raw |  | 5.2751E6 | 1.4938E6 |  |  |  | 4.2829E8 | 1.4347E9 | 1.578E9 | 3.3235E7 | 3.1822E6 |  | 6.4482E6 | 17 | 0 | 1 | 1 | 0 | 0 | 0 | 3 | 5 | 4 | 1 | 1 | 0 | 1 | 50 | 69 | Carbamidomethylation | C7:Carbamidomethylation:1000.00;C8:Carbamidomethylation:1000.00;C13:Carbamidomethylation:1000.00 | PEAKS DB |
| K.TGVDIQC(+57.02)C(+57.02)STDNC(+57.02)NPFPTR.K | Y | 94.74 | 2240.9248 | 19 | 1.1 | 1121.4709 | 2 | 44.69 | 3 | F3:24754 | NaNaKA16\_F11.raw |  | 1.6333E8 | 7.7958E7 | 7.513E7 | 6.0306E6 | 5.3603E8 | 3.7554E10 | 5.9156E10 | 6.2024E10 | 3.6074E9 | 8.3057E7 | 2.6489E7 | 3.5028E8 | 108 | 0 | 2 | 2 | 2 | 2 | 2 | 19 | 35 | 30 | 3 | 2 | 2 | 7 | 50 | 68 | Carbamidomethylation | C7:Carbamidomethylation:1000.00;C8:Carbamidomethylation:1000.00;C13:Carbamidomethylation:1000.00 | PEAKS DB |
| R.VDLGC(+57.02)AATC(+57.02)PTVK.T | N | 93.61 | 1390.6584 | 13 | 6.1 | 696.3372 | 2 | 26.78 | 7 | F7:8603 | NaNaKA16\_F3.raw |  |  |  | 1.5246E7 | 1.5932E6 | 5.0659E7 | 1.9121E8 | 2.2473E10 | 6.1192E10 | 1.9939E9 | 5.3549E6 |  | 0 | 56 | 0 | 0 | 0 | 1 | 1 | 1 | 2 | 13 | 32 | 5 | 1 | 0 | 0 | 37 | 49 | Carbamidomethylation | C5:Carbamidomethylation:1000.00;C9:Carbamidomethylation:1000.00 | PEAKS DB |
| T.GVDIQC(+57.02)C(+57.02)STDNC(+57.02)NPFPTR.K | Y | 89.23 | 2139.8772 | 18 | 4.9 | 1070.9458 | 2 | 42.08 | 7 | F7:20869 | NaNaKA16\_F3.raw |  |  |  |  |  |  | 1.8625E7 | 1.2871E7 | 2.0018E7 |  |  |  |  | 5 | 0 | 0 | 0 | 0 | 0 | 0 | 1 | 1 | 3 | 0 | 0 | 0 | 0 | 51 | 68 | Carbamidomethylation | C6:Carbamidomethylation:1000.00;C7:Carbamidomethylation:1000.00;C12:Carbamidomethylation:1000.00 | PEAKS DB |
| G.VDIQC(+57.02)C(+57.02)STDNC(+57.02)NPFPTR.K | Y | 86.06 | 2082.8557 | 17 | 5.7 | 1042.4355 | 2 | 40.37 | 8 | F8:21741 | NaNaKA16\_F4.raw |  |  |  |  |  |  | 9.4539E6 | 2.0548E7 | 4.7809E7 |  |  |  |  | 4 | 0 | 0 | 0 | 0 | 0 | 0 | 1 | 1 | 2 | 0 | 0 | 0 | 0 | 52 | 68 | Carbamidomethylation | C5:Carbamidomethylation:1000.00;C6:Carbamidomethylation:1000.00;C11:Carbamidomethylation:1000.00 | PEAKS DB |
| D.IQC(+57.02)C(+57.02)STDNC(+57.02)NPFPTR.K | Y | 84.44 | 1868.7604 | 15 | 0.1 | 935.3876 | 2 | 26.32 | 9 | F9:12175 | NaNaKA16\_F5.raw | 8.3412E6 |  |  |  |  | 6.6128E6 | 1.3076E6 | 2.197E8 | 3.1221E8 |  |  |  |  | 9 | 1 | 0 | 0 | 0 | 0 | 1 | 1 | 3 | 3 | 0 | 0 | 0 | 0 | 54 | 68 | Carbamidomethylation | C3:Carbamidomethylation:1000.00;C4:Carbamidomethylation:1000.00;C9:Carbamidomethylation:1000.00 | PEAKS DB |
| V.DIQC(+57.02)C(+57.02)STDNC(+57.02)NPFPTR.K | Y | 83.87 | 1983.7874 | 16 | 6.2 | 992.9019 | 2 | 37.25 | 8 | F8:19226 | NaNaKA16\_F4.raw |  |  |  |  |  | 8.8806E6 | 1.0298E7 | 1.017E7 | 5.0018E8 | 3.4519E7 |  |  |  | 9 | 0 | 0 | 0 | 0 | 0 | 1 | 1 | 1 | 4 | 2 | 0 | 0 | 0 | 53 | 68 | Carbamidomethylation | C4:Carbamidomethylation:1000.00;C5:Carbamidomethylation:1000.00;C10:Carbamidomethylation:1000.00 | PEAKS DB |
| Q.C(+57.02)C(+57.02)STDNC(+57.02)NPFPTR.K | N | 81.03 | 1627.6178 | 13 | 6.1 | 814.8168 | 2 | 20.86 | 8 | F8:6556 | NaNaKA16\_F4.raw | 4.0032E5 |  |  |  |  | 2.1346E6 | 1.8581E7 | 2.2172E6 | 1.8648E7 | 7.3726E5 |  |  |  | 12 | 1 | 0 | 0 | 0 | 0 | 1 | 4 | 2 | 3 | 1 | 0 | 0 | 0 | 56 | 68 | Carbamidomethylation | C1:Carbamidomethylation:1000.00;C2:Carbamidomethylation:1000.00;C7:Carbamidomethylation:1000.00 | PEAKS DB |
| R.C(+57.02)FITPDITSK.D | N | 75.82 | 1180.5798 | 10 | 7.7 | 591.2988 | 2 | 43.13 | 7 | F7:21737 | NaNaKA16\_F3.raw |  | 2.2482E7 | 8.6587E7 | 7.912E7 | 7.0808E6 | 5.0807E8 | 6.1225E10 | 8.5353E10 | 1.2307E11 | 5.4499E9 | 1.0861E8 | 6.3864E7 | 2.9206E8 | 82 | 0 | 4 | 2 | 4 | 2 | 4 | 9 | 21 | 14 | 13 | 2 | 2 | 5 | 3 | 12 | Carbamidomethylation | C1:Carbamidomethylation:1000.00 | PEAKS DB |
| V.KTGVDIQC(+57.02)C(+57.02)STDNC(+57.02)NPFPTR.K | Y | 73.92 | 2369.0198 | 20 | -0.3 | 1185.5168 | 2 | 30.58 | 9 | F9:15182 | NaNaKA16\_F5.raw |  |  |  |  |  |  | 4.0817E8 | 1.4066E9 | 1.5575E9 |  |  |  |  | 5 | 0 | 0 | 0 | 0 | 0 | 0 | 1 | 2 | 2 | 0 | 0 | 0 | 0 | 49 | 68 | Carbamidomethylation | C8:Carbamidomethylation:1000.00;C9:Carbamidomethylation:1000.00;C14:Carbamidomethylation:1000.00 | PEAKS DB |
| C.STDNC(+57.02)NPFPTR.K | N | 69.23 | 1307.5564 | 11 | 4.8 | 654.7853 | 2 | 11.47 | 7 | F7:1955 | NaNaKA16\_F3.raw |  |  |  |  |  | 3.2217E6 | 1.3046E7 | 2.4922E6 | 1.0115E7 |  |  |  |  | 5 | 0 | 0 | 0 | 0 | 0 | 1 | 1 | 1 | 2 | 0 | 0 | 0 | 0 | 58 | 68 | Carbamidomethylation | C5:Carbamidomethylation:1000.00 | PEAKS DB |
| IRC(+57.02)FITPDITSK.D | N | 67.07 | 1449.7650 | 12 | 0.1 | 484.2623 | 3 | 34.24 | 9 | F9:18401 | NaNaKA16\_F5.raw |  |  |  |  |  |  | 2.4051E7 | 7.2194E7 | 8.0035E7 |  |  |  |  | 10 | 0 | 0 | 0 | 0 | 0 | 0 | 3 | 4 | 3 | 0 | 0 | 0 | 0 | 1 | 12 | Carbamidomethylation | C3:Carbamidomethylation:1000.00 | PEAKS DB |
| C.C(+57.02)STDNC(+57.02)NPFPTR.K | N | 66.15 | 1467.5872 | 12 | 0.1 | 734.8009 | 2 | 20.98 | 6 | F6:8194 | NaNaKA16\_F2.raw | 3.5509E6 |  |  |  |  | 1.6348E6 | 1.0498E7 |  | 1.4874E7 |  |  |  |  | 5 | 1 | 0 | 0 | 0 | 0 | 1 | 2 | 0 | 1 | 0 | 0 | 0 | 0 | 57 | 68 | Carbamidomethylation | C1:Carbamidomethylation:1000.00;C6:Carbamidomethylation:1000.00 | PEAKS DB |
| K.TGVDIQC(+57.02)C(+57.02)STDNC(+57.02)NPFPTRKRP | Y | 58.58 | 2622.1738 | 22 | 5.5 | 656.5510 | 4 | 25.48 | 7 | F7:7710 | NaNaKA16\_F3.raw |  |  |  |  |  |  | 0 |  | 1.7072E7 |  |  |  |  | 2 | 0 | 0 | 0 | 0 | 0 | 0 | 0 | 0 | 2 | 0 | 0 | 0 | 0 | 50 | 71 | Carbamidomethylation | C7:Carbamidomethylation:1000.00;C8:Carbamidomethylation:1000.00;C13:Carbamidomethylation:1000.00 | PEAKS DB |
| R.C(+57.02)FITPDITSKD.C | N | 53.03 | 1295.6067 | 11 | 0.2 | 648.8107 | 2 | 44.47 | 9 | F9:26660 | NaNaKA16\_F5.raw |  |  |  |  |  |  | 7.9006E5 | 3.8625E6 | 1.7287E7 |  |  |  |  | 6 | 0 | 0 | 0 | 0 | 0 | 0 | 1 | 2 | 3 | 0 | 0 | 0 | 0 | 3 | 13 | Carbamidomethylation | C1:Carbamidomethylation:1000.00 | PEAKS DB |
| V.DIQC(+57.02)C(+57.02)STDNC(+57.02)NPFPTRK.R | Y | 52.18 | 2111.8823 | 17 | 0.2 | 704.9682 | 3 | 22.55 | 9 | F9:9549 | NaNaKA16\_F5.raw |  |  |  |  |  |  |  |  | 3.3337E6 |  |  |  |  | 1 | 0 | 0 | 0 | 0 | 0 | 0 | 0 | 0 | 1 | 0 | 0 | 0 | 0 | 53 | 69 | Carbamidomethylation | C4:Carbamidomethylation:1000.00;C5:Carbamidomethylation:1000.00;C10:Carbamidomethylation:1000.00 | PEAKS DB |
| K.TGVDIQC(+57.02)C(+57.02)STDNC(+57.02)NPFPT.R | Y | 50.82 | 2084.8237 | 18 | 7.4 | 1043.4213 | 2 | 61.82 | 8 | F8:39511 | NaNaKA16\_F4.raw |  |  |  |  |  |  | 1.4461E8 | 1.6598E8 | 1.2556E9 | 1.4388E8 |  |  |  | 4 | 0 | 0 | 0 | 0 | 0 | 0 | 1 | 1 | 1 | 1 | 0 | 0 | 0 | 50 | 67 | Carbamidomethylation | C7:Carbamidomethylation:1000.00;C8:Carbamidomethylation:1000.00;C13:Carbamidomethylation:1000.00 | PEAKS DB |
| K.TWC(+57.02)DAFC(+57.02)SIR.G | N | 50.20 | 1314.5486 | 10 | 0.4 | 658.2819 | 2 | 55.05 | 9 | F9:35981 | NaNaKA16\_F5.raw |  |  |  |  |  |  |  |  | 1.2559E7 |  |  |  |  | 1 | 0 | 0 | 0 | 0 | 0 | 0 | 0 | 0 | 1 | 0 | 0 | 0 | 0 | 24 | 33 | Carbamidomethylation | C3:Carbamidomethylation:1000.00;C7:Carbamidomethylation:1000.00 | PEAKS DB |
| K.DC(+57.02)PNGHVC(+57.02)YTK.T | N | 50.09 | 1349.5493 | 11 | -0.1 | 450.8570 | 3 | 15.59 | 9 | F9:3959 | NaNaKA16\_F5.raw |  |  |  |  |  |  |  |  | 0 |  |  |  |  | 0 | 0 | 0 | 0 | 0 | 0 | 0 | 0 | 0 | 0 | 0 | 0 | 0 | 0 | 13 | 23 | Carbamidomethylation | C2:Carbamidomethylation:1000.00;C8:Carbamidomethylation:1000.00 | PEAKS DB |
| D.LGC(+57.02)AATC(+57.02)PTVK.T | N | 49.50 | 1176.5631 | 11 | 4.9 | 589.2886 | 2 | 11.80 | 8 | F8:1936 | NaNaKA16\_F4.raw |  |  |  |  |  |  |  | 1.7287E5 |  |  |  |  |  | 1 | 0 | 0 | 0 | 0 | 0 | 0 | 0 | 1 | 0 | 0 | 0 | 0 | 0 | 39 | 49 | Carbamidomethylation | C3:Carbamidomethylation:1000.00;C7:Carbamidomethylation:1000.00 | PEAKS DB |
| I.RC(+57.02)FITPDITSK.D | N | 48.93 | 1336.6809 | 11 | 0.7 | 446.5679 | 3 | 25.75 | 9 | F9:12007 | NaNaKA16\_F5.raw |  |  |  |  |  |  |  |  | 8.2828E6 |  |  |  |  | 1 | 0 | 0 | 0 | 0 | 0 | 0 | 0 | 0 | 1 | 0 | 0 | 0 | 0 | 2 | 12 | Carbamidomethylation | C2:Carbamidomethylation:1000.00 | PEAKS DB |
| I.QC(+57.02)C(+57.02)STDNC(+57.02)NPFPTR.K | Y | 47.30 | 1755.6764 | 14 | 1.0 | 878.8463 | 2 | 22.00 | 1 | F1:7265 | NaNaKA16\_F1.raw | 8.1149E5 |  |  |  |  |  |  |  |  |  |  |  |  | 1 | 1 | 0 | 0 | 0 | 0 | 0 | 0 | 0 | 0 | 0 | 0 | 0 | 0 | 55 | 68 | Carbamidomethylation | C2:Carbamidomethylation:1000.00;C3:Carbamidomethylation:1000.00;C8:Carbamidomethylation:1000.00 | PEAKS DB |
| C.FITPDITSK.D | N | 45.76 | 1020.5491 | 9 | -2.0 | 511.2808 | 2 | 35.97 | 9 | F9:20225 | NaNaKA16\_F5.raw |  |  |  |  |  |  |  | 2.3378E8 | 1.8E8 |  |  |  |  | 3 | 0 | 0 | 0 | 0 | 0 | 0 | 0 | 2 | 1 | 0 | 0 | 0 | 0 | 4 | 12 |  |  | PEAKS DB |
| R.VDLGC(+57.02)AATC(+57.02)PTV.K | N | 45.13 | 1262.5635 | 12 | -0.9 | 632.2885 | 2 | 50.91 | 10 | F10:33445 | NaNaKA16\_F6.raw |  |  |  |  |  |  | 1.1303E7 |  |  | 5.2132E7 |  |  |  | 2 | 0 | 0 | 0 | 0 | 0 | 0 | 1 | 0 | 0 | 1 | 0 | 0 | 0 | 37 | 48 | Carbamidomethylation | C5:Carbamidomethylation:1000.00;C9:Carbamidomethylation:1000.00 | PEAKS DB |
| T.DNC(+57.02)NPFPTR.K | N | 44.94 | 1119.4767 | 9 | 0.7 | 560.7460 | 2 | 23.18 | 6 | F6:10002 | NaNaKA16\_F2.raw |  |  |  |  |  | 3.0548E5 |  |  |  |  |  |  |  | 1 | 0 | 0 | 0 | 0 | 0 | 1 | 0 | 0 | 0 | 0 | 0 | 0 | 0 | 60 | 68 | Carbamidomethylation | C3:Carbamidomethylation:1000.00 | PEAKS DB |
| A.TC(+57.02)PTVKTGVDIQC(+57.02)C(+57.02)STDNC(+57.02)NPFPTR.K | Y | 44.77 | 2927.2671 | 25 | -7.1 | 976.7509 | 3 | 63.76 | 8 | F8:41290 | NaNaKA16\_F4.raw |  |  |  |  |  |  |  | 7.0141E6 |  |  |  |  |  | 1 | 0 | 0 | 0 | 0 | 0 | 0 | 0 | 1 | 0 | 0 | 0 | 0 | 0 | 44 | 68 | Carbamidomethylation | C2:Carbamidomethylation:1000.00;C13:Carbamidomethylation:1000.00;C14:Carbamidomethylation:1000.00;C19:Carbamidomethylation:1000.00 | PEAKS DB |
| K.RVDLGC(+57.02)AATC(+57.02)PTVK.T | N | 44.70 | 1546.7595 | 14 | -0.8 | 516.5934 | 3 | 18.27 | 9 | F9:5871 | NaNaKA16\_F5.raw |  |  |  |  |  |  |  | 1.5478E5 | 2.2798E6 |  |  |  |  | 2 | 0 | 0 | 0 | 0 | 0 | 0 | 0 | 1 | 1 | 0 | 0 | 0 | 0 | 36 | 49 | Carbamidomethylation | C6:Carbamidomethylation:1000.00;C10:Carbamidomethylation:1000.00 | PEAKS DB |
| F.ITPDITSK.D | N | 42.90 | 873.4807 | 8 | 4.9 | 437.7476 | 2 | 11.46 | 7 | F7:1934 | NaNaKA16\_F3.raw |  |  |  |  |  |  | 1.0681E6 |  |  |  |  |  |  | 1 | 0 | 0 | 0 | 0 | 0 | 0 | 1 | 0 | 0 | 0 | 0 | 0 | 0 | 5 | 12 |  |  | PEAKS DB |
| V.KTGVDIQC(+57.02)C(+57.02)STDNC(+57.02)NPFPTRK.R | Y | 42.67 | 2497.1147 | 21 | 5.2 | 625.2859 | 4 | 20.10 | 8 | F8:6040 | NaNaKA16\_F4.raw |  |  |  |  |  |  |  | 1.4274E6 |  |  |  |  |  | 1 | 0 | 0 | 0 | 0 | 0 | 0 | 0 | 1 | 0 | 0 | 0 | 0 | 0 | 49 | 69 | Carbamidomethylation | C8:Carbamidomethylation:1000.00;C9:Carbamidomethylation:1000.00;C14:Carbamidomethylation:1000.00 | PEAKS DB |
| D.NC(+57.02)NPFPTR.K | N | 41.96 | 1004.4498 | 8 | -0.2 | 503.2321 | 2 | 43.27 | 9 | F9:25860 | NaNaKA16\_F5.raw |  |  |  |  |  |  |  |  | 0 |  |  |  |  | 0 | 0 | 0 | 0 | 0 | 0 | 0 | 0 | 0 | 0 | 0 | 0 | 0 | 0 | 61 | 68 | Carbamidomethylation | C2:Carbamidomethylation:1000.00 | PEAKS DB |
| total 31 peptides |  |  |  |  |  |  |  |  |  |  |  |  |  |  |  |  |  |  |  |  |  |  |  |  |  |  |  |  |  |  |  |  |  |  |  |  |  |  |  |  |  |  |  |
| --- | --- | --- | --- | --- | --- | --- | --- | --- | --- | --- | --- | --- | --- | --- | --- | --- | --- | --- | --- | --- | --- | --- | --- | --- | --- | --- | --- | --- | --- | --- | --- | --- | --- | --- | --- | --- | --- | --- | --- | --- | --- | --- | --- |

P01391.1

back to list

  

| Protein Coverage
| Supporting Peptides
|

Protein Coverage:

Supporting Peptides:

| Peptide | Uniq | -10lgP | Mass | Length | ppm | m/z | z | RT | Fraction | Scan | Source File | Area F1 | Area F10 | Area F11 | Area F12 | Area F13 | Area F2 | Area F3 | Area F4 | Area F5 | Area F6 | Area F7 | Area F8 | Area F9 | #Feature | #Feature F1 | #Feature F10 | #Feature F11 | #Feature F12 | #Feature F13 | #Feature F2 | #Feature F3 | #Feature F4 | #Feature F5 | #Feature F6 | #Feature F7 | #Feature F8 | #Feature F9 | Start | End | PTM | AScore | Found By |
| --- | --- | --- | --- | --- | --- | --- | --- | --- | --- | --- | --- | --- | --- | --- | --- | --- | --- | --- | --- | --- | --- | --- | --- | --- | --- | --- | --- | --- | --- | --- | --- | --- | --- | --- | --- | --- | --- | --- | --- | --- | --- | --- | --- |
| R.C(+57.02)FITPDITSKDC(+57.02)PNGHVC(+57.02)YTK.T | N | 97.70 | 2512.1184 | 21 | -0.2 | 838.3799 | 3 | 32.91 | 9 | F9:17528 | NaNaKA16\_F5.raw |  |  |  |  |  |  | 1.3652E7 | 5.3048E7 | 1.1015E8 |  |  |  |  | 10 | 0 | 0 | 0 | 0 | 0 | 0 | 3 | 4 | 3 | 0 | 0 | 0 | 0 | 3 | 23 | Carbamidomethylation | C1:Carbamidomethylation:1000.00;C12:Carbamidomethylation:1000.00;C18:Carbamidomethylation:1000.00 | PEAKS DB |
| K.TGVDIQC(+57.02)C(+57.02)STDNC(+57.02)NPFPTRK.R | Y | 97.32 | 2369.0198 | 20 | 5.0 | 1185.5167 | 2 | 30.34 | 8 | F8:13936 | NaNaKA16\_F4.raw |  | 5.2751E6 | 1.4938E6 |  |  |  | 4.2829E8 | 1.4347E9 | 1.578E9 | 3.3235E7 | 3.1822E6 |  | 6.4482E6 | 17 | 0 | 1 | 1 | 0 | 0 | 0 | 3 | 5 | 4 | 1 | 1 | 0 | 1 | 50 | 69 | Carbamidomethylation | C7:Carbamidomethylation:1000.00;C8:Carbamidomethylation:1000.00;C13:Carbamidomethylation:1000.00 | PEAKS DB |
| K.TGVDIQC(+57.02)C(+57.02)STDNC(+57.02)NPFPTR.K | Y | 94.74 | 2240.9248 | 19 | 1.1 | 1121.4709 | 2 | 44.69 | 3 | F3:24754 | NaNaKA16\_F11.raw |  | 1.6333E8 | 7.7958E7 | 7.513E7 | 6.0306E6 | 5.3603E8 | 3.7554E10 | 5.9156E10 | 6.2024E10 | 3.6074E9 | 8.3057E7 | 2.6489E7 | 3.5028E8 | 108 | 0 | 2 | 2 | 2 | 2 | 2 | 19 | 35 | 30 | 3 | 2 | 2 | 7 | 50 | 68 | Carbamidomethylation | C7:Carbamidomethylation:1000.00;C8:Carbamidomethylation:1000.00;C13:Carbamidomethylation:1000.00 | PEAKS DB |
| R.VDLGC(+57.02)AATC(+57.02)PTVK.T | N | 93.61 | 1390.6584 | 13 | 6.1 | 696.3372 | 2 | 26.78 | 7 | F7:8603 | NaNaKA16\_F3.raw |  |  |  | 1.5246E7 | 1.5932E6 | 5.0659E7 | 1.9121E8 | 2.2473E10 | 6.1192E10 | 1.9939E9 | 5.3549E6 |  | 0 | 56 | 0 | 0 | 0 | 1 | 1 | 1 | 2 | 13 | 32 | 5 | 1 | 0 | 0 | 37 | 49 | Carbamidomethylation | C5:Carbamidomethylation:1000.00;C9:Carbamidomethylation:1000.00 | PEAKS DB |
| T.GVDIQC(+57.02)C(+57.02)STDNC(+57.02)NPFPTR.K | Y | 89.23 | 2139.8772 | 18 | 4.9 | 1070.9458 | 2 | 42.08 | 7 | F7:20869 | NaNaKA16\_F3.raw |  |  |  |  |  |  | 1.8625E7 | 1.2871E7 | 2.0018E7 |  |  |  |  | 5 | 0 | 0 | 0 | 0 | 0 | 0 | 1 | 1 | 3 | 0 | 0 | 0 | 0 | 51 | 68 | Carbamidomethylation | C6:Carbamidomethylation:1000.00;C7:Carbamidomethylation:1000.00;C12:Carbamidomethylation:1000.00 | PEAKS DB |
| G.VDIQC(+57.02)C(+57.02)STDNC(+57.02)NPFPTR.K | Y | 86.06 | 2082.8557 | 17 | 5.7 | 1042.4355 | 2 | 40.37 | 8 | F8:21741 | NaNaKA16\_F4.raw |  |  |  |  |  |  | 9.4539E6 | 2.0548E7 | 4.7809E7 |  |  |  |  | 4 | 0 | 0 | 0 | 0 | 0 | 0 | 1 | 1 | 2 | 0 | 0 | 0 | 0 | 52 | 68 | Carbamidomethylation | C5:Carbamidomethylation:1000.00;C6:Carbamidomethylation:1000.00;C11:Carbamidomethylation:1000.00 | PEAKS DB |
| D.IQC(+57.02)C(+57.02)STDNC(+57.02)NPFPTR.K | Y | 84.44 | 1868.7604 | 15 | 0.1 | 935.3876 | 2 | 26.32 | 9 | F9:12175 | NaNaKA16\_F5.raw | 8.3412E6 |  |  |  |  | 6.6128E6 | 1.3076E6 | 2.197E8 | 3.1221E8 |  |  |  |  | 9 | 1 | 0 | 0 | 0 | 0 | 1 | 1 | 3 | 3 | 0 | 0 | 0 | 0 | 54 | 68 | Carbamidomethylation | C3:Carbamidomethylation:1000.00;C4:Carbamidomethylation:1000.00;C9:Carbamidomethylation:1000.00 | PEAKS DB |
| V.DIQC(+57.02)C(+57.02)STDNC(+57.02)NPFPTR.K | Y | 83.87 | 1983.7874 | 16 | 6.2 | 992.9019 | 2 | 37.25 | 8 | F8:19226 | NaNaKA16\_F4.raw |  |  |  |  |  | 8.8806E6 | 1.0298E7 | 1.017E7 | 5.0018E8 | 3.4519E7 |  |  |  | 9 | 0 | 0 | 0 | 0 | 0 | 1 | 1 | 1 | 4 | 2 | 0 | 0 | 0 | 53 | 68 | Carbamidomethylation | C4:Carbamidomethylation:1000.00;C5:Carbamidomethylation:1000.00;C10:Carbamidomethylation:1000.00 | PEAKS DB |
| Q.C(+57.02)C(+57.02)STDNC(+57.02)NPFPTR.K | N | 81.03 | 1627.6178 | 13 | 6.1 | 814.8168 | 2 | 20.86 | 8 | F8:6556 | NaNaKA16\_F4.raw | 4.0032E5 |  |  |  |  | 2.1346E6 | 1.8581E7 | 2.2172E6 | 1.8648E7 | 7.3726E5 |  |  |  | 12 | 1 | 0 | 0 | 0 | 0 | 1 | 4 | 2 | 3 | 1 | 0 | 0 | 0 | 56 | 68 | Carbamidomethylation | C1:Carbamidomethylation:1000.00;C2:Carbamidomethylation:1000.00;C7:Carbamidomethylation:1000.00 | PEAKS DB |
| R.C(+57.02)FITPDITSK.D | N | 75.82 | 1180.5798 | 10 | 7.7 | 591.2988 | 2 | 43.13 | 7 | F7:21737 | NaNaKA16\_F3.raw |  | 2.2482E7 | 8.6587E7 | 7.912E7 | 7.0808E6 | 5.0807E8 | 6.1225E10 | 8.5353E10 | 1.2307E11 | 5.4499E9 | 1.0861E8 | 6.3864E7 | 2.9206E8 | 82 | 0 | 4 | 2 | 4 | 2 | 4 | 9 | 21 | 14 | 13 | 2 | 2 | 5 | 3 | 12 | Carbamidomethylation | C1:Carbamidomethylation:1000.00 | PEAKS DB |
| V.KTGVDIQC(+57.02)C(+57.02)STDNC(+57.02)NPFPTR.K | Y | 73.92 | 2369.0198 | 20 | -0.3 | 1185.5168 | 2 | 30.58 | 9 | F9:15182 | NaNaKA16\_F5.raw |  |  |  |  |  |  | 4.0817E8 | 1.4066E9 | 1.5575E9 |  |  |  |  | 5 | 0 | 0 | 0 | 0 | 0 | 0 | 1 | 2 | 2 | 0 | 0 | 0 | 0 | 49 | 68 | Carbamidomethylation | C8:Carbamidomethylation:1000.00;C9:Carbamidomethylation:1000.00;C14:Carbamidomethylation:1000.00 | PEAKS DB |
| C.STDNC(+57.02)NPFPTR.K | N | 69.23 | 1307.5564 | 11 | 4.8 | 654.7853 | 2 | 11.47 | 7 | F7:1955 | NaNaKA16\_F3.raw |  |  |  |  |  | 3.2217E6 | 1.3046E7 | 2.4922E6 | 1.0115E7 |  |  |  |  | 5 | 0 | 0 | 0 | 0 | 0 | 1 | 1 | 1 | 2 | 0 | 0 | 0 | 0 | 58 | 68 | Carbamidomethylation | C5:Carbamidomethylation:1000.00 | PEAKS DB |
| IRC(+57.02)FITPDITSK.D | N | 67.07 | 1449.7650 | 12 | 0.1 | 484.2623 | 3 | 34.24 | 9 | F9:18401 | NaNaKA16\_F5.raw |  |  |  |  |  |  | 2.4051E7 | 7.2194E7 | 8.0035E7 |  |  |  |  | 10 | 0 | 0 | 0 | 0 | 0 | 0 | 3 | 4 | 3 | 0 | 0 | 0 | 0 | 1 | 12 | Carbamidomethylation | C3:Carbamidomethylation:1000.00 | PEAKS DB |
| C.C(+57.02)STDNC(+57.02)NPFPTR.K | N | 66.15 | 1467.5872 | 12 | 0.1 | 734.8009 | 2 | 20.98 | 6 | F6:8194 | NaNaKA16\_F2.raw | 3.5509E6 |  |  |  |  | 1.6348E6 | 1.0498E7 |  | 1.4874E7 |  |  |  |  | 5 | 1 | 0 | 0 | 0 | 0 | 1 | 2 | 0 | 1 | 0 | 0 | 0 | 0 | 57 | 68 | Carbamidomethylation | C1:Carbamidomethylation:1000.00;C6:Carbamidomethylation:1000.00 | PEAKS DB |
| K.TGVDIQC(+57.02)C(+57.02)STDNC(+57.02)NPFPTRKRP | Y | 58.58 | 2622.1738 | 22 | 5.5 | 656.5510 | 4 | 25.48 | 7 | F7:7710 | NaNaKA16\_F3.raw |  |  |  |  |  |  | 0 |  | 1.7072E7 |  |  |  |  | 2 | 0 | 0 | 0 | 0 | 0 | 0 | 0 | 0 | 2 | 0 | 0 | 0 | 0 | 50 | 71 | Carbamidomethylation | C7:Carbamidomethylation:1000.00;C8:Carbamidomethylation:1000.00;C13:Carbamidomethylation:1000.00 | PEAKS DB |
| R.C(+57.02)FITPDITSKD.C | N | 53.03 | 1295.6067 | 11 | 0.2 | 648.8107 | 2 | 44.47 | 9 | F9:26660 | NaNaKA16\_F5.raw |  |  |  |  |  |  | 7.9006E5 | 3.8625E6 | 1.7287E7 |  |  |  |  | 6 | 0 | 0 | 0 | 0 | 0 | 0 | 1 | 2 | 3 | 0 | 0 | 0 | 0 | 3 | 13 | Carbamidomethylation | C1:Carbamidomethylation:1000.00 | PEAKS DB |
| V.DIQC(+57.02)C(+57.02)STDNC(+57.02)NPFPTRK.R | Y | 52.18 | 2111.8823 | 17 | 0.2 | 704.9682 | 3 | 22.55 | 9 | F9:9549 | NaNaKA16\_F5.raw |  |  |  |  |  |  |  |  | 3.3337E6 |  |  |  |  | 1 | 0 | 0 | 0 | 0 | 0 | 0 | 0 | 0 | 1 | 0 | 0 | 0 | 0 | 53 | 69 | Carbamidomethylation | C4:Carbamidomethylation:1000.00;C5:Carbamidomethylation:1000.00;C10:Carbamidomethylation:1000.00 | PEAKS DB |
| K.TGVDIQC(+57.02)C(+57.02)STDNC(+57.02)NPFPT.R | Y | 50.82 | 2084.8237 | 18 | 7.4 | 1043.4213 | 2 | 61.82 | 8 | F8:39511 | NaNaKA16\_F4.raw |  |  |  |  |  |  | 1.4461E8 | 1.6598E8 | 1.2556E9 | 1.4388E8 |  |  |  | 4 | 0 | 0 | 0 | 0 | 0 | 0 | 1 | 1 | 1 | 1 | 0 | 0 | 0 | 50 | 67 | Carbamidomethylation | C7:Carbamidomethylation:1000.00;C8:Carbamidomethylation:1000.00;C13:Carbamidomethylation:1000.00 | PEAKS DB |
| K.TWC(+57.02)DAFC(+57.02)SIR.G | N | 50.20 | 1314.5486 | 10 | 0.4 | 658.2819 | 2 | 55.05 | 9 | F9:35981 | NaNaKA16\_F5.raw |  |  |  |  |  |  |  |  | 1.2559E7 |  |  |  |  | 1 | 0 | 0 | 0 | 0 | 0 | 0 | 0 | 0 | 1 | 0 | 0 | 0 | 0 | 24 | 33 | Carbamidomethylation | C3:Carbamidomethylation:1000.00;C7:Carbamidomethylation:1000.00 | PEAKS DB |
| K.DC(+57.02)PNGHVC(+57.02)YTK.T | N | 50.09 | 1349.5493 | 11 | -0.1 | 450.8570 | 3 | 15.59 | 9 | F9:3959 | NaNaKA16\_F5.raw |  |  |  |  |  |  |  |  | 0 |  |  |  |  | 0 | 0 | 0 | 0 | 0 | 0 | 0 | 0 | 0 | 0 | 0 | 0 | 0 | 0 | 13 | 23 | Carbamidomethylation | C2:Carbamidomethylation:1000.00;C8:Carbamidomethylation:1000.00 | PEAKS DB |
| D.LGC(+57.02)AATC(+57.02)PTVK.T | N | 49.50 | 1176.5631 | 11 | 4.9 | 589.2886 | 2 | 11.80 | 8 | F8:1936 | NaNaKA16\_F4.raw |  |  |  |  |  |  |  | 1.7287E5 |  |  |  |  |  | 1 | 0 | 0 | 0 | 0 | 0 | 0 | 0 | 1 | 0 | 0 | 0 | 0 | 0 | 39 | 49 | Carbamidomethylation | C3:Carbamidomethylation:1000.00;C7:Carbamidomethylation:1000.00 | PEAKS DB |
| I.RC(+57.02)FITPDITSK.D | N | 48.93 | 1336.6809 | 11 | 0.7 | 446.5679 | 3 | 25.75 | 9 | F9:12007 | NaNaKA16\_F5.raw |  |  |  |  |  |  |  |  | 8.2828E6 |  |  |  |  | 1 | 0 | 0 | 0 | 0 | 0 | 0 | 0 | 0 | 1 | 0 | 0 | 0 | 0 | 2 | 12 | Carbamidomethylation | C2:Carbamidomethylation:1000.00 | PEAKS DB |
| I.QC(+57.02)C(+57.02)STDNC(+57.02)NPFPTR.K | Y | 47.30 | 1755.6764 | 14 | 1.0 | 878.8463 | 2 | 22.00 | 1 | F1:7265 | NaNaKA16\_F1.raw | 8.1149E5 |  |  |  |  |  |  |  |  |  |  |  |  | 1 | 1 | 0 | 0 | 0 | 0 | 0 | 0 | 0 | 0 | 0 | 0 | 0 | 0 | 55 | 68 | Carbamidomethylation | C2:Carbamidomethylation:1000.00;C3:Carbamidomethylation:1000.00;C8:Carbamidomethylation:1000.00 | PEAKS DB |
| C.FITPDITSK.D | N | 45.76 | 1020.5491 | 9 | -2.0 | 511.2808 | 2 | 35.97 | 9 | F9:20225 | NaNaKA16\_F5.raw |  |  |  |  |  |  |  | 2.3378E8 | 1.8E8 |  |  |  |  | 3 | 0 | 0 | 0 | 0 | 0 | 0 | 0 | 2 | 1 | 0 | 0 | 0 | 0 | 4 | 12 |  |  | PEAKS DB |
| R.VDLGC(+57.02)AATC(+57.02)PTV.K | N | 45.13 | 1262.5635 | 12 | -0.9 | 632.2885 | 2 | 50.91 | 10 | F10:33445 | NaNaKA16\_F6.raw |  |  |  |  |  |  | 1.1303E7 |  |  | 5.2132E7 |  |  |  | 2 | 0 | 0 | 0 | 0 | 0 | 0 | 1 | 0 | 0 | 1 | 0 | 0 | 0 | 37 | 48 | Carbamidomethylation | C5:Carbamidomethylation:1000.00;C9:Carbamidomethylation:1000.00 | PEAKS DB |
| T.DNC(+57.02)NPFPTR.K | N | 44.94 | 1119.4767 | 9 | 0.7 | 560.7460 | 2 | 23.18 | 6 | F6:10002 | NaNaKA16\_F2.raw |  |  |  |  |  | 3.0548E5 |  |  |  |  |  |  |  | 1 | 0 | 0 | 0 | 0 | 0 | 1 | 0 | 0 | 0 | 0 | 0 | 0 | 0 | 60 | 68 | Carbamidomethylation | C3:Carbamidomethylation:1000.00 | PEAKS DB |
| A.TC(+57.02)PTVKTGVDIQC(+57.02)C(+57.02)STDNC(+57.02)NPFPTR.K | Y | 44.77 | 2927.2671 | 25 | -7.1 | 976.7509 | 3 | 63.76 | 8 | F8:41290 | NaNaKA16\_F4.raw |  |  |  |  |  |  |  | 7.0141E6 |  |  |  |  |  | 1 | 0 | 0 | 0 | 0 | 0 | 0 | 0 | 1 | 0 | 0 | 0 | 0 | 0 | 44 | 68 | Carbamidomethylation | C2:Carbamidomethylation:1000.00;C13:Carbamidomethylation:1000.00;C14:Carbamidomethylation:1000.00;C19:Carbamidomethylation:1000.00 | PEAKS DB |
| K.RVDLGC(+57.02)AATC(+57.02)PTVK.T | N | 44.70 | 1546.7595 | 14 | -0.8 | 516.5934 | 3 | 18.27 | 9 | F9:5871 | NaNaKA16\_F5.raw |  |  |  |  |  |  |  | 1.5478E5 | 2.2798E6 |  |  |  |  | 2 | 0 | 0 | 0 | 0 | 0 | 0 | 0 | 1 | 1 | 0 | 0 | 0 | 0 | 36 | 49 | Carbamidomethylation | C6:Carbamidomethylation:1000.00;C10:Carbamidomethylation:1000.00 | PEAKS DB |
| F.ITPDITSK.D | N | 42.90 | 873.4807 | 8 | 4.9 | 437.7476 | 2 | 11.46 | 7 | F7:1934 | NaNaKA16\_F3.raw |  |  |  |  |  |  | 1.0681E6 |  |  |  |  |  |  | 1 | 0 | 0 | 0 | 0 | 0 | 0 | 1 | 0 | 0 | 0 | 0 | 0 | 0 | 5 | 12 |  |  | PEAKS DB |
| V.KTGVDIQC(+57.02)C(+57.02)STDNC(+57.02)NPFPTRK.R | Y | 42.67 | 2497.1147 | 21 | 5.2 | 625.2859 | 4 | 20.10 | 8 | F8:6040 | NaNaKA16\_F4.raw |  |  |  |  |  |  |  | 1.4274E6 |  |  |  |  |  | 1 | 0 | 0 | 0 | 0 | 0 | 0 | 0 | 1 | 0 | 0 | 0 | 0 | 0 | 49 | 69 | Carbamidomethylation | C8:Carbamidomethylation:1000.00;C9:Carbamidomethylation:1000.00;C14:Carbamidomethylation:1000.00 | PEAKS DB |
| D.NC(+57.02)NPFPTR.K | N | 41.96 | 1004.4498 | 8 | -0.2 | 503.2321 | 2 | 43.27 | 9 | F9:25860 | NaNaKA16\_F5.raw |  |  |  |  |  |  |  |  | 0 |  |  |  |  | 0 | 0 | 0 | 0 | 0 | 0 | 0 | 0 | 0 | 0 | 0 | 0 | 0 | 0 | 61 | 68 | Carbamidomethylation | C2:Carbamidomethylation:1000.00 | PEAKS DB |
| total 31 peptides |  |  |  |  |  |  |  |  |  |  |  |  |  |  |  |  |  |  |  |  |  |  |  |  |  |  |  |  |  |  |  |  |  |  |  |  |  |  |  |  |  |  |  |
| --- | --- | --- | --- | --- | --- | --- | --- | --- | --- | --- | --- | --- | --- | --- | --- | --- | --- | --- | --- | --- | --- | --- | --- | --- | --- | --- | --- | --- | --- | --- | --- | --- | --- | --- | --- | --- | --- | --- | --- | --- | --- | --- | --- |

1LXH

back to list

  

| Protein Coverage
| Supporting Peptides
|

Protein Coverage:

Supporting Peptides:

| Peptide | Uniq | -10lgP | Mass | Length | ppm | m/z | z | RT | Fraction | Scan | Source File | Area F1 | Area F10 | Area F11 | Area F12 | Area F13 | Area F2 | Area F3 | Area F4 | Area F5 | Area F6 | Area F7 | Area F8 | Area F9 | #Feature | #Feature F1 | #Feature F10 | #Feature F11 | #Feature F12 | #Feature F13 | #Feature F2 | #Feature F3 | #Feature F4 | #Feature F5 | #Feature F6 | #Feature F7 | #Feature F8 | #Feature F9 | Start | End | PTM | AScore | Found By |
| --- | --- | --- | --- | --- | --- | --- | --- | --- | --- | --- | --- | --- | --- | --- | --- | --- | --- | --- | --- | --- | --- | --- | --- | --- | --- | --- | --- | --- | --- | --- | --- | --- | --- | --- | --- | --- | --- | --- | --- | --- | --- | --- | --- |
| R.C(+57.02)FITPDITSKDC(+57.02)PNGHVC(+57.02)YTK.T | N | 97.70 | 2512.1184 | 21 | -0.2 | 838.3799 | 3 | 32.91 | 9 | F9:17528 | NaNaKA16\_F5.raw |  |  |  |  |  |  | 1.3652E7 | 5.3048E7 | 1.1015E8 |  |  |  |  | 10 | 0 | 0 | 0 | 0 | 0 | 0 | 3 | 4 | 3 | 0 | 0 | 0 | 0 | 3 | 23 | Carbamidomethylation | C1:Carbamidomethylation:1000.00;C12:Carbamidomethylation:1000.00;C18:Carbamidomethylation:1000.00 | PEAKS DB |
| K.TGVDIQC(+57.02)C(+57.02)STDNC(+57.02)NPFPTRK.R | Y | 97.32 | 2369.0198 | 20 | 5.0 | 1185.5167 | 2 | 30.34 | 8 | F8:13936 | NaNaKA16\_F4.raw |  | 5.2751E6 | 1.4938E6 |  |  |  | 4.2829E8 | 1.4347E9 | 1.578E9 | 3.3235E7 | 3.1822E6 |  | 6.4482E6 | 17 | 0 | 1 | 1 | 0 | 0 | 0 | 3 | 5 | 4 | 1 | 1 | 0 | 1 | 50 | 69 | Carbamidomethylation | C7:Carbamidomethylation:1000.00;C8:Carbamidomethylation:1000.00;C13:Carbamidomethylation:1000.00 | PEAKS DB |
| K.TGVDIQC(+57.02)C(+57.02)STDNC(+57.02)NPFPTR.K | Y | 94.74 | 2240.9248 | 19 | 1.1 | 1121.4709 | 2 | 44.69 | 3 | F3:24754 | NaNaKA16\_F11.raw |  | 1.6333E8 | 7.7958E7 | 7.513E7 | 6.0306E6 | 5.3603E8 | 3.7554E10 | 5.9156E10 | 6.2024E10 | 3.6074E9 | 8.3057E7 | 2.6489E7 | 3.5028E8 | 108 | 0 | 2 | 2 | 2 | 2 | 2 | 19 | 35 | 30 | 3 | 2 | 2 | 7 | 50 | 68 | Carbamidomethylation | C7:Carbamidomethylation:1000.00;C8:Carbamidomethylation:1000.00;C13:Carbamidomethylation:1000.00 | PEAKS DB |
| R.VDLGC(+57.02)AATC(+57.02)PTVK.T | N | 93.61 | 1390.6584 | 13 | 6.1 | 696.3372 | 2 | 26.78 | 7 | F7:8603 | NaNaKA16\_F3.raw |  |  |  | 1.5246E7 | 1.5932E6 | 5.0659E7 | 1.9121E8 | 2.2473E10 | 6.1192E10 | 1.9939E9 | 5.3549E6 |  | 0 | 56 | 0 | 0 | 0 | 1 | 1 | 1 | 2 | 13 | 32 | 5 | 1 | 0 | 0 | 37 | 49 | Carbamidomethylation | C5:Carbamidomethylation:1000.00;C9:Carbamidomethylation:1000.00 | PEAKS DB |
| T.GVDIQC(+57.02)C(+57.02)STDNC(+57.02)NPFPTR.K | Y | 89.23 | 2139.8772 | 18 | 4.9 | 1070.9458 | 2 | 42.08 | 7 | F7:20869 | NaNaKA16\_F3.raw |  |  |  |  |  |  | 1.8625E7 | 1.2871E7 | 2.0018E7 |  |  |  |  | 5 | 0 | 0 | 0 | 0 | 0 | 0 | 1 | 1 | 3 | 0 | 0 | 0 | 0 | 51 | 68 | Carbamidomethylation | C6:Carbamidomethylation:1000.00;C7:Carbamidomethylation:1000.00;C12:Carbamidomethylation:1000.00 | PEAKS DB |
| G.VDIQC(+57.02)C(+57.02)STDNC(+57.02)NPFPTR.K | Y | 86.06 | 2082.8557 | 17 | 5.7 | 1042.4355 | 2 | 40.37 | 8 | F8:21741 | NaNaKA16\_F4.raw |  |  |  |  |  |  | 9.4539E6 | 2.0548E7 | 4.7809E7 |  |  |  |  | 4 | 0 | 0 | 0 | 0 | 0 | 0 | 1 | 1 | 2 | 0 | 0 | 0 | 0 | 52 | 68 | Carbamidomethylation | C5:Carbamidomethylation:1000.00;C6:Carbamidomethylation:1000.00;C11:Carbamidomethylation:1000.00 | PEAKS DB |
| D.IQC(+57.02)C(+57.02)STDNC(+57.02)NPFPTR.K | Y | 84.44 | 1868.7604 | 15 | 0.1 | 935.3876 | 2 | 26.32 | 9 | F9:12175 | NaNaKA16\_F5.raw | 8.3412E6 |  |  |  |  | 6.6128E6 | 1.3076E6 | 2.197E8 | 3.1221E8 |  |  |  |  | 9 | 1 | 0 | 0 | 0 | 0 | 1 | 1 | 3 | 3 | 0 | 0 | 0 | 0 | 54 | 68 | Carbamidomethylation | C3:Carbamidomethylation:1000.00;C4:Carbamidomethylation:1000.00;C9:Carbamidomethylation:1000.00 | PEAKS DB |
| V.DIQC(+57.02)C(+57.02)STDNC(+57.02)NPFPTR.K | Y | 83.87 | 1983.7874 | 16 | 6.2 | 992.9019 | 2 | 37.25 | 8 | F8:19226 | NaNaKA16\_F4.raw |  |  |  |  |  | 8.8806E6 | 1.0298E7 | 1.017E7 | 5.0018E8 | 3.4519E7 |  |  |  | 9 | 0 | 0 | 0 | 0 | 0 | 1 | 1 | 1 | 4 | 2 | 0 | 0 | 0 | 53 | 68 | Carbamidomethylation | C4:Carbamidomethylation:1000.00;C5:Carbamidomethylation:1000.00;C10:Carbamidomethylation:1000.00 | PEAKS DB |
| Q.C(+57.02)C(+57.02)STDNC(+57.02)NPFPTR.K | N | 81.03 | 1627.6178 | 13 | 6.1 | 814.8168 | 2 | 20.86 | 8 | F8:6556 | NaNaKA16\_F4.raw | 4.0032E5 |  |  |  |  | 2.1346E6 | 1.8581E7 | 2.2172E6 | 1.8648E7 | 7.3726E5 |  |  |  | 12 | 1 | 0 | 0 | 0 | 0 | 1 | 4 | 2 | 3 | 1 | 0 | 0 | 0 | 56 | 68 | Carbamidomethylation | C1:Carbamidomethylation:1000.00;C2:Carbamidomethylation:1000.00;C7:Carbamidomethylation:1000.00 | PEAKS DB |
| R.C(+57.02)FITPDITSK.D | N | 75.82 | 1180.5798 | 10 | 7.7 | 591.2988 | 2 | 43.13 | 7 | F7:21737 | NaNaKA16\_F3.raw |  | 2.2482E7 | 8.6587E7 | 7.912E7 | 7.0808E6 | 5.0807E8 | 6.1225E10 | 8.5353E10 | 1.2307E11 | 5.4499E9 | 1.0861E8 | 6.3864E7 | 2.9206E8 | 82 | 0 | 4 | 2 | 4 | 2 | 4 | 9 | 21 | 14 | 13 | 2 | 2 | 5 | 3 | 12 | Carbamidomethylation | C1:Carbamidomethylation:1000.00 | PEAKS DB |
| V.KTGVDIQC(+57.02)C(+57.02)STDNC(+57.02)NPFPTR.K | Y | 73.92 | 2369.0198 | 20 | -0.3 | 1185.5168 | 2 | 30.58 | 9 | F9:15182 | NaNaKA16\_F5.raw |  |  |  |  |  |  | 4.0817E8 | 1.4066E9 | 1.5575E9 |  |  |  |  | 5 | 0 | 0 | 0 | 0 | 0 | 0 | 1 | 2 | 2 | 0 | 0 | 0 | 0 | 49 | 68 | Carbamidomethylation | C8:Carbamidomethylation:1000.00;C9:Carbamidomethylation:1000.00;C14:Carbamidomethylation:1000.00 | PEAKS DB |
| C.STDNC(+57.02)NPFPTR.K | N | 69.23 | 1307.5564 | 11 | 4.8 | 654.7853 | 2 | 11.47 | 7 | F7:1955 | NaNaKA16\_F3.raw |  |  |  |  |  | 3.2217E6 | 1.3046E7 | 2.4922E6 | 1.0115E7 |  |  |  |  | 5 | 0 | 0 | 0 | 0 | 0 | 1 | 1 | 1 | 2 | 0 | 0 | 0 | 0 | 58 | 68 | Carbamidomethylation | C5:Carbamidomethylation:1000.00 | PEAKS DB |
| IRC(+57.02)FITPDITSK.D | N | 67.07 | 1449.7650 | 12 | 0.1 | 484.2623 | 3 | 34.24 | 9 | F9:18401 | NaNaKA16\_F5.raw |  |  |  |  |  |  | 2.4051E7 | 7.2194E7 | 8.0035E7 |  |  |  |  | 10 | 0 | 0 | 0 | 0 | 0 | 0 | 3 | 4 | 3 | 0 | 0 | 0 | 0 | 1 | 12 | Carbamidomethylation | C3:Carbamidomethylation:1000.00 | PEAKS DB |
| C.C(+57.02)STDNC(+57.02)NPFPTR.K | N | 66.15 | 1467.5872 | 12 | 0.1 | 734.8009 | 2 | 20.98 | 6 | F6:8194 | NaNaKA16\_F2.raw | 3.5509E6 |  |  |  |  | 1.6348E6 | 1.0498E7 |  | 1.4874E7 |  |  |  |  | 5 | 1 | 0 | 0 | 0 | 0 | 1 | 2 | 0 | 1 | 0 | 0 | 0 | 0 | 57 | 68 | Carbamidomethylation | C1:Carbamidomethylation:1000.00;C6:Carbamidomethylation:1000.00 | PEAKS DB |
| K.TGVDIQC(+57.02)C(+57.02)STDNC(+57.02)NPFPTRKRP | Y | 58.58 | 2622.1738 | 22 | 5.5 | 656.5510 | 4 | 25.48 | 7 | F7:7710 | NaNaKA16\_F3.raw |  |  |  |  |  |  | 0 |  | 1.7072E7 |  |  |  |  | 2 | 0 | 0 | 0 | 0 | 0 | 0 | 0 | 0 | 2 | 0 | 0 | 0 | 0 | 50 | 71 | Carbamidomethylation | C7:Carbamidomethylation:1000.00;C8:Carbamidomethylation:1000.00;C13:Carbamidomethylation:1000.00 | PEAKS DB |
| R.C(+57.02)FITPDITSKD.C | N | 53.03 | 1295.6067 | 11 | 0.2 | 648.8107 | 2 | 44.47 | 9 | F9:26660 | NaNaKA16\_F5.raw |  |  |  |  |  |  | 7.9006E5 | 3.8625E6 | 1.7287E7 |  |  |  |  | 6 | 0 | 0 | 0 | 0 | 0 | 0 | 1 | 2 | 3 | 0 | 0 | 0 | 0 | 3 | 13 | Carbamidomethylation | C1:Carbamidomethylation:1000.00 | PEAKS DB |
| V.DIQC(+57.02)C(+57.02)STDNC(+57.02)NPFPTRK.R | Y | 52.18 | 2111.8823 | 17 | 0.2 | 704.9682 | 3 | 22.55 | 9 | F9:9549 | NaNaKA16\_F5.raw |  |  |  |  |  |  |  |  | 3.3337E6 |  |  |  |  | 1 | 0 | 0 | 0 | 0 | 0 | 0 | 0 | 0 | 1 | 0 | 0 | 0 | 0 | 53 | 69 | Carbamidomethylation | C4:Carbamidomethylation:1000.00;C5:Carbamidomethylation:1000.00;C10:Carbamidomethylation:1000.00 | PEAKS DB |
| K.TGVDIQC(+57.02)C(+57.02)STDNC(+57.02)NPFPT.R | Y | 50.82 | 2084.8237 | 18 | 7.4 | 1043.4213 | 2 | 61.82 | 8 | F8:39511 | NaNaKA16\_F4.raw |  |  |  |  |  |  | 1.4461E8 | 1.6598E8 | 1.2556E9 | 1.4388E8 |  |  |  | 4 | 0 | 0 | 0 | 0 | 0 | 0 | 1 | 1 | 1 | 1 | 0 | 0 | 0 | 50 | 67 | Carbamidomethylation | C7:Carbamidomethylation:1000.00;C8:Carbamidomethylation:1000.00;C13:Carbamidomethylation:1000.00 | PEAKS DB |
| K.TWC(+57.02)DAFC(+57.02)SIR.G | N | 50.20 | 1314.5486 | 10 | 0.4 | 658.2819 | 2 | 55.05 | 9 | F9:35981 | NaNaKA16\_F5.raw |  |  |  |  |  |  |  |  | 1.2559E7 |  |  |  |  | 1 | 0 | 0 | 0 | 0 | 0 | 0 | 0 | 0 | 1 | 0 | 0 | 0 | 0 | 24 | 33 | Carbamidomethylation | C3:Carbamidomethylation:1000.00;C7:Carbamidomethylation:1000.00 | PEAKS DB |
| K.DC(+57.02)PNGHVC(+57.02)YTK.T | N | 50.09 | 1349.5493 | 11 | -0.1 | 450.8570 | 3 | 15.59 | 9 | F9:3959 | NaNaKA16\_F5.raw |  |  |  |  |  |  |  |  | 0 |  |  |  |  | 0 | 0 | 0 | 0 | 0 | 0 | 0 | 0 | 0 | 0 | 0 | 0 | 0 | 0 | 13 | 23 | Carbamidomethylation | C2:Carbamidomethylation:1000.00;C8:Carbamidomethylation:1000.00 | PEAKS DB |
| D.LGC(+57.02)AATC(+57.02)PTVK.T | N | 49.50 | 1176.5631 | 11 | 4.9 | 589.2886 | 2 | 11.80 | 8 | F8:1936 | NaNaKA16\_F4.raw |  |  |  |  |  |  |  | 1.7287E5 |  |  |  |  |  | 1 | 0 | 0 | 0 | 0 | 0 | 0 | 0 | 1 | 0 | 0 | 0 | 0 | 0 | 39 | 49 | Carbamidomethylation | C3:Carbamidomethylation:1000.00;C7:Carbamidomethylation:1000.00 | PEAKS DB |
| I.RC(+57.02)FITPDITSK.D | N | 48.93 | 1336.6809 | 11 | 0.7 | 446.5679 | 3 | 25.75 | 9 | F9:12007 | NaNaKA16\_F5.raw |  |  |  |  |  |  |  |  | 8.2828E6 |  |  |  |  | 1 | 0 | 0 | 0 | 0 | 0 | 0 | 0 | 0 | 1 | 0 | 0 | 0 | 0 | 2 | 12 | Carbamidomethylation | C2:Carbamidomethylation:1000.00 | PEAKS DB |
| I.QC(+57.02)C(+57.02)STDNC(+57.02)NPFPTR.K | Y | 47.30 | 1755.6764 | 14 | 1.0 | 878.8463 | 2 | 22.00 | 1 | F1:7265 | NaNaKA16\_F1.raw | 8.1149E5 |  |  |  |  |  |  |  |  |  |  |  |  | 1 | 1 | 0 | 0 | 0 | 0 | 0 | 0 | 0 | 0 | 0 | 0 | 0 | 0 | 55 | 68 | Carbamidomethylation | C2:Carbamidomethylation:1000.00;C3:Carbamidomethylation:1000.00;C8:Carbamidomethylation:1000.00 | PEAKS DB |
| C.FITPDITSK.D | N | 45.76 | 1020.5491 | 9 | -2.0 | 511.2808 | 2 | 35.97 | 9 | F9:20225 | NaNaKA16\_F5.raw |  |  |  |  |  |  |  | 2.3378E8 | 1.8E8 |  |  |  |  | 3 | 0 | 0 | 0 | 0 | 0 | 0 | 0 | 2 | 1 | 0 | 0 | 0 | 0 | 4 | 12 |  |  | PEAKS DB |
| R.VDLGC(+57.02)AATC(+57.02)PTV.K | N | 45.13 | 1262.5635 | 12 | -0.9 | 632.2885 | 2 | 50.91 | 10 | F10:33445 | NaNaKA16\_F6.raw |  |  |  |  |  |  | 1.1303E7 |  |  | 5.2132E7 |  |  |  | 2 | 0 | 0 | 0 | 0 | 0 | 0 | 1 | 0 | 0 | 1 | 0 | 0 | 0 | 37 | 48 | Carbamidomethylation | C5:Carbamidomethylation:1000.00;C9:Carbamidomethylation:1000.00 | PEAKS DB |
| T.DNC(+57.02)NPFPTR.K | N | 44.94 | 1119.4767 | 9 | 0.7 | 560.7460 | 2 | 23.18 | 6 | F6:10002 | NaNaKA16\_F2.raw |  |  |  |  |  | 3.0548E5 |  |  |  |  |  |  |  | 1 | 0 | 0 | 0 | 0 | 0 | 1 | 0 | 0 | 0 | 0 | 0 | 0 | 0 | 60 | 68 | Carbamidomethylation | C3:Carbamidomethylation:1000.00 | PEAKS DB |
| A.TC(+57.02)PTVKTGVDIQC(+57.02)C(+57.02)STDNC(+57.02)NPFPTR.K | Y | 44.77 | 2927.2671 | 25 | -7.1 | 976.7509 | 3 | 63.76 | 8 | F8:41290 | NaNaKA16\_F4.raw |  |  |  |  |  |  |  | 7.0141E6 |  |  |  |  |  | 1 | 0 | 0 | 0 | 0 | 0 | 0 | 0 | 1 | 0 | 0 | 0 | 0 | 0 | 44 | 68 | Carbamidomethylation | C2:Carbamidomethylation:1000.00;C13:Carbamidomethylation:1000.00;C14:Carbamidomethylation:1000.00;C19:Carbamidomethylation:1000.00 | PEAKS DB |
| K.RVDLGC(+57.02)AATC(+57.02)PTVK.T | N | 44.70 | 1546.7595 | 14 | -0.8 | 516.5934 | 3 | 18.27 | 9 | F9:5871 | NaNaKA16\_F5.raw |  |  |  |  |  |  |  | 1.5478E5 | 2.2798E6 |  |  |  |  | 2 | 0 | 0 | 0 | 0 | 0 | 0 | 0 | 1 | 1 | 0 | 0 | 0 | 0 | 36 | 49 | Carbamidomethylation | C6:Carbamidomethylation:1000.00;C10:Carbamidomethylation:1000.00 | PEAKS DB |
| F.ITPDITSK.D | N | 42.90 | 873.4807 | 8 | 4.9 | 437.7476 | 2 | 11.46 | 7 | F7:1934 | NaNaKA16\_F3.raw |  |  |  |  |  |  | 1.0681E6 |  |  |  |  |  |  | 1 | 0 | 0 | 0 | 0 | 0 | 0 | 1 | 0 | 0 | 0 | 0 | 0 | 0 | 5 | 12 |  |  | PEAKS DB |
| V.KTGVDIQC(+57.02)C(+57.02)STDNC(+57.02)NPFPTRK.R | Y | 42.67 | 2497.1147 | 21 | 5.2 | 625.2859 | 4 | 20.10 | 8 | F8:6040 | NaNaKA16\_F4.raw |  |  |  |  |  |  |  | 1.4274E6 |  |  |  |  |  | 1 | 0 | 0 | 0 | 0 | 0 | 0 | 0 | 1 | 0 | 0 | 0 | 0 | 0 | 49 | 69 | Carbamidomethylation | C8:Carbamidomethylation:1000.00;C9:Carbamidomethylation:1000.00;C14:Carbamidomethylation:1000.00 | PEAKS DB |
| D.NC(+57.02)NPFPTR.K | N | 41.96 | 1004.4498 | 8 | -0.2 | 503.2321 | 2 | 43.27 | 9 | F9:25860 | NaNaKA16\_F5.raw |  |  |  |  |  |  |  |  | 0 |  |  |  |  | 0 | 0 | 0 | 0 | 0 | 0 | 0 | 0 | 0 | 0 | 0 | 0 | 0 | 0 | 61 | 68 | Carbamidomethylation | C2:Carbamidomethylation:1000.00 | PEAKS DB |
| total 31 peptides |  |  |  |  |  |  |  |  |  |  |  |  |  |  |  |  |  |  |  |  |  |  |  |  |  |  |  |  |  |  |  |  |  |  |  |  |  |  |  |  |  |  |  |
| --- | --- | --- | --- | --- | --- | --- | --- | --- | --- | --- | --- | --- | --- | --- | --- | --- | --- | --- | --- | --- | --- | --- | --- | --- | --- | --- | --- | --- | --- | --- | --- | --- | --- | --- | --- | --- | --- | --- | --- | --- | --- | --- | --- |

1LXG

back to list

  

| Protein Coverage
| Supporting Peptides
|

Protein Coverage:

Supporting Peptides:

| Peptide | Uniq | -10lgP | Mass | Length | ppm | m/z | z | RT | Fraction | Scan | Source File | Area F1 | Area F10 | Area F11 | Area F12 | Area F13 | Area F2 | Area F3 | Area F4 | Area F5 | Area F6 | Area F7 | Area F8 | Area F9 | #Feature | #Feature F1 | #Feature F10 | #Feature F11 | #Feature F12 | #Feature F13 | #Feature F2 | #Feature F3 | #Feature F4 | #Feature F5 | #Feature F6 | #Feature F7 | #Feature F8 | #Feature F9 | Start | End | PTM | AScore | Found By |
| --- | --- | --- | --- | --- | --- | --- | --- | --- | --- | --- | --- | --- | --- | --- | --- | --- | --- | --- | --- | --- | --- | --- | --- | --- | --- | --- | --- | --- | --- | --- | --- | --- | --- | --- | --- | --- | --- | --- | --- | --- | --- | --- | --- |
| R.C(+57.02)FITPDITSKDC(+57.02)PNGHVC(+57.02)YTK.T | N | 97.70 | 2512.1184 | 21 | -0.2 | 838.3799 | 3 | 32.91 | 9 | F9:17528 | NaNaKA16\_F5.raw |  |  |  |  |  |  | 1.3652E7 | 5.3048E7 | 1.1015E8 |  |  |  |  | 10 | 0 | 0 | 0 | 0 | 0 | 0 | 3 | 4 | 3 | 0 | 0 | 0 | 0 | 3 | 23 | Carbamidomethylation | C1:Carbamidomethylation:1000.00;C12:Carbamidomethylation:1000.00;C18:Carbamidomethylation:1000.00 | PEAKS DB |
| K.TGVDIQC(+57.02)C(+57.02)STDNC(+57.02)NPFPTRK.R | Y | 97.32 | 2369.0198 | 20 | 5.0 | 1185.5167 | 2 | 30.34 | 8 | F8:13936 | NaNaKA16\_F4.raw |  | 5.2751E6 | 1.4938E6 |  |  |  | 4.2829E8 | 1.4347E9 | 1.578E9 | 3.3235E7 | 3.1822E6 |  | 6.4482E6 | 17 | 0 | 1 | 1 | 0 | 0 | 0 | 3 | 5 | 4 | 1 | 1 | 0 | 1 | 50 | 69 | Carbamidomethylation | C7:Carbamidomethylation:1000.00;C8:Carbamidomethylation:1000.00;C13:Carbamidomethylation:1000.00 | PEAKS DB |
| K.TGVDIQC(+57.02)C(+57.02)STDNC(+57.02)NPFPTR.K | Y | 94.74 | 2240.9248 | 19 | 1.1 | 1121.4709 | 2 | 44.69 | 3 | F3:24754 | NaNaKA16\_F11.raw |  | 1.6333E8 | 7.7958E7 | 7.513E7 | 6.0306E6 | 5.3603E8 | 3.7554E10 | 5.9156E10 | 6.2024E10 | 3.6074E9 | 8.3057E7 | 2.6489E7 | 3.5028E8 | 108 | 0 | 2 | 2 | 2 | 2 | 2 | 19 | 35 | 30 | 3 | 2 | 2 | 7 | 50 | 68 | Carbamidomethylation | C7:Carbamidomethylation:1000.00;C8:Carbamidomethylation:1000.00;C13:Carbamidomethylation:1000.00 | PEAKS DB |
| R.VDLGC(+57.02)AATC(+57.02)PTVK.T | N | 93.61 | 1390.6584 | 13 | 6.1 | 696.3372 | 2 | 26.78 | 7 | F7:8603 | NaNaKA16\_F3.raw |  |  |  | 1.5246E7 | 1.5932E6 | 5.0659E7 | 1.9121E8 | 2.2473E10 | 6.1192E10 | 1.9939E9 | 5.3549E6 |  | 0 | 56 | 0 | 0 | 0 | 1 | 1 | 1 | 2 | 13 | 32 | 5 | 1 | 0 | 0 | 37 | 49 | Carbamidomethylation | C5:Carbamidomethylation:1000.00;C9:Carbamidomethylation:1000.00 | PEAKS DB |
| T.GVDIQC(+57.02)C(+57.02)STDNC(+57.02)NPFPTR.K | Y | 89.23 | 2139.8772 | 18 | 4.9 | 1070.9458 | 2 | 42.08 | 7 | F7:20869 | NaNaKA16\_F3.raw |  |  |  |  |  |  | 1.8625E7 | 1.2871E7 | 2.0018E7 |  |  |  |  | 5 | 0 | 0 | 0 | 0 | 0 | 0 | 1 | 1 | 3 | 0 | 0 | 0 | 0 | 51 | 68 | Carbamidomethylation | C6:Carbamidomethylation:1000.00;C7:Carbamidomethylation:1000.00;C12:Carbamidomethylation:1000.00 | PEAKS DB |
| G.VDIQC(+57.02)C(+57.02)STDNC(+57.02)NPFPTR.K | Y | 86.06 | 2082.8557 | 17 | 5.7 | 1042.4355 | 2 | 40.37 | 8 | F8:21741 | NaNaKA16\_F4.raw |  |  |  |  |  |  | 9.4539E6 | 2.0548E7 | 4.7809E7 |  |  |  |  | 4 | 0 | 0 | 0 | 0 | 0 | 0 | 1 | 1 | 2 | 0 | 0 | 0 | 0 | 52 | 68 | Carbamidomethylation | C5:Carbamidomethylation:1000.00;C6:Carbamidomethylation:1000.00;C11:Carbamidomethylation:1000.00 | PEAKS DB |
| D.IQC(+57.02)C(+57.02)STDNC(+57.02)NPFPTR.K | Y | 84.44 | 1868.7604 | 15 | 0.1 | 935.3876 | 2 | 26.32 | 9 | F9:12175 | NaNaKA16\_F5.raw | 8.3412E6 |  |  |  |  | 6.6128E6 | 1.3076E6 | 2.197E8 | 3.1221E8 |  |  |  |  | 9 | 1 | 0 | 0 | 0 | 0 | 1 | 1 | 3 | 3 | 0 | 0 | 0 | 0 | 54 | 68 | Carbamidomethylation | C3:Carbamidomethylation:1000.00;C4:Carbamidomethylation:1000.00;C9:Carbamidomethylation:1000.00 | PEAKS DB |
| V.DIQC(+57.02)C(+57.02)STDNC(+57.02)NPFPTR.K | Y | 83.87 | 1983.7874 | 16 | 6.2 | 992.9019 | 2 | 37.25 | 8 | F8:19226 | NaNaKA16\_F4.raw |  |  |  |  |  | 8.8806E6 | 1.0298E7 | 1.017E7 | 5.0018E8 | 3.4519E7 |  |  |  | 9 | 0 | 0 | 0 | 0 | 0 | 1 | 1 | 1 | 4 | 2 | 0 | 0 | 0 | 53 | 68 | Carbamidomethylation | C4:Carbamidomethylation:1000.00;C5:Carbamidomethylation:1000.00;C10:Carbamidomethylation:1000.00 | PEAKS DB |
| Q.C(+57.02)C(+57.02)STDNC(+57.02)NPFPTR.K | N | 81.03 | 1627.6178 | 13 | 6.1 | 814.8168 | 2 | 20.86 | 8 | F8:6556 | NaNaKA16\_F4.raw | 4.0032E5 |  |  |  |  | 2.1346E6 | 1.8581E7 | 2.2172E6 | 1.8648E7 | 7.3726E5 |  |  |  | 12 | 1 | 0 | 0 | 0 | 0 | 1 | 4 | 2 | 3 | 1 | 0 | 0 | 0 | 56 | 68 | Carbamidomethylation | C1:Carbamidomethylation:1000.00;C2:Carbamidomethylation:1000.00;C7:Carbamidomethylation:1000.00 | PEAKS DB |
| R.C(+57.02)FITPDITSK.D | N | 75.82 | 1180.5798 | 10 | 7.7 | 591.2988 | 2 | 43.13 | 7 | F7:21737 | NaNaKA16\_F3.raw |  | 2.2482E7 | 8.6587E7 | 7.912E7 | 7.0808E6 | 5.0807E8 | 6.1225E10 | 8.5353E10 | 1.2307E11 | 5.4499E9 | 1.0861E8 | 6.3864E7 | 2.9206E8 | 82 | 0 | 4 | 2 | 4 | 2 | 4 | 9 | 21 | 14 | 13 | 2 | 2 | 5 | 3 | 12 | Carbamidomethylation | C1:Carbamidomethylation:1000.00 | PEAKS DB |
| V.KTGVDIQC(+57.02)C(+57.02)STDNC(+57.02)NPFPTR.K | Y | 73.92 | 2369.0198 | 20 | -0.3 | 1185.5168 | 2 | 30.58 | 9 | F9:15182 | NaNaKA16\_F5.raw |  |  |  |  |  |  | 4.0817E8 | 1.4066E9 | 1.5575E9 |  |  |  |  | 5 | 0 | 0 | 0 | 0 | 0 | 0 | 1 | 2 | 2 | 0 | 0 | 0 | 0 | 49 | 68 | Carbamidomethylation | C8:Carbamidomethylation:1000.00;C9:Carbamidomethylation:1000.00;C14:Carbamidomethylation:1000.00 | PEAKS DB |
| C.STDNC(+57.02)NPFPTR.K | N | 69.23 | 1307.5564 | 11 | 4.8 | 654.7853 | 2 | 11.47 | 7 | F7:1955 | NaNaKA16\_F3.raw |  |  |  |  |  | 3.2217E6 | 1.3046E7 | 2.4922E6 | 1.0115E7 |  |  |  |  | 5 | 0 | 0 | 0 | 0 | 0 | 1 | 1 | 1 | 2 | 0 | 0 | 0 | 0 | 58 | 68 | Carbamidomethylation | C5:Carbamidomethylation:1000.00 | PEAKS DB |
| IRC(+57.02)FITPDITSK.D | N | 67.07 | 1449.7650 | 12 | 0.1 | 484.2623 | 3 | 34.24 | 9 | F9:18401 | NaNaKA16\_F5.raw |  |  |  |  |  |  | 2.4051E7 | 7.2194E7 | 8.0035E7 |  |  |  |  | 10 | 0 | 0 | 0 | 0 | 0 | 0 | 3 | 4 | 3 | 0 | 0 | 0 | 0 | 1 | 12 | Carbamidomethylation | C3:Carbamidomethylation:1000.00 | PEAKS DB |
| C.C(+57.02)STDNC(+57.02)NPFPTR.K | N | 66.15 | 1467.5872 | 12 | 0.1 | 734.8009 | 2 | 20.98 | 6 | F6:8194 | NaNaKA16\_F2.raw | 3.5509E6 |  |  |  |  | 1.6348E6 | 1.0498E7 |  | 1.4874E7 |  |  |  |  | 5 | 1 | 0 | 0 | 0 | 0 | 1 | 2 | 0 | 1 | 0 | 0 | 0 | 0 | 57 | 68 | Carbamidomethylation | C1:Carbamidomethylation:1000.00;C6:Carbamidomethylation:1000.00 | PEAKS DB |
| K.TGVDIQC(+57.02)C(+57.02)STDNC(+57.02)NPFPTRKRP | Y | 58.58 | 2622.1738 | 22 | 5.5 | 656.5510 | 4 | 25.48 | 7 | F7:7710 | NaNaKA16\_F3.raw |  |  |  |  |  |  | 0 |  | 1.7072E7 |  |  |  |  | 2 | 0 | 0 | 0 | 0 | 0 | 0 | 0 | 0 | 2 | 0 | 0 | 0 | 0 | 50 | 71 | Carbamidomethylation | C7:Carbamidomethylation:1000.00;C8:Carbamidomethylation:1000.00;C13:Carbamidomethylation:1000.00 | PEAKS DB |
| R.C(+57.02)FITPDITSKD.C | N | 53.03 | 1295.6067 | 11 | 0.2 | 648.8107 | 2 | 44.47 | 9 | F9:26660 | NaNaKA16\_F5.raw |  |  |  |  |  |  | 7.9006E5 | 3.8625E6 | 1.7287E7 |  |  |  |  | 6 | 0 | 0 | 0 | 0 | 0 | 0 | 1 | 2 | 3 | 0 | 0 | 0 | 0 | 3 | 13 | Carbamidomethylation | C1:Carbamidomethylation:1000.00 | PEAKS DB |
| V.DIQC(+57.02)C(+57.02)STDNC(+57.02)NPFPTRK.R | Y | 52.18 | 2111.8823 | 17 | 0.2 | 704.9682 | 3 | 22.55 | 9 | F9:9549 | NaNaKA16\_F5.raw |  |  |  |  |  |  |  |  | 3.3337E6 |  |  |  |  | 1 | 0 | 0 | 0 | 0 | 0 | 0 | 0 | 0 | 1 | 0 | 0 | 0 | 0 | 53 | 69 | Carbamidomethylation | C4:Carbamidomethylation:1000.00;C5:Carbamidomethylation:1000.00;C10:Carbamidomethylation:1000.00 | PEAKS DB |
| K.TGVDIQC(+57.02)C(+57.02)STDNC(+57.02)NPFPT.R | Y | 50.82 | 2084.8237 | 18 | 7.4 | 1043.4213 | 2 | 61.82 | 8 | F8:39511 | NaNaKA16\_F4.raw |  |  |  |  |  |  | 1.4461E8 | 1.6598E8 | 1.2556E9 | 1.4388E8 |  |  |  | 4 | 0 | 0 | 0 | 0 | 0 | 0 | 1 | 1 | 1 | 1 | 0 | 0 | 0 | 50 | 67 | Carbamidomethylation | C7:Carbamidomethylation:1000.00;C8:Carbamidomethylation:1000.00;C13:Carbamidomethylation:1000.00 | PEAKS DB |
| K.TWC(+57.02)DAFC(+57.02)SIR.G | N | 50.20 | 1314.5486 | 10 | 0.4 | 658.2819 | 2 | 55.05 | 9 | F9:35981 | NaNaKA16\_F5.raw |  |  |  |  |  |  |  |  | 1.2559E7 |  |  |  |  | 1 | 0 | 0 | 0 | 0 | 0 | 0 | 0 | 0 | 1 | 0 | 0 | 0 | 0 | 24 | 33 | Carbamidomethylation | C3:Carbamidomethylation:1000.00;C7:Carbamidomethylation:1000.00 | PEAKS DB |
| K.DC(+57.02)PNGHVC(+57.02)YTK.T | N | 50.09 | 1349.5493 | 11 | -0.1 | 450.8570 | 3 | 15.59 | 9 | F9:3959 | NaNaKA16\_F5.raw |  |  |  |  |  |  |  |  | 0 |  |  |  |  | 0 | 0 | 0 | 0 | 0 | 0 | 0 | 0 | 0 | 0 | 0 | 0 | 0 | 0 | 13 | 23 | Carbamidomethylation | C2:Carbamidomethylation:1000.00;C8:Carbamidomethylation:1000.00 | PEAKS DB |
| D.LGC(+57.02)AATC(+57.02)PTVK.T | N | 49.50 | 1176.5631 | 11 | 4.9 | 589.2886 | 2 | 11.80 | 8 | F8:1936 | NaNaKA16\_F4.raw |  |  |  |  |  |  |  | 1.7287E5 |  |  |  |  |  | 1 | 0 | 0 | 0 | 0 | 0 | 0 | 0 | 1 | 0 | 0 | 0 | 0 | 0 | 39 | 49 | Carbamidomethylation | C3:Carbamidomethylation:1000.00;C7:Carbamidomethylation:1000.00 | PEAKS DB |
| I.RC(+57.02)FITPDITSK.D | N | 48.93 | 1336.6809 | 11 | 0.7 | 446.5679 | 3 | 25.75 | 9 | F9:12007 | NaNaKA16\_F5.raw |  |  |  |  |  |  |  |  | 8.2828E6 |  |  |  |  | 1 | 0 | 0 | 0 | 0 | 0 | 0 | 0 | 0 | 1 | 0 | 0 | 0 | 0 | 2 | 12 | Carbamidomethylation | C2:Carbamidomethylation:1000.00 | PEAKS DB |
| I.QC(+57.02)C(+57.02)STDNC(+57.02)NPFPTR.K | Y | 47.30 | 1755.6764 | 14 | 1.0 | 878.8463 | 2 | 22.00 | 1 | F1:7265 | NaNaKA16\_F1.raw | 8.1149E5 |  |  |  |  |  |  |  |  |  |  |  |  | 1 | 1 | 0 | 0 | 0 | 0 | 0 | 0 | 0 | 0 | 0 | 0 | 0 | 0 | 55 | 68 | Carbamidomethylation | C2:Carbamidomethylation:1000.00;C3:Carbamidomethylation:1000.00;C8:Carbamidomethylation:1000.00 | PEAKS DB |
| C.FITPDITSK.D | N | 45.76 | 1020.5491 | 9 | -2.0 | 511.2808 | 2 | 35.97 | 9 | F9:20225 | NaNaKA16\_F5.raw |  |  |  |  |  |  |  | 2.3378E8 | 1.8E8 |  |  |  |  | 3 | 0 | 0 | 0 | 0 | 0 | 0 | 0 | 2 | 1 | 0 | 0 | 0 | 0 | 4 | 12 |  |  | PEAKS DB |
| R.VDLGC(+57.02)AATC(+57.02)PTV.K | N | 45.13 | 1262.5635 | 12 | -0.9 | 632.2885 | 2 | 50.91 | 10 | F10:33445 | NaNaKA16\_F6.raw |  |  |  |  |  |  | 1.1303E7 |  |  | 5.2132E7 |  |  |  | 2 | 0 | 0 | 0 | 0 | 0 | 0 | 1 | 0 | 0 | 1 | 0 | 0 | 0 | 37 | 48 | Carbamidomethylation | C5:Carbamidomethylation:1000.00;C9:Carbamidomethylation:1000.00 | PEAKS DB |
| T.DNC(+57.02)NPFPTR.K | N | 44.94 | 1119.4767 | 9 | 0.7 | 560.7460 | 2 | 23.18 | 6 | F6:10002 | NaNaKA16\_F2.raw |  |  |  |  |  | 3.0548E5 |  |  |  |  |  |  |  | 1 | 0 | 0 | 0 | 0 | 0 | 1 | 0 | 0 | 0 | 0 | 0 | 0 | 0 | 60 | 68 | Carbamidomethylation | C3:Carbamidomethylation:1000.00 | PEAKS DB |
| A.TC(+57.02)PTVKTGVDIQC(+57.02)C(+57.02)STDNC(+57.02)NPFPTR.K | Y | 44.77 | 2927.2671 | 25 | -7.1 | 976.7509 | 3 | 63.76 | 8 | F8:41290 | NaNaKA16\_F4.raw |  |  |  |  |  |  |  | 7.0141E6 |  |  |  |  |  | 1 | 0 | 0 | 0 | 0 | 0 | 0 | 0 | 1 | 0 | 0 | 0 | 0 | 0 | 44 | 68 | Carbamidomethylation | C2:Carbamidomethylation:1000.00;C13:Carbamidomethylation:1000.00;C14:Carbamidomethylation:1000.00;C19:Carbamidomethylation:1000.00 | PEAKS DB |
| K.RVDLGC(+57.02)AATC(+57.02)PTVK.T | N | 44.70 | 1546.7595 | 14 | -0.8 | 516.5934 | 3 | 18.27 | 9 | F9:5871 | NaNaKA16\_F5.raw |  |  |  |  |  |  |  | 1.5478E5 | 2.2798E6 |  |  |  |  | 2 | 0 | 0 | 0 | 0 | 0 | 0 | 0 | 1 | 1 | 0 | 0 | 0 | 0 | 36 | 49 | Carbamidomethylation | C6:Carbamidomethylation:1000.00;C10:Carbamidomethylation:1000.00 | PEAKS DB |
| F.ITPDITSK.D | N | 42.90 | 873.4807 | 8 | 4.9 | 437.7476 | 2 | 11.46 | 7 | F7:1934 | NaNaKA16\_F3.raw |  |  |  |  |  |  | 1.0681E6 |  |  |  |  |  |  | 1 | 0 | 0 | 0 | 0 | 0 | 0 | 1 | 0 | 0 | 0 | 0 | 0 | 0 | 5 | 12 |  |  | PEAKS DB |
| V.KTGVDIQC(+57.02)C(+57.02)STDNC(+57.02)NPFPTRK.R | Y | 42.67 | 2497.1147 | 21 | 5.2 | 625.2859 | 4 | 20.10 | 8 | F8:6040 | NaNaKA16\_F4.raw |  |  |  |  |  |  |  | 1.4274E6 |  |  |  |  |  | 1 | 0 | 0 | 0 | 0 | 0 | 0 | 0 | 1 | 0 | 0 | 0 | 0 | 0 | 49 | 69 | Carbamidomethylation | C8:Carbamidomethylation:1000.00;C9:Carbamidomethylation:1000.00;C14:Carbamidomethylation:1000.00 | PEAKS DB |
| D.NC(+57.02)NPFPTR.K | N | 41.96 | 1004.4498 | 8 | -0.2 | 503.2321 | 2 | 43.27 | 9 | F9:25860 | NaNaKA16\_F5.raw |  |  |  |  |  |  |  |  | 0 |  |  |  |  | 0 | 0 | 0 | 0 | 0 | 0 | 0 | 0 | 0 | 0 | 0 | 0 | 0 | 0 | 61 | 68 | Carbamidomethylation | C2:Carbamidomethylation:1000.00 | PEAKS DB |
| total 31 peptides |  |  |  |  |  |  |  |  |  |  |  |  |  |  |  |  |  |  |  |  |  |  |  |  |  |  |  |  |  |  |  |  |  |  |  |  |  |  |  |  |  |  |  |
| --- | --- | --- | --- | --- | --- | --- | --- | --- | --- | --- | --- | --- | --- | --- | --- | --- | --- | --- | --- | --- | --- | --- | --- | --- | --- | --- | --- | --- | --- | --- | --- | --- | --- | --- | --- | --- | --- | --- | --- | --- | --- | --- | --- |

AAS94269.1

back to list

  

| Protein Coverage
| Supporting Peptides
|

Protein Coverage:

Supporting Peptides:

| Peptide | Uniq | -10lgP | Mass | Length | ppm | m/z | z | RT | Fraction | Scan | Source File | Area F1 | Area F10 | Area F11 | Area F12 | Area F13 | Area F2 | Area F3 | Area F4 | Area F5 | Area F6 | Area F7 | Area F8 | Area F9 | #Feature | #Feature F1 | #Feature F10 | #Feature F11 | #Feature F12 | #Feature F13 | #Feature F2 | #Feature F3 | #Feature F4 | #Feature F5 | #Feature F6 | #Feature F7 | #Feature F8 | #Feature F9 | Start | End | PTM | AScore | Found By |
| --- | --- | --- | --- | --- | --- | --- | --- | --- | --- | --- | --- | --- | --- | --- | --- | --- | --- | --- | --- | --- | --- | --- | --- | --- | --- | --- | --- | --- | --- | --- | --- | --- | --- | --- | --- | --- | --- | --- | --- | --- | --- | --- | --- |
| R.GIDSSHWNSYC(+57.02)TETDTFIK.A | N | 103.21 | 2259.9744 | 19 | 0.4 | 1130.9949 | 2 | 51.96 | 13 | F13:29726 | NaNaKA16\_F9.raw |  | 3.1367E8 | 1.2423E7 | 7.064E6 |  |  |  |  |  | 2.5623E9 | 1.1361E7 | 1.4907E7 | 3.8727E8 | 19 | 0 | 2 | 2 | 1 | 0 | 0 | 0 | 0 | 0 | 8 | 1 | 1 | 4 | 193 | 211 | Carbamidomethylation | C11:Carbamidomethylation:1000.00 | PEAKS DB |
| R.EDHPVHNLGEHSVC(+57.02)DSVSAWVTK.T | N | 97.91 | 2602.1870 | 23 | 0.9 | 868.4037 | 3 | 37.09 | 12 | F12:20537 | NaNaKA16\_F8.raw |  | 2.7036E8 | 6.8592E6 | 4.8528E6 |  |  |  |  |  | 4.7202E9 | 1.5853E7 | 7.7354E7 | 8.6437E8 | 30 | 0 | 4 | 3 | 1 | 0 | 0 | 0 | 0 | 0 | 11 | 3 | 3 | 5 | 126 | 148 | Carbamidomethylation | C14:Carbamidomethylation:1000.00 | PEAKS DB |
| K.TTATDIKGNTVTVMENVNLDNKVYK.Q | N | 89.49 | 2767.4062 | 25 | 0.1 | 923.4761 | 3 | 46.76 | 10 | F10:30493 | NaNaKA16\_F6.raw |  | 2.6398E6 |  |  |  |  |  |  |  | 6.2672E7 |  |  |  | 3 | 0 | 1 | 0 | 0 | 0 | 0 | 0 | 0 | 0 | 2 | 0 | 0 | 0 | 149 | 173 |  |  | PEAKS DB |
| N.LGEHSVC(+57.02)DSVSAWVTK.T | N | 86.52 | 1773.8356 | 16 | 0.4 | 887.9254 | 2 | 46.38 | 2 | F2:26570 | NaNaKA16\_F10.raw |  | 1.1807E8 |  |  |  |  |  |  |  | 2.8318E6 |  |  |  | 4 | 0 | 3 | 0 | 0 | 0 | 0 | 0 | 0 | 0 | 1 | 0 | 0 | 0 | 133 | 148 | Carbamidomethylation | C7:Carbamidomethylation:1000.00 | PEAKS DB |
| E.DHPVHNLGEHSVC(+57.02)DSVSAWVTK.T | N | 84.83 | 2473.1445 | 22 | -0.1 | 825.3887 | 3 | 37.67 | 10 | F10:21732 | NaNaKA16\_F6.raw |  |  |  |  |  |  |  |  |  | 1.5802E8 |  | 4.8658E6 | 5.1183E7 | 8 | 0 | 0 | 0 | 0 | 0 | 0 | 0 | 0 | 0 | 3 | 0 | 1 | 4 | 127 | 148 | Carbamidomethylation | C13:Carbamidomethylation:1000.00 | PEAKS DB |
| K.GNTVTVMENVNLDNK.V | N | 83.96 | 1646.7933 | 15 | 0.6 | 824.4044 | 2 | 40.84 | 13 | F13:21057 | NaNaKA16\_F9.raw |  | 5.4691E7 | 2.4265E6 | 5.4824E6 |  |  |  |  |  | 2.2522E9 | 4.1291E6 | 4.3163E6 | 1.0123E8 | 18 | 0 | 2 | 1 | 1 | 0 | 0 | 0 | 0 | 0 | 9 | 2 | 1 | 2 | 156 | 170 |  |  | PEAKS DB |
| K.GNTVTVM(+15.99)ENVNLDNK.V | N | 81.84 | 1662.7883 | 15 | -0.2 | 832.4012 | 2 | 39.33 | 10 | F10:25249 | NaNaKA16\_F6.raw |  |  |  |  |  |  |  |  |  | 3.5104E8 |  |  |  | 11 | 0 | 0 | 0 | 0 | 0 | 0 | 0 | 0 | 0 | 11 | 0 | 0 | 0 | 156 | 170 | Oxidation (M) | M7:Oxidation (M):1000.00 | PEAKS DB |
| K.ALTMEGNQASWR.F | N | 80.49 | 1362.6350 | 12 | 0.5 | 682.3251 | 2 | 33.00 | 11 | F11:18088 | NaNaKA16\_F7.raw |  | 1.1026E6 |  | 1.0384E7 | 1.8628E5 |  |  |  |  | 3.7415E9 | 5.9758E6 | 6.0181E6 | 1.0381E7 | 10 | 0 | 1 | 0 | 1 | 1 | 0 | 0 | 0 | 0 | 3 | 1 | 1 | 2 | 212 | 223 |  |  | PEAKS DB |
| K.ALTM(+15.99)EGNQASWR.F | N | 77.54 | 1378.6299 | 12 | -0.1 | 690.3221 | 2 | 19.80 | 10 | F10:7091 | NaNaKA16\_F6.raw |  |  |  |  |  |  |  |  |  | 3.2429E8 |  |  |  | 6 | 0 | 0 | 0 | 0 | 0 | 0 | 0 | 0 | 0 | 6 | 0 | 0 | 0 | 212 | 223 | Oxidation (M) | M4:Oxidation (M):1000.00 | PEAKS DB |
| R.IDTAC(+57.02)VC(+57.02)VITK.K | N | 77.37 | 1278.6312 | 11 | 0.1 | 640.3229 | 2 | 33.63 | 10 | F10:18937 | NaNaKA16\_F6.raw |  | 4.3837E5 |  | 8.3453E6 |  | 2.1819E6 |  |  |  | 1.6909E9 | 4.6903E6 |  | 9.8784E6 | 8 | 0 | 1 | 0 | 1 | 0 | 1 | 0 | 0 | 0 | 2 | 1 | 0 | 2 | 227 | 237 | Carbamidomethylation | C5:Carbamidomethylation:1000.00;C7:Carbamidomethylation:1000.00 | PEAKS DB |
| N.TVTVMENVNLDNK.V | N | 76.68 | 1475.7290 | 13 | -0.1 | 738.8717 | 2 | 38.38 | 10 | F10:22544 | NaNaKA16\_F6.raw |  |  |  |  |  |  |  |  |  | 5.0544E7 |  |  |  | 1 | 0 | 0 | 0 | 0 | 0 | 0 | 0 | 0 | 0 | 1 | 0 | 0 | 0 | 158 | 170 |  |  | PEAKS DB |
| D.HPVHNLGEHSVC(+57.02)DSVSAWVTK.T | N | 76.11 | 2358.1174 | 21 | -0.2 | 590.5365 | 4 | 31.29 | 13 | F13:13388 | NaNaKA16\_F9.raw |  | 3.1467E6 |  |  |  |  |  |  |  | 6.048E7 |  | 4.5368E6 | 1.0948E7 | 5 | 0 | 1 | 0 | 0 | 0 | 0 | 0 | 0 | 0 | 2 | 0 | 1 | 1 | 128 | 148 | Carbamidomethylation | C12:Carbamidomethylation:1000.00 | PEAKS DB |
| P.VHNLGEHSVC(+57.02)DSVSAWVTK.T | N | 74.21 | 2124.0059 | 19 | 0.2 | 709.0094 | 3 | 38.53 | 2 | F2:19930 | NaNaKA16\_F10.raw |  | 6.7215E6 |  |  |  |  |  |  |  |  |  |  | 2.2348E6 | 2 | 0 | 1 | 0 | 0 | 0 | 0 | 0 | 0 | 0 | 0 | 0 | 0 | 1 | 130 | 148 | Carbamidomethylation | C10:Carbamidomethylation:1000.00 | PEAKS DB |
| K.TTATDIKGNTVTVMENVNLDNK.V | N | 72.26 | 2377.1794 | 22 | 0.7 | 793.4010 | 3 | 46.61 | 13 | F13:25988 | NaNaKA16\_F9.raw |  | 5.7149E7 |  |  |  |  |  |  |  | 9.9295E7 | 1.6066E6 |  | 7.6356E6 | 5 | 0 | 1 | 0 | 0 | 0 | 0 | 0 | 0 | 0 | 2 | 1 | 0 | 1 | 149 | 170 |  |  | PEAKS DB |
| K.GNTVTVMENVNLDNKVYK.Q | N | 68.33 | 2037.0200 | 18 | 0.0 | 680.0140 | 3 | 40.94 | 10 | F10:25126 | NaNaKA16\_F6.raw |  | 1.592E6 |  |  |  |  |  |  |  | 1.2605E7 |  |  | 9.7102E5 | 4 | 0 | 1 | 0 | 0 | 0 | 0 | 0 | 0 | 0 | 2 | 0 | 0 | 1 | 156 | 173 |  |  | PEAKS DB |
| H.NLGEHSVC(+57.02)DSVSAWVTK.T | N | 64.92 | 1887.8785 | 17 | 0.6 | 944.9471 | 2 | 47.73 | 2 | F2:27755 | NaNaKA16\_F10.raw |  | 2.3917E6 |  |  |  |  |  |  |  |  |  |  |  | 1 | 0 | 1 | 0 | 0 | 0 | 0 | 0 | 0 | 0 | 0 | 0 | 0 | 0 | 132 | 148 | Carbamidomethylation | C8:Carbamidomethylation:1000.00 | PEAKS DB |
| R.FIRIDTAC(+57.02)VC(+57.02)VITK.K | N | 63.81 | 1694.8848 | 14 | -0.9 | 565.9684 | 3 | 50.26 | 10 | F10:33096 | NaNaKA16\_F6.raw |  |  |  |  |  |  |  |  |  | 2.9114E6 |  |  |  | 1 | 0 | 0 | 0 | 0 | 0 | 0 | 0 | 0 | 0 | 1 | 0 | 0 | 0 | 224 | 237 | Carbamidomethylation | C8:Carbamidomethylation:1000.00;C10:Carbamidomethylation:1000.00 | PEAKS DB |
| N.SYC(+57.02)TETDTFIK.A | N | 61.32 | 1363.5966 | 11 | 0.1 | 682.8056 | 2 | 33.68 | 10 | F10:18512 | NaNaKA16\_F6.raw |  |  |  | 2.51E6 |  |  |  |  |  | 6.55E8 |  |  | 2.6358E6 | 3 | 0 | 0 | 0 | 1 | 0 | 0 | 0 | 0 | 0 | 1 | 0 | 0 | 1 | 201 | 211 | Carbamidomethylation | C3:Carbamidomethylation:1000.00 | PEAKS DB |
| I.RIDTAC(+57.02)VC(+57.02)VITK.K | N | 59.60 | 1434.7323 | 12 | -0.1 | 718.3734 | 2 | 24.70 | 6 | F6:11037 | NaNaKA16\_F2.raw |  |  |  |  |  | 1.966E7 |  | 1.6159E6 | 4.9245E5 |  |  |  |  | 5 | 0 | 0 | 0 | 0 | 0 | 2 | 0 | 2 | 1 | 0 | 0 | 0 | 0 | 226 | 237 | Carbamidomethylation | C6:Carbamidomethylation:1000.00;C8:Carbamidomethylation:1000.00 | PEAKS DB |
| K.VYKQYFFETK.C | Y | 58.39 | 1351.6812 | 10 | 0.5 | 451.5679 | 3 | 28.15 | 10 | F10:14035 | NaNaKA16\_F6.raw |  |  |  |  |  |  |  |  |  | 7.2994E6 |  |  |  | 1 | 0 | 0 | 0 | 0 | 0 | 0 | 0 | 0 | 0 | 1 | 0 | 0 | 0 | 171 | 180 |  |  | PEAKS DB |
| R.FIRIDTAC(+57.02)VC(+57.02)VITKK.T | N | 56.47 | 1822.9797 | 15 | -0.4 | 456.7520 | 4 | 38.55 | 10 | F10:22920 | NaNaKA16\_F6.raw |  |  |  |  |  |  |  |  |  | 3.6578E6 |  |  |  | 1 | 0 | 0 | 0 | 0 | 0 | 0 | 0 | 0 | 0 | 1 | 0 | 0 | 0 | 224 | 238 | Carbamidomethylation | C8:Carbamidomethylation:1000.00;C10:Carbamidomethylation:1000.00 | PEAKS DB |
| K.TTATDIKGNTVTVM(+15.99)ENVNLDNK.V | N | 55.48 | 2393.1743 | 22 | 0.9 | 798.7328 | 3 | 32.04 | 10 | F10:17489 | NaNaKA16\_F6.raw |  |  |  |  |  |  |  |  |  | 6.6667E6 |  |  |  | 1 | 0 | 0 | 0 | 0 | 0 | 0 | 0 | 0 | 0 | 1 | 0 | 0 | 0 | 149 | 170 | Oxidation (M) | M14:Oxidation (M):1000.00 | PEAKS DB |
| R.GIDSSHWNSY.C | N | 54.59 | 1164.4836 | 10 | 0.3 | 583.2493 | 2 | 32.17 | 10 | F10:17444 | NaNaKA16\_F6.raw |  |  |  |  |  |  |  |  |  | 1.7494E7 |  |  |  | 1 | 0 | 0 | 0 | 0 | 0 | 0 | 0 | 0 | 0 | 1 | 0 | 0 | 0 | 193 | 202 |  |  | PEAKS DB |
| K.QYFFETK.C | Y | 54.39 | 961.4545 | 7 | 0.7 | 481.7349 | 2 | 34.22 | 9 | F9:18329 | NaNaKA16\_F5.raw |  |  |  | 2.1402E6 |  |  |  |  | 2.8298E6 | 1.9246E9 |  |  | 7.0917E5 | 6 | 0 | 0 | 0 | 1 | 0 | 0 | 0 | 0 | 1 | 3 | 0 | 0 | 1 | 174 | 180 |  |  | PEAKS DB |
| K.REDHPVHNLGEHSVC(+57.02)DSVSAWVTK.T | Y | 46.52 | 2758.2881 | 24 | -1.2 | 690.5785 | 4 | 33.98 | 10 | F10:19035 | NaNaKA16\_F6.raw |  |  |  |  |  |  |  |  |  | 0 |  |  |  | 0 | 0 | 0 | 0 | 0 | 0 | 0 | 0 | 0 | 0 | 0 | 0 | 0 | 0 | 125 | 148 | Carbamidomethylation | C15:Carbamidomethylation:1000.00 | PEAKS DB |
| K.GNTVTVMENVNL.D | N | 42.39 | 1289.6285 | 12 | 0.5 | 645.8219 | 2 | 65.97 | 10 | F10:46212 | NaNaKA16\_F6.raw |  |  |  |  |  |  |  |  |  | 1.2191E7 |  |  |  | 1 | 0 | 0 | 0 | 0 | 0 | 0 | 0 | 0 | 0 | 1 | 0 | 0 | 0 | 156 | 167 |  |  | PEAKS DB |
| D.SSHWNSYC(+57.02)TETDTFIK.A | N | 42.21 | 1974.8418 | 16 | 0.0 | 659.2878 | 3 | 38.84 | 10 | F10:22969 | NaNaKA16\_F6.raw |  |  |  |  |  |  |  |  |  | 1.017E7 |  |  |  | 1 | 0 | 0 | 0 | 0 | 0 | 0 | 0 | 0 | 0 | 1 | 0 | 0 | 0 | 196 | 211 | Carbamidomethylation | C8:Carbamidomethylation:1000.00 | PEAKS DB |
| total 27 peptides |  |  |  |  |  |  |  |  |  |  |  |  |  |  |  |  |  |  |  |  |  |  |  |  |  |  |  |  |  |  |  |  |  |  |  |  |  |  |  |  |  |  |  |
| --- | --- | --- | --- | --- | --- | --- | --- | --- | --- | --- | --- | --- | --- | --- | --- | --- | --- | --- | --- | --- | --- | --- | --- | --- | --- | --- | --- | --- | --- | --- | --- | --- | --- | --- | --- | --- | --- | --- | --- | --- | --- | --- | --- |

Q5YF89.1

back to list

  

| Protein Coverage
| Supporting Peptides
|

Protein Coverage:

Supporting Peptides:

| Peptide | Uniq | -10lgP | Mass | Length | ppm | m/z | z | RT | Fraction | Scan | Source File | Area F1 | Area F10 | Area F11 | Area F12 | Area F13 | Area F2 | Area F3 | Area F4 | Area F5 | Area F6 | Area F7 | Area F8 | Area F9 | #Feature | #Feature F1 | #Feature F10 | #Feature F11 | #Feature F12 | #Feature F13 | #Feature F2 | #Feature F3 | #Feature F4 | #Feature F5 | #Feature F6 | #Feature F7 | #Feature F8 | #Feature F9 | Start | End | PTM | AScore | Found By |
| --- | --- | --- | --- | --- | --- | --- | --- | --- | --- | --- | --- | --- | --- | --- | --- | --- | --- | --- | --- | --- | --- | --- | --- | --- | --- | --- | --- | --- | --- | --- | --- | --- | --- | --- | --- | --- | --- | --- | --- | --- | --- | --- | --- |
| R.GIDSSHWNSYC(+57.02)TETDTFIK.A | N | 103.21 | 2259.9744 | 19 | 0.4 | 1130.9949 | 2 | 51.96 | 13 | F13:29726 | NaNaKA16\_F9.raw |  | 3.1367E8 | 1.2423E7 | 7.064E6 |  |  |  |  |  | 2.5623E9 | 1.1361E7 | 1.4907E7 | 3.8727E8 | 19 | 0 | 2 | 2 | 1 | 0 | 0 | 0 | 0 | 0 | 8 | 1 | 1 | 4 | 193 | 211 | Carbamidomethylation | C11:Carbamidomethylation:1000.00 | PEAKS DB |
| R.EDHPVHNLGEHSVC(+57.02)DSVSAWVTK.T | N | 97.91 | 2602.1870 | 23 | 0.9 | 868.4037 | 3 | 37.09 | 12 | F12:20537 | NaNaKA16\_F8.raw |  | 2.7036E8 | 6.8592E6 | 4.8528E6 |  |  |  |  |  | 4.7202E9 | 1.5853E7 | 7.7354E7 | 8.6437E8 | 30 | 0 | 4 | 3 | 1 | 0 | 0 | 0 | 0 | 0 | 11 | 3 | 3 | 5 | 126 | 148 | Carbamidomethylation | C14:Carbamidomethylation:1000.00 | PEAKS DB |
| K.TTATDIKGNTVTVMENVNLDNKVYK.Q | N | 89.49 | 2767.4062 | 25 | 0.1 | 923.4761 | 3 | 46.76 | 10 | F10:30493 | NaNaKA16\_F6.raw |  | 2.6398E6 |  |  |  |  |  |  |  | 6.2672E7 |  |  |  | 3 | 0 | 1 | 0 | 0 | 0 | 0 | 0 | 0 | 0 | 2 | 0 | 0 | 0 | 149 | 173 |  |  | PEAKS DB |
| N.LGEHSVC(+57.02)DSVSAWVTK.T | N | 86.52 | 1773.8356 | 16 | 0.4 | 887.9254 | 2 | 46.38 | 2 | F2:26570 | NaNaKA16\_F10.raw |  | 1.1807E8 |  |  |  |  |  |  |  | 2.8318E6 |  |  |  | 4 | 0 | 3 | 0 | 0 | 0 | 0 | 0 | 0 | 0 | 1 | 0 | 0 | 0 | 133 | 148 | Carbamidomethylation | C7:Carbamidomethylation:1000.00 | PEAKS DB |
| E.DHPVHNLGEHSVC(+57.02)DSVSAWVTK.T | N | 84.83 | 2473.1445 | 22 | -0.1 | 825.3887 | 3 | 37.67 | 10 | F10:21732 | NaNaKA16\_F6.raw |  |  |  |  |  |  |  |  |  | 1.5802E8 |  | 4.8658E6 | 5.1183E7 | 8 | 0 | 0 | 0 | 0 | 0 | 0 | 0 | 0 | 0 | 3 | 0 | 1 | 4 | 127 | 148 | Carbamidomethylation | C13:Carbamidomethylation:1000.00 | PEAKS DB |
| K.GNTVTVMENVNLDNK.V | N | 83.96 | 1646.7933 | 15 | 0.6 | 824.4044 | 2 | 40.84 | 13 | F13:21057 | NaNaKA16\_F9.raw |  | 5.4691E7 | 2.4265E6 | 5.4824E6 |  |  |  |  |  | 2.2522E9 | 4.1291E6 | 4.3163E6 | 1.0123E8 | 18 | 0 | 2 | 1 | 1 | 0 | 0 | 0 | 0 | 0 | 9 | 2 | 1 | 2 | 156 | 170 |  |  | PEAKS DB |
| K.GNTVTVM(+15.99)ENVNLDNK.V | N | 81.84 | 1662.7883 | 15 | -0.2 | 832.4012 | 2 | 39.33 | 10 | F10:25249 | NaNaKA16\_F6.raw |  |  |  |  |  |  |  |  |  | 3.5104E8 |  |  |  | 11 | 0 | 0 | 0 | 0 | 0 | 0 | 0 | 0 | 0 | 11 | 0 | 0 | 0 | 156 | 170 | Oxidation (M) | M7:Oxidation (M):1000.00 | PEAKS DB |
| K.ALTMEGNQASWR.F | N | 80.49 | 1362.6350 | 12 | 0.5 | 682.3251 | 2 | 33.00 | 11 | F11:18088 | NaNaKA16\_F7.raw |  | 1.1026E6 |  | 1.0384E7 | 1.8628E5 |  |  |  |  | 3.7415E9 | 5.9758E6 | 6.0181E6 | 1.0381E7 | 10 | 0 | 1 | 0 | 1 | 1 | 0 | 0 | 0 | 0 | 3 | 1 | 1 | 2 | 212 | 223 |  |  | PEAKS DB |
| K.ALTM(+15.99)EGNQASWR.F | N | 77.54 | 1378.6299 | 12 | -0.1 | 690.3221 | 2 | 19.80 | 10 | F10:7091 | NaNaKA16\_F6.raw |  |  |  |  |  |  |  |  |  | 3.2429E8 |  |  |  | 6 | 0 | 0 | 0 | 0 | 0 | 0 | 0 | 0 | 0 | 6 | 0 | 0 | 0 | 212 | 223 | Oxidation (M) | M4:Oxidation (M):1000.00 | PEAKS DB |
| R.IDTAC(+57.02)VC(+57.02)VITK.K | N | 77.37 | 1278.6312 | 11 | 0.1 | 640.3229 | 2 | 33.63 | 10 | F10:18937 | NaNaKA16\_F6.raw |  | 4.3837E5 |  | 8.3453E6 |  | 2.1819E6 |  |  |  | 1.6909E9 | 4.6903E6 |  | 9.8784E6 | 8 | 0 | 1 | 0 | 1 | 0 | 1 | 0 | 0 | 0 | 2 | 1 | 0 | 2 | 227 | 237 | Carbamidomethylation | C5:Carbamidomethylation:1000.00;C7:Carbamidomethylation:1000.00 | PEAKS DB |
| N.TVTVMENVNLDNK.V | N | 76.68 | 1475.7290 | 13 | -0.1 | 738.8717 | 2 | 38.38 | 10 | F10:22544 | NaNaKA16\_F6.raw |  |  |  |  |  |  |  |  |  | 5.0544E7 |  |  |  | 1 | 0 | 0 | 0 | 0 | 0 | 0 | 0 | 0 | 0 | 1 | 0 | 0 | 0 | 158 | 170 |  |  | PEAKS DB |
| D.HPVHNLGEHSVC(+57.02)DSVSAWVTK.T | N | 76.11 | 2358.1174 | 21 | -0.2 | 590.5365 | 4 | 31.29 | 13 | F13:13388 | NaNaKA16\_F9.raw |  | 3.1467E6 |  |  |  |  |  |  |  | 6.048E7 |  | 4.5368E6 | 1.0948E7 | 5 | 0 | 1 | 0 | 0 | 0 | 0 | 0 | 0 | 0 | 2 | 0 | 1 | 1 | 128 | 148 | Carbamidomethylation | C12:Carbamidomethylation:1000.00 | PEAKS DB |
| P.VHNLGEHSVC(+57.02)DSVSAWVTK.T | N | 74.21 | 2124.0059 | 19 | 0.2 | 709.0094 | 3 | 38.53 | 2 | F2:19930 | NaNaKA16\_F10.raw |  | 6.7215E6 |  |  |  |  |  |  |  |  |  |  | 2.2348E6 | 2 | 0 | 1 | 0 | 0 | 0 | 0 | 0 | 0 | 0 | 0 | 0 | 0 | 1 | 130 | 148 | Carbamidomethylation | C10:Carbamidomethylation:1000.00 | PEAKS DB |
| K.TTATDIKGNTVTVMENVNLDNK.V | N | 72.26 | 2377.1794 | 22 | 0.7 | 793.4010 | 3 | 46.61 | 13 | F13:25988 | NaNaKA16\_F9.raw |  | 5.7149E7 |  |  |  |  |  |  |  | 9.9295E7 | 1.6066E6 |  | 7.6356E6 | 5 | 0 | 1 | 0 | 0 | 0 | 0 | 0 | 0 | 0 | 2 | 1 | 0 | 1 | 149 | 170 |  |  | PEAKS DB |
| K.GNTVTVMENVNLDNKVYK.Q | N | 68.33 | 2037.0200 | 18 | 0.0 | 680.0140 | 3 | 40.94 | 10 | F10:25126 | NaNaKA16\_F6.raw |  | 1.592E6 |  |  |  |  |  |  |  | 1.2605E7 |  |  | 9.7102E5 | 4 | 0 | 1 | 0 | 0 | 0 | 0 | 0 | 0 | 0 | 2 | 0 | 0 | 1 | 156 | 173 |  |  | PEAKS DB |
| H.NLGEHSVC(+57.02)DSVSAWVTK.T | N | 64.92 | 1887.8785 | 17 | 0.6 | 944.9471 | 2 | 47.73 | 2 | F2:27755 | NaNaKA16\_F10.raw |  | 2.3917E6 |  |  |  |  |  |  |  |  |  |  |  | 1 | 0 | 1 | 0 | 0 | 0 | 0 | 0 | 0 | 0 | 0 | 0 | 0 | 0 | 132 | 148 | Carbamidomethylation | C8:Carbamidomethylation:1000.00 | PEAKS DB |
| R.FIRIDTAC(+57.02)VC(+57.02)VITK.K | N | 63.81 | 1694.8848 | 14 | -0.9 | 565.9684 | 3 | 50.26 | 10 | F10:33096 | NaNaKA16\_F6.raw |  |  |  |  |  |  |  |  |  | 2.9114E6 |  |  |  | 1 | 0 | 0 | 0 | 0 | 0 | 0 | 0 | 0 | 0 | 1 | 0 | 0 | 0 | 224 | 237 | Carbamidomethylation | C8:Carbamidomethylation:1000.00;C10:Carbamidomethylation:1000.00 | PEAKS DB |
| N.SYC(+57.02)TETDTFIK.A | N | 61.32 | 1363.5966 | 11 | 0.1 | 682.8056 | 2 | 33.68 | 10 | F10:18512 | NaNaKA16\_F6.raw |  |  |  | 2.51E6 |  |  |  |  |  | 6.55E8 |  |  | 2.6358E6 | 3 | 0 | 0 | 0 | 1 | 0 | 0 | 0 | 0 | 0 | 1 | 0 | 0 | 1 | 201 | 211 | Carbamidomethylation | C3:Carbamidomethylation:1000.00 | PEAKS DB |
| I.RIDTAC(+57.02)VC(+57.02)VITK.K | N | 59.60 | 1434.7323 | 12 | -0.1 | 718.3734 | 2 | 24.70 | 6 | F6:11037 | NaNaKA16\_F2.raw |  |  |  |  |  | 1.966E7 |  | 1.6159E6 | 4.9245E5 |  |  |  |  | 5 | 0 | 0 | 0 | 0 | 0 | 2 | 0 | 2 | 1 | 0 | 0 | 0 | 0 | 226 | 237 | Carbamidomethylation | C6:Carbamidomethylation:1000.00;C8:Carbamidomethylation:1000.00 | PEAKS DB |
| K.VYKQYFFETK.C | Y | 58.39 | 1351.6812 | 10 | 0.5 | 451.5679 | 3 | 28.15 | 10 | F10:14035 | NaNaKA16\_F6.raw |  |  |  |  |  |  |  |  |  | 7.2994E6 |  |  |  | 1 | 0 | 0 | 0 | 0 | 0 | 0 | 0 | 0 | 0 | 1 | 0 | 0 | 0 | 171 | 180 |  |  | PEAKS DB |
| R.FIRIDTAC(+57.02)VC(+57.02)VITKK.T | N | 56.47 | 1822.9797 | 15 | -0.4 | 456.7520 | 4 | 38.55 | 10 | F10:22920 | NaNaKA16\_F6.raw |  |  |  |  |  |  |  |  |  | 3.6578E6 |  |  |  | 1 | 0 | 0 | 0 | 0 | 0 | 0 | 0 | 0 | 0 | 1 | 0 | 0 | 0 | 224 | 238 | Carbamidomethylation | C8:Carbamidomethylation:1000.00;C10:Carbamidomethylation:1000.00 | PEAKS DB |
| K.TTATDIKGNTVTVM(+15.99)ENVNLDNK.V | N | 55.48 | 2393.1743 | 22 | 0.9 | 798.7328 | 3 | 32.04 | 10 | F10:17489 | NaNaKA16\_F6.raw |  |  |  |  |  |  |  |  |  | 6.6667E6 |  |  |  | 1 | 0 | 0 | 0 | 0 | 0 | 0 | 0 | 0 | 0 | 1 | 0 | 0 | 0 | 149 | 170 | Oxidation (M) | M14:Oxidation (M):1000.00 | PEAKS DB |
| R.GIDSSHWNSY.C | N | 54.59 | 1164.4836 | 10 | 0.3 | 583.2493 | 2 | 32.17 | 10 | F10:17444 | NaNaKA16\_F6.raw |  |  |  |  |  |  |  |  |  | 1.7494E7 |  |  |  | 1 | 0 | 0 | 0 | 0 | 0 | 0 | 0 | 0 | 0 | 1 | 0 | 0 | 0 | 193 | 202 |  |  | PEAKS DB |
| K.QYFFETK.C | Y | 54.39 | 961.4545 | 7 | 0.7 | 481.7349 | 2 | 34.22 | 9 | F9:18329 | NaNaKA16\_F5.raw |  |  |  | 2.1402E6 |  |  |  |  | 2.8298E6 | 1.9246E9 |  |  | 7.0917E5 | 6 | 0 | 0 | 0 | 1 | 0 | 0 | 0 | 0 | 1 | 3 | 0 | 0 | 1 | 174 | 180 |  |  | PEAKS DB |
| K.REDHPVHNLGEHSVC(+57.02)DSVSAWVTK.T | Y | 46.52 | 2758.2881 | 24 | -1.2 | 690.5785 | 4 | 33.98 | 10 | F10:19035 | NaNaKA16\_F6.raw |  |  |  |  |  |  |  |  |  | 0 |  |  |  | 0 | 0 | 0 | 0 | 0 | 0 | 0 | 0 | 0 | 0 | 0 | 0 | 0 | 0 | 125 | 148 | Carbamidomethylation | C15:Carbamidomethylation:1000.00 | PEAKS DB |
| K.GNTVTVMENVNL.D | N | 42.39 | 1289.6285 | 12 | 0.5 | 645.8219 | 2 | 65.97 | 10 | F10:46212 | NaNaKA16\_F6.raw |  |  |  |  |  |  |  |  |  | 1.2191E7 |  |  |  | 1 | 0 | 0 | 0 | 0 | 0 | 0 | 0 | 0 | 0 | 1 | 0 | 0 | 0 | 156 | 167 |  |  | PEAKS DB |
| D.SSHWNSYC(+57.02)TETDTFIK.A | N | 42.21 | 1974.8418 | 16 | 0.0 | 659.2878 | 3 | 38.84 | 10 | F10:22969 | NaNaKA16\_F6.raw |  |  |  |  |  |  |  |  |  | 1.017E7 |  |  |  | 1 | 0 | 0 | 0 | 0 | 0 | 0 | 0 | 0 | 0 | 1 | 0 | 0 | 0 | 196 | 211 | Carbamidomethylation | C8:Carbamidomethylation:1000.00 | PEAKS DB |
| total 27 peptides |  |  |  |  |  |  |  |  |  |  |  |  |  |  |  |  |  |  |  |  |  |  |  |  |  |  |  |  |  |  |  |  |  |  |  |  |  |  |  |  |  |  |  |
| --- | --- | --- | --- | --- | --- | --- | --- | --- | --- | --- | --- | --- | --- | --- | --- | --- | --- | --- | --- | --- | --- | --- | --- | --- | --- | --- | --- | --- | --- | --- | --- | --- | --- | --- | --- | --- | --- | --- | --- | --- | --- | --- | --- |

Q9PVK7.1

back to list

  

| Protein Coverage
| Supporting Peptides
|

Protein Coverage:

Supporting Peptides:

| Peptide | Uniq | -10lgP | Mass | Length | ppm | m/z | z | RT | Fraction | Scan | Source File | Area F1 | Area F10 | Area F11 | Area F12 | Area F13 | Area F2 | Area F3 | Area F4 | Area F5 | Area F6 | Area F7 | Area F8 | Area F9 | #Feature | #Feature F1 | #Feature F10 | #Feature F11 | #Feature F12 | #Feature F13 | #Feature F2 | #Feature F3 | #Feature F4 | #Feature F5 | #Feature F6 | #Feature F7 | #Feature F8 | #Feature F9 | Start | End | PTM | AScore | Found By |
| --- | --- | --- | --- | --- | --- | --- | --- | --- | --- | --- | --- | --- | --- | --- | --- | --- | --- | --- | --- | --- | --- | --- | --- | --- | --- | --- | --- | --- | --- | --- | --- | --- | --- | --- | --- | --- | --- | --- | --- | --- | --- | --- | --- |
| R.AAKDDC(+57.02)DLPELC(+57.02)TGQSAEC(+57.02)PTDVFQR.N | N | 91.95 | 2982.2793 | 26 | 1.7 | 995.1021 | 3 | 54.73 | 9 | F9:35402 | NaNaKA16\_F5.raw |  |  |  |  | 1.7077E8 |  |  |  | 1.5002E8 | 4.1894E7 |  |  |  | 4 | 0 | 0 | 0 | 0 | 1 | 0 | 0 | 0 | 2 | 1 | 0 | 0 | 0 | 456 | 481 | Carbamidomethylation | C6:Carbamidomethylation:1000.00;C12:Carbamidomethylation:1000.00;C19:Carbamidomethylation:1000.00 | PEAKS DB |
| K.DDC(+57.02)DLPELC(+57.02)TGQSAEC(+57.02)PTDVFQR.N | N | 90.56 | 2712.1101 | 23 | 1.2 | 1357.0640 | 2 | 72.09 | 9 | F9:50509 | NaNaKA16\_F5.raw |  |  |  |  | 9.0813E7 |  |  |  | 4.1609E7 | 6.0829E6 |  |  |  | 6 | 0 | 0 | 0 | 0 | 2 | 0 | 0 | 0 | 2 | 2 | 0 | 0 | 0 | 459 | 481 | Carbamidomethylation | C3:Carbamidomethylation:1000.00;C9:Carbamidomethylation:1000.00;C16:Carbamidomethylation:1000.00 | PEAKS DB |
| K.LQGEIYFIEPLK.I | Y | 79.49 | 1448.7915 | 12 | -0.4 | 725.4027 | 2 | 71.80 | 12 | F12:49303 | NaNaKA16\_F8.raw |  | 7.7381E6 | 9.8751E5 | 1.0165E6 |  |  |  |  |  |  | 1.432E7 | 9.2872E7 | 2.2471E7 | 6 | 0 | 1 | 1 | 1 | 0 | 0 | 0 | 0 | 0 | 0 | 1 | 1 | 1 | 124 | 135 |  |  | PEAKS DB |
| K.C(+57.02)PIMTNQC(+57.02)IALR.G | N | 77.27 | 1475.7047 | 12 | 0.2 | 738.8598 | 2 | 38.95 | 9 | F9:22183 | NaNaKA16\_F5.raw |  |  | 5.0717E5 | 3.2472E5 | 1.9097E8 |  |  |  | 2.1736E8 | 4.0241E7 |  | 3.7147E6 | 4.6756E6 | 12 | 0 | 0 | 1 | 1 | 3 | 0 | 0 | 0 | 2 | 2 | 0 | 2 | 1 | 497 | 508 | Carbamidomethylation | C1:Carbamidomethylation:1000.00;C8:Carbamidomethylation:1000.00 | PEAKS DB |
| R.DPSYGMVEPGTK.C | N | 77.06 | 1279.5754 | 12 | 0.6 | 640.7954 | 2 | 29.06 | 10 | F10:14931 | NaNaKA16\_F6.raw |  |  |  |  |  |  |  |  |  | 4.4389E8 |  |  |  | 1 | 0 | 0 | 0 | 0 | 0 | 0 | 0 | 0 | 0 | 1 | 0 | 0 | 0 | 570 | 581 |  |  | PEAKS DB |
| K.C(+57.02)PIM(+15.99)TNQC(+57.02)IALR.G | N | 75.90 | 1491.6996 | 12 | -0.8 | 746.8564 | 2 | 29.62 | 9 | F9:14662 | NaNaKA16\_F5.raw |  |  |  |  | 3.2733E7 |  |  |  | 8.8589E6 | 2.9095E6 |  |  |  | 7 | 0 | 0 | 0 | 0 | 4 | 0 | 0 | 0 | 2 | 1 | 0 | 0 | 0 | 497 | 508 | Carbamidomethylation; Oxidation (M) | C1:Carbamidomethylation:1000.00;M4:Oxidation (M):1000.00;C8:Carbamidomethylation:1000.00 | PEAKS DB |
| R.NSMIC(+57.02)NC(+57.02)SISPR.D | N | 75.66 | 1437.6163 | 12 | 0.1 | 719.8155 | 2 | 24.50 | 5 | F5:7209 | NaNaKA16\_F13.raw |  |  |  |  | 6.4703E7 |  |  |  | 2.8886E6 | 2.2986E6 | 1.2892E5 |  |  | 4 | 0 | 0 | 0 | 0 | 1 | 0 | 0 | 0 | 1 | 1 | 1 | 0 | 0 | 558 | 569 | Carbamidomethylation | C5:Carbamidomethylation:1000.00;C7:Carbamidomethylation:1000.00 | PEAKS DB |
| G.TPVGLAYIGSIC(+57.02)NPK.T | N | 73.67 | 1588.8282 | 15 | 0.1 | 795.4215 | 2 | 60.14 | 12 | F12:39283 | NaNaKA16\_F8.raw |  |  |  |  |  |  |  |  |  |  | 1.0459E6 | 4.5255E6 |  | 2 | 0 | 0 | 0 | 0 | 0 | 0 | 0 | 0 | 0 | 0 | 1 | 1 | 0 | 293 | 307 | Carbamidomethylation | C12:Carbamidomethylation:1000.00 | PEAKS DB |
| N.GTPVGLAYIGSIC(+57.02)NPK.T | N | 73.11 | 1645.8497 | 16 | 0.4 | 823.9325 | 2 | 60.77 | 11 | F11:39960 | NaNaKA16\_F7.raw |  |  |  |  | 1.4933E6 |  |  |  |  |  | 3.1956E6 | 2.0263E7 |  | 3 | 0 | 0 | 0 | 0 | 1 | 0 | 0 | 0 | 0 | 0 | 1 | 1 | 0 | 292 | 307 | Carbamidomethylation | C13:Carbamidomethylation:1000.00 | PEAKS DB |
| R.DPSYGM(+15.99)VEPGTK.C | N | 69.32 | 1295.5703 | 12 | -0.5 | 648.7921 | 2 | 28.08 | 10 | F10:14794 | NaNaKA16\_F6.raw |  |  |  |  |  |  |  |  |  | 6.1916E7 |  |  |  | 9 | 0 | 0 | 0 | 0 | 0 | 0 | 0 | 0 | 0 | 9 | 0 | 0 | 0 | 570 | 581 | Oxidation (M) | M6:Oxidation (M):1000.00 | PEAKS DB |
| K.TSAAVVQDYSK.S | Y | 68.03 | 1167.5771 | 11 | 0.0 | 584.7958 | 2 | 17.55 | 5 | F5:3663 | NaNaKA16\_F13.raw |  |  |  |  | 1.026E7 |  |  |  |  |  |  |  |  | 2 | 0 | 0 | 0 | 0 | 2 | 0 | 0 | 0 | 0 | 0 | 0 | 0 | 0 | 308 | 318 |  |  | PEAKS DB |
| R.TKPAYQFSSC(+57.02)SVR.E | N | 67.55 | 1529.7296 | 13 | -0.1 | 765.8720 | 2 | 12.28 | 5 | F5:2284 | NaNaKA16\_F13.raw |  |  |  |  | 2.3942E8 |  |  |  |  |  |  |  |  | 2 | 0 | 0 | 0 | 0 | 2 | 0 | 0 | 0 | 0 | 0 | 0 | 0 | 0 | 358 | 370 | Carbamidomethylation | C10:Carbamidomethylation:1000.00 | PEAKS DB |
| K.ATLDLFGEWR.E | N | 65.68 | 1206.6033 | 10 | 0.9 | 604.3094 | 2 | 78.42 | 2 | F2:53296 | NaNaKA16\_F10.raw |  | 2.2448E6 |  | 3.9536E5 | 1.1467E7 |  |  |  |  | 5.6872E6 | 6.3509E5 | 1.6325E6 | 1.5953E6 | 8 | 0 | 1 | 0 | 1 | 2 | 0 | 0 | 0 | 0 | 1 | 1 | 1 | 1 | 260 | 269 |  |  | PEAKS DB |
| R.NSM(+15.99)IC(+57.02)NC(+57.02)SISPR.D | N | 64.85 | 1453.6112 | 12 | 0.3 | 727.8131 | 2 | 22.81 | 5 | F5:7058 | NaNaKA16\_F13.raw |  |  |  |  | 7.9998E6 |  |  |  |  |  |  |  |  | 3 | 0 | 0 | 0 | 0 | 3 | 0 | 0 | 0 | 0 | 0 | 0 | 0 | 0 | 558 | 569 | Oxidation (M); Carbamidomethylation | M3:Oxidation (M):1000.00;C5:Carbamidomethylation:1000.00;C7:Carbamidomethylation:1000.00 | PEAKS DB |
| R.RTKPAYQFSSC(+57.02)SVR.E | N | 61.84 | 1685.8307 | 14 | 1.0 | 562.9514 | 3 | 11.85 | 5 | F5:1803 | NaNaKA16\_F13.raw |  |  |  |  | 1.7851E6 |  |  |  |  |  |  |  |  | 1 | 0 | 0 | 0 | 0 | 1 | 0 | 0 | 0 | 0 | 0 | 0 | 0 | 0 | 357 | 370 | Carbamidomethylation | C11:Carbamidomethylation:1000.00 | PEAKS DB |
| A.DSSAVISAC(+57.02)DGLK.G | N | 60.11 | 1321.6184 | 13 | 0.4 | 661.8168 | 2 | 33.21 | 10 | F10:19042 | NaNaKA16\_F6.raw |  |  |  |  |  |  |  |  |  | 6.9505E6 |  |  |  | 1 | 0 | 0 | 0 | 0 | 0 | 0 | 0 | 0 | 0 | 1 | 0 | 0 | 0 | 107 | 119 | Carbamidomethylation | C9:Carbamidomethylation:1000.00 | PEAKS DB |
| K.RNSMIC(+57.02)NC(+57.02)SISPR.D | N | 59.91 | 1593.7174 | 13 | 0.1 | 532.2465 | 3 | 12.17 | 5 | F5:2067 | NaNaKA16\_F13.raw |  |  |  |  | 7.3377E5 |  |  |  |  |  |  |  |  | 1 | 0 | 0 | 0 | 0 | 1 | 0 | 0 | 0 | 0 | 0 | 0 | 0 | 0 | 557 | 569 | Carbamidomethylation | C6:Carbamidomethylation:1000.00;C8:Carbamidomethylation:1000.00 | PEAKS DB |
| C.TGQSAEC(+57.02)PTDVFQR.N | N | 57.94 | 1594.7046 | 14 | 0.9 | 798.3603 | 2 | 31.39 | 5 | F5:11052 | NaNaKA16\_F13.raw |  |  |  |  | 8.9511E5 |  |  |  |  |  |  |  |  | 1 | 0 | 0 | 0 | 0 | 1 | 0 | 0 | 0 | 0 | 0 | 0 | 0 | 0 | 468 | 481 | Carbamidomethylation | C7:Carbamidomethylation:1000.00 | PEAKS DB |
| R.DSC(+57.02)FTLNQR.T | N | 57.18 | 1139.5029 | 9 | 0.1 | 570.7588 | 2 | 26.24 | 5 | F5:8465 | NaNaKA16\_F13.raw |  |  |  |  | 2.8674E8 |  |  |  |  | 8.7317E5 |  |  |  | 2 | 0 | 0 | 0 | 0 | 1 | 0 | 0 | 0 | 0 | 1 | 0 | 0 | 0 | 517 | 525 | Carbamidomethylation | C3:Carbamidomethylation:1000.00 | PEAKS DB |
| R.MVAITMAHEMGHNLGMNHDK.G | Y | 56.24 | 2236.0010 | 20 | 0.1 | 560.0076 | 4 | 33.19 | 12 | F12:17336 | NaNaKA16\_F8.raw |  |  |  |  |  |  |  |  |  |  |  | 3.3512E6 |  | 1 | 0 | 0 | 0 | 0 | 0 | 0 | 0 | 0 | 0 | 0 | 0 | 1 | 0 | 322 | 341 |  |  | PEAKS DB |
| R.MVAITMAHEMGH.N | N | 55.46 | 1326.5883 | 12 | 0.8 | 664.3019 | 2 | 33.28 | 5 | F5:12224 | NaNaKA16\_F13.raw |  |  |  |  | 4.5918E6 |  |  |  |  |  |  |  |  | 1 | 0 | 0 | 0 | 0 | 1 | 0 | 0 | 0 | 0 | 0 | 0 | 0 | 0 | 322 | 333 |  |  | PEAKS DB |
| W.SNINEINVQSDVK.A | Y | 55.08 | 1458.7314 | 13 | 0.2 | 730.3731 | 2 | 27.42 | 5 | F5:8945 | NaNaKA16\_F13.raw |  |  |  |  | 1.8809E6 |  |  |  |  |  |  |  |  | 1 | 0 | 0 | 0 | 0 | 1 | 0 | 0 | 0 | 0 | 0 | 0 | 0 | 0 | 247 | 259 |  |  | PEAKS DB |
| K.PAYQFSSC(+57.02)SVR.E | N | 53.21 | 1300.5870 | 11 | 0.2 | 651.3009 | 2 | 25.11 | 5 | F5:7381 | NaNaKA16\_F13.raw |  |  |  |  | 5.016E7 |  |  |  |  |  |  |  |  | 1 | 0 | 0 | 0 | 0 | 1 | 0 | 0 | 0 | 0 | 0 | 0 | 0 | 0 | 360 | 370 | Carbamidomethylation | C8:Carbamidomethylation:1000.00 | PEAKS DB |
| K.TSLLTNTPEQDR.Y | Y | 51.47 | 1373.6787 | 12 | 0.4 | 687.8469 | 2 | 23.22 | 5 | F5:6477 | NaNaKA16\_F13.raw |  |  |  |  | 5.9896E5 |  |  |  |  |  |  |  |  | 1 | 0 | 0 | 0 | 0 | 1 | 0 | 0 | 0 | 0 | 0 | 0 | 0 | 0 | 176 | 187 |  |  | PEAKS DB |
| K.C(+57.02)GDGMVC(+57.02)SNR.Q | N | 51.31 | 1154.4268 | 10 | 1.1 | 578.2213 | 2 | 11.85 | 5 | F5:1806 | NaNaKA16\_F13.raw |  |  |  |  | 4.5755E5 |  |  |  |  |  |  |  |  | 1 | 0 | 0 | 0 | 0 | 1 | 0 | 0 | 0 | 0 | 0 | 0 | 0 | 0 | 582 | 591 | Carbamidomethylation | C1:Carbamidomethylation:1000.00;C7:Carbamidomethylation:1000.00 | PEAKS DB |
| N.INEINVQSDVK.A | Y | 50.36 | 1257.6565 | 11 | 0.0 | 629.8355 | 2 | 23.51 | 5 | F5:6491 | NaNaKA16\_F13.raw |  |  |  |  | 2.41E6 |  |  |  |  |  |  |  |  | 1 | 0 | 0 | 0 | 0 | 1 | 0 | 0 | 0 | 0 | 0 | 0 | 0 | 0 | 249 | 259 |  |  | PEAKS DB |
| Q.SAEC(+57.02)PTDVFQR.N | N | 49.48 | 1308.5768 | 11 | 0.4 | 655.2960 | 2 | 32.04 | 5 | F5:11429 | NaNaKA16\_F13.raw |  |  |  |  | 1.3941E7 |  |  |  |  |  |  |  |  | 1 | 0 | 0 | 0 | 0 | 1 | 0 | 0 | 0 | 0 | 0 | 0 | 0 | 0 | 471 | 481 | Carbamidomethylation | C4:Carbamidomethylation:1000.00 | PEAKS DB |
| Y.MVVDNIMYR.H | Y | 48.66 | 1139.5468 | 9 | 0.0 | 570.7806 | 2 | 44.92 | 5 | F5:18068 | NaNaKA16\_F13.raw |  |  |  |  | 7.0995E7 |  |  |  |  |  |  |  |  | 1 | 0 | 0 | 0 | 0 | 1 | 0 | 0 | 0 | 0 | 0 | 0 | 0 | 0 | 199 | 207 |  |  | PEAKS DB |
| S.NINEINVQSDVK.A | Y | 48.17 | 1371.6993 | 12 | 0.3 | 686.8572 | 2 | 27.44 | 5 | F5:8958 | NaNaKA16\_F13.raw |  |  |  |  | 1.1123E6 |  |  |  |  |  |  |  |  | 1 | 0 | 0 | 0 | 0 | 1 | 0 | 0 | 0 | 0 | 0 | 0 | 0 | 0 | 248 | 259 |  |  | PEAKS DB |
| N.SMIC(+57.02)NC(+57.02)SISPR.D | N | 47.00 | 1323.5734 | 11 | 3.9 | 662.7966 | 2 | 23.55 | 5 | F5:6539 | NaNaKA16\_F13.raw |  |  |  |  | 0 |  |  |  |  |  |  |  |  | 0 | 0 | 0 | 0 | 0 | 0 | 0 | 0 | 0 | 0 | 0 | 0 | 0 | 0 | 559 | 569 | Carbamidomethylation | C4:Carbamidomethylation:1000.00;C6:Carbamidomethylation:1000.00 | PEAKS DB |
| K.LQHEAQC(+57.02)DSEEC(+57.02)C(+57.02)EK.C | N | 46.38 | 1921.7240 | 15 | 0.0 | 641.5820 | 3 | 11.83 | 5 | F5:1808 | NaNaKA16\_F13.raw |  |  |  |  | 2.9375E5 |  |  |  |  |  |  |  |  | 1 | 0 | 0 | 0 | 0 | 1 | 0 | 0 | 0 | 0 | 0 | 0 | 0 | 0 | 430 | 444 | Carbamidomethylation | C7:Carbamidomethylation:1000.00;C12:Carbamidomethylation:1000.00;C13:Carbamidomethylation:1000.00 | PEAKS DB |
| R.DRPQC(+57.02)ILNKPLST.D | N | 46.29 | 1540.8031 | 13 | -0.4 | 514.6081 | 3 | 27.16 | 4 | F4:11109 | NaNaKA16\_F12.raw |  |  |  | 7.0589E5 |  |  |  | 8.268E5 |  |  |  |  |  | 2 | 0 | 0 | 0 | 1 | 0 | 0 | 0 | 1 | 0 | 0 | 0 | 0 | 0 | 379 | 391 | Carbamidomethylation | C5:Carbamidomethylation:1000.00 | PEAKS DB |
| K.C(+57.02)GDGM(+15.99)VC(+57.02)SNR.Q | N | 45.27 | 1170.4216 | 10 | 0.3 | 586.2183 | 2 | 11.84 | 5 | F5:1815 | NaNaKA16\_F13.raw |  |  |  |  | 9.9702E4 |  |  |  |  |  |  |  |  | 1 | 0 | 0 | 0 | 0 | 1 | 0 | 0 | 0 | 0 | 0 | 0 | 0 | 0 | 582 | 591 | Carbamidomethylation; Oxidation (M) | C1:Carbamidomethylation:1000.00;M5:Oxidation (M):1000.00;C7:Carbamidomethylation:1000.00 | PEAKS DB |
| Y.MVVDNIM(+15.99)YR.H | Y | 41.81 | 1155.5416 | 9 | -0.2 | 578.7780 | 2 | 32.88 | 5 | F5:12042 | NaNaKA16\_F13.raw |  |  |  |  | 6.8639E6 |  |  |  |  |  |  |  |  | 1 | 0 | 0 | 0 | 0 | 1 | 0 | 0 | 0 | 0 | 0 | 0 | 0 | 0 | 199 | 207 | Oxidation (M) | M7:Oxidation (M):95.35 | PEAKS DB |
| total 34 peptides |  |  |  |  |  |  |  |  |  |  |  |  |  |  |  |  |  |  |  |  |  |  |  |  |  |  |  |  |  |  |  |  |  |  |  |  |  |  |  |  |  |  |  |
| --- | --- | --- | --- | --- | --- | --- | --- | --- | --- | --- | --- | --- | --- | --- | --- | --- | --- | --- | --- | --- | --- | --- | --- | --- | --- | --- | --- | --- | --- | --- | --- | --- | --- | --- | --- | --- | --- | --- | --- | --- | --- | --- | --- |

AAF00693.1

back to list

  

| Protein Coverage
| Supporting Peptides
|

Protein Coverage:

Supporting Peptides:

| Peptide | Uniq | -10lgP | Mass | Length | ppm | m/z | z | RT | Fraction | Scan | Source File | Area F1 | Area F10 | Area F11 | Area F12 | Area F13 | Area F2 | Area F3 | Area F4 | Area F5 | Area F6 | Area F7 | Area F8 | Area F9 | #Feature | #Feature F1 | #Feature F10 | #Feature F11 | #Feature F12 | #Feature F13 | #Feature F2 | #Feature F3 | #Feature F4 | #Feature F5 | #Feature F6 | #Feature F7 | #Feature F8 | #Feature F9 | Start | End | PTM | AScore | Found By |
| --- | --- | --- | --- | --- | --- | --- | --- | --- | --- | --- | --- | --- | --- | --- | --- | --- | --- | --- | --- | --- | --- | --- | --- | --- | --- | --- | --- | --- | --- | --- | --- | --- | --- | --- | --- | --- | --- | --- | --- | --- | --- | --- | --- |
| R.AAKDDC(+57.02)DLPELC(+57.02)TGQSAEC(+57.02)PTDVFQR.N | N | 91.95 | 2982.2793 | 26 | 1.7 | 995.1021 | 3 | 54.73 | 9 | F9:35402 | NaNaKA16\_F5.raw |  |  |  |  | 1.7077E8 |  |  |  | 1.5002E8 | 4.1894E7 |  |  |  | 4 | 0 | 0 | 0 | 0 | 1 | 0 | 0 | 0 | 2 | 1 | 0 | 0 | 0 | 456 | 481 | Carbamidomethylation | C6:Carbamidomethylation:1000.00;C12:Carbamidomethylation:1000.00;C19:Carbamidomethylation:1000.00 | PEAKS DB |
| K.DDC(+57.02)DLPELC(+57.02)TGQSAEC(+57.02)PTDVFQR.N | N | 90.56 | 2712.1101 | 23 | 1.2 | 1357.0640 | 2 | 72.09 | 9 | F9:50509 | NaNaKA16\_F5.raw |  |  |  |  | 9.0813E7 |  |  |  | 4.1609E7 | 6.0829E6 |  |  |  | 6 | 0 | 0 | 0 | 0 | 2 | 0 | 0 | 0 | 2 | 2 | 0 | 0 | 0 | 459 | 481 | Carbamidomethylation | C3:Carbamidomethylation:1000.00;C9:Carbamidomethylation:1000.00;C16:Carbamidomethylation:1000.00 | PEAKS DB |
| K.LQGEIYFIEPLK.I | Y | 79.49 | 1448.7915 | 12 | -0.4 | 725.4027 | 2 | 71.80 | 12 | F12:49303 | NaNaKA16\_F8.raw |  | 7.7381E6 | 9.8751E5 | 1.0165E6 |  |  |  |  |  |  | 1.432E7 | 9.2872E7 | 2.2471E7 | 6 | 0 | 1 | 1 | 1 | 0 | 0 | 0 | 0 | 0 | 0 | 1 | 1 | 1 | 124 | 135 |  |  | PEAKS DB |
| K.C(+57.02)PIMTNQC(+57.02)IALR.G | N | 77.27 | 1475.7047 | 12 | 0.2 | 738.8598 | 2 | 38.95 | 9 | F9:22183 | NaNaKA16\_F5.raw |  |  | 5.0717E5 | 3.2472E5 | 1.9097E8 |  |  |  | 2.1736E8 | 4.0241E7 |  | 3.7147E6 | 4.6756E6 | 12 | 0 | 0 | 1 | 1 | 3 | 0 | 0 | 0 | 2 | 2 | 0 | 2 | 1 | 497 | 508 | Carbamidomethylation | C1:Carbamidomethylation:1000.00;C8:Carbamidomethylation:1000.00 | PEAKS DB |
| R.DPSYGMVEPGTK.C | N | 77.06 | 1279.5754 | 12 | 0.6 | 640.7954 | 2 | 29.06 | 10 | F10:14931 | NaNaKA16\_F6.raw |  |  |  |  |  |  |  |  |  | 4.4389E8 |  |  |  | 1 | 0 | 0 | 0 | 0 | 0 | 0 | 0 | 0 | 0 | 1 | 0 | 0 | 0 | 570 | 581 |  |  | PEAKS DB |
| K.C(+57.02)PIM(+15.99)TNQC(+57.02)IALR.G | N | 75.90 | 1491.6996 | 12 | -0.8 | 746.8564 | 2 | 29.62 | 9 | F9:14662 | NaNaKA16\_F5.raw |  |  |  |  | 3.2733E7 |  |  |  | 8.8589E6 | 2.9095E6 |  |  |  | 7 | 0 | 0 | 0 | 0 | 4 | 0 | 0 | 0 | 2 | 1 | 0 | 0 | 0 | 497 | 508 | Carbamidomethylation; Oxidation (M) | C1:Carbamidomethylation:1000.00;M4:Oxidation (M):1000.00;C8:Carbamidomethylation:1000.00 | PEAKS DB |
| R.NSMIC(+57.02)NC(+57.02)SISPR.D | N | 75.66 | 1437.6163 | 12 | 0.1 | 719.8155 | 2 | 24.50 | 5 | F5:7209 | NaNaKA16\_F13.raw |  |  |  |  | 6.4703E7 |  |  |  | 2.8886E6 | 2.2986E6 | 1.2892E5 |  |  | 4 | 0 | 0 | 0 | 0 | 1 | 0 | 0 | 0 | 1 | 1 | 1 | 0 | 0 | 558 | 569 | Carbamidomethylation | C5:Carbamidomethylation:1000.00;C7:Carbamidomethylation:1000.00 | PEAKS DB |
| G.TPVGLAYIGSIC(+57.02)NPK.T | N | 73.67 | 1588.8282 | 15 | 0.1 | 795.4215 | 2 | 60.14 | 12 | F12:39283 | NaNaKA16\_F8.raw |  |  |  |  |  |  |  |  |  |  | 1.0459E6 | 4.5255E6 |  | 2 | 0 | 0 | 0 | 0 | 0 | 0 | 0 | 0 | 0 | 0 | 1 | 1 | 0 | 293 | 307 | Carbamidomethylation | C12:Carbamidomethylation:1000.00 | PEAKS DB |
| N.GTPVGLAYIGSIC(+57.02)NPK.T | N | 73.11 | 1645.8497 | 16 | 0.4 | 823.9325 | 2 | 60.77 | 11 | F11:39960 | NaNaKA16\_F7.raw |  |  |  |  | 1.4933E6 |  |  |  |  |  | 3.1956E6 | 2.0263E7 |  | 3 | 0 | 0 | 0 | 0 | 1 | 0 | 0 | 0 | 0 | 0 | 1 | 1 | 0 | 292 | 307 | Carbamidomethylation | C13:Carbamidomethylation:1000.00 | PEAKS DB |
| R.DPSYGM(+15.99)VEPGTK.C | N | 69.32 | 1295.5703 | 12 | -0.5 | 648.7921 | 2 | 28.08 | 10 | F10:14794 | NaNaKA16\_F6.raw |  |  |  |  |  |  |  |  |  | 6.1916E7 |  |  |  | 9 | 0 | 0 | 0 | 0 | 0 | 0 | 0 | 0 | 0 | 9 | 0 | 0 | 0 | 570 | 581 | Oxidation (M) | M6:Oxidation (M):1000.00 | PEAKS DB |
| K.TSAAVVQDYSK.S | Y | 68.03 | 1167.5771 | 11 | 0.0 | 584.7958 | 2 | 17.55 | 5 | F5:3663 | NaNaKA16\_F13.raw |  |  |  |  | 1.026E7 |  |  |  |  |  |  |  |  | 2 | 0 | 0 | 0 | 0 | 2 | 0 | 0 | 0 | 0 | 0 | 0 | 0 | 0 | 308 | 318 |  |  | PEAKS DB |
| R.TKPAYQFSSC(+57.02)SVR.E | N | 67.55 | 1529.7296 | 13 | -0.1 | 765.8720 | 2 | 12.28 | 5 | F5:2284 | NaNaKA16\_F13.raw |  |  |  |  | 2.3942E8 |  |  |  |  |  |  |  |  | 2 | 0 | 0 | 0 | 0 | 2 | 0 | 0 | 0 | 0 | 0 | 0 | 0 | 0 | 358 | 370 | Carbamidomethylation | C10:Carbamidomethylation:1000.00 | PEAKS DB |
| K.ATLDLFGEWR.E | N | 65.68 | 1206.6033 | 10 | 0.9 | 604.3094 | 2 | 78.42 | 2 | F2:53296 | NaNaKA16\_F10.raw |  | 2.2448E6 |  | 3.9536E5 | 1.1467E7 |  |  |  |  | 5.6872E6 | 6.3509E5 | 1.6325E6 | 1.5953E6 | 8 | 0 | 1 | 0 | 1 | 2 | 0 | 0 | 0 | 0 | 1 | 1 | 1 | 1 | 260 | 269 |  |  | PEAKS DB |
| R.NSM(+15.99)IC(+57.02)NC(+57.02)SISPR.D | N | 64.85 | 1453.6112 | 12 | 0.3 | 727.8131 | 2 | 22.81 | 5 | F5:7058 | NaNaKA16\_F13.raw |  |  |  |  | 7.9998E6 |  |  |  |  |  |  |  |  | 3 | 0 | 0 | 0 | 0 | 3 | 0 | 0 | 0 | 0 | 0 | 0 | 0 | 0 | 558 | 569 | Oxidation (M); Carbamidomethylation | M3:Oxidation (M):1000.00;C5:Carbamidomethylation:1000.00;C7:Carbamidomethylation:1000.00 | PEAKS DB |
| R.RTKPAYQFSSC(+57.02)SVR.E | N | 61.84 | 1685.8307 | 14 | 1.0 | 562.9514 | 3 | 11.85 | 5 | F5:1803 | NaNaKA16\_F13.raw |  |  |  |  | 1.7851E6 |  |  |  |  |  |  |  |  | 1 | 0 | 0 | 0 | 0 | 1 | 0 | 0 | 0 | 0 | 0 | 0 | 0 | 0 | 357 | 370 | Carbamidomethylation | C11:Carbamidomethylation:1000.00 | PEAKS DB |
| A.DSSAVISAC(+57.02)DGLK.G | N | 60.11 | 1321.6184 | 13 | 0.4 | 661.8168 | 2 | 33.21 | 10 | F10:19042 | NaNaKA16\_F6.raw |  |  |  |  |  |  |  |  |  | 6.9505E6 |  |  |  | 1 | 0 | 0 | 0 | 0 | 0 | 0 | 0 | 0 | 0 | 1 | 0 | 0 | 0 | 107 | 119 | Carbamidomethylation | C9:Carbamidomethylation:1000.00 | PEAKS DB |
| K.RNSMIC(+57.02)NC(+57.02)SISPR.D | N | 59.91 | 1593.7174 | 13 | 0.1 | 532.2465 | 3 | 12.17 | 5 | F5:2067 | NaNaKA16\_F13.raw |  |  |  |  | 7.3377E5 |  |  |  |  |  |  |  |  | 1 | 0 | 0 | 0 | 0 | 1 | 0 | 0 | 0 | 0 | 0 | 0 | 0 | 0 | 557 | 569 | Carbamidomethylation | C6:Carbamidomethylation:1000.00;C8:Carbamidomethylation:1000.00 | PEAKS DB |
| C.TGQSAEC(+57.02)PTDVFQR.N | N | 57.94 | 1594.7046 | 14 | 0.9 | 798.3603 | 2 | 31.39 | 5 | F5:11052 | NaNaKA16\_F13.raw |  |  |  |  | 8.9511E5 |  |  |  |  |  |  |  |  | 1 | 0 | 0 | 0 | 0 | 1 | 0 | 0 | 0 | 0 | 0 | 0 | 0 | 0 | 468 | 481 | Carbamidomethylation | C7:Carbamidomethylation:1000.00 | PEAKS DB |
| R.DSC(+57.02)FTLNQR.T | N | 57.18 | 1139.5029 | 9 | 0.1 | 570.7588 | 2 | 26.24 | 5 | F5:8465 | NaNaKA16\_F13.raw |  |  |  |  | 2.8674E8 |  |  |  |  | 8.7317E5 |  |  |  | 2 | 0 | 0 | 0 | 0 | 1 | 0 | 0 | 0 | 0 | 1 | 0 | 0 | 0 | 517 | 525 | Carbamidomethylation | C3:Carbamidomethylation:1000.00 | PEAKS DB |
| R.MVAITMAHEMGHNLGMNHDK.G | Y | 56.24 | 2236.0010 | 20 | 0.1 | 560.0076 | 4 | 33.19 | 12 | F12:17336 | NaNaKA16\_F8.raw |  |  |  |  |  |  |  |  |  |  |  | 3.3512E6 |  | 1 | 0 | 0 | 0 | 0 | 0 | 0 | 0 | 0 | 0 | 0 | 0 | 1 | 0 | 322 | 341 |  |  | PEAKS DB |
| R.MVAITMAHEMGH.N | N | 55.46 | 1326.5883 | 12 | 0.8 | 664.3019 | 2 | 33.28 | 5 | F5:12224 | NaNaKA16\_F13.raw |  |  |  |  | 4.5918E6 |  |  |  |  |  |  |  |  | 1 | 0 | 0 | 0 | 0 | 1 | 0 | 0 | 0 | 0 | 0 | 0 | 0 | 0 | 322 | 333 |  |  | PEAKS DB |
| W.SNINEINVQSDVK.A | Y | 55.08 | 1458.7314 | 13 | 0.2 | 730.3731 | 2 | 27.42 | 5 | F5:8945 | NaNaKA16\_F13.raw |  |  |  |  | 1.8809E6 |  |  |  |  |  |  |  |  | 1 | 0 | 0 | 0 | 0 | 1 | 0 | 0 | 0 | 0 | 0 | 0 | 0 | 0 | 247 | 259 |  |  | PEAKS DB |
| K.PAYQFSSC(+57.02)SVR.E | N | 53.21 | 1300.5870 | 11 | 0.2 | 651.3009 | 2 | 25.11 | 5 | F5:7381 | NaNaKA16\_F13.raw |  |  |  |  | 5.016E7 |  |  |  |  |  |  |  |  | 1 | 0 | 0 | 0 | 0 | 1 | 0 | 0 | 0 | 0 | 0 | 0 | 0 | 0 | 360 | 370 | Carbamidomethylation | C8:Carbamidomethylation:1000.00 | PEAKS DB |
| K.TSLLTNTPEQDR.Y | Y | 51.47 | 1373.6787 | 12 | 0.4 | 687.8469 | 2 | 23.22 | 5 | F5:6477 | NaNaKA16\_F13.raw |  |  |  |  | 5.9896E5 |  |  |  |  |  |  |  |  | 1 | 0 | 0 | 0 | 0 | 1 | 0 | 0 | 0 | 0 | 0 | 0 | 0 | 0 | 176 | 187 |  |  | PEAKS DB |
| K.C(+57.02)GDGMVC(+57.02)SNR.Q | N | 51.31 | 1154.4268 | 10 | 1.1 | 578.2213 | 2 | 11.85 | 5 | F5:1806 | NaNaKA16\_F13.raw |  |  |  |  | 4.5755E5 |  |  |  |  |  |  |  |  | 1 | 0 | 0 | 0 | 0 | 1 | 0 | 0 | 0 | 0 | 0 | 0 | 0 | 0 | 582 | 591 | Carbamidomethylation | C1:Carbamidomethylation:1000.00;C7:Carbamidomethylation:1000.00 | PEAKS DB |
| N.INEINVQSDVK.A | Y | 50.36 | 1257.6565 | 11 | 0.0 | 629.8355 | 2 | 23.51 | 5 | F5:6491 | NaNaKA16\_F13.raw |  |  |  |  | 2.41E6 |  |  |  |  |  |  |  |  | 1 | 0 | 0 | 0 | 0 | 1 | 0 | 0 | 0 | 0 | 0 | 0 | 0 | 0 | 249 | 259 |  |  | PEAKS DB |
| Q.SAEC(+57.02)PTDVFQR.N | N | 49.48 | 1308.5768 | 11 | 0.4 | 655.2960 | 2 | 32.04 | 5 | F5:11429 | NaNaKA16\_F13.raw |  |  |  |  | 1.3941E7 |  |  |  |  |  |  |  |  | 1 | 0 | 0 | 0 | 0 | 1 | 0 | 0 | 0 | 0 | 0 | 0 | 0 | 0 | 471 | 481 | Carbamidomethylation | C4:Carbamidomethylation:1000.00 | PEAKS DB |
| Y.MVVDNIMYR.H | Y | 48.66 | 1139.5468 | 9 | 0.0 | 570.7806 | 2 | 44.92 | 5 | F5:18068 | NaNaKA16\_F13.raw |  |  |  |  | 7.0995E7 |  |  |  |  |  |  |  |  | 1 | 0 | 0 | 0 | 0 | 1 | 0 | 0 | 0 | 0 | 0 | 0 | 0 | 0 | 199 | 207 |  |  | PEAKS DB |
| S.NINEINVQSDVK.A | Y | 48.17 | 1371.6993 | 12 | 0.3 | 686.8572 | 2 | 27.44 | 5 | F5:8958 | NaNaKA16\_F13.raw |  |  |  |  | 1.1123E6 |  |  |  |  |  |  |  |  | 1 | 0 | 0 | 0 | 0 | 1 | 0 | 0 | 0 | 0 | 0 | 0 | 0 | 0 | 248 | 259 |  |  | PEAKS DB |
| N.SMIC(+57.02)NC(+57.02)SISPR.D | N | 47.00 | 1323.5734 | 11 | 3.9 | 662.7966 | 2 | 23.55 | 5 | F5:6539 | NaNaKA16\_F13.raw |  |  |  |  | 0 |  |  |  |  |  |  |  |  | 0 | 0 | 0 | 0 | 0 | 0 | 0 | 0 | 0 | 0 | 0 | 0 | 0 | 0 | 559 | 569 | Carbamidomethylation | C4:Carbamidomethylation:1000.00;C6:Carbamidomethylation:1000.00 | PEAKS DB |
| K.LQHEAQC(+57.02)DSEEC(+57.02)C(+57.02)EK.C | N | 46.38 | 1921.7240 | 15 | 0.0 | 641.5820 | 3 | 11.83 | 5 | F5:1808 | NaNaKA16\_F13.raw |  |  |  |  | 2.9375E5 |  |  |  |  |  |  |  |  | 1 | 0 | 0 | 0 | 0 | 1 | 0 | 0 | 0 | 0 | 0 | 0 | 0 | 0 | 430 | 444 | Carbamidomethylation | C7:Carbamidomethylation:1000.00;C12:Carbamidomethylation:1000.00;C13:Carbamidomethylation:1000.00 | PEAKS DB |
| R.DRPQC(+57.02)ILNKPLST.D | N | 46.29 | 1540.8031 | 13 | -0.4 | 514.6081 | 3 | 27.16 | 4 | F4:11109 | NaNaKA16\_F12.raw |  |  |  | 7.0589E5 |  |  |  | 8.268E5 |  |  |  |  |  | 2 | 0 | 0 | 0 | 1 | 0 | 0 | 0 | 1 | 0 | 0 | 0 | 0 | 0 | 379 | 391 | Carbamidomethylation | C5:Carbamidomethylation:1000.00 | PEAKS DB |
| K.C(+57.02)GDGM(+15.99)VC(+57.02)SNR.Q | N | 45.27 | 1170.4216 | 10 | 0.3 | 586.2183 | 2 | 11.84 | 5 | F5:1815 | NaNaKA16\_F13.raw |  |  |  |  | 9.9702E4 |  |  |  |  |  |  |  |  | 1 | 0 | 0 | 0 | 0 | 1 | 0 | 0 | 0 | 0 | 0 | 0 | 0 | 0 | 582 | 591 | Carbamidomethylation; Oxidation (M) | C1:Carbamidomethylation:1000.00;M5:Oxidation (M):1000.00;C7:Carbamidomethylation:1000.00 | PEAKS DB |
| Y.MVVDNIM(+15.99)YR.H | Y | 41.81 | 1155.5416 | 9 | -0.2 | 578.7780 | 2 | 32.88 | 5 | F5:12042 | NaNaKA16\_F13.raw |  |  |  |  | 6.8639E6 |  |  |  |  |  |  |  |  | 1 | 0 | 0 | 0 | 0 | 1 | 0 | 0 | 0 | 0 | 0 | 0 | 0 | 0 | 199 | 207 | Oxidation (M) | M7:Oxidation (M):95.35 | PEAKS DB |
| total 34 peptides |  |  |  |  |  |  |  |  |  |  |  |  |  |  |  |  |  |  |  |  |  |  |  |  |  |  |  |  |  |  |  |  |  |  |  |  |  |  |  |  |  |  |  |
| --- | --- | --- | --- | --- | --- | --- | --- | --- | --- | --- | --- | --- | --- | --- | --- | --- | --- | --- | --- | --- | --- | --- | --- | --- | --- | --- | --- | --- | --- | --- | --- | --- | --- | --- | --- | --- | --- | --- | --- | --- | --- | --- | --- |

XP\_026544671.1

back to list

  

| Protein Coverage
| Supporting Peptides
|

Protein Coverage:

Supporting Peptides:

| Peptide | Uniq | -10lgP | Mass | Length | ppm | m/z | z | RT | Fraction | Scan | Source File | Area F1 | Area F10 | Area F11 | Area F12 | Area F13 | Area F2 | Area F3 | Area F4 | Area F5 | Area F6 | Area F7 | Area F8 | Area F9 | #Feature | #Feature F1 | #Feature F10 | #Feature F11 | #Feature F12 | #Feature F13 | #Feature F2 | #Feature F3 | #Feature F4 | #Feature F5 | #Feature F6 | #Feature F7 | #Feature F8 | #Feature F9 | Start | End | PTM | AScore | Found By |
| --- | --- | --- | --- | --- | --- | --- | --- | --- | --- | --- | --- | --- | --- | --- | --- | --- | --- | --- | --- | --- | --- | --- | --- | --- | --- | --- | --- | --- | --- | --- | --- | --- | --- | --- | --- | --- | --- | --- | --- | --- | --- | --- | --- |
| K.GGAYSYIESHPWQAGIFVK.N | N | 101.78 | 2109.0320 | 19 | -0.3 | 1055.5229 | 2 | 65.05 | 4 | F4:42731 | NaNaKA16\_F12.raw |  | 5.8839E6 | 1.1656E7 | 5.0772E8 |  |  |  |  |  |  |  | 0 | 1.9031E6 | 10 | 0 | 1 | 2 | 6 | 0 | 0 | 0 | 0 | 0 | 0 | 0 | 0 | 1 | 292 | 310 |  |  | PEAKS DB |
| I.KGGAYSYIESHPWQAGIFVK.N | N | 92.59 | 2237.1270 | 20 | 0.8 | 746.7169 | 3 | 55.07 | 4 | F4:34289 | NaNaKA16\_F12.raw |  |  | 2.0972E6 | 3.4652E7 |  |  |  |  |  |  |  |  |  | 3 | 0 | 0 | 1 | 2 | 0 | 0 | 0 | 0 | 0 | 0 | 0 | 0 | 0 | 291 | 310 |  |  | PEAKS DB |
| Q.LPDWTEC(+57.02)EVSGYGK.H | N | 89.52 | 1639.7188 | 14 | 0.4 | 820.8669 | 2 | 53.18 | 4 | F4:33245 | NaNaKA16\_F12.raw |  | 2.4114E6 | 1.7456E7 | 5.2898E8 | 5.0846E5 |  |  |  |  |  |  |  |  | 6 | 0 | 1 | 1 | 3 | 1 | 0 | 0 | 0 | 0 | 0 | 0 | 0 | 0 | 416 | 429 | Carbamidomethylation | C7:Carbamidomethylation:1000.00 | PEAKS DB |
| Y.SYIESHPWQAGIFVK.N | N | 86.86 | 1760.8885 | 15 | 0.0 | 881.4515 | 2 | 54.37 | 4 | F4:33882 | NaNaKA16\_F12.raw |  |  |  | 1.4376E7 |  |  |  |  |  |  |  |  |  | 2 | 0 | 0 | 0 | 2 | 0 | 0 | 0 | 0 | 0 | 0 | 0 | 0 | 0 | 296 | 310 |  |  | PEAKS DB |
| K.ALYSPNLFIC(+57.02)FC(+57.02)PPGFSGK.F | N | 84.91 | 2174.0330 | 19 | 0.6 | 1088.0244 | 2 | 95.42 | 12 | F12:68966 | NaNaKA16\_F8.raw |  | 3.6583E5 |  |  |  |  |  |  |  | 1.5307E7 | 5.8251E7 | 1.0308E7 | 4.354E6 | 9 | 0 | 1 | 0 | 0 | 0 | 0 | 0 | 0 | 0 | 2 | 2 | 2 | 2 | 77 | 95 | Carbamidomethylation | C10:Carbamidomethylation:1000.00;C12:Carbamidomethylation:1000.00 | PEAKS DB |
| R.TVTENMLC(+57.02)AGDTR.Q | N | 84.34 | 1466.6494 | 13 | -0.1 | 734.3319 | 2 | 30.20 | 4 | F4:13667 | NaNaKA16\_F12.raw |  |  |  | 9.0057E7 |  |  |  |  |  |  |  |  |  | 2 | 0 | 0 | 0 | 2 | 0 | 0 | 0 | 0 | 0 | 0 | 0 | 0 | 0 | 463 | 475 | Carbamidomethylation | C8:Carbamidomethylation:1000.00 | PEAKS DB |
| R.GTWSTTESGAEC(+57.02)VNWK.T | N | 79.36 | 1811.7784 | 16 | 0.6 | 906.8970 | 2 | 40.11 | 11 | F11:23748 | NaNaKA16\_F7.raw |  |  |  | 3.6435E6 |  |  |  |  |  | 2.669E7 | 9.2931E6 |  |  | 3 | 0 | 0 | 0 | 1 | 0 | 0 | 0 | 0 | 0 | 1 | 1 | 0 | 0 | 115 | 130 | Carbamidomethylation | C12:Carbamidomethylation:1000.00 | PEAKS DB |
| R.LYPDSFC(+57.02)TSAR.L | N | 69.84 | 1315.5867 | 11 | 0.6 | 658.8010 | 2 | 33.08 | 4 | F4:16532 | NaNaKA16\_F12.raw |  |  |  | 5.6231E8 | 6.5311E5 |  |  |  |  |  |  |  |  | 2 | 0 | 0 | 0 | 1 | 1 | 0 | 0 | 0 | 0 | 0 | 0 | 0 | 0 | 448 | 458 | Carbamidomethylation | C7:Carbamidomethylation:1000.00 | PEAKS DB |
| Q.LGLGDHNYC(+57.02)R.N | N | 69.80 | 1203.5455 | 10 | 0.7 | 602.7805 | 2 | 12.35 | 11 | F11:2053 | NaNaKA16\_F7.raw |  |  |  |  |  |  |  |  |  | 2.6837E5 | 1.9952E6 |  |  | 3 | 0 | 0 | 0 | 0 | 0 | 0 | 0 | 0 | 0 | 1 | 2 | 0 | 0 | 150 | 159 | Carbamidomethylation | C9:Carbamidomethylation:1000.00 | PEAKS DB |
| R.TVTENM(+15.99)LC(+57.02)AGDTR.Q | N | 67.27 | 1482.6443 | 13 | 0.9 | 742.3301 | 2 | 28.18 | 4 | F4:11849 | NaNaKA16\_F12.raw |  |  |  | 6.8651E6 |  |  |  |  |  |  |  |  |  | 5 | 0 | 0 | 0 | 5 | 0 | 0 | 0 | 0 | 0 | 0 | 0 | 0 | 0 | 463 | 475 | Oxidation (M); Carbamidomethylation | M6:Oxidation (M):1000.00;C8:Carbamidomethylation:1000.00 | PEAKS DB |
| Q.IKGGAYSYIESHPWQAGIFVK.N | N | 64.84 | 2350.2109 | 21 | 0.7 | 588.5604 | 4 | 57.69 | 4 | F4:36700 | NaNaKA16\_F12.raw |  |  | 1.8752E6 | 1.979E7 |  |  |  |  |  |  |  |  |  | 3 | 0 | 0 | 1 | 2 | 0 | 0 | 0 | 0 | 0 | 0 | 0 | 0 | 0 | 290 | 310 |  |  | PEAKS DB |
| K.HEMSSPFHSER.L | Y | 63.71 | 1342.5724 | 11 | -0.1 | 448.5314 | 3 | 11.45 | 4 | F4:1812 | NaNaKA16\_F12.raw |  |  |  | 1.6186E6 |  |  |  |  |  |  |  |  |  | 1 | 0 | 0 | 0 | 1 | 0 | 0 | 0 | 0 | 0 | 0 | 0 | 0 | 0 | 430 | 440 |  |  | PEAKS DB |
| K.SSKPWC(+57.02)HVLK.R | N | 63.39 | 1240.6387 | 10 | 0.1 | 414.5535 | 3 | 12.10 | 11 | F11:1845 | NaNaKA16\_F7.raw |  |  |  |  |  |  |  |  |  |  | 2.6656E6 |  |  | 1 | 0 | 0 | 0 | 0 | 0 | 0 | 0 | 0 | 0 | 0 | 1 | 0 | 0 | 164 | 173 | Carbamidomethylation | C6:Carbamidomethylation:1000.00 | PEAKS DB |
| K.HEM(+15.99)SSPFHSER.L | Y | 63.34 | 1358.5674 | 11 | -0.2 | 453.8630 | 3 | 11.45 | 4 | F4:1807 | NaNaKA16\_F12.raw |  |  |  | 4.1513E5 |  |  |  |  |  |  |  |  |  | 1 | 0 | 0 | 0 | 1 | 0 | 0 | 0 | 0 | 0 | 0 | 0 | 0 | 0 | 430 | 440 | Oxidation (M) | M3:Oxidation (M):1000.00 | PEAKS DB |
| N.LFIC(+57.02)FC(+57.02)PPGFSGK.F | N | 61.36 | 1528.7206 | 13 | -0.1 | 765.3675 | 2 | 80.11 | 10 | F10:58024 | NaNaKA16\_F6.raw |  |  |  |  |  |  |  |  |  | 1.3335E7 | 7.5039E6 |  |  | 3 | 0 | 0 | 0 | 0 | 0 | 0 | 0 | 0 | 0 | 1 | 2 | 0 | 0 | 83 | 95 | Carbamidomethylation | C4:Carbamidomethylation:1000.00;C6:Carbamidomethylation:1000.00 | PEAKS DB |
| F.IC(+57.02)FC(+57.02)PPGFSGK.F | N | 58.86 | 1268.5681 | 11 | 0.1 | 635.2914 | 2 | 51.30 | 10 | F10:33995 | NaNaKA16\_F6.raw |  |  |  |  |  |  |  |  |  | 1.0459E7 | 5.3422E6 | 2.3238E6 |  | 3 | 0 | 0 | 0 | 0 | 0 | 0 | 0 | 0 | 0 | 1 | 1 | 1 | 0 | 85 | 95 | Carbamidomethylation | C2:Carbamidomethylation:1000.00;C4:Carbamidomethylation:1000.00 | PEAKS DB |
| R.TLDNDIALLK.L | N | 56.75 | 1114.6234 | 10 | 0.2 | 558.3191 | 2 | 53.18 | 4 | F4:32689 | NaNaKA16\_F12.raw |  |  | 4.6197E6 | 4.3448E8 |  |  |  |  |  |  |  |  |  | 3 | 0 | 0 | 1 | 2 | 0 | 0 | 0 | 0 | 0 | 0 | 0 | 0 | 0 | 380 | 389 |  |  | PEAKS DB |
| K.GGAYSYIESHPWQAGIFVKNR.G | N | 56.73 | 2379.1760 | 21 | 0.6 | 595.8016 | 4 | 55.04 | 4 | F4:34397 | NaNaKA16\_F12.raw |  |  |  | 1.2222E6 |  |  |  |  |  |  |  |  |  | 1 | 0 | 0 | 0 | 1 | 0 | 0 | 0 | 0 | 0 | 0 | 0 | 0 | 0 | 292 | 312 |  |  | PEAKS DB |
| Y.SPNLFIC(+57.02)FC(+57.02)PPGFSGK.F | N | 49.42 | 1826.8484 | 16 | 1.4 | 914.4327 | 2 | 84.61 | 12 | F12:60077 | NaNaKA16\_F8.raw |  |  |  |  |  |  |  |  |  |  | 5.8546E5 | 7.1533E5 |  | 2 | 0 | 0 | 0 | 0 | 0 | 0 | 0 | 0 | 0 | 0 | 1 | 1 | 0 | 80 | 95 | Carbamidomethylation | C7:Carbamidomethylation:1000.00;C9:Carbamidomethylation:1000.00 | PEAKS DB |
| R.TLDNDIALLKLK.H | N | 47.35 | 1355.8024 | 12 | 0.6 | 452.9417 | 3 | 55.08 | 4 | F4:34569 | NaNaKA16\_F12.raw |  |  |  | 8.5072E5 |  |  |  |  |  |  |  |  |  | 1 | 0 | 0 | 0 | 1 | 0 | 0 | 0 | 0 | 0 | 0 | 0 | 0 | 0 | 380 | 391 |  |  | PEAKS DB |
| Y.IESHPWQAGIFVK.N | N | 45.33 | 1510.7932 | 13 | 0.4 | 504.6052 | 3 | 44.51 | 4 | F4:25555 | NaNaKA16\_F12.raw |  |  |  | 7.3497E5 |  |  |  |  |  |  |  |  |  | 1 | 0 | 0 | 0 | 1 | 0 | 0 | 0 | 0 | 0 | 0 | 0 | 0 | 0 | 298 | 310 |  |  | PEAKS DB |
| K.QAYNWKESWLR.R | Y | 43.79 | 1479.7258 | 11 | 0.4 | 494.2494 | 3 | 39.94 | 11 | F11:23494 | NaNaKA16\_F7.raw |  |  |  |  |  |  |  |  |  |  | 8.9589E5 |  |  | 1 | 0 | 0 | 0 | 0 | 0 | 0 | 0 | 0 | 0 | 0 | 1 | 0 | 0 | 27 | 37 |  |  | PEAKS DB |
| I.ESHPWQAGIFVK.N | N | 42.01 | 1397.7091 | 12 | -1.2 | 699.8610 | 2 | 39.12 | 13 | F13:19537 | NaNaKA16\_F9.raw |  |  |  |  |  |  |  |  |  |  |  |  | 6.7594E5 | 1 | 0 | 0 | 0 | 0 | 0 | 0 | 0 | 0 | 0 | 0 | 0 | 0 | 1 | 299 | 310 |  |  | PEAKS DB |
| total 23 peptides |  |  |  |  |  |  |  |  |  |  |  |  |  |  |  |  |  |  |  |  |  |  |  |  |  |  |  |  |  |  |  |  |  |  |  |  |  |  |  |  |  |  |  |
| --- | --- | --- | --- | --- | --- | --- | --- | --- | --- | --- | --- | --- | --- | --- | --- | --- | --- | --- | --- | --- | --- | --- | --- | --- | --- | --- | --- | --- | --- | --- | --- | --- | --- | --- | --- | --- | --- | --- | --- | --- | --- | --- | --- |

ACH73168.1

back to list

  

| Protein Coverage
| Supporting Peptides
|

Protein Coverage:

Supporting Peptides:

| Peptide | Uniq | -10lgP | Mass | Length | ppm | m/z | z | RT | Fraction | Scan | Source File | Area F1 | Area F10 | Area F11 | Area F12 | Area F13 | Area F2 | Area F3 | Area F4 | Area F5 | Area F6 | Area F7 | Area F8 | Area F9 | #Feature | #Feature F1 | #Feature F10 | #Feature F11 | #Feature F12 | #Feature F13 | #Feature F2 | #Feature F3 | #Feature F4 | #Feature F5 | #Feature F6 | #Feature F7 | #Feature F8 | #Feature F9 | Start | End | PTM | AScore | Found By |
| --- | --- | --- | --- | --- | --- | --- | --- | --- | --- | --- | --- | --- | --- | --- | --- | --- | --- | --- | --- | --- | --- | --- | --- | --- | --- | --- | --- | --- | --- | --- | --- | --- | --- | --- | --- | --- | --- | --- | --- | --- | --- | --- | --- |
| K.IGC(+57.02)GENLFMSSQPYAWSR.V | Y | 91.49 | 2101.9351 | 18 | 0.0 | 1051.9749 | 2 | 72.04 | 3 | F3:46337 | NaNaKA16\_F11.raw |  | 1.3602E7 | 3.9929E8 | 3.8506E7 |  |  |  |  |  |  |  |  |  | 6 | 0 | 2 | 2 | 2 | 0 | 0 | 0 | 0 | 0 | 0 | 0 | 0 | 0 | 90 | 107 | Carbamidomethylation | C3:Carbamidomethylation:1000.00 | PEAKS DB |
| K.YLYVC(+57.02)QYC(+57.02)PAGNIIGSIATPYK.S | N | 91.36 | 2550.2288 | 22 | 1.1 | 1276.1230 | 2 | 83.73 | 3 | F3:56509 | NaNaKA16\_F11.raw |  | 2.391E7 | 7.701E7 | 4.861E7 |  |  |  |  |  |  |  |  |  | 8 | 0 | 2 | 4 | 2 | 0 | 0 | 0 | 0 | 0 | 0 | 0 | 0 | 0 | 158 | 179 | Carbamidomethylation | C5:Carbamidomethylation:1000.00;C8:Carbamidomethylation:1000.00 | PEAKS DB |
| R.NMLQMEWNSNAAQNAK.R | N | 89.24 | 1848.8247 | 16 | -0.1 | 925.4196 | 2 | 49.03 | 3 | F3:28088 | NaNaKA16\_F11.raw |  | 9.3217E7 | 1.2893E9 | 8.3194E7 |  |  |  |  |  |  |  |  |  | 10 | 0 | 1 | 7 | 2 | 0 | 0 | 0 | 0 | 0 | 0 | 0 | 0 | 0 | 54 | 69 |  |  | PEAKS DB |
| R.NMLQM(+15.99)EWNSNAAQNAK.R | N | 83.61 | 1864.8196 | 16 | 0.5 | 933.4175 | 2 | 35.34 | 3 | F3:18162 | NaNaKA16\_F11.raw |  |  | 2.1836E8 | 1.6881E7 |  |  |  |  |  |  |  |  |  | 9 | 0 | 0 | 6 | 3 | 0 | 0 | 0 | 0 | 0 | 0 | 0 | 0 | 0 | 54 | 69 | Oxidation (M) | M5:Oxidation (M):78.06 | PEAKS DB |
| R.NM(+15.99)LQMEWNSNAAQNAK.R | N | 82.80 | 1864.8196 | 16 | 2.7 | 933.4196 | 2 | 48.27 | 3 | F3:27735 | NaNaKA16\_F11.raw |  |  | 2.3687E8 | 8.0538E6 |  |  |  |  |  |  |  |  |  | 9 | 0 | 0 | 8 | 1 | 0 | 0 | 0 | 0 | 0 | 0 | 0 | 0 | 0 | 54 | 69 | Oxidation (M) | M2:Oxidation (M):91.37 | PEAKS DB |
| Q.YC(+57.02)PAGNIIGSIATPYK.S | N | 81.36 | 1723.8604 | 16 | 0.9 | 862.9382 | 2 | 63.36 | 3 | F3:39220 | NaNaKA16\_F11.raw |  |  | 2.5562E7 |  |  |  |  |  |  |  |  |  |  | 2 | 0 | 0 | 2 | 0 | 0 | 0 | 0 | 0 | 0 | 0 | 0 | 0 | 0 | 164 | 179 | Carbamidomethylation | C2:Carbamidomethylation:1000.00 | PEAKS DB |
| H.YTQIVWYNSHLLGC(+57.02)GAAK.C | Y | 80.75 | 2080.0200 | 18 | -0.1 | 1041.0172 | 2 | 55.41 | 3 | F3:32205 | NaNaKA16\_F11.raw |  |  | 2.0551E7 | 1.9181E6 |  |  |  |  |  |  |  |  |  | 3 | 0 | 0 | 2 | 1 | 0 | 0 | 0 | 0 | 0 | 0 | 0 | 0 | 0 | 135 | 152 | Carbamidomethylation | C14:Carbamidomethylation:1000.00 | PEAKS DB |
| K.IGC(+57.02)GENLFM(+15.99)SSQPYAWSR.V | Y | 79.45 | 2117.9299 | 18 | 1.3 | 1059.9736 | 2 | 64.65 | 3 | F3:40437 | NaNaKA16\_F11.raw |  | 1.0083E8 | 8.4481E8 | 6.9317E7 |  |  |  |  |  |  |  |  |  | 9 | 0 | 2 | 3 | 4 | 0 | 0 | 0 | 0 | 0 | 0 | 0 | 0 | 0 | 90 | 107 | Carbamidomethylation; Oxidation (M) | C3:Carbamidomethylation:1000.00;M9:Oxidation (M):1000.00 | PEAKS DB |
| C.QYC(+57.02)PAGNIIGSIATPYK.S | N | 76.59 | 1851.9188 | 17 | 0.8 | 926.9674 | 2 | 64.28 | 3 | F3:39934 | NaNaKA16\_F11.raw |  |  | 6.9974E6 |  |  |  |  |  |  |  |  |  |  | 3 | 0 | 0 | 3 | 0 | 0 | 0 | 0 | 0 | 0 | 0 | 0 | 0 | 0 | 163 | 179 | Carbamidomethylation | C3:Carbamidomethylation:1000.00 | PEAKS DB |
| Y.C(+57.02)PAGNIIGSIATPYK.S | N | 75.44 | 1560.7970 | 15 | 0.4 | 781.4061 | 2 | 55.64 | 3 | F3:32530 | NaNaKA16\_F11.raw |  |  | 1.6565E7 | 2.6149E6 |  |  |  |  |  |  |  |  |  | 5 | 0 | 0 | 3 | 2 | 0 | 0 | 0 | 0 | 0 | 0 | 0 | 0 | 0 | 165 | 179 | Carbamidomethylation | C1:Carbamidomethylation:1000.00 | PEAKS DB |
| K.FVYGVGANPPGSVIGH.Y | N | 74.39 | 1569.7939 | 16 | 0.1 | 785.9044 | 2 | 54.74 | 4 | F4:34170 | NaNaKA16\_F12.raw |  | 1.3128E6 | 2.8221E7 | 6.0658E6 |  |  |  |  |  |  |  |  |  | 3 | 0 | 1 | 1 | 1 | 0 | 0 | 0 | 0 | 0 | 0 | 0 | 0 | 0 | 119 | 134 |  |  | PEAKS DB |
| R.NMLQMEWNSNAAQNAKR.W | N | 70.72 | 2004.9258 | 17 | -0.1 | 669.3158 | 3 | 36.44 | 3 | F3:17827 | NaNaKA16\_F11.raw |  |  | 4.2399E6 |  |  |  |  |  |  |  |  |  |  | 1 | 0 | 0 | 1 | 0 | 0 | 0 | 0 | 0 | 0 | 0 | 0 | 0 | 0 | 54 | 70 |  |  | PEAKS DB |
| Q.IVWYNSHLLGC(+57.02)GAAK.C | Y | 66.82 | 1687.8505 | 15 | -0.1 | 563.6240 | 3 | 45.96 | 3 | F3:25859 | NaNaKA16\_F11.raw |  |  | 1.167E7 |  |  |  |  |  |  |  |  |  |  | 2 | 0 | 0 | 2 | 0 | 0 | 0 | 0 | 0 | 0 | 0 | 0 | 0 | 0 | 138 | 152 | Carbamidomethylation | C11:Carbamidomethylation:1000.00 | PEAKS DB |
| K.SGPPC(+57.02)GDC(+57.02)PSAC(+57.02)VNGLC(+57.02)TNPC(+57.02)K.H | N | 64.25 | 2406.9482 | 22 | 2.2 | 803.3251 | 3 | 33.04 | 3 | F3:14934 | NaNaKA16\_F11.raw |  |  | 4.4345E7 | 3.0856E6 |  |  |  |  |  |  |  |  |  | 2 | 0 | 0 | 1 | 1 | 0 | 0 | 0 | 0 | 0 | 0 | 0 | 0 | 0 | 180 | 201 | Carbamidomethylation | C5:Carbamidomethylation:1000.00;C8:Carbamidomethylation:1000.00;C12:Carbamidomethylation:1000.00;C17:Carbamidomethylation:1000.00;C21:Carbamidomethylation:1000.00 | PEAKS DB |
| N.PPGSVIGHYTQIVWYNSHLLGC(+57.02)GAAK.C | Y | 63.95 | 2824.4119 | 26 | 1.2 | 707.1111 | 4 | 68.85 | 3 | F3:43871 | NaNaKA16\_F11.raw |  | 2.0238E6 | 6.4925E6 |  |  |  |  |  |  |  |  |  |  | 2 | 0 | 1 | 1 | 0 | 0 | 0 | 0 | 0 | 0 | 0 | 0 | 0 | 0 | 127 | 152 | Carbamidomethylation | C22:Carbamidomethylation:1000.00 | PEAKS DB |
| N.MLQMEWNSNAAQNAK.R | N | 61.96 | 1734.7817 | 15 | 0.2 | 868.3983 | 2 | 42.10 | 3 | F3:22578 | NaNaKA16\_F11.raw |  |  | 1.0377E7 |  |  |  |  |  |  |  |  |  |  | 1 | 0 | 0 | 1 | 0 | 0 | 0 | 0 | 0 | 0 | 0 | 0 | 0 | 0 | 55 | 69 |  |  | PEAKS DB |
| Y.VC(+57.02)QYC(+57.02)PAGNIIGSIATPYK.S | N | 60.72 | 2111.0181 | 19 | 0.5 | 1056.5168 | 2 | 67.50 | 3 | F3:42701 | NaNaKA16\_F11.raw |  |  | 3.3686E6 | 2.1087E6 |  |  |  |  |  |  |  |  |  | 2 | 0 | 0 | 1 | 1 | 0 | 0 | 0 | 0 | 0 | 0 | 0 | 0 | 0 | 161 | 179 | Carbamidomethylation | C2:Carbamidomethylation:1000.00;C5:Carbamidomethylation:1000.00 | PEAKS DB |
| A.GNIIGSIATPYK.S | N | 58.43 | 1232.6765 | 12 | -0.2 | 617.3454 | 2 | 46.96 | 3 | F3:26760 | NaNaKA16\_F11.raw |  |  | 2.4959E6 |  |  |  |  |  |  |  |  |  |  | 1 | 0 | 0 | 1 | 0 | 0 | 0 | 0 | 0 | 0 | 0 | 0 | 0 | 0 | 168 | 179 |  |  | PEAKS DB |
| K.QNAC(+57.02)QTEWMK.S | Y | 58.40 | 1294.5435 | 10 | 0.1 | 648.2791 | 2 | 41.31 | 3 | F3:21944 | NaNaKA16\_F11.raw |  |  | 4.4811E5 |  |  |  |  |  |  |  |  |  |  | 1 | 0 | 0 | 1 | 0 | 0 | 0 | 0 | 0 | 0 | 0 | 0 | 0 | 0 | 215 | 224 | Carbamidomethylation | C4:Carbamidomethylation:1000.00 | PEAKS DB |
| K.FVYGVGANPPGSVIGHY.T | N | 57.66 | 1732.8573 | 17 | 1.1 | 867.4369 | 2 | 61.67 | 3 | F3:37669 | NaNaKA16\_F11.raw |  |  | 5.3586E6 |  |  |  |  |  |  |  |  |  |  | 1 | 0 | 0 | 1 | 0 | 0 | 0 | 0 | 0 | 0 | 0 | 0 | 0 | 0 | 119 | 135 |  |  | PEAKS DB |
| C.PAGNIIGSIATPYK.S | N | 56.00 | 1400.7664 | 14 | 0.0 | 701.3904 | 2 | 51.58 | 3 | F3:29077 | NaNaKA16\_F11.raw |  |  | 7.112E5 |  |  |  |  |  |  |  |  |  |  | 1 | 0 | 0 | 1 | 0 | 0 | 0 | 0 | 0 | 0 | 0 | 0 | 0 | 0 | 166 | 179 |  |  | PEAKS DB |
| K.FVYGVGANPPGSVIGHYTQIVWYNSHLLGC(+57.02)GAAK.C | Y | 54.12 | 3631.8035 | 34 | 0.9 | 908.9590 | 4 | 82.67 | 4 | F4:57037 | NaNaKA16\_F12.raw |  | 5.2429E6 |  | 1.58E7 |  |  |  |  |  |  |  |  |  | 2 | 0 | 1 | 0 | 1 | 0 | 0 | 0 | 0 | 0 | 0 | 0 | 0 | 0 | 119 | 152 | Carbamidomethylation | C30:Carbamidomethylation:1000.00 | PEAKS DB |
| R.VIQSWYDENKK.F | N | 54.01 | 1408.6986 | 11 | -0.3 | 705.3564 | 2 | 11.80 | 3 | F3:2163 | NaNaKA16\_F11.raw |  |  | 6.7786E6 | 1.1269E6 |  |  |  |  |  |  |  |  |  | 3 | 0 | 0 | 2 | 1 | 0 | 0 | 0 | 0 | 0 | 0 | 0 | 0 | 0 | 108 | 118 |  |  | PEAKS DB |
| R.C(+57.02)SFAHSPPHLR.T | N | 52.64 | 1307.6193 | 11 | 0.9 | 436.8807 | 3 | 11.47 | 4 | F4:1824 | NaNaKA16\_F12.raw |  |  |  | 3.3923E5 |  |  |  |  |  |  |  |  |  | 1 | 0 | 0 | 0 | 1 | 0 | 0 | 0 | 0 | 0 | 0 | 0 | 0 | 0 | 75 | 85 | Carbamidomethylation | C1:Carbamidomethylation:1000.00 | PEAKS DB |
| K.YLYVC(+57.02)QYC(+57.02)PAGN.I | N | 48.44 | 1506.6272 | 12 | 0.2 | 754.3210 | 2 | 57.46 | 3 | F3:34059 | NaNaKA16\_F11.raw |  |  | 1.5296E7 | 4.5046E6 |  |  |  |  |  |  |  |  |  | 2 | 0 | 0 | 1 | 1 | 0 | 0 | 0 | 0 | 0 | 0 | 0 | 0 | 0 | 158 | 169 | Carbamidomethylation | C5:Carbamidomethylation:1000.00;C8:Carbamidomethylation:1000.00 | PEAKS DB |
| Y.TQIVWYNSHLLGC(+57.02)GAAK.C | Y | 46.64 | 1916.9567 | 17 | 1.2 | 639.9936 | 3 | 50.10 | 3 | F3:28369 | NaNaKA16\_F11.raw |  |  | 0 |  |  |  |  |  |  |  |  |  |  | 0 | 0 | 0 | 0 | 0 | 0 | 0 | 0 | 0 | 0 | 0 | 0 | 0 | 0 | 136 | 152 | Carbamidomethylation | C13:Carbamidomethylation:1000.00 | PEAKS DB |
| K.FVYGVGANPPGSVIGHYTQ.I | N | 45.19 | 1961.9635 | 19 | 1.6 | 981.9906 | 2 | 60.19 | 3 | F3:36550 | NaNaKA16\_F11.raw |  |  | 4.6839E6 |  |  |  |  |  |  |  |  |  |  | 1 | 0 | 0 | 1 | 0 | 0 | 0 | 0 | 0 | 0 | 0 | 0 | 0 | 0 | 119 | 137 |  |  | PEAKS DB |
| total 27 peptides |  |  |  |  |  |  |  |  |  |  |  |  |  |  |  |  |  |  |  |  |  |  |  |  |  |  |  |  |  |  |  |  |  |  |  |  |  |  |  |  |  |  |  |
| --- | --- | --- | --- | --- | --- | --- | --- | --- | --- | --- | --- | --- | --- | --- | --- | --- | --- | --- | --- | --- | --- | --- | --- | --- | --- | --- | --- | --- | --- | --- | --- | --- | --- | --- | --- | --- | --- | --- | --- | --- | --- | --- | --- |

P84808.2

back to list

  

| Protein Coverage
| Supporting Peptides
|

Protein Coverage:

Supporting Peptides:

| Peptide | Uniq | -10lgP | Mass | Length | ppm | m/z | z | RT | Fraction | Scan | Source File | Area F1 | Area F10 | Area F11 | Area F12 | Area F13 | Area F2 | Area F3 | Area F4 | Area F5 | Area F6 | Area F7 | Area F8 | Area F9 | #Feature | #Feature F1 | #Feature F10 | #Feature F11 | #Feature F12 | #Feature F13 | #Feature F2 | #Feature F3 | #Feature F4 | #Feature F5 | #Feature F6 | #Feature F7 | #Feature F8 | #Feature F9 | Start | End | PTM | AScore | Found By |
| --- | --- | --- | --- | --- | --- | --- | --- | --- | --- | --- | --- | --- | --- | --- | --- | --- | --- | --- | --- | --- | --- | --- | --- | --- | --- | --- | --- | --- | --- | --- | --- | --- | --- | --- | --- | --- | --- | --- | --- | --- | --- | --- | --- |
| K.IGC(+57.02)GENLFMSSQPYAWSR.V | Y | 91.49 | 2101.9351 | 18 | 0.0 | 1051.9749 | 2 | 72.04 | 3 | F3:46337 | NaNaKA16\_F11.raw |  | 1.3602E7 | 3.9929E8 | 3.8506E7 |  |  |  |  |  |  |  |  |  | 6 | 0 | 2 | 2 | 2 | 0 | 0 | 0 | 0 | 0 | 0 | 0 | 0 | 0 | 90 | 107 | Carbamidomethylation | C3:Carbamidomethylation:1000.00 | PEAKS DB |
| K.YLYVC(+57.02)QYC(+57.02)PAGNIIGSIATPYK.S | N | 91.36 | 2550.2288 | 22 | 1.1 | 1276.1230 | 2 | 83.73 | 3 | F3:56509 | NaNaKA16\_F11.raw |  | 2.391E7 | 7.701E7 | 4.861E7 |  |  |  |  |  |  |  |  |  | 8 | 0 | 2 | 4 | 2 | 0 | 0 | 0 | 0 | 0 | 0 | 0 | 0 | 0 | 158 | 179 | Carbamidomethylation | C5:Carbamidomethylation:1000.00;C8:Carbamidomethylation:1000.00 | PEAKS DB |
| R.NMLQMEWNSNAAQNAK.R | N | 89.24 | 1848.8247 | 16 | -0.1 | 925.4196 | 2 | 49.03 | 3 | F3:28088 | NaNaKA16\_F11.raw |  | 9.3217E7 | 1.2893E9 | 8.3194E7 |  |  |  |  |  |  |  |  |  | 10 | 0 | 1 | 7 | 2 | 0 | 0 | 0 | 0 | 0 | 0 | 0 | 0 | 0 | 54 | 69 |  |  | PEAKS DB |
| R.NMLQM(+15.99)EWNSNAAQNAK.R | N | 83.61 | 1864.8196 | 16 | 0.5 | 933.4175 | 2 | 35.34 | 3 | F3:18162 | NaNaKA16\_F11.raw |  |  | 2.1836E8 | 1.6881E7 |  |  |  |  |  |  |  |  |  | 9 | 0 | 0 | 6 | 3 | 0 | 0 | 0 | 0 | 0 | 0 | 0 | 0 | 0 | 54 | 69 | Oxidation (M) | M5:Oxidation (M):78.06 | PEAKS DB |
| R.NM(+15.99)LQMEWNSNAAQNAK.R | N | 82.80 | 1864.8196 | 16 | 2.7 | 933.4196 | 2 | 48.27 | 3 | F3:27735 | NaNaKA16\_F11.raw |  |  | 2.3687E8 | 8.0538E6 |  |  |  |  |  |  |  |  |  | 9 | 0 | 0 | 8 | 1 | 0 | 0 | 0 | 0 | 0 | 0 | 0 | 0 | 0 | 54 | 69 | Oxidation (M) | M2:Oxidation (M):91.37 | PEAKS DB |
| Q.YC(+57.02)PAGNIIGSIATPYK.S | N | 81.36 | 1723.8604 | 16 | 0.9 | 862.9382 | 2 | 63.36 | 3 | F3:39220 | NaNaKA16\_F11.raw |  |  | 2.5562E7 |  |  |  |  |  |  |  |  |  |  | 2 | 0 | 0 | 2 | 0 | 0 | 0 | 0 | 0 | 0 | 0 | 0 | 0 | 0 | 164 | 179 | Carbamidomethylation | C2:Carbamidomethylation:1000.00 | PEAKS DB |
| H.YTQIVWYNSHLLGC(+57.02)GAAK.C | Y | 80.75 | 2080.0200 | 18 | -0.1 | 1041.0172 | 2 | 55.41 | 3 | F3:32205 | NaNaKA16\_F11.raw |  |  | 2.0551E7 | 1.9181E6 |  |  |  |  |  |  |  |  |  | 3 | 0 | 0 | 2 | 1 | 0 | 0 | 0 | 0 | 0 | 0 | 0 | 0 | 0 | 135 | 152 | Carbamidomethylation | C14:Carbamidomethylation:1000.00 | PEAKS DB |
| K.IGC(+57.02)GENLFM(+15.99)SSQPYAWSR.V | Y | 79.45 | 2117.9299 | 18 | 1.3 | 1059.9736 | 2 | 64.65 | 3 | F3:40437 | NaNaKA16\_F11.raw |  | 1.0083E8 | 8.4481E8 | 6.9317E7 |  |  |  |  |  |  |  |  |  | 9 | 0 | 2 | 3 | 4 | 0 | 0 | 0 | 0 | 0 | 0 | 0 | 0 | 0 | 90 | 107 | Carbamidomethylation; Oxidation (M) | C3:Carbamidomethylation:1000.00;M9:Oxidation (M):1000.00 | PEAKS DB |
| C.QYC(+57.02)PAGNIIGSIATPYK.S | N | 76.59 | 1851.9188 | 17 | 0.8 | 926.9674 | 2 | 64.28 | 3 | F3:39934 | NaNaKA16\_F11.raw |  |  | 6.9974E6 |  |  |  |  |  |  |  |  |  |  | 3 | 0 | 0 | 3 | 0 | 0 | 0 | 0 | 0 | 0 | 0 | 0 | 0 | 0 | 163 | 179 | Carbamidomethylation | C3:Carbamidomethylation:1000.00 | PEAKS DB |
| Y.C(+57.02)PAGNIIGSIATPYK.S | N | 75.44 | 1560.7970 | 15 | 0.4 | 781.4061 | 2 | 55.64 | 3 | F3:32530 | NaNaKA16\_F11.raw |  |  | 1.6565E7 | 2.6149E6 |  |  |  |  |  |  |  |  |  | 5 | 0 | 0 | 3 | 2 | 0 | 0 | 0 | 0 | 0 | 0 | 0 | 0 | 0 | 165 | 179 | Carbamidomethylation | C1:Carbamidomethylation:1000.00 | PEAKS DB |
| K.FVYGVGANPPGSVIGH.Y | N | 74.39 | 1569.7939 | 16 | 0.1 | 785.9044 | 2 | 54.74 | 4 | F4:34170 | NaNaKA16\_F12.raw |  | 1.3128E6 | 2.8221E7 | 6.0658E6 |  |  |  |  |  |  |  |  |  | 3 | 0 | 1 | 1 | 1 | 0 | 0 | 0 | 0 | 0 | 0 | 0 | 0 | 0 | 119 | 134 |  |  | PEAKS DB |
| R.NMLQMEWNSNAAQNAKR.W | N | 70.72 | 2004.9258 | 17 | -0.1 | 669.3158 | 3 | 36.44 | 3 | F3:17827 | NaNaKA16\_F11.raw |  |  | 4.2399E6 |  |  |  |  |  |  |  |  |  |  | 1 | 0 | 0 | 1 | 0 | 0 | 0 | 0 | 0 | 0 | 0 | 0 | 0 | 0 | 54 | 70 |  |  | PEAKS DB |
| Q.IVWYNSHLLGC(+57.02)GAAK.C | Y | 66.82 | 1687.8505 | 15 | -0.1 | 563.6240 | 3 | 45.96 | 3 | F3:25859 | NaNaKA16\_F11.raw |  |  | 1.167E7 |  |  |  |  |  |  |  |  |  |  | 2 | 0 | 0 | 2 | 0 | 0 | 0 | 0 | 0 | 0 | 0 | 0 | 0 | 0 | 138 | 152 | Carbamidomethylation | C11:Carbamidomethylation:1000.00 | PEAKS DB |
| K.SGPPC(+57.02)GDC(+57.02)PSAC(+57.02)VNGLC(+57.02)TNPC(+57.02)K.H | N | 64.25 | 2406.9482 | 22 | 2.2 | 803.3251 | 3 | 33.04 | 3 | F3:14934 | NaNaKA16\_F11.raw |  |  | 4.4345E7 | 3.0856E6 |  |  |  |  |  |  |  |  |  | 2 | 0 | 0 | 1 | 1 | 0 | 0 | 0 | 0 | 0 | 0 | 0 | 0 | 0 | 180 | 201 | Carbamidomethylation | C5:Carbamidomethylation:1000.00;C8:Carbamidomethylation:1000.00;C12:Carbamidomethylation:1000.00;C17:Carbamidomethylation:1000.00;C21:Carbamidomethylation:1000.00 | PEAKS DB |
| N.PPGSVIGHYTQIVWYNSHLLGC(+57.02)GAAK.C | Y | 63.95 | 2824.4119 | 26 | 1.2 | 707.1111 | 4 | 68.85 | 3 | F3:43871 | NaNaKA16\_F11.raw |  | 2.0238E6 | 6.4925E6 |  |  |  |  |  |  |  |  |  |  | 2 | 0 | 1 | 1 | 0 | 0 | 0 | 0 | 0 | 0 | 0 | 0 | 0 | 0 | 127 | 152 | Carbamidomethylation | C22:Carbamidomethylation:1000.00 | PEAKS DB |
| N.MLQMEWNSNAAQNAK.R | N | 61.96 | 1734.7817 | 15 | 0.2 | 868.3983 | 2 | 42.10 | 3 | F3:22578 | NaNaKA16\_F11.raw |  |  | 1.0377E7 |  |  |  |  |  |  |  |  |  |  | 1 | 0 | 0 | 1 | 0 | 0 | 0 | 0 | 0 | 0 | 0 | 0 | 0 | 0 | 55 | 69 |  |  | PEAKS DB |
| Y.VC(+57.02)QYC(+57.02)PAGNIIGSIATPYK.S | N | 60.72 | 2111.0181 | 19 | 0.5 | 1056.5168 | 2 | 67.50 | 3 | F3:42701 | NaNaKA16\_F11.raw |  |  | 3.3686E6 | 2.1087E6 |  |  |  |  |  |  |  |  |  | 2 | 0 | 0 | 1 | 1 | 0 | 0 | 0 | 0 | 0 | 0 | 0 | 0 | 0 | 161 | 179 | Carbamidomethylation | C2:Carbamidomethylation:1000.00;C5:Carbamidomethylation:1000.00 | PEAKS DB |
| A.GNIIGSIATPYK.S | N | 58.43 | 1232.6765 | 12 | -0.2 | 617.3454 | 2 | 46.96 | 3 | F3:26760 | NaNaKA16\_F11.raw |  |  | 2.4959E6 |  |  |  |  |  |  |  |  |  |  | 1 | 0 | 0 | 1 | 0 | 0 | 0 | 0 | 0 | 0 | 0 | 0 | 0 | 0 | 168 | 179 |  |  | PEAKS DB |
| K.QNAC(+57.02)QTEWMK.S | Y | 58.40 | 1294.5435 | 10 | 0.1 | 648.2791 | 2 | 41.31 | 3 | F3:21944 | NaNaKA16\_F11.raw |  |  | 4.4811E5 |  |  |  |  |  |  |  |  |  |  | 1 | 0 | 0 | 1 | 0 | 0 | 0 | 0 | 0 | 0 | 0 | 0 | 0 | 0 | 215 | 224 | Carbamidomethylation | C4:Carbamidomethylation:1000.00 | PEAKS DB |
| K.FVYGVGANPPGSVIGHY.T | N | 57.66 | 1732.8573 | 17 | 1.1 | 867.4369 | 2 | 61.67 | 3 | F3:37669 | NaNaKA16\_F11.raw |  |  | 5.3586E6 |  |  |  |  |  |  |  |  |  |  | 1 | 0 | 0 | 1 | 0 | 0 | 0 | 0 | 0 | 0 | 0 | 0 | 0 | 0 | 119 | 135 |  |  | PEAKS DB |
| C.PAGNIIGSIATPYK.S | N | 56.00 | 1400.7664 | 14 | 0.0 | 701.3904 | 2 | 51.58 | 3 | F3:29077 | NaNaKA16\_F11.raw |  |  | 7.112E5 |  |  |  |  |  |  |  |  |  |  | 1 | 0 | 0 | 1 | 0 | 0 | 0 | 0 | 0 | 0 | 0 | 0 | 0 | 0 | 166 | 179 |  |  | PEAKS DB |
| K.FVYGVGANPPGSVIGHYTQIVWYNSHLLGC(+57.02)GAAK.C | Y | 54.12 | 3631.8035 | 34 | 0.9 | 908.9590 | 4 | 82.67 | 4 | F4:57037 | NaNaKA16\_F12.raw |  | 5.2429E6 |  | 1.58E7 |  |  |  |  |  |  |  |  |  | 2 | 0 | 1 | 0 | 1 | 0 | 0 | 0 | 0 | 0 | 0 | 0 | 0 | 0 | 119 | 152 | Carbamidomethylation | C30:Carbamidomethylation:1000.00 | PEAKS DB |
| R.VIQSWYDENKK.F | N | 54.01 | 1408.6986 | 11 | -0.3 | 705.3564 | 2 | 11.80 | 3 | F3:2163 | NaNaKA16\_F11.raw |  |  | 6.7786E6 | 1.1269E6 |  |  |  |  |  |  |  |  |  | 3 | 0 | 0 | 2 | 1 | 0 | 0 | 0 | 0 | 0 | 0 | 0 | 0 | 0 | 108 | 118 |  |  | PEAKS DB |
| R.C(+57.02)SFAHSPPHLR.T | N | 52.64 | 1307.6193 | 11 | 0.9 | 436.8807 | 3 | 11.47 | 4 | F4:1824 | NaNaKA16\_F12.raw |  |  |  | 3.3923E5 |  |  |  |  |  |  |  |  |  | 1 | 0 | 0 | 0 | 1 | 0 | 0 | 0 | 0 | 0 | 0 | 0 | 0 | 0 | 75 | 85 | Carbamidomethylation | C1:Carbamidomethylation:1000.00 | PEAKS DB |
| K.YLYVC(+57.02)QYC(+57.02)PAGN.I | N | 48.44 | 1506.6272 | 12 | 0.2 | 754.3210 | 2 | 57.46 | 3 | F3:34059 | NaNaKA16\_F11.raw |  |  | 1.5296E7 | 4.5046E6 |  |  |  |  |  |  |  |  |  | 2 | 0 | 0 | 1 | 1 | 0 | 0 | 0 | 0 | 0 | 0 | 0 | 0 | 0 | 158 | 169 | Carbamidomethylation | C5:Carbamidomethylation:1000.00;C8:Carbamidomethylation:1000.00 | PEAKS DB |
| Y.TQIVWYNSHLLGC(+57.02)GAAK.C | Y | 46.64 | 1916.9567 | 17 | 1.2 | 639.9936 | 3 | 50.10 | 3 | F3:28369 | NaNaKA16\_F11.raw |  |  | 0 |  |  |  |  |  |  |  |  |  |  | 0 | 0 | 0 | 0 | 0 | 0 | 0 | 0 | 0 | 0 | 0 | 0 | 0 | 0 | 136 | 152 | Carbamidomethylation | C13:Carbamidomethylation:1000.00 | PEAKS DB |
| K.FVYGVGANPPGSVIGHYTQ.I | N | 45.19 | 1961.9635 | 19 | 1.6 | 981.9906 | 2 | 60.19 | 3 | F3:36550 | NaNaKA16\_F11.raw |  |  | 4.6839E6 |  |  |  |  |  |  |  |  |  |  | 1 | 0 | 0 | 1 | 0 | 0 | 0 | 0 | 0 | 0 | 0 | 0 | 0 | 0 | 119 | 137 |  |  | PEAKS DB |
| total 27 peptides |  |  |  |  |  |  |  |  |  |  |  |  |  |  |  |  |  |  |  |  |  |  |  |  |  |  |  |  |  |  |  |  |  |  |  |  |  |  |  |  |  |  |  |
| --- | --- | --- | --- | --- | --- | --- | --- | --- | --- | --- | --- | --- | --- | --- | --- | --- | --- | --- | --- | --- | --- | --- | --- | --- | --- | --- | --- | --- | --- | --- | --- | --- | --- | --- | --- | --- | --- | --- | --- | --- | --- | --- | --- |

AHZ08822.1

back to list

  

| Protein Coverage
| Supporting Peptides
|

Protein Coverage:

Supporting Peptides:

| Peptide | Uniq | -10lgP | Mass | Length | ppm | m/z | z | RT | Fraction | Scan | Source File | Area F1 | Area F10 | Area F11 | Area F12 | Area F13 | Area F2 | Area F3 | Area F4 | Area F5 | Area F6 | Area F7 | Area F8 | Area F9 | #Feature | #Feature F1 | #Feature F10 | #Feature F11 | #Feature F12 | #Feature F13 | #Feature F2 | #Feature F3 | #Feature F4 | #Feature F5 | #Feature F6 | #Feature F7 | #Feature F8 | #Feature F9 | Start | End | PTM | AScore | Found By |
| --- | --- | --- | --- | --- | --- | --- | --- | --- | --- | --- | --- | --- | --- | --- | --- | --- | --- | --- | --- | --- | --- | --- | --- | --- | --- | --- | --- | --- | --- | --- | --- | --- | --- | --- | --- | --- | --- | --- | --- | --- | --- | --- | --- |
| K.IGC(+57.02)GENLFMSSQPYAWSR.V | Y | 91.49 | 2101.9351 | 18 | 0.0 | 1051.9749 | 2 | 72.04 | 3 | F3:46337 | NaNaKA16\_F11.raw |  | 1.3602E7 | 3.9929E8 | 3.8506E7 |  |  |  |  |  |  |  |  |  | 6 | 0 | 2 | 2 | 2 | 0 | 0 | 0 | 0 | 0 | 0 | 0 | 0 | 0 | 90 | 107 | Carbamidomethylation | C3:Carbamidomethylation:1000.00 | PEAKS DB |
| K.YLYVC(+57.02)QYC(+57.02)PAGNIIGSIATPYK.S | N | 91.36 | 2550.2288 | 22 | 1.1 | 1276.1230 | 2 | 83.73 | 3 | F3:56509 | NaNaKA16\_F11.raw |  | 2.391E7 | 7.701E7 | 4.861E7 |  |  |  |  |  |  |  |  |  | 8 | 0 | 2 | 4 | 2 | 0 | 0 | 0 | 0 | 0 | 0 | 0 | 0 | 0 | 158 | 179 | Carbamidomethylation | C5:Carbamidomethylation:1000.00;C8:Carbamidomethylation:1000.00 | PEAKS DB |
| R.NMLQMEWNSNAAQNAK.R | N | 89.24 | 1848.8247 | 16 | -0.1 | 925.4196 | 2 | 49.03 | 3 | F3:28088 | NaNaKA16\_F11.raw |  | 9.3217E7 | 1.2893E9 | 8.3194E7 |  |  |  |  |  |  |  |  |  | 10 | 0 | 1 | 7 | 2 | 0 | 0 | 0 | 0 | 0 | 0 | 0 | 0 | 0 | 54 | 69 |  |  | PEAKS DB |
| R.NMLQM(+15.99)EWNSNAAQNAK.R | N | 83.61 | 1864.8196 | 16 | 0.5 | 933.4175 | 2 | 35.34 | 3 | F3:18162 | NaNaKA16\_F11.raw |  |  | 2.1836E8 | 1.6881E7 |  |  |  |  |  |  |  |  |  | 9 | 0 | 0 | 6 | 3 | 0 | 0 | 0 | 0 | 0 | 0 | 0 | 0 | 0 | 54 | 69 | Oxidation (M) | M5:Oxidation (M):78.06 | PEAKS DB |
| R.NM(+15.99)LQMEWNSNAAQNAK.R | N | 82.80 | 1864.8196 | 16 | 2.7 | 933.4196 | 2 | 48.27 | 3 | F3:27735 | NaNaKA16\_F11.raw |  |  | 2.3687E8 | 8.0538E6 |  |  |  |  |  |  |  |  |  | 9 | 0 | 0 | 8 | 1 | 0 | 0 | 0 | 0 | 0 | 0 | 0 | 0 | 0 | 54 | 69 | Oxidation (M) | M2:Oxidation (M):91.37 | PEAKS DB |
| Q.YC(+57.02)PAGNIIGSIATPYK.S | N | 81.36 | 1723.8604 | 16 | 0.9 | 862.9382 | 2 | 63.36 | 3 | F3:39220 | NaNaKA16\_F11.raw |  |  | 2.5562E7 |  |  |  |  |  |  |  |  |  |  | 2 | 0 | 0 | 2 | 0 | 0 | 0 | 0 | 0 | 0 | 0 | 0 | 0 | 0 | 164 | 179 | Carbamidomethylation | C2:Carbamidomethylation:1000.00 | PEAKS DB |
| H.YTQIVWYNSHLLGC(+57.02)GAAK.C | Y | 80.75 | 2080.0200 | 18 | -0.1 | 1041.0172 | 2 | 55.41 | 3 | F3:32205 | NaNaKA16\_F11.raw |  |  | 2.0551E7 | 1.9181E6 |  |  |  |  |  |  |  |  |  | 3 | 0 | 0 | 2 | 1 | 0 | 0 | 0 | 0 | 0 | 0 | 0 | 0 | 0 | 135 | 152 | Carbamidomethylation | C14:Carbamidomethylation:1000.00 | PEAKS DB |
| K.IGC(+57.02)GENLFM(+15.99)SSQPYAWSR.V | Y | 79.45 | 2117.9299 | 18 | 1.3 | 1059.9736 | 2 | 64.65 | 3 | F3:40437 | NaNaKA16\_F11.raw |  | 1.0083E8 | 8.4481E8 | 6.9317E7 |  |  |  |  |  |  |  |  |  | 9 | 0 | 2 | 3 | 4 | 0 | 0 | 0 | 0 | 0 | 0 | 0 | 0 | 0 | 90 | 107 | Carbamidomethylation; Oxidation (M) | C3:Carbamidomethylation:1000.00;M9:Oxidation (M):1000.00 | PEAKS DB |
| C.QYC(+57.02)PAGNIIGSIATPYK.S | N | 76.59 | 1851.9188 | 17 | 0.8 | 926.9674 | 2 | 64.28 | 3 | F3:39934 | NaNaKA16\_F11.raw |  |  | 6.9974E6 |  |  |  |  |  |  |  |  |  |  | 3 | 0 | 0 | 3 | 0 | 0 | 0 | 0 | 0 | 0 | 0 | 0 | 0 | 0 | 163 | 179 | Carbamidomethylation | C3:Carbamidomethylation:1000.00 | PEAKS DB |
| Y.C(+57.02)PAGNIIGSIATPYK.S | N | 75.44 | 1560.7970 | 15 | 0.4 | 781.4061 | 2 | 55.64 | 3 | F3:32530 | NaNaKA16\_F11.raw |  |  | 1.6565E7 | 2.6149E6 |  |  |  |  |  |  |  |  |  | 5 | 0 | 0 | 3 | 2 | 0 | 0 | 0 | 0 | 0 | 0 | 0 | 0 | 0 | 165 | 179 | Carbamidomethylation | C1:Carbamidomethylation:1000.00 | PEAKS DB |
| K.FVYGVGANPPGSVIGH.Y | N | 74.39 | 1569.7939 | 16 | 0.1 | 785.9044 | 2 | 54.74 | 4 | F4:34170 | NaNaKA16\_F12.raw |  | 1.3128E6 | 2.8221E7 | 6.0658E6 |  |  |  |  |  |  |  |  |  | 3 | 0 | 1 | 1 | 1 | 0 | 0 | 0 | 0 | 0 | 0 | 0 | 0 | 0 | 119 | 134 |  |  | PEAKS DB |
| R.NMLQMEWNSNAAQNAKR.W | N | 70.72 | 2004.9258 | 17 | -0.1 | 669.3158 | 3 | 36.44 | 3 | F3:17827 | NaNaKA16\_F11.raw |  |  | 4.2399E6 |  |  |  |  |  |  |  |  |  |  | 1 | 0 | 0 | 1 | 0 | 0 | 0 | 0 | 0 | 0 | 0 | 0 | 0 | 0 | 54 | 70 |  |  | PEAKS DB |
| Q.IVWYNSHLLGC(+57.02)GAAK.C | Y | 66.82 | 1687.8505 | 15 | -0.1 | 563.6240 | 3 | 45.96 | 3 | F3:25859 | NaNaKA16\_F11.raw |  |  | 1.167E7 |  |  |  |  |  |  |  |  |  |  | 2 | 0 | 0 | 2 | 0 | 0 | 0 | 0 | 0 | 0 | 0 | 0 | 0 | 0 | 138 | 152 | Carbamidomethylation | C11:Carbamidomethylation:1000.00 | PEAKS DB |
| K.SGPPC(+57.02)GDC(+57.02)PSAC(+57.02)VNGLC(+57.02)TNPC(+57.02)K.H | N | 64.25 | 2406.9482 | 22 | 2.2 | 803.3251 | 3 | 33.04 | 3 | F3:14934 | NaNaKA16\_F11.raw |  |  | 4.4345E7 | 3.0856E6 |  |  |  |  |  |  |  |  |  | 2 | 0 | 0 | 1 | 1 | 0 | 0 | 0 | 0 | 0 | 0 | 0 | 0 | 0 | 180 | 201 | Carbamidomethylation | C5:Carbamidomethylation:1000.00;C8:Carbamidomethylation:1000.00;C12:Carbamidomethylation:1000.00;C17:Carbamidomethylation:1000.00;C21:Carbamidomethylation:1000.00 | PEAKS DB |
| N.PPGSVIGHYTQIVWYNSHLLGC(+57.02)GAAK.C | Y | 63.95 | 2824.4119 | 26 | 1.2 | 707.1111 | 4 | 68.85 | 3 | F3:43871 | NaNaKA16\_F11.raw |  | 2.0238E6 | 6.4925E6 |  |  |  |  |  |  |  |  |  |  | 2 | 0 | 1 | 1 | 0 | 0 | 0 | 0 | 0 | 0 | 0 | 0 | 0 | 0 | 127 | 152 | Carbamidomethylation | C22:Carbamidomethylation:1000.00 | PEAKS DB |
| N.MLQMEWNSNAAQNAK.R | N | 61.96 | 1734.7817 | 15 | 0.2 | 868.3983 | 2 | 42.10 | 3 | F3:22578 | NaNaKA16\_F11.raw |  |  | 1.0377E7 |  |  |  |  |  |  |  |  |  |  | 1 | 0 | 0 | 1 | 0 | 0 | 0 | 0 | 0 | 0 | 0 | 0 | 0 | 0 | 55 | 69 |  |  | PEAKS DB |
| Y.VC(+57.02)QYC(+57.02)PAGNIIGSIATPYK.S | N | 60.72 | 2111.0181 | 19 | 0.5 | 1056.5168 | 2 | 67.50 | 3 | F3:42701 | NaNaKA16\_F11.raw |  |  | 3.3686E6 | 2.1087E6 |  |  |  |  |  |  |  |  |  | 2 | 0 | 0 | 1 | 1 | 0 | 0 | 0 | 0 | 0 | 0 | 0 | 0 | 0 | 161 | 179 | Carbamidomethylation | C2:Carbamidomethylation:1000.00;C5:Carbamidomethylation:1000.00 | PEAKS DB |
| A.GNIIGSIATPYK.S | N | 58.43 | 1232.6765 | 12 | -0.2 | 617.3454 | 2 | 46.96 | 3 | F3:26760 | NaNaKA16\_F11.raw |  |  | 2.4959E6 |  |  |  |  |  |  |  |  |  |  | 1 | 0 | 0 | 1 | 0 | 0 | 0 | 0 | 0 | 0 | 0 | 0 | 0 | 0 | 168 | 179 |  |  | PEAKS DB |
| K.QNAC(+57.02)QTEWMK.S | Y | 58.40 | 1294.5435 | 10 | 0.1 | 648.2791 | 2 | 41.31 | 3 | F3:21944 | NaNaKA16\_F11.raw |  |  | 4.4811E5 |  |  |  |  |  |  |  |  |  |  | 1 | 0 | 0 | 1 | 0 | 0 | 0 | 0 | 0 | 0 | 0 | 0 | 0 | 0 | 215 | 224 | Carbamidomethylation | C4:Carbamidomethylation:1000.00 | PEAKS DB |
| K.FVYGVGANPPGSVIGHY.T | N | 57.66 | 1732.8573 | 17 | 1.1 | 867.4369 | 2 | 61.67 | 3 | F3:37669 | NaNaKA16\_F11.raw |  |  | 5.3586E6 |  |  |  |  |  |  |  |  |  |  | 1 | 0 | 0 | 1 | 0 | 0 | 0 | 0 | 0 | 0 | 0 | 0 | 0 | 0 | 119 | 135 |  |  | PEAKS DB |
| C.PAGNIIGSIATPYK.S | N | 56.00 | 1400.7664 | 14 | 0.0 | 701.3904 | 2 | 51.58 | 3 | F3:29077 | NaNaKA16\_F11.raw |  |  | 7.112E5 |  |  |  |  |  |  |  |  |  |  | 1 | 0 | 0 | 1 | 0 | 0 | 0 | 0 | 0 | 0 | 0 | 0 | 0 | 0 | 166 | 179 |  |  | PEAKS DB |
| K.FVYGVGANPPGSVIGHYTQIVWYNSHLLGC(+57.02)GAAK.C | Y | 54.12 | 3631.8035 | 34 | 0.9 | 908.9590 | 4 | 82.67 | 4 | F4:57037 | NaNaKA16\_F12.raw |  | 5.2429E6 |  | 1.58E7 |  |  |  |  |  |  |  |  |  | 2 | 0 | 1 | 0 | 1 | 0 | 0 | 0 | 0 | 0 | 0 | 0 | 0 | 0 | 119 | 152 | Carbamidomethylation | C30:Carbamidomethylation:1000.00 | PEAKS DB |
| R.VIQSWYDENKK.F | N | 54.01 | 1408.6986 | 11 | -0.3 | 705.3564 | 2 | 11.80 | 3 | F3:2163 | NaNaKA16\_F11.raw |  |  | 6.7786E6 | 1.1269E6 |  |  |  |  |  |  |  |  |  | 3 | 0 | 0 | 2 | 1 | 0 | 0 | 0 | 0 | 0 | 0 | 0 | 0 | 0 | 108 | 118 |  |  | PEAKS DB |
| R.C(+57.02)SFAHSPPHLR.T | N | 52.64 | 1307.6193 | 11 | 0.9 | 436.8807 | 3 | 11.47 | 4 | F4:1824 | NaNaKA16\_F12.raw |  |  |  | 3.3923E5 |  |  |  |  |  |  |  |  |  | 1 | 0 | 0 | 0 | 1 | 0 | 0 | 0 | 0 | 0 | 0 | 0 | 0 | 0 | 75 | 85 | Carbamidomethylation | C1:Carbamidomethylation:1000.00 | PEAKS DB |
| K.YLYVC(+57.02)QYC(+57.02)PAGN.I | N | 48.44 | 1506.6272 | 12 | 0.2 | 754.3210 | 2 | 57.46 | 3 | F3:34059 | NaNaKA16\_F11.raw |  |  | 1.5296E7 | 4.5046E6 |  |  |  |  |  |  |  |  |  | 2 | 0 | 0 | 1 | 1 | 0 | 0 | 0 | 0 | 0 | 0 | 0 | 0 | 0 | 158 | 169 | Carbamidomethylation | C5:Carbamidomethylation:1000.00;C8:Carbamidomethylation:1000.00 | PEAKS DB |
| Y.TQIVWYNSHLLGC(+57.02)GAAK.C | Y | 46.64 | 1916.9567 | 17 | 1.2 | 639.9936 | 3 | 50.10 | 3 | F3:28369 | NaNaKA16\_F11.raw |  |  | 0 |  |  |  |  |  |  |  |  |  |  | 0 | 0 | 0 | 0 | 0 | 0 | 0 | 0 | 0 | 0 | 0 | 0 | 0 | 0 | 136 | 152 | Carbamidomethylation | C13:Carbamidomethylation:1000.00 | PEAKS DB |
| K.FVYGVGANPPGSVIGHYTQ.I | N | 45.19 | 1961.9635 | 19 | 1.6 | 981.9906 | 2 | 60.19 | 3 | F3:36550 | NaNaKA16\_F11.raw |  |  | 4.6839E6 |  |  |  |  |  |  |  |  |  |  | 1 | 0 | 0 | 1 | 0 | 0 | 0 | 0 | 0 | 0 | 0 | 0 | 0 | 0 | 119 | 137 |  |  | PEAKS DB |
| total 27 peptides |  |  |  |  |  |  |  |  |  |  |  |  |  |  |  |  |  |  |  |  |  |  |  |  |  |  |  |  |  |  |  |  |  |  |  |  |  |  |  |  |  |  |  |
| --- | --- | --- | --- | --- | --- | --- | --- | --- | --- | --- | --- | --- | --- | --- | --- | --- | --- | --- | --- | --- | --- | --- | --- | --- | --- | --- | --- | --- | --- | --- | --- | --- | --- | --- | --- | --- | --- | --- | --- | --- | --- | --- | --- |

XP\_034291085.1

back to list

  

| Protein Coverage
| Supporting Peptides
|

Protein Coverage:

Supporting Peptides:

| Peptide | Uniq | -10lgP | Mass | Length | ppm | m/z | z | RT | Fraction | Scan | Source File | Area F1 | Area F10 | Area F11 | Area F12 | Area F13 | Area F2 | Area F3 | Area F4 | Area F5 | Area F6 | Area F7 | Area F8 | Area F9 | #Feature | #Feature F1 | #Feature F10 | #Feature F11 | #Feature F12 | #Feature F13 | #Feature F2 | #Feature F3 | #Feature F4 | #Feature F5 | #Feature F6 | #Feature F7 | #Feature F8 | #Feature F9 | Start | End | PTM | AScore | Found By |
| --- | --- | --- | --- | --- | --- | --- | --- | --- | --- | --- | --- | --- | --- | --- | --- | --- | --- | --- | --- | --- | --- | --- | --- | --- | --- | --- | --- | --- | --- | --- | --- | --- | --- | --- | --- | --- | --- | --- | --- | --- | --- | --- | --- |
| K.GGAYSYIESHPWQAGIFVK.N | N | 101.78 | 2109.0320 | 19 | -0.3 | 1055.5229 | 2 | 65.05 | 4 | F4:42731 | NaNaKA16\_F12.raw |  | 5.8839E6 | 1.1656E7 | 5.0772E8 |  |  |  |  |  |  |  | 0 | 1.9031E6 | 10 | 0 | 1 | 2 | 6 | 0 | 0 | 0 | 0 | 0 | 0 | 0 | 0 | 1 | 315 | 333 |  |  | PEAKS DB |
| I.KGGAYSYIESHPWQAGIFVK.N | N | 92.59 | 2237.1270 | 20 | 0.8 | 746.7169 | 3 | 55.07 | 4 | F4:34289 | NaNaKA16\_F12.raw |  |  | 2.0972E6 | 3.4652E7 |  |  |  |  |  |  |  |  |  | 3 | 0 | 0 | 1 | 2 | 0 | 0 | 0 | 0 | 0 | 0 | 0 | 0 | 0 | 314 | 333 |  |  | PEAKS DB |
| Y.SYIESHPWQAGIFVK.N | N | 86.86 | 1760.8885 | 15 | 0.0 | 881.4515 | 2 | 54.37 | 4 | F4:33882 | NaNaKA16\_F12.raw |  |  |  | 1.4376E7 |  |  |  |  |  |  |  |  |  | 2 | 0 | 0 | 0 | 2 | 0 | 0 | 0 | 0 | 0 | 0 | 0 | 0 | 0 | 319 | 333 |  |  | PEAKS DB |
| K.ALYSPNLFIC(+57.02)FC(+57.02)PPGFSGK.F | N | 84.91 | 2174.0330 | 19 | 0.6 | 1088.0244 | 2 | 95.42 | 12 | F12:68966 | NaNaKA16\_F8.raw |  | 3.6583E5 |  |  |  |  |  |  |  | 1.5307E7 | 5.8251E7 | 1.0308E7 | 4.354E6 | 9 | 0 | 1 | 0 | 0 | 0 | 0 | 0 | 0 | 0 | 2 | 2 | 2 | 2 | 101 | 119 | Carbamidomethylation | C10:Carbamidomethylation:1000.00;C12:Carbamidomethylation:1000.00 | PEAKS DB |
| R.TVTENMLC(+57.02)AGDTR.Q | N | 84.34 | 1466.6494 | 13 | -0.1 | 734.3319 | 2 | 30.20 | 4 | F4:13667 | NaNaKA16\_F12.raw |  |  |  | 9.0057E7 |  |  |  |  |  |  |  |  |  | 2 | 0 | 0 | 0 | 2 | 0 | 0 | 0 | 0 | 0 | 0 | 0 | 0 | 0 | 486 | 498 | Carbamidomethylation | C8:Carbamidomethylation:1000.00 | PEAKS DB |
| K.LIGIISWGVGC(+57.02)GK.K | N | 71.96 | 1358.7380 | 13 | -0.9 | 680.3757 | 2 | 73.70 | 4 | F4:50270 | NaNaKA16\_F12.raw |  | 8.0282E6 | 2.5854E7 | 1.9381E8 |  |  |  |  |  |  |  | 3.9035E5 | 1.3461E5 | 8 | 0 | 1 | 1 | 4 | 0 | 0 | 0 | 0 | 0 | 0 | 0 | 1 | 1 | 522 | 534 | Carbamidomethylation | C11:Carbamidomethylation:1000.00 | PEAKS DB |
| R.LYPDSFC(+57.02)TSAR.L | N | 69.84 | 1315.5867 | 11 | 0.6 | 658.8010 | 2 | 33.08 | 4 | F4:16532 | NaNaKA16\_F12.raw |  |  |  | 5.6231E8 | 6.5311E5 |  |  |  |  |  |  |  |  | 2 | 0 | 0 | 0 | 1 | 1 | 0 | 0 | 0 | 0 | 0 | 0 | 0 | 0 | 471 | 481 | Carbamidomethylation | C7:Carbamidomethylation:1000.00 | PEAKS DB |
| Q.LGLGDHNYC(+57.02)R.N | N | 69.80 | 1203.5455 | 10 | 0.7 | 602.7805 | 2 | 12.35 | 11 | F11:2053 | NaNaKA16\_F7.raw |  |  |  |  |  |  |  |  |  | 2.6837E5 | 1.9952E6 |  |  | 3 | 0 | 0 | 0 | 0 | 0 | 0 | 0 | 0 | 0 | 1 | 2 | 0 | 0 | 173 | 182 | Carbamidomethylation | C9:Carbamidomethylation:1000.00 | PEAKS DB |
| R.TVTENM(+15.99)LC(+57.02)AGDTR.Q | N | 67.27 | 1482.6443 | 13 | 0.9 | 742.3301 | 2 | 28.18 | 4 | F4:11849 | NaNaKA16\_F12.raw |  |  |  | 6.8651E6 |  |  |  |  |  |  |  |  |  | 5 | 0 | 0 | 0 | 5 | 0 | 0 | 0 | 0 | 0 | 0 | 0 | 0 | 0 | 486 | 498 | Oxidation (M); Carbamidomethylation | M6:Oxidation (M):1000.00;C8:Carbamidomethylation:1000.00 | PEAKS DB |
| Q.IKGGAYSYIESHPWQAGIFVK.N | N | 64.84 | 2350.2109 | 21 | 0.7 | 588.5604 | 4 | 57.69 | 4 | F4:36700 | NaNaKA16\_F12.raw |  |  | 1.8752E6 | 1.979E7 |  |  |  |  |  |  |  |  |  | 3 | 0 | 0 | 1 | 2 | 0 | 0 | 0 | 0 | 0 | 0 | 0 | 0 | 0 | 313 | 333 |  |  | PEAKS DB |
| R.VKSSVTEQIFQVER.Y | Y | 63.06 | 1648.8784 | 14 | -0.2 | 550.6333 | 3 | 35.31 | 4 | F4:17952 | NaNaKA16\_F12.raw |  |  |  | 8.9659E6 |  |  |  |  |  |  |  |  |  | 2 | 0 | 0 | 0 | 2 | 0 | 0 | 0 | 0 | 0 | 0 | 0 | 0 | 0 | 379 | 392 |  |  | PEAKS DB |
| N.LFIC(+57.02)FC(+57.02)PPGFSGK.F | N | 61.36 | 1528.7206 | 13 | -0.1 | 765.3675 | 2 | 80.11 | 10 | F10:58024 | NaNaKA16\_F6.raw |  |  |  |  |  |  |  |  |  | 1.3335E7 | 7.5039E6 |  |  | 3 | 0 | 0 | 0 | 0 | 0 | 0 | 0 | 0 | 0 | 1 | 2 | 0 | 0 | 107 | 119 | Carbamidomethylation | C4:Carbamidomethylation:1000.00;C6:Carbamidomethylation:1000.00 | PEAKS DB |
| M.KLIGIISWGVGC(+57.02)GK.K | N | 60.37 | 1486.8330 | 14 | -0.3 | 496.6181 | 3 | 58.22 | 4 | F4:37010 | NaNaKA16\_F12.raw |  |  |  | 6.5528E5 |  |  |  |  |  |  |  |  |  | 1 | 0 | 0 | 0 | 1 | 0 | 0 | 0 | 0 | 0 | 0 | 0 | 0 | 0 | 521 | 534 | Carbamidomethylation | C12:Carbamidomethylation:1000.00 | PEAKS DB |
| K.SSVTEQIFQVER.Y | Y | 59.86 | 1421.7151 | 12 | 0.8 | 711.8654 | 2 | 49.80 | 4 | F4:30233 | NaNaKA16\_F12.raw |  | 0 | 1.4032E6 | 4.0689E8 |  |  |  |  |  |  |  |  |  | 4 | 0 | 0 | 1 | 3 | 0 | 0 | 0 | 0 | 0 | 0 | 0 | 0 | 0 | 381 | 392 |  |  | PEAKS DB |
| F.IC(+57.02)FC(+57.02)PPGFSGK.F | N | 58.86 | 1268.5681 | 11 | 0.1 | 635.2914 | 2 | 51.30 | 10 | F10:33995 | NaNaKA16\_F6.raw |  |  |  |  |  |  |  |  |  | 1.0459E7 | 5.3422E6 | 2.3238E6 |  | 3 | 0 | 0 | 0 | 0 | 0 | 0 | 0 | 0 | 0 | 1 | 1 | 1 | 0 | 109 | 119 | Carbamidomethylation | C2:Carbamidomethylation:1000.00;C4:Carbamidomethylation:1000.00 | PEAKS DB |
| R.TLDNDIALLK.L | N | 56.75 | 1114.6234 | 10 | 0.2 | 558.3191 | 2 | 53.18 | 4 | F4:32689 | NaNaKA16\_F12.raw |  |  | 4.6197E6 | 4.3448E8 |  |  |  |  |  |  |  |  |  | 3 | 0 | 0 | 1 | 2 | 0 | 0 | 0 | 0 | 0 | 0 | 0 | 0 | 0 | 403 | 412 |  |  | PEAKS DB |
| K.GGAYSYIESHPWQAGIFVKNR.G | N | 56.73 | 2379.1760 | 21 | 0.6 | 595.8016 | 4 | 55.04 | 4 | F4:34397 | NaNaKA16\_F12.raw |  |  |  | 1.2222E6 |  |  |  |  |  |  |  |  |  | 1 | 0 | 0 | 0 | 1 | 0 | 0 | 0 | 0 | 0 | 0 | 0 | 0 | 0 | 315 | 335 |  |  | PEAKS DB |
| C.DVPAC(+57.02)STC(+57.02)GLR.K | N | 54.02 | 1234.5435 | 11 | -0.2 | 618.2789 | 2 | 11.74 | 4 | F4:2088 | NaNaKA16\_F12.raw |  |  |  | 3.2897E6 |  |  |  |  |  |  |  |  |  | 1 | 0 | 0 | 0 | 1 | 0 | 0 | 0 | 0 | 0 | 0 | 0 | 0 | 0 | 294 | 304 | Carbamidomethylation | C5:Carbamidomethylation:1000.00;C8:Carbamidomethylation:1000.00 | PEAKS DB |
| Y.SPNLFIC(+57.02)FC(+57.02)PPGFSGK.F | N | 49.42 | 1826.8484 | 16 | 1.4 | 914.4327 | 2 | 84.61 | 12 | F12:60077 | NaNaKA16\_F8.raw |  |  |  |  |  |  |  |  |  |  | 5.8546E5 | 7.1533E5 |  | 2 | 0 | 0 | 0 | 0 | 0 | 0 | 0 | 0 | 0 | 0 | 1 | 1 | 0 | 104 | 119 | Carbamidomethylation | C7:Carbamidomethylation:1000.00;C9:Carbamidomethylation:1000.00 | PEAKS DB |
| R.TLDNDIALLKLK.S | N | 47.35 | 1355.8024 | 12 | 0.6 | 452.9417 | 3 | 55.08 | 4 | F4:34569 | NaNaKA16\_F12.raw |  |  |  | 8.5072E5 |  |  |  |  |  |  |  |  |  | 1 | 0 | 0 | 0 | 1 | 0 | 0 | 0 | 0 | 0 | 0 | 0 | 0 | 0 | 403 | 414 |  |  | PEAKS DB |
| Y.IESHPWQAGIFVK.N | N | 45.33 | 1510.7932 | 13 | 0.4 | 504.6052 | 3 | 44.51 | 4 | F4:25555 | NaNaKA16\_F12.raw |  |  |  | 7.3497E5 |  |  |  |  |  |  |  |  |  | 1 | 0 | 0 | 0 | 1 | 0 | 0 | 0 | 0 | 0 | 0 | 0 | 0 | 0 | 321 | 333 |  |  | PEAKS DB |
| I.ESHPWQAGIFVK.N | N | 42.01 | 1397.7091 | 12 | -1.2 | 699.8610 | 2 | 39.12 | 13 | F13:19537 | NaNaKA16\_F9.raw |  |  |  |  |  |  |  |  |  |  |  |  | 6.7594E5 | 1 | 0 | 0 | 0 | 0 | 0 | 0 | 0 | 0 | 0 | 0 | 0 | 0 | 1 | 322 | 333 |  |  | PEAKS DB |
| total 22 peptides |  |  |  |  |  |  |  |  |  |  |  |  |  |  |  |  |  |  |  |  |  |  |  |  |  |  |  |  |  |  |  |  |  |  |  |  |  |  |  |  |  |  |  |
| --- | --- | --- | --- | --- | --- | --- | --- | --- | --- | --- | --- | --- | --- | --- | --- | --- | --- | --- | --- | --- | --- | --- | --- | --- | --- | --- | --- | --- | --- | --- | --- | --- | --- | --- | --- | --- | --- | --- | --- | --- | --- | --- | --- |

XP\_034291087.1

back to list

  

| Protein Coverage
| Supporting Peptides
|

Protein Coverage:

Supporting Peptides:

| Peptide | Uniq | -10lgP | Mass | Length | ppm | m/z | z | RT | Fraction | Scan | Source File | Area F1 | Area F10 | Area F11 | Area F12 | Area F13 | Area F2 | Area F3 | Area F4 | Area F5 | Area F6 | Area F7 | Area F8 | Area F9 | #Feature | #Feature F1 | #Feature F10 | #Feature F11 | #Feature F12 | #Feature F13 | #Feature F2 | #Feature F3 | #Feature F4 | #Feature F5 | #Feature F6 | #Feature F7 | #Feature F8 | #Feature F9 | Start | End | PTM | AScore | Found By |
| --- | --- | --- | --- | --- | --- | --- | --- | --- | --- | --- | --- | --- | --- | --- | --- | --- | --- | --- | --- | --- | --- | --- | --- | --- | --- | --- | --- | --- | --- | --- | --- | --- | --- | --- | --- | --- | --- | --- | --- | --- | --- | --- | --- |
| K.GGAYSYIESHPWQAGIFVK.N | N | 101.78 | 2109.0320 | 19 | -0.3 | 1055.5229 | 2 | 65.05 | 4 | F4:42731 | NaNaKA16\_F12.raw |  | 5.8839E6 | 1.1656E7 | 5.0772E8 |  |  |  |  |  |  |  | 0 | 1.9031E6 | 10 | 0 | 1 | 2 | 6 | 0 | 0 | 0 | 0 | 0 | 0 | 0 | 0 | 1 | 315 | 333 |  |  | PEAKS DB |
| I.KGGAYSYIESHPWQAGIFVK.N | N | 92.59 | 2237.1270 | 20 | 0.8 | 746.7169 | 3 | 55.07 | 4 | F4:34289 | NaNaKA16\_F12.raw |  |  | 2.0972E6 | 3.4652E7 |  |  |  |  |  |  |  |  |  | 3 | 0 | 0 | 1 | 2 | 0 | 0 | 0 | 0 | 0 | 0 | 0 | 0 | 0 | 314 | 333 |  |  | PEAKS DB |
| Y.SYIESHPWQAGIFVK.N | N | 86.86 | 1760.8885 | 15 | 0.0 | 881.4515 | 2 | 54.37 | 4 | F4:33882 | NaNaKA16\_F12.raw |  |  |  | 1.4376E7 |  |  |  |  |  |  |  |  |  | 2 | 0 | 0 | 0 | 2 | 0 | 0 | 0 | 0 | 0 | 0 | 0 | 0 | 0 | 319 | 333 |  |  | PEAKS DB |
| K.ALYSPNLFIC(+57.02)FC(+57.02)PPGFSGK.F | N | 84.91 | 2174.0330 | 19 | 0.6 | 1088.0244 | 2 | 95.42 | 12 | F12:68966 | NaNaKA16\_F8.raw |  | 3.6583E5 |  |  |  |  |  |  |  | 1.5307E7 | 5.8251E7 | 1.0308E7 | 4.354E6 | 9 | 0 | 1 | 0 | 0 | 0 | 0 | 0 | 0 | 0 | 2 | 2 | 2 | 2 | 101 | 119 | Carbamidomethylation | C10:Carbamidomethylation:1000.00;C12:Carbamidomethylation:1000.00 | PEAKS DB |
| R.TVTENMLC(+57.02)AGDTR.Q | N | 84.34 | 1466.6494 | 13 | -0.1 | 734.3319 | 2 | 30.20 | 4 | F4:13667 | NaNaKA16\_F12.raw |  |  |  | 9.0057E7 |  |  |  |  |  |  |  |  |  | 2 | 0 | 0 | 0 | 2 | 0 | 0 | 0 | 0 | 0 | 0 | 0 | 0 | 0 | 486 | 498 | Carbamidomethylation | C8:Carbamidomethylation:1000.00 | PEAKS DB |
| K.LIGIISWGVGC(+57.02)GK.K | N | 71.96 | 1358.7380 | 13 | -0.9 | 680.3757 | 2 | 73.70 | 4 | F4:50270 | NaNaKA16\_F12.raw |  | 8.0282E6 | 2.5854E7 | 1.9381E8 |  |  |  |  |  |  |  | 3.9035E5 | 1.3461E5 | 8 | 0 | 1 | 1 | 4 | 0 | 0 | 0 | 0 | 0 | 0 | 0 | 1 | 1 | 522 | 534 | Carbamidomethylation | C11:Carbamidomethylation:1000.00 | PEAKS DB |
| R.LYPDSFC(+57.02)TSAR.L | N | 69.84 | 1315.5867 | 11 | 0.6 | 658.8010 | 2 | 33.08 | 4 | F4:16532 | NaNaKA16\_F12.raw |  |  |  | 5.6231E8 | 6.5311E5 |  |  |  |  |  |  |  |  | 2 | 0 | 0 | 0 | 1 | 1 | 0 | 0 | 0 | 0 | 0 | 0 | 0 | 0 | 471 | 481 | Carbamidomethylation | C7:Carbamidomethylation:1000.00 | PEAKS DB |
| Q.LGLGDHNYC(+57.02)R.N | N | 69.80 | 1203.5455 | 10 | 0.7 | 602.7805 | 2 | 12.35 | 11 | F11:2053 | NaNaKA16\_F7.raw |  |  |  |  |  |  |  |  |  | 2.6837E5 | 1.9952E6 |  |  | 3 | 0 | 0 | 0 | 0 | 0 | 0 | 0 | 0 | 0 | 1 | 2 | 0 | 0 | 173 | 182 | Carbamidomethylation | C9:Carbamidomethylation:1000.00 | PEAKS DB |
| R.TVTENM(+15.99)LC(+57.02)AGDTR.Q | N | 67.27 | 1482.6443 | 13 | 0.9 | 742.3301 | 2 | 28.18 | 4 | F4:11849 | NaNaKA16\_F12.raw |  |  |  | 6.8651E6 |  |  |  |  |  |  |  |  |  | 5 | 0 | 0 | 0 | 5 | 0 | 0 | 0 | 0 | 0 | 0 | 0 | 0 | 0 | 486 | 498 | Oxidation (M); Carbamidomethylation | M6:Oxidation (M):1000.00;C8:Carbamidomethylation:1000.00 | PEAKS DB |
| Q.IKGGAYSYIESHPWQAGIFVK.N | N | 64.84 | 2350.2109 | 21 | 0.7 | 588.5604 | 4 | 57.69 | 4 | F4:36700 | NaNaKA16\_F12.raw |  |  | 1.8752E6 | 1.979E7 |  |  |  |  |  |  |  |  |  | 3 | 0 | 0 | 1 | 2 | 0 | 0 | 0 | 0 | 0 | 0 | 0 | 0 | 0 | 313 | 333 |  |  | PEAKS DB |
| R.VKSSVTEQIFQVER.Y | Y | 63.06 | 1648.8784 | 14 | -0.2 | 550.6333 | 3 | 35.31 | 4 | F4:17952 | NaNaKA16\_F12.raw |  |  |  | 8.9659E6 |  |  |  |  |  |  |  |  |  | 2 | 0 | 0 | 0 | 2 | 0 | 0 | 0 | 0 | 0 | 0 | 0 | 0 | 0 | 379 | 392 |  |  | PEAKS DB |
| N.LFIC(+57.02)FC(+57.02)PPGFSGK.F | N | 61.36 | 1528.7206 | 13 | -0.1 | 765.3675 | 2 | 80.11 | 10 | F10:58024 | NaNaKA16\_F6.raw |  |  |  |  |  |  |  |  |  | 1.3335E7 | 7.5039E6 |  |  | 3 | 0 | 0 | 0 | 0 | 0 | 0 | 0 | 0 | 0 | 1 | 2 | 0 | 0 | 107 | 119 | Carbamidomethylation | C4:Carbamidomethylation:1000.00;C6:Carbamidomethylation:1000.00 | PEAKS DB |
| M.KLIGIISWGVGC(+57.02)GK.K | N | 60.37 | 1486.8330 | 14 | -0.3 | 496.6181 | 3 | 58.22 | 4 | F4:37010 | NaNaKA16\_F12.raw |  |  |  | 6.5528E5 |  |  |  |  |  |  |  |  |  | 1 | 0 | 0 | 0 | 1 | 0 | 0 | 0 | 0 | 0 | 0 | 0 | 0 | 0 | 521 | 534 | Carbamidomethylation | C12:Carbamidomethylation:1000.00 | PEAKS DB |
| K.SSVTEQIFQVER.Y | Y | 59.86 | 1421.7151 | 12 | 0.8 | 711.8654 | 2 | 49.80 | 4 | F4:30233 | NaNaKA16\_F12.raw |  | 0 | 1.4032E6 | 4.0689E8 |  |  |  |  |  |  |  |  |  | 4 | 0 | 0 | 1 | 3 | 0 | 0 | 0 | 0 | 0 | 0 | 0 | 0 | 0 | 381 | 392 |  |  | PEAKS DB |
| F.IC(+57.02)FC(+57.02)PPGFSGK.F | N | 58.86 | 1268.5681 | 11 | 0.1 | 635.2914 | 2 | 51.30 | 10 | F10:33995 | NaNaKA16\_F6.raw |  |  |  |  |  |  |  |  |  | 1.0459E7 | 5.3422E6 | 2.3238E6 |  | 3 | 0 | 0 | 0 | 0 | 0 | 0 | 0 | 0 | 0 | 1 | 1 | 1 | 0 | 109 | 119 | Carbamidomethylation | C2:Carbamidomethylation:1000.00;C4:Carbamidomethylation:1000.00 | PEAKS DB |
| R.TLDNDIALLK.L | N | 56.75 | 1114.6234 | 10 | 0.2 | 558.3191 | 2 | 53.18 | 4 | F4:32689 | NaNaKA16\_F12.raw |  |  | 4.6197E6 | 4.3448E8 |  |  |  |  |  |  |  |  |  | 3 | 0 | 0 | 1 | 2 | 0 | 0 | 0 | 0 | 0 | 0 | 0 | 0 | 0 | 403 | 412 |  |  | PEAKS DB |
| K.GGAYSYIESHPWQAGIFVKNR.G | N | 56.73 | 2379.1760 | 21 | 0.6 | 595.8016 | 4 | 55.04 | 4 | F4:34397 | NaNaKA16\_F12.raw |  |  |  | 1.2222E6 |  |  |  |  |  |  |  |  |  | 1 | 0 | 0 | 0 | 1 | 0 | 0 | 0 | 0 | 0 | 0 | 0 | 0 | 0 | 315 | 335 |  |  | PEAKS DB |
| C.DVPAC(+57.02)STC(+57.02)GLR.K | N | 54.02 | 1234.5435 | 11 | -0.2 | 618.2789 | 2 | 11.74 | 4 | F4:2088 | NaNaKA16\_F12.raw |  |  |  | 3.2897E6 |  |  |  |  |  |  |  |  |  | 1 | 0 | 0 | 0 | 1 | 0 | 0 | 0 | 0 | 0 | 0 | 0 | 0 | 0 | 294 | 304 | Carbamidomethylation | C5:Carbamidomethylation:1000.00;C8:Carbamidomethylation:1000.00 | PEAKS DB |
| Y.SPNLFIC(+57.02)FC(+57.02)PPGFSGK.F | N | 49.42 | 1826.8484 | 16 | 1.4 | 914.4327 | 2 | 84.61 | 12 | F12:60077 | NaNaKA16\_F8.raw |  |  |  |  |  |  |  |  |  |  | 5.8546E5 | 7.1533E5 |  | 2 | 0 | 0 | 0 | 0 | 0 | 0 | 0 | 0 | 0 | 0 | 1 | 1 | 0 | 104 | 119 | Carbamidomethylation | C7:Carbamidomethylation:1000.00;C9:Carbamidomethylation:1000.00 | PEAKS DB |
| R.TLDNDIALLKLK.S | N | 47.35 | 1355.8024 | 12 | 0.6 | 452.9417 | 3 | 55.08 | 4 | F4:34569 | NaNaKA16\_F12.raw |  |  |  | 8.5072E5 |  |  |  |  |  |  |  |  |  | 1 | 0 | 0 | 0 | 1 | 0 | 0 | 0 | 0 | 0 | 0 | 0 | 0 | 0 | 403 | 414 |  |  | PEAKS DB |
| Y.IESHPWQAGIFVK.N | N | 45.33 | 1510.7932 | 13 | 0.4 | 504.6052 | 3 | 44.51 | 4 | F4:25555 | NaNaKA16\_F12.raw |  |  |  | 7.3497E5 |  |  |  |  |  |  |  |  |  | 1 | 0 | 0 | 0 | 1 | 0 | 0 | 0 | 0 | 0 | 0 | 0 | 0 | 0 | 321 | 333 |  |  | PEAKS DB |
| I.ESHPWQAGIFVK.N | N | 42.01 | 1397.7091 | 12 | -1.2 | 699.8610 | 2 | 39.12 | 13 | F13:19537 | NaNaKA16\_F9.raw |  |  |  |  |  |  |  |  |  |  |  |  | 6.7594E5 | 1 | 0 | 0 | 0 | 0 | 0 | 0 | 0 | 0 | 0 | 0 | 0 | 0 | 1 | 322 | 333 |  |  | PEAKS DB |
| total 22 peptides |  |  |  |  |  |  |  |  |  |  |  |  |  |  |  |  |  |  |  |  |  |  |  |  |  |  |  |  |  |  |  |  |  |  |  |  |  |  |  |  |  |  |  |
| --- | --- | --- | --- | --- | --- | --- | --- | --- | --- | --- | --- | --- | --- | --- | --- | --- | --- | --- | --- | --- | --- | --- | --- | --- | --- | --- | --- | --- | --- | --- | --- | --- | --- | --- | --- | --- | --- | --- | --- | --- | --- | --- | --- |

XP\_034291086.1

back to list

  

| Protein Coverage
| Supporting Peptides
|

Protein Coverage:

Supporting Peptides:

| Peptide | Uniq | -10lgP | Mass | Length | ppm | m/z | z | RT | Fraction | Scan | Source File | Area F1 | Area F10 | Area F11 | Area F12 | Area F13 | Area F2 | Area F3 | Area F4 | Area F5 | Area F6 | Area F7 | Area F8 | Area F9 | #Feature | #Feature F1 | #Feature F10 | #Feature F11 | #Feature F12 | #Feature F13 | #Feature F2 | #Feature F3 | #Feature F4 | #Feature F5 | #Feature F6 | #Feature F7 | #Feature F8 | #Feature F9 | Start | End | PTM | AScore | Found By |
| --- | --- | --- | --- | --- | --- | --- | --- | --- | --- | --- | --- | --- | --- | --- | --- | --- | --- | --- | --- | --- | --- | --- | --- | --- | --- | --- | --- | --- | --- | --- | --- | --- | --- | --- | --- | --- | --- | --- | --- | --- | --- | --- | --- |
| K.GGAYSYIESHPWQAGIFVK.N | N | 101.78 | 2109.0320 | 19 | -0.3 | 1055.5229 | 2 | 65.05 | 4 | F4:42731 | NaNaKA16\_F12.raw |  | 5.8839E6 | 1.1656E7 | 5.0772E8 |  |  |  |  |  |  |  | 0 | 1.9031E6 | 10 | 0 | 1 | 2 | 6 | 0 | 0 | 0 | 0 | 0 | 0 | 0 | 0 | 1 | 315 | 333 |  |  | PEAKS DB |
| I.KGGAYSYIESHPWQAGIFVK.N | N | 92.59 | 2237.1270 | 20 | 0.8 | 746.7169 | 3 | 55.07 | 4 | F4:34289 | NaNaKA16\_F12.raw |  |  | 2.0972E6 | 3.4652E7 |  |  |  |  |  |  |  |  |  | 3 | 0 | 0 | 1 | 2 | 0 | 0 | 0 | 0 | 0 | 0 | 0 | 0 | 0 | 314 | 333 |  |  | PEAKS DB |
| Y.SYIESHPWQAGIFVK.N | N | 86.86 | 1760.8885 | 15 | 0.0 | 881.4515 | 2 | 54.37 | 4 | F4:33882 | NaNaKA16\_F12.raw |  |  |  | 1.4376E7 |  |  |  |  |  |  |  |  |  | 2 | 0 | 0 | 0 | 2 | 0 | 0 | 0 | 0 | 0 | 0 | 0 | 0 | 0 | 319 | 333 |  |  | PEAKS DB |
| K.ALYSPNLFIC(+57.02)FC(+57.02)PPGFSGK.F | N | 84.91 | 2174.0330 | 19 | 0.6 | 1088.0244 | 2 | 95.42 | 12 | F12:68966 | NaNaKA16\_F8.raw |  | 3.6583E5 |  |  |  |  |  |  |  | 1.5307E7 | 5.8251E7 | 1.0308E7 | 4.354E6 | 9 | 0 | 1 | 0 | 0 | 0 | 0 | 0 | 0 | 0 | 2 | 2 | 2 | 2 | 101 | 119 | Carbamidomethylation | C10:Carbamidomethylation:1000.00;C12:Carbamidomethylation:1000.00 | PEAKS DB |
| R.TVTENMLC(+57.02)AGDTR.Q | N | 84.34 | 1466.6494 | 13 | -0.1 | 734.3319 | 2 | 30.20 | 4 | F4:13667 | NaNaKA16\_F12.raw |  |  |  | 9.0057E7 |  |  |  |  |  |  |  |  |  | 2 | 0 | 0 | 0 | 2 | 0 | 0 | 0 | 0 | 0 | 0 | 0 | 0 | 0 | 486 | 498 | Carbamidomethylation | C8:Carbamidomethylation:1000.00 | PEAKS DB |
| K.LIGIISWGVGC(+57.02)GK.K | N | 71.96 | 1358.7380 | 13 | -0.9 | 680.3757 | 2 | 73.70 | 4 | F4:50270 | NaNaKA16\_F12.raw |  | 8.0282E6 | 2.5854E7 | 1.9381E8 |  |  |  |  |  |  |  | 3.9035E5 | 1.3461E5 | 8 | 0 | 1 | 1 | 4 | 0 | 0 | 0 | 0 | 0 | 0 | 0 | 1 | 1 | 522 | 534 | Carbamidomethylation | C11:Carbamidomethylation:1000.00 | PEAKS DB |
| R.LYPDSFC(+57.02)TSAR.L | N | 69.84 | 1315.5867 | 11 | 0.6 | 658.8010 | 2 | 33.08 | 4 | F4:16532 | NaNaKA16\_F12.raw |  |  |  | 5.6231E8 | 6.5311E5 |  |  |  |  |  |  |  |  | 2 | 0 | 0 | 0 | 1 | 1 | 0 | 0 | 0 | 0 | 0 | 0 | 0 | 0 | 471 | 481 | Carbamidomethylation | C7:Carbamidomethylation:1000.00 | PEAKS DB |
| Q.LGLGDHNYC(+57.02)R.N | N | 69.80 | 1203.5455 | 10 | 0.7 | 602.7805 | 2 | 12.35 | 11 | F11:2053 | NaNaKA16\_F7.raw |  |  |  |  |  |  |  |  |  | 2.6837E5 | 1.9952E6 |  |  | 3 | 0 | 0 | 0 | 0 | 0 | 0 | 0 | 0 | 0 | 1 | 2 | 0 | 0 | 173 | 182 | Carbamidomethylation | C9:Carbamidomethylation:1000.00 | PEAKS DB |
| R.TVTENM(+15.99)LC(+57.02)AGDTR.Q | N | 67.27 | 1482.6443 | 13 | 0.9 | 742.3301 | 2 | 28.18 | 4 | F4:11849 | NaNaKA16\_F12.raw |  |  |  | 6.8651E6 |  |  |  |  |  |  |  |  |  | 5 | 0 | 0 | 0 | 5 | 0 | 0 | 0 | 0 | 0 | 0 | 0 | 0 | 0 | 486 | 498 | Oxidation (M); Carbamidomethylation | M6:Oxidation (M):1000.00;C8:Carbamidomethylation:1000.00 | PEAKS DB |
| Q.IKGGAYSYIESHPWQAGIFVK.N | N | 64.84 | 2350.2109 | 21 | 0.7 | 588.5604 | 4 | 57.69 | 4 | F4:36700 | NaNaKA16\_F12.raw |  |  | 1.8752E6 | 1.979E7 |  |  |  |  |  |  |  |  |  | 3 | 0 | 0 | 1 | 2 | 0 | 0 | 0 | 0 | 0 | 0 | 0 | 0 | 0 | 313 | 333 |  |  | PEAKS DB |
| R.VKSSVTEQIFQVER.Y | Y | 63.06 | 1648.8784 | 14 | -0.2 | 550.6333 | 3 | 35.31 | 4 | F4:17952 | NaNaKA16\_F12.raw |  |  |  | 8.9659E6 |  |  |  |  |  |  |  |  |  | 2 | 0 | 0 | 0 | 2 | 0 | 0 | 0 | 0 | 0 | 0 | 0 | 0 | 0 | 379 | 392 |  |  | PEAKS DB |
| N.LFIC(+57.02)FC(+57.02)PPGFSGK.F | N | 61.36 | 1528.7206 | 13 | -0.1 | 765.3675 | 2 | 80.11 | 10 | F10:58024 | NaNaKA16\_F6.raw |  |  |  |  |  |  |  |  |  | 1.3335E7 | 7.5039E6 |  |  | 3 | 0 | 0 | 0 | 0 | 0 | 0 | 0 | 0 | 0 | 1 | 2 | 0 | 0 | 107 | 119 | Carbamidomethylation | C4:Carbamidomethylation:1000.00;C6:Carbamidomethylation:1000.00 | PEAKS DB |
| M.KLIGIISWGVGC(+57.02)GK.K | N | 60.37 | 1486.8330 | 14 | -0.3 | 496.6181 | 3 | 58.22 | 4 | F4:37010 | NaNaKA16\_F12.raw |  |  |  | 6.5528E5 |  |  |  |  |  |  |  |  |  | 1 | 0 | 0 | 0 | 1 | 0 | 0 | 0 | 0 | 0 | 0 | 0 | 0 | 0 | 521 | 534 | Carbamidomethylation | C12:Carbamidomethylation:1000.00 | PEAKS DB |
| K.SSVTEQIFQVER.Y | Y | 59.86 | 1421.7151 | 12 | 0.8 | 711.8654 | 2 | 49.80 | 4 | F4:30233 | NaNaKA16\_F12.raw |  | 0 | 1.4032E6 | 4.0689E8 |  |  |  |  |  |  |  |  |  | 4 | 0 | 0 | 1 | 3 | 0 | 0 | 0 | 0 | 0 | 0 | 0 | 0 | 0 | 381 | 392 |  |  | PEAKS DB |
| F.IC(+57.02)FC(+57.02)PPGFSGK.F | N | 58.86 | 1268.5681 | 11 | 0.1 | 635.2914 | 2 | 51.30 | 10 | F10:33995 | NaNaKA16\_F6.raw |  |  |  |  |  |  |  |  |  | 1.0459E7 | 5.3422E6 | 2.3238E6 |  | 3 | 0 | 0 | 0 | 0 | 0 | 0 | 0 | 0 | 0 | 1 | 1 | 1 | 0 | 109 | 119 | Carbamidomethylation | C2:Carbamidomethylation:1000.00;C4:Carbamidomethylation:1000.00 | PEAKS DB |
| R.TLDNDIALLK.L | N | 56.75 | 1114.6234 | 10 | 0.2 | 558.3191 | 2 | 53.18 | 4 | F4:32689 | NaNaKA16\_F12.raw |  |  | 4.6197E6 | 4.3448E8 |  |  |  |  |  |  |  |  |  | 3 | 0 | 0 | 1 | 2 | 0 | 0 | 0 | 0 | 0 | 0 | 0 | 0 | 0 | 403 | 412 |  |  | PEAKS DB |
| K.GGAYSYIESHPWQAGIFVKNR.G | N | 56.73 | 2379.1760 | 21 | 0.6 | 595.8016 | 4 | 55.04 | 4 | F4:34397 | NaNaKA16\_F12.raw |  |  |  | 1.2222E6 |  |  |  |  |  |  |  |  |  | 1 | 0 | 0 | 0 | 1 | 0 | 0 | 0 | 0 | 0 | 0 | 0 | 0 | 0 | 315 | 335 |  |  | PEAKS DB |
| C.DVPAC(+57.02)STC(+57.02)GLR.K | N | 54.02 | 1234.5435 | 11 | -0.2 | 618.2789 | 2 | 11.74 | 4 | F4:2088 | NaNaKA16\_F12.raw |  |  |  | 3.2897E6 |  |  |  |  |  |  |  |  |  | 1 | 0 | 0 | 0 | 1 | 0 | 0 | 0 | 0 | 0 | 0 | 0 | 0 | 0 | 294 | 304 | Carbamidomethylation | C5:Carbamidomethylation:1000.00;C8:Carbamidomethylation:1000.00 | PEAKS DB |
| Y.SPNLFIC(+57.02)FC(+57.02)PPGFSGK.F | N | 49.42 | 1826.8484 | 16 | 1.4 | 914.4327 | 2 | 84.61 | 12 | F12:60077 | NaNaKA16\_F8.raw |  |  |  |  |  |  |  |  |  |  | 5.8546E5 | 7.1533E5 |  | 2 | 0 | 0 | 0 | 0 | 0 | 0 | 0 | 0 | 0 | 0 | 1 | 1 | 0 | 104 | 119 | Carbamidomethylation | C7:Carbamidomethylation:1000.00;C9:Carbamidomethylation:1000.00 | PEAKS DB |
| R.TLDNDIALLKLK.S | N | 47.35 | 1355.8024 | 12 | 0.6 | 452.9417 | 3 | 55.08 | 4 | F4:34569 | NaNaKA16\_F12.raw |  |  |  | 8.5072E5 |  |  |  |  |  |  |  |  |  | 1 | 0 | 0 | 0 | 1 | 0 | 0 | 0 | 0 | 0 | 0 | 0 | 0 | 0 | 403 | 414 |  |  | PEAKS DB |
| Y.IESHPWQAGIFVK.N | N | 45.33 | 1510.7932 | 13 | 0.4 | 504.6052 | 3 | 44.51 | 4 | F4:25555 | NaNaKA16\_F12.raw |  |  |  | 7.3497E5 |  |  |  |  |  |  |  |  |  | 1 | 0 | 0 | 0 | 1 | 0 | 0 | 0 | 0 | 0 | 0 | 0 | 0 | 0 | 321 | 333 |  |  | PEAKS DB |
| I.ESHPWQAGIFVK.N | N | 42.01 | 1397.7091 | 12 | -1.2 | 699.8610 | 2 | 39.12 | 13 | F13:19537 | NaNaKA16\_F9.raw |  |  |  |  |  |  |  |  |  |  |  |  | 6.7594E5 | 1 | 0 | 0 | 0 | 0 | 0 | 0 | 0 | 0 | 0 | 0 | 0 | 0 | 1 | 322 | 333 |  |  | PEAKS DB |
| total 22 peptides |  |  |  |  |  |  |  |  |  |  |  |  |  |  |  |  |  |  |  |  |  |  |  |  |  |  |  |  |  |  |  |  |  |  |  |  |  |  |  |  |  |  |  |
| --- | --- | --- | --- | --- | --- | --- | --- | --- | --- | --- | --- | --- | --- | --- | --- | --- | --- | --- | --- | --- | --- | --- | --- | --- | --- | --- | --- | --- | --- | --- | --- | --- | --- | --- | --- | --- | --- | --- | --- | --- | --- | --- | --- |

3PRX

back to list

  

| Protein Coverage
| Supporting Peptides
|

Protein Coverage:

Supporting Peptides:

| Peptide | Uniq | -10lgP | Mass | Length | ppm | m/z | z | RT | Fraction | Scan | Source File | Area F1 | Area F10 | Area F11 | Area F12 | Area F13 | Area F2 | Area F3 | Area F4 | Area F5 | Area F6 | Area F7 | Area F8 | Area F9 | #Feature | #Feature F1 | #Feature F10 | #Feature F11 | #Feature F12 | #Feature F13 | #Feature F2 | #Feature F3 | #Feature F4 | #Feature F5 | #Feature F6 | #Feature F7 | #Feature F8 | #Feature F9 | Start | End | PTM | AScore | Found By |
| --- | --- | --- | --- | --- | --- | --- | --- | --- | --- | --- | --- | --- | --- | --- | --- | --- | --- | --- | --- | --- | --- | --- | --- | --- | --- | --- | --- | --- | --- | --- | --- | --- | --- | --- | --- | --- | --- | --- | --- | --- | --- | --- | --- |
| R.KC(+57.02)C(+57.02)EDVMHENPMGYTC(+57.02)EK.R | Y | 83.50 | 2286.8835 | 18 | 0.7 | 763.3023 | 3 | 20.65 | 9 | F9:7758 | NaNaKA16\_F5.raw |  |  |  |  |  |  |  |  | 5.1572E7 |  |  |  |  | 2 | 0 | 0 | 0 | 0 | 0 | 0 | 0 | 0 | 2 | 0 | 0 | 0 | 0 | 676 | 693 | Carbamidomethylation | C2:Carbamidomethylation:1000.00;C3:Carbamidomethylation:1000.00;C16:Carbamidomethylation:1000.00 | PEAKS DB |
| K.C(+57.02)C(+57.02)EDVMHENPMGYTC(+57.02)EK.R | Y | 83.21 | 2158.7886 | 17 | 0.2 | 720.6036 | 3 | 31.20 | 9 | F9:15937 | NaNaKA16\_F5.raw |  |  |  |  |  |  |  |  | 5.0681E7 |  |  |  |  | 2 | 0 | 0 | 0 | 0 | 0 | 0 | 0 | 0 | 2 | 0 | 0 | 0 | 0 | 677 | 693 | Carbamidomethylation | C1:Carbamidomethylation:1000.00;C2:Carbamidomethylation:1000.00;C15:Carbamidomethylation:1000.00 | PEAKS DB |
| K.VNDDYLIWGSR.S | Y | 78.30 | 1336.6411 | 11 | 0.2 | 669.3279 | 2 | 59.90 | 4 | F4:38472 | NaNaKA16\_F12.raw |  |  |  | 6.8624E6 | 3.781E6 |  |  |  |  |  |  |  |  | 2 | 0 | 0 | 0 | 1 | 1 | 0 | 0 | 0 | 0 | 0 | 0 | 0 | 0 | 1577 | 1587 |  |  | PEAKS DB |
| K.AC(+57.02)ETNVDYVYK.T | Y | 69.83 | 1360.5969 | 11 | 1.0 | 681.3064 | 2 | 23.73 | 5 | F5:6621 | NaNaKA16\_F13.raw |  |  |  |  | 5.6014E6 |  |  |  |  |  |  |  |  | 1 | 0 | 0 | 0 | 0 | 1 | 0 | 0 | 0 | 0 | 0 | 0 | 0 | 0 | 1515 | 1525 | Carbamidomethylation | C2:Carbamidomethylation:1000.00 | PEAKS DB |
| R.DDNEDGFIADSDIISR.S | Y | 69.48 | 1780.7751 | 16 | 1.0 | 891.3958 | 2 | 68.58 | 5 | F5:27981 | NaNaKA16\_F13.raw |  |  |  |  | 1.4552E6 |  |  |  |  |  |  |  |  | 2 | 0 | 0 | 0 | 0 | 2 | 0 | 0 | 0 | 0 | 0 | 0 | 0 | 0 | 733 | 748 |  |  | PEAKS DB |
| R.QNQYVVVQVTGPQVR.L | N | 69.18 | 1713.9163 | 15 | 0.5 | 857.9658 | 2 | 44.31 | 5 | F5:17897 | NaNaKA16\_F13.raw |  |  |  | 1.095E6 | 1.2725E6 |  |  |  |  |  |  | 0 |  | 3 | 0 | 0 | 0 | 1 | 2 | 0 | 0 | 0 | 0 | 0 | 0 | 0 | 0 | 100 | 114 |  |  | PEAKS DB |
| K.QLDIFVHDFPR.K | N | 68.50 | 1385.7091 | 11 | 0.5 | 462.9105 | 3 | 58.12 | 2 | F2:35492 | NaNaKA16\_F10.raw |  | 4.8609E6 |  | 4.087E6 |  |  |  |  |  |  |  |  |  | 3 | 0 | 1 | 0 | 2 | 0 | 0 | 0 | 0 | 0 | 0 | 0 | 0 | 0 | 53 | 63 |  |  | PEAKS DB |
| K.GDNLIQMPGAAMK.I | Y | 68.40 | 1344.6530 | 13 | 0.9 | 673.3344 | 2 | 49.77 | 5 | F5:20116 | NaNaKA16\_F13.raw |  |  |  |  | 3.458E6 |  |  |  |  |  |  |  |  | 1 | 0 | 0 | 0 | 0 | 1 | 0 | 0 | 0 | 0 | 0 | 0 | 0 | 0 | 552 | 564 |  |  | PEAKS DB |
| G.ALYTLITPAVLR.T | N | 67.00 | 1329.8020 | 12 | 0.9 | 665.9089 | 2 | 76.05 | 2 | F2:51268 | NaNaKA16\_F10.raw |  | 1.5845E7 | 2.1186E6 | 5.6852E6 | 3.5213E6 |  |  |  |  |  |  | 6.0544E5 | 4.0982E5 | 6 | 0 | 1 | 1 | 1 | 1 | 0 | 0 | 0 | 0 | 0 | 0 | 1 | 1 | 23 | 34 |  |  | PEAKS DB |
| R.INYENALLAR.T | Y | 66.45 | 1175.6299 | 10 | 0.9 | 588.8228 | 2 | 45.80 | 5 | F5:18665 | NaNaKA16\_F13.raw |  |  |  | 1.5955E6 | 3.825E6 |  |  |  |  |  |  |  |  | 2 | 0 | 0 | 0 | 1 | 1 | 0 | 0 | 0 | 0 | 0 | 0 | 0 | 0 | 1292 | 1301 |  |  | PEAKS DB |
| K.LNQDITVTASGDGK.A | Y | 66.30 | 1417.7048 | 14 | 0.8 | 709.8603 | 2 | 20.53 | 5 | F5:5136 | NaNaKA16\_F13.raw |  |  |  |  | 2.7531E6 |  |  |  |  |  |  |  |  | 1 | 0 | 0 | 0 | 0 | 1 | 0 | 0 | 0 | 0 | 0 | 0 | 0 | 0 | 1307 | 1320 |  |  | PEAKS DB |
| R.VELLYNPAFC(+57.02)SASTK.G | Y | 65.11 | 1698.8286 | 15 | 0.9 | 850.4224 | 2 | 59.57 | 5 | F5:24606 | NaNaKA16\_F13.raw |  |  |  | 2.9621E5 | 4.7366E6 |  |  |  |  |  |  |  |  | 2 | 0 | 0 | 0 | 1 | 1 | 0 | 0 | 0 | 0 | 0 | 0 | 0 | 0 | 848 | 862 | Carbamidomethylation | C10:Carbamidomethylation:1000.00 | PEAKS DB |
| K.DLTEEPNSQGISSK.T | Y | 64.83 | 1503.7052 | 14 | 0.2 | 752.8600 | 2 | 21.48 | 5 | F5:5531 | NaNaKA16\_F13.raw |  |  |  |  | 5.0484E6 |  |  |  |  |  |  |  |  | 1 | 0 | 0 | 0 | 0 | 1 | 0 | 0 | 0 | 0 | 0 | 0 | 0 | 0 | 761 | 774 |  |  | PEAKS DB |
| K.ASVQEALWSDGVR.K | Y | 64.04 | 1416.6997 | 13 | 0.6 | 709.3575 | 2 | 50.23 | 5 | F5:20313 | NaNaKA16\_F13.raw |  |  |  |  | 3.3991E6 |  |  |  |  |  |  |  |  | 1 | 0 | 0 | 0 | 0 | 1 | 0 | 0 | 0 | 0 | 0 | 0 | 0 | 0 | 898 | 910 |  |  | PEAKS DB |
| K.DTC(+57.02)MGTLVVK.G | N | 63.96 | 1122.5414 | 10 | 1.1 | 562.2786 | 2 | 36.58 | 5 | F5:14251 | NaNaKA16\_F13.raw |  |  |  |  | 3.5107E6 |  |  |  |  |  |  |  |  | 1 | 0 | 0 | 0 | 0 | 1 | 0 | 0 | 0 | 0 | 0 | 0 | 0 | 0 | 542 | 551 | Carbamidomethylation | C3:Carbamidomethylation:1000.00 | PEAKS DB |
| K.GVGGTQLEVIK.A | Y | 62.39 | 1099.6237 | 11 | -0.4 | 550.8189 | 2 | 32.10 | 5 | F5:11471 | NaNaKA16\_F13.raw |  |  |  |  | 3.5972E6 |  |  |  |  |  |  |  |  | 1 | 0 | 0 | 0 | 0 | 1 | 0 | 0 | 0 | 0 | 0 | 0 | 0 | 0 | 936 | 946 |  |  | PEAKS DB |
| K.HFEVGFIQPGSVK.V | N | 61.71 | 1443.7510 | 13 | 0.1 | 722.8828 | 2 | 45.04 | 5 | F5:18328 | NaNaKA16\_F13.raw |  |  |  | 7.8322E5 | 1.9589E6 |  |  |  |  |  |  |  |  | 3 | 0 | 0 | 0 | 1 | 2 | 0 | 0 | 0 | 0 | 0 | 0 | 0 | 0 | 1445 | 1457 |  |  | PEAKS DB |
| K.YFTYLILNK.G | N | 59.48 | 1173.6433 | 9 | 0.6 | 587.8293 | 2 | 64.63 | 4 | F4:42455 | NaNaKA16\_F12.raw |  |  |  | 3.9819E5 | 3.5813E5 |  |  |  |  |  |  |  |  | 2 | 0 | 0 | 0 | 1 | 1 | 0 | 0 | 0 | 0 | 0 | 0 | 0 | 0 | 478 | 486 |  |  | PEAKS DB |
| R.WPHEDEC(+57.02)QEEEFQK.L | Y | 56.35 | 1889.7526 | 14 | 0.4 | 630.9250 | 3 | 23.79 | 4 | F4:8642 | NaNaKA16\_F12.raw |  |  |  | 4.157E6 | 3.5558E6 |  |  |  |  |  |  |  |  | 2 | 0 | 0 | 0 | 1 | 1 | 0 | 0 | 0 | 0 | 0 | 0 | 0 | 0 | 1610 | 1623 | Carbamidomethylation | C7:Carbamidomethylation:1000.00 | PEAKS DB |
| K.VYSYYNLDEK.C | N | 53.87 | 1292.5924 | 10 | -0.2 | 647.3033 | 2 | 35.45 | 5 | F5:13480 | NaNaKA16\_F13.raw |  |  |  |  | 6.2293E6 |  |  |  |  |  |  |  |  | 1 | 0 | 0 | 0 | 0 | 1 | 0 | 0 | 0 | 0 | 0 | 0 | 0 | 0 | 1458 | 1467 |  |  | PEAKS DB |
| R.IEEQDGNDIYVMDVLEVIK.Q | Y | 53.58 | 2221.0823 | 19 | 0.4 | 1111.5488 | 2 | 104.84 | 4 | F4:72376 | NaNaKA16\_F12.raw |  |  |  | 9.1078E5 |  |  |  |  |  |  |  |  |  | 1 | 0 | 0 | 0 | 1 | 0 | 0 | 0 | 0 | 0 | 0 | 0 | 0 | 0 | 1531 | 1549 |  |  | PEAKS DB |
| K.C(+57.02)C(+57.02)EDVMHENPM(+15.99)GYTC(+57.02)EK.R | Y | 52.94 | 2174.7837 | 17 | 0.3 | 725.9354 | 3 | 19.23 | 9 | F9:6612 | NaNaKA16\_F5.raw |  |  |  |  |  |  |  |  | 4.2545E6 |  |  |  |  | 1 | 0 | 0 | 0 | 0 | 0 | 0 | 0 | 0 | 1 | 0 | 0 | 0 | 0 | 677 | 693 | Carbamidomethylation; Oxidation (M) | C1:Carbamidomethylation:1000.00;C2:Carbamidomethylation:1000.00;M11:Oxidation (M):62.41;C15:Carbamidomethylation:1000.00 | PEAKS DB |
| R.IDVPLQIEK.A | Y | 52.36 | 1053.6069 | 9 | 1.3 | 527.8114 | 2 | 47.84 | 4 | F4:28430 | NaNaKA16\_F12.raw |  |  |  | 8.3958E6 | 6.968E6 |  |  |  |  |  |  |  |  | 2 | 0 | 0 | 0 | 1 | 1 | 0 | 0 | 0 | 0 | 0 | 0 | 0 | 0 | 1506 | 1514 |  |  | PEAKS DB |
| K.GDNLIQM(+15.99)PGAAMK.I | Y | 51.92 | 1360.6479 | 13 | -0.1 | 681.3312 | 2 | 30.12 | 5 | F5:10521 | NaNaKA16\_F13.raw |  |  |  |  | 7.1208E5 |  |  |  |  |  |  |  |  | 1 | 0 | 0 | 0 | 0 | 1 | 0 | 0 | 0 | 0 | 0 | 0 | 0 | 0 | 552 | 564 | Oxidation (M) | M7:Oxidation (M):35.03 | PEAKS DB |
| K.YVLPSFEVR.L | N | 51.15 | 1108.5917 | 9 | 0.2 | 555.3032 | 2 | 58.46 | 4 | F4:37268 | NaNaKA16\_F12.raw |  |  |  | 1.8594E6 | 2.5932E6 |  |  |  |  |  |  |  |  | 2 | 0 | 0 | 0 | 1 | 1 | 0 | 0 | 0 | 0 | 0 | 0 | 0 | 0 | 219 | 227 |  |  | PEAKS DB |
| K.LDDRVPDTEIETK.I | Y | 48.94 | 1529.7573 | 13 | 0.1 | 510.9265 | 3 | 24.23 | 5 | F5:6922 | NaNaKA16\_F13.raw |  |  |  |  | 2.685E5 |  |  |  |  |  |  |  |  | 1 | 0 | 0 | 0 | 0 | 1 | 0 | 0 | 0 | 0 | 0 | 0 | 0 | 0 | 950 | 962 |  |  | PEAKS DB |
| R.AVPFVIVPLEQGLHDVEIK.A | Y | 48.75 | 2102.1775 | 19 | 0.1 | 701.7332 | 3 | 86.03 | 4 | F4:59879 | NaNaKA16\_F12.raw |  |  |  | 5.8004E5 |  |  |  |  |  |  |  |  |  | 1 | 0 | 0 | 0 | 1 | 0 | 0 | 0 | 0 | 0 | 0 | 0 | 0 | 0 | 879 | 897 |  |  | PEAKS DB |
| H.ITPDLIPSFR.F | N | 47.01 | 1157.6444 | 10 | 0.6 | 579.8298 | 2 | 65.39 | 5 | F5:26870 | NaNaKA16\_F13.raw |  |  |  |  | 4.6001E5 |  |  |  |  |  |  |  |  | 1 | 0 | 0 | 0 | 0 | 1 | 0 | 0 | 0 | 0 | 0 | 0 | 0 | 0 | 510 | 519 |  |  | PEAKS DB |
| R.IPIIDGDGK.A | Y | 46.67 | 926.5073 | 9 | 0.6 | 464.2612 | 2 | 33.77 | 5 | F5:12591 | NaNaKA16\_F13.raw |  |  |  |  | 5.1851E6 |  |  |  |  |  |  |  |  | 1 | 0 | 0 | 0 | 0 | 1 | 0 | 0 | 0 | 0 | 0 | 0 | 0 | 0 | 283 | 291 |  |  | PEAKS DB |
| K.GIYTPGSPVLYR.V | N | 46.58 | 1321.7030 | 12 | -0.9 | 661.8582 | 2 | 48.12 | 5 | F5:19514 | NaNaKA16\_F13.raw |  |  |  |  | 0 |  |  |  |  |  |  |  |  | 0 | 0 | 0 | 0 | 0 | 0 | 0 | 0 | 0 | 0 | 0 | 0 | 0 | 0 | 135 | 146 |  |  | PEAKS DB |
| E.DGFIADSDIISR.S | Y | 45.89 | 1307.6357 | 12 | 0.4 | 654.8254 | 2 | 61.00 | 5 | F5:25193 | NaNaKA16\_F13.raw |  |  |  |  | 1.257E6 |  |  |  |  |  |  |  |  | 2 | 0 | 0 | 0 | 0 | 2 | 0 | 0 | 0 | 0 | 0 | 0 | 0 | 0 | 737 | 748 |  |  | PEAKS DB |
| W.VVLAVSFTPTK.G | Y | 45.67 | 1160.6804 | 11 | 0.8 | 581.3480 | 2 | 52.57 | 5 | F5:21349 | NaNaKA16\_F13.raw |  |  |  |  | 9.857E4 |  |  |  |  |  |  |  |  | 1 | 0 | 0 | 0 | 0 | 1 | 0 | 0 | 0 | 0 | 0 | 0 | 0 | 0 | 788 | 798 |  |  | PEAKS DB |
| K.GIC(+57.02)VAEPYEIR.V | Y | 45.63 | 1305.6387 | 11 | 0.3 | 653.8268 | 2 | 44.92 | 5 | F5:18246 | NaNaKA16\_F13.raw |  |  |  |  | 6.0645E6 |  |  |  |  |  |  |  |  | 1 | 0 | 0 | 0 | 0 | 1 | 0 | 0 | 0 | 0 | 0 | 0 | 0 | 0 | 799 | 809 | Carbamidomethylation | C3:Carbamidomethylation:1000.00 | PEAKS DB |
| K.IWDTIEK.S | N | 43.23 | 903.4702 | 7 | 0.6 | 452.7426 | 2 | 29.33 | 5 | F5:9813 | NaNaKA16\_F13.raw |  |  |  |  | 4.4002E6 |  |  |  |  |  |  |  |  | 1 | 0 | 0 | 0 | 0 | 1 | 0 | 0 | 0 | 0 | 0 | 0 | 0 | 0 | 598 | 604 |  |  | PEAKS DB |
| K.VAVIIYLNK.V | Y | 42.64 | 1031.6379 | 9 | 0.3 | 516.8264 | 2 | 50.89 | 5 | F5:20439 | NaNaKA16\_F13.raw |  |  |  |  | 2.7389E5 |  |  |  |  |  |  |  |  | 1 | 0 | 0 | 0 | 0 | 1 | 0 | 0 | 0 | 0 | 0 | 0 | 0 | 0 | 1421 | 1429 |  |  | PEAKS DB |
| total 35 peptides |  |  |  |  |  |  |  |  |  |  |  |  |  |  |  |  |  |  |  |  |  |  |  |  |  |  |  |  |  |  |  |  |  |  |  |  |  |  |  |  |  |  |  |
| --- | --- | --- | --- | --- | --- | --- | --- | --- | --- | --- | --- | --- | --- | --- | --- | --- | --- | --- | --- | --- | --- | --- | --- | --- | --- | --- | --- | --- | --- | --- | --- | --- | --- | --- | --- | --- | --- | --- | --- | --- | --- | --- | --- |

3PVM

back to list

  

| Protein Coverage
| Supporting Peptides
|

Protein Coverage:

Supporting Peptides:

| Peptide | Uniq | -10lgP | Mass | Length | ppm | m/z | z | RT | Fraction | Scan | Source File | Area F1 | Area F10 | Area F11 | Area F12 | Area F13 | Area F2 | Area F3 | Area F4 | Area F5 | Area F6 | Area F7 | Area F8 | Area F9 | #Feature | #Feature F1 | #Feature F10 | #Feature F11 | #Feature F12 | #Feature F13 | #Feature F2 | #Feature F3 | #Feature F4 | #Feature F5 | #Feature F6 | #Feature F7 | #Feature F8 | #Feature F9 | Start | End | PTM | AScore | Found By |
| --- | --- | --- | --- | --- | --- | --- | --- | --- | --- | --- | --- | --- | --- | --- | --- | --- | --- | --- | --- | --- | --- | --- | --- | --- | --- | --- | --- | --- | --- | --- | --- | --- | --- | --- | --- | --- | --- | --- | --- | --- | --- | --- | --- |
| R.KC(+57.02)C(+57.02)EDVMHENPMGYTC(+57.02)EK.R | Y | 83.50 | 2286.8835 | 18 | 0.7 | 763.3023 | 3 | 20.65 | 9 | F9:7758 | NaNaKA16\_F5.raw |  |  |  |  |  |  |  |  | 5.1572E7 |  |  |  |  | 2 | 0 | 0 | 0 | 0 | 0 | 0 | 0 | 0 | 2 | 0 | 0 | 0 | 0 | 676 | 693 | Carbamidomethylation | C2:Carbamidomethylation:1000.00;C3:Carbamidomethylation:1000.00;C16:Carbamidomethylation:1000.00 | PEAKS DB |
| K.C(+57.02)C(+57.02)EDVMHENPMGYTC(+57.02)EK.R | Y | 83.21 | 2158.7886 | 17 | 0.2 | 720.6036 | 3 | 31.20 | 9 | F9:15937 | NaNaKA16\_F5.raw |  |  |  |  |  |  |  |  | 5.0681E7 |  |  |  |  | 2 | 0 | 0 | 0 | 0 | 0 | 0 | 0 | 0 | 2 | 0 | 0 | 0 | 0 | 677 | 693 | Carbamidomethylation | C1:Carbamidomethylation:1000.00;C2:Carbamidomethylation:1000.00;C15:Carbamidomethylation:1000.00 | PEAKS DB |
| K.VNDDYLIWGSR.S | Y | 78.30 | 1336.6411 | 11 | 0.2 | 669.3279 | 2 | 59.90 | 4 | F4:38472 | NaNaKA16\_F12.raw |  |  |  | 6.8624E6 | 3.781E6 |  |  |  |  |  |  |  |  | 2 | 0 | 0 | 0 | 1 | 1 | 0 | 0 | 0 | 0 | 0 | 0 | 0 | 0 | 1577 | 1587 |  |  | PEAKS DB |
| K.AC(+57.02)ETNVDYVYK.T | Y | 69.83 | 1360.5969 | 11 | 1.0 | 681.3064 | 2 | 23.73 | 5 | F5:6621 | NaNaKA16\_F13.raw |  |  |  |  | 5.6014E6 |  |  |  |  |  |  |  |  | 1 | 0 | 0 | 0 | 0 | 1 | 0 | 0 | 0 | 0 | 0 | 0 | 0 | 0 | 1515 | 1525 | Carbamidomethylation | C2:Carbamidomethylation:1000.00 | PEAKS DB |
| R.DDNEDGFIADSDIISR.S | Y | 69.48 | 1780.7751 | 16 | 1.0 | 891.3958 | 2 | 68.58 | 5 | F5:27981 | NaNaKA16\_F13.raw |  |  |  |  | 1.4552E6 |  |  |  |  |  |  |  |  | 2 | 0 | 0 | 0 | 0 | 2 | 0 | 0 | 0 | 0 | 0 | 0 | 0 | 0 | 733 | 748 |  |  | PEAKS DB |
| R.QNQYVVVQVTGPQVR.L | N | 69.18 | 1713.9163 | 15 | 0.5 | 857.9658 | 2 | 44.31 | 5 | F5:17897 | NaNaKA16\_F13.raw |  |  |  | 1.095E6 | 1.2725E6 |  |  |  |  |  |  | 0 |  | 3 | 0 | 0 | 0 | 1 | 2 | 0 | 0 | 0 | 0 | 0 | 0 | 0 | 0 | 100 | 114 |  |  | PEAKS DB |
| K.QLDIFVHDFPR.K | N | 68.50 | 1385.7091 | 11 | 0.5 | 462.9105 | 3 | 58.12 | 2 | F2:35492 | NaNaKA16\_F10.raw |  | 4.8609E6 |  | 4.087E6 |  |  |  |  |  |  |  |  |  | 3 | 0 | 1 | 0 | 2 | 0 | 0 | 0 | 0 | 0 | 0 | 0 | 0 | 0 | 53 | 63 |  |  | PEAKS DB |
| K.GDNLIQMPGAAMK.I | Y | 68.40 | 1344.6530 | 13 | 0.9 | 673.3344 | 2 | 49.77 | 5 | F5:20116 | NaNaKA16\_F13.raw |  |  |  |  | 3.458E6 |  |  |  |  |  |  |  |  | 1 | 0 | 0 | 0 | 0 | 1 | 0 | 0 | 0 | 0 | 0 | 0 | 0 | 0 | 552 | 564 |  |  | PEAKS DB |
| G.ALYTLITPAVLR.T | N | 67.00 | 1329.8020 | 12 | 0.9 | 665.9089 | 2 | 76.05 | 2 | F2:51268 | NaNaKA16\_F10.raw |  | 1.5845E7 | 2.1186E6 | 5.6852E6 | 3.5213E6 |  |  |  |  |  |  | 6.0544E5 | 4.0982E5 | 6 | 0 | 1 | 1 | 1 | 1 | 0 | 0 | 0 | 0 | 0 | 0 | 1 | 1 | 23 | 34 |  |  | PEAKS DB |
| R.INYENALLAR.T | Y | 66.45 | 1175.6299 | 10 | 0.9 | 588.8228 | 2 | 45.80 | 5 | F5:18665 | NaNaKA16\_F13.raw |  |  |  | 1.5955E6 | 3.825E6 |  |  |  |  |  |  |  |  | 2 | 0 | 0 | 0 | 1 | 1 | 0 | 0 | 0 | 0 | 0 | 0 | 0 | 0 | 1292 | 1301 |  |  | PEAKS DB |
| K.LNQDITVTASGDGK.A | Y | 66.30 | 1417.7048 | 14 | 0.8 | 709.8603 | 2 | 20.53 | 5 | F5:5136 | NaNaKA16\_F13.raw |  |  |  |  | 2.7531E6 |  |  |  |  |  |  |  |  | 1 | 0 | 0 | 0 | 0 | 1 | 0 | 0 | 0 | 0 | 0 | 0 | 0 | 0 | 1307 | 1320 |  |  | PEAKS DB |
| R.VELLYNPAFC(+57.02)SASTK.G | Y | 65.11 | 1698.8286 | 15 | 0.9 | 850.4224 | 2 | 59.57 | 5 | F5:24606 | NaNaKA16\_F13.raw |  |  |  | 2.9621E5 | 4.7366E6 |  |  |  |  |  |  |  |  | 2 | 0 | 0 | 0 | 1 | 1 | 0 | 0 | 0 | 0 | 0 | 0 | 0 | 0 | 848 | 862 | Carbamidomethylation | C10:Carbamidomethylation:1000.00 | PEAKS DB |
| K.DLTEEPNSQGISSK.T | Y | 64.83 | 1503.7052 | 14 | 0.2 | 752.8600 | 2 | 21.48 | 5 | F5:5531 | NaNaKA16\_F13.raw |  |  |  |  | 5.0484E6 |  |  |  |  |  |  |  |  | 1 | 0 | 0 | 0 | 0 | 1 | 0 | 0 | 0 | 0 | 0 | 0 | 0 | 0 | 761 | 774 |  |  | PEAKS DB |
| K.ASVQEALWSDGVR.K | Y | 64.04 | 1416.6997 | 13 | 0.6 | 709.3575 | 2 | 50.23 | 5 | F5:20313 | NaNaKA16\_F13.raw |  |  |  |  | 3.3991E6 |  |  |  |  |  |  |  |  | 1 | 0 | 0 | 0 | 0 | 1 | 0 | 0 | 0 | 0 | 0 | 0 | 0 | 0 | 898 | 910 |  |  | PEAKS DB |
| K.DTC(+57.02)MGTLVVK.G | N | 63.96 | 1122.5414 | 10 | 1.1 | 562.2786 | 2 | 36.58 | 5 | F5:14251 | NaNaKA16\_F13.raw |  |  |  |  | 3.5107E6 |  |  |  |  |  |  |  |  | 1 | 0 | 0 | 0 | 0 | 1 | 0 | 0 | 0 | 0 | 0 | 0 | 0 | 0 | 542 | 551 | Carbamidomethylation | C3:Carbamidomethylation:1000.00 | PEAKS DB |
| K.GVGGTQLEVIK.A | Y | 62.39 | 1099.6237 | 11 | -0.4 | 550.8189 | 2 | 32.10 | 5 | F5:11471 | NaNaKA16\_F13.raw |  |  |  |  | 3.5972E6 |  |  |  |  |  |  |  |  | 1 | 0 | 0 | 0 | 0 | 1 | 0 | 0 | 0 | 0 | 0 | 0 | 0 | 0 | 936 | 946 |  |  | PEAKS DB |
| K.HFEVGFIQPGSVK.V | N | 61.71 | 1443.7510 | 13 | 0.1 | 722.8828 | 2 | 45.04 | 5 | F5:18328 | NaNaKA16\_F13.raw |  |  |  | 7.8322E5 | 1.9589E6 |  |  |  |  |  |  |  |  | 3 | 0 | 0 | 0 | 1 | 2 | 0 | 0 | 0 | 0 | 0 | 0 | 0 | 0 | 1445 | 1457 |  |  | PEAKS DB |
| K.YFTYLILNK.G | N | 59.48 | 1173.6433 | 9 | 0.6 | 587.8293 | 2 | 64.63 | 4 | F4:42455 | NaNaKA16\_F12.raw |  |  |  | 3.9819E5 | 3.5813E5 |  |  |  |  |  |  |  |  | 2 | 0 | 0 | 0 | 1 | 1 | 0 | 0 | 0 | 0 | 0 | 0 | 0 | 0 | 478 | 486 |  |  | PEAKS DB |
| R.WPHEDEC(+57.02)QEEEFQK.L | Y | 56.35 | 1889.7526 | 14 | 0.4 | 630.9250 | 3 | 23.79 | 4 | F4:8642 | NaNaKA16\_F12.raw |  |  |  | 4.157E6 | 3.5558E6 |  |  |  |  |  |  |  |  | 2 | 0 | 0 | 0 | 1 | 1 | 0 | 0 | 0 | 0 | 0 | 0 | 0 | 0 | 1610 | 1623 | Carbamidomethylation | C7:Carbamidomethylation:1000.00 | PEAKS DB |
| K.VYSYYNLDEK.C | N | 53.87 | 1292.5924 | 10 | -0.2 | 647.3033 | 2 | 35.45 | 5 | F5:13480 | NaNaKA16\_F13.raw |  |  |  |  | 6.2293E6 |  |  |  |  |  |  |  |  | 1 | 0 | 0 | 0 | 0 | 1 | 0 | 0 | 0 | 0 | 0 | 0 | 0 | 0 | 1458 | 1467 |  |  | PEAKS DB |
| R.IEEQDGNDIYVMDVLEVIK.Q | Y | 53.58 | 2221.0823 | 19 | 0.4 | 1111.5488 | 2 | 104.84 | 4 | F4:72376 | NaNaKA16\_F12.raw |  |  |  | 9.1078E5 |  |  |  |  |  |  |  |  |  | 1 | 0 | 0 | 0 | 1 | 0 | 0 | 0 | 0 | 0 | 0 | 0 | 0 | 0 | 1531 | 1549 |  |  | PEAKS DB |
| K.C(+57.02)C(+57.02)EDVMHENPM(+15.99)GYTC(+57.02)EK.R | Y | 52.94 | 2174.7837 | 17 | 0.3 | 725.9354 | 3 | 19.23 | 9 | F9:6612 | NaNaKA16\_F5.raw |  |  |  |  |  |  |  |  | 4.2545E6 |  |  |  |  | 1 | 0 | 0 | 0 | 0 | 0 | 0 | 0 | 0 | 1 | 0 | 0 | 0 | 0 | 677 | 693 | Carbamidomethylation; Oxidation (M) | C1:Carbamidomethylation:1000.00;C2:Carbamidomethylation:1000.00;M11:Oxidation (M):62.41;C15:Carbamidomethylation:1000.00 | PEAKS DB |
| R.IDVPLQIEK.A | Y | 52.36 | 1053.6069 | 9 | 1.3 | 527.8114 | 2 | 47.84 | 4 | F4:28430 | NaNaKA16\_F12.raw |  |  |  | 8.3958E6 | 6.968E6 |  |  |  |  |  |  |  |  | 2 | 0 | 0 | 0 | 1 | 1 | 0 | 0 | 0 | 0 | 0 | 0 | 0 | 0 | 1506 | 1514 |  |  | PEAKS DB |
| K.GDNLIQM(+15.99)PGAAMK.I | Y | 51.92 | 1360.6479 | 13 | -0.1 | 681.3312 | 2 | 30.12 | 5 | F5:10521 | NaNaKA16\_F13.raw |  |  |  |  | 7.1208E5 |  |  |  |  |  |  |  |  | 1 | 0 | 0 | 0 | 0 | 1 | 0 | 0 | 0 | 0 | 0 | 0 | 0 | 0 | 552 | 564 | Oxidation (M) | M7:Oxidation (M):35.03 | PEAKS DB |
| K.YVLPSFEVR.L | N | 51.15 | 1108.5917 | 9 | 0.2 | 555.3032 | 2 | 58.46 | 4 | F4:37268 | NaNaKA16\_F12.raw |  |  |  | 1.8594E6 | 2.5932E6 |  |  |  |  |  |  |  |  | 2 | 0 | 0 | 0 | 1 | 1 | 0 | 0 | 0 | 0 | 0 | 0 | 0 | 0 | 219 | 227 |  |  | PEAKS DB |
| K.LDDRVPDTEIETK.I | Y | 48.94 | 1529.7573 | 13 | 0.1 | 510.9265 | 3 | 24.23 | 5 | F5:6922 | NaNaKA16\_F13.raw |  |  |  |  | 2.685E5 |  |  |  |  |  |  |  |  | 1 | 0 | 0 | 0 | 0 | 1 | 0 | 0 | 0 | 0 | 0 | 0 | 0 | 0 | 950 | 962 |  |  | PEAKS DB |
| R.AVPFVIVPLEQGLHDVEIK.A | Y | 48.75 | 2102.1775 | 19 | 0.1 | 701.7332 | 3 | 86.03 | 4 | F4:59879 | NaNaKA16\_F12.raw |  |  |  | 5.8004E5 |  |  |  |  |  |  |  |  |  | 1 | 0 | 0 | 0 | 1 | 0 | 0 | 0 | 0 | 0 | 0 | 0 | 0 | 0 | 879 | 897 |  |  | PEAKS DB |
| H.ITPDLIPSFR.F | N | 47.01 | 1157.6444 | 10 | 0.6 | 579.8298 | 2 | 65.39 | 5 | F5:26870 | NaNaKA16\_F13.raw |  |  |  |  | 4.6001E5 |  |  |  |  |  |  |  |  | 1 | 0 | 0 | 0 | 0 | 1 | 0 | 0 | 0 | 0 | 0 | 0 | 0 | 0 | 510 | 519 |  |  | PEAKS DB |
| R.IPIIDGDGK.A | Y | 46.67 | 926.5073 | 9 | 0.6 | 464.2612 | 2 | 33.77 | 5 | F5:12591 | NaNaKA16\_F13.raw |  |  |  |  | 5.1851E6 |  |  |  |  |  |  |  |  | 1 | 0 | 0 | 0 | 0 | 1 | 0 | 0 | 0 | 0 | 0 | 0 | 0 | 0 | 283 | 291 |  |  | PEAKS DB |
| K.GIYTPGSPVLYR.V | N | 46.58 | 1321.7030 | 12 | -0.9 | 661.8582 | 2 | 48.12 | 5 | F5:19514 | NaNaKA16\_F13.raw |  |  |  |  | 0 |  |  |  |  |  |  |  |  | 0 | 0 | 0 | 0 | 0 | 0 | 0 | 0 | 0 | 0 | 0 | 0 | 0 | 0 | 135 | 146 |  |  | PEAKS DB |
| E.DGFIADSDIISR.S | Y | 45.89 | 1307.6357 | 12 | 0.4 | 654.8254 | 2 | 61.00 | 5 | F5:25193 | NaNaKA16\_F13.raw |  |  |  |  | 1.257E6 |  |  |  |  |  |  |  |  | 2 | 0 | 0 | 0 | 0 | 2 | 0 | 0 | 0 | 0 | 0 | 0 | 0 | 0 | 737 | 748 |  |  | PEAKS DB |
| W.VVLAVSFTPTK.G | Y | 45.67 | 1160.6804 | 11 | 0.8 | 581.3480 | 2 | 52.57 | 5 | F5:21349 | NaNaKA16\_F13.raw |  |  |  |  | 9.857E4 |  |  |  |  |  |  |  |  | 1 | 0 | 0 | 0 | 0 | 1 | 0 | 0 | 0 | 0 | 0 | 0 | 0 | 0 | 788 | 798 |  |  | PEAKS DB |
| K.GIC(+57.02)VAEPYEIR.V | Y | 45.63 | 1305.6387 | 11 | 0.3 | 653.8268 | 2 | 44.92 | 5 | F5:18246 | NaNaKA16\_F13.raw |  |  |  |  | 6.0645E6 |  |  |  |  |  |  |  |  | 1 | 0 | 0 | 0 | 0 | 1 | 0 | 0 | 0 | 0 | 0 | 0 | 0 | 0 | 799 | 809 | Carbamidomethylation | C3:Carbamidomethylation:1000.00 | PEAKS DB |
| K.IWDTIEK.S | N | 43.23 | 903.4702 | 7 | 0.6 | 452.7426 | 2 | 29.33 | 5 | F5:9813 | NaNaKA16\_F13.raw |  |  |  |  | 4.4002E6 |  |  |  |  |  |  |  |  | 1 | 0 | 0 | 0 | 0 | 1 | 0 | 0 | 0 | 0 | 0 | 0 | 0 | 0 | 598 | 604 |  |  | PEAKS DB |
| K.VAVIIYLNK.V | Y | 42.64 | 1031.6379 | 9 | 0.3 | 516.8264 | 2 | 50.89 | 5 | F5:20439 | NaNaKA16\_F13.raw |  |  |  |  | 2.7389E5 |  |  |  |  |  |  |  |  | 1 | 0 | 0 | 0 | 0 | 1 | 0 | 0 | 0 | 0 | 0 | 0 | 0 | 0 | 1421 | 1429 |  |  | PEAKS DB |
| total 35 peptides |  |  |  |  |  |  |  |  |  |  |  |  |  |  |  |  |  |  |  |  |  |  |  |  |  |  |  |  |  |  |  |  |  |  |  |  |  |  |  |  |  |  |  |
| --- | --- | --- | --- | --- | --- | --- | --- | --- | --- | --- | --- | --- | --- | --- | --- | --- | --- | --- | --- | --- | --- | --- | --- | --- | --- | --- | --- | --- | --- | --- | --- | --- | --- | --- | --- | --- | --- | --- | --- | --- | --- | --- | --- |

Q91132.1

back to list

  

| Protein Coverage
| Supporting Peptides
|

Protein Coverage:

Supporting Peptides:

| Peptide | Uniq | -10lgP | Mass | Length | ppm | m/z | z | RT | Fraction | Scan | Source File | Area F1 | Area F10 | Area F11 | Area F12 | Area F13 | Area F2 | Area F3 | Area F4 | Area F5 | Area F6 | Area F7 | Area F8 | Area F9 | #Feature | #Feature F1 | #Feature F10 | #Feature F11 | #Feature F12 | #Feature F13 | #Feature F2 | #Feature F3 | #Feature F4 | #Feature F5 | #Feature F6 | #Feature F7 | #Feature F8 | #Feature F9 | Start | End | PTM | AScore | Found By |
| --- | --- | --- | --- | --- | --- | --- | --- | --- | --- | --- | --- | --- | --- | --- | --- | --- | --- | --- | --- | --- | --- | --- | --- | --- | --- | --- | --- | --- | --- | --- | --- | --- | --- | --- | --- | --- | --- | --- | --- | --- | --- | --- | --- |
| R.KC(+57.02)C(+57.02)EDVMHENPMGYTC(+57.02)EK.R | Y | 83.50 | 2286.8835 | 18 | 0.7 | 763.3023 | 3 | 20.65 | 9 | F9:7758 | NaNaKA16\_F5.raw |  |  |  |  |  |  |  |  | 5.1572E7 |  |  |  |  | 2 | 0 | 0 | 0 | 0 | 0 | 0 | 0 | 0 | 2 | 0 | 0 | 0 | 0 | 676 | 693 | Carbamidomethylation | C2:Carbamidomethylation:1000.00;C3:Carbamidomethylation:1000.00;C16:Carbamidomethylation:1000.00 | PEAKS DB |
| K.C(+57.02)C(+57.02)EDVMHENPMGYTC(+57.02)EK.R | Y | 83.21 | 2158.7886 | 17 | 0.2 | 720.6036 | 3 | 31.20 | 9 | F9:15937 | NaNaKA16\_F5.raw |  |  |  |  |  |  |  |  | 5.0681E7 |  |  |  |  | 2 | 0 | 0 | 0 | 0 | 0 | 0 | 0 | 0 | 2 | 0 | 0 | 0 | 0 | 677 | 693 | Carbamidomethylation | C1:Carbamidomethylation:1000.00;C2:Carbamidomethylation:1000.00;C15:Carbamidomethylation:1000.00 | PEAKS DB |
| K.VNDDYLIWGSR.S | Y | 78.30 | 1336.6411 | 11 | 0.2 | 669.3279 | 2 | 59.90 | 4 | F4:38472 | NaNaKA16\_F12.raw |  |  |  | 6.8624E6 | 3.781E6 |  |  |  |  |  |  |  |  | 2 | 0 | 0 | 0 | 1 | 1 | 0 | 0 | 0 | 0 | 0 | 0 | 0 | 0 | 1577 | 1587 |  |  | PEAKS DB |
| K.AC(+57.02)ETNVDYVYK.T | Y | 69.83 | 1360.5969 | 11 | 1.0 | 681.3064 | 2 | 23.73 | 5 | F5:6621 | NaNaKA16\_F13.raw |  |  |  |  | 5.6014E6 |  |  |  |  |  |  |  |  | 1 | 0 | 0 | 0 | 0 | 1 | 0 | 0 | 0 | 0 | 0 | 0 | 0 | 0 | 1515 | 1525 | Carbamidomethylation | C2:Carbamidomethylation:1000.00 | PEAKS DB |
| R.DDNEDGFIADSDIISR.S | Y | 69.48 | 1780.7751 | 16 | 1.0 | 891.3958 | 2 | 68.58 | 5 | F5:27981 | NaNaKA16\_F13.raw |  |  |  |  | 1.4552E6 |  |  |  |  |  |  |  |  | 2 | 0 | 0 | 0 | 0 | 2 | 0 | 0 | 0 | 0 | 0 | 0 | 0 | 0 | 733 | 748 |  |  | PEAKS DB |
| R.QNQYVVVQVTGPQVR.L | N | 69.18 | 1713.9163 | 15 | 0.5 | 857.9658 | 2 | 44.31 | 5 | F5:17897 | NaNaKA16\_F13.raw |  |  |  | 1.095E6 | 1.2725E6 |  |  |  |  |  |  | 0 |  | 3 | 0 | 0 | 0 | 1 | 2 | 0 | 0 | 0 | 0 | 0 | 0 | 0 | 0 | 100 | 114 |  |  | PEAKS DB |
| K.QLDIFVHDFPR.K | N | 68.50 | 1385.7091 | 11 | 0.5 | 462.9105 | 3 | 58.12 | 2 | F2:35492 | NaNaKA16\_F10.raw |  | 4.8609E6 |  | 4.087E6 |  |  |  |  |  |  |  |  |  | 3 | 0 | 1 | 0 | 2 | 0 | 0 | 0 | 0 | 0 | 0 | 0 | 0 | 0 | 53 | 63 |  |  | PEAKS DB |
| K.GDNLIQMPGAAMK.I | Y | 68.40 | 1344.6530 | 13 | 0.9 | 673.3344 | 2 | 49.77 | 5 | F5:20116 | NaNaKA16\_F13.raw |  |  |  |  | 3.458E6 |  |  |  |  |  |  |  |  | 1 | 0 | 0 | 0 | 0 | 1 | 0 | 0 | 0 | 0 | 0 | 0 | 0 | 0 | 552 | 564 |  |  | PEAKS DB |
| G.ALYTLITPAVLR.T | N | 67.00 | 1329.8020 | 12 | 0.9 | 665.9089 | 2 | 76.05 | 2 | F2:51268 | NaNaKA16\_F10.raw |  | 1.5845E7 | 2.1186E6 | 5.6852E6 | 3.5213E6 |  |  |  |  |  |  | 6.0544E5 | 4.0982E5 | 6 | 0 | 1 | 1 | 1 | 1 | 0 | 0 | 0 | 0 | 0 | 0 | 1 | 1 | 23 | 34 |  |  | PEAKS DB |
| R.INYENALLAR.T | Y | 66.45 | 1175.6299 | 10 | 0.9 | 588.8228 | 2 | 45.80 | 5 | F5:18665 | NaNaKA16\_F13.raw |  |  |  | 1.5955E6 | 3.825E6 |  |  |  |  |  |  |  |  | 2 | 0 | 0 | 0 | 1 | 1 | 0 | 0 | 0 | 0 | 0 | 0 | 0 | 0 | 1292 | 1301 |  |  | PEAKS DB |
| K.LNQDITVTASGDGK.A | Y | 66.30 | 1417.7048 | 14 | 0.8 | 709.8603 | 2 | 20.53 | 5 | F5:5136 | NaNaKA16\_F13.raw |  |  |  |  | 2.7531E6 |  |  |  |  |  |  |  |  | 1 | 0 | 0 | 0 | 0 | 1 | 0 | 0 | 0 | 0 | 0 | 0 | 0 | 0 | 1307 | 1320 |  |  | PEAKS DB |
| R.VELLYNPAFC(+57.02)SASTK.G | Y | 65.11 | 1698.8286 | 15 | 0.9 | 850.4224 | 2 | 59.57 | 5 | F5:24606 | NaNaKA16\_F13.raw |  |  |  | 2.9621E5 | 4.7366E6 |  |  |  |  |  |  |  |  | 2 | 0 | 0 | 0 | 1 | 1 | 0 | 0 | 0 | 0 | 0 | 0 | 0 | 0 | 848 | 862 | Carbamidomethylation | C10:Carbamidomethylation:1000.00 | PEAKS DB |
| K.DLTEEPNSQGISSK.T | Y | 64.83 | 1503.7052 | 14 | 0.2 | 752.8600 | 2 | 21.48 | 5 | F5:5531 | NaNaKA16\_F13.raw |  |  |  |  | 5.0484E6 |  |  |  |  |  |  |  |  | 1 | 0 | 0 | 0 | 0 | 1 | 0 | 0 | 0 | 0 | 0 | 0 | 0 | 0 | 761 | 774 |  |  | PEAKS DB |
| K.ASVQEALWSDGVR.K | Y | 64.04 | 1416.6997 | 13 | 0.6 | 709.3575 | 2 | 50.23 | 5 | F5:20313 | NaNaKA16\_F13.raw |  |  |  |  | 3.3991E6 |  |  |  |  |  |  |  |  | 1 | 0 | 0 | 0 | 0 | 1 | 0 | 0 | 0 | 0 | 0 | 0 | 0 | 0 | 898 | 910 |  |  | PEAKS DB |
| K.DTC(+57.02)MGTLVVK.G | N | 63.96 | 1122.5414 | 10 | 1.1 | 562.2786 | 2 | 36.58 | 5 | F5:14251 | NaNaKA16\_F13.raw |  |  |  |  | 3.5107E6 |  |  |  |  |  |  |  |  | 1 | 0 | 0 | 0 | 0 | 1 | 0 | 0 | 0 | 0 | 0 | 0 | 0 | 0 | 542 | 551 | Carbamidomethylation | C3:Carbamidomethylation:1000.00 | PEAKS DB |
| K.GVGGTQLEVIK.A | Y | 62.39 | 1099.6237 | 11 | -0.4 | 550.8189 | 2 | 32.10 | 5 | F5:11471 | NaNaKA16\_F13.raw |  |  |  |  | 3.5972E6 |  |  |  |  |  |  |  |  | 1 | 0 | 0 | 0 | 0 | 1 | 0 | 0 | 0 | 0 | 0 | 0 | 0 | 0 | 936 | 946 |  |  | PEAKS DB |
| K.HFEVGFIQPGSVK.V | N | 61.71 | 1443.7510 | 13 | 0.1 | 722.8828 | 2 | 45.04 | 5 | F5:18328 | NaNaKA16\_F13.raw |  |  |  | 7.8322E5 | 1.9589E6 |  |  |  |  |  |  |  |  | 3 | 0 | 0 | 0 | 1 | 2 | 0 | 0 | 0 | 0 | 0 | 0 | 0 | 0 | 1445 | 1457 |  |  | PEAKS DB |
| K.YFTYLILNK.G | N | 59.48 | 1173.6433 | 9 | 0.6 | 587.8293 | 2 | 64.63 | 4 | F4:42455 | NaNaKA16\_F12.raw |  |  |  | 3.9819E5 | 3.5813E5 |  |  |  |  |  |  |  |  | 2 | 0 | 0 | 0 | 1 | 1 | 0 | 0 | 0 | 0 | 0 | 0 | 0 | 0 | 478 | 486 |  |  | PEAKS DB |
| R.WPHEDEC(+57.02)QEEEFQK.L | Y | 56.35 | 1889.7526 | 14 | 0.4 | 630.9250 | 3 | 23.79 | 4 | F4:8642 | NaNaKA16\_F12.raw |  |  |  | 4.157E6 | 3.5558E6 |  |  |  |  |  |  |  |  | 2 | 0 | 0 | 0 | 1 | 1 | 0 | 0 | 0 | 0 | 0 | 0 | 0 | 0 | 1610 | 1623 | Carbamidomethylation | C7:Carbamidomethylation:1000.00 | PEAKS DB |
| K.VYSYYNLDEK.C | N | 53.87 | 1292.5924 | 10 | -0.2 | 647.3033 | 2 | 35.45 | 5 | F5:13480 | NaNaKA16\_F13.raw |  |  |  |  | 6.2293E6 |  |  |  |  |  |  |  |  | 1 | 0 | 0 | 0 | 0 | 1 | 0 | 0 | 0 | 0 | 0 | 0 | 0 | 0 | 1458 | 1467 |  |  | PEAKS DB |
| R.IEEQDGNDIYVMDVLEVIK.Q | Y | 53.58 | 2221.0823 | 19 | 0.4 | 1111.5488 | 2 | 104.84 | 4 | F4:72376 | NaNaKA16\_F12.raw |  |  |  | 9.1078E5 |  |  |  |  |  |  |  |  |  | 1 | 0 | 0 | 0 | 1 | 0 | 0 | 0 | 0 | 0 | 0 | 0 | 0 | 0 | 1531 | 1549 |  |  | PEAKS DB |
| K.C(+57.02)C(+57.02)EDVMHENPM(+15.99)GYTC(+57.02)EK.R | Y | 52.94 | 2174.7837 | 17 | 0.3 | 725.9354 | 3 | 19.23 | 9 | F9:6612 | NaNaKA16\_F5.raw |  |  |  |  |  |  |  |  | 4.2545E6 |  |  |  |  | 1 | 0 | 0 | 0 | 0 | 0 | 0 | 0 | 0 | 1 | 0 | 0 | 0 | 0 | 677 | 693 | Carbamidomethylation; Oxidation (M) | C1:Carbamidomethylation:1000.00;C2:Carbamidomethylation:1000.00;M11:Oxidation (M):62.41;C15:Carbamidomethylation:1000.00 | PEAKS DB |
| R.IDVPLQIEK.A | Y | 52.36 | 1053.6069 | 9 | 1.3 | 527.8114 | 2 | 47.84 | 4 | F4:28430 | NaNaKA16\_F12.raw |  |  |  | 8.3958E6 | 6.968E6 |  |  |  |  |  |  |  |  | 2 | 0 | 0 | 0 | 1 | 1 | 0 | 0 | 0 | 0 | 0 | 0 | 0 | 0 | 1506 | 1514 |  |  | PEAKS DB |
| K.GDNLIQM(+15.99)PGAAMK.I | Y | 51.92 | 1360.6479 | 13 | -0.1 | 681.3312 | 2 | 30.12 | 5 | F5:10521 | NaNaKA16\_F13.raw |  |  |  |  | 7.1208E5 |  |  |  |  |  |  |  |  | 1 | 0 | 0 | 0 | 0 | 1 | 0 | 0 | 0 | 0 | 0 | 0 | 0 | 0 | 552 | 564 | Oxidation (M) | M7:Oxidation (M):35.03 | PEAKS DB |
| K.YVLPSFEVR.L | N | 51.15 | 1108.5917 | 9 | 0.2 | 555.3032 | 2 | 58.46 | 4 | F4:37268 | NaNaKA16\_F12.raw |  |  |  | 1.8594E6 | 2.5932E6 |  |  |  |  |  |  |  |  | 2 | 0 | 0 | 0 | 1 | 1 | 0 | 0 | 0 | 0 | 0 | 0 | 0 | 0 | 219 | 227 |  |  | PEAKS DB |
| K.LDDRVPDTEIETK.I | Y | 48.94 | 1529.7573 | 13 | 0.1 | 510.9265 | 3 | 24.23 | 5 | F5:6922 | NaNaKA16\_F13.raw |  |  |  |  | 2.685E5 |  |  |  |  |  |  |  |  | 1 | 0 | 0 | 0 | 0 | 1 | 0 | 0 | 0 | 0 | 0 | 0 | 0 | 0 | 950 | 962 |  |  | PEAKS DB |
| R.AVPFVIVPLEQGLHDVEIK.A | Y | 48.75 | 2102.1775 | 19 | 0.1 | 701.7332 | 3 | 86.03 | 4 | F4:59879 | NaNaKA16\_F12.raw |  |  |  | 5.8004E5 |  |  |  |  |  |  |  |  |  | 1 | 0 | 0 | 0 | 1 | 0 | 0 | 0 | 0 | 0 | 0 | 0 | 0 | 0 | 879 | 897 |  |  | PEAKS DB |
| H.ITPDLIPSFR.F | N | 47.01 | 1157.6444 | 10 | 0.6 | 579.8298 | 2 | 65.39 | 5 | F5:26870 | NaNaKA16\_F13.raw |  |  |  |  | 4.6001E5 |  |  |  |  |  |  |  |  | 1 | 0 | 0 | 0 | 0 | 1 | 0 | 0 | 0 | 0 | 0 | 0 | 0 | 0 | 510 | 519 |  |  | PEAKS DB |
| R.IPIIDGDGK.A | Y | 46.67 | 926.5073 | 9 | 0.6 | 464.2612 | 2 | 33.77 | 5 | F5:12591 | NaNaKA16\_F13.raw |  |  |  |  | 5.1851E6 |  |  |  |  |  |  |  |  | 1 | 0 | 0 | 0 | 0 | 1 | 0 | 0 | 0 | 0 | 0 | 0 | 0 | 0 | 283 | 291 |  |  | PEAKS DB |
| K.GIYTPGSPVLYR.V | N | 46.58 | 1321.7030 | 12 | -0.9 | 661.8582 | 2 | 48.12 | 5 | F5:19514 | NaNaKA16\_F13.raw |  |  |  |  | 0 |  |  |  |  |  |  |  |  | 0 | 0 | 0 | 0 | 0 | 0 | 0 | 0 | 0 | 0 | 0 | 0 | 0 | 0 | 135 | 146 |  |  | PEAKS DB |
| E.DGFIADSDIISR.S | Y | 45.89 | 1307.6357 | 12 | 0.4 | 654.8254 | 2 | 61.00 | 5 | F5:25193 | NaNaKA16\_F13.raw |  |  |  |  | 1.257E6 |  |  |  |  |  |  |  |  | 2 | 0 | 0 | 0 | 0 | 2 | 0 | 0 | 0 | 0 | 0 | 0 | 0 | 0 | 737 | 748 |  |  | PEAKS DB |
| W.VVLAVSFTPTK.G | Y | 45.67 | 1160.6804 | 11 | 0.8 | 581.3480 | 2 | 52.57 | 5 | F5:21349 | NaNaKA16\_F13.raw |  |  |  |  | 9.857E4 |  |  |  |  |  |  |  |  | 1 | 0 | 0 | 0 | 0 | 1 | 0 | 0 | 0 | 0 | 0 | 0 | 0 | 0 | 788 | 798 |  |  | PEAKS DB |
| K.GIC(+57.02)VAEPYEIR.V | Y | 45.63 | 1305.6387 | 11 | 0.3 | 653.8268 | 2 | 44.92 | 5 | F5:18246 | NaNaKA16\_F13.raw |  |  |  |  | 6.0645E6 |  |  |  |  |  |  |  |  | 1 | 0 | 0 | 0 | 0 | 1 | 0 | 0 | 0 | 0 | 0 | 0 | 0 | 0 | 799 | 809 | Carbamidomethylation | C3:Carbamidomethylation:1000.00 | PEAKS DB |
| K.IWDTIEK.S | N | 43.23 | 903.4702 | 7 | 0.6 | 452.7426 | 2 | 29.33 | 5 | F5:9813 | NaNaKA16\_F13.raw |  |  |  |  | 4.4002E6 |  |  |  |  |  |  |  |  | 1 | 0 | 0 | 0 | 0 | 1 | 0 | 0 | 0 | 0 | 0 | 0 | 0 | 0 | 598 | 604 |  |  | PEAKS DB |
| K.VAVIIYLNK.V | Y | 42.64 | 1031.6379 | 9 | 0.3 | 516.8264 | 2 | 50.89 | 5 | F5:20439 | NaNaKA16\_F13.raw |  |  |  |  | 2.7389E5 |  |  |  |  |  |  |  |  | 1 | 0 | 0 | 0 | 0 | 1 | 0 | 0 | 0 | 0 | 0 | 0 | 0 | 0 | 1421 | 1429 |  |  | PEAKS DB |
| total 35 peptides |  |  |  |  |  |  |  |  |  |  |  |  |  |  |  |  |  |  |  |  |  |  |  |  |  |  |  |  |  |  |  |  |  |  |  |  |  |  |  |  |  |  |  |
| --- | --- | --- | --- | --- | --- | --- | --- | --- | --- | --- | --- | --- | --- | --- | --- | --- | --- | --- | --- | --- | --- | --- | --- | --- | --- | --- | --- | --- | --- | --- | --- | --- | --- | --- | --- | --- | --- | --- | --- | --- | --- | --- | --- |

AAA68989.1

back to list

  

| Protein Coverage
| Supporting Peptides
|

Protein Coverage:

Supporting Peptides:

| Peptide | Uniq | -10lgP | Mass | Length | ppm | m/z | z | RT | Fraction | Scan | Source File | Area F1 | Area F10 | Area F11 | Area F12 | Area F13 | Area F2 | Area F3 | Area F4 | Area F5 | Area F6 | Area F7 | Area F8 | Area F9 | #Feature | #Feature F1 | #Feature F10 | #Feature F11 | #Feature F12 | #Feature F13 | #Feature F2 | #Feature F3 | #Feature F4 | #Feature F5 | #Feature F6 | #Feature F7 | #Feature F8 | #Feature F9 | Start | End | PTM | AScore | Found By |
| --- | --- | --- | --- | --- | --- | --- | --- | --- | --- | --- | --- | --- | --- | --- | --- | --- | --- | --- | --- | --- | --- | --- | --- | --- | --- | --- | --- | --- | --- | --- | --- | --- | --- | --- | --- | --- | --- | --- | --- | --- | --- | --- | --- |
| R.KC(+57.02)C(+57.02)EDVMHENPMGYTC(+57.02)EK.R | Y | 83.50 | 2286.8835 | 18 | 0.7 | 763.3023 | 3 | 20.65 | 9 | F9:7758 | NaNaKA16\_F5.raw |  |  |  |  |  |  |  |  | 5.1572E7 |  |  |  |  | 2 | 0 | 0 | 0 | 0 | 0 | 0 | 0 | 0 | 2 | 0 | 0 | 0 | 0 | 676 | 693 | Carbamidomethylation | C2:Carbamidomethylation:1000.00;C3:Carbamidomethylation:1000.00;C16:Carbamidomethylation:1000.00 | PEAKS DB |
| K.C(+57.02)C(+57.02)EDVMHENPMGYTC(+57.02)EK.R | Y | 83.21 | 2158.7886 | 17 | 0.2 | 720.6036 | 3 | 31.20 | 9 | F9:15937 | NaNaKA16\_F5.raw |  |  |  |  |  |  |  |  | 5.0681E7 |  |  |  |  | 2 | 0 | 0 | 0 | 0 | 0 | 0 | 0 | 0 | 2 | 0 | 0 | 0 | 0 | 677 | 693 | Carbamidomethylation | C1:Carbamidomethylation:1000.00;C2:Carbamidomethylation:1000.00;C15:Carbamidomethylation:1000.00 | PEAKS DB |
| K.VNDDYLIWGSR.S | Y | 78.30 | 1336.6411 | 11 | 0.2 | 669.3279 | 2 | 59.90 | 4 | F4:38472 | NaNaKA16\_F12.raw |  |  |  | 6.8624E6 | 3.781E6 |  |  |  |  |  |  |  |  | 2 | 0 | 0 | 0 | 1 | 1 | 0 | 0 | 0 | 0 | 0 | 0 | 0 | 0 | 1577 | 1587 |  |  | PEAKS DB |
| K.AC(+57.02)ETNVDYVYK.T | Y | 69.83 | 1360.5969 | 11 | 1.0 | 681.3064 | 2 | 23.73 | 5 | F5:6621 | NaNaKA16\_F13.raw |  |  |  |  | 5.6014E6 |  |  |  |  |  |  |  |  | 1 | 0 | 0 | 0 | 0 | 1 | 0 | 0 | 0 | 0 | 0 | 0 | 0 | 0 | 1515 | 1525 | Carbamidomethylation | C2:Carbamidomethylation:1000.00 | PEAKS DB |
| R.DDNEDGFIADSDIISR.S | Y | 69.48 | 1780.7751 | 16 | 1.0 | 891.3958 | 2 | 68.58 | 5 | F5:27981 | NaNaKA16\_F13.raw |  |  |  |  | 1.4552E6 |  |  |  |  |  |  |  |  | 2 | 0 | 0 | 0 | 0 | 2 | 0 | 0 | 0 | 0 | 0 | 0 | 0 | 0 | 733 | 748 |  |  | PEAKS DB |
| R.QNQYVVVQVTGPQVR.L | N | 69.18 | 1713.9163 | 15 | 0.5 | 857.9658 | 2 | 44.31 | 5 | F5:17897 | NaNaKA16\_F13.raw |  |  |  | 1.095E6 | 1.2725E6 |  |  |  |  |  |  | 0 |  | 3 | 0 | 0 | 0 | 1 | 2 | 0 | 0 | 0 | 0 | 0 | 0 | 0 | 0 | 100 | 114 |  |  | PEAKS DB |
| K.QLDIFVHDFPR.K | N | 68.50 | 1385.7091 | 11 | 0.5 | 462.9105 | 3 | 58.12 | 2 | F2:35492 | NaNaKA16\_F10.raw |  | 4.8609E6 |  | 4.087E6 |  |  |  |  |  |  |  |  |  | 3 | 0 | 1 | 0 | 2 | 0 | 0 | 0 | 0 | 0 | 0 | 0 | 0 | 0 | 53 | 63 |  |  | PEAKS DB |
| K.GDNLIQMPGAAMK.I | Y | 68.40 | 1344.6530 | 13 | 0.9 | 673.3344 | 2 | 49.77 | 5 | F5:20116 | NaNaKA16\_F13.raw |  |  |  |  | 3.458E6 |  |  |  |  |  |  |  |  | 1 | 0 | 0 | 0 | 0 | 1 | 0 | 0 | 0 | 0 | 0 | 0 | 0 | 0 | 552 | 564 |  |  | PEAKS DB |
| G.ALYTLITPAVLR.T | N | 67.00 | 1329.8020 | 12 | 0.9 | 665.9089 | 2 | 76.05 | 2 | F2:51268 | NaNaKA16\_F10.raw |  | 1.5845E7 | 2.1186E6 | 5.6852E6 | 3.5213E6 |  |  |  |  |  |  | 6.0544E5 | 4.0982E5 | 6 | 0 | 1 | 1 | 1 | 1 | 0 | 0 | 0 | 0 | 0 | 0 | 1 | 1 | 23 | 34 |  |  | PEAKS DB |
| R.INYENALLAR.T | Y | 66.45 | 1175.6299 | 10 | 0.9 | 588.8228 | 2 | 45.80 | 5 | F5:18665 | NaNaKA16\_F13.raw |  |  |  | 1.5955E6 | 3.825E6 |  |  |  |  |  |  |  |  | 2 | 0 | 0 | 0 | 1 | 1 | 0 | 0 | 0 | 0 | 0 | 0 | 0 | 0 | 1292 | 1301 |  |  | PEAKS DB |
| K.LNQDITVTASGDGK.A | Y | 66.30 | 1417.7048 | 14 | 0.8 | 709.8603 | 2 | 20.53 | 5 | F5:5136 | NaNaKA16\_F13.raw |  |  |  |  | 2.7531E6 |  |  |  |  |  |  |  |  | 1 | 0 | 0 | 0 | 0 | 1 | 0 | 0 | 0 | 0 | 0 | 0 | 0 | 0 | 1307 | 1320 |  |  | PEAKS DB |
| R.VELLYNPAFC(+57.02)SASTK.G | Y | 65.11 | 1698.8286 | 15 | 0.9 | 850.4224 | 2 | 59.57 | 5 | F5:24606 | NaNaKA16\_F13.raw |  |  |  | 2.9621E5 | 4.7366E6 |  |  |  |  |  |  |  |  | 2 | 0 | 0 | 0 | 1 | 1 | 0 | 0 | 0 | 0 | 0 | 0 | 0 | 0 | 848 | 862 | Carbamidomethylation | C10:Carbamidomethylation:1000.00 | PEAKS DB |
| K.DLTEEPNSQGISSK.T | Y | 64.83 | 1503.7052 | 14 | 0.2 | 752.8600 | 2 | 21.48 | 5 | F5:5531 | NaNaKA16\_F13.raw |  |  |  |  | 5.0484E6 |  |  |  |  |  |  |  |  | 1 | 0 | 0 | 0 | 0 | 1 | 0 | 0 | 0 | 0 | 0 | 0 | 0 | 0 | 761 | 774 |  |  | PEAKS DB |
| K.ASVQEALWSDGVR.K | Y | 64.04 | 1416.6997 | 13 | 0.6 | 709.3575 | 2 | 50.23 | 5 | F5:20313 | NaNaKA16\_F13.raw |  |  |  |  | 3.3991E6 |  |  |  |  |  |  |  |  | 1 | 0 | 0 | 0 | 0 | 1 | 0 | 0 | 0 | 0 | 0 | 0 | 0 | 0 | 898 | 910 |  |  | PEAKS DB |
| K.DTC(+57.02)MGTLVVK.G | N | 63.96 | 1122.5414 | 10 | 1.1 | 562.2786 | 2 | 36.58 | 5 | F5:14251 | NaNaKA16\_F13.raw |  |  |  |  | 3.5107E6 |  |  |  |  |  |  |  |  | 1 | 0 | 0 | 0 | 0 | 1 | 0 | 0 | 0 | 0 | 0 | 0 | 0 | 0 | 542 | 551 | Carbamidomethylation | C3:Carbamidomethylation:1000.00 | PEAKS DB |
| K.GVGGTQLEVIK.A | Y | 62.39 | 1099.6237 | 11 | -0.4 | 550.8189 | 2 | 32.10 | 5 | F5:11471 | NaNaKA16\_F13.raw |  |  |  |  | 3.5972E6 |  |  |  |  |  |  |  |  | 1 | 0 | 0 | 0 | 0 | 1 | 0 | 0 | 0 | 0 | 0 | 0 | 0 | 0 | 936 | 946 |  |  | PEAKS DB |
| K.HFEVGFIQPGSVK.V | N | 61.71 | 1443.7510 | 13 | 0.1 | 722.8828 | 2 | 45.04 | 5 | F5:18328 | NaNaKA16\_F13.raw |  |  |  | 7.8322E5 | 1.9589E6 |  |  |  |  |  |  |  |  | 3 | 0 | 0 | 0 | 1 | 2 | 0 | 0 | 0 | 0 | 0 | 0 | 0 | 0 | 1445 | 1457 |  |  | PEAKS DB |
| K.YFTYLILNK.G | N | 59.48 | 1173.6433 | 9 | 0.6 | 587.8293 | 2 | 64.63 | 4 | F4:42455 | NaNaKA16\_F12.raw |  |  |  | 3.9819E5 | 3.5813E5 |  |  |  |  |  |  |  |  | 2 | 0 | 0 | 0 | 1 | 1 | 0 | 0 | 0 | 0 | 0 | 0 | 0 | 0 | 478 | 486 |  |  | PEAKS DB |
| R.WPHEDEC(+57.02)QEEEFQK.L | Y | 56.35 | 1889.7526 | 14 | 0.4 | 630.9250 | 3 | 23.79 | 4 | F4:8642 | NaNaKA16\_F12.raw |  |  |  | 4.157E6 | 3.5558E6 |  |  |  |  |  |  |  |  | 2 | 0 | 0 | 0 | 1 | 1 | 0 | 0 | 0 | 0 | 0 | 0 | 0 | 0 | 1610 | 1623 | Carbamidomethylation | C7:Carbamidomethylation:1000.00 | PEAKS DB |
| K.VYSYYNLDEK.C | N | 53.87 | 1292.5924 | 10 | -0.2 | 647.3033 | 2 | 35.45 | 5 | F5:13480 | NaNaKA16\_F13.raw |  |  |  |  | 6.2293E6 |  |  |  |  |  |  |  |  | 1 | 0 | 0 | 0 | 0 | 1 | 0 | 0 | 0 | 0 | 0 | 0 | 0 | 0 | 1458 | 1467 |  |  | PEAKS DB |
| R.IEEQDGNDIYVMDVLEVIK.Q | Y | 53.58 | 2221.0823 | 19 | 0.4 | 1111.5488 | 2 | 104.84 | 4 | F4:72376 | NaNaKA16\_F12.raw |  |  |  | 9.1078E5 |  |  |  |  |  |  |  |  |  | 1 | 0 | 0 | 0 | 1 | 0 | 0 | 0 | 0 | 0 | 0 | 0 | 0 | 0 | 1531 | 1549 |  |  | PEAKS DB |
| K.C(+57.02)C(+57.02)EDVMHENPM(+15.99)GYTC(+57.02)EK.R | Y | 52.94 | 2174.7837 | 17 | 0.3 | 725.9354 | 3 | 19.23 | 9 | F9:6612 | NaNaKA16\_F5.raw |  |  |  |  |  |  |  |  | 4.2545E6 |  |  |  |  | 1 | 0 | 0 | 0 | 0 | 0 | 0 | 0 | 0 | 1 | 0 | 0 | 0 | 0 | 677 | 693 | Carbamidomethylation; Oxidation (M) | C1:Carbamidomethylation:1000.00;C2:Carbamidomethylation:1000.00;M11:Oxidation (M):62.41;C15:Carbamidomethylation:1000.00 | PEAKS DB |
| R.IDVPLQIEK.A | Y | 52.36 | 1053.6069 | 9 | 1.3 | 527.8114 | 2 | 47.84 | 4 | F4:28430 | NaNaKA16\_F12.raw |  |  |  | 8.3958E6 | 6.968E6 |  |  |  |  |  |  |  |  | 2 | 0 | 0 | 0 | 1 | 1 | 0 | 0 | 0 | 0 | 0 | 0 | 0 | 0 | 1506 | 1514 |  |  | PEAKS DB |
| K.GDNLIQM(+15.99)PGAAMK.I | Y | 51.92 | 1360.6479 | 13 | -0.1 | 681.3312 | 2 | 30.12 | 5 | F5:10521 | NaNaKA16\_F13.raw |  |  |  |  | 7.1208E5 |  |  |  |  |  |  |  |  | 1 | 0 | 0 | 0 | 0 | 1 | 0 | 0 | 0 | 0 | 0 | 0 | 0 | 0 | 552 | 564 | Oxidation (M) | M7:Oxidation (M):35.03 | PEAKS DB |
| K.YVLPSFEVR.L | N | 51.15 | 1108.5917 | 9 | 0.2 | 555.3032 | 2 | 58.46 | 4 | F4:37268 | NaNaKA16\_F12.raw |  |  |  | 1.8594E6 | 2.5932E6 |  |  |  |  |  |  |  |  | 2 | 0 | 0 | 0 | 1 | 1 | 0 | 0 | 0 | 0 | 0 | 0 | 0 | 0 | 219 | 227 |  |  | PEAKS DB |
| K.LDDRVPDTEIETK.I | Y | 48.94 | 1529.7573 | 13 | 0.1 | 510.9265 | 3 | 24.23 | 5 | F5:6922 | NaNaKA16\_F13.raw |  |  |  |  | 2.685E5 |  |  |  |  |  |  |  |  | 1 | 0 | 0 | 0 | 0 | 1 | 0 | 0 | 0 | 0 | 0 | 0 | 0 | 0 | 950 | 962 |  |  | PEAKS DB |
| R.AVPFVIVPLEQGLHDVEIK.A | Y | 48.75 | 2102.1775 | 19 | 0.1 | 701.7332 | 3 | 86.03 | 4 | F4:59879 | NaNaKA16\_F12.raw |  |  |  | 5.8004E5 |  |  |  |  |  |  |  |  |  | 1 | 0 | 0 | 0 | 1 | 0 | 0 | 0 | 0 | 0 | 0 | 0 | 0 | 0 | 879 | 897 |  |  | PEAKS DB |
| H.ITPDLIPSFR.F | N | 47.01 | 1157.6444 | 10 | 0.6 | 579.8298 | 2 | 65.39 | 5 | F5:26870 | NaNaKA16\_F13.raw |  |  |  |  | 4.6001E5 |  |  |  |  |  |  |  |  | 1 | 0 | 0 | 0 | 0 | 1 | 0 | 0 | 0 | 0 | 0 | 0 | 0 | 0 | 510 | 519 |  |  | PEAKS DB |
| R.IPIIDGDGK.A | Y | 46.67 | 926.5073 | 9 | 0.6 | 464.2612 | 2 | 33.77 | 5 | F5:12591 | NaNaKA16\_F13.raw |  |  |  |  | 5.1851E6 |  |  |  |  |  |  |  |  | 1 | 0 | 0 | 0 | 0 | 1 | 0 | 0 | 0 | 0 | 0 | 0 | 0 | 0 | 283 | 291 |  |  | PEAKS DB |
| K.GIYTPGSPVLYR.V | N | 46.58 | 1321.7030 | 12 | -0.9 | 661.8582 | 2 | 48.12 | 5 | F5:19514 | NaNaKA16\_F13.raw |  |  |  |  | 0 |  |  |  |  |  |  |  |  | 0 | 0 | 0 | 0 | 0 | 0 | 0 | 0 | 0 | 0 | 0 | 0 | 0 | 0 | 135 | 146 |  |  | PEAKS DB |
| E.DGFIADSDIISR.S | Y | 45.89 | 1307.6357 | 12 | 0.4 | 654.8254 | 2 | 61.00 | 5 | F5:25193 | NaNaKA16\_F13.raw |  |  |  |  | 1.257E6 |  |  |  |  |  |  |  |  | 2 | 0 | 0 | 0 | 0 | 2 | 0 | 0 | 0 | 0 | 0 | 0 | 0 | 0 | 737 | 748 |  |  | PEAKS DB |
| W.VVLAVSFTPTK.G | Y | 45.67 | 1160.6804 | 11 | 0.8 | 581.3480 | 2 | 52.57 | 5 | F5:21349 | NaNaKA16\_F13.raw |  |  |  |  | 9.857E4 |  |  |  |  |  |  |  |  | 1 | 0 | 0 | 0 | 0 | 1 | 0 | 0 | 0 | 0 | 0 | 0 | 0 | 0 | 788 | 798 |  |  | PEAKS DB |
| K.GIC(+57.02)VAEPYEIR.V | Y | 45.63 | 1305.6387 | 11 | 0.3 | 653.8268 | 2 | 44.92 | 5 | F5:18246 | NaNaKA16\_F13.raw |  |  |  |  | 6.0645E6 |  |  |  |  |  |  |  |  | 1 | 0 | 0 | 0 | 0 | 1 | 0 | 0 | 0 | 0 | 0 | 0 | 0 | 0 | 799 | 809 | Carbamidomethylation | C3:Carbamidomethylation:1000.00 | PEAKS DB |
| K.IWDTIEK.S | N | 43.23 | 903.4702 | 7 | 0.6 | 452.7426 | 2 | 29.33 | 5 | F5:9813 | NaNaKA16\_F13.raw |  |  |  |  | 4.4002E6 |  |  |  |  |  |  |  |  | 1 | 0 | 0 | 0 | 0 | 1 | 0 | 0 | 0 | 0 | 0 | 0 | 0 | 0 | 598 | 604 |  |  | PEAKS DB |
| K.VAVIIYLNK.V | Y | 42.64 | 1031.6379 | 9 | 0.3 | 516.8264 | 2 | 50.89 | 5 | F5:20439 | NaNaKA16\_F13.raw |  |  |  |  | 2.7389E5 |  |  |  |  |  |  |  |  | 1 | 0 | 0 | 0 | 0 | 1 | 0 | 0 | 0 | 0 | 0 | 0 | 0 | 0 | 1421 | 1429 |  |  | PEAKS DB |
| total 35 peptides |  |  |  |  |  |  |  |  |  |  |  |  |  |  |  |  |  |  |  |  |  |  |  |  |  |  |  |  |  |  |  |  |  |  |  |  |  |  |  |  |  |  |  |
| --- | --- | --- | --- | --- | --- | --- | --- | --- | --- | --- | --- | --- | --- | --- | --- | --- | --- | --- | --- | --- | --- | --- | --- | --- | --- | --- | --- | --- | --- | --- | --- | --- | --- | --- | --- | --- | --- | --- | --- | --- | --- | --- | --- |

CAI46845.1

back to list

  

| Protein Coverage
| Supporting Peptides
|

Protein Coverage:

Supporting Peptides:

| Peptide | Uniq | -10lgP | Mass | Length | ppm | m/z | z | RT | Fraction | Scan | Source File | Area F1 | Area F10 | Area F11 | Area F12 | Area F13 | Area F2 | Area F3 | Area F4 | Area F5 | Area F6 | Area F7 | Area F8 | Area F9 | #Feature | #Feature F1 | #Feature F10 | #Feature F11 | #Feature F12 | #Feature F13 | #Feature F2 | #Feature F3 | #Feature F4 | #Feature F5 | #Feature F6 | #Feature F7 | #Feature F8 | #Feature F9 | Start | End | PTM | AScore | Found By |
| --- | --- | --- | --- | --- | --- | --- | --- | --- | --- | --- | --- | --- | --- | --- | --- | --- | --- | --- | --- | --- | --- | --- | --- | --- | --- | --- | --- | --- | --- | --- | --- | --- | --- | --- | --- | --- | --- | --- | --- | --- | --- | --- | --- |
| R.KC(+57.02)C(+57.02)EDVMHENPMGYTC(+57.02)EK.R | Y | 83.50 | 2286.8835 | 18 | 0.7 | 763.3023 | 3 | 20.65 | 9 | F9:7758 | NaNaKA16\_F5.raw |  |  |  |  |  |  |  |  | 5.1572E7 |  |  |  |  | 2 | 0 | 0 | 0 | 0 | 0 | 0 | 0 | 0 | 2 | 0 | 0 | 0 | 0 | 676 | 693 | Carbamidomethylation | C2:Carbamidomethylation:1000.00;C3:Carbamidomethylation:1000.00;C16:Carbamidomethylation:1000.00 | PEAKS DB |
| K.C(+57.02)C(+57.02)EDVMHENPMGYTC(+57.02)EK.R | Y | 83.21 | 2158.7886 | 17 | 0.2 | 720.6036 | 3 | 31.20 | 9 | F9:15937 | NaNaKA16\_F5.raw |  |  |  |  |  |  |  |  | 5.0681E7 |  |  |  |  | 2 | 0 | 0 | 0 | 0 | 0 | 0 | 0 | 0 | 2 | 0 | 0 | 0 | 0 | 677 | 693 | Carbamidomethylation | C1:Carbamidomethylation:1000.00;C2:Carbamidomethylation:1000.00;C15:Carbamidomethylation:1000.00 | PEAKS DB |
| K.VNDDYLIWGSR.S | Y | 78.30 | 1336.6411 | 11 | 0.2 | 669.3279 | 2 | 59.90 | 4 | F4:38472 | NaNaKA16\_F12.raw |  |  |  | 6.8624E6 | 3.781E6 |  |  |  |  |  |  |  |  | 2 | 0 | 0 | 0 | 1 | 1 | 0 | 0 | 0 | 0 | 0 | 0 | 0 | 0 | 1577 | 1587 |  |  | PEAKS DB |
| K.AC(+57.02)ETNVDYVYK.T | Y | 69.83 | 1360.5969 | 11 | 1.0 | 681.3064 | 2 | 23.73 | 5 | F5:6621 | NaNaKA16\_F13.raw |  |  |  |  | 5.6014E6 |  |  |  |  |  |  |  |  | 1 | 0 | 0 | 0 | 0 | 1 | 0 | 0 | 0 | 0 | 0 | 0 | 0 | 0 | 1515 | 1525 | Carbamidomethylation | C2:Carbamidomethylation:1000.00 | PEAKS DB |
| R.DDNEDGFIADSDIISR.S | Y | 69.48 | 1780.7751 | 16 | 1.0 | 891.3958 | 2 | 68.58 | 5 | F5:27981 | NaNaKA16\_F13.raw |  |  |  |  | 1.4552E6 |  |  |  |  |  |  |  |  | 2 | 0 | 0 | 0 | 0 | 2 | 0 | 0 | 0 | 0 | 0 | 0 | 0 | 0 | 733 | 748 |  |  | PEAKS DB |
| R.QNQYVVVQVTGPQVR.L | N | 69.18 | 1713.9163 | 15 | 0.5 | 857.9658 | 2 | 44.31 | 5 | F5:17897 | NaNaKA16\_F13.raw |  |  |  | 1.095E6 | 1.2725E6 |  |  |  |  |  |  | 0 |  | 3 | 0 | 0 | 0 | 1 | 2 | 0 | 0 | 0 | 0 | 0 | 0 | 0 | 0 | 100 | 114 |  |  | PEAKS DB |
| K.QLDIFVHDFPR.K | N | 68.50 | 1385.7091 | 11 | 0.5 | 462.9105 | 3 | 58.12 | 2 | F2:35492 | NaNaKA16\_F10.raw |  | 4.8609E6 |  | 4.087E6 |  |  |  |  |  |  |  |  |  | 3 | 0 | 1 | 0 | 2 | 0 | 0 | 0 | 0 | 0 | 0 | 0 | 0 | 0 | 53 | 63 |  |  | PEAKS DB |
| K.GDNLIQMPGAAMK.I | Y | 68.40 | 1344.6530 | 13 | 0.9 | 673.3344 | 2 | 49.77 | 5 | F5:20116 | NaNaKA16\_F13.raw |  |  |  |  | 3.458E6 |  |  |  |  |  |  |  |  | 1 | 0 | 0 | 0 | 0 | 1 | 0 | 0 | 0 | 0 | 0 | 0 | 0 | 0 | 552 | 564 |  |  | PEAKS DB |
| G.ALYTLITPAVLR.T | N | 67.00 | 1329.8020 | 12 | 0.9 | 665.9089 | 2 | 76.05 | 2 | F2:51268 | NaNaKA16\_F10.raw |  | 1.5845E7 | 2.1186E6 | 5.6852E6 | 3.5213E6 |  |  |  |  |  |  | 6.0544E5 | 4.0982E5 | 6 | 0 | 1 | 1 | 1 | 1 | 0 | 0 | 0 | 0 | 0 | 0 | 1 | 1 | 23 | 34 |  |  | PEAKS DB |
| R.INYENALLAR.T | Y | 66.45 | 1175.6299 | 10 | 0.9 | 588.8228 | 2 | 45.80 | 5 | F5:18665 | NaNaKA16\_F13.raw |  |  |  | 1.5955E6 | 3.825E6 |  |  |  |  |  |  |  |  | 2 | 0 | 0 | 0 | 1 | 1 | 0 | 0 | 0 | 0 | 0 | 0 | 0 | 0 | 1292 | 1301 |  |  | PEAKS DB |
| K.LNQDITVTASGDGK.A | Y | 66.30 | 1417.7048 | 14 | 0.8 | 709.8603 | 2 | 20.53 | 5 | F5:5136 | NaNaKA16\_F13.raw |  |  |  |  | 2.7531E6 |  |  |  |  |  |  |  |  | 1 | 0 | 0 | 0 | 0 | 1 | 0 | 0 | 0 | 0 | 0 | 0 | 0 | 0 | 1307 | 1320 |  |  | PEAKS DB |
| R.VELLYNPAFC(+57.02)SASTK.G | Y | 65.11 | 1698.8286 | 15 | 0.9 | 850.4224 | 2 | 59.57 | 5 | F5:24606 | NaNaKA16\_F13.raw |  |  |  | 2.9621E5 | 4.7366E6 |  |  |  |  |  |  |  |  | 2 | 0 | 0 | 0 | 1 | 1 | 0 | 0 | 0 | 0 | 0 | 0 | 0 | 0 | 848 | 862 | Carbamidomethylation | C10:Carbamidomethylation:1000.00 | PEAKS DB |
| K.DLTEEPNSQGISSK.T | Y | 64.83 | 1503.7052 | 14 | 0.2 | 752.8600 | 2 | 21.48 | 5 | F5:5531 | NaNaKA16\_F13.raw |  |  |  |  | 5.0484E6 |  |  |  |  |  |  |  |  | 1 | 0 | 0 | 0 | 0 | 1 | 0 | 0 | 0 | 0 | 0 | 0 | 0 | 0 | 761 | 774 |  |  | PEAKS DB |
| K.ASVQEALWSDGVR.K | Y | 64.04 | 1416.6997 | 13 | 0.6 | 709.3575 | 2 | 50.23 | 5 | F5:20313 | NaNaKA16\_F13.raw |  |  |  |  | 3.3991E6 |  |  |  |  |  |  |  |  | 1 | 0 | 0 | 0 | 0 | 1 | 0 | 0 | 0 | 0 | 0 | 0 | 0 | 0 | 898 | 910 |  |  | PEAKS DB |
| K.DTC(+57.02)MGTLVVK.G | N | 63.96 | 1122.5414 | 10 | 1.1 | 562.2786 | 2 | 36.58 | 5 | F5:14251 | NaNaKA16\_F13.raw |  |  |  |  | 3.5107E6 |  |  |  |  |  |  |  |  | 1 | 0 | 0 | 0 | 0 | 1 | 0 | 0 | 0 | 0 | 0 | 0 | 0 | 0 | 542 | 551 | Carbamidomethylation | C3:Carbamidomethylation:1000.00 | PEAKS DB |
| K.GVGGTQLEVIK.A | Y | 62.39 | 1099.6237 | 11 | -0.4 | 550.8189 | 2 | 32.10 | 5 | F5:11471 | NaNaKA16\_F13.raw |  |  |  |  | 3.5972E6 |  |  |  |  |  |  |  |  | 1 | 0 | 0 | 0 | 0 | 1 | 0 | 0 | 0 | 0 | 0 | 0 | 0 | 0 | 936 | 946 |  |  | PEAKS DB |
| K.HFEVGFIQPGSVK.V | N | 61.71 | 1443.7510 | 13 | 0.1 | 722.8828 | 2 | 45.04 | 5 | F5:18328 | NaNaKA16\_F13.raw |  |  |  | 7.8322E5 | 1.9589E6 |  |  |  |  |  |  |  |  | 3 | 0 | 0 | 0 | 1 | 2 | 0 | 0 | 0 | 0 | 0 | 0 | 0 | 0 | 1445 | 1457 |  |  | PEAKS DB |
| K.YFTYLILNK.G | N | 59.48 | 1173.6433 | 9 | 0.6 | 587.8293 | 2 | 64.63 | 4 | F4:42455 | NaNaKA16\_F12.raw |  |  |  | 3.9819E5 | 3.5813E5 |  |  |  |  |  |  |  |  | 2 | 0 | 0 | 0 | 1 | 1 | 0 | 0 | 0 | 0 | 0 | 0 | 0 | 0 | 478 | 486 |  |  | PEAKS DB |
| R.WPHEDEC(+57.02)QEEEFQK.L | Y | 56.35 | 1889.7526 | 14 | 0.4 | 630.9250 | 3 | 23.79 | 4 | F4:8642 | NaNaKA16\_F12.raw |  |  |  | 4.157E6 | 3.5558E6 |  |  |  |  |  |  |  |  | 2 | 0 | 0 | 0 | 1 | 1 | 0 | 0 | 0 | 0 | 0 | 0 | 0 | 0 | 1610 | 1623 | Carbamidomethylation | C7:Carbamidomethylation:1000.00 | PEAKS DB |
| K.VYSYYNLDEK.C | N | 53.87 | 1292.5924 | 10 | -0.2 | 647.3033 | 2 | 35.45 | 5 | F5:13480 | NaNaKA16\_F13.raw |  |  |  |  | 6.2293E6 |  |  |  |  |  |  |  |  | 1 | 0 | 0 | 0 | 0 | 1 | 0 | 0 | 0 | 0 | 0 | 0 | 0 | 0 | 1458 | 1467 |  |  | PEAKS DB |
| R.IEEQDGNDIYVMDVLEVIK.Q | Y | 53.58 | 2221.0823 | 19 | 0.4 | 1111.5488 | 2 | 104.84 | 4 | F4:72376 | NaNaKA16\_F12.raw |  |  |  | 9.1078E5 |  |  |  |  |  |  |  |  |  | 1 | 0 | 0 | 0 | 1 | 0 | 0 | 0 | 0 | 0 | 0 | 0 | 0 | 0 | 1531 | 1549 |  |  | PEAKS DB |
| K.C(+57.02)C(+57.02)EDVMHENPM(+15.99)GYTC(+57.02)EK.R | Y | 52.94 | 2174.7837 | 17 | 0.3 | 725.9354 | 3 | 19.23 | 9 | F9:6612 | NaNaKA16\_F5.raw |  |  |  |  |  |  |  |  | 4.2545E6 |  |  |  |  | 1 | 0 | 0 | 0 | 0 | 0 | 0 | 0 | 0 | 1 | 0 | 0 | 0 | 0 | 677 | 693 | Carbamidomethylation; Oxidation (M) | C1:Carbamidomethylation:1000.00;C2:Carbamidomethylation:1000.00;M11:Oxidation (M):62.41;C15:Carbamidomethylation:1000.00 | PEAKS DB |
| R.IDVPLQIEK.A | Y | 52.36 | 1053.6069 | 9 | 1.3 | 527.8114 | 2 | 47.84 | 4 | F4:28430 | NaNaKA16\_F12.raw |  |  |  | 8.3958E6 | 6.968E6 |  |  |  |  |  |  |  |  | 2 | 0 | 0 | 0 | 1 | 1 | 0 | 0 | 0 | 0 | 0 | 0 | 0 | 0 | 1506 | 1514 |  |  | PEAKS DB |
| K.GDNLIQM(+15.99)PGAAMK.I | Y | 51.92 | 1360.6479 | 13 | -0.1 | 681.3312 | 2 | 30.12 | 5 | F5:10521 | NaNaKA16\_F13.raw |  |  |  |  | 7.1208E5 |  |  |  |  |  |  |  |  | 1 | 0 | 0 | 0 | 0 | 1 | 0 | 0 | 0 | 0 | 0 | 0 | 0 | 0 | 552 | 564 | Oxidation (M) | M7:Oxidation (M):35.03 | PEAKS DB |
| K.YVLPSFEVR.L | N | 51.15 | 1108.5917 | 9 | 0.2 | 555.3032 | 2 | 58.46 | 4 | F4:37268 | NaNaKA16\_F12.raw |  |  |  | 1.8594E6 | 2.5932E6 |  |  |  |  |  |  |  |  | 2 | 0 | 0 | 0 | 1 | 1 | 0 | 0 | 0 | 0 | 0 | 0 | 0 | 0 | 219 | 227 |  |  | PEAKS DB |
| K.LDDRVPDTEIETK.I | Y | 48.94 | 1529.7573 | 13 | 0.1 | 510.9265 | 3 | 24.23 | 5 | F5:6922 | NaNaKA16\_F13.raw |  |  |  |  | 2.685E5 |  |  |  |  |  |  |  |  | 1 | 0 | 0 | 0 | 0 | 1 | 0 | 0 | 0 | 0 | 0 | 0 | 0 | 0 | 950 | 962 |  |  | PEAKS DB |
| R.AVPFVIVPLEQGLHDVEIK.A | Y | 48.75 | 2102.1775 | 19 | 0.1 | 701.7332 | 3 | 86.03 | 4 | F4:59879 | NaNaKA16\_F12.raw |  |  |  | 5.8004E5 |  |  |  |  |  |  |  |  |  | 1 | 0 | 0 | 0 | 1 | 0 | 0 | 0 | 0 | 0 | 0 | 0 | 0 | 0 | 879 | 897 |  |  | PEAKS DB |
| H.ITPDLIPSFR.F | N | 47.01 | 1157.6444 | 10 | 0.6 | 579.8298 | 2 | 65.39 | 5 | F5:26870 | NaNaKA16\_F13.raw |  |  |  |  | 4.6001E5 |  |  |  |  |  |  |  |  | 1 | 0 | 0 | 0 | 0 | 1 | 0 | 0 | 0 | 0 | 0 | 0 | 0 | 0 | 510 | 519 |  |  | PEAKS DB |
| R.IPIIDGDGK.A | Y | 46.67 | 926.5073 | 9 | 0.6 | 464.2612 | 2 | 33.77 | 5 | F5:12591 | NaNaKA16\_F13.raw |  |  |  |  | 5.1851E6 |  |  |  |  |  |  |  |  | 1 | 0 | 0 | 0 | 0 | 1 | 0 | 0 | 0 | 0 | 0 | 0 | 0 | 0 | 283 | 291 |  |  | PEAKS DB |
| K.GIYTPGSPVLYR.V | N | 46.58 | 1321.7030 | 12 | -0.9 | 661.8582 | 2 | 48.12 | 5 | F5:19514 | NaNaKA16\_F13.raw |  |  |  |  | 0 |  |  |  |  |  |  |  |  | 0 | 0 | 0 | 0 | 0 | 0 | 0 | 0 | 0 | 0 | 0 | 0 | 0 | 0 | 135 | 146 |  |  | PEAKS DB |
| E.DGFIADSDIISR.S | Y | 45.89 | 1307.6357 | 12 | 0.4 | 654.8254 | 2 | 61.00 | 5 | F5:25193 | NaNaKA16\_F13.raw |  |  |  |  | 1.257E6 |  |  |  |  |  |  |  |  | 2 | 0 | 0 | 0 | 0 | 2 | 0 | 0 | 0 | 0 | 0 | 0 | 0 | 0 | 737 | 748 |  |  | PEAKS DB |
| W.VVLAVSFTPTK.G | Y | 45.67 | 1160.6804 | 11 | 0.8 | 581.3480 | 2 | 52.57 | 5 | F5:21349 | NaNaKA16\_F13.raw |  |  |  |  | 9.857E4 |  |  |  |  |  |  |  |  | 1 | 0 | 0 | 0 | 0 | 1 | 0 | 0 | 0 | 0 | 0 | 0 | 0 | 0 | 788 | 798 |  |  | PEAKS DB |
| K.GIC(+57.02)VAEPYEIR.V | Y | 45.63 | 1305.6387 | 11 | 0.3 | 653.8268 | 2 | 44.92 | 5 | F5:18246 | NaNaKA16\_F13.raw |  |  |  |  | 6.0645E6 |  |  |  |  |  |  |  |  | 1 | 0 | 0 | 0 | 0 | 1 | 0 | 0 | 0 | 0 | 0 | 0 | 0 | 0 | 799 | 809 | Carbamidomethylation | C3:Carbamidomethylation:1000.00 | PEAKS DB |
| K.IWDTIEK.S | N | 43.23 | 903.4702 | 7 | 0.6 | 452.7426 | 2 | 29.33 | 5 | F5:9813 | NaNaKA16\_F13.raw |  |  |  |  | 4.4002E6 |  |  |  |  |  |  |  |  | 1 | 0 | 0 | 0 | 0 | 1 | 0 | 0 | 0 | 0 | 0 | 0 | 0 | 0 | 598 | 604 |  |  | PEAKS DB |
| K.VAVIIYLNK.V | Y | 42.64 | 1031.6379 | 9 | 0.3 | 516.8264 | 2 | 50.89 | 5 | F5:20439 | NaNaKA16\_F13.raw |  |  |  |  | 2.7389E5 |  |  |  |  |  |  |  |  | 1 | 0 | 0 | 0 | 0 | 1 | 0 | 0 | 0 | 0 | 0 | 0 | 0 | 0 | 1421 | 1429 |  |  | PEAKS DB |
| total 35 peptides |  |  |  |  |  |  |  |  |  |  |  |  |  |  |  |  |  |  |  |  |  |  |  |  |  |  |  |  |  |  |  |  |  |  |  |  |  |  |  |  |  |  |  |
| --- | --- | --- | --- | --- | --- | --- | --- | --- | --- | --- | --- | --- | --- | --- | --- | --- | --- | --- | --- | --- | --- | --- | --- | --- | --- | --- | --- | --- | --- | --- | --- | --- | --- | --- | --- | --- | --- | --- | --- | --- | --- | --- | --- |

6I2X

back to list

  

| Protein Coverage
| Supporting Peptides
|

Protein Coverage:

Supporting Peptides:

| Peptide | Uniq | -10lgP | Mass | Length | ppm | m/z | z | RT | Fraction | Scan | Source File | Area F1 | Area F10 | Area F11 | Area F12 | Area F13 | Area F2 | Area F3 | Area F4 | Area F5 | Area F6 | Area F7 | Area F8 | Area F9 | #Feature | #Feature F1 | #Feature F10 | #Feature F11 | #Feature F12 | #Feature F13 | #Feature F2 | #Feature F3 | #Feature F4 | #Feature F5 | #Feature F6 | #Feature F7 | #Feature F8 | #Feature F9 | Start | End | PTM | AScore | Found By |
| --- | --- | --- | --- | --- | --- | --- | --- | --- | --- | --- | --- | --- | --- | --- | --- | --- | --- | --- | --- | --- | --- | --- | --- | --- | --- | --- | --- | --- | --- | --- | --- | --- | --- | --- | --- | --- | --- | --- | --- | --- | --- | --- | --- |
| R.KC(+57.02)C(+57.02)EDVMHENPMGYTC(+57.02)EK.R | Y | 83.50 | 2286.8835 | 18 | 0.7 | 763.3023 | 3 | 20.65 | 9 | F9:7758 | NaNaKA16\_F5.raw |  |  |  |  |  |  |  |  | 5.1572E7 |  |  |  |  | 2 | 0 | 0 | 0 | 0 | 0 | 0 | 0 | 0 | 2 | 0 | 0 | 0 | 0 | 676 | 693 | Carbamidomethylation | C2:Carbamidomethylation:1000.00;C3:Carbamidomethylation:1000.00;C16:Carbamidomethylation:1000.00 | PEAKS DB |
| K.C(+57.02)C(+57.02)EDVMHENPMGYTC(+57.02)EK.R | Y | 83.21 | 2158.7886 | 17 | 0.2 | 720.6036 | 3 | 31.20 | 9 | F9:15937 | NaNaKA16\_F5.raw |  |  |  |  |  |  |  |  | 5.0681E7 |  |  |  |  | 2 | 0 | 0 | 0 | 0 | 0 | 0 | 0 | 0 | 2 | 0 | 0 | 0 | 0 | 677 | 693 | Carbamidomethylation | C1:Carbamidomethylation:1000.00;C2:Carbamidomethylation:1000.00;C15:Carbamidomethylation:1000.00 | PEAKS DB |
| K.VNDDYLIWGSR.S | Y | 78.30 | 1336.6411 | 11 | 0.2 | 669.3279 | 2 | 59.90 | 4 | F4:38472 | NaNaKA16\_F12.raw |  |  |  | 6.8624E6 | 3.781E6 |  |  |  |  |  |  |  |  | 2 | 0 | 0 | 0 | 1 | 1 | 0 | 0 | 0 | 0 | 0 | 0 | 0 | 0 | 1577 | 1587 |  |  | PEAKS DB |
| K.AC(+57.02)ETNVDYVYK.T | Y | 69.83 | 1360.5969 | 11 | 1.0 | 681.3064 | 2 | 23.73 | 5 | F5:6621 | NaNaKA16\_F13.raw |  |  |  |  | 5.6014E6 |  |  |  |  |  |  |  |  | 1 | 0 | 0 | 0 | 0 | 1 | 0 | 0 | 0 | 0 | 0 | 0 | 0 | 0 | 1515 | 1525 | Carbamidomethylation | C2:Carbamidomethylation:1000.00 | PEAKS DB |
| R.DDNEDGFIADSDIISR.S | Y | 69.48 | 1780.7751 | 16 | 1.0 | 891.3958 | 2 | 68.58 | 5 | F5:27981 | NaNaKA16\_F13.raw |  |  |  |  | 1.4552E6 |  |  |  |  |  |  |  |  | 2 | 0 | 0 | 0 | 0 | 2 | 0 | 0 | 0 | 0 | 0 | 0 | 0 | 0 | 733 | 748 |  |  | PEAKS DB |
| R.QNQYVVVQVTGPQVR.L | N | 69.18 | 1713.9163 | 15 | 0.5 | 857.9658 | 2 | 44.31 | 5 | F5:17897 | NaNaKA16\_F13.raw |  |  |  | 1.095E6 | 1.2725E6 |  |  |  |  |  |  | 0 |  | 3 | 0 | 0 | 0 | 1 | 2 | 0 | 0 | 0 | 0 | 0 | 0 | 0 | 0 | 100 | 114 |  |  | PEAKS DB |
| K.QLDIFVHDFPR.K | N | 68.50 | 1385.7091 | 11 | 0.5 | 462.9105 | 3 | 58.12 | 2 | F2:35492 | NaNaKA16\_F10.raw |  | 4.8609E6 |  | 4.087E6 |  |  |  |  |  |  |  |  |  | 3 | 0 | 1 | 0 | 2 | 0 | 0 | 0 | 0 | 0 | 0 | 0 | 0 | 0 | 53 | 63 |  |  | PEAKS DB |
| K.GDNLIQMPGAAMK.I | Y | 68.40 | 1344.6530 | 13 | 0.9 | 673.3344 | 2 | 49.77 | 5 | F5:20116 | NaNaKA16\_F13.raw |  |  |  |  | 3.458E6 |  |  |  |  |  |  |  |  | 1 | 0 | 0 | 0 | 0 | 1 | 0 | 0 | 0 | 0 | 0 | 0 | 0 | 0 | 552 | 564 |  |  | PEAKS DB |
| G.ALYTLITPAVLR.T | N | 67.00 | 1329.8020 | 12 | 0.9 | 665.9089 | 2 | 76.05 | 2 | F2:51268 | NaNaKA16\_F10.raw |  | 1.5845E7 | 2.1186E6 | 5.6852E6 | 3.5213E6 |  |  |  |  |  |  | 6.0544E5 | 4.0982E5 | 6 | 0 | 1 | 1 | 1 | 1 | 0 | 0 | 0 | 0 | 0 | 0 | 1 | 1 | 23 | 34 |  |  | PEAKS DB |
| R.INYENALLAR.T | Y | 66.45 | 1175.6299 | 10 | 0.9 | 588.8228 | 2 | 45.80 | 5 | F5:18665 | NaNaKA16\_F13.raw |  |  |  | 1.5955E6 | 3.825E6 |  |  |  |  |  |  |  |  | 2 | 0 | 0 | 0 | 1 | 1 | 0 | 0 | 0 | 0 | 0 | 0 | 0 | 0 | 1292 | 1301 |  |  | PEAKS DB |
| K.LNQDITVTASGDGK.A | Y | 66.30 | 1417.7048 | 14 | 0.8 | 709.8603 | 2 | 20.53 | 5 | F5:5136 | NaNaKA16\_F13.raw |  |  |  |  | 2.7531E6 |  |  |  |  |  |  |  |  | 1 | 0 | 0 | 0 | 0 | 1 | 0 | 0 | 0 | 0 | 0 | 0 | 0 | 0 | 1307 | 1320 |  |  | PEAKS DB |
| R.VELLYNPAFC(+57.02)SASTK.G | Y | 65.11 | 1698.8286 | 15 | 0.9 | 850.4224 | 2 | 59.57 | 5 | F5:24606 | NaNaKA16\_F13.raw |  |  |  | 2.9621E5 | 4.7366E6 |  |  |  |  |  |  |  |  | 2 | 0 | 0 | 0 | 1 | 1 | 0 | 0 | 0 | 0 | 0 | 0 | 0 | 0 | 848 | 862 | Carbamidomethylation | C10:Carbamidomethylation:1000.00 | PEAKS DB |
| K.DLTEEPNSQGISSK.T | Y | 64.83 | 1503.7052 | 14 | 0.2 | 752.8600 | 2 | 21.48 | 5 | F5:5531 | NaNaKA16\_F13.raw |  |  |  |  | 5.0484E6 |  |  |  |  |  |  |  |  | 1 | 0 | 0 | 0 | 0 | 1 | 0 | 0 | 0 | 0 | 0 | 0 | 0 | 0 | 761 | 774 |  |  | PEAKS DB |
| K.ASVQEALWSDGVR.K | Y | 64.04 | 1416.6997 | 13 | 0.6 | 709.3575 | 2 | 50.23 | 5 | F5:20313 | NaNaKA16\_F13.raw |  |  |  |  | 3.3991E6 |  |  |  |  |  |  |  |  | 1 | 0 | 0 | 0 | 0 | 1 | 0 | 0 | 0 | 0 | 0 | 0 | 0 | 0 | 898 | 910 |  |  | PEAKS DB |
| K.DTC(+57.02)MGTLVVK.G | N | 63.96 | 1122.5414 | 10 | 1.1 | 562.2786 | 2 | 36.58 | 5 | F5:14251 | NaNaKA16\_F13.raw |  |  |  |  | 3.5107E6 |  |  |  |  |  |  |  |  | 1 | 0 | 0 | 0 | 0 | 1 | 0 | 0 | 0 | 0 | 0 | 0 | 0 | 0 | 542 | 551 | Carbamidomethylation | C3:Carbamidomethylation:1000.00 | PEAKS DB |
| K.GVGGTQLEVIK.A | Y | 62.39 | 1099.6237 | 11 | -0.4 | 550.8189 | 2 | 32.10 | 5 | F5:11471 | NaNaKA16\_F13.raw |  |  |  |  | 3.5972E6 |  |  |  |  |  |  |  |  | 1 | 0 | 0 | 0 | 0 | 1 | 0 | 0 | 0 | 0 | 0 | 0 | 0 | 0 | 936 | 946 |  |  | PEAKS DB |
| K.HFEVGFIQPGSVK.V | N | 61.71 | 1443.7510 | 13 | 0.1 | 722.8828 | 2 | 45.04 | 5 | F5:18328 | NaNaKA16\_F13.raw |  |  |  | 7.8322E5 | 1.9589E6 |  |  |  |  |  |  |  |  | 3 | 0 | 0 | 0 | 1 | 2 | 0 | 0 | 0 | 0 | 0 | 0 | 0 | 0 | 1445 | 1457 |  |  | PEAKS DB |
| K.YFTYLILNK.G | N | 59.48 | 1173.6433 | 9 | 0.6 | 587.8293 | 2 | 64.63 | 4 | F4:42455 | NaNaKA16\_F12.raw |  |  |  | 3.9819E5 | 3.5813E5 |  |  |  |  |  |  |  |  | 2 | 0 | 0 | 0 | 1 | 1 | 0 | 0 | 0 | 0 | 0 | 0 | 0 | 0 | 478 | 486 |  |  | PEAKS DB |
| R.WPHEDEC(+57.02)QEEEFQK.L | Y | 56.35 | 1889.7526 | 14 | 0.4 | 630.9250 | 3 | 23.79 | 4 | F4:8642 | NaNaKA16\_F12.raw |  |  |  | 4.157E6 | 3.5558E6 |  |  |  |  |  |  |  |  | 2 | 0 | 0 | 0 | 1 | 1 | 0 | 0 | 0 | 0 | 0 | 0 | 0 | 0 | 1610 | 1623 | Carbamidomethylation | C7:Carbamidomethylation:1000.00 | PEAKS DB |
| K.VYSYYNLDEK.C | N | 53.87 | 1292.5924 | 10 | -0.2 | 647.3033 | 2 | 35.45 | 5 | F5:13480 | NaNaKA16\_F13.raw |  |  |  |  | 6.2293E6 |  |  |  |  |  |  |  |  | 1 | 0 | 0 | 0 | 0 | 1 | 0 | 0 | 0 | 0 | 0 | 0 | 0 | 0 | 1458 | 1467 |  |  | PEAKS DB |
| R.IEEQDGNDIYVMDVLEVIK.Q | Y | 53.58 | 2221.0823 | 19 | 0.4 | 1111.5488 | 2 | 104.84 | 4 | F4:72376 | NaNaKA16\_F12.raw |  |  |  | 9.1078E5 |  |  |  |  |  |  |  |  |  | 1 | 0 | 0 | 0 | 1 | 0 | 0 | 0 | 0 | 0 | 0 | 0 | 0 | 0 | 1531 | 1549 |  |  | PEAKS DB |
| K.C(+57.02)C(+57.02)EDVMHENPM(+15.99)GYTC(+57.02)EK.R | Y | 52.94 | 2174.7837 | 17 | 0.3 | 725.9354 | 3 | 19.23 | 9 | F9:6612 | NaNaKA16\_F5.raw |  |  |  |  |  |  |  |  | 4.2545E6 |  |  |  |  | 1 | 0 | 0 | 0 | 0 | 0 | 0 | 0 | 0 | 1 | 0 | 0 | 0 | 0 | 677 | 693 | Carbamidomethylation; Oxidation (M) | C1:Carbamidomethylation:1000.00;C2:Carbamidomethylation:1000.00;M11:Oxidation (M):62.41;C15:Carbamidomethylation:1000.00 | PEAKS DB |
| R.IDVPLQIEK.A | Y | 52.36 | 1053.6069 | 9 | 1.3 | 527.8114 | 2 | 47.84 | 4 | F4:28430 | NaNaKA16\_F12.raw |  |  |  | 8.3958E6 | 6.968E6 |  |  |  |  |  |  |  |  | 2 | 0 | 0 | 0 | 1 | 1 | 0 | 0 | 0 | 0 | 0 | 0 | 0 | 0 | 1506 | 1514 |  |  | PEAKS DB |
| K.GDNLIQM(+15.99)PGAAMK.I | Y | 51.92 | 1360.6479 | 13 | -0.1 | 681.3312 | 2 | 30.12 | 5 | F5:10521 | NaNaKA16\_F13.raw |  |  |  |  | 7.1208E5 |  |  |  |  |  |  |  |  | 1 | 0 | 0 | 0 | 0 | 1 | 0 | 0 | 0 | 0 | 0 | 0 | 0 | 0 | 552 | 564 | Oxidation (M) | M7:Oxidation (M):35.03 | PEAKS DB |
| K.YVLPSFEVR.L | N | 51.15 | 1108.5917 | 9 | 0.2 | 555.3032 | 2 | 58.46 | 4 | F4:37268 | NaNaKA16\_F12.raw |  |  |  | 1.8594E6 | 2.5932E6 |  |  |  |  |  |  |  |  | 2 | 0 | 0 | 0 | 1 | 1 | 0 | 0 | 0 | 0 | 0 | 0 | 0 | 0 | 219 | 227 |  |  | PEAKS DB |
| K.LDDRVPDTEIETK.I | Y | 48.94 | 1529.7573 | 13 | 0.1 | 510.9265 | 3 | 24.23 | 5 | F5:6922 | NaNaKA16\_F13.raw |  |  |  |  | 2.685E5 |  |  |  |  |  |  |  |  | 1 | 0 | 0 | 0 | 0 | 1 | 0 | 0 | 0 | 0 | 0 | 0 | 0 | 0 | 950 | 962 |  |  | PEAKS DB |
| R.AVPFVIVPLEQGLHDVEIK.A | Y | 48.75 | 2102.1775 | 19 | 0.1 | 701.7332 | 3 | 86.03 | 4 | F4:59879 | NaNaKA16\_F12.raw |  |  |  | 5.8004E5 |  |  |  |  |  |  |  |  |  | 1 | 0 | 0 | 0 | 1 | 0 | 0 | 0 | 0 | 0 | 0 | 0 | 0 | 0 | 879 | 897 |  |  | PEAKS DB |
| H.ITPDLIPSFR.F | N | 47.01 | 1157.6444 | 10 | 0.6 | 579.8298 | 2 | 65.39 | 5 | F5:26870 | NaNaKA16\_F13.raw |  |  |  |  | 4.6001E5 |  |  |  |  |  |  |  |  | 1 | 0 | 0 | 0 | 0 | 1 | 0 | 0 | 0 | 0 | 0 | 0 | 0 | 0 | 510 | 519 |  |  | PEAKS DB |
| R.IPIIDGDGK.A | Y | 46.67 | 926.5073 | 9 | 0.6 | 464.2612 | 2 | 33.77 | 5 | F5:12591 | NaNaKA16\_F13.raw |  |  |  |  | 5.1851E6 |  |  |  |  |  |  |  |  | 1 | 0 | 0 | 0 | 0 | 1 | 0 | 0 | 0 | 0 | 0 | 0 | 0 | 0 | 283 | 291 |  |  | PEAKS DB |
| K.GIYTPGSPVLYR.V | N | 46.58 | 1321.7030 | 12 | -0.9 | 661.8582 | 2 | 48.12 | 5 | F5:19514 | NaNaKA16\_F13.raw |  |  |  |  | 0 |  |  |  |  |  |  |  |  | 0 | 0 | 0 | 0 | 0 | 0 | 0 | 0 | 0 | 0 | 0 | 0 | 0 | 0 | 135 | 146 |  |  | PEAKS DB |
| E.DGFIADSDIISR.S | Y | 45.89 | 1307.6357 | 12 | 0.4 | 654.8254 | 2 | 61.00 | 5 | F5:25193 | NaNaKA16\_F13.raw |  |  |  |  | 1.257E6 |  |  |  |  |  |  |  |  | 2 | 0 | 0 | 0 | 0 | 2 | 0 | 0 | 0 | 0 | 0 | 0 | 0 | 0 | 737 | 748 |  |  | PEAKS DB |
| W.VVLAVSFTPTK.G | Y | 45.67 | 1160.6804 | 11 | 0.8 | 581.3480 | 2 | 52.57 | 5 | F5:21349 | NaNaKA16\_F13.raw |  |  |  |  | 9.857E4 |  |  |  |  |  |  |  |  | 1 | 0 | 0 | 0 | 0 | 1 | 0 | 0 | 0 | 0 | 0 | 0 | 0 | 0 | 788 | 798 |  |  | PEAKS DB |
| K.GIC(+57.02)VAEPYEIR.V | Y | 45.63 | 1305.6387 | 11 | 0.3 | 653.8268 | 2 | 44.92 | 5 | F5:18246 | NaNaKA16\_F13.raw |  |  |  |  | 6.0645E6 |  |  |  |  |  |  |  |  | 1 | 0 | 0 | 0 | 0 | 1 | 0 | 0 | 0 | 0 | 0 | 0 | 0 | 0 | 799 | 809 | Carbamidomethylation | C3:Carbamidomethylation:1000.00 | PEAKS DB |
| K.IWDTIEK.S | N | 43.23 | 903.4702 | 7 | 0.6 | 452.7426 | 2 | 29.33 | 5 | F5:9813 | NaNaKA16\_F13.raw |  |  |  |  | 4.4002E6 |  |  |  |  |  |  |  |  | 1 | 0 | 0 | 0 | 0 | 1 | 0 | 0 | 0 | 0 | 0 | 0 | 0 | 0 | 598 | 604 |  |  | PEAKS DB |
| K.VAVIIYLNK.V | Y | 42.64 | 1031.6379 | 9 | 0.3 | 516.8264 | 2 | 50.89 | 5 | F5:20439 | NaNaKA16\_F13.raw |  |  |  |  | 2.7389E5 |  |  |  |  |  |  |  |  | 1 | 0 | 0 | 0 | 0 | 1 | 0 | 0 | 0 | 0 | 0 | 0 | 0 | 0 | 1421 | 1429 |  |  | PEAKS DB |
| total 35 peptides |  |  |  |  |  |  |  |  |  |  |  |  |  |  |  |  |  |  |  |  |  |  |  |  |  |  |  |  |  |  |  |  |  |  |  |  |  |  |  |  |  |  |  |
| --- | --- | --- | --- | --- | --- | --- | --- | --- | --- | --- | --- | --- | --- | --- | --- | --- | --- | --- | --- | --- | --- | --- | --- | --- | --- | --- | --- | --- | --- | --- | --- | --- | --- | --- | --- | --- | --- | --- | --- | --- | --- | --- | --- |

I51018

back to list

  

| Protein Coverage
| Supporting Peptides
|

Protein Coverage:

Supporting Peptides:

| Peptide | Uniq | -10lgP | Mass | Length | ppm | m/z | z | RT | Fraction | Scan | Source File | Area F1 | Area F10 | Area F11 | Area F12 | Area F13 | Area F2 | Area F3 | Area F4 | Area F5 | Area F6 | Area F7 | Area F8 | Area F9 | #Feature | #Feature F1 | #Feature F10 | #Feature F11 | #Feature F12 | #Feature F13 | #Feature F2 | #Feature F3 | #Feature F4 | #Feature F5 | #Feature F6 | #Feature F7 | #Feature F8 | #Feature F9 | Start | End | PTM | AScore | Found By |
| --- | --- | --- | --- | --- | --- | --- | --- | --- | --- | --- | --- | --- | --- | --- | --- | --- | --- | --- | --- | --- | --- | --- | --- | --- | --- | --- | --- | --- | --- | --- | --- | --- | --- | --- | --- | --- | --- | --- | --- | --- | --- | --- | --- |
| R.KC(+57.02)C(+57.02)EDVMHENPMGYTC(+57.02)EK.R | Y | 83.50 | 2286.8835 | 18 | 0.7 | 763.3023 | 3 | 20.65 | 9 | F9:7758 | NaNaKA16\_F5.raw |  |  |  |  |  |  |  |  | 5.1572E7 |  |  |  |  | 2 | 0 | 0 | 0 | 0 | 0 | 0 | 0 | 0 | 2 | 0 | 0 | 0 | 0 | 676 | 693 | Carbamidomethylation | C2:Carbamidomethylation:1000.00;C3:Carbamidomethylation:1000.00;C16:Carbamidomethylation:1000.00 | PEAKS DB |
| K.C(+57.02)C(+57.02)EDVMHENPMGYTC(+57.02)EK.R | Y | 83.21 | 2158.7886 | 17 | 0.2 | 720.6036 | 3 | 31.20 | 9 | F9:15937 | NaNaKA16\_F5.raw |  |  |  |  |  |  |  |  | 5.0681E7 |  |  |  |  | 2 | 0 | 0 | 0 | 0 | 0 | 0 | 0 | 0 | 2 | 0 | 0 | 0 | 0 | 677 | 693 | Carbamidomethylation | C1:Carbamidomethylation:1000.00;C2:Carbamidomethylation:1000.00;C15:Carbamidomethylation:1000.00 | PEAKS DB |
| K.VNDDYLIWGSR.S | Y | 78.30 | 1336.6411 | 11 | 0.2 | 669.3279 | 2 | 59.90 | 4 | F4:38472 | NaNaKA16\_F12.raw |  |  |  | 6.8624E6 | 3.781E6 |  |  |  |  |  |  |  |  | 2 | 0 | 0 | 0 | 1 | 1 | 0 | 0 | 0 | 0 | 0 | 0 | 0 | 0 | 1577 | 1587 |  |  | PEAKS DB |
| K.AC(+57.02)ETNVDYVYK.T | Y | 69.83 | 1360.5969 | 11 | 1.0 | 681.3064 | 2 | 23.73 | 5 | F5:6621 | NaNaKA16\_F13.raw |  |  |  |  | 5.6014E6 |  |  |  |  |  |  |  |  | 1 | 0 | 0 | 0 | 0 | 1 | 0 | 0 | 0 | 0 | 0 | 0 | 0 | 0 | 1515 | 1525 | Carbamidomethylation | C2:Carbamidomethylation:1000.00 | PEAKS DB |
| R.DDNEDGFIADSDIISR.S | Y | 69.48 | 1780.7751 | 16 | 1.0 | 891.3958 | 2 | 68.58 | 5 | F5:27981 | NaNaKA16\_F13.raw |  |  |  |  | 1.4552E6 |  |  |  |  |  |  |  |  | 2 | 0 | 0 | 0 | 0 | 2 | 0 | 0 | 0 | 0 | 0 | 0 | 0 | 0 | 733 | 748 |  |  | PEAKS DB |
| R.QNQYVVVQVTGPQVR.L | N | 69.18 | 1713.9163 | 15 | 0.5 | 857.9658 | 2 | 44.31 | 5 | F5:17897 | NaNaKA16\_F13.raw |  |  |  | 1.095E6 | 1.2725E6 |  |  |  |  |  |  | 0 |  | 3 | 0 | 0 | 0 | 1 | 2 | 0 | 0 | 0 | 0 | 0 | 0 | 0 | 0 | 100 | 114 |  |  | PEAKS DB |
| K.QLDIFVHDFPR.K | N | 68.50 | 1385.7091 | 11 | 0.5 | 462.9105 | 3 | 58.12 | 2 | F2:35492 | NaNaKA16\_F10.raw |  | 4.8609E6 |  | 4.087E6 |  |  |  |  |  |  |  |  |  | 3 | 0 | 1 | 0 | 2 | 0 | 0 | 0 | 0 | 0 | 0 | 0 | 0 | 0 | 53 | 63 |  |  | PEAKS DB |
| K.GDNLIQMPGAAMK.I | Y | 68.40 | 1344.6530 | 13 | 0.9 | 673.3344 | 2 | 49.77 | 5 | F5:20116 | NaNaKA16\_F13.raw |  |  |  |  | 3.458E6 |  |  |  |  |  |  |  |  | 1 | 0 | 0 | 0 | 0 | 1 | 0 | 0 | 0 | 0 | 0 | 0 | 0 | 0 | 552 | 564 |  |  | PEAKS DB |
| G.ALYTLITPAVLR.T | N | 67.00 | 1329.8020 | 12 | 0.9 | 665.9089 | 2 | 76.05 | 2 | F2:51268 | NaNaKA16\_F10.raw |  | 1.5845E7 | 2.1186E6 | 5.6852E6 | 3.5213E6 |  |  |  |  |  |  | 6.0544E5 | 4.0982E5 | 6 | 0 | 1 | 1 | 1 | 1 | 0 | 0 | 0 | 0 | 0 | 0 | 1 | 1 | 23 | 34 |  |  | PEAKS DB |
| R.INYENALLAR.T | Y | 66.45 | 1175.6299 | 10 | 0.9 | 588.8228 | 2 | 45.80 | 5 | F5:18665 | NaNaKA16\_F13.raw |  |  |  | 1.5955E6 | 3.825E6 |  |  |  |  |  |  |  |  | 2 | 0 | 0 | 0 | 1 | 1 | 0 | 0 | 0 | 0 | 0 | 0 | 0 | 0 | 1292 | 1301 |  |  | PEAKS DB |
| K.LNQDITVTASGDGK.A | Y | 66.30 | 1417.7048 | 14 | 0.8 | 709.8603 | 2 | 20.53 | 5 | F5:5136 | NaNaKA16\_F13.raw |  |  |  |  | 2.7531E6 |  |  |  |  |  |  |  |  | 1 | 0 | 0 | 0 | 0 | 1 | 0 | 0 | 0 | 0 | 0 | 0 | 0 | 0 | 1307 | 1320 |  |  | PEAKS DB |
| R.VELLYNPAFC(+57.02)SASTK.G | Y | 65.11 | 1698.8286 | 15 | 0.9 | 850.4224 | 2 | 59.57 | 5 | F5:24606 | NaNaKA16\_F13.raw |  |  |  | 2.9621E5 | 4.7366E6 |  |  |  |  |  |  |  |  | 2 | 0 | 0 | 0 | 1 | 1 | 0 | 0 | 0 | 0 | 0 | 0 | 0 | 0 | 848 | 862 | Carbamidomethylation | C10:Carbamidomethylation:1000.00 | PEAKS DB |
| K.DLTEEPNSQGISSK.T | Y | 64.83 | 1503.7052 | 14 | 0.2 | 752.8600 | 2 | 21.48 | 5 | F5:5531 | NaNaKA16\_F13.raw |  |  |  |  | 5.0484E6 |  |  |  |  |  |  |  |  | 1 | 0 | 0 | 0 | 0 | 1 | 0 | 0 | 0 | 0 | 0 | 0 | 0 | 0 | 761 | 774 |  |  | PEAKS DB |
| K.ASVQEALWSDGVR.K | Y | 64.04 | 1416.6997 | 13 | 0.6 | 709.3575 | 2 | 50.23 | 5 | F5:20313 | NaNaKA16\_F13.raw |  |  |  |  | 3.3991E6 |  |  |  |  |  |  |  |  | 1 | 0 | 0 | 0 | 0 | 1 | 0 | 0 | 0 | 0 | 0 | 0 | 0 | 0 | 898 | 910 |  |  | PEAKS DB |
| K.DTC(+57.02)MGTLVVK.G | N | 63.96 | 1122.5414 | 10 | 1.1 | 562.2786 | 2 | 36.58 | 5 | F5:14251 | NaNaKA16\_F13.raw |  |  |  |  | 3.5107E6 |  |  |  |  |  |  |  |  | 1 | 0 | 0 | 0 | 0 | 1 | 0 | 0 | 0 | 0 | 0 | 0 | 0 | 0 | 542 | 551 | Carbamidomethylation | C3:Carbamidomethylation:1000.00 | PEAKS DB |
| K.GVGGTQLEVIK.A | Y | 62.39 | 1099.6237 | 11 | -0.4 | 550.8189 | 2 | 32.10 | 5 | F5:11471 | NaNaKA16\_F13.raw |  |  |  |  | 3.5972E6 |  |  |  |  |  |  |  |  | 1 | 0 | 0 | 0 | 0 | 1 | 0 | 0 | 0 | 0 | 0 | 0 | 0 | 0 | 936 | 946 |  |  | PEAKS DB |
| K.HFEVGFIQPGSVK.V | N | 61.71 | 1443.7510 | 13 | 0.1 | 722.8828 | 2 | 45.04 | 5 | F5:18328 | NaNaKA16\_F13.raw |  |  |  | 7.8322E5 | 1.9589E6 |  |  |  |  |  |  |  |  | 3 | 0 | 0 | 0 | 1 | 2 | 0 | 0 | 0 | 0 | 0 | 0 | 0 | 0 | 1445 | 1457 |  |  | PEAKS DB |
| K.YFTYLILNK.G | N | 59.48 | 1173.6433 | 9 | 0.6 | 587.8293 | 2 | 64.63 | 4 | F4:42455 | NaNaKA16\_F12.raw |  |  |  | 3.9819E5 | 3.5813E5 |  |  |  |  |  |  |  |  | 2 | 0 | 0 | 0 | 1 | 1 | 0 | 0 | 0 | 0 | 0 | 0 | 0 | 0 | 478 | 486 |  |  | PEAKS DB |
| R.WPHEDEC(+57.02)QEEEFQK.L | Y | 56.35 | 1889.7526 | 14 | 0.4 | 630.9250 | 3 | 23.79 | 4 | F4:8642 | NaNaKA16\_F12.raw |  |  |  | 4.157E6 | 3.5558E6 |  |  |  |  |  |  |  |  | 2 | 0 | 0 | 0 | 1 | 1 | 0 | 0 | 0 | 0 | 0 | 0 | 0 | 0 | 1610 | 1623 | Carbamidomethylation | C7:Carbamidomethylation:1000.00 | PEAKS DB |
| K.VYSYYNLDEK.C | N | 53.87 | 1292.5924 | 10 | -0.2 | 647.3033 | 2 | 35.45 | 5 | F5:13480 | NaNaKA16\_F13.raw |  |  |  |  | 6.2293E6 |  |  |  |  |  |  |  |  | 1 | 0 | 0 | 0 | 0 | 1 | 0 | 0 | 0 | 0 | 0 | 0 | 0 | 0 | 1458 | 1467 |  |  | PEAKS DB |
| R.IEEQDGNDIYVMDVLEVIK.Q | Y | 53.58 | 2221.0823 | 19 | 0.4 | 1111.5488 | 2 | 104.84 | 4 | F4:72376 | NaNaKA16\_F12.raw |  |  |  | 9.1078E5 |  |  |  |  |  |  |  |  |  | 1 | 0 | 0 | 0 | 1 | 0 | 0 | 0 | 0 | 0 | 0 | 0 | 0 | 0 | 1531 | 1549 |  |  | PEAKS DB |
| K.C(+57.02)C(+57.02)EDVMHENPM(+15.99)GYTC(+57.02)EK.R | Y | 52.94 | 2174.7837 | 17 | 0.3 | 725.9354 | 3 | 19.23 | 9 | F9:6612 | NaNaKA16\_F5.raw |  |  |  |  |  |  |  |  | 4.2545E6 |  |  |  |  | 1 | 0 | 0 | 0 | 0 | 0 | 0 | 0 | 0 | 1 | 0 | 0 | 0 | 0 | 677 | 693 | Carbamidomethylation; Oxidation (M) | C1:Carbamidomethylation:1000.00;C2:Carbamidomethylation:1000.00;M11:Oxidation (M):62.41;C15:Carbamidomethylation:1000.00 | PEAKS DB |
| R.IDVPLQIEK.A | Y | 52.36 | 1053.6069 | 9 | 1.3 | 527.8114 | 2 | 47.84 | 4 | F4:28430 | NaNaKA16\_F12.raw |  |  |  | 8.3958E6 | 6.968E6 |  |  |  |  |  |  |  |  | 2 | 0 | 0 | 0 | 1 | 1 | 0 | 0 | 0 | 0 | 0 | 0 | 0 | 0 | 1506 | 1514 |  |  | PEAKS DB |
| K.GDNLIQM(+15.99)PGAAMK.I | Y | 51.92 | 1360.6479 | 13 | -0.1 | 681.3312 | 2 | 30.12 | 5 | F5:10521 | NaNaKA16\_F13.raw |  |  |  |  | 7.1208E5 |  |  |  |  |  |  |  |  | 1 | 0 | 0 | 0 | 0 | 1 | 0 | 0 | 0 | 0 | 0 | 0 | 0 | 0 | 552 | 564 | Oxidation (M) | M7:Oxidation (M):35.03 | PEAKS DB |
| K.YVLPSFEVR.L | N | 51.15 | 1108.5917 | 9 | 0.2 | 555.3032 | 2 | 58.46 | 4 | F4:37268 | NaNaKA16\_F12.raw |  |  |  | 1.8594E6 | 2.5932E6 |  |  |  |  |  |  |  |  | 2 | 0 | 0 | 0 | 1 | 1 | 0 | 0 | 0 | 0 | 0 | 0 | 0 | 0 | 219 | 227 |  |  | PEAKS DB |
| K.LDDRVPDTEIETK.I | Y | 48.94 | 1529.7573 | 13 | 0.1 | 510.9265 | 3 | 24.23 | 5 | F5:6922 | NaNaKA16\_F13.raw |  |  |  |  | 2.685E5 |  |  |  |  |  |  |  |  | 1 | 0 | 0 | 0 | 0 | 1 | 0 | 0 | 0 | 0 | 0 | 0 | 0 | 0 | 950 | 962 |  |  | PEAKS DB |
| R.AVPFVIVPLEQGLHDVEIK.A | Y | 48.75 | 2102.1775 | 19 | 0.1 | 701.7332 | 3 | 86.03 | 4 | F4:59879 | NaNaKA16\_F12.raw |  |  |  | 5.8004E5 |  |  |  |  |  |  |  |  |  | 1 | 0 | 0 | 0 | 1 | 0 | 0 | 0 | 0 | 0 | 0 | 0 | 0 | 0 | 879 | 897 |  |  | PEAKS DB |
| H.ITPDLIPSFR.F | N | 47.01 | 1157.6444 | 10 | 0.6 | 579.8298 | 2 | 65.39 | 5 | F5:26870 | NaNaKA16\_F13.raw |  |  |  |  | 4.6001E5 |  |  |  |  |  |  |  |  | 1 | 0 | 0 | 0 | 0 | 1 | 0 | 0 | 0 | 0 | 0 | 0 | 0 | 0 | 510 | 519 |  |  | PEAKS DB |
| R.IPIIDGDGK.A | Y | 46.67 | 926.5073 | 9 | 0.6 | 464.2612 | 2 | 33.77 | 5 | F5:12591 | NaNaKA16\_F13.raw |  |  |  |  | 5.1851E6 |  |  |  |  |  |  |  |  | 1 | 0 | 0 | 0 | 0 | 1 | 0 | 0 | 0 | 0 | 0 | 0 | 0 | 0 | 283 | 291 |  |  | PEAKS DB |
| K.GIYTPGSPVLYR.V | N | 46.58 | 1321.7030 | 12 | -0.9 | 661.8582 | 2 | 48.12 | 5 | F5:19514 | NaNaKA16\_F13.raw |  |  |  |  | 0 |  |  |  |  |  |  |  |  | 0 | 0 | 0 | 0 | 0 | 0 | 0 | 0 | 0 | 0 | 0 | 0 | 0 | 0 | 135 | 146 |  |  | PEAKS DB |
| E.DGFIADSDIISR.S | Y | 45.89 | 1307.6357 | 12 | 0.4 | 654.8254 | 2 | 61.00 | 5 | F5:25193 | NaNaKA16\_F13.raw |  |  |  |  | 1.257E6 |  |  |  |  |  |  |  |  | 2 | 0 | 0 | 0 | 0 | 2 | 0 | 0 | 0 | 0 | 0 | 0 | 0 | 0 | 737 | 748 |  |  | PEAKS DB |
| W.VVLAVSFTPTK.G | Y | 45.67 | 1160.6804 | 11 | 0.8 | 581.3480 | 2 | 52.57 | 5 | F5:21349 | NaNaKA16\_F13.raw |  |  |  |  | 9.857E4 |  |  |  |  |  |  |  |  | 1 | 0 | 0 | 0 | 0 | 1 | 0 | 0 | 0 | 0 | 0 | 0 | 0 | 0 | 788 | 798 |  |  | PEAKS DB |
| K.GIC(+57.02)VAEPYEIR.V | Y | 45.63 | 1305.6387 | 11 | 0.3 | 653.8268 | 2 | 44.92 | 5 | F5:18246 | NaNaKA16\_F13.raw |  |  |  |  | 6.0645E6 |  |  |  |  |  |  |  |  | 1 | 0 | 0 | 0 | 0 | 1 | 0 | 0 | 0 | 0 | 0 | 0 | 0 | 0 | 799 | 809 | Carbamidomethylation | C3:Carbamidomethylation:1000.00 | PEAKS DB |
| K.IWDTIEK.S | N | 43.23 | 903.4702 | 7 | 0.6 | 452.7426 | 2 | 29.33 | 5 | F5:9813 | NaNaKA16\_F13.raw |  |  |  |  | 4.4002E6 |  |  |  |  |  |  |  |  | 1 | 0 | 0 | 0 | 0 | 1 | 0 | 0 | 0 | 0 | 0 | 0 | 0 | 0 | 598 | 604 |  |  | PEAKS DB |
| K.VAVIIYLNK.V | Y | 42.64 | 1031.6379 | 9 | 0.3 | 516.8264 | 2 | 50.89 | 5 | F5:20439 | NaNaKA16\_F13.raw |  |  |  |  | 2.7389E5 |  |  |  |  |  |  |  |  | 1 | 0 | 0 | 0 | 0 | 1 | 0 | 0 | 0 | 0 | 0 | 0 | 0 | 0 | 1421 | 1429 |  |  | PEAKS DB |
| total 35 peptides |  |  |  |  |  |  |  |  |  |  |  |  |  |  |  |  |  |  |  |  |  |  |  |  |  |  |  |  |  |  |  |  |  |  |  |  |  |  |  |  |  |  |  |
| --- | --- | --- | --- | --- | --- | --- | --- | --- | --- | --- | --- | --- | --- | --- | --- | --- | --- | --- | --- | --- | --- | --- | --- | --- | --- | --- | --- | --- | --- | --- | --- | --- | --- | --- | --- | --- | --- | --- | --- | --- | --- | --- | --- |

ACN50006.1

back to list

  

| Protein Coverage
| Supporting Peptides
|

Protein Coverage:

Supporting Peptides:

| Peptide | Uniq | -10lgP | Mass | Length | ppm | m/z | z | RT | Fraction | Scan | Source File | Area F1 | Area F10 | Area F11 | Area F12 | Area F13 | Area F2 | Area F3 | Area F4 | Area F5 | Area F6 | Area F7 | Area F8 | Area F9 | #Feature | #Feature F1 | #Feature F10 | #Feature F11 | #Feature F12 | #Feature F13 | #Feature F2 | #Feature F3 | #Feature F4 | #Feature F5 | #Feature F6 | #Feature F7 | #Feature F8 | #Feature F9 | Start | End | PTM | AScore | Found By |
| --- | --- | --- | --- | --- | --- | --- | --- | --- | --- | --- | --- | --- | --- | --- | --- | --- | --- | --- | --- | --- | --- | --- | --- | --- | --- | --- | --- | --- | --- | --- | --- | --- | --- | --- | --- | --- | --- | --- | --- | --- | --- | --- | --- |
| R.AAKDDC(+57.02)DLPELC(+57.02)TGQSAEC(+57.02)PTDVFQR.N | N | 91.95 | 2982.2793 | 26 | 1.7 | 995.1021 | 3 | 54.73 | 9 | F9:35402 | NaNaKA16\_F5.raw |  |  |  |  | 1.7077E8 |  |  |  | 1.5002E8 | 4.1894E7 |  |  |  | 4 | 0 | 0 | 0 | 0 | 1 | 0 | 0 | 0 | 2 | 1 | 0 | 0 | 0 | 468 | 493 | Carbamidomethylation | C6:Carbamidomethylation:1000.00;C12:Carbamidomethylation:1000.00;C19:Carbamidomethylation:1000.00 | PEAKS DB |
| K.DDC(+57.02)DLPELC(+57.02)TGQSAEC(+57.02)PTDVFQR.N | N | 90.56 | 2712.1101 | 23 | 1.2 | 1357.0640 | 2 | 72.09 | 9 | F9:50509 | NaNaKA16\_F5.raw |  |  |  |  | 9.0813E7 |  |  |  | 4.1609E7 | 6.0829E6 |  |  |  | 6 | 0 | 0 | 0 | 0 | 2 | 0 | 0 | 0 | 2 | 2 | 0 | 0 | 0 | 471 | 493 | Carbamidomethylation | C3:Carbamidomethylation:1000.00;C9:Carbamidomethylation:1000.00;C16:Carbamidomethylation:1000.00 | PEAKS DB |
| R.DPNYGMVEPGTK.C | N | 79.93 | 1306.5863 | 12 | 0.1 | 654.3005 | 2 | 28.66 | 10 | F10:14262 | NaNaKA16\_F6.raw |  |  |  |  | 4.8233E8 |  |  |  | 1.8661E7 | 4.1689E7 |  |  |  | 3 | 0 | 0 | 0 | 0 | 1 | 0 | 0 | 0 | 1 | 1 | 0 | 0 | 0 | 583 | 594 |  |  | PEAKS DB |
| R.DPNYGM(+15.99)VEPGTK.C | N | 77.87 | 1322.5813 | 12 | -0.3 | 662.2977 | 2 | 26.33 | 5 | F5:10060 | NaNaKA16\_F13.raw |  |  |  |  | 9.0917E7 |  |  |  |  |  |  |  |  | 5 | 0 | 0 | 0 | 0 | 5 | 0 | 0 | 0 | 0 | 0 | 0 | 0 | 0 | 583 | 594 | Oxidation (M) | M6:Oxidation (M):1000.00 | PEAKS DB |
| K.C(+57.02)PIMTNQC(+57.02)IALR.G | N | 77.27 | 1475.7047 | 12 | 0.2 | 738.8598 | 2 | 38.95 | 9 | F9:22183 | NaNaKA16\_F5.raw |  |  | 5.0717E5 | 3.2472E5 | 1.9097E8 |  |  |  | 2.1736E8 | 4.0241E7 |  | 3.7147E6 | 4.6756E6 | 12 | 0 | 0 | 1 | 1 | 3 | 0 | 0 | 0 | 2 | 2 | 0 | 2 | 1 | 510 | 521 | Carbamidomethylation | C1:Carbamidomethylation:1000.00;C8:Carbamidomethylation:1000.00 | PEAKS DB |
| K.C(+57.02)PIM(+15.99)TNQC(+57.02)IALR.G | N | 75.90 | 1491.6996 | 12 | -0.8 | 746.8564 | 2 | 29.62 | 9 | F9:14662 | NaNaKA16\_F5.raw |  |  |  |  | 3.2733E7 |  |  |  | 8.8589E6 | 2.9095E6 |  |  |  | 7 | 0 | 0 | 0 | 0 | 4 | 0 | 0 | 0 | 2 | 1 | 0 | 0 | 0 | 510 | 521 | Carbamidomethylation; Oxidation (M) | C1:Carbamidomethylation:1000.00;M4:Oxidation (M):1000.00;C8:Carbamidomethylation:1000.00 | PEAKS DB |
| R.NSMIC(+57.02)NC(+57.02)SISPR.D | N | 75.66 | 1437.6163 | 12 | 0.1 | 719.8155 | 2 | 24.50 | 5 | F5:7209 | NaNaKA16\_F13.raw |  |  |  |  | 6.4703E7 |  |  |  | 2.8886E6 | 2.2986E6 | 1.2892E5 |  |  | 4 | 0 | 0 | 0 | 0 | 1 | 0 | 0 | 0 | 1 | 1 | 1 | 0 | 0 | 571 | 582 | Carbamidomethylation | C5:Carbamidomethylation:1000.00;C7:Carbamidomethylation:1000.00 | PEAKS DB |
| G.TPVGLAYIGSIC(+57.02)NPK.T | N | 73.67 | 1588.8282 | 15 | 0.1 | 795.4215 | 2 | 60.14 | 12 | F12:39283 | NaNaKA16\_F8.raw |  |  |  |  |  |  |  |  |  |  | 1.0459E6 | 4.5255E6 |  | 2 | 0 | 0 | 0 | 0 | 0 | 0 | 0 | 0 | 0 | 0 | 1 | 1 | 0 | 305 | 319 | Carbamidomethylation | C12:Carbamidomethylation:1000.00 | PEAKS DB |
| N.GTPVGLAYIGSIC(+57.02)NPK.T | N | 73.11 | 1645.8497 | 16 | 0.4 | 823.9325 | 2 | 60.77 | 11 | F11:39960 | NaNaKA16\_F7.raw |  |  |  |  | 1.4933E6 |  |  |  |  |  | 3.1956E6 | 2.0263E7 |  | 3 | 0 | 0 | 0 | 0 | 1 | 0 | 0 | 0 | 0 | 0 | 1 | 1 | 0 | 304 | 319 | Carbamidomethylation | C13:Carbamidomethylation:1000.00 | PEAKS DB |
| R.TKPAYQFSSC(+57.02)SVR.E | N | 67.55 | 1529.7296 | 13 | -0.1 | 765.8720 | 2 | 12.28 | 5 | F5:2284 | NaNaKA16\_F13.raw |  |  |  |  | 2.3942E8 |  |  |  |  |  |  |  |  | 2 | 0 | 0 | 0 | 0 | 2 | 0 | 0 | 0 | 0 | 0 | 0 | 0 | 0 | 370 | 382 | Carbamidomethylation | C10:Carbamidomethylation:1000.00 | PEAKS DB |
| R.NSM(+15.99)IC(+57.02)NC(+57.02)SISPR.D | N | 64.85 | 1453.6112 | 12 | 0.3 | 727.8131 | 2 | 22.81 | 5 | F5:7058 | NaNaKA16\_F13.raw |  |  |  |  | 7.9998E6 |  |  |  |  |  |  |  |  | 3 | 0 | 0 | 0 | 0 | 3 | 0 | 0 | 0 | 0 | 0 | 0 | 0 | 0 | 571 | 582 | Oxidation (M); Carbamidomethylation | M3:Oxidation (M):1000.00;C5:Carbamidomethylation:1000.00;C7:Carbamidomethylation:1000.00 | PEAKS DB |
| R.RTKPAYQFSSC(+57.02)SVR.E | N | 61.84 | 1685.8307 | 14 | 1.0 | 562.9514 | 3 | 11.85 | 5 | F5:1803 | NaNaKA16\_F13.raw |  |  |  |  | 1.7851E6 |  |  |  |  |  |  |  |  | 1 | 0 | 0 | 0 | 0 | 1 | 0 | 0 | 0 | 0 | 0 | 0 | 0 | 0 | 369 | 382 | Carbamidomethylation | C11:Carbamidomethylation:1000.00 | PEAKS DB |
| D.PNYGMVEPGTK.C | N | 60.45 | 1191.5593 | 11 | 1.0 | 596.7875 | 2 | 20.63 | 5 | F5:5320 | NaNaKA16\_F13.raw |  |  |  |  | 1.9113E6 |  |  |  |  |  |  |  |  | 1 | 0 | 0 | 0 | 0 | 1 | 0 | 0 | 0 | 0 | 0 | 0 | 0 | 0 | 584 | 594 |  |  | PEAKS DB |
| A.DSSAVISAC(+57.02)DGLK.G | N | 60.11 | 1321.6184 | 13 | 0.4 | 661.8168 | 2 | 33.21 | 10 | F10:19042 | NaNaKA16\_F6.raw |  |  |  |  |  |  |  |  |  | 6.9505E6 |  |  |  | 1 | 0 | 0 | 0 | 0 | 0 | 0 | 0 | 0 | 0 | 1 | 0 | 0 | 0 | 119 | 131 | Carbamidomethylation | C9:Carbamidomethylation:1000.00 | PEAKS DB |
| R.RNSMIC(+57.02)NC(+57.02)SISPR.D | N | 59.91 | 1593.7174 | 13 | 0.1 | 532.2465 | 3 | 12.17 | 5 | F5:2067 | NaNaKA16\_F13.raw |  |  |  |  | 7.3377E5 |  |  |  |  |  |  |  |  | 1 | 0 | 0 | 0 | 0 | 1 | 0 | 0 | 0 | 0 | 0 | 0 | 0 | 0 | 570 | 582 | Carbamidomethylation | C6:Carbamidomethylation:1000.00;C8:Carbamidomethylation:1000.00 | PEAKS DB |
| C.TGQSAEC(+57.02)PTDVFQR.N | N | 57.94 | 1594.7046 | 14 | 0.9 | 798.3603 | 2 | 31.39 | 5 | F5:11052 | NaNaKA16\_F13.raw |  |  |  |  | 8.9511E5 |  |  |  |  |  |  |  |  | 1 | 0 | 0 | 0 | 0 | 1 | 0 | 0 | 0 | 0 | 0 | 0 | 0 | 0 | 480 | 493 | Carbamidomethylation | C7:Carbamidomethylation:1000.00 | PEAKS DB |
| R.DSC(+57.02)FTLNQR.T | N | 57.18 | 1139.5029 | 9 | 0.1 | 570.7588 | 2 | 26.24 | 5 | F5:8465 | NaNaKA16\_F13.raw |  |  |  |  | 2.8674E8 |  |  |  |  | 8.7317E5 |  |  |  | 2 | 0 | 0 | 0 | 0 | 1 | 0 | 0 | 0 | 0 | 1 | 0 | 0 | 0 | 530 | 538 | Carbamidomethylation | C3:Carbamidomethylation:1000.00 | PEAKS DB |
| R.NGLPC(+57.02)QNNQGYC(+57.02)YNGK.C | N | 55.77 | 1885.7836 | 16 | 0.8 | 943.8998 | 2 | 19.60 | 9 | F9:6919 | NaNaKA16\_F5.raw |  |  |  |  | 1.7165E6 |  |  |  | 6.1944E6 | 1.2392E6 |  |  |  | 3 | 0 | 0 | 0 | 0 | 1 | 0 | 0 | 0 | 1 | 1 | 0 | 0 | 0 | 494 | 509 | Carbamidomethylation | C5:Carbamidomethylation:1000.00;C12:Carbamidomethylation:1000.00 | PEAKS DB |
| R.MVAITMAHEMGH.N | N | 55.46 | 1326.5883 | 12 | 0.8 | 664.3019 | 2 | 33.28 | 5 | F5:12224 | NaNaKA16\_F13.raw |  |  |  |  | 4.5918E6 |  |  |  |  |  |  |  |  | 1 | 0 | 0 | 0 | 0 | 1 | 0 | 0 | 0 | 0 | 0 | 0 | 0 | 0 | 334 | 345 |  |  | PEAKS DB |
| K.PAYQFSSC(+57.02)SVR.E | N | 53.21 | 1300.5870 | 11 | 0.2 | 651.3009 | 2 | 25.11 | 5 | F5:7381 | NaNaKA16\_F13.raw |  |  |  |  | 5.016E7 |  |  |  |  |  |  |  |  | 1 | 0 | 0 | 0 | 0 | 1 | 0 | 0 | 0 | 0 | 0 | 0 | 0 | 0 | 372 | 382 | Carbamidomethylation | C8:Carbamidomethylation:1000.00 | PEAKS DB |
| K.C(+57.02)GDGMVC(+57.02)SNR.Q | N | 51.31 | 1154.4268 | 10 | 1.1 | 578.2213 | 2 | 11.85 | 5 | F5:1806 | NaNaKA16\_F13.raw |  |  |  |  | 4.5755E5 |  |  |  |  |  |  |  |  | 1 | 0 | 0 | 0 | 0 | 1 | 0 | 0 | 0 | 0 | 0 | 0 | 0 | 0 | 595 | 604 | Carbamidomethylation | C1:Carbamidomethylation:1000.00;C7:Carbamidomethylation:1000.00 | PEAKS DB |
| Q.SAEC(+57.02)PTDVFQR.N | N | 49.48 | 1308.5768 | 11 | 0.4 | 655.2960 | 2 | 32.04 | 5 | F5:11429 | NaNaKA16\_F13.raw |  |  |  |  | 1.3941E7 |  |  |  |  |  |  |  |  | 1 | 0 | 0 | 0 | 0 | 1 | 0 | 0 | 0 | 0 | 0 | 0 | 0 | 0 | 483 | 493 | Carbamidomethylation | C4:Carbamidomethylation:1000.00 | PEAKS DB |
| N.SMIC(+57.02)NC(+57.02)SISPR.D | N | 47.00 | 1323.5734 | 11 | 3.9 | 662.7966 | 2 | 23.55 | 5 | F5:6539 | NaNaKA16\_F13.raw |  |  |  |  | 0 |  |  |  |  |  |  |  |  | 0 | 0 | 0 | 0 | 0 | 0 | 0 | 0 | 0 | 0 | 0 | 0 | 0 | 0 | 572 | 582 | Carbamidomethylation | C4:Carbamidomethylation:1000.00;C6:Carbamidomethylation:1000.00 | PEAKS DB |
| K.LQHEAQC(+57.02)DSEEC(+57.02)C(+57.02)EK.C | N | 46.38 | 1921.7240 | 15 | 0.0 | 641.5820 | 3 | 11.83 | 5 | F5:1808 | NaNaKA16\_F13.raw |  |  |  |  | 2.9375E5 |  |  |  |  |  |  |  |  | 1 | 0 | 0 | 0 | 0 | 1 | 0 | 0 | 0 | 0 | 0 | 0 | 0 | 0 | 442 | 456 | Carbamidomethylation | C7:Carbamidomethylation:1000.00;C12:Carbamidomethylation:1000.00;C13:Carbamidomethylation:1000.00 | PEAKS DB |
| R.DRPQC(+57.02)ILNKPLST.D | N | 46.29 | 1540.8031 | 13 | -0.4 | 514.6081 | 3 | 27.16 | 4 | F4:11109 | NaNaKA16\_F12.raw |  |  |  | 7.0589E5 |  |  |  | 8.268E5 |  |  |  |  |  | 2 | 0 | 0 | 0 | 1 | 0 | 0 | 0 | 1 | 0 | 0 | 0 | 0 | 0 | 391 | 403 | Carbamidomethylation | C5:Carbamidomethylation:1000.00 | PEAKS DB |
| K.C(+57.02)GDGM(+15.99)VC(+57.02)SNR.Q | N | 45.27 | 1170.4216 | 10 | 0.3 | 586.2183 | 2 | 11.84 | 5 | F5:1815 | NaNaKA16\_F13.raw |  |  |  |  | 9.9702E4 |  |  |  |  |  |  |  |  | 1 | 0 | 0 | 0 | 0 | 1 | 0 | 0 | 0 | 0 | 0 | 0 | 0 | 0 | 595 | 604 | Carbamidomethylation; Oxidation (M) | C1:Carbamidomethylation:1000.00;M5:Oxidation (M):1000.00;C7:Carbamidomethylation:1000.00 | PEAKS DB |
| R.DPNYGMVEPGTKC(+57.02)GDGMVC(+57.02)SNR.Q | Y | 43.23 | 2443.0024 | 22 | -0.7 | 815.3408 | 3 | 34.89 | 5 | F5:13136 | NaNaKA16\_F13.raw |  |  |  |  | 0 |  |  |  |  |  |  |  |  | 0 | 0 | 0 | 0 | 0 | 0 | 0 | 0 | 0 | 0 | 0 | 0 | 0 | 0 | 583 | 604 | Carbamidomethylation | C13:Carbamidomethylation:1000.00;C19:Carbamidomethylation:1000.00 | PEAKS DB |
| total 27 peptides |  |  |  |  |  |  |  |  |  |  |  |  |  |  |  |  |  |  |  |  |  |  |  |  |  |  |  |  |  |  |  |  |  |  |  |  |  |  |  |  |  |  |  |
| --- | --- | --- | --- | --- | --- | --- | --- | --- | --- | --- | --- | --- | --- | --- | --- | --- | --- | --- | --- | --- | --- | --- | --- | --- | --- | --- | --- | --- | --- | --- | --- | --- | --- | --- | --- | --- | --- | --- | --- | --- | --- | --- | --- |

D3TTC2.1

back to list

  

| Protein Coverage
| Supporting Peptides
|

Protein Coverage:

Supporting Peptides:

| Peptide | Uniq | -10lgP | Mass | Length | ppm | m/z | z | RT | Fraction | Scan | Source File | Area F1 | Area F10 | Area F11 | Area F12 | Area F13 | Area F2 | Area F3 | Area F4 | Area F5 | Area F6 | Area F7 | Area F8 | Area F9 | #Feature | #Feature F1 | #Feature F10 | #Feature F11 | #Feature F12 | #Feature F13 | #Feature F2 | #Feature F3 | #Feature F4 | #Feature F5 | #Feature F6 | #Feature F7 | #Feature F8 | #Feature F9 | Start | End | PTM | AScore | Found By |
| --- | --- | --- | --- | --- | --- | --- | --- | --- | --- | --- | --- | --- | --- | --- | --- | --- | --- | --- | --- | --- | --- | --- | --- | --- | --- | --- | --- | --- | --- | --- | --- | --- | --- | --- | --- | --- | --- | --- | --- | --- | --- | --- | --- |
| R.AAKDDC(+57.02)DLPELC(+57.02)TGQSAEC(+57.02)PTDVFQR.N | N | 91.95 | 2982.2793 | 26 | 1.7 | 995.1021 | 3 | 54.73 | 9 | F9:35402 | NaNaKA16\_F5.raw |  |  |  |  | 1.7077E8 |  |  |  | 1.5002E8 | 4.1894E7 |  |  |  | 4 | 0 | 0 | 0 | 0 | 1 | 0 | 0 | 0 | 2 | 1 | 0 | 0 | 0 | 468 | 493 | Carbamidomethylation | C6:Carbamidomethylation:1000.00;C12:Carbamidomethylation:1000.00;C19:Carbamidomethylation:1000.00 | PEAKS DB |
| K.DDC(+57.02)DLPELC(+57.02)TGQSAEC(+57.02)PTDVFQR.N | N | 90.56 | 2712.1101 | 23 | 1.2 | 1357.0640 | 2 | 72.09 | 9 | F9:50509 | NaNaKA16\_F5.raw |  |  |  |  | 9.0813E7 |  |  |  | 4.1609E7 | 6.0829E6 |  |  |  | 6 | 0 | 0 | 0 | 0 | 2 | 0 | 0 | 0 | 2 | 2 | 0 | 0 | 0 | 471 | 493 | Carbamidomethylation | C3:Carbamidomethylation:1000.00;C9:Carbamidomethylation:1000.00;C16:Carbamidomethylation:1000.00 | PEAKS DB |
| R.DPNYGMVEPGTK.C | N | 79.93 | 1306.5863 | 12 | 0.1 | 654.3005 | 2 | 28.66 | 10 | F10:14262 | NaNaKA16\_F6.raw |  |  |  |  | 4.8233E8 |  |  |  | 1.8661E7 | 4.1689E7 |  |  |  | 3 | 0 | 0 | 0 | 0 | 1 | 0 | 0 | 0 | 1 | 1 | 0 | 0 | 0 | 583 | 594 |  |  | PEAKS DB |
| R.DPNYGM(+15.99)VEPGTK.C | N | 77.87 | 1322.5813 | 12 | -0.3 | 662.2977 | 2 | 26.33 | 5 | F5:10060 | NaNaKA16\_F13.raw |  |  |  |  | 9.0917E7 |  |  |  |  |  |  |  |  | 5 | 0 | 0 | 0 | 0 | 5 | 0 | 0 | 0 | 0 | 0 | 0 | 0 | 0 | 583 | 594 | Oxidation (M) | M6:Oxidation (M):1000.00 | PEAKS DB |
| K.C(+57.02)PIMTNQC(+57.02)IALR.G | N | 77.27 | 1475.7047 | 12 | 0.2 | 738.8598 | 2 | 38.95 | 9 | F9:22183 | NaNaKA16\_F5.raw |  |  | 5.0717E5 | 3.2472E5 | 1.9097E8 |  |  |  | 2.1736E8 | 4.0241E7 |  | 3.7147E6 | 4.6756E6 | 12 | 0 | 0 | 1 | 1 | 3 | 0 | 0 | 0 | 2 | 2 | 0 | 2 | 1 | 510 | 521 | Carbamidomethylation | C1:Carbamidomethylation:1000.00;C8:Carbamidomethylation:1000.00 | PEAKS DB |
| K.C(+57.02)PIM(+15.99)TNQC(+57.02)IALR.G | N | 75.90 | 1491.6996 | 12 | -0.8 | 746.8564 | 2 | 29.62 | 9 | F9:14662 | NaNaKA16\_F5.raw |  |  |  |  | 3.2733E7 |  |  |  | 8.8589E6 | 2.9095E6 |  |  |  | 7 | 0 | 0 | 0 | 0 | 4 | 0 | 0 | 0 | 2 | 1 | 0 | 0 | 0 | 510 | 521 | Carbamidomethylation; Oxidation (M) | C1:Carbamidomethylation:1000.00;M4:Oxidation (M):1000.00;C8:Carbamidomethylation:1000.00 | PEAKS DB |
| R.NSMIC(+57.02)NC(+57.02)SISPR.D | N | 75.66 | 1437.6163 | 12 | 0.1 | 719.8155 | 2 | 24.50 | 5 | F5:7209 | NaNaKA16\_F13.raw |  |  |  |  | 6.4703E7 |  |  |  | 2.8886E6 | 2.2986E6 | 1.2892E5 |  |  | 4 | 0 | 0 | 0 | 0 | 1 | 0 | 0 | 0 | 1 | 1 | 1 | 0 | 0 | 571 | 582 | Carbamidomethylation | C5:Carbamidomethylation:1000.00;C7:Carbamidomethylation:1000.00 | PEAKS DB |
| G.TPVGLAYIGSIC(+57.02)NPK.T | N | 73.67 | 1588.8282 | 15 | 0.1 | 795.4215 | 2 | 60.14 | 12 | F12:39283 | NaNaKA16\_F8.raw |  |  |  |  |  |  |  |  |  |  | 1.0459E6 | 4.5255E6 |  | 2 | 0 | 0 | 0 | 0 | 0 | 0 | 0 | 0 | 0 | 0 | 1 | 1 | 0 | 305 | 319 | Carbamidomethylation | C12:Carbamidomethylation:1000.00 | PEAKS DB |
| N.GTPVGLAYIGSIC(+57.02)NPK.T | N | 73.11 | 1645.8497 | 16 | 0.4 | 823.9325 | 2 | 60.77 | 11 | F11:39960 | NaNaKA16\_F7.raw |  |  |  |  | 1.4933E6 |  |  |  |  |  | 3.1956E6 | 2.0263E7 |  | 3 | 0 | 0 | 0 | 0 | 1 | 0 | 0 | 0 | 0 | 0 | 1 | 1 | 0 | 304 | 319 | Carbamidomethylation | C13:Carbamidomethylation:1000.00 | PEAKS DB |
| R.TKPAYQFSSC(+57.02)SVR.E | N | 67.55 | 1529.7296 | 13 | -0.1 | 765.8720 | 2 | 12.28 | 5 | F5:2284 | NaNaKA16\_F13.raw |  |  |  |  | 2.3942E8 |  |  |  |  |  |  |  |  | 2 | 0 | 0 | 0 | 0 | 2 | 0 | 0 | 0 | 0 | 0 | 0 | 0 | 0 | 370 | 382 | Carbamidomethylation | C10:Carbamidomethylation:1000.00 | PEAKS DB |
| R.NSM(+15.99)IC(+57.02)NC(+57.02)SISPR.D | N | 64.85 | 1453.6112 | 12 | 0.3 | 727.8131 | 2 | 22.81 | 5 | F5:7058 | NaNaKA16\_F13.raw |  |  |  |  | 7.9998E6 |  |  |  |  |  |  |  |  | 3 | 0 | 0 | 0 | 0 | 3 | 0 | 0 | 0 | 0 | 0 | 0 | 0 | 0 | 571 | 582 | Oxidation (M); Carbamidomethylation | M3:Oxidation (M):1000.00;C5:Carbamidomethylation:1000.00;C7:Carbamidomethylation:1000.00 | PEAKS DB |
| R.RTKPAYQFSSC(+57.02)SVR.E | N | 61.84 | 1685.8307 | 14 | 1.0 | 562.9514 | 3 | 11.85 | 5 | F5:1803 | NaNaKA16\_F13.raw |  |  |  |  | 1.7851E6 |  |  |  |  |  |  |  |  | 1 | 0 | 0 | 0 | 0 | 1 | 0 | 0 | 0 | 0 | 0 | 0 | 0 | 0 | 369 | 382 | Carbamidomethylation | C11:Carbamidomethylation:1000.00 | PEAKS DB |
| D.PNYGMVEPGTK.C | N | 60.45 | 1191.5593 | 11 | 1.0 | 596.7875 | 2 | 20.63 | 5 | F5:5320 | NaNaKA16\_F13.raw |  |  |  |  | 1.9113E6 |  |  |  |  |  |  |  |  | 1 | 0 | 0 | 0 | 0 | 1 | 0 | 0 | 0 | 0 | 0 | 0 | 0 | 0 | 584 | 594 |  |  | PEAKS DB |
| A.DSSAVISAC(+57.02)DGLK.G | N | 60.11 | 1321.6184 | 13 | 0.4 | 661.8168 | 2 | 33.21 | 10 | F10:19042 | NaNaKA16\_F6.raw |  |  |  |  |  |  |  |  |  | 6.9505E6 |  |  |  | 1 | 0 | 0 | 0 | 0 | 0 | 0 | 0 | 0 | 0 | 1 | 0 | 0 | 0 | 119 | 131 | Carbamidomethylation | C9:Carbamidomethylation:1000.00 | PEAKS DB |
| R.RNSMIC(+57.02)NC(+57.02)SISPR.D | N | 59.91 | 1593.7174 | 13 | 0.1 | 532.2465 | 3 | 12.17 | 5 | F5:2067 | NaNaKA16\_F13.raw |  |  |  |  | 7.3377E5 |  |  |  |  |  |  |  |  | 1 | 0 | 0 | 0 | 0 | 1 | 0 | 0 | 0 | 0 | 0 | 0 | 0 | 0 | 570 | 582 | Carbamidomethylation | C6:Carbamidomethylation:1000.00;C8:Carbamidomethylation:1000.00 | PEAKS DB |
| C.TGQSAEC(+57.02)PTDVFQR.N | N | 57.94 | 1594.7046 | 14 | 0.9 | 798.3603 | 2 | 31.39 | 5 | F5:11052 | NaNaKA16\_F13.raw |  |  |  |  | 8.9511E5 |  |  |  |  |  |  |  |  | 1 | 0 | 0 | 0 | 0 | 1 | 0 | 0 | 0 | 0 | 0 | 0 | 0 | 0 | 480 | 493 | Carbamidomethylation | C7:Carbamidomethylation:1000.00 | PEAKS DB |
| R.DSC(+57.02)FTLNQR.T | N | 57.18 | 1139.5029 | 9 | 0.1 | 570.7588 | 2 | 26.24 | 5 | F5:8465 | NaNaKA16\_F13.raw |  |  |  |  | 2.8674E8 |  |  |  |  | 8.7317E5 |  |  |  | 2 | 0 | 0 | 0 | 0 | 1 | 0 | 0 | 0 | 0 | 1 | 0 | 0 | 0 | 530 | 538 | Carbamidomethylation | C3:Carbamidomethylation:1000.00 | PEAKS DB |
| R.NGLPC(+57.02)QNNQGYC(+57.02)YNGK.C | N | 55.77 | 1885.7836 | 16 | 0.8 | 943.8998 | 2 | 19.60 | 9 | F9:6919 | NaNaKA16\_F5.raw |  |  |  |  | 1.7165E6 |  |  |  | 6.1944E6 | 1.2392E6 |  |  |  | 3 | 0 | 0 | 0 | 0 | 1 | 0 | 0 | 0 | 1 | 1 | 0 | 0 | 0 | 494 | 509 | Carbamidomethylation | C5:Carbamidomethylation:1000.00;C12:Carbamidomethylation:1000.00 | PEAKS DB |
| R.MVAITMAHEMGH.N | N | 55.46 | 1326.5883 | 12 | 0.8 | 664.3019 | 2 | 33.28 | 5 | F5:12224 | NaNaKA16\_F13.raw |  |  |  |  | 4.5918E6 |  |  |  |  |  |  |  |  | 1 | 0 | 0 | 0 | 0 | 1 | 0 | 0 | 0 | 0 | 0 | 0 | 0 | 0 | 334 | 345 |  |  | PEAKS DB |
| K.PAYQFSSC(+57.02)SVR.E | N | 53.21 | 1300.5870 | 11 | 0.2 | 651.3009 | 2 | 25.11 | 5 | F5:7381 | NaNaKA16\_F13.raw |  |  |  |  | 5.016E7 |  |  |  |  |  |  |  |  | 1 | 0 | 0 | 0 | 0 | 1 | 0 | 0 | 0 | 0 | 0 | 0 | 0 | 0 | 372 | 382 | Carbamidomethylation | C8:Carbamidomethylation:1000.00 | PEAKS DB |
| K.C(+57.02)GDGMVC(+57.02)SNR.Q | N | 51.31 | 1154.4268 | 10 | 1.1 | 578.2213 | 2 | 11.85 | 5 | F5:1806 | NaNaKA16\_F13.raw |  |  |  |  | 4.5755E5 |  |  |  |  |  |  |  |  | 1 | 0 | 0 | 0 | 0 | 1 | 0 | 0 | 0 | 0 | 0 | 0 | 0 | 0 | 595 | 604 | Carbamidomethylation | C1:Carbamidomethylation:1000.00;C7:Carbamidomethylation:1000.00 | PEAKS DB |
| Q.SAEC(+57.02)PTDVFQR.N | N | 49.48 | 1308.5768 | 11 | 0.4 | 655.2960 | 2 | 32.04 | 5 | F5:11429 | NaNaKA16\_F13.raw |  |  |  |  | 1.3941E7 |  |  |  |  |  |  |  |  | 1 | 0 | 0 | 0 | 0 | 1 | 0 | 0 | 0 | 0 | 0 | 0 | 0 | 0 | 483 | 493 | Carbamidomethylation | C4:Carbamidomethylation:1000.00 | PEAKS DB |
| N.SMIC(+57.02)NC(+57.02)SISPR.D | N | 47.00 | 1323.5734 | 11 | 3.9 | 662.7966 | 2 | 23.55 | 5 | F5:6539 | NaNaKA16\_F13.raw |  |  |  |  | 0 |  |  |  |  |  |  |  |  | 0 | 0 | 0 | 0 | 0 | 0 | 0 | 0 | 0 | 0 | 0 | 0 | 0 | 0 | 572 | 582 | Carbamidomethylation | C4:Carbamidomethylation:1000.00;C6:Carbamidomethylation:1000.00 | PEAKS DB |
| K.LQHEAQC(+57.02)DSEEC(+57.02)C(+57.02)EK.C | N | 46.38 | 1921.7240 | 15 | 0.0 | 641.5820 | 3 | 11.83 | 5 | F5:1808 | NaNaKA16\_F13.raw |  |  |  |  | 2.9375E5 |  |  |  |  |  |  |  |  | 1 | 0 | 0 | 0 | 0 | 1 | 0 | 0 | 0 | 0 | 0 | 0 | 0 | 0 | 442 | 456 | Carbamidomethylation | C7:Carbamidomethylation:1000.00;C12:Carbamidomethylation:1000.00;C13:Carbamidomethylation:1000.00 | PEAKS DB |
| R.DRPQC(+57.02)ILNKPLST.D | N | 46.29 | 1540.8031 | 13 | -0.4 | 514.6081 | 3 | 27.16 | 4 | F4:11109 | NaNaKA16\_F12.raw |  |  |  | 7.0589E5 |  |  |  | 8.268E5 |  |  |  |  |  | 2 | 0 | 0 | 0 | 1 | 0 | 0 | 0 | 1 | 0 | 0 | 0 | 0 | 0 | 391 | 403 | Carbamidomethylation | C5:Carbamidomethylation:1000.00 | PEAKS DB |
| K.C(+57.02)GDGM(+15.99)VC(+57.02)SNR.Q | N | 45.27 | 1170.4216 | 10 | 0.3 | 586.2183 | 2 | 11.84 | 5 | F5:1815 | NaNaKA16\_F13.raw |  |  |  |  | 9.9702E4 |  |  |  |  |  |  |  |  | 1 | 0 | 0 | 0 | 0 | 1 | 0 | 0 | 0 | 0 | 0 | 0 | 0 | 0 | 595 | 604 | Carbamidomethylation; Oxidation (M) | C1:Carbamidomethylation:1000.00;M5:Oxidation (M):1000.00;C7:Carbamidomethylation:1000.00 | PEAKS DB |
| R.DPNYGMVEPGTKC(+57.02)GDGMVC(+57.02)SNR.Q | Y | 43.23 | 2443.0024 | 22 | -0.7 | 815.3408 | 3 | 34.89 | 5 | F5:13136 | NaNaKA16\_F13.raw |  |  |  |  | 0 |  |  |  |  |  |  |  |  | 0 | 0 | 0 | 0 | 0 | 0 | 0 | 0 | 0 | 0 | 0 | 0 | 0 | 0 | 583 | 604 | Carbamidomethylation | C13:Carbamidomethylation:1000.00;C19:Carbamidomethylation:1000.00 | PEAKS DB |
| total 27 peptides |  |  |  |  |  |  |  |  |  |  |  |  |  |  |  |  |  |  |  |  |  |  |  |  |  |  |  |  |  |  |  |  |  |  |  |  |  |  |  |  |  |  |  |
| --- | --- | --- | --- | --- | --- | --- | --- | --- | --- | --- | --- | --- | --- | --- | --- | --- | --- | --- | --- | --- | --- | --- | --- | --- | --- | --- | --- | --- | --- | --- | --- | --- | --- | --- | --- | --- | --- | --- | --- | --- | --- | --- | --- |

P82942.1

back to list

  

| Protein Coverage
| Supporting Peptides
|

Protein Coverage:

Supporting Peptides:

| Peptide | Uniq | -10lgP | Mass | Length | ppm | m/z | z | RT | Fraction | Scan | Source File | Area F1 | Area F10 | Area F11 | Area F12 | Area F13 | Area F2 | Area F3 | Area F4 | Area F5 | Area F6 | Area F7 | Area F8 | Area F9 | #Feature | #Feature F1 | #Feature F10 | #Feature F11 | #Feature F12 | #Feature F13 | #Feature F2 | #Feature F3 | #Feature F4 | #Feature F5 | #Feature F6 | #Feature F7 | #Feature F8 | #Feature F9 | Start | End | PTM | AScore | Found By |
| --- | --- | --- | --- | --- | --- | --- | --- | --- | --- | --- | --- | --- | --- | --- | --- | --- | --- | --- | --- | --- | --- | --- | --- | --- | --- | --- | --- | --- | --- | --- | --- | --- | --- | --- | --- | --- | --- | --- | --- | --- | --- | --- | --- |
| K.HDC(+57.02)DLPELC(+57.02)TGQSAEC(+57.02)PTDSLQR.N | Y | 96.36 | 2688.1213 | 23 | 1.5 | 897.0491 | 3 | 43.20 | 9 | F9:25555 | NaNaKA16\_F5.raw |  | 1.636E6 |  | 4.7159E6 | 1.008E6 |  |  |  | 3.2481E8 | 1.0386E9 | 1.5129E6 |  |  | 10 | 0 | 1 | 0 | 1 | 1 | 0 | 0 | 0 | 4 | 2 | 1 | 0 | 0 | 262 | 284 | Carbamidomethylation | C3:Carbamidomethylation:1000.00;C9:Carbamidomethylation:1000.00;C16:Carbamidomethylation:1000.00 | PEAKS DB |
| K.C(+57.02)PTLTNQC(+57.02)IALLGPHFTVSPK.G | Y | 92.22 | 2353.1921 | 21 | 0.7 | 785.4052 | 3 | 63.06 | 13 | F13:39142 | NaNaKA16\_F9.raw |  | 0 | 1.6577E6 | 4.7406E6 |  |  |  |  | 1.1748E7 | 1.7565E9 | 5.2146E7 |  | 7.1814E6 | 8 | 0 | 0 | 1 | 1 | 0 | 0 | 0 | 0 | 1 | 3 | 1 | 0 | 1 | 301 | 321 | Carbamidomethylation | C1:Carbamidomethylation:1000.00;C8:Carbamidomethylation:1000.00 | PEAKS DB |
| R.TAPAFQFSSC(+57.02)SIR.D | N | 80.42 | 1470.6925 | 13 | 0.6 | 736.3539 | 2 | 46.94 | 4 | F4:27572 | NaNaKA16\_F12.raw |  | 7.7695E6 | 7.0106E6 | 6.7741E6 | 7.3303E5 |  |  |  |  |  |  |  | 2.6381E6 | 5 | 0 | 1 | 1 | 1 | 1 | 0 | 0 | 0 | 0 | 0 | 0 | 0 | 1 | 178 | 190 | Carbamidomethylation | C10:Carbamidomethylation:1000.00 | PEAKS DB |
| D.LPELC(+57.02)TGQSAEC(+57.02)PTDSLQR.N | N | 78.21 | 2160.9780 | 19 | 0.3 | 1081.4966 | 2 | 44.94 | 10 | F10:28461 | NaNaKA16\_F6.raw |  |  |  |  |  |  |  |  |  | 2.2687E6 |  |  |  | 1 | 0 | 0 | 0 | 0 | 0 | 0 | 0 | 0 | 0 | 1 | 0 | 0 | 0 | 266 | 284 | Carbamidomethylation | C5:Carbamidomethylation:1000.00;C12:Carbamidomethylation:1000.00 | PEAKS DB |
| K.C(+57.02)PTLTNQC(+57.02)IALLGPH.F | Y | 73.45 | 1693.8280 | 15 | 1.1 | 847.9222 | 2 | 60.70 | 10 | F10:41651 | NaNaKA16\_F6.raw |  |  |  |  |  |  |  |  | 4.295E6 | 5.2604E6 |  |  |  | 3 | 0 | 0 | 0 | 0 | 0 | 0 | 0 | 0 | 2 | 1 | 0 | 0 | 0 | 301 | 315 | Carbamidomethylation | C1:Carbamidomethylation:1000.00;C8:Carbamidomethylation:1000.00 | PEAKS DB |
| N.QC(+57.02)IALLGPHFTVSPK.G | Y | 68.85 | 1666.8865 | 15 | 1.2 | 556.6368 | 3 | 49.77 | 10 | F10:32665 | NaNaKA16\_F6.raw |  |  |  |  |  |  |  |  |  | 5.0752E6 |  |  |  | 1 | 0 | 0 | 0 | 0 | 0 | 0 | 0 | 0 | 0 | 1 | 0 | 0 | 0 | 307 | 321 | Carbamidomethylation | C2:Carbamidomethylation:1000.00 | PEAKS DB |
| R.VYEMINAVNTK.F | N | 67.13 | 1280.6434 | 11 | -0.4 | 641.3287 | 2 | 34.28 | 4 | F4:17180 | NaNaKA16\_F12.raw |  |  |  | 2.766E6 | 2.8909E6 |  |  |  |  |  |  |  |  | 2 | 0 | 0 | 0 | 1 | 1 | 0 | 0 | 0 | 0 | 0 | 0 | 0 | 0 | 41 | 51 |  |  | PEAKS DB |
| P.TLTNQC(+57.02)IALLGPHFTVSPK.G | Y | 63.50 | 2096.1089 | 19 | 0.3 | 699.7104 | 3 | 59.91 | 10 | F10:40892 | NaNaKA16\_F6.raw |  |  |  |  |  |  |  |  |  | 1.6477E7 |  |  |  | 1 | 0 | 0 | 0 | 0 | 0 | 0 | 0 | 0 | 0 | 1 | 0 | 0 | 0 | 303 | 321 | Carbamidomethylation | C6:Carbamidomethylation:1000.00 | PEAKS DB |
| R.YYNYDKPAIK.I | Y | 61.87 | 1273.6343 | 10 | 0.3 | 637.8246 | 2 | 12.17 | 5 | F5:2066 | NaNaKA16\_F13.raw |  |  |  |  | 2.5131E6 |  |  |  |  |  |  |  |  | 2 | 0 | 0 | 0 | 0 | 2 | 0 | 0 | 0 | 0 | 0 | 0 | 0 | 0 | 29 | 38 |  |  | PEAKS DB |
| Q.C(+57.02)IALLGPHFTVSPK.G | Y | 61.06 | 1538.8279 | 14 | 0.0 | 513.9499 | 3 | 50.06 | 10 | F10:32795 | NaNaKA16\_F6.raw |  |  |  |  |  |  |  |  |  | 4.7258E6 |  |  |  | 1 | 0 | 0 | 0 | 0 | 0 | 0 | 0 | 0 | 0 | 1 | 0 | 0 | 0 | 308 | 321 | Carbamidomethylation | C1:Carbamidomethylation:1000.00 | PEAKS DB |
| C.PTLTNQC(+57.02)IALLGPHFTVSPK.G | Y | 59.80 | 2193.1616 | 20 | 0.3 | 732.0613 | 3 | 62.40 | 10 | F10:43172 | NaNaKA16\_F6.raw |  |  |  |  |  |  |  |  |  | 2.4828E6 |  |  |  | 1 | 0 | 0 | 0 | 0 | 0 | 0 | 0 | 0 | 0 | 1 | 0 | 0 | 0 | 302 | 321 | Carbamidomethylation | C7:Carbamidomethylation:1000.00 | PEAKS DB |
| K.GQC(+57.02)VDVQTAY | N | 56.58 | 1139.4917 | 10 | 0.2 | 570.7532 | 2 | 32.85 | 10 | F10:17935 | NaNaKA16\_F6.raw |  |  |  |  |  |  |  |  |  | 2.4491E8 |  |  |  | 1 | 0 | 0 | 0 | 0 | 0 | 0 | 0 | 0 | 0 | 1 | 0 | 0 | 0 | 392 | 401 | Carbamidomethylation | C3:Carbamidomethylation:1000.00 | PEAKS DB |
| R.DYQEYLLR.D | Y | 55.85 | 1098.5345 | 8 | 0.1 | 550.2746 | 2 | 52.54 | 4 | F4:32349 | NaNaKA16\_F12.raw |  | 4.2836E6 | 6.5338E6 | 1.251E7 | 2.2766E6 |  |  |  |  |  |  |  |  | 5 | 0 | 1 | 1 | 2 | 1 | 0 | 0 | 0 | 0 | 0 | 0 | 0 | 0 | 191 | 198 |  |  | PEAKS DB |
| C.IALLGPHFTVSPK.G | Y | 55.81 | 1378.7972 | 13 | 0.2 | 460.6064 | 3 | 46.93 | 10 | F10:30261 | NaNaKA16\_F6.raw |  |  |  |  |  |  |  |  |  | 2.2132E7 |  |  |  | 2 | 0 | 0 | 0 | 0 | 0 | 0 | 0 | 0 | 0 | 2 | 0 | 0 | 0 | 309 | 321 |  |  | PEAKS DB |
| K.GC(+57.02)FDLNMR.G | N | 52.79 | 1011.4266 | 8 | -0.1 | 506.7205 | 2 | 36.97 | 10 | F10:21218 | NaNaKA16\_F6.raw |  |  |  |  |  |  |  |  |  | 9.5883E8 |  |  |  | 1 | 0 | 0 | 0 | 0 | 0 | 0 | 0 | 0 | 0 | 1 | 0 | 0 | 0 | 322 | 329 | Carbamidomethylation | C2:Carbamidomethylation:1000.00 | PEAKS DB |
| T.LTNQC(+57.02)IALLGPHFTVSPK.G | Y | 49.25 | 1995.0612 | 18 | 0.5 | 666.0280 | 3 | 55.31 | 10 | F10:37057 | NaNaKA16\_F6.raw |  |  |  |  |  |  |  |  |  | 0 |  |  |  | 0 | 0 | 0 | 0 | 0 | 0 | 0 | 0 | 0 | 0 | 0 | 0 | 0 | 0 | 304 | 321 | Carbamidomethylation | C5:Carbamidomethylation:1000.00 | PEAKS DB |
| R.DRPQC(+57.02)ILNKPLST.D | N | 46.29 | 1540.8031 | 13 | -0.4 | 514.6081 | 3 | 27.16 | 4 | F4:11109 | NaNaKA16\_F12.raw |  |  |  | 7.0589E5 |  |  |  | 8.268E5 |  |  |  |  |  | 2 | 0 | 0 | 0 | 1 | 0 | 0 | 0 | 1 | 0 | 0 | 0 | 0 | 0 | 199 | 211 | Carbamidomethylation | C5:Carbamidomethylation:1000.00 | PEAKS DB |
| K.C(+57.02)PTLTNQC(+57.02)IALLGPHF.T | Y | 43.82 | 1840.8964 | 16 | 0.4 | 921.4558 | 2 | 105.10 | 10 | F10:78175 | NaNaKA16\_F6.raw |  |  |  |  |  |  |  |  |  | 7.0352E5 |  |  |  | 1 | 0 | 0 | 0 | 0 | 0 | 0 | 0 | 0 | 0 | 1 | 0 | 0 | 0 | 301 | 316 | Carbamidomethylation | C1:Carbamidomethylation:1000.00;C8:Carbamidomethylation:1000.00 | PEAKS DB |
| total 18 peptides |  |  |  |  |  |  |  |  |  |  |  |  |  |  |  |  |  |  |  |  |  |  |  |  |  |  |  |  |  |  |  |  |  |  |  |  |  |  |  |  |  |  |  |
| --- | --- | --- | --- | --- | --- | --- | --- | --- | --- | --- | --- | --- | --- | --- | --- | --- | --- | --- | --- | --- | --- | --- | --- | --- | --- | --- | --- | --- | --- | --- | --- | --- | --- | --- | --- | --- | --- | --- | --- | --- | --- | --- | --- |

764177A

back to list

  

| Protein Coverage
| Supporting Peptides
|

Protein Coverage:

Supporting Peptides:

| Peptide | Uniq | -10lgP | Mass | Length | ppm | m/z | z | RT | Fraction | Scan | Source File | Area F1 | Area F10 | Area F11 | Area F12 | Area F13 | Area F2 | Area F3 | Area F4 | Area F5 | Area F6 | Area F7 | Area F8 | Area F9 | #Feature | #Feature F1 | #Feature F10 | #Feature F11 | #Feature F12 | #Feature F13 | #Feature F2 | #Feature F3 | #Feature F4 | #Feature F5 | #Feature F6 | #Feature F7 | #Feature F8 | #Feature F9 | Start | End | PTM | AScore | Found By |
| --- | --- | --- | --- | --- | --- | --- | --- | --- | --- | --- | --- | --- | --- | --- | --- | --- | --- | --- | --- | --- | --- | --- | --- | --- | --- | --- | --- | --- | --- | --- | --- | --- | --- | --- | --- | --- | --- | --- | --- | --- | --- | --- | --- |
| R.C(+57.02)FITPDITSKDC(+57.02)PNGHVC(+57.02)YTK.T | N | 97.70 | 2512.1184 | 21 | -0.2 | 838.3799 | 3 | 32.91 | 9 | F9:17528 | NaNaKA16\_F5.raw |  |  |  |  |  |  | 1.3652E7 | 5.3048E7 | 1.1015E8 |  |  |  |  | 10 | 0 | 0 | 0 | 0 | 0 | 0 | 3 | 4 | 3 | 0 | 0 | 0 | 0 | 3 | 23 | Carbamidomethylation | C1:Carbamidomethylation:1000.00;C12:Carbamidomethylation:1000.00;C18:Carbamidomethylation:1000.00 | PEAKS DB |
| R.VDLGC(+57.02)AATC(+57.02)PTVR.T | N | 95.85 | 1418.6646 | 13 | 4.9 | 710.3395 | 2 | 28.78 | 7 | F7:10382 | NaNaKA16\_F3.raw |  |  | 9.9972E6 | 7.1823E7 | 6.7682E6 | 6.8283E8 | 2.2422E9 | 8.9511E10 | 1.0067E11 | 4.7969E9 | 2.1963E7 | 1.6215E7 | 4.704E6 | 103 | 0 | 0 | 1 | 2 | 1 | 2 | 8 | 40 | 41 | 5 | 1 | 1 | 1 | 37 | 49 | Carbamidomethylation | C5:Carbamidomethylation:1000.00;C9:Carbamidomethylation:1000.00 | PEAKS DB |
| K.RVDLGC(+57.02)AATC(+57.02)PTVR.T | N | 81.66 | 1574.7657 | 14 | 0.8 | 788.3908 | 2 | 21.92 | 9 | F9:8931 | NaNaKA16\_F5.raw |  |  |  |  | 2.0401E6 | 1.4583E8 | 2.0841E8 | 1.8696E9 | 1.6734E10 | 1.4455E9 | 2.6335E7 |  | 1.2378E6 | 55 | 0 | 0 | 0 | 0 | 1 | 3 | 10 | 13 | 21 | 4 | 2 | 0 | 1 | 36 | 49 | Carbamidomethylation | C6:Carbamidomethylation:1000.00;C10:Carbamidomethylation:1000.00 | PEAKS DB |
| R.C(+57.02)FITPDITSK.D | N | 75.82 | 1180.5798 | 10 | 7.7 | 591.2988 | 2 | 43.13 | 7 | F7:21737 | NaNaKA16\_F3.raw |  | 2.2482E7 | 8.6587E7 | 7.912E7 | 7.0808E6 | 5.0807E8 | 6.1225E10 | 8.5353E10 | 1.2307E11 | 5.4499E9 | 1.0861E8 | 6.3864E7 | 2.9206E8 | 82 | 0 | 4 | 2 | 4 | 2 | 4 | 9 | 21 | 14 | 13 | 2 | 2 | 5 | 3 | 12 | Carbamidomethylation | C1:Carbamidomethylation:1000.00 | PEAKS DB |
| K.TWC(+57.02)DGFC(+57.02)SSR.G | N | 68.51 | 1274.4808 | 10 | 5.8 | 638.2482 | 2 | 29.20 | 7 | F7:11885 | NaNaKA16\_F3.raw |  |  |  | 1.908E6 | 1.8858E6 | 1.7769E8 | 6.7555E8 | 3.8045E10 | 6.7549E9 | 1.7828E9 |  |  |  | 60 | 0 | 0 | 0 | 1 | 2 | 2 | 4 | 27 | 21 | 3 | 0 | 0 | 0 | 24 | 33 | Carbamidomethylation | C3:Carbamidomethylation:1000.00;C7:Carbamidomethylation:1000.00 | PEAKS DB |
| IRC(+57.02)FITPDITSK.D | N | 67.07 | 1449.7650 | 12 | 0.1 | 484.2623 | 3 | 34.24 | 9 | F9:18401 | NaNaKA16\_F5.raw |  |  |  |  |  |  | 2.4051E7 | 7.2194E7 | 8.0035E7 |  |  |  |  | 10 | 0 | 0 | 0 | 0 | 0 | 0 | 3 | 4 | 3 | 0 | 0 | 0 | 0 | 1 | 12 | Carbamidomethylation | C3:Carbamidomethylation:1000.00 | PEAKS DB |
| D.LGC(+57.02)AATC(+57.02)PTVR.T | N | 53.82 | 1204.5692 | 11 | 5.0 | 603.2917 | 2 | 11.90 | 8 | F8:2032 | NaNaKA16\_F4.raw |  |  |  |  |  |  | 5.7193E5 | 2.9789E6 | 1.0924E6 |  |  |  |  | 5 | 0 | 0 | 0 | 0 | 0 | 0 | 1 | 3 | 1 | 0 | 0 | 0 | 0 | 39 | 49 | Carbamidomethylation | C3:Carbamidomethylation:1000.00;C7:Carbamidomethylation:1000.00 | PEAKS DB |
| R.C(+57.02)FITPDITSKD.C | N | 53.03 | 1295.6067 | 11 | 0.2 | 648.8107 | 2 | 44.47 | 9 | F9:26660 | NaNaKA16\_F5.raw |  |  |  |  |  |  | 7.9006E5 | 3.8625E6 | 1.7287E7 |  |  |  |  | 6 | 0 | 0 | 0 | 0 | 0 | 0 | 1 | 2 | 3 | 0 | 0 | 0 | 0 | 3 | 13 | Carbamidomethylation | C1:Carbamidomethylation:1000.00 | PEAKS DB |
| K.DC(+57.02)PNGHVC(+57.02)YTK.T | N | 50.09 | 1349.5493 | 11 | -0.1 | 450.8570 | 3 | 15.59 | 9 | F9:3959 | NaNaKA16\_F5.raw |  |  |  |  |  |  |  |  | 0 |  |  |  |  | 0 | 0 | 0 | 0 | 0 | 0 | 0 | 0 | 0 | 0 | 0 | 0 | 0 | 0 | 13 | 23 | Carbamidomethylation | C2:Carbamidomethylation:1000.00;C8:Carbamidomethylation:1000.00 | PEAKS DB |
| R.TGVDIQC(+57.02)C(+57.02)STDDC(+57.02)PFPTR.K | Y | 49.86 | 2127.8660 | 18 | 5.3 | 1064.9406 | 2 | 51.93 | 7 | F7:29166 | NaNaKA16\_F3.raw |  |  |  |  |  |  | 0 |  |  |  |  |  |  | 0 | 0 | 0 | 0 | 0 | 0 | 0 | 0 | 0 | 0 | 0 | 0 | 0 | 0 | 50 | 67 | Carbamidomethylation | C7:Carbamidomethylation:1000.00;C8:Carbamidomethylation:1000.00;C13:Carbamidomethylation:1000.00 | PEAKS DB |
| I.RC(+57.02)FITPDITSK.D | N | 48.93 | 1336.6809 | 11 | 0.7 | 446.5679 | 3 | 25.75 | 9 | F9:12007 | NaNaKA16\_F5.raw |  |  |  |  |  |  |  |  | 8.2828E6 |  |  |  |  | 1 | 0 | 0 | 0 | 0 | 0 | 0 | 0 | 0 | 1 | 0 | 0 | 0 | 0 | 2 | 12 | Carbamidomethylation | C2:Carbamidomethylation:1000.00 | PEAKS DB |
| K.DC(+57.02)PNGHVC(+57.02)YTKTWC(+57.02)DGFC(+57.02)SSR.G | N | 47.50 | 2606.0195 | 21 | 5.3 | 652.5123 | 4 | 32.55 | 7 | F7:13432 | NaNaKA16\_F3.raw |  |  |  |  |  |  | 2.079E6 |  | 1.2197E6 |  |  |  |  | 2 | 0 | 0 | 0 | 0 | 0 | 0 | 1 | 0 | 1 | 0 | 0 | 0 | 0 | 13 | 33 | Carbamidomethylation | C2:Carbamidomethylation:1000.00;C8:Carbamidomethylation:1000.00;C14:Carbamidomethylation:1000.00;C18:Carbamidomethylation:1000.00 | PEAKS DB |
| C.FITPDITSK.D | N | 45.76 | 1020.5491 | 9 | -2.0 | 511.2808 | 2 | 35.97 | 9 | F9:20225 | NaNaKA16\_F5.raw |  |  |  |  |  |  |  | 2.3378E8 | 1.8E8 |  |  |  |  | 3 | 0 | 0 | 0 | 0 | 0 | 0 | 0 | 2 | 1 | 0 | 0 | 0 | 0 | 4 | 12 |  |  | PEAKS DB |
| R.VDLGC(+57.02)AATC(+57.02)PTV.R | N | 45.13 | 1262.5635 | 12 | -0.9 | 632.2885 | 2 | 50.91 | 10 | F10:33445 | NaNaKA16\_F6.raw |  |  |  |  |  |  | 1.1303E7 |  |  | 5.2132E7 |  |  |  | 2 | 0 | 0 | 0 | 0 | 0 | 0 | 1 | 0 | 0 | 1 | 0 | 0 | 0 | 37 | 48 | Carbamidomethylation | C5:Carbamidomethylation:1000.00;C9:Carbamidomethylation:1000.00 | PEAKS DB |
| T.KTWC(+57.02)DGFC(+57.02)SSR.G | N | 45.02 | 1402.5758 | 11 | 4.8 | 468.5323 | 3 | 12.27 | 8 | F8:2350 | NaNaKA16\_F4.raw |  |  |  |  |  |  |  | 1.5491E5 |  |  |  |  |  | 1 | 0 | 0 | 0 | 0 | 0 | 0 | 0 | 1 | 0 | 0 | 0 | 0 | 0 | 23 | 33 | Carbamidomethylation | C4:Carbamidomethylation:1000.00;C8:Carbamidomethylation:1000.00 | PEAKS DB |
| F.ITPDITSK.D | N | 42.90 | 873.4807 | 8 | 4.9 | 437.7476 | 2 | 11.46 | 7 | F7:1934 | NaNaKA16\_F3.raw |  |  |  |  |  |  | 1.0681E6 |  |  |  |  |  |  | 1 | 0 | 0 | 0 | 0 | 0 | 0 | 1 | 0 | 0 | 0 | 0 | 0 | 0 | 5 | 12 |  |  | PEAKS DB |
| V.DLGC(+57.02)AATC(+57.02)PTVR.T | N | 41.93 | 1319.5962 | 12 | 0.1 | 660.8054 | 2 | 23.57 | 6 | F6:10177 | NaNaKA16\_F2.raw |  |  |  |  |  | 4.9836E5 |  |  |  |  |  |  |  | 1 | 0 | 0 | 0 | 0 | 0 | 1 | 0 | 0 | 0 | 0 | 0 | 0 | 0 | 38 | 49 | Carbamidomethylation | C4:Carbamidomethylation:1000.00;C8:Carbamidomethylation:1000.00 | PEAKS DB |
| total 17 peptides |  |  |  |  |  |  |  |  |  |  |  |  |  |  |  |  |  |  |  |  |  |  |  |  |  |  |  |  |  |  |  |  |  |  |  |  |  |  |  |  |  |  |  |
| --- | --- | --- | --- | --- | --- | --- | --- | --- | --- | --- | --- | --- | --- | --- | --- | --- | --- | --- | --- | --- | --- | --- | --- | --- | --- | --- | --- | --- | --- | --- | --- | --- | --- | --- | --- | --- | --- | --- | --- | --- | --- | --- | --- |

P25668.1

back to list

  

| Protein Coverage
| Supporting Peptides
|

Protein Coverage:

Supporting Peptides:

| Peptide | Uniq | -10lgP | Mass | Length | ppm | m/z | z | RT | Fraction | Scan | Source File | Area F1 | Area F10 | Area F11 | Area F12 | Area F13 | Area F2 | Area F3 | Area F4 | Area F5 | Area F6 | Area F7 | Area F8 | Area F9 | #Feature | #Feature F1 | #Feature F10 | #Feature F11 | #Feature F12 | #Feature F13 | #Feature F2 | #Feature F3 | #Feature F4 | #Feature F5 | #Feature F6 | #Feature F7 | #Feature F8 | #Feature F9 | Start | End | PTM | AScore | Found By |
| --- | --- | --- | --- | --- | --- | --- | --- | --- | --- | --- | --- | --- | --- | --- | --- | --- | --- | --- | --- | --- | --- | --- | --- | --- | --- | --- | --- | --- | --- | --- | --- | --- | --- | --- | --- | --- | --- | --- | --- | --- | --- | --- | --- |
| R.C(+57.02)FITPDITSKDC(+57.02)PNGHVC(+57.02)YTK.T | N | 97.70 | 2512.1184 | 21 | -0.2 | 838.3799 | 3 | 32.91 | 9 | F9:17528 | NaNaKA16\_F5.raw |  |  |  |  |  |  | 1.3652E7 | 5.3048E7 | 1.1015E8 |  |  |  |  | 10 | 0 | 0 | 0 | 0 | 0 | 0 | 3 | 4 | 3 | 0 | 0 | 0 | 0 | 3 | 23 | Carbamidomethylation | C1:Carbamidomethylation:1000.00;C12:Carbamidomethylation:1000.00;C18:Carbamidomethylation:1000.00 | PEAKS DB |
| R.VDLGC(+57.02)AATC(+57.02)PTVR.T | N | 95.85 | 1418.6646 | 13 | 4.9 | 710.3395 | 2 | 28.78 | 7 | F7:10382 | NaNaKA16\_F3.raw |  |  | 9.9972E6 | 7.1823E7 | 6.7682E6 | 6.8283E8 | 2.2422E9 | 8.9511E10 | 1.0067E11 | 4.7969E9 | 2.1963E7 | 1.6215E7 | 4.704E6 | 103 | 0 | 0 | 1 | 2 | 1 | 2 | 8 | 40 | 41 | 5 | 1 | 1 | 1 | 37 | 49 | Carbamidomethylation | C5:Carbamidomethylation:1000.00;C9:Carbamidomethylation:1000.00 | PEAKS DB |
| K.RVDLGC(+57.02)AATC(+57.02)PTVR.T | N | 81.66 | 1574.7657 | 14 | 0.8 | 788.3908 | 2 | 21.92 | 9 | F9:8931 | NaNaKA16\_F5.raw |  |  |  |  | 2.0401E6 | 1.4583E8 | 2.0841E8 | 1.8696E9 | 1.6734E10 | 1.4455E9 | 2.6335E7 |  | 1.2378E6 | 55 | 0 | 0 | 0 | 0 | 1 | 3 | 10 | 13 | 21 | 4 | 2 | 0 | 1 | 36 | 49 | Carbamidomethylation | C6:Carbamidomethylation:1000.00;C10:Carbamidomethylation:1000.00 | PEAKS DB |
| R.C(+57.02)FITPDITSK.D | N | 75.82 | 1180.5798 | 10 | 7.7 | 591.2988 | 2 | 43.13 | 7 | F7:21737 | NaNaKA16\_F3.raw |  | 2.2482E7 | 8.6587E7 | 7.912E7 | 7.0808E6 | 5.0807E8 | 6.1225E10 | 8.5353E10 | 1.2307E11 | 5.4499E9 | 1.0861E8 | 6.3864E7 | 2.9206E8 | 82 | 0 | 4 | 2 | 4 | 2 | 4 | 9 | 21 | 14 | 13 | 2 | 2 | 5 | 3 | 12 | Carbamidomethylation | C1:Carbamidomethylation:1000.00 | PEAKS DB |
| IRC(+57.02)FITPDITSK.D | N | 67.07 | 1449.7650 | 12 | 0.1 | 484.2623 | 3 | 34.24 | 9 | F9:18401 | NaNaKA16\_F5.raw |  |  |  |  |  |  | 2.4051E7 | 7.2194E7 | 8.0035E7 |  |  |  |  | 10 | 0 | 0 | 0 | 0 | 0 | 0 | 3 | 4 | 3 | 0 | 0 | 0 | 0 | 1 | 12 | Carbamidomethylation | C3:Carbamidomethylation:1000.00 | PEAKS DB |
| R.TGVDIQC(+57.02)C(+57.02)STDDC(+57.02)DPFPTR.K | N | 64.22 | 2242.8928 | 19 | -1.9 | 1122.4456 | 2 | 55.16 | 8 | F8:34181 | NaNaKA16\_F4.raw |  |  |  |  |  |  | 2.6424E7 | 1.4048E8 | 6.7036E7 | 2.7519E7 |  |  |  | 8 | 0 | 0 | 0 | 0 | 0 | 0 | 1 | 3 | 3 | 1 | 0 | 0 | 0 | 50 | 68 | Carbamidomethylation | C7:Carbamidomethylation:1000.00;C8:Carbamidomethylation:1000.00;C13:Carbamidomethylation:1000.00 | PEAKS DB |
| K.TWC(+57.02)DGFC(+57.02)SIR.G | Y | 58.43 | 1300.5328 | 10 | -2.3 | 651.2722 | 2 | 47.10 | 9 | F9:29057 | NaNaKA16\_F5.raw |  | 5.247E6 |  |  |  | 5.4239E6 | 8.3364E9 | 2.3412E10 | 1.7299E10 | 5.9718E8 |  |  | 2.0334E7 | 38 | 0 | 1 | 0 | 0 | 0 | 1 | 3 | 14 | 13 | 5 | 0 | 0 | 1 | 24 | 33 | Carbamidomethylation | C3:Carbamidomethylation:1000.00;C7:Carbamidomethylation:1000.00 | PEAKS DB |
| D.LGC(+57.02)AATC(+57.02)PTVR.T | N | 53.82 | 1204.5692 | 11 | 5.0 | 603.2917 | 2 | 11.90 | 8 | F8:2032 | NaNaKA16\_F4.raw |  |  |  |  |  |  | 5.7193E5 | 2.9789E6 | 1.0924E6 |  |  |  |  | 5 | 0 | 0 | 0 | 0 | 0 | 0 | 1 | 3 | 1 | 0 | 0 | 0 | 0 | 39 | 49 | Carbamidomethylation | C3:Carbamidomethylation:1000.00;C7:Carbamidomethylation:1000.00 | PEAKS DB |
| R.C(+57.02)FITPDITSKD.C | N | 53.03 | 1295.6067 | 11 | 0.2 | 648.8107 | 2 | 44.47 | 9 | F9:26660 | NaNaKA16\_F5.raw |  |  |  |  |  |  | 7.9006E5 | 3.8625E6 | 1.7287E7 |  |  |  |  | 6 | 0 | 0 | 0 | 0 | 0 | 0 | 1 | 2 | 3 | 0 | 0 | 0 | 0 | 3 | 13 | Carbamidomethylation | C1:Carbamidomethylation:1000.00 | PEAKS DB |
| T.KTWC(+57.02)DGFC(+57.02)SIR.G | Y | 51.45 | 1428.6278 | 11 | 5.0 | 715.3209 | 2 | 30.42 | 8 | F8:13777 | NaNaKA16\_F4.raw |  |  |  |  |  |  |  | 1.2676E7 |  |  |  |  |  | 2 | 0 | 0 | 0 | 0 | 0 | 0 | 0 | 2 | 0 | 0 | 0 | 0 | 0 | 23 | 33 | Carbamidomethylation | C4:Carbamidomethylation:1000.00;C8:Carbamidomethylation:1000.00 | PEAKS DB |
| K.DC(+57.02)PNGHVC(+57.02)YTK.T | N | 50.09 | 1349.5493 | 11 | -0.1 | 450.8570 | 3 | 15.59 | 9 | F9:3959 | NaNaKA16\_F5.raw |  |  |  |  |  |  |  |  | 0 |  |  |  |  | 0 | 0 | 0 | 0 | 0 | 0 | 0 | 0 | 0 | 0 | 0 | 0 | 0 | 0 | 13 | 23 | Carbamidomethylation | C2:Carbamidomethylation:1000.00;C8:Carbamidomethylation:1000.00 | PEAKS DB |
| I.RC(+57.02)FITPDITSK.D | N | 48.93 | 1336.6809 | 11 | 0.7 | 446.5679 | 3 | 25.75 | 9 | F9:12007 | NaNaKA16\_F5.raw |  |  |  |  |  |  |  |  | 8.2828E6 |  |  |  |  | 1 | 0 | 0 | 0 | 0 | 0 | 0 | 0 | 0 | 1 | 0 | 0 | 0 | 0 | 2 | 12 | Carbamidomethylation | C2:Carbamidomethylation:1000.00 | PEAKS DB |
| C.FITPDITSK.D | N | 45.76 | 1020.5491 | 9 | -2.0 | 511.2808 | 2 | 35.97 | 9 | F9:20225 | NaNaKA16\_F5.raw |  |  |  |  |  |  |  | 2.3378E8 | 1.8E8 |  |  |  |  | 3 | 0 | 0 | 0 | 0 | 0 | 0 | 0 | 2 | 1 | 0 | 0 | 0 | 0 | 4 | 12 |  |  | PEAKS DB |
| R.VDLGC(+57.02)AATC(+57.02)PTV.R | N | 45.13 | 1262.5635 | 12 | -0.9 | 632.2885 | 2 | 50.91 | 10 | F10:33445 | NaNaKA16\_F6.raw |  |  |  |  |  |  | 1.1303E7 |  |  | 5.2132E7 |  |  |  | 2 | 0 | 0 | 0 | 0 | 0 | 0 | 1 | 0 | 0 | 1 | 0 | 0 | 0 | 37 | 48 | Carbamidomethylation | C5:Carbamidomethylation:1000.00;C9:Carbamidomethylation:1000.00 | PEAKS DB |
| F.ITPDITSK.D | N | 42.90 | 873.4807 | 8 | 4.9 | 437.7476 | 2 | 11.46 | 7 | F7:1934 | NaNaKA16\_F3.raw |  |  |  |  |  |  | 1.0681E6 |  |  |  |  |  |  | 1 | 0 | 0 | 0 | 0 | 0 | 0 | 1 | 0 | 0 | 0 | 0 | 0 | 0 | 5 | 12 |  |  | PEAKS DB |
| V.DLGC(+57.02)AATC(+57.02)PTVR.T | N | 41.93 | 1319.5962 | 12 | 0.1 | 660.8054 | 2 | 23.57 | 6 | F6:10177 | NaNaKA16\_F2.raw |  |  |  |  |  | 4.9836E5 |  |  |  |  |  |  |  | 1 | 0 | 0 | 0 | 0 | 0 | 1 | 0 | 0 | 0 | 0 | 0 | 0 | 0 | 38 | 49 | Carbamidomethylation | C4:Carbamidomethylation:1000.00;C8:Carbamidomethylation:1000.00 | PEAKS DB |
| total 16 peptides |  |  |  |  |  |  |  |  |  |  |  |  |  |  |  |  |  |  |  |  |  |  |  |  |  |  |  |  |  |  |  |  |  |  |  |  |  |  |  |  |  |  |  |
| --- | --- | --- | --- | --- | --- | --- | --- | --- | --- | --- | --- | --- | --- | --- | --- | --- | --- | --- | --- | --- | --- | --- | --- | --- | --- | --- | --- | --- | --- | --- | --- | --- | --- | --- | --- | --- | --- | --- | --- | --- | --- | --- | --- |

P25672.1

back to list

  

| Protein Coverage
| Supporting Peptides
|

Protein Coverage:

Supporting Peptides:

| Peptide | Uniq | -10lgP | Mass | Length | ppm | m/z | z | RT | Fraction | Scan | Source File | Area F1 | Area F10 | Area F11 | Area F12 | Area F13 | Area F2 | Area F3 | Area F4 | Area F5 | Area F6 | Area F7 | Area F8 | Area F9 | #Feature | #Feature F1 | #Feature F10 | #Feature F11 | #Feature F12 | #Feature F13 | #Feature F2 | #Feature F3 | #Feature F4 | #Feature F5 | #Feature F6 | #Feature F7 | #Feature F8 | #Feature F9 | Start | End | PTM | AScore | Found By |
| --- | --- | --- | --- | --- | --- | --- | --- | --- | --- | --- | --- | --- | --- | --- | --- | --- | --- | --- | --- | --- | --- | --- | --- | --- | --- | --- | --- | --- | --- | --- | --- | --- | --- | --- | --- | --- | --- | --- | --- | --- | --- | --- | --- |
| R.C(+57.02)FITPDITSKDC(+57.02)PNGHVC(+57.02)YTK.T | N | 97.70 | 2512.1184 | 21 | -0.2 | 838.3799 | 3 | 32.91 | 9 | F9:17528 | NaNaKA16\_F5.raw |  |  |  |  |  |  | 1.3652E7 | 5.3048E7 | 1.1015E8 |  |  |  |  | 10 | 0 | 0 | 0 | 0 | 0 | 0 | 3 | 4 | 3 | 0 | 0 | 0 | 0 | 3 | 23 | Carbamidomethylation | C1:Carbamidomethylation:1000.00;C12:Carbamidomethylation:1000.00;C18:Carbamidomethylation:1000.00 | PEAKS DB |
| R.VDLGC(+57.02)AATC(+57.02)PTVK.T | N | 93.61 | 1390.6584 | 13 | 6.1 | 696.3372 | 2 | 26.78 | 7 | F7:8603 | NaNaKA16\_F3.raw |  |  |  | 1.5246E7 | 1.5932E6 | 5.0659E7 | 1.9121E8 | 2.2473E10 | 6.1192E10 | 1.9939E9 | 5.3549E6 |  | 0 | 56 | 0 | 0 | 0 | 1 | 1 | 1 | 2 | 13 | 32 | 5 | 1 | 0 | 0 | 37 | 49 | Carbamidomethylation | C5:Carbamidomethylation:1000.00;C9:Carbamidomethylation:1000.00 | PEAKS DB |
| G.ERVDLGC(+57.02)AATC(+57.02)PTVK.T | Y | 86.02 | 1675.8021 | 15 | -0.2 | 838.9081 | 2 | 20.70 | 9 | F9:7862 | NaNaKA16\_F5.raw |  |  |  |  |  |  |  | 1.6699E6 | 1.2973E8 | 2.0806E7 |  |  |  | 5 | 0 | 0 | 0 | 0 | 0 | 0 | 0 | 1 | 2 | 2 | 0 | 0 | 0 | 35 | 49 | Carbamidomethylation | C7:Carbamidomethylation:1000.00;C11:Carbamidomethylation:1000.00 | PEAKS DB |
| R.C(+57.02)FITPDITSK.D | N | 75.82 | 1180.5798 | 10 | 7.7 | 591.2988 | 2 | 43.13 | 7 | F7:21737 | NaNaKA16\_F3.raw |  | 2.2482E7 | 8.6587E7 | 7.912E7 | 7.0808E6 | 5.0807E8 | 6.1225E10 | 8.5353E10 | 1.2307E11 | 5.4499E9 | 1.0861E8 | 6.3864E7 | 2.9206E8 | 82 | 0 | 4 | 2 | 4 | 2 | 4 | 9 | 21 | 14 | 13 | 2 | 2 | 5 | 3 | 12 | Carbamidomethylation | C1:Carbamidomethylation:1000.00 | PEAKS DB |
| IRC(+57.02)FITPDITSK.D | N | 67.07 | 1449.7650 | 12 | 0.1 | 484.2623 | 3 | 34.24 | 9 | F9:18401 | NaNaKA16\_F5.raw |  |  |  |  |  |  | 2.4051E7 | 7.2194E7 | 8.0035E7 |  |  |  |  | 10 | 0 | 0 | 0 | 0 | 0 | 0 | 3 | 4 | 3 | 0 | 0 | 0 | 0 | 1 | 12 | Carbamidomethylation | C3:Carbamidomethylation:1000.00 | PEAKS DB |
| K.TGVDIQC(+57.02)C(+57.02)STDDC(+57.02)DPFPTR.K | N | 64.22 | 2242.8928 | 19 | -1.9 | 1122.4456 | 2 | 55.16 | 8 | F8:34181 | NaNaKA16\_F4.raw |  |  |  |  |  |  | 2.6424E7 | 1.4048E8 | 6.7036E7 | 2.7519E7 |  |  |  | 8 | 0 | 0 | 0 | 0 | 0 | 0 | 1 | 3 | 3 | 1 | 0 | 0 | 0 | 50 | 68 | Carbamidomethylation | C7:Carbamidomethylation:1000.00;C8:Carbamidomethylation:1000.00;C13:Carbamidomethylation:1000.00 | PEAKS DB |
| K.TWC(+57.02)DGFC(+57.02)R.I | Y | 58.38 | 1100.4167 | 8 | 5.0 | 551.2156 | 2 | 29.88 | 7 | F7:11237 | NaNaKA16\_F3.raw |  |  |  |  |  | 1.1563E7 | 9.3448E7 | 4.4944E9 | 1.1459E10 | 2.7255E8 |  |  |  | 27 | 0 | 0 | 0 | 0 | 0 | 2 | 2 | 8 | 14 | 1 | 0 | 0 | 0 | 24 | 31 | Carbamidomethylation | C3:Carbamidomethylation:1000.00;C7:Carbamidomethylation:1000.00 | PEAKS DB |
| R.GERVDLGC(+57.02)AATC(+57.02)PTVK.T | Y | 54.17 | 1732.8236 | 16 | -0.5 | 578.6149 | 3 | 20.46 | 9 | F9:7831 | NaNaKA16\_F5.raw |  |  |  |  |  |  |  | 2.0096E6 | 9.0307E6 |  |  |  |  | 3 | 0 | 0 | 0 | 0 | 0 | 0 | 0 | 1 | 2 | 0 | 0 | 0 | 0 | 34 | 49 | Carbamidomethylation | C8:Carbamidomethylation:1000.00;C12:Carbamidomethylation:1000.00 | PEAKS DB |
| R.C(+57.02)FITPDITSKD.C | N | 53.03 | 1295.6067 | 11 | 0.2 | 648.8107 | 2 | 44.47 | 9 | F9:26660 | NaNaKA16\_F5.raw |  |  |  |  |  |  | 7.9006E5 | 3.8625E6 | 1.7287E7 |  |  |  |  | 6 | 0 | 0 | 0 | 0 | 0 | 0 | 1 | 2 | 3 | 0 | 0 | 0 | 0 | 3 | 13 | Carbamidomethylation | C1:Carbamidomethylation:1000.00 | PEAKS DB |
| K.DC(+57.02)PNGHVC(+57.02)YTK.T | N | 50.09 | 1349.5493 | 11 | -0.1 | 450.8570 | 3 | 15.59 | 9 | F9:3959 | NaNaKA16\_F5.raw |  |  |  |  |  |  |  |  | 0 |  |  |  |  | 0 | 0 | 0 | 0 | 0 | 0 | 0 | 0 | 0 | 0 | 0 | 0 | 0 | 0 | 13 | 23 | Carbamidomethylation | C2:Carbamidomethylation:1000.00;C8:Carbamidomethylation:1000.00 | PEAKS DB |
| D.LGC(+57.02)AATC(+57.02)PTVK.T | N | 49.50 | 1176.5631 | 11 | 4.9 | 589.2886 | 2 | 11.80 | 8 | F8:1936 | NaNaKA16\_F4.raw |  |  |  |  |  |  |  | 1.7287E5 |  |  |  |  |  | 1 | 0 | 0 | 0 | 0 | 0 | 0 | 0 | 1 | 0 | 0 | 0 | 0 | 0 | 39 | 49 | Carbamidomethylation | C3:Carbamidomethylation:1000.00;C7:Carbamidomethylation:1000.00 | PEAKS DB |
| I.RC(+57.02)FITPDITSK.D | N | 48.93 | 1336.6809 | 11 | 0.7 | 446.5679 | 3 | 25.75 | 9 | F9:12007 | NaNaKA16\_F5.raw |  |  |  |  |  |  |  |  | 8.2828E6 |  |  |  |  | 1 | 0 | 0 | 0 | 0 | 0 | 0 | 0 | 0 | 1 | 0 | 0 | 0 | 0 | 2 | 12 | Carbamidomethylation | C2:Carbamidomethylation:1000.00 | PEAKS DB |
| C.FITPDITSK.D | N | 45.76 | 1020.5491 | 9 | -2.0 | 511.2808 | 2 | 35.97 | 9 | F9:20225 | NaNaKA16\_F5.raw |  |  |  |  |  |  |  | 2.3378E8 | 1.8E8 |  |  |  |  | 3 | 0 | 0 | 0 | 0 | 0 | 0 | 0 | 2 | 1 | 0 | 0 | 0 | 0 | 4 | 12 |  |  | PEAKS DB |
| R.VDLGC(+57.02)AATC(+57.02)PTV.K | N | 45.13 | 1262.5635 | 12 | -0.9 | 632.2885 | 2 | 50.91 | 10 | F10:33445 | NaNaKA16\_F6.raw |  |  |  |  |  |  | 1.1303E7 |  |  | 5.2132E7 |  |  |  | 2 | 0 | 0 | 0 | 0 | 0 | 0 | 1 | 0 | 0 | 1 | 0 | 0 | 0 | 37 | 48 | Carbamidomethylation | C5:Carbamidomethylation:1000.00;C9:Carbamidomethylation:1000.00 | PEAKS DB |
| E.RVDLGC(+57.02)AATC(+57.02)PTVK.T | N | 44.70 | 1546.7595 | 14 | -0.8 | 516.5934 | 3 | 18.27 | 9 | F9:5871 | NaNaKA16\_F5.raw |  |  |  |  |  |  |  | 1.5478E5 | 2.2798E6 |  |  |  |  | 2 | 0 | 0 | 0 | 0 | 0 | 0 | 0 | 1 | 1 | 0 | 0 | 0 | 0 | 36 | 49 | Carbamidomethylation | C6:Carbamidomethylation:1000.00;C10:Carbamidomethylation:1000.00 | PEAKS DB |
| F.ITPDITSK.D | N | 42.90 | 873.4807 | 8 | 4.9 | 437.7476 | 2 | 11.46 | 7 | F7:1934 | NaNaKA16\_F3.raw |  |  |  |  |  |  | 1.0681E6 |  |  |  |  |  |  | 1 | 0 | 0 | 0 | 0 | 0 | 0 | 1 | 0 | 0 | 0 | 0 | 0 | 0 | 5 | 12 |  |  | PEAKS DB |
| total 16 peptides |  |  |  |  |  |  |  |  |  |  |  |  |  |  |  |  |  |  |  |  |  |  |  |  |  |  |  |  |  |  |  |  |  |  |  |  |  |  |  |  |  |  |  |
| --- | --- | --- | --- | --- | --- | --- | --- | --- | --- | --- | --- | --- | --- | --- | --- | --- | --- | --- | --- | --- | --- | --- | --- | --- | --- | --- | --- | --- | --- | --- | --- | --- | --- | --- | --- | --- | --- | --- | --- | --- | --- | --- | --- |

PSNJ3K

back to list

  

| Protein Coverage
| Supporting Peptides
|

Protein Coverage:

Supporting Peptides:

| Peptide | Uniq | -10lgP | Mass | Length | ppm | m/z | z | RT | Fraction | Scan | Source File | Area F1 | Area F10 | Area F11 | Area F12 | Area F13 | Area F2 | Area F3 | Area F4 | Area F5 | Area F6 | Area F7 | Area F8 | Area F9 | #Feature | #Feature F1 | #Feature F10 | #Feature F11 | #Feature F12 | #Feature F13 | #Feature F2 | #Feature F3 | #Feature F4 | #Feature F5 | #Feature F6 | #Feature F7 | #Feature F8 | #Feature F9 | Start | End | PTM | AScore | Found By |
| --- | --- | --- | --- | --- | --- | --- | --- | --- | --- | --- | --- | --- | --- | --- | --- | --- | --- | --- | --- | --- | --- | --- | --- | --- | --- | --- | --- | --- | --- | --- | --- | --- | --- | --- | --- | --- | --- | --- | --- | --- | --- | --- | --- |
| K.ISGC(+57.02)WPYFKTYSYEC(+57.02)SQGTLTC(+57.02)K.G | N | 89.48 | 2835.2341 | 23 | -1.5 | 946.0839 | 3 | 57.58 | 11 | F11:37368 | NaNaKA16\_F7.raw |  |  |  |  |  |  |  |  |  | 1.3653E7 | 7.0731E7 | 1.9929E7 | 6.9253E6 | 5 | 0 | 0 | 0 | 0 | 0 | 0 | 0 | 0 | 0 | 1 | 2 | 1 | 1 | 57 | 79 | Carbamidomethylation | C4:Carbamidomethylation:1000.00;C15:Carbamidomethylation:1000.00;C22:Carbamidomethylation:1000.00 | PEAKS DB |
| K.TYSYEC(+57.02)SQGTLTC(+57.02)K.G | N | 89.30 | 1696.7073 | 14 | 0.3 | 849.3611 | 2 | 21.20 | 9 | F9:9021 | NaNaKA16\_F5.raw |  | 7.5988E5 |  | 1.6097E7 | 6.3725E6 | 1.1263E6 |  | 2.9128E6 | 6.1482E8 | 3.4541E10 | 2.2713E9 | 9.1534E6 | 4.1844E8 | 46 | 0 | 1 | 0 | 3 | 1 | 1 | 0 | 1 | 4 | 18 | 3 | 2 | 12 | 66 | 79 | Carbamidomethylation | C6:Carbamidomethylation:1000.00;C13:Carbamidomethylation:1000.00 | PEAKS DB |
| K.ISGC(+57.02)WPYFK.T | N | 70.32 | 1156.5375 | 9 | 0.1 | 579.2761 | 2 | 68.82 | 11 | F11:46583 | NaNaKA16\_F7.raw |  | 3.6915E8 | 1.4139E8 | 6.9856E7 | 2.3823E6 |  |  | 3.6353E6 | 3.5126E8 | 2.1014E10 | 1.2727E11 | 1.9979E10 | 1.2073E10 | 71 | 0 | 1 | 1 | 3 | 1 | 0 | 0 | 1 | 1 | 15 | 17 | 10 | 21 | 57 | 65 | Carbamidomethylation | C4:Carbamidomethylation:1000.00 | PEAKS DB |
| E.AEKISGC(+57.02)WPYFK.T | N | 69.57 | 1484.7122 | 12 | 0.0 | 743.3633 | 2 | 40.74 | 11 | F11:24212 | NaNaKA16\_F7.raw |  |  |  |  |  |  |  |  |  | 9.0796E6 | 5.2934E7 | 1.0921E7 |  | 9 | 0 | 0 | 0 | 0 | 0 | 0 | 0 | 0 | 0 | 2 | 5 | 2 | 0 | 54 | 65 | Carbamidomethylation | C7:Carbamidomethylation:1000.00 | PEAKS DB |
| R.GGSGTPVDDLDR.C | N | 67.81 | 1187.5417 | 12 | 0.8 | 594.7786 | 2 | 19.63 | 10 | F10:6988 | NaNaKA16\_F6.raw |  |  |  |  |  |  |  |  |  | 5.7224E7 | 2.6509E9 | 3.9395E6 |  | 4 | 0 | 0 | 0 | 0 | 0 | 0 | 0 | 0 | 0 | 2 | 1 | 1 | 0 | 31 | 42 |  |  | PEAKS DB |
| T.YSYEC(+57.02)SQGTLTC(+57.02)K.G | N | 65.61 | 1595.6595 | 13 | -0.2 | 798.8369 | 2 | 19.78 | 10 | F10:7072 | NaNaKA16\_F6.raw |  |  |  |  |  |  |  |  |  | 9.5485E6 |  |  |  | 1 | 0 | 0 | 0 | 0 | 0 | 0 | 0 | 0 | 0 | 1 | 0 | 0 | 0 | 67 | 79 | Carbamidomethylation | C5:Carbamidomethylation:1000.00;C12:Carbamidomethylation:1000.00 | PEAKS DB |
| Y.SYEC(+57.02)SQGTLTC(+57.02)K.G | N | 65.06 | 1432.5963 | 12 | 0.7 | 717.3060 | 2 | 11.79 | 10 | F10:1902 | NaNaKA16\_F6.raw |  |  |  |  |  |  |  |  |  | 7.186E5 |  |  |  | 1 | 0 | 0 | 0 | 0 | 0 | 0 | 0 | 0 | 0 | 1 | 0 | 0 | 0 | 68 | 79 | Carbamidomethylation | C4:Carbamidomethylation:1000.00;C11:Carbamidomethylation:1000.00 | PEAKS DB |
| G.GSGTPVDDLDR.C | N | 60.74 | 1130.5204 | 11 | -0.3 | 566.2673 | 2 | 21.08 | 11 | F11:8172 | NaNaKA16\_F7.raw |  |  |  |  |  |  |  |  |  | 1.4052E5 | 1.2671E7 |  |  | 2 | 0 | 0 | 0 | 0 | 0 | 0 | 0 | 0 | 0 | 1 | 1 | 0 | 0 | 32 | 42 |  |  | PEAKS DB |
| N.FADYGC(+57.02)YC(+57.02)GR.G | N | 57.15 | 1267.4750 | 10 | 0.8 | 634.7452 | 2 | 25.42 | 11 | F11:11882 | NaNaKA16\_F7.raw |  |  |  |  |  |  |  |  |  | 2.1506E6 | 1.834E7 |  |  | 2 | 0 | 0 | 0 | 0 | 0 | 0 | 0 | 0 | 0 | 1 | 1 | 0 | 0 | 21 | 30 | Carbamidomethylation | C6:Carbamidomethylation:1000.00;C8:Carbamidomethylation:1000.00 | PEAKS DB |
| Y.FKTYSYEC(+57.02)SQGTLTC(+57.02)K.G | N | 54.26 | 1971.8706 | 16 | 0.6 | 658.2979 | 3 | 23.41 | 11 | F11:10060 | NaNaKA16\_F7.raw |  |  |  |  |  |  |  |  |  | 3.532E6 | 1.7096E6 |  |  | 2 | 0 | 0 | 0 | 0 | 0 | 0 | 0 | 0 | 0 | 1 | 1 | 0 | 0 | 64 | 79 | Carbamidomethylation | C8:Carbamidomethylation:1000.00;C15:Carbamidomethylation:1000.00 | PEAKS DB |
| R.LAAIC(+57.02)FAGAPYN.N | N | 53.90 | 1266.6067 | 12 | 0.8 | 634.3111 | 2 | 70.83 | 11 | F11:49747 | NaNaKA16\_F7.raw |  | 2.6932E8 | 6.3964E7 |  |  |  |  |  | 3.6922E8 | 7.2309E9 | 3.8533E9 | 2.4096E9 | 1.5572E9 | 11 | 0 | 1 | 1 | 0 | 0 | 0 | 0 | 0 | 1 | 2 | 3 | 2 | 1 | 95 | 106 | Carbamidomethylation | C5:Carbamidomethylation:1000.00 | PEAKS DB |
| I.SGC(+57.02)WPYFK.T | N | 53.87 | 1043.4535 | 8 | -1.4 | 522.7333 | 2 | 44.38 | 11 | F11:27176 | NaNaKA16\_F7.raw |  |  |  |  |  |  |  |  |  | 8.1441E6 | 5.2634E6 |  |  | 2 | 0 | 0 | 0 | 0 | 0 | 0 | 0 | 0 | 0 | 1 | 1 | 0 | 0 | 58 | 65 | Carbamidomethylation | C3:Carbamidomethylation:1000.00 | PEAKS DB |
| R.SWWNFADYGC(+57.02)YC(+57.02)GR.G | Y | 52.83 | 1840.7086 | 14 | 1.2 | 921.3627 | 2 | 83.49 | 11 | F11:58616 | NaNaKA16\_F7.raw |  |  |  |  |  |  |  |  |  |  | 5.6525E6 |  |  | 2 | 0 | 0 | 0 | 0 | 0 | 0 | 0 | 0 | 0 | 0 | 2 | 0 | 0 | 17 | 30 | Carbamidomethylation | C10:Carbamidomethylation:1000.00;C12:Carbamidomethylation:1000.00 | PEAKS DB |
| E.KISGC(+57.02)WPYFK.T | N | 51.04 | 1284.6324 | 10 | 0.2 | 643.3236 | 2 | 38.26 | 11 | F11:22081 | NaNaKA16\_F7.raw |  |  |  |  |  |  |  |  |  |  | 3.8432E6 |  |  | 2 | 0 | 0 | 0 | 0 | 0 | 0 | 0 | 0 | 0 | 0 | 2 | 0 | 0 | 56 | 65 | Carbamidomethylation | C5:Carbamidomethylation:1000.00 | PEAKS DB |
| G.SGTPVDDLDR.C | N | 45.52 | 1073.4989 | 10 | 0.9 | 537.7572 | 2 | 20.23 | 11 | F11:7497 | NaNaKA16\_F7.raw |  |  |  |  |  |  |  |  |  |  | 1.1072E7 |  |  | 1 | 0 | 0 | 0 | 0 | 0 | 0 | 0 | 0 | 0 | 0 | 1 | 0 | 0 | 33 | 42 |  |  | PEAKS DB |
| K.TYSYEC(+57.02)SQGTLTC(+57.02)KG.G | N | 45.40 | 1753.7288 | 15 | 1.4 | 877.8729 | 2 | 21.92 | 10 | F10:8643 | NaNaKA16\_F6.raw |  |  |  |  |  |  |  |  |  | 0 |  |  |  | 0 | 0 | 0 | 0 | 0 | 0 | 0 | 0 | 0 | 0 | 0 | 0 | 0 | 0 | 66 | 80 | Carbamidomethylation | C6:Carbamidomethylation:1000.00;C13:Carbamidomethylation:1000.00 | PEAKS DB |
| S.WWNFADYGC(+57.02)YC(+57.02)GRGGSGTPVDDLDR.C | Y | 45.25 | 2923.2078 | 25 | 0.2 | 975.4101 | 3 | 93.13 | 13 | F13:64247 | NaNaKA16\_F9.raw |  |  |  |  |  |  |  |  |  |  |  |  | 5.4823E7 | 1 | 0 | 0 | 0 | 0 | 0 | 0 | 0 | 0 | 0 | 0 | 0 | 0 | 1 | 18 | 42 | Carbamidomethylation | C9:Carbamidomethylation:1000.00;C11:Carbamidomethylation:1000.00 | PEAKS DB |
| total 17 peptides |  |  |  |  |  |  |  |  |  |  |  |  |  |  |  |  |  |  |  |  |  |  |  |  |  |  |  |  |  |  |  |  |  |  |  |  |  |  |  |  |  |  |  |
| --- | --- | --- | --- | --- | --- | --- | --- | --- | --- | --- | --- | --- | --- | --- | --- | --- | --- | --- | --- | --- | --- | --- | --- | --- | --- | --- | --- | --- | --- | --- | --- | --- | --- | --- | --- | --- | --- | --- | --- | --- | --- | --- | --- |

0508173A

back to list

  

| Protein Coverage
| Supporting Peptides
|

Protein Coverage:

Supporting Peptides:

| Peptide | Uniq | -10lgP | Mass | Length | ppm | m/z | z | RT | Fraction | Scan | Source File | Area F1 | Area F10 | Area F11 | Area F12 | Area F13 | Area F2 | Area F3 | Area F4 | Area F5 | Area F6 | Area F7 | Area F8 | Area F9 | #Feature | #Feature F1 | #Feature F10 | #Feature F11 | #Feature F12 | #Feature F13 | #Feature F2 | #Feature F3 | #Feature F4 | #Feature F5 | #Feature F6 | #Feature F7 | #Feature F8 | #Feature F9 | Start | End | PTM | AScore | Found By |
| --- | --- | --- | --- | --- | --- | --- | --- | --- | --- | --- | --- | --- | --- | --- | --- | --- | --- | --- | --- | --- | --- | --- | --- | --- | --- | --- | --- | --- | --- | --- | --- | --- | --- | --- | --- | --- | --- | --- | --- | --- | --- | --- | --- |
| K.ISGC(+57.02)WPYFKTYSYEC(+57.02)SQGTLTC(+57.02)K.G | N | 89.48 | 2835.2341 | 23 | -1.5 | 946.0839 | 3 | 57.58 | 11 | F11:37368 | NaNaKA16\_F7.raw |  |  |  |  |  |  |  |  |  | 1.3653E7 | 7.0731E7 | 1.9929E7 | 6.9253E6 | 5 | 0 | 0 | 0 | 0 | 0 | 0 | 0 | 0 | 0 | 1 | 2 | 1 | 1 | 57 | 79 | Carbamidomethylation | C4:Carbamidomethylation:1000.00;C15:Carbamidomethylation:1000.00;C22:Carbamidomethylation:1000.00 | PEAKS DB |
| K.TYSYEC(+57.02)SQGTLTC(+57.02)K.G | N | 89.30 | 1696.7073 | 14 | 0.3 | 849.3611 | 2 | 21.20 | 9 | F9:9021 | NaNaKA16\_F5.raw |  | 7.5988E5 |  | 1.6097E7 | 6.3725E6 | 1.1263E6 |  | 2.9128E6 | 6.1482E8 | 3.4541E10 | 2.2713E9 | 9.1534E6 | 4.1844E8 | 46 | 0 | 1 | 0 | 3 | 1 | 1 | 0 | 1 | 4 | 18 | 3 | 2 | 12 | 66 | 79 | Carbamidomethylation | C6:Carbamidomethylation:1000.00;C13:Carbamidomethylation:1000.00 | PEAKS DB |
| K.ISGC(+57.02)WPYFK.T | N | 70.32 | 1156.5375 | 9 | 0.1 | 579.2761 | 2 | 68.82 | 11 | F11:46583 | NaNaKA16\_F7.raw |  | 3.6915E8 | 1.4139E8 | 6.9856E7 | 2.3823E6 |  |  | 3.6353E6 | 3.5126E8 | 2.1014E10 | 1.2727E11 | 1.9979E10 | 1.2073E10 | 71 | 0 | 1 | 1 | 3 | 1 | 0 | 0 | 1 | 1 | 15 | 17 | 10 | 21 | 57 | 65 | Carbamidomethylation | C4:Carbamidomethylation:1000.00 | PEAKS DB |
| R.GGSGTPVDDLDR.C | N | 67.81 | 1187.5417 | 12 | 0.8 | 594.7786 | 2 | 19.63 | 10 | F10:6988 | NaNaKA16\_F6.raw |  |  |  |  |  |  |  |  |  | 5.7224E7 | 2.6509E9 | 3.9395E6 |  | 4 | 0 | 0 | 0 | 0 | 0 | 0 | 0 | 0 | 0 | 2 | 1 | 1 | 0 | 31 | 42 |  |  | PEAKS DB |
| NLYQFKNMIKC(+57.02)TVPSR.S | N | 66.48 | 1998.0179 | 16 | 0.3 | 667.0134 | 3 | 40.68 | 12 | F12:23498 | NaNaKA16\_F8.raw |  |  |  |  |  |  |  |  |  |  |  | 3.4886E6 |  | 1 | 0 | 0 | 0 | 0 | 0 | 0 | 0 | 0 | 0 | 0 | 0 | 1 | 0 | 1 | 16 | Carbamidomethylation | C11:Carbamidomethylation:1000.00 | PEAKS DB |
| T.YSYEC(+57.02)SQGTLTC(+57.02)K.G | N | 65.61 | 1595.6595 | 13 | -0.2 | 798.8369 | 2 | 19.78 | 10 | F10:7072 | NaNaKA16\_F6.raw |  |  |  |  |  |  |  |  |  | 9.5485E6 |  |  |  | 1 | 0 | 0 | 0 | 0 | 0 | 0 | 0 | 0 | 0 | 1 | 0 | 0 | 0 | 67 | 79 | Carbamidomethylation | C5:Carbamidomethylation:1000.00;C12:Carbamidomethylation:1000.00 | PEAKS DB |
| Y.SYEC(+57.02)SQGTLTC(+57.02)K.G | N | 65.06 | 1432.5963 | 12 | 0.7 | 717.3060 | 2 | 11.79 | 10 | F10:1902 | NaNaKA16\_F6.raw |  |  |  |  |  |  |  |  |  | 7.186E5 |  |  |  | 1 | 0 | 0 | 0 | 0 | 0 | 0 | 0 | 0 | 0 | 1 | 0 | 0 | 0 | 68 | 79 | Carbamidomethylation | C4:Carbamidomethylation:1000.00;C11:Carbamidomethylation:1000.00 | PEAKS DB |
| G.GSGTPVDDLDR.C | N | 60.74 | 1130.5204 | 11 | -0.3 | 566.2673 | 2 | 21.08 | 11 | F11:8172 | NaNaKA16\_F7.raw |  |  |  |  |  |  |  |  |  | 1.4052E5 | 1.2671E7 |  |  | 2 | 0 | 0 | 0 | 0 | 0 | 0 | 0 | 0 | 0 | 1 | 1 | 0 | 0 | 32 | 42 |  |  | PEAKS DB |
| NLYQFKNMIK.C | N | 55.84 | 1297.6853 | 10 | 0.6 | 649.8503 | 2 | 34.48 | 11 | F11:19260 | NaNaKA16\_F7.raw |  |  |  |  |  |  |  |  |  | 2.422E7 | 1.1905E8 | 3.5531E7 | 5.6701E7 | 7 | 0 | 0 | 0 | 0 | 0 | 0 | 0 | 0 | 0 | 2 | 2 | 1 | 2 | 1 | 10 |  |  | PEAKS DB |
| Y.FKTYSYEC(+57.02)SQGTLTC(+57.02)K.G | N | 54.26 | 1971.8706 | 16 | 0.6 | 658.2979 | 3 | 23.41 | 11 | F11:10060 | NaNaKA16\_F7.raw |  |  |  |  |  |  |  |  |  | 3.532E6 | 1.7096E6 |  |  | 2 | 0 | 0 | 0 | 0 | 0 | 0 | 0 | 0 | 0 | 1 | 1 | 0 | 0 | 64 | 79 | Carbamidomethylation | C8:Carbamidomethylation:1000.00;C15:Carbamidomethylation:1000.00 | PEAKS DB |
| R.LAAIC(+57.02)FAGAPYN.N | N | 53.90 | 1266.6067 | 12 | 0.8 | 634.3111 | 2 | 70.83 | 11 | F11:49747 | NaNaKA16\_F7.raw |  | 2.6932E8 | 6.3964E7 |  |  |  |  |  | 3.6922E8 | 7.2309E9 | 3.8533E9 | 2.4096E9 | 1.5572E9 | 11 | 0 | 1 | 1 | 0 | 0 | 0 | 0 | 0 | 1 | 2 | 3 | 2 | 1 | 95 | 106 | Carbamidomethylation | C5:Carbamidomethylation:1000.00 | PEAKS DB |
| I.SGC(+57.02)WPYFK.T | N | 53.87 | 1043.4535 | 8 | -1.4 | 522.7333 | 2 | 44.38 | 11 | F11:27176 | NaNaKA16\_F7.raw |  |  |  |  |  |  |  |  |  | 8.1441E6 | 5.2634E6 |  |  | 2 | 0 | 0 | 0 | 0 | 0 | 0 | 0 | 0 | 0 | 1 | 1 | 0 | 0 | 58 | 65 | Carbamidomethylation | C3:Carbamidomethylation:1000.00 | PEAKS DB |
| E.AGKISGC(+57.02)WPYFK.T | Y | 53.67 | 1412.6910 | 12 | 0.2 | 707.3530 | 2 | 38.00 | 11 | F11:21893 | NaNaKA16\_F7.raw |  |  |  |  |  |  |  |  |  |  | 2.0025E7 |  |  | 2 | 0 | 0 | 0 | 0 | 0 | 0 | 0 | 0 | 0 | 0 | 2 | 0 | 0 | 54 | 65 | Carbamidomethylation | C7:Carbamidomethylation:1000.00 | PEAKS DB |
| G.KISGC(+57.02)WPYFK.T | N | 51.04 | 1284.6324 | 10 | 0.2 | 643.3236 | 2 | 38.26 | 11 | F11:22081 | NaNaKA16\_F7.raw |  |  |  |  |  |  |  |  |  |  | 3.8432E6 |  |  | 2 | 0 | 0 | 0 | 0 | 0 | 0 | 0 | 0 | 0 | 0 | 2 | 0 | 0 | 56 | 65 | Carbamidomethylation | C5:Carbamidomethylation:1000.00 | PEAKS DB |
| K.NMIKC(+57.02)TVPSR.S | N | 48.35 | 1204.6056 | 10 | 0.1 | 603.3101 | 2 | 12.09 | 11 | F11:1822 | NaNaKA16\_F7.raw |  |  |  |  |  |  |  |  |  |  | 1.7697E7 |  |  | 2 | 0 | 0 | 0 | 0 | 0 | 0 | 0 | 0 | 0 | 0 | 2 | 0 | 0 | 7 | 16 | Carbamidomethylation | C5:Carbamidomethylation:1000.00 | PEAKS DB |
| N.EAGKISGC(+57.02)WPYFK.T | Y | 47.71 | 1541.7336 | 13 | 1.4 | 514.9192 | 3 | 41.78 | 11 | F11:24729 | NaNaKA16\_F7.raw |  |  |  |  |  |  |  |  |  |  | 1.5282E6 |  |  | 1 | 0 | 0 | 0 | 0 | 0 | 0 | 0 | 0 | 0 | 0 | 1 | 0 | 0 | 53 | 65 | Carbamidomethylation | C8:Carbamidomethylation:1000.00 | PEAKS DB |
| NLYQFKNM(+15.99)IK.C | N | 46.70 | 1313.6802 | 10 | 0.4 | 657.8476 | 2 | 22.54 | 11 | F11:9423 | NaNaKA16\_F7.raw |  |  |  |  |  |  |  |  |  |  | 2.8165E6 |  |  | 1 | 0 | 0 | 0 | 0 | 0 | 0 | 0 | 0 | 0 | 0 | 1 | 0 | 0 | 1 | 10 | Oxidation (M) | M8:Oxidation (M):1000.00 | PEAKS DB |
| G.SGTPVDDLDR.C | N | 45.52 | 1073.4989 | 10 | 0.9 | 537.7572 | 2 | 20.23 | 11 | F11:7497 | NaNaKA16\_F7.raw |  |  |  |  |  |  |  |  |  |  | 1.1072E7 |  |  | 1 | 0 | 0 | 0 | 0 | 0 | 0 | 0 | 0 | 0 | 0 | 1 | 0 | 0 | 33 | 42 |  |  | PEAKS DB |
| K.TYSYEC(+57.02)SQGTLTC(+57.02)KG.D | N | 45.40 | 1753.7288 | 15 | 1.4 | 877.8729 | 2 | 21.92 | 10 | F10:8643 | NaNaKA16\_F6.raw |  |  |  |  |  |  |  |  |  | 0 |  |  |  | 0 | 0 | 0 | 0 | 0 | 0 | 0 | 0 | 0 | 0 | 0 | 0 | 0 | 0 | 66 | 80 | Carbamidomethylation | C6:Carbamidomethylation:1000.00;C13:Carbamidomethylation:1000.00 | PEAKS DB |
| R.LAAIC(+57.02)FAGAPYNNDNYNINLK.A | Y | 42.31 | 2355.1318 | 21 | 0.3 | 786.0515 | 3 | 71.00 | 13 | F13:46181 | NaNaKA16\_F9.raw |  |  |  |  |  |  |  |  |  |  |  |  | 3.7844E6 | 1 | 0 | 0 | 0 | 0 | 0 | 0 | 0 | 0 | 0 | 0 | 0 | 0 | 1 | 95 | 115 | Carbamidomethylation | C5:Carbamidomethylation:1000.00 | PEAKS DB |
| R.SWLDFANYGC(+57.02)YC(+57.02)GRGGSGTPVDDLDR.C | Y | 41.95 | 2937.2446 | 26 | -6.4 | 980.0825 | 3 | 95.85 | 13 | F13:66499 | NaNaKA16\_F9.raw |  |  |  |  |  |  |  |  |  |  |  |  | 1.1072E7 | 1 | 0 | 0 | 0 | 0 | 0 | 0 | 0 | 0 | 0 | 0 | 0 | 0 | 1 | 17 | 42 | Carbamidomethylation | C10:Carbamidomethylation:1000.00;C12:Carbamidomethylation:1000.00 | PEAKS DB |
| total 21 peptides |  |  |  |  |  |  |  |  |  |  |  |  |  |  |  |  |  |  |  |  |  |  |  |  |  |  |  |  |  |  |  |  |  |  |  |  |  |  |  |  |  |  |  |
| --- | --- | --- | --- | --- | --- | --- | --- | --- | --- | --- | --- | --- | --- | --- | --- | --- | --- | --- | --- | --- | --- | --- | --- | --- | --- | --- | --- | --- | --- | --- | --- | --- | --- | --- | --- | --- | --- | --- | --- | --- | --- | --- | --- |

P25498.1

back to list

  

| Protein Coverage
| Supporting Peptides
|

Protein Coverage:

Supporting Peptides:

| Peptide | Uniq | -10lgP | Mass | Length | ppm | m/z | z | RT | Fraction | Scan | Source File | Area F1 | Area F10 | Area F11 | Area F12 | Area F13 | Area F2 | Area F3 | Area F4 | Area F5 | Area F6 | Area F7 | Area F8 | Area F9 | #Feature | #Feature F1 | #Feature F10 | #Feature F11 | #Feature F12 | #Feature F13 | #Feature F2 | #Feature F3 | #Feature F4 | #Feature F5 | #Feature F6 | #Feature F7 | #Feature F8 | #Feature F9 | Start | End | PTM | AScore | Found By |
| --- | --- | --- | --- | --- | --- | --- | --- | --- | --- | --- | --- | --- | --- | --- | --- | --- | --- | --- | --- | --- | --- | --- | --- | --- | --- | --- | --- | --- | --- | --- | --- | --- | --- | --- | --- | --- | --- | --- | --- | --- | --- | --- | --- |
| K.ISGC(+57.02)WPYFKTYSYEC(+57.02)SQGTLTC(+57.02)K.G | N | 89.48 | 2835.2341 | 23 | -1.5 | 946.0839 | 3 | 57.58 | 11 | F11:37368 | NaNaKA16\_F7.raw |  |  |  |  |  |  |  |  |  | 1.3653E7 | 7.0731E7 | 1.9929E7 | 6.9253E6 | 5 | 0 | 0 | 0 | 0 | 0 | 0 | 0 | 0 | 0 | 1 | 2 | 1 | 1 | 57 | 79 | Carbamidomethylation | C4:Carbamidomethylation:1000.00;C15:Carbamidomethylation:1000.00;C22:Carbamidomethylation:1000.00 | PEAKS DB |
| K.TYSYEC(+57.02)SQGTLTC(+57.02)K.G | N | 89.30 | 1696.7073 | 14 | 0.3 | 849.3611 | 2 | 21.20 | 9 | F9:9021 | NaNaKA16\_F5.raw |  | 7.5988E5 |  | 1.6097E7 | 6.3725E6 | 1.1263E6 |  | 2.9128E6 | 6.1482E8 | 3.4541E10 | 2.2713E9 | 9.1534E6 | 4.1844E8 | 46 | 0 | 1 | 0 | 3 | 1 | 1 | 0 | 1 | 4 | 18 | 3 | 2 | 12 | 66 | 79 | Carbamidomethylation | C6:Carbamidomethylation:1000.00;C13:Carbamidomethylation:1000.00 | PEAKS DB |
| K.ISGC(+57.02)WPYFK.T | N | 70.32 | 1156.5375 | 9 | 0.1 | 579.2761 | 2 | 68.82 | 11 | F11:46583 | NaNaKA16\_F7.raw |  | 3.6915E8 | 1.4139E8 | 6.9856E7 | 2.3823E6 |  |  | 3.6353E6 | 3.5126E8 | 2.1014E10 | 1.2727E11 | 1.9979E10 | 1.2073E10 | 71 | 0 | 1 | 1 | 3 | 1 | 0 | 0 | 1 | 1 | 15 | 17 | 10 | 21 | 57 | 65 | Carbamidomethylation | C4:Carbamidomethylation:1000.00 | PEAKS DB |
| R.GGSGTPVDDLDR.C | N | 67.81 | 1187.5417 | 12 | 0.8 | 594.7786 | 2 | 19.63 | 10 | F10:6988 | NaNaKA16\_F6.raw |  |  |  |  |  |  |  |  |  | 5.7224E7 | 2.6509E9 | 3.9395E6 |  | 4 | 0 | 0 | 0 | 0 | 0 | 0 | 0 | 0 | 0 | 2 | 1 | 1 | 0 | 31 | 42 |  |  | PEAKS DB |
| NLYQFKNMIKC(+57.02)TVPSR.S | N | 66.48 | 1998.0179 | 16 | 0.3 | 667.0134 | 3 | 40.68 | 12 | F12:23498 | NaNaKA16\_F8.raw |  |  |  |  |  |  |  |  |  |  |  | 3.4886E6 |  | 1 | 0 | 0 | 0 | 0 | 0 | 0 | 0 | 0 | 0 | 0 | 0 | 1 | 0 | 1 | 16 | Carbamidomethylation | C11:Carbamidomethylation:1000.00 | PEAKS DB |
| T.YSYEC(+57.02)SQGTLTC(+57.02)K.G | N | 65.61 | 1595.6595 | 13 | -0.2 | 798.8369 | 2 | 19.78 | 10 | F10:7072 | NaNaKA16\_F6.raw |  |  |  |  |  |  |  |  |  | 9.5485E6 |  |  |  | 1 | 0 | 0 | 0 | 0 | 0 | 0 | 0 | 0 | 0 | 1 | 0 | 0 | 0 | 67 | 79 | Carbamidomethylation | C5:Carbamidomethylation:1000.00;C12:Carbamidomethylation:1000.00 | PEAKS DB |
| Y.SYEC(+57.02)SQGTLTC(+57.02)K.G | N | 65.06 | 1432.5963 | 12 | 0.7 | 717.3060 | 2 | 11.79 | 10 | F10:1902 | NaNaKA16\_F6.raw |  |  |  |  |  |  |  |  |  | 7.186E5 |  |  |  | 1 | 0 | 0 | 0 | 0 | 0 | 0 | 0 | 0 | 0 | 1 | 0 | 0 | 0 | 68 | 79 | Carbamidomethylation | C4:Carbamidomethylation:1000.00;C11:Carbamidomethylation:1000.00 | PEAKS DB |
| G.GSGTPVDDLDR.C | N | 60.74 | 1130.5204 | 11 | -0.3 | 566.2673 | 2 | 21.08 | 11 | F11:8172 | NaNaKA16\_F7.raw |  |  |  |  |  |  |  |  |  | 1.4052E5 | 1.2671E7 |  |  | 2 | 0 | 0 | 0 | 0 | 0 | 0 | 0 | 0 | 0 | 1 | 1 | 0 | 0 | 32 | 42 |  |  | PEAKS DB |
| NLYQFKNMIK.C | N | 55.84 | 1297.6853 | 10 | 0.6 | 649.8503 | 2 | 34.48 | 11 | F11:19260 | NaNaKA16\_F7.raw |  |  |  |  |  |  |  |  |  | 2.422E7 | 1.1905E8 | 3.5531E7 | 5.6701E7 | 7 | 0 | 0 | 0 | 0 | 0 | 0 | 0 | 0 | 0 | 2 | 2 | 1 | 2 | 1 | 10 |  |  | PEAKS DB |
| Y.FKTYSYEC(+57.02)SQGTLTC(+57.02)K.G | N | 54.26 | 1971.8706 | 16 | 0.6 | 658.2979 | 3 | 23.41 | 11 | F11:10060 | NaNaKA16\_F7.raw |  |  |  |  |  |  |  |  |  | 3.532E6 | 1.7096E6 |  |  | 2 | 0 | 0 | 0 | 0 | 0 | 0 | 0 | 0 | 0 | 1 | 1 | 0 | 0 | 64 | 79 | Carbamidomethylation | C8:Carbamidomethylation:1000.00;C15:Carbamidomethylation:1000.00 | PEAKS DB |
| R.LAAIC(+57.02)FAGAPYN.N | N | 53.90 | 1266.6067 | 12 | 0.8 | 634.3111 | 2 | 70.83 | 11 | F11:49747 | NaNaKA16\_F7.raw |  | 2.6932E8 | 6.3964E7 |  |  |  |  |  | 3.6922E8 | 7.2309E9 | 3.8533E9 | 2.4096E9 | 1.5572E9 | 11 | 0 | 1 | 1 | 0 | 0 | 0 | 0 | 0 | 1 | 2 | 3 | 2 | 1 | 95 | 106 | Carbamidomethylation | C5:Carbamidomethylation:1000.00 | PEAKS DB |
| I.SGC(+57.02)WPYFK.T | N | 53.87 | 1043.4535 | 8 | -1.4 | 522.7333 | 2 | 44.38 | 11 | F11:27176 | NaNaKA16\_F7.raw |  |  |  |  |  |  |  |  |  | 8.1441E6 | 5.2634E6 |  |  | 2 | 0 | 0 | 0 | 0 | 0 | 0 | 0 | 0 | 0 | 1 | 1 | 0 | 0 | 58 | 65 | Carbamidomethylation | C3:Carbamidomethylation:1000.00 | PEAKS DB |
| E.AGKISGC(+57.02)WPYFK.T | Y | 53.67 | 1412.6910 | 12 | 0.2 | 707.3530 | 2 | 38.00 | 11 | F11:21893 | NaNaKA16\_F7.raw |  |  |  |  |  |  |  |  |  |  | 2.0025E7 |  |  | 2 | 0 | 0 | 0 | 0 | 0 | 0 | 0 | 0 | 0 | 0 | 2 | 0 | 0 | 54 | 65 | Carbamidomethylation | C7:Carbamidomethylation:1000.00 | PEAKS DB |
| G.KISGC(+57.02)WPYFK.T | N | 51.04 | 1284.6324 | 10 | 0.2 | 643.3236 | 2 | 38.26 | 11 | F11:22081 | NaNaKA16\_F7.raw |  |  |  |  |  |  |  |  |  |  | 3.8432E6 |  |  | 2 | 0 | 0 | 0 | 0 | 0 | 0 | 0 | 0 | 0 | 0 | 2 | 0 | 0 | 56 | 65 | Carbamidomethylation | C5:Carbamidomethylation:1000.00 | PEAKS DB |
| K.NMIKC(+57.02)TVPSR.S | N | 48.35 | 1204.6056 | 10 | 0.1 | 603.3101 | 2 | 12.09 | 11 | F11:1822 | NaNaKA16\_F7.raw |  |  |  |  |  |  |  |  |  |  | 1.7697E7 |  |  | 2 | 0 | 0 | 0 | 0 | 0 | 0 | 0 | 0 | 0 | 0 | 2 | 0 | 0 | 7 | 16 | Carbamidomethylation | C5:Carbamidomethylation:1000.00 | PEAKS DB |
| N.EAGKISGC(+57.02)WPYFK.T | Y | 47.71 | 1541.7336 | 13 | 1.4 | 514.9192 | 3 | 41.78 | 11 | F11:24729 | NaNaKA16\_F7.raw |  |  |  |  |  |  |  |  |  |  | 1.5282E6 |  |  | 1 | 0 | 0 | 0 | 0 | 0 | 0 | 0 | 0 | 0 | 0 | 1 | 0 | 0 | 53 | 65 | Carbamidomethylation | C8:Carbamidomethylation:1000.00 | PEAKS DB |
| NLYQFKNM(+15.99)IK.C | N | 46.70 | 1313.6802 | 10 | 0.4 | 657.8476 | 2 | 22.54 | 11 | F11:9423 | NaNaKA16\_F7.raw |  |  |  |  |  |  |  |  |  |  | 2.8165E6 |  |  | 1 | 0 | 0 | 0 | 0 | 0 | 0 | 0 | 0 | 0 | 0 | 1 | 0 | 0 | 1 | 10 | Oxidation (M) | M8:Oxidation (M):1000.00 | PEAKS DB |
| G.SGTPVDDLDR.C | N | 45.52 | 1073.4989 | 10 | 0.9 | 537.7572 | 2 | 20.23 | 11 | F11:7497 | NaNaKA16\_F7.raw |  |  |  |  |  |  |  |  |  |  | 1.1072E7 |  |  | 1 | 0 | 0 | 0 | 0 | 0 | 0 | 0 | 0 | 0 | 0 | 1 | 0 | 0 | 33 | 42 |  |  | PEAKS DB |
| K.TYSYEC(+57.02)SQGTLTC(+57.02)KG.D | N | 45.40 | 1753.7288 | 15 | 1.4 | 877.8729 | 2 | 21.92 | 10 | F10:8643 | NaNaKA16\_F6.raw |  |  |  |  |  |  |  |  |  | 0 |  |  |  | 0 | 0 | 0 | 0 | 0 | 0 | 0 | 0 | 0 | 0 | 0 | 0 | 0 | 0 | 66 | 80 | Carbamidomethylation | C6:Carbamidomethylation:1000.00;C13:Carbamidomethylation:1000.00 | PEAKS DB |
| R.LAAIC(+57.02)FAGAPYNNDNYNINLK.A | Y | 42.31 | 2355.1318 | 21 | 0.3 | 786.0515 | 3 | 71.00 | 13 | F13:46181 | NaNaKA16\_F9.raw |  |  |  |  |  |  |  |  |  |  |  |  | 3.7844E6 | 1 | 0 | 0 | 0 | 0 | 0 | 0 | 0 | 0 | 0 | 0 | 0 | 0 | 1 | 95 | 115 | Carbamidomethylation | C5:Carbamidomethylation:1000.00 | PEAKS DB |
| R.SWLDFANYGC(+57.02)YC(+57.02)GRGGSGTPVDDLDR.C | Y | 41.95 | 2937.2446 | 26 | -6.4 | 980.0825 | 3 | 95.85 | 13 | F13:66499 | NaNaKA16\_F9.raw |  |  |  |  |  |  |  |  |  |  |  |  | 1.1072E7 | 1 | 0 | 0 | 0 | 0 | 0 | 0 | 0 | 0 | 0 | 0 | 0 | 0 | 1 | 17 | 42 | Carbamidomethylation | C10:Carbamidomethylation:1000.00;C12:Carbamidomethylation:1000.00 | PEAKS DB |
| total 21 peptides |  |  |  |  |  |  |  |  |  |  |  |  |  |  |  |  |  |  |  |  |  |  |  |  |  |  |  |  |  |  |  |  |  |  |  |  |  |  |  |  |  |  |  |
| --- | --- | --- | --- | --- | --- | --- | --- | --- | --- | --- | --- | --- | --- | --- | --- | --- | --- | --- | --- | --- | --- | --- | --- | --- | --- | --- | --- | --- | --- | --- | --- | --- | --- | --- | --- | --- | --- | --- | --- | --- | --- | --- | --- |

P82464.1

back to list

  

| Protein Coverage
| Supporting Peptides
|

Protein Coverage:

Supporting Peptides:

| Peptide | Uniq | -10lgP | Mass | Length | ppm | m/z | z | RT | Fraction | Scan | Source File | Area F1 | Area F10 | Area F11 | Area F12 | Area F13 | Area F2 | Area F3 | Area F4 | Area F5 | Area F6 | Area F7 | Area F8 | Area F9 | #Feature | #Feature F1 | #Feature F10 | #Feature F11 | #Feature F12 | #Feature F13 | #Feature F2 | #Feature F3 | #Feature F4 | #Feature F5 | #Feature F6 | #Feature F7 | #Feature F8 | #Feature F9 | Start | End | PTM | AScore | Found By |
| --- | --- | --- | --- | --- | --- | --- | --- | --- | --- | --- | --- | --- | --- | --- | --- | --- | --- | --- | --- | --- | --- | --- | --- | --- | --- | --- | --- | --- | --- | --- | --- | --- | --- | --- | --- | --- | --- | --- | --- | --- | --- | --- | --- |
| R.GC(+57.02)TFTC(+57.02)PELRPTGIYVYC(+57.02)C(+57.02)R.R | Y | 93.20 | 2509.1011 | 20 | 2.4 | 1255.5608 | 2 | 54.83 | 11 | F11:35150 | NaNaKA16\_F7.raw |  | 8.0424E6 | 3.6818E6 |  |  |  |  |  | 7.2338E6 | 1.7518E8 | 4.5165E9 | 7.8194E7 | 3.3354E7 | 14 | 0 | 1 | 1 | 0 | 0 | 0 | 0 | 0 | 1 | 1 | 7 | 2 | 1 | 40 | 59 | Carbamidomethylation | C2:Carbamidomethylation:1000.00;C6:Carbamidomethylation:1000.00;C18:Carbamidomethylation:1000.00;C19:Carbamidomethylation:1000.00 | PEAKS DB |
| R.TSETTEIC(+57.02)PDSWYFC(+57.02)YK.I | Y | 80.65 | 2185.8972 | 17 | 0.6 | 1093.9565 | 2 | 69.45 | 11 | F11:47497 | NaNaKA16\_F7.raw |  |  |  |  |  |  |  |  |  | 1.0809E8 | 7.9719E8 | 4.2967E6 | 7.6956E6 | 10 | 0 | 0 | 0 | 0 | 0 | 0 | 0 | 0 | 0 | 3 | 5 | 1 | 1 | 10 | 26 | Carbamidomethylation | C8:Carbamidomethylation:1000.00;C15:Carbamidomethylation:1000.00 | PEAKS DB |
| F.TC(+57.02)PELRPTGIYVYC(+57.02)C(+57.02)R.R | Y | 73.49 | 2043.9329 | 16 | 0.0 | 682.3182 | 3 | 40.54 | 11 | F11:24086 | NaNaKA16\_F7.raw |  |  |  |  |  |  |  |  |  | 3.7008E6 | 9.5751E7 |  |  | 3 | 0 | 0 | 0 | 0 | 0 | 0 | 0 | 0 | 0 | 1 | 2 | 0 | 0 | 44 | 59 | Carbamidomethylation | C2:Carbamidomethylation:1000.00;C14:Carbamidomethylation:1000.00;C15:Carbamidomethylation:1000.00 | PEAKS DB |
| R.PTGIYVYC(+57.02)C(+57.02)R.R | Y | 72.07 | 1287.5740 | 10 | 0.6 | 644.7947 | 2 | 31.86 | 11 | F11:16896 | NaNaKA16\_F7.raw |  |  |  |  |  |  |  |  |  | 9.518E6 | 1.4426E8 |  |  | 2 | 0 | 0 | 0 | 0 | 0 | 0 | 0 | 0 | 0 | 1 | 1 | 0 | 0 | 50 | 59 | Carbamidomethylation | C8:Carbamidomethylation:1000.00;C9:Carbamidomethylation:1000.00 | PEAKS DB |
| R.GC(+57.02)TFTC(+57.02)PELRPTGIYVY.C | Y | 70.60 | 2032.9386 | 17 | 0.4 | 1017.4770 | 2 | 69.56 | 9 | F9:48347 | NaNaKA16\_F5.raw |  |  |  |  |  |  |  |  | 4.2507E6 | 6.6478E7 | 1.6776E9 | 1.2673E7 | 8.2441E6 | 6 | 0 | 0 | 0 | 0 | 0 | 0 | 0 | 0 | 1 | 1 | 2 | 1 | 1 | 40 | 56 | Carbamidomethylation | C2:Carbamidomethylation:1000.00;C6:Carbamidomethylation:1000.00 | PEAKS DB |
| R.GC(+57.02)TFTC(+57.02)PELRPTGIYVYC(+57.02).C | Y | 67.43 | 2192.9692 | 18 | -0.3 | 1097.4916 | 2 | 68.75 | 11 | F11:46523 | NaNaKA16\_F7.raw |  |  |  |  |  |  |  |  |  |  | 3.7504E8 | 3.4122E6 |  | 2 | 0 | 0 | 0 | 0 | 0 | 0 | 0 | 0 | 0 | 0 | 1 | 1 | 0 | 40 | 57 | Carbamidomethylation | C2:Carbamidomethylation:1000.00;C6:Carbamidomethylation:1000.00;C18:Carbamidomethylation:1000.00 | PEAKS DB |
| R.GC(+57.02)TFTC(+57.02)PELRPTGIY.V | Y | 63.58 | 1770.8069 | 15 | 0.3 | 886.4110 | 2 | 57.41 | 10 | F10:38814 | NaNaKA16\_F6.raw |  |  |  |  |  |  |  |  |  | 1.3319E7 |  |  |  | 1 | 0 | 0 | 0 | 0 | 0 | 0 | 0 | 0 | 0 | 1 | 0 | 0 | 0 | 40 | 54 | Carbamidomethylation | C2:Carbamidomethylation:1000.00;C6:Carbamidomethylation:1000.00 | PEAKS DB |
| C.TFTC(+57.02)PELRPTGIYVYC(+57.02)C(+57.02)R.R | Y | 63.50 | 2292.0488 | 18 | -0.3 | 765.0233 | 3 | 53.86 | 11 | F11:34667 | NaNaKA16\_F7.raw |  |  |  |  |  |  |  |  |  |  | 0 |  |  | 0 | 0 | 0 | 0 | 0 | 0 | 0 | 0 | 0 | 0 | 0 | 0 | 0 | 0 | 42 | 59 | Carbamidomethylation | C4:Carbamidomethylation:1000.00;C16:Carbamidomethylation:1000.00;C17:Carbamidomethylation:1000.00 | PEAKS DB |
| P.ELRPTGIYVYC(+57.02)C(+57.02)R.R | Y | 62.65 | 1685.8018 | 13 | -0.8 | 562.9407 | 3 | 36.04 | 11 | F11:20468 | NaNaKA16\_F7.raw |  |  |  |  |  |  |  |  | 4.1728E6 | 3.0509E7 | 1.9498E7 | 6.7525E6 | 1.6307E6 | 7 | 0 | 0 | 0 | 0 | 0 | 0 | 0 | 0 | 1 | 2 | 2 | 1 | 1 | 47 | 59 | Carbamidomethylation | C11:Carbamidomethylation:1000.00;C12:Carbamidomethylation:1000.00 | PEAKS DB |
| K.ISLADGNDVR.I | N | 62.61 | 1058.5356 | 10 | -0.5 | 530.2748 | 2 | 22.28 | 11 | F11:9363 | NaNaKA16\_F7.raw |  |  |  |  |  |  |  |  |  | 3.5268E6 | 5.5622E7 |  |  | 4 | 0 | 0 | 0 | 0 | 0 | 0 | 0 | 0 | 0 | 2 | 2 | 0 | 0 | 27 | 36 |  |  | PEAKS DB |
| TIC(+57.02)YNHLTR.T | Y | 59.52 | 1176.5709 | 9 | -0.3 | 589.2925 | 2 | 12.11 | 11 | F11:1854 | NaNaKA16\_F7.raw |  |  |  |  |  |  |  |  |  | 5.0884E5 | 1.9464E7 |  |  | 3 | 0 | 0 | 0 | 0 | 0 | 0 | 0 | 0 | 0 | 1 | 2 | 0 | 0 | 1 | 9 | Carbamidomethylation | C3:Carbamidomethylation:1000.00 | PEAKS DB |
| L.RPTGIYVYC(+57.02)C(+57.02)R.R | Y | 51.69 | 1443.6750 | 11 | 0.8 | 482.2327 | 3 | 19.57 | 11 | F11:7076 | NaNaKA16\_F7.raw |  |  |  |  |  |  |  |  |  |  | 1.1449E6 |  |  | 1 | 0 | 0 | 0 | 0 | 0 | 0 | 0 | 0 | 0 | 0 | 1 | 0 | 0 | 49 | 59 | Carbamidomethylation | C9:Carbamidomethylation:1000.00;C10:Carbamidomethylation:1000.00 | PEAKS DB |
| P.TGIYVYC(+57.02)C(+57.02)R.R | Y | 46.97 | 1190.5212 | 9 | 0.2 | 596.2680 | 2 | 28.61 | 11 | F11:14302 | NaNaKA16\_F7.raw |  |  |  |  |  |  |  |  |  |  | 7.0149E5 |  |  | 1 | 0 | 0 | 0 | 0 | 0 | 0 | 0 | 0 | 0 | 0 | 1 | 0 | 0 | 51 | 59 | Carbamidomethylation | C7:Carbamidomethylation:1000.00;C8:Carbamidomethylation:1000.00 | PEAKS DB |
| R.GC(+57.02)TFTC(+57.02)PELR.P | N | 45.94 | 1239.5376 | 10 | 0.0 | 620.7761 | 2 | 30.33 | 10 | F10:15872 | NaNaKA16\_F6.raw |  |  |  |  |  |  |  |  |  | 1.838E7 |  |  |  | 1 | 0 | 0 | 0 | 0 | 0 | 0 | 0 | 0 | 0 | 1 | 0 | 0 | 0 | 40 | 49 | Carbamidomethylation | C2:Carbamidomethylation:1000.00;C6:Carbamidomethylation:1000.00 | PEAKS DB |
| T.GIYVYC(+57.02)C(+57.02)R.R | Y | 43.24 | 1089.4735 | 8 | 0.6 | 545.7444 | 2 | 25.37 | 11 | F11:11525 | NaNaKA16\_F7.raw |  |  |  |  |  |  |  |  |  |  | 2.1848E6 |  |  | 1 | 0 | 0 | 0 | 0 | 0 | 0 | 0 | 0 | 0 | 0 | 1 | 0 | 0 | 52 | 59 | Carbamidomethylation | C6:Carbamidomethylation:1000.00;C7:Carbamidomethylation:1000.00 | PEAKS DB |
| T.C(+57.02)PELRPTGIYVYC(+57.02)C(+57.02)R.R | Y | 42.68 | 1942.8851 | 15 | 1.6 | 648.6367 | 3 | 39.71 | 11 | F11:23366 | NaNaKA16\_F7.raw |  |  |  |  |  |  |  |  |  |  | 0 |  |  | 0 | 0 | 0 | 0 | 0 | 0 | 0 | 0 | 0 | 0 | 0 | 0 | 0 | 0 | 45 | 59 | Carbamidomethylation | C1:Carbamidomethylation:1000.00;C13:Carbamidomethylation:1000.00;C14:Carbamidomethylation:1000.00 | PEAKS DB |
| E.TTEIC(+57.02)PDSWYFC(+57.02)YK.I | N | 42.51 | 1868.7749 | 14 | 1.2 | 935.3958 | 2 | 68.81 | 10 | F10:48624 | NaNaKA16\_F6.raw |  |  |  |  |  |  |  |  |  | 1.3848E6 |  |  |  | 1 | 0 | 0 | 0 | 0 | 0 | 0 | 0 | 0 | 0 | 1 | 0 | 0 | 0 | 13 | 26 | Carbamidomethylation | C5:Carbamidomethylation:1000.00;C12:Carbamidomethylation:1000.00 | PEAKS DB |
| total 17 peptides |  |  |  |  |  |  |  |  |  |  |  |  |  |  |  |  |  |  |  |  |  |  |  |  |  |  |  |  |  |  |  |  |  |  |  |  |  |  |  |  |  |  |  |
| --- | --- | --- | --- | --- | --- | --- | --- | --- | --- | --- | --- | --- | --- | --- | --- | --- | --- | --- | --- | --- | --- | --- | --- | --- | --- | --- | --- | --- | --- | --- | --- | --- | --- | --- | --- | --- | --- | --- | --- | --- | --- | --- | --- |

1T37

back to list

  

| Protein Coverage
| Supporting Peptides
|

Protein Coverage:

Supporting Peptides:

| Peptide | Uniq | -10lgP | Mass | Length | ppm | m/z | z | RT | Fraction | Scan | Source File | Area F1 | Area F10 | Area F11 | Area F12 | Area F13 | Area F2 | Area F3 | Area F4 | Area F5 | Area F6 | Area F7 | Area F8 | Area F9 | #Feature | #Feature F1 | #Feature F10 | #Feature F11 | #Feature F12 | #Feature F13 | #Feature F2 | #Feature F3 | #Feature F4 | #Feature F5 | #Feature F6 | #Feature F7 | #Feature F8 | #Feature F9 | Start | End | PTM | AScore | Found By |
| --- | --- | --- | --- | --- | --- | --- | --- | --- | --- | --- | --- | --- | --- | --- | --- | --- | --- | --- | --- | --- | --- | --- | --- | --- | --- | --- | --- | --- | --- | --- | --- | --- | --- | --- | --- | --- | --- | --- | --- | --- | --- | --- | --- |
| R.LAAIC(+57.02)FAGAPYNDDNYNIDLK.A | Y | 92.87 | 2357.0999 | 21 | -0.9 | 1179.5562 | 2 | 77.04 | 13 | F13:51637 | NaNaKA16\_F9.raw |  | 3.6037E7 |  | 3.3667E7 |  |  |  |  |  | 1.9932E9 | 2.5595E10 | 8.2815E9 | 1.462E10 | 68 | 0 | 7 | 0 | 5 | 0 | 0 | 0 | 0 | 0 | 11 | 16 | 12 | 17 | 95 | 115 | Carbamidomethylation | C5:Carbamidomethylation:1000.00 | PEAKS DB |
| G.APYNDDNYNIDLK.A | Y | 80.92 | 1553.6997 | 13 | 1.1 | 777.8580 | 2 | 40.11 | 11 | F11:24310 | NaNaKA16\_F7.raw |  |  |  |  |  |  |  |  |  | 1.285E9 | 1.4557E9 | 5.7971E7 | 7.0516E7 | 19 | 0 | 0 | 0 | 0 | 0 | 0 | 0 | 0 | 0 | 5 | 5 | 5 | 4 | 103 | 115 |  |  | PEAKS DB |
| F.AGAPYNDDNYNIDLK.A | Y | 77.81 | 1681.7583 | 15 | 0.1 | 841.8865 | 2 | 42.69 | 13 | F13:22802 | NaNaKA16\_F9.raw |  |  |  |  |  |  |  |  |  | 1.3878E8 | 7.6515E7 | 1.16E7 | 3.0896E8 | 16 | 0 | 0 | 0 | 0 | 0 | 0 | 0 | 0 | 0 | 4 | 3 | 3 | 6 | 101 | 115 |  |  | PEAKS DB |
| K.GDNNAC(+57.02)AASVC(+57.02)DC(+57.02)DR.L | N | 70.22 | 1683.6035 | 15 | 0.2 | 842.8092 | 2 | 12.08 | 11 | F11:1839 | NaNaKA16\_F7.raw |  |  |  |  |  |  |  |  |  | 6.3052E6 | 1.0666E6 | 0 |  | 2 | 0 | 0 | 0 | 0 | 0 | 0 | 0 | 0 | 0 | 1 | 1 | 0 | 0 | 80 | 94 | Carbamidomethylation | C6:Carbamidomethylation:1000.00;C11:Carbamidomethylation:1000.00;C13:Carbamidomethylation:1000.00 | PEAKS DB |
| C.FAGAPYNDDNYNIDLK.A | Y | 69.06 | 1828.8268 | 16 | 0.1 | 915.4208 | 2 | 54.79 | 13 | F13:31983 | NaNaKA16\_F9.raw |  |  |  |  |  |  |  |  |  | 4.2561E6 | 5.8811E5 |  | 5.6426E7 | 5 | 0 | 0 | 0 | 0 | 0 | 0 | 0 | 0 | 0 | 1 | 1 | 0 | 3 | 100 | 115 |  |  | PEAKS DB |
| K.GGSGTPVDDLDR.C | N | 67.81 | 1187.5417 | 12 | 0.8 | 594.7786 | 2 | 19.63 | 10 | F10:6988 | NaNaKA16\_F6.raw |  |  |  |  |  |  |  |  |  | 5.7224E7 | 2.6509E9 | 3.9395E6 |  | 4 | 0 | 0 | 0 | 0 | 0 | 0 | 0 | 0 | 0 | 2 | 1 | 1 | 0 | 31 | 42 |  |  | PEAKS DB |
| D.RLAAIC(+57.02)FAGAPYNDDNYNIDLK.A | Y | 63.91 | 2513.2009 | 22 | 0.9 | 838.7416 | 3 | 64.79 | 12 | F12:43839 | NaNaKA16\_F8.raw |  |  |  |  |  |  |  |  |  |  |  | 1.7196E7 | 3.8048E6 | 3 | 0 | 0 | 0 | 0 | 0 | 0 | 0 | 0 | 0 | 0 | 0 | 2 | 1 | 94 | 115 | Carbamidomethylation | C6:Carbamidomethylation:1000.00 | PEAKS DB |
| G.GSGTPVDDLDR.C | N | 60.74 | 1130.5204 | 11 | -0.3 | 566.2673 | 2 | 21.08 | 11 | F11:8172 | NaNaKA16\_F7.raw |  |  |  |  |  |  |  |  |  | 1.4052E5 | 1.2671E7 |  |  | 2 | 0 | 0 | 0 | 0 | 0 | 0 | 0 | 0 | 0 | 1 | 1 | 0 | 0 | 32 | 42 |  |  | PEAKS DB |
| R.LAAIC(+57.02)FAGAPYN.D | N | 53.90 | 1266.6067 | 12 | 0.8 | 634.3111 | 2 | 70.83 | 11 | F11:49747 | NaNaKA16\_F7.raw |  | 2.6932E8 | 6.3964E7 |  |  |  |  |  | 3.6922E8 | 7.2309E9 | 3.8533E9 | 2.4096E9 | 1.5572E9 | 11 | 0 | 1 | 1 | 0 | 0 | 0 | 0 | 0 | 1 | 2 | 3 | 2 | 1 | 95 | 106 | Carbamidomethylation | C5:Carbamidomethylation:1000.00 | PEAKS DB |
| P.YNDDNYNIDLK.A | Y | 49.28 | 1385.6099 | 11 | 0.1 | 693.8123 | 2 | 36.17 | 10 | F10:20793 | NaNaKA16\_F6.raw |  |  |  |  |  |  |  |  |  | 2.4481E6 |  |  |  | 1 | 0 | 0 | 0 | 0 | 0 | 0 | 0 | 0 | 0 | 1 | 0 | 0 | 0 | 105 | 115 |  |  | PEAKS DB |
| N.DDNYNIDLK.A | Y | 48.54 | 1108.5037 | 9 | -0.4 | 555.2589 | 2 | 37.39 | 10 | F10:21951 | NaNaKA16\_F6.raw |  |  |  |  |  |  |  |  |  | 9.0684E5 |  |  |  | 1 | 0 | 0 | 0 | 0 | 0 | 0 | 0 | 0 | 0 | 1 | 0 | 0 | 0 | 107 | 115 |  |  | PEAKS DB |
| G.SGTPVDDLDR.C | N | 45.52 | 1073.4989 | 10 | 0.9 | 537.7572 | 2 | 20.23 | 11 | F11:7497 | NaNaKA16\_F7.raw |  |  |  |  |  |  |  |  |  |  | 1.1072E7 |  |  | 1 | 0 | 0 | 0 | 0 | 0 | 0 | 0 | 0 | 0 | 0 | 1 | 0 | 0 | 33 | 42 |  |  | PEAKS DB |
| R.LAAIC(+57.02)FAGAPYNDDNYNIDLKAR.C | Y | 43.98 | 2584.2380 | 23 | 0.0 | 862.4199 | 3 | 66.94 | 12 | F12:45096 | NaNaKA16\_F8.raw |  |  |  |  |  |  |  |  |  |  | 4.0557E5 | 6.9771E5 |  | 2 | 0 | 0 | 0 | 0 | 0 | 0 | 0 | 0 | 0 | 0 | 1 | 1 | 0 | 95 | 117 | Carbamidomethylation | C5:Carbamidomethylation:1000.00 | PEAKS DB |
| total 13 peptides |  |  |  |  |  |  |  |  |  |  |  |  |  |  |  |  |  |  |  |  |  |  |  |  |  |  |  |  |  |  |  |  |  |  |  |  |  |  |  |  |  |  |  |
| --- | --- | --- | --- | --- | --- | --- | --- | --- | --- | --- | --- | --- | --- | --- | --- | --- | --- | --- | --- | --- | --- | --- | --- | --- | --- | --- | --- | --- | --- | --- | --- | --- | --- | --- | --- | --- | --- | --- | --- | --- | --- | --- | --- |

1ZM6

back to list

  

| Protein Coverage
| Supporting Peptides
|

Protein Coverage:

Supporting Peptides:

| Peptide | Uniq | -10lgP | Mass | Length | ppm | m/z | z | RT | Fraction | Scan | Source File | Area F1 | Area F10 | Area F11 | Area F12 | Area F13 | Area F2 | Area F3 | Area F4 | Area F5 | Area F6 | Area F7 | Area F8 | Area F9 | #Feature | #Feature F1 | #Feature F10 | #Feature F11 | #Feature F12 | #Feature F13 | #Feature F2 | #Feature F3 | #Feature F4 | #Feature F5 | #Feature F6 | #Feature F7 | #Feature F8 | #Feature F9 | Start | End | PTM | AScore | Found By |
| --- | --- | --- | --- | --- | --- | --- | --- | --- | --- | --- | --- | --- | --- | --- | --- | --- | --- | --- | --- | --- | --- | --- | --- | --- | --- | --- | --- | --- | --- | --- | --- | --- | --- | --- | --- | --- | --- | --- | --- | --- | --- | --- | --- |
| R.LAAIC(+57.02)FAGAPYNDDNYNIDLK.A | Y | 92.87 | 2357.0999 | 21 | -0.9 | 1179.5562 | 2 | 77.04 | 13 | F13:51637 | NaNaKA16\_F9.raw |  | 3.6037E7 |  | 3.3667E7 |  |  |  |  |  | 1.9932E9 | 2.5595E10 | 8.2815E9 | 1.462E10 | 68 | 0 | 7 | 0 | 5 | 0 | 0 | 0 | 0 | 0 | 11 | 16 | 12 | 17 | 95 | 115 | Carbamidomethylation | C5:Carbamidomethylation:1000.00 | PEAKS DB |
| G.APYNDDNYNIDLK.A | Y | 80.92 | 1553.6997 | 13 | 1.1 | 777.8580 | 2 | 40.11 | 11 | F11:24310 | NaNaKA16\_F7.raw |  |  |  |  |  |  |  |  |  | 1.285E9 | 1.4557E9 | 5.7971E7 | 7.0516E7 | 19 | 0 | 0 | 0 | 0 | 0 | 0 | 0 | 0 | 0 | 5 | 5 | 5 | 4 | 103 | 115 |  |  | PEAKS DB |
| F.AGAPYNDDNYNIDLK.A | Y | 77.81 | 1681.7583 | 15 | 0.1 | 841.8865 | 2 | 42.69 | 13 | F13:22802 | NaNaKA16\_F9.raw |  |  |  |  |  |  |  |  |  | 1.3878E8 | 7.6515E7 | 1.16E7 | 3.0896E8 | 16 | 0 | 0 | 0 | 0 | 0 | 0 | 0 | 0 | 0 | 4 | 3 | 3 | 6 | 101 | 115 |  |  | PEAKS DB |
| K.GDNNAC(+57.02)AASVC(+57.02)DC(+57.02)DR.L | N | 70.22 | 1683.6035 | 15 | 0.2 | 842.8092 | 2 | 12.08 | 11 | F11:1839 | NaNaKA16\_F7.raw |  |  |  |  |  |  |  |  |  | 6.3052E6 | 1.0666E6 | 0 |  | 2 | 0 | 0 | 0 | 0 | 0 | 0 | 0 | 0 | 0 | 1 | 1 | 0 | 0 | 80 | 94 | Carbamidomethylation | C6:Carbamidomethylation:1000.00;C11:Carbamidomethylation:1000.00;C13:Carbamidomethylation:1000.00 | PEAKS DB |
| C.FAGAPYNDDNYNIDLK.A | Y | 69.06 | 1828.8268 | 16 | 0.1 | 915.4208 | 2 | 54.79 | 13 | F13:31983 | NaNaKA16\_F9.raw |  |  |  |  |  |  |  |  |  | 4.2561E6 | 5.8811E5 |  | 5.6426E7 | 5 | 0 | 0 | 0 | 0 | 0 | 0 | 0 | 0 | 0 | 1 | 1 | 0 | 3 | 100 | 115 |  |  | PEAKS DB |
| K.GGSGTPVDDLDR.C | N | 67.81 | 1187.5417 | 12 | 0.8 | 594.7786 | 2 | 19.63 | 10 | F10:6988 | NaNaKA16\_F6.raw |  |  |  |  |  |  |  |  |  | 5.7224E7 | 2.6509E9 | 3.9395E6 |  | 4 | 0 | 0 | 0 | 0 | 0 | 0 | 0 | 0 | 0 | 2 | 1 | 1 | 0 | 31 | 42 |  |  | PEAKS DB |
| D.RLAAIC(+57.02)FAGAPYNDDNYNIDLK.A | Y | 63.91 | 2513.2009 | 22 | 0.9 | 838.7416 | 3 | 64.79 | 12 | F12:43839 | NaNaKA16\_F8.raw |  |  |  |  |  |  |  |  |  |  |  | 1.7196E7 | 3.8048E6 | 3 | 0 | 0 | 0 | 0 | 0 | 0 | 0 | 0 | 0 | 0 | 0 | 2 | 1 | 94 | 115 | Carbamidomethylation | C6:Carbamidomethylation:1000.00 | PEAKS DB |
| G.GSGTPVDDLDR.C | N | 60.74 | 1130.5204 | 11 | -0.3 | 566.2673 | 2 | 21.08 | 11 | F11:8172 | NaNaKA16\_F7.raw |  |  |  |  |  |  |  |  |  | 1.4052E5 | 1.2671E7 |  |  | 2 | 0 | 0 | 0 | 0 | 0 | 0 | 0 | 0 | 0 | 1 | 1 | 0 | 0 | 32 | 42 |  |  | PEAKS DB |
| R.LAAIC(+57.02)FAGAPYN.D | N | 53.90 | 1266.6067 | 12 | 0.8 | 634.3111 | 2 | 70.83 | 11 | F11:49747 | NaNaKA16\_F7.raw |  | 2.6932E8 | 6.3964E7 |  |  |  |  |  | 3.6922E8 | 7.2309E9 | 3.8533E9 | 2.4096E9 | 1.5572E9 | 11 | 0 | 1 | 1 | 0 | 0 | 0 | 0 | 0 | 1 | 2 | 3 | 2 | 1 | 95 | 106 | Carbamidomethylation | C5:Carbamidomethylation:1000.00 | PEAKS DB |
| P.YNDDNYNIDLK.A | Y | 49.28 | 1385.6099 | 11 | 0.1 | 693.8123 | 2 | 36.17 | 10 | F10:20793 | NaNaKA16\_F6.raw |  |  |  |  |  |  |  |  |  | 2.4481E6 |  |  |  | 1 | 0 | 0 | 0 | 0 | 0 | 0 | 0 | 0 | 0 | 1 | 0 | 0 | 0 | 105 | 115 |  |  | PEAKS DB |
| N.DDNYNIDLK.A | Y | 48.54 | 1108.5037 | 9 | -0.4 | 555.2589 | 2 | 37.39 | 10 | F10:21951 | NaNaKA16\_F6.raw |  |  |  |  |  |  |  |  |  | 9.0684E5 |  |  |  | 1 | 0 | 0 | 0 | 0 | 0 | 0 | 0 | 0 | 0 | 1 | 0 | 0 | 0 | 107 | 115 |  |  | PEAKS DB |
| G.SGTPVDDLDR.C | N | 45.52 | 1073.4989 | 10 | 0.9 | 537.7572 | 2 | 20.23 | 11 | F11:7497 | NaNaKA16\_F7.raw |  |  |  |  |  |  |  |  |  |  | 1.1072E7 |  |  | 1 | 0 | 0 | 0 | 0 | 0 | 0 | 0 | 0 | 0 | 0 | 1 | 0 | 0 | 33 | 42 |  |  | PEAKS DB |
| R.LAAIC(+57.02)FAGAPYNDDNYNIDLKAR.C | Y | 43.98 | 2584.2380 | 23 | 0.0 | 862.4199 | 3 | 66.94 | 12 | F12:45096 | NaNaKA16\_F8.raw |  |  |  |  |  |  |  |  |  |  | 4.0557E5 | 6.9771E5 |  | 2 | 0 | 0 | 0 | 0 | 0 | 0 | 0 | 0 | 0 | 0 | 1 | 1 | 0 | 95 | 117 | Carbamidomethylation | C5:Carbamidomethylation:1000.00 | PEAKS DB |
| total 13 peptides |  |  |  |  |  |  |  |  |  |  |  |  |  |  |  |  |  |  |  |  |  |  |  |  |  |  |  |  |  |  |  |  |  |  |  |  |  |  |  |  |  |  |  |
| --- | --- | --- | --- | --- | --- | --- | --- | --- | --- | --- | --- | --- | --- | --- | --- | --- | --- | --- | --- | --- | --- | --- | --- | --- | --- | --- | --- | --- | --- | --- | --- | --- | --- | --- | --- | --- | --- | --- | --- | --- | --- | --- | --- |

XP\_029140080.1

back to list

  

| Protein Coverage
| Supporting Peptides
|

Protein Coverage:

Supporting Peptides:

| Peptide | Uniq | -10lgP | Mass | Length | ppm | m/z | z | RT | Fraction | Scan | Source File | Area F1 | Area F10 | Area F11 | Area F12 | Area F13 | Area F2 | Area F3 | Area F4 | Area F5 | Area F6 | Area F7 | Area F8 | Area F9 | #Feature | #Feature F1 | #Feature F10 | #Feature F11 | #Feature F12 | #Feature F13 | #Feature F2 | #Feature F3 | #Feature F4 | #Feature F5 | #Feature F6 | #Feature F7 | #Feature F8 | #Feature F9 | Start | End | PTM | AScore | Found By |
| --- | --- | --- | --- | --- | --- | --- | --- | --- | --- | --- | --- | --- | --- | --- | --- | --- | --- | --- | --- | --- | --- | --- | --- | --- | --- | --- | --- | --- | --- | --- | --- | --- | --- | --- | --- | --- | --- | --- | --- | --- | --- | --- | --- |
| Q.LPDWTEC(+57.02)EVSGYGK.H | N | 89.52 | 1639.7188 | 14 | 0.4 | 820.8669 | 2 | 53.18 | 4 | F4:33245 | NaNaKA16\_F12.raw |  | 2.4114E6 | 1.7456E7 | 5.2898E8 | 5.0846E5 |  |  |  |  |  |  |  |  | 6 | 0 | 1 | 1 | 3 | 1 | 0 | 0 | 0 | 0 | 0 | 0 | 0 | 0 | 436 | 449 | Carbamidomethylation | C7:Carbamidomethylation:1000.00 | PEAKS DB |
| Q.ALYSPNLFIC(+57.02)FC(+57.02)PPGFSGK.F | N | 84.91 | 2174.0330 | 19 | 0.6 | 1088.0244 | 2 | 95.42 | 12 | F12:68966 | NaNaKA16\_F8.raw |  | 3.6583E5 |  |  |  |  |  |  |  | 1.5307E7 | 5.8251E7 | 1.0308E7 | 4.354E6 | 9 | 0 | 1 | 0 | 0 | 0 | 0 | 0 | 0 | 0 | 2 | 2 | 2 | 2 | 99 | 117 | Carbamidomethylation | C10:Carbamidomethylation:1000.00;C12:Carbamidomethylation:1000.00 | PEAKS DB |
| R.TVTENMLC(+57.02)AGDTR.H | N | 84.34 | 1466.6494 | 13 | -0.1 | 734.3319 | 2 | 30.20 | 4 | F4:13667 | NaNaKA16\_F12.raw |  |  |  | 9.0057E7 |  |  |  |  |  |  |  |  |  | 2 | 0 | 0 | 0 | 2 | 0 | 0 | 0 | 0 | 0 | 0 | 0 | 0 | 0 | 483 | 495 | Carbamidomethylation | C8:Carbamidomethylation:1000.00 | PEAKS DB |
| R.AVC(+57.02)YQDIGDTYR.G | Y | 79.54 | 1459.6401 | 12 | 0.7 | 730.8278 | 2 | 35.07 | 11 | F11:19729 | NaNaKA16\_F7.raw |  | 1.2319E6 | 1.3702E6 | 9.6234E6 |  |  |  |  |  | 2.3912E7 | 7.3676E6 |  | 1.527E6 | 6 | 0 | 1 | 1 | 1 | 0 | 0 | 0 | 0 | 0 | 1 | 1 | 0 | 1 | 125 | 136 | Carbamidomethylation | C3:Carbamidomethylation:1000.00 | PEAKS DB |
| R.GTWSTTESGAEC(+57.02)VNWK.T | N | 79.36 | 1811.7784 | 16 | 0.6 | 906.8970 | 2 | 40.11 | 11 | F11:23748 | NaNaKA16\_F7.raw |  |  |  | 3.6435E6 |  |  |  |  |  | 2.669E7 | 9.2931E6 |  |  | 3 | 0 | 0 | 0 | 1 | 0 | 0 | 0 | 0 | 0 | 1 | 1 | 0 | 0 | 137 | 152 | Carbamidomethylation | C12:Carbamidomethylation:1000.00 | PEAKS DB |
| L.LGLGDHNYC(+57.02)R.N | N | 69.80 | 1203.5455 | 10 | 0.7 | 602.7805 | 2 | 12.35 | 11 | F11:2053 | NaNaKA16\_F7.raw |  |  |  |  |  |  |  |  |  | 2.6837E5 | 1.9952E6 |  |  | 3 | 0 | 0 | 0 | 0 | 0 | 0 | 0 | 0 | 0 | 1 | 2 | 0 | 0 | 171 | 180 | Carbamidomethylation | C9:Carbamidomethylation:1000.00 | PEAKS DB |
| R.TVTENM(+15.99)LC(+57.02)AGDTR.H | N | 67.27 | 1482.6443 | 13 | 0.9 | 742.3301 | 2 | 28.18 | 4 | F4:11849 | NaNaKA16\_F12.raw |  |  |  | 6.8651E6 |  |  |  |  |  |  |  |  |  | 5 | 0 | 0 | 0 | 5 | 0 | 0 | 0 | 0 | 0 | 0 | 0 | 0 | 0 | 483 | 495 | Oxidation (M); Carbamidomethylation | M6:Oxidation (M):1000.00;C8:Carbamidomethylation:1000.00 | PEAKS DB |
| K.SSKPWC(+57.02)HVLK.K | N | 63.39 | 1240.6387 | 10 | 0.1 | 414.5535 | 3 | 12.10 | 11 | F11:1845 | NaNaKA16\_F7.raw |  |  |  |  |  |  |  |  |  |  | 2.6656E6 |  |  | 1 | 0 | 0 | 0 | 0 | 0 | 0 | 0 | 0 | 0 | 0 | 1 | 0 | 0 | 185 | 194 | Carbamidomethylation | C6:Carbamidomethylation:1000.00 | PEAKS DB |
| N.LFIC(+57.02)FC(+57.02)PPGFSGK.F | N | 61.36 | 1528.7206 | 13 | -0.1 | 765.3675 | 2 | 80.11 | 10 | F10:58024 | NaNaKA16\_F6.raw |  |  |  |  |  |  |  |  |  | 1.3335E7 | 7.5039E6 |  |  | 3 | 0 | 0 | 0 | 0 | 0 | 0 | 0 | 0 | 0 | 1 | 2 | 0 | 0 | 105 | 117 | Carbamidomethylation | C4:Carbamidomethylation:1000.00;C6:Carbamidomethylation:1000.00 | PEAKS DB |
| F.IC(+57.02)FC(+57.02)PPGFSGK.F | N | 58.86 | 1268.5681 | 11 | 0.1 | 635.2914 | 2 | 51.30 | 10 | F10:33995 | NaNaKA16\_F6.raw |  |  |  |  |  |  |  |  |  | 1.0459E7 | 5.3422E6 | 2.3238E6 |  | 3 | 0 | 0 | 0 | 0 | 0 | 0 | 0 | 0 | 0 | 1 | 1 | 1 | 0 | 107 | 117 | Carbamidomethylation | C2:Carbamidomethylation:1000.00;C4:Carbamidomethylation:1000.00 | PEAKS DB |
| R.TLDNDIALLK.L | N | 56.75 | 1114.6234 | 10 | 0.2 | 558.3191 | 2 | 53.18 | 4 | F4:32689 | NaNaKA16\_F12.raw |  |  | 4.6197E6 | 4.3448E8 |  |  |  |  |  |  |  |  |  | 3 | 0 | 0 | 1 | 2 | 0 | 0 | 0 | 0 | 0 | 0 | 0 | 0 | 0 | 400 | 409 |  |  | PEAKS DB |
| Y.SPNLFIC(+57.02)FC(+57.02)PPGFSGK.F | N | 49.42 | 1826.8484 | 16 | 1.4 | 914.4327 | 2 | 84.61 | 12 | F12:60077 | NaNaKA16\_F8.raw |  |  |  |  |  |  |  |  |  |  | 5.8546E5 | 7.1533E5 |  | 2 | 0 | 0 | 0 | 0 | 0 | 0 | 0 | 0 | 0 | 0 | 1 | 1 | 0 | 102 | 117 | Carbamidomethylation | C7:Carbamidomethylation:1000.00;C9:Carbamidomethylation:1000.00 | PEAKS DB |
| R.TLDNDIALLKLK.E | N | 47.35 | 1355.8024 | 12 | 0.6 | 452.9417 | 3 | 55.08 | 4 | F4:34569 | NaNaKA16\_F12.raw |  |  |  | 8.5072E5 |  |  |  |  |  |  |  |  |  | 1 | 0 | 0 | 0 | 1 | 0 | 0 | 0 | 0 | 0 | 0 | 0 | 0 | 0 | 400 | 411 |  |  | PEAKS DB |
| total 13 peptides |  |  |  |  |  |  |  |  |  |  |  |  |  |  |  |  |  |  |  |  |  |  |  |  |  |  |  |  |  |  |  |  |  |  |  |  |  |  |  |  |  |  |  |
| --- | --- | --- | --- | --- | --- | --- | --- | --- | --- | --- | --- | --- | --- | --- | --- | --- | --- | --- | --- | --- | --- | --- | --- | --- | --- | --- | --- | --- | --- | --- | --- | --- | --- | --- | --- | --- | --- | --- | --- | --- | --- | --- | --- |

JAI11009.1

back to list

  

| Protein Coverage
| Supporting Peptides
|

Protein Coverage:

Supporting Peptides:

| Peptide | Uniq | -10lgP | Mass | Length | ppm | m/z | z | RT | Fraction | Scan | Source File | Area F1 | Area F10 | Area F11 | Area F12 | Area F13 | Area F2 | Area F3 | Area F4 | Area F5 | Area F6 | Area F7 | Area F8 | Area F9 | #Feature | #Feature F1 | #Feature F10 | #Feature F11 | #Feature F12 | #Feature F13 | #Feature F2 | #Feature F3 | #Feature F4 | #Feature F5 | #Feature F6 | #Feature F7 | #Feature F8 | #Feature F9 | Start | End | PTM | AScore | Found By |
| --- | --- | --- | --- | --- | --- | --- | --- | --- | --- | --- | --- | --- | --- | --- | --- | --- | --- | --- | --- | --- | --- | --- | --- | --- | --- | --- | --- | --- | --- | --- | --- | --- | --- | --- | --- | --- | --- | --- | --- | --- | --- | --- | --- |
| Q.LPDWTEC(+57.02)EVSGYGK.H | N | 89.52 | 1639.7188 | 14 | 0.4 | 820.8669 | 2 | 53.18 | 4 | F4:33245 | NaNaKA16\_F12.raw |  | 2.4114E6 | 1.7456E7 | 5.2898E8 | 5.0846E5 |  |  |  |  |  |  |  |  | 6 | 0 | 1 | 1 | 3 | 1 | 0 | 0 | 0 | 0 | 0 | 0 | 0 | 0 | 437 | 450 | Carbamidomethylation | C7:Carbamidomethylation:1000.00 | PEAKS DB |
| Q.ALYSPNLFIC(+57.02)FC(+57.02)PPGFSGK.F | N | 84.91 | 2174.0330 | 19 | 0.6 | 1088.0244 | 2 | 95.42 | 12 | F12:68966 | NaNaKA16\_F8.raw |  | 3.6583E5 |  |  |  |  |  |  |  | 1.5307E7 | 5.8251E7 | 1.0308E7 | 4.354E6 | 9 | 0 | 1 | 0 | 0 | 0 | 0 | 0 | 0 | 0 | 2 | 2 | 2 | 2 | 99 | 117 | Carbamidomethylation | C10:Carbamidomethylation:1000.00;C12:Carbamidomethylation:1000.00 | PEAKS DB |
| R.TVTENMLC(+57.02)AGDTR.H | N | 84.34 | 1466.6494 | 13 | -0.1 | 734.3319 | 2 | 30.20 | 4 | F4:13667 | NaNaKA16\_F12.raw |  |  |  | 9.0057E7 |  |  |  |  |  |  |  |  |  | 2 | 0 | 0 | 0 | 2 | 0 | 0 | 0 | 0 | 0 | 0 | 0 | 0 | 0 | 484 | 496 | Carbamidomethylation | C8:Carbamidomethylation:1000.00 | PEAKS DB |
| R.AVC(+57.02)YQDIGDTYR.G | Y | 79.54 | 1459.6401 | 12 | 0.7 | 730.8278 | 2 | 35.07 | 11 | F11:19729 | NaNaKA16\_F7.raw |  | 1.2319E6 | 1.3702E6 | 9.6234E6 |  |  |  |  |  | 2.3912E7 | 7.3676E6 |  | 1.527E6 | 6 | 0 | 1 | 1 | 1 | 0 | 0 | 0 | 0 | 0 | 1 | 1 | 0 | 1 | 125 | 136 | Carbamidomethylation | C3:Carbamidomethylation:1000.00 | PEAKS DB |
| R.GTWSTTESGAEC(+57.02)VNWK.T | N | 79.36 | 1811.7784 | 16 | 0.6 | 906.8970 | 2 | 40.11 | 11 | F11:23748 | NaNaKA16\_F7.raw |  |  |  | 3.6435E6 |  |  |  |  |  | 2.669E7 | 9.2931E6 |  |  | 3 | 0 | 0 | 0 | 1 | 0 | 0 | 0 | 0 | 0 | 1 | 1 | 0 | 0 | 137 | 152 | Carbamidomethylation | C12:Carbamidomethylation:1000.00 | PEAKS DB |
| L.LGLGDHNYC(+57.02)R.N | N | 69.80 | 1203.5455 | 10 | 0.7 | 602.7805 | 2 | 12.35 | 11 | F11:2053 | NaNaKA16\_F7.raw |  |  |  |  |  |  |  |  |  | 2.6837E5 | 1.9952E6 |  |  | 3 | 0 | 0 | 0 | 0 | 0 | 0 | 0 | 0 | 0 | 1 | 2 | 0 | 0 | 171 | 180 | Carbamidomethylation | C9:Carbamidomethylation:1000.00 | PEAKS DB |
| R.TVTENM(+15.99)LC(+57.02)AGDTR.H | N | 67.27 | 1482.6443 | 13 | 0.9 | 742.3301 | 2 | 28.18 | 4 | F4:11849 | NaNaKA16\_F12.raw |  |  |  | 6.8651E6 |  |  |  |  |  |  |  |  |  | 5 | 0 | 0 | 0 | 5 | 0 | 0 | 0 | 0 | 0 | 0 | 0 | 0 | 0 | 484 | 496 | Oxidation (M); Carbamidomethylation | M6:Oxidation (M):1000.00;C8:Carbamidomethylation:1000.00 | PEAKS DB |
| K.SSKPWC(+57.02)HVLK.K | N | 63.39 | 1240.6387 | 10 | 0.1 | 414.5535 | 3 | 12.10 | 11 | F11:1845 | NaNaKA16\_F7.raw |  |  |  |  |  |  |  |  |  |  | 2.6656E6 |  |  | 1 | 0 | 0 | 0 | 0 | 0 | 0 | 0 | 0 | 0 | 0 | 1 | 0 | 0 | 185 | 194 | Carbamidomethylation | C6:Carbamidomethylation:1000.00 | PEAKS DB |
| N.LFIC(+57.02)FC(+57.02)PPGFSGK.F | N | 61.36 | 1528.7206 | 13 | -0.1 | 765.3675 | 2 | 80.11 | 10 | F10:58024 | NaNaKA16\_F6.raw |  |  |  |  |  |  |  |  |  | 1.3335E7 | 7.5039E6 |  |  | 3 | 0 | 0 | 0 | 0 | 0 | 0 | 0 | 0 | 0 | 1 | 2 | 0 | 0 | 105 | 117 | Carbamidomethylation | C4:Carbamidomethylation:1000.00;C6:Carbamidomethylation:1000.00 | PEAKS DB |
| F.IC(+57.02)FC(+57.02)PPGFSGK.F | N | 58.86 | 1268.5681 | 11 | 0.1 | 635.2914 | 2 | 51.30 | 10 | F10:33995 | NaNaKA16\_F6.raw |  |  |  |  |  |  |  |  |  | 1.0459E7 | 5.3422E6 | 2.3238E6 |  | 3 | 0 | 0 | 0 | 0 | 0 | 0 | 0 | 0 | 0 | 1 | 1 | 1 | 0 | 107 | 117 | Carbamidomethylation | C2:Carbamidomethylation:1000.00;C4:Carbamidomethylation:1000.00 | PEAKS DB |
| R.TLDNDIALLK.L | N | 56.75 | 1114.6234 | 10 | 0.2 | 558.3191 | 2 | 53.18 | 4 | F4:32689 | NaNaKA16\_F12.raw |  |  | 4.6197E6 | 4.3448E8 |  |  |  |  |  |  |  |  |  | 3 | 0 | 0 | 1 | 2 | 0 | 0 | 0 | 0 | 0 | 0 | 0 | 0 | 0 | 401 | 410 |  |  | PEAKS DB |
| Y.SPNLFIC(+57.02)FC(+57.02)PPGFSGK.F | N | 49.42 | 1826.8484 | 16 | 1.4 | 914.4327 | 2 | 84.61 | 12 | F12:60077 | NaNaKA16\_F8.raw |  |  |  |  |  |  |  |  |  |  | 5.8546E5 | 7.1533E5 |  | 2 | 0 | 0 | 0 | 0 | 0 | 0 | 0 | 0 | 0 | 0 | 1 | 1 | 0 | 102 | 117 | Carbamidomethylation | C7:Carbamidomethylation:1000.00;C9:Carbamidomethylation:1000.00 | PEAKS DB |
| R.TLDNDIALLKLK.E | N | 47.35 | 1355.8024 | 12 | 0.6 | 452.9417 | 3 | 55.08 | 4 | F4:34569 | NaNaKA16\_F12.raw |  |  |  | 8.5072E5 |  |  |  |  |  |  |  |  |  | 1 | 0 | 0 | 0 | 1 | 0 | 0 | 0 | 0 | 0 | 0 | 0 | 0 | 0 | 401 | 412 |  |  | PEAKS DB |
| total 13 peptides |  |  |  |  |  |  |  |  |  |  |  |  |  |  |  |  |  |  |  |  |  |  |  |  |  |  |  |  |  |  |  |  |  |  |  |  |  |  |  |  |  |  |  |
| --- | --- | --- | --- | --- | --- | --- | --- | --- | --- | --- | --- | --- | --- | --- | --- | --- | --- | --- | --- | --- | --- | --- | --- | --- | --- | --- | --- | --- | --- | --- | --- | --- | --- | --- | --- | --- | --- | --- | --- | --- | --- | --- | --- |

ADG02948.1

back to list

  

| Protein Coverage
| Supporting Peptides
|

Protein Coverage:

Supporting Peptides:

| Peptide | Uniq | -10lgP | Mass | Length | ppm | m/z | z | RT | Fraction | Scan | Source File | Area F1 | Area F10 | Area F11 | Area F12 | Area F13 | Area F2 | Area F3 | Area F4 | Area F5 | Area F6 | Area F7 | Area F8 | Area F9 | #Feature | #Feature F1 | #Feature F10 | #Feature F11 | #Feature F12 | #Feature F13 | #Feature F2 | #Feature F3 | #Feature F4 | #Feature F5 | #Feature F6 | #Feature F7 | #Feature F8 | #Feature F9 | Start | End | PTM | AScore | Found By |
| --- | --- | --- | --- | --- | --- | --- | --- | --- | --- | --- | --- | --- | --- | --- | --- | --- | --- | --- | --- | --- | --- | --- | --- | --- | --- | --- | --- | --- | --- | --- | --- | --- | --- | --- | --- | --- | --- | --- | --- | --- | --- | --- | --- |
| R.VAKDDC(+57.02)DLPELC(+57.02)TGQSAEC(+57.02)PTDSLQR.N | Y | 95.50 | 2964.2898 | 26 | 0.7 | 989.1046 | 3 | 43.19 | 9 | F9:25736 | NaNaKA16\_F5.raw |  |  |  |  |  |  |  |  | 5.0308E7 | 1.4807E8 |  |  |  | 2 | 0 | 0 | 0 | 0 | 0 | 0 | 0 | 0 | 1 | 1 | 0 | 0 | 0 | 451 | 476 | Carbamidomethylation | C6:Carbamidomethylation:1000.00;C12:Carbamidomethylation:1000.00;C19:Carbamidomethylation:1000.00 | PEAKS DB |
| R.TAPAFQFSSC(+57.02)SIR.E | N | 80.42 | 1470.6925 | 13 | 0.6 | 736.3539 | 2 | 46.94 | 4 | F4:27572 | NaNaKA16\_F12.raw |  | 7.7695E6 | 7.0106E6 | 6.7741E6 | 7.3303E5 |  |  |  |  |  |  |  | 2.6381E6 | 5 | 0 | 1 | 1 | 1 | 1 | 0 | 0 | 0 | 0 | 0 | 0 | 0 | 1 | 370 | 382 | Carbamidomethylation | C10:Carbamidomethylation:1000.00 | PEAKS DB |
| D.LPELC(+57.02)TGQSAEC(+57.02)PTDSLQR.N | N | 78.21 | 2160.9780 | 19 | 0.3 | 1081.4966 | 2 | 44.94 | 10 | F10:28461 | NaNaKA16\_F6.raw |  |  |  |  |  |  |  |  |  | 2.2687E6 |  |  |  | 1 | 0 | 0 | 0 | 0 | 0 | 0 | 0 | 0 | 0 | 1 | 0 | 0 | 0 | 458 | 476 | Carbamidomethylation | C5:Carbamidomethylation:1000.00;C12:Carbamidomethylation:1000.00 | PEAKS DB |
| K.DDC(+57.02)DLPELC(+57.02)TGQSAEC(+57.02)PTDSLQR.N | Y | 68.03 | 2666.0894 | 23 | -1.2 | 1334.0503 | 2 | 58.77 | 10 | F10:39904 | NaNaKA16\_F6.raw |  |  |  | 6.6603E6 |  | 3.9675E6 | 5.5621E5 |  | 6.4952E6 | 2.3844E7 |  |  |  | 9 | 0 | 0 | 0 | 2 | 0 | 2 | 1 | 0 | 2 | 2 | 0 | 0 | 0 | 454 | 476 | Carbamidomethylation | C3:Carbamidomethylation:1000.00;C9:Carbamidomethylation:1000.00;C16:Carbamidomethylation:1000.00 | PEAKS DB |
| R.VYEMINAVNTK.F | N | 67.13 | 1280.6434 | 11 | -0.4 | 641.3287 | 2 | 34.28 | 4 | F4:17180 | NaNaKA16\_F12.raw |  |  |  | 2.766E6 | 2.8909E6 |  |  |  |  |  |  |  |  | 2 | 0 | 0 | 0 | 1 | 1 | 0 | 0 | 0 | 0 | 0 | 0 | 0 | 0 | 232 | 242 |  |  | PEAKS DB |
| A.DSSAVISAC(+57.02)DGLK.G | N | 60.11 | 1321.6184 | 13 | 0.4 | 661.8168 | 2 | 33.21 | 10 | F10:19042 | NaNaKA16\_F6.raw |  |  |  |  |  |  |  |  |  | 6.9505E6 |  |  |  | 1 | 0 | 0 | 0 | 0 | 0 | 0 | 0 | 0 | 0 | 1 | 0 | 0 | 0 | 119 | 131 | Carbamidomethylation | C9:Carbamidomethylation:1000.00 | PEAKS DB |
| K.GQC(+57.02)VDVQTAY | N | 56.58 | 1139.4917 | 10 | 0.2 | 570.7532 | 2 | 32.85 | 10 | F10:17935 | NaNaKA16\_F6.raw |  |  |  |  |  |  |  |  |  | 2.4491E8 |  |  |  | 1 | 0 | 0 | 0 | 0 | 0 | 0 | 0 | 0 | 0 | 1 | 0 | 0 | 0 | 584 | 593 | Carbamidomethylation | C3:Carbamidomethylation:1000.00 | PEAKS DB |
| H.IALIGLEIWSNK.D | N | 54.66 | 1355.7812 | 12 | 0.3 | 678.8981 | 2 | 85.43 | 4 | F4:59423 | NaNaKA16\_F12.raw |  |  | 7.965E5 | 2.2382E6 |  |  |  |  |  |  |  |  |  | 2 | 0 | 0 | 1 | 1 | 0 | 0 | 0 | 0 | 0 | 0 | 0 | 0 | 0 | 250 | 261 |  |  | PEAKS DB |
| K.GC(+57.02)FDLNMR.G | N | 52.79 | 1011.4266 | 8 | -0.1 | 506.7205 | 2 | 36.97 | 10 | F10:21218 | NaNaKA16\_F6.raw |  |  |  |  |  |  |  |  |  | 9.5883E8 |  |  |  | 1 | 0 | 0 | 0 | 0 | 0 | 0 | 0 | 0 | 0 | 1 | 0 | 0 | 0 | 514 | 521 | Carbamidomethylation | C2:Carbamidomethylation:1000.00 | PEAKS DB |
| A.LIGLEIWSNK.D | N | 47.34 | 1171.6600 | 10 | 0.4 | 586.8375 | 2 | 69.88 | 4 | F4:46518 | NaNaKA16\_F12.raw |  |  |  | 3.0353E5 |  |  |  |  |  |  |  |  |  | 1 | 0 | 0 | 0 | 1 | 0 | 0 | 0 | 0 | 0 | 0 | 0 | 0 | 0 | 252 | 261 |  |  | PEAKS DB |
| L.IGLEIWSNK.D | N | 46.89 | 1058.5760 | 9 | -0.3 | 530.2952 | 2 | 56.37 | 4 | F4:35622 | NaNaKA16\_F12.raw |  |  |  | 6.5535E5 |  |  |  |  |  | 0 |  |  |  | 1 | 0 | 0 | 0 | 1 | 0 | 0 | 0 | 0 | 0 | 0 | 0 | 0 | 0 | 253 | 261 |  |  | PEAKS DB |
| R.DRPQC(+57.02)ILNKPLST.D | N | 46.29 | 1540.8031 | 13 | -0.4 | 514.6081 | 3 | 27.16 | 4 | F4:11109 | NaNaKA16\_F12.raw |  |  |  | 7.0589E5 |  |  |  | 8.268E5 |  |  |  |  |  | 2 | 0 | 0 | 0 | 1 | 0 | 0 | 0 | 1 | 0 | 0 | 0 | 0 | 0 | 391 | 403 | Carbamidomethylation | C5:Carbamidomethylation:1000.00 | PEAKS DB |
| I.HIALIGLEIWSNK.D | Y | 45.00 | 1492.8402 | 13 | 0.2 | 498.6208 | 3 | 70.93 | 3 | F3:45643 | NaNaKA16\_F11.raw |  | 1.7705E6 | 1.4324E6 |  |  |  |  |  |  |  |  |  |  | 2 | 0 | 1 | 1 | 0 | 0 | 0 | 0 | 0 | 0 | 0 | 0 | 0 | 0 | 249 | 261 |  |  | PEAKS DB |
| total 13 peptides |  |  |  |  |  |  |  |  |  |  |  |  |  |  |  |  |  |  |  |  |  |  |  |  |  |  |  |  |  |  |  |  |  |  |  |  |  |  |  |  |  |  |  |
| --- | --- | --- | --- | --- | --- | --- | --- | --- | --- | --- | --- | --- | --- | --- | --- | --- | --- | --- | --- | --- | --- | --- | --- | --- | --- | --- | --- | --- | --- | --- | --- | --- | --- | --- | --- | --- | --- | --- | --- | --- | --- | --- | --- |

D6PXE8.1

back to list

  

| Protein Coverage
| Supporting Peptides
|

Protein Coverage:

Supporting Peptides:

| Peptide | Uniq | -10lgP | Mass | Length | ppm | m/z | z | RT | Fraction | Scan | Source File | Area F1 | Area F10 | Area F11 | Area F12 | Area F13 | Area F2 | Area F3 | Area F4 | Area F5 | Area F6 | Area F7 | Area F8 | Area F9 | #Feature | #Feature F1 | #Feature F10 | #Feature F11 | #Feature F12 | #Feature F13 | #Feature F2 | #Feature F3 | #Feature F4 | #Feature F5 | #Feature F6 | #Feature F7 | #Feature F8 | #Feature F9 | Start | End | PTM | AScore | Found By |
| --- | --- | --- | --- | --- | --- | --- | --- | --- | --- | --- | --- | --- | --- | --- | --- | --- | --- | --- | --- | --- | --- | --- | --- | --- | --- | --- | --- | --- | --- | --- | --- | --- | --- | --- | --- | --- | --- | --- | --- | --- | --- | --- | --- |
| R.VAKDDC(+57.02)DLPELC(+57.02)TGQSAEC(+57.02)PTDSLQR.N | Y | 95.50 | 2964.2898 | 26 | 0.7 | 989.1046 | 3 | 43.19 | 9 | F9:25736 | NaNaKA16\_F5.raw |  |  |  |  |  |  |  |  | 5.0308E7 | 1.4807E8 |  |  |  | 2 | 0 | 0 | 0 | 0 | 0 | 0 | 0 | 0 | 1 | 1 | 0 | 0 | 0 | 451 | 476 | Carbamidomethylation | C6:Carbamidomethylation:1000.00;C12:Carbamidomethylation:1000.00;C19:Carbamidomethylation:1000.00 | PEAKS DB |
| R.TAPAFQFSSC(+57.02)SIR.E | N | 80.42 | 1470.6925 | 13 | 0.6 | 736.3539 | 2 | 46.94 | 4 | F4:27572 | NaNaKA16\_F12.raw |  | 7.7695E6 | 7.0106E6 | 6.7741E6 | 7.3303E5 |  |  |  |  |  |  |  | 2.6381E6 | 5 | 0 | 1 | 1 | 1 | 1 | 0 | 0 | 0 | 0 | 0 | 0 | 0 | 1 | 370 | 382 | Carbamidomethylation | C10:Carbamidomethylation:1000.00 | PEAKS DB |
| D.LPELC(+57.02)TGQSAEC(+57.02)PTDSLQR.N | N | 78.21 | 2160.9780 | 19 | 0.3 | 1081.4966 | 2 | 44.94 | 10 | F10:28461 | NaNaKA16\_F6.raw |  |  |  |  |  |  |  |  |  | 2.2687E6 |  |  |  | 1 | 0 | 0 | 0 | 0 | 0 | 0 | 0 | 0 | 0 | 1 | 0 | 0 | 0 | 458 | 476 | Carbamidomethylation | C5:Carbamidomethylation:1000.00;C12:Carbamidomethylation:1000.00 | PEAKS DB |
| K.DDC(+57.02)DLPELC(+57.02)TGQSAEC(+57.02)PTDSLQR.N | Y | 68.03 | 2666.0894 | 23 | -1.2 | 1334.0503 | 2 | 58.77 | 10 | F10:39904 | NaNaKA16\_F6.raw |  |  |  | 6.6603E6 |  | 3.9675E6 | 5.5621E5 |  | 6.4952E6 | 2.3844E7 |  |  |  | 9 | 0 | 0 | 0 | 2 | 0 | 2 | 1 | 0 | 2 | 2 | 0 | 0 | 0 | 454 | 476 | Carbamidomethylation | C3:Carbamidomethylation:1000.00;C9:Carbamidomethylation:1000.00;C16:Carbamidomethylation:1000.00 | PEAKS DB |
| R.VYEMINAVNTK.F | N | 67.13 | 1280.6434 | 11 | -0.4 | 641.3287 | 2 | 34.28 | 4 | F4:17180 | NaNaKA16\_F12.raw |  |  |  | 2.766E6 | 2.8909E6 |  |  |  |  |  |  |  |  | 2 | 0 | 0 | 0 | 1 | 1 | 0 | 0 | 0 | 0 | 0 | 0 | 0 | 0 | 232 | 242 |  |  | PEAKS DB |
| A.DSSAVISAC(+57.02)DGLK.G | N | 60.11 | 1321.6184 | 13 | 0.4 | 661.8168 | 2 | 33.21 | 10 | F10:19042 | NaNaKA16\_F6.raw |  |  |  |  |  |  |  |  |  | 6.9505E6 |  |  |  | 1 | 0 | 0 | 0 | 0 | 0 | 0 | 0 | 0 | 0 | 1 | 0 | 0 | 0 | 119 | 131 | Carbamidomethylation | C9:Carbamidomethylation:1000.00 | PEAKS DB |
| K.GQC(+57.02)VDVQTAY | N | 56.58 | 1139.4917 | 10 | 0.2 | 570.7532 | 2 | 32.85 | 10 | F10:17935 | NaNaKA16\_F6.raw |  |  |  |  |  |  |  |  |  | 2.4491E8 |  |  |  | 1 | 0 | 0 | 0 | 0 | 0 | 0 | 0 | 0 | 0 | 1 | 0 | 0 | 0 | 584 | 593 | Carbamidomethylation | C3:Carbamidomethylation:1000.00 | PEAKS DB |
| H.IALIGLEIWSNK.D | N | 54.66 | 1355.7812 | 12 | 0.3 | 678.8981 | 2 | 85.43 | 4 | F4:59423 | NaNaKA16\_F12.raw |  |  | 7.965E5 | 2.2382E6 |  |  |  |  |  |  |  |  |  | 2 | 0 | 0 | 1 | 1 | 0 | 0 | 0 | 0 | 0 | 0 | 0 | 0 | 0 | 250 | 261 |  |  | PEAKS DB |
| K.GC(+57.02)FDLNMR.G | N | 52.79 | 1011.4266 | 8 | -0.1 | 506.7205 | 2 | 36.97 | 10 | F10:21218 | NaNaKA16\_F6.raw |  |  |  |  |  |  |  |  |  | 9.5883E8 |  |  |  | 1 | 0 | 0 | 0 | 0 | 0 | 0 | 0 | 0 | 0 | 1 | 0 | 0 | 0 | 514 | 521 | Carbamidomethylation | C2:Carbamidomethylation:1000.00 | PEAKS DB |
| A.LIGLEIWSNK.D | N | 47.34 | 1171.6600 | 10 | 0.4 | 586.8375 | 2 | 69.88 | 4 | F4:46518 | NaNaKA16\_F12.raw |  |  |  | 3.0353E5 |  |  |  |  |  |  |  |  |  | 1 | 0 | 0 | 0 | 1 | 0 | 0 | 0 | 0 | 0 | 0 | 0 | 0 | 0 | 252 | 261 |  |  | PEAKS DB |
| L.IGLEIWSNK.D | N | 46.89 | 1058.5760 | 9 | -0.3 | 530.2952 | 2 | 56.37 | 4 | F4:35622 | NaNaKA16\_F12.raw |  |  |  | 6.5535E5 |  |  |  |  |  | 0 |  |  |  | 1 | 0 | 0 | 0 | 1 | 0 | 0 | 0 | 0 | 0 | 0 | 0 | 0 | 0 | 253 | 261 |  |  | PEAKS DB |
| R.DRPQC(+57.02)ILNKPLST.D | N | 46.29 | 1540.8031 | 13 | -0.4 | 514.6081 | 3 | 27.16 | 4 | F4:11109 | NaNaKA16\_F12.raw |  |  |  | 7.0589E5 |  |  |  | 8.268E5 |  |  |  |  |  | 2 | 0 | 0 | 0 | 1 | 0 | 0 | 0 | 1 | 0 | 0 | 0 | 0 | 0 | 391 | 403 | Carbamidomethylation | C5:Carbamidomethylation:1000.00 | PEAKS DB |
| I.HIALIGLEIWSNK.D | Y | 45.00 | 1492.8402 | 13 | 0.2 | 498.6208 | 3 | 70.93 | 3 | F3:45643 | NaNaKA16\_F11.raw |  | 1.7705E6 | 1.4324E6 |  |  |  |  |  |  |  |  |  |  | 2 | 0 | 1 | 1 | 0 | 0 | 0 | 0 | 0 | 0 | 0 | 0 | 0 | 0 | 249 | 261 |  |  | PEAKS DB |
| total 13 peptides |  |  |  |  |  |  |  |  |  |  |  |  |  |  |  |  |  |  |  |  |  |  |  |  |  |  |  |  |  |  |  |  |  |  |  |  |  |  |  |  |  |  |  |
| --- | --- | --- | --- | --- | --- | --- | --- | --- | --- | --- | --- | --- | --- | --- | --- | --- | --- | --- | --- | --- | --- | --- | --- | --- | --- | --- | --- | --- | --- | --- | --- | --- | --- | --- | --- | --- | --- | --- | --- | --- | --- | --- | --- |

ACN50005.1

back to list

  

| Protein Coverage
| Supporting Peptides
|

Protein Coverage:

Supporting Peptides:

| Peptide | Uniq | -10lgP | Mass | Length | ppm | m/z | z | RT | Fraction | Scan | Source File | Area F1 | Area F10 | Area F11 | Area F12 | Area F13 | Area F2 | Area F3 | Area F4 | Area F5 | Area F6 | Area F7 | Area F8 | Area F9 | #Feature | #Feature F1 | #Feature F10 | #Feature F11 | #Feature F12 | #Feature F13 | #Feature F2 | #Feature F3 | #Feature F4 | #Feature F5 | #Feature F6 | #Feature F7 | #Feature F8 | #Feature F9 | Start | End | PTM | AScore | Found By |
| --- | --- | --- | --- | --- | --- | --- | --- | --- | --- | --- | --- | --- | --- | --- | --- | --- | --- | --- | --- | --- | --- | --- | --- | --- | --- | --- | --- | --- | --- | --- | --- | --- | --- | --- | --- | --- | --- | --- | --- | --- | --- | --- | --- |
| R.VAKDDC(+57.02)DLPELC(+57.02)TGQSAEC(+57.02)PTDSLQR.N | Y | 95.50 | 2964.2898 | 26 | 0.7 | 989.1046 | 3 | 43.19 | 9 | F9:25736 | NaNaKA16\_F5.raw |  |  |  |  |  |  |  |  | 5.0308E7 | 1.4807E8 |  |  |  | 2 | 0 | 0 | 0 | 0 | 0 | 0 | 0 | 0 | 1 | 1 | 0 | 0 | 0 | 451 | 476 | Carbamidomethylation | C6:Carbamidomethylation:1000.00;C12:Carbamidomethylation:1000.00;C19:Carbamidomethylation:1000.00 | PEAKS DB |
| R.TAPAFQFSSC(+57.02)SIR.E | N | 80.42 | 1470.6925 | 13 | 0.6 | 736.3539 | 2 | 46.94 | 4 | F4:27572 | NaNaKA16\_F12.raw |  | 7.7695E6 | 7.0106E6 | 6.7741E6 | 7.3303E5 |  |  |  |  |  |  |  | 2.6381E6 | 5 | 0 | 1 | 1 | 1 | 1 | 0 | 0 | 0 | 0 | 0 | 0 | 0 | 1 | 370 | 382 | Carbamidomethylation | C10:Carbamidomethylation:1000.00 | PEAKS DB |
| D.LPELC(+57.02)TGQSAEC(+57.02)PTDSLQR.N | N | 78.21 | 2160.9780 | 19 | 0.3 | 1081.4966 | 2 | 44.94 | 10 | F10:28461 | NaNaKA16\_F6.raw |  |  |  |  |  |  |  |  |  | 2.2687E6 |  |  |  | 1 | 0 | 0 | 0 | 0 | 0 | 0 | 0 | 0 | 0 | 1 | 0 | 0 | 0 | 458 | 476 | Carbamidomethylation | C5:Carbamidomethylation:1000.00;C12:Carbamidomethylation:1000.00 | PEAKS DB |
| K.DDC(+57.02)DLPELC(+57.02)TGQSAEC(+57.02)PTDSLQR.N | Y | 68.03 | 2666.0894 | 23 | -1.2 | 1334.0503 | 2 | 58.77 | 10 | F10:39904 | NaNaKA16\_F6.raw |  |  |  | 6.6603E6 |  | 3.9675E6 | 5.5621E5 |  | 6.4952E6 | 2.3844E7 |  |  |  | 9 | 0 | 0 | 0 | 2 | 0 | 2 | 1 | 0 | 2 | 2 | 0 | 0 | 0 | 454 | 476 | Carbamidomethylation | C3:Carbamidomethylation:1000.00;C9:Carbamidomethylation:1000.00;C16:Carbamidomethylation:1000.00 | PEAKS DB |
| R.VYEMINAVNTK.F | N | 67.13 | 1280.6434 | 11 | -0.4 | 641.3287 | 2 | 34.28 | 4 | F4:17180 | NaNaKA16\_F12.raw |  |  |  | 2.766E6 | 2.8909E6 |  |  |  |  |  |  |  |  | 2 | 0 | 0 | 0 | 1 | 1 | 0 | 0 | 0 | 0 | 0 | 0 | 0 | 0 | 232 | 242 |  |  | PEAKS DB |
| A.DSSAVISAC(+57.02)DGLK.G | N | 60.11 | 1321.6184 | 13 | 0.4 | 661.8168 | 2 | 33.21 | 10 | F10:19042 | NaNaKA16\_F6.raw |  |  |  |  |  |  |  |  |  | 6.9505E6 |  |  |  | 1 | 0 | 0 | 0 | 0 | 0 | 0 | 0 | 0 | 0 | 1 | 0 | 0 | 0 | 119 | 131 | Carbamidomethylation | C9:Carbamidomethylation:1000.00 | PEAKS DB |
| K.GQC(+57.02)VDVQTAY | N | 56.58 | 1139.4917 | 10 | 0.2 | 570.7532 | 2 | 32.85 | 10 | F10:17935 | NaNaKA16\_F6.raw |  |  |  |  |  |  |  |  |  | 2.4491E8 |  |  |  | 1 | 0 | 0 | 0 | 0 | 0 | 0 | 0 | 0 | 0 | 1 | 0 | 0 | 0 | 584 | 593 | Carbamidomethylation | C3:Carbamidomethylation:1000.00 | PEAKS DB |
| H.IALIGLEIWSNK.D | N | 54.66 | 1355.7812 | 12 | 0.3 | 678.8981 | 2 | 85.43 | 4 | F4:59423 | NaNaKA16\_F12.raw |  |  | 7.965E5 | 2.2382E6 |  |  |  |  |  |  |  |  |  | 2 | 0 | 0 | 1 | 1 | 0 | 0 | 0 | 0 | 0 | 0 | 0 | 0 | 0 | 250 | 261 |  |  | PEAKS DB |
| K.GC(+57.02)FDLNMR.G | N | 52.79 | 1011.4266 | 8 | -0.1 | 506.7205 | 2 | 36.97 | 10 | F10:21218 | NaNaKA16\_F6.raw |  |  |  |  |  |  |  |  |  | 9.5883E8 |  |  |  | 1 | 0 | 0 | 0 | 0 | 0 | 0 | 0 | 0 | 0 | 1 | 0 | 0 | 0 | 514 | 521 | Carbamidomethylation | C2:Carbamidomethylation:1000.00 | PEAKS DB |
| A.LIGLEIWSNK.D | N | 47.34 | 1171.6600 | 10 | 0.4 | 586.8375 | 2 | 69.88 | 4 | F4:46518 | NaNaKA16\_F12.raw |  |  |  | 3.0353E5 |  |  |  |  |  |  |  |  |  | 1 | 0 | 0 | 0 | 1 | 0 | 0 | 0 | 0 | 0 | 0 | 0 | 0 | 0 | 252 | 261 |  |  | PEAKS DB |
| L.IGLEIWSNK.D | N | 46.89 | 1058.5760 | 9 | -0.3 | 530.2952 | 2 | 56.37 | 4 | F4:35622 | NaNaKA16\_F12.raw |  |  |  | 6.5535E5 |  |  |  |  |  | 0 |  |  |  | 1 | 0 | 0 | 0 | 1 | 0 | 0 | 0 | 0 | 0 | 0 | 0 | 0 | 0 | 253 | 261 |  |  | PEAKS DB |
| R.DRPQC(+57.02)ILNKPLST.D | N | 46.29 | 1540.8031 | 13 | -0.4 | 514.6081 | 3 | 27.16 | 4 | F4:11109 | NaNaKA16\_F12.raw |  |  |  | 7.0589E5 |  |  |  | 8.268E5 |  |  |  |  |  | 2 | 0 | 0 | 0 | 1 | 0 | 0 | 0 | 1 | 0 | 0 | 0 | 0 | 0 | 391 | 403 | Carbamidomethylation | C5:Carbamidomethylation:1000.00 | PEAKS DB |
| I.HIALIGLEIWSNK.D | Y | 45.00 | 1492.8402 | 13 | 0.2 | 498.6208 | 3 | 70.93 | 3 | F3:45643 | NaNaKA16\_F11.raw |  | 1.7705E6 | 1.4324E6 |  |  |  |  |  |  |  |  |  |  | 2 | 0 | 1 | 1 | 0 | 0 | 0 | 0 | 0 | 0 | 0 | 0 | 0 | 0 | 249 | 261 |  |  | PEAKS DB |
| total 13 peptides |  |  |  |  |  |  |  |  |  |  |  |  |  |  |  |  |  |  |  |  |  |  |  |  |  |  |  |  |  |  |  |  |  |  |  |  |  |  |  |  |  |  |  |
| --- | --- | --- | --- | --- | --- | --- | --- | --- | --- | --- | --- | --- | --- | --- | --- | --- | --- | --- | --- | --- | --- | --- | --- | --- | --- | --- | --- | --- | --- | --- | --- | --- | --- | --- | --- | --- | --- | --- | --- | --- | --- | --- | --- |

D3TTC1.1

back to list

  

| Protein Coverage
| Supporting Peptides
|

Protein Coverage:

Supporting Peptides:

| Peptide | Uniq | -10lgP | Mass | Length | ppm | m/z | z | RT | Fraction | Scan | Source File | Area F1 | Area F10 | Area F11 | Area F12 | Area F13 | Area F2 | Area F3 | Area F4 | Area F5 | Area F6 | Area F7 | Area F8 | Area F9 | #Feature | #Feature F1 | #Feature F10 | #Feature F11 | #Feature F12 | #Feature F13 | #Feature F2 | #Feature F3 | #Feature F4 | #Feature F5 | #Feature F6 | #Feature F7 | #Feature F8 | #Feature F9 | Start | End | PTM | AScore | Found By |
| --- | --- | --- | --- | --- | --- | --- | --- | --- | --- | --- | --- | --- | --- | --- | --- | --- | --- | --- | --- | --- | --- | --- | --- | --- | --- | --- | --- | --- | --- | --- | --- | --- | --- | --- | --- | --- | --- | --- | --- | --- | --- | --- | --- |
| R.VAKDDC(+57.02)DLPELC(+57.02)TGQSAEC(+57.02)PTDSLQR.N | Y | 95.50 | 2964.2898 | 26 | 0.7 | 989.1046 | 3 | 43.19 | 9 | F9:25736 | NaNaKA16\_F5.raw |  |  |  |  |  |  |  |  | 5.0308E7 | 1.4807E8 |  |  |  | 2 | 0 | 0 | 0 | 0 | 0 | 0 | 0 | 0 | 1 | 1 | 0 | 0 | 0 | 451 | 476 | Carbamidomethylation | C6:Carbamidomethylation:1000.00;C12:Carbamidomethylation:1000.00;C19:Carbamidomethylation:1000.00 | PEAKS DB |
| R.TAPAFQFSSC(+57.02)SIR.E | N | 80.42 | 1470.6925 | 13 | 0.6 | 736.3539 | 2 | 46.94 | 4 | F4:27572 | NaNaKA16\_F12.raw |  | 7.7695E6 | 7.0106E6 | 6.7741E6 | 7.3303E5 |  |  |  |  |  |  |  | 2.6381E6 | 5 | 0 | 1 | 1 | 1 | 1 | 0 | 0 | 0 | 0 | 0 | 0 | 0 | 1 | 370 | 382 | Carbamidomethylation | C10:Carbamidomethylation:1000.00 | PEAKS DB |
| D.LPELC(+57.02)TGQSAEC(+57.02)PTDSLQR.N | N | 78.21 | 2160.9780 | 19 | 0.3 | 1081.4966 | 2 | 44.94 | 10 | F10:28461 | NaNaKA16\_F6.raw |  |  |  |  |  |  |  |  |  | 2.2687E6 |  |  |  | 1 | 0 | 0 | 0 | 0 | 0 | 0 | 0 | 0 | 0 | 1 | 0 | 0 | 0 | 458 | 476 | Carbamidomethylation | C5:Carbamidomethylation:1000.00;C12:Carbamidomethylation:1000.00 | PEAKS DB |
| K.DDC(+57.02)DLPELC(+57.02)TGQSAEC(+57.02)PTDSLQR.N | Y | 68.03 | 2666.0894 | 23 | -1.2 | 1334.0503 | 2 | 58.77 | 10 | F10:39904 | NaNaKA16\_F6.raw |  |  |  | 6.6603E6 |  | 3.9675E6 | 5.5621E5 |  | 6.4952E6 | 2.3844E7 |  |  |  | 9 | 0 | 0 | 0 | 2 | 0 | 2 | 1 | 0 | 2 | 2 | 0 | 0 | 0 | 454 | 476 | Carbamidomethylation | C3:Carbamidomethylation:1000.00;C9:Carbamidomethylation:1000.00;C16:Carbamidomethylation:1000.00 | PEAKS DB |
| R.VYEMINAVNTK.F | N | 67.13 | 1280.6434 | 11 | -0.4 | 641.3287 | 2 | 34.28 | 4 | F4:17180 | NaNaKA16\_F12.raw |  |  |  | 2.766E6 | 2.8909E6 |  |  |  |  |  |  |  |  | 2 | 0 | 0 | 0 | 1 | 1 | 0 | 0 | 0 | 0 | 0 | 0 | 0 | 0 | 232 | 242 |  |  | PEAKS DB |
| A.DSSAVISAC(+57.02)DGLK.G | N | 60.11 | 1321.6184 | 13 | 0.4 | 661.8168 | 2 | 33.21 | 10 | F10:19042 | NaNaKA16\_F6.raw |  |  |  |  |  |  |  |  |  | 6.9505E6 |  |  |  | 1 | 0 | 0 | 0 | 0 | 0 | 0 | 0 | 0 | 0 | 1 | 0 | 0 | 0 | 119 | 131 | Carbamidomethylation | C9:Carbamidomethylation:1000.00 | PEAKS DB |
| K.GQC(+57.02)VDVQTAY | N | 56.58 | 1139.4917 | 10 | 0.2 | 570.7532 | 2 | 32.85 | 10 | F10:17935 | NaNaKA16\_F6.raw |  |  |  |  |  |  |  |  |  | 2.4491E8 |  |  |  | 1 | 0 | 0 | 0 | 0 | 0 | 0 | 0 | 0 | 0 | 1 | 0 | 0 | 0 | 584 | 593 | Carbamidomethylation | C3:Carbamidomethylation:1000.00 | PEAKS DB |
| H.IALIGLEIWSNK.D | N | 54.66 | 1355.7812 | 12 | 0.3 | 678.8981 | 2 | 85.43 | 4 | F4:59423 | NaNaKA16\_F12.raw |  |  | 7.965E5 | 2.2382E6 |  |  |  |  |  |  |  |  |  | 2 | 0 | 0 | 1 | 1 | 0 | 0 | 0 | 0 | 0 | 0 | 0 | 0 | 0 | 250 | 261 |  |  | PEAKS DB |
| K.GC(+57.02)FDLNMR.G | N | 52.79 | 1011.4266 | 8 | -0.1 | 506.7205 | 2 | 36.97 | 10 | F10:21218 | NaNaKA16\_F6.raw |  |  |  |  |  |  |  |  |  | 9.5883E8 |  |  |  | 1 | 0 | 0 | 0 | 0 | 0 | 0 | 0 | 0 | 0 | 1 | 0 | 0 | 0 | 514 | 521 | Carbamidomethylation | C2:Carbamidomethylation:1000.00 | PEAKS DB |
| A.LIGLEIWSNK.D | N | 47.34 | 1171.6600 | 10 | 0.4 | 586.8375 | 2 | 69.88 | 4 | F4:46518 | NaNaKA16\_F12.raw |  |  |  | 3.0353E5 |  |  |  |  |  |  |  |  |  | 1 | 0 | 0 | 0 | 1 | 0 | 0 | 0 | 0 | 0 | 0 | 0 | 0 | 0 | 252 | 261 |  |  | PEAKS DB |
| L.IGLEIWSNK.D | N | 46.89 | 1058.5760 | 9 | -0.3 | 530.2952 | 2 | 56.37 | 4 | F4:35622 | NaNaKA16\_F12.raw |  |  |  | 6.5535E5 |  |  |  |  |  | 0 |  |  |  | 1 | 0 | 0 | 0 | 1 | 0 | 0 | 0 | 0 | 0 | 0 | 0 | 0 | 0 | 253 | 261 |  |  | PEAKS DB |
| R.DRPQC(+57.02)ILNKPLST.D | N | 46.29 | 1540.8031 | 13 | -0.4 | 514.6081 | 3 | 27.16 | 4 | F4:11109 | NaNaKA16\_F12.raw |  |  |  | 7.0589E5 |  |  |  | 8.268E5 |  |  |  |  |  | 2 | 0 | 0 | 0 | 1 | 0 | 0 | 0 | 1 | 0 | 0 | 0 | 0 | 0 | 391 | 403 | Carbamidomethylation | C5:Carbamidomethylation:1000.00 | PEAKS DB |
| I.HIALIGLEIWSNK.D | Y | 45.00 | 1492.8402 | 13 | 0.2 | 498.6208 | 3 | 70.93 | 3 | F3:45643 | NaNaKA16\_F11.raw |  | 1.7705E6 | 1.4324E6 |  |  |  |  |  |  |  |  |  |  | 2 | 0 | 1 | 1 | 0 | 0 | 0 | 0 | 0 | 0 | 0 | 0 | 0 | 0 | 249 | 261 |  |  | PEAKS DB |
| total 13 peptides |  |  |  |  |  |  |  |  |  |  |  |  |  |  |  |  |  |  |  |  |  |  |  |  |  |  |  |  |  |  |  |  |  |  |  |  |  |  |  |  |  |  |  |
| --- | --- | --- | --- | --- | --- | --- | --- | --- | --- | --- | --- | --- | --- | --- | --- | --- | --- | --- | --- | --- | --- | --- | --- | --- | --- | --- | --- | --- | --- | --- | --- | --- | --- | --- | --- | --- | --- | --- | --- | --- | --- | --- | --- |

P60044.1

back to list

  

| Protein Coverage
| Supporting Peptides
|

Protein Coverage:

Supporting Peptides:

| Peptide | Uniq | -10lgP | Mass | Length | ppm | m/z | z | RT | Fraction | Scan | Source File | Area F1 | Area F10 | Area F11 | Area F12 | Area F13 | Area F2 | Area F3 | Area F4 | Area F5 | Area F6 | Area F7 | Area F8 | Area F9 | #Feature | #Feature F1 | #Feature F10 | #Feature F11 | #Feature F12 | #Feature F13 | #Feature F2 | #Feature F3 | #Feature F4 | #Feature F5 | #Feature F6 | #Feature F7 | #Feature F8 | #Feature F9 | Start | End | PTM | AScore | Found By |
| --- | --- | --- | --- | --- | --- | --- | --- | --- | --- | --- | --- | --- | --- | --- | --- | --- | --- | --- | --- | --- | --- | --- | --- | --- | --- | --- | --- | --- | --- | --- | --- | --- | --- | --- | --- | --- | --- | --- | --- | --- | --- | --- | --- |
| S.RSWWDFADYGC(+57.02)YC(+57.02)GR.G | N | 77.58 | 1997.7937 | 15 | 1.2 | 666.9393 | 3 | 70.64 | 11 | F11:48142 | NaNaKA16\_F7.raw |  |  |  |  |  |  |  |  |  |  | 3.4823E7 | 9.6445E6 |  | 4 | 0 | 0 | 0 | 0 | 0 | 0 | 0 | 0 | 0 | 0 | 2 | 2 | 0 | 23 | 37 | Carbamidomethylation | C11:Carbamidomethylation:1000.00;C13:Carbamidomethylation:1000.00 | PEAKS DB |
| R.SWWDFADYGC(+57.02)YC(+57.02)GR.G | N | 77.10 | 1841.6926 | 14 | 2.4 | 921.8558 | 2 | 86.60 | 11 | F11:61115 | NaNaKA16\_F7.raw |  | 2.3849E7 | 3.1182E6 | 1.2353E7 | 9.152E5 |  |  |  | 6.0399E6 | 1.4952E9 | 5.4032E10 | 1.5192E10 | 2.1735E9 | 46 | 0 | 2 | 1 | 2 | 1 | 0 | 0 | 0 | 1 | 3 | 17 | 6 | 13 | 24 | 37 | Carbamidomethylation | C10:Carbamidomethylation:1000.00;C12:Carbamidomethylation:1000.00 | PEAKS DB |
| R.GGSGTPVDDLDR.C | N | 67.81 | 1187.5417 | 12 | 0.8 | 594.7786 | 2 | 19.63 | 10 | F10:6988 | NaNaKA16\_F6.raw |  |  |  |  |  |  |  |  |  | 5.7224E7 | 2.6509E9 | 3.9395E6 |  | 4 | 0 | 0 | 0 | 0 | 0 | 0 | 0 | 0 | 0 | 2 | 1 | 1 | 0 | 38 | 49 |  |  | PEAKS DB |
| R.SWWDFADYGC(+57.02)YC(+57.02)GRG.G | N | 61.68 | 1898.7141 | 15 | 1.5 | 950.3657 | 2 | 84.85 | 11 | F11:59757 | NaNaKA16\_F7.raw |  |  |  |  |  |  |  |  |  |  | 1.1046E7 |  |  | 1 | 0 | 0 | 0 | 0 | 0 | 0 | 0 | 0 | 0 | 0 | 1 | 0 | 0 | 24 | 38 | Carbamidomethylation | C10:Carbamidomethylation:1000.00;C12:Carbamidomethylation:1000.00 | PEAKS DB |
| G.GSGTPVDDLDR.C | N | 60.74 | 1130.5204 | 11 | -0.3 | 566.2673 | 2 | 21.08 | 11 | F11:8172 | NaNaKA16\_F7.raw |  |  |  |  |  |  |  |  |  | 1.4052E5 | 1.2671E7 |  |  | 2 | 0 | 0 | 0 | 0 | 0 | 0 | 0 | 0 | 0 | 1 | 1 | 0 | 0 | 39 | 49 |  |  | PEAKS DB |
| R.LAAIC(+57.02)FAGAPYNDNNYNIDLQ.A | Y | 58.95 | 2356.0793 | 21 | 4.1 | 1179.0518 | 2 | 118.61 | 11 | F11:86921 | NaNaKA16\_F7.raw |  |  |  |  |  |  |  |  |  |  | 0 |  | 1.489E6 | 1 | 0 | 0 | 0 | 0 | 0 | 0 | 0 | 0 | 0 | 0 | 0 | 0 | 1 | 102 | 122 | Carbamidomethylation | C5:Carbamidomethylation:1000.00 | PEAKS DB |
| D.FADYGC(+57.02)YC(+57.02)GR.G | N | 57.15 | 1267.4750 | 10 | 0.8 | 634.7452 | 2 | 25.42 | 11 | F11:11882 | NaNaKA16\_F7.raw |  |  |  |  |  |  |  |  |  | 2.1506E6 | 1.834E7 |  |  | 2 | 0 | 0 | 0 | 0 | 0 | 0 | 0 | 0 | 0 | 1 | 1 | 0 | 0 | 28 | 37 | Carbamidomethylation | C6:Carbamidomethylation:1000.00;C8:Carbamidomethylation:1000.00 | PEAKS DB |
| R.SWWDFADYGC(+57.02)YC(+57.02)GRGGSGTPVDDLDR.C | N | 56.86 | 3011.2239 | 26 | 0.6 | 1004.7491 | 3 | 81.63 | 11 | F11:57027 | NaNaKA16\_F7.raw |  |  |  |  |  |  |  |  |  |  | 4.9169E6 |  |  | 1 | 0 | 0 | 0 | 0 | 0 | 0 | 0 | 0 | 0 | 0 | 1 | 0 | 0 | 24 | 49 | Carbamidomethylation | C10:Carbamidomethylation:1000.00;C12:Carbamidomethylation:1000.00 | PEAKS DB |
| S.WWDFADYGC(+57.02)YC(+57.02)GR.G | N | 56.83 | 1754.6605 | 13 | 0.8 | 878.3382 | 2 | 78.92 | 12 | F12:55446 | NaNaKA16\_F8.raw |  |  |  |  |  |  |  |  |  |  | 1.3475E7 | 3.6699E6 |  | 2 | 0 | 0 | 0 | 0 | 0 | 0 | 0 | 0 | 0 | 0 | 1 | 1 | 0 | 25 | 37 | Carbamidomethylation | C9:Carbamidomethylation:1000.00;C11:Carbamidomethylation:1000.00 | PEAKS DB |
| R.LAAIC(+57.02)FAGAPYN.D | N | 53.90 | 1266.6067 | 12 | 0.8 | 634.3111 | 2 | 70.83 | 11 | F11:49747 | NaNaKA16\_F7.raw |  | 2.6932E8 | 6.3964E7 |  |  |  |  |  | 3.6922E8 | 7.2309E9 | 3.8533E9 | 2.4096E9 | 1.5572E9 | 11 | 0 | 1 | 1 | 0 | 0 | 0 | 0 | 0 | 1 | 2 | 3 | 2 | 1 | 102 | 113 | Carbamidomethylation | C5:Carbamidomethylation:1000.00 | PEAKS DB |
| W.WDFADYGC(+57.02)YC(+57.02)GR.G | N | 50.34 | 1568.5813 | 12 | 0.5 | 785.2983 | 2 | 61.41 | 11 | F11:40293 | NaNaKA16\_F7.raw |  |  |  |  |  |  |  |  |  |  | 5.6691E6 |  |  | 1 | 0 | 0 | 0 | 0 | 0 | 0 | 0 | 0 | 0 | 0 | 1 | 0 | 0 | 26 | 37 | Carbamidomethylation | C8:Carbamidomethylation:1000.00;C10:Carbamidomethylation:1000.00 | PEAKS DB |
| W.DFADYGC(+57.02)YC(+57.02)GR.G | N | 49.85 | 1382.5020 | 11 | 0.3 | 692.2585 | 2 | 40.50 | 10 | F10:24767 | NaNaKA16\_F6.raw |  |  |  |  |  |  |  |  | 5.0674E6 | 1.1493E7 | 1.4257E6 |  |  | 3 | 0 | 0 | 0 | 0 | 0 | 0 | 0 | 0 | 1 | 1 | 1 | 0 | 0 | 27 | 37 | Carbamidomethylation | C7:Carbamidomethylation:1000.00;C9:Carbamidomethylation:1000.00 | PEAKS DB |
| S.C(+57.02)TVPSRSWWDFADYGC(+57.02)YC(+57.02)GR.G | N | 48.21 | 2542.0251 | 20 | -0.3 | 848.3488 | 3 | 69.09 | 11 | F11:46749 | NaNaKA16\_F7.raw |  |  |  |  |  |  |  |  |  |  | 6.2414E6 |  |  | 1 | 0 | 0 | 0 | 0 | 0 | 0 | 0 | 0 | 0 | 0 | 1 | 0 | 0 | 18 | 37 | Carbamidomethylation | C1:Carbamidomethylation:1000.00;C16:Carbamidomethylation:1000.00;C18:Carbamidomethylation:1000.00 | PEAKS DB |
| R.SWWDFADYGC(+57.02).Y | N | 45.71 | 1305.4761 | 10 | 0.8 | 653.7458 | 2 | 109.86 | 11 | F11:80150 | NaNaKA16\_F7.raw |  |  |  |  |  |  |  |  |  |  | 6.5059E6 |  |  | 1 | 0 | 0 | 0 | 0 | 0 | 0 | 0 | 0 | 0 | 0 | 1 | 0 | 0 | 24 | 33 | Carbamidomethylation | C10:Carbamidomethylation:1000.00 | PEAKS DB |
| G.SGTPVDDLDR.C | N | 45.52 | 1073.4989 | 10 | 0.9 | 537.7572 | 2 | 20.23 | 11 | F11:7497 | NaNaKA16\_F7.raw |  |  |  |  |  |  |  |  |  |  | 1.1072E7 |  |  | 1 | 0 | 0 | 0 | 0 | 0 | 0 | 0 | 0 | 0 | 0 | 1 | 0 | 0 | 40 | 49 |  |  | PEAKS DB |
| R.SWWDFADYGC(+57.02)Y.C | N | 44.81 | 1468.5394 | 11 | 0.1 | 735.2771 | 2 | 111.77 | 10 | F10:82113 | NaNaKA16\_F6.raw |  |  |  |  |  |  |  |  |  | 1.9586E7 |  | 1.4496E7 |  | 2 | 0 | 0 | 0 | 0 | 0 | 0 | 0 | 0 | 0 | 1 | 0 | 1 | 0 | 24 | 34 | Carbamidomethylation | C10:Carbamidomethylation:1000.00 | PEAKS DB |
| total 16 peptides |  |  |  |  |  |  |  |  |  |  |  |  |  |  |  |  |  |  |  |  |  |  |  |  |  |  |  |  |  |  |  |  |  |  |  |  |  |  |  |  |  |  |  |
| --- | --- | --- | --- | --- | --- | --- | --- | --- | --- | --- | --- | --- | --- | --- | --- | --- | --- | --- | --- | --- | --- | --- | --- | --- | --- | --- | --- | --- | --- | --- | --- | --- | --- | --- | --- | --- | --- | --- | --- | --- | --- | --- | --- |

AAR00254.1

back to list

  

| Protein Coverage
| Supporting Peptides
|

Protein Coverage:

Supporting Peptides:

| Peptide | Uniq | -10lgP | Mass | Length | ppm | m/z | z | RT | Fraction | Scan | Source File | Area F1 | Area F10 | Area F11 | Area F12 | Area F13 | Area F2 | Area F3 | Area F4 | Area F5 | Area F6 | Area F7 | Area F8 | Area F9 | #Feature | #Feature F1 | #Feature F10 | #Feature F11 | #Feature F12 | #Feature F13 | #Feature F2 | #Feature F3 | #Feature F4 | #Feature F5 | #Feature F6 | #Feature F7 | #Feature F8 | #Feature F9 | Start | End | PTM | AScore | Found By |
| --- | --- | --- | --- | --- | --- | --- | --- | --- | --- | --- | --- | --- | --- | --- | --- | --- | --- | --- | --- | --- | --- | --- | --- | --- | --- | --- | --- | --- | --- | --- | --- | --- | --- | --- | --- | --- | --- | --- | --- | --- | --- | --- | --- |
| S.RSWWDFADYGC(+57.02)YC(+57.02)GR.G | N | 77.58 | 1997.7937 | 15 | 1.2 | 666.9393 | 3 | 70.64 | 11 | F11:48142 | NaNaKA16\_F7.raw |  |  |  |  |  |  |  |  |  |  | 3.4823E7 | 9.6445E6 |  | 4 | 0 | 0 | 0 | 0 | 0 | 0 | 0 | 0 | 0 | 0 | 2 | 2 | 0 | 23 | 37 | Carbamidomethylation | C11:Carbamidomethylation:1000.00;C13:Carbamidomethylation:1000.00 | PEAKS DB |
| R.SWWDFADYGC(+57.02)YC(+57.02)GR.G | N | 77.10 | 1841.6926 | 14 | 2.4 | 921.8558 | 2 | 86.60 | 11 | F11:61115 | NaNaKA16\_F7.raw |  | 2.3849E7 | 3.1182E6 | 1.2353E7 | 9.152E5 |  |  |  | 6.0399E6 | 1.4952E9 | 5.4032E10 | 1.5192E10 | 2.1735E9 | 46 | 0 | 2 | 1 | 2 | 1 | 0 | 0 | 0 | 1 | 3 | 17 | 6 | 13 | 24 | 37 | Carbamidomethylation | C10:Carbamidomethylation:1000.00;C12:Carbamidomethylation:1000.00 | PEAKS DB |
| R.GGSGTPVDDLDR.C | N | 67.81 | 1187.5417 | 12 | 0.8 | 594.7786 | 2 | 19.63 | 10 | F10:6988 | NaNaKA16\_F6.raw |  |  |  |  |  |  |  |  |  | 5.7224E7 | 2.6509E9 | 3.9395E6 |  | 4 | 0 | 0 | 0 | 0 | 0 | 0 | 0 | 0 | 0 | 2 | 1 | 1 | 0 | 38 | 49 |  |  | PEAKS DB |
| R.SWWDFADYGC(+57.02)YC(+57.02)GRG.G | N | 61.68 | 1898.7141 | 15 | 1.5 | 950.3657 | 2 | 84.85 | 11 | F11:59757 | NaNaKA16\_F7.raw |  |  |  |  |  |  |  |  |  |  | 1.1046E7 |  |  | 1 | 0 | 0 | 0 | 0 | 0 | 0 | 0 | 0 | 0 | 0 | 1 | 0 | 0 | 24 | 38 | Carbamidomethylation | C10:Carbamidomethylation:1000.00;C12:Carbamidomethylation:1000.00 | PEAKS DB |
| G.GSGTPVDDLDR.C | N | 60.74 | 1130.5204 | 11 | -0.3 | 566.2673 | 2 | 21.08 | 11 | F11:8172 | NaNaKA16\_F7.raw |  |  |  |  |  |  |  |  |  | 1.4052E5 | 1.2671E7 |  |  | 2 | 0 | 0 | 0 | 0 | 0 | 0 | 0 | 0 | 0 | 1 | 1 | 0 | 0 | 39 | 49 |  |  | PEAKS DB |
| R.LAAIC(+57.02)FAGAPYNDNNYNIDLQ.A | Y | 58.95 | 2356.0793 | 21 | 4.1 | 1179.0518 | 2 | 118.61 | 11 | F11:86921 | NaNaKA16\_F7.raw |  |  |  |  |  |  |  |  |  |  | 0 |  | 1.489E6 | 1 | 0 | 0 | 0 | 0 | 0 | 0 | 0 | 0 | 0 | 0 | 0 | 0 | 1 | 102 | 122 | Carbamidomethylation | C5:Carbamidomethylation:1000.00 | PEAKS DB |
| D.FADYGC(+57.02)YC(+57.02)GR.G | N | 57.15 | 1267.4750 | 10 | 0.8 | 634.7452 | 2 | 25.42 | 11 | F11:11882 | NaNaKA16\_F7.raw |  |  |  |  |  |  |  |  |  | 2.1506E6 | 1.834E7 |  |  | 2 | 0 | 0 | 0 | 0 | 0 | 0 | 0 | 0 | 0 | 1 | 1 | 0 | 0 | 28 | 37 | Carbamidomethylation | C6:Carbamidomethylation:1000.00;C8:Carbamidomethylation:1000.00 | PEAKS DB |
| R.SWWDFADYGC(+57.02)YC(+57.02)GRGGSGTPVDDLDR.C | N | 56.86 | 3011.2239 | 26 | 0.6 | 1004.7491 | 3 | 81.63 | 11 | F11:57027 | NaNaKA16\_F7.raw |  |  |  |  |  |  |  |  |  |  | 4.9169E6 |  |  | 1 | 0 | 0 | 0 | 0 | 0 | 0 | 0 | 0 | 0 | 0 | 1 | 0 | 0 | 24 | 49 | Carbamidomethylation | C10:Carbamidomethylation:1000.00;C12:Carbamidomethylation:1000.00 | PEAKS DB |
| S.WWDFADYGC(+57.02)YC(+57.02)GR.G | N | 56.83 | 1754.6605 | 13 | 0.8 | 878.3382 | 2 | 78.92 | 12 | F12:55446 | NaNaKA16\_F8.raw |  |  |  |  |  |  |  |  |  |  | 1.3475E7 | 3.6699E6 |  | 2 | 0 | 0 | 0 | 0 | 0 | 0 | 0 | 0 | 0 | 0 | 1 | 1 | 0 | 25 | 37 | Carbamidomethylation | C9:Carbamidomethylation:1000.00;C11:Carbamidomethylation:1000.00 | PEAKS DB |
| R.LAAIC(+57.02)FAGAPYN.D | N | 53.90 | 1266.6067 | 12 | 0.8 | 634.3111 | 2 | 70.83 | 11 | F11:49747 | NaNaKA16\_F7.raw |  | 2.6932E8 | 6.3964E7 |  |  |  |  |  | 3.6922E8 | 7.2309E9 | 3.8533E9 | 2.4096E9 | 1.5572E9 | 11 | 0 | 1 | 1 | 0 | 0 | 0 | 0 | 0 | 1 | 2 | 3 | 2 | 1 | 102 | 113 | Carbamidomethylation | C5:Carbamidomethylation:1000.00 | PEAKS DB |
| W.WDFADYGC(+57.02)YC(+57.02)GR.G | N | 50.34 | 1568.5813 | 12 | 0.5 | 785.2983 | 2 | 61.41 | 11 | F11:40293 | NaNaKA16\_F7.raw |  |  |  |  |  |  |  |  |  |  | 5.6691E6 |  |  | 1 | 0 | 0 | 0 | 0 | 0 | 0 | 0 | 0 | 0 | 0 | 1 | 0 | 0 | 26 | 37 | Carbamidomethylation | C8:Carbamidomethylation:1000.00;C10:Carbamidomethylation:1000.00 | PEAKS DB |
| W.DFADYGC(+57.02)YC(+57.02)GR.G | N | 49.85 | 1382.5020 | 11 | 0.3 | 692.2585 | 2 | 40.50 | 10 | F10:24767 | NaNaKA16\_F6.raw |  |  |  |  |  |  |  |  | 5.0674E6 | 1.1493E7 | 1.4257E6 |  |  | 3 | 0 | 0 | 0 | 0 | 0 | 0 | 0 | 0 | 1 | 1 | 1 | 0 | 0 | 27 | 37 | Carbamidomethylation | C7:Carbamidomethylation:1000.00;C9:Carbamidomethylation:1000.00 | PEAKS DB |
| S.C(+57.02)TVPSRSWWDFADYGC(+57.02)YC(+57.02)GR.G | N | 48.21 | 2542.0251 | 20 | -0.3 | 848.3488 | 3 | 69.09 | 11 | F11:46749 | NaNaKA16\_F7.raw |  |  |  |  |  |  |  |  |  |  | 6.2414E6 |  |  | 1 | 0 | 0 | 0 | 0 | 0 | 0 | 0 | 0 | 0 | 0 | 1 | 0 | 0 | 18 | 37 | Carbamidomethylation | C1:Carbamidomethylation:1000.00;C16:Carbamidomethylation:1000.00;C18:Carbamidomethylation:1000.00 | PEAKS DB |
| R.SWWDFADYGC(+57.02).Y | N | 45.71 | 1305.4761 | 10 | 0.8 | 653.7458 | 2 | 109.86 | 11 | F11:80150 | NaNaKA16\_F7.raw |  |  |  |  |  |  |  |  |  |  | 6.5059E6 |  |  | 1 | 0 | 0 | 0 | 0 | 0 | 0 | 0 | 0 | 0 | 0 | 1 | 0 | 0 | 24 | 33 | Carbamidomethylation | C10:Carbamidomethylation:1000.00 | PEAKS DB |
| G.SGTPVDDLDR.C | N | 45.52 | 1073.4989 | 10 | 0.9 | 537.7572 | 2 | 20.23 | 11 | F11:7497 | NaNaKA16\_F7.raw |  |  |  |  |  |  |  |  |  |  | 1.1072E7 |  |  | 1 | 0 | 0 | 0 | 0 | 0 | 0 | 0 | 0 | 0 | 0 | 1 | 0 | 0 | 40 | 49 |  |  | PEAKS DB |
| R.SWWDFADYGC(+57.02)Y.C | N | 44.81 | 1468.5394 | 11 | 0.1 | 735.2771 | 2 | 111.77 | 10 | F10:82113 | NaNaKA16\_F6.raw |  |  |  |  |  |  |  |  |  | 1.9586E7 |  | 1.4496E7 |  | 2 | 0 | 0 | 0 | 0 | 0 | 0 | 0 | 0 | 0 | 1 | 0 | 1 | 0 | 24 | 34 | Carbamidomethylation | C10:Carbamidomethylation:1000.00 | PEAKS DB |
| total 16 peptides |  |  |  |  |  |  |  |  |  |  |  |  |  |  |  |  |  |  |  |  |  |  |  |  |  |  |  |  |  |  |  |  |  |  |  |  |  |  |  |  |  |  |  |
| --- | --- | --- | --- | --- | --- | --- | --- | --- | --- | --- | --- | --- | --- | --- | --- | --- | --- | --- | --- | --- | --- | --- | --- | --- | --- | --- | --- | --- | --- | --- | --- | --- | --- | --- | --- | --- | --- | --- | --- | --- | --- | --- | --- |

1XXW

back to list

  

| Protein Coverage
| Supporting Peptides
|

Protein Coverage:

Supporting Peptides:

| Peptide | Uniq | -10lgP | Mass | Length | ppm | m/z | z | RT | Fraction | Scan | Source File | Area F1 | Area F10 | Area F11 | Area F12 | Area F13 | Area F2 | Area F3 | Area F4 | Area F5 | Area F6 | Area F7 | Area F8 | Area F9 | #Feature | #Feature F1 | #Feature F10 | #Feature F11 | #Feature F12 | #Feature F13 | #Feature F2 | #Feature F3 | #Feature F4 | #Feature F5 | #Feature F6 | #Feature F7 | #Feature F8 | #Feature F9 | Start | End | PTM | AScore | Found By |
| --- | --- | --- | --- | --- | --- | --- | --- | --- | --- | --- | --- | --- | --- | --- | --- | --- | --- | --- | --- | --- | --- | --- | --- | --- | --- | --- | --- | --- | --- | --- | --- | --- | --- | --- | --- | --- | --- | --- | --- | --- | --- | --- | --- |
| S.RSWWDFADYGC(+57.02)YC(+57.02)GR.G | N | 77.58 | 1997.7937 | 15 | 1.2 | 666.9393 | 3 | 70.64 | 11 | F11:48142 | NaNaKA16\_F7.raw |  |  |  |  |  |  |  |  |  |  | 3.4823E7 | 9.6445E6 |  | 4 | 0 | 0 | 0 | 0 | 0 | 0 | 0 | 0 | 0 | 0 | 2 | 2 | 0 | 16 | 30 | Carbamidomethylation | C11:Carbamidomethylation:1000.00;C13:Carbamidomethylation:1000.00 | PEAKS DB |
| R.SWWDFADYGC(+57.02)YC(+57.02)GR.G | N | 77.10 | 1841.6926 | 14 | 2.4 | 921.8558 | 2 | 86.60 | 11 | F11:61115 | NaNaKA16\_F7.raw |  | 2.3849E7 | 3.1182E6 | 1.2353E7 | 9.152E5 |  |  |  | 6.0399E6 | 1.4952E9 | 5.4032E10 | 1.5192E10 | 2.1735E9 | 46 | 0 | 2 | 1 | 2 | 1 | 0 | 0 | 0 | 1 | 3 | 17 | 6 | 13 | 17 | 30 | Carbamidomethylation | C10:Carbamidomethylation:1000.00;C12:Carbamidomethylation:1000.00 | PEAKS DB |
| R.GGSGTPVDDLDR.C | N | 67.81 | 1187.5417 | 12 | 0.8 | 594.7786 | 2 | 19.63 | 10 | F10:6988 | NaNaKA16\_F6.raw |  |  |  |  |  |  |  |  |  | 5.7224E7 | 2.6509E9 | 3.9395E6 |  | 4 | 0 | 0 | 0 | 0 | 0 | 0 | 0 | 0 | 0 | 2 | 1 | 1 | 0 | 31 | 42 |  |  | PEAKS DB |
| R.SWWDFADYGC(+57.02)YC(+57.02)GRG.G | N | 61.68 | 1898.7141 | 15 | 1.5 | 950.3657 | 2 | 84.85 | 11 | F11:59757 | NaNaKA16\_F7.raw |  |  |  |  |  |  |  |  |  |  | 1.1046E7 |  |  | 1 | 0 | 0 | 0 | 0 | 0 | 0 | 0 | 0 | 0 | 0 | 1 | 0 | 0 | 17 | 31 | Carbamidomethylation | C10:Carbamidomethylation:1000.00;C12:Carbamidomethylation:1000.00 | PEAKS DB |
| G.GSGTPVDDLDR.C | N | 60.74 | 1130.5204 | 11 | -0.3 | 566.2673 | 2 | 21.08 | 11 | F11:8172 | NaNaKA16\_F7.raw |  |  |  |  |  |  |  |  |  | 1.4052E5 | 1.2671E7 |  |  | 2 | 0 | 0 | 0 | 0 | 0 | 0 | 0 | 0 | 0 | 1 | 1 | 0 | 0 | 32 | 42 |  |  | PEAKS DB |
| R.LAAIC(+57.02)FAGAPYNDNNYNIDLQ.A | Y | 58.95 | 2356.0793 | 21 | 4.1 | 1179.0518 | 2 | 118.61 | 11 | F11:86921 | NaNaKA16\_F7.raw |  |  |  |  |  |  |  |  |  |  | 0 |  | 1.489E6 | 1 | 0 | 0 | 0 | 0 | 0 | 0 | 0 | 0 | 0 | 0 | 0 | 0 | 1 | 95 | 115 | Carbamidomethylation | C5:Carbamidomethylation:1000.00 | PEAKS DB |
| D.FADYGC(+57.02)YC(+57.02)GR.G | N | 57.15 | 1267.4750 | 10 | 0.8 | 634.7452 | 2 | 25.42 | 11 | F11:11882 | NaNaKA16\_F7.raw |  |  |  |  |  |  |  |  |  | 2.1506E6 | 1.834E7 |  |  | 2 | 0 | 0 | 0 | 0 | 0 | 0 | 0 | 0 | 0 | 1 | 1 | 0 | 0 | 21 | 30 | Carbamidomethylation | C6:Carbamidomethylation:1000.00;C8:Carbamidomethylation:1000.00 | PEAKS DB |
| R.SWWDFADYGC(+57.02)YC(+57.02)GRGGSGTPVDDLDR.C | N | 56.86 | 3011.2239 | 26 | 0.6 | 1004.7491 | 3 | 81.63 | 11 | F11:57027 | NaNaKA16\_F7.raw |  |  |  |  |  |  |  |  |  |  | 4.9169E6 |  |  | 1 | 0 | 0 | 0 | 0 | 0 | 0 | 0 | 0 | 0 | 0 | 1 | 0 | 0 | 17 | 42 | Carbamidomethylation | C10:Carbamidomethylation:1000.00;C12:Carbamidomethylation:1000.00 | PEAKS DB |
| S.WWDFADYGC(+57.02)YC(+57.02)GR.G | N | 56.83 | 1754.6605 | 13 | 0.8 | 878.3382 | 2 | 78.92 | 12 | F12:55446 | NaNaKA16\_F8.raw |  |  |  |  |  |  |  |  |  |  | 1.3475E7 | 3.6699E6 |  | 2 | 0 | 0 | 0 | 0 | 0 | 0 | 0 | 0 | 0 | 0 | 1 | 1 | 0 | 18 | 30 | Carbamidomethylation | C9:Carbamidomethylation:1000.00;C11:Carbamidomethylation:1000.00 | PEAKS DB |
| R.LAAIC(+57.02)FAGAPYN.D | N | 53.90 | 1266.6067 | 12 | 0.8 | 634.3111 | 2 | 70.83 | 11 | F11:49747 | NaNaKA16\_F7.raw |  | 2.6932E8 | 6.3964E7 |  |  |  |  |  | 3.6922E8 | 7.2309E9 | 3.8533E9 | 2.4096E9 | 1.5572E9 | 11 | 0 | 1 | 1 | 0 | 0 | 0 | 0 | 0 | 1 | 2 | 3 | 2 | 1 | 95 | 106 | Carbamidomethylation | C5:Carbamidomethylation:1000.00 | PEAKS DB |
| W.WDFADYGC(+57.02)YC(+57.02)GR.G | N | 50.34 | 1568.5813 | 12 | 0.5 | 785.2983 | 2 | 61.41 | 11 | F11:40293 | NaNaKA16\_F7.raw |  |  |  |  |  |  |  |  |  |  | 5.6691E6 |  |  | 1 | 0 | 0 | 0 | 0 | 0 | 0 | 0 | 0 | 0 | 0 | 1 | 0 | 0 | 19 | 30 | Carbamidomethylation | C8:Carbamidomethylation:1000.00;C10:Carbamidomethylation:1000.00 | PEAKS DB |
| W.DFADYGC(+57.02)YC(+57.02)GR.G | N | 49.85 | 1382.5020 | 11 | 0.3 | 692.2585 | 2 | 40.50 | 10 | F10:24767 | NaNaKA16\_F6.raw |  |  |  |  |  |  |  |  | 5.0674E6 | 1.1493E7 | 1.4257E6 |  |  | 3 | 0 | 0 | 0 | 0 | 0 | 0 | 0 | 0 | 1 | 1 | 1 | 0 | 0 | 20 | 30 | Carbamidomethylation | C7:Carbamidomethylation:1000.00;C9:Carbamidomethylation:1000.00 | PEAKS DB |
| S.C(+57.02)TVPSRSWWDFADYGC(+57.02)YC(+57.02)GR.G | N | 48.21 | 2542.0251 | 20 | -0.3 | 848.3488 | 3 | 69.09 | 11 | F11:46749 | NaNaKA16\_F7.raw |  |  |  |  |  |  |  |  |  |  | 6.2414E6 |  |  | 1 | 0 | 0 | 0 | 0 | 0 | 0 | 0 | 0 | 0 | 0 | 1 | 0 | 0 | 11 | 30 | Carbamidomethylation | C1:Carbamidomethylation:1000.00;C16:Carbamidomethylation:1000.00;C18:Carbamidomethylation:1000.00 | PEAKS DB |
| R.SWWDFADYGC(+57.02).Y | N | 45.71 | 1305.4761 | 10 | 0.8 | 653.7458 | 2 | 109.86 | 11 | F11:80150 | NaNaKA16\_F7.raw |  |  |  |  |  |  |  |  |  |  | 6.5059E6 |  |  | 1 | 0 | 0 | 0 | 0 | 0 | 0 | 0 | 0 | 0 | 0 | 1 | 0 | 0 | 17 | 26 | Carbamidomethylation | C10:Carbamidomethylation:1000.00 | PEAKS DB |
| G.SGTPVDDLDR.C | N | 45.52 | 1073.4989 | 10 | 0.9 | 537.7572 | 2 | 20.23 | 11 | F11:7497 | NaNaKA16\_F7.raw |  |  |  |  |  |  |  |  |  |  | 1.1072E7 |  |  | 1 | 0 | 0 | 0 | 0 | 0 | 0 | 0 | 0 | 0 | 0 | 1 | 0 | 0 | 33 | 42 |  |  | PEAKS DB |
| R.SWWDFADYGC(+57.02)Y.C | N | 44.81 | 1468.5394 | 11 | 0.1 | 735.2771 | 2 | 111.77 | 10 | F10:82113 | NaNaKA16\_F6.raw |  |  |  |  |  |  |  |  |  | 1.9586E7 |  | 1.4496E7 |  | 2 | 0 | 0 | 0 | 0 | 0 | 0 | 0 | 0 | 0 | 1 | 0 | 1 | 0 | 17 | 27 | Carbamidomethylation | C10:Carbamidomethylation:1000.00 | PEAKS DB |
| total 16 peptides |  |  |  |  |  |  |  |  |  |  |  |  |  |  |  |  |  |  |  |  |  |  |  |  |  |  |  |  |  |  |  |  |  |  |  |  |  |  |  |  |  |  |  |
| --- | --- | --- | --- | --- | --- | --- | --- | --- | --- | --- | --- | --- | --- | --- | --- | --- | --- | --- | --- | --- | --- | --- | --- | --- | --- | --- | --- | --- | --- | --- | --- | --- | --- | --- | --- | --- | --- | --- | --- | --- | --- | --- | --- |

1S6B

back to list

  

| Protein Coverage
| Supporting Peptides
|

Protein Coverage:

Supporting Peptides:

| Peptide | Uniq | -10lgP | Mass | Length | ppm | m/z | z | RT | Fraction | Scan | Source File | Area F1 | Area F10 | Area F11 | Area F12 | Area F13 | Area F2 | Area F3 | Area F4 | Area F5 | Area F6 | Area F7 | Area F8 | Area F9 | #Feature | #Feature F1 | #Feature F10 | #Feature F11 | #Feature F12 | #Feature F13 | #Feature F2 | #Feature F3 | #Feature F4 | #Feature F5 | #Feature F6 | #Feature F7 | #Feature F8 | #Feature F9 | Start | End | PTM | AScore | Found By |
| --- | --- | --- | --- | --- | --- | --- | --- | --- | --- | --- | --- | --- | --- | --- | --- | --- | --- | --- | --- | --- | --- | --- | --- | --- | --- | --- | --- | --- | --- | --- | --- | --- | --- | --- | --- | --- | --- | --- | --- | --- | --- | --- | --- |
| S.RSWWDFADYGC(+57.02)YC(+57.02)GR.G | N | 77.58 | 1997.7937 | 15 | 1.2 | 666.9393 | 3 | 70.64 | 11 | F11:48142 | NaNaKA16\_F7.raw |  |  |  |  |  |  |  |  |  |  | 3.4823E7 | 9.6445E6 |  | 4 | 0 | 0 | 0 | 0 | 0 | 0 | 0 | 0 | 0 | 0 | 2 | 2 | 0 | 16 | 30 | Carbamidomethylation | C11:Carbamidomethylation:1000.00;C13:Carbamidomethylation:1000.00 | PEAKS DB |
| R.SWWDFADYGC(+57.02)YC(+57.02)GR.G | N | 77.10 | 1841.6926 | 14 | 2.4 | 921.8558 | 2 | 86.60 | 11 | F11:61115 | NaNaKA16\_F7.raw |  | 2.3849E7 | 3.1182E6 | 1.2353E7 | 9.152E5 |  |  |  | 6.0399E6 | 1.4952E9 | 5.4032E10 | 1.5192E10 | 2.1735E9 | 46 | 0 | 2 | 1 | 2 | 1 | 0 | 0 | 0 | 1 | 3 | 17 | 6 | 13 | 17 | 30 | Carbamidomethylation | C10:Carbamidomethylation:1000.00;C12:Carbamidomethylation:1000.00 | PEAKS DB |
| R.GGSGTPVDDLDR.C | N | 67.81 | 1187.5417 | 12 | 0.8 | 594.7786 | 2 | 19.63 | 10 | F10:6988 | NaNaKA16\_F6.raw |  |  |  |  |  |  |  |  |  | 5.7224E7 | 2.6509E9 | 3.9395E6 |  | 4 | 0 | 0 | 0 | 0 | 0 | 0 | 0 | 0 | 0 | 2 | 1 | 1 | 0 | 31 | 42 |  |  | PEAKS DB |
| R.SWWDFADYGC(+57.02)YC(+57.02)GRG.G | N | 61.68 | 1898.7141 | 15 | 1.5 | 950.3657 | 2 | 84.85 | 11 | F11:59757 | NaNaKA16\_F7.raw |  |  |  |  |  |  |  |  |  |  | 1.1046E7 |  |  | 1 | 0 | 0 | 0 | 0 | 0 | 0 | 0 | 0 | 0 | 0 | 1 | 0 | 0 | 17 | 31 | Carbamidomethylation | C10:Carbamidomethylation:1000.00;C12:Carbamidomethylation:1000.00 | PEAKS DB |
| G.GSGTPVDDLDR.C | N | 60.74 | 1130.5204 | 11 | -0.3 | 566.2673 | 2 | 21.08 | 11 | F11:8172 | NaNaKA16\_F7.raw |  |  |  |  |  |  |  |  |  | 1.4052E5 | 1.2671E7 |  |  | 2 | 0 | 0 | 0 | 0 | 0 | 0 | 0 | 0 | 0 | 1 | 1 | 0 | 0 | 32 | 42 |  |  | PEAKS DB |
| R.LAAIC(+57.02)FAGAPYNDNNYNIDLQ.A | Y | 58.95 | 2356.0793 | 21 | 4.1 | 1179.0518 | 2 | 118.61 | 11 | F11:86921 | NaNaKA16\_F7.raw |  |  |  |  |  |  |  |  |  |  | 0 |  | 1.489E6 | 1 | 0 | 0 | 0 | 0 | 0 | 0 | 0 | 0 | 0 | 0 | 0 | 0 | 1 | 95 | 115 | Carbamidomethylation | C5:Carbamidomethylation:1000.00 | PEAKS DB |
| D.FADYGC(+57.02)YC(+57.02)GR.G | N | 57.15 | 1267.4750 | 10 | 0.8 | 634.7452 | 2 | 25.42 | 11 | F11:11882 | NaNaKA16\_F7.raw |  |  |  |  |  |  |  |  |  | 2.1506E6 | 1.834E7 |  |  | 2 | 0 | 0 | 0 | 0 | 0 | 0 | 0 | 0 | 0 | 1 | 1 | 0 | 0 | 21 | 30 | Carbamidomethylation | C6:Carbamidomethylation:1000.00;C8:Carbamidomethylation:1000.00 | PEAKS DB |
| R.SWWDFADYGC(+57.02)YC(+57.02)GRGGSGTPVDDLDR.C | N | 56.86 | 3011.2239 | 26 | 0.6 | 1004.7491 | 3 | 81.63 | 11 | F11:57027 | NaNaKA16\_F7.raw |  |  |  |  |  |  |  |  |  |  | 4.9169E6 |  |  | 1 | 0 | 0 | 0 | 0 | 0 | 0 | 0 | 0 | 0 | 0 | 1 | 0 | 0 | 17 | 42 | Carbamidomethylation | C10:Carbamidomethylation:1000.00;C12:Carbamidomethylation:1000.00 | PEAKS DB |
| S.WWDFADYGC(+57.02)YC(+57.02)GR.G | N | 56.83 | 1754.6605 | 13 | 0.8 | 878.3382 | 2 | 78.92 | 12 | F12:55446 | NaNaKA16\_F8.raw |  |  |  |  |  |  |  |  |  |  | 1.3475E7 | 3.6699E6 |  | 2 | 0 | 0 | 0 | 0 | 0 | 0 | 0 | 0 | 0 | 0 | 1 | 1 | 0 | 18 | 30 | Carbamidomethylation | C9:Carbamidomethylation:1000.00;C11:Carbamidomethylation:1000.00 | PEAKS DB |
| R.LAAIC(+57.02)FAGAPYN.D | N | 53.90 | 1266.6067 | 12 | 0.8 | 634.3111 | 2 | 70.83 | 11 | F11:49747 | NaNaKA16\_F7.raw |  | 2.6932E8 | 6.3964E7 |  |  |  |  |  | 3.6922E8 | 7.2309E9 | 3.8533E9 | 2.4096E9 | 1.5572E9 | 11 | 0 | 1 | 1 | 0 | 0 | 0 | 0 | 0 | 1 | 2 | 3 | 2 | 1 | 95 | 106 | Carbamidomethylation | C5:Carbamidomethylation:1000.00 | PEAKS DB |
| W.WDFADYGC(+57.02)YC(+57.02)GR.G | N | 50.34 | 1568.5813 | 12 | 0.5 | 785.2983 | 2 | 61.41 | 11 | F11:40293 | NaNaKA16\_F7.raw |  |  |  |  |  |  |  |  |  |  | 5.6691E6 |  |  | 1 | 0 | 0 | 0 | 0 | 0 | 0 | 0 | 0 | 0 | 0 | 1 | 0 | 0 | 19 | 30 | Carbamidomethylation | C8:Carbamidomethylation:1000.00;C10:Carbamidomethylation:1000.00 | PEAKS DB |
| W.DFADYGC(+57.02)YC(+57.02)GR.G | N | 49.85 | 1382.5020 | 11 | 0.3 | 692.2585 | 2 | 40.50 | 10 | F10:24767 | NaNaKA16\_F6.raw |  |  |  |  |  |  |  |  | 5.0674E6 | 1.1493E7 | 1.4257E6 |  |  | 3 | 0 | 0 | 0 | 0 | 0 | 0 | 0 | 0 | 1 | 1 | 1 | 0 | 0 | 20 | 30 | Carbamidomethylation | C7:Carbamidomethylation:1000.00;C9:Carbamidomethylation:1000.00 | PEAKS DB |
| S.C(+57.02)TVPSRSWWDFADYGC(+57.02)YC(+57.02)GR.G | N | 48.21 | 2542.0251 | 20 | -0.3 | 848.3488 | 3 | 69.09 | 11 | F11:46749 | NaNaKA16\_F7.raw |  |  |  |  |  |  |  |  |  |  | 6.2414E6 |  |  | 1 | 0 | 0 | 0 | 0 | 0 | 0 | 0 | 0 | 0 | 0 | 1 | 0 | 0 | 11 | 30 | Carbamidomethylation | C1:Carbamidomethylation:1000.00;C16:Carbamidomethylation:1000.00;C18:Carbamidomethylation:1000.00 | PEAKS DB |
| R.SWWDFADYGC(+57.02).Y | N | 45.71 | 1305.4761 | 10 | 0.8 | 653.7458 | 2 | 109.86 | 11 | F11:80150 | NaNaKA16\_F7.raw |  |  |  |  |  |  |  |  |  |  | 6.5059E6 |  |  | 1 | 0 | 0 | 0 | 0 | 0 | 0 | 0 | 0 | 0 | 0 | 1 | 0 | 0 | 17 | 26 | Carbamidomethylation | C10:Carbamidomethylation:1000.00 | PEAKS DB |
| G.SGTPVDDLDR.C | N | 45.52 | 1073.4989 | 10 | 0.9 | 537.7572 | 2 | 20.23 | 11 | F11:7497 | NaNaKA16\_F7.raw |  |  |  |  |  |  |  |  |  |  | 1.1072E7 |  |  | 1 | 0 | 0 | 0 | 0 | 0 | 0 | 0 | 0 | 0 | 0 | 1 | 0 | 0 | 33 | 42 |  |  | PEAKS DB |
| R.SWWDFADYGC(+57.02)Y.C | N | 44.81 | 1468.5394 | 11 | 0.1 | 735.2771 | 2 | 111.77 | 10 | F10:82113 | NaNaKA16\_F6.raw |  |  |  |  |  |  |  |  |  | 1.9586E7 |  | 1.4496E7 |  | 2 | 0 | 0 | 0 | 0 | 0 | 0 | 0 | 0 | 0 | 1 | 0 | 1 | 0 | 17 | 27 | Carbamidomethylation | C10:Carbamidomethylation:1000.00 | PEAKS DB |
| total 16 peptides |  |  |  |  |  |  |  |  |  |  |  |  |  |  |  |  |  |  |  |  |  |  |  |  |  |  |  |  |  |  |  |  |  |  |  |  |  |  |  |  |  |  |  |
| --- | --- | --- | --- | --- | --- | --- | --- | --- | --- | --- | --- | --- | --- | --- | --- | --- | --- | --- | --- | --- | --- | --- | --- | --- | --- | --- | --- | --- | --- | --- | --- | --- | --- | --- | --- | --- | --- | --- | --- | --- | --- | --- | --- |

2RD4

back to list

  

| Protein Coverage
| Supporting Peptides
|

Protein Coverage:

Supporting Peptides:

| Peptide | Uniq | -10lgP | Mass | Length | ppm | m/z | z | RT | Fraction | Scan | Source File | Area F1 | Area F10 | Area F11 | Area F12 | Area F13 | Area F2 | Area F3 | Area F4 | Area F5 | Area F6 | Area F7 | Area F8 | Area F9 | #Feature | #Feature F1 | #Feature F10 | #Feature F11 | #Feature F12 | #Feature F13 | #Feature F2 | #Feature F3 | #Feature F4 | #Feature F5 | #Feature F6 | #Feature F7 | #Feature F8 | #Feature F9 | Start | End | PTM | AScore | Found By |
| --- | --- | --- | --- | --- | --- | --- | --- | --- | --- | --- | --- | --- | --- | --- | --- | --- | --- | --- | --- | --- | --- | --- | --- | --- | --- | --- | --- | --- | --- | --- | --- | --- | --- | --- | --- | --- | --- | --- | --- | --- | --- | --- | --- |
| S.RSWWDFADYGC(+57.02)YC(+57.02)GR.G | N | 77.58 | 1997.7937 | 15 | 1.2 | 666.9393 | 3 | 70.64 | 11 | F11:48142 | NaNaKA16\_F7.raw |  |  |  |  |  |  |  |  |  |  | 3.4823E7 | 9.6445E6 |  | 4 | 0 | 0 | 0 | 0 | 0 | 0 | 0 | 0 | 0 | 0 | 2 | 2 | 0 | 16 | 30 | Carbamidomethylation | C11:Carbamidomethylation:1000.00;C13:Carbamidomethylation:1000.00 | PEAKS DB |
| R.SWWDFADYGC(+57.02)YC(+57.02)GR.G | N | 77.10 | 1841.6926 | 14 | 2.4 | 921.8558 | 2 | 86.60 | 11 | F11:61115 | NaNaKA16\_F7.raw |  | 2.3849E7 | 3.1182E6 | 1.2353E7 | 9.152E5 |  |  |  | 6.0399E6 | 1.4952E9 | 5.4032E10 | 1.5192E10 | 2.1735E9 | 46 | 0 | 2 | 1 | 2 | 1 | 0 | 0 | 0 | 1 | 3 | 17 | 6 | 13 | 17 | 30 | Carbamidomethylation | C10:Carbamidomethylation:1000.00;C12:Carbamidomethylation:1000.00 | PEAKS DB |
| R.GGSGTPVDDLDR.C | N | 67.81 | 1187.5417 | 12 | 0.8 | 594.7786 | 2 | 19.63 | 10 | F10:6988 | NaNaKA16\_F6.raw |  |  |  |  |  |  |  |  |  | 5.7224E7 | 2.6509E9 | 3.9395E6 |  | 4 | 0 | 0 | 0 | 0 | 0 | 0 | 0 | 0 | 0 | 2 | 1 | 1 | 0 | 31 | 42 |  |  | PEAKS DB |
| R.SWWDFADYGC(+57.02)YC(+57.02)GRG.G | N | 61.68 | 1898.7141 | 15 | 1.5 | 950.3657 | 2 | 84.85 | 11 | F11:59757 | NaNaKA16\_F7.raw |  |  |  |  |  |  |  |  |  |  | 1.1046E7 |  |  | 1 | 0 | 0 | 0 | 0 | 0 | 0 | 0 | 0 | 0 | 0 | 1 | 0 | 0 | 17 | 31 | Carbamidomethylation | C10:Carbamidomethylation:1000.00;C12:Carbamidomethylation:1000.00 | PEAKS DB |
| G.GSGTPVDDLDR.C | N | 60.74 | 1130.5204 | 11 | -0.3 | 566.2673 | 2 | 21.08 | 11 | F11:8172 | NaNaKA16\_F7.raw |  |  |  |  |  |  |  |  |  | 1.4052E5 | 1.2671E7 |  |  | 2 | 0 | 0 | 0 | 0 | 0 | 0 | 0 | 0 | 0 | 1 | 1 | 0 | 0 | 32 | 42 |  |  | PEAKS DB |
| R.LAAIC(+57.02)FAGAPYNDNNYNIDLQ.A | Y | 58.95 | 2356.0793 | 21 | 4.1 | 1179.0518 | 2 | 118.61 | 11 | F11:86921 | NaNaKA16\_F7.raw |  |  |  |  |  |  |  |  |  |  | 0 |  | 1.489E6 | 1 | 0 | 0 | 0 | 0 | 0 | 0 | 0 | 0 | 0 | 0 | 0 | 0 | 1 | 95 | 115 | Carbamidomethylation | C5:Carbamidomethylation:1000.00 | PEAKS DB |
| D.FADYGC(+57.02)YC(+57.02)GR.G | N | 57.15 | 1267.4750 | 10 | 0.8 | 634.7452 | 2 | 25.42 | 11 | F11:11882 | NaNaKA16\_F7.raw |  |  |  |  |  |  |  |  |  | 2.1506E6 | 1.834E7 |  |  | 2 | 0 | 0 | 0 | 0 | 0 | 0 | 0 | 0 | 0 | 1 | 1 | 0 | 0 | 21 | 30 | Carbamidomethylation | C6:Carbamidomethylation:1000.00;C8:Carbamidomethylation:1000.00 | PEAKS DB |
| R.SWWDFADYGC(+57.02)YC(+57.02)GRGGSGTPVDDLDR.C | N | 56.86 | 3011.2239 | 26 | 0.6 | 1004.7491 | 3 | 81.63 | 11 | F11:57027 | NaNaKA16\_F7.raw |  |  |  |  |  |  |  |  |  |  | 4.9169E6 |  |  | 1 | 0 | 0 | 0 | 0 | 0 | 0 | 0 | 0 | 0 | 0 | 1 | 0 | 0 | 17 | 42 | Carbamidomethylation | C10:Carbamidomethylation:1000.00;C12:Carbamidomethylation:1000.00 | PEAKS DB |
| S.WWDFADYGC(+57.02)YC(+57.02)GR.G | N | 56.83 | 1754.6605 | 13 | 0.8 | 878.3382 | 2 | 78.92 | 12 | F12:55446 | NaNaKA16\_F8.raw |  |  |  |  |  |  |  |  |  |  | 1.3475E7 | 3.6699E6 |  | 2 | 0 | 0 | 0 | 0 | 0 | 0 | 0 | 0 | 0 | 0 | 1 | 1 | 0 | 18 | 30 | Carbamidomethylation | C9:Carbamidomethylation:1000.00;C11:Carbamidomethylation:1000.00 | PEAKS DB |
| R.LAAIC(+57.02)FAGAPYN.D | N | 53.90 | 1266.6067 | 12 | 0.8 | 634.3111 | 2 | 70.83 | 11 | F11:49747 | NaNaKA16\_F7.raw |  | 2.6932E8 | 6.3964E7 |  |  |  |  |  | 3.6922E8 | 7.2309E9 | 3.8533E9 | 2.4096E9 | 1.5572E9 | 11 | 0 | 1 | 1 | 0 | 0 | 0 | 0 | 0 | 1 | 2 | 3 | 2 | 1 | 95 | 106 | Carbamidomethylation | C5:Carbamidomethylation:1000.00 | PEAKS DB |
| W.WDFADYGC(+57.02)YC(+57.02)GR.G | N | 50.34 | 1568.5813 | 12 | 0.5 | 785.2983 | 2 | 61.41 | 11 | F11:40293 | NaNaKA16\_F7.raw |  |  |  |  |  |  |  |  |  |  | 5.6691E6 |  |  | 1 | 0 | 0 | 0 | 0 | 0 | 0 | 0 | 0 | 0 | 0 | 1 | 0 | 0 | 19 | 30 | Carbamidomethylation | C8:Carbamidomethylation:1000.00;C10:Carbamidomethylation:1000.00 | PEAKS DB |
| W.DFADYGC(+57.02)YC(+57.02)GR.G | N | 49.85 | 1382.5020 | 11 | 0.3 | 692.2585 | 2 | 40.50 | 10 | F10:24767 | NaNaKA16\_F6.raw |  |  |  |  |  |  |  |  | 5.0674E6 | 1.1493E7 | 1.4257E6 |  |  | 3 | 0 | 0 | 0 | 0 | 0 | 0 | 0 | 0 | 1 | 1 | 1 | 0 | 0 | 20 | 30 | Carbamidomethylation | C7:Carbamidomethylation:1000.00;C9:Carbamidomethylation:1000.00 | PEAKS DB |
| S.C(+57.02)TVPSRSWWDFADYGC(+57.02)YC(+57.02)GR.G | N | 48.21 | 2542.0251 | 20 | -0.3 | 848.3488 | 3 | 69.09 | 11 | F11:46749 | NaNaKA16\_F7.raw |  |  |  |  |  |  |  |  |  |  | 6.2414E6 |  |  | 1 | 0 | 0 | 0 | 0 | 0 | 0 | 0 | 0 | 0 | 0 | 1 | 0 | 0 | 11 | 30 | Carbamidomethylation | C1:Carbamidomethylation:1000.00;C16:Carbamidomethylation:1000.00;C18:Carbamidomethylation:1000.00 | PEAKS DB |
| R.SWWDFADYGC(+57.02).Y | N | 45.71 | 1305.4761 | 10 | 0.8 | 653.7458 | 2 | 109.86 | 11 | F11:80150 | NaNaKA16\_F7.raw |  |  |  |  |  |  |  |  |  |  | 6.5059E6 |  |  | 1 | 0 | 0 | 0 | 0 | 0 | 0 | 0 | 0 | 0 | 0 | 1 | 0 | 0 | 17 | 26 | Carbamidomethylation | C10:Carbamidomethylation:1000.00 | PEAKS DB |
| G.SGTPVDDLDR.C | N | 45.52 | 1073.4989 | 10 | 0.9 | 537.7572 | 2 | 20.23 | 11 | F11:7497 | NaNaKA16\_F7.raw |  |  |  |  |  |  |  |  |  |  | 1.1072E7 |  |  | 1 | 0 | 0 | 0 | 0 | 0 | 0 | 0 | 0 | 0 | 0 | 1 | 0 | 0 | 33 | 42 |  |  | PEAKS DB |
| R.SWWDFADYGC(+57.02)Y.C | N | 44.81 | 1468.5394 | 11 | 0.1 | 735.2771 | 2 | 111.77 | 10 | F10:82113 | NaNaKA16\_F6.raw |  |  |  |  |  |  |  |  |  | 1.9586E7 |  | 1.4496E7 |  | 2 | 0 | 0 | 0 | 0 | 0 | 0 | 0 | 0 | 0 | 1 | 0 | 1 | 0 | 17 | 27 | Carbamidomethylation | C10:Carbamidomethylation:1000.00 | PEAKS DB |
| total 16 peptides |  |  |  |  |  |  |  |  |  |  |  |  |  |  |  |  |  |  |  |  |  |  |  |  |  |  |  |  |  |  |  |  |  |  |  |  |  |  |  |  |  |  |  |
| --- | --- | --- | --- | --- | --- | --- | --- | --- | --- | --- | --- | --- | --- | --- | --- | --- | --- | --- | --- | --- | --- | --- | --- | --- | --- | --- | --- | --- | --- | --- | --- | --- | --- | --- | --- | --- | --- | --- | --- | --- | --- | --- | --- |

JAA75025.1

back to list

  

| Protein Coverage
| Supporting Peptides
|

Protein Coverage:

Supporting Peptides:

| Peptide | Uniq | -10lgP | Mass | Length | ppm | m/z | z | RT | Fraction | Scan | Source File | Area F1 | Area F10 | Area F11 | Area F12 | Area F13 | Area F2 | Area F3 | Area F4 | Area F5 | Area F6 | Area F7 | Area F8 | Area F9 | #Feature | #Feature F1 | #Feature F10 | #Feature F11 | #Feature F12 | #Feature F13 | #Feature F2 | #Feature F3 | #Feature F4 | #Feature F5 | #Feature F6 | #Feature F7 | #Feature F8 | #Feature F9 | Start | End | PTM | AScore | Found By |
| --- | --- | --- | --- | --- | --- | --- | --- | --- | --- | --- | --- | --- | --- | --- | --- | --- | --- | --- | --- | --- | --- | --- | --- | --- | --- | --- | --- | --- | --- | --- | --- | --- | --- | --- | --- | --- | --- | --- | --- | --- | --- | --- | --- |
| D.YGC(+57.02)YC(+57.02)GPGGSGTPVDDLDR.C | Y | 89.23 | 2044.8254 | 19 | -0.4 | 1023.4196 | 2 | 42.89 | 13 | F13:22904 | NaNaKA16\_F9.raw |  |  |  |  |  |  |  |  |  | 3.6643E7 |  |  | 1.535E7 | 3 | 0 | 0 | 0 | 0 | 0 | 0 | 0 | 0 | 0 | 2 | 0 | 0 | 1 | 52 | 70 | Carbamidomethylation | C3:Carbamidomethylation:1000.00;C5:Carbamidomethylation:1000.00 | PEAKS DB |
| M.DYGC(+57.02)YC(+57.02)GPGGSGTPVDDLDR.C | Y | 85.76 | 2159.8523 | 20 | -2.3 | 1080.9309 | 2 | 50.66 | 10 | F10:34442 | NaNaKA16\_F6.raw |  | 5.8775E6 | 2.4285E6 | 2.4039E6 |  |  |  |  | 1.2439E7 | 7.7573E9 | 1.9455E7 | 6.9427E6 | 1.1867E7 | 19 | 0 | 1 | 1 | 1 | 0 | 0 | 0 | 0 | 1 | 11 | 1 | 2 | 1 | 51 | 70 | Carbamidomethylation | C4:Carbamidomethylation:1000.00;C6:Carbamidomethylation:1000.00 | PEAKS DB |
| C.YC(+57.02)GPGGSGTPVDDLDR.C | Y | 84.89 | 1664.7100 | 16 | 0.9 | 833.3630 | 2 | 34.13 | 13 | F13:15430 | NaNaKA16\_F9.raw |  |  |  |  |  |  |  |  |  | 1.7463E8 | 1.9571E6 | 8.391E5 | 5.506E6 | 6 | 0 | 0 | 0 | 0 | 0 | 0 | 0 | 0 | 0 | 3 | 1 | 1 | 1 | 55 | 70 | Carbamidomethylation | C2:Carbamidomethylation:1000.00 | PEAKS DB |
| Y.C(+57.02)GPGGSGTPVDDLDR.C | Y | 83.55 | 1501.6467 | 15 | -0.3 | 751.8304 | 2 | 25.73 | 10 | F10:12021 | NaNaKA16\_F6.raw |  |  |  |  |  |  |  |  |  | 1.6307E8 | 4.9153E5 |  | 3.7447E6 | 4 | 0 | 0 | 0 | 0 | 0 | 0 | 0 | 0 | 0 | 2 | 1 | 0 | 1 | 56 | 70 | Carbamidomethylation | C1:Carbamidomethylation:1000.00 | PEAKS DB |
| Y.GC(+57.02)YC(+57.02)GPGGSGTPVDDLDR.C | Y | 82.65 | 1881.7621 | 18 | 0.2 | 941.8885 | 2 | 35.82 | 13 | F13:16810 | NaNaKA16\_F9.raw |  |  |  |  |  |  |  |  |  | 1.02E8 |  |  | 1.5202E7 | 3 | 0 | 0 | 0 | 0 | 0 | 0 | 0 | 0 | 0 | 2 | 0 | 0 | 1 | 53 | 70 | Carbamidomethylation | C2:Carbamidomethylation:1000.00;C4:Carbamidomethylation:1000.00 | PEAKS DB |
| G.C(+57.02)YC(+57.02)GPGGSGTPVDDLDR.C | Y | 75.89 | 1824.7407 | 17 | -0.1 | 913.3776 | 2 | 35.67 | 13 | F13:16718 | NaNaKA16\_F9.raw |  |  |  |  |  |  |  |  |  | 6.2401E6 |  |  | 8.775E6 | 4 | 0 | 0 | 0 | 0 | 0 | 0 | 0 | 0 | 0 | 3 | 0 | 0 | 1 | 54 | 70 | Carbamidomethylation | C1:Carbamidomethylation:1000.00;C3:Carbamidomethylation:1000.00 | PEAKS DB |
| P.GGSGTPVDDLDR.C | N | 67.81 | 1187.5417 | 12 | 0.8 | 594.7786 | 2 | 19.63 | 10 | F10:6988 | NaNaKA16\_F6.raw |  |  |  |  |  |  |  |  |  | 5.7224E7 | 2.6509E9 | 3.9395E6 |  | 4 | 0 | 0 | 0 | 0 | 0 | 0 | 0 | 0 | 0 | 2 | 1 | 1 | 0 | 59 | 70 |  |  | PEAKS DB |
| G.GSGTPVDDLDR.C | N | 60.74 | 1130.5204 | 11 | -0.3 | 566.2673 | 2 | 21.08 | 11 | F11:8172 | NaNaKA16\_F7.raw |  |  |  |  |  |  |  |  |  | 1.4052E5 | 1.2671E7 |  |  | 2 | 0 | 0 | 0 | 0 | 0 | 0 | 0 | 0 | 0 | 1 | 1 | 0 | 0 | 60 | 70 |  |  | PEAKS DB |
| G.SGTPVDDLDR.C | N | 45.52 | 1073.4989 | 10 | 0.9 | 537.7572 | 2 | 20.23 | 11 | F11:7497 | NaNaKA16\_F7.raw |  |  |  |  |  |  |  |  |  |  | 1.1072E7 |  |  | 1 | 0 | 0 | 0 | 0 | 0 | 0 | 0 | 0 | 0 | 0 | 1 | 0 | 0 | 61 | 70 |  |  | PEAKS DB |
| total 9 peptides |  |  |  |  |  |  |  |  |  |  |  |  |  |  |  |  |  |  |  |  |  |  |  |  |  |  |  |  |  |  |  |  |  |  |  |  |  |  |  |  |  |  |  |
| --- | --- | --- | --- | --- | --- | --- | --- | --- | --- | --- | --- | --- | --- | --- | --- | --- | --- | --- | --- | --- | --- | --- | --- | --- | --- | --- | --- | --- | --- | --- | --- | --- | --- | --- | --- | --- | --- | --- | --- | --- | --- | --- | --- |

ABN72541.1

back to list

  

| Protein Coverage
| Supporting Peptides
|

Protein Coverage:

Supporting Peptides:

| Peptide | Uniq | -10lgP | Mass | Length | ppm | m/z | z | RT | Fraction | Scan | Source File | Area F1 | Area F10 | Area F11 | Area F12 | Area F13 | Area F2 | Area F3 | Area F4 | Area F5 | Area F6 | Area F7 | Area F8 | Area F9 | #Feature | #Feature F1 | #Feature F10 | #Feature F11 | #Feature F12 | #Feature F13 | #Feature F2 | #Feature F3 | #Feature F4 | #Feature F5 | #Feature F6 | #Feature F7 | #Feature F8 | #Feature F9 | Start | End | PTM | AScore | Found By |
| --- | --- | --- | --- | --- | --- | --- | --- | --- | --- | --- | --- | --- | --- | --- | --- | --- | --- | --- | --- | --- | --- | --- | --- | --- | --- | --- | --- | --- | --- | --- | --- | --- | --- | --- | --- | --- | --- | --- | --- | --- | --- | --- | --- |
| K.LGVHNVHVHYEDEQIR.V | N | 101.62 | 1943.9602 | 16 | 0.2 | 486.9974 | 4 | 17.09 | 4 | F4:3865 | NaNaKA16\_F12.raw |  |  |  | 6.3497E8 |  |  |  |  |  |  |  |  |  | 7 | 0 | 0 | 0 | 7 | 0 | 0 | 0 | 0 | 0 | 0 | 0 | 0 | 0 | 107 | 122 |  |  | PEAKS DB |
| K.LGVHNVHVHYEDEQIRVPK.E | N | 95.24 | 2268.1763 | 19 | 0.0 | 568.0513 | 4 | 23.37 | 4 | F4:8250 | NaNaKA16\_F12.raw |  |  |  | 1.3694E8 |  |  |  |  |  |  |  |  |  | 3 | 0 | 0 | 0 | 3 | 0 | 0 | 0 | 0 | 0 | 0 | 0 | 0 | 0 | 107 | 125 |  |  | PEAKS DB |
| R.FPC(+57.02)AQLLEPGVYTK.V | N | 90.02 | 1621.8174 | 14 | 0.7 | 811.9165 | 2 | 63.25 | 4 | F4:41641 | NaNaKA16\_F12.raw |  |  | 4.8534E6 | 6.8218E8 | 1.5279E6 |  |  |  |  |  |  |  |  | 10 | 0 | 0 | 1 | 8 | 1 | 0 | 0 | 0 | 0 | 0 | 0 | 0 | 0 | 254 | 267 | Carbamidomethylation | C3:Carbamidomethylation:1000.00 | PEAKS DB |
| Q.LKLGVHNVHVHYEDEQIR.V | Y | 71.23 | 2185.1392 | 18 | 0.9 | 547.2925 | 4 | 22.98 | 4 | F4:7749 | NaNaKA16\_F12.raw |  |  |  | 4.1304E6 |  |  |  |  |  |  |  |  |  | 1 | 0 | 0 | 0 | 1 | 0 | 0 | 0 | 0 | 0 | 0 | 0 | 0 | 0 | 105 | 122 |  |  | PEAKS DB |
| G.QIQGIVSWGR.F | N | 61.28 | 1142.6196 | 10 | -0.6 | 572.3168 | 2 | 83.53 | 4 | F4:57766 | NaNaKA16\_F12.raw |  |  | 3.0835E6 | 3.6982E8 |  |  |  |  |  |  |  |  |  | 3 | 0 | 0 | 1 | 2 | 0 | 0 | 0 | 0 | 0 | 0 | 0 | 0 | 0 | 244 | 253 |  |  | PEAKS DB |
| P.C(+57.02)AQLLEPGVYTK.V | N | 56.21 | 1377.6962 | 12 | 0.9 | 689.8560 | 2 | 38.93 | 4 | F4:20939 | NaNaKA16\_F12.raw |  |  |  | 2.6297E6 |  |  |  |  |  |  |  |  |  | 1 | 0 | 0 | 0 | 1 | 0 | 0 | 0 | 0 | 0 | 0 | 0 | 0 | 0 | 256 | 267 | Carbamidomethylation | C1:Carbamidomethylation:1000.00 | PEAKS DB |
| D.GQIQGIVSWGR.F | N | 51.14 | 1199.6411 | 11 | 0.9 | 600.8284 | 2 | 49.57 | 4 | F4:29910 | NaNaKA16\_F12.raw |  |  |  | 3.2311E6 |  |  |  |  |  |  |  |  |  | 1 | 0 | 0 | 0 | 1 | 0 | 0 | 0 | 0 | 0 | 0 | 0 | 0 | 0 | 243 | 253 |  |  | PEAKS DB |
| C.AQLLEPGVYTK.V | N | 44.01 | 1217.6655 | 11 | 0.3 | 609.8402 | 2 | 35.96 | 4 | F4:18428 | NaNaKA16\_F12.raw |  |  |  | 4.2348E6 |  |  |  |  |  |  |  |  |  | 1 | 0 | 0 | 0 | 1 | 0 | 0 | 0 | 0 | 0 | 0 | 0 | 0 | 0 | 257 | 267 |  |  | PEAKS DB |
| total 8 peptides |  |  |  |  |  |  |  |  |  |  |  |  |  |  |  |  |  |  |  |  |  |  |  |  |  |  |  |  |  |  |  |  |  |  |  |  |  |  |  |  |  |  |  |
| --- | --- | --- | --- | --- | --- | --- | --- | --- | --- | --- | --- | --- | --- | --- | --- | --- | --- | --- | --- | --- | --- | --- | --- | --- | --- | --- | --- | --- | --- | --- | --- | --- | --- | --- | --- | --- | --- | --- | --- | --- | --- | --- | --- |

ABN72545.1

back to list

  

| Protein Coverage
| Supporting Peptides
|

Protein Coverage:

Supporting Peptides:

| Peptide | Uniq | -10lgP | Mass | Length | ppm | m/z | z | RT | Fraction | Scan | Source File | Area F1 | Area F10 | Area F11 | Area F12 | Area F13 | Area F2 | Area F3 | Area F4 | Area F5 | Area F6 | Area F7 | Area F8 | Area F9 | #Feature | #Feature F1 | #Feature F10 | #Feature F11 | #Feature F12 | #Feature F13 | #Feature F2 | #Feature F3 | #Feature F4 | #Feature F5 | #Feature F6 | #Feature F7 | #Feature F8 | #Feature F9 | Start | End | PTM | AScore | Found By |
| --- | --- | --- | --- | --- | --- | --- | --- | --- | --- | --- | --- | --- | --- | --- | --- | --- | --- | --- | --- | --- | --- | --- | --- | --- | --- | --- | --- | --- | --- | --- | --- | --- | --- | --- | --- | --- | --- | --- | --- | --- | --- | --- | --- |
| K.LGVHNVHVHYEDEQIR.V | N | 101.62 | 1943.9602 | 16 | 0.2 | 486.9974 | 4 | 17.09 | 4 | F4:3865 | NaNaKA16\_F12.raw |  |  |  | 6.3497E8 |  |  |  |  |  |  |  |  |  | 7 | 0 | 0 | 0 | 7 | 0 | 0 | 0 | 0 | 0 | 0 | 0 | 0 | 0 | 107 | 122 |  |  | PEAKS DB |
| K.LGVHNVHVHYEDEQIRVPK.E | N | 95.24 | 2268.1763 | 19 | 0.0 | 568.0513 | 4 | 23.37 | 4 | F4:8250 | NaNaKA16\_F12.raw |  |  |  | 1.3694E8 |  |  |  |  |  |  |  |  |  | 3 | 0 | 0 | 0 | 3 | 0 | 0 | 0 | 0 | 0 | 0 | 0 | 0 | 0 | 107 | 125 |  |  | PEAKS DB |
| R.FPC(+57.02)AQLLEPGVYTK.V | N | 90.02 | 1621.8174 | 14 | 0.7 | 811.9165 | 2 | 63.25 | 4 | F4:41641 | NaNaKA16\_F12.raw |  |  | 4.8534E6 | 6.8218E8 | 1.5279E6 |  |  |  |  |  |  |  |  | 10 | 0 | 0 | 1 | 8 | 1 | 0 | 0 | 0 | 0 | 0 | 0 | 0 | 0 | 254 | 267 | Carbamidomethylation | C3:Carbamidomethylation:1000.00 | PEAKS DB |
| Q.LKLGVHNVHVHYEDEQIR.V | Y | 71.23 | 2185.1392 | 18 | 0.9 | 547.2925 | 4 | 22.98 | 4 | F4:7749 | NaNaKA16\_F12.raw |  |  |  | 4.1304E6 |  |  |  |  |  |  |  |  |  | 1 | 0 | 0 | 0 | 1 | 0 | 0 | 0 | 0 | 0 | 0 | 0 | 0 | 0 | 105 | 122 |  |  | PEAKS DB |
| G.QIQGIVSWGR.F | N | 61.28 | 1142.6196 | 10 | -0.6 | 572.3168 | 2 | 83.53 | 4 | F4:57766 | NaNaKA16\_F12.raw |  |  | 3.0835E6 | 3.6982E8 |  |  |  |  |  |  |  |  |  | 3 | 0 | 0 | 1 | 2 | 0 | 0 | 0 | 0 | 0 | 0 | 0 | 0 | 0 | 244 | 253 |  |  | PEAKS DB |
| P.C(+57.02)AQLLEPGVYTK.V | N | 56.21 | 1377.6962 | 12 | 0.9 | 689.8560 | 2 | 38.93 | 4 | F4:20939 | NaNaKA16\_F12.raw |  |  |  | 2.6297E6 |  |  |  |  |  |  |  |  |  | 1 | 0 | 0 | 0 | 1 | 0 | 0 | 0 | 0 | 0 | 0 | 0 | 0 | 0 | 256 | 267 | Carbamidomethylation | C1:Carbamidomethylation:1000.00 | PEAKS DB |
| D.GQIQGIVSWGR.F | N | 51.14 | 1199.6411 | 11 | 0.9 | 600.8284 | 2 | 49.57 | 4 | F4:29910 | NaNaKA16\_F12.raw |  |  |  | 3.2311E6 |  |  |  |  |  |  |  |  |  | 1 | 0 | 0 | 0 | 1 | 0 | 0 | 0 | 0 | 0 | 0 | 0 | 0 | 0 | 243 | 253 |  |  | PEAKS DB |
| C.AQLLEPGVYTK.V | N | 44.01 | 1217.6655 | 11 | 0.3 | 609.8402 | 2 | 35.96 | 4 | F4:18428 | NaNaKA16\_F12.raw |  |  |  | 4.2348E6 |  |  |  |  |  |  |  |  |  | 1 | 0 | 0 | 0 | 1 | 0 | 0 | 0 | 0 | 0 | 0 | 0 | 0 | 0 | 257 | 267 |  |  | PEAKS DB |
| total 8 peptides |  |  |  |  |  |  |  |  |  |  |  |  |  |  |  |  |  |  |  |  |  |  |  |  |  |  |  |  |  |  |  |  |  |  |  |  |  |  |  |  |  |  |  |
| --- | --- | --- | --- | --- | --- | --- | --- | --- | --- | --- | --- | --- | --- | --- | --- | --- | --- | --- | --- | --- | --- | --- | --- | --- | --- | --- | --- | --- | --- | --- | --- | --- | --- | --- | --- | --- | --- | --- | --- | --- | --- | --- | --- |

A8QL57.1

back to list

  

| Protein Coverage
| Supporting Peptides
|

Protein Coverage:

Supporting Peptides:

| Peptide | Uniq | -10lgP | Mass | Length | ppm | m/z | z | RT | Fraction | Scan | Source File | Area F1 | Area F10 | Area F11 | Area F12 | Area F13 | Area F2 | Area F3 | Area F4 | Area F5 | Area F6 | Area F7 | Area F8 | Area F9 | #Feature | #Feature F1 | #Feature F10 | #Feature F11 | #Feature F12 | #Feature F13 | #Feature F2 | #Feature F3 | #Feature F4 | #Feature F5 | #Feature F6 | #Feature F7 | #Feature F8 | #Feature F9 | Start | End | PTM | AScore | Found By |
| --- | --- | --- | --- | --- | --- | --- | --- | --- | --- | --- | --- | --- | --- | --- | --- | --- | --- | --- | --- | --- | --- | --- | --- | --- | --- | --- | --- | --- | --- | --- | --- | --- | --- | --- | --- | --- | --- | --- | --- | --- | --- | --- | --- |
| K.LGVHNVHVHYEDEQIR.V | N | 101.62 | 1943.9602 | 16 | 0.2 | 486.9974 | 4 | 17.09 | 4 | F4:3865 | NaNaKA16\_F12.raw |  |  |  | 6.3497E8 |  |  |  |  |  |  |  |  |  | 7 | 0 | 0 | 0 | 7 | 0 | 0 | 0 | 0 | 0 | 0 | 0 | 0 | 0 | 107 | 122 |  |  | PEAKS DB |
| K.LGVHNVHVHYEDEQIRVPK.E | N | 95.24 | 2268.1763 | 19 | 0.0 | 568.0513 | 4 | 23.37 | 4 | F4:8250 | NaNaKA16\_F12.raw |  |  |  | 1.3694E8 |  |  |  |  |  |  |  |  |  | 3 | 0 | 0 | 0 | 3 | 0 | 0 | 0 | 0 | 0 | 0 | 0 | 0 | 0 | 107 | 125 |  |  | PEAKS DB |
| R.FPC(+57.02)AQLLEPGVYTK.V | N | 90.02 | 1621.8174 | 14 | 0.7 | 811.9165 | 2 | 63.25 | 4 | F4:41641 | NaNaKA16\_F12.raw |  |  | 4.8534E6 | 6.8218E8 | 1.5279E6 |  |  |  |  |  |  |  |  | 10 | 0 | 0 | 1 | 8 | 1 | 0 | 0 | 0 | 0 | 0 | 0 | 0 | 0 | 254 | 267 | Carbamidomethylation | C3:Carbamidomethylation:1000.00 | PEAKS DB |
| Q.LKLGVHNVHVHYEDEQIR.V | Y | 71.23 | 2185.1392 | 18 | 0.9 | 547.2925 | 4 | 22.98 | 4 | F4:7749 | NaNaKA16\_F12.raw |  |  |  | 4.1304E6 |  |  |  |  |  |  |  |  |  | 1 | 0 | 0 | 0 | 1 | 0 | 0 | 0 | 0 | 0 | 0 | 0 | 0 | 0 | 105 | 122 |  |  | PEAKS DB |
| G.QIQGIVSWGR.F | N | 61.28 | 1142.6196 | 10 | -0.6 | 572.3168 | 2 | 83.53 | 4 | F4:57766 | NaNaKA16\_F12.raw |  |  | 3.0835E6 | 3.6982E8 |  |  |  |  |  |  |  |  |  | 3 | 0 | 0 | 1 | 2 | 0 | 0 | 0 | 0 | 0 | 0 | 0 | 0 | 0 | 244 | 253 |  |  | PEAKS DB |
| P.C(+57.02)AQLLEPGVYTK.V | N | 56.21 | 1377.6962 | 12 | 0.9 | 689.8560 | 2 | 38.93 | 4 | F4:20939 | NaNaKA16\_F12.raw |  |  |  | 2.6297E6 |  |  |  |  |  |  |  |  |  | 1 | 0 | 0 | 0 | 1 | 0 | 0 | 0 | 0 | 0 | 0 | 0 | 0 | 0 | 256 | 267 | Carbamidomethylation | C1:Carbamidomethylation:1000.00 | PEAKS DB |
| D.GQIQGIVSWGR.F | N | 51.14 | 1199.6411 | 11 | 0.9 | 600.8284 | 2 | 49.57 | 4 | F4:29910 | NaNaKA16\_F12.raw |  |  |  | 3.2311E6 |  |  |  |  |  |  |  |  |  | 1 | 0 | 0 | 0 | 1 | 0 | 0 | 0 | 0 | 0 | 0 | 0 | 0 | 0 | 243 | 253 |  |  | PEAKS DB |
| C.AQLLEPGVYTK.V | N | 44.01 | 1217.6655 | 11 | 0.3 | 609.8402 | 2 | 35.96 | 4 | F4:18428 | NaNaKA16\_F12.raw |  |  |  | 4.2348E6 |  |  |  |  |  |  |  |  |  | 1 | 0 | 0 | 0 | 1 | 0 | 0 | 0 | 0 | 0 | 0 | 0 | 0 | 0 | 257 | 267 |  |  | PEAKS DB |
| total 8 peptides |  |  |  |  |  |  |  |  |  |  |  |  |  |  |  |  |  |  |  |  |  |  |  |  |  |  |  |  |  |  |  |  |  |  |  |  |  |  |  |  |  |  |  |
| --- | --- | --- | --- | --- | --- | --- | --- | --- | --- | --- | --- | --- | --- | --- | --- | --- | --- | --- | --- | --- | --- | --- | --- | --- | --- | --- | --- | --- | --- | --- | --- | --- | --- | --- | --- | --- | --- | --- | --- | --- | --- | --- | --- |

A8QL53.1

back to list

  

| Protein Coverage
| Supporting Peptides
|

Protein Coverage:

Supporting Peptides:

| Peptide | Uniq | -10lgP | Mass | Length | ppm | m/z | z | RT | Fraction | Scan | Source File | Area F1 | Area F10 | Area F11 | Area F12 | Area F13 | Area F2 | Area F3 | Area F4 | Area F5 | Area F6 | Area F7 | Area F8 | Area F9 | #Feature | #Feature F1 | #Feature F10 | #Feature F11 | #Feature F12 | #Feature F13 | #Feature F2 | #Feature F3 | #Feature F4 | #Feature F5 | #Feature F6 | #Feature F7 | #Feature F8 | #Feature F9 | Start | End | PTM | AScore | Found By |
| --- | --- | --- | --- | --- | --- | --- | --- | --- | --- | --- | --- | --- | --- | --- | --- | --- | --- | --- | --- | --- | --- | --- | --- | --- | --- | --- | --- | --- | --- | --- | --- | --- | --- | --- | --- | --- | --- | --- | --- | --- | --- | --- | --- |
| K.LGVHNVHVHYEDEQIR.V | N | 101.62 | 1943.9602 | 16 | 0.2 | 486.9974 | 4 | 17.09 | 4 | F4:3865 | NaNaKA16\_F12.raw |  |  |  | 6.3497E8 |  |  |  |  |  |  |  |  |  | 7 | 0 | 0 | 0 | 7 | 0 | 0 | 0 | 0 | 0 | 0 | 0 | 0 | 0 | 107 | 122 |  |  | PEAKS DB |
| K.LGVHNVHVHYEDEQIRVPK.E | N | 95.24 | 2268.1763 | 19 | 0.0 | 568.0513 | 4 | 23.37 | 4 | F4:8250 | NaNaKA16\_F12.raw |  |  |  | 1.3694E8 |  |  |  |  |  |  |  |  |  | 3 | 0 | 0 | 0 | 3 | 0 | 0 | 0 | 0 | 0 | 0 | 0 | 0 | 0 | 107 | 125 |  |  | PEAKS DB |
| R.FPC(+57.02)AQLLEPGVYTK.V | N | 90.02 | 1621.8174 | 14 | 0.7 | 811.9165 | 2 | 63.25 | 4 | F4:41641 | NaNaKA16\_F12.raw |  |  | 4.8534E6 | 6.8218E8 | 1.5279E6 |  |  |  |  |  |  |  |  | 10 | 0 | 0 | 1 | 8 | 1 | 0 | 0 | 0 | 0 | 0 | 0 | 0 | 0 | 254 | 267 | Carbamidomethylation | C3:Carbamidomethylation:1000.00 | PEAKS DB |
| Q.LKLGVHNVHVHYEDEQIR.V | Y | 71.23 | 2185.1392 | 18 | 0.9 | 547.2925 | 4 | 22.98 | 4 | F4:7749 | NaNaKA16\_F12.raw |  |  |  | 4.1304E6 |  |  |  |  |  |  |  |  |  | 1 | 0 | 0 | 0 | 1 | 0 | 0 | 0 | 0 | 0 | 0 | 0 | 0 | 0 | 105 | 122 |  |  | PEAKS DB |
| G.QIQGIVSWGR.F | N | 61.28 | 1142.6196 | 10 | -0.6 | 572.3168 | 2 | 83.53 | 4 | F4:57766 | NaNaKA16\_F12.raw |  |  | 3.0835E6 | 3.6982E8 |  |  |  |  |  |  |  |  |  | 3 | 0 | 0 | 1 | 2 | 0 | 0 | 0 | 0 | 0 | 0 | 0 | 0 | 0 | 244 | 253 |  |  | PEAKS DB |
| P.C(+57.02)AQLLEPGVYTK.V | N | 56.21 | 1377.6962 | 12 | 0.9 | 689.8560 | 2 | 38.93 | 4 | F4:20939 | NaNaKA16\_F12.raw |  |  |  | 2.6297E6 |  |  |  |  |  |  |  |  |  | 1 | 0 | 0 | 0 | 1 | 0 | 0 | 0 | 0 | 0 | 0 | 0 | 0 | 0 | 256 | 267 | Carbamidomethylation | C1:Carbamidomethylation:1000.00 | PEAKS DB |
| D.GQIQGIVSWGR.F | N | 51.14 | 1199.6411 | 11 | 0.9 | 600.8284 | 2 | 49.57 | 4 | F4:29910 | NaNaKA16\_F12.raw |  |  |  | 3.2311E6 |  |  |  |  |  |  |  |  |  | 1 | 0 | 0 | 0 | 1 | 0 | 0 | 0 | 0 | 0 | 0 | 0 | 0 | 0 | 243 | 253 |  |  | PEAKS DB |
| C.AQLLEPGVYTK.V | N | 44.01 | 1217.6655 | 11 | 0.3 | 609.8402 | 2 | 35.96 | 4 | F4:18428 | NaNaKA16\_F12.raw |  |  |  | 4.2348E6 |  |  |  |  |  |  |  |  |  | 1 | 0 | 0 | 0 | 1 | 0 | 0 | 0 | 0 | 0 | 0 | 0 | 0 | 0 | 257 | 267 |  |  | PEAKS DB |
| total 8 peptides |  |  |  |  |  |  |  |  |  |  |  |  |  |  |  |  |  |  |  |  |  |  |  |  |  |  |  |  |  |  |  |  |  |  |  |  |  |  |  |  |  |  |  |
| --- | --- | --- | --- | --- | --- | --- | --- | --- | --- | --- | --- | --- | --- | --- | --- | --- | --- | --- | --- | --- | --- | --- | --- | --- | --- | --- | --- | --- | --- | --- | --- | --- | --- | --- | --- | --- | --- | --- | --- | --- | --- | --- | --- |

XP\_026522175.1

back to list

  

| Protein Coverage
| Supporting Peptides
|

Protein Coverage:

Supporting Peptides:

| Peptide | Uniq | -10lgP | Mass | Length | ppm | m/z | z | RT | Fraction | Scan | Source File | Area F1 | Area F10 | Area F11 | Area F12 | Area F13 | Area F2 | Area F3 | Area F4 | Area F5 | Area F6 | Area F7 | Area F8 | Area F9 | #Feature | #Feature F1 | #Feature F10 | #Feature F11 | #Feature F12 | #Feature F13 | #Feature F2 | #Feature F3 | #Feature F4 | #Feature F5 | #Feature F6 | #Feature F7 | #Feature F8 | #Feature F9 | Start | End | PTM | AScore | Found By |
| --- | --- | --- | --- | --- | --- | --- | --- | --- | --- | --- | --- | --- | --- | --- | --- | --- | --- | --- | --- | --- | --- | --- | --- | --- | --- | --- | --- | --- | --- | --- | --- | --- | --- | --- | --- | --- | --- | --- | --- | --- | --- | --- | --- |
| R.LGVHNVHVHYEDEQIR.V | N | 101.62 | 1943.9602 | 16 | 0.2 | 486.9974 | 4 | 17.09 | 4 | F4:3865 | NaNaKA16\_F12.raw |  |  |  | 6.3497E8 |  |  |  |  |  |  |  |  |  | 7 | 0 | 0 | 0 | 7 | 0 | 0 | 0 | 0 | 0 | 0 | 0 | 0 | 0 | 107 | 122 |  |  | PEAKS DB |
| R.LGVHNVHVHYEDEQIRVPK.E | N | 95.24 | 2268.1763 | 19 | 0.0 | 568.0513 | 4 | 23.37 | 4 | F4:8250 | NaNaKA16\_F12.raw |  |  |  | 1.3694E8 |  |  |  |  |  |  |  |  |  | 3 | 0 | 0 | 0 | 3 | 0 | 0 | 0 | 0 | 0 | 0 | 0 | 0 | 0 | 107 | 125 |  |  | PEAKS DB |
| R.FPC(+57.02)AQLLEPGVYTK.V | N | 90.02 | 1621.8174 | 14 | 0.7 | 811.9165 | 2 | 63.25 | 4 | F4:41641 | NaNaKA16\_F12.raw |  |  | 4.8534E6 | 6.8218E8 | 1.5279E6 |  |  |  |  |  |  |  |  | 10 | 0 | 0 | 1 | 8 | 1 | 0 | 0 | 0 | 0 | 0 | 0 | 0 | 0 | 254 | 267 | Carbamidomethylation | C3:Carbamidomethylation:1000.00 | PEAKS DB |
| G.QIQGIVSWGR.F | N | 61.28 | 1142.6196 | 10 | -0.6 | 572.3168 | 2 | 83.53 | 4 | F4:57766 | NaNaKA16\_F12.raw |  |  | 3.0835E6 | 3.6982E8 |  |  |  |  |  |  |  |  |  | 3 | 0 | 0 | 1 | 2 | 0 | 0 | 0 | 0 | 0 | 0 | 0 | 0 | 0 | 244 | 253 |  |  | PEAKS DB |
| P.C(+57.02)AQLLEPGVYTK.V | N | 56.21 | 1377.6962 | 12 | 0.9 | 689.8560 | 2 | 38.93 | 4 | F4:20939 | NaNaKA16\_F12.raw |  |  |  | 2.6297E6 |  |  |  |  |  |  |  |  |  | 1 | 0 | 0 | 0 | 1 | 0 | 0 | 0 | 0 | 0 | 0 | 0 | 0 | 0 | 256 | 267 | Carbamidomethylation | C1:Carbamidomethylation:1000.00 | PEAKS DB |
| N.YSEHIAPLSLPSNPPR.M | Y | 51.85 | 1776.9158 | 16 | -0.2 | 593.3124 | 3 | 41.69 | 4 | F4:23304 | NaNaKA16\_F12.raw |  |  |  | 1.7578E6 |  |  |  |  |  |  |  |  |  | 1 | 0 | 0 | 0 | 1 | 0 | 0 | 0 | 0 | 0 | 0 | 0 | 0 | 0 | 154 | 169 |  |  | PEAKS DB |
| N.GQIQGIVSWGR.F | N | 51.14 | 1199.6411 | 11 | 0.9 | 600.8284 | 2 | 49.57 | 4 | F4:29910 | NaNaKA16\_F12.raw |  |  |  | 3.2311E6 |  |  |  |  |  |  |  |  |  | 1 | 0 | 0 | 0 | 1 | 0 | 0 | 0 | 0 | 0 | 0 | 0 | 0 | 0 | 243 | 253 |  |  | PEAKS DB |
| C.AQLLEPGVYTK.V | N | 44.01 | 1217.6655 | 11 | 0.3 | 609.8402 | 2 | 35.96 | 4 | F4:18428 | NaNaKA16\_F12.raw |  |  |  | 4.2348E6 |  |  |  |  |  |  |  |  |  | 1 | 0 | 0 | 0 | 1 | 0 | 0 | 0 | 0 | 0 | 0 | 0 | 0 | 0 | 257 | 267 |  |  | PEAKS DB |
| total 8 peptides |  |  |  |  |  |  |  |  |  |  |  |  |  |  |  |  |  |  |  |  |  |  |  |  |  |  |  |  |  |  |  |  |  |  |  |  |  |  |  |  |  |  |  |
| --- | --- | --- | --- | --- | --- | --- | --- | --- | --- | --- | --- | --- | --- | --- | --- | --- | --- | --- | --- | --- | --- | --- | --- | --- | --- | --- | --- | --- | --- | --- | --- | --- | --- | --- | --- | --- | --- | --- | --- | --- | --- | --- | --- |

XP\_026522176.1

back to list

  

| Protein Coverage
| Supporting Peptides
|

Protein Coverage:

Supporting Peptides:

| Peptide | Uniq | -10lgP | Mass | Length | ppm | m/z | z | RT | Fraction | Scan | Source File | Area F1 | Area F10 | Area F11 | Area F12 | Area F13 | Area F2 | Area F3 | Area F4 | Area F5 | Area F6 | Area F7 | Area F8 | Area F9 | #Feature | #Feature F1 | #Feature F10 | #Feature F11 | #Feature F12 | #Feature F13 | #Feature F2 | #Feature F3 | #Feature F4 | #Feature F5 | #Feature F6 | #Feature F7 | #Feature F8 | #Feature F9 | Start | End | PTM | AScore | Found By |
| --- | --- | --- | --- | --- | --- | --- | --- | --- | --- | --- | --- | --- | --- | --- | --- | --- | --- | --- | --- | --- | --- | --- | --- | --- | --- | --- | --- | --- | --- | --- | --- | --- | --- | --- | --- | --- | --- | --- | --- | --- | --- | --- | --- |
| R.LGVHNVHVHYEDEQIR.V | N | 101.62 | 1943.9602 | 16 | 0.2 | 486.9974 | 4 | 17.09 | 4 | F4:3865 | NaNaKA16\_F12.raw |  |  |  | 6.3497E8 |  |  |  |  |  |  |  |  |  | 7 | 0 | 0 | 0 | 7 | 0 | 0 | 0 | 0 | 0 | 0 | 0 | 0 | 0 | 84 | 99 |  |  | PEAKS DB |
| R.LGVHNVHVHYEDEQIRVPK.E | N | 95.24 | 2268.1763 | 19 | 0.0 | 568.0513 | 4 | 23.37 | 4 | F4:8250 | NaNaKA16\_F12.raw |  |  |  | 1.3694E8 |  |  |  |  |  |  |  |  |  | 3 | 0 | 0 | 0 | 3 | 0 | 0 | 0 | 0 | 0 | 0 | 0 | 0 | 0 | 84 | 102 |  |  | PEAKS DB |
| R.FPC(+57.02)AQLLEPGVYTK.V | N | 90.02 | 1621.8174 | 14 | 0.7 | 811.9165 | 2 | 63.25 | 4 | F4:41641 | NaNaKA16\_F12.raw |  |  | 4.8534E6 | 6.8218E8 | 1.5279E6 |  |  |  |  |  |  |  |  | 10 | 0 | 0 | 1 | 8 | 1 | 0 | 0 | 0 | 0 | 0 | 0 | 0 | 0 | 231 | 244 | Carbamidomethylation | C3:Carbamidomethylation:1000.00 | PEAKS DB |
| G.QIQGIVSWGR.F | N | 61.28 | 1142.6196 | 10 | -0.6 | 572.3168 | 2 | 83.53 | 4 | F4:57766 | NaNaKA16\_F12.raw |  |  | 3.0835E6 | 3.6982E8 |  |  |  |  |  |  |  |  |  | 3 | 0 | 0 | 1 | 2 | 0 | 0 | 0 | 0 | 0 | 0 | 0 | 0 | 0 | 221 | 230 |  |  | PEAKS DB |
| P.C(+57.02)AQLLEPGVYTK.V | N | 56.21 | 1377.6962 | 12 | 0.9 | 689.8560 | 2 | 38.93 | 4 | F4:20939 | NaNaKA16\_F12.raw |  |  |  | 2.6297E6 |  |  |  |  |  |  |  |  |  | 1 | 0 | 0 | 0 | 1 | 0 | 0 | 0 | 0 | 0 | 0 | 0 | 0 | 0 | 233 | 244 | Carbamidomethylation | C1:Carbamidomethylation:1000.00 | PEAKS DB |
| N.YSEHIAPLSLPSNPPR.M | Y | 51.85 | 1776.9158 | 16 | -0.2 | 593.3124 | 3 | 41.69 | 4 | F4:23304 | NaNaKA16\_F12.raw |  |  |  | 1.7578E6 |  |  |  |  |  |  |  |  |  | 1 | 0 | 0 | 0 | 1 | 0 | 0 | 0 | 0 | 0 | 0 | 0 | 0 | 0 | 131 | 146 |  |  | PEAKS DB |
| N.GQIQGIVSWGR.F | N | 51.14 | 1199.6411 | 11 | 0.9 | 600.8284 | 2 | 49.57 | 4 | F4:29910 | NaNaKA16\_F12.raw |  |  |  | 3.2311E6 |  |  |  |  |  |  |  |  |  | 1 | 0 | 0 | 0 | 1 | 0 | 0 | 0 | 0 | 0 | 0 | 0 | 0 | 0 | 220 | 230 |  |  | PEAKS DB |
| C.AQLLEPGVYTK.V | N | 44.01 | 1217.6655 | 11 | 0.3 | 609.8402 | 2 | 35.96 | 4 | F4:18428 | NaNaKA16\_F12.raw |  |  |  | 4.2348E6 |  |  |  |  |  |  |  |  |  | 1 | 0 | 0 | 0 | 1 | 0 | 0 | 0 | 0 | 0 | 0 | 0 | 0 | 0 | 234 | 244 |  |  | PEAKS DB |
| total 8 peptides |  |  |  |  |  |  |  |  |  |  |  |  |  |  |  |  |  |  |  |  |  |  |  |  |  |  |  |  |  |  |  |  |  |  |  |  |  |  |  |  |  |  |  |
| --- | --- | --- | --- | --- | --- | --- | --- | --- | --- | --- | --- | --- | --- | --- | --- | --- | --- | --- | --- | --- | --- | --- | --- | --- | --- | --- | --- | --- | --- | --- | --- | --- | --- | --- | --- | --- | --- | --- | --- | --- | --- | --- | --- |

XP\_026522177.1

back to list

  

| Protein Coverage
| Supporting Peptides
|

Protein Coverage:

Supporting Peptides:

| Peptide | Uniq | -10lgP | Mass | Length | ppm | m/z | z | RT | Fraction | Scan | Source File | Area F1 | Area F10 | Area F11 | Area F12 | Area F13 | Area F2 | Area F3 | Area F4 | Area F5 | Area F6 | Area F7 | Area F8 | Area F9 | #Feature | #Feature F1 | #Feature F10 | #Feature F11 | #Feature F12 | #Feature F13 | #Feature F2 | #Feature F3 | #Feature F4 | #Feature F5 | #Feature F6 | #Feature F7 | #Feature F8 | #Feature F9 | Start | End | PTM | AScore | Found By |
| --- | --- | --- | --- | --- | --- | --- | --- | --- | --- | --- | --- | --- | --- | --- | --- | --- | --- | --- | --- | --- | --- | --- | --- | --- | --- | --- | --- | --- | --- | --- | --- | --- | --- | --- | --- | --- | --- | --- | --- | --- | --- | --- | --- |
| R.LGVHNVHVHYEDEQIR.V | N | 101.62 | 1943.9602 | 16 | 0.2 | 486.9974 | 4 | 17.09 | 4 | F4:3865 | NaNaKA16\_F12.raw |  |  |  | 6.3497E8 |  |  |  |  |  |  |  |  |  | 7 | 0 | 0 | 0 | 7 | 0 | 0 | 0 | 0 | 0 | 0 | 0 | 0 | 0 | 75 | 90 |  |  | PEAKS DB |
| R.LGVHNVHVHYEDEQIRVPK.E | N | 95.24 | 2268.1763 | 19 | 0.0 | 568.0513 | 4 | 23.37 | 4 | F4:8250 | NaNaKA16\_F12.raw |  |  |  | 1.3694E8 |  |  |  |  |  |  |  |  |  | 3 | 0 | 0 | 0 | 3 | 0 | 0 | 0 | 0 | 0 | 0 | 0 | 0 | 0 | 75 | 93 |  |  | PEAKS DB |
| R.FPC(+57.02)AQLLEPGVYTK.V | N | 90.02 | 1621.8174 | 14 | 0.7 | 811.9165 | 2 | 63.25 | 4 | F4:41641 | NaNaKA16\_F12.raw |  |  | 4.8534E6 | 6.8218E8 | 1.5279E6 |  |  |  |  |  |  |  |  | 10 | 0 | 0 | 1 | 8 | 1 | 0 | 0 | 0 | 0 | 0 | 0 | 0 | 0 | 222 | 235 | Carbamidomethylation | C3:Carbamidomethylation:1000.00 | PEAKS DB |
| G.QIQGIVSWGR.F | N | 61.28 | 1142.6196 | 10 | -0.6 | 572.3168 | 2 | 83.53 | 4 | F4:57766 | NaNaKA16\_F12.raw |  |  | 3.0835E6 | 3.6982E8 |  |  |  |  |  |  |  |  |  | 3 | 0 | 0 | 1 | 2 | 0 | 0 | 0 | 0 | 0 | 0 | 0 | 0 | 0 | 212 | 221 |  |  | PEAKS DB |
| P.C(+57.02)AQLLEPGVYTK.V | N | 56.21 | 1377.6962 | 12 | 0.9 | 689.8560 | 2 | 38.93 | 4 | F4:20939 | NaNaKA16\_F12.raw |  |  |  | 2.6297E6 |  |  |  |  |  |  |  |  |  | 1 | 0 | 0 | 0 | 1 | 0 | 0 | 0 | 0 | 0 | 0 | 0 | 0 | 0 | 224 | 235 | Carbamidomethylation | C1:Carbamidomethylation:1000.00 | PEAKS DB |
| N.YSEHIAPLSLPSNPPR.M | Y | 51.85 | 1776.9158 | 16 | -0.2 | 593.3124 | 3 | 41.69 | 4 | F4:23304 | NaNaKA16\_F12.raw |  |  |  | 1.7578E6 |  |  |  |  |  |  |  |  |  | 1 | 0 | 0 | 0 | 1 | 0 | 0 | 0 | 0 | 0 | 0 | 0 | 0 | 0 | 122 | 137 |  |  | PEAKS DB |
| N.GQIQGIVSWGR.F | N | 51.14 | 1199.6411 | 11 | 0.9 | 600.8284 | 2 | 49.57 | 4 | F4:29910 | NaNaKA16\_F12.raw |  |  |  | 3.2311E6 |  |  |  |  |  |  |  |  |  | 1 | 0 | 0 | 0 | 1 | 0 | 0 | 0 | 0 | 0 | 0 | 0 | 0 | 0 | 211 | 221 |  |  | PEAKS DB |
| C.AQLLEPGVYTK.V | N | 44.01 | 1217.6655 | 11 | 0.3 | 609.8402 | 2 | 35.96 | 4 | F4:18428 | NaNaKA16\_F12.raw |  |  |  | 4.2348E6 |  |  |  |  |  |  |  |  |  | 1 | 0 | 0 | 0 | 1 | 0 | 0 | 0 | 0 | 0 | 0 | 0 | 0 | 0 | 225 | 235 |  |  | PEAKS DB |
| total 8 peptides |  |  |  |  |  |  |  |  |  |  |  |  |  |  |  |  |  |  |  |  |  |  |  |  |  |  |  |  |  |  |  |  |  |  |  |  |  |  |  |  |  |  |  |
| --- | --- | --- | --- | --- | --- | --- | --- | --- | --- | --- | --- | --- | --- | --- | --- | --- | --- | --- | --- | --- | --- | --- | --- | --- | --- | --- | --- | --- | --- | --- | --- | --- | --- | --- | --- | --- | --- | --- | --- | --- | --- | --- | --- |

P82463.1

back to list

  

| Protein Coverage
| Supporting Peptides
|

Protein Coverage:

Supporting Peptides:

| Peptide | Uniq | -10lgP | Mass | Length | ppm | m/z | z | RT | Fraction | Scan | Source File | Area F1 | Area F10 | Area F11 | Area F12 | Area F13 | Area F2 | Area F3 | Area F4 | Area F5 | Area F6 | Area F7 | Area F8 | Area F9 | #Feature | #Feature F1 | #Feature F10 | #Feature F11 | #Feature F12 | #Feature F13 | #Feature F2 | #Feature F3 | #Feature F4 | #Feature F5 | #Feature F6 | #Feature F7 | #Feature F8 | #Feature F9 | Start | End | PTM | AScore | Found By |
| --- | --- | --- | --- | --- | --- | --- | --- | --- | --- | --- | --- | --- | --- | --- | --- | --- | --- | --- | --- | --- | --- | --- | --- | --- | --- | --- | --- | --- | --- | --- | --- | --- | --- | --- | --- | --- | --- | --- | --- | --- | --- | --- | --- |
| K.SIFGVTTEDC(+57.02)PDGQNLC(+57.02)FK.R | Y | 97.83 | 2186.9612 | 19 | -0.6 | 1094.4872 | 2 | 66.71 | 9 | F9:45775 | NaNaKA16\_F5.raw |  | 6.3064E6 | 2.4346E6 |  |  |  |  | 2.938E6 | 1.407E10 | 1.3055E8 | 3.8362E6 | 2.3887E6 | 1.6324E7 | 21 | 0 | 1 | 1 | 0 | 0 | 0 | 0 | 1 | 11 | 3 | 1 | 1 | 2 | 8 | 26 | Carbamidomethylation | C10:Carbamidomethylation:1000.00;C17:Carbamidomethylation:1000.00 | PEAKS DB |
| K.SIFGVTTEDC(+57.02)PDGQNLC(+57.02)FKR.W | Y | 91.00 | 2343.0623 | 20 | 0.8 | 782.0287 | 3 | 52.75 | 9 | F9:33810 | NaNaKA16\_F5.raw |  |  |  |  |  |  |  |  | 1.8626E7 |  |  |  |  | 1 | 0 | 0 | 0 | 0 | 0 | 0 | 0 | 0 | 1 | 0 | 0 | 0 | 0 | 8 | 27 | Carbamidomethylation | C10:Carbamidomethylation:1000.00;C17:Carbamidomethylation:1000.00 | PEAKS DB |
| R.GC(+57.02)AATC(+57.02)PIAENRDVIEC(+57.02)C(+57.02)STDK.C | Y | 82.94 | 2526.0608 | 22 | 0.1 | 843.0276 | 3 | 26.01 | 9 | F9:12076 | NaNaKA16\_F5.raw |  |  |  |  |  |  |  |  | 1.1949E8 |  |  |  |  | 3 | 0 | 0 | 0 | 0 | 0 | 0 | 0 | 0 | 3 | 0 | 0 | 0 | 0 | 41 | 62 | Carbamidomethylation | C2:Carbamidomethylation:1000.00;C6:Carbamidomethylation:1000.00;C17:Carbamidomethylation:1000.00;C18:Carbamidomethylation:1000.00 | PEAKS DB |
| R.GC(+57.02)AATC(+57.02)PIAENR.D | Y | 69.11 | 1318.5758 | 12 | 0.5 | 660.2955 | 2 | 11.86 | 9 | F9:1876 | NaNaKA16\_F5.raw |  |  |  |  |  |  |  |  | 2.4558E6 |  |  |  |  | 1 | 0 | 0 | 0 | 0 | 0 | 0 | 0 | 0 | 1 | 0 | 0 | 0 | 0 | 41 | 52 | Carbamidomethylation | C2:Carbamidomethylation:1000.00;C6:Carbamidomethylation:1000.00 | PEAKS DB |
| R.WHMIVPGR.Y | Y | 69.06 | 994.5171 | 8 | -1.8 | 498.2649 | 2 | 31.00 | 9 | F9:16710 | NaNaKA16\_F5.raw |  |  |  | 3.3475E5 |  |  |  |  | 4.6031E9 | 4.7328E7 | 2.2059E6 |  |  | 25 | 0 | 0 | 0 | 1 | 0 | 0 | 0 | 0 | 21 | 2 | 1 | 0 | 0 | 28 | 35 |  |  | PEAKS DB |
| F.GVTTEDC(+57.02)PDGQNLC(+57.02)FK.R | Y | 67.79 | 1839.7767 | 16 | 0.1 | 920.8958 | 2 | 34.49 | 9 | F9:18426 | NaNaKA16\_F5.raw |  |  |  |  |  |  |  |  | 8.7884E6 |  |  |  |  | 1 | 0 | 0 | 0 | 0 | 0 | 0 | 0 | 0 | 1 | 0 | 0 | 0 | 0 | 11 | 26 | Carbamidomethylation | C7:Carbamidomethylation:1000.00;C14:Carbamidomethylation:1000.00 | PEAKS DB |
| R.WHM(+15.99)IVPGR.Y | Y | 63.81 | 1010.5120 | 8 | 0.2 | 506.2634 | 2 | 18.00 | 9 | F9:4372 | NaNaKA16\_F5.raw |  |  |  |  |  |  |  |  | 4.2675E8 | 4.9641E5 |  |  |  | 12 | 0 | 0 | 0 | 0 | 0 | 0 | 0 | 0 | 11 | 1 | 0 | 0 | 0 | 28 | 35 | Oxidation (M) | M3:Oxidation (M):1000.00 | PEAKS DB |
| K.RWHMIVPGR.Y | Y | 54.49 | 1150.6182 | 9 | 0.4 | 384.5468 | 3 | 18.86 | 9 | F9:6666 | NaNaKA16\_F5.raw |  |  |  |  |  |  |  |  | 1.434E6 |  |  |  |  | 2 | 0 | 0 | 0 | 0 | 0 | 0 | 0 | 0 | 2 | 0 | 0 | 0 | 0 | 27 | 35 |  |  | PEAKS DB |
| I.FGVTTEDC(+57.02)PDGQNLC(+57.02)FK.R | Y | 42.20 | 1986.8451 | 17 | 1.0 | 994.4308 | 2 | 50.65 | 9 | F9:32237 | NaNaKA16\_F5.raw |  |  |  |  |  |  |  |  | 1.0969E6 |  |  |  |  | 1 | 0 | 0 | 0 | 0 | 0 | 0 | 0 | 0 | 1 | 0 | 0 | 0 | 0 | 10 | 26 | Carbamidomethylation | C8:Carbamidomethylation:1000.00;C15:Carbamidomethylation:1000.00 | PEAKS DB |
| total 9 peptides |  |  |  |  |  |  |  |  |  |  |  |  |  |  |  |  |  |  |  |  |  |  |  |  |  |  |  |  |  |  |  |  |  |  |  |  |  |  |  |  |  |  |  |
| --- | --- | --- | --- | --- | --- | --- | --- | --- | --- | --- | --- | --- | --- | --- | --- | --- | --- | --- | --- | --- | --- | --- | --- | --- | --- | --- | --- | --- | --- | --- | --- | --- | --- | --- | --- | --- | --- | --- | --- | --- | --- | --- | --- |

P25674.1

back to list

  

| Protein Coverage
| Supporting Peptides
|

Protein Coverage:

Supporting Peptides:

| Peptide | Uniq | -10lgP | Mass | Length | ppm | m/z | z | RT | Fraction | Scan | Source File | Area F1 | Area F10 | Area F11 | Area F12 | Area F13 | Area F2 | Area F3 | Area F4 | Area F5 | Area F6 | Area F7 | Area F8 | Area F9 | #Feature | #Feature F1 | #Feature F10 | #Feature F11 | #Feature F12 | #Feature F13 | #Feature F2 | #Feature F3 | #Feature F4 | #Feature F5 | #Feature F6 | #Feature F7 | #Feature F8 | #Feature F9 | Start | End | PTM | AScore | Found By |
| --- | --- | --- | --- | --- | --- | --- | --- | --- | --- | --- | --- | --- | --- | --- | --- | --- | --- | --- | --- | --- | --- | --- | --- | --- | --- | --- | --- | --- | --- | --- | --- | --- | --- | --- | --- | --- | --- | --- | --- | --- | --- | --- | --- |
| R.VDLGC(+57.02)AATC(+57.02)PTVK.P | N | 93.61 | 1390.6584 | 13 | 6.1 | 696.3372 | 2 | 26.78 | 7 | F7:8603 | NaNaKA16\_F3.raw |  |  |  | 1.5246E7 | 1.5932E6 | 5.0659E7 | 1.9121E8 | 2.2473E10 | 6.1192E10 | 1.9939E9 | 5.3549E6 |  | 0 | 56 | 0 | 0 | 0 | 1 | 1 | 1 | 2 | 13 | 32 | 5 | 1 | 0 | 0 | 37 | 49 | Carbamidomethylation | C5:Carbamidomethylation:1000.00;C9:Carbamidomethylation:1000.00 | PEAKS DB |
| K.C(+57.02)C(+57.02)STDNC(+57.02)NPFPTR.K | N | 81.03 | 1627.6178 | 13 | 6.1 | 814.8168 | 2 | 20.86 | 8 | F8:6556 | NaNaKA16\_F4.raw | 4.0032E5 |  |  |  |  | 2.1346E6 | 1.8581E7 | 2.2172E6 | 1.8648E7 | 7.3726E5 |  |  |  | 12 | 1 | 0 | 0 | 0 | 0 | 1 | 4 | 2 | 3 | 1 | 0 | 0 | 0 | 56 | 68 | Carbamidomethylation | C1:Carbamidomethylation:1000.00;C2:Carbamidomethylation:1000.00;C7:Carbamidomethylation:1000.00 | PEAKS DB |
| C.STDNC(+57.02)NPFPTR.K | N | 69.23 | 1307.5564 | 11 | 4.8 | 654.7853 | 2 | 11.47 | 7 | F7:1955 | NaNaKA16\_F3.raw |  |  |  |  |  | 3.2217E6 | 1.3046E7 | 2.4922E6 | 1.0115E7 |  |  |  |  | 5 | 0 | 0 | 0 | 0 | 0 | 1 | 1 | 1 | 2 | 0 | 0 | 0 | 0 | 58 | 68 | Carbamidomethylation | C5:Carbamidomethylation:1000.00 | PEAKS DB |
| C.C(+57.02)STDNC(+57.02)NPFPTR.K | N | 66.15 | 1467.5872 | 12 | 0.1 | 734.8009 | 2 | 20.98 | 6 | F6:8194 | NaNaKA16\_F2.raw | 3.5509E6 |  |  |  |  | 1.6348E6 | 1.0498E7 |  | 1.4874E7 |  |  |  |  | 5 | 1 | 0 | 0 | 0 | 0 | 1 | 2 | 0 | 1 | 0 | 0 | 0 | 0 | 57 | 68 | Carbamidomethylation | C1:Carbamidomethylation:1000.00;C6:Carbamidomethylation:1000.00 | PEAKS DB |
| R.VDLGC(+57.02)AATC(+57.02)PTV.K | N | 45.13 | 1262.5635 | 12 | -0.9 | 632.2885 | 2 | 50.91 | 10 | F10:33445 | NaNaKA16\_F6.raw |  |  |  |  |  |  | 1.1303E7 |  |  | 5.2132E7 |  |  |  | 2 | 0 | 0 | 0 | 0 | 0 | 0 | 1 | 0 | 0 | 1 | 0 | 0 | 0 | 37 | 48 | Carbamidomethylation | C5:Carbamidomethylation:1000.00;C9:Carbamidomethylation:1000.00 | PEAKS DB |
| T.DNC(+57.02)NPFPTR.K | N | 44.94 | 1119.4767 | 9 | 0.7 | 560.7460 | 2 | 23.18 | 6 | F6:10002 | NaNaKA16\_F2.raw |  |  |  |  |  | 3.0548E5 |  |  |  |  |  |  |  | 1 | 0 | 0 | 0 | 0 | 0 | 1 | 0 | 0 | 0 | 0 | 0 | 0 | 0 | 60 | 68 | Carbamidomethylation | C3:Carbamidomethylation:1000.00 | PEAKS DB |
| K.RVDLGC(+57.02)AATC(+57.02)PTVK.P | N | 44.70 | 1546.7595 | 14 | -0.8 | 516.5934 | 3 | 18.27 | 9 | F9:5871 | NaNaKA16\_F5.raw |  |  |  |  |  |  |  | 1.5478E5 | 2.2798E6 |  |  |  |  | 2 | 0 | 0 | 0 | 0 | 0 | 0 | 0 | 1 | 1 | 0 | 0 | 0 | 0 | 36 | 49 | Carbamidomethylation | C6:Carbamidomethylation:1000.00;C10:Carbamidomethylation:1000.00 | PEAKS DB |
| G.VDIKC(+57.02)C(+57.02)STDNC(+57.02)NPFPTR.K | Y | 42.24 | 2082.8921 | 17 | -4.8 | 695.2978 | 3 | 40.50 | 7 | F7:19795 | NaNaKA16\_F3.raw |  |  |  |  |  |  | 4.6388E6 |  |  |  |  |  |  | 1 | 0 | 0 | 0 | 0 | 0 | 0 | 1 | 0 | 0 | 0 | 0 | 0 | 0 | 52 | 68 | Carbamidomethylation | C5:Carbamidomethylation:1000.00;C6:Carbamidomethylation:1000.00;C11:Carbamidomethylation:1000.00 | PEAKS DB |
| D.NC(+57.02)NPFPTR.K | N | 41.96 | 1004.4498 | 8 | -0.2 | 503.2321 | 2 | 43.27 | 9 | F9:25860 | NaNaKA16\_F5.raw |  |  |  |  |  |  |  |  | 0 |  |  |  |  | 0 | 0 | 0 | 0 | 0 | 0 | 0 | 0 | 0 | 0 | 0 | 0 | 0 | 0 | 61 | 68 | Carbamidomethylation | C2:Carbamidomethylation:1000.00 | PEAKS DB |
| total 9 peptides |  |  |  |  |  |  |  |  |  |  |  |  |  |  |  |  |  |  |  |  |  |  |  |  |  |  |  |  |  |  |  |  |  |  |  |  |  |  |  |  |  |  |  |
| --- | --- | --- | --- | --- | --- | --- | --- | --- | --- | --- | --- | --- | --- | --- | --- | --- | --- | --- | --- | --- | --- | --- | --- | --- | --- | --- | --- | --- | --- | --- | --- | --- | --- | --- | --- | --- | --- | --- | --- | --- | --- | --- | --- |

P0DI91.1

back to list

  

| Protein Coverage
| Supporting Peptides
|

Protein Coverage:

Supporting Peptides:

| Peptide | Uniq | -10lgP | Mass | Length | ppm | m/z | z | RT | Fraction | Scan | Source File | Area F1 | Area F10 | Area F11 | Area F12 | Area F13 | Area F2 | Area F3 | Area F4 | Area F5 | Area F6 | Area F7 | Area F8 | Area F9 | #Feature | #Feature F1 | #Feature F10 | #Feature F11 | #Feature F12 | #Feature F13 | #Feature F2 | #Feature F3 | #Feature F4 | #Feature F5 | #Feature F6 | #Feature F7 | #Feature F8 | #Feature F9 | Start | End | PTM | AScore | Found By |
| --- | --- | --- | --- | --- | --- | --- | --- | --- | --- | --- | --- | --- | --- | --- | --- | --- | --- | --- | --- | --- | --- | --- | --- | --- | --- | --- | --- | --- | --- | --- | --- | --- | --- | --- | --- | --- | --- | --- | --- | --- | --- | --- | --- |
| R.RSPLEEC(+57.02)FQQNDYEEFLEIAR.N | Y | 83.05 | 2672.2175 | 21 | 1.4 | 891.7477 | 3 | 78.80 | 4 | F4:53897 | NaNaKA16\_F12.raw |  |  |  | 2.2899E7 |  |  |  |  |  | 1.3716E6 |  |  |  | 2 | 0 | 0 | 0 | 1 | 0 | 0 | 0 | 0 | 0 | 1 | 0 | 0 | 0 | 4 | 24 | Carbamidomethylation | C7:Carbamidomethylation:1000.00 | PEAKS DB |
| K.REIQALC(+57.02)YPSIK.K | N | 74.57 | 1476.7759 | 12 | -0.3 | 493.2658 | 3 | 32.85 | 4 | F4:15922 | NaNaKA16\_F12.raw |  |  |  | 1.3566E7 | 2.8894E6 |  |  |  |  |  |  |  | 2.7998E5 | 4 | 0 | 0 | 0 | 2 | 1 | 0 | 0 | 0 | 0 | 0 | 0 | 0 | 1 | 83 | 94 | Carbamidomethylation | C7:Carbamidomethylation:1000.00 | PEAKS DB |
| R.EIQALC(+57.02)YPSIK.K | N | 73.42 | 1320.6747 | 11 | -0.2 | 661.3445 | 2 | 47.89 | 3 | F3:27602 | NaNaKA16\_F11.raw |  | 0 | 1.5106E7 | 3.3878E7 | 2.1775E7 |  |  |  |  |  |  |  | 3.2256E6 | 4 | 0 | 0 | 1 | 1 | 1 | 0 | 0 | 0 | 0 | 0 | 0 | 0 | 1 | 84 | 94 | Carbamidomethylation | C6:Carbamidomethylation:1000.00 | PEAKS DB |
| R.SPLEEC(+57.02)FQQNDYEEFLEIAR.N | Y | 69.73 | 2516.1165 | 20 | 0.9 | 839.7135 | 3 | 88.96 | 4 | F4:62291 | NaNaKA16\_F12.raw |  |  |  | 1.3696E6 |  |  |  |  |  | 4.3724E5 |  |  | 7.453E5 | 3 | 0 | 0 | 0 | 1 | 0 | 0 | 0 | 0 | 0 | 1 | 0 | 0 | 1 | 5 | 24 | Carbamidomethylation | C6:Carbamidomethylation:1000.00 | PEAKS DB |
| K.KFWEADGIHGGK.V | N | 66.46 | 1343.6622 | 12 | -0.8 | 448.8943 | 3 | 17.58 | 5 | F5:3842 | NaNaKA16\_F13.raw |  |  |  |  | 2.6107E6 |  |  |  |  |  |  |  |  | 2 | 0 | 0 | 0 | 0 | 2 | 0 | 0 | 0 | 0 | 0 | 0 | 0 | 0 | 58 | 69 |  |  | PEAKS DB |
| N.DLSLIHDLPK.R | N | 62.72 | 1149.6393 | 10 | 1.4 | 575.8277 | 2 | 52.52 | 3 | F3:29824 | NaNaKA16\_F11.raw |  | 1.6899E6 | 7.7612E6 | 4.7179E6 |  |  |  |  |  |  |  |  |  | 4 | 0 | 1 | 1 | 2 | 0 | 0 | 0 | 0 | 0 | 0 | 0 | 0 | 0 | 73 | 82 |  |  | PEAKS DB |
| K.FWEADGIHGGK.V | N | 57.27 | 1215.5673 | 11 | -0.2 | 608.7908 | 2 | 22.40 | 5 | F5:5921 | NaNaKA16\_F13.raw |  |  |  |  | 1.8542E7 |  |  |  |  |  |  |  |  | 2 | 0 | 0 | 0 | 0 | 2 | 0 | 0 | 0 | 0 | 0 | 0 | 0 | 0 | 59 | 69 |  |  | PEAKS DB |
| E.DSSYHLSFIESLK.S | Y | 54.67 | 1524.7460 | 13 | -0.4 | 509.2557 | 3 | 60.89 | 2 | F2:38089 | NaNaKA16\_F10.raw |  | 1.0135E6 |  |  |  |  |  |  |  |  |  |  |  | 1 | 0 | 1 | 0 | 0 | 0 | 0 | 0 | 0 | 0 | 0 | 0 | 0 | 0 | 36 | 48 |  |  | PEAKS DB |
| K.SDALFSYEK.K | N | 53.51 | 1058.4919 | 9 | 1.2 | 530.2539 | 2 | 36.12 | 5 | F5:13813 | NaNaKA16\_F13.raw |  |  |  | 1.7934E6 | 2.9321E7 |  |  |  |  |  |  |  |  | 2 | 0 | 0 | 0 | 1 | 1 | 0 | 0 | 0 | 0 | 0 | 0 | 0 | 0 | 49 | 57 |  |  | PEAKS DB |
| R.RSPLEEC(+57.02)FQQN.D | N | 46.24 | 1406.6249 | 11 | 0.2 | 704.3199 | 2 | 30.98 | 6 | F6:16775 | NaNaKA16\_F2.raw |  |  |  |  |  | 5.3696E6 |  |  |  |  |  |  |  | 1 | 0 | 0 | 0 | 0 | 0 | 1 | 0 | 0 | 0 | 0 | 0 | 0 | 0 | 4 | 14 | Carbamidomethylation | C7:Carbamidomethylation:1000.00 | PEAKS DB |
| R.RSPLEEC(+57.02)FQQNDYEEF.L | Y | 43.99 | 2089.8687 | 16 | 0.5 | 1045.9421 | 2 | 63.35 | 9 | F9:43147 | NaNaKA16\_F5.raw |  |  |  |  |  |  |  |  | 2.7322E6 |  |  |  |  | 1 | 0 | 0 | 0 | 0 | 0 | 0 | 0 | 0 | 1 | 0 | 0 | 0 | 0 | 4 | 19 | Carbamidomethylation | C7:Carbamidomethylation:1000.00 | PEAKS DB |
| total 11 peptides |  |  |  |  |  |  |  |  |  |  |  |  |  |  |  |  |  |  |  |  |  |  |  |  |  |  |  |  |  |  |  |  |  |  |  |  |  |  |  |  |  |  |  |
| --- | --- | --- | --- | --- | --- | --- | --- | --- | --- | --- | --- | --- | --- | --- | --- | --- | --- | --- | --- | --- | --- | --- | --- | --- | --- | --- | --- | --- | --- | --- | --- | --- | --- | --- | --- | --- | --- | --- | --- | --- | --- | --- | --- |

P86540.2

back to list

  

| Protein Coverage
| Supporting Peptides
|

Protein Coverage:

Supporting Peptides:

| Peptide | Uniq | -10lgP | Mass | Length | ppm | m/z | z | RT | Fraction | Scan | Source File | Area F1 | Area F10 | Area F11 | Area F12 | Area F13 | Area F2 | Area F3 | Area F4 | Area F5 | Area F6 | Area F7 | Area F8 | Area F9 | #Feature | #Feature F1 | #Feature F10 | #Feature F11 | #Feature F12 | #Feature F13 | #Feature F2 | #Feature F3 | #Feature F4 | #Feature F5 | #Feature F6 | #Feature F7 | #Feature F8 | #Feature F9 | Start | End | PTM | AScore | Found By |
| --- | --- | --- | --- | --- | --- | --- | --- | --- | --- | --- | --- | --- | --- | --- | --- | --- | --- | --- | --- | --- | --- | --- | --- | --- | --- | --- | --- | --- | --- | --- | --- | --- | --- | --- | --- | --- | --- | --- | --- | --- | --- | --- | --- |
| K.LIPLAYKTC(+57.02)PAGK.D | N | 79.70 | 1430.7955 | 13 | 0.0 | 716.4050 | 2 | 25.87 | 11 | F11:12139 | NaNaKA16\_F7.raw |  | 9.7323E4 |  |  |  |  |  |  | 0 | 4.3516E7 | 4.1837E7 | 2.4864E6 |  | 7 | 0 | 1 | 0 | 0 | 0 | 0 | 0 | 0 | 0 | 2 | 2 | 2 | 0 | 6 | 18 | Carbamidomethylation | C9:Carbamidomethylation:1000.00 | PEAKS DB |
| K.NSLLVKYEC(+57.02)C(+57.02)NTDRC(+57.02)N | N | 75.75 | 2044.8765 | 16 | 0.3 | 682.6329 | 3 | 23.00 | 11 | F11:9699 | NaNaKA16\_F7.raw |  |  |  |  |  |  |  |  | 9.8402E5 |  | 2.1018E7 | 1.6302E6 |  | 4 | 0 | 0 | 0 | 0 | 0 | 0 | 0 | 0 | 1 | 0 | 2 | 1 | 0 | 45 | 60 | Carbamidomethylation | C9:Carbamidomethylation:1000.00;C10:Carbamidomethylation:1000.00;C15:Carbamidomethylation:1000.00 | PEAKS DB |
| K.NSLLVKYEC(+57.02)C(+57.02)NTDR.C | N | 74.45 | 1770.8029 | 14 | 0.3 | 591.2751 | 3 | 21.35 | 9 | F9:8563 | NaNaKA16\_F5.raw |  |  |  |  |  |  |  |  | 6.2524E6 | 9.9017E6 | 4.0334E7 | 1.719E6 |  | 7 | 0 | 0 | 0 | 0 | 0 | 0 | 0 | 0 | 2 | 2 | 2 | 1 | 0 | 45 | 58 | Carbamidomethylation | C9:Carbamidomethylation:1000.00;C10:Carbamidomethylation:1000.00 | PEAKS DB |
| R.GC(+57.02)IDVC(+57.02)PKNSLLVK.Y | N | 68.20 | 1601.8269 | 14 | 1.2 | 801.9217 | 2 | 32.93 | 11 | F11:17854 | NaNaKA16\_F7.raw |  |  |  | 3.4688E6 |  |  |  |  | 2.4226E7 | 4.8159E7 | 3.5284E7 | 1.3913E8 | 3.2893E6 | 11 | 0 | 0 | 0 | 1 | 0 | 0 | 0 | 0 | 2 | 2 | 3 | 2 | 1 | 37 | 50 | Carbamidomethylation | C2:Carbamidomethylation:1000.00;C6:Carbamidomethylation:1000.00 | PEAKS DB |
| K.MYMVSDKTVPVK.R | Y | 66.57 | 1396.7095 | 12 | 0.4 | 699.3623 | 2 | 24.45 | 10 | F10:10888 | NaNaKA16\_F6.raw |  |  |  |  |  |  |  |  |  | 5.2696E6 | 8.0968E5 |  |  | 9 | 0 | 0 | 0 | 0 | 0 | 0 | 0 | 0 | 0 | 6 | 3 | 0 | 0 | 24 | 35 |  |  | PEAKS DB |
| K.C(+57.02)NKLIPLAYK.T | N | 64.76 | 1218.6794 | 10 | 0.0 | 610.3470 | 2 | 27.82 | 11 | F11:13604 | NaNaKA16\_F7.raw |  |  |  |  |  |  |  |  |  | 4.8462E6 | 2.3967E7 | 1.338E6 |  | 6 | 0 | 0 | 0 | 0 | 0 | 0 | 0 | 0 | 0 | 2 | 2 | 2 | 0 | 3 | 12 | Carbamidomethylation | C1:Carbamidomethylation:1000.00 | PEAKS DB |
| K.YEC(+57.02)C(+57.02)NTDR.C | N | 48.64 | 1116.3965 | 8 | 0.5 | 559.2058 | 2 | 11.28 | 2 | F2:1740 | NaNaKA16\_F10.raw |  | 8.8663E5 |  |  |  |  |  |  |  |  |  | 7.7742E5 |  | 2 | 0 | 1 | 0 | 0 | 0 | 0 | 0 | 0 | 0 | 0 | 0 | 1 | 0 | 51 | 58 | Carbamidomethylation | C3:Carbamidomethylation:1000.00;C4:Carbamidomethylation:1000.00 | PEAKS DB |
| K.MYMVSDKTVPVKR.G | Y | 46.93 | 1552.8105 | 13 | -0.3 | 389.2098 | 4 | 17.80 | 11 | F11:5501 | NaNaKA16\_F7.raw |  |  |  |  |  |  |  |  |  |  | 3.3127E5 |  |  | 1 | 0 | 0 | 0 | 0 | 0 | 0 | 0 | 0 | 0 | 0 | 1 | 0 | 0 | 24 | 36 |  |  | PEAKS DB |
| R.GC(+57.02)IDVC(+57.02)PK.N | N | 46.03 | 947.4205 | 8 | -0.5 | 474.7173 | 2 | 11.54 | 12 | F12:1895 | NaNaKA16\_F8.raw |  |  | 1.5891E7 |  |  |  | 6.084E5 |  | 2.0697E6 | 6.3919E7 | 1.8242E6 | 6.8843E6 |  | 6 | 0 | 0 | 1 | 0 | 0 | 0 | 1 | 0 | 1 | 1 | 1 | 1 | 0 | 37 | 44 | Carbamidomethylation | C2:Carbamidomethylation:1000.00;C6:Carbamidomethylation:1000.00 | PEAKS DB |
| K.LIPLAYK.T | N | 44.45 | 816.5109 | 7 | 0.5 | 409.2629 | 2 | 54.15 | 9 | F9:34957 | NaNaKA16\_F5.raw |  |  |  |  |  | 3.0941E7 |  | 2.6175E7 | 2.3481E7 | 0 | 9.2494E6 | 0 | 0 | 12 | 0 | 0 | 0 | 0 | 0 | 1 | 0 | 2 | 7 | 0 | 2 | 0 | 0 | 6 | 12 |  |  | PEAKS DB |
| total 10 peptides |  |  |  |  |  |  |  |  |  |  |  |  |  |  |  |  |  |  |  |  |  |  |  |  |  |  |  |  |  |  |  |  |  |  |  |  |  |  |  |  |  |  |  |
| --- | --- | --- | --- | --- | --- | --- | --- | --- | --- | --- | --- | --- | --- | --- | --- | --- | --- | --- | --- | --- | --- | --- | --- | --- | --- | --- | --- | --- | --- | --- | --- | --- | --- | --- | --- | --- | --- | --- | --- | --- | --- | --- | --- |

P01440.1

back to list

  

| Protein Coverage
| Supporting Peptides
|

Protein Coverage:

Supporting Peptides:

| Peptide | Uniq | -10lgP | Mass | Length | ppm | m/z | z | RT | Fraction | Scan | Source File | Area F1 | Area F10 | Area F11 | Area F12 | Area F13 | Area F2 | Area F3 | Area F4 | Area F5 | Area F6 | Area F7 | Area F8 | Area F9 | #Feature | #Feature F1 | #Feature F10 | #Feature F11 | #Feature F12 | #Feature F13 | #Feature F2 | #Feature F3 | #Feature F4 | #Feature F5 | #Feature F6 | #Feature F7 | #Feature F8 | #Feature F9 | Start | End | PTM | AScore | Found By |
| --- | --- | --- | --- | --- | --- | --- | --- | --- | --- | --- | --- | --- | --- | --- | --- | --- | --- | --- | --- | --- | --- | --- | --- | --- | --- | --- | --- | --- | --- | --- | --- | --- | --- | --- | --- | --- | --- | --- | --- | --- | --- | --- | --- |
| K.NLC(+57.02)YKMYMVATPK.V | N | 76.09 | 1617.7717 | 13 | 1.0 | 540.2651 | 3 | 40.76 | 3 | F3:21520 | NaNaKA16\_F11.raw |  | 1.1161E8 | 8.8933E7 | 2.7026E6 |  |  |  |  |  |  |  | 6.0171E6 | 1.0433E7 | 13 | 0 | 4 | 3 | 2 | 0 | 0 | 0 | 0 | 0 | 0 | 0 | 2 | 2 | 19 | 31 | Carbamidomethylation | C3:Carbamidomethylation:1000.00 | PEAKS DB |
| K.LVPLFYKTC(+57.02)PAGK.N | N | 73.66 | 1492.8112 | 13 | -0.3 | 498.6109 | 3 | 36.55 | 10 | F10:21078 | NaNaKA16\_F6.raw |  | 1.9305E6 | 6.2426E6 | 1.2125E7 |  |  |  |  |  | 1.1688E7 | 5.7221E5 | 1.8629E7 |  | 10 | 0 | 1 | 2 | 2 | 0 | 0 | 0 | 0 | 0 | 2 | 1 | 2 | 0 | 6 | 18 | Carbamidomethylation | C9:Carbamidomethylation:1000.00 | PEAKS DB |
| K.MYMVATPKVPVK.R | N | 69.79 | 1362.7404 | 12 | 0.3 | 682.3777 | 2 | 33.08 | 3 | F3:15035 | NaNaKA16\_F11.raw |  | 1.4824E6 | 1.7787E7 | 5.0904E6 |  |  |  |  | 0 | 3.8618E6 |  | 5.7667E6 | 1.0364E7 | 10 | 0 | 1 | 2 | 2 | 0 | 0 | 0 | 0 | 0 | 1 | 0 | 2 | 2 | 24 | 35 |  |  | PEAKS DB |
| K.LVPLFYKTC(+57.02)PAGKNLC(+57.02)YK.M | N | 68.71 | 2171.1272 | 18 | 0.0 | 543.7891 | 4 | 39.14 | 2 | F2:20436 | NaNaKA16\_F10.raw |  | 1.2494E7 | 5.7358E6 |  |  |  |  |  |  |  |  |  |  | 4 | 0 | 2 | 2 | 0 | 0 | 0 | 0 | 0 | 0 | 0 | 0 | 0 | 0 | 6 | 23 | Carbamidomethylation | C9:Carbamidomethylation:1000.00;C16:Carbamidomethylation:1000.00 | PEAKS DB |
| K.C(+57.02)NKLVPLFYK.T | N | 68.01 | 1280.6951 | 10 | -0.1 | 641.3547 | 2 | 39.35 | 4 | F4:21415 | NaNaKA16\_F12.raw |  | 3.2793E5 |  | 5.2282E6 |  |  |  |  |  |  |  | 7.4866E6 | 7.2675E6 | 7 | 0 | 1 | 0 | 2 | 0 | 0 | 0 | 0 | 0 | 0 | 0 | 2 | 2 | 3 | 12 | Carbamidomethylation | C1:Carbamidomethylation:1000.00 | PEAKS DB |
| K.MYMVATPK.V | N | 57.98 | 939.4558 | 8 | 0.2 | 470.7353 | 2 | 80.14 | 2 | F2:54827 | NaNaKA16\_F10.raw | 6.1524E5 | 4.1543E8 | 7.6807E8 | 9.3976E6 | 8.1538E6 | 3.4343E5 |  |  |  | 5.559E7 | 3.0059E7 |  | 1.5397E7 | 31 | 1 | 10 | 12 | 2 | 1 | 1 | 0 | 0 | 0 | 1 | 1 | 0 | 2 | 24 | 31 |  |  | PEAKS DB |
| R.GC(+57.02)IDVC(+57.02)PKSSLVLK.Y | Y | 57.12 | 1574.8160 | 14 | -0.3 | 525.9458 | 3 | 33.98 | 4 | F4:16775 | NaNaKA16\_F12.raw |  |  |  | 3.3882E6 |  |  |  |  |  |  |  | 1.4076E6 |  | 2 | 0 | 0 | 0 | 1 | 0 | 0 | 0 | 0 | 0 | 0 | 0 | 1 | 0 | 37 | 50 | Carbamidomethylation | C2:Carbamidomethylation:1000.00;C6:Carbamidomethylation:1000.00 | PEAKS DB |
| K.MYMVATPKVPVKR.G | N | 55.52 | 1518.8414 | 13 | 0.7 | 507.2881 | 3 | 22.16 | 3 | F3:6654 | NaNaKA16\_F11.raw |  | 4.2443E5 | 6.4334E5 |  |  |  |  |  |  |  |  | 7.6552E5 |  | 3 | 0 | 1 | 1 | 0 | 0 | 0 | 0 | 0 | 0 | 0 | 0 | 1 | 0 | 24 | 36 |  |  | PEAKS DB |
| K.YVC(+57.02)C(+57.02)NTDR.C | N | 50.92 | 1086.4222 | 8 | -0.2 | 544.2183 | 2 | 11.37 | 12 | F12:1787 | NaNaKA16\_F8.raw |  | 2.1124E6 | 2.9778E6 | 1.3296E6 |  |  |  |  |  | 1.5915E6 |  | 1.7945E6 |  | 5 | 0 | 1 | 1 | 1 | 0 | 0 | 0 | 0 | 0 | 1 | 0 | 1 | 0 | 51 | 58 | Carbamidomethylation | C3:Carbamidomethylation:1000.00;C4:Carbamidomethylation:1000.00 | PEAKS DB |
| K.MYM(+15.99)VATPK.V | N | 50.67 | 955.4507 | 8 | 0.9 | 478.7330 | 2 | 12.45 | 9 | F9:2420 | NaNaKA16\_F5.raw |  | 0 | 1.5678E8 |  |  |  |  |  | 2.9325E5 | 1.4671E7 | 4.6131E6 | 3.2608E5 | 4.1111E6 | 10 | 0 | 0 | 3 | 0 | 0 | 0 | 0 | 0 | 1 | 2 | 2 | 1 | 1 | 24 | 31 | Oxidation (M) | M3:Oxidation (M):39.76 | PEAKS DB |
| K.M(+15.99)YMVATPK.V | N | 50.53 | 955.4507 | 8 | -1.4 | 478.7319 | 2 | 23.80 | 3 | F3:7731 | NaNaKA16\_F11.raw |  | 4.99E7 | 1.1486E8 |  |  |  |  |  |  | 1.4502E7 |  |  |  | 5 | 0 | 1 | 2 | 0 | 0 | 0 | 0 | 0 | 0 | 2 | 0 | 0 | 0 | 24 | 31 | Oxidation (M) | M1:Oxidation (M):40.21 | PEAKS DB |
| K.M(+15.99)YM(+15.99)VATPK.V | N | 49.54 | 971.4456 | 8 | -0.9 | 486.7296 | 2 | 18.43 | 3 | F3:3370 | NaNaKA16\_F11.raw |  | 2.4924E6 | 7.1227E6 |  |  |  |  |  |  | 6.7927E6 |  |  |  | 5 | 0 | 1 | 2 | 0 | 0 | 0 | 0 | 0 | 0 | 2 | 0 | 0 | 0 | 24 | 31 | Oxidation (M) | M1:Oxidation (M):1000.00;M3:Oxidation (M):1000.00 | PEAKS DB |
| K.SSLVLKYVC(+57.02)C(+57.02)NTDRC(+57.02)N | Y | 48.34 | 1987.8914 | 16 | 0.9 | 994.9539 | 2 | 34.13 | 2 | F2:16403 | NaNaKA16\_F10.raw |  | 9.2923E5 |  |  |  |  |  |  |  |  |  |  |  | 1 | 0 | 1 | 0 | 0 | 0 | 0 | 0 | 0 | 0 | 0 | 0 | 0 | 0 | 45 | 60 | Carbamidomethylation | C9:Carbamidomethylation:1000.00;C10:Carbamidomethylation:1000.00;C15:Carbamidomethylation:1000.00 | PEAKS DB |
| K.NLC(+57.02)YKM(+15.99)YMVATPK.V | N | 48.00 | 1633.7666 | 13 | 0.4 | 817.8909 | 2 | 40.76 | 3 | F3:21375 | NaNaKA16\_F11.raw |  |  | 1.9576E6 |  |  |  |  |  |  |  |  |  |  | 1 | 0 | 0 | 1 | 0 | 0 | 0 | 0 | 0 | 0 | 0 | 0 | 0 | 0 | 19 | 31 | Carbamidomethylation; Oxidation (M) | C3:Carbamidomethylation:1000.00;M6:Oxidation (M):40.21 | PEAKS DB |
| R.GC(+57.02)IDVC(+57.02)PK.S | N | 46.03 | 947.4205 | 8 | -0.5 | 474.7173 | 2 | 11.54 | 12 | F12:1895 | NaNaKA16\_F8.raw |  |  | 1.5891E7 |  |  |  | 6.084E5 |  | 2.0697E6 | 6.3919E7 | 1.8242E6 | 6.8843E6 |  | 6 | 0 | 0 | 1 | 0 | 0 | 0 | 1 | 0 | 1 | 1 | 1 | 1 | 0 | 37 | 44 | Carbamidomethylation | C2:Carbamidomethylation:1000.00;C6:Carbamidomethylation:1000.00 | PEAKS DB |
| K.TC(+57.02)PAGKNLC(+57.02)YK.M | N | 44.80 | 1310.6111 | 11 | 0.6 | 437.8779 | 3 | 11.38 | 12 | F12:1793 | NaNaKA16\_F8.raw |  |  |  |  |  |  |  |  |  |  |  | 2.5665E5 |  | 1 | 0 | 0 | 0 | 0 | 0 | 0 | 0 | 0 | 0 | 0 | 0 | 1 | 0 | 13 | 23 | Carbamidomethylation | C2:Carbamidomethylation:1000.00;C9:Carbamidomethylation:1000.00 | PEAKS DB |
| K.MYM(+15.99)VATPKVPVK.R | N | 43.62 | 1378.7352 | 12 | -0.7 | 690.3744 | 2 | 26.28 | 12 | F12:11654 | NaNaKA16\_F8.raw |  |  |  |  |  |  |  |  |  |  |  | 5.4527E5 |  | 1 | 0 | 0 | 0 | 0 | 0 | 0 | 0 | 0 | 0 | 0 | 0 | 1 | 0 | 24 | 35 | Oxidation (M) | M3:Oxidation (M):25.86 | PEAKS DB |
| total 17 peptides |  |  |  |  |  |  |  |  |  |  |  |  |  |  |  |  |  |  |  |  |  |  |  |  |  |  |  |  |  |  |  |  |  |  |  |  |  |  |  |  |  |  |  |
| --- | --- | --- | --- | --- | --- | --- | --- | --- | --- | --- | --- | --- | --- | --- | --- | --- | --- | --- | --- | --- | --- | --- | --- | --- | --- | --- | --- | --- | --- | --- | --- | --- | --- | --- | --- | --- | --- | --- | --- | --- | --- | --- | --- |

5H7W

back to list

  

| Protein Coverage
| Supporting Peptides
|

Protein Coverage:

Supporting Peptides:

| Peptide | Uniq | -10lgP | Mass | Length | ppm | m/z | z | RT | Fraction | Scan | Source File | Area F1 | Area F10 | Area F11 | Area F12 | Area F13 | Area F2 | Area F3 | Area F4 | Area F5 | Area F6 | Area F7 | Area F8 | Area F9 | #Feature | #Feature F1 | #Feature F10 | #Feature F11 | #Feature F12 | #Feature F13 | #Feature F2 | #Feature F3 | #Feature F4 | #Feature F5 | #Feature F6 | #Feature F7 | #Feature F8 | #Feature F9 | Start | End | PTM | AScore | Found By |
| --- | --- | --- | --- | --- | --- | --- | --- | --- | --- | --- | --- | --- | --- | --- | --- | --- | --- | --- | --- | --- | --- | --- | --- | --- | --- | --- | --- | --- | --- | --- | --- | --- | --- | --- | --- | --- | --- | --- | --- | --- | --- | --- | --- |
| R.HGQGTGELLQVSGIK.V | N | 77.57 | 1522.8103 | 15 | 0.5 | 762.4128 | 2 | 32.78 | 5 | F5:12050 | NaNaKA16\_F13.raw |  |  |  | 4.2458E5 | 5.0296E6 |  |  |  |  |  |  |  |  | 3 | 0 | 0 | 0 | 1 | 2 | 0 | 0 | 0 | 0 | 0 | 0 | 0 | 0 | 418 | 432 |  |  | PEAKS DB |
| R.VVSLNVLC(+57.02)TEC(+57.02)R.V | Y | 73.80 | 1448.7115 | 12 | 0.0 | 725.3630 | 2 | 49.09 | 10 | F10:32110 | NaNaKA16\_F6.raw |  |  |  |  | 2.3522E6 |  |  |  |  | 7.2729E6 |  |  |  | 2 | 0 | 0 | 0 | 0 | 1 | 0 | 0 | 0 | 0 | 1 | 0 | 0 | 0 | 445 | 456 | Carbamidomethylation | C8:Carbamidomethylation:1000.00;C11:Carbamidomethylation:1000.00 | PEAKS DB |
| R.FHEC(+57.02)NLGNLIC(+57.02)DAVVYNNLR.H | N | 72.86 | 2420.1365 | 20 | 0.2 | 807.7196 | 3 | 76.91 | 13 | F13:50987 | NaNaKA16\_F9.raw |  |  |  |  |  |  |  |  |  |  |  | 5.2029E6 | 2.2685E7 | 3 | 0 | 0 | 0 | 0 | 0 | 0 | 0 | 0 | 0 | 0 | 0 | 1 | 2 | 330 | 349 | Carbamidomethylation | C4:Carbamidomethylation:1000.00;C11:Carbamidomethylation:1000.00 | PEAKS DB |
| R.VPTYVPLEMEK.T | N | 72.07 | 1304.6686 | 11 | 1.1 | 653.3423 | 2 | 50.51 | 5 | F5:20383 | NaNaKA16\_F13.raw |  |  | 0 | 1.0366E6 | 6.7478E6 |  |  |  |  |  |  |  |  | 2 | 0 | 0 | 0 | 1 | 1 | 0 | 0 | 0 | 0 | 0 | 0 | 0 | 0 | 457 | 467 |  |  | PEAKS DB |
| R.QVPVVQAYAFGK.Y | N | 68.51 | 1305.7081 | 12 | -0.2 | 653.8612 | 2 | 81.22 | 5 | F5:33335 | NaNaKA16\_F13.raw |  |  |  | 4.7302E5 | 4.1666E6 |  |  |  |  |  |  |  |  | 3 | 0 | 0 | 0 | 1 | 2 | 0 | 0 | 0 | 0 | 0 | 0 | 0 | 0 | 249 | 260 |  |  | PEAKS DB |
| E.LTILHTNDVHAR.L | N | 61.22 | 1388.7524 | 12 | -0.8 | 463.9244 | 3 | 12.08 | 5 | F5:1993 | NaNaKA16\_F13.raw |  |  |  |  | 9.2667E5 |  |  |  |  |  |  |  |  | 1 | 0 | 0 | 0 | 0 | 1 | 0 | 0 | 0 | 0 | 0 | 0 | 0 | 0 | 5 | 16 |  |  | PEAKS DB |
| K.ETPVLSNPGPYLEFR.D | N | 55.50 | 1717.8674 | 15 | -0.2 | 859.9409 | 2 | 68.90 | 5 | F5:28106 | NaNaKA16\_F13.raw |  |  |  |  | 1.3217E6 |  |  |  |  |  |  |  |  | 1 | 0 | 0 | 0 | 0 | 1 | 0 | 0 | 0 | 0 | 0 | 0 | 0 | 0 | 155 | 169 |  |  | PEAKS DB |
| K.VVYDLSQKPGK.R | Y | 51.28 | 1232.6764 | 11 | 0.5 | 411.8996 | 3 | 12.16 | 5 | F5:2047 | NaNaKA16\_F13.raw |  |  |  |  | 4.4733E4 |  |  |  |  |  |  |  |  | 1 | 0 | 0 | 0 | 0 | 1 | 0 | 0 | 0 | 0 | 0 | 0 | 0 | 0 | 433 | 443 |  |  | PEAKS DB |
| K.VQLQNYSSQEIGR.T | Y | 49.47 | 1520.7583 | 13 | 0.1 | 761.3865 | 2 | 29.54 | 5 | F5:9993 | NaNaKA16\_F13.raw |  |  |  |  | 3.2134E5 |  |  |  |  |  |  |  |  | 1 | 0 | 0 | 0 | 0 | 1 | 0 | 0 | 0 | 0 | 0 | 0 | 0 | 0 | 304 | 316 |  |  | PEAKS DB |
| R.VPTYVPLEM(+15.99)EK.T | N | 47.95 | 1320.6635 | 11 | 0.2 | 661.3391 | 2 | 38.01 | 5 | F5:14849 | NaNaKA16\_F13.raw |  |  |  |  | 6.2061E5 |  |  |  |  |  |  |  |  | 1 | 0 | 0 | 0 | 0 | 1 | 0 | 0 | 0 | 0 | 0 | 0 | 0 | 0 | 457 | 467 | Oxidation (M) | M9:Oxidation (M):1000.00 | PEAKS DB |
| K.VGIIGYTTK.E | N | 46.40 | 950.5436 | 9 | 0.7 | 476.2794 | 2 | 30.07 | 5 | F5:10298 | NaNaKA16\_F13.raw |  |  |  |  | 6.7595E6 |  |  |  |  |  |  |  |  | 1 | 0 | 0 | 0 | 0 | 1 | 0 | 0 | 0 | 0 | 0 | 0 | 0 | 0 | 146 | 154 |  |  | PEAKS DB |
| N.ISGYILPYK.I | N | 45.32 | 1052.5906 | 9 | 0.8 | 527.3030 | 2 | 46.37 | 5 | F5:18970 | NaNaKA16\_F13.raw |  |  |  |  | 5.2097E6 |  |  |  |  |  |  |  |  | 1 | 0 | 0 | 0 | 0 | 1 | 0 | 0 | 0 | 0 | 0 | 0 | 0 | 0 | 129 | 137 |  |  | PEAKS DB |
| total 12 peptides |  |  |  |  |  |  |  |  |  |  |  |  |  |  |  |  |  |  |  |  |  |  |  |  |  |  |  |  |  |  |  |  |  |  |  |  |  |  |  |  |  |  |  |
| --- | --- | --- | --- | --- | --- | --- | --- | --- | --- | --- | --- | --- | --- | --- | --- | --- | --- | --- | --- | --- | --- | --- | --- | --- | --- | --- | --- | --- | --- | --- | --- | --- | --- | --- | --- | --- | --- | --- | --- | --- | --- | --- | --- |

A0A2I4HXH5.1

back to list

  

| Protein Coverage
| Supporting Peptides
|

Protein Coverage:

Supporting Peptides:

| Peptide | Uniq | -10lgP | Mass | Length | ppm | m/z | z | RT | Fraction | Scan | Source File | Area F1 | Area F10 | Area F11 | Area F12 | Area F13 | Area F2 | Area F3 | Area F4 | Area F5 | Area F6 | Area F7 | Area F8 | Area F9 | #Feature | #Feature F1 | #Feature F10 | #Feature F11 | #Feature F12 | #Feature F13 | #Feature F2 | #Feature F3 | #Feature F4 | #Feature F5 | #Feature F6 | #Feature F7 | #Feature F8 | #Feature F9 | Start | End | PTM | AScore | Found By |
| --- | --- | --- | --- | --- | --- | --- | --- | --- | --- | --- | --- | --- | --- | --- | --- | --- | --- | --- | --- | --- | --- | --- | --- | --- | --- | --- | --- | --- | --- | --- | --- | --- | --- | --- | --- | --- | --- | --- | --- | --- | --- | --- | --- |
| R.HGQGTGELLQVSGIK.V | N | 77.57 | 1522.8103 | 15 | 0.5 | 762.4128 | 2 | 32.78 | 5 | F5:12050 | NaNaKA16\_F13.raw |  |  |  | 4.2458E5 | 5.0296E6 |  |  |  |  |  |  |  |  | 3 | 0 | 0 | 0 | 1 | 2 | 0 | 0 | 0 | 0 | 0 | 0 | 0 | 0 | 418 | 432 |  |  | PEAKS DB |
| R.VVSLNVLC(+57.02)TEC(+57.02)R.V | Y | 73.80 | 1448.7115 | 12 | 0.0 | 725.3630 | 2 | 49.09 | 10 | F10:32110 | NaNaKA16\_F6.raw |  |  |  |  | 2.3522E6 |  |  |  |  | 7.2729E6 |  |  |  | 2 | 0 | 0 | 0 | 0 | 1 | 0 | 0 | 0 | 0 | 1 | 0 | 0 | 0 | 445 | 456 | Carbamidomethylation | C8:Carbamidomethylation:1000.00;C11:Carbamidomethylation:1000.00 | PEAKS DB |
| R.FHEC(+57.02)NLGNLIC(+57.02)DAVVYNNLR.H | N | 72.86 | 2420.1365 | 20 | 0.2 | 807.7196 | 3 | 76.91 | 13 | F13:50987 | NaNaKA16\_F9.raw |  |  |  |  |  |  |  |  |  |  |  | 5.2029E6 | 2.2685E7 | 3 | 0 | 0 | 0 | 0 | 0 | 0 | 0 | 0 | 0 | 0 | 0 | 1 | 2 | 330 | 349 | Carbamidomethylation | C4:Carbamidomethylation:1000.00;C11:Carbamidomethylation:1000.00 | PEAKS DB |
| R.VPTYVPLEMEK.T | N | 72.07 | 1304.6686 | 11 | 1.1 | 653.3423 | 2 | 50.51 | 5 | F5:20383 | NaNaKA16\_F13.raw |  |  | 0 | 1.0366E6 | 6.7478E6 |  |  |  |  |  |  |  |  | 2 | 0 | 0 | 0 | 1 | 1 | 0 | 0 | 0 | 0 | 0 | 0 | 0 | 0 | 457 | 467 |  |  | PEAKS DB |
| R.QVPVVQAYAFGK.Y | N | 68.51 | 1305.7081 | 12 | -0.2 | 653.8612 | 2 | 81.22 | 5 | F5:33335 | NaNaKA16\_F13.raw |  |  |  | 4.7302E5 | 4.1666E6 |  |  |  |  |  |  |  |  | 3 | 0 | 0 | 0 | 1 | 2 | 0 | 0 | 0 | 0 | 0 | 0 | 0 | 0 | 249 | 260 |  |  | PEAKS DB |
| E.LTILHTNDVHAR.L | N | 61.22 | 1388.7524 | 12 | -0.8 | 463.9244 | 3 | 12.08 | 5 | F5:1993 | NaNaKA16\_F13.raw |  |  |  |  | 9.2667E5 |  |  |  |  |  |  |  |  | 1 | 0 | 0 | 0 | 0 | 1 | 0 | 0 | 0 | 0 | 0 | 0 | 0 | 0 | 5 | 16 |  |  | PEAKS DB |
| K.ETPVLSNPGPYLEFR.D | N | 55.50 | 1717.8674 | 15 | -0.2 | 859.9409 | 2 | 68.90 | 5 | F5:28106 | NaNaKA16\_F13.raw |  |  |  |  | 1.3217E6 |  |  |  |  |  |  |  |  | 1 | 0 | 0 | 0 | 0 | 1 | 0 | 0 | 0 | 0 | 0 | 0 | 0 | 0 | 155 | 169 |  |  | PEAKS DB |
| K.VVYDLSQKPGK.R | Y | 51.28 | 1232.6764 | 11 | 0.5 | 411.8996 | 3 | 12.16 | 5 | F5:2047 | NaNaKA16\_F13.raw |  |  |  |  | 4.4733E4 |  |  |  |  |  |  |  |  | 1 | 0 | 0 | 0 | 0 | 1 | 0 | 0 | 0 | 0 | 0 | 0 | 0 | 0 | 433 | 443 |  |  | PEAKS DB |
| K.VQLQNYSSQEIGR.T | Y | 49.47 | 1520.7583 | 13 | 0.1 | 761.3865 | 2 | 29.54 | 5 | F5:9993 | NaNaKA16\_F13.raw |  |  |  |  | 3.2134E5 |  |  |  |  |  |  |  |  | 1 | 0 | 0 | 0 | 0 | 1 | 0 | 0 | 0 | 0 | 0 | 0 | 0 | 0 | 304 | 316 |  |  | PEAKS DB |
| R.VPTYVPLEM(+15.99)EK.T | N | 47.95 | 1320.6635 | 11 | 0.2 | 661.3391 | 2 | 38.01 | 5 | F5:14849 | NaNaKA16\_F13.raw |  |  |  |  | 6.2061E5 |  |  |  |  |  |  |  |  | 1 | 0 | 0 | 0 | 0 | 1 | 0 | 0 | 0 | 0 | 0 | 0 | 0 | 0 | 457 | 467 | Oxidation (M) | M9:Oxidation (M):1000.00 | PEAKS DB |
| K.VGIIGYTTK.E | N | 46.40 | 950.5436 | 9 | 0.7 | 476.2794 | 2 | 30.07 | 5 | F5:10298 | NaNaKA16\_F13.raw |  |  |  |  | 6.7595E6 |  |  |  |  |  |  |  |  | 1 | 0 | 0 | 0 | 0 | 1 | 0 | 0 | 0 | 0 | 0 | 0 | 0 | 0 | 146 | 154 |  |  | PEAKS DB |
| N.ISGYILPYK.I | N | 45.32 | 1052.5906 | 9 | 0.8 | 527.3030 | 2 | 46.37 | 5 | F5:18970 | NaNaKA16\_F13.raw |  |  |  |  | 5.2097E6 |  |  |  |  |  |  |  |  | 1 | 0 | 0 | 0 | 0 | 1 | 0 | 0 | 0 | 0 | 0 | 0 | 0 | 0 | 129 | 137 |  |  | PEAKS DB |
| total 12 peptides |  |  |  |  |  |  |  |  |  |  |  |  |  |  |  |  |  |  |  |  |  |  |  |  |  |  |  |  |  |  |  |  |  |  |  |  |  |  |  |  |  |  |  |
| --- | --- | --- | --- | --- | --- | --- | --- | --- | --- | --- | --- | --- | --- | --- | --- | --- | --- | --- | --- | --- | --- | --- | --- | --- | --- | --- | --- | --- | --- | --- | --- | --- | --- | --- | --- | --- | --- | --- | --- | --- | --- | --- | --- |

P19859.1

back to list

  

| Protein Coverage
| Supporting Peptides
|

Protein Coverage:

Supporting Peptides:

| Peptide | Uniq | -10lgP | Mass | Length | ppm | m/z | z | RT | Fraction | Scan | Source File | Area F1 | Area F10 | Area F11 | Area F12 | Area F13 | Area F2 | Area F3 | Area F4 | Area F5 | Area F6 | Area F7 | Area F8 | Area F9 | #Feature | #Feature F1 | #Feature F10 | #Feature F11 | #Feature F12 | #Feature F13 | #Feature F2 | #Feature F3 | #Feature F4 | #Feature F5 | #Feature F6 | #Feature F7 | #Feature F8 | #Feature F9 | Start | End | PTM | AScore | Found By |
| --- | --- | --- | --- | --- | --- | --- | --- | --- | --- | --- | --- | --- | --- | --- | --- | --- | --- | --- | --- | --- | --- | --- | --- | --- | --- | --- | --- | --- | --- | --- | --- | --- | --- | --- | --- | --- | --- | --- | --- | --- | --- | --- | --- |
| R.FC(+57.02)ELAPSAGSC(+57.02)FGFVSSYYYNR.Y | Y | 88.81 | 2581.1042 | 22 | 1.4 | 861.3766 | 3 | 80.30 | 9 | F9:58071 | NaNaKA16\_F5.raw |  |  |  |  |  |  |  | 9.1242E6 | 3.1298E8 | 7.8424E6 | 2.7271E7 | 1.4564E7 | 8.7373E6 | 15 | 0 | 0 | 0 | 0 | 0 | 0 | 0 | 2 | 6 | 1 | 2 | 2 | 2 | 4 | 25 | Carbamidomethylation | C2:Carbamidomethylation:1000.00;C11:Carbamidomethylation:1000.00 | PEAKS DB |
| C.FGFVSSYYYNR.Y | Y | 66.32 | 1401.6353 | 11 | 0.4 | 701.8252 | 2 | 55.40 | 9 | F9:35924 | NaNaKA16\_F5.raw |  |  |  |  |  |  |  |  | 7.7088E7 |  |  |  |  | 1 | 0 | 0 | 0 | 0 | 0 | 0 | 0 | 0 | 1 | 0 | 0 | 0 | 0 | 15 | 25 |  |  | PEAKS DB |
| F.GFVSSYYYNR.Y | Y | 57.18 | 1254.5669 | 10 | -1.2 | 628.2866 | 2 | 35.39 | 8 | F8:17699 | NaNaKA16\_F4.raw |  |  |  |  |  |  | 3.3057E7 | 2.894E7 |  |  |  |  |  | 2 | 0 | 0 | 0 | 0 | 0 | 0 | 1 | 1 | 0 | 0 | 0 | 0 | 0 | 16 | 25 |  |  | PEAKS DB |
| R.YSNTC(+57.02)HSFTY.S | Y | 55.37 | 1278.4976 | 10 | 0.0 | 640.2560 | 2 | 21.02 | 9 | F9:8253 | NaNaKA16\_F5.raw |  |  |  |  |  |  |  |  | 1.0479E7 |  |  |  |  | 1 | 0 | 0 | 0 | 0 | 0 | 0 | 0 | 0 | 1 | 0 | 0 | 0 | 0 | 26 | 35 | Carbamidomethylation | C5:Carbamidomethylation:1000.00 | PEAKS DB |
| R.FC(+57.02)ELAPSAGSC(+57.02)FGF.V | Y | 54.88 | 1548.6377 | 14 | 0.9 | 775.3268 | 2 | 86.97 | 9 | F9:63427 | NaNaKA16\_F5.raw |  |  |  |  |  |  |  |  | 3.4844E8 |  |  |  |  | 1 | 0 | 0 | 0 | 0 | 0 | 0 | 0 | 0 | 1 | 0 | 0 | 0 | 0 | 4 | 17 | Carbamidomethylation | C2:Carbamidomethylation:1000.00;C11:Carbamidomethylation:1000.00 | PEAKS DB |
| S.AGSC(+57.02)FGFVSSYYYNR.Y | Y | 54.26 | 1776.7566 | 15 | 0.3 | 889.3858 | 2 | 61.53 | 9 | F9:41371 | NaNaKA16\_F5.raw |  |  |  |  |  |  |  |  | 9.3714E6 |  |  |  |  | 1 | 0 | 0 | 0 | 0 | 0 | 0 | 0 | 0 | 1 | 0 | 0 | 0 | 0 | 11 | 25 | Carbamidomethylation | C4:Carbamidomethylation:1000.00 | PEAKS DB |
| L.APSAGSC(+57.02)FGFVSSYYYNR.Y | Y | 53.17 | 2031.8784 | 18 | 0.6 | 1016.9471 | 2 | 64.10 | 9 | F9:43764 | NaNaKA16\_F5.raw |  |  |  |  |  |  |  |  | 2.531E6 |  |  |  |  | 1 | 0 | 0 | 0 | 0 | 0 | 0 | 0 | 0 | 1 | 0 | 0 | 0 | 0 | 8 | 25 | Carbamidomethylation | C7:Carbamidomethylation:1000.00 | PEAKS DB |
| R.FC(+57.02)ELAPSAGSC(+57.02)F.G | N | 51.69 | 1344.5479 | 12 | 0.6 | 673.2816 | 2 | 63.59 | 9 | F9:43111 | NaNaKA16\_F5.raw |  |  |  |  |  |  |  |  | 1.5951E8 |  |  |  |  | 1 | 0 | 0 | 0 | 0 | 0 | 0 | 0 | 0 | 1 | 0 | 0 | 0 | 0 | 4 | 15 | Carbamidomethylation | C2:Carbamidomethylation:1000.00;C11:Carbamidomethylation:1000.00 | PEAKS DB |
| G.FVSSYYYNR.Y | Y | 49.56 | 1197.5454 | 9 | 5.5 | 599.7801 | 2 | 26.39 | 8 | F8:10833 | NaNaKA16\_F4.raw |  |  |  |  |  |  |  | 2.4207E6 | 6.1561E6 |  |  |  |  | 2 | 0 | 0 | 0 | 0 | 0 | 0 | 0 | 1 | 1 | 0 | 0 | 0 | 0 | 17 | 25 |  |  | PEAKS DB |
| R.FC(+57.02)ELAPSAGSC(+57.02)FGFVSSY.Y | Y | 45.14 | 1984.8335 | 18 | 0.8 | 993.4248 | 2 | 91.33 | 9 | F9:67008 | NaNaKA16\_F5.raw |  |  |  |  |  |  |  |  | 4.9023E7 |  |  |  |  | 1 | 0 | 0 | 0 | 0 | 0 | 0 | 0 | 0 | 1 | 0 | 0 | 0 | 0 | 4 | 21 | Carbamidomethylation | C2:Carbamidomethylation:1000.00;C11:Carbamidomethylation:1000.00 | PEAKS DB |
| R.YSNTC(+57.02)HSFTYSGC(+57.02).G | Y | 44.20 | 1582.5817 | 13 | 0.6 | 792.2986 | 2 | 21.90 | 9 | F9:8790 | NaNaKA16\_F5.raw |  |  |  |  |  |  |  |  | 1.9651E7 |  |  |  |  | 1 | 0 | 0 | 0 | 0 | 0 | 0 | 0 | 0 | 1 | 0 | 0 | 0 | 0 | 26 | 38 | Carbamidomethylation | C5:Carbamidomethylation:1000.00;C13:Carbamidomethylation:1000.00 | PEAKS DB |
| P.RFC(+57.02)ELAPSAGSC(+57.02)FGFVSSYYYNR.Y | Y | 42.03 | 2737.2053 | 23 | -0.9 | 913.4082 | 3 | 67.15 | 11 | F11:45247 | NaNaKA16\_F7.raw |  |  |  |  |  |  |  |  |  |  | 2.4763E6 |  |  | 1 | 0 | 0 | 0 | 0 | 0 | 0 | 0 | 0 | 0 | 0 | 1 | 0 | 0 | 3 | 25 | Carbamidomethylation | C3:Carbamidomethylation:1000.00;C12:Carbamidomethylation:1000.00 | PEAKS DB |
| total 12 peptides |  |  |  |  |  |  |  |  |  |  |  |  |  |  |  |  |  |  |  |  |  |  |  |  |  |  |  |  |  |  |  |  |  |  |  |  |  |  |  |  |  |  |  |
| --- | --- | --- | --- | --- | --- | --- | --- | --- | --- | --- | --- | --- | --- | --- | --- | --- | --- | --- | --- | --- | --- | --- | --- | --- | --- | --- | --- | --- | --- | --- | --- | --- | --- | --- | --- | --- | --- | --- | --- | --- | --- | --- | --- |

1702215A

back to list

  

| Protein Coverage
| Supporting Peptides
|

Protein Coverage:

Supporting Peptides:

| Peptide | Uniq | -10lgP | Mass | Length | ppm | m/z | z | RT | Fraction | Scan | Source File | Area F1 | Area F10 | Area F11 | Area F12 | Area F13 | Area F2 | Area F3 | Area F4 | Area F5 | Area F6 | Area F7 | Area F8 | Area F9 | #Feature | #Feature F1 | #Feature F10 | #Feature F11 | #Feature F12 | #Feature F13 | #Feature F2 | #Feature F3 | #Feature F4 | #Feature F5 | #Feature F6 | #Feature F7 | #Feature F8 | #Feature F9 | Start | End | PTM | AScore | Found By |
| --- | --- | --- | --- | --- | --- | --- | --- | --- | --- | --- | --- | --- | --- | --- | --- | --- | --- | --- | --- | --- | --- | --- | --- | --- | --- | --- | --- | --- | --- | --- | --- | --- | --- | --- | --- | --- | --- | --- | --- | --- | --- | --- | --- |
| R.FC(+57.02)ELAPSAGSC(+57.02)FGFVSSYYYNR.Y | Y | 88.81 | 2581.1042 | 22 | 1.4 | 861.3766 | 3 | 80.30 | 9 | F9:58071 | NaNaKA16\_F5.raw |  |  |  |  |  |  |  | 9.1242E6 | 3.1298E8 | 7.8424E6 | 2.7271E7 | 1.4564E7 | 8.7373E6 | 15 | 0 | 0 | 0 | 0 | 0 | 0 | 0 | 2 | 6 | 1 | 2 | 2 | 2 | 4 | 25 | Carbamidomethylation | C2:Carbamidomethylation:1000.00;C11:Carbamidomethylation:1000.00 | PEAKS DB |
| C.FGFVSSYYYNR.Y | Y | 66.32 | 1401.6353 | 11 | 0.4 | 701.8252 | 2 | 55.40 | 9 | F9:35924 | NaNaKA16\_F5.raw |  |  |  |  |  |  |  |  | 7.7088E7 |  |  |  |  | 1 | 0 | 0 | 0 | 0 | 0 | 0 | 0 | 0 | 1 | 0 | 0 | 0 | 0 | 15 | 25 |  |  | PEAKS DB |
| F.GFVSSYYYNR.Y | Y | 57.18 | 1254.5669 | 10 | -1.2 | 628.2866 | 2 | 35.39 | 8 | F8:17699 | NaNaKA16\_F4.raw |  |  |  |  |  |  | 3.3057E7 | 2.894E7 |  |  |  |  |  | 2 | 0 | 0 | 0 | 0 | 0 | 0 | 1 | 1 | 0 | 0 | 0 | 0 | 0 | 16 | 25 |  |  | PEAKS DB |
| R.YSNTC(+57.02)HSFTY.S | Y | 55.37 | 1278.4976 | 10 | 0.0 | 640.2560 | 2 | 21.02 | 9 | F9:8253 | NaNaKA16\_F5.raw |  |  |  |  |  |  |  |  | 1.0479E7 |  |  |  |  | 1 | 0 | 0 | 0 | 0 | 0 | 0 | 0 | 0 | 1 | 0 | 0 | 0 | 0 | 26 | 35 | Carbamidomethylation | C5:Carbamidomethylation:1000.00 | PEAKS DB |
| R.FC(+57.02)ELAPSAGSC(+57.02)FGF.V | Y | 54.88 | 1548.6377 | 14 | 0.9 | 775.3268 | 2 | 86.97 | 9 | F9:63427 | NaNaKA16\_F5.raw |  |  |  |  |  |  |  |  | 3.4844E8 |  |  |  |  | 1 | 0 | 0 | 0 | 0 | 0 | 0 | 0 | 0 | 1 | 0 | 0 | 0 | 0 | 4 | 17 | Carbamidomethylation | C2:Carbamidomethylation:1000.00;C11:Carbamidomethylation:1000.00 | PEAKS DB |
| S.AGSC(+57.02)FGFVSSYYYNR.Y | Y | 54.26 | 1776.7566 | 15 | 0.3 | 889.3858 | 2 | 61.53 | 9 | F9:41371 | NaNaKA16\_F5.raw |  |  |  |  |  |  |  |  | 9.3714E6 |  |  |  |  | 1 | 0 | 0 | 0 | 0 | 0 | 0 | 0 | 0 | 1 | 0 | 0 | 0 | 0 | 11 | 25 | Carbamidomethylation | C4:Carbamidomethylation:1000.00 | PEAKS DB |
| L.APSAGSC(+57.02)FGFVSSYYYNR.Y | Y | 53.17 | 2031.8784 | 18 | 0.6 | 1016.9471 | 2 | 64.10 | 9 | F9:43764 | NaNaKA16\_F5.raw |  |  |  |  |  |  |  |  | 2.531E6 |  |  |  |  | 1 | 0 | 0 | 0 | 0 | 0 | 0 | 0 | 0 | 1 | 0 | 0 | 0 | 0 | 8 | 25 | Carbamidomethylation | C7:Carbamidomethylation:1000.00 | PEAKS DB |
| R.FC(+57.02)ELAPSAGSC(+57.02)F.G | N | 51.69 | 1344.5479 | 12 | 0.6 | 673.2816 | 2 | 63.59 | 9 | F9:43111 | NaNaKA16\_F5.raw |  |  |  |  |  |  |  |  | 1.5951E8 |  |  |  |  | 1 | 0 | 0 | 0 | 0 | 0 | 0 | 0 | 0 | 1 | 0 | 0 | 0 | 0 | 4 | 15 | Carbamidomethylation | C2:Carbamidomethylation:1000.00;C11:Carbamidomethylation:1000.00 | PEAKS DB |
| G.FVSSYYYNR.Y | Y | 49.56 | 1197.5454 | 9 | 5.5 | 599.7801 | 2 | 26.39 | 8 | F8:10833 | NaNaKA16\_F4.raw |  |  |  |  |  |  |  | 2.4207E6 | 6.1561E6 |  |  |  |  | 2 | 0 | 0 | 0 | 0 | 0 | 0 | 0 | 1 | 1 | 0 | 0 | 0 | 0 | 17 | 25 |  |  | PEAKS DB |
| R.FC(+57.02)ELAPSAGSC(+57.02)FGFVSSY.Y | Y | 45.14 | 1984.8335 | 18 | 0.8 | 993.4248 | 2 | 91.33 | 9 | F9:67008 | NaNaKA16\_F5.raw |  |  |  |  |  |  |  |  | 4.9023E7 |  |  |  |  | 1 | 0 | 0 | 0 | 0 | 0 | 0 | 0 | 0 | 1 | 0 | 0 | 0 | 0 | 4 | 21 | Carbamidomethylation | C2:Carbamidomethylation:1000.00;C11:Carbamidomethylation:1000.00 | PEAKS DB |
| R.YSNTC(+57.02)HSFTYSGC(+57.02).G | Y | 44.20 | 1582.5817 | 13 | 0.6 | 792.2986 | 2 | 21.90 | 9 | F9:8790 | NaNaKA16\_F5.raw |  |  |  |  |  |  |  |  | 1.9651E7 |  |  |  |  | 1 | 0 | 0 | 0 | 0 | 0 | 0 | 0 | 0 | 1 | 0 | 0 | 0 | 0 | 26 | 38 | Carbamidomethylation | C5:Carbamidomethylation:1000.00;C13:Carbamidomethylation:1000.00 | PEAKS DB |
| P.RFC(+57.02)ELAPSAGSC(+57.02)FGFVSSYYYNR.Y | Y | 42.03 | 2737.2053 | 23 | -0.9 | 913.4082 | 3 | 67.15 | 11 | F11:45247 | NaNaKA16\_F7.raw |  |  |  |  |  |  |  |  |  |  | 2.4763E6 |  |  | 1 | 0 | 0 | 0 | 0 | 0 | 0 | 0 | 0 | 0 | 0 | 1 | 0 | 0 | 3 | 25 | Carbamidomethylation | C3:Carbamidomethylation:1000.00;C12:Carbamidomethylation:1000.00 | PEAKS DB |
| total 12 peptides |  |  |  |  |  |  |  |  |  |  |  |  |  |  |  |  |  |  |  |  |  |  |  |  |  |  |  |  |  |  |  |  |  |  |  |  |  |  |  |  |  |  |  |
| --- | --- | --- | --- | --- | --- | --- | --- | --- | --- | --- | --- | --- | --- | --- | --- | --- | --- | --- | --- | --- | --- | --- | --- | --- | --- | --- | --- | --- | --- | --- | --- | --- | --- | --- | --- | --- | --- | --- | --- | --- | --- | --- | --- |

ETE68810.1

back to list

  

| Protein Coverage
| Supporting Peptides
|

Protein Coverage:

Supporting Peptides:

| Peptide | Uniq | -10lgP | Mass | Length | ppm | m/z | z | RT | Fraction | Scan | Source File | Area F1 | Area F10 | Area F11 | Area F12 | Area F13 | Area F2 | Area F3 | Area F4 | Area F5 | Area F6 | Area F7 | Area F8 | Area F9 | #Feature | #Feature F1 | #Feature F10 | #Feature F11 | #Feature F12 | #Feature F13 | #Feature F2 | #Feature F3 | #Feature F4 | #Feature F5 | #Feature F6 | #Feature F7 | #Feature F8 | #Feature F9 | Start | End | PTM | AScore | Found By |
| --- | --- | --- | --- | --- | --- | --- | --- | --- | --- | --- | --- | --- | --- | --- | --- | --- | --- | --- | --- | --- | --- | --- | --- | --- | --- | --- | --- | --- | --- | --- | --- | --- | --- | --- | --- | --- | --- | --- | --- | --- | --- | --- | --- |
| K.NSC(+57.02)PPVVETFGDTAK.L | Y | 83.83 | 1620.7454 | 15 | 0.4 | 811.3803 | 2 | 37.81 | 6 | F6:22302 | NaNaKA16\_F2.raw |  |  |  | 3.7961E6 | 2.8507E7 | 2.9105E7 |  | 2.4421E6 | 3.064E6 | 4.1372E6 |  |  |  | 8 | 0 | 0 | 0 | 1 | 2 | 2 | 0 | 1 | 1 | 1 | 0 | 0 | 0 | 197 | 211 | Carbamidomethylation | C3:Carbamidomethylation:1000.00 | PEAKS DB |
| R.LVILGFPC(+57.02)NQFGK.Q | N | 75.66 | 1491.7908 | 13 | 0.9 | 746.9033 | 2 | 75.14 | 4 | F4:50923 | NaNaKA16\_F12.raw |  | 1.9324E7 | 8.891E6 | 1.0383E7 | 1.972E7 |  |  |  |  |  | 2.0275E6 | 1.0176E7 | 4.4683E6 | 7 | 0 | 1 | 1 | 1 | 1 | 0 | 0 | 0 | 0 | 0 | 1 | 1 | 1 | 137 | 149 | Carbamidomethylation | C8:Carbamidomethylation:1000.00 | PEAKS DB |
| K.QEPGQNSEILQGIK.H | N | 72.65 | 1539.7893 | 14 | 1.0 | 770.9027 | 2 | 36.16 | 5 | F5:14005 | NaNaKA16\_F13.raw |  |  |  | 3.6181E6 | 2.8478E7 |  |  |  |  |  |  |  |  | 3 | 0 | 0 | 0 | 1 | 2 | 0 | 0 | 0 | 0 | 0 | 0 | 0 | 0 | 150 | 163 |  |  | PEAKS DB |
| L.VILGFPC(+57.02)NQFGK.Q | N | 63.26 | 1378.7067 | 12 | 0.9 | 690.3612 | 2 | 60.52 | 12 | F12:39695 | NaNaKA16\_F8.raw |  | 3.3914E6 |  |  |  |  |  |  |  | 2.4189E6 | 2.1917E6 | 3.4327E6 | 4.8393E6 | 5 | 0 | 1 | 0 | 0 | 0 | 0 | 0 | 0 | 0 | 1 | 1 | 1 | 1 | 138 | 149 | Carbamidomethylation | C7:Carbamidomethylation:1000.00 | PEAKS DB |
| L.KNSC(+57.02)PPVVETFGDTAK.L | Y | 58.26 | 1748.8403 | 16 | 0.7 | 583.9545 | 3 | 24.33 | 6 | F6:10585 | NaNaKA16\_F2.raw |  |  |  |  |  | 2.3512E6 |  |  |  |  |  |  |  | 1 | 0 | 0 | 0 | 0 | 0 | 1 | 0 | 0 | 0 | 0 | 0 | 0 | 0 | 196 | 211 | Carbamidomethylation | C4:Carbamidomethylation:1000.00 | PEAKS DB |
| T.ELNALQNSLK.T | Y | 58.17 | 1128.6139 | 10 | -0.1 | 565.3141 | 2 | 31.56 | 6 | F6:17301 | NaNaKA16\_F2.raw |  |  |  |  |  | 1.2474E7 |  |  |  |  |  |  |  | 1 | 0 | 0 | 0 | 0 | 0 | 1 | 0 | 0 | 0 | 0 | 0 | 0 | 0 | 124 | 133 |  |  | PEAKS DB |
| K.FLVNPQGKPVMR.W | N | 57.92 | 1384.7649 | 12 | 1.1 | 462.5961 | 3 | 26.92 | 5 | F5:8608 | NaNaKA16\_F13.raw |  |  |  |  | 1.9299E6 |  |  |  |  |  |  |  |  | 1 | 0 | 0 | 0 | 0 | 1 | 0 | 0 | 0 | 0 | 0 | 0 | 0 | 0 | 229 | 240 |  |  | PEAKS DB |
| C.SYAPNSQPIPDR.A | N | 52.88 | 1343.6470 | 12 | 0.3 | 672.8310 | 2 | 19.74 | 5 | F5:4861 | NaNaKA16\_F13.raw |  |  |  |  | 1.9768E6 | 8.8175E5 |  |  |  |  |  |  |  | 2 | 0 | 0 | 0 | 0 | 1 | 1 | 0 | 0 | 0 | 0 | 0 | 0 | 0 | 90 | 101 |  |  | PEAKS DB |
| K.NSC(+57.02)PPVVETFG.D | N | 51.85 | 1205.5387 | 11 | 0.2 | 603.7767 | 2 | 53.03 | 6 | F6:34639 | NaNaKA16\_F2.raw |  |  |  |  |  | 5.7957E7 | 1.0144E7 |  |  | 3.2539E6 |  |  |  | 3 | 0 | 0 | 0 | 0 | 0 | 1 | 1 | 0 | 0 | 1 | 0 | 0 | 0 | 197 | 207 | Carbamidomethylation | C3:Carbamidomethylation:1000.00 | PEAKS DB |
| K.LFWSPLK.I | N | 50.13 | 889.5062 | 7 | -0.4 | 445.7602 | 2 | 61.25 | 2 | F2:38338 | NaNaKA16\_F10.raw |  | 1.0564E7 | 2.3049E7 | 4.474E6 | 9.3271E6 |  |  |  |  | 8.0536E6 | 4.602E6 |  |  | 6 | 0 | 1 | 1 | 1 | 1 | 0 | 0 | 0 | 0 | 1 | 1 | 0 | 0 | 212 | 218 |  |  | PEAKS DB |
| total 10 peptides |  |  |  |  |  |  |  |  |  |  |  |  |  |  |  |  |  |  |  |  |  |  |  |  |  |  |  |  |  |  |  |  |  |  |  |  |  |  |  |  |  |  |  |
| --- | --- | --- | --- | --- | --- | --- | --- | --- | --- | --- | --- | --- | --- | --- | --- | --- | --- | --- | --- | --- | --- | --- | --- | --- | --- | --- | --- | --- | --- | --- | --- | --- | --- | --- | --- | --- | --- | --- | --- | --- | --- | --- | --- |

XP\_026570202.1

back to list

  

| Protein Coverage
| Supporting Peptides
|

Protein Coverage:

Supporting Peptides:

| Peptide | Uniq | -10lgP | Mass | Length | ppm | m/z | z | RT | Fraction | Scan | Source File | Area F1 | Area F10 | Area F11 | Area F12 | Area F13 | Area F2 | Area F3 | Area F4 | Area F5 | Area F6 | Area F7 | Area F8 | Area F9 | #Feature | #Feature F1 | #Feature F10 | #Feature F11 | #Feature F12 | #Feature F13 | #Feature F2 | #Feature F3 | #Feature F4 | #Feature F5 | #Feature F6 | #Feature F7 | #Feature F8 | #Feature F9 | Start | End | PTM | AScore | Found By |
| --- | --- | --- | --- | --- | --- | --- | --- | --- | --- | --- | --- | --- | --- | --- | --- | --- | --- | --- | --- | --- | --- | --- | --- | --- | --- | --- | --- | --- | --- | --- | --- | --- | --- | --- | --- | --- | --- | --- | --- | --- | --- | --- | --- |
| R.ALNEAC(+57.02)ESVIQTAC(+57.02)K.H | Y | 79.78 | 1692.7811 | 15 | 0.1 | 847.3979 | 2 | 33.28 | 4 | F4:16250 | NaNaKA16\_F12.raw |  |  | 7.8004E5 | 2.1456E6 |  |  |  |  |  |  |  |  |  | 2 | 0 | 0 | 1 | 1 | 0 | 0 | 0 | 0 | 0 | 0 | 0 | 0 | 0 | 488 | 502 | Carbamidomethylation | C6:Carbamidomethylation:1000.00;C14:Carbamidomethylation:1000.00 | PEAKS DB |
| R.LSYLLMC(+57.02)LESAVHR.G | Y | 66.53 | 1690.8535 | 14 | 0.4 | 564.6254 | 3 | 75.96 | 4 | F4:51604 | NaNaKA16\_F12.raw |  | 1.2498E6 | 5.0919E5 | 5.032E5 |  |  |  |  |  |  |  |  |  | 4 | 0 | 2 | 1 | 1 | 0 | 0 | 0 | 0 | 0 | 0 | 0 | 0 | 0 | 384 | 397 | Carbamidomethylation | C7:Carbamidomethylation:1000.00 | PEAKS DB |
| T.EMMDPELDYTLMR.V | Y | 65.34 | 1642.7041 | 13 | 0.2 | 822.3595 | 2 | 71.71 | 10 | F10:50951 | NaNaKA16\_F6.raw |  |  |  |  |  |  |  |  |  | 5.7591E6 |  |  |  | 1 | 0 | 0 | 0 | 0 | 0 | 0 | 0 | 0 | 0 | 1 | 0 | 0 | 0 | 856 | 868 |  |  | PEAKS DB |
| R.QVSSEC(+57.02)QGEMLDYR.R | Y | 65.27 | 1700.7134 | 14 | 0.5 | 851.3644 | 2 | 30.29 | 4 | F4:13595 | NaNaKA16\_F12.raw |  |  |  | 9.4592E5 |  |  |  |  |  |  |  |  |  | 1 | 0 | 0 | 0 | 1 | 0 | 0 | 0 | 0 | 0 | 0 | 0 | 0 | 0 | 400 | 413 | Carbamidomethylation | C6:Carbamidomethylation:1000.00 | PEAKS DB |
| K.MTAIIFSDYR.L | Y | 62.66 | 1215.5958 | 10 | 0.0 | 608.8052 | 2 | 58.13 | 4 | F4:36942 | NaNaKA16\_F12.raw |  | 9.3455E5 | 1.2514E6 | 8.0173E5 |  |  |  |  |  |  |  |  |  | 3 | 0 | 1 | 1 | 1 | 0 | 0 | 0 | 0 | 0 | 0 | 0 | 0 | 0 | 209 | 218 |  |  | PEAKS DB |
| M.DPELDYTLMR.V | Y | 58.88 | 1251.5806 | 10 | 0.0 | 626.7975 | 2 | 58.42 | 9 | F9:38833 | NaNaKA16\_F5.raw |  |  |  |  |  |  |  |  | 2.3119E6 |  |  |  |  | 1 | 0 | 0 | 0 | 0 | 0 | 0 | 0 | 0 | 1 | 0 | 0 | 0 | 0 | 859 | 868 |  |  | PEAKS DB |
| R.SGDPMILSC(+57.02)LMEHLYTEK.M | Y | 54.83 | 2122.9736 | 18 | 0.2 | 708.6653 | 3 | 87.32 | 4 | F4:60933 | NaNaKA16\_F12.raw |  |  |  | 1.0515E6 |  |  |  |  |  |  |  |  |  | 1 | 0 | 0 | 0 | 1 | 0 | 0 | 0 | 0 | 0 | 0 | 0 | 0 | 0 | 506 | 523 | Carbamidomethylation | C9:Carbamidomethylation:1000.00 | PEAKS DB |
| R.LLELQYFISR.D | Y | 53.33 | 1280.7129 | 10 | 0.6 | 641.3641 | 2 | 79.15 | 4 | F4:54084 | NaNaKA16\_F12.raw |  |  |  | 1.3142E6 |  |  |  |  |  |  |  |  |  | 1 | 0 | 0 | 0 | 1 | 0 | 0 | 0 | 0 | 0 | 0 | 0 | 0 | 0 | 532 | 541 |  |  | PEAKS DB |
| P.ELDYTLMR.V | Y | 50.52 | 1039.5009 | 8 | -0.5 | 520.7574 | 2 | 44.47 | 9 | F9:26860 | NaNaKA16\_F5.raw |  |  |  |  |  |  |  |  | 5.1797E6 |  |  |  |  | 2 | 0 | 0 | 0 | 0 | 0 | 0 | 0 | 0 | 2 | 0 | 0 | 0 | 0 | 861 | 868 |  |  | PEAKS DB |
| R.IEMWSFAAK.V | Y | 48.70 | 1081.5266 | 9 | 0.2 | 541.7707 | 2 | 59.06 | 2 | F2:36380 | NaNaKA16\_F10.raw |  | 4.9011E5 |  |  |  |  |  |  |  |  |  |  |  | 1 | 0 | 1 | 0 | 0 | 0 | 0 | 0 | 0 | 0 | 0 | 0 | 0 | 0 | 1102 | 1110 |  |  | PEAKS DB |
| R.VAELSSDDFHLDR.H | Y | 48.48 | 1502.7001 | 13 | -0.1 | 501.9072 | 3 | 38.95 | 4 | F4:20893 | NaNaKA16\_F12.raw |  |  |  | 3.427E5 |  |  |  |  |  |  |  |  |  | 1 | 0 | 0 | 0 | 1 | 0 | 0 | 0 | 0 | 0 | 0 | 0 | 0 | 0 | 284 | 296 |  |  | PEAKS DB |
| R.LSYLLM(+15.99)C(+57.02)LESAVHR.G | Y | 42.33 | 1706.8484 | 14 | 0.2 | 569.9568 | 3 | 71.41 | 3 | F3:45998 | NaNaKA16\_F11.raw |  |  | 3.4744E5 |  |  |  |  |  |  |  |  |  |  | 1 | 0 | 0 | 1 | 0 | 0 | 0 | 0 | 0 | 0 | 0 | 0 | 0 | 0 | 384 | 397 | Oxidation (M); Carbamidomethylation | M6:Oxidation (M):1000.00;C7:Carbamidomethylation:1000.00 | PEAKS DB |
| total 12 peptides |  |  |  |  |  |  |  |  |  |  |  |  |  |  |  |  |  |  |  |  |  |  |  |  |  |  |  |  |  |  |  |  |  |  |  |  |  |  |  |  |  |  |  |
| --- | --- | --- | --- | --- | --- | --- | --- | --- | --- | --- | --- | --- | --- | --- | --- | --- | --- | --- | --- | --- | --- | --- | --- | --- | --- | --- | --- | --- | --- | --- | --- | --- | --- | --- | --- | --- | --- | --- | --- | --- | --- | --- | --- |

Q9W6W9.1

back to list

  

| Protein Coverage
| Supporting Peptides
|

Protein Coverage:

Supporting Peptides:

| Peptide | Uniq | -10lgP | Mass | Length | ppm | m/z | z | RT | Fraction | Scan | Source File | Area F1 | Area F10 | Area F11 | Area F12 | Area F13 | Area F2 | Area F3 | Area F4 | Area F5 | Area F6 | Area F7 | Area F8 | Area F9 | #Feature | #Feature F1 | #Feature F10 | #Feature F11 | #Feature F12 | #Feature F13 | #Feature F2 | #Feature F3 | #Feature F4 | #Feature F5 | #Feature F6 | #Feature F7 | #Feature F8 | #Feature F9 | Start | End | PTM | AScore | Found By |
| --- | --- | --- | --- | --- | --- | --- | --- | --- | --- | --- | --- | --- | --- | --- | --- | --- | --- | --- | --- | --- | --- | --- | --- | --- | --- | --- | --- | --- | --- | --- | --- | --- | --- | --- | --- | --- | --- | --- | --- | --- | --- | --- | --- |
| K.SSLLVKYVC(+57.02)C(+57.02)NTDRC(+57.02)N | N | 73.90 | 1987.8914 | 16 | 0.9 | 663.6383 | 3 | 34.50 | 3 | F3:16042 | NaNaKA16\_F11.raw |  | 4.3691E6 | 4.3984E7 |  |  |  |  |  |  |  |  |  |  | 3 | 0 | 1 | 2 | 0 | 0 | 0 | 0 | 0 | 0 | 0 | 0 | 0 | 0 | 66 | 81 | Carbamidomethylation | C9:Carbamidomethylation:1000.00;C10:Carbamidomethylation:1000.00;C15:Carbamidomethylation:1000.00 | PEAKS DB |
| K.LVPLFYKTC(+57.02)PAGK.N | N | 73.66 | 1492.8112 | 13 | -0.3 | 498.6109 | 3 | 36.55 | 10 | F10:21078 | NaNaKA16\_F6.raw |  | 1.9305E6 | 6.2426E6 | 1.2125E7 |  |  |  |  |  | 1.1688E7 | 5.7221E5 | 1.8629E7 |  | 10 | 0 | 1 | 2 | 2 | 0 | 0 | 0 | 0 | 0 | 2 | 1 | 2 | 0 | 27 | 39 | Carbamidomethylation | C9:Carbamidomethylation:1000.00 | PEAKS DB |
| K.SSLLVKYVC(+57.02)C(+57.02)NTDR.C | N | 70.96 | 1713.8179 | 14 | 0.6 | 857.9167 | 2 | 34.00 | 3 | F3:15457 | NaNaKA16\_F11.raw |  | 5.0554E6 | 1.1527E8 | 1.6508E6 |  |  |  |  |  |  |  | 0 | 3.3481E6 | 7 | 0 | 2 | 2 | 1 | 0 | 0 | 0 | 0 | 0 | 0 | 0 | 0 | 2 | 66 | 79 | Carbamidomethylation | C9:Carbamidomethylation:1000.00;C10:Carbamidomethylation:1000.00 | PEAKS DB |
| K.LVPLFYKTC(+57.02)PAGKNLC(+57.02)YK.M | N | 68.71 | 2171.1272 | 18 | 0.0 | 543.7891 | 4 | 39.14 | 2 | F2:20436 | NaNaKA16\_F10.raw |  | 1.2494E7 | 5.7358E6 |  |  |  |  |  |  |  |  |  |  | 4 | 0 | 2 | 2 | 0 | 0 | 0 | 0 | 0 | 0 | 0 | 0 | 0 | 0 | 27 | 44 | Carbamidomethylation | C9:Carbamidomethylation:1000.00;C16:Carbamidomethylation:1000.00 | PEAKS DB |
| K.C(+57.02)NKLVPLFYK.T | N | 68.01 | 1280.6951 | 10 | -0.1 | 641.3547 | 2 | 39.35 | 4 | F4:21415 | NaNaKA16\_F12.raw |  | 3.2793E5 |  | 5.2282E6 |  |  |  |  |  |  |  | 7.4866E6 | 7.2675E6 | 7 | 0 | 1 | 0 | 2 | 0 | 0 | 0 | 0 | 0 | 0 | 0 | 2 | 2 | 24 | 33 | Carbamidomethylation | C1:Carbamidomethylation:1000.00 | PEAKS DB |
| R.GC(+57.02)IDVC(+57.02)PKSSLLVK.Y | N | 65.71 | 1574.8160 | 14 | 0.6 | 788.4158 | 2 | 32.88 | 3 | F3:14936 | NaNaKA16\_F11.raw |  |  | 2.0322E7 |  |  |  |  |  |  |  |  |  |  | 2 | 0 | 0 | 2 | 0 | 0 | 0 | 0 | 0 | 0 | 0 | 0 | 0 | 0 | 58 | 71 | Carbamidomethylation | C2:Carbamidomethylation:1000.00;C6:Carbamidomethylation:1000.00 | PEAKS DB |
| K.YVC(+57.02)C(+57.02)NTDR.C | N | 50.92 | 1086.4222 | 8 | -0.2 | 544.2183 | 2 | 11.37 | 12 | F12:1787 | NaNaKA16\_F8.raw |  | 2.1124E6 | 2.9778E6 | 1.3296E6 |  |  |  |  |  | 1.5915E6 |  | 1.7945E6 |  | 5 | 0 | 1 | 1 | 1 | 0 | 0 | 0 | 0 | 0 | 1 | 0 | 1 | 0 | 72 | 79 | Carbamidomethylation | C3:Carbamidomethylation:1000.00;C4:Carbamidomethylation:1000.00 | PEAKS DB |
| R.GC(+57.02)IDVC(+57.02)PK.S | N | 46.03 | 947.4205 | 8 | -0.5 | 474.7173 | 2 | 11.54 | 12 | F12:1895 | NaNaKA16\_F8.raw |  |  | 1.5891E7 |  |  |  | 6.084E5 |  | 2.0697E6 | 6.3919E7 | 1.8242E6 | 6.8843E6 |  | 6 | 0 | 0 | 1 | 0 | 0 | 0 | 1 | 0 | 1 | 1 | 1 | 1 | 0 | 58 | 65 | Carbamidomethylation | C2:Carbamidomethylation:1000.00;C6:Carbamidomethylation:1000.00 | PEAKS DB |
| K.TC(+57.02)PAGKNLC(+57.02)YK.M | N | 44.80 | 1310.6111 | 11 | 0.6 | 437.8779 | 3 | 11.38 | 12 | F12:1793 | NaNaKA16\_F8.raw |  |  |  |  |  |  |  |  |  |  |  | 2.5665E5 |  | 1 | 0 | 0 | 0 | 0 | 0 | 0 | 0 | 0 | 0 | 0 | 0 | 1 | 0 | 34 | 44 | Carbamidomethylation | C2:Carbamidomethylation:1000.00;C9:Carbamidomethylation:1000.00 | PEAKS DB |
| K.MFM(+15.99)VSNLTVPVKR.G | Y | 42.32 | 1536.8157 | 13 | -0.3 | 385.2111 | 4 | 24.96 | 10 | F10:11084 | NaNaKA16\_F6.raw |  |  |  |  |  |  |  |  |  | 3.5601E5 |  |  |  | 1 | 0 | 0 | 0 | 0 | 0 | 0 | 0 | 0 | 0 | 1 | 0 | 0 | 0 | 45 | 57 | Oxidation (M) | M3:Oxidation (M):0.00 | PEAKS DB |
| total 10 peptides |  |  |  |  |  |  |  |  |  |  |  |  |  |  |  |  |  |  |  |  |  |  |  |  |  |  |  |  |  |  |  |  |  |  |  |  |  |  |  |  |  |  |  |
| --- | --- | --- | --- | --- | --- | --- | --- | --- | --- | --- | --- | --- | --- | --- | --- | --- | --- | --- | --- | --- | --- | --- | --- | --- | --- | --- | --- | --- | --- | --- | --- | --- | --- | --- | --- | --- | --- | --- | --- | --- | --- | --- | --- |

CAB42057.1

back to list

  

| Protein Coverage
| Supporting Peptides
|

Protein Coverage:

Supporting Peptides:

| Peptide | Uniq | -10lgP | Mass | Length | ppm | m/z | z | RT | Fraction | Scan | Source File | Area F1 | Area F10 | Area F11 | Area F12 | Area F13 | Area F2 | Area F3 | Area F4 | Area F5 | Area F6 | Area F7 | Area F8 | Area F9 | #Feature | #Feature F1 | #Feature F10 | #Feature F11 | #Feature F12 | #Feature F13 | #Feature F2 | #Feature F3 | #Feature F4 | #Feature F5 | #Feature F6 | #Feature F7 | #Feature F8 | #Feature F9 | Start | End | PTM | AScore | Found By |
| --- | --- | --- | --- | --- | --- | --- | --- | --- | --- | --- | --- | --- | --- | --- | --- | --- | --- | --- | --- | --- | --- | --- | --- | --- | --- | --- | --- | --- | --- | --- | --- | --- | --- | --- | --- | --- | --- | --- | --- | --- | --- | --- | --- |
| K.SSLLVKYVC(+57.02)C(+57.02)NTDRC(+57.02)N | N | 73.90 | 1987.8914 | 16 | 0.9 | 663.6383 | 3 | 34.50 | 3 | F3:16042 | NaNaKA16\_F11.raw |  | 4.3691E6 | 4.3984E7 |  |  |  |  |  |  |  |  |  |  | 3 | 0 | 1 | 2 | 0 | 0 | 0 | 0 | 0 | 0 | 0 | 0 | 0 | 0 | 66 | 81 | Carbamidomethylation | C9:Carbamidomethylation:1000.00;C10:Carbamidomethylation:1000.00;C15:Carbamidomethylation:1000.00 | PEAKS DB |
| K.LVPLFYKTC(+57.02)PAGK.N | N | 73.66 | 1492.8112 | 13 | -0.3 | 498.6109 | 3 | 36.55 | 10 | F10:21078 | NaNaKA16\_F6.raw |  | 1.9305E6 | 6.2426E6 | 1.2125E7 |  |  |  |  |  | 1.1688E7 | 5.7221E5 | 1.8629E7 |  | 10 | 0 | 1 | 2 | 2 | 0 | 0 | 0 | 0 | 0 | 2 | 1 | 2 | 0 | 27 | 39 | Carbamidomethylation | C9:Carbamidomethylation:1000.00 | PEAKS DB |
| K.SSLLVKYVC(+57.02)C(+57.02)NTDR.C | N | 70.96 | 1713.8179 | 14 | 0.6 | 857.9167 | 2 | 34.00 | 3 | F3:15457 | NaNaKA16\_F11.raw |  | 5.0554E6 | 1.1527E8 | 1.6508E6 |  |  |  |  |  |  |  | 0 | 3.3481E6 | 7 | 0 | 2 | 2 | 1 | 0 | 0 | 0 | 0 | 0 | 0 | 0 | 0 | 2 | 66 | 79 | Carbamidomethylation | C9:Carbamidomethylation:1000.00;C10:Carbamidomethylation:1000.00 | PEAKS DB |
| K.LVPLFYKTC(+57.02)PAGKNLC(+57.02)YK.M | N | 68.71 | 2171.1272 | 18 | 0.0 | 543.7891 | 4 | 39.14 | 2 | F2:20436 | NaNaKA16\_F10.raw |  | 1.2494E7 | 5.7358E6 |  |  |  |  |  |  |  |  |  |  | 4 | 0 | 2 | 2 | 0 | 0 | 0 | 0 | 0 | 0 | 0 | 0 | 0 | 0 | 27 | 44 | Carbamidomethylation | C9:Carbamidomethylation:1000.00;C16:Carbamidomethylation:1000.00 | PEAKS DB |
| K.C(+57.02)NKLVPLFYK.T | N | 68.01 | 1280.6951 | 10 | -0.1 | 641.3547 | 2 | 39.35 | 4 | F4:21415 | NaNaKA16\_F12.raw |  | 3.2793E5 |  | 5.2282E6 |  |  |  |  |  |  |  | 7.4866E6 | 7.2675E6 | 7 | 0 | 1 | 0 | 2 | 0 | 0 | 0 | 0 | 0 | 0 | 0 | 2 | 2 | 24 | 33 | Carbamidomethylation | C1:Carbamidomethylation:1000.00 | PEAKS DB |
| R.GC(+57.02)IDVC(+57.02)PKSSLLVK.Y | N | 65.71 | 1574.8160 | 14 | 0.6 | 788.4158 | 2 | 32.88 | 3 | F3:14936 | NaNaKA16\_F11.raw |  |  | 2.0322E7 |  |  |  |  |  |  |  |  |  |  | 2 | 0 | 0 | 2 | 0 | 0 | 0 | 0 | 0 | 0 | 0 | 0 | 0 | 0 | 58 | 71 | Carbamidomethylation | C2:Carbamidomethylation:1000.00;C6:Carbamidomethylation:1000.00 | PEAKS DB |
| K.YVC(+57.02)C(+57.02)NTDR.C | N | 50.92 | 1086.4222 | 8 | -0.2 | 544.2183 | 2 | 11.37 | 12 | F12:1787 | NaNaKA16\_F8.raw |  | 2.1124E6 | 2.9778E6 | 1.3296E6 |  |  |  |  |  | 1.5915E6 |  | 1.7945E6 |  | 5 | 0 | 1 | 1 | 1 | 0 | 0 | 0 | 0 | 0 | 1 | 0 | 1 | 0 | 72 | 79 | Carbamidomethylation | C3:Carbamidomethylation:1000.00;C4:Carbamidomethylation:1000.00 | PEAKS DB |
| R.GC(+57.02)IDVC(+57.02)PK.S | N | 46.03 | 947.4205 | 8 | -0.5 | 474.7173 | 2 | 11.54 | 12 | F12:1895 | NaNaKA16\_F8.raw |  |  | 1.5891E7 |  |  |  | 6.084E5 |  | 2.0697E6 | 6.3919E7 | 1.8242E6 | 6.8843E6 |  | 6 | 0 | 0 | 1 | 0 | 0 | 0 | 1 | 0 | 1 | 1 | 1 | 1 | 0 | 58 | 65 | Carbamidomethylation | C2:Carbamidomethylation:1000.00;C6:Carbamidomethylation:1000.00 | PEAKS DB |
| K.TC(+57.02)PAGKNLC(+57.02)YK.M | N | 44.80 | 1310.6111 | 11 | 0.6 | 437.8779 | 3 | 11.38 | 12 | F12:1793 | NaNaKA16\_F8.raw |  |  |  |  |  |  |  |  |  |  |  | 2.5665E5 |  | 1 | 0 | 0 | 0 | 0 | 0 | 0 | 0 | 0 | 0 | 0 | 0 | 1 | 0 | 34 | 44 | Carbamidomethylation | C2:Carbamidomethylation:1000.00;C9:Carbamidomethylation:1000.00 | PEAKS DB |
| K.MFM(+15.99)VSNLTVPVKR.G | Y | 42.32 | 1536.8157 | 13 | -0.3 | 385.2111 | 4 | 24.96 | 10 | F10:11084 | NaNaKA16\_F6.raw |  |  |  |  |  |  |  |  |  | 3.5601E5 |  |  |  | 1 | 0 | 0 | 0 | 0 | 0 | 0 | 0 | 0 | 0 | 1 | 0 | 0 | 0 | 45 | 57 | Oxidation (M) | M3:Oxidation (M):0.00 | PEAKS DB |
| total 10 peptides |  |  |  |  |  |  |  |  |  |  |  |  |  |  |  |  |  |  |  |  |  |  |  |  |  |  |  |  |  |  |  |  |  |  |  |  |  |  |  |  |  |  |  |
| --- | --- | --- | --- | --- | --- | --- | --- | --- | --- | --- | --- | --- | --- | --- | --- | --- | --- | --- | --- | --- | --- | --- | --- | --- | --- | --- | --- | --- | --- | --- | --- | --- | --- | --- | --- | --- | --- | --- | --- | --- | --- | --- | --- |

P82885.1

back to list

  

| Protein Coverage
| Supporting Peptides
|

Protein Coverage:

Supporting Peptides:

| Peptide | Uniq | -10lgP | Mass | Length | ppm | m/z | z | RT | Fraction | Scan | Source File | Area F1 | Area F10 | Area F11 | Area F12 | Area F13 | Area F2 | Area F3 | Area F4 | Area F5 | Area F6 | Area F7 | Area F8 | Area F9 | #Feature | #Feature F1 | #Feature F10 | #Feature F11 | #Feature F12 | #Feature F13 | #Feature F2 | #Feature F3 | #Feature F4 | #Feature F5 | #Feature F6 | #Feature F7 | #Feature F8 | #Feature F9 | Start | End | PTM | AScore | Found By |
| --- | --- | --- | --- | --- | --- | --- | --- | --- | --- | --- | --- | --- | --- | --- | --- | --- | --- | --- | --- | --- | --- | --- | --- | --- | --- | --- | --- | --- | --- | --- | --- | --- | --- | --- | --- | --- | --- | --- | --- | --- | --- | --- | --- |
| K.ADVTFDSNTAFESLVVSPDKK.T | Y | 94.59 | 2269.1113 | 21 | -0.4 | 1135.5625 | 2 | 66.19 | 2 | F2:42503 | NaNaKA16\_F10.raw |  | 2.8885E8 | 3.556E6 | 2.6478E6 |  |  |  |  |  | 2.1479E6 |  |  |  | 6 | 0 | 3 | 1 | 1 | 0 | 0 | 0 | 0 | 0 | 1 | 0 | 0 | 0 | 9 | 29 |  |  | PEAKS DB |
| R.FDGSPC(+57.02)VLGSPGFR.S | Y | 81.12 | 1494.6925 | 14 | 1.3 | 748.3545 | 2 | 52.39 | 2 | F2:30743 | NaNaKA16\_F10.raw |  | 1.3035E8 | 8.1299E6 | 1.7629E6 |  |  |  |  | 5.5091E6 | 1.2638E6 |  |  | 5.3893E5 | 8 | 0 | 3 | 1 | 1 | 0 | 0 | 0 | 0 | 1 | 1 | 0 | 0 | 1 | 47 | 60 | Carbamidomethylation | C6:Carbamidomethylation:1000.00 | PEAKS DB |
| K.TVENVGVSQVAPDNPER.F | Y | 72.54 | 1809.8856 | 17 | 0.3 | 905.9504 | 2 | 28.99 | 4 | F4:12723 | NaNaKA16\_F12.raw | 4.4967E5 |  |  | 2.8451E6 |  |  |  |  | 1.0607E6 |  |  |  |  | 3 | 1 | 0 | 0 | 1 | 0 | 0 | 0 | 0 | 1 | 0 | 0 | 0 | 0 | 30 | 46 |  |  | PEAKS DB |
| K.TVENVGVSQVAPDNPERFDGSPC(+57.02)VLGSPGFR.S | Y | 68.97 | 3286.5676 | 31 | 0.6 | 1096.5305 | 3 | 61.34 | 2 | F2:38663 | NaNaKA16\_F10.raw |  | 4.7404E7 |  |  |  |  |  |  |  |  |  |  |  | 1 | 0 | 1 | 0 | 0 | 0 | 0 | 0 | 0 | 0 | 0 | 0 | 0 | 0 | 30 | 60 | Carbamidomethylation | C23:Carbamidomethylation:1000.00 | PEAKS DB |
| K.ADVTFDSNTAFESLVVSPDK.K | Y | 67.96 | 2141.0164 | 20 | 0.4 | 1071.5159 | 2 | 79.78 | 2 | F2:54526 | NaNaKA16\_F10.raw |  | 1.937E6 |  |  |  |  |  |  |  |  |  |  |  | 1 | 0 | 1 | 0 | 0 | 0 | 0 | 0 | 0 | 0 | 0 | 0 | 0 | 0 | 9 | 28 |  |  | PEAKS DB |
| R.EWAVGLAGK.S | Y | 43.22 | 929.4970 | 9 | -0.6 | 465.7555 | 2 | 37.09 | 4 | F4:19505 | NaNaKA16\_F12.raw |  |  |  | 4.4943E5 |  |  |  |  | 6.5516E5 |  |  |  |  | 2 | 0 | 0 | 0 | 1 | 0 | 0 | 0 | 0 | 1 | 0 | 0 | 0 | 0 | 75 | 83 |  |  | PEAKS DB |
| total 6 peptides |  |  |  |  |  |  |  |  |  |  |  |  |  |  |  |  |  |  |  |  |  |  |  |  |  |  |  |  |  |  |  |  |  |  |  |  |  |  |  |  |  |  |  |
| --- | --- | --- | --- | --- | --- | --- | --- | --- | --- | --- | --- | --- | --- | --- | --- | --- | --- | --- | --- | --- | --- | --- | --- | --- | --- | --- | --- | --- | --- | --- | --- | --- | --- | --- | --- | --- | --- | --- | --- | --- | --- | --- | --- |

P01427.1

back to list

  

| Protein Coverage
| Supporting Peptides
|

Protein Coverage:

Supporting Peptides:

| Peptide | Uniq | -10lgP | Mass | Length | ppm | m/z | z | RT | Fraction | Scan | Source File | Area F1 | Area F10 | Area F11 | Area F12 | Area F13 | Area F2 | Area F3 | Area F4 | Area F5 | Area F6 | Area F7 | Area F8 | Area F9 | #Feature | #Feature F1 | #Feature F10 | #Feature F11 | #Feature F12 | #Feature F13 | #Feature F2 | #Feature F3 | #Feature F4 | #Feature F5 | #Feature F6 | #Feature F7 | #Feature F8 | #Feature F9 | Start | End | PTM | AScore | Found By |
| --- | --- | --- | --- | --- | --- | --- | --- | --- | --- | --- | --- | --- | --- | --- | --- | --- | --- | --- | --- | --- | --- | --- | --- | --- | --- | --- | --- | --- | --- | --- | --- | --- | --- | --- | --- | --- | --- | --- | --- | --- | --- | --- | --- |
| LEC(+57.02)HNQQSSQPPTTK.T | Y | 90.54 | 1753.8053 | 15 | -2.8 | 877.9075 | 2 | 11.84 | 6 | F6:1921 | NaNaKA16\_F2.raw | 3.9815E7 |  |  |  |  | 3.3625E8 | 2.7134E6 |  |  |  |  |  |  | 7 | 2 | 0 | 0 | 0 | 0 | 3 | 2 | 0 | 0 | 0 | 0 | 0 | 0 | 1 | 15 | Carbamidomethylation | C3:Carbamidomethylation:1000.00 | PEAKS DB |
| K.VKPGVNLNC(+57.02)C(+57.02)R.T | Y | 73.50 | 1315.6489 | 11 | 4.7 | 658.8315 | 2 | 11.28 | 7 | F7:1787 | NaNaKA16\_F3.raw | 8.7631E6 |  |  |  |  | 2.55E8 | 6.5001E6 | 8.5404E4 |  |  |  |  |  | 10 | 2 | 0 | 0 | 0 | 0 | 5 | 2 | 1 | 0 | 0 | 0 | 0 | 0 | 45 | 55 | Carbamidomethylation | C9:Carbamidomethylation:1000.00;C10:Carbamidomethylation:1000.00 | PEAKS DB |
| K.PGVNLNC(+57.02)C(+57.02)R.T | Y | 61.71 | 1088.4856 | 9 | -3.5 | 545.2482 | 2 | 11.84 | 6 | F6:2180 | NaNaKA16\_F2.raw | 6.794E4 |  |  |  |  | 4.9207E6 |  |  |  |  |  |  |  | 3 | 1 | 0 | 0 | 0 | 0 | 2 | 0 | 0 | 0 | 0 | 0 | 0 | 0 | 47 | 55 | Carbamidomethylation | C7:Carbamidomethylation:1000.00;C8:Carbamidomethylation:1000.00 | PEAKS DB |
| K.VKPGVNLNC(+57.02).C | Y | 60.37 | 999.5172 | 9 | 0.2 | 500.7659 | 2 | 12.45 | 6 | F6:2465 | NaNaKA16\_F2.raw |  |  |  |  |  | 1.6082E6 |  |  |  |  |  |  |  | 1 | 0 | 0 | 0 | 0 | 0 | 1 | 0 | 0 | 0 | 0 | 0 | 0 | 0 | 45 | 53 | Carbamidomethylation | C9:Carbamidomethylation:1000.00 | PEAKS DB |
| K.TC(+57.02)SGETNC(+57.02)YK.K | Y | 54.62 | 1218.4645 | 10 | -2.4 | 610.2380 | 2 | 11.84 | 6 | F6:1942 | NaNaKA16\_F2.raw | 2.3803E6 |  |  |  |  | 5.9614E6 |  |  |  |  |  |  |  | 2 | 1 | 0 | 0 | 0 | 0 | 1 | 0 | 0 | 0 | 0 | 0 | 0 | 0 | 16 | 25 | Carbamidomethylation | C2:Carbamidomethylation:1000.00;C8:Carbamidomethylation:1000.00 | PEAKS DB |
| K.VKPGVNLNC(+57.02)C(+57.02).R | Y | 51.22 | 1159.5479 | 10 | 0.8 | 580.7817 | 2 | 12.51 | 6 | F6:2512 | NaNaKA16\_F2.raw |  |  |  |  |  | 9.6916E5 |  |  |  |  |  |  |  | 1 | 0 | 0 | 0 | 0 | 0 | 1 | 0 | 0 | 0 | 0 | 0 | 0 | 0 | 45 | 54 | Carbamidomethylation | C9:Carbamidomethylation:1000.00;C10:Carbamidomethylation:1000.00 | PEAKS DB |
| K.WWSDHRGTIIER.G | Y | 50.82 | 1554.7692 | 12 | 0.6 | 389.6998 | 4 | 33.34 | 6 | F6:18624 | NaNaKA16\_F2.raw |  |  |  |  |  | 3.5624E6 |  |  |  |  |  |  |  | 1 | 0 | 0 | 0 | 0 | 0 | 1 | 0 | 0 | 0 | 0 | 0 | 0 | 0 | 27 | 38 |  |  | PEAKS DB |
| K.KWWSDHR.G | Y | 48.51 | 1013.4832 | 7 | -0.3 | 507.7487 | 2 | 14.91 | 6 | F6:3847 | NaNaKA16\_F2.raw |  |  |  |  |  | 1.42E6 |  |  |  |  |  |  |  | 1 | 0 | 0 | 0 | 0 | 0 | 1 | 0 | 0 | 0 | 0 | 0 | 0 | 0 | 26 | 32 |  |  | PEAKS DB |
| total 8 peptides |  |  |  |  |  |  |  |  |  |  |  |  |  |  |  |  |  |  |  |  |  |  |  |  |  |  |  |  |  |  |  |  |  |  |  |  |  |  |  |  |  |  |  |
| --- | --- | --- | --- | --- | --- | --- | --- | --- | --- | --- | --- | --- | --- | --- | --- | --- | --- | --- | --- | --- | --- | --- | --- | --- | --- | --- | --- | --- | --- | --- | --- | --- | --- | --- | --- | --- | --- | --- | --- | --- | --- | --- | --- |

1NOR

back to list

  

| Protein Coverage
| Supporting Peptides
|

Protein Coverage:

Supporting Peptides:

| Peptide | Uniq | -10lgP | Mass | Length | ppm | m/z | z | RT | Fraction | Scan | Source File | Area F1 | Area F10 | Area F11 | Area F12 | Area F13 | Area F2 | Area F3 | Area F4 | Area F5 | Area F6 | Area F7 | Area F8 | Area F9 | #Feature | #Feature F1 | #Feature F10 | #Feature F11 | #Feature F12 | #Feature F13 | #Feature F2 | #Feature F3 | #Feature F4 | #Feature F5 | #Feature F6 | #Feature F7 | #Feature F8 | #Feature F9 | Start | End | PTM | AScore | Found By |
| --- | --- | --- | --- | --- | --- | --- | --- | --- | --- | --- | --- | --- | --- | --- | --- | --- | --- | --- | --- | --- | --- | --- | --- | --- | --- | --- | --- | --- | --- | --- | --- | --- | --- | --- | --- | --- | --- | --- | --- | --- | --- | --- | --- |
| LEC(+57.02)HNQQSSQPPTTK.T | Y | 90.54 | 1753.8053 | 15 | -2.8 | 877.9075 | 2 | 11.84 | 6 | F6:1921 | NaNaKA16\_F2.raw | 3.9815E7 |  |  |  |  | 3.3625E8 | 2.7134E6 |  |  |  |  |  |  | 7 | 2 | 0 | 0 | 0 | 0 | 3 | 2 | 0 | 0 | 0 | 0 | 0 | 0 | 1 | 15 | Carbamidomethylation | C3:Carbamidomethylation:1000.00 | PEAKS DB |
| K.VKPGVNLNC(+57.02)C(+57.02)R.T | Y | 73.50 | 1315.6489 | 11 | 4.7 | 658.8315 | 2 | 11.28 | 7 | F7:1787 | NaNaKA16\_F3.raw | 8.7631E6 |  |  |  |  | 2.55E8 | 6.5001E6 | 8.5404E4 |  |  |  |  |  | 10 | 2 | 0 | 0 | 0 | 0 | 5 | 2 | 1 | 0 | 0 | 0 | 0 | 0 | 45 | 55 | Carbamidomethylation | C9:Carbamidomethylation:1000.00;C10:Carbamidomethylation:1000.00 | PEAKS DB |
| K.PGVNLNC(+57.02)C(+57.02)R.T | Y | 61.71 | 1088.4856 | 9 | -3.5 | 545.2482 | 2 | 11.84 | 6 | F6:2180 | NaNaKA16\_F2.raw | 6.794E4 |  |  |  |  | 4.9207E6 |  |  |  |  |  |  |  | 3 | 1 | 0 | 0 | 0 | 0 | 2 | 0 | 0 | 0 | 0 | 0 | 0 | 0 | 47 | 55 | Carbamidomethylation | C7:Carbamidomethylation:1000.00;C8:Carbamidomethylation:1000.00 | PEAKS DB |
| K.VKPGVNLNC(+57.02).C | Y | 60.37 | 999.5172 | 9 | 0.2 | 500.7659 | 2 | 12.45 | 6 | F6:2465 | NaNaKA16\_F2.raw |  |  |  |  |  | 1.6082E6 |  |  |  |  |  |  |  | 1 | 0 | 0 | 0 | 0 | 0 | 1 | 0 | 0 | 0 | 0 | 0 | 0 | 0 | 45 | 53 | Carbamidomethylation | C9:Carbamidomethylation:1000.00 | PEAKS DB |
| K.TC(+57.02)SGETNC(+57.02)YK.K | Y | 54.62 | 1218.4645 | 10 | -2.4 | 610.2380 | 2 | 11.84 | 6 | F6:1942 | NaNaKA16\_F2.raw | 2.3803E6 |  |  |  |  | 5.9614E6 |  |  |  |  |  |  |  | 2 | 1 | 0 | 0 | 0 | 0 | 1 | 0 | 0 | 0 | 0 | 0 | 0 | 0 | 16 | 25 | Carbamidomethylation | C2:Carbamidomethylation:1000.00;C8:Carbamidomethylation:1000.00 | PEAKS DB |
| K.VKPGVNLNC(+57.02)C(+57.02).R | Y | 51.22 | 1159.5479 | 10 | 0.8 | 580.7817 | 2 | 12.51 | 6 | F6:2512 | NaNaKA16\_F2.raw |  |  |  |  |  | 9.6916E5 |  |  |  |  |  |  |  | 1 | 0 | 0 | 0 | 0 | 0 | 1 | 0 | 0 | 0 | 0 | 0 | 0 | 0 | 45 | 54 | Carbamidomethylation | C9:Carbamidomethylation:1000.00;C10:Carbamidomethylation:1000.00 | PEAKS DB |
| K.WWSDHRGTIIER.G | Y | 50.82 | 1554.7692 | 12 | 0.6 | 389.6998 | 4 | 33.34 | 6 | F6:18624 | NaNaKA16\_F2.raw |  |  |  |  |  | 3.5624E6 |  |  |  |  |  |  |  | 1 | 0 | 0 | 0 | 0 | 0 | 1 | 0 | 0 | 0 | 0 | 0 | 0 | 0 | 27 | 38 |  |  | PEAKS DB |
| K.KWWSDHR.G | Y | 48.51 | 1013.4832 | 7 | -0.3 | 507.7487 | 2 | 14.91 | 6 | F6:3847 | NaNaKA16\_F2.raw |  |  |  |  |  | 1.42E6 |  |  |  |  |  |  |  | 1 | 0 | 0 | 0 | 0 | 0 | 1 | 0 | 0 | 0 | 0 | 0 | 0 | 0 | 26 | 32 |  |  | PEAKS DB |
| total 8 peptides |  |  |  |  |  |  |  |  |  |  |  |  |  |  |  |  |  |  |  |  |  |  |  |  |  |  |  |  |  |  |  |  |  |  |  |  |  |  |  |  |  |  |  |
| --- | --- | --- | --- | --- | --- | --- | --- | --- | --- | --- | --- | --- | --- | --- | --- | --- | --- | --- | --- | --- | --- | --- | --- | --- | --- | --- | --- | --- | --- | --- | --- | --- | --- | --- | --- | --- | --- | --- | --- | --- | --- | --- | --- |

2MJ4

back to list

  

| Protein Coverage
| Supporting Peptides
|

Protein Coverage:

Supporting Peptides:

| Peptide | Uniq | -10lgP | Mass | Length | ppm | m/z | z | RT | Fraction | Scan | Source File | Area F1 | Area F10 | Area F11 | Area F12 | Area F13 | Area F2 | Area F3 | Area F4 | Area F5 | Area F6 | Area F7 | Area F8 | Area F9 | #Feature | #Feature F1 | #Feature F10 | #Feature F11 | #Feature F12 | #Feature F13 | #Feature F2 | #Feature F3 | #Feature F4 | #Feature F5 | #Feature F6 | #Feature F7 | #Feature F8 | #Feature F9 | Start | End | PTM | AScore | Found By |
| --- | --- | --- | --- | --- | --- | --- | --- | --- | --- | --- | --- | --- | --- | --- | --- | --- | --- | --- | --- | --- | --- | --- | --- | --- | --- | --- | --- | --- | --- | --- | --- | --- | --- | --- | --- | --- | --- | --- | --- | --- | --- | --- | --- |
| LEC(+57.02)HNQQSSQPPTTK.T | Y | 90.54 | 1753.8053 | 15 | -2.8 | 877.9075 | 2 | 11.84 | 6 | F6:1921 | NaNaKA16\_F2.raw | 3.9815E7 |  |  |  |  | 3.3625E8 | 2.7134E6 |  |  |  |  |  |  | 7 | 2 | 0 | 0 | 0 | 0 | 3 | 2 | 0 | 0 | 0 | 0 | 0 | 0 | 1 | 15 | Carbamidomethylation | C3:Carbamidomethylation:1000.00 | PEAKS DB |
| K.VKPGVNLNC(+57.02)C(+57.02)R.T | Y | 73.50 | 1315.6489 | 11 | 4.7 | 658.8315 | 2 | 11.28 | 7 | F7:1787 | NaNaKA16\_F3.raw | 8.7631E6 |  |  |  |  | 2.55E8 | 6.5001E6 | 8.5404E4 |  |  |  |  |  | 10 | 2 | 0 | 0 | 0 | 0 | 5 | 2 | 1 | 0 | 0 | 0 | 0 | 0 | 45 | 55 | Carbamidomethylation | C9:Carbamidomethylation:1000.00;C10:Carbamidomethylation:1000.00 | PEAKS DB |
| K.PGVNLNC(+57.02)C(+57.02)R.T | Y | 61.71 | 1088.4856 | 9 | -3.5 | 545.2482 | 2 | 11.84 | 6 | F6:2180 | NaNaKA16\_F2.raw | 6.794E4 |  |  |  |  | 4.9207E6 |  |  |  |  |  |  |  | 3 | 1 | 0 | 0 | 0 | 0 | 2 | 0 | 0 | 0 | 0 | 0 | 0 | 0 | 47 | 55 | Carbamidomethylation | C7:Carbamidomethylation:1000.00;C8:Carbamidomethylation:1000.00 | PEAKS DB |
| K.VKPGVNLNC(+57.02).C | Y | 60.37 | 999.5172 | 9 | 0.2 | 500.7659 | 2 | 12.45 | 6 | F6:2465 | NaNaKA16\_F2.raw |  |  |  |  |  | 1.6082E6 |  |  |  |  |  |  |  | 1 | 0 | 0 | 0 | 0 | 0 | 1 | 0 | 0 | 0 | 0 | 0 | 0 | 0 | 45 | 53 | Carbamidomethylation | C9:Carbamidomethylation:1000.00 | PEAKS DB |
| K.TC(+57.02)SGETNC(+57.02)YK.K | Y | 54.62 | 1218.4645 | 10 | -2.4 | 610.2380 | 2 | 11.84 | 6 | F6:1942 | NaNaKA16\_F2.raw | 2.3803E6 |  |  |  |  | 5.9614E6 |  |  |  |  |  |  |  | 2 | 1 | 0 | 0 | 0 | 0 | 1 | 0 | 0 | 0 | 0 | 0 | 0 | 0 | 16 | 25 | Carbamidomethylation | C2:Carbamidomethylation:1000.00;C8:Carbamidomethylation:1000.00 | PEAKS DB |
| K.VKPGVNLNC(+57.02)C(+57.02).R | Y | 51.22 | 1159.5479 | 10 | 0.8 | 580.7817 | 2 | 12.51 | 6 | F6:2512 | NaNaKA16\_F2.raw |  |  |  |  |  | 9.6916E5 |  |  |  |  |  |  |  | 1 | 0 | 0 | 0 | 0 | 0 | 1 | 0 | 0 | 0 | 0 | 0 | 0 | 0 | 45 | 54 | Carbamidomethylation | C9:Carbamidomethylation:1000.00;C10:Carbamidomethylation:1000.00 | PEAKS DB |
| K.WWSDHRGTIIER.G | Y | 50.82 | 1554.7692 | 12 | 0.6 | 389.6998 | 4 | 33.34 | 6 | F6:18624 | NaNaKA16\_F2.raw |  |  |  |  |  | 3.5624E6 |  |  |  |  |  |  |  | 1 | 0 | 0 | 0 | 0 | 0 | 1 | 0 | 0 | 0 | 0 | 0 | 0 | 0 | 27 | 38 |  |  | PEAKS DB |
| K.KWWSDHR.G | Y | 48.51 | 1013.4832 | 7 | -0.3 | 507.7487 | 2 | 14.91 | 6 | F6:3847 | NaNaKA16\_F2.raw |  |  |  |  |  | 1.42E6 |  |  |  |  |  |  |  | 1 | 0 | 0 | 0 | 0 | 0 | 1 | 0 | 0 | 0 | 0 | 0 | 0 | 0 | 26 | 32 |  |  | PEAKS DB |
| total 8 peptides |  |  |  |  |  |  |  |  |  |  |  |  |  |  |  |  |  |  |  |  |  |  |  |  |  |  |  |  |  |  |  |  |  |  |  |  |  |  |  |  |  |  |  |
| --- | --- | --- | --- | --- | --- | --- | --- | --- | --- | --- | --- | --- | --- | --- | --- | --- | --- | --- | --- | --- | --- | --- | --- | --- | --- | --- | --- | --- | --- | --- | --- | --- | --- | --- | --- | --- | --- | --- | --- | --- | --- | --- | --- |

JAG67188.1

back to list

  

| Protein Coverage
| Supporting Peptides
|

Protein Coverage:

Supporting Peptides:

| Peptide | Uniq | -10lgP | Mass | Length | ppm | m/z | z | RT | Fraction | Scan | Source File | Area F1 | Area F10 | Area F11 | Area F12 | Area F13 | Area F2 | Area F3 | Area F4 | Area F5 | Area F6 | Area F7 | Area F8 | Area F9 | #Feature | #Feature F1 | #Feature F10 | #Feature F11 | #Feature F12 | #Feature F13 | #Feature F2 | #Feature F3 | #Feature F4 | #Feature F5 | #Feature F6 | #Feature F7 | #Feature F8 | #Feature F9 | Start | End | PTM | AScore | Found By |
| --- | --- | --- | --- | --- | --- | --- | --- | --- | --- | --- | --- | --- | --- | --- | --- | --- | --- | --- | --- | --- | --- | --- | --- | --- | --- | --- | --- | --- | --- | --- | --- | --- | --- | --- | --- | --- | --- | --- | --- | --- | --- | --- | --- |
| R.HGQGTGELLQVSGIK.V | N | 77.57 | 1522.8103 | 15 | 0.5 | 762.4128 | 2 | 32.78 | 5 | F5:12050 | NaNaKA16\_F13.raw |  |  |  | 4.2458E5 | 5.0296E6 |  |  |  |  |  |  |  |  | 3 | 0 | 0 | 0 | 1 | 2 | 0 | 0 | 0 | 0 | 0 | 0 | 0 | 0 | 457 | 471 |  |  | PEAKS DB |
| R.FHEC(+57.02)NLGNLIC(+57.02)DAVVYNNLR.H | N | 72.86 | 2420.1365 | 20 | 0.2 | 807.7196 | 3 | 76.91 | 13 | F13:50987 | NaNaKA16\_F9.raw |  |  |  |  |  |  |  |  |  |  |  | 5.2029E6 | 2.2685E7 | 3 | 0 | 0 | 0 | 0 | 0 | 0 | 0 | 0 | 0 | 0 | 0 | 1 | 2 | 369 | 388 | Carbamidomethylation | C4:Carbamidomethylation:1000.00;C11:Carbamidomethylation:1000.00 | PEAKS DB |
| R.VPTYVPLEMEK.S | N | 72.07 | 1304.6686 | 11 | 1.1 | 653.3423 | 2 | 50.51 | 5 | F5:20383 | NaNaKA16\_F13.raw |  |  | 0 | 1.0366E6 | 6.7478E6 |  |  |  |  |  |  |  |  | 2 | 0 | 0 | 0 | 1 | 1 | 0 | 0 | 0 | 0 | 0 | 0 | 0 | 0 | 496 | 506 |  |  | PEAKS DB |
| R.QVPVVQAYAFGK.Y | N | 68.51 | 1305.7081 | 12 | -0.2 | 653.8612 | 2 | 81.22 | 5 | F5:33335 | NaNaKA16\_F13.raw |  |  |  | 4.7302E5 | 4.1666E6 |  |  |  |  |  |  |  |  | 3 | 0 | 0 | 0 | 1 | 2 | 0 | 0 | 0 | 0 | 0 | 0 | 0 | 0 | 288 | 299 |  |  | PEAKS DB |
| E.LTILHTNDVHAR.V | N | 61.22 | 1388.7524 | 12 | -0.8 | 463.9244 | 3 | 12.08 | 5 | F5:1993 | NaNaKA16\_F13.raw |  |  |  |  | 9.2667E5 |  |  |  |  |  |  |  |  | 1 | 0 | 0 | 0 | 0 | 1 | 0 | 0 | 0 | 0 | 0 | 0 | 0 | 0 | 44 | 55 |  |  | PEAKS DB |
| K.ETPVLSNPGPYLEFR.D | N | 55.50 | 1717.8674 | 15 | -0.2 | 859.9409 | 2 | 68.90 | 5 | F5:28106 | NaNaKA16\_F13.raw |  |  |  |  | 1.3217E6 |  |  |  |  |  |  |  |  | 1 | 0 | 0 | 0 | 0 | 1 | 0 | 0 | 0 | 0 | 0 | 0 | 0 | 0 | 194 | 208 |  |  | PEAKS DB |
| R.EVVQFMNSLR.Y | Y | 51.27 | 1221.6176 | 10 | -0.2 | 611.8159 | 2 | 54.42 | 5 | F5:22037 | NaNaKA16\_F13.raw |  |  |  |  | 1.1341E6 |  |  |  |  |  |  |  |  | 1 | 0 | 0 | 0 | 0 | 1 | 0 | 0 | 0 | 0 | 0 | 0 | 0 | 0 | 114 | 123 |  |  | PEAKS DB |
| R.VPTYVPLEM(+15.99)EK.S | N | 47.95 | 1320.6635 | 11 | 0.2 | 661.3391 | 2 | 38.01 | 5 | F5:14849 | NaNaKA16\_F13.raw |  |  |  |  | 6.2061E5 |  |  |  |  |  |  |  |  | 1 | 0 | 0 | 0 | 0 | 1 | 0 | 0 | 0 | 0 | 0 | 0 | 0 | 0 | 496 | 506 | Oxidation (M) | M9:Oxidation (M):1000.00 | PEAKS DB |
| K.VGIIGYTTK.E | N | 46.40 | 950.5436 | 9 | 0.7 | 476.2794 | 2 | 30.07 | 5 | F5:10298 | NaNaKA16\_F13.raw |  |  |  |  | 6.7595E6 |  |  |  |  |  |  |  |  | 1 | 0 | 0 | 0 | 0 | 1 | 0 | 0 | 0 | 0 | 0 | 0 | 0 | 0 | 185 | 193 |  |  | PEAKS DB |
| N.ISGYILPYK.I | N | 45.32 | 1052.5906 | 9 | 0.8 | 527.3030 | 2 | 46.37 | 5 | F5:18970 | NaNaKA16\_F13.raw |  |  |  |  | 5.2097E6 |  |  |  |  |  |  |  |  | 1 | 0 | 0 | 0 | 0 | 1 | 0 | 0 | 0 | 0 | 0 | 0 | 0 | 0 | 168 | 176 |  |  | PEAKS DB |
| total 10 peptides |  |  |  |  |  |  |  |  |  |  |  |  |  |  |  |  |  |  |  |  |  |  |  |  |  |  |  |  |  |  |  |  |  |  |  |  |  |  |  |  |  |  |  |
| --- | --- | --- | --- | --- | --- | --- | --- | --- | --- | --- | --- | --- | --- | --- | --- | --- | --- | --- | --- | --- | --- | --- | --- | --- | --- | --- | --- | --- | --- | --- | --- | --- | --- | --- | --- | --- | --- | --- | --- | --- | --- | --- | --- |

APB88857.1

back to list

  

| Protein Coverage
| Supporting Peptides
|

Protein Coverage:

Supporting Peptides:

| Peptide | Uniq | -10lgP | Mass | Length | ppm | m/z | z | RT | Fraction | Scan | Source File | Area F1 | Area F10 | Area F11 | Area F12 | Area F13 | Area F2 | Area F3 | Area F4 | Area F5 | Area F6 | Area F7 | Area F8 | Area F9 | #Feature | #Feature F1 | #Feature F10 | #Feature F11 | #Feature F12 | #Feature F13 | #Feature F2 | #Feature F3 | #Feature F4 | #Feature F5 | #Feature F6 | #Feature F7 | #Feature F8 | #Feature F9 | Start | End | PTM | AScore | Found By |
| --- | --- | --- | --- | --- | --- | --- | --- | --- | --- | --- | --- | --- | --- | --- | --- | --- | --- | --- | --- | --- | --- | --- | --- | --- | --- | --- | --- | --- | --- | --- | --- | --- | --- | --- | --- | --- | --- | --- | --- | --- | --- | --- | --- |
| T.RLC(+57.02)LSDYSIFSETIEIC(+57.02)PDGHNFC(+57.02)FK.K | Y | 79.97 | 3207.4463 | 26 | -1.6 | 802.8676 | 4 | 81.23 | 13 | F13:56229 | NaNaKA16\_F9.raw |  |  |  |  |  |  |  |  |  |  |  |  | 6.2243E7 | 2 | 0 | 0 | 0 | 0 | 0 | 0 | 0 | 0 | 0 | 0 | 0 | 0 | 2 | 22 | 47 | Carbamidomethylation | C3:Carbamidomethylation:1000.00;C17:Carbamidomethylation:1000.00;C24:Carbamidomethylation:1000.00 | PEAKS DB |
| F.SETIEIC(+57.02)PDGHNFC(+57.02)FK.K | Y | 77.77 | 1952.8396 | 16 | -1.2 | 977.4259 | 2 | 49.68 | 10 | F10:32514 | NaNaKA16\_F6.raw |  |  |  |  |  |  |  |  |  | 5.3752E7 |  |  | 3.3568E7 | 4 | 0 | 0 | 0 | 0 | 0 | 0 | 0 | 0 | 0 | 2 | 0 | 0 | 2 | 32 | 47 | Carbamidomethylation | C7:Carbamidomethylation:1000.00;C14:Carbamidomethylation:1000.00 | PEAKS DB |
| S.ETIEIC(+57.02)PDGHNFC(+57.02)FK.K | Y | 62.63 | 1865.8076 | 15 | 0.0 | 622.9432 | 3 | 50.23 | 10 | F10:33009 | NaNaKA16\_F6.raw |  |  |  |  |  |  |  |  |  | 1.5094E7 |  |  |  | 2 | 0 | 0 | 0 | 0 | 0 | 0 | 0 | 0 | 0 | 2 | 0 | 0 | 0 | 33 | 47 | Carbamidomethylation | C6:Carbamidomethylation:1000.00;C13:Carbamidomethylation:1000.00 | PEAKS DB |
| C.LSDYSIFSETIEIC(+57.02)PDGHNFC(+57.02)FK.K | Y | 61.37 | 2778.2305 | 23 | 0.8 | 927.0848 | 3 | 83.94 | 13 | F13:56808 | NaNaKA16\_F9.raw |  |  |  |  |  |  |  |  |  |  |  |  | 1.0126E7 | 1 | 0 | 0 | 0 | 0 | 0 | 0 | 0 | 0 | 0 | 0 | 0 | 0 | 1 | 25 | 47 | Carbamidomethylation | C14:Carbamidomethylation:1000.00;C21:Carbamidomethylation:1000.00 | PEAKS DB |
| R.LC(+57.02)LSDYSIFSETIEIC(+57.02)PDGHNFC(+57.02)FK.K | Y | 54.85 | 3051.3452 | 25 | 0.3 | 1018.1226 | 3 | 92.48 | 13 | F13:64193 | NaNaKA16\_F9.raw |  |  |  |  |  |  |  |  |  |  |  |  | 1.7346E7 | 1 | 0 | 0 | 0 | 0 | 0 | 0 | 0 | 0 | 0 | 0 | 0 | 0 | 1 | 23 | 47 | Carbamidomethylation | C2:Carbamidomethylation:1000.00;C16:Carbamidomethylation:1000.00;C23:Carbamidomethylation:1000.00 | PEAKS DB |
| K.GITRLPWVIR.G | N | 53.94 | 1209.7346 | 10 | 0.7 | 404.2524 | 3 | 50.16 | 12 | F12:31448 | NaNaKA16\_F8.raw |  |  |  |  |  |  |  |  |  |  |  | 4.8367E6 | 4.8796E6 | 2 | 0 | 0 | 0 | 0 | 0 | 0 | 0 | 0 | 0 | 0 | 0 | 1 | 1 | 52 | 61 |  |  | PEAKS DB |
| Y.SIFSETIEIC(+57.02)PDGHNFC(+57.02)FK.K | Y | 53.11 | 2300.0242 | 19 | 1.0 | 767.6827 | 3 | 72.97 | 13 | F13:47475 | NaNaKA16\_F9.raw |  |  |  |  |  |  |  |  |  |  |  | 7.4795E6 | 9.9901E6 | 2 | 0 | 0 | 0 | 0 | 0 | 0 | 0 | 0 | 0 | 0 | 0 | 1 | 1 | 29 | 47 | Carbamidomethylation | C10:Carbamidomethylation:1000.00;C17:Carbamidomethylation:1000.00 | PEAKS DB |
| S.IFSETIEIC(+57.02)PDGHNFC(+57.02)FK.K | Y | 48.14 | 2212.9922 | 18 | 0.6 | 738.6718 | 3 | 65.16 | 12 | F12:43580 | NaNaKA16\_F8.raw |  |  |  |  |  |  |  |  |  |  |  | 6.4302E6 |  | 1 | 0 | 0 | 0 | 0 | 0 | 0 | 0 | 0 | 0 | 0 | 0 | 1 | 0 | 30 | 47 | Carbamidomethylation | C9:Carbamidomethylation:1000.00;C16:Carbamidomethylation:1000.00 | PEAKS DB |
| S.DYSIFSETIEIC(+57.02)PDGHNFC(+57.02)FK.K | Y | 47.17 | 2578.1145 | 21 | 0.6 | 860.3793 | 3 | 86.30 | 12 | F12:61577 | NaNaKA16\_F8.raw |  |  |  |  |  |  |  |  |  |  |  | 2.9844E6 |  | 1 | 0 | 0 | 0 | 0 | 0 | 0 | 0 | 0 | 0 | 0 | 0 | 1 | 0 | 27 | 47 | Carbamidomethylation | C12:Carbamidomethylation:1000.00;C19:Carbamidomethylation:1000.00 | PEAKS DB |
| I.TRLPWVIR.G | N | 42.46 | 1039.6290 | 8 | -0.3 | 520.8217 | 2 | 38.07 | 12 | F12:21421 | NaNaKA16\_F8.raw |  |  |  |  |  |  |  |  |  |  |  | 1.3591E7 |  | 1 | 0 | 0 | 0 | 0 | 0 | 0 | 0 | 0 | 0 | 0 | 0 | 1 | 0 | 54 | 61 |  |  | PEAKS DB |
| total 10 peptides |  |  |  |  |  |  |  |  |  |  |  |  |  |  |  |  |  |  |  |  |  |  |  |  |  |  |  |  |  |  |  |  |  |  |  |  |  |  |  |  |  |  |  |
| --- | --- | --- | --- | --- | --- | --- | --- | --- | --- | --- | --- | --- | --- | --- | --- | --- | --- | --- | --- | --- | --- | --- | --- | --- | --- | --- | --- | --- | --- | --- | --- | --- | --- | --- | --- | --- | --- | --- | --- | --- | --- | --- | --- |

CAB45156.1

back to list

  

| Protein Coverage
| Supporting Peptides
|

Protein Coverage:

Supporting Peptides:

| Peptide | Uniq | -10lgP | Mass | Length | ppm | m/z | z | RT | Fraction | Scan | Source File | Area F1 | Area F10 | Area F11 | Area F12 | Area F13 | Area F2 | Area F3 | Area F4 | Area F5 | Area F6 | Area F7 | Area F8 | Area F9 | #Feature | #Feature F1 | #Feature F10 | #Feature F11 | #Feature F12 | #Feature F13 | #Feature F2 | #Feature F3 | #Feature F4 | #Feature F5 | #Feature F6 | #Feature F7 | #Feature F8 | #Feature F9 | Start | End | PTM | AScore | Found By |
| --- | --- | --- | --- | --- | --- | --- | --- | --- | --- | --- | --- | --- | --- | --- | --- | --- | --- | --- | --- | --- | --- | --- | --- | --- | --- | --- | --- | --- | --- | --- | --- | --- | --- | --- | --- | --- | --- | --- | --- | --- | --- | --- | --- |
| T.RLC(+57.02)LSDYSIFSETIEIC(+57.02)PDGHNFC(+57.02)FK.K | Y | 79.97 | 3207.4463 | 26 | -1.6 | 802.8676 | 4 | 81.23 | 13 | F13:56229 | NaNaKA16\_F9.raw |  |  |  |  |  |  |  |  |  |  |  |  | 6.2243E7 | 2 | 0 | 0 | 0 | 0 | 0 | 0 | 0 | 0 | 0 | 0 | 0 | 0 | 2 | 22 | 47 | Carbamidomethylation | C3:Carbamidomethylation:1000.00;C17:Carbamidomethylation:1000.00;C24:Carbamidomethylation:1000.00 | PEAKS DB |
| F.SETIEIC(+57.02)PDGHNFC(+57.02)FK.K | Y | 77.77 | 1952.8396 | 16 | -1.2 | 977.4259 | 2 | 49.68 | 10 | F10:32514 | NaNaKA16\_F6.raw |  |  |  |  |  |  |  |  |  | 5.3752E7 |  |  | 3.3568E7 | 4 | 0 | 0 | 0 | 0 | 0 | 0 | 0 | 0 | 0 | 2 | 0 | 0 | 2 | 32 | 47 | Carbamidomethylation | C7:Carbamidomethylation:1000.00;C14:Carbamidomethylation:1000.00 | PEAKS DB |
| S.ETIEIC(+57.02)PDGHNFC(+57.02)FK.K | Y | 62.63 | 1865.8076 | 15 | 0.0 | 622.9432 | 3 | 50.23 | 10 | F10:33009 | NaNaKA16\_F6.raw |  |  |  |  |  |  |  |  |  | 1.5094E7 |  |  |  | 2 | 0 | 0 | 0 | 0 | 0 | 0 | 0 | 0 | 0 | 2 | 0 | 0 | 0 | 33 | 47 | Carbamidomethylation | C6:Carbamidomethylation:1000.00;C13:Carbamidomethylation:1000.00 | PEAKS DB |
| C.LSDYSIFSETIEIC(+57.02)PDGHNFC(+57.02)FK.K | Y | 61.37 | 2778.2305 | 23 | 0.8 | 927.0848 | 3 | 83.94 | 13 | F13:56808 | NaNaKA16\_F9.raw |  |  |  |  |  |  |  |  |  |  |  |  | 1.0126E7 | 1 | 0 | 0 | 0 | 0 | 0 | 0 | 0 | 0 | 0 | 0 | 0 | 0 | 1 | 25 | 47 | Carbamidomethylation | C14:Carbamidomethylation:1000.00;C21:Carbamidomethylation:1000.00 | PEAKS DB |
| R.LC(+57.02)LSDYSIFSETIEIC(+57.02)PDGHNFC(+57.02)FK.K | Y | 54.85 | 3051.3452 | 25 | 0.3 | 1018.1226 | 3 | 92.48 | 13 | F13:64193 | NaNaKA16\_F9.raw |  |  |  |  |  |  |  |  |  |  |  |  | 1.7346E7 | 1 | 0 | 0 | 0 | 0 | 0 | 0 | 0 | 0 | 0 | 0 | 0 | 0 | 1 | 23 | 47 | Carbamidomethylation | C2:Carbamidomethylation:1000.00;C16:Carbamidomethylation:1000.00;C23:Carbamidomethylation:1000.00 | PEAKS DB |
| K.GITRLPWVIR.G | N | 53.94 | 1209.7346 | 10 | 0.7 | 404.2524 | 3 | 50.16 | 12 | F12:31448 | NaNaKA16\_F8.raw |  |  |  |  |  |  |  |  |  |  |  | 4.8367E6 | 4.8796E6 | 2 | 0 | 0 | 0 | 0 | 0 | 0 | 0 | 0 | 0 | 0 | 0 | 1 | 1 | 52 | 61 |  |  | PEAKS DB |
| Y.SIFSETIEIC(+57.02)PDGHNFC(+57.02)FK.K | Y | 53.11 | 2300.0242 | 19 | 1.0 | 767.6827 | 3 | 72.97 | 13 | F13:47475 | NaNaKA16\_F9.raw |  |  |  |  |  |  |  |  |  |  |  | 7.4795E6 | 9.9901E6 | 2 | 0 | 0 | 0 | 0 | 0 | 0 | 0 | 0 | 0 | 0 | 0 | 1 | 1 | 29 | 47 | Carbamidomethylation | C10:Carbamidomethylation:1000.00;C17:Carbamidomethylation:1000.00 | PEAKS DB |
| S.IFSETIEIC(+57.02)PDGHNFC(+57.02)FK.K | Y | 48.14 | 2212.9922 | 18 | 0.6 | 738.6718 | 3 | 65.16 | 12 | F12:43580 | NaNaKA16\_F8.raw |  |  |  |  |  |  |  |  |  |  |  | 6.4302E6 |  | 1 | 0 | 0 | 0 | 0 | 0 | 0 | 0 | 0 | 0 | 0 | 0 | 1 | 0 | 30 | 47 | Carbamidomethylation | C9:Carbamidomethylation:1000.00;C16:Carbamidomethylation:1000.00 | PEAKS DB |
| S.DYSIFSETIEIC(+57.02)PDGHNFC(+57.02)FK.K | Y | 47.17 | 2578.1145 | 21 | 0.6 | 860.3793 | 3 | 86.30 | 12 | F12:61577 | NaNaKA16\_F8.raw |  |  |  |  |  |  |  |  |  |  |  | 2.9844E6 |  | 1 | 0 | 0 | 0 | 0 | 0 | 0 | 0 | 0 | 0 | 0 | 0 | 1 | 0 | 27 | 47 | Carbamidomethylation | C12:Carbamidomethylation:1000.00;C19:Carbamidomethylation:1000.00 | PEAKS DB |
| I.TRLPWVIR.G | N | 42.46 | 1039.6290 | 8 | -0.3 | 520.8217 | 2 | 38.07 | 12 | F12:21421 | NaNaKA16\_F8.raw |  |  |  |  |  |  |  |  |  |  |  | 1.3591E7 |  | 1 | 0 | 0 | 0 | 0 | 0 | 0 | 0 | 0 | 0 | 0 | 0 | 1 | 0 | 54 | 61 |  |  | PEAKS DB |
| total 10 peptides |  |  |  |  |  |  |  |  |  |  |  |  |  |  |  |  |  |  |  |  |  |  |  |  |  |  |  |  |  |  |  |  |  |  |  |  |  |  |  |  |  |  |  |
| --- | --- | --- | --- | --- | --- | --- | --- | --- | --- | --- | --- | --- | --- | --- | --- | --- | --- | --- | --- | --- | --- | --- | --- | --- | --- | --- | --- | --- | --- | --- | --- | --- | --- | --- | --- | --- | --- | --- | --- | --- | --- | --- | --- |

Q9W717.1

back to list

  

| Protein Coverage
| Supporting Peptides
|

Protein Coverage:

Supporting Peptides:

| Peptide | Uniq | -10lgP | Mass | Length | ppm | m/z | z | RT | Fraction | Scan | Source File | Area F1 | Area F10 | Area F11 | Area F12 | Area F13 | Area F2 | Area F3 | Area F4 | Area F5 | Area F6 | Area F7 | Area F8 | Area F9 | #Feature | #Feature F1 | #Feature F10 | #Feature F11 | #Feature F12 | #Feature F13 | #Feature F2 | #Feature F3 | #Feature F4 | #Feature F5 | #Feature F6 | #Feature F7 | #Feature F8 | #Feature F9 | Start | End | PTM | AScore | Found By |
| --- | --- | --- | --- | --- | --- | --- | --- | --- | --- | --- | --- | --- | --- | --- | --- | --- | --- | --- | --- | --- | --- | --- | --- | --- | --- | --- | --- | --- | --- | --- | --- | --- | --- | --- | --- | --- | --- | --- | --- | --- | --- | --- | --- |
| T.RLC(+57.02)LSDYSIFSETIEIC(+57.02)PDGHNFC(+57.02)FK.K | Y | 79.97 | 3207.4463 | 26 | -1.6 | 802.8676 | 4 | 81.23 | 13 | F13:56229 | NaNaKA16\_F9.raw |  |  |  |  |  |  |  |  |  |  |  |  | 6.2243E7 | 2 | 0 | 0 | 0 | 0 | 0 | 0 | 0 | 0 | 0 | 0 | 0 | 0 | 2 | 22 | 47 | Carbamidomethylation | C3:Carbamidomethylation:1000.00;C17:Carbamidomethylation:1000.00;C24:Carbamidomethylation:1000.00 | PEAKS DB |
| F.SETIEIC(+57.02)PDGHNFC(+57.02)FK.K | Y | 77.77 | 1952.8396 | 16 | -1.2 | 977.4259 | 2 | 49.68 | 10 | F10:32514 | NaNaKA16\_F6.raw |  |  |  |  |  |  |  |  |  | 5.3752E7 |  |  | 3.3568E7 | 4 | 0 | 0 | 0 | 0 | 0 | 0 | 0 | 0 | 0 | 2 | 0 | 0 | 2 | 32 | 47 | Carbamidomethylation | C7:Carbamidomethylation:1000.00;C14:Carbamidomethylation:1000.00 | PEAKS DB |
| S.ETIEIC(+57.02)PDGHNFC(+57.02)FK.K | Y | 62.63 | 1865.8076 | 15 | 0.0 | 622.9432 | 3 | 50.23 | 10 | F10:33009 | NaNaKA16\_F6.raw |  |  |  |  |  |  |  |  |  | 1.5094E7 |  |  |  | 2 | 0 | 0 | 0 | 0 | 0 | 0 | 0 | 0 | 0 | 2 | 0 | 0 | 0 | 33 | 47 | Carbamidomethylation | C6:Carbamidomethylation:1000.00;C13:Carbamidomethylation:1000.00 | PEAKS DB |
| C.LSDYSIFSETIEIC(+57.02)PDGHNFC(+57.02)FK.K | Y | 61.37 | 2778.2305 | 23 | 0.8 | 927.0848 | 3 | 83.94 | 13 | F13:56808 | NaNaKA16\_F9.raw |  |  |  |  |  |  |  |  |  |  |  |  | 1.0126E7 | 1 | 0 | 0 | 0 | 0 | 0 | 0 | 0 | 0 | 0 | 0 | 0 | 0 | 1 | 25 | 47 | Carbamidomethylation | C14:Carbamidomethylation:1000.00;C21:Carbamidomethylation:1000.00 | PEAKS DB |
| R.LC(+57.02)LSDYSIFSETIEIC(+57.02)PDGHNFC(+57.02)FK.K | Y | 54.85 | 3051.3452 | 25 | 0.3 | 1018.1226 | 3 | 92.48 | 13 | F13:64193 | NaNaKA16\_F9.raw |  |  |  |  |  |  |  |  |  |  |  |  | 1.7346E7 | 1 | 0 | 0 | 0 | 0 | 0 | 0 | 0 | 0 | 0 | 0 | 0 | 0 | 1 | 23 | 47 | Carbamidomethylation | C2:Carbamidomethylation:1000.00;C16:Carbamidomethylation:1000.00;C23:Carbamidomethylation:1000.00 | PEAKS DB |
| K.GITRLPWVIR.G | N | 53.94 | 1209.7346 | 10 | 0.7 | 404.2524 | 3 | 50.16 | 12 | F12:31448 | NaNaKA16\_F8.raw |  |  |  |  |  |  |  |  |  |  |  | 4.8367E6 | 4.8796E6 | 2 | 0 | 0 | 0 | 0 | 0 | 0 | 0 | 0 | 0 | 0 | 0 | 1 | 1 | 52 | 61 |  |  | PEAKS DB |
| Y.SIFSETIEIC(+57.02)PDGHNFC(+57.02)FK.K | Y | 53.11 | 2300.0242 | 19 | 1.0 | 767.6827 | 3 | 72.97 | 13 | F13:47475 | NaNaKA16\_F9.raw |  |  |  |  |  |  |  |  |  |  |  | 7.4795E6 | 9.9901E6 | 2 | 0 | 0 | 0 | 0 | 0 | 0 | 0 | 0 | 0 | 0 | 0 | 1 | 1 | 29 | 47 | Carbamidomethylation | C10:Carbamidomethylation:1000.00;C17:Carbamidomethylation:1000.00 | PEAKS DB |
| S.IFSETIEIC(+57.02)PDGHNFC(+57.02)FK.K | Y | 48.14 | 2212.9922 | 18 | 0.6 | 738.6718 | 3 | 65.16 | 12 | F12:43580 | NaNaKA16\_F8.raw |  |  |  |  |  |  |  |  |  |  |  | 6.4302E6 |  | 1 | 0 | 0 | 0 | 0 | 0 | 0 | 0 | 0 | 0 | 0 | 0 | 1 | 0 | 30 | 47 | Carbamidomethylation | C9:Carbamidomethylation:1000.00;C16:Carbamidomethylation:1000.00 | PEAKS DB |
| S.DYSIFSETIEIC(+57.02)PDGHNFC(+57.02)FK.K | Y | 47.17 | 2578.1145 | 21 | 0.6 | 860.3793 | 3 | 86.30 | 12 | F12:61577 | NaNaKA16\_F8.raw |  |  |  |  |  |  |  |  |  |  |  | 2.9844E6 |  | 1 | 0 | 0 | 0 | 0 | 0 | 0 | 0 | 0 | 0 | 0 | 0 | 1 | 0 | 27 | 47 | Carbamidomethylation | C12:Carbamidomethylation:1000.00;C19:Carbamidomethylation:1000.00 | PEAKS DB |
| I.TRLPWVIR.G | N | 42.46 | 1039.6290 | 8 | -0.3 | 520.8217 | 2 | 38.07 | 12 | F12:21421 | NaNaKA16\_F8.raw |  |  |  |  |  |  |  |  |  |  |  | 1.3591E7 |  | 1 | 0 | 0 | 0 | 0 | 0 | 0 | 0 | 0 | 0 | 0 | 0 | 1 | 0 | 54 | 61 |  |  | PEAKS DB |
| total 10 peptides |  |  |  |  |  |  |  |  |  |  |  |  |  |  |  |  |  |  |  |  |  |  |  |  |  |  |  |  |  |  |  |  |  |  |  |  |  |  |  |  |  |  |  |
| --- | --- | --- | --- | --- | --- | --- | --- | --- | --- | --- | --- | --- | --- | --- | --- | --- | --- | --- | --- | --- | --- | --- | --- | --- | --- | --- | --- | --- | --- | --- | --- | --- | --- | --- | --- | --- | --- | --- | --- | --- | --- | --- | --- |

AAB25732.1

back to list

  

| Protein Coverage
| Supporting Peptides
|

Protein Coverage:

Supporting Peptides:

| Peptide | Uniq | -10lgP | Mass | Length | ppm | m/z | z | RT | Fraction | Scan | Source File | Area F1 | Area F10 | Area F11 | Area F12 | Area F13 | Area F2 | Area F3 | Area F4 | Area F5 | Area F6 | Area F7 | Area F8 | Area F9 | #Feature | #Feature F1 | #Feature F10 | #Feature F11 | #Feature F12 | #Feature F13 | #Feature F2 | #Feature F3 | #Feature F4 | #Feature F5 | #Feature F6 | #Feature F7 | #Feature F8 | #Feature F9 | Start | End | PTM | AScore | Found By |
| --- | --- | --- | --- | --- | --- | --- | --- | --- | --- | --- | --- | --- | --- | --- | --- | --- | --- | --- | --- | --- | --- | --- | --- | --- | --- | --- | --- | --- | --- | --- | --- | --- | --- | --- | --- | --- | --- | --- | --- | --- | --- | --- | --- |
| K.MFMMSDLTIPVKR.G | N | 78.37 | 1567.7925 | 13 | 0.7 | 784.9041 | 2 | 64.37 | 13 | F13:40261 | NaNaKA16\_F9.raw |  | 4.2403E9 | 1.3439E7 | 1.7372E6 | 3.8642E6 |  |  |  |  | 5.9217E5 | 1.0397E7 | 3.2403E7 | 3.9215E7 | 33 | 0 | 9 | 4 | 2 | 3 | 0 | 0 | 0 | 0 | 1 | 4 | 6 | 4 | 24 | 36 |  |  | PEAKS DB |
| K.MFM(+15.99)MSDLTIPVKR.G | N | 73.58 | 1583.7874 | 13 | -3.6 | 528.9345 | 3 | 64.32 | 2 | F2:41277 | NaNaKA16\_F10.raw |  | 6.4476E8 | 1.4987E6 | 3.4668E5 | 1.3565E5 |  |  |  |  |  | 1.7098E6 | 4.1492E7 | 7.8309E6 | 19 | 0 | 4 | 1 | 1 | 1 | 0 | 0 | 0 | 0 | 0 | 1 | 6 | 5 | 24 | 36 | Oxidation (M) | M3:Oxidation (M):54.01 | PEAKS DB |
| K.MFMM(+15.99)SDLTIPVKR.G | N | 73.15 | 1583.7874 | 13 | -0.7 | 528.9360 | 3 | 50.11 | 2 | F2:28972 | NaNaKA16\_F10.raw |  | 2.9709E8 |  |  |  |  |  |  |  |  | 4.1034E6 | 6.2939E6 | 0 | 11 | 0 | 6 | 0 | 0 | 0 | 0 | 0 | 0 | 0 | 0 | 2 | 3 | 0 | 24 | 36 | Oxidation (M) | M4:Oxidation (M):33.98 | PEAKS DB |
| K.M(+15.99)FMMSDLTIPVKR.G | N | 72.36 | 1583.7874 | 13 | 0.2 | 528.9365 | 3 | 56.84 | 2 | F2:34425 | NaNaKA16\_F10.raw |  | 2.8654E8 | 1.319E6 |  |  |  |  |  |  |  |  | 2.7338E6 | 4.1047E6 | 7 | 0 | 2 | 1 | 0 | 0 | 0 | 0 | 0 | 0 | 0 | 0 | 2 | 2 | 24 | 36 | Oxidation (M) | M1:Oxidation (M):39.76 | PEAKS DB |
| K.MFMMSDLTIPVK.R | N | 71.68 | 1411.6914 | 12 | 0.8 | 706.8535 | 2 | 79.29 | 3 | F3:53820 | NaNaKA16\_F11.raw |  | 1.5688E9 | 1.881E7 | 2.977E6 | 1.2231E6 |  |  |  |  | 9.2248E5 | 1.3238E7 | 3.1707E7 | 1.643E7 | 16 | 0 | 2 | 2 | 2 | 1 | 0 | 0 | 0 | 0 | 1 | 4 | 3 | 1 | 24 | 35 |  |  | PEAKS DB |
| K.MFMM(+15.99)SDLTIPVK.R | N | 68.52 | 1427.6863 | 12 | 0.4 | 714.8507 | 2 | 65.23 | 2 | F2:42286 | NaNaKA16\_F10.raw |  | 6.9158E7 |  |  |  |  |  |  |  |  |  | 5.2718E6 |  | 3 | 0 | 2 | 0 | 0 | 0 | 0 | 0 | 0 | 0 | 0 | 0 | 1 | 0 | 24 | 35 | Oxidation (M) | M4:Oxidation (M):40.00 | PEAKS DB |
| K.M(+15.99)FMMSDLTIPVK.R | N | 67.62 | 1427.6863 | 12 | 1.3 | 714.8513 | 2 | 72.40 | 2 | F2:48081 | NaNaKA16\_F10.raw |  | 2.2728E8 |  |  |  |  |  |  |  |  |  | 2.5292E6 | 1.6964E6 | 4 | 0 | 2 | 0 | 0 | 0 | 0 | 0 | 0 | 0 | 0 | 0 | 1 | 1 | 24 | 35 | Oxidation (M) | M1:Oxidation (M):45.01 | PEAKS DB |
| K.M(+15.99)FM(+15.99)MSDLTIPVKR.G | N | 67.36 | 1599.7822 | 13 | -0.1 | 534.2679 | 3 | 52.56 | 2 | F2:30572 | NaNaKA16\_F10.raw |  | 1.3826E7 |  |  |  |  |  |  |  |  |  |  |  | 4 | 0 | 4 | 0 | 0 | 0 | 0 | 0 | 0 | 0 | 0 | 0 | 0 | 0 | 24 | 36 | Oxidation (M) | M1:Oxidation (M):77.24;M3:Oxidation (M):33.98 | PEAKS DB |
| K.MFM(+15.99)MSDLTIPVK.R | N | 66.69 | 1427.6863 | 12 | -1.0 | 714.8497 | 2 | 80.39 | 2 | F2:54977 | NaNaKA16\_F10.raw |  | 2.2728E8 | 2.7181E6 |  | 1.0282E6 |  |  |  |  |  | 1.1732E6 | 1.7882E7 | 2.4723E6 | 10 | 0 | 2 | 1 | 0 | 1 | 0 | 0 | 0 | 0 | 0 | 2 | 2 | 2 | 24 | 35 | Oxidation (M) | M3:Oxidation (M):40.00 | PEAKS DB |
| F.MMSDLTIPVK.R | N | 61.69 | 1133.5824 | 10 | -0.7 | 567.7981 | 2 | 48.83 | 2 | F2:28577 | NaNaKA16\_F10.raw |  | 1.0581E7 |  |  |  |  |  |  |  |  |  |  |  | 1 | 0 | 1 | 0 | 0 | 0 | 0 | 0 | 0 | 0 | 0 | 0 | 0 | 0 | 26 | 35 |  |  | PEAKS DB |
| K.SNLLVKYVC(+57.02)C(+57.02)NTDRC(+57.02)N | Y | 59.74 | 2014.9023 | 16 | -0.1 | 1008.4584 | 2 | 33.22 | 2 | F2:15712 | NaNaKA16\_F10.raw |  | 6.926E6 |  |  |  |  |  |  |  |  |  |  |  | 1 | 0 | 1 | 0 | 0 | 0 | 0 | 0 | 0 | 0 | 0 | 0 | 0 | 0 | 45 | 60 | Carbamidomethylation | C9:Carbamidomethylation:1000.00;C10:Carbamidomethylation:1000.00;C15:Carbamidomethylation:1000.00 | PEAKS DB |
| K.MFM(+15.99)M(+15.99)SDLTIPVKR.G | N | 53.05 | 1599.7822 | 13 | 0.1 | 534.2681 | 3 | 41.44 | 2 | F2:22207 | NaNaKA16\_F10.raw |  | 2.4835E7 |  |  |  |  |  |  |  |  |  |  |  | 6 | 0 | 6 | 0 | 0 | 0 | 0 | 0 | 0 | 0 | 0 | 0 | 0 | 0 | 24 | 36 | Oxidation (M) | M3:Oxidation (M):32.28;M4:Oxidation (M):47.09 | PEAKS DB |
| K.M(+15.99)FMM(+15.99)SDLTIPVK.R | N | 52.06 | 1443.6812 | 12 | -0.4 | 722.8475 | 2 | 79.59 | 2 | F2:55048 | NaNaKA16\_F10.raw |  | 3.8316E6 |  |  |  |  |  |  |  |  |  |  |  | 1 | 0 | 1 | 0 | 0 | 0 | 0 | 0 | 0 | 0 | 0 | 0 | 0 | 0 | 24 | 35 | Oxidation (M) | M1:Oxidation (M):38.40;M4:Oxidation (M):33.98 | PEAKS DB |
| K.YVC(+57.02)C(+57.02)NTDR.C | N | 50.92 | 1086.4222 | 8 | -0.2 | 544.2183 | 2 | 11.37 | 12 | F12:1787 | NaNaKA16\_F8.raw |  | 2.1124E6 | 2.9778E6 | 1.3296E6 |  |  |  |  |  | 1.5915E6 |  | 1.7945E6 |  | 5 | 0 | 1 | 1 | 1 | 0 | 0 | 0 | 0 | 0 | 1 | 0 | 1 | 0 | 51 | 58 | Carbamidomethylation | C3:Carbamidomethylation:1000.00;C4:Carbamidomethylation:1000.00 | PEAKS DB |
| K.M(+15.99)FM(+15.99)M(+15.99)SDLTIPVKR.G | N | 49.67 | 1615.7772 | 13 | -0.6 | 539.5994 | 3 | 64.71 | 2 | F2:41281 | NaNaKA16\_F10.raw |  | 3.6921E5 |  |  |  |  |  |  |  |  |  |  |  | 1 | 0 | 1 | 0 | 0 | 0 | 0 | 0 | 0 | 0 | 0 | 0 | 0 | 0 | 24 | 36 | Oxidation (M) | M1:Oxidation (M):1000.00;M3:Oxidation (M):1000.00;M4:Oxidation (M):1000.00 | PEAKS DB |
| R.GC(+57.02)IDVC(+57.02)PKSNLLVK.Y | Y | 48.08 | 1601.8269 | 14 | 0.9 | 534.9501 | 3 | 32.72 | 12 | F12:16982 | NaNaKA16\_F8.raw |  |  |  |  |  |  |  |  |  | 1.1895E7 |  | 1.0889E8 |  | 2 | 0 | 0 | 0 | 0 | 0 | 0 | 0 | 0 | 0 | 1 | 0 | 1 | 0 | 37 | 50 | Carbamidomethylation | C2:Carbamidomethylation:1000.00;C6:Carbamidomethylation:1000.00 | PEAKS DB |
| K.M(+15.99)FM(+15.99)MSDLTIPVK.R | N | 46.34 | 1443.6812 | 12 | -0.7 | 722.8474 | 2 | 80.40 | 2 | F2:55048 | NaNaKA16\_F10.raw |  | 9.9748E6 |  |  |  |  |  |  |  |  |  |  |  | 3 | 0 | 3 | 0 | 0 | 0 | 0 | 0 | 0 | 0 | 0 | 0 | 0 | 0 | 24 | 35 | Oxidation (M) | M1:Oxidation (M):56.34;M3:Oxidation (M):40.00 | PEAKS DB |
| R.GC(+57.02)IDVC(+57.02)PK.S | N | 46.03 | 947.4205 | 8 | -0.5 | 474.7173 | 2 | 11.54 | 12 | F12:1895 | NaNaKA16\_F8.raw |  |  | 1.5891E7 |  |  |  | 6.084E5 |  | 2.0697E6 | 6.3919E7 | 1.8242E6 | 6.8843E6 |  | 6 | 0 | 0 | 1 | 0 | 0 | 0 | 1 | 0 | 1 | 1 | 1 | 1 | 0 | 37 | 44 | Carbamidomethylation | C2:Carbamidomethylation:1000.00;C6:Carbamidomethylation:1000.00 | PEAKS DB |
| K.TC(+57.02)PAGKNLC(+57.02)YK.M | N | 44.80 | 1310.6111 | 11 | 0.6 | 437.8779 | 3 | 11.38 | 12 | F12:1793 | NaNaKA16\_F8.raw |  |  |  |  |  |  |  |  |  |  |  | 2.5665E5 |  | 1 | 0 | 0 | 0 | 0 | 0 | 0 | 0 | 0 | 0 | 0 | 0 | 1 | 0 | 13 | 23 | Carbamidomethylation | C2:Carbamidomethylation:1000.00;C9:Carbamidomethylation:1000.00 | PEAKS DB |
| K.SNLLVKYVC(+57.02)C(+57.02)NTDR.C | Y | 44.53 | 1740.8287 | 14 | 0.3 | 871.4219 | 2 | 32.22 | 13 | F13:14152 | NaNaKA16\_F9.raw |  |  |  |  |  |  |  |  |  |  |  |  | 6.0589E6 | 1 | 0 | 0 | 0 | 0 | 0 | 0 | 0 | 0 | 0 | 0 | 0 | 0 | 1 | 45 | 58 | Carbamidomethylation | C9:Carbamidomethylation:1000.00;C10:Carbamidomethylation:1000.00 | PEAKS DB |
| total 20 peptides |  |  |  |  |  |  |  |  |  |  |  |  |  |  |  |  |  |  |  |  |  |  |  |  |  |  |  |  |  |  |  |  |  |  |  |  |  |  |  |  |  |  |  |
| --- | --- | --- | --- | --- | --- | --- | --- | --- | --- | --- | --- | --- | --- | --- | --- | --- | --- | --- | --- | --- | --- | --- | --- | --- | --- | --- | --- | --- | --- | --- | --- | --- | --- | --- | --- | --- | --- | --- | --- | --- | --- | --- | --- |

XP\_026575983.1

back to list

  

| Protein Coverage
| Supporting Peptides
|

Protein Coverage:

Supporting Peptides:

| Peptide | Uniq | -10lgP | Mass | Length | ppm | m/z | z | RT | Fraction | Scan | Source File | Area F1 | Area F10 | Area F11 | Area F12 | Area F13 | Area F2 | Area F3 | Area F4 | Area F5 | Area F6 | Area F7 | Area F8 | Area F9 | #Feature | #Feature F1 | #Feature F10 | #Feature F11 | #Feature F12 | #Feature F13 | #Feature F2 | #Feature F3 | #Feature F4 | #Feature F5 | #Feature F6 | #Feature F7 | #Feature F8 | #Feature F9 | Start | End | PTM | AScore | Found By |
| --- | --- | --- | --- | --- | --- | --- | --- | --- | --- | --- | --- | --- | --- | --- | --- | --- | --- | --- | --- | --- | --- | --- | --- | --- | --- | --- | --- | --- | --- | --- | --- | --- | --- | --- | --- | --- | --- | --- | --- | --- | --- | --- | --- |
| K.SNVGIGIPESIDWR.D | Y | 78.34 | 1541.7838 | 14 | 0.1 | 771.8993 | 2 | 70.19 | 4 | F4:46856 | NaNaKA16\_F12.raw |  |  | 1.2185E5 | 2.1799E7 |  |  |  |  |  |  |  |  |  | 2 | 0 | 0 | 1 | 1 | 0 | 0 | 0 | 0 | 0 | 0 | 0 | 0 | 0 | 115 | 128 |  |  | PEAKS DB |
| K.ENGMDYWLVK.N | N | 68.67 | 1253.5751 | 10 | 0.4 | 627.7950 | 2 | 61.07 | 4 | F4:39425 | NaNaKA16\_F12.raw |  |  |  | 4.1193E6 |  |  |  |  |  |  |  |  |  | 1 | 0 | 0 | 0 | 1 | 0 | 0 | 0 | 0 | 0 | 0 | 0 | 0 | 0 | 294 | 303 |  |  | PEAKS DB |
| N.VGIGIPESIDWR.D | Y | 60.83 | 1340.7089 | 12 | -0.3 | 671.3615 | 2 | 71.29 | 4 | F4:47876 | NaNaKA16\_F12.raw |  |  |  | 1.4762E7 |  |  |  |  |  |  |  |  |  | 1 | 0 | 0 | 0 | 1 | 0 | 0 | 0 | 0 | 0 | 0 | 0 | 0 | 0 | 117 | 128 |  |  | PEAKS DB |
| K.NVQFIALHNLE.Y | Y | 57.39 | 1296.6826 | 11 | 0.5 | 649.3489 | 2 | 60.01 | 12 | F12:39273 | NaNaKA16\_F8.raw |  |  |  |  |  |  |  |  |  |  | 1.7428E6 | 3.131E6 |  | 2 | 0 | 0 | 0 | 0 | 0 | 0 | 0 | 0 | 0 | 0 | 1 | 1 | 0 | 60 | 70 |  |  | PEAKS DB |
| V.GIGIPESIDWR.D | Y | 55.43 | 1241.6404 | 11 | 0.5 | 621.8278 | 2 | 65.64 | 4 | F4:43184 | NaNaKA16\_F12.raw |  |  |  | 2.0063E6 |  |  |  |  |  |  |  |  |  | 1 | 0 | 0 | 0 | 1 | 0 | 0 | 0 | 0 | 0 | 0 | 0 | 0 | 0 | 118 | 128 |  |  | PEAKS DB |
| F.SAVGALEAQLK.L | Y | 53.15 | 1085.6080 | 11 | 0.8 | 543.8117 | 2 | 40.64 | 4 | F4:22485 | NaNaKA16\_F12.raw |  |  |  | 5.635E6 |  |  |  |  |  |  |  |  |  | 1 | 0 | 0 | 0 | 1 | 0 | 0 | 0 | 0 | 0 | 0 | 0 | 0 | 0 | 149 | 159 |  |  | PEAKS DB |
| K.GINSEASYPYK.A | Y | 44.03 | 1227.5771 | 11 | 0.3 | 614.7960 | 2 | 19.35 | 5 | F5:4472 | NaNaKA16\_F13.raw |  |  |  |  | 1.333E5 |  |  |  |  |  |  |  |  | 1 | 0 | 0 | 0 | 0 | 1 | 0 | 0 | 0 | 0 | 0 | 0 | 0 | 0 | 202 | 212 |  |  | PEAKS DB |
| K.NVQFIALHNLEYSLGLH.S | Y | 43.16 | 1967.0265 | 17 | 1.1 | 656.6835 | 3 | 83.79 | 2 | F2:57904 | NaNaKA16\_F10.raw |  | 6.3277E5 |  |  |  |  |  |  |  |  |  |  |  | 1 | 0 | 1 | 0 | 0 | 0 | 0 | 0 | 0 | 0 | 0 | 0 | 0 | 0 | 60 | 76 |  |  | PEAKS DB |
| total 8 peptides |  |  |  |  |  |  |  |  |  |  |  |  |  |  |  |  |  |  |  |  |  |  |  |  |  |  |  |  |  |  |  |  |  |  |  |  |  |  |  |  |  |  |  |
| --- | --- | --- | --- | --- | --- | --- | --- | --- | --- | --- | --- | --- | --- | --- | --- | --- | --- | --- | --- | --- | --- | --- | --- | --- | --- | --- | --- | --- | --- | --- | --- | --- | --- | --- | --- | --- | --- | --- | --- | --- | --- | --- | --- |

XP\_026575982.1

back to list

  

| Protein Coverage
| Supporting Peptides
|

Protein Coverage:

Supporting Peptides:

| Peptide | Uniq | -10lgP | Mass | Length | ppm | m/z | z | RT | Fraction | Scan | Source File | Area F1 | Area F10 | Area F11 | Area F12 | Area F13 | Area F2 | Area F3 | Area F4 | Area F5 | Area F6 | Area F7 | Area F8 | Area F9 | #Feature | #Feature F1 | #Feature F10 | #Feature F11 | #Feature F12 | #Feature F13 | #Feature F2 | #Feature F3 | #Feature F4 | #Feature F5 | #Feature F6 | #Feature F7 | #Feature F8 | #Feature F9 | Start | End | PTM | AScore | Found By |
| --- | --- | --- | --- | --- | --- | --- | --- | --- | --- | --- | --- | --- | --- | --- | --- | --- | --- | --- | --- | --- | --- | --- | --- | --- | --- | --- | --- | --- | --- | --- | --- | --- | --- | --- | --- | --- | --- | --- | --- | --- | --- | --- | --- |
| K.SNVGIGIPESIDWR.D | Y | 78.34 | 1541.7838 | 14 | 0.1 | 771.8993 | 2 | 70.19 | 4 | F4:46856 | NaNaKA16\_F12.raw |  |  | 1.2185E5 | 2.1799E7 |  |  |  |  |  |  |  |  |  | 2 | 0 | 0 | 1 | 1 | 0 | 0 | 0 | 0 | 0 | 0 | 0 | 0 | 0 | 115 | 128 |  |  | PEAKS DB |
| K.ENGMDYWLVK.N | N | 68.67 | 1253.5751 | 10 | 0.4 | 627.7950 | 2 | 61.07 | 4 | F4:39425 | NaNaKA16\_F12.raw |  |  |  | 4.1193E6 |  |  |  |  |  |  |  |  |  | 1 | 0 | 0 | 0 | 1 | 0 | 0 | 0 | 0 | 0 | 0 | 0 | 0 | 0 | 294 | 303 |  |  | PEAKS DB |
| N.VGIGIPESIDWR.D | Y | 60.83 | 1340.7089 | 12 | -0.3 | 671.3615 | 2 | 71.29 | 4 | F4:47876 | NaNaKA16\_F12.raw |  |  |  | 1.4762E7 |  |  |  |  |  |  |  |  |  | 1 | 0 | 0 | 0 | 1 | 0 | 0 | 0 | 0 | 0 | 0 | 0 | 0 | 0 | 117 | 128 |  |  | PEAKS DB |
| K.NVQFIALHNLE.Y | Y | 57.39 | 1296.6826 | 11 | 0.5 | 649.3489 | 2 | 60.01 | 12 | F12:39273 | NaNaKA16\_F8.raw |  |  |  |  |  |  |  |  |  |  | 1.7428E6 | 3.131E6 |  | 2 | 0 | 0 | 0 | 0 | 0 | 0 | 0 | 0 | 0 | 0 | 1 | 1 | 0 | 60 | 70 |  |  | PEAKS DB |
| V.GIGIPESIDWR.D | Y | 55.43 | 1241.6404 | 11 | 0.5 | 621.8278 | 2 | 65.64 | 4 | F4:43184 | NaNaKA16\_F12.raw |  |  |  | 2.0063E6 |  |  |  |  |  |  |  |  |  | 1 | 0 | 0 | 0 | 1 | 0 | 0 | 0 | 0 | 0 | 0 | 0 | 0 | 0 | 118 | 128 |  |  | PEAKS DB |
| F.SAVGALEAQLK.L | Y | 53.15 | 1085.6080 | 11 | 0.8 | 543.8117 | 2 | 40.64 | 4 | F4:22485 | NaNaKA16\_F12.raw |  |  |  | 5.635E6 |  |  |  |  |  |  |  |  |  | 1 | 0 | 0 | 0 | 1 | 0 | 0 | 0 | 0 | 0 | 0 | 0 | 0 | 0 | 149 | 159 |  |  | PEAKS DB |
| K.GINSEASYPYK.A | Y | 44.03 | 1227.5771 | 11 | 0.3 | 614.7960 | 2 | 19.35 | 5 | F5:4472 | NaNaKA16\_F13.raw |  |  |  |  | 1.333E5 |  |  |  |  |  |  |  |  | 1 | 0 | 0 | 0 | 0 | 1 | 0 | 0 | 0 | 0 | 0 | 0 | 0 | 0 | 202 | 212 |  |  | PEAKS DB |
| K.NVQFIALHNLEYSLGLH.S | Y | 43.16 | 1967.0265 | 17 | 1.1 | 656.6835 | 3 | 83.79 | 2 | F2:57904 | NaNaKA16\_F10.raw |  | 6.3277E5 |  |  |  |  |  |  |  |  |  |  |  | 1 | 0 | 1 | 0 | 0 | 0 | 0 | 0 | 0 | 0 | 0 | 0 | 0 | 0 | 60 | 76 |  |  | PEAKS DB |
| total 8 peptides |  |  |  |  |  |  |  |  |  |  |  |  |  |  |  |  |  |  |  |  |  |  |  |  |  |  |  |  |  |  |  |  |  |  |  |  |  |  |  |  |  |  |  |
| --- | --- | --- | --- | --- | --- | --- | --- | --- | --- | --- | --- | --- | --- | --- | --- | --- | --- | --- | --- | --- | --- | --- | --- | --- | --- | --- | --- | --- | --- | --- | --- | --- | --- | --- | --- | --- | --- | --- | --- | --- | --- | --- | --- |

4GFY

back to list

  

| Protein Coverage
| Supporting Peptides
|

Protein Coverage:

Supporting Peptides:

| Peptide | Uniq | -10lgP | Mass | Length | ppm | m/z | z | RT | Fraction | Scan | Source File | Area F1 | Area F10 | Area F11 | Area F12 | Area F13 | Area F2 | Area F3 | Area F4 | Area F5 | Area F6 | Area F7 | Area F8 | Area F9 | #Feature | #Feature F1 | #Feature F10 | #Feature F11 | #Feature F12 | #Feature F13 | #Feature F2 | #Feature F3 | #Feature F4 | #Feature F5 | #Feature F6 | #Feature F7 | #Feature F8 | #Feature F9 | Start | End | PTM | AScore | Found By |
| --- | --- | --- | --- | --- | --- | --- | --- | --- | --- | --- | --- | --- | --- | --- | --- | --- | --- | --- | --- | --- | --- | --- | --- | --- | --- | --- | --- | --- | --- | --- | --- | --- | --- | --- | --- | --- | --- | --- | --- | --- | --- | --- | --- |
| K.LAIPSYSSYGC(+57.02)YC(+57.02)GWGGK.G | Y | 88.95 | 2024.8760 | 18 | 1.0 | 1013.4462 | 2 | 65.94 | 4 | F4:43325 | NaNaKA16\_F12.raw |  |  |  | 5.4816E7 |  |  |  |  |  |  |  |  |  | 2 | 0 | 0 | 0 | 2 | 0 | 0 | 0 | 0 | 0 | 0 | 0 | 0 | 0 | 16 | 33 | Carbamidomethylation | C11:Carbamidomethylation:1000.00;C13:Carbamidomethylation:1000.00 | PEAKS DB |
| K.KYMLYPDFLC(+57.02)K.G | Y | 78.94 | 1476.7145 | 11 | 0.6 | 739.3649 | 2 | 58.50 | 4 | F4:37246 | NaNaKA16\_F12.raw |  |  |  | 3.9698E7 |  |  |  |  |  |  |  |  |  | 2 | 0 | 0 | 0 | 2 | 0 | 0 | 0 | 0 | 0 | 0 | 0 | 0 | 0 | 106 | 116 | Carbamidomethylation | C10:Carbamidomethylation:1000.00 | PEAKS DB |
| R.C(+57.02)C(+57.02)FVHDC(+57.02)C(+57.02)YGNLPDC(+57.02)NPK.S | N | 74.08 | 2314.8687 | 18 | 0.4 | 772.6305 | 3 | 32.68 | 4 | F4:15898 | NaNaKA16\_F12.raw |  |  |  | 5.4885E7 |  |  |  |  |  |  |  |  |  | 2 | 0 | 0 | 0 | 2 | 0 | 0 | 0 | 0 | 0 | 0 | 0 | 0 | 0 | 43 | 60 | Carbamidomethylation | C1:Carbamidomethylation:1000.00;C2:Carbamidomethylation:1000.00;C7:Carbamidomethylation:1000.00;C8:Carbamidomethylation:1000.00;C15:Carbamidomethylation:1000.00 | PEAKS DB |
| K.YM(+15.99)LYPDFLC(+57.02)K.G | N | 68.84 | 1364.6145 | 10 | 0.1 | 683.3146 | 2 | 68.38 | 4 | F4:45614 | NaNaKA16\_F12.raw |  |  |  | 8.5839E6 |  |  |  |  |  |  |  |  |  | 4 | 0 | 0 | 0 | 4 | 0 | 0 | 0 | 0 | 0 | 0 | 0 | 0 | 0 | 107 | 116 | Oxidation (M); Carbamidomethylation | M2:Oxidation (M):1000.00;C9:Carbamidomethylation:1000.00 | PEAKS DB |
| K.YMLYPDFLC(+57.02)K.G | N | 67.92 | 1348.6195 | 10 | 0.7 | 675.3175 | 2 | 75.32 | 4 | F4:51022 | NaNaKA16\_F12.raw |  | 6.0196E5 | 6.6727E5 | 5.9248E7 |  |  |  |  |  | 4.1845E5 |  |  |  | 4 | 0 | 1 | 1 | 1 | 0 | 0 | 0 | 0 | 0 | 1 | 0 | 0 | 0 | 107 | 116 | Carbamidomethylation | C9:Carbamidomethylation:1000.00 | PEAKS DB |
| K.KYM(+15.99)LYPDFLC(+57.02)K.G | Y | 62.57 | 1492.7095 | 11 | 0.2 | 498.5772 | 3 | 52.20 | 4 | F4:32001 | NaNaKA16\_F12.raw |  |  |  | 4.3437E6 |  |  |  |  |  |  |  |  |  | 1 | 0 | 0 | 0 | 1 | 0 | 0 | 0 | 0 | 0 | 0 | 0 | 0 | 0 | 106 | 116 | Oxidation (M); Carbamidomethylation | M3:Oxidation (M):1000.00;C10:Carbamidomethylation:1000.00 | PEAKS DB |
| total 6 peptides |  |  |  |  |  |  |  |  |  |  |  |  |  |  |  |  |  |  |  |  |  |  |  |  |  |  |  |  |  |  |  |  |  |  |  |  |  |  |  |  |  |  |  |
| --- | --- | --- | --- | --- | --- | --- | --- | --- | --- | --- | --- | --- | --- | --- | --- | --- | --- | --- | --- | --- | --- | --- | --- | --- | --- | --- | --- | --- | --- | --- | --- | --- | --- | --- | --- | --- | --- | --- | --- | --- | --- | --- | --- |

1SV9

back to list

  

| Protein Coverage
| Supporting Peptides
|

Protein Coverage:

Supporting Peptides:

| Peptide | Uniq | -10lgP | Mass | Length | ppm | m/z | z | RT | Fraction | Scan | Source File | Area F1 | Area F10 | Area F11 | Area F12 | Area F13 | Area F2 | Area F3 | Area F4 | Area F5 | Area F6 | Area F7 | Area F8 | Area F9 | #Feature | #Feature F1 | #Feature F10 | #Feature F11 | #Feature F12 | #Feature F13 | #Feature F2 | #Feature F3 | #Feature F4 | #Feature F5 | #Feature F6 | #Feature F7 | #Feature F8 | #Feature F9 | Start | End | PTM | AScore | Found By |
| --- | --- | --- | --- | --- | --- | --- | --- | --- | --- | --- | --- | --- | --- | --- | --- | --- | --- | --- | --- | --- | --- | --- | --- | --- | --- | --- | --- | --- | --- | --- | --- | --- | --- | --- | --- | --- | --- | --- | --- | --- | --- | --- | --- |
| K.LAIPSYSSYGC(+57.02)YC(+57.02)GWGGK.G | Y | 88.95 | 2024.8760 | 18 | 1.0 | 1013.4462 | 2 | 65.94 | 4 | F4:43325 | NaNaKA16\_F12.raw |  |  |  | 5.4816E7 |  |  |  |  |  |  |  |  |  | 2 | 0 | 0 | 0 | 2 | 0 | 0 | 0 | 0 | 0 | 0 | 0 | 0 | 0 | 16 | 33 | Carbamidomethylation | C11:Carbamidomethylation:1000.00;C13:Carbamidomethylation:1000.00 | PEAKS DB |
| K.KYMLYPDFLC(+57.02)K.G | Y | 78.94 | 1476.7145 | 11 | 0.6 | 739.3649 | 2 | 58.50 | 4 | F4:37246 | NaNaKA16\_F12.raw |  |  |  | 3.9698E7 |  |  |  |  |  |  |  |  |  | 2 | 0 | 0 | 0 | 2 | 0 | 0 | 0 | 0 | 0 | 0 | 0 | 0 | 0 | 106 | 116 | Carbamidomethylation | C10:Carbamidomethylation:1000.00 | PEAKS DB |
| R.C(+57.02)C(+57.02)FVHDC(+57.02)C(+57.02)YGNLPDC(+57.02)NPK.S | N | 74.08 | 2314.8687 | 18 | 0.4 | 772.6305 | 3 | 32.68 | 4 | F4:15898 | NaNaKA16\_F12.raw |  |  |  | 5.4885E7 |  |  |  |  |  |  |  |  |  | 2 | 0 | 0 | 0 | 2 | 0 | 0 | 0 | 0 | 0 | 0 | 0 | 0 | 0 | 43 | 60 | Carbamidomethylation | C1:Carbamidomethylation:1000.00;C2:Carbamidomethylation:1000.00;C7:Carbamidomethylation:1000.00;C8:Carbamidomethylation:1000.00;C15:Carbamidomethylation:1000.00 | PEAKS DB |
| K.YM(+15.99)LYPDFLC(+57.02)K.G | N | 68.84 | 1364.6145 | 10 | 0.1 | 683.3146 | 2 | 68.38 | 4 | F4:45614 | NaNaKA16\_F12.raw |  |  |  | 8.5839E6 |  |  |  |  |  |  |  |  |  | 4 | 0 | 0 | 0 | 4 | 0 | 0 | 0 | 0 | 0 | 0 | 0 | 0 | 0 | 107 | 116 | Oxidation (M); Carbamidomethylation | M2:Oxidation (M):1000.00;C9:Carbamidomethylation:1000.00 | PEAKS DB |
| K.YMLYPDFLC(+57.02)K.G | N | 67.92 | 1348.6195 | 10 | 0.7 | 675.3175 | 2 | 75.32 | 4 | F4:51022 | NaNaKA16\_F12.raw |  | 6.0196E5 | 6.6727E5 | 5.9248E7 |  |  |  |  |  | 4.1845E5 |  |  |  | 4 | 0 | 1 | 1 | 1 | 0 | 0 | 0 | 0 | 0 | 1 | 0 | 0 | 0 | 107 | 116 | Carbamidomethylation | C9:Carbamidomethylation:1000.00 | PEAKS DB |
| K.KYM(+15.99)LYPDFLC(+57.02)K.G | Y | 62.57 | 1492.7095 | 11 | 0.2 | 498.5772 | 3 | 52.20 | 4 | F4:32001 | NaNaKA16\_F12.raw |  |  |  | 4.3437E6 |  |  |  |  |  |  |  |  |  | 1 | 0 | 0 | 0 | 1 | 0 | 0 | 0 | 0 | 0 | 0 | 0 | 0 | 0 | 106 | 116 | Oxidation (M); Carbamidomethylation | M3:Oxidation (M):1000.00;C10:Carbamidomethylation:1000.00 | PEAKS DB |
| total 6 peptides |  |  |  |  |  |  |  |  |  |  |  |  |  |  |  |  |  |  |  |  |  |  |  |  |  |  |  |  |  |  |  |  |  |  |  |  |  |  |  |  |  |  |  |
| --- | --- | --- | --- | --- | --- | --- | --- | --- | --- | --- | --- | --- | --- | --- | --- | --- | --- | --- | --- | --- | --- | --- | --- | --- | --- | --- | --- | --- | --- | --- | --- | --- | --- | --- | --- | --- | --- | --- | --- | --- | --- | --- | --- |

3G8F

back to list

  

| Protein Coverage
| Supporting Peptides
|

Protein Coverage:

Supporting Peptides:

| Peptide | Uniq | -10lgP | Mass | Length | ppm | m/z | z | RT | Fraction | Scan | Source File | Area F1 | Area F10 | Area F11 | Area F12 | Area F13 | Area F2 | Area F3 | Area F4 | Area F5 | Area F6 | Area F7 | Area F8 | Area F9 | #Feature | #Feature F1 | #Feature F10 | #Feature F11 | #Feature F12 | #Feature F13 | #Feature F2 | #Feature F3 | #Feature F4 | #Feature F5 | #Feature F6 | #Feature F7 | #Feature F8 | #Feature F9 | Start | End | PTM | AScore | Found By |
| --- | --- | --- | --- | --- | --- | --- | --- | --- | --- | --- | --- | --- | --- | --- | --- | --- | --- | --- | --- | --- | --- | --- | --- | --- | --- | --- | --- | --- | --- | --- | --- | --- | --- | --- | --- | --- | --- | --- | --- | --- | --- | --- | --- |
| K.LAIPSYSSYGC(+57.02)YC(+57.02)GWGGK.G | Y | 88.95 | 2024.8760 | 18 | 1.0 | 1013.4462 | 2 | 65.94 | 4 | F4:43325 | NaNaKA16\_F12.raw |  |  |  | 5.4816E7 |  |  |  |  |  |  |  |  |  | 2 | 0 | 0 | 0 | 2 | 0 | 0 | 0 | 0 | 0 | 0 | 0 | 0 | 0 | 16 | 33 | Carbamidomethylation | C11:Carbamidomethylation:1000.00;C13:Carbamidomethylation:1000.00 | PEAKS DB |
| K.KYMLYPDFLC(+57.02)K.G | Y | 78.94 | 1476.7145 | 11 | 0.6 | 739.3649 | 2 | 58.50 | 4 | F4:37246 | NaNaKA16\_F12.raw |  |  |  | 3.9698E7 |  |  |  |  |  |  |  |  |  | 2 | 0 | 0 | 0 | 2 | 0 | 0 | 0 | 0 | 0 | 0 | 0 | 0 | 0 | 106 | 116 | Carbamidomethylation | C10:Carbamidomethylation:1000.00 | PEAKS DB |
| R.C(+57.02)C(+57.02)FVHDC(+57.02)C(+57.02)YGNLPDC(+57.02)NPK.S | N | 74.08 | 2314.8687 | 18 | 0.4 | 772.6305 | 3 | 32.68 | 4 | F4:15898 | NaNaKA16\_F12.raw |  |  |  | 5.4885E7 |  |  |  |  |  |  |  |  |  | 2 | 0 | 0 | 0 | 2 | 0 | 0 | 0 | 0 | 0 | 0 | 0 | 0 | 0 | 43 | 60 | Carbamidomethylation | C1:Carbamidomethylation:1000.00;C2:Carbamidomethylation:1000.00;C7:Carbamidomethylation:1000.00;C8:Carbamidomethylation:1000.00;C15:Carbamidomethylation:1000.00 | PEAKS DB |
| K.YM(+15.99)LYPDFLC(+57.02)K.G | N | 68.84 | 1364.6145 | 10 | 0.1 | 683.3146 | 2 | 68.38 | 4 | F4:45614 | NaNaKA16\_F12.raw |  |  |  | 8.5839E6 |  |  |  |  |  |  |  |  |  | 4 | 0 | 0 | 0 | 4 | 0 | 0 | 0 | 0 | 0 | 0 | 0 | 0 | 0 | 107 | 116 | Oxidation (M); Carbamidomethylation | M2:Oxidation (M):1000.00;C9:Carbamidomethylation:1000.00 | PEAKS DB |
| K.YMLYPDFLC(+57.02)K.G | N | 67.92 | 1348.6195 | 10 | 0.7 | 675.3175 | 2 | 75.32 | 4 | F4:51022 | NaNaKA16\_F12.raw |  | 6.0196E5 | 6.6727E5 | 5.9248E7 |  |  |  |  |  | 4.1845E5 |  |  |  | 4 | 0 | 1 | 1 | 1 | 0 | 0 | 0 | 0 | 0 | 1 | 0 | 0 | 0 | 107 | 116 | Carbamidomethylation | C9:Carbamidomethylation:1000.00 | PEAKS DB |
| K.KYM(+15.99)LYPDFLC(+57.02)K.G | Y | 62.57 | 1492.7095 | 11 | 0.2 | 498.5772 | 3 | 52.20 | 4 | F4:32001 | NaNaKA16\_F12.raw |  |  |  | 4.3437E6 |  |  |  |  |  |  |  |  |  | 1 | 0 | 0 | 0 | 1 | 0 | 0 | 0 | 0 | 0 | 0 | 0 | 0 | 0 | 106 | 116 | Oxidation (M); Carbamidomethylation | M3:Oxidation (M):1000.00;C10:Carbamidomethylation:1000.00 | PEAKS DB |
| total 6 peptides |  |  |  |  |  |  |  |  |  |  |  |  |  |  |  |  |  |  |  |  |  |  |  |  |  |  |  |  |  |  |  |  |  |  |  |  |  |  |  |  |  |  |  |
| --- | --- | --- | --- | --- | --- | --- | --- | --- | --- | --- | --- | --- | --- | --- | --- | --- | --- | --- | --- | --- | --- | --- | --- | --- | --- | --- | --- | --- | --- | --- | --- | --- | --- | --- | --- | --- | --- | --- | --- | --- | --- | --- | --- |

1TK4

back to list

  

| Protein Coverage
| Supporting Peptides
|

Protein Coverage:

Supporting Peptides:

| Peptide | Uniq | -10lgP | Mass | Length | ppm | m/z | z | RT | Fraction | Scan | Source File | Area F1 | Area F10 | Area F11 | Area F12 | Area F13 | Area F2 | Area F3 | Area F4 | Area F5 | Area F6 | Area F7 | Area F8 | Area F9 | #Feature | #Feature F1 | #Feature F10 | #Feature F11 | #Feature F12 | #Feature F13 | #Feature F2 | #Feature F3 | #Feature F4 | #Feature F5 | #Feature F6 | #Feature F7 | #Feature F8 | #Feature F9 | Start | End | PTM | AScore | Found By |
| --- | --- | --- | --- | --- | --- | --- | --- | --- | --- | --- | --- | --- | --- | --- | --- | --- | --- | --- | --- | --- | --- | --- | --- | --- | --- | --- | --- | --- | --- | --- | --- | --- | --- | --- | --- | --- | --- | --- | --- | --- | --- | --- | --- |
| K.LAIPSYSSYGC(+57.02)YC(+57.02)GWGGK.G | Y | 88.95 | 2024.8760 | 18 | 1.0 | 1013.4462 | 2 | 65.94 | 4 | F4:43325 | NaNaKA16\_F12.raw |  |  |  | 5.4816E7 |  |  |  |  |  |  |  |  |  | 2 | 0 | 0 | 0 | 2 | 0 | 0 | 0 | 0 | 0 | 0 | 0 | 0 | 0 | 16 | 33 | Carbamidomethylation | C11:Carbamidomethylation:1000.00;C13:Carbamidomethylation:1000.00 | PEAKS DB |
| K.KYMLYPDFLC(+57.02)K.G | Y | 78.94 | 1476.7145 | 11 | 0.6 | 739.3649 | 2 | 58.50 | 4 | F4:37246 | NaNaKA16\_F12.raw |  |  |  | 3.9698E7 |  |  |  |  |  |  |  |  |  | 2 | 0 | 0 | 0 | 2 | 0 | 0 | 0 | 0 | 0 | 0 | 0 | 0 | 0 | 106 | 116 | Carbamidomethylation | C10:Carbamidomethylation:1000.00 | PEAKS DB |
| R.C(+57.02)C(+57.02)FVHDC(+57.02)C(+57.02)YGNLPDC(+57.02)NPK.S | N | 74.08 | 2314.8687 | 18 | 0.4 | 772.6305 | 3 | 32.68 | 4 | F4:15898 | NaNaKA16\_F12.raw |  |  |  | 5.4885E7 |  |  |  |  |  |  |  |  |  | 2 | 0 | 0 | 0 | 2 | 0 | 0 | 0 | 0 | 0 | 0 | 0 | 0 | 0 | 43 | 60 | Carbamidomethylation | C1:Carbamidomethylation:1000.00;C2:Carbamidomethylation:1000.00;C7:Carbamidomethylation:1000.00;C8:Carbamidomethylation:1000.00;C15:Carbamidomethylation:1000.00 | PEAKS DB |
| K.YM(+15.99)LYPDFLC(+57.02)K.G | N | 68.84 | 1364.6145 | 10 | 0.1 | 683.3146 | 2 | 68.38 | 4 | F4:45614 | NaNaKA16\_F12.raw |  |  |  | 8.5839E6 |  |  |  |  |  |  |  |  |  | 4 | 0 | 0 | 0 | 4 | 0 | 0 | 0 | 0 | 0 | 0 | 0 | 0 | 0 | 107 | 116 | Oxidation (M); Carbamidomethylation | M2:Oxidation (M):1000.00;C9:Carbamidomethylation:1000.00 | PEAKS DB |
| K.YMLYPDFLC(+57.02)K.G | N | 67.92 | 1348.6195 | 10 | 0.7 | 675.3175 | 2 | 75.32 | 4 | F4:51022 | NaNaKA16\_F12.raw |  | 6.0196E5 | 6.6727E5 | 5.9248E7 |  |  |  |  |  | 4.1845E5 |  |  |  | 4 | 0 | 1 | 1 | 1 | 0 | 0 | 0 | 0 | 0 | 1 | 0 | 0 | 0 | 107 | 116 | Carbamidomethylation | C9:Carbamidomethylation:1000.00 | PEAKS DB |
| K.KYM(+15.99)LYPDFLC(+57.02)K.G | Y | 62.57 | 1492.7095 | 11 | 0.2 | 498.5772 | 3 | 52.20 | 4 | F4:32001 | NaNaKA16\_F12.raw |  |  |  | 4.3437E6 |  |  |  |  |  |  |  |  |  | 1 | 0 | 0 | 0 | 1 | 0 | 0 | 0 | 0 | 0 | 0 | 0 | 0 | 0 | 106 | 116 | Oxidation (M); Carbamidomethylation | M3:Oxidation (M):1000.00;C10:Carbamidomethylation:1000.00 | PEAKS DB |
| total 6 peptides |  |  |  |  |  |  |  |  |  |  |  |  |  |  |  |  |  |  |  |  |  |  |  |  |  |  |  |  |  |  |  |  |  |  |  |  |  |  |  |  |  |  |  |
| --- | --- | --- | --- | --- | --- | --- | --- | --- | --- | --- | --- | --- | --- | --- | --- | --- | --- | --- | --- | --- | --- | --- | --- | --- | --- | --- | --- | --- | --- | --- | --- | --- | --- | --- | --- | --- | --- | --- | --- | --- | --- | --- | --- |

2QVD

back to list

  

| Protein Coverage
| Supporting Peptides
|

Protein Coverage:

Supporting Peptides:

| Peptide | Uniq | -10lgP | Mass | Length | ppm | m/z | z | RT | Fraction | Scan | Source File | Area F1 | Area F10 | Area F11 | Area F12 | Area F13 | Area F2 | Area F3 | Area F4 | Area F5 | Area F6 | Area F7 | Area F8 | Area F9 | #Feature | #Feature F1 | #Feature F10 | #Feature F11 | #Feature F12 | #Feature F13 | #Feature F2 | #Feature F3 | #Feature F4 | #Feature F5 | #Feature F6 | #Feature F7 | #Feature F8 | #Feature F9 | Start | End | PTM | AScore | Found By |
| --- | --- | --- | --- | --- | --- | --- | --- | --- | --- | --- | --- | --- | --- | --- | --- | --- | --- | --- | --- | --- | --- | --- | --- | --- | --- | --- | --- | --- | --- | --- | --- | --- | --- | --- | --- | --- | --- | --- | --- | --- | --- | --- | --- |
| K.LAIPSYSSYGC(+57.02)YC(+57.02)GWGGK.G | Y | 88.95 | 2024.8760 | 18 | 1.0 | 1013.4462 | 2 | 65.94 | 4 | F4:43325 | NaNaKA16\_F12.raw |  |  |  | 5.4816E7 |  |  |  |  |  |  |  |  |  | 2 | 0 | 0 | 0 | 2 | 0 | 0 | 0 | 0 | 0 | 0 | 0 | 0 | 0 | 16 | 33 | Carbamidomethylation | C11:Carbamidomethylation:1000.00;C13:Carbamidomethylation:1000.00 | PEAKS DB |
| K.KYMLYPDFLC(+57.02)K.G | Y | 78.94 | 1476.7145 | 11 | 0.6 | 739.3649 | 2 | 58.50 | 4 | F4:37246 | NaNaKA16\_F12.raw |  |  |  | 3.9698E7 |  |  |  |  |  |  |  |  |  | 2 | 0 | 0 | 0 | 2 | 0 | 0 | 0 | 0 | 0 | 0 | 0 | 0 | 0 | 106 | 116 | Carbamidomethylation | C10:Carbamidomethylation:1000.00 | PEAKS DB |
| R.C(+57.02)C(+57.02)FVHDC(+57.02)C(+57.02)YGNLPDC(+57.02)NPK.S | N | 74.08 | 2314.8687 | 18 | 0.4 | 772.6305 | 3 | 32.68 | 4 | F4:15898 | NaNaKA16\_F12.raw |  |  |  | 5.4885E7 |  |  |  |  |  |  |  |  |  | 2 | 0 | 0 | 0 | 2 | 0 | 0 | 0 | 0 | 0 | 0 | 0 | 0 | 0 | 43 | 60 | Carbamidomethylation | C1:Carbamidomethylation:1000.00;C2:Carbamidomethylation:1000.00;C7:Carbamidomethylation:1000.00;C8:Carbamidomethylation:1000.00;C15:Carbamidomethylation:1000.00 | PEAKS DB |
| K.YM(+15.99)LYPDFLC(+57.02)K.G | N | 68.84 | 1364.6145 | 10 | 0.1 | 683.3146 | 2 | 68.38 | 4 | F4:45614 | NaNaKA16\_F12.raw |  |  |  | 8.5839E6 |  |  |  |  |  |  |  |  |  | 4 | 0 | 0 | 0 | 4 | 0 | 0 | 0 | 0 | 0 | 0 | 0 | 0 | 0 | 107 | 116 | Oxidation (M); Carbamidomethylation | M2:Oxidation (M):1000.00;C9:Carbamidomethylation:1000.00 | PEAKS DB |
| K.YMLYPDFLC(+57.02)K.G | N | 67.92 | 1348.6195 | 10 | 0.7 | 675.3175 | 2 | 75.32 | 4 | F4:51022 | NaNaKA16\_F12.raw |  | 6.0196E5 | 6.6727E5 | 5.9248E7 |  |  |  |  |  | 4.1845E5 |  |  |  | 4 | 0 | 1 | 1 | 1 | 0 | 0 | 0 | 0 | 0 | 1 | 0 | 0 | 0 | 107 | 116 | Carbamidomethylation | C9:Carbamidomethylation:1000.00 | PEAKS DB |
| K.KYM(+15.99)LYPDFLC(+57.02)K.G | Y | 62.57 | 1492.7095 | 11 | 0.2 | 498.5772 | 3 | 52.20 | 4 | F4:32001 | NaNaKA16\_F12.raw |  |  |  | 4.3437E6 |  |  |  |  |  |  |  |  |  | 1 | 0 | 0 | 0 | 1 | 0 | 0 | 0 | 0 | 0 | 0 | 0 | 0 | 0 | 106 | 116 | Oxidation (M); Carbamidomethylation | M3:Oxidation (M):1000.00;C10:Carbamidomethylation:1000.00 | PEAKS DB |
| total 6 peptides |  |  |  |  |  |  |  |  |  |  |  |  |  |  |  |  |  |  |  |  |  |  |  |  |  |  |  |  |  |  |  |  |  |  |  |  |  |  |  |  |  |  |  |
| --- | --- | --- | --- | --- | --- | --- | --- | --- | --- | --- | --- | --- | --- | --- | --- | --- | --- | --- | --- | --- | --- | --- | --- | --- | --- | --- | --- | --- | --- | --- | --- | --- | --- | --- | --- | --- | --- | --- | --- | --- | --- | --- | --- |

4EIX

back to list

  

| Protein Coverage
| Supporting Peptides
|

Protein Coverage:

Supporting Peptides:

| Peptide | Uniq | -10lgP | Mass | Length | ppm | m/z | z | RT | Fraction | Scan | Source File | Area F1 | Area F10 | Area F11 | Area F12 | Area F13 | Area F2 | Area F3 | Area F4 | Area F5 | Area F6 | Area F7 | Area F8 | Area F9 | #Feature | #Feature F1 | #Feature F10 | #Feature F11 | #Feature F12 | #Feature F13 | #Feature F2 | #Feature F3 | #Feature F4 | #Feature F5 | #Feature F6 | #Feature F7 | #Feature F8 | #Feature F9 | Start | End | PTM | AScore | Found By |
| --- | --- | --- | --- | --- | --- | --- | --- | --- | --- | --- | --- | --- | --- | --- | --- | --- | --- | --- | --- | --- | --- | --- | --- | --- | --- | --- | --- | --- | --- | --- | --- | --- | --- | --- | --- | --- | --- | --- | --- | --- | --- | --- | --- |
| K.LAIPSYSSYGC(+57.02)YC(+57.02)GWGGK.G | Y | 88.95 | 2024.8760 | 18 | 1.0 | 1013.4462 | 2 | 65.94 | 4 | F4:43325 | NaNaKA16\_F12.raw |  |  |  | 5.4816E7 |  |  |  |  |  |  |  |  |  | 2 | 0 | 0 | 0 | 2 | 0 | 0 | 0 | 0 | 0 | 0 | 0 | 0 | 0 | 16 | 33 | Carbamidomethylation | C11:Carbamidomethylation:1000.00;C13:Carbamidomethylation:1000.00 | PEAKS DB |
| K.KYMLYPDFLC(+57.02)K.G | Y | 78.94 | 1476.7145 | 11 | 0.6 | 739.3649 | 2 | 58.50 | 4 | F4:37246 | NaNaKA16\_F12.raw |  |  |  | 3.9698E7 |  |  |  |  |  |  |  |  |  | 2 | 0 | 0 | 0 | 2 | 0 | 0 | 0 | 0 | 0 | 0 | 0 | 0 | 0 | 106 | 116 | Carbamidomethylation | C10:Carbamidomethylation:1000.00 | PEAKS DB |
| R.C(+57.02)C(+57.02)FVHDC(+57.02)C(+57.02)YGNLPDC(+57.02)NPK.S | N | 74.08 | 2314.8687 | 18 | 0.4 | 772.6305 | 3 | 32.68 | 4 | F4:15898 | NaNaKA16\_F12.raw |  |  |  | 5.4885E7 |  |  |  |  |  |  |  |  |  | 2 | 0 | 0 | 0 | 2 | 0 | 0 | 0 | 0 | 0 | 0 | 0 | 0 | 0 | 43 | 60 | Carbamidomethylation | C1:Carbamidomethylation:1000.00;C2:Carbamidomethylation:1000.00;C7:Carbamidomethylation:1000.00;C8:Carbamidomethylation:1000.00;C15:Carbamidomethylation:1000.00 | PEAKS DB |
| K.YM(+15.99)LYPDFLC(+57.02)K.G | N | 68.84 | 1364.6145 | 10 | 0.1 | 683.3146 | 2 | 68.38 | 4 | F4:45614 | NaNaKA16\_F12.raw |  |  |  | 8.5839E6 |  |  |  |  |  |  |  |  |  | 4 | 0 | 0 | 0 | 4 | 0 | 0 | 0 | 0 | 0 | 0 | 0 | 0 | 0 | 107 | 116 | Oxidation (M); Carbamidomethylation | M2:Oxidation (M):1000.00;C9:Carbamidomethylation:1000.00 | PEAKS DB |
| K.YMLYPDFLC(+57.02)K.G | N | 67.92 | 1348.6195 | 10 | 0.7 | 675.3175 | 2 | 75.32 | 4 | F4:51022 | NaNaKA16\_F12.raw |  | 6.0196E5 | 6.6727E5 | 5.9248E7 |  |  |  |  |  | 4.1845E5 |  |  |  | 4 | 0 | 1 | 1 | 1 | 0 | 0 | 0 | 0 | 0 | 1 | 0 | 0 | 0 | 107 | 116 | Carbamidomethylation | C9:Carbamidomethylation:1000.00 | PEAKS DB |
| K.KYM(+15.99)LYPDFLC(+57.02)K.G | Y | 62.57 | 1492.7095 | 11 | 0.2 | 498.5772 | 3 | 52.20 | 4 | F4:32001 | NaNaKA16\_F12.raw |  |  |  | 4.3437E6 |  |  |  |  |  |  |  |  |  | 1 | 0 | 0 | 0 | 1 | 0 | 0 | 0 | 0 | 0 | 0 | 0 | 0 | 0 | 106 | 116 | Oxidation (M); Carbamidomethylation | M3:Oxidation (M):1000.00;C10:Carbamidomethylation:1000.00 | PEAKS DB |
| total 6 peptides |  |  |  |  |  |  |  |  |  |  |  |  |  |  |  |  |  |  |  |  |  |  |  |  |  |  |  |  |  |  |  |  |  |  |  |  |  |  |  |  |  |  |  |
| --- | --- | --- | --- | --- | --- | --- | --- | --- | --- | --- | --- | --- | --- | --- | --- | --- | --- | --- | --- | --- | --- | --- | --- | --- | --- | --- | --- | --- | --- | --- | --- | --- | --- | --- | --- | --- | --- | --- | --- | --- | --- | --- | --- |

3H1X

back to list

  

| Protein Coverage
| Supporting Peptides
|

Protein Coverage:

Supporting Peptides:

| Peptide | Uniq | -10lgP | Mass | Length | ppm | m/z | z | RT | Fraction | Scan | Source File | Area F1 | Area F10 | Area F11 | Area F12 | Area F13 | Area F2 | Area F3 | Area F4 | Area F5 | Area F6 | Area F7 | Area F8 | Area F9 | #Feature | #Feature F1 | #Feature F10 | #Feature F11 | #Feature F12 | #Feature F13 | #Feature F2 | #Feature F3 | #Feature F4 | #Feature F5 | #Feature F6 | #Feature F7 | #Feature F8 | #Feature F9 | Start | End | PTM | AScore | Found By |
| --- | --- | --- | --- | --- | --- | --- | --- | --- | --- | --- | --- | --- | --- | --- | --- | --- | --- | --- | --- | --- | --- | --- | --- | --- | --- | --- | --- | --- | --- | --- | --- | --- | --- | --- | --- | --- | --- | --- | --- | --- | --- | --- | --- |
| K.LAIPSYSSYGC(+57.02)YC(+57.02)GWGGK.G | Y | 88.95 | 2024.8760 | 18 | 1.0 | 1013.4462 | 2 | 65.94 | 4 | F4:43325 | NaNaKA16\_F12.raw |  |  |  | 5.4816E7 |  |  |  |  |  |  |  |  |  | 2 | 0 | 0 | 0 | 2 | 0 | 0 | 0 | 0 | 0 | 0 | 0 | 0 | 0 | 16 | 33 | Carbamidomethylation | C11:Carbamidomethylation:1000.00;C13:Carbamidomethylation:1000.00 | PEAKS DB |
| K.KYMLYPDFLC(+57.02)K.G | Y | 78.94 | 1476.7145 | 11 | 0.6 | 739.3649 | 2 | 58.50 | 4 | F4:37246 | NaNaKA16\_F12.raw |  |  |  | 3.9698E7 |  |  |  |  |  |  |  |  |  | 2 | 0 | 0 | 0 | 2 | 0 | 0 | 0 | 0 | 0 | 0 | 0 | 0 | 0 | 106 | 116 | Carbamidomethylation | C10:Carbamidomethylation:1000.00 | PEAKS DB |
| R.C(+57.02)C(+57.02)FVHDC(+57.02)C(+57.02)YGNLPDC(+57.02)NPK.S | N | 74.08 | 2314.8687 | 18 | 0.4 | 772.6305 | 3 | 32.68 | 4 | F4:15898 | NaNaKA16\_F12.raw |  |  |  | 5.4885E7 |  |  |  |  |  |  |  |  |  | 2 | 0 | 0 | 0 | 2 | 0 | 0 | 0 | 0 | 0 | 0 | 0 | 0 | 0 | 43 | 60 | Carbamidomethylation | C1:Carbamidomethylation:1000.00;C2:Carbamidomethylation:1000.00;C7:Carbamidomethylation:1000.00;C8:Carbamidomethylation:1000.00;C15:Carbamidomethylation:1000.00 | PEAKS DB |
| K.YM(+15.99)LYPDFLC(+57.02)K.G | N | 68.84 | 1364.6145 | 10 | 0.1 | 683.3146 | 2 | 68.38 | 4 | F4:45614 | NaNaKA16\_F12.raw |  |  |  | 8.5839E6 |  |  |  |  |  |  |  |  |  | 4 | 0 | 0 | 0 | 4 | 0 | 0 | 0 | 0 | 0 | 0 | 0 | 0 | 0 | 107 | 116 | Oxidation (M); Carbamidomethylation | M2:Oxidation (M):1000.00;C9:Carbamidomethylation:1000.00 | PEAKS DB |
| K.YMLYPDFLC(+57.02)K.G | N | 67.92 | 1348.6195 | 10 | 0.7 | 675.3175 | 2 | 75.32 | 4 | F4:51022 | NaNaKA16\_F12.raw |  | 6.0196E5 | 6.6727E5 | 5.9248E7 |  |  |  |  |  | 4.1845E5 |  |  |  | 4 | 0 | 1 | 1 | 1 | 0 | 0 | 0 | 0 | 0 | 1 | 0 | 0 | 0 | 107 | 116 | Carbamidomethylation | C9:Carbamidomethylation:1000.00 | PEAKS DB |
| K.KYM(+15.99)LYPDFLC(+57.02)K.G | Y | 62.57 | 1492.7095 | 11 | 0.2 | 498.5772 | 3 | 52.20 | 4 | F4:32001 | NaNaKA16\_F12.raw |  |  |  | 4.3437E6 |  |  |  |  |  |  |  |  |  | 1 | 0 | 0 | 0 | 1 | 0 | 0 | 0 | 0 | 0 | 0 | 0 | 0 | 0 | 106 | 116 | Oxidation (M); Carbamidomethylation | M3:Oxidation (M):1000.00;C10:Carbamidomethylation:1000.00 | PEAKS DB |
| total 6 peptides |  |  |  |  |  |  |  |  |  |  |  |  |  |  |  |  |  |  |  |  |  |  |  |  |  |  |  |  |  |  |  |  |  |  |  |  |  |  |  |  |  |  |  |
| --- | --- | --- | --- | --- | --- | --- | --- | --- | --- | --- | --- | --- | --- | --- | --- | --- | --- | --- | --- | --- | --- | --- | --- | --- | --- | --- | --- | --- | --- | --- | --- | --- | --- | --- | --- | --- | --- | --- | --- | --- | --- | --- | --- |

2B17

back to list

  

| Protein Coverage
| Supporting Peptides
|

Protein Coverage:

Supporting Peptides:

| Peptide | Uniq | -10lgP | Mass | Length | ppm | m/z | z | RT | Fraction | Scan | Source File | Area F1 | Area F10 | Area F11 | Area F12 | Area F13 | Area F2 | Area F3 | Area F4 | Area F5 | Area F6 | Area F7 | Area F8 | Area F9 | #Feature | #Feature F1 | #Feature F10 | #Feature F11 | #Feature F12 | #Feature F13 | #Feature F2 | #Feature F3 | #Feature F4 | #Feature F5 | #Feature F6 | #Feature F7 | #Feature F8 | #Feature F9 | Start | End | PTM | AScore | Found By |
| --- | --- | --- | --- | --- | --- | --- | --- | --- | --- | --- | --- | --- | --- | --- | --- | --- | --- | --- | --- | --- | --- | --- | --- | --- | --- | --- | --- | --- | --- | --- | --- | --- | --- | --- | --- | --- | --- | --- | --- | --- | --- | --- | --- |
| K.LAIPSYSSYGC(+57.02)YC(+57.02)GWGGK.G | Y | 88.95 | 2024.8760 | 18 | 1.0 | 1013.4462 | 2 | 65.94 | 4 | F4:43325 | NaNaKA16\_F12.raw |  |  |  | 5.4816E7 |  |  |  |  |  |  |  |  |  | 2 | 0 | 0 | 0 | 2 | 0 | 0 | 0 | 0 | 0 | 0 | 0 | 0 | 0 | 16 | 33 | Carbamidomethylation | C11:Carbamidomethylation:1000.00;C13:Carbamidomethylation:1000.00 | PEAKS DB |
| K.KYMLYPDFLC(+57.02)K.G | Y | 78.94 | 1476.7145 | 11 | 0.6 | 739.3649 | 2 | 58.50 | 4 | F4:37246 | NaNaKA16\_F12.raw |  |  |  | 3.9698E7 |  |  |  |  |  |  |  |  |  | 2 | 0 | 0 | 0 | 2 | 0 | 0 | 0 | 0 | 0 | 0 | 0 | 0 | 0 | 106 | 116 | Carbamidomethylation | C10:Carbamidomethylation:1000.00 | PEAKS DB |
| R.C(+57.02)C(+57.02)FVHDC(+57.02)C(+57.02)YGNLPDC(+57.02)NPK.S | N | 74.08 | 2314.8687 | 18 | 0.4 | 772.6305 | 3 | 32.68 | 4 | F4:15898 | NaNaKA16\_F12.raw |  |  |  | 5.4885E7 |  |  |  |  |  |  |  |  |  | 2 | 0 | 0 | 0 | 2 | 0 | 0 | 0 | 0 | 0 | 0 | 0 | 0 | 0 | 43 | 60 | Carbamidomethylation | C1:Carbamidomethylation:1000.00;C2:Carbamidomethylation:1000.00;C7:Carbamidomethylation:1000.00;C8:Carbamidomethylation:1000.00;C15:Carbamidomethylation:1000.00 | PEAKS DB |
| K.YM(+15.99)LYPDFLC(+57.02)K.G | N | 68.84 | 1364.6145 | 10 | 0.1 | 683.3146 | 2 | 68.38 | 4 | F4:45614 | NaNaKA16\_F12.raw |  |  |  | 8.5839E6 |  |  |  |  |  |  |  |  |  | 4 | 0 | 0 | 0 | 4 | 0 | 0 | 0 | 0 | 0 | 0 | 0 | 0 | 0 | 107 | 116 | Oxidation (M); Carbamidomethylation | M2:Oxidation (M):1000.00;C9:Carbamidomethylation:1000.00 | PEAKS DB |
| K.YMLYPDFLC(+57.02)K.G | N | 67.92 | 1348.6195 | 10 | 0.7 | 675.3175 | 2 | 75.32 | 4 | F4:51022 | NaNaKA16\_F12.raw |  | 6.0196E5 | 6.6727E5 | 5.9248E7 |  |  |  |  |  | 4.1845E5 |  |  |  | 4 | 0 | 1 | 1 | 1 | 0 | 0 | 0 | 0 | 0 | 1 | 0 | 0 | 0 | 107 | 116 | Carbamidomethylation | C9:Carbamidomethylation:1000.00 | PEAKS DB |
| K.KYM(+15.99)LYPDFLC(+57.02)K.G | Y | 62.57 | 1492.7095 | 11 | 0.2 | 498.5772 | 3 | 52.20 | 4 | F4:32001 | NaNaKA16\_F12.raw |  |  |  | 4.3437E6 |  |  |  |  |  |  |  |  |  | 1 | 0 | 0 | 0 | 1 | 0 | 0 | 0 | 0 | 0 | 0 | 0 | 0 | 0 | 106 | 116 | Oxidation (M); Carbamidomethylation | M3:Oxidation (M):1000.00;C10:Carbamidomethylation:1000.00 | PEAKS DB |
| total 6 peptides |  |  |  |  |  |  |  |  |  |  |  |  |  |  |  |  |  |  |  |  |  |  |  |  |  |  |  |  |  |  |  |  |  |  |  |  |  |  |  |  |  |  |  |
| --- | --- | --- | --- | --- | --- | --- | --- | --- | --- | --- | --- | --- | --- | --- | --- | --- | --- | --- | --- | --- | --- | --- | --- | --- | --- | --- | --- | --- | --- | --- | --- | --- | --- | --- | --- | --- | --- | --- | --- | --- | --- | --- | --- |

2ARM

back to list

  

| Protein Coverage
| Supporting Peptides
|

Protein Coverage:

Supporting Peptides:

| Peptide | Uniq | -10lgP | Mass | Length | ppm | m/z | z | RT | Fraction | Scan | Source File | Area F1 | Area F10 | Area F11 | Area F12 | Area F13 | Area F2 | Area F3 | Area F4 | Area F5 | Area F6 | Area F7 | Area F8 | Area F9 | #Feature | #Feature F1 | #Feature F10 | #Feature F11 | #Feature F12 | #Feature F13 | #Feature F2 | #Feature F3 | #Feature F4 | #Feature F5 | #Feature F6 | #Feature F7 | #Feature F8 | #Feature F9 | Start | End | PTM | AScore | Found By |
| --- | --- | --- | --- | --- | --- | --- | --- | --- | --- | --- | --- | --- | --- | --- | --- | --- | --- | --- | --- | --- | --- | --- | --- | --- | --- | --- | --- | --- | --- | --- | --- | --- | --- | --- | --- | --- | --- | --- | --- | --- | --- | --- | --- |
| K.LAIPSYSSYGC(+57.02)YC(+57.02)GWGGK.G | Y | 88.95 | 2024.8760 | 18 | 1.0 | 1013.4462 | 2 | 65.94 | 4 | F4:43325 | NaNaKA16\_F12.raw |  |  |  | 5.4816E7 |  |  |  |  |  |  |  |  |  | 2 | 0 | 0 | 0 | 2 | 0 | 0 | 0 | 0 | 0 | 0 | 0 | 0 | 0 | 16 | 33 | Carbamidomethylation | C11:Carbamidomethylation:1000.00;C13:Carbamidomethylation:1000.00 | PEAKS DB |
| K.KYMLYPDFLC(+57.02)K.G | Y | 78.94 | 1476.7145 | 11 | 0.6 | 739.3649 | 2 | 58.50 | 4 | F4:37246 | NaNaKA16\_F12.raw |  |  |  | 3.9698E7 |  |  |  |  |  |  |  |  |  | 2 | 0 | 0 | 0 | 2 | 0 | 0 | 0 | 0 | 0 | 0 | 0 | 0 | 0 | 106 | 116 | Carbamidomethylation | C10:Carbamidomethylation:1000.00 | PEAKS DB |
| R.C(+57.02)C(+57.02)FVHDC(+57.02)C(+57.02)YGNLPDC(+57.02)NPK.S | N | 74.08 | 2314.8687 | 18 | 0.4 | 772.6305 | 3 | 32.68 | 4 | F4:15898 | NaNaKA16\_F12.raw |  |  |  | 5.4885E7 |  |  |  |  |  |  |  |  |  | 2 | 0 | 0 | 0 | 2 | 0 | 0 | 0 | 0 | 0 | 0 | 0 | 0 | 0 | 43 | 60 | Carbamidomethylation | C1:Carbamidomethylation:1000.00;C2:Carbamidomethylation:1000.00;C7:Carbamidomethylation:1000.00;C8:Carbamidomethylation:1000.00;C15:Carbamidomethylation:1000.00 | PEAKS DB |
| K.YM(+15.99)LYPDFLC(+57.02)K.G | N | 68.84 | 1364.6145 | 10 | 0.1 | 683.3146 | 2 | 68.38 | 4 | F4:45614 | NaNaKA16\_F12.raw |  |  |  | 8.5839E6 |  |  |  |  |  |  |  |  |  | 4 | 0 | 0 | 0 | 4 | 0 | 0 | 0 | 0 | 0 | 0 | 0 | 0 | 0 | 107 | 116 | Oxidation (M); Carbamidomethylation | M2:Oxidation (M):1000.00;C9:Carbamidomethylation:1000.00 | PEAKS DB |
| K.YMLYPDFLC(+57.02)K.G | N | 67.92 | 1348.6195 | 10 | 0.7 | 675.3175 | 2 | 75.32 | 4 | F4:51022 | NaNaKA16\_F12.raw |  | 6.0196E5 | 6.6727E5 | 5.9248E7 |  |  |  |  |  | 4.1845E5 |  |  |  | 4 | 0 | 1 | 1 | 1 | 0 | 0 | 0 | 0 | 0 | 1 | 0 | 0 | 0 | 107 | 116 | Carbamidomethylation | C9:Carbamidomethylation:1000.00 | PEAKS DB |
| K.KYM(+15.99)LYPDFLC(+57.02)K.G | Y | 62.57 | 1492.7095 | 11 | 0.2 | 498.5772 | 3 | 52.20 | 4 | F4:32001 | NaNaKA16\_F12.raw |  |  |  | 4.3437E6 |  |  |  |  |  |  |  |  |  | 1 | 0 | 0 | 0 | 1 | 0 | 0 | 0 | 0 | 0 | 0 | 0 | 0 | 0 | 106 | 116 | Oxidation (M); Carbamidomethylation | M3:Oxidation (M):1000.00;C10:Carbamidomethylation:1000.00 | PEAKS DB |
| total 6 peptides |  |  |  |  |  |  |  |  |  |  |  |  |  |  |  |  |  |  |  |  |  |  |  |  |  |  |  |  |  |  |  |  |  |  |  |  |  |  |  |  |  |  |  |
| --- | --- | --- | --- | --- | --- | --- | --- | --- | --- | --- | --- | --- | --- | --- | --- | --- | --- | --- | --- | --- | --- | --- | --- | --- | --- | --- | --- | --- | --- | --- | --- | --- | --- | --- | --- | --- | --- | --- | --- | --- | --- | --- | --- |

1TG4

back to list

  

| Protein Coverage
| Supporting Peptides
|

Protein Coverage:

Supporting Peptides:

| Peptide | Uniq | -10lgP | Mass | Length | ppm | m/z | z | RT | Fraction | Scan | Source File | Area F1 | Area F10 | Area F11 | Area F12 | Area F13 | Area F2 | Area F3 | Area F4 | Area F5 | Area F6 | Area F7 | Area F8 | Area F9 | #Feature | #Feature F1 | #Feature F10 | #Feature F11 | #Feature F12 | #Feature F13 | #Feature F2 | #Feature F3 | #Feature F4 | #Feature F5 | #Feature F6 | #Feature F7 | #Feature F8 | #Feature F9 | Start | End | PTM | AScore | Found By |
| --- | --- | --- | --- | --- | --- | --- | --- | --- | --- | --- | --- | --- | --- | --- | --- | --- | --- | --- | --- | --- | --- | --- | --- | --- | --- | --- | --- | --- | --- | --- | --- | --- | --- | --- | --- | --- | --- | --- | --- | --- | --- | --- | --- |
| K.LAIPSYSSYGC(+57.02)YC(+57.02)GWGGK.G | Y | 88.95 | 2024.8760 | 18 | 1.0 | 1013.4462 | 2 | 65.94 | 4 | F4:43325 | NaNaKA16\_F12.raw |  |  |  | 5.4816E7 |  |  |  |  |  |  |  |  |  | 2 | 0 | 0 | 0 | 2 | 0 | 0 | 0 | 0 | 0 | 0 | 0 | 0 | 0 | 16 | 33 | Carbamidomethylation | C11:Carbamidomethylation:1000.00;C13:Carbamidomethylation:1000.00 | PEAKS DB |
| K.KYMLYPDFLC(+57.02)K.G | Y | 78.94 | 1476.7145 | 11 | 0.6 | 739.3649 | 2 | 58.50 | 4 | F4:37246 | NaNaKA16\_F12.raw |  |  |  | 3.9698E7 |  |  |  |  |  |  |  |  |  | 2 | 0 | 0 | 0 | 2 | 0 | 0 | 0 | 0 | 0 | 0 | 0 | 0 | 0 | 106 | 116 | Carbamidomethylation | C10:Carbamidomethylation:1000.00 | PEAKS DB |
| R.C(+57.02)C(+57.02)FVHDC(+57.02)C(+57.02)YGNLPDC(+57.02)NPK.S | N | 74.08 | 2314.8687 | 18 | 0.4 | 772.6305 | 3 | 32.68 | 4 | F4:15898 | NaNaKA16\_F12.raw |  |  |  | 5.4885E7 |  |  |  |  |  |  |  |  |  | 2 | 0 | 0 | 0 | 2 | 0 | 0 | 0 | 0 | 0 | 0 | 0 | 0 | 0 | 43 | 60 | Carbamidomethylation | C1:Carbamidomethylation:1000.00;C2:Carbamidomethylation:1000.00;C7:Carbamidomethylation:1000.00;C8:Carbamidomethylation:1000.00;C15:Carbamidomethylation:1000.00 | PEAKS DB |
| K.YM(+15.99)LYPDFLC(+57.02)K.G | N | 68.84 | 1364.6145 | 10 | 0.1 | 683.3146 | 2 | 68.38 | 4 | F4:45614 | NaNaKA16\_F12.raw |  |  |  | 8.5839E6 |  |  |  |  |  |  |  |  |  | 4 | 0 | 0 | 0 | 4 | 0 | 0 | 0 | 0 | 0 | 0 | 0 | 0 | 0 | 107 | 116 | Oxidation (M); Carbamidomethylation | M2:Oxidation (M):1000.00;C9:Carbamidomethylation:1000.00 | PEAKS DB |
| K.YMLYPDFLC(+57.02)K.G | N | 67.92 | 1348.6195 | 10 | 0.7 | 675.3175 | 2 | 75.32 | 4 | F4:51022 | NaNaKA16\_F12.raw |  | 6.0196E5 | 6.6727E5 | 5.9248E7 |  |  |  |  |  | 4.1845E5 |  |  |  | 4 | 0 | 1 | 1 | 1 | 0 | 0 | 0 | 0 | 0 | 1 | 0 | 0 | 0 | 107 | 116 | Carbamidomethylation | C9:Carbamidomethylation:1000.00 | PEAKS DB |
| K.KYM(+15.99)LYPDFLC(+57.02)K.G | Y | 62.57 | 1492.7095 | 11 | 0.2 | 498.5772 | 3 | 52.20 | 4 | F4:32001 | NaNaKA16\_F12.raw |  |  |  | 4.3437E6 |  |  |  |  |  |  |  |  |  | 1 | 0 | 0 | 0 | 1 | 0 | 0 | 0 | 0 | 0 | 0 | 0 | 0 | 0 | 106 | 116 | Oxidation (M); Carbamidomethylation | M3:Oxidation (M):1000.00;C10:Carbamidomethylation:1000.00 | PEAKS DB |
| total 6 peptides |  |  |  |  |  |  |  |  |  |  |  |  |  |  |  |  |  |  |  |  |  |  |  |  |  |  |  |  |  |  |  |  |  |  |  |  |  |  |  |  |  |  |  |
| --- | --- | --- | --- | --- | --- | --- | --- | --- | --- | --- | --- | --- | --- | --- | --- | --- | --- | --- | --- | --- | --- | --- | --- | --- | --- | --- | --- | --- | --- | --- | --- | --- | --- | --- | --- | --- | --- | --- | --- | --- | --- | --- | --- |

1SQZ

back to list

  

| Protein Coverage
| Supporting Peptides
|

Protein Coverage:

Supporting Peptides:

| Peptide | Uniq | -10lgP | Mass | Length | ppm | m/z | z | RT | Fraction | Scan | Source File | Area F1 | Area F10 | Area F11 | Area F12 | Area F13 | Area F2 | Area F3 | Area F4 | Area F5 | Area F6 | Area F7 | Area F8 | Area F9 | #Feature | #Feature F1 | #Feature F10 | #Feature F11 | #Feature F12 | #Feature F13 | #Feature F2 | #Feature F3 | #Feature F4 | #Feature F5 | #Feature F6 | #Feature F7 | #Feature F8 | #Feature F9 | Start | End | PTM | AScore | Found By |
| --- | --- | --- | --- | --- | --- | --- | --- | --- | --- | --- | --- | --- | --- | --- | --- | --- | --- | --- | --- | --- | --- | --- | --- | --- | --- | --- | --- | --- | --- | --- | --- | --- | --- | --- | --- | --- | --- | --- | --- | --- | --- | --- | --- |
| K.LAIPSYSSYGC(+57.02)YC(+57.02)GWGGK.G | Y | 88.95 | 2024.8760 | 18 | 1.0 | 1013.4462 | 2 | 65.94 | 4 | F4:43325 | NaNaKA16\_F12.raw |  |  |  | 5.4816E7 |  |  |  |  |  |  |  |  |  | 2 | 0 | 0 | 0 | 2 | 0 | 0 | 0 | 0 | 0 | 0 | 0 | 0 | 0 | 16 | 33 | Carbamidomethylation | C11:Carbamidomethylation:1000.00;C13:Carbamidomethylation:1000.00 | PEAKS DB |
| K.KYMLYPDFLC(+57.02)K.G | Y | 78.94 | 1476.7145 | 11 | 0.6 | 739.3649 | 2 | 58.50 | 4 | F4:37246 | NaNaKA16\_F12.raw |  |  |  | 3.9698E7 |  |  |  |  |  |  |  |  |  | 2 | 0 | 0 | 0 | 2 | 0 | 0 | 0 | 0 | 0 | 0 | 0 | 0 | 0 | 106 | 116 | Carbamidomethylation | C10:Carbamidomethylation:1000.00 | PEAKS DB |
| R.C(+57.02)C(+57.02)FVHDC(+57.02)C(+57.02)YGNLPDC(+57.02)NPK.S | N | 74.08 | 2314.8687 | 18 | 0.4 | 772.6305 | 3 | 32.68 | 4 | F4:15898 | NaNaKA16\_F12.raw |  |  |  | 5.4885E7 |  |  |  |  |  |  |  |  |  | 2 | 0 | 0 | 0 | 2 | 0 | 0 | 0 | 0 | 0 | 0 | 0 | 0 | 0 | 43 | 60 | Carbamidomethylation | C1:Carbamidomethylation:1000.00;C2:Carbamidomethylation:1000.00;C7:Carbamidomethylation:1000.00;C8:Carbamidomethylation:1000.00;C15:Carbamidomethylation:1000.00 | PEAKS DB |
| K.YM(+15.99)LYPDFLC(+57.02)K.G | N | 68.84 | 1364.6145 | 10 | 0.1 | 683.3146 | 2 | 68.38 | 4 | F4:45614 | NaNaKA16\_F12.raw |  |  |  | 8.5839E6 |  |  |  |  |  |  |  |  |  | 4 | 0 | 0 | 0 | 4 | 0 | 0 | 0 | 0 | 0 | 0 | 0 | 0 | 0 | 107 | 116 | Oxidation (M); Carbamidomethylation | M2:Oxidation (M):1000.00;C9:Carbamidomethylation:1000.00 | PEAKS DB |
| K.YMLYPDFLC(+57.02)K.G | N | 67.92 | 1348.6195 | 10 | 0.7 | 675.3175 | 2 | 75.32 | 4 | F4:51022 | NaNaKA16\_F12.raw |  | 6.0196E5 | 6.6727E5 | 5.9248E7 |  |  |  |  |  | 4.1845E5 |  |  |  | 4 | 0 | 1 | 1 | 1 | 0 | 0 | 0 | 0 | 0 | 1 | 0 | 0 | 0 | 107 | 116 | Carbamidomethylation | C9:Carbamidomethylation:1000.00 | PEAKS DB |
| K.KYM(+15.99)LYPDFLC(+57.02)K.G | Y | 62.57 | 1492.7095 | 11 | 0.2 | 498.5772 | 3 | 52.20 | 4 | F4:32001 | NaNaKA16\_F12.raw |  |  |  | 4.3437E6 |  |  |  |  |  |  |  |  |  | 1 | 0 | 0 | 0 | 1 | 0 | 0 | 0 | 0 | 0 | 0 | 0 | 0 | 0 | 106 | 116 | Oxidation (M); Carbamidomethylation | M3:Oxidation (M):1000.00;C10:Carbamidomethylation:1000.00 | PEAKS DB |
| total 6 peptides |  |  |  |  |  |  |  |  |  |  |  |  |  |  |  |  |  |  |  |  |  |  |  |  |  |  |  |  |  |  |  |  |  |  |  |  |  |  |  |  |  |  |  |
| --- | --- | --- | --- | --- | --- | --- | --- | --- | --- | --- | --- | --- | --- | --- | --- | --- | --- | --- | --- | --- | --- | --- | --- | --- | --- | --- | --- | --- | --- | --- | --- | --- | --- | --- | --- | --- | --- | --- | --- | --- | --- | --- | --- |

3FG5

back to list

  

| Protein Coverage
| Supporting Peptides
|

Protein Coverage:

Supporting Peptides:

| Peptide | Uniq | -10lgP | Mass | Length | ppm | m/z | z | RT | Fraction | Scan | Source File | Area F1 | Area F10 | Area F11 | Area F12 | Area F13 | Area F2 | Area F3 | Area F4 | Area F5 | Area F6 | Area F7 | Area F8 | Area F9 | #Feature | #Feature F1 | #Feature F10 | #Feature F11 | #Feature F12 | #Feature F13 | #Feature F2 | #Feature F3 | #Feature F4 | #Feature F5 | #Feature F6 | #Feature F7 | #Feature F8 | #Feature F9 | Start | End | PTM | AScore | Found By |
| --- | --- | --- | --- | --- | --- | --- | --- | --- | --- | --- | --- | --- | --- | --- | --- | --- | --- | --- | --- | --- | --- | --- | --- | --- | --- | --- | --- | --- | --- | --- | --- | --- | --- | --- | --- | --- | --- | --- | --- | --- | --- | --- | --- |
| K.LAIPSYSSYGC(+57.02)YC(+57.02)GWGGK.G | Y | 88.95 | 2024.8760 | 18 | 1.0 | 1013.4462 | 2 | 65.94 | 4 | F4:43325 | NaNaKA16\_F12.raw |  |  |  | 5.4816E7 |  |  |  |  |  |  |  |  |  | 2 | 0 | 0 | 0 | 2 | 0 | 0 | 0 | 0 | 0 | 0 | 0 | 0 | 0 | 16 | 33 | Carbamidomethylation | C11:Carbamidomethylation:1000.00;C13:Carbamidomethylation:1000.00 | PEAKS DB |
| K.KYMLYPDFLC(+57.02)K.G | Y | 78.94 | 1476.7145 | 11 | 0.6 | 739.3649 | 2 | 58.50 | 4 | F4:37246 | NaNaKA16\_F12.raw |  |  |  | 3.9698E7 |  |  |  |  |  |  |  |  |  | 2 | 0 | 0 | 0 | 2 | 0 | 0 | 0 | 0 | 0 | 0 | 0 | 0 | 0 | 106 | 116 | Carbamidomethylation | C10:Carbamidomethylation:1000.00 | PEAKS DB |
| R.C(+57.02)C(+57.02)FVHDC(+57.02)C(+57.02)YGNLPDC(+57.02)NPK.S | N | 74.08 | 2314.8687 | 18 | 0.4 | 772.6305 | 3 | 32.68 | 4 | F4:15898 | NaNaKA16\_F12.raw |  |  |  | 5.4885E7 |  |  |  |  |  |  |  |  |  | 2 | 0 | 0 | 0 | 2 | 0 | 0 | 0 | 0 | 0 | 0 | 0 | 0 | 0 | 43 | 60 | Carbamidomethylation | C1:Carbamidomethylation:1000.00;C2:Carbamidomethylation:1000.00;C7:Carbamidomethylation:1000.00;C8:Carbamidomethylation:1000.00;C15:Carbamidomethylation:1000.00 | PEAKS DB |
| K.YM(+15.99)LYPDFLC(+57.02)K.G | N | 68.84 | 1364.6145 | 10 | 0.1 | 683.3146 | 2 | 68.38 | 4 | F4:45614 | NaNaKA16\_F12.raw |  |  |  | 8.5839E6 |  |  |  |  |  |  |  |  |  | 4 | 0 | 0 | 0 | 4 | 0 | 0 | 0 | 0 | 0 | 0 | 0 | 0 | 0 | 107 | 116 | Oxidation (M); Carbamidomethylation | M2:Oxidation (M):1000.00;C9:Carbamidomethylation:1000.00 | PEAKS DB |
| K.YMLYPDFLC(+57.02)K.G | N | 67.92 | 1348.6195 | 10 | 0.7 | 675.3175 | 2 | 75.32 | 4 | F4:51022 | NaNaKA16\_F12.raw |  | 6.0196E5 | 6.6727E5 | 5.9248E7 |  |  |  |  |  | 4.1845E5 |  |  |  | 4 | 0 | 1 | 1 | 1 | 0 | 0 | 0 | 0 | 0 | 1 | 0 | 0 | 0 | 107 | 116 | Carbamidomethylation | C9:Carbamidomethylation:1000.00 | PEAKS DB |
| K.KYM(+15.99)LYPDFLC(+57.02)K.G | Y | 62.57 | 1492.7095 | 11 | 0.2 | 498.5772 | 3 | 52.20 | 4 | F4:32001 | NaNaKA16\_F12.raw |  |  |  | 4.3437E6 |  |  |  |  |  |  |  |  |  | 1 | 0 | 0 | 0 | 1 | 0 | 0 | 0 | 0 | 0 | 0 | 0 | 0 | 0 | 106 | 116 | Oxidation (M); Carbamidomethylation | M3:Oxidation (M):1000.00;C10:Carbamidomethylation:1000.00 | PEAKS DB |
| total 6 peptides |  |  |  |  |  |  |  |  |  |  |  |  |  |  |  |  |  |  |  |  |  |  |  |  |  |  |  |  |  |  |  |  |  |  |  |  |  |  |  |  |  |  |  |
| --- | --- | --- | --- | --- | --- | --- | --- | --- | --- | --- | --- | --- | --- | --- | --- | --- | --- | --- | --- | --- | --- | --- | --- | --- | --- | --- | --- | --- | --- | --- | --- | --- | --- | --- | --- | --- | --- | --- | --- | --- | --- | --- | --- |

1ZWP

back to list

  

| Protein Coverage
| Supporting Peptides
|

Protein Coverage:

Supporting Peptides:

| Peptide | Uniq | -10lgP | Mass | Length | ppm | m/z | z | RT | Fraction | Scan | Source File | Area F1 | Area F10 | Area F11 | Area F12 | Area F13 | Area F2 | Area F3 | Area F4 | Area F5 | Area F6 | Area F7 | Area F8 | Area F9 | #Feature | #Feature F1 | #Feature F10 | #Feature F11 | #Feature F12 | #Feature F13 | #Feature F2 | #Feature F3 | #Feature F4 | #Feature F5 | #Feature F6 | #Feature F7 | #Feature F8 | #Feature F9 | Start | End | PTM | AScore | Found By |
| --- | --- | --- | --- | --- | --- | --- | --- | --- | --- | --- | --- | --- | --- | --- | --- | --- | --- | --- | --- | --- | --- | --- | --- | --- | --- | --- | --- | --- | --- | --- | --- | --- | --- | --- | --- | --- | --- | --- | --- | --- | --- | --- | --- |
| K.LAIPSYSSYGC(+57.02)YC(+57.02)GWGGK.G | Y | 88.95 | 2024.8760 | 18 | 1.0 | 1013.4462 | 2 | 65.94 | 4 | F4:43325 | NaNaKA16\_F12.raw |  |  |  | 5.4816E7 |  |  |  |  |  |  |  |  |  | 2 | 0 | 0 | 0 | 2 | 0 | 0 | 0 | 0 | 0 | 0 | 0 | 0 | 0 | 16 | 33 | Carbamidomethylation | C11:Carbamidomethylation:1000.00;C13:Carbamidomethylation:1000.00 | PEAKS DB |
| K.KYMLYPDFLC(+57.02)K.G | Y | 78.94 | 1476.7145 | 11 | 0.6 | 739.3649 | 2 | 58.50 | 4 | F4:37246 | NaNaKA16\_F12.raw |  |  |  | 3.9698E7 |  |  |  |  |  |  |  |  |  | 2 | 0 | 0 | 0 | 2 | 0 | 0 | 0 | 0 | 0 | 0 | 0 | 0 | 0 | 106 | 116 | Carbamidomethylation | C10:Carbamidomethylation:1000.00 | PEAKS DB |
| R.C(+57.02)C(+57.02)FVHDC(+57.02)C(+57.02)YGNLPDC(+57.02)NPK.S | N | 74.08 | 2314.8687 | 18 | 0.4 | 772.6305 | 3 | 32.68 | 4 | F4:15898 | NaNaKA16\_F12.raw |  |  |  | 5.4885E7 |  |  |  |  |  |  |  |  |  | 2 | 0 | 0 | 0 | 2 | 0 | 0 | 0 | 0 | 0 | 0 | 0 | 0 | 0 | 43 | 60 | Carbamidomethylation | C1:Carbamidomethylation:1000.00;C2:Carbamidomethylation:1000.00;C7:Carbamidomethylation:1000.00;C8:Carbamidomethylation:1000.00;C15:Carbamidomethylation:1000.00 | PEAKS DB |
| K.YM(+15.99)LYPDFLC(+57.02)K.G | N | 68.84 | 1364.6145 | 10 | 0.1 | 683.3146 | 2 | 68.38 | 4 | F4:45614 | NaNaKA16\_F12.raw |  |  |  | 8.5839E6 |  |  |  |  |  |  |  |  |  | 4 | 0 | 0 | 0 | 4 | 0 | 0 | 0 | 0 | 0 | 0 | 0 | 0 | 0 | 107 | 116 | Oxidation (M); Carbamidomethylation | M2:Oxidation (M):1000.00;C9:Carbamidomethylation:1000.00 | PEAKS DB |
| K.YMLYPDFLC(+57.02)K.G | N | 67.92 | 1348.6195 | 10 | 0.7 | 675.3175 | 2 | 75.32 | 4 | F4:51022 | NaNaKA16\_F12.raw |  | 6.0196E5 | 6.6727E5 | 5.9248E7 |  |  |  |  |  | 4.1845E5 |  |  |  | 4 | 0 | 1 | 1 | 1 | 0 | 0 | 0 | 0 | 0 | 1 | 0 | 0 | 0 | 107 | 116 | Carbamidomethylation | C9:Carbamidomethylation:1000.00 | PEAKS DB |
| K.KYM(+15.99)LYPDFLC(+57.02)K.G | Y | 62.57 | 1492.7095 | 11 | 0.2 | 498.5772 | 3 | 52.20 | 4 | F4:32001 | NaNaKA16\_F12.raw |  |  |  | 4.3437E6 |  |  |  |  |  |  |  |  |  | 1 | 0 | 0 | 0 | 1 | 0 | 0 | 0 | 0 | 0 | 0 | 0 | 0 | 0 | 106 | 116 | Oxidation (M); Carbamidomethylation | M3:Oxidation (M):1000.00;C10:Carbamidomethylation:1000.00 | PEAKS DB |
| total 6 peptides |  |  |  |  |  |  |  |  |  |  |  |  |  |  |  |  |  |  |  |  |  |  |  |  |  |  |  |  |  |  |  |  |  |  |  |  |  |  |  |  |  |  |  |
| --- | --- | --- | --- | --- | --- | --- | --- | --- | --- | --- | --- | --- | --- | --- | --- | --- | --- | --- | --- | --- | --- | --- | --- | --- | --- | --- | --- | --- | --- | --- | --- | --- | --- | --- | --- | --- | --- | --- | --- | --- | --- | --- | --- |

2QU9

back to list

  

| Protein Coverage
| Supporting Peptides
|

Protein Coverage:

Supporting Peptides:

| Peptide | Uniq | -10lgP | Mass | Length | ppm | m/z | z | RT | Fraction | Scan | Source File | Area F1 | Area F10 | Area F11 | Area F12 | Area F13 | Area F2 | Area F3 | Area F4 | Area F5 | Area F6 | Area F7 | Area F8 | Area F9 | #Feature | #Feature F1 | #Feature F10 | #Feature F11 | #Feature F12 | #Feature F13 | #Feature F2 | #Feature F3 | #Feature F4 | #Feature F5 | #Feature F6 | #Feature F7 | #Feature F8 | #Feature F9 | Start | End | PTM | AScore | Found By |
| --- | --- | --- | --- | --- | --- | --- | --- | --- | --- | --- | --- | --- | --- | --- | --- | --- | --- | --- | --- | --- | --- | --- | --- | --- | --- | --- | --- | --- | --- | --- | --- | --- | --- | --- | --- | --- | --- | --- | --- | --- | --- | --- | --- |
| K.LAIPSYSSYGC(+57.02)YC(+57.02)GWGGK.G | Y | 88.95 | 2024.8760 | 18 | 1.0 | 1013.4462 | 2 | 65.94 | 4 | F4:43325 | NaNaKA16\_F12.raw |  |  |  | 5.4816E7 |  |  |  |  |  |  |  |  |  | 2 | 0 | 0 | 0 | 2 | 0 | 0 | 0 | 0 | 0 | 0 | 0 | 0 | 0 | 16 | 33 | Carbamidomethylation | C11:Carbamidomethylation:1000.00;C13:Carbamidomethylation:1000.00 | PEAKS DB |
| K.KYMLYPDFLC(+57.02)K.G | Y | 78.94 | 1476.7145 | 11 | 0.6 | 739.3649 | 2 | 58.50 | 4 | F4:37246 | NaNaKA16\_F12.raw |  |  |  | 3.9698E7 |  |  |  |  |  |  |  |  |  | 2 | 0 | 0 | 0 | 2 | 0 | 0 | 0 | 0 | 0 | 0 | 0 | 0 | 0 | 106 | 116 | Carbamidomethylation | C10:Carbamidomethylation:1000.00 | PEAKS DB |
| R.C(+57.02)C(+57.02)FVHDC(+57.02)C(+57.02)YGNLPDC(+57.02)NPK.S | N | 74.08 | 2314.8687 | 18 | 0.4 | 772.6305 | 3 | 32.68 | 4 | F4:15898 | NaNaKA16\_F12.raw |  |  |  | 5.4885E7 |  |  |  |  |  |  |  |  |  | 2 | 0 | 0 | 0 | 2 | 0 | 0 | 0 | 0 | 0 | 0 | 0 | 0 | 0 | 43 | 60 | Carbamidomethylation | C1:Carbamidomethylation:1000.00;C2:Carbamidomethylation:1000.00;C7:Carbamidomethylation:1000.00;C8:Carbamidomethylation:1000.00;C15:Carbamidomethylation:1000.00 | PEAKS DB |
| K.YM(+15.99)LYPDFLC(+57.02)K.G | N | 68.84 | 1364.6145 | 10 | 0.1 | 683.3146 | 2 | 68.38 | 4 | F4:45614 | NaNaKA16\_F12.raw |  |  |  | 8.5839E6 |  |  |  |  |  |  |  |  |  | 4 | 0 | 0 | 0 | 4 | 0 | 0 | 0 | 0 | 0 | 0 | 0 | 0 | 0 | 107 | 116 | Oxidation (M); Carbamidomethylation | M2:Oxidation (M):1000.00;C9:Carbamidomethylation:1000.00 | PEAKS DB |
| K.YMLYPDFLC(+57.02)K.G | N | 67.92 | 1348.6195 | 10 | 0.7 | 675.3175 | 2 | 75.32 | 4 | F4:51022 | NaNaKA16\_F12.raw |  | 6.0196E5 | 6.6727E5 | 5.9248E7 |  |  |  |  |  | 4.1845E5 |  |  |  | 4 | 0 | 1 | 1 | 1 | 0 | 0 | 0 | 0 | 0 | 1 | 0 | 0 | 0 | 107 | 116 | Carbamidomethylation | C9:Carbamidomethylation:1000.00 | PEAKS DB |
| K.KYM(+15.99)LYPDFLC(+57.02)K.G | Y | 62.57 | 1492.7095 | 11 | 0.2 | 498.5772 | 3 | 52.20 | 4 | F4:32001 | NaNaKA16\_F12.raw |  |  |  | 4.3437E6 |  |  |  |  |  |  |  |  |  | 1 | 0 | 0 | 0 | 1 | 0 | 0 | 0 | 0 | 0 | 0 | 0 | 0 | 0 | 106 | 116 | Oxidation (M); Carbamidomethylation | M3:Oxidation (M):1000.00;C10:Carbamidomethylation:1000.00 | PEAKS DB |
| total 6 peptides |  |  |  |  |  |  |  |  |  |  |  |  |  |  |  |  |  |  |  |  |  |  |  |  |  |  |  |  |  |  |  |  |  |  |  |  |  |  |  |  |  |  |  |
| --- | --- | --- | --- | --- | --- | --- | --- | --- | --- | --- | --- | --- | --- | --- | --- | --- | --- | --- | --- | --- | --- | --- | --- | --- | --- | --- | --- | --- | --- | --- | --- | --- | --- | --- | --- | --- | --- | --- | --- | --- | --- | --- | --- |

1Y38

back to list

  

| Protein Coverage
| Supporting Peptides
|

Protein Coverage:

Supporting Peptides:

| Peptide | Uniq | -10lgP | Mass | Length | ppm | m/z | z | RT | Fraction | Scan | Source File | Area F1 | Area F10 | Area F11 | Area F12 | Area F13 | Area F2 | Area F3 | Area F4 | Area F5 | Area F6 | Area F7 | Area F8 | Area F9 | #Feature | #Feature F1 | #Feature F10 | #Feature F11 | #Feature F12 | #Feature F13 | #Feature F2 | #Feature F3 | #Feature F4 | #Feature F5 | #Feature F6 | #Feature F7 | #Feature F8 | #Feature F9 | Start | End | PTM | AScore | Found By |
| --- | --- | --- | --- | --- | --- | --- | --- | --- | --- | --- | --- | --- | --- | --- | --- | --- | --- | --- | --- | --- | --- | --- | --- | --- | --- | --- | --- | --- | --- | --- | --- | --- | --- | --- | --- | --- | --- | --- | --- | --- | --- | --- | --- |
| K.LAIPSYSSYGC(+57.02)YC(+57.02)GWGGK.G | Y | 88.95 | 2024.8760 | 18 | 1.0 | 1013.4462 | 2 | 65.94 | 4 | F4:43325 | NaNaKA16\_F12.raw |  |  |  | 5.4816E7 |  |  |  |  |  |  |  |  |  | 2 | 0 | 0 | 0 | 2 | 0 | 0 | 0 | 0 | 0 | 0 | 0 | 0 | 0 | 16 | 33 | Carbamidomethylation | C11:Carbamidomethylation:1000.00;C13:Carbamidomethylation:1000.00 | PEAKS DB |
| K.KYMLYPDFLC(+57.02)K.G | Y | 78.94 | 1476.7145 | 11 | 0.6 | 739.3649 | 2 | 58.50 | 4 | F4:37246 | NaNaKA16\_F12.raw |  |  |  | 3.9698E7 |  |  |  |  |  |  |  |  |  | 2 | 0 | 0 | 0 | 2 | 0 | 0 | 0 | 0 | 0 | 0 | 0 | 0 | 0 | 106 | 116 | Carbamidomethylation | C10:Carbamidomethylation:1000.00 | PEAKS DB |
| R.C(+57.02)C(+57.02)FVHDC(+57.02)C(+57.02)YGNLPDC(+57.02)NPK.S | N | 74.08 | 2314.8687 | 18 | 0.4 | 772.6305 | 3 | 32.68 | 4 | F4:15898 | NaNaKA16\_F12.raw |  |  |  | 5.4885E7 |  |  |  |  |  |  |  |  |  | 2 | 0 | 0 | 0 | 2 | 0 | 0 | 0 | 0 | 0 | 0 | 0 | 0 | 0 | 43 | 60 | Carbamidomethylation | C1:Carbamidomethylation:1000.00;C2:Carbamidomethylation:1000.00;C7:Carbamidomethylation:1000.00;C8:Carbamidomethylation:1000.00;C15:Carbamidomethylation:1000.00 | PEAKS DB |
| K.YM(+15.99)LYPDFLC(+57.02)K.G | N | 68.84 | 1364.6145 | 10 | 0.1 | 683.3146 | 2 | 68.38 | 4 | F4:45614 | NaNaKA16\_F12.raw |  |  |  | 8.5839E6 |  |  |  |  |  |  |  |  |  | 4 | 0 | 0 | 0 | 4 | 0 | 0 | 0 | 0 | 0 | 0 | 0 | 0 | 0 | 107 | 116 | Oxidation (M); Carbamidomethylation | M2:Oxidation (M):1000.00;C9:Carbamidomethylation:1000.00 | PEAKS DB |
| K.YMLYPDFLC(+57.02)K.G | N | 67.92 | 1348.6195 | 10 | 0.7 | 675.3175 | 2 | 75.32 | 4 | F4:51022 | NaNaKA16\_F12.raw |  | 6.0196E5 | 6.6727E5 | 5.9248E7 |  |  |  |  |  | 4.1845E5 |  |  |  | 4 | 0 | 1 | 1 | 1 | 0 | 0 | 0 | 0 | 0 | 1 | 0 | 0 | 0 | 107 | 116 | Carbamidomethylation | C9:Carbamidomethylation:1000.00 | PEAKS DB |
| K.KYM(+15.99)LYPDFLC(+57.02)K.G | Y | 62.57 | 1492.7095 | 11 | 0.2 | 498.5772 | 3 | 52.20 | 4 | F4:32001 | NaNaKA16\_F12.raw |  |  |  | 4.3437E6 |  |  |  |  |  |  |  |  |  | 1 | 0 | 0 | 0 | 1 | 0 | 0 | 0 | 0 | 0 | 0 | 0 | 0 | 0 | 106 | 116 | Oxidation (M); Carbamidomethylation | M3:Oxidation (M):1000.00;C10:Carbamidomethylation:1000.00 | PEAKS DB |
| total 6 peptides |  |  |  |  |  |  |  |  |  |  |  |  |  |  |  |  |  |  |  |  |  |  |  |  |  |  |  |  |  |  |  |  |  |  |  |  |  |  |  |  |  |  |  |
| --- | --- | --- | --- | --- | --- | --- | --- | --- | --- | --- | --- | --- | --- | --- | --- | --- | --- | --- | --- | --- | --- | --- | --- | --- | --- | --- | --- | --- | --- | --- | --- | --- | --- | --- | --- | --- | --- | --- | --- | --- | --- | --- | --- |

4GLD

back to list

  

| Protein Coverage
| Supporting Peptides
|

Protein Coverage:

Supporting Peptides:

| Peptide | Uniq | -10lgP | Mass | Length | ppm | m/z | z | RT | Fraction | Scan | Source File | Area F1 | Area F10 | Area F11 | Area F12 | Area F13 | Area F2 | Area F3 | Area F4 | Area F5 | Area F6 | Area F7 | Area F8 | Area F9 | #Feature | #Feature F1 | #Feature F10 | #Feature F11 | #Feature F12 | #Feature F13 | #Feature F2 | #Feature F3 | #Feature F4 | #Feature F5 | #Feature F6 | #Feature F7 | #Feature F8 | #Feature F9 | Start | End | PTM | AScore | Found By |
| --- | --- | --- | --- | --- | --- | --- | --- | --- | --- | --- | --- | --- | --- | --- | --- | --- | --- | --- | --- | --- | --- | --- | --- | --- | --- | --- | --- | --- | --- | --- | --- | --- | --- | --- | --- | --- | --- | --- | --- | --- | --- | --- | --- |
| K.LAIPSYSSYGC(+57.02)YC(+57.02)GWGGK.G | Y | 88.95 | 2024.8760 | 18 | 1.0 | 1013.4462 | 2 | 65.94 | 4 | F4:43325 | NaNaKA16\_F12.raw |  |  |  | 5.4816E7 |  |  |  |  |  |  |  |  |  | 2 | 0 | 0 | 0 | 2 | 0 | 0 | 0 | 0 | 0 | 0 | 0 | 0 | 0 | 16 | 33 | Carbamidomethylation | C11:Carbamidomethylation:1000.00;C13:Carbamidomethylation:1000.00 | PEAKS DB |
| K.KYMLYPDFLC(+57.02)K.G | Y | 78.94 | 1476.7145 | 11 | 0.6 | 739.3649 | 2 | 58.50 | 4 | F4:37246 | NaNaKA16\_F12.raw |  |  |  | 3.9698E7 |  |  |  |  |  |  |  |  |  | 2 | 0 | 0 | 0 | 2 | 0 | 0 | 0 | 0 | 0 | 0 | 0 | 0 | 0 | 106 | 116 | Carbamidomethylation | C10:Carbamidomethylation:1000.00 | PEAKS DB |
| R.C(+57.02)C(+57.02)FVHDC(+57.02)C(+57.02)YGNLPDC(+57.02)NPK.S | N | 74.08 | 2314.8687 | 18 | 0.4 | 772.6305 | 3 | 32.68 | 4 | F4:15898 | NaNaKA16\_F12.raw |  |  |  | 5.4885E7 |  |  |  |  |  |  |  |  |  | 2 | 0 | 0 | 0 | 2 | 0 | 0 | 0 | 0 | 0 | 0 | 0 | 0 | 0 | 43 | 60 | Carbamidomethylation | C1:Carbamidomethylation:1000.00;C2:Carbamidomethylation:1000.00;C7:Carbamidomethylation:1000.00;C8:Carbamidomethylation:1000.00;C15:Carbamidomethylation:1000.00 | PEAKS DB |
| K.YM(+15.99)LYPDFLC(+57.02)K.G | N | 68.84 | 1364.6145 | 10 | 0.1 | 683.3146 | 2 | 68.38 | 4 | F4:45614 | NaNaKA16\_F12.raw |  |  |  | 8.5839E6 |  |  |  |  |  |  |  |  |  | 4 | 0 | 0 | 0 | 4 | 0 | 0 | 0 | 0 | 0 | 0 | 0 | 0 | 0 | 107 | 116 | Oxidation (M); Carbamidomethylation | M2:Oxidation (M):1000.00;C9:Carbamidomethylation:1000.00 | PEAKS DB |
| K.YMLYPDFLC(+57.02)K.G | N | 67.92 | 1348.6195 | 10 | 0.7 | 675.3175 | 2 | 75.32 | 4 | F4:51022 | NaNaKA16\_F12.raw |  | 6.0196E5 | 6.6727E5 | 5.9248E7 |  |  |  |  |  | 4.1845E5 |  |  |  | 4 | 0 | 1 | 1 | 1 | 0 | 0 | 0 | 0 | 0 | 1 | 0 | 0 | 0 | 107 | 116 | Carbamidomethylation | C9:Carbamidomethylation:1000.00 | PEAKS DB |
| K.KYM(+15.99)LYPDFLC(+57.02)K.G | Y | 62.57 | 1492.7095 | 11 | 0.2 | 498.5772 | 3 | 52.20 | 4 | F4:32001 | NaNaKA16\_F12.raw |  |  |  | 4.3437E6 |  |  |  |  |  |  |  |  |  | 1 | 0 | 0 | 0 | 1 | 0 | 0 | 0 | 0 | 0 | 0 | 0 | 0 | 0 | 106 | 116 | Oxidation (M); Carbamidomethylation | M3:Oxidation (M):1000.00;C10:Carbamidomethylation:1000.00 | PEAKS DB |
| total 6 peptides |  |  |  |  |  |  |  |  |  |  |  |  |  |  |  |  |  |  |  |  |  |  |  |  |  |  |  |  |  |  |  |  |  |  |  |  |  |  |  |  |  |  |  |
| --- | --- | --- | --- | --- | --- | --- | --- | --- | --- | --- | --- | --- | --- | --- | --- | --- | --- | --- | --- | --- | --- | --- | --- | --- | --- | --- | --- | --- | --- | --- | --- | --- | --- | --- | --- | --- | --- | --- | --- | --- | --- | --- | --- |

4FGA

back to list

  

| Protein Coverage
| Supporting Peptides
|

Protein Coverage:

Supporting Peptides:

| Peptide | Uniq | -10lgP | Mass | Length | ppm | m/z | z | RT | Fraction | Scan | Source File | Area F1 | Area F10 | Area F11 | Area F12 | Area F13 | Area F2 | Area F3 | Area F4 | Area F5 | Area F6 | Area F7 | Area F8 | Area F9 | #Feature | #Feature F1 | #Feature F10 | #Feature F11 | #Feature F12 | #Feature F13 | #Feature F2 | #Feature F3 | #Feature F4 | #Feature F5 | #Feature F6 | #Feature F7 | #Feature F8 | #Feature F9 | Start | End | PTM | AScore | Found By |
| --- | --- | --- | --- | --- | --- | --- | --- | --- | --- | --- | --- | --- | --- | --- | --- | --- | --- | --- | --- | --- | --- | --- | --- | --- | --- | --- | --- | --- | --- | --- | --- | --- | --- | --- | --- | --- | --- | --- | --- | --- | --- | --- | --- |
| K.LAIPSYSSYGC(+57.02)YC(+57.02)GWGGK.G | Y | 88.95 | 2024.8760 | 18 | 1.0 | 1013.4462 | 2 | 65.94 | 4 | F4:43325 | NaNaKA16\_F12.raw |  |  |  | 5.4816E7 |  |  |  |  |  |  |  |  |  | 2 | 0 | 0 | 0 | 2 | 0 | 0 | 0 | 0 | 0 | 0 | 0 | 0 | 0 | 16 | 33 | Carbamidomethylation | C11:Carbamidomethylation:1000.00;C13:Carbamidomethylation:1000.00 | PEAKS DB |
| K.KYMLYPDFLC(+57.02)K.G | Y | 78.94 | 1476.7145 | 11 | 0.6 | 739.3649 | 2 | 58.50 | 4 | F4:37246 | NaNaKA16\_F12.raw |  |  |  | 3.9698E7 |  |  |  |  |  |  |  |  |  | 2 | 0 | 0 | 0 | 2 | 0 | 0 | 0 | 0 | 0 | 0 | 0 | 0 | 0 | 106 | 116 | Carbamidomethylation | C10:Carbamidomethylation:1000.00 | PEAKS DB |
| R.C(+57.02)C(+57.02)FVHDC(+57.02)C(+57.02)YGNLPDC(+57.02)NPK.S | N | 74.08 | 2314.8687 | 18 | 0.4 | 772.6305 | 3 | 32.68 | 4 | F4:15898 | NaNaKA16\_F12.raw |  |  |  | 5.4885E7 |  |  |  |  |  |  |  |  |  | 2 | 0 | 0 | 0 | 2 | 0 | 0 | 0 | 0 | 0 | 0 | 0 | 0 | 0 | 43 | 60 | Carbamidomethylation | C1:Carbamidomethylation:1000.00;C2:Carbamidomethylation:1000.00;C7:Carbamidomethylation:1000.00;C8:Carbamidomethylation:1000.00;C15:Carbamidomethylation:1000.00 | PEAKS DB |
| K.YM(+15.99)LYPDFLC(+57.02)K.G | N | 68.84 | 1364.6145 | 10 | 0.1 | 683.3146 | 2 | 68.38 | 4 | F4:45614 | NaNaKA16\_F12.raw |  |  |  | 8.5839E6 |  |  |  |  |  |  |  |  |  | 4 | 0 | 0 | 0 | 4 | 0 | 0 | 0 | 0 | 0 | 0 | 0 | 0 | 0 | 107 | 116 | Oxidation (M); Carbamidomethylation | M2:Oxidation (M):1000.00;C9:Carbamidomethylation:1000.00 | PEAKS DB |
| K.YMLYPDFLC(+57.02)K.G | N | 67.92 | 1348.6195 | 10 | 0.7 | 675.3175 | 2 | 75.32 | 4 | F4:51022 | NaNaKA16\_F12.raw |  | 6.0196E5 | 6.6727E5 | 5.9248E7 |  |  |  |  |  | 4.1845E5 |  |  |  | 4 | 0 | 1 | 1 | 1 | 0 | 0 | 0 | 0 | 0 | 1 | 0 | 0 | 0 | 107 | 116 | Carbamidomethylation | C9:Carbamidomethylation:1000.00 | PEAKS DB |
| K.KYM(+15.99)LYPDFLC(+57.02)K.G | Y | 62.57 | 1492.7095 | 11 | 0.2 | 498.5772 | 3 | 52.20 | 4 | F4:32001 | NaNaKA16\_F12.raw |  |  |  | 4.3437E6 |  |  |  |  |  |  |  |  |  | 1 | 0 | 0 | 0 | 1 | 0 | 0 | 0 | 0 | 0 | 0 | 0 | 0 | 0 | 106 | 116 | Oxidation (M); Carbamidomethylation | M3:Oxidation (M):1000.00;C10:Carbamidomethylation:1000.00 | PEAKS DB |
| total 6 peptides |  |  |  |  |  |  |  |  |  |  |  |  |  |  |  |  |  |  |  |  |  |  |  |  |  |  |  |  |  |  |  |  |  |  |  |  |  |  |  |  |  |  |  |
| --- | --- | --- | --- | --- | --- | --- | --- | --- | --- | --- | --- | --- | --- | --- | --- | --- | --- | --- | --- | --- | --- | --- | --- | --- | --- | --- | --- | --- | --- | --- | --- | --- | --- | --- | --- | --- | --- | --- | --- | --- | --- | --- | --- |

1ZR8

back to list

  

| Protein Coverage
| Supporting Peptides
|

Protein Coverage:

Supporting Peptides:

| Peptide | Uniq | -10lgP | Mass | Length | ppm | m/z | z | RT | Fraction | Scan | Source File | Area F1 | Area F10 | Area F11 | Area F12 | Area F13 | Area F2 | Area F3 | Area F4 | Area F5 | Area F6 | Area F7 | Area F8 | Area F9 | #Feature | #Feature F1 | #Feature F10 | #Feature F11 | #Feature F12 | #Feature F13 | #Feature F2 | #Feature F3 | #Feature F4 | #Feature F5 | #Feature F6 | #Feature F7 | #Feature F8 | #Feature F9 | Start | End | PTM | AScore | Found By |
| --- | --- | --- | --- | --- | --- | --- | --- | --- | --- | --- | --- | --- | --- | --- | --- | --- | --- | --- | --- | --- | --- | --- | --- | --- | --- | --- | --- | --- | --- | --- | --- | --- | --- | --- | --- | --- | --- | --- | --- | --- | --- | --- | --- |
| K.LAIPSYSSYGC(+57.02)YC(+57.02)GWGGK.G | Y | 88.95 | 2024.8760 | 18 | 1.0 | 1013.4462 | 2 | 65.94 | 4 | F4:43325 | NaNaKA16\_F12.raw |  |  |  | 5.4816E7 |  |  |  |  |  |  |  |  |  | 2 | 0 | 0 | 0 | 2 | 0 | 0 | 0 | 0 | 0 | 0 | 0 | 0 | 0 | 16 | 33 | Carbamidomethylation | C11:Carbamidomethylation:1000.00;C13:Carbamidomethylation:1000.00 | PEAKS DB |
| K.KYMLYPDFLC(+57.02)K.G | Y | 78.94 | 1476.7145 | 11 | 0.6 | 739.3649 | 2 | 58.50 | 4 | F4:37246 | NaNaKA16\_F12.raw |  |  |  | 3.9698E7 |  |  |  |  |  |  |  |  |  | 2 | 0 | 0 | 0 | 2 | 0 | 0 | 0 | 0 | 0 | 0 | 0 | 0 | 0 | 106 | 116 | Carbamidomethylation | C10:Carbamidomethylation:1000.00 | PEAKS DB |
| R.C(+57.02)C(+57.02)FVHDC(+57.02)C(+57.02)YGNLPDC(+57.02)NPK.S | N | 74.08 | 2314.8687 | 18 | 0.4 | 772.6305 | 3 | 32.68 | 4 | F4:15898 | NaNaKA16\_F12.raw |  |  |  | 5.4885E7 |  |  |  |  |  |  |  |  |  | 2 | 0 | 0 | 0 | 2 | 0 | 0 | 0 | 0 | 0 | 0 | 0 | 0 | 0 | 43 | 60 | Carbamidomethylation | C1:Carbamidomethylation:1000.00;C2:Carbamidomethylation:1000.00;C7:Carbamidomethylation:1000.00;C8:Carbamidomethylation:1000.00;C15:Carbamidomethylation:1000.00 | PEAKS DB |
| K.YM(+15.99)LYPDFLC(+57.02)K.G | N | 68.84 | 1364.6145 | 10 | 0.1 | 683.3146 | 2 | 68.38 | 4 | F4:45614 | NaNaKA16\_F12.raw |  |  |  | 8.5839E6 |  |  |  |  |  |  |  |  |  | 4 | 0 | 0 | 0 | 4 | 0 | 0 | 0 | 0 | 0 | 0 | 0 | 0 | 0 | 107 | 116 | Oxidation (M); Carbamidomethylation | M2:Oxidation (M):1000.00;C9:Carbamidomethylation:1000.00 | PEAKS DB |
| K.YMLYPDFLC(+57.02)K.G | N | 67.92 | 1348.6195 | 10 | 0.7 | 675.3175 | 2 | 75.32 | 4 | F4:51022 | NaNaKA16\_F12.raw |  | 6.0196E5 | 6.6727E5 | 5.9248E7 |  |  |  |  |  | 4.1845E5 |  |  |  | 4 | 0 | 1 | 1 | 1 | 0 | 0 | 0 | 0 | 0 | 1 | 0 | 0 | 0 | 107 | 116 | Carbamidomethylation | C9:Carbamidomethylation:1000.00 | PEAKS DB |
| K.KYM(+15.99)LYPDFLC(+57.02)K.G | Y | 62.57 | 1492.7095 | 11 | 0.2 | 498.5772 | 3 | 52.20 | 4 | F4:32001 | NaNaKA16\_F12.raw |  |  |  | 4.3437E6 |  |  |  |  |  |  |  |  |  | 1 | 0 | 0 | 0 | 1 | 0 | 0 | 0 | 0 | 0 | 0 | 0 | 0 | 0 | 106 | 116 | Oxidation (M); Carbamidomethylation | M3:Oxidation (M):1000.00;C10:Carbamidomethylation:1000.00 | PEAKS DB |
| total 6 peptides |  |  |  |  |  |  |  |  |  |  |  |  |  |  |  |  |  |  |  |  |  |  |  |  |  |  |  |  |  |  |  |  |  |  |  |  |  |  |  |  |  |  |  |
| --- | --- | --- | --- | --- | --- | --- | --- | --- | --- | --- | --- | --- | --- | --- | --- | --- | --- | --- | --- | --- | --- | --- | --- | --- | --- | --- | --- | --- | --- | --- | --- | --- | --- | --- | --- | --- | --- | --- | --- | --- | --- | --- | --- |

1TP2

back to list

  

| Protein Coverage
| Supporting Peptides
|

Protein Coverage:

Supporting Peptides:

| Peptide | Uniq | -10lgP | Mass | Length | ppm | m/z | z | RT | Fraction | Scan | Source File | Area F1 | Area F10 | Area F11 | Area F12 | Area F13 | Area F2 | Area F3 | Area F4 | Area F5 | Area F6 | Area F7 | Area F8 | Area F9 | #Feature | #Feature F1 | #Feature F10 | #Feature F11 | #Feature F12 | #Feature F13 | #Feature F2 | #Feature F3 | #Feature F4 | #Feature F5 | #Feature F6 | #Feature F7 | #Feature F8 | #Feature F9 | Start | End | PTM | AScore | Found By |
| --- | --- | --- | --- | --- | --- | --- | --- | --- | --- | --- | --- | --- | --- | --- | --- | --- | --- | --- | --- | --- | --- | --- | --- | --- | --- | --- | --- | --- | --- | --- | --- | --- | --- | --- | --- | --- | --- | --- | --- | --- | --- | --- | --- |
| K.LAIPSYSSYGC(+57.02)YC(+57.02)GWGGK.G | Y | 88.95 | 2024.8760 | 18 | 1.0 | 1013.4462 | 2 | 65.94 | 4 | F4:43325 | NaNaKA16\_F12.raw |  |  |  | 5.4816E7 |  |  |  |  |  |  |  |  |  | 2 | 0 | 0 | 0 | 2 | 0 | 0 | 0 | 0 | 0 | 0 | 0 | 0 | 0 | 16 | 33 | Carbamidomethylation | C11:Carbamidomethylation:1000.00;C13:Carbamidomethylation:1000.00 | PEAKS DB |
| K.KYMLYPDFLC(+57.02)K.G | Y | 78.94 | 1476.7145 | 11 | 0.6 | 739.3649 | 2 | 58.50 | 4 | F4:37246 | NaNaKA16\_F12.raw |  |  |  | 3.9698E7 |  |  |  |  |  |  |  |  |  | 2 | 0 | 0 | 0 | 2 | 0 | 0 | 0 | 0 | 0 | 0 | 0 | 0 | 0 | 106 | 116 | Carbamidomethylation | C10:Carbamidomethylation:1000.00 | PEAKS DB |
| R.C(+57.02)C(+57.02)FVHDC(+57.02)C(+57.02)YGNLPDC(+57.02)NPK.S | N | 74.08 | 2314.8687 | 18 | 0.4 | 772.6305 | 3 | 32.68 | 4 | F4:15898 | NaNaKA16\_F12.raw |  |  |  | 5.4885E7 |  |  |  |  |  |  |  |  |  | 2 | 0 | 0 | 0 | 2 | 0 | 0 | 0 | 0 | 0 | 0 | 0 | 0 | 0 | 43 | 60 | Carbamidomethylation | C1:Carbamidomethylation:1000.00;C2:Carbamidomethylation:1000.00;C7:Carbamidomethylation:1000.00;C8:Carbamidomethylation:1000.00;C15:Carbamidomethylation:1000.00 | PEAKS DB |
| K.YM(+15.99)LYPDFLC(+57.02)K.G | N | 68.84 | 1364.6145 | 10 | 0.1 | 683.3146 | 2 | 68.38 | 4 | F4:45614 | NaNaKA16\_F12.raw |  |  |  | 8.5839E6 |  |  |  |  |  |  |  |  |  | 4 | 0 | 0 | 0 | 4 | 0 | 0 | 0 | 0 | 0 | 0 | 0 | 0 | 0 | 107 | 116 | Oxidation (M); Carbamidomethylation | M2:Oxidation (M):1000.00;C9:Carbamidomethylation:1000.00 | PEAKS DB |
| K.YMLYPDFLC(+57.02)K.G | N | 67.92 | 1348.6195 | 10 | 0.7 | 675.3175 | 2 | 75.32 | 4 | F4:51022 | NaNaKA16\_F12.raw |  | 6.0196E5 | 6.6727E5 | 5.9248E7 |  |  |  |  |  | 4.1845E5 |  |  |  | 4 | 0 | 1 | 1 | 1 | 0 | 0 | 0 | 0 | 0 | 1 | 0 | 0 | 0 | 107 | 116 | Carbamidomethylation | C9:Carbamidomethylation:1000.00 | PEAKS DB |
| K.KYM(+15.99)LYPDFLC(+57.02)K.G | Y | 62.57 | 1492.7095 | 11 | 0.2 | 498.5772 | 3 | 52.20 | 4 | F4:32001 | NaNaKA16\_F12.raw |  |  |  | 4.3437E6 |  |  |  |  |  |  |  |  |  | 1 | 0 | 0 | 0 | 1 | 0 | 0 | 0 | 0 | 0 | 0 | 0 | 0 | 0 | 106 | 116 | Oxidation (M); Carbamidomethylation | M3:Oxidation (M):1000.00;C10:Carbamidomethylation:1000.00 | PEAKS DB |
| total 6 peptides |  |  |  |  |  |  |  |  |  |  |  |  |  |  |  |  |  |  |  |  |  |  |  |  |  |  |  |  |  |  |  |  |  |  |  |  |  |  |  |  |  |  |  |
| --- | --- | --- | --- | --- | --- | --- | --- | --- | --- | --- | --- | --- | --- | --- | --- | --- | --- | --- | --- | --- | --- | --- | --- | --- | --- | --- | --- | --- | --- | --- | --- | --- | --- | --- | --- | --- | --- | --- | --- | --- | --- | --- | --- |

1Q7A

back to list

  

| Protein Coverage
| Supporting Peptides
|

Protein Coverage:

Supporting Peptides:

| Peptide | Uniq | -10lgP | Mass | Length | ppm | m/z | z | RT | Fraction | Scan | Source File | Area F1 | Area F10 | Area F11 | Area F12 | Area F13 | Area F2 | Area F3 | Area F4 | Area F5 | Area F6 | Area F7 | Area F8 | Area F9 | #Feature | #Feature F1 | #Feature F10 | #Feature F11 | #Feature F12 | #Feature F13 | #Feature F2 | #Feature F3 | #Feature F4 | #Feature F5 | #Feature F6 | #Feature F7 | #Feature F8 | #Feature F9 | Start | End | PTM | AScore | Found By |
| --- | --- | --- | --- | --- | --- | --- | --- | --- | --- | --- | --- | --- | --- | --- | --- | --- | --- | --- | --- | --- | --- | --- | --- | --- | --- | --- | --- | --- | --- | --- | --- | --- | --- | --- | --- | --- | --- | --- | --- | --- | --- | --- | --- |
| K.LAIPSYSSYGC(+57.02)YC(+57.02)GWGGK.G | Y | 88.95 | 2024.8760 | 18 | 1.0 | 1013.4462 | 2 | 65.94 | 4 | F4:43325 | NaNaKA16\_F12.raw |  |  |  | 5.4816E7 |  |  |  |  |  |  |  |  |  | 2 | 0 | 0 | 0 | 2 | 0 | 0 | 0 | 0 | 0 | 0 | 0 | 0 | 0 | 16 | 33 | Carbamidomethylation | C11:Carbamidomethylation:1000.00;C13:Carbamidomethylation:1000.00 | PEAKS DB |
| K.KYMLYPDFLC(+57.02)K.G | Y | 78.94 | 1476.7145 | 11 | 0.6 | 739.3649 | 2 | 58.50 | 4 | F4:37246 | NaNaKA16\_F12.raw |  |  |  | 3.9698E7 |  |  |  |  |  |  |  |  |  | 2 | 0 | 0 | 0 | 2 | 0 | 0 | 0 | 0 | 0 | 0 | 0 | 0 | 0 | 106 | 116 | Carbamidomethylation | C10:Carbamidomethylation:1000.00 | PEAKS DB |
| R.C(+57.02)C(+57.02)FVHDC(+57.02)C(+57.02)YGNLPDC(+57.02)NPK.S | N | 74.08 | 2314.8687 | 18 | 0.4 | 772.6305 | 3 | 32.68 | 4 | F4:15898 | NaNaKA16\_F12.raw |  |  |  | 5.4885E7 |  |  |  |  |  |  |  |  |  | 2 | 0 | 0 | 0 | 2 | 0 | 0 | 0 | 0 | 0 | 0 | 0 | 0 | 0 | 43 | 60 | Carbamidomethylation | C1:Carbamidomethylation:1000.00;C2:Carbamidomethylation:1000.00;C7:Carbamidomethylation:1000.00;C8:Carbamidomethylation:1000.00;C15:Carbamidomethylation:1000.00 | PEAKS DB |
| K.YM(+15.99)LYPDFLC(+57.02)K.G | N | 68.84 | 1364.6145 | 10 | 0.1 | 683.3146 | 2 | 68.38 | 4 | F4:45614 | NaNaKA16\_F12.raw |  |  |  | 8.5839E6 |  |  |  |  |  |  |  |  |  | 4 | 0 | 0 | 0 | 4 | 0 | 0 | 0 | 0 | 0 | 0 | 0 | 0 | 0 | 107 | 116 | Oxidation (M); Carbamidomethylation | M2:Oxidation (M):1000.00;C9:Carbamidomethylation:1000.00 | PEAKS DB |
| K.YMLYPDFLC(+57.02)K.G | N | 67.92 | 1348.6195 | 10 | 0.7 | 675.3175 | 2 | 75.32 | 4 | F4:51022 | NaNaKA16\_F12.raw |  | 6.0196E5 | 6.6727E5 | 5.9248E7 |  |  |  |  |  | 4.1845E5 |  |  |  | 4 | 0 | 1 | 1 | 1 | 0 | 0 | 0 | 0 | 0 | 1 | 0 | 0 | 0 | 107 | 116 | Carbamidomethylation | C9:Carbamidomethylation:1000.00 | PEAKS DB |
| K.KYM(+15.99)LYPDFLC(+57.02)K.G | Y | 62.57 | 1492.7095 | 11 | 0.2 | 498.5772 | 3 | 52.20 | 4 | F4:32001 | NaNaKA16\_F12.raw |  |  |  | 4.3437E6 |  |  |  |  |  |  |  |  |  | 1 | 0 | 0 | 0 | 1 | 0 | 0 | 0 | 0 | 0 | 0 | 0 | 0 | 0 | 106 | 116 | Oxidation (M); Carbamidomethylation | M3:Oxidation (M):1000.00;C10:Carbamidomethylation:1000.00 | PEAKS DB |
| total 6 peptides |  |  |  |  |  |  |  |  |  |  |  |  |  |  |  |  |  |  |  |  |  |  |  |  |  |  |  |  |  |  |  |  |  |  |  |  |  |  |  |  |  |  |  |
| --- | --- | --- | --- | --- | --- | --- | --- | --- | --- | --- | --- | --- | --- | --- | --- | --- | --- | --- | --- | --- | --- | --- | --- | --- | --- | --- | --- | --- | --- | --- | --- | --- | --- | --- | --- | --- | --- | --- | --- | --- | --- | --- | --- |

1TH6

back to list

  

| Protein Coverage
| Supporting Peptides
|

Protein Coverage:

Supporting Peptides:

| Peptide | Uniq | -10lgP | Mass | Length | ppm | m/z | z | RT | Fraction | Scan | Source File | Area F1 | Area F10 | Area F11 | Area F12 | Area F13 | Area F2 | Area F3 | Area F4 | Area F5 | Area F6 | Area F7 | Area F8 | Area F9 | #Feature | #Feature F1 | #Feature F10 | #Feature F11 | #Feature F12 | #Feature F13 | #Feature F2 | #Feature F3 | #Feature F4 | #Feature F5 | #Feature F6 | #Feature F7 | #Feature F8 | #Feature F9 | Start | End | PTM | AScore | Found By |
| --- | --- | --- | --- | --- | --- | --- | --- | --- | --- | --- | --- | --- | --- | --- | --- | --- | --- | --- | --- | --- | --- | --- | --- | --- | --- | --- | --- | --- | --- | --- | --- | --- | --- | --- | --- | --- | --- | --- | --- | --- | --- | --- | --- |
| K.LAIPSYSSYGC(+57.02)YC(+57.02)GWGGK.G | Y | 88.95 | 2024.8760 | 18 | 1.0 | 1013.4462 | 2 | 65.94 | 4 | F4:43325 | NaNaKA16\_F12.raw |  |  |  | 5.4816E7 |  |  |  |  |  |  |  |  |  | 2 | 0 | 0 | 0 | 2 | 0 | 0 | 0 | 0 | 0 | 0 | 0 | 0 | 0 | 16 | 33 | Carbamidomethylation | C11:Carbamidomethylation:1000.00;C13:Carbamidomethylation:1000.00 | PEAKS DB |
| K.KYMLYPDFLC(+57.02)K.G | Y | 78.94 | 1476.7145 | 11 | 0.6 | 739.3649 | 2 | 58.50 | 4 | F4:37246 | NaNaKA16\_F12.raw |  |  |  | 3.9698E7 |  |  |  |  |  |  |  |  |  | 2 | 0 | 0 | 0 | 2 | 0 | 0 | 0 | 0 | 0 | 0 | 0 | 0 | 0 | 106 | 116 | Carbamidomethylation | C10:Carbamidomethylation:1000.00 | PEAKS DB |
| R.C(+57.02)C(+57.02)FVHDC(+57.02)C(+57.02)YGNLPDC(+57.02)NPK.S | N | 74.08 | 2314.8687 | 18 | 0.4 | 772.6305 | 3 | 32.68 | 4 | F4:15898 | NaNaKA16\_F12.raw |  |  |  | 5.4885E7 |  |  |  |  |  |  |  |  |  | 2 | 0 | 0 | 0 | 2 | 0 | 0 | 0 | 0 | 0 | 0 | 0 | 0 | 0 | 43 | 60 | Carbamidomethylation | C1:Carbamidomethylation:1000.00;C2:Carbamidomethylation:1000.00;C7:Carbamidomethylation:1000.00;C8:Carbamidomethylation:1000.00;C15:Carbamidomethylation:1000.00 | PEAKS DB |
| K.YM(+15.99)LYPDFLC(+57.02)K.G | N | 68.84 | 1364.6145 | 10 | 0.1 | 683.3146 | 2 | 68.38 | 4 | F4:45614 | NaNaKA16\_F12.raw |  |  |  | 8.5839E6 |  |  |  |  |  |  |  |  |  | 4 | 0 | 0 | 0 | 4 | 0 | 0 | 0 | 0 | 0 | 0 | 0 | 0 | 0 | 107 | 116 | Oxidation (M); Carbamidomethylation | M2:Oxidation (M):1000.00;C9:Carbamidomethylation:1000.00 | PEAKS DB |
| K.YMLYPDFLC(+57.02)K.G | N | 67.92 | 1348.6195 | 10 | 0.7 | 675.3175 | 2 | 75.32 | 4 | F4:51022 | NaNaKA16\_F12.raw |  | 6.0196E5 | 6.6727E5 | 5.9248E7 |  |  |  |  |  | 4.1845E5 |  |  |  | 4 | 0 | 1 | 1 | 1 | 0 | 0 | 0 | 0 | 0 | 1 | 0 | 0 | 0 | 107 | 116 | Carbamidomethylation | C9:Carbamidomethylation:1000.00 | PEAKS DB |
| K.KYM(+15.99)LYPDFLC(+57.02)K.G | Y | 62.57 | 1492.7095 | 11 | 0.2 | 498.5772 | 3 | 52.20 | 4 | F4:32001 | NaNaKA16\_F12.raw |  |  |  | 4.3437E6 |  |  |  |  |  |  |  |  |  | 1 | 0 | 0 | 0 | 1 | 0 | 0 | 0 | 0 | 0 | 0 | 0 | 0 | 0 | 106 | 116 | Oxidation (M); Carbamidomethylation | M3:Oxidation (M):1000.00;C10:Carbamidomethylation:1000.00 | PEAKS DB |
| total 6 peptides |  |  |  |  |  |  |  |  |  |  |  |  |  |  |  |  |  |  |  |  |  |  |  |  |  |  |  |  |  |  |  |  |  |  |  |  |  |  |  |  |  |  |  |
| --- | --- | --- | --- | --- | --- | --- | --- | --- | --- | --- | --- | --- | --- | --- | --- | --- | --- | --- | --- | --- | --- | --- | --- | --- | --- | --- | --- | --- | --- | --- | --- | --- | --- | --- | --- | --- | --- | --- | --- | --- | --- | --- | --- |

4HMB

back to list

  

| Protein Coverage
| Supporting Peptides
|

Protein Coverage:

Supporting Peptides:

| Peptide | Uniq | -10lgP | Mass | Length | ppm | m/z | z | RT | Fraction | Scan | Source File | Area F1 | Area F10 | Area F11 | Area F12 | Area F13 | Area F2 | Area F3 | Area F4 | Area F5 | Area F6 | Area F7 | Area F8 | Area F9 | #Feature | #Feature F1 | #Feature F10 | #Feature F11 | #Feature F12 | #Feature F13 | #Feature F2 | #Feature F3 | #Feature F4 | #Feature F5 | #Feature F6 | #Feature F7 | #Feature F8 | #Feature F9 | Start | End | PTM | AScore | Found By |
| --- | --- | --- | --- | --- | --- | --- | --- | --- | --- | --- | --- | --- | --- | --- | --- | --- | --- | --- | --- | --- | --- | --- | --- | --- | --- | --- | --- | --- | --- | --- | --- | --- | --- | --- | --- | --- | --- | --- | --- | --- | --- | --- | --- |
| K.LAIPSYSSYGC(+57.02)YC(+57.02)GWGGK.G | Y | 88.95 | 2024.8760 | 18 | 1.0 | 1013.4462 | 2 | 65.94 | 4 | F4:43325 | NaNaKA16\_F12.raw |  |  |  | 5.4816E7 |  |  |  |  |  |  |  |  |  | 2 | 0 | 0 | 0 | 2 | 0 | 0 | 0 | 0 | 0 | 0 | 0 | 0 | 0 | 16 | 33 | Carbamidomethylation | C11:Carbamidomethylation:1000.00;C13:Carbamidomethylation:1000.00 | PEAKS DB |
| K.KYMLYPDFLC(+57.02)K.G | Y | 78.94 | 1476.7145 | 11 | 0.6 | 739.3649 | 2 | 58.50 | 4 | F4:37246 | NaNaKA16\_F12.raw |  |  |  | 3.9698E7 |  |  |  |  |  |  |  |  |  | 2 | 0 | 0 | 0 | 2 | 0 | 0 | 0 | 0 | 0 | 0 | 0 | 0 | 0 | 106 | 116 | Carbamidomethylation | C10:Carbamidomethylation:1000.00 | PEAKS DB |
| R.C(+57.02)C(+57.02)FVHDC(+57.02)C(+57.02)YGNLPDC(+57.02)NPK.S | N | 74.08 | 2314.8687 | 18 | 0.4 | 772.6305 | 3 | 32.68 | 4 | F4:15898 | NaNaKA16\_F12.raw |  |  |  | 5.4885E7 |  |  |  |  |  |  |  |  |  | 2 | 0 | 0 | 0 | 2 | 0 | 0 | 0 | 0 | 0 | 0 | 0 | 0 | 0 | 43 | 60 | Carbamidomethylation | C1:Carbamidomethylation:1000.00;C2:Carbamidomethylation:1000.00;C7:Carbamidomethylation:1000.00;C8:Carbamidomethylation:1000.00;C15:Carbamidomethylation:1000.00 | PEAKS DB |
| K.YM(+15.99)LYPDFLC(+57.02)K.G | N | 68.84 | 1364.6145 | 10 | 0.1 | 683.3146 | 2 | 68.38 | 4 | F4:45614 | NaNaKA16\_F12.raw |  |  |  | 8.5839E6 |  |  |  |  |  |  |  |  |  | 4 | 0 | 0 | 0 | 4 | 0 | 0 | 0 | 0 | 0 | 0 | 0 | 0 | 0 | 107 | 116 | Oxidation (M); Carbamidomethylation | M2:Oxidation (M):1000.00;C9:Carbamidomethylation:1000.00 | PEAKS DB |
| K.YMLYPDFLC(+57.02)K.G | N | 67.92 | 1348.6195 | 10 | 0.7 | 675.3175 | 2 | 75.32 | 4 | F4:51022 | NaNaKA16\_F12.raw |  | 6.0196E5 | 6.6727E5 | 5.9248E7 |  |  |  |  |  | 4.1845E5 |  |  |  | 4 | 0 | 1 | 1 | 1 | 0 | 0 | 0 | 0 | 0 | 1 | 0 | 0 | 0 | 107 | 116 | Carbamidomethylation | C9:Carbamidomethylation:1000.00 | PEAKS DB |
| K.KYM(+15.99)LYPDFLC(+57.02)K.G | Y | 62.57 | 1492.7095 | 11 | 0.2 | 498.5772 | 3 | 52.20 | 4 | F4:32001 | NaNaKA16\_F12.raw |  |  |  | 4.3437E6 |  |  |  |  |  |  |  |  |  | 1 | 0 | 0 | 0 | 1 | 0 | 0 | 0 | 0 | 0 | 0 | 0 | 0 | 0 | 106 | 116 | Oxidation (M); Carbamidomethylation | M3:Oxidation (M):1000.00;C10:Carbamidomethylation:1000.00 | PEAKS DB |
| total 6 peptides |  |  |  |  |  |  |  |  |  |  |  |  |  |  |  |  |  |  |  |  |  |  |  |  |  |  |  |  |  |  |  |  |  |  |  |  |  |  |  |  |  |  |  |
| --- | --- | --- | --- | --- | --- | --- | --- | --- | --- | --- | --- | --- | --- | --- | --- | --- | --- | --- | --- | --- | --- | --- | --- | --- | --- | --- | --- | --- | --- | --- | --- | --- | --- | --- | --- | --- | --- | --- | --- | --- | --- | --- | --- |

3CBI

back to list

  

| Protein Coverage
| Supporting Peptides
|

Protein Coverage:

Supporting Peptides:

| Peptide | Uniq | -10lgP | Mass | Length | ppm | m/z | z | RT | Fraction | Scan | Source File | Area F1 | Area F10 | Area F11 | Area F12 | Area F13 | Area F2 | Area F3 | Area F4 | Area F5 | Area F6 | Area F7 | Area F8 | Area F9 | #Feature | #Feature F1 | #Feature F10 | #Feature F11 | #Feature F12 | #Feature F13 | #Feature F2 | #Feature F3 | #Feature F4 | #Feature F5 | #Feature F6 | #Feature F7 | #Feature F8 | #Feature F9 | Start | End | PTM | AScore | Found By |
| --- | --- | --- | --- | --- | --- | --- | --- | --- | --- | --- | --- | --- | --- | --- | --- | --- | --- | --- | --- | --- | --- | --- | --- | --- | --- | --- | --- | --- | --- | --- | --- | --- | --- | --- | --- | --- | --- | --- | --- | --- | --- | --- | --- |
| K.LAIPSYSSYGC(+57.02)YC(+57.02)GWGGK.G | Y | 88.95 | 2024.8760 | 18 | 1.0 | 1013.4462 | 2 | 65.94 | 4 | F4:43325 | NaNaKA16\_F12.raw |  |  |  | 5.4816E7 |  |  |  |  |  |  |  |  |  | 2 | 0 | 0 | 0 | 2 | 0 | 0 | 0 | 0 | 0 | 0 | 0 | 0 | 0 | 16 | 33 | Carbamidomethylation | C11:Carbamidomethylation:1000.00;C13:Carbamidomethylation:1000.00 | PEAKS DB |
| K.KYMLYPDFLC(+57.02)K.G | Y | 78.94 | 1476.7145 | 11 | 0.6 | 739.3649 | 2 | 58.50 | 4 | F4:37246 | NaNaKA16\_F12.raw |  |  |  | 3.9698E7 |  |  |  |  |  |  |  |  |  | 2 | 0 | 0 | 0 | 2 | 0 | 0 | 0 | 0 | 0 | 0 | 0 | 0 | 0 | 106 | 116 | Carbamidomethylation | C10:Carbamidomethylation:1000.00 | PEAKS DB |
| R.C(+57.02)C(+57.02)FVHDC(+57.02)C(+57.02)YGNLPDC(+57.02)NPK.S | N | 74.08 | 2314.8687 | 18 | 0.4 | 772.6305 | 3 | 32.68 | 4 | F4:15898 | NaNaKA16\_F12.raw |  |  |  | 5.4885E7 |  |  |  |  |  |  |  |  |  | 2 | 0 | 0 | 0 | 2 | 0 | 0 | 0 | 0 | 0 | 0 | 0 | 0 | 0 | 43 | 60 | Carbamidomethylation | C1:Carbamidomethylation:1000.00;C2:Carbamidomethylation:1000.00;C7:Carbamidomethylation:1000.00;C8:Carbamidomethylation:1000.00;C15:Carbamidomethylation:1000.00 | PEAKS DB |
| K.YM(+15.99)LYPDFLC(+57.02)K.G | N | 68.84 | 1364.6145 | 10 | 0.1 | 683.3146 | 2 | 68.38 | 4 | F4:45614 | NaNaKA16\_F12.raw |  |  |  | 8.5839E6 |  |  |  |  |  |  |  |  |  | 4 | 0 | 0 | 0 | 4 | 0 | 0 | 0 | 0 | 0 | 0 | 0 | 0 | 0 | 107 | 116 | Oxidation (M); Carbamidomethylation | M2:Oxidation (M):1000.00;C9:Carbamidomethylation:1000.00 | PEAKS DB |
| K.YMLYPDFLC(+57.02)K.G | N | 67.92 | 1348.6195 | 10 | 0.7 | 675.3175 | 2 | 75.32 | 4 | F4:51022 | NaNaKA16\_F12.raw |  | 6.0196E5 | 6.6727E5 | 5.9248E7 |  |  |  |  |  | 4.1845E5 |  |  |  | 4 | 0 | 1 | 1 | 1 | 0 | 0 | 0 | 0 | 0 | 1 | 0 | 0 | 0 | 107 | 116 | Carbamidomethylation | C9:Carbamidomethylation:1000.00 | PEAKS DB |
| K.KYM(+15.99)LYPDFLC(+57.02)K.G | Y | 62.57 | 1492.7095 | 11 | 0.2 | 498.5772 | 3 | 52.20 | 4 | F4:32001 | NaNaKA16\_F12.raw |  |  |  | 4.3437E6 |  |  |  |  |  |  |  |  |  | 1 | 0 | 0 | 0 | 1 | 0 | 0 | 0 | 0 | 0 | 0 | 0 | 0 | 0 | 106 | 116 | Oxidation (M); Carbamidomethylation | M3:Oxidation (M):1000.00;C10:Carbamidomethylation:1000.00 | PEAKS DB |
| total 6 peptides |  |  |  |  |  |  |  |  |  |  |  |  |  |  |  |  |  |  |  |  |  |  |  |  |  |  |  |  |  |  |  |  |  |  |  |  |  |  |  |  |  |  |  |
| --- | --- | --- | --- | --- | --- | --- | --- | --- | --- | --- | --- | --- | --- | --- | --- | --- | --- | --- | --- | --- | --- | --- | --- | --- | --- | --- | --- | --- | --- | --- | --- | --- | --- | --- | --- | --- | --- | --- | --- | --- | --- | --- | --- |

P59071.1

back to list

  

| Protein Coverage
| Supporting Peptides
|

Protein Coverage:

Supporting Peptides:

| Peptide | Uniq | -10lgP | Mass | Length | ppm | m/z | z | RT | Fraction | Scan | Source File | Area F1 | Area F10 | Area F11 | Area F12 | Area F13 | Area F2 | Area F3 | Area F4 | Area F5 | Area F6 | Area F7 | Area F8 | Area F9 | #Feature | #Feature F1 | #Feature F10 | #Feature F11 | #Feature F12 | #Feature F13 | #Feature F2 | #Feature F3 | #Feature F4 | #Feature F5 | #Feature F6 | #Feature F7 | #Feature F8 | #Feature F9 | Start | End | PTM | AScore | Found By |
| --- | --- | --- | --- | --- | --- | --- | --- | --- | --- | --- | --- | --- | --- | --- | --- | --- | --- | --- | --- | --- | --- | --- | --- | --- | --- | --- | --- | --- | --- | --- | --- | --- | --- | --- | --- | --- | --- | --- | --- | --- | --- | --- | --- |
| K.LAIPSYSSYGC(+57.02)YC(+57.02)GWGGK.G | Y | 88.95 | 2024.8760 | 18 | 1.0 | 1013.4462 | 2 | 65.94 | 4 | F4:43325 | NaNaKA16\_F12.raw |  |  |  | 5.4816E7 |  |  |  |  |  |  |  |  |  | 2 | 0 | 0 | 0 | 2 | 0 | 0 | 0 | 0 | 0 | 0 | 0 | 0 | 0 | 16 | 33 | Carbamidomethylation | C11:Carbamidomethylation:1000.00;C13:Carbamidomethylation:1000.00 | PEAKS DB |
| K.KYMLYPDFLC(+57.02)K.G | Y | 78.94 | 1476.7145 | 11 | 0.6 | 739.3649 | 2 | 58.50 | 4 | F4:37246 | NaNaKA16\_F12.raw |  |  |  | 3.9698E7 |  |  |  |  |  |  |  |  |  | 2 | 0 | 0 | 0 | 2 | 0 | 0 | 0 | 0 | 0 | 0 | 0 | 0 | 0 | 106 | 116 | Carbamidomethylation | C10:Carbamidomethylation:1000.00 | PEAKS DB |
| R.C(+57.02)C(+57.02)FVHDC(+57.02)C(+57.02)YGNLPDC(+57.02)NPK.S | N | 74.08 | 2314.8687 | 18 | 0.4 | 772.6305 | 3 | 32.68 | 4 | F4:15898 | NaNaKA16\_F12.raw |  |  |  | 5.4885E7 |  |  |  |  |  |  |  |  |  | 2 | 0 | 0 | 0 | 2 | 0 | 0 | 0 | 0 | 0 | 0 | 0 | 0 | 0 | 43 | 60 | Carbamidomethylation | C1:Carbamidomethylation:1000.00;C2:Carbamidomethylation:1000.00;C7:Carbamidomethylation:1000.00;C8:Carbamidomethylation:1000.00;C15:Carbamidomethylation:1000.00 | PEAKS DB |
| K.YM(+15.99)LYPDFLC(+57.02)K.G | N | 68.84 | 1364.6145 | 10 | 0.1 | 683.3146 | 2 | 68.38 | 4 | F4:45614 | NaNaKA16\_F12.raw |  |  |  | 8.5839E6 |  |  |  |  |  |  |  |  |  | 4 | 0 | 0 | 0 | 4 | 0 | 0 | 0 | 0 | 0 | 0 | 0 | 0 | 0 | 107 | 116 | Oxidation (M); Carbamidomethylation | M2:Oxidation (M):1000.00;C9:Carbamidomethylation:1000.00 | PEAKS DB |
| K.YMLYPDFLC(+57.02)K.G | N | 67.92 | 1348.6195 | 10 | 0.7 | 675.3175 | 2 | 75.32 | 4 | F4:51022 | NaNaKA16\_F12.raw |  | 6.0196E5 | 6.6727E5 | 5.9248E7 |  |  |  |  |  | 4.1845E5 |  |  |  | 4 | 0 | 1 | 1 | 1 | 0 | 0 | 0 | 0 | 0 | 1 | 0 | 0 | 0 | 107 | 116 | Carbamidomethylation | C9:Carbamidomethylation:1000.00 | PEAKS DB |
| K.KYM(+15.99)LYPDFLC(+57.02)K.G | Y | 62.57 | 1492.7095 | 11 | 0.2 | 498.5772 | 3 | 52.20 | 4 | F4:32001 | NaNaKA16\_F12.raw |  |  |  | 4.3437E6 |  |  |  |  |  |  |  |  |  | 1 | 0 | 0 | 0 | 1 | 0 | 0 | 0 | 0 | 0 | 0 | 0 | 0 | 0 | 106 | 116 | Oxidation (M); Carbamidomethylation | M3:Oxidation (M):1000.00;C10:Carbamidomethylation:1000.00 | PEAKS DB |
| total 6 peptides |  |  |  |  |  |  |  |  |  |  |  |  |  |  |  |  |  |  |  |  |  |  |  |  |  |  |  |  |  |  |  |  |  |  |  |  |  |  |  |  |  |  |  |
| --- | --- | --- | --- | --- | --- | --- | --- | --- | --- | --- | --- | --- | --- | --- | --- | --- | --- | --- | --- | --- | --- | --- | --- | --- | --- | --- | --- | --- | --- | --- | --- | --- | --- | --- | --- | --- | --- | --- | --- | --- | --- | --- | --- |

2QUE

back to list

  

| Protein Coverage
| Supporting Peptides
|

Protein Coverage:

Supporting Peptides:

| Peptide | Uniq | -10lgP | Mass | Length | ppm | m/z | z | RT | Fraction | Scan | Source File | Area F1 | Area F10 | Area F11 | Area F12 | Area F13 | Area F2 | Area F3 | Area F4 | Area F5 | Area F6 | Area F7 | Area F8 | Area F9 | #Feature | #Feature F1 | #Feature F10 | #Feature F11 | #Feature F12 | #Feature F13 | #Feature F2 | #Feature F3 | #Feature F4 | #Feature F5 | #Feature F6 | #Feature F7 | #Feature F8 | #Feature F9 | Start | End | PTM | AScore | Found By |
| --- | --- | --- | --- | --- | --- | --- | --- | --- | --- | --- | --- | --- | --- | --- | --- | --- | --- | --- | --- | --- | --- | --- | --- | --- | --- | --- | --- | --- | --- | --- | --- | --- | --- | --- | --- | --- | --- | --- | --- | --- | --- | --- | --- |
| K.LAIPSYSSYGC(+57.02)YC(+57.02)GWGGK.G | Y | 88.95 | 2024.8760 | 18 | 1.0 | 1013.4462 | 2 | 65.94 | 4 | F4:43325 | NaNaKA16\_F12.raw |  |  |  | 5.4816E7 |  |  |  |  |  |  |  |  |  | 2 | 0 | 0 | 0 | 2 | 0 | 0 | 0 | 0 | 0 | 0 | 0 | 0 | 0 | 16 | 33 | Carbamidomethylation | C11:Carbamidomethylation:1000.00;C13:Carbamidomethylation:1000.00 | PEAKS DB |
| K.KYMLYPDFLC(+57.02)K.G | Y | 78.94 | 1476.7145 | 11 | 0.6 | 739.3649 | 2 | 58.50 | 4 | F4:37246 | NaNaKA16\_F12.raw |  |  |  | 3.9698E7 |  |  |  |  |  |  |  |  |  | 2 | 0 | 0 | 0 | 2 | 0 | 0 | 0 | 0 | 0 | 0 | 0 | 0 | 0 | 106 | 116 | Carbamidomethylation | C10:Carbamidomethylation:1000.00 | PEAKS DB |
| R.C(+57.02)C(+57.02)FVHDC(+57.02)C(+57.02)YGNLPDC(+57.02)NPK.S | N | 74.08 | 2314.8687 | 18 | 0.4 | 772.6305 | 3 | 32.68 | 4 | F4:15898 | NaNaKA16\_F12.raw |  |  |  | 5.4885E7 |  |  |  |  |  |  |  |  |  | 2 | 0 | 0 | 0 | 2 | 0 | 0 | 0 | 0 | 0 | 0 | 0 | 0 | 0 | 43 | 60 | Carbamidomethylation | C1:Carbamidomethylation:1000.00;C2:Carbamidomethylation:1000.00;C7:Carbamidomethylation:1000.00;C8:Carbamidomethylation:1000.00;C15:Carbamidomethylation:1000.00 | PEAKS DB |
| K.YM(+15.99)LYPDFLC(+57.02)K.G | N | 68.84 | 1364.6145 | 10 | 0.1 | 683.3146 | 2 | 68.38 | 4 | F4:45614 | NaNaKA16\_F12.raw |  |  |  | 8.5839E6 |  |  |  |  |  |  |  |  |  | 4 | 0 | 0 | 0 | 4 | 0 | 0 | 0 | 0 | 0 | 0 | 0 | 0 | 0 | 107 | 116 | Oxidation (M); Carbamidomethylation | M2:Oxidation (M):1000.00;C9:Carbamidomethylation:1000.00 | PEAKS DB |
| K.YMLYPDFLC(+57.02)K.G | N | 67.92 | 1348.6195 | 10 | 0.7 | 675.3175 | 2 | 75.32 | 4 | F4:51022 | NaNaKA16\_F12.raw |  | 6.0196E5 | 6.6727E5 | 5.9248E7 |  |  |  |  |  | 4.1845E5 |  |  |  | 4 | 0 | 1 | 1 | 1 | 0 | 0 | 0 | 0 | 0 | 1 | 0 | 0 | 0 | 107 | 116 | Carbamidomethylation | C9:Carbamidomethylation:1000.00 | PEAKS DB |
| K.KYM(+15.99)LYPDFLC(+57.02)K.G | Y | 62.57 | 1492.7095 | 11 | 0.2 | 498.5772 | 3 | 52.20 | 4 | F4:32001 | NaNaKA16\_F12.raw |  |  |  | 4.3437E6 |  |  |  |  |  |  |  |  |  | 1 | 0 | 0 | 0 | 1 | 0 | 0 | 0 | 0 | 0 | 0 | 0 | 0 | 0 | 106 | 116 | Oxidation (M); Carbamidomethylation | M3:Oxidation (M):1000.00;C10:Carbamidomethylation:1000.00 | PEAKS DB |
| total 6 peptides |  |  |  |  |  |  |  |  |  |  |  |  |  |  |  |  |  |  |  |  |  |  |  |  |  |  |  |  |  |  |  |  |  |  |  |  |  |  |  |  |  |  |  |
| --- | --- | --- | --- | --- | --- | --- | --- | --- | --- | --- | --- | --- | --- | --- | --- | --- | --- | --- | --- | --- | --- | --- | --- | --- | --- | --- | --- | --- | --- | --- | --- | --- | --- | --- | --- | --- | --- | --- | --- | --- | --- | --- | --- |

3FO7

back to list

  

| Protein Coverage
| Supporting Peptides
|

Protein Coverage:

Supporting Peptides:

| Peptide | Uniq | -10lgP | Mass | Length | ppm | m/z | z | RT | Fraction | Scan | Source File | Area F1 | Area F10 | Area F11 | Area F12 | Area F13 | Area F2 | Area F3 | Area F4 | Area F5 | Area F6 | Area F7 | Area F8 | Area F9 | #Feature | #Feature F1 | #Feature F10 | #Feature F11 | #Feature F12 | #Feature F13 | #Feature F2 | #Feature F3 | #Feature F4 | #Feature F5 | #Feature F6 | #Feature F7 | #Feature F8 | #Feature F9 | Start | End | PTM | AScore | Found By |
| --- | --- | --- | --- | --- | --- | --- | --- | --- | --- | --- | --- | --- | --- | --- | --- | --- | --- | --- | --- | --- | --- | --- | --- | --- | --- | --- | --- | --- | --- | --- | --- | --- | --- | --- | --- | --- | --- | --- | --- | --- | --- | --- | --- |
| K.LAIPSYSSYGC(+57.02)YC(+57.02)GWGGK.G | Y | 88.95 | 2024.8760 | 18 | 1.0 | 1013.4462 | 2 | 65.94 | 4 | F4:43325 | NaNaKA16\_F12.raw |  |  |  | 5.4816E7 |  |  |  |  |  |  |  |  |  | 2 | 0 | 0 | 0 | 2 | 0 | 0 | 0 | 0 | 0 | 0 | 0 | 0 | 0 | 16 | 33 | Carbamidomethylation | C11:Carbamidomethylation:1000.00;C13:Carbamidomethylation:1000.00 | PEAKS DB |
| K.KYMLYPDFLC(+57.02)K.G | Y | 78.94 | 1476.7145 | 11 | 0.6 | 739.3649 | 2 | 58.50 | 4 | F4:37246 | NaNaKA16\_F12.raw |  |  |  | 3.9698E7 |  |  |  |  |  |  |  |  |  | 2 | 0 | 0 | 0 | 2 | 0 | 0 | 0 | 0 | 0 | 0 | 0 | 0 | 0 | 106 | 116 | Carbamidomethylation | C10:Carbamidomethylation:1000.00 | PEAKS DB |
| R.C(+57.02)C(+57.02)FVHDC(+57.02)C(+57.02)YGNLPDC(+57.02)NPK.S | N | 74.08 | 2314.8687 | 18 | 0.4 | 772.6305 | 3 | 32.68 | 4 | F4:15898 | NaNaKA16\_F12.raw |  |  |  | 5.4885E7 |  |  |  |  |  |  |  |  |  | 2 | 0 | 0 | 0 | 2 | 0 | 0 | 0 | 0 | 0 | 0 | 0 | 0 | 0 | 43 | 60 | Carbamidomethylation | C1:Carbamidomethylation:1000.00;C2:Carbamidomethylation:1000.00;C7:Carbamidomethylation:1000.00;C8:Carbamidomethylation:1000.00;C15:Carbamidomethylation:1000.00 | PEAKS DB |
| K.YM(+15.99)LYPDFLC(+57.02)K.G | N | 68.84 | 1364.6145 | 10 | 0.1 | 683.3146 | 2 | 68.38 | 4 | F4:45614 | NaNaKA16\_F12.raw |  |  |  | 8.5839E6 |  |  |  |  |  |  |  |  |  | 4 | 0 | 0 | 0 | 4 | 0 | 0 | 0 | 0 | 0 | 0 | 0 | 0 | 0 | 107 | 116 | Oxidation (M); Carbamidomethylation | M2:Oxidation (M):1000.00;C9:Carbamidomethylation:1000.00 | PEAKS DB |
| K.YMLYPDFLC(+57.02)K.G | N | 67.92 | 1348.6195 | 10 | 0.7 | 675.3175 | 2 | 75.32 | 4 | F4:51022 | NaNaKA16\_F12.raw |  | 6.0196E5 | 6.6727E5 | 5.9248E7 |  |  |  |  |  | 4.1845E5 |  |  |  | 4 | 0 | 1 | 1 | 1 | 0 | 0 | 0 | 0 | 0 | 1 | 0 | 0 | 0 | 107 | 116 | Carbamidomethylation | C9:Carbamidomethylation:1000.00 | PEAKS DB |
| K.KYM(+15.99)LYPDFLC(+57.02)K.G | Y | 62.57 | 1492.7095 | 11 | 0.2 | 498.5772 | 3 | 52.20 | 4 | F4:32001 | NaNaKA16\_F12.raw |  |  |  | 4.3437E6 |  |  |  |  |  |  |  |  |  | 1 | 0 | 0 | 0 | 1 | 0 | 0 | 0 | 0 | 0 | 0 | 0 | 0 | 0 | 106 | 116 | Oxidation (M); Carbamidomethylation | M3:Oxidation (M):1000.00;C10:Carbamidomethylation:1000.00 | PEAKS DB |
| total 6 peptides |  |  |  |  |  |  |  |  |  |  |  |  |  |  |  |  |  |  |  |  |  |  |  |  |  |  |  |  |  |  |  |  |  |  |  |  |  |  |  |  |  |  |  |
| --- | --- | --- | --- | --- | --- | --- | --- | --- | --- | --- | --- | --- | --- | --- | --- | --- | --- | --- | --- | --- | --- | --- | --- | --- | --- | --- | --- | --- | --- | --- | --- | --- | --- | --- | --- | --- | --- | --- | --- | --- | --- | --- | --- |

AAZ53186.1

back to list

  

| Protein Coverage
| Supporting Peptides
|

Protein Coverage:

Supporting Peptides:

| Peptide | Uniq | -10lgP | Mass | Length | ppm | m/z | z | RT | Fraction | Scan | Source File | Area F1 | Area F10 | Area F11 | Area F12 | Area F13 | Area F2 | Area F3 | Area F4 | Area F5 | Area F6 | Area F7 | Area F8 | Area F9 | #Feature | #Feature F1 | #Feature F10 | #Feature F11 | #Feature F12 | #Feature F13 | #Feature F2 | #Feature F3 | #Feature F4 | #Feature F5 | #Feature F6 | #Feature F7 | #Feature F8 | #Feature F9 | Start | End | PTM | AScore | Found By |
| --- | --- | --- | --- | --- | --- | --- | --- | --- | --- | --- | --- | --- | --- | --- | --- | --- | --- | --- | --- | --- | --- | --- | --- | --- | --- | --- | --- | --- | --- | --- | --- | --- | --- | --- | --- | --- | --- | --- | --- | --- | --- | --- | --- |
| K.LAIPSYSSYGC(+57.02)YC(+57.02)GWGGK.G | Y | 88.95 | 2024.8760 | 18 | 1.0 | 1013.4462 | 2 | 65.94 | 4 | F4:43325 | NaNaKA16\_F12.raw |  |  |  | 5.4816E7 |  |  |  |  |  |  |  |  |  | 2 | 0 | 0 | 0 | 2 | 0 | 0 | 0 | 0 | 0 | 0 | 0 | 0 | 0 | 32 | 49 | Carbamidomethylation | C11:Carbamidomethylation:1000.00;C13:Carbamidomethylation:1000.00 | PEAKS DB |
| K.KYMLYPDFLC(+57.02)K.G | Y | 78.94 | 1476.7145 | 11 | 0.6 | 739.3649 | 2 | 58.50 | 4 | F4:37246 | NaNaKA16\_F12.raw |  |  |  | 3.9698E7 |  |  |  |  |  |  |  |  |  | 2 | 0 | 0 | 0 | 2 | 0 | 0 | 0 | 0 | 0 | 0 | 0 | 0 | 0 | 122 | 132 | Carbamidomethylation | C10:Carbamidomethylation:1000.00 | PEAKS DB |
| R.C(+57.02)C(+57.02)FVHDC(+57.02)C(+57.02)YGNLPDC(+57.02)NPK.S | N | 74.08 | 2314.8687 | 18 | 0.4 | 772.6305 | 3 | 32.68 | 4 | F4:15898 | NaNaKA16\_F12.raw |  |  |  | 5.4885E7 |  |  |  |  |  |  |  |  |  | 2 | 0 | 0 | 0 | 2 | 0 | 0 | 0 | 0 | 0 | 0 | 0 | 0 | 0 | 59 | 76 | Carbamidomethylation | C1:Carbamidomethylation:1000.00;C2:Carbamidomethylation:1000.00;C7:Carbamidomethylation:1000.00;C8:Carbamidomethylation:1000.00;C15:Carbamidomethylation:1000.00 | PEAKS DB |
| K.YM(+15.99)LYPDFLC(+57.02)K.G | N | 68.84 | 1364.6145 | 10 | 0.1 | 683.3146 | 2 | 68.38 | 4 | F4:45614 | NaNaKA16\_F12.raw |  |  |  | 8.5839E6 |  |  |  |  |  |  |  |  |  | 4 | 0 | 0 | 0 | 4 | 0 | 0 | 0 | 0 | 0 | 0 | 0 | 0 | 0 | 123 | 132 | Oxidation (M); Carbamidomethylation | M2:Oxidation (M):1000.00;C9:Carbamidomethylation:1000.00 | PEAKS DB |
| K.YMLYPDFLC(+57.02)K.G | N | 67.92 | 1348.6195 | 10 | 0.7 | 675.3175 | 2 | 75.32 | 4 | F4:51022 | NaNaKA16\_F12.raw |  | 6.0196E5 | 6.6727E5 | 5.9248E7 |  |  |  |  |  | 4.1845E5 |  |  |  | 4 | 0 | 1 | 1 | 1 | 0 | 0 | 0 | 0 | 0 | 1 | 0 | 0 | 0 | 123 | 132 | Carbamidomethylation | C9:Carbamidomethylation:1000.00 | PEAKS DB |
| K.KYM(+15.99)LYPDFLC(+57.02)K.G | Y | 62.57 | 1492.7095 | 11 | 0.2 | 498.5772 | 3 | 52.20 | 4 | F4:32001 | NaNaKA16\_F12.raw |  |  |  | 4.3437E6 |  |  |  |  |  |  |  |  |  | 1 | 0 | 0 | 0 | 1 | 0 | 0 | 0 | 0 | 0 | 0 | 0 | 0 | 0 | 122 | 132 | Oxidation (M); Carbamidomethylation | M3:Oxidation (M):1000.00;C10:Carbamidomethylation:1000.00 | PEAKS DB |
| total 6 peptides |  |  |  |  |  |  |  |  |  |  |  |  |  |  |  |  |  |  |  |  |  |  |  |  |  |  |  |  |  |  |  |  |  |  |  |  |  |  |  |  |  |  |  |
| --- | --- | --- | --- | --- | --- | --- | --- | --- | --- | --- | --- | --- | --- | --- | --- | --- | --- | --- | --- | --- | --- | --- | --- | --- | --- | --- | --- | --- | --- | --- | --- | --- | --- | --- | --- | --- | --- | --- | --- | --- | --- | --- | --- |

AAZ53179.1

back to list

  

| Protein Coverage
| Supporting Peptides
|

Protein Coverage:

Supporting Peptides:

| Peptide | Uniq | -10lgP | Mass | Length | ppm | m/z | z | RT | Fraction | Scan | Source File | Area F1 | Area F10 | Area F11 | Area F12 | Area F13 | Area F2 | Area F3 | Area F4 | Area F5 | Area F6 | Area F7 | Area F8 | Area F9 | #Feature | #Feature F1 | #Feature F10 | #Feature F11 | #Feature F12 | #Feature F13 | #Feature F2 | #Feature F3 | #Feature F4 | #Feature F5 | #Feature F6 | #Feature F7 | #Feature F8 | #Feature F9 | Start | End | PTM | AScore | Found By |
| --- | --- | --- | --- | --- | --- | --- | --- | --- | --- | --- | --- | --- | --- | --- | --- | --- | --- | --- | --- | --- | --- | --- | --- | --- | --- | --- | --- | --- | --- | --- | --- | --- | --- | --- | --- | --- | --- | --- | --- | --- | --- | --- | --- |
| K.LAIPSYSSYGC(+57.02)YC(+57.02)GWGGK.G | Y | 88.95 | 2024.8760 | 18 | 1.0 | 1013.4462 | 2 | 65.94 | 4 | F4:43325 | NaNaKA16\_F12.raw |  |  |  | 5.4816E7 |  |  |  |  |  |  |  |  |  | 2 | 0 | 0 | 0 | 2 | 0 | 0 | 0 | 0 | 0 | 0 | 0 | 0 | 0 | 32 | 49 | Carbamidomethylation | C11:Carbamidomethylation:1000.00;C13:Carbamidomethylation:1000.00 | PEAKS DB |
| K.KYMLYPDFLC(+57.02)K.G | Y | 78.94 | 1476.7145 | 11 | 0.6 | 739.3649 | 2 | 58.50 | 4 | F4:37246 | NaNaKA16\_F12.raw |  |  |  | 3.9698E7 |  |  |  |  |  |  |  |  |  | 2 | 0 | 0 | 0 | 2 | 0 | 0 | 0 | 0 | 0 | 0 | 0 | 0 | 0 | 122 | 132 | Carbamidomethylation | C10:Carbamidomethylation:1000.00 | PEAKS DB |
| R.C(+57.02)C(+57.02)FVHDC(+57.02)C(+57.02)YGNLPDC(+57.02)NPK.S | N | 74.08 | 2314.8687 | 18 | 0.4 | 772.6305 | 3 | 32.68 | 4 | F4:15898 | NaNaKA16\_F12.raw |  |  |  | 5.4885E7 |  |  |  |  |  |  |  |  |  | 2 | 0 | 0 | 0 | 2 | 0 | 0 | 0 | 0 | 0 | 0 | 0 | 0 | 0 | 59 | 76 | Carbamidomethylation | C1:Carbamidomethylation:1000.00;C2:Carbamidomethylation:1000.00;C7:Carbamidomethylation:1000.00;C8:Carbamidomethylation:1000.00;C15:Carbamidomethylation:1000.00 | PEAKS DB |
| K.YM(+15.99)LYPDFLC(+57.02)K.G | N | 68.84 | 1364.6145 | 10 | 0.1 | 683.3146 | 2 | 68.38 | 4 | F4:45614 | NaNaKA16\_F12.raw |  |  |  | 8.5839E6 |  |  |  |  |  |  |  |  |  | 4 | 0 | 0 | 0 | 4 | 0 | 0 | 0 | 0 | 0 | 0 | 0 | 0 | 0 | 123 | 132 | Oxidation (M); Carbamidomethylation | M2:Oxidation (M):1000.00;C9:Carbamidomethylation:1000.00 | PEAKS DB |
| K.YMLYPDFLC(+57.02)K.G | N | 67.92 | 1348.6195 | 10 | 0.7 | 675.3175 | 2 | 75.32 | 4 | F4:51022 | NaNaKA16\_F12.raw |  | 6.0196E5 | 6.6727E5 | 5.9248E7 |  |  |  |  |  | 4.1845E5 |  |  |  | 4 | 0 | 1 | 1 | 1 | 0 | 0 | 0 | 0 | 0 | 1 | 0 | 0 | 0 | 123 | 132 | Carbamidomethylation | C9:Carbamidomethylation:1000.00 | PEAKS DB |
| K.KYM(+15.99)LYPDFLC(+57.02)K.G | Y | 62.57 | 1492.7095 | 11 | 0.2 | 498.5772 | 3 | 52.20 | 4 | F4:32001 | NaNaKA16\_F12.raw |  |  |  | 4.3437E6 |  |  |  |  |  |  |  |  |  | 1 | 0 | 0 | 0 | 1 | 0 | 0 | 0 | 0 | 0 | 0 | 0 | 0 | 0 | 122 | 132 | Oxidation (M); Carbamidomethylation | M3:Oxidation (M):1000.00;C10:Carbamidomethylation:1000.00 | PEAKS DB |
| total 6 peptides |  |  |  |  |  |  |  |  |  |  |  |  |  |  |  |  |  |  |  |  |  |  |  |  |  |  |  |  |  |  |  |  |  |  |  |  |  |  |  |  |  |  |  |
| --- | --- | --- | --- | --- | --- | --- | --- | --- | --- | --- | --- | --- | --- | --- | --- | --- | --- | --- | --- | --- | --- | --- | --- | --- | --- | --- | --- | --- | --- | --- | --- | --- | --- | --- | --- | --- | --- | --- | --- | --- | --- | --- | --- |

5GZ4

back to list

  

| Protein Coverage
| Supporting Peptides
|

Protein Coverage:

Supporting Peptides:

| Peptide | Uniq | -10lgP | Mass | Length | ppm | m/z | z | RT | Fraction | Scan | Source File | Area F1 | Area F10 | Area F11 | Area F12 | Area F13 | Area F2 | Area F3 | Area F4 | Area F5 | Area F6 | Area F7 | Area F8 | Area F9 | #Feature | #Feature F1 | #Feature F10 | #Feature F11 | #Feature F12 | #Feature F13 | #Feature F2 | #Feature F3 | #Feature F4 | #Feature F5 | #Feature F6 | #Feature F7 | #Feature F8 | #Feature F9 | Start | End | PTM | AScore | Found By |
| --- | --- | --- | --- | --- | --- | --- | --- | --- | --- | --- | --- | --- | --- | --- | --- | --- | --- | --- | --- | --- | --- | --- | --- | --- | --- | --- | --- | --- | --- | --- | --- | --- | --- | --- | --- | --- | --- | --- | --- | --- | --- | --- | --- |
| R.MANVLC(+57.02)SC(+57.02)SEDC(+57.02)LTK.K | N | 76.27 | 1786.7358 | 15 | 5.5 | 894.3754 | 2 | 37.96 | 8 | F8:19800 | NaNaKA16\_F4.raw |  |  |  |  |  |  | 2.2299E6 | 1.4838E7 | 4.3252E6 |  |  |  |  | 3 | 0 | 0 | 0 | 0 | 0 | 0 | 1 | 1 | 1 | 0 | 0 | 0 | 0 | 65 | 79 | Carbamidomethylation | C6:Carbamidomethylation:1000.00;C8:Carbamidomethylation:1000.00;C12:Carbamidomethylation:1000.00 | PEAKS DB |
| R.MANVLC(+57.02)SC(+57.02)SEDC(+57.02)LTKK.D | N | 64.42 | 1914.8308 | 16 | 5.8 | 639.2845 | 3 | 25.10 | 8 | F8:9795 | NaNaKA16\_F4.raw |  |  |  |  |  |  |  | 4.6506E6 |  |  |  |  |  | 1 | 0 | 0 | 0 | 0 | 0 | 0 | 0 | 1 | 0 | 0 | 0 | 0 | 0 | 65 | 80 | Carbamidomethylation | C6:Carbamidomethylation:1000.00;C8:Carbamidomethylation:1000.00;C12:Carbamidomethylation:1000.00 | PEAKS DB |
| K.NPFYNPSPAK.E | Y | 62.89 | 1133.5505 | 10 | 0.5 | 567.7828 | 2 | 24.80 | 5 | F5:7428 | NaNaKA16\_F13.raw |  |  |  |  | 1.5571E6 |  |  |  |  |  |  |  |  | 1 | 0 | 0 | 0 | 0 | 1 | 0 | 0 | 0 | 0 | 0 | 0 | 0 | 0 | 501 | 510 |  |  | PEAKS DB |
| K.FGPVSGQVIK.S | Y | 62.30 | 1030.5811 | 10 | -0.3 | 516.2977 | 2 | 31.02 | 5 | F5:10994 | NaNaKA16\_F13.raw |  |  |  |  | 1.4827E6 |  |  |  |  |  |  |  |  | 1 | 0 | 0 | 0 | 0 | 1 | 0 | 0 | 0 | 0 | 0 | 0 | 0 | 0 | 288 | 297 |  |  | PEAKS DB |
| K.YC(+57.02)SGGTHGYDNEFK.S | N | 55.74 | 1633.6467 | 14 | 0.9 | 545.5567 | 3 | 11.99 | 5 | F5:1916 | NaNaKA16\_F13.raw |  |  |  |  | 4.2751E5 |  |  |  |  |  |  |  |  | 1 | 0 | 0 | 0 | 0 | 1 | 0 | 0 | 0 | 0 | 0 | 0 | 0 | 0 | 433 | 446 | Carbamidomethylation | C2:Carbamidomethylation:1000.00 | PEAKS DB |
| K.DFYTFDSEAIVK.K | N | 52.96 | 1433.6714 | 12 | 0.4 | 717.8433 | 2 | 71.19 | 5 | F5:28900 | NaNaKA16\_F13.raw |  |  |  |  | 5.5404E5 |  |  |  |  |  |  |  |  | 1 | 0 | 0 | 0 | 0 | 1 | 0 | 0 | 0 | 0 | 0 | 0 | 0 | 0 | 370 | 381 |  |  | PEAKS DB |
| total 6 peptides |  |  |  |  |  |  |  |  |  |  |  |  |  |  |  |  |  |  |  |  |  |  |  |  |  |  |  |  |  |  |  |  |  |  |  |  |  |  |  |  |  |  |  |
| --- | --- | --- | --- | --- | --- | --- | --- | --- | --- | --- | --- | --- | --- | --- | --- | --- | --- | --- | --- | --- | --- | --- | --- | --- | --- | --- | --- | --- | --- | --- | --- | --- | --- | --- | --- | --- | --- | --- | --- | --- | --- | --- | --- |

A0A2D0TC04.1

back to list

  

| Protein Coverage
| Supporting Peptides
|

Protein Coverage:

Supporting Peptides:

| Peptide | Uniq | -10lgP | Mass | Length | ppm | m/z | z | RT | Fraction | Scan | Source File | Area F1 | Area F10 | Area F11 | Area F12 | Area F13 | Area F2 | Area F3 | Area F4 | Area F5 | Area F6 | Area F7 | Area F8 | Area F9 | #Feature | #Feature F1 | #Feature F10 | #Feature F11 | #Feature F12 | #Feature F13 | #Feature F2 | #Feature F3 | #Feature F4 | #Feature F5 | #Feature F6 | #Feature F7 | #Feature F8 | #Feature F9 | Start | End | PTM | AScore | Found By |
| --- | --- | --- | --- | --- | --- | --- | --- | --- | --- | --- | --- | --- | --- | --- | --- | --- | --- | --- | --- | --- | --- | --- | --- | --- | --- | --- | --- | --- | --- | --- | --- | --- | --- | --- | --- | --- | --- | --- | --- | --- | --- | --- | --- |
| R.MANVLC(+57.02)SC(+57.02)SEDC(+57.02)LTK.K | N | 76.27 | 1786.7358 | 15 | 5.5 | 894.3754 | 2 | 37.96 | 8 | F8:19800 | NaNaKA16\_F4.raw |  |  |  |  |  |  | 2.2299E6 | 1.4838E7 | 4.3252E6 |  |  |  |  | 3 | 0 | 0 | 0 | 0 | 0 | 0 | 1 | 1 | 1 | 0 | 0 | 0 | 0 | 65 | 79 | Carbamidomethylation | C6:Carbamidomethylation:1000.00;C8:Carbamidomethylation:1000.00;C12:Carbamidomethylation:1000.00 | PEAKS DB |
| R.MANVLC(+57.02)SC(+57.02)SEDC(+57.02)LTKK.D | N | 64.42 | 1914.8308 | 16 | 5.8 | 639.2845 | 3 | 25.10 | 8 | F8:9795 | NaNaKA16\_F4.raw |  |  |  |  |  |  |  | 4.6506E6 |  |  |  |  |  | 1 | 0 | 0 | 0 | 0 | 0 | 0 | 0 | 1 | 0 | 0 | 0 | 0 | 0 | 65 | 80 | Carbamidomethylation | C6:Carbamidomethylation:1000.00;C8:Carbamidomethylation:1000.00;C12:Carbamidomethylation:1000.00 | PEAKS DB |
| K.NPFYNPSPAK.E | Y | 62.89 | 1133.5505 | 10 | 0.5 | 567.7828 | 2 | 24.80 | 5 | F5:7428 | NaNaKA16\_F13.raw |  |  |  |  | 1.5571E6 |  |  |  |  |  |  |  |  | 1 | 0 | 0 | 0 | 0 | 1 | 0 | 0 | 0 | 0 | 0 | 0 | 0 | 0 | 501 | 510 |  |  | PEAKS DB |
| K.FGPVSGQVIK.S | Y | 62.30 | 1030.5811 | 10 | -0.3 | 516.2977 | 2 | 31.02 | 5 | F5:10994 | NaNaKA16\_F13.raw |  |  |  |  | 1.4827E6 |  |  |  |  |  |  |  |  | 1 | 0 | 0 | 0 | 0 | 1 | 0 | 0 | 0 | 0 | 0 | 0 | 0 | 0 | 288 | 297 |  |  | PEAKS DB |
| K.YC(+57.02)SGGTHGYDNEFK.S | N | 55.74 | 1633.6467 | 14 | 0.9 | 545.5567 | 3 | 11.99 | 5 | F5:1916 | NaNaKA16\_F13.raw |  |  |  |  | 4.2751E5 |  |  |  |  |  |  |  |  | 1 | 0 | 0 | 0 | 0 | 1 | 0 | 0 | 0 | 0 | 0 | 0 | 0 | 0 | 433 | 446 | Carbamidomethylation | C2:Carbamidomethylation:1000.00 | PEAKS DB |
| K.DFYTFDSEAIVK.K | N | 52.96 | 1433.6714 | 12 | 0.4 | 717.8433 | 2 | 71.19 | 5 | F5:28900 | NaNaKA16\_F13.raw |  |  |  |  | 5.5404E5 |  |  |  |  |  |  |  |  | 1 | 0 | 0 | 0 | 0 | 1 | 0 | 0 | 0 | 0 | 0 | 0 | 0 | 0 | 370 | 381 |  |  | PEAKS DB |
| total 6 peptides |  |  |  |  |  |  |  |  |  |  |  |  |  |  |  |  |  |  |  |  |  |  |  |  |  |  |  |  |  |  |  |  |  |  |  |  |  |  |  |  |  |  |  |
| --- | --- | --- | --- | --- | --- | --- | --- | --- | --- | --- | --- | --- | --- | --- | --- | --- | --- | --- | --- | --- | --- | --- | --- | --- | --- | --- | --- | --- | --- | --- | --- | --- | --- | --- | --- | --- | --- | --- | --- | --- | --- | --- | --- |

5GZ5

back to list

  

| Protein Coverage
| Supporting Peptides
|

Protein Coverage:

Supporting Peptides:

| Peptide | Uniq | -10lgP | Mass | Length | ppm | m/z | z | RT | Fraction | Scan | Source File | Area F1 | Area F10 | Area F11 | Area F12 | Area F13 | Area F2 | Area F3 | Area F4 | Area F5 | Area F6 | Area F7 | Area F8 | Area F9 | #Feature | #Feature F1 | #Feature F10 | #Feature F11 | #Feature F12 | #Feature F13 | #Feature F2 | #Feature F3 | #Feature F4 | #Feature F5 | #Feature F6 | #Feature F7 | #Feature F8 | #Feature F9 | Start | End | PTM | AScore | Found By |
| --- | --- | --- | --- | --- | --- | --- | --- | --- | --- | --- | --- | --- | --- | --- | --- | --- | --- | --- | --- | --- | --- | --- | --- | --- | --- | --- | --- | --- | --- | --- | --- | --- | --- | --- | --- | --- | --- | --- | --- | --- | --- | --- | --- |
| R.MANVLC(+57.02)SC(+57.02)SEDC(+57.02)LTK.K | N | 76.27 | 1786.7358 | 15 | 5.5 | 894.3754 | 2 | 37.96 | 8 | F8:19800 | NaNaKA16\_F4.raw |  |  |  |  |  |  | 2.2299E6 | 1.4838E7 | 4.3252E6 |  |  |  |  | 3 | 0 | 0 | 0 | 0 | 0 | 0 | 1 | 1 | 1 | 0 | 0 | 0 | 0 | 65 | 79 | Carbamidomethylation | C6:Carbamidomethylation:1000.00;C8:Carbamidomethylation:1000.00;C12:Carbamidomethylation:1000.00 | PEAKS DB |
| R.MANVLC(+57.02)SC(+57.02)SEDC(+57.02)LTKK.D | N | 64.42 | 1914.8308 | 16 | 5.8 | 639.2845 | 3 | 25.10 | 8 | F8:9795 | NaNaKA16\_F4.raw |  |  |  |  |  |  |  | 4.6506E6 |  |  |  |  |  | 1 | 0 | 0 | 0 | 0 | 0 | 0 | 0 | 1 | 0 | 0 | 0 | 0 | 0 | 65 | 80 | Carbamidomethylation | C6:Carbamidomethylation:1000.00;C8:Carbamidomethylation:1000.00;C12:Carbamidomethylation:1000.00 | PEAKS DB |
| K.NPFYNPSPAK.E | Y | 62.89 | 1133.5505 | 10 | 0.5 | 567.7828 | 2 | 24.80 | 5 | F5:7428 | NaNaKA16\_F13.raw |  |  |  |  | 1.5571E6 |  |  |  |  |  |  |  |  | 1 | 0 | 0 | 0 | 0 | 1 | 0 | 0 | 0 | 0 | 0 | 0 | 0 | 0 | 501 | 510 |  |  | PEAKS DB |
| K.FGPVSGQVIK.S | Y | 62.30 | 1030.5811 | 10 | -0.3 | 516.2977 | 2 | 31.02 | 5 | F5:10994 | NaNaKA16\_F13.raw |  |  |  |  | 1.4827E6 |  |  |  |  |  |  |  |  | 1 | 0 | 0 | 0 | 0 | 1 | 0 | 0 | 0 | 0 | 0 | 0 | 0 | 0 | 288 | 297 |  |  | PEAKS DB |
| K.YC(+57.02)SGGTHGYDNEFK.S | N | 55.74 | 1633.6467 | 14 | 0.9 | 545.5567 | 3 | 11.99 | 5 | F5:1916 | NaNaKA16\_F13.raw |  |  |  |  | 4.2751E5 |  |  |  |  |  |  |  |  | 1 | 0 | 0 | 0 | 0 | 1 | 0 | 0 | 0 | 0 | 0 | 0 | 0 | 0 | 433 | 446 | Carbamidomethylation | C2:Carbamidomethylation:1000.00 | PEAKS DB |
| K.DFYTFDSEAIVK.K | N | 52.96 | 1433.6714 | 12 | 0.4 | 717.8433 | 2 | 71.19 | 5 | F5:28900 | NaNaKA16\_F13.raw |  |  |  |  | 5.5404E5 |  |  |  |  |  |  |  |  | 1 | 0 | 0 | 0 | 0 | 1 | 0 | 0 | 0 | 0 | 0 | 0 | 0 | 0 | 370 | 381 |  |  | PEAKS DB |
| total 6 peptides |  |  |  |  |  |  |  |  |  |  |  |  |  |  |  |  |  |  |  |  |  |  |  |  |  |  |  |  |  |  |  |  |  |  |  |  |  |  |  |  |  |  |  |
| --- | --- | --- | --- | --- | --- | --- | --- | --- | --- | --- | --- | --- | --- | --- | --- | --- | --- | --- | --- | --- | --- | --- | --- | --- | --- | --- | --- | --- | --- | --- | --- | --- | --- | --- | --- | --- | --- | --- | --- | --- | --- | --- | --- |

XP\_026541908.1

back to list

  

| Protein Coverage
| Supporting Peptides
|

Protein Coverage:

Supporting Peptides:

| Peptide | Uniq | -10lgP | Mass | Length | ppm | m/z | z | RT | Fraction | Scan | Source File | Area F1 | Area F10 | Area F11 | Area F12 | Area F13 | Area F2 | Area F3 | Area F4 | Area F5 | Area F6 | Area F7 | Area F8 | Area F9 | #Feature | #Feature F1 | #Feature F10 | #Feature F11 | #Feature F12 | #Feature F13 | #Feature F2 | #Feature F3 | #Feature F4 | #Feature F5 | #Feature F6 | #Feature F7 | #Feature F8 | #Feature F9 | Start | End | PTM | AScore | Found By |
| --- | --- | --- | --- | --- | --- | --- | --- | --- | --- | --- | --- | --- | --- | --- | --- | --- | --- | --- | --- | --- | --- | --- | --- | --- | --- | --- | --- | --- | --- | --- | --- | --- | --- | --- | --- | --- | --- | --- | --- | --- | --- | --- | --- |
| R.LVILGFPC(+57.02)NQFGK.Q | N | 75.66 | 1491.7908 | 13 | 0.9 | 746.9033 | 2 | 75.14 | 4 | F4:50923 | NaNaKA16\_F12.raw |  | 1.9324E7 | 8.891E6 | 1.0383E7 | 1.972E7 |  |  |  |  |  | 2.0275E6 | 1.0176E7 | 4.4683E6 | 7 | 0 | 1 | 1 | 1 | 1 | 0 | 0 | 0 | 0 | 0 | 1 | 1 | 1 | 124 | 136 | Carbamidomethylation | C8:Carbamidomethylation:1000.00 | PEAKS DB |
| K.QEPGQNSEILQGIK.Y | N | 72.65 | 1539.7893 | 14 | 1.0 | 770.9027 | 2 | 36.16 | 5 | F5:14005 | NaNaKA16\_F13.raw |  |  |  | 3.6181E6 | 2.8478E7 |  |  |  |  |  |  |  |  | 3 | 0 | 0 | 0 | 1 | 2 | 0 | 0 | 0 | 0 | 0 | 0 | 0 | 0 | 137 | 150 |  |  | PEAKS DB |
| R.PGGGYVPNFQLFQK.G | Y | 67.77 | 1550.7881 | 14 | 0.4 | 776.4016 | 2 | 67.84 | 10 | F10:47726 | NaNaKA16\_F6.raw |  |  |  | 1.2069E6 | 3.3174E6 |  |  |  | 1.1691E7 | 3.9836E6 |  |  |  | 4 | 0 | 0 | 0 | 1 | 1 | 0 | 0 | 0 | 1 | 1 | 0 | 0 | 0 | 154 | 167 |  |  | PEAKS DB |
| L.VILGFPC(+57.02)NQFGK.Q | N | 63.26 | 1378.7067 | 12 | 0.9 | 690.3612 | 2 | 60.52 | 12 | F12:39695 | NaNaKA16\_F8.raw |  | 3.3914E6 |  |  |  |  |  |  |  | 2.4189E6 | 2.1917E6 | 3.4327E6 | 4.8393E6 | 5 | 0 | 1 | 0 | 0 | 0 | 0 | 0 | 0 | 0 | 1 | 1 | 1 | 1 | 125 | 136 | Carbamidomethylation | C7:Carbamidomethylation:1000.00 | PEAKS DB |
| K.FLVNPQGKPVMR.W | N | 57.92 | 1384.7649 | 12 | 1.1 | 462.5961 | 3 | 26.92 | 5 | F5:8608 | NaNaKA16\_F13.raw |  |  |  |  | 1.9299E6 |  |  |  |  |  |  |  |  | 1 | 0 | 0 | 0 | 0 | 1 | 0 | 0 | 0 | 0 | 0 | 0 | 0 | 0 | 216 | 227 |  |  | PEAKS DB |
| C.SYAPNSQPIPDR.G | N | 52.88 | 1343.6470 | 12 | 0.3 | 672.8310 | 2 | 19.74 | 5 | F5:4861 | NaNaKA16\_F13.raw |  |  |  |  | 1.9768E6 | 8.8175E5 |  |  |  |  |  |  |  | 2 | 0 | 0 | 0 | 0 | 1 | 1 | 0 | 0 | 0 | 0 | 0 | 0 | 0 | 47 | 58 |  |  | PEAKS DB |
| K.NSC(+57.02)PPVVETFG.D | N | 51.85 | 1205.5387 | 11 | 0.2 | 603.7767 | 2 | 53.03 | 6 | F6:34639 | NaNaKA16\_F2.raw |  |  |  |  |  | 5.7957E7 | 1.0144E7 |  |  | 3.2539E6 |  |  |  | 3 | 0 | 0 | 0 | 0 | 0 | 1 | 1 | 0 | 0 | 1 | 0 | 0 | 0 | 184 | 194 | Carbamidomethylation | C3:Carbamidomethylation:1000.00 | PEAKS DB |
| R.LFWSPLK.I | N | 50.13 | 889.5062 | 7 | -0.4 | 445.7602 | 2 | 61.25 | 2 | F2:38338 | NaNaKA16\_F10.raw |  | 1.0564E7 | 2.3049E7 | 4.474E6 | 9.3271E6 |  |  |  |  | 8.0536E6 | 4.602E6 |  |  | 6 | 0 | 1 | 1 | 1 | 1 | 0 | 0 | 0 | 0 | 1 | 1 | 0 | 0 | 199 | 205 |  |  | PEAKS DB |
| total 8 peptides |  |  |  |  |  |  |  |  |  |  |  |  |  |  |  |  |  |  |  |  |  |  |  |  |  |  |  |  |  |  |  |  |  |  |  |  |  |  |  |  |  |  |  |
| --- | --- | --- | --- | --- | --- | --- | --- | --- | --- | --- | --- | --- | --- | --- | --- | --- | --- | --- | --- | --- | --- | --- | --- | --- | --- | --- | --- | --- | --- | --- | --- | --- | --- | --- | --- | --- | --- | --- | --- | --- | --- | --- | --- |

P20229.1

back to list

  

| Protein Coverage
| Supporting Peptides
|

Protein Coverage:

Supporting Peptides:

| Peptide | Uniq | -10lgP | Mass | Length | ppm | m/z | z | RT | Fraction | Scan | Source File | Area F1 | Area F10 | Area F11 | Area F12 | Area F13 | Area F2 | Area F3 | Area F4 | Area F5 | Area F6 | Area F7 | Area F8 | Area F9 | #Feature | #Feature F1 | #Feature F10 | #Feature F11 | #Feature F12 | #Feature F13 | #Feature F2 | #Feature F3 | #Feature F4 | #Feature F5 | #Feature F6 | #Feature F7 | #Feature F8 | #Feature F9 | Start | End | PTM | AScore | Found By |
| --- | --- | --- | --- | --- | --- | --- | --- | --- | --- | --- | --- | --- | --- | --- | --- | --- | --- | --- | --- | --- | --- | --- | --- | --- | --- | --- | --- | --- | --- | --- | --- | --- | --- | --- | --- | --- | --- | --- | --- | --- | --- | --- | --- |
| RPGFC(+57.02)ELPAAK.G | Y | 72.25 | 1244.6335 | 11 | 0.2 | 415.8852 | 3 | 25.83 | 6 | F6:12043 | NaNaKA16\_F2.raw |  |  |  |  |  | 1.8481E10 | 6.6671E6 | 1.2942E5 | 7.7812E4 |  |  |  |  | 18 | 0 | 0 | 0 | 0 | 0 | 13 | 3 | 1 | 1 | 0 | 0 | 0 | 0 | 1 | 11 | Carbamidomethylation | C5:Carbamidomethylation:1000.00 | PEAKS DB |
| R.PGFC(+57.02)ELPAAK.G | Y | 69.38 | 1088.5325 | 10 | -0.4 | 545.2733 | 2 | 30.14 | 6 | F6:15808 | NaNaKA16\_F2.raw |  |  |  |  |  | 5.4631E7 |  |  |  |  |  |  |  | 1 | 0 | 0 | 0 | 0 | 0 | 1 | 0 | 0 | 0 | 0 | 0 | 0 | 0 | 2 | 11 | Carbamidomethylation | C4:Carbamidomethylation:1000.00 | PEAKS DB |
| K.AHKPAFYYNK.D | Y | 67.89 | 1237.6244 | 10 | -3.0 | 413.5475 | 3 | 11.83 | 6 | F6:1936 | NaNaKA16\_F2.raw |  |  |  |  |  | 7.8577E6 |  |  |  |  |  |  |  | 1 | 0 | 0 | 0 | 0 | 0 | 1 | 0 | 0 | 0 | 0 | 0 | 0 | 0 | 16 | 25 |  |  | PEAKS DB |
| P.GFC(+57.02)ELPAAK.G | Y | 63.36 | 991.4797 | 9 | 0.5 | 496.7473 | 2 | 17.61 | 6 | F6:5902 | NaNaKA16\_F2.raw | 4.9737E5 |  |  |  |  | 1.1731E9 |  |  |  |  |  |  |  | 3 | 1 | 0 | 0 | 0 | 0 | 2 | 0 | 0 | 0 | 0 | 0 | 0 | 0 | 3 | 11 | Carbamidomethylation | C3:Carbamidomethylation:1000.00 | PEAKS DB |
| G.FC(+57.02)ELPAAK.G | Y | 56.52 | 934.4582 | 8 | 0.0 | 468.2364 | 2 | 18.99 | 6 | F6:7115 | NaNaKA16\_F2.raw |  |  |  |  |  | 7.1119E7 |  |  |  |  |  |  |  | 4 | 0 | 0 | 0 | 0 | 0 | 4 | 0 | 0 | 0 | 0 | 0 | 0 | 0 | 4 | 11 | Carbamidomethylation | C2:Carbamidomethylation:1000.00 | PEAKS DB |
| RPGFC(+57.02)ELPAAKGLC(+57.02)K.A | Y | 51.83 | 1702.8646 | 15 | 0.1 | 426.7235 | 4 | 25.88 | 6 | F6:12144 | NaNaKA16\_F2.raw |  |  |  |  |  | 2.0513E6 |  |  |  |  |  |  |  | 2 | 0 | 0 | 0 | 0 | 0 | 2 | 0 | 0 | 0 | 0 | 0 | 0 | 0 | 1 | 15 | Carbamidomethylation | C5:Carbamidomethylation:1000.00;C14:Carbamidomethylation:1000.00 | PEAKS DB |
| RPGFC(+57.02)ELPAAKG.L | Y | 49.32 | 1301.6550 | 12 | -0.7 | 651.8344 | 2 | 18.25 | 6 | F6:5806 | NaNaKA16\_F2.raw |  |  |  |  |  | 1.9208E8 |  |  |  |  |  |  |  | 2 | 0 | 0 | 0 | 0 | 0 | 2 | 0 | 0 | 0 | 0 | 0 | 0 | 0 | 1 | 12 | Carbamidomethylation | C5:Carbamidomethylation:1000.00 | PEAKS DB |
| K.AHKPAFYY.N | Y | 47.54 | 995.4865 | 8 | 0.8 | 498.7509 | 2 | 12.31 | 6 | F6:2289 | NaNaKA16\_F2.raw |  |  |  |  |  | 1.4654E7 |  |  |  |  |  |  |  | 1 | 0 | 0 | 0 | 0 | 0 | 1 | 0 | 0 | 0 | 0 | 0 | 0 | 0 | 16 | 23 |  |  | PEAKS DB |
| F.C(+57.02)ELPAAK.G | Y | 42.14 | 787.3898 | 7 | -0.3 | 394.7021 | 2 | 17.61 | 6 | F6:5910 | NaNaKA16\_F2.raw |  |  |  |  |  | 4.1672E7 |  |  |  |  |  |  |  | 1 | 0 | 0 | 0 | 0 | 0 | 1 | 0 | 0 | 0 | 0 | 0 | 0 | 0 | 5 | 11 | Carbamidomethylation | C1:Carbamidomethylation:1000.00 | PEAKS DB |
| total 9 peptides |  |  |  |  |  |  |  |  |  |  |  |  |  |  |  |  |  |  |  |  |  |  |  |  |  |  |  |  |  |  |  |  |  |  |  |  |  |  |  |  |  |  |  |
| --- | --- | --- | --- | --- | --- | --- | --- | --- | --- | --- | --- | --- | --- | --- | --- | --- | --- | --- | --- | --- | --- | --- | --- | --- | --- | --- | --- | --- | --- | --- | --- | --- | --- | --- | --- | --- | --- | --- | --- | --- | --- | --- | --- |

XP\_034258146.1

back to list

  

| Protein Coverage
| Supporting Peptides
|

Protein Coverage:

Supporting Peptides:

| Peptide | Uniq | -10lgP | Mass | Length | ppm | m/z | z | RT | Fraction | Scan | Source File | Area F1 | Area F10 | Area F11 | Area F12 | Area F13 | Area F2 | Area F3 | Area F4 | Area F5 | Area F6 | Area F7 | Area F8 | Area F9 | #Feature | #Feature F1 | #Feature F10 | #Feature F11 | #Feature F12 | #Feature F13 | #Feature F2 | #Feature F3 | #Feature F4 | #Feature F5 | #Feature F6 | #Feature F7 | #Feature F8 | #Feature F9 | Start | End | PTM | AScore | Found By |
| --- | --- | --- | --- | --- | --- | --- | --- | --- | --- | --- | --- | --- | --- | --- | --- | --- | --- | --- | --- | --- | --- | --- | --- | --- | --- | --- | --- | --- | --- | --- | --- | --- | --- | --- | --- | --- | --- | --- | --- | --- | --- | --- | --- |
| R.LVILGFPC(+57.02)NQFGK.Q | N | 75.66 | 1491.7908 | 13 | 0.9 | 746.9033 | 2 | 75.14 | 4 | F4:50923 | NaNaKA16\_F12.raw |  | 1.9324E7 | 8.891E6 | 1.0383E7 | 1.972E7 |  |  |  |  |  | 2.0275E6 | 1.0176E7 | 4.4683E6 | 7 | 0 | 1 | 1 | 1 | 1 | 0 | 0 | 0 | 0 | 0 | 1 | 1 | 1 | 124 | 136 | Carbamidomethylation | C8:Carbamidomethylation:1000.00 | PEAKS DB |
| K.QEPGQNSEILQGIK.Y | N | 72.65 | 1539.7893 | 14 | 1.0 | 770.9027 | 2 | 36.16 | 5 | F5:14005 | NaNaKA16\_F13.raw |  |  |  | 3.6181E6 | 2.8478E7 |  |  |  |  |  |  |  |  | 3 | 0 | 0 | 0 | 1 | 2 | 0 | 0 | 0 | 0 | 0 | 0 | 0 | 0 | 137 | 150 |  |  | PEAKS DB |
| L.VILGFPC(+57.02)NQFGK.Q | N | 63.26 | 1378.7067 | 12 | 0.9 | 690.3612 | 2 | 60.52 | 12 | F12:39695 | NaNaKA16\_F8.raw |  | 3.3914E6 |  |  |  |  |  |  |  | 2.4189E6 | 2.1917E6 | 3.4327E6 | 4.8393E6 | 5 | 0 | 1 | 0 | 0 | 0 | 0 | 0 | 0 | 0 | 1 | 1 | 1 | 1 | 125 | 136 | Carbamidomethylation | C7:Carbamidomethylation:1000.00 | PEAKS DB |
| K.NSC(+57.02)PPVVETFG.D | N | 51.85 | 1205.5387 | 11 | 0.2 | 603.7767 | 2 | 53.03 | 6 | F6:34639 | NaNaKA16\_F2.raw |  |  |  |  |  | 5.7957E7 | 1.0144E7 |  |  | 3.2539E6 |  |  |  | 3 | 0 | 0 | 0 | 0 | 0 | 1 | 1 | 0 | 0 | 1 | 0 | 0 | 0 | 184 | 194 | Carbamidomethylation | C3:Carbamidomethylation:1000.00 | PEAKS DB |
| S.ITGDYIAFR.N | Y | 43.42 | 1054.5447 | 9 | 4.7 | 528.2793 | 2 | 43.17 | 8 | F8:23978 | NaNaKA16\_F4.raw |  |  |  |  |  |  |  | 1.2476E5 |  |  |  |  |  | 1 | 0 | 0 | 0 | 0 | 0 | 0 | 0 | 1 | 0 | 0 | 0 | 0 | 0 | 79 | 87 |  |  | PEAKS DB |
| H.DYGALSITGDYIAFR.N | Y | 43.37 | 1660.8096 | 15 | 1.4 | 831.4132 | 2 | 87.62 | 5 | F5:35334 | NaNaKA16\_F13.raw |  |  |  |  | 6.8309E5 |  |  |  |  |  |  |  |  | 1 | 0 | 0 | 0 | 0 | 1 | 0 | 0 | 0 | 0 | 0 | 0 | 0 | 0 | 73 | 87 |  |  | PEAKS DB |
| total 6 peptides |  |  |  |  |  |  |  |  |  |  |  |  |  |  |  |  |  |  |  |  |  |  |  |  |  |  |  |  |  |  |  |  |  |  |  |  |  |  |  |  |  |  |  |
| --- | --- | --- | --- | --- | --- | --- | --- | --- | --- | --- | --- | --- | --- | --- | --- | --- | --- | --- | --- | --- | --- | --- | --- | --- | --- | --- | --- | --- | --- | --- | --- | --- | --- | --- | --- | --- | --- | --- | --- | --- | --- | --- | --- |

1UG4

back to list

  

| Protein Coverage
| Supporting Peptides
|

Protein Coverage:

Supporting Peptides:

| Peptide | Uniq | -10lgP | Mass | Length | ppm | m/z | z | RT | Fraction | Scan | Source File | Area F1 | Area F10 | Area F11 | Area F12 | Area F13 | Area F2 | Area F3 | Area F4 | Area F5 | Area F6 | Area F7 | Area F8 | Area F9 | #Feature | #Feature F1 | #Feature F10 | #Feature F11 | #Feature F12 | #Feature F13 | #Feature F2 | #Feature F3 | #Feature F4 | #Feature F5 | #Feature F6 | #Feature F7 | #Feature F8 | #Feature F9 | Start | End | PTM | AScore | Found By |
| --- | --- | --- | --- | --- | --- | --- | --- | --- | --- | --- | --- | --- | --- | --- | --- | --- | --- | --- | --- | --- | --- | --- | --- | --- | --- | --- | --- | --- | --- | --- | --- | --- | --- | --- | --- | --- | --- | --- | --- | --- | --- | --- | --- |
| K.SSLLVKYVC(+57.02)C(+57.02)NTDRC(+57.02)N | N | 73.90 | 1987.8914 | 16 | 0.9 | 663.6383 | 3 | 34.50 | 3 | F3:16042 | NaNaKA16\_F11.raw |  | 4.3691E6 | 4.3984E7 |  |  |  |  |  |  |  |  |  |  | 3 | 0 | 1 | 2 | 0 | 0 | 0 | 0 | 0 | 0 | 0 | 0 | 0 | 0 | 45 | 60 | Carbamidomethylation | C9:Carbamidomethylation:1000.00;C10:Carbamidomethylation:1000.00;C15:Carbamidomethylation:1000.00 | PEAKS DB |
| K.SSLLVKYVC(+57.02)C(+57.02)NTDR.C | N | 70.96 | 1713.8179 | 14 | 0.6 | 857.9167 | 2 | 34.00 | 3 | F3:15457 | NaNaKA16\_F11.raw |  | 5.0554E6 | 1.1527E8 | 1.6508E6 |  |  |  |  |  |  |  | 0 | 3.3481E6 | 7 | 0 | 2 | 2 | 1 | 0 | 0 | 0 | 0 | 0 | 0 | 0 | 0 | 2 | 45 | 58 | Carbamidomethylation | C9:Carbamidomethylation:1000.00;C10:Carbamidomethylation:1000.00 | PEAKS DB |
| R.GC(+57.02)IDVC(+57.02)PKSSLLVK.Y | N | 65.71 | 1574.8160 | 14 | 0.6 | 788.4158 | 2 | 32.88 | 3 | F3:14936 | NaNaKA16\_F11.raw |  |  | 2.0322E7 |  |  |  |  |  |  |  |  |  |  | 2 | 0 | 0 | 2 | 0 | 0 | 0 | 0 | 0 | 0 | 0 | 0 | 0 | 0 | 37 | 50 | Carbamidomethylation | C2:Carbamidomethylation:1000.00;C6:Carbamidomethylation:1000.00 | PEAKS DB |
| K.C(+57.02)NQLIPPFYK.T | Y | 52.30 | 1278.6431 | 10 | 1.2 | 640.3296 | 2 | 54.59 | 3 | F3:31482 | NaNaKA16\_F11.raw |  |  | 2.358E6 |  |  |  |  |  |  |  |  |  |  | 1 | 0 | 0 | 1 | 0 | 0 | 0 | 0 | 0 | 0 | 0 | 0 | 0 | 0 | 3 | 12 | Carbamidomethylation | C1:Carbamidomethylation:1000.00 | PEAKS DB |
| K.YVC(+57.02)C(+57.02)NTDR.C | N | 50.92 | 1086.4222 | 8 | -0.2 | 544.2183 | 2 | 11.37 | 12 | F12:1787 | NaNaKA16\_F8.raw |  | 2.1124E6 | 2.9778E6 | 1.3296E6 |  |  |  |  |  | 1.5915E6 |  | 1.7945E6 |  | 5 | 0 | 1 | 1 | 1 | 0 | 0 | 0 | 0 | 0 | 1 | 0 | 1 | 0 | 51 | 58 | Carbamidomethylation | C3:Carbamidomethylation:1000.00;C4:Carbamidomethylation:1000.00 | PEAKS DB |
| R.GC(+57.02)IDVC(+57.02)PK.S | N | 46.03 | 947.4205 | 8 | -0.5 | 474.7173 | 2 | 11.54 | 12 | F12:1895 | NaNaKA16\_F8.raw |  |  | 1.5891E7 |  |  |  | 6.084E5 |  | 2.0697E6 | 6.3919E7 | 1.8242E6 | 6.8843E6 |  | 6 | 0 | 0 | 1 | 0 | 0 | 0 | 1 | 0 | 1 | 1 | 1 | 1 | 0 | 37 | 44 | Carbamidomethylation | C2:Carbamidomethylation:1000.00;C6:Carbamidomethylation:1000.00 | PEAKS DB |
| total 6 peptides |  |  |  |  |  |  |  |  |  |  |  |  |  |  |  |  |  |  |  |  |  |  |  |  |  |  |  |  |  |  |  |  |  |  |  |  |  |  |  |  |  |  |  |
| --- | --- | --- | --- | --- | --- | --- | --- | --- | --- | --- | --- | --- | --- | --- | --- | --- | --- | --- | --- | --- | --- | --- | --- | --- | --- | --- | --- | --- | --- | --- | --- | --- | --- | --- | --- | --- | --- | --- | --- | --- | --- | --- | --- |

ADN67586.1

back to list

  

| Protein Coverage
| Supporting Peptides
|

Protein Coverage:

Supporting Peptides:

| Peptide | Uniq | -10lgP | Mass | Length | ppm | m/z | z | RT | Fraction | Scan | Source File | Area F1 | Area F10 | Area F11 | Area F12 | Area F13 | Area F2 | Area F3 | Area F4 | Area F5 | Area F6 | Area F7 | Area F8 | Area F9 | #Feature | #Feature F1 | #Feature F10 | #Feature F11 | #Feature F12 | #Feature F13 | #Feature F2 | #Feature F3 | #Feature F4 | #Feature F5 | #Feature F6 | #Feature F7 | #Feature F8 | #Feature F9 | Start | End | PTM | AScore | Found By |
| --- | --- | --- | --- | --- | --- | --- | --- | --- | --- | --- | --- | --- | --- | --- | --- | --- | --- | --- | --- | --- | --- | --- | --- | --- | --- | --- | --- | --- | --- | --- | --- | --- | --- | --- | --- | --- | --- | --- | --- | --- | --- | --- | --- |
| K.SSLLVKYVC(+57.02)C(+57.02)NTDRC(+57.02)N | N | 73.90 | 1987.8914 | 16 | 0.9 | 663.6383 | 3 | 34.50 | 3 | F3:16042 | NaNaKA16\_F11.raw |  | 4.3691E6 | 4.3984E7 |  |  |  |  |  |  |  |  |  |  | 3 | 0 | 1 | 2 | 0 | 0 | 0 | 0 | 0 | 0 | 0 | 0 | 0 | 0 | 47 | 62 | Carbamidomethylation | C9:Carbamidomethylation:1000.00;C10:Carbamidomethylation:1000.00;C15:Carbamidomethylation:1000.00 | PEAKS DB |
| K.SSLLVKYVC(+57.02)C(+57.02)NTDR.C | N | 70.96 | 1713.8179 | 14 | 0.6 | 857.9167 | 2 | 34.00 | 3 | F3:15457 | NaNaKA16\_F11.raw |  | 5.0554E6 | 1.1527E8 | 1.6508E6 |  |  |  |  |  |  |  | 0 | 3.3481E6 | 7 | 0 | 2 | 2 | 1 | 0 | 0 | 0 | 0 | 0 | 0 | 0 | 0 | 2 | 47 | 60 | Carbamidomethylation | C9:Carbamidomethylation:1000.00;C10:Carbamidomethylation:1000.00 | PEAKS DB |
| R.GC(+57.02)IDVC(+57.02)PKSSLLVK.Y | N | 65.71 | 1574.8160 | 14 | 0.6 | 788.4158 | 2 | 32.88 | 3 | F3:14936 | NaNaKA16\_F11.raw |  |  | 2.0322E7 |  |  |  |  |  |  |  |  |  |  | 2 | 0 | 0 | 2 | 0 | 0 | 0 | 0 | 0 | 0 | 0 | 0 | 0 | 0 | 39 | 52 | Carbamidomethylation | C2:Carbamidomethylation:1000.00;C6:Carbamidomethylation:1000.00 | PEAKS DB |
| K.C(+57.02)NQLIPPFYK.T | Y | 52.30 | 1278.6431 | 10 | 1.2 | 640.3296 | 2 | 54.59 | 3 | F3:31482 | NaNaKA16\_F11.raw |  |  | 2.358E6 |  |  |  |  |  |  |  |  |  |  | 1 | 0 | 0 | 1 | 0 | 0 | 0 | 0 | 0 | 0 | 0 | 0 | 0 | 0 | 5 | 14 | Carbamidomethylation | C1:Carbamidomethylation:1000.00 | PEAKS DB |
| K.YVC(+57.02)C(+57.02)NTDR.C | N | 50.92 | 1086.4222 | 8 | -0.2 | 544.2183 | 2 | 11.37 | 12 | F12:1787 | NaNaKA16\_F8.raw |  | 2.1124E6 | 2.9778E6 | 1.3296E6 |  |  |  |  |  | 1.5915E6 |  | 1.7945E6 |  | 5 | 0 | 1 | 1 | 1 | 0 | 0 | 0 | 0 | 0 | 1 | 0 | 1 | 0 | 53 | 60 | Carbamidomethylation | C3:Carbamidomethylation:1000.00;C4:Carbamidomethylation:1000.00 | PEAKS DB |
| R.GC(+57.02)IDVC(+57.02)PK.S | N | 46.03 | 947.4205 | 8 | -0.5 | 474.7173 | 2 | 11.54 | 12 | F12:1895 | NaNaKA16\_F8.raw |  |  | 1.5891E7 |  |  |  | 6.084E5 |  | 2.0697E6 | 6.3919E7 | 1.8242E6 | 6.8843E6 |  | 6 | 0 | 0 | 1 | 0 | 0 | 0 | 1 | 0 | 1 | 1 | 1 | 1 | 0 | 39 | 46 | Carbamidomethylation | C2:Carbamidomethylation:1000.00;C6:Carbamidomethylation:1000.00 | PEAKS DB |
| total 6 peptides |  |  |  |  |  |  |  |  |  |  |  |  |  |  |  |  |  |  |  |  |  |  |  |  |  |  |  |  |  |  |  |  |  |  |  |  |  |  |  |  |  |  |  |
| --- | --- | --- | --- | --- | --- | --- | --- | --- | --- | --- | --- | --- | --- | --- | --- | --- | --- | --- | --- | --- | --- | --- | --- | --- | --- | --- | --- | --- | --- | --- | --- | --- | --- | --- | --- | --- | --- | --- | --- | --- | --- | --- | --- |

2207174C

back to list

  

| Protein Coverage
| Supporting Peptides
|

Protein Coverage:

Supporting Peptides:

| Peptide | Uniq | -10lgP | Mass | Length | ppm | m/z | z | RT | Fraction | Scan | Source File | Area F1 | Area F10 | Area F11 | Area F12 | Area F13 | Area F2 | Area F3 | Area F4 | Area F5 | Area F6 | Area F7 | Area F8 | Area F9 | #Feature | #Feature F1 | #Feature F10 | #Feature F11 | #Feature F12 | #Feature F13 | #Feature F2 | #Feature F3 | #Feature F4 | #Feature F5 | #Feature F6 | #Feature F7 | #Feature F8 | #Feature F9 | Start | End | PTM | AScore | Found By |
| --- | --- | --- | --- | --- | --- | --- | --- | --- | --- | --- | --- | --- | --- | --- | --- | --- | --- | --- | --- | --- | --- | --- | --- | --- | --- | --- | --- | --- | --- | --- | --- | --- | --- | --- | --- | --- | --- | --- | --- | --- | --- | --- | --- |
| K.SSLLVKYVC(+57.02)C(+57.02)NTDRC(+57.02)N | N | 73.90 | 1987.8914 | 16 | 0.9 | 663.6383 | 3 | 34.50 | 3 | F3:16042 | NaNaKA16\_F11.raw |  | 4.3691E6 | 4.3984E7 |  |  |  |  |  |  |  |  |  |  | 3 | 0 | 1 | 2 | 0 | 0 | 0 | 0 | 0 | 0 | 0 | 0 | 0 | 0 | 66 | 81 | Carbamidomethylation | C9:Carbamidomethylation:1000.00;C10:Carbamidomethylation:1000.00;C15:Carbamidomethylation:1000.00 | PEAKS DB |
| K.SSLLVKYVC(+57.02)C(+57.02)NTDR.C | N | 70.96 | 1713.8179 | 14 | 0.6 | 857.9167 | 2 | 34.00 | 3 | F3:15457 | NaNaKA16\_F11.raw |  | 5.0554E6 | 1.1527E8 | 1.6508E6 |  |  |  |  |  |  |  | 0 | 3.3481E6 | 7 | 0 | 2 | 2 | 1 | 0 | 0 | 0 | 0 | 0 | 0 | 0 | 0 | 2 | 66 | 79 | Carbamidomethylation | C9:Carbamidomethylation:1000.00;C10:Carbamidomethylation:1000.00 | PEAKS DB |
| R.GC(+57.02)IDVC(+57.02)PKSSLLVK.Y | N | 65.71 | 1574.8160 | 14 | 0.6 | 788.4158 | 2 | 32.88 | 3 | F3:14936 | NaNaKA16\_F11.raw |  |  | 2.0322E7 |  |  |  |  |  |  |  |  |  |  | 2 | 0 | 0 | 2 | 0 | 0 | 0 | 0 | 0 | 0 | 0 | 0 | 0 | 0 | 58 | 71 | Carbamidomethylation | C2:Carbamidomethylation:1000.00;C6:Carbamidomethylation:1000.00 | PEAKS DB |
| K.C(+57.02)NQLIPPFYK.T | Y | 52.30 | 1278.6431 | 10 | 1.2 | 640.3296 | 2 | 54.59 | 3 | F3:31482 | NaNaKA16\_F11.raw |  |  | 2.358E6 |  |  |  |  |  |  |  |  |  |  | 1 | 0 | 0 | 1 | 0 | 0 | 0 | 0 | 0 | 0 | 0 | 0 | 0 | 0 | 24 | 33 | Carbamidomethylation | C1:Carbamidomethylation:1000.00 | PEAKS DB |
| K.YVC(+57.02)C(+57.02)NTDR.C | N | 50.92 | 1086.4222 | 8 | -0.2 | 544.2183 | 2 | 11.37 | 12 | F12:1787 | NaNaKA16\_F8.raw |  | 2.1124E6 | 2.9778E6 | 1.3296E6 |  |  |  |  |  | 1.5915E6 |  | 1.7945E6 |  | 5 | 0 | 1 | 1 | 1 | 0 | 0 | 0 | 0 | 0 | 1 | 0 | 1 | 0 | 72 | 79 | Carbamidomethylation | C3:Carbamidomethylation:1000.00;C4:Carbamidomethylation:1000.00 | PEAKS DB |
| R.GC(+57.02)IDVC(+57.02)PK.S | N | 46.03 | 947.4205 | 8 | -0.5 | 474.7173 | 2 | 11.54 | 12 | F12:1895 | NaNaKA16\_F8.raw |  |  | 1.5891E7 |  |  |  | 6.084E5 |  | 2.0697E6 | 6.3919E7 | 1.8242E6 | 6.8843E6 |  | 6 | 0 | 0 | 1 | 0 | 0 | 0 | 1 | 0 | 1 | 1 | 1 | 1 | 0 | 58 | 65 | Carbamidomethylation | C2:Carbamidomethylation:1000.00;C6:Carbamidomethylation:1000.00 | PEAKS DB |
| total 6 peptides |  |  |  |  |  |  |  |  |  |  |  |  |  |  |  |  |  |  |  |  |  |  |  |  |  |  |  |  |  |  |  |  |  |  |  |  |  |  |  |  |  |  |  |
| --- | --- | --- | --- | --- | --- | --- | --- | --- | --- | --- | --- | --- | --- | --- | --- | --- | --- | --- | --- | --- | --- | --- | --- | --- | --- | --- | --- | --- | --- | --- | --- | --- | --- | --- | --- | --- | --- | --- | --- | --- | --- | --- | --- |

CAB42058.1

back to list

  

| Protein Coverage
| Supporting Peptides
|

Protein Coverage:

Supporting Peptides:

| Peptide | Uniq | -10lgP | Mass | Length | ppm | m/z | z | RT | Fraction | Scan | Source File | Area F1 | Area F10 | Area F11 | Area F12 | Area F13 | Area F2 | Area F3 | Area F4 | Area F5 | Area F6 | Area F7 | Area F8 | Area F9 | #Feature | #Feature F1 | #Feature F10 | #Feature F11 | #Feature F12 | #Feature F13 | #Feature F2 | #Feature F3 | #Feature F4 | #Feature F5 | #Feature F6 | #Feature F7 | #Feature F8 | #Feature F9 | Start | End | PTM | AScore | Found By |
| --- | --- | --- | --- | --- | --- | --- | --- | --- | --- | --- | --- | --- | --- | --- | --- | --- | --- | --- | --- | --- | --- | --- | --- | --- | --- | --- | --- | --- | --- | --- | --- | --- | --- | --- | --- | --- | --- | --- | --- | --- | --- | --- | --- |
| K.SSLLVKYVC(+57.02)C(+57.02)NTDRC(+57.02)N | N | 73.90 | 1987.8914 | 16 | 0.9 | 663.6383 | 3 | 34.50 | 3 | F3:16042 | NaNaKA16\_F11.raw |  | 4.3691E6 | 4.3984E7 |  |  |  |  |  |  |  |  |  |  | 3 | 0 | 1 | 2 | 0 | 0 | 0 | 0 | 0 | 0 | 0 | 0 | 0 | 0 | 66 | 81 | Carbamidomethylation | C9:Carbamidomethylation:1000.00;C10:Carbamidomethylation:1000.00;C15:Carbamidomethylation:1000.00 | PEAKS DB |
| K.SSLLVKYVC(+57.02)C(+57.02)NTDR.C | N | 70.96 | 1713.8179 | 14 | 0.6 | 857.9167 | 2 | 34.00 | 3 | F3:15457 | NaNaKA16\_F11.raw |  | 5.0554E6 | 1.1527E8 | 1.6508E6 |  |  |  |  |  |  |  | 0 | 3.3481E6 | 7 | 0 | 2 | 2 | 1 | 0 | 0 | 0 | 0 | 0 | 0 | 0 | 0 | 2 | 66 | 79 | Carbamidomethylation | C9:Carbamidomethylation:1000.00;C10:Carbamidomethylation:1000.00 | PEAKS DB |
| R.GC(+57.02)IDVC(+57.02)PKSSLLVK.Y | N | 65.71 | 1574.8160 | 14 | 0.6 | 788.4158 | 2 | 32.88 | 3 | F3:14936 | NaNaKA16\_F11.raw |  |  | 2.0322E7 |  |  |  |  |  |  |  |  |  |  | 2 | 0 | 0 | 2 | 0 | 0 | 0 | 0 | 0 | 0 | 0 | 0 | 0 | 0 | 58 | 71 | Carbamidomethylation | C2:Carbamidomethylation:1000.00;C6:Carbamidomethylation:1000.00 | PEAKS DB |
| K.C(+57.02)NQLIPPFYK.T | Y | 52.30 | 1278.6431 | 10 | 1.2 | 640.3296 | 2 | 54.59 | 3 | F3:31482 | NaNaKA16\_F11.raw |  |  | 2.358E6 |  |  |  |  |  |  |  |  |  |  | 1 | 0 | 0 | 1 | 0 | 0 | 0 | 0 | 0 | 0 | 0 | 0 | 0 | 0 | 24 | 33 | Carbamidomethylation | C1:Carbamidomethylation:1000.00 | PEAKS DB |
| K.YVC(+57.02)C(+57.02)NTDR.C | N | 50.92 | 1086.4222 | 8 | -0.2 | 544.2183 | 2 | 11.37 | 12 | F12:1787 | NaNaKA16\_F8.raw |  | 2.1124E6 | 2.9778E6 | 1.3296E6 |  |  |  |  |  | 1.5915E6 |  | 1.7945E6 |  | 5 | 0 | 1 | 1 | 1 | 0 | 0 | 0 | 0 | 0 | 1 | 0 | 1 | 0 | 72 | 79 | Carbamidomethylation | C3:Carbamidomethylation:1000.00;C4:Carbamidomethylation:1000.00 | PEAKS DB |
| R.GC(+57.02)IDVC(+57.02)PK.S | N | 46.03 | 947.4205 | 8 | -0.5 | 474.7173 | 2 | 11.54 | 12 | F12:1895 | NaNaKA16\_F8.raw |  |  | 1.5891E7 |  |  |  | 6.084E5 |  | 2.0697E6 | 6.3919E7 | 1.8242E6 | 6.8843E6 |  | 6 | 0 | 0 | 1 | 0 | 0 | 0 | 1 | 0 | 1 | 1 | 1 | 1 | 0 | 58 | 65 | Carbamidomethylation | C2:Carbamidomethylation:1000.00;C6:Carbamidomethylation:1000.00 | PEAKS DB |
| total 6 peptides |  |  |  |  |  |  |  |  |  |  |  |  |  |  |  |  |  |  |  |  |  |  |  |  |  |  |  |  |  |  |  |  |  |  |  |  |  |  |  |  |  |  |  |
| --- | --- | --- | --- | --- | --- | --- | --- | --- | --- | --- | --- | --- | --- | --- | --- | --- | --- | --- | --- | --- | --- | --- | --- | --- | --- | --- | --- | --- | --- | --- | --- | --- | --- | --- | --- | --- | --- | --- | --- | --- | --- | --- | --- |

AAB86638.1

back to list

  

| Protein Coverage
| Supporting Peptides
|

Protein Coverage:

Supporting Peptides:

| Peptide | Uniq | -10lgP | Mass | Length | ppm | m/z | z | RT | Fraction | Scan | Source File | Area F1 | Area F10 | Area F11 | Area F12 | Area F13 | Area F2 | Area F3 | Area F4 | Area F5 | Area F6 | Area F7 | Area F8 | Area F9 | #Feature | #Feature F1 | #Feature F10 | #Feature F11 | #Feature F12 | #Feature F13 | #Feature F2 | #Feature F3 | #Feature F4 | #Feature F5 | #Feature F6 | #Feature F7 | #Feature F8 | #Feature F9 | Start | End | PTM | AScore | Found By |
| --- | --- | --- | --- | --- | --- | --- | --- | --- | --- | --- | --- | --- | --- | --- | --- | --- | --- | --- | --- | --- | --- | --- | --- | --- | --- | --- | --- | --- | --- | --- | --- | --- | --- | --- | --- | --- | --- | --- | --- | --- | --- | --- | --- |
| K.SSLLVKYVC(+57.02)C(+57.02)NTDRC(+57.02)N | N | 73.90 | 1987.8914 | 16 | 0.9 | 663.6383 | 3 | 34.50 | 3 | F3:16042 | NaNaKA16\_F11.raw |  | 4.3691E6 | 4.3984E7 |  |  |  |  |  |  |  |  |  |  | 3 | 0 | 1 | 2 | 0 | 0 | 0 | 0 | 0 | 0 | 0 | 0 | 0 | 0 | 66 | 81 | Carbamidomethylation | C9:Carbamidomethylation:1000.00;C10:Carbamidomethylation:1000.00;C15:Carbamidomethylation:1000.00 | PEAKS DB |
| K.SSLLVKYVC(+57.02)C(+57.02)NTDR.C | N | 70.96 | 1713.8179 | 14 | 0.6 | 857.9167 | 2 | 34.00 | 3 | F3:15457 | NaNaKA16\_F11.raw |  | 5.0554E6 | 1.1527E8 | 1.6508E6 |  |  |  |  |  |  |  | 0 | 3.3481E6 | 7 | 0 | 2 | 2 | 1 | 0 | 0 | 0 | 0 | 0 | 0 | 0 | 0 | 2 | 66 | 79 | Carbamidomethylation | C9:Carbamidomethylation:1000.00;C10:Carbamidomethylation:1000.00 | PEAKS DB |
| R.GC(+57.02)IDVC(+57.02)PKSSLLVK.Y | N | 65.71 | 1574.8160 | 14 | 0.6 | 788.4158 | 2 | 32.88 | 3 | F3:14936 | NaNaKA16\_F11.raw |  |  | 2.0322E7 |  |  |  |  |  |  |  |  |  |  | 2 | 0 | 0 | 2 | 0 | 0 | 0 | 0 | 0 | 0 | 0 | 0 | 0 | 0 | 58 | 71 | Carbamidomethylation | C2:Carbamidomethylation:1000.00;C6:Carbamidomethylation:1000.00 | PEAKS DB |
| K.C(+57.02)NQLIPPFYK.T | Y | 52.30 | 1278.6431 | 10 | 1.2 | 640.3296 | 2 | 54.59 | 3 | F3:31482 | NaNaKA16\_F11.raw |  |  | 2.358E6 |  |  |  |  |  |  |  |  |  |  | 1 | 0 | 0 | 1 | 0 | 0 | 0 | 0 | 0 | 0 | 0 | 0 | 0 | 0 | 24 | 33 | Carbamidomethylation | C1:Carbamidomethylation:1000.00 | PEAKS DB |
| K.YVC(+57.02)C(+57.02)NTDR.C | N | 50.92 | 1086.4222 | 8 | -0.2 | 544.2183 | 2 | 11.37 | 12 | F12:1787 | NaNaKA16\_F8.raw |  | 2.1124E6 | 2.9778E6 | 1.3296E6 |  |  |  |  |  | 1.5915E6 |  | 1.7945E6 |  | 5 | 0 | 1 | 1 | 1 | 0 | 0 | 0 | 0 | 0 | 1 | 0 | 1 | 0 | 72 | 79 | Carbamidomethylation | C3:Carbamidomethylation:1000.00;C4:Carbamidomethylation:1000.00 | PEAKS DB |
| R.GC(+57.02)IDVC(+57.02)PK.S | N | 46.03 | 947.4205 | 8 | -0.5 | 474.7173 | 2 | 11.54 | 12 | F12:1895 | NaNaKA16\_F8.raw |  |  | 1.5891E7 |  |  |  | 6.084E5 |  | 2.0697E6 | 6.3919E7 | 1.8242E6 | 6.8843E6 |  | 6 | 0 | 0 | 1 | 0 | 0 | 0 | 1 | 0 | 1 | 1 | 1 | 1 | 0 | 58 | 65 | Carbamidomethylation | C2:Carbamidomethylation:1000.00;C6:Carbamidomethylation:1000.00 | PEAKS DB |
| total 6 peptides |  |  |  |  |  |  |  |  |  |  |  |  |  |  |  |  |  |  |  |  |  |  |  |  |  |  |  |  |  |  |  |  |  |  |  |  |  |  |  |  |  |  |  |
| --- | --- | --- | --- | --- | --- | --- | --- | --- | --- | --- | --- | --- | --- | --- | --- | --- | --- | --- | --- | --- | --- | --- | --- | --- | --- | --- | --- | --- | --- | --- | --- | --- | --- | --- | --- | --- | --- | --- | --- | --- | --- | --- | --- |

CAA07687.1

back to list

  

| Protein Coverage
| Supporting Peptides
|

Protein Coverage:

Supporting Peptides:

| Peptide | Uniq | -10lgP | Mass | Length | ppm | m/z | z | RT | Fraction | Scan | Source File | Area F1 | Area F10 | Area F11 | Area F12 | Area F13 | Area F2 | Area F3 | Area F4 | Area F5 | Area F6 | Area F7 | Area F8 | Area F9 | #Feature | #Feature F1 | #Feature F10 | #Feature F11 | #Feature F12 | #Feature F13 | #Feature F2 | #Feature F3 | #Feature F4 | #Feature F5 | #Feature F6 | #Feature F7 | #Feature F8 | #Feature F9 | Start | End | PTM | AScore | Found By |
| --- | --- | --- | --- | --- | --- | --- | --- | --- | --- | --- | --- | --- | --- | --- | --- | --- | --- | --- | --- | --- | --- | --- | --- | --- | --- | --- | --- | --- | --- | --- | --- | --- | --- | --- | --- | --- | --- | --- | --- | --- | --- | --- | --- |
| K.SSLLVKYVC(+57.02)C(+57.02)NTDRC(+57.02)N | N | 73.90 | 1987.8914 | 16 | 0.9 | 663.6383 | 3 | 34.50 | 3 | F3:16042 | NaNaKA16\_F11.raw |  | 4.3691E6 | 4.3984E7 |  |  |  |  |  |  |  |  |  |  | 3 | 0 | 1 | 2 | 0 | 0 | 0 | 0 | 0 | 0 | 0 | 0 | 0 | 0 | 66 | 81 | Carbamidomethylation | C9:Carbamidomethylation:1000.00;C10:Carbamidomethylation:1000.00;C15:Carbamidomethylation:1000.00 | PEAKS DB |
| K.SSLLVKYVC(+57.02)C(+57.02)NTDR.C | N | 70.96 | 1713.8179 | 14 | 0.6 | 857.9167 | 2 | 34.00 | 3 | F3:15457 | NaNaKA16\_F11.raw |  | 5.0554E6 | 1.1527E8 | 1.6508E6 |  |  |  |  |  |  |  | 0 | 3.3481E6 | 7 | 0 | 2 | 2 | 1 | 0 | 0 | 0 | 0 | 0 | 0 | 0 | 0 | 2 | 66 | 79 | Carbamidomethylation | C9:Carbamidomethylation:1000.00;C10:Carbamidomethylation:1000.00 | PEAKS DB |
| R.GC(+57.02)IDVC(+57.02)PKSSLLVK.Y | N | 65.71 | 1574.8160 | 14 | 0.6 | 788.4158 | 2 | 32.88 | 3 | F3:14936 | NaNaKA16\_F11.raw |  |  | 2.0322E7 |  |  |  |  |  |  |  |  |  |  | 2 | 0 | 0 | 2 | 0 | 0 | 0 | 0 | 0 | 0 | 0 | 0 | 0 | 0 | 58 | 71 | Carbamidomethylation | C2:Carbamidomethylation:1000.00;C6:Carbamidomethylation:1000.00 | PEAKS DB |
| K.C(+57.02)NQLIPPFYK.T | Y | 52.30 | 1278.6431 | 10 | 1.2 | 640.3296 | 2 | 54.59 | 3 | F3:31482 | NaNaKA16\_F11.raw |  |  | 2.358E6 |  |  |  |  |  |  |  |  |  |  | 1 | 0 | 0 | 1 | 0 | 0 | 0 | 0 | 0 | 0 | 0 | 0 | 0 | 0 | 24 | 33 | Carbamidomethylation | C1:Carbamidomethylation:1000.00 | PEAKS DB |
| K.YVC(+57.02)C(+57.02)NTDR.C | N | 50.92 | 1086.4222 | 8 | -0.2 | 544.2183 | 2 | 11.37 | 12 | F12:1787 | NaNaKA16\_F8.raw |  | 2.1124E6 | 2.9778E6 | 1.3296E6 |  |  |  |  |  | 1.5915E6 |  | 1.7945E6 |  | 5 | 0 | 1 | 1 | 1 | 0 | 0 | 0 | 0 | 0 | 1 | 0 | 1 | 0 | 72 | 79 | Carbamidomethylation | C3:Carbamidomethylation:1000.00;C4:Carbamidomethylation:1000.00 | PEAKS DB |
| R.GC(+57.02)IDVC(+57.02)PK.S | N | 46.03 | 947.4205 | 8 | -0.5 | 474.7173 | 2 | 11.54 | 12 | F12:1895 | NaNaKA16\_F8.raw |  |  | 1.5891E7 |  |  |  | 6.084E5 |  | 2.0697E6 | 6.3919E7 | 1.8242E6 | 6.8843E6 |  | 6 | 0 | 0 | 1 | 0 | 0 | 0 | 1 | 0 | 1 | 1 | 1 | 1 | 0 | 58 | 65 | Carbamidomethylation | C2:Carbamidomethylation:1000.00;C6:Carbamidomethylation:1000.00 | PEAKS DB |
| total 6 peptides |  |  |  |  |  |  |  |  |  |  |  |  |  |  |  |  |  |  |  |  |  |  |  |  |  |  |  |  |  |  |  |  |  |  |  |  |  |  |  |  |  |  |  |
| --- | --- | --- | --- | --- | --- | --- | --- | --- | --- | --- | --- | --- | --- | --- | --- | --- | --- | --- | --- | --- | --- | --- | --- | --- | --- | --- | --- | --- | --- | --- | --- | --- | --- | --- | --- | --- | --- | --- | --- | --- | --- | --- | --- |

P80245.2

back to list

  

| Protein Coverage
| Supporting Peptides
|

Protein Coverage:

Supporting Peptides:

| Peptide | Uniq | -10lgP | Mass | Length | ppm | m/z | z | RT | Fraction | Scan | Source File | Area F1 | Area F10 | Area F11 | Area F12 | Area F13 | Area F2 | Area F3 | Area F4 | Area F5 | Area F6 | Area F7 | Area F8 | Area F9 | #Feature | #Feature F1 | #Feature F10 | #Feature F11 | #Feature F12 | #Feature F13 | #Feature F2 | #Feature F3 | #Feature F4 | #Feature F5 | #Feature F6 | #Feature F7 | #Feature F8 | #Feature F9 | Start | End | PTM | AScore | Found By |
| --- | --- | --- | --- | --- | --- | --- | --- | --- | --- | --- | --- | --- | --- | --- | --- | --- | --- | --- | --- | --- | --- | --- | --- | --- | --- | --- | --- | --- | --- | --- | --- | --- | --- | --- | --- | --- | --- | --- | --- | --- | --- | --- | --- |
| K.SSLLVKYVC(+57.02)C(+57.02)NTDRC(+57.02)N | N | 73.90 | 1987.8914 | 16 | 0.9 | 663.6383 | 3 | 34.50 | 3 | F3:16042 | NaNaKA16\_F11.raw |  | 4.3691E6 | 4.3984E7 |  |  |  |  |  |  |  |  |  |  | 3 | 0 | 1 | 2 | 0 | 0 | 0 | 0 | 0 | 0 | 0 | 0 | 0 | 0 | 66 | 81 | Carbamidomethylation | C9:Carbamidomethylation:1000.00;C10:Carbamidomethylation:1000.00;C15:Carbamidomethylation:1000.00 | PEAKS DB |
| K.SSLLVKYVC(+57.02)C(+57.02)NTDR.C | N | 70.96 | 1713.8179 | 14 | 0.6 | 857.9167 | 2 | 34.00 | 3 | F3:15457 | NaNaKA16\_F11.raw |  | 5.0554E6 | 1.1527E8 | 1.6508E6 |  |  |  |  |  |  |  | 0 | 3.3481E6 | 7 | 0 | 2 | 2 | 1 | 0 | 0 | 0 | 0 | 0 | 0 | 0 | 0 | 2 | 66 | 79 | Carbamidomethylation | C9:Carbamidomethylation:1000.00;C10:Carbamidomethylation:1000.00 | PEAKS DB |
| R.GC(+57.02)IDVC(+57.02)PKSSLLVK.Y | N | 65.71 | 1574.8160 | 14 | 0.6 | 788.4158 | 2 | 32.88 | 3 | F3:14936 | NaNaKA16\_F11.raw |  |  | 2.0322E7 |  |  |  |  |  |  |  |  |  |  | 2 | 0 | 0 | 2 | 0 | 0 | 0 | 0 | 0 | 0 | 0 | 0 | 0 | 0 | 58 | 71 | Carbamidomethylation | C2:Carbamidomethylation:1000.00;C6:Carbamidomethylation:1000.00 | PEAKS DB |
| K.C(+57.02)NQLIPPFYK.T | Y | 52.30 | 1278.6431 | 10 | 1.2 | 640.3296 | 2 | 54.59 | 3 | F3:31482 | NaNaKA16\_F11.raw |  |  | 2.358E6 |  |  |  |  |  |  |  |  |  |  | 1 | 0 | 0 | 1 | 0 | 0 | 0 | 0 | 0 | 0 | 0 | 0 | 0 | 0 | 24 | 33 | Carbamidomethylation | C1:Carbamidomethylation:1000.00 | PEAKS DB |
| K.YVC(+57.02)C(+57.02)NTDR.C | N | 50.92 | 1086.4222 | 8 | -0.2 | 544.2183 | 2 | 11.37 | 12 | F12:1787 | NaNaKA16\_F8.raw |  | 2.1124E6 | 2.9778E6 | 1.3296E6 |  |  |  |  |  | 1.5915E6 |  | 1.7945E6 |  | 5 | 0 | 1 | 1 | 1 | 0 | 0 | 0 | 0 | 0 | 1 | 0 | 1 | 0 | 72 | 79 | Carbamidomethylation | C3:Carbamidomethylation:1000.00;C4:Carbamidomethylation:1000.00 | PEAKS DB |
| R.GC(+57.02)IDVC(+57.02)PK.S | N | 46.03 | 947.4205 | 8 | -0.5 | 474.7173 | 2 | 11.54 | 12 | F12:1895 | NaNaKA16\_F8.raw |  |  | 1.5891E7 |  |  |  | 6.084E5 |  | 2.0697E6 | 6.3919E7 | 1.8242E6 | 6.8843E6 |  | 6 | 0 | 0 | 1 | 0 | 0 | 0 | 1 | 0 | 1 | 1 | 1 | 1 | 0 | 58 | 65 | Carbamidomethylation | C2:Carbamidomethylation:1000.00;C6:Carbamidomethylation:1000.00 | PEAKS DB |
| total 6 peptides |  |  |  |  |  |  |  |  |  |  |  |  |  |  |  |  |  |  |  |  |  |  |  |  |  |  |  |  |  |  |  |  |  |  |  |  |  |  |  |  |  |  |  |
| --- | --- | --- | --- | --- | --- | --- | --- | --- | --- | --- | --- | --- | --- | --- | --- | --- | --- | --- | --- | --- | --- | --- | --- | --- | --- | --- | --- | --- | --- | --- | --- | --- | --- | --- | --- | --- | --- | --- | --- | --- | --- | --- | --- |

CAA90966.1

back to list

  

| Protein Coverage
| Supporting Peptides
|

Protein Coverage:

Supporting Peptides:

| Peptide | Uniq | -10lgP | Mass | Length | ppm | m/z | z | RT | Fraction | Scan | Source File | Area F1 | Area F10 | Area F11 | Area F12 | Area F13 | Area F2 | Area F3 | Area F4 | Area F5 | Area F6 | Area F7 | Area F8 | Area F9 | #Feature | #Feature F1 | #Feature F10 | #Feature F11 | #Feature F12 | #Feature F13 | #Feature F2 | #Feature F3 | #Feature F4 | #Feature F5 | #Feature F6 | #Feature F7 | #Feature F8 | #Feature F9 | Start | End | PTM | AScore | Found By |
| --- | --- | --- | --- | --- | --- | --- | --- | --- | --- | --- | --- | --- | --- | --- | --- | --- | --- | --- | --- | --- | --- | --- | --- | --- | --- | --- | --- | --- | --- | --- | --- | --- | --- | --- | --- | --- | --- | --- | --- | --- | --- | --- | --- |
| K.SSLLVKYVC(+57.02)C(+57.02)NTDRC(+57.02)N | N | 73.90 | 1987.8914 | 16 | 0.9 | 663.6383 | 3 | 34.50 | 3 | F3:16042 | NaNaKA16\_F11.raw |  | 4.3691E6 | 4.3984E7 |  |  |  |  |  |  |  |  |  |  | 3 | 0 | 1 | 2 | 0 | 0 | 0 | 0 | 0 | 0 | 0 | 0 | 0 | 0 | 66 | 81 | Carbamidomethylation | C9:Carbamidomethylation:1000.00;C10:Carbamidomethylation:1000.00;C15:Carbamidomethylation:1000.00 | PEAKS DB |
| K.SSLLVKYVC(+57.02)C(+57.02)NTDR.C | N | 70.96 | 1713.8179 | 14 | 0.6 | 857.9167 | 2 | 34.00 | 3 | F3:15457 | NaNaKA16\_F11.raw |  | 5.0554E6 | 1.1527E8 | 1.6508E6 |  |  |  |  |  |  |  | 0 | 3.3481E6 | 7 | 0 | 2 | 2 | 1 | 0 | 0 | 0 | 0 | 0 | 0 | 0 | 0 | 2 | 66 | 79 | Carbamidomethylation | C9:Carbamidomethylation:1000.00;C10:Carbamidomethylation:1000.00 | PEAKS DB |
| R.GC(+57.02)IDVC(+57.02)PKSSLLVK.Y | N | 65.71 | 1574.8160 | 14 | 0.6 | 788.4158 | 2 | 32.88 | 3 | F3:14936 | NaNaKA16\_F11.raw |  |  | 2.0322E7 |  |  |  |  |  |  |  |  |  |  | 2 | 0 | 0 | 2 | 0 | 0 | 0 | 0 | 0 | 0 | 0 | 0 | 0 | 0 | 58 | 71 | Carbamidomethylation | C2:Carbamidomethylation:1000.00;C6:Carbamidomethylation:1000.00 | PEAKS DB |
| K.C(+57.02)NQLIPPFYK.T | Y | 52.30 | 1278.6431 | 10 | 1.2 | 640.3296 | 2 | 54.59 | 3 | F3:31482 | NaNaKA16\_F11.raw |  |  | 2.358E6 |  |  |  |  |  |  |  |  |  |  | 1 | 0 | 0 | 1 | 0 | 0 | 0 | 0 | 0 | 0 | 0 | 0 | 0 | 0 | 24 | 33 | Carbamidomethylation | C1:Carbamidomethylation:1000.00 | PEAKS DB |
| K.YVC(+57.02)C(+57.02)NTDR.C | N | 50.92 | 1086.4222 | 8 | -0.2 | 544.2183 | 2 | 11.37 | 12 | F12:1787 | NaNaKA16\_F8.raw |  | 2.1124E6 | 2.9778E6 | 1.3296E6 |  |  |  |  |  | 1.5915E6 |  | 1.7945E6 |  | 5 | 0 | 1 | 1 | 1 | 0 | 0 | 0 | 0 | 0 | 1 | 0 | 1 | 0 | 72 | 79 | Carbamidomethylation | C3:Carbamidomethylation:1000.00;C4:Carbamidomethylation:1000.00 | PEAKS DB |
| R.GC(+57.02)IDVC(+57.02)PK.S | N | 46.03 | 947.4205 | 8 | -0.5 | 474.7173 | 2 | 11.54 | 12 | F12:1895 | NaNaKA16\_F8.raw |  |  | 1.5891E7 |  |  |  | 6.084E5 |  | 2.0697E6 | 6.3919E7 | 1.8242E6 | 6.8843E6 |  | 6 | 0 | 0 | 1 | 0 | 0 | 0 | 1 | 0 | 1 | 1 | 1 | 1 | 0 | 58 | 65 | Carbamidomethylation | C2:Carbamidomethylation:1000.00;C6:Carbamidomethylation:1000.00 | PEAKS DB |
| total 6 peptides |  |  |  |  |  |  |  |  |  |  |  |  |  |  |  |  |  |  |  |  |  |  |  |  |  |  |  |  |  |  |  |  |  |  |  |  |  |  |  |  |  |  |  |
| --- | --- | --- | --- | --- | --- | --- | --- | --- | --- | --- | --- | --- | --- | --- | --- | --- | --- | --- | --- | --- | --- | --- | --- | --- | --- | --- | --- | --- | --- | --- | --- | --- | --- | --- | --- | --- | --- | --- | --- | --- | --- | --- | --- |

CAA69977.1

back to list

  

| Protein Coverage
| Supporting Peptides
|

Protein Coverage:

Supporting Peptides:

| Peptide | Uniq | -10lgP | Mass | Length | ppm | m/z | z | RT | Fraction | Scan | Source File | Area F1 | Area F10 | Area F11 | Area F12 | Area F13 | Area F2 | Area F3 | Area F4 | Area F5 | Area F6 | Area F7 | Area F8 | Area F9 | #Feature | #Feature F1 | #Feature F10 | #Feature F11 | #Feature F12 | #Feature F13 | #Feature F2 | #Feature F3 | #Feature F4 | #Feature F5 | #Feature F6 | #Feature F7 | #Feature F8 | #Feature F9 | Start | End | PTM | AScore | Found By |
| --- | --- | --- | --- | --- | --- | --- | --- | --- | --- | --- | --- | --- | --- | --- | --- | --- | --- | --- | --- | --- | --- | --- | --- | --- | --- | --- | --- | --- | --- | --- | --- | --- | --- | --- | --- | --- | --- | --- | --- | --- | --- | --- | --- |
| K.SSLLVKYVC(+57.02)C(+57.02)NTDRC(+57.02)N.N | N | 73.90 | 1987.8914 | 16 | 0.9 | 663.6383 | 3 | 34.50 | 3 | F3:16042 | NaNaKA16\_F11.raw |  | 4.3691E6 | 4.3984E7 |  |  |  |  |  |  |  |  |  |  | 3 | 0 | 1 | 2 | 0 | 0 | 0 | 0 | 0 | 0 | 0 | 0 | 0 | 0 | 66 | 81 | Carbamidomethylation | C9:Carbamidomethylation:1000.00;C10:Carbamidomethylation:1000.00;C15:Carbamidomethylation:1000.00 | PEAKS DB |
| K.SSLLVKYVC(+57.02)C(+57.02)NTDR.C | N | 70.96 | 1713.8179 | 14 | 0.6 | 857.9167 | 2 | 34.00 | 3 | F3:15457 | NaNaKA16\_F11.raw |  | 5.0554E6 | 1.1527E8 | 1.6508E6 |  |  |  |  |  |  |  | 0 | 3.3481E6 | 7 | 0 | 2 | 2 | 1 | 0 | 0 | 0 | 0 | 0 | 0 | 0 | 0 | 2 | 66 | 79 | Carbamidomethylation | C9:Carbamidomethylation:1000.00;C10:Carbamidomethylation:1000.00 | PEAKS DB |
| R.GC(+57.02)IDVC(+57.02)PKSSLLVK.Y | N | 65.71 | 1574.8160 | 14 | 0.6 | 788.4158 | 2 | 32.88 | 3 | F3:14936 | NaNaKA16\_F11.raw |  |  | 2.0322E7 |  |  |  |  |  |  |  |  |  |  | 2 | 0 | 0 | 2 | 0 | 0 | 0 | 0 | 0 | 0 | 0 | 0 | 0 | 0 | 58 | 71 | Carbamidomethylation | C2:Carbamidomethylation:1000.00;C6:Carbamidomethylation:1000.00 | PEAKS DB |
| K.C(+57.02)NQLIPPFYK.T | Y | 52.30 | 1278.6431 | 10 | 1.2 | 640.3296 | 2 | 54.59 | 3 | F3:31482 | NaNaKA16\_F11.raw |  |  | 2.358E6 |  |  |  |  |  |  |  |  |  |  | 1 | 0 | 0 | 1 | 0 | 0 | 0 | 0 | 0 | 0 | 0 | 0 | 0 | 0 | 24 | 33 | Carbamidomethylation | C1:Carbamidomethylation:1000.00 | PEAKS DB |
| K.YVC(+57.02)C(+57.02)NTDR.C | N | 50.92 | 1086.4222 | 8 | -0.2 | 544.2183 | 2 | 11.37 | 12 | F12:1787 | NaNaKA16\_F8.raw |  | 2.1124E6 | 2.9778E6 | 1.3296E6 |  |  |  |  |  | 1.5915E6 |  | 1.7945E6 |  | 5 | 0 | 1 | 1 | 1 | 0 | 0 | 0 | 0 | 0 | 1 | 0 | 1 | 0 | 72 | 79 | Carbamidomethylation | C3:Carbamidomethylation:1000.00;C4:Carbamidomethylation:1000.00 | PEAKS DB |
| R.GC(+57.02)IDVC(+57.02)PK.S | N | 46.03 | 947.4205 | 8 | -0.5 | 474.7173 | 2 | 11.54 | 12 | F12:1895 | NaNaKA16\_F8.raw |  |  | 1.5891E7 |  |  |  | 6.084E5 |  | 2.0697E6 | 6.3919E7 | 1.8242E6 | 6.8843E6 |  | 6 | 0 | 0 | 1 | 0 | 0 | 0 | 1 | 0 | 1 | 1 | 1 | 1 | 0 | 58 | 65 | Carbamidomethylation | C2:Carbamidomethylation:1000.00;C6:Carbamidomethylation:1000.00 | PEAKS DB |
| total 6 peptides |  |  |  |  |  |  |  |  |  |  |  |  |  |  |  |  |  |  |  |  |  |  |  |  |  |  |  |  |  |  |  |  |  |  |  |  |  |  |  |  |  |  |  |
| --- | --- | --- | --- | --- | --- | --- | --- | --- | --- | --- | --- | --- | --- | --- | --- | --- | --- | --- | --- | --- | --- | --- | --- | --- | --- | --- | --- | --- | --- | --- | --- | --- | --- | --- | --- | --- | --- | --- | --- | --- | --- | --- | --- |

Q98965.1

back to list

  

| Protein Coverage
| Supporting Peptides
|

Protein Coverage:

Supporting Peptides:

| Peptide | Uniq | -10lgP | Mass | Length | ppm | m/z | z | RT | Fraction | Scan | Source File | Area F1 | Area F10 | Area F11 | Area F12 | Area F13 | Area F2 | Area F3 | Area F4 | Area F5 | Area F6 | Area F7 | Area F8 | Area F9 | #Feature | #Feature F1 | #Feature F10 | #Feature F11 | #Feature F12 | #Feature F13 | #Feature F2 | #Feature F3 | #Feature F4 | #Feature F5 | #Feature F6 | #Feature F7 | #Feature F8 | #Feature F9 | Start | End | PTM | AScore | Found By |
| --- | --- | --- | --- | --- | --- | --- | --- | --- | --- | --- | --- | --- | --- | --- | --- | --- | --- | --- | --- | --- | --- | --- | --- | --- | --- | --- | --- | --- | --- | --- | --- | --- | --- | --- | --- | --- | --- | --- | --- | --- | --- | --- | --- |
| K.SSLLVKYVC(+57.02)C(+57.02)NTDRC(+57.02)N.N | N | 73.90 | 1987.8914 | 16 | 0.9 | 663.6383 | 3 | 34.50 | 3 | F3:16042 | NaNaKA16\_F11.raw |  | 4.3691E6 | 4.3984E7 |  |  |  |  |  |  |  |  |  |  | 3 | 0 | 1 | 2 | 0 | 0 | 0 | 0 | 0 | 0 | 0 | 0 | 0 | 0 | 66 | 81 | Carbamidomethylation | C9:Carbamidomethylation:1000.00;C10:Carbamidomethylation:1000.00;C15:Carbamidomethylation:1000.00 | PEAKS DB |
| K.SSLLVKYVC(+57.02)C(+57.02)NTDR.C | N | 70.96 | 1713.8179 | 14 | 0.6 | 857.9167 | 2 | 34.00 | 3 | F3:15457 | NaNaKA16\_F11.raw |  | 5.0554E6 | 1.1527E8 | 1.6508E6 |  |  |  |  |  |  |  | 0 | 3.3481E6 | 7 | 0 | 2 | 2 | 1 | 0 | 0 | 0 | 0 | 0 | 0 | 0 | 0 | 2 | 66 | 79 | Carbamidomethylation | C9:Carbamidomethylation:1000.00;C10:Carbamidomethylation:1000.00 | PEAKS DB |
| R.GC(+57.02)IDVC(+57.02)PKSSLLVK.Y | N | 65.71 | 1574.8160 | 14 | 0.6 | 788.4158 | 2 | 32.88 | 3 | F3:14936 | NaNaKA16\_F11.raw |  |  | 2.0322E7 |  |  |  |  |  |  |  |  |  |  | 2 | 0 | 0 | 2 | 0 | 0 | 0 | 0 | 0 | 0 | 0 | 0 | 0 | 0 | 58 | 71 | Carbamidomethylation | C2:Carbamidomethylation:1000.00;C6:Carbamidomethylation:1000.00 | PEAKS DB |
| K.C(+57.02)NQLIPPFYK.T | Y | 52.30 | 1278.6431 | 10 | 1.2 | 640.3296 | 2 | 54.59 | 3 | F3:31482 | NaNaKA16\_F11.raw |  |  | 2.358E6 |  |  |  |  |  |  |  |  |  |  | 1 | 0 | 0 | 1 | 0 | 0 | 0 | 0 | 0 | 0 | 0 | 0 | 0 | 0 | 24 | 33 | Carbamidomethylation | C1:Carbamidomethylation:1000.00 | PEAKS DB |
| K.YVC(+57.02)C(+57.02)NTDR.C | N | 50.92 | 1086.4222 | 8 | -0.2 | 544.2183 | 2 | 11.37 | 12 | F12:1787 | NaNaKA16\_F8.raw |  | 2.1124E6 | 2.9778E6 | 1.3296E6 |  |  |  |  |  | 1.5915E6 |  | 1.7945E6 |  | 5 | 0 | 1 | 1 | 1 | 0 | 0 | 0 | 0 | 0 | 1 | 0 | 1 | 0 | 72 | 79 | Carbamidomethylation | C3:Carbamidomethylation:1000.00;C4:Carbamidomethylation:1000.00 | PEAKS DB |
| R.GC(+57.02)IDVC(+57.02)PK.S | N | 46.03 | 947.4205 | 8 | -0.5 | 474.7173 | 2 | 11.54 | 12 | F12:1895 | NaNaKA16\_F8.raw |  |  | 1.5891E7 |  |  |  | 6.084E5 |  | 2.0697E6 | 6.3919E7 | 1.8242E6 | 6.8843E6 |  | 6 | 0 | 0 | 1 | 0 | 0 | 0 | 1 | 0 | 1 | 1 | 1 | 1 | 0 | 58 | 65 | Carbamidomethylation | C2:Carbamidomethylation:1000.00;C6:Carbamidomethylation:1000.00 | PEAKS DB |
| total 6 peptides |  |  |  |  |  |  |  |  |  |  |  |  |  |  |  |  |  |  |  |  |  |  |  |  |  |  |  |  |  |  |  |  |  |  |  |  |  |  |  |  |  |  |  |
| --- | --- | --- | --- | --- | --- | --- | --- | --- | --- | --- | --- | --- | --- | --- | --- | --- | --- | --- | --- | --- | --- | --- | --- | --- | --- | --- | --- | --- | --- | --- | --- | --- | --- | --- | --- | --- | --- | --- | --- | --- | --- | --- | --- |

CAA55333.1

back to list

  

| Protein Coverage
| Supporting Peptides
|

Protein Coverage:

Supporting Peptides:

| Peptide | Uniq | -10lgP | Mass | Length | ppm | m/z | z | RT | Fraction | Scan | Source File | Area F1 | Area F10 | Area F11 | Area F12 | Area F13 | Area F2 | Area F3 | Area F4 | Area F5 | Area F6 | Area F7 | Area F8 | Area F9 | #Feature | #Feature F1 | #Feature F10 | #Feature F11 | #Feature F12 | #Feature F13 | #Feature F2 | #Feature F3 | #Feature F4 | #Feature F5 | #Feature F6 | #Feature F7 | #Feature F8 | #Feature F9 | Start | End | PTM | AScore | Found By |
| --- | --- | --- | --- | --- | --- | --- | --- | --- | --- | --- | --- | --- | --- | --- | --- | --- | --- | --- | --- | --- | --- | --- | --- | --- | --- | --- | --- | --- | --- | --- | --- | --- | --- | --- | --- | --- | --- | --- | --- | --- | --- | --- | --- |
| R.DSVLAQYIFELSR.R | Y | 69.25 | 1539.7932 | 13 | 0.9 | 770.9045 | 2 | 100.63 | 4 | F4:70292 | NaNaKA16\_F12.raw |  |  |  | 1.0257E6 |  |  |  |  |  |  |  |  |  | 1 | 0 | 0 | 0 | 1 | 0 | 0 | 0 | 0 | 0 | 0 | 0 | 0 | 0 | 160 | 172 |  |  | PEAKS DB |
| K.ESFLFTLTR.N | N | 61.74 | 1112.5865 | 9 | 0.9 | 557.3010 | 2 | 70.76 | 4 | F4:47352 | NaNaKA16\_F12.raw |  | 9.6337E5 |  | 1.0016E7 |  |  |  |  |  |  |  |  |  | 2 | 0 | 1 | 0 | 1 | 0 | 0 | 0 | 0 | 0 | 0 | 0 | 0 | 0 | 356 | 364 |  |  | PEAKS DB |
| R.AALSQYVC(+57.02)EHK.D | N | 61.07 | 1304.6183 | 11 | 0.1 | 653.3165 | 2 | 12.43 | 5 | F5:2258 | NaNaKA16\_F13.raw |  |  |  |  | 1.4233E6 |  |  |  |  |  |  |  |  | 2 | 0 | 0 | 0 | 0 | 2 | 0 | 0 | 0 | 0 | 0 | 0 | 0 | 0 | 286 | 296 | Carbamidomethylation | C8:Carbamidomethylation:1000.00 | PEAKS DB |
| K.ILETC(+57.02)C(+57.02)AEADKDAC(+57.02)IHEK.A | Y | 55.71 | 2161.9441 | 18 | 0.9 | 721.6559 | 3 | 16.34 | 5 | F5:3387 | NaNaKA16\_F13.raw |  |  |  |  | 8.0793E5 |  |  |  |  |  |  |  |  | 1 | 0 | 0 | 0 | 0 | 1 | 0 | 0 | 0 | 0 | 0 | 0 | 0 | 0 | 192 | 209 | Carbamidomethylation | C5:Carbamidomethylation:1000.00;C6:Carbamidomethylation:1000.00;C14:Carbamidomethylation:1000.00 | PEAKS DB |
| K.LSAEIIELHK.K | Y | 51.62 | 1151.6550 | 10 | 0.1 | 576.8348 | 2 | 34.25 | 5 | F5:12846 | NaNaKA16\_F13.raw |  |  |  |  | 1.2882E6 |  |  |  |  |  |  |  |  | 2 | 0 | 0 | 0 | 0 | 2 | 0 | 0 | 0 | 0 | 0 | 0 | 0 | 0 | 69 | 78 |  |  | PEAKS DB |
| R.RYPTALSVVILESTK.T | Y | 50.77 | 1675.9508 | 15 | 0.7 | 559.6580 | 3 | 59.32 | 4 | F4:37972 | NaNaKA16\_F12.raw |  |  |  | 1.8323E6 |  |  |  |  |  |  |  |  |  | 1 | 0 | 0 | 0 | 1 | 0 | 0 | 0 | 0 | 0 | 0 | 0 | 0 | 0 | 173 | 187 |  |  | PEAKS DB |
| R.YPTALSVVILESTK.T | Y | 46.54 | 1519.8497 | 14 | 1.3 | 760.9331 | 2 | 75.67 | 4 | F4:51451 | NaNaKA16\_F12.raw |  |  |  | 5.6387E5 |  |  |  |  |  |  |  |  |  | 1 | 0 | 0 | 0 | 1 | 0 | 0 | 0 | 0 | 0 | 0 | 0 | 0 | 0 | 174 | 187 |  |  | PEAKS DB |
| R.SPDLPPPSEEILK.E | Y | 43.64 | 1420.7449 | 13 | 1.0 | 711.3804 | 2 | 49.60 | 5 | F5:19999 | NaNaKA16\_F13.raw |  |  |  |  | 2.2776E6 |  |  |  |  |  |  |  |  | 1 | 0 | 0 | 0 | 0 | 1 | 0 | 0 | 0 | 0 | 0 | 0 | 0 | 0 | 327 | 339 |  |  | PEAKS DB |
| total 8 peptides |  |  |  |  |  |  |  |  |  |  |  |  |  |  |  |  |  |  |  |  |  |  |  |  |  |  |  |  |  |  |  |  |  |  |  |  |  |  |  |  |  |  |  |
| --- | --- | --- | --- | --- | --- | --- | --- | --- | --- | --- | --- | --- | --- | --- | --- | --- | --- | --- | --- | --- | --- | --- | --- | --- | --- | --- | --- | --- | --- | --- | --- | --- | --- | --- | --- | --- | --- | --- | --- | --- | --- | --- | --- |

S59517

back to list

  

| Protein Coverage
| Supporting Peptides
|

Protein Coverage:

Supporting Peptides:

| Peptide | Uniq | -10lgP | Mass | Length | ppm | m/z | z | RT | Fraction | Scan | Source File | Area F1 | Area F10 | Area F11 | Area F12 | Area F13 | Area F2 | Area F3 | Area F4 | Area F5 | Area F6 | Area F7 | Area F8 | Area F9 | #Feature | #Feature F1 | #Feature F10 | #Feature F11 | #Feature F12 | #Feature F13 | #Feature F2 | #Feature F3 | #Feature F4 | #Feature F5 | #Feature F6 | #Feature F7 | #Feature F8 | #Feature F9 | Start | End | PTM | AScore | Found By |
| --- | --- | --- | --- | --- | --- | --- | --- | --- | --- | --- | --- | --- | --- | --- | --- | --- | --- | --- | --- | --- | --- | --- | --- | --- | --- | --- | --- | --- | --- | --- | --- | --- | --- | --- | --- | --- | --- | --- | --- | --- | --- | --- | --- |
| R.DSVLAQYIFELSR.R | Y | 69.25 | 1539.7932 | 13 | 0.9 | 770.9045 | 2 | 100.63 | 4 | F4:70292 | NaNaKA16\_F12.raw |  |  |  | 1.0257E6 |  |  |  |  |  |  |  |  |  | 1 | 0 | 0 | 0 | 1 | 0 | 0 | 0 | 0 | 0 | 0 | 0 | 0 | 0 | 160 | 172 |  |  | PEAKS DB |
| K.ESFLFTLTR.N | N | 61.74 | 1112.5865 | 9 | 0.9 | 557.3010 | 2 | 70.76 | 4 | F4:47352 | NaNaKA16\_F12.raw |  | 9.6337E5 |  | 1.0016E7 |  |  |  |  |  |  |  |  |  | 2 | 0 | 1 | 0 | 1 | 0 | 0 | 0 | 0 | 0 | 0 | 0 | 0 | 0 | 356 | 364 |  |  | PEAKS DB |
| R.AALSQYVC(+57.02)EHK.D | N | 61.07 | 1304.6183 | 11 | 0.1 | 653.3165 | 2 | 12.43 | 5 | F5:2258 | NaNaKA16\_F13.raw |  |  |  |  | 1.4233E6 |  |  |  |  |  |  |  |  | 2 | 0 | 0 | 0 | 0 | 2 | 0 | 0 | 0 | 0 | 0 | 0 | 0 | 0 | 286 | 296 | Carbamidomethylation | C8:Carbamidomethylation:1000.00 | PEAKS DB |
| K.ILETC(+57.02)C(+57.02)AEADKDAC(+57.02)IHEK.A | Y | 55.71 | 2161.9441 | 18 | 0.9 | 721.6559 | 3 | 16.34 | 5 | F5:3387 | NaNaKA16\_F13.raw |  |  |  |  | 8.0793E5 |  |  |  |  |  |  |  |  | 1 | 0 | 0 | 0 | 0 | 1 | 0 | 0 | 0 | 0 | 0 | 0 | 0 | 0 | 192 | 209 | Carbamidomethylation | C5:Carbamidomethylation:1000.00;C6:Carbamidomethylation:1000.00;C14:Carbamidomethylation:1000.00 | PEAKS DB |
| K.LSAEIIELHK.K | Y | 51.62 | 1151.6550 | 10 | 0.1 | 576.8348 | 2 | 34.25 | 5 | F5:12846 | NaNaKA16\_F13.raw |  |  |  |  | 1.2882E6 |  |  |  |  |  |  |  |  | 2 | 0 | 0 | 0 | 0 | 2 | 0 | 0 | 0 | 0 | 0 | 0 | 0 | 0 | 69 | 78 |  |  | PEAKS DB |
| R.RYPTALSVVILESTK.T | Y | 50.77 | 1675.9508 | 15 | 0.7 | 559.6580 | 3 | 59.32 | 4 | F4:37972 | NaNaKA16\_F12.raw |  |  |  | 1.8323E6 |  |  |  |  |  |  |  |  |  | 1 | 0 | 0 | 0 | 1 | 0 | 0 | 0 | 0 | 0 | 0 | 0 | 0 | 0 | 173 | 187 |  |  | PEAKS DB |
| R.YPTALSVVILESTK.T | Y | 46.54 | 1519.8497 | 14 | 1.3 | 760.9331 | 2 | 75.67 | 4 | F4:51451 | NaNaKA16\_F12.raw |  |  |  | 5.6387E5 |  |  |  |  |  |  |  |  |  | 1 | 0 | 0 | 0 | 1 | 0 | 0 | 0 | 0 | 0 | 0 | 0 | 0 | 0 | 174 | 187 |  |  | PEAKS DB |
| R.SPDLPPPSEEILK.E | Y | 43.64 | 1420.7449 | 13 | 1.0 | 711.3804 | 2 | 49.60 | 5 | F5:19999 | NaNaKA16\_F13.raw |  |  |  |  | 2.2776E6 |  |  |  |  |  |  |  |  | 1 | 0 | 0 | 0 | 0 | 1 | 0 | 0 | 0 | 0 | 0 | 0 | 0 | 0 | 327 | 339 |  |  | PEAKS DB |
| total 8 peptides |  |  |  |  |  |  |  |  |  |  |  |  |  |  |  |  |  |  |  |  |  |  |  |  |  |  |  |  |  |  |  |  |  |  |  |  |  |  |  |  |  |  |  |
| --- | --- | --- | --- | --- | --- | --- | --- | --- | --- | --- | --- | --- | --- | --- | --- | --- | --- | --- | --- | --- | --- | --- | --- | --- | --- | --- | --- | --- | --- | --- | --- | --- | --- | --- | --- | --- | --- | --- | --- | --- | --- | --- | --- |

JAS05092.1

back to list

  

| Protein Coverage
| Supporting Peptides
|

Protein Coverage:

Supporting Peptides:

| Peptide | Uniq | -10lgP | Mass | Length | ppm | m/z | z | RT | Fraction | Scan | Source File | Area F1 | Area F10 | Area F11 | Area F12 | Area F13 | Area F2 | Area F3 | Area F4 | Area F5 | Area F6 | Area F7 | Area F8 | Area F9 | #Feature | #Feature F1 | #Feature F10 | #Feature F11 | #Feature F12 | #Feature F13 | #Feature F2 | #Feature F3 | #Feature F4 | #Feature F5 | #Feature F6 | #Feature F7 | #Feature F8 | #Feature F9 | Start | End | PTM | AScore | Found By |
| --- | --- | --- | --- | --- | --- | --- | --- | --- | --- | --- | --- | --- | --- | --- | --- | --- | --- | --- | --- | --- | --- | --- | --- | --- | --- | --- | --- | --- | --- | --- | --- | --- | --- | --- | --- | --- | --- | --- | --- | --- | --- | --- | --- |
| R.DPDYGMVEPGTK.C | N | 79.87 | 1307.5703 | 12 | 0.4 | 654.7927 | 2 | 33.63 | 5 | F5:12378 | NaNaKA16\_F13.raw |  |  |  |  | 3.4025E6 |  |  |  |  |  |  |  |  | 2 | 0 | 0 | 0 | 0 | 2 | 0 | 0 | 0 | 0 | 0 | 0 | 0 | 0 | 583 | 594 |  |  | PEAKS DB |
| A.DSSAVISAC(+57.02)DGLK.G | N | 60.11 | 1321.6184 | 13 | 0.4 | 661.8168 | 2 | 33.21 | 10 | F10:19042 | NaNaKA16\_F6.raw |  |  |  |  |  |  |  |  |  | 6.9505E6 |  |  |  | 1 | 0 | 0 | 0 | 0 | 0 | 0 | 0 | 0 | 0 | 1 | 0 | 0 | 0 | 119 | 131 | Carbamidomethylation | C9:Carbamidomethylation:1000.00 | PEAKS DB |
| R.AAKDDC(+57.02)DLPEFC(+57.02)TGR.S | Y | 58.01 | 1753.7400 | 15 | -0.2 | 877.8771 | 2 | 29.51 | 10 | F10:15073 | NaNaKA16\_F6.raw |  |  |  |  |  |  |  |  |  | 2.1613E7 |  |  |  | 1 | 0 | 0 | 0 | 0 | 0 | 0 | 0 | 0 | 0 | 1 | 0 | 0 | 0 | 468 | 482 | Carbamidomethylation | C6:Carbamidomethylation:1000.00;C12:Carbamidomethylation:1000.00 | PEAKS DB |
| K.C(+57.02)GDGMVC(+57.02)SNR.Q | N | 51.31 | 1154.4268 | 10 | 1.1 | 578.2213 | 2 | 11.85 | 5 | F5:1806 | NaNaKA16\_F13.raw |  |  |  |  | 4.5755E5 |  |  |  |  |  |  |  |  | 1 | 0 | 0 | 0 | 0 | 1 | 0 | 0 | 0 | 0 | 0 | 0 | 0 | 0 | 595 | 604 | Carbamidomethylation | C1:Carbamidomethylation:1000.00;C7:Carbamidomethylation:1000.00 | PEAKS DB |
| K.LQHEAQC(+57.02)DSEEC(+57.02)C(+57.02)EK.C | N | 46.38 | 1921.7240 | 15 | 0.0 | 641.5820 | 3 | 11.83 | 5 | F5:1808 | NaNaKA16\_F13.raw |  |  |  |  | 2.9375E5 |  |  |  |  |  |  |  |  | 1 | 0 | 0 | 0 | 0 | 1 | 0 | 0 | 0 | 0 | 0 | 0 | 0 | 0 | 443 | 457 | Carbamidomethylation | C7:Carbamidomethylation:1000.00;C12:Carbamidomethylation:1000.00;C13:Carbamidomethylation:1000.00 | PEAKS DB |
| R.DRPQC(+57.02)ILNKPLST.D | N | 46.29 | 1540.8031 | 13 | -0.4 | 514.6081 | 3 | 27.16 | 4 | F4:11109 | NaNaKA16\_F12.raw |  |  |  | 7.0589E5 |  |  |  | 8.268E5 |  |  |  |  |  | 2 | 0 | 0 | 0 | 1 | 0 | 0 | 0 | 1 | 0 | 0 | 0 | 0 | 0 | 392 | 404 | Carbamidomethylation | C5:Carbamidomethylation:1000.00 | PEAKS DB |
| K.C(+57.02)GDGM(+15.99)VC(+57.02)SNR.Q | N | 45.27 | 1170.4216 | 10 | 0.3 | 586.2183 | 2 | 11.84 | 5 | F5:1815 | NaNaKA16\_F13.raw |  |  |  |  | 9.9702E4 |  |  |  |  |  |  |  |  | 1 | 0 | 0 | 0 | 0 | 1 | 0 | 0 | 0 | 0 | 0 | 0 | 0 | 0 | 595 | 604 | Carbamidomethylation; Oxidation (M) | C1:Carbamidomethylation:1000.00;M5:Oxidation (M):1000.00;C7:Carbamidomethylation:1000.00 | PEAKS DB |
| total 7 peptides |  |  |  |  |  |  |  |  |  |  |  |  |  |  |  |  |  |  |  |  |  |  |  |  |  |  |  |  |  |  |  |  |  |  |  |  |  |  |  |  |  |  |  |
| --- | --- | --- | --- | --- | --- | --- | --- | --- | --- | --- | --- | --- | --- | --- | --- | --- | --- | --- | --- | --- | --- | --- | --- | --- | --- | --- | --- | --- | --- | --- | --- | --- | --- | --- | --- | --- | --- | --- | --- | --- | --- | --- | --- |

P49122.1

back to list

  

| Protein Coverage
| Supporting Peptides
|

Protein Coverage:

Supporting Peptides:

| Peptide | Uniq | -10lgP | Mass | Length | ppm | m/z | z | RT | Fraction | Scan | Source File | Area F1 | Area F10 | Area F11 | Area F12 | Area F13 | Area F2 | Area F3 | Area F4 | Area F5 | Area F6 | Area F7 | Area F8 | Area F9 | #Feature | #Feature F1 | #Feature F10 | #Feature F11 | #Feature F12 | #Feature F13 | #Feature F2 | #Feature F3 | #Feature F4 | #Feature F5 | #Feature F6 | #Feature F7 | #Feature F8 | #Feature F9 | Start | End | PTM | AScore | Found By |
| --- | --- | --- | --- | --- | --- | --- | --- | --- | --- | --- | --- | --- | --- | --- | --- | --- | --- | --- | --- | --- | --- | --- | --- | --- | --- | --- | --- | --- | --- | --- | --- | --- | --- | --- | --- | --- | --- | --- | --- | --- | --- | --- | --- |
| K.C(+57.02)HNTQLPFIYNTC(+57.02)PEGK.N | Y | 86.07 | 2077.9351 | 17 | 1.0 | 693.6530 | 3 | 41.97 | 3 | F3:22380 | NaNaKA16\_F11.raw |  | 3.8675E6 | 4.1549E7 |  |  |  |  |  |  |  |  |  |  | 4 | 0 | 1 | 3 | 0 | 0 | 0 | 0 | 0 | 0 | 0 | 0 | 0 | 0 | 24 | 40 | Carbamidomethylation | C1:Carbamidomethylation:1000.00;C13:Carbamidomethylation:1000.00 | PEAKS DB |
| K.FPLKFPVK.R | N | 54.70 | 974.5953 | 8 | 0.0 | 488.3049 | 2 | 39.56 | 13 | F13:19983 | NaNaKA16\_F9.raw |  | 1.6603E7 | 4.0114E6 | 1.1085E7 |  |  |  |  |  | 5.1109E6 | 5.3917E6 | 2.3367E8 | 9.727E8 | 7 | 0 | 1 | 1 | 1 | 0 | 0 | 0 | 0 | 0 | 1 | 1 | 1 | 1 | 50 | 57 |  |  | PEAKS DB |
| K.NLC(+57.02)FKATLK.F | N | 53.76 | 1093.5953 | 9 | 0.6 | 547.8053 | 2 | 11.47 | 13 | F13:1926 | NaNaKA16\_F9.raw |  |  |  |  |  |  |  |  |  |  |  |  | 1.6934E5 | 1 | 0 | 0 | 0 | 0 | 0 | 0 | 0 | 0 | 0 | 0 | 0 | 0 | 1 | 41 | 49 | Carbamidomethylation | C3:Carbamidomethylation:1000.00 | PEAKS DB |
| L.KFPLKFPVK.R | N | 53.60 | 1102.6902 | 9 | 0.2 | 552.3525 | 2 | 23.75 | 13 | F13:7063 | NaNaKA16\_F9.raw |  |  |  |  |  |  |  |  |  |  |  |  | 6.4443E6 | 1 | 0 | 0 | 0 | 0 | 0 | 0 | 0 | 0 | 0 | 0 | 0 | 0 | 1 | 49 | 57 |  |  | PEAKS DB |
| total 4 peptides |  |  |  |  |  |  |  |  |  |  |  |  |  |  |  |  |  |  |  |  |  |  |  |  |  |  |  |  |  |  |  |  |  |  |  |  |  |  |  |  |  |  |  |
| --- | --- | --- | --- | --- | --- | --- | --- | --- | --- | --- | --- | --- | --- | --- | --- | --- | --- | --- | --- | --- | --- | --- | --- | --- | --- | --- | --- | --- | --- | --- | --- | --- | --- | --- | --- | --- | --- | --- | --- | --- | --- | --- | --- |

CAA77017.1

back to list

  

| Protein Coverage
| Supporting Peptides
|

Protein Coverage:

Supporting Peptides:

| Peptide | Uniq | -10lgP | Mass | Length | ppm | m/z | z | RT | Fraction | Scan | Source File | Area F1 | Area F10 | Area F11 | Area F12 | Area F13 | Area F2 | Area F3 | Area F4 | Area F5 | Area F6 | Area F7 | Area F8 | Area F9 | #Feature | #Feature F1 | #Feature F10 | #Feature F11 | #Feature F12 | #Feature F13 | #Feature F2 | #Feature F3 | #Feature F4 | #Feature F5 | #Feature F6 | #Feature F7 | #Feature F8 | #Feature F9 | Start | End | PTM | AScore | Found By |
| --- | --- | --- | --- | --- | --- | --- | --- | --- | --- | --- | --- | --- | --- | --- | --- | --- | --- | --- | --- | --- | --- | --- | --- | --- | --- | --- | --- | --- | --- | --- | --- | --- | --- | --- | --- | --- | --- | --- | --- | --- | --- | --- | --- |
| K.C(+57.02)HNTQLPFIYNTC(+57.02)PEGK.N | Y | 86.07 | 2077.9351 | 17 | 1.0 | 693.6530 | 3 | 41.97 | 3 | F3:22380 | NaNaKA16\_F11.raw |  | 3.8675E6 | 4.1549E7 |  |  |  |  |  |  |  |  |  |  | 4 | 0 | 1 | 3 | 0 | 0 | 0 | 0 | 0 | 0 | 0 | 0 | 0 | 0 | 24 | 40 | Carbamidomethylation | C1:Carbamidomethylation:1000.00;C13:Carbamidomethylation:1000.00 | PEAKS DB |
| K.FPLKFPVK.R | N | 54.70 | 974.5953 | 8 | 0.0 | 488.3049 | 2 | 39.56 | 13 | F13:19983 | NaNaKA16\_F9.raw |  | 1.6603E7 | 4.0114E6 | 1.1085E7 |  |  |  |  |  | 5.1109E6 | 5.3917E6 | 2.3367E8 | 9.727E8 | 7 | 0 | 1 | 1 | 1 | 0 | 0 | 0 | 0 | 0 | 1 | 1 | 1 | 1 | 50 | 57 |  |  | PEAKS DB |
| K.NLC(+57.02)FKATLK.F | N | 53.76 | 1093.5953 | 9 | 0.6 | 547.8053 | 2 | 11.47 | 13 | F13:1926 | NaNaKA16\_F9.raw |  |  |  |  |  |  |  |  |  |  |  |  | 1.6934E5 | 1 | 0 | 0 | 0 | 0 | 0 | 0 | 0 | 0 | 0 | 0 | 0 | 0 | 1 | 41 | 49 | Carbamidomethylation | C3:Carbamidomethylation:1000.00 | PEAKS DB |
| L.KFPLKFPVK.R | N | 53.60 | 1102.6902 | 9 | 0.2 | 552.3525 | 2 | 23.75 | 13 | F13:7063 | NaNaKA16\_F9.raw |  |  |  |  |  |  |  |  |  |  |  |  | 6.4443E6 | 1 | 0 | 0 | 0 | 0 | 0 | 0 | 0 | 0 | 0 | 0 | 0 | 0 | 1 | 49 | 57 |  |  | PEAKS DB |
| total 4 peptides |  |  |  |  |  |  |  |  |  |  |  |  |  |  |  |  |  |  |  |  |  |  |  |  |  |  |  |  |  |  |  |  |  |  |  |  |  |  |  |  |  |  |  |
| --- | --- | --- | --- | --- | --- | --- | --- | --- | --- | --- | --- | --- | --- | --- | --- | --- | --- | --- | --- | --- | --- | --- | --- | --- | --- | --- | --- | --- | --- | --- | --- | --- | --- | --- | --- | --- | --- | --- | --- | --- | --- | --- | --- |

CAA90967.1

back to list

  

| Protein Coverage
| Supporting Peptides
|

Protein Coverage:

Supporting Peptides:

| Peptide | Uniq | -10lgP | Mass | Length | ppm | m/z | z | RT | Fraction | Scan | Source File | Area F1 | Area F10 | Area F11 | Area F12 | Area F13 | Area F2 | Area F3 | Area F4 | Area F5 | Area F6 | Area F7 | Area F8 | Area F9 | #Feature | #Feature F1 | #Feature F10 | #Feature F11 | #Feature F12 | #Feature F13 | #Feature F2 | #Feature F3 | #Feature F4 | #Feature F5 | #Feature F6 | #Feature F7 | #Feature F8 | #Feature F9 | Start | End | PTM | AScore | Found By |
| --- | --- | --- | --- | --- | --- | --- | --- | --- | --- | --- | --- | --- | --- | --- | --- | --- | --- | --- | --- | --- | --- | --- | --- | --- | --- | --- | --- | --- | --- | --- | --- | --- | --- | --- | --- | --- | --- | --- | --- | --- | --- | --- | --- |
| K.C(+57.02)HNTQLPFIYNTC(+57.02)PEGK.N | Y | 86.07 | 2077.9351 | 17 | 1.0 | 693.6530 | 3 | 41.97 | 3 | F3:22380 | NaNaKA16\_F11.raw |  | 3.8675E6 | 4.1549E7 |  |  |  |  |  |  |  |  |  |  | 4 | 0 | 1 | 3 | 0 | 0 | 0 | 0 | 0 | 0 | 0 | 0 | 0 | 0 | 24 | 40 | Carbamidomethylation | C1:Carbamidomethylation:1000.00;C13:Carbamidomethylation:1000.00 | PEAKS DB |
| K.FPLKFPVK.R | N | 54.70 | 974.5953 | 8 | 0.0 | 488.3049 | 2 | 39.56 | 13 | F13:19983 | NaNaKA16\_F9.raw |  | 1.6603E7 | 4.0114E6 | 1.1085E7 |  |  |  |  |  | 5.1109E6 | 5.3917E6 | 2.3367E8 | 9.727E8 | 7 | 0 | 1 | 1 | 1 | 0 | 0 | 0 | 0 | 0 | 1 | 1 | 1 | 1 | 50 | 57 |  |  | PEAKS DB |
| K.NLC(+57.02)FKATLK.F | N | 53.76 | 1093.5953 | 9 | 0.6 | 547.8053 | 2 | 11.47 | 13 | F13:1926 | NaNaKA16\_F9.raw |  |  |  |  |  |  |  |  |  |  |  |  | 1.6934E5 | 1 | 0 | 0 | 0 | 0 | 0 | 0 | 0 | 0 | 0 | 0 | 0 | 0 | 1 | 41 | 49 | Carbamidomethylation | C3:Carbamidomethylation:1000.00 | PEAKS DB |
| L.KFPLKFPVK.R | N | 53.60 | 1102.6902 | 9 | 0.2 | 552.3525 | 2 | 23.75 | 13 | F13:7063 | NaNaKA16\_F9.raw |  |  |  |  |  |  |  |  |  |  |  |  | 6.4443E6 | 1 | 0 | 0 | 0 | 0 | 0 | 0 | 0 | 0 | 0 | 0 | 0 | 0 | 1 | 49 | 57 |  |  | PEAKS DB |
| total 4 peptides |  |  |  |  |  |  |  |  |  |  |  |  |  |  |  |  |  |  |  |  |  |  |  |  |  |  |  |  |  |  |  |  |  |  |  |  |  |  |  |  |  |  |  |
| --- | --- | --- | --- | --- | --- | --- | --- | --- | --- | --- | --- | --- | --- | --- | --- | --- | --- | --- | --- | --- | --- | --- | --- | --- | --- | --- | --- | --- | --- | --- | --- | --- | --- | --- | --- | --- | --- | --- | --- | --- | --- | --- | --- |

XP\_026581281.1

back to list

  

| Protein Coverage
| Supporting Peptides
|

Protein Coverage:

Supporting Peptides:

| Peptide | Uniq | -10lgP | Mass | Length | ppm | m/z | z | RT | Fraction | Scan | Source File | Area F1 | Area F10 | Area F11 | Area F12 | Area F13 | Area F2 | Area F3 | Area F4 | Area F5 | Area F6 | Area F7 | Area F8 | Area F9 | #Feature | #Feature F1 | #Feature F10 | #Feature F11 | #Feature F12 | #Feature F13 | #Feature F2 | #Feature F3 | #Feature F4 | #Feature F5 | #Feature F6 | #Feature F7 | #Feature F8 | #Feature F9 | Start | End | PTM | AScore | Found By |
| --- | --- | --- | --- | --- | --- | --- | --- | --- | --- | --- | --- | --- | --- | --- | --- | --- | --- | --- | --- | --- | --- | --- | --- | --- | --- | --- | --- | --- | --- | --- | --- | --- | --- | --- | --- | --- | --- | --- | --- | --- | --- | --- | --- |
| S.AVTIFGESAGAASVGMHLLSTQSR.G | Y | 64.96 | 2389.2061 | 24 | 0.8 | 797.4099 | 3 | 74.57 | 3 | F3:48871 | NaNaKA16\_F11.raw |  | 1.5157E6 | 2.4175E6 | 2.4449E6 |  |  |  |  |  |  |  |  |  | 3 | 0 | 1 | 1 | 1 | 0 | 0 | 0 | 0 | 0 | 0 | 0 | 0 | 0 | 224 | 247 |  |  | PEAKS DB |
| R.AQIC(+57.02)AFWNHFLPK.L | N | 62.23 | 1630.8079 | 13 | 0.8 | 544.6104 | 3 | 69.52 | 3 | F3:44588 | NaNaKA16\_F11.raw |  |  | 9.0724E5 | 2.7007E6 |  |  |  |  |  |  |  |  |  | 2 | 0 | 0 | 1 | 1 | 0 | 0 | 0 | 0 | 0 | 0 | 0 | 0 | 0 | 549 | 561 | Carbamidomethylation | C4:Carbamidomethylation:1000.00 | PEAKS DB |
| Y.SGAASLDVYDGR.F | N | 59.74 | 1209.5625 | 12 | 0.9 | 605.7891 | 2 | 28.23 | 5 | F5:9342 | NaNaKA16\_F13.raw |  |  |  |  | 7.7144E5 |  |  |  |  |  |  |  |  | 1 | 0 | 0 | 0 | 0 | 1 | 0 | 0 | 0 | 0 | 0 | 0 | 0 | 0 | 153 | 164 |  |  | PEAKS DB |
| K.VYAYLFDHR.A | N | 53.79 | 1182.5822 | 9 | 0.8 | 592.2988 | 2 | 36.66 | 5 | F5:14316 | NaNaKA16\_F13.raw |  |  |  |  | 4.8237E6 |  |  |  |  |  |  |  |  | 2 | 0 | 0 | 0 | 0 | 2 | 0 | 0 | 0 | 0 | 0 | 0 | 0 | 0 | 449 | 457 |  |  | PEAKS DB |
| G.ESAGAASVGMHLLSTQSR.G | N | 53.24 | 1800.8788 | 18 | 0.5 | 601.3005 | 3 | 37.20 | 2 | F2:18899 | NaNaKA16\_F10.raw |  | 5.5667E6 |  |  |  |  |  |  |  |  |  |  | 1.2248E6 | 2 | 0 | 1 | 0 | 0 | 0 | 0 | 0 | 0 | 0 | 0 | 0 | 0 | 1 | 230 | 247 |  |  | PEAKS DB |
| F.LGVPFAEPPVGR.M | Y | 45.46 | 1237.6819 | 12 | 0.1 | 619.8483 | 2 | 56.17 | 5 | F5:22990 | NaNaKA16\_F13.raw |  |  |  |  | 2.3712E6 |  |  |  |  |  |  |  |  | 1 | 0 | 0 | 0 | 0 | 1 | 0 | 0 | 0 | 0 | 0 | 0 | 0 | 0 | 62 | 73 |  |  | PEAKS DB |
| total 6 peptides |  |  |  |  |  |  |  |  |  |  |  |  |  |  |  |  |  |  |  |  |  |  |  |  |  |  |  |  |  |  |  |  |  |  |  |  |  |  |  |  |  |  |  |
| --- | --- | --- | --- | --- | --- | --- | --- | --- | --- | --- | --- | --- | --- | --- | --- | --- | --- | --- | --- | --- | --- | --- | --- | --- | --- | --- | --- | --- | --- | --- | --- | --- | --- | --- | --- | --- | --- | --- | --- | --- | --- | --- | --- |

XP\_026561288.1

back to list

  

| Protein Coverage
| Supporting Peptides
|

Protein Coverage:

Supporting Peptides:

| Peptide | Uniq | -10lgP | Mass | Length | ppm | m/z | z | RT | Fraction | Scan | Source File | Area F1 | Area F10 | Area F11 | Area F12 | Area F13 | Area F2 | Area F3 | Area F4 | Area F5 | Area F6 | Area F7 | Area F8 | Area F9 | #Feature | #Feature F1 | #Feature F10 | #Feature F11 | #Feature F12 | #Feature F13 | #Feature F2 | #Feature F3 | #Feature F4 | #Feature F5 | #Feature F6 | #Feature F7 | #Feature F8 | #Feature F9 | Start | End | PTM | AScore | Found By |
| --- | --- | --- | --- | --- | --- | --- | --- | --- | --- | --- | --- | --- | --- | --- | --- | --- | --- | --- | --- | --- | --- | --- | --- | --- | --- | --- | --- | --- | --- | --- | --- | --- | --- | --- | --- | --- | --- | --- | --- | --- | --- | --- | --- |
| R.MANVLC(+57.02)SC(+57.02)SEDC(+57.02)LTK.K | N | 76.27 | 1786.7358 | 15 | 5.5 | 894.3754 | 2 | 37.96 | 8 | F8:19800 | NaNaKA16\_F4.raw |  |  |  |  |  |  | 2.2299E6 | 1.4838E7 | 4.3252E6 |  |  |  |  | 3 | 0 | 0 | 0 | 0 | 0 | 0 | 1 | 1 | 1 | 0 | 0 | 0 | 0 | 88 | 102 | Carbamidomethylation | C6:Carbamidomethylation:1000.00;C8:Carbamidomethylation:1000.00;C12:Carbamidomethylation:1000.00 | PEAKS DB |
| R.MANVLC(+57.02)SC(+57.02)SEDC(+57.02)LTKK.D | N | 64.42 | 1914.8308 | 16 | 5.8 | 639.2845 | 3 | 25.10 | 8 | F8:9795 | NaNaKA16\_F4.raw |  |  |  |  |  |  |  | 4.6506E6 |  |  |  |  |  | 1 | 0 | 0 | 0 | 0 | 0 | 0 | 0 | 1 | 0 | 0 | 0 | 0 | 0 | 88 | 103 | Carbamidomethylation | C6:Carbamidomethylation:1000.00;C8:Carbamidomethylation:1000.00;C12:Carbamidomethylation:1000.00 | PEAKS DB |
| K.YC(+57.02)SGGTHGYDNEFK.S | N | 55.74 | 1633.6467 | 14 | 0.9 | 545.5567 | 3 | 11.99 | 5 | F5:1916 | NaNaKA16\_F13.raw |  |  |  |  | 4.2751E5 |  |  |  |  |  |  |  |  | 1 | 0 | 0 | 0 | 0 | 1 | 0 | 0 | 0 | 0 | 0 | 0 | 0 | 0 | 456 | 469 | Carbamidomethylation | C2:Carbamidomethylation:1000.00 | PEAKS DB |
| K.DFYTFDSEAIVK.N | N | 52.96 | 1433.6714 | 12 | 0.4 | 717.8433 | 2 | 71.19 | 5 | F5:28900 | NaNaKA16\_F13.raw |  |  |  |  | 5.5404E5 |  |  |  |  |  |  |  |  | 1 | 0 | 0 | 0 | 0 | 1 | 0 | 0 | 0 | 0 | 0 | 0 | 0 | 0 | 393 | 404 |  |  | PEAKS DB |
| E.AC(+57.02)C(+57.02)WDYQDTC(+57.02)VLPTQSWSC(+57.02)NK.L | Y | 45.73 | 2678.0659 | 21 | 6.3 | 893.6968 | 3 | 66.86 | 8 | F8:43778 | NaNaKA16\_F4.raw |  |  |  |  |  |  |  | 6.225E6 |  |  |  |  |  | 1 | 0 | 0 | 0 | 0 | 0 | 0 | 0 | 1 | 0 | 0 | 0 | 0 | 0 | 60 | 80 | Carbamidomethylation | C2:Carbamidomethylation:1000.00;C3:Carbamidomethylation:1000.00;C10:Carbamidomethylation:1000.00;C19:Carbamidomethylation:1000.00 | PEAKS DB |
| total 5 peptides |  |  |  |  |  |  |  |  |  |  |  |  |  |  |  |  |  |  |  |  |  |  |  |  |  |  |  |  |  |  |  |  |  |  |  |  |  |  |  |  |  |  |  |
| --- | --- | --- | --- | --- | --- | --- | --- | --- | --- | --- | --- | --- | --- | --- | --- | --- | --- | --- | --- | --- | --- | --- | --- | --- | --- | --- | --- | --- | --- | --- | --- | --- | --- | --- | --- | --- | --- | --- | --- | --- | --- | --- | --- |

XP\_026561287.1

back to list

  

| Protein Coverage
| Supporting Peptides
|

Protein Coverage:

Supporting Peptides:

| Peptide | Uniq | -10lgP | Mass | Length | ppm | m/z | z | RT | Fraction | Scan | Source File | Area F1 | Area F10 | Area F11 | Area F12 | Area F13 | Area F2 | Area F3 | Area F4 | Area F5 | Area F6 | Area F7 | Area F8 | Area F9 | #Feature | #Feature F1 | #Feature F10 | #Feature F11 | #Feature F12 | #Feature F13 | #Feature F2 | #Feature F3 | #Feature F4 | #Feature F5 | #Feature F6 | #Feature F7 | #Feature F8 | #Feature F9 | Start | End | PTM | AScore | Found By |
| --- | --- | --- | --- | --- | --- | --- | --- | --- | --- | --- | --- | --- | --- | --- | --- | --- | --- | --- | --- | --- | --- | --- | --- | --- | --- | --- | --- | --- | --- | --- | --- | --- | --- | --- | --- | --- | --- | --- | --- | --- | --- | --- | --- |
| R.MANVLC(+57.02)SC(+57.02)SEDC(+57.02)LTK.K | N | 76.27 | 1786.7358 | 15 | 5.5 | 894.3754 | 2 | 37.96 | 8 | F8:19800 | NaNaKA16\_F4.raw |  |  |  |  |  |  | 2.2299E6 | 1.4838E7 | 4.3252E6 |  |  |  |  | 3 | 0 | 0 | 0 | 0 | 0 | 0 | 1 | 1 | 1 | 0 | 0 | 0 | 0 | 88 | 102 | Carbamidomethylation | C6:Carbamidomethylation:1000.00;C8:Carbamidomethylation:1000.00;C12:Carbamidomethylation:1000.00 | PEAKS DB |
| R.MANVLC(+57.02)SC(+57.02)SEDC(+57.02)LTKK.D | N | 64.42 | 1914.8308 | 16 | 5.8 | 639.2845 | 3 | 25.10 | 8 | F8:9795 | NaNaKA16\_F4.raw |  |  |  |  |  |  |  | 4.6506E6 |  |  |  |  |  | 1 | 0 | 0 | 0 | 0 | 0 | 0 | 0 | 1 | 0 | 0 | 0 | 0 | 0 | 88 | 103 | Carbamidomethylation | C6:Carbamidomethylation:1000.00;C8:Carbamidomethylation:1000.00;C12:Carbamidomethylation:1000.00 | PEAKS DB |
| K.YC(+57.02)SGGTHGYDNEFK.S | N | 55.74 | 1633.6467 | 14 | 0.9 | 545.5567 | 3 | 11.99 | 5 | F5:1916 | NaNaKA16\_F13.raw |  |  |  |  | 4.2751E5 |  |  |  |  |  |  |  |  | 1 | 0 | 0 | 0 | 0 | 1 | 0 | 0 | 0 | 0 | 0 | 0 | 0 | 0 | 456 | 469 | Carbamidomethylation | C2:Carbamidomethylation:1000.00 | PEAKS DB |
| K.DFYTFDSEAIVK.N | N | 52.96 | 1433.6714 | 12 | 0.4 | 717.8433 | 2 | 71.19 | 5 | F5:28900 | NaNaKA16\_F13.raw |  |  |  |  | 5.5404E5 |  |  |  |  |  |  |  |  | 1 | 0 | 0 | 0 | 0 | 1 | 0 | 0 | 0 | 0 | 0 | 0 | 0 | 0 | 393 | 404 |  |  | PEAKS DB |
| E.AC(+57.02)C(+57.02)WDYQDTC(+57.02)VLPTQSWSC(+57.02)NK.L | Y | 45.73 | 2678.0659 | 21 | 6.3 | 893.6968 | 3 | 66.86 | 8 | F8:43778 | NaNaKA16\_F4.raw |  |  |  |  |  |  |  | 6.225E6 |  |  |  |  |  | 1 | 0 | 0 | 0 | 0 | 0 | 0 | 0 | 1 | 0 | 0 | 0 | 0 | 0 | 60 | 80 | Carbamidomethylation | C2:Carbamidomethylation:1000.00;C3:Carbamidomethylation:1000.00;C10:Carbamidomethylation:1000.00;C19:Carbamidomethylation:1000.00 | PEAKS DB |
| total 5 peptides |  |  |  |  |  |  |  |  |  |  |  |  |  |  |  |  |  |  |  |  |  |  |  |  |  |  |  |  |  |  |  |  |  |  |  |  |  |  |  |  |  |  |  |
| --- | --- | --- | --- | --- | --- | --- | --- | --- | --- | --- | --- | --- | --- | --- | --- | --- | --- | --- | --- | --- | --- | --- | --- | --- | --- | --- | --- | --- | --- | --- | --- | --- | --- | --- | --- | --- | --- | --- | --- | --- | --- | --- | --- |

XP\_026561289.1

back to list

  

| Protein Coverage
| Supporting Peptides
|

Protein Coverage:

Supporting Peptides:

| Peptide | Uniq | -10lgP | Mass | Length | ppm | m/z | z | RT | Fraction | Scan | Source File | Area F1 | Area F10 | Area F11 | Area F12 | Area F13 | Area F2 | Area F3 | Area F4 | Area F5 | Area F6 | Area F7 | Area F8 | Area F9 | #Feature | #Feature F1 | #Feature F10 | #Feature F11 | #Feature F12 | #Feature F13 | #Feature F2 | #Feature F3 | #Feature F4 | #Feature F5 | #Feature F6 | #Feature F7 | #Feature F8 | #Feature F9 | Start | End | PTM | AScore | Found By |
| --- | --- | --- | --- | --- | --- | --- | --- | --- | --- | --- | --- | --- | --- | --- | --- | --- | --- | --- | --- | --- | --- | --- | --- | --- | --- | --- | --- | --- | --- | --- | --- | --- | --- | --- | --- | --- | --- | --- | --- | --- | --- | --- | --- |
| R.MANVLC(+57.02)SC(+57.02)SEDC(+57.02)LTK.K | N | 76.27 | 1786.7358 | 15 | 5.5 | 894.3754 | 2 | 37.96 | 8 | F8:19800 | NaNaKA16\_F4.raw |  |  |  |  |  |  | 2.2299E6 | 1.4838E7 | 4.3252E6 |  |  |  |  | 3 | 0 | 0 | 0 | 0 | 0 | 0 | 1 | 1 | 1 | 0 | 0 | 0 | 0 | 88 | 102 | Carbamidomethylation | C6:Carbamidomethylation:1000.00;C8:Carbamidomethylation:1000.00;C12:Carbamidomethylation:1000.00 | PEAKS DB |
| R.MANVLC(+57.02)SC(+57.02)SEDC(+57.02)LTKK.D | N | 64.42 | 1914.8308 | 16 | 5.8 | 639.2845 | 3 | 25.10 | 8 | F8:9795 | NaNaKA16\_F4.raw |  |  |  |  |  |  |  | 4.6506E6 |  |  |  |  |  | 1 | 0 | 0 | 0 | 0 | 0 | 0 | 0 | 1 | 0 | 0 | 0 | 0 | 0 | 88 | 103 | Carbamidomethylation | C6:Carbamidomethylation:1000.00;C8:Carbamidomethylation:1000.00;C12:Carbamidomethylation:1000.00 | PEAKS DB |
| K.YC(+57.02)SGGTHGYDNEFK.S | N | 55.74 | 1633.6467 | 14 | 0.9 | 545.5567 | 3 | 11.99 | 5 | F5:1916 | NaNaKA16\_F13.raw |  |  |  |  | 4.2751E5 |  |  |  |  |  |  |  |  | 1 | 0 | 0 | 0 | 0 | 1 | 0 | 0 | 0 | 0 | 0 | 0 | 0 | 0 | 456 | 469 | Carbamidomethylation | C2:Carbamidomethylation:1000.00 | PEAKS DB |
| K.DFYTFDSEAIVK.N | N | 52.96 | 1433.6714 | 12 | 0.4 | 717.8433 | 2 | 71.19 | 5 | F5:28900 | NaNaKA16\_F13.raw |  |  |  |  | 5.5404E5 |  |  |  |  |  |  |  |  | 1 | 0 | 0 | 0 | 0 | 1 | 0 | 0 | 0 | 0 | 0 | 0 | 0 | 0 | 393 | 404 |  |  | PEAKS DB |
| E.AC(+57.02)C(+57.02)WDYQDTC(+57.02)VLPTQSWSC(+57.02)NK.L | Y | 45.73 | 2678.0659 | 21 | 6.3 | 893.6968 | 3 | 66.86 | 8 | F8:43778 | NaNaKA16\_F4.raw |  |  |  |  |  |  |  | 6.225E6 |  |  |  |  |  | 1 | 0 | 0 | 0 | 0 | 0 | 0 | 0 | 1 | 0 | 0 | 0 | 0 | 0 | 60 | 80 | Carbamidomethylation | C2:Carbamidomethylation:1000.00;C3:Carbamidomethylation:1000.00;C10:Carbamidomethylation:1000.00;C19:Carbamidomethylation:1000.00 | PEAKS DB |
| total 5 peptides |  |  |  |  |  |  |  |  |  |  |  |  |  |  |  |  |  |  |  |  |  |  |  |  |  |  |  |  |  |  |  |  |  |  |  |  |  |  |  |  |  |  |  |
| --- | --- | --- | --- | --- | --- | --- | --- | --- | --- | --- | --- | --- | --- | --- | --- | --- | --- | --- | --- | --- | --- | --- | --- | --- | --- | --- | --- | --- | --- | --- | --- | --- | --- | --- | --- | --- | --- | --- | --- | --- | --- | --- | --- |

XP\_026561284.1

back to list

  

| Protein Coverage
| Supporting Peptides
|

Protein Coverage:

Supporting Peptides:

| Peptide | Uniq | -10lgP | Mass | Length | ppm | m/z | z | RT | Fraction | Scan | Source File | Area F1 | Area F10 | Area F11 | Area F12 | Area F13 | Area F2 | Area F3 | Area F4 | Area F5 | Area F6 | Area F7 | Area F8 | Area F9 | #Feature | #Feature F1 | #Feature F10 | #Feature F11 | #Feature F12 | #Feature F13 | #Feature F2 | #Feature F3 | #Feature F4 | #Feature F5 | #Feature F6 | #Feature F7 | #Feature F8 | #Feature F9 | Start | End | PTM | AScore | Found By |
| --- | --- | --- | --- | --- | --- | --- | --- | --- | --- | --- | --- | --- | --- | --- | --- | --- | --- | --- | --- | --- | --- | --- | --- | --- | --- | --- | --- | --- | --- | --- | --- | --- | --- | --- | --- | --- | --- | --- | --- | --- | --- | --- | --- |
| R.MANVLC(+57.02)SC(+57.02)SEDC(+57.02)LTK.K | N | 76.27 | 1786.7358 | 15 | 5.5 | 894.3754 | 2 | 37.96 | 8 | F8:19800 | NaNaKA16\_F4.raw |  |  |  |  |  |  | 2.2299E6 | 1.4838E7 | 4.3252E6 |  |  |  |  | 3 | 0 | 0 | 0 | 0 | 0 | 0 | 1 | 1 | 1 | 0 | 0 | 0 | 0 | 124 | 138 | Carbamidomethylation | C6:Carbamidomethylation:1000.00;C8:Carbamidomethylation:1000.00;C12:Carbamidomethylation:1000.00 | PEAKS DB |
| R.MANVLC(+57.02)SC(+57.02)SEDC(+57.02)LTKK.D | N | 64.42 | 1914.8308 | 16 | 5.8 | 639.2845 | 3 | 25.10 | 8 | F8:9795 | NaNaKA16\_F4.raw |  |  |  |  |  |  |  | 4.6506E6 |  |  |  |  |  | 1 | 0 | 0 | 0 | 0 | 0 | 0 | 0 | 1 | 0 | 0 | 0 | 0 | 0 | 124 | 139 | Carbamidomethylation | C6:Carbamidomethylation:1000.00;C8:Carbamidomethylation:1000.00;C12:Carbamidomethylation:1000.00 | PEAKS DB |
| K.YC(+57.02)SGGTHGYDNEFK.S | N | 55.74 | 1633.6467 | 14 | 0.9 | 545.5567 | 3 | 11.99 | 5 | F5:1916 | NaNaKA16\_F13.raw |  |  |  |  | 4.2751E5 |  |  |  |  |  |  |  |  | 1 | 0 | 0 | 0 | 0 | 1 | 0 | 0 | 0 | 0 | 0 | 0 | 0 | 0 | 492 | 505 | Carbamidomethylation | C2:Carbamidomethylation:1000.00 | PEAKS DB |
| K.DFYTFDSEAIVK.N | N | 52.96 | 1433.6714 | 12 | 0.4 | 717.8433 | 2 | 71.19 | 5 | F5:28900 | NaNaKA16\_F13.raw |  |  |  |  | 5.5404E5 |  |  |  |  |  |  |  |  | 1 | 0 | 0 | 0 | 0 | 1 | 0 | 0 | 0 | 0 | 0 | 0 | 0 | 0 | 429 | 440 |  |  | PEAKS DB |
| E.AC(+57.02)C(+57.02)WDYQDTC(+57.02)VLPTQSWSC(+57.02)NK.L | Y | 45.73 | 2678.0659 | 21 | 6.3 | 893.6968 | 3 | 66.86 | 8 | F8:43778 | NaNaKA16\_F4.raw |  |  |  |  |  |  |  | 6.225E6 |  |  |  |  |  | 1 | 0 | 0 | 0 | 0 | 0 | 0 | 0 | 1 | 0 | 0 | 0 | 0 | 0 | 96 | 116 | Carbamidomethylation | C2:Carbamidomethylation:1000.00;C3:Carbamidomethylation:1000.00;C10:Carbamidomethylation:1000.00;C19:Carbamidomethylation:1000.00 | PEAKS DB |
| total 5 peptides |  |  |  |  |  |  |  |  |  |  |  |  |  |  |  |  |  |  |  |  |  |  |  |  |  |  |  |  |  |  |  |  |  |  |  |  |  |  |  |  |  |  |  |
| --- | --- | --- | --- | --- | --- | --- | --- | --- | --- | --- | --- | --- | --- | --- | --- | --- | --- | --- | --- | --- | --- | --- | --- | --- | --- | --- | --- | --- | --- | --- | --- | --- | --- | --- | --- | --- | --- | --- | --- | --- | --- | --- | --- |

XP\_026561286.1

back to list

  

| Protein Coverage
| Supporting Peptides
|

Protein Coverage:

Supporting Peptides:

| Peptide | Uniq | -10lgP | Mass | Length | ppm | m/z | z | RT | Fraction | Scan | Source File | Area F1 | Area F10 | Area F11 | Area F12 | Area F13 | Area F2 | Area F3 | Area F4 | Area F5 | Area F6 | Area F7 | Area F8 | Area F9 | #Feature | #Feature F1 | #Feature F10 | #Feature F11 | #Feature F12 | #Feature F13 | #Feature F2 | #Feature F3 | #Feature F4 | #Feature F5 | #Feature F6 | #Feature F7 | #Feature F8 | #Feature F9 | Start | End | PTM | AScore | Found By |
| --- | --- | --- | --- | --- | --- | --- | --- | --- | --- | --- | --- | --- | --- | --- | --- | --- | --- | --- | --- | --- | --- | --- | --- | --- | --- | --- | --- | --- | --- | --- | --- | --- | --- | --- | --- | --- | --- | --- | --- | --- | --- | --- | --- |
| R.MANVLC(+57.02)SC(+57.02)SEDC(+57.02)LTK.K | N | 76.27 | 1786.7358 | 15 | 5.5 | 894.3754 | 2 | 37.96 | 8 | F8:19800 | NaNaKA16\_F4.raw |  |  |  |  |  |  | 2.2299E6 | 1.4838E7 | 4.3252E6 |  |  |  |  | 3 | 0 | 0 | 0 | 0 | 0 | 0 | 1 | 1 | 1 | 0 | 0 | 0 | 0 | 124 | 138 | Carbamidomethylation | C6:Carbamidomethylation:1000.00;C8:Carbamidomethylation:1000.00;C12:Carbamidomethylation:1000.00 | PEAKS DB |
| R.MANVLC(+57.02)SC(+57.02)SEDC(+57.02)LTKK.D | N | 64.42 | 1914.8308 | 16 | 5.8 | 639.2845 | 3 | 25.10 | 8 | F8:9795 | NaNaKA16\_F4.raw |  |  |  |  |  |  |  | 4.6506E6 |  |  |  |  |  | 1 | 0 | 0 | 0 | 0 | 0 | 0 | 0 | 1 | 0 | 0 | 0 | 0 | 0 | 124 | 139 | Carbamidomethylation | C6:Carbamidomethylation:1000.00;C8:Carbamidomethylation:1000.00;C12:Carbamidomethylation:1000.00 | PEAKS DB |
| K.YC(+57.02)SGGTHGYDNEFK.S | N | 55.74 | 1633.6467 | 14 | 0.9 | 545.5567 | 3 | 11.99 | 5 | F5:1916 | NaNaKA16\_F13.raw |  |  |  |  | 4.2751E5 |  |  |  |  |  |  |  |  | 1 | 0 | 0 | 0 | 0 | 1 | 0 | 0 | 0 | 0 | 0 | 0 | 0 | 0 | 492 | 505 | Carbamidomethylation | C2:Carbamidomethylation:1000.00 | PEAKS DB |
| K.DFYTFDSEAIVK.N | N | 52.96 | 1433.6714 | 12 | 0.4 | 717.8433 | 2 | 71.19 | 5 | F5:28900 | NaNaKA16\_F13.raw |  |  |  |  | 5.5404E5 |  |  |  |  |  |  |  |  | 1 | 0 | 0 | 0 | 0 | 1 | 0 | 0 | 0 | 0 | 0 | 0 | 0 | 0 | 429 | 440 |  |  | PEAKS DB |
| E.AC(+57.02)C(+57.02)WDYQDTC(+57.02)VLPTQSWSC(+57.02)NK.L | Y | 45.73 | 2678.0659 | 21 | 6.3 | 893.6968 | 3 | 66.86 | 8 | F8:43778 | NaNaKA16\_F4.raw |  |  |  |  |  |  |  | 6.225E6 |  |  |  |  |  | 1 | 0 | 0 | 0 | 0 | 0 | 0 | 0 | 1 | 0 | 0 | 0 | 0 | 0 | 96 | 116 | Carbamidomethylation | C2:Carbamidomethylation:1000.00;C3:Carbamidomethylation:1000.00;C10:Carbamidomethylation:1000.00;C19:Carbamidomethylation:1000.00 | PEAKS DB |
| total 5 peptides |  |  |  |  |  |  |  |  |  |  |  |  |  |  |  |  |  |  |  |  |  |  |  |  |  |  |  |  |  |  |  |  |  |  |  |  |  |  |  |  |  |  |  |
| --- | --- | --- | --- | --- | --- | --- | --- | --- | --- | --- | --- | --- | --- | --- | --- | --- | --- | --- | --- | --- | --- | --- | --- | --- | --- | --- | --- | --- | --- | --- | --- | --- | --- | --- | --- | --- | --- | --- | --- | --- | --- | --- | --- |

AJB84504.1

back to list

  

| Protein Coverage
| Supporting Peptides
|

Protein Coverage:

Supporting Peptides:

| Peptide | Uniq | -10lgP | Mass | Length | ppm | m/z | z | RT | Fraction | Scan | Source File | Area F1 | Area F10 | Area F11 | Area F12 | Area F13 | Area F2 | Area F3 | Area F4 | Area F5 | Area F6 | Area F7 | Area F8 | Area F9 | #Feature | #Feature F1 | #Feature F10 | #Feature F11 | #Feature F12 | #Feature F13 | #Feature F2 | #Feature F3 | #Feature F4 | #Feature F5 | #Feature F6 | #Feature F7 | #Feature F8 | #Feature F9 | Start | End | PTM | AScore | Found By |
| --- | --- | --- | --- | --- | --- | --- | --- | --- | --- | --- | --- | --- | --- | --- | --- | --- | --- | --- | --- | --- | --- | --- | --- | --- | --- | --- | --- | --- | --- | --- | --- | --- | --- | --- | --- | --- | --- | --- | --- | --- | --- | --- | --- |
| G.FPC(+57.02)AQILEPGVYTK.V | N | 90.02 | 1621.8174 | 14 | 0.7 | 811.9165 | 2 | 63.25 | 4 | F4:41641 | NaNaKA16\_F12.raw |  |  | 4.8534E6 | 6.8218E8 | 1.5279E6 |  |  |  |  |  |  |  |  | 10 | 0 | 0 | 1 | 8 | 1 | 0 | 0 | 0 | 0 | 0 | 0 | 0 | 0 | 223 | 236 | Carbamidomethylation | C3:Carbamidomethylation:1000.00 | PEAKS DB |
| G.GFPC(+57.02)AQILEPGVYTK.V | Y | 73.73 | 1678.8389 | 15 | -0.2 | 840.4265 | 2 | 67.68 | 4 | F4:44810 | NaNaKA16\_F12.raw |  |  |  | 8.0725E6 |  |  |  |  |  |  |  |  |  | 1 | 0 | 0 | 0 | 1 | 0 | 0 | 0 | 0 | 0 | 0 | 0 | 0 | 0 | 222 | 236 | Carbamidomethylation | C4:Carbamidomethylation:1000.00 | PEAKS DB |
| P.C(+57.02)AQILEPGVYTK.V | N | 56.21 | 1377.6962 | 12 | 0.9 | 689.8560 | 2 | 38.93 | 4 | F4:20939 | NaNaKA16\_F12.raw |  |  |  | 2.6297E6 |  |  |  |  |  |  |  |  |  | 1 | 0 | 0 | 0 | 1 | 0 | 0 | 0 | 0 | 0 | 0 | 0 | 0 | 0 | 225 | 236 | Carbamidomethylation | C1:Carbamidomethylation:1000.00 | PEAKS DB |
| C.AQILEPGVYTK.V | N | 44.01 | 1217.6655 | 11 | 0.3 | 609.8402 | 2 | 35.96 | 4 | F4:18428 | NaNaKA16\_F12.raw |  |  |  | 4.2348E6 |  |  |  |  |  |  |  |  |  | 1 | 0 | 0 | 0 | 1 | 0 | 0 | 0 | 0 | 0 | 0 | 0 | 0 | 0 | 226 | 236 |  |  | PEAKS DB |
| total 4 peptides |  |  |  |  |  |  |  |  |  |  |  |  |  |  |  |  |  |  |  |  |  |  |  |  |  |  |  |  |  |  |  |  |  |  |  |  |  |  |  |  |  |  |  |
| --- | --- | --- | --- | --- | --- | --- | --- | --- | --- | --- | --- | --- | --- | --- | --- | --- | --- | --- | --- | --- | --- | --- | --- | --- | --- | --- | --- | --- | --- | --- | --- | --- | --- | --- | --- | --- | --- | --- | --- | --- | --- | --- | --- |

4QWW

back to list

  

| Protein Coverage
| Supporting Peptides
|

Protein Coverage:

Supporting Peptides:

| Peptide | Uniq | -10lgP | Mass | Length | ppm | m/z | z | RT | Fraction | Scan | Source File | Area F1 | Area F10 | Area F11 | Area F12 | Area F13 | Area F2 | Area F3 | Area F4 | Area F5 | Area F6 | Area F7 | Area F8 | Area F9 | #Feature | #Feature F1 | #Feature F10 | #Feature F11 | #Feature F12 | #Feature F13 | #Feature F2 | #Feature F3 | #Feature F4 | #Feature F5 | #Feature F6 | #Feature F7 | #Feature F8 | #Feature F9 | Start | End | PTM | AScore | Found By |
| --- | --- | --- | --- | --- | --- | --- | --- | --- | --- | --- | --- | --- | --- | --- | --- | --- | --- | --- | --- | --- | --- | --- | --- | --- | --- | --- | --- | --- | --- | --- | --- | --- | --- | --- | --- | --- | --- | --- | --- | --- | --- | --- | --- |
| R.AQIC(+57.02)AFWNHFLPK.L | N | 62.23 | 1630.8079 | 13 | 0.8 | 544.6104 | 3 | 69.52 | 3 | F3:44588 | NaNaKA16\_F11.raw |  |  | 9.0724E5 | 2.7007E6 |  |  |  |  |  |  |  |  |  | 2 | 0 | 0 | 1 | 1 | 0 | 0 | 0 | 0 | 0 | 0 | 0 | 0 | 0 | 518 | 530 | Carbamidomethylation | C4:Carbamidomethylation:1000.00 | PEAKS DB |
| K.LLNATVDPPR.A | Y | 60.70 | 1094.6084 | 10 | 0.6 | 548.3118 | 2 | 27.22 | 5 | F5:8897 | NaNaKA16\_F13.raw |  |  |  |  | 4.4219E6 |  |  |  |  |  |  |  |  | 1 | 0 | 0 | 0 | 0 | 1 | 0 | 0 | 0 | 0 | 0 | 0 | 0 | 0 | 531 | 540 |  |  | PEAKS DB |
| Y.SGAASLDVYDGR.F | N | 59.74 | 1209.5625 | 12 | 0.9 | 605.7891 | 2 | 28.23 | 5 | F5:9342 | NaNaKA16\_F13.raw |  |  |  |  | 7.7144E5 |  |  |  |  |  |  |  |  | 1 | 0 | 0 | 0 | 0 | 1 | 0 | 0 | 0 | 0 | 0 | 0 | 0 | 0 | 122 | 133 |  |  | PEAKS DB |
| K.VYAYLFDHR.A | N | 53.79 | 1182.5822 | 9 | 0.8 | 592.2988 | 2 | 36.66 | 5 | F5:14316 | NaNaKA16\_F13.raw |  |  |  |  | 4.8237E6 |  |  |  |  |  |  |  |  | 2 | 0 | 0 | 0 | 0 | 2 | 0 | 0 | 0 | 0 | 0 | 0 | 0 | 0 | 418 | 426 |  |  | PEAKS DB |
| G.ESAGAASVGMHLLSTQSR.T | N | 53.24 | 1800.8788 | 18 | 0.5 | 601.3005 | 3 | 37.20 | 2 | F2:18899 | NaNaKA16\_F10.raw |  | 5.5667E6 |  |  |  |  |  |  |  |  |  |  | 1.2248E6 | 2 | 0 | 1 | 0 | 0 | 0 | 0 | 0 | 0 | 0 | 0 | 0 | 0 | 1 | 199 | 216 |  |  | PEAKS DB |
| K.LLNATVDPPRA.D | Y | 51.91 | 1165.6455 | 11 | -0.1 | 583.8300 | 2 | 32.03 | 5 | F5:11512 | NaNaKA16\_F13.raw |  |  |  |  | 7.4587E6 |  |  |  |  |  |  |  |  | 1 | 0 | 0 | 0 | 0 | 1 | 0 | 0 | 0 | 0 | 0 | 0 | 0 | 0 | 531 | 541 |  |  | PEAKS DB |
| total 6 peptides |  |  |  |  |  |  |  |  |  |  |  |  |  |  |  |  |  |  |  |  |  |  |  |  |  |  |  |  |  |  |  |  |  |  |  |  |  |  |  |  |  |  |  |
| --- | --- | --- | --- | --- | --- | --- | --- | --- | --- | --- | --- | --- | --- | --- | --- | --- | --- | --- | --- | --- | --- | --- | --- | --- | --- | --- | --- | --- | --- | --- | --- | --- | --- | --- | --- | --- | --- | --- | --- | --- | --- | --- | --- |

AAC59905.1

back to list

  

| Protein Coverage
| Supporting Peptides
|

Protein Coverage:

Supporting Peptides:

| Peptide | Uniq | -10lgP | Mass | Length | ppm | m/z | z | RT | Fraction | Scan | Source File | Area F1 | Area F10 | Area F11 | Area F12 | Area F13 | Area F2 | Area F3 | Area F4 | Area F5 | Area F6 | Area F7 | Area F8 | Area F9 | #Feature | #Feature F1 | #Feature F10 | #Feature F11 | #Feature F12 | #Feature F13 | #Feature F2 | #Feature F3 | #Feature F4 | #Feature F5 | #Feature F6 | #Feature F7 | #Feature F8 | #Feature F9 | Start | End | PTM | AScore | Found By |
| --- | --- | --- | --- | --- | --- | --- | --- | --- | --- | --- | --- | --- | --- | --- | --- | --- | --- | --- | --- | --- | --- | --- | --- | --- | --- | --- | --- | --- | --- | --- | --- | --- | --- | --- | --- | --- | --- | --- | --- | --- | --- | --- | --- |
| R.AQIC(+57.02)AFWNHFLPK.L | N | 62.23 | 1630.8079 | 13 | 0.8 | 544.6104 | 3 | 69.52 | 3 | F3:44588 | NaNaKA16\_F11.raw |  |  | 9.0724E5 | 2.7007E6 |  |  |  |  |  |  |  |  |  | 2 | 0 | 0 | 1 | 1 | 0 | 0 | 0 | 0 | 0 | 0 | 0 | 0 | 0 | 549 | 561 | Carbamidomethylation | C4:Carbamidomethylation:1000.00 | PEAKS DB |
| K.LLNATVDPPR.A | Y | 60.70 | 1094.6084 | 10 | 0.6 | 548.3118 | 2 | 27.22 | 5 | F5:8897 | NaNaKA16\_F13.raw |  |  |  |  | 4.4219E6 |  |  |  |  |  |  |  |  | 1 | 0 | 0 | 0 | 0 | 1 | 0 | 0 | 0 | 0 | 0 | 0 | 0 | 0 | 562 | 571 |  |  | PEAKS DB |
| Y.SGAASLDVYDGR.F | N | 59.74 | 1209.5625 | 12 | 0.9 | 605.7891 | 2 | 28.23 | 5 | F5:9342 | NaNaKA16\_F13.raw |  |  |  |  | 7.7144E5 |  |  |  |  |  |  |  |  | 1 | 0 | 0 | 0 | 0 | 1 | 0 | 0 | 0 | 0 | 0 | 0 | 0 | 0 | 153 | 164 |  |  | PEAKS DB |
| K.VYAYLFDHR.A | N | 53.79 | 1182.5822 | 9 | 0.8 | 592.2988 | 2 | 36.66 | 5 | F5:14316 | NaNaKA16\_F13.raw |  |  |  |  | 4.8237E6 |  |  |  |  |  |  |  |  | 2 | 0 | 0 | 0 | 0 | 2 | 0 | 0 | 0 | 0 | 0 | 0 | 0 | 0 | 449 | 457 |  |  | PEAKS DB |
| G.ESAGAASVGMHLLSTQSR.T | N | 53.24 | 1800.8788 | 18 | 0.5 | 601.3005 | 3 | 37.20 | 2 | F2:18899 | NaNaKA16\_F10.raw |  | 5.5667E6 |  |  |  |  |  |  |  |  |  |  | 1.2248E6 | 2 | 0 | 1 | 0 | 0 | 0 | 0 | 0 | 0 | 0 | 0 | 0 | 0 | 1 | 230 | 247 |  |  | PEAKS DB |
| K.LLNATVDPPRA.D | Y | 51.91 | 1165.6455 | 11 | -0.1 | 583.8300 | 2 | 32.03 | 5 | F5:11512 | NaNaKA16\_F13.raw |  |  |  |  | 7.4587E6 |  |  |  |  |  |  |  |  | 1 | 0 | 0 | 0 | 0 | 1 | 0 | 0 | 0 | 0 | 0 | 0 | 0 | 0 | 562 | 572 |  |  | PEAKS DB |
| total 6 peptides |  |  |  |  |  |  |  |  |  |  |  |  |  |  |  |  |  |  |  |  |  |  |  |  |  |  |  |  |  |  |  |  |  |  |  |  |  |  |  |  |  |  |  |
| --- | --- | --- | --- | --- | --- | --- | --- | --- | --- | --- | --- | --- | --- | --- | --- | --- | --- | --- | --- | --- | --- | --- | --- | --- | --- | --- | --- | --- | --- | --- | --- | --- | --- | --- | --- | --- | --- | --- | --- | --- | --- | --- | --- |

P00600.1

back to list

  

| Protein Coverage
| Supporting Peptides
|

Protein Coverage:

Supporting Peptides:

| Peptide | Uniq | -10lgP | Mass | Length | ppm | m/z | z | RT | Fraction | Scan | Source File | Area F1 | Area F10 | Area F11 | Area F12 | Area F13 | Area F2 | Area F3 | Area F4 | Area F5 | Area F6 | Area F7 | Area F8 | Area F9 | #Feature | #Feature F1 | #Feature F10 | #Feature F11 | #Feature F12 | #Feature F13 | #Feature F2 | #Feature F3 | #Feature F4 | #Feature F5 | #Feature F6 | #Feature F7 | #Feature F8 | #Feature F9 | Start | End | PTM | AScore | Found By |
| --- | --- | --- | --- | --- | --- | --- | --- | --- | --- | --- | --- | --- | --- | --- | --- | --- | --- | --- | --- | --- | --- | --- | --- | --- | --- | --- | --- | --- | --- | --- | --- | --- | --- | --- | --- | --- | --- | --- | --- | --- | --- | --- | --- |
| R.GGSGTPVDDLDR.C | N | 67.81 | 1187.5417 | 12 | 0.8 | 594.7786 | 2 | 19.63 | 10 | F10:6988 | NaNaKA16\_F6.raw |  |  |  |  |  |  |  |  |  | 5.7224E7 | 2.6509E9 | 3.9395E6 |  | 4 | 0 | 0 | 0 | 0 | 0 | 0 | 0 | 0 | 0 | 2 | 1 | 1 | 0 | 31 | 42 |  |  | PEAKS DB |
| K.ISGC(+57.02)WPYIK.T | Y | 67.75 | 1122.5532 | 9 | 2.8 | 562.2855 | 2 | 51.79 | 13 | F13:29736 | NaNaKA16\_F9.raw |  | 3.7449E8 | 7.0956E7 | 5.0959E7 | 2.0327E6 |  |  | 3.4961E5 | 1.7447E8 | 1.5647E10 | 3.8482E9 | 3.7982E9 | 1.0131E11 | 42 | 0 | 2 | 2 | 1 | 1 | 0 | 0 | 1 | 2 | 5 | 5 | 5 | 18 | 57 | 65 | Carbamidomethylation | C4:Carbamidomethylation:1000.00 | PEAKS DB |
| G.GSGTPVDDLDR.C | N | 60.74 | 1130.5204 | 11 | -0.3 | 566.2673 | 2 | 21.08 | 11 | F11:8172 | NaNaKA16\_F7.raw |  |  |  |  |  |  |  |  |  | 1.4052E5 | 1.2671E7 |  |  | 2 | 0 | 0 | 0 | 0 | 0 | 0 | 0 | 0 | 0 | 1 | 1 | 0 | 0 | 32 | 42 |  |  | PEAKS DB |
| E.AEKISGC(+57.02)WPYIK.T | Y | 59.04 | 1450.7278 | 12 | 0.5 | 726.3715 | 2 | 40.43 | 13 | F13:20797 | NaNaKA16\_F9.raw |  |  |  |  |  |  |  |  |  |  |  |  | 7.6834E6 | 2 | 0 | 0 | 0 | 0 | 0 | 0 | 0 | 0 | 0 | 0 | 0 | 0 | 2 | 54 | 65 | Carbamidomethylation | C7:Carbamidomethylation:1000.00 | PEAKS DB |
| G.SGTPVDDLDR.C | N | 45.52 | 1073.4989 | 10 | 0.9 | 537.7572 | 2 | 20.23 | 11 | F11:7497 | NaNaKA16\_F7.raw |  |  |  |  |  |  |  |  |  |  | 1.1072E7 |  |  | 1 | 0 | 0 | 0 | 0 | 0 | 0 | 0 | 0 | 0 | 0 | 1 | 0 | 0 | 33 | 42 |  |  | PEAKS DB |
| total 5 peptides |  |  |  |  |  |  |  |  |  |  |  |  |  |  |  |  |  |  |  |  |  |  |  |  |  |  |  |  |  |  |  |  |  |  |  |  |  |  |  |  |  |  |  |
| --- | --- | --- | --- | --- | --- | --- | --- | --- | --- | --- | --- | --- | --- | --- | --- | --- | --- | --- | --- | --- | --- | --- | --- | --- | --- | --- | --- | --- | --- | --- | --- | --- | --- | --- | --- | --- | --- | --- | --- | --- | --- | --- | --- |

P00601.1

back to list

  

| Protein Coverage
| Supporting Peptides
|

Protein Coverage:

Supporting Peptides:

| Peptide | Uniq | -10lgP | Mass | Length | ppm | m/z | z | RT | Fraction | Scan | Source File | Area F1 | Area F10 | Area F11 | Area F12 | Area F13 | Area F2 | Area F3 | Area F4 | Area F5 | Area F6 | Area F7 | Area F8 | Area F9 | #Feature | #Feature F1 | #Feature F10 | #Feature F11 | #Feature F12 | #Feature F13 | #Feature F2 | #Feature F3 | #Feature F4 | #Feature F5 | #Feature F6 | #Feature F7 | #Feature F8 | #Feature F9 | Start | End | PTM | AScore | Found By |
| --- | --- | --- | --- | --- | --- | --- | --- | --- | --- | --- | --- | --- | --- | --- | --- | --- | --- | --- | --- | --- | --- | --- | --- | --- | --- | --- | --- | --- | --- | --- | --- | --- | --- | --- | --- | --- | --- | --- | --- | --- | --- | --- | --- |
| R.GGSGTPVDDLDR.C | N | 67.81 | 1187.5417 | 12 | 0.8 | 594.7786 | 2 | 19.63 | 10 | F10:6988 | NaNaKA16\_F6.raw |  |  |  |  |  |  |  |  |  | 5.7224E7 | 2.6509E9 | 3.9395E6 |  | 4 | 0 | 0 | 0 | 0 | 0 | 0 | 0 | 0 | 0 | 2 | 1 | 1 | 0 | 31 | 42 |  |  | PEAKS DB |
| K.ISGC(+57.02)WPYIK.T | Y | 67.75 | 1122.5532 | 9 | 2.8 | 562.2855 | 2 | 51.79 | 13 | F13:29736 | NaNaKA16\_F9.raw |  | 3.7449E8 | 7.0956E7 | 5.0959E7 | 2.0327E6 |  |  | 3.4961E5 | 1.7447E8 | 1.5647E10 | 3.8482E9 | 3.7982E9 | 1.0131E11 | 42 | 0 | 2 | 2 | 1 | 1 | 0 | 0 | 1 | 2 | 5 | 5 | 5 | 18 | 57 | 65 | Carbamidomethylation | C4:Carbamidomethylation:1000.00 | PEAKS DB |
| G.GSGTPVDDLDR.C | N | 60.74 | 1130.5204 | 11 | -0.3 | 566.2673 | 2 | 21.08 | 11 | F11:8172 | NaNaKA16\_F7.raw |  |  |  |  |  |  |  |  |  | 1.4052E5 | 1.2671E7 |  |  | 2 | 0 | 0 | 0 | 0 | 0 | 0 | 0 | 0 | 0 | 1 | 1 | 0 | 0 | 32 | 42 |  |  | PEAKS DB |
| E.AEKISGC(+57.02)WPYIK.T | Y | 59.04 | 1450.7278 | 12 | 0.5 | 726.3715 | 2 | 40.43 | 13 | F13:20797 | NaNaKA16\_F9.raw |  |  |  |  |  |  |  |  |  |  |  |  | 7.6834E6 | 2 | 0 | 0 | 0 | 0 | 0 | 0 | 0 | 0 | 0 | 0 | 0 | 0 | 2 | 54 | 65 | Carbamidomethylation | C7:Carbamidomethylation:1000.00 | PEAKS DB |
| G.SGTPVDDLDR.C | N | 45.52 | 1073.4989 | 10 | 0.9 | 537.7572 | 2 | 20.23 | 11 | F11:7497 | NaNaKA16\_F7.raw |  |  |  |  |  |  |  |  |  |  | 1.1072E7 |  |  | 1 | 0 | 0 | 0 | 0 | 0 | 0 | 0 | 0 | 0 | 0 | 1 | 0 | 0 | 33 | 42 |  |  | PEAKS DB |
| total 5 peptides |  |  |  |  |  |  |  |  |  |  |  |  |  |  |  |  |  |  |  |  |  |  |  |  |  |  |  |  |  |  |  |  |  |  |  |  |  |  |  |  |  |  |  |
| --- | --- | --- | --- | --- | --- | --- | --- | --- | --- | --- | --- | --- | --- | --- | --- | --- | --- | --- | --- | --- | --- | --- | --- | --- | --- | --- | --- | --- | --- | --- | --- | --- | --- | --- | --- | --- | --- | --- | --- | --- | --- | --- | --- |

XP\_013911763.1

back to list

  

| Protein Coverage
| Supporting Peptides
|

Protein Coverage:

Supporting Peptides:

| Peptide | Uniq | -10lgP | Mass | Length | ppm | m/z | z | RT | Fraction | Scan | Source File | Area F1 | Area F10 | Area F11 | Area F12 | Area F13 | Area F2 | Area F3 | Area F4 | Area F5 | Area F6 | Area F7 | Area F8 | Area F9 | #Feature | #Feature F1 | #Feature F10 | #Feature F11 | #Feature F12 | #Feature F13 | #Feature F2 | #Feature F3 | #Feature F4 | #Feature F5 | #Feature F6 | #Feature F7 | #Feature F8 | #Feature F9 | Start | End | PTM | AScore | Found By |
| --- | --- | --- | --- | --- | --- | --- | --- | --- | --- | --- | --- | --- | --- | --- | --- | --- | --- | --- | --- | --- | --- | --- | --- | --- | --- | --- | --- | --- | --- | --- | --- | --- | --- | --- | --- | --- | --- | --- | --- | --- | --- | --- | --- |
| R.MEWYPEAASNAER.W | N | 77.76 | 1552.6616 | 13 | 0.7 | 518.5615 | 3 | 44.66 | 4 | F4:25052 | NaNaKA16\_F12.raw |  |  | 1.7754E7 | 3.6E10 | 1.0543E7 |  |  |  |  |  |  |  |  | 15 | 0 | 0 | 1 | 12 | 2 | 0 | 0 | 0 | 0 | 0 | 0 | 0 | 0 | 57 | 69 |  |  | PEAKS DB |
| R.M(+15.99)EWYPEAASNAER.W | N | 75.60 | 1568.6565 | 13 | 1.2 | 785.3364 | 2 | 31.92 | 4 | F4:18885 | NaNaKA16\_F12.raw |  |  |  | 4.1615E9 |  |  |  |  |  |  |  |  |  | 21 | 0 | 0 | 0 | 21 | 0 | 0 | 0 | 0 | 0 | 0 | 0 | 0 | 0 | 57 | 69 | Oxidation (M) | M1:Oxidation (M):1000.00 | PEAKS DB |
| L.RMEWYPEAASNAER.W | Y | 55.40 | 1708.7627 | 14 | 0.1 | 570.5949 | 3 | 30.68 | 4 | F4:14194 | NaNaKA16\_F12.raw |  |  |  | 1.1323E7 |  |  |  |  |  |  |  |  |  | 1 | 0 | 0 | 0 | 1 | 0 | 0 | 0 | 0 | 0 | 0 | 0 | 0 | 0 | 56 | 69 |  |  | PEAKS DB |
| M.EWYPEAASNAER.W | N | 53.15 | 1421.6211 | 12 | 0.6 | 711.8182 | 2 | 30.28 | 4 | F4:13787 | NaNaKA16\_F12.raw |  |  |  | 1.7579E7 |  |  |  |  |  |  |  |  |  | 1 | 0 | 0 | 0 | 1 | 0 | 0 | 0 | 0 | 0 | 0 | 0 | 0 | 0 | 58 | 69 |  |  | PEAKS DB |
| total 4 peptides |  |  |  |  |  |  |  |  |  |  |  |  |  |  |  |  |  |  |  |  |  |  |  |  |  |  |  |  |  |  |  |  |  |  |  |  |  |  |  |  |  |  |  |
| --- | --- | --- | --- | --- | --- | --- | --- | --- | --- | --- | --- | --- | --- | --- | --- | --- | --- | --- | --- | --- | --- | --- | --- | --- | --- | --- | --- | --- | --- | --- | --- | --- | --- | --- | --- | --- | --- | --- | --- | --- | --- | --- | --- |

XP\_032069584.1

back to list

  

| Protein Coverage
| Supporting Peptides
|

Protein Coverage:

Supporting Peptides:

| Peptide | Uniq | -10lgP | Mass | Length | ppm | m/z | z | RT | Fraction | Scan | Source File | Area F1 | Area F10 | Area F11 | Area F12 | Area F13 | Area F2 | Area F3 | Area F4 | Area F5 | Area F6 | Area F7 | Area F8 | Area F9 | #Feature | #Feature F1 | #Feature F10 | #Feature F11 | #Feature F12 | #Feature F13 | #Feature F2 | #Feature F3 | #Feature F4 | #Feature F5 | #Feature F6 | #Feature F7 | #Feature F8 | #Feature F9 | Start | End | PTM | AScore | Found By |
| --- | --- | --- | --- | --- | --- | --- | --- | --- | --- | --- | --- | --- | --- | --- | --- | --- | --- | --- | --- | --- | --- | --- | --- | --- | --- | --- | --- | --- | --- | --- | --- | --- | --- | --- | --- | --- | --- | --- | --- | --- | --- | --- | --- |
| R.MEWYPEAASNAER.W | N | 77.76 | 1552.6616 | 13 | 0.7 | 518.5615 | 3 | 44.66 | 4 | F4:25052 | NaNaKA16\_F12.raw |  |  | 1.7754E7 | 3.6E10 | 1.0543E7 |  |  |  |  |  |  |  |  | 15 | 0 | 0 | 1 | 12 | 2 | 0 | 0 | 0 | 0 | 0 | 0 | 0 | 0 | 57 | 69 |  |  | PEAKS DB |
| R.M(+15.99)EWYPEAASNAER.W | N | 75.60 | 1568.6565 | 13 | 1.2 | 785.3364 | 2 | 31.92 | 4 | F4:18885 | NaNaKA16\_F12.raw |  |  |  | 4.1615E9 |  |  |  |  |  |  |  |  |  | 21 | 0 | 0 | 0 | 21 | 0 | 0 | 0 | 0 | 0 | 0 | 0 | 0 | 0 | 57 | 69 | Oxidation (M) | M1:Oxidation (M):1000.00 | PEAKS DB |
| L.RMEWYPEAASNAER.W | Y | 55.40 | 1708.7627 | 14 | 0.1 | 570.5949 | 3 | 30.68 | 4 | F4:14194 | NaNaKA16\_F12.raw |  |  |  | 1.1323E7 |  |  |  |  |  |  |  |  |  | 1 | 0 | 0 | 0 | 1 | 0 | 0 | 0 | 0 | 0 | 0 | 0 | 0 | 0 | 56 | 69 |  |  | PEAKS DB |
| M.EWYPEAASNAER.W | N | 53.15 | 1421.6211 | 12 | 0.6 | 711.8182 | 2 | 30.28 | 4 | F4:13787 | NaNaKA16\_F12.raw |  |  |  | 1.7579E7 |  |  |  |  |  |  |  |  |  | 1 | 0 | 0 | 0 | 1 | 0 | 0 | 0 | 0 | 0 | 0 | 0 | 0 | 0 | 58 | 69 |  |  | PEAKS DB |
| total 4 peptides |  |  |  |  |  |  |  |  |  |  |  |  |  |  |  |  |  |  |  |  |  |  |  |  |  |  |  |  |  |  |  |  |  |  |  |  |  |  |  |  |  |  |  |
| --- | --- | --- | --- | --- | --- | --- | --- | --- | --- | --- | --- | --- | --- | --- | --- | --- | --- | --- | --- | --- | --- | --- | --- | --- | --- | --- | --- | --- | --- | --- | --- | --- | --- | --- | --- | --- | --- | --- | --- | --- | --- | --- | --- |

XP\_026579406.1

back to list

  

| Protein Coverage
| Supporting Peptides
|

Protein Coverage:

Supporting Peptides:

| Peptide | Uniq | -10lgP | Mass | Length | ppm | m/z | z | RT | Fraction | Scan | Source File | Area F1 | Area F10 | Area F11 | Area F12 | Area F13 | Area F2 | Area F3 | Area F4 | Area F5 | Area F6 | Area F7 | Area F8 | Area F9 | #Feature | #Feature F1 | #Feature F10 | #Feature F11 | #Feature F12 | #Feature F13 | #Feature F2 | #Feature F3 | #Feature F4 | #Feature F5 | #Feature F6 | #Feature F7 | #Feature F8 | #Feature F9 | Start | End | PTM | AScore | Found By |
| --- | --- | --- | --- | --- | --- | --- | --- | --- | --- | --- | --- | --- | --- | --- | --- | --- | --- | --- | --- | --- | --- | --- | --- | --- | --- | --- | --- | --- | --- | --- | --- | --- | --- | --- | --- | --- | --- | --- | --- | --- | --- | --- | --- |
| K.FTYGGC(+57.02)AGNANNFK.K | Y | 83.49 | 1519.6514 | 14 | 0.1 | 760.8330 | 2 | 24.59 | 9 | F9:11113 | NaNaKA16\_F5.raw |  |  |  |  |  |  |  |  | 5.8819E7 | 6.0965E7 |  |  |  | 2 | 0 | 0 | 0 | 0 | 0 | 0 | 0 | 0 | 1 | 1 | 0 | 0 | 0 | 170 | 183 | Carbamidomethylation | C6:Carbamidomethylation:1000.00 | PEAKS DB |
| L.FEGSC(+57.02)SIFPNDSQR.C | Y | 75.18 | 1642.7046 | 14 | 0.2 | 822.3597 | 2 | 40.72 | 10 | F10:24722 | NaNaKA16\_F6.raw |  |  |  |  |  |  |  |  | 5.6415E5 | 1.0068E7 |  |  |  | 2 | 0 | 0 | 0 | 0 | 0 | 0 | 0 | 0 | 1 | 1 | 0 | 0 | 0 | 34 | 47 | Carbamidomethylation | C5:Carbamidomethylation:1000.00 | PEAKS DB |
| G.LFEGSC(+57.02)SIFPNDSQR.C | Y | 64.56 | 1755.7886 | 15 | 0.3 | 878.9018 | 2 | 55.75 | 10 | F10:37299 | NaNaKA16\_F6.raw |  |  |  |  |  |  |  |  |  | 4.6458E7 |  |  |  | 1 | 0 | 0 | 0 | 0 | 0 | 0 | 0 | 0 | 0 | 1 | 0 | 0 | 0 | 33 | 47 | Carbamidomethylation | C6:Carbamidomethylation:1000.00 | PEAKS DB |
| total 3 peptides |  |  |  |  |  |  |  |  |  |  |  |  |  |  |  |  |  |  |  |  |  |  |  |  |  |  |  |  |  |  |  |  |  |  |  |  |  |  |  |  |  |  |  |
| --- | --- | --- | --- | --- | --- | --- | --- | --- | --- | --- | --- | --- | --- | --- | --- | --- | --- | --- | --- | --- | --- | --- | --- | --- | --- | --- | --- | --- | --- | --- | --- | --- | --- | --- | --- | --- | --- | --- | --- | --- | --- | --- | --- |

XP\_026579408.1

back to list

  

| Protein Coverage
| Supporting Peptides
|

Protein Coverage:

Supporting Peptides:

| Peptide | Uniq | -10lgP | Mass | Length | ppm | m/z | z | RT | Fraction | Scan | Source File | Area F1 | Area F10 | Area F11 | Area F12 | Area F13 | Area F2 | Area F3 | Area F4 | Area F5 | Area F6 | Area F7 | Area F8 | Area F9 | #Feature | #Feature F1 | #Feature F10 | #Feature F11 | #Feature F12 | #Feature F13 | #Feature F2 | #Feature F3 | #Feature F4 | #Feature F5 | #Feature F6 | #Feature F7 | #Feature F8 | #Feature F9 | Start | End | PTM | AScore | Found By |
| --- | --- | --- | --- | --- | --- | --- | --- | --- | --- | --- | --- | --- | --- | --- | --- | --- | --- | --- | --- | --- | --- | --- | --- | --- | --- | --- | --- | --- | --- | --- | --- | --- | --- | --- | --- | --- | --- | --- | --- | --- | --- | --- | --- |
| K.FTYGGC(+57.02)AGNANNFK.K | Y | 83.49 | 1519.6514 | 14 | 0.1 | 760.8330 | 2 | 24.59 | 9 | F9:11113 | NaNaKA16\_F5.raw |  |  |  |  |  |  |  |  | 5.8819E7 | 6.0965E7 |  |  |  | 2 | 0 | 0 | 0 | 0 | 0 | 0 | 0 | 0 | 1 | 1 | 0 | 0 | 0 | 170 | 183 | Carbamidomethylation | C6:Carbamidomethylation:1000.00 | PEAKS DB |
| L.FEGSC(+57.02)SIFPNDSQR.C | Y | 75.18 | 1642.7046 | 14 | 0.2 | 822.3597 | 2 | 40.72 | 10 | F10:24722 | NaNaKA16\_F6.raw |  |  |  |  |  |  |  |  | 5.6415E5 | 1.0068E7 |  |  |  | 2 | 0 | 0 | 0 | 0 | 0 | 0 | 0 | 0 | 1 | 1 | 0 | 0 | 0 | 34 | 47 | Carbamidomethylation | C5:Carbamidomethylation:1000.00 | PEAKS DB |
| G.LFEGSC(+57.02)SIFPNDSQR.C | Y | 64.56 | 1755.7886 | 15 | 0.3 | 878.9018 | 2 | 55.75 | 10 | F10:37299 | NaNaKA16\_F6.raw |  |  |  |  |  |  |  |  |  | 4.6458E7 |  |  |  | 1 | 0 | 0 | 0 | 0 | 0 | 0 | 0 | 0 | 0 | 1 | 0 | 0 | 0 | 33 | 47 | Carbamidomethylation | C6:Carbamidomethylation:1000.00 | PEAKS DB |
| total 3 peptides |  |  |  |  |  |  |  |  |  |  |  |  |  |  |  |  |  |  |  |  |  |  |  |  |  |  |  |  |  |  |  |  |  |  |  |  |  |  |  |  |  |  |  |
| --- | --- | --- | --- | --- | --- | --- | --- | --- | --- | --- | --- | --- | --- | --- | --- | --- | --- | --- | --- | --- | --- | --- | --- | --- | --- | --- | --- | --- | --- | --- | --- | --- | --- | --- | --- | --- | --- | --- | --- | --- | --- | --- | --- |

XP\_026579405.1

back to list

  

| Protein Coverage
| Supporting Peptides
|

Protein Coverage:

Supporting Peptides:

| Peptide | Uniq | -10lgP | Mass | Length | ppm | m/z | z | RT | Fraction | Scan | Source File | Area F1 | Area F10 | Area F11 | Area F12 | Area F13 | Area F2 | Area F3 | Area F4 | Area F5 | Area F6 | Area F7 | Area F8 | Area F9 | #Feature | #Feature F1 | #Feature F10 | #Feature F11 | #Feature F12 | #Feature F13 | #Feature F2 | #Feature F3 | #Feature F4 | #Feature F5 | #Feature F6 | #Feature F7 | #Feature F8 | #Feature F9 | Start | End | PTM | AScore | Found By |
| --- | --- | --- | --- | --- | --- | --- | --- | --- | --- | --- | --- | --- | --- | --- | --- | --- | --- | --- | --- | --- | --- | --- | --- | --- | --- | --- | --- | --- | --- | --- | --- | --- | --- | --- | --- | --- | --- | --- | --- | --- | --- | --- | --- |
| K.FTYGGC(+57.02)AGNANNFK.K | Y | 83.49 | 1519.6514 | 14 | 0.1 | 760.8330 | 2 | 24.59 | 9 | F9:11113 | NaNaKA16\_F5.raw |  |  |  |  |  |  |  |  | 5.8819E7 | 6.0965E7 |  |  |  | 2 | 0 | 0 | 0 | 0 | 0 | 0 | 0 | 0 | 1 | 1 | 0 | 0 | 0 | 170 | 183 | Carbamidomethylation | C6:Carbamidomethylation:1000.00 | PEAKS DB |
| L.FEGSC(+57.02)SIFPNDSQR.C | Y | 75.18 | 1642.7046 | 14 | 0.2 | 822.3597 | 2 | 40.72 | 10 | F10:24722 | NaNaKA16\_F6.raw |  |  |  |  |  |  |  |  | 5.6415E5 | 1.0068E7 |  |  |  | 2 | 0 | 0 | 0 | 0 | 0 | 0 | 0 | 0 | 1 | 1 | 0 | 0 | 0 | 34 | 47 | Carbamidomethylation | C5:Carbamidomethylation:1000.00 | PEAKS DB |
| G.LFEGSC(+57.02)SIFPNDSQR.C | Y | 64.56 | 1755.7886 | 15 | 0.3 | 878.9018 | 2 | 55.75 | 10 | F10:37299 | NaNaKA16\_F6.raw |  |  |  |  |  |  |  |  |  | 4.6458E7 |  |  |  | 1 | 0 | 0 | 0 | 0 | 0 | 0 | 0 | 0 | 0 | 1 | 0 | 0 | 0 | 33 | 47 | Carbamidomethylation | C6:Carbamidomethylation:1000.00 | PEAKS DB |
| total 3 peptides |  |  |  |  |  |  |  |  |  |  |  |  |  |  |  |  |  |  |  |  |  |  |  |  |  |  |  |  |  |  |  |  |  |  |  |  |  |  |  |  |  |  |  |
| --- | --- | --- | --- | --- | --- | --- | --- | --- | --- | --- | --- | --- | --- | --- | --- | --- | --- | --- | --- | --- | --- | --- | --- | --- | --- | --- | --- | --- | --- | --- | --- | --- | --- | --- | --- | --- | --- | --- | --- | --- | --- | --- | --- |

XP\_026579404.1

back to list

  

| Protein Coverage
| Supporting Peptides
|

Protein Coverage:

Supporting Peptides:

| Peptide | Uniq | -10lgP | Mass | Length | ppm | m/z | z | RT | Fraction | Scan | Source File | Area F1 | Area F10 | Area F11 | Area F12 | Area F13 | Area F2 | Area F3 | Area F4 | Area F5 | Area F6 | Area F7 | Area F8 | Area F9 | #Feature | #Feature F1 | #Feature F10 | #Feature F11 | #Feature F12 | #Feature F13 | #Feature F2 | #Feature F3 | #Feature F4 | #Feature F5 | #Feature F6 | #Feature F7 | #Feature F8 | #Feature F9 | Start | End | PTM | AScore | Found By |
| --- | --- | --- | --- | --- | --- | --- | --- | --- | --- | --- | --- | --- | --- | --- | --- | --- | --- | --- | --- | --- | --- | --- | --- | --- | --- | --- | --- | --- | --- | --- | --- | --- | --- | --- | --- | --- | --- | --- | --- | --- | --- | --- | --- |
| K.FTYGGC(+57.02)AGNANNFK.K | Y | 83.49 | 1519.6514 | 14 | 0.1 | 760.8330 | 2 | 24.59 | 9 | F9:11113 | NaNaKA16\_F5.raw |  |  |  |  |  |  |  |  | 5.8819E7 | 6.0965E7 |  |  |  | 2 | 0 | 0 | 0 | 0 | 0 | 0 | 0 | 0 | 1 | 1 | 0 | 0 | 0 | 170 | 183 | Carbamidomethylation | C6:Carbamidomethylation:1000.00 | PEAKS DB |
| L.FEGSC(+57.02)SIFPNDSQR.C | Y | 75.18 | 1642.7046 | 14 | 0.2 | 822.3597 | 2 | 40.72 | 10 | F10:24722 | NaNaKA16\_F6.raw |  |  |  |  |  |  |  |  | 5.6415E5 | 1.0068E7 |  |  |  | 2 | 0 | 0 | 0 | 0 | 0 | 0 | 0 | 0 | 1 | 1 | 0 | 0 | 0 | 34 | 47 | Carbamidomethylation | C5:Carbamidomethylation:1000.00 | PEAKS DB |
| G.LFEGSC(+57.02)SIFPNDSQR.C | Y | 64.56 | 1755.7886 | 15 | 0.3 | 878.9018 | 2 | 55.75 | 10 | F10:37299 | NaNaKA16\_F6.raw |  |  |  |  |  |  |  |  |  | 4.6458E7 |  |  |  | 1 | 0 | 0 | 0 | 0 | 0 | 0 | 0 | 0 | 0 | 1 | 0 | 0 | 0 | 33 | 47 | Carbamidomethylation | C6:Carbamidomethylation:1000.00 | PEAKS DB |
| total 3 peptides |  |  |  |  |  |  |  |  |  |  |  |  |  |  |  |  |  |  |  |  |  |  |  |  |  |  |  |  |  |  |  |  |  |  |  |  |  |  |  |  |  |  |  |
| --- | --- | --- | --- | --- | --- | --- | --- | --- | --- | --- | --- | --- | --- | --- | --- | --- | --- | --- | --- | --- | --- | --- | --- | --- | --- | --- | --- | --- | --- | --- | --- | --- | --- | --- | --- | --- | --- | --- | --- | --- | --- | --- | --- |

XP\_026579409.1

back to list

  

| Protein Coverage
| Supporting Peptides
|

Protein Coverage:

Supporting Peptides:

| Peptide | Uniq | -10lgP | Mass | Length | ppm | m/z | z | RT | Fraction | Scan | Source File | Area F1 | Area F10 | Area F11 | Area F12 | Area F13 | Area F2 | Area F3 | Area F4 | Area F5 | Area F6 | Area F7 | Area F8 | Area F9 | #Feature | #Feature F1 | #Feature F10 | #Feature F11 | #Feature F12 | #Feature F13 | #Feature F2 | #Feature F3 | #Feature F4 | #Feature F5 | #Feature F6 | #Feature F7 | #Feature F8 | #Feature F9 | Start | End | PTM | AScore | Found By |
| --- | --- | --- | --- | --- | --- | --- | --- | --- | --- | --- | --- | --- | --- | --- | --- | --- | --- | --- | --- | --- | --- | --- | --- | --- | --- | --- | --- | --- | --- | --- | --- | --- | --- | --- | --- | --- | --- | --- | --- | --- | --- | --- | --- |
| K.FTYGGC(+57.02)AGNANNFK.K | Y | 83.49 | 1519.6514 | 14 | 0.1 | 760.8330 | 2 | 24.59 | 9 | F9:11113 | NaNaKA16\_F5.raw |  |  |  |  |  |  |  |  | 5.8819E7 | 6.0965E7 |  |  |  | 2 | 0 | 0 | 0 | 0 | 0 | 0 | 0 | 0 | 1 | 1 | 0 | 0 | 0 | 170 | 183 | Carbamidomethylation | C6:Carbamidomethylation:1000.00 | PEAKS DB |
| L.FEGSC(+57.02)SIFPNDSQR.C | Y | 75.18 | 1642.7046 | 14 | 0.2 | 822.3597 | 2 | 40.72 | 10 | F10:24722 | NaNaKA16\_F6.raw |  |  |  |  |  |  |  |  | 5.6415E5 | 1.0068E7 |  |  |  | 2 | 0 | 0 | 0 | 0 | 0 | 0 | 0 | 0 | 1 | 1 | 0 | 0 | 0 | 34 | 47 | Carbamidomethylation | C5:Carbamidomethylation:1000.00 | PEAKS DB |
| G.LFEGSC(+57.02)SIFPNDSQR.C | Y | 64.56 | 1755.7886 | 15 | 0.3 | 878.9018 | 2 | 55.75 | 10 | F10:37299 | NaNaKA16\_F6.raw |  |  |  |  |  |  |  |  |  | 4.6458E7 |  |  |  | 1 | 0 | 0 | 0 | 0 | 0 | 0 | 0 | 0 | 0 | 1 | 0 | 0 | 0 | 33 | 47 | Carbamidomethylation | C6:Carbamidomethylation:1000.00 | PEAKS DB |
| total 3 peptides |  |  |  |  |  |  |  |  |  |  |  |  |  |  |  |  |  |  |  |  |  |  |  |  |  |  |  |  |  |  |  |  |  |  |  |  |  |  |  |  |  |  |  |
| --- | --- | --- | --- | --- | --- | --- | --- | --- | --- | --- | --- | --- | --- | --- | --- | --- | --- | --- | --- | --- | --- | --- | --- | --- | --- | --- | --- | --- | --- | --- | --- | --- | --- | --- | --- | --- | --- | --- | --- | --- | --- | --- | --- |

2M99

back to list

  

| Protein Coverage
| Supporting Peptides
|

Protein Coverage:

Supporting Peptides:

| Peptide | Uniq | -10lgP | Mass | Length | ppm | m/z | z | RT | Fraction | Scan | Source File | Area F1 | Area F10 | Area F11 | Area F12 | Area F13 | Area F2 | Area F3 | Area F4 | Area F5 | Area F6 | Area F7 | Area F8 | Area F9 | #Feature | #Feature F1 | #Feature F10 | #Feature F11 | #Feature F12 | #Feature F13 | #Feature F2 | #Feature F3 | #Feature F4 | #Feature F5 | #Feature F6 | #Feature F7 | #Feature F8 | #Feature F9 | Start | End | PTM | AScore | Found By |
| --- | --- | --- | --- | --- | --- | --- | --- | --- | --- | --- | --- | --- | --- | --- | --- | --- | --- | --- | --- | --- | --- | --- | --- | --- | --- | --- | --- | --- | --- | --- | --- | --- | --- | --- | --- | --- | --- | --- | --- | --- | --- | --- | --- |
| Q.YSNTC(+57.02)HSFTYSGC(+57.02)GGNANR.F | Y | 88.06 | 2151.8486 | 19 | -0.4 | 1076.9312 | 2 | 11.90 | 9 | F9:1956 | NaNaKA16\_F5.raw |  |  |  |  |  |  |  | 7.3152E5 | 1.3863E8 |  | 4.2021E5 |  |  | 3 | 0 | 0 | 0 | 0 | 0 | 0 | 0 | 1 | 1 | 0 | 1 | 0 | 0 | 26 | 44 | Carbamidomethylation | C5:Carbamidomethylation:1000.00;C13:Carbamidomethylation:1000.00 | PEAKS DB |
| N.TC(+57.02)HSFTYSGC(+57.02)GGNANR.F | Y | 56.15 | 1787.7104 | 16 | 0.4 | 596.9110 | 3 | 11.84 | 9 | F9:1871 | NaNaKA16\_F5.raw |  |  |  |  |  |  |  |  | 5.9811E5 |  |  |  |  | 1 | 0 | 0 | 0 | 0 | 0 | 0 | 0 | 0 | 1 | 0 | 0 | 0 | 0 | 29 | 44 | Carbamidomethylation | C2:Carbamidomethylation:1000.00;C10:Carbamidomethylation:1000.00 | PEAKS DB |
| R.FC(+57.02)ELAPSAGSC(+57.02)F.A | N | 51.69 | 1344.5479 | 12 | 0.6 | 673.2816 | 2 | 63.59 | 9 | F9:43111 | NaNaKA16\_F5.raw |  |  |  |  |  |  |  |  | 1.5951E8 |  |  |  |  | 1 | 0 | 0 | 0 | 0 | 0 | 0 | 0 | 0 | 1 | 0 | 0 | 0 | 0 | 4 | 15 | Carbamidomethylation | C2:Carbamidomethylation:1000.00;C11:Carbamidomethylation:1000.00 | PEAKS DB |
| H.SFTYSGC(+57.02)GGNANR.F | Y | 45.38 | 1389.5731 | 13 | 0.0 | 695.7938 | 2 | 11.92 | 9 | F9:1980 | NaNaKA16\_F5.raw |  |  |  |  |  |  |  |  | 2.6632E5 |  |  |  |  | 1 | 0 | 0 | 0 | 0 | 0 | 0 | 0 | 0 | 1 | 0 | 0 | 0 | 0 | 32 | 44 | Carbamidomethylation | C7:Carbamidomethylation:1000.00 | PEAKS DB |
| total 4 peptides |  |  |  |  |  |  |  |  |  |  |  |  |  |  |  |  |  |  |  |  |  |  |  |  |  |  |  |  |  |  |  |  |  |  |  |  |  |  |  |  |  |  |  |
| --- | --- | --- | --- | --- | --- | --- | --- | --- | --- | --- | --- | --- | --- | --- | --- | --- | --- | --- | --- | --- | --- | --- | --- | --- | --- | --- | --- | --- | --- | --- | --- | --- | --- | --- | --- | --- | --- | --- | --- | --- | --- | --- | --- |

CAE51866.1

back to list

  

| Protein Coverage
| Supporting Peptides
|

Protein Coverage:

Supporting Peptides:

| Peptide | Uniq | -10lgP | Mass | Length | ppm | m/z | z | RT | Fraction | Scan | Source File | Area F1 | Area F10 | Area F11 | Area F12 | Area F13 | Area F2 | Area F3 | Area F4 | Area F5 | Area F6 | Area F7 | Area F8 | Area F9 | #Feature | #Feature F1 | #Feature F10 | #Feature F11 | #Feature F12 | #Feature F13 | #Feature F2 | #Feature F3 | #Feature F4 | #Feature F5 | #Feature F6 | #Feature F7 | #Feature F8 | #Feature F9 | Start | End | PTM | AScore | Found By |
| --- | --- | --- | --- | --- | --- | --- | --- | --- | --- | --- | --- | --- | --- | --- | --- | --- | --- | --- | --- | --- | --- | --- | --- | --- | --- | --- | --- | --- | --- | --- | --- | --- | --- | --- | --- | --- | --- | --- | --- | --- | --- | --- | --- |
| Q.YSNTC(+57.02)HSFTYSGC(+57.02)GGNANR.F | Y | 88.06 | 2151.8486 | 19 | -0.4 | 1076.9312 | 2 | 11.90 | 9 | F9:1956 | NaNaKA16\_F5.raw |  |  |  |  |  |  |  | 7.3152E5 | 1.3863E8 |  | 4.2021E5 |  |  | 3 | 0 | 0 | 0 | 0 | 0 | 0 | 0 | 1 | 1 | 0 | 1 | 0 | 0 | 50 | 68 | Carbamidomethylation | C5:Carbamidomethylation:1000.00;C13:Carbamidomethylation:1000.00 | PEAKS DB |
| N.TC(+57.02)HSFTYSGC(+57.02)GGNANR.F | Y | 56.15 | 1787.7104 | 16 | 0.4 | 596.9110 | 3 | 11.84 | 9 | F9:1871 | NaNaKA16\_F5.raw |  |  |  |  |  |  |  |  | 5.9811E5 |  |  |  |  | 1 | 0 | 0 | 0 | 0 | 0 | 0 | 0 | 0 | 1 | 0 | 0 | 0 | 0 | 53 | 68 | Carbamidomethylation | C2:Carbamidomethylation:1000.00;C10:Carbamidomethylation:1000.00 | PEAKS DB |
| R.FC(+57.02)ELAPSAGSC(+57.02)F.A | N | 51.69 | 1344.5479 | 12 | 0.6 | 673.2816 | 2 | 63.59 | 9 | F9:43111 | NaNaKA16\_F5.raw |  |  |  |  |  |  |  |  | 1.5951E8 |  |  |  |  | 1 | 0 | 0 | 0 | 0 | 0 | 0 | 0 | 0 | 1 | 0 | 0 | 0 | 0 | 28 | 39 | Carbamidomethylation | C2:Carbamidomethylation:1000.00;C11:Carbamidomethylation:1000.00 | PEAKS DB |
| H.SFTYSGC(+57.02)GGNANR.F | Y | 45.38 | 1389.5731 | 13 | 0.0 | 695.7938 | 2 | 11.92 | 9 | F9:1980 | NaNaKA16\_F5.raw |  |  |  |  |  |  |  |  | 2.6632E5 |  |  |  |  | 1 | 0 | 0 | 0 | 0 | 0 | 0 | 0 | 0 | 1 | 0 | 0 | 0 | 0 | 56 | 68 | Carbamidomethylation | C7:Carbamidomethylation:1000.00 | PEAKS DB |
| total 4 peptides |  |  |  |  |  |  |  |  |  |  |  |  |  |  |  |  |  |  |  |  |  |  |  |  |  |  |  |  |  |  |  |  |  |  |  |  |  |  |  |  |  |  |  |
| --- | --- | --- | --- | --- | --- | --- | --- | --- | --- | --- | --- | --- | --- | --- | --- | --- | --- | --- | --- | --- | --- | --- | --- | --- | --- | --- | --- | --- | --- | --- | --- | --- | --- | --- | --- | --- | --- | --- | --- | --- | --- | --- | --- |

Q5ZPJ7.1

back to list

  

| Protein Coverage
| Supporting Peptides
|

Protein Coverage:

Supporting Peptides:

| Peptide | Uniq | -10lgP | Mass | Length | ppm | m/z | z | RT | Fraction | Scan | Source File | Area F1 | Area F10 | Area F11 | Area F12 | Area F13 | Area F2 | Area F3 | Area F4 | Area F5 | Area F6 | Area F7 | Area F8 | Area F9 | #Feature | #Feature F1 | #Feature F10 | #Feature F11 | #Feature F12 | #Feature F13 | #Feature F2 | #Feature F3 | #Feature F4 | #Feature F5 | #Feature F6 | #Feature F7 | #Feature F8 | #Feature F9 | Start | End | PTM | AScore | Found By |
| --- | --- | --- | --- | --- | --- | --- | --- | --- | --- | --- | --- | --- | --- | --- | --- | --- | --- | --- | --- | --- | --- | --- | --- | --- | --- | --- | --- | --- | --- | --- | --- | --- | --- | --- | --- | --- | --- | --- | --- | --- | --- | --- | --- |
| Q.YSNTC(+57.02)HSFTYSGC(+57.02)GGNANR.F | Y | 88.06 | 2151.8486 | 19 | -0.4 | 1076.9312 | 2 | 11.90 | 9 | F9:1956 | NaNaKA16\_F5.raw |  |  |  |  |  |  |  | 7.3152E5 | 1.3863E8 |  | 4.2021E5 |  |  | 3 | 0 | 0 | 0 | 0 | 0 | 0 | 0 | 1 | 1 | 0 | 1 | 0 | 0 | 50 | 68 | Carbamidomethylation | C5:Carbamidomethylation:1000.00;C13:Carbamidomethylation:1000.00 | PEAKS DB |
| N.TC(+57.02)HSFTYSGC(+57.02)GGNANR.F | Y | 56.15 | 1787.7104 | 16 | 0.4 | 596.9110 | 3 | 11.84 | 9 | F9:1871 | NaNaKA16\_F5.raw |  |  |  |  |  |  |  |  | 5.9811E5 |  |  |  |  | 1 | 0 | 0 | 0 | 0 | 0 | 0 | 0 | 0 | 1 | 0 | 0 | 0 | 0 | 53 | 68 | Carbamidomethylation | C2:Carbamidomethylation:1000.00;C10:Carbamidomethylation:1000.00 | PEAKS DB |
| R.FC(+57.02)ELAPSAGSC(+57.02)F.A | N | 51.69 | 1344.5479 | 12 | 0.6 | 673.2816 | 2 | 63.59 | 9 | F9:43111 | NaNaKA16\_F5.raw |  |  |  |  |  |  |  |  | 1.5951E8 |  |  |  |  | 1 | 0 | 0 | 0 | 0 | 0 | 0 | 0 | 0 | 1 | 0 | 0 | 0 | 0 | 28 | 39 | Carbamidomethylation | C2:Carbamidomethylation:1000.00;C11:Carbamidomethylation:1000.00 | PEAKS DB |
| H.SFTYSGC(+57.02)GGNANR.F | Y | 45.38 | 1389.5731 | 13 | 0.0 | 695.7938 | 2 | 11.92 | 9 | F9:1980 | NaNaKA16\_F5.raw |  |  |  |  |  |  |  |  | 2.6632E5 |  |  |  |  | 1 | 0 | 0 | 0 | 0 | 0 | 0 | 0 | 0 | 1 | 0 | 0 | 0 | 0 | 56 | 68 | Carbamidomethylation | C7:Carbamidomethylation:1000.00 | PEAKS DB |
| total 4 peptides |  |  |  |  |  |  |  |  |  |  |  |  |  |  |  |  |  |  |  |  |  |  |  |  |  |  |  |  |  |  |  |  |  |  |  |  |  |  |  |  |  |  |  |
| --- | --- | --- | --- | --- | --- | --- | --- | --- | --- | --- | --- | --- | --- | --- | --- | --- | --- | --- | --- | --- | --- | --- | --- | --- | --- | --- | --- | --- | --- | --- | --- | --- | --- | --- | --- | --- | --- | --- | --- | --- | --- | --- | --- |

CAE51867.1

back to list

  

| Protein Coverage
| Supporting Peptides
|

Protein Coverage:

Supporting Peptides:

| Peptide | Uniq | -10lgP | Mass | Length | ppm | m/z | z | RT | Fraction | Scan | Source File | Area F1 | Area F10 | Area F11 | Area F12 | Area F13 | Area F2 | Area F3 | Area F4 | Area F5 | Area F6 | Area F7 | Area F8 | Area F9 | #Feature | #Feature F1 | #Feature F10 | #Feature F11 | #Feature F12 | #Feature F13 | #Feature F2 | #Feature F3 | #Feature F4 | #Feature F5 | #Feature F6 | #Feature F7 | #Feature F8 | #Feature F9 | Start | End | PTM | AScore | Found By |
| --- | --- | --- | --- | --- | --- | --- | --- | --- | --- | --- | --- | --- | --- | --- | --- | --- | --- | --- | --- | --- | --- | --- | --- | --- | --- | --- | --- | --- | --- | --- | --- | --- | --- | --- | --- | --- | --- | --- | --- | --- | --- | --- | --- |
| Q.YSNTC(+57.02)HSFTYSGC(+57.02)GGNANR.F | Y | 88.06 | 2151.8486 | 19 | -0.4 | 1076.9312 | 2 | 11.90 | 9 | F9:1956 | NaNaKA16\_F5.raw |  |  |  |  |  |  |  | 7.3152E5 | 1.3863E8 |  | 4.2021E5 |  |  | 3 | 0 | 0 | 0 | 0 | 0 | 0 | 0 | 1 | 1 | 0 | 1 | 0 | 0 | 50 | 68 | Carbamidomethylation | C5:Carbamidomethylation:1000.00;C13:Carbamidomethylation:1000.00 | PEAKS DB |
| N.TC(+57.02)HSFTYSGC(+57.02)GGNANR.F | Y | 56.15 | 1787.7104 | 16 | 0.4 | 596.9110 | 3 | 11.84 | 9 | F9:1871 | NaNaKA16\_F5.raw |  |  |  |  |  |  |  |  | 5.9811E5 |  |  |  |  | 1 | 0 | 0 | 0 | 0 | 0 | 0 | 0 | 0 | 1 | 0 | 0 | 0 | 0 | 53 | 68 | Carbamidomethylation | C2:Carbamidomethylation:1000.00;C10:Carbamidomethylation:1000.00 | PEAKS DB |
| R.FC(+57.02)ELAPSAGSC(+57.02)F.A | N | 51.69 | 1344.5479 | 12 | 0.6 | 673.2816 | 2 | 63.59 | 9 | F9:43111 | NaNaKA16\_F5.raw |  |  |  |  |  |  |  |  | 1.5951E8 |  |  |  |  | 1 | 0 | 0 | 0 | 0 | 0 | 0 | 0 | 0 | 1 | 0 | 0 | 0 | 0 | 28 | 39 | Carbamidomethylation | C2:Carbamidomethylation:1000.00;C11:Carbamidomethylation:1000.00 | PEAKS DB |
| H.SFTYSGC(+57.02)GGNANR.F | Y | 45.38 | 1389.5731 | 13 | 0.0 | 695.7938 | 2 | 11.92 | 9 | F9:1980 | NaNaKA16\_F5.raw |  |  |  |  |  |  |  |  | 2.6632E5 |  |  |  |  | 1 | 0 | 0 | 0 | 0 | 0 | 0 | 0 | 0 | 1 | 0 | 0 | 0 | 0 | 56 | 68 | Carbamidomethylation | C7:Carbamidomethylation:1000.00 | PEAKS DB |
| total 4 peptides |  |  |  |  |  |  |  |  |  |  |  |  |  |  |  |  |  |  |  |  |  |  |  |  |  |  |  |  |  |  |  |  |  |  |  |  |  |  |  |  |  |  |  |
| --- | --- | --- | --- | --- | --- | --- | --- | --- | --- | --- | --- | --- | --- | --- | --- | --- | --- | --- | --- | --- | --- | --- | --- | --- | --- | --- | --- | --- | --- | --- | --- | --- | --- | --- | --- | --- | --- | --- | --- | --- | --- | --- | --- |

JAB52939.1

back to list

  

| Protein Coverage
| Supporting Peptides
|

Protein Coverage:

Supporting Peptides:

| Peptide | Uniq | -10lgP | Mass | Length | ppm | m/z | z | RT | Fraction | Scan | Source File | Area F1 | Area F10 | Area F11 | Area F12 | Area F13 | Area F2 | Area F3 | Area F4 | Area F5 | Area F6 | Area F7 | Area F8 | Area F9 | #Feature | #Feature F1 | #Feature F10 | #Feature F11 | #Feature F12 | #Feature F13 | #Feature F2 | #Feature F3 | #Feature F4 | #Feature F5 | #Feature F6 | #Feature F7 | #Feature F8 | #Feature F9 | Start | End | PTM | AScore | Found By |
| --- | --- | --- | --- | --- | --- | --- | --- | --- | --- | --- | --- | --- | --- | --- | --- | --- | --- | --- | --- | --- | --- | --- | --- | --- | --- | --- | --- | --- | --- | --- | --- | --- | --- | --- | --- | --- | --- | --- | --- | --- | --- | --- | --- |
| K.TLFEITVPLSHGPK.P | Y | 67.52 | 1537.8503 | 14 | -0.1 | 513.6240 | 3 | 58.66 | 2 | F2:36076 | NaNaKA16\_F10.raw |  | 9.1753E6 |  |  |  |  |  |  |  |  |  | 1.5718E6 | 2.8923E6 | 4 | 0 | 2 | 0 | 0 | 0 | 0 | 0 | 0 | 0 | 0 | 0 | 1 | 1 | 185 | 198 |  |  | PEAKS DB |
| K.C(+57.02)IC(+57.02)EC(+57.02)VESPNK.C | Y | 61.53 | 1394.5629 | 11 | 0.3 | 698.2889 | 2 | 47.51 | 6 | F6:30331 | NaNaKA16\_F2.raw |  |  |  |  |  | 4.592E5 |  |  |  |  |  |  |  | 1 | 0 | 0 | 0 | 0 | 0 | 1 | 0 | 0 | 0 | 0 | 0 | 0 | 0 | 360 | 370 | Carbamidomethylation | C1:Carbamidomethylation:1000.00;C3:Carbamidomethylation:1000.00;C5:Carbamidomethylation:1000.00 | PEAKS DB |
| K.LLPSSC(+57.02)GLNKEFDEEK.C | Y | 60.34 | 1864.8876 | 16 | 4.8 | 622.6364 | 3 | 33.83 | 7 | F7:14388 | NaNaKA16\_F3.raw |  |  |  |  |  |  | 1.4164E5 |  |  |  |  |  |  | 1 | 0 | 0 | 0 | 0 | 0 | 0 | 1 | 0 | 0 | 0 | 0 | 0 | 0 | 325 | 340 | Carbamidomethylation | C6:Carbamidomethylation:1000.00 | PEAKS DB |
| K.LLPSSC(+57.02)GLNK.E | Y | 54.98 | 1087.5696 | 10 | -0.1 | 544.7920 | 2 | 19.39 | 6 | F6:6810 | NaNaKA16\_F2.raw |  |  |  |  |  | 2.1852E6 |  |  |  |  |  |  |  | 1 | 0 | 0 | 0 | 0 | 0 | 1 | 0 | 0 | 0 | 0 | 0 | 0 | 0 | 325 | 334 | Carbamidomethylation | C6:Carbamidomethylation:1000.00 | PEAKS DB |
| K.ELDEETC(+57.02)QC(+57.02)VC(+57.02)K.G | Y | 48.15 | 1569.6110 | 12 | 0.1 | 785.8129 | 2 | 12.37 | 6 | F6:2413 | NaNaKA16\_F2.raw |  |  |  |  |  | 8.8422E4 |  |  |  |  |  |  |  | 1 | 0 | 0 | 0 | 0 | 0 | 1 | 0 | 0 | 0 | 0 | 0 | 0 | 0 | 287 | 298 | Carbamidomethylation | C7:Carbamidomethylation:1000.00;C9:Carbamidomethylation:1000.00;C11:Carbamidomethylation:1000.00 | PEAKS DB |
| total 5 peptides |  |  |  |  |  |  |  |  |  |  |  |  |  |  |  |  |  |  |  |  |  |  |  |  |  |  |  |  |  |  |  |  |  |  |  |  |  |  |  |  |  |  |  |
| --- | --- | --- | --- | --- | --- | --- | --- | --- | --- | --- | --- | --- | --- | --- | --- | --- | --- | --- | --- | --- | --- | --- | --- | --- | --- | --- | --- | --- | --- | --- | --- | --- | --- | --- | --- | --- | --- | --- | --- | --- | --- | --- | --- |

JAI08988.1

back to list

  

| Protein Coverage
| Supporting Peptides
|

Protein Coverage:

Supporting Peptides:

| Peptide | Uniq | -10lgP | Mass | Length | ppm | m/z | z | RT | Fraction | Scan | Source File | Area F1 | Area F10 | Area F11 | Area F12 | Area F13 | Area F2 | Area F3 | Area F4 | Area F5 | Area F6 | Area F7 | Area F8 | Area F9 | #Feature | #Feature F1 | #Feature F10 | #Feature F11 | #Feature F12 | #Feature F13 | #Feature F2 | #Feature F3 | #Feature F4 | #Feature F5 | #Feature F6 | #Feature F7 | #Feature F8 | #Feature F9 | Start | End | PTM | AScore | Found By |
| --- | --- | --- | --- | --- | --- | --- | --- | --- | --- | --- | --- | --- | --- | --- | --- | --- | --- | --- | --- | --- | --- | --- | --- | --- | --- | --- | --- | --- | --- | --- | --- | --- | --- | --- | --- | --- | --- | --- | --- | --- | --- | --- | --- |
| K.TLFEITVPLSHGPK.P | Y | 67.52 | 1537.8503 | 14 | -0.1 | 513.6240 | 3 | 58.66 | 2 | F2:36076 | NaNaKA16\_F10.raw |  | 9.1753E6 |  |  |  |  |  |  |  |  |  | 1.5718E6 | 2.8923E6 | 4 | 0 | 2 | 0 | 0 | 0 | 0 | 0 | 0 | 0 | 0 | 0 | 1 | 1 | 185 | 198 |  |  | PEAKS DB |
| K.C(+57.02)IC(+57.02)EC(+57.02)VESPNK.C | Y | 61.53 | 1394.5629 | 11 | 0.3 | 698.2889 | 2 | 47.51 | 6 | F6:30331 | NaNaKA16\_F2.raw |  |  |  |  |  | 4.592E5 |  |  |  |  |  |  |  | 1 | 0 | 0 | 0 | 0 | 0 | 1 | 0 | 0 | 0 | 0 | 0 | 0 | 0 | 360 | 370 | Carbamidomethylation | C1:Carbamidomethylation:1000.00;C3:Carbamidomethylation:1000.00;C5:Carbamidomethylation:1000.00 | PEAKS DB |
| K.LLPSSC(+57.02)GLNKEFDEEK.C | Y | 60.34 | 1864.8876 | 16 | 4.8 | 622.6364 | 3 | 33.83 | 7 | F7:14388 | NaNaKA16\_F3.raw |  |  |  |  |  |  | 1.4164E5 |  |  |  |  |  |  | 1 | 0 | 0 | 0 | 0 | 0 | 0 | 1 | 0 | 0 | 0 | 0 | 0 | 0 | 325 | 340 | Carbamidomethylation | C6:Carbamidomethylation:1000.00 | PEAKS DB |
| K.LLPSSC(+57.02)GLNK.E | Y | 54.98 | 1087.5696 | 10 | -0.1 | 544.7920 | 2 | 19.39 | 6 | F6:6810 | NaNaKA16\_F2.raw |  |  |  |  |  | 2.1852E6 |  |  |  |  |  |  |  | 1 | 0 | 0 | 0 | 0 | 0 | 1 | 0 | 0 | 0 | 0 | 0 | 0 | 0 | 325 | 334 | Carbamidomethylation | C6:Carbamidomethylation:1000.00 | PEAKS DB |
| K.ELDEETC(+57.02)QC(+57.02)VC(+57.02)K.G | Y | 48.15 | 1569.6110 | 12 | 0.1 | 785.8129 | 2 | 12.37 | 6 | F6:2413 | NaNaKA16\_F2.raw |  |  |  |  |  | 8.8422E4 |  |  |  |  |  |  |  | 1 | 0 | 0 | 0 | 0 | 0 | 1 | 0 | 0 | 0 | 0 | 0 | 0 | 0 | 287 | 298 | Carbamidomethylation | C7:Carbamidomethylation:1000.00;C9:Carbamidomethylation:1000.00;C11:Carbamidomethylation:1000.00 | PEAKS DB |
| total 5 peptides |  |  |  |  |  |  |  |  |  |  |  |  |  |  |  |  |  |  |  |  |  |  |  |  |  |  |  |  |  |  |  |  |  |  |  |  |  |  |  |  |  |  |  |
| --- | --- | --- | --- | --- | --- | --- | --- | --- | --- | --- | --- | --- | --- | --- | --- | --- | --- | --- | --- | --- | --- | --- | --- | --- | --- | --- | --- | --- | --- | --- | --- | --- | --- | --- | --- | --- | --- | --- | --- | --- | --- | --- | --- |

JAI09156.1

back to list

  

| Protein Coverage
| Supporting Peptides
|

Protein Coverage:

Supporting Peptides:

| Peptide | Uniq | -10lgP | Mass | Length | ppm | m/z | z | RT | Fraction | Scan | Source File | Area F1 | Area F10 | Area F11 | Area F12 | Area F13 | Area F2 | Area F3 | Area F4 | Area F5 | Area F6 | Area F7 | Area F8 | Area F9 | #Feature | #Feature F1 | #Feature F10 | #Feature F11 | #Feature F12 | #Feature F13 | #Feature F2 | #Feature F3 | #Feature F4 | #Feature F5 | #Feature F6 | #Feature F7 | #Feature F8 | #Feature F9 | Start | End | PTM | AScore | Found By |
| --- | --- | --- | --- | --- | --- | --- | --- | --- | --- | --- | --- | --- | --- | --- | --- | --- | --- | --- | --- | --- | --- | --- | --- | --- | --- | --- | --- | --- | --- | --- | --- | --- | --- | --- | --- | --- | --- | --- | --- | --- | --- | --- | --- |
| K.TLFEITVPLSHGPK.P | Y | 67.52 | 1537.8503 | 14 | -0.1 | 513.6240 | 3 | 58.66 | 2 | F2:36076 | NaNaKA16\_F10.raw |  | 9.1753E6 |  |  |  |  |  |  |  |  |  | 1.5718E6 | 2.8923E6 | 4 | 0 | 2 | 0 | 0 | 0 | 0 | 0 | 0 | 0 | 0 | 0 | 1 | 1 | 185 | 198 |  |  | PEAKS DB |
| K.C(+57.02)IC(+57.02)EC(+57.02)VESPNK.C | Y | 61.53 | 1394.5629 | 11 | 0.3 | 698.2889 | 2 | 47.51 | 6 | F6:30331 | NaNaKA16\_F2.raw |  |  |  |  |  | 4.592E5 |  |  |  |  |  |  |  | 1 | 0 | 0 | 0 | 0 | 0 | 1 | 0 | 0 | 0 | 0 | 0 | 0 | 0 | 360 | 370 | Carbamidomethylation | C1:Carbamidomethylation:1000.00;C3:Carbamidomethylation:1000.00;C5:Carbamidomethylation:1000.00 | PEAKS DB |
| K.LLPSSC(+57.02)GLNKEFDEEK.C | Y | 60.34 | 1864.8876 | 16 | 4.8 | 622.6364 | 3 | 33.83 | 7 | F7:14388 | NaNaKA16\_F3.raw |  |  |  |  |  |  | 1.4164E5 |  |  |  |  |  |  | 1 | 0 | 0 | 0 | 0 | 0 | 0 | 1 | 0 | 0 | 0 | 0 | 0 | 0 | 325 | 340 | Carbamidomethylation | C6:Carbamidomethylation:1000.00 | PEAKS DB |
| K.LLPSSC(+57.02)GLNK.E | Y | 54.98 | 1087.5696 | 10 | -0.1 | 544.7920 | 2 | 19.39 | 6 | F6:6810 | NaNaKA16\_F2.raw |  |  |  |  |  | 2.1852E6 |  |  |  |  |  |  |  | 1 | 0 | 0 | 0 | 0 | 0 | 1 | 0 | 0 | 0 | 0 | 0 | 0 | 0 | 325 | 334 | Carbamidomethylation | C6:Carbamidomethylation:1000.00 | PEAKS DB |
| K.ELDEETC(+57.02)QC(+57.02)VC(+57.02)K.G | Y | 48.15 | 1569.6110 | 12 | 0.1 | 785.8129 | 2 | 12.37 | 6 | F6:2413 | NaNaKA16\_F2.raw |  |  |  |  |  | 8.8422E4 |  |  |  |  |  |  |  | 1 | 0 | 0 | 0 | 0 | 0 | 1 | 0 | 0 | 0 | 0 | 0 | 0 | 0 | 287 | 298 | Carbamidomethylation | C7:Carbamidomethylation:1000.00;C9:Carbamidomethylation:1000.00;C11:Carbamidomethylation:1000.00 | PEAKS DB |
| total 5 peptides |  |  |  |  |  |  |  |  |  |  |  |  |  |  |  |  |  |  |  |  |  |  |  |  |  |  |  |  |  |  |  |  |  |  |  |  |  |  |  |  |  |  |  |
| --- | --- | --- | --- | --- | --- | --- | --- | --- | --- | --- | --- | --- | --- | --- | --- | --- | --- | --- | --- | --- | --- | --- | --- | --- | --- | --- | --- | --- | --- | --- | --- | --- | --- | --- | --- | --- | --- | --- | --- | --- | --- | --- | --- |

JAS04977.1

back to list

  

| Protein Coverage
| Supporting Peptides
|

Protein Coverage:

Supporting Peptides:

| Peptide | Uniq | -10lgP | Mass | Length | ppm | m/z | z | RT | Fraction | Scan | Source File | Area F1 | Area F10 | Area F11 | Area F12 | Area F13 | Area F2 | Area F3 | Area F4 | Area F5 | Area F6 | Area F7 | Area F8 | Area F9 | #Feature | #Feature F1 | #Feature F10 | #Feature F11 | #Feature F12 | #Feature F13 | #Feature F2 | #Feature F3 | #Feature F4 | #Feature F5 | #Feature F6 | #Feature F7 | #Feature F8 | #Feature F9 | Start | End | PTM | AScore | Found By |
| --- | --- | --- | --- | --- | --- | --- | --- | --- | --- | --- | --- | --- | --- | --- | --- | --- | --- | --- | --- | --- | --- | --- | --- | --- | --- | --- | --- | --- | --- | --- | --- | --- | --- | --- | --- | --- | --- | --- | --- | --- | --- | --- | --- |
| K.TLFEITVPLSHGPK.P | Y | 67.52 | 1537.8503 | 14 | -0.1 | 513.6240 | 3 | 58.66 | 2 | F2:36076 | NaNaKA16\_F10.raw |  | 9.1753E6 |  |  |  |  |  |  |  |  |  | 1.5718E6 | 2.8923E6 | 4 | 0 | 2 | 0 | 0 | 0 | 0 | 0 | 0 | 0 | 0 | 0 | 1 | 1 | 185 | 198 |  |  | PEAKS DB |
| K.C(+57.02)IC(+57.02)EC(+57.02)VESPNK.C | Y | 61.53 | 1394.5629 | 11 | 0.3 | 698.2889 | 2 | 47.51 | 6 | F6:30331 | NaNaKA16\_F2.raw |  |  |  |  |  | 4.592E5 |  |  |  |  |  |  |  | 1 | 0 | 0 | 0 | 0 | 0 | 1 | 0 | 0 | 0 | 0 | 0 | 0 | 0 | 360 | 370 | Carbamidomethylation | C1:Carbamidomethylation:1000.00;C3:Carbamidomethylation:1000.00;C5:Carbamidomethylation:1000.00 | PEAKS DB |
| K.LLPSSC(+57.02)GLNKEFDEEK.C | Y | 60.34 | 1864.8876 | 16 | 4.8 | 622.6364 | 3 | 33.83 | 7 | F7:14388 | NaNaKA16\_F3.raw |  |  |  |  |  |  | 1.4164E5 |  |  |  |  |  |  | 1 | 0 | 0 | 0 | 0 | 0 | 0 | 1 | 0 | 0 | 0 | 0 | 0 | 0 | 325 | 340 | Carbamidomethylation | C6:Carbamidomethylation:1000.00 | PEAKS DB |
| K.LLPSSC(+57.02)GLNK.E | Y | 54.98 | 1087.5696 | 10 | -0.1 | 544.7920 | 2 | 19.39 | 6 | F6:6810 | NaNaKA16\_F2.raw |  |  |  |  |  | 2.1852E6 |  |  |  |  |  |  |  | 1 | 0 | 0 | 0 | 0 | 0 | 1 | 0 | 0 | 0 | 0 | 0 | 0 | 0 | 325 | 334 | Carbamidomethylation | C6:Carbamidomethylation:1000.00 | PEAKS DB |
| K.ELDEETC(+57.02)QC(+57.02)VC(+57.02)K.G | Y | 48.15 | 1569.6110 | 12 | 0.1 | 785.8129 | 2 | 12.37 | 6 | F6:2413 | NaNaKA16\_F2.raw |  |  |  |  |  | 8.8422E4 |  |  |  |  |  |  |  | 1 | 0 | 0 | 0 | 0 | 0 | 1 | 0 | 0 | 0 | 0 | 0 | 0 | 0 | 287 | 298 | Carbamidomethylation | C7:Carbamidomethylation:1000.00;C9:Carbamidomethylation:1000.00;C11:Carbamidomethylation:1000.00 | PEAKS DB |
| total 5 peptides |  |  |  |  |  |  |  |  |  |  |  |  |  |  |  |  |  |  |  |  |  |  |  |  |  |  |  |  |  |  |  |  |  |  |  |  |  |  |  |  |  |  |  |
| --- | --- | --- | --- | --- | --- | --- | --- | --- | --- | --- | --- | --- | --- | --- | --- | --- | --- | --- | --- | --- | --- | --- | --- | --- | --- | --- | --- | --- | --- | --- | --- | --- | --- | --- | --- | --- | --- | --- | --- | --- | --- | --- | --- |

JAC95037.1

back to list

  

| Protein Coverage
| Supporting Peptides
|

Protein Coverage:

Supporting Peptides:

| Peptide | Uniq | -10lgP | Mass | Length | ppm | m/z | z | RT | Fraction | Scan | Source File | Area F1 | Area F10 | Area F11 | Area F12 | Area F13 | Area F2 | Area F3 | Area F4 | Area F5 | Area F6 | Area F7 | Area F8 | Area F9 | #Feature | #Feature F1 | #Feature F10 | #Feature F11 | #Feature F12 | #Feature F13 | #Feature F2 | #Feature F3 | #Feature F4 | #Feature F5 | #Feature F6 | #Feature F7 | #Feature F8 | #Feature F9 | Start | End | PTM | AScore | Found By |
| --- | --- | --- | --- | --- | --- | --- | --- | --- | --- | --- | --- | --- | --- | --- | --- | --- | --- | --- | --- | --- | --- | --- | --- | --- | --- | --- | --- | --- | --- | --- | --- | --- | --- | --- | --- | --- | --- | --- | --- | --- | --- | --- | --- |
| K.TLFEITVPLSHGPK.P | Y | 67.52 | 1537.8503 | 14 | -0.1 | 513.6240 | 3 | 58.66 | 2 | F2:36076 | NaNaKA16\_F10.raw |  | 9.1753E6 |  |  |  |  |  |  |  |  |  | 1.5718E6 | 2.8923E6 | 4 | 0 | 2 | 0 | 0 | 0 | 0 | 0 | 0 | 0 | 0 | 0 | 1 | 1 | 185 | 198 |  |  | PEAKS DB |
| K.C(+57.02)IC(+57.02)EC(+57.02)VESPNK.C | Y | 61.53 | 1394.5629 | 11 | 0.3 | 698.2889 | 2 | 47.51 | 6 | F6:30331 | NaNaKA16\_F2.raw |  |  |  |  |  | 4.592E5 |  |  |  |  |  |  |  | 1 | 0 | 0 | 0 | 0 | 0 | 1 | 0 | 0 | 0 | 0 | 0 | 0 | 0 | 360 | 370 | Carbamidomethylation | C1:Carbamidomethylation:1000.00;C3:Carbamidomethylation:1000.00;C5:Carbamidomethylation:1000.00 | PEAKS DB |
| K.LLPSSC(+57.02)GLNKEFDEEK.C | Y | 60.34 | 1864.8876 | 16 | 4.8 | 622.6364 | 3 | 33.83 | 7 | F7:14388 | NaNaKA16\_F3.raw |  |  |  |  |  |  | 1.4164E5 |  |  |  |  |  |  | 1 | 0 | 0 | 0 | 0 | 0 | 0 | 1 | 0 | 0 | 0 | 0 | 0 | 0 | 325 | 340 | Carbamidomethylation | C6:Carbamidomethylation:1000.00 | PEAKS DB |
| K.LLPSSC(+57.02)GLNK.E | Y | 54.98 | 1087.5696 | 10 | -0.1 | 544.7920 | 2 | 19.39 | 6 | F6:6810 | NaNaKA16\_F2.raw |  |  |  |  |  | 2.1852E6 |  |  |  |  |  |  |  | 1 | 0 | 0 | 0 | 0 | 0 | 1 | 0 | 0 | 0 | 0 | 0 | 0 | 0 | 325 | 334 | Carbamidomethylation | C6:Carbamidomethylation:1000.00 | PEAKS DB |
| K.ELDEETC(+57.02)QC(+57.02)VC(+57.02)K.G | Y | 48.15 | 1569.6110 | 12 | 0.1 | 785.8129 | 2 | 12.37 | 6 | F6:2413 | NaNaKA16\_F2.raw |  |  |  |  |  | 8.8422E4 |  |  |  |  |  |  |  | 1 | 0 | 0 | 0 | 0 | 0 | 1 | 0 | 0 | 0 | 0 | 0 | 0 | 0 | 287 | 298 | Carbamidomethylation | C7:Carbamidomethylation:1000.00;C9:Carbamidomethylation:1000.00;C11:Carbamidomethylation:1000.00 | PEAKS DB |
| total 5 peptides |  |  |  |  |  |  |  |  |  |  |  |  |  |  |  |  |  |  |  |  |  |  |  |  |  |  |  |  |  |  |  |  |  |  |  |  |  |  |  |  |  |  |  |
| --- | --- | --- | --- | --- | --- | --- | --- | --- | --- | --- | --- | --- | --- | --- | --- | --- | --- | --- | --- | --- | --- | --- | --- | --- | --- | --- | --- | --- | --- | --- | --- | --- | --- | --- | --- | --- | --- | --- | --- | --- | --- | --- | --- |

XP\_034298588.1

back to list

  

| Protein Coverage
| Supporting Peptides
|

Protein Coverage:

Supporting Peptides:

| Peptide | Uniq | -10lgP | Mass | Length | ppm | m/z | z | RT | Fraction | Scan | Source File | Area F1 | Area F10 | Area F11 | Area F12 | Area F13 | Area F2 | Area F3 | Area F4 | Area F5 | Area F6 | Area F7 | Area F8 | Area F9 | #Feature | #Feature F1 | #Feature F10 | #Feature F11 | #Feature F12 | #Feature F13 | #Feature F2 | #Feature F3 | #Feature F4 | #Feature F5 | #Feature F6 | #Feature F7 | #Feature F8 | #Feature F9 | Start | End | PTM | AScore | Found By |
| --- | --- | --- | --- | --- | --- | --- | --- | --- | --- | --- | --- | --- | --- | --- | --- | --- | --- | --- | --- | --- | --- | --- | --- | --- | --- | --- | --- | --- | --- | --- | --- | --- | --- | --- | --- | --- | --- | --- | --- | --- | --- | --- | --- |
| K.TLFEITVPLSHGPK.P | Y | 67.52 | 1537.8503 | 14 | -0.1 | 513.6240 | 3 | 58.66 | 2 | F2:36076 | NaNaKA16\_F10.raw |  | 9.1753E6 |  |  |  |  |  |  |  |  |  | 1.5718E6 | 2.8923E6 | 4 | 0 | 2 | 0 | 0 | 0 | 0 | 0 | 0 | 0 | 0 | 0 | 1 | 1 | 185 | 198 |  |  | PEAKS DB |
| K.C(+57.02)IC(+57.02)EC(+57.02)VESPNK.C | Y | 61.53 | 1394.5629 | 11 | 0.3 | 698.2889 | 2 | 47.51 | 6 | F6:30331 | NaNaKA16\_F2.raw |  |  |  |  |  | 4.592E5 |  |  |  |  |  |  |  | 1 | 0 | 0 | 0 | 0 | 0 | 1 | 0 | 0 | 0 | 0 | 0 | 0 | 0 | 360 | 370 | Carbamidomethylation | C1:Carbamidomethylation:1000.00;C3:Carbamidomethylation:1000.00;C5:Carbamidomethylation:1000.00 | PEAKS DB |
| K.LLPSSC(+57.02)GLNKEFDEEK.C | Y | 60.34 | 1864.8876 | 16 | 4.8 | 622.6364 | 3 | 33.83 | 7 | F7:14388 | NaNaKA16\_F3.raw |  |  |  |  |  |  | 1.4164E5 |  |  |  |  |  |  | 1 | 0 | 0 | 0 | 0 | 0 | 0 | 1 | 0 | 0 | 0 | 0 | 0 | 0 | 325 | 340 | Carbamidomethylation | C6:Carbamidomethylation:1000.00 | PEAKS DB |
| K.LLPSSC(+57.02)GLNK.E | Y | 54.98 | 1087.5696 | 10 | -0.1 | 544.7920 | 2 | 19.39 | 6 | F6:6810 | NaNaKA16\_F2.raw |  |  |  |  |  | 2.1852E6 |  |  |  |  |  |  |  | 1 | 0 | 0 | 0 | 0 | 0 | 1 | 0 | 0 | 0 | 0 | 0 | 0 | 0 | 325 | 334 | Carbamidomethylation | C6:Carbamidomethylation:1000.00 | PEAKS DB |
| K.ELDEETC(+57.02)QC(+57.02)VC(+57.02)K.G | Y | 48.15 | 1569.6110 | 12 | 0.1 | 785.8129 | 2 | 12.37 | 6 | F6:2413 | NaNaKA16\_F2.raw |  |  |  |  |  | 8.8422E4 |  |  |  |  |  |  |  | 1 | 0 | 0 | 0 | 0 | 0 | 1 | 0 | 0 | 0 | 0 | 0 | 0 | 0 | 287 | 298 | Carbamidomethylation | C7:Carbamidomethylation:1000.00;C9:Carbamidomethylation:1000.00;C11:Carbamidomethylation:1000.00 | PEAKS DB |
| total 5 peptides |  |  |  |  |  |  |  |  |  |  |  |  |  |  |  |  |  |  |  |  |  |  |  |  |  |  |  |  |  |  |  |  |  |  |  |  |  |  |  |  |  |  |  |
| --- | --- | --- | --- | --- | --- | --- | --- | --- | --- | --- | --- | --- | --- | --- | --- | --- | --- | --- | --- | --- | --- | --- | --- | --- | --- | --- | --- | --- | --- | --- | --- | --- | --- | --- | --- | --- | --- | --- | --- | --- | --- | --- | --- |

XP\_026522979.1

back to list

  

| Protein Coverage
| Supporting Peptides
|

Protein Coverage:

Supporting Peptides:

| Peptide | Uniq | -10lgP | Mass | Length | ppm | m/z | z | RT | Fraction | Scan | Source File | Area F1 | Area F10 | Area F11 | Area F12 | Area F13 | Area F2 | Area F3 | Area F4 | Area F5 | Area F6 | Area F7 | Area F8 | Area F9 | #Feature | #Feature F1 | #Feature F10 | #Feature F11 | #Feature F12 | #Feature F13 | #Feature F2 | #Feature F3 | #Feature F4 | #Feature F5 | #Feature F6 | #Feature F7 | #Feature F8 | #Feature F9 | Start | End | PTM | AScore | Found By |
| --- | --- | --- | --- | --- | --- | --- | --- | --- | --- | --- | --- | --- | --- | --- | --- | --- | --- | --- | --- | --- | --- | --- | --- | --- | --- | --- | --- | --- | --- | --- | --- | --- | --- | --- | --- | --- | --- | --- | --- | --- | --- | --- | --- |
| K.TLFEITVPLSHGPK.P | Y | 67.52 | 1537.8503 | 14 | -0.1 | 513.6240 | 3 | 58.66 | 2 | F2:36076 | NaNaKA16\_F10.raw |  | 9.1753E6 |  |  |  |  |  |  |  |  |  | 1.5718E6 | 2.8923E6 | 4 | 0 | 2 | 0 | 0 | 0 | 0 | 0 | 0 | 0 | 0 | 0 | 1 | 1 | 185 | 198 |  |  | PEAKS DB |
| K.C(+57.02)IC(+57.02)EC(+57.02)VESPNK.C | Y | 61.53 | 1394.5629 | 11 | 0.3 | 698.2889 | 2 | 47.51 | 6 | F6:30331 | NaNaKA16\_F2.raw |  |  |  |  |  | 4.592E5 |  |  |  |  |  |  |  | 1 | 0 | 0 | 0 | 0 | 0 | 1 | 0 | 0 | 0 | 0 | 0 | 0 | 0 | 360 | 370 | Carbamidomethylation | C1:Carbamidomethylation:1000.00;C3:Carbamidomethylation:1000.00;C5:Carbamidomethylation:1000.00 | PEAKS DB |
| K.LLPSSC(+57.02)GLNKEFDEEK.C | Y | 60.34 | 1864.8876 | 16 | 4.8 | 622.6364 | 3 | 33.83 | 7 | F7:14388 | NaNaKA16\_F3.raw |  |  |  |  |  |  | 1.4164E5 |  |  |  |  |  |  | 1 | 0 | 0 | 0 | 0 | 0 | 0 | 1 | 0 | 0 | 0 | 0 | 0 | 0 | 325 | 340 | Carbamidomethylation | C6:Carbamidomethylation:1000.00 | PEAKS DB |
| K.LLPSSC(+57.02)GLNK.E | Y | 54.98 | 1087.5696 | 10 | -0.1 | 544.7920 | 2 | 19.39 | 6 | F6:6810 | NaNaKA16\_F2.raw |  |  |  |  |  | 2.1852E6 |  |  |  |  |  |  |  | 1 | 0 | 0 | 0 | 0 | 0 | 1 | 0 | 0 | 0 | 0 | 0 | 0 | 0 | 325 | 334 | Carbamidomethylation | C6:Carbamidomethylation:1000.00 | PEAKS DB |
| K.ELDEETC(+57.02)QC(+57.02)VC(+57.02)K.G | Y | 48.15 | 1569.6110 | 12 | 0.1 | 785.8129 | 2 | 12.37 | 6 | F6:2413 | NaNaKA16\_F2.raw |  |  |  |  |  | 8.8422E4 |  |  |  |  |  |  |  | 1 | 0 | 0 | 0 | 0 | 0 | 1 | 0 | 0 | 0 | 0 | 0 | 0 | 0 | 287 | 298 | Carbamidomethylation | C7:Carbamidomethylation:1000.00;C9:Carbamidomethylation:1000.00;C11:Carbamidomethylation:1000.00 | PEAKS DB |
| total 5 peptides |  |  |  |  |  |  |  |  |  |  |  |  |  |  |  |  |  |  |  |  |  |  |  |  |  |  |  |  |  |  |  |  |  |  |  |  |  |  |  |  |  |  |  |
| --- | --- | --- | --- | --- | --- | --- | --- | --- | --- | --- | --- | --- | --- | --- | --- | --- | --- | --- | --- | --- | --- | --- | --- | --- | --- | --- | --- | --- | --- | --- | --- | --- | --- | --- | --- | --- | --- | --- | --- | --- | --- | --- | --- |

ETE60014.1

back to list

  

| Protein Coverage
| Supporting Peptides
|

Protein Coverage:

Supporting Peptides:

| Peptide | Uniq | -10lgP | Mass | Length | ppm | m/z | z | RT | Fraction | Scan | Source File | Area F1 | Area F10 | Area F11 | Area F12 | Area F13 | Area F2 | Area F3 | Area F4 | Area F5 | Area F6 | Area F7 | Area F8 | Area F9 | #Feature | #Feature F1 | #Feature F10 | #Feature F11 | #Feature F12 | #Feature F13 | #Feature F2 | #Feature F3 | #Feature F4 | #Feature F5 | #Feature F6 | #Feature F7 | #Feature F8 | #Feature F9 | Start | End | PTM | AScore | Found By |
| --- | --- | --- | --- | --- | --- | --- | --- | --- | --- | --- | --- | --- | --- | --- | --- | --- | --- | --- | --- | --- | --- | --- | --- | --- | --- | --- | --- | --- | --- | --- | --- | --- | --- | --- | --- | --- | --- | --- | --- | --- | --- | --- | --- |
| K.TLFEITVPLSHGPK.P | Y | 67.52 | 1537.8503 | 14 | -0.1 | 513.6240 | 3 | 58.66 | 2 | F2:36076 | NaNaKA16\_F10.raw |  | 9.1753E6 |  |  |  |  |  |  |  |  |  | 1.5718E6 | 2.8923E6 | 4 | 0 | 2 | 0 | 0 | 0 | 0 | 0 | 0 | 0 | 0 | 0 | 1 | 1 | 185 | 198 |  |  | PEAKS DB |
| K.C(+57.02)IC(+57.02)EC(+57.02)VESPNK.C | Y | 61.53 | 1394.5629 | 11 | 0.3 | 698.2889 | 2 | 47.51 | 6 | F6:30331 | NaNaKA16\_F2.raw |  |  |  |  |  | 4.592E5 |  |  |  |  |  |  |  | 1 | 0 | 0 | 0 | 0 | 0 | 1 | 0 | 0 | 0 | 0 | 0 | 0 | 0 | 360 | 370 | Carbamidomethylation | C1:Carbamidomethylation:1000.00;C3:Carbamidomethylation:1000.00;C5:Carbamidomethylation:1000.00 | PEAKS DB |
| K.LLPSSC(+57.02)GLNKEFDEEK.C | Y | 60.34 | 1864.8876 | 16 | 4.8 | 622.6364 | 3 | 33.83 | 7 | F7:14388 | NaNaKA16\_F3.raw |  |  |  |  |  |  | 1.4164E5 |  |  |  |  |  |  | 1 | 0 | 0 | 0 | 0 | 0 | 0 | 1 | 0 | 0 | 0 | 0 | 0 | 0 | 325 | 340 | Carbamidomethylation | C6:Carbamidomethylation:1000.00 | PEAKS DB |
| K.LLPSSC(+57.02)GLNK.E | Y | 54.98 | 1087.5696 | 10 | -0.1 | 544.7920 | 2 | 19.39 | 6 | F6:6810 | NaNaKA16\_F2.raw |  |  |  |  |  | 2.1852E6 |  |  |  |  |  |  |  | 1 | 0 | 0 | 0 | 0 | 0 | 1 | 0 | 0 | 0 | 0 | 0 | 0 | 0 | 325 | 334 | Carbamidomethylation | C6:Carbamidomethylation:1000.00 | PEAKS DB |
| K.ELDEETC(+57.02)QC(+57.02)VC(+57.02)K.G | Y | 48.15 | 1569.6110 | 12 | 0.1 | 785.8129 | 2 | 12.37 | 6 | F6:2413 | NaNaKA16\_F2.raw |  |  |  |  |  | 8.8422E4 |  |  |  |  |  |  |  | 1 | 0 | 0 | 0 | 0 | 0 | 1 | 0 | 0 | 0 | 0 | 0 | 0 | 0 | 287 | 298 | Carbamidomethylation | C7:Carbamidomethylation:1000.00;C9:Carbamidomethylation:1000.00;C11:Carbamidomethylation:1000.00 | PEAKS DB |
| total 5 peptides |  |  |  |  |  |  |  |  |  |  |  |  |  |  |  |  |  |  |  |  |  |  |  |  |  |  |  |  |  |  |  |  |  |  |  |  |  |  |  |  |  |  |  |
| --- | --- | --- | --- | --- | --- | --- | --- | --- | --- | --- | --- | --- | --- | --- | --- | --- | --- | --- | --- | --- | --- | --- | --- | --- | --- | --- | --- | --- | --- | --- | --- | --- | --- | --- | --- | --- | --- | --- | --- | --- | --- | --- | --- |

XP\_026555459.1

back to list

  

| Protein Coverage
| Supporting Peptides
|

Protein Coverage:

Supporting Peptides:

| Peptide | Uniq | -10lgP | Mass | Length | ppm | m/z | z | RT | Fraction | Scan | Source File | Area F1 | Area F10 | Area F11 | Area F12 | Area F13 | Area F2 | Area F3 | Area F4 | Area F5 | Area F6 | Area F7 | Area F8 | Area F9 | #Feature | #Feature F1 | #Feature F10 | #Feature F11 | #Feature F12 | #Feature F13 | #Feature F2 | #Feature F3 | #Feature F4 | #Feature F5 | #Feature F6 | #Feature F7 | #Feature F8 | #Feature F9 | Start | End | PTM | AScore | Found By |
| --- | --- | --- | --- | --- | --- | --- | --- | --- | --- | --- | --- | --- | --- | --- | --- | --- | --- | --- | --- | --- | --- | --- | --- | --- | --- | --- | --- | --- | --- | --- | --- | --- | --- | --- | --- | --- | --- | --- | --- | --- | --- | --- | --- |
| K.TLFEITVPLSHGPK.P | Y | 67.52 | 1537.8503 | 14 | -0.1 | 513.6240 | 3 | 58.66 | 2 | F2:36076 | NaNaKA16\_F10.raw |  | 9.1753E6 |  |  |  |  |  |  |  |  |  | 1.5718E6 | 2.8923E6 | 4 | 0 | 2 | 0 | 0 | 0 | 0 | 0 | 0 | 0 | 0 | 0 | 1 | 1 | 185 | 198 |  |  | PEAKS DB |
| K.C(+57.02)IC(+57.02)EC(+57.02)VESPNK.C | Y | 61.53 | 1394.5629 | 11 | 0.3 | 698.2889 | 2 | 47.51 | 6 | F6:30331 | NaNaKA16\_F2.raw |  |  |  |  |  | 4.592E5 |  |  |  |  |  |  |  | 1 | 0 | 0 | 0 | 0 | 0 | 1 | 0 | 0 | 0 | 0 | 0 | 0 | 0 | 360 | 370 | Carbamidomethylation | C1:Carbamidomethylation:1000.00;C3:Carbamidomethylation:1000.00;C5:Carbamidomethylation:1000.00 | PEAKS DB |
| K.LLPSSC(+57.02)GLNKEFDEEK.C | Y | 60.34 | 1864.8876 | 16 | 4.8 | 622.6364 | 3 | 33.83 | 7 | F7:14388 | NaNaKA16\_F3.raw |  |  |  |  |  |  | 1.4164E5 |  |  |  |  |  |  | 1 | 0 | 0 | 0 | 0 | 0 | 0 | 1 | 0 | 0 | 0 | 0 | 0 | 0 | 325 | 340 | Carbamidomethylation | C6:Carbamidomethylation:1000.00 | PEAKS DB |
| K.LLPSSC(+57.02)GLNK.E | Y | 54.98 | 1087.5696 | 10 | -0.1 | 544.7920 | 2 | 19.39 | 6 | F6:6810 | NaNaKA16\_F2.raw |  |  |  |  |  | 2.1852E6 |  |  |  |  |  |  |  | 1 | 0 | 0 | 0 | 0 | 0 | 1 | 0 | 0 | 0 | 0 | 0 | 0 | 0 | 325 | 334 | Carbamidomethylation | C6:Carbamidomethylation:1000.00 | PEAKS DB |
| K.ELDEETC(+57.02)QC(+57.02)VC(+57.02)K.G | Y | 48.15 | 1569.6110 | 12 | 0.1 | 785.8129 | 2 | 12.37 | 6 | F6:2413 | NaNaKA16\_F2.raw |  |  |  |  |  | 8.8422E4 |  |  |  |  |  |  |  | 1 | 0 | 0 | 0 | 0 | 0 | 1 | 0 | 0 | 0 | 0 | 0 | 0 | 0 | 287 | 298 | Carbamidomethylation | C7:Carbamidomethylation:1000.00;C9:Carbamidomethylation:1000.00;C11:Carbamidomethylation:1000.00 | PEAKS DB |
| total 5 peptides |  |  |  |  |  |  |  |  |  |  |  |  |  |  |  |  |  |  |  |  |  |  |  |  |  |  |  |  |  |  |  |  |  |  |  |  |  |  |  |  |  |  |  |
| --- | --- | --- | --- | --- | --- | --- | --- | --- | --- | --- | --- | --- | --- | --- | --- | --- | --- | --- | --- | --- | --- | --- | --- | --- | --- | --- | --- | --- | --- | --- | --- | --- | --- | --- | --- | --- | --- | --- | --- | --- | --- | --- | --- |

LAA17506.1

back to list

  

| Protein Coverage
| Supporting Peptides
|

Protein Coverage:

Supporting Peptides:

| Peptide | Uniq | -10lgP | Mass | Length | ppm | m/z | z | RT | Fraction | Scan | Source File | Area F1 | Area F10 | Area F11 | Area F12 | Area F13 | Area F2 | Area F3 | Area F4 | Area F5 | Area F6 | Area F7 | Area F8 | Area F9 | #Feature | #Feature F1 | #Feature F10 | #Feature F11 | #Feature F12 | #Feature F13 | #Feature F2 | #Feature F3 | #Feature F4 | #Feature F5 | #Feature F6 | #Feature F7 | #Feature F8 | #Feature F9 | Start | End | PTM | AScore | Found By |
| --- | --- | --- | --- | --- | --- | --- | --- | --- | --- | --- | --- | --- | --- | --- | --- | --- | --- | --- | --- | --- | --- | --- | --- | --- | --- | --- | --- | --- | --- | --- | --- | --- | --- | --- | --- | --- | --- | --- | --- | --- | --- | --- | --- |
| K.TLFEITVPLSHGPK.P | Y | 67.52 | 1537.8503 | 14 | -0.1 | 513.6240 | 3 | 58.66 | 2 | F2:36076 | NaNaKA16\_F10.raw |  | 9.1753E6 |  |  |  |  |  |  |  |  |  | 1.5718E6 | 2.8923E6 | 4 | 0 | 2 | 0 | 0 | 0 | 0 | 0 | 0 | 0 | 0 | 0 | 1 | 1 | 238 | 251 |  |  | PEAKS DB |
| K.C(+57.02)IC(+57.02)EC(+57.02)VESPNK.C | Y | 61.53 | 1394.5629 | 11 | 0.3 | 698.2889 | 2 | 47.51 | 6 | F6:30331 | NaNaKA16\_F2.raw |  |  |  |  |  | 4.592E5 |  |  |  |  |  |  |  | 1 | 0 | 0 | 0 | 0 | 0 | 1 | 0 | 0 | 0 | 0 | 0 | 0 | 0 | 413 | 423 | Carbamidomethylation | C1:Carbamidomethylation:1000.00;C3:Carbamidomethylation:1000.00;C5:Carbamidomethylation:1000.00 | PEAKS DB |
| K.LLPSSC(+57.02)GLNKEFDEEK.C | Y | 60.34 | 1864.8876 | 16 | 4.8 | 622.6364 | 3 | 33.83 | 7 | F7:14388 | NaNaKA16\_F3.raw |  |  |  |  |  |  | 1.4164E5 |  |  |  |  |  |  | 1 | 0 | 0 | 0 | 0 | 0 | 0 | 1 | 0 | 0 | 0 | 0 | 0 | 0 | 378 | 393 | Carbamidomethylation | C6:Carbamidomethylation:1000.00 | PEAKS DB |
| K.LLPSSC(+57.02)GLNK.E | Y | 54.98 | 1087.5696 | 10 | -0.1 | 544.7920 | 2 | 19.39 | 6 | F6:6810 | NaNaKA16\_F2.raw |  |  |  |  |  | 2.1852E6 |  |  |  |  |  |  |  | 1 | 0 | 0 | 0 | 0 | 0 | 1 | 0 | 0 | 0 | 0 | 0 | 0 | 0 | 378 | 387 | Carbamidomethylation | C6:Carbamidomethylation:1000.00 | PEAKS DB |
| K.ELDEETC(+57.02)QC(+57.02)VC(+57.02)K.G | Y | 48.15 | 1569.6110 | 12 | 0.1 | 785.8129 | 2 | 12.37 | 6 | F6:2413 | NaNaKA16\_F2.raw |  |  |  |  |  | 8.8422E4 |  |  |  |  |  |  |  | 1 | 0 | 0 | 0 | 0 | 0 | 1 | 0 | 0 | 0 | 0 | 0 | 0 | 0 | 340 | 351 | Carbamidomethylation | C7:Carbamidomethylation:1000.00;C9:Carbamidomethylation:1000.00;C11:Carbamidomethylation:1000.00 | PEAKS DB |
| total 5 peptides |  |  |  |  |  |  |  |  |  |  |  |  |  |  |  |  |  |  |  |  |  |  |  |  |  |  |  |  |  |  |  |  |  |  |  |  |  |  |  |  |  |  |  |
| --- | --- | --- | --- | --- | --- | --- | --- | --- | --- | --- | --- | --- | --- | --- | --- | --- | --- | --- | --- | --- | --- | --- | --- | --- | --- | --- | --- | --- | --- | --- | --- | --- | --- | --- | --- | --- | --- | --- | --- | --- | --- | --- | --- |

LAB17921.1

back to list

  

| Protein Coverage
| Supporting Peptides
|

Protein Coverage:

Supporting Peptides:

| Peptide | Uniq | -10lgP | Mass | Length | ppm | m/z | z | RT | Fraction | Scan | Source File | Area F1 | Area F10 | Area F11 | Area F12 | Area F13 | Area F2 | Area F3 | Area F4 | Area F5 | Area F6 | Area F7 | Area F8 | Area F9 | #Feature | #Feature F1 | #Feature F10 | #Feature F11 | #Feature F12 | #Feature F13 | #Feature F2 | #Feature F3 | #Feature F4 | #Feature F5 | #Feature F6 | #Feature F7 | #Feature F8 | #Feature F9 | Start | End | PTM | AScore | Found By |
| --- | --- | --- | --- | --- | --- | --- | --- | --- | --- | --- | --- | --- | --- | --- | --- | --- | --- | --- | --- | --- | --- | --- | --- | --- | --- | --- | --- | --- | --- | --- | --- | --- | --- | --- | --- | --- | --- | --- | --- | --- | --- | --- | --- |
| K.TLFEITVPLSHGPK.P | Y | 67.52 | 1537.8503 | 14 | -0.1 | 513.6240 | 3 | 58.66 | 2 | F2:36076 | NaNaKA16\_F10.raw |  | 9.1753E6 |  |  |  |  |  |  |  |  |  | 1.5718E6 | 2.8923E6 | 4 | 0 | 2 | 0 | 0 | 0 | 0 | 0 | 0 | 0 | 0 | 0 | 1 | 1 | 242 | 255 |  |  | PEAKS DB |
| K.C(+57.02)IC(+57.02)EC(+57.02)VESPNK.C | Y | 61.53 | 1394.5629 | 11 | 0.3 | 698.2889 | 2 | 47.51 | 6 | F6:30331 | NaNaKA16\_F2.raw |  |  |  |  |  | 4.592E5 |  |  |  |  |  |  |  | 1 | 0 | 0 | 0 | 0 | 0 | 1 | 0 | 0 | 0 | 0 | 0 | 0 | 0 | 417 | 427 | Carbamidomethylation | C1:Carbamidomethylation:1000.00;C3:Carbamidomethylation:1000.00;C5:Carbamidomethylation:1000.00 | PEAKS DB |
| K.LLPSSC(+57.02)GLNKEFDEEK.C | Y | 60.34 | 1864.8876 | 16 | 4.8 | 622.6364 | 3 | 33.83 | 7 | F7:14388 | NaNaKA16\_F3.raw |  |  |  |  |  |  | 1.4164E5 |  |  |  |  |  |  | 1 | 0 | 0 | 0 | 0 | 0 | 0 | 1 | 0 | 0 | 0 | 0 | 0 | 0 | 382 | 397 | Carbamidomethylation | C6:Carbamidomethylation:1000.00 | PEAKS DB |
| K.LLPSSC(+57.02)GLNK.E | Y | 54.98 | 1087.5696 | 10 | -0.1 | 544.7920 | 2 | 19.39 | 6 | F6:6810 | NaNaKA16\_F2.raw |  |  |  |  |  | 2.1852E6 |  |  |  |  |  |  |  | 1 | 0 | 0 | 0 | 0 | 0 | 1 | 0 | 0 | 0 | 0 | 0 | 0 | 0 | 382 | 391 | Carbamidomethylation | C6:Carbamidomethylation:1000.00 | PEAKS DB |
| K.ELDEETC(+57.02)QC(+57.02)VC(+57.02)K.G | Y | 48.15 | 1569.6110 | 12 | 0.1 | 785.8129 | 2 | 12.37 | 6 | F6:2413 | NaNaKA16\_F2.raw |  |  |  |  |  | 8.8422E4 |  |  |  |  |  |  |  | 1 | 0 | 0 | 0 | 0 | 0 | 1 | 0 | 0 | 0 | 0 | 0 | 0 | 0 | 344 | 355 | Carbamidomethylation | C7:Carbamidomethylation:1000.00;C9:Carbamidomethylation:1000.00;C11:Carbamidomethylation:1000.00 | PEAKS DB |
| total 5 peptides |  |  |  |  |  |  |  |  |  |  |  |  |  |  |  |  |  |  |  |  |  |  |  |  |  |  |  |  |  |  |  |  |  |  |  |  |  |  |  |  |  |  |  |
| --- | --- | --- | --- | --- | --- | --- | --- | --- | --- | --- | --- | --- | --- | --- | --- | --- | --- | --- | --- | --- | --- | --- | --- | --- | --- | --- | --- | --- | --- | --- | --- | --- | --- | --- | --- | --- | --- | --- | --- | --- | --- | --- | --- |

XP\_034298590.1

back to list

  

| Protein Coverage
| Supporting Peptides
|

Protein Coverage:

Supporting Peptides:

| Peptide | Uniq | -10lgP | Mass | Length | ppm | m/z | z | RT | Fraction | Scan | Source File | Area F1 | Area F10 | Area F11 | Area F12 | Area F13 | Area F2 | Area F3 | Area F4 | Area F5 | Area F6 | Area F7 | Area F8 | Area F9 | #Feature | #Feature F1 | #Feature F10 | #Feature F11 | #Feature F12 | #Feature F13 | #Feature F2 | #Feature F3 | #Feature F4 | #Feature F5 | #Feature F6 | #Feature F7 | #Feature F8 | #Feature F9 | Start | End | PTM | AScore | Found By |
| --- | --- | --- | --- | --- | --- | --- | --- | --- | --- | --- | --- | --- | --- | --- | --- | --- | --- | --- | --- | --- | --- | --- | --- | --- | --- | --- | --- | --- | --- | --- | --- | --- | --- | --- | --- | --- | --- | --- | --- | --- | --- | --- | --- |
| K.TLFEITVPLSHGPK.P | Y | 67.52 | 1537.8503 | 14 | -0.1 | 513.6240 | 3 | 58.66 | 2 | F2:36076 | NaNaKA16\_F10.raw |  | 9.1753E6 |  |  |  |  |  |  |  |  |  | 1.5718E6 | 2.8923E6 | 4 | 0 | 2 | 0 | 0 | 0 | 0 | 0 | 0 | 0 | 0 | 0 | 1 | 1 | 116 | 129 |  |  | PEAKS DB |
| K.C(+57.02)IC(+57.02)EC(+57.02)VESPNK.C | Y | 61.53 | 1394.5629 | 11 | 0.3 | 698.2889 | 2 | 47.51 | 6 | F6:30331 | NaNaKA16\_F2.raw |  |  |  |  |  | 4.592E5 |  |  |  |  |  |  |  | 1 | 0 | 0 | 0 | 0 | 0 | 1 | 0 | 0 | 0 | 0 | 0 | 0 | 0 | 291 | 301 | Carbamidomethylation | C1:Carbamidomethylation:1000.00;C3:Carbamidomethylation:1000.00;C5:Carbamidomethylation:1000.00 | PEAKS DB |
| K.LLPSSC(+57.02)GLNKEFDEEK.C | Y | 60.34 | 1864.8876 | 16 | 4.8 | 622.6364 | 3 | 33.83 | 7 | F7:14388 | NaNaKA16\_F3.raw |  |  |  |  |  |  | 1.4164E5 |  |  |  |  |  |  | 1 | 0 | 0 | 0 | 0 | 0 | 0 | 1 | 0 | 0 | 0 | 0 | 0 | 0 | 256 | 271 | Carbamidomethylation | C6:Carbamidomethylation:1000.00 | PEAKS DB |
| K.LLPSSC(+57.02)GLNK.E | Y | 54.98 | 1087.5696 | 10 | -0.1 | 544.7920 | 2 | 19.39 | 6 | F6:6810 | NaNaKA16\_F2.raw |  |  |  |  |  | 2.1852E6 |  |  |  |  |  |  |  | 1 | 0 | 0 | 0 | 0 | 0 | 1 | 0 | 0 | 0 | 0 | 0 | 0 | 0 | 256 | 265 | Carbamidomethylation | C6:Carbamidomethylation:1000.00 | PEAKS DB |
| K.ELDEETC(+57.02)QC(+57.02)VC(+57.02)K.G | Y | 48.15 | 1569.6110 | 12 | 0.1 | 785.8129 | 2 | 12.37 | 6 | F6:2413 | NaNaKA16\_F2.raw |  |  |  |  |  | 8.8422E4 |  |  |  |  |  |  |  | 1 | 0 | 0 | 0 | 0 | 0 | 1 | 0 | 0 | 0 | 0 | 0 | 0 | 0 | 218 | 229 | Carbamidomethylation | C7:Carbamidomethylation:1000.00;C9:Carbamidomethylation:1000.00;C11:Carbamidomethylation:1000.00 | PEAKS DB |
| total 5 peptides |  |  |  |  |  |  |  |  |  |  |  |  |  |  |  |  |  |  |  |  |  |  |  |  |  |  |  |  |  |  |  |  |  |  |  |  |  |  |  |  |  |  |  |
| --- | --- | --- | --- | --- | --- | --- | --- | --- | --- | --- | --- | --- | --- | --- | --- | --- | --- | --- | --- | --- | --- | --- | --- | --- | --- | --- | --- | --- | --- | --- | --- | --- | --- | --- | --- | --- | --- | --- | --- | --- | --- | --- | --- |

XP\_034298589.1

back to list

  

| Protein Coverage
| Supporting Peptides
|

Protein Coverage:

Supporting Peptides:

| Peptide | Uniq | -10lgP | Mass | Length | ppm | m/z | z | RT | Fraction | Scan | Source File | Area F1 | Area F10 | Area F11 | Area F12 | Area F13 | Area F2 | Area F3 | Area F4 | Area F5 | Area F6 | Area F7 | Area F8 | Area F9 | #Feature | #Feature F1 | #Feature F10 | #Feature F11 | #Feature F12 | #Feature F13 | #Feature F2 | #Feature F3 | #Feature F4 | #Feature F5 | #Feature F6 | #Feature F7 | #Feature F8 | #Feature F9 | Start | End | PTM | AScore | Found By |
| --- | --- | --- | --- | --- | --- | --- | --- | --- | --- | --- | --- | --- | --- | --- | --- | --- | --- | --- | --- | --- | --- | --- | --- | --- | --- | --- | --- | --- | --- | --- | --- | --- | --- | --- | --- | --- | --- | --- | --- | --- | --- | --- | --- |
| K.TLFEITVPLSHGPK.P | Y | 67.52 | 1537.8503 | 14 | -0.1 | 513.6240 | 3 | 58.66 | 2 | F2:36076 | NaNaKA16\_F10.raw |  | 9.1753E6 |  |  |  |  |  |  |  |  |  | 1.5718E6 | 2.8923E6 | 4 | 0 | 2 | 0 | 0 | 0 | 0 | 0 | 0 | 0 | 0 | 0 | 1 | 1 | 141 | 154 |  |  | PEAKS DB |
| K.C(+57.02)IC(+57.02)EC(+57.02)VESPNK.C | Y | 61.53 | 1394.5629 | 11 | 0.3 | 698.2889 | 2 | 47.51 | 6 | F6:30331 | NaNaKA16\_F2.raw |  |  |  |  |  | 4.592E5 |  |  |  |  |  |  |  | 1 | 0 | 0 | 0 | 0 | 0 | 1 | 0 | 0 | 0 | 0 | 0 | 0 | 0 | 316 | 326 | Carbamidomethylation | C1:Carbamidomethylation:1000.00;C3:Carbamidomethylation:1000.00;C5:Carbamidomethylation:1000.00 | PEAKS DB |
| K.LLPSSC(+57.02)GLNKEFDEEK.C | Y | 60.34 | 1864.8876 | 16 | 4.8 | 622.6364 | 3 | 33.83 | 7 | F7:14388 | NaNaKA16\_F3.raw |  |  |  |  |  |  | 1.4164E5 |  |  |  |  |  |  | 1 | 0 | 0 | 0 | 0 | 0 | 0 | 1 | 0 | 0 | 0 | 0 | 0 | 0 | 281 | 296 | Carbamidomethylation | C6:Carbamidomethylation:1000.00 | PEAKS DB |
| K.LLPSSC(+57.02)GLNK.E | Y | 54.98 | 1087.5696 | 10 | -0.1 | 544.7920 | 2 | 19.39 | 6 | F6:6810 | NaNaKA16\_F2.raw |  |  |  |  |  | 2.1852E6 |  |  |  |  |  |  |  | 1 | 0 | 0 | 0 | 0 | 0 | 1 | 0 | 0 | 0 | 0 | 0 | 0 | 0 | 281 | 290 | Carbamidomethylation | C6:Carbamidomethylation:1000.00 | PEAKS DB |
| K.ELDEETC(+57.02)QC(+57.02)VC(+57.02)K.G | Y | 48.15 | 1569.6110 | 12 | 0.1 | 785.8129 | 2 | 12.37 | 6 | F6:2413 | NaNaKA16\_F2.raw |  |  |  |  |  | 8.8422E4 |  |  |  |  |  |  |  | 1 | 0 | 0 | 0 | 0 | 0 | 1 | 0 | 0 | 0 | 0 | 0 | 0 | 0 | 243 | 254 | Carbamidomethylation | C7:Carbamidomethylation:1000.00;C9:Carbamidomethylation:1000.00;C11:Carbamidomethylation:1000.00 | PEAKS DB |
| total 5 peptides |  |  |  |  |  |  |  |  |  |  |  |  |  |  |  |  |  |  |  |  |  |  |  |  |  |  |  |  |  |  |  |  |  |  |  |  |  |  |  |  |  |  |  |
| --- | --- | --- | --- | --- | --- | --- | --- | --- | --- | --- | --- | --- | --- | --- | --- | --- | --- | --- | --- | --- | --- | --- | --- | --- | --- | --- | --- | --- | --- | --- | --- | --- | --- | --- | --- | --- | --- | --- | --- | --- | --- | --- | --- |

JAC96561.1

back to list

  

| Protein Coverage
| Supporting Peptides
|

Protein Coverage:

Supporting Peptides:

| Peptide | Uniq | -10lgP | Mass | Length | ppm | m/z | z | RT | Fraction | Scan | Source File | Area F1 | Area F10 | Area F11 | Area F12 | Area F13 | Area F2 | Area F3 | Area F4 | Area F5 | Area F6 | Area F7 | Area F8 | Area F9 | #Feature | #Feature F1 | #Feature F10 | #Feature F11 | #Feature F12 | #Feature F13 | #Feature F2 | #Feature F3 | #Feature F4 | #Feature F5 | #Feature F6 | #Feature F7 | #Feature F8 | #Feature F9 | Start | End | PTM | AScore | Found By |
| --- | --- | --- | --- | --- | --- | --- | --- | --- | --- | --- | --- | --- | --- | --- | --- | --- | --- | --- | --- | --- | --- | --- | --- | --- | --- | --- | --- | --- | --- | --- | --- | --- | --- | --- | --- | --- | --- | --- | --- | --- | --- | --- | --- |
| K.TLFEITVPLSHGPK.P | Y | 67.52 | 1537.8503 | 14 | -0.1 | 513.6240 | 3 | 58.66 | 2 | F2:36076 | NaNaKA16\_F10.raw |  | 9.1753E6 |  |  |  |  |  |  |  |  |  | 1.5718E6 | 2.8923E6 | 4 | 0 | 2 | 0 | 0 | 0 | 0 | 0 | 0 | 0 | 0 | 0 | 1 | 1 | 185 | 198 |  |  | PEAKS DB |
| K.C(+57.02)IC(+57.02)EC(+57.02)VESPNK.C | Y | 61.53 | 1394.5629 | 11 | 0.3 | 698.2889 | 2 | 47.51 | 6 | F6:30331 | NaNaKA16\_F2.raw |  |  |  |  |  | 4.592E5 |  |  |  |  |  |  |  | 1 | 0 | 0 | 0 | 0 | 0 | 1 | 0 | 0 | 0 | 0 | 0 | 0 | 0 | 360 | 370 | Carbamidomethylation | C1:Carbamidomethylation:1000.00;C3:Carbamidomethylation:1000.00;C5:Carbamidomethylation:1000.00 | PEAKS DB |
| K.LLPSSC(+57.02)GLNKEFDEEK.C | Y | 60.34 | 1864.8876 | 16 | 4.8 | 622.6364 | 3 | 33.83 | 7 | F7:14388 | NaNaKA16\_F3.raw |  |  |  |  |  |  | 1.4164E5 |  |  |  |  |  |  | 1 | 0 | 0 | 0 | 0 | 0 | 0 | 1 | 0 | 0 | 0 | 0 | 0 | 0 | 325 | 340 | Carbamidomethylation | C6:Carbamidomethylation:1000.00 | PEAKS DB |
| K.LLPSSC(+57.02)GLNK.E | Y | 54.98 | 1087.5696 | 10 | -0.1 | 544.7920 | 2 | 19.39 | 6 | F6:6810 | NaNaKA16\_F2.raw |  |  |  |  |  | 2.1852E6 |  |  |  |  |  |  |  | 1 | 0 | 0 | 0 | 0 | 0 | 1 | 0 | 0 | 0 | 0 | 0 | 0 | 0 | 325 | 334 | Carbamidomethylation | C6:Carbamidomethylation:1000.00 | PEAKS DB |
| K.ELDEETC(+57.02)QC(+57.02)VC(+57.02)K.G | Y | 48.15 | 1569.6110 | 12 | 0.1 | 785.8129 | 2 | 12.37 | 6 | F6:2413 | NaNaKA16\_F2.raw |  |  |  |  |  | 8.8422E4 |  |  |  |  |  |  |  | 1 | 0 | 0 | 0 | 0 | 0 | 1 | 0 | 0 | 0 | 0 | 0 | 0 | 0 | 287 | 298 | Carbamidomethylation | C7:Carbamidomethylation:1000.00;C9:Carbamidomethylation:1000.00;C11:Carbamidomethylation:1000.00 | PEAKS DB |
| total 5 peptides |  |  |  |  |  |  |  |  |  |  |  |  |  |  |  |  |  |  |  |  |  |  |  |  |  |  |  |  |  |  |  |  |  |  |  |  |  |  |  |  |  |  |  |
| --- | --- | --- | --- | --- | --- | --- | --- | --- | --- | --- | --- | --- | --- | --- | --- | --- | --- | --- | --- | --- | --- | --- | --- | --- | --- | --- | --- | --- | --- | --- | --- | --- | --- | --- | --- | --- | --- | --- | --- | --- | --- | --- | --- |

XP\_029139565.1

back to list

  

| Protein Coverage
| Supporting Peptides
|

Protein Coverage:

Supporting Peptides:

| Peptide | Uniq | -10lgP | Mass | Length | ppm | m/z | z | RT | Fraction | Scan | Source File | Area F1 | Area F10 | Area F11 | Area F12 | Area F13 | Area F2 | Area F3 | Area F4 | Area F5 | Area F6 | Area F7 | Area F8 | Area F9 | #Feature | #Feature F1 | #Feature F10 | #Feature F11 | #Feature F12 | #Feature F13 | #Feature F2 | #Feature F3 | #Feature F4 | #Feature F5 | #Feature F6 | #Feature F7 | #Feature F8 | #Feature F9 | Start | End | PTM | AScore | Found By |
| --- | --- | --- | --- | --- | --- | --- | --- | --- | --- | --- | --- | --- | --- | --- | --- | --- | --- | --- | --- | --- | --- | --- | --- | --- | --- | --- | --- | --- | --- | --- | --- | --- | --- | --- | --- | --- | --- | --- | --- | --- | --- | --- | --- |
| K.TLFEITVPLSHGPK.P | Y | 67.52 | 1537.8503 | 14 | -0.1 | 513.6240 | 3 | 58.66 | 2 | F2:36076 | NaNaKA16\_F10.raw |  | 9.1753E6 |  |  |  |  |  |  |  |  |  | 1.5718E6 | 2.8923E6 | 4 | 0 | 2 | 0 | 0 | 0 | 0 | 0 | 0 | 0 | 0 | 0 | 1 | 1 | 186 | 199 |  |  | PEAKS DB |
| K.C(+57.02)IC(+57.02)EC(+57.02)VESPNK.C | Y | 61.53 | 1394.5629 | 11 | 0.3 | 698.2889 | 2 | 47.51 | 6 | F6:30331 | NaNaKA16\_F2.raw |  |  |  |  |  | 4.592E5 |  |  |  |  |  |  |  | 1 | 0 | 0 | 0 | 0 | 0 | 1 | 0 | 0 | 0 | 0 | 0 | 0 | 0 | 361 | 371 | Carbamidomethylation | C1:Carbamidomethylation:1000.00;C3:Carbamidomethylation:1000.00;C5:Carbamidomethylation:1000.00 | PEAKS DB |
| K.LLPSSC(+57.02)GLNKEFDEEK.C | Y | 60.34 | 1864.8876 | 16 | 4.8 | 622.6364 | 3 | 33.83 | 7 | F7:14388 | NaNaKA16\_F3.raw |  |  |  |  |  |  | 1.4164E5 |  |  |  |  |  |  | 1 | 0 | 0 | 0 | 0 | 0 | 0 | 1 | 0 | 0 | 0 | 0 | 0 | 0 | 326 | 341 | Carbamidomethylation | C6:Carbamidomethylation:1000.00 | PEAKS DB |
| K.LLPSSC(+57.02)GLNK.E | Y | 54.98 | 1087.5696 | 10 | -0.1 | 544.7920 | 2 | 19.39 | 6 | F6:6810 | NaNaKA16\_F2.raw |  |  |  |  |  | 2.1852E6 |  |  |  |  |  |  |  | 1 | 0 | 0 | 0 | 0 | 0 | 1 | 0 | 0 | 0 | 0 | 0 | 0 | 0 | 326 | 335 | Carbamidomethylation | C6:Carbamidomethylation:1000.00 | PEAKS DB |
| K.ELDEETC(+57.02)QC(+57.02)VC(+57.02)K.G | Y | 48.15 | 1569.6110 | 12 | 0.1 | 785.8129 | 2 | 12.37 | 6 | F6:2413 | NaNaKA16\_F2.raw |  |  |  |  |  | 8.8422E4 |  |  |  |  |  |  |  | 1 | 0 | 0 | 0 | 0 | 0 | 1 | 0 | 0 | 0 | 0 | 0 | 0 | 0 | 288 | 299 | Carbamidomethylation | C7:Carbamidomethylation:1000.00;C9:Carbamidomethylation:1000.00;C11:Carbamidomethylation:1000.00 | PEAKS DB |
| total 5 peptides |  |  |  |  |  |  |  |  |  |  |  |  |  |  |  |  |  |  |  |  |  |  |  |  |  |  |  |  |  |  |  |  |  |  |  |  |  |  |  |  |  |  |  |
| --- | --- | --- | --- | --- | --- | --- | --- | --- | --- | --- | --- | --- | --- | --- | --- | --- | --- | --- | --- | --- | --- | --- | --- | --- | --- | --- | --- | --- | --- | --- | --- | --- | --- | --- | --- | --- | --- | --- | --- | --- | --- | --- | --- |

JAI10614.1

back to list

  

| Protein Coverage
| Supporting Peptides
|

Protein Coverage:

Supporting Peptides:

| Peptide | Uniq | -10lgP | Mass | Length | ppm | m/z | z | RT | Fraction | Scan | Source File | Area F1 | Area F10 | Area F11 | Area F12 | Area F13 | Area F2 | Area F3 | Area F4 | Area F5 | Area F6 | Area F7 | Area F8 | Area F9 | #Feature | #Feature F1 | #Feature F10 | #Feature F11 | #Feature F12 | #Feature F13 | #Feature F2 | #Feature F3 | #Feature F4 | #Feature F5 | #Feature F6 | #Feature F7 | #Feature F8 | #Feature F9 | Start | End | PTM | AScore | Found By |
| --- | --- | --- | --- | --- | --- | --- | --- | --- | --- | --- | --- | --- | --- | --- | --- | --- | --- | --- | --- | --- | --- | --- | --- | --- | --- | --- | --- | --- | --- | --- | --- | --- | --- | --- | --- | --- | --- | --- | --- | --- | --- | --- | --- |
| K.TLFEITVPLSHGPK.P | Y | 67.52 | 1537.8503 | 14 | -0.1 | 513.6240 | 3 | 58.66 | 2 | F2:36076 | NaNaKA16\_F10.raw |  | 9.1753E6 |  |  |  |  |  |  |  |  |  | 1.5718E6 | 2.8923E6 | 4 | 0 | 2 | 0 | 0 | 0 | 0 | 0 | 0 | 0 | 0 | 0 | 1 | 1 | 185 | 198 |  |  | PEAKS DB |
| K.C(+57.02)IC(+57.02)EC(+57.02)VESPNK.C | Y | 61.53 | 1394.5629 | 11 | 0.3 | 698.2889 | 2 | 47.51 | 6 | F6:30331 | NaNaKA16\_F2.raw |  |  |  |  |  | 4.592E5 |  |  |  |  |  |  |  | 1 | 0 | 0 | 0 | 0 | 0 | 1 | 0 | 0 | 0 | 0 | 0 | 0 | 0 | 360 | 370 | Carbamidomethylation | C1:Carbamidomethylation:1000.00;C3:Carbamidomethylation:1000.00;C5:Carbamidomethylation:1000.00 | PEAKS DB |
| K.LLPSSC(+57.02)GLNKEFDEEK.C | Y | 60.34 | 1864.8876 | 16 | 4.8 | 622.6364 | 3 | 33.83 | 7 | F7:14388 | NaNaKA16\_F3.raw |  |  |  |  |  |  | 1.4164E5 |  |  |  |  |  |  | 1 | 0 | 0 | 0 | 0 | 0 | 0 | 1 | 0 | 0 | 0 | 0 | 0 | 0 | 325 | 340 | Carbamidomethylation | C6:Carbamidomethylation:1000.00 | PEAKS DB |
| K.LLPSSC(+57.02)GLNK.E | Y | 54.98 | 1087.5696 | 10 | -0.1 | 544.7920 | 2 | 19.39 | 6 | F6:6810 | NaNaKA16\_F2.raw |  |  |  |  |  | 2.1852E6 |  |  |  |  |  |  |  | 1 | 0 | 0 | 0 | 0 | 0 | 1 | 0 | 0 | 0 | 0 | 0 | 0 | 0 | 325 | 334 | Carbamidomethylation | C6:Carbamidomethylation:1000.00 | PEAKS DB |
| K.ELDEETC(+57.02)QC(+57.02)VC(+57.02)K.G | Y | 48.15 | 1569.6110 | 12 | 0.1 | 785.8129 | 2 | 12.37 | 6 | F6:2413 | NaNaKA16\_F2.raw |  |  |  |  |  | 8.8422E4 |  |  |  |  |  |  |  | 1 | 0 | 0 | 0 | 0 | 0 | 1 | 0 | 0 | 0 | 0 | 0 | 0 | 0 | 287 | 298 | Carbamidomethylation | C7:Carbamidomethylation:1000.00;C9:Carbamidomethylation:1000.00;C11:Carbamidomethylation:1000.00 | PEAKS DB |
| total 5 peptides |  |  |  |  |  |  |  |  |  |  |  |  |  |  |  |  |  |  |  |  |  |  |  |  |  |  |  |  |  |  |  |  |  |  |  |  |  |  |  |  |  |  |  |
| --- | --- | --- | --- | --- | --- | --- | --- | --- | --- | --- | --- | --- | --- | --- | --- | --- | --- | --- | --- | --- | --- | --- | --- | --- | --- | --- | --- | --- | --- | --- | --- | --- | --- | --- | --- | --- | --- | --- | --- | --- | --- | --- | --- |

JAV48556.1

back to list

  

| Protein Coverage
| Supporting Peptides
|

Protein Coverage:

Supporting Peptides:

| Peptide | Uniq | -10lgP | Mass | Length | ppm | m/z | z | RT | Fraction | Scan | Source File | Area F1 | Area F10 | Area F11 | Area F12 | Area F13 | Area F2 | Area F3 | Area F4 | Area F5 | Area F6 | Area F7 | Area F8 | Area F9 | #Feature | #Feature F1 | #Feature F10 | #Feature F11 | #Feature F12 | #Feature F13 | #Feature F2 | #Feature F3 | #Feature F4 | #Feature F5 | #Feature F6 | #Feature F7 | #Feature F8 | #Feature F9 | Start | End | PTM | AScore | Found By |
| --- | --- | --- | --- | --- | --- | --- | --- | --- | --- | --- | --- | --- | --- | --- | --- | --- | --- | --- | --- | --- | --- | --- | --- | --- | --- | --- | --- | --- | --- | --- | --- | --- | --- | --- | --- | --- | --- | --- | --- | --- | --- | --- | --- |
| K.TLFEITVPLSHGPK.P | Y | 67.52 | 1537.8503 | 14 | -0.1 | 513.6240 | 3 | 58.66 | 2 | F2:36076 | NaNaKA16\_F10.raw |  | 9.1753E6 |  |  |  |  |  |  |  |  |  | 1.5718E6 | 2.8923E6 | 4 | 0 | 2 | 0 | 0 | 0 | 0 | 0 | 0 | 0 | 0 | 0 | 1 | 1 | 185 | 198 |  |  | PEAKS DB |
| K.C(+57.02)IC(+57.02)EC(+57.02)VESPNK.C | Y | 61.53 | 1394.5629 | 11 | 0.3 | 698.2889 | 2 | 47.51 | 6 | F6:30331 | NaNaKA16\_F2.raw |  |  |  |  |  | 4.592E5 |  |  |  |  |  |  |  | 1 | 0 | 0 | 0 | 0 | 0 | 1 | 0 | 0 | 0 | 0 | 0 | 0 | 0 | 360 | 370 | Carbamidomethylation | C1:Carbamidomethylation:1000.00;C3:Carbamidomethylation:1000.00;C5:Carbamidomethylation:1000.00 | PEAKS DB |
| K.LLPSSC(+57.02)GLNKEFDEEK.C | Y | 60.34 | 1864.8876 | 16 | 4.8 | 622.6364 | 3 | 33.83 | 7 | F7:14388 | NaNaKA16\_F3.raw |  |  |  |  |  |  | 1.4164E5 |  |  |  |  |  |  | 1 | 0 | 0 | 0 | 0 | 0 | 0 | 1 | 0 | 0 | 0 | 0 | 0 | 0 | 325 | 340 | Carbamidomethylation | C6:Carbamidomethylation:1000.00 | PEAKS DB |
| K.LLPSSC(+57.02)GLNK.E | Y | 54.98 | 1087.5696 | 10 | -0.1 | 544.7920 | 2 | 19.39 | 6 | F6:6810 | NaNaKA16\_F2.raw |  |  |  |  |  | 2.1852E6 |  |  |  |  |  |  |  | 1 | 0 | 0 | 0 | 0 | 0 | 1 | 0 | 0 | 0 | 0 | 0 | 0 | 0 | 325 | 334 | Carbamidomethylation | C6:Carbamidomethylation:1000.00 | PEAKS DB |
| K.ELDEETC(+57.02)QC(+57.02)VC(+57.02)K.G | Y | 48.15 | 1569.6110 | 12 | 0.1 | 785.8129 | 2 | 12.37 | 6 | F6:2413 | NaNaKA16\_F2.raw |  |  |  |  |  | 8.8422E4 |  |  |  |  |  |  |  | 1 | 0 | 0 | 0 | 0 | 0 | 1 | 0 | 0 | 0 | 0 | 0 | 0 | 0 | 287 | 298 | Carbamidomethylation | C7:Carbamidomethylation:1000.00;C9:Carbamidomethylation:1000.00;C11:Carbamidomethylation:1000.00 | PEAKS DB |
| total 5 peptides |  |  |  |  |  |  |  |  |  |  |  |  |  |  |  |  |  |  |  |  |  |  |  |  |  |  |  |  |  |  |  |  |  |  |  |  |  |  |  |  |  |  |  |
| --- | --- | --- | --- | --- | --- | --- | --- | --- | --- | --- | --- | --- | --- | --- | --- | --- | --- | --- | --- | --- | --- | --- | --- | --- | --- | --- | --- | --- | --- | --- | --- | --- | --- | --- | --- | --- | --- | --- | --- | --- | --- | --- | --- |

XP\_013912093.1

back to list

  

| Protein Coverage
| Supporting Peptides
|

Protein Coverage:

Supporting Peptides:

| Peptide | Uniq | -10lgP | Mass | Length | ppm | m/z | z | RT | Fraction | Scan | Source File | Area F1 | Area F10 | Area F11 | Area F12 | Area F13 | Area F2 | Area F3 | Area F4 | Area F5 | Area F6 | Area F7 | Area F8 | Area F9 | #Feature | #Feature F1 | #Feature F10 | #Feature F11 | #Feature F12 | #Feature F13 | #Feature F2 | #Feature F3 | #Feature F4 | #Feature F5 | #Feature F6 | #Feature F7 | #Feature F8 | #Feature F9 | Start | End | PTM | AScore | Found By |
| --- | --- | --- | --- | --- | --- | --- | --- | --- | --- | --- | --- | --- | --- | --- | --- | --- | --- | --- | --- | --- | --- | --- | --- | --- | --- | --- | --- | --- | --- | --- | --- | --- | --- | --- | --- | --- | --- | --- | --- | --- | --- | --- | --- |
| K.TLFEITVPLSHGPK.P | Y | 67.52 | 1537.8503 | 14 | -0.1 | 513.6240 | 3 | 58.66 | 2 | F2:36076 | NaNaKA16\_F10.raw |  | 9.1753E6 |  |  |  |  |  |  |  |  |  | 1.5718E6 | 2.8923E6 | 4 | 0 | 2 | 0 | 0 | 0 | 0 | 0 | 0 | 0 | 0 | 0 | 1 | 1 | 185 | 198 |  |  | PEAKS DB |
| K.C(+57.02)IC(+57.02)EC(+57.02)VESPNK.C | Y | 61.53 | 1394.5629 | 11 | 0.3 | 698.2889 | 2 | 47.51 | 6 | F6:30331 | NaNaKA16\_F2.raw |  |  |  |  |  | 4.592E5 |  |  |  |  |  |  |  | 1 | 0 | 0 | 0 | 0 | 0 | 1 | 0 | 0 | 0 | 0 | 0 | 0 | 0 | 360 | 370 | Carbamidomethylation | C1:Carbamidomethylation:1000.00;C3:Carbamidomethylation:1000.00;C5:Carbamidomethylation:1000.00 | PEAKS DB |
| K.LLPSSC(+57.02)GLNKEFDEEK.C | Y | 60.34 | 1864.8876 | 16 | 4.8 | 622.6364 | 3 | 33.83 | 7 | F7:14388 | NaNaKA16\_F3.raw |  |  |  |  |  |  | 1.4164E5 |  |  |  |  |  |  | 1 | 0 | 0 | 0 | 0 | 0 | 0 | 1 | 0 | 0 | 0 | 0 | 0 | 0 | 325 | 340 | Carbamidomethylation | C6:Carbamidomethylation:1000.00 | PEAKS DB |
| K.LLPSSC(+57.02)GLNK.E | Y | 54.98 | 1087.5696 | 10 | -0.1 | 544.7920 | 2 | 19.39 | 6 | F6:6810 | NaNaKA16\_F2.raw |  |  |  |  |  | 2.1852E6 |  |  |  |  |  |  |  | 1 | 0 | 0 | 0 | 0 | 0 | 1 | 0 | 0 | 0 | 0 | 0 | 0 | 0 | 325 | 334 | Carbamidomethylation | C6:Carbamidomethylation:1000.00 | PEAKS DB |
| K.ELDEETC(+57.02)QC(+57.02)VC(+57.02)K.G | Y | 48.15 | 1569.6110 | 12 | 0.1 | 785.8129 | 2 | 12.37 | 6 | F6:2413 | NaNaKA16\_F2.raw |  |  |  |  |  | 8.8422E4 |  |  |  |  |  |  |  | 1 | 0 | 0 | 0 | 0 | 0 | 1 | 0 | 0 | 0 | 0 | 0 | 0 | 0 | 287 | 298 | Carbamidomethylation | C7:Carbamidomethylation:1000.00;C9:Carbamidomethylation:1000.00;C11:Carbamidomethylation:1000.00 | PEAKS DB |
| total 5 peptides |  |  |  |  |  |  |  |  |  |  |  |  |  |  |  |  |  |  |  |  |  |  |  |  |  |  |  |  |  |  |  |  |  |  |  |  |  |  |  |  |  |  |  |
| --- | --- | --- | --- | --- | --- | --- | --- | --- | --- | --- | --- | --- | --- | --- | --- | --- | --- | --- | --- | --- | --- | --- | --- | --- | --- | --- | --- | --- | --- | --- | --- | --- | --- | --- | --- | --- | --- | --- | --- | --- | --- | --- | --- |

JAC94973.1

back to list

  

| Protein Coverage
| Supporting Peptides
|

Protein Coverage:

Supporting Peptides:

| Peptide | Uniq | -10lgP | Mass | Length | ppm | m/z | z | RT | Fraction | Scan | Source File | Area F1 | Area F10 | Area F11 | Area F12 | Area F13 | Area F2 | Area F3 | Area F4 | Area F5 | Area F6 | Area F7 | Area F8 | Area F9 | #Feature | #Feature F1 | #Feature F10 | #Feature F11 | #Feature F12 | #Feature F13 | #Feature F2 | #Feature F3 | #Feature F4 | #Feature F5 | #Feature F6 | #Feature F7 | #Feature F8 | #Feature F9 | Start | End | PTM | AScore | Found By |
| --- | --- | --- | --- | --- | --- | --- | --- | --- | --- | --- | --- | --- | --- | --- | --- | --- | --- | --- | --- | --- | --- | --- | --- | --- | --- | --- | --- | --- | --- | --- | --- | --- | --- | --- | --- | --- | --- | --- | --- | --- | --- | --- | --- |
| K.TLFEITVPLSHGPK.P | Y | 67.52 | 1537.8503 | 14 | -0.1 | 513.6240 | 3 | 58.66 | 2 | F2:36076 | NaNaKA16\_F10.raw |  | 9.1753E6 |  |  |  |  |  |  |  |  |  | 1.5718E6 | 2.8923E6 | 4 | 0 | 2 | 0 | 0 | 0 | 0 | 0 | 0 | 0 | 0 | 0 | 1 | 1 | 116 | 129 |  |  | PEAKS DB |
| K.C(+57.02)IC(+57.02)EC(+57.02)VESPNK.C | Y | 61.53 | 1394.5629 | 11 | 0.3 | 698.2889 | 2 | 47.51 | 6 | F6:30331 | NaNaKA16\_F2.raw |  |  |  |  |  | 4.592E5 |  |  |  |  |  |  |  | 1 | 0 | 0 | 0 | 0 | 0 | 1 | 0 | 0 | 0 | 0 | 0 | 0 | 0 | 291 | 301 | Carbamidomethylation | C1:Carbamidomethylation:1000.00;C3:Carbamidomethylation:1000.00;C5:Carbamidomethylation:1000.00 | PEAKS DB |
| K.LLPSSC(+57.02)GLNKEFDEEK.C | Y | 60.34 | 1864.8876 | 16 | 4.8 | 622.6364 | 3 | 33.83 | 7 | F7:14388 | NaNaKA16\_F3.raw |  |  |  |  |  |  | 1.4164E5 |  |  |  |  |  |  | 1 | 0 | 0 | 0 | 0 | 0 | 0 | 1 | 0 | 0 | 0 | 0 | 0 | 0 | 256 | 271 | Carbamidomethylation | C6:Carbamidomethylation:1000.00 | PEAKS DB |
| K.LLPSSC(+57.02)GLNK.E | Y | 54.98 | 1087.5696 | 10 | -0.1 | 544.7920 | 2 | 19.39 | 6 | F6:6810 | NaNaKA16\_F2.raw |  |  |  |  |  | 2.1852E6 |  |  |  |  |  |  |  | 1 | 0 | 0 | 0 | 0 | 0 | 1 | 0 | 0 | 0 | 0 | 0 | 0 | 0 | 256 | 265 | Carbamidomethylation | C6:Carbamidomethylation:1000.00 | PEAKS DB |
| K.ELDEETC(+57.02)QC(+57.02)VC(+57.02)K.G | Y | 48.15 | 1569.6110 | 12 | 0.1 | 785.8129 | 2 | 12.37 | 6 | F6:2413 | NaNaKA16\_F2.raw |  |  |  |  |  | 8.8422E4 |  |  |  |  |  |  |  | 1 | 0 | 0 | 0 | 0 | 0 | 1 | 0 | 0 | 0 | 0 | 0 | 0 | 0 | 218 | 229 | Carbamidomethylation | C7:Carbamidomethylation:1000.00;C9:Carbamidomethylation:1000.00;C11:Carbamidomethylation:1000.00 | PEAKS DB |
| total 5 peptides |  |  |  |  |  |  |  |  |  |  |  |  |  |  |  |  |  |  |  |  |  |  |  |  |  |  |  |  |  |  |  |  |  |  |  |  |  |  |  |  |  |  |  |
| --- | --- | --- | --- | --- | --- | --- | --- | --- | --- | --- | --- | --- | --- | --- | --- | --- | --- | --- | --- | --- | --- | --- | --- | --- | --- | --- | --- | --- | --- | --- | --- | --- | --- | --- | --- | --- | --- | --- | --- | --- | --- | --- | --- |

SMD28268.1

back to list

  

| Protein Coverage
| Supporting Peptides
|

Protein Coverage:

Supporting Peptides:

| Peptide | Uniq | -10lgP | Mass | Length | ppm | m/z | z | RT | Fraction | Scan | Source File | Area F1 | Area F10 | Area F11 | Area F12 | Area F13 | Area F2 | Area F3 | Area F4 | Area F5 | Area F6 | Area F7 | Area F8 | Area F9 | #Feature | #Feature F1 | #Feature F10 | #Feature F11 | #Feature F12 | #Feature F13 | #Feature F2 | #Feature F3 | #Feature F4 | #Feature F5 | #Feature F6 | #Feature F7 | #Feature F8 | #Feature F9 | Start | End | PTM | AScore | Found By |
| --- | --- | --- | --- | --- | --- | --- | --- | --- | --- | --- | --- | --- | --- | --- | --- | --- | --- | --- | --- | --- | --- | --- | --- | --- | --- | --- | --- | --- | --- | --- | --- | --- | --- | --- | --- | --- | --- | --- | --- | --- | --- | --- | --- |
| K.TLFEITVPLSHGPK.P | Y | 67.52 | 1537.8503 | 14 | -0.1 | 513.6240 | 3 | 58.66 | 2 | F2:36076 | NaNaKA16\_F10.raw |  | 9.1753E6 |  |  |  |  |  |  |  |  |  | 1.5718E6 | 2.8923E6 | 4 | 0 | 2 | 0 | 0 | 0 | 0 | 0 | 0 | 0 | 0 | 0 | 1 | 1 | 185 | 198 |  |  | PEAKS DB |
| K.C(+57.02)IC(+57.02)EC(+57.02)VESPNK.C | Y | 61.53 | 1394.5629 | 11 | 0.3 | 698.2889 | 2 | 47.51 | 6 | F6:30331 | NaNaKA16\_F2.raw |  |  |  |  |  | 4.592E5 |  |  |  |  |  |  |  | 1 | 0 | 0 | 0 | 0 | 0 | 1 | 0 | 0 | 0 | 0 | 0 | 0 | 0 | 360 | 370 | Carbamidomethylation | C1:Carbamidomethylation:1000.00;C3:Carbamidomethylation:1000.00;C5:Carbamidomethylation:1000.00 | PEAKS DB |
| K.LLPSSC(+57.02)GLNKEFDEEK.C | Y | 60.34 | 1864.8876 | 16 | 4.8 | 622.6364 | 3 | 33.83 | 7 | F7:14388 | NaNaKA16\_F3.raw |  |  |  |  |  |  | 1.4164E5 |  |  |  |  |  |  | 1 | 0 | 0 | 0 | 0 | 0 | 0 | 1 | 0 | 0 | 0 | 0 | 0 | 0 | 325 | 340 | Carbamidomethylation | C6:Carbamidomethylation:1000.00 | PEAKS DB |
| K.LLPSSC(+57.02)GLNK.E | Y | 54.98 | 1087.5696 | 10 | -0.1 | 544.7920 | 2 | 19.39 | 6 | F6:6810 | NaNaKA16\_F2.raw |  |  |  |  |  | 2.1852E6 |  |  |  |  |  |  |  | 1 | 0 | 0 | 0 | 0 | 0 | 1 | 0 | 0 | 0 | 0 | 0 | 0 | 0 | 325 | 334 | Carbamidomethylation | C6:Carbamidomethylation:1000.00 | PEAKS DB |
| K.ELDEETC(+57.02)QC(+57.02)VC(+57.02)K.G | Y | 48.15 | 1569.6110 | 12 | 0.1 | 785.8129 | 2 | 12.37 | 6 | F6:2413 | NaNaKA16\_F2.raw |  |  |  |  |  | 8.8422E4 |  |  |  |  |  |  |  | 1 | 0 | 0 | 0 | 0 | 0 | 1 | 0 | 0 | 0 | 0 | 0 | 0 | 0 | 287 | 298 | Carbamidomethylation | C7:Carbamidomethylation:1000.00;C9:Carbamidomethylation:1000.00;C11:Carbamidomethylation:1000.00 | PEAKS DB |
| total 5 peptides |  |  |  |  |  |  |  |  |  |  |  |  |  |  |  |  |  |  |  |  |  |  |  |  |  |  |  |  |  |  |  |  |  |  |  |  |  |  |  |  |  |  |  |
| --- | --- | --- | --- | --- | --- | --- | --- | --- | --- | --- | --- | --- | --- | --- | --- | --- | --- | --- | --- | --- | --- | --- | --- | --- | --- | --- | --- | --- | --- | --- | --- | --- | --- | --- | --- | --- | --- | --- | --- | --- | --- | --- | --- |

JAA95033.1

back to list

  

| Protein Coverage
| Supporting Peptides
|

Protein Coverage:

Supporting Peptides:

| Peptide | Uniq | -10lgP | Mass | Length | ppm | m/z | z | RT | Fraction | Scan | Source File | Area F1 | Area F10 | Area F11 | Area F12 | Area F13 | Area F2 | Area F3 | Area F4 | Area F5 | Area F6 | Area F7 | Area F8 | Area F9 | #Feature | #Feature F1 | #Feature F10 | #Feature F11 | #Feature F12 | #Feature F13 | #Feature F2 | #Feature F3 | #Feature F4 | #Feature F5 | #Feature F6 | #Feature F7 | #Feature F8 | #Feature F9 | Start | End | PTM | AScore | Found By |
| --- | --- | --- | --- | --- | --- | --- | --- | --- | --- | --- | --- | --- | --- | --- | --- | --- | --- | --- | --- | --- | --- | --- | --- | --- | --- | --- | --- | --- | --- | --- | --- | --- | --- | --- | --- | --- | --- | --- | --- | --- | --- | --- | --- |
| K.TLFEITVPLSHGPK.P | Y | 67.52 | 1537.8503 | 14 | -0.1 | 513.6240 | 3 | 58.66 | 2 | F2:36076 | NaNaKA16\_F10.raw |  | 9.1753E6 |  |  |  |  |  |  |  |  |  | 1.5718E6 | 2.8923E6 | 4 | 0 | 2 | 0 | 0 | 0 | 0 | 0 | 0 | 0 | 0 | 0 | 1 | 1 | 185 | 198 |  |  | PEAKS DB |
| K.C(+57.02)IC(+57.02)EC(+57.02)VESPNK.C | Y | 61.53 | 1394.5629 | 11 | 0.3 | 698.2889 | 2 | 47.51 | 6 | F6:30331 | NaNaKA16\_F2.raw |  |  |  |  |  | 4.592E5 |  |  |  |  |  |  |  | 1 | 0 | 0 | 0 | 0 | 0 | 1 | 0 | 0 | 0 | 0 | 0 | 0 | 0 | 360 | 370 | Carbamidomethylation | C1:Carbamidomethylation:1000.00;C3:Carbamidomethylation:1000.00;C5:Carbamidomethylation:1000.00 | PEAKS DB |
| K.LLPSSC(+57.02)GLNKEFDEEK.C | Y | 60.34 | 1864.8876 | 16 | 4.8 | 622.6364 | 3 | 33.83 | 7 | F7:14388 | NaNaKA16\_F3.raw |  |  |  |  |  |  | 1.4164E5 |  |  |  |  |  |  | 1 | 0 | 0 | 0 | 0 | 0 | 0 | 1 | 0 | 0 | 0 | 0 | 0 | 0 | 325 | 340 | Carbamidomethylation | C6:Carbamidomethylation:1000.00 | PEAKS DB |
| K.LLPSSC(+57.02)GLNK.E | Y | 54.98 | 1087.5696 | 10 | -0.1 | 544.7920 | 2 | 19.39 | 6 | F6:6810 | NaNaKA16\_F2.raw |  |  |  |  |  | 2.1852E6 |  |  |  |  |  |  |  | 1 | 0 | 0 | 0 | 0 | 0 | 1 | 0 | 0 | 0 | 0 | 0 | 0 | 0 | 325 | 334 | Carbamidomethylation | C6:Carbamidomethylation:1000.00 | PEAKS DB |
| K.ELDEETC(+57.02)QC(+57.02)VC(+57.02)K.G | Y | 48.15 | 1569.6110 | 12 | 0.1 | 785.8129 | 2 | 12.37 | 6 | F6:2413 | NaNaKA16\_F2.raw |  |  |  |  |  | 8.8422E4 |  |  |  |  |  |  |  | 1 | 0 | 0 | 0 | 0 | 0 | 1 | 0 | 0 | 0 | 0 | 0 | 0 | 0 | 287 | 298 | Carbamidomethylation | C7:Carbamidomethylation:1000.00;C9:Carbamidomethylation:1000.00;C11:Carbamidomethylation:1000.00 | PEAKS DB |
| total 5 peptides |  |  |  |  |  |  |  |  |  |  |  |  |  |  |  |  |  |  |  |  |  |  |  |  |  |  |  |  |  |  |  |  |  |  |  |  |  |  |  |  |  |  |  |
| --- | --- | --- | --- | --- | --- | --- | --- | --- | --- | --- | --- | --- | --- | --- | --- | --- | --- | --- | --- | --- | --- | --- | --- | --- | --- | --- | --- | --- | --- | --- | --- | --- | --- | --- | --- | --- | --- | --- | --- | --- | --- | --- | --- |

LAB17920.1

back to list

  

| Protein Coverage
| Supporting Peptides
|

Protein Coverage:

Supporting Peptides:

| Peptide | Uniq | -10lgP | Mass | Length | ppm | m/z | z | RT | Fraction | Scan | Source File | Area F1 | Area F10 | Area F11 | Area F12 | Area F13 | Area F2 | Area F3 | Area F4 | Area F5 | Area F6 | Area F7 | Area F8 | Area F9 | #Feature | #Feature F1 | #Feature F10 | #Feature F11 | #Feature F12 | #Feature F13 | #Feature F2 | #Feature F3 | #Feature F4 | #Feature F5 | #Feature F6 | #Feature F7 | #Feature F8 | #Feature F9 | Start | End | PTM | AScore | Found By |
| --- | --- | --- | --- | --- | --- | --- | --- | --- | --- | --- | --- | --- | --- | --- | --- | --- | --- | --- | --- | --- | --- | --- | --- | --- | --- | --- | --- | --- | --- | --- | --- | --- | --- | --- | --- | --- | --- | --- | --- | --- | --- | --- | --- |
| K.TLFEITVPLSHGPK.P | Y | 67.52 | 1537.8503 | 14 | -0.1 | 513.6240 | 3 | 58.66 | 2 | F2:36076 | NaNaKA16\_F10.raw |  | 9.1753E6 |  |  |  |  |  |  |  |  |  | 1.5718E6 | 2.8923E6 | 4 | 0 | 2 | 0 | 0 | 0 | 0 | 0 | 0 | 0 | 0 | 0 | 1 | 1 | 53 | 66 |  |  | PEAKS DB |
| K.C(+57.02)IC(+57.02)EC(+57.02)VESPNK.C | Y | 61.53 | 1394.5629 | 11 | 0.3 | 698.2889 | 2 | 47.51 | 6 | F6:30331 | NaNaKA16\_F2.raw |  |  |  |  |  | 4.592E5 |  |  |  |  |  |  |  | 1 | 0 | 0 | 0 | 0 | 0 | 1 | 0 | 0 | 0 | 0 | 0 | 0 | 0 | 228 | 238 | Carbamidomethylation | C1:Carbamidomethylation:1000.00;C3:Carbamidomethylation:1000.00;C5:Carbamidomethylation:1000.00 | PEAKS DB |
| K.LLPSSC(+57.02)GLNKEFDEEK.C | Y | 60.34 | 1864.8876 | 16 | 4.8 | 622.6364 | 3 | 33.83 | 7 | F7:14388 | NaNaKA16\_F3.raw |  |  |  |  |  |  | 1.4164E5 |  |  |  |  |  |  | 1 | 0 | 0 | 0 | 0 | 0 | 0 | 1 | 0 | 0 | 0 | 0 | 0 | 0 | 193 | 208 | Carbamidomethylation | C6:Carbamidomethylation:1000.00 | PEAKS DB |
| K.LLPSSC(+57.02)GLNK.E | Y | 54.98 | 1087.5696 | 10 | -0.1 | 544.7920 | 2 | 19.39 | 6 | F6:6810 | NaNaKA16\_F2.raw |  |  |  |  |  | 2.1852E6 |  |  |  |  |  |  |  | 1 | 0 | 0 | 0 | 0 | 0 | 1 | 0 | 0 | 0 | 0 | 0 | 0 | 0 | 193 | 202 | Carbamidomethylation | C6:Carbamidomethylation:1000.00 | PEAKS DB |
| K.ELDEETC(+57.02)QC(+57.02)VC(+57.02)K.G | Y | 48.15 | 1569.6110 | 12 | 0.1 | 785.8129 | 2 | 12.37 | 6 | F6:2413 | NaNaKA16\_F2.raw |  |  |  |  |  | 8.8422E4 |  |  |  |  |  |  |  | 1 | 0 | 0 | 0 | 0 | 0 | 1 | 0 | 0 | 0 | 0 | 0 | 0 | 0 | 155 | 166 | Carbamidomethylation | C7:Carbamidomethylation:1000.00;C9:Carbamidomethylation:1000.00;C11:Carbamidomethylation:1000.00 | PEAKS DB |
| total 5 peptides |  |  |  |  |  |  |  |  |  |  |  |  |  |  |  |  |  |  |  |  |  |  |  |  |  |  |  |  |  |  |  |  |  |  |  |  |  |  |  |  |  |  |  |
| --- | --- | --- | --- | --- | --- | --- | --- | --- | --- | --- | --- | --- | --- | --- | --- | --- | --- | --- | --- | --- | --- | --- | --- | --- | --- | --- | --- | --- | --- | --- | --- | --- | --- | --- | --- | --- | --- | --- | --- | --- | --- | --- | --- |

XP\_026575343.1

back to list

  

| Protein Coverage
| Supporting Peptides
|

Protein Coverage:

Supporting Peptides:

| Peptide | Uniq | -10lgP | Mass | Length | ppm | m/z | z | RT | Fraction | Scan | Source File | Area F1 | Area F10 | Area F11 | Area F12 | Area F13 | Area F2 | Area F3 | Area F4 | Area F5 | Area F6 | Area F7 | Area F8 | Area F9 | #Feature | #Feature F1 | #Feature F10 | #Feature F11 | #Feature F12 | #Feature F13 | #Feature F2 | #Feature F3 | #Feature F4 | #Feature F5 | #Feature F6 | #Feature F7 | #Feature F8 | #Feature F9 | Start | End | PTM | AScore | Found By |
| --- | --- | --- | --- | --- | --- | --- | --- | --- | --- | --- | --- | --- | --- | --- | --- | --- | --- | --- | --- | --- | --- | --- | --- | --- | --- | --- | --- | --- | --- | --- | --- | --- | --- | --- | --- | --- | --- | --- | --- | --- | --- | --- | --- |
| R.QLVPIGLSEFALDIR.M | Y | 75.78 | 1669.9402 | 15 | 0.1 | 557.6541 | 3 | 95.82 | 4 | F4:67305 | NaNaKA16\_F12.raw |  |  |  | 2.3291E6 |  |  |  |  |  |  |  |  |  | 2 | 0 | 0 | 0 | 2 | 0 | 0 | 0 | 0 | 0 | 0 | 0 | 0 | 0 | 55 | 69 |  |  | PEAKS DB |
| K.YFVLQVHYGDVGVFK.D | Y | 72.55 | 1769.9141 | 15 | -0.4 | 590.9784 | 3 | 68.48 | 4 | F4:45599 | NaNaKA16\_F12.raw |  |  |  | 1.0363E6 |  |  |  |  |  |  |  |  |  | 1 | 0 | 0 | 0 | 1 | 0 | 0 | 0 | 0 | 0 | 0 | 0 | 0 | 0 | 168 | 182 |  |  | PEAKS DB |
| R.LPVEEEAYVVDFKPH.A | Y | 50.34 | 1770.8828 | 15 | 0.8 | 591.3020 | 3 | 55.69 | 4 | F4:35066 | NaNaKA16\_F12.raw |  |  |  | 2.2836E6 |  |  |  |  |  |  |  |  |  | 1 | 0 | 0 | 0 | 1 | 0 | 0 | 0 | 0 | 0 | 0 | 0 | 0 | 0 | 88 | 102 |  |  | PEAKS DB |
| total 3 peptides |  |  |  |  |  |  |  |  |  |  |  |  |  |  |  |  |  |  |  |  |  |  |  |  |  |  |  |  |  |  |  |  |  |  |  |  |  |  |  |  |  |  |  |
| --- | --- | --- | --- | --- | --- | --- | --- | --- | --- | --- | --- | --- | --- | --- | --- | --- | --- | --- | --- | --- | --- | --- | --- | --- | --- | --- | --- | --- | --- | --- | --- | --- | --- | --- | --- | --- | --- | --- | --- | --- | --- | --- | --- |

XP\_026575334.1

back to list

  

| Protein Coverage
| Supporting Peptides
|

Protein Coverage:

Supporting Peptides:

| Peptide | Uniq | -10lgP | Mass | Length | ppm | m/z | z | RT | Fraction | Scan | Source File | Area F1 | Area F10 | Area F11 | Area F12 | Area F13 | Area F2 | Area F3 | Area F4 | Area F5 | Area F6 | Area F7 | Area F8 | Area F9 | #Feature | #Feature F1 | #Feature F10 | #Feature F11 | #Feature F12 | #Feature F13 | #Feature F2 | #Feature F3 | #Feature F4 | #Feature F5 | #Feature F6 | #Feature F7 | #Feature F8 | #Feature F9 | Start | End | PTM | AScore | Found By |
| --- | --- | --- | --- | --- | --- | --- | --- | --- | --- | --- | --- | --- | --- | --- | --- | --- | --- | --- | --- | --- | --- | --- | --- | --- | --- | --- | --- | --- | --- | --- | --- | --- | --- | --- | --- | --- | --- | --- | --- | --- | --- | --- | --- |
| R.QLVPIGLSEFALDIR.M | Y | 75.78 | 1669.9402 | 15 | 0.1 | 557.6541 | 3 | 95.82 | 4 | F4:67305 | NaNaKA16\_F12.raw |  |  |  | 2.3291E6 |  |  |  |  |  |  |  |  |  | 2 | 0 | 0 | 0 | 2 | 0 | 0 | 0 | 0 | 0 | 0 | 0 | 0 | 0 | 55 | 69 |  |  | PEAKS DB |
| K.YFVLQVHYGDVGVFK.D | Y | 72.55 | 1769.9141 | 15 | -0.4 | 590.9784 | 3 | 68.48 | 4 | F4:45599 | NaNaKA16\_F12.raw |  |  |  | 1.0363E6 |  |  |  |  |  |  |  |  |  | 1 | 0 | 0 | 0 | 1 | 0 | 0 | 0 | 0 | 0 | 0 | 0 | 0 | 0 | 168 | 182 |  |  | PEAKS DB |
| R.LPVEEEAYVVDFKPH.A | Y | 50.34 | 1770.8828 | 15 | 0.8 | 591.3020 | 3 | 55.69 | 4 | F4:35066 | NaNaKA16\_F12.raw |  |  |  | 2.2836E6 |  |  |  |  |  |  |  |  |  | 1 | 0 | 0 | 0 | 1 | 0 | 0 | 0 | 0 | 0 | 0 | 0 | 0 | 0 | 88 | 102 |  |  | PEAKS DB |
| total 3 peptides |  |  |  |  |  |  |  |  |  |  |  |  |  |  |  |  |  |  |  |  |  |  |  |  |  |  |  |  |  |  |  |  |  |  |  |  |  |  |  |  |  |  |  |
| --- | --- | --- | --- | --- | --- | --- | --- | --- | --- | --- | --- | --- | --- | --- | --- | --- | --- | --- | --- | --- | --- | --- | --- | --- | --- | --- | --- | --- | --- | --- | --- | --- | --- | --- | --- | --- | --- | --- | --- | --- | --- | --- | --- |

XP\_026575314.1

back to list

  

| Protein Coverage
| Supporting Peptides
|

Protein Coverage:

Supporting Peptides:

| Peptide | Uniq | -10lgP | Mass | Length | ppm | m/z | z | RT | Fraction | Scan | Source File | Area F1 | Area F10 | Area F11 | Area F12 | Area F13 | Area F2 | Area F3 | Area F4 | Area F5 | Area F6 | Area F7 | Area F8 | Area F9 | #Feature | #Feature F1 | #Feature F10 | #Feature F11 | #Feature F12 | #Feature F13 | #Feature F2 | #Feature F3 | #Feature F4 | #Feature F5 | #Feature F6 | #Feature F7 | #Feature F8 | #Feature F9 | Start | End | PTM | AScore | Found By |
| --- | --- | --- | --- | --- | --- | --- | --- | --- | --- | --- | --- | --- | --- | --- | --- | --- | --- | --- | --- | --- | --- | --- | --- | --- | --- | --- | --- | --- | --- | --- | --- | --- | --- | --- | --- | --- | --- | --- | --- | --- | --- | --- | --- |
| R.QLVPIGLSEFALDIR.M | Y | 75.78 | 1669.9402 | 15 | 0.1 | 557.6541 | 3 | 95.82 | 4 | F4:67305 | NaNaKA16\_F12.raw |  |  |  | 2.3291E6 |  |  |  |  |  |  |  |  |  | 2 | 0 | 0 | 0 | 2 | 0 | 0 | 0 | 0 | 0 | 0 | 0 | 0 | 0 | 55 | 69 |  |  | PEAKS DB |
| K.YFVLQVHYGDVGVFK.D | Y | 72.55 | 1769.9141 | 15 | -0.4 | 590.9784 | 3 | 68.48 | 4 | F4:45599 | NaNaKA16\_F12.raw |  |  |  | 1.0363E6 |  |  |  |  |  |  |  |  |  | 1 | 0 | 0 | 0 | 1 | 0 | 0 | 0 | 0 | 0 | 0 | 0 | 0 | 0 | 168 | 182 |  |  | PEAKS DB |
| R.LPVEEEAYVVDFKPH.A | Y | 50.34 | 1770.8828 | 15 | 0.8 | 591.3020 | 3 | 55.69 | 4 | F4:35066 | NaNaKA16\_F12.raw |  |  |  | 2.2836E6 |  |  |  |  |  |  |  |  |  | 1 | 0 | 0 | 0 | 1 | 0 | 0 | 0 | 0 | 0 | 0 | 0 | 0 | 0 | 88 | 102 |  |  | PEAKS DB |
| total 3 peptides |  |  |  |  |  |  |  |  |  |  |  |  |  |  |  |  |  |  |  |  |  |  |  |  |  |  |  |  |  |  |  |  |  |  |  |  |  |  |  |  |  |  |  |
| --- | --- | --- | --- | --- | --- | --- | --- | --- | --- | --- | --- | --- | --- | --- | --- | --- | --- | --- | --- | --- | --- | --- | --- | --- | --- | --- | --- | --- | --- | --- | --- | --- | --- | --- | --- | --- | --- | --- | --- | --- | --- | --- | --- |

XP\_026575325.1

back to list

  

| Protein Coverage
| Supporting Peptides
|

Protein Coverage:

Supporting Peptides:

| Peptide | Uniq | -10lgP | Mass | Length | ppm | m/z | z | RT | Fraction | Scan | Source File | Area F1 | Area F10 | Area F11 | Area F12 | Area F13 | Area F2 | Area F3 | Area F4 | Area F5 | Area F6 | Area F7 | Area F8 | Area F9 | #Feature | #Feature F1 | #Feature F10 | #Feature F11 | #Feature F12 | #Feature F13 | #Feature F2 | #Feature F3 | #Feature F4 | #Feature F5 | #Feature F6 | #Feature F7 | #Feature F8 | #Feature F9 | Start | End | PTM | AScore | Found By |
| --- | --- | --- | --- | --- | --- | --- | --- | --- | --- | --- | --- | --- | --- | --- | --- | --- | --- | --- | --- | --- | --- | --- | --- | --- | --- | --- | --- | --- | --- | --- | --- | --- | --- | --- | --- | --- | --- | --- | --- | --- | --- | --- | --- |
| R.QLVPIGLSEFALDIR.M | Y | 75.78 | 1669.9402 | 15 | 0.1 | 557.6541 | 3 | 95.82 | 4 | F4:67305 | NaNaKA16\_F12.raw |  |  |  | 2.3291E6 |  |  |  |  |  |  |  |  |  | 2 | 0 | 0 | 0 | 2 | 0 | 0 | 0 | 0 | 0 | 0 | 0 | 0 | 0 | 55 | 69 |  |  | PEAKS DB |
| K.YFVLQVHYGDVGVFK.D | Y | 72.55 | 1769.9141 | 15 | -0.4 | 590.9784 | 3 | 68.48 | 4 | F4:45599 | NaNaKA16\_F12.raw |  |  |  | 1.0363E6 |  |  |  |  |  |  |  |  |  | 1 | 0 | 0 | 0 | 1 | 0 | 0 | 0 | 0 | 0 | 0 | 0 | 0 | 0 | 168 | 182 |  |  | PEAKS DB |
| R.LPVEEEAYVVDFKPH.A | Y | 50.34 | 1770.8828 | 15 | 0.8 | 591.3020 | 3 | 55.69 | 4 | F4:35066 | NaNaKA16\_F12.raw |  |  |  | 2.2836E6 |  |  |  |  |  |  |  |  |  | 1 | 0 | 0 | 0 | 1 | 0 | 0 | 0 | 0 | 0 | 0 | 0 | 0 | 0 | 88 | 102 |  |  | PEAKS DB |
| total 3 peptides |  |  |  |  |  |  |  |  |  |  |  |  |  |  |  |  |  |  |  |  |  |  |  |  |  |  |  |  |  |  |  |  |  |  |  |  |  |  |  |  |  |  |  |
| --- | --- | --- | --- | --- | --- | --- | --- | --- | --- | --- | --- | --- | --- | --- | --- | --- | --- | --- | --- | --- | --- | --- | --- | --- | --- | --- | --- | --- | --- | --- | --- | --- | --- | --- | --- | --- | --- | --- | --- | --- | --- | --- | --- |

XP\_026575319.1

back to list

  

| Protein Coverage
| Supporting Peptides
|

Protein Coverage:

Supporting Peptides:

| Peptide | Uniq | -10lgP | Mass | Length | ppm | m/z | z | RT | Fraction | Scan | Source File | Area F1 | Area F10 | Area F11 | Area F12 | Area F13 | Area F2 | Area F3 | Area F4 | Area F5 | Area F6 | Area F7 | Area F8 | Area F9 | #Feature | #Feature F1 | #Feature F10 | #Feature F11 | #Feature F12 | #Feature F13 | #Feature F2 | #Feature F3 | #Feature F4 | #Feature F5 | #Feature F6 | #Feature F7 | #Feature F8 | #Feature F9 | Start | End | PTM | AScore | Found By |
| --- | --- | --- | --- | --- | --- | --- | --- | --- | --- | --- | --- | --- | --- | --- | --- | --- | --- | --- | --- | --- | --- | --- | --- | --- | --- | --- | --- | --- | --- | --- | --- | --- | --- | --- | --- | --- | --- | --- | --- | --- | --- | --- | --- |
| R.QLVPIGLSEFALDIR.M | Y | 75.78 | 1669.9402 | 15 | 0.1 | 557.6541 | 3 | 95.82 | 4 | F4:67305 | NaNaKA16\_F12.raw |  |  |  | 2.3291E6 |  |  |  |  |  |  |  |  |  | 2 | 0 | 0 | 0 | 2 | 0 | 0 | 0 | 0 | 0 | 0 | 0 | 0 | 0 | 55 | 69 |  |  | PEAKS DB |
| K.YFVLQVHYGDVGVFK.D | Y | 72.55 | 1769.9141 | 15 | -0.4 | 590.9784 | 3 | 68.48 | 4 | F4:45599 | NaNaKA16\_F12.raw |  |  |  | 1.0363E6 |  |  |  |  |  |  |  |  |  | 1 | 0 | 0 | 0 | 1 | 0 | 0 | 0 | 0 | 0 | 0 | 0 | 0 | 0 | 168 | 182 |  |  | PEAKS DB |
| R.LPVEEEAYVVDFKPH.A | Y | 50.34 | 1770.8828 | 15 | 0.8 | 591.3020 | 3 | 55.69 | 4 | F4:35066 | NaNaKA16\_F12.raw |  |  |  | 2.2836E6 |  |  |  |  |  |  |  |  |  | 1 | 0 | 0 | 0 | 1 | 0 | 0 | 0 | 0 | 0 | 0 | 0 | 0 | 0 | 88 | 102 |  |  | PEAKS DB |
| total 3 peptides |  |  |  |  |  |  |  |  |  |  |  |  |  |  |  |  |  |  |  |  |  |  |  |  |  |  |  |  |  |  |  |  |  |  |  |  |  |  |  |  |  |  |  |
| --- | --- | --- | --- | --- | --- | --- | --- | --- | --- | --- | --- | --- | --- | --- | --- | --- | --- | --- | --- | --- | --- | --- | --- | --- | --- | --- | --- | --- | --- | --- | --- | --- | --- | --- | --- | --- | --- | --- | --- | --- | --- | --- | --- |

XP\_026570451.1

back to list

  

| Protein Coverage
| Supporting Peptides
|

Protein Coverage:

Supporting Peptides:

| Peptide | Uniq | -10lgP | Mass | Length | ppm | m/z | z | RT | Fraction | Scan | Source File | Area F1 | Area F10 | Area F11 | Area F12 | Area F13 | Area F2 | Area F3 | Area F4 | Area F5 | Area F6 | Area F7 | Area F8 | Area F9 | #Feature | #Feature F1 | #Feature F10 | #Feature F11 | #Feature F12 | #Feature F13 | #Feature F2 | #Feature F3 | #Feature F4 | #Feature F5 | #Feature F6 | #Feature F7 | #Feature F8 | #Feature F9 | Start | End | PTM | AScore | Found By |
| --- | --- | --- | --- | --- | --- | --- | --- | --- | --- | --- | --- | --- | --- | --- | --- | --- | --- | --- | --- | --- | --- | --- | --- | --- | --- | --- | --- | --- | --- | --- | --- | --- | --- | --- | --- | --- | --- | --- | --- | --- | --- | --- | --- |
| K.FASFIDKVR.F | N | 65.27 | 1081.5920 | 9 | 3.5 | 541.8023 | 2 | 29.71 | 8 | F8:13159 | NaNaKA16\_F4.raw |  |  |  | 5.894E5 | 7.4963E5 | 0 |  | 1.9656E6 | 2.1554E6 | 2.1372E6 |  |  |  | 5 | 0 | 0 | 0 | 1 | 1 | 0 | 0 | 1 | 1 | 1 | 0 | 0 | 0 | 182 | 190 |  |  | PEAKS DB |
| K.LALDIEIATYR.K | N | 64.60 | 1276.7026 | 11 | 0.8 | 639.3591 | 2 | 69.21 | 11 | F11:46918 | NaNaKA16\_F7.raw |  |  |  |  |  | 1.118E6 |  |  | 1.7861E6 | 1.6805E6 | 6.0957E5 | 8.3228E5 | 2.295E6 | 6 | 0 | 0 | 0 | 0 | 0 | 1 | 0 | 0 | 1 | 1 | 1 | 1 | 1 | 464 | 474 |  |  | PEAKS DB |
| R.TEAESWYQTK.Y | Y | 53.60 | 1241.5564 | 10 | -0.5 | 621.7852 | 2 | 22.28 | 5 | F5:6009 | NaNaKA16\_F13.raw |  |  |  |  | 1.1357E6 |  |  |  |  |  |  |  |  | 1 | 0 | 0 | 0 | 0 | 1 | 0 | 0 | 0 | 0 | 0 | 0 | 0 | 0 | 358 | 367 |  |  | PEAKS DB |
| K.YEELQVTAGR.H | Y | 44.80 | 1164.5775 | 10 | 0.0 | 583.2960 | 2 | 24.04 | 5 | F5:6769 | NaNaKA16\_F13.raw |  |  |  |  | 3.6427E5 |  |  |  |  |  |  |  |  | 1 | 0 | 0 | 0 | 0 | 1 | 0 | 0 | 0 | 0 | 0 | 0 | 0 | 0 | 368 | 377 |  |  | PEAKS DB |
| total 4 peptides |  |  |  |  |  |  |  |  |  |  |  |  |  |  |  |  |  |  |  |  |  |  |  |  |  |  |  |  |  |  |  |  |  |  |  |  |  |  |  |  |  |  |  |
| --- | --- | --- | --- | --- | --- | --- | --- | --- | --- | --- | --- | --- | --- | --- | --- | --- | --- | --- | --- | --- | --- | --- | --- | --- | --- | --- | --- | --- | --- | --- | --- | --- | --- | --- | --- | --- | --- | --- | --- | --- | --- | --- | --- |

AAM51550.1

back to list

  

| Protein Coverage
| Supporting Peptides
|

Protein Coverage:

Supporting Peptides:

| Peptide | Uniq | -10lgP | Mass | Length | ppm | m/z | z | RT | Fraction | Scan | Source File | Area F1 | Area F10 | Area F11 | Area F12 | Area F13 | Area F2 | Area F3 | Area F4 | Area F5 | Area F6 | Area F7 | Area F8 | Area F9 | #Feature | #Feature F1 | #Feature F10 | #Feature F11 | #Feature F12 | #Feature F13 | #Feature F2 | #Feature F3 | #Feature F4 | #Feature F5 | #Feature F6 | #Feature F7 | #Feature F8 | #Feature F9 | Start | End | PTM | AScore | Found By |
| --- | --- | --- | --- | --- | --- | --- | --- | --- | --- | --- | --- | --- | --- | --- | --- | --- | --- | --- | --- | --- | --- | --- | --- | --- | --- | --- | --- | --- | --- | --- | --- | --- | --- | --- | --- | --- | --- | --- | --- | --- | --- | --- | --- |
| R.VYEMVNALNTMYR.R | Y | 67.38 | 1602.7534 | 13 | 0.8 | 802.3846 | 2 | 65.11 | 5 | F5:26842 | NaNaKA16\_F13.raw |  |  |  |  | 1.0623E6 |  |  |  |  |  |  |  |  | 1 | 0 | 0 | 0 | 0 | 1 | 0 | 0 | 0 | 0 | 0 | 0 | 0 | 0 | 232 | 244 |  |  | PEAKS DB |
| Q.ATLDLFGEWR.E | N | 65.68 | 1206.6033 | 10 | 0.9 | 604.3094 | 2 | 78.42 | 2 | F2:53296 | NaNaKA16\_F10.raw |  | 2.2448E6 |  | 3.9536E5 | 1.1467E7 |  |  |  |  | 5.6872E6 | 6.3509E5 | 1.6325E6 | 1.5953E6 | 8 | 0 | 1 | 0 | 1 | 2 | 0 | 0 | 0 | 0 | 1 | 1 | 1 | 1 | 272 | 281 |  |  | PEAKS DB |
| R.DRPQC(+57.02)ILNKPSR.K | Y | 65.30 | 1482.7725 | 12 | 0.3 | 495.2649 | 3 | 11.84 | 5 | F5:1801 | NaNaKA16\_F13.raw |  |  |  |  | 2.8122E6 |  |  |  |  |  |  |  |  | 1 | 0 | 0 | 0 | 0 | 1 | 0 | 0 | 0 | 0 | 0 | 0 | 0 | 0 | 391 | 402 | Carbamidomethylation | C5:Carbamidomethylation:1000.00 | PEAKS DB |
| A.DSSAVISAC(+57.02)DGLK.G | N | 60.11 | 1321.6184 | 13 | 0.4 | 661.8168 | 2 | 33.21 | 10 | F10:19042 | NaNaKA16\_F6.raw |  |  |  |  |  |  |  |  |  | 6.9505E6 |  |  |  | 1 | 0 | 0 | 0 | 0 | 0 | 0 | 0 | 0 | 0 | 1 | 0 | 0 | 0 | 119 | 131 | Carbamidomethylation | C9:Carbamidomethylation:1000.00 | PEAKS DB |
| total 4 peptides |  |  |  |  |  |  |  |  |  |  |  |  |  |  |  |  |  |  |  |  |  |  |  |  |  |  |  |  |  |  |  |  |  |  |  |  |  |  |  |  |  |  |  |
| --- | --- | --- | --- | --- | --- | --- | --- | --- | --- | --- | --- | --- | --- | --- | --- | --- | --- | --- | --- | --- | --- | --- | --- | --- | --- | --- | --- | --- | --- | --- | --- | --- | --- | --- | --- | --- | --- | --- | --- | --- | --- | --- | --- |

Q10749.3

back to list

  

| Protein Coverage
| Supporting Peptides
|

Protein Coverage:

Supporting Peptides:

| Peptide | Uniq | -10lgP | Mass | Length | ppm | m/z | z | RT | Fraction | Scan | Source File | Area F1 | Area F10 | Area F11 | Area F12 | Area F13 | Area F2 | Area F3 | Area F4 | Area F5 | Area F6 | Area F7 | Area F8 | Area F9 | #Feature | #Feature F1 | #Feature F10 | #Feature F11 | #Feature F12 | #Feature F13 | #Feature F2 | #Feature F3 | #Feature F4 | #Feature F5 | #Feature F6 | #Feature F7 | #Feature F8 | #Feature F9 | Start | End | PTM | AScore | Found By |
| --- | --- | --- | --- | --- | --- | --- | --- | --- | --- | --- | --- | --- | --- | --- | --- | --- | --- | --- | --- | --- | --- | --- | --- | --- | --- | --- | --- | --- | --- | --- | --- | --- | --- | --- | --- | --- | --- | --- | --- | --- | --- | --- | --- |
| R.VYEMVNALNTMYR.R | Y | 67.38 | 1602.7534 | 13 | 0.8 | 802.3846 | 2 | 65.11 | 5 | F5:26842 | NaNaKA16\_F13.raw |  |  |  |  | 1.0623E6 |  |  |  |  |  |  |  |  | 1 | 0 | 0 | 0 | 0 | 1 | 0 | 0 | 0 | 0 | 0 | 0 | 0 | 0 | 232 | 244 |  |  | PEAKS DB |
| Q.ATLDLFGEWR.E | N | 65.68 | 1206.6033 | 10 | 0.9 | 604.3094 | 2 | 78.42 | 2 | F2:53296 | NaNaKA16\_F10.raw |  | 2.2448E6 |  | 3.9536E5 | 1.1467E7 |  |  |  |  | 5.6872E6 | 6.3509E5 | 1.6325E6 | 1.5953E6 | 8 | 0 | 1 | 0 | 1 | 2 | 0 | 0 | 0 | 0 | 1 | 1 | 1 | 1 | 272 | 281 |  |  | PEAKS DB |
| R.DRPQC(+57.02)ILNKPSR.K | Y | 65.30 | 1482.7725 | 12 | 0.3 | 495.2649 | 3 | 11.84 | 5 | F5:1801 | NaNaKA16\_F13.raw |  |  |  |  | 2.8122E6 |  |  |  |  |  |  |  |  | 1 | 0 | 0 | 0 | 0 | 1 | 0 | 0 | 0 | 0 | 0 | 0 | 0 | 0 | 391 | 402 | Carbamidomethylation | C5:Carbamidomethylation:1000.00 | PEAKS DB |
| A.DSSAVISAC(+57.02)DGLK.G | N | 60.11 | 1321.6184 | 13 | 0.4 | 661.8168 | 2 | 33.21 | 10 | F10:19042 | NaNaKA16\_F6.raw |  |  |  |  |  |  |  |  |  | 6.9505E6 |  |  |  | 1 | 0 | 0 | 0 | 0 | 0 | 0 | 0 | 0 | 0 | 1 | 0 | 0 | 0 | 119 | 131 | Carbamidomethylation | C9:Carbamidomethylation:1000.00 | PEAKS DB |
| total 4 peptides |  |  |  |  |  |  |  |  |  |  |  |  |  |  |  |  |  |  |  |  |  |  |  |  |  |  |  |  |  |  |  |  |  |  |  |  |  |  |  |  |  |  |  |
| --- | --- | --- | --- | --- | --- | --- | --- | --- | --- | --- | --- | --- | --- | --- | --- | --- | --- | --- | --- | --- | --- | --- | --- | --- | --- | --- | --- | --- | --- | --- | --- | --- | --- | --- | --- | --- | --- | --- | --- | --- | --- | --- | --- |

P18964.1

back to list

  

| Protein Coverage
| Supporting Peptides
|

Protein Coverage:

Supporting Peptides:

| Peptide | Uniq | -10lgP | Mass | Length | ppm | m/z | z | RT | Fraction | Scan | Source File | Area F1 | Area F10 | Area F11 | Area F12 | Area F13 | Area F2 | Area F3 | Area F4 | Area F5 | Area F6 | Area F7 | Area F8 | Area F9 | #Feature | #Feature F1 | #Feature F10 | #Feature F11 | #Feature F12 | #Feature F13 | #Feature F2 | #Feature F3 | #Feature F4 | #Feature F5 | #Feature F6 | #Feature F7 | #Feature F8 | #Feature F9 | Start | End | PTM | AScore | Found By |
| --- | --- | --- | --- | --- | --- | --- | --- | --- | --- | --- | --- | --- | --- | --- | --- | --- | --- | --- | --- | --- | --- | --- | --- | --- | --- | --- | --- | --- | --- | --- | --- | --- | --- | --- | --- | --- | --- | --- | --- | --- | --- | --- | --- |
| K.WC(+57.02)EPLYPWVPADSR.T | Y | 78.81 | 1774.8137 | 14 | 0.9 | 888.4149 | 2 | 77.31 | 4 | F4:52756 | NaNaKA16\_F12.raw |  |  | 3.2408E5 | 3.1766E6 |  |  |  |  |  |  |  |  |  | 2 | 0 | 0 | 1 | 1 | 0 | 0 | 0 | 0 | 0 | 0 | 0 | 0 | 0 | 151 | 164 | Carbamidomethylation | C2:Carbamidomethylation:1000.00 | PEAKS DB |
| K.ISTTEDTYPDVPHC(+57.02)TNIFIVK.H | Y | 69.19 | 2449.1836 | 21 | -0.4 | 817.4015 | 3 | 59.42 | 4 | F4:38046 | NaNaKA16\_F12.raw |  |  |  | 3.898E6 |  |  |  |  |  |  |  |  |  | 1 | 0 | 0 | 0 | 1 | 0 | 0 | 0 | 0 | 0 | 0 | 0 | 0 | 0 | 128 | 148 | Carbamidomethylation | C14:Carbamidomethylation:1000.00 | PEAKS DB |
| R.RPVTYSTHIAPVSLPSR.S | Y | 54.67 | 1880.0267 | 17 | 0.5 | 627.6832 | 3 | 28.52 | 4 | F4:12074 | NaNaKA16\_F12.raw |  |  |  | 7.8153E6 |  |  |  |  |  |  |  |  |  | 2 | 0 | 0 | 0 | 2 | 0 | 0 | 0 | 0 | 0 | 0 | 0 | 0 | 0 | 96 | 112 |  |  | PEAKS DB |
| K.FPNGLDKDIMLIR.L | Y | 47.58 | 1530.8228 | 13 | 0.2 | 511.2816 | 3 | 62.71 | 4 | F4:40623 | NaNaKA16\_F12.raw |  |  |  | 5.0748E5 |  |  |  |  |  |  |  |  |  | 1 | 0 | 0 | 0 | 1 | 0 | 0 | 0 | 0 | 0 | 0 | 0 | 0 | 0 | 81 | 93 |  |  | PEAKS DB |
| total 4 peptides |  |  |  |  |  |  |  |  |  |  |  |  |  |  |  |  |  |  |  |  |  |  |  |  |  |  |  |  |  |  |  |  |  |  |  |  |  |  |  |  |  |  |  |
| --- | --- | --- | --- | --- | --- | --- | --- | --- | --- | --- | --- | --- | --- | --- | --- | --- | --- | --- | --- | --- | --- | --- | --- | --- | --- | --- | --- | --- | --- | --- | --- | --- | --- | --- | --- | --- | --- | --- | --- | --- | --- | --- | --- |

P18965.2

back to list

  

| Protein Coverage
| Supporting Peptides
|

Protein Coverage:

Supporting Peptides:

| Peptide | Uniq | -10lgP | Mass | Length | ppm | m/z | z | RT | Fraction | Scan | Source File | Area F1 | Area F10 | Area F11 | Area F12 | Area F13 | Area F2 | Area F3 | Area F4 | Area F5 | Area F6 | Area F7 | Area F8 | Area F9 | #Feature | #Feature F1 | #Feature F10 | #Feature F11 | #Feature F12 | #Feature F13 | #Feature F2 | #Feature F3 | #Feature F4 | #Feature F5 | #Feature F6 | #Feature F7 | #Feature F8 | #Feature F9 | Start | End | PTM | AScore | Found By |
| --- | --- | --- | --- | --- | --- | --- | --- | --- | --- | --- | --- | --- | --- | --- | --- | --- | --- | --- | --- | --- | --- | --- | --- | --- | --- | --- | --- | --- | --- | --- | --- | --- | --- | --- | --- | --- | --- | --- | --- | --- | --- | --- | --- |
| K.WC(+57.02)EPLYPWVPADSR.T | Y | 78.81 | 1774.8137 | 14 | 0.9 | 888.4149 | 2 | 77.31 | 4 | F4:52756 | NaNaKA16\_F12.raw |  |  | 3.2408E5 | 3.1766E6 |  |  |  |  |  |  |  |  |  | 2 | 0 | 0 | 1 | 1 | 0 | 0 | 0 | 0 | 0 | 0 | 0 | 0 | 0 | 175 | 188 | Carbamidomethylation | C2:Carbamidomethylation:1000.00 | PEAKS DB |
| K.ISTTEDTYPDVPHC(+57.02)TNIFIVK.H | Y | 69.19 | 2449.1836 | 21 | -0.4 | 817.4015 | 3 | 59.42 | 4 | F4:38046 | NaNaKA16\_F12.raw |  |  |  | 3.898E6 |  |  |  |  |  |  |  |  |  | 1 | 0 | 0 | 0 | 1 | 0 | 0 | 0 | 0 | 0 | 0 | 0 | 0 | 0 | 152 | 172 | Carbamidomethylation | C14:Carbamidomethylation:1000.00 | PEAKS DB |
| R.RPVTYSTHIAPVSLPSR.S | Y | 54.67 | 1880.0267 | 17 | 0.5 | 627.6832 | 3 | 28.52 | 4 | F4:12074 | NaNaKA16\_F12.raw |  |  |  | 7.8153E6 |  |  |  |  |  |  |  |  |  | 2 | 0 | 0 | 0 | 2 | 0 | 0 | 0 | 0 | 0 | 0 | 0 | 0 | 0 | 120 | 136 |  |  | PEAKS DB |
| K.FPNGLDKDIMLIR.L | Y | 47.58 | 1530.8228 | 13 | 0.2 | 511.2816 | 3 | 62.71 | 4 | F4:40623 | NaNaKA16\_F12.raw |  |  |  | 5.0748E5 |  |  |  |  |  |  |  |  |  | 1 | 0 | 0 | 0 | 1 | 0 | 0 | 0 | 0 | 0 | 0 | 0 | 0 | 0 | 105 | 117 |  |  | PEAKS DB |
| total 4 peptides |  |  |  |  |  |  |  |  |  |  |  |  |  |  |  |  |  |  |  |  |  |  |  |  |  |  |  |  |  |  |  |  |  |  |  |  |  |  |  |  |  |  |  |
| --- | --- | --- | --- | --- | --- | --- | --- | --- | --- | --- | --- | --- | --- | --- | --- | --- | --- | --- | --- | --- | --- | --- | --- | --- | --- | --- | --- | --- | --- | --- | --- | --- | --- | --- | --- | --- | --- | --- | --- | --- | --- | --- | --- |

ADP88558.1

back to list

  

| Protein Coverage
| Supporting Peptides
|

Protein Coverage:

Supporting Peptides:

| Peptide | Uniq | -10lgP | Mass | Length | ppm | m/z | z | RT | Fraction | Scan | Source File | Area F1 | Area F10 | Area F11 | Area F12 | Area F13 | Area F2 | Area F3 | Area F4 | Area F5 | Area F6 | Area F7 | Area F8 | Area F9 | #Feature | #Feature F1 | #Feature F10 | #Feature F11 | #Feature F12 | #Feature F13 | #Feature F2 | #Feature F3 | #Feature F4 | #Feature F5 | #Feature F6 | #Feature F7 | #Feature F8 | #Feature F9 | Start | End | PTM | AScore | Found By |
| --- | --- | --- | --- | --- | --- | --- | --- | --- | --- | --- | --- | --- | --- | --- | --- | --- | --- | --- | --- | --- | --- | --- | --- | --- | --- | --- | --- | --- | --- | --- | --- | --- | --- | --- | --- | --- | --- | --- | --- | --- | --- | --- | --- |
| K.WC(+57.02)EPLYPWVPADSR.T | Y | 78.81 | 1774.8137 | 14 | 0.9 | 888.4149 | 2 | 77.31 | 4 | F4:52756 | NaNaKA16\_F12.raw |  |  | 3.2408E5 | 3.1766E6 |  |  |  |  |  |  |  |  |  | 2 | 0 | 0 | 1 | 1 | 0 | 0 | 0 | 0 | 0 | 0 | 0 | 0 | 0 | 175 | 188 | Carbamidomethylation | C2:Carbamidomethylation:1000.00 | PEAKS DB |
| K.ISTTEDTYPDVPHC(+57.02)TNIFIVK.H | Y | 69.19 | 2449.1836 | 21 | -0.4 | 817.4015 | 3 | 59.42 | 4 | F4:38046 | NaNaKA16\_F12.raw |  |  |  | 3.898E6 |  |  |  |  |  |  |  |  |  | 1 | 0 | 0 | 0 | 1 | 0 | 0 | 0 | 0 | 0 | 0 | 0 | 0 | 0 | 152 | 172 | Carbamidomethylation | C14:Carbamidomethylation:1000.00 | PEAKS DB |
| R.RPVTYSTHIAPVSLPSR.S | Y | 54.67 | 1880.0267 | 17 | 0.5 | 627.6832 | 3 | 28.52 | 4 | F4:12074 | NaNaKA16\_F12.raw |  |  |  | 7.8153E6 |  |  |  |  |  |  |  |  |  | 2 | 0 | 0 | 0 | 2 | 0 | 0 | 0 | 0 | 0 | 0 | 0 | 0 | 0 | 120 | 136 |  |  | PEAKS DB |
| K.FPNGLDKDIMLIR.L | Y | 47.58 | 1530.8228 | 13 | 0.2 | 511.2816 | 3 | 62.71 | 4 | F4:40623 | NaNaKA16\_F12.raw |  |  |  | 5.0748E5 |  |  |  |  |  |  |  |  |  | 1 | 0 | 0 | 0 | 1 | 0 | 0 | 0 | 0 | 0 | 0 | 0 | 0 | 0 | 105 | 117 |  |  | PEAKS DB |
| total 4 peptides |  |  |  |  |  |  |  |  |  |  |  |  |  |  |  |  |  |  |  |  |  |  |  |  |  |  |  |  |  |  |  |  |  |  |  |  |  |  |  |  |  |  |  |
| --- | --- | --- | --- | --- | --- | --- | --- | --- | --- | --- | --- | --- | --- | --- | --- | --- | --- | --- | --- | --- | --- | --- | --- | --- | --- | --- | --- | --- | --- | --- | --- | --- | --- | --- | --- | --- | --- | --- | --- | --- | --- | --- | --- |

LAA86163.1

back to list

  

| Protein Coverage
| Supporting Peptides
|

Protein Coverage:

Supporting Peptides:

| Peptide | Uniq | -10lgP | Mass | Length | ppm | m/z | z | RT | Fraction | Scan | Source File | Area F1 | Area F10 | Area F11 | Area F12 | Area F13 | Area F2 | Area F3 | Area F4 | Area F5 | Area F6 | Area F7 | Area F8 | Area F9 | #Feature | #Feature F1 | #Feature F10 | #Feature F11 | #Feature F12 | #Feature F13 | #Feature F2 | #Feature F3 | #Feature F4 | #Feature F5 | #Feature F6 | #Feature F7 | #Feature F8 | #Feature F9 | Start | End | PTM | AScore | Found By |
| --- | --- | --- | --- | --- | --- | --- | --- | --- | --- | --- | --- | --- | --- | --- | --- | --- | --- | --- | --- | --- | --- | --- | --- | --- | --- | --- | --- | --- | --- | --- | --- | --- | --- | --- | --- | --- | --- | --- | --- | --- | --- | --- | --- |
| R.C(+57.02)LETFGEQPLSK.K | Y | 71.04 | 1407.6704 | 12 | -0.1 | 704.8424 | 2 | 37.43 | 9 | F9:21037 | NaNaKA16\_F5.raw |  |  |  |  |  |  |  |  | 9.5826E6 |  |  |  |  | 2 | 0 | 0 | 0 | 0 | 0 | 0 | 0 | 0 | 2 | 0 | 0 | 0 | 0 | 193 | 204 | Carbamidomethylation | C1:Carbamidomethylation:1000.00 | PEAKS DB |
| K.TTAIVGNLPSSR.D | Y | 59.87 | 1214.6619 | 12 | -0.7 | 608.3378 | 2 | 27.46 | 10 | F10:13494 | NaNaKA16\_F6.raw |  |  |  |  |  |  |  |  |  | 5.8183E6 |  |  |  | 1 | 0 | 0 | 0 | 0 | 0 | 0 | 0 | 0 | 0 | 1 | 0 | 0 | 0 | 453 | 464 |  |  | PEAKS DB |
| K.DGQSISLVLK.I | Y | 58.68 | 1058.5972 | 10 | -0.3 | 530.3057 | 2 | 56.75 | 6 | F6:37699 | NaNaKA16\_F2.raw |  |  |  |  |  | 1.6308E7 | 1.6776E6 | 2.4729E5 |  | 1.2797E6 |  |  |  | 7 | 0 | 0 | 0 | 0 | 0 | 2 | 3 | 1 | 0 | 1 | 0 | 0 | 0 | 354 | 363 |  |  | PEAKS DB |
| R.VC(+57.02)IENEDSTSAVIVR.A | Y | 52.64 | 1690.8196 | 15 | 0.5 | 846.4175 | 2 | 37.00 | 10 | F10:21436 | NaNaKA16\_F6.raw |  |  |  |  |  |  |  |  |  | 6.7885E6 |  |  |  | 1 | 0 | 0 | 0 | 0 | 0 | 0 | 0 | 0 | 0 | 1 | 0 | 0 | 0 | 123 | 137 | Carbamidomethylation | C2:Carbamidomethylation:1000.00 | PEAKS DB |
| total 4 peptides |  |  |  |  |  |  |  |  |  |  |  |  |  |  |  |  |  |  |  |  |  |  |  |  |  |  |  |  |  |  |  |  |  |  |  |  |  |  |  |  |  |  |  |
| --- | --- | --- | --- | --- | --- | --- | --- | --- | --- | --- | --- | --- | --- | --- | --- | --- | --- | --- | --- | --- | --- | --- | --- | --- | --- | --- | --- | --- | --- | --- | --- | --- | --- | --- | --- | --- | --- | --- | --- | --- | --- | --- | --- |

JAA75028.1

back to list

  

| Protein Coverage
| Supporting Peptides
|

Protein Coverage:

Supporting Peptides:

| Peptide | Uniq | -10lgP | Mass | Length | ppm | m/z | z | RT | Fraction | Scan | Source File | Area F1 | Area F10 | Area F11 | Area F12 | Area F13 | Area F2 | Area F3 | Area F4 | Area F5 | Area F6 | Area F7 | Area F8 | Area F9 | #Feature | #Feature F1 | #Feature F10 | #Feature F11 | #Feature F12 | #Feature F13 | #Feature F2 | #Feature F3 | #Feature F4 | #Feature F5 | #Feature F6 | #Feature F7 | #Feature F8 | #Feature F9 | Start | End | PTM | AScore | Found By |
| --- | --- | --- | --- | --- | --- | --- | --- | --- | --- | --- | --- | --- | --- | --- | --- | --- | --- | --- | --- | --- | --- | --- | --- | --- | --- | --- | --- | --- | --- | --- | --- | --- | --- | --- | --- | --- | --- | --- | --- | --- | --- | --- | --- |
| E.GGSGTPVDDLDR.C | N | 67.81 | 1187.5417 | 12 | 0.8 | 594.7786 | 2 | 19.63 | 10 | F10:6988 | NaNaKA16\_F6.raw |  |  |  |  |  |  |  |  |  | 5.7224E7 | 2.6509E9 | 3.9395E6 |  | 4 | 0 | 0 | 0 | 0 | 0 | 0 | 0 | 0 | 0 | 2 | 1 | 1 | 0 | 59 | 70 |  |  | PEAKS DB |
| G.GSGTPVDDLDR.C | N | 60.74 | 1130.5204 | 11 | -0.3 | 566.2673 | 2 | 21.08 | 11 | F11:8172 | NaNaKA16\_F7.raw |  |  |  |  |  |  |  |  |  | 1.4052E5 | 1.2671E7 |  |  | 2 | 0 | 0 | 0 | 0 | 0 | 0 | 0 | 0 | 0 | 1 | 1 | 0 | 0 | 60 | 70 |  |  | PEAKS DB |
| M.DYGC(+57.02)YC(+57.02)GEGGSGTPVDDLDR.C | Y | 53.94 | 2191.8423 | 20 | -8.0 | 1096.9197 | 2 | 53.56 | 10 | F10:35748 | NaNaKA16\_F6.raw |  |  |  |  |  |  |  |  |  | 7.4633E6 |  |  |  | 2 | 0 | 0 | 0 | 0 | 0 | 0 | 0 | 0 | 0 | 2 | 0 | 0 | 0 | 51 | 70 | Carbamidomethylation | C4:Carbamidomethylation:1000.00;C6:Carbamidomethylation:1000.00 | PEAKS DB |
| G.SGTPVDDLDR.C | N | 45.52 | 1073.4989 | 10 | 0.9 | 537.7572 | 2 | 20.23 | 11 | F11:7497 | NaNaKA16\_F7.raw |  |  |  |  |  |  |  |  |  |  | 1.1072E7 |  |  | 1 | 0 | 0 | 0 | 0 | 0 | 0 | 0 | 0 | 0 | 0 | 1 | 0 | 0 | 61 | 70 |  |  | PEAKS DB |
| total 4 peptides |  |  |  |  |  |  |  |  |  |  |  |  |  |  |  |  |  |  |  |  |  |  |  |  |  |  |  |  |  |  |  |  |  |  |  |  |  |  |  |  |  |  |  |
| --- | --- | --- | --- | --- | --- | --- | --- | --- | --- | --- | --- | --- | --- | --- | --- | --- | --- | --- | --- | --- | --- | --- | --- | --- | --- | --- | --- | --- | --- | --- | --- | --- | --- | --- | --- | --- | --- | --- | --- | --- | --- | --- | --- |

AAZ39880.1

back to list

  

| Protein Coverage
| Supporting Peptides
|

Protein Coverage:

Supporting Peptides:

| Peptide | Uniq | -10lgP | Mass | Length | ppm | m/z | z | RT | Fraction | Scan | Source File | Area F1 | Area F10 | Area F11 | Area F12 | Area F13 | Area F2 | Area F3 | Area F4 | Area F5 | Area F6 | Area F7 | Area F8 | Area F9 | #Feature | #Feature F1 | #Feature F10 | #Feature F11 | #Feature F12 | #Feature F13 | #Feature F2 | #Feature F3 | #Feature F4 | #Feature F5 | #Feature F6 | #Feature F7 | #Feature F8 | #Feature F9 | Start | End | PTM | AScore | Found By |
| --- | --- | --- | --- | --- | --- | --- | --- | --- | --- | --- | --- | --- | --- | --- | --- | --- | --- | --- | --- | --- | --- | --- | --- | --- | --- | --- | --- | --- | --- | --- | --- | --- | --- | --- | --- | --- | --- | --- | --- | --- | --- | --- | --- |
| R.FLTEHNPEC(+57.02)IINPPLR.T | Y | 78.43 | 1948.9829 | 16 | -0.1 | 650.6682 | 3 | 50.62 | 4 | F4:30756 | NaNaKA16\_F12.raw |  |  |  | 4.0513E6 |  |  |  |  |  |  |  |  |  | 1 | 0 | 0 | 0 | 1 | 0 | 0 | 0 | 0 | 0 | 0 | 0 | 0 | 0 | 386 | 401 | Carbamidomethylation | C9:Carbamidomethylation:1000.00 | PEAKS DB |
| R.TDIVSPPAC(+57.02)GNELLER.G | Y | 65.09 | 1769.8618 | 16 | 1.0 | 885.9391 | 2 | 51.94 | 4 | F4:32003 | NaNaKA16\_F12.raw |  |  |  | 2.786E6 |  |  |  |  |  |  |  |  |  | 1 | 0 | 0 | 0 | 1 | 0 | 0 | 0 | 0 | 0 | 0 | 0 | 0 | 0 | 402 | 417 | Carbamidomethylation | C9:Carbamidomethylation:1000.00 | PEAKS DB |
| N.VTLDLFGEWR.K | N | 61.73 | 1234.6346 | 10 | 0.5 | 618.3249 | 2 | 82.47 | 11 | F11:57626 | NaNaKA16\_F7.raw |  |  |  |  |  |  |  |  |  |  | 1.9987E5 | 4.7038E5 |  | 2 | 0 | 0 | 0 | 0 | 0 | 0 | 0 | 0 | 0 | 0 | 1 | 1 | 0 | 270 | 279 |  |  | PEAKS DB |
| total 3 peptides |  |  |  |  |  |  |  |  |  |  |  |  |  |  |  |  |  |  |  |  |  |  |  |  |  |  |  |  |  |  |  |  |  |  |  |  |  |  |  |  |  |  |  |
| --- | --- | --- | --- | --- | --- | --- | --- | --- | --- | --- | --- | --- | --- | --- | --- | --- | --- | --- | --- | --- | --- | --- | --- | --- | --- | --- | --- | --- | --- | --- | --- | --- | --- | --- | --- | --- | --- | --- | --- | --- | --- | --- | --- |

B8K1W0.1

back to list

  

| Protein Coverage
| Supporting Peptides
|

Protein Coverage:

Supporting Peptides:

| Peptide | Uniq | -10lgP | Mass | Length | ppm | m/z | z | RT | Fraction | Scan | Source File | Area F1 | Area F10 | Area F11 | Area F12 | Area F13 | Area F2 | Area F3 | Area F4 | Area F5 | Area F6 | Area F7 | Area F8 | Area F9 | #Feature | #Feature F1 | #Feature F10 | #Feature F11 | #Feature F12 | #Feature F13 | #Feature F2 | #Feature F3 | #Feature F4 | #Feature F5 | #Feature F6 | #Feature F7 | #Feature F8 | #Feature F9 | Start | End | PTM | AScore | Found By |
| --- | --- | --- | --- | --- | --- | --- | --- | --- | --- | --- | --- | --- | --- | --- | --- | --- | --- | --- | --- | --- | --- | --- | --- | --- | --- | --- | --- | --- | --- | --- | --- | --- | --- | --- | --- | --- | --- | --- | --- | --- | --- | --- | --- |
| R.FLTEHNPEC(+57.02)IINPPLR.T | Y | 78.43 | 1948.9829 | 16 | -0.1 | 650.6682 | 3 | 50.62 | 4 | F4:30756 | NaNaKA16\_F12.raw |  |  |  | 4.0513E6 |  |  |  |  |  |  |  |  |  | 1 | 0 | 0 | 0 | 1 | 0 | 0 | 0 | 0 | 0 | 0 | 0 | 0 | 0 | 386 | 401 | Carbamidomethylation | C9:Carbamidomethylation:1000.00 | PEAKS DB |
| R.TDIVSPPAC(+57.02)GNELLER.G | Y | 65.09 | 1769.8618 | 16 | 1.0 | 885.9391 | 2 | 51.94 | 4 | F4:32003 | NaNaKA16\_F12.raw |  |  |  | 2.786E6 |  |  |  |  |  |  |  |  |  | 1 | 0 | 0 | 0 | 1 | 0 | 0 | 0 | 0 | 0 | 0 | 0 | 0 | 0 | 402 | 417 | Carbamidomethylation | C9:Carbamidomethylation:1000.00 | PEAKS DB |
| N.VTLDLFGEWR.K | N | 61.73 | 1234.6346 | 10 | 0.5 | 618.3249 | 2 | 82.47 | 11 | F11:57626 | NaNaKA16\_F7.raw |  |  |  |  |  |  |  |  |  |  | 1.9987E5 | 4.7038E5 |  | 2 | 0 | 0 | 0 | 0 | 0 | 0 | 0 | 0 | 0 | 0 | 1 | 1 | 0 | 270 | 279 |  |  | PEAKS DB |
| total 3 peptides |  |  |  |  |  |  |  |  |  |  |  |  |  |  |  |  |  |  |  |  |  |  |  |  |  |  |  |  |  |  |  |  |  |  |  |  |  |  |  |  |  |  |  |
| --- | --- | --- | --- | --- | --- | --- | --- | --- | --- | --- | --- | --- | --- | --- | --- | --- | --- | --- | --- | --- | --- | --- | --- | --- | --- | --- | --- | --- | --- | --- | --- | --- | --- | --- | --- | --- | --- | --- | --- | --- | --- | --- | --- |

Q9W727.1

back to list

  

| Protein Coverage
| Supporting Peptides
|

Protein Coverage:

Supporting Peptides:

| Peptide | Uniq | -10lgP | Mass | Length | ppm | m/z | z | RT | Fraction | Scan | Source File | Area F1 | Area F10 | Area F11 | Area F12 | Area F13 | Area F2 | Area F3 | Area F4 | Area F5 | Area F6 | Area F7 | Area F8 | Area F9 | #Feature | #Feature F1 | #Feature F10 | #Feature F11 | #Feature F12 | #Feature F13 | #Feature F2 | #Feature F3 | #Feature F4 | #Feature F5 | #Feature F6 | #Feature F7 | #Feature F8 | #Feature F9 | Start | End | PTM | AScore | Found By |
| --- | --- | --- | --- | --- | --- | --- | --- | --- | --- | --- | --- | --- | --- | --- | --- | --- | --- | --- | --- | --- | --- | --- | --- | --- | --- | --- | --- | --- | --- | --- | --- | --- | --- | --- | --- | --- | --- | --- | --- | --- | --- | --- | --- |
| K.ISLADGNDVR.I | N | 62.61 | 1058.5356 | 10 | -0.5 | 530.2748 | 2 | 22.28 | 11 | F11:9363 | NaNaKA16\_F7.raw |  |  |  |  |  |  |  |  |  | 3.5268E6 | 5.5622E7 |  |  | 4 | 0 | 0 | 0 | 0 | 0 | 0 | 0 | 0 | 0 | 2 | 2 | 0 | 0 | 48 | 57 |  |  | PEAKS DB |
| R.TPETTEIC(+57.02)PDSWYFC(+57.02)YK.I | Y | 57.52 | 2195.9180 | 17 | -0.8 | 1098.9653 | 2 | 71.57 | 10 | F10:50882 | NaNaKA16\_F6.raw |  |  |  |  |  |  |  |  |  | 1.6607E6 |  |  |  | 1 | 0 | 0 | 0 | 0 | 0 | 0 | 0 | 0 | 0 | 1 | 0 | 0 | 0 | 31 | 47 | Carbamidomethylation | C8:Carbamidomethylation:1000.00;C15:Carbamidomethylation:1000.00 | PEAKS DB |
| R.GC(+57.02)TFTC(+57.02)PELR.P | N | 45.94 | 1239.5376 | 10 | 0.0 | 620.7761 | 2 | 30.33 | 10 | F10:15872 | NaNaKA16\_F6.raw |  |  |  |  |  |  |  |  |  | 1.838E7 |  |  |  | 1 | 0 | 0 | 0 | 0 | 0 | 0 | 0 | 0 | 0 | 1 | 0 | 0 | 0 | 61 | 70 | Carbamidomethylation | C2:Carbamidomethylation:1000.00;C6:Carbamidomethylation:1000.00 | PEAKS DB |
| E.TTEIC(+57.02)PDSWYFC(+57.02)YK.I | N | 42.51 | 1868.7749 | 14 | 1.2 | 935.3958 | 2 | 68.81 | 10 | F10:48624 | NaNaKA16\_F6.raw |  |  |  |  |  |  |  |  |  | 1.3848E6 |  |  |  | 1 | 0 | 0 | 0 | 0 | 0 | 0 | 0 | 0 | 0 | 1 | 0 | 0 | 0 | 34 | 47 | Carbamidomethylation | C5:Carbamidomethylation:1000.00;C12:Carbamidomethylation:1000.00 | PEAKS DB |
| total 4 peptides |  |  |  |  |  |  |  |  |  |  |  |  |  |  |  |  |  |  |  |  |  |  |  |  |  |  |  |  |  |  |  |  |  |  |  |  |  |  |  |  |  |  |  |
| --- | --- | --- | --- | --- | --- | --- | --- | --- | --- | --- | --- | --- | --- | --- | --- | --- | --- | --- | --- | --- | --- | --- | --- | --- | --- | --- | --- | --- | --- | --- | --- | --- | --- | --- | --- | --- | --- | --- | --- | --- | --- | --- | --- |

Q9DEQ3.1

back to list

  

| Protein Coverage
| Supporting Peptides
|

Protein Coverage:

Supporting Peptides:

| Peptide | Uniq | -10lgP | Mass | Length | ppm | m/z | z | RT | Fraction | Scan | Source File | Area F1 | Area F10 | Area F11 | Area F12 | Area F13 | Area F2 | Area F3 | Area F4 | Area F5 | Area F6 | Area F7 | Area F8 | Area F9 | #Feature | #Feature F1 | #Feature F10 | #Feature F11 | #Feature F12 | #Feature F13 | #Feature F2 | #Feature F3 | #Feature F4 | #Feature F5 | #Feature F6 | #Feature F7 | #Feature F8 | #Feature F9 | Start | End | PTM | AScore | Found By |
| --- | --- | --- | --- | --- | --- | --- | --- | --- | --- | --- | --- | --- | --- | --- | --- | --- | --- | --- | --- | --- | --- | --- | --- | --- | --- | --- | --- | --- | --- | --- | --- | --- | --- | --- | --- | --- | --- | --- | --- | --- | --- | --- | --- |
| K.ISLADGNDVR.I | N | 62.61 | 1058.5356 | 10 | -0.5 | 530.2748 | 2 | 22.28 | 11 | F11:9363 | NaNaKA16\_F7.raw |  |  |  |  |  |  |  |  |  | 3.5268E6 | 5.5622E7 |  |  | 4 | 0 | 0 | 0 | 0 | 0 | 0 | 0 | 0 | 0 | 2 | 2 | 0 | 0 | 48 | 57 |  |  | PEAKS DB |
| R.TPETTEIC(+57.02)PDSWYFC(+57.02)YK.I | Y | 57.52 | 2195.9180 | 17 | -0.8 | 1098.9653 | 2 | 71.57 | 10 | F10:50882 | NaNaKA16\_F6.raw |  |  |  |  |  |  |  |  |  | 1.6607E6 |  |  |  | 1 | 0 | 0 | 0 | 0 | 0 | 0 | 0 | 0 | 0 | 1 | 0 | 0 | 0 | 31 | 47 | Carbamidomethylation | C8:Carbamidomethylation:1000.00;C15:Carbamidomethylation:1000.00 | PEAKS DB |
| R.GC(+57.02)TFTC(+57.02)PELR.P | N | 45.94 | 1239.5376 | 10 | 0.0 | 620.7761 | 2 | 30.33 | 10 | F10:15872 | NaNaKA16\_F6.raw |  |  |  |  |  |  |  |  |  | 1.838E7 |  |  |  | 1 | 0 | 0 | 0 | 0 | 0 | 0 | 0 | 0 | 0 | 1 | 0 | 0 | 0 | 61 | 70 | Carbamidomethylation | C2:Carbamidomethylation:1000.00;C6:Carbamidomethylation:1000.00 | PEAKS DB |
| E.TTEIC(+57.02)PDSWYFC(+57.02)YK.I | N | 42.51 | 1868.7749 | 14 | 1.2 | 935.3958 | 2 | 68.81 | 10 | F10:48624 | NaNaKA16\_F6.raw |  |  |  |  |  |  |  |  |  | 1.3848E6 |  |  |  | 1 | 0 | 0 | 0 | 0 | 0 | 0 | 0 | 0 | 0 | 1 | 0 | 0 | 0 | 34 | 47 | Carbamidomethylation | C5:Carbamidomethylation:1000.00;C12:Carbamidomethylation:1000.00 | PEAKS DB |
| total 4 peptides |  |  |  |  |  |  |  |  |  |  |  |  |  |  |  |  |  |  |  |  |  |  |  |  |  |  |  |  |  |  |  |  |  |  |  |  |  |  |  |  |  |  |  |
| --- | --- | --- | --- | --- | --- | --- | --- | --- | --- | --- | --- | --- | --- | --- | --- | --- | --- | --- | --- | --- | --- | --- | --- | --- | --- | --- | --- | --- | --- | --- | --- | --- | --- | --- | --- | --- | --- | --- | --- | --- | --- | --- | --- |

CAB50691.1

back to list

  

| Protein Coverage
| Supporting Peptides
|

Protein Coverage:

Supporting Peptides:

| Peptide | Uniq | -10lgP | Mass | Length | ppm | m/z | z | RT | Fraction | Scan | Source File | Area F1 | Area F10 | Area F11 | Area F12 | Area F13 | Area F2 | Area F3 | Area F4 | Area F5 | Area F6 | Area F7 | Area F8 | Area F9 | #Feature | #Feature F1 | #Feature F10 | #Feature F11 | #Feature F12 | #Feature F13 | #Feature F2 | #Feature F3 | #Feature F4 | #Feature F5 | #Feature F6 | #Feature F7 | #Feature F8 | #Feature F9 | Start | End | PTM | AScore | Found By |
| --- | --- | --- | --- | --- | --- | --- | --- | --- | --- | --- | --- | --- | --- | --- | --- | --- | --- | --- | --- | --- | --- | --- | --- | --- | --- | --- | --- | --- | --- | --- | --- | --- | --- | --- | --- | --- | --- | --- | --- | --- | --- | --- | --- |
| K.ISLADGNDVR.I | N | 62.61 | 1058.5356 | 10 | -0.5 | 530.2748 | 2 | 22.28 | 11 | F11:9363 | NaNaKA16\_F7.raw |  |  |  |  |  |  |  |  |  | 3.5268E6 | 5.5622E7 |  |  | 4 | 0 | 0 | 0 | 0 | 0 | 0 | 0 | 0 | 0 | 2 | 2 | 0 | 0 | 48 | 57 |  |  | PEAKS DB |
| R.TPETTEIC(+57.02)PDSWYFC(+57.02)YK.I | Y | 57.52 | 2195.9180 | 17 | -0.8 | 1098.9653 | 2 | 71.57 | 10 | F10:50882 | NaNaKA16\_F6.raw |  |  |  |  |  |  |  |  |  | 1.6607E6 |  |  |  | 1 | 0 | 0 | 0 | 0 | 0 | 0 | 0 | 0 | 0 | 1 | 0 | 0 | 0 | 31 | 47 | Carbamidomethylation | C8:Carbamidomethylation:1000.00;C15:Carbamidomethylation:1000.00 | PEAKS DB |
| R.GC(+57.02)TFTC(+57.02)PELR.P | N | 45.94 | 1239.5376 | 10 | 0.0 | 620.7761 | 2 | 30.33 | 10 | F10:15872 | NaNaKA16\_F6.raw |  |  |  |  |  |  |  |  |  | 1.838E7 |  |  |  | 1 | 0 | 0 | 0 | 0 | 0 | 0 | 0 | 0 | 0 | 1 | 0 | 0 | 0 | 61 | 70 | Carbamidomethylation | C2:Carbamidomethylation:1000.00;C6:Carbamidomethylation:1000.00 | PEAKS DB |
| E.TTEIC(+57.02)PDSWYFC(+57.02)YK.I | N | 42.51 | 1868.7749 | 14 | 1.2 | 935.3958 | 2 | 68.81 | 10 | F10:48624 | NaNaKA16\_F6.raw |  |  |  |  |  |  |  |  |  | 1.3848E6 |  |  |  | 1 | 0 | 0 | 0 | 0 | 0 | 0 | 0 | 0 | 0 | 1 | 0 | 0 | 0 | 34 | 47 | Carbamidomethylation | C5:Carbamidomethylation:1000.00;C12:Carbamidomethylation:1000.00 | PEAKS DB |
| total 4 peptides |  |  |  |  |  |  |  |  |  |  |  |  |  |  |  |  |  |  |  |  |  |  |  |  |  |  |  |  |  |  |  |  |  |  |  |  |  |  |  |  |  |  |  |
| --- | --- | --- | --- | --- | --- | --- | --- | --- | --- | --- | --- | --- | --- | --- | --- | --- | --- | --- | --- | --- | --- | --- | --- | --- | --- | --- | --- | --- | --- | --- | --- | --- | --- | --- | --- | --- | --- | --- | --- | --- | --- | --- | --- |

CAC08183.1

back to list

  

| Protein Coverage
| Supporting Peptides
|

Protein Coverage:

Supporting Peptides:

| Peptide | Uniq | -10lgP | Mass | Length | ppm | m/z | z | RT | Fraction | Scan | Source File | Area F1 | Area F10 | Area F11 | Area F12 | Area F13 | Area F2 | Area F3 | Area F4 | Area F5 | Area F6 | Area F7 | Area F8 | Area F9 | #Feature | #Feature F1 | #Feature F10 | #Feature F11 | #Feature F12 | #Feature F13 | #Feature F2 | #Feature F3 | #Feature F4 | #Feature F5 | #Feature F6 | #Feature F7 | #Feature F8 | #Feature F9 | Start | End | PTM | AScore | Found By |
| --- | --- | --- | --- | --- | --- | --- | --- | --- | --- | --- | --- | --- | --- | --- | --- | --- | --- | --- | --- | --- | --- | --- | --- | --- | --- | --- | --- | --- | --- | --- | --- | --- | --- | --- | --- | --- | --- | --- | --- | --- | --- | --- | --- |
| K.ISLADGNDVR.I | N | 62.61 | 1058.5356 | 10 | -0.5 | 530.2748 | 2 | 22.28 | 11 | F11:9363 | NaNaKA16\_F7.raw |  |  |  |  |  |  |  |  |  | 3.5268E6 | 5.5622E7 |  |  | 4 | 0 | 0 | 0 | 0 | 0 | 0 | 0 | 0 | 0 | 2 | 2 | 0 | 0 | 48 | 57 |  |  | PEAKS DB |
| R.TPETTEIC(+57.02)PDSWYFC(+57.02)YK.I | Y | 57.52 | 2195.9180 | 17 | -0.8 | 1098.9653 | 2 | 71.57 | 10 | F10:50882 | NaNaKA16\_F6.raw |  |  |  |  |  |  |  |  |  | 1.6607E6 |  |  |  | 1 | 0 | 0 | 0 | 0 | 0 | 0 | 0 | 0 | 0 | 1 | 0 | 0 | 0 | 31 | 47 | Carbamidomethylation | C8:Carbamidomethylation:1000.00;C15:Carbamidomethylation:1000.00 | PEAKS DB |
| R.GC(+57.02)TFTC(+57.02)PELR.P | N | 45.94 | 1239.5376 | 10 | 0.0 | 620.7761 | 2 | 30.33 | 10 | F10:15872 | NaNaKA16\_F6.raw |  |  |  |  |  |  |  |  |  | 1.838E7 |  |  |  | 1 | 0 | 0 | 0 | 0 | 0 | 0 | 0 | 0 | 0 | 1 | 0 | 0 | 0 | 61 | 70 | Carbamidomethylation | C2:Carbamidomethylation:1000.00;C6:Carbamidomethylation:1000.00 | PEAKS DB |
| E.TTEIC(+57.02)PDSWYFC(+57.02)YK.I | N | 42.51 | 1868.7749 | 14 | 1.2 | 935.3958 | 2 | 68.81 | 10 | F10:48624 | NaNaKA16\_F6.raw |  |  |  |  |  |  |  |  |  | 1.3848E6 |  |  |  | 1 | 0 | 0 | 0 | 0 | 0 | 0 | 0 | 0 | 0 | 1 | 0 | 0 | 0 | 34 | 47 | Carbamidomethylation | C5:Carbamidomethylation:1000.00;C12:Carbamidomethylation:1000.00 | PEAKS DB |
| total 4 peptides |  |  |  |  |  |  |  |  |  |  |  |  |  |  |  |  |  |  |  |  |  |  |  |  |  |  |  |  |  |  |  |  |  |  |  |  |  |  |  |  |  |  |  |
| --- | --- | --- | --- | --- | --- | --- | --- | --- | --- | --- | --- | --- | --- | --- | --- | --- | --- | --- | --- | --- | --- | --- | --- | --- | --- | --- | --- | --- | --- | --- | --- | --- | --- | --- | --- | --- | --- | --- | --- | --- | --- | --- | --- |

XP\_032075406.1

back to list

  

| Protein Coverage
| Supporting Peptides
|

Protein Coverage:

Supporting Peptides:

| Peptide | Uniq | -10lgP | Mass | Length | ppm | m/z | z | RT | Fraction | Scan | Source File | Area F1 | Area F10 | Area F11 | Area F12 | Area F13 | Area F2 | Area F3 | Area F4 | Area F5 | Area F6 | Area F7 | Area F8 | Area F9 | #Feature | #Feature F1 | #Feature F10 | #Feature F11 | #Feature F12 | #Feature F13 | #Feature F2 | #Feature F3 | #Feature F4 | #Feature F5 | #Feature F6 | #Feature F7 | #Feature F8 | #Feature F9 | Start | End | PTM | AScore | Found By |
| --- | --- | --- | --- | --- | --- | --- | --- | --- | --- | --- | --- | --- | --- | --- | --- | --- | --- | --- | --- | --- | --- | --- | --- | --- | --- | --- | --- | --- | --- | --- | --- | --- | --- | --- | --- | --- | --- | --- | --- | --- | --- | --- | --- |
| K.GMNLHDYGMLLPC(+57.02)GIDK.F | Y | 84.67 | 1932.8896 | 17 | 1.6 | 967.4536 | 2 | 65.84 | 9 | F9:45212 | NaNaKA16\_F5.raw |  |  |  |  |  |  |  |  | 3.28E7 |  |  |  |  | 2 | 0 | 0 | 0 | 0 | 0 | 0 | 0 | 0 | 2 | 0 | 0 | 0 | 0 | 163 | 179 | Carbamidomethylation | C13:Carbamidomethylation:1000.00 | PEAKS DB |
| R.MDIC(+57.02)ETHLHWHTVAK.E | Y | 79.85 | 1876.8712 | 15 | -0.1 | 626.6310 | 3 | 22.06 | 9 | F9:9168 | NaNaKA16\_F5.raw |  |  |  |  |  |  |  |  | 1.6968E7 |  |  |  |  | 2 | 0 | 0 | 0 | 0 | 0 | 0 | 0 | 0 | 2 | 0 | 0 | 0 | 0 | 142 | 156 | Carbamidomethylation | C4:Carbamidomethylation:1000.00 | PEAKS DB |
| total 2 peptides |  |  |  |  |  |  |  |  |  |  |  |  |  |  |  |  |  |  |  |  |  |  |  |  |  |  |  |  |  |  |  |  |  |  |  |  |  |  |  |  |  |  |  |
| --- | --- | --- | --- | --- | --- | --- | --- | --- | --- | --- | --- | --- | --- | --- | --- | --- | --- | --- | --- | --- | --- | --- | --- | --- | --- | --- | --- | --- | --- | --- | --- | --- | --- | --- | --- | --- | --- | --- | --- | --- | --- | --- | --- |

XP\_013912685.1

back to list

  

| Protein Coverage
| Supporting Peptides
|

Protein Coverage:

Supporting Peptides:

| Peptide | Uniq | -10lgP | Mass | Length | ppm | m/z | z | RT | Fraction | Scan | Source File | Area F1 | Area F10 | Area F11 | Area F12 | Area F13 | Area F2 | Area F3 | Area F4 | Area F5 | Area F6 | Area F7 | Area F8 | Area F9 | #Feature | #Feature F1 | #Feature F10 | #Feature F11 | #Feature F12 | #Feature F13 | #Feature F2 | #Feature F3 | #Feature F4 | #Feature F5 | #Feature F6 | #Feature F7 | #Feature F8 | #Feature F9 | Start | End | PTM | AScore | Found By |
| --- | --- | --- | --- | --- | --- | --- | --- | --- | --- | --- | --- | --- | --- | --- | --- | --- | --- | --- | --- | --- | --- | --- | --- | --- | --- | --- | --- | --- | --- | --- | --- | --- | --- | --- | --- | --- | --- | --- | --- | --- | --- | --- | --- |
| K.GMNLHDYGMLLPC(+57.02)GIDK.F | Y | 84.67 | 1932.8896 | 17 | 1.6 | 967.4536 | 2 | 65.84 | 9 | F9:45212 | NaNaKA16\_F5.raw |  |  |  |  |  |  |  |  | 3.28E7 |  |  |  |  | 2 | 0 | 0 | 0 | 0 | 0 | 0 | 0 | 0 | 2 | 0 | 0 | 0 | 0 | 163 | 179 | Carbamidomethylation | C13:Carbamidomethylation:1000.00 | PEAKS DB |
| R.MDIC(+57.02)ETHLHWHTVAK.E | Y | 79.85 | 1876.8712 | 15 | -0.1 | 626.6310 | 3 | 22.06 | 9 | F9:9168 | NaNaKA16\_F5.raw |  |  |  |  |  |  |  |  | 1.6968E7 |  |  |  |  | 2 | 0 | 0 | 0 | 0 | 0 | 0 | 0 | 0 | 2 | 0 | 0 | 0 | 0 | 142 | 156 | Carbamidomethylation | C4:Carbamidomethylation:1000.00 | PEAKS DB |
| total 2 peptides |  |  |  |  |  |  |  |  |  |  |  |  |  |  |  |  |  |  |  |  |  |  |  |  |  |  |  |  |  |  |  |  |  |  |  |  |  |  |  |  |  |  |  |
| --- | --- | --- | --- | --- | --- | --- | --- | --- | --- | --- | --- | --- | --- | --- | --- | --- | --- | --- | --- | --- | --- | --- | --- | --- | --- | --- | --- | --- | --- | --- | --- | --- | --- | --- | --- | --- | --- | --- | --- | --- | --- | --- | --- |

LAB26479.1

back to list

  

| Protein Coverage
| Supporting Peptides
|

Protein Coverage:

Supporting Peptides:

| Peptide | Uniq | -10lgP | Mass | Length | ppm | m/z | z | RT | Fraction | Scan | Source File | Area F1 | Area F10 | Area F11 | Area F12 | Area F13 | Area F2 | Area F3 | Area F4 | Area F5 | Area F6 | Area F7 | Area F8 | Area F9 | #Feature | #Feature F1 | #Feature F10 | #Feature F11 | #Feature F12 | #Feature F13 | #Feature F2 | #Feature F3 | #Feature F4 | #Feature F5 | #Feature F6 | #Feature F7 | #Feature F8 | #Feature F9 | Start | End | PTM | AScore | Found By |
| --- | --- | --- | --- | --- | --- | --- | --- | --- | --- | --- | --- | --- | --- | --- | --- | --- | --- | --- | --- | --- | --- | --- | --- | --- | --- | --- | --- | --- | --- | --- | --- | --- | --- | --- | --- | --- | --- | --- | --- | --- | --- | --- | --- |
| K.GMNLHDYGMLLPC(+57.02)GIDK.F | Y | 84.67 | 1932.8896 | 17 | 1.6 | 967.4536 | 2 | 65.84 | 9 | F9:45212 | NaNaKA16\_F5.raw |  |  |  |  |  |  |  |  | 3.28E7 |  |  |  |  | 2 | 0 | 0 | 0 | 0 | 0 | 0 | 0 | 0 | 2 | 0 | 0 | 0 | 0 | 163 | 179 | Carbamidomethylation | C13:Carbamidomethylation:1000.00 | PEAKS DB |
| R.MDIC(+57.02)ETHLHWHTVAK.E | Y | 79.85 | 1876.8712 | 15 | -0.1 | 626.6310 | 3 | 22.06 | 9 | F9:9168 | NaNaKA16\_F5.raw |  |  |  |  |  |  |  |  | 1.6968E7 |  |  |  |  | 2 | 0 | 0 | 0 | 0 | 0 | 0 | 0 | 0 | 2 | 0 | 0 | 0 | 0 | 142 | 156 | Carbamidomethylation | C4:Carbamidomethylation:1000.00 | PEAKS DB |
| total 2 peptides |  |  |  |  |  |  |  |  |  |  |  |  |  |  |  |  |  |  |  |  |  |  |  |  |  |  |  |  |  |  |  |  |  |  |  |  |  |  |  |  |  |  |  |
| --- | --- | --- | --- | --- | --- | --- | --- | --- | --- | --- | --- | --- | --- | --- | --- | --- | --- | --- | --- | --- | --- | --- | --- | --- | --- | --- | --- | --- | --- | --- | --- | --- | --- | --- | --- | --- | --- | --- | --- | --- | --- | --- | --- |

XP\_026519907.1

back to list

  

| Protein Coverage
| Supporting Peptides
|

Protein Coverage:

Supporting Peptides:

| Peptide | Uniq | -10lgP | Mass | Length | ppm | m/z | z | RT | Fraction | Scan | Source File | Area F1 | Area F10 | Area F11 | Area F12 | Area F13 | Area F2 | Area F3 | Area F4 | Area F5 | Area F6 | Area F7 | Area F8 | Area F9 | #Feature | #Feature F1 | #Feature F10 | #Feature F11 | #Feature F12 | #Feature F13 | #Feature F2 | #Feature F3 | #Feature F4 | #Feature F5 | #Feature F6 | #Feature F7 | #Feature F8 | #Feature F9 | Start | End | PTM | AScore | Found By |
| --- | --- | --- | --- | --- | --- | --- | --- | --- | --- | --- | --- | --- | --- | --- | --- | --- | --- | --- | --- | --- | --- | --- | --- | --- | --- | --- | --- | --- | --- | --- | --- | --- | --- | --- | --- | --- | --- | --- | --- | --- | --- | --- | --- |
| K.GMNLHDYGMLLPC(+57.02)GIDK.F | Y | 84.67 | 1932.8896 | 17 | 1.6 | 967.4536 | 2 | 65.84 | 9 | F9:45212 | NaNaKA16\_F5.raw |  |  |  |  |  |  |  |  | 3.28E7 |  |  |  |  | 2 | 0 | 0 | 0 | 0 | 0 | 0 | 0 | 0 | 2 | 0 | 0 | 0 | 0 | 163 | 179 | Carbamidomethylation | C13:Carbamidomethylation:1000.00 | PEAKS DB |
| R.MDIC(+57.02)ETHLHWHTVAK.E | Y | 79.85 | 1876.8712 | 15 | -0.1 | 626.6310 | 3 | 22.06 | 9 | F9:9168 | NaNaKA16\_F5.raw |  |  |  |  |  |  |  |  | 1.6968E7 |  |  |  |  | 2 | 0 | 0 | 0 | 0 | 0 | 0 | 0 | 0 | 2 | 0 | 0 | 0 | 0 | 142 | 156 | Carbamidomethylation | C4:Carbamidomethylation:1000.00 | PEAKS DB |
| total 2 peptides |  |  |  |  |  |  |  |  |  |  |  |  |  |  |  |  |  |  |  |  |  |  |  |  |  |  |  |  |  |  |  |  |  |  |  |  |  |  |  |  |  |  |  |
| --- | --- | --- | --- | --- | --- | --- | --- | --- | --- | --- | --- | --- | --- | --- | --- | --- | --- | --- | --- | --- | --- | --- | --- | --- | --- | --- | --- | --- | --- | --- | --- | --- | --- | --- | --- | --- | --- | --- | --- | --- | --- | --- | --- |

XP\_026556422.1

back to list

  

| Protein Coverage
| Supporting Peptides
|

Protein Coverage:

Supporting Peptides:

| Peptide | Uniq | -10lgP | Mass | Length | ppm | m/z | z | RT | Fraction | Scan | Source File | Area F1 | Area F10 | Area F11 | Area F12 | Area F13 | Area F2 | Area F3 | Area F4 | Area F5 | Area F6 | Area F7 | Area F8 | Area F9 | #Feature | #Feature F1 | #Feature F10 | #Feature F11 | #Feature F12 | #Feature F13 | #Feature F2 | #Feature F3 | #Feature F4 | #Feature F5 | #Feature F6 | #Feature F7 | #Feature F8 | #Feature F9 | Start | End | PTM | AScore | Found By |
| --- | --- | --- | --- | --- | --- | --- | --- | --- | --- | --- | --- | --- | --- | --- | --- | --- | --- | --- | --- | --- | --- | --- | --- | --- | --- | --- | --- | --- | --- | --- | --- | --- | --- | --- | --- | --- | --- | --- | --- | --- | --- | --- | --- |
| K.GMNLHDYGMLLPC(+57.02)GIDK.F | Y | 84.67 | 1932.8896 | 17 | 1.6 | 967.4536 | 2 | 65.84 | 9 | F9:45212 | NaNaKA16\_F5.raw |  |  |  |  |  |  |  |  | 3.28E7 |  |  |  |  | 2 | 0 | 0 | 0 | 0 | 0 | 0 | 0 | 0 | 2 | 0 | 0 | 0 | 0 | 163 | 179 | Carbamidomethylation | C13:Carbamidomethylation:1000.00 | PEAKS DB |
| R.MDIC(+57.02)ETHLHWHTVAK.E | Y | 79.85 | 1876.8712 | 15 | -0.1 | 626.6310 | 3 | 22.06 | 9 | F9:9168 | NaNaKA16\_F5.raw |  |  |  |  |  |  |  |  | 1.6968E7 |  |  |  |  | 2 | 0 | 0 | 0 | 0 | 0 | 0 | 0 | 0 | 2 | 0 | 0 | 0 | 0 | 142 | 156 | Carbamidomethylation | C4:Carbamidomethylation:1000.00 | PEAKS DB |
| total 2 peptides |  |  |  |  |  |  |  |  |  |  |  |  |  |  |  |  |  |  |  |  |  |  |  |  |  |  |  |  |  |  |  |  |  |  |  |  |  |  |  |  |  |  |  |
| --- | --- | --- | --- | --- | --- | --- | --- | --- | --- | --- | --- | --- | --- | --- | --- | --- | --- | --- | --- | --- | --- | --- | --- | --- | --- | --- | --- | --- | --- | --- | --- | --- | --- | --- | --- | --- | --- | --- | --- | --- | --- | --- | --- |

XP\_015676850.1

back to list

  

| Protein Coverage
| Supporting Peptides
|

Protein Coverage:

Supporting Peptides:

| Peptide | Uniq | -10lgP | Mass | Length | ppm | m/z | z | RT | Fraction | Scan | Source File | Area F1 | Area F10 | Area F11 | Area F12 | Area F13 | Area F2 | Area F3 | Area F4 | Area F5 | Area F6 | Area F7 | Area F8 | Area F9 | #Feature | #Feature F1 | #Feature F10 | #Feature F11 | #Feature F12 | #Feature F13 | #Feature F2 | #Feature F3 | #Feature F4 | #Feature F5 | #Feature F6 | #Feature F7 | #Feature F8 | #Feature F9 | Start | End | PTM | AScore | Found By |
| --- | --- | --- | --- | --- | --- | --- | --- | --- | --- | --- | --- | --- | --- | --- | --- | --- | --- | --- | --- | --- | --- | --- | --- | --- | --- | --- | --- | --- | --- | --- | --- | --- | --- | --- | --- | --- | --- | --- | --- | --- | --- | --- | --- |
| K.GMNLHDYGMLLPC(+57.02)GIDK.F | Y | 84.67 | 1932.8896 | 17 | 1.6 | 967.4536 | 2 | 65.84 | 9 | F9:45212 | NaNaKA16\_F5.raw |  |  |  |  |  |  |  |  | 3.28E7 |  |  |  |  | 2 | 0 | 0 | 0 | 0 | 0 | 0 | 0 | 0 | 2 | 0 | 0 | 0 | 0 | 163 | 179 | Carbamidomethylation | C13:Carbamidomethylation:1000.00 | PEAKS DB |
| K.MDIC(+57.02)ETHLHWHTVAK.E | Y | 79.85 | 1876.8712 | 15 | -0.1 | 626.6310 | 3 | 22.06 | 9 | F9:9168 | NaNaKA16\_F5.raw |  |  |  |  |  |  |  |  | 1.6968E7 |  |  |  |  | 2 | 0 | 0 | 0 | 0 | 0 | 0 | 0 | 0 | 2 | 0 | 0 | 0 | 0 | 142 | 156 | Carbamidomethylation | C4:Carbamidomethylation:1000.00 | PEAKS DB |
| total 2 peptides |  |  |  |  |  |  |  |  |  |  |  |  |  |  |  |  |  |  |  |  |  |  |  |  |  |  |  |  |  |  |  |  |  |  |  |  |  |  |  |  |  |  |  |
| --- | --- | --- | --- | --- | --- | --- | --- | --- | --- | --- | --- | --- | --- | --- | --- | --- | --- | --- | --- | --- | --- | --- | --- | --- | --- | --- | --- | --- | --- | --- | --- | --- | --- | --- | --- | --- | --- | --- | --- | --- | --- | --- | --- |

JAV51273.1

back to list

  

| Protein Coverage
| Supporting Peptides
|

Protein Coverage:

Supporting Peptides:

| Peptide | Uniq | -10lgP | Mass | Length | ppm | m/z | z | RT | Fraction | Scan | Source File | Area F1 | Area F10 | Area F11 | Area F12 | Area F13 | Area F2 | Area F3 | Area F4 | Area F5 | Area F6 | Area F7 | Area F8 | Area F9 | #Feature | #Feature F1 | #Feature F10 | #Feature F11 | #Feature F12 | #Feature F13 | #Feature F2 | #Feature F3 | #Feature F4 | #Feature F5 | #Feature F6 | #Feature F7 | #Feature F8 | #Feature F9 | Start | End | PTM | AScore | Found By |
| --- | --- | --- | --- | --- | --- | --- | --- | --- | --- | --- | --- | --- | --- | --- | --- | --- | --- | --- | --- | --- | --- | --- | --- | --- | --- | --- | --- | --- | --- | --- | --- | --- | --- | --- | --- | --- | --- | --- | --- | --- | --- | --- | --- |
| K.GMNLHDYGMLLPC(+57.02)GIDK.F | Y | 84.67 | 1932.8896 | 17 | 1.6 | 967.4536 | 2 | 65.84 | 9 | F9:45212 | NaNaKA16\_F5.raw |  |  |  |  |  |  |  |  | 3.28E7 |  |  |  |  | 2 | 0 | 0 | 0 | 0 | 0 | 0 | 0 | 0 | 2 | 0 | 0 | 0 | 0 | 163 | 179 | Carbamidomethylation | C13:Carbamidomethylation:1000.00 | PEAKS DB |
| R.MDIC(+57.02)ETHLHWHTVAK.E | Y | 79.85 | 1876.8712 | 15 | -0.1 | 626.6310 | 3 | 22.06 | 9 | F9:9168 | NaNaKA16\_F5.raw |  |  |  |  |  |  |  |  | 1.6968E7 |  |  |  |  | 2 | 0 | 0 | 0 | 0 | 0 | 0 | 0 | 0 | 2 | 0 | 0 | 0 | 0 | 142 | 156 | Carbamidomethylation | C4:Carbamidomethylation:1000.00 | PEAKS DB |
| total 2 peptides |  |  |  |  |  |  |  |  |  |  |  |  |  |  |  |  |  |  |  |  |  |  |  |  |  |  |  |  |  |  |  |  |  |  |  |  |  |  |  |  |  |  |  |
| --- | --- | --- | --- | --- | --- | --- | --- | --- | --- | --- | --- | --- | --- | --- | --- | --- | --- | --- | --- | --- | --- | --- | --- | --- | --- | --- | --- | --- | --- | --- | --- | --- | --- | --- | --- | --- | --- | --- | --- | --- | --- | --- | --- |

AFJ49397.1

back to list

  

| Protein Coverage
| Supporting Peptides
|

Protein Coverage:

Supporting Peptides:

| Peptide | Uniq | -10lgP | Mass | Length | ppm | m/z | z | RT | Fraction | Scan | Source File | Area F1 | Area F10 | Area F11 | Area F12 | Area F13 | Area F2 | Area F3 | Area F4 | Area F5 | Area F6 | Area F7 | Area F8 | Area F9 | #Feature | #Feature F1 | #Feature F10 | #Feature F11 | #Feature F12 | #Feature F13 | #Feature F2 | #Feature F3 | #Feature F4 | #Feature F5 | #Feature F6 | #Feature F7 | #Feature F8 | #Feature F9 | Start | End | PTM | AScore | Found By |
| --- | --- | --- | --- | --- | --- | --- | --- | --- | --- | --- | --- | --- | --- | --- | --- | --- | --- | --- | --- | --- | --- | --- | --- | --- | --- | --- | --- | --- | --- | --- | --- | --- | --- | --- | --- | --- | --- | --- | --- | --- | --- | --- | --- |
| K.GMNLHDYGMLLPC(+57.02)GIDK.F | Y | 84.67 | 1932.8896 | 17 | 1.6 | 967.4536 | 2 | 65.84 | 9 | F9:45212 | NaNaKA16\_F5.raw |  |  |  |  |  |  |  |  | 3.28E7 |  |  |  |  | 2 | 0 | 0 | 0 | 0 | 0 | 0 | 0 | 0 | 2 | 0 | 0 | 0 | 0 | 163 | 179 | Carbamidomethylation | C13:Carbamidomethylation:1000.00 | PEAKS DB |
| K.MDIC(+57.02)ETHLHWHTVAK.E | Y | 79.85 | 1876.8712 | 15 | -0.1 | 626.6310 | 3 | 22.06 | 9 | F9:9168 | NaNaKA16\_F5.raw |  |  |  |  |  |  |  |  | 1.6968E7 |  |  |  |  | 2 | 0 | 0 | 0 | 0 | 0 | 0 | 0 | 0 | 2 | 0 | 0 | 0 | 0 | 142 | 156 | Carbamidomethylation | C4:Carbamidomethylation:1000.00 | PEAKS DB |
| total 2 peptides |  |  |  |  |  |  |  |  |  |  |  |  |  |  |  |  |  |  |  |  |  |  |  |  |  |  |  |  |  |  |  |  |  |  |  |  |  |  |  |  |  |  |  |
| --- | --- | --- | --- | --- | --- | --- | --- | --- | --- | --- | --- | --- | --- | --- | --- | --- | --- | --- | --- | --- | --- | --- | --- | --- | --- | --- | --- | --- | --- | --- | --- | --- | --- | --- | --- | --- | --- | --- | --- | --- | --- | --- | --- |

JAG47493.1

back to list

  

| Protein Coverage
| Supporting Peptides
|

Protein Coverage:

Supporting Peptides:

| Peptide | Uniq | -10lgP | Mass | Length | ppm | m/z | z | RT | Fraction | Scan | Source File | Area F1 | Area F10 | Area F11 | Area F12 | Area F13 | Area F2 | Area F3 | Area F4 | Area F5 | Area F6 | Area F7 | Area F8 | Area F9 | #Feature | #Feature F1 | #Feature F10 | #Feature F11 | #Feature F12 | #Feature F13 | #Feature F2 | #Feature F3 | #Feature F4 | #Feature F5 | #Feature F6 | #Feature F7 | #Feature F8 | #Feature F9 | Start | End | PTM | AScore | Found By |
| --- | --- | --- | --- | --- | --- | --- | --- | --- | --- | --- | --- | --- | --- | --- | --- | --- | --- | --- | --- | --- | --- | --- | --- | --- | --- | --- | --- | --- | --- | --- | --- | --- | --- | --- | --- | --- | --- | --- | --- | --- | --- | --- | --- |
| K.GMNLHDYGMLLPC(+57.02)GIDK.F | Y | 84.67 | 1932.8896 | 17 | 1.6 | 967.4536 | 2 | 65.84 | 9 | F9:45212 | NaNaKA16\_F5.raw |  |  |  |  |  |  |  |  | 3.28E7 |  |  |  |  | 2 | 0 | 0 | 0 | 0 | 0 | 0 | 0 | 0 | 2 | 0 | 0 | 0 | 0 | 163 | 179 | Carbamidomethylation | C13:Carbamidomethylation:1000.00 | PEAKS DB |
| K.MDIC(+57.02)ETHLHWHTVAK.E | Y | 79.85 | 1876.8712 | 15 | -0.1 | 626.6310 | 3 | 22.06 | 9 | F9:9168 | NaNaKA16\_F5.raw |  |  |  |  |  |  |  |  | 1.6968E7 |  |  |  |  | 2 | 0 | 0 | 0 | 0 | 0 | 0 | 0 | 0 | 2 | 0 | 0 | 0 | 0 | 142 | 156 | Carbamidomethylation | C4:Carbamidomethylation:1000.00 | PEAKS DB |
| total 2 peptides |  |  |  |  |  |  |  |  |  |  |  |  |  |  |  |  |  |  |  |  |  |  |  |  |  |  |  |  |  |  |  |  |  |  |  |  |  |  |  |  |  |  |  |
| --- | --- | --- | --- | --- | --- | --- | --- | --- | --- | --- | --- | --- | --- | --- | --- | --- | --- | --- | --- | --- | --- | --- | --- | --- | --- | --- | --- | --- | --- | --- | --- | --- | --- | --- | --- | --- | --- | --- | --- | --- | --- | --- | --- |

JAG45861.1

back to list

  

| Protein Coverage
| Supporting Peptides
|

Protein Coverage:

Supporting Peptides:

| Peptide | Uniq | -10lgP | Mass | Length | ppm | m/z | z | RT | Fraction | Scan | Source File | Area F1 | Area F10 | Area F11 | Area F12 | Area F13 | Area F2 | Area F3 | Area F4 | Area F5 | Area F6 | Area F7 | Area F8 | Area F9 | #Feature | #Feature F1 | #Feature F10 | #Feature F11 | #Feature F12 | #Feature F13 | #Feature F2 | #Feature F3 | #Feature F4 | #Feature F5 | #Feature F6 | #Feature F7 | #Feature F8 | #Feature F9 | Start | End | PTM | AScore | Found By |
| --- | --- | --- | --- | --- | --- | --- | --- | --- | --- | --- | --- | --- | --- | --- | --- | --- | --- | --- | --- | --- | --- | --- | --- | --- | --- | --- | --- | --- | --- | --- | --- | --- | --- | --- | --- | --- | --- | --- | --- | --- | --- | --- | --- |
| K.GMNLHDYGMLLPC(+57.02)GIDK.F | Y | 84.67 | 1932.8896 | 17 | 1.6 | 967.4536 | 2 | 65.84 | 9 | F9:45212 | NaNaKA16\_F5.raw |  |  |  |  |  |  |  |  | 3.28E7 |  |  |  |  | 2 | 0 | 0 | 0 | 0 | 0 | 0 | 0 | 0 | 2 | 0 | 0 | 0 | 0 | 163 | 179 | Carbamidomethylation | C13:Carbamidomethylation:1000.00 | PEAKS DB |
| K.MDIC(+57.02)ETHLHWHTVAK.E | Y | 79.85 | 1876.8712 | 15 | -0.1 | 626.6310 | 3 | 22.06 | 9 | F9:9168 | NaNaKA16\_F5.raw |  |  |  |  |  |  |  |  | 1.6968E7 |  |  |  |  | 2 | 0 | 0 | 0 | 0 | 0 | 0 | 0 | 0 | 2 | 0 | 0 | 0 | 0 | 142 | 156 | Carbamidomethylation | C4:Carbamidomethylation:1000.00 | PEAKS DB |
| total 2 peptides |  |  |  |  |  |  |  |  |  |  |  |  |  |  |  |  |  |  |  |  |  |  |  |  |  |  |  |  |  |  |  |  |  |  |  |  |  |  |  |  |  |  |  |
| --- | --- | --- | --- | --- | --- | --- | --- | --- | --- | --- | --- | --- | --- | --- | --- | --- | --- | --- | --- | --- | --- | --- | --- | --- | --- | --- | --- | --- | --- | --- | --- | --- | --- | --- | --- | --- | --- | --- | --- | --- | --- | --- | --- |

JAI14367.1

back to list

  

| Protein Coverage
| Supporting Peptides
|

Protein Coverage:

Supporting Peptides:

| Peptide | Uniq | -10lgP | Mass | Length | ppm | m/z | z | RT | Fraction | Scan | Source File | Area F1 | Area F10 | Area F11 | Area F12 | Area F13 | Area F2 | Area F3 | Area F4 | Area F5 | Area F6 | Area F7 | Area F8 | Area F9 | #Feature | #Feature F1 | #Feature F10 | #Feature F11 | #Feature F12 | #Feature F13 | #Feature F2 | #Feature F3 | #Feature F4 | #Feature F5 | #Feature F6 | #Feature F7 | #Feature F8 | #Feature F9 | Start | End | PTM | AScore | Found By |
| --- | --- | --- | --- | --- | --- | --- | --- | --- | --- | --- | --- | --- | --- | --- | --- | --- | --- | --- | --- | --- | --- | --- | --- | --- | --- | --- | --- | --- | --- | --- | --- | --- | --- | --- | --- | --- | --- | --- | --- | --- | --- | --- | --- |
| K.GMNLHDYGMLLPC(+57.02)GIDK.F | Y | 84.67 | 1932.8896 | 17 | 1.6 | 967.4536 | 2 | 65.84 | 9 | F9:45212 | NaNaKA16\_F5.raw |  |  |  |  |  |  |  |  | 3.28E7 |  |  |  |  | 2 | 0 | 0 | 0 | 0 | 0 | 0 | 0 | 0 | 2 | 0 | 0 | 0 | 0 | 163 | 179 | Carbamidomethylation | C13:Carbamidomethylation:1000.00 | PEAKS DB |
| K.MDIC(+57.02)ETHLHWHTVAK.E | Y | 79.85 | 1876.8712 | 15 | -0.1 | 626.6310 | 3 | 22.06 | 9 | F9:9168 | NaNaKA16\_F5.raw |  |  |  |  |  |  |  |  | 1.6968E7 |  |  |  |  | 2 | 0 | 0 | 0 | 0 | 0 | 0 | 0 | 0 | 2 | 0 | 0 | 0 | 0 | 142 | 156 | Carbamidomethylation | C4:Carbamidomethylation:1000.00 | PEAKS DB |
| total 2 peptides |  |  |  |  |  |  |  |  |  |  |  |  |  |  |  |  |  |  |  |  |  |  |  |  |  |  |  |  |  |  |  |  |  |  |  |  |  |  |  |  |  |  |  |
| --- | --- | --- | --- | --- | --- | --- | --- | --- | --- | --- | --- | --- | --- | --- | --- | --- | --- | --- | --- | --- | --- | --- | --- | --- | --- | --- | --- | --- | --- | --- | --- | --- | --- | --- | --- | --- | --- | --- | --- | --- | --- | --- | --- |

JAA97793.1

back to list

  

| Protein Coverage
| Supporting Peptides
|

Protein Coverage:

Supporting Peptides:

| Peptide | Uniq | -10lgP | Mass | Length | ppm | m/z | z | RT | Fraction | Scan | Source File | Area F1 | Area F10 | Area F11 | Area F12 | Area F13 | Area F2 | Area F3 | Area F4 | Area F5 | Area F6 | Area F7 | Area F8 | Area F9 | #Feature | #Feature F1 | #Feature F10 | #Feature F11 | #Feature F12 | #Feature F13 | #Feature F2 | #Feature F3 | #Feature F4 | #Feature F5 | #Feature F6 | #Feature F7 | #Feature F8 | #Feature F9 | Start | End | PTM | AScore | Found By |
| --- | --- | --- | --- | --- | --- | --- | --- | --- | --- | --- | --- | --- | --- | --- | --- | --- | --- | --- | --- | --- | --- | --- | --- | --- | --- | --- | --- | --- | --- | --- | --- | --- | --- | --- | --- | --- | --- | --- | --- | --- | --- | --- | --- |
| K.GMNLHDYGMLLPC(+57.02)GIDK.F | Y | 84.67 | 1932.8896 | 17 | 1.6 | 967.4536 | 2 | 65.84 | 9 | F9:45212 | NaNaKA16\_F5.raw |  |  |  |  |  |  |  |  | 3.28E7 |  |  |  |  | 2 | 0 | 0 | 0 | 0 | 0 | 0 | 0 | 0 | 2 | 0 | 0 | 0 | 0 | 163 | 179 | Carbamidomethylation | C13:Carbamidomethylation:1000.00 | PEAKS DB |
| K.MDIC(+57.02)ETHLHWHTVAK.E | Y | 79.85 | 1876.8712 | 15 | -0.1 | 626.6310 | 3 | 22.06 | 9 | F9:9168 | NaNaKA16\_F5.raw |  |  |  |  |  |  |  |  | 1.6968E7 |  |  |  |  | 2 | 0 | 0 | 0 | 0 | 0 | 0 | 0 | 0 | 2 | 0 | 0 | 0 | 0 | 142 | 156 | Carbamidomethylation | C4:Carbamidomethylation:1000.00 | PEAKS DB |
| total 2 peptides |  |  |  |  |  |  |  |  |  |  |  |  |  |  |  |  |  |  |  |  |  |  |  |  |  |  |  |  |  |  |  |  |  |  |  |  |  |  |  |  |  |  |  |
| --- | --- | --- | --- | --- | --- | --- | --- | --- | --- | --- | --- | --- | --- | --- | --- | --- | --- | --- | --- | --- | --- | --- | --- | --- | --- | --- | --- | --- | --- | --- | --- | --- | --- | --- | --- | --- | --- | --- | --- | --- | --- | --- | --- |

XP\_034283168.1

back to list

  

| Protein Coverage
| Supporting Peptides
|

Protein Coverage:

Supporting Peptides:

| Peptide | Uniq | -10lgP | Mass | Length | ppm | m/z | z | RT | Fraction | Scan | Source File | Area F1 | Area F10 | Area F11 | Area F12 | Area F13 | Area F2 | Area F3 | Area F4 | Area F5 | Area F6 | Area F7 | Area F8 | Area F9 | #Feature | #Feature F1 | #Feature F10 | #Feature F11 | #Feature F12 | #Feature F13 | #Feature F2 | #Feature F3 | #Feature F4 | #Feature F5 | #Feature F6 | #Feature F7 | #Feature F8 | #Feature F9 | Start | End | PTM | AScore | Found By |
| --- | --- | --- | --- | --- | --- | --- | --- | --- | --- | --- | --- | --- | --- | --- | --- | --- | --- | --- | --- | --- | --- | --- | --- | --- | --- | --- | --- | --- | --- | --- | --- | --- | --- | --- | --- | --- | --- | --- | --- | --- | --- | --- | --- |
| K.GMNLHDYGMLLPC(+57.02)GIDK.F | Y | 84.67 | 1932.8896 | 17 | 1.6 | 967.4536 | 2 | 65.84 | 9 | F9:45212 | NaNaKA16\_F5.raw |  |  |  |  |  |  |  |  | 3.28E7 |  |  |  |  | 2 | 0 | 0 | 0 | 0 | 0 | 0 | 0 | 0 | 2 | 0 | 0 | 0 | 0 | 163 | 179 | Carbamidomethylation | C13:Carbamidomethylation:1000.00 | PEAKS DB |
| R.MDIC(+57.02)ETHLHWHTVAK.E | Y | 79.85 | 1876.8712 | 15 | -0.1 | 626.6310 | 3 | 22.06 | 9 | F9:9168 | NaNaKA16\_F5.raw |  |  |  |  |  |  |  |  | 1.6968E7 |  |  |  |  | 2 | 0 | 0 | 0 | 0 | 0 | 0 | 0 | 0 | 2 | 0 | 0 | 0 | 0 | 142 | 156 | Carbamidomethylation | C4:Carbamidomethylation:1000.00 | PEAKS DB |
| total 2 peptides |  |  |  |  |  |  |  |  |  |  |  |  |  |  |  |  |  |  |  |  |  |  |  |  |  |  |  |  |  |  |  |  |  |  |  |  |  |  |  |  |  |  |  |
| --- | --- | --- | --- | --- | --- | --- | --- | --- | --- | --- | --- | --- | --- | --- | --- | --- | --- | --- | --- | --- | --- | --- | --- | --- | --- | --- | --- | --- | --- | --- | --- | --- | --- | --- | --- | --- | --- | --- | --- | --- | --- | --- | --- |

XP\_026519906.1

back to list

  

| Protein Coverage
| Supporting Peptides
|

Protein Coverage:

Supporting Peptides:

| Peptide | Uniq | -10lgP | Mass | Length | ppm | m/z | z | RT | Fraction | Scan | Source File | Area F1 | Area F10 | Area F11 | Area F12 | Area F13 | Area F2 | Area F3 | Area F4 | Area F5 | Area F6 | Area F7 | Area F8 | Area F9 | #Feature | #Feature F1 | #Feature F10 | #Feature F11 | #Feature F12 | #Feature F13 | #Feature F2 | #Feature F3 | #Feature F4 | #Feature F5 | #Feature F6 | #Feature F7 | #Feature F8 | #Feature F9 | Start | End | PTM | AScore | Found By |
| --- | --- | --- | --- | --- | --- | --- | --- | --- | --- | --- | --- | --- | --- | --- | --- | --- | --- | --- | --- | --- | --- | --- | --- | --- | --- | --- | --- | --- | --- | --- | --- | --- | --- | --- | --- | --- | --- | --- | --- | --- | --- | --- | --- |
| K.GMNLHDYGMLLPC(+57.02)GIDK.F | Y | 84.67 | 1932.8896 | 17 | 1.6 | 967.4536 | 2 | 65.84 | 9 | F9:45212 | NaNaKA16\_F5.raw |  |  |  |  |  |  |  |  | 3.28E7 |  |  |  |  | 2 | 0 | 0 | 0 | 0 | 0 | 0 | 0 | 0 | 2 | 0 | 0 | 0 | 0 | 163 | 179 | Carbamidomethylation | C13:Carbamidomethylation:1000.00 | PEAKS DB |
| R.MDIC(+57.02)ETHLHWHTVAK.E | Y | 79.85 | 1876.8712 | 15 | -0.1 | 626.6310 | 3 | 22.06 | 9 | F9:9168 | NaNaKA16\_F5.raw |  |  |  |  |  |  |  |  | 1.6968E7 |  |  |  |  | 2 | 0 | 0 | 0 | 0 | 0 | 0 | 0 | 0 | 2 | 0 | 0 | 0 | 0 | 142 | 156 | Carbamidomethylation | C4:Carbamidomethylation:1000.00 | PEAKS DB |
| total 2 peptides |  |  |  |  |  |  |  |  |  |  |  |  |  |  |  |  |  |  |  |  |  |  |  |  |  |  |  |  |  |  |  |  |  |  |  |  |  |  |  |  |  |  |  |
| --- | --- | --- | --- | --- | --- | --- | --- | --- | --- | --- | --- | --- | --- | --- | --- | --- | --- | --- | --- | --- | --- | --- | --- | --- | --- | --- | --- | --- | --- | --- | --- | --- | --- | --- | --- | --- | --- | --- | --- | --- | --- | --- | --- |

XP\_026556420.1

back to list

  

| Protein Coverage
| Supporting Peptides
|

Protein Coverage:

Supporting Peptides:

| Peptide | Uniq | -10lgP | Mass | Length | ppm | m/z | z | RT | Fraction | Scan | Source File | Area F1 | Area F10 | Area F11 | Area F12 | Area F13 | Area F2 | Area F3 | Area F4 | Area F5 | Area F6 | Area F7 | Area F8 | Area F9 | #Feature | #Feature F1 | #Feature F10 | #Feature F11 | #Feature F12 | #Feature F13 | #Feature F2 | #Feature F3 | #Feature F4 | #Feature F5 | #Feature F6 | #Feature F7 | #Feature F8 | #Feature F9 | Start | End | PTM | AScore | Found By |
| --- | --- | --- | --- | --- | --- | --- | --- | --- | --- | --- | --- | --- | --- | --- | --- | --- | --- | --- | --- | --- | --- | --- | --- | --- | --- | --- | --- | --- | --- | --- | --- | --- | --- | --- | --- | --- | --- | --- | --- | --- | --- | --- | --- |
| K.GMNLHDYGMLLPC(+57.02)GIDK.F | Y | 84.67 | 1932.8896 | 17 | 1.6 | 967.4536 | 2 | 65.84 | 9 | F9:45212 | NaNaKA16\_F5.raw |  |  |  |  |  |  |  |  | 3.28E7 |  |  |  |  | 2 | 0 | 0 | 0 | 0 | 0 | 0 | 0 | 0 | 2 | 0 | 0 | 0 | 0 | 163 | 179 | Carbamidomethylation | C13:Carbamidomethylation:1000.00 | PEAKS DB |
| R.MDIC(+57.02)ETHLHWHTVAK.E | Y | 79.85 | 1876.8712 | 15 | -0.1 | 626.6310 | 3 | 22.06 | 9 | F9:9168 | NaNaKA16\_F5.raw |  |  |  |  |  |  |  |  | 1.6968E7 |  |  |  |  | 2 | 0 | 0 | 0 | 0 | 0 | 0 | 0 | 0 | 2 | 0 | 0 | 0 | 0 | 142 | 156 | Carbamidomethylation | C4:Carbamidomethylation:1000.00 | PEAKS DB |
| total 2 peptides |  |  |  |  |  |  |  |  |  |  |  |  |  |  |  |  |  |  |  |  |  |  |  |  |  |  |  |  |  |  |  |  |  |  |  |  |  |  |  |  |  |  |  |
| --- | --- | --- | --- | --- | --- | --- | --- | --- | --- | --- | --- | --- | --- | --- | --- | --- | --- | --- | --- | --- | --- | --- | --- | --- | --- | --- | --- | --- | --- | --- | --- | --- | --- | --- | --- | --- | --- | --- | --- | --- | --- | --- | --- |

XP\_013912686.1

back to list

  

| Protein Coverage
| Supporting Peptides
|

Protein Coverage:

Supporting Peptides:

| Peptide | Uniq | -10lgP | Mass | Length | ppm | m/z | z | RT | Fraction | Scan | Source File | Area F1 | Area F10 | Area F11 | Area F12 | Area F13 | Area F2 | Area F3 | Area F4 | Area F5 | Area F6 | Area F7 | Area F8 | Area F9 | #Feature | #Feature F1 | #Feature F10 | #Feature F11 | #Feature F12 | #Feature F13 | #Feature F2 | #Feature F3 | #Feature F4 | #Feature F5 | #Feature F6 | #Feature F7 | #Feature F8 | #Feature F9 | Start | End | PTM | AScore | Found By |
| --- | --- | --- | --- | --- | --- | --- | --- | --- | --- | --- | --- | --- | --- | --- | --- | --- | --- | --- | --- | --- | --- | --- | --- | --- | --- | --- | --- | --- | --- | --- | --- | --- | --- | --- | --- | --- | --- | --- | --- | --- | --- | --- | --- |
| K.GMNLHDYGMLLPC(+57.02)GIDK.F | Y | 84.67 | 1932.8896 | 17 | 1.6 | 967.4536 | 2 | 65.84 | 9 | F9:45212 | NaNaKA16\_F5.raw |  |  |  |  |  |  |  |  | 3.28E7 |  |  |  |  | 2 | 0 | 0 | 0 | 0 | 0 | 0 | 0 | 0 | 2 | 0 | 0 | 0 | 0 | 163 | 179 | Carbamidomethylation | C13:Carbamidomethylation:1000.00 | PEAKS DB |
| R.MDIC(+57.02)ETHLHWHTVAK.E | Y | 79.85 | 1876.8712 | 15 | -0.1 | 626.6310 | 3 | 22.06 | 9 | F9:9168 | NaNaKA16\_F5.raw |  |  |  |  |  |  |  |  | 1.6968E7 |  |  |  |  | 2 | 0 | 0 | 0 | 0 | 0 | 0 | 0 | 0 | 2 | 0 | 0 | 0 | 0 | 142 | 156 | Carbamidomethylation | C4:Carbamidomethylation:1000.00 | PEAKS DB |
| total 2 peptides |  |  |  |  |  |  |  |  |  |  |  |  |  |  |  |  |  |  |  |  |  |  |  |  |  |  |  |  |  |  |  |  |  |  |  |  |  |  |  |  |  |  |  |
| --- | --- | --- | --- | --- | --- | --- | --- | --- | --- | --- | --- | --- | --- | --- | --- | --- | --- | --- | --- | --- | --- | --- | --- | --- | --- | --- | --- | --- | --- | --- | --- | --- | --- | --- | --- | --- | --- | --- | --- | --- | --- | --- | --- |

XP\_034283217.1

back to list

  

| Protein Coverage
| Supporting Peptides
|

Protein Coverage:

Supporting Peptides:

| Peptide | Uniq | -10lgP | Mass | Length | ppm | m/z | z | RT | Fraction | Scan | Source File | Area F1 | Area F10 | Area F11 | Area F12 | Area F13 | Area F2 | Area F3 | Area F4 | Area F5 | Area F6 | Area F7 | Area F8 | Area F9 | #Feature | #Feature F1 | #Feature F10 | #Feature F11 | #Feature F12 | #Feature F13 | #Feature F2 | #Feature F3 | #Feature F4 | #Feature F5 | #Feature F6 | #Feature F7 | #Feature F8 | #Feature F9 | Start | End | PTM | AScore | Found By |
| --- | --- | --- | --- | --- | --- | --- | --- | --- | --- | --- | --- | --- | --- | --- | --- | --- | --- | --- | --- | --- | --- | --- | --- | --- | --- | --- | --- | --- | --- | --- | --- | --- | --- | --- | --- | --- | --- | --- | --- | --- | --- | --- | --- |
| K.GMNLHDYGMLLPC(+57.02)GIDK.F | Y | 84.67 | 1932.8896 | 17 | 1.6 | 967.4536 | 2 | 65.84 | 9 | F9:45212 | NaNaKA16\_F5.raw |  |  |  |  |  |  |  |  | 3.28E7 |  |  |  |  | 2 | 0 | 0 | 0 | 0 | 0 | 0 | 0 | 0 | 2 | 0 | 0 | 0 | 0 | 163 | 179 | Carbamidomethylation | C13:Carbamidomethylation:1000.00 | PEAKS DB |
| R.MDIC(+57.02)ETHLHWHTVAK.E | Y | 79.85 | 1876.8712 | 15 | -0.1 | 626.6310 | 3 | 22.06 | 9 | F9:9168 | NaNaKA16\_F5.raw |  |  |  |  |  |  |  |  | 1.6968E7 |  |  |  |  | 2 | 0 | 0 | 0 | 0 | 0 | 0 | 0 | 0 | 2 | 0 | 0 | 0 | 0 | 142 | 156 | Carbamidomethylation | C4:Carbamidomethylation:1000.00 | PEAKS DB |
| total 2 peptides |  |  |  |  |  |  |  |  |  |  |  |  |  |  |  |  |  |  |  |  |  |  |  |  |  |  |  |  |  |  |  |  |  |  |  |  |  |  |  |  |  |  |  |
| --- | --- | --- | --- | --- | --- | --- | --- | --- | --- | --- | --- | --- | --- | --- | --- | --- | --- | --- | --- | --- | --- | --- | --- | --- | --- | --- | --- | --- | --- | --- | --- | --- | --- | --- | --- | --- | --- | --- | --- | --- | --- | --- | --- |

XP\_015676849.1

back to list

  

| Protein Coverage
| Supporting Peptides
|

Protein Coverage:

Supporting Peptides:

| Peptide | Uniq | -10lgP | Mass | Length | ppm | m/z | z | RT | Fraction | Scan | Source File | Area F1 | Area F10 | Area F11 | Area F12 | Area F13 | Area F2 | Area F3 | Area F4 | Area F5 | Area F6 | Area F7 | Area F8 | Area F9 | #Feature | #Feature F1 | #Feature F10 | #Feature F11 | #Feature F12 | #Feature F13 | #Feature F2 | #Feature F3 | #Feature F4 | #Feature F5 | #Feature F6 | #Feature F7 | #Feature F8 | #Feature F9 | Start | End | PTM | AScore | Found By |
| --- | --- | --- | --- | --- | --- | --- | --- | --- | --- | --- | --- | --- | --- | --- | --- | --- | --- | --- | --- | --- | --- | --- | --- | --- | --- | --- | --- | --- | --- | --- | --- | --- | --- | --- | --- | --- | --- | --- | --- | --- | --- | --- | --- |
| K.GMNLHDYGMLLPC(+57.02)GIDK.F | Y | 84.67 | 1932.8896 | 17 | 1.6 | 967.4536 | 2 | 65.84 | 9 | F9:45212 | NaNaKA16\_F5.raw |  |  |  |  |  |  |  |  | 3.28E7 |  |  |  |  | 2 | 0 | 0 | 0 | 0 | 0 | 0 | 0 | 0 | 2 | 0 | 0 | 0 | 0 | 163 | 179 | Carbamidomethylation | C13:Carbamidomethylation:1000.00 | PEAKS DB |
| K.MDIC(+57.02)ETHLHWHTVAK.E | Y | 79.85 | 1876.8712 | 15 | -0.1 | 626.6310 | 3 | 22.06 | 9 | F9:9168 | NaNaKA16\_F5.raw |  |  |  |  |  |  |  |  | 1.6968E7 |  |  |  |  | 2 | 0 | 0 | 0 | 0 | 0 | 0 | 0 | 0 | 2 | 0 | 0 | 0 | 0 | 142 | 156 | Carbamidomethylation | C4:Carbamidomethylation:1000.00 | PEAKS DB |
| total 2 peptides |  |  |  |  |  |  |  |  |  |  |  |  |  |  |  |  |  |  |  |  |  |  |  |  |  |  |  |  |  |  |  |  |  |  |  |  |  |  |  |  |  |  |  |
| --- | --- | --- | --- | --- | --- | --- | --- | --- | --- | --- | --- | --- | --- | --- | --- | --- | --- | --- | --- | --- | --- | --- | --- | --- | --- | --- | --- | --- | --- | --- | --- | --- | --- | --- | --- | --- | --- | --- | --- | --- | --- | --- | --- |

JAI10297.1

back to list

  

| Protein Coverage
| Supporting Peptides
|

Protein Coverage:

Supporting Peptides:

| Peptide | Uniq | -10lgP | Mass | Length | ppm | m/z | z | RT | Fraction | Scan | Source File | Area F1 | Area F10 | Area F11 | Area F12 | Area F13 | Area F2 | Area F3 | Area F4 | Area F5 | Area F6 | Area F7 | Area F8 | Area F9 | #Feature | #Feature F1 | #Feature F10 | #Feature F11 | #Feature F12 | #Feature F13 | #Feature F2 | #Feature F3 | #Feature F4 | #Feature F5 | #Feature F6 | #Feature F7 | #Feature F8 | #Feature F9 | Start | End | PTM | AScore | Found By |
| --- | --- | --- | --- | --- | --- | --- | --- | --- | --- | --- | --- | --- | --- | --- | --- | --- | --- | --- | --- | --- | --- | --- | --- | --- | --- | --- | --- | --- | --- | --- | --- | --- | --- | --- | --- | --- | --- | --- | --- | --- | --- | --- | --- |
| K.GMNLHDYGMLLPC(+57.02)GIDK.F | Y | 84.67 | 1932.8896 | 17 | 1.6 | 967.4536 | 2 | 65.84 | 9 | F9:45212 | NaNaKA16\_F5.raw |  |  |  |  |  |  |  |  | 3.28E7 |  |  |  |  | 2 | 0 | 0 | 0 | 0 | 0 | 0 | 0 | 0 | 2 | 0 | 0 | 0 | 0 | 163 | 179 | Carbamidomethylation | C13:Carbamidomethylation:1000.00 | PEAKS DB |
| R.MDIC(+57.02)ETHLHWHTVAK.E | Y | 79.85 | 1876.8712 | 15 | -0.1 | 626.6310 | 3 | 22.06 | 9 | F9:9168 | NaNaKA16\_F5.raw |  |  |  |  |  |  |  |  | 1.6968E7 |  |  |  |  | 2 | 0 | 0 | 0 | 0 | 0 | 0 | 0 | 0 | 2 | 0 | 0 | 0 | 0 | 142 | 156 | Carbamidomethylation | C4:Carbamidomethylation:1000.00 | PEAKS DB |
| total 2 peptides |  |  |  |  |  |  |  |  |  |  |  |  |  |  |  |  |  |  |  |  |  |  |  |  |  |  |  |  |  |  |  |  |  |  |  |  |  |  |  |  |  |  |  |
| --- | --- | --- | --- | --- | --- | --- | --- | --- | --- | --- | --- | --- | --- | --- | --- | --- | --- | --- | --- | --- | --- | --- | --- | --- | --- | --- | --- | --- | --- | --- | --- | --- | --- | --- | --- | --- | --- | --- | --- | --- | --- | --- | --- |

JAG68744.1

back to list

  

| Protein Coverage
| Supporting Peptides
|

Protein Coverage:

Supporting Peptides:

| Peptide | Uniq | -10lgP | Mass | Length | ppm | m/z | z | RT | Fraction | Scan | Source File | Area F1 | Area F10 | Area F11 | Area F12 | Area F13 | Area F2 | Area F3 | Area F4 | Area F5 | Area F6 | Area F7 | Area F8 | Area F9 | #Feature | #Feature F1 | #Feature F10 | #Feature F11 | #Feature F12 | #Feature F13 | #Feature F2 | #Feature F3 | #Feature F4 | #Feature F5 | #Feature F6 | #Feature F7 | #Feature F8 | #Feature F9 | Start | End | PTM | AScore | Found By |
| --- | --- | --- | --- | --- | --- | --- | --- | --- | --- | --- | --- | --- | --- | --- | --- | --- | --- | --- | --- | --- | --- | --- | --- | --- | --- | --- | --- | --- | --- | --- | --- | --- | --- | --- | --- | --- | --- | --- | --- | --- | --- | --- | --- |
| K.GMNLHDYGMLLPC(+57.02)GIDK.F | Y | 84.67 | 1932.8896 | 17 | 1.6 | 967.4536 | 2 | 65.84 | 9 | F9:45212 | NaNaKA16\_F5.raw |  |  |  |  |  |  |  |  | 3.28E7 |  |  |  |  | 2 | 0 | 0 | 0 | 0 | 0 | 0 | 0 | 0 | 2 | 0 | 0 | 0 | 0 | 163 | 179 | Carbamidomethylation | C13:Carbamidomethylation:1000.00 | PEAKS DB |
| R.MDIC(+57.02)ETHLHWHTVAK.E | Y | 79.85 | 1876.8712 | 15 | -0.1 | 626.6310 | 3 | 22.06 | 9 | F9:9168 | NaNaKA16\_F5.raw |  |  |  |  |  |  |  |  | 1.6968E7 |  |  |  |  | 2 | 0 | 0 | 0 | 0 | 0 | 0 | 0 | 0 | 2 | 0 | 0 | 0 | 0 | 142 | 156 | Carbamidomethylation | C4:Carbamidomethylation:1000.00 | PEAKS DB |
| total 2 peptides |  |  |  |  |  |  |  |  |  |  |  |  |  |  |  |  |  |  |  |  |  |  |  |  |  |  |  |  |  |  |  |  |  |  |  |  |  |  |  |  |  |  |  |
| --- | --- | --- | --- | --- | --- | --- | --- | --- | --- | --- | --- | --- | --- | --- | --- | --- | --- | --- | --- | --- | --- | --- | --- | --- | --- | --- | --- | --- | --- | --- | --- | --- | --- | --- | --- | --- | --- | --- | --- | --- | --- | --- | --- |

ETE71713.1

back to list

  

| Protein Coverage
| Supporting Peptides
|

Protein Coverage:

Supporting Peptides:

| Peptide | Uniq | -10lgP | Mass | Length | ppm | m/z | z | RT | Fraction | Scan | Source File | Area F1 | Area F10 | Area F11 | Area F12 | Area F13 | Area F2 | Area F3 | Area F4 | Area F5 | Area F6 | Area F7 | Area F8 | Area F9 | #Feature | #Feature F1 | #Feature F10 | #Feature F11 | #Feature F12 | #Feature F13 | #Feature F2 | #Feature F3 | #Feature F4 | #Feature F5 | #Feature F6 | #Feature F7 | #Feature F8 | #Feature F9 | Start | End | PTM | AScore | Found By |
| --- | --- | --- | --- | --- | --- | --- | --- | --- | --- | --- | --- | --- | --- | --- | --- | --- | --- | --- | --- | --- | --- | --- | --- | --- | --- | --- | --- | --- | --- | --- | --- | --- | --- | --- | --- | --- | --- | --- | --- | --- | --- | --- | --- |
| K.GMNLHDYGMLLPC(+57.02)GIDK.F | Y | 84.67 | 1932.8896 | 17 | 1.6 | 967.4536 | 2 | 65.84 | 9 | F9:45212 | NaNaKA16\_F5.raw |  |  |  |  |  |  |  |  | 3.28E7 |  |  |  |  | 2 | 0 | 0 | 0 | 0 | 0 | 0 | 0 | 0 | 2 | 0 | 0 | 0 | 0 | 155 | 171 | Carbamidomethylation | C13:Carbamidomethylation:1000.00 | PEAKS DB |
| R.MDIC(+57.02)ETHLHWHTVAK.E | Y | 79.85 | 1876.8712 | 15 | -0.1 | 626.6310 | 3 | 22.06 | 9 | F9:9168 | NaNaKA16\_F5.raw |  |  |  |  |  |  |  |  | 1.6968E7 |  |  |  |  | 2 | 0 | 0 | 0 | 0 | 0 | 0 | 0 | 0 | 2 | 0 | 0 | 0 | 0 | 134 | 148 | Carbamidomethylation | C4:Carbamidomethylation:1000.00 | PEAKS DB |
| total 2 peptides |  |  |  |  |  |  |  |  |  |  |  |  |  |  |  |  |  |  |  |  |  |  |  |  |  |  |  |  |  |  |  |  |  |  |  |  |  |  |  |  |  |  |  |
| --- | --- | --- | --- | --- | --- | --- | --- | --- | --- | --- | --- | --- | --- | --- | --- | --- | --- | --- | --- | --- | --- | --- | --- | --- | --- | --- | --- | --- | --- | --- | --- | --- | --- | --- | --- | --- | --- | --- | --- | --- | --- | --- | --- |

XP\_026556851.1

back to list

  

| Protein Coverage
| Supporting Peptides
|

Protein Coverage:

Supporting Peptides:

| Peptide | Uniq | -10lgP | Mass | Length | ppm | m/z | z | RT | Fraction | Scan | Source File | Area F1 | Area F10 | Area F11 | Area F12 | Area F13 | Area F2 | Area F3 | Area F4 | Area F5 | Area F6 | Area F7 | Area F8 | Area F9 | #Feature | #Feature F1 | #Feature F10 | #Feature F11 | #Feature F12 | #Feature F13 | #Feature F2 | #Feature F3 | #Feature F4 | #Feature F5 | #Feature F6 | #Feature F7 | #Feature F8 | #Feature F9 | Start | End | PTM | AScore | Found By |
| --- | --- | --- | --- | --- | --- | --- | --- | --- | --- | --- | --- | --- | --- | --- | --- | --- | --- | --- | --- | --- | --- | --- | --- | --- | --- | --- | --- | --- | --- | --- | --- | --- | --- | --- | --- | --- | --- | --- | --- | --- | --- | --- | --- |
| R.NPDGAEVPWC(+57.02)FTTNPNIR.I | Y | 71.09 | 2086.9531 | 18 | -0.7 | 1044.4832 | 2 | 67.95 | 10 | F10:47791 | NaNaKA16\_F6.raw |  |  |  |  |  |  |  |  |  | 5.5425E6 |  |  |  | 2 | 0 | 0 | 0 | 0 | 0 | 0 | 0 | 0 | 0 | 2 | 0 | 0 | 0 | 343 | 360 | Carbamidomethylation | C10:Carbamidomethylation:1000.00 | PEAKS DB |
| R.GTVNTIWSGIEC(+57.02)QR.W | Y | 54.12 | 1619.7726 | 14 | -0.8 | 810.8929 | 2 | 54.83 | 10 | F10:36806 | NaNaKA16\_F6.raw |  |  |  |  |  |  |  |  |  | 9.9884E6 |  |  |  | 1 | 0 | 0 | 0 | 0 | 0 | 0 | 0 | 0 | 0 | 1 | 0 | 0 | 0 | 302 | 315 | Carbamidomethylation | C12:Carbamidomethylation:1000.00 | PEAKS DB |
| K.IVVGVIIPGR.G | Y | 51.24 | 1021.6648 | 10 | 0.5 | 511.8399 | 2 | 54.45 | 13 | F13:31672 | NaNaKA16\_F9.raw |  | 1.8048E6 |  |  |  |  |  |  |  |  |  | 0 | 0 | 1 | 0 | 1 | 0 | 0 | 0 | 0 | 0 | 0 | 0 | 0 | 0 | 0 | 0 | 677 | 686 |  |  | PEAKS DB |
| K.LSRPAVSNNAVAIIR.L | Y | 46.51 | 1579.9158 | 15 | 1.2 | 527.6465 | 3 | 29.81 | 4 | F4:13118 | NaNaKA16\_F12.raw |  |  |  | 1.5704E5 |  |  |  |  |  |  |  |  |  | 1 | 0 | 0 | 0 | 1 | 0 | 0 | 0 | 0 | 0 | 0 | 0 | 0 | 0 | 575 | 589 |  |  | PEAKS DB |
| S.AVIETTEC(+57.02)IQGQGEGYR.G | Y | 43.56 | 1909.8839 | 17 | 0.5 | 955.9497 | 2 | 32.89 | 10 | F10:18132 | NaNaKA16\_F6.raw |  |  |  |  |  |  |  |  |  | 1.1889E6 |  |  |  | 1 | 0 | 0 | 0 | 0 | 0 | 0 | 0 | 0 | 0 | 1 | 0 | 0 | 0 | 285 | 301 | Carbamidomethylation | C8:Carbamidomethylation:1000.00 | PEAKS DB |
| total 5 peptides |  |  |  |  |  |  |  |  |  |  |  |  |  |  |  |  |  |  |  |  |  |  |  |  |  |  |  |  |  |  |  |  |  |  |  |  |  |  |  |  |  |  |  |
| --- | --- | --- | --- | --- | --- | --- | --- | --- | --- | --- | --- | --- | --- | --- | --- | --- | --- | --- | --- | --- | --- | --- | --- | --- | --- | --- | --- | --- | --- | --- | --- | --- | --- | --- | --- | --- | --- | --- | --- | --- | --- | --- | --- |

XP\_026556843.1

back to list

  

| Protein Coverage
| Supporting Peptides
|

Protein Coverage:

Supporting Peptides:

| Peptide | Uniq | -10lgP | Mass | Length | ppm | m/z | z | RT | Fraction | Scan | Source File | Area F1 | Area F10 | Area F11 | Area F12 | Area F13 | Area F2 | Area F3 | Area F4 | Area F5 | Area F6 | Area F7 | Area F8 | Area F9 | #Feature | #Feature F1 | #Feature F10 | #Feature F11 | #Feature F12 | #Feature F13 | #Feature F2 | #Feature F3 | #Feature F4 | #Feature F5 | #Feature F6 | #Feature F7 | #Feature F8 | #Feature F9 | Start | End | PTM | AScore | Found By |
| --- | --- | --- | --- | --- | --- | --- | --- | --- | --- | --- | --- | --- | --- | --- | --- | --- | --- | --- | --- | --- | --- | --- | --- | --- | --- | --- | --- | --- | --- | --- | --- | --- | --- | --- | --- | --- | --- | --- | --- | --- | --- | --- | --- |
| R.NPDGAEVPWC(+57.02)FTTNPNIR.I | Y | 71.09 | 2086.9531 | 18 | -0.7 | 1044.4832 | 2 | 67.95 | 10 | F10:47791 | NaNaKA16\_F6.raw |  |  |  |  |  |  |  |  |  | 5.5425E6 |  |  |  | 2 | 0 | 0 | 0 | 0 | 0 | 0 | 0 | 0 | 0 | 2 | 0 | 0 | 0 | 348 | 365 | Carbamidomethylation | C10:Carbamidomethylation:1000.00 | PEAKS DB |
| R.GTVNTIWSGIEC(+57.02)QR.W | Y | 54.12 | 1619.7726 | 14 | -0.8 | 810.8929 | 2 | 54.83 | 10 | F10:36806 | NaNaKA16\_F6.raw |  |  |  |  |  |  |  |  |  | 9.9884E6 |  |  |  | 1 | 0 | 0 | 0 | 0 | 0 | 0 | 0 | 0 | 0 | 1 | 0 | 0 | 0 | 307 | 320 | Carbamidomethylation | C12:Carbamidomethylation:1000.00 | PEAKS DB |
| K.IVVGVIIPGR.G | Y | 51.24 | 1021.6648 | 10 | 0.5 | 511.8399 | 2 | 54.45 | 13 | F13:31672 | NaNaKA16\_F9.raw |  | 1.8048E6 |  |  |  |  |  |  |  |  |  | 0 | 0 | 1 | 0 | 1 | 0 | 0 | 0 | 0 | 0 | 0 | 0 | 0 | 0 | 0 | 0 | 682 | 691 |  |  | PEAKS DB |
| K.LSRPAVSNNAVAIIR.L | Y | 46.51 | 1579.9158 | 15 | 1.2 | 527.6465 | 3 | 29.81 | 4 | F4:13118 | NaNaKA16\_F12.raw |  |  |  | 1.5704E5 |  |  |  |  |  |  |  |  |  | 1 | 0 | 0 | 0 | 1 | 0 | 0 | 0 | 0 | 0 | 0 | 0 | 0 | 0 | 580 | 594 |  |  | PEAKS DB |
| S.AVIETTEC(+57.02)IQGQGEGYR.G | Y | 43.56 | 1909.8839 | 17 | 0.5 | 955.9497 | 2 | 32.89 | 10 | F10:18132 | NaNaKA16\_F6.raw |  |  |  |  |  |  |  |  |  | 1.1889E6 |  |  |  | 1 | 0 | 0 | 0 | 0 | 0 | 0 | 0 | 0 | 0 | 1 | 0 | 0 | 0 | 290 | 306 | Carbamidomethylation | C8:Carbamidomethylation:1000.00 | PEAKS DB |
| total 5 peptides |  |  |  |  |  |  |  |  |  |  |  |  |  |  |  |  |  |  |  |  |  |  |  |  |  |  |  |  |  |  |  |  |  |  |  |  |  |  |  |  |  |  |  |
| --- | --- | --- | --- | --- | --- | --- | --- | --- | --- | --- | --- | --- | --- | --- | --- | --- | --- | --- | --- | --- | --- | --- | --- | --- | --- | --- | --- | --- | --- | --- | --- | --- | --- | --- | --- | --- | --- | --- | --- | --- | --- | --- | --- |

1COD

back to list

  

| Protein Coverage
| Supporting Peptides
|

Protein Coverage:

Supporting Peptides:

| Peptide | Uniq | -10lgP | Mass | Length | ppm | m/z | z | RT | Fraction | Scan | Source File | Area F1 | Area F10 | Area F11 | Area F12 | Area F13 | Area F2 | Area F3 | Area F4 | Area F5 | Area F6 | Area F7 | Area F8 | Area F9 | #Feature | #Feature F1 | #Feature F10 | #Feature F11 | #Feature F12 | #Feature F13 | #Feature F2 | #Feature F3 | #Feature F4 | #Feature F5 | #Feature F6 | #Feature F7 | #Feature F8 | #Feature F9 | Start | End | PTM | AScore | Found By |
| --- | --- | --- | --- | --- | --- | --- | --- | --- | --- | --- | --- | --- | --- | --- | --- | --- | --- | --- | --- | --- | --- | --- | --- | --- | --- | --- | --- | --- | --- | --- | --- | --- | --- | --- | --- | --- | --- | --- | --- | --- | --- | --- | --- |
| LEC(+57.02)HNQQSSQTPTTTGC(+57.02)SGGETNC(+57.02)YK.K | Y | 81.34 | 2944.2021 | 26 | -3.1 | 982.4050 | 3 | 11.84 | 6 | F6:2150 | NaNaKA16\_F2.raw | 1.1842E6 |  |  |  |  | 1.0244E8 |  |  |  |  |  |  |  | 2 | 1 | 0 | 0 | 0 | 0 | 1 | 0 | 0 | 0 | 0 | 0 | 0 | 0 | 1 | 26 | Carbamidomethylation | C3:Carbamidomethylation:1000.00;C17:Carbamidomethylation:1000.00;C24:Carbamidomethylation:1000.00 | PEAKS DB |
| K.NGIEINC(+57.02)C(+57.02)TTDR.C | Y | 73.30 | 1451.6133 | 12 | 0.2 | 726.8141 | 2 | 20.42 | 1 | F1:6421 | NaNaKA16\_F1.raw | 8.7813E6 |  |  |  |  | 1.338E7 |  |  |  |  |  |  |  | 2 | 1 | 0 | 0 | 0 | 0 | 1 | 0 | 0 | 0 | 0 | 0 | 0 | 0 | 48 | 59 | Carbamidomethylation | C7:Carbamidomethylation:1000.00;C8:Carbamidomethylation:1000.00 | PEAKS DB |
| N.GIEINC(+57.02)C(+57.02)TTDR.C | N | 48.70 | 1337.5704 | 11 | -0.5 | 669.7922 | 2 | 18.63 | 6 | F6:6296 | NaNaKA16\_F2.raw |  |  |  |  |  | 4.8926E5 |  |  |  |  |  |  |  | 1 | 0 | 0 | 0 | 0 | 0 | 1 | 0 | 0 | 0 | 0 | 0 | 0 | 0 | 49 | 59 | Carbamidomethylation | C6:Carbamidomethylation:1000.00;C7:Carbamidomethylation:1000.00 | PEAKS DB |
| total 3 peptides |  |  |  |  |  |  |  |  |  |  |  |  |  |  |  |  |  |  |  |  |  |  |  |  |  |  |  |  |  |  |  |  |  |  |  |  |  |  |  |  |  |  |  |
| --- | --- | --- | --- | --- | --- | --- | --- | --- | --- | --- | --- | --- | --- | --- | --- | --- | --- | --- | --- | --- | --- | --- | --- | --- | --- | --- | --- | --- | --- | --- | --- | --- | --- | --- | --- | --- | --- | --- | --- | --- | --- | --- | --- |

1V6P

back to list

  

| Protein Coverage
| Supporting Peptides
|

Protein Coverage:

Supporting Peptides:

| Peptide | Uniq | -10lgP | Mass | Length | ppm | m/z | z | RT | Fraction | Scan | Source File | Area F1 | Area F10 | Area F11 | Area F12 | Area F13 | Area F2 | Area F3 | Area F4 | Area F5 | Area F6 | Area F7 | Area F8 | Area F9 | #Feature | #Feature F1 | #Feature F10 | #Feature F11 | #Feature F12 | #Feature F13 | #Feature F2 | #Feature F3 | #Feature F4 | #Feature F5 | #Feature F6 | #Feature F7 | #Feature F8 | #Feature F9 | Start | End | PTM | AScore | Found By |
| --- | --- | --- | --- | --- | --- | --- | --- | --- | --- | --- | --- | --- | --- | --- | --- | --- | --- | --- | --- | --- | --- | --- | --- | --- | --- | --- | --- | --- | --- | --- | --- | --- | --- | --- | --- | --- | --- | --- | --- | --- | --- | --- | --- |
| LEC(+57.02)HNQQSSQTPTTTGC(+57.02)SGGETNC(+57.02)YK.K | Y | 81.34 | 2944.2021 | 26 | -3.1 | 982.4050 | 3 | 11.84 | 6 | F6:2150 | NaNaKA16\_F2.raw | 1.1842E6 |  |  |  |  | 1.0244E8 |  |  |  |  |  |  |  | 2 | 1 | 0 | 0 | 0 | 0 | 1 | 0 | 0 | 0 | 0 | 0 | 0 | 0 | 1 | 26 | Carbamidomethylation | C3:Carbamidomethylation:1000.00;C17:Carbamidomethylation:1000.00;C24:Carbamidomethylation:1000.00 | PEAKS DB |
| K.NGIEINC(+57.02)C(+57.02)TTDR.C | Y | 73.30 | 1451.6133 | 12 | 0.2 | 726.8141 | 2 | 20.42 | 1 | F1:6421 | NaNaKA16\_F1.raw | 8.7813E6 |  |  |  |  | 1.338E7 |  |  |  |  |  |  |  | 2 | 1 | 0 | 0 | 0 | 0 | 1 | 0 | 0 | 0 | 0 | 0 | 0 | 0 | 48 | 59 | Carbamidomethylation | C7:Carbamidomethylation:1000.00;C8:Carbamidomethylation:1000.00 | PEAKS DB |
| N.GIEINC(+57.02)C(+57.02)TTDR.C | N | 48.70 | 1337.5704 | 11 | -0.5 | 669.7922 | 2 | 18.63 | 6 | F6:6296 | NaNaKA16\_F2.raw |  |  |  |  |  | 4.8926E5 |  |  |  |  |  |  |  | 1 | 0 | 0 | 0 | 0 | 0 | 1 | 0 | 0 | 0 | 0 | 0 | 0 | 0 | 49 | 59 | Carbamidomethylation | C6:Carbamidomethylation:1000.00;C7:Carbamidomethylation:1000.00 | PEAKS DB |
| total 3 peptides |  |  |  |  |  |  |  |  |  |  |  |  |  |  |  |  |  |  |  |  |  |  |  |  |  |  |  |  |  |  |  |  |  |  |  |  |  |  |  |  |  |  |  |
| --- | --- | --- | --- | --- | --- | --- | --- | --- | --- | --- | --- | --- | --- | --- | --- | --- | --- | --- | --- | --- | --- | --- | --- | --- | --- | --- | --- | --- | --- | --- | --- | --- | --- | --- | --- | --- | --- | --- | --- | --- | --- | --- | --- |

1COE

back to list

  

| Protein Coverage
| Supporting Peptides
|

Protein Coverage:

Supporting Peptides:

| Peptide | Uniq | -10lgP | Mass | Length | ppm | m/z | z | RT | Fraction | Scan | Source File | Area F1 | Area F10 | Area F11 | Area F12 | Area F13 | Area F2 | Area F3 | Area F4 | Area F5 | Area F6 | Area F7 | Area F8 | Area F9 | #Feature | #Feature F1 | #Feature F10 | #Feature F11 | #Feature F12 | #Feature F13 | #Feature F2 | #Feature F3 | #Feature F4 | #Feature F5 | #Feature F6 | #Feature F7 | #Feature F8 | #Feature F9 | Start | End | PTM | AScore | Found By |
| --- | --- | --- | --- | --- | --- | --- | --- | --- | --- | --- | --- | --- | --- | --- | --- | --- | --- | --- | --- | --- | --- | --- | --- | --- | --- | --- | --- | --- | --- | --- | --- | --- | --- | --- | --- | --- | --- | --- | --- | --- | --- | --- | --- |
| LEC(+57.02)HNQQSSQTPTTTGC(+57.02)SGGETNC(+57.02)YK.K | Y | 81.34 | 2944.2021 | 26 | -3.1 | 982.4050 | 3 | 11.84 | 6 | F6:2150 | NaNaKA16\_F2.raw | 1.1842E6 |  |  |  |  | 1.0244E8 |  |  |  |  |  |  |  | 2 | 1 | 0 | 0 | 0 | 0 | 1 | 0 | 0 | 0 | 0 | 0 | 0 | 0 | 1 | 26 | Carbamidomethylation | C3:Carbamidomethylation:1000.00;C17:Carbamidomethylation:1000.00;C24:Carbamidomethylation:1000.00 | PEAKS DB |
| K.NGIEINC(+57.02)C(+57.02)TTDR.C | Y | 73.30 | 1451.6133 | 12 | 0.2 | 726.8141 | 2 | 20.42 | 1 | F1:6421 | NaNaKA16\_F1.raw | 8.7813E6 |  |  |  |  | 1.338E7 |  |  |  |  |  |  |  | 2 | 1 | 0 | 0 | 0 | 0 | 1 | 0 | 0 | 0 | 0 | 0 | 0 | 0 | 48 | 59 | Carbamidomethylation | C7:Carbamidomethylation:1000.00;C8:Carbamidomethylation:1000.00 | PEAKS DB |
| N.GIEINC(+57.02)C(+57.02)TTDR.C | N | 48.70 | 1337.5704 | 11 | -0.5 | 669.7922 | 2 | 18.63 | 6 | F6:6296 | NaNaKA16\_F2.raw |  |  |  |  |  | 4.8926E5 |  |  |  |  |  |  |  | 1 | 0 | 0 | 0 | 0 | 0 | 1 | 0 | 0 | 0 | 0 | 0 | 0 | 0 | 49 | 59 | Carbamidomethylation | C6:Carbamidomethylation:1000.00;C7:Carbamidomethylation:1000.00 | PEAKS DB |
| total 3 peptides |  |  |  |  |  |  |  |  |  |  |  |  |  |  |  |  |  |  |  |  |  |  |  |  |  |  |  |  |  |  |  |  |  |  |  |  |  |  |  |  |  |  |  |
| --- | --- | --- | --- | --- | --- | --- | --- | --- | --- | --- | --- | --- | --- | --- | --- | --- | --- | --- | --- | --- | --- | --- | --- | --- | --- | --- | --- | --- | --- | --- | --- | --- | --- | --- | --- | --- | --- | --- | --- | --- | --- | --- | --- |

AAB25735.1

back to list

  

| Protein Coverage
| Supporting Peptides
|

Protein Coverage:

Supporting Peptides:

| Peptide | Uniq | -10lgP | Mass | Length | ppm | m/z | z | RT | Fraction | Scan | Source File | Area F1 | Area F10 | Area F11 | Area F12 | Area F13 | Area F2 | Area F3 | Area F4 | Area F5 | Area F6 | Area F7 | Area F8 | Area F9 | #Feature | #Feature F1 | #Feature F10 | #Feature F11 | #Feature F12 | #Feature F13 | #Feature F2 | #Feature F3 | #Feature F4 | #Feature F5 | #Feature F6 | #Feature F7 | #Feature F8 | #Feature F9 | Start | End | PTM | AScore | Found By |
| --- | --- | --- | --- | --- | --- | --- | --- | --- | --- | --- | --- | --- | --- | --- | --- | --- | --- | --- | --- | --- | --- | --- | --- | --- | --- | --- | --- | --- | --- | --- | --- | --- | --- | --- | --- | --- | --- | --- | --- | --- | --- | --- | --- |
| LEC(+57.02)HNQQSSQTPTTTGC(+57.02)SGGETNC(+57.02)YK.K | Y | 81.34 | 2944.2021 | 26 | -3.1 | 982.4050 | 3 | 11.84 | 6 | F6:2150 | NaNaKA16\_F2.raw | 1.1842E6 |  |  |  |  | 1.0244E8 |  |  |  |  |  |  |  | 2 | 1 | 0 | 0 | 0 | 0 | 1 | 0 | 0 | 0 | 0 | 0 | 0 | 0 | 1 | 26 | Carbamidomethylation | C3:Carbamidomethylation:1000.00;C17:Carbamidomethylation:1000.00;C24:Carbamidomethylation:1000.00 | PEAKS DB |
| K.NGIEINC(+57.02)C(+57.02)TTDR.C | Y | 73.30 | 1451.6133 | 12 | 0.2 | 726.8141 | 2 | 20.42 | 1 | F1:6421 | NaNaKA16\_F1.raw | 8.7813E6 |  |  |  |  | 1.338E7 |  |  |  |  |  |  |  | 2 | 1 | 0 | 0 | 0 | 0 | 1 | 0 | 0 | 0 | 0 | 0 | 0 | 0 | 48 | 59 | Carbamidomethylation | C7:Carbamidomethylation:1000.00;C8:Carbamidomethylation:1000.00 | PEAKS DB |
| N.GIEINC(+57.02)C(+57.02)TTDR.C | N | 48.70 | 1337.5704 | 11 | -0.5 | 669.7922 | 2 | 18.63 | 6 | F6:6296 | NaNaKA16\_F2.raw |  |  |  |  |  | 4.8926E5 |  |  |  |  |  |  |  | 1 | 0 | 0 | 0 | 0 | 0 | 1 | 0 | 0 | 0 | 0 | 0 | 0 | 0 | 49 | 59 | Carbamidomethylation | C6:Carbamidomethylation:1000.00;C7:Carbamidomethylation:1000.00 | PEAKS DB |
| total 3 peptides |  |  |  |  |  |  |  |  |  |  |  |  |  |  |  |  |  |  |  |  |  |  |  |  |  |  |  |  |  |  |  |  |  |  |  |  |  |  |  |  |  |  |  |
| --- | --- | --- | --- | --- | --- | --- | --- | --- | --- | --- | --- | --- | --- | --- | --- | --- | --- | --- | --- | --- | --- | --- | --- | --- | --- | --- | --- | --- | --- | --- | --- | --- | --- | --- | --- | --- | --- | --- | --- | --- | --- | --- | --- |

ADN67584.1

back to list

  

| Protein Coverage
| Supporting Peptides
|

Protein Coverage:

Supporting Peptides:

| Peptide | Uniq | -10lgP | Mass | Length | ppm | m/z | z | RT | Fraction | Scan | Source File | Area F1 | Area F10 | Area F11 | Area F12 | Area F13 | Area F2 | Area F3 | Area F4 | Area F5 | Area F6 | Area F7 | Area F8 | Area F9 | #Feature | #Feature F1 | #Feature F10 | #Feature F11 | #Feature F12 | #Feature F13 | #Feature F2 | #Feature F3 | #Feature F4 | #Feature F5 | #Feature F6 | #Feature F7 | #Feature F8 | #Feature F9 | Start | End | PTM | AScore | Found By |
| --- | --- | --- | --- | --- | --- | --- | --- | --- | --- | --- | --- | --- | --- | --- | --- | --- | --- | --- | --- | --- | --- | --- | --- | --- | --- | --- | --- | --- | --- | --- | --- | --- | --- | --- | --- | --- | --- | --- | --- | --- | --- | --- | --- |
| T.LEC(+57.02)HNQQSSQTPTTTGC(+57.02)SGGETNC(+57.02)YK.K | Y | 81.34 | 2944.2021 | 26 | -3.1 | 982.4050 | 3 | 11.84 | 6 | F6:2150 | NaNaKA16\_F2.raw | 1.1842E6 |  |  |  |  | 1.0244E8 |  |  |  |  |  |  |  | 2 | 1 | 0 | 0 | 0 | 0 | 1 | 0 | 0 | 0 | 0 | 0 | 0 | 0 | 3 | 28 | Carbamidomethylation | C3:Carbamidomethylation:1000.00;C17:Carbamidomethylation:1000.00;C24:Carbamidomethylation:1000.00 | PEAKS DB |
| K.NGIEINC(+57.02)C(+57.02)TTDR.C | Y | 73.30 | 1451.6133 | 12 | 0.2 | 726.8141 | 2 | 20.42 | 1 | F1:6421 | NaNaKA16\_F1.raw | 8.7813E6 |  |  |  |  | 1.338E7 |  |  |  |  |  |  |  | 2 | 1 | 0 | 0 | 0 | 0 | 1 | 0 | 0 | 0 | 0 | 0 | 0 | 0 | 50 | 61 | Carbamidomethylation | C7:Carbamidomethylation:1000.00;C8:Carbamidomethylation:1000.00 | PEAKS DB |
| N.GIEINC(+57.02)C(+57.02)TTDR.C | N | 48.70 | 1337.5704 | 11 | -0.5 | 669.7922 | 2 | 18.63 | 6 | F6:6296 | NaNaKA16\_F2.raw |  |  |  |  |  | 4.8926E5 |  |  |  |  |  |  |  | 1 | 0 | 0 | 0 | 0 | 0 | 1 | 0 | 0 | 0 | 0 | 0 | 0 | 0 | 51 | 61 | Carbamidomethylation | C6:Carbamidomethylation:1000.00;C7:Carbamidomethylation:1000.00 | PEAKS DB |
| total 3 peptides |  |  |  |  |  |  |  |  |  |  |  |  |  |  |  |  |  |  |  |  |  |  |  |  |  |  |  |  |  |  |  |  |  |  |  |  |  |  |  |  |  |  |  |
| --- | --- | --- | --- | --- | --- | --- | --- | --- | --- | --- | --- | --- | --- | --- | --- | --- | --- | --- | --- | --- | --- | --- | --- | --- | --- | --- | --- | --- | --- | --- | --- | --- | --- | --- | --- | --- | --- | --- | --- | --- | --- | --- | --- |

AAB36932.1

back to list

  

| Protein Coverage
| Supporting Peptides
|

Protein Coverage:

Supporting Peptides:

| Peptide | Uniq | -10lgP | Mass | Length | ppm | m/z | z | RT | Fraction | Scan | Source File | Area F1 | Area F10 | Area F11 | Area F12 | Area F13 | Area F2 | Area F3 | Area F4 | Area F5 | Area F6 | Area F7 | Area F8 | Area F9 | #Feature | #Feature F1 | #Feature F10 | #Feature F11 | #Feature F12 | #Feature F13 | #Feature F2 | #Feature F3 | #Feature F4 | #Feature F5 | #Feature F6 | #Feature F7 | #Feature F8 | #Feature F9 | Start | End | PTM | AScore | Found By |
| --- | --- | --- | --- | --- | --- | --- | --- | --- | --- | --- | --- | --- | --- | --- | --- | --- | --- | --- | --- | --- | --- | --- | --- | --- | --- | --- | --- | --- | --- | --- | --- | --- | --- | --- | --- | --- | --- | --- | --- | --- | --- | --- | --- |
| T.LEC(+57.02)HNQQSSQTPTTTGC(+57.02)SGGETNC(+57.02)YK.K | Y | 81.34 | 2944.2021 | 26 | -3.1 | 982.4050 | 3 | 11.84 | 6 | F6:2150 | NaNaKA16\_F2.raw | 1.1842E6 |  |  |  |  | 1.0244E8 |  |  |  |  |  |  |  | 2 | 1 | 0 | 0 | 0 | 0 | 1 | 0 | 0 | 0 | 0 | 0 | 0 | 0 | 22 | 47 | Carbamidomethylation | C3:Carbamidomethylation:1000.00;C17:Carbamidomethylation:1000.00;C24:Carbamidomethylation:1000.00 | PEAKS DB |
| K.NGIEINC(+57.02)C(+57.02)TTDR.C | Y | 73.30 | 1451.6133 | 12 | 0.2 | 726.8141 | 2 | 20.42 | 1 | F1:6421 | NaNaKA16\_F1.raw | 8.7813E6 |  |  |  |  | 1.338E7 |  |  |  |  |  |  |  | 2 | 1 | 0 | 0 | 0 | 0 | 1 | 0 | 0 | 0 | 0 | 0 | 0 | 0 | 69 | 80 | Carbamidomethylation | C7:Carbamidomethylation:1000.00;C8:Carbamidomethylation:1000.00 | PEAKS DB |
| N.GIEINC(+57.02)C(+57.02)TTDR.C | N | 48.70 | 1337.5704 | 11 | -0.5 | 669.7922 | 2 | 18.63 | 6 | F6:6296 | NaNaKA16\_F2.raw |  |  |  |  |  | 4.8926E5 |  |  |  |  |  |  |  | 1 | 0 | 0 | 0 | 0 | 0 | 1 | 0 | 0 | 0 | 0 | 0 | 0 | 0 | 70 | 80 | Carbamidomethylation | C6:Carbamidomethylation:1000.00;C7:Carbamidomethylation:1000.00 | PEAKS DB |
| total 3 peptides |  |  |  |  |  |  |  |  |  |  |  |  |  |  |  |  |  |  |  |  |  |  |  |  |  |  |  |  |  |  |  |  |  |  |  |  |  |  |  |  |  |  |  |
| --- | --- | --- | --- | --- | --- | --- | --- | --- | --- | --- | --- | --- | --- | --- | --- | --- | --- | --- | --- | --- | --- | --- | --- | --- | --- | --- | --- | --- | --- | --- | --- | --- | --- | --- | --- | --- | --- | --- | --- | --- | --- | --- | --- |

Q9PTT0.1

back to list

  

| Protein Coverage
| Supporting Peptides
|

Protein Coverage:

Supporting Peptides:

| Peptide | Uniq | -10lgP | Mass | Length | ppm | m/z | z | RT | Fraction | Scan | Source File | Area F1 | Area F10 | Area F11 | Area F12 | Area F13 | Area F2 | Area F3 | Area F4 | Area F5 | Area F6 | Area F7 | Area F8 | Area F9 | #Feature | #Feature F1 | #Feature F10 | #Feature F11 | #Feature F12 | #Feature F13 | #Feature F2 | #Feature F3 | #Feature F4 | #Feature F5 | #Feature F6 | #Feature F7 | #Feature F8 | #Feature F9 | Start | End | PTM | AScore | Found By |
| --- | --- | --- | --- | --- | --- | --- | --- | --- | --- | --- | --- | --- | --- | --- | --- | --- | --- | --- | --- | --- | --- | --- | --- | --- | --- | --- | --- | --- | --- | --- | --- | --- | --- | --- | --- | --- | --- | --- | --- | --- | --- | --- | --- |
| T.LEC(+57.02)HNQQSSQTPTTTGC(+57.02)SGGETNC(+57.02)YK.K | Y | 81.34 | 2944.2021 | 26 | -3.1 | 982.4050 | 3 | 11.84 | 6 | F6:2150 | NaNaKA16\_F2.raw | 1.1842E6 |  |  |  |  | 1.0244E8 |  |  |  |  |  |  |  | 2 | 1 | 0 | 0 | 0 | 0 | 1 | 0 | 0 | 0 | 0 | 0 | 0 | 0 | 22 | 47 | Carbamidomethylation | C3:Carbamidomethylation:1000.00;C17:Carbamidomethylation:1000.00;C24:Carbamidomethylation:1000.00 | PEAKS DB |
| K.NGIEINC(+57.02)C(+57.02)TTDR.C | Y | 73.30 | 1451.6133 | 12 | 0.2 | 726.8141 | 2 | 20.42 | 1 | F1:6421 | NaNaKA16\_F1.raw | 8.7813E6 |  |  |  |  | 1.338E7 |  |  |  |  |  |  |  | 2 | 1 | 0 | 0 | 0 | 0 | 1 | 0 | 0 | 0 | 0 | 0 | 0 | 0 | 69 | 80 | Carbamidomethylation | C7:Carbamidomethylation:1000.00;C8:Carbamidomethylation:1000.00 | PEAKS DB |
| N.GIEINC(+57.02)C(+57.02)TTDR.C | N | 48.70 | 1337.5704 | 11 | -0.5 | 669.7922 | 2 | 18.63 | 6 | F6:6296 | NaNaKA16\_F2.raw |  |  |  |  |  | 4.8926E5 |  |  |  |  |  |  |  | 1 | 0 | 0 | 0 | 0 | 0 | 1 | 0 | 0 | 0 | 0 | 0 | 0 | 0 | 70 | 80 | Carbamidomethylation | C6:Carbamidomethylation:1000.00;C7:Carbamidomethylation:1000.00 | PEAKS DB |
| total 3 peptides |  |  |  |  |  |  |  |  |  |  |  |  |  |  |  |  |  |  |  |  |  |  |  |  |  |  |  |  |  |  |  |  |  |  |  |  |  |  |  |  |  |  |  |
| --- | --- | --- | --- | --- | --- | --- | --- | --- | --- | --- | --- | --- | --- | --- | --- | --- | --- | --- | --- | --- | --- | --- | --- | --- | --- | --- | --- | --- | --- | --- | --- | --- | --- | --- | --- | --- | --- | --- | --- | --- | --- | --- | --- |

P60770.1

back to list

  

| Protein Coverage
| Supporting Peptides
|

Protein Coverage:

Supporting Peptides:

| Peptide | Uniq | -10lgP | Mass | Length | ppm | m/z | z | RT | Fraction | Scan | Source File | Area F1 | Area F10 | Area F11 | Area F12 | Area F13 | Area F2 | Area F3 | Area F4 | Area F5 | Area F6 | Area F7 | Area F8 | Area F9 | #Feature | #Feature F1 | #Feature F10 | #Feature F11 | #Feature F12 | #Feature F13 | #Feature F2 | #Feature F3 | #Feature F4 | #Feature F5 | #Feature F6 | #Feature F7 | #Feature F8 | #Feature F9 | Start | End | PTM | AScore | Found By |
| --- | --- | --- | --- | --- | --- | --- | --- | --- | --- | --- | --- | --- | --- | --- | --- | --- | --- | --- | --- | --- | --- | --- | --- | --- | --- | --- | --- | --- | --- | --- | --- | --- | --- | --- | --- | --- | --- | --- | --- | --- | --- | --- | --- |
| T.LEC(+57.02)HNQQSSQTPTTTGC(+57.02)SGGETNC(+57.02)YK.K | Y | 81.34 | 2944.2021 | 26 | -3.1 | 982.4050 | 3 | 11.84 | 6 | F6:2150 | NaNaKA16\_F2.raw | 1.1842E6 |  |  |  |  | 1.0244E8 |  |  |  |  |  |  |  | 2 | 1 | 0 | 0 | 0 | 0 | 1 | 0 | 0 | 0 | 0 | 0 | 0 | 0 | 22 | 47 | Carbamidomethylation | C3:Carbamidomethylation:1000.00;C17:Carbamidomethylation:1000.00;C24:Carbamidomethylation:1000.00 | PEAKS DB |
| K.NGIEINC(+57.02)C(+57.02)TTDR.C | Y | 73.30 | 1451.6133 | 12 | 0.2 | 726.8141 | 2 | 20.42 | 1 | F1:6421 | NaNaKA16\_F1.raw | 8.7813E6 |  |  |  |  | 1.338E7 |  |  |  |  |  |  |  | 2 | 1 | 0 | 0 | 0 | 0 | 1 | 0 | 0 | 0 | 0 | 0 | 0 | 0 | 69 | 80 | Carbamidomethylation | C7:Carbamidomethylation:1000.00;C8:Carbamidomethylation:1000.00 | PEAKS DB |
| N.GIEINC(+57.02)C(+57.02)TTDR.C | N | 48.70 | 1337.5704 | 11 | -0.5 | 669.7922 | 2 | 18.63 | 6 | F6:6296 | NaNaKA16\_F2.raw |  |  |  |  |  | 4.8926E5 |  |  |  |  |  |  |  | 1 | 0 | 0 | 0 | 0 | 0 | 1 | 0 | 0 | 0 | 0 | 0 | 0 | 0 | 70 | 80 | Carbamidomethylation | C6:Carbamidomethylation:1000.00;C7:Carbamidomethylation:1000.00 | PEAKS DB |
| total 3 peptides |  |  |  |  |  |  |  |  |  |  |  |  |  |  |  |  |  |  |  |  |  |  |  |  |  |  |  |  |  |  |  |  |  |  |  |  |  |  |  |  |  |  |  |
| --- | --- | --- | --- | --- | --- | --- | --- | --- | --- | --- | --- | --- | --- | --- | --- | --- | --- | --- | --- | --- | --- | --- | --- | --- | --- | --- | --- | --- | --- | --- | --- | --- | --- | --- | --- | --- | --- | --- | --- | --- | --- | --- | --- |

P60771.2

back to list

  

| Protein Coverage
| Supporting Peptides
|

Protein Coverage:

Supporting Peptides:

| Peptide | Uniq | -10lgP | Mass | Length | ppm | m/z | z | RT | Fraction | Scan | Source File | Area F1 | Area F10 | Area F11 | Area F12 | Area F13 | Area F2 | Area F3 | Area F4 | Area F5 | Area F6 | Area F7 | Area F8 | Area F9 | #Feature | #Feature F1 | #Feature F10 | #Feature F11 | #Feature F12 | #Feature F13 | #Feature F2 | #Feature F3 | #Feature F4 | #Feature F5 | #Feature F6 | #Feature F7 | #Feature F8 | #Feature F9 | Start | End | PTM | AScore | Found By |
| --- | --- | --- | --- | --- | --- | --- | --- | --- | --- | --- | --- | --- | --- | --- | --- | --- | --- | --- | --- | --- | --- | --- | --- | --- | --- | --- | --- | --- | --- | --- | --- | --- | --- | --- | --- | --- | --- | --- | --- | --- | --- | --- | --- |
| T.LEC(+57.02)HNQQSSQTPTTTGC(+57.02)SGGETNC(+57.02)YK.K | Y | 81.34 | 2944.2021 | 26 | -3.1 | 982.4050 | 3 | 11.84 | 6 | F6:2150 | NaNaKA16\_F2.raw | 1.1842E6 |  |  |  |  | 1.0244E8 |  |  |  |  |  |  |  | 2 | 1 | 0 | 0 | 0 | 0 | 1 | 0 | 0 | 0 | 0 | 0 | 0 | 0 | 22 | 47 | Carbamidomethylation | C3:Carbamidomethylation:1000.00;C17:Carbamidomethylation:1000.00;C24:Carbamidomethylation:1000.00 | PEAKS DB |
| K.NGIEINC(+57.02)C(+57.02)TTDR.C | Y | 73.30 | 1451.6133 | 12 | 0.2 | 726.8141 | 2 | 20.42 | 1 | F1:6421 | NaNaKA16\_F1.raw | 8.7813E6 |  |  |  |  | 1.338E7 |  |  |  |  |  |  |  | 2 | 1 | 0 | 0 | 0 | 0 | 1 | 0 | 0 | 0 | 0 | 0 | 0 | 0 | 69 | 80 | Carbamidomethylation | C7:Carbamidomethylation:1000.00;C8:Carbamidomethylation:1000.00 | PEAKS DB |
| N.GIEINC(+57.02)C(+57.02)TTDR.C | N | 48.70 | 1337.5704 | 11 | -0.5 | 669.7922 | 2 | 18.63 | 6 | F6:6296 | NaNaKA16\_F2.raw |  |  |  |  |  | 4.8926E5 |  |  |  |  |  |  |  | 1 | 0 | 0 | 0 | 0 | 0 | 1 | 0 | 0 | 0 | 0 | 0 | 0 | 0 | 70 | 80 | Carbamidomethylation | C6:Carbamidomethylation:1000.00;C7:Carbamidomethylation:1000.00 | PEAKS DB |
| total 3 peptides |  |  |  |  |  |  |  |  |  |  |  |  |  |  |  |  |  |  |  |  |  |  |  |  |  |  |  |  |  |  |  |  |  |  |  |  |  |  |  |  |  |  |  |
| --- | --- | --- | --- | --- | --- | --- | --- | --- | --- | --- | --- | --- | --- | --- | --- | --- | --- | --- | --- | --- | --- | --- | --- | --- | --- | --- | --- | --- | --- | --- | --- | --- | --- | --- | --- | --- | --- | --- | --- | --- | --- | --- | --- |

AAY63884.1

back to list

  

| Protein Coverage
| Supporting Peptides
|

Protein Coverage:

Supporting Peptides:

| Peptide | Uniq | -10lgP | Mass | Length | ppm | m/z | z | RT | Fraction | Scan | Source File | Area F1 | Area F10 | Area F11 | Area F12 | Area F13 | Area F2 | Area F3 | Area F4 | Area F5 | Area F6 | Area F7 | Area F8 | Area F9 | #Feature | #Feature F1 | #Feature F10 | #Feature F11 | #Feature F12 | #Feature F13 | #Feature F2 | #Feature F3 | #Feature F4 | #Feature F5 | #Feature F6 | #Feature F7 | #Feature F8 | #Feature F9 | Start | End | PTM | AScore | Found By |
| --- | --- | --- | --- | --- | --- | --- | --- | --- | --- | --- | --- | --- | --- | --- | --- | --- | --- | --- | --- | --- | --- | --- | --- | --- | --- | --- | --- | --- | --- | --- | --- | --- | --- | --- | --- | --- | --- | --- | --- | --- | --- | --- | --- |
| T.LEC(+57.02)HNQQSSQTPTTTGC(+57.02)SGGETNC(+57.02)YK.K | Y | 81.34 | 2944.2021 | 26 | -3.1 | 982.4050 | 3 | 11.84 | 6 | F6:2150 | NaNaKA16\_F2.raw | 1.1842E6 |  |  |  |  | 1.0244E8 |  |  |  |  |  |  |  | 2 | 1 | 0 | 0 | 0 | 0 | 1 | 0 | 0 | 0 | 0 | 0 | 0 | 0 | 22 | 47 | Carbamidomethylation | C3:Carbamidomethylation:1000.00;C17:Carbamidomethylation:1000.00;C24:Carbamidomethylation:1000.00 | PEAKS DB |
| K.NGIEINC(+57.02)C(+57.02)TTDR.C | Y | 73.30 | 1451.6133 | 12 | 0.2 | 726.8141 | 2 | 20.42 | 1 | F1:6421 | NaNaKA16\_F1.raw | 8.7813E6 |  |  |  |  | 1.338E7 |  |  |  |  |  |  |  | 2 | 1 | 0 | 0 | 0 | 0 | 1 | 0 | 0 | 0 | 0 | 0 | 0 | 0 | 69 | 80 | Carbamidomethylation | C7:Carbamidomethylation:1000.00;C8:Carbamidomethylation:1000.00 | PEAKS DB |
| N.GIEINC(+57.02)C(+57.02)TTDR.C | N | 48.70 | 1337.5704 | 11 | -0.5 | 669.7922 | 2 | 18.63 | 6 | F6:6296 | NaNaKA16\_F2.raw |  |  |  |  |  | 4.8926E5 |  |  |  |  |  |  |  | 1 | 0 | 0 | 0 | 0 | 0 | 1 | 0 | 0 | 0 | 0 | 0 | 0 | 0 | 70 | 80 | Carbamidomethylation | C6:Carbamidomethylation:1000.00;C7:Carbamidomethylation:1000.00 | PEAKS DB |
| total 3 peptides |  |  |  |  |  |  |  |  |  |  |  |  |  |  |  |  |  |  |  |  |  |  |  |  |  |  |  |  |  |  |  |  |  |  |  |  |  |  |  |  |  |  |  |
| --- | --- | --- | --- | --- | --- | --- | --- | --- | --- | --- | --- | --- | --- | --- | --- | --- | --- | --- | --- | --- | --- | --- | --- | --- | --- | --- | --- | --- | --- | --- | --- | --- | --- | --- | --- | --- | --- | --- | --- | --- | --- | --- | --- |

AAB36930.1

back to list

  

| Protein Coverage
| Supporting Peptides
|

Protein Coverage:

Supporting Peptides:

| Peptide | Uniq | -10lgP | Mass | Length | ppm | m/z | z | RT | Fraction | Scan | Source File | Area F1 | Area F10 | Area F11 | Area F12 | Area F13 | Area F2 | Area F3 | Area F4 | Area F5 | Area F6 | Area F7 | Area F8 | Area F9 | #Feature | #Feature F1 | #Feature F10 | #Feature F11 | #Feature F12 | #Feature F13 | #Feature F2 | #Feature F3 | #Feature F4 | #Feature F5 | #Feature F6 | #Feature F7 | #Feature F8 | #Feature F9 | Start | End | PTM | AScore | Found By |
| --- | --- | --- | --- | --- | --- | --- | --- | --- | --- | --- | --- | --- | --- | --- | --- | --- | --- | --- | --- | --- | --- | --- | --- | --- | --- | --- | --- | --- | --- | --- | --- | --- | --- | --- | --- | --- | --- | --- | --- | --- | --- | --- | --- |
| T.LEC(+57.02)HNQQSSQTPTTTGC(+57.02)SGGETNC(+57.02)YK.K | Y | 81.34 | 2944.2021 | 26 | -3.1 | 982.4050 | 3 | 11.84 | 6 | F6:2150 | NaNaKA16\_F2.raw | 1.1842E6 |  |  |  |  | 1.0244E8 |  |  |  |  |  |  |  | 2 | 1 | 0 | 0 | 0 | 0 | 1 | 0 | 0 | 0 | 0 | 0 | 0 | 0 | 22 | 47 | Carbamidomethylation | C3:Carbamidomethylation:1000.00;C17:Carbamidomethylation:1000.00;C24:Carbamidomethylation:1000.00 | PEAKS DB |
| K.NGIEINC(+57.02)C(+57.02)TTDR.C | Y | 73.30 | 1451.6133 | 12 | 0.2 | 726.8141 | 2 | 20.42 | 1 | F1:6421 | NaNaKA16\_F1.raw | 8.7813E6 |  |  |  |  | 1.338E7 |  |  |  |  |  |  |  | 2 | 1 | 0 | 0 | 0 | 0 | 1 | 0 | 0 | 0 | 0 | 0 | 0 | 0 | 69 | 80 | Carbamidomethylation | C7:Carbamidomethylation:1000.00;C8:Carbamidomethylation:1000.00 | PEAKS DB |
| N.GIEINC(+57.02)C(+57.02)TTDR.C | N | 48.70 | 1337.5704 | 11 | -0.5 | 669.7922 | 2 | 18.63 | 6 | F6:6296 | NaNaKA16\_F2.raw |  |  |  |  |  | 4.8926E5 |  |  |  |  |  |  |  | 1 | 0 | 0 | 0 | 0 | 0 | 1 | 0 | 0 | 0 | 0 | 0 | 0 | 0 | 70 | 80 | Carbamidomethylation | C6:Carbamidomethylation:1000.00;C7:Carbamidomethylation:1000.00 | PEAKS DB |
| total 3 peptides |  |  |  |  |  |  |  |  |  |  |  |  |  |  |  |  |  |  |  |  |  |  |  |  |  |  |  |  |  |  |  |  |  |  |  |  |  |  |  |  |  |  |  |
| --- | --- | --- | --- | --- | --- | --- | --- | --- | --- | --- | --- | --- | --- | --- | --- | --- | --- | --- | --- | --- | --- | --- | --- | --- | --- | --- | --- | --- | --- | --- | --- | --- | --- | --- | --- | --- | --- | --- | --- | --- | --- | --- | --- |

AAB03222.1

back to list

  

| Protein Coverage
| Supporting Peptides
|

Protein Coverage:

Supporting Peptides:

| Peptide | Uniq | -10lgP | Mass | Length | ppm | m/z | z | RT | Fraction | Scan | Source File | Area F1 | Area F10 | Area F11 | Area F12 | Area F13 | Area F2 | Area F3 | Area F4 | Area F5 | Area F6 | Area F7 | Area F8 | Area F9 | #Feature | #Feature F1 | #Feature F10 | #Feature F11 | #Feature F12 | #Feature F13 | #Feature F2 | #Feature F3 | #Feature F4 | #Feature F5 | #Feature F6 | #Feature F7 | #Feature F8 | #Feature F9 | Start | End | PTM | AScore | Found By |
| --- | --- | --- | --- | --- | --- | --- | --- | --- | --- | --- | --- | --- | --- | --- | --- | --- | --- | --- | --- | --- | --- | --- | --- | --- | --- | --- | --- | --- | --- | --- | --- | --- | --- | --- | --- | --- | --- | --- | --- | --- | --- | --- | --- |
| T.LEC(+57.02)HNQQSSQTPTTTGC(+57.02)SGGETNC(+57.02)YK.K | Y | 81.34 | 2944.2021 | 26 | -3.1 | 982.4050 | 3 | 11.84 | 6 | F6:2150 | NaNaKA16\_F2.raw | 1.1842E6 |  |  |  |  | 1.0244E8 |  |  |  |  |  |  |  | 2 | 1 | 0 | 0 | 0 | 0 | 1 | 0 | 0 | 0 | 0 | 0 | 0 | 0 | 22 | 47 | Carbamidomethylation | C3:Carbamidomethylation:1000.00;C17:Carbamidomethylation:1000.00;C24:Carbamidomethylation:1000.00 | PEAKS DB |
| K.NGIEINC(+57.02)C(+57.02)TTDR.C | Y | 73.30 | 1451.6133 | 12 | 0.2 | 726.8141 | 2 | 20.42 | 1 | F1:6421 | NaNaKA16\_F1.raw | 8.7813E6 |  |  |  |  | 1.338E7 |  |  |  |  |  |  |  | 2 | 1 | 0 | 0 | 0 | 0 | 1 | 0 | 0 | 0 | 0 | 0 | 0 | 0 | 69 | 80 | Carbamidomethylation | C7:Carbamidomethylation:1000.00;C8:Carbamidomethylation:1000.00 | PEAKS DB |
| N.GIEINC(+57.02)C(+57.02)TTDR.C | N | 48.70 | 1337.5704 | 11 | -0.5 | 669.7922 | 2 | 18.63 | 6 | F6:6296 | NaNaKA16\_F2.raw |  |  |  |  |  | 4.8926E5 |  |  |  |  |  |  |  | 1 | 0 | 0 | 0 | 0 | 0 | 1 | 0 | 0 | 0 | 0 | 0 | 0 | 0 | 70 | 80 | Carbamidomethylation | C6:Carbamidomethylation:1000.00;C7:Carbamidomethylation:1000.00 | PEAKS DB |
| total 3 peptides |  |  |  |  |  |  |  |  |  |  |  |  |  |  |  |  |  |  |  |  |  |  |  |  |  |  |  |  |  |  |  |  |  |  |  |  |  |  |  |  |  |  |  |
| --- | --- | --- | --- | --- | --- | --- | --- | --- | --- | --- | --- | --- | --- | --- | --- | --- | --- | --- | --- | --- | --- | --- | --- | --- | --- | --- | --- | --- | --- | --- | --- | --- | --- | --- | --- | --- | --- | --- | --- | --- | --- | --- | --- |

CAA73097.2

back to list

  

| Protein Coverage
| Supporting Peptides
|

Protein Coverage:

Supporting Peptides:

| Peptide | Uniq | -10lgP | Mass | Length | ppm | m/z | z | RT | Fraction | Scan | Source File | Area F1 | Area F10 | Area F11 | Area F12 | Area F13 | Area F2 | Area F3 | Area F4 | Area F5 | Area F6 | Area F7 | Area F8 | Area F9 | #Feature | #Feature F1 | #Feature F10 | #Feature F11 | #Feature F12 | #Feature F13 | #Feature F2 | #Feature F3 | #Feature F4 | #Feature F5 | #Feature F6 | #Feature F7 | #Feature F8 | #Feature F9 | Start | End | PTM | AScore | Found By |
| --- | --- | --- | --- | --- | --- | --- | --- | --- | --- | --- | --- | --- | --- | --- | --- | --- | --- | --- | --- | --- | --- | --- | --- | --- | --- | --- | --- | --- | --- | --- | --- | --- | --- | --- | --- | --- | --- | --- | --- | --- | --- | --- | --- |
| T.LEC(+57.02)HNQQSSQTPTTTGC(+57.02)SGGETNC(+57.02)YK.K | Y | 81.34 | 2944.2021 | 26 | -3.1 | 982.4050 | 3 | 11.84 | 6 | F6:2150 | NaNaKA16\_F2.raw | 1.1842E6 |  |  |  |  | 1.0244E8 |  |  |  |  |  |  |  | 2 | 1 | 0 | 0 | 0 | 0 | 1 | 0 | 0 | 0 | 0 | 0 | 0 | 0 | 22 | 47 | Carbamidomethylation | C3:Carbamidomethylation:1000.00;C17:Carbamidomethylation:1000.00;C24:Carbamidomethylation:1000.00 | PEAKS DB |
| K.NGIEINC(+57.02)C(+57.02)TTDR.C | Y | 73.30 | 1451.6133 | 12 | 0.2 | 726.8141 | 2 | 20.42 | 1 | F1:6421 | NaNaKA16\_F1.raw | 8.7813E6 |  |  |  |  | 1.338E7 |  |  |  |  |  |  |  | 2 | 1 | 0 | 0 | 0 | 0 | 1 | 0 | 0 | 0 | 0 | 0 | 0 | 0 | 69 | 80 | Carbamidomethylation | C7:Carbamidomethylation:1000.00;C8:Carbamidomethylation:1000.00 | PEAKS DB |
| N.GIEINC(+57.02)C(+57.02)TTDR.C | N | 48.70 | 1337.5704 | 11 | -0.5 | 669.7922 | 2 | 18.63 | 6 | F6:6296 | NaNaKA16\_F2.raw |  |  |  |  |  | 4.8926E5 |  |  |  |  |  |  |  | 1 | 0 | 0 | 0 | 0 | 0 | 1 | 0 | 0 | 0 | 0 | 0 | 0 | 0 | 70 | 80 | Carbamidomethylation | C6:Carbamidomethylation:1000.00;C7:Carbamidomethylation:1000.00 | PEAKS DB |
| total 3 peptides |  |  |  |  |  |  |  |  |  |  |  |  |  |  |  |  |  |  |  |  |  |  |  |  |  |  |  |  |  |  |  |  |  |  |  |  |  |  |  |  |  |  |  |
| --- | --- | --- | --- | --- | --- | --- | --- | --- | --- | --- | --- | --- | --- | --- | --- | --- | --- | --- | --- | --- | --- | --- | --- | --- | --- | --- | --- | --- | --- | --- | --- | --- | --- | --- | --- | --- | --- | --- | --- | --- | --- | --- | --- |

AAB03221.1

back to list

  

| Protein Coverage
| Supporting Peptides
|

Protein Coverage:

Supporting Peptides:

| Peptide | Uniq | -10lgP | Mass | Length | ppm | m/z | z | RT | Fraction | Scan | Source File | Area F1 | Area F10 | Area F11 | Area F12 | Area F13 | Area F2 | Area F3 | Area F4 | Area F5 | Area F6 | Area F7 | Area F8 | Area F9 | #Feature | #Feature F1 | #Feature F10 | #Feature F11 | #Feature F12 | #Feature F13 | #Feature F2 | #Feature F3 | #Feature F4 | #Feature F5 | #Feature F6 | #Feature F7 | #Feature F8 | #Feature F9 | Start | End | PTM | AScore | Found By |
| --- | --- | --- | --- | --- | --- | --- | --- | --- | --- | --- | --- | --- | --- | --- | --- | --- | --- | --- | --- | --- | --- | --- | --- | --- | --- | --- | --- | --- | --- | --- | --- | --- | --- | --- | --- | --- | --- | --- | --- | --- | --- | --- | --- |
| T.LEC(+57.02)HNQQSSQTPTTTGC(+57.02)SGGETNC(+57.02)YK.K | Y | 81.34 | 2944.2021 | 26 | -3.1 | 982.4050 | 3 | 11.84 | 6 | F6:2150 | NaNaKA16\_F2.raw | 1.1842E6 |  |  |  |  | 1.0244E8 |  |  |  |  |  |  |  | 2 | 1 | 0 | 0 | 0 | 0 | 1 | 0 | 0 | 0 | 0 | 0 | 0 | 0 | 22 | 47 | Carbamidomethylation | C3:Carbamidomethylation:1000.00;C17:Carbamidomethylation:1000.00;C24:Carbamidomethylation:1000.00 | PEAKS DB |
| K.NGIEINC(+57.02)C(+57.02)TTDR.C | Y | 73.30 | 1451.6133 | 12 | 0.2 | 726.8141 | 2 | 20.42 | 1 | F1:6421 | NaNaKA16\_F1.raw | 8.7813E6 |  |  |  |  | 1.338E7 |  |  |  |  |  |  |  | 2 | 1 | 0 | 0 | 0 | 0 | 1 | 0 | 0 | 0 | 0 | 0 | 0 | 0 | 69 | 80 | Carbamidomethylation | C7:Carbamidomethylation:1000.00;C8:Carbamidomethylation:1000.00 | PEAKS DB |
| N.GIEINC(+57.02)C(+57.02)TTDR.C | N | 48.70 | 1337.5704 | 11 | -0.5 | 669.7922 | 2 | 18.63 | 6 | F6:6296 | NaNaKA16\_F2.raw |  |  |  |  |  | 4.8926E5 |  |  |  |  |  |  |  | 1 | 0 | 0 | 0 | 0 | 0 | 1 | 0 | 0 | 0 | 0 | 0 | 0 | 0 | 70 | 80 | Carbamidomethylation | C6:Carbamidomethylation:1000.00;C7:Carbamidomethylation:1000.00 | PEAKS DB |
| total 3 peptides |  |  |  |  |  |  |  |  |  |  |  |  |  |  |  |  |  |  |  |  |  |  |  |  |  |  |  |  |  |  |  |  |  |  |  |  |  |  |  |  |  |  |  |
| --- | --- | --- | --- | --- | --- | --- | --- | --- | --- | --- | --- | --- | --- | --- | --- | --- | --- | --- | --- | --- | --- | --- | --- | --- | --- | --- | --- | --- | --- | --- | --- | --- | --- | --- | --- | --- | --- | --- | --- | --- | --- | --- | --- |

ADN67574.1

back to list

  

| Protein Coverage
| Supporting Peptides
|

Protein Coverage:

Supporting Peptides:

| Peptide | Uniq | -10lgP | Mass | Length | ppm | m/z | z | RT | Fraction | Scan | Source File | Area F1 | Area F10 | Area F11 | Area F12 | Area F13 | Area F2 | Area F3 | Area F4 | Area F5 | Area F6 | Area F7 | Area F8 | Area F9 | #Feature | #Feature F1 | #Feature F10 | #Feature F11 | #Feature F12 | #Feature F13 | #Feature F2 | #Feature F3 | #Feature F4 | #Feature F5 | #Feature F6 | #Feature F7 | #Feature F8 | #Feature F9 | Start | End | PTM | AScore | Found By |
| --- | --- | --- | --- | --- | --- | --- | --- | --- | --- | --- | --- | --- | --- | --- | --- | --- | --- | --- | --- | --- | --- | --- | --- | --- | --- | --- | --- | --- | --- | --- | --- | --- | --- | --- | --- | --- | --- | --- | --- | --- | --- | --- | --- |
| T.LEC(+57.02)HNQQSSQTPTTTGC(+57.02)SGGETNC(+57.02)YK.K | Y | 81.34 | 2944.2021 | 26 | -3.1 | 982.4050 | 3 | 11.84 | 6 | F6:2150 | NaNaKA16\_F2.raw | 1.1842E6 |  |  |  |  | 1.0244E8 |  |  |  |  |  |  |  | 2 | 1 | 0 | 0 | 0 | 0 | 1 | 0 | 0 | 0 | 0 | 0 | 0 | 0 | 22 | 47 | Carbamidomethylation | C3:Carbamidomethylation:1000.00;C17:Carbamidomethylation:1000.00;C24:Carbamidomethylation:1000.00 | PEAKS DB |
| K.NGIEINC(+57.02)C(+57.02)TTDR.C | Y | 73.30 | 1451.6133 | 12 | 0.2 | 726.8141 | 2 | 20.42 | 1 | F1:6421 | NaNaKA16\_F1.raw | 8.7813E6 |  |  |  |  | 1.338E7 |  |  |  |  |  |  |  | 2 | 1 | 0 | 0 | 0 | 0 | 1 | 0 | 0 | 0 | 0 | 0 | 0 | 0 | 69 | 80 | Carbamidomethylation | C7:Carbamidomethylation:1000.00;C8:Carbamidomethylation:1000.00 | PEAKS DB |
| N.GIEINC(+57.02)C(+57.02)TTDR.C | N | 48.70 | 1337.5704 | 11 | -0.5 | 669.7922 | 2 | 18.63 | 6 | F6:6296 | NaNaKA16\_F2.raw |  |  |  |  |  | 4.8926E5 |  |  |  |  |  |  |  | 1 | 0 | 0 | 0 | 0 | 0 | 1 | 0 | 0 | 0 | 0 | 0 | 0 | 0 | 70 | 80 | Carbamidomethylation | C6:Carbamidomethylation:1000.00;C7:Carbamidomethylation:1000.00 | PEAKS DB |
| total 3 peptides |  |  |  |  |  |  |  |  |  |  |  |  |  |  |  |  |  |  |  |  |  |  |  |  |  |  |  |  |  |  |  |  |  |  |  |  |  |  |  |  |  |  |  |
| --- | --- | --- | --- | --- | --- | --- | --- | --- | --- | --- | --- | --- | --- | --- | --- | --- | --- | --- | --- | --- | --- | --- | --- | --- | --- | --- | --- | --- | --- | --- | --- | --- | --- | --- | --- | --- | --- | --- | --- | --- | --- | --- | --- |

AAB03223.1

back to list

  

| Protein Coverage
| Supporting Peptides
|

Protein Coverage:

Supporting Peptides:

| Peptide | Uniq | -10lgP | Mass | Length | ppm | m/z | z | RT | Fraction | Scan | Source File | Area F1 | Area F10 | Area F11 | Area F12 | Area F13 | Area F2 | Area F3 | Area F4 | Area F5 | Area F6 | Area F7 | Area F8 | Area F9 | #Feature | #Feature F1 | #Feature F10 | #Feature F11 | #Feature F12 | #Feature F13 | #Feature F2 | #Feature F3 | #Feature F4 | #Feature F5 | #Feature F6 | #Feature F7 | #Feature F8 | #Feature F9 | Start | End | PTM | AScore | Found By |
| --- | --- | --- | --- | --- | --- | --- | --- | --- | --- | --- | --- | --- | --- | --- | --- | --- | --- | --- | --- | --- | --- | --- | --- | --- | --- | --- | --- | --- | --- | --- | --- | --- | --- | --- | --- | --- | --- | --- | --- | --- | --- | --- | --- |
| T.LEC(+57.02)HNQQSSQTPTTTGC(+57.02)SGGETNC(+57.02)YK.K | Y | 81.34 | 2944.2021 | 26 | -3.1 | 982.4050 | 3 | 11.84 | 6 | F6:2150 | NaNaKA16\_F2.raw | 1.1842E6 |  |  |  |  | 1.0244E8 |  |  |  |  |  |  |  | 2 | 1 | 0 | 0 | 0 | 0 | 1 | 0 | 0 | 0 | 0 | 0 | 0 | 0 | 22 | 47 | Carbamidomethylation | C3:Carbamidomethylation:1000.00;C17:Carbamidomethylation:1000.00;C24:Carbamidomethylation:1000.00 | PEAKS DB |
| K.NGIEINC(+57.02)C(+57.02)TTDR.C | Y | 73.30 | 1451.6133 | 12 | 0.2 | 726.8141 | 2 | 20.42 | 1 | F1:6421 | NaNaKA16\_F1.raw | 8.7813E6 |  |  |  |  | 1.338E7 |  |  |  |  |  |  |  | 2 | 1 | 0 | 0 | 0 | 0 | 1 | 0 | 0 | 0 | 0 | 0 | 0 | 0 | 69 | 80 | Carbamidomethylation | C7:Carbamidomethylation:1000.00;C8:Carbamidomethylation:1000.00 | PEAKS DB |
| N.GIEINC(+57.02)C(+57.02)TTDR.C | N | 48.70 | 1337.5704 | 11 | -0.5 | 669.7922 | 2 | 18.63 | 6 | F6:6296 | NaNaKA16\_F2.raw |  |  |  |  |  | 4.8926E5 |  |  |  |  |  |  |  | 1 | 0 | 0 | 0 | 0 | 0 | 1 | 0 | 0 | 0 | 0 | 0 | 0 | 0 | 70 | 80 | Carbamidomethylation | C6:Carbamidomethylation:1000.00;C7:Carbamidomethylation:1000.00 | PEAKS DB |
| total 3 peptides |  |  |  |  |  |  |  |  |  |  |  |  |  |  |  |  |  |  |  |  |  |  |  |  |  |  |  |  |  |  |  |  |  |  |  |  |  |  |  |  |  |  |  |
| --- | --- | --- | --- | --- | --- | --- | --- | --- | --- | --- | --- | --- | --- | --- | --- | --- | --- | --- | --- | --- | --- | --- | --- | --- | --- | --- | --- | --- | --- | --- | --- | --- | --- | --- | --- | --- | --- | --- | --- | --- | --- | --- | --- |

AAB36931.1

back to list

  

| Protein Coverage
| Supporting Peptides
|

Protein Coverage:

Supporting Peptides:

| Peptide | Uniq | -10lgP | Mass | Length | ppm | m/z | z | RT | Fraction | Scan | Source File | Area F1 | Area F10 | Area F11 | Area F12 | Area F13 | Area F2 | Area F3 | Area F4 | Area F5 | Area F6 | Area F7 | Area F8 | Area F9 | #Feature | #Feature F1 | #Feature F10 | #Feature F11 | #Feature F12 | #Feature F13 | #Feature F2 | #Feature F3 | #Feature F4 | #Feature F5 | #Feature F6 | #Feature F7 | #Feature F8 | #Feature F9 | Start | End | PTM | AScore | Found By |
| --- | --- | --- | --- | --- | --- | --- | --- | --- | --- | --- | --- | --- | --- | --- | --- | --- | --- | --- | --- | --- | --- | --- | --- | --- | --- | --- | --- | --- | --- | --- | --- | --- | --- | --- | --- | --- | --- | --- | --- | --- | --- | --- | --- |
| T.LEC(+57.02)HNQQSSQTPTTTGC(+57.02)SGGETNC(+57.02)YK.K | Y | 81.34 | 2944.2021 | 26 | -3.1 | 982.4050 | 3 | 11.84 | 6 | F6:2150 | NaNaKA16\_F2.raw | 1.1842E6 |  |  |  |  | 1.0244E8 |  |  |  |  |  |  |  | 2 | 1 | 0 | 0 | 0 | 0 | 1 | 0 | 0 | 0 | 0 | 0 | 0 | 0 | 22 | 47 | Carbamidomethylation | C3:Carbamidomethylation:1000.00;C17:Carbamidomethylation:1000.00;C24:Carbamidomethylation:1000.00 | PEAKS DB |
| K.NGIEINC(+57.02)C(+57.02)TTDR.C | Y | 73.30 | 1451.6133 | 12 | 0.2 | 726.8141 | 2 | 20.42 | 1 | F1:6421 | NaNaKA16\_F1.raw | 8.7813E6 |  |  |  |  | 1.338E7 |  |  |  |  |  |  |  | 2 | 1 | 0 | 0 | 0 | 0 | 1 | 0 | 0 | 0 | 0 | 0 | 0 | 0 | 69 | 80 | Carbamidomethylation | C7:Carbamidomethylation:1000.00;C8:Carbamidomethylation:1000.00 | PEAKS DB |
| N.GIEINC(+57.02)C(+57.02)TTDR.C | N | 48.70 | 1337.5704 | 11 | -0.5 | 669.7922 | 2 | 18.63 | 6 | F6:6296 | NaNaKA16\_F2.raw |  |  |  |  |  | 4.8926E5 |  |  |  |  |  |  |  | 1 | 0 | 0 | 0 | 0 | 0 | 1 | 0 | 0 | 0 | 0 | 0 | 0 | 0 | 70 | 80 | Carbamidomethylation | C6:Carbamidomethylation:1000.00;C7:Carbamidomethylation:1000.00 | PEAKS DB |
| total 3 peptides |  |  |  |  |  |  |  |  |  |  |  |  |  |  |  |  |  |  |  |  |  |  |  |  |  |  |  |  |  |  |  |  |  |  |  |  |  |  |  |  |  |  |  |
| --- | --- | --- | --- | --- | --- | --- | --- | --- | --- | --- | --- | --- | --- | --- | --- | --- | --- | --- | --- | --- | --- | --- | --- | --- | --- | --- | --- | --- | --- | --- | --- | --- | --- | --- | --- | --- | --- | --- | --- | --- | --- | --- | --- |

AAB01538.1

back to list

  

| Protein Coverage
| Supporting Peptides
|

Protein Coverage:

Supporting Peptides:

| Peptide | Uniq | -10lgP | Mass | Length | ppm | m/z | z | RT | Fraction | Scan | Source File | Area F1 | Area F10 | Area F11 | Area F12 | Area F13 | Area F2 | Area F3 | Area F4 | Area F5 | Area F6 | Area F7 | Area F8 | Area F9 | #Feature | #Feature F1 | #Feature F10 | #Feature F11 | #Feature F12 | #Feature F13 | #Feature F2 | #Feature F3 | #Feature F4 | #Feature F5 | #Feature F6 | #Feature F7 | #Feature F8 | #Feature F9 | Start | End | PTM | AScore | Found By |
| --- | --- | --- | --- | --- | --- | --- | --- | --- | --- | --- | --- | --- | --- | --- | --- | --- | --- | --- | --- | --- | --- | --- | --- | --- | --- | --- | --- | --- | --- | --- | --- | --- | --- | --- | --- | --- | --- | --- | --- | --- | --- | --- | --- |
| T.LEC(+57.02)HNQQSSQTPTTTGC(+57.02)SGGETNC(+57.02)YK.K | Y | 81.34 | 2944.2021 | 26 | -3.1 | 982.4050 | 3 | 11.84 | 6 | F6:2150 | NaNaKA16\_F2.raw | 1.1842E6 |  |  |  |  | 1.0244E8 |  |  |  |  |  |  |  | 2 | 1 | 0 | 0 | 0 | 0 | 1 | 0 | 0 | 0 | 0 | 0 | 0 | 0 | 22 | 47 | Carbamidomethylation | C3:Carbamidomethylation:1000.00;C17:Carbamidomethylation:1000.00;C24:Carbamidomethylation:1000.00 | PEAKS DB |
| K.NGIEINC(+57.02)C(+57.02)TTDR.C | Y | 73.30 | 1451.6133 | 12 | 0.2 | 726.8141 | 2 | 20.42 | 1 | F1:6421 | NaNaKA16\_F1.raw | 8.7813E6 |  |  |  |  | 1.338E7 |  |  |  |  |  |  |  | 2 | 1 | 0 | 0 | 0 | 0 | 1 | 0 | 0 | 0 | 0 | 0 | 0 | 0 | 69 | 80 | Carbamidomethylation | C7:Carbamidomethylation:1000.00;C8:Carbamidomethylation:1000.00 | PEAKS DB |
| N.GIEINC(+57.02)C(+57.02)TTDR.C | N | 48.70 | 1337.5704 | 11 | -0.5 | 669.7922 | 2 | 18.63 | 6 | F6:6296 | NaNaKA16\_F2.raw |  |  |  |  |  | 4.8926E5 |  |  |  |  |  |  |  | 1 | 0 | 0 | 0 | 0 | 0 | 1 | 0 | 0 | 0 | 0 | 0 | 0 | 0 | 70 | 80 | Carbamidomethylation | C6:Carbamidomethylation:1000.00;C7:Carbamidomethylation:1000.00 | PEAKS DB |
| total 3 peptides |  |  |  |  |  |  |  |  |  |  |  |  |  |  |  |  |  |  |  |  |  |  |  |  |  |  |  |  |  |  |  |  |  |  |  |  |  |  |  |  |  |  |  |
| --- | --- | --- | --- | --- | --- | --- | --- | --- | --- | --- | --- | --- | --- | --- | --- | --- | --- | --- | --- | --- | --- | --- | --- | --- | --- | --- | --- | --- | --- | --- | --- | --- | --- | --- | --- | --- | --- | --- | --- | --- | --- | --- | --- |

AAF21774.1

back to list

  

| Protein Coverage
| Supporting Peptides
|

Protein Coverage:

Supporting Peptides:

| Peptide | Uniq | -10lgP | Mass | Length | ppm | m/z | z | RT | Fraction | Scan | Source File | Area F1 | Area F10 | Area F11 | Area F12 | Area F13 | Area F2 | Area F3 | Area F4 | Area F5 | Area F6 | Area F7 | Area F8 | Area F9 | #Feature | #Feature F1 | #Feature F10 | #Feature F11 | #Feature F12 | #Feature F13 | #Feature F2 | #Feature F3 | #Feature F4 | #Feature F5 | #Feature F6 | #Feature F7 | #Feature F8 | #Feature F9 | Start | End | PTM | AScore | Found By |
| --- | --- | --- | --- | --- | --- | --- | --- | --- | --- | --- | --- | --- | --- | --- | --- | --- | --- | --- | --- | --- | --- | --- | --- | --- | --- | --- | --- | --- | --- | --- | --- | --- | --- | --- | --- | --- | --- | --- | --- | --- | --- | --- | --- |
| T.LEC(+57.02)HNQQSSQTPTTTGC(+57.02)SGGETNC(+57.02)YK.K | Y | 81.34 | 2944.2021 | 26 | -3.1 | 982.4050 | 3 | 11.84 | 6 | F6:2150 | NaNaKA16\_F2.raw | 1.1842E6 |  |  |  |  | 1.0244E8 |  |  |  |  |  |  |  | 2 | 1 | 0 | 0 | 0 | 0 | 1 | 0 | 0 | 0 | 0 | 0 | 0 | 0 | 22 | 47 | Carbamidomethylation | C3:Carbamidomethylation:1000.00;C17:Carbamidomethylation:1000.00;C24:Carbamidomethylation:1000.00 | PEAKS DB |
| K.NGIEINC(+57.02)C(+57.02)TTDR.C | Y | 73.30 | 1451.6133 | 12 | 0.2 | 726.8141 | 2 | 20.42 | 1 | F1:6421 | NaNaKA16\_F1.raw | 8.7813E6 |  |  |  |  | 1.338E7 |  |  |  |  |  |  |  | 2 | 1 | 0 | 0 | 0 | 0 | 1 | 0 | 0 | 0 | 0 | 0 | 0 | 0 | 69 | 80 | Carbamidomethylation | C7:Carbamidomethylation:1000.00;C8:Carbamidomethylation:1000.00 | PEAKS DB |
| N.GIEINC(+57.02)C(+57.02)TTDR.C | N | 48.70 | 1337.5704 | 11 | -0.5 | 669.7922 | 2 | 18.63 | 6 | F6:6296 | NaNaKA16\_F2.raw |  |  |  |  |  | 4.8926E5 |  |  |  |  |  |  |  | 1 | 0 | 0 | 0 | 0 | 0 | 1 | 0 | 0 | 0 | 0 | 0 | 0 | 0 | 70 | 80 | Carbamidomethylation | C6:Carbamidomethylation:1000.00;C7:Carbamidomethylation:1000.00 | PEAKS DB |
| total 3 peptides |  |  |  |  |  |  |  |  |  |  |  |  |  |  |  |  |  |  |  |  |  |  |  |  |  |  |  |  |  |  |  |  |  |  |  |  |  |  |  |  |  |  |  |
| --- | --- | --- | --- | --- | --- | --- | --- | --- | --- | --- | --- | --- | --- | --- | --- | --- | --- | --- | --- | --- | --- | --- | --- | --- | --- | --- | --- | --- | --- | --- | --- | --- | --- | --- | --- | --- | --- | --- | --- | --- | --- | --- | --- |

AAR33035.1

back to list

  

| Protein Coverage
| Supporting Peptides
|

Protein Coverage:

Supporting Peptides:

| Peptide | Uniq | -10lgP | Mass | Length | ppm | m/z | z | RT | Fraction | Scan | Source File | Area F1 | Area F10 | Area F11 | Area F12 | Area F13 | Area F2 | Area F3 | Area F4 | Area F5 | Area F6 | Area F7 | Area F8 | Area F9 | #Feature | #Feature F1 | #Feature F10 | #Feature F11 | #Feature F12 | #Feature F13 | #Feature F2 | #Feature F3 | #Feature F4 | #Feature F5 | #Feature F6 | #Feature F7 | #Feature F8 | #Feature F9 | Start | End | PTM | AScore | Found By |
| --- | --- | --- | --- | --- | --- | --- | --- | --- | --- | --- | --- | --- | --- | --- | --- | --- | --- | --- | --- | --- | --- | --- | --- | --- | --- | --- | --- | --- | --- | --- | --- | --- | --- | --- | --- | --- | --- | --- | --- | --- | --- | --- | --- |
| T.LEC(+57.02)HNQQSSQTPTTTGC(+57.02)SGGETNC(+57.02)YK.K | Y | 81.34 | 2944.2021 | 26 | -3.1 | 982.4050 | 3 | 11.84 | 6 | F6:2150 | NaNaKA16\_F2.raw | 1.1842E6 |  |  |  |  | 1.0244E8 |  |  |  |  |  |  |  | 2 | 1 | 0 | 0 | 0 | 0 | 1 | 0 | 0 | 0 | 0 | 0 | 0 | 0 | 22 | 47 | Carbamidomethylation | C3:Carbamidomethylation:1000.00;C17:Carbamidomethylation:1000.00;C24:Carbamidomethylation:1000.00 | PEAKS DB |
| K.NGIEINC(+57.02)C(+57.02)TTDR.C | Y | 73.30 | 1451.6133 | 12 | 0.2 | 726.8141 | 2 | 20.42 | 1 | F1:6421 | NaNaKA16\_F1.raw | 8.7813E6 |  |  |  |  | 1.338E7 |  |  |  |  |  |  |  | 2 | 1 | 0 | 0 | 0 | 0 | 1 | 0 | 0 | 0 | 0 | 0 | 0 | 0 | 69 | 80 | Carbamidomethylation | C7:Carbamidomethylation:1000.00;C8:Carbamidomethylation:1000.00 | PEAKS DB |
| N.GIEINC(+57.02)C(+57.02)TTDR.C | N | 48.70 | 1337.5704 | 11 | -0.5 | 669.7922 | 2 | 18.63 | 6 | F6:6296 | NaNaKA16\_F2.raw |  |  |  |  |  | 4.8926E5 |  |  |  |  |  |  |  | 1 | 0 | 0 | 0 | 0 | 0 | 1 | 0 | 0 | 0 | 0 | 0 | 0 | 0 | 70 | 80 | Carbamidomethylation | C6:Carbamidomethylation:1000.00;C7:Carbamidomethylation:1000.00 | PEAKS DB |
| total 3 peptides |  |  |  |  |  |  |  |  |  |  |  |  |  |  |  |  |  |  |  |  |  |  |  |  |  |  |  |  |  |  |  |  |  |  |  |  |  |  |  |  |  |  |  |
| --- | --- | --- | --- | --- | --- | --- | --- | --- | --- | --- | --- | --- | --- | --- | --- | --- | --- | --- | --- | --- | --- | --- | --- | --- | --- | --- | --- | --- | --- | --- | --- | --- | --- | --- | --- | --- | --- | --- | --- | --- | --- | --- | --- |

LAB46845.1

back to list

  

| Protein Coverage
| Supporting Peptides
|

Protein Coverage:

Supporting Peptides:

| Peptide | Uniq | -10lgP | Mass | Length | ppm | m/z | z | RT | Fraction | Scan | Source File | Area F1 | Area F10 | Area F11 | Area F12 | Area F13 | Area F2 | Area F3 | Area F4 | Area F5 | Area F6 | Area F7 | Area F8 | Area F9 | #Feature | #Feature F1 | #Feature F10 | #Feature F11 | #Feature F12 | #Feature F13 | #Feature F2 | #Feature F3 | #Feature F4 | #Feature F5 | #Feature F6 | #Feature F7 | #Feature F8 | #Feature F9 | Start | End | PTM | AScore | Found By |
| --- | --- | --- | --- | --- | --- | --- | --- | --- | --- | --- | --- | --- | --- | --- | --- | --- | --- | --- | --- | --- | --- | --- | --- | --- | --- | --- | --- | --- | --- | --- | --- | --- | --- | --- | --- | --- | --- | --- | --- | --- | --- | --- | --- |
| A.GGSGTPVDDLDR.C | N | 67.81 | 1187.5417 | 12 | 0.8 | 594.7786 | 2 | 19.63 | 10 | F10:6988 | NaNaKA16\_F6.raw |  |  |  |  |  |  |  |  |  | 5.7224E7 | 2.6509E9 | 3.9395E6 |  | 4 | 0 | 0 | 0 | 0 | 0 | 0 | 0 | 0 | 0 | 2 | 1 | 1 | 0 | 25 | 36 |  |  | PEAKS DB |
| G.GSGTPVDDLDR.C | N | 60.74 | 1130.5204 | 11 | -0.3 | 566.2673 | 2 | 21.08 | 11 | F11:8172 | NaNaKA16\_F7.raw |  |  |  |  |  |  |  |  |  | 1.4052E5 | 1.2671E7 |  |  | 2 | 0 | 0 | 0 | 0 | 0 | 0 | 0 | 0 | 0 | 1 | 1 | 0 | 0 | 26 | 36 |  |  | PEAKS DB |
| R.SAWDFTNYGC(+57.02)YC(+57.02)GAGGSGTPVDDLDR.C | Y | 46.35 | 2840.1443 | 26 | 4.4 | 947.7262 | 3 | 105.93 | 13 | F13:74891 | NaNaKA16\_F9.raw |  |  |  |  |  |  |  |  |  |  |  |  | 4.6144E7 | 1 | 0 | 0 | 0 | 0 | 0 | 0 | 0 | 0 | 0 | 0 | 0 | 0 | 1 | 11 | 36 | Carbamidomethylation | C10:Carbamidomethylation:1000.00;C12:Carbamidomethylation:1000.00 | PEAKS DB |
| G.SGTPVDDLDR.C | N | 45.52 | 1073.4989 | 10 | 0.9 | 537.7572 | 2 | 20.23 | 11 | F11:7497 | NaNaKA16\_F7.raw |  |  |  |  |  |  |  |  |  |  | 1.1072E7 |  |  | 1 | 0 | 0 | 0 | 0 | 0 | 0 | 0 | 0 | 0 | 0 | 1 | 0 | 0 | 27 | 36 |  |  | PEAKS DB |
| total 4 peptides |  |  |  |  |  |  |  |  |  |  |  |  |  |  |  |  |  |  |  |  |  |  |  |  |  |  |  |  |  |  |  |  |  |  |  |  |  |  |  |  |  |  |  |
| --- | --- | --- | --- | --- | --- | --- | --- | --- | --- | --- | --- | --- | --- | --- | --- | --- | --- | --- | --- | --- | --- | --- | --- | --- | --- | --- | --- | --- | --- | --- | --- | --- | --- | --- | --- | --- | --- | --- | --- | --- | --- | --- | --- |

LAA90412.1

back to list

  

| Protein Coverage
| Supporting Peptides
|

Protein Coverage:

Supporting Peptides:

| Peptide | Uniq | -10lgP | Mass | Length | ppm | m/z | z | RT | Fraction | Scan | Source File | Area F1 | Area F10 | Area F11 | Area F12 | Area F13 | Area F2 | Area F3 | Area F4 | Area F5 | Area F6 | Area F7 | Area F8 | Area F9 | #Feature | #Feature F1 | #Feature F10 | #Feature F11 | #Feature F12 | #Feature F13 | #Feature F2 | #Feature F3 | #Feature F4 | #Feature F5 | #Feature F6 | #Feature F7 | #Feature F8 | #Feature F9 | Start | End | PTM | AScore | Found By |
| --- | --- | --- | --- | --- | --- | --- | --- | --- | --- | --- | --- | --- | --- | --- | --- | --- | --- | --- | --- | --- | --- | --- | --- | --- | --- | --- | --- | --- | --- | --- | --- | --- | --- | --- | --- | --- | --- | --- | --- | --- | --- | --- | --- |
| A.GGSGTPVDDLDR.C | N | 67.81 | 1187.5417 | 12 | 0.8 | 594.7786 | 2 | 19.63 | 10 | F10:6988 | NaNaKA16\_F6.raw |  |  |  |  |  |  |  |  |  | 5.7224E7 | 2.6509E9 | 3.9395E6 |  | 4 | 0 | 0 | 0 | 0 | 0 | 0 | 0 | 0 | 0 | 2 | 1 | 1 | 0 | 44 | 55 |  |  | PEAKS DB |
| G.GSGTPVDDLDR.C | N | 60.74 | 1130.5204 | 11 | -0.3 | 566.2673 | 2 | 21.08 | 11 | F11:8172 | NaNaKA16\_F7.raw |  |  |  |  |  |  |  |  |  | 1.4052E5 | 1.2671E7 |  |  | 2 | 0 | 0 | 0 | 0 | 0 | 0 | 0 | 0 | 0 | 1 | 1 | 0 | 0 | 45 | 55 |  |  | PEAKS DB |
| R.SAWDFTNYGC(+57.02)YC(+57.02)GAGGSGTPVDDLDR.C | Y | 46.35 | 2840.1443 | 26 | 4.4 | 947.7262 | 3 | 105.93 | 13 | F13:74891 | NaNaKA16\_F9.raw |  |  |  |  |  |  |  |  |  |  |  |  | 4.6144E7 | 1 | 0 | 0 | 0 | 0 | 0 | 0 | 0 | 0 | 0 | 0 | 0 | 0 | 1 | 30 | 55 | Carbamidomethylation | C10:Carbamidomethylation:1000.00;C12:Carbamidomethylation:1000.00 | PEAKS DB |
| G.SGTPVDDLDR.C | N | 45.52 | 1073.4989 | 10 | 0.9 | 537.7572 | 2 | 20.23 | 11 | F11:7497 | NaNaKA16\_F7.raw |  |  |  |  |  |  |  |  |  |  | 1.1072E7 |  |  | 1 | 0 | 0 | 0 | 0 | 0 | 0 | 0 | 0 | 0 | 0 | 1 | 0 | 0 | 46 | 55 |  |  | PEAKS DB |
| total 4 peptides |  |  |  |  |  |  |  |  |  |  |  |  |  |  |  |  |  |  |  |  |  |  |  |  |  |  |  |  |  |  |  |  |  |  |  |  |  |  |  |  |  |  |  |
| --- | --- | --- | --- | --- | --- | --- | --- | --- | --- | --- | --- | --- | --- | --- | --- | --- | --- | --- | --- | --- | --- | --- | --- | --- | --- | --- | --- | --- | --- | --- | --- | --- | --- | --- | --- | --- | --- | --- | --- | --- | --- | --- | --- |

LAA90421.1

back to list

  

| Protein Coverage
| Supporting Peptides
|

Protein Coverage:

Supporting Peptides:

| Peptide | Uniq | -10lgP | Mass | Length | ppm | m/z | z | RT | Fraction | Scan | Source File | Area F1 | Area F10 | Area F11 | Area F12 | Area F13 | Area F2 | Area F3 | Area F4 | Area F5 | Area F6 | Area F7 | Area F8 | Area F9 | #Feature | #Feature F1 | #Feature F10 | #Feature F11 | #Feature F12 | #Feature F13 | #Feature F2 | #Feature F3 | #Feature F4 | #Feature F5 | #Feature F6 | #Feature F7 | #Feature F8 | #Feature F9 | Start | End | PTM | AScore | Found By |
| --- | --- | --- | --- | --- | --- | --- | --- | --- | --- | --- | --- | --- | --- | --- | --- | --- | --- | --- | --- | --- | --- | --- | --- | --- | --- | --- | --- | --- | --- | --- | --- | --- | --- | --- | --- | --- | --- | --- | --- | --- | --- | --- | --- |
| A.GGSGTPVDDLDR.C | N | 67.81 | 1187.5417 | 12 | 0.8 | 594.7786 | 2 | 19.63 | 10 | F10:6988 | NaNaKA16\_F6.raw |  |  |  |  |  |  |  |  |  | 5.7224E7 | 2.6509E9 | 3.9395E6 |  | 4 | 0 | 0 | 0 | 0 | 0 | 0 | 0 | 0 | 0 | 2 | 1 | 1 | 0 | 44 | 55 |  |  | PEAKS DB |
| G.GSGTPVDDLDR.C | N | 60.74 | 1130.5204 | 11 | -0.3 | 566.2673 | 2 | 21.08 | 11 | F11:8172 | NaNaKA16\_F7.raw |  |  |  |  |  |  |  |  |  | 1.4052E5 | 1.2671E7 |  |  | 2 | 0 | 0 | 0 | 0 | 0 | 0 | 0 | 0 | 0 | 1 | 1 | 0 | 0 | 45 | 55 |  |  | PEAKS DB |
| R.SAWDFTNYGC(+57.02)YC(+57.02)GAGGSGTPVDDLDR.C | Y | 46.35 | 2840.1443 | 26 | 4.4 | 947.7262 | 3 | 105.93 | 13 | F13:74891 | NaNaKA16\_F9.raw |  |  |  |  |  |  |  |  |  |  |  |  | 4.6144E7 | 1 | 0 | 0 | 0 | 0 | 0 | 0 | 0 | 0 | 0 | 0 | 0 | 0 | 1 | 30 | 55 | Carbamidomethylation | C10:Carbamidomethylation:1000.00;C12:Carbamidomethylation:1000.00 | PEAKS DB |
| G.SGTPVDDLDR.C | N | 45.52 | 1073.4989 | 10 | 0.9 | 537.7572 | 2 | 20.23 | 11 | F11:7497 | NaNaKA16\_F7.raw |  |  |  |  |  |  |  |  |  |  | 1.1072E7 |  |  | 1 | 0 | 0 | 0 | 0 | 0 | 0 | 0 | 0 | 0 | 0 | 1 | 0 | 0 | 46 | 55 |  |  | PEAKS DB |
| total 4 peptides |  |  |  |  |  |  |  |  |  |  |  |  |  |  |  |  |  |  |  |  |  |  |  |  |  |  |  |  |  |  |  |  |  |  |  |  |  |  |  |  |  |  |  |
| --- | --- | --- | --- | --- | --- | --- | --- | --- | --- | --- | --- | --- | --- | --- | --- | --- | --- | --- | --- | --- | --- | --- | --- | --- | --- | --- | --- | --- | --- | --- | --- | --- | --- | --- | --- | --- | --- | --- | --- | --- | --- | --- | --- |

LAB46837.1

back to list

  

| Protein Coverage
| Supporting Peptides
|

Protein Coverage:

Supporting Peptides:

| Peptide | Uniq | -10lgP | Mass | Length | ppm | m/z | z | RT | Fraction | Scan | Source File | Area F1 | Area F10 | Area F11 | Area F12 | Area F13 | Area F2 | Area F3 | Area F4 | Area F5 | Area F6 | Area F7 | Area F8 | Area F9 | #Feature | #Feature F1 | #Feature F10 | #Feature F11 | #Feature F12 | #Feature F13 | #Feature F2 | #Feature F3 | #Feature F4 | #Feature F5 | #Feature F6 | #Feature F7 | #Feature F8 | #Feature F9 | Start | End | PTM | AScore | Found By |
| --- | --- | --- | --- | --- | --- | --- | --- | --- | --- | --- | --- | --- | --- | --- | --- | --- | --- | --- | --- | --- | --- | --- | --- | --- | --- | --- | --- | --- | --- | --- | --- | --- | --- | --- | --- | --- | --- | --- | --- | --- | --- | --- | --- |
| A.GGSGTPVDDLDR.C | N | 67.81 | 1187.5417 | 12 | 0.8 | 594.7786 | 2 | 19.63 | 10 | F10:6988 | NaNaKA16\_F6.raw |  |  |  |  |  |  |  |  |  | 5.7224E7 | 2.6509E9 | 3.9395E6 |  | 4 | 0 | 0 | 0 | 0 | 0 | 0 | 0 | 0 | 0 | 2 | 1 | 1 | 0 | 25 | 36 |  |  | PEAKS DB |
| G.GSGTPVDDLDR.C | N | 60.74 | 1130.5204 | 11 | -0.3 | 566.2673 | 2 | 21.08 | 11 | F11:8172 | NaNaKA16\_F7.raw |  |  |  |  |  |  |  |  |  | 1.4052E5 | 1.2671E7 |  |  | 2 | 0 | 0 | 0 | 0 | 0 | 0 | 0 | 0 | 0 | 1 | 1 | 0 | 0 | 26 | 36 |  |  | PEAKS DB |
| R.SAWDFTNYGC(+57.02)YC(+57.02)GAGGSGTPVDDLDR.C | Y | 46.35 | 2840.1443 | 26 | 4.4 | 947.7262 | 3 | 105.93 | 13 | F13:74891 | NaNaKA16\_F9.raw |  |  |  |  |  |  |  |  |  |  |  |  | 4.6144E7 | 1 | 0 | 0 | 0 | 0 | 0 | 0 | 0 | 0 | 0 | 0 | 0 | 0 | 1 | 11 | 36 | Carbamidomethylation | C10:Carbamidomethylation:1000.00;C12:Carbamidomethylation:1000.00 | PEAKS DB |
| G.SGTPVDDLDR.C | N | 45.52 | 1073.4989 | 10 | 0.9 | 537.7572 | 2 | 20.23 | 11 | F11:7497 | NaNaKA16\_F7.raw |  |  |  |  |  |  |  |  |  |  | 1.1072E7 |  |  | 1 | 0 | 0 | 0 | 0 | 0 | 0 | 0 | 0 | 0 | 0 | 1 | 0 | 0 | 27 | 36 |  |  | PEAKS DB |
| total 4 peptides |  |  |  |  |  |  |  |  |  |  |  |  |  |  |  |  |  |  |  |  |  |  |  |  |  |  |  |  |  |  |  |  |  |  |  |  |  |  |  |  |  |  |  |
| --- | --- | --- | --- | --- | --- | --- | --- | --- | --- | --- | --- | --- | --- | --- | --- | --- | --- | --- | --- | --- | --- | --- | --- | --- | --- | --- | --- | --- | --- | --- | --- | --- | --- | --- | --- | --- | --- | --- | --- | --- | --- | --- | --- |

LAB46855.1

back to list

  

| Protein Coverage
| Supporting Peptides
|

Protein Coverage:

Supporting Peptides:

| Peptide | Uniq | -10lgP | Mass | Length | ppm | m/z | z | RT | Fraction | Scan | Source File | Area F1 | Area F10 | Area F11 | Area F12 | Area F13 | Area F2 | Area F3 | Area F4 | Area F5 | Area F6 | Area F7 | Area F8 | Area F9 | #Feature | #Feature F1 | #Feature F10 | #Feature F11 | #Feature F12 | #Feature F13 | #Feature F2 | #Feature F3 | #Feature F4 | #Feature F5 | #Feature F6 | #Feature F7 | #Feature F8 | #Feature F9 | Start | End | PTM | AScore | Found By |
| --- | --- | --- | --- | --- | --- | --- | --- | --- | --- | --- | --- | --- | --- | --- | --- | --- | --- | --- | --- | --- | --- | --- | --- | --- | --- | --- | --- | --- | --- | --- | --- | --- | --- | --- | --- | --- | --- | --- | --- | --- | --- | --- | --- |
| A.GGSGTPVDDLDR.C | N | 67.81 | 1187.5417 | 12 | 0.8 | 594.7786 | 2 | 19.63 | 10 | F10:6988 | NaNaKA16\_F6.raw |  |  |  |  |  |  |  |  |  | 5.7224E7 | 2.6509E9 | 3.9395E6 |  | 4 | 0 | 0 | 0 | 0 | 0 | 0 | 0 | 0 | 0 | 2 | 1 | 1 | 0 | 25 | 36 |  |  | PEAKS DB |
| G.GSGTPVDDLDR.C | N | 60.74 | 1130.5204 | 11 | -0.3 | 566.2673 | 2 | 21.08 | 11 | F11:8172 | NaNaKA16\_F7.raw |  |  |  |  |  |  |  |  |  | 1.4052E5 | 1.2671E7 |  |  | 2 | 0 | 0 | 0 | 0 | 0 | 0 | 0 | 0 | 0 | 1 | 1 | 0 | 0 | 26 | 36 |  |  | PEAKS DB |
| R.SAWDFTNYGC(+57.02)YC(+57.02)GAGGSGTPVDDLDR.C | Y | 46.35 | 2840.1443 | 26 | 4.4 | 947.7262 | 3 | 105.93 | 13 | F13:74891 | NaNaKA16\_F9.raw |  |  |  |  |  |  |  |  |  |  |  |  | 4.6144E7 | 1 | 0 | 0 | 0 | 0 | 0 | 0 | 0 | 0 | 0 | 0 | 0 | 0 | 1 | 11 | 36 | Carbamidomethylation | C10:Carbamidomethylation:1000.00;C12:Carbamidomethylation:1000.00 | PEAKS DB |
| G.SGTPVDDLDR.C | N | 45.52 | 1073.4989 | 10 | 0.9 | 537.7572 | 2 | 20.23 | 11 | F11:7497 | NaNaKA16\_F7.raw |  |  |  |  |  |  |  |  |  |  | 1.1072E7 |  |  | 1 | 0 | 0 | 0 | 0 | 0 | 0 | 0 | 0 | 0 | 0 | 1 | 0 | 0 | 27 | 36 |  |  | PEAKS DB |
| total 4 peptides |  |  |  |  |  |  |  |  |  |  |  |  |  |  |  |  |  |  |  |  |  |  |  |  |  |  |  |  |  |  |  |  |  |  |  |  |  |  |  |  |  |  |  |
| --- | --- | --- | --- | --- | --- | --- | --- | --- | --- | --- | --- | --- | --- | --- | --- | --- | --- | --- | --- | --- | --- | --- | --- | --- | --- | --- | --- | --- | --- | --- | --- | --- | --- | --- | --- | --- | --- | --- | --- | --- | --- | --- | --- |

XP\_032067281.1

back to list

  

| Protein Coverage
| Supporting Peptides
|

Protein Coverage:

Supporting Peptides:

| Peptide | Uniq | -10lgP | Mass | Length | ppm | m/z | z | RT | Fraction | Scan | Source File | Area F1 | Area F10 | Area F11 | Area F12 | Area F13 | Area F2 | Area F3 | Area F4 | Area F5 | Area F6 | Area F7 | Area F8 | Area F9 | #Feature | #Feature F1 | #Feature F10 | #Feature F11 | #Feature F12 | #Feature F13 | #Feature F2 | #Feature F3 | #Feature F4 | #Feature F5 | #Feature F6 | #Feature F7 | #Feature F8 | #Feature F9 | Start | End | PTM | AScore | Found By |
| --- | --- | --- | --- | --- | --- | --- | --- | --- | --- | --- | --- | --- | --- | --- | --- | --- | --- | --- | --- | --- | --- | --- | --- | --- | --- | --- | --- | --- | --- | --- | --- | --- | --- | --- | --- | --- | --- | --- | --- | --- | --- | --- | --- |
| K.FASFIDKVR.F | N | 65.27 | 1081.5920 | 9 | 3.5 | 541.8023 | 2 | 29.71 | 8 | F8:13159 | NaNaKA16\_F4.raw |  |  |  | 5.894E5 | 7.4963E5 | 0 |  | 1.9656E6 | 2.1554E6 | 2.1372E6 |  |  |  | 5 | 0 | 0 | 0 | 1 | 1 | 0 | 0 | 1 | 1 | 1 | 0 | 0 | 0 | 161 | 169 |  |  | PEAKS DB |
| K.LALDIEIATYR.K | N | 64.60 | 1276.7026 | 11 | 0.8 | 639.3591 | 2 | 69.21 | 11 | F11:46918 | NaNaKA16\_F7.raw |  |  |  |  |  | 1.118E6 |  |  | 1.7861E6 | 1.6805E6 | 6.0957E5 | 8.3228E5 | 2.295E6 | 6 | 0 | 0 | 0 | 0 | 0 | 1 | 0 | 0 | 1 | 1 | 1 | 1 | 1 | 444 | 454 |  |  | PEAKS DB |
| K.YEELQVSVGR.H | Y | 51.57 | 1178.5931 | 10 | 5.3 | 590.3040 | 2 | 30.47 | 7 | F7:11700 | NaNaKA16\_F3.raw |  |  |  |  |  |  | 3.5293E6 |  |  |  |  |  |  | 1 | 0 | 0 | 0 | 0 | 0 | 0 | 1 | 0 | 0 | 0 | 0 | 0 | 0 | 348 | 357 |  |  | PEAKS DB |
| total 3 peptides |  |  |  |  |  |  |  |  |  |  |  |  |  |  |  |  |  |  |  |  |  |  |  |  |  |  |  |  |  |  |  |  |  |  |  |  |  |  |  |  |  |  |  |
| --- | --- | --- | --- | --- | --- | --- | --- | --- | --- | --- | --- | --- | --- | --- | --- | --- | --- | --- | --- | --- | --- | --- | --- | --- | --- | --- | --- | --- | --- | --- | --- | --- | --- | --- | --- | --- | --- | --- | --- | --- | --- | --- | --- |

XP\_013915209.1

back to list

  

| Protein Coverage
| Supporting Peptides
|

Protein Coverage:

Supporting Peptides:

| Peptide | Uniq | -10lgP | Mass | Length | ppm | m/z | z | RT | Fraction | Scan | Source File | Area F1 | Area F10 | Area F11 | Area F12 | Area F13 | Area F2 | Area F3 | Area F4 | Area F5 | Area F6 | Area F7 | Area F8 | Area F9 | #Feature | #Feature F1 | #Feature F10 | #Feature F11 | #Feature F12 | #Feature F13 | #Feature F2 | #Feature F3 | #Feature F4 | #Feature F5 | #Feature F6 | #Feature F7 | #Feature F8 | #Feature F9 | Start | End | PTM | AScore | Found By |
| --- | --- | --- | --- | --- | --- | --- | --- | --- | --- | --- | --- | --- | --- | --- | --- | --- | --- | --- | --- | --- | --- | --- | --- | --- | --- | --- | --- | --- | --- | --- | --- | --- | --- | --- | --- | --- | --- | --- | --- | --- | --- | --- | --- |
| K.FASFIDKVR.F | N | 65.27 | 1081.5920 | 9 | 3.5 | 541.8023 | 2 | 29.71 | 8 | F8:13159 | NaNaKA16\_F4.raw |  |  |  | 5.894E5 | 7.4963E5 | 0 |  | 1.9656E6 | 2.1554E6 | 2.1372E6 |  |  |  | 5 | 0 | 0 | 0 | 1 | 1 | 0 | 0 | 1 | 1 | 1 | 0 | 0 | 0 | 161 | 169 |  |  | PEAKS DB |
| K.LALDIEIATYR.K | N | 64.60 | 1276.7026 | 11 | 0.8 | 639.3591 | 2 | 69.21 | 11 | F11:46918 | NaNaKA16\_F7.raw |  |  |  |  |  | 1.118E6 |  |  | 1.7861E6 | 1.6805E6 | 6.0957E5 | 8.3228E5 | 2.295E6 | 6 | 0 | 0 | 0 | 0 | 0 | 1 | 0 | 0 | 1 | 1 | 1 | 1 | 1 | 444 | 454 |  |  | PEAKS DB |
| K.YEELQVSVGR.H | Y | 51.57 | 1178.5931 | 10 | 5.3 | 590.3040 | 2 | 30.47 | 7 | F7:11700 | NaNaKA16\_F3.raw |  |  |  |  |  |  | 3.5293E6 |  |  |  |  |  |  | 1 | 0 | 0 | 0 | 0 | 0 | 0 | 1 | 0 | 0 | 0 | 0 | 0 | 0 | 348 | 357 |  |  | PEAKS DB |
| total 3 peptides |  |  |  |  |  |  |  |  |  |  |  |  |  |  |  |  |  |  |  |  |  |  |  |  |  |  |  |  |  |  |  |  |  |  |  |  |  |  |  |  |  |  |  |
| --- | --- | --- | --- | --- | --- | --- | --- | --- | --- | --- | --- | --- | --- | --- | --- | --- | --- | --- | --- | --- | --- | --- | --- | --- | --- | --- | --- | --- | --- | --- | --- | --- | --- | --- | --- | --- | --- | --- | --- | --- | --- | --- | --- |

P86368.1

back to list

  

| Protein Coverage
| Supporting Peptides
|

Protein Coverage:

Supporting Peptides:

| Peptide | Uniq | -10lgP | Mass | Length | ppm | m/z | z | RT | Fraction | Scan | Source File | Area F1 | Area F10 | Area F11 | Area F12 | Area F13 | Area F2 | Area F3 | Area F4 | Area F5 | Area F6 | Area F7 | Area F8 | Area F9 | #Feature | #Feature F1 | #Feature F10 | #Feature F11 | #Feature F12 | #Feature F13 | #Feature F2 | #Feature F3 | #Feature F4 | #Feature F5 | #Feature F6 | #Feature F7 | #Feature F8 | #Feature F9 | Start | End | PTM | AScore | Found By |
| --- | --- | --- | --- | --- | --- | --- | --- | --- | --- | --- | --- | --- | --- | --- | --- | --- | --- | --- | --- | --- | --- | --- | --- | --- | --- | --- | --- | --- | --- | --- | --- | --- | --- | --- | --- | --- | --- | --- | --- | --- | --- | --- | --- |
| R.C(+57.02)C(+57.02)FVHDC(+57.02)C(+57.02)YGNLPDC(+57.02)NPK.S | N | 74.08 | 2314.8687 | 18 | 0.4 | 772.6305 | 3 | 32.68 | 4 | F4:15898 | NaNaKA16\_F12.raw |  |  |  | 5.4885E7 |  |  |  |  |  |  |  |  |  | 2 | 0 | 0 | 0 | 2 | 0 | 0 | 0 | 0 | 0 | 0 | 0 | 0 | 0 | 43 | 60 | Carbamidomethylation | C1:Carbamidomethylation:1000.00;C2:Carbamidomethylation:1000.00;C7:Carbamidomethylation:1000.00;C8:Carbamidomethylation:1000.00;C15:Carbamidomethylation:1000.00 | PEAKS DB |
| I.YM(+15.99)LYPDFLC(+57.02)K.G | N | 68.84 | 1364.6145 | 10 | 0.1 | 683.3146 | 2 | 68.38 | 4 | F4:45614 | NaNaKA16\_F12.raw |  |  |  | 8.5839E6 |  |  |  |  |  |  |  |  |  | 4 | 0 | 0 | 0 | 4 | 0 | 0 | 0 | 0 | 0 | 0 | 0 | 0 | 0 | 107 | 116 | Oxidation (M); Carbamidomethylation | M2:Oxidation (M):1000.00;C9:Carbamidomethylation:1000.00 | PEAKS DB |
| I.YMLYPDFLC(+57.02)K.G | N | 67.92 | 1348.6195 | 10 | 0.7 | 675.3175 | 2 | 75.32 | 4 | F4:51022 | NaNaKA16\_F12.raw |  | 6.0196E5 | 6.6727E5 | 5.9248E7 |  |  |  |  |  | 4.1845E5 |  |  |  | 4 | 0 | 1 | 1 | 1 | 0 | 0 | 0 | 0 | 0 | 1 | 0 | 0 | 0 | 107 | 116 | Carbamidomethylation | C9:Carbamidomethylation:1000.00 | PEAKS DB |
| K.IYMLYPDFLC(+57.02)K.G | Y | 45.34 | 1461.7036 | 11 | 0.7 | 731.8596 | 2 | 82.77 | 11 | F11:57956 | NaNaKA16\_F7.raw |  |  |  |  |  |  |  |  |  |  | 2.1087E5 |  |  | 1 | 0 | 0 | 0 | 0 | 0 | 0 | 0 | 0 | 0 | 0 | 1 | 0 | 0 | 106 | 116 | Carbamidomethylation | C10:Carbamidomethylation:1000.00 | PEAKS DB |
| total 4 peptides |  |  |  |  |  |  |  |  |  |  |  |  |  |  |  |  |  |  |  |  |  |  |  |  |  |  |  |  |  |  |  |  |  |  |  |  |  |  |  |  |  |  |  |
| --- | --- | --- | --- | --- | --- | --- | --- | --- | --- | --- | --- | --- | --- | --- | --- | --- | --- | --- | --- | --- | --- | --- | --- | --- | --- | --- | --- | --- | --- | --- | --- | --- | --- | --- | --- | --- | --- | --- | --- | --- | --- | --- | --- |

JAG66642.1

back to list

  

| Protein Coverage
| Supporting Peptides
|

Protein Coverage:

Supporting Peptides:

| Peptide | Uniq | -10lgP | Mass | Length | ppm | m/z | z | RT | Fraction | Scan | Source File | Area F1 | Area F10 | Area F11 | Area F12 | Area F13 | Area F2 | Area F3 | Area F4 | Area F5 | Area F6 | Area F7 | Area F8 | Area F9 | #Feature | #Feature F1 | #Feature F10 | #Feature F11 | #Feature F12 | #Feature F13 | #Feature F2 | #Feature F3 | #Feature F4 | #Feature F5 | #Feature F6 | #Feature F7 | #Feature F8 | #Feature F9 | Start | End | PTM | AScore | Found By |
| --- | --- | --- | --- | --- | --- | --- | --- | --- | --- | --- | --- | --- | --- | --- | --- | --- | --- | --- | --- | --- | --- | --- | --- | --- | --- | --- | --- | --- | --- | --- | --- | --- | --- | --- | --- | --- | --- | --- | --- | --- | --- | --- | --- |
| K.TITLEVEPSDTIENVK.A | Y | 71.38 | 1786.9200 | 16 | 1.1 | 894.4683 | 2 | 56.97 | 1 | F1:22100 | NaNaKA16\_F1.raw | 1.7352E7 |  |  | 8.563E5 |  |  |  |  | 7.9338E5 |  |  |  |  | 3 | 1 | 0 | 0 | 1 | 0 | 0 | 0 | 0 | 1 | 0 | 0 | 0 | 0 | 12 | 27 |  |  | PEAKS DB |
| K.ESTLHLVLR.L | Y | 62.86 | 1066.6135 | 9 | 0.3 | 534.3142 | 2 | 28.01 | 9 | F9:13605 | NaNaKA16\_F5.raw |  |  |  |  |  |  |  |  | 2.5029E5 |  |  |  |  | 1 | 0 | 0 | 0 | 0 | 0 | 0 | 0 | 0 | 1 | 0 | 0 | 0 | 0 | 64 | 72 |  |  | PEAKS DB |
| R.TLSDYNIQK.E | Y | 56.48 | 1080.5452 | 9 | 1.0 | 541.2804 | 2 | 19.39 | 1 | F1:5953 | NaNaKA16\_F1.raw | 3.0489E6 |  |  |  |  |  |  |  |  |  |  |  |  | 2 | 2 | 0 | 0 | 0 | 0 | 0 | 0 | 0 | 0 | 0 | 0 | 0 | 0 | 55 | 63 |  |  | PEAKS DB |
| K.IQDKEGIPPDQQR.L | Y | 42.00 | 1522.7739 | 13 | -0.1 | 508.5985 | 3 | 11.71 | 1 | F1:2169 | NaNaKA16\_F1.raw | 1.117E6 |  |  |  |  |  |  |  |  |  |  |  |  | 1 | 1 | 0 | 0 | 0 | 0 | 0 | 0 | 0 | 0 | 0 | 0 | 0 | 0 | 30 | 42 |  |  | PEAKS DB |
| total 4 peptides |  |  |  |  |  |  |  |  |  |  |  |  |  |  |  |  |  |  |  |  |  |  |  |  |  |  |  |  |  |  |  |  |  |  |  |  |  |  |  |  |  |  |  |
| --- | --- | --- | --- | --- | --- | --- | --- | --- | --- | --- | --- | --- | --- | --- | --- | --- | --- | --- | --- | --- | --- | --- | --- | --- | --- | --- | --- | --- | --- | --- | --- | --- | --- | --- | --- | --- | --- | --- | --- | --- | --- | --- | --- |

JAB53407.1

back to list

  

| Protein Coverage
| Supporting Peptides
|

Protein Coverage:

Supporting Peptides:

| Peptide | Uniq | -10lgP | Mass | Length | ppm | m/z | z | RT | Fraction | Scan | Source File | Area F1 | Area F10 | Area F11 | Area F12 | Area F13 | Area F2 | Area F3 | Area F4 | Area F5 | Area F6 | Area F7 | Area F8 | Area F9 | #Feature | #Feature F1 | #Feature F10 | #Feature F11 | #Feature F12 | #Feature F13 | #Feature F2 | #Feature F3 | #Feature F4 | #Feature F5 | #Feature F6 | #Feature F7 | #Feature F8 | #Feature F9 | Start | End | PTM | AScore | Found By |
| --- | --- | --- | --- | --- | --- | --- | --- | --- | --- | --- | --- | --- | --- | --- | --- | --- | --- | --- | --- | --- | --- | --- | --- | --- | --- | --- | --- | --- | --- | --- | --- | --- | --- | --- | --- | --- | --- | --- | --- | --- | --- | --- | --- |
| K.TITLEVEPSDTIENVK.A | Y | 71.38 | 1786.9200 | 16 | 1.1 | 894.4683 | 2 | 56.97 | 1 | F1:22100 | NaNaKA16\_F1.raw | 1.7352E7 |  |  | 8.563E5 |  |  |  |  | 7.9338E5 |  |  |  |  | 3 | 1 | 0 | 0 | 1 | 0 | 0 | 0 | 0 | 1 | 0 | 0 | 0 | 0 | 12 | 27 |  |  | PEAKS DB |
| K.ESTLHLVLR.L | Y | 62.86 | 1066.6135 | 9 | 0.3 | 534.3142 | 2 | 28.01 | 9 | F9:13605 | NaNaKA16\_F5.raw |  |  |  |  |  |  |  |  | 2.5029E5 |  |  |  |  | 1 | 0 | 0 | 0 | 0 | 0 | 0 | 0 | 0 | 1 | 0 | 0 | 0 | 0 | 64 | 72 |  |  | PEAKS DB |
| R.TLSDYNIQK.E | Y | 56.48 | 1080.5452 | 9 | 1.0 | 541.2804 | 2 | 19.39 | 1 | F1:5953 | NaNaKA16\_F1.raw | 3.0489E6 |  |  |  |  |  |  |  |  |  |  |  |  | 2 | 2 | 0 | 0 | 0 | 0 | 0 | 0 | 0 | 0 | 0 | 0 | 0 | 0 | 55 | 63 |  |  | PEAKS DB |
| K.IQDKEGIPPDQQR.L | Y | 42.00 | 1522.7739 | 13 | -0.1 | 508.5985 | 3 | 11.71 | 1 | F1:2169 | NaNaKA16\_F1.raw | 1.117E6 |  |  |  |  |  |  |  |  |  |  |  |  | 1 | 1 | 0 | 0 | 0 | 0 | 0 | 0 | 0 | 0 | 0 | 0 | 0 | 0 | 30 | 42 |  |  | PEAKS DB |
| total 4 peptides |  |  |  |  |  |  |  |  |  |  |  |  |  |  |  |  |  |  |  |  |  |  |  |  |  |  |  |  |  |  |  |  |  |  |  |  |  |  |  |  |  |  |  |
| --- | --- | --- | --- | --- | --- | --- | --- | --- | --- | --- | --- | --- | --- | --- | --- | --- | --- | --- | --- | --- | --- | --- | --- | --- | --- | --- | --- | --- | --- | --- | --- | --- | --- | --- | --- | --- | --- | --- | --- | --- | --- | --- | --- |

JAI11580.1

back to list

  

| Protein Coverage
| Supporting Peptides
|

Protein Coverage:

Supporting Peptides:

| Peptide | Uniq | -10lgP | Mass | Length | ppm | m/z | z | RT | Fraction | Scan | Source File | Area F1 | Area F10 | Area F11 | Area F12 | Area F13 | Area F2 | Area F3 | Area F4 | Area F5 | Area F6 | Area F7 | Area F8 | Area F9 | #Feature | #Feature F1 | #Feature F10 | #Feature F11 | #Feature F12 | #Feature F13 | #Feature F2 | #Feature F3 | #Feature F4 | #Feature F5 | #Feature F6 | #Feature F7 | #Feature F8 | #Feature F9 | Start | End | PTM | AScore | Found By |
| --- | --- | --- | --- | --- | --- | --- | --- | --- | --- | --- | --- | --- | --- | --- | --- | --- | --- | --- | --- | --- | --- | --- | --- | --- | --- | --- | --- | --- | --- | --- | --- | --- | --- | --- | --- | --- | --- | --- | --- | --- | --- | --- | --- |
| K.TITLEVEPSDTIENVK.A | Y | 71.38 | 1786.9200 | 16 | 1.1 | 894.4683 | 2 | 56.97 | 1 | F1:22100 | NaNaKA16\_F1.raw | 1.7352E7 |  |  | 8.563E5 |  |  |  |  | 7.9338E5 |  |  |  |  | 3 | 1 | 0 | 0 | 1 | 0 | 0 | 0 | 0 | 1 | 0 | 0 | 0 | 0 | 12 | 27 |  |  | PEAKS DB |
| K.ESTLHLVLR.L | Y | 62.86 | 1066.6135 | 9 | 0.3 | 534.3142 | 2 | 28.01 | 9 | F9:13605 | NaNaKA16\_F5.raw |  |  |  |  |  |  |  |  | 2.5029E5 |  |  |  |  | 1 | 0 | 0 | 0 | 0 | 0 | 0 | 0 | 0 | 1 | 0 | 0 | 0 | 0 | 64 | 72 |  |  | PEAKS DB |
| R.TLSDYNIQK.E | Y | 56.48 | 1080.5452 | 9 | 1.0 | 541.2804 | 2 | 19.39 | 1 | F1:5953 | NaNaKA16\_F1.raw | 3.0489E6 |  |  |  |  |  |  |  |  |  |  |  |  | 2 | 2 | 0 | 0 | 0 | 0 | 0 | 0 | 0 | 0 | 0 | 0 | 0 | 0 | 55 | 63 |  |  | PEAKS DB |
| K.IQDKEGIPPDQQR.L | Y | 42.00 | 1522.7739 | 13 | -0.1 | 508.5985 | 3 | 11.71 | 1 | F1:2169 | NaNaKA16\_F1.raw | 1.117E6 |  |  |  |  |  |  |  |  |  |  |  |  | 1 | 1 | 0 | 0 | 0 | 0 | 0 | 0 | 0 | 0 | 0 | 0 | 0 | 0 | 30 | 42 |  |  | PEAKS DB |
| total 4 peptides |  |  |  |  |  |  |  |  |  |  |  |  |  |  |  |  |  |  |  |  |  |  |  |  |  |  |  |  |  |  |  |  |  |  |  |  |  |  |  |  |  |  |  |
| --- | --- | --- | --- | --- | --- | --- | --- | --- | --- | --- | --- | --- | --- | --- | --- | --- | --- | --- | --- | --- | --- | --- | --- | --- | --- | --- | --- | --- | --- | --- | --- | --- | --- | --- | --- | --- | --- | --- | --- | --- | --- | --- | --- |

XP\_015686894.1

back to list

  

| Protein Coverage
| Supporting Peptides
|

Protein Coverage:

Supporting Peptides:

| Peptide | Uniq | -10lgP | Mass | Length | ppm | m/z | z | RT | Fraction | Scan | Source File | Area F1 | Area F10 | Area F11 | Area F12 | Area F13 | Area F2 | Area F3 | Area F4 | Area F5 | Area F6 | Area F7 | Area F8 | Area F9 | #Feature | #Feature F1 | #Feature F10 | #Feature F11 | #Feature F12 | #Feature F13 | #Feature F2 | #Feature F3 | #Feature F4 | #Feature F5 | #Feature F6 | #Feature F7 | #Feature F8 | #Feature F9 | Start | End | PTM | AScore | Found By |
| --- | --- | --- | --- | --- | --- | --- | --- | --- | --- | --- | --- | --- | --- | --- | --- | --- | --- | --- | --- | --- | --- | --- | --- | --- | --- | --- | --- | --- | --- | --- | --- | --- | --- | --- | --- | --- | --- | --- | --- | --- | --- | --- | --- |
| K.TITLEVEPSDTIENVK.A | Y | 71.38 | 1786.9200 | 16 | 1.1 | 894.4683 | 2 | 56.97 | 1 | F1:22100 | NaNaKA16\_F1.raw | 1.7352E7 |  |  | 8.563E5 |  |  |  |  | 7.9338E5 |  |  |  |  | 3 | 1 | 0 | 0 | 1 | 0 | 0 | 0 | 0 | 1 | 0 | 0 | 0 | 0 | 12 | 27 |  |  | PEAKS DB |
| K.ESTLHLVLR.L | Y | 62.86 | 1066.6135 | 9 | 0.3 | 534.3142 | 2 | 28.01 | 9 | F9:13605 | NaNaKA16\_F5.raw |  |  |  |  |  |  |  |  | 2.5029E5 |  |  |  |  | 1 | 0 | 0 | 0 | 0 | 0 | 0 | 0 | 0 | 1 | 0 | 0 | 0 | 0 | 64 | 72 |  |  | PEAKS DB |
| R.TLSDYNIQK.E | Y | 56.48 | 1080.5452 | 9 | 1.0 | 541.2804 | 2 | 19.39 | 1 | F1:5953 | NaNaKA16\_F1.raw | 3.0489E6 |  |  |  |  |  |  |  |  |  |  |  |  | 2 | 2 | 0 | 0 | 0 | 0 | 0 | 0 | 0 | 0 | 0 | 0 | 0 | 0 | 55 | 63 |  |  | PEAKS DB |
| K.IQDKEGIPPDQQR.L | Y | 42.00 | 1522.7739 | 13 | -0.1 | 508.5985 | 3 | 11.71 | 1 | F1:2169 | NaNaKA16\_F1.raw | 1.117E6 |  |  |  |  |  |  |  |  |  |  |  |  | 1 | 1 | 0 | 0 | 0 | 0 | 0 | 0 | 0 | 0 | 0 | 0 | 0 | 0 | 30 | 42 |  |  | PEAKS DB |
| total 4 peptides |  |  |  |  |  |  |  |  |  |  |  |  |  |  |  |  |  |  |  |  |  |  |  |  |  |  |  |  |  |  |  |  |  |  |  |  |  |  |  |  |  |  |  |
| --- | --- | --- | --- | --- | --- | --- | --- | --- | --- | --- | --- | --- | --- | --- | --- | --- | --- | --- | --- | --- | --- | --- | --- | --- | --- | --- | --- | --- | --- | --- | --- | --- | --- | --- | --- | --- | --- | --- | --- | --- | --- | --- | --- |

XP\_007432078.1

back to list

  

| Protein Coverage
| Supporting Peptides
|

Protein Coverage:

Supporting Peptides:

| Peptide | Uniq | -10lgP | Mass | Length | ppm | m/z | z | RT | Fraction | Scan | Source File | Area F1 | Area F10 | Area F11 | Area F12 | Area F13 | Area F2 | Area F3 | Area F4 | Area F5 | Area F6 | Area F7 | Area F8 | Area F9 | #Feature | #Feature F1 | #Feature F10 | #Feature F11 | #Feature F12 | #Feature F13 | #Feature F2 | #Feature F3 | #Feature F4 | #Feature F5 | #Feature F6 | #Feature F7 | #Feature F8 | #Feature F9 | Start | End | PTM | AScore | Found By |
| --- | --- | --- | --- | --- | --- | --- | --- | --- | --- | --- | --- | --- | --- | --- | --- | --- | --- | --- | --- | --- | --- | --- | --- | --- | --- | --- | --- | --- | --- | --- | --- | --- | --- | --- | --- | --- | --- | --- | --- | --- | --- | --- | --- |
| K.TITLEVEPSDTIENVK.A | Y | 71.38 | 1786.9200 | 16 | 1.1 | 894.4683 | 2 | 56.97 | 1 | F1:22100 | NaNaKA16\_F1.raw | 1.7352E7 |  |  | 8.563E5 |  |  |  |  | 7.9338E5 |  |  |  |  | 3 | 1 | 0 | 0 | 1 | 0 | 0 | 0 | 0 | 1 | 0 | 0 | 0 | 0 | 12 | 27 |  |  | PEAKS DB |
| K.ESTLHLVLR.L | Y | 62.86 | 1066.6135 | 9 | 0.3 | 534.3142 | 2 | 28.01 | 9 | F9:13605 | NaNaKA16\_F5.raw |  |  |  |  |  |  |  |  | 2.5029E5 |  |  |  |  | 1 | 0 | 0 | 0 | 0 | 0 | 0 | 0 | 0 | 1 | 0 | 0 | 0 | 0 | 64 | 72 |  |  | PEAKS DB |
| R.TLSDYNIQK.E | Y | 56.48 | 1080.5452 | 9 | 1.0 | 541.2804 | 2 | 19.39 | 1 | F1:5953 | NaNaKA16\_F1.raw | 3.0489E6 |  |  |  |  |  |  |  |  |  |  |  |  | 2 | 2 | 0 | 0 | 0 | 0 | 0 | 0 | 0 | 0 | 0 | 0 | 0 | 0 | 55 | 63 |  |  | PEAKS DB |
| K.IQDKEGIPPDQQR.L | Y | 42.00 | 1522.7739 | 13 | -0.1 | 508.5985 | 3 | 11.71 | 1 | F1:2169 | NaNaKA16\_F1.raw | 1.117E6 |  |  |  |  |  |  |  |  |  |  |  |  | 1 | 1 | 0 | 0 | 0 | 0 | 0 | 0 | 0 | 0 | 0 | 0 | 0 | 0 | 30 | 42 |  |  | PEAKS DB |
| total 4 peptides |  |  |  |  |  |  |  |  |  |  |  |  |  |  |  |  |  |  |  |  |  |  |  |  |  |  |  |  |  |  |  |  |  |  |  |  |  |  |  |  |  |  |  |
| --- | --- | --- | --- | --- | --- | --- | --- | --- | --- | --- | --- | --- | --- | --- | --- | --- | --- | --- | --- | --- | --- | --- | --- | --- | --- | --- | --- | --- | --- | --- | --- | --- | --- | --- | --- | --- | --- | --- | --- | --- | --- | --- | --- |

JAV49286.1

back to list

  

| Protein Coverage
| Supporting Peptides
|

Protein Coverage:

Supporting Peptides:

| Peptide | Uniq | -10lgP | Mass | Length | ppm | m/z | z | RT | Fraction | Scan | Source File | Area F1 | Area F10 | Area F11 | Area F12 | Area F13 | Area F2 | Area F3 | Area F4 | Area F5 | Area F6 | Area F7 | Area F8 | Area F9 | #Feature | #Feature F1 | #Feature F10 | #Feature F11 | #Feature F12 | #Feature F13 | #Feature F2 | #Feature F3 | #Feature F4 | #Feature F5 | #Feature F6 | #Feature F7 | #Feature F8 | #Feature F9 | Start | End | PTM | AScore | Found By |
| --- | --- | --- | --- | --- | --- | --- | --- | --- | --- | --- | --- | --- | --- | --- | --- | --- | --- | --- | --- | --- | --- | --- | --- | --- | --- | --- | --- | --- | --- | --- | --- | --- | --- | --- | --- | --- | --- | --- | --- | --- | --- | --- | --- |
| K.TITLEVEPSDTIENVK.A | Y | 71.38 | 1786.9200 | 16 | 1.1 | 894.4683 | 2 | 56.97 | 1 | F1:22100 | NaNaKA16\_F1.raw | 1.7352E7 |  |  | 8.563E5 |  |  |  |  | 7.9338E5 |  |  |  |  | 3 | 1 | 0 | 0 | 1 | 0 | 0 | 0 | 0 | 1 | 0 | 0 | 0 | 0 | 12 | 27 |  |  | PEAKS DB |
| K.ESTLHLVLR.L | Y | 62.86 | 1066.6135 | 9 | 0.3 | 534.3142 | 2 | 28.01 | 9 | F9:13605 | NaNaKA16\_F5.raw |  |  |  |  |  |  |  |  | 2.5029E5 |  |  |  |  | 1 | 0 | 0 | 0 | 0 | 0 | 0 | 0 | 0 | 1 | 0 | 0 | 0 | 0 | 64 | 72 |  |  | PEAKS DB |
| R.TLSDYNIQK.E | Y | 56.48 | 1080.5452 | 9 | 1.0 | 541.2804 | 2 | 19.39 | 1 | F1:5953 | NaNaKA16\_F1.raw | 3.0489E6 |  |  |  |  |  |  |  |  |  |  |  |  | 2 | 2 | 0 | 0 | 0 | 0 | 0 | 0 | 0 | 0 | 0 | 0 | 0 | 0 | 55 | 63 |  |  | PEAKS DB |
| K.IQDKEGIPPDQQR.L | Y | 42.00 | 1522.7739 | 13 | -0.1 | 508.5985 | 3 | 11.71 | 1 | F1:2169 | NaNaKA16\_F1.raw | 1.117E6 |  |  |  |  |  |  |  |  |  |  |  |  | 1 | 1 | 0 | 0 | 0 | 0 | 0 | 0 | 0 | 0 | 0 | 0 | 0 | 0 | 30 | 42 |  |  | PEAKS DB |
| total 4 peptides |  |  |  |  |  |  |  |  |  |  |  |  |  |  |  |  |  |  |  |  |  |  |  |  |  |  |  |  |  |  |  |  |  |  |  |  |  |  |  |  |  |  |  |
| --- | --- | --- | --- | --- | --- | --- | --- | --- | --- | --- | --- | --- | --- | --- | --- | --- | --- | --- | --- | --- | --- | --- | --- | --- | --- | --- | --- | --- | --- | --- | --- | --- | --- | --- | --- | --- | --- | --- | --- | --- | --- | --- | --- |

LAA53283.1

back to list

  

| Protein Coverage
| Supporting Peptides
|

Protein Coverage:

Supporting Peptides:

| Peptide | Uniq | -10lgP | Mass | Length | ppm | m/z | z | RT | Fraction | Scan | Source File | Area F1 | Area F10 | Area F11 | Area F12 | Area F13 | Area F2 | Area F3 | Area F4 | Area F5 | Area F6 | Area F7 | Area F8 | Area F9 | #Feature | #Feature F1 | #Feature F10 | #Feature F11 | #Feature F12 | #Feature F13 | #Feature F2 | #Feature F3 | #Feature F4 | #Feature F5 | #Feature F6 | #Feature F7 | #Feature F8 | #Feature F9 | Start | End | PTM | AScore | Found By |
| --- | --- | --- | --- | --- | --- | --- | --- | --- | --- | --- | --- | --- | --- | --- | --- | --- | --- | --- | --- | --- | --- | --- | --- | --- | --- | --- | --- | --- | --- | --- | --- | --- | --- | --- | --- | --- | --- | --- | --- | --- | --- | --- | --- |
| K.TITLEVEPSDTIENVK.A | Y | 71.38 | 1786.9200 | 16 | 1.1 | 894.4683 | 2 | 56.97 | 1 | F1:22100 | NaNaKA16\_F1.raw | 1.7352E7 |  |  | 8.563E5 |  |  |  |  | 7.9338E5 |  |  |  |  | 3 | 1 | 0 | 0 | 1 | 0 | 0 | 0 | 0 | 1 | 0 | 0 | 0 | 0 | 12 | 27 |  |  | PEAKS DB |
| K.ESTLHLVLR.L | Y | 62.86 | 1066.6135 | 9 | 0.3 | 534.3142 | 2 | 28.01 | 9 | F9:13605 | NaNaKA16\_F5.raw |  |  |  |  |  |  |  |  | 2.5029E5 |  |  |  |  | 1 | 0 | 0 | 0 | 0 | 0 | 0 | 0 | 0 | 1 | 0 | 0 | 0 | 0 | 64 | 72 |  |  | PEAKS DB |
| R.TLSDYNIQK.E | Y | 56.48 | 1080.5452 | 9 | 1.0 | 541.2804 | 2 | 19.39 | 1 | F1:5953 | NaNaKA16\_F1.raw | 3.0489E6 |  |  |  |  |  |  |  |  |  |  |  |  | 2 | 2 | 0 | 0 | 0 | 0 | 0 | 0 | 0 | 0 | 0 | 0 | 0 | 0 | 55 | 63 |  |  | PEAKS DB |
| K.IQDKEGIPPDQQR.L | Y | 42.00 | 1522.7739 | 13 | -0.1 | 508.5985 | 3 | 11.71 | 1 | F1:2169 | NaNaKA16\_F1.raw | 1.117E6 |  |  |  |  |  |  |  |  |  |  |  |  | 1 | 1 | 0 | 0 | 0 | 0 | 0 | 0 | 0 | 0 | 0 | 0 | 0 | 0 | 30 | 42 |  |  | PEAKS DB |
| total 4 peptides |  |  |  |  |  |  |  |  |  |  |  |  |  |  |  |  |  |  |  |  |  |  |  |  |  |  |  |  |  |  |  |  |  |  |  |  |  |  |  |  |  |  |  |
| --- | --- | --- | --- | --- | --- | --- | --- | --- | --- | --- | --- | --- | --- | --- | --- | --- | --- | --- | --- | --- | --- | --- | --- | --- | --- | --- | --- | --- | --- | --- | --- | --- | --- | --- | --- | --- | --- | --- | --- | --- | --- | --- | --- |

XP\_015686900.1

back to list

  

| Protein Coverage
| Supporting Peptides
|

Protein Coverage:

Supporting Peptides:

| Peptide | Uniq | -10lgP | Mass | Length | ppm | m/z | z | RT | Fraction | Scan | Source File | Area F1 | Area F10 | Area F11 | Area F12 | Area F13 | Area F2 | Area F3 | Area F4 | Area F5 | Area F6 | Area F7 | Area F8 | Area F9 | #Feature | #Feature F1 | #Feature F10 | #Feature F11 | #Feature F12 | #Feature F13 | #Feature F2 | #Feature F3 | #Feature F4 | #Feature F5 | #Feature F6 | #Feature F7 | #Feature F8 | #Feature F9 | Start | End | PTM | AScore | Found By |
| --- | --- | --- | --- | --- | --- | --- | --- | --- | --- | --- | --- | --- | --- | --- | --- | --- | --- | --- | --- | --- | --- | --- | --- | --- | --- | --- | --- | --- | --- | --- | --- | --- | --- | --- | --- | --- | --- | --- | --- | --- | --- | --- | --- |
| K.TITLEVEPSDTIENVK.A | Y | 71.38 | 1786.9200 | 16 | 1.1 | 894.4683 | 2 | 56.97 | 1 | F1:22100 | NaNaKA16\_F1.raw | 1.7352E7 |  |  | 8.563E5 |  |  |  |  | 7.9338E5 |  |  |  |  | 3 | 1 | 0 | 0 | 1 | 0 | 0 | 0 | 0 | 1 | 0 | 0 | 0 | 0 | 12 | 27 |  |  | PEAKS DB |
| K.ESTLHLVLR.L | Y | 62.86 | 1066.6135 | 9 | 0.3 | 534.3142 | 2 | 28.01 | 9 | F9:13605 | NaNaKA16\_F5.raw |  |  |  |  |  |  |  |  | 2.5029E5 |  |  |  |  | 1 | 0 | 0 | 0 | 0 | 0 | 0 | 0 | 0 | 1 | 0 | 0 | 0 | 0 | 64 | 72 |  |  | PEAKS DB |
| R.TLSDYNIQK.E | Y | 56.48 | 1080.5452 | 9 | 1.0 | 541.2804 | 2 | 19.39 | 1 | F1:5953 | NaNaKA16\_F1.raw | 3.0489E6 |  |  |  |  |  |  |  |  |  |  |  |  | 2 | 2 | 0 | 0 | 0 | 0 | 0 | 0 | 0 | 0 | 0 | 0 | 0 | 0 | 55 | 63 |  |  | PEAKS DB |
| K.IQDKEGIPPDQQR.L | Y | 42.00 | 1522.7739 | 13 | -0.1 | 508.5985 | 3 | 11.71 | 1 | F1:2169 | NaNaKA16\_F1.raw | 1.117E6 |  |  |  |  |  |  |  |  |  |  |  |  | 1 | 1 | 0 | 0 | 0 | 0 | 0 | 0 | 0 | 0 | 0 | 0 | 0 | 0 | 30 | 42 |  |  | PEAKS DB |
| total 4 peptides |  |  |  |  |  |  |  |  |  |  |  |  |  |  |  |  |  |  |  |  |  |  |  |  |  |  |  |  |  |  |  |  |  |  |  |  |  |  |  |  |  |  |  |
| --- | --- | --- | --- | --- | --- | --- | --- | --- | --- | --- | --- | --- | --- | --- | --- | --- | --- | --- | --- | --- | --- | --- | --- | --- | --- | --- | --- | --- | --- | --- | --- | --- | --- | --- | --- | --- | --- | --- | --- | --- | --- | --- | --- |

XP\_007432077.1

back to list

  

| Protein Coverage
| Supporting Peptides
|

Protein Coverage:

Supporting Peptides:

| Peptide | Uniq | -10lgP | Mass | Length | ppm | m/z | z | RT | Fraction | Scan | Source File | Area F1 | Area F10 | Area F11 | Area F12 | Area F13 | Area F2 | Area F3 | Area F4 | Area F5 | Area F6 | Area F7 | Area F8 | Area F9 | #Feature | #Feature F1 | #Feature F10 | #Feature F11 | #Feature F12 | #Feature F13 | #Feature F2 | #Feature F3 | #Feature F4 | #Feature F5 | #Feature F6 | #Feature F7 | #Feature F8 | #Feature F9 | Start | End | PTM | AScore | Found By |
| --- | --- | --- | --- | --- | --- | --- | --- | --- | --- | --- | --- | --- | --- | --- | --- | --- | --- | --- | --- | --- | --- | --- | --- | --- | --- | --- | --- | --- | --- | --- | --- | --- | --- | --- | --- | --- | --- | --- | --- | --- | --- | --- | --- |
| K.TITLEVEPSDTIENVK.A | Y | 71.38 | 1786.9200 | 16 | 1.1 | 894.4683 | 2 | 56.97 | 1 | F1:22100 | NaNaKA16\_F1.raw | 1.7352E7 |  |  | 8.563E5 |  |  |  |  | 7.9338E5 |  |  |  |  | 3 | 1 | 0 | 0 | 1 | 0 | 0 | 0 | 0 | 1 | 0 | 0 | 0 | 0 | 12 | 27 |  |  | PEAKS DB |
| K.ESTLHLVLR.L | Y | 62.86 | 1066.6135 | 9 | 0.3 | 534.3142 | 2 | 28.01 | 9 | F9:13605 | NaNaKA16\_F5.raw |  |  |  |  |  |  |  |  | 2.5029E5 |  |  |  |  | 1 | 0 | 0 | 0 | 0 | 0 | 0 | 0 | 0 | 1 | 0 | 0 | 0 | 0 | 64 | 72 |  |  | PEAKS DB |
| R.TLSDYNIQK.E | Y | 56.48 | 1080.5452 | 9 | 1.0 | 541.2804 | 2 | 19.39 | 1 | F1:5953 | NaNaKA16\_F1.raw | 3.0489E6 |  |  |  |  |  |  |  |  |  |  |  |  | 2 | 2 | 0 | 0 | 0 | 0 | 0 | 0 | 0 | 0 | 0 | 0 | 0 | 0 | 55 | 63 |  |  | PEAKS DB |
| K.IQDKEGIPPDQQR.L | Y | 42.00 | 1522.7739 | 13 | -0.1 | 508.5985 | 3 | 11.71 | 1 | F1:2169 | NaNaKA16\_F1.raw | 1.117E6 |  |  |  |  |  |  |  |  |  |  |  |  | 1 | 1 | 0 | 0 | 0 | 0 | 0 | 0 | 0 | 0 | 0 | 0 | 0 | 0 | 30 | 42 |  |  | PEAKS DB |
| total 4 peptides |  |  |  |  |  |  |  |  |  |  |  |  |  |  |  |  |  |  |  |  |  |  |  |  |  |  |  |  |  |  |  |  |  |  |  |  |  |  |  |  |  |  |  |
| --- | --- | --- | --- | --- | --- | --- | --- | --- | --- | --- | --- | --- | --- | --- | --- | --- | --- | --- | --- | --- | --- | --- | --- | --- | --- | --- | --- | --- | --- | --- | --- | --- | --- | --- | --- | --- | --- | --- | --- | --- | --- | --- | --- |

XP\_026551822.1

back to list

  

| Protein Coverage
| Supporting Peptides
|

Protein Coverage:

Supporting Peptides:

| Peptide | Uniq | -10lgP | Mass | Length | ppm | m/z | z | RT | Fraction | Scan | Source File | Area F1 | Area F10 | Area F11 | Area F12 | Area F13 | Area F2 | Area F3 | Area F4 | Area F5 | Area F6 | Area F7 | Area F8 | Area F9 | #Feature | #Feature F1 | #Feature F10 | #Feature F11 | #Feature F12 | #Feature F13 | #Feature F2 | #Feature F3 | #Feature F4 | #Feature F5 | #Feature F6 | #Feature F7 | #Feature F8 | #Feature F9 | Start | End | PTM | AScore | Found By |
| --- | --- | --- | --- | --- | --- | --- | --- | --- | --- | --- | --- | --- | --- | --- | --- | --- | --- | --- | --- | --- | --- | --- | --- | --- | --- | --- | --- | --- | --- | --- | --- | --- | --- | --- | --- | --- | --- | --- | --- | --- | --- | --- | --- |
| K.TITLEVEPSDTIENVK.A | Y | 71.38 | 1786.9200 | 16 | 1.1 | 894.4683 | 2 | 56.97 | 1 | F1:22100 | NaNaKA16\_F1.raw | 1.7352E7 |  |  | 8.563E5 |  |  |  |  | 7.9338E5 |  |  |  |  | 3 | 1 | 0 | 0 | 1 | 0 | 0 | 0 | 0 | 1 | 0 | 0 | 0 | 0 | 12 | 27 |  |  | PEAKS DB |
| K.ESTLHLVLR.L | Y | 62.86 | 1066.6135 | 9 | 0.3 | 534.3142 | 2 | 28.01 | 9 | F9:13605 | NaNaKA16\_F5.raw |  |  |  |  |  |  |  |  | 2.5029E5 |  |  |  |  | 1 | 0 | 0 | 0 | 0 | 0 | 0 | 0 | 0 | 1 | 0 | 0 | 0 | 0 | 64 | 72 |  |  | PEAKS DB |
| R.TLSDYNIQK.E | Y | 56.48 | 1080.5452 | 9 | 1.0 | 541.2804 | 2 | 19.39 | 1 | F1:5953 | NaNaKA16\_F1.raw | 3.0489E6 |  |  |  |  |  |  |  |  |  |  |  |  | 2 | 2 | 0 | 0 | 0 | 0 | 0 | 0 | 0 | 0 | 0 | 0 | 0 | 0 | 55 | 63 |  |  | PEAKS DB |
| K.IQDKEGIPPDQQR.L | Y | 42.00 | 1522.7739 | 13 | -0.1 | 508.5985 | 3 | 11.71 | 1 | F1:2169 | NaNaKA16\_F1.raw | 1.117E6 |  |  |  |  |  |  |  |  |  |  |  |  | 1 | 1 | 0 | 0 | 0 | 0 | 0 | 0 | 0 | 0 | 0 | 0 | 0 | 0 | 30 | 42 |  |  | PEAKS DB |
| total 4 peptides |  |  |  |  |  |  |  |  |  |  |  |  |  |  |  |  |  |  |  |  |  |  |  |  |  |  |  |  |  |  |  |  |  |  |  |  |  |  |  |  |  |  |  |
| --- | --- | --- | --- | --- | --- | --- | --- | --- | --- | --- | --- | --- | --- | --- | --- | --- | --- | --- | --- | --- | --- | --- | --- | --- | --- | --- | --- | --- | --- | --- | --- | --- | --- | --- | --- | --- | --- | --- | --- | --- | --- | --- | --- |

AFJ51304.1

back to list

  

| Protein Coverage
| Supporting Peptides
|

Protein Coverage:

Supporting Peptides:

| Peptide | Uniq | -10lgP | Mass | Length | ppm | m/z | z | RT | Fraction | Scan | Source File | Area F1 | Area F10 | Area F11 | Area F12 | Area F13 | Area F2 | Area F3 | Area F4 | Area F5 | Area F6 | Area F7 | Area F8 | Area F9 | #Feature | #Feature F1 | #Feature F10 | #Feature F11 | #Feature F12 | #Feature F13 | #Feature F2 | #Feature F3 | #Feature F4 | #Feature F5 | #Feature F6 | #Feature F7 | #Feature F8 | #Feature F9 | Start | End | PTM | AScore | Found By |
| --- | --- | --- | --- | --- | --- | --- | --- | --- | --- | --- | --- | --- | --- | --- | --- | --- | --- | --- | --- | --- | --- | --- | --- | --- | --- | --- | --- | --- | --- | --- | --- | --- | --- | --- | --- | --- | --- | --- | --- | --- | --- | --- | --- |
| K.TITLEVEPSDTIENVK.A | Y | 71.38 | 1786.9200 | 16 | 1.1 | 894.4683 | 2 | 56.97 | 1 | F1:22100 | NaNaKA16\_F1.raw | 1.7352E7 |  |  | 8.563E5 |  |  |  |  | 7.9338E5 |  |  |  |  | 3 | 1 | 0 | 0 | 1 | 0 | 0 | 0 | 0 | 1 | 0 | 0 | 0 | 0 | 12 | 27 |  |  | PEAKS DB |
| K.ESTLHLVLR.L | Y | 62.86 | 1066.6135 | 9 | 0.3 | 534.3142 | 2 | 28.01 | 9 | F9:13605 | NaNaKA16\_F5.raw |  |  |  |  |  |  |  |  | 2.5029E5 |  |  |  |  | 1 | 0 | 0 | 0 | 0 | 0 | 0 | 0 | 0 | 1 | 0 | 0 | 0 | 0 | 64 | 72 |  |  | PEAKS DB |
| R.TLSDYNIQK.E | Y | 56.48 | 1080.5452 | 9 | 1.0 | 541.2804 | 2 | 19.39 | 1 | F1:5953 | NaNaKA16\_F1.raw | 3.0489E6 |  |  |  |  |  |  |  |  |  |  |  |  | 2 | 2 | 0 | 0 | 0 | 0 | 0 | 0 | 0 | 0 | 0 | 0 | 0 | 0 | 55 | 63 |  |  | PEAKS DB |
| K.IQDKEGIPPDQQR.L | Y | 42.00 | 1522.7739 | 13 | -0.1 | 508.5985 | 3 | 11.71 | 1 | F1:2169 | NaNaKA16\_F1.raw | 1.117E6 |  |  |  |  |  |  |  |  |  |  |  |  | 1 | 1 | 0 | 0 | 0 | 0 | 0 | 0 | 0 | 0 | 0 | 0 | 0 | 0 | 30 | 42 |  |  | PEAKS DB |
| total 4 peptides |  |  |  |  |  |  |  |  |  |  |  |  |  |  |  |  |  |  |  |  |  |  |  |  |  |  |  |  |  |  |  |  |  |  |  |  |  |  |  |  |  |  |  |
| --- | --- | --- | --- | --- | --- | --- | --- | --- | --- | --- | --- | --- | --- | --- | --- | --- | --- | --- | --- | --- | --- | --- | --- | --- | --- | --- | --- | --- | --- | --- | --- | --- | --- | --- | --- | --- | --- | --- | --- | --- | --- | --- | --- |

XP\_034283249.1

back to list

  

| Protein Coverage
| Supporting Peptides
|

Protein Coverage:

Supporting Peptides:

| Peptide | Uniq | -10lgP | Mass | Length | ppm | m/z | z | RT | Fraction | Scan | Source File | Area F1 | Area F10 | Area F11 | Area F12 | Area F13 | Area F2 | Area F3 | Area F4 | Area F5 | Area F6 | Area F7 | Area F8 | Area F9 | #Feature | #Feature F1 | #Feature F10 | #Feature F11 | #Feature F12 | #Feature F13 | #Feature F2 | #Feature F3 | #Feature F4 | #Feature F5 | #Feature F6 | #Feature F7 | #Feature F8 | #Feature F9 | Start | End | PTM | AScore | Found By |
| --- | --- | --- | --- | --- | --- | --- | --- | --- | --- | --- | --- | --- | --- | --- | --- | --- | --- | --- | --- | --- | --- | --- | --- | --- | --- | --- | --- | --- | --- | --- | --- | --- | --- | --- | --- | --- | --- | --- | --- | --- | --- | --- | --- |
| K.TITLEVEPSDTIENVK.A | Y | 71.38 | 1786.9200 | 16 | 1.1 | 894.4683 | 2 | 56.97 | 1 | F1:22100 | NaNaKA16\_F1.raw | 1.7352E7 |  |  | 8.563E5 |  |  |  |  | 7.9338E5 |  |  |  |  | 3 | 1 | 0 | 0 | 1 | 0 | 0 | 0 | 0 | 1 | 0 | 0 | 0 | 0 | 12 | 27 |  |  | PEAKS DB |
| K.ESTLHLVLR.L | Y | 62.86 | 1066.6135 | 9 | 0.3 | 534.3142 | 2 | 28.01 | 9 | F9:13605 | NaNaKA16\_F5.raw |  |  |  |  |  |  |  |  | 2.5029E5 |  |  |  |  | 1 | 0 | 0 | 0 | 0 | 0 | 0 | 0 | 0 | 1 | 0 | 0 | 0 | 0 | 64 | 72 |  |  | PEAKS DB |
| R.TLSDYNIQK.E | Y | 56.48 | 1080.5452 | 9 | 1.0 | 541.2804 | 2 | 19.39 | 1 | F1:5953 | NaNaKA16\_F1.raw | 3.0489E6 |  |  |  |  |  |  |  |  |  |  |  |  | 2 | 2 | 0 | 0 | 0 | 0 | 0 | 0 | 0 | 0 | 0 | 0 | 0 | 0 | 55 | 63 |  |  | PEAKS DB |
| K.IQDKEGIPPDQQR.L | Y | 42.00 | 1522.7739 | 13 | -0.1 | 508.5985 | 3 | 11.71 | 1 | F1:2169 | NaNaKA16\_F1.raw | 1.117E6 |  |  |  |  |  |  |  |  |  |  |  |  | 1 | 1 | 0 | 0 | 0 | 0 | 0 | 0 | 0 | 0 | 0 | 0 | 0 | 0 | 30 | 42 |  |  | PEAKS DB |
| total 4 peptides |  |  |  |  |  |  |  |  |  |  |  |  |  |  |  |  |  |  |  |  |  |  |  |  |  |  |  |  |  |  |  |  |  |  |  |  |  |  |  |  |  |  |  |
| --- | --- | --- | --- | --- | --- | --- | --- | --- | --- | --- | --- | --- | --- | --- | --- | --- | --- | --- | --- | --- | --- | --- | --- | --- | --- | --- | --- | --- | --- | --- | --- | --- | --- | --- | --- | --- | --- | --- | --- | --- | --- | --- | --- |

XP\_026551821.1

back to list

  

| Protein Coverage
| Supporting Peptides
|

Protein Coverage:

Supporting Peptides:

| Peptide | Uniq | -10lgP | Mass | Length | ppm | m/z | z | RT | Fraction | Scan | Source File | Area F1 | Area F10 | Area F11 | Area F12 | Area F13 | Area F2 | Area F3 | Area F4 | Area F5 | Area F6 | Area F7 | Area F8 | Area F9 | #Feature | #Feature F1 | #Feature F10 | #Feature F11 | #Feature F12 | #Feature F13 | #Feature F2 | #Feature F3 | #Feature F4 | #Feature F5 | #Feature F6 | #Feature F7 | #Feature F8 | #Feature F9 | Start | End | PTM | AScore | Found By |
| --- | --- | --- | --- | --- | --- | --- | --- | --- | --- | --- | --- | --- | --- | --- | --- | --- | --- | --- | --- | --- | --- | --- | --- | --- | --- | --- | --- | --- | --- | --- | --- | --- | --- | --- | --- | --- | --- | --- | --- | --- | --- | --- | --- |
| K.TITLEVEPSDTIENVK.A | Y | 71.38 | 1786.9200 | 16 | 1.1 | 894.4683 | 2 | 56.97 | 1 | F1:22100 | NaNaKA16\_F1.raw | 1.7352E7 |  |  | 8.563E5 |  |  |  |  | 7.9338E5 |  |  |  |  | 3 | 1 | 0 | 0 | 1 | 0 | 0 | 0 | 0 | 1 | 0 | 0 | 0 | 0 | 12 | 27 |  |  | PEAKS DB |
| K.ESTLHLVLR.L | Y | 62.86 | 1066.6135 | 9 | 0.3 | 534.3142 | 2 | 28.01 | 9 | F9:13605 | NaNaKA16\_F5.raw |  |  |  |  |  |  |  |  | 2.5029E5 |  |  |  |  | 1 | 0 | 0 | 0 | 0 | 0 | 0 | 0 | 0 | 1 | 0 | 0 | 0 | 0 | 64 | 72 |  |  | PEAKS DB |
| R.TLSDYNIQK.E | Y | 56.48 | 1080.5452 | 9 | 1.0 | 541.2804 | 2 | 19.39 | 1 | F1:5953 | NaNaKA16\_F1.raw | 3.0489E6 |  |  |  |  |  |  |  |  |  |  |  |  | 2 | 2 | 0 | 0 | 0 | 0 | 0 | 0 | 0 | 0 | 0 | 0 | 0 | 0 | 55 | 63 |  |  | PEAKS DB |
| K.IQDKEGIPPDQQR.L | Y | 42.00 | 1522.7739 | 13 | -0.1 | 508.5985 | 3 | 11.71 | 1 | F1:2169 | NaNaKA16\_F1.raw | 1.117E6 |  |  |  |  |  |  |  |  |  |  |  |  | 1 | 1 | 0 | 0 | 0 | 0 | 0 | 0 | 0 | 0 | 0 | 0 | 0 | 0 | 30 | 42 |  |  | PEAKS DB |
| total 4 peptides |  |  |  |  |  |  |  |  |  |  |  |  |  |  |  |  |  |  |  |  |  |  |  |  |  |  |  |  |  |  |  |  |  |  |  |  |  |  |  |  |  |  |  |
| --- | --- | --- | --- | --- | --- | --- | --- | --- | --- | --- | --- | --- | --- | --- | --- | --- | --- | --- | --- | --- | --- | --- | --- | --- | --- | --- | --- | --- | --- | --- | --- | --- | --- | --- | --- | --- | --- | --- | --- | --- | --- | --- | --- |

JAA95751.1

back to list

  

| Protein Coverage
| Supporting Peptides
|

Protein Coverage:

Supporting Peptides:

| Peptide | Uniq | -10lgP | Mass | Length | ppm | m/z | z | RT | Fraction | Scan | Source File | Area F1 | Area F10 | Area F11 | Area F12 | Area F13 | Area F2 | Area F3 | Area F4 | Area F5 | Area F6 | Area F7 | Area F8 | Area F9 | #Feature | #Feature F1 | #Feature F10 | #Feature F11 | #Feature F12 | #Feature F13 | #Feature F2 | #Feature F3 | #Feature F4 | #Feature F5 | #Feature F6 | #Feature F7 | #Feature F8 | #Feature F9 | Start | End | PTM | AScore | Found By |
| --- | --- | --- | --- | --- | --- | --- | --- | --- | --- | --- | --- | --- | --- | --- | --- | --- | --- | --- | --- | --- | --- | --- | --- | --- | --- | --- | --- | --- | --- | --- | --- | --- | --- | --- | --- | --- | --- | --- | --- | --- | --- | --- | --- |
| K.TITLEVEPSDTIENVK.A | Y | 71.38 | 1786.9200 | 16 | 1.1 | 894.4683 | 2 | 56.97 | 1 | F1:22100 | NaNaKA16\_F1.raw | 1.7352E7 |  |  | 8.563E5 |  |  |  |  | 7.9338E5 |  |  |  |  | 3 | 1 | 0 | 0 | 1 | 0 | 0 | 0 | 0 | 1 | 0 | 0 | 0 | 0 | 12 | 27 |  |  | PEAKS DB |
| K.ESTLHLVLR.L | Y | 62.86 | 1066.6135 | 9 | 0.3 | 534.3142 | 2 | 28.01 | 9 | F9:13605 | NaNaKA16\_F5.raw |  |  |  |  |  |  |  |  | 2.5029E5 |  |  |  |  | 1 | 0 | 0 | 0 | 0 | 0 | 0 | 0 | 0 | 1 | 0 | 0 | 0 | 0 | 64 | 72 |  |  | PEAKS DB |
| R.TLSDYNIQK.E | Y | 56.48 | 1080.5452 | 9 | 1.0 | 541.2804 | 2 | 19.39 | 1 | F1:5953 | NaNaKA16\_F1.raw | 3.0489E6 |  |  |  |  |  |  |  |  |  |  |  |  | 2 | 2 | 0 | 0 | 0 | 0 | 0 | 0 | 0 | 0 | 0 | 0 | 0 | 0 | 55 | 63 |  |  | PEAKS DB |
| K.IQDKEGIPPDQQR.L | Y | 42.00 | 1522.7739 | 13 | -0.1 | 508.5985 | 3 | 11.71 | 1 | F1:2169 | NaNaKA16\_F1.raw | 1.117E6 |  |  |  |  |  |  |  |  |  |  |  |  | 1 | 1 | 0 | 0 | 0 | 0 | 0 | 0 | 0 | 0 | 0 | 0 | 0 | 0 | 30 | 42 |  |  | PEAKS DB |
| total 4 peptides |  |  |  |  |  |  |  |  |  |  |  |  |  |  |  |  |  |  |  |  |  |  |  |  |  |  |  |  |  |  |  |  |  |  |  |  |  |  |  |  |  |  |  |
| --- | --- | --- | --- | --- | --- | --- | --- | --- | --- | --- | --- | --- | --- | --- | --- | --- | --- | --- | --- | --- | --- | --- | --- | --- | --- | --- | --- | --- | --- | --- | --- | --- | --- | --- | --- | --- | --- | --- | --- | --- | --- | --- | --- |

XP\_032072309.1

back to list

  

| Protein Coverage
| Supporting Peptides
|

Protein Coverage:

Supporting Peptides:

| Peptide | Uniq | -10lgP | Mass | Length | ppm | m/z | z | RT | Fraction | Scan | Source File | Area F1 | Area F10 | Area F11 | Area F12 | Area F13 | Area F2 | Area F3 | Area F4 | Area F5 | Area F6 | Area F7 | Area F8 | Area F9 | #Feature | #Feature F1 | #Feature F10 | #Feature F11 | #Feature F12 | #Feature F13 | #Feature F2 | #Feature F3 | #Feature F4 | #Feature F5 | #Feature F6 | #Feature F7 | #Feature F8 | #Feature F9 | Start | End | PTM | AScore | Found By |
| --- | --- | --- | --- | --- | --- | --- | --- | --- | --- | --- | --- | --- | --- | --- | --- | --- | --- | --- | --- | --- | --- | --- | --- | --- | --- | --- | --- | --- | --- | --- | --- | --- | --- | --- | --- | --- | --- | --- | --- | --- | --- | --- | --- |
| K.TITLEVEPSDTIENVK.A | Y | 71.38 | 1786.9200 | 16 | 1.1 | 894.4683 | 2 | 56.97 | 1 | F1:22100 | NaNaKA16\_F1.raw | 1.7352E7 |  |  | 8.563E5 |  |  |  |  | 7.9338E5 |  |  |  |  | 3 | 1 | 0 | 0 | 1 | 0 | 0 | 0 | 0 | 1 | 0 | 0 | 0 | 0 | 12 | 27 |  |  | PEAKS DB |
| K.ESTLHLVLR.L | Y | 62.86 | 1066.6135 | 9 | 0.3 | 534.3142 | 2 | 28.01 | 9 | F9:13605 | NaNaKA16\_F5.raw |  |  |  |  |  |  |  |  | 2.5029E5 |  |  |  |  | 1 | 0 | 0 | 0 | 0 | 0 | 0 | 0 | 0 | 1 | 0 | 0 | 0 | 0 | 64 | 72 |  |  | PEAKS DB |
| R.TLSDYNIQK.E | Y | 56.48 | 1080.5452 | 9 | 1.0 | 541.2804 | 2 | 19.39 | 1 | F1:5953 | NaNaKA16\_F1.raw | 3.0489E6 |  |  |  |  |  |  |  |  |  |  |  |  | 2 | 2 | 0 | 0 | 0 | 0 | 0 | 0 | 0 | 0 | 0 | 0 | 0 | 0 | 55 | 63 |  |  | PEAKS DB |
| K.IQDKEGIPPDQQR.L | Y | 42.00 | 1522.7739 | 13 | -0.1 | 508.5985 | 3 | 11.71 | 1 | F1:2169 | NaNaKA16\_F1.raw | 1.117E6 |  |  |  |  |  |  |  |  |  |  |  |  | 1 | 1 | 0 | 0 | 0 | 0 | 0 | 0 | 0 | 0 | 0 | 0 | 0 | 0 | 30 | 42 |  |  | PEAKS DB |
| total 4 peptides |  |  |  |  |  |  |  |  |  |  |  |  |  |  |  |  |  |  |  |  |  |  |  |  |  |  |  |  |  |  |  |  |  |  |  |  |  |  |  |  |  |  |  |
| --- | --- | --- | --- | --- | --- | --- | --- | --- | --- | --- | --- | --- | --- | --- | --- | --- | --- | --- | --- | --- | --- | --- | --- | --- | --- | --- | --- | --- | --- | --- | --- | --- | --- | --- | --- | --- | --- | --- | --- | --- | --- | --- | --- |

XP\_026532495.1

back to list

  

| Protein Coverage
| Supporting Peptides
|

Protein Coverage:

Supporting Peptides:

| Peptide | Uniq | -10lgP | Mass | Length | ppm | m/z | z | RT | Fraction | Scan | Source File | Area F1 | Area F10 | Area F11 | Area F12 | Area F13 | Area F2 | Area F3 | Area F4 | Area F5 | Area F6 | Area F7 | Area F8 | Area F9 | #Feature | #Feature F1 | #Feature F10 | #Feature F11 | #Feature F12 | #Feature F13 | #Feature F2 | #Feature F3 | #Feature F4 | #Feature F5 | #Feature F6 | #Feature F7 | #Feature F8 | #Feature F9 | Start | End | PTM | AScore | Found By |
| --- | --- | --- | --- | --- | --- | --- | --- | --- | --- | --- | --- | --- | --- | --- | --- | --- | --- | --- | --- | --- | --- | --- | --- | --- | --- | --- | --- | --- | --- | --- | --- | --- | --- | --- | --- | --- | --- | --- | --- | --- | --- | --- | --- |
| K.TITLEVEPSDTIENVK.A | Y | 71.38 | 1786.9200 | 16 | 1.1 | 894.4683 | 2 | 56.97 | 1 | F1:22100 | NaNaKA16\_F1.raw | 1.7352E7 |  |  | 8.563E5 |  |  |  |  | 7.9338E5 |  |  |  |  | 3 | 1 | 0 | 0 | 1 | 0 | 0 | 0 | 0 | 1 | 0 | 0 | 0 | 0 | 12 | 27 |  |  | PEAKS DB |
| K.ESTLHLVLR.L | Y | 62.86 | 1066.6135 | 9 | 0.3 | 534.3142 | 2 | 28.01 | 9 | F9:13605 | NaNaKA16\_F5.raw |  |  |  |  |  |  |  |  | 2.5029E5 |  |  |  |  | 1 | 0 | 0 | 0 | 0 | 0 | 0 | 0 | 0 | 1 | 0 | 0 | 0 | 0 | 64 | 72 |  |  | PEAKS DB |
| R.TLSDYNIQK.E | Y | 56.48 | 1080.5452 | 9 | 1.0 | 541.2804 | 2 | 19.39 | 1 | F1:5953 | NaNaKA16\_F1.raw | 3.0489E6 |  |  |  |  |  |  |  |  |  |  |  |  | 2 | 2 | 0 | 0 | 0 | 0 | 0 | 0 | 0 | 0 | 0 | 0 | 0 | 0 | 55 | 63 |  |  | PEAKS DB |
| K.IQDKEGIPPDQQR.L | Y | 42.00 | 1522.7739 | 13 | -0.1 | 508.5985 | 3 | 11.71 | 1 | F1:2169 | NaNaKA16\_F1.raw | 1.117E6 |  |  |  |  |  |  |  |  |  |  |  |  | 1 | 1 | 0 | 0 | 0 | 0 | 0 | 0 | 0 | 0 | 0 | 0 | 0 | 0 | 30 | 42 |  |  | PEAKS DB |
| total 4 peptides |  |  |  |  |  |  |  |  |  |  |  |  |  |  |  |  |  |  |  |  |  |  |  |  |  |  |  |  |  |  |  |  |  |  |  |  |  |  |  |  |  |  |  |
| --- | --- | --- | --- | --- | --- | --- | --- | --- | --- | --- | --- | --- | --- | --- | --- | --- | --- | --- | --- | --- | --- | --- | --- | --- | --- | --- | --- | --- | --- | --- | --- | --- | --- | --- | --- | --- | --- | --- | --- | --- | --- | --- | --- |

XP\_026532494.1

back to list

  

| Protein Coverage
| Supporting Peptides
|

Protein Coverage:

Supporting Peptides:

| Peptide | Uniq | -10lgP | Mass | Length | ppm | m/z | z | RT | Fraction | Scan | Source File | Area F1 | Area F10 | Area F11 | Area F12 | Area F13 | Area F2 | Area F3 | Area F4 | Area F5 | Area F6 | Area F7 | Area F8 | Area F9 | #Feature | #Feature F1 | #Feature F10 | #Feature F11 | #Feature F12 | #Feature F13 | #Feature F2 | #Feature F3 | #Feature F4 | #Feature F5 | #Feature F6 | #Feature F7 | #Feature F8 | #Feature F9 | Start | End | PTM | AScore | Found By |
| --- | --- | --- | --- | --- | --- | --- | --- | --- | --- | --- | --- | --- | --- | --- | --- | --- | --- | --- | --- | --- | --- | --- | --- | --- | --- | --- | --- | --- | --- | --- | --- | --- | --- | --- | --- | --- | --- | --- | --- | --- | --- | --- | --- |
| K.TITLEVEPSDTIENVK.A | Y | 71.38 | 1786.9200 | 16 | 1.1 | 894.4683 | 2 | 56.97 | 1 | F1:22100 | NaNaKA16\_F1.raw | 1.7352E7 |  |  | 8.563E5 |  |  |  |  | 7.9338E5 |  |  |  |  | 3 | 1 | 0 | 0 | 1 | 0 | 0 | 0 | 0 | 1 | 0 | 0 | 0 | 0 | 12 | 27 |  |  | PEAKS DB |
| K.ESTLHLVLR.L | Y | 62.86 | 1066.6135 | 9 | 0.3 | 534.3142 | 2 | 28.01 | 9 | F9:13605 | NaNaKA16\_F5.raw |  |  |  |  |  |  |  |  | 2.5029E5 |  |  |  |  | 1 | 0 | 0 | 0 | 0 | 0 | 0 | 0 | 0 | 1 | 0 | 0 | 0 | 0 | 64 | 72 |  |  | PEAKS DB |
| R.TLSDYNIQK.E | Y | 56.48 | 1080.5452 | 9 | 1.0 | 541.2804 | 2 | 19.39 | 1 | F1:5953 | NaNaKA16\_F1.raw | 3.0489E6 |  |  |  |  |  |  |  |  |  |  |  |  | 2 | 2 | 0 | 0 | 0 | 0 | 0 | 0 | 0 | 0 | 0 | 0 | 0 | 0 | 55 | 63 |  |  | PEAKS DB |
| K.IQDKEGIPPDQQR.L | Y | 42.00 | 1522.7739 | 13 | -0.1 | 508.5985 | 3 | 11.71 | 1 | F1:2169 | NaNaKA16\_F1.raw | 1.117E6 |  |  |  |  |  |  |  |  |  |  |  |  | 1 | 1 | 0 | 0 | 0 | 0 | 0 | 0 | 0 | 0 | 0 | 0 | 0 | 0 | 30 | 42 |  |  | PEAKS DB |
| total 4 peptides |  |  |  |  |  |  |  |  |  |  |  |  |  |  |  |  |  |  |  |  |  |  |  |  |  |  |  |  |  |  |  |  |  |  |  |  |  |  |  |  |  |  |  |
| --- | --- | --- | --- | --- | --- | --- | --- | --- | --- | --- | --- | --- | --- | --- | --- | --- | --- | --- | --- | --- | --- | --- | --- | --- | --- | --- | --- | --- | --- | --- | --- | --- | --- | --- | --- | --- | --- | --- | --- | --- | --- | --- | --- |

JAI09470.1

back to list

  

| Protein Coverage
| Supporting Peptides
|

Protein Coverage:

Supporting Peptides:

| Peptide | Uniq | -10lgP | Mass | Length | ppm | m/z | z | RT | Fraction | Scan | Source File | Area F1 | Area F10 | Area F11 | Area F12 | Area F13 | Area F2 | Area F3 | Area F4 | Area F5 | Area F6 | Area F7 | Area F8 | Area F9 | #Feature | #Feature F1 | #Feature F10 | #Feature F11 | #Feature F12 | #Feature F13 | #Feature F2 | #Feature F3 | #Feature F4 | #Feature F5 | #Feature F6 | #Feature F7 | #Feature F8 | #Feature F9 | Start | End | PTM | AScore | Found By |
| --- | --- | --- | --- | --- | --- | --- | --- | --- | --- | --- | --- | --- | --- | --- | --- | --- | --- | --- | --- | --- | --- | --- | --- | --- | --- | --- | --- | --- | --- | --- | --- | --- | --- | --- | --- | --- | --- | --- | --- | --- | --- | --- | --- |
| K.TITLEVEPSDTIENVK.A | Y | 71.38 | 1786.9200 | 16 | 1.1 | 894.4683 | 2 | 56.97 | 1 | F1:22100 | NaNaKA16\_F1.raw | 1.7352E7 |  |  | 8.563E5 |  |  |  |  | 7.9338E5 |  |  |  |  | 3 | 1 | 0 | 0 | 1 | 0 | 0 | 0 | 0 | 1 | 0 | 0 | 0 | 0 | 12 | 27 |  |  | PEAKS DB |
| K.ESTLHLVLR.L | Y | 62.86 | 1066.6135 | 9 | 0.3 | 534.3142 | 2 | 28.01 | 9 | F9:13605 | NaNaKA16\_F5.raw |  |  |  |  |  |  |  |  | 2.5029E5 |  |  |  |  | 1 | 0 | 0 | 0 | 0 | 0 | 0 | 0 | 0 | 1 | 0 | 0 | 0 | 0 | 64 | 72 |  |  | PEAKS DB |
| R.TLSDYNIQK.E | Y | 56.48 | 1080.5452 | 9 | 1.0 | 541.2804 | 2 | 19.39 | 1 | F1:5953 | NaNaKA16\_F1.raw | 3.0489E6 |  |  |  |  |  |  |  |  |  |  |  |  | 2 | 2 | 0 | 0 | 0 | 0 | 0 | 0 | 0 | 0 | 0 | 0 | 0 | 0 | 55 | 63 |  |  | PEAKS DB |
| K.IQDKEGIPPDQQR.L | Y | 42.00 | 1522.7739 | 13 | -0.1 | 508.5985 | 3 | 11.71 | 1 | F1:2169 | NaNaKA16\_F1.raw | 1.117E6 |  |  |  |  |  |  |  |  |  |  |  |  | 1 | 1 | 0 | 0 | 0 | 0 | 0 | 0 | 0 | 0 | 0 | 0 | 0 | 0 | 30 | 42 |  |  | PEAKS DB |
| total 4 peptides |  |  |  |  |  |  |  |  |  |  |  |  |  |  |  |  |  |  |  |  |  |  |  |  |  |  |  |  |  |  |  |  |  |  |  |  |  |  |  |  |  |  |  |
| --- | --- | --- | --- | --- | --- | --- | --- | --- | --- | --- | --- | --- | --- | --- | --- | --- | --- | --- | --- | --- | --- | --- | --- | --- | --- | --- | --- | --- | --- | --- | --- | --- | --- | --- | --- | --- | --- | --- | --- | --- | --- | --- | --- |

XP\_032072311.1

back to list

  

| Protein Coverage
| Supporting Peptides
|

Protein Coverage:

Supporting Peptides:

| Peptide | Uniq | -10lgP | Mass | Length | ppm | m/z | z | RT | Fraction | Scan | Source File | Area F1 | Area F10 | Area F11 | Area F12 | Area F13 | Area F2 | Area F3 | Area F4 | Area F5 | Area F6 | Area F7 | Area F8 | Area F9 | #Feature | #Feature F1 | #Feature F10 | #Feature F11 | #Feature F12 | #Feature F13 | #Feature F2 | #Feature F3 | #Feature F4 | #Feature F5 | #Feature F6 | #Feature F7 | #Feature F8 | #Feature F9 | Start | End | PTM | AScore | Found By |
| --- | --- | --- | --- | --- | --- | --- | --- | --- | --- | --- | --- | --- | --- | --- | --- | --- | --- | --- | --- | --- | --- | --- | --- | --- | --- | --- | --- | --- | --- | --- | --- | --- | --- | --- | --- | --- | --- | --- | --- | --- | --- | --- | --- |
| K.TITLEVEPSDTIENVK.A | Y | 71.38 | 1786.9200 | 16 | 1.1 | 894.4683 | 2 | 56.97 | 1 | F1:22100 | NaNaKA16\_F1.raw | 1.7352E7 |  |  | 8.563E5 |  |  |  |  | 7.9338E5 |  |  |  |  | 3 | 1 | 0 | 0 | 1 | 0 | 0 | 0 | 0 | 1 | 0 | 0 | 0 | 0 | 12 | 27 |  |  | PEAKS DB |
| K.ESTLHLVLR.L | Y | 62.86 | 1066.6135 | 9 | 0.3 | 534.3142 | 2 | 28.01 | 9 | F9:13605 | NaNaKA16\_F5.raw |  |  |  |  |  |  |  |  | 2.5029E5 |  |  |  |  | 1 | 0 | 0 | 0 | 0 | 0 | 0 | 0 | 0 | 1 | 0 | 0 | 0 | 0 | 64 | 72 |  |  | PEAKS DB |
| R.TLSDYNIQK.E | Y | 56.48 | 1080.5452 | 9 | 1.0 | 541.2804 | 2 | 19.39 | 1 | F1:5953 | NaNaKA16\_F1.raw | 3.0489E6 |  |  |  |  |  |  |  |  |  |  |  |  | 2 | 2 | 0 | 0 | 0 | 0 | 0 | 0 | 0 | 0 | 0 | 0 | 0 | 0 | 55 | 63 |  |  | PEAKS DB |
| K.IQDKEGIPPDQQR.L | Y | 42.00 | 1522.7739 | 13 | -0.1 | 508.5985 | 3 | 11.71 | 1 | F1:2169 | NaNaKA16\_F1.raw | 1.117E6 |  |  |  |  |  |  |  |  |  |  |  |  | 1 | 1 | 0 | 0 | 0 | 0 | 0 | 0 | 0 | 0 | 0 | 0 | 0 | 0 | 30 | 42 |  |  | PEAKS DB |
| total 4 peptides |  |  |  |  |  |  |  |  |  |  |  |  |  |  |  |  |  |  |  |  |  |  |  |  |  |  |  |  |  |  |  |  |  |  |  |  |  |  |  |  |  |  |  |
| --- | --- | --- | --- | --- | --- | --- | --- | --- | --- | --- | --- | --- | --- | --- | --- | --- | --- | --- | --- | --- | --- | --- | --- | --- | --- | --- | --- | --- | --- | --- | --- | --- | --- | --- | --- | --- | --- | --- | --- | --- | --- | --- | --- |

XP\_034283248.1

back to list

  

| Protein Coverage
| Supporting Peptides
|

Protein Coverage:

Supporting Peptides:

| Peptide | Uniq | -10lgP | Mass | Length | ppm | m/z | z | RT | Fraction | Scan | Source File | Area F1 | Area F10 | Area F11 | Area F12 | Area F13 | Area F2 | Area F3 | Area F4 | Area F5 | Area F6 | Area F7 | Area F8 | Area F9 | #Feature | #Feature F1 | #Feature F10 | #Feature F11 | #Feature F12 | #Feature F13 | #Feature F2 | #Feature F3 | #Feature F4 | #Feature F5 | #Feature F6 | #Feature F7 | #Feature F8 | #Feature F9 | Start | End | PTM | AScore | Found By |
| --- | --- | --- | --- | --- | --- | --- | --- | --- | --- | --- | --- | --- | --- | --- | --- | --- | --- | --- | --- | --- | --- | --- | --- | --- | --- | --- | --- | --- | --- | --- | --- | --- | --- | --- | --- | --- | --- | --- | --- | --- | --- | --- | --- |
| K.TITLEVEPSDTIENVK.A | Y | 71.38 | 1786.9200 | 16 | 1.1 | 894.4683 | 2 | 56.97 | 1 | F1:22100 | NaNaKA16\_F1.raw | 1.7352E7 |  |  | 8.563E5 |  |  |  |  | 7.9338E5 |  |  |  |  | 3 | 1 | 0 | 0 | 1 | 0 | 0 | 0 | 0 | 1 | 0 | 0 | 0 | 0 | 12 | 27 |  |  | PEAKS DB |
| K.ESTLHLVLR.L | Y | 62.86 | 1066.6135 | 9 | 0.3 | 534.3142 | 2 | 28.01 | 9 | F9:13605 | NaNaKA16\_F5.raw |  |  |  |  |  |  |  |  | 2.5029E5 |  |  |  |  | 1 | 0 | 0 | 0 | 0 | 0 | 0 | 0 | 0 | 1 | 0 | 0 | 0 | 0 | 64 | 72 |  |  | PEAKS DB |
| R.TLSDYNIQK.E | Y | 56.48 | 1080.5452 | 9 | 1.0 | 541.2804 | 2 | 19.39 | 1 | F1:5953 | NaNaKA16\_F1.raw | 3.0489E6 |  |  |  |  |  |  |  |  |  |  |  |  | 2 | 2 | 0 | 0 | 0 | 0 | 0 | 0 | 0 | 0 | 0 | 0 | 0 | 0 | 55 | 63 |  |  | PEAKS DB |
| K.IQDKEGIPPDQQR.L | Y | 42.00 | 1522.7739 | 13 | -0.1 | 508.5985 | 3 | 11.71 | 1 | F1:2169 | NaNaKA16\_F1.raw | 1.117E6 |  |  |  |  |  |  |  |  |  |  |  |  | 1 | 1 | 0 | 0 | 0 | 0 | 0 | 0 | 0 | 0 | 0 | 0 | 0 | 0 | 30 | 42 |  |  | PEAKS DB |
| total 4 peptides |  |  |  |  |  |  |  |  |  |  |  |  |  |  |  |  |  |  |  |  |  |  |  |  |  |  |  |  |  |  |  |  |  |  |  |  |  |  |  |  |  |  |  |
| --- | --- | --- | --- | --- | --- | --- | --- | --- | --- | --- | --- | --- | --- | --- | --- | --- | --- | --- | --- | --- | --- | --- | --- | --- | --- | --- | --- | --- | --- | --- | --- | --- | --- | --- | --- | --- | --- | --- | --- | --- | --- | --- | --- |

LAB39333.1

back to list

  

| Protein Coverage
| Supporting Peptides
|

Protein Coverage:

Supporting Peptides:

| Peptide | Uniq | -10lgP | Mass | Length | ppm | m/z | z | RT | Fraction | Scan | Source File | Area F1 | Area F10 | Area F11 | Area F12 | Area F13 | Area F2 | Area F3 | Area F4 | Area F5 | Area F6 | Area F7 | Area F8 | Area F9 | #Feature | #Feature F1 | #Feature F10 | #Feature F11 | #Feature F12 | #Feature F13 | #Feature F2 | #Feature F3 | #Feature F4 | #Feature F5 | #Feature F6 | #Feature F7 | #Feature F8 | #Feature F9 | Start | End | PTM | AScore | Found By |
| --- | --- | --- | --- | --- | --- | --- | --- | --- | --- | --- | --- | --- | --- | --- | --- | --- | --- | --- | --- | --- | --- | --- | --- | --- | --- | --- | --- | --- | --- | --- | --- | --- | --- | --- | --- | --- | --- | --- | --- | --- | --- | --- | --- |
| K.TITLEVEPSDTIENVK.A | Y | 71.38 | 1786.9200 | 16 | 1.1 | 894.4683 | 2 | 56.97 | 1 | F1:22100 | NaNaKA16\_F1.raw | 1.7352E7 |  |  | 8.563E5 |  |  |  |  | 7.9338E5 |  |  |  |  | 3 | 1 | 0 | 0 | 1 | 0 | 0 | 0 | 0 | 1 | 0 | 0 | 0 | 0 | 12 | 27 |  |  | PEAKS DB |
| K.ESTLHLVLR.L | Y | 62.86 | 1066.6135 | 9 | 0.3 | 534.3142 | 2 | 28.01 | 9 | F9:13605 | NaNaKA16\_F5.raw |  |  |  |  |  |  |  |  | 2.5029E5 |  |  |  |  | 1 | 0 | 0 | 0 | 0 | 0 | 0 | 0 | 0 | 1 | 0 | 0 | 0 | 0 | 64 | 72 |  |  | PEAKS DB |
| R.TLSDYNIQK.E | Y | 56.48 | 1080.5452 | 9 | 1.0 | 541.2804 | 2 | 19.39 | 1 | F1:5953 | NaNaKA16\_F1.raw | 3.0489E6 |  |  |  |  |  |  |  |  |  |  |  |  | 2 | 2 | 0 | 0 | 0 | 0 | 0 | 0 | 0 | 0 | 0 | 0 | 0 | 0 | 55 | 63 |  |  | PEAKS DB |
| K.IQDKEGIPPDQQR.L | Y | 42.00 | 1522.7739 | 13 | -0.1 | 508.5985 | 3 | 11.71 | 1 | F1:2169 | NaNaKA16\_F1.raw | 1.117E6 |  |  |  |  |  |  |  |  |  |  |  |  | 1 | 1 | 0 | 0 | 0 | 0 | 0 | 0 | 0 | 0 | 0 | 0 | 0 | 0 | 30 | 42 |  |  | PEAKS DB |
| total 4 peptides |  |  |  |  |  |  |  |  |  |  |  |  |  |  |  |  |  |  |  |  |  |  |  |  |  |  |  |  |  |  |  |  |  |  |  |  |  |  |  |  |  |  |  |
| --- | --- | --- | --- | --- | --- | --- | --- | --- | --- | --- | --- | --- | --- | --- | --- | --- | --- | --- | --- | --- | --- | --- | --- | --- | --- | --- | --- | --- | --- | --- | --- | --- | --- | --- | --- | --- | --- | --- | --- | --- | --- | --- | --- |

LAB39331.1

back to list

  

| Protein Coverage
| Supporting Peptides
|

Protein Coverage:

Supporting Peptides:

| Peptide | Uniq | -10lgP | Mass | Length | ppm | m/z | z | RT | Fraction | Scan | Source File | Area F1 | Area F10 | Area F11 | Area F12 | Area F13 | Area F2 | Area F3 | Area F4 | Area F5 | Area F6 | Area F7 | Area F8 | Area F9 | #Feature | #Feature F1 | #Feature F10 | #Feature F11 | #Feature F12 | #Feature F13 | #Feature F2 | #Feature F3 | #Feature F4 | #Feature F5 | #Feature F6 | #Feature F7 | #Feature F8 | #Feature F9 | Start | End | PTM | AScore | Found By |
| --- | --- | --- | --- | --- | --- | --- | --- | --- | --- | --- | --- | --- | --- | --- | --- | --- | --- | --- | --- | --- | --- | --- | --- | --- | --- | --- | --- | --- | --- | --- | --- | --- | --- | --- | --- | --- | --- | --- | --- | --- | --- | --- | --- |
| K.TITLEVEPSDTIENVK.A | Y | 71.38 | 1786.9200 | 16 | 1.1 | 894.4683 | 2 | 56.97 | 1 | F1:22100 | NaNaKA16\_F1.raw | 1.7352E7 |  |  | 8.563E5 |  |  |  |  | 7.9338E5 |  |  |  |  | 3 | 1 | 0 | 0 | 1 | 0 | 0 | 0 | 0 | 1 | 0 | 0 | 0 | 0 | 25 | 40 |  |  | PEAKS DB |
| K.ESTLHLVLR.L | Y | 62.86 | 1066.6135 | 9 | 0.3 | 534.3142 | 2 | 28.01 | 9 | F9:13605 | NaNaKA16\_F5.raw |  |  |  |  |  |  |  |  | 2.5029E5 |  |  |  |  | 1 | 0 | 0 | 0 | 0 | 0 | 0 | 0 | 0 | 1 | 0 | 0 | 0 | 0 | 77 | 85 |  |  | PEAKS DB |
| R.TLSDYNIQK.E | Y | 56.48 | 1080.5452 | 9 | 1.0 | 541.2804 | 2 | 19.39 | 1 | F1:5953 | NaNaKA16\_F1.raw | 3.0489E6 |  |  |  |  |  |  |  |  |  |  |  |  | 2 | 2 | 0 | 0 | 0 | 0 | 0 | 0 | 0 | 0 | 0 | 0 | 0 | 0 | 68 | 76 |  |  | PEAKS DB |
| K.IQDKEGIPPDQQR.L | Y | 42.00 | 1522.7739 | 13 | -0.1 | 508.5985 | 3 | 11.71 | 1 | F1:2169 | NaNaKA16\_F1.raw | 1.117E6 |  |  |  |  |  |  |  |  |  |  |  |  | 1 | 1 | 0 | 0 | 0 | 0 | 0 | 0 | 0 | 0 | 0 | 0 | 0 | 0 | 43 | 55 |  |  | PEAKS DB |
| total 4 peptides |  |  |  |  |  |  |  |  |  |  |  |  |  |  |  |  |  |  |  |  |  |  |  |  |  |  |  |  |  |  |  |  |  |  |  |  |  |  |  |  |  |  |  |
| --- | --- | --- | --- | --- | --- | --- | --- | --- | --- | --- | --- | --- | --- | --- | --- | --- | --- | --- | --- | --- | --- | --- | --- | --- | --- | --- | --- | --- | --- | --- | --- | --- | --- | --- | --- | --- | --- | --- | --- | --- | --- | --- | --- |

LAA53282.1

back to list

  

| Protein Coverage
| Supporting Peptides
|

Protein Coverage:

Supporting Peptides:

| Peptide | Uniq | -10lgP | Mass | Length | ppm | m/z | z | RT | Fraction | Scan | Source File | Area F1 | Area F10 | Area F11 | Area F12 | Area F13 | Area F2 | Area F3 | Area F4 | Area F5 | Area F6 | Area F7 | Area F8 | Area F9 | #Feature | #Feature F1 | #Feature F10 | #Feature F11 | #Feature F12 | #Feature F13 | #Feature F2 | #Feature F3 | #Feature F4 | #Feature F5 | #Feature F6 | #Feature F7 | #Feature F8 | #Feature F9 | Start | End | PTM | AScore | Found By |
| --- | --- | --- | --- | --- | --- | --- | --- | --- | --- | --- | --- | --- | --- | --- | --- | --- | --- | --- | --- | --- | --- | --- | --- | --- | --- | --- | --- | --- | --- | --- | --- | --- | --- | --- | --- | --- | --- | --- | --- | --- | --- | --- | --- |
| K.TITLEVEPSDTIENVK.A | Y | 71.38 | 1786.9200 | 16 | 1.1 | 894.4683 | 2 | 56.97 | 1 | F1:22100 | NaNaKA16\_F1.raw | 1.7352E7 |  |  | 8.563E5 |  |  |  |  | 7.9338E5 |  |  |  |  | 3 | 1 | 0 | 0 | 1 | 0 | 0 | 0 | 0 | 1 | 0 | 0 | 0 | 0 | 35 | 50 |  |  | PEAKS DB |
| K.ESTLHLVLR.L | Y | 62.86 | 1066.6135 | 9 | 0.3 | 534.3142 | 2 | 28.01 | 9 | F9:13605 | NaNaKA16\_F5.raw |  |  |  |  |  |  |  |  | 2.5029E5 |  |  |  |  | 1 | 0 | 0 | 0 | 0 | 0 | 0 | 0 | 0 | 1 | 0 | 0 | 0 | 0 | 87 | 95 |  |  | PEAKS DB |
| R.TLSDYNIQK.E | Y | 56.48 | 1080.5452 | 9 | 1.0 | 541.2804 | 2 | 19.39 | 1 | F1:5953 | NaNaKA16\_F1.raw | 3.0489E6 |  |  |  |  |  |  |  |  |  |  |  |  | 2 | 2 | 0 | 0 | 0 | 0 | 0 | 0 | 0 | 0 | 0 | 0 | 0 | 0 | 78 | 86 |  |  | PEAKS DB |
| K.IQDKEGIPPDQQR.L | Y | 42.00 | 1522.7739 | 13 | -0.1 | 508.5985 | 3 | 11.71 | 1 | F1:2169 | NaNaKA16\_F1.raw | 1.117E6 |  |  |  |  |  |  |  |  |  |  |  |  | 1 | 1 | 0 | 0 | 0 | 0 | 0 | 0 | 0 | 0 | 0 | 0 | 0 | 0 | 53 | 65 |  |  | PEAKS DB |
| total 4 peptides |  |  |  |  |  |  |  |  |  |  |  |  |  |  |  |  |  |  |  |  |  |  |  |  |  |  |  |  |  |  |  |  |  |  |  |  |  |  |  |  |  |  |  |
| --- | --- | --- | --- | --- | --- | --- | --- | --- | --- | --- | --- | --- | --- | --- | --- | --- | --- | --- | --- | --- | --- | --- | --- | --- | --- | --- | --- | --- | --- | --- | --- | --- | --- | --- | --- | --- | --- | --- | --- | --- | --- | --- | --- |

XP\_013929631.1

back to list

  

| Protein Coverage
| Supporting Peptides
|

Protein Coverage:

Supporting Peptides:

| Peptide | Uniq | -10lgP | Mass | Length | ppm | m/z | z | RT | Fraction | Scan | Source File | Area F1 | Area F10 | Area F11 | Area F12 | Area F13 | Area F2 | Area F3 | Area F4 | Area F5 | Area F6 | Area F7 | Area F8 | Area F9 | #Feature | #Feature F1 | #Feature F10 | #Feature F11 | #Feature F12 | #Feature F13 | #Feature F2 | #Feature F3 | #Feature F4 | #Feature F5 | #Feature F6 | #Feature F7 | #Feature F8 | #Feature F9 | Start | End | PTM | AScore | Found By |
| --- | --- | --- | --- | --- | --- | --- | --- | --- | --- | --- | --- | --- | --- | --- | --- | --- | --- | --- | --- | --- | --- | --- | --- | --- | --- | --- | --- | --- | --- | --- | --- | --- | --- | --- | --- | --- | --- | --- | --- | --- | --- | --- | --- |
| K.TITLEVEPSDTIENVK.A | Y | 71.38 | 1786.9200 | 16 | 1.1 | 894.4683 | 2 | 56.97 | 1 | F1:22100 | NaNaKA16\_F1.raw | 1.7352E7 |  |  | 8.563E5 |  |  |  |  | 7.9338E5 |  |  |  |  | 3 | 1 | 0 | 0 | 1 | 0 | 0 | 0 | 0 | 1 | 0 | 0 | 0 | 0 | 12 | 27 |  |  | PEAKS DB |
| K.ESTLHLVLR.L | Y | 62.86 | 1066.6135 | 9 | 0.3 | 534.3142 | 2 | 28.01 | 9 | F9:13605 | NaNaKA16\_F5.raw |  |  |  |  |  |  |  |  | 2.5029E5 |  |  |  |  | 1 | 0 | 0 | 0 | 0 | 0 | 0 | 0 | 0 | 1 | 0 | 0 | 0 | 0 | 64 | 72 |  |  | PEAKS DB |
| R.TLSDYNIQK.E | Y | 56.48 | 1080.5452 | 9 | 1.0 | 541.2804 | 2 | 19.39 | 1 | F1:5953 | NaNaKA16\_F1.raw | 3.0489E6 |  |  |  |  |  |  |  |  |  |  |  |  | 2 | 2 | 0 | 0 | 0 | 0 | 0 | 0 | 0 | 0 | 0 | 0 | 0 | 0 | 55 | 63 |  |  | PEAKS DB |
| K.IQDKEGIPPDQQR.L | Y | 42.00 | 1522.7739 | 13 | -0.1 | 508.5985 | 3 | 11.71 | 1 | F1:2169 | NaNaKA16\_F1.raw | 1.117E6 |  |  |  |  |  |  |  |  |  |  |  |  | 1 | 1 | 0 | 0 | 0 | 0 | 0 | 0 | 0 | 0 | 0 | 0 | 0 | 0 | 30 | 42 |  |  | PEAKS DB |
| total 4 peptides |  |  |  |  |  |  |  |  |  |  |  |  |  |  |  |  |  |  |  |  |  |  |  |  |  |  |  |  |  |  |  |  |  |  |  |  |  |  |  |  |  |  |  |
| --- | --- | --- | --- | --- | --- | --- | --- | --- | --- | --- | --- | --- | --- | --- | --- | --- | --- | --- | --- | --- | --- | --- | --- | --- | --- | --- | --- | --- | --- | --- | --- | --- | --- | --- | --- | --- | --- | --- | --- | --- | --- | --- | --- |

XP\_013929630.1

back to list

  

| Protein Coverage
| Supporting Peptides
|

Protein Coverage:

Supporting Peptides:

| Peptide | Uniq | -10lgP | Mass | Length | ppm | m/z | z | RT | Fraction | Scan | Source File | Area F1 | Area F10 | Area F11 | Area F12 | Area F13 | Area F2 | Area F3 | Area F4 | Area F5 | Area F6 | Area F7 | Area F8 | Area F9 | #Feature | #Feature F1 | #Feature F10 | #Feature F11 | #Feature F12 | #Feature F13 | #Feature F2 | #Feature F3 | #Feature F4 | #Feature F5 | #Feature F6 | #Feature F7 | #Feature F8 | #Feature F9 | Start | End | PTM | AScore | Found By |
| --- | --- | --- | --- | --- | --- | --- | --- | --- | --- | --- | --- | --- | --- | --- | --- | --- | --- | --- | --- | --- | --- | --- | --- | --- | --- | --- | --- | --- | --- | --- | --- | --- | --- | --- | --- | --- | --- | --- | --- | --- | --- | --- | --- |
| K.TITLEVEPSDTIENVK.A | Y | 71.38 | 1786.9200 | 16 | 1.1 | 894.4683 | 2 | 56.97 | 1 | F1:22100 | NaNaKA16\_F1.raw | 1.7352E7 |  |  | 8.563E5 |  |  |  |  | 7.9338E5 |  |  |  |  | 3 | 1 | 0 | 0 | 1 | 0 | 0 | 0 | 0 | 1 | 0 | 0 | 0 | 0 | 12 | 27 |  |  | PEAKS DB |
| K.ESTLHLVLR.L | Y | 62.86 | 1066.6135 | 9 | 0.3 | 534.3142 | 2 | 28.01 | 9 | F9:13605 | NaNaKA16\_F5.raw |  |  |  |  |  |  |  |  | 2.5029E5 |  |  |  |  | 1 | 0 | 0 | 0 | 0 | 0 | 0 | 0 | 0 | 1 | 0 | 0 | 0 | 0 | 64 | 72 |  |  | PEAKS DB |
| R.TLSDYNIQK.E | Y | 56.48 | 1080.5452 | 9 | 1.0 | 541.2804 | 2 | 19.39 | 1 | F1:5953 | NaNaKA16\_F1.raw | 3.0489E6 |  |  |  |  |  |  |  |  |  |  |  |  | 2 | 2 | 0 | 0 | 0 | 0 | 0 | 0 | 0 | 0 | 0 | 0 | 0 | 0 | 55 | 63 |  |  | PEAKS DB |
| K.IQDKEGIPPDQQR.L | Y | 42.00 | 1522.7739 | 13 | -0.1 | 508.5985 | 3 | 11.71 | 1 | F1:2169 | NaNaKA16\_F1.raw | 1.117E6 |  |  |  |  |  |  |  |  |  |  |  |  | 1 | 1 | 0 | 0 | 0 | 0 | 0 | 0 | 0 | 0 | 0 | 0 | 0 | 0 | 30 | 42 |  |  | PEAKS DB |
| total 4 peptides |  |  |  |  |  |  |  |  |  |  |  |  |  |  |  |  |  |  |  |  |  |  |  |  |  |  |  |  |  |  |  |  |  |  |  |  |  |  |  |  |  |  |  |
| --- | --- | --- | --- | --- | --- | --- | --- | --- | --- | --- | --- | --- | --- | --- | --- | --- | --- | --- | --- | --- | --- | --- | --- | --- | --- | --- | --- | --- | --- | --- | --- | --- | --- | --- | --- | --- | --- | --- | --- | --- | --- | --- | --- |

JAI09497.1

back to list

  

| Protein Coverage
| Supporting Peptides
|

Protein Coverage:

Supporting Peptides:

| Peptide | Uniq | -10lgP | Mass | Length | ppm | m/z | z | RT | Fraction | Scan | Source File | Area F1 | Area F10 | Area F11 | Area F12 | Area F13 | Area F2 | Area F3 | Area F4 | Area F5 | Area F6 | Area F7 | Area F8 | Area F9 | #Feature | #Feature F1 | #Feature F10 | #Feature F11 | #Feature F12 | #Feature F13 | #Feature F2 | #Feature F3 | #Feature F4 | #Feature F5 | #Feature F6 | #Feature F7 | #Feature F8 | #Feature F9 | Start | End | PTM | AScore | Found By |
| --- | --- | --- | --- | --- | --- | --- | --- | --- | --- | --- | --- | --- | --- | --- | --- | --- | --- | --- | --- | --- | --- | --- | --- | --- | --- | --- | --- | --- | --- | --- | --- | --- | --- | --- | --- | --- | --- | --- | --- | --- | --- | --- | --- |
| K.TITLEVEPSDTIENVK.A | Y | 71.38 | 1786.9200 | 16 | 1.1 | 894.4683 | 2 | 56.97 | 1 | F1:22100 | NaNaKA16\_F1.raw | 1.7352E7 |  |  | 8.563E5 |  |  |  |  | 7.9338E5 |  |  |  |  | 3 | 1 | 0 | 0 | 1 | 0 | 0 | 0 | 0 | 1 | 0 | 0 | 0 | 0 | 12 | 27 |  |  | PEAKS DB |
| K.ESTLHLVLR.L | Y | 62.86 | 1066.6135 | 9 | 0.3 | 534.3142 | 2 | 28.01 | 9 | F9:13605 | NaNaKA16\_F5.raw |  |  |  |  |  |  |  |  | 2.5029E5 |  |  |  |  | 1 | 0 | 0 | 0 | 0 | 0 | 0 | 0 | 0 | 1 | 0 | 0 | 0 | 0 | 64 | 72 |  |  | PEAKS DB |
| R.TLSDYNIQK.E | Y | 56.48 | 1080.5452 | 9 | 1.0 | 541.2804 | 2 | 19.39 | 1 | F1:5953 | NaNaKA16\_F1.raw | 3.0489E6 |  |  |  |  |  |  |  |  |  |  |  |  | 2 | 2 | 0 | 0 | 0 | 0 | 0 | 0 | 0 | 0 | 0 | 0 | 0 | 0 | 55 | 63 |  |  | PEAKS DB |
| K.IQDKEGIPPDQQR.L | Y | 42.00 | 1522.7739 | 13 | -0.1 | 508.5985 | 3 | 11.71 | 1 | F1:2169 | NaNaKA16\_F1.raw | 1.117E6 |  |  |  |  |  |  |  |  |  |  |  |  | 1 | 1 | 0 | 0 | 0 | 0 | 0 | 0 | 0 | 0 | 0 | 0 | 0 | 0 | 30 | 42 |  |  | PEAKS DB |
| total 4 peptides |  |  |  |  |  |  |  |  |  |  |  |  |  |  |  |  |  |  |  |  |  |  |  |  |  |  |  |  |  |  |  |  |  |  |  |  |  |  |  |  |  |  |  |
| --- | --- | --- | --- | --- | --- | --- | --- | --- | --- | --- | --- | --- | --- | --- | --- | --- | --- | --- | --- | --- | --- | --- | --- | --- | --- | --- | --- | --- | --- | --- | --- | --- | --- | --- | --- | --- | --- | --- | --- | --- | --- | --- | --- |

JAB53439.1

back to list

  

| Protein Coverage
| Supporting Peptides
|

Protein Coverage:

Supporting Peptides:

| Peptide | Uniq | -10lgP | Mass | Length | ppm | m/z | z | RT | Fraction | Scan | Source File | Area F1 | Area F10 | Area F11 | Area F12 | Area F13 | Area F2 | Area F3 | Area F4 | Area F5 | Area F6 | Area F7 | Area F8 | Area F9 | #Feature | #Feature F1 | #Feature F10 | #Feature F11 | #Feature F12 | #Feature F13 | #Feature F2 | #Feature F3 | #Feature F4 | #Feature F5 | #Feature F6 | #Feature F7 | #Feature F8 | #Feature F9 | Start | End | PTM | AScore | Found By |
| --- | --- | --- | --- | --- | --- | --- | --- | --- | --- | --- | --- | --- | --- | --- | --- | --- | --- | --- | --- | --- | --- | --- | --- | --- | --- | --- | --- | --- | --- | --- | --- | --- | --- | --- | --- | --- | --- | --- | --- | --- | --- | --- | --- |
| K.TITLEVEPSDTIENVK.A | Y | 71.38 | 1786.9200 | 16 | 1.1 | 894.4683 | 2 | 56.97 | 1 | F1:22100 | NaNaKA16\_F1.raw | 1.7352E7 |  |  | 8.563E5 |  |  |  |  | 7.9338E5 |  |  |  |  | 3 | 1 | 0 | 0 | 1 | 0 | 0 | 0 | 0 | 1 | 0 | 0 | 0 | 0 | 12 | 27 |  |  | PEAKS DB |
| K.ESTLHLVLR.L | Y | 62.86 | 1066.6135 | 9 | 0.3 | 534.3142 | 2 | 28.01 | 9 | F9:13605 | NaNaKA16\_F5.raw |  |  |  |  |  |  |  |  | 2.5029E5 |  |  |  |  | 1 | 0 | 0 | 0 | 0 | 0 | 0 | 0 | 0 | 1 | 0 | 0 | 0 | 0 | 64 | 72 |  |  | PEAKS DB |
| R.TLSDYNIQK.E | Y | 56.48 | 1080.5452 | 9 | 1.0 | 541.2804 | 2 | 19.39 | 1 | F1:5953 | NaNaKA16\_F1.raw | 3.0489E6 |  |  |  |  |  |  |  |  |  |  |  |  | 2 | 2 | 0 | 0 | 0 | 0 | 0 | 0 | 0 | 0 | 0 | 0 | 0 | 0 | 55 | 63 |  |  | PEAKS DB |
| K.IQDKEGIPPDQQR.L | Y | 42.00 | 1522.7739 | 13 | -0.1 | 508.5985 | 3 | 11.71 | 1 | F1:2169 | NaNaKA16\_F1.raw | 1.117E6 |  |  |  |  |  |  |  |  |  |  |  |  | 1 | 1 | 0 | 0 | 0 | 0 | 0 | 0 | 0 | 0 | 0 | 0 | 0 | 0 | 30 | 42 |  |  | PEAKS DB |
| total 4 peptides |  |  |  |  |  |  |  |  |  |  |  |  |  |  |  |  |  |  |  |  |  |  |  |  |  |  |  |  |  |  |  |  |  |  |  |  |  |  |  |  |  |  |  |
| --- | --- | --- | --- | --- | --- | --- | --- | --- | --- | --- | --- | --- | --- | --- | --- | --- | --- | --- | --- | --- | --- | --- | --- | --- | --- | --- | --- | --- | --- | --- | --- | --- | --- | --- | --- | --- | --- | --- | --- | --- | --- | --- | --- |

JAI11613.1

back to list

  

| Protein Coverage
| Supporting Peptides
|

Protein Coverage:

Supporting Peptides:

| Peptide | Uniq | -10lgP | Mass | Length | ppm | m/z | z | RT | Fraction | Scan | Source File | Area F1 | Area F10 | Area F11 | Area F12 | Area F13 | Area F2 | Area F3 | Area F4 | Area F5 | Area F6 | Area F7 | Area F8 | Area F9 | #Feature | #Feature F1 | #Feature F10 | #Feature F11 | #Feature F12 | #Feature F13 | #Feature F2 | #Feature F3 | #Feature F4 | #Feature F5 | #Feature F6 | #Feature F7 | #Feature F8 | #Feature F9 | Start | End | PTM | AScore | Found By |
| --- | --- | --- | --- | --- | --- | --- | --- | --- | --- | --- | --- | --- | --- | --- | --- | --- | --- | --- | --- | --- | --- | --- | --- | --- | --- | --- | --- | --- | --- | --- | --- | --- | --- | --- | --- | --- | --- | --- | --- | --- | --- | --- | --- |
| K.TITLEVEPSDTIENVK.A | Y | 71.38 | 1786.9200 | 16 | 1.1 | 894.4683 | 2 | 56.97 | 1 | F1:22100 | NaNaKA16\_F1.raw | 1.7352E7 |  |  | 8.563E5 |  |  |  |  | 7.9338E5 |  |  |  |  | 3 | 1 | 0 | 0 | 1 | 0 | 0 | 0 | 0 | 1 | 0 | 0 | 0 | 0 | 12 | 27 |  |  | PEAKS DB |
| K.ESTLHLVLR.L | Y | 62.86 | 1066.6135 | 9 | 0.3 | 534.3142 | 2 | 28.01 | 9 | F9:13605 | NaNaKA16\_F5.raw |  |  |  |  |  |  |  |  | 2.5029E5 |  |  |  |  | 1 | 0 | 0 | 0 | 0 | 0 | 0 | 0 | 0 | 1 | 0 | 0 | 0 | 0 | 64 | 72 |  |  | PEAKS DB |
| R.TLSDYNIQK.E | Y | 56.48 | 1080.5452 | 9 | 1.0 | 541.2804 | 2 | 19.39 | 1 | F1:5953 | NaNaKA16\_F1.raw | 3.0489E6 |  |  |  |  |  |  |  |  |  |  |  |  | 2 | 2 | 0 | 0 | 0 | 0 | 0 | 0 | 0 | 0 | 0 | 0 | 0 | 0 | 55 | 63 |  |  | PEAKS DB |
| K.IQDKEGIPPDQQR.L | Y | 42.00 | 1522.7739 | 13 | -0.1 | 508.5985 | 3 | 11.71 | 1 | F1:2169 | NaNaKA16\_F1.raw | 1.117E6 |  |  |  |  |  |  |  |  |  |  |  |  | 1 | 1 | 0 | 0 | 0 | 0 | 0 | 0 | 0 | 0 | 0 | 0 | 0 | 0 | 30 | 42 |  |  | PEAKS DB |
| total 4 peptides |  |  |  |  |  |  |  |  |  |  |  |  |  |  |  |  |  |  |  |  |  |  |  |  |  |  |  |  |  |  |  |  |  |  |  |  |  |  |  |  |  |  |  |
| --- | --- | --- | --- | --- | --- | --- | --- | --- | --- | --- | --- | --- | --- | --- | --- | --- | --- | --- | --- | --- | --- | --- | --- | --- | --- | --- | --- | --- | --- | --- | --- | --- | --- | --- | --- | --- | --- | --- | --- | --- | --- | --- | --- |

AFJ51273.1

back to list

  

| Protein Coverage
| Supporting Peptides
|

Protein Coverage:

Supporting Peptides:

| Peptide | Uniq | -10lgP | Mass | Length | ppm | m/z | z | RT | Fraction | Scan | Source File | Area F1 | Area F10 | Area F11 | Area F12 | Area F13 | Area F2 | Area F3 | Area F4 | Area F5 | Area F6 | Area F7 | Area F8 | Area F9 | #Feature | #Feature F1 | #Feature F10 | #Feature F11 | #Feature F12 | #Feature F13 | #Feature F2 | #Feature F3 | #Feature F4 | #Feature F5 | #Feature F6 | #Feature F7 | #Feature F8 | #Feature F9 | Start | End | PTM | AScore | Found By |
| --- | --- | --- | --- | --- | --- | --- | --- | --- | --- | --- | --- | --- | --- | --- | --- | --- | --- | --- | --- | --- | --- | --- | --- | --- | --- | --- | --- | --- | --- | --- | --- | --- | --- | --- | --- | --- | --- | --- | --- | --- | --- | --- | --- |
| K.TITLEVEPSDTIENVK.A | Y | 71.38 | 1786.9200 | 16 | 1.1 | 894.4683 | 2 | 56.97 | 1 | F1:22100 | NaNaKA16\_F1.raw | 1.7352E7 |  |  | 8.563E5 |  |  |  |  | 7.9338E5 |  |  |  |  | 3 | 1 | 0 | 0 | 1 | 0 | 0 | 0 | 0 | 1 | 0 | 0 | 0 | 0 | 12 | 27 |  |  | PEAKS DB |
| K.ESTLHLVLR.L | Y | 62.86 | 1066.6135 | 9 | 0.3 | 534.3142 | 2 | 28.01 | 9 | F9:13605 | NaNaKA16\_F5.raw |  |  |  |  |  |  |  |  | 2.5029E5 |  |  |  |  | 1 | 0 | 0 | 0 | 0 | 0 | 0 | 0 | 0 | 1 | 0 | 0 | 0 | 0 | 64 | 72 |  |  | PEAKS DB |
| R.TLSDYNIQK.E | Y | 56.48 | 1080.5452 | 9 | 1.0 | 541.2804 | 2 | 19.39 | 1 | F1:5953 | NaNaKA16\_F1.raw | 3.0489E6 |  |  |  |  |  |  |  |  |  |  |  |  | 2 | 2 | 0 | 0 | 0 | 0 | 0 | 0 | 0 | 0 | 0 | 0 | 0 | 0 | 55 | 63 |  |  | PEAKS DB |
| K.IQDKEGIPPDQQR.L | Y | 42.00 | 1522.7739 | 13 | -0.1 | 508.5985 | 3 | 11.71 | 1 | F1:2169 | NaNaKA16\_F1.raw | 1.117E6 |  |  |  |  |  |  |  |  |  |  |  |  | 1 | 1 | 0 | 0 | 0 | 0 | 0 | 0 | 0 | 0 | 0 | 0 | 0 | 0 | 30 | 42 |  |  | PEAKS DB |
| total 4 peptides |  |  |  |  |  |  |  |  |  |  |  |  |  |  |  |  |  |  |  |  |  |  |  |  |  |  |  |  |  |  |  |  |  |  |  |  |  |  |  |  |  |  |  |
| --- | --- | --- | --- | --- | --- | --- | --- | --- | --- | --- | --- | --- | --- | --- | --- | --- | --- | --- | --- | --- | --- | --- | --- | --- | --- | --- | --- | --- | --- | --- | --- | --- | --- | --- | --- | --- | --- | --- | --- | --- | --- | --- | --- |

JAG66673.1

back to list

  

| Protein Coverage
| Supporting Peptides
|

Protein Coverage:

Supporting Peptides:

| Peptide | Uniq | -10lgP | Mass | Length | ppm | m/z | z | RT | Fraction | Scan | Source File | Area F1 | Area F10 | Area F11 | Area F12 | Area F13 | Area F2 | Area F3 | Area F4 | Area F5 | Area F6 | Area F7 | Area F8 | Area F9 | #Feature | #Feature F1 | #Feature F10 | #Feature F11 | #Feature F12 | #Feature F13 | #Feature F2 | #Feature F3 | #Feature F4 | #Feature F5 | #Feature F6 | #Feature F7 | #Feature F8 | #Feature F9 | Start | End | PTM | AScore | Found By |
| --- | --- | --- | --- | --- | --- | --- | --- | --- | --- | --- | --- | --- | --- | --- | --- | --- | --- | --- | --- | --- | --- | --- | --- | --- | --- | --- | --- | --- | --- | --- | --- | --- | --- | --- | --- | --- | --- | --- | --- | --- | --- | --- | --- |
| K.TITLEVEPSDTIENVK.A | Y | 71.38 | 1786.9200 | 16 | 1.1 | 894.4683 | 2 | 56.97 | 1 | F1:22100 | NaNaKA16\_F1.raw | 1.7352E7 |  |  | 8.563E5 |  |  |  |  | 7.9338E5 |  |  |  |  | 3 | 1 | 0 | 0 | 1 | 0 | 0 | 0 | 0 | 1 | 0 | 0 | 0 | 0 | 12 | 27 |  |  | PEAKS DB |
| K.ESTLHLVLR.L | Y | 62.86 | 1066.6135 | 9 | 0.3 | 534.3142 | 2 | 28.01 | 9 | F9:13605 | NaNaKA16\_F5.raw |  |  |  |  |  |  |  |  | 2.5029E5 |  |  |  |  | 1 | 0 | 0 | 0 | 0 | 0 | 0 | 0 | 0 | 1 | 0 | 0 | 0 | 0 | 64 | 72 |  |  | PEAKS DB |
| R.TLSDYNIQK.E | Y | 56.48 | 1080.5452 | 9 | 1.0 | 541.2804 | 2 | 19.39 | 1 | F1:5953 | NaNaKA16\_F1.raw | 3.0489E6 |  |  |  |  |  |  |  |  |  |  |  |  | 2 | 2 | 0 | 0 | 0 | 0 | 0 | 0 | 0 | 0 | 0 | 0 | 0 | 0 | 55 | 63 |  |  | PEAKS DB |
| K.IQDKEGIPPDQQR.L | Y | 42.00 | 1522.7739 | 13 | -0.1 | 508.5985 | 3 | 11.71 | 1 | F1:2169 | NaNaKA16\_F1.raw | 1.117E6 |  |  |  |  |  |  |  |  |  |  |  |  | 1 | 1 | 0 | 0 | 0 | 0 | 0 | 0 | 0 | 0 | 0 | 0 | 0 | 0 | 30 | 42 |  |  | PEAKS DB |
| total 4 peptides |  |  |  |  |  |  |  |  |  |  |  |  |  |  |  |  |  |  |  |  |  |  |  |  |  |  |  |  |  |  |  |  |  |  |  |  |  |  |  |  |  |  |  |
| --- | --- | --- | --- | --- | --- | --- | --- | --- | --- | --- | --- | --- | --- | --- | --- | --- | --- | --- | --- | --- | --- | --- | --- | --- | --- | --- | --- | --- | --- | --- | --- | --- | --- | --- | --- | --- | --- | --- | --- | --- | --- | --- | --- |

JAA95781.1

back to list

  

| Protein Coverage
| Supporting Peptides
|

Protein Coverage:

Supporting Peptides:

| Peptide | Uniq | -10lgP | Mass | Length | ppm | m/z | z | RT | Fraction | Scan | Source File | Area F1 | Area F10 | Area F11 | Area F12 | Area F13 | Area F2 | Area F3 | Area F4 | Area F5 | Area F6 | Area F7 | Area F8 | Area F9 | #Feature | #Feature F1 | #Feature F10 | #Feature F11 | #Feature F12 | #Feature F13 | #Feature F2 | #Feature F3 | #Feature F4 | #Feature F5 | #Feature F6 | #Feature F7 | #Feature F8 | #Feature F9 | Start | End | PTM | AScore | Found By |
| --- | --- | --- | --- | --- | --- | --- | --- | --- | --- | --- | --- | --- | --- | --- | --- | --- | --- | --- | --- | --- | --- | --- | --- | --- | --- | --- | --- | --- | --- | --- | --- | --- | --- | --- | --- | --- | --- | --- | --- | --- | --- | --- | --- |
| K.TITLEVEPSDTIENVK.A | Y | 71.38 | 1786.9200 | 16 | 1.1 | 894.4683 | 2 | 56.97 | 1 | F1:22100 | NaNaKA16\_F1.raw | 1.7352E7 |  |  | 8.563E5 |  |  |  |  | 7.9338E5 |  |  |  |  | 3 | 1 | 0 | 0 | 1 | 0 | 0 | 0 | 0 | 1 | 0 | 0 | 0 | 0 | 12 | 27 |  |  | PEAKS DB |
| K.ESTLHLVLR.L | Y | 62.86 | 1066.6135 | 9 | 0.3 | 534.3142 | 2 | 28.01 | 9 | F9:13605 | NaNaKA16\_F5.raw |  |  |  |  |  |  |  |  | 2.5029E5 |  |  |  |  | 1 | 0 | 0 | 0 | 0 | 0 | 0 | 0 | 0 | 1 | 0 | 0 | 0 | 0 | 64 | 72 |  |  | PEAKS DB |
| R.TLSDYNIQK.E | Y | 56.48 | 1080.5452 | 9 | 1.0 | 541.2804 | 2 | 19.39 | 1 | F1:5953 | NaNaKA16\_F1.raw | 3.0489E6 |  |  |  |  |  |  |  |  |  |  |  |  | 2 | 2 | 0 | 0 | 0 | 0 | 0 | 0 | 0 | 0 | 0 | 0 | 0 | 0 | 55 | 63 |  |  | PEAKS DB |
| K.IQDKEGIPPDQQR.L | Y | 42.00 | 1522.7739 | 13 | -0.1 | 508.5985 | 3 | 11.71 | 1 | F1:2169 | NaNaKA16\_F1.raw | 1.117E6 |  |  |  |  |  |  |  |  |  |  |  |  | 1 | 1 | 0 | 0 | 0 | 0 | 0 | 0 | 0 | 0 | 0 | 0 | 0 | 0 | 30 | 42 |  |  | PEAKS DB |
| total 4 peptides |  |  |  |  |  |  |  |  |  |  |  |  |  |  |  |  |  |  |  |  |  |  |  |  |  |  |  |  |  |  |  |  |  |  |  |  |  |  |  |  |  |  |  |
| --- | --- | --- | --- | --- | --- | --- | --- | --- | --- | --- | --- | --- | --- | --- | --- | --- | --- | --- | --- | --- | --- | --- | --- | --- | --- | --- | --- | --- | --- | --- | --- | --- | --- | --- | --- | --- | --- | --- | --- | --- | --- | --- | --- |

JAB53440.1

back to list

  

| Protein Coverage
| Supporting Peptides
|

Protein Coverage:

Supporting Peptides:

| Peptide | Uniq | -10lgP | Mass | Length | ppm | m/z | z | RT | Fraction | Scan | Source File | Area F1 | Area F10 | Area F11 | Area F12 | Area F13 | Area F2 | Area F3 | Area F4 | Area F5 | Area F6 | Area F7 | Area F8 | Area F9 | #Feature | #Feature F1 | #Feature F10 | #Feature F11 | #Feature F12 | #Feature F13 | #Feature F2 | #Feature F3 | #Feature F4 | #Feature F5 | #Feature F6 | #Feature F7 | #Feature F8 | #Feature F9 | Start | End | PTM | AScore | Found By |
| --- | --- | --- | --- | --- | --- | --- | --- | --- | --- | --- | --- | --- | --- | --- | --- | --- | --- | --- | --- | --- | --- | --- | --- | --- | --- | --- | --- | --- | --- | --- | --- | --- | --- | --- | --- | --- | --- | --- | --- | --- | --- | --- | --- |
| K.TITLEVEPSDTIENVK.A | Y | 71.38 | 1786.9200 | 16 | 1.1 | 894.4683 | 2 | 56.97 | 1 | F1:22100 | NaNaKA16\_F1.raw | 1.7352E7 |  |  | 8.563E5 |  |  |  |  | 7.9338E5 |  |  |  |  | 3 | 1 | 0 | 0 | 1 | 0 | 0 | 0 | 0 | 1 | 0 | 0 | 0 | 0 | 12 | 27 |  |  | PEAKS DB |
| K.ESTLHLVLR.L | Y | 62.86 | 1066.6135 | 9 | 0.3 | 534.3142 | 2 | 28.01 | 9 | F9:13605 | NaNaKA16\_F5.raw |  |  |  |  |  |  |  |  | 2.5029E5 |  |  |  |  | 1 | 0 | 0 | 0 | 0 | 0 | 0 | 0 | 0 | 1 | 0 | 0 | 0 | 0 | 64 | 72 |  |  | PEAKS DB |
| R.TLSDYNIQK.E | Y | 56.48 | 1080.5452 | 9 | 1.0 | 541.2804 | 2 | 19.39 | 1 | F1:5953 | NaNaKA16\_F1.raw | 3.0489E6 |  |  |  |  |  |  |  |  |  |  |  |  | 2 | 2 | 0 | 0 | 0 | 0 | 0 | 0 | 0 | 0 | 0 | 0 | 0 | 0 | 55 | 63 |  |  | PEAKS DB |
| K.IQDKEGIPPDQQR.L | Y | 42.00 | 1522.7739 | 13 | -0.1 | 508.5985 | 3 | 11.71 | 1 | F1:2169 | NaNaKA16\_F1.raw | 1.117E6 |  |  |  |  |  |  |  |  |  |  |  |  | 1 | 1 | 0 | 0 | 0 | 0 | 0 | 0 | 0 | 0 | 0 | 0 | 0 | 0 | 30 | 42 |  |  | PEAKS DB |
| total 4 peptides |  |  |  |  |  |  |  |  |  |  |  |  |  |  |  |  |  |  |  |  |  |  |  |  |  |  |  |  |  |  |  |  |  |  |  |  |  |  |  |  |  |  |  |
| --- | --- | --- | --- | --- | --- | --- | --- | --- | --- | --- | --- | --- | --- | --- | --- | --- | --- | --- | --- | --- | --- | --- | --- | --- | --- | --- | --- | --- | --- | --- | --- | --- | --- | --- | --- | --- | --- | --- | --- | --- | --- | --- | --- |

AAG17445.1

back to list

  

| Protein Coverage
| Supporting Peptides
|

Protein Coverage:

Supporting Peptides:

| Peptide | Uniq | -10lgP | Mass | Length | ppm | m/z | z | RT | Fraction | Scan | Source File | Area F1 | Area F10 | Area F11 | Area F12 | Area F13 | Area F2 | Area F3 | Area F4 | Area F5 | Area F6 | Area F7 | Area F8 | Area F9 | #Feature | #Feature F1 | #Feature F10 | #Feature F11 | #Feature F12 | #Feature F13 | #Feature F2 | #Feature F3 | #Feature F4 | #Feature F5 | #Feature F6 | #Feature F7 | #Feature F8 | #Feature F9 | Start | End | PTM | AScore | Found By |
| --- | --- | --- | --- | --- | --- | --- | --- | --- | --- | --- | --- | --- | --- | --- | --- | --- | --- | --- | --- | --- | --- | --- | --- | --- | --- | --- | --- | --- | --- | --- | --- | --- | --- | --- | --- | --- | --- | --- | --- | --- | --- | --- | --- |
| K.TITLEVEPSDTIENVK.A | Y | 71.38 | 1786.9200 | 16 | 1.1 | 894.4683 | 2 | 56.97 | 1 | F1:22100 | NaNaKA16\_F1.raw | 1.7352E7 |  |  | 8.563E5 |  |  |  |  | 7.9338E5 |  |  |  |  | 3 | 1 | 0 | 0 | 1 | 0 | 0 | 0 | 0 | 1 | 0 | 0 | 0 | 0 | 12 | 27 |  |  | PEAKS DB |
| K.ESTLHLVLR.L | Y | 62.86 | 1066.6135 | 9 | 0.3 | 534.3142 | 2 | 28.01 | 9 | F9:13605 | NaNaKA16\_F5.raw |  |  |  |  |  |  |  |  | 2.5029E5 |  |  |  |  | 1 | 0 | 0 | 0 | 0 | 0 | 0 | 0 | 0 | 1 | 0 | 0 | 0 | 0 | 64 | 72 |  |  | PEAKS DB |
| R.TLSDYNIQK.E | Y | 56.48 | 1080.5452 | 9 | 1.0 | 541.2804 | 2 | 19.39 | 1 | F1:5953 | NaNaKA16\_F1.raw | 3.0489E6 |  |  |  |  |  |  |  |  |  |  |  |  | 2 | 2 | 0 | 0 | 0 | 0 | 0 | 0 | 0 | 0 | 0 | 0 | 0 | 0 | 55 | 63 |  |  | PEAKS DB |
| K.IQDKEGIPPDQQR.L | Y | 42.00 | 1522.7739 | 13 | -0.1 | 508.5985 | 3 | 11.71 | 1 | F1:2169 | NaNaKA16\_F1.raw | 1.117E6 |  |  |  |  |  |  |  |  |  |  |  |  | 1 | 1 | 0 | 0 | 0 | 0 | 0 | 0 | 0 | 0 | 0 | 0 | 0 | 0 | 30 | 42 |  |  | PEAKS DB |
| total 4 peptides |  |  |  |  |  |  |  |  |  |  |  |  |  |  |  |  |  |  |  |  |  |  |  |  |  |  |  |  |  |  |  |  |  |  |  |  |  |  |  |  |  |  |  |
| --- | --- | --- | --- | --- | --- | --- | --- | --- | --- | --- | --- | --- | --- | --- | --- | --- | --- | --- | --- | --- | --- | --- | --- | --- | --- | --- | --- | --- | --- | --- | --- | --- | --- | --- | --- | --- | --- | --- | --- | --- | --- | --- | --- |

JAI10738.1

back to list

  

| Protein Coverage
| Supporting Peptides
|

Protein Coverage:

Supporting Peptides:

| Peptide | Uniq | -10lgP | Mass | Length | ppm | m/z | z | RT | Fraction | Scan | Source File | Area F1 | Area F10 | Area F11 | Area F12 | Area F13 | Area F2 | Area F3 | Area F4 | Area F5 | Area F6 | Area F7 | Area F8 | Area F9 | #Feature | #Feature F1 | #Feature F10 | #Feature F11 | #Feature F12 | #Feature F13 | #Feature F2 | #Feature F3 | #Feature F4 | #Feature F5 | #Feature F6 | #Feature F7 | #Feature F8 | #Feature F9 | Start | End | PTM | AScore | Found By |
| --- | --- | --- | --- | --- | --- | --- | --- | --- | --- | --- | --- | --- | --- | --- | --- | --- | --- | --- | --- | --- | --- | --- | --- | --- | --- | --- | --- | --- | --- | --- | --- | --- | --- | --- | --- | --- | --- | --- | --- | --- | --- | --- | --- |
| K.TITLEVEPSDTIENVK.A | Y | 71.38 | 1786.9200 | 16 | 1.1 | 894.4683 | 2 | 56.97 | 1 | F1:22100 | NaNaKA16\_F1.raw | 1.7352E7 |  |  | 8.563E5 |  |  |  |  | 7.9338E5 |  |  |  |  | 3 | 1 | 0 | 0 | 1 | 0 | 0 | 0 | 0 | 1 | 0 | 0 | 0 | 0 | 12 | 27 |  |  | PEAKS DB |
| K.ESTLHLVLR.L | Y | 62.86 | 1066.6135 | 9 | 0.3 | 534.3142 | 2 | 28.01 | 9 | F9:13605 | NaNaKA16\_F5.raw |  |  |  |  |  |  |  |  | 2.5029E5 |  |  |  |  | 1 | 0 | 0 | 0 | 0 | 0 | 0 | 0 | 0 | 1 | 0 | 0 | 0 | 0 | 64 | 72 |  |  | PEAKS DB |
| R.TLSDYNIQK.E | Y | 56.48 | 1080.5452 | 9 | 1.0 | 541.2804 | 2 | 19.39 | 1 | F1:5953 | NaNaKA16\_F1.raw | 3.0489E6 |  |  |  |  |  |  |  |  |  |  |  |  | 2 | 2 | 0 | 0 | 0 | 0 | 0 | 0 | 0 | 0 | 0 | 0 | 0 | 0 | 55 | 63 |  |  | PEAKS DB |
| K.IQDKEGIPPDQQR.L | Y | 42.00 | 1522.7739 | 13 | -0.1 | 508.5985 | 3 | 11.71 | 1 | F1:2169 | NaNaKA16\_F1.raw | 1.117E6 |  |  |  |  |  |  |  |  |  |  |  |  | 1 | 1 | 0 | 0 | 0 | 0 | 0 | 0 | 0 | 0 | 0 | 0 | 0 | 0 | 30 | 42 |  |  | PEAKS DB |
| total 4 peptides |  |  |  |  |  |  |  |  |  |  |  |  |  |  |  |  |  |  |  |  |  |  |  |  |  |  |  |  |  |  |  |  |  |  |  |  |  |  |  |  |  |  |  |
| --- | --- | --- | --- | --- | --- | --- | --- | --- | --- | --- | --- | --- | --- | --- | --- | --- | --- | --- | --- | --- | --- | --- | --- | --- | --- | --- | --- | --- | --- | --- | --- | --- | --- | --- | --- | --- | --- | --- | --- | --- | --- | --- | --- |

XP\_013928637.1

back to list

  

| Protein Coverage
| Supporting Peptides
|

Protein Coverage:

Supporting Peptides:

| Peptide | Uniq | -10lgP | Mass | Length | ppm | m/z | z | RT | Fraction | Scan | Source File | Area F1 | Area F10 | Area F11 | Area F12 | Area F13 | Area F2 | Area F3 | Area F4 | Area F5 | Area F6 | Area F7 | Area F8 | Area F9 | #Feature | #Feature F1 | #Feature F10 | #Feature F11 | #Feature F12 | #Feature F13 | #Feature F2 | #Feature F3 | #Feature F4 | #Feature F5 | #Feature F6 | #Feature F7 | #Feature F8 | #Feature F9 | Start | End | PTM | AScore | Found By |
| --- | --- | --- | --- | --- | --- | --- | --- | --- | --- | --- | --- | --- | --- | --- | --- | --- | --- | --- | --- | --- | --- | --- | --- | --- | --- | --- | --- | --- | --- | --- | --- | --- | --- | --- | --- | --- | --- | --- | --- | --- | --- | --- | --- |
| K.TITLEVEPSDTIENVK.A | Y | 71.38 | 1786.9200 | 16 | 1.1 | 894.4683 | 2 | 56.97 | 1 | F1:22100 | NaNaKA16\_F1.raw | 1.7352E7 |  |  | 8.563E5 |  |  |  |  | 7.9338E5 |  |  |  |  | 3 | 1 | 0 | 0 | 1 | 0 | 0 | 0 | 0 | 1 | 0 | 0 | 0 | 0 | 12 | 27 |  |  | PEAKS DB |
| K.ESTLHLVLR.L | Y | 62.86 | 1066.6135 | 9 | 0.3 | 534.3142 | 2 | 28.01 | 9 | F9:13605 | NaNaKA16\_F5.raw |  |  |  |  |  |  |  |  | 2.5029E5 |  |  |  |  | 1 | 0 | 0 | 0 | 0 | 0 | 0 | 0 | 0 | 1 | 0 | 0 | 0 | 0 | 64 | 72 |  |  | PEAKS DB |
| R.TLSDYNIQK.E | Y | 56.48 | 1080.5452 | 9 | 1.0 | 541.2804 | 2 | 19.39 | 1 | F1:5953 | NaNaKA16\_F1.raw | 3.0489E6 |  |  |  |  |  |  |  |  |  |  |  |  | 2 | 2 | 0 | 0 | 0 | 0 | 0 | 0 | 0 | 0 | 0 | 0 | 0 | 0 | 55 | 63 |  |  | PEAKS DB |
| K.IQDKEGIPPDQQR.L | Y | 42.00 | 1522.7739 | 13 | -0.1 | 508.5985 | 3 | 11.71 | 1 | F1:2169 | NaNaKA16\_F1.raw | 1.117E6 |  |  |  |  |  |  |  |  |  |  |  |  | 1 | 1 | 0 | 0 | 0 | 0 | 0 | 0 | 0 | 0 | 0 | 0 | 0 | 0 | 30 | 42 |  |  | PEAKS DB |
| total 4 peptides |  |  |  |  |  |  |  |  |  |  |  |  |  |  |  |  |  |  |  |  |  |  |  |  |  |  |  |  |  |  |  |  |  |  |  |  |  |  |  |  |  |  |  |
| --- | --- | --- | --- | --- | --- | --- | --- | --- | --- | --- | --- | --- | --- | --- | --- | --- | --- | --- | --- | --- | --- | --- | --- | --- | --- | --- | --- | --- | --- | --- | --- | --- | --- | --- | --- | --- | --- | --- | --- | --- | --- | --- | --- |

AFJ51941.1

back to list

  

| Protein Coverage
| Supporting Peptides
|

Protein Coverage:

Supporting Peptides:

| Peptide | Uniq | -10lgP | Mass | Length | ppm | m/z | z | RT | Fraction | Scan | Source File | Area F1 | Area F10 | Area F11 | Area F12 | Area F13 | Area F2 | Area F3 | Area F4 | Area F5 | Area F6 | Area F7 | Area F8 | Area F9 | #Feature | #Feature F1 | #Feature F10 | #Feature F11 | #Feature F12 | #Feature F13 | #Feature F2 | #Feature F3 | #Feature F4 | #Feature F5 | #Feature F6 | #Feature F7 | #Feature F8 | #Feature F9 | Start | End | PTM | AScore | Found By |
| --- | --- | --- | --- | --- | --- | --- | --- | --- | --- | --- | --- | --- | --- | --- | --- | --- | --- | --- | --- | --- | --- | --- | --- | --- | --- | --- | --- | --- | --- | --- | --- | --- | --- | --- | --- | --- | --- | --- | --- | --- | --- | --- | --- |
| K.TITLEVEPSDTIENVK.A | Y | 71.38 | 1786.9200 | 16 | 1.1 | 894.4683 | 2 | 56.97 | 1 | F1:22100 | NaNaKA16\_F1.raw | 1.7352E7 |  |  | 8.563E5 |  |  |  |  | 7.9338E5 |  |  |  |  | 3 | 1 | 0 | 0 | 1 | 0 | 0 | 0 | 0 | 1 | 0 | 0 | 0 | 0 | 12 | 27 |  |  | PEAKS DB |
| K.ESTLHLVLR.L | Y | 62.86 | 1066.6135 | 9 | 0.3 | 534.3142 | 2 | 28.01 | 9 | F9:13605 | NaNaKA16\_F5.raw |  |  |  |  |  |  |  |  | 2.5029E5 |  |  |  |  | 1 | 0 | 0 | 0 | 0 | 0 | 0 | 0 | 0 | 1 | 0 | 0 | 0 | 0 | 64 | 72 |  |  | PEAKS DB |
| R.TLSDYNIQK.E | Y | 56.48 | 1080.5452 | 9 | 1.0 | 541.2804 | 2 | 19.39 | 1 | F1:5953 | NaNaKA16\_F1.raw | 3.0489E6 |  |  |  |  |  |  |  |  |  |  |  |  | 2 | 2 | 0 | 0 | 0 | 0 | 0 | 0 | 0 | 0 | 0 | 0 | 0 | 0 | 55 | 63 |  |  | PEAKS DB |
| K.IQDKEGIPPDQQR.L | Y | 42.00 | 1522.7739 | 13 | -0.1 | 508.5985 | 3 | 11.71 | 1 | F1:2169 | NaNaKA16\_F1.raw | 1.117E6 |  |  |  |  |  |  |  |  |  |  |  |  | 1 | 1 | 0 | 0 | 0 | 0 | 0 | 0 | 0 | 0 | 0 | 0 | 0 | 0 | 30 | 42 |  |  | PEAKS DB |
| total 4 peptides |  |  |  |  |  |  |  |  |  |  |  |  |  |  |  |  |  |  |  |  |  |  |  |  |  |  |  |  |  |  |  |  |  |  |  |  |  |  |  |  |  |  |  |
| --- | --- | --- | --- | --- | --- | --- | --- | --- | --- | --- | --- | --- | --- | --- | --- | --- | --- | --- | --- | --- | --- | --- | --- | --- | --- | --- | --- | --- | --- | --- | --- | --- | --- | --- | --- | --- | --- | --- | --- | --- | --- | --- | --- |

JAB53023.1

back to list

  

| Protein Coverage
| Supporting Peptides
|

Protein Coverage:

Supporting Peptides:

| Peptide | Uniq | -10lgP | Mass | Length | ppm | m/z | z | RT | Fraction | Scan | Source File | Area F1 | Area F10 | Area F11 | Area F12 | Area F13 | Area F2 | Area F3 | Area F4 | Area F5 | Area F6 | Area F7 | Area F8 | Area F9 | #Feature | #Feature F1 | #Feature F10 | #Feature F11 | #Feature F12 | #Feature F13 | #Feature F2 | #Feature F3 | #Feature F4 | #Feature F5 | #Feature F6 | #Feature F7 | #Feature F8 | #Feature F9 | Start | End | PTM | AScore | Found By |
| --- | --- | --- | --- | --- | --- | --- | --- | --- | --- | --- | --- | --- | --- | --- | --- | --- | --- | --- | --- | --- | --- | --- | --- | --- | --- | --- | --- | --- | --- | --- | --- | --- | --- | --- | --- | --- | --- | --- | --- | --- | --- | --- | --- |
| K.TITLEVEPSDTIENVK.A | Y | 71.38 | 1786.9200 | 16 | 1.1 | 894.4683 | 2 | 56.97 | 1 | F1:22100 | NaNaKA16\_F1.raw | 1.7352E7 |  |  | 8.563E5 |  |  |  |  | 7.9338E5 |  |  |  |  | 3 | 1 | 0 | 0 | 1 | 0 | 0 | 0 | 0 | 1 | 0 | 0 | 0 | 0 | 12 | 27 |  |  | PEAKS DB |
| K.ESTLHLVLR.L | Y | 62.86 | 1066.6135 | 9 | 0.3 | 534.3142 | 2 | 28.01 | 9 | F9:13605 | NaNaKA16\_F5.raw |  |  |  |  |  |  |  |  | 2.5029E5 |  |  |  |  | 1 | 0 | 0 | 0 | 0 | 0 | 0 | 0 | 0 | 1 | 0 | 0 | 0 | 0 | 64 | 72 |  |  | PEAKS DB |
| R.TLSDYNIQK.E | Y | 56.48 | 1080.5452 | 9 | 1.0 | 541.2804 | 2 | 19.39 | 1 | F1:5953 | NaNaKA16\_F1.raw | 3.0489E6 |  |  |  |  |  |  |  |  |  |  |  |  | 2 | 2 | 0 | 0 | 0 | 0 | 0 | 0 | 0 | 0 | 0 | 0 | 0 | 0 | 55 | 63 |  |  | PEAKS DB |
| K.IQDKEGIPPDQQR.L | Y | 42.00 | 1522.7739 | 13 | -0.1 | 508.5985 | 3 | 11.71 | 1 | F1:2169 | NaNaKA16\_F1.raw | 1.117E6 |  |  |  |  |  |  |  |  |  |  |  |  | 1 | 1 | 0 | 0 | 0 | 0 | 0 | 0 | 0 | 0 | 0 | 0 | 0 | 0 | 30 | 42 |  |  | PEAKS DB |
| total 4 peptides |  |  |  |  |  |  |  |  |  |  |  |  |  |  |  |  |  |  |  |  |  |  |  |  |  |  |  |  |  |  |  |  |  |  |  |  |  |  |  |  |  |  |  |
| --- | --- | --- | --- | --- | --- | --- | --- | --- | --- | --- | --- | --- | --- | --- | --- | --- | --- | --- | --- | --- | --- | --- | --- | --- | --- | --- | --- | --- | --- | --- | --- | --- | --- | --- | --- | --- | --- | --- | --- | --- | --- | --- | --- |

LAA22167.1

back to list

  

| Protein Coverage
| Supporting Peptides
|

Protein Coverage:

Supporting Peptides:

| Peptide | Uniq | -10lgP | Mass | Length | ppm | m/z | z | RT | Fraction | Scan | Source File | Area F1 | Area F10 | Area F11 | Area F12 | Area F13 | Area F2 | Area F3 | Area F4 | Area F5 | Area F6 | Area F7 | Area F8 | Area F9 | #Feature | #Feature F1 | #Feature F10 | #Feature F11 | #Feature F12 | #Feature F13 | #Feature F2 | #Feature F3 | #Feature F4 | #Feature F5 | #Feature F6 | #Feature F7 | #Feature F8 | #Feature F9 | Start | End | PTM | AScore | Found By |
| --- | --- | --- | --- | --- | --- | --- | --- | --- | --- | --- | --- | --- | --- | --- | --- | --- | --- | --- | --- | --- | --- | --- | --- | --- | --- | --- | --- | --- | --- | --- | --- | --- | --- | --- | --- | --- | --- | --- | --- | --- | --- | --- | --- |
| K.NVQAEEMVEFSSGLK.G | Y | 72.67 | 1666.7872 | 15 | 0.7 | 834.4015 | 2 | 57.47 | 1 | F1:22310 | NaNaKA16\_F1.raw | 6.2448E6 |  |  |  |  |  |  |  |  |  |  |  |  | 2 | 2 | 0 | 0 | 0 | 0 | 0 | 0 | 0 | 0 | 0 | 0 | 0 | 0 | 107 | 121 |  |  | PEAKS DB |
| K.HALIIYDDLSK.Q | Y | 67.93 | 1286.6870 | 11 | 0.3 | 644.3510 | 2 | 34.90 | 1 | F1:15224 | NaNaKA16\_F1.raw | 1.6656E6 |  |  |  |  |  |  |  |  |  |  |  |  | 1 | 1 | 0 | 0 | 0 | 0 | 0 | 0 | 0 | 0 | 0 | 0 | 0 | 0 | 324 | 334 |  |  | PEAKS DB |
| R.TGAIVDVPVGEELLGR.V | Y | 43.34 | 1623.8832 | 16 | 0.7 | 812.9495 | 2 | 71.89 | 1 | F1:26457 | NaNaKA16\_F1.raw | 8.0145E5 |  |  |  |  |  |  |  |  |  |  |  |  | 1 | 1 | 0 | 0 | 0 | 0 | 0 | 0 | 0 | 0 | 0 | 0 | 0 | 0 | 152 | 167 |  |  | PEAKS DB |
| total 3 peptides |  |  |  |  |  |  |  |  |  |  |  |  |  |  |  |  |  |  |  |  |  |  |  |  |  |  |  |  |  |  |  |  |  |  |  |  |  |  |  |  |  |  |  |
| --- | --- | --- | --- | --- | --- | --- | --- | --- | --- | --- | --- | --- | --- | --- | --- | --- | --- | --- | --- | --- | --- | --- | --- | --- | --- | --- | --- | --- | --- | --- | --- | --- | --- | --- | --- | --- | --- | --- | --- | --- | --- | --- | --- |

XP\_007444287.1

back to list

  

| Protein Coverage
| Supporting Peptides
|

Protein Coverage:

Supporting Peptides:

| Peptide | Uniq | -10lgP | Mass | Length | ppm | m/z | z | RT | Fraction | Scan | Source File | Area F1 | Area F10 | Area F11 | Area F12 | Area F13 | Area F2 | Area F3 | Area F4 | Area F5 | Area F6 | Area F7 | Area F8 | Area F9 | #Feature | #Feature F1 | #Feature F10 | #Feature F11 | #Feature F12 | #Feature F13 | #Feature F2 | #Feature F3 | #Feature F4 | #Feature F5 | #Feature F6 | #Feature F7 | #Feature F8 | #Feature F9 | Start | End | PTM | AScore | Found By |
| --- | --- | --- | --- | --- | --- | --- | --- | --- | --- | --- | --- | --- | --- | --- | --- | --- | --- | --- | --- | --- | --- | --- | --- | --- | --- | --- | --- | --- | --- | --- | --- | --- | --- | --- | --- | --- | --- | --- | --- | --- | --- | --- | --- |
| K.NVQAEEMVEFSSGLK.G | Y | 72.67 | 1666.7872 | 15 | 0.7 | 834.4015 | 2 | 57.47 | 1 | F1:22310 | NaNaKA16\_F1.raw | 6.2448E6 |  |  |  |  |  |  |  |  |  |  |  |  | 2 | 2 | 0 | 0 | 0 | 0 | 0 | 0 | 0 | 0 | 0 | 0 | 0 | 0 | 89 | 103 |  |  | PEAKS DB |
| K.HALIIYDDLSK.Q | Y | 67.93 | 1286.6870 | 11 | 0.3 | 644.3510 | 2 | 34.90 | 1 | F1:15224 | NaNaKA16\_F1.raw | 1.6656E6 |  |  |  |  |  |  |  |  |  |  |  |  | 1 | 1 | 0 | 0 | 0 | 0 | 0 | 0 | 0 | 0 | 0 | 0 | 0 | 0 | 306 | 316 |  |  | PEAKS DB |
| R.TGAIVDVPVGEELLGR.V | Y | 43.34 | 1623.8832 | 16 | 0.7 | 812.9495 | 2 | 71.89 | 1 | F1:26457 | NaNaKA16\_F1.raw | 8.0145E5 |  |  |  |  |  |  |  |  |  |  |  |  | 1 | 1 | 0 | 0 | 0 | 0 | 0 | 0 | 0 | 0 | 0 | 0 | 0 | 0 | 134 | 149 |  |  | PEAKS DB |
| total 3 peptides |  |  |  |  |  |  |  |  |  |  |  |  |  |  |  |  |  |  |  |  |  |  |  |  |  |  |  |  |  |  |  |  |  |  |  |  |  |  |  |  |  |  |  |
| --- | --- | --- | --- | --- | --- | --- | --- | --- | --- | --- | --- | --- | --- | --- | --- | --- | --- | --- | --- | --- | --- | --- | --- | --- | --- | --- | --- | --- | --- | --- | --- | --- | --- | --- | --- | --- | --- | --- | --- | --- | --- | --- | --- |

XP\_026569669.1

back to list

  

| Protein Coverage
| Supporting Peptides
|

Protein Coverage:

Supporting Peptides:

| Peptide | Uniq | -10lgP | Mass | Length | ppm | m/z | z | RT | Fraction | Scan | Source File | Area F1 | Area F10 | Area F11 | Area F12 | Area F13 | Area F2 | Area F3 | Area F4 | Area F5 | Area F6 | Area F7 | Area F8 | Area F9 | #Feature | #Feature F1 | #Feature F10 | #Feature F11 | #Feature F12 | #Feature F13 | #Feature F2 | #Feature F3 | #Feature F4 | #Feature F5 | #Feature F6 | #Feature F7 | #Feature F8 | #Feature F9 | Start | End | PTM | AScore | Found By |
| --- | --- | --- | --- | --- | --- | --- | --- | --- | --- | --- | --- | --- | --- | --- | --- | --- | --- | --- | --- | --- | --- | --- | --- | --- | --- | --- | --- | --- | --- | --- | --- | --- | --- | --- | --- | --- | --- | --- | --- | --- | --- | --- | --- |
| K.NVQAEEMVEFSSGLK.G | Y | 72.67 | 1666.7872 | 15 | 0.7 | 834.4015 | 2 | 57.47 | 1 | F1:22310 | NaNaKA16\_F1.raw | 6.2448E6 |  |  |  |  |  |  |  |  |  |  |  |  | 2 | 2 | 0 | 0 | 0 | 0 | 0 | 0 | 0 | 0 | 0 | 0 | 0 | 0 | 89 | 103 |  |  | PEAKS DB |
| K.HALIIYDDLSK.Q | Y | 67.93 | 1286.6870 | 11 | 0.3 | 644.3510 | 2 | 34.90 | 1 | F1:15224 | NaNaKA16\_F1.raw | 1.6656E6 |  |  |  |  |  |  |  |  |  |  |  |  | 1 | 1 | 0 | 0 | 0 | 0 | 0 | 0 | 0 | 0 | 0 | 0 | 0 | 0 | 306 | 316 |  |  | PEAKS DB |
| R.TGAIVDVPVGEELLGR.V | Y | 43.34 | 1623.8832 | 16 | 0.7 | 812.9495 | 2 | 71.89 | 1 | F1:26457 | NaNaKA16\_F1.raw | 8.0145E5 |  |  |  |  |  |  |  |  |  |  |  |  | 1 | 1 | 0 | 0 | 0 | 0 | 0 | 0 | 0 | 0 | 0 | 0 | 0 | 0 | 134 | 149 |  |  | PEAKS DB |
| total 3 peptides |  |  |  |  |  |  |  |  |  |  |  |  |  |  |  |  |  |  |  |  |  |  |  |  |  |  |  |  |  |  |  |  |  |  |  |  |  |  |  |  |  |  |  |
| --- | --- | --- | --- | --- | --- | --- | --- | --- | --- | --- | --- | --- | --- | --- | --- | --- | --- | --- | --- | --- | --- | --- | --- | --- | --- | --- | --- | --- | --- | --- | --- | --- | --- | --- | --- | --- | --- | --- | --- | --- | --- | --- | --- |

JAB53920.1

back to list

  

| Protein Coverage
| Supporting Peptides
|

Protein Coverage:

Supporting Peptides:

| Peptide | Uniq | -10lgP | Mass | Length | ppm | m/z | z | RT | Fraction | Scan | Source File | Area F1 | Area F10 | Area F11 | Area F12 | Area F13 | Area F2 | Area F3 | Area F4 | Area F5 | Area F6 | Area F7 | Area F8 | Area F9 | #Feature | #Feature F1 | #Feature F10 | #Feature F11 | #Feature F12 | #Feature F13 | #Feature F2 | #Feature F3 | #Feature F4 | #Feature F5 | #Feature F6 | #Feature F7 | #Feature F8 | #Feature F9 | Start | End | PTM | AScore | Found By |
| --- | --- | --- | --- | --- | --- | --- | --- | --- | --- | --- | --- | --- | --- | --- | --- | --- | --- | --- | --- | --- | --- | --- | --- | --- | --- | --- | --- | --- | --- | --- | --- | --- | --- | --- | --- | --- | --- | --- | --- | --- | --- | --- | --- |
| K.NVQAEEMVEFSSGLK.G | Y | 72.67 | 1666.7872 | 15 | 0.7 | 834.4015 | 2 | 57.47 | 1 | F1:22310 | NaNaKA16\_F1.raw | 6.2448E6 |  |  |  |  |  |  |  |  |  |  |  |  | 2 | 2 | 0 | 0 | 0 | 0 | 0 | 0 | 0 | 0 | 0 | 0 | 0 | 0 | 89 | 103 |  |  | PEAKS DB |
| K.HALIIYDDLSK.Q | Y | 67.93 | 1286.6870 | 11 | 0.3 | 644.3510 | 2 | 34.90 | 1 | F1:15224 | NaNaKA16\_F1.raw | 1.6656E6 |  |  |  |  |  |  |  |  |  |  |  |  | 1 | 1 | 0 | 0 | 0 | 0 | 0 | 0 | 0 | 0 | 0 | 0 | 0 | 0 | 306 | 316 |  |  | PEAKS DB |
| R.TGAIVDVPVGEELLGR.V | Y | 43.34 | 1623.8832 | 16 | 0.7 | 812.9495 | 2 | 71.89 | 1 | F1:26457 | NaNaKA16\_F1.raw | 8.0145E5 |  |  |  |  |  |  |  |  |  |  |  |  | 1 | 1 | 0 | 0 | 0 | 0 | 0 | 0 | 0 | 0 | 0 | 0 | 0 | 0 | 134 | 149 |  |  | PEAKS DB |
| total 3 peptides |  |  |  |  |  |  |  |  |  |  |  |  |  |  |  |  |  |  |  |  |  |  |  |  |  |  |  |  |  |  |  |  |  |  |  |  |  |  |  |  |  |  |  |
| --- | --- | --- | --- | --- | --- | --- | --- | --- | --- | --- | --- | --- | --- | --- | --- | --- | --- | --- | --- | --- | --- | --- | --- | --- | --- | --- | --- | --- | --- | --- | --- | --- | --- | --- | --- | --- | --- | --- | --- | --- | --- | --- | --- |

XP\_026544199.1

back to list

  

| Protein Coverage
| Supporting Peptides
|

Protein Coverage:

Supporting Peptides:

| Peptide | Uniq | -10lgP | Mass | Length | ppm | m/z | z | RT | Fraction | Scan | Source File | Area F1 | Area F10 | Area F11 | Area F12 | Area F13 | Area F2 | Area F3 | Area F4 | Area F5 | Area F6 | Area F7 | Area F8 | Area F9 | #Feature | #Feature F1 | #Feature F10 | #Feature F11 | #Feature F12 | #Feature F13 | #Feature F2 | #Feature F3 | #Feature F4 | #Feature F5 | #Feature F6 | #Feature F7 | #Feature F8 | #Feature F9 | Start | End | PTM | AScore | Found By |
| --- | --- | --- | --- | --- | --- | --- | --- | --- | --- | --- | --- | --- | --- | --- | --- | --- | --- | --- | --- | --- | --- | --- | --- | --- | --- | --- | --- | --- | --- | --- | --- | --- | --- | --- | --- | --- | --- | --- | --- | --- | --- | --- | --- |
| K.NVQAEEMVEFSSGLK.G | Y | 72.67 | 1666.7872 | 15 | 0.7 | 834.4015 | 2 | 57.47 | 1 | F1:22310 | NaNaKA16\_F1.raw | 6.2448E6 |  |  |  |  |  |  |  |  |  |  |  |  | 2 | 2 | 0 | 0 | 0 | 0 | 0 | 0 | 0 | 0 | 0 | 0 | 0 | 0 | 89 | 103 |  |  | PEAKS DB |
| K.HALIIYDDLSK.Q | Y | 67.93 | 1286.6870 | 11 | 0.3 | 644.3510 | 2 | 34.90 | 1 | F1:15224 | NaNaKA16\_F1.raw | 1.6656E6 |  |  |  |  |  |  |  |  |  |  |  |  | 1 | 1 | 0 | 0 | 0 | 0 | 0 | 0 | 0 | 0 | 0 | 0 | 0 | 0 | 306 | 316 |  |  | PEAKS DB |
| R.TGAIVDVPVGEELLGR.V | Y | 43.34 | 1623.8832 | 16 | 0.7 | 812.9495 | 2 | 71.89 | 1 | F1:26457 | NaNaKA16\_F1.raw | 8.0145E5 |  |  |  |  |  |  |  |  |  |  |  |  | 1 | 1 | 0 | 0 | 0 | 0 | 0 | 0 | 0 | 0 | 0 | 0 | 0 | 0 | 134 | 149 |  |  | PEAKS DB |
| total 3 peptides |  |  |  |  |  |  |  |  |  |  |  |  |  |  |  |  |  |  |  |  |  |  |  |  |  |  |  |  |  |  |  |  |  |  |  |  |  |  |  |  |  |  |  |
| --- | --- | --- | --- | --- | --- | --- | --- | --- | --- | --- | --- | --- | --- | --- | --- | --- | --- | --- | --- | --- | --- | --- | --- | --- | --- | --- | --- | --- | --- | --- | --- | --- | --- | --- | --- | --- | --- | --- | --- | --- | --- | --- | --- |

JAG47406.1

back to list

  

| Protein Coverage
| Supporting Peptides
|

Protein Coverage:

Supporting Peptides:

| Peptide | Uniq | -10lgP | Mass | Length | ppm | m/z | z | RT | Fraction | Scan | Source File | Area F1 | Area F10 | Area F11 | Area F12 | Area F13 | Area F2 | Area F3 | Area F4 | Area F5 | Area F6 | Area F7 | Area F8 | Area F9 | #Feature | #Feature F1 | #Feature F10 | #Feature F11 | #Feature F12 | #Feature F13 | #Feature F2 | #Feature F3 | #Feature F4 | #Feature F5 | #Feature F6 | #Feature F7 | #Feature F8 | #Feature F9 | Start | End | PTM | AScore | Found By |
| --- | --- | --- | --- | --- | --- | --- | --- | --- | --- | --- | --- | --- | --- | --- | --- | --- | --- | --- | --- | --- | --- | --- | --- | --- | --- | --- | --- | --- | --- | --- | --- | --- | --- | --- | --- | --- | --- | --- | --- | --- | --- | --- | --- |
| K.NVQAEEMVEFSSGLK.G | Y | 72.67 | 1666.7872 | 15 | 0.7 | 834.4015 | 2 | 57.47 | 1 | F1:22310 | NaNaKA16\_F1.raw | 6.2448E6 |  |  |  |  |  |  |  |  |  |  |  |  | 2 | 2 | 0 | 0 | 0 | 0 | 0 | 0 | 0 | 0 | 0 | 0 | 0 | 0 | 89 | 103 |  |  | PEAKS DB |
| K.HALIIYDDLSK.Q | Y | 67.93 | 1286.6870 | 11 | 0.3 | 644.3510 | 2 | 34.90 | 1 | F1:15224 | NaNaKA16\_F1.raw | 1.6656E6 |  |  |  |  |  |  |  |  |  |  |  |  | 1 | 1 | 0 | 0 | 0 | 0 | 0 | 0 | 0 | 0 | 0 | 0 | 0 | 0 | 306 | 316 |  |  | PEAKS DB |
| R.TGAIVDVPVGEELLGR.V | Y | 43.34 | 1623.8832 | 16 | 0.7 | 812.9495 | 2 | 71.89 | 1 | F1:26457 | NaNaKA16\_F1.raw | 8.0145E5 |  |  |  |  |  |  |  |  |  |  |  |  | 1 | 1 | 0 | 0 | 0 | 0 | 0 | 0 | 0 | 0 | 0 | 0 | 0 | 0 | 134 | 149 |  |  | PEAKS DB |
| total 3 peptides |  |  |  |  |  |  |  |  |  |  |  |  |  |  |  |  |  |  |  |  |  |  |  |  |  |  |  |  |  |  |  |  |  |  |  |  |  |  |  |  |  |  |  |
| --- | --- | --- | --- | --- | --- | --- | --- | --- | --- | --- | --- | --- | --- | --- | --- | --- | --- | --- | --- | --- | --- | --- | --- | --- | --- | --- | --- | --- | --- | --- | --- | --- | --- | --- | --- | --- | --- | --- | --- | --- | --- | --- | --- |

JAI14245.1

back to list

  

| Protein Coverage
| Supporting Peptides
|

Protein Coverage:

Supporting Peptides:

| Peptide | Uniq | -10lgP | Mass | Length | ppm | m/z | z | RT | Fraction | Scan | Source File | Area F1 | Area F10 | Area F11 | Area F12 | Area F13 | Area F2 | Area F3 | Area F4 | Area F5 | Area F6 | Area F7 | Area F8 | Area F9 | #Feature | #Feature F1 | #Feature F10 | #Feature F11 | #Feature F12 | #Feature F13 | #Feature F2 | #Feature F3 | #Feature F4 | #Feature F5 | #Feature F6 | #Feature F7 | #Feature F8 | #Feature F9 | Start | End | PTM | AScore | Found By |
| --- | --- | --- | --- | --- | --- | --- | --- | --- | --- | --- | --- | --- | --- | --- | --- | --- | --- | --- | --- | --- | --- | --- | --- | --- | --- | --- | --- | --- | --- | --- | --- | --- | --- | --- | --- | --- | --- | --- | --- | --- | --- | --- | --- |
| K.NVQAEEMVEFSSGLK.G | Y | 72.67 | 1666.7872 | 15 | 0.7 | 834.4015 | 2 | 57.47 | 1 | F1:22310 | NaNaKA16\_F1.raw | 6.2448E6 |  |  |  |  |  |  |  |  |  |  |  |  | 2 | 2 | 0 | 0 | 0 | 0 | 0 | 0 | 0 | 0 | 0 | 0 | 0 | 0 | 89 | 103 |  |  | PEAKS DB |
| K.HALIIYDDLSK.Q | Y | 67.93 | 1286.6870 | 11 | 0.3 | 644.3510 | 2 | 34.90 | 1 | F1:15224 | NaNaKA16\_F1.raw | 1.6656E6 |  |  |  |  |  |  |  |  |  |  |  |  | 1 | 1 | 0 | 0 | 0 | 0 | 0 | 0 | 0 | 0 | 0 | 0 | 0 | 0 | 306 | 316 |  |  | PEAKS DB |
| R.TGAIVDVPVGEELLGR.V | Y | 43.34 | 1623.8832 | 16 | 0.7 | 812.9495 | 2 | 71.89 | 1 | F1:26457 | NaNaKA16\_F1.raw | 8.0145E5 |  |  |  |  |  |  |  |  |  |  |  |  | 1 | 1 | 0 | 0 | 0 | 0 | 0 | 0 | 0 | 0 | 0 | 0 | 0 | 0 | 134 | 149 |  |  | PEAKS DB |
| total 3 peptides |  |  |  |  |  |  |  |  |  |  |  |  |  |  |  |  |  |  |  |  |  |  |  |  |  |  |  |  |  |  |  |  |  |  |  |  |  |  |  |  |  |  |  |
| --- | --- | --- | --- | --- | --- | --- | --- | --- | --- | --- | --- | --- | --- | --- | --- | --- | --- | --- | --- | --- | --- | --- | --- | --- | --- | --- | --- | --- | --- | --- | --- | --- | --- | --- | --- | --- | --- | --- | --- | --- | --- | --- | --- |

JAG45747.1

back to list

  

| Protein Coverage
| Supporting Peptides
|

Protein Coverage:

Supporting Peptides:

| Peptide | Uniq | -10lgP | Mass | Length | ppm | m/z | z | RT | Fraction | Scan | Source File | Area F1 | Area F10 | Area F11 | Area F12 | Area F13 | Area F2 | Area F3 | Area F4 | Area F5 | Area F6 | Area F7 | Area F8 | Area F9 | #Feature | #Feature F1 | #Feature F10 | #Feature F11 | #Feature F12 | #Feature F13 | #Feature F2 | #Feature F3 | #Feature F4 | #Feature F5 | #Feature F6 | #Feature F7 | #Feature F8 | #Feature F9 | Start | End | PTM | AScore | Found By |
| --- | --- | --- | --- | --- | --- | --- | --- | --- | --- | --- | --- | --- | --- | --- | --- | --- | --- | --- | --- | --- | --- | --- | --- | --- | --- | --- | --- | --- | --- | --- | --- | --- | --- | --- | --- | --- | --- | --- | --- | --- | --- | --- | --- |
| K.NVQAEEMVEFSSGLK.G | Y | 72.67 | 1666.7872 | 15 | 0.7 | 834.4015 | 2 | 57.47 | 1 | F1:22310 | NaNaKA16\_F1.raw | 6.2448E6 |  |  |  |  |  |  |  |  |  |  |  |  | 2 | 2 | 0 | 0 | 0 | 0 | 0 | 0 | 0 | 0 | 0 | 0 | 0 | 0 | 89 | 103 |  |  | PEAKS DB |
| K.HALIIYDDLSK.Q | Y | 67.93 | 1286.6870 | 11 | 0.3 | 644.3510 | 2 | 34.90 | 1 | F1:15224 | NaNaKA16\_F1.raw | 1.6656E6 |  |  |  |  |  |  |  |  |  |  |  |  | 1 | 1 | 0 | 0 | 0 | 0 | 0 | 0 | 0 | 0 | 0 | 0 | 0 | 0 | 306 | 316 |  |  | PEAKS DB |
| R.TGAIVDVPVGEELLGR.V | Y | 43.34 | 1623.8832 | 16 | 0.7 | 812.9495 | 2 | 71.89 | 1 | F1:26457 | NaNaKA16\_F1.raw | 8.0145E5 |  |  |  |  |  |  |  |  |  |  |  |  | 1 | 1 | 0 | 0 | 0 | 0 | 0 | 0 | 0 | 0 | 0 | 0 | 0 | 0 | 134 | 149 |  |  | PEAKS DB |
| total 3 peptides |  |  |  |  |  |  |  |  |  |  |  |  |  |  |  |  |  |  |  |  |  |  |  |  |  |  |  |  |  |  |  |  |  |  |  |  |  |  |  |  |  |  |  |
| --- | --- | --- | --- | --- | --- | --- | --- | --- | --- | --- | --- | --- | --- | --- | --- | --- | --- | --- | --- | --- | --- | --- | --- | --- | --- | --- | --- | --- | --- | --- | --- | --- | --- | --- | --- | --- | --- | --- | --- | --- | --- | --- | --- |

JAA97708.1

back to list

  

| Protein Coverage
| Supporting Peptides
|

Protein Coverage:

Supporting Peptides:

| Peptide | Uniq | -10lgP | Mass | Length | ppm | m/z | z | RT | Fraction | Scan | Source File | Area F1 | Area F10 | Area F11 | Area F12 | Area F13 | Area F2 | Area F3 | Area F4 | Area F5 | Area F6 | Area F7 | Area F8 | Area F9 | #Feature | #Feature F1 | #Feature F10 | #Feature F11 | #Feature F12 | #Feature F13 | #Feature F2 | #Feature F3 | #Feature F4 | #Feature F5 | #Feature F6 | #Feature F7 | #Feature F8 | #Feature F9 | Start | End | PTM | AScore | Found By |
| --- | --- | --- | --- | --- | --- | --- | --- | --- | --- | --- | --- | --- | --- | --- | --- | --- | --- | --- | --- | --- | --- | --- | --- | --- | --- | --- | --- | --- | --- | --- | --- | --- | --- | --- | --- | --- | --- | --- | --- | --- | --- | --- | --- |
| K.NVQAEEMVEFSSGLK.G | Y | 72.67 | 1666.7872 | 15 | 0.7 | 834.4015 | 2 | 57.47 | 1 | F1:22310 | NaNaKA16\_F1.raw | 6.2448E6 |  |  |  |  |  |  |  |  |  |  |  |  | 2 | 2 | 0 | 0 | 0 | 0 | 0 | 0 | 0 | 0 | 0 | 0 | 0 | 0 | 89 | 103 |  |  | PEAKS DB |
| K.HALIIYDDLSK.Q | Y | 67.93 | 1286.6870 | 11 | 0.3 | 644.3510 | 2 | 34.90 | 1 | F1:15224 | NaNaKA16\_F1.raw | 1.6656E6 |  |  |  |  |  |  |  |  |  |  |  |  | 1 | 1 | 0 | 0 | 0 | 0 | 0 | 0 | 0 | 0 | 0 | 0 | 0 | 0 | 306 | 316 |  |  | PEAKS DB |
| R.TGAIVDVPVGEELLGR.V | Y | 43.34 | 1623.8832 | 16 | 0.7 | 812.9495 | 2 | 71.89 | 1 | F1:26457 | NaNaKA16\_F1.raw | 8.0145E5 |  |  |  |  |  |  |  |  |  |  |  |  | 1 | 1 | 0 | 0 | 0 | 0 | 0 | 0 | 0 | 0 | 0 | 0 | 0 | 0 | 134 | 149 |  |  | PEAKS DB |
| total 3 peptides |  |  |  |  |  |  |  |  |  |  |  |  |  |  |  |  |  |  |  |  |  |  |  |  |  |  |  |  |  |  |  |  |  |  |  |  |  |  |  |  |  |  |  |
| --- | --- | --- | --- | --- | --- | --- | --- | --- | --- | --- | --- | --- | --- | --- | --- | --- | --- | --- | --- | --- | --- | --- | --- | --- | --- | --- | --- | --- | --- | --- | --- | --- | --- | --- | --- | --- | --- | --- | --- | --- | --- | --- | --- |

XP\_013922009.1

back to list

  

| Protein Coverage
| Supporting Peptides
|

Protein Coverage:

Supporting Peptides:

| Peptide | Uniq | -10lgP | Mass | Length | ppm | m/z | z | RT | Fraction | Scan | Source File | Area F1 | Area F10 | Area F11 | Area F12 | Area F13 | Area F2 | Area F3 | Area F4 | Area F5 | Area F6 | Area F7 | Area F8 | Area F9 | #Feature | #Feature F1 | #Feature F10 | #Feature F11 | #Feature F12 | #Feature F13 | #Feature F2 | #Feature F3 | #Feature F4 | #Feature F5 | #Feature F6 | #Feature F7 | #Feature F8 | #Feature F9 | Start | End | PTM | AScore | Found By |
| --- | --- | --- | --- | --- | --- | --- | --- | --- | --- | --- | --- | --- | --- | --- | --- | --- | --- | --- | --- | --- | --- | --- | --- | --- | --- | --- | --- | --- | --- | --- | --- | --- | --- | --- | --- | --- | --- | --- | --- | --- | --- | --- | --- |
| K.NVQAEEMVEFSSGLK.G | Y | 72.67 | 1666.7872 | 15 | 0.7 | 834.4015 | 2 | 57.47 | 1 | F1:22310 | NaNaKA16\_F1.raw | 6.2448E6 |  |  |  |  |  |  |  |  |  |  |  |  | 2 | 2 | 0 | 0 | 0 | 0 | 0 | 0 | 0 | 0 | 0 | 0 | 0 | 0 | 77 | 91 |  |  | PEAKS DB |
| K.HALIIYDDLSK.Q | Y | 67.93 | 1286.6870 | 11 | 0.3 | 644.3510 | 2 | 34.90 | 1 | F1:15224 | NaNaKA16\_F1.raw | 1.6656E6 |  |  |  |  |  |  |  |  |  |  |  |  | 1 | 1 | 0 | 0 | 0 | 0 | 0 | 0 | 0 | 0 | 0 | 0 | 0 | 0 | 294 | 304 |  |  | PEAKS DB |
| R.TGAIVDVPVGEELLGR.V | Y | 43.34 | 1623.8832 | 16 | 0.7 | 812.9495 | 2 | 71.89 | 1 | F1:26457 | NaNaKA16\_F1.raw | 8.0145E5 |  |  |  |  |  |  |  |  |  |  |  |  | 1 | 1 | 0 | 0 | 0 | 0 | 0 | 0 | 0 | 0 | 0 | 0 | 0 | 0 | 122 | 137 |  |  | PEAKS DB |
| total 3 peptides |  |  |  |  |  |  |  |  |  |  |  |  |  |  |  |  |  |  |  |  |  |  |  |  |  |  |  |  |  |  |  |  |  |  |  |  |  |  |  |  |  |  |  |
| --- | --- | --- | --- | --- | --- | --- | --- | --- | --- | --- | --- | --- | --- | --- | --- | --- | --- | --- | --- | --- | --- | --- | --- | --- | --- | --- | --- | --- | --- | --- | --- | --- | --- | --- | --- | --- | --- | --- | --- | --- | --- | --- | --- |

XP\_032069128.1

back to list

  

| Protein Coverage
| Supporting Peptides
|

Protein Coverage:

Supporting Peptides:

| Peptide | Uniq | -10lgP | Mass | Length | ppm | m/z | z | RT | Fraction | Scan | Source File | Area F1 | Area F10 | Area F11 | Area F12 | Area F13 | Area F2 | Area F3 | Area F4 | Area F5 | Area F6 | Area F7 | Area F8 | Area F9 | #Feature | #Feature F1 | #Feature F10 | #Feature F11 | #Feature F12 | #Feature F13 | #Feature F2 | #Feature F3 | #Feature F4 | #Feature F5 | #Feature F6 | #Feature F7 | #Feature F8 | #Feature F9 | Start | End | PTM | AScore | Found By |
| --- | --- | --- | --- | --- | --- | --- | --- | --- | --- | --- | --- | --- | --- | --- | --- | --- | --- | --- | --- | --- | --- | --- | --- | --- | --- | --- | --- | --- | --- | --- | --- | --- | --- | --- | --- | --- | --- | --- | --- | --- | --- | --- | --- |
| K.NVQAEEMVEFSSGLK.G | Y | 72.67 | 1666.7872 | 15 | 0.7 | 834.4015 | 2 | 57.47 | 1 | F1:22310 | NaNaKA16\_F1.raw | 6.2448E6 |  |  |  |  |  |  |  |  |  |  |  |  | 2 | 2 | 0 | 0 | 0 | 0 | 0 | 0 | 0 | 0 | 0 | 0 | 0 | 0 | 89 | 103 |  |  | PEAKS DB |
| K.HALIIYDDLSK.Q | Y | 67.93 | 1286.6870 | 11 | 0.3 | 644.3510 | 2 | 34.90 | 1 | F1:15224 | NaNaKA16\_F1.raw | 1.6656E6 |  |  |  |  |  |  |  |  |  |  |  |  | 1 | 1 | 0 | 0 | 0 | 0 | 0 | 0 | 0 | 0 | 0 | 0 | 0 | 0 | 306 | 316 |  |  | PEAKS DB |
| R.TGAIVDVPVGEELLGR.V | Y | 43.34 | 1623.8832 | 16 | 0.7 | 812.9495 | 2 | 71.89 | 1 | F1:26457 | NaNaKA16\_F1.raw | 8.0145E5 |  |  |  |  |  |  |  |  |  |  |  |  | 1 | 1 | 0 | 0 | 0 | 0 | 0 | 0 | 0 | 0 | 0 | 0 | 0 | 0 | 134 | 149 |  |  | PEAKS DB |
| total 3 peptides |  |  |  |  |  |  |  |  |  |  |  |  |  |  |  |  |  |  |  |  |  |  |  |  |  |  |  |  |  |  |  |  |  |  |  |  |  |  |  |  |  |  |  |
| --- | --- | --- | --- | --- | --- | --- | --- | --- | --- | --- | --- | --- | --- | --- | --- | --- | --- | --- | --- | --- | --- | --- | --- | --- | --- | --- | --- | --- | --- | --- | --- | --- | --- | --- | --- | --- | --- | --- | --- | --- | --- | --- | --- |

D5LMJ3.1

back to list

  

| Protein Coverage
| Supporting Peptides
|

Protein Coverage:

Supporting Peptides:

| Peptide | Uniq | -10lgP | Mass | Length | ppm | m/z | z | RT | Fraction | Scan | Source File | Area F1 | Area F10 | Area F11 | Area F12 | Area F13 | Area F2 | Area F3 | Area F4 | Area F5 | Area F6 | Area F7 | Area F8 | Area F9 | #Feature | #Feature F1 | #Feature F10 | #Feature F11 | #Feature F12 | #Feature F13 | #Feature F2 | #Feature F3 | #Feature F4 | #Feature F5 | #Feature F6 | #Feature F7 | #Feature F8 | #Feature F9 | Start | End | PTM | AScore | Found By |
| --- | --- | --- | --- | --- | --- | --- | --- | --- | --- | --- | --- | --- | --- | --- | --- | --- | --- | --- | --- | --- | --- | --- | --- | --- | --- | --- | --- | --- | --- | --- | --- | --- | --- | --- | --- | --- | --- | --- | --- | --- | --- | --- | --- |
| R.VYEMVNYLNTK.Y | Y | 76.05 | 1372.6697 | 11 | 0.9 | 687.3427 | 2 | 49.71 | 3 | F3:28127 | NaNaKA16\_F11.raw |  | 5.6566E7 | 1.1334E8 | 7.86E7 | 2.6505E5 |  |  |  |  |  |  |  |  | 5 | 0 | 1 | 1 | 2 | 1 | 0 | 0 | 0 | 0 | 0 | 0 | 0 | 0 | 231 | 241 |  |  | PEAKS DB |
| R.VYEM(+15.99)VNYLNTK.Y | Y | 60.35 | 1388.6646 | 11 | 0.6 | 695.3400 | 2 | 31.58 | 4 | F4:14688 | NaNaKA16\_F12.raw |  |  |  | 2.73E6 |  |  |  |  |  |  |  |  |  | 1 | 0 | 0 | 0 | 1 | 0 | 0 | 0 | 0 | 0 | 0 | 0 | 0 | 0 | 231 | 241 | Oxidation (M) | M4:Oxidation (M):1000.00 | PEAKS DB |
| W.GSVAVVQDYSR.R | Y | 45.46 | 1179.5884 | 11 | 0.2 | 590.8016 | 2 | 21.25 | 5 | F5:5450 | NaNaKA16\_F13.raw |  |  |  |  | 1.2356E5 |  |  |  |  |  |  |  |  | 1 | 0 | 0 | 0 | 0 | 1 | 0 | 0 | 0 | 0 | 0 | 0 | 0 | 0 | 317 | 327 |  |  | PEAKS DB |
| R.VYEMVNYLNTKYR.R | Y | 42.80 | 1691.8341 | 13 | 0.1 | 564.9520 | 3 | 45.94 | 4 | F4:26761 | NaNaKA16\_F12.raw |  |  |  | 2.3007E5 |  |  |  |  |  |  |  |  |  | 1 | 0 | 0 | 0 | 1 | 0 | 0 | 0 | 0 | 0 | 0 | 0 | 0 | 0 | 231 | 243 |  |  | PEAKS DB |
| total 4 peptides |  |  |  |  |  |  |  |  |  |  |  |  |  |  |  |  |  |  |  |  |  |  |  |  |  |  |  |  |  |  |  |  |  |  |  |  |  |  |  |  |  |  |  |
| --- | --- | --- | --- | --- | --- | --- | --- | --- | --- | --- | --- | --- | --- | --- | --- | --- | --- | --- | --- | --- | --- | --- | --- | --- | --- | --- | --- | --- | --- | --- | --- | --- | --- | --- | --- | --- | --- | --- | --- | --- | --- | --- | --- |

ADF43026.1

back to list

  

| Protein Coverage
| Supporting Peptides
|

Protein Coverage:

Supporting Peptides:

| Peptide | Uniq | -10lgP | Mass | Length | ppm | m/z | z | RT | Fraction | Scan | Source File | Area F1 | Area F10 | Area F11 | Area F12 | Area F13 | Area F2 | Area F3 | Area F4 | Area F5 | Area F6 | Area F7 | Area F8 | Area F9 | #Feature | #Feature F1 | #Feature F10 | #Feature F11 | #Feature F12 | #Feature F13 | #Feature F2 | #Feature F3 | #Feature F4 | #Feature F5 | #Feature F6 | #Feature F7 | #Feature F8 | #Feature F9 | Start | End | PTM | AScore | Found By |
| --- | --- | --- | --- | --- | --- | --- | --- | --- | --- | --- | --- | --- | --- | --- | --- | --- | --- | --- | --- | --- | --- | --- | --- | --- | --- | --- | --- | --- | --- | --- | --- | --- | --- | --- | --- | --- | --- | --- | --- | --- | --- | --- | --- |
| R.VYEMVNYLNTK.Y | Y | 76.05 | 1372.6697 | 11 | 0.9 | 687.3427 | 2 | 49.71 | 3 | F3:28127 | NaNaKA16\_F11.raw |  | 5.6566E7 | 1.1334E8 | 7.86E7 | 2.6505E5 |  |  |  |  |  |  |  |  | 5 | 0 | 1 | 1 | 2 | 1 | 0 | 0 | 0 | 0 | 0 | 0 | 0 | 0 | 231 | 241 |  |  | PEAKS DB |
| R.VYEM(+15.99)VNYLNTK.Y | Y | 60.35 | 1388.6646 | 11 | 0.6 | 695.3400 | 2 | 31.58 | 4 | F4:14688 | NaNaKA16\_F12.raw |  |  |  | 2.73E6 |  |  |  |  |  |  |  |  |  | 1 | 0 | 0 | 0 | 1 | 0 | 0 | 0 | 0 | 0 | 0 | 0 | 0 | 0 | 231 | 241 | Oxidation (M) | M4:Oxidation (M):1000.00 | PEAKS DB |
| W.GSVAVVQDYSR.R | Y | 45.46 | 1179.5884 | 11 | 0.2 | 590.8016 | 2 | 21.25 | 5 | F5:5450 | NaNaKA16\_F13.raw |  |  |  |  | 1.2356E5 |  |  |  |  |  |  |  |  | 1 | 0 | 0 | 0 | 0 | 1 | 0 | 0 | 0 | 0 | 0 | 0 | 0 | 0 | 317 | 327 |  |  | PEAKS DB |
| R.VYEMVNYLNTKYR.R | Y | 42.80 | 1691.8341 | 13 | 0.1 | 564.9520 | 3 | 45.94 | 4 | F4:26761 | NaNaKA16\_F12.raw |  |  |  | 2.3007E5 |  |  |  |  |  |  |  |  |  | 1 | 0 | 0 | 0 | 1 | 0 | 0 | 0 | 0 | 0 | 0 | 0 | 0 | 0 | 231 | 243 |  |  | PEAKS DB |
| total 4 peptides |  |  |  |  |  |  |  |  |  |  |  |  |  |  |  |  |  |  |  |  |  |  |  |  |  |  |  |  |  |  |  |  |  |  |  |  |  |  |  |  |  |  |  |
| --- | --- | --- | --- | --- | --- | --- | --- | --- | --- | --- | --- | --- | --- | --- | --- | --- | --- | --- | --- | --- | --- | --- | --- | --- | --- | --- | --- | --- | --- | --- | --- | --- | --- | --- | --- | --- | --- | --- | --- | --- | --- | --- | --- |

ACF21000.1

back to list

  

| Protein Coverage
| Supporting Peptides
|

Protein Coverage:

Supporting Peptides:

| Peptide | Uniq | -10lgP | Mass | Length | ppm | m/z | z | RT | Fraction | Scan | Source File | Area F1 | Area F10 | Area F11 | Area F12 | Area F13 | Area F2 | Area F3 | Area F4 | Area F5 | Area F6 | Area F7 | Area F8 | Area F9 | #Feature | #Feature F1 | #Feature F10 | #Feature F11 | #Feature F12 | #Feature F13 | #Feature F2 | #Feature F3 | #Feature F4 | #Feature F5 | #Feature F6 | #Feature F7 | #Feature F8 | #Feature F9 | Start | End | PTM | AScore | Found By |
| --- | --- | --- | --- | --- | --- | --- | --- | --- | --- | --- | --- | --- | --- | --- | --- | --- | --- | --- | --- | --- | --- | --- | --- | --- | --- | --- | --- | --- | --- | --- | --- | --- | --- | --- | --- | --- | --- | --- | --- | --- | --- | --- | --- |
| Y.YFFGESPPVLVLTC(+57.02)K.S | Y | 64.06 | 1755.8905 | 15 | 0.3 | 878.9528 | 2 | 83.86 | 4 | F4:58111 | NaNaKA16\_F12.raw |  |  |  | 1.8725E6 |  |  |  |  |  |  |  |  |  | 1 | 0 | 0 | 0 | 1 | 0 | 0 | 0 | 0 | 0 | 0 | 0 | 0 | 0 | 107 | 121 | Carbamidomethylation | C14:Carbamidomethylation:1000.00 | PEAKS DB |
| C.TGYYFFGESPPVLVLTC(+57.02)K.S | Y | 62.06 | 2077.0229 | 18 | 0.3 | 1039.5190 | 2 | 91.83 | 4 | F4:64506 | NaNaKA16\_F12.raw |  |  |  | 1.724E6 |  |  |  |  |  |  |  |  |  | 1 | 0 | 0 | 0 | 1 | 0 | 0 | 0 | 0 | 0 | 0 | 0 | 0 | 0 | 104 | 121 | Carbamidomethylation | C17:Carbamidomethylation:1000.00 | PEAKS DB |
| F.FGESPPVLVLTC(+57.02)K.S | Y | 61.85 | 1445.7588 | 13 | 0.4 | 723.8870 | 2 | 61.00 | 10 | F10:41816 | NaNaKA16\_F6.raw |  |  |  |  |  |  |  |  | 3.4892E6 | 1.1315E6 |  |  |  | 2 | 0 | 0 | 0 | 0 | 0 | 0 | 0 | 0 | 1 | 1 | 0 | 0 | 0 | 109 | 121 | Carbamidomethylation | C12:Carbamidomethylation:1000.00 | PEAKS DB |
| Y.FFGESPPVLVLTC(+57.02)K.S | Y | 56.41 | 1592.8271 | 14 | 0.4 | 797.4211 | 2 | 75.32 | 4 | F4:50993 | NaNaKA16\_F12.raw |  |  |  | 2.1589E6 |  |  |  |  |  |  |  |  |  | 1 | 0 | 0 | 0 | 1 | 0 | 0 | 0 | 0 | 0 | 0 | 0 | 0 | 0 | 108 | 121 | Carbamidomethylation | C13:Carbamidomethylation:1000.00 | PEAKS DB |
| total 4 peptides |  |  |  |  |  |  |  |  |  |  |  |  |  |  |  |  |  |  |  |  |  |  |  |  |  |  |  |  |  |  |  |  |  |  |  |  |  |  |  |  |  |  |  |
| --- | --- | --- | --- | --- | --- | --- | --- | --- | --- | --- | --- | --- | --- | --- | --- | --- | --- | --- | --- | --- | --- | --- | --- | --- | --- | --- | --- | --- | --- | --- | --- | --- | --- | --- | --- | --- | --- | --- | --- | --- | --- | --- | --- |

B6S2X0.1

back to list

  

| Protein Coverage
| Supporting Peptides
|

Protein Coverage:

Supporting Peptides:

| Peptide | Uniq | -10lgP | Mass | Length | ppm | m/z | z | RT | Fraction | Scan | Source File | Area F1 | Area F10 | Area F11 | Area F12 | Area F13 | Area F2 | Area F3 | Area F4 | Area F5 | Area F6 | Area F7 | Area F8 | Area F9 | #Feature | #Feature F1 | #Feature F10 | #Feature F11 | #Feature F12 | #Feature F13 | #Feature F2 | #Feature F3 | #Feature F4 | #Feature F5 | #Feature F6 | #Feature F7 | #Feature F8 | #Feature F9 | Start | End | PTM | AScore | Found By |
| --- | --- | --- | --- | --- | --- | --- | --- | --- | --- | --- | --- | --- | --- | --- | --- | --- | --- | --- | --- | --- | --- | --- | --- | --- | --- | --- | --- | --- | --- | --- | --- | --- | --- | --- | --- | --- | --- | --- | --- | --- | --- | --- | --- |
| Y.YFFGESPPVLVLTC(+57.02)K.S | Y | 64.06 | 1755.8905 | 15 | 0.3 | 878.9528 | 2 | 83.86 | 4 | F4:58111 | NaNaKA16\_F12.raw |  |  |  | 1.8725E6 |  |  |  |  |  |  |  |  |  | 1 | 0 | 0 | 0 | 1 | 0 | 0 | 0 | 0 | 0 | 0 | 0 | 0 | 0 | 107 | 121 | Carbamidomethylation | C14:Carbamidomethylation:1000.00 | PEAKS DB |
| C.TGYYFFGESPPVLVLTC(+57.02)K.S | Y | 62.06 | 2077.0229 | 18 | 0.3 | 1039.5190 | 2 | 91.83 | 4 | F4:64506 | NaNaKA16\_F12.raw |  |  |  | 1.724E6 |  |  |  |  |  |  |  |  |  | 1 | 0 | 0 | 0 | 1 | 0 | 0 | 0 | 0 | 0 | 0 | 0 | 0 | 0 | 104 | 121 | Carbamidomethylation | C17:Carbamidomethylation:1000.00 | PEAKS DB |
| F.FGESPPVLVLTC(+57.02)K.S | Y | 61.85 | 1445.7588 | 13 | 0.4 | 723.8870 | 2 | 61.00 | 10 | F10:41816 | NaNaKA16\_F6.raw |  |  |  |  |  |  |  |  | 3.4892E6 | 1.1315E6 |  |  |  | 2 | 0 | 0 | 0 | 0 | 0 | 0 | 0 | 0 | 1 | 1 | 0 | 0 | 0 | 109 | 121 | Carbamidomethylation | C12:Carbamidomethylation:1000.00 | PEAKS DB |
| Y.FFGESPPVLVLTC(+57.02)K.S | Y | 56.41 | 1592.8271 | 14 | 0.4 | 797.4211 | 2 | 75.32 | 4 | F4:50993 | NaNaKA16\_F12.raw |  |  |  | 2.1589E6 |  |  |  |  |  |  |  |  |  | 1 | 0 | 0 | 0 | 1 | 0 | 0 | 0 | 0 | 0 | 0 | 0 | 0 | 0 | 108 | 121 | Carbamidomethylation | C13:Carbamidomethylation:1000.00 | PEAKS DB |
| total 4 peptides |  |  |  |  |  |  |  |  |  |  |  |  |  |  |  |  |  |  |  |  |  |  |  |  |  |  |  |  |  |  |  |  |  |  |  |  |  |  |  |  |  |  |  |
| --- | --- | --- | --- | --- | --- | --- | --- | --- | --- | --- | --- | --- | --- | --- | --- | --- | --- | --- | --- | --- | --- | --- | --- | --- | --- | --- | --- | --- | --- | --- | --- | --- | --- | --- | --- | --- | --- | --- | --- | --- | --- | --- | --- |

JAG47405.1

back to list

  

| Protein Coverage
| Supporting Peptides
|

Protein Coverage:

Supporting Peptides:

| Peptide | Uniq | -10lgP | Mass | Length | ppm | m/z | z | RT | Fraction | Scan | Source File | Area F1 | Area F10 | Area F11 | Area F12 | Area F13 | Area F2 | Area F3 | Area F4 | Area F5 | Area F6 | Area F7 | Area F8 | Area F9 | #Feature | #Feature F1 | #Feature F10 | #Feature F11 | #Feature F12 | #Feature F13 | #Feature F2 | #Feature F3 | #Feature F4 | #Feature F5 | #Feature F6 | #Feature F7 | #Feature F8 | #Feature F9 | Start | End | PTM | AScore | Found By |
| --- | --- | --- | --- | --- | --- | --- | --- | --- | --- | --- | --- | --- | --- | --- | --- | --- | --- | --- | --- | --- | --- | --- | --- | --- | --- | --- | --- | --- | --- | --- | --- | --- | --- | --- | --- | --- | --- | --- | --- | --- | --- | --- | --- |
| R.IMNVIGEPIDER.G | Y | 54.69 | 1384.7020 | 12 | 0.1 | 693.3583 | 2 | 47.41 | 1 | F1:19045 | NaNaKA16\_F1.raw | 1.9988E6 |  |  |  |  |  |  |  |  |  |  |  |  | 2 | 2 | 0 | 0 | 0 | 0 | 0 | 0 | 0 | 0 | 0 | 0 | 0 | 0 | 146 | 157 |  |  | PEAKS DB |
| K.VVDLLAPYAK.G | Y | 54.59 | 1087.6277 | 10 | 0.3 | 544.8213 | 2 | 48.85 | 1 | F1:20321 | NaNaKA16\_F1.raw | 1.4349E6 |  |  |  |  |  |  |  |  |  |  |  |  | 2 | 2 | 0 | 0 | 0 | 0 | 0 | 0 | 0 | 0 | 0 | 0 | 0 | 0 | 191 | 200 |  |  | PEAKS DB |
| R.FTQAGSEVSALLGR.I | Y | 48.99 | 1434.7467 | 14 | 0.8 | 718.3812 | 2 | 49.83 | 1 | F1:21345 | NaNaKA16\_F1.raw | 2.5822E5 |  |  |  |  |  |  |  |  |  |  |  |  | 1 | 1 | 0 | 0 | 0 | 0 | 0 | 0 | 0 | 0 | 0 | 0 | 0 | 0 | 313 | 326 |  |  | PEAKS DB |
| total 3 peptides |  |  |  |  |  |  |  |  |  |  |  |  |  |  |  |  |  |  |  |  |  |  |  |  |  |  |  |  |  |  |  |  |  |  |  |  |  |  |  |  |  |  |  |
| --- | --- | --- | --- | --- | --- | --- | --- | --- | --- | --- | --- | --- | --- | --- | --- | --- | --- | --- | --- | --- | --- | --- | --- | --- | --- | --- | --- | --- | --- | --- | --- | --- | --- | --- | --- | --- | --- | --- | --- | --- | --- | --- | --- |

JAA97707.1

back to list

  

| Protein Coverage
| Supporting Peptides
|

Protein Coverage:

Supporting Peptides:

| Peptide | Uniq | -10lgP | Mass | Length | ppm | m/z | z | RT | Fraction | Scan | Source File | Area F1 | Area F10 | Area F11 | Area F12 | Area F13 | Area F2 | Area F3 | Area F4 | Area F5 | Area F6 | Area F7 | Area F8 | Area F9 | #Feature | #Feature F1 | #Feature F10 | #Feature F11 | #Feature F12 | #Feature F13 | #Feature F2 | #Feature F3 | #Feature F4 | #Feature F5 | #Feature F6 | #Feature F7 | #Feature F8 | #Feature F9 | Start | End | PTM | AScore | Found By |
| --- | --- | --- | --- | --- | --- | --- | --- | --- | --- | --- | --- | --- | --- | --- | --- | --- | --- | --- | --- | --- | --- | --- | --- | --- | --- | --- | --- | --- | --- | --- | --- | --- | --- | --- | --- | --- | --- | --- | --- | --- | --- | --- | --- |
| R.IMNVIGEPIDER.G | Y | 54.69 | 1384.7020 | 12 | 0.1 | 693.3583 | 2 | 47.41 | 1 | F1:19045 | NaNaKA16\_F1.raw | 1.9988E6 |  |  |  |  |  |  |  |  |  |  |  |  | 2 | 2 | 0 | 0 | 0 | 0 | 0 | 0 | 0 | 0 | 0 | 0 | 0 | 0 | 146 | 157 |  |  | PEAKS DB |
| K.VVDLLAPYAK.G | Y | 54.59 | 1087.6277 | 10 | 0.3 | 544.8213 | 2 | 48.85 | 1 | F1:20321 | NaNaKA16\_F1.raw | 1.4349E6 |  |  |  |  |  |  |  |  |  |  |  |  | 2 | 2 | 0 | 0 | 0 | 0 | 0 | 0 | 0 | 0 | 0 | 0 | 0 | 0 | 191 | 200 |  |  | PEAKS DB |
| R.FTQAGSEVSALLGR.I | Y | 48.99 | 1434.7467 | 14 | 0.8 | 718.3812 | 2 | 49.83 | 1 | F1:21345 | NaNaKA16\_F1.raw | 2.5822E5 |  |  |  |  |  |  |  |  |  |  |  |  | 1 | 1 | 0 | 0 | 0 | 0 | 0 | 0 | 0 | 0 | 0 | 0 | 0 | 0 | 313 | 326 |  |  | PEAKS DB |
| total 3 peptides |  |  |  |  |  |  |  |  |  |  |  |  |  |  |  |  |  |  |  |  |  |  |  |  |  |  |  |  |  |  |  |  |  |  |  |  |  |  |  |  |  |  |  |
| --- | --- | --- | --- | --- | --- | --- | --- | --- | --- | --- | --- | --- | --- | --- | --- | --- | --- | --- | --- | --- | --- | --- | --- | --- | --- | --- | --- | --- | --- | --- | --- | --- | --- | --- | --- | --- | --- | --- | --- | --- | --- | --- | --- |

JAG45746.1

back to list

  

| Protein Coverage
| Supporting Peptides
|

Protein Coverage:

Supporting Peptides:

| Peptide | Uniq | -10lgP | Mass | Length | ppm | m/z | z | RT | Fraction | Scan | Source File | Area F1 | Area F10 | Area F11 | Area F12 | Area F13 | Area F2 | Area F3 | Area F4 | Area F5 | Area F6 | Area F7 | Area F8 | Area F9 | #Feature | #Feature F1 | #Feature F10 | #Feature F11 | #Feature F12 | #Feature F13 | #Feature F2 | #Feature F3 | #Feature F4 | #Feature F5 | #Feature F6 | #Feature F7 | #Feature F8 | #Feature F9 | Start | End | PTM | AScore | Found By |
| --- | --- | --- | --- | --- | --- | --- | --- | --- | --- | --- | --- | --- | --- | --- | --- | --- | --- | --- | --- | --- | --- | --- | --- | --- | --- | --- | --- | --- | --- | --- | --- | --- | --- | --- | --- | --- | --- | --- | --- | --- | --- | --- | --- |
| R.IMNVIGEPIDER.G | Y | 54.69 | 1384.7020 | 12 | 0.1 | 693.3583 | 2 | 47.41 | 1 | F1:19045 | NaNaKA16\_F1.raw | 1.9988E6 |  |  |  |  |  |  |  |  |  |  |  |  | 2 | 2 | 0 | 0 | 0 | 0 | 0 | 0 | 0 | 0 | 0 | 0 | 0 | 0 | 146 | 157 |  |  | PEAKS DB |
| K.VVDLLAPYAK.G | Y | 54.59 | 1087.6277 | 10 | 0.3 | 544.8213 | 2 | 48.85 | 1 | F1:20321 | NaNaKA16\_F1.raw | 1.4349E6 |  |  |  |  |  |  |  |  |  |  |  |  | 2 | 2 | 0 | 0 | 0 | 0 | 0 | 0 | 0 | 0 | 0 | 0 | 0 | 0 | 191 | 200 |  |  | PEAKS DB |
| R.FTQAGSEVSALLGR.I | Y | 48.99 | 1434.7467 | 14 | 0.8 | 718.3812 | 2 | 49.83 | 1 | F1:21345 | NaNaKA16\_F1.raw | 2.5822E5 |  |  |  |  |  |  |  |  |  |  |  |  | 1 | 1 | 0 | 0 | 0 | 0 | 0 | 0 | 0 | 0 | 0 | 0 | 0 | 0 | 313 | 326 |  |  | PEAKS DB |
| total 3 peptides |  |  |  |  |  |  |  |  |  |  |  |  |  |  |  |  |  |  |  |  |  |  |  |  |  |  |  |  |  |  |  |  |  |  |  |  |  |  |  |  |  |  |  |
| --- | --- | --- | --- | --- | --- | --- | --- | --- | --- | --- | --- | --- | --- | --- | --- | --- | --- | --- | --- | --- | --- | --- | --- | --- | --- | --- | --- | --- | --- | --- | --- | --- | --- | --- | --- | --- | --- | --- | --- | --- | --- | --- | --- |

JAV51185.1

back to list

  

| Protein Coverage
| Supporting Peptides
|

Protein Coverage:

Supporting Peptides:

| Peptide | Uniq | -10lgP | Mass | Length | ppm | m/z | z | RT | Fraction | Scan | Source File | Area F1 | Area F10 | Area F11 | Area F12 | Area F13 | Area F2 | Area F3 | Area F4 | Area F5 | Area F6 | Area F7 | Area F8 | Area F9 | #Feature | #Feature F1 | #Feature F10 | #Feature F11 | #Feature F12 | #Feature F13 | #Feature F2 | #Feature F3 | #Feature F4 | #Feature F5 | #Feature F6 | #Feature F7 | #Feature F8 | #Feature F9 | Start | End | PTM | AScore | Found By |
| --- | --- | --- | --- | --- | --- | --- | --- | --- | --- | --- | --- | --- | --- | --- | --- | --- | --- | --- | --- | --- | --- | --- | --- | --- | --- | --- | --- | --- | --- | --- | --- | --- | --- | --- | --- | --- | --- | --- | --- | --- | --- | --- | --- |
| R.IMNVIGEPIDER.G | Y | 54.69 | 1384.7020 | 12 | 0.1 | 693.3583 | 2 | 47.41 | 1 | F1:19045 | NaNaKA16\_F1.raw | 1.9988E6 |  |  |  |  |  |  |  |  |  |  |  |  | 2 | 2 | 0 | 0 | 0 | 0 | 0 | 0 | 0 | 0 | 0 | 0 | 0 | 0 | 146 | 157 |  |  | PEAKS DB |
| K.VVDLLAPYAK.G | Y | 54.59 | 1087.6277 | 10 | 0.3 | 544.8213 | 2 | 48.85 | 1 | F1:20321 | NaNaKA16\_F1.raw | 1.4349E6 |  |  |  |  |  |  |  |  |  |  |  |  | 2 | 2 | 0 | 0 | 0 | 0 | 0 | 0 | 0 | 0 | 0 | 0 | 0 | 0 | 191 | 200 |  |  | PEAKS DB |
| R.FTQAGSEVSALLGR.I | Y | 48.99 | 1434.7467 | 14 | 0.8 | 718.3812 | 2 | 49.83 | 1 | F1:21345 | NaNaKA16\_F1.raw | 2.5822E5 |  |  |  |  |  |  |  |  |  |  |  |  | 1 | 1 | 0 | 0 | 0 | 0 | 0 | 0 | 0 | 0 | 0 | 0 | 0 | 0 | 313 | 326 |  |  | PEAKS DB |
| total 3 peptides |  |  |  |  |  |  |  |  |  |  |  |  |  |  |  |  |  |  |  |  |  |  |  |  |  |  |  |  |  |  |  |  |  |  |  |  |  |  |  |  |  |  |  |
| --- | --- | --- | --- | --- | --- | --- | --- | --- | --- | --- | --- | --- | --- | --- | --- | --- | --- | --- | --- | --- | --- | --- | --- | --- | --- | --- | --- | --- | --- | --- | --- | --- | --- | --- | --- | --- | --- | --- | --- | --- | --- | --- | --- |

AFJ49478.1

back to list

  

| Protein Coverage
| Supporting Peptides
|

Protein Coverage:

Supporting Peptides:

| Peptide | Uniq | -10lgP | Mass | Length | ppm | m/z | z | RT | Fraction | Scan | Source File | Area F1 | Area F10 | Area F11 | Area F12 | Area F13 | Area F2 | Area F3 | Area F4 | Area F5 | Area F6 | Area F7 | Area F8 | Area F9 | #Feature | #Feature F1 | #Feature F10 | #Feature F11 | #Feature F12 | #Feature F13 | #Feature F2 | #Feature F3 | #Feature F4 | #Feature F5 | #Feature F6 | #Feature F7 | #Feature F8 | #Feature F9 | Start | End | PTM | AScore | Found By |
| --- | --- | --- | --- | --- | --- | --- | --- | --- | --- | --- | --- | --- | --- | --- | --- | --- | --- | --- | --- | --- | --- | --- | --- | --- | --- | --- | --- | --- | --- | --- | --- | --- | --- | --- | --- | --- | --- | --- | --- | --- | --- | --- | --- |
| R.IMNVIGEPIDER.G | Y | 54.69 | 1384.7020 | 12 | 0.1 | 693.3583 | 2 | 47.41 | 1 | F1:19045 | NaNaKA16\_F1.raw | 1.9988E6 |  |  |  |  |  |  |  |  |  |  |  |  | 2 | 2 | 0 | 0 | 0 | 0 | 0 | 0 | 0 | 0 | 0 | 0 | 0 | 0 | 146 | 157 |  |  | PEAKS DB |
| K.VVDLLAPYAK.G | Y | 54.59 | 1087.6277 | 10 | 0.3 | 544.8213 | 2 | 48.85 | 1 | F1:20321 | NaNaKA16\_F1.raw | 1.4349E6 |  |  |  |  |  |  |  |  |  |  |  |  | 2 | 2 | 0 | 0 | 0 | 0 | 0 | 0 | 0 | 0 | 0 | 0 | 0 | 0 | 191 | 200 |  |  | PEAKS DB |
| R.FTQAGSEVSALLGR.I | Y | 48.99 | 1434.7467 | 14 | 0.8 | 718.3812 | 2 | 49.83 | 1 | F1:21345 | NaNaKA16\_F1.raw | 2.5822E5 |  |  |  |  |  |  |  |  |  |  |  |  | 1 | 1 | 0 | 0 | 0 | 0 | 0 | 0 | 0 | 0 | 0 | 0 | 0 | 0 | 313 | 326 |  |  | PEAKS DB |
| total 3 peptides |  |  |  |  |  |  |  |  |  |  |  |  |  |  |  |  |  |  |  |  |  |  |  |  |  |  |  |  |  |  |  |  |  |  |  |  |  |  |  |  |  |  |  |
| --- | --- | --- | --- | --- | --- | --- | --- | --- | --- | --- | --- | --- | --- | --- | --- | --- | --- | --- | --- | --- | --- | --- | --- | --- | --- | --- | --- | --- | --- | --- | --- | --- | --- | --- | --- | --- | --- | --- | --- | --- | --- | --- | --- |

JAI14244.1

back to list

  

| Protein Coverage
| Supporting Peptides
|

Protein Coverage:

Supporting Peptides:

| Peptide | Uniq | -10lgP | Mass | Length | ppm | m/z | z | RT | Fraction | Scan | Source File | Area F1 | Area F10 | Area F11 | Area F12 | Area F13 | Area F2 | Area F3 | Area F4 | Area F5 | Area F6 | Area F7 | Area F8 | Area F9 | #Feature | #Feature F1 | #Feature F10 | #Feature F11 | #Feature F12 | #Feature F13 | #Feature F2 | #Feature F3 | #Feature F4 | #Feature F5 | #Feature F6 | #Feature F7 | #Feature F8 | #Feature F9 | Start | End | PTM | AScore | Found By |
| --- | --- | --- | --- | --- | --- | --- | --- | --- | --- | --- | --- | --- | --- | --- | --- | --- | --- | --- | --- | --- | --- | --- | --- | --- | --- | --- | --- | --- | --- | --- | --- | --- | --- | --- | --- | --- | --- | --- | --- | --- | --- | --- | --- |
| R.IMNVIGEPIDER.G | Y | 54.69 | 1384.7020 | 12 | 0.1 | 693.3583 | 2 | 47.41 | 1 | F1:19045 | NaNaKA16\_F1.raw | 1.9988E6 |  |  |  |  |  |  |  |  |  |  |  |  | 2 | 2 | 0 | 0 | 0 | 0 | 0 | 0 | 0 | 0 | 0 | 0 | 0 | 0 | 146 | 157 |  |  | PEAKS DB |
| K.VVDLLAPYAK.G | Y | 54.59 | 1087.6277 | 10 | 0.3 | 544.8213 | 2 | 48.85 | 1 | F1:20321 | NaNaKA16\_F1.raw | 1.4349E6 |  |  |  |  |  |  |  |  |  |  |  |  | 2 | 2 | 0 | 0 | 0 | 0 | 0 | 0 | 0 | 0 | 0 | 0 | 0 | 0 | 191 | 200 |  |  | PEAKS DB |
| R.FTQAGSEVSALLGR.I | Y | 48.99 | 1434.7467 | 14 | 0.8 | 718.3812 | 2 | 49.83 | 1 | F1:21345 | NaNaKA16\_F1.raw | 2.5822E5 |  |  |  |  |  |  |  |  |  |  |  |  | 1 | 1 | 0 | 0 | 0 | 0 | 0 | 0 | 0 | 0 | 0 | 0 | 0 | 0 | 313 | 326 |  |  | PEAKS DB |
| total 3 peptides |  |  |  |  |  |  |  |  |  |  |  |  |  |  |  |  |  |  |  |  |  |  |  |  |  |  |  |  |  |  |  |  |  |  |  |  |  |  |  |  |  |  |  |
| --- | --- | --- | --- | --- | --- | --- | --- | --- | --- | --- | --- | --- | --- | --- | --- | --- | --- | --- | --- | --- | --- | --- | --- | --- | --- | --- | --- | --- | --- | --- | --- | --- | --- | --- | --- | --- | --- | --- | --- | --- | --- | --- | --- |

XP\_025032248.1

back to list

  

| Protein Coverage
| Supporting Peptides
|

Protein Coverage:

Supporting Peptides:

| Peptide | Uniq | -10lgP | Mass | Length | ppm | m/z | z | RT | Fraction | Scan | Source File | Area F1 | Area F10 | Area F11 | Area F12 | Area F13 | Area F2 | Area F3 | Area F4 | Area F5 | Area F6 | Area F7 | Area F8 | Area F9 | #Feature | #Feature F1 | #Feature F10 | #Feature F11 | #Feature F12 | #Feature F13 | #Feature F2 | #Feature F3 | #Feature F4 | #Feature F5 | #Feature F6 | #Feature F7 | #Feature F8 | #Feature F9 | Start | End | PTM | AScore | Found By |
| --- | --- | --- | --- | --- | --- | --- | --- | --- | --- | --- | --- | --- | --- | --- | --- | --- | --- | --- | --- | --- | --- | --- | --- | --- | --- | --- | --- | --- | --- | --- | --- | --- | --- | --- | --- | --- | --- | --- | --- | --- | --- | --- | --- |
| R.EPGC(+57.02)GC(+57.02)C(+57.02)LTC(+57.02)ALQEGQPC(+57.02)GVYTER.C | Y | 65.15 | 2801.1335 | 24 | 0.6 | 934.7190 | 3 | 54.05 | 9 | F9:34846 | NaNaKA16\_F5.raw |  |  |  |  |  |  |  |  | 5.9367E7 |  |  |  |  | 2 | 0 | 0 | 0 | 0 | 0 | 0 | 0 | 0 | 2 | 0 | 0 | 0 | 0 | 147 | 170 | Carbamidomethylation | C4:Carbamidomethylation:1000.00;C6:Carbamidomethylation:1000.00;C7:Carbamidomethylation:1000.00;C10:Carbamidomethylation:1000.00;C18:Carbamidomethylation:1000.00 | PEAKS DB |
| C.ALQEGQPC(+57.02)GVYTER.C | N | 51.25 | 1606.7410 | 14 | 0.7 | 804.3783 | 2 | 20.76 | 9 | F9:8058 | NaNaKA16\_F5.raw |  |  |  |  |  |  |  |  | 2.4162E6 |  |  |  |  | 1 | 0 | 0 | 0 | 0 | 0 | 0 | 0 | 0 | 1 | 0 | 0 | 0 | 0 | 157 | 170 | Carbamidomethylation | C8:Carbamidomethylation:1000.00 | PEAKS DB |
| K.PLQALLDGK.G | N | 49.53 | 953.5546 | 9 | 0.0 | 477.7845 | 2 | 36.75 | 9 | F9:20556 | NaNaKA16\_F5.raw |  |  |  |  |  |  |  |  | 1.0511E7 |  |  |  |  | 1 | 0 | 0 | 0 | 0 | 0 | 0 | 0 | 0 | 1 | 0 | 0 | 0 | 0 | 186 | 194 |  |  | PEAKS DB |
| C.LTC(+57.02)ALQEGQPC(+57.02)GVYTER.C | Y | 46.53 | 1980.9033 | 17 | 0.4 | 991.4593 | 2 | 35.17 | 9 | F9:19212 | NaNaKA16\_F5.raw |  |  |  |  |  |  |  |  | 0 |  |  |  |  | 0 | 0 | 0 | 0 | 0 | 0 | 0 | 0 | 0 | 0 | 0 | 0 | 0 | 0 | 154 | 170 | Carbamidomethylation | C3:Carbamidomethylation:1000.00;C11:Carbamidomethylation:1000.00 | PEAKS DB |
| K.ILNVLSPR.G | N | 45.28 | 910.5600 | 8 | 0.0 | 456.2873 | 2 | 37.44 | 10 | F10:22021 | NaNaKA16\_F6.raw |  |  |  |  |  |  |  |  |  | 8.1117E5 |  |  |  | 1 | 0 | 0 | 0 | 0 | 0 | 0 | 0 | 0 | 0 | 1 | 0 | 0 | 0 | 318 | 325 |  |  | PEAKS DB |
| total 5 peptides |  |  |  |  |  |  |  |  |  |  |  |  |  |  |  |  |  |  |  |  |  |  |  |  |  |  |  |  |  |  |  |  |  |  |  |  |  |  |  |  |  |  |  |
| --- | --- | --- | --- | --- | --- | --- | --- | --- | --- | --- | --- | --- | --- | --- | --- | --- | --- | --- | --- | --- | --- | --- | --- | --- | --- | --- | --- | --- | --- | --- | --- | --- | --- | --- | --- | --- | --- | --- | --- | --- | --- | --- | --- |

XP\_013925299.1

back to list

  

| Protein Coverage
| Supporting Peptides
|

Protein Coverage:

Supporting Peptides:

| Peptide | Uniq | -10lgP | Mass | Length | ppm | m/z | z | RT | Fraction | Scan | Source File | Area F1 | Area F10 | Area F11 | Area F12 | Area F13 | Area F2 | Area F3 | Area F4 | Area F5 | Area F6 | Area F7 | Area F8 | Area F9 | #Feature | #Feature F1 | #Feature F10 | #Feature F11 | #Feature F12 | #Feature F13 | #Feature F2 | #Feature F3 | #Feature F4 | #Feature F5 | #Feature F6 | #Feature F7 | #Feature F8 | #Feature F9 | Start | End | PTM | AScore | Found By |
| --- | --- | --- | --- | --- | --- | --- | --- | --- | --- | --- | --- | --- | --- | --- | --- | --- | --- | --- | --- | --- | --- | --- | --- | --- | --- | --- | --- | --- | --- | --- | --- | --- | --- | --- | --- | --- | --- | --- | --- | --- | --- | --- | --- |
| R.EPGC(+57.02)GC(+57.02)C(+57.02)LTC(+57.02)ALQEGQPC(+57.02)GVYTER.C | Y | 65.15 | 2801.1335 | 24 | 0.6 | 934.7190 | 3 | 54.05 | 9 | F9:34846 | NaNaKA16\_F5.raw |  |  |  |  |  |  |  |  | 5.9367E7 |  |  |  |  | 2 | 0 | 0 | 0 | 0 | 0 | 0 | 0 | 0 | 2 | 0 | 0 | 0 | 0 | 56 | 79 | Carbamidomethylation | C4:Carbamidomethylation:1000.00;C6:Carbamidomethylation:1000.00;C7:Carbamidomethylation:1000.00;C10:Carbamidomethylation:1000.00;C18:Carbamidomethylation:1000.00 | PEAKS DB |
| C.ALQEGQPC(+57.02)GVYTER.C | N | 51.25 | 1606.7410 | 14 | 0.7 | 804.3783 | 2 | 20.76 | 9 | F9:8058 | NaNaKA16\_F5.raw |  |  |  |  |  |  |  |  | 2.4162E6 |  |  |  |  | 1 | 0 | 0 | 0 | 0 | 0 | 0 | 0 | 0 | 1 | 0 | 0 | 0 | 0 | 66 | 79 | Carbamidomethylation | C8:Carbamidomethylation:1000.00 | PEAKS DB |
| K.PLQALLDGK.G | N | 49.53 | 953.5546 | 9 | 0.0 | 477.7845 | 2 | 36.75 | 9 | F9:20556 | NaNaKA16\_F5.raw |  |  |  |  |  |  |  |  | 1.0511E7 |  |  |  |  | 1 | 0 | 0 | 0 | 0 | 0 | 0 | 0 | 0 | 1 | 0 | 0 | 0 | 0 | 95 | 103 |  |  | PEAKS DB |
| C.LTC(+57.02)ALQEGQPC(+57.02)GVYTER.C | Y | 46.53 | 1980.9033 | 17 | 0.4 | 991.4593 | 2 | 35.17 | 9 | F9:19212 | NaNaKA16\_F5.raw |  |  |  |  |  |  |  |  | 0 |  |  |  |  | 0 | 0 | 0 | 0 | 0 | 0 | 0 | 0 | 0 | 0 | 0 | 0 | 0 | 0 | 63 | 79 | Carbamidomethylation | C3:Carbamidomethylation:1000.00;C11:Carbamidomethylation:1000.00 | PEAKS DB |
| K.ILNVLSPR.G | N | 45.28 | 910.5600 | 8 | 0.0 | 456.2873 | 2 | 37.44 | 10 | F10:22021 | NaNaKA16\_F6.raw |  |  |  |  |  |  |  |  |  | 8.1117E5 |  |  |  | 1 | 0 | 0 | 0 | 0 | 0 | 0 | 0 | 0 | 0 | 1 | 0 | 0 | 0 | 229 | 236 |  |  | PEAKS DB |
| total 5 peptides |  |  |  |  |  |  |  |  |  |  |  |  |  |  |  |  |  |  |  |  |  |  |  |  |  |  |  |  |  |  |  |  |  |  |  |  |  |  |  |  |  |  |  |
| --- | --- | --- | --- | --- | --- | --- | --- | --- | --- | --- | --- | --- | --- | --- | --- | --- | --- | --- | --- | --- | --- | --- | --- | --- | --- | --- | --- | --- | --- | --- | --- | --- | --- | --- | --- | --- | --- | --- | --- | --- | --- | --- | --- |

XP\_032093683.1

back to list

  

| Protein Coverage
| Supporting Peptides
|

Protein Coverage:

Supporting Peptides:

| Peptide | Uniq | -10lgP | Mass | Length | ppm | m/z | z | RT | Fraction | Scan | Source File | Area F1 | Area F10 | Area F11 | Area F12 | Area F13 | Area F2 | Area F3 | Area F4 | Area F5 | Area F6 | Area F7 | Area F8 | Area F9 | #Feature | #Feature F1 | #Feature F10 | #Feature F11 | #Feature F12 | #Feature F13 | #Feature F2 | #Feature F3 | #Feature F4 | #Feature F5 | #Feature F6 | #Feature F7 | #Feature F8 | #Feature F9 | Start | End | PTM | AScore | Found By |
| --- | --- | --- | --- | --- | --- | --- | --- | --- | --- | --- | --- | --- | --- | --- | --- | --- | --- | --- | --- | --- | --- | --- | --- | --- | --- | --- | --- | --- | --- | --- | --- | --- | --- | --- | --- | --- | --- | --- | --- | --- | --- | --- | --- |
| R.EPGC(+57.02)GC(+57.02)C(+57.02)LTC(+57.02)ALQEGQPC(+57.02)GVYTER.C | Y | 65.15 | 2801.1335 | 24 | 0.6 | 934.7190 | 3 | 54.05 | 9 | F9:34846 | NaNaKA16\_F5.raw |  |  |  |  |  |  |  |  | 5.9367E7 |  |  |  |  | 2 | 0 | 0 | 0 | 0 | 0 | 0 | 0 | 0 | 2 | 0 | 0 | 0 | 0 | 56 | 79 | Carbamidomethylation | C4:Carbamidomethylation:1000.00;C6:Carbamidomethylation:1000.00;C7:Carbamidomethylation:1000.00;C10:Carbamidomethylation:1000.00;C18:Carbamidomethylation:1000.00 | PEAKS DB |
| C.ALQEGQPC(+57.02)GVYTER.C | N | 51.25 | 1606.7410 | 14 | 0.7 | 804.3783 | 2 | 20.76 | 9 | F9:8058 | NaNaKA16\_F5.raw |  |  |  |  |  |  |  |  | 2.4162E6 |  |  |  |  | 1 | 0 | 0 | 0 | 0 | 0 | 0 | 0 | 0 | 1 | 0 | 0 | 0 | 0 | 66 | 79 | Carbamidomethylation | C8:Carbamidomethylation:1000.00 | PEAKS DB |
| K.PLQALLDGK.G | N | 49.53 | 953.5546 | 9 | 0.0 | 477.7845 | 2 | 36.75 | 9 | F9:20556 | NaNaKA16\_F5.raw |  |  |  |  |  |  |  |  | 1.0511E7 |  |  |  |  | 1 | 0 | 0 | 0 | 0 | 0 | 0 | 0 | 0 | 1 | 0 | 0 | 0 | 0 | 95 | 103 |  |  | PEAKS DB |
| C.LTC(+57.02)ALQEGQPC(+57.02)GVYTER.C | Y | 46.53 | 1980.9033 | 17 | 0.4 | 991.4593 | 2 | 35.17 | 9 | F9:19212 | NaNaKA16\_F5.raw |  |  |  |  |  |  |  |  | 0 |  |  |  |  | 0 | 0 | 0 | 0 | 0 | 0 | 0 | 0 | 0 | 0 | 0 | 0 | 0 | 0 | 63 | 79 | Carbamidomethylation | C3:Carbamidomethylation:1000.00;C11:Carbamidomethylation:1000.00 | PEAKS DB |
| K.ILNVLSPR.G | N | 45.28 | 910.5600 | 8 | 0.0 | 456.2873 | 2 | 37.44 | 10 | F10:22021 | NaNaKA16\_F6.raw |  |  |  |  |  |  |  |  |  | 8.1117E5 |  |  |  | 1 | 0 | 0 | 0 | 0 | 0 | 0 | 0 | 0 | 0 | 1 | 0 | 0 | 0 | 226 | 233 |  |  | PEAKS DB |
| total 5 peptides |  |  |  |  |  |  |  |  |  |  |  |  |  |  |  |  |  |  |  |  |  |  |  |  |  |  |  |  |  |  |  |  |  |  |  |  |  |  |  |  |  |  |  |
| --- | --- | --- | --- | --- | --- | --- | --- | --- | --- | --- | --- | --- | --- | --- | --- | --- | --- | --- | --- | --- | --- | --- | --- | --- | --- | --- | --- | --- | --- | --- | --- | --- | --- | --- | --- | --- | --- | --- | --- | --- | --- | --- | --- |

XP\_015680080.1

back to list

  

| Protein Coverage
| Supporting Peptides
|

Protein Coverage:

Supporting Peptides:

| Peptide | Uniq | -10lgP | Mass | Length | ppm | m/z | z | RT | Fraction | Scan | Source File | Area F1 | Area F10 | Area F11 | Area F12 | Area F13 | Area F2 | Area F3 | Area F4 | Area F5 | Area F6 | Area F7 | Area F8 | Area F9 | #Feature | #Feature F1 | #Feature F10 | #Feature F11 | #Feature F12 | #Feature F13 | #Feature F2 | #Feature F3 | #Feature F4 | #Feature F5 | #Feature F6 | #Feature F7 | #Feature F8 | #Feature F9 | Start | End | PTM | AScore | Found By |
| --- | --- | --- | --- | --- | --- | --- | --- | --- | --- | --- | --- | --- | --- | --- | --- | --- | --- | --- | --- | --- | --- | --- | --- | --- | --- | --- | --- | --- | --- | --- | --- | --- | --- | --- | --- | --- | --- | --- | --- | --- | --- | --- | --- |
| R.EPGC(+57.02)GC(+57.02)C(+57.02)LTC(+57.02)ALQEGQPC(+57.02)GVYTER.C | Y | 65.15 | 2801.1335 | 24 | 0.6 | 934.7190 | 3 | 54.05 | 9 | F9:34846 | NaNaKA16\_F5.raw |  |  |  |  |  |  |  |  | 5.9367E7 |  |  |  |  | 2 | 0 | 0 | 0 | 0 | 0 | 0 | 0 | 0 | 2 | 0 | 0 | 0 | 0 | 54 | 77 | Carbamidomethylation | C4:Carbamidomethylation:1000.00;C6:Carbamidomethylation:1000.00;C7:Carbamidomethylation:1000.00;C10:Carbamidomethylation:1000.00;C18:Carbamidomethylation:1000.00 | PEAKS DB |
| C.ALQEGQPC(+57.02)GVYTER.C | N | 51.25 | 1606.7410 | 14 | 0.7 | 804.3783 | 2 | 20.76 | 9 | F9:8058 | NaNaKA16\_F5.raw |  |  |  |  |  |  |  |  | 2.4162E6 |  |  |  |  | 1 | 0 | 0 | 0 | 0 | 0 | 0 | 0 | 0 | 1 | 0 | 0 | 0 | 0 | 64 | 77 | Carbamidomethylation | C8:Carbamidomethylation:1000.00 | PEAKS DB |
| K.PLQALLDGK.G | N | 49.53 | 953.5546 | 9 | 0.0 | 477.7845 | 2 | 36.75 | 9 | F9:20556 | NaNaKA16\_F5.raw |  |  |  |  |  |  |  |  | 1.0511E7 |  |  |  |  | 1 | 0 | 0 | 0 | 0 | 0 | 0 | 0 | 0 | 1 | 0 | 0 | 0 | 0 | 93 | 101 |  |  | PEAKS DB |
| C.LTC(+57.02)ALQEGQPC(+57.02)GVYTER.C | Y | 46.53 | 1980.9033 | 17 | 0.4 | 991.4593 | 2 | 35.17 | 9 | F9:19212 | NaNaKA16\_F5.raw |  |  |  |  |  |  |  |  | 0 |  |  |  |  | 0 | 0 | 0 | 0 | 0 | 0 | 0 | 0 | 0 | 0 | 0 | 0 | 0 | 0 | 61 | 77 | Carbamidomethylation | C3:Carbamidomethylation:1000.00;C11:Carbamidomethylation:1000.00 | PEAKS DB |
| K.ILNVLSPR.G | N | 45.28 | 910.5600 | 8 | 0.0 | 456.2873 | 2 | 37.44 | 10 | F10:22021 | NaNaKA16\_F6.raw |  |  |  |  |  |  |  |  |  | 8.1117E5 |  |  |  | 1 | 0 | 0 | 0 | 0 | 0 | 0 | 0 | 0 | 0 | 1 | 0 | 0 | 0 | 225 | 232 |  |  | PEAKS DB |
| total 5 peptides |  |  |  |  |  |  |  |  |  |  |  |  |  |  |  |  |  |  |  |  |  |  |  |  |  |  |  |  |  |  |  |  |  |  |  |  |  |  |  |  |  |  |  |
| --- | --- | --- | --- | --- | --- | --- | --- | --- | --- | --- | --- | --- | --- | --- | --- | --- | --- | --- | --- | --- | --- | --- | --- | --- | --- | --- | --- | --- | --- | --- | --- | --- | --- | --- | --- | --- | --- | --- | --- | --- | --- | --- | --- |

XP\_026571876.1

back to list

  

| Protein Coverage
| Supporting Peptides
|

Protein Coverage:

Supporting Peptides:

| Peptide | Uniq | -10lgP | Mass | Length | ppm | m/z | z | RT | Fraction | Scan | Source File | Area F1 | Area F10 | Area F11 | Area F12 | Area F13 | Area F2 | Area F3 | Area F4 | Area F5 | Area F6 | Area F7 | Area F8 | Area F9 | #Feature | #Feature F1 | #Feature F10 | #Feature F11 | #Feature F12 | #Feature F13 | #Feature F2 | #Feature F3 | #Feature F4 | #Feature F5 | #Feature F6 | #Feature F7 | #Feature F8 | #Feature F9 | Start | End | PTM | AScore | Found By |
| --- | --- | --- | --- | --- | --- | --- | --- | --- | --- | --- | --- | --- | --- | --- | --- | --- | --- | --- | --- | --- | --- | --- | --- | --- | --- | --- | --- | --- | --- | --- | --- | --- | --- | --- | --- | --- | --- | --- | --- | --- | --- | --- | --- |
| K.ITANLC(+57.02)MLGALK.C | N | 73.92 | 1303.6992 | 12 | -0.5 | 652.8566 | 2 | 62.39 | 9 | F9:42245 | NaNaKA16\_F5.raw |  |  |  |  |  |  |  |  | 5.2097E6 |  |  |  |  | 1 | 0 | 0 | 0 | 0 | 0 | 0 | 0 | 0 | 1 | 0 | 0 | 0 | 0 | 930 | 941 | Carbamidomethylation | C6:Carbamidomethylation:1000.00 | PEAKS DB |
| K.VVLSGTEC(+57.02)LC(+57.02)VC(+57.02)R.A | Y | 61.14 | 1551.7207 | 13 | 8.1 | 776.8698 | 2 | 40.78 | 8 | F8:22141 | NaNaKA16\_F4.raw |  |  |  |  |  |  |  | 2.542E5 |  |  |  |  |  | 1 | 0 | 0 | 0 | 0 | 0 | 0 | 0 | 1 | 0 | 0 | 0 | 0 | 0 | 556 | 568 | Carbamidomethylation | C8:Carbamidomethylation:1000.00;C10:Carbamidomethylation:1000.00;C12:Carbamidomethylation:1000.00 | PEAKS DB |
| R.KITANLC(+57.02)MLGALK.C | N | 58.28 | 1431.7942 | 13 | 0.2 | 478.2721 | 3 | 47.11 | 9 | F9:28940 | NaNaKA16\_F5.raw |  |  |  |  |  |  |  |  | 9.6699E5 |  |  |  |  | 1 | 0 | 0 | 0 | 0 | 0 | 0 | 0 | 0 | 1 | 0 | 0 | 0 | 0 | 929 | 941 | Carbamidomethylation | C7:Carbamidomethylation:1000.00 | PEAKS DB |
| total 3 peptides |  |  |  |  |  |  |  |  |  |  |  |  |  |  |  |  |  |  |  |  |  |  |  |  |  |  |  |  |  |  |  |  |  |  |  |  |  |  |  |  |  |  |  |
| --- | --- | --- | --- | --- | --- | --- | --- | --- | --- | --- | --- | --- | --- | --- | --- | --- | --- | --- | --- | --- | --- | --- | --- | --- | --- | --- | --- | --- | --- | --- | --- | --- | --- | --- | --- | --- | --- | --- | --- | --- | --- | --- | --- |

XP\_026558105.1

back to list

  

| Protein Coverage
| Supporting Peptides
|

Protein Coverage:

Supporting Peptides:

| Peptide | Uniq | -10lgP | Mass | Length | ppm | m/z | z | RT | Fraction | Scan | Source File | Area F1 | Area F10 | Area F11 | Area F12 | Area F13 | Area F2 | Area F3 | Area F4 | Area F5 | Area F6 | Area F7 | Area F8 | Area F9 | #Feature | #Feature F1 | #Feature F10 | #Feature F11 | #Feature F12 | #Feature F13 | #Feature F2 | #Feature F3 | #Feature F4 | #Feature F5 | #Feature F6 | #Feature F7 | #Feature F8 | #Feature F9 | Start | End | PTM | AScore | Found By |
| --- | --- | --- | --- | --- | --- | --- | --- | --- | --- | --- | --- | --- | --- | --- | --- | --- | --- | --- | --- | --- | --- | --- | --- | --- | --- | --- | --- | --- | --- | --- | --- | --- | --- | --- | --- | --- | --- | --- | --- | --- | --- | --- | --- |
| R.ALEQC(+57.02)KPM(+15.99)VAEC(+57.02)PELVR.E | Y | 70.41 | 2044.9744 | 17 | -0.5 | 1023.4940 | 2 | 33.02 | 9 | F9:17611 | NaNaKA16\_F5.raw |  |  |  |  |  |  |  |  | 2.241E8 |  |  |  |  | 2 | 0 | 0 | 0 | 0 | 0 | 0 | 0 | 0 | 2 | 0 | 0 | 0 | 0 | 39 | 55 | Carbamidomethylation; Oxidation (M) | C5:Carbamidomethylation:1000.00;M8:Oxidation (M):1000.00;C12:Carbamidomethylation:1000.00 | PEAKS DB |
| C.ALQEGQPC(+57.02)GVYTER.C | N | 51.25 | 1606.7410 | 14 | 0.7 | 804.3783 | 2 | 20.76 | 9 | F9:8058 | NaNaKA16\_F5.raw |  |  |  |  |  |  |  |  | 2.4162E6 |  |  |  |  | 1 | 0 | 0 | 0 | 0 | 0 | 0 | 0 | 0 | 1 | 0 | 0 | 0 | 0 | 66 | 79 | Carbamidomethylation | C8:Carbamidomethylation:1000.00 | PEAKS DB |
| K.PLQALLDGK.G | N | 49.53 | 953.5546 | 9 | 0.0 | 477.7845 | 2 | 36.75 | 9 | F9:20556 | NaNaKA16\_F5.raw |  |  |  |  |  |  |  |  | 1.0511E7 |  |  |  |  | 1 | 0 | 0 | 0 | 0 | 0 | 0 | 0 | 0 | 1 | 0 | 0 | 0 | 0 | 95 | 103 |  |  | PEAKS DB |
| K.ILNVLSPR.G | N | 45.28 | 910.5600 | 8 | 0.0 | 456.2873 | 2 | 37.44 | 10 | F10:22021 | NaNaKA16\_F6.raw |  |  |  |  |  |  |  |  |  | 8.1117E5 |  |  |  | 1 | 0 | 0 | 0 | 0 | 0 | 0 | 0 | 0 | 0 | 1 | 0 | 0 | 0 | 228 | 235 |  |  | PEAKS DB |
| total 4 peptides |  |  |  |  |  |  |  |  |  |  |  |  |  |  |  |  |  |  |  |  |  |  |  |  |  |  |  |  |  |  |  |  |  |  |  |  |  |  |  |  |  |  |  |
| --- | --- | --- | --- | --- | --- | --- | --- | --- | --- | --- | --- | --- | --- | --- | --- | --- | --- | --- | --- | --- | --- | --- | --- | --- | --- | --- | --- | --- | --- | --- | --- | --- | --- | --- | --- | --- | --- | --- | --- | --- | --- | --- | --- |

AXL96618.1

back to list

  

| Protein Coverage
| Supporting Peptides
|

Protein Coverage:

Supporting Peptides:

| Peptide | Uniq | -10lgP | Mass | Length | ppm | m/z | z | RT | Fraction | Scan | Source File | Area F1 | Area F10 | Area F11 | Area F12 | Area F13 | Area F2 | Area F3 | Area F4 | Area F5 | Area F6 | Area F7 | Area F8 | Area F9 | #Feature | #Feature F1 | #Feature F10 | #Feature F11 | #Feature F12 | #Feature F13 | #Feature F2 | #Feature F3 | #Feature F4 | #Feature F5 | #Feature F6 | #Feature F7 | #Feature F8 | #Feature F9 | Start | End | PTM | AScore | Found By |
| --- | --- | --- | --- | --- | --- | --- | --- | --- | --- | --- | --- | --- | --- | --- | --- | --- | --- | --- | --- | --- | --- | --- | --- | --- | --- | --- | --- | --- | --- | --- | --- | --- | --- | --- | --- | --- | --- | --- | --- | --- | --- | --- | --- |
| R.AQIC(+57.02)AFWNHFLPK.L | N | 62.23 | 1630.8079 | 13 | 0.8 | 544.6104 | 3 | 69.52 | 3 | F3:44588 | NaNaKA16\_F11.raw |  |  | 9.0724E5 | 2.7007E6 |  |  |  |  |  |  |  |  |  | 2 | 0 | 0 | 1 | 1 | 0 | 0 | 0 | 0 | 0 | 0 | 0 | 0 | 0 | 548 | 560 | Carbamidomethylation | C4:Carbamidomethylation:1000.00 | PEAKS DB |
| K.VYAYLFDHR.A | N | 53.79 | 1182.5822 | 9 | 0.8 | 592.2988 | 2 | 36.66 | 5 | F5:14316 | NaNaKA16\_F13.raw |  |  |  |  | 4.8237E6 |  |  |  |  |  |  |  |  | 2 | 0 | 0 | 0 | 0 | 2 | 0 | 0 | 0 | 0 | 0 | 0 | 0 | 0 | 449 | 457 |  |  | PEAKS DB |
| K.AVTIFGESAGAASVGMH.L | Y | 45.30 | 1603.7664 | 17 | 1.0 | 802.8912 | 2 | 59.66 | 5 | F5:24642 | NaNaKA16\_F13.raw |  |  |  |  | 1.3261E5 |  |  |  |  |  |  |  |  | 1 | 0 | 0 | 0 | 0 | 1 | 0 | 0 | 0 | 0 | 0 | 0 | 0 | 0 | 224 | 240 |  |  | PEAKS DB |
| total 3 peptides |  |  |  |  |  |  |  |  |  |  |  |  |  |  |  |  |  |  |  |  |  |  |  |  |  |  |  |  |  |  |  |  |  |  |  |  |  |  |  |  |  |  |  |
| --- | --- | --- | --- | --- | --- | --- | --- | --- | --- | --- | --- | --- | --- | --- | --- | --- | --- | --- | --- | --- | --- | --- | --- | --- | --- | --- | --- | --- | --- | --- | --- | --- | --- | --- | --- | --- | --- | --- | --- | --- | --- | --- | --- |

JAC96629.1

back to list

  

| Protein Coverage
| Supporting Peptides
|

Protein Coverage:

Supporting Peptides:

| Peptide | Uniq | -10lgP | Mass | Length | ppm | m/z | z | RT | Fraction | Scan | Source File | Area F1 | Area F10 | Area F11 | Area F12 | Area F13 | Area F2 | Area F3 | Area F4 | Area F5 | Area F6 | Area F7 | Area F8 | Area F9 | #Feature | #Feature F1 | #Feature F10 | #Feature F11 | #Feature F12 | #Feature F13 | #Feature F2 | #Feature F3 | #Feature F4 | #Feature F5 | #Feature F6 | #Feature F7 | #Feature F8 | #Feature F9 | Start | End | PTM | AScore | Found By |
| --- | --- | --- | --- | --- | --- | --- | --- | --- | --- | --- | --- | --- | --- | --- | --- | --- | --- | --- | --- | --- | --- | --- | --- | --- | --- | --- | --- | --- | --- | --- | --- | --- | --- | --- | --- | --- | --- | --- | --- | --- | --- | --- | --- |
| R.AQIC(+57.02)AFWNHFLPK.L | N | 62.23 | 1630.8079 | 13 | 0.8 | 544.6104 | 3 | 69.52 | 3 | F3:44588 | NaNaKA16\_F11.raw |  |  | 9.0724E5 | 2.7007E6 |  |  |  |  |  |  |  |  |  | 2 | 0 | 0 | 1 | 1 | 0 | 0 | 0 | 0 | 0 | 0 | 0 | 0 | 0 | 551 | 563 | Carbamidomethylation | C4:Carbamidomethylation:1000.00 | PEAKS DB |
| K.VYAYLFDHR.A | N | 53.79 | 1182.5822 | 9 | 0.8 | 592.2988 | 2 | 36.66 | 5 | F5:14316 | NaNaKA16\_F13.raw |  |  |  |  | 4.8237E6 |  |  |  |  |  |  |  |  | 2 | 0 | 0 | 0 | 0 | 2 | 0 | 0 | 0 | 0 | 0 | 0 | 0 | 0 | 451 | 459 |  |  | PEAKS DB |
| K.AVTIFGESAGAASVGMH.L | Y | 45.30 | 1603.7664 | 17 | 1.0 | 802.8912 | 2 | 59.66 | 5 | F5:24642 | NaNaKA16\_F13.raw |  |  |  |  | 1.3261E5 |  |  |  |  |  |  |  |  | 1 | 0 | 0 | 0 | 0 | 1 | 0 | 0 | 0 | 0 | 0 | 0 | 0 | 0 | 226 | 242 |  |  | PEAKS DB |
| total 3 peptides |  |  |  |  |  |  |  |  |  |  |  |  |  |  |  |  |  |  |  |  |  |  |  |  |  |  |  |  |  |  |  |  |  |  |  |  |  |  |  |  |  |  |  |
| --- | --- | --- | --- | --- | --- | --- | --- | --- | --- | --- | --- | --- | --- | --- | --- | --- | --- | --- | --- | --- | --- | --- | --- | --- | --- | --- | --- | --- | --- | --- | --- | --- | --- | --- | --- | --- | --- | --- | --- | --- | --- | --- | --- |

AHY20009.1

back to list

  

| Protein Coverage
| Supporting Peptides
|

Protein Coverage:

Supporting Peptides:

| Peptide | Uniq | -10lgP | Mass | Length | ppm | m/z | z | RT | Fraction | Scan | Source File | Area F1 | Area F10 | Area F11 | Area F12 | Area F13 | Area F2 | Area F3 | Area F4 | Area F5 | Area F6 | Area F7 | Area F8 | Area F9 | #Feature | #Feature F1 | #Feature F10 | #Feature F11 | #Feature F12 | #Feature F13 | #Feature F2 | #Feature F3 | #Feature F4 | #Feature F5 | #Feature F6 | #Feature F7 | #Feature F8 | #Feature F9 | Start | End | PTM | AScore | Found By |
| --- | --- | --- | --- | --- | --- | --- | --- | --- | --- | --- | --- | --- | --- | --- | --- | --- | --- | --- | --- | --- | --- | --- | --- | --- | --- | --- | --- | --- | --- | --- | --- | --- | --- | --- | --- | --- | --- | --- | --- | --- | --- | --- | --- |
| R.AQIC(+57.02)AFWNHFLPK.L | N | 62.23 | 1630.8079 | 13 | 0.8 | 544.6104 | 3 | 69.52 | 3 | F3:44588 | NaNaKA16\_F11.raw |  |  | 9.0724E5 | 2.7007E6 |  |  |  |  |  |  |  |  |  | 2 | 0 | 0 | 1 | 1 | 0 | 0 | 0 | 0 | 0 | 0 | 0 | 0 | 0 | 551 | 563 | Carbamidomethylation | C4:Carbamidomethylation:1000.00 | PEAKS DB |
| K.VYAYLFDHR.A | N | 53.79 | 1182.5822 | 9 | 0.8 | 592.2988 | 2 | 36.66 | 5 | F5:14316 | NaNaKA16\_F13.raw |  |  |  |  | 4.8237E6 |  |  |  |  |  |  |  |  | 2 | 0 | 0 | 0 | 0 | 2 | 0 | 0 | 0 | 0 | 0 | 0 | 0 | 0 | 451 | 459 |  |  | PEAKS DB |
| K.AVTIFGESAGAASVGMH.L | Y | 45.30 | 1603.7664 | 17 | 1.0 | 802.8912 | 2 | 59.66 | 5 | F5:24642 | NaNaKA16\_F13.raw |  |  |  |  | 1.3261E5 |  |  |  |  |  |  |  |  | 1 | 0 | 0 | 0 | 0 | 1 | 0 | 0 | 0 | 0 | 0 | 0 | 0 | 0 | 226 | 242 |  |  | PEAKS DB |
| total 3 peptides |  |  |  |  |  |  |  |  |  |  |  |  |  |  |  |  |  |  |  |  |  |  |  |  |  |  |  |  |  |  |  |  |  |  |  |  |  |  |  |  |  |  |  |
| --- | --- | --- | --- | --- | --- | --- | --- | --- | --- | --- | --- | --- | --- | --- | --- | --- | --- | --- | --- | --- | --- | --- | --- | --- | --- | --- | --- | --- | --- | --- | --- | --- | --- | --- | --- | --- | --- | --- | --- | --- | --- | --- | --- |

ASU45022.1

back to list

  

| Protein Coverage
| Supporting Peptides
|

Protein Coverage:

Supporting Peptides:

| Peptide | Uniq | -10lgP | Mass | Length | ppm | m/z | z | RT | Fraction | Scan | Source File | Area F1 | Area F10 | Area F11 | Area F12 | Area F13 | Area F2 | Area F3 | Area F4 | Area F5 | Area F6 | Area F7 | Area F8 | Area F9 | #Feature | #Feature F1 | #Feature F10 | #Feature F11 | #Feature F12 | #Feature F13 | #Feature F2 | #Feature F3 | #Feature F4 | #Feature F5 | #Feature F6 | #Feature F7 | #Feature F8 | #Feature F9 | Start | End | PTM | AScore | Found By |
| --- | --- | --- | --- | --- | --- | --- | --- | --- | --- | --- | --- | --- | --- | --- | --- | --- | --- | --- | --- | --- | --- | --- | --- | --- | --- | --- | --- | --- | --- | --- | --- | --- | --- | --- | --- | --- | --- | --- | --- | --- | --- | --- | --- |
| R.AQIC(+57.02)AFWNHFLPK.L | N | 62.23 | 1630.8079 | 13 | 0.8 | 544.6104 | 3 | 69.52 | 3 | F3:44588 | NaNaKA16\_F11.raw |  |  | 9.0724E5 | 2.7007E6 |  |  |  |  |  |  |  |  |  | 2 | 0 | 0 | 1 | 1 | 0 | 0 | 0 | 0 | 0 | 0 | 0 | 0 | 0 | 541 | 553 | Carbamidomethylation | C4:Carbamidomethylation:1000.00 | PEAKS DB |
| K.VYAYLFDHR.A | N | 53.79 | 1182.5822 | 9 | 0.8 | 592.2988 | 2 | 36.66 | 5 | F5:14316 | NaNaKA16\_F13.raw |  |  |  |  | 4.8237E6 |  |  |  |  |  |  |  |  | 2 | 0 | 0 | 0 | 0 | 2 | 0 | 0 | 0 | 0 | 0 | 0 | 0 | 0 | 441 | 449 |  |  | PEAKS DB |
| K.AVTIFGESAGAASVGMH.L | Y | 45.30 | 1603.7664 | 17 | 1.0 | 802.8912 | 2 | 59.66 | 5 | F5:24642 | NaNaKA16\_F13.raw |  |  |  |  | 1.3261E5 |  |  |  |  |  |  |  |  | 1 | 0 | 0 | 0 | 0 | 1 | 0 | 0 | 0 | 0 | 0 | 0 | 0 | 0 | 216 | 232 |  |  | PEAKS DB |
| total 3 peptides |  |  |  |  |  |  |  |  |  |  |  |  |  |  |  |  |  |  |  |  |  |  |  |  |  |  |  |  |  |  |  |  |  |  |  |  |  |  |  |  |  |  |  |
| --- | --- | --- | --- | --- | --- | --- | --- | --- | --- | --- | --- | --- | --- | --- | --- | --- | --- | --- | --- | --- | --- | --- | --- | --- | --- | --- | --- | --- | --- | --- | --- | --- | --- | --- | --- | --- | --- | --- | --- | --- | --- | --- | --- |

XP\_029140716.1

back to list

  

| Protein Coverage
| Supporting Peptides
|

Protein Coverage:

Supporting Peptides:

| Peptide | Uniq | -10lgP | Mass | Length | ppm | m/z | z | RT | Fraction | Scan | Source File | Area F1 | Area F10 | Area F11 | Area F12 | Area F13 | Area F2 | Area F3 | Area F4 | Area F5 | Area F6 | Area F7 | Area F8 | Area F9 | #Feature | #Feature F1 | #Feature F10 | #Feature F11 | #Feature F12 | #Feature F13 | #Feature F2 | #Feature F3 | #Feature F4 | #Feature F5 | #Feature F6 | #Feature F7 | #Feature F8 | #Feature F9 | Start | End | PTM | AScore | Found By |
| --- | --- | --- | --- | --- | --- | --- | --- | --- | --- | --- | --- | --- | --- | --- | --- | --- | --- | --- | --- | --- | --- | --- | --- | --- | --- | --- | --- | --- | --- | --- | --- | --- | --- | --- | --- | --- | --- | --- | --- | --- | --- | --- | --- |
| K.ITANLC(+57.02)MLGALK.C | N | 73.92 | 1303.6992 | 12 | -0.5 | 652.8566 | 2 | 62.39 | 9 | F9:42245 | NaNaKA16\_F5.raw |  |  |  |  |  |  |  |  | 5.2097E6 |  |  |  |  | 1 | 0 | 0 | 0 | 0 | 0 | 0 | 0 | 0 | 1 | 0 | 0 | 0 | 0 | 907 | 918 | Carbamidomethylation | C6:Carbamidomethylation:1000.00 | PEAKS DB |
| R.KITANLC(+57.02)MLGALK.C | N | 58.28 | 1431.7942 | 13 | 0.2 | 478.2721 | 3 | 47.11 | 9 | F9:28940 | NaNaKA16\_F5.raw |  |  |  |  |  |  |  |  | 9.6699E5 |  |  |  |  | 1 | 0 | 0 | 0 | 0 | 0 | 0 | 0 | 0 | 1 | 0 | 0 | 0 | 0 | 906 | 918 | Carbamidomethylation | C7:Carbamidomethylation:1000.00 | PEAKS DB |
| N.TDIVILHEGSC(+57.02)PN | Y | 57.60 | 1453.6871 | 13 | 0.2 | 727.8510 | 2 | 39.23 | 9 | F9:22795 | NaNaKA16\_F5.raw |  |  |  |  |  |  |  |  | 2.402E6 |  |  |  |  | 1 | 0 | 0 | 0 | 0 | 0 | 0 | 0 | 0 | 1 | 0 | 0 | 0 | 0 | 922 | 934 | Carbamidomethylation | C11:Carbamidomethylation:1000.00 | PEAKS DB |
| total 3 peptides |  |  |  |  |  |  |  |  |  |  |  |  |  |  |  |  |  |  |  |  |  |  |  |  |  |  |  |  |  |  |  |  |  |  |  |  |  |  |  |  |  |  |  |
| --- | --- | --- | --- | --- | --- | --- | --- | --- | --- | --- | --- | --- | --- | --- | --- | --- | --- | --- | --- | --- | --- | --- | --- | --- | --- | --- | --- | --- | --- | --- | --- | --- | --- | --- | --- | --- | --- | --- | --- | --- | --- | --- | --- |

XP\_026522231.1

back to list

  

| Protein Coverage
| Supporting Peptides
|

Protein Coverage:

Supporting Peptides:

| Peptide | Uniq | -10lgP | Mass | Length | ppm | m/z | z | RT | Fraction | Scan | Source File | Area F1 | Area F10 | Area F11 | Area F12 | Area F13 | Area F2 | Area F3 | Area F4 | Area F5 | Area F6 | Area F7 | Area F8 | Area F9 | #Feature | #Feature F1 | #Feature F10 | #Feature F11 | #Feature F12 | #Feature F13 | #Feature F2 | #Feature F3 | #Feature F4 | #Feature F5 | #Feature F6 | #Feature F7 | #Feature F8 | #Feature F9 | Start | End | PTM | AScore | Found By |
| --- | --- | --- | --- | --- | --- | --- | --- | --- | --- | --- | --- | --- | --- | --- | --- | --- | --- | --- | --- | --- | --- | --- | --- | --- | --- | --- | --- | --- | --- | --- | --- | --- | --- | --- | --- | --- | --- | --- | --- | --- | --- | --- | --- |
| K.ITANLC(+57.02)MLGALK.C | N | 73.92 | 1303.6992 | 12 | -0.5 | 652.8566 | 2 | 62.39 | 9 | F9:42245 | NaNaKA16\_F5.raw |  |  |  |  |  |  |  |  | 5.2097E6 |  |  |  |  | 1 | 0 | 0 | 0 | 0 | 0 | 0 | 0 | 0 | 1 | 0 | 0 | 0 | 0 | 930 | 941 | Carbamidomethylation | C6:Carbamidomethylation:1000.00 | PEAKS DB |
| R.KITANLC(+57.02)MLGALK.C | N | 58.28 | 1431.7942 | 13 | 0.2 | 478.2721 | 3 | 47.11 | 9 | F9:28940 | NaNaKA16\_F5.raw |  |  |  |  |  |  |  |  | 9.6699E5 |  |  |  |  | 1 | 0 | 0 | 0 | 0 | 0 | 0 | 0 | 0 | 1 | 0 | 0 | 0 | 0 | 929 | 941 | Carbamidomethylation | C7:Carbamidomethylation:1000.00 | PEAKS DB |
| K.TDIVILHEGSC(+57.02)PN | Y | 57.60 | 1453.6871 | 13 | 0.2 | 727.8510 | 2 | 39.23 | 9 | F9:22795 | NaNaKA16\_F5.raw |  |  |  |  |  |  |  |  | 2.402E6 |  |  |  |  | 1 | 0 | 0 | 0 | 0 | 0 | 0 | 0 | 0 | 1 | 0 | 0 | 0 | 0 | 945 | 957 | Carbamidomethylation | C11:Carbamidomethylation:1000.00 | PEAKS DB |
| total 3 peptides |  |  |  |  |  |  |  |  |  |  |  |  |  |  |  |  |  |  |  |  |  |  |  |  |  |  |  |  |  |  |  |  |  |  |  |  |  |  |  |  |  |  |  |
| --- | --- | --- | --- | --- | --- | --- | --- | --- | --- | --- | --- | --- | --- | --- | --- | --- | --- | --- | --- | --- | --- | --- | --- | --- | --- | --- | --- | --- | --- | --- | --- | --- | --- | --- | --- | --- | --- | --- | --- | --- | --- | --- | --- |

XP\_032070501.1

back to list

  

| Protein Coverage
| Supporting Peptides
|

Protein Coverage:

Supporting Peptides:

| Peptide | Uniq | -10lgP | Mass | Length | ppm | m/z | z | RT | Fraction | Scan | Source File | Area F1 | Area F10 | Area F11 | Area F12 | Area F13 | Area F2 | Area F3 | Area F4 | Area F5 | Area F6 | Area F7 | Area F8 | Area F9 | #Feature | #Feature F1 | #Feature F10 | #Feature F11 | #Feature F12 | #Feature F13 | #Feature F2 | #Feature F3 | #Feature F4 | #Feature F5 | #Feature F6 | #Feature F7 | #Feature F8 | #Feature F9 | Start | End | PTM | AScore | Found By |
| --- | --- | --- | --- | --- | --- | --- | --- | --- | --- | --- | --- | --- | --- | --- | --- | --- | --- | --- | --- | --- | --- | --- | --- | --- | --- | --- | --- | --- | --- | --- | --- | --- | --- | --- | --- | --- | --- | --- | --- | --- | --- | --- | --- |
| K.ITANLC(+57.02)MLGALK.C | N | 73.92 | 1303.6992 | 12 | -0.5 | 652.8566 | 2 | 62.39 | 9 | F9:42245 | NaNaKA16\_F5.raw |  |  |  |  |  |  |  |  | 5.2097E6 |  |  |  |  | 1 | 0 | 0 | 0 | 0 | 0 | 0 | 0 | 0 | 1 | 0 | 0 | 0 | 0 | 907 | 918 | Carbamidomethylation | C6:Carbamidomethylation:1000.00 | PEAKS DB |
| R.KITANLC(+57.02)MLGALK.C | N | 58.28 | 1431.7942 | 13 | 0.2 | 478.2721 | 3 | 47.11 | 9 | F9:28940 | NaNaKA16\_F5.raw |  |  |  |  |  |  |  |  | 9.6699E5 |  |  |  |  | 1 | 0 | 0 | 0 | 0 | 0 | 0 | 0 | 0 | 1 | 0 | 0 | 0 | 0 | 906 | 918 | Carbamidomethylation | C7:Carbamidomethylation:1000.00 | PEAKS DB |
| N.TDIVILHEGSC(+57.02)PN | Y | 57.60 | 1453.6871 | 13 | 0.2 | 727.8510 | 2 | 39.23 | 9 | F9:22795 | NaNaKA16\_F5.raw |  |  |  |  |  |  |  |  | 2.402E6 |  |  |  |  | 1 | 0 | 0 | 0 | 0 | 0 | 0 | 0 | 0 | 1 | 0 | 0 | 0 | 0 | 922 | 934 | Carbamidomethylation | C11:Carbamidomethylation:1000.00 | PEAKS DB |
| total 3 peptides |  |  |  |  |  |  |  |  |  |  |  |  |  |  |  |  |  |  |  |  |  |  |  |  |  |  |  |  |  |  |  |  |  |  |  |  |  |  |  |  |  |  |  |
| --- | --- | --- | --- | --- | --- | --- | --- | --- | --- | --- | --- | --- | --- | --- | --- | --- | --- | --- | --- | --- | --- | --- | --- | --- | --- | --- | --- | --- | --- | --- | --- | --- | --- | --- | --- | --- | --- | --- | --- | --- | --- | --- | --- |

AAB32582.1

back to list

  

| Protein Coverage
| Supporting Peptides
|

Protein Coverage:

Supporting Peptides:

| Peptide | Uniq | -10lgP | Mass | Length | ppm | m/z | z | RT | Fraction | Scan | Source File | Area F1 | Area F10 | Area F11 | Area F12 | Area F13 | Area F2 | Area F3 | Area F4 | Area F5 | Area F6 | Area F7 | Area F8 | Area F9 | #Feature | #Feature F1 | #Feature F10 | #Feature F11 | #Feature F12 | #Feature F13 | #Feature F2 | #Feature F3 | #Feature F4 | #Feature F5 | #Feature F6 | #Feature F7 | #Feature F8 | #Feature F9 | Start | End | PTM | AScore | Found By |
| --- | --- | --- | --- | --- | --- | --- | --- | --- | --- | --- | --- | --- | --- | --- | --- | --- | --- | --- | --- | --- | --- | --- | --- | --- | --- | --- | --- | --- | --- | --- | --- | --- | --- | --- | --- | --- | --- | --- | --- | --- | --- | --- | --- |
| K.LGSFDVNVGQNTYVR.G | Y | 85.27 | 1667.8267 | 15 | 1.5 | 834.9219 | 2 | 53.32 | 2 | F2:31321 | NaNaKA16\_F10.raw |  | 2.4785E7 | 9.3413E6 | 4.4857E6 |  |  |  |  |  |  |  |  | 0 | 3 | 0 | 1 | 1 | 1 | 0 | 0 | 0 | 0 | 0 | 0 | 0 | 0 | 0 | 56 | 70 |  |  | PEAKS DB |
| K.EMYPGDIAYNIK.G | Y | 65.81 | 1412.6646 | 12 | 0.2 | 707.3397 | 2 | 52.62 | 4 | F4:32516 | NaNaKA16\_F12.raw |  | 5.6975E6 | 5.1901E6 | 3.4246E6 |  |  |  |  |  |  |  |  |  | 3 | 0 | 1 | 1 | 1 | 0 | 0 | 0 | 0 | 0 | 0 | 0 | 0 | 0 | 133 | 144 |  |  | PEAKS DB |
| K.EM(+15.99)YPGDIAYNIK.G | Y | 45.15 | 1428.6594 | 12 | 0.4 | 715.3373 | 2 | 43.64 | 3 | F3:23989 | NaNaKA16\_F11.raw |  |  | 7.7933E5 |  |  |  |  |  |  |  |  |  |  | 1 | 0 | 0 | 1 | 0 | 0 | 0 | 0 | 0 | 0 | 0 | 0 | 0 | 0 | 133 | 144 | Oxidation (M) | M2:Oxidation (M):1000.00 | PEAKS DB |
| total 3 peptides |  |  |  |  |  |  |  |  |  |  |  |  |  |  |  |  |  |  |  |  |  |  |  |  |  |  |  |  |  |  |  |  |  |  |  |  |  |  |  |  |  |  |  |
| --- | --- | --- | --- | --- | --- | --- | --- | --- | --- | --- | --- | --- | --- | --- | --- | --- | --- | --- | --- | --- | --- | --- | --- | --- | --- | --- | --- | --- | --- | --- | --- | --- | --- | --- | --- | --- | --- | --- | --- | --- | --- | --- | --- |

Q7LZI1.1

back to list

  

| Protein Coverage
| Supporting Peptides
|

Protein Coverage:

Supporting Peptides:

| Peptide | Uniq | -10lgP | Mass | Length | ppm | m/z | z | RT | Fraction | Scan | Source File | Area F1 | Area F10 | Area F11 | Area F12 | Area F13 | Area F2 | Area F3 | Area F4 | Area F5 | Area F6 | Area F7 | Area F8 | Area F9 | #Feature | #Feature F1 | #Feature F10 | #Feature F11 | #Feature F12 | #Feature F13 | #Feature F2 | #Feature F3 | #Feature F4 | #Feature F5 | #Feature F6 | #Feature F7 | #Feature F8 | #Feature F9 | Start | End | PTM | AScore | Found By |
| --- | --- | --- | --- | --- | --- | --- | --- | --- | --- | --- | --- | --- | --- | --- | --- | --- | --- | --- | --- | --- | --- | --- | --- | --- | --- | --- | --- | --- | --- | --- | --- | --- | --- | --- | --- | --- | --- | --- | --- | --- | --- | --- | --- |
| K.LGSFDVNVGQNTYVR.G | Y | 85.27 | 1667.8267 | 15 | 1.5 | 834.9219 | 2 | 53.32 | 2 | F2:31321 | NaNaKA16\_F10.raw |  | 2.4785E7 | 9.3413E6 | 4.4857E6 |  |  |  |  |  |  |  |  | 0 | 3 | 0 | 1 | 1 | 1 | 0 | 0 | 0 | 0 | 0 | 0 | 0 | 0 | 0 | 56 | 70 |  |  | PEAKS DB |
| K.EMYPGDIAYNIK.G | Y | 65.81 | 1412.6646 | 12 | 0.2 | 707.3397 | 2 | 52.62 | 4 | F4:32516 | NaNaKA16\_F12.raw |  | 5.6975E6 | 5.1901E6 | 3.4246E6 |  |  |  |  |  |  |  |  |  | 3 | 0 | 1 | 1 | 1 | 0 | 0 | 0 | 0 | 0 | 0 | 0 | 0 | 0 | 133 | 144 |  |  | PEAKS DB |
| K.EM(+15.99)YPGDIAYNIK.G | Y | 45.15 | 1428.6594 | 12 | 0.4 | 715.3373 | 2 | 43.64 | 3 | F3:23989 | NaNaKA16\_F11.raw |  |  | 7.7933E5 |  |  |  |  |  |  |  |  |  |  | 1 | 0 | 0 | 1 | 0 | 0 | 0 | 0 | 0 | 0 | 0 | 0 | 0 | 0 | 133 | 144 | Oxidation (M) | M2:Oxidation (M):1000.00 | PEAKS DB |
| total 3 peptides |  |  |  |  |  |  |  |  |  |  |  |  |  |  |  |  |  |  |  |  |  |  |  |  |  |  |  |  |  |  |  |  |  |  |  |  |  |  |  |  |  |  |  |
| --- | --- | --- | --- | --- | --- | --- | --- | --- | --- | --- | --- | --- | --- | --- | --- | --- | --- | --- | --- | --- | --- | --- | --- | --- | --- | --- | --- | --- | --- | --- | --- | --- | --- | --- | --- | --- | --- | --- | --- | --- | --- | --- | --- |

ETE59846.1

back to list

  

| Protein Coverage
| Supporting Peptides
|

Protein Coverage:

Supporting Peptides:

| Peptide | Uniq | -10lgP | Mass | Length | ppm | m/z | z | RT | Fraction | Scan | Source File | Area F1 | Area F10 | Area F11 | Area F12 | Area F13 | Area F2 | Area F3 | Area F4 | Area F5 | Area F6 | Area F7 | Area F8 | Area F9 | #Feature | #Feature F1 | #Feature F10 | #Feature F11 | #Feature F12 | #Feature F13 | #Feature F2 | #Feature F3 | #Feature F4 | #Feature F5 | #Feature F6 | #Feature F7 | #Feature F8 | #Feature F9 | Start | End | PTM | AScore | Found By |
| --- | --- | --- | --- | --- | --- | --- | --- | --- | --- | --- | --- | --- | --- | --- | --- | --- | --- | --- | --- | --- | --- | --- | --- | --- | --- | --- | --- | --- | --- | --- | --- | --- | --- | --- | --- | --- | --- | --- | --- | --- | --- | --- | --- |
| K.ESFLFTLTR.N | N | 61.74 | 1112.5865 | 9 | 0.9 | 557.3010 | 2 | 70.76 | 4 | F4:47352 | NaNaKA16\_F12.raw |  | 9.6337E5 |  | 1.0016E7 |  |  |  |  |  |  |  |  |  | 2 | 0 | 1 | 0 | 1 | 0 | 0 | 0 | 0 | 0 | 0 | 0 | 0 | 0 | 327 | 335 |  |  | PEAKS DB |
| R.AALSQYVC(+57.02)EHK.D | N | 61.07 | 1304.6183 | 11 | 0.1 | 653.3165 | 2 | 12.43 | 5 | F5:2258 | NaNaKA16\_F13.raw |  |  |  |  | 1.4233E6 |  |  |  |  |  |  |  |  | 2 | 0 | 0 | 0 | 0 | 2 | 0 | 0 | 0 | 0 | 0 | 0 | 0 | 0 | 257 | 267 | Carbamidomethylation | C8:Carbamidomethylation:1000.00 | PEAKS DB |
| K.LIDLELLHK.Y | Y | 48.33 | 1092.6543 | 9 | -0.4 | 547.3342 | 2 | 51.36 | 4 | F4:31335 | NaNaKA16\_F12.raw |  |  |  | 8.1726E6 |  |  |  |  |  |  |  |  |  | 1 | 0 | 0 | 0 | 1 | 0 | 0 | 0 | 0 | 0 | 0 | 0 | 0 | 0 | 343 | 351 |  |  | PEAKS DB |
| total 3 peptides |  |  |  |  |  |  |  |  |  |  |  |  |  |  |  |  |  |  |  |  |  |  |  |  |  |  |  |  |  |  |  |  |  |  |  |  |  |  |  |  |  |  |  |
| --- | --- | --- | --- | --- | --- | --- | --- | --- | --- | --- | --- | --- | --- | --- | --- | --- | --- | --- | --- | --- | --- | --- | --- | --- | --- | --- | --- | --- | --- | --- | --- | --- | --- | --- | --- | --- | --- | --- | --- | --- | --- | --- | --- |

XP\_026555558.1

back to list

  

| Protein Coverage
| Supporting Peptides
|

Protein Coverage:

Supporting Peptides:

| Peptide | Uniq | -10lgP | Mass | Length | ppm | m/z | z | RT | Fraction | Scan | Source File | Area F1 | Area F10 | Area F11 | Area F12 | Area F13 | Area F2 | Area F3 | Area F4 | Area F5 | Area F6 | Area F7 | Area F8 | Area F9 | #Feature | #Feature F1 | #Feature F10 | #Feature F11 | #Feature F12 | #Feature F13 | #Feature F2 | #Feature F3 | #Feature F4 | #Feature F5 | #Feature F6 | #Feature F7 | #Feature F8 | #Feature F9 | Start | End | PTM | AScore | Found By |
| --- | --- | --- | --- | --- | --- | --- | --- | --- | --- | --- | --- | --- | --- | --- | --- | --- | --- | --- | --- | --- | --- | --- | --- | --- | --- | --- | --- | --- | --- | --- | --- | --- | --- | --- | --- | --- | --- | --- | --- | --- | --- | --- | --- |
| N.VVQEALIPIIPDYK.C | N | 71.29 | 1596.9126 | 14 | 0.6 | 799.4641 | 2 | 76.22 | 4 | F4:51790 | NaNaKA16\_F12.raw |  | 7.806E5 | 3.194E6 | 8.6522E6 |  |  |  |  |  |  |  |  |  | 4 | 0 | 1 | 1 | 2 | 0 | 0 | 0 | 0 | 0 | 0 | 0 | 0 | 0 | 449 | 462 |  |  | PEAKS DB |
| R.SPDEDEQPWC(+57.02)YFIK.D | N | 53.69 | 1812.7665 | 14 | 0.0 | 907.3905 | 2 | 80.04 | 2 | F2:54723 | NaNaKA16\_F10.raw |  | 2.0014E6 |  |  |  |  |  |  |  |  |  |  |  | 1 | 0 | 1 | 0 | 0 | 0 | 0 | 0 | 0 | 0 | 0 | 0 | 0 | 0 | 235 | 248 | Carbamidomethylation | C10:Carbamidomethylation:1000.00 | PEAKS DB |
| A.EISENMFC(+57.02)AGYLDGR.S | Y | 52.55 | 1760.7498 | 15 | 0.7 | 881.3828 | 2 | 64.89 | 4 | F4:42607 | NaNaKA16\_F12.raw |  |  |  | 1.2567E6 |  |  |  |  |  |  |  |  |  | 1 | 0 | 0 | 0 | 1 | 0 | 0 | 0 | 0 | 0 | 0 | 0 | 0 | 0 | 472 | 486 | Carbamidomethylation | C8:Carbamidomethylation:1000.00 | PEAKS DB |
| total 3 peptides |  |  |  |  |  |  |  |  |  |  |  |  |  |  |  |  |  |  |  |  |  |  |  |  |  |  |  |  |  |  |  |  |  |  |  |  |  |  |  |  |  |  |  |
| --- | --- | --- | --- | --- | --- | --- | --- | --- | --- | --- | --- | --- | --- | --- | --- | --- | --- | --- | --- | --- | --- | --- | --- | --- | --- | --- | --- | --- | --- | --- | --- | --- | --- | --- | --- | --- | --- | --- | --- | --- | --- | --- | --- |

XP\_034282171.1

back to list

  

| Protein Coverage
| Supporting Peptides
|

Protein Coverage:

Supporting Peptides:

| Peptide | Uniq | -10lgP | Mass | Length | ppm | m/z | z | RT | Fraction | Scan | Source File | Area F1 | Area F10 | Area F11 | Area F12 | Area F13 | Area F2 | Area F3 | Area F4 | Area F5 | Area F6 | Area F7 | Area F8 | Area F9 | #Feature | #Feature F1 | #Feature F10 | #Feature F11 | #Feature F12 | #Feature F13 | #Feature F2 | #Feature F3 | #Feature F4 | #Feature F5 | #Feature F6 | #Feature F7 | #Feature F8 | #Feature F9 | Start | End | PTM | AScore | Found By |
| --- | --- | --- | --- | --- | --- | --- | --- | --- | --- | --- | --- | --- | --- | --- | --- | --- | --- | --- | --- | --- | --- | --- | --- | --- | --- | --- | --- | --- | --- | --- | --- | --- | --- | --- | --- | --- | --- | --- | --- | --- | --- | --- | --- |
| N.VVQEALIPIIPDYK.C | N | 71.29 | 1596.9126 | 14 | 0.6 | 799.4641 | 2 | 76.22 | 4 | F4:51790 | NaNaKA16\_F12.raw |  | 7.806E5 | 3.194E6 | 8.6522E6 |  |  |  |  |  |  |  |  |  | 4 | 0 | 1 | 1 | 2 | 0 | 0 | 0 | 0 | 0 | 0 | 0 | 0 | 0 | 315 | 328 |  |  | PEAKS DB |
| R.SPDEDEQPWC(+57.02)YFIK.D | N | 53.69 | 1812.7665 | 14 | 0.0 | 907.3905 | 2 | 80.04 | 2 | F2:54723 | NaNaKA16\_F10.raw |  | 2.0014E6 |  |  |  |  |  |  |  |  |  |  |  | 1 | 0 | 1 | 0 | 0 | 0 | 0 | 0 | 0 | 0 | 0 | 0 | 0 | 0 | 101 | 114 | Carbamidomethylation | C10:Carbamidomethylation:1000.00 | PEAKS DB |
| A.EISENMFC(+57.02)AGYLDGR.S | Y | 52.55 | 1760.7498 | 15 | 0.7 | 881.3828 | 2 | 64.89 | 4 | F4:42607 | NaNaKA16\_F12.raw |  |  |  | 1.2567E6 |  |  |  |  |  |  |  |  |  | 1 | 0 | 0 | 0 | 1 | 0 | 0 | 0 | 0 | 0 | 0 | 0 | 0 | 0 | 338 | 352 | Carbamidomethylation | C8:Carbamidomethylation:1000.00 | PEAKS DB |
| total 3 peptides |  |  |  |  |  |  |  |  |  |  |  |  |  |  |  |  |  |  |  |  |  |  |  |  |  |  |  |  |  |  |  |  |  |  |  |  |  |  |  |  |  |  |  |
| --- | --- | --- | --- | --- | --- | --- | --- | --- | --- | --- | --- | --- | --- | --- | --- | --- | --- | --- | --- | --- | --- | --- | --- | --- | --- | --- | --- | --- | --- | --- | --- | --- | --- | --- | --- | --- | --- | --- | --- | --- | --- | --- | --- |

1CDT

back to list

  

| Protein Coverage
| Supporting Peptides
|

Protein Coverage:

Supporting Peptides:

| Peptide | Uniq | -10lgP | Mass | Length | ppm | m/z | z | RT | Fraction | Scan | Source File | Area F1 | Area F10 | Area F11 | Area F12 | Area F13 | Area F2 | Area F3 | Area F4 | Area F5 | Area F6 | Area F7 | Area F8 | Area F9 | #Feature | #Feature F1 | #Feature F10 | #Feature F11 | #Feature F12 | #Feature F13 | #Feature F2 | #Feature F3 | #Feature F4 | #Feature F5 | #Feature F6 | #Feature F7 | #Feature F8 | #Feature F9 | Start | End | PTM | AScore | Found By |
| --- | --- | --- | --- | --- | --- | --- | --- | --- | --- | --- | --- | --- | --- | --- | --- | --- | --- | --- | --- | --- | --- | --- | --- | --- | --- | --- | --- | --- | --- | --- | --- | --- | --- | --- | --- | --- | --- | --- | --- | --- | --- | --- | --- |
| K.C(+57.02)NKLIPIAYK.T | N | 64.76 | 1218.6794 | 10 | 0.0 | 610.3470 | 2 | 27.82 | 11 | F11:13604 | NaNaKA16\_F7.raw |  |  |  |  |  |  |  |  |  | 4.8462E6 | 2.3967E7 | 1.338E6 |  | 6 | 0 | 0 | 0 | 0 | 0 | 0 | 0 | 0 | 0 | 2 | 2 | 2 | 0 | 3 | 12 | Carbamidomethylation | C1:Carbamidomethylation:1000.00 | PEAKS DB |
| K.LIPIAYKTC(+57.02)PEGK.N | Y | 51.73 | 1488.8010 | 13 | -0.2 | 745.4077 | 2 | 37.95 | 10 | F10:22266 | NaNaKA16\_F6.raw |  | 2.4904E8 |  |  |  |  |  |  | 2.7013E6 | 5.7501E7 | 1.6839E6 | 3.5494E6 | 1.9896E6 | 14 | 0 | 8 | 0 | 0 | 0 | 0 | 0 | 0 | 1 | 2 | 1 | 1 | 1 | 6 | 18 | Carbamidomethylation | C9:Carbamidomethylation:1000.00 | PEAKS DB |
| K.LIPIAYK.T | N | 44.45 | 816.5109 | 7 | 0.5 | 409.2629 | 2 | 54.15 | 9 | F9:34957 | NaNaKA16\_F5.raw |  |  |  |  |  | 3.0941E7 |  | 2.6175E7 | 2.3481E7 | 0 | 9.2494E6 | 0 | 0 | 12 | 0 | 0 | 0 | 0 | 0 | 1 | 0 | 2 | 7 | 0 | 2 | 0 | 0 | 6 | 12 |  |  | PEAKS DB |
| total 3 peptides |  |  |  |  |  |  |  |  |  |  |  |  |  |  |  |  |  |  |  |  |  |  |  |  |  |  |  |  |  |  |  |  |  |  |  |  |  |  |  |  |  |  |  |
| --- | --- | --- | --- | --- | --- | --- | --- | --- | --- | --- | --- | --- | --- | --- | --- | --- | --- | --- | --- | --- | --- | --- | --- | --- | --- | --- | --- | --- | --- | --- | --- | --- | --- | --- | --- | --- | --- | --- | --- | --- | --- | --- | --- |

P01452.1

back to list

  

| Protein Coverage
| Supporting Peptides
|

Protein Coverage:

Supporting Peptides:

| Peptide | Uniq | -10lgP | Mass | Length | ppm | m/z | z | RT | Fraction | Scan | Source File | Area F1 | Area F10 | Area F11 | Area F12 | Area F13 | Area F2 | Area F3 | Area F4 | Area F5 | Area F6 | Area F7 | Area F8 | Area F9 | #Feature | #Feature F1 | #Feature F10 | #Feature F11 | #Feature F12 | #Feature F13 | #Feature F2 | #Feature F3 | #Feature F4 | #Feature F5 | #Feature F6 | #Feature F7 | #Feature F8 | #Feature F9 | Start | End | PTM | AScore | Found By |
| --- | --- | --- | --- | --- | --- | --- | --- | --- | --- | --- | --- | --- | --- | --- | --- | --- | --- | --- | --- | --- | --- | --- | --- | --- | --- | --- | --- | --- | --- | --- | --- | --- | --- | --- | --- | --- | --- | --- | --- | --- | --- | --- | --- |
| K.C(+57.02)NKLIPIAYK.T | N | 64.76 | 1218.6794 | 10 | 0.0 | 610.3470 | 2 | 27.82 | 11 | F11:13604 | NaNaKA16\_F7.raw |  |  |  |  |  |  |  |  |  | 4.8462E6 | 2.3967E7 | 1.338E6 |  | 6 | 0 | 0 | 0 | 0 | 0 | 0 | 0 | 0 | 0 | 2 | 2 | 2 | 0 | 3 | 12 | Carbamidomethylation | C1:Carbamidomethylation:1000.00 | PEAKS DB |
| K.LIPIAYKTC(+57.02)PEGK.N | Y | 51.73 | 1488.8010 | 13 | -0.2 | 745.4077 | 2 | 37.95 | 10 | F10:22266 | NaNaKA16\_F6.raw |  | 2.4904E8 |  |  |  |  |  |  | 2.7013E6 | 5.7501E7 | 1.6839E6 | 3.5494E6 | 1.9896E6 | 14 | 0 | 8 | 0 | 0 | 0 | 0 | 0 | 0 | 1 | 2 | 1 | 1 | 1 | 6 | 18 | Carbamidomethylation | C9:Carbamidomethylation:1000.00 | PEAKS DB |
| K.LIPIAYK.T | N | 44.45 | 816.5109 | 7 | 0.5 | 409.2629 | 2 | 54.15 | 9 | F9:34957 | NaNaKA16\_F5.raw |  |  |  |  |  | 3.0941E7 |  | 2.6175E7 | 2.3481E7 | 0 | 9.2494E6 | 0 | 0 | 12 | 0 | 0 | 0 | 0 | 0 | 1 | 0 | 2 | 7 | 0 | 2 | 0 | 0 | 6 | 12 |  |  | PEAKS DB |
| total 3 peptides |  |  |  |  |  |  |  |  |  |  |  |  |  |  |  |  |  |  |  |  |  |  |  |  |  |  |  |  |  |  |  |  |  |  |  |  |  |  |  |  |  |  |  |
| --- | --- | --- | --- | --- | --- | --- | --- | --- | --- | --- | --- | --- | --- | --- | --- | --- | --- | --- | --- | --- | --- | --- | --- | --- | --- | --- | --- | --- | --- | --- | --- | --- | --- | --- | --- | --- | --- | --- | --- | --- | --- | --- | --- |

XP\_013925940.1

back to list

  

| Protein Coverage
| Supporting Peptides
|

Protein Coverage:

Supporting Peptides:

| Peptide | Uniq | -10lgP | Mass | Length | ppm | m/z | z | RT | Fraction | Scan | Source File | Area F1 | Area F10 | Area F11 | Area F12 | Area F13 | Area F2 | Area F3 | Area F4 | Area F5 | Area F6 | Area F7 | Area F8 | Area F9 | #Feature | #Feature F1 | #Feature F10 | #Feature F11 | #Feature F12 | #Feature F13 | #Feature F2 | #Feature F3 | #Feature F4 | #Feature F5 | #Feature F6 | #Feature F7 | #Feature F8 | #Feature F9 | Start | End | PTM | AScore | Found By |
| --- | --- | --- | --- | --- | --- | --- | --- | --- | --- | --- | --- | --- | --- | --- | --- | --- | --- | --- | --- | --- | --- | --- | --- | --- | --- | --- | --- | --- | --- | --- | --- | --- | --- | --- | --- | --- | --- | --- | --- | --- | --- | --- | --- |
| I.TLDNDIMLIK.L | N | 66.86 | 1174.6267 | 10 | 0.5 | 588.3209 | 2 | 56.32 | 9 | F9:36929 | NaNaKA16\_F5.raw | 1.0093E6 |  |  | 2.61E6 | 4.6175E6 | 4.0613E7 | 4.4662E6 | 1.8032E6 | 4.6095E6 | 3.5083E6 |  |  |  | 10 | 1 | 0 | 0 | 1 | 1 | 1 | 1 | 2 | 2 | 1 | 0 | 0 | 0 | 105 | 114 |  |  | PEAKS DB |
| I.TLDNDIM(+15.99)LIK.L | N | 54.91 | 1190.6217 | 10 | 0.0 | 596.3181 | 2 | 48.16 | 6 | F6:30625 | NaNaKA16\_F2.raw |  |  |  |  |  | 1.1327E7 |  |  |  |  |  |  |  | 2 | 0 | 0 | 0 | 0 | 0 | 2 | 0 | 0 | 0 | 0 | 0 | 0 | 0 | 105 | 114 | Oxidation (M) | M7:Oxidation (M):1000.00 | PEAKS DB |
| G.ELQGIVSWGR.G | Y | 48.20 | 1143.6036 | 10 | 0.1 | 572.8091 | 2 | 48.96 | 4 | F4:29286 | NaNaKA16\_F12.raw |  |  |  | 3.8043E5 |  |  |  |  |  |  |  |  |  | 1 | 0 | 0 | 0 | 1 | 0 | 0 | 0 | 0 | 0 | 0 | 0 | 0 | 0 | 211 | 220 |  |  | PEAKS DB |
| L.DNDIMLIK.L | N | 42.58 | 960.4950 | 8 | 0.7 | 481.2551 | 2 | 48.07 | 6 | F6:30522 | NaNaKA16\_F2.raw |  |  |  |  |  | 8.6792E5 |  |  |  |  |  |  |  | 1 | 0 | 0 | 0 | 0 | 0 | 1 | 0 | 0 | 0 | 0 | 0 | 0 | 0 | 107 | 114 |  |  | PEAKS DB |
| total 4 peptides |  |  |  |  |  |  |  |  |  |  |  |  |  |  |  |  |  |  |  |  |  |  |  |  |  |  |  |  |  |  |  |  |  |  |  |  |  |  |  |  |  |  |  |
| --- | --- | --- | --- | --- | --- | --- | --- | --- | --- | --- | --- | --- | --- | --- | --- | --- | --- | --- | --- | --- | --- | --- | --- | --- | --- | --- | --- | --- | --- | --- | --- | --- | --- | --- | --- | --- | --- | --- | --- | --- | --- | --- | --- |

XP\_029139862.1

back to list

  

| Protein Coverage
| Supporting Peptides
|

Protein Coverage:

Supporting Peptides:

| Peptide | Uniq | -10lgP | Mass | Length | ppm | m/z | z | RT | Fraction | Scan | Source File | Area F1 | Area F10 | Area F11 | Area F12 | Area F13 | Area F2 | Area F3 | Area F4 | Area F5 | Area F6 | Area F7 | Area F8 | Area F9 | #Feature | #Feature F1 | #Feature F10 | #Feature F11 | #Feature F12 | #Feature F13 | #Feature F2 | #Feature F3 | #Feature F4 | #Feature F5 | #Feature F6 | #Feature F7 | #Feature F8 | #Feature F9 | Start | End | PTM | AScore | Found By |
| --- | --- | --- | --- | --- | --- | --- | --- | --- | --- | --- | --- | --- | --- | --- | --- | --- | --- | --- | --- | --- | --- | --- | --- | --- | --- | --- | --- | --- | --- | --- | --- | --- | --- | --- | --- | --- | --- | --- | --- | --- | --- | --- | --- |
| R.TLDNDIMLIK.L | N | 66.86 | 1174.6267 | 10 | 0.5 | 588.3209 | 2 | 56.32 | 9 | F9:36929 | NaNaKA16\_F5.raw | 1.0093E6 |  |  | 2.61E6 | 4.6175E6 | 4.0613E7 | 4.4662E6 | 1.8032E6 | 4.6095E6 | 3.5083E6 |  |  |  | 10 | 1 | 0 | 0 | 1 | 1 | 1 | 1 | 2 | 2 | 1 | 0 | 0 | 0 | 150 | 159 |  |  | PEAKS DB |
| R.TLDNDIM(+15.99)LIK.L | N | 54.91 | 1190.6217 | 10 | 0.0 | 596.3181 | 2 | 48.16 | 6 | F6:30625 | NaNaKA16\_F2.raw |  |  |  |  |  | 1.1327E7 |  |  |  |  |  |  |  | 2 | 0 | 0 | 0 | 0 | 0 | 2 | 0 | 0 | 0 | 0 | 0 | 0 | 0 | 150 | 159 | Oxidation (M) | M7:Oxidation (M):1000.00 | PEAKS DB |
| G.ELQGIVSWGR.G | Y | 48.20 | 1143.6036 | 10 | 0.1 | 572.8091 | 2 | 48.96 | 4 | F4:29286 | NaNaKA16\_F12.raw |  |  |  | 3.8043E5 |  |  |  |  |  |  |  |  |  | 1 | 0 | 0 | 0 | 1 | 0 | 0 | 0 | 0 | 0 | 0 | 0 | 0 | 0 | 256 | 265 |  |  | PEAKS DB |
| L.DNDIMLIK.L | N | 42.58 | 960.4950 | 8 | 0.7 | 481.2551 | 2 | 48.07 | 6 | F6:30522 | NaNaKA16\_F2.raw |  |  |  |  |  | 8.6792E5 |  |  |  |  |  |  |  | 1 | 0 | 0 | 0 | 0 | 0 | 1 | 0 | 0 | 0 | 0 | 0 | 0 | 0 | 152 | 159 |  |  | PEAKS DB |
| total 4 peptides |  |  |  |  |  |  |  |  |  |  |  |  |  |  |  |  |  |  |  |  |  |  |  |  |  |  |  |  |  |  |  |  |  |  |  |  |  |  |  |  |  |  |  |
| --- | --- | --- | --- | --- | --- | --- | --- | --- | --- | --- | --- | --- | --- | --- | --- | --- | --- | --- | --- | --- | --- | --- | --- | --- | --- | --- | --- | --- | --- | --- | --- | --- | --- | --- | --- | --- | --- | --- | --- | --- | --- | --- | --- |

XP\_015670838.1

back to list

  

| Protein Coverage
| Supporting Peptides
|

Protein Coverage:

Supporting Peptides:

| Peptide | Uniq | -10lgP | Mass | Length | ppm | m/z | z | RT | Fraction | Scan | Source File | Area F1 | Area F10 | Area F11 | Area F12 | Area F13 | Area F2 | Area F3 | Area F4 | Area F5 | Area F6 | Area F7 | Area F8 | Area F9 | #Feature | #Feature F1 | #Feature F10 | #Feature F11 | #Feature F12 | #Feature F13 | #Feature F2 | #Feature F3 | #Feature F4 | #Feature F5 | #Feature F6 | #Feature F7 | #Feature F8 | #Feature F9 | Start | End | PTM | AScore | Found By |
| --- | --- | --- | --- | --- | --- | --- | --- | --- | --- | --- | --- | --- | --- | --- | --- | --- | --- | --- | --- | --- | --- | --- | --- | --- | --- | --- | --- | --- | --- | --- | --- | --- | --- | --- | --- | --- | --- | --- | --- | --- | --- | --- | --- |
| R.TLDNDIMLIK.L | N | 66.86 | 1174.6267 | 10 | 0.5 | 588.3209 | 2 | 56.32 | 9 | F9:36929 | NaNaKA16\_F5.raw | 1.0093E6 |  |  | 2.61E6 | 4.6175E6 | 4.0613E7 | 4.4662E6 | 1.8032E6 | 4.6095E6 | 3.5083E6 |  |  |  | 10 | 1 | 0 | 0 | 1 | 1 | 1 | 1 | 2 | 2 | 1 | 0 | 0 | 0 | 105 | 114 |  |  | PEAKS DB |
| R.TLDNDIM(+15.99)LIK.L | N | 54.91 | 1190.6217 | 10 | 0.0 | 596.3181 | 2 | 48.16 | 6 | F6:30625 | NaNaKA16\_F2.raw |  |  |  |  |  | 1.1327E7 |  |  |  |  |  |  |  | 2 | 0 | 0 | 0 | 0 | 0 | 2 | 0 | 0 | 0 | 0 | 0 | 0 | 0 | 105 | 114 | Oxidation (M) | M7:Oxidation (M):1000.00 | PEAKS DB |
| G.ELQGIVSWGR.G | Y | 48.20 | 1143.6036 | 10 | 0.1 | 572.8091 | 2 | 48.96 | 4 | F4:29286 | NaNaKA16\_F12.raw |  |  |  | 3.8043E5 |  |  |  |  |  |  |  |  |  | 1 | 0 | 0 | 0 | 1 | 0 | 0 | 0 | 0 | 0 | 0 | 0 | 0 | 0 | 211 | 220 |  |  | PEAKS DB |
| L.DNDIMLIK.L | N | 42.58 | 960.4950 | 8 | 0.7 | 481.2551 | 2 | 48.07 | 6 | F6:30522 | NaNaKA16\_F2.raw |  |  |  |  |  | 8.6792E5 |  |  |  |  |  |  |  | 1 | 0 | 0 | 0 | 0 | 0 | 1 | 0 | 0 | 0 | 0 | 0 | 0 | 0 | 107 | 114 |  |  | PEAKS DB |
| total 4 peptides |  |  |  |  |  |  |  |  |  |  |  |  |  |  |  |  |  |  |  |  |  |  |  |  |  |  |  |  |  |  |  |  |  |  |  |  |  |  |  |  |  |  |  |
| --- | --- | --- | --- | --- | --- | --- | --- | --- | --- | --- | --- | --- | --- | --- | --- | --- | --- | --- | --- | --- | --- | --- | --- | --- | --- | --- | --- | --- | --- | --- | --- | --- | --- | --- | --- | --- | --- | --- | --- | --- | --- | --- | --- |

ETE63009.1

back to list

  

| Protein Coverage
| Supporting Peptides
|

Protein Coverage:

Supporting Peptides:

| Peptide | Uniq | -10lgP | Mass | Length | ppm | m/z | z | RT | Fraction | Scan | Source File | Area F1 | Area F10 | Area F11 | Area F12 | Area F13 | Area F2 | Area F3 | Area F4 | Area F5 | Area F6 | Area F7 | Area F8 | Area F9 | #Feature | #Feature F1 | #Feature F10 | #Feature F11 | #Feature F12 | #Feature F13 | #Feature F2 | #Feature F3 | #Feature F4 | #Feature F5 | #Feature F6 | #Feature F7 | #Feature F8 | #Feature F9 | Start | End | PTM | AScore | Found By |
| --- | --- | --- | --- | --- | --- | --- | --- | --- | --- | --- | --- | --- | --- | --- | --- | --- | --- | --- | --- | --- | --- | --- | --- | --- | --- | --- | --- | --- | --- | --- | --- | --- | --- | --- | --- | --- | --- | --- | --- | --- | --- | --- | --- |
| R.QGEALNQLELQFTR.C | Y | 84.93 | 1645.8423 | 14 | 1.2 | 823.9294 | 2 | 65.43 | 3 | F3:40903 | NaNaKA16\_F11.raw |  | 4.1542E7 | 1.6447E7 | 4.5596E6 |  |  |  |  |  |  |  |  |  | 6 | 0 | 2 | 2 | 2 | 0 | 0 | 0 | 0 | 0 | 0 | 0 | 0 | 0 | 136 | 149 |  |  | PEAKS DB |
| K.SQFITAFC(+57.02)SSQR.R | N | 53.09 | 1430.6613 | 12 | 0.0 | 716.3379 | 2 | 41.78 | 2 | F2:22780 | NaNaKA16\_F10.raw |  | 3.9839E6 | 4.2283E6 |  |  |  |  |  |  |  |  |  |  | 2 | 0 | 1 | 1 | 0 | 0 | 0 | 0 | 0 | 0 | 0 | 0 | 0 | 0 | 239 | 250 | Carbamidomethylation | C8:Carbamidomethylation:1000.00 | PEAKS DB |
| total 2 peptides |  |  |  |  |  |  |  |  |  |  |  |  |  |  |  |  |  |  |  |  |  |  |  |  |  |  |  |  |  |  |  |  |  |  |  |  |  |  |  |  |  |  |  |
| --- | --- | --- | --- | --- | --- | --- | --- | --- | --- | --- | --- | --- | --- | --- | --- | --- | --- | --- | --- | --- | --- | --- | --- | --- | --- | --- | --- | --- | --- | --- | --- | --- | --- | --- | --- | --- | --- | --- | --- | --- | --- | --- | --- |

XP\_034280930.1

back to list

  

| Protein Coverage
| Supporting Peptides
|

Protein Coverage:

Supporting Peptides:

| Peptide | Uniq | -10lgP | Mass | Length | ppm | m/z | z | RT | Fraction | Scan | Source File | Area F1 | Area F10 | Area F11 | Area F12 | Area F13 | Area F2 | Area F3 | Area F4 | Area F5 | Area F6 | Area F7 | Area F8 | Area F9 | #Feature | #Feature F1 | #Feature F10 | #Feature F11 | #Feature F12 | #Feature F13 | #Feature F2 | #Feature F3 | #Feature F4 | #Feature F5 | #Feature F6 | #Feature F7 | #Feature F8 | #Feature F9 | Start | End | PTM | AScore | Found By |
| --- | --- | --- | --- | --- | --- | --- | --- | --- | --- | --- | --- | --- | --- | --- | --- | --- | --- | --- | --- | --- | --- | --- | --- | --- | --- | --- | --- | --- | --- | --- | --- | --- | --- | --- | --- | --- | --- | --- | --- | --- | --- | --- | --- |
| R.QGEALNQLELQFTR.C | Y | 84.93 | 1645.8423 | 14 | 1.2 | 823.9294 | 2 | 65.43 | 3 | F3:40903 | NaNaKA16\_F11.raw |  | 4.1542E7 | 1.6447E7 | 4.5596E6 |  |  |  |  |  |  |  |  |  | 6 | 0 | 2 | 2 | 2 | 0 | 0 | 0 | 0 | 0 | 0 | 0 | 0 | 0 | 156 | 169 |  |  | PEAKS DB |
| K.SQFITAFC(+57.02)SSQR.R | N | 53.09 | 1430.6613 | 12 | 0.0 | 716.3379 | 2 | 41.78 | 2 | F2:22780 | NaNaKA16\_F10.raw |  | 3.9839E6 | 4.2283E6 |  |  |  |  |  |  |  |  |  |  | 2 | 0 | 1 | 1 | 0 | 0 | 0 | 0 | 0 | 0 | 0 | 0 | 0 | 0 | 290 | 301 | Carbamidomethylation | C8:Carbamidomethylation:1000.00 | PEAKS DB |
| total 2 peptides |  |  |  |  |  |  |  |  |  |  |  |  |  |  |  |  |  |  |  |  |  |  |  |  |  |  |  |  |  |  |  |  |  |  |  |  |  |  |  |  |  |  |  |
| --- | --- | --- | --- | --- | --- | --- | --- | --- | --- | --- | --- | --- | --- | --- | --- | --- | --- | --- | --- | --- | --- | --- | --- | --- | --- | --- | --- | --- | --- | --- | --- | --- | --- | --- | --- | --- | --- | --- | --- | --- | --- | --- | --- |

ETE67452.1

back to list

  

| Protein Coverage
| Supporting Peptides
|

Protein Coverage:

Supporting Peptides:

| Peptide | Uniq | -10lgP | Mass | Length | ppm | m/z | z | RT | Fraction | Scan | Source File | Area F1 | Area F10 | Area F11 | Area F12 | Area F13 | Area F2 | Area F3 | Area F4 | Area F5 | Area F6 | Area F7 | Area F8 | Area F9 | #Feature | #Feature F1 | #Feature F10 | #Feature F11 | #Feature F12 | #Feature F13 | #Feature F2 | #Feature F3 | #Feature F4 | #Feature F5 | #Feature F6 | #Feature F7 | #Feature F8 | #Feature F9 | Start | End | PTM | AScore | Found By |
| --- | --- | --- | --- | --- | --- | --- | --- | --- | --- | --- | --- | --- | --- | --- | --- | --- | --- | --- | --- | --- | --- | --- | --- | --- | --- | --- | --- | --- | --- | --- | --- | --- | --- | --- | --- | --- | --- | --- | --- | --- | --- | --- | --- |
| K.NVGREEMEQIFR.M | Y | 63.24 | 1506.7249 | 12 | 1.4 | 503.2496 | 3 | 38.31 | 3 | F3:19369 | NaNaKA16\_F11.raw |  |  | 1.6125E6 | 9.1581E5 |  |  |  |  |  |  |  |  |  | 2 | 0 | 0 | 1 | 1 | 0 | 0 | 0 | 0 | 0 | 0 | 0 | 0 | 0 | 242 | 253 |  |  | PEAKS DB |
| K.YLHYDPITSR.Q | Y | 62.44 | 1263.6248 | 10 | -0.1 | 632.8196 | 2 | 23.18 | 10 | F10:10782 | NaNaKA16\_F6.raw |  |  |  |  |  |  |  |  |  | 7.4787E7 |  |  |  | 3 | 0 | 0 | 0 | 0 | 0 | 0 | 0 | 0 | 0 | 3 | 0 | 0 | 0 | 28 | 37 |  |  | PEAKS DB |
| R.YLNIEFC(+57.02)LR.H | Y | 49.96 | 1226.6117 | 9 | -0.2 | 614.3130 | 2 | 67.95 | 10 | F10:48613 | NaNaKA16\_F6.raw |  |  |  |  |  |  |  |  |  | 5.7078E6 |  |  |  | 1 | 0 | 0 | 0 | 0 | 0 | 0 | 0 | 0 | 0 | 1 | 0 | 0 | 0 | 112 | 120 | Carbamidomethylation | C7:Carbamidomethylation:1000.00 | PEAKS DB |
| total 3 peptides |  |  |  |  |  |  |  |  |  |  |  |  |  |  |  |  |  |  |  |  |  |  |  |  |  |  |  |  |  |  |  |  |  |  |  |  |  |  |  |  |  |  |  |
| --- | --- | --- | --- | --- | --- | --- | --- | --- | --- | --- | --- | --- | --- | --- | --- | --- | --- | --- | --- | --- | --- | --- | --- | --- | --- | --- | --- | --- | --- | --- | --- | --- | --- | --- | --- | --- | --- | --- | --- | --- | --- | --- | --- |

XP\_026576072.1

back to list

  

| Protein Coverage
| Supporting Peptides
|

Protein Coverage:

Supporting Peptides:

| Peptide | Uniq | -10lgP | Mass | Length | ppm | m/z | z | RT | Fraction | Scan | Source File | Area F1 | Area F10 | Area F11 | Area F12 | Area F13 | Area F2 | Area F3 | Area F4 | Area F5 | Area F6 | Area F7 | Area F8 | Area F9 | #Feature | #Feature F1 | #Feature F10 | #Feature F11 | #Feature F12 | #Feature F13 | #Feature F2 | #Feature F3 | #Feature F4 | #Feature F5 | #Feature F6 | #Feature F7 | #Feature F8 | #Feature F9 | Start | End | PTM | AScore | Found By |
| --- | --- | --- | --- | --- | --- | --- | --- | --- | --- | --- | --- | --- | --- | --- | --- | --- | --- | --- | --- | --- | --- | --- | --- | --- | --- | --- | --- | --- | --- | --- | --- | --- | --- | --- | --- | --- | --- | --- | --- | --- | --- | --- | --- |
| R.SYLGHVSLLR.R | Y | 70.48 | 1143.6400 | 10 | 1.0 | 572.8279 | 2 | 35.15 | 4 | F4:17845 | NaNaKA16\_F12.raw |  | 1.3801E5 |  | 5.5659E6 |  |  |  |  |  |  |  |  |  | 3 | 0 | 1 | 0 | 2 | 0 | 0 | 0 | 0 | 0 | 0 | 0 | 0 | 0 | 322 | 331 |  |  | PEAKS DB |
| K.SQFITAFC(+57.02)SSQR.R | N | 53.09 | 1430.6613 | 12 | 0.0 | 716.3379 | 2 | 41.78 | 2 | F2:22780 | NaNaKA16\_F10.raw |  | 3.9839E6 | 4.2283E6 |  |  |  |  |  |  |  |  |  |  | 2 | 0 | 1 | 1 | 0 | 0 | 0 | 0 | 0 | 0 | 0 | 0 | 0 | 0 | 286 | 297 | Carbamidomethylation | C8:Carbamidomethylation:1000.00 | PEAKS DB |
| total 2 peptides |  |  |  |  |  |  |  |  |  |  |  |  |  |  |  |  |  |  |  |  |  |  |  |  |  |  |  |  |  |  |  |  |  |  |  |  |  |  |  |  |  |  |  |
| --- | --- | --- | --- | --- | --- | --- | --- | --- | --- | --- | --- | --- | --- | --- | --- | --- | --- | --- | --- | --- | --- | --- | --- | --- | --- | --- | --- | --- | --- | --- | --- | --- | --- | --- | --- | --- | --- | --- | --- | --- | --- | --- | --- |

XP\_026576070.1

back to list

  

| Protein Coverage
| Supporting Peptides
|

Protein Coverage:

Supporting Peptides:

| Peptide | Uniq | -10lgP | Mass | Length | ppm | m/z | z | RT | Fraction | Scan | Source File | Area F1 | Area F10 | Area F11 | Area F12 | Area F13 | Area F2 | Area F3 | Area F4 | Area F5 | Area F6 | Area F7 | Area F8 | Area F9 | #Feature | #Feature F1 | #Feature F10 | #Feature F11 | #Feature F12 | #Feature F13 | #Feature F2 | #Feature F3 | #Feature F4 | #Feature F5 | #Feature F6 | #Feature F7 | #Feature F8 | #Feature F9 | Start | End | PTM | AScore | Found By |
| --- | --- | --- | --- | --- | --- | --- | --- | --- | --- | --- | --- | --- | --- | --- | --- | --- | --- | --- | --- | --- | --- | --- | --- | --- | --- | --- | --- | --- | --- | --- | --- | --- | --- | --- | --- | --- | --- | --- | --- | --- | --- | --- | --- |
| R.SYLGHVSLLR.R | Y | 70.48 | 1143.6400 | 10 | 1.0 | 572.8279 | 2 | 35.15 | 4 | F4:17845 | NaNaKA16\_F12.raw |  | 1.3801E5 |  | 5.5659E6 |  |  |  |  |  |  |  |  |  | 3 | 0 | 1 | 0 | 2 | 0 | 0 | 0 | 0 | 0 | 0 | 0 | 0 | 0 | 323 | 332 |  |  | PEAKS DB |
| K.SQFITAFC(+57.02)SSQR.R | N | 53.09 | 1430.6613 | 12 | 0.0 | 716.3379 | 2 | 41.78 | 2 | F2:22780 | NaNaKA16\_F10.raw |  | 3.9839E6 | 4.2283E6 |  |  |  |  |  |  |  |  |  |  | 2 | 0 | 1 | 1 | 0 | 0 | 0 | 0 | 0 | 0 | 0 | 0 | 0 | 0 | 287 | 298 | Carbamidomethylation | C8:Carbamidomethylation:1000.00 | PEAKS DB |
| total 2 peptides |  |  |  |  |  |  |  |  |  |  |  |  |  |  |  |  |  |  |  |  |  |  |  |  |  |  |  |  |  |  |  |  |  |  |  |  |  |  |  |  |  |  |  |
| --- | --- | --- | --- | --- | --- | --- | --- | --- | --- | --- | --- | --- | --- | --- | --- | --- | --- | --- | --- | --- | --- | --- | --- | --- | --- | --- | --- | --- | --- | --- | --- | --- | --- | --- | --- | --- | --- | --- | --- | --- | --- | --- | --- |

JAG67228.1

back to list

  

| Protein Coverage
| Supporting Peptides
|

Protein Coverage:

Supporting Peptides:

| Peptide | Uniq | -10lgP | Mass | Length | ppm | m/z | z | RT | Fraction | Scan | Source File | Area F1 | Area F10 | Area F11 | Area F12 | Area F13 | Area F2 | Area F3 | Area F4 | Area F5 | Area F6 | Area F7 | Area F8 | Area F9 | #Feature | #Feature F1 | #Feature F10 | #Feature F11 | #Feature F12 | #Feature F13 | #Feature F2 | #Feature F3 | #Feature F4 | #Feature F5 | #Feature F6 | #Feature F7 | #Feature F8 | #Feature F9 | Start | End | PTM | AScore | Found By |
| --- | --- | --- | --- | --- | --- | --- | --- | --- | --- | --- | --- | --- | --- | --- | --- | --- | --- | --- | --- | --- | --- | --- | --- | --- | --- | --- | --- | --- | --- | --- | --- | --- | --- | --- | --- | --- | --- | --- | --- | --- | --- | --- | --- |
| K.IANIVFANSGLK.M | N | 53.29 | 1245.7081 | 12 | -0.5 | 623.8610 | 2 | 51.01 | 5 | F5:20652 | NaNaKA16\_F13.raw |  |  |  |  | 7.0934E5 |  |  |  |  |  |  |  |  | 1 | 0 | 0 | 0 | 0 | 1 | 0 | 0 | 0 | 0 | 0 | 0 | 0 | 0 | 109 | 120 |  |  | PEAKS DB |
| R.SENIYVSQLLQK.A | Y | 52.64 | 1420.7561 | 12 | 0.9 | 711.3860 | 2 | 55.38 | 5 | F5:22493 | NaNaKA16\_F13.raw |  |  |  |  | 5.1493E5 |  |  |  |  |  |  |  |  | 1 | 0 | 0 | 0 | 0 | 1 | 0 | 0 | 0 | 0 | 0 | 0 | 0 | 0 | 329 | 340 |  |  | PEAKS DB |
| K.FTAEAETDLKEPLK.E | N | 48.53 | 1590.8141 | 14 | 0.3 | 531.2788 | 3 | 30.97 | 5 | F5:10833 | NaNaKA16\_F13.raw |  |  |  |  | 3.3213E5 |  |  |  |  |  |  |  |  | 1 | 0 | 0 | 0 | 0 | 1 | 0 | 0 | 0 | 0 | 0 | 0 | 0 | 0 | 295 | 308 |  |  | PEAKS DB |
| R.NSEVFQC(+57.02)PVK.N | N | 48.20 | 1206.5703 | 10 | 1.0 | 604.2930 | 2 | 22.94 | 5 | F5:6259 | NaNaKA16\_F13.raw |  |  |  |  | 3.6129E5 |  |  |  |  |  |  |  |  | 1 | 0 | 0 | 0 | 0 | 1 | 0 | 0 | 0 | 0 | 0 | 0 | 0 | 0 | 129 | 138 | Carbamidomethylation | C7:Carbamidomethylation:1000.00 | PEAKS DB |
| total 4 peptides |  |  |  |  |  |  |  |  |  |  |  |  |  |  |  |  |  |  |  |  |  |  |  |  |  |  |  |  |  |  |  |  |  |  |  |  |  |  |  |  |  |  |  |
| --- | --- | --- | --- | --- | --- | --- | --- | --- | --- | --- | --- | --- | --- | --- | --- | --- | --- | --- | --- | --- | --- | --- | --- | --- | --- | --- | --- | --- | --- | --- | --- | --- | --- | --- | --- | --- | --- | --- | --- | --- | --- | --- | --- |

XP\_034295297.1

back to list

  

| Protein Coverage
| Supporting Peptides
|

Protein Coverage:

Supporting Peptides:

| Peptide | Uniq | -10lgP | Mass | Length | ppm | m/z | z | RT | Fraction | Scan | Source File | Area F1 | Area F10 | Area F11 | Area F12 | Area F13 | Area F2 | Area F3 | Area F4 | Area F5 | Area F6 | Area F7 | Area F8 | Area F9 | #Feature | #Feature F1 | #Feature F10 | #Feature F11 | #Feature F12 | #Feature F13 | #Feature F2 | #Feature F3 | #Feature F4 | #Feature F5 | #Feature F6 | #Feature F7 | #Feature F8 | #Feature F9 | Start | End | PTM | AScore | Found By |
| --- | --- | --- | --- | --- | --- | --- | --- | --- | --- | --- | --- | --- | --- | --- | --- | --- | --- | --- | --- | --- | --- | --- | --- | --- | --- | --- | --- | --- | --- | --- | --- | --- | --- | --- | --- | --- | --- | --- | --- | --- | --- | --- | --- |
| K.IANIVFANSGLK.M | N | 53.29 | 1245.7081 | 12 | -0.5 | 623.8610 | 2 | 51.01 | 5 | F5:20652 | NaNaKA16\_F13.raw |  |  |  |  | 7.0934E5 |  |  |  |  |  |  |  |  | 1 | 0 | 0 | 0 | 0 | 1 | 0 | 0 | 0 | 0 | 0 | 0 | 0 | 0 | 109 | 120 |  |  | PEAKS DB |
| R.SENIYVSQLLQK.A | Y | 52.64 | 1420.7561 | 12 | 0.9 | 711.3860 | 2 | 55.38 | 5 | F5:22493 | NaNaKA16\_F13.raw |  |  |  |  | 5.1493E5 |  |  |  |  |  |  |  |  | 1 | 0 | 0 | 0 | 0 | 1 | 0 | 0 | 0 | 0 | 0 | 0 | 0 | 0 | 329 | 340 |  |  | PEAKS DB |
| K.FTAEAETDLKEPLK.E | N | 48.53 | 1590.8141 | 14 | 0.3 | 531.2788 | 3 | 30.97 | 5 | F5:10833 | NaNaKA16\_F13.raw |  |  |  |  | 3.3213E5 |  |  |  |  |  |  |  |  | 1 | 0 | 0 | 0 | 0 | 1 | 0 | 0 | 0 | 0 | 0 | 0 | 0 | 0 | 295 | 308 |  |  | PEAKS DB |
| R.NSEVFQC(+57.02)PVK.N | N | 48.20 | 1206.5703 | 10 | 1.0 | 604.2930 | 2 | 22.94 | 5 | F5:6259 | NaNaKA16\_F13.raw |  |  |  |  | 3.6129E5 |  |  |  |  |  |  |  |  | 1 | 0 | 0 | 0 | 0 | 1 | 0 | 0 | 0 | 0 | 0 | 0 | 0 | 0 | 129 | 138 | Carbamidomethylation | C7:Carbamidomethylation:1000.00 | PEAKS DB |
| total 4 peptides |  |  |  |  |  |  |  |  |  |  |  |  |  |  |  |  |  |  |  |  |  |  |  |  |  |  |  |  |  |  |  |  |  |  |  |  |  |  |  |  |  |  |  |
| --- | --- | --- | --- | --- | --- | --- | --- | --- | --- | --- | --- | --- | --- | --- | --- | --- | --- | --- | --- | --- | --- | --- | --- | --- | --- | --- | --- | --- | --- | --- | --- | --- | --- | --- | --- | --- | --- | --- | --- | --- | --- | --- | --- |

P82462.1

back to list

  

| Protein Coverage
| Supporting Peptides
|

Protein Coverage:

Supporting Peptides:

| Peptide | Uniq | -10lgP | Mass | Length | ppm | m/z | z | RT | Fraction | Scan | Source File | Area F1 | Area F10 | Area F11 | Area F12 | Area F13 | Area F2 | Area F3 | Area F4 | Area F5 | Area F6 | Area F7 | Area F8 | Area F9 | #Feature | #Feature F1 | #Feature F10 | #Feature F11 | #Feature F12 | #Feature F13 | #Feature F2 | #Feature F3 | #Feature F4 | #Feature F5 | #Feature F6 | #Feature F7 | #Feature F8 | #Feature F9 | Start | End | PTM | AScore | Found By |
| --- | --- | --- | --- | --- | --- | --- | --- | --- | --- | --- | --- | --- | --- | --- | --- | --- | --- | --- | --- | --- | --- | --- | --- | --- | --- | --- | --- | --- | --- | --- | --- | --- | --- | --- | --- | --- | --- | --- | --- | --- | --- | --- | --- |
| K.FLFSETTETC(+57.02)PDGQNVC(+57.02)FNQAHLIYPGK.Y | Y | 74.46 | 3272.4907 | 28 | 0.8 | 1091.8384 | 3 | 69.74 | 11 | F11:47367 | NaNaKA16\_F7.raw |  |  |  |  |  |  |  |  |  |  | 4.7405E7 | 4.4988E7 |  | 2 | 0 | 0 | 0 | 0 | 0 | 0 | 0 | 0 | 0 | 0 | 1 | 1 | 0 | 8 | 35 | Carbamidomethylation | C10:Carbamidomethylation:1000.00;C17:Carbamidomethylation:1000.00 | PEAKS DB |
| total 1 peptides |  |  |  |  |  |  |  |  |  |  |  |  |  |  |  |  |  |  |  |  |  |  |  |  |  |  |  |  |  |  |  |  |  |  |  |  |  |  |  |  |  |  |  |
| --- | --- | --- | --- | --- | --- | --- | --- | --- | --- | --- | --- | --- | --- | --- | --- | --- | --- | --- | --- | --- | --- | --- | --- | --- | --- | --- | --- | --- | --- | --- | --- | --- | --- | --- | --- | --- | --- | --- | --- | --- | --- | --- | --- |

BAN08536.1

back to list

  

| Protein Coverage
| Supporting Peptides
|

Protein Coverage:

Supporting Peptides:

| Peptide | Uniq | -10lgP | Mass | Length | ppm | m/z | z | RT | Fraction | Scan | Source File | Area F1 | Area F10 | Area F11 | Area F12 | Area F13 | Area F2 | Area F3 | Area F4 | Area F5 | Area F6 | Area F7 | Area F8 | Area F9 | #Feature | #Feature F1 | #Feature F10 | #Feature F11 | #Feature F12 | #Feature F13 | #Feature F2 | #Feature F3 | #Feature F4 | #Feature F5 | #Feature F6 | #Feature F7 | #Feature F8 | #Feature F9 | Start | End | PTM | AScore | Found By |
| --- | --- | --- | --- | --- | --- | --- | --- | --- | --- | --- | --- | --- | --- | --- | --- | --- | --- | --- | --- | --- | --- | --- | --- | --- | --- | --- | --- | --- | --- | --- | --- | --- | --- | --- | --- | --- | --- | --- | --- | --- | --- | --- | --- |
| R.SAAIC(+57.02)FAGAPYNK.E | Y | 76.75 | 1368.6495 | 13 | 0.2 | 685.3322 | 2 | 36.97 | 10 | F10:21587 | NaNaKA16\_F6.raw |  |  |  |  |  |  |  |  |  | 7.9588E6 |  |  | 1.268E6 | 2 | 0 | 0 | 0 | 0 | 0 | 0 | 0 | 0 | 0 | 1 | 0 | 0 | 1 | 127 | 139 | Carbamidomethylation | C5:Carbamidomethylation:1000.00 | PEAKS DB |
| R.NLLQFSNMIK.C | N | 56.92 | 1206.6431 | 10 | 0.5 | 604.3291 | 2 | 65.68 | 2 | F2:42174 | NaNaKA16\_F10.raw |  | 8.2085E6 | 1.7232E6 |  |  |  |  |  |  |  |  |  |  | 2 | 0 | 1 | 1 | 0 | 0 | 0 | 0 | 0 | 0 | 0 | 0 | 0 | 0 | 27 | 36 |  |  | PEAKS DB |
| total 2 peptides |  |  |  |  |  |  |  |  |  |  |  |  |  |  |  |  |  |  |  |  |  |  |  |  |  |  |  |  |  |  |  |  |  |  |  |  |  |  |  |  |  |  |  |
| --- | --- | --- | --- | --- | --- | --- | --- | --- | --- | --- | --- | --- | --- | --- | --- | --- | --- | --- | --- | --- | --- | --- | --- | --- | --- | --- | --- | --- | --- | --- | --- | --- | --- | --- | --- | --- | --- | --- | --- | --- | --- | --- | --- |

BAM76245.1

back to list

  

| Protein Coverage
| Supporting Peptides
|

Protein Coverage:

Supporting Peptides:

| Peptide | Uniq | -10lgP | Mass | Length | ppm | m/z | z | RT | Fraction | Scan | Source File | Area F1 | Area F10 | Area F11 | Area F12 | Area F13 | Area F2 | Area F3 | Area F4 | Area F5 | Area F6 | Area F7 | Area F8 | Area F9 | #Feature | #Feature F1 | #Feature F10 | #Feature F11 | #Feature F12 | #Feature F13 | #Feature F2 | #Feature F3 | #Feature F4 | #Feature F5 | #Feature F6 | #Feature F7 | #Feature F8 | #Feature F9 | Start | End | PTM | AScore | Found By |
| --- | --- | --- | --- | --- | --- | --- | --- | --- | --- | --- | --- | --- | --- | --- | --- | --- | --- | --- | --- | --- | --- | --- | --- | --- | --- | --- | --- | --- | --- | --- | --- | --- | --- | --- | --- | --- | --- | --- | --- | --- | --- | --- | --- |
| R.SAAIC(+57.02)FAGAPYNK.E | Y | 76.75 | 1368.6495 | 13 | 0.2 | 685.3322 | 2 | 36.97 | 10 | F10:21587 | NaNaKA16\_F6.raw |  |  |  |  |  |  |  |  |  | 7.9588E6 |  |  | 1.268E6 | 2 | 0 | 0 | 0 | 0 | 0 | 0 | 0 | 0 | 0 | 1 | 0 | 0 | 1 | 127 | 139 | Carbamidomethylation | C5:Carbamidomethylation:1000.00 | PEAKS DB |
| R.NLLQFSNMIK.C | N | 56.92 | 1206.6431 | 10 | 0.5 | 604.3291 | 2 | 65.68 | 2 | F2:42174 | NaNaKA16\_F10.raw |  | 8.2085E6 | 1.7232E6 |  |  |  |  |  |  |  |  |  |  | 2 | 0 | 1 | 1 | 0 | 0 | 0 | 0 | 0 | 0 | 0 | 0 | 0 | 0 | 27 | 36 |  |  | PEAKS DB |
| total 2 peptides |  |  |  |  |  |  |  |  |  |  |  |  |  |  |  |  |  |  |  |  |  |  |  |  |  |  |  |  |  |  |  |  |  |  |  |  |  |  |  |  |  |  |  |
| --- | --- | --- | --- | --- | --- | --- | --- | --- | --- | --- | --- | --- | --- | --- | --- | --- | --- | --- | --- | --- | --- | --- | --- | --- | --- | --- | --- | --- | --- | --- | --- | --- | --- | --- | --- | --- | --- | --- | --- | --- | --- | --- | --- |

XP\_034292109.1

back to list

  

| Protein Coverage
| Supporting Peptides
|

Protein Coverage:

Supporting Peptides:

| Peptide | Uniq | -10lgP | Mass | Length | ppm | m/z | z | RT | Fraction | Scan | Source File | Area F1 | Area F10 | Area F11 | Area F12 | Area F13 | Area F2 | Area F3 | Area F4 | Area F5 | Area F6 | Area F7 | Area F8 | Area F9 | #Feature | #Feature F1 | #Feature F10 | #Feature F11 | #Feature F12 | #Feature F13 | #Feature F2 | #Feature F3 | #Feature F4 | #Feature F5 | #Feature F6 | #Feature F7 | #Feature F8 | #Feature F9 | Start | End | PTM | AScore | Found By |
| --- | --- | --- | --- | --- | --- | --- | --- | --- | --- | --- | --- | --- | --- | --- | --- | --- | --- | --- | --- | --- | --- | --- | --- | --- | --- | --- | --- | --- | --- | --- | --- | --- | --- | --- | --- | --- | --- | --- | --- | --- | --- | --- | --- |
| K.EC(+57.02)PLGSHAC(+57.02)GPC(+57.02)LPQFVEDR.E | Y | 82.45 | 2328.0085 | 20 | 5.3 | 777.0103 | 3 | 51.07 | 7 | F7:28442 | NaNaKA16\_F3.raw |  |  |  |  |  | 1.2208E7 | 3.9264E7 | 4.6158E7 | 4.2897E6 |  |  |  |  | 6 | 0 | 0 | 0 | 0 | 0 | 1 | 2 | 2 | 1 | 0 | 0 | 0 | 0 | 56 | 75 | Carbamidomethylation | C2:Carbamidomethylation:1000.00;C9:Carbamidomethylation:1000.00;C12:Carbamidomethylation:1000.00 | PEAKS DB |
| R.KEC(+57.02)PLGSHAC(+57.02)GPC(+57.02)LPQFVEDR.E | Y | 42.19 | 2456.1035 | 21 | -0.3 | 615.0330 | 4 | 42.77 | 6 | F6:26226 | NaNaKA16\_F2.raw |  |  |  |  |  | 8.3877E5 |  |  |  |  |  |  |  | 1 | 0 | 0 | 0 | 0 | 0 | 1 | 0 | 0 | 0 | 0 | 0 | 0 | 0 | 55 | 75 | Carbamidomethylation | C3:Carbamidomethylation:1000.00;C10:Carbamidomethylation:1000.00;C13:Carbamidomethylation:1000.00 | PEAKS DB |
| total 2 peptides |  |  |  |  |  |  |  |  |  |  |  |  |  |  |  |  |  |  |  |  |  |  |  |  |  |  |  |  |  |  |  |  |  |  |  |  |  |  |  |  |  |  |  |
| --- | --- | --- | --- | --- | --- | --- | --- | --- | --- | --- | --- | --- | --- | --- | --- | --- | --- | --- | --- | --- | --- | --- | --- | --- | --- | --- | --- | --- | --- | --- | --- | --- | --- | --- | --- | --- | --- | --- | --- | --- | --- | --- | --- |

JAB53674.1

back to list

  

| Protein Coverage
| Supporting Peptides
|

Protein Coverage:

Supporting Peptides:

| Peptide | Uniq | -10lgP | Mass | Length | ppm | m/z | z | RT | Fraction | Scan | Source File | Area F1 | Area F10 | Area F11 | Area F12 | Area F13 | Area F2 | Area F3 | Area F4 | Area F5 | Area F6 | Area F7 | Area F8 | Area F9 | #Feature | #Feature F1 | #Feature F10 | #Feature F11 | #Feature F12 | #Feature F13 | #Feature F2 | #Feature F3 | #Feature F4 | #Feature F5 | #Feature F6 | #Feature F7 | #Feature F8 | #Feature F9 | Start | End | PTM | AScore | Found By |
| --- | --- | --- | --- | --- | --- | --- | --- | --- | --- | --- | --- | --- | --- | --- | --- | --- | --- | --- | --- | --- | --- | --- | --- | --- | --- | --- | --- | --- | --- | --- | --- | --- | --- | --- | --- | --- | --- | --- | --- | --- | --- | --- | --- |
| R.SVEEAIPAVC(+57.02)K.T | Y | 59.34 | 1201.6012 | 11 | 0.8 | 601.8083 | 2 | 28.40 | 10 | F10:14244 | NaNaKA16\_F6.raw |  |  |  |  |  |  |  |  |  | 1.8435E6 |  |  |  | 1 | 0 | 0 | 0 | 0 | 0 | 0 | 0 | 0 | 0 | 1 | 0 | 0 | 0 | 85 | 95 | Carbamidomethylation | C10:Carbamidomethylation:1000.00 | PEAKS DB |
| R.SQIDPTSANFLIWPPC(+57.02)VEVK.R | Y | 55.24 | 2300.1511 | 20 | -0.4 | 1151.0824 | 2 | 93.15 | 12 | F12:67124 | NaNaKA16\_F8.raw |  |  |  |  |  |  |  |  |  |  |  | 1.4123E7 |  | 2 | 0 | 0 | 0 | 0 | 0 | 0 | 0 | 0 | 0 | 0 | 0 | 2 | 0 | 106 | 125 | Carbamidomethylation | C16:Carbamidomethylation:1000.00 | PEAKS DB |
| R.TVIYEIPR.S | Y | 41.82 | 989.5546 | 8 | -0.1 | 495.7845 | 2 | 37.13 | 10 | F10:21430 | NaNaKA16\_F6.raw |  |  |  |  |  |  |  |  |  | 3.051E6 |  |  |  | 1 | 0 | 0 | 0 | 0 | 0 | 0 | 0 | 0 | 0 | 1 | 0 | 0 | 0 | 98 | 105 |  |  | PEAKS DB |
| total 3 peptides |  |  |  |  |  |  |  |  |  |  |  |  |  |  |  |  |  |  |  |  |  |  |  |  |  |  |  |  |  |  |  |  |  |  |  |  |  |  |  |  |  |  |  |
| --- | --- | --- | --- | --- | --- | --- | --- | --- | --- | --- | --- | --- | --- | --- | --- | --- | --- | --- | --- | --- | --- | --- | --- | --- | --- | --- | --- | --- | --- | --- | --- | --- | --- | --- | --- | --- | --- | --- | --- | --- | --- | --- | --- |

LAB42085.1

back to list

  

| Protein Coverage
| Supporting Peptides
|

Protein Coverage:

Supporting Peptides:

| Peptide | Uniq | -10lgP | Mass | Length | ppm | m/z | z | RT | Fraction | Scan | Source File | Area F1 | Area F10 | Area F11 | Area F12 | Area F13 | Area F2 | Area F3 | Area F4 | Area F5 | Area F6 | Area F7 | Area F8 | Area F9 | #Feature | #Feature F1 | #Feature F10 | #Feature F11 | #Feature F12 | #Feature F13 | #Feature F2 | #Feature F3 | #Feature F4 | #Feature F5 | #Feature F6 | #Feature F7 | #Feature F8 | #Feature F9 | Start | End | PTM | AScore | Found By |
| --- | --- | --- | --- | --- | --- | --- | --- | --- | --- | --- | --- | --- | --- | --- | --- | --- | --- | --- | --- | --- | --- | --- | --- | --- | --- | --- | --- | --- | --- | --- | --- | --- | --- | --- | --- | --- | --- | --- | --- | --- | --- | --- | --- |
| R.SVEEAIPAVC(+57.02)K.T | Y | 59.34 | 1201.6012 | 11 | 0.8 | 601.8083 | 2 | 28.40 | 10 | F10:14244 | NaNaKA16\_F6.raw |  |  |  |  |  |  |  |  |  | 1.8435E6 |  |  |  | 1 | 0 | 0 | 0 | 0 | 0 | 0 | 0 | 0 | 0 | 1 | 0 | 0 | 0 | 118 | 128 | Carbamidomethylation | C10:Carbamidomethylation:1000.00 | PEAKS DB |
| R.SQIDPTSANFLIWPPC(+57.02)VEVK.R | Y | 55.24 | 2300.1511 | 20 | -0.4 | 1151.0824 | 2 | 93.15 | 12 | F12:67124 | NaNaKA16\_F8.raw |  |  |  |  |  |  |  |  |  |  |  | 1.4123E7 |  | 2 | 0 | 0 | 0 | 0 | 0 | 0 | 0 | 0 | 0 | 0 | 0 | 2 | 0 | 139 | 158 | Carbamidomethylation | C16:Carbamidomethylation:1000.00 | PEAKS DB |
| R.TVIYEIPR.S | Y | 41.82 | 989.5546 | 8 | -0.1 | 495.7845 | 2 | 37.13 | 10 | F10:21430 | NaNaKA16\_F6.raw |  |  |  |  |  |  |  |  |  | 3.051E6 |  |  |  | 1 | 0 | 0 | 0 | 0 | 0 | 0 | 0 | 0 | 0 | 1 | 0 | 0 | 0 | 131 | 138 |  |  | PEAKS DB |
| total 3 peptides |  |  |  |  |  |  |  |  |  |  |  |  |  |  |  |  |  |  |  |  |  |  |  |  |  |  |  |  |  |  |  |  |  |  |  |  |  |  |  |  |  |  |  |
| --- | --- | --- | --- | --- | --- | --- | --- | --- | --- | --- | --- | --- | --- | --- | --- | --- | --- | --- | --- | --- | --- | --- | --- | --- | --- | --- | --- | --- | --- | --- | --- | --- | --- | --- | --- | --- | --- | --- | --- | --- | --- | --- | --- |

LAB42082.1

back to list

  

| Protein Coverage
| Supporting Peptides
|

Protein Coverage:

Supporting Peptides:

| Peptide | Uniq | -10lgP | Mass | Length | ppm | m/z | z | RT | Fraction | Scan | Source File | Area F1 | Area F10 | Area F11 | Area F12 | Area F13 | Area F2 | Area F3 | Area F4 | Area F5 | Area F6 | Area F7 | Area F8 | Area F9 | #Feature | #Feature F1 | #Feature F10 | #Feature F11 | #Feature F12 | #Feature F13 | #Feature F2 | #Feature F3 | #Feature F4 | #Feature F5 | #Feature F6 | #Feature F7 | #Feature F8 | #Feature F9 | Start | End | PTM | AScore | Found By |
| --- | --- | --- | --- | --- | --- | --- | --- | --- | --- | --- | --- | --- | --- | --- | --- | --- | --- | --- | --- | --- | --- | --- | --- | --- | --- | --- | --- | --- | --- | --- | --- | --- | --- | --- | --- | --- | --- | --- | --- | --- | --- | --- | --- |
| R.SVEEAIPAVC(+57.02)K.T | Y | 59.34 | 1201.6012 | 11 | 0.8 | 601.8083 | 2 | 28.40 | 10 | F10:14244 | NaNaKA16\_F6.raw |  |  |  |  |  |  |  |  |  | 1.8435E6 |  |  |  | 1 | 0 | 0 | 0 | 0 | 0 | 0 | 0 | 0 | 0 | 1 | 0 | 0 | 0 | 118 | 128 | Carbamidomethylation | C10:Carbamidomethylation:1000.00 | PEAKS DB |
| R.SQIDPTSANFLIWPPC(+57.02)VEVK.R | Y | 55.24 | 2300.1511 | 20 | -0.4 | 1151.0824 | 2 | 93.15 | 12 | F12:67124 | NaNaKA16\_F8.raw |  |  |  |  |  |  |  |  |  |  |  | 1.4123E7 |  | 2 | 0 | 0 | 0 | 0 | 0 | 0 | 0 | 0 | 0 | 0 | 0 | 2 | 0 | 139 | 158 | Carbamidomethylation | C16:Carbamidomethylation:1000.00 | PEAKS DB |
| R.TVIYEIPR.S | Y | 41.82 | 989.5546 | 8 | -0.1 | 495.7845 | 2 | 37.13 | 10 | F10:21430 | NaNaKA16\_F6.raw |  |  |  |  |  |  |  |  |  | 3.051E6 |  |  |  | 1 | 0 | 0 | 0 | 0 | 0 | 0 | 0 | 0 | 0 | 1 | 0 | 0 | 0 | 131 | 138 |  |  | PEAKS DB |
| total 3 peptides |  |  |  |  |  |  |  |  |  |  |  |  |  |  |  |  |  |  |  |  |  |  |  |  |  |  |  |  |  |  |  |  |  |  |  |  |  |  |  |  |  |  |  |
| --- | --- | --- | --- | --- | --- | --- | --- | --- | --- | --- | --- | --- | --- | --- | --- | --- | --- | --- | --- | --- | --- | --- | --- | --- | --- | --- | --- | --- | --- | --- | --- | --- | --- | --- | --- | --- | --- | --- | --- | --- | --- | --- | --- |

XP\_026560089.1

back to list

  

| Protein Coverage
| Supporting Peptides
|

Protein Coverage:

Supporting Peptides:

| Peptide | Uniq | -10lgP | Mass | Length | ppm | m/z | z | RT | Fraction | Scan | Source File | Area F1 | Area F10 | Area F11 | Area F12 | Area F13 | Area F2 | Area F3 | Area F4 | Area F5 | Area F6 | Area F7 | Area F8 | Area F9 | #Feature | #Feature F1 | #Feature F10 | #Feature F11 | #Feature F12 | #Feature F13 | #Feature F2 | #Feature F3 | #Feature F4 | #Feature F5 | #Feature F6 | #Feature F7 | #Feature F8 | #Feature F9 | Start | End | PTM | AScore | Found By |
| --- | --- | --- | --- | --- | --- | --- | --- | --- | --- | --- | --- | --- | --- | --- | --- | --- | --- | --- | --- | --- | --- | --- | --- | --- | --- | --- | --- | --- | --- | --- | --- | --- | --- | --- | --- | --- | --- | --- | --- | --- | --- | --- | --- |
| R.SVEEAIPAVC(+57.02)K.T | Y | 59.34 | 1201.6012 | 11 | 0.8 | 601.8083 | 2 | 28.40 | 10 | F10:14244 | NaNaKA16\_F6.raw |  |  |  |  |  |  |  |  |  | 1.8435E6 |  |  |  | 1 | 0 | 0 | 0 | 0 | 0 | 0 | 0 | 0 | 0 | 1 | 0 | 0 | 0 | 86 | 96 | Carbamidomethylation | C10:Carbamidomethylation:1000.00 | PEAKS DB |
| R.SQIDPTSANFLIWPPC(+57.02)VEVK.R | Y | 55.24 | 2300.1511 | 20 | -0.4 | 1151.0824 | 2 | 93.15 | 12 | F12:67124 | NaNaKA16\_F8.raw |  |  |  |  |  |  |  |  |  |  |  | 1.4123E7 |  | 2 | 0 | 0 | 0 | 0 | 0 | 0 | 0 | 0 | 0 | 0 | 0 | 2 | 0 | 107 | 126 | Carbamidomethylation | C16:Carbamidomethylation:1000.00 | PEAKS DB |
| R.TVIYEIPR.S | Y | 41.82 | 989.5546 | 8 | -0.1 | 495.7845 | 2 | 37.13 | 10 | F10:21430 | NaNaKA16\_F6.raw |  |  |  |  |  |  |  |  |  | 3.051E6 |  |  |  | 1 | 0 | 0 | 0 | 0 | 0 | 0 | 0 | 0 | 0 | 1 | 0 | 0 | 0 | 99 | 106 |  |  | PEAKS DB |
| total 3 peptides |  |  |  |  |  |  |  |  |  |  |  |  |  |  |  |  |  |  |  |  |  |  |  |  |  |  |  |  |  |  |  |  |  |  |  |  |  |  |  |  |  |  |  |
| --- | --- | --- | --- | --- | --- | --- | --- | --- | --- | --- | --- | --- | --- | --- | --- | --- | --- | --- | --- | --- | --- | --- | --- | --- | --- | --- | --- | --- | --- | --- | --- | --- | --- | --- | --- | --- | --- | --- | --- | --- | --- | --- | --- |

XP\_026560087.1

back to list

  

| Protein Coverage
| Supporting Peptides
|

Protein Coverage:

Supporting Peptides:

| Peptide | Uniq | -10lgP | Mass | Length | ppm | m/z | z | RT | Fraction | Scan | Source File | Area F1 | Area F10 | Area F11 | Area F12 | Area F13 | Area F2 | Area F3 | Area F4 | Area F5 | Area F6 | Area F7 | Area F8 | Area F9 | #Feature | #Feature F1 | #Feature F10 | #Feature F11 | #Feature F12 | #Feature F13 | #Feature F2 | #Feature F3 | #Feature F4 | #Feature F5 | #Feature F6 | #Feature F7 | #Feature F8 | #Feature F9 | Start | End | PTM | AScore | Found By |
| --- | --- | --- | --- | --- | --- | --- | --- | --- | --- | --- | --- | --- | --- | --- | --- | --- | --- | --- | --- | --- | --- | --- | --- | --- | --- | --- | --- | --- | --- | --- | --- | --- | --- | --- | --- | --- | --- | --- | --- | --- | --- | --- | --- |
| R.SVEEAIPAVC(+57.02)K.T | Y | 59.34 | 1201.6012 | 11 | 0.8 | 601.8083 | 2 | 28.40 | 10 | F10:14244 | NaNaKA16\_F6.raw |  |  |  |  |  |  |  |  |  | 1.8435E6 |  |  |  | 1 | 0 | 0 | 0 | 0 | 0 | 0 | 0 | 0 | 0 | 1 | 0 | 0 | 0 | 86 | 96 | Carbamidomethylation | C10:Carbamidomethylation:1000.00 | PEAKS DB |
| R.SQIDPTSANFLIWPPC(+57.02)VEVK.R | Y | 55.24 | 2300.1511 | 20 | -0.4 | 1151.0824 | 2 | 93.15 | 12 | F12:67124 | NaNaKA16\_F8.raw |  |  |  |  |  |  |  |  |  |  |  | 1.4123E7 |  | 2 | 0 | 0 | 0 | 0 | 0 | 0 | 0 | 0 | 0 | 0 | 0 | 2 | 0 | 107 | 126 | Carbamidomethylation | C16:Carbamidomethylation:1000.00 | PEAKS DB |
| R.TVIYEIPR.S | Y | 41.82 | 989.5546 | 8 | -0.1 | 495.7845 | 2 | 37.13 | 10 | F10:21430 | NaNaKA16\_F6.raw |  |  |  |  |  |  |  |  |  | 3.051E6 |  |  |  | 1 | 0 | 0 | 0 | 0 | 0 | 0 | 0 | 0 | 0 | 1 | 0 | 0 | 0 | 99 | 106 |  |  | PEAKS DB |
| total 3 peptides |  |  |  |  |  |  |  |  |  |  |  |  |  |  |  |  |  |  |  |  |  |  |  |  |  |  |  |  |  |  |  |  |  |  |  |  |  |  |  |  |  |  |  |
| --- | --- | --- | --- | --- | --- | --- | --- | --- | --- | --- | --- | --- | --- | --- | --- | --- | --- | --- | --- | --- | --- | --- | --- | --- | --- | --- | --- | --- | --- | --- | --- | --- | --- | --- | --- | --- | --- | --- | --- | --- | --- | --- | --- |

LAA51478.1

back to list

  

| Protein Coverage
| Supporting Peptides
|

Protein Coverage:

Supporting Peptides:

| Peptide | Uniq | -10lgP | Mass | Length | ppm | m/z | z | RT | Fraction | Scan | Source File | Area F1 | Area F10 | Area F11 | Area F12 | Area F13 | Area F2 | Area F3 | Area F4 | Area F5 | Area F6 | Area F7 | Area F8 | Area F9 | #Feature | #Feature F1 | #Feature F10 | #Feature F11 | #Feature F12 | #Feature F13 | #Feature F2 | #Feature F3 | #Feature F4 | #Feature F5 | #Feature F6 | #Feature F7 | #Feature F8 | #Feature F9 | Start | End | PTM | AScore | Found By |
| --- | --- | --- | --- | --- | --- | --- | --- | --- | --- | --- | --- | --- | --- | --- | --- | --- | --- | --- | --- | --- | --- | --- | --- | --- | --- | --- | --- | --- | --- | --- | --- | --- | --- | --- | --- | --- | --- | --- | --- | --- | --- | --- | --- |
| R.SVEEAIPAVC(+57.02)K.T | Y | 59.34 | 1201.6012 | 11 | 0.8 | 601.8083 | 2 | 28.40 | 10 | F10:14244 | NaNaKA16\_F6.raw |  |  |  |  |  |  |  |  |  | 1.8435E6 |  |  |  | 1 | 0 | 0 | 0 | 0 | 0 | 0 | 0 | 0 | 0 | 1 | 0 | 0 | 0 | 9 | 19 | Carbamidomethylation | C10:Carbamidomethylation:1000.00 | PEAKS DB |
| R.SQIDPTSANFLIWPPC(+57.02)VEVK.R | Y | 55.24 | 2300.1511 | 20 | -0.4 | 1151.0824 | 2 | 93.15 | 12 | F12:67124 | NaNaKA16\_F8.raw |  |  |  |  |  |  |  |  |  |  |  | 1.4123E7 |  | 2 | 0 | 0 | 0 | 0 | 0 | 0 | 0 | 0 | 0 | 0 | 0 | 2 | 0 | 30 | 49 | Carbamidomethylation | C16:Carbamidomethylation:1000.00 | PEAKS DB |
| R.TVIYEIPR.S | Y | 41.82 | 989.5546 | 8 | -0.1 | 495.7845 | 2 | 37.13 | 10 | F10:21430 | NaNaKA16\_F6.raw |  |  |  |  |  |  |  |  |  | 3.051E6 |  |  |  | 1 | 0 | 0 | 0 | 0 | 0 | 0 | 0 | 0 | 0 | 1 | 0 | 0 | 0 | 22 | 29 |  |  | PEAKS DB |
| total 3 peptides |  |  |  |  |  |  |  |  |  |  |  |  |  |  |  |  |  |  |  |  |  |  |  |  |  |  |  |  |  |  |  |  |  |  |  |  |  |  |  |  |  |  |  |
| --- | --- | --- | --- | --- | --- | --- | --- | --- | --- | --- | --- | --- | --- | --- | --- | --- | --- | --- | --- | --- | --- | --- | --- | --- | --- | --- | --- | --- | --- | --- | --- | --- | --- | --- | --- | --- | --- | --- | --- | --- | --- | --- | --- |

XP\_034286468.1

back to list

  

| Protein Coverage
| Supporting Peptides
|

Protein Coverage:

Supporting Peptides:

| Peptide | Uniq | -10lgP | Mass | Length | ppm | m/z | z | RT | Fraction | Scan | Source File | Area F1 | Area F10 | Area F11 | Area F12 | Area F13 | Area F2 | Area F3 | Area F4 | Area F5 | Area F6 | Area F7 | Area F8 | Area F9 | #Feature | #Feature F1 | #Feature F10 | #Feature F11 | #Feature F12 | #Feature F13 | #Feature F2 | #Feature F3 | #Feature F4 | #Feature F5 | #Feature F6 | #Feature F7 | #Feature F8 | #Feature F9 | Start | End | PTM | AScore | Found By |
| --- | --- | --- | --- | --- | --- | --- | --- | --- | --- | --- | --- | --- | --- | --- | --- | --- | --- | --- | --- | --- | --- | --- | --- | --- | --- | --- | --- | --- | --- | --- | --- | --- | --- | --- | --- | --- | --- | --- | --- | --- | --- | --- | --- |
| R.SVEEAIPAVC(+57.02)K.T | Y | 59.34 | 1201.6012 | 11 | 0.8 | 601.8083 | 2 | 28.40 | 10 | F10:14244 | NaNaKA16\_F6.raw |  |  |  |  |  |  |  |  |  | 1.8435E6 |  |  |  | 1 | 0 | 0 | 0 | 0 | 0 | 0 | 0 | 0 | 0 | 1 | 0 | 0 | 0 | 50 | 60 | Carbamidomethylation | C10:Carbamidomethylation:1000.00 | PEAKS DB |
| R.SQIDPTSANFLIWPPC(+57.02)VEVK.R | Y | 55.24 | 2300.1511 | 20 | -0.4 | 1151.0824 | 2 | 93.15 | 12 | F12:67124 | NaNaKA16\_F8.raw |  |  |  |  |  |  |  |  |  |  |  | 1.4123E7 |  | 2 | 0 | 0 | 0 | 0 | 0 | 0 | 0 | 0 | 0 | 0 | 0 | 2 | 0 | 71 | 90 | Carbamidomethylation | C16:Carbamidomethylation:1000.00 | PEAKS DB |
| R.TVIYEIPR.S | Y | 41.82 | 989.5546 | 8 | -0.1 | 495.7845 | 2 | 37.13 | 10 | F10:21430 | NaNaKA16\_F6.raw |  |  |  |  |  |  |  |  |  | 3.051E6 |  |  |  | 1 | 0 | 0 | 0 | 0 | 0 | 0 | 0 | 0 | 0 | 1 | 0 | 0 | 0 | 63 | 70 |  |  | PEAKS DB |
| total 3 peptides |  |  |  |  |  |  |  |  |  |  |  |  |  |  |  |  |  |  |  |  |  |  |  |  |  |  |  |  |  |  |  |  |  |  |  |  |  |  |  |  |  |  |  |
| --- | --- | --- | --- | --- | --- | --- | --- | --- | --- | --- | --- | --- | --- | --- | --- | --- | --- | --- | --- | --- | --- | --- | --- | --- | --- | --- | --- | --- | --- | --- | --- | --- | --- | --- | --- | --- | --- | --- | --- | --- | --- | --- | --- |

XP\_026560088.1

back to list

  

| Protein Coverage
| Supporting Peptides
|

Protein Coverage:

Supporting Peptides:

| Peptide | Uniq | -10lgP | Mass | Length | ppm | m/z | z | RT | Fraction | Scan | Source File | Area F1 | Area F10 | Area F11 | Area F12 | Area F13 | Area F2 | Area F3 | Area F4 | Area F5 | Area F6 | Area F7 | Area F8 | Area F9 | #Feature | #Feature F1 | #Feature F10 | #Feature F11 | #Feature F12 | #Feature F13 | #Feature F2 | #Feature F3 | #Feature F4 | #Feature F5 | #Feature F6 | #Feature F7 | #Feature F8 | #Feature F9 | Start | End | PTM | AScore | Found By |
| --- | --- | --- | --- | --- | --- | --- | --- | --- | --- | --- | --- | --- | --- | --- | --- | --- | --- | --- | --- | --- | --- | --- | --- | --- | --- | --- | --- | --- | --- | --- | --- | --- | --- | --- | --- | --- | --- | --- | --- | --- | --- | --- | --- |
| R.SVEEAIPAVC(+57.02)K.T | Y | 59.34 | 1201.6012 | 11 | 0.8 | 601.8083 | 2 | 28.40 | 10 | F10:14244 | NaNaKA16\_F6.raw |  |  |  |  |  |  |  |  |  | 1.8435E6 |  |  |  | 1 | 0 | 0 | 0 | 0 | 0 | 0 | 0 | 0 | 0 | 1 | 0 | 0 | 0 | 86 | 96 | Carbamidomethylation | C10:Carbamidomethylation:1000.00 | PEAKS DB |
| R.SQIDPTSANFLIWPPC(+57.02)VEVK.R | Y | 55.24 | 2300.1511 | 20 | -0.4 | 1151.0824 | 2 | 93.15 | 12 | F12:67124 | NaNaKA16\_F8.raw |  |  |  |  |  |  |  |  |  |  |  | 1.4123E7 |  | 2 | 0 | 0 | 0 | 0 | 0 | 0 | 0 | 0 | 0 | 0 | 0 | 2 | 0 | 107 | 126 | Carbamidomethylation | C16:Carbamidomethylation:1000.00 | PEAKS DB |
| R.TVIYEIPR.S | Y | 41.82 | 989.5546 | 8 | -0.1 | 495.7845 | 2 | 37.13 | 10 | F10:21430 | NaNaKA16\_F6.raw |  |  |  |  |  |  |  |  |  | 3.051E6 |  |  |  | 1 | 0 | 0 | 0 | 0 | 0 | 0 | 0 | 0 | 0 | 1 | 0 | 0 | 0 | 99 | 106 |  |  | PEAKS DB |
| total 3 peptides |  |  |  |  |  |  |  |  |  |  |  |  |  |  |  |  |  |  |  |  |  |  |  |  |  |  |  |  |  |  |  |  |  |  |  |  |  |  |  |  |  |  |  |
| --- | --- | --- | --- | --- | --- | --- | --- | --- | --- | --- | --- | --- | --- | --- | --- | --- | --- | --- | --- | --- | --- | --- | --- | --- | --- | --- | --- | --- | --- | --- | --- | --- | --- | --- | --- | --- | --- | --- | --- | --- | --- | --- | --- |

AFJ50955.1

back to list

  

| Protein Coverage
| Supporting Peptides
|

Protein Coverage:

Supporting Peptides:

| Peptide | Uniq | -10lgP | Mass | Length | ppm | m/z | z | RT | Fraction | Scan | Source File | Area F1 | Area F10 | Area F11 | Area F12 | Area F13 | Area F2 | Area F3 | Area F4 | Area F5 | Area F6 | Area F7 | Area F8 | Area F9 | #Feature | #Feature F1 | #Feature F10 | #Feature F11 | #Feature F12 | #Feature F13 | #Feature F2 | #Feature F3 | #Feature F4 | #Feature F5 | #Feature F6 | #Feature F7 | #Feature F8 | #Feature F9 | Start | End | PTM | AScore | Found By |
| --- | --- | --- | --- | --- | --- | --- | --- | --- | --- | --- | --- | --- | --- | --- | --- | --- | --- | --- | --- | --- | --- | --- | --- | --- | --- | --- | --- | --- | --- | --- | --- | --- | --- | --- | --- | --- | --- | --- | --- | --- | --- | --- | --- |
| R.SVEEAIPAVC(+57.02)K.T | Y | 59.34 | 1201.6012 | 11 | 0.8 | 601.8083 | 2 | 28.40 | 10 | F10:14244 | NaNaKA16\_F6.raw |  |  |  |  |  |  |  |  |  | 1.8435E6 |  |  |  | 1 | 0 | 0 | 0 | 0 | 0 | 0 | 0 | 0 | 0 | 1 | 0 | 0 | 0 | 87 | 97 | Carbamidomethylation | C10:Carbamidomethylation:1000.00 | PEAKS DB |
| R.SQIDPTSANFLIWPPC(+57.02)VEVK.R | Y | 55.24 | 2300.1511 | 20 | -0.4 | 1151.0824 | 2 | 93.15 | 12 | F12:67124 | NaNaKA16\_F8.raw |  |  |  |  |  |  |  |  |  |  |  | 1.4123E7 |  | 2 | 0 | 0 | 0 | 0 | 0 | 0 | 0 | 0 | 0 | 0 | 0 | 2 | 0 | 108 | 127 | Carbamidomethylation | C16:Carbamidomethylation:1000.00 | PEAKS DB |
| R.TVIYEIPR.S | Y | 41.82 | 989.5546 | 8 | -0.1 | 495.7845 | 2 | 37.13 | 10 | F10:21430 | NaNaKA16\_F6.raw |  |  |  |  |  |  |  |  |  | 3.051E6 |  |  |  | 1 | 0 | 0 | 0 | 0 | 0 | 0 | 0 | 0 | 0 | 1 | 0 | 0 | 0 | 100 | 107 |  |  | PEAKS DB |
| total 3 peptides |  |  |  |  |  |  |  |  |  |  |  |  |  |  |  |  |  |  |  |  |  |  |  |  |  |  |  |  |  |  |  |  |  |  |  |  |  |  |  |  |  |  |  |
| --- | --- | --- | --- | --- | --- | --- | --- | --- | --- | --- | --- | --- | --- | --- | --- | --- | --- | --- | --- | --- | --- | --- | --- | --- | --- | --- | --- | --- | --- | --- | --- | --- | --- | --- | --- | --- | --- | --- | --- | --- | --- | --- | --- |

JAI12034.1

back to list

  

| Protein Coverage
| Supporting Peptides
|

Protein Coverage:

Supporting Peptides:

| Peptide | Uniq | -10lgP | Mass | Length | ppm | m/z | z | RT | Fraction | Scan | Source File | Area F1 | Area F10 | Area F11 | Area F12 | Area F13 | Area F2 | Area F3 | Area F4 | Area F5 | Area F6 | Area F7 | Area F8 | Area F9 | #Feature | #Feature F1 | #Feature F10 | #Feature F11 | #Feature F12 | #Feature F13 | #Feature F2 | #Feature F3 | #Feature F4 | #Feature F5 | #Feature F6 | #Feature F7 | #Feature F8 | #Feature F9 | Start | End | PTM | AScore | Found By |
| --- | --- | --- | --- | --- | --- | --- | --- | --- | --- | --- | --- | --- | --- | --- | --- | --- | --- | --- | --- | --- | --- | --- | --- | --- | --- | --- | --- | --- | --- | --- | --- | --- | --- | --- | --- | --- | --- | --- | --- | --- | --- | --- | --- |
| R.SVEEAIPAVC(+57.02)K.T | Y | 59.34 | 1201.6012 | 11 | 0.8 | 601.8083 | 2 | 28.40 | 10 | F10:14244 | NaNaKA16\_F6.raw |  |  |  |  |  |  |  |  |  | 1.8435E6 |  |  |  | 1 | 0 | 0 | 0 | 0 | 0 | 0 | 0 | 0 | 0 | 1 | 0 | 0 | 0 | 87 | 97 | Carbamidomethylation | C10:Carbamidomethylation:1000.00 | PEAKS DB |
| R.SQIDPTSANFLIWPPC(+57.02)VEVK.R | Y | 55.24 | 2300.1511 | 20 | -0.4 | 1151.0824 | 2 | 93.15 | 12 | F12:67124 | NaNaKA16\_F8.raw |  |  |  |  |  |  |  |  |  |  |  | 1.4123E7 |  | 2 | 0 | 0 | 0 | 0 | 0 | 0 | 0 | 0 | 0 | 0 | 0 | 2 | 0 | 108 | 127 | Carbamidomethylation | C16:Carbamidomethylation:1000.00 | PEAKS DB |
| R.TVIYEIPR.S | Y | 41.82 | 989.5546 | 8 | -0.1 | 495.7845 | 2 | 37.13 | 10 | F10:21430 | NaNaKA16\_F6.raw |  |  |  |  |  |  |  |  |  | 3.051E6 |  |  |  | 1 | 0 | 0 | 0 | 0 | 0 | 0 | 0 | 0 | 0 | 1 | 0 | 0 | 0 | 100 | 107 |  |  | PEAKS DB |
| total 3 peptides |  |  |  |  |  |  |  |  |  |  |  |  |  |  |  |  |  |  |  |  |  |  |  |  |  |  |  |  |  |  |  |  |  |  |  |  |  |  |  |  |  |  |  |
| --- | --- | --- | --- | --- | --- | --- | --- | --- | --- | --- | --- | --- | --- | --- | --- | --- | --- | --- | --- | --- | --- | --- | --- | --- | --- | --- | --- | --- | --- | --- | --- | --- | --- | --- | --- | --- | --- | --- | --- | --- | --- | --- | --- |

XP\_015676418.1

back to list

  

| Protein Coverage
| Supporting Peptides
|

Protein Coverage:

Supporting Peptides:

| Peptide | Uniq | -10lgP | Mass | Length | ppm | m/z | z | RT | Fraction | Scan | Source File | Area F1 | Area F10 | Area F11 | Area F12 | Area F13 | Area F2 | Area F3 | Area F4 | Area F5 | Area F6 | Area F7 | Area F8 | Area F9 | #Feature | #Feature F1 | #Feature F10 | #Feature F11 | #Feature F12 | #Feature F13 | #Feature F2 | #Feature F3 | #Feature F4 | #Feature F5 | #Feature F6 | #Feature F7 | #Feature F8 | #Feature F9 | Start | End | PTM | AScore | Found By |
| --- | --- | --- | --- | --- | --- | --- | --- | --- | --- | --- | --- | --- | --- | --- | --- | --- | --- | --- | --- | --- | --- | --- | --- | --- | --- | --- | --- | --- | --- | --- | --- | --- | --- | --- | --- | --- | --- | --- | --- | --- | --- | --- | --- |
| R.SVEEAIPAVC(+57.02)K.T | Y | 59.34 | 1201.6012 | 11 | 0.8 | 601.8083 | 2 | 28.40 | 10 | F10:14244 | NaNaKA16\_F6.raw |  |  |  |  |  |  |  |  |  | 1.8435E6 |  |  |  | 1 | 0 | 0 | 0 | 0 | 0 | 0 | 0 | 0 | 0 | 1 | 0 | 0 | 0 | 87 | 97 | Carbamidomethylation | C10:Carbamidomethylation:1000.00 | PEAKS DB |
| R.SQIDPTSANFLIWPPC(+57.02)VEVK.R | Y | 55.24 | 2300.1511 | 20 | -0.4 | 1151.0824 | 2 | 93.15 | 12 | F12:67124 | NaNaKA16\_F8.raw |  |  |  |  |  |  |  |  |  |  |  | 1.4123E7 |  | 2 | 0 | 0 | 0 | 0 | 0 | 0 | 0 | 0 | 0 | 0 | 0 | 2 | 0 | 108 | 127 | Carbamidomethylation | C16:Carbamidomethylation:1000.00 | PEAKS DB |
| R.TVIYEIPR.S | Y | 41.82 | 989.5546 | 8 | -0.1 | 495.7845 | 2 | 37.13 | 10 | F10:21430 | NaNaKA16\_F6.raw |  |  |  |  |  |  |  |  |  | 3.051E6 |  |  |  | 1 | 0 | 0 | 0 | 0 | 0 | 0 | 0 | 0 | 0 | 1 | 0 | 0 | 0 | 100 | 107 |  |  | PEAKS DB |
| total 3 peptides |  |  |  |  |  |  |  |  |  |  |  |  |  |  |  |  |  |  |  |  |  |  |  |  |  |  |  |  |  |  |  |  |  |  |  |  |  |  |  |  |  |  |  |
| --- | --- | --- | --- | --- | --- | --- | --- | --- | --- | --- | --- | --- | --- | --- | --- | --- | --- | --- | --- | --- | --- | --- | --- | --- | --- | --- | --- | --- | --- | --- | --- | --- | --- | --- | --- | --- | --- | --- | --- | --- | --- | --- | --- |

LAA51474.1

back to list

  

| Protein Coverage
| Supporting Peptides
|

Protein Coverage:

Supporting Peptides:

| Peptide | Uniq | -10lgP | Mass | Length | ppm | m/z | z | RT | Fraction | Scan | Source File | Area F1 | Area F10 | Area F11 | Area F12 | Area F13 | Area F2 | Area F3 | Area F4 | Area F5 | Area F6 | Area F7 | Area F8 | Area F9 | #Feature | #Feature F1 | #Feature F10 | #Feature F11 | #Feature F12 | #Feature F13 | #Feature F2 | #Feature F3 | #Feature F4 | #Feature F5 | #Feature F6 | #Feature F7 | #Feature F8 | #Feature F9 | Start | End | PTM | AScore | Found By |
| --- | --- | --- | --- | --- | --- | --- | --- | --- | --- | --- | --- | --- | --- | --- | --- | --- | --- | --- | --- | --- | --- | --- | --- | --- | --- | --- | --- | --- | --- | --- | --- | --- | --- | --- | --- | --- | --- | --- | --- | --- | --- | --- | --- |
| R.SVEEAIPAVC(+57.02)K.T | Y | 59.34 | 1201.6012 | 11 | 0.8 | 601.8083 | 2 | 28.40 | 10 | F10:14244 | NaNaKA16\_F6.raw |  |  |  |  |  |  |  |  |  | 1.8435E6 |  |  |  | 1 | 0 | 0 | 0 | 0 | 0 | 0 | 0 | 0 | 0 | 1 | 0 | 0 | 0 | 9 | 19 | Carbamidomethylation | C10:Carbamidomethylation:1000.00 | PEAKS DB |
| R.SQIDPTSANFLIWPPC(+57.02)VEVK.R | Y | 55.24 | 2300.1511 | 20 | -0.4 | 1151.0824 | 2 | 93.15 | 12 | F12:67124 | NaNaKA16\_F8.raw |  |  |  |  |  |  |  |  |  |  |  | 1.4123E7 |  | 2 | 0 | 0 | 0 | 0 | 0 | 0 | 0 | 0 | 0 | 0 | 0 | 2 | 0 | 30 | 49 | Carbamidomethylation | C16:Carbamidomethylation:1000.00 | PEAKS DB |
| R.TVIYEIPR.S | Y | 41.82 | 989.5546 | 8 | -0.1 | 495.7845 | 2 | 37.13 | 10 | F10:21430 | NaNaKA16\_F6.raw |  |  |  |  |  |  |  |  |  | 3.051E6 |  |  |  | 1 | 0 | 0 | 0 | 0 | 0 | 0 | 0 | 0 | 0 | 1 | 0 | 0 | 0 | 22 | 29 |  |  | PEAKS DB |
| total 3 peptides |  |  |  |  |  |  |  |  |  |  |  |  |  |  |  |  |  |  |  |  |  |  |  |  |  |  |  |  |  |  |  |  |  |  |  |  |  |  |  |  |  |  |  |
| --- | --- | --- | --- | --- | --- | --- | --- | --- | --- | --- | --- | --- | --- | --- | --- | --- | --- | --- | --- | --- | --- | --- | --- | --- | --- | --- | --- | --- | --- | --- | --- | --- | --- | --- | --- | --- | --- | --- | --- | --- | --- | --- | --- |

XP\_007426296.1

back to list

  

| Protein Coverage
| Supporting Peptides
|

Protein Coverage:

Supporting Peptides:

| Peptide | Uniq | -10lgP | Mass | Length | ppm | m/z | z | RT | Fraction | Scan | Source File | Area F1 | Area F10 | Area F11 | Area F12 | Area F13 | Area F2 | Area F3 | Area F4 | Area F5 | Area F6 | Area F7 | Area F8 | Area F9 | #Feature | #Feature F1 | #Feature F10 | #Feature F11 | #Feature F12 | #Feature F13 | #Feature F2 | #Feature F3 | #Feature F4 | #Feature F5 | #Feature F6 | #Feature F7 | #Feature F8 | #Feature F9 | Start | End | PTM | AScore | Found By |
| --- | --- | --- | --- | --- | --- | --- | --- | --- | --- | --- | --- | --- | --- | --- | --- | --- | --- | --- | --- | --- | --- | --- | --- | --- | --- | --- | --- | --- | --- | --- | --- | --- | --- | --- | --- | --- | --- | --- | --- | --- | --- | --- | --- |
| R.SVEEAIPAVC(+57.02)K.T | Y | 59.34 | 1201.6012 | 11 | 0.8 | 601.8083 | 2 | 28.40 | 10 | F10:14244 | NaNaKA16\_F6.raw |  |  |  |  |  |  |  |  |  | 1.8435E6 |  |  |  | 1 | 0 | 0 | 0 | 0 | 0 | 0 | 0 | 0 | 0 | 1 | 0 | 0 | 0 | 87 | 97 | Carbamidomethylation | C10:Carbamidomethylation:1000.00 | PEAKS DB |
| R.SQIDPTSANFLIWPPC(+57.02)VEVK.R | Y | 55.24 | 2300.1511 | 20 | -0.4 | 1151.0824 | 2 | 93.15 | 12 | F12:67124 | NaNaKA16\_F8.raw |  |  |  |  |  |  |  |  |  |  |  | 1.4123E7 |  | 2 | 0 | 0 | 0 | 0 | 0 | 0 | 0 | 0 | 0 | 0 | 0 | 2 | 0 | 108 | 127 | Carbamidomethylation | C16:Carbamidomethylation:1000.00 | PEAKS DB |
| R.TVIYEIPR.S | Y | 41.82 | 989.5546 | 8 | -0.1 | 495.7845 | 2 | 37.13 | 10 | F10:21430 | NaNaKA16\_F6.raw |  |  |  |  |  |  |  |  |  | 3.051E6 |  |  |  | 1 | 0 | 0 | 0 | 0 | 0 | 0 | 0 | 0 | 0 | 1 | 0 | 0 | 0 | 100 | 107 |  |  | PEAKS DB |
| total 3 peptides |  |  |  |  |  |  |  |  |  |  |  |  |  |  |  |  |  |  |  |  |  |  |  |  |  |  |  |  |  |  |  |  |  |  |  |  |  |  |  |  |  |  |  |
| --- | --- | --- | --- | --- | --- | --- | --- | --- | --- | --- | --- | --- | --- | --- | --- | --- | --- | --- | --- | --- | --- | --- | --- | --- | --- | --- | --- | --- | --- | --- | --- | --- | --- | --- | --- | --- | --- | --- | --- | --- | --- | --- | --- |

XP\_025021193.1

back to list

  

| Protein Coverage
| Supporting Peptides
|

Protein Coverage:

Supporting Peptides:

| Peptide | Uniq | -10lgP | Mass | Length | ppm | m/z | z | RT | Fraction | Scan | Source File | Area F1 | Area F10 | Area F11 | Area F12 | Area F13 | Area F2 | Area F3 | Area F4 | Area F5 | Area F6 | Area F7 | Area F8 | Area F9 | #Feature | #Feature F1 | #Feature F10 | #Feature F11 | #Feature F12 | #Feature F13 | #Feature F2 | #Feature F3 | #Feature F4 | #Feature F5 | #Feature F6 | #Feature F7 | #Feature F8 | #Feature F9 | Start | End | PTM | AScore | Found By |
| --- | --- | --- | --- | --- | --- | --- | --- | --- | --- | --- | --- | --- | --- | --- | --- | --- | --- | --- | --- | --- | --- | --- | --- | --- | --- | --- | --- | --- | --- | --- | --- | --- | --- | --- | --- | --- | --- | --- | --- | --- | --- | --- | --- |
| R.SVEEAIPAVC(+57.02)K.T | Y | 59.34 | 1201.6012 | 11 | 0.8 | 601.8083 | 2 | 28.40 | 10 | F10:14244 | NaNaKA16\_F6.raw |  |  |  |  |  |  |  |  |  | 1.8435E6 |  |  |  | 1 | 0 | 0 | 0 | 0 | 0 | 0 | 0 | 0 | 0 | 1 | 0 | 0 | 0 | 72 | 82 | Carbamidomethylation | C10:Carbamidomethylation:1000.00 | PEAKS DB |
| R.SQIDPTSANFLIWPPC(+57.02)VEVK.R | Y | 55.24 | 2300.1511 | 20 | -0.4 | 1151.0824 | 2 | 93.15 | 12 | F12:67124 | NaNaKA16\_F8.raw |  |  |  |  |  |  |  |  |  |  |  | 1.4123E7 |  | 2 | 0 | 0 | 0 | 0 | 0 | 0 | 0 | 0 | 0 | 0 | 0 | 2 | 0 | 93 | 112 | Carbamidomethylation | C16:Carbamidomethylation:1000.00 | PEAKS DB |
| R.TVIYEIPR.S | Y | 41.82 | 989.5546 | 8 | -0.1 | 495.7845 | 2 | 37.13 | 10 | F10:21430 | NaNaKA16\_F6.raw |  |  |  |  |  |  |  |  |  | 3.051E6 |  |  |  | 1 | 0 | 0 | 0 | 0 | 0 | 0 | 0 | 0 | 0 | 1 | 0 | 0 | 0 | 85 | 92 |  |  | PEAKS DB |
| total 3 peptides |  |  |  |  |  |  |  |  |  |  |  |  |  |  |  |  |  |  |  |  |  |  |  |  |  |  |  |  |  |  |  |  |  |  |  |  |  |  |  |  |  |  |  |
| --- | --- | --- | --- | --- | --- | --- | --- | --- | --- | --- | --- | --- | --- | --- | --- | --- | --- | --- | --- | --- | --- | --- | --- | --- | --- | --- | --- | --- | --- | --- | --- | --- | --- | --- | --- | --- | --- | --- | --- | --- | --- | --- | --- |

XP\_015743230.1

back to list

  

| Protein Coverage
| Supporting Peptides
|

Protein Coverage:

Supporting Peptides:

| Peptide | Uniq | -10lgP | Mass | Length | ppm | m/z | z | RT | Fraction | Scan | Source File | Area F1 | Area F10 | Area F11 | Area F12 | Area F13 | Area F2 | Area F3 | Area F4 | Area F5 | Area F6 | Area F7 | Area F8 | Area F9 | #Feature | #Feature F1 | #Feature F10 | #Feature F11 | #Feature F12 | #Feature F13 | #Feature F2 | #Feature F3 | #Feature F4 | #Feature F5 | #Feature F6 | #Feature F7 | #Feature F8 | #Feature F9 | Start | End | PTM | AScore | Found By |
| --- | --- | --- | --- | --- | --- | --- | --- | --- | --- | --- | --- | --- | --- | --- | --- | --- | --- | --- | --- | --- | --- | --- | --- | --- | --- | --- | --- | --- | --- | --- | --- | --- | --- | --- | --- | --- | --- | --- | --- | --- | --- | --- | --- |
| R.SVEEAIPAVC(+57.02)K.T | Y | 59.34 | 1201.6012 | 11 | 0.8 | 601.8083 | 2 | 28.40 | 10 | F10:14244 | NaNaKA16\_F6.raw |  |  |  |  |  |  |  |  |  | 1.8435E6 |  |  |  | 1 | 0 | 0 | 0 | 0 | 0 | 0 | 0 | 0 | 0 | 1 | 0 | 0 | 0 | 87 | 97 | Carbamidomethylation | C10:Carbamidomethylation:1000.00 | PEAKS DB |
| R.SQIDPTSANFLIWPPC(+57.02)VEVK.R | Y | 55.24 | 2300.1511 | 20 | -0.4 | 1151.0824 | 2 | 93.15 | 12 | F12:67124 | NaNaKA16\_F8.raw |  |  |  |  |  |  |  |  |  |  |  | 1.4123E7 |  | 2 | 0 | 0 | 0 | 0 | 0 | 0 | 0 | 0 | 0 | 0 | 0 | 2 | 0 | 108 | 127 | Carbamidomethylation | C16:Carbamidomethylation:1000.00 | PEAKS DB |
| R.TVIYEIPR.S | Y | 41.82 | 989.5546 | 8 | -0.1 | 495.7845 | 2 | 37.13 | 10 | F10:21430 | NaNaKA16\_F6.raw |  |  |  |  |  |  |  |  |  | 3.051E6 |  |  |  | 1 | 0 | 0 | 0 | 0 | 0 | 0 | 0 | 0 | 0 | 1 | 0 | 0 | 0 | 100 | 107 |  |  | PEAKS DB |
| total 3 peptides |  |  |  |  |  |  |  |  |  |  |  |  |  |  |  |  |  |  |  |  |  |  |  |  |  |  |  |  |  |  |  |  |  |  |  |  |  |  |  |  |  |  |  |
| --- | --- | --- | --- | --- | --- | --- | --- | --- | --- | --- | --- | --- | --- | --- | --- | --- | --- | --- | --- | --- | --- | --- | --- | --- | --- | --- | --- | --- | --- | --- | --- | --- | --- | --- | --- | --- | --- | --- | --- | --- | --- | --- | --- |

XP\_026540879.1

back to list

  

| Protein Coverage
| Supporting Peptides
|

Protein Coverage:

Supporting Peptides:

| Peptide | Uniq | -10lgP | Mass | Length | ppm | m/z | z | RT | Fraction | Scan | Source File | Area F1 | Area F10 | Area F11 | Area F12 | Area F13 | Area F2 | Area F3 | Area F4 | Area F5 | Area F6 | Area F7 | Area F8 | Area F9 | #Feature | #Feature F1 | #Feature F10 | #Feature F11 | #Feature F12 | #Feature F13 | #Feature F2 | #Feature F3 | #Feature F4 | #Feature F5 | #Feature F6 | #Feature F7 | #Feature F8 | #Feature F9 | Start | End | PTM | AScore | Found By |
| --- | --- | --- | --- | --- | --- | --- | --- | --- | --- | --- | --- | --- | --- | --- | --- | --- | --- | --- | --- | --- | --- | --- | --- | --- | --- | --- | --- | --- | --- | --- | --- | --- | --- | --- | --- | --- | --- | --- | --- | --- | --- | --- | --- |
| R.SVEEAIPAVC(+57.02)K.T | Y | 59.34 | 1201.6012 | 11 | 0.8 | 601.8083 | 2 | 28.40 | 10 | F10:14244 | NaNaKA16\_F6.raw |  |  |  |  |  |  |  |  |  | 1.8435E6 |  |  |  | 1 | 0 | 0 | 0 | 0 | 0 | 0 | 0 | 0 | 0 | 1 | 0 | 0 | 0 | 93 | 103 | Carbamidomethylation | C10:Carbamidomethylation:1000.00 | PEAKS DB |
| R.SQIDPTSANFLIWPPC(+57.02)VEVK.R | Y | 55.24 | 2300.1511 | 20 | -0.4 | 1151.0824 | 2 | 93.15 | 12 | F12:67124 | NaNaKA16\_F8.raw |  |  |  |  |  |  |  |  |  |  |  | 1.4123E7 |  | 2 | 0 | 0 | 0 | 0 | 0 | 0 | 0 | 0 | 0 | 0 | 0 | 2 | 0 | 114 | 133 | Carbamidomethylation | C16:Carbamidomethylation:1000.00 | PEAKS DB |
| R.TVIYEIPR.S | Y | 41.82 | 989.5546 | 8 | -0.1 | 495.7845 | 2 | 37.13 | 10 | F10:21430 | NaNaKA16\_F6.raw |  |  |  |  |  |  |  |  |  | 3.051E6 |  |  |  | 1 | 0 | 0 | 0 | 0 | 0 | 0 | 0 | 0 | 0 | 1 | 0 | 0 | 0 | 106 | 113 |  |  | PEAKS DB |
| total 3 peptides |  |  |  |  |  |  |  |  |  |  |  |  |  |  |  |  |  |  |  |  |  |  |  |  |  |  |  |  |  |  |  |  |  |  |  |  |  |  |  |  |  |  |  |
| --- | --- | --- | --- | --- | --- | --- | --- | --- | --- | --- | --- | --- | --- | --- | --- | --- | --- | --- | --- | --- | --- | --- | --- | --- | --- | --- | --- | --- | --- | --- | --- | --- | --- | --- | --- | --- | --- | --- | --- | --- | --- | --- | --- |

ETE63349.1

back to list

  

| Protein Coverage
| Supporting Peptides
|

Protein Coverage:

Supporting Peptides:

| Peptide | Uniq | -10lgP | Mass | Length | ppm | m/z | z | RT | Fraction | Scan | Source File | Area F1 | Area F10 | Area F11 | Area F12 | Area F13 | Area F2 | Area F3 | Area F4 | Area F5 | Area F6 | Area F7 | Area F8 | Area F9 | #Feature | #Feature F1 | #Feature F10 | #Feature F11 | #Feature F12 | #Feature F13 | #Feature F2 | #Feature F3 | #Feature F4 | #Feature F5 | #Feature F6 | #Feature F7 | #Feature F8 | #Feature F9 | Start | End | PTM | AScore | Found By |
| --- | --- | --- | --- | --- | --- | --- | --- | --- | --- | --- | --- | --- | --- | --- | --- | --- | --- | --- | --- | --- | --- | --- | --- | --- | --- | --- | --- | --- | --- | --- | --- | --- | --- | --- | --- | --- | --- | --- | --- | --- | --- | --- | --- |
| K.C(+57.02)GIGLNDYGLSFK.V | Y | 67.09 | 1442.6864 | 13 | -0.2 | 722.3503 | 2 | 66.05 | 4 | F4:43579 | NaNaKA16\_F12.raw |  |  |  | 1.3331E6 |  |  |  |  |  |  |  |  |  | 1 | 0 | 0 | 0 | 1 | 0 | 0 | 0 | 0 | 0 | 0 | 0 | 0 | 0 | 113 | 125 | Carbamidomethylation | C1:Carbamidomethylation:1000.00 | PEAKS DB |
| K.C(+57.02)FYPPTSVNIHSR.K | Y | 43.53 | 1576.7456 | 13 | -0.1 | 526.5891 | 3 | 32.35 | 4 | F4:15444 | NaNaKA16\_F12.raw |  |  |  | 6.0638E5 |  |  |  |  |  |  |  |  |  | 1 | 0 | 0 | 0 | 1 | 0 | 0 | 0 | 0 | 0 | 0 | 0 | 0 | 0 | 44 | 56 | Carbamidomethylation | C1:Carbamidomethylation:1000.00 | PEAKS DB |
| total 2 peptides |  |  |  |  |  |  |  |  |  |  |  |  |  |  |  |  |  |  |  |  |  |  |  |  |  |  |  |  |  |  |  |  |  |  |  |  |  |  |  |  |  |  |  |
| --- | --- | --- | --- | --- | --- | --- | --- | --- | --- | --- | --- | --- | --- | --- | --- | --- | --- | --- | --- | --- | --- | --- | --- | --- | --- | --- | --- | --- | --- | --- | --- | --- | --- | --- | --- | --- | --- | --- | --- | --- | --- | --- | --- |

ETE61477.1

back to list

  

| Protein Coverage
| Supporting Peptides
|

Protein Coverage:

Supporting Peptides:

| Peptide | Uniq | -10lgP | Mass | Length | ppm | m/z | z | RT | Fraction | Scan | Source File | Area F1 | Area F10 | Area F11 | Area F12 | Area F13 | Area F2 | Area F3 | Area F4 | Area F5 | Area F6 | Area F7 | Area F8 | Area F9 | #Feature | #Feature F1 | #Feature F10 | #Feature F11 | #Feature F12 | #Feature F13 | #Feature F2 | #Feature F3 | #Feature F4 | #Feature F5 | #Feature F6 | #Feature F7 | #Feature F8 | #Feature F9 | Start | End | PTM | AScore | Found By |
| --- | --- | --- | --- | --- | --- | --- | --- | --- | --- | --- | --- | --- | --- | --- | --- | --- | --- | --- | --- | --- | --- | --- | --- | --- | --- | --- | --- | --- | --- | --- | --- | --- | --- | --- | --- | --- | --- | --- | --- | --- | --- | --- | --- |
| N.VVQEALIPIIPDYK.C | N | 71.29 | 1596.9126 | 14 | 0.6 | 799.4641 | 2 | 76.22 | 4 | F4:51790 | NaNaKA16\_F12.raw |  | 7.806E5 | 3.194E6 | 8.6522E6 |  |  |  |  |  |  |  |  |  | 4 | 0 | 1 | 1 | 2 | 0 | 0 | 0 | 0 | 0 | 0 | 0 | 0 | 0 | 58 | 71 |  |  | PEAKS DB |
| R.FIQPIC(+57.02)LPEASMSFPDYYK.C | Y | 54.41 | 2305.0798 | 19 | 1.1 | 769.3680 | 3 | 90.19 | 4 | F4:63215 | NaNaKA16\_F12.raw |  |  |  | 6.87E5 |  |  |  |  |  |  |  |  |  | 1 | 0 | 0 | 0 | 1 | 0 | 0 | 0 | 0 | 0 | 0 | 0 | 0 | 0 | 21 | 39 | Carbamidomethylation | C6:Carbamidomethylation:1000.00 | PEAKS DB |
| total 2 peptides |  |  |  |  |  |  |  |  |  |  |  |  |  |  |  |  |  |  |  |  |  |  |  |  |  |  |  |  |  |  |  |  |  |  |  |  |  |  |  |  |  |  |  |
| --- | --- | --- | --- | --- | --- | --- | --- | --- | --- | --- | --- | --- | --- | --- | --- | --- | --- | --- | --- | --- | --- | --- | --- | --- | --- | --- | --- | --- | --- | --- | --- | --- | --- | --- | --- | --- | --- | --- | --- | --- | --- | --- | --- |

JAS05124.1

back to list

  

| Protein Coverage
| Supporting Peptides
|

Protein Coverage:

Supporting Peptides:

| Peptide | Uniq | -10lgP | Mass | Length | ppm | m/z | z | RT | Fraction | Scan | Source File | Area F1 | Area F10 | Area F11 | Area F12 | Area F13 | Area F2 | Area F3 | Area F4 | Area F5 | Area F6 | Area F7 | Area F8 | Area F9 | #Feature | #Feature F1 | #Feature F10 | #Feature F11 | #Feature F12 | #Feature F13 | #Feature F2 | #Feature F3 | #Feature F4 | #Feature F5 | #Feature F6 | #Feature F7 | #Feature F8 | #Feature F9 | Start | End | PTM | AScore | Found By |
| --- | --- | --- | --- | --- | --- | --- | --- | --- | --- | --- | --- | --- | --- | --- | --- | --- | --- | --- | --- | --- | --- | --- | --- | --- | --- | --- | --- | --- | --- | --- | --- | --- | --- | --- | --- | --- | --- | --- | --- | --- | --- | --- | --- |
| R.SWWDFADYGC(+57.02).Y | N | 45.71 | 1305.4761 | 10 | 0.8 | 653.7458 | 2 | 109.86 | 11 | F11:80150 | NaNaKA16\_F7.raw |  |  |  |  |  |  |  |  |  |  | 6.5059E6 |  |  | 1 | 0 | 0 | 0 | 0 | 0 | 0 | 0 | 0 | 0 | 0 | 1 | 0 | 0 | 43 | 52 | Carbamidomethylation | C10:Carbamidomethylation:1000.00 | PEAKS DB |
| R.SWWDFADYGC(+57.02)Y.C | N | 44.81 | 1468.5394 | 11 | 0.1 | 735.2771 | 2 | 111.77 | 10 | F10:82113 | NaNaKA16\_F6.raw |  |  |  |  |  |  |  |  |  | 1.9586E7 |  | 1.4496E7 |  | 2 | 0 | 0 | 0 | 0 | 0 | 0 | 0 | 0 | 0 | 1 | 0 | 1 | 0 | 43 | 53 | Carbamidomethylation | C10:Carbamidomethylation:1000.00 | PEAKS DB |
| R.SWWDFADYGC(+57.02)YC(+57.02)GSGGSGTPVDDLDK.C | Y | 43.00 | 2914.1487 | 26 | 4.7 | 729.5479 | 4 | 82.68 | 11 | F11:57840 | NaNaKA16\_F7.raw |  |  |  |  |  |  |  |  |  |  | 5.5123E7 |  |  | 2 | 0 | 0 | 0 | 0 | 0 | 0 | 0 | 0 | 0 | 0 | 2 | 0 | 0 | 43 | 68 | Carbamidomethylation | C10:Carbamidomethylation:1000.00;C12:Carbamidomethylation:1000.00 | PEAKS DB |
| total 3 peptides |  |  |  |  |  |  |  |  |  |  |  |  |  |  |  |  |  |  |  |  |  |  |  |  |  |  |  |  |  |  |  |  |  |  |  |  |  |  |  |  |  |  |  |
| --- | --- | --- | --- | --- | --- | --- | --- | --- | --- | --- | --- | --- | --- | --- | --- | --- | --- | --- | --- | --- | --- | --- | --- | --- | --- | --- | --- | --- | --- | --- | --- | --- | --- | --- | --- | --- | --- | --- | --- | --- | --- | --- | --- |

JAS05123.1

back to list

  

| Protein Coverage
| Supporting Peptides
|

Protein Coverage:

Supporting Peptides:

| Peptide | Uniq | -10lgP | Mass | Length | ppm | m/z | z | RT | Fraction | Scan | Source File | Area F1 | Area F10 | Area F11 | Area F12 | Area F13 | Area F2 | Area F3 | Area F4 | Area F5 | Area F6 | Area F7 | Area F8 | Area F9 | #Feature | #Feature F1 | #Feature F10 | #Feature F11 | #Feature F12 | #Feature F13 | #Feature F2 | #Feature F3 | #Feature F4 | #Feature F5 | #Feature F6 | #Feature F7 | #Feature F8 | #Feature F9 | Start | End | PTM | AScore | Found By |
| --- | --- | --- | --- | --- | --- | --- | --- | --- | --- | --- | --- | --- | --- | --- | --- | --- | --- | --- | --- | --- | --- | --- | --- | --- | --- | --- | --- | --- | --- | --- | --- | --- | --- | --- | --- | --- | --- | --- | --- | --- | --- | --- | --- |
| R.SWWDFADYGC(+57.02).Y | N | 45.71 | 1305.4761 | 10 | 0.8 | 653.7458 | 2 | 109.86 | 11 | F11:80150 | NaNaKA16\_F7.raw |  |  |  |  |  |  |  |  |  |  | 6.5059E6 |  |  | 1 | 0 | 0 | 0 | 0 | 0 | 0 | 0 | 0 | 0 | 0 | 1 | 0 | 0 | 43 | 52 | Carbamidomethylation | C10:Carbamidomethylation:1000.00 | PEAKS DB |
| R.SWWDFADYGC(+57.02)Y.C | N | 44.81 | 1468.5394 | 11 | 0.1 | 735.2771 | 2 | 111.77 | 10 | F10:82113 | NaNaKA16\_F6.raw |  |  |  |  |  |  |  |  |  | 1.9586E7 |  | 1.4496E7 |  | 2 | 0 | 0 | 0 | 0 | 0 | 0 | 0 | 0 | 0 | 1 | 0 | 1 | 0 | 43 | 53 | Carbamidomethylation | C10:Carbamidomethylation:1000.00 | PEAKS DB |
| R.SWWDFADYGC(+57.02)YC(+57.02)GSGGSGTPVDDLDK.C | Y | 43.00 | 2914.1487 | 26 | 4.7 | 729.5479 | 4 | 82.68 | 11 | F11:57840 | NaNaKA16\_F7.raw |  |  |  |  |  |  |  |  |  |  | 5.5123E7 |  |  | 2 | 0 | 0 | 0 | 0 | 0 | 0 | 0 | 0 | 0 | 0 | 2 | 0 | 0 | 43 | 68 | Carbamidomethylation | C10:Carbamidomethylation:1000.00;C12:Carbamidomethylation:1000.00 | PEAKS DB |
| total 3 peptides |  |  |  |  |  |  |  |  |  |  |  |  |  |  |  |  |  |  |  |  |  |  |  |  |  |  |  |  |  |  |  |  |  |  |  |  |  |  |  |  |  |  |  |
| --- | --- | --- | --- | --- | --- | --- | --- | --- | --- | --- | --- | --- | --- | --- | --- | --- | --- | --- | --- | --- | --- | --- | --- | --- | --- | --- | --- | --- | --- | --- | --- | --- | --- | --- | --- | --- | --- | --- | --- | --- | --- | --- | --- |

AET85561.1

back to list

  

| Protein Coverage
| Supporting Peptides
|

Protein Coverage:

Supporting Peptides:

| Peptide | Uniq | -10lgP | Mass | Length | ppm | m/z | z | RT | Fraction | Scan | Source File | Area F1 | Area F10 | Area F11 | Area F12 | Area F13 | Area F2 | Area F3 | Area F4 | Area F5 | Area F6 | Area F7 | Area F8 | Area F9 | #Feature | #Feature F1 | #Feature F10 | #Feature F11 | #Feature F12 | #Feature F13 | #Feature F2 | #Feature F3 | #Feature F4 | #Feature F5 | #Feature F6 | #Feature F7 | #Feature F8 | #Feature F9 | Start | End | PTM | AScore | Found By |
| --- | --- | --- | --- | --- | --- | --- | --- | --- | --- | --- | --- | --- | --- | --- | --- | --- | --- | --- | --- | --- | --- | --- | --- | --- | --- | --- | --- | --- | --- | --- | --- | --- | --- | --- | --- | --- | --- | --- | --- | --- | --- | --- | --- |
| R.SWWDFADYGC(+57.02).Y | N | 45.71 | 1305.4761 | 10 | 0.8 | 653.7458 | 2 | 109.86 | 11 | F11:80150 | NaNaKA16\_F7.raw |  |  |  |  |  |  |  |  |  |  | 6.5059E6 |  |  | 1 | 0 | 0 | 0 | 0 | 0 | 0 | 0 | 0 | 0 | 0 | 1 | 0 | 0 | 16 | 25 | Carbamidomethylation | C10:Carbamidomethylation:1000.00 | PEAKS DB |
| R.SWWDFADYGC(+57.02)Y.C | N | 44.81 | 1468.5394 | 11 | 0.1 | 735.2771 | 2 | 111.77 | 10 | F10:82113 | NaNaKA16\_F6.raw |  |  |  |  |  |  |  |  |  | 1.9586E7 |  | 1.4496E7 |  | 2 | 0 | 0 | 0 | 0 | 0 | 0 | 0 | 0 | 0 | 1 | 0 | 1 | 0 | 16 | 26 | Carbamidomethylation | C10:Carbamidomethylation:1000.00 | PEAKS DB |
| R.SWWDFADYGC(+57.02)YC(+57.02)GSGGSGTPVDDLDK.C | Y | 43.00 | 2914.1487 | 26 | 4.7 | 729.5479 | 4 | 82.68 | 11 | F11:57840 | NaNaKA16\_F7.raw |  |  |  |  |  |  |  |  |  |  | 5.5123E7 |  |  | 2 | 0 | 0 | 0 | 0 | 0 | 0 | 0 | 0 | 0 | 0 | 2 | 0 | 0 | 16 | 41 | Carbamidomethylation | C10:Carbamidomethylation:1000.00;C12:Carbamidomethylation:1000.00 | PEAKS DB |
| total 3 peptides |  |  |  |  |  |  |  |  |  |  |  |  |  |  |  |  |  |  |  |  |  |  |  |  |  |  |  |  |  |  |  |  |  |  |  |  |  |  |  |  |  |  |  |
| --- | --- | --- | --- | --- | --- | --- | --- | --- | --- | --- | --- | --- | --- | --- | --- | --- | --- | --- | --- | --- | --- | --- | --- | --- | --- | --- | --- | --- | --- | --- | --- | --- | --- | --- | --- | --- | --- | --- | --- | --- | --- | --- | --- |

JAS05125.1

back to list

  

| Protein Coverage
| Supporting Peptides
|

Protein Coverage:

Supporting Peptides:

| Peptide | Uniq | -10lgP | Mass | Length | ppm | m/z | z | RT | Fraction | Scan | Source File | Area F1 | Area F10 | Area F11 | Area F12 | Area F13 | Area F2 | Area F3 | Area F4 | Area F5 | Area F6 | Area F7 | Area F8 | Area F9 | #Feature | #Feature F1 | #Feature F10 | #Feature F11 | #Feature F12 | #Feature F13 | #Feature F2 | #Feature F3 | #Feature F4 | #Feature F5 | #Feature F6 | #Feature F7 | #Feature F8 | #Feature F9 | Start | End | PTM | AScore | Found By |
| --- | --- | --- | --- | --- | --- | --- | --- | --- | --- | --- | --- | --- | --- | --- | --- | --- | --- | --- | --- | --- | --- | --- | --- | --- | --- | --- | --- | --- | --- | --- | --- | --- | --- | --- | --- | --- | --- | --- | --- | --- | --- | --- | --- |
| R.SWWDFADYGC(+57.02).Y | N | 45.71 | 1305.4761 | 10 | 0.8 | 653.7458 | 2 | 109.86 | 11 | F11:80150 | NaNaKA16\_F7.raw |  |  |  |  |  |  |  |  |  |  | 6.5059E6 |  |  | 1 | 0 | 0 | 0 | 0 | 0 | 0 | 0 | 0 | 0 | 0 | 1 | 0 | 0 | 43 | 52 | Carbamidomethylation | C10:Carbamidomethylation:1000.00 | PEAKS DB |
| R.SWWDFADYGC(+57.02)Y.C | N | 44.81 | 1468.5394 | 11 | 0.1 | 735.2771 | 2 | 111.77 | 10 | F10:82113 | NaNaKA16\_F6.raw |  |  |  |  |  |  |  |  |  | 1.9586E7 |  | 1.4496E7 |  | 2 | 0 | 0 | 0 | 0 | 0 | 0 | 0 | 0 | 0 | 1 | 0 | 1 | 0 | 43 | 53 | Carbamidomethylation | C10:Carbamidomethylation:1000.00 | PEAKS DB |
| R.SWWDFADYGC(+57.02)YC(+57.02)GSGGSGTPVDDLDK.C | Y | 43.00 | 2914.1487 | 26 | 4.7 | 729.5479 | 4 | 82.68 | 11 | F11:57840 | NaNaKA16\_F7.raw |  |  |  |  |  |  |  |  |  |  | 5.5123E7 |  |  | 2 | 0 | 0 | 0 | 0 | 0 | 0 | 0 | 0 | 0 | 0 | 2 | 0 | 0 | 43 | 68 | Carbamidomethylation | C10:Carbamidomethylation:1000.00;C12:Carbamidomethylation:1000.00 | PEAKS DB |
| total 3 peptides |  |  |  |  |  |  |  |  |  |  |  |  |  |  |  |  |  |  |  |  |  |  |  |  |  |  |  |  |  |  |  |  |  |  |  |  |  |  |  |  |  |  |  |
| --- | --- | --- | --- | --- | --- | --- | --- | --- | --- | --- | --- | --- | --- | --- | --- | --- | --- | --- | --- | --- | --- | --- | --- | --- | --- | --- | --- | --- | --- | --- | --- | --- | --- | --- | --- | --- | --- | --- | --- | --- | --- | --- | --- |

JAS05128.1

back to list

  

| Protein Coverage
| Supporting Peptides
|

Protein Coverage:

Supporting Peptides:

| Peptide | Uniq | -10lgP | Mass | Length | ppm | m/z | z | RT | Fraction | Scan | Source File | Area F1 | Area F10 | Area F11 | Area F12 | Area F13 | Area F2 | Area F3 | Area F4 | Area F5 | Area F6 | Area F7 | Area F8 | Area F9 | #Feature | #Feature F1 | #Feature F10 | #Feature F11 | #Feature F12 | #Feature F13 | #Feature F2 | #Feature F3 | #Feature F4 | #Feature F5 | #Feature F6 | #Feature F7 | #Feature F8 | #Feature F9 | Start | End | PTM | AScore | Found By |
| --- | --- | --- | --- | --- | --- | --- | --- | --- | --- | --- | --- | --- | --- | --- | --- | --- | --- | --- | --- | --- | --- | --- | --- | --- | --- | --- | --- | --- | --- | --- | --- | --- | --- | --- | --- | --- | --- | --- | --- | --- | --- | --- | --- |
| R.SWWDFADYGC(+57.02).Y | N | 45.71 | 1305.4761 | 10 | 0.8 | 653.7458 | 2 | 109.86 | 11 | F11:80150 | NaNaKA16\_F7.raw |  |  |  |  |  |  |  |  |  |  | 6.5059E6 |  |  | 1 | 0 | 0 | 0 | 0 | 0 | 0 | 0 | 0 | 0 | 0 | 1 | 0 | 0 | 43 | 52 | Carbamidomethylation | C10:Carbamidomethylation:1000.00 | PEAKS DB |
| R.SWWDFADYGC(+57.02)Y.C | N | 44.81 | 1468.5394 | 11 | 0.1 | 735.2771 | 2 | 111.77 | 10 | F10:82113 | NaNaKA16\_F6.raw |  |  |  |  |  |  |  |  |  | 1.9586E7 |  | 1.4496E7 |  | 2 | 0 | 0 | 0 | 0 | 0 | 0 | 0 | 0 | 0 | 1 | 0 | 1 | 0 | 43 | 53 | Carbamidomethylation | C10:Carbamidomethylation:1000.00 | PEAKS DB |
| R.SWWDFADYGC(+57.02)YC(+57.02)GSGGSGTPVDDLDK.C | Y | 43.00 | 2914.1487 | 26 | 4.7 | 729.5479 | 4 | 82.68 | 11 | F11:57840 | NaNaKA16\_F7.raw |  |  |  |  |  |  |  |  |  |  | 5.5123E7 |  |  | 2 | 0 | 0 | 0 | 0 | 0 | 0 | 0 | 0 | 0 | 0 | 2 | 0 | 0 | 43 | 68 | Carbamidomethylation | C10:Carbamidomethylation:1000.00;C12:Carbamidomethylation:1000.00 | PEAKS DB |
| total 3 peptides |  |  |  |  |  |  |  |  |  |  |  |  |  |  |  |  |  |  |  |  |  |  |  |  |  |  |  |  |  |  |  |  |  |  |  |  |  |  |  |  |  |  |  |
| --- | --- | --- | --- | --- | --- | --- | --- | --- | --- | --- | --- | --- | --- | --- | --- | --- | --- | --- | --- | --- | --- | --- | --- | --- | --- | --- | --- | --- | --- | --- | --- | --- | --- | --- | --- | --- | --- | --- | --- | --- | --- | --- | --- |

JAS05127.1

back to list

  

| Protein Coverage
| Supporting Peptides
|

Protein Coverage:

Supporting Peptides:

| Peptide | Uniq | -10lgP | Mass | Length | ppm | m/z | z | RT | Fraction | Scan | Source File | Area F1 | Area F10 | Area F11 | Area F12 | Area F13 | Area F2 | Area F3 | Area F4 | Area F5 | Area F6 | Area F7 | Area F8 | Area F9 | #Feature | #Feature F1 | #Feature F10 | #Feature F11 | #Feature F12 | #Feature F13 | #Feature F2 | #Feature F3 | #Feature F4 | #Feature F5 | #Feature F6 | #Feature F7 | #Feature F8 | #Feature F9 | Start | End | PTM | AScore | Found By |
| --- | --- | --- | --- | --- | --- | --- | --- | --- | --- | --- | --- | --- | --- | --- | --- | --- | --- | --- | --- | --- | --- | --- | --- | --- | --- | --- | --- | --- | --- | --- | --- | --- | --- | --- | --- | --- | --- | --- | --- | --- | --- | --- | --- |
| R.SWWDFADYGC(+57.02).Y | N | 45.71 | 1305.4761 | 10 | 0.8 | 653.7458 | 2 | 109.86 | 11 | F11:80150 | NaNaKA16\_F7.raw |  |  |  |  |  |  |  |  |  |  | 6.5059E6 |  |  | 1 | 0 | 0 | 0 | 0 | 0 | 0 | 0 | 0 | 0 | 0 | 1 | 0 | 0 | 43 | 52 | Carbamidomethylation | C10:Carbamidomethylation:1000.00 | PEAKS DB |
| R.SWWDFADYGC(+57.02)Y.C | N | 44.81 | 1468.5394 | 11 | 0.1 | 735.2771 | 2 | 111.77 | 10 | F10:82113 | NaNaKA16\_F6.raw |  |  |  |  |  |  |  |  |  | 1.9586E7 |  | 1.4496E7 |  | 2 | 0 | 0 | 0 | 0 | 0 | 0 | 0 | 0 | 0 | 1 | 0 | 1 | 0 | 43 | 53 | Carbamidomethylation | C10:Carbamidomethylation:1000.00 | PEAKS DB |
| R.SWWDFADYGC(+57.02)YC(+57.02)GSGGSGTPVDDLDK.C | Y | 43.00 | 2914.1487 | 26 | 4.7 | 729.5479 | 4 | 82.68 | 11 | F11:57840 | NaNaKA16\_F7.raw |  |  |  |  |  |  |  |  |  |  | 5.5123E7 |  |  | 2 | 0 | 0 | 0 | 0 | 0 | 0 | 0 | 0 | 0 | 0 | 2 | 0 | 0 | 43 | 68 | Carbamidomethylation | C10:Carbamidomethylation:1000.00;C12:Carbamidomethylation:1000.00 | PEAKS DB |
| total 3 peptides |  |  |  |  |  |  |  |  |  |  |  |  |  |  |  |  |  |  |  |  |  |  |  |  |  |  |  |  |  |  |  |  |  |  |  |  |  |  |  |  |  |  |  |
| --- | --- | --- | --- | --- | --- | --- | --- | --- | --- | --- | --- | --- | --- | --- | --- | --- | --- | --- | --- | --- | --- | --- | --- | --- | --- | --- | --- | --- | --- | --- | --- | --- | --- | --- | --- | --- | --- | --- | --- | --- | --- | --- | --- |

JAS05126.1

back to list

  

| Protein Coverage
| Supporting Peptides
|

Protein Coverage:

Supporting Peptides:

| Peptide | Uniq | -10lgP | Mass | Length | ppm | m/z | z | RT | Fraction | Scan | Source File | Area F1 | Area F10 | Area F11 | Area F12 | Area F13 | Area F2 | Area F3 | Area F4 | Area F5 | Area F6 | Area F7 | Area F8 | Area F9 | #Feature | #Feature F1 | #Feature F10 | #Feature F11 | #Feature F12 | #Feature F13 | #Feature F2 | #Feature F3 | #Feature F4 | #Feature F5 | #Feature F6 | #Feature F7 | #Feature F8 | #Feature F9 | Start | End | PTM | AScore | Found By |
| --- | --- | --- | --- | --- | --- | --- | --- | --- | --- | --- | --- | --- | --- | --- | --- | --- | --- | --- | --- | --- | --- | --- | --- | --- | --- | --- | --- | --- | --- | --- | --- | --- | --- | --- | --- | --- | --- | --- | --- | --- | --- | --- | --- |
| R.SWWDFADYGC(+57.02).Y | N | 45.71 | 1305.4761 | 10 | 0.8 | 653.7458 | 2 | 109.86 | 11 | F11:80150 | NaNaKA16\_F7.raw |  |  |  |  |  |  |  |  |  |  | 6.5059E6 |  |  | 1 | 0 | 0 | 0 | 0 | 0 | 0 | 0 | 0 | 0 | 0 | 1 | 0 | 0 | 43 | 52 | Carbamidomethylation | C10:Carbamidomethylation:1000.00 | PEAKS DB |
| R.SWWDFADYGC(+57.02)Y.C | N | 44.81 | 1468.5394 | 11 | 0.1 | 735.2771 | 2 | 111.77 | 10 | F10:82113 | NaNaKA16\_F6.raw |  |  |  |  |  |  |  |  |  | 1.9586E7 |  | 1.4496E7 |  | 2 | 0 | 0 | 0 | 0 | 0 | 0 | 0 | 0 | 0 | 1 | 0 | 1 | 0 | 43 | 53 | Carbamidomethylation | C10:Carbamidomethylation:1000.00 | PEAKS DB |
| R.SWWDFADYGC(+57.02)YC(+57.02)GSGGSGTPVDDLDK.C | Y | 43.00 | 2914.1487 | 26 | 4.7 | 729.5479 | 4 | 82.68 | 11 | F11:57840 | NaNaKA16\_F7.raw |  |  |  |  |  |  |  |  |  |  | 5.5123E7 |  |  | 2 | 0 | 0 | 0 | 0 | 0 | 0 | 0 | 0 | 0 | 0 | 2 | 0 | 0 | 43 | 68 | Carbamidomethylation | C10:Carbamidomethylation:1000.00;C12:Carbamidomethylation:1000.00 | PEAKS DB |
| total 3 peptides |  |  |  |  |  |  |  |  |  |  |  |  |  |  |  |  |  |  |  |  |  |  |  |  |  |  |  |  |  |  |  |  |  |  |  |  |  |  |  |  |  |  |  |
| --- | --- | --- | --- | --- | --- | --- | --- | --- | --- | --- | --- | --- | --- | --- | --- | --- | --- | --- | --- | --- | --- | --- | --- | --- | --- | --- | --- | --- | --- | --- | --- | --- | --- | --- | --- | --- | --- | --- | --- | --- | --- | --- | --- |

JAB52812.1

back to list

  

| Protein Coverage
| Supporting Peptides
|

Protein Coverage:

Supporting Peptides:

| Peptide | Uniq | -10lgP | Mass | Length | ppm | m/z | z | RT | Fraction | Scan | Source File | Area F1 | Area F10 | Area F11 | Area F12 | Area F13 | Area F2 | Area F3 | Area F4 | Area F5 | Area F6 | Area F7 | Area F8 | Area F9 | #Feature | #Feature F1 | #Feature F10 | #Feature F11 | #Feature F12 | #Feature F13 | #Feature F2 | #Feature F3 | #Feature F4 | #Feature F5 | #Feature F6 | #Feature F7 | #Feature F8 | #Feature F9 | Start | End | PTM | AScore | Found By |
| --- | --- | --- | --- | --- | --- | --- | --- | --- | --- | --- | --- | --- | --- | --- | --- | --- | --- | --- | --- | --- | --- | --- | --- | --- | --- | --- | --- | --- | --- | --- | --- | --- | --- | --- | --- | --- | --- | --- | --- | --- | --- | --- | --- |
| R.SWWDFADYGC(+57.02).Y | N | 45.71 | 1305.4761 | 10 | 0.8 | 653.7458 | 2 | 109.86 | 11 | F11:80150 | NaNaKA16\_F7.raw |  |  |  |  |  |  |  |  |  |  | 6.5059E6 |  |  | 1 | 0 | 0 | 0 | 0 | 0 | 0 | 0 | 0 | 0 | 0 | 1 | 0 | 0 | 41 | 50 | Carbamidomethylation | C10:Carbamidomethylation:1000.00 | PEAKS DB |
| R.SWWDFADYGC(+57.02)Y.C | N | 44.81 | 1468.5394 | 11 | 0.1 | 735.2771 | 2 | 111.77 | 10 | F10:82113 | NaNaKA16\_F6.raw |  |  |  |  |  |  |  |  |  | 1.9586E7 |  | 1.4496E7 |  | 2 | 0 | 0 | 0 | 0 | 0 | 0 | 0 | 0 | 0 | 1 | 0 | 1 | 0 | 41 | 51 | Carbamidomethylation | C10:Carbamidomethylation:1000.00 | PEAKS DB |
| R.SWWDFADYGC(+57.02)YC(+57.02)GSGGSGTPVDDLDK.C | Y | 43.00 | 2914.1487 | 26 | 4.7 | 729.5479 | 4 | 82.68 | 11 | F11:57840 | NaNaKA16\_F7.raw |  |  |  |  |  |  |  |  |  |  | 5.5123E7 |  |  | 2 | 0 | 0 | 0 | 0 | 0 | 0 | 0 | 0 | 0 | 0 | 2 | 0 | 0 | 41 | 66 | Carbamidomethylation | C10:Carbamidomethylation:1000.00;C12:Carbamidomethylation:1000.00 | PEAKS DB |
| total 3 peptides |  |  |  |  |  |  |  |  |  |  |  |  |  |  |  |  |  |  |  |  |  |  |  |  |  |  |  |  |  |  |  |  |  |  |  |  |  |  |  |  |  |  |  |
| --- | --- | --- | --- | --- | --- | --- | --- | --- | --- | --- | --- | --- | --- | --- | --- | --- | --- | --- | --- | --- | --- | --- | --- | --- | --- | --- | --- | --- | --- | --- | --- | --- | --- | --- | --- | --- | --- | --- | --- | --- | --- | --- | --- |

JAB52803.1

back to list

  

| Protein Coverage
| Supporting Peptides
|

Protein Coverage:

Supporting Peptides:

| Peptide | Uniq | -10lgP | Mass | Length | ppm | m/z | z | RT | Fraction | Scan | Source File | Area F1 | Area F10 | Area F11 | Area F12 | Area F13 | Area F2 | Area F3 | Area F4 | Area F5 | Area F6 | Area F7 | Area F8 | Area F9 | #Feature | #Feature F1 | #Feature F10 | #Feature F11 | #Feature F12 | #Feature F13 | #Feature F2 | #Feature F3 | #Feature F4 | #Feature F5 | #Feature F6 | #Feature F7 | #Feature F8 | #Feature F9 | Start | End | PTM | AScore | Found By |
| --- | --- | --- | --- | --- | --- | --- | --- | --- | --- | --- | --- | --- | --- | --- | --- | --- | --- | --- | --- | --- | --- | --- | --- | --- | --- | --- | --- | --- | --- | --- | --- | --- | --- | --- | --- | --- | --- | --- | --- | --- | --- | --- | --- |
| R.SWWDFADYGC(+57.02).Y | N | 45.71 | 1305.4761 | 10 | 0.8 | 653.7458 | 2 | 109.86 | 11 | F11:80150 | NaNaKA16\_F7.raw |  |  |  |  |  |  |  |  |  |  | 6.5059E6 |  |  | 1 | 0 | 0 | 0 | 0 | 0 | 0 | 0 | 0 | 0 | 0 | 1 | 0 | 0 | 43 | 52 | Carbamidomethylation | C10:Carbamidomethylation:1000.00 | PEAKS DB |
| R.SWWDFADYGC(+57.02)Y.C | N | 44.81 | 1468.5394 | 11 | 0.1 | 735.2771 | 2 | 111.77 | 10 | F10:82113 | NaNaKA16\_F6.raw |  |  |  |  |  |  |  |  |  | 1.9586E7 |  | 1.4496E7 |  | 2 | 0 | 0 | 0 | 0 | 0 | 0 | 0 | 0 | 0 | 1 | 0 | 1 | 0 | 43 | 53 | Carbamidomethylation | C10:Carbamidomethylation:1000.00 | PEAKS DB |
| R.SWWDFADYGC(+57.02)YC(+57.02)GSGGSGTPVDDLDK.C | Y | 43.00 | 2914.1487 | 26 | 4.7 | 729.5479 | 4 | 82.68 | 11 | F11:57840 | NaNaKA16\_F7.raw |  |  |  |  |  |  |  |  |  |  | 5.5123E7 |  |  | 2 | 0 | 0 | 0 | 0 | 0 | 0 | 0 | 0 | 0 | 0 | 2 | 0 | 0 | 43 | 68 | Carbamidomethylation | C10:Carbamidomethylation:1000.00;C12:Carbamidomethylation:1000.00 | PEAKS DB |
| total 3 peptides |  |  |  |  |  |  |  |  |  |  |  |  |  |  |  |  |  |  |  |  |  |  |  |  |  |  |  |  |  |  |  |  |  |  |  |  |  |  |  |  |  |  |  |
| --- | --- | --- | --- | --- | --- | --- | --- | --- | --- | --- | --- | --- | --- | --- | --- | --- | --- | --- | --- | --- | --- | --- | --- | --- | --- | --- | --- | --- | --- | --- | --- | --- | --- | --- | --- | --- | --- | --- | --- | --- | --- | --- | --- |

JAS05033.1

back to list

  

| Protein Coverage
| Supporting Peptides
|

Protein Coverage:

Supporting Peptides:

| Peptide | Uniq | -10lgP | Mass | Length | ppm | m/z | z | RT | Fraction | Scan | Source File | Area F1 | Area F10 | Area F11 | Area F12 | Area F13 | Area F2 | Area F3 | Area F4 | Area F5 | Area F6 | Area F7 | Area F8 | Area F9 | #Feature | #Feature F1 | #Feature F10 | #Feature F11 | #Feature F12 | #Feature F13 | #Feature F2 | #Feature F3 | #Feature F4 | #Feature F5 | #Feature F6 | #Feature F7 | #Feature F8 | #Feature F9 | Start | End | PTM | AScore | Found By |
| --- | --- | --- | --- | --- | --- | --- | --- | --- | --- | --- | --- | --- | --- | --- | --- | --- | --- | --- | --- | --- | --- | --- | --- | --- | --- | --- | --- | --- | --- | --- | --- | --- | --- | --- | --- | --- | --- | --- | --- | --- | --- | --- | --- |
| R.SWWDFADYGC(+57.02).Y | N | 45.71 | 1305.4761 | 10 | 0.8 | 653.7458 | 2 | 109.86 | 11 | F11:80150 | NaNaKA16\_F7.raw |  |  |  |  |  |  |  |  |  |  | 6.5059E6 |  |  | 1 | 0 | 0 | 0 | 0 | 0 | 0 | 0 | 0 | 0 | 0 | 1 | 0 | 0 | 43 | 52 | Carbamidomethylation | C10:Carbamidomethylation:1000.00 | PEAKS DB |
| R.SWWDFADYGC(+57.02)Y.C | N | 44.81 | 1468.5394 | 11 | 0.1 | 735.2771 | 2 | 111.77 | 10 | F10:82113 | NaNaKA16\_F6.raw |  |  |  |  |  |  |  |  |  | 1.9586E7 |  | 1.4496E7 |  | 2 | 0 | 0 | 0 | 0 | 0 | 0 | 0 | 0 | 0 | 1 | 0 | 1 | 0 | 43 | 53 | Carbamidomethylation | C10:Carbamidomethylation:1000.00 | PEAKS DB |
| R.SWWDFADYGC(+57.02)YC(+57.02)GSGGSGTPVDDLDK.C | Y | 43.00 | 2914.1487 | 26 | 4.7 | 729.5479 | 4 | 82.68 | 11 | F11:57840 | NaNaKA16\_F7.raw |  |  |  |  |  |  |  |  |  |  | 5.5123E7 |  |  | 2 | 0 | 0 | 0 | 0 | 0 | 0 | 0 | 0 | 0 | 0 | 2 | 0 | 0 | 43 | 68 | Carbamidomethylation | C10:Carbamidomethylation:1000.00;C12:Carbamidomethylation:1000.00 | PEAKS DB |
| total 3 peptides |  |  |  |  |  |  |  |  |  |  |  |  |  |  |  |  |  |  |  |  |  |  |  |  |  |  |  |  |  |  |  |  |  |  |  |  |  |  |  |  |  |  |  |
| --- | --- | --- | --- | --- | --- | --- | --- | --- | --- | --- | --- | --- | --- | --- | --- | --- | --- | --- | --- | --- | --- | --- | --- | --- | --- | --- | --- | --- | --- | --- | --- | --- | --- | --- | --- | --- | --- | --- | --- | --- | --- | --- | --- |

JAI09044.1

back to list

  

| Protein Coverage
| Supporting Peptides
|

Protein Coverage:

Supporting Peptides:

| Peptide | Uniq | -10lgP | Mass | Length | ppm | m/z | z | RT | Fraction | Scan | Source File | Area F1 | Area F10 | Area F11 | Area F12 | Area F13 | Area F2 | Area F3 | Area F4 | Area F5 | Area F6 | Area F7 | Area F8 | Area F9 | #Feature | #Feature F1 | #Feature F10 | #Feature F11 | #Feature F12 | #Feature F13 | #Feature F2 | #Feature F3 | #Feature F4 | #Feature F5 | #Feature F6 | #Feature F7 | #Feature F8 | #Feature F9 | Start | End | PTM | AScore | Found By |
| --- | --- | --- | --- | --- | --- | --- | --- | --- | --- | --- | --- | --- | --- | --- | --- | --- | --- | --- | --- | --- | --- | --- | --- | --- | --- | --- | --- | --- | --- | --- | --- | --- | --- | --- | --- | --- | --- | --- | --- | --- | --- | --- | --- |
| R.SWWDFADYGC(+57.02).Y | N | 45.71 | 1305.4761 | 10 | 0.8 | 653.7458 | 2 | 109.86 | 11 | F11:80150 | NaNaKA16\_F7.raw |  |  |  |  |  |  |  |  |  |  | 6.5059E6 |  |  | 1 | 0 | 0 | 0 | 0 | 0 | 0 | 0 | 0 | 0 | 0 | 1 | 0 | 0 | 43 | 52 | Carbamidomethylation | C10:Carbamidomethylation:1000.00 | PEAKS DB |
| R.SWWDFADYGC(+57.02)Y.C | N | 44.81 | 1468.5394 | 11 | 0.1 | 735.2771 | 2 | 111.77 | 10 | F10:82113 | NaNaKA16\_F6.raw |  |  |  |  |  |  |  |  |  | 1.9586E7 |  | 1.4496E7 |  | 2 | 0 | 0 | 0 | 0 | 0 | 0 | 0 | 0 | 0 | 1 | 0 | 1 | 0 | 43 | 53 | Carbamidomethylation | C10:Carbamidomethylation:1000.00 | PEAKS DB |
| R.SWWDFADYGC(+57.02)YC(+57.02)GSGGSGTPVDDLDK.C | Y | 43.00 | 2914.1487 | 26 | 4.7 | 729.5479 | 4 | 82.68 | 11 | F11:57840 | NaNaKA16\_F7.raw |  |  |  |  |  |  |  |  |  |  | 5.5123E7 |  |  | 2 | 0 | 0 | 0 | 0 | 0 | 0 | 0 | 0 | 0 | 0 | 2 | 0 | 0 | 43 | 68 | Carbamidomethylation | C10:Carbamidomethylation:1000.00;C12:Carbamidomethylation:1000.00 | PEAKS DB |
| total 3 peptides |  |  |  |  |  |  |  |  |  |  |  |  |  |  |  |  |  |  |  |  |  |  |  |  |  |  |  |  |  |  |  |  |  |  |  |  |  |  |  |  |  |  |  |
| --- | --- | --- | --- | --- | --- | --- | --- | --- | --- | --- | --- | --- | --- | --- | --- | --- | --- | --- | --- | --- | --- | --- | --- | --- | --- | --- | --- | --- | --- | --- | --- | --- | --- | --- | --- | --- | --- | --- | --- | --- | --- | --- | --- |

JAS05025.1

back to list

  

| Protein Coverage
| Supporting Peptides
|

Protein Coverage:

Supporting Peptides:

| Peptide | Uniq | -10lgP | Mass | Length | ppm | m/z | z | RT | Fraction | Scan | Source File | Area F1 | Area F10 | Area F11 | Area F12 | Area F13 | Area F2 | Area F3 | Area F4 | Area F5 | Area F6 | Area F7 | Area F8 | Area F9 | #Feature | #Feature F1 | #Feature F10 | #Feature F11 | #Feature F12 | #Feature F13 | #Feature F2 | #Feature F3 | #Feature F4 | #Feature F5 | #Feature F6 | #Feature F7 | #Feature F8 | #Feature F9 | Start | End | PTM | AScore | Found By |
| --- | --- | --- | --- | --- | --- | --- | --- | --- | --- | --- | --- | --- | --- | --- | --- | --- | --- | --- | --- | --- | --- | --- | --- | --- | --- | --- | --- | --- | --- | --- | --- | --- | --- | --- | --- | --- | --- | --- | --- | --- | --- | --- | --- |
| R.SWWDFADYGC(+57.02).Y | N | 45.71 | 1305.4761 | 10 | 0.8 | 653.7458 | 2 | 109.86 | 11 | F11:80150 | NaNaKA16\_F7.raw |  |  |  |  |  |  |  |  |  |  | 6.5059E6 |  |  | 1 | 0 | 0 | 0 | 0 | 0 | 0 | 0 | 0 | 0 | 0 | 1 | 0 | 0 | 43 | 52 | Carbamidomethylation | C10:Carbamidomethylation:1000.00 | PEAKS DB |
| R.SWWDFADYGC(+57.02)Y.C | N | 44.81 | 1468.5394 | 11 | 0.1 | 735.2771 | 2 | 111.77 | 10 | F10:82113 | NaNaKA16\_F6.raw |  |  |  |  |  |  |  |  |  | 1.9586E7 |  | 1.4496E7 |  | 2 | 0 | 0 | 0 | 0 | 0 | 0 | 0 | 0 | 0 | 1 | 0 | 1 | 0 | 43 | 53 | Carbamidomethylation | C10:Carbamidomethylation:1000.00 | PEAKS DB |
| R.SWWDFADYGC(+57.02)YC(+57.02)GSGGSGTPVDDLDK.C | Y | 43.00 | 2914.1487 | 26 | 4.7 | 729.5479 | 4 | 82.68 | 11 | F11:57840 | NaNaKA16\_F7.raw |  |  |  |  |  |  |  |  |  |  | 5.5123E7 |  |  | 2 | 0 | 0 | 0 | 0 | 0 | 0 | 0 | 0 | 0 | 0 | 2 | 0 | 0 | 43 | 68 | Carbamidomethylation | C10:Carbamidomethylation:1000.00;C12:Carbamidomethylation:1000.00 | PEAKS DB |
| total 3 peptides |  |  |  |  |  |  |  |  |  |  |  |  |  |  |  |  |  |  |  |  |  |  |  |  |  |  |  |  |  |  |  |  |  |  |  |  |  |  |  |  |  |  |  |
| --- | --- | --- | --- | --- | --- | --- | --- | --- | --- | --- | --- | --- | --- | --- | --- | --- | --- | --- | --- | --- | --- | --- | --- | --- | --- | --- | --- | --- | --- | --- | --- | --- | --- | --- | --- | --- | --- | --- | --- | --- | --- | --- | --- |

JAS05019.1

back to list

  

| Protein Coverage
| Supporting Peptides
|

Protein Coverage:

Supporting Peptides:

| Peptide | Uniq | -10lgP | Mass | Length | ppm | m/z | z | RT | Fraction | Scan | Source File | Area F1 | Area F10 | Area F11 | Area F12 | Area F13 | Area F2 | Area F3 | Area F4 | Area F5 | Area F6 | Area F7 | Area F8 | Area F9 | #Feature | #Feature F1 | #Feature F10 | #Feature F11 | #Feature F12 | #Feature F13 | #Feature F2 | #Feature F3 | #Feature F4 | #Feature F5 | #Feature F6 | #Feature F7 | #Feature F8 | #Feature F9 | Start | End | PTM | AScore | Found By |
| --- | --- | --- | --- | --- | --- | --- | --- | --- | --- | --- | --- | --- | --- | --- | --- | --- | --- | --- | --- | --- | --- | --- | --- | --- | --- | --- | --- | --- | --- | --- | --- | --- | --- | --- | --- | --- | --- | --- | --- | --- | --- | --- | --- |
| R.SWWDFADYGC(+57.02).Y | N | 45.71 | 1305.4761 | 10 | 0.8 | 653.7458 | 2 | 109.86 | 11 | F11:80150 | NaNaKA16\_F7.raw |  |  |  |  |  |  |  |  |  |  | 6.5059E6 |  |  | 1 | 0 | 0 | 0 | 0 | 0 | 0 | 0 | 0 | 0 | 0 | 1 | 0 | 0 | 43 | 52 | Carbamidomethylation | C10:Carbamidomethylation:1000.00 | PEAKS DB |
| R.SWWDFADYGC(+57.02)Y.C | N | 44.81 | 1468.5394 | 11 | 0.1 | 735.2771 | 2 | 111.77 | 10 | F10:82113 | NaNaKA16\_F6.raw |  |  |  |  |  |  |  |  |  | 1.9586E7 |  | 1.4496E7 |  | 2 | 0 | 0 | 0 | 0 | 0 | 0 | 0 | 0 | 0 | 1 | 0 | 1 | 0 | 43 | 53 | Carbamidomethylation | C10:Carbamidomethylation:1000.00 | PEAKS DB |
| R.SWWDFADYGC(+57.02)YC(+57.02)GSGGSGTPVDDLDK.C | Y | 43.00 | 2914.1487 | 26 | 4.7 | 729.5479 | 4 | 82.68 | 11 | F11:57840 | NaNaKA16\_F7.raw |  |  |  |  |  |  |  |  |  |  | 5.5123E7 |  |  | 2 | 0 | 0 | 0 | 0 | 0 | 0 | 0 | 0 | 0 | 0 | 2 | 0 | 0 | 43 | 68 | Carbamidomethylation | C10:Carbamidomethylation:1000.00;C12:Carbamidomethylation:1000.00 | PEAKS DB |
| total 3 peptides |  |  |  |  |  |  |  |  |  |  |  |  |  |  |  |  |  |  |  |  |  |  |  |  |  |  |  |  |  |  |  |  |  |  |  |  |  |  |  |  |  |  |  |
| --- | --- | --- | --- | --- | --- | --- | --- | --- | --- | --- | --- | --- | --- | --- | --- | --- | --- | --- | --- | --- | --- | --- | --- | --- | --- | --- | --- | --- | --- | --- | --- | --- | --- | --- | --- | --- | --- | --- | --- | --- | --- | --- | --- |

JAI09030.1

back to list

  

| Protein Coverage
| Supporting Peptides
|

Protein Coverage:

Supporting Peptides:

| Peptide | Uniq | -10lgP | Mass | Length | ppm | m/z | z | RT | Fraction | Scan | Source File | Area F1 | Area F10 | Area F11 | Area F12 | Area F13 | Area F2 | Area F3 | Area F4 | Area F5 | Area F6 | Area F7 | Area F8 | Area F9 | #Feature | #Feature F1 | #Feature F10 | #Feature F11 | #Feature F12 | #Feature F13 | #Feature F2 | #Feature F3 | #Feature F4 | #Feature F5 | #Feature F6 | #Feature F7 | #Feature F8 | #Feature F9 | Start | End | PTM | AScore | Found By |
| --- | --- | --- | --- | --- | --- | --- | --- | --- | --- | --- | --- | --- | --- | --- | --- | --- | --- | --- | --- | --- | --- | --- | --- | --- | --- | --- | --- | --- | --- | --- | --- | --- | --- | --- | --- | --- | --- | --- | --- | --- | --- | --- | --- |
| R.SWWDFADYGC(+57.02).Y | N | 45.71 | 1305.4761 | 10 | 0.8 | 653.7458 | 2 | 109.86 | 11 | F11:80150 | NaNaKA16\_F7.raw |  |  |  |  |  |  |  |  |  |  | 6.5059E6 |  |  | 1 | 0 | 0 | 0 | 0 | 0 | 0 | 0 | 0 | 0 | 0 | 1 | 0 | 0 | 43 | 52 | Carbamidomethylation | C10:Carbamidomethylation:1000.00 | PEAKS DB |
| R.SWWDFADYGC(+57.02)Y.C | N | 44.81 | 1468.5394 | 11 | 0.1 | 735.2771 | 2 | 111.77 | 10 | F10:82113 | NaNaKA16\_F6.raw |  |  |  |  |  |  |  |  |  | 1.9586E7 |  | 1.4496E7 |  | 2 | 0 | 0 | 0 | 0 | 0 | 0 | 0 | 0 | 0 | 1 | 0 | 1 | 0 | 43 | 53 | Carbamidomethylation | C10:Carbamidomethylation:1000.00 | PEAKS DB |
| R.SWWDFADYGC(+57.02)YC(+57.02)GSGGSGTPVDDLDK.C | Y | 43.00 | 2914.1487 | 26 | 4.7 | 729.5479 | 4 | 82.68 | 11 | F11:57840 | NaNaKA16\_F7.raw |  |  |  |  |  |  |  |  |  |  | 5.5123E7 |  |  | 2 | 0 | 0 | 0 | 0 | 0 | 0 | 0 | 0 | 0 | 0 | 2 | 0 | 0 | 43 | 68 | Carbamidomethylation | C10:Carbamidomethylation:1000.00;C12:Carbamidomethylation:1000.00 | PEAKS DB |
| total 3 peptides |  |  |  |  |  |  |  |  |  |  |  |  |  |  |  |  |  |  |  |  |  |  |  |  |  |  |  |  |  |  |  |  |  |  |  |  |  |  |  |  |  |  |  |
| --- | --- | --- | --- | --- | --- | --- | --- | --- | --- | --- | --- | --- | --- | --- | --- | --- | --- | --- | --- | --- | --- | --- | --- | --- | --- | --- | --- | --- | --- | --- | --- | --- | --- | --- | --- | --- | --- | --- | --- | --- | --- | --- | --- |

JAI09036.1

back to list

  

| Protein Coverage
| Supporting Peptides
|

Protein Coverage:

Supporting Peptides:

| Peptide | Uniq | -10lgP | Mass | Length | ppm | m/z | z | RT | Fraction | Scan | Source File | Area F1 | Area F10 | Area F11 | Area F12 | Area F13 | Area F2 | Area F3 | Area F4 | Area F5 | Area F6 | Area F7 | Area F8 | Area F9 | #Feature | #Feature F1 | #Feature F10 | #Feature F11 | #Feature F12 | #Feature F13 | #Feature F2 | #Feature F3 | #Feature F4 | #Feature F5 | #Feature F6 | #Feature F7 | #Feature F8 | #Feature F9 | Start | End | PTM | AScore | Found By |
| --- | --- | --- | --- | --- | --- | --- | --- | --- | --- | --- | --- | --- | --- | --- | --- | --- | --- | --- | --- | --- | --- | --- | --- | --- | --- | --- | --- | --- | --- | --- | --- | --- | --- | --- | --- | --- | --- | --- | --- | --- | --- | --- | --- |
| R.SWWDFADYGC(+57.02).Y | N | 45.71 | 1305.4761 | 10 | 0.8 | 653.7458 | 2 | 109.86 | 11 | F11:80150 | NaNaKA16\_F7.raw |  |  |  |  |  |  |  |  |  |  | 6.5059E6 |  |  | 1 | 0 | 0 | 0 | 0 | 0 | 0 | 0 | 0 | 0 | 0 | 1 | 0 | 0 | 43 | 52 | Carbamidomethylation | C10:Carbamidomethylation:1000.00 | PEAKS DB |
| R.SWWDFADYGC(+57.02)Y.C | N | 44.81 | 1468.5394 | 11 | 0.1 | 735.2771 | 2 | 111.77 | 10 | F10:82113 | NaNaKA16\_F6.raw |  |  |  |  |  |  |  |  |  | 1.9586E7 |  | 1.4496E7 |  | 2 | 0 | 0 | 0 | 0 | 0 | 0 | 0 | 0 | 0 | 1 | 0 | 1 | 0 | 43 | 53 | Carbamidomethylation | C10:Carbamidomethylation:1000.00 | PEAKS DB |
| R.SWWDFADYGC(+57.02)YC(+57.02)GSGGSGTPVDDLDK.C | Y | 43.00 | 2914.1487 | 26 | 4.7 | 729.5479 | 4 | 82.68 | 11 | F11:57840 | NaNaKA16\_F7.raw |  |  |  |  |  |  |  |  |  |  | 5.5123E7 |  |  | 2 | 0 | 0 | 0 | 0 | 0 | 0 | 0 | 0 | 0 | 0 | 2 | 0 | 0 | 43 | 68 | Carbamidomethylation | C10:Carbamidomethylation:1000.00;C12:Carbamidomethylation:1000.00 | PEAKS DB |
| total 3 peptides |  |  |  |  |  |  |  |  |  |  |  |  |  |  |  |  |  |  |  |  |  |  |  |  |  |  |  |  |  |  |  |  |  |  |  |  |  |  |  |  |  |  |  |
| --- | --- | --- | --- | --- | --- | --- | --- | --- | --- | --- | --- | --- | --- | --- | --- | --- | --- | --- | --- | --- | --- | --- | --- | --- | --- | --- | --- | --- | --- | --- | --- | --- | --- | --- | --- | --- | --- | --- | --- | --- | --- | --- | --- |

JAB52789.1

back to list

  

| Protein Coverage
| Supporting Peptides
|

Protein Coverage:

Supporting Peptides:

| Peptide | Uniq | -10lgP | Mass | Length | ppm | m/z | z | RT | Fraction | Scan | Source File | Area F1 | Area F10 | Area F11 | Area F12 | Area F13 | Area F2 | Area F3 | Area F4 | Area F5 | Area F6 | Area F7 | Area F8 | Area F9 | #Feature | #Feature F1 | #Feature F10 | #Feature F11 | #Feature F12 | #Feature F13 | #Feature F2 | #Feature F3 | #Feature F4 | #Feature F5 | #Feature F6 | #Feature F7 | #Feature F8 | #Feature F9 | Start | End | PTM | AScore | Found By |
| --- | --- | --- | --- | --- | --- | --- | --- | --- | --- | --- | --- | --- | --- | --- | --- | --- | --- | --- | --- | --- | --- | --- | --- | --- | --- | --- | --- | --- | --- | --- | --- | --- | --- | --- | --- | --- | --- | --- | --- | --- | --- | --- | --- |
| R.SWWDFADYGC(+57.02).Y | N | 45.71 | 1305.4761 | 10 | 0.8 | 653.7458 | 2 | 109.86 | 11 | F11:80150 | NaNaKA16\_F7.raw |  |  |  |  |  |  |  |  |  |  | 6.5059E6 |  |  | 1 | 0 | 0 | 0 | 0 | 0 | 0 | 0 | 0 | 0 | 0 | 1 | 0 | 0 | 43 | 52 | Carbamidomethylation | C10:Carbamidomethylation:1000.00 | PEAKS DB |
| R.SWWDFADYGC(+57.02)Y.C | N | 44.81 | 1468.5394 | 11 | 0.1 | 735.2771 | 2 | 111.77 | 10 | F10:82113 | NaNaKA16\_F6.raw |  |  |  |  |  |  |  |  |  | 1.9586E7 |  | 1.4496E7 |  | 2 | 0 | 0 | 0 | 0 | 0 | 0 | 0 | 0 | 0 | 1 | 0 | 1 | 0 | 43 | 53 | Carbamidomethylation | C10:Carbamidomethylation:1000.00 | PEAKS DB |
| R.SWWDFADYGC(+57.02)YC(+57.02)GSGGSGTPVDDLDK.C | Y | 43.00 | 2914.1487 | 26 | 4.7 | 729.5479 | 4 | 82.68 | 11 | F11:57840 | NaNaKA16\_F7.raw |  |  |  |  |  |  |  |  |  |  | 5.5123E7 |  |  | 2 | 0 | 0 | 0 | 0 | 0 | 0 | 0 | 0 | 0 | 0 | 2 | 0 | 0 | 43 | 68 | Carbamidomethylation | C10:Carbamidomethylation:1000.00;C12:Carbamidomethylation:1000.00 | PEAKS DB |
| total 3 peptides |  |  |  |  |  |  |  |  |  |  |  |  |  |  |  |  |  |  |  |  |  |  |  |  |  |  |  |  |  |  |  |  |  |  |  |  |  |  |  |  |  |  |  |
| --- | --- | --- | --- | --- | --- | --- | --- | --- | --- | --- | --- | --- | --- | --- | --- | --- | --- | --- | --- | --- | --- | --- | --- | --- | --- | --- | --- | --- | --- | --- | --- | --- | --- | --- | --- | --- | --- | --- | --- | --- | --- | --- | --- |

JAS05020.1

back to list

  

| Protein Coverage
| Supporting Peptides
|

Protein Coverage:

Supporting Peptides:

| Peptide | Uniq | -10lgP | Mass | Length | ppm | m/z | z | RT | Fraction | Scan | Source File | Area F1 | Area F10 | Area F11 | Area F12 | Area F13 | Area F2 | Area F3 | Area F4 | Area F5 | Area F6 | Area F7 | Area F8 | Area F9 | #Feature | #Feature F1 | #Feature F10 | #Feature F11 | #Feature F12 | #Feature F13 | #Feature F2 | #Feature F3 | #Feature F4 | #Feature F5 | #Feature F6 | #Feature F7 | #Feature F8 | #Feature F9 | Start | End | PTM | AScore | Found By |
| --- | --- | --- | --- | --- | --- | --- | --- | --- | --- | --- | --- | --- | --- | --- | --- | --- | --- | --- | --- | --- | --- | --- | --- | --- | --- | --- | --- | --- | --- | --- | --- | --- | --- | --- | --- | --- | --- | --- | --- | --- | --- | --- | --- |
| R.SWWDFADYGC(+57.02).Y | N | 45.71 | 1305.4761 | 10 | 0.8 | 653.7458 | 2 | 109.86 | 11 | F11:80150 | NaNaKA16\_F7.raw |  |  |  |  |  |  |  |  |  |  | 6.5059E6 |  |  | 1 | 0 | 0 | 0 | 0 | 0 | 0 | 0 | 0 | 0 | 0 | 1 | 0 | 0 | 43 | 52 | Carbamidomethylation | C10:Carbamidomethylation:1000.00 | PEAKS DB |
| R.SWWDFADYGC(+57.02)Y.C | N | 44.81 | 1468.5394 | 11 | 0.1 | 735.2771 | 2 | 111.77 | 10 | F10:82113 | NaNaKA16\_F6.raw |  |  |  |  |  |  |  |  |  | 1.9586E7 |  | 1.4496E7 |  | 2 | 0 | 0 | 0 | 0 | 0 | 0 | 0 | 0 | 0 | 1 | 0 | 1 | 0 | 43 | 53 | Carbamidomethylation | C10:Carbamidomethylation:1000.00 | PEAKS DB |
| R.SWWDFADYGC(+57.02)YC(+57.02)GSGGSGTPVDDLDK.C | Y | 43.00 | 2914.1487 | 26 | 4.7 | 729.5479 | 4 | 82.68 | 11 | F11:57840 | NaNaKA16\_F7.raw |  |  |  |  |  |  |  |  |  |  | 5.5123E7 |  |  | 2 | 0 | 0 | 0 | 0 | 0 | 0 | 0 | 0 | 0 | 0 | 2 | 0 | 0 | 43 | 68 | Carbamidomethylation | C10:Carbamidomethylation:1000.00;C12:Carbamidomethylation:1000.00 | PEAKS DB |
| total 3 peptides |  |  |  |  |  |  |  |  |  |  |  |  |  |  |  |  |  |  |  |  |  |  |  |  |  |  |  |  |  |  |  |  |  |  |  |  |  |  |  |  |  |  |  |
| --- | --- | --- | --- | --- | --- | --- | --- | --- | --- | --- | --- | --- | --- | --- | --- | --- | --- | --- | --- | --- | --- | --- | --- | --- | --- | --- | --- | --- | --- | --- | --- | --- | --- | --- | --- | --- | --- | --- | --- | --- | --- | --- | --- |

JAI09031.1

back to list

  

| Protein Coverage
| Supporting Peptides
|

Protein Coverage:

Supporting Peptides:

| Peptide | Uniq | -10lgP | Mass | Length | ppm | m/z | z | RT | Fraction | Scan | Source File | Area F1 | Area F10 | Area F11 | Area F12 | Area F13 | Area F2 | Area F3 | Area F4 | Area F5 | Area F6 | Area F7 | Area F8 | Area F9 | #Feature | #Feature F1 | #Feature F10 | #Feature F11 | #Feature F12 | #Feature F13 | #Feature F2 | #Feature F3 | #Feature F4 | #Feature F5 | #Feature F6 | #Feature F7 | #Feature F8 | #Feature F9 | Start | End | PTM | AScore | Found By |
| --- | --- | --- | --- | --- | --- | --- | --- | --- | --- | --- | --- | --- | --- | --- | --- | --- | --- | --- | --- | --- | --- | --- | --- | --- | --- | --- | --- | --- | --- | --- | --- | --- | --- | --- | --- | --- | --- | --- | --- | --- | --- | --- | --- |
| R.SWWDFADYGC(+57.02).Y | N | 45.71 | 1305.4761 | 10 | 0.8 | 653.7458 | 2 | 109.86 | 11 | F11:80150 | NaNaKA16\_F7.raw |  |  |  |  |  |  |  |  |  |  | 6.5059E6 |  |  | 1 | 0 | 0 | 0 | 0 | 0 | 0 | 0 | 0 | 0 | 0 | 1 | 0 | 0 | 43 | 52 | Carbamidomethylation | C10:Carbamidomethylation:1000.00 | PEAKS DB |
| R.SWWDFADYGC(+57.02)Y.C | N | 44.81 | 1468.5394 | 11 | 0.1 | 735.2771 | 2 | 111.77 | 10 | F10:82113 | NaNaKA16\_F6.raw |  |  |  |  |  |  |  |  |  | 1.9586E7 |  | 1.4496E7 |  | 2 | 0 | 0 | 0 | 0 | 0 | 0 | 0 | 0 | 0 | 1 | 0 | 1 | 0 | 43 | 53 | Carbamidomethylation | C10:Carbamidomethylation:1000.00 | PEAKS DB |
| R.SWWDFADYGC(+57.02)YC(+57.02)GSGGSGTPVDDLDK.C | Y | 43.00 | 2914.1487 | 26 | 4.7 | 729.5479 | 4 | 82.68 | 11 | F11:57840 | NaNaKA16\_F7.raw |  |  |  |  |  |  |  |  |  |  | 5.5123E7 |  |  | 2 | 0 | 0 | 0 | 0 | 0 | 0 | 0 | 0 | 0 | 0 | 2 | 0 | 0 | 43 | 68 | Carbamidomethylation | C10:Carbamidomethylation:1000.00;C12:Carbamidomethylation:1000.00 | PEAKS DB |
| total 3 peptides |  |  |  |  |  |  |  |  |  |  |  |  |  |  |  |  |  |  |  |  |  |  |  |  |  |  |  |  |  |  |  |  |  |  |  |  |  |  |  |  |  |  |  |
| --- | --- | --- | --- | --- | --- | --- | --- | --- | --- | --- | --- | --- | --- | --- | --- | --- | --- | --- | --- | --- | --- | --- | --- | --- | --- | --- | --- | --- | --- | --- | --- | --- | --- | --- | --- | --- | --- | --- | --- | --- | --- | --- | --- |

JAB52781.1

back to list

  

| Protein Coverage
| Supporting Peptides
|

Protein Coverage:

Supporting Peptides:

| Peptide | Uniq | -10lgP | Mass | Length | ppm | m/z | z | RT | Fraction | Scan | Source File | Area F1 | Area F10 | Area F11 | Area F12 | Area F13 | Area F2 | Area F3 | Area F4 | Area F5 | Area F6 | Area F7 | Area F8 | Area F9 | #Feature | #Feature F1 | #Feature F10 | #Feature F11 | #Feature F12 | #Feature F13 | #Feature F2 | #Feature F3 | #Feature F4 | #Feature F5 | #Feature F6 | #Feature F7 | #Feature F8 | #Feature F9 | Start | End | PTM | AScore | Found By |
| --- | --- | --- | --- | --- | --- | --- | --- | --- | --- | --- | --- | --- | --- | --- | --- | --- | --- | --- | --- | --- | --- | --- | --- | --- | --- | --- | --- | --- | --- | --- | --- | --- | --- | --- | --- | --- | --- | --- | --- | --- | --- | --- | --- |
| R.SWWDFADYGC(+57.02).Y | N | 45.71 | 1305.4761 | 10 | 0.8 | 653.7458 | 2 | 109.86 | 11 | F11:80150 | NaNaKA16\_F7.raw |  |  |  |  |  |  |  |  |  |  | 6.5059E6 |  |  | 1 | 0 | 0 | 0 | 0 | 0 | 0 | 0 | 0 | 0 | 0 | 1 | 0 | 0 | 43 | 52 | Carbamidomethylation | C10:Carbamidomethylation:1000.00 | PEAKS DB |
| R.SWWDFADYGC(+57.02)Y.C | N | 44.81 | 1468.5394 | 11 | 0.1 | 735.2771 | 2 | 111.77 | 10 | F10:82113 | NaNaKA16\_F6.raw |  |  |  |  |  |  |  |  |  | 1.9586E7 |  | 1.4496E7 |  | 2 | 0 | 0 | 0 | 0 | 0 | 0 | 0 | 0 | 0 | 1 | 0 | 1 | 0 | 43 | 53 | Carbamidomethylation | C10:Carbamidomethylation:1000.00 | PEAKS DB |
| R.SWWDFADYGC(+57.02)YC(+57.02)GSGGSGTPVDDLDK.C | Y | 43.00 | 2914.1487 | 26 | 4.7 | 729.5479 | 4 | 82.68 | 11 | F11:57840 | NaNaKA16\_F7.raw |  |  |  |  |  |  |  |  |  |  | 5.5123E7 |  |  | 2 | 0 | 0 | 0 | 0 | 0 | 0 | 0 | 0 | 0 | 0 | 2 | 0 | 0 | 43 | 68 | Carbamidomethylation | C10:Carbamidomethylation:1000.00;C12:Carbamidomethylation:1000.00 | PEAKS DB |
| total 3 peptides |  |  |  |  |  |  |  |  |  |  |  |  |  |  |  |  |  |  |  |  |  |  |  |  |  |  |  |  |  |  |  |  |  |  |  |  |  |  |  |  |  |  |  |
| --- | --- | --- | --- | --- | --- | --- | --- | --- | --- | --- | --- | --- | --- | --- | --- | --- | --- | --- | --- | --- | --- | --- | --- | --- | --- | --- | --- | --- | --- | --- | --- | --- | --- | --- | --- | --- | --- | --- | --- | --- | --- | --- | --- |

ETE59007.1

back to list

  

| Protein Coverage
| Supporting Peptides
|

Protein Coverage:

Supporting Peptides:

| Peptide | Uniq | -10lgP | Mass | Length | ppm | m/z | z | RT | Fraction | Scan | Source File | Area F1 | Area F10 | Area F11 | Area F12 | Area F13 | Area F2 | Area F3 | Area F4 | Area F5 | Area F6 | Area F7 | Area F8 | Area F9 | #Feature | #Feature F1 | #Feature F10 | #Feature F11 | #Feature F12 | #Feature F13 | #Feature F2 | #Feature F3 | #Feature F4 | #Feature F5 | #Feature F6 | #Feature F7 | #Feature F8 | #Feature F9 | Start | End | PTM | AScore | Found By |
| --- | --- | --- | --- | --- | --- | --- | --- | --- | --- | --- | --- | --- | --- | --- | --- | --- | --- | --- | --- | --- | --- | --- | --- | --- | --- | --- | --- | --- | --- | --- | --- | --- | --- | --- | --- | --- | --- | --- | --- | --- | --- | --- | --- |
| Q.DLYDAPINHPYVQK.A | Y | 60.45 | 1671.8257 | 14 | 0.8 | 558.2830 | 3 | 37.41 | 4 | F4:19732 | NaNaKA16\_F12.raw |  |  |  | 3.1543E6 |  |  |  |  |  |  |  |  |  | 2 | 0 | 0 | 0 | 2 | 0 | 0 | 0 | 0 | 0 | 0 | 0 | 0 | 0 | 13 | 26 |  |  | PEAKS DB |
| Q.YNLDNEESANYFK.V | Y | 43.01 | 1605.6947 | 13 | -6.3 | 803.8495 | 2 | 43.62 | 4 | F4:24676 | NaNaKA16\_F12.raw |  |  |  | 6.8453E6 |  |  |  |  |  |  |  |  |  | 1 | 0 | 0 | 0 | 1 | 0 | 0 | 0 | 0 | 0 | 0 | 0 | 0 | 0 | 35 | 47 |  |  | PEAKS DB |
| total 2 peptides |  |  |  |  |  |  |  |  |  |  |  |  |  |  |  |  |  |  |  |  |  |  |  |  |  |  |  |  |  |  |  |  |  |  |  |  |  |  |  |  |  |  |  |
| --- | --- | --- | --- | --- | --- | --- | --- | --- | --- | --- | --- | --- | --- | --- | --- | --- | --- | --- | --- | --- | --- | --- | --- | --- | --- | --- | --- | --- | --- | --- | --- | --- | --- | --- | --- | --- | --- | --- | --- | --- | --- | --- | --- |

ETE65528.1

back to list

  

| Protein Coverage
| Supporting Peptides
|

Protein Coverage:

Supporting Peptides:

| Peptide | Uniq | -10lgP | Mass | Length | ppm | m/z | z | RT | Fraction | Scan | Source File | Area F1 | Area F10 | Area F11 | Area F12 | Area F13 | Area F2 | Area F3 | Area F4 | Area F5 | Area F6 | Area F7 | Area F8 | Area F9 | #Feature | #Feature F1 | #Feature F10 | #Feature F11 | #Feature F12 | #Feature F13 | #Feature F2 | #Feature F3 | #Feature F4 | #Feature F5 | #Feature F6 | #Feature F7 | #Feature F8 | #Feature F9 | Start | End | PTM | AScore | Found By |
| --- | --- | --- | --- | --- | --- | --- | --- | --- | --- | --- | --- | --- | --- | --- | --- | --- | --- | --- | --- | --- | --- | --- | --- | --- | --- | --- | --- | --- | --- | --- | --- | --- | --- | --- | --- | --- | --- | --- | --- | --- | --- | --- | --- |
| K.WLIFQMAQK.Q | N | 56.71 | 1163.6161 | 9 | 0.5 | 582.8156 | 2 | 72.93 | 3 | F3:47124 | NaNaKA16\_F11.raw |  | 1.6779E6 | 1.2456E6 | 1.6279E6 |  |  |  |  |  |  | 3.0604E6 | 8.2771E6 | 8.1378E5 | 6 | 0 | 1 | 1 | 1 | 0 | 0 | 0 | 0 | 0 | 0 | 1 | 1 | 1 | 162 | 170 |  |  | PEAKS DB |
| K.GNYLPMQC(+57.02)HSSSGY.C | Y | 44.91 | 1599.6447 | 14 | 0.9 | 800.8303 | 2 | 34.42 | 6 | F6:19369 | NaNaKA16\_F2.raw |  |  |  |  |  | 1.6385E6 |  |  |  |  |  |  |  | 1 | 0 | 0 | 0 | 0 | 0 | 1 | 0 | 0 | 0 | 0 | 0 | 0 | 0 | 203 | 216 | Carbamidomethylation | C8:Carbamidomethylation:1000.00 | PEAKS DB |
| total 2 peptides |  |  |  |  |  |  |  |  |  |  |  |  |  |  |  |  |  |  |  |  |  |  |  |  |  |  |  |  |  |  |  |  |  |  |  |  |  |  |  |  |  |  |  |
| --- | --- | --- | --- | --- | --- | --- | --- | --- | --- | --- | --- | --- | --- | --- | --- | --- | --- | --- | --- | --- | --- | --- | --- | --- | --- | --- | --- | --- | --- | --- | --- | --- | --- | --- | --- | --- | --- | --- | --- | --- | --- | --- | --- |

XP\_026560868.1

back to list

  

| Protein Coverage
| Supporting Peptides
|

Protein Coverage:

Supporting Peptides:

| Peptide | Uniq | -10lgP | Mass | Length | ppm | m/z | z | RT | Fraction | Scan | Source File | Area F1 | Area F10 | Area F11 | Area F12 | Area F13 | Area F2 | Area F3 | Area F4 | Area F5 | Area F6 | Area F7 | Area F8 | Area F9 | #Feature | #Feature F1 | #Feature F10 | #Feature F11 | #Feature F12 | #Feature F13 | #Feature F2 | #Feature F3 | #Feature F4 | #Feature F5 | #Feature F6 | #Feature F7 | #Feature F8 | #Feature F9 | Start | End | PTM | AScore | Found By |
| --- | --- | --- | --- | --- | --- | --- | --- | --- | --- | --- | --- | --- | --- | --- | --- | --- | --- | --- | --- | --- | --- | --- | --- | --- | --- | --- | --- | --- | --- | --- | --- | --- | --- | --- | --- | --- | --- | --- | --- | --- | --- | --- | --- |
| K.DPNTGFLYNLEPLALEK.A | Y | 69.80 | 1932.9833 | 17 | -0.4 | 967.4985 | 2 | 94.92 | 2 | F2:66309 | NaNaKA16\_F10.raw |  | 1.0243E6 |  |  |  |  |  |  |  |  |  |  |  | 1 | 0 | 1 | 0 | 0 | 0 | 0 | 0 | 0 | 0 | 0 | 0 | 0 | 0 | 932 | 948 |  |  | PEAKS DB |
| K.WGFC(+57.02)VNEAGQR.E | Y | 42.42 | 1322.5826 | 11 | -0.1 | 662.2985 | 2 | 42.60 | 9 | F9:25257 | NaNaKA16\_F5.raw |  |  |  |  |  |  |  |  | 0 |  |  |  |  | 0 | 0 | 0 | 0 | 0 | 0 | 0 | 0 | 0 | 0 | 0 | 0 | 0 | 0 | 1929 | 1939 | Carbamidomethylation | C4:Carbamidomethylation:1000.00 | PEAKS DB |
| total 2 peptides |  |  |  |  |  |  |  |  |  |  |  |  |  |  |  |  |  |  |  |  |  |  |  |  |  |  |  |  |  |  |  |  |  |  |  |  |  |  |  |  |  |  |  |
| --- | --- | --- | --- | --- | --- | --- | --- | --- | --- | --- | --- | --- | --- | --- | --- | --- | --- | --- | --- | --- | --- | --- | --- | --- | --- | --- | --- | --- | --- | --- | --- | --- | --- | --- | --- | --- | --- | --- | --- | --- | --- | --- | --- |

XP\_026531033.1

back to list

  

| Protein Coverage
| Supporting Peptides
|

Protein Coverage:

Supporting Peptides:

| Peptide | Uniq | -10lgP | Mass | Length | ppm | m/z | z | RT | Fraction | Scan | Source File | Area F1 | Area F10 | Area F11 | Area F12 | Area F13 | Area F2 | Area F3 | Area F4 | Area F5 | Area F6 | Area F7 | Area F8 | Area F9 | #Feature | #Feature F1 | #Feature F10 | #Feature F11 | #Feature F12 | #Feature F13 | #Feature F2 | #Feature F3 | #Feature F4 | #Feature F5 | #Feature F6 | #Feature F7 | #Feature F8 | #Feature F9 | Start | End | PTM | AScore | Found By |
| --- | --- | --- | --- | --- | --- | --- | --- | --- | --- | --- | --- | --- | --- | --- | --- | --- | --- | --- | --- | --- | --- | --- | --- | --- | --- | --- | --- | --- | --- | --- | --- | --- | --- | --- | --- | --- | --- | --- | --- | --- | --- | --- | --- |
| K.DPNTGFLYNLEPLALEK.A | Y | 69.80 | 1932.9833 | 17 | -0.4 | 967.4985 | 2 | 94.92 | 2 | F2:66309 | NaNaKA16\_F10.raw |  | 1.0243E6 |  |  |  |  |  |  |  |  |  |  |  | 1 | 0 | 1 | 0 | 0 | 0 | 0 | 0 | 0 | 0 | 0 | 0 | 0 | 0 | 965 | 981 |  |  | PEAKS DB |
| K.WGFC(+57.02)VNEAGQR.E | Y | 42.42 | 1322.5826 | 11 | -0.1 | 662.2985 | 2 | 42.60 | 9 | F9:25257 | NaNaKA16\_F5.raw |  |  |  |  |  |  |  |  | 0 |  |  |  |  | 0 | 0 | 0 | 0 | 0 | 0 | 0 | 0 | 0 | 0 | 0 | 0 | 0 | 0 | 1962 | 1972 | Carbamidomethylation | C4:Carbamidomethylation:1000.00 | PEAKS DB |
| total 2 peptides |  |  |  |  |  |  |  |  |  |  |  |  |  |  |  |  |  |  |  |  |  |  |  |  |  |  |  |  |  |  |  |  |  |  |  |  |  |  |  |  |  |  |  |
| --- | --- | --- | --- | --- | --- | --- | --- | --- | --- | --- | --- | --- | --- | --- | --- | --- | --- | --- | --- | --- | --- | --- | --- | --- | --- | --- | --- | --- | --- | --- | --- | --- | --- | --- | --- | --- | --- | --- | --- | --- | --- | --- | --- |

ETE64374.1

back to list

  

| Protein Coverage
| Supporting Peptides
|

Protein Coverage:

Supporting Peptides:

| Peptide | Uniq | -10lgP | Mass | Length | ppm | m/z | z | RT | Fraction | Scan | Source File | Area F1 | Area F10 | Area F11 | Area F12 | Area F13 | Area F2 | Area F3 | Area F4 | Area F5 | Area F6 | Area F7 | Area F8 | Area F9 | #Feature | #Feature F1 | #Feature F10 | #Feature F11 | #Feature F12 | #Feature F13 | #Feature F2 | #Feature F3 | #Feature F4 | #Feature F5 | #Feature F6 | #Feature F7 | #Feature F8 | #Feature F9 | Start | End | PTM | AScore | Found By |
| --- | --- | --- | --- | --- | --- | --- | --- | --- | --- | --- | --- | --- | --- | --- | --- | --- | --- | --- | --- | --- | --- | --- | --- | --- | --- | --- | --- | --- | --- | --- | --- | --- | --- | --- | --- | --- | --- | --- | --- | --- | --- | --- | --- |
| K.DPNTGFLYNLEPLALEK.A | Y | 69.80 | 1932.9833 | 17 | -0.4 | 967.4985 | 2 | 94.92 | 2 | F2:66309 | NaNaKA16\_F10.raw |  | 1.0243E6 |  |  |  |  |  |  |  |  |  |  |  | 1 | 0 | 1 | 0 | 0 | 0 | 0 | 0 | 0 | 0 | 0 | 0 | 0 | 0 | 733 | 749 |  |  | PEAKS DB |
| K.WGFC(+57.02)VNEAGQR.E | Y | 42.42 | 1322.5826 | 11 | -0.1 | 662.2985 | 2 | 42.60 | 9 | F9:25257 | NaNaKA16\_F5.raw |  |  |  |  |  |  |  |  | 0 |  |  |  |  | 0 | 0 | 0 | 0 | 0 | 0 | 0 | 0 | 0 | 0 | 0 | 0 | 0 | 0 | 1626 | 1636 | Carbamidomethylation | C4:Carbamidomethylation:1000.00 | PEAKS DB |
| total 2 peptides |  |  |  |  |  |  |  |  |  |  |  |  |  |  |  |  |  |  |  |  |  |  |  |  |  |  |  |  |  |  |  |  |  |  |  |  |  |  |  |  |  |  |  |
| --- | --- | --- | --- | --- | --- | --- | --- | --- | --- | --- | --- | --- | --- | --- | --- | --- | --- | --- | --- | --- | --- | --- | --- | --- | --- | --- | --- | --- | --- | --- | --- | --- | --- | --- | --- | --- | --- | --- | --- | --- | --- | --- | --- |

XP\_034284419.1

back to list

  

| Protein Coverage
| Supporting Peptides
|

Protein Coverage:

Supporting Peptides:

| Peptide | Uniq | -10lgP | Mass | Length | ppm | m/z | z | RT | Fraction | Scan | Source File | Area F1 | Area F10 | Area F11 | Area F12 | Area F13 | Area F2 | Area F3 | Area F4 | Area F5 | Area F6 | Area F7 | Area F8 | Area F9 | #Feature | #Feature F1 | #Feature F10 | #Feature F11 | #Feature F12 | #Feature F13 | #Feature F2 | #Feature F3 | #Feature F4 | #Feature F5 | #Feature F6 | #Feature F7 | #Feature F8 | #Feature F9 | Start | End | PTM | AScore | Found By |
| --- | --- | --- | --- | --- | --- | --- | --- | --- | --- | --- | --- | --- | --- | --- | --- | --- | --- | --- | --- | --- | --- | --- | --- | --- | --- | --- | --- | --- | --- | --- | --- | --- | --- | --- | --- | --- | --- | --- | --- | --- | --- | --- | --- |
| K.DPNTGFLYNLEPLALEK.A | Y | 69.80 | 1932.9833 | 17 | -0.4 | 967.4985 | 2 | 94.92 | 2 | F2:66309 | NaNaKA16\_F10.raw |  | 1.0243E6 |  |  |  |  |  |  |  |  |  |  |  | 1 | 0 | 1 | 0 | 0 | 0 | 0 | 0 | 0 | 0 | 0 | 0 | 0 | 0 | 932 | 948 |  |  | PEAKS DB |
| K.WGFC(+57.02)VNEAGQR.E | Y | 42.42 | 1322.5826 | 11 | -0.1 | 662.2985 | 2 | 42.60 | 9 | F9:25257 | NaNaKA16\_F5.raw |  |  |  |  |  |  |  |  | 0 |  |  |  |  | 0 | 0 | 0 | 0 | 0 | 0 | 0 | 0 | 0 | 0 | 0 | 0 | 0 | 0 | 1929 | 1939 | Carbamidomethylation | C4:Carbamidomethylation:1000.00 | PEAKS DB |
| total 2 peptides |  |  |  |  |  |  |  |  |  |  |  |  |  |  |  |  |  |  |  |  |  |  |  |  |  |  |  |  |  |  |  |  |  |  |  |  |  |  |  |  |  |  |  |
| --- | --- | --- | --- | --- | --- | --- | --- | --- | --- | --- | --- | --- | --- | --- | --- | --- | --- | --- | --- | --- | --- | --- | --- | --- | --- | --- | --- | --- | --- | --- | --- | --- | --- | --- | --- | --- | --- | --- | --- | --- | --- | --- | --- |

XP\_032090066.1

back to list

  

| Protein Coverage
| Supporting Peptides
|

Protein Coverage:

Supporting Peptides:

| Peptide | Uniq | -10lgP | Mass | Length | ppm | m/z | z | RT | Fraction | Scan | Source File | Area F1 | Area F10 | Area F11 | Area F12 | Area F13 | Area F2 | Area F3 | Area F4 | Area F5 | Area F6 | Area F7 | Area F8 | Area F9 | #Feature | #Feature F1 | #Feature F10 | #Feature F11 | #Feature F12 | #Feature F13 | #Feature F2 | #Feature F3 | #Feature F4 | #Feature F5 | #Feature F6 | #Feature F7 | #Feature F8 | #Feature F9 | Start | End | PTM | AScore | Found By |
| --- | --- | --- | --- | --- | --- | --- | --- | --- | --- | --- | --- | --- | --- | --- | --- | --- | --- | --- | --- | --- | --- | --- | --- | --- | --- | --- | --- | --- | --- | --- | --- | --- | --- | --- | --- | --- | --- | --- | --- | --- | --- | --- | --- |
| K.SQFITAFC(+57.02)SSQR.R | N | 53.09 | 1430.6613 | 12 | 0.0 | 716.3379 | 2 | 41.78 | 2 | F2:22780 | NaNaKA16\_F10.raw |  | 3.9839E6 | 4.2283E6 |  |  |  |  |  |  |  |  |  |  | 2 | 0 | 1 | 1 | 0 | 0 | 0 | 0 | 0 | 0 | 0 | 0 | 0 | 0 | 285 | 296 | Carbamidomethylation | C8:Carbamidomethylation:1000.00 | PEAKS DB |
| R.C(+57.02)C(+57.02)QLSEDQRLPC(+57.02)ASNEWEK.S | Y | 47.26 | 2409.0146 | 19 | -3.3 | 804.0095 | 3 | 34.83 | 10 | F10:19694 | NaNaKA16\_F6.raw |  |  |  | 1.5737E6 |  |  |  |  |  | 0 |  |  |  | 1 | 0 | 0 | 0 | 1 | 0 | 0 | 0 | 0 | 0 | 0 | 0 | 0 | 0 | 165 | 183 | Carbamidomethylation | C1:Carbamidomethylation:1000.00;C2:Carbamidomethylation:1000.00;C12:Carbamidomethylation:1000.00 | PEAKS DB |
| total 2 peptides |  |  |  |  |  |  |  |  |  |  |  |  |  |  |  |  |  |  |  |  |  |  |  |  |  |  |  |  |  |  |  |  |  |  |  |  |  |  |  |  |  |  |  |
| --- | --- | --- | --- | --- | --- | --- | --- | --- | --- | --- | --- | --- | --- | --- | --- | --- | --- | --- | --- | --- | --- | --- | --- | --- | --- | --- | --- | --- | --- | --- | --- | --- | --- | --- | --- | --- | --- | --- | --- | --- | --- | --- | --- |

XP\_032090065.1

back to list

  

| Protein Coverage
| Supporting Peptides
|

Protein Coverage:

Supporting Peptides:

| Peptide | Uniq | -10lgP | Mass | Length | ppm | m/z | z | RT | Fraction | Scan | Source File | Area F1 | Area F10 | Area F11 | Area F12 | Area F13 | Area F2 | Area F3 | Area F4 | Area F5 | Area F6 | Area F7 | Area F8 | Area F9 | #Feature | #Feature F1 | #Feature F10 | #Feature F11 | #Feature F12 | #Feature F13 | #Feature F2 | #Feature F3 | #Feature F4 | #Feature F5 | #Feature F6 | #Feature F7 | #Feature F8 | #Feature F9 | Start | End | PTM | AScore | Found By |
| --- | --- | --- | --- | --- | --- | --- | --- | --- | --- | --- | --- | --- | --- | --- | --- | --- | --- | --- | --- | --- | --- | --- | --- | --- | --- | --- | --- | --- | --- | --- | --- | --- | --- | --- | --- | --- | --- | --- | --- | --- | --- | --- | --- |
| K.SQFITAFC(+57.02)SSQR.R | N | 53.09 | 1430.6613 | 12 | 0.0 | 716.3379 | 2 | 41.78 | 2 | F2:22780 | NaNaKA16\_F10.raw |  | 3.9839E6 | 4.2283E6 |  |  |  |  |  |  |  |  |  |  | 2 | 0 | 1 | 1 | 0 | 0 | 0 | 0 | 0 | 0 | 0 | 0 | 0 | 0 | 295 | 306 | Carbamidomethylation | C8:Carbamidomethylation:1000.00 | PEAKS DB |
| R.C(+57.02)C(+57.02)QLSEDQRLPC(+57.02)ASNEWEK.S | Y | 47.26 | 2409.0146 | 19 | -3.3 | 804.0095 | 3 | 34.83 | 10 | F10:19694 | NaNaKA16\_F6.raw |  |  |  | 1.5737E6 |  |  |  |  |  | 0 |  |  |  | 1 | 0 | 0 | 0 | 1 | 0 | 0 | 0 | 0 | 0 | 0 | 0 | 0 | 0 | 165 | 183 | Carbamidomethylation | C1:Carbamidomethylation:1000.00;C2:Carbamidomethylation:1000.00;C12:Carbamidomethylation:1000.00 | PEAKS DB |
| total 2 peptides |  |  |  |  |  |  |  |  |  |  |  |  |  |  |  |  |  |  |  |  |  |  |  |  |  |  |  |  |  |  |  |  |  |  |  |  |  |  |  |  |  |  |  |
| --- | --- | --- | --- | --- | --- | --- | --- | --- | --- | --- | --- | --- | --- | --- | --- | --- | --- | --- | --- | --- | --- | --- | --- | --- | --- | --- | --- | --- | --- | --- | --- | --- | --- | --- | --- | --- | --- | --- | --- | --- | --- | --- | --- |

JAA74930.1

back to list

  

| Protein Coverage
| Supporting Peptides
|

Protein Coverage:

Supporting Peptides:

| Peptide | Uniq | -10lgP | Mass | Length | ppm | m/z | z | RT | Fraction | Scan | Source File | Area F1 | Area F10 | Area F11 | Area F12 | Area F13 | Area F2 | Area F3 | Area F4 | Area F5 | Area F6 | Area F7 | Area F8 | Area F9 | #Feature | #Feature F1 | #Feature F10 | #Feature F11 | #Feature F12 | #Feature F13 | #Feature F2 | #Feature F3 | #Feature F4 | #Feature F5 | #Feature F6 | #Feature F7 | #Feature F8 | #Feature F9 | Start | End | PTM | AScore | Found By |
| --- | --- | --- | --- | --- | --- | --- | --- | --- | --- | --- | --- | --- | --- | --- | --- | --- | --- | --- | --- | --- | --- | --- | --- | --- | --- | --- | --- | --- | --- | --- | --- | --- | --- | --- | --- | --- | --- | --- | --- | --- | --- | --- | --- |
| R.VDLGC(+57.02)AATC(+57.02)PTTDK.T | Y | 47.27 | 1507.6647 | 14 | 8.1 | 754.8457 | 2 | 23.07 | 9 | F9:10080 | NaNaKA16\_F5.raw |  |  |  |  |  |  |  |  | 8.4235E5 |  |  |  |  | 1 | 0 | 0 | 0 | 0 | 0 | 0 | 0 | 0 | 1 | 0 | 0 | 0 | 0 | 60 | 73 | Carbamidomethylation | C5:Carbamidomethylation:1000.00;C9:Carbamidomethylation:1000.00 | PEAKS DB |
| total 1 peptides |  |  |  |  |  |  |  |  |  |  |  |  |  |  |  |  |  |  |  |  |  |  |  |  |  |  |  |  |  |  |  |  |  |  |  |  |  |  |  |  |  |  |  |
| --- | --- | --- | --- | --- | --- | --- | --- | --- | --- | --- | --- | --- | --- | --- | --- | --- | --- | --- | --- | --- | --- | --- | --- | --- | --- | --- | --- | --- | --- | --- | --- | --- | --- | --- | --- | --- | --- | --- | --- | --- | --- | --- | --- |

XP\_026544110.1

back to list

  

| Protein Coverage
| Supporting Peptides
|

Protein Coverage:

Supporting Peptides:

| Peptide | Uniq | -10lgP | Mass | Length | ppm | m/z | z | RT | Fraction | Scan | Source File | Area F1 | Area F10 | Area F11 | Area F12 | Area F13 | Area F2 | Area F3 | Area F4 | Area F5 | Area F6 | Area F7 | Area F8 | Area F9 | #Feature | #Feature F1 | #Feature F10 | #Feature F11 | #Feature F12 | #Feature F13 | #Feature F2 | #Feature F3 | #Feature F4 | #Feature F5 | #Feature F6 | #Feature F7 | #Feature F8 | #Feature F9 | Start | End | PTM | AScore | Found By |
| --- | --- | --- | --- | --- | --- | --- | --- | --- | --- | --- | --- | --- | --- | --- | --- | --- | --- | --- | --- | --- | --- | --- | --- | --- | --- | --- | --- | --- | --- | --- | --- | --- | --- | --- | --- | --- | --- | --- | --- | --- | --- | --- | --- |
| K.YIMHPDYSVFNPTENDIVLIK.L | Y | 60.56 | 2507.2407 | 21 | 1.3 | 836.7553 | 3 | 77.64 | 4 | F4:52998 | NaNaKA16\_F12.raw |  |  |  | 1.4523E7 |  |  |  |  |  |  |  |  |  | 1 | 0 | 0 | 0 | 1 | 0 | 0 | 0 | 0 | 0 | 0 | 0 | 0 | 0 | 378 | 398 |  |  | PEAKS DB |
| R.SPDEDEQPWC(+57.02)YFIK.D | N | 53.69 | 1812.7665 | 14 | 0.0 | 907.3905 | 2 | 80.04 | 2 | F2:54723 | NaNaKA16\_F10.raw |  | 2.0014E6 |  |  |  |  |  |  |  |  |  |  |  | 1 | 0 | 1 | 0 | 0 | 0 | 0 | 0 | 0 | 0 | 0 | 0 | 0 | 0 | 235 | 248 | Carbamidomethylation | C10:Carbamidomethylation:1000.00 | PEAKS DB |
| total 2 peptides |  |  |  |  |  |  |  |  |  |  |  |  |  |  |  |  |  |  |  |  |  |  |  |  |  |  |  |  |  |  |  |  |  |  |  |  |  |  |  |  |  |  |  |
| --- | --- | --- | --- | --- | --- | --- | --- | --- | --- | --- | --- | --- | --- | --- | --- | --- | --- | --- | --- | --- | --- | --- | --- | --- | --- | --- | --- | --- | --- | --- | --- | --- | --- | --- | --- | --- | --- | --- | --- | --- | --- | --- | --- |

P0DI84.1

back to list

  

| Protein Coverage
| Supporting Peptides
|

Protein Coverage:

Supporting Peptides:

| Peptide | Uniq | -10lgP | Mass | Length | ppm | m/z | z | RT | Fraction | Scan | Source File | Area F1 | Area F10 | Area F11 | Area F12 | Area F13 | Area F2 | Area F3 | Area F4 | Area F5 | Area F6 | Area F7 | Area F8 | Area F9 | #Feature | #Feature F1 | #Feature F10 | #Feature F11 | #Feature F12 | #Feature F13 | #Feature F2 | #Feature F3 | #Feature F4 | #Feature F5 | #Feature F6 | #Feature F7 | #Feature F8 | #Feature F9 | Start | End | PTM | AScore | Found By |
| --- | --- | --- | --- | --- | --- | --- | --- | --- | --- | --- | --- | --- | --- | --- | --- | --- | --- | --- | --- | --- | --- | --- | --- | --- | --- | --- | --- | --- | --- | --- | --- | --- | --- | --- | --- | --- | --- | --- | --- | --- | --- | --- | --- |
| K.HVVVVGAGMSGLSAAYVLAGAGHK.V | Y | 65.98 | 2250.1943 | 24 | 0.0 | 563.5558 | 4 | 52.57 | 2 | F2:30863 | NaNaKA16\_F10.raw |  | 1.7954E6 |  |  |  |  |  |  |  |  |  |  |  | 1 | 0 | 1 | 0 | 0 | 0 | 0 | 0 | 0 | 0 | 0 | 0 | 0 | 0 | 33 | 56 |  |  | PEAKS DB |
| total 1 peptides |  |  |  |  |  |  |  |  |  |  |  |  |  |  |  |  |  |  |  |  |  |  |  |  |  |  |  |  |  |  |  |  |  |  |  |  |  |  |  |  |  |  |  |
| --- | --- | --- | --- | --- | --- | --- | --- | --- | --- | --- | --- | --- | --- | --- | --- | --- | --- | --- | --- | --- | --- | --- | --- | --- | --- | --- | --- | --- | --- | --- | --- | --- | --- | --- | --- | --- | --- | --- | --- | --- | --- | --- | --- |

3KVE

back to list

  

| Protein Coverage
| Supporting Peptides
|

Protein Coverage:

Supporting Peptides:

| Peptide | Uniq | -10lgP | Mass | Length | ppm | m/z | z | RT | Fraction | Scan | Source File | Area F1 | Area F10 | Area F11 | Area F12 | Area F13 | Area F2 | Area F3 | Area F4 | Area F5 | Area F6 | Area F7 | Area F8 | Area F9 | #Feature | #Feature F1 | #Feature F10 | #Feature F11 | #Feature F12 | #Feature F13 | #Feature F2 | #Feature F3 | #Feature F4 | #Feature F5 | #Feature F6 | #Feature F7 | #Feature F8 | #Feature F9 | Start | End | PTM | AScore | Found By |
| --- | --- | --- | --- | --- | --- | --- | --- | --- | --- | --- | --- | --- | --- | --- | --- | --- | --- | --- | --- | --- | --- | --- | --- | --- | --- | --- | --- | --- | --- | --- | --- | --- | --- | --- | --- | --- | --- | --- | --- | --- | --- | --- | --- |
| K.HVVVVGAGMSGLSAAYVLAGAGHK.V | Y | 65.98 | 2250.1943 | 24 | 0.0 | 563.5558 | 4 | 52.57 | 2 | F2:30863 | NaNaKA16\_F10.raw |  | 1.7954E6 |  |  |  |  |  |  |  |  |  |  |  | 1 | 0 | 1 | 0 | 0 | 0 | 0 | 0 | 0 | 0 | 0 | 0 | 0 | 0 | 35 | 58 |  |  | PEAKS DB |
| total 1 peptides |  |  |  |  |  |  |  |  |  |  |  |  |  |  |  |  |  |  |  |  |  |  |  |  |  |  |  |  |  |  |  |  |  |  |  |  |  |  |  |  |  |  |  |
| --- | --- | --- | --- | --- | --- | --- | --- | --- | --- | --- | --- | --- | --- | --- | --- | --- | --- | --- | --- | --- | --- | --- | --- | --- | --- | --- | --- | --- | --- | --- | --- | --- | --- | --- | --- | --- | --- | --- | --- | --- | --- | --- | --- |

JAA75029.1

back to list

  

| Protein Coverage
| Supporting Peptides
|

Protein Coverage:

Supporting Peptides:

| Peptide | Uniq | -10lgP | Mass | Length | ppm | m/z | z | RT | Fraction | Scan | Source File | Area F1 | Area F10 | Area F11 | Area F12 | Area F13 | Area F2 | Area F3 | Area F4 | Area F5 | Area F6 | Area F7 | Area F8 | Area F9 | #Feature | #Feature F1 | #Feature F10 | #Feature F11 | #Feature F12 | #Feature F13 | #Feature F2 | #Feature F3 | #Feature F4 | #Feature F5 | #Feature F6 | #Feature F7 | #Feature F8 | #Feature F9 | Start | End | PTM | AScore | Found By |
| --- | --- | --- | --- | --- | --- | --- | --- | --- | --- | --- | --- | --- | --- | --- | --- | --- | --- | --- | --- | --- | --- | --- | --- | --- | --- | --- | --- | --- | --- | --- | --- | --- | --- | --- | --- | --- | --- | --- | --- | --- | --- | --- | --- |
| R.SALDYMDYGC(+57.02)YC(+57.02)GKGDSGTPVDDLDR.C | Y | 48.14 | 2929.1841 | 26 | -1.3 | 1465.5974 | 2 | 104.51 | 13 | F13:74071 | NaNaKA16\_F9.raw |  |  |  |  |  |  |  |  |  |  |  |  | 1.751E6 | 1 | 0 | 0 | 0 | 0 | 0 | 0 | 0 | 0 | 0 | 0 | 0 | 0 | 1 | 45 | 70 | Carbamidomethylation | C10:Carbamidomethylation:1000.00;C12:Carbamidomethylation:1000.00 | PEAKS DB |
| D.SGTPVDDLDR.C | N | 45.52 | 1073.4989 | 10 | 0.9 | 537.7572 | 2 | 20.23 | 11 | F11:7497 | NaNaKA16\_F7.raw |  |  |  |  |  |  |  |  |  |  | 1.1072E7 |  |  | 1 | 0 | 0 | 0 | 0 | 0 | 0 | 0 | 0 | 0 | 0 | 1 | 0 | 0 | 61 | 70 |  |  | PEAKS DB |
| total 2 peptides |  |  |  |  |  |  |  |  |  |  |  |  |  |  |  |  |  |  |  |  |  |  |  |  |  |  |  |  |  |  |  |  |  |  |  |  |  |  |  |  |  |  |  |
| --- | --- | --- | --- | --- | --- | --- | --- | --- | --- | --- | --- | --- | --- | --- | --- | --- | --- | --- | --- | --- | --- | --- | --- | --- | --- | --- | --- | --- | --- | --- | --- | --- | --- | --- | --- | --- | --- | --- | --- | --- | --- | --- | --- |

ADI47614.1

back to list

  

| Protein Coverage
| Supporting Peptides
|

Protein Coverage:

Supporting Peptides:

| Peptide | Uniq | -10lgP | Mass | Length | ppm | m/z | z | RT | Fraction | Scan | Source File | Area F1 | Area F10 | Area F11 | Area F12 | Area F13 | Area F2 | Area F3 | Area F4 | Area F5 | Area F6 | Area F7 | Area F8 | Area F9 | #Feature | #Feature F1 | #Feature F10 | #Feature F11 | #Feature F12 | #Feature F13 | #Feature F2 | #Feature F3 | #Feature F4 | #Feature F5 | #Feature F6 | #Feature F7 | #Feature F8 | #Feature F9 | Start | End | PTM | AScore | Found By |
| --- | --- | --- | --- | --- | --- | --- | --- | --- | --- | --- | --- | --- | --- | --- | --- | --- | --- | --- | --- | --- | --- | --- | --- | --- | --- | --- | --- | --- | --- | --- | --- | --- | --- | --- | --- | --- | --- | --- | --- | --- | --- | --- | --- |
| K.VTLDLFGEWR.Q | N | 61.73 | 1234.6346 | 10 | 0.5 | 618.3249 | 2 | 82.47 | 11 | F11:57626 | NaNaKA16\_F7.raw |  |  |  |  |  |  |  |  |  |  | 1.9987E5 | 4.7038E5 |  | 2 | 0 | 0 | 0 | 0 | 0 | 0 | 0 | 0 | 0 | 0 | 1 | 1 | 0 | 166 | 175 |  |  | PEAKS DB |
| R.LYC(+57.02)FDNLPEHK.N | Y | 44.50 | 1434.6602 | 11 | 0.7 | 479.2277 | 3 | 34.30 | 10 | F10:19382 | NaNaKA16\_F6.raw |  |  |  |  |  |  |  |  |  | 1.1234E6 |  |  |  | 1 | 0 | 0 | 0 | 0 | 0 | 0 | 0 | 0 | 0 | 1 | 0 | 0 | 0 | 460 | 470 | Carbamidomethylation | C3:Carbamidomethylation:1000.00 | PEAKS DB |
| total 2 peptides |  |  |  |  |  |  |  |  |  |  |  |  |  |  |  |  |  |  |  |  |  |  |  |  |  |  |  |  |  |  |  |  |  |  |  |  |  |  |  |  |  |  |  |
| --- | --- | --- | --- | --- | --- | --- | --- | --- | --- | --- | --- | --- | --- | --- | --- | --- | --- | --- | --- | --- | --- | --- | --- | --- | --- | --- | --- | --- | --- | --- | --- | --- | --- | --- | --- | --- | --- | --- | --- | --- | --- | --- | --- |

P0DMT2.1

back to list

  

| Protein Coverage
| Supporting Peptides
|

Protein Coverage:

Supporting Peptides:

| Peptide | Uniq | -10lgP | Mass | Length | ppm | m/z | z | RT | Fraction | Scan | Source File | Area F1 | Area F10 | Area F11 | Area F12 | Area F13 | Area F2 | Area F3 | Area F4 | Area F5 | Area F6 | Area F7 | Area F8 | Area F9 | #Feature | #Feature F1 | #Feature F10 | #Feature F11 | #Feature F12 | #Feature F13 | #Feature F2 | #Feature F3 | #Feature F4 | #Feature F5 | #Feature F6 | #Feature F7 | #Feature F8 | #Feature F9 | Start | End | PTM | AScore | Found By |
| --- | --- | --- | --- | --- | --- | --- | --- | --- | --- | --- | --- | --- | --- | --- | --- | --- | --- | --- | --- | --- | --- | --- | --- | --- | --- | --- | --- | --- | --- | --- | --- | --- | --- | --- | --- | --- | --- | --- | --- | --- | --- | --- | --- |
| K.SPFPSYTSYGC(+57.02)FC(+57.02)GGGER.G | Y | 83.46 | 2027.8142 | 18 | 0.0 | 1014.9144 | 2 | 56.41 | 12 | F12:36093 | NaNaKA16\_F8.raw |  |  |  |  |  |  |  |  |  |  | 1.7369E6 | 1.2456E7 |  | 2 | 0 | 0 | 0 | 0 | 0 | 0 | 0 | 0 | 0 | 0 | 1 | 1 | 0 | 16 | 33 | Carbamidomethylation | C11:Carbamidomethylation:1000.00;C13:Carbamidomethylation:1000.00 | PEAKS DB |
| total 1 peptides |  |  |  |  |  |  |  |  |  |  |  |  |  |  |  |  |  |  |  |  |  |  |  |  |  |  |  |  |  |  |  |  |  |  |  |  |  |  |  |  |  |  |  |
| --- | --- | --- | --- | --- | --- | --- | --- | --- | --- | --- | --- | --- | --- | --- | --- | --- | --- | --- | --- | --- | --- | --- | --- | --- | --- | --- | --- | --- | --- | --- | --- | --- | --- | --- | --- | --- | --- | --- | --- | --- | --- | --- | --- |

3BJW

back to list

  

| Protein Coverage
| Supporting Peptides
|

Protein Coverage:

Supporting Peptides:

| Peptide | Uniq | -10lgP | Mass | Length | ppm | m/z | z | RT | Fraction | Scan | Source File | Area F1 | Area F10 | Area F11 | Area F12 | Area F13 | Area F2 | Area F3 | Area F4 | Area F5 | Area F6 | Area F7 | Area F8 | Area F9 | #Feature | #Feature F1 | #Feature F10 | #Feature F11 | #Feature F12 | #Feature F13 | #Feature F2 | #Feature F3 | #Feature F4 | #Feature F5 | #Feature F6 | #Feature F7 | #Feature F8 | #Feature F9 | Start | End | PTM | AScore | Found By |
| --- | --- | --- | --- | --- | --- | --- | --- | --- | --- | --- | --- | --- | --- | --- | --- | --- | --- | --- | --- | --- | --- | --- | --- | --- | --- | --- | --- | --- | --- | --- | --- | --- | --- | --- | --- | --- | --- | --- | --- | --- | --- | --- | --- |
| K.SPFPSYTSYGC(+57.02)FC(+57.02)GGGER.G | Y | 83.46 | 2027.8142 | 18 | 0.0 | 1014.9144 | 2 | 56.41 | 12 | F12:36093 | NaNaKA16\_F8.raw |  |  |  |  |  |  |  |  |  |  | 1.7369E6 | 1.2456E7 |  | 2 | 0 | 0 | 0 | 0 | 0 | 0 | 0 | 0 | 0 | 0 | 1 | 1 | 0 | 16 | 33 | Carbamidomethylation | C11:Carbamidomethylation:1000.00;C13:Carbamidomethylation:1000.00 | PEAKS DB |
| total 1 peptides |  |  |  |  |  |  |  |  |  |  |  |  |  |  |  |  |  |  |  |  |  |  |  |  |  |  |  |  |  |  |  |  |  |  |  |  |  |  |  |  |  |  |  |
| --- | --- | --- | --- | --- | --- | --- | --- | --- | --- | --- | --- | --- | --- | --- | --- | --- | --- | --- | --- | --- | --- | --- | --- | --- | --- | --- | --- | --- | --- | --- | --- | --- | --- | --- | --- | --- | --- | --- | --- | --- | --- | --- | --- |

2QHD

back to list

  

| Protein Coverage
| Supporting Peptides
|

Protein Coverage:

Supporting Peptides:

| Peptide | Uniq | -10lgP | Mass | Length | ppm | m/z | z | RT | Fraction | Scan | Source File | Area F1 | Area F10 | Area F11 | Area F12 | Area F13 | Area F2 | Area F3 | Area F4 | Area F5 | Area F6 | Area F7 | Area F8 | Area F9 | #Feature | #Feature F1 | #Feature F10 | #Feature F11 | #Feature F12 | #Feature F13 | #Feature F2 | #Feature F3 | #Feature F4 | #Feature F5 | #Feature F6 | #Feature F7 | #Feature F8 | #Feature F9 | Start | End | PTM | AScore | Found By |
| --- | --- | --- | --- | --- | --- | --- | --- | --- | --- | --- | --- | --- | --- | --- | --- | --- | --- | --- | --- | --- | --- | --- | --- | --- | --- | --- | --- | --- | --- | --- | --- | --- | --- | --- | --- | --- | --- | --- | --- | --- | --- | --- | --- |
| K.SPFPSYTSYGC(+57.02)FC(+57.02)GGGER.G | Y | 83.46 | 2027.8142 | 18 | 0.0 | 1014.9144 | 2 | 56.41 | 12 | F12:36093 | NaNaKA16\_F8.raw |  |  |  |  |  |  |  |  |  |  | 1.7369E6 | 1.2456E7 |  | 2 | 0 | 0 | 0 | 0 | 0 | 0 | 0 | 0 | 0 | 0 | 1 | 1 | 0 | 16 | 33 | Carbamidomethylation | C11:Carbamidomethylation:1000.00;C13:Carbamidomethylation:1000.00 | PEAKS DB |
| total 1 peptides |  |  |  |  |  |  |  |  |  |  |  |  |  |  |  |  |  |  |  |  |  |  |  |  |  |  |  |  |  |  |  |  |  |  |  |  |  |  |  |  |  |  |  |
| --- | --- | --- | --- | --- | --- | --- | --- | --- | --- | --- | --- | --- | --- | --- | --- | --- | --- | --- | --- | --- | --- | --- | --- | --- | --- | --- | --- | --- | --- | --- | --- | --- | --- | --- | --- | --- | --- | --- | --- | --- | --- | --- | --- |

P48650.1

back to list

  

| Protein Coverage
| Supporting Peptides
|

Protein Coverage:

Supporting Peptides:

| Peptide | Uniq | -10lgP | Mass | Length | ppm | m/z | z | RT | Fraction | Scan | Source File | Area F1 | Area F10 | Area F11 | Area F12 | Area F13 | Area F2 | Area F3 | Area F4 | Area F5 | Area F6 | Area F7 | Area F8 | Area F9 | #Feature | #Feature F1 | #Feature F10 | #Feature F11 | #Feature F12 | #Feature F13 | #Feature F2 | #Feature F3 | #Feature F4 | #Feature F5 | #Feature F6 | #Feature F7 | #Feature F8 | #Feature F9 | Start | End | PTM | AScore | Found By |
| --- | --- | --- | --- | --- | --- | --- | --- | --- | --- | --- | --- | --- | --- | --- | --- | --- | --- | --- | --- | --- | --- | --- | --- | --- | --- | --- | --- | --- | --- | --- | --- | --- | --- | --- | --- | --- | --- | --- | --- | --- | --- | --- | --- |
| K.SPFPSYTSYGC(+57.02)FC(+57.02)GGGER.G | Y | 83.46 | 2027.8142 | 18 | 0.0 | 1014.9144 | 2 | 56.41 | 12 | F12:36093 | NaNaKA16\_F8.raw |  |  |  |  |  |  |  |  |  |  | 1.7369E6 | 1.2456E7 |  | 2 | 0 | 0 | 0 | 0 | 0 | 0 | 0 | 0 | 0 | 0 | 1 | 1 | 0 | 16 | 33 | Carbamidomethylation | C11:Carbamidomethylation:1000.00;C13:Carbamidomethylation:1000.00 | PEAKS DB |
| total 1 peptides |  |  |  |  |  |  |  |  |  |  |  |  |  |  |  |  |  |  |  |  |  |  |  |  |  |  |  |  |  |  |  |  |  |  |  |  |  |  |  |  |  |  |  |
| --- | --- | --- | --- | --- | --- | --- | --- | --- | --- | --- | --- | --- | --- | --- | --- | --- | --- | --- | --- | --- | --- | --- | --- | --- | --- | --- | --- | --- | --- | --- | --- | --- | --- | --- | --- | --- | --- | --- | --- | --- | --- | --- | --- |

2QHE

back to list

  

| Protein Coverage
| Supporting Peptides
|

Protein Coverage:

Supporting Peptides:

| Peptide | Uniq | -10lgP | Mass | Length | ppm | m/z | z | RT | Fraction | Scan | Source File | Area F1 | Area F10 | Area F11 | Area F12 | Area F13 | Area F2 | Area F3 | Area F4 | Area F5 | Area F6 | Area F7 | Area F8 | Area F9 | #Feature | #Feature F1 | #Feature F10 | #Feature F11 | #Feature F12 | #Feature F13 | #Feature F2 | #Feature F3 | #Feature F4 | #Feature F5 | #Feature F6 | #Feature F7 | #Feature F8 | #Feature F9 | Start | End | PTM | AScore | Found By |
| --- | --- | --- | --- | --- | --- | --- | --- | --- | --- | --- | --- | --- | --- | --- | --- | --- | --- | --- | --- | --- | --- | --- | --- | --- | --- | --- | --- | --- | --- | --- | --- | --- | --- | --- | --- | --- | --- | --- | --- | --- | --- | --- | --- |
| K.SPFPSYTSYGC(+57.02)FC(+57.02)GGGER.G | Y | 83.46 | 2027.8142 | 18 | 0.0 | 1014.9144 | 2 | 56.41 | 12 | F12:36093 | NaNaKA16\_F8.raw |  |  |  |  |  |  |  |  |  |  | 1.7369E6 | 1.2456E7 |  | 2 | 0 | 0 | 0 | 0 | 0 | 0 | 0 | 0 | 0 | 0 | 1 | 1 | 0 | 16 | 33 | Carbamidomethylation | C11:Carbamidomethylation:1000.00;C13:Carbamidomethylation:1000.00 | PEAKS DB |
| total 1 peptides |  |  |  |  |  |  |  |  |  |  |  |  |  |  |  |  |  |  |  |  |  |  |  |  |  |  |  |  |  |  |  |  |  |  |  |  |  |  |  |  |  |  |  |
| --- | --- | --- | --- | --- | --- | --- | --- | --- | --- | --- | --- | --- | --- | --- | --- | --- | --- | --- | --- | --- | --- | --- | --- | --- | --- | --- | --- | --- | --- | --- | --- | --- | --- | --- | --- | --- | --- | --- | --- | --- | --- | --- | --- |

XP\_026571379.1

back to list

  

| Protein Coverage
| Supporting Peptides
|

Protein Coverage:

Supporting Peptides:

| Peptide | Uniq | -10lgP | Mass | Length | ppm | m/z | z | RT | Fraction | Scan | Source File | Area F1 | Area F10 | Area F11 | Area F12 | Area F13 | Area F2 | Area F3 | Area F4 | Area F5 | Area F6 | Area F7 | Area F8 | Area F9 | #Feature | #Feature F1 | #Feature F10 | #Feature F11 | #Feature F12 | #Feature F13 | #Feature F2 | #Feature F3 | #Feature F4 | #Feature F5 | #Feature F6 | #Feature F7 | #Feature F8 | #Feature F9 | Start | End | PTM | AScore | Found By |
| --- | --- | --- | --- | --- | --- | --- | --- | --- | --- | --- | --- | --- | --- | --- | --- | --- | --- | --- | --- | --- | --- | --- | --- | --- | --- | --- | --- | --- | --- | --- | --- | --- | --- | --- | --- | --- | --- | --- | --- | --- | --- | --- | --- |
| K.WNLPSPEC(+57.02)K.K | Y | 59.14 | 1129.5226 | 9 | 0.7 | 565.7690 | 2 | 39.67 | 4 | F4:21853 | NaNaKA16\_F12.raw |  | 1.8284E6 | 1.4947E7 | 7.0028E6 |  |  |  |  |  |  |  |  | 4.4868E6 | 4 | 0 | 1 | 1 | 1 | 0 | 0 | 0 | 0 | 0 | 0 | 0 | 0 | 1 | 209 | 217 | Carbamidomethylation | C8:Carbamidomethylation:1000.00 | PEAKS DB |
| S.DC(+57.02)PRPVLPPHSSLR.G | Y | 47.89 | 1629.8409 | 14 | 1.0 | 544.2881 | 3 | 11.80 | 3 | F3:2167 | NaNaKA16\_F11.raw |  |  | 5.4229E5 |  |  |  |  |  |  |  |  |  |  | 1 | 0 | 0 | 1 | 0 | 0 | 0 | 0 | 0 | 0 | 0 | 0 | 0 | 0 | 27 | 40 | Carbamidomethylation | C2:Carbamidomethylation:1000.00 | PEAKS DB |
| total 2 peptides |  |  |  |  |  |  |  |  |  |  |  |  |  |  |  |  |  |  |  |  |  |  |  |  |  |  |  |  |  |  |  |  |  |  |  |  |  |  |  |  |  |  |  |
| --- | --- | --- | --- | --- | --- | --- | --- | --- | --- | --- | --- | --- | --- | --- | --- | --- | --- | --- | --- | --- | --- | --- | --- | --- | --- | --- | --- | --- | --- | --- | --- | --- | --- | --- | --- | --- | --- | --- | --- | --- | --- | --- | --- |

XP\_032085287.1

back to list

  

| Protein Coverage
| Supporting Peptides
|

Protein Coverage:

Supporting Peptides:

| Peptide | Uniq | -10lgP | Mass | Length | ppm | m/z | z | RT | Fraction | Scan | Source File | Area F1 | Area F10 | Area F11 | Area F12 | Area F13 | Area F2 | Area F3 | Area F4 | Area F5 | Area F6 | Area F7 | Area F8 | Area F9 | #Feature | #Feature F1 | #Feature F10 | #Feature F11 | #Feature F12 | #Feature F13 | #Feature F2 | #Feature F3 | #Feature F4 | #Feature F5 | #Feature F6 | #Feature F7 | #Feature F8 | #Feature F9 | Start | End | PTM | AScore | Found By |
| --- | --- | --- | --- | --- | --- | --- | --- | --- | --- | --- | --- | --- | --- | --- | --- | --- | --- | --- | --- | --- | --- | --- | --- | --- | --- | --- | --- | --- | --- | --- | --- | --- | --- | --- | --- | --- | --- | --- | --- | --- | --- | --- | --- |
| R.NLLQFSNMIK.C | N | 56.92 | 1206.6431 | 10 | 0.5 | 604.3291 | 2 | 65.68 | 2 | F2:42174 | NaNaKA16\_F10.raw |  | 8.2085E6 | 1.7232E6 |  |  |  |  |  |  |  |  |  |  | 2 | 0 | 1 | 1 | 0 | 0 | 0 | 0 | 0 | 0 | 0 | 0 | 0 | 0 | 28 | 37 |  |  | PEAKS DB |
| R.SAAIC(+57.02)FAGAPYNEEYKK.L | Y | 50.34 | 1917.8931 | 17 | 0.3 | 640.3052 | 3 | 34.35 | 2 | F2:16469 | NaNaKA16\_F10.raw |  | 1.8111E6 |  |  |  |  |  |  |  |  |  |  |  | 1 | 0 | 1 | 0 | 0 | 0 | 0 | 0 | 0 | 0 | 0 | 0 | 0 | 0 | 128 | 144 | Carbamidomethylation | C5:Carbamidomethylation:1000.00 | PEAKS DB |
| total 2 peptides |  |  |  |  |  |  |  |  |  |  |  |  |  |  |  |  |  |  |  |  |  |  |  |  |  |  |  |  |  |  |  |  |  |  |  |  |  |  |  |  |  |  |  |
| --- | --- | --- | --- | --- | --- | --- | --- | --- | --- | --- | --- | --- | --- | --- | --- | --- | --- | --- | --- | --- | --- | --- | --- | --- | --- | --- | --- | --- | --- | --- | --- | --- | --- | --- | --- | --- | --- | --- | --- | --- | --- | --- | --- |

JAG46496.1

back to list

  

| Protein Coverage
| Supporting Peptides
|

Protein Coverage:

Supporting Peptides:

| Peptide | Uniq | -10lgP | Mass | Length | ppm | m/z | z | RT | Fraction | Scan | Source File | Area F1 | Area F10 | Area F11 | Area F12 | Area F13 | Area F2 | Area F3 | Area F4 | Area F5 | Area F6 | Area F7 | Area F8 | Area F9 | #Feature | #Feature F1 | #Feature F10 | #Feature F11 | #Feature F12 | #Feature F13 | #Feature F2 | #Feature F3 | #Feature F4 | #Feature F5 | #Feature F6 | #Feature F7 | #Feature F8 | #Feature F9 | Start | End | PTM | AScore | Found By |
| --- | --- | --- | --- | --- | --- | --- | --- | --- | --- | --- | --- | --- | --- | --- | --- | --- | --- | --- | --- | --- | --- | --- | --- | --- | --- | --- | --- | --- | --- | --- | --- | --- | --- | --- | --- | --- | --- | --- | --- | --- | --- | --- | --- |
| K.WMEDSDVEDLRK.E | Y | 58.39 | 1521.6769 | 12 | 0.3 | 508.2330 | 3 | 33.02 | 4 | F4:16009 | NaNaKA16\_F12.raw |  |  |  | 2.3013E6 |  |  |  |  |  |  |  |  |  | 1 | 0 | 0 | 0 | 1 | 0 | 0 | 0 | 0 | 0 | 0 | 0 | 0 | 0 | 237 | 248 |  |  | PEAKS DB |
| K.LMATNDIYEAK.A | N | 47.30 | 1267.6118 | 11 | 0.5 | 634.8135 | 2 | 29.68 | 4 | F4:13304 | NaNaKA16\_F12.raw |  |  |  | 1.2791E6 |  |  |  |  |  |  |  |  |  | 1 | 0 | 0 | 0 | 1 | 0 | 0 | 0 | 0 | 0 | 0 | 0 | 0 | 0 | 319 | 329 |  |  | PEAKS DB |
| total 2 peptides |  |  |  |  |  |  |  |  |  |  |  |  |  |  |  |  |  |  |  |  |  |  |  |  |  |  |  |  |  |  |  |  |  |  |  |  |  |  |  |  |  |  |  |
| --- | --- | --- | --- | --- | --- | --- | --- | --- | --- | --- | --- | --- | --- | --- | --- | --- | --- | --- | --- | --- | --- | --- | --- | --- | --- | --- | --- | --- | --- | --- | --- | --- | --- | --- | --- | --- | --- | --- | --- | --- | --- | --- | --- |

ACD43466.1

back to list

  

| Protein Coverage
| Supporting Peptides
|

Protein Coverage:

Supporting Peptides:

| Peptide | Uniq | -10lgP | Mass | Length | ppm | m/z | z | RT | Fraction | Scan | Source File | Area F1 | Area F10 | Area F11 | Area F12 | Area F13 | Area F2 | Area F3 | Area F4 | Area F5 | Area F6 | Area F7 | Area F8 | Area F9 | #Feature | #Feature F1 | #Feature F10 | #Feature F11 | #Feature F12 | #Feature F13 | #Feature F2 | #Feature F3 | #Feature F4 | #Feature F5 | #Feature F6 | #Feature F7 | #Feature F8 | #Feature F9 | Start | End | PTM | AScore | Found By |
| --- | --- | --- | --- | --- | --- | --- | --- | --- | --- | --- | --- | --- | --- | --- | --- | --- | --- | --- | --- | --- | --- | --- | --- | --- | --- | --- | --- | --- | --- | --- | --- | --- | --- | --- | --- | --- | --- | --- | --- | --- | --- | --- | --- |
| G.NLFQFGEMILEK.T | Y | 58.42 | 1467.7432 | 12 | 0.9 | 734.8795 | 2 | 93.40 | 4 | F4:65640 | NaNaKA16\_F12.raw |  |  |  | 1.3999E6 |  |  |  |  |  |  |  |  |  | 1 | 0 | 0 | 0 | 1 | 0 | 0 | 0 | 0 | 0 | 0 | 0 | 0 | 0 | 17 | 28 |  |  | PEAKS DB |
| R.AAAIC(+57.02)LGQNVNTYDK.N | Y | 46.79 | 1636.7878 | 15 | 1.1 | 819.4021 | 2 | 36.24 | 4 | F4:18606 | NaNaKA16\_F12.raw |  |  |  | 1.3298E6 |  |  |  |  |  |  |  |  |  | 1 | 0 | 0 | 0 | 1 | 0 | 0 | 0 | 0 | 0 | 0 | 0 | 0 | 0 | 107 | 121 | Carbamidomethylation | C5:Carbamidomethylation:1000.00 | PEAKS DB |
| total 2 peptides |  |  |  |  |  |  |  |  |  |  |  |  |  |  |  |  |  |  |  |  |  |  |  |  |  |  |  |  |  |  |  |  |  |  |  |  |  |  |  |  |  |  |  |
| --- | --- | --- | --- | --- | --- | --- | --- | --- | --- | --- | --- | --- | --- | --- | --- | --- | --- | --- | --- | --- | --- | --- | --- | --- | --- | --- | --- | --- | --- | --- | --- | --- | --- | --- | --- | --- | --- | --- | --- | --- | --- | --- | --- |

P31100.1

back to list

  

| Protein Coverage
| Supporting Peptides
|

Protein Coverage:

Supporting Peptides:

| Peptide | Uniq | -10lgP | Mass | Length | ppm | m/z | z | RT | Fraction | Scan | Source File | Area F1 | Area F10 | Area F11 | Area F12 | Area F13 | Area F2 | Area F3 | Area F4 | Area F5 | Area F6 | Area F7 | Area F8 | Area F9 | #Feature | #Feature F1 | #Feature F10 | #Feature F11 | #Feature F12 | #Feature F13 | #Feature F2 | #Feature F3 | #Feature F4 | #Feature F5 | #Feature F6 | #Feature F7 | #Feature F8 | #Feature F9 | Start | End | PTM | AScore | Found By |
| --- | --- | --- | --- | --- | --- | --- | --- | --- | --- | --- | --- | --- | --- | --- | --- | --- | --- | --- | --- | --- | --- | --- | --- | --- | --- | --- | --- | --- | --- | --- | --- | --- | --- | --- | --- | --- | --- | --- | --- | --- | --- | --- | --- |
| G.NLFQFGEMILEK.T | Y | 58.42 | 1467.7432 | 12 | 0.9 | 734.8795 | 2 | 93.40 | 4 | F4:65640 | NaNaKA16\_F12.raw |  |  |  | 1.3999E6 |  |  |  |  |  |  |  |  |  | 1 | 0 | 0 | 0 | 1 | 0 | 0 | 0 | 0 | 0 | 0 | 0 | 0 | 0 | 17 | 28 |  |  | PEAKS DB |
| R.AAAIC(+57.02)LGQNVNTYDK.N | Y | 46.79 | 1636.7878 | 15 | 1.1 | 819.4021 | 2 | 36.24 | 4 | F4:18606 | NaNaKA16\_F12.raw |  |  |  | 1.3298E6 |  |  |  |  |  |  |  |  |  | 1 | 0 | 0 | 0 | 1 | 0 | 0 | 0 | 0 | 0 | 0 | 0 | 0 | 0 | 107 | 121 | Carbamidomethylation | C5:Carbamidomethylation:1000.00 | PEAKS DB |
| total 2 peptides |  |  |  |  |  |  |  |  |  |  |  |  |  |  |  |  |  |  |  |  |  |  |  |  |  |  |  |  |  |  |  |  |  |  |  |  |  |  |  |  |  |  |  |
| --- | --- | --- | --- | --- | --- | --- | --- | --- | --- | --- | --- | --- | --- | --- | --- | --- | --- | --- | --- | --- | --- | --- | --- | --- | --- | --- | --- | --- | --- | --- | --- | --- | --- | --- | --- | --- | --- | --- | --- | --- | --- | --- | --- |

CAA48457.1

back to list

  

| Protein Coverage
| Supporting Peptides
|

Protein Coverage:

Supporting Peptides:

| Peptide | Uniq | -10lgP | Mass | Length | ppm | m/z | z | RT | Fraction | Scan | Source File | Area F1 | Area F10 | Area F11 | Area F12 | Area F13 | Area F2 | Area F3 | Area F4 | Area F5 | Area F6 | Area F7 | Area F8 | Area F9 | #Feature | #Feature F1 | #Feature F10 | #Feature F11 | #Feature F12 | #Feature F13 | #Feature F2 | #Feature F3 | #Feature F4 | #Feature F5 | #Feature F6 | #Feature F7 | #Feature F8 | #Feature F9 | Start | End | PTM | AScore | Found By |
| --- | --- | --- | --- | --- | --- | --- | --- | --- | --- | --- | --- | --- | --- | --- | --- | --- | --- | --- | --- | --- | --- | --- | --- | --- | --- | --- | --- | --- | --- | --- | --- | --- | --- | --- | --- | --- | --- | --- | --- | --- | --- | --- | --- |
| G.NLFQFGEMILEK.T | Y | 58.42 | 1467.7432 | 12 | 0.9 | 734.8795 | 2 | 93.40 | 4 | F4:65640 | NaNaKA16\_F12.raw |  |  |  | 1.3999E6 |  |  |  |  |  |  |  |  |  | 1 | 0 | 0 | 0 | 1 | 0 | 0 | 0 | 0 | 0 | 0 | 0 | 0 | 0 | 17 | 28 |  |  | PEAKS DB |
| R.AAAIC(+57.02)LGQNVNTYDK.N | Y | 46.79 | 1636.7878 | 15 | 1.1 | 819.4021 | 2 | 36.24 | 4 | F4:18606 | NaNaKA16\_F12.raw |  |  |  | 1.3298E6 |  |  |  |  |  |  |  |  |  | 1 | 0 | 0 | 0 | 1 | 0 | 0 | 0 | 0 | 0 | 0 | 0 | 0 | 0 | 107 | 121 | Carbamidomethylation | C5:Carbamidomethylation:1000.00 | PEAKS DB |
| total 2 peptides |  |  |  |  |  |  |  |  |  |  |  |  |  |  |  |  |  |  |  |  |  |  |  |  |  |  |  |  |  |  |  |  |  |  |  |  |  |  |  |  |  |  |  |
| --- | --- | --- | --- | --- | --- | --- | --- | --- | --- | --- | --- | --- | --- | --- | --- | --- | --- | --- | --- | --- | --- | --- | --- | --- | --- | --- | --- | --- | --- | --- | --- | --- | --- | --- | --- | --- | --- | --- | --- | --- | --- | --- | --- |

S29299

back to list

  

| Protein Coverage
| Supporting Peptides
|

Protein Coverage:

Supporting Peptides:

| Peptide | Uniq | -10lgP | Mass | Length | ppm | m/z | z | RT | Fraction | Scan | Source File | Area F1 | Area F10 | Area F11 | Area F12 | Area F13 | Area F2 | Area F3 | Area F4 | Area F5 | Area F6 | Area F7 | Area F8 | Area F9 | #Feature | #Feature F1 | #Feature F10 | #Feature F11 | #Feature F12 | #Feature F13 | #Feature F2 | #Feature F3 | #Feature F4 | #Feature F5 | #Feature F6 | #Feature F7 | #Feature F8 | #Feature F9 | Start | End | PTM | AScore | Found By |
| --- | --- | --- | --- | --- | --- | --- | --- | --- | --- | --- | --- | --- | --- | --- | --- | --- | --- | --- | --- | --- | --- | --- | --- | --- | --- | --- | --- | --- | --- | --- | --- | --- | --- | --- | --- | --- | --- | --- | --- | --- | --- | --- | --- |
| G.NLFQFGEMILEK.T | Y | 58.42 | 1467.7432 | 12 | 0.9 | 734.8795 | 2 | 93.40 | 4 | F4:65640 | NaNaKA16\_F12.raw |  |  |  | 1.3999E6 |  |  |  |  |  |  |  |  |  | 1 | 0 | 0 | 0 | 1 | 0 | 0 | 0 | 0 | 0 | 0 | 0 | 0 | 0 | 17 | 28 |  |  | PEAKS DB |
| R.AAAIC(+57.02)LGQNVNTYDK.N | Y | 46.79 | 1636.7878 | 15 | 1.1 | 819.4021 | 2 | 36.24 | 4 | F4:18606 | NaNaKA16\_F12.raw |  |  |  | 1.3298E6 |  |  |  |  |  |  |  |  |  | 1 | 0 | 0 | 0 | 1 | 0 | 0 | 0 | 0 | 0 | 0 | 0 | 0 | 0 | 107 | 121 | Carbamidomethylation | C5:Carbamidomethylation:1000.00 | PEAKS DB |
| total 2 peptides |  |  |  |  |  |  |  |  |  |  |  |  |  |  |  |  |  |  |  |  |  |  |  |  |  |  |  |  |  |  |  |  |  |  |  |  |  |  |  |  |  |  |  |
| --- | --- | --- | --- | --- | --- | --- | --- | --- | --- | --- | --- | --- | --- | --- | --- | --- | --- | --- | --- | --- | --- | --- | --- | --- | --- | --- | --- | --- | --- | --- | --- | --- | --- | --- | --- | --- | --- | --- | --- | --- | --- | --- | --- |

AAZ53184.1

back to list

  

| Protein Coverage
| Supporting Peptides
|

Protein Coverage:

Supporting Peptides:

| Peptide | Uniq | -10lgP | Mass | Length | ppm | m/z | z | RT | Fraction | Scan | Source File | Area F1 | Area F10 | Area F11 | Area F12 | Area F13 | Area F2 | Area F3 | Area F4 | Area F5 | Area F6 | Area F7 | Area F8 | Area F9 | #Feature | #Feature F1 | #Feature F10 | #Feature F11 | #Feature F12 | #Feature F13 | #Feature F2 | #Feature F3 | #Feature F4 | #Feature F5 | #Feature F6 | #Feature F7 | #Feature F8 | #Feature F9 | Start | End | PTM | AScore | Found By |
| --- | --- | --- | --- | --- | --- | --- | --- | --- | --- | --- | --- | --- | --- | --- | --- | --- | --- | --- | --- | --- | --- | --- | --- | --- | --- | --- | --- | --- | --- | --- | --- | --- | --- | --- | --- | --- | --- | --- | --- | --- | --- | --- | --- |
| G.NLFQFGEMILEK.T | Y | 58.42 | 1467.7432 | 12 | 0.9 | 734.8795 | 2 | 93.40 | 4 | F4:65640 | NaNaKA16\_F12.raw |  |  |  | 1.3999E6 |  |  |  |  |  |  |  |  |  | 1 | 0 | 0 | 0 | 1 | 0 | 0 | 0 | 0 | 0 | 0 | 0 | 0 | 0 | 17 | 28 |  |  | PEAKS DB |
| R.AAAIC(+57.02)LGQNVNTYDK.N | Y | 46.79 | 1636.7878 | 15 | 1.1 | 819.4021 | 2 | 36.24 | 4 | F4:18606 | NaNaKA16\_F12.raw |  |  |  | 1.3298E6 |  |  |  |  |  |  |  |  |  | 1 | 0 | 0 | 0 | 1 | 0 | 0 | 0 | 0 | 0 | 0 | 0 | 0 | 0 | 107 | 121 | Carbamidomethylation | C5:Carbamidomethylation:1000.00 | PEAKS DB |
| total 2 peptides |  |  |  |  |  |  |  |  |  |  |  |  |  |  |  |  |  |  |  |  |  |  |  |  |  |  |  |  |  |  |  |  |  |  |  |  |  |  |  |  |  |  |  |
| --- | --- | --- | --- | --- | --- | --- | --- | --- | --- | --- | --- | --- | --- | --- | --- | --- | --- | --- | --- | --- | --- | --- | --- | --- | --- | --- | --- | --- | --- | --- | --- | --- | --- | --- | --- | --- | --- | --- | --- | --- | --- | --- | --- |

ACD43468.1

back to list

  

| Protein Coverage
| Supporting Peptides
|

Protein Coverage:

Supporting Peptides:

| Peptide | Uniq | -10lgP | Mass | Length | ppm | m/z | z | RT | Fraction | Scan | Source File | Area F1 | Area F10 | Area F11 | Area F12 | Area F13 | Area F2 | Area F3 | Area F4 | Area F5 | Area F6 | Area F7 | Area F8 | Area F9 | #Feature | #Feature F1 | #Feature F10 | #Feature F11 | #Feature F12 | #Feature F13 | #Feature F2 | #Feature F3 | #Feature F4 | #Feature F5 | #Feature F6 | #Feature F7 | #Feature F8 | #Feature F9 | Start | End | PTM | AScore | Found By |
| --- | --- | --- | --- | --- | --- | --- | --- | --- | --- | --- | --- | --- | --- | --- | --- | --- | --- | --- | --- | --- | --- | --- | --- | --- | --- | --- | --- | --- | --- | --- | --- | --- | --- | --- | --- | --- | --- | --- | --- | --- | --- | --- | --- |
| G.NLFQFGEMILEK.T | Y | 58.42 | 1467.7432 | 12 | 0.9 | 734.8795 | 2 | 93.40 | 4 | F4:65640 | NaNaKA16\_F12.raw |  |  |  | 1.3999E6 |  |  |  |  |  |  |  |  |  | 1 | 0 | 0 | 0 | 1 | 0 | 0 | 0 | 0 | 0 | 0 | 0 | 0 | 0 | 17 | 28 |  |  | PEAKS DB |
| R.AAAIC(+57.02)LGQNVNTYDK.N | Y | 46.79 | 1636.7878 | 15 | 1.1 | 819.4021 | 2 | 36.24 | 4 | F4:18606 | NaNaKA16\_F12.raw |  |  |  | 1.3298E6 |  |  |  |  |  |  |  |  |  | 1 | 0 | 0 | 0 | 1 | 0 | 0 | 0 | 0 | 0 | 0 | 0 | 0 | 0 | 107 | 121 | Carbamidomethylation | C5:Carbamidomethylation:1000.00 | PEAKS DB |
| total 2 peptides |  |  |  |  |  |  |  |  |  |  |  |  |  |  |  |  |  |  |  |  |  |  |  |  |  |  |  |  |  |  |  |  |  |  |  |  |  |  |  |  |  |  |  |
| --- | --- | --- | --- | --- | --- | --- | --- | --- | --- | --- | --- | --- | --- | --- | --- | --- | --- | --- | --- | --- | --- | --- | --- | --- | --- | --- | --- | --- | --- | --- | --- | --- | --- | --- | --- | --- | --- | --- | --- | --- | --- | --- | --- |

AXL95289.1

back to list

  

| Protein Coverage
| Supporting Peptides
|

Protein Coverage:

Supporting Peptides:

| Peptide | Uniq | -10lgP | Mass | Length | ppm | m/z | z | RT | Fraction | Scan | Source File | Area F1 | Area F10 | Area F11 | Area F12 | Area F13 | Area F2 | Area F3 | Area F4 | Area F5 | Area F6 | Area F7 | Area F8 | Area F9 | #Feature | #Feature F1 | #Feature F10 | #Feature F11 | #Feature F12 | #Feature F13 | #Feature F2 | #Feature F3 | #Feature F4 | #Feature F5 | #Feature F6 | #Feature F7 | #Feature F8 | #Feature F9 | Start | End | PTM | AScore | Found By |
| --- | --- | --- | --- | --- | --- | --- | --- | --- | --- | --- | --- | --- | --- | --- | --- | --- | --- | --- | --- | --- | --- | --- | --- | --- | --- | --- | --- | --- | --- | --- | --- | --- | --- | --- | --- | --- | --- | --- | --- | --- | --- | --- | --- |
| S.VSPTASNM(+15.99)LK.M | N | 56.73 | 1062.5380 | 10 | 0.7 | 532.2766 | 2 | 11.45 | 4 | F4:1800 | NaNaKA16\_F12.raw |  |  |  | 1.2798E7 |  |  |  |  |  |  |  |  |  | 1 | 0 | 0 | 0 | 1 | 0 | 0 | 0 | 0 | 0 | 0 | 0 | 0 | 0 | 47 | 56 | Oxidation (M) | M8:Oxidation (M):1000.00 | PEAKS DB |
| S.VSPTASNMLK.M | N | 54.02 | 1046.5430 | 10 | -0.7 | 524.2784 | 2 | 11.74 | 4 | F4:2287 | NaNaKA16\_F12.raw |  |  |  | 1.7067E8 |  |  |  |  |  |  |  |  |  | 2 | 0 | 0 | 0 | 2 | 0 | 0 | 0 | 0 | 0 | 0 | 0 | 0 | 0 | 47 | 56 |  |  | PEAKS DB |
| Q.QSNC(+57.02)QDDWIK.S | Y | 50.04 | 1292.5455 | 10 | -0.2 | 647.2799 | 2 | 44.84 | 4 | F4:26184 | NaNaKA16\_F12.raw |  |  |  | 2.2338E8 |  |  |  |  |  |  |  |  |  | 3 | 0 | 0 | 0 | 3 | 0 | 0 | 0 | 0 | 0 | 0 | 0 | 0 | 0 | 216 | 225 | Carbamidomethylation | C4:Carbamidomethylation:1000.00 | PEAKS DB |
| total 3 peptides |  |  |  |  |  |  |  |  |  |  |  |  |  |  |  |  |  |  |  |  |  |  |  |  |  |  |  |  |  |  |  |  |  |  |  |  |  |  |  |  |  |  |  |
| --- | --- | --- | --- | --- | --- | --- | --- | --- | --- | --- | --- | --- | --- | --- | --- | --- | --- | --- | --- | --- | --- | --- | --- | --- | --- | --- | --- | --- | --- | --- | --- | --- | --- | --- | --- | --- | --- | --- | --- | --- | --- | --- | --- |

XP\_034262239.1

back to list

  

| Protein Coverage
| Supporting Peptides
|

Protein Coverage:

Supporting Peptides:

| Peptide | Uniq | -10lgP | Mass | Length | ppm | m/z | z | RT | Fraction | Scan | Source File | Area F1 | Area F10 | Area F11 | Area F12 | Area F13 | Area F2 | Area F3 | Area F4 | Area F5 | Area F6 | Area F7 | Area F8 | Area F9 | #Feature | #Feature F1 | #Feature F10 | #Feature F11 | #Feature F12 | #Feature F13 | #Feature F2 | #Feature F3 | #Feature F4 | #Feature F5 | #Feature F6 | #Feature F7 | #Feature F8 | #Feature F9 | Start | End | PTM | AScore | Found By |
| --- | --- | --- | --- | --- | --- | --- | --- | --- | --- | --- | --- | --- | --- | --- | --- | --- | --- | --- | --- | --- | --- | --- | --- | --- | --- | --- | --- | --- | --- | --- | --- | --- | --- | --- | --- | --- | --- | --- | --- | --- | --- | --- | --- |
| R.LEDEEEMNAELTAK.K | Y | 52.69 | 1620.7189 | 14 | 0.6 | 811.3672 | 2 | 34.79 | 1 | F1:14100 | NaNaKA16\_F1.raw | 4.3764E5 |  |  |  |  |  |  |  |  |  |  |  |  | 1 | 1 | 0 | 0 | 0 | 0 | 0 | 0 | 0 | 0 | 0 | 0 | 0 | 0 | 640 | 653 |  |  | PEAKS DB |
| R.LEEAGGATSVQIEMNK.K | Y | 49.97 | 1675.8087 | 16 | 1.0 | 838.9125 | 2 | 34.20 | 1 | F1:13672 | NaNaKA16\_F1.raw | 1.2438E6 |  |  |  |  |  |  |  |  |  |  |  |  | 1 | 1 | 0 | 0 | 0 | 0 | 0 | 0 | 0 | 0 | 0 | 0 | 0 | 0 | 864 | 879 |  |  | PEAKS DB |
| total 2 peptides |  |  |  |  |  |  |  |  |  |  |  |  |  |  |  |  |  |  |  |  |  |  |  |  |  |  |  |  |  |  |  |  |  |  |  |  |  |  |  |  |  |  |  |
| --- | --- | --- | --- | --- | --- | --- | --- | --- | --- | --- | --- | --- | --- | --- | --- | --- | --- | --- | --- | --- | --- | --- | --- | --- | --- | --- | --- | --- | --- | --- | --- | --- | --- | --- | --- | --- | --- | --- | --- | --- | --- | --- | --- |

XP\_032092515.1

back to list

  

| Protein Coverage
| Supporting Peptides
|

Protein Coverage:

Supporting Peptides:

| Peptide | Uniq | -10lgP | Mass | Length | ppm | m/z | z | RT | Fraction | Scan | Source File | Area F1 | Area F10 | Area F11 | Area F12 | Area F13 | Area F2 | Area F3 | Area F4 | Area F5 | Area F6 | Area F7 | Area F8 | Area F9 | #Feature | #Feature F1 | #Feature F10 | #Feature F11 | #Feature F12 | #Feature F13 | #Feature F2 | #Feature F3 | #Feature F4 | #Feature F5 | #Feature F6 | #Feature F7 | #Feature F8 | #Feature F9 | Start | End | PTM | AScore | Found By |
| --- | --- | --- | --- | --- | --- | --- | --- | --- | --- | --- | --- | --- | --- | --- | --- | --- | --- | --- | --- | --- | --- | --- | --- | --- | --- | --- | --- | --- | --- | --- | --- | --- | --- | --- | --- | --- | --- | --- | --- | --- | --- | --- | --- |
| R.LEDEEEMNAELTAK.K | Y | 52.69 | 1620.7189 | 14 | 0.6 | 811.3672 | 2 | 34.79 | 1 | F1:14100 | NaNaKA16\_F1.raw | 4.3764E5 |  |  |  |  |  |  |  |  |  |  |  |  | 1 | 1 | 0 | 0 | 0 | 0 | 0 | 0 | 0 | 0 | 0 | 0 | 0 | 0 | 929 | 942 |  |  | PEAKS DB |
| R.LEEAGGATSVQIEMNK.K | Y | 49.97 | 1675.8087 | 16 | 1.0 | 838.9125 | 2 | 34.20 | 1 | F1:13672 | NaNaKA16\_F1.raw | 1.2438E6 |  |  |  |  |  |  |  |  |  |  |  |  | 1 | 1 | 0 | 0 | 0 | 0 | 0 | 0 | 0 | 0 | 0 | 0 | 0 | 0 | 1153 | 1168 |  |  | PEAKS DB |
| total 2 peptides |  |  |  |  |  |  |  |  |  |  |  |  |  |  |  |  |  |  |  |  |  |  |  |  |  |  |  |  |  |  |  |  |  |  |  |  |  |  |  |  |  |  |  |
| --- | --- | --- | --- | --- | --- | --- | --- | --- | --- | --- | --- | --- | --- | --- | --- | --- | --- | --- | --- | --- | --- | --- | --- | --- | --- | --- | --- | --- | --- | --- | --- | --- | --- | --- | --- | --- | --- | --- | --- | --- | --- | --- | --- |

XP\_026538839.1

back to list

  

| Protein Coverage
| Supporting Peptides
|

Protein Coverage:

Supporting Peptides:

| Peptide | Uniq | -10lgP | Mass | Length | ppm | m/z | z | RT | Fraction | Scan | Source File | Area F1 | Area F10 | Area F11 | Area F12 | Area F13 | Area F2 | Area F3 | Area F4 | Area F5 | Area F6 | Area F7 | Area F8 | Area F9 | #Feature | #Feature F1 | #Feature F10 | #Feature F11 | #Feature F12 | #Feature F13 | #Feature F2 | #Feature F3 | #Feature F4 | #Feature F5 | #Feature F6 | #Feature F7 | #Feature F8 | #Feature F9 | Start | End | PTM | AScore | Found By |
| --- | --- | --- | --- | --- | --- | --- | --- | --- | --- | --- | --- | --- | --- | --- | --- | --- | --- | --- | --- | --- | --- | --- | --- | --- | --- | --- | --- | --- | --- | --- | --- | --- | --- | --- | --- | --- | --- | --- | --- | --- | --- | --- | --- |
| R.LEDEEEMNAELTAK.K | Y | 52.69 | 1620.7189 | 14 | 0.6 | 811.3672 | 2 | 34.79 | 1 | F1:14100 | NaNaKA16\_F1.raw | 4.3764E5 |  |  |  |  |  |  |  |  |  |  |  |  | 1 | 1 | 0 | 0 | 0 | 0 | 0 | 0 | 0 | 0 | 0 | 0 | 0 | 0 | 929 | 942 |  |  | PEAKS DB |
| R.LEEAGGATSVQIEMNK.K | Y | 49.97 | 1675.8087 | 16 | 1.0 | 838.9125 | 2 | 34.20 | 1 | F1:13672 | NaNaKA16\_F1.raw | 1.2438E6 |  |  |  |  |  |  |  |  |  |  |  |  | 1 | 1 | 0 | 0 | 0 | 0 | 0 | 0 | 0 | 0 | 0 | 0 | 0 | 0 | 1153 | 1168 |  |  | PEAKS DB |
| total 2 peptides |  |  |  |  |  |  |  |  |  |  |  |  |  |  |  |  |  |  |  |  |  |  |  |  |  |  |  |  |  |  |  |  |  |  |  |  |  |  |  |  |  |  |  |
| --- | --- | --- | --- | --- | --- | --- | --- | --- | --- | --- | --- | --- | --- | --- | --- | --- | --- | --- | --- | --- | --- | --- | --- | --- | --- | --- | --- | --- | --- | --- | --- | --- | --- | --- | --- | --- | --- | --- | --- | --- | --- | --- | --- |

XP\_015677713.1

back to list

  

| Protein Coverage
| Supporting Peptides
|

Protein Coverage:

Supporting Peptides:

| Peptide | Uniq | -10lgP | Mass | Length | ppm | m/z | z | RT | Fraction | Scan | Source File | Area F1 | Area F10 | Area F11 | Area F12 | Area F13 | Area F2 | Area F3 | Area F4 | Area F5 | Area F6 | Area F7 | Area F8 | Area F9 | #Feature | #Feature F1 | #Feature F10 | #Feature F11 | #Feature F12 | #Feature F13 | #Feature F2 | #Feature F3 | #Feature F4 | #Feature F5 | #Feature F6 | #Feature F7 | #Feature F8 | #Feature F9 | Start | End | PTM | AScore | Found By |
| --- | --- | --- | --- | --- | --- | --- | --- | --- | --- | --- | --- | --- | --- | --- | --- | --- | --- | --- | --- | --- | --- | --- | --- | --- | --- | --- | --- | --- | --- | --- | --- | --- | --- | --- | --- | --- | --- | --- | --- | --- | --- | --- | --- |
| R.LEDEEEMNAELTAK.K | Y | 52.69 | 1620.7189 | 14 | 0.6 | 811.3672 | 2 | 34.79 | 1 | F1:14100 | NaNaKA16\_F1.raw | 4.3764E5 |  |  |  |  |  |  |  |  |  |  |  |  | 1 | 1 | 0 | 0 | 0 | 0 | 0 | 0 | 0 | 0 | 0 | 0 | 0 | 0 | 930 | 943 |  |  | PEAKS DB |
| R.LEEAGGATSVQIEMNK.K | Y | 49.97 | 1675.8087 | 16 | 1.0 | 838.9125 | 2 | 34.20 | 1 | F1:13672 | NaNaKA16\_F1.raw | 1.2438E6 |  |  |  |  |  |  |  |  |  |  |  |  | 1 | 1 | 0 | 0 | 0 | 0 | 0 | 0 | 0 | 0 | 0 | 0 | 0 | 0 | 1154 | 1169 |  |  | PEAKS DB |
| total 2 peptides |  |  |  |  |  |  |  |  |  |  |  |  |  |  |  |  |  |  |  |  |  |  |  |  |  |  |  |  |  |  |  |  |  |  |  |  |  |  |  |  |  |  |  |
| --- | --- | --- | --- | --- | --- | --- | --- | --- | --- | --- | --- | --- | --- | --- | --- | --- | --- | --- | --- | --- | --- | --- | --- | --- | --- | --- | --- | --- | --- | --- | --- | --- | --- | --- | --- | --- | --- | --- | --- | --- | --- | --- | --- |

ETE67243.1

back to list

  

| Protein Coverage
| Supporting Peptides
|

Protein Coverage:

Supporting Peptides:

| Peptide | Uniq | -10lgP | Mass | Length | ppm | m/z | z | RT | Fraction | Scan | Source File | Area F1 | Area F10 | Area F11 | Area F12 | Area F13 | Area F2 | Area F3 | Area F4 | Area F5 | Area F6 | Area F7 | Area F8 | Area F9 | #Feature | #Feature F1 | #Feature F10 | #Feature F11 | #Feature F12 | #Feature F13 | #Feature F2 | #Feature F3 | #Feature F4 | #Feature F5 | #Feature F6 | #Feature F7 | #Feature F8 | #Feature F9 | Start | End | PTM | AScore | Found By |
| --- | --- | --- | --- | --- | --- | --- | --- | --- | --- | --- | --- | --- | --- | --- | --- | --- | --- | --- | --- | --- | --- | --- | --- | --- | --- | --- | --- | --- | --- | --- | --- | --- | --- | --- | --- | --- | --- | --- | --- | --- | --- | --- | --- |
| R.LGLDPTMAC(+57.02)QK.V | Y | 44.93 | 1232.5894 | 11 | 0.4 | 617.3022 | 2 | 34.45 | 4 | F4:17128 | NaNaKA16\_F12.raw |  |  |  | 1.6499E5 |  |  |  |  |  |  |  |  |  | 1 | 0 | 0 | 0 | 1 | 0 | 0 | 0 | 0 | 0 | 0 | 0 | 0 | 0 | 374 | 384 | Carbamidomethylation | C9:Carbamidomethylation:1000.00 | PEAKS DB |
| total 1 peptides |  |  |  |  |  |  |  |  |  |  |  |  |  |  |  |  |  |  |  |  |  |  |  |  |  |  |  |  |  |  |  |  |  |  |  |  |  |  |  |  |  |  |  |
| --- | --- | --- | --- | --- | --- | --- | --- | --- | --- | --- | --- | --- | --- | --- | --- | --- | --- | --- | --- | --- | --- | --- | --- | --- | --- | --- | --- | --- | --- | --- | --- | --- | --- | --- | --- | --- | --- | --- | --- | --- | --- | --- | --- |

XP\_032079951.1

back to list

  

| Protein Coverage
| Supporting Peptides
|

Protein Coverage:

Supporting Peptides:

| Peptide | Uniq | -10lgP | Mass | Length | ppm | m/z | z | RT | Fraction | Scan | Source File | Area F1 | Area F10 | Area F11 | Area F12 | Area F13 | Area F2 | Area F3 | Area F4 | Area F5 | Area F6 | Area F7 | Area F8 | Area F9 | #Feature | #Feature F1 | #Feature F10 | #Feature F11 | #Feature F12 | #Feature F13 | #Feature F2 | #Feature F3 | #Feature F4 | #Feature F5 | #Feature F6 | #Feature F7 | #Feature F8 | #Feature F9 | Start | End | PTM | AScore | Found By |
| --- | --- | --- | --- | --- | --- | --- | --- | --- | --- | --- | --- | --- | --- | --- | --- | --- | --- | --- | --- | --- | --- | --- | --- | --- | --- | --- | --- | --- | --- | --- | --- | --- | --- | --- | --- | --- | --- | --- | --- | --- | --- | --- | --- |
| R.LGLDPTMAC(+57.02)QK.V | Y | 44.93 | 1232.5894 | 11 | 0.4 | 617.3022 | 2 | 34.45 | 4 | F4:17128 | NaNaKA16\_F12.raw |  |  |  | 1.6499E5 |  |  |  |  |  |  |  |  |  | 1 | 0 | 0 | 0 | 1 | 0 | 0 | 0 | 0 | 0 | 0 | 0 | 0 | 0 | 285 | 295 | Carbamidomethylation | C9:Carbamidomethylation:1000.00 | PEAKS DB |
| total 1 peptides |  |  |  |  |  |  |  |  |  |  |  |  |  |  |  |  |  |  |  |  |  |  |  |  |  |  |  |  |  |  |  |  |  |  |  |  |  |  |  |  |  |  |  |
| --- | --- | --- | --- | --- | --- | --- | --- | --- | --- | --- | --- | --- | --- | --- | --- | --- | --- | --- | --- | --- | --- | --- | --- | --- | --- | --- | --- | --- | --- | --- | --- | --- | --- | --- | --- | --- | --- | --- | --- | --- | --- | --- | --- |

XP\_034298591.1

back to list

  

| Protein Coverage
| Supporting Peptides
|

Protein Coverage:

Supporting Peptides:

| Peptide | Uniq | -10lgP | Mass | Length | ppm | m/z | z | RT | Fraction | Scan | Source File | Area F1 | Area F10 | Area F11 | Area F12 | Area F13 | Area F2 | Area F3 | Area F4 | Area F5 | Area F6 | Area F7 | Area F8 | Area F9 | #Feature | #Feature F1 | #Feature F10 | #Feature F11 | #Feature F12 | #Feature F13 | #Feature F2 | #Feature F3 | #Feature F4 | #Feature F5 | #Feature F6 | #Feature F7 | #Feature F8 | #Feature F9 | Start | End | PTM | AScore | Found By |
| --- | --- | --- | --- | --- | --- | --- | --- | --- | --- | --- | --- | --- | --- | --- | --- | --- | --- | --- | --- | --- | --- | --- | --- | --- | --- | --- | --- | --- | --- | --- | --- | --- | --- | --- | --- | --- | --- | --- | --- | --- | --- | --- | --- |
| R.LGLDPTMAC(+57.02)QK.V | Y | 44.93 | 1232.5894 | 11 | 0.4 | 617.3022 | 2 | 34.45 | 4 | F4:17128 | NaNaKA16\_F12.raw |  |  |  | 1.6499E5 |  |  |  |  |  |  |  |  |  | 1 | 0 | 0 | 0 | 1 | 0 | 0 | 0 | 0 | 0 | 0 | 0 | 0 | 0 | 285 | 295 | Carbamidomethylation | C9:Carbamidomethylation:1000.00 | PEAKS DB |
| total 1 peptides |  |  |  |  |  |  |  |  |  |  |  |  |  |  |  |  |  |  |  |  |  |  |  |  |  |  |  |  |  |  |  |  |  |  |  |  |  |  |  |  |  |  |  |
| --- | --- | --- | --- | --- | --- | --- | --- | --- | --- | --- | --- | --- | --- | --- | --- | --- | --- | --- | --- | --- | --- | --- | --- | --- | --- | --- | --- | --- | --- | --- | --- | --- | --- | --- | --- | --- | --- | --- | --- | --- | --- | --- | --- |

XP\_026555457.1

back to list

  

| Protein Coverage
| Supporting Peptides
|

Protein Coverage:

Supporting Peptides:

| Peptide | Uniq | -10lgP | Mass | Length | ppm | m/z | z | RT | Fraction | Scan | Source File | Area F1 | Area F10 | Area F11 | Area F12 | Area F13 | Area F2 | Area F3 | Area F4 | Area F5 | Area F6 | Area F7 | Area F8 | Area F9 | #Feature | #Feature F1 | #Feature F10 | #Feature F11 | #Feature F12 | #Feature F13 | #Feature F2 | #Feature F3 | #Feature F4 | #Feature F5 | #Feature F6 | #Feature F7 | #Feature F8 | #Feature F9 | Start | End | PTM | AScore | Found By |
| --- | --- | --- | --- | --- | --- | --- | --- | --- | --- | --- | --- | --- | --- | --- | --- | --- | --- | --- | --- | --- | --- | --- | --- | --- | --- | --- | --- | --- | --- | --- | --- | --- | --- | --- | --- | --- | --- | --- | --- | --- | --- | --- | --- |
| R.LGLDPTMAC(+57.02)QK.V | Y | 44.93 | 1232.5894 | 11 | 0.4 | 617.3022 | 2 | 34.45 | 4 | F4:17128 | NaNaKA16\_F12.raw |  |  |  | 1.6499E5 |  |  |  |  |  |  |  |  |  | 1 | 0 | 0 | 0 | 1 | 0 | 0 | 0 | 0 | 0 | 0 | 0 | 0 | 0 | 285 | 295 | Carbamidomethylation | C9:Carbamidomethylation:1000.00 | PEAKS DB |
| total 1 peptides |  |  |  |  |  |  |  |  |  |  |  |  |  |  |  |  |  |  |  |  |  |  |  |  |  |  |  |  |  |  |  |  |  |  |  |  |  |  |  |  |  |  |  |
| --- | --- | --- | --- | --- | --- | --- | --- | --- | --- | --- | --- | --- | --- | --- | --- | --- | --- | --- | --- | --- | --- | --- | --- | --- | --- | --- | --- | --- | --- | --- | --- | --- | --- | --- | --- | --- | --- | --- | --- | --- | --- | --- | --- |

XP\_013912092.1

back to list

  

| Protein Coverage
| Supporting Peptides
|

Protein Coverage:

Supporting Peptides:

| Peptide | Uniq | -10lgP | Mass | Length | ppm | m/z | z | RT | Fraction | Scan | Source File | Area F1 | Area F10 | Area F11 | Area F12 | Area F13 | Area F2 | Area F3 | Area F4 | Area F5 | Area F6 | Area F7 | Area F8 | Area F9 | #Feature | #Feature F1 | #Feature F10 | #Feature F11 | #Feature F12 | #Feature F13 | #Feature F2 | #Feature F3 | #Feature F4 | #Feature F5 | #Feature F6 | #Feature F7 | #Feature F8 | #Feature F9 | Start | End | PTM | AScore | Found By |
| --- | --- | --- | --- | --- | --- | --- | --- | --- | --- | --- | --- | --- | --- | --- | --- | --- | --- | --- | --- | --- | --- | --- | --- | --- | --- | --- | --- | --- | --- | --- | --- | --- | --- | --- | --- | --- | --- | --- | --- | --- | --- | --- | --- |
| R.LGLDPTMAC(+57.02)QK.V | Y | 44.93 | 1232.5894 | 11 | 0.4 | 617.3022 | 2 | 34.45 | 4 | F4:17128 | NaNaKA16\_F12.raw |  |  |  | 1.6499E5 |  |  |  |  |  |  |  |  |  | 1 | 0 | 0 | 0 | 1 | 0 | 0 | 0 | 0 | 0 | 0 | 0 | 0 | 0 | 16 | 26 | Carbamidomethylation | C9:Carbamidomethylation:1000.00 | PEAKS DB |
| total 1 peptides |  |  |  |  |  |  |  |  |  |  |  |  |  |  |  |  |  |  |  |  |  |  |  |  |  |  |  |  |  |  |  |  |  |  |  |  |  |  |  |  |  |  |  |
| --- | --- | --- | --- | --- | --- | --- | --- | --- | --- | --- | --- | --- | --- | --- | --- | --- | --- | --- | --- | --- | --- | --- | --- | --- | --- | --- | --- | --- | --- | --- | --- | --- | --- | --- | --- | --- | --- | --- | --- | --- | --- | --- | --- |

Q9YGJ6.1

back to list

  

| Protein Coverage
| Supporting Peptides
|

Protein Coverage:

Supporting Peptides:

| Peptide | Uniq | -10lgP | Mass | Length | ppm | m/z | z | RT | Fraction | Scan | Source File | Area F1 | Area F10 | Area F11 | Area F12 | Area F13 | Area F2 | Area F3 | Area F4 | Area F5 | Area F6 | Area F7 | Area F8 | Area F9 | #Feature | #Feature F1 | #Feature F10 | #Feature F11 | #Feature F12 | #Feature F13 | #Feature F2 | #Feature F3 | #Feature F4 | #Feature F5 | #Feature F6 | #Feature F7 | #Feature F8 | #Feature F9 | Start | End | PTM | AScore | Found By |
| --- | --- | --- | --- | --- | --- | --- | --- | --- | --- | --- | --- | --- | --- | --- | --- | --- | --- | --- | --- | --- | --- | --- | --- | --- | --- | --- | --- | --- | --- | --- | --- | --- | --- | --- | --- | --- | --- | --- | --- | --- | --- | --- | --- |
| T.LEC(+57.02)HNQQSSETPTTTGC(+57.02)SGGETNC(+57.02)YK.K | Y | 51.33 | 2945.1863 | 26 | 6.0 | 982.7419 | 3 | 11.71 | 1 | F1:2367 | NaNaKA16\_F1.raw | 1.582E8 |  |  |  |  |  |  |  |  |  |  |  |  | 1 | 1 | 0 | 0 | 0 | 0 | 0 | 0 | 0 | 0 | 0 | 0 | 0 | 0 | 22 | 47 | Carbamidomethylation | C3:Carbamidomethylation:1000.00;C17:Carbamidomethylation:1000.00;C24:Carbamidomethylation:1000.00 | PEAKS DB |
| K.GIEINC(+57.02)C(+57.02)TTDR.C | N | 48.70 | 1337.5704 | 11 | -0.5 | 669.7922 | 2 | 18.63 | 6 | F6:6296 | NaNaKA16\_F2.raw |  |  |  |  |  | 4.8926E5 |  |  |  |  |  |  |  | 1 | 0 | 0 | 0 | 0 | 0 | 1 | 0 | 0 | 0 | 0 | 0 | 0 | 0 | 70 | 80 | Carbamidomethylation | C6:Carbamidomethylation:1000.00;C7:Carbamidomethylation:1000.00 | PEAKS DB |
| total 2 peptides |  |  |  |  |  |  |  |  |  |  |  |  |  |  |  |  |  |  |  |  |  |  |  |  |  |  |  |  |  |  |  |  |  |  |  |  |  |  |  |  |  |  |  |
| --- | --- | --- | --- | --- | --- | --- | --- | --- | --- | --- | --- | --- | --- | --- | --- | --- | --- | --- | --- | --- | --- | --- | --- | --- | --- | --- | --- | --- | --- | --- | --- | --- | --- | --- | --- | --- | --- | --- | --- | --- | --- | --- | --- |

O57326.1

back to list

  

| Protein Coverage
| Supporting Peptides
|

Protein Coverage:

Supporting Peptides:

| Peptide | Uniq | -10lgP | Mass | Length | ppm | m/z | z | RT | Fraction | Scan | Source File | Area F1 | Area F10 | Area F11 | Area F12 | Area F13 | Area F2 | Area F3 | Area F4 | Area F5 | Area F6 | Area F7 | Area F8 | Area F9 | #Feature | #Feature F1 | #Feature F10 | #Feature F11 | #Feature F12 | #Feature F13 | #Feature F2 | #Feature F3 | #Feature F4 | #Feature F5 | #Feature F6 | #Feature F7 | #Feature F8 | #Feature F9 | Start | End | PTM | AScore | Found By |
| --- | --- | --- | --- | --- | --- | --- | --- | --- | --- | --- | --- | --- | --- | --- | --- | --- | --- | --- | --- | --- | --- | --- | --- | --- | --- | --- | --- | --- | --- | --- | --- | --- | --- | --- | --- | --- | --- | --- | --- | --- | --- | --- | --- |
| T.LEC(+57.02)HNQQSSETPTTTGC(+57.02)SGGETNC(+57.02)YK.K | Y | 51.33 | 2945.1863 | 26 | 6.0 | 982.7419 | 3 | 11.71 | 1 | F1:2367 | NaNaKA16\_F1.raw | 1.582E8 |  |  |  |  |  |  |  |  |  |  |  |  | 1 | 1 | 0 | 0 | 0 | 0 | 0 | 0 | 0 | 0 | 0 | 0 | 0 | 0 | 22 | 47 | Carbamidomethylation | C3:Carbamidomethylation:1000.00;C17:Carbamidomethylation:1000.00;C24:Carbamidomethylation:1000.00 | PEAKS DB |
| K.GIEINC(+57.02)C(+57.02)TTDR.C | N | 48.70 | 1337.5704 | 11 | -0.5 | 669.7922 | 2 | 18.63 | 6 | F6:6296 | NaNaKA16\_F2.raw |  |  |  |  |  | 4.8926E5 |  |  |  |  |  |  |  | 1 | 0 | 0 | 0 | 0 | 0 | 1 | 0 | 0 | 0 | 0 | 0 | 0 | 0 | 70 | 80 | Carbamidomethylation | C6:Carbamidomethylation:1000.00;C7:Carbamidomethylation:1000.00 | PEAKS DB |
| total 2 peptides |  |  |  |  |  |  |  |  |  |  |  |  |  |  |  |  |  |  |  |  |  |  |  |  |  |  |  |  |  |  |  |  |  |  |  |  |  |  |  |  |  |  |  |
| --- | --- | --- | --- | --- | --- | --- | --- | --- | --- | --- | --- | --- | --- | --- | --- | --- | --- | --- | --- | --- | --- | --- | --- | --- | --- | --- | --- | --- | --- | --- | --- | --- | --- | --- | --- | --- | --- | --- | --- | --- | --- | --- | --- |

AAC69915.1

back to list

  

| Protein Coverage
| Supporting Peptides
|

Protein Coverage:

Supporting Peptides:

| Peptide | Uniq | -10lgP | Mass | Length | ppm | m/z | z | RT | Fraction | Scan | Source File | Area F1 | Area F10 | Area F11 | Area F12 | Area F13 | Area F2 | Area F3 | Area F4 | Area F5 | Area F6 | Area F7 | Area F8 | Area F9 | #Feature | #Feature F1 | #Feature F10 | #Feature F11 | #Feature F12 | #Feature F13 | #Feature F2 | #Feature F3 | #Feature F4 | #Feature F5 | #Feature F6 | #Feature F7 | #Feature F8 | #Feature F9 | Start | End | PTM | AScore | Found By |
| --- | --- | --- | --- | --- | --- | --- | --- | --- | --- | --- | --- | --- | --- | --- | --- | --- | --- | --- | --- | --- | --- | --- | --- | --- | --- | --- | --- | --- | --- | --- | --- | --- | --- | --- | --- | --- | --- | --- | --- | --- | --- | --- | --- |
| T.LEC(+57.02)HNQQSSETPTTTGC(+57.02)SGGETNC(+57.02)YK.K | Y | 51.33 | 2945.1863 | 26 | 6.0 | 982.7419 | 3 | 11.71 | 1 | F1:2367 | NaNaKA16\_F1.raw | 1.582E8 |  |  |  |  |  |  |  |  |  |  |  |  | 1 | 1 | 0 | 0 | 0 | 0 | 0 | 0 | 0 | 0 | 0 | 0 | 0 | 0 | 22 | 47 | Carbamidomethylation | C3:Carbamidomethylation:1000.00;C17:Carbamidomethylation:1000.00;C24:Carbamidomethylation:1000.00 | PEAKS DB |
| K.GIEINC(+57.02)C(+57.02)TTDR.C | N | 48.70 | 1337.5704 | 11 | -0.5 | 669.7922 | 2 | 18.63 | 6 | F6:6296 | NaNaKA16\_F2.raw |  |  |  |  |  | 4.8926E5 |  |  |  |  |  |  |  | 1 | 0 | 0 | 0 | 0 | 0 | 1 | 0 | 0 | 0 | 0 | 0 | 0 | 0 | 70 | 80 | Carbamidomethylation | C6:Carbamidomethylation:1000.00;C7:Carbamidomethylation:1000.00 | PEAKS DB |
| total 2 peptides |  |  |  |  |  |  |  |  |  |  |  |  |  |  |  |  |  |  |  |  |  |  |  |  |  |  |  |  |  |  |  |  |  |  |  |  |  |  |  |  |  |  |  |
| --- | --- | --- | --- | --- | --- | --- | --- | --- | --- | --- | --- | --- | --- | --- | --- | --- | --- | --- | --- | --- | --- | --- | --- | --- | --- | --- | --- | --- | --- | --- | --- | --- | --- | --- | --- | --- | --- | --- | --- | --- | --- | --- | --- |

AAD08812.1

back to list

  

| Protein Coverage
| Supporting Peptides
|

Protein Coverage:

Supporting Peptides:

| Peptide | Uniq | -10lgP | Mass | Length | ppm | m/z | z | RT | Fraction | Scan | Source File | Area F1 | Area F10 | Area F11 | Area F12 | Area F13 | Area F2 | Area F3 | Area F4 | Area F5 | Area F6 | Area F7 | Area F8 | Area F9 | #Feature | #Feature F1 | #Feature F10 | #Feature F11 | #Feature F12 | #Feature F13 | #Feature F2 | #Feature F3 | #Feature F4 | #Feature F5 | #Feature F6 | #Feature F7 | #Feature F8 | #Feature F9 | Start | End | PTM | AScore | Found By |
| --- | --- | --- | --- | --- | --- | --- | --- | --- | --- | --- | --- | --- | --- | --- | --- | --- | --- | --- | --- | --- | --- | --- | --- | --- | --- | --- | --- | --- | --- | --- | --- | --- | --- | --- | --- | --- | --- | --- | --- | --- | --- | --- | --- |
| T.LEC(+57.02)HNQQSSETPTTTGC(+57.02)SGGETNC(+57.02)YK.K | Y | 51.33 | 2945.1863 | 26 | 6.0 | 982.7419 | 3 | 11.71 | 1 | F1:2367 | NaNaKA16\_F1.raw | 1.582E8 |  |  |  |  |  |  |  |  |  |  |  |  | 1 | 1 | 0 | 0 | 0 | 0 | 0 | 0 | 0 | 0 | 0 | 0 | 0 | 0 | 22 | 47 | Carbamidomethylation | C3:Carbamidomethylation:1000.00;C17:Carbamidomethylation:1000.00;C24:Carbamidomethylation:1000.00 | PEAKS DB |
| K.GIEINC(+57.02)C(+57.02)TTDR.C | N | 48.70 | 1337.5704 | 11 | -0.5 | 669.7922 | 2 | 18.63 | 6 | F6:6296 | NaNaKA16\_F2.raw |  |  |  |  |  | 4.8926E5 |  |  |  |  |  |  |  | 1 | 0 | 0 | 0 | 0 | 0 | 1 | 0 | 0 | 0 | 0 | 0 | 0 | 0 | 70 | 80 | Carbamidomethylation | C6:Carbamidomethylation:1000.00;C7:Carbamidomethylation:1000.00 | PEAKS DB |
| total 2 peptides |  |  |  |  |  |  |  |  |  |  |  |  |  |  |  |  |  |  |  |  |  |  |  |  |  |  |  |  |  |  |  |  |  |  |  |  |  |  |  |  |  |  |  |
| --- | --- | --- | --- | --- | --- | --- | --- | --- | --- | --- | --- | --- | --- | --- | --- | --- | --- | --- | --- | --- | --- | --- | --- | --- | --- | --- | --- | --- | --- | --- | --- | --- | --- | --- | --- | --- | --- | --- | --- | --- | --- | --- | --- |

Q53B55.1

back to list

  

| Protein Coverage
| Supporting Peptides
|

Protein Coverage:

Supporting Peptides:

| Peptide | Uniq | -10lgP | Mass | Length | ppm | m/z | z | RT | Fraction | Scan | Source File | Area F1 | Area F10 | Area F11 | Area F12 | Area F13 | Area F2 | Area F3 | Area F4 | Area F5 | Area F6 | Area F7 | Area F8 | Area F9 | #Feature | #Feature F1 | #Feature F10 | #Feature F11 | #Feature F12 | #Feature F13 | #Feature F2 | #Feature F3 | #Feature F4 | #Feature F5 | #Feature F6 | #Feature F7 | #Feature F8 | #Feature F9 | Start | End | PTM | AScore | Found By |
| --- | --- | --- | --- | --- | --- | --- | --- | --- | --- | --- | --- | --- | --- | --- | --- | --- | --- | --- | --- | --- | --- | --- | --- | --- | --- | --- | --- | --- | --- | --- | --- | --- | --- | --- | --- | --- | --- | --- | --- | --- | --- | --- | --- |
| R.VDLGC(+57.02)AATC(+57.02)PIVK.P | Y | 44.43 | 1402.6948 | 13 | 0.5 | 702.3550 | 2 | 39.33 | 9 | F9:22998 | NaNaKA16\_F5.raw |  |  |  |  |  |  |  |  | 2.7231E6 |  |  |  |  | 1 | 0 | 0 | 0 | 0 | 0 | 0 | 0 | 0 | 1 | 0 | 0 | 0 | 0 | 62 | 74 | Carbamidomethylation | C5:Carbamidomethylation:1000.00;C9:Carbamidomethylation:1000.00 | PEAKS DB |
| total 1 peptides |  |  |  |  |  |  |  |  |  |  |  |  |  |  |  |  |  |  |  |  |  |  |  |  |  |  |  |  |  |  |  |  |  |  |  |  |  |  |  |  |  |  |  |
| --- | --- | --- | --- | --- | --- | --- | --- | --- | --- | --- | --- | --- | --- | --- | --- | --- | --- | --- | --- | --- | --- | --- | --- | --- | --- | --- | --- | --- | --- | --- | --- | --- | --- | --- | --- | --- | --- | --- | --- | --- | --- | --- | --- |

AAT97253.1

back to list

  

| Protein Coverage
| Supporting Peptides
|

Protein Coverage:

Supporting Peptides:

| Peptide | Uniq | -10lgP | Mass | Length | ppm | m/z | z | RT | Fraction | Scan | Source File | Area F1 | Area F10 | Area F11 | Area F12 | Area F13 | Area F2 | Area F3 | Area F4 | Area F5 | Area F6 | Area F7 | Area F8 | Area F9 | #Feature | #Feature F1 | #Feature F10 | #Feature F11 | #Feature F12 | #Feature F13 | #Feature F2 | #Feature F3 | #Feature F4 | #Feature F5 | #Feature F6 | #Feature F7 | #Feature F8 | #Feature F9 | Start | End | PTM | AScore | Found By |
| --- | --- | --- | --- | --- | --- | --- | --- | --- | --- | --- | --- | --- | --- | --- | --- | --- | --- | --- | --- | --- | --- | --- | --- | --- | --- | --- | --- | --- | --- | --- | --- | --- | --- | --- | --- | --- | --- | --- | --- | --- | --- | --- | --- |
| R.VDLGC(+57.02)AATC(+57.02)PIVK.P | Y | 44.43 | 1402.6948 | 13 | 0.5 | 702.3550 | 2 | 39.33 | 9 | F9:22998 | NaNaKA16\_F5.raw |  |  |  |  |  |  |  |  | 2.7231E6 |  |  |  |  | 1 | 0 | 0 | 0 | 0 | 0 | 0 | 0 | 0 | 1 | 0 | 0 | 0 | 0 | 62 | 74 | Carbamidomethylation | C5:Carbamidomethylation:1000.00;C9:Carbamidomethylation:1000.00 | PEAKS DB |
| total 1 peptides |  |  |  |  |  |  |  |  |  |  |  |  |  |  |  |  |  |  |  |  |  |  |  |  |  |  |  |  |  |  |  |  |  |  |  |  |  |  |  |  |  |  |  |
| --- | --- | --- | --- | --- | --- | --- | --- | --- | --- | --- | --- | --- | --- | --- | --- | --- | --- | --- | --- | --- | --- | --- | --- | --- | --- | --- | --- | --- | --- | --- | --- | --- | --- | --- | --- | --- | --- | --- | --- | --- | --- | --- | --- |

P01387.1

back to list

  

| Protein Coverage
| Supporting Peptides
|

Protein Coverage:

Supporting Peptides:

| Peptide | Uniq | -10lgP | Mass | Length | ppm | m/z | z | RT | Fraction | Scan | Source File | Area F1 | Area F10 | Area F11 | Area F12 | Area F13 | Area F2 | Area F3 | Area F4 | Area F5 | Area F6 | Area F7 | Area F8 | Area F9 | #Feature | #Feature F1 | #Feature F10 | #Feature F11 | #Feature F12 | #Feature F13 | #Feature F2 | #Feature F3 | #Feature F4 | #Feature F5 | #Feature F6 | #Feature F7 | #Feature F8 | #Feature F9 | Start | End | PTM | AScore | Found By |
| --- | --- | --- | --- | --- | --- | --- | --- | --- | --- | --- | --- | --- | --- | --- | --- | --- | --- | --- | --- | --- | --- | --- | --- | --- | --- | --- | --- | --- | --- | --- | --- | --- | --- | --- | --- | --- | --- | --- | --- | --- | --- | --- | --- |
| R.VDLGC(+57.02)AATC(+57.02)PIVK.P | Y | 44.43 | 1402.6948 | 13 | 0.5 | 702.3550 | 2 | 39.33 | 9 | F9:22998 | NaNaKA16\_F5.raw |  |  |  |  |  |  |  |  | 2.7231E6 |  |  |  |  | 1 | 0 | 0 | 0 | 0 | 0 | 0 | 0 | 0 | 1 | 0 | 0 | 0 | 0 | 38 | 50 | Carbamidomethylation | C5:Carbamidomethylation:1000.00;C9:Carbamidomethylation:1000.00 | PEAKS DB |
| total 1 peptides |  |  |  |  |  |  |  |  |  |  |  |  |  |  |  |  |  |  |  |  |  |  |  |  |  |  |  |  |  |  |  |  |  |  |  |  |  |  |  |  |  |  |  |
| --- | --- | --- | --- | --- | --- | --- | --- | --- | --- | --- | --- | --- | --- | --- | --- | --- | --- | --- | --- | --- | --- | --- | --- | --- | --- | --- | --- | --- | --- | --- | --- | --- | --- | --- | --- | --- | --- | --- | --- | --- | --- | --- | --- |

Q53B53.1

back to list

  

| Protein Coverage
| Supporting Peptides
|

Protein Coverage:

Supporting Peptides:

| Peptide | Uniq | -10lgP | Mass | Length | ppm | m/z | z | RT | Fraction | Scan | Source File | Area F1 | Area F10 | Area F11 | Area F12 | Area F13 | Area F2 | Area F3 | Area F4 | Area F5 | Area F6 | Area F7 | Area F8 | Area F9 | #Feature | #Feature F1 | #Feature F10 | #Feature F11 | #Feature F12 | #Feature F13 | #Feature F2 | #Feature F3 | #Feature F4 | #Feature F5 | #Feature F6 | #Feature F7 | #Feature F8 | #Feature F9 | Start | End | PTM | AScore | Found By |
| --- | --- | --- | --- | --- | --- | --- | --- | --- | --- | --- | --- | --- | --- | --- | --- | --- | --- | --- | --- | --- | --- | --- | --- | --- | --- | --- | --- | --- | --- | --- | --- | --- | --- | --- | --- | --- | --- | --- | --- | --- | --- | --- | --- |
| R.VDLGC(+57.02)AATC(+57.02)PIVK.P | Y | 44.43 | 1402.6948 | 13 | 0.5 | 702.3550 | 2 | 39.33 | 9 | F9:22998 | NaNaKA16\_F5.raw |  |  |  |  |  |  |  |  | 2.7231E6 |  |  |  |  | 1 | 0 | 0 | 0 | 0 | 0 | 0 | 0 | 0 | 1 | 0 | 0 | 0 | 0 | 58 | 70 | Carbamidomethylation | C5:Carbamidomethylation:1000.00;C9:Carbamidomethylation:1000.00 | PEAKS DB |
| total 1 peptides |  |  |  |  |  |  |  |  |  |  |  |  |  |  |  |  |  |  |  |  |  |  |  |  |  |  |  |  |  |  |  |  |  |  |  |  |  |  |  |  |  |  |  |
| --- | --- | --- | --- | --- | --- | --- | --- | --- | --- | --- | --- | --- | --- | --- | --- | --- | --- | --- | --- | --- | --- | --- | --- | --- | --- | --- | --- | --- | --- | --- | --- | --- | --- | --- | --- | --- | --- | --- | --- | --- | --- | --- | --- |

AAT97255.1

back to list

  

| Protein Coverage
| Supporting Peptides
|

Protein Coverage:

Supporting Peptides:

| Peptide | Uniq | -10lgP | Mass | Length | ppm | m/z | z | RT | Fraction | Scan | Source File | Area F1 | Area F10 | Area F11 | Area F12 | Area F13 | Area F2 | Area F3 | Area F4 | Area F5 | Area F6 | Area F7 | Area F8 | Area F9 | #Feature | #Feature F1 | #Feature F10 | #Feature F11 | #Feature F12 | #Feature F13 | #Feature F2 | #Feature F3 | #Feature F4 | #Feature F5 | #Feature F6 | #Feature F7 | #Feature F8 | #Feature F9 | Start | End | PTM | AScore | Found By |
| --- | --- | --- | --- | --- | --- | --- | --- | --- | --- | --- | --- | --- | --- | --- | --- | --- | --- | --- | --- | --- | --- | --- | --- | --- | --- | --- | --- | --- | --- | --- | --- | --- | --- | --- | --- | --- | --- | --- | --- | --- | --- | --- | --- |
| R.VDLGC(+57.02)AATC(+57.02)PIVK.P | Y | 44.43 | 1402.6948 | 13 | 0.5 | 702.3550 | 2 | 39.33 | 9 | F9:22998 | NaNaKA16\_F5.raw |  |  |  |  |  |  |  |  | 2.7231E6 |  |  |  |  | 1 | 0 | 0 | 0 | 0 | 0 | 0 | 0 | 0 | 1 | 0 | 0 | 0 | 0 | 58 | 70 | Carbamidomethylation | C5:Carbamidomethylation:1000.00;C9:Carbamidomethylation:1000.00 | PEAKS DB |
| total 1 peptides |  |  |  |  |  |  |  |  |  |  |  |  |  |  |  |  |  |  |  |  |  |  |  |  |  |  |  |  |  |  |  |  |  |  |  |  |  |  |  |  |  |  |  |
| --- | --- | --- | --- | --- | --- | --- | --- | --- | --- | --- | --- | --- | --- | --- | --- | --- | --- | --- | --- | --- | --- | --- | --- | --- | --- | --- | --- | --- | --- | --- | --- | --- | --- | --- | --- | --- | --- | --- | --- | --- | --- | --- | --- |

AAZ39881.1

back to list

  

| Protein Coverage
| Supporting Peptides
|

Protein Coverage:

Supporting Peptides:

| Peptide | Uniq | -10lgP | Mass | Length | ppm | m/z | z | RT | Fraction | Scan | Source File | Area F1 | Area F10 | Area F11 | Area F12 | Area F13 | Area F2 | Area F3 | Area F4 | Area F5 | Area F6 | Area F7 | Area F8 | Area F9 | #Feature | #Feature F1 | #Feature F10 | #Feature F11 | #Feature F12 | #Feature F13 | #Feature F2 | #Feature F3 | #Feature F4 | #Feature F5 | #Feature F6 | #Feature F7 | #Feature F8 | #Feature F9 | Start | End | PTM | AScore | Found By |
| --- | --- | --- | --- | --- | --- | --- | --- | --- | --- | --- | --- | --- | --- | --- | --- | --- | --- | --- | --- | --- | --- | --- | --- | --- | --- | --- | --- | --- | --- | --- | --- | --- | --- | --- | --- | --- | --- | --- | --- | --- | --- | --- | --- |
| K.SHDNALLFTDMR.F | Y | 53.98 | 1418.6613 | 12 | 0.4 | 473.8945 | 3 | 41.71 | 4 | F4:23364 | NaNaKA16\_F12.raw |  |  |  | 7.5878E4 |  |  |  |  |  |  |  |  |  | 1 | 0 | 0 | 0 | 1 | 0 | 0 | 0 | 0 | 0 | 0 | 0 | 0 | 0 | 282 | 293 |  |  | PEAKS DB |
| total 1 peptides |  |  |  |  |  |  |  |  |  |  |  |  |  |  |  |  |  |  |  |  |  |  |  |  |  |  |  |  |  |  |  |  |  |  |  |  |  |  |  |  |  |  |  |
| --- | --- | --- | --- | --- | --- | --- | --- | --- | --- | --- | --- | --- | --- | --- | --- | --- | --- | --- | --- | --- | --- | --- | --- | --- | --- | --- | --- | --- | --- | --- | --- | --- | --- | --- | --- | --- | --- | --- | --- | --- | --- | --- | --- |

AAB22477.1

back to list

  

| Protein Coverage
| Supporting Peptides
|

Protein Coverage:

Supporting Peptides:

| Peptide | Uniq | -10lgP | Mass | Length | ppm | m/z | z | RT | Fraction | Scan | Source File | Area F1 | Area F10 | Area F11 | Area F12 | Area F13 | Area F2 | Area F3 | Area F4 | Area F5 | Area F6 | Area F7 | Area F8 | Area F9 | #Feature | #Feature F1 | #Feature F10 | #Feature F11 | #Feature F12 | #Feature F13 | #Feature F2 | #Feature F3 | #Feature F4 | #Feature F5 | #Feature F6 | #Feature F7 | #Feature F8 | #Feature F9 | Start | End | PTM | AScore | Found By |
| --- | --- | --- | --- | --- | --- | --- | --- | --- | --- | --- | --- | --- | --- | --- | --- | --- | --- | --- | --- | --- | --- | --- | --- | --- | --- | --- | --- | --- | --- | --- | --- | --- | --- | --- | --- | --- | --- | --- | --- | --- | --- | --- | --- |
| K.SHDNALLFTDMR.F | Y | 53.98 | 1418.6613 | 12 | 0.4 | 473.8945 | 3 | 41.71 | 4 | F4:23364 | NaNaKA16\_F12.raw |  |  |  | 7.5878E4 |  |  |  |  |  |  |  |  |  | 1 | 0 | 0 | 0 | 1 | 0 | 0 | 0 | 0 | 0 | 0 | 0 | 0 | 0 | 94 | 105 |  |  | PEAKS DB |
| total 1 peptides |  |  |  |  |  |  |  |  |  |  |  |  |  |  |  |  |  |  |  |  |  |  |  |  |  |  |  |  |  |  |  |  |  |  |  |  |  |  |  |  |  |  |  |
| --- | --- | --- | --- | --- | --- | --- | --- | --- | --- | --- | --- | --- | --- | --- | --- | --- | --- | --- | --- | --- | --- | --- | --- | --- | --- | --- | --- | --- | --- | --- | --- | --- | --- | --- | --- | --- | --- | --- | --- | --- | --- | --- | --- |

CAL69604.1

back to list

  

| Protein Coverage
| Supporting Peptides
|

Protein Coverage:

Supporting Peptides:

| Peptide | Uniq | -10lgP | Mass | Length | ppm | m/z | z | RT | Fraction | Scan | Source File | Area F1 | Area F10 | Area F11 | Area F12 | Area F13 | Area F2 | Area F3 | Area F4 | Area F5 | Area F6 | Area F7 | Area F8 | Area F9 | #Feature | #Feature F1 | #Feature F10 | #Feature F11 | #Feature F12 | #Feature F13 | #Feature F2 | #Feature F3 | #Feature F4 | #Feature F5 | #Feature F6 | #Feature F7 | #Feature F8 | #Feature F9 | Start | End | PTM | AScore | Found By |
| --- | --- | --- | --- | --- | --- | --- | --- | --- | --- | --- | --- | --- | --- | --- | --- | --- | --- | --- | --- | --- | --- | --- | --- | --- | --- | --- | --- | --- | --- | --- | --- | --- | --- | --- | --- | --- | --- | --- | --- | --- | --- | --- | --- |
| K.FC(+57.02)YLPADPGEC(+57.02)MAYIR.S | Y | 57.54 | 1961.8474 | 16 | 5.8 | 981.9314 | 2 | 72.26 | 8 | F8:48595 | NaNaKA16\_F4.raw |  |  |  |  |  |  |  | 1.1621E6 |  |  |  |  |  | 1 | 0 | 0 | 0 | 0 | 0 | 0 | 0 | 1 | 0 | 0 | 0 | 0 | 0 | 30 | 45 | Carbamidomethylation | C2:Carbamidomethylation:1000.00;C11:Carbamidomethylation:1000.00 | PEAKS DB |
| total 1 peptides |  |  |  |  |  |  |  |  |  |  |  |  |  |  |  |  |  |  |  |  |  |  |  |  |  |  |  |  |  |  |  |  |  |  |  |  |  |  |  |  |  |  |  |
| --- | --- | --- | --- | --- | --- | --- | --- | --- | --- | --- | --- | --- | --- | --- | --- | --- | --- | --- | --- | --- | --- | --- | --- | --- | --- | --- | --- | --- | --- | --- | --- | --- | --- | --- | --- | --- | --- | --- | --- | --- | --- | --- | --- |

A8Y7N6.1

back to list

  

| Protein Coverage
| Supporting Peptides
|

Protein Coverage:

Supporting Peptides:

| Peptide | Uniq | -10lgP | Mass | Length | ppm | m/z | z | RT | Fraction | Scan | Source File | Area F1 | Area F10 | Area F11 | Area F12 | Area F13 | Area F2 | Area F3 | Area F4 | Area F5 | Area F6 | Area F7 | Area F8 | Area F9 | #Feature | #Feature F1 | #Feature F10 | #Feature F11 | #Feature F12 | #Feature F13 | #Feature F2 | #Feature F3 | #Feature F4 | #Feature F5 | #Feature F6 | #Feature F7 | #Feature F8 | #Feature F9 | Start | End | PTM | AScore | Found By |
| --- | --- | --- | --- | --- | --- | --- | --- | --- | --- | --- | --- | --- | --- | --- | --- | --- | --- | --- | --- | --- | --- | --- | --- | --- | --- | --- | --- | --- | --- | --- | --- | --- | --- | --- | --- | --- | --- | --- | --- | --- | --- | --- | --- |
| K.FC(+57.02)YLPADPGEC(+57.02)MAYIR.S | Y | 57.54 | 1961.8474 | 16 | 5.8 | 981.9314 | 2 | 72.26 | 8 | F8:48595 | NaNaKA16\_F4.raw |  |  |  |  |  |  |  | 1.1621E6 |  |  |  |  |  | 1 | 0 | 0 | 0 | 0 | 0 | 0 | 0 | 1 | 0 | 0 | 0 | 0 | 0 | 30 | 45 | Carbamidomethylation | C2:Carbamidomethylation:1000.00;C11:Carbamidomethylation:1000.00 | PEAKS DB |
| total 1 peptides |  |  |  |  |  |  |  |  |  |  |  |  |  |  |  |  |  |  |  |  |  |  |  |  |  |  |  |  |  |  |  |  |  |  |  |  |  |  |  |  |  |  |  |
| --- | --- | --- | --- | --- | --- | --- | --- | --- | --- | --- | --- | --- | --- | --- | --- | --- | --- | --- | --- | --- | --- | --- | --- | --- | --- | --- | --- | --- | --- | --- | --- | --- | --- | --- | --- | --- | --- | --- | --- | --- | --- | --- | --- |

AFD04724.1

back to list

  

| Protein Coverage
| Supporting Peptides
|

Protein Coverage:

Supporting Peptides:

| Peptide | Uniq | -10lgP | Mass | Length | ppm | m/z | z | RT | Fraction | Scan | Source File | Area F1 | Area F10 | Area F11 | Area F12 | Area F13 | Area F2 | Area F3 | Area F4 | Area F5 | Area F6 | Area F7 | Area F8 | Area F9 | #Feature | #Feature F1 | #Feature F10 | #Feature F11 | #Feature F12 | #Feature F13 | #Feature F2 | #Feature F3 | #Feature F4 | #Feature F5 | #Feature F6 | #Feature F7 | #Feature F8 | #Feature F9 | Start | End | PTM | AScore | Found By |
| --- | --- | --- | --- | --- | --- | --- | --- | --- | --- | --- | --- | --- | --- | --- | --- | --- | --- | --- | --- | --- | --- | --- | --- | --- | --- | --- | --- | --- | --- | --- | --- | --- | --- | --- | --- | --- | --- | --- | --- | --- | --- | --- | --- |
| K.FC(+57.02)YLPADPGEC(+57.02)MAYIR.S | Y | 57.54 | 1961.8474 | 16 | 5.8 | 981.9314 | 2 | 72.26 | 8 | F8:48595 | NaNaKA16\_F4.raw |  |  |  |  |  |  |  | 1.1621E6 |  |  |  |  |  | 1 | 0 | 0 | 0 | 0 | 0 | 0 | 0 | 1 | 0 | 0 | 0 | 0 | 0 | 30 | 45 | Carbamidomethylation | C2:Carbamidomethylation:1000.00;C11:Carbamidomethylation:1000.00 | PEAKS DB |
| total 1 peptides |  |  |  |  |  |  |  |  |  |  |  |  |  |  |  |  |  |  |  |  |  |  |  |  |  |  |  |  |  |  |  |  |  |  |  |  |  |  |  |  |  |  |  |
| --- | --- | --- | --- | --- | --- | --- | --- | --- | --- | --- | --- | --- | --- | --- | --- | --- | --- | --- | --- | --- | --- | --- | --- | --- | --- | --- | --- | --- | --- | --- | --- | --- | --- | --- | --- | --- | --- | --- | --- | --- | --- | --- | --- |

CAL69605.1

back to list

  

| Protein Coverage
| Supporting Peptides
|

Protein Coverage:

Supporting Peptides:

| Peptide | Uniq | -10lgP | Mass | Length | ppm | m/z | z | RT | Fraction | Scan | Source File | Area F1 | Area F10 | Area F11 | Area F12 | Area F13 | Area F2 | Area F3 | Area F4 | Area F5 | Area F6 | Area F7 | Area F8 | Area F9 | #Feature | #Feature F1 | #Feature F10 | #Feature F11 | #Feature F12 | #Feature F13 | #Feature F2 | #Feature F3 | #Feature F4 | #Feature F5 | #Feature F6 | #Feature F7 | #Feature F8 | #Feature F9 | Start | End | PTM | AScore | Found By |
| --- | --- | --- | --- | --- | --- | --- | --- | --- | --- | --- | --- | --- | --- | --- | --- | --- | --- | --- | --- | --- | --- | --- | --- | --- | --- | --- | --- | --- | --- | --- | --- | --- | --- | --- | --- | --- | --- | --- | --- | --- | --- | --- | --- |
| K.FC(+57.02)YLPADPGEC(+57.02)MAYIR.S | Y | 57.54 | 1961.8474 | 16 | 5.8 | 981.9314 | 2 | 72.26 | 8 | F8:48595 | NaNaKA16\_F4.raw |  |  |  |  |  |  |  | 1.1621E6 |  |  |  |  |  | 1 | 0 | 0 | 0 | 0 | 0 | 0 | 0 | 1 | 0 | 0 | 0 | 0 | 0 | 30 | 45 | Carbamidomethylation | C2:Carbamidomethylation:1000.00;C11:Carbamidomethylation:1000.00 | PEAKS DB |
| total 1 peptides |  |  |  |  |  |  |  |  |  |  |  |  |  |  |  |  |  |  |  |  |  |  |  |  |  |  |  |  |  |  |  |  |  |  |  |  |  |  |  |  |  |  |  |
| --- | --- | --- | --- | --- | --- | --- | --- | --- | --- | --- | --- | --- | --- | --- | --- | --- | --- | --- | --- | --- | --- | --- | --- | --- | --- | --- | --- | --- | --- | --- | --- | --- | --- | --- | --- | --- | --- | --- | --- | --- | --- | --- | --- |

A8Y7N7.1

back to list

  

| Protein Coverage
| Supporting Peptides
|

Protein Coverage:

Supporting Peptides:

| Peptide | Uniq | -10lgP | Mass | Length | ppm | m/z | z | RT | Fraction | Scan | Source File | Area F1 | Area F10 | Area F11 | Area F12 | Area F13 | Area F2 | Area F3 | Area F4 | Area F5 | Area F6 | Area F7 | Area F8 | Area F9 | #Feature | #Feature F1 | #Feature F10 | #Feature F11 | #Feature F12 | #Feature F13 | #Feature F2 | #Feature F3 | #Feature F4 | #Feature F5 | #Feature F6 | #Feature F7 | #Feature F8 | #Feature F9 | Start | End | PTM | AScore | Found By |
| --- | --- | --- | --- | --- | --- | --- | --- | --- | --- | --- | --- | --- | --- | --- | --- | --- | --- | --- | --- | --- | --- | --- | --- | --- | --- | --- | --- | --- | --- | --- | --- | --- | --- | --- | --- | --- | --- | --- | --- | --- | --- | --- | --- |
| K.FC(+57.02)YLPADPGEC(+57.02)MAYIR.S | Y | 57.54 | 1961.8474 | 16 | 5.8 | 981.9314 | 2 | 72.26 | 8 | F8:48595 | NaNaKA16\_F4.raw |  |  |  |  |  |  |  | 1.1621E6 |  |  |  |  |  | 1 | 0 | 0 | 0 | 0 | 0 | 0 | 0 | 1 | 0 | 0 | 0 | 0 | 0 | 30 | 45 | Carbamidomethylation | C2:Carbamidomethylation:1000.00;C11:Carbamidomethylation:1000.00 | PEAKS DB |
| total 1 peptides |  |  |  |  |  |  |  |  |  |  |  |  |  |  |  |  |  |  |  |  |  |  |  |  |  |  |  |  |  |  |  |  |  |  |  |  |  |  |  |  |  |  |  |
| --- | --- | --- | --- | --- | --- | --- | --- | --- | --- | --- | --- | --- | --- | --- | --- | --- | --- | --- | --- | --- | --- | --- | --- | --- | --- | --- | --- | --- | --- | --- | --- | --- | --- | --- | --- | --- | --- | --- | --- | --- | --- | --- | --- |

ABD24042.1

back to list

  

| Protein Coverage
| Supporting Peptides
|

Protein Coverage:

Supporting Peptides:

| Peptide | Uniq | -10lgP | Mass | Length | ppm | m/z | z | RT | Fraction | Scan | Source File | Area F1 | Area F10 | Area F11 | Area F12 | Area F13 | Area F2 | Area F3 | Area F4 | Area F5 | Area F6 | Area F7 | Area F8 | Area F9 | #Feature | #Feature F1 | #Feature F10 | #Feature F11 | #Feature F12 | #Feature F13 | #Feature F2 | #Feature F3 | #Feature F4 | #Feature F5 | #Feature F6 | #Feature F7 | #Feature F8 | #Feature F9 | Start | End | PTM | AScore | Found By |
| --- | --- | --- | --- | --- | --- | --- | --- | --- | --- | --- | --- | --- | --- | --- | --- | --- | --- | --- | --- | --- | --- | --- | --- | --- | --- | --- | --- | --- | --- | --- | --- | --- | --- | --- | --- | --- | --- | --- | --- | --- | --- | --- | --- |
| K.FC(+57.02)YLPADPGEC(+57.02)MAYIR.S | Y | 57.54 | 1961.8474 | 16 | 5.8 | 981.9314 | 2 | 72.26 | 8 | F8:48595 | NaNaKA16\_F4.raw |  |  |  |  |  |  |  | 1.1621E6 |  |  |  |  |  | 1 | 0 | 0 | 0 | 0 | 0 | 0 | 0 | 1 | 0 | 0 | 0 | 0 | 0 | 30 | 45 | Carbamidomethylation | C2:Carbamidomethylation:1000.00;C11:Carbamidomethylation:1000.00 | PEAKS DB |
| total 1 peptides |  |  |  |  |  |  |  |  |  |  |  |  |  |  |  |  |  |  |  |  |  |  |  |  |  |  |  |  |  |  |  |  |  |  |  |  |  |  |  |  |  |  |  |
| --- | --- | --- | --- | --- | --- | --- | --- | --- | --- | --- | --- | --- | --- | --- | --- | --- | --- | --- | --- | --- | --- | --- | --- | --- | --- | --- | --- | --- | --- | --- | --- | --- | --- | --- | --- | --- | --- | --- | --- | --- | --- | --- | --- |

Q2ES48.1

back to list

  

| Protein Coverage
| Supporting Peptides
|

Protein Coverage:

Supporting Peptides:

| Peptide | Uniq | -10lgP | Mass | Length | ppm | m/z | z | RT | Fraction | Scan | Source File | Area F1 | Area F10 | Area F11 | Area F12 | Area F13 | Area F2 | Area F3 | Area F4 | Area F5 | Area F6 | Area F7 | Area F8 | Area F9 | #Feature | #Feature F1 | #Feature F10 | #Feature F11 | #Feature F12 | #Feature F13 | #Feature F2 | #Feature F3 | #Feature F4 | #Feature F5 | #Feature F6 | #Feature F7 | #Feature F8 | #Feature F9 | Start | End | PTM | AScore | Found By |
| --- | --- | --- | --- | --- | --- | --- | --- | --- | --- | --- | --- | --- | --- | --- | --- | --- | --- | --- | --- | --- | --- | --- | --- | --- | --- | --- | --- | --- | --- | --- | --- | --- | --- | --- | --- | --- | --- | --- | --- | --- | --- | --- | --- |
| K.FC(+57.02)YLPADPGEC(+57.02)MAYIR.S | Y | 57.54 | 1961.8474 | 16 | 5.8 | 981.9314 | 2 | 72.26 | 8 | F8:48595 | NaNaKA16\_F4.raw |  |  |  |  |  |  |  | 1.1621E6 |  |  |  |  |  | 1 | 0 | 0 | 0 | 0 | 0 | 0 | 0 | 1 | 0 | 0 | 0 | 0 | 0 | 30 | 45 | Carbamidomethylation | C2:Carbamidomethylation:1000.00;C11:Carbamidomethylation:1000.00 | PEAKS DB |
| total 1 peptides |  |  |  |  |  |  |  |  |  |  |  |  |  |  |  |  |  |  |  |  |  |  |  |  |  |  |  |  |  |  |  |  |  |  |  |  |  |  |  |  |  |  |  |
| --- | --- | --- | --- | --- | --- | --- | --- | --- | --- | --- | --- | --- | --- | --- | --- | --- | --- | --- | --- | --- | --- | --- | --- | --- | --- | --- | --- | --- | --- | --- | --- | --- | --- | --- | --- | --- | --- | --- | --- | --- | --- | --- | --- |

XP\_032084681.1

back to list

  

| Protein Coverage
| Supporting Peptides
|

Protein Coverage:

Supporting Peptides:

| Peptide | Uniq | -10lgP | Mass | Length | ppm | m/z | z | RT | Fraction | Scan | Source File | Area F1 | Area F10 | Area F11 | Area F12 | Area F13 | Area F2 | Area F3 | Area F4 | Area F5 | Area F6 | Area F7 | Area F8 | Area F9 | #Feature | #Feature F1 | #Feature F10 | #Feature F11 | #Feature F12 | #Feature F13 | #Feature F2 | #Feature F3 | #Feature F4 | #Feature F5 | #Feature F6 | #Feature F7 | #Feature F8 | #Feature F9 | Start | End | PTM | AScore | Found By |
| --- | --- | --- | --- | --- | --- | --- | --- | --- | --- | --- | --- | --- | --- | --- | --- | --- | --- | --- | --- | --- | --- | --- | --- | --- | --- | --- | --- | --- | --- | --- | --- | --- | --- | --- | --- | --- | --- | --- | --- | --- | --- | --- | --- |
| R.DDC(+57.02)DFPELC(+57.02)TGR.S | Y | 55.95 | 1483.5708 | 12 | 0.7 | 742.7932 | 2 | 50.53 | 10 | F10:33305 | NaNaKA16\_F6.raw |  |  |  |  | 6.4023E5 |  |  |  |  | 2.2329E7 |  |  |  | 2 | 0 | 0 | 0 | 0 | 1 | 0 | 0 | 0 | 0 | 1 | 0 | 0 | 0 | 473 | 484 | Carbamidomethylation | C3:Carbamidomethylation:1000.00;C9:Carbamidomethylation:1000.00 | PEAKS DB |
| total 1 peptides |  |  |  |  |  |  |  |  |  |  |  |  |  |  |  |  |  |  |  |  |  |  |  |  |  |  |  |  |  |  |  |  |  |  |  |  |  |  |  |  |  |  |  |
| --- | --- | --- | --- | --- | --- | --- | --- | --- | --- | --- | --- | --- | --- | --- | --- | --- | --- | --- | --- | --- | --- | --- | --- | --- | --- | --- | --- | --- | --- | --- | --- | --- | --- | --- | --- | --- | --- | --- | --- | --- | --- | --- | --- |

XP\_013922279.1

back to list

  

| Protein Coverage
| Supporting Peptides
|

Protein Coverage:

Supporting Peptides:

| Peptide | Uniq | -10lgP | Mass | Length | ppm | m/z | z | RT | Fraction | Scan | Source File | Area F1 | Area F10 | Area F11 | Area F12 | Area F13 | Area F2 | Area F3 | Area F4 | Area F5 | Area F6 | Area F7 | Area F8 | Area F9 | #Feature | #Feature F1 | #Feature F10 | #Feature F11 | #Feature F12 | #Feature F13 | #Feature F2 | #Feature F3 | #Feature F4 | #Feature F5 | #Feature F6 | #Feature F7 | #Feature F8 | #Feature F9 | Start | End | PTM | AScore | Found By |
| --- | --- | --- | --- | --- | --- | --- | --- | --- | --- | --- | --- | --- | --- | --- | --- | --- | --- | --- | --- | --- | --- | --- | --- | --- | --- | --- | --- | --- | --- | --- | --- | --- | --- | --- | --- | --- | --- | --- | --- | --- | --- | --- | --- |
| R.DDC(+57.02)DFPELC(+57.02)TGR.S | Y | 55.95 | 1483.5708 | 12 | 0.7 | 742.7932 | 2 | 50.53 | 10 | F10:33305 | NaNaKA16\_F6.raw |  |  |  |  | 6.4023E5 |  |  |  |  | 2.2329E7 |  |  |  | 2 | 0 | 0 | 0 | 0 | 1 | 0 | 0 | 0 | 0 | 1 | 0 | 0 | 0 | 25 | 36 | Carbamidomethylation | C3:Carbamidomethylation:1000.00;C9:Carbamidomethylation:1000.00 | PEAKS DB |
| total 1 peptides |  |  |  |  |  |  |  |  |  |  |  |  |  |  |  |  |  |  |  |  |  |  |  |  |  |  |  |  |  |  |  |  |  |  |  |  |  |  |  |  |  |  |  |
| --- | --- | --- | --- | --- | --- | --- | --- | --- | --- | --- | --- | --- | --- | --- | --- | --- | --- | --- | --- | --- | --- | --- | --- | --- | --- | --- | --- | --- | --- | --- | --- | --- | --- | --- | --- | --- | --- | --- | --- | --- | --- | --- | --- |

P25679.2

back to list

  

| Protein Coverage
| Supporting Peptides
|

Protein Coverage:

Supporting Peptides:

| Peptide | Uniq | -10lgP | Mass | Length | ppm | m/z | z | RT | Fraction | Scan | Source File | Area F1 | Area F10 | Area F11 | Area F12 | Area F13 | Area F2 | Area F3 | Area F4 | Area F5 | Area F6 | Area F7 | Area F8 | Area F9 | #Feature | #Feature F1 | #Feature F10 | #Feature F11 | #Feature F12 | #Feature F13 | #Feature F2 | #Feature F3 | #Feature F4 | #Feature F5 | #Feature F6 | #Feature F7 | #Feature F8 | #Feature F9 | Start | End | PTM | AScore | Found By |
| --- | --- | --- | --- | --- | --- | --- | --- | --- | --- | --- | --- | --- | --- | --- | --- | --- | --- | --- | --- | --- | --- | --- | --- | --- | --- | --- | --- | --- | --- | --- | --- | --- | --- | --- | --- | --- | --- | --- | --- | --- | --- | --- | --- |
| LTC(+57.02)LNC(+57.02)PEMFC(+57.02)GK.F | Y | 71.90 | 1628.6819 | 13 | 0.7 | 815.3488 | 2 | 46.10 | 10 | F10:29493 | NaNaKA16\_F6.raw |  |  |  |  |  |  |  |  |  | 1.8887E7 |  |  |  | 1 | 0 | 0 | 0 | 0 | 0 | 0 | 0 | 0 | 0 | 1 | 0 | 0 | 0 | 1 | 13 | Carbamidomethylation | C3:Carbamidomethylation:1000.00;C6:Carbamidomethylation:1000.00;C11:Carbamidomethylation:1000.00 | PEAKS DB |
| total 1 peptides |  |  |  |  |  |  |  |  |  |  |  |  |  |  |  |  |  |  |  |  |  |  |  |  |  |  |  |  |  |  |  |  |  |  |  |  |  |  |  |  |  |  |  |
| --- | --- | --- | --- | --- | --- | --- | --- | --- | --- | --- | --- | --- | --- | --- | --- | --- | --- | --- | --- | --- | --- | --- | --- | --- | --- | --- | --- | --- | --- | --- | --- | --- | --- | --- | --- | --- | --- | --- | --- | --- | --- | --- | --- |

P60814.1

back to list

  

| Protein Coverage
| Supporting Peptides
|

Protein Coverage:

Supporting Peptides:

| Peptide | Uniq | -10lgP | Mass | Length | ppm | m/z | z | RT | Fraction | Scan | Source File | Area F1 | Area F10 | Area F11 | Area F12 | Area F13 | Area F2 | Area F3 | Area F4 | Area F5 | Area F6 | Area F7 | Area F8 | Area F9 | #Feature | #Feature F1 | #Feature F10 | #Feature F11 | #Feature F12 | #Feature F13 | #Feature F2 | #Feature F3 | #Feature F4 | #Feature F5 | #Feature F6 | #Feature F7 | #Feature F8 | #Feature F9 | Start | End | PTM | AScore | Found By |
| --- | --- | --- | --- | --- | --- | --- | --- | --- | --- | --- | --- | --- | --- | --- | --- | --- | --- | --- | --- | --- | --- | --- | --- | --- | --- | --- | --- | --- | --- | --- | --- | --- | --- | --- | --- | --- | --- | --- | --- | --- | --- | --- | --- |
| T.LTC(+57.02)LNC(+57.02)PEMFC(+57.02)GK.F | Y | 71.90 | 1628.6819 | 13 | 0.7 | 815.3488 | 2 | 46.10 | 10 | F10:29493 | NaNaKA16\_F6.raw |  |  |  |  |  |  |  |  |  | 1.8887E7 |  |  |  | 1 | 0 | 0 | 0 | 0 | 0 | 0 | 0 | 0 | 0 | 1 | 0 | 0 | 0 | 22 | 34 | Carbamidomethylation | C3:Carbamidomethylation:1000.00;C6:Carbamidomethylation:1000.00;C11:Carbamidomethylation:1000.00 | PEAKS DB |
| total 1 peptides |  |  |  |  |  |  |  |  |  |  |  |  |  |  |  |  |  |  |  |  |  |  |  |  |  |  |  |  |  |  |  |  |  |  |  |  |  |  |  |  |  |  |  |
| --- | --- | --- | --- | --- | --- | --- | --- | --- | --- | --- | --- | --- | --- | --- | --- | --- | --- | --- | --- | --- | --- | --- | --- | --- | --- | --- | --- | --- | --- | --- | --- | --- | --- | --- | --- | --- | --- | --- | --- | --- | --- | --- | --- |

CAA06888.1

back to list

  

| Protein Coverage
| Supporting Peptides
|

Protein Coverage:

Supporting Peptides:

| Peptide | Uniq | -10lgP | Mass | Length | ppm | m/z | z | RT | Fraction | Scan | Source File | Area F1 | Area F10 | Area F11 | Area F12 | Area F13 | Area F2 | Area F3 | Area F4 | Area F5 | Area F6 | Area F7 | Area F8 | Area F9 | #Feature | #Feature F1 | #Feature F10 | #Feature F11 | #Feature F12 | #Feature F13 | #Feature F2 | #Feature F3 | #Feature F4 | #Feature F5 | #Feature F6 | #Feature F7 | #Feature F8 | #Feature F9 | Start | End | PTM | AScore | Found By |
| --- | --- | --- | --- | --- | --- | --- | --- | --- | --- | --- | --- | --- | --- | --- | --- | --- | --- | --- | --- | --- | --- | --- | --- | --- | --- | --- | --- | --- | --- | --- | --- | --- | --- | --- | --- | --- | --- | --- | --- | --- | --- | --- | --- |
| T.LTC(+57.02)LNC(+57.02)PEMFC(+57.02)GK.F | Y | 71.90 | 1628.6819 | 13 | 0.7 | 815.3488 | 2 | 46.10 | 10 | F10:29493 | NaNaKA16\_F6.raw |  |  |  |  |  |  |  |  |  | 1.8887E7 |  |  |  | 1 | 0 | 0 | 0 | 0 | 0 | 0 | 0 | 0 | 0 | 1 | 0 | 0 | 0 | 22 | 34 | Carbamidomethylation | C3:Carbamidomethylation:1000.00;C6:Carbamidomethylation:1000.00;C11:Carbamidomethylation:1000.00 | PEAKS DB |
| total 1 peptides |  |  |  |  |  |  |  |  |  |  |  |  |  |  |  |  |  |  |  |  |  |  |  |  |  |  |  |  |  |  |  |  |  |  |  |  |  |  |  |  |  |  |  |
| --- | --- | --- | --- | --- | --- | --- | --- | --- | --- | --- | --- | --- | --- | --- | --- | --- | --- | --- | --- | --- | --- | --- | --- | --- | --- | --- | --- | --- | --- | --- | --- | --- | --- | --- | --- | --- | --- | --- | --- | --- | --- | --- | --- |

2MJ0

back to list

  

| Protein Coverage
| Supporting Peptides
|

Protein Coverage:

Supporting Peptides:

| Peptide | Uniq | -10lgP | Mass | Length | ppm | m/z | z | RT | Fraction | Scan | Source File | Area F1 | Area F10 | Area F11 | Area F12 | Area F13 | Area F2 | Area F3 | Area F4 | Area F5 | Area F6 | Area F7 | Area F8 | Area F9 | #Feature | #Feature F1 | #Feature F10 | #Feature F11 | #Feature F12 | #Feature F13 | #Feature F2 | #Feature F3 | #Feature F4 | #Feature F5 | #Feature F6 | #Feature F7 | #Feature F8 | #Feature F9 | Start | End | PTM | AScore | Found By |
| --- | --- | --- | --- | --- | --- | --- | --- | --- | --- | --- | --- | --- | --- | --- | --- | --- | --- | --- | --- | --- | --- | --- | --- | --- | --- | --- | --- | --- | --- | --- | --- | --- | --- | --- | --- | --- | --- | --- | --- | --- | --- | --- | --- |
| M.LTC(+57.02)LNC(+57.02)PEMFC(+57.02)GK.F | Y | 71.90 | 1628.6819 | 13 | 0.7 | 815.3488 | 2 | 46.10 | 10 | F10:29493 | NaNaKA16\_F6.raw |  |  |  |  |  |  |  |  |  | 1.8887E7 |  |  |  | 1 | 0 | 0 | 0 | 0 | 0 | 0 | 0 | 0 | 0 | 1 | 0 | 0 | 0 | 2 | 14 | Carbamidomethylation | C3:Carbamidomethylation:1000.00;C6:Carbamidomethylation:1000.00;C11:Carbamidomethylation:1000.00 | PEAKS DB |
| total 1 peptides |  |  |  |  |  |  |  |  |  |  |  |  |  |  |  |  |  |  |  |  |  |  |  |  |  |  |  |  |  |  |  |  |  |  |  |  |  |  |  |  |  |  |  |
| --- | --- | --- | --- | --- | --- | --- | --- | --- | --- | --- | --- | --- | --- | --- | --- | --- | --- | --- | --- | --- | --- | --- | --- | --- | --- | --- | --- | --- | --- | --- | --- | --- | --- | --- | --- | --- | --- | --- | --- | --- | --- | --- | --- |

P82935.2

back to list

  

| Protein Coverage
| Supporting Peptides
|

Protein Coverage:

Supporting Peptides:

| Peptide | Uniq | -10lgP | Mass | Length | ppm | m/z | z | RT | Fraction | Scan | Source File | Area F1 | Area F10 | Area F11 | Area F12 | Area F13 | Area F2 | Area F3 | Area F4 | Area F5 | Area F6 | Area F7 | Area F8 | Area F9 | #Feature | #Feature F1 | #Feature F10 | #Feature F11 | #Feature F12 | #Feature F13 | #Feature F2 | #Feature F3 | #Feature F4 | #Feature F5 | #Feature F6 | #Feature F7 | #Feature F8 | #Feature F9 | Start | End | PTM | AScore | Found By |
| --- | --- | --- | --- | --- | --- | --- | --- | --- | --- | --- | --- | --- | --- | --- | --- | --- | --- | --- | --- | --- | --- | --- | --- | --- | --- | --- | --- | --- | --- | --- | --- | --- | --- | --- | --- | --- | --- | --- | --- | --- | --- | --- | --- |
| T.LTC(+57.02)LNC(+57.02)PEMFC(+57.02)GK.F | Y | 71.90 | 1628.6819 | 13 | 0.7 | 815.3488 | 2 | 46.10 | 10 | F10:29493 | NaNaKA16\_F6.raw |  |  |  |  |  |  |  |  |  | 1.8887E7 |  |  |  | 1 | 0 | 0 | 0 | 0 | 0 | 0 | 0 | 0 | 0 | 1 | 0 | 0 | 0 | 22 | 34 | Carbamidomethylation | C3:Carbamidomethylation:1000.00;C6:Carbamidomethylation:1000.00;C11:Carbamidomethylation:1000.00 | PEAKS DB |
| total 1 peptides |  |  |  |  |  |  |  |  |  |  |  |  |  |  |  |  |  |  |  |  |  |  |  |  |  |  |  |  |  |  |  |  |  |  |  |  |  |  |  |  |  |  |  |
| --- | --- | --- | --- | --- | --- | --- | --- | --- | --- | --- | --- | --- | --- | --- | --- | --- | --- | --- | --- | --- | --- | --- | --- | --- | --- | --- | --- | --- | --- | --- | --- | --- | --- | --- | --- | --- | --- | --- | --- | --- | --- | --- | --- |

CAA04578.1

back to list

  

| Protein Coverage
| Supporting Peptides
|

Protein Coverage:

Supporting Peptides:

| Peptide | Uniq | -10lgP | Mass | Length | ppm | m/z | z | RT | Fraction | Scan | Source File | Area F1 | Area F10 | Area F11 | Area F12 | Area F13 | Area F2 | Area F3 | Area F4 | Area F5 | Area F6 | Area F7 | Area F8 | Area F9 | #Feature | #Feature F1 | #Feature F10 | #Feature F11 | #Feature F12 | #Feature F13 | #Feature F2 | #Feature F3 | #Feature F4 | #Feature F5 | #Feature F6 | #Feature F7 | #Feature F8 | #Feature F9 | Start | End | PTM | AScore | Found By |
| --- | --- | --- | --- | --- | --- | --- | --- | --- | --- | --- | --- | --- | --- | --- | --- | --- | --- | --- | --- | --- | --- | --- | --- | --- | --- | --- | --- | --- | --- | --- | --- | --- | --- | --- | --- | --- | --- | --- | --- | --- | --- | --- | --- |
| T.LTC(+57.02)LNC(+57.02)PEMFC(+57.02)GK.F | Y | 71.90 | 1628.6819 | 13 | 0.7 | 815.3488 | 2 | 46.10 | 10 | F10:29493 | NaNaKA16\_F6.raw |  |  |  |  |  |  |  |  |  | 1.8887E7 |  |  |  | 1 | 0 | 0 | 0 | 0 | 0 | 0 | 0 | 0 | 0 | 1 | 0 | 0 | 0 | 22 | 34 | Carbamidomethylation | C3:Carbamidomethylation:1000.00;C6:Carbamidomethylation:1000.00;C11:Carbamidomethylation:1000.00 | PEAKS DB |
| total 1 peptides |  |  |  |  |  |  |  |  |  |  |  |  |  |  |  |  |  |  |  |  |  |  |  |  |  |  |  |  |  |  |  |  |  |  |  |  |  |  |  |  |  |  |  |
| --- | --- | --- | --- | --- | --- | --- | --- | --- | --- | --- | --- | --- | --- | --- | --- | --- | --- | --- | --- | --- | --- | --- | --- | --- | --- | --- | --- | --- | --- | --- | --- | --- | --- | --- | --- | --- | --- | --- | --- | --- | --- | --- | --- |

AAL87467.1

back to list

  

| Protein Coverage
| Supporting Peptides
|

Protein Coverage:

Supporting Peptides:

| Peptide | Uniq | -10lgP | Mass | Length | ppm | m/z | z | RT | Fraction | Scan | Source File | Area F1 | Area F10 | Area F11 | Area F12 | Area F13 | Area F2 | Area F3 | Area F4 | Area F5 | Area F6 | Area F7 | Area F8 | Area F9 | #Feature | #Feature F1 | #Feature F10 | #Feature F11 | #Feature F12 | #Feature F13 | #Feature F2 | #Feature F3 | #Feature F4 | #Feature F5 | #Feature F6 | #Feature F7 | #Feature F8 | #Feature F9 | Start | End | PTM | AScore | Found By |
| --- | --- | --- | --- | --- | --- | --- | --- | --- | --- | --- | --- | --- | --- | --- | --- | --- | --- | --- | --- | --- | --- | --- | --- | --- | --- | --- | --- | --- | --- | --- | --- | --- | --- | --- | --- | --- | --- | --- | --- | --- | --- | --- | --- |
| T.LTC(+57.02)LNC(+57.02)PEMFC(+57.02)GK.F | Y | 71.90 | 1628.6819 | 13 | 0.7 | 815.3488 | 2 | 46.10 | 10 | F10:29493 | NaNaKA16\_F6.raw |  |  |  |  |  |  |  |  |  | 1.8887E7 |  |  |  | 1 | 0 | 0 | 0 | 0 | 0 | 0 | 0 | 0 | 0 | 1 | 0 | 0 | 0 | 22 | 34 | Carbamidomethylation | C3:Carbamidomethylation:1000.00;C6:Carbamidomethylation:1000.00;C11:Carbamidomethylation:1000.00 | PEAKS DB |
| total 1 peptides |  |  |  |  |  |  |  |  |  |  |  |  |  |  |  |  |  |  |  |  |  |  |  |  |  |  |  |  |  |  |  |  |  |  |  |  |  |  |  |  |  |  |  |
| --- | --- | --- | --- | --- | --- | --- | --- | --- | --- | --- | --- | --- | --- | --- | --- | --- | --- | --- | --- | --- | --- | --- | --- | --- | --- | --- | --- | --- | --- | --- | --- | --- | --- | --- | --- | --- | --- | --- | --- | --- | --- | --- | --- |

O42255.1

back to list

  

| Protein Coverage
| Supporting Peptides
|

Protein Coverage:

Supporting Peptides:

| Peptide | Uniq | -10lgP | Mass | Length | ppm | m/z | z | RT | Fraction | Scan | Source File | Area F1 | Area F10 | Area F11 | Area F12 | Area F13 | Area F2 | Area F3 | Area F4 | Area F5 | Area F6 | Area F7 | Area F8 | Area F9 | #Feature | #Feature F1 | #Feature F10 | #Feature F11 | #Feature F12 | #Feature F13 | #Feature F2 | #Feature F3 | #Feature F4 | #Feature F5 | #Feature F6 | #Feature F7 | #Feature F8 | #Feature F9 | Start | End | PTM | AScore | Found By |
| --- | --- | --- | --- | --- | --- | --- | --- | --- | --- | --- | --- | --- | --- | --- | --- | --- | --- | --- | --- | --- | --- | --- | --- | --- | --- | --- | --- | --- | --- | --- | --- | --- | --- | --- | --- | --- | --- | --- | --- | --- | --- | --- | --- |
| T.LTC(+57.02)LNC(+57.02)PEMFC(+57.02)GK.F | Y | 71.90 | 1628.6819 | 13 | 0.7 | 815.3488 | 2 | 46.10 | 10 | F10:29493 | NaNaKA16\_F6.raw |  |  |  |  |  |  |  |  |  | 1.8887E7 |  |  |  | 1 | 0 | 0 | 0 | 0 | 0 | 0 | 0 | 0 | 0 | 1 | 0 | 0 | 0 | 22 | 34 | Carbamidomethylation | C3:Carbamidomethylation:1000.00;C6:Carbamidomethylation:1000.00;C11:Carbamidomethylation:1000.00 | PEAKS DB |
| total 1 peptides |  |  |  |  |  |  |  |  |  |  |  |  |  |  |  |  |  |  |  |  |  |  |  |  |  |  |  |  |  |  |  |  |  |  |  |  |  |  |  |  |  |  |  |
| --- | --- | --- | --- | --- | --- | --- | --- | --- | --- | --- | --- | --- | --- | --- | --- | --- | --- | --- | --- | --- | --- | --- | --- | --- | --- | --- | --- | --- | --- | --- | --- | --- | --- | --- | --- | --- | --- | --- | --- | --- | --- | --- | --- |

AAB87415.1

back to list

  

| Protein Coverage
| Supporting Peptides
|

Protein Coverage:

Supporting Peptides:

| Peptide | Uniq | -10lgP | Mass | Length | ppm | m/z | z | RT | Fraction | Scan | Source File | Area F1 | Area F10 | Area F11 | Area F12 | Area F13 | Area F2 | Area F3 | Area F4 | Area F5 | Area F6 | Area F7 | Area F8 | Area F9 | #Feature | #Feature F1 | #Feature F10 | #Feature F11 | #Feature F12 | #Feature F13 | #Feature F2 | #Feature F3 | #Feature F4 | #Feature F5 | #Feature F6 | #Feature F7 | #Feature F8 | #Feature F9 | Start | End | PTM | AScore | Found By |
| --- | --- | --- | --- | --- | --- | --- | --- | --- | --- | --- | --- | --- | --- | --- | --- | --- | --- | --- | --- | --- | --- | --- | --- | --- | --- | --- | --- | --- | --- | --- | --- | --- | --- | --- | --- | --- | --- | --- | --- | --- | --- | --- | --- |
| T.LTC(+57.02)LNC(+57.02)PEMFC(+57.02)GK.F | Y | 71.90 | 1628.6819 | 13 | 0.7 | 815.3488 | 2 | 46.10 | 10 | F10:29493 | NaNaKA16\_F6.raw |  |  |  |  |  |  |  |  |  | 1.8887E7 |  |  |  | 1 | 0 | 0 | 0 | 0 | 0 | 0 | 0 | 0 | 0 | 1 | 0 | 0 | 0 | 22 | 34 | Carbamidomethylation | C3:Carbamidomethylation:1000.00;C6:Carbamidomethylation:1000.00;C11:Carbamidomethylation:1000.00 | PEAKS DB |
| total 1 peptides |  |  |  |  |  |  |  |  |  |  |  |  |  |  |  |  |  |  |  |  |  |  |  |  |  |  |  |  |  |  |  |  |  |  |  |  |  |  |  |  |  |  |  |
| --- | --- | --- | --- | --- | --- | --- | --- | --- | --- | --- | --- | --- | --- | --- | --- | --- | --- | --- | --- | --- | --- | --- | --- | --- | --- | --- | --- | --- | --- | --- | --- | --- | --- | --- | --- | --- | --- | --- | --- | --- | --- | --- | --- |

AAB87416.1

back to list

  

| Protein Coverage
| Supporting Peptides
|

Protein Coverage:

Supporting Peptides:

| Peptide | Uniq | -10lgP | Mass | Length | ppm | m/z | z | RT | Fraction | Scan | Source File | Area F1 | Area F10 | Area F11 | Area F12 | Area F13 | Area F2 | Area F3 | Area F4 | Area F5 | Area F6 | Area F7 | Area F8 | Area F9 | #Feature | #Feature F1 | #Feature F10 | #Feature F11 | #Feature F12 | #Feature F13 | #Feature F2 | #Feature F3 | #Feature F4 | #Feature F5 | #Feature F6 | #Feature F7 | #Feature F8 | #Feature F9 | Start | End | PTM | AScore | Found By |
| --- | --- | --- | --- | --- | --- | --- | --- | --- | --- | --- | --- | --- | --- | --- | --- | --- | --- | --- | --- | --- | --- | --- | --- | --- | --- | --- | --- | --- | --- | --- | --- | --- | --- | --- | --- | --- | --- | --- | --- | --- | --- | --- | --- |
| T.LTC(+57.02)LNC(+57.02)PEMFC(+57.02)GK.F | Y | 71.90 | 1628.6819 | 13 | 0.7 | 815.3488 | 2 | 46.10 | 10 | F10:29493 | NaNaKA16\_F6.raw |  |  |  |  |  |  |  |  |  | 1.8887E7 |  |  |  | 1 | 0 | 0 | 0 | 0 | 0 | 0 | 0 | 0 | 0 | 1 | 0 | 0 | 0 | 22 | 34 | Carbamidomethylation | C3:Carbamidomethylation:1000.00;C6:Carbamidomethylation:1000.00;C11:Carbamidomethylation:1000.00 | PEAKS DB |
| total 1 peptides |  |  |  |  |  |  |  |  |  |  |  |  |  |  |  |  |  |  |  |  |  |  |  |  |  |  |  |  |  |  |  |  |  |  |  |  |  |  |  |  |  |  |  |
| --- | --- | --- | --- | --- | --- | --- | --- | --- | --- | --- | --- | --- | --- | --- | --- | --- | --- | --- | --- | --- | --- | --- | --- | --- | --- | --- | --- | --- | --- | --- | --- | --- | --- | --- | --- | --- | --- | --- | --- | --- | --- | --- | --- |

O42256.1

back to list

  

| Protein Coverage
| Supporting Peptides
|

Protein Coverage:

Supporting Peptides:

| Peptide | Uniq | -10lgP | Mass | Length | ppm | m/z | z | RT | Fraction | Scan | Source File | Area F1 | Area F10 | Area F11 | Area F12 | Area F13 | Area F2 | Area F3 | Area F4 | Area F5 | Area F6 | Area F7 | Area F8 | Area F9 | #Feature | #Feature F1 | #Feature F10 | #Feature F11 | #Feature F12 | #Feature F13 | #Feature F2 | #Feature F3 | #Feature F4 | #Feature F5 | #Feature F6 | #Feature F7 | #Feature F8 | #Feature F9 | Start | End | PTM | AScore | Found By |
| --- | --- | --- | --- | --- | --- | --- | --- | --- | --- | --- | --- | --- | --- | --- | --- | --- | --- | --- | --- | --- | --- | --- | --- | --- | --- | --- | --- | --- | --- | --- | --- | --- | --- | --- | --- | --- | --- | --- | --- | --- | --- | --- | --- |
| T.LTC(+57.02)LNC(+57.02)PEMFC(+57.02)GK.F | Y | 71.90 | 1628.6819 | 13 | 0.7 | 815.3488 | 2 | 46.10 | 10 | F10:29493 | NaNaKA16\_F6.raw |  |  |  |  |  |  |  |  |  | 1.8887E7 |  |  |  | 1 | 0 | 0 | 0 | 0 | 0 | 0 | 0 | 0 | 0 | 1 | 0 | 0 | 0 | 22 | 34 | Carbamidomethylation | C3:Carbamidomethylation:1000.00;C6:Carbamidomethylation:1000.00;C11:Carbamidomethylation:1000.00 | PEAKS DB |
| total 1 peptides |  |  |  |  |  |  |  |  |  |  |  |  |  |  |  |  |  |  |  |  |  |  |  |  |  |  |  |  |  |  |  |  |  |  |  |  |  |  |  |  |  |  |  |
| --- | --- | --- | --- | --- | --- | --- | --- | --- | --- | --- | --- | --- | --- | --- | --- | --- | --- | --- | --- | --- | --- | --- | --- | --- | --- | --- | --- | --- | --- | --- | --- | --- | --- | --- | --- | --- | --- | --- | --- | --- | --- | --- | --- |

CAA10530.1

back to list

  

| Protein Coverage
| Supporting Peptides
|

Protein Coverage:

Supporting Peptides:

| Peptide | Uniq | -10lgP | Mass | Length | ppm | m/z | z | RT | Fraction | Scan | Source File | Area F1 | Area F10 | Area F11 | Area F12 | Area F13 | Area F2 | Area F3 | Area F4 | Area F5 | Area F6 | Area F7 | Area F8 | Area F9 | #Feature | #Feature F1 | #Feature F10 | #Feature F11 | #Feature F12 | #Feature F13 | #Feature F2 | #Feature F3 | #Feature F4 | #Feature F5 | #Feature F6 | #Feature F7 | #Feature F8 | #Feature F9 | Start | End | PTM | AScore | Found By |
| --- | --- | --- | --- | --- | --- | --- | --- | --- | --- | --- | --- | --- | --- | --- | --- | --- | --- | --- | --- | --- | --- | --- | --- | --- | --- | --- | --- | --- | --- | --- | --- | --- | --- | --- | --- | --- | --- | --- | --- | --- | --- | --- | --- |
| T.LTC(+57.02)LNC(+57.02)PEMFC(+57.02)GK.F | Y | 71.90 | 1628.6819 | 13 | 0.7 | 815.3488 | 2 | 46.10 | 10 | F10:29493 | NaNaKA16\_F6.raw |  |  |  |  |  |  |  |  |  | 1.8887E7 |  |  |  | 1 | 0 | 0 | 0 | 0 | 0 | 0 | 0 | 0 | 0 | 1 | 0 | 0 | 0 | 22 | 34 | Carbamidomethylation | C3:Carbamidomethylation:1000.00;C6:Carbamidomethylation:1000.00;C11:Carbamidomethylation:1000.00 | PEAKS DB |
| total 1 peptides |  |  |  |  |  |  |  |  |  |  |  |  |  |  |  |  |  |  |  |  |  |  |  |  |  |  |  |  |  |  |  |  |  |  |  |  |  |  |  |  |  |  |  |
| --- | --- | --- | --- | --- | --- | --- | --- | --- | --- | --- | --- | --- | --- | --- | --- | --- | --- | --- | --- | --- | --- | --- | --- | --- | --- | --- | --- | --- | --- | --- | --- | --- | --- | --- | --- | --- | --- | --- | --- | --- | --- | --- | --- |

Q9YGI4.1

back to list

  

| Protein Coverage
| Supporting Peptides
|

Protein Coverage:

Supporting Peptides:

| Peptide | Uniq | -10lgP | Mass | Length | ppm | m/z | z | RT | Fraction | Scan | Source File | Area F1 | Area F10 | Area F11 | Area F12 | Area F13 | Area F2 | Area F3 | Area F4 | Area F5 | Area F6 | Area F7 | Area F8 | Area F9 | #Feature | #Feature F1 | #Feature F10 | #Feature F11 | #Feature F12 | #Feature F13 | #Feature F2 | #Feature F3 | #Feature F4 | #Feature F5 | #Feature F6 | #Feature F7 | #Feature F8 | #Feature F9 | Start | End | PTM | AScore | Found By |
| --- | --- | --- | --- | --- | --- | --- | --- | --- | --- | --- | --- | --- | --- | --- | --- | --- | --- | --- | --- | --- | --- | --- | --- | --- | --- | --- | --- | --- | --- | --- | --- | --- | --- | --- | --- | --- | --- | --- | --- | --- | --- | --- | --- |
| T.LTC(+57.02)LNC(+57.02)PEMFC(+57.02)GK.F | Y | 71.90 | 1628.6819 | 13 | 0.7 | 815.3488 | 2 | 46.10 | 10 | F10:29493 | NaNaKA16\_F6.raw |  |  |  |  |  |  |  |  |  | 1.8887E7 |  |  |  | 1 | 0 | 0 | 0 | 0 | 0 | 0 | 0 | 0 | 0 | 1 | 0 | 0 | 0 | 22 | 34 | Carbamidomethylation | C3:Carbamidomethylation:1000.00;C6:Carbamidomethylation:1000.00;C11:Carbamidomethylation:1000.00 | PEAKS DB |
| total 1 peptides |  |  |  |  |  |  |  |  |  |  |  |  |  |  |  |  |  |  |  |  |  |  |  |  |  |  |  |  |  |  |  |  |  |  |  |  |  |  |  |  |  |  |  |
| --- | --- | --- | --- | --- | --- | --- | --- | --- | --- | --- | --- | --- | --- | --- | --- | --- | --- | --- | --- | --- | --- | --- | --- | --- | --- | --- | --- | --- | --- | --- | --- | --- | --- | --- | --- | --- | --- | --- | --- | --- | --- | --- | --- |

AAL87466.1

back to list

  

| Protein Coverage
| Supporting Peptides
|

Protein Coverage:

Supporting Peptides:

| Peptide | Uniq | -10lgP | Mass | Length | ppm | m/z | z | RT | Fraction | Scan | Source File | Area F1 | Area F10 | Area F11 | Area F12 | Area F13 | Area F2 | Area F3 | Area F4 | Area F5 | Area F6 | Area F7 | Area F8 | Area F9 | #Feature | #Feature F1 | #Feature F10 | #Feature F11 | #Feature F12 | #Feature F13 | #Feature F2 | #Feature F3 | #Feature F4 | #Feature F5 | #Feature F6 | #Feature F7 | #Feature F8 | #Feature F9 | Start | End | PTM | AScore | Found By |
| --- | --- | --- | --- | --- | --- | --- | --- | --- | --- | --- | --- | --- | --- | --- | --- | --- | --- | --- | --- | --- | --- | --- | --- | --- | --- | --- | --- | --- | --- | --- | --- | --- | --- | --- | --- | --- | --- | --- | --- | --- | --- | --- | --- |
| T.LTC(+57.02)LNC(+57.02)PEMFC(+57.02)GK.F | Y | 71.90 | 1628.6819 | 13 | 0.7 | 815.3488 | 2 | 46.10 | 10 | F10:29493 | NaNaKA16\_F6.raw |  |  |  |  |  |  |  |  |  | 1.8887E7 |  |  |  | 1 | 0 | 0 | 0 | 0 | 0 | 0 | 0 | 0 | 0 | 1 | 0 | 0 | 0 | 22 | 34 | Carbamidomethylation | C3:Carbamidomethylation:1000.00;C6:Carbamidomethylation:1000.00;C11:Carbamidomethylation:1000.00 | PEAKS DB |
| total 1 peptides |  |  |  |  |  |  |  |  |  |  |  |  |  |  |  |  |  |  |  |  |  |  |  |  |  |  |  |  |  |  |  |  |  |  |  |  |  |  |  |  |  |  |  |
| --- | --- | --- | --- | --- | --- | --- | --- | --- | --- | --- | --- | --- | --- | --- | --- | --- | --- | --- | --- | --- | --- | --- | --- | --- | --- | --- | --- | --- | --- | --- | --- | --- | --- | --- | --- | --- | --- | --- | --- | --- | --- | --- | --- |

AAD39353.1

back to list

  

| Protein Coverage
| Supporting Peptides
|

Protein Coverage:

Supporting Peptides:

| Peptide | Uniq | -10lgP | Mass | Length | ppm | m/z | z | RT | Fraction | Scan | Source File | Area F1 | Area F10 | Area F11 | Area F12 | Area F13 | Area F2 | Area F3 | Area F4 | Area F5 | Area F6 | Area F7 | Area F8 | Area F9 | #Feature | #Feature F1 | #Feature F10 | #Feature F11 | #Feature F12 | #Feature F13 | #Feature F2 | #Feature F3 | #Feature F4 | #Feature F5 | #Feature F6 | #Feature F7 | #Feature F8 | #Feature F9 | Start | End | PTM | AScore | Found By |
| --- | --- | --- | --- | --- | --- | --- | --- | --- | --- | --- | --- | --- | --- | --- | --- | --- | --- | --- | --- | --- | --- | --- | --- | --- | --- | --- | --- | --- | --- | --- | --- | --- | --- | --- | --- | --- | --- | --- | --- | --- | --- | --- | --- |
| T.LTC(+57.02)LNC(+57.02)PEMFC(+57.02)GK.F | Y | 71.90 | 1628.6819 | 13 | 0.7 | 815.3488 | 2 | 46.10 | 10 | F10:29493 | NaNaKA16\_F6.raw |  |  |  |  |  |  |  |  |  | 1.8887E7 |  |  |  | 1 | 0 | 0 | 0 | 0 | 0 | 0 | 0 | 0 | 0 | 1 | 0 | 0 | 0 | 22 | 34 | Carbamidomethylation | C3:Carbamidomethylation:1000.00;C6:Carbamidomethylation:1000.00;C11:Carbamidomethylation:1000.00 | PEAKS DB |
| total 1 peptides |  |  |  |  |  |  |  |  |  |  |  |  |  |  |  |  |  |  |  |  |  |  |  |  |  |  |  |  |  |  |  |  |  |  |  |  |  |  |  |  |  |  |  |
| --- | --- | --- | --- | --- | --- | --- | --- | --- | --- | --- | --- | --- | --- | --- | --- | --- | --- | --- | --- | --- | --- | --- | --- | --- | --- | --- | --- | --- | --- | --- | --- | --- | --- | --- | --- | --- | --- | --- | --- | --- | --- | --- | --- |

Q802B3.2

back to list

  

| Protein Coverage
| Supporting Peptides
|

Protein Coverage:

Supporting Peptides:

| Peptide | Uniq | -10lgP | Mass | Length | ppm | m/z | z | RT | Fraction | Scan | Source File | Area F1 | Area F10 | Area F11 | Area F12 | Area F13 | Area F2 | Area F3 | Area F4 | Area F5 | Area F6 | Area F7 | Area F8 | Area F9 | #Feature | #Feature F1 | #Feature F10 | #Feature F11 | #Feature F12 | #Feature F13 | #Feature F2 | #Feature F3 | #Feature F4 | #Feature F5 | #Feature F6 | #Feature F7 | #Feature F8 | #Feature F9 | Start | End | PTM | AScore | Found By |
| --- | --- | --- | --- | --- | --- | --- | --- | --- | --- | --- | --- | --- | --- | --- | --- | --- | --- | --- | --- | --- | --- | --- | --- | --- | --- | --- | --- | --- | --- | --- | --- | --- | --- | --- | --- | --- | --- | --- | --- | --- | --- | --- | --- |
| T.LTC(+57.02)LNC(+57.02)PEMFC(+57.02)GK.F | Y | 71.90 | 1628.6819 | 13 | 0.7 | 815.3488 | 2 | 46.10 | 10 | F10:29493 | NaNaKA16\_F6.raw |  |  |  |  |  |  |  |  |  | 1.8887E7 |  |  |  | 1 | 0 | 0 | 0 | 0 | 0 | 0 | 0 | 0 | 0 | 1 | 0 | 0 | 0 | 22 | 34 | Carbamidomethylation | C3:Carbamidomethylation:1000.00;C6:Carbamidomethylation:1000.00;C11:Carbamidomethylation:1000.00 | PEAKS DB |
| total 1 peptides |  |  |  |  |  |  |  |  |  |  |  |  |  |  |  |  |  |  |  |  |  |  |  |  |  |  |  |  |  |  |  |  |  |  |  |  |  |  |  |  |  |  |  |
| --- | --- | --- | --- | --- | --- | --- | --- | --- | --- | --- | --- | --- | --- | --- | --- | --- | --- | --- | --- | --- | --- | --- | --- | --- | --- | --- | --- | --- | --- | --- | --- | --- | --- | --- | --- | --- | --- | --- | --- | --- | --- | --- | --- |

AAD39354.1

back to list

  

| Protein Coverage
| Supporting Peptides
|

Protein Coverage:

Supporting Peptides:

| Peptide | Uniq | -10lgP | Mass | Length | ppm | m/z | z | RT | Fraction | Scan | Source File | Area F1 | Area F10 | Area F11 | Area F12 | Area F13 | Area F2 | Area F3 | Area F4 | Area F5 | Area F6 | Area F7 | Area F8 | Area F9 | #Feature | #Feature F1 | #Feature F10 | #Feature F11 | #Feature F12 | #Feature F13 | #Feature F2 | #Feature F3 | #Feature F4 | #Feature F5 | #Feature F6 | #Feature F7 | #Feature F8 | #Feature F9 | Start | End | PTM | AScore | Found By |
| --- | --- | --- | --- | --- | --- | --- | --- | --- | --- | --- | --- | --- | --- | --- | --- | --- | --- | --- | --- | --- | --- | --- | --- | --- | --- | --- | --- | --- | --- | --- | --- | --- | --- | --- | --- | --- | --- | --- | --- | --- | --- | --- | --- |
| T.LTC(+57.02)LNC(+57.02)PEMFC(+57.02)GK.F | Y | 71.90 | 1628.6819 | 13 | 0.7 | 815.3488 | 2 | 46.10 | 10 | F10:29493 | NaNaKA16\_F6.raw |  |  |  |  |  |  |  |  |  | 1.8887E7 |  |  |  | 1 | 0 | 0 | 0 | 0 | 0 | 0 | 0 | 0 | 0 | 1 | 0 | 0 | 0 | 22 | 34 | Carbamidomethylation | C3:Carbamidomethylation:1000.00;C6:Carbamidomethylation:1000.00;C11:Carbamidomethylation:1000.00 | PEAKS DB |
| total 1 peptides |  |  |  |  |  |  |  |  |  |  |  |  |  |  |  |  |  |  |  |  |  |  |  |  |  |  |  |  |  |  |  |  |  |  |  |  |  |  |  |  |  |  |  |
| --- | --- | --- | --- | --- | --- | --- | --- | --- | --- | --- | --- | --- | --- | --- | --- | --- | --- | --- | --- | --- | --- | --- | --- | --- | --- | --- | --- | --- | --- | --- | --- | --- | --- | --- | --- | --- | --- | --- | --- | --- | --- | --- | --- |

Q9W7I3.1

back to list

  

| Protein Coverage
| Supporting Peptides
|

Protein Coverage:

Supporting Peptides:

| Peptide | Uniq | -10lgP | Mass | Length | ppm | m/z | z | RT | Fraction | Scan | Source File | Area F1 | Area F10 | Area F11 | Area F12 | Area F13 | Area F2 | Area F3 | Area F4 | Area F5 | Area F6 | Area F7 | Area F8 | Area F9 | #Feature | #Feature F1 | #Feature F10 | #Feature F11 | #Feature F12 | #Feature F13 | #Feature F2 | #Feature F3 | #Feature F4 | #Feature F5 | #Feature F6 | #Feature F7 | #Feature F8 | #Feature F9 | Start | End | PTM | AScore | Found By |
| --- | --- | --- | --- | --- | --- | --- | --- | --- | --- | --- | --- | --- | --- | --- | --- | --- | --- | --- | --- | --- | --- | --- | --- | --- | --- | --- | --- | --- | --- | --- | --- | --- | --- | --- | --- | --- | --- | --- | --- | --- | --- | --- | --- |
| T.LTC(+57.02)LNC(+57.02)PEMFC(+57.02)GK.F | Y | 71.90 | 1628.6819 | 13 | 0.7 | 815.3488 | 2 | 46.10 | 10 | F10:29493 | NaNaKA16\_F6.raw |  |  |  |  |  |  |  |  |  | 1.8887E7 |  |  |  | 1 | 0 | 0 | 0 | 0 | 0 | 0 | 0 | 0 | 0 | 1 | 0 | 0 | 0 | 22 | 34 | Carbamidomethylation | C3:Carbamidomethylation:1000.00;C6:Carbamidomethylation:1000.00;C11:Carbamidomethylation:1000.00 | PEAKS DB |
| total 1 peptides |  |  |  |  |  |  |  |  |  |  |  |  |  |  |  |  |  |  |  |  |  |  |  |  |  |  |  |  |  |  |  |  |  |  |  |  |  |  |  |  |  |  |  |
| --- | --- | --- | --- | --- | --- | --- | --- | --- | --- | --- | --- | --- | --- | --- | --- | --- | --- | --- | --- | --- | --- | --- | --- | --- | --- | --- | --- | --- | --- | --- | --- | --- | --- | --- | --- | --- | --- | --- | --- | --- | --- | --- | --- |

AAL87465.1

back to list

  

| Protein Coverage
| Supporting Peptides
|

Protein Coverage:

Supporting Peptides:

| Peptide | Uniq | -10lgP | Mass | Length | ppm | m/z | z | RT | Fraction | Scan | Source File | Area F1 | Area F10 | Area F11 | Area F12 | Area F13 | Area F2 | Area F3 | Area F4 | Area F5 | Area F6 | Area F7 | Area F8 | Area F9 | #Feature | #Feature F1 | #Feature F10 | #Feature F11 | #Feature F12 | #Feature F13 | #Feature F2 | #Feature F3 | #Feature F4 | #Feature F5 | #Feature F6 | #Feature F7 | #Feature F8 | #Feature F9 | Start | End | PTM | AScore | Found By |
| --- | --- | --- | --- | --- | --- | --- | --- | --- | --- | --- | --- | --- | --- | --- | --- | --- | --- | --- | --- | --- | --- | --- | --- | --- | --- | --- | --- | --- | --- | --- | --- | --- | --- | --- | --- | --- | --- | --- | --- | --- | --- | --- | --- |
| T.LTC(+57.02)LNC(+57.02)PEMFC(+57.02)GK.F | Y | 71.90 | 1628.6819 | 13 | 0.7 | 815.3488 | 2 | 46.10 | 10 | F10:29493 | NaNaKA16\_F6.raw |  |  |  |  |  |  |  |  |  | 1.8887E7 |  |  |  | 1 | 0 | 0 | 0 | 0 | 0 | 0 | 0 | 0 | 0 | 1 | 0 | 0 | 0 | 22 | 34 | Carbamidomethylation | C3:Carbamidomethylation:1000.00;C6:Carbamidomethylation:1000.00;C11:Carbamidomethylation:1000.00 | PEAKS DB |
| total 1 peptides |  |  |  |  |  |  |  |  |  |  |  |  |  |  |  |  |  |  |  |  |  |  |  |  |  |  |  |  |  |  |  |  |  |  |  |  |  |  |  |  |  |  |  |
| --- | --- | --- | --- | --- | --- | --- | --- | --- | --- | --- | --- | --- | --- | --- | --- | --- | --- | --- | --- | --- | --- | --- | --- | --- | --- | --- | --- | --- | --- | --- | --- | --- | --- | --- | --- | --- | --- | --- | --- | --- | --- | --- | --- |

C0HJW9.2

back to list

  

| Protein Coverage
| Supporting Peptides
|

Protein Coverage:

Supporting Peptides:

| Peptide | Uniq | -10lgP | Mass | Length | ppm | m/z | z | RT | Fraction | Scan | Source File | Area F1 | Area F10 | Area F11 | Area F12 | Area F13 | Area F2 | Area F3 | Area F4 | Area F5 | Area F6 | Area F7 | Area F8 | Area F9 | #Feature | #Feature F1 | #Feature F10 | #Feature F11 | #Feature F12 | #Feature F13 | #Feature F2 | #Feature F3 | #Feature F4 | #Feature F5 | #Feature F6 | #Feature F7 | #Feature F8 | #Feature F9 | Start | End | PTM | AScore | Found By |
| --- | --- | --- | --- | --- | --- | --- | --- | --- | --- | --- | --- | --- | --- | --- | --- | --- | --- | --- | --- | --- | --- | --- | --- | --- | --- | --- | --- | --- | --- | --- | --- | --- | --- | --- | --- | --- | --- | --- | --- | --- | --- | --- | --- |
| LTC(+57.02)LNC(+57.02)PEMFC(+57.02)GK.F | Y | 71.90 | 1628.6819 | 13 | 0.7 | 815.3488 | 2 | 46.10 | 10 | F10:29493 | NaNaKA16\_F6.raw |  |  |  |  |  |  |  |  |  | 1.8887E7 |  |  |  | 1 | 0 | 0 | 0 | 0 | 0 | 0 | 0 | 0 | 0 | 1 | 0 | 0 | 0 | 1 | 13 | Carbamidomethylation | C3:Carbamidomethylation:1000.00;C6:Carbamidomethylation:1000.00;C11:Carbamidomethylation:1000.00 | PEAKS DB |
| total 1 peptides |  |  |  |  |  |  |  |  |  |  |  |  |  |  |  |  |  |  |  |  |  |  |  |  |  |  |  |  |  |  |  |  |  |  |  |  |  |  |  |  |  |  |  |
| --- | --- | --- | --- | --- | --- | --- | --- | --- | --- | --- | --- | --- | --- | --- | --- | --- | --- | --- | --- | --- | --- | --- | --- | --- | --- | --- | --- | --- | --- | --- | --- | --- | --- | --- | --- | --- | --- | --- | --- | --- | --- | --- | --- |

E5L0E4.1

back to list

  

| Protein Coverage
| Supporting Peptides
|

Protein Coverage:

Supporting Peptides:

| Peptide | Uniq | -10lgP | Mass | Length | ppm | m/z | z | RT | Fraction | Scan | Source File | Area F1 | Area F10 | Area F11 | Area F12 | Area F13 | Area F2 | Area F3 | Area F4 | Area F5 | Area F6 | Area F7 | Area F8 | Area F9 | #Feature | #Feature F1 | #Feature F10 | #Feature F11 | #Feature F12 | #Feature F13 | #Feature F2 | #Feature F3 | #Feature F4 | #Feature F5 | #Feature F6 | #Feature F7 | #Feature F8 | #Feature F9 | Start | End | PTM | AScore | Found By |
| --- | --- | --- | --- | --- | --- | --- | --- | --- | --- | --- | --- | --- | --- | --- | --- | --- | --- | --- | --- | --- | --- | --- | --- | --- | --- | --- | --- | --- | --- | --- | --- | --- | --- | --- | --- | --- | --- | --- | --- | --- | --- | --- | --- |
| K.TSTYIAPLSLPSSPPR.V | Y | 71.71 | 1685.8988 | 16 | 0.9 | 843.9575 | 2 | 59.90 | 2 | F2:37072 | NaNaKA16\_F10.raw |  | 3.9103E6 | 3.6573E6 | 1.9316E6 |  |  |  |  |  |  |  |  |  | 4 | 0 | 1 | 2 | 1 | 0 | 0 | 0 | 0 | 0 | 0 | 0 | 0 | 0 | 120 | 135 |  |  | PEAKS DB |
| total 1 peptides |  |  |  |  |  |  |  |  |  |  |  |  |  |  |  |  |  |  |  |  |  |  |  |  |  |  |  |  |  |  |  |  |  |  |  |  |  |  |  |  |  |  |  |
| --- | --- | --- | --- | --- | --- | --- | --- | --- | --- | --- | --- | --- | --- | --- | --- | --- | --- | --- | --- | --- | --- | --- | --- | --- | --- | --- | --- | --- | --- | --- | --- | --- | --- | --- | --- | --- | --- | --- | --- | --- | --- | --- | --- |

ADP88560.1

back to list

  

| Protein Coverage
| Supporting Peptides
|

Protein Coverage:

Supporting Peptides:

| Peptide | Uniq | -10lgP | Mass | Length | ppm | m/z | z | RT | Fraction | Scan | Source File | Area F1 | Area F10 | Area F11 | Area F12 | Area F13 | Area F2 | Area F3 | Area F4 | Area F5 | Area F6 | Area F7 | Area F8 | Area F9 | #Feature | #Feature F1 | #Feature F10 | #Feature F11 | #Feature F12 | #Feature F13 | #Feature F2 | #Feature F3 | #Feature F4 | #Feature F5 | #Feature F6 | #Feature F7 | #Feature F8 | #Feature F9 | Start | End | PTM | AScore | Found By |
| --- | --- | --- | --- | --- | --- | --- | --- | --- | --- | --- | --- | --- | --- | --- | --- | --- | --- | --- | --- | --- | --- | --- | --- | --- | --- | --- | --- | --- | --- | --- | --- | --- | --- | --- | --- | --- | --- | --- | --- | --- | --- | --- | --- |
| K.TSTYIAPLSLPSSPPR.V | Y | 71.71 | 1685.8988 | 16 | 0.9 | 843.9575 | 2 | 59.90 | 2 | F2:37072 | NaNaKA16\_F10.raw |  | 3.9103E6 | 3.6573E6 | 1.9316E6 |  |  |  |  |  |  |  |  |  | 4 | 0 | 1 | 2 | 1 | 0 | 0 | 0 | 0 | 0 | 0 | 0 | 0 | 0 | 120 | 135 |  |  | PEAKS DB |
| total 1 peptides |  |  |  |  |  |  |  |  |  |  |  |  |  |  |  |  |  |  |  |  |  |  |  |  |  |  |  |  |  |  |  |  |  |  |  |  |  |  |  |  |  |  |  |
| --- | --- | --- | --- | --- | --- | --- | --- | --- | --- | --- | --- | --- | --- | --- | --- | --- | --- | --- | --- | --- | --- | --- | --- | --- | --- | --- | --- | --- | --- | --- | --- | --- | --- | --- | --- | --- | --- | --- | --- | --- | --- | --- | --- |

ETE73990.1

back to list

  

| Protein Coverage
| Supporting Peptides
|

Protein Coverage:

Supporting Peptides:

| Peptide | Uniq | -10lgP | Mass | Length | ppm | m/z | z | RT | Fraction | Scan | Source File | Area F1 | Area F10 | Area F11 | Area F12 | Area F13 | Area F2 | Area F3 | Area F4 | Area F5 | Area F6 | Area F7 | Area F8 | Area F9 | #Feature | #Feature F1 | #Feature F10 | #Feature F11 | #Feature F12 | #Feature F13 | #Feature F2 | #Feature F3 | #Feature F4 | #Feature F5 | #Feature F6 | #Feature F7 | #Feature F8 | #Feature F9 | Start | End | PTM | AScore | Found By |
| --- | --- | --- | --- | --- | --- | --- | --- | --- | --- | --- | --- | --- | --- | --- | --- | --- | --- | --- | --- | --- | --- | --- | --- | --- | --- | --- | --- | --- | --- | --- | --- | --- | --- | --- | --- | --- | --- | --- | --- | --- | --- | --- | --- |
| K.LNPDTLIGQVNLK.R | Y | 71.44 | 1423.8035 | 13 | 0.8 | 712.9095 | 2 | 62.87 | 2 | F2:39695 | NaNaKA16\_F10.raw |  | 7.3137E6 |  |  |  |  |  |  |  |  |  |  | 4.6581E6 | 2 | 0 | 1 | 0 | 0 | 0 | 0 | 0 | 0 | 0 | 0 | 0 | 0 | 1 | 499 | 511 |  |  | PEAKS DB |
| total 1 peptides |  |  |  |  |  |  |  |  |  |  |  |  |  |  |  |  |  |  |  |  |  |  |  |  |  |  |  |  |  |  |  |  |  |  |  |  |  |  |  |  |  |  |  |
| --- | --- | --- | --- | --- | --- | --- | --- | --- | --- | --- | --- | --- | --- | --- | --- | --- | --- | --- | --- | --- | --- | --- | --- | --- | --- | --- | --- | --- | --- | --- | --- | --- | --- | --- | --- | --- | --- | --- | --- | --- | --- | --- | --- |

XP\_032078632.1

back to list

  

| Protein Coverage
| Supporting Peptides
|

Protein Coverage:

Supporting Peptides:

| Peptide | Uniq | -10lgP | Mass | Length | ppm | m/z | z | RT | Fraction | Scan | Source File | Area F1 | Area F10 | Area F11 | Area F12 | Area F13 | Area F2 | Area F3 | Area F4 | Area F5 | Area F6 | Area F7 | Area F8 | Area F9 | #Feature | #Feature F1 | #Feature F10 | #Feature F11 | #Feature F12 | #Feature F13 | #Feature F2 | #Feature F3 | #Feature F4 | #Feature F5 | #Feature F6 | #Feature F7 | #Feature F8 | #Feature F9 | Start | End | PTM | AScore | Found By |
| --- | --- | --- | --- | --- | --- | --- | --- | --- | --- | --- | --- | --- | --- | --- | --- | --- | --- | --- | --- | --- | --- | --- | --- | --- | --- | --- | --- | --- | --- | --- | --- | --- | --- | --- | --- | --- | --- | --- | --- | --- | --- | --- | --- |
| K.LNPDTLIGQVNLK.G | Y | 71.44 | 1423.8035 | 13 | 0.8 | 712.9095 | 2 | 62.87 | 2 | F2:39695 | NaNaKA16\_F10.raw |  | 7.3137E6 |  |  |  |  |  |  |  |  |  |  | 4.6581E6 | 2 | 0 | 1 | 0 | 0 | 0 | 0 | 0 | 0 | 0 | 0 | 0 | 0 | 1 | 46 | 58 |  |  | PEAKS DB |
| total 1 peptides |  |  |  |  |  |  |  |  |  |  |  |  |  |  |  |  |  |  |  |  |  |  |  |  |  |  |  |  |  |  |  |  |  |  |  |  |  |  |  |  |  |  |  |
| --- | --- | --- | --- | --- | --- | --- | --- | --- | --- | --- | --- | --- | --- | --- | --- | --- | --- | --- | --- | --- | --- | --- | --- | --- | --- | --- | --- | --- | --- | --- | --- | --- | --- | --- | --- | --- | --- | --- | --- | --- | --- | --- | --- |

XP\_013911097.1

back to list

  

| Protein Coverage
| Supporting Peptides
|

Protein Coverage:

Supporting Peptides:

| Peptide | Uniq | -10lgP | Mass | Length | ppm | m/z | z | RT | Fraction | Scan | Source File | Area F1 | Area F10 | Area F11 | Area F12 | Area F13 | Area F2 | Area F3 | Area F4 | Area F5 | Area F6 | Area F7 | Area F8 | Area F9 | #Feature | #Feature F1 | #Feature F10 | #Feature F11 | #Feature F12 | #Feature F13 | #Feature F2 | #Feature F3 | #Feature F4 | #Feature F5 | #Feature F6 | #Feature F7 | #Feature F8 | #Feature F9 | Start | End | PTM | AScore | Found By |
| --- | --- | --- | --- | --- | --- | --- | --- | --- | --- | --- | --- | --- | --- | --- | --- | --- | --- | --- | --- | --- | --- | --- | --- | --- | --- | --- | --- | --- | --- | --- | --- | --- | --- | --- | --- | --- | --- | --- | --- | --- | --- | --- | --- |
| K.LNPDTLIGQVNLK.G | Y | 71.44 | 1423.8035 | 13 | 0.8 | 712.9095 | 2 | 62.87 | 2 | F2:39695 | NaNaKA16\_F10.raw |  | 7.3137E6 |  |  |  |  |  |  |  |  |  |  | 4.6581E6 | 2 | 0 | 1 | 0 | 0 | 0 | 0 | 0 | 0 | 0 | 0 | 0 | 0 | 1 | 46 | 58 |  |  | PEAKS DB |
| total 1 peptides |  |  |  |  |  |  |  |  |  |  |  |  |  |  |  |  |  |  |  |  |  |  |  |  |  |  |  |  |  |  |  |  |  |  |  |  |  |  |  |  |  |  |  |
| --- | --- | --- | --- | --- | --- | --- | --- | --- | --- | --- | --- | --- | --- | --- | --- | --- | --- | --- | --- | --- | --- | --- | --- | --- | --- | --- | --- | --- | --- | --- | --- | --- | --- | --- | --- | --- | --- | --- | --- | --- | --- | --- | --- |

XP\_013911096.1

back to list

  

| Protein Coverage
| Supporting Peptides
|

Protein Coverage:

Supporting Peptides:

| Peptide | Uniq | -10lgP | Mass | Length | ppm | m/z | z | RT | Fraction | Scan | Source File | Area F1 | Area F10 | Area F11 | Area F12 | Area F13 | Area F2 | Area F3 | Area F4 | Area F5 | Area F6 | Area F7 | Area F8 | Area F9 | #Feature | #Feature F1 | #Feature F10 | #Feature F11 | #Feature F12 | #Feature F13 | #Feature F2 | #Feature F3 | #Feature F4 | #Feature F5 | #Feature F6 | #Feature F7 | #Feature F8 | #Feature F9 | Start | End | PTM | AScore | Found By |
| --- | --- | --- | --- | --- | --- | --- | --- | --- | --- | --- | --- | --- | --- | --- | --- | --- | --- | --- | --- | --- | --- | --- | --- | --- | --- | --- | --- | --- | --- | --- | --- | --- | --- | --- | --- | --- | --- | --- | --- | --- | --- | --- | --- |
| K.LNPDTLIGQVNLK.G | Y | 71.44 | 1423.8035 | 13 | 0.8 | 712.9095 | 2 | 62.87 | 2 | F2:39695 | NaNaKA16\_F10.raw |  | 7.3137E6 |  |  |  |  |  |  |  |  |  |  | 4.6581E6 | 2 | 0 | 1 | 0 | 0 | 0 | 0 | 0 | 0 | 0 | 0 | 0 | 0 | 1 | 46 | 58 |  |  | PEAKS DB |
| total 1 peptides |  |  |  |  |  |  |  |  |  |  |  |  |  |  |  |  |  |  |  |  |  |  |  |  |  |  |  |  |  |  |  |  |  |  |  |  |  |  |  |  |  |  |  |
| --- | --- | --- | --- | --- | --- | --- | --- | --- | --- | --- | --- | --- | --- | --- | --- | --- | --- | --- | --- | --- | --- | --- | --- | --- | --- | --- | --- | --- | --- | --- | --- | --- | --- | --- | --- | --- | --- | --- | --- | --- | --- | --- | --- |

XP\_013911098.1

back to list

  

| Protein Coverage
| Supporting Peptides
|

Protein Coverage:

Supporting Peptides:

| Peptide | Uniq | -10lgP | Mass | Length | ppm | m/z | z | RT | Fraction | Scan | Source File | Area F1 | Area F10 | Area F11 | Area F12 | Area F13 | Area F2 | Area F3 | Area F4 | Area F5 | Area F6 | Area F7 | Area F8 | Area F9 | #Feature | #Feature F1 | #Feature F10 | #Feature F11 | #Feature F12 | #Feature F13 | #Feature F2 | #Feature F3 | #Feature F4 | #Feature F5 | #Feature F6 | #Feature F7 | #Feature F8 | #Feature F9 | Start | End | PTM | AScore | Found By |
| --- | --- | --- | --- | --- | --- | --- | --- | --- | --- | --- | --- | --- | --- | --- | --- | --- | --- | --- | --- | --- | --- | --- | --- | --- | --- | --- | --- | --- | --- | --- | --- | --- | --- | --- | --- | --- | --- | --- | --- | --- | --- | --- | --- |
| K.LNPDTLIGQVNLK.G | Y | 71.44 | 1423.8035 | 13 | 0.8 | 712.9095 | 2 | 62.87 | 2 | F2:39695 | NaNaKA16\_F10.raw |  | 7.3137E6 |  |  |  |  |  |  |  |  |  |  | 4.6581E6 | 2 | 0 | 1 | 0 | 0 | 0 | 0 | 0 | 0 | 0 | 0 | 0 | 0 | 1 | 46 | 58 |  |  | PEAKS DB |
| total 1 peptides |  |  |  |  |  |  |  |  |  |  |  |  |  |  |  |  |  |  |  |  |  |  |  |  |  |  |  |  |  |  |  |  |  |  |  |  |  |  |  |  |  |  |  |
| --- | --- | --- | --- | --- | --- | --- | --- | --- | --- | --- | --- | --- | --- | --- | --- | --- | --- | --- | --- | --- | --- | --- | --- | --- | --- | --- | --- | --- | --- | --- | --- | --- | --- | --- | --- | --- | --- | --- | --- | --- | --- | --- | --- |

XP\_026526159.1

back to list

  

| Protein Coverage
| Supporting Peptides
|

Protein Coverage:

Supporting Peptides:

| Peptide | Uniq | -10lgP | Mass | Length | ppm | m/z | z | RT | Fraction | Scan | Source File | Area F1 | Area F10 | Area F11 | Area F12 | Area F13 | Area F2 | Area F3 | Area F4 | Area F5 | Area F6 | Area F7 | Area F8 | Area F9 | #Feature | #Feature F1 | #Feature F10 | #Feature F11 | #Feature F12 | #Feature F13 | #Feature F2 | #Feature F3 | #Feature F4 | #Feature F5 | #Feature F6 | #Feature F7 | #Feature F8 | #Feature F9 | Start | End | PTM | AScore | Found By |
| --- | --- | --- | --- | --- | --- | --- | --- | --- | --- | --- | --- | --- | --- | --- | --- | --- | --- | --- | --- | --- | --- | --- | --- | --- | --- | --- | --- | --- | --- | --- | --- | --- | --- | --- | --- | --- | --- | --- | --- | --- | --- | --- | --- |
| K.LNPDTLIGQVNLK.R | Y | 71.44 | 1423.8035 | 13 | 0.8 | 712.9095 | 2 | 62.87 | 2 | F2:39695 | NaNaKA16\_F10.raw |  | 7.3137E6 |  |  |  |  |  |  |  |  |  |  | 4.6581E6 | 2 | 0 | 1 | 0 | 0 | 0 | 0 | 0 | 0 | 0 | 0 | 0 | 0 | 1 | 46 | 58 |  |  | PEAKS DB |
| total 1 peptides |  |  |  |  |  |  |  |  |  |  |  |  |  |  |  |  |  |  |  |  |  |  |  |  |  |  |  |  |  |  |  |  |  |  |  |  |  |  |  |  |  |  |  |
| --- | --- | --- | --- | --- | --- | --- | --- | --- | --- | --- | --- | --- | --- | --- | --- | --- | --- | --- | --- | --- | --- | --- | --- | --- | --- | --- | --- | --- | --- | --- | --- | --- | --- | --- | --- | --- | --- | --- | --- | --- | --- | --- | --- |

XP\_026526158.1

back to list

  

| Protein Coverage
| Supporting Peptides
|

Protein Coverage:

Supporting Peptides:

| Peptide | Uniq | -10lgP | Mass | Length | ppm | m/z | z | RT | Fraction | Scan | Source File | Area F1 | Area F10 | Area F11 | Area F12 | Area F13 | Area F2 | Area F3 | Area F4 | Area F5 | Area F6 | Area F7 | Area F8 | Area F9 | #Feature | #Feature F1 | #Feature F10 | #Feature F11 | #Feature F12 | #Feature F13 | #Feature F2 | #Feature F3 | #Feature F4 | #Feature F5 | #Feature F6 | #Feature F7 | #Feature F8 | #Feature F9 | Start | End | PTM | AScore | Found By |
| --- | --- | --- | --- | --- | --- | --- | --- | --- | --- | --- | --- | --- | --- | --- | --- | --- | --- | --- | --- | --- | --- | --- | --- | --- | --- | --- | --- | --- | --- | --- | --- | --- | --- | --- | --- | --- | --- | --- | --- | --- | --- | --- | --- |
| K.LNPDTLIGQVNLK.R | Y | 71.44 | 1423.8035 | 13 | 0.8 | 712.9095 | 2 | 62.87 | 2 | F2:39695 | NaNaKA16\_F10.raw |  | 7.3137E6 |  |  |  |  |  |  |  |  |  |  | 4.6581E6 | 2 | 0 | 1 | 0 | 0 | 0 | 0 | 0 | 0 | 0 | 0 | 0 | 0 | 1 | 46 | 58 |  |  | PEAKS DB |
| total 1 peptides |  |  |  |  |  |  |  |  |  |  |  |  |  |  |  |  |  |  |  |  |  |  |  |  |  |  |  |  |  |  |  |  |  |  |  |  |  |  |  |  |  |  |  |
| --- | --- | --- | --- | --- | --- | --- | --- | --- | --- | --- | --- | --- | --- | --- | --- | --- | --- | --- | --- | --- | --- | --- | --- | --- | --- | --- | --- | --- | --- | --- | --- | --- | --- | --- | --- | --- | --- | --- | --- | --- | --- | --- | --- |

XP\_026526157.1

back to list

  

| Protein Coverage
| Supporting Peptides
|

Protein Coverage:

Supporting Peptides:

| Peptide | Uniq | -10lgP | Mass | Length | ppm | m/z | z | RT | Fraction | Scan | Source File | Area F1 | Area F10 | Area F11 | Area F12 | Area F13 | Area F2 | Area F3 | Area F4 | Area F5 | Area F6 | Area F7 | Area F8 | Area F9 | #Feature | #Feature F1 | #Feature F10 | #Feature F11 | #Feature F12 | #Feature F13 | #Feature F2 | #Feature F3 | #Feature F4 | #Feature F5 | #Feature F6 | #Feature F7 | #Feature F8 | #Feature F9 | Start | End | PTM | AScore | Found By |
| --- | --- | --- | --- | --- | --- | --- | --- | --- | --- | --- | --- | --- | --- | --- | --- | --- | --- | --- | --- | --- | --- | --- | --- | --- | --- | --- | --- | --- | --- | --- | --- | --- | --- | --- | --- | --- | --- | --- | --- | --- | --- | --- | --- |
| K.LNPDTLIGQVNLK.R | Y | 71.44 | 1423.8035 | 13 | 0.8 | 712.9095 | 2 | 62.87 | 2 | F2:39695 | NaNaKA16\_F10.raw |  | 7.3137E6 |  |  |  |  |  |  |  |  |  |  | 4.6581E6 | 2 | 0 | 1 | 0 | 0 | 0 | 0 | 0 | 0 | 0 | 0 | 0 | 0 | 1 | 46 | 58 |  |  | PEAKS DB |
| total 1 peptides |  |  |  |  |  |  |  |  |  |  |  |  |  |  |  |  |  |  |  |  |  |  |  |  |  |  |  |  |  |  |  |  |  |  |  |  |  |  |  |  |  |  |  |
| --- | --- | --- | --- | --- | --- | --- | --- | --- | --- | --- | --- | --- | --- | --- | --- | --- | --- | --- | --- | --- | --- | --- | --- | --- | --- | --- | --- | --- | --- | --- | --- | --- | --- | --- | --- | --- | --- | --- | --- | --- | --- | --- | --- |

ETE67110.1

back to list

  

| Protein Coverage
| Supporting Peptides
|

Protein Coverage:

Supporting Peptides:

| Peptide | Uniq | -10lgP | Mass | Length | ppm | m/z | z | RT | Fraction | Scan | Source File | Area F1 | Area F10 | Area F11 | Area F12 | Area F13 | Area F2 | Area F3 | Area F4 | Area F5 | Area F6 | Area F7 | Area F8 | Area F9 | #Feature | #Feature F1 | #Feature F10 | #Feature F11 | #Feature F12 | #Feature F13 | #Feature F2 | #Feature F3 | #Feature F4 | #Feature F5 | #Feature F6 | #Feature F7 | #Feature F8 | #Feature F9 | Start | End | PTM | AScore | Found By |
| --- | --- | --- | --- | --- | --- | --- | --- | --- | --- | --- | --- | --- | --- | --- | --- | --- | --- | --- | --- | --- | --- | --- | --- | --- | --- | --- | --- | --- | --- | --- | --- | --- | --- | --- | --- | --- | --- | --- | --- | --- | --- | --- | --- |
| R.MHLETILQELK.A | Y | 70.63 | 1353.7327 | 11 | 0.8 | 677.8741 | 2 | 62.78 | 3 | F3:38731 | NaNaKA16\_F11.raw |  | 1.0165E6 | 1.4658E6 |  |  |  |  |  |  |  |  |  |  | 4 | 0 | 2 | 2 | 0 | 0 | 0 | 0 | 0 | 0 | 0 | 0 | 0 | 0 | 74 | 84 |  |  | PEAKS DB |
| total 1 peptides |  |  |  |  |  |  |  |  |  |  |  |  |  |  |  |  |  |  |  |  |  |  |  |  |  |  |  |  |  |  |  |  |  |  |  |  |  |  |  |  |  |  |  |
| --- | --- | --- | --- | --- | --- | --- | --- | --- | --- | --- | --- | --- | --- | --- | --- | --- | --- | --- | --- | --- | --- | --- | --- | --- | --- | --- | --- | --- | --- | --- | --- | --- | --- | --- | --- | --- | --- | --- | --- | --- | --- | --- | --- |

JAB53705.1

back to list

  

| Protein Coverage
| Supporting Peptides
|

Protein Coverage:

Supporting Peptides:

| Peptide | Uniq | -10lgP | Mass | Length | ppm | m/z | z | RT | Fraction | Scan | Source File | Area F1 | Area F10 | Area F11 | Area F12 | Area F13 | Area F2 | Area F3 | Area F4 | Area F5 | Area F6 | Area F7 | Area F8 | Area F9 | #Feature | #Feature F1 | #Feature F10 | #Feature F11 | #Feature F12 | #Feature F13 | #Feature F2 | #Feature F3 | #Feature F4 | #Feature F5 | #Feature F6 | #Feature F7 | #Feature F8 | #Feature F9 | Start | End | PTM | AScore | Found By |
| --- | --- | --- | --- | --- | --- | --- | --- | --- | --- | --- | --- | --- | --- | --- | --- | --- | --- | --- | --- | --- | --- | --- | --- | --- | --- | --- | --- | --- | --- | --- | --- | --- | --- | --- | --- | --- | --- | --- | --- | --- | --- | --- | --- |
| R.FHLGTPVASTQFVTK.D | Y | 47.35 | 1631.8671 | 15 | 1.0 | 544.9635 | 3 | 44.16 | 4 | F4:25169 | NaNaKA16\_F12.raw |  |  |  | 1.0968E6 |  |  |  |  |  |  |  |  |  | 1 | 0 | 0 | 0 | 1 | 0 | 0 | 0 | 0 | 0 | 0 | 0 | 0 | 0 | 184 | 198 |  |  | PEAKS DB |
| K.YVLLMVDPDAPSR.S | Y | 46.37 | 1474.7490 | 13 | 0.1 | 738.3818 | 2 | 65.12 | 4 | F4:42632 | NaNaKA16\_F12.raw |  |  |  | 4.9917E5 |  |  |  |  |  |  |  |  |  | 1 | 0 | 0 | 0 | 1 | 0 | 0 | 0 | 0 | 0 | 0 | 0 | 0 | 0 | 89 | 101 |  |  | PEAKS DB |
| total 2 peptides |  |  |  |  |  |  |  |  |  |  |  |  |  |  |  |  |  |  |  |  |  |  |  |  |  |  |  |  |  |  |  |  |  |  |  |  |  |  |  |  |  |  |  |
| --- | --- | --- | --- | --- | --- | --- | --- | --- | --- | --- | --- | --- | --- | --- | --- | --- | --- | --- | --- | --- | --- | --- | --- | --- | --- | --- | --- | --- | --- | --- | --- | --- | --- | --- | --- | --- | --- | --- | --- | --- | --- | --- | --- |

XP\_026539772.1

back to list

  

| Protein Coverage
| Supporting Peptides
|

Protein Coverage:

Supporting Peptides:

| Peptide | Uniq | -10lgP | Mass | Length | ppm | m/z | z | RT | Fraction | Scan | Source File | Area F1 | Area F10 | Area F11 | Area F12 | Area F13 | Area F2 | Area F3 | Area F4 | Area F5 | Area F6 | Area F7 | Area F8 | Area F9 | #Feature | #Feature F1 | #Feature F10 | #Feature F11 | #Feature F12 | #Feature F13 | #Feature F2 | #Feature F3 | #Feature F4 | #Feature F5 | #Feature F6 | #Feature F7 | #Feature F8 | #Feature F9 | Start | End | PTM | AScore | Found By |
| --- | --- | --- | --- | --- | --- | --- | --- | --- | --- | --- | --- | --- | --- | --- | --- | --- | --- | --- | --- | --- | --- | --- | --- | --- | --- | --- | --- | --- | --- | --- | --- | --- | --- | --- | --- | --- | --- | --- | --- | --- | --- | --- | --- |
| R.FHLGTPVASTQFVTK.D | Y | 47.35 | 1631.8671 | 15 | 1.0 | 544.9635 | 3 | 44.16 | 4 | F4:25169 | NaNaKA16\_F12.raw |  |  |  | 1.0968E6 |  |  |  |  |  |  |  |  |  | 1 | 0 | 0 | 0 | 1 | 0 | 0 | 0 | 0 | 0 | 0 | 0 | 0 | 0 | 184 | 198 |  |  | PEAKS DB |
| K.YVLIMVDPDAPSR.S | Y | 46.37 | 1474.7490 | 13 | 0.1 | 738.3818 | 2 | 65.12 | 4 | F4:42632 | NaNaKA16\_F12.raw |  |  |  | 4.9917E5 |  |  |  |  |  |  |  |  |  | 1 | 0 | 0 | 0 | 1 | 0 | 0 | 0 | 0 | 0 | 0 | 0 | 0 | 0 | 89 | 101 |  |  | PEAKS DB |
| total 2 peptides |  |  |  |  |  |  |  |  |  |  |  |  |  |  |  |  |  |  |  |  |  |  |  |  |  |  |  |  |  |  |  |  |  |  |  |  |  |  |  |  |  |  |  |
| --- | --- | --- | --- | --- | --- | --- | --- | --- | --- | --- | --- | --- | --- | --- | --- | --- | --- | --- | --- | --- | --- | --- | --- | --- | --- | --- | --- | --- | --- | --- | --- | --- | --- | --- | --- | --- | --- | --- | --- | --- | --- | --- | --- |

XP\_026539773.1

back to list

  

| Protein Coverage
| Supporting Peptides
|

Protein Coverage:

Supporting Peptides:

| Peptide | Uniq | -10lgP | Mass | Length | ppm | m/z | z | RT | Fraction | Scan | Source File | Area F1 | Area F10 | Area F11 | Area F12 | Area F13 | Area F2 | Area F3 | Area F4 | Area F5 | Area F6 | Area F7 | Area F8 | Area F9 | #Feature | #Feature F1 | #Feature F10 | #Feature F11 | #Feature F12 | #Feature F13 | #Feature F2 | #Feature F3 | #Feature F4 | #Feature F5 | #Feature F6 | #Feature F7 | #Feature F8 | #Feature F9 | Start | End | PTM | AScore | Found By |
| --- | --- | --- | --- | --- | --- | --- | --- | --- | --- | --- | --- | --- | --- | --- | --- | --- | --- | --- | --- | --- | --- | --- | --- | --- | --- | --- | --- | --- | --- | --- | --- | --- | --- | --- | --- | --- | --- | --- | --- | --- | --- | --- | --- |
| R.FHLGTPVASTQFVTK.D | Y | 47.35 | 1631.8671 | 15 | 1.0 | 544.9635 | 3 | 44.16 | 4 | F4:25169 | NaNaKA16\_F12.raw |  |  |  | 1.0968E6 |  |  |  |  |  |  |  |  |  | 1 | 0 | 0 | 0 | 1 | 0 | 0 | 0 | 0 | 0 | 0 | 0 | 0 | 0 | 184 | 198 |  |  | PEAKS DB |
| K.YVLIMVDPDAPSR.S | Y | 46.37 | 1474.7490 | 13 | 0.1 | 738.3818 | 2 | 65.12 | 4 | F4:42632 | NaNaKA16\_F12.raw |  |  |  | 4.9917E5 |  |  |  |  |  |  |  |  |  | 1 | 0 | 0 | 0 | 1 | 0 | 0 | 0 | 0 | 0 | 0 | 0 | 0 | 0 | 89 | 101 |  |  | PEAKS DB |
| total 2 peptides |  |  |  |  |  |  |  |  |  |  |  |  |  |  |  |  |  |  |  |  |  |  |  |  |  |  |  |  |  |  |  |  |  |  |  |  |  |  |  |  |  |  |  |
| --- | --- | --- | --- | --- | --- | --- | --- | --- | --- | --- | --- | --- | --- | --- | --- | --- | --- | --- | --- | --- | --- | --- | --- | --- | --- | --- | --- | --- | --- | --- | --- | --- | --- | --- | --- | --- | --- | --- | --- | --- | --- | --- | --- |

XP\_026570986.1

back to list

  

| Protein Coverage
| Supporting Peptides
|

Protein Coverage:

Supporting Peptides:

| Peptide | Uniq | -10lgP | Mass | Length | ppm | m/z | z | RT | Fraction | Scan | Source File | Area F1 | Area F10 | Area F11 | Area F12 | Area F13 | Area F2 | Area F3 | Area F4 | Area F5 | Area F6 | Area F7 | Area F8 | Area F9 | #Feature | #Feature F1 | #Feature F10 | #Feature F11 | #Feature F12 | #Feature F13 | #Feature F2 | #Feature F3 | #Feature F4 | #Feature F5 | #Feature F6 | #Feature F7 | #Feature F8 | #Feature F9 | Start | End | PTM | AScore | Found By |
| --- | --- | --- | --- | --- | --- | --- | --- | --- | --- | --- | --- | --- | --- | --- | --- | --- | --- | --- | --- | --- | --- | --- | --- | --- | --- | --- | --- | --- | --- | --- | --- | --- | --- | --- | --- | --- | --- | --- | --- | --- | --- | --- | --- |
| R.FHLGTPVASTQFVTK.D | Y | 47.35 | 1631.8671 | 15 | 1.0 | 544.9635 | 3 | 44.16 | 4 | F4:25169 | NaNaKA16\_F12.raw |  |  |  | 1.0968E6 |  |  |  |  |  |  |  |  |  | 1 | 0 | 0 | 0 | 1 | 0 | 0 | 0 | 0 | 0 | 0 | 0 | 0 | 0 | 184 | 198 |  |  | PEAKS DB |
| K.YVLIMVDPDAPSR.S | Y | 46.37 | 1474.7490 | 13 | 0.1 | 738.3818 | 2 | 65.12 | 4 | F4:42632 | NaNaKA16\_F12.raw |  |  |  | 4.9917E5 |  |  |  |  |  |  |  |  |  | 1 | 0 | 0 | 0 | 1 | 0 | 0 | 0 | 0 | 0 | 0 | 0 | 0 | 0 | 89 | 101 |  |  | PEAKS DB |
| total 2 peptides |  |  |  |  |  |  |  |  |  |  |  |  |  |  |  |  |  |  |  |  |  |  |  |  |  |  |  |  |  |  |  |  |  |  |  |  |  |  |  |  |  |  |  |
| --- | --- | --- | --- | --- | --- | --- | --- | --- | --- | --- | --- | --- | --- | --- | --- | --- | --- | --- | --- | --- | --- | --- | --- | --- | --- | --- | --- | --- | --- | --- | --- | --- | --- | --- | --- | --- | --- | --- | --- | --- | --- | --- | --- |

XP\_026570987.1

back to list

  

| Protein Coverage
| Supporting Peptides
|

Protein Coverage:

Supporting Peptides:

| Peptide | Uniq | -10lgP | Mass | Length | ppm | m/z | z | RT | Fraction | Scan | Source File | Area F1 | Area F10 | Area F11 | Area F12 | Area F13 | Area F2 | Area F3 | Area F4 | Area F5 | Area F6 | Area F7 | Area F8 | Area F9 | #Feature | #Feature F1 | #Feature F10 | #Feature F11 | #Feature F12 | #Feature F13 | #Feature F2 | #Feature F3 | #Feature F4 | #Feature F5 | #Feature F6 | #Feature F7 | #Feature F8 | #Feature F9 | Start | End | PTM | AScore | Found By |
| --- | --- | --- | --- | --- | --- | --- | --- | --- | --- | --- | --- | --- | --- | --- | --- | --- | --- | --- | --- | --- | --- | --- | --- | --- | --- | --- | --- | --- | --- | --- | --- | --- | --- | --- | --- | --- | --- | --- | --- | --- | --- | --- | --- |
| R.FHLGTPVASTQFVTK.D | Y | 47.35 | 1631.8671 | 15 | 1.0 | 544.9635 | 3 | 44.16 | 4 | F4:25169 | NaNaKA16\_F12.raw |  |  |  | 1.0968E6 |  |  |  |  |  |  |  |  |  | 1 | 0 | 0 | 0 | 1 | 0 | 0 | 0 | 0 | 0 | 0 | 0 | 0 | 0 | 184 | 198 |  |  | PEAKS DB |
| K.YVLIMVDPDAPSR.S | Y | 46.37 | 1474.7490 | 13 | 0.1 | 738.3818 | 2 | 65.12 | 4 | F4:42632 | NaNaKA16\_F12.raw |  |  |  | 4.9917E5 |  |  |  |  |  |  |  |  |  | 1 | 0 | 0 | 0 | 1 | 0 | 0 | 0 | 0 | 0 | 0 | 0 | 0 | 0 | 89 | 101 |  |  | PEAKS DB |
| total 2 peptides |  |  |  |  |  |  |  |  |  |  |  |  |  |  |  |  |  |  |  |  |  |  |  |  |  |  |  |  |  |  |  |  |  |  |  |  |  |  |  |  |  |  |  |
| --- | --- | --- | --- | --- | --- | --- | --- | --- | --- | --- | --- | --- | --- | --- | --- | --- | --- | --- | --- | --- | --- | --- | --- | --- | --- | --- | --- | --- | --- | --- | --- | --- | --- | --- | --- | --- | --- | --- | --- | --- | --- | --- | --- |

JAC94989.1

back to list

  

| Protein Coverage
| Supporting Peptides
|

Protein Coverage:

Supporting Peptides:

| Peptide | Uniq | -10lgP | Mass | Length | ppm | m/z | z | RT | Fraction | Scan | Source File | Area F1 | Area F10 | Area F11 | Area F12 | Area F13 | Area F2 | Area F3 | Area F4 | Area F5 | Area F6 | Area F7 | Area F8 | Area F9 | #Feature | #Feature F1 | #Feature F10 | #Feature F11 | #Feature F12 | #Feature F13 | #Feature F2 | #Feature F3 | #Feature F4 | #Feature F5 | #Feature F6 | #Feature F7 | #Feature F8 | #Feature F9 | Start | End | PTM | AScore | Found By |
| --- | --- | --- | --- | --- | --- | --- | --- | --- | --- | --- | --- | --- | --- | --- | --- | --- | --- | --- | --- | --- | --- | --- | --- | --- | --- | --- | --- | --- | --- | --- | --- | --- | --- | --- | --- | --- | --- | --- | --- | --- | --- | --- | --- |
| K.QNSGTYNNQYMILDTK.K | N | 46.65 | 1888.8625 | 16 | -0.1 | 945.4385 | 2 | 44.83 | 5 | F5:18100 | NaNaKA16\_F13.raw |  |  |  |  | 7.159E5 |  |  |  |  |  |  |  |  | 1 | 0 | 0 | 0 | 0 | 1 | 0 | 0 | 0 | 0 | 0 | 0 | 0 | 0 | 351 | 366 |  |  | PEAKS DB |
| K.QVVPESLLAWER.V | Y | 45.42 | 1425.7616 | 12 | 0.0 | 713.8881 | 2 | 101.54 | 5 | F5:38599 | NaNaKA16\_F13.raw |  |  |  |  | 1.7683E4 |  |  |  |  |  |  |  |  | 1 | 0 | 0 | 0 | 0 | 1 | 0 | 0 | 0 | 0 | 0 | 0 | 0 | 0 | 319 | 330 |  |  | PEAKS DB |
| total 2 peptides |  |  |  |  |  |  |  |  |  |  |  |  |  |  |  |  |  |  |  |  |  |  |  |  |  |  |  |  |  |  |  |  |  |  |  |  |  |  |  |  |  |  |  |
| --- | --- | --- | --- | --- | --- | --- | --- | --- | --- | --- | --- | --- | --- | --- | --- | --- | --- | --- | --- | --- | --- | --- | --- | --- | --- | --- | --- | --- | --- | --- | --- | --- | --- | --- | --- | --- | --- | --- | --- | --- | --- | --- | --- |

JAB52813.1

back to list

  

| Protein Coverage
| Supporting Peptides
|

Protein Coverage:

Supporting Peptides:

| Peptide | Uniq | -10lgP | Mass | Length | ppm | m/z | z | RT | Fraction | Scan | Source File | Area F1 | Area F10 | Area F11 | Area F12 | Area F13 | Area F2 | Area F3 | Area F4 | Area F5 | Area F6 | Area F7 | Area F8 | Area F9 | #Feature | #Feature F1 | #Feature F10 | #Feature F11 | #Feature F12 | #Feature F13 | #Feature F2 | #Feature F3 | #Feature F4 | #Feature F5 | #Feature F6 | #Feature F7 | #Feature F8 | #Feature F9 | Start | End | PTM | AScore | Found By |
| --- | --- | --- | --- | --- | --- | --- | --- | --- | --- | --- | --- | --- | --- | --- | --- | --- | --- | --- | --- | --- | --- | --- | --- | --- | --- | --- | --- | --- | --- | --- | --- | --- | --- | --- | --- | --- | --- | --- | --- | --- | --- | --- | --- |
| A.DYGC(+57.02)YC(+57.02)GFGGSGTPVDQLDR.C | Y | 69.00 | 2222.8997 | 20 | 1.0 | 1112.4583 | 2 | 63.32 | 2 | F2:40217 | NaNaKA16\_F10.raw |  | 2.1439E6 |  |  |  |  |  |  |  |  |  |  | 1.8341E6 | 2 | 0 | 1 | 0 | 0 | 0 | 0 | 0 | 0 | 0 | 0 | 0 | 0 | 1 | 49 | 68 | Carbamidomethylation | C4:Carbamidomethylation:1000.00;C6:Carbamidomethylation:1000.00 | PEAKS DB |
| total 1 peptides |  |  |  |  |  |  |  |  |  |  |  |  |  |  |  |  |  |  |  |  |  |  |  |  |  |  |  |  |  |  |  |  |  |  |  |  |  |  |  |  |  |  |  |
| --- | --- | --- | --- | --- | --- | --- | --- | --- | --- | --- | --- | --- | --- | --- | --- | --- | --- | --- | --- | --- | --- | --- | --- | --- | --- | --- | --- | --- | --- | --- | --- | --- | --- | --- | --- | --- | --- | --- | --- | --- | --- | --- | --- |

JAI09045.1

back to list

  

| Protein Coverage
| Supporting Peptides
|

Protein Coverage:

Supporting Peptides:

| Peptide | Uniq | -10lgP | Mass | Length | ppm | m/z | z | RT | Fraction | Scan | Source File | Area F1 | Area F10 | Area F11 | Area F12 | Area F13 | Area F2 | Area F3 | Area F4 | Area F5 | Area F6 | Area F7 | Area F8 | Area F9 | #Feature | #Feature F1 | #Feature F10 | #Feature F11 | #Feature F12 | #Feature F13 | #Feature F2 | #Feature F3 | #Feature F4 | #Feature F5 | #Feature F6 | #Feature F7 | #Feature F8 | #Feature F9 | Start | End | PTM | AScore | Found By |
| --- | --- | --- | --- | --- | --- | --- | --- | --- | --- | --- | --- | --- | --- | --- | --- | --- | --- | --- | --- | --- | --- | --- | --- | --- | --- | --- | --- | --- | --- | --- | --- | --- | --- | --- | --- | --- | --- | --- | --- | --- | --- | --- | --- |
| A.DYGC(+57.02)YC(+57.02)GFGGSGTPVDQLDR.C | Y | 69.00 | 2222.8997 | 20 | 1.0 | 1112.4583 | 2 | 63.32 | 2 | F2:40217 | NaNaKA16\_F10.raw |  | 2.1439E6 |  |  |  |  |  |  |  |  |  |  | 1.8341E6 | 2 | 0 | 1 | 0 | 0 | 0 | 0 | 0 | 0 | 0 | 0 | 0 | 0 | 1 | 49 | 68 | Carbamidomethylation | C4:Carbamidomethylation:1000.00;C6:Carbamidomethylation:1000.00 | PEAKS DB |
| total 1 peptides |  |  |  |  |  |  |  |  |  |  |  |  |  |  |  |  |  |  |  |  |  |  |  |  |  |  |  |  |  |  |  |  |  |  |  |  |  |  |  |  |  |  |  |
| --- | --- | --- | --- | --- | --- | --- | --- | --- | --- | --- | --- | --- | --- | --- | --- | --- | --- | --- | --- | --- | --- | --- | --- | --- | --- | --- | --- | --- | --- | --- | --- | --- | --- | --- | --- | --- | --- | --- | --- | --- | --- | --- | --- |

JAS05034.1

back to list

  

| Protein Coverage
| Supporting Peptides
|

Protein Coverage:

Supporting Peptides:

| Peptide | Uniq | -10lgP | Mass | Length | ppm | m/z | z | RT | Fraction | Scan | Source File | Area F1 | Area F10 | Area F11 | Area F12 | Area F13 | Area F2 | Area F3 | Area F4 | Area F5 | Area F6 | Area F7 | Area F8 | Area F9 | #Feature | #Feature F1 | #Feature F10 | #Feature F11 | #Feature F12 | #Feature F13 | #Feature F2 | #Feature F3 | #Feature F4 | #Feature F5 | #Feature F6 | #Feature F7 | #Feature F8 | #Feature F9 | Start | End | PTM | AScore | Found By |
| --- | --- | --- | --- | --- | --- | --- | --- | --- | --- | --- | --- | --- | --- | --- | --- | --- | --- | --- | --- | --- | --- | --- | --- | --- | --- | --- | --- | --- | --- | --- | --- | --- | --- | --- | --- | --- | --- | --- | --- | --- | --- | --- | --- |
| A.DYGC(+57.02)YC(+57.02)GFGGSGTPVDQLDR.C | Y | 69.00 | 2222.8997 | 20 | 1.0 | 1112.4583 | 2 | 63.32 | 2 | F2:40217 | NaNaKA16\_F10.raw |  | 2.1439E6 |  |  |  |  |  |  |  |  |  |  | 1.8341E6 | 2 | 0 | 1 | 0 | 0 | 0 | 0 | 0 | 0 | 0 | 0 | 0 | 0 | 1 | 49 | 68 | Carbamidomethylation | C4:Carbamidomethylation:1000.00;C6:Carbamidomethylation:1000.00 | PEAKS DB |
| total 1 peptides |  |  |  |  |  |  |  |  |  |  |  |  |  |  |  |  |  |  |  |  |  |  |  |  |  |  |  |  |  |  |  |  |  |  |  |  |  |  |  |  |  |  |  |
| --- | --- | --- | --- | --- | --- | --- | --- | --- | --- | --- | --- | --- | --- | --- | --- | --- | --- | --- | --- | --- | --- | --- | --- | --- | --- | --- | --- | --- | --- | --- | --- | --- | --- | --- | --- | --- | --- | --- | --- | --- | --- | --- | --- |

JAS05141.1

back to list

  

| Protein Coverage
| Supporting Peptides
|

Protein Coverage:

Supporting Peptides:

| Peptide | Uniq | -10lgP | Mass | Length | ppm | m/z | z | RT | Fraction | Scan | Source File | Area F1 | Area F10 | Area F11 | Area F12 | Area F13 | Area F2 | Area F3 | Area F4 | Area F5 | Area F6 | Area F7 | Area F8 | Area F9 | #Feature | #Feature F1 | #Feature F10 | #Feature F11 | #Feature F12 | #Feature F13 | #Feature F2 | #Feature F3 | #Feature F4 | #Feature F5 | #Feature F6 | #Feature F7 | #Feature F8 | #Feature F9 | Start | End | PTM | AScore | Found By |
| --- | --- | --- | --- | --- | --- | --- | --- | --- | --- | --- | --- | --- | --- | --- | --- | --- | --- | --- | --- | --- | --- | --- | --- | --- | --- | --- | --- | --- | --- | --- | --- | --- | --- | --- | --- | --- | --- | --- | --- | --- | --- | --- | --- |
| A.DYGC(+57.02)YC(+57.02)GFGGSGTPVDQLDR.C | Y | 69.00 | 2222.8997 | 20 | 1.0 | 1112.4583 | 2 | 63.32 | 2 | F2:40217 | NaNaKA16\_F10.raw |  | 2.1439E6 |  |  |  |  |  |  |  |  |  |  | 1.8341E6 | 2 | 0 | 1 | 0 | 0 | 0 | 0 | 0 | 0 | 0 | 0 | 0 | 0 | 1 | 49 | 68 | Carbamidomethylation | C4:Carbamidomethylation:1000.00;C6:Carbamidomethylation:1000.00 | PEAKS DB |
| total 1 peptides |  |  |  |  |  |  |  |  |  |  |  |  |  |  |  |  |  |  |  |  |  |  |  |  |  |  |  |  |  |  |  |  |  |  |  |  |  |  |  |  |  |  |  |
| --- | --- | --- | --- | --- | --- | --- | --- | --- | --- | --- | --- | --- | --- | --- | --- | --- | --- | --- | --- | --- | --- | --- | --- | --- | --- | --- | --- | --- | --- | --- | --- | --- | --- | --- | --- | --- | --- | --- | --- | --- | --- | --- | --- |

XP\_026558006.1

back to list

  

| Protein Coverage
| Supporting Peptides
|

Protein Coverage:

Supporting Peptides:

| Peptide | Uniq | -10lgP | Mass | Length | ppm | m/z | z | RT | Fraction | Scan | Source File | Area F1 | Area F10 | Area F11 | Area F12 | Area F13 | Area F2 | Area F3 | Area F4 | Area F5 | Area F6 | Area F7 | Area F8 | Area F9 | #Feature | #Feature F1 | #Feature F10 | #Feature F11 | #Feature F12 | #Feature F13 | #Feature F2 | #Feature F3 | #Feature F4 | #Feature F5 | #Feature F6 | #Feature F7 | #Feature F8 | #Feature F9 | Start | End | PTM | AScore | Found By |
| --- | --- | --- | --- | --- | --- | --- | --- | --- | --- | --- | --- | --- | --- | --- | --- | --- | --- | --- | --- | --- | --- | --- | --- | --- | --- | --- | --- | --- | --- | --- | --- | --- | --- | --- | --- | --- | --- | --- | --- | --- | --- | --- | --- |
| K.GSSPNC(+57.02)YHFYFPAQETC(+57.02)PVKPALGLITYR.I | Y | 68.99 | 3372.6060 | 29 | 1.5 | 844.1600 | 4 | 69.38 | 2 | F2:45476 | NaNaKA16\_F10.raw |  | 9.5542E6 |  |  |  |  |  |  |  |  |  |  |  | 1 | 0 | 1 | 0 | 0 | 0 | 0 | 0 | 0 | 0 | 0 | 0 | 0 | 0 | 89 | 117 | Carbamidomethylation | C6:Carbamidomethylation:1000.00;C17:Carbamidomethylation:1000.00 | PEAKS DB |
| total 1 peptides |  |  |  |  |  |  |  |  |  |  |  |  |  |  |  |  |  |  |  |  |  |  |  |  |  |  |  |  |  |  |  |  |  |  |  |  |  |  |  |  |  |  |  |
| --- | --- | --- | --- | --- | --- | --- | --- | --- | --- | --- | --- | --- | --- | --- | --- | --- | --- | --- | --- | --- | --- | --- | --- | --- | --- | --- | --- | --- | --- | --- | --- | --- | --- | --- | --- | --- | --- | --- | --- | --- | --- | --- | --- |

XP\_032084613.1

back to list

  

| Protein Coverage
| Supporting Peptides
|

Protein Coverage:

Supporting Peptides:

| Peptide | Uniq | -10lgP | Mass | Length | ppm | m/z | z | RT | Fraction | Scan | Source File | Area F1 | Area F10 | Area F11 | Area F12 | Area F13 | Area F2 | Area F3 | Area F4 | Area F5 | Area F6 | Area F7 | Area F8 | Area F9 | #Feature | #Feature F1 | #Feature F10 | #Feature F11 | #Feature F12 | #Feature F13 | #Feature F2 | #Feature F3 | #Feature F4 | #Feature F5 | #Feature F6 | #Feature F7 | #Feature F8 | #Feature F9 | Start | End | PTM | AScore | Found By |
| --- | --- | --- | --- | --- | --- | --- | --- | --- | --- | --- | --- | --- | --- | --- | --- | --- | --- | --- | --- | --- | --- | --- | --- | --- | --- | --- | --- | --- | --- | --- | --- | --- | --- | --- | --- | --- | --- | --- | --- | --- | --- | --- | --- |
| F.AVAEPQIAMFC(+57.02)GK.L | Y | 51.18 | 1420.6842 | 13 | -9.2 | 711.3428 | 2 | 51.26 | 2 | F2:29587 | NaNaKA16\_F10.raw |  | 0 |  |  |  |  |  |  |  |  |  |  |  | 0 | 0 | 0 | 0 | 0 | 0 | 0 | 0 | 0 | 0 | 0 | 0 | 0 | 0 | 56 | 68 | Carbamidomethylation | C11:Carbamidomethylation:1000.00 | PEAKS DB |
| total 1 peptides |  |  |  |  |  |  |  |  |  |  |  |  |  |  |  |  |  |  |  |  |  |  |  |  |  |  |  |  |  |  |  |  |  |  |  |  |  |  |  |  |  |  |  |
| --- | --- | --- | --- | --- | --- | --- | --- | --- | --- | --- | --- | --- | --- | --- | --- | --- | --- | --- | --- | --- | --- | --- | --- | --- | --- | --- | --- | --- | --- | --- | --- | --- | --- | --- | --- | --- | --- | --- | --- | --- | --- | --- | --- |

XP\_032084612.1

back to list

  

| Protein Coverage
| Supporting Peptides
|

Protein Coverage:

Supporting Peptides:

| Peptide | Uniq | -10lgP | Mass | Length | ppm | m/z | z | RT | Fraction | Scan | Source File | Area F1 | Area F10 | Area F11 | Area F12 | Area F13 | Area F2 | Area F3 | Area F4 | Area F5 | Area F6 | Area F7 | Area F8 | Area F9 | #Feature | #Feature F1 | #Feature F10 | #Feature F11 | #Feature F12 | #Feature F13 | #Feature F2 | #Feature F3 | #Feature F4 | #Feature F5 | #Feature F6 | #Feature F7 | #Feature F8 | #Feature F9 | Start | End | PTM | AScore | Found By |
| --- | --- | --- | --- | --- | --- | --- | --- | --- | --- | --- | --- | --- | --- | --- | --- | --- | --- | --- | --- | --- | --- | --- | --- | --- | --- | --- | --- | --- | --- | --- | --- | --- | --- | --- | --- | --- | --- | --- | --- | --- | --- | --- | --- |
| F.AVAEPQIAMFC(+57.02)GK.L | Y | 51.18 | 1420.6842 | 13 | -9.2 | 711.3428 | 2 | 51.26 | 2 | F2:29587 | NaNaKA16\_F10.raw |  | 0 |  |  |  |  |  |  |  |  |  |  |  | 0 | 0 | 0 | 0 | 0 | 0 | 0 | 0 | 0 | 0 | 0 | 0 | 0 | 0 | 56 | 68 | Carbamidomethylation | C11:Carbamidomethylation:1000.00 | PEAKS DB |
| total 1 peptides |  |  |  |  |  |  |  |  |  |  |  |  |  |  |  |  |  |  |  |  |  |  |  |  |  |  |  |  |  |  |  |  |  |  |  |  |  |  |  |  |  |  |  |
| --- | --- | --- | --- | --- | --- | --- | --- | --- | --- | --- | --- | --- | --- | --- | --- | --- | --- | --- | --- | --- | --- | --- | --- | --- | --- | --- | --- | --- | --- | --- | --- | --- | --- | --- | --- | --- | --- | --- | --- | --- | --- | --- | --- |

XP\_032084615.1

back to list

  

| Protein Coverage
| Supporting Peptides
|

Protein Coverage:

Supporting Peptides:

| Peptide | Uniq | -10lgP | Mass | Length | ppm | m/z | z | RT | Fraction | Scan | Source File | Area F1 | Area F10 | Area F11 | Area F12 | Area F13 | Area F2 | Area F3 | Area F4 | Area F5 | Area F6 | Area F7 | Area F8 | Area F9 | #Feature | #Feature F1 | #Feature F10 | #Feature F11 | #Feature F12 | #Feature F13 | #Feature F2 | #Feature F3 | #Feature F4 | #Feature F5 | #Feature F6 | #Feature F7 | #Feature F8 | #Feature F9 | Start | End | PTM | AScore | Found By |
| --- | --- | --- | --- | --- | --- | --- | --- | --- | --- | --- | --- | --- | --- | --- | --- | --- | --- | --- | --- | --- | --- | --- | --- | --- | --- | --- | --- | --- | --- | --- | --- | --- | --- | --- | --- | --- | --- | --- | --- | --- | --- | --- | --- |
| F.AVAEPQIAMFC(+57.02)GK.L | Y | 51.18 | 1420.6842 | 13 | -9.2 | 711.3428 | 2 | 51.26 | 2 | F2:29587 | NaNaKA16\_F10.raw |  | 0 |  |  |  |  |  |  |  |  |  |  |  | 0 | 0 | 0 | 0 | 0 | 0 | 0 | 0 | 0 | 0 | 0 | 0 | 0 | 0 | 56 | 68 | Carbamidomethylation | C11:Carbamidomethylation:1000.00 | PEAKS DB |
| total 1 peptides |  |  |  |  |  |  |  |  |  |  |  |  |  |  |  |  |  |  |  |  |  |  |  |  |  |  |  |  |  |  |  |  |  |  |  |  |  |  |  |  |  |  |  |
| --- | --- | --- | --- | --- | --- | --- | --- | --- | --- | --- | --- | --- | --- | --- | --- | --- | --- | --- | --- | --- | --- | --- | --- | --- | --- | --- | --- | --- | --- | --- | --- | --- | --- | --- | --- | --- | --- | --- | --- | --- | --- | --- | --- |

XP\_032084614.1

back to list

  

| Protein Coverage
| Supporting Peptides
|

Protein Coverage:

Supporting Peptides:

| Peptide | Uniq | -10lgP | Mass | Length | ppm | m/z | z | RT | Fraction | Scan | Source File | Area F1 | Area F10 | Area F11 | Area F12 | Area F13 | Area F2 | Area F3 | Area F4 | Area F5 | Area F6 | Area F7 | Area F8 | Area F9 | #Feature | #Feature F1 | #Feature F10 | #Feature F11 | #Feature F12 | #Feature F13 | #Feature F2 | #Feature F3 | #Feature F4 | #Feature F5 | #Feature F6 | #Feature F7 | #Feature F8 | #Feature F9 | Start | End | PTM | AScore | Found By |
| --- | --- | --- | --- | --- | --- | --- | --- | --- | --- | --- | --- | --- | --- | --- | --- | --- | --- | --- | --- | --- | --- | --- | --- | --- | --- | --- | --- | --- | --- | --- | --- | --- | --- | --- | --- | --- | --- | --- | --- | --- | --- | --- | --- |
| F.AVAEPQIAMFC(+57.02)GK.L | Y | 51.18 | 1420.6842 | 13 | -9.2 | 711.3428 | 2 | 51.26 | 2 | F2:29587 | NaNaKA16\_F10.raw |  | 0 |  |  |  |  |  |  |  |  |  |  |  | 0 | 0 | 0 | 0 | 0 | 0 | 0 | 0 | 0 | 0 | 0 | 0 | 0 | 0 | 56 | 68 | Carbamidomethylation | C11:Carbamidomethylation:1000.00 | PEAKS DB |
| total 1 peptides |  |  |  |  |  |  |  |  |  |  |  |  |  |  |  |  |  |  |  |  |  |  |  |  |  |  |  |  |  |  |  |  |  |  |  |  |  |  |  |  |  |  |  |
| --- | --- | --- | --- | --- | --- | --- | --- | --- | --- | --- | --- | --- | --- | --- | --- | --- | --- | --- | --- | --- | --- | --- | --- | --- | --- | --- | --- | --- | --- | --- | --- | --- | --- | --- | --- | --- | --- | --- | --- | --- | --- | --- | --- |

XP\_026534403.1

back to list

  

| Protein Coverage
| Supporting Peptides
|

Protein Coverage:

Supporting Peptides:

| Peptide | Uniq | -10lgP | Mass | Length | ppm | m/z | z | RT | Fraction | Scan | Source File | Area F1 | Area F10 | Area F11 | Area F12 | Area F13 | Area F2 | Area F3 | Area F4 | Area F5 | Area F6 | Area F7 | Area F8 | Area F9 | #Feature | #Feature F1 | #Feature F10 | #Feature F11 | #Feature F12 | #Feature F13 | #Feature F2 | #Feature F3 | #Feature F4 | #Feature F5 | #Feature F6 | #Feature F7 | #Feature F8 | #Feature F9 | Start | End | PTM | AScore | Found By |
| --- | --- | --- | --- | --- | --- | --- | --- | --- | --- | --- | --- | --- | --- | --- | --- | --- | --- | --- | --- | --- | --- | --- | --- | --- | --- | --- | --- | --- | --- | --- | --- | --- | --- | --- | --- | --- | --- | --- | --- | --- | --- | --- | --- |
| F.AVAEPQIAMFC(+57.02)GK.L | Y | 51.18 | 1420.6842 | 13 | -9.2 | 711.3428 | 2 | 51.26 | 2 | F2:29587 | NaNaKA16\_F10.raw |  | 0 |  |  |  |  |  |  |  |  |  |  |  | 0 | 0 | 0 | 0 | 0 | 0 | 0 | 0 | 0 | 0 | 0 | 0 | 0 | 0 | 56 | 68 | Carbamidomethylation | C11:Carbamidomethylation:1000.00 | PEAKS DB |
| total 1 peptides |  |  |  |  |  |  |  |  |  |  |  |  |  |  |  |  |  |  |  |  |  |  |  |  |  |  |  |  |  |  |  |  |  |  |  |  |  |  |  |  |  |  |  |
| --- | --- | --- | --- | --- | --- | --- | --- | --- | --- | --- | --- | --- | --- | --- | --- | --- | --- | --- | --- | --- | --- | --- | --- | --- | --- | --- | --- | --- | --- | --- | --- | --- | --- | --- | --- | --- | --- | --- | --- | --- | --- | --- | --- |

XP\_026534402.1

back to list

  

| Protein Coverage
| Supporting Peptides
|

Protein Coverage:

Supporting Peptides:

| Peptide | Uniq | -10lgP | Mass | Length | ppm | m/z | z | RT | Fraction | Scan | Source File | Area F1 | Area F10 | Area F11 | Area F12 | Area F13 | Area F2 | Area F3 | Area F4 | Area F5 | Area F6 | Area F7 | Area F8 | Area F9 | #Feature | #Feature F1 | #Feature F10 | #Feature F11 | #Feature F12 | #Feature F13 | #Feature F2 | #Feature F3 | #Feature F4 | #Feature F5 | #Feature F6 | #Feature F7 | #Feature F8 | #Feature F9 | Start | End | PTM | AScore | Found By |
| --- | --- | --- | --- | --- | --- | --- | --- | --- | --- | --- | --- | --- | --- | --- | --- | --- | --- | --- | --- | --- | --- | --- | --- | --- | --- | --- | --- | --- | --- | --- | --- | --- | --- | --- | --- | --- | --- | --- | --- | --- | --- | --- | --- |
| F.AVAEPQIAMFC(+57.02)GK.L | Y | 51.18 | 1420.6842 | 13 | -9.2 | 711.3428 | 2 | 51.26 | 2 | F2:29587 | NaNaKA16\_F10.raw |  | 0 |  |  |  |  |  |  |  |  |  |  |  | 0 | 0 | 0 | 0 | 0 | 0 | 0 | 0 | 0 | 0 | 0 | 0 | 0 | 0 | 56 | 68 | Carbamidomethylation | C11:Carbamidomethylation:1000.00 | PEAKS DB |
| total 1 peptides |  |  |  |  |  |  |  |  |  |  |  |  |  |  |  |  |  |  |  |  |  |  |  |  |  |  |  |  |  |  |  |  |  |  |  |  |  |  |  |  |  |  |  |
| --- | --- | --- | --- | --- | --- | --- | --- | --- | --- | --- | --- | --- | --- | --- | --- | --- | --- | --- | --- | --- | --- | --- | --- | --- | --- | --- | --- | --- | --- | --- | --- | --- | --- | --- | --- | --- | --- | --- | --- | --- | --- | --- | --- |

XP\_026564759.1

back to list

  

| Protein Coverage
| Supporting Peptides
|

Protein Coverage:

Supporting Peptides:

| Peptide | Uniq | -10lgP | Mass | Length | ppm | m/z | z | RT | Fraction | Scan | Source File | Area F1 | Area F10 | Area F11 | Area F12 | Area F13 | Area F2 | Area F3 | Area F4 | Area F5 | Area F6 | Area F7 | Area F8 | Area F9 | #Feature | #Feature F1 | #Feature F10 | #Feature F11 | #Feature F12 | #Feature F13 | #Feature F2 | #Feature F3 | #Feature F4 | #Feature F5 | #Feature F6 | #Feature F7 | #Feature F8 | #Feature F9 | Start | End | PTM | AScore | Found By |
| --- | --- | --- | --- | --- | --- | --- | --- | --- | --- | --- | --- | --- | --- | --- | --- | --- | --- | --- | --- | --- | --- | --- | --- | --- | --- | --- | --- | --- | --- | --- | --- | --- | --- | --- | --- | --- | --- | --- | --- | --- | --- | --- | --- |
| F.AVAEPQIAMFC(+57.02)GK.L | Y | 51.18 | 1420.6842 | 13 | -9.2 | 711.3428 | 2 | 51.26 | 2 | F2:29587 | NaNaKA16\_F10.raw |  | 0 |  |  |  |  |  |  |  |  |  |  |  | 0 | 0 | 0 | 0 | 0 | 0 | 0 | 0 | 0 | 0 | 0 | 0 | 0 | 0 | 56 | 68 | Carbamidomethylation | C11:Carbamidomethylation:1000.00 | PEAKS DB |
| total 1 peptides |  |  |  |  |  |  |  |  |  |  |  |  |  |  |  |  |  |  |  |  |  |  |  |  |  |  |  |  |  |  |  |  |  |  |  |  |  |  |  |  |  |  |  |
| --- | --- | --- | --- | --- | --- | --- | --- | --- | --- | --- | --- | --- | --- | --- | --- | --- | --- | --- | --- | --- | --- | --- | --- | --- | --- | --- | --- | --- | --- | --- | --- | --- | --- | --- | --- | --- | --- | --- | --- | --- | --- | --- | --- |

XP\_026564758.1

back to list

  

| Protein Coverage
| Supporting Peptides
|

Protein Coverage:

Supporting Peptides:

| Peptide | Uniq | -10lgP | Mass | Length | ppm | m/z | z | RT | Fraction | Scan | Source File | Area F1 | Area F10 | Area F11 | Area F12 | Area F13 | Area F2 | Area F3 | Area F4 | Area F5 | Area F6 | Area F7 | Area F8 | Area F9 | #Feature | #Feature F1 | #Feature F10 | #Feature F11 | #Feature F12 | #Feature F13 | #Feature F2 | #Feature F3 | #Feature F4 | #Feature F5 | #Feature F6 | #Feature F7 | #Feature F8 | #Feature F9 | Start | End | PTM | AScore | Found By |
| --- | --- | --- | --- | --- | --- | --- | --- | --- | --- | --- | --- | --- | --- | --- | --- | --- | --- | --- | --- | --- | --- | --- | --- | --- | --- | --- | --- | --- | --- | --- | --- | --- | --- | --- | --- | --- | --- | --- | --- | --- | --- | --- | --- |
| F.AVAEPQIAMFC(+57.02)GK.L | Y | 51.18 | 1420.6842 | 13 | -9.2 | 711.3428 | 2 | 51.26 | 2 | F2:29587 | NaNaKA16\_F10.raw |  | 0 |  |  |  |  |  |  |  |  |  |  |  | 0 | 0 | 0 | 0 | 0 | 0 | 0 | 0 | 0 | 0 | 0 | 0 | 0 | 0 | 56 | 68 | Carbamidomethylation | C11:Carbamidomethylation:1000.00 | PEAKS DB |
| total 1 peptides |  |  |  |  |  |  |  |  |  |  |  |  |  |  |  |  |  |  |  |  |  |  |  |  |  |  |  |  |  |  |  |  |  |  |  |  |  |  |  |  |  |  |  |
| --- | --- | --- | --- | --- | --- | --- | --- | --- | --- | --- | --- | --- | --- | --- | --- | --- | --- | --- | --- | --- | --- | --- | --- | --- | --- | --- | --- | --- | --- | --- | --- | --- | --- | --- | --- | --- | --- | --- | --- | --- | --- | --- | --- |

XP\_026534405.1

back to list

  

| Protein Coverage
| Supporting Peptides
|

Protein Coverage:

Supporting Peptides:

| Peptide | Uniq | -10lgP | Mass | Length | ppm | m/z | z | RT | Fraction | Scan | Source File | Area F1 | Area F10 | Area F11 | Area F12 | Area F13 | Area F2 | Area F3 | Area F4 | Area F5 | Area F6 | Area F7 | Area F8 | Area F9 | #Feature | #Feature F1 | #Feature F10 | #Feature F11 | #Feature F12 | #Feature F13 | #Feature F2 | #Feature F3 | #Feature F4 | #Feature F5 | #Feature F6 | #Feature F7 | #Feature F8 | #Feature F9 | Start | End | PTM | AScore | Found By |
| --- | --- | --- | --- | --- | --- | --- | --- | --- | --- | --- | --- | --- | --- | --- | --- | --- | --- | --- | --- | --- | --- | --- | --- | --- | --- | --- | --- | --- | --- | --- | --- | --- | --- | --- | --- | --- | --- | --- | --- | --- | --- | --- | --- |
| F.AVAEPQIAMFC(+57.02)GK.L | Y | 51.18 | 1420.6842 | 13 | -9.2 | 711.3428 | 2 | 51.26 | 2 | F2:29587 | NaNaKA16\_F10.raw |  | 0 |  |  |  |  |  |  |  |  |  |  |  | 0 | 0 | 0 | 0 | 0 | 0 | 0 | 0 | 0 | 0 | 0 | 0 | 0 | 0 | 56 | 68 | Carbamidomethylation | C11:Carbamidomethylation:1000.00 | PEAKS DB |
| total 1 peptides |  |  |  |  |  |  |  |  |  |  |  |  |  |  |  |  |  |  |  |  |  |  |  |  |  |  |  |  |  |  |  |  |  |  |  |  |  |  |  |  |  |  |  |
| --- | --- | --- | --- | --- | --- | --- | --- | --- | --- | --- | --- | --- | --- | --- | --- | --- | --- | --- | --- | --- | --- | --- | --- | --- | --- | --- | --- | --- | --- | --- | --- | --- | --- | --- | --- | --- | --- | --- | --- | --- | --- | --- | --- |

XP\_026534404.1

back to list

  

| Protein Coverage
| Supporting Peptides
|

Protein Coverage:

Supporting Peptides:

| Peptide | Uniq | -10lgP | Mass | Length | ppm | m/z | z | RT | Fraction | Scan | Source File | Area F1 | Area F10 | Area F11 | Area F12 | Area F13 | Area F2 | Area F3 | Area F4 | Area F5 | Area F6 | Area F7 | Area F8 | Area F9 | #Feature | #Feature F1 | #Feature F10 | #Feature F11 | #Feature F12 | #Feature F13 | #Feature F2 | #Feature F3 | #Feature F4 | #Feature F5 | #Feature F6 | #Feature F7 | #Feature F8 | #Feature F9 | Start | End | PTM | AScore | Found By |
| --- | --- | --- | --- | --- | --- | --- | --- | --- | --- | --- | --- | --- | --- | --- | --- | --- | --- | --- | --- | --- | --- | --- | --- | --- | --- | --- | --- | --- | --- | --- | --- | --- | --- | --- | --- | --- | --- | --- | --- | --- | --- | --- | --- |
| F.AVAEPQIAMFC(+57.02)GK.L | Y | 51.18 | 1420.6842 | 13 | -9.2 | 711.3428 | 2 | 51.26 | 2 | F2:29587 | NaNaKA16\_F10.raw |  | 0 |  |  |  |  |  |  |  |  |  |  |  | 0 | 0 | 0 | 0 | 0 | 0 | 0 | 0 | 0 | 0 | 0 | 0 | 0 | 0 | 56 | 68 | Carbamidomethylation | C11:Carbamidomethylation:1000.00 | PEAKS DB |
| total 1 peptides |  |  |  |  |  |  |  |  |  |  |  |  |  |  |  |  |  |  |  |  |  |  |  |  |  |  |  |  |  |  |  |  |  |  |  |  |  |  |  |  |  |  |  |
| --- | --- | --- | --- | --- | --- | --- | --- | --- | --- | --- | --- | --- | --- | --- | --- | --- | --- | --- | --- | --- | --- | --- | --- | --- | --- | --- | --- | --- | --- | --- | --- | --- | --- | --- | --- | --- | --- | --- | --- | --- | --- | --- | --- |

XP\_026564761.1

back to list

  

| Protein Coverage
| Supporting Peptides
|

Protein Coverage:

Supporting Peptides:

| Peptide | Uniq | -10lgP | Mass | Length | ppm | m/z | z | RT | Fraction | Scan | Source File | Area F1 | Area F10 | Area F11 | Area F12 | Area F13 | Area F2 | Area F3 | Area F4 | Area F5 | Area F6 | Area F7 | Area F8 | Area F9 | #Feature | #Feature F1 | #Feature F10 | #Feature F11 | #Feature F12 | #Feature F13 | #Feature F2 | #Feature F3 | #Feature F4 | #Feature F5 | #Feature F6 | #Feature F7 | #Feature F8 | #Feature F9 | Start | End | PTM | AScore | Found By |
| --- | --- | --- | --- | --- | --- | --- | --- | --- | --- | --- | --- | --- | --- | --- | --- | --- | --- | --- | --- | --- | --- | --- | --- | --- | --- | --- | --- | --- | --- | --- | --- | --- | --- | --- | --- | --- | --- | --- | --- | --- | --- | --- | --- |
| F.AVAEPQIAMFC(+57.02)GK.L | Y | 51.18 | 1420.6842 | 13 | -9.2 | 711.3428 | 2 | 51.26 | 2 | F2:29587 | NaNaKA16\_F10.raw |  | 0 |  |  |  |  |  |  |  |  |  |  |  | 0 | 0 | 0 | 0 | 0 | 0 | 0 | 0 | 0 | 0 | 0 | 0 | 0 | 0 | 56 | 68 | Carbamidomethylation | C11:Carbamidomethylation:1000.00 | PEAKS DB |
| total 1 peptides |  |  |  |  |  |  |  |  |  |  |  |  |  |  |  |  |  |  |  |  |  |  |  |  |  |  |  |  |  |  |  |  |  |  |  |  |  |  |  |  |  |  |  |
| --- | --- | --- | --- | --- | --- | --- | --- | --- | --- | --- | --- | --- | --- | --- | --- | --- | --- | --- | --- | --- | --- | --- | --- | --- | --- | --- | --- | --- | --- | --- | --- | --- | --- | --- | --- | --- | --- | --- | --- | --- | --- | --- | --- |

XP\_026564760.1

back to list

  

| Protein Coverage
| Supporting Peptides
|

Protein Coverage:

Supporting Peptides:

| Peptide | Uniq | -10lgP | Mass | Length | ppm | m/z | z | RT | Fraction | Scan | Source File | Area F1 | Area F10 | Area F11 | Area F12 | Area F13 | Area F2 | Area F3 | Area F4 | Area F5 | Area F6 | Area F7 | Area F8 | Area F9 | #Feature | #Feature F1 | #Feature F10 | #Feature F11 | #Feature F12 | #Feature F13 | #Feature F2 | #Feature F3 | #Feature F4 | #Feature F5 | #Feature F6 | #Feature F7 | #Feature F8 | #Feature F9 | Start | End | PTM | AScore | Found By |
| --- | --- | --- | --- | --- | --- | --- | --- | --- | --- | --- | --- | --- | --- | --- | --- | --- | --- | --- | --- | --- | --- | --- | --- | --- | --- | --- | --- | --- | --- | --- | --- | --- | --- | --- | --- | --- | --- | --- | --- | --- | --- | --- | --- |
| F.AVAEPQIAMFC(+57.02)GK.L | Y | 51.18 | 1420.6842 | 13 | -9.2 | 711.3428 | 2 | 51.26 | 2 | F2:29587 | NaNaKA16\_F10.raw |  | 0 |  |  |  |  |  |  |  |  |  |  |  | 0 | 0 | 0 | 0 | 0 | 0 | 0 | 0 | 0 | 0 | 0 | 0 | 0 | 0 | 56 | 68 | Carbamidomethylation | C11:Carbamidomethylation:1000.00 | PEAKS DB |
| total 1 peptides |  |  |  |  |  |  |  |  |  |  |  |  |  |  |  |  |  |  |  |  |  |  |  |  |  |  |  |  |  |  |  |  |  |  |  |  |  |  |  |  |  |  |  |
| --- | --- | --- | --- | --- | --- | --- | --- | --- | --- | --- | --- | --- | --- | --- | --- | --- | --- | --- | --- | --- | --- | --- | --- | --- | --- | --- | --- | --- | --- | --- | --- | --- | --- | --- | --- | --- | --- | --- | --- | --- | --- | --- | --- |

LAA59850.1

back to list

  

| Protein Coverage
| Supporting Peptides
|

Protein Coverage:

Supporting Peptides:

| Peptide | Uniq | -10lgP | Mass | Length | ppm | m/z | z | RT | Fraction | Scan | Source File | Area F1 | Area F10 | Area F11 | Area F12 | Area F13 | Area F2 | Area F3 | Area F4 | Area F5 | Area F6 | Area F7 | Area F8 | Area F9 | #Feature | #Feature F1 | #Feature F10 | #Feature F11 | #Feature F12 | #Feature F13 | #Feature F2 | #Feature F3 | #Feature F4 | #Feature F5 | #Feature F6 | #Feature F7 | #Feature F8 | #Feature F9 | Start | End | PTM | AScore | Found By |
| --- | --- | --- | --- | --- | --- | --- | --- | --- | --- | --- | --- | --- | --- | --- | --- | --- | --- | --- | --- | --- | --- | --- | --- | --- | --- | --- | --- | --- | --- | --- | --- | --- | --- | --- | --- | --- | --- | --- | --- | --- | --- | --- | --- |
| F.AVAEPQIAMFC(+57.02)GK.L | Y | 51.18 | 1420.6842 | 13 | -9.2 | 711.3428 | 2 | 51.26 | 2 | F2:29587 | NaNaKA16\_F10.raw |  | 0 |  |  |  |  |  |  |  |  |  |  |  | 0 | 0 | 0 | 0 | 0 | 0 | 0 | 0 | 0 | 0 | 0 | 0 | 0 | 0 | 30 | 42 | Carbamidomethylation | C11:Carbamidomethylation:1000.00 | PEAKS DB |
| total 1 peptides |  |  |  |  |  |  |  |  |  |  |  |  |  |  |  |  |  |  |  |  |  |  |  |  |  |  |  |  |  |  |  |  |  |  |  |  |  |  |  |  |  |  |  |
| --- | --- | --- | --- | --- | --- | --- | --- | --- | --- | --- | --- | --- | --- | --- | --- | --- | --- | --- | --- | --- | --- | --- | --- | --- | --- | --- | --- | --- | --- | --- | --- | --- | --- | --- | --- | --- | --- | --- | --- | --- | --- | --- | --- |

ETE57873.1

back to list

  

| Protein Coverage
| Supporting Peptides
|

Protein Coverage:

Supporting Peptides:

| Peptide | Uniq | -10lgP | Mass | Length | ppm | m/z | z | RT | Fraction | Scan | Source File | Area F1 | Area F10 | Area F11 | Area F12 | Area F13 | Area F2 | Area F3 | Area F4 | Area F5 | Area F6 | Area F7 | Area F8 | Area F9 | #Feature | #Feature F1 | #Feature F10 | #Feature F11 | #Feature F12 | #Feature F13 | #Feature F2 | #Feature F3 | #Feature F4 | #Feature F5 | #Feature F6 | #Feature F7 | #Feature F8 | #Feature F9 | Start | End | PTM | AScore | Found By |
| --- | --- | --- | --- | --- | --- | --- | --- | --- | --- | --- | --- | --- | --- | --- | --- | --- | --- | --- | --- | --- | --- | --- | --- | --- | --- | --- | --- | --- | --- | --- | --- | --- | --- | --- | --- | --- | --- | --- | --- | --- | --- | --- | --- |
| A.QVTQSGPVSVAIAGR.T | Y | 67.63 | 1468.7998 | 15 | 0.4 | 735.4075 | 2 | 59.76 | 4 | F4:38474 | NaNaKA16\_F12.raw |  |  |  | 8.0605E5 |  |  |  |  |  |  |  |  |  | 1 | 0 | 0 | 0 | 1 | 0 | 0 | 0 | 0 | 0 | 0 | 0 | 0 | 0 | 33 | 47 |  |  | PEAKS DB |
| total 1 peptides |  |  |  |  |  |  |  |  |  |  |  |  |  |  |  |  |  |  |  |  |  |  |  |  |  |  |  |  |  |  |  |  |  |  |  |  |  |  |  |  |  |  |  |
| --- | --- | --- | --- | --- | --- | --- | --- | --- | --- | --- | --- | --- | --- | --- | --- | --- | --- | --- | --- | --- | --- | --- | --- | --- | --- | --- | --- | --- | --- | --- | --- | --- | --- | --- | --- | --- | --- | --- | --- | --- | --- | --- | --- |

E3P6P4.1

back to list

  

| Protein Coverage
| Supporting Peptides
|

Protein Coverage:

Supporting Peptides:

| Peptide | Uniq | -10lgP | Mass | Length | ppm | m/z | z | RT | Fraction | Scan | Source File | Area F1 | Area F10 | Area F11 | Area F12 | Area F13 | Area F2 | Area F3 | Area F4 | Area F5 | Area F6 | Area F7 | Area F8 | Area F9 | #Feature | #Feature F1 | #Feature F10 | #Feature F11 | #Feature F12 | #Feature F13 | #Feature F2 | #Feature F3 | #Feature F4 | #Feature F5 | #Feature F6 | #Feature F7 | #Feature F8 | #Feature F9 | Start | End | PTM | AScore | Found By |
| --- | --- | --- | --- | --- | --- | --- | --- | --- | --- | --- | --- | --- | --- | --- | --- | --- | --- | --- | --- | --- | --- | --- | --- | --- | --- | --- | --- | --- | --- | --- | --- | --- | --- | --- | --- | --- | --- | --- | --- | --- | --- | --- | --- |
| K.YYLMMELVK.T | Y | 65.98 | 1188.5923 | 9 | 3.8 | 595.3057 | 2 | 71.44 | 2 | F2:47172 | NaNaKA16\_F10.raw |  | 1.0741E8 | 5.9073E5 |  |  |  |  |  |  |  |  |  | 8.0349E5 | 3 | 0 | 1 | 1 | 0 | 0 | 0 | 0 | 0 | 0 | 0 | 0 | 0 | 1 | 80 | 88 |  |  | PEAKS DB |
| K.YYLMM(+15.99)ELVK.T | Y | 58.06 | 1204.5872 | 9 | 0.1 | 603.3009 | 2 | 71.52 | 2 | F2:47461 | NaNaKA16\_F10.raw |  | 6.0185E6 |  |  |  |  |  |  |  |  |  |  |  | 1 | 0 | 1 | 0 | 0 | 0 | 0 | 0 | 0 | 0 | 0 | 0 | 0 | 0 | 80 | 88 | Oxidation (M) | M5:Oxidation (M):33.98 | PEAKS DB |
| K.YYLM(+15.99)MELVK.T | Y | 45.07 | 1204.5872 | 9 | 0.1 | 603.3009 | 2 | 71.52 | 2 | F2:47228 | NaNaKA16\_F10.raw |  | 6.0185E6 |  |  |  |  |  |  |  |  |  |  | 9.6788E5 | 2 | 0 | 1 | 0 | 0 | 0 | 0 | 0 | 0 | 0 | 0 | 0 | 0 | 1 | 80 | 88 | Oxidation (M) | M4:Oxidation (M):21.94 | PEAKS DB |
| total 3 peptides |  |  |  |  |  |  |  |  |  |  |  |  |  |  |  |  |  |  |  |  |  |  |  |  |  |  |  |  |  |  |  |  |  |  |  |  |  |  |  |  |  |  |  |
| --- | --- | --- | --- | --- | --- | --- | --- | --- | --- | --- | --- | --- | --- | --- | --- | --- | --- | --- | --- | --- | --- | --- | --- | --- | --- | --- | --- | --- | --- | --- | --- | --- | --- | --- | --- | --- | --- | --- | --- | --- | --- | --- | --- |

ACR83850.1

back to list

  

| Protein Coverage
| Supporting Peptides
|

Protein Coverage:

Supporting Peptides:

| Peptide | Uniq | -10lgP | Mass | Length | ppm | m/z | z | RT | Fraction | Scan | Source File | Area F1 | Area F10 | Area F11 | Area F12 | Area F13 | Area F2 | Area F3 | Area F4 | Area F5 | Area F6 | Area F7 | Area F8 | Area F9 | #Feature | #Feature F1 | #Feature F10 | #Feature F11 | #Feature F12 | #Feature F13 | #Feature F2 | #Feature F3 | #Feature F4 | #Feature F5 | #Feature F6 | #Feature F7 | #Feature F8 | #Feature F9 | Start | End | PTM | AScore | Found By |
| --- | --- | --- | --- | --- | --- | --- | --- | --- | --- | --- | --- | --- | --- | --- | --- | --- | --- | --- | --- | --- | --- | --- | --- | --- | --- | --- | --- | --- | --- | --- | --- | --- | --- | --- | --- | --- | --- | --- | --- | --- | --- | --- | --- |
| K.YYLMMELVK.T | Y | 65.98 | 1188.5923 | 9 | 3.8 | 595.3057 | 2 | 71.44 | 2 | F2:47172 | NaNaKA16\_F10.raw |  | 1.0741E8 | 5.9073E5 |  |  |  |  |  |  |  |  |  | 8.0349E5 | 3 | 0 | 1 | 1 | 0 | 0 | 0 | 0 | 0 | 0 | 0 | 0 | 0 | 1 | 80 | 88 |  |  | PEAKS DB |
| K.YYLMM(+15.99)ELVK.T | Y | 58.06 | 1204.5872 | 9 | 0.1 | 603.3009 | 2 | 71.52 | 2 | F2:47461 | NaNaKA16\_F10.raw |  | 6.0185E6 |  |  |  |  |  |  |  |  |  |  |  | 1 | 0 | 1 | 0 | 0 | 0 | 0 | 0 | 0 | 0 | 0 | 0 | 0 | 0 | 80 | 88 | Oxidation (M) | M5:Oxidation (M):33.98 | PEAKS DB |
| K.YYLM(+15.99)MELVK.T | Y | 45.07 | 1204.5872 | 9 | 0.1 | 603.3009 | 2 | 71.52 | 2 | F2:47228 | NaNaKA16\_F10.raw |  | 6.0185E6 |  |  |  |  |  |  |  |  |  |  | 9.6788E5 | 2 | 0 | 1 | 0 | 0 | 0 | 0 | 0 | 0 | 0 | 0 | 0 | 0 | 1 | 80 | 88 | Oxidation (M) | M4:Oxidation (M):21.94 | PEAKS DB |
| total 3 peptides |  |  |  |  |  |  |  |  |  |  |  |  |  |  |  |  |  |  |  |  |  |  |  |  |  |  |  |  |  |  |  |  |  |  |  |  |  |  |  |  |  |  |  |
| --- | --- | --- | --- | --- | --- | --- | --- | --- | --- | --- | --- | --- | --- | --- | --- | --- | --- | --- | --- | --- | --- | --- | --- | --- | --- | --- | --- | --- | --- | --- | --- | --- | --- | --- | --- | --- | --- | --- | --- | --- | --- | --- | --- |

JAS03127.1

back to list

  

| Protein Coverage
| Supporting Peptides
|

Protein Coverage:

Supporting Peptides:

| Peptide | Uniq | -10lgP | Mass | Length | ppm | m/z | z | RT | Fraction | Scan | Source File | Area F1 | Area F10 | Area F11 | Area F12 | Area F13 | Area F2 | Area F3 | Area F4 | Area F5 | Area F6 | Area F7 | Area F8 | Area F9 | #Feature | #Feature F1 | #Feature F10 | #Feature F11 | #Feature F12 | #Feature F13 | #Feature F2 | #Feature F3 | #Feature F4 | #Feature F5 | #Feature F6 | #Feature F7 | #Feature F8 | #Feature F9 | Start | End | PTM | AScore | Found By |
| --- | --- | --- | --- | --- | --- | --- | --- | --- | --- | --- | --- | --- | --- | --- | --- | --- | --- | --- | --- | --- | --- | --- | --- | --- | --- | --- | --- | --- | --- | --- | --- | --- | --- | --- | --- | --- | --- | --- | --- | --- | --- | --- | --- |
| K.SC(+57.02)EPFIYHGC(+57.02)PGNR.N | Y | 64.42 | 1692.7136 | 14 | 0.3 | 565.2453 | 3 | 20.59 | 10 | F10:7927 | NaNaKA16\_F6.raw |  |  |  |  |  |  |  |  |  | 1.7968E7 |  |  |  | 1 | 0 | 0 | 0 | 0 | 0 | 0 | 0 | 0 | 0 | 1 | 0 | 0 | 0 | 48 | 61 | Carbamidomethylation | C2:Carbamidomethylation:1000.00;C10:Carbamidomethylation:1000.00 | PEAKS DB |
| total 1 peptides |  |  |  |  |  |  |  |  |  |  |  |  |  |  |  |  |  |  |  |  |  |  |  |  |  |  |  |  |  |  |  |  |  |  |  |  |  |  |  |  |  |  |  |
| --- | --- | --- | --- | --- | --- | --- | --- | --- | --- | --- | --- | --- | --- | --- | --- | --- | --- | --- | --- | --- | --- | --- | --- | --- | --- | --- | --- | --- | --- | --- | --- | --- | --- | --- | --- | --- | --- | --- | --- | --- | --- | --- | --- |

S68801

back to list

  

| Protein Coverage
| Supporting Peptides
|

Protein Coverage:

Supporting Peptides:

| Peptide | Uniq | -10lgP | Mass | Length | ppm | m/z | z | RT | Fraction | Scan | Source File | Area F1 | Area F10 | Area F11 | Area F12 | Area F13 | Area F2 | Area F3 | Area F4 | Area F5 | Area F6 | Area F7 | Area F8 | Area F9 | #Feature | #Feature F1 | #Feature F10 | #Feature F11 | #Feature F12 | #Feature F13 | #Feature F2 | #Feature F3 | #Feature F4 | #Feature F5 | #Feature F6 | #Feature F7 | #Feature F8 | #Feature F9 | Start | End | PTM | AScore | Found By |
| --- | --- | --- | --- | --- | --- | --- | --- | --- | --- | --- | --- | --- | --- | --- | --- | --- | --- | --- | --- | --- | --- | --- | --- | --- | --- | --- | --- | --- | --- | --- | --- | --- | --- | --- | --- | --- | --- | --- | --- | --- | --- | --- | --- |
| L.ETQLLLGVVK.D | Y | 44.12 | 1098.6648 | 10 | 1.3 | 550.3404 | 2 | 54.20 | 4 | F4:33688 | NaNaKA16\_F12.raw |  |  |  | 2.7542E5 |  |  |  |  |  |  |  |  |  | 1 | 0 | 0 | 0 | 1 | 0 | 0 | 0 | 0 | 0 | 0 | 0 | 0 | 0 | 56 | 65 |  |  | PEAKS DB |
| total 1 peptides |  |  |  |  |  |  |  |  |  |  |  |  |  |  |  |  |  |  |  |  |  |  |  |  |  |  |  |  |  |  |  |  |  |  |  |  |  |  |  |  |  |  |  |
| --- | --- | --- | --- | --- | --- | --- | --- | --- | --- | --- | --- | --- | --- | --- | --- | --- | --- | --- | --- | --- | --- | --- | --- | --- | --- | --- | --- | --- | --- | --- | --- | --- | --- | --- | --- | --- | --- | --- | --- | --- | --- | --- | --- |

XP\_026524106.1

back to list

  

| Protein Coverage
| Supporting Peptides
|

Protein Coverage:

Supporting Peptides:

| Peptide | Uniq | -10lgP | Mass | Length | ppm | m/z | z | RT | Fraction | Scan | Source File | Area F1 | Area F10 | Area F11 | Area F12 | Area F13 | Area F2 | Area F3 | Area F4 | Area F5 | Area F6 | Area F7 | Area F8 | Area F9 | #Feature | #Feature F1 | #Feature F10 | #Feature F11 | #Feature F12 | #Feature F13 | #Feature F2 | #Feature F3 | #Feature F4 | #Feature F5 | #Feature F6 | #Feature F7 | #Feature F8 | #Feature F9 | Start | End | PTM | AScore | Found By |
| --- | --- | --- | --- | --- | --- | --- | --- | --- | --- | --- | --- | --- | --- | --- | --- | --- | --- | --- | --- | --- | --- | --- | --- | --- | --- | --- | --- | --- | --- | --- | --- | --- | --- | --- | --- | --- | --- | --- | --- | --- | --- | --- | --- |
| R.LITMEEFIR.S | Y | 46.77 | 1150.6056 | 9 | 0.4 | 576.3103 | 2 | 67.32 | 4 | F4:44606 | NaNaKA16\_F12.raw |  |  | 6.5993E5 | 6.3396E5 |  |  |  |  |  |  |  |  |  | 2 | 0 | 0 | 1 | 1 | 0 | 0 | 0 | 0 | 0 | 0 | 0 | 0 | 0 | 319 | 327 |  |  | PEAKS DB |
| total 1 peptides |  |  |  |  |  |  |  |  |  |  |  |  |  |  |  |  |  |  |  |  |  |  |  |  |  |  |  |  |  |  |  |  |  |  |  |  |  |  |  |  |  |  |  |
| --- | --- | --- | --- | --- | --- | --- | --- | --- | --- | --- | --- | --- | --- | --- | --- | --- | --- | --- | --- | --- | --- | --- | --- | --- | --- | --- | --- | --- | --- | --- | --- | --- | --- | --- | --- | --- | --- | --- | --- | --- | --- | --- | --- |

XP\_026562300.1

back to list

  

| Protein Coverage
| Supporting Peptides
|

Protein Coverage:

Supporting Peptides:

| Peptide | Uniq | -10lgP | Mass | Length | ppm | m/z | z | RT | Fraction | Scan | Source File | Area F1 | Area F10 | Area F11 | Area F12 | Area F13 | Area F2 | Area F3 | Area F4 | Area F5 | Area F6 | Area F7 | Area F8 | Area F9 | #Feature | #Feature F1 | #Feature F10 | #Feature F11 | #Feature F12 | #Feature F13 | #Feature F2 | #Feature F3 | #Feature F4 | #Feature F5 | #Feature F6 | #Feature F7 | #Feature F8 | #Feature F9 | Start | End | PTM | AScore | Found By |
| --- | --- | --- | --- | --- | --- | --- | --- | --- | --- | --- | --- | --- | --- | --- | --- | --- | --- | --- | --- | --- | --- | --- | --- | --- | --- | --- | --- | --- | --- | --- | --- | --- | --- | --- | --- | --- | --- | --- | --- | --- | --- | --- | --- |
| R.LITMEEFIR.S | Y | 46.77 | 1150.6056 | 9 | 0.4 | 576.3103 | 2 | 67.32 | 4 | F4:44606 | NaNaKA16\_F12.raw |  |  | 6.5993E5 | 6.3396E5 |  |  |  |  |  |  |  |  |  | 2 | 0 | 0 | 1 | 1 | 0 | 0 | 0 | 0 | 0 | 0 | 0 | 0 | 0 | 319 | 327 |  |  | PEAKS DB |
| total 1 peptides |  |  |  |  |  |  |  |  |  |  |  |  |  |  |  |  |  |  |  |  |  |  |  |  |  |  |  |  |  |  |  |  |  |  |  |  |  |  |  |  |  |  |  |
| --- | --- | --- | --- | --- | --- | --- | --- | --- | --- | --- | --- | --- | --- | --- | --- | --- | --- | --- | --- | --- | --- | --- | --- | --- | --- | --- | --- | --- | --- | --- | --- | --- | --- | --- | --- | --- | --- | --- | --- | --- | --- | --- | --- |

XP\_026562299.1

back to list

  

| Protein Coverage
| Supporting Peptides
|

Protein Coverage:

Supporting Peptides:

| Peptide | Uniq | -10lgP | Mass | Length | ppm | m/z | z | RT | Fraction | Scan | Source File | Area F1 | Area F10 | Area F11 | Area F12 | Area F13 | Area F2 | Area F3 | Area F4 | Area F5 | Area F6 | Area F7 | Area F8 | Area F9 | #Feature | #Feature F1 | #Feature F10 | #Feature F11 | #Feature F12 | #Feature F13 | #Feature F2 | #Feature F3 | #Feature F4 | #Feature F5 | #Feature F6 | #Feature F7 | #Feature F8 | #Feature F9 | Start | End | PTM | AScore | Found By |
| --- | --- | --- | --- | --- | --- | --- | --- | --- | --- | --- | --- | --- | --- | --- | --- | --- | --- | --- | --- | --- | --- | --- | --- | --- | --- | --- | --- | --- | --- | --- | --- | --- | --- | --- | --- | --- | --- | --- | --- | --- | --- | --- | --- |
| R.LITMEEFIR.S | Y | 46.77 | 1150.6056 | 9 | 0.4 | 576.3103 | 2 | 67.32 | 4 | F4:44606 | NaNaKA16\_F12.raw |  |  | 6.5993E5 | 6.3396E5 |  |  |  |  |  |  |  |  |  | 2 | 0 | 0 | 1 | 1 | 0 | 0 | 0 | 0 | 0 | 0 | 0 | 0 | 0 | 319 | 327 |  |  | PEAKS DB |
| total 1 peptides |  |  |  |  |  |  |  |  |  |  |  |  |  |  |  |  |  |  |  |  |  |  |  |  |  |  |  |  |  |  |  |  |  |  |  |  |  |  |  |  |  |  |  |
| --- | --- | --- | --- | --- | --- | --- | --- | --- | --- | --- | --- | --- | --- | --- | --- | --- | --- | --- | --- | --- | --- | --- | --- | --- | --- | --- | --- | --- | --- | --- | --- | --- | --- | --- | --- | --- | --- | --- | --- | --- | --- | --- | --- |

XP\_034262406.1

back to list

  

| Protein Coverage
| Supporting Peptides
|

Protein Coverage:

Supporting Peptides:

| Peptide | Uniq | -10lgP | Mass | Length | ppm | m/z | z | RT | Fraction | Scan | Source File | Area F1 | Area F10 | Area F11 | Area F12 | Area F13 | Area F2 | Area F3 | Area F4 | Area F5 | Area F6 | Area F7 | Area F8 | Area F9 | #Feature | #Feature F1 | #Feature F10 | #Feature F11 | #Feature F12 | #Feature F13 | #Feature F2 | #Feature F3 | #Feature F4 | #Feature F5 | #Feature F6 | #Feature F7 | #Feature F8 | #Feature F9 | Start | End | PTM | AScore | Found By |
| --- | --- | --- | --- | --- | --- | --- | --- | --- | --- | --- | --- | --- | --- | --- | --- | --- | --- | --- | --- | --- | --- | --- | --- | --- | --- | --- | --- | --- | --- | --- | --- | --- | --- | --- | --- | --- | --- | --- | --- | --- | --- | --- | --- |
| R.SSDNDFLIFK.P | Y | 62.29 | 1184.5713 | 10 | 0.1 | 593.2930 | 2 | 64.20 | 4 | F4:42212 | NaNaKA16\_F12.raw |  |  |  | 1.462E7 |  |  |  |  |  |  |  |  |  | 1 | 0 | 0 | 0 | 1 | 0 | 0 | 0 | 0 | 0 | 0 | 0 | 0 | 0 | 73 | 82 |  |  | PEAKS DB |
| total 1 peptides |  |  |  |  |  |  |  |  |  |  |  |  |  |  |  |  |  |  |  |  |  |  |  |  |  |  |  |  |  |  |  |  |  |  |  |  |  |  |  |  |  |  |  |
| --- | --- | --- | --- | --- | --- | --- | --- | --- | --- | --- | --- | --- | --- | --- | --- | --- | --- | --- | --- | --- | --- | --- | --- | --- | --- | --- | --- | --- | --- | --- | --- | --- | --- | --- | --- | --- | --- | --- | --- | --- | --- | --- | --- |

XP\_015678953.1

back to list

  

| Protein Coverage
| Supporting Peptides
|

Protein Coverage:

Supporting Peptides:

| Peptide | Uniq | -10lgP | Mass | Length | ppm | m/z | z | RT | Fraction | Scan | Source File | Area F1 | Area F10 | Area F11 | Area F12 | Area F13 | Area F2 | Area F3 | Area F4 | Area F5 | Area F6 | Area F7 | Area F8 | Area F9 | #Feature | #Feature F1 | #Feature F10 | #Feature F11 | #Feature F12 | #Feature F13 | #Feature F2 | #Feature F3 | #Feature F4 | #Feature F5 | #Feature F6 | #Feature F7 | #Feature F8 | #Feature F9 | Start | End | PTM | AScore | Found By |
| --- | --- | --- | --- | --- | --- | --- | --- | --- | --- | --- | --- | --- | --- | --- | --- | --- | --- | --- | --- | --- | --- | --- | --- | --- | --- | --- | --- | --- | --- | --- | --- | --- | --- | --- | --- | --- | --- | --- | --- | --- | --- | --- | --- |
| Q.SSDNDFLIFK.S | Y | 62.29 | 1184.5713 | 10 | 0.1 | 593.2930 | 2 | 64.20 | 4 | F4:42212 | NaNaKA16\_F12.raw |  |  |  | 1.462E7 |  |  |  |  |  |  |  |  |  | 1 | 0 | 0 | 0 | 1 | 0 | 0 | 0 | 0 | 0 | 0 | 0 | 0 | 0 | 75 | 84 |  |  | PEAKS DB |
| total 1 peptides |  |  |  |  |  |  |  |  |  |  |  |  |  |  |  |  |  |  |  |  |  |  |  |  |  |  |  |  |  |  |  |  |  |  |  |  |  |  |  |  |  |  |  |
| --- | --- | --- | --- | --- | --- | --- | --- | --- | --- | --- | --- | --- | --- | --- | --- | --- | --- | --- | --- | --- | --- | --- | --- | --- | --- | --- | --- | --- | --- | --- | --- | --- | --- | --- | --- | --- | --- | --- | --- | --- | --- | --- | --- |

JAG66558.1

back to list

  

| Protein Coverage
| Supporting Peptides
|

Protein Coverage:

Supporting Peptides:

| Peptide | Uniq | -10lgP | Mass | Length | ppm | m/z | z | RT | Fraction | Scan | Source File | Area F1 | Area F10 | Area F11 | Area F12 | Area F13 | Area F2 | Area F3 | Area F4 | Area F5 | Area F6 | Area F7 | Area F8 | Area F9 | #Feature | #Feature F1 | #Feature F10 | #Feature F11 | #Feature F12 | #Feature F13 | #Feature F2 | #Feature F3 | #Feature F4 | #Feature F5 | #Feature F6 | #Feature F7 | #Feature F8 | #Feature F9 | Start | End | PTM | AScore | Found By |
| --- | --- | --- | --- | --- | --- | --- | --- | --- | --- | --- | --- | --- | --- | --- | --- | --- | --- | --- | --- | --- | --- | --- | --- | --- | --- | --- | --- | --- | --- | --- | --- | --- | --- | --- | --- | --- | --- | --- | --- | --- | --- | --- | --- |
| R.SSDNDFLIFK.T | Y | 62.29 | 1184.5713 | 10 | 0.1 | 593.2930 | 2 | 64.20 | 4 | F4:42212 | NaNaKA16\_F12.raw |  |  |  | 1.462E7 |  |  |  |  |  |  |  |  |  | 1 | 0 | 0 | 0 | 1 | 0 | 0 | 0 | 0 | 0 | 0 | 0 | 0 | 0 | 75 | 84 |  |  | PEAKS DB |
| total 1 peptides |  |  |  |  |  |  |  |  |  |  |  |  |  |  |  |  |  |  |  |  |  |  |  |  |  |  |  |  |  |  |  |  |  |  |  |  |  |  |  |  |  |  |  |
| --- | --- | --- | --- | --- | --- | --- | --- | --- | --- | --- | --- | --- | --- | --- | --- | --- | --- | --- | --- | --- | --- | --- | --- | --- | --- | --- | --- | --- | --- | --- | --- | --- | --- | --- | --- | --- | --- | --- | --- | --- | --- | --- | --- |

XP\_026545516.1

back to list

  

| Protein Coverage
| Supporting Peptides
|

Protein Coverage:

Supporting Peptides:

| Peptide | Uniq | -10lgP | Mass | Length | ppm | m/z | z | RT | Fraction | Scan | Source File | Area F1 | Area F10 | Area F11 | Area F12 | Area F13 | Area F2 | Area F3 | Area F4 | Area F5 | Area F6 | Area F7 | Area F8 | Area F9 | #Feature | #Feature F1 | #Feature F10 | #Feature F11 | #Feature F12 | #Feature F13 | #Feature F2 | #Feature F3 | #Feature F4 | #Feature F5 | #Feature F6 | #Feature F7 | #Feature F8 | #Feature F9 | Start | End | PTM | AScore | Found By |
| --- | --- | --- | --- | --- | --- | --- | --- | --- | --- | --- | --- | --- | --- | --- | --- | --- | --- | --- | --- | --- | --- | --- | --- | --- | --- | --- | --- | --- | --- | --- | --- | --- | --- | --- | --- | --- | --- | --- | --- | --- | --- | --- | --- |
| R.SSDNDFLIFK.D | Y | 62.29 | 1184.5713 | 10 | 0.1 | 593.2930 | 2 | 64.20 | 4 | F4:42212 | NaNaKA16\_F12.raw |  |  |  | 1.462E7 |  |  |  |  |  |  |  |  |  | 1 | 0 | 0 | 0 | 1 | 0 | 0 | 0 | 0 | 0 | 0 | 0 | 0 | 0 | 75 | 84 |  |  | PEAKS DB |
| total 1 peptides |  |  |  |  |  |  |  |  |  |  |  |  |  |  |  |  |  |  |  |  |  |  |  |  |  |  |  |  |  |  |  |  |  |  |  |  |  |  |  |  |  |  |  |
| --- | --- | --- | --- | --- | --- | --- | --- | --- | --- | --- | --- | --- | --- | --- | --- | --- | --- | --- | --- | --- | --- | --- | --- | --- | --- | --- | --- | --- | --- | --- | --- | --- | --- | --- | --- | --- | --- | --- | --- | --- | --- | --- | --- |

ETE56526.1

back to list

  

| Protein Coverage
| Supporting Peptides
|

Protein Coverage:

Supporting Peptides:

| Peptide | Uniq | -10lgP | Mass | Length | ppm | m/z | z | RT | Fraction | Scan | Source File | Area F1 | Area F10 | Area F11 | Area F12 | Area F13 | Area F2 | Area F3 | Area F4 | Area F5 | Area F6 | Area F7 | Area F8 | Area F9 | #Feature | #Feature F1 | #Feature F10 | #Feature F11 | #Feature F12 | #Feature F13 | #Feature F2 | #Feature F3 | #Feature F4 | #Feature F5 | #Feature F6 | #Feature F7 | #Feature F8 | #Feature F9 | Start | End | PTM | AScore | Found By |
| --- | --- | --- | --- | --- | --- | --- | --- | --- | --- | --- | --- | --- | --- | --- | --- | --- | --- | --- | --- | --- | --- | --- | --- | --- | --- | --- | --- | --- | --- | --- | --- | --- | --- | --- | --- | --- | --- | --- | --- | --- | --- | --- | --- |
| R.SSDNDFLIFK.N | Y | 62.29 | 1184.5713 | 10 | 0.1 | 593.2930 | 2 | 64.20 | 4 | F4:42212 | NaNaKA16\_F12.raw |  |  |  | 1.462E7 |  |  |  |  |  |  |  |  |  | 1 | 0 | 0 | 0 | 1 | 0 | 0 | 0 | 0 | 0 | 0 | 0 | 0 | 0 | 52 | 61 |  |  | PEAKS DB |
| total 1 peptides |  |  |  |  |  |  |  |  |  |  |  |  |  |  |  |  |  |  |  |  |  |  |  |  |  |  |  |  |  |  |  |  |  |  |  |  |  |  |  |  |  |  |  |
| --- | --- | --- | --- | --- | --- | --- | --- | --- | --- | --- | --- | --- | --- | --- | --- | --- | --- | --- | --- | --- | --- | --- | --- | --- | --- | --- | --- | --- | --- | --- | --- | --- | --- | --- | --- | --- | --- | --- | --- | --- | --- | --- | --- |

XP\_034261801.1

back to list

  

| Protein Coverage
| Supporting Peptides
|

Protein Coverage:

Supporting Peptides:

| Peptide | Uniq | -10lgP | Mass | Length | ppm | m/z | z | RT | Fraction | Scan | Source File | Area F1 | Area F10 | Area F11 | Area F12 | Area F13 | Area F2 | Area F3 | Area F4 | Area F5 | Area F6 | Area F7 | Area F8 | Area F9 | #Feature | #Feature F1 | #Feature F10 | #Feature F11 | #Feature F12 | #Feature F13 | #Feature F2 | #Feature F3 | #Feature F4 | #Feature F5 | #Feature F6 | #Feature F7 | #Feature F8 | #Feature F9 | Start | End | PTM | AScore | Found By |
| --- | --- | --- | --- | --- | --- | --- | --- | --- | --- | --- | --- | --- | --- | --- | --- | --- | --- | --- | --- | --- | --- | --- | --- | --- | --- | --- | --- | --- | --- | --- | --- | --- | --- | --- | --- | --- | --- | --- | --- | --- | --- | --- | --- |
| R.SSDNDFLIFK.E | Y | 62.29 | 1184.5713 | 10 | 0.1 | 593.2930 | 2 | 64.20 | 4 | F4:42212 | NaNaKA16\_F12.raw |  |  |  | 1.462E7 |  |  |  |  |  |  |  |  |  | 1 | 0 | 0 | 0 | 1 | 0 | 0 | 0 | 0 | 0 | 0 | 0 | 0 | 0 | 75 | 84 |  |  | PEAKS DB |
| total 1 peptides |  |  |  |  |  |  |  |  |  |  |  |  |  |  |  |  |  |  |  |  |  |  |  |  |  |  |  |  |  |  |  |  |  |  |  |  |  |  |  |  |  |  |  |
| --- | --- | --- | --- | --- | --- | --- | --- | --- | --- | --- | --- | --- | --- | --- | --- | --- | --- | --- | --- | --- | --- | --- | --- | --- | --- | --- | --- | --- | --- | --- | --- | --- | --- | --- | --- | --- | --- | --- | --- | --- | --- | --- | --- |

XP\_026572193.1

back to list

  

| Protein Coverage
| Supporting Peptides
|

Protein Coverage:

Supporting Peptides:

| Peptide | Uniq | -10lgP | Mass | Length | ppm | m/z | z | RT | Fraction | Scan | Source File | Area F1 | Area F10 | Area F11 | Area F12 | Area F13 | Area F2 | Area F3 | Area F4 | Area F5 | Area F6 | Area F7 | Area F8 | Area F9 | #Feature | #Feature F1 | #Feature F10 | #Feature F11 | #Feature F12 | #Feature F13 | #Feature F2 | #Feature F3 | #Feature F4 | #Feature F5 | #Feature F6 | #Feature F7 | #Feature F8 | #Feature F9 | Start | End | PTM | AScore | Found By |
| --- | --- | --- | --- | --- | --- | --- | --- | --- | --- | --- | --- | --- | --- | --- | --- | --- | --- | --- | --- | --- | --- | --- | --- | --- | --- | --- | --- | --- | --- | --- | --- | --- | --- | --- | --- | --- | --- | --- | --- | --- | --- | --- | --- |
| R.SSDNDFLIFK.D | Y | 62.29 | 1184.5713 | 10 | 0.1 | 593.2930 | 2 | 64.20 | 4 | F4:42212 | NaNaKA16\_F12.raw |  |  |  | 1.462E7 |  |  |  |  |  |  |  |  |  | 1 | 0 | 0 | 0 | 1 | 0 | 0 | 0 | 0 | 0 | 0 | 0 | 0 | 0 | 75 | 84 |  |  | PEAKS DB |
| total 1 peptides |  |  |  |  |  |  |  |  |  |  |  |  |  |  |  |  |  |  |  |  |  |  |  |  |  |  |  |  |  |  |  |  |  |  |  |  |  |  |  |  |  |  |  |
| --- | --- | --- | --- | --- | --- | --- | --- | --- | --- | --- | --- | --- | --- | --- | --- | --- | --- | --- | --- | --- | --- | --- | --- | --- | --- | --- | --- | --- | --- | --- | --- | --- | --- | --- | --- | --- | --- | --- | --- | --- | --- | --- | --- |

XP\_015678948.1

back to list

  

| Protein Coverage
| Supporting Peptides
|

Protein Coverage:

Supporting Peptides:

| Peptide | Uniq | -10lgP | Mass | Length | ppm | m/z | z | RT | Fraction | Scan | Source File | Area F1 | Area F10 | Area F11 | Area F12 | Area F13 | Area F2 | Area F3 | Area F4 | Area F5 | Area F6 | Area F7 | Area F8 | Area F9 | #Feature | #Feature F1 | #Feature F10 | #Feature F11 | #Feature F12 | #Feature F13 | #Feature F2 | #Feature F3 | #Feature F4 | #Feature F5 | #Feature F6 | #Feature F7 | #Feature F8 | #Feature F9 | Start | End | PTM | AScore | Found By |
| --- | --- | --- | --- | --- | --- | --- | --- | --- | --- | --- | --- | --- | --- | --- | --- | --- | --- | --- | --- | --- | --- | --- | --- | --- | --- | --- | --- | --- | --- | --- | --- | --- | --- | --- | --- | --- | --- | --- | --- | --- | --- | --- | --- |
| R.SSDNDFLIFK.D | Y | 62.29 | 1184.5713 | 10 | 0.1 | 593.2930 | 2 | 64.20 | 4 | F4:42212 | NaNaKA16\_F12.raw |  |  |  | 1.462E7 |  |  |  |  |  |  |  |  |  | 1 | 0 | 0 | 0 | 1 | 0 | 0 | 0 | 0 | 0 | 0 | 0 | 0 | 0 | 75 | 84 |  |  | PEAKS DB |
| total 1 peptides |  |  |  |  |  |  |  |  |  |  |  |  |  |  |  |  |  |  |  |  |  |  |  |  |  |  |  |  |  |  |  |  |  |  |  |  |  |  |  |  |  |  |  |
| --- | --- | --- | --- | --- | --- | --- | --- | --- | --- | --- | --- | --- | --- | --- | --- | --- | --- | --- | --- | --- | --- | --- | --- | --- | --- | --- | --- | --- | --- | --- | --- | --- | --- | --- | --- | --- | --- | --- | --- | --- | --- | --- | --- |

XP\_015680851.2

back to list

  

| Protein Coverage
| Supporting Peptides
|

Protein Coverage:

Supporting Peptides:

| Peptide | Uniq | -10lgP | Mass | Length | ppm | m/z | z | RT | Fraction | Scan | Source File | Area F1 | Area F10 | Area F11 | Area F12 | Area F13 | Area F2 | Area F3 | Area F4 | Area F5 | Area F6 | Area F7 | Area F8 | Area F9 | #Feature | #Feature F1 | #Feature F10 | #Feature F11 | #Feature F12 | #Feature F13 | #Feature F2 | #Feature F3 | #Feature F4 | #Feature F5 | #Feature F6 | #Feature F7 | #Feature F8 | #Feature F9 | Start | End | PTM | AScore | Found By |
| --- | --- | --- | --- | --- | --- | --- | --- | --- | --- | --- | --- | --- | --- | --- | --- | --- | --- | --- | --- | --- | --- | --- | --- | --- | --- | --- | --- | --- | --- | --- | --- | --- | --- | --- | --- | --- | --- | --- | --- | --- | --- | --- | --- |
| K.LTC(+57.02)NFQVWSR.P | Y | 61.93 | 1309.6238 | 10 | -1.1 | 655.8184 | 2 | 47.95 | 2 | F2:27930 | NaNaKA16\_F10.raw |  | 1.9911E7 |  |  |  |  |  |  |  |  |  |  |  | 1 | 0 | 1 | 0 | 0 | 0 | 0 | 0 | 0 | 0 | 0 | 0 | 0 | 0 | 116 | 125 | Carbamidomethylation | C3:Carbamidomethylation:1000.00 | PEAKS DB |
| total 1 peptides |  |  |  |  |  |  |  |  |  |  |  |  |  |  |  |  |  |  |  |  |  |  |  |  |  |  |  |  |  |  |  |  |  |  |  |  |  |  |  |  |  |  |  |
| --- | --- | --- | --- | --- | --- | --- | --- | --- | --- | --- | --- | --- | --- | --- | --- | --- | --- | --- | --- | --- | --- | --- | --- | --- | --- | --- | --- | --- | --- | --- | --- | --- | --- | --- | --- | --- | --- | --- | --- | --- | --- | --- | --- |

XP\_026553567.1

back to list

  

| Protein Coverage
| Supporting Peptides
|

Protein Coverage:

Supporting Peptides:

| Peptide | Uniq | -10lgP | Mass | Length | ppm | m/z | z | RT | Fraction | Scan | Source File | Area F1 | Area F10 | Area F11 | Area F12 | Area F13 | Area F2 | Area F3 | Area F4 | Area F5 | Area F6 | Area F7 | Area F8 | Area F9 | #Feature | #Feature F1 | #Feature F10 | #Feature F11 | #Feature F12 | #Feature F13 | #Feature F2 | #Feature F3 | #Feature F4 | #Feature F5 | #Feature F6 | #Feature F7 | #Feature F8 | #Feature F9 | Start | End | PTM | AScore | Found By |
| --- | --- | --- | --- | --- | --- | --- | --- | --- | --- | --- | --- | --- | --- | --- | --- | --- | --- | --- | --- | --- | --- | --- | --- | --- | --- | --- | --- | --- | --- | --- | --- | --- | --- | --- | --- | --- | --- | --- | --- | --- | --- | --- | --- |
| K.KNQLLHWQAVGNPR.C | Y | 44.56 | 1659.8958 | 14 | 0.0 | 554.3058 | 3 | 20.07 | 9 | F9:7331 | NaNaKA16\_F5.raw |  |  |  |  |  |  |  |  | 5.4621E5 |  |  |  |  | 1 | 0 | 0 | 0 | 0 | 0 | 0 | 0 | 0 | 1 | 0 | 0 | 0 | 0 | 229 | 242 |  |  | PEAKS DB |
| total 1 peptides |  |  |  |  |  |  |  |  |  |  |  |  |  |  |  |  |  |  |  |  |  |  |  |  |  |  |  |  |  |  |  |  |  |  |  |  |  |  |  |  |  |  |  |
| --- | --- | --- | --- | --- | --- | --- | --- | --- | --- | --- | --- | --- | --- | --- | --- | --- | --- | --- | --- | --- | --- | --- | --- | --- | --- | --- | --- | --- | --- | --- | --- | --- | --- | --- | --- | --- | --- | --- | --- | --- | --- | --- | --- |

XP\_013928420.1

back to list

  

| Protein Coverage
| Supporting Peptides
|

Protein Coverage:

Supporting Peptides:

| Peptide | Uniq | -10lgP | Mass | Length | ppm | m/z | z | RT | Fraction | Scan | Source File | Area F1 | Area F10 | Area F11 | Area F12 | Area F13 | Area F2 | Area F3 | Area F4 | Area F5 | Area F6 | Area F7 | Area F8 | Area F9 | #Feature | #Feature F1 | #Feature F10 | #Feature F11 | #Feature F12 | #Feature F13 | #Feature F2 | #Feature F3 | #Feature F4 | #Feature F5 | #Feature F6 | #Feature F7 | #Feature F8 | #Feature F9 | Start | End | PTM | AScore | Found By |
| --- | --- | --- | --- | --- | --- | --- | --- | --- | --- | --- | --- | --- | --- | --- | --- | --- | --- | --- | --- | --- | --- | --- | --- | --- | --- | --- | --- | --- | --- | --- | --- | --- | --- | --- | --- | --- | --- | --- | --- | --- | --- | --- | --- |
| K.SIGLDITVNC(+57.02)QTLTSK.C | Y | 60.77 | 1748.8978 | 16 | 0.9 | 875.4570 | 2 | 60.29 | 6 | F6:40580 | NaNaKA16\_F2.raw |  |  |  |  |  | 7.5948E5 |  |  |  |  |  |  |  | 1 | 0 | 0 | 0 | 0 | 0 | 1 | 0 | 0 | 0 | 0 | 0 | 0 | 0 | 49 | 64 | Carbamidomethylation | C10:Carbamidomethylation:1000.00 | PEAKS DB |
| total 1 peptides |  |  |  |  |  |  |  |  |  |  |  |  |  |  |  |  |  |  |  |  |  |  |  |  |  |  |  |  |  |  |  |  |  |  |  |  |  |  |  |  |  |  |  |
| --- | --- | --- | --- | --- | --- | --- | --- | --- | --- | --- | --- | --- | --- | --- | --- | --- | --- | --- | --- | --- | --- | --- | --- | --- | --- | --- | --- | --- | --- | --- | --- | --- | --- | --- | --- | --- | --- | --- | --- | --- | --- | --- | --- |

ETE70163.1

back to list

  

| Protein Coverage
| Supporting Peptides
|

Protein Coverage:

Supporting Peptides:

| Peptide | Uniq | -10lgP | Mass | Length | ppm | m/z | z | RT | Fraction | Scan | Source File | Area F1 | Area F10 | Area F11 | Area F12 | Area F13 | Area F2 | Area F3 | Area F4 | Area F5 | Area F6 | Area F7 | Area F8 | Area F9 | #Feature | #Feature F1 | #Feature F10 | #Feature F11 | #Feature F12 | #Feature F13 | #Feature F2 | #Feature F3 | #Feature F4 | #Feature F5 | #Feature F6 | #Feature F7 | #Feature F8 | #Feature F9 | Start | End | PTM | AScore | Found By |
| --- | --- | --- | --- | --- | --- | --- | --- | --- | --- | --- | --- | --- | --- | --- | --- | --- | --- | --- | --- | --- | --- | --- | --- | --- | --- | --- | --- | --- | --- | --- | --- | --- | --- | --- | --- | --- | --- | --- | --- | --- | --- | --- | --- |
| K.SIGLDITVNC(+57.02)QTLTSK.C | Y | 60.77 | 1748.8978 | 16 | 0.9 | 875.4570 | 2 | 60.29 | 6 | F6:40580 | NaNaKA16\_F2.raw |  |  |  |  |  | 7.5948E5 |  |  |  |  |  |  |  | 1 | 0 | 0 | 0 | 0 | 0 | 1 | 0 | 0 | 0 | 0 | 0 | 0 | 0 | 49 | 64 | Carbamidomethylation | C10:Carbamidomethylation:1000.00 | PEAKS DB |
| total 1 peptides |  |  |  |  |  |  |  |  |  |  |  |  |  |  |  |  |  |  |  |  |  |  |  |  |  |  |  |  |  |  |  |  |  |  |  |  |  |  |  |  |  |  |  |
| --- | --- | --- | --- | --- | --- | --- | --- | --- | --- | --- | --- | --- | --- | --- | --- | --- | --- | --- | --- | --- | --- | --- | --- | --- | --- | --- | --- | --- | --- | --- | --- | --- | --- | --- | --- | --- | --- | --- | --- | --- | --- | --- | --- |

XP\_032073624.1

back to list

  

| Protein Coverage
| Supporting Peptides
|

Protein Coverage:

Supporting Peptides:

| Peptide | Uniq | -10lgP | Mass | Length | ppm | m/z | z | RT | Fraction | Scan | Source File | Area F1 | Area F10 | Area F11 | Area F12 | Area F13 | Area F2 | Area F3 | Area F4 | Area F5 | Area F6 | Area F7 | Area F8 | Area F9 | #Feature | #Feature F1 | #Feature F10 | #Feature F11 | #Feature F12 | #Feature F13 | #Feature F2 | #Feature F3 | #Feature F4 | #Feature F5 | #Feature F6 | #Feature F7 | #Feature F8 | #Feature F9 | Start | End | PTM | AScore | Found By |
| --- | --- | --- | --- | --- | --- | --- | --- | --- | --- | --- | --- | --- | --- | --- | --- | --- | --- | --- | --- | --- | --- | --- | --- | --- | --- | --- | --- | --- | --- | --- | --- | --- | --- | --- | --- | --- | --- | --- | --- | --- | --- | --- | --- |
| K.VTFGC(+57.02)DYGFR.I | Y | 60.71 | 1220.5284 | 10 | 0.2 | 611.2716 | 2 | 46.60 | 3 | F3:26349 | NaNaKA16\_F11.raw |  | 1.2432E7 | 8.5152E7 | 3.2948E6 |  |  |  |  |  |  |  |  |  | 3 | 0 | 1 | 1 | 1 | 0 | 0 | 0 | 0 | 0 | 0 | 0 | 0 | 0 | 117 | 126 | Carbamidomethylation | C5:Carbamidomethylation:1000.00 | PEAKS DB |
| total 1 peptides |  |  |  |  |  |  |  |  |  |  |  |  |  |  |  |  |  |  |  |  |  |  |  |  |  |  |  |  |  |  |  |  |  |  |  |  |  |  |  |  |  |  |  |
| --- | --- | --- | --- | --- | --- | --- | --- | --- | --- | --- | --- | --- | --- | --- | --- | --- | --- | --- | --- | --- | --- | --- | --- | --- | --- | --- | --- | --- | --- | --- | --- | --- | --- | --- | --- | --- | --- | --- | --- | --- | --- | --- | --- |

XP\_032073625.1

back to list

  

| Protein Coverage
| Supporting Peptides
|

Protein Coverage:

Supporting Peptides:

| Peptide | Uniq | -10lgP | Mass | Length | ppm | m/z | z | RT | Fraction | Scan | Source File | Area F1 | Area F10 | Area F11 | Area F12 | Area F13 | Area F2 | Area F3 | Area F4 | Area F5 | Area F6 | Area F7 | Area F8 | Area F9 | #Feature | #Feature F1 | #Feature F10 | #Feature F11 | #Feature F12 | #Feature F13 | #Feature F2 | #Feature F3 | #Feature F4 | #Feature F5 | #Feature F6 | #Feature F7 | #Feature F8 | #Feature F9 | Start | End | PTM | AScore | Found By |
| --- | --- | --- | --- | --- | --- | --- | --- | --- | --- | --- | --- | --- | --- | --- | --- | --- | --- | --- | --- | --- | --- | --- | --- | --- | --- | --- | --- | --- | --- | --- | --- | --- | --- | --- | --- | --- | --- | --- | --- | --- | --- | --- | --- |
| K.VTFGC(+57.02)DYGFR.I | Y | 60.71 | 1220.5284 | 10 | 0.2 | 611.2716 | 2 | 46.60 | 3 | F3:26349 | NaNaKA16\_F11.raw |  | 1.2432E7 | 8.5152E7 | 3.2948E6 |  |  |  |  |  |  |  |  |  | 3 | 0 | 1 | 1 | 1 | 0 | 0 | 0 | 0 | 0 | 0 | 0 | 0 | 0 | 117 | 126 | Carbamidomethylation | C5:Carbamidomethylation:1000.00 | PEAKS DB |
| total 1 peptides |  |  |  |  |  |  |  |  |  |  |  |  |  |  |  |  |  |  |  |  |  |  |  |  |  |  |  |  |  |  |  |  |  |  |  |  |  |  |  |  |  |  |  |
| --- | --- | --- | --- | --- | --- | --- | --- | --- | --- | --- | --- | --- | --- | --- | --- | --- | --- | --- | --- | --- | --- | --- | --- | --- | --- | --- | --- | --- | --- | --- | --- | --- | --- | --- | --- | --- | --- | --- | --- | --- | --- | --- | --- |

XP\_032073626.1

back to list

  

| Protein Coverage
| Supporting Peptides
|

Protein Coverage:

Supporting Peptides:

| Peptide | Uniq | -10lgP | Mass | Length | ppm | m/z | z | RT | Fraction | Scan | Source File | Area F1 | Area F10 | Area F11 | Area F12 | Area F13 | Area F2 | Area F3 | Area F4 | Area F5 | Area F6 | Area F7 | Area F8 | Area F9 | #Feature | #Feature F1 | #Feature F10 | #Feature F11 | #Feature F12 | #Feature F13 | #Feature F2 | #Feature F3 | #Feature F4 | #Feature F5 | #Feature F6 | #Feature F7 | #Feature F8 | #Feature F9 | Start | End | PTM | AScore | Found By |
| --- | --- | --- | --- | --- | --- | --- | --- | --- | --- | --- | --- | --- | --- | --- | --- | --- | --- | --- | --- | --- | --- | --- | --- | --- | --- | --- | --- | --- | --- | --- | --- | --- | --- | --- | --- | --- | --- | --- | --- | --- | --- | --- | --- |
| K.VTFGC(+57.02)DYGFR.I | Y | 60.71 | 1220.5284 | 10 | 0.2 | 611.2716 | 2 | 46.60 | 3 | F3:26349 | NaNaKA16\_F11.raw |  | 1.2432E7 | 8.5152E7 | 3.2948E6 |  |  |  |  |  |  |  |  |  | 3 | 0 | 1 | 1 | 1 | 0 | 0 | 0 | 0 | 0 | 0 | 0 | 0 | 0 | 117 | 126 | Carbamidomethylation | C5:Carbamidomethylation:1000.00 | PEAKS DB |
| total 1 peptides |  |  |  |  |  |  |  |  |  |  |  |  |  |  |  |  |  |  |  |  |  |  |  |  |  |  |  |  |  |  |  |  |  |  |  |  |  |  |  |  |  |  |  |
| --- | --- | --- | --- | --- | --- | --- | --- | --- | --- | --- | --- | --- | --- | --- | --- | --- | --- | --- | --- | --- | --- | --- | --- | --- | --- | --- | --- | --- | --- | --- | --- | --- | --- | --- | --- | --- | --- | --- | --- | --- | --- | --- | --- |

ETE72142.1

back to list

  

| Protein Coverage
| Supporting Peptides
|

Protein Coverage:

Supporting Peptides:

| Peptide | Uniq | -10lgP | Mass | Length | ppm | m/z | z | RT | Fraction | Scan | Source File | Area F1 | Area F10 | Area F11 | Area F12 | Area F13 | Area F2 | Area F3 | Area F4 | Area F5 | Area F6 | Area F7 | Area F8 | Area F9 | #Feature | #Feature F1 | #Feature F10 | #Feature F11 | #Feature F12 | #Feature F13 | #Feature F2 | #Feature F3 | #Feature F4 | #Feature F5 | #Feature F6 | #Feature F7 | #Feature F8 | #Feature F9 | Start | End | PTM | AScore | Found By |
| --- | --- | --- | --- | --- | --- | --- | --- | --- | --- | --- | --- | --- | --- | --- | --- | --- | --- | --- | --- | --- | --- | --- | --- | --- | --- | --- | --- | --- | --- | --- | --- | --- | --- | --- | --- | --- | --- | --- | --- | --- | --- | --- | --- |
| K.VGIKPGSTYYQLTAIK.E | Y | 42.50 | 1737.9664 | 16 | -0.1 | 580.3293 | 3 | 43.57 | 4 | F4:24771 | NaNaKA16\_F12.raw |  |  |  | 0 |  |  |  |  |  |  |  |  |  | 0 | 0 | 0 | 0 | 0 | 0 | 0 | 0 | 0 | 0 | 0 | 0 | 0 | 0 | 103 | 118 |  |  | PEAKS DB |
| total 1 peptides |  |  |  |  |  |  |  |  |  |  |  |  |  |  |  |  |  |  |  |  |  |  |  |  |  |  |  |  |  |  |  |  |  |  |  |  |  |  |  |  |  |  |  |
| --- | --- | --- | --- | --- | --- | --- | --- | --- | --- | --- | --- | --- | --- | --- | --- | --- | --- | --- | --- | --- | --- | --- | --- | --- | --- | --- | --- | --- | --- | --- | --- | --- | --- | --- | --- | --- | --- | --- | --- | --- | --- | --- | --- |

LAB36464.1

back to list

  

| Protein Coverage
| Supporting Peptides
|

Protein Coverage:

Supporting Peptides:

| Peptide | Uniq | -10lgP | Mass | Length | ppm | m/z | z | RT | Fraction | Scan | Source File | Area F1 | Area F10 | Area F11 | Area F12 | Area F13 | Area F2 | Area F3 | Area F4 | Area F5 | Area F6 | Area F7 | Area F8 | Area F9 | #Feature | #Feature F1 | #Feature F10 | #Feature F11 | #Feature F12 | #Feature F13 | #Feature F2 | #Feature F3 | #Feature F4 | #Feature F5 | #Feature F6 | #Feature F7 | #Feature F8 | #Feature F9 | Start | End | PTM | AScore | Found By |
| --- | --- | --- | --- | --- | --- | --- | --- | --- | --- | --- | --- | --- | --- | --- | --- | --- | --- | --- | --- | --- | --- | --- | --- | --- | --- | --- | --- | --- | --- | --- | --- | --- | --- | --- | --- | --- | --- | --- | --- | --- | --- | --- | --- |
| K.RPLHLHNQELSFLVHTWDLEGR.K | Y | 59.68 | 2696.3936 | 22 | 0.4 | 675.1060 | 4 | 64.77 | 12 | F12:43187 | NaNaKA16\_F8.raw |  |  |  |  |  |  |  |  |  |  |  | 2.7719E6 |  | 1 | 0 | 0 | 0 | 0 | 0 | 0 | 0 | 0 | 0 | 0 | 0 | 1 | 0 | 89 | 110 |  |  | PEAKS DB |
| total 1 peptides |  |  |  |  |  |  |  |  |  |  |  |  |  |  |  |  |  |  |  |  |  |  |  |  |  |  |  |  |  |  |  |  |  |  |  |  |  |  |  |  |  |  |  |
| --- | --- | --- | --- | --- | --- | --- | --- | --- | --- | --- | --- | --- | --- | --- | --- | --- | --- | --- | --- | --- | --- | --- | --- | --- | --- | --- | --- | --- | --- | --- | --- | --- | --- | --- | --- | --- | --- | --- | --- | --- | --- | --- | --- |

XP\_015686076.1

back to list

  

| Protein Coverage
| Supporting Peptides
|

Protein Coverage:

Supporting Peptides:

| Peptide | Uniq | -10lgP | Mass | Length | ppm | m/z | z | RT | Fraction | Scan | Source File | Area F1 | Area F10 | Area F11 | Area F12 | Area F13 | Area F2 | Area F3 | Area F4 | Area F5 | Area F6 | Area F7 | Area F8 | Area F9 | #Feature | #Feature F1 | #Feature F10 | #Feature F11 | #Feature F12 | #Feature F13 | #Feature F2 | #Feature F3 | #Feature F4 | #Feature F5 | #Feature F6 | #Feature F7 | #Feature F8 | #Feature F9 | Start | End | PTM | AScore | Found By |
| --- | --- | --- | --- | --- | --- | --- | --- | --- | --- | --- | --- | --- | --- | --- | --- | --- | --- | --- | --- | --- | --- | --- | --- | --- | --- | --- | --- | --- | --- | --- | --- | --- | --- | --- | --- | --- | --- | --- | --- | --- | --- | --- | --- |
| R.SYGLFSYATR.S | Y | 59.64 | 1163.5610 | 10 | 0.4 | 582.7880 | 2 | 52.44 | 4 | F4:32236 | NaNaKA16\_F12.raw |  |  |  | 1.2509E7 |  |  |  |  |  |  |  |  |  | 1 | 0 | 0 | 0 | 1 | 0 | 0 | 0 | 0 | 0 | 0 | 0 | 0 | 0 | 43 | 52 |  |  | PEAKS DB |
| total 1 peptides |  |  |  |  |  |  |  |  |  |  |  |  |  |  |  |  |  |  |  |  |  |  |  |  |  |  |  |  |  |  |  |  |  |  |  |  |  |  |  |  |  |  |  |
| --- | --- | --- | --- | --- | --- | --- | --- | --- | --- | --- | --- | --- | --- | --- | --- | --- | --- | --- | --- | --- | --- | --- | --- | --- | --- | --- | --- | --- | --- | --- | --- | --- | --- | --- | --- | --- | --- | --- | --- | --- | --- | --- | --- |

XP\_034262785.1

back to list

  

| Protein Coverage
| Supporting Peptides
|

Protein Coverage:

Supporting Peptides:

| Peptide | Uniq | -10lgP | Mass | Length | ppm | m/z | z | RT | Fraction | Scan | Source File | Area F1 | Area F10 | Area F11 | Area F12 | Area F13 | Area F2 | Area F3 | Area F4 | Area F5 | Area F6 | Area F7 | Area F8 | Area F9 | #Feature | #Feature F1 | #Feature F10 | #Feature F11 | #Feature F12 | #Feature F13 | #Feature F2 | #Feature F3 | #Feature F4 | #Feature F5 | #Feature F6 | #Feature F7 | #Feature F8 | #Feature F9 | Start | End | PTM | AScore | Found By |
| --- | --- | --- | --- | --- | --- | --- | --- | --- | --- | --- | --- | --- | --- | --- | --- | --- | --- | --- | --- | --- | --- | --- | --- | --- | --- | --- | --- | --- | --- | --- | --- | --- | --- | --- | --- | --- | --- | --- | --- | --- | --- | --- | --- |
| R.LDPAHIELSPMQIR.T | Y | 58.50 | 1618.8501 | 14 | 0.5 | 540.6243 | 3 | 56.64 | 3 | F3:33454 | NaNaKA16\_F11.raw |  | 1.4943E6 | 8.1117E5 |  |  |  |  |  |  |  |  |  |  | 3 | 0 | 2 | 1 | 0 | 0 | 0 | 0 | 0 | 0 | 0 | 0 | 0 | 0 | 976 | 989 |  |  | PEAKS DB |
| total 1 peptides |  |  |  |  |  |  |  |  |  |  |  |  |  |  |  |  |  |  |  |  |  |  |  |  |  |  |  |  |  |  |  |  |  |  |  |  |  |  |  |  |  |  |  |
| --- | --- | --- | --- | --- | --- | --- | --- | --- | --- | --- | --- | --- | --- | --- | --- | --- | --- | --- | --- | --- | --- | --- | --- | --- | --- | --- | --- | --- | --- | --- | --- | --- | --- | --- | --- | --- | --- | --- | --- | --- | --- | --- | --- |

LAB27223.1

back to list

  

| Protein Coverage
| Supporting Peptides
|

Protein Coverage:

Supporting Peptides:

| Peptide | Uniq | -10lgP | Mass | Length | ppm | m/z | z | RT | Fraction | Scan | Source File | Area F1 | Area F10 | Area F11 | Area F12 | Area F13 | Area F2 | Area F3 | Area F4 | Area F5 | Area F6 | Area F7 | Area F8 | Area F9 | #Feature | #Feature F1 | #Feature F10 | #Feature F11 | #Feature F12 | #Feature F13 | #Feature F2 | #Feature F3 | #Feature F4 | #Feature F5 | #Feature F6 | #Feature F7 | #Feature F8 | #Feature F9 | Start | End | PTM | AScore | Found By |
| --- | --- | --- | --- | --- | --- | --- | --- | --- | --- | --- | --- | --- | --- | --- | --- | --- | --- | --- | --- | --- | --- | --- | --- | --- | --- | --- | --- | --- | --- | --- | --- | --- | --- | --- | --- | --- | --- | --- | --- | --- | --- | --- | --- |
| R.LDPAHIELSPMQIR.T | Y | 58.50 | 1618.8501 | 14 | 0.5 | 540.6243 | 3 | 56.64 | 3 | F3:33454 | NaNaKA16\_F11.raw |  | 1.4943E6 | 8.1117E5 |  |  |  |  |  |  |  |  |  |  | 3 | 0 | 2 | 1 | 0 | 0 | 0 | 0 | 0 | 0 | 0 | 0 | 0 | 0 | 975 | 988 |  |  | PEAKS DB |
| total 1 peptides |  |  |  |  |  |  |  |  |  |  |  |  |  |  |  |  |  |  |  |  |  |  |  |  |  |  |  |  |  |  |  |  |  |  |  |  |  |  |  |  |  |  |  |
| --- | --- | --- | --- | --- | --- | --- | --- | --- | --- | --- | --- | --- | --- | --- | --- | --- | --- | --- | --- | --- | --- | --- | --- | --- | --- | --- | --- | --- | --- | --- | --- | --- | --- | --- | --- | --- | --- | --- | --- | --- | --- | --- | --- |

XP\_026537394.1

back to list

  

| Protein Coverage
| Supporting Peptides
|

Protein Coverage:

Supporting Peptides:

| Peptide | Uniq | -10lgP | Mass | Length | ppm | m/z | z | RT | Fraction | Scan | Source File | Area F1 | Area F10 | Area F11 | Area F12 | Area F13 | Area F2 | Area F3 | Area F4 | Area F5 | Area F6 | Area F7 | Area F8 | Area F9 | #Feature | #Feature F1 | #Feature F10 | #Feature F11 | #Feature F12 | #Feature F13 | #Feature F2 | #Feature F3 | #Feature F4 | #Feature F5 | #Feature F6 | #Feature F7 | #Feature F8 | #Feature F9 | Start | End | PTM | AScore | Found By |
| --- | --- | --- | --- | --- | --- | --- | --- | --- | --- | --- | --- | --- | --- | --- | --- | --- | --- | --- | --- | --- | --- | --- | --- | --- | --- | --- | --- | --- | --- | --- | --- | --- | --- | --- | --- | --- | --- | --- | --- | --- | --- | --- | --- |
| R.LDPAHIELSPMQIR.T | Y | 58.50 | 1618.8501 | 14 | 0.5 | 540.6243 | 3 | 56.64 | 3 | F3:33454 | NaNaKA16\_F11.raw |  | 1.4943E6 | 8.1117E5 |  |  |  |  |  |  |  |  |  |  | 3 | 0 | 2 | 1 | 0 | 0 | 0 | 0 | 0 | 0 | 0 | 0 | 0 | 0 | 961 | 974 |  |  | PEAKS DB |
| total 1 peptides |  |  |  |  |  |  |  |  |  |  |  |  |  |  |  |  |  |  |  |  |  |  |  |  |  |  |  |  |  |  |  |  |  |  |  |  |  |  |  |  |  |  |  |
| --- | --- | --- | --- | --- | --- | --- | --- | --- | --- | --- | --- | --- | --- | --- | --- | --- | --- | --- | --- | --- | --- | --- | --- | --- | --- | --- | --- | --- | --- | --- | --- | --- | --- | --- | --- | --- | --- | --- | --- | --- | --- | --- | --- |

XP\_026537257.1

back to list

  

| Protein Coverage
| Supporting Peptides
|

Protein Coverage:

Supporting Peptides:

| Peptide | Uniq | -10lgP | Mass | Length | ppm | m/z | z | RT | Fraction | Scan | Source File | Area F1 | Area F10 | Area F11 | Area F12 | Area F13 | Area F2 | Area F3 | Area F4 | Area F5 | Area F6 | Area F7 | Area F8 | Area F9 | #Feature | #Feature F1 | #Feature F10 | #Feature F11 | #Feature F12 | #Feature F13 | #Feature F2 | #Feature F3 | #Feature F4 | #Feature F5 | #Feature F6 | #Feature F7 | #Feature F8 | #Feature F9 | Start | End | PTM | AScore | Found By |
| --- | --- | --- | --- | --- | --- | --- | --- | --- | --- | --- | --- | --- | --- | --- | --- | --- | --- | --- | --- | --- | --- | --- | --- | --- | --- | --- | --- | --- | --- | --- | --- | --- | --- | --- | --- | --- | --- | --- | --- | --- | --- | --- | --- |
| R.LDPAHIELSPMQIR.T | Y | 58.50 | 1618.8501 | 14 | 0.5 | 540.6243 | 3 | 56.64 | 3 | F3:33454 | NaNaKA16\_F11.raw |  | 1.4943E6 | 8.1117E5 |  |  |  |  |  |  |  |  |  |  | 3 | 0 | 2 | 1 | 0 | 0 | 0 | 0 | 0 | 0 | 0 | 0 | 0 | 0 | 961 | 974 |  |  | PEAKS DB |
| total 1 peptides |  |  |  |  |  |  |  |  |  |  |  |  |  |  |  |  |  |  |  |  |  |  |  |  |  |  |  |  |  |  |  |  |  |  |  |  |  |  |  |  |  |  |  |
| --- | --- | --- | --- | --- | --- | --- | --- | --- | --- | --- | --- | --- | --- | --- | --- | --- | --- | --- | --- | --- | --- | --- | --- | --- | --- | --- | --- | --- | --- | --- | --- | --- | --- | --- | --- | --- | --- | --- | --- | --- | --- | --- | --- |

XP\_026552070.1

back to list

  

| Protein Coverage
| Supporting Peptides
|

Protein Coverage:

Supporting Peptides:

| Peptide | Uniq | -10lgP | Mass | Length | ppm | m/z | z | RT | Fraction | Scan | Source File | Area F1 | Area F10 | Area F11 | Area F12 | Area F13 | Area F2 | Area F3 | Area F4 | Area F5 | Area F6 | Area F7 | Area F8 | Area F9 | #Feature | #Feature F1 | #Feature F10 | #Feature F11 | #Feature F12 | #Feature F13 | #Feature F2 | #Feature F3 | #Feature F4 | #Feature F5 | #Feature F6 | #Feature F7 | #Feature F8 | #Feature F9 | Start | End | PTM | AScore | Found By |
| --- | --- | --- | --- | --- | --- | --- | --- | --- | --- | --- | --- | --- | --- | --- | --- | --- | --- | --- | --- | --- | --- | --- | --- | --- | --- | --- | --- | --- | --- | --- | --- | --- | --- | --- | --- | --- | --- | --- | --- | --- | --- | --- | --- |
| R.LDPAHIELSPMQIR.T | Y | 58.50 | 1618.8501 | 14 | 0.5 | 540.6243 | 3 | 56.64 | 3 | F3:33454 | NaNaKA16\_F11.raw |  | 1.4943E6 | 8.1117E5 |  |  |  |  |  |  |  |  |  |  | 3 | 0 | 2 | 1 | 0 | 0 | 0 | 0 | 0 | 0 | 0 | 0 | 0 | 0 | 961 | 974 |  |  | PEAKS DB |
| total 1 peptides |  |  |  |  |  |  |  |  |  |  |  |  |  |  |  |  |  |  |  |  |  |  |  |  |  |  |  |  |  |  |  |  |  |  |  |  |  |  |  |  |  |  |  |
| --- | --- | --- | --- | --- | --- | --- | --- | --- | --- | --- | --- | --- | --- | --- | --- | --- | --- | --- | --- | --- | --- | --- | --- | --- | --- | --- | --- | --- | --- | --- | --- | --- | --- | --- | --- | --- | --- | --- | --- | --- | --- | --- | --- |

LAB59085.1

back to list

  

| Protein Coverage
| Supporting Peptides
|

Protein Coverage:

Supporting Peptides:

| Peptide | Uniq | -10lgP | Mass | Length | ppm | m/z | z | RT | Fraction | Scan | Source File | Area F1 | Area F10 | Area F11 | Area F12 | Area F13 | Area F2 | Area F3 | Area F4 | Area F5 | Area F6 | Area F7 | Area F8 | Area F9 | #Feature | #Feature F1 | #Feature F10 | #Feature F11 | #Feature F12 | #Feature F13 | #Feature F2 | #Feature F3 | #Feature F4 | #Feature F5 | #Feature F6 | #Feature F7 | #Feature F8 | #Feature F9 | Start | End | PTM | AScore | Found By |
| --- | --- | --- | --- | --- | --- | --- | --- | --- | --- | --- | --- | --- | --- | --- | --- | --- | --- | --- | --- | --- | --- | --- | --- | --- | --- | --- | --- | --- | --- | --- | --- | --- | --- | --- | --- | --- | --- | --- | --- | --- | --- | --- | --- |
| R.LDPAHIELSPMQIR.T | Y | 58.50 | 1618.8501 | 14 | 0.5 | 540.6243 | 3 | 56.64 | 3 | F3:33454 | NaNaKA16\_F11.raw |  | 1.4943E6 | 8.1117E5 |  |  |  |  |  |  |  |  |  |  | 3 | 0 | 2 | 1 | 0 | 0 | 0 | 0 | 0 | 0 | 0 | 0 | 0 | 0 | 310 | 323 |  |  | PEAKS DB |
| total 1 peptides |  |  |  |  |  |  |  |  |  |  |  |  |  |  |  |  |  |  |  |  |  |  |  |  |  |  |  |  |  |  |  |  |  |  |  |  |  |  |  |  |  |  |  |
| --- | --- | --- | --- | --- | --- | --- | --- | --- | --- | --- | --- | --- | --- | --- | --- | --- | --- | --- | --- | --- | --- | --- | --- | --- | --- | --- | --- | --- | --- | --- | --- | --- | --- | --- | --- | --- | --- | --- | --- | --- | --- | --- | --- |

LAB59082.1

back to list

  

| Protein Coverage
| Supporting Peptides
|

Protein Coverage:

Supporting Peptides:

| Peptide | Uniq | -10lgP | Mass | Length | ppm | m/z | z | RT | Fraction | Scan | Source File | Area F1 | Area F10 | Area F11 | Area F12 | Area F13 | Area F2 | Area F3 | Area F4 | Area F5 | Area F6 | Area F7 | Area F8 | Area F9 | #Feature | #Feature F1 | #Feature F10 | #Feature F11 | #Feature F12 | #Feature F13 | #Feature F2 | #Feature F3 | #Feature F4 | #Feature F5 | #Feature F6 | #Feature F7 | #Feature F8 | #Feature F9 | Start | End | PTM | AScore | Found By |
| --- | --- | --- | --- | --- | --- | --- | --- | --- | --- | --- | --- | --- | --- | --- | --- | --- | --- | --- | --- | --- | --- | --- | --- | --- | --- | --- | --- | --- | --- | --- | --- | --- | --- | --- | --- | --- | --- | --- | --- | --- | --- | --- | --- |
| R.LDPAHIELSPMQIR.T | Y | 58.50 | 1618.8501 | 14 | 0.5 | 540.6243 | 3 | 56.64 | 3 | F3:33454 | NaNaKA16\_F11.raw |  | 1.4943E6 | 8.1117E5 |  |  |  |  |  |  |  |  |  |  | 3 | 0 | 2 | 1 | 0 | 0 | 0 | 0 | 0 | 0 | 0 | 0 | 0 | 0 | 450 | 463 |  |  | PEAKS DB |
| total 1 peptides |  |  |  |  |  |  |  |  |  |  |  |  |  |  |  |  |  |  |  |  |  |  |  |  |  |  |  |  |  |  |  |  |  |  |  |  |  |  |  |  |  |  |  |
| --- | --- | --- | --- | --- | --- | --- | --- | --- | --- | --- | --- | --- | --- | --- | --- | --- | --- | --- | --- | --- | --- | --- | --- | --- | --- | --- | --- | --- | --- | --- | --- | --- | --- | --- | --- | --- | --- | --- | --- | --- | --- | --- | --- |

LAA55510.1

back to list

  

| Protein Coverage
| Supporting Peptides
|

Protein Coverage:

Supporting Peptides:

| Peptide | Uniq | -10lgP | Mass | Length | ppm | m/z | z | RT | Fraction | Scan | Source File | Area F1 | Area F10 | Area F11 | Area F12 | Area F13 | Area F2 | Area F3 | Area F4 | Area F5 | Area F6 | Area F7 | Area F8 | Area F9 | #Feature | #Feature F1 | #Feature F10 | #Feature F11 | #Feature F12 | #Feature F13 | #Feature F2 | #Feature F3 | #Feature F4 | #Feature F5 | #Feature F6 | #Feature F7 | #Feature F8 | #Feature F9 | Start | End | PTM | AScore | Found By |
| --- | --- | --- | --- | --- | --- | --- | --- | --- | --- | --- | --- | --- | --- | --- | --- | --- | --- | --- | --- | --- | --- | --- | --- | --- | --- | --- | --- | --- | --- | --- | --- | --- | --- | --- | --- | --- | --- | --- | --- | --- | --- | --- | --- |
| R.LDPAHIELSPMQIR.T | Y | 58.50 | 1618.8501 | 14 | 0.5 | 540.6243 | 3 | 56.64 | 3 | F3:33454 | NaNaKA16\_F11.raw |  | 1.4943E6 | 8.1117E5 |  |  |  |  |  |  |  |  |  |  | 3 | 0 | 2 | 1 | 0 | 0 | 0 | 0 | 0 | 0 | 0 | 0 | 0 | 0 | 469 | 482 |  |  | PEAKS DB |
| total 1 peptides |  |  |  |  |  |  |  |  |  |  |  |  |  |  |  |  |  |  |  |  |  |  |  |  |  |  |  |  |  |  |  |  |  |  |  |  |  |  |  |  |  |  |  |
| --- | --- | --- | --- | --- | --- | --- | --- | --- | --- | --- | --- | --- | --- | --- | --- | --- | --- | --- | --- | --- | --- | --- | --- | --- | --- | --- | --- | --- | --- | --- | --- | --- | --- | --- | --- | --- | --- | --- | --- | --- | --- | --- | --- |

LAB59086.1

back to list

  

| Protein Coverage
| Supporting Peptides
|

Protein Coverage:

Supporting Peptides:

| Peptide | Uniq | -10lgP | Mass | Length | ppm | m/z | z | RT | Fraction | Scan | Source File | Area F1 | Area F10 | Area F11 | Area F12 | Area F13 | Area F2 | Area F3 | Area F4 | Area F5 | Area F6 | Area F7 | Area F8 | Area F9 | #Feature | #Feature F1 | #Feature F10 | #Feature F11 | #Feature F12 | #Feature F13 | #Feature F2 | #Feature F3 | #Feature F4 | #Feature F5 | #Feature F6 | #Feature F7 | #Feature F8 | #Feature F9 | Start | End | PTM | AScore | Found By |
| --- | --- | --- | --- | --- | --- | --- | --- | --- | --- | --- | --- | --- | --- | --- | --- | --- | --- | --- | --- | --- | --- | --- | --- | --- | --- | --- | --- | --- | --- | --- | --- | --- | --- | --- | --- | --- | --- | --- | --- | --- | --- | --- | --- |
| R.LDPAHIELSPMQIR.T | Y | 58.50 | 1618.8501 | 14 | 0.5 | 540.6243 | 3 | 56.64 | 3 | F3:33454 | NaNaKA16\_F11.raw |  | 1.4943E6 | 8.1117E5 |  |  |  |  |  |  |  |  |  |  | 3 | 0 | 2 | 1 | 0 | 0 | 0 | 0 | 0 | 0 | 0 | 0 | 0 | 0 | 305 | 318 |  |  | PEAKS DB |
| total 1 peptides |  |  |  |  |  |  |  |  |  |  |  |  |  |  |  |  |  |  |  |  |  |  |  |  |  |  |  |  |  |  |  |  |  |  |  |  |  |  |  |  |  |  |  |
| --- | --- | --- | --- | --- | --- | --- | --- | --- | --- | --- | --- | --- | --- | --- | --- | --- | --- | --- | --- | --- | --- | --- | --- | --- | --- | --- | --- | --- | --- | --- | --- | --- | --- | --- | --- | --- | --- | --- | --- | --- | --- | --- | --- |

ADI47719.1

back to list

  

| Protein Coverage
| Supporting Peptides
|

Protein Coverage:

Supporting Peptides:

| Peptide | Uniq | -10lgP | Mass | Length | ppm | m/z | z | RT | Fraction | Scan | Source File | Area F1 | Area F10 | Area F11 | Area F12 | Area F13 | Area F2 | Area F3 | Area F4 | Area F5 | Area F6 | Area F7 | Area F8 | Area F9 | #Feature | #Feature F1 | #Feature F10 | #Feature F11 | #Feature F12 | #Feature F13 | #Feature F2 | #Feature F3 | #Feature F4 | #Feature F5 | #Feature F6 | #Feature F7 | #Feature F8 | #Feature F9 | Start | End | PTM | AScore | Found By |
| --- | --- | --- | --- | --- | --- | --- | --- | --- | --- | --- | --- | --- | --- | --- | --- | --- | --- | --- | --- | --- | --- | --- | --- | --- | --- | --- | --- | --- | --- | --- | --- | --- | --- | --- | --- | --- | --- | --- | --- | --- | --- | --- | --- |
| R.DLINVVSSSSDTLR.S | Y | 57.47 | 1504.7733 | 14 | -0.3 | 753.3937 | 2 | 64.92 | 4 | F4:42619 | NaNaKA16\_F12.raw |  |  |  | 8.4323E5 |  |  |  |  |  |  |  |  |  | 1 | 0 | 0 | 0 | 1 | 0 | 0 | 0 | 0 | 0 | 0 | 0 | 0 | 0 | 85 | 98 |  |  | PEAKS DB |
| total 1 peptides |  |  |  |  |  |  |  |  |  |  |  |  |  |  |  |  |  |  |  |  |  |  |  |  |  |  |  |  |  |  |  |  |  |  |  |  |  |  |  |  |  |  |  |
| --- | --- | --- | --- | --- | --- | --- | --- | --- | --- | --- | --- | --- | --- | --- | --- | --- | --- | --- | --- | --- | --- | --- | --- | --- | --- | --- | --- | --- | --- | --- | --- | --- | --- | --- | --- | --- | --- | --- | --- | --- | --- | --- | --- |

ADI47718.1

back to list

  

| Protein Coverage
| Supporting Peptides
|

Protein Coverage:

Supporting Peptides:

| Peptide | Uniq | -10lgP | Mass | Length | ppm | m/z | z | RT | Fraction | Scan | Source File | Area F1 | Area F10 | Area F11 | Area F12 | Area F13 | Area F2 | Area F3 | Area F4 | Area F5 | Area F6 | Area F7 | Area F8 | Area F9 | #Feature | #Feature F1 | #Feature F10 | #Feature F11 | #Feature F12 | #Feature F13 | #Feature F2 | #Feature F3 | #Feature F4 | #Feature F5 | #Feature F6 | #Feature F7 | #Feature F8 | #Feature F9 | Start | End | PTM | AScore | Found By |
| --- | --- | --- | --- | --- | --- | --- | --- | --- | --- | --- | --- | --- | --- | --- | --- | --- | --- | --- | --- | --- | --- | --- | --- | --- | --- | --- | --- | --- | --- | --- | --- | --- | --- | --- | --- | --- | --- | --- | --- | --- | --- | --- | --- |
| R.DLINVVSSSSDTLR.S | Y | 57.47 | 1504.7733 | 14 | -0.3 | 753.3937 | 2 | 64.92 | 4 | F4:42619 | NaNaKA16\_F12.raw |  |  |  | 8.4323E5 |  |  |  |  |  |  |  |  |  | 1 | 0 | 0 | 0 | 1 | 0 | 0 | 0 | 0 | 0 | 0 | 0 | 0 | 0 | 18 | 31 |  |  | PEAKS DB |
| total 1 peptides |  |  |  |  |  |  |  |  |  |  |  |  |  |  |  |  |  |  |  |  |  |  |  |  |  |  |  |  |  |  |  |  |  |  |  |  |  |  |  |  |  |  |  |
| --- | --- | --- | --- | --- | --- | --- | --- | --- | --- | --- | --- | --- | --- | --- | --- | --- | --- | --- | --- | --- | --- | --- | --- | --- | --- | --- | --- | --- | --- | --- | --- | --- | --- | --- | --- | --- | --- | --- | --- | --- | --- | --- | --- |

JAA95034.1

back to list

  

| Protein Coverage
| Supporting Peptides
|

Protein Coverage:

Supporting Peptides:

| Peptide | Uniq | -10lgP | Mass | Length | ppm | m/z | z | RT | Fraction | Scan | Source File | Area F1 | Area F10 | Area F11 | Area F12 | Area F13 | Area F2 | Area F3 | Area F4 | Area F5 | Area F6 | Area F7 | Area F8 | Area F9 | #Feature | #Feature F1 | #Feature F10 | #Feature F11 | #Feature F12 | #Feature F13 | #Feature F2 | #Feature F3 | #Feature F4 | #Feature F5 | #Feature F6 | #Feature F7 | #Feature F8 | #Feature F9 | Start | End | PTM | AScore | Found By |
| --- | --- | --- | --- | --- | --- | --- | --- | --- | --- | --- | --- | --- | --- | --- | --- | --- | --- | --- | --- | --- | --- | --- | --- | --- | --- | --- | --- | --- | --- | --- | --- | --- | --- | --- | --- | --- | --- | --- | --- | --- | --- | --- | --- |
| K.TDEFQLHTNVNDGTEFGGSIYQK.V | Y | 57.46 | 2599.1826 | 23 | 0.6 | 867.4020 | 3 | 53.88 | 1 | F1:20872 | NaNaKA16\_F1.raw | 3.2728E6 |  |  |  |  |  |  |  |  |  |  |  |  | 1 | 1 | 0 | 0 | 0 | 0 | 0 | 0 | 0 | 0 | 0 | 0 | 0 | 0 | 175 | 197 |  |  | PEAKS DB |
| total 1 peptides |  |  |  |  |  |  |  |  |  |  |  |  |  |  |  |  |  |  |  |  |  |  |  |  |  |  |  |  |  |  |  |  |  |  |  |  |  |  |  |  |  |  |  |
| --- | --- | --- | --- | --- | --- | --- | --- | --- | --- | --- | --- | --- | --- | --- | --- | --- | --- | --- | --- | --- | --- | --- | --- | --- | --- | --- | --- | --- | --- | --- | --- | --- | --- | --- | --- | --- | --- | --- | --- | --- | --- | --- | --- |

XP\_032066440.1

back to list

  

| Protein Coverage
| Supporting Peptides
|

Protein Coverage:

Supporting Peptides:

| Peptide | Uniq | -10lgP | Mass | Length | ppm | m/z | z | RT | Fraction | Scan | Source File | Area F1 | Area F10 | Area F11 | Area F12 | Area F13 | Area F2 | Area F3 | Area F4 | Area F5 | Area F6 | Area F7 | Area F8 | Area F9 | #Feature | #Feature F1 | #Feature F10 | #Feature F11 | #Feature F12 | #Feature F13 | #Feature F2 | #Feature F3 | #Feature F4 | #Feature F5 | #Feature F6 | #Feature F7 | #Feature F8 | #Feature F9 | Start | End | PTM | AScore | Found By |
| --- | --- | --- | --- | --- | --- | --- | --- | --- | --- | --- | --- | --- | --- | --- | --- | --- | --- | --- | --- | --- | --- | --- | --- | --- | --- | --- | --- | --- | --- | --- | --- | --- | --- | --- | --- | --- | --- | --- | --- | --- | --- | --- | --- |
| K.TDEFQLHTNVNDGTEFGGSIYQK.V | Y | 57.46 | 2599.1826 | 23 | 0.6 | 867.4020 | 3 | 53.88 | 1 | F1:20872 | NaNaKA16\_F1.raw | 3.2728E6 |  |  |  |  |  |  |  |  |  |  |  |  | 1 | 1 | 0 | 0 | 0 | 0 | 0 | 0 | 0 | 0 | 0 | 0 | 0 | 0 | 217 | 239 |  |  | PEAKS DB |
| total 1 peptides |  |  |  |  |  |  |  |  |  |  |  |  |  |  |  |  |  |  |  |  |  |  |  |  |  |  |  |  |  |  |  |  |  |  |  |  |  |  |  |  |  |  |  |
| --- | --- | --- | --- | --- | --- | --- | --- | --- | --- | --- | --- | --- | --- | --- | --- | --- | --- | --- | --- | --- | --- | --- | --- | --- | --- | --- | --- | --- | --- | --- | --- | --- | --- | --- | --- | --- | --- | --- | --- | --- | --- | --- | --- |

XP\_015680353.1

back to list

  

| Protein Coverage
| Supporting Peptides
|

Protein Coverage:

Supporting Peptides:

| Peptide | Uniq | -10lgP | Mass | Length | ppm | m/z | z | RT | Fraction | Scan | Source File | Area F1 | Area F10 | Area F11 | Area F12 | Area F13 | Area F2 | Area F3 | Area F4 | Area F5 | Area F6 | Area F7 | Area F8 | Area F9 | #Feature | #Feature F1 | #Feature F10 | #Feature F11 | #Feature F12 | #Feature F13 | #Feature F2 | #Feature F3 | #Feature F4 | #Feature F5 | #Feature F6 | #Feature F7 | #Feature F8 | #Feature F9 | Start | End | PTM | AScore | Found By |
| --- | --- | --- | --- | --- | --- | --- | --- | --- | --- | --- | --- | --- | --- | --- | --- | --- | --- | --- | --- | --- | --- | --- | --- | --- | --- | --- | --- | --- | --- | --- | --- | --- | --- | --- | --- | --- | --- | --- | --- | --- | --- | --- | --- |
| K.FNQC(+57.02)GTC(+57.02)TTFGEC(+57.02)HVIK.N | Y | 54.64 | 2057.8757 | 17 | -0.1 | 686.9658 | 3 | 25.86 | 4 | F4:9996 | NaNaKA16\_F12.raw |  |  |  | 5.9741E5 |  |  |  |  |  |  |  |  |  | 1 | 0 | 0 | 0 | 1 | 0 | 0 | 0 | 0 | 0 | 0 | 0 | 0 | 0 | 166 | 182 | Carbamidomethylation | C4:Carbamidomethylation:1000.00;C7:Carbamidomethylation:1000.00;C13:Carbamidomethylation:1000.00 | PEAKS DB |
| total 1 peptides |  |  |  |  |  |  |  |  |  |  |  |  |  |  |  |  |  |  |  |  |  |  |  |  |  |  |  |  |  |  |  |  |  |  |  |  |  |  |  |  |  |  |  |
| --- | --- | --- | --- | --- | --- | --- | --- | --- | --- | --- | --- | --- | --- | --- | --- | --- | --- | --- | --- | --- | --- | --- | --- | --- | --- | --- | --- | --- | --- | --- | --- | --- | --- | --- | --- | --- | --- | --- | --- | --- | --- | --- | --- |

XP\_032073085.1

back to list

  

| Protein Coverage
| Supporting Peptides
|

Protein Coverage:

Supporting Peptides:

| Peptide | Uniq | -10lgP | Mass | Length | ppm | m/z | z | RT | Fraction | Scan | Source File | Area F1 | Area F10 | Area F11 | Area F12 | Area F13 | Area F2 | Area F3 | Area F4 | Area F5 | Area F6 | Area F7 | Area F8 | Area F9 | #Feature | #Feature F1 | #Feature F10 | #Feature F11 | #Feature F12 | #Feature F13 | #Feature F2 | #Feature F3 | #Feature F4 | #Feature F5 | #Feature F6 | #Feature F7 | #Feature F8 | #Feature F9 | Start | End | PTM | AScore | Found By |
| --- | --- | --- | --- | --- | --- | --- | --- | --- | --- | --- | --- | --- | --- | --- | --- | --- | --- | --- | --- | --- | --- | --- | --- | --- | --- | --- | --- | --- | --- | --- | --- | --- | --- | --- | --- | --- | --- | --- | --- | --- | --- | --- | --- |
| K.FNQC(+57.02)GTC(+57.02)TTFGEC(+57.02)HVIK.N | Y | 54.64 | 2057.8757 | 17 | -0.1 | 686.9658 | 3 | 25.86 | 4 | F4:9996 | NaNaKA16\_F12.raw |  |  |  | 5.9741E5 |  |  |  |  |  |  |  |  |  | 1 | 0 | 0 | 0 | 1 | 0 | 0 | 0 | 0 | 0 | 0 | 0 | 0 | 0 | 164 | 180 | Carbamidomethylation | C4:Carbamidomethylation:1000.00;C7:Carbamidomethylation:1000.00;C13:Carbamidomethylation:1000.00 | PEAKS DB |
| total 1 peptides |  |  |  |  |  |  |  |  |  |  |  |  |  |  |  |  |  |  |  |  |  |  |  |  |  |  |  |  |  |  |  |  |  |  |  |  |  |  |  |  |  |  |  |
| --- | --- | --- | --- | --- | --- | --- | --- | --- | --- | --- | --- | --- | --- | --- | --- | --- | --- | --- | --- | --- | --- | --- | --- | --- | --- | --- | --- | --- | --- | --- | --- | --- | --- | --- | --- | --- | --- | --- | --- | --- | --- | --- | --- |

JAA97490.1

back to list

  

| Protein Coverage
| Supporting Peptides
|

Protein Coverage:

Supporting Peptides:

| Peptide | Uniq | -10lgP | Mass | Length | ppm | m/z | z | RT | Fraction | Scan | Source File | Area F1 | Area F10 | Area F11 | Area F12 | Area F13 | Area F2 | Area F3 | Area F4 | Area F5 | Area F6 | Area F7 | Area F8 | Area F9 | #Feature | #Feature F1 | #Feature F10 | #Feature F11 | #Feature F12 | #Feature F13 | #Feature F2 | #Feature F3 | #Feature F4 | #Feature F5 | #Feature F6 | #Feature F7 | #Feature F8 | #Feature F9 | Start | End | PTM | AScore | Found By |
| --- | --- | --- | --- | --- | --- | --- | --- | --- | --- | --- | --- | --- | --- | --- | --- | --- | --- | --- | --- | --- | --- | --- | --- | --- | --- | --- | --- | --- | --- | --- | --- | --- | --- | --- | --- | --- | --- | --- | --- | --- | --- | --- | --- |
| K.FNQC(+57.02)GTC(+57.02)TTFGEC(+57.02)HVIK.N | Y | 54.64 | 2057.8757 | 17 | -0.1 | 686.9658 | 3 | 25.86 | 4 | F4:9996 | NaNaKA16\_F12.raw |  |  |  | 5.9741E5 |  |  |  |  |  |  |  |  |  | 1 | 0 | 0 | 0 | 1 | 0 | 0 | 0 | 0 | 0 | 0 | 0 | 0 | 0 | 166 | 182 | Carbamidomethylation | C4:Carbamidomethylation:1000.00;C7:Carbamidomethylation:1000.00;C13:Carbamidomethylation:1000.00 | PEAKS DB |
| total 1 peptides |  |  |  |  |  |  |  |  |  |  |  |  |  |  |  |  |  |  |  |  |  |  |  |  |  |  |  |  |  |  |  |  |  |  |  |  |  |  |  |  |  |  |  |
| --- | --- | --- | --- | --- | --- | --- | --- | --- | --- | --- | --- | --- | --- | --- | --- | --- | --- | --- | --- | --- | --- | --- | --- | --- | --- | --- | --- | --- | --- | --- | --- | --- | --- | --- | --- | --- | --- | --- | --- | --- | --- | --- | --- |

JAG45445.1

back to list

  

| Protein Coverage
| Supporting Peptides
|

Protein Coverage:

Supporting Peptides:

| Peptide | Uniq | -10lgP | Mass | Length | ppm | m/z | z | RT | Fraction | Scan | Source File | Area F1 | Area F10 | Area F11 | Area F12 | Area F13 | Area F2 | Area F3 | Area F4 | Area F5 | Area F6 | Area F7 | Area F8 | Area F9 | #Feature | #Feature F1 | #Feature F10 | #Feature F11 | #Feature F12 | #Feature F13 | #Feature F2 | #Feature F3 | #Feature F4 | #Feature F5 | #Feature F6 | #Feature F7 | #Feature F8 | #Feature F9 | Start | End | PTM | AScore | Found By |
| --- | --- | --- | --- | --- | --- | --- | --- | --- | --- | --- | --- | --- | --- | --- | --- | --- | --- | --- | --- | --- | --- | --- | --- | --- | --- | --- | --- | --- | --- | --- | --- | --- | --- | --- | --- | --- | --- | --- | --- | --- | --- | --- | --- |
| K.FNQC(+57.02)GTC(+57.02)TTFGEC(+57.02)HVIK.N | Y | 54.64 | 2057.8757 | 17 | -0.1 | 686.9658 | 3 | 25.86 | 4 | F4:9996 | NaNaKA16\_F12.raw |  |  |  | 5.9741E5 |  |  |  |  |  |  |  |  |  | 1 | 0 | 0 | 0 | 1 | 0 | 0 | 0 | 0 | 0 | 0 | 0 | 0 | 0 | 166 | 182 | Carbamidomethylation | C4:Carbamidomethylation:1000.00;C7:Carbamidomethylation:1000.00;C13:Carbamidomethylation:1000.00 | PEAKS DB |
| total 1 peptides |  |  |  |  |  |  |  |  |  |  |  |  |  |  |  |  |  |  |  |  |  |  |  |  |  |  |  |  |  |  |  |  |  |  |  |  |  |  |  |  |  |  |  |
| --- | --- | --- | --- | --- | --- | --- | --- | --- | --- | --- | --- | --- | --- | --- | --- | --- | --- | --- | --- | --- | --- | --- | --- | --- | --- | --- | --- | --- | --- | --- | --- | --- | --- | --- | --- | --- | --- | --- | --- | --- | --- | --- | --- |

XP\_034289001.1

back to list

  

| Protein Coverage
| Supporting Peptides
|

Protein Coverage:

Supporting Peptides:

| Peptide | Uniq | -10lgP | Mass | Length | ppm | m/z | z | RT | Fraction | Scan | Source File | Area F1 | Area F10 | Area F11 | Area F12 | Area F13 | Area F2 | Area F3 | Area F4 | Area F5 | Area F6 | Area F7 | Area F8 | Area F9 | #Feature | #Feature F1 | #Feature F10 | #Feature F11 | #Feature F12 | #Feature F13 | #Feature F2 | #Feature F3 | #Feature F4 | #Feature F5 | #Feature F6 | #Feature F7 | #Feature F8 | #Feature F9 | Start | End | PTM | AScore | Found By |
| --- | --- | --- | --- | --- | --- | --- | --- | --- | --- | --- | --- | --- | --- | --- | --- | --- | --- | --- | --- | --- | --- | --- | --- | --- | --- | --- | --- | --- | --- | --- | --- | --- | --- | --- | --- | --- | --- | --- | --- | --- | --- | --- | --- |
| K.FNQC(+57.02)GTC(+57.02)TTFGEC(+57.02)HVIK.N | Y | 54.64 | 2057.8757 | 17 | -0.1 | 686.9658 | 3 | 25.86 | 4 | F4:9996 | NaNaKA16\_F12.raw |  |  |  | 5.9741E5 |  |  |  |  |  |  |  |  |  | 1 | 0 | 0 | 0 | 1 | 0 | 0 | 0 | 0 | 0 | 0 | 0 | 0 | 0 | 166 | 182 | Carbamidomethylation | C4:Carbamidomethylation:1000.00;C7:Carbamidomethylation:1000.00;C13:Carbamidomethylation:1000.00 | PEAKS DB |
| total 1 peptides |  |  |  |  |  |  |  |  |  |  |  |  |  |  |  |  |  |  |  |  |  |  |  |  |  |  |  |  |  |  |  |  |  |  |  |  |  |  |  |  |  |  |  |
| --- | --- | --- | --- | --- | --- | --- | --- | --- | --- | --- | --- | --- | --- | --- | --- | --- | --- | --- | --- | --- | --- | --- | --- | --- | --- | --- | --- | --- | --- | --- | --- | --- | --- | --- | --- | --- | --- | --- | --- | --- | --- | --- | --- |

XP\_026520698.1

back to list

  

| Protein Coverage
| Supporting Peptides
|

Protein Coverage:

Supporting Peptides:

| Peptide | Uniq | -10lgP | Mass | Length | ppm | m/z | z | RT | Fraction | Scan | Source File | Area F1 | Area F10 | Area F11 | Area F12 | Area F13 | Area F2 | Area F3 | Area F4 | Area F5 | Area F6 | Area F7 | Area F8 | Area F9 | #Feature | #Feature F1 | #Feature F10 | #Feature F11 | #Feature F12 | #Feature F13 | #Feature F2 | #Feature F3 | #Feature F4 | #Feature F5 | #Feature F6 | #Feature F7 | #Feature F8 | #Feature F9 | Start | End | PTM | AScore | Found By |
| --- | --- | --- | --- | --- | --- | --- | --- | --- | --- | --- | --- | --- | --- | --- | --- | --- | --- | --- | --- | --- | --- | --- | --- | --- | --- | --- | --- | --- | --- | --- | --- | --- | --- | --- | --- | --- | --- | --- | --- | --- | --- | --- | --- |
| K.FNQC(+57.02)GTC(+57.02)TTFGEC(+57.02)HVIK.N | Y | 54.64 | 2057.8757 | 17 | -0.1 | 686.9658 | 3 | 25.86 | 4 | F4:9996 | NaNaKA16\_F12.raw |  |  |  | 5.9741E5 |  |  |  |  |  |  |  |  |  | 1 | 0 | 0 | 0 | 1 | 0 | 0 | 0 | 0 | 0 | 0 | 0 | 0 | 0 | 166 | 182 | Carbamidomethylation | C4:Carbamidomethylation:1000.00;C7:Carbamidomethylation:1000.00;C13:Carbamidomethylation:1000.00 | PEAKS DB |
| total 1 peptides |  |  |  |  |  |  |  |  |  |  |  |  |  |  |  |  |  |  |  |  |  |  |  |  |  |  |  |  |  |  |  |  |  |  |  |  |  |  |  |  |  |  |  |
| --- | --- | --- | --- | --- | --- | --- | --- | --- | --- | --- | --- | --- | --- | --- | --- | --- | --- | --- | --- | --- | --- | --- | --- | --- | --- | --- | --- | --- | --- | --- | --- | --- | --- | --- | --- | --- | --- | --- | --- | --- | --- | --- | --- |

LAA68286.1

back to list

  

| Protein Coverage
| Supporting Peptides
|

Protein Coverage:

Supporting Peptides:

| Peptide | Uniq | -10lgP | Mass | Length | ppm | m/z | z | RT | Fraction | Scan | Source File | Area F1 | Area F10 | Area F11 | Area F12 | Area F13 | Area F2 | Area F3 | Area F4 | Area F5 | Area F6 | Area F7 | Area F8 | Area F9 | #Feature | #Feature F1 | #Feature F10 | #Feature F11 | #Feature F12 | #Feature F13 | #Feature F2 | #Feature F3 | #Feature F4 | #Feature F5 | #Feature F6 | #Feature F7 | #Feature F8 | #Feature F9 | Start | End | PTM | AScore | Found By |
| --- | --- | --- | --- | --- | --- | --- | --- | --- | --- | --- | --- | --- | --- | --- | --- | --- | --- | --- | --- | --- | --- | --- | --- | --- | --- | --- | --- | --- | --- | --- | --- | --- | --- | --- | --- | --- | --- | --- | --- | --- | --- | --- | --- |
| K.FNQC(+57.02)GTC(+57.02)TTFGEC(+57.02)HVIK.N | Y | 54.64 | 2057.8757 | 17 | -0.1 | 686.9658 | 3 | 25.86 | 4 | F4:9996 | NaNaKA16\_F12.raw |  |  |  | 5.9741E5 |  |  |  |  |  |  |  |  |  | 1 | 0 | 0 | 0 | 1 | 0 | 0 | 0 | 0 | 0 | 0 | 0 | 0 | 0 | 185 | 201 | Carbamidomethylation | C4:Carbamidomethylation:1000.00;C7:Carbamidomethylation:1000.00;C13:Carbamidomethylation:1000.00 | PEAKS DB |
| total 1 peptides |  |  |  |  |  |  |  |  |  |  |  |  |  |  |  |  |  |  |  |  |  |  |  |  |  |  |  |  |  |  |  |  |  |  |  |  |  |  |  |  |  |  |  |
| --- | --- | --- | --- | --- | --- | --- | --- | --- | --- | --- | --- | --- | --- | --- | --- | --- | --- | --- | --- | --- | --- | --- | --- | --- | --- | --- | --- | --- | --- | --- | --- | --- | --- | --- | --- | --- | --- | --- | --- | --- | --- | --- | --- |

XP\_026553211.1

back to list

  

| Protein Coverage
| Supporting Peptides
|

Protein Coverage:

Supporting Peptides:

| Peptide | Uniq | -10lgP | Mass | Length | ppm | m/z | z | RT | Fraction | Scan | Source File | Area F1 | Area F10 | Area F11 | Area F12 | Area F13 | Area F2 | Area F3 | Area F4 | Area F5 | Area F6 | Area F7 | Area F8 | Area F9 | #Feature | #Feature F1 | #Feature F10 | #Feature F11 | #Feature F12 | #Feature F13 | #Feature F2 | #Feature F3 | #Feature F4 | #Feature F5 | #Feature F6 | #Feature F7 | #Feature F8 | #Feature F9 | Start | End | PTM | AScore | Found By |
| --- | --- | --- | --- | --- | --- | --- | --- | --- | --- | --- | --- | --- | --- | --- | --- | --- | --- | --- | --- | --- | --- | --- | --- | --- | --- | --- | --- | --- | --- | --- | --- | --- | --- | --- | --- | --- | --- | --- | --- | --- | --- | --- | --- |
| K.FNQC(+57.02)GTC(+57.02)TTFGEC(+57.02)HVIK.N | Y | 54.64 | 2057.8757 | 17 | -0.1 | 686.9658 | 3 | 25.86 | 4 | F4:9996 | NaNaKA16\_F12.raw |  |  |  | 5.9741E5 |  |  |  |  |  |  |  |  |  | 1 | 0 | 0 | 0 | 1 | 0 | 0 | 0 | 0 | 0 | 0 | 0 | 0 | 0 | 172 | 188 | Carbamidomethylation | C4:Carbamidomethylation:1000.00;C7:Carbamidomethylation:1000.00;C13:Carbamidomethylation:1000.00 | PEAKS DB |
| total 1 peptides |  |  |  |  |  |  |  |  |  |  |  |  |  |  |  |  |  |  |  |  |  |  |  |  |  |  |  |  |  |  |  |  |  |  |  |  |  |  |  |  |  |  |  |
| --- | --- | --- | --- | --- | --- | --- | --- | --- | --- | --- | --- | --- | --- | --- | --- | --- | --- | --- | --- | --- | --- | --- | --- | --- | --- | --- | --- | --- | --- | --- | --- | --- | --- | --- | --- | --- | --- | --- | --- | --- | --- | --- | --- |

XP\_032074969.1

back to list

  

| Protein Coverage
| Supporting Peptides
|

Protein Coverage:

Supporting Peptides:

| Peptide | Uniq | -10lgP | Mass | Length | ppm | m/z | z | RT | Fraction | Scan | Source File | Area F1 | Area F10 | Area F11 | Area F12 | Area F13 | Area F2 | Area F3 | Area F4 | Area F5 | Area F6 | Area F7 | Area F8 | Area F9 | #Feature | #Feature F1 | #Feature F10 | #Feature F11 | #Feature F12 | #Feature F13 | #Feature F2 | #Feature F3 | #Feature F4 | #Feature F5 | #Feature F6 | #Feature F7 | #Feature F8 | #Feature F9 | Start | End | PTM | AScore | Found By |
| --- | --- | --- | --- | --- | --- | --- | --- | --- | --- | --- | --- | --- | --- | --- | --- | --- | --- | --- | --- | --- | --- | --- | --- | --- | --- | --- | --- | --- | --- | --- | --- | --- | --- | --- | --- | --- | --- | --- | --- | --- | --- | --- | --- |
| K.FNQC(+57.02)GTC(+57.02)TTFGEC(+57.02)HVIK.N | Y | 54.64 | 2057.8757 | 17 | -0.1 | 686.9658 | 3 | 25.86 | 4 | F4:9996 | NaNaKA16\_F12.raw |  |  |  | 5.9741E5 |  |  |  |  |  |  |  |  |  | 1 | 0 | 0 | 0 | 1 | 0 | 0 | 0 | 0 | 0 | 0 | 0 | 0 | 0 | 174 | 190 | Carbamidomethylation | C4:Carbamidomethylation:1000.00;C7:Carbamidomethylation:1000.00;C13:Carbamidomethylation:1000.00 | PEAKS DB |
| total 1 peptides |  |  |  |  |  |  |  |  |  |  |  |  |  |  |  |  |  |  |  |  |  |  |  |  |  |  |  |  |  |  |  |  |  |  |  |  |  |  |  |  |  |  |  |
| --- | --- | --- | --- | --- | --- | --- | --- | --- | --- | --- | --- | --- | --- | --- | --- | --- | --- | --- | --- | --- | --- | --- | --- | --- | --- | --- | --- | --- | --- | --- | --- | --- | --- | --- | --- | --- | --- | --- | --- | --- | --- | --- | --- |

XP\_026553213.1

back to list

  

| Protein Coverage
| Supporting Peptides
|

Protein Coverage:

Supporting Peptides:

| Peptide | Uniq | -10lgP | Mass | Length | ppm | m/z | z | RT | Fraction | Scan | Source File | Area F1 | Area F10 | Area F11 | Area F12 | Area F13 | Area F2 | Area F3 | Area F4 | Area F5 | Area F6 | Area F7 | Area F8 | Area F9 | #Feature | #Feature F1 | #Feature F10 | #Feature F11 | #Feature F12 | #Feature F13 | #Feature F2 | #Feature F3 | #Feature F4 | #Feature F5 | #Feature F6 | #Feature F7 | #Feature F8 | #Feature F9 | Start | End | PTM | AScore | Found By |
| --- | --- | --- | --- | --- | --- | --- | --- | --- | --- | --- | --- | --- | --- | --- | --- | --- | --- | --- | --- | --- | --- | --- | --- | --- | --- | --- | --- | --- | --- | --- | --- | --- | --- | --- | --- | --- | --- | --- | --- | --- | --- | --- | --- |
| K.FNQC(+57.02)GTC(+57.02)TTFGEC(+57.02)HVIK.N | Y | 54.64 | 2057.8757 | 17 | -0.1 | 686.9658 | 3 | 25.86 | 4 | F4:9996 | NaNaKA16\_F12.raw |  |  |  | 5.9741E5 |  |  |  |  |  |  |  |  |  | 1 | 0 | 0 | 0 | 1 | 0 | 0 | 0 | 0 | 0 | 0 | 0 | 0 | 0 | 166 | 182 | Carbamidomethylation | C4:Carbamidomethylation:1000.00;C7:Carbamidomethylation:1000.00;C13:Carbamidomethylation:1000.00 | PEAKS DB |
| total 1 peptides |  |  |  |  |  |  |  |  |  |  |  |  |  |  |  |  |  |  |  |  |  |  |  |  |  |  |  |  |  |  |  |  |  |  |  |  |  |  |  |  |  |  |  |
| --- | --- | --- | --- | --- | --- | --- | --- | --- | --- | --- | --- | --- | --- | --- | --- | --- | --- | --- | --- | --- | --- | --- | --- | --- | --- | --- | --- | --- | --- | --- | --- | --- | --- | --- | --- | --- | --- | --- | --- | --- | --- | --- | --- |

XP\_034289002.1

back to list

  

| Protein Coverage
| Supporting Peptides
|

Protein Coverage:

Supporting Peptides:

| Peptide | Uniq | -10lgP | Mass | Length | ppm | m/z | z | RT | Fraction | Scan | Source File | Area F1 | Area F10 | Area F11 | Area F12 | Area F13 | Area F2 | Area F3 | Area F4 | Area F5 | Area F6 | Area F7 | Area F8 | Area F9 | #Feature | #Feature F1 | #Feature F10 | #Feature F11 | #Feature F12 | #Feature F13 | #Feature F2 | #Feature F3 | #Feature F4 | #Feature F5 | #Feature F6 | #Feature F7 | #Feature F8 | #Feature F9 | Start | End | PTM | AScore | Found By |
| --- | --- | --- | --- | --- | --- | --- | --- | --- | --- | --- | --- | --- | --- | --- | --- | --- | --- | --- | --- | --- | --- | --- | --- | --- | --- | --- | --- | --- | --- | --- | --- | --- | --- | --- | --- | --- | --- | --- | --- | --- | --- | --- | --- |
| K.FNQC(+57.02)GTC(+57.02)TTFGEC(+57.02)HVIK.N | Y | 54.64 | 2057.8757 | 17 | -0.1 | 686.9658 | 3 | 25.86 | 4 | F4:9996 | NaNaKA16\_F12.raw |  |  |  | 5.9741E5 |  |  |  |  |  |  |  |  |  | 1 | 0 | 0 | 0 | 1 | 0 | 0 | 0 | 0 | 0 | 0 | 0 | 0 | 0 | 152 | 168 | Carbamidomethylation | C4:Carbamidomethylation:1000.00;C7:Carbamidomethylation:1000.00;C13:Carbamidomethylation:1000.00 | PEAKS DB |
| total 1 peptides |  |  |  |  |  |  |  |  |  |  |  |  |  |  |  |  |  |  |  |  |  |  |  |  |  |  |  |  |  |  |  |  |  |  |  |  |  |  |  |  |  |  |  |
| --- | --- | --- | --- | --- | --- | --- | --- | --- | --- | --- | --- | --- | --- | --- | --- | --- | --- | --- | --- | --- | --- | --- | --- | --- | --- | --- | --- | --- | --- | --- | --- | --- | --- | --- | --- | --- | --- | --- | --- | --- | --- | --- | --- |

LAB21704.1

back to list

  

| Protein Coverage
| Supporting Peptides
|

Protein Coverage:

Supporting Peptides:

| Peptide | Uniq | -10lgP | Mass | Length | ppm | m/z | z | RT | Fraction | Scan | Source File | Area F1 | Area F10 | Area F11 | Area F12 | Area F13 | Area F2 | Area F3 | Area F4 | Area F5 | Area F6 | Area F7 | Area F8 | Area F9 | #Feature | #Feature F1 | #Feature F10 | #Feature F11 | #Feature F12 | #Feature F13 | #Feature F2 | #Feature F3 | #Feature F4 | #Feature F5 | #Feature F6 | #Feature F7 | #Feature F8 | #Feature F9 | Start | End | PTM | AScore | Found By |
| --- | --- | --- | --- | --- | --- | --- | --- | --- | --- | --- | --- | --- | --- | --- | --- | --- | --- | --- | --- | --- | --- | --- | --- | --- | --- | --- | --- | --- | --- | --- | --- | --- | --- | --- | --- | --- | --- | --- | --- | --- | --- | --- | --- |
| K.FNQC(+57.02)GTC(+57.02)TTFGEC(+57.02)HVIK.N | Y | 54.64 | 2057.8757 | 17 | -0.1 | 686.9658 | 3 | 25.86 | 4 | F4:9996 | NaNaKA16\_F12.raw |  |  |  | 5.9741E5 |  |  |  |  |  |  |  |  |  | 1 | 0 | 0 | 0 | 1 | 0 | 0 | 0 | 0 | 0 | 0 | 0 | 0 | 0 | 105 | 121 | Carbamidomethylation | C4:Carbamidomethylation:1000.00;C7:Carbamidomethylation:1000.00;C13:Carbamidomethylation:1000.00 | PEAKS DB |
| total 1 peptides |  |  |  |  |  |  |  |  |  |  |  |  |  |  |  |  |  |  |  |  |  |  |  |  |  |  |  |  |  |  |  |  |  |  |  |  |  |  |  |  |  |  |  |
| --- | --- | --- | --- | --- | --- | --- | --- | --- | --- | --- | --- | --- | --- | --- | --- | --- | --- | --- | --- | --- | --- | --- | --- | --- | --- | --- | --- | --- | --- | --- | --- | --- | --- | --- | --- | --- | --- | --- | --- | --- | --- | --- | --- |

XP\_015685975.1

back to list

  

| Protein Coverage
| Supporting Peptides
|

Protein Coverage:

Supporting Peptides:

| Peptide | Uniq | -10lgP | Mass | Length | ppm | m/z | z | RT | Fraction | Scan | Source File | Area F1 | Area F10 | Area F11 | Area F12 | Area F13 | Area F2 | Area F3 | Area F4 | Area F5 | Area F6 | Area F7 | Area F8 | Area F9 | #Feature | #Feature F1 | #Feature F10 | #Feature F11 | #Feature F12 | #Feature F13 | #Feature F2 | #Feature F3 | #Feature F4 | #Feature F5 | #Feature F6 | #Feature F7 | #Feature F8 | #Feature F9 | Start | End | PTM | AScore | Found By |
| --- | --- | --- | --- | --- | --- | --- | --- | --- | --- | --- | --- | --- | --- | --- | --- | --- | --- | --- | --- | --- | --- | --- | --- | --- | --- | --- | --- | --- | --- | --- | --- | --- | --- | --- | --- | --- | --- | --- | --- | --- | --- | --- | --- |
| K.FNQC(+57.02)GTC(+57.02)TTFGEC(+57.02)HVIK.N | Y | 54.64 | 2057.8757 | 17 | -0.1 | 686.9658 | 3 | 25.86 | 4 | F4:9996 | NaNaKA16\_F12.raw |  |  |  | 5.9741E5 |  |  |  |  |  |  |  |  |  | 1 | 0 | 0 | 0 | 1 | 0 | 0 | 0 | 0 | 0 | 0 | 0 | 0 | 0 | 83 | 99 | Carbamidomethylation | C4:Carbamidomethylation:1000.00;C7:Carbamidomethylation:1000.00;C13:Carbamidomethylation:1000.00 | PEAKS DB |
| total 1 peptides |  |  |  |  |  |  |  |  |  |  |  |  |  |  |  |  |  |  |  |  |  |  |  |  |  |  |  |  |  |  |  |  |  |  |  |  |  |  |  |  |  |  |  |
| --- | --- | --- | --- | --- | --- | --- | --- | --- | --- | --- | --- | --- | --- | --- | --- | --- | --- | --- | --- | --- | --- | --- | --- | --- | --- | --- | --- | --- | --- | --- | --- | --- | --- | --- | --- | --- | --- | --- | --- | --- | --- | --- | --- |

LAA25473.1

back to list

  

| Protein Coverage
| Supporting Peptides
|

Protein Coverage:

Supporting Peptides:

| Peptide | Uniq | -10lgP | Mass | Length | ppm | m/z | z | RT | Fraction | Scan | Source File | Area F1 | Area F10 | Area F11 | Area F12 | Area F13 | Area F2 | Area F3 | Area F4 | Area F5 | Area F6 | Area F7 | Area F8 | Area F9 | #Feature | #Feature F1 | #Feature F10 | #Feature F11 | #Feature F12 | #Feature F13 | #Feature F2 | #Feature F3 | #Feature F4 | #Feature F5 | #Feature F6 | #Feature F7 | #Feature F8 | #Feature F9 | Start | End | PTM | AScore | Found By |
| --- | --- | --- | --- | --- | --- | --- | --- | --- | --- | --- | --- | --- | --- | --- | --- | --- | --- | --- | --- | --- | --- | --- | --- | --- | --- | --- | --- | --- | --- | --- | --- | --- | --- | --- | --- | --- | --- | --- | --- | --- | --- | --- | --- |
| K.FNQC(+57.02)GTC(+57.02)TTFGEC(+57.02)HVIK.N | Y | 54.64 | 2057.8757 | 17 | -0.1 | 686.9658 | 3 | 25.86 | 4 | F4:9996 | NaNaKA16\_F12.raw |  |  |  | 5.9741E5 |  |  |  |  |  |  |  |  |  | 1 | 0 | 0 | 0 | 1 | 0 | 0 | 0 | 0 | 0 | 0 | 0 | 0 | 0 | 150 | 166 | Carbamidomethylation | C4:Carbamidomethylation:1000.00;C7:Carbamidomethylation:1000.00;C13:Carbamidomethylation:1000.00 | PEAKS DB |
| total 1 peptides |  |  |  |  |  |  |  |  |  |  |  |  |  |  |  |  |  |  |  |  |  |  |  |  |  |  |  |  |  |  |  |  |  |  |  |  |  |  |  |  |  |  |  |
[truncated: 195,672 more chars]
