# Supplementary material for: Venomics of the Enigmatic Andaman Cobra (Naja sagittifera) and the Preclinical Failure of Indian Antivenoms in Andaman and Nicobar Islands
Source: Front Pharmacol. 2021 Oct 25;12:768210. doi: 10.3389/fphar.2021.768210 (PMC8573199; doi:10.3389/fphar.2021.768210)
Supplement: Supplementary file 1 [file DataSheet3.ZIP › Naja sagittifera/Naja sagittifera.html]

proteins


Summary

  

# 1. Notes

# 2. Result Statistics

**Figure 1.**
False discovery rate (FDR) curve. X axis is the number of peptide-spectrum matches (PSM) being kept. Y axis is the corresponding FDR.

  


  

**Figure 2.**
PSM score distribution.  **(a)**
Distribution of PEAKS peptide score;  **(b)**
Scatterplot of PEAKS peptide score versus precursor mass error.

**(a)**

**(b)**

**Table 1.**
Statistics of data.


|  |  |  |  |  |  |  |  |  |  |  |  |
| --- | --- | --- | --- | --- | --- | --- | --- | --- | --- | --- | --- |
|  | #Scans | | #Features | Identified | | | #Peptides | #Sequences | #Proteins^\*^ | | |
|  | MS1 | MS2 |  | #PSMs | #Scans | #Features |  |  | Groups | All | Top |
| Total | 195476 | 902153 | 1316548 | 26897 | 25407 | 73409 | 1506 | 1325 | 196 | 1449 | 811 |
| F1 | 15414 | 38456 | 62280 | 16 | 15 | 2064 | 8 | 8 | 7 | 29 | 27 |
| F10 | 15588 | 65435 | 79983 | 2859 | 2672 | 438 | 125 | 95 | 40 | 228 | 89 |
| F11 | 13469 | 74243 | 117089 | 3769 | 3562 | 8858 | 250 | 209 | 68 | 376 | 216 |
| F12 | 12729 | 73051 | 109824 | 5020 | 4763 | 8193 | 465 | 413 | 107 | 625 | 299 |
| F13 | 13508 | 68720 | 93662 | 2470 | 2355 | 730 | 442 | 393 | 76 | 373 | 218 |
| F14 | 12938 | 75405 | 103552 | 4713 | 4476 | 9436 | 718 | 633 | 113 | 776 | 460 |
| F2 | 14673 | 58625 | 90277 | 355 | 341 | 8053 | 81 | 80 | 15 | 69 | 44 |
| F3 | 15392 | 43045 | 59681 | 154 | 146 | 1832 | 41 | 41 | 21 | 84 | 63 |
| F4 | 16834 | 31050 | 43327 | 369 | 365 | 1768 | 81 | 76 | 43 | 186 | 112 |
| F5 | 11976 | 76010 | 116882 | 653 | 622 | 158 | 89 | 82 | 43 | 180 | 125 |
| F6 | 13075 | 69581 | 116236 | 479 | 419 | 149 | 73 | 63 | 40 | 146 | 90 |
| F7 | 12516 | 75450 | 111990 | 1401 | 1337 | 7744 | 193 | 171 | 62 | 299 | 215 |
| F8 | 13519 | 78973 | 109425 | 2069 | 1939 | 11036 | 180 | 155 | 53 | 271 | 165 |
| F9 | 13845 | 74109 | 102340 | 2570 | 2395 | 12950 | 181 | 144 | 66 | 301 | 173 |

^\* proteins with significant peptides are used in counts.^

**Figure 3.**
Sample overlap for Proteins and Peptides (up to 8 samples).  **(a)**
All Proteins;  **(b)**
Top Proteins;  **(c)**
Peptides;

**(a)**

**Do not support more than 8 samples**

**(b)**

**Do not support more than 8 samples**

**(c)**

**Do not support more than 8 samples**

**Figure 4.**
Distribution of peptide feature detection.  **(a)**
Feature m/z distribution;  **(b)**
Feature RT distribution.

**(a)**

**(b)**

**Figure 5.**
Distribution of identified peptide features.  **(a)**
Feature abundance distribution;  **(b)**
 *De novo*
sequencing validation.

**(a)**

**(b)**

**Table 2.**
Result filtration parameters.

|  |  |
| --- | --- |
| Peptide -10lgP | ≥37.6 |
| PTM Ascore | ≥0 |
| Protein -10lgP | ≥20 |
| Proteins unique peptides | ≥1 |
| De novo score(%) | ≥50% |


**Table 3.**
Statistics of filtered result.

|  |  |
| --- | --- |
| FDR (Peptide-Spectrum Matches) | 0.1% |
| FDR (Peptide Sequences) | 1.5% |
| FDR (Protein Group) | 11.8% |
| De Novo Only Spectra | 137524 |

**Table 4.**
PTM profile.

| Name | ∆Mass | Position | #PSM | -10lgP | Abundance | AScore || Oxidation | 15.99 | M | 7521 | 95.06 | 9.34E7 | 1000.00 |
| Carbamidomethyl | 57.02 | C | 7042 | 100.92 | 2.61E7 | 1000.00 |

# 3. Experiment Control

**Figure 6.**
Precursor mass error of peptide-spectrum matches (PSM) in filtered result.  **(a)**
Distribution of precursor mass error in ppm;  **(b)**
Scatterplot of precursor m/z versus precursor mass error in ppm.

**(a)**

**(b)**

**Table 5.**
Number of identified peptides in each sample by the number of missed cleavages.

|  |  |  |  |  |  |
| --- | --- | --- | --- | --- | --- |
| Missed Cleavages | 0 | 1 | 2 | 3 | 4+ |
| F1 | 6 | 2 | 0 | 0 | 0 |
| F10 | 76 | 34 | 15 | 0 | 0 |
| F11 | 171 | 64 | 15 | 0 | 0 |
| F12 | 384 | 76 | 5 | 0 | 0 |
| F13 | 389 | 51 | 2 | 0 | 0 |
| F14 | 642 | 74 | 2 | 0 | 0 |
| F2 | 64 | 16 | 1 | 0 | 0 |
| F3 | 38 | 3 | 0 | 0 | 0 |
| F4 | 77 | 4 | 0 | 0 | 0 |
| F5 | 76 | 11 | 2 | 0 | 0 || ... |

# 4. Other Information

**Table 6.**
Search parameters.

Search Engine Name: PEAKS  
Parent Mass Error Tolerance: 10.0 ppm  
Fragment Mass Error Tolerance: 0.6 Da  
Precursor Mass Search Type: monoisotopic  
Enzyme: Trypsin  
Max Missed Cleavages: 2  
Digest Mode: Semispecific  
Fixed Modifications:   
  Carbamidomethylation: 57.02  
Variable Modifications:   
  Oxidation (M): 15.99  
Max Variable PTM Per Peptide: 3  
Database: NCBI\_Serpentes\_NR  
Taxon: All  
Contaminant Database: cRAP\_contaminants  
Searched Entry: 410049  
FDR Estimation: Enabled  
Merge Options: no merge  
Precursor Options: corrected  
Charge Options: no correction  
Filter Charge: 2 - 8  
Process: true  
Associate chimera: yes

**Table 7.**
Instrument parameters.

Fractions: NaSa01\_F1.raw, NaSa01\_F10.raw, NaSa01\_F11.raw, NaSa0  
1\_F12.raw, NaSa01\_F13.raw, NaSa01\_F14.raw, NaSa01\_F2.raw, NaS  
a01\_F3.raw, NaSa01\_F4.raw, NaSa01\_F5.raw, NaSa01\_F6.raw, NaSa  
01\_F7.raw, NaSa01\_F8.raw, NaSa01\_F9.raw  
Ion Source: ESI(nano-spray)  
Fragmentation Mode: CID, CAD(y and b ions)  
MS Scan Mode: FT-ICR/Orbitrap  
MS/MS Scan Mode: FT-ICR/Orbitrap

  

Protein List

  

Protein Accession Contains:

Protein Description Contains:

Protein Sample Area >=

Protein PTM Contains:

| Protein Group | Protein ID | Accession | -10lgP | Coverage (%) | Coverage (%) F1 | Coverage (%) F10 | Coverage (%) F11 | Coverage (%) F12 | Coverage (%) F13 | Coverage (%) F14 | Coverage (%) F2 | Coverage (%) F3 | Coverage (%) F4 | Coverage (%) F5 | Coverage (%) F6 | Coverage (%) F7 | Coverage (%) F8 | Coverage (%) F9 | Area F1 | Area F10 | Area F11 | Area F12 | Area F13 | Area F14 | Area F2 | Area F3 | Area F4 | Area F5 | Area F6 | Area F7 | Area F8 | Area F9 | #Peptides | #Unique | #Spec F1 | #Spec F10 | #Spec F11 | #Spec F12 | #Spec F13 | #Spec F14 | #Spec F2 | #Spec F3 | #Spec F4 | #Spec F5 | #Spec F6 | #Spec F7 | #Spec F8 | #Spec F9 | PTM | Avg. Mass | Description |
| --- | --- | --- | --- | --- | --- | --- | --- | --- | --- | --- | --- | --- | --- | --- | --- | --- | --- | --- | --- | --- | --- | --- | --- | --- | --- | --- | --- | --- | --- | --- | --- | --- | --- | --- | --- | --- | --- | --- | --- | --- | --- | --- | --- | --- | --- | --- | --- | --- | --- | --- | --- |
| 5 | 4 | 5Z2G | 417.14 | 63 | 0 | 3 | 0 | 26 | 48 | 62 | 0 | 2 | 2 | 0 | 0 | 3 | 3 | 0 |  | 2.7707E6 |  | 1.8098E7 | 6.7903E8 | 7.3192E9 |  |  |  |  |  | 7.9457E5 | 5.5981E5 |  | 85 | 38 | 0 | 3 | 0 | 34 | 254 | 1231 | 0 | 1 | 3 | 0 | 0 | 1 | 2 | 0 | Y | 57963 | Chain B, L-amino acid oxidase |
| 5 | 5 | A8QL58.2 | 417.14 | 63 | 0 | 3 | 0 | 26 | 48 | 62 | 0 | 2 | 2 | 0 | 0 | 3 | 3 | 0 |  | 2.7707E6 |  | 1.8098E7 | 6.7903E8 | 7.3192E9 |  |  |  |  |  | 7.9457E5 | 5.5981E5 |  | 85 | 38 | 0 | 3 | 0 | 34 | 254 | 1231 | 0 | 1 | 3 | 0 | 0 | 1 | 2 | 0 | Y | 57963 | RecName: Full=L-amino-acid oxidase; Short=LAO; Short=NA-LAAO; Flags: Precursor |
| 5 | 6 | AVX27607.1 | 417.14 | 63 | 0 | 3 | 0 | 26 | 48 | 62 | 0 | 2 | 2 | 0 | 0 | 3 | 3 | 0 |  | 2.7707E6 |  | 1.8098E7 | 6.7903E8 | 7.3192E9 |  |  |  |  |  | 7.9457E5 | 5.5981E5 |  | 85 | 38 | 0 | 3 | 0 | 34 | 254 | 1231 | 0 | 1 | 3 | 0 | 0 | 1 | 2 | 0 | Y | 57963 | L-amino acid oxidase, partial [Naja atra] |
| 18 | 1 | A0A2I4HXH5.1 | 385.55 | 70 | 0 | 0 | 3 | 22 | 67 | 70 | 0 | 0 | 0 | 3 | 2 | 2 | 4 | 8 |  |  |  |  | 4.5751E8 | 8.6909E8 |  |  |  |  |  |  |  |  | 62 | 5 | 0 | 0 | 1 | 12 | 279 | 641 | 0 | 0 | 0 | 1 | 2 | 1 | 4 | 14 | Y | 58198 | RecName: Full=Snake venom 5'-nucleotidase; Short=5'-NT; AltName: Full=Ecto-5'-nucleotidase; Flags: Precursor |
| 18 | 2 | 5H7W | 385.55 | 70 | 0 | 0 | 3 | 22 | 67 | 70 | 0 | 0 | 0 | 3 | 2 | 2 | 4 | 8 |  |  |  |  | 4.5751E8 | 8.6909E8 |  |  |  |  |  |  |  |  | 62 | 5 | 0 | 0 | 1 | 12 | 279 | 641 | 0 | 0 | 0 | 1 | 2 | 1 | 4 | 14 | Y | 58198 | Chain B, venom 5'-nucleotidase |
| 28 | 17 | 3PRX | 383.14 | 43 | 0 | 0 | 3 | 8 | 22 | 41 | 1 | 2 | 2 | 1 | 0 | 1 | 0 | 0 |  |  | 1.0191E6 | 1.2629E8 | 1.7995E8 | 1.9831E9 |  | 1.0485E7 | 6.8715E7 | 1.8154E7 |  | 1.7364E6 |  |  | 80 | 46 | 0 | 0 | 3 | 36 | 86 | 384 | 1 | 4 | 17 | 6 | 0 | 1 | 0 | 0 | Y | 184517 | Chain D, Cobra Venom Factor |
| 28 | 18 | 3PVM | 383.14 | 43 | 0 | 0 | 3 | 8 | 22 | 41 | 1 | 2 | 2 | 1 | 0 | 1 | 0 | 0 |  |  | 1.0191E6 | 1.2629E8 | 1.7995E8 | 1.9831E9 |  | 1.0485E7 | 6.8715E7 | 1.8154E7 |  | 1.7364E6 |  |  | 80 | 46 | 0 | 0 | 3 | 36 | 86 | 384 | 1 | 4 | 17 | 6 | 0 | 1 | 0 | 0 | Y | 184517 | Chain D, Cobra Venom Factor |
| 28 | 19 | AAA68989.1 | 383.14 | 43 | 0 | 0 | 3 | 8 | 22 | 41 | 1 | 2 | 2 | 1 | 0 | 1 | 0 | 0 |  |  | 1.0191E6 | 1.2629E8 | 1.7995E8 | 1.9831E9 |  | 1.0485E7 | 6.8715E7 | 1.8154E7 |  | 1.7364E6 |  |  | 80 | 46 | 0 | 0 | 3 | 36 | 86 | 384 | 1 | 4 | 17 | 6 | 0 | 1 | 0 | 0 | Y | 184517 | cobra venom factor precursor [Naja kaouthia] |
| 28 | 20 | Q91132.1 | 383.14 | 43 | 0 | 0 | 3 | 8 | 22 | 41 | 1 | 2 | 2 | 1 | 0 | 1 | 0 | 0 |  |  | 1.0191E6 | 1.2629E8 | 1.7995E8 | 1.9831E9 |  | 1.0485E7 | 6.8715E7 | 1.8154E7 |  | 1.7364E6 |  |  | 80 | 46 | 0 | 0 | 3 | 36 | 86 | 384 | 1 | 4 | 17 | 6 | 0 | 1 | 0 | 0 | Y | 184517 | RecName: Full=Cobra venom factor; Short=CVF; Short=CVFk; AltName: Full=Complement C3 homolog; Contains: RecName: Full=Cobra venom factor alpha chain; Contains: RecName: Full=Cobra venom factor gamma chain; Contains: RecName: Full=C... |
| 28 | 21 | CAI46845.1 | 383.14 | 43 | 0 | 0 | 3 | 8 | 22 | 41 | 1 | 2 | 2 | 1 | 0 | 1 | 0 | 0 |  |  | 1.0191E6 | 1.2629E8 | 1.7995E8 | 1.9831E9 |  | 1.0485E7 | 6.8715E7 | 1.8154E7 |  | 1.7364E6 |  |  | 80 | 46 | 0 | 0 | 3 | 36 | 86 | 384 | 1 | 4 | 17 | 6 | 0 | 1 | 0 | 0 | Y | 184517 | unnamed protein product [Naja naja] |
| 28 | 22 | 6I2X | 383.14 | 43 | 0 | 0 | 3 | 8 | 22 | 41 | 1 | 2 | 2 | 1 | 0 | 1 | 0 | 0 |  |  | 1.0191E6 | 1.2629E8 | 1.7995E8 | 1.9831E9 |  | 1.0485E7 | 6.8715E7 | 1.8154E7 |  | 1.7364E6 |  |  | 80 | 46 | 0 | 0 | 3 | 36 | 86 | 384 | 1 | 4 | 17 | 6 | 0 | 1 | 0 | 0 | Y | 184517 | Chain B, Cobra venom factor |
| 28 | 23 | I51018 | 383.14 | 43 | 0 | 0 | 3 | 8 | 22 | 41 | 1 | 2 | 2 | 1 | 0 | 1 | 0 | 0 |  |  | 1.0191E6 | 1.2629E8 | 1.7995E8 | 1.9831E9 |  | 1.0485E7 | 6.8715E7 | 1.8154E7 |  | 1.7364E6 |  |  | 80 | 46 | 0 | 0 | 3 | 36 | 86 | 384 | 1 | 4 | 17 | 6 | 0 | 1 | 0 | 0 | Y | 184517 | cobra venom factor precursor - monocled cobra |
| 4 | 47 | 2GIZ | 374.95 | 96 | 0 | 8 | 31 | 96 | 53 | 45 | 0 | 0 | 0 | 0 | 0 | 12 | 0 | 0 |  | 6.539E5 | 1.7427E7 | 9.3815E10 | 2.4237E8 | 1.8735E7 |  |  |  |  |  | 7.6318E6 |  |  | 80 | 60 | 0 | 2 | 7 | 2324 | 81 | 20 | 0 | 0 | 0 | 0 | 0 | 2 | 0 | 0 | Y | 24954 | Chain B, Structural And Functional Analysis Of Natrin, A Member Of Crisp-3 Family Blocks A Variety Of Ion Channels |
| 4 | 48 | 1XX5 | 374.95 | 96 | 0 | 8 | 31 | 96 | 53 | 45 | 0 | 0 | 0 | 0 | 0 | 12 | 0 | 0 |  | 6.539E5 | 1.7427E7 | 9.3815E10 | 2.4237E8 | 1.8735E7 |  |  |  |  |  | 7.6318E6 |  |  | 80 | 60 | 0 | 2 | 7 | 2324 | 81 | 20 | 0 | 0 | 0 | 0 | 0 | 2 | 0 | 0 | Y | 24954 | Chain C, Natrin 1 |
| 4 | 49 | 3MZ8 | 374.95 | 96 | 0 | 8 | 31 | 96 | 53 | 45 | 0 | 0 | 0 | 0 | 0 | 12 | 0 | 0 |  | 6.539E5 | 1.7427E7 | 9.3815E10 | 2.4237E8 | 1.8735E7 |  |  |  |  |  | 7.6318E6 |  |  | 80 | 60 | 0 | 2 | 7 | 2324 | 81 | 20 | 0 | 0 | 0 | 0 | 0 | 2 | 0 | 0 | Y | 24954 | Chain B, Crystal Structure Of Zinc-Bound Natrin From Naja Atra |
| 4 | 51 | Q7T1K6.1 | 374.95 | 89 | 0 | 8 | 29 | 89 | 49 | 41 | 0 | 0 | 0 | 0 | 0 | 11 | 0 | 0 |  | 6.539E5 | 1.7427E7 | 9.3815E10 | 2.4237E8 | 1.8735E7 |  |  |  |  |  | 7.6318E6 |  |  | 80 | 60 | 0 | 2 | 7 | 2324 | 81 | 20 | 0 | 0 | 0 | 0 | 0 | 2 | 0 | 0 | Y | 26882 | RecName: Full=Cysteine-rich venom protein natrin-1; AltName: Full=Cysteine-rich venom protein 1; AltName: Full=NA-CRVP1; AltName: Full=Protein G2a; Flags: Precursor |
| 4 | 52 | AAP85301.1 | 374.95 | 89 | 0 | 8 | 29 | 89 | 49 | 41 | 0 | 0 | 0 | 0 | 0 | 11 | 0 | 0 |  | 6.539E5 | 1.7427E7 | 9.3815E10 | 2.4237E8 | 1.8735E7 |  |  |  |  |  | 7.6318E6 |  |  | 80 | 60 | 0 | 2 | 7 | 2324 | 81 | 20 | 0 | 0 | 0 | 0 | 0 | 2 | 0 | 0 | Y | 26882 | natrin [Naja atra] |
| 24 | 7 | JAS05143.1 | 364.20 | 54 | 0 | 0 | 3 | 19 | 47 | 54 | 0 | 0 | 0 | 3 | 2 | 2 | 3 | 7 |  |  |  |  | 1.8927E6 |  |  |  |  |  |  |  |  |  | 53 | 1 | 0 | 0 | 1 | 10 | 185 | 470 | 0 | 0 | 0 | 1 | 2 | 1 | 4 | 14 | Y | 63012 | ecto-5'-nucleotidase [Micrurus tener] |
| 24 | 8 | JAS05036.1 | 364.20 | 54 | 0 | 0 | 3 | 19 | 47 | 54 | 0 | 0 | 0 | 3 | 2 | 2 | 3 | 7 |  |  |  |  | 1.8927E6 |  |  |  |  |  |  |  |  |  | 53 | 1 | 0 | 0 | 1 | 10 | 185 | 470 | 0 | 0 | 0 | 1 | 2 | 1 | 4 | 14 | Y | 62983 | Ecto-5'-nucleotidase 1c [Micrurus fulvius] |
| 24 | 9 | JAS05037.1 | 364.20 | 54 | 0 | 0 | 3 | 19 | 47 | 54 | 0 | 0 | 0 | 3 | 2 | 2 | 3 | 7 |  |  |  |  | 1.8927E6 |  |  |  |  |  |  |  |  |  | 53 | 1 | 0 | 0 | 1 | 10 | 185 | 470 | 0 | 0 | 0 | 1 | 2 | 1 | 4 | 14 | Y | 63025 | Ecto-5'-nucleotidase 1b [Micrurus fulvius] |
| 24 | 10 | JAI09047.1 | 364.20 | 54 | 0 | 0 | 3 | 19 | 47 | 54 | 0 | 0 | 0 | 3 | 2 | 2 | 3 | 7 |  |  |  |  | 1.8927E6 |  |  |  |  |  |  |  |  |  | 53 | 1 | 0 | 0 | 1 | 10 | 185 | 470 | 0 | 0 | 0 | 1 | 2 | 1 | 4 | 14 | Y | 62983 | Ecto-5'-nucleotidase 1c [Micrurus fulvius] |
| 24 | 11 | JAB52815.1 | 364.20 | 54 | 0 | 0 | 3 | 19 | 47 | 54 | 0 | 0 | 0 | 3 | 2 | 2 | 3 | 7 |  |  |  |  | 1.8927E6 |  |  |  |  |  |  |  |  |  | 53 | 1 | 0 | 0 | 1 | 10 | 185 | 470 | 0 | 0 | 0 | 1 | 2 | 1 | 4 | 14 | Y | 62983 | Ecto-5'-nucleotidase 1 [Micrurus fulvius] |
| 24 | 12 | JAI09048.1 | 364.20 | 54 | 0 | 0 | 3 | 19 | 47 | 54 | 0 | 0 | 0 | 3 | 2 | 2 | 3 | 7 |  |  |  |  | 1.8927E6 |  |  |  |  |  |  |  |  |  | 53 | 1 | 0 | 0 | 1 | 10 | 185 | 470 | 0 | 0 | 0 | 1 | 2 | 1 | 4 | 14 | Y | 63025 | Ecto-5'-nucleotidase 1b [Micrurus fulvius] |
| 24 | 13 | JAI09049.1 | 364.20 | 54 | 0 | 0 | 3 | 19 | 47 | 54 | 0 | 0 | 0 | 3 | 2 | 2 | 3 | 7 |  |  |  |  | 1.8927E6 |  |  |  |  |  |  |  |  |  | 53 | 1 | 0 | 0 | 1 | 10 | 185 | 470 | 0 | 0 | 0 | 1 | 2 | 1 | 4 | 14 | Y | 63025 | Ecto-5'-nucleotidase 1a [Micrurus fulvius] |
| 24 | 14 | JAS05038.1 | 364.20 | 54 | 0 | 0 | 3 | 19 | 47 | 54 | 0 | 0 | 0 | 3 | 2 | 2 | 3 | 7 |  |  |  |  | 1.8927E6 |  |  |  |  |  |  |  |  |  | 53 | 1 | 0 | 0 | 1 | 10 | 185 | 470 | 0 | 0 | 0 | 1 | 2 | 1 | 4 | 14 | Y | 63025 | Ecto-5'-nucleotidase 1a [Micrurus fulvius] |
| 22 | 15 | Q9PVK7.1 | 360.97 | 44 | 0 | 0 | 8 | 4 | 11 | 39 | 0 | 3 | 0 | 8 | 4 | 4 | 4 | 4 |  |  |  |  | 2.3112E6 | 1.0258E10 |  |  |  |  |  |  |  |  | 48 | 8 | 0 | 0 | 4 | 4 | 19 | 723 | 0 | 2 | 0 | 5 | 5 | 6 | 4 | 4 | Y | 67662 | RecName: Full=Zinc metalloproteinase-disintegrin-like cobrin; AltName: Full=Snake venom metalloproteinase; Short=SVMP; Flags: Precursor |
| 22 | 16 | AAF00693.1 | 360.97 | 44 | 0 | 0 | 8 | 4 | 11 | 39 | 0 | 3 | 0 | 8 | 4 | 4 | 4 | 4 |  |  |  |  | 2.3112E6 | 1.0258E10 |  |  |  |  |  |  |  |  | 48 | 8 | 0 | 0 | 4 | 4 | 19 | 723 | 0 | 2 | 0 | 5 | 5 | 6 | 4 | 4 | Y | 67662 | cobrin precursor [Naja naja] |
| 45 | 29 | ACN50006.1 | 341.03 | 38 | 0 | 0 | 7 | 2 | 6 | 35 | 0 | 3 | 0 | 8 | 2 | 2 | 4 | 2 |  |  |  |  |  | 1.8498E7 |  |  |  | 1.1625E6 |  |  | 3.2642E6 |  | 40 | 3 | 0 | 0 | 2 | 1 | 13 | 370 | 0 | 2 | 0 | 5 | 3 | 2 | 5 | 2 | Y | 69181 | atragin precursor, partial [Naja atra] |
| 45 | 30 | D3TTC2.1 | 341.03 | 38 | 0 | 0 | 7 | 2 | 6 | 35 | 0 | 3 | 0 | 8 | 2 | 2 | 4 | 2 |  |  |  |  |  | 1.8498E7 |  |  |  | 1.1625E6 |  |  | 3.2642E6 |  | 40 | 3 | 0 | 0 | 2 | 1 | 13 | 370 | 0 | 2 | 0 | 5 | 3 | 2 | 5 | 2 | Y | 69181 | RecName: Full=Zinc metalloproteinase-disintegrin-like atragin; AltName: Full=Snake venom metalloproteinase; Short=SVMP; Flags: Precursor |
| 8 | 55 | D5LMJ3.1 | 324.05 | 30 | 0 | 10 | 20 | 25 | 15 | 15 | 0 | 3 | 2 | 0 | 0 | 0 | 0 | 0 |  | 6.0464E7 | 9.2604E9 | 3.708E9 | 3.2029E8 | 1.826E8 |  |  | 1.8152E7 |  |  |  |  |  | 31 | 28 | 0 | 19 | 677 | 380 | 54 | 33 | 0 | 2 | 6 | 0 | 0 | 0 | 0 | 0 | Y | 68254 | RecName: Full=Zinc metalloproteinase-disintegrin-like atrase-A; AltName: Full=Snake venom metalloproteinase; Short=SVMP; Flags: Precursor |
| 8 | 56 | ADF43026.1 | 324.05 | 30 | 0 | 10 | 20 | 25 | 15 | 15 | 0 | 3 | 2 | 0 | 0 | 0 | 0 | 0 |  | 6.0464E7 | 9.2604E9 | 3.708E9 | 3.2029E8 | 1.826E8 |  |  | 1.8152E7 |  |  |  |  |  | 31 | 28 | 0 | 19 | 677 | 380 | 54 | 33 | 0 | 2 | 6 | 0 | 0 | 0 | 0 | 0 | Y | 68254 | metalloproteinase atrase A [Naja atra] |
| 49 | 132 | ACH73168.1 | 319.84 | 71 | 0 | 24 | 65 | 61 | 7 | 7 | 0 | 0 | 0 | 0 | 0 | 0 | 0 | 24 |  | 2.9504E7 | 1.6432E10 | 4.9952E8 | 2.2797E6 | 1.3086E6 |  |  |  |  |  |  |  | 7.9528E6 | 36 | 33 | 0 | 9 | 331 | 57 | 2 | 2 | 0 | 0 | 0 | 0 | 0 | 0 | 0 | 6 | Y | 26216 | kaouthin-2 precursor [Naja kaouthia] |
| 49 | 133 | P84808.2 | 319.84 | 71 | 0 | 24 | 65 | 61 | 7 | 7 | 0 | 0 | 0 | 0 | 0 | 0 | 0 | 24 |  | 2.9504E7 | 1.6432E10 | 4.9952E8 | 2.2797E6 | 1.3086E6 |  |  |  |  |  |  |  | 7.9528E6 | 36 | 33 | 0 | 9 | 331 | 57 | 2 | 2 | 0 | 0 | 0 | 0 | 0 | 0 | 0 | 6 | Y | 26216 | RecName: Full=Cysteine-rich venom protein kaouthin-2; AltName: Full=Cysteine-rich venom protein 23; Short=CRVP-23k; Flags: Precursor |
| 49 | 134 | AHZ08822.1 | 319.84 | 71 | 0 | 24 | 65 | 61 | 7 | 7 | 0 | 0 | 0 | 0 | 0 | 0 | 0 | 24 |  | 2.9504E7 | 1.6432E10 | 4.9952E8 | 2.2797E6 | 1.3086E6 |  |  |  |  |  |  |  | 7.9528E6 | 36 | 33 | 0 | 9 | 331 | 57 | 2 | 2 | 0 | 0 | 0 | 0 | 0 | 0 | 0 | 6 | Y | 26216 | cysteine-rich secretory protein [Micropechis ikaheca] |
| 35 | 28 | P82942.1 | 317.86 | 61 | 0 | 0 | 8 | 31 | 50 | 45 | 0 | 0 | 5 | 0 | 5 | 24 | 21 | 3 |  |  | 1.0913E6 | 1.4904E8 | 2.2681E9 | 5.4733E8 |  |  |  |  | 3.1018E6 | 5.9158E8 | 7.1274E7 |  | 35 | 17 | 0 | 0 | 3 | 44 | 275 | 86 | 0 | 0 | 1 | 0 | 1 | 38 | 19 | 1 | Y | 44493 | RecName: Full=Hemorrhagic metalloproteinase-disintegrin-like kaouthiagin; AltName: Full=Snake venom metalloproteinase; Short=SVMP |
| 40 | 36 | JAG67188.1 | 315.97 | 34 | 0 | 0 | 0 | 11 | 30 | 34 | 0 | 0 | 0 | 3 | 0 | 0 | 3 | 7 |  |  |  |  | 4.3E5 | 4.1793E6 |  |  |  |  |  |  |  |  | 26 | 1 | 0 | 0 | 0 | 7 | 145 | 293 | 0 | 0 | 0 | 1 | 0 | 0 | 4 | 14 | Y | 64759 | Snake venom 5'-nucleotidase [Boiga irregularis] |
| 53 | 124 | AXL95287.1 | 308.56 | 26 | 0 | 0 | 0 | 5 | 10 | 26 | 0 | 0 | 0 | 0 | 0 | 0 | 0 | 0 |  |  |  |  |  | 1.5989E8 |  |  |  |  |  |  |  |  | 33 | 1 | 0 | 0 | 0 | 8 | 41 | 328 | 0 | 0 | 0 | 0 | 0 | 0 | 0 | 0 | Y | 58594 | L-amino acid oxidase 1, partial [Spilotes sulphureus] |
| 9 | 257 | P01446.1 | 291.30 | 100 | 0 | 27 | 37 | 23 | 0 | 12 | 0 | 0 | 0 | 0 | 0 | 100 | 100 | 47 |  |  |  |  |  |  |  |  |  |  |  | 7.4956E7 | 1.2098E9 |  | 22 | 4 | 0 | 39 | 7 | 2 | 0 | 1 | 0 | 0 | 0 | 0 | 0 | 219 | 411 | 12 | Y | 6717 | RecName: Full=Cytotoxin 3; Short=CX3; AltName: Full=Toxin CM-7 |
| 9 | 258 | 6RC7 | 291.30 | 100 | 0 | 27 | 37 | 23 | 0 | 12 | 0 | 0 | 0 | 0 | 0 | 100 | 100 | 47 |  |  |  |  |  |  |  |  |  |  |  | 7.4956E7 | 1.2098E9 |  | 22 | 4 | 0 | 39 | 7 | 2 | 0 | 1 | 0 | 0 | 0 | 0 | 0 | 219 | 411 | 12 | Y | 6717 | Chain A, Cytotoxin 3 |
| 57 | 233 | JAC95028.1 | 283.56 | 17 | 0 | 0 | 0 | 5 | 7 | 17 | 0 | 0 | 0 | 0 | 0 | 0 | 0 | 0 |  |  |  |  |  | 2.8702E6 |  |  |  |  |  |  |  |  | 21 | 1 | 0 | 0 | 0 | 8 | 37 | 275 | 0 | 0 | 0 | 0 | 0 | 0 | 0 | 0 | Y | 58544 | L-amino acid oxidase [Pantherophis guttatus] |
| 48 | 361 | P82885.1 | 283.09 | 73 | 0 | 56 | 73 | 56 | 48 | 48 | 0 | 13 | 12 | 0 | 19 | 31 | 19 | 35 |  | 4.1242E8 | 8.971E9 | 2.3986E8 | 4.0412E7 | 4.1224E7 |  | 1.7236E5 | 4.8282E5 |  | 7.2734E6 | 6.9626E7 | 1.604E6 | 7.9139E6 | 19 | 18 | 0 | 31 | 336 | 32 | 14 | 10 | 0 | 2 | 1 | 0 | 2 | 9 | 1 | 3 | Y | 12038 | RecName: Full=Thaicobrin |
| 50 | 592 | P82462.1 | 282.74 | 88 | 0 | 0 | 43 | 43 | 0 | 0 | 0 | 0 | 15 | 43 | 43 | 88 | 85 | 46 |  |  | 2.0449E7 | 1.1485E7 |  |  |  |  |  | 3.9373E7 | 6.4432E6 | 4.4053E10 | 6.1326E9 | 4.7454E8 | 23 | 18 | 0 | 0 | 2 | 2 | 0 | 0 | 0 | 0 | 2 | 2 | 1 | 314 | 69 | 12 | Y | 7366 | RecName: Full=Muscarinic toxin-like protein 1; Short=MTLP-1 |
| 96 | 25 | 5GZ4 | 280.62 | 35 | 0 | 0 | 0 | 1 | 25 | 35 | 0 | 0 | 2 | 0 | 0 | 0 | 0 | 0 |  |  |  | 0 | 1.8457E7 | 6.3614E7 |  |  | 3.4819E6 |  |  |  |  |  | 23 | 14 | 0 | 0 | 0 | 1 | 30 | 75 | 0 | 0 | 3 | 0 | 0 | 0 | 0 | 0 | Y | 94616 | Chain A, Snake Venom Phosphodiesterase (pde) |
| 96 | 26 | A0A2D0TC04.1 | 280.62 | 35 | 0 | 0 | 0 | 1 | 25 | 35 | 0 | 0 | 2 | 0 | 0 | 0 | 0 | 0 |  |  |  | 0 | 1.8457E7 | 6.3614E7 |  |  | 3.4819E6 |  |  |  |  |  | 23 | 14 | 0 | 0 | 0 | 1 | 30 | 75 | 0 | 0 | 3 | 0 | 0 | 0 | 0 | 0 | Y | 94616 | RecName: Full=Venom phosphodiesterase; Short=PDE |
| 96 | 27 | 5GZ5 | 280.62 | 35 | 0 | 0 | 0 | 1 | 25 | 35 | 0 | 0 | 2 | 0 | 0 | 0 | 0 | 0 |  |  |  | 0 | 1.8457E7 | 6.3614E7 |  |  | 3.4819E6 |  |  |  |  |  | 23 | 14 | 0 | 0 | 0 | 1 | 30 | 75 | 0 | 0 | 3 | 0 | 0 | 0 | 0 | 0 | Y | 94616 | Chain A, Snake Venom Phosphodiesterase (pde) |
| 3 | 309 | AAB25732.1 | 275.86 | 92 | 0 | 88 | 57 | 48 | 22 | 22 | 0 | 0 | 13 | 22 | 23 | 70 | 48 | 67 |  | 7.2667E7 |  |  |  |  |  |  |  |  |  | 1.4718E5 |  | 1.6796E7 | 27 | 4 | 0 | 2557 | 1832 | 1127 | 417 | 301 | 0 | 0 | 1 | 14 | 141 | 390 | 737 | 1967 | Y | 6701 | cardiotoxin isoform 1, cytotoxin isoform 1, CTX-1 [Naja naja=Formosan cobra, ssp. atra, venom, Peptide, 60 aa] |
| 47 | 102 | 3K7N | 272.20 | 38 | 0 | 6 | 10 | 22 | 30 | 33 | 0 | 0 | 6 | 0 | 0 | 13 | 16 | 3 |  |  |  |  | 4.1409E5 |  |  |  |  |  |  |  |  |  | 27 | 1 | 0 | 2 | 36 | 67 | 210 | 65 | 0 | 0 | 18 | 0 | 0 | 18 | 9 | 1 | Y | 44191 | Chain A, Structures Of Two Elapid Snake Venom Metalloproteases With Distinct Activities Highlight The Disulfide Patterns In The D Domain Of Adamalysin Family Proteins |
| 33 | 300 | P14541.1 | 269.90 | 97 | 0 | 21 | 18 | 18 | 18 | 18 | 19 | 18 | 18 | 18 | 18 | 19 | 47 | 97 |  |  |  |  |  |  |  |  |  |  |  |  | 3.2872E7 | 1.0825E9 | 18 | 1 | 0 | 32 | 2 | 6 | 3 | 1 | 6 | 14 | 13 | 12 | 6 | 16 | 85 | 283 | Y | 6994 | RecName: Full=Cytotoxin homolog; AltName: Full=Cytotoxin-like basic protein; Short=CLBP |
| 103 | 94 | C3NJ | 268.22 | 13 | 0 | 0 | 0 | 1 | 4 | 12 | 1 | 1 | 0 | 0 | 0 | 0 | 0 | 0 |  |  |  |  |  | 6.2278E6 |  |  |  |  |  |  |  |  | 21 | 1 | 0 | 0 | 0 | 3 | 17 | 90 | 1 | 1 | 0 | 0 | 0 | 0 | 0 | 0 | Y | 184926 | complement C3 precursor - monocled cobra |
| 103 | 95 | Q01833.1 | 268.22 | 13 | 0 | 0 | 0 | 1 | 4 | 12 | 1 | 1 | 0 | 0 | 0 | 0 | 0 | 0 |  |  |  |  |  | 6.2278E6 |  |  |  |  |  |  |  |  | 21 | 1 | 0 | 0 | 0 | 3 | 17 | 90 | 1 | 1 | 0 | 0 | 0 | 0 | 0 | 0 | Y | 184926 | RecName: Full=Complement C3; Contains: RecName: Full=Complement C3 beta chain; Contains: RecName: Full=Complement C3 alpha chain; Contains: RecName: Full=C3a anaphylatoxin; Contains: RecName: Full=Complement C3b alpha' chain; Contai... |
| 103 | 96 | AAA49385.1 | 268.22 | 13 | 0 | 0 | 0 | 1 | 4 | 12 | 1 | 1 | 0 | 0 | 0 | 0 | 0 | 0 |  |  |  |  |  | 6.2278E6 |  |  |  |  |  |  |  |  | 21 | 1 | 0 | 0 | 0 | 3 | 17 | 90 | 1 | 1 | 0 | 0 | 0 | 0 | 0 | 0 | Y | 184926 | complement component C3 [Naja naja] |
| 52 | 408 | AAF82186.1 | 262.80 | 65 | 0 | 34 | 24 | 34 | 10 | 14 | 0 | 0 | 0 | 10 | 27 | 34 | 55 | 44 |  |  |  |  |  |  |  |  |  |  |  |  | 3.3766E7 |  | 19 | 1 | 0 | 6 | 9 | 7 | 8 | 6 | 0 | 0 | 0 | 1 | 3 | 50 | 266 | 34 | Y | 16097 | acidic phospholipase A2 [Naja sputatrix] |
| 52 | 409 | Q9I900.1 | 262.80 | 65 | 0 | 34 | 24 | 34 | 10 | 14 | 0 | 0 | 0 | 10 | 27 | 34 | 55 | 44 |  |  |  |  |  |  |  |  |  |  |  |  | 3.3766E7 |  | 19 | 1 | 0 | 6 | 9 | 7 | 8 | 6 | 0 | 0 | 0 | 1 | 3 | 50 | 266 | 34 | Y | 16097 | RecName: Full=Acidic phospholipase A2 D; Short=svPLA2; AltName: Full=APLA; AltName: Full=Phosphatidylcholine 2-acylhydrolase; Flags: Precursor |
| 76 | 600 | XP\_034258146.1 | 260.75 | 22 | 0 | 5 | 11 | 14 | 22 | 22 | 0 | 0 | 0 | 5 | 5 | 5 | 15 | 5 |  |  |  | 6.6915E5 | 2.8909E8 | 1.907E9 |  |  |  |  |  |  | 1.9563E6 |  | 16 | 10 | 0 | 2 | 4 | 5 | 51 | 129 | 0 | 0 | 0 | 1 | 2 | 4 | 7 | 5 | Y | 28406 | glutathione peroxidase 3 [Pantherophis guttatus] |
| 86 | 341 | P0DI91.1 | 260.03 | 78 | 0 | 0 | 22 | 44 | 66 | 78 | 0 | 0 | 0 | 0 | 0 | 0 | 0 | 0 |  |  | 1.5723E6 | 6.0794E6 | 2.5094E7 | 3.2226E8 |  |  |  |  |  |  |  |  | 15 | 5 | 0 | 0 | 4 | 7 | 28 | 150 | 0 | 0 | 0 | 0 | 0 | 0 | 0 | 0 | Y | 11216 | RecName: Full=L-amino-acid oxidase; Short=LAAO; Short=LAO |
| 89 | 145 | XP\_026544671.1 | 257.18 | 23 | 0 | 17 | 17 | 19 | 13 | 9 | 0 | 0 | 2 | 0 | 4 | 10 | 9 | 4 |  |  |  |  |  |  |  |  |  |  |  | 1.0505E7 |  |  | 14 | 1 | 0 | 16 | 24 | 44 | 13 | 3 | 0 | 0 | 1 | 0 | 8 | 48 | 5 | 2 | Y | 60656 | tissue-type plasminogen activator, partial [Notechis scutatus] |
| 42 | 370 | 1POB | 255.48 | 85 | 0 | 31 | 30 | 49 | 19 | 18 | 0 | 0 | 0 | 19 | 42 | 42 | 77 | 31 |  |  |  | 5.4292E6 |  |  |  |  |  |  |  |  |  |  | 16 | 1 | 0 | 8 | 12 | 11 | 9 | 4 | 0 | 0 | 0 | 3 | 7 | 55 | 324 | 26 | Y | 13144 | Chain B, PHOSPHOLIPASE A2 |
| 42 | 371 | 1POA | 255.48 | 85 | 0 | 31 | 30 | 49 | 19 | 18 | 0 | 0 | 0 | 19 | 42 | 42 | 77 | 31 |  |  |  | 5.4292E6 |  |  |  |  |  |  |  |  |  |  | 16 | 1 | 0 | 8 | 12 | 11 | 9 | 4 | 0 | 0 | 0 | 3 | 7 | 55 | 324 | 26 | Y | 13144 | Chain A, PHOSPHOLIPASE A2 |
| 71 | 141 | XP\_026546198.1 | 255.41 | 44 | 0 | 0 | 11 | 31 | 42 | 34 | 0 | 0 | 3 | 5 | 5 | 5 | 11 | 5 |  |  |  |  | 4.6886E8 | 5.0932E6 |  |  | 4.3246E5 |  |  |  |  |  | 18 | 1 | 0 | 0 | 3 | 33 | 142 | 18 | 0 | 0 | 1 | 1 | 2 | 1 | 2 | 1 | Y | 31377 | endonuclease domain-containing 1 protein-like isoform X2 [Notechis scutatus] |
| 71 | 142 | XP\_026546199.1 | 255.41 | 44 | 0 | 0 | 11 | 31 | 42 | 34 | 0 | 0 | 3 | 5 | 5 | 5 | 11 | 5 |  |  |  |  | 4.6886E8 | 5.0932E6 |  |  | 4.3246E5 |  |  |  |  |  | 18 | 1 | 0 | 0 | 3 | 33 | 142 | 18 | 0 | 0 | 1 | 1 | 2 | 1 | 2 | 1 | Y | 31377 | endonuclease domain-containing 1 protein-like isoform X2 [Notechis scutatus] |
| 71 | 143 | XP\_026546197.1 | 255.41 | 44 | 0 | 0 | 11 | 31 | 42 | 34 | 0 | 0 | 3 | 5 | 5 | 5 | 11 | 5 |  |  |  |  | 4.6886E8 | 5.0932E6 |  |  | 4.3246E5 |  |  |  |  |  | 18 | 1 | 0 | 0 | 3 | 33 | 142 | 18 | 0 | 0 | 1 | 1 | 2 | 1 | 2 | 1 | Y | 31377 | endonuclease domain-containing 1 protein-like isoform X2 [Notechis scutatus] |
| 71 | 144 | XP\_026546196.1 | 255.41 | 42 | 0 | 0 | 10 | 29 | 40 | 32 | 0 | 0 | 3 | 5 | 5 | 5 | 10 | 5 |  |  |  |  | 4.6886E8 | 5.0932E6 |  |  | 4.3246E5 |  |  |  |  |  | 18 | 1 | 0 | 0 | 3 | 33 | 142 | 18 | 0 | 0 | 1 | 1 | 2 | 1 | 2 | 1 | Y | 33047 | endonuclease domain-containing 1 protein-like isoform X1 [Notechis scutatus] |
| 133 | 86 | CAA55333.1 | 254.01 | 35 | 0 | 0 | 0 | 2 | 7 | 34 | 0 | 0 | 0 | 0 | 0 | 0 | 0 | 0 |  |  |  | 1.0464E5 | 2.5788E6 | 7.8771E7 |  |  |  |  |  |  |  |  | 18 | 17 | 0 | 0 | 0 | 1 | 7 | 49 | 0 | 0 | 0 | 0 | 0 | 0 | 0 | 0 | Y | 69799 | cobra serum albumin [Naja naja] |
| 133 | 87 | S59517 | 254.01 | 35 | 0 | 0 | 0 | 2 | 7 | 34 | 0 | 0 | 0 | 0 | 0 | 0 | 0 | 0 |  |  |  | 1.0464E5 | 2.5788E6 | 7.8771E7 |  |  |  |  |  |  |  |  | 18 | 17 | 0 | 0 | 0 | 1 | 7 | 49 | 0 | 0 | 0 | 0 | 0 | 0 | 0 | 0 | Y | 69799 | serum albumin precursor - monocled cobra |
| 87 | 230 | XP\_034291087.1 | 252.70 | 20 | 0 | 15 | 15 | 18 | 14 | 6 | 0 | 0 | 2 | 0 | 3 | 5 | 6 | 6 |  | 8.347E7 | 1.017E8 | 9.6392E7 | 5.4956E6 | 3.7275E5 |  |  |  |  |  |  | 1.646E5 | 1.1739E6 | 15 | 4 | 0 | 28 | 34 | 51 | 13 | 3 | 0 | 0 | 1 | 0 | 8 | 28 | 5 | 3 | Y | 63129 | tissue-type plasminogen activator [Pantherophis guttatus] |
| 87 | 231 | XP\_034291085.1 | 252.70 | 20 | 0 | 15 | 15 | 18 | 14 | 6 | 0 | 0 | 2 | 0 | 3 | 5 | 6 | 6 |  | 8.347E7 | 1.017E8 | 9.6392E7 | 5.4956E6 | 3.7275E5 |  |  |  |  |  |  | 1.646E5 | 1.1739E6 | 15 | 4 | 0 | 28 | 34 | 51 | 13 | 3 | 0 | 0 | 1 | 0 | 8 | 28 | 5 | 3 | Y | 63129 | tissue-type plasminogen activator [Pantherophis guttatus] |
| 87 | 232 | XP\_034291086.1 | 252.70 | 20 | 0 | 15 | 15 | 18 | 14 | 6 | 0 | 0 | 2 | 0 | 3 | 5 | 6 | 6 |  | 8.347E7 | 1.017E8 | 9.6392E7 | 5.4956E6 | 3.7275E5 |  |  |  |  |  |  | 1.646E5 | 1.1739E6 | 15 | 4 | 0 | 28 | 34 | 51 | 13 | 3 | 0 | 0 | 1 | 0 | 8 | 28 | 5 | 3 | Y | 63129 | tissue-type plasminogen activator [Pantherophis guttatus] |
| 81 | 483 | ETE56723.1 | 249.25 | 61 | 0 | 0 | 0 | 51 | 56 | 38 | 0 | 0 | 0 | 0 | 0 | 0 | 0 | 13 |  |  |  | 2.7309E7 | 5.5151E8 | 2.9423E6 |  |  |  |  |  |  |  |  | 21 | 3 | 0 | 0 | 0 | 36 | 139 | 11 | 0 | 0 | 0 | 0 | 0 | 0 | 0 | 2 | Y | 18083 | Endonuclease domain-containing 1 protein [Ophiophagus hannah] |
| 84 | 121 | ETE68810.1 | 244.77 | 41 | 0 | 5 | 10 | 16 | 24 | 39 | 0 | 0 | 0 | 5 | 5 | 8 | 10 | 5 |  |  |  | 1.1558E6 | 8.6172E7 | 6.579E7 |  |  |  |  |  |  |  |  | 16 | 3 | 0 | 2 | 4 | 6 | 40 | 105 | 0 | 0 | 0 | 1 | 2 | 6 | 6 | 5 | Y | 29588 | Glutathione peroxidase 3, partial [Ophiophagus hannah] |
| 85 | 488 | P49122.1 | 243.68 | 45 | 0 | 30 | 39 | 39 | 0 | 0 | 0 | 22 | 21 | 21 | 30 | 32 | 30 | 41 |  | 3.0385E8 | 5.5153E9 | 1.9058E8 |  |  |  | 3.3627E6 | 2.1531E6 | 8.7169E6 | 6.8957E6 | 2.0445E7 | 1.87E7 | 8.8187E7 | 18 | 12 | 0 | 20 | 76 | 26 | 0 | 0 | 0 | 6 | 4 | 3 | 4 | 11 | 9 | 23 | Y | 9086 | RecName: Full=Cytotoxin 7; AltName: Full=Cardiotoxin-7; Short=CTX7; Short=Ctx-7; AltName: Full=Cardiotoxin-like basic protein 2; Short=CLBP2; Flags: Precursor |
| 85 | 489 | CAA90967.1 | 243.68 | 45 | 0 | 30 | 39 | 39 | 0 | 0 | 0 | 22 | 21 | 21 | 30 | 32 | 30 | 41 |  | 3.0385E8 | 5.5153E9 | 1.9058E8 |  |  |  | 3.3627E6 | 2.1531E6 | 8.7169E6 | 6.8957E6 | 2.0445E7 | 1.87E7 | 8.8187E7 | 18 | 12 | 0 | 20 | 76 | 26 | 0 | 0 | 0 | 6 | 4 | 3 | 4 | 11 | 9 | 23 | Y | 9086 | cardiotoxin 7 [Naja naja] |
| 85 | 490 | CAA77017.1 | 243.68 | 45 | 0 | 30 | 39 | 39 | 0 | 0 | 0 | 22 | 21 | 21 | 30 | 32 | 30 | 41 |  | 3.0385E8 | 5.5153E9 | 1.9058E8 |  |  |  | 3.3627E6 | 2.1531E6 | 8.7169E6 | 6.8957E6 | 2.0445E7 | 1.87E7 | 8.8187E7 | 18 | 12 | 0 | 20 | 76 | 26 | 0 | 0 | 0 | 6 | 4 | 3 | 4 | 11 | 9 | 23 | Y | 9086 | cardiotoxin 7 precursor [Naja atra] |
| 78 | 234 | XP\_026541908.1 | 235.88 | 32 | 0 | 5 | 11 | 16 | 21 | 30 | 0 | 0 | 0 | 10 | 5 | 14 | 11 | 5 |  |  |  | 1.3787E6 | 2.3027E8 | 1.1209E9 |  |  |  | 1.1813E7 |  | 5.021E7 |  |  | 16 | 3 | 0 | 2 | 4 | 5 | 42 | 121 | 0 | 0 | 0 | 3 | 2 | 9 | 6 | 5 | Y | 28574 | glutathione peroxidase 3 isoform X1 [Notechis scutatus] |
| 78 | 235 | XP\_026541916.1 | 235.88 | 36 | 0 | 6 | 12 | 19 | 24 | 35 | 0 | 0 | 0 | 12 | 5 | 16 | 12 | 6 |  |  |  | 1.3787E6 | 2.3027E8 | 1.1209E9 |  |  |  | 1.1813E7 |  | 5.021E7 |  |  | 16 | 3 | 0 | 2 | 4 | 5 | 42 | 121 | 0 | 0 | 0 | 3 | 2 | 9 | 6 | 5 | Y | 25191 | glutathione peroxidase 3 isoform X2 [Notechis scutatus] |
| 78 | 242 | XP\_026552407.1 | 235.88 | 36 | 0 | 6 | 12 | 19 | 24 | 35 | 0 | 0 | 0 | 12 | 5 | 16 | 12 | 6 |  |  |  | 1.3787E6 | 2.3027E8 | 1.1209E9 |  |  |  | 1.1813E7 |  | 5.021E7 |  |  | 16 | 3 | 0 | 2 | 4 | 5 | 42 | 121 | 0 | 0 | 0 | 3 | 2 | 9 | 6 | 5 | Y | 25168 | glutathione peroxidase 3 isoform X2 [Pseudonaja textilis] |
| 78 | 243 | XP\_026552406.1 | 235.88 | 32 | 0 | 5 | 11 | 16 | 21 | 30 | 0 | 0 | 0 | 10 | 5 | 14 | 11 | 5 |  |  |  | 1.3787E6 | 2.3027E8 | 1.1209E9 |  |  |  | 1.1813E7 |  | 5.021E7 |  |  | 16 | 3 | 0 | 2 | 4 | 5 | 42 | 121 | 0 | 0 | 0 | 3 | 2 | 9 | 6 | 5 | Y | 28619 | glutathione peroxidase 3 isoform X1 [Pseudonaja textilis] |
| 93 | 316 | XP\_029140080.1 | 233.21 | 17 | 0 | 13 | 13 | 15 | 9 | 8 | 0 | 0 | 0 | 0 | 6 | 10 | 11 | 6 |  | 4.2652E6 | 1.0449E7 | 1.23E7 | 1.1923E6 | 6.1065E5 |  |  |  |  | 4.6972E6 | 8.5238E7 | 3.4801E5 | 1.4118E6 | 11 | 1 | 0 | 10 | 18 | 30 | 8 | 3 | 0 | 0 | 0 | 0 | 9 | 46 | 8 | 3 | Y | 62665 | tissue-type plasminogen activator [Protobothrops mucrosquamatus] |
| 93 | 317 | JAI11009.1 | 233.21 | 18 | 0 | 13 | 13 | 16 | 9 | 8 | 0 | 0 | 0 | 0 | 6 | 11 | 12 | 6 |  | 4.2652E6 | 1.0449E7 | 1.23E7 | 1.1923E6 | 6.1065E5 |  |  |  |  | 4.6972E6 | 8.5238E7 | 3.4801E5 | 1.4118E6 | 11 | 1 | 0 | 10 | 18 | 30 | 8 | 3 | 0 | 0 | 0 | 0 | 9 | 46 | 8 | 3 | Y | 59716 | tissue-type plasminogen activator [Crotalus adamanteus] |
| 152 | 103 | XP\_026535930.1 | 229.43 | 17 | 0 | 0 | 6 | 9 | 0 | 1 | 0 | 0 | 0 | 0 | 1 | 0 | 1 | 4 |  |  | 1.5663E7 | 9.0084E6 |  | 2.5942E5 |  |  |  |  | 5.4593E5 |  | 4.3746E6 | 1.7729E7 | 14 | 14 | 0 | 0 | 8 | 13 | 0 | 1 | 0 | 0 | 0 | 0 | 1 | 0 | 2 | 10 | Y | 132789 | Golgi apparatus protein 1 [Notechis scutatus] |
| 152 | 104 | XP\_026570202.1 | 229.43 | 17 | 0 | 0 | 6 | 9 | 0 | 1 | 0 | 0 | 0 | 0 | 1 | 0 | 1 | 4 |  |  | 1.5663E7 | 9.0084E6 |  | 2.5942E5 |  |  |  |  | 5.4593E5 |  | 4.3746E6 | 1.7729E7 | 14 | 14 | 0 | 0 | 8 | 13 | 0 | 1 | 0 | 0 | 0 | 0 | 1 | 0 | 2 | 10 | Y | 133145 | Golgi apparatus protein 1 [Pseudonaja textilis] |
| 60 | 164 | ABN72539.1 | 222.14 | 20 | 0 | 0 | 0 | 2 | 8 | 20 | 0 | 0 | 0 | 0 | 0 | 0 | 0 | 0 |  |  |  |  |  | 0 |  |  |  |  |  |  |  |  | 17 | 1 | 0 | 0 | 0 | 1 | 31 | 245 | 0 | 0 | 0 | 0 | 0 | 0 | 0 | 0 | Y | 58811 | L-amino acid oxidase [Bungarus multicinctus] |
| 60 | 165 | A8QL51.1 | 222.14 | 20 | 0 | 0 | 0 | 2 | 8 | 20 | 0 | 0 | 0 | 0 | 0 | 0 | 0 | 0 |  |  |  |  |  | 0 |  |  |  |  |  |  |  |  | 17 | 1 | 0 | 0 | 0 | 1 | 31 | 245 | 0 | 0 | 0 | 0 | 0 | 0 | 0 | 0 | Y | 58811 | RecName: Full=L-amino-acid oxidase; Short=Bm-LAAO; Short=LAO; Flags: Precursor |
| 30 | 890 | P82463.1 | 219.84 | 92 | 0 | 42 | 29 | 42 | 0 | 12 | 0 | 0 | 0 | 92 | 77 | 42 | 42 | 29 |  | 2.7994E6 | 2.389E6 | 6.4607E6 |  |  |  |  |  | 3.8249E10 | 1.8196E8 | 2.6228E7 | 1.411E7 | 8.1596E6 | 11 | 9 | 0 | 3 | 1 | 7 | 0 | 1 | 0 | 0 | 0 | 465 | 50 | 9 | 5 | 1 | Y | 7298 | RecName: Full=Muscarinic toxin-like protein 2; Short=MTLP-2 |
| 44 | 187 | AAM51550.1 | 214.69 | 12 | 0 | 0 | 2 | 2 | 4 | 12 | 0 | 0 | 0 | 0 | 0 | 0 | 0 | 2 |  |  |  |  | 1.5899E9 | 2.0831E9 |  |  |  |  |  |  |  |  | 12 | 9 | 0 | 0 | 2 | 3 | 128 | 281 | 0 | 0 | 0 | 0 | 0 | 0 | 0 | 2 | Y | 68176 | mocarhagin 1 [Naja mossambica] |
| 44 | 188 | Q10749.3 | 214.69 | 12 | 0 | 0 | 2 | 2 | 4 | 12 | 0 | 0 | 0 | 0 | 0 | 0 | 0 | 2 |  |  |  |  | 1.5899E9 | 2.0831E9 |  |  |  |  |  |  |  |  | 12 | 9 | 0 | 0 | 2 | 3 | 128 | 281 | 0 | 0 | 0 | 0 | 0 | 0 | 0 | 2 | Y | 68176 | RecName: Full=Snake venom metalloproteinase-disintegrin-like mocarhagin; Short=MOC; Short=Mocarhagin-1; Short=SVMP; AltName: Full=Zinc metalloproteinase mocarhagin; Flags: Precursor |
| 75 | 837 | 1JE9 | 214.55 | 80 | 18 | 0 | 0 | 0 | 0 | 0 | 80 | 54 | 18 | 0 | 0 | 18 | 0 | 0 |  |  |  |  |  |  | 3.965E7 | 7.7122E5 |  |  |  |  |  |  | 9 | 1 | 1 | 0 | 0 | 0 | 0 | 0 | 188 | 13 | 1 | 0 | 0 | 1 | 0 | 0 | Y | 6859 | Chain A, Nmr Solution Structure Of Nt2 |
| 75 | 838 | P59276.1 | 214.55 | 80 | 18 | 0 | 0 | 0 | 0 | 0 | 80 | 54 | 18 | 0 | 0 | 18 | 0 | 0 |  |  |  |  |  |  | 3.965E7 | 7.7122E5 |  |  |  |  |  |  | 9 | 1 | 1 | 0 | 0 | 0 | 0 | 0 | 188 | 13 | 1 | 0 | 0 | 1 | 0 | 0 | Y | 6859 | RecName: Full=Cobrotoxin-c; Short=CBT-c; AltName: Full=Short neurotoxin II; Short=NT2 |
| 107 | 841 | Q9W727.1 | 207.94 | 62 | 0 | 20 | 20 | 36 | 0 | 0 | 0 | 0 | 16 | 36 | 50 | 62 | 36 | 20 |  | 1.7723E6 | 2.8638E6 | 3.1748E6 |  |  |  |  | 6.803E5 | 1.4053E8 | 9.5891E9 | 2.1299E8 | 4.5307E7 | 1.097E7 | 10 | 6 | 0 | 1 | 2 | 2 | 0 | 0 | 0 | 0 | 5 | 13 | 50 | 20 | 8 | 3 | Y | 9934 | RecName: Full=Muscarinic toxin-like protein; Flags: Precursor |
| 107 | 842 | Q9DEQ3.1 | 207.94 | 62 | 0 | 20 | 20 | 36 | 0 | 0 | 0 | 0 | 16 | 36 | 50 | 62 | 36 | 20 |  | 1.7723E6 | 2.8638E6 | 3.1748E6 |  |  |  |  | 6.803E5 | 1.4053E8 | 9.5891E9 | 2.1299E8 | 4.5307E7 | 1.097E7 | 10 | 6 | 0 | 1 | 2 | 2 | 0 | 0 | 0 | 0 | 5 | 13 | 50 | 20 | 8 | 3 | Y | 9962 | RecName: Full=Neurotoxin homolog NL1; Flags: Precursor |
| 107 | 843 | CAB50691.1 | 207.94 | 62 | 0 | 20 | 20 | 36 | 0 | 0 | 0 | 0 | 16 | 36 | 50 | 62 | 36 | 20 |  | 1.7723E6 | 2.8638E6 | 3.1748E6 |  |  |  |  | 6.803E5 | 1.4053E8 | 9.5891E9 | 2.1299E8 | 4.5307E7 | 1.097E7 | 10 | 6 | 0 | 1 | 2 | 2 | 0 | 0 | 0 | 0 | 5 | 13 | 50 | 20 | 8 | 3 | Y | 9934 | snake venom [Bungarus multicinctus multicinctus] |
| 107 | 844 | CAC08183.1 | 207.94 | 62 | 0 | 20 | 20 | 36 | 0 | 0 | 0 | 0 | 16 | 36 | 50 | 62 | 36 | 20 |  | 1.7723E6 | 2.8638E6 | 3.1748E6 |  |  |  |  | 6.803E5 | 1.4053E8 | 9.5891E9 | 2.1299E8 | 4.5307E7 | 1.097E7 | 10 | 6 | 0 | 1 | 2 | 2 | 0 | 0 | 0 | 0 | 5 | 13 | 50 | 20 | 8 | 3 | Y | 9962 | unnamed protein product (plasmid) [Naja atra] |
| 79 | 789 | XP\_026522175.1 | 206.93 | 15 | 0 | 0 | 8 | 15 | 10 | 10 | 0 | 0 | 0 | 0 | 0 | 0 | 0 | 0 |  |  |  | 3.8557E6 |  |  |  |  |  |  |  |  |  |  | 7 | 1 | 0 | 0 | 4 | 205 | 13 | 9 | 0 | 0 | 0 | 0 | 0 | 0 | 0 | 0 | Y | 31530 | snake venom serine protease NaSP isoform X1 [Notechis scutatus] |
| 79 | 790 | XP\_026522177.1 | 206.93 | 17 | 0 | 0 | 9 | 17 | 12 | 12 | 0 | 0 | 0 | 0 | 0 | 0 | 0 | 0 |  |  |  | 3.8557E6 |  |  |  |  |  |  |  |  |  |  | 7 | 1 | 0 | 0 | 4 | 205 | 13 | 9 | 0 | 0 | 0 | 0 | 0 | 0 | 0 | 0 | Y | 28056 | serine protease harobin isoform X3 [Notechis scutatus] |
| 79 | 791 | XP\_026522176.1 | 206.93 | 16 | 0 | 0 | 9 | 16 | 11 | 11 | 0 | 0 | 0 | 0 | 0 | 0 | 0 | 0 |  |  |  | 3.8557E6 |  |  |  |  |  |  |  |  |  |  | 7 | 1 | 0 | 0 | 4 | 205 | 13 | 9 | 0 | 0 | 0 | 0 | 0 | 0 | 0 | 0 | Y | 28988 | serine protease harobin isoform X2 [Notechis scutatus] |
| 79 | 976 | ABN72545.1 | 206.93 | 15 | 0 | 0 | 9 | 15 | 11 | 11 | 0 | 0 | 0 | 0 | 0 | 0 | 0 | 0 |  |  |  | 3.8557E6 |  |  |  |  |  |  |  |  |  |  | 7 | 1 | 0 | 0 | 4 | 205 | 13 | 9 | 0 | 0 | 0 | 0 | 0 | 0 | 0 | 0 | Y | 31010 | putative serine protease, partial [Bungarus multicinctus] |
| 79 | 977 | A8QL57.1 | 206.93 | 15 | 0 | 0 | 9 | 15 | 11 | 11 | 0 | 0 | 0 | 0 | 0 | 0 | 0 | 0 |  |  |  | 3.8557E6 |  |  |  |  |  |  |  |  |  |  | 7 | 1 | 0 | 0 | 4 | 205 | 13 | 9 | 0 | 0 | 0 | 0 | 0 | 0 | 0 | 0 | Y | 31010 | RecName: Full=Snake venom serine protease BmSP; Short=SVSP; Flags: Precursor |
| 79 | 986 | ABN72541.1 | 206.93 | 15 | 0 | 0 | 9 | 15 | 11 | 11 | 0 | 0 | 0 | 0 | 0 | 0 | 0 | 0 |  |  |  | 3.8557E6 |  |  |  |  |  |  |  |  |  |  | 7 | 1 | 0 | 0 | 4 | 205 | 13 | 9 | 0 | 0 | 0 | 0 | 0 | 0 | 0 | 0 | Y | 31137 | putative serine protease, partial [Naja atra] |
| 79 | 987 | A8QL53.1 | 206.93 | 15 | 0 | 0 | 9 | 15 | 11 | 11 | 0 | 0 | 0 | 0 | 0 | 0 | 0 | 0 |  |  |  | 3.8557E6 |  |  |  |  |  |  |  |  |  |  | 7 | 1 | 0 | 0 | 4 | 205 | 13 | 9 | 0 | 0 | 0 | 0 | 0 | 0 | 0 | 0 | Y | 31137 | RecName: Full=Snake venom serine protease NaSP; Short=SVSP; Flags: Precursor |
| 79 | 1050 | XP\_026574628.1 | 206.93 | 17 | 0 | 0 | 9 | 17 | 12 | 12 | 0 | 0 | 0 | 0 | 0 | 0 | 0 | 0 |  |  |  | 3.8557E6 |  |  |  |  |  |  |  |  |  |  | 7 | 1 | 0 | 0 | 4 | 205 | 13 | 9 | 0 | 0 | 0 | 0 | 0 | 0 | 0 | 0 | Y | 28072 | serine protease harobin isoform X2 [Pseudonaja textilis] |
| 79 | 1121 | XP\_026574627.1 | 206.93 | 16 | 0 | 0 | 9 | 16 | 11 | 11 | 0 | 0 | 0 | 0 | 0 | 0 | 0 | 0 |  |  |  | 3.8557E6 |  |  |  |  |  |  |  |  |  |  | 7 | 1 | 0 | 0 | 4 | 205 | 13 | 9 | 0 | 0 | 0 | 0 | 0 | 0 | 0 | 0 | Y | 28966 | serine protease harobin isoform X1 [Pseudonaja textilis] |
| 153 | 146 | XP\_026524834.1 | 200.49 | 26 | 0 | 0 | 0 | 0 | 0 | 26 | 0 | 0 | 0 | 0 | 0 | 0 | 0 | 0 |  |  |  |  |  | 5.7889E7 |  |  |  |  |  |  |  |  | 11 | 11 | 0 | 0 | 0 | 0 | 0 | 30 | 0 | 0 | 0 | 0 | 0 | 0 | 0 | 0 | Y | 54680 | hyaluronidase-2-like isoform X1 [Notechis scutatus] |
| 153 | 147 | XP\_026524835.1 | 200.49 | 26 | 0 | 0 | 0 | 0 | 0 | 26 | 0 | 0 | 0 | 0 | 0 | 0 | 0 | 0 |  |  |  |  |  | 5.7889E7 |  |  |  |  |  |  |  |  | 11 | 11 | 0 | 0 | 0 | 0 | 0 | 30 | 0 | 0 | 0 | 0 | 0 | 0 | 0 | 0 | Y | 54568 | hyaluronidase-2-like isoform X2 [Notechis scutatus] |
| 112 | 1053 | 1T37 | 198.60 | 42 | 0 | 0 | 10 | 10 | 0 | 10 | 0 | 0 | 0 | 0 | 10 | 10 | 42 | 18 |  |  |  |  |  |  |  |  |  |  |  |  | 1.629E9 | 2.4885E7 | 8 | 4 | 0 | 0 | 2 | 1 | 0 | 1 | 0 | 0 | 0 | 0 | 1 | 1 | 82 | 4 | Y | 13162 | Chain A, Phospholipase A2 Isoform 3 |
| 112 | 1054 | 1ZM6 | 198.60 | 42 | 0 | 0 | 10 | 10 | 0 | 10 | 0 | 0 | 0 | 0 | 10 | 10 | 42 | 18 |  |  |  |  |  |  |  |  |  |  |  |  | 1.629E9 | 2.4885E7 | 8 | 4 | 0 | 0 | 2 | 1 | 0 | 1 | 0 | 0 | 0 | 0 | 1 | 1 | 82 | 4 | Y | 13162 | Chain A, Phospholipase A2 Isoform 3 |
| 150 | 297 | JAC94989.1 | 193.88 | 14 | 0 | 0 | 0 | 2 | 4 | 14 | 0 | 0 | 2 | 0 | 0 | 0 | 0 | 0 |  |  |  | 1.5221E6 | 1.9871E6 | 5.3908E7 |  |  |  |  |  |  |  |  | 7 | 2 | 0 | 0 | 0 | 1 | 4 | 28 | 0 | 0 | 2 | 0 | 0 | 0 | 0 | 0 | Y | 63907 | Phospholipase B [Opheodrys aestivus] |
| 99 | 658 | AXL96651.1 | 182.77 | 6 | 0 | 0 | 3 | 2 | 4 | 5 | 0 | 0 | 0 | 0 | 0 | 1 | 1 | 2 |  |  |  |  | 4.8225E8 | 4.5214E7 |  |  |  |  |  |  |  |  | 8 | 3 | 0 | 0 | 5 | 4 | 30 | 52 | 0 | 0 | 0 | 0 | 0 | 2 | 1 | 1 | Y | 68041 | metalloproteinase 35, partial [Ahaetulla prasina] |
| 99 | 659 | AXL96665.1 | 182.77 | 6 | 0 | 0 | 3 | 2 | 4 | 5 | 0 | 0 | 0 | 0 | 0 | 1 | 1 | 2 |  |  |  |  | 4.8225E8 | 4.5214E7 |  |  |  |  |  |  |  |  | 8 | 3 | 0 | 0 | 5 | 4 | 30 | 52 | 0 | 0 | 0 | 0 | 0 | 2 | 1 | 1 | Y | 68148 | metalloproteinase 37, partial [Ahaetulla prasina] |
| 99 | 660 | AXL96660.1 | 182.77 | 6 | 0 | 0 | 3 | 2 | 4 | 5 | 0 | 0 | 0 | 0 | 0 | 1 | 1 | 2 |  |  |  |  | 4.8225E8 | 4.5214E7 |  |  |  |  |  |  |  |  | 8 | 3 | 0 | 0 | 5 | 4 | 30 | 52 | 0 | 0 | 0 | 0 | 0 | 2 | 1 | 1 | Y | 68120 | metalloproteinase 31, partial [Ahaetulla prasina] |
| 99 | 661 | AXL96668.1 | 182.77 | 6 | 0 | 0 | 3 | 2 | 4 | 5 | 0 | 0 | 0 | 0 | 0 | 1 | 1 | 2 |  |  |  |  | 4.8225E8 | 4.5214E7 |  |  |  |  |  |  |  |  | 8 | 3 | 0 | 0 | 5 | 4 | 30 | 52 | 0 | 0 | 0 | 0 | 0 | 2 | 1 | 1 | Y | 68144 | metalloproteinase 17, partial [Ahaetulla prasina] |
| 99 | 662 | AXL96656.1 | 182.77 | 6 | 0 | 0 | 3 | 2 | 4 | 5 | 0 | 0 | 0 | 0 | 0 | 1 | 1 | 2 |  |  |  |  | 4.8225E8 | 4.5214E7 |  |  |  |  |  |  |  |  | 8 | 3 | 0 | 0 | 5 | 4 | 30 | 52 | 0 | 0 | 0 | 0 | 0 | 2 | 1 | 1 | Y | 68152 | metalloproteinase 22, partial [Ahaetulla prasina] |
| 106 | 301 | JAA74859.1 | 182.48 | 18 | 0 | 0 | 5 | 6 | 5 | 7 | 0 | 4 | 0 | 3 | 3 | 0 | 3 | 3 |  |  | 1.8228E6 |  |  | 4.3158E7 |  |  |  |  |  |  |  |  | 8 | 2 | 0 | 0 | 7 | 3 | 10 | 83 | 0 | 2 | 0 | 3 | 3 | 0 | 2 | 2 | Y | 53521 | SVMP-Hop-46, partial [Hoplocephalus bungaroides] |
| 157 | 166 | XP\_026581281.1 | 181.47 | 22 | 0 | 0 | 0 | 2 | 1 | 19 | 0 | 0 | 0 | 0 | 0 | 0 | 0 | 0 |  |  |  |  |  | 4.6246E6 |  |  |  |  |  |  |  |  | 9 | 2 | 0 | 0 | 0 | 2 | 4 | 23 | 0 | 0 | 0 | 0 | 0 | 0 | 0 | 0 | Y | 67027 | acetylcholinesterase, partial [Pseudonaja textilis] |
| 163 | 796 | 4GFY | 180.96 | 45 | 0 | 0 | 0 | 45 | 0 | 26 | 0 | 0 | 0 | 0 | 0 | 0 | 0 | 0 |  |  |  | 3.2805E7 |  |  |  |  |  |  |  |  |  |  | 6 | 3 | 0 | 0 | 0 | 22 | 0 | 3 | 0 | 0 | 0 | 0 | 0 | 0 | 0 | 0 | Y | 13611 | Chain A, Phospholipase A2 Vrv-pl-viiia |
| 163 | 797 | 2QU9 | 180.96 | 45 | 0 | 0 | 0 | 45 | 0 | 26 | 0 | 0 | 0 | 0 | 0 | 0 | 0 | 0 |  |  |  | 3.2805E7 |  |  |  |  |  |  |  |  |  |  | 6 | 3 | 0 | 0 | 0 | 22 | 0 | 3 | 0 | 0 | 0 | 0 | 0 | 0 | 0 | 0 | Y | 13611 | Chain A, Phospholipase A2 Vrv-pl-viiia |
| 163 | 798 | 1SV9 | 180.96 | 45 | 0 | 0 | 0 | 45 | 0 | 26 | 0 | 0 | 0 | 0 | 0 | 0 | 0 | 0 |  |  |  | 3.2805E7 |  |  |  |  |  |  |  |  |  |  | 6 | 3 | 0 | 0 | 0 | 22 | 0 | 3 | 0 | 0 | 0 | 0 | 0 | 0 | 0 | 0 | Y | 13611 | Chain A, Phospholipase A2 |
| 163 | 799 | 3G8F | 180.96 | 45 | 0 | 0 | 0 | 45 | 0 | 26 | 0 | 0 | 0 | 0 | 0 | 0 | 0 | 0 |  |  |  | 3.2805E7 |  |  |  |  |  |  |  |  |  |  | 6 | 3 | 0 | 0 | 0 | 22 | 0 | 3 | 0 | 0 | 0 | 0 | 0 | 0 | 0 | 0 | Y | 13611 | Chain A, Phospholipase A2 Vrv-pl-viiia |
| 163 | 800 | 1Y38 | 180.96 | 45 | 0 | 0 | 0 | 45 | 0 | 26 | 0 | 0 | 0 | 0 | 0 | 0 | 0 | 0 |  |  |  | 3.2805E7 |  |  |  |  |  |  |  |  |  |  | 6 | 3 | 0 | 0 | 0 | 22 | 0 | 3 | 0 | 0 | 0 | 0 | 0 | 0 | 0 | 0 | Y | 13611 | Chain B, Phospholipase A2 Vrv-pl-viiia |
| 163 | 801 | 1TK4 | 180.96 | 45 | 0 | 0 | 0 | 45 | 0 | 26 | 0 | 0 | 0 | 0 | 0 | 0 | 0 | 0 |  |  |  | 3.2805E7 |  |  |  |  |  |  |  |  |  |  | 6 | 3 | 0 | 0 | 0 | 22 | 0 | 3 | 0 | 0 | 0 | 0 | 0 | 0 | 0 | 0 | Y | 13611 | Chain A, Phospholipase A2 Vrv-pl-viiia |
| 163 | 802 | 2QVD | 180.96 | 45 | 0 | 0 | 0 | 45 | 0 | 26 | 0 | 0 | 0 | 0 | 0 | 0 | 0 | 0 |  |  |  | 3.2805E7 |  |  |  |  |  |  |  |  |  |  | 6 | 3 | 0 | 0 | 0 | 22 | 0 | 3 | 0 | 0 | 0 | 0 | 0 | 0 | 0 | 0 | Y | 13611 | Chain A, Phospholipase A2 Vrv-pl-viiia |
| 163 | 803 | 4GLD | 180.96 | 45 | 0 | 0 | 0 | 45 | 0 | 26 | 0 | 0 | 0 | 0 | 0 | 0 | 0 | 0 |  |  |  | 3.2805E7 |  |  |  |  |  |  |  |  |  |  | 6 | 3 | 0 | 0 | 0 | 22 | 0 | 3 | 0 | 0 | 0 | 0 | 0 | 0 | 0 | 0 | Y | 13611 | Chain A, Phospholipase A2 Vrv-pl-viiia |
| 163 | 804 | 4EIX | 180.96 | 45 | 0 | 0 | 0 | 45 | 0 | 26 | 0 | 0 | 0 | 0 | 0 | 0 | 0 | 0 |  |  |  | 3.2805E7 |  |  |  |  |  |  |  |  |  |  | 6 | 3 | 0 | 0 | 0 | 22 | 0 | 3 | 0 | 0 | 0 | 0 | 0 | 0 | 0 | 0 | Y | 13611 | Chain A, Phospholipase A2 Vrv-pl-viiia |
| 163 | 805 | 3H1X | 180.96 | 45 | 0 | 0 | 0 | 45 | 0 | 26 | 0 | 0 | 0 | 0 | 0 | 0 | 0 | 0 |  |  |  | 3.2805E7 |  |  |  |  |  |  |  |  |  |  | 6 | 3 | 0 | 0 | 0 | 22 | 0 | 3 | 0 | 0 | 0 | 0 | 0 | 0 | 0 | 0 | Y | 13611 | Chain A, Simultaneous Inhibition Of Anti-Coagulation And Inflammation: Crystal Structure Of Phospholipase A2 Complexed With Indomethacin At 1.4 A Resolution Reveals The Presence Of The New Common Ligand Binding Site |
| 163 | 806 | 2B17 | 180.96 | 45 | 0 | 0 | 0 | 45 | 0 | 26 | 0 | 0 | 0 | 0 | 0 | 0 | 0 | 0 |  |  |  | 3.2805E7 |  |  |  |  |  |  |  |  |  |  | 6 | 3 | 0 | 0 | 0 | 22 | 0 | 3 | 0 | 0 | 0 | 0 | 0 | 0 | 0 | 0 | Y | 13611 | Chain A, Specific Binding Of Non-Steroidal Anti-Inflammatory Drugs (Nsaids) To Phospholipase A2: Crystal Structure Of The Complex Formed Between Phospholipase A2 And Diclofenac At 2.7 A Resolution: |
| 163 | 807 | 4FGA | 180.96 | 45 | 0 | 0 | 0 | 45 | 0 | 26 | 0 | 0 | 0 | 0 | 0 | 0 | 0 | 0 |  |  |  | 3.2805E7 |  |  |  |  |  |  |  |  |  |  | 6 | 3 | 0 | 0 | 0 | 22 | 0 | 3 | 0 | 0 | 0 | 0 | 0 | 0 | 0 | 0 | Y | 13611 | Chain A, Phospholipase A2 Vrv-pl-viiia |
| 163 | 808 | 2ARM | 180.96 | 45 | 0 | 0 | 0 | 45 | 0 | 26 | 0 | 0 | 0 | 0 | 0 | 0 | 0 | 0 |  |  |  | 3.2805E7 |  |  |  |  |  |  |  |  |  |  | 6 | 3 | 0 | 0 | 0 | 22 | 0 | 3 | 0 | 0 | 0 | 0 | 0 | 0 | 0 | 0 | Y | 13611 | Chain A, Phospholipase A2 Vrv-pl-viiia |
| 163 | 809 | 1ZR8 | 180.96 | 45 | 0 | 0 | 0 | 45 | 0 | 26 | 0 | 0 | 0 | 0 | 0 | 0 | 0 | 0 |  |  |  | 3.2805E7 |  |  |  |  |  |  |  |  |  |  | 6 | 3 | 0 | 0 | 0 | 22 | 0 | 3 | 0 | 0 | 0 | 0 | 0 | 0 | 0 | 0 | Y | 13611 | Chain A, Phospholipase A2 Vrv-pl-viiia |
| 163 | 810 | 1TG4 | 180.96 | 45 | 0 | 0 | 0 | 45 | 0 | 26 | 0 | 0 | 0 | 0 | 0 | 0 | 0 | 0 |  |  |  | 3.2805E7 |  |  |  |  |  |  |  |  |  |  | 6 | 3 | 0 | 0 | 0 | 22 | 0 | 3 | 0 | 0 | 0 | 0 | 0 | 0 | 0 | 0 | Y | 13611 | Chain A, Phospholipase A2 |
| 163 | 811 | 1SQZ | 180.96 | 45 | 0 | 0 | 0 | 45 | 0 | 26 | 0 | 0 | 0 | 0 | 0 | 0 | 0 | 0 |  |  |  | 3.2805E7 |  |  |  |  |  |  |  |  |  |  | 6 | 3 | 0 | 0 | 0 | 22 | 0 | 3 | 0 | 0 | 0 | 0 | 0 | 0 | 0 | 0 | Y | 13611 | Chain A, Phospholipase A2 |
| 163 | 812 | 3FG5 | 180.96 | 45 | 0 | 0 | 0 | 45 | 0 | 26 | 0 | 0 | 0 | 0 | 0 | 0 | 0 | 0 |  |  |  | 3.2805E7 |  |  |  |  |  |  |  |  |  |  | 6 | 3 | 0 | 0 | 0 | 22 | 0 | 3 | 0 | 0 | 0 | 0 | 0 | 0 | 0 | 0 | Y | 13611 | Chain A, Crystal Structure Determination Of A Ternary Complex Of Phospholipase A2 With A Pentapetide Flsyk And Ajmaline At 2.5 A Resolution |
| 163 | 813 | 1TP2 | 180.96 | 45 | 0 | 0 | 0 | 45 | 0 | 26 | 0 | 0 | 0 | 0 | 0 | 0 | 0 | 0 |  |  |  | 3.2805E7 |  |  |  |  |  |  |  |  |  |  | 6 | 3 | 0 | 0 | 0 | 22 | 0 | 3 | 0 | 0 | 0 | 0 | 0 | 0 | 0 | 0 | Y | 13611 | Chain B, Phospholipase A2 Vrv-pl-viiia |
| 163 | 814 | 1Q7A | 180.96 | 45 | 0 | 0 | 0 | 45 | 0 | 26 | 0 | 0 | 0 | 0 | 0 | 0 | 0 | 0 |  |  |  | 3.2805E7 |  |  |  |  |  |  |  |  |  |  | 6 | 3 | 0 | 0 | 0 | 22 | 0 | 3 | 0 | 0 | 0 | 0 | 0 | 0 | 0 | 0 | Y | 13611 | Chain A, Phospholipase A2 Vrv-pl-viiia |
| 163 | 815 | 1TH6 | 180.96 | 45 | 0 | 0 | 0 | 45 | 0 | 26 | 0 | 0 | 0 | 0 | 0 | 0 | 0 | 0 |  |  |  | 3.2805E7 |  |  |  |  |  |  |  |  |  |  | 6 | 3 | 0 | 0 | 0 | 22 | 0 | 3 | 0 | 0 | 0 | 0 | 0 | 0 | 0 | 0 | Y | 13611 | Chain A, Phospholipase A2 |
| 163 | 816 | 4HMB | 180.96 | 45 | 0 | 0 | 0 | 45 | 0 | 26 | 0 | 0 | 0 | 0 | 0 | 0 | 0 | 0 |  |  |  | 3.2805E7 |  |  |  |  |  |  |  |  |  |  | 6 | 3 | 0 | 0 | 0 | 22 | 0 | 3 | 0 | 0 | 0 | 0 | 0 | 0 | 0 | 0 | Y | 13611 | Chain A, Phospholipase A2 Vrv-pl-viiia |
| 163 | 817 | 3CBI | 180.96 | 45 | 0 | 0 | 0 | 45 | 0 | 26 | 0 | 0 | 0 | 0 | 0 | 0 | 0 | 0 |  |  |  | 3.2805E7 |  |  |  |  |  |  |  |  |  |  | 6 | 3 | 0 | 0 | 0 | 22 | 0 | 3 | 0 | 0 | 0 | 0 | 0 | 0 | 0 | 0 | Y | 13611 | Chain D, Crystal Structure Of The Ternary Complex Of Phospholipase A2 With Ajmaline And Anisic Acid At 3.1 A Resolution |
| 163 | 818 | 1OYF | 180.96 | 45 | 0 | 0 | 0 | 45 | 0 | 26 | 0 | 0 | 0 | 0 | 0 | 0 | 0 | 0 |  |  |  | 3.2805E7 |  |  |  |  |  |  |  |  |  |  | 6 | 3 | 0 | 0 | 0 | 22 | 0 | 3 | 0 | 0 | 0 | 0 | 0 | 0 | 0 | 0 | Y | 13639 | Chain B, Phospholipase A2 |
| 163 | 819 | 2QUE | 180.96 | 45 | 0 | 0 | 0 | 45 | 0 | 26 | 0 | 0 | 0 | 0 | 0 | 0 | 0 | 0 |  |  |  | 3.2805E7 |  |  |  |  |  |  |  |  |  |  | 6 | 3 | 0 | 0 | 0 | 22 | 0 | 3 | 0 | 0 | 0 | 0 | 0 | 0 | 0 | 0 | Y | 13611 | Chain A, Phospholipase A2 Vrv-pl-viiia |
| 163 | 820 | 1ZWP | 180.96 | 45 | 0 | 0 | 0 | 45 | 0 | 26 | 0 | 0 | 0 | 0 | 0 | 0 | 0 | 0 |  |  |  | 3.2805E7 |  |  |  |  |  |  |  |  |  |  | 6 | 3 | 0 | 0 | 0 | 22 | 0 | 3 | 0 | 0 | 0 | 0 | 0 | 0 | 0 | 0 | Y | 13611 | Chain A, Phospholipase A2 Vrv-pl-viiia |
| 163 | 821 | 3FO7 | 180.96 | 45 | 0 | 0 | 0 | 45 | 0 | 26 | 0 | 0 | 0 | 0 | 0 | 0 | 0 | 0 |  |  |  | 3.2805E7 |  |  |  |  |  |  |  |  |  |  | 6 | 3 | 0 | 0 | 0 | 22 | 0 | 3 | 0 | 0 | 0 | 0 | 0 | 0 | 0 | 0 | Y | 13611 | Chain A, Simultaneous Inhibition Of Anti-Coagulation And Inflammation: Crystal Structure Of Phospholipase A2 Complexed With Indomethacin At 1.4 A Resolution Reveals The Presence Of The New Common Ligand Binding Site |
| 163 | 822 | AAZ53186.1 | 180.96 | 39 | 0 | 0 | 0 | 39 | 0 | 23 | 0 | 0 | 0 | 0 | 0 | 0 | 0 | 0 |  |  |  | 3.2805E7 |  |  |  |  |  |  |  |  |  |  | 6 | 3 | 0 | 0 | 0 | 22 | 0 | 3 | 0 | 0 | 0 | 0 | 0 | 0 | 0 | 0 | Y | 15353 | phospholipase A2 [Daboia russelii limitis] |
| 163 | 823 | AAZ53179.1 | 180.96 | 39 | 0 | 0 | 0 | 39 | 0 | 23 | 0 | 0 | 0 | 0 | 0 | 0 | 0 | 0 |  |  |  | 3.2805E7 |  |  |  |  |  |  |  |  |  |  | 6 | 3 | 0 | 0 | 0 | 22 | 0 | 3 | 0 | 0 | 0 | 0 | 0 | 0 | 0 | 0 | Y | 15381 | phospholipase A2 [Daboia siamensis] |
| 177 | 389 | LAA27322.1 | 180.03 | 13 | 0 | 0 | 0 | 0 | 0 | 11 | 0 | 0 | 2 | 0 | 0 | 0 | 0 | 0 |  |  |  |  |  | 2.3974E7 |  |  |  |  |  |  |  |  | 6 | 1 | 0 | 0 | 0 | 0 | 0 | 16 | 0 | 0 | 2 | 0 | 0 | 0 | 0 | 0 | Y | 63374 | hypothetical protein [Micrurus lemniscatus carvalhoi] |
| 177 | 395 | LAA73439.1 | 180.03 | 13 | 0 | 0 | 0 | 0 | 0 | 11 | 0 | 0 | 2 | 0 | 0 | 0 | 0 | 0 |  |  |  |  |  | 2.3974E7 |  |  |  |  |  |  |  |  | 6 | 1 | 0 | 0 | 0 | 0 | 0 | 16 | 0 | 0 | 2 | 0 | 0 | 0 | 0 | 0 | Y | 63079 | hypothetical protein [Micrurus lemniscatus lemniscatus] |
| 173 | 477 | P0DI84.1 | 176.02 | 14 | 0 | 0 | 0 | 0 | 3 | 14 | 0 | 0 | 0 | 0 | 0 | 0 | 0 | 0 |  |  |  |  |  | 1.5605E8 |  |  |  |  |  |  |  |  | 9 | 1 | 0 | 0 | 0 | 0 | 1 | 19 | 0 | 0 | 0 | 0 | 0 | 0 | 0 | 0 | Y | 54748 | RecName: Full=L-amino-acid oxidase; Short=LAO; Short=VAA-LAAO I |
| 173 | 478 | 3KVE | 176.02 | 14 | 0 | 0 | 0 | 0 | 3 | 14 | 0 | 0 | 0 | 0 | 0 | 0 | 0 | 0 |  |  |  |  |  | 1.5605E8 |  |  |  |  |  |  |  |  | 9 | 1 | 0 | 0 | 0 | 0 | 1 | 19 | 0 | 0 | 0 | 0 | 0 | 0 | 0 | 0 | Y | 54933 | Chain D, Structure Of Native L-Amino Acid Oxidase From Vipera Ammodytes Ammodytes: Stabilization Of The Quaternary Structure By Divalent Ions And Structural Changes In The Dynamic Active Site |
| 37 | 1542 | XP\_013911763.1 | 168.64 | 10 | 0 | 0 | 5 | 10 | 5 | 5 | 0 | 0 | 0 | 0 | 0 | 0 | 0 | 0 |  |  |  | 1.8272E8 |  |  |  |  |  |  |  |  |  |  | 7 | 3 | 0 | 0 | 2 | 476 | 20 | 8 | 0 | 0 | 0 | 0 | 0 | 0 | 0 | 0 | Y | 27138 | PREDICTED: cysteine-rich venom protein TEL1-like [Thamnophis sirtalis] |
| 37 | 1614 | XP\_032069584.1 | 168.64 | 10 | 0 | 0 | 5 | 10 | 5 | 5 | 0 | 0 | 0 | 0 | 0 | 0 | 0 | 0 |  |  |  | 1.8272E8 |  |  |  |  |  |  |  |  |  |  | 7 | 3 | 0 | 0 | 2 | 476 | 20 | 8 | 0 | 0 | 0 | 0 | 0 | 0 | 0 | 0 | Y | 27051 | cysteine-rich venom protein TEL1-like [Thamnophis elegans] |
| 158 | 1014 | P86368.1 | 167.63 | 39 | 0 | 0 | 0 | 24 | 9 | 39 | 0 | 0 | 0 | 0 | 0 | 0 | 0 | 0 |  |  |  | 1.8441E5 | 1.2227E6 | 2.9436E7 |  |  |  |  |  |  |  |  | 5 | 2 | 0 | 0 | 0 | 14 | 2 | 13 | 0 | 0 | 0 | 0 | 0 | 0 | 0 | 0 | Y | 13687 | RecName: Full=Basic phospholipase A2 3; Short=PLA23; Short=svPLA2; AltName: Full=Phosphatidylcholine 2-acylhydrolase |
| 179 | 498 | AAS94269.1 | 167.52 | 33 | 0 | 13 | 0 | 11 | 0 | 0 | 0 | 0 | 0 | 0 | 0 | 0 | 16 | 29 |  | 9.701E5 |  | 7.7906E5 |  |  |  |  |  |  |  |  | 2.3954E6 | 5.715E7 | 5 | 5 | 0 | 3 | 0 | 2 | 0 | 0 | 0 | 0 | 0 | 0 | 0 | 0 | 3 | 11 | Y | 27030 | nerve growth factor II [Naja sputatrix] |
| 179 | 499 | Q5YF89.1 | 167.52 | 33 | 0 | 13 | 0 | 11 | 0 | 0 | 0 | 0 | 0 | 0 | 0 | 0 | 16 | 29 |  | 9.701E5 |  | 7.7906E5 |  |  |  |  |  |  |  |  | 2.3954E6 | 5.715E7 | 5 | 5 | 0 | 3 | 0 | 2 | 0 | 0 | 0 | 0 | 0 | 0 | 0 | 0 | 3 | 11 | Y | 27030 | RecName: Full=Venom nerve growth factor 2; Short=v-NGF-2; Short=vNGF-2; AltName: Full=Nerve growth factor II; Flags: Precursor |
| 179 | 593 | P01140.2 | 167.52 | 69 | 0 | 27 | 0 | 23 | 0 | 0 | 0 | 0 | 0 | 0 | 0 | 0 | 33 | 59 |  | 9.701E5 |  | 7.7906E5 |  |  |  |  |  |  |  |  | 2.3954E6 | 5.715E7 | 5 | 5 | 0 | 3 | 0 | 2 | 0 | 0 | 0 | 0 | 0 | 0 | 0 | 0 | 3 | 11 | Y | 13023 | RecName: Full=Venom nerve growth factor; Short=v-NGF; Short=vNGF |
| 145 | 611 | ACR83850.1 | 166.50 | 38 | 0 | 21 | 0 | 0 | 0 | 0 | 0 | 0 | 0 | 0 | 0 | 0 | 0 | 35 |  | 1.1303E8 |  |  |  |  |  |  |  |  |  |  |  | 2.0546E8 | 6 | 6 | 0 | 15 | 0 | 0 | 0 | 0 | 0 | 0 | 0 | 0 | 0 | 0 | 0 | 23 | Y | 15772 | cystatin precursor [Naja kaouthia] |
| 145 | 612 | E3P6P4.1 | 166.50 | 38 | 0 | 21 | 0 | 0 | 0 | 0 | 0 | 0 | 0 | 0 | 0 | 0 | 0 | 35 |  | 1.1303E8 |  |  |  |  |  |  |  |  |  |  |  | 2.0546E8 | 6 | 6 | 0 | 15 | 0 | 0 | 0 | 0 | 0 | 0 | 0 | 0 | 0 | 0 | 0 | 23 | Y | 15772 | RecName: Full=Cystatin; Flags: Precursor |
| 144 | 365 | JAS05092.1 | 165.32 | 13 | 0 | 0 | 0 | 3 | 9 | 8 | 0 | 0 | 0 | 0 | 0 | 0 | 0 | 0 |  |  |  |  | 5.2347E7 | 1.1976E7 |  |  |  |  |  |  |  |  | 6 | 2 | 0 | 0 | 0 | 2 | 21 | 11 | 0 | 0 | 0 | 0 | 0 | 0 | 0 | 0 | Y | 68997 | Metalloproteinase type III 2 [Micrurus tener] |
| 108 | 859 | 2CTX | 165.18 | 59 | 0 | 41 | 41 | 59 | 59 | 41 | 0 | 14 | 59 | 59 | 32 | 59 | 59 | 41 |  | 3.1989E6 | 1.2538E6 | 1.652E7 | 2.9518E6 | 1.2399E7 |  |  | 1.2051E8 | 3.3128E7 |  | 4.2361E6 | 7.1424E6 | 1.221E7 | 4 | 2 | 0 | 4 | 3 | 7 | 6 | 13 | 0 | 1 | 43 | 13 | 5 | 5 | 9 | 8 | Y | 7831 | Chain A, ALPHA-COBRATOXIN |
| 108 | 860 | P01391.1 | 165.18 | 59 | 0 | 41 | 41 | 59 | 59 | 41 | 0 | 14 | 59 | 59 | 32 | 59 | 59 | 41 |  | 3.1989E6 | 1.2538E6 | 1.652E7 | 2.9518E6 | 1.2399E7 |  |  | 1.2051E8 | 3.3128E7 |  | 4.2361E6 | 7.1424E6 | 1.221E7 | 4 | 2 | 0 | 4 | 3 | 7 | 6 | 13 | 0 | 1 | 43 | 13 | 5 | 5 | 9 | 8 | Y | 7831 | RecName: Full=Alpha-cobratoxin; Short=Alpha-CbT; Short=alpha-CT; Short=alpha-Cbtx; AltName: Full=Alpha-elapitoxin-Nk2a; Short=Alpha-EPTX-Nk2a; AltName: Full=Long neurotoxin 1; AltName: Full=Siamensis 3 |
| 108 | 861 | 1YI5 | 165.18 | 59 | 0 | 41 | 41 | 59 | 59 | 41 | 0 | 14 | 59 | 59 | 32 | 59 | 59 | 41 |  | 3.1989E6 | 1.2538E6 | 1.652E7 | 2.9518E6 | 1.2399E7 |  |  | 1.2051E8 | 3.3128E7 |  | 4.2361E6 | 7.1424E6 | 1.221E7 | 4 | 2 | 0 | 4 | 3 | 7 | 6 | 13 | 0 | 1 | 43 | 13 | 5 | 5 | 9 | 8 | Y | 7831 | Chain J, Long Neurotoxin 1 |
| 108 | 862 | 1LXH | 165.18 | 59 | 0 | 41 | 41 | 59 | 59 | 41 | 0 | 14 | 59 | 59 | 32 | 59 | 59 | 41 |  | 3.1989E6 | 1.2538E6 | 1.652E7 | 2.9518E6 | 1.2399E7 |  |  | 1.2051E8 | 3.3128E7 |  | 4.2361E6 | 7.1424E6 | 1.221E7 | 4 | 2 | 0 | 4 | 3 | 7 | 6 | 13 | 0 | 1 | 43 | 13 | 5 | 5 | 9 | 8 | Y | 7831 | Chain A, LONG NEUROTOXIN 1 |
| 108 | 863 | 1LXG | 165.18 | 59 | 0 | 41 | 41 | 59 | 59 | 41 | 0 | 14 | 59 | 59 | 32 | 59 | 59 | 41 |  | 3.1989E6 | 1.2538E6 | 1.652E7 | 2.9518E6 | 1.2399E7 |  |  | 1.2051E8 | 3.3128E7 |  | 4.2361E6 | 7.1424E6 | 1.221E7 | 4 | 2 | 0 | 4 | 3 | 7 | 6 | 13 | 0 | 1 | 43 | 13 | 5 | 5 | 9 | 8 | Y | 7831 | Chain A, Long neurotoxin 1 |
| 108 | 864 | 4AEA | 165.18 | 59 | 0 | 41 | 41 | 59 | 59 | 41 | 0 | 14 | 59 | 59 | 32 | 59 | 59 | 41 |  | 3.1989E6 | 1.2538E6 | 1.652E7 | 2.9518E6 | 1.2399E7 |  |  | 1.2051E8 | 3.3128E7 |  | 4.2361E6 | 7.1424E6 | 1.221E7 | 4 | 2 | 0 | 4 | 3 | 7 | 6 | 13 | 0 | 1 | 43 | 13 | 5 | 5 | 9 | 8 | Y | 7831 | Chain B, Long Neurotoxin 1 |
| 108 | 865 | 1CTX | 165.18 | 59 | 0 | 41 | 41 | 59 | 59 | 41 | 0 | 14 | 59 | 59 | 32 | 59 | 59 | 41 |  | 3.1989E6 | 1.2538E6 | 1.652E7 | 2.9518E6 | 1.2399E7 |  |  | 1.2051E8 | 3.3128E7 |  | 4.2361E6 | 7.1424E6 | 1.221E7 | 4 | 2 | 0 | 4 | 3 | 7 | 6 | 13 | 0 | 1 | 43 | 13 | 5 | 5 | 9 | 8 | Y | 7831 | Chain A, ALPHA-COBRATOXIN |
| 180 | 138 | XP\_026568326.1 | 164.24 | 16 | 0 | 0 | 0 | 0 | 10 | 16 | 0 | 0 | 0 | 0 | 0 | 0 | 0 | 0 |  |  |  |  | 3.3716E6 | 6.8546E6 |  |  |  |  |  |  |  |  | 7 | 7 | 0 | 0 | 0 | 0 | 5 | 11 | 0 | 0 | 0 | 0 | 0 | 0 | 0 | 0 | N | 72187 | endoplasmic reticulum chaperone BiP [Pseudonaja textilis] |
| 155 | 722 | CAB45156.1 | 164.05 | 52 | 0 | 0 | 0 | 0 | 0 | 0 | 0 | 0 | 14 | 0 | 0 | 9 | 52 | 30 |  |  |  |  |  |  |  |  | 3.2308E5 |  |  |  | 1.598E8 | 4.585E7 | 7 | 6 | 0 | 0 | 0 | 0 | 0 | 0 | 0 | 0 | 1 | 0 | 0 | 1 | 25 | 5 | Y | 9695 | neurotoxin-like protein [Naja atra] |
| 155 | 723 | Q9W717.1 | 164.05 | 52 | 0 | 0 | 0 | 0 | 0 | 0 | 0 | 0 | 14 | 0 | 0 | 9 | 52 | 30 |  |  |  |  |  |  |  |  | 3.2308E5 |  |  |  | 1.598E8 | 4.585E7 | 7 | 6 | 0 | 0 | 0 | 0 | 0 | 0 | 0 | 0 | 1 | 0 | 0 | 1 | 25 | 5 | Y | 9695 | RecName: Full=Neurotoxin-like protein NTL2; Flags: Precursor |
| 155 | 724 | APB88857.1 | 164.05 | 52 | 0 | 0 | 0 | 0 | 0 | 0 | 0 | 0 | 14 | 0 | 0 | 9 | 52 | 30 |  |  |  |  |  |  |  |  | 3.2308E5 |  |  |  | 1.598E8 | 4.585E7 | 7 | 6 | 0 | 0 | 0 | 0 | 0 | 0 | 0 | 0 | 1 | 0 | 0 | 1 | 25 | 5 | Y | 9695 | neurotoxin-like protein [Naja naja] |
| 217 | 510 | XP\_026530096.1 | 162.48 | 14 | 0 | 0 | 0 | 6 | 0 | 11 | 0 | 0 | 0 | 0 | 0 | 0 | 0 | 0 |  |  |  | 8.604E5 |  | 1.4894E7 |  |  |  |  |  |  |  |  | 5 | 5 | 0 | 0 | 0 | 2 | 0 | 9 | 0 | 0 | 0 | 0 | 0 | 0 | 0 | 0 | N | 48986 | protein disulfide-isomerase A6 [Notechis scutatus] |
| 217 | 513 | ETE67495.1 | 162.48 | 15 | 0 | 0 | 0 | 6 | 0 | 12 | 0 | 0 | 0 | 0 | 0 | 0 | 0 | 0 |  |  |  | 8.604E5 |  | 1.4894E7 |  |  |  |  |  |  |  |  | 5 | 5 | 0 | 0 | 0 | 2 | 0 | 9 | 0 | 0 | 0 | 0 | 0 | 0 | 0 | 0 | N | 45796 | Protein disulfide-isomerase A6, partial [Ophiophagus hannah] |
| 149 | 406 | ABQ01132.1 | 161.42 | 13 | 0 | 0 | 0 | 3 | 5 | 8 | 0 | 3 | 0 | 0 | 0 | 0 | 0 | 0 |  |  |  |  | 5.0409E8 | 1.3552E9 |  |  |  |  |  |  |  |  | 7 | 1 | 0 | 0 | 0 | 2 | 14 | 8 | 0 | 2 | 0 | 0 | 0 | 0 | 0 | 0 | Y | 68090 | carinatease-1 [Tropidechis carinatus] |
| 175 | 399 | JAI08992.1 | 158.06 | 10 | 0 | 0 | 0 | 3 | 3 | 6 | 0 | 0 | 0 | 0 | 0 | 0 | 0 | 0 |  |  |  |  |  | 1.1544E7 |  |  |  |  |  |  |  |  | 6 | 1 | 0 | 0 | 0 | 2 | 5 | 5 | 0 | 0 | 0 | 0 | 0 | 0 | 0 | 0 | Y | 69037 | Metalloproteinase (type III) 1 [Micrurus fulvius] |
| 175 | 400 | JAB52758.1 | 158.06 | 10 | 0 | 0 | 0 | 3 | 3 | 6 | 0 | 0 | 0 | 0 | 0 | 0 | 0 | 0 |  |  |  |  |  | 1.1544E7 |  |  |  |  |  |  |  |  | 6 | 1 | 0 | 0 | 0 | 2 | 5 | 5 | 0 | 0 | 0 | 0 | 0 | 0 | 0 | 0 | Y | 69037 | Snake venom metalloproteinase 1 [Micrurus fulvius] |
| 175 | 401 | JAS04981.1 | 158.06 | 10 | 0 | 0 | 0 | 3 | 3 | 6 | 0 | 0 | 0 | 0 | 0 | 0 | 0 | 0 |  |  |  |  |  | 1.1544E7 |  |  |  |  |  |  |  |  | 6 | 1 | 0 | 0 | 0 | 2 | 5 | 5 | 0 | 0 | 0 | 0 | 0 | 0 | 0 | 0 | Y | 69037 | Metalloproteinase type III 1 [Micrurus fulvius] |
| 175 | 402 | JAS05093.1 | 158.06 | 10 | 0 | 0 | 0 | 3 | 3 | 6 | 0 | 0 | 0 | 0 | 0 | 0 | 0 | 0 |  |  |  |  |  | 1.1544E7 |  |  |  |  |  |  |  |  | 6 | 1 | 0 | 0 | 0 | 2 | 5 | 5 | 0 | 0 | 0 | 0 | 0 | 0 | 0 | 0 | Y | 69138 | Metalloproteinase type III 2 [Micrurus tener] |
| 110 | 1786 | ADN67584.1 | 155.73 | 61 | 59 | 0 | 0 | 0 | 0 | 0 | 59 | 61 | 59 | 41 | 0 | 0 | 0 | 0 | 2.6655E8 |  |  |  |  |  | 2.8883E7 | 2.1676E6 | 4.5532E7 | 3.2829E5 |  |  |  |  | 4 | 2 | 6 | 0 | 0 | 0 | 0 | 0 | 4 | 74 | 11 | 1 | 0 | 0 | 0 | 0 | Y | 7221 | three-finger toxin precursor, partial [Naja atra] |
| 110 | 1787 | AAB36932.1 | 155.73 | 47 | 46 | 0 | 0 | 0 | 0 | 0 | 46 | 47 | 46 | 31 | 0 | 0 | 0 | 0 | 2.6655E8 |  |  |  |  |  | 2.8883E7 | 2.1676E6 | 4.5532E7 | 3.2829E5 |  |  |  |  | 4 | 2 | 6 | 0 | 0 | 0 | 0 | 0 | 4 | 74 | 11 | 1 | 0 | 0 | 0 | 0 | Y | 9262 | cobrotoxin''' [Naja atra] |
| 110 | 1788 | AAB03221.1 | 155.73 | 47 | 46 | 0 | 0 | 0 | 0 | 0 | 46 | 47 | 46 | 31 | 0 | 0 | 0 | 0 | 2.6655E8 |  |  |  |  |  | 2.8883E7 | 2.1676E6 | 4.5532E7 | 3.2829E5 |  |  |  |  | 4 | 2 | 6 | 0 | 0 | 0 | 0 | 0 | 4 | 74 | 11 | 1 | 0 | 0 | 0 | 0 | Y | 9262 | cobrotoxin' [Naja atra] |
| 110 | 1789 | ADN67574.1 | 155.73 | 47 | 46 | 0 | 0 | 0 | 0 | 0 | 46 | 47 | 46 | 31 | 0 | 0 | 0 | 0 | 2.6655E8 |  |  |  |  |  | 2.8883E7 | 2.1676E6 | 4.5532E7 | 3.2829E5 |  |  |  |  | 4 | 2 | 6 | 0 | 0 | 0 | 0 | 0 | 4 | 74 | 11 | 1 | 0 | 0 | 0 | 0 | Y | 9262 | three-finger toxin precursor [Naja atra] |
| 110 | 1790 | AAB03223.1 | 155.73 | 47 | 46 | 0 | 0 | 0 | 0 | 0 | 46 | 47 | 46 | 31 | 0 | 0 | 0 | 0 | 2.6655E8 |  |  |  |  |  | 2.8883E7 | 2.1676E6 | 4.5532E7 | 3.2829E5 |  |  |  |  | 4 | 2 | 6 | 0 | 0 | 0 | 0 | 0 | 4 | 74 | 11 | 1 | 0 | 0 | 0 | 0 | Y | 9262 | cobrotoxin''' [Naja atra] |
| 110 | 1791 | Q9PTT0.1 | 155.73 | 47 | 46 | 0 | 0 | 0 | 0 | 0 | 46 | 47 | 46 | 31 | 0 | 0 | 0 | 0 | 2.6655E8 |  |  |  |  |  | 2.8883E7 | 2.1676E6 | 4.5532E7 | 3.2829E5 |  |  |  |  | 4 | 2 | 6 | 0 | 0 | 0 | 0 | 0 | 4 | 74 | 11 | 1 | 0 | 0 | 0 | 0 | Y | 9262 | RecName: Full=Cobrotoxin homolog; AltName: Full=Short neurotoxin; Flags: Precursor |
| 110 | 1792 | P60770.1 | 155.73 | 47 | 46 | 0 | 0 | 0 | 0 | 0 | 46 | 47 | 46 | 31 | 0 | 0 | 0 | 0 | 2.6655E8 |  |  |  |  |  | 2.8883E7 | 2.1676E6 | 4.5532E7 | 3.2829E5 |  |  |  |  | 4 | 2 | 6 | 0 | 0 | 0 | 0 | 0 | 4 | 74 | 11 | 1 | 0 | 0 | 0 | 0 | Y | 9262 | RecName: Full=Cobrotoxin; Short=CBT; AltName: Full=Atratoxin; AltName: Full=Short neurotoxin 1; Flags: Precursor |
| 110 | 1793 | P60771.2 | 155.73 | 47 | 46 | 0 | 0 | 0 | 0 | 0 | 46 | 47 | 46 | 31 | 0 | 0 | 0 | 0 | 2.6655E8 |  |  |  |  |  | 2.8883E7 | 2.1676E6 | 4.5532E7 | 3.2829E5 |  |  |  |  | 4 | 2 | 6 | 0 | 0 | 0 | 0 | 0 | 4 | 74 | 11 | 1 | 0 | 0 | 0 | 0 | Y | 9262 | RecName: Full=Cobrotoxin; Short=CBT; AltName: Full=Short neurotoxin I; Short=NT1; Flags: Precursor |
| 110 | 1794 | AAB36931.1 | 155.73 | 47 | 46 | 0 | 0 | 0 | 0 | 0 | 46 | 47 | 46 | 31 | 0 | 0 | 0 | 0 | 2.6655E8 |  |  |  |  |  | 2.8883E7 | 2.1676E6 | 4.5532E7 | 3.2829E5 |  |  |  |  | 4 | 2 | 6 | 0 | 0 | 0 | 0 | 0 | 4 | 74 | 11 | 1 | 0 | 0 | 0 | 0 | Y | 9262 | cobrotoxin'' [Naja atra] |
| 110 | 1795 | AAB01538.1 | 155.73 | 47 | 46 | 0 | 0 | 0 | 0 | 0 | 46 | 47 | 46 | 31 | 0 | 0 | 0 | 0 | 2.6655E8 |  |  |  |  |  | 2.8883E7 | 2.1676E6 | 4.5532E7 | 3.2829E5 |  |  |  |  | 4 | 2 | 6 | 0 | 0 | 0 | 0 | 0 | 4 | 74 | 11 | 1 | 0 | 0 | 0 | 0 | Y | 9262 | cobrotoxin [Naja atra] |
| 110 | 1796 | AAY63884.1 | 155.73 | 47 | 46 | 0 | 0 | 0 | 0 | 0 | 46 | 47 | 46 | 31 | 0 | 0 | 0 | 0 | 2.6655E8 |  |  |  |  |  | 2.8883E7 | 2.1676E6 | 4.5532E7 | 3.2829E5 |  |  |  |  | 4 | 2 | 6 | 0 | 0 | 0 | 0 | 0 | 4 | 74 | 11 | 1 | 0 | 0 | 0 | 0 | Y | 9262 | neurotoxin [Naja kaouthia] |
| 110 | 1797 | AAB36930.1 | 155.73 | 47 | 46 | 0 | 0 | 0 | 0 | 0 | 46 | 47 | 46 | 31 | 0 | 0 | 0 | 0 | 2.6655E8 |  |  |  |  |  | 2.8883E7 | 2.1676E6 | 4.5532E7 | 3.2829E5 |  |  |  |  | 4 | 2 | 6 | 0 | 0 | 0 | 0 | 0 | 4 | 74 | 11 | 1 | 0 | 0 | 0 | 0 | Y | 9262 | cobrotoxin' [Naja atra] |
| 110 | 1798 | AAB03222.1 | 155.73 | 47 | 46 | 0 | 0 | 0 | 0 | 0 | 46 | 47 | 46 | 31 | 0 | 0 | 0 | 0 | 2.6655E8 |  |  |  |  |  | 2.8883E7 | 2.1676E6 | 4.5532E7 | 3.2829E5 |  |  |  |  | 4 | 2 | 6 | 0 | 0 | 0 | 0 | 0 | 4 | 74 | 11 | 1 | 0 | 0 | 0 | 0 | Y | 9262 | cobrotoxin'' [Naja atra] |
| 110 | 1799 | AAF21774.1 | 155.73 | 47 | 46 | 0 | 0 | 0 | 0 | 0 | 46 | 47 | 46 | 31 | 0 | 0 | 0 | 0 | 2.6655E8 |  |  |  |  |  | 2.8883E7 | 2.1676E6 | 4.5532E7 | 3.2829E5 |  |  |  |  | 4 | 2 | 6 | 0 | 0 | 0 | 0 | 0 | 4 | 74 | 11 | 1 | 0 | 0 | 0 | 0 | Y | 9262 | neurotoxin preprotein precursor [Naja naja] |
| 110 | 1800 | AAR33035.1 | 155.73 | 47 | 46 | 0 | 0 | 0 | 0 | 0 | 46 | 47 | 46 | 31 | 0 | 0 | 0 | 0 | 2.6655E8 |  |  |  |  |  | 2.8883E7 | 2.1676E6 | 4.5532E7 | 3.2829E5 |  |  |  |  | 4 | 2 | 6 | 0 | 0 | 0 | 0 | 0 | 4 | 74 | 11 | 1 | 0 | 0 | 0 | 0 | Y | 9262 | atratoxin [Naja atra] |
| 110 | 1801 | CAA73097.2 | 155.73 | 47 | 46 | 0 | 0 | 0 | 0 | 0 | 46 | 47 | 46 | 31 | 0 | 0 | 0 | 0 | 2.6655E8 |  |  |  |  |  | 2.8883E7 | 2.1676E6 | 4.5532E7 | 3.2829E5 |  |  |  |  | 4 | 2 | 6 | 0 | 0 | 0 | 0 | 0 | 4 | 74 | 11 | 1 | 0 | 0 | 0 | 0 | Y | 9262 | cobrotoxin [Naja atra] |
| 110 | 1802 | 1COD | 155.73 | 63 | 61 | 0 | 0 | 0 | 0 | 0 | 61 | 63 | 61 | 42 | 0 | 0 | 0 | 0 | 2.6655E8 |  |  |  |  |  | 2.8883E7 | 2.1676E6 | 4.5532E7 | 3.2829E5 |  |  |  |  | 4 | 2 | 6 | 0 | 0 | 0 | 0 | 0 | 4 | 74 | 11 | 1 | 0 | 0 | 0 | 0 | Y | 6957 | Chain A, COBROTOXIN |
| 110 | 1803 | 1V6P | 155.73 | 63 | 61 | 0 | 0 | 0 | 0 | 0 | 61 | 63 | 61 | 42 | 0 | 0 | 0 | 0 | 2.6655E8 |  |  |  |  |  | 2.8883E7 | 2.1676E6 | 4.5532E7 | 3.2829E5 |  |  |  |  | 4 | 2 | 6 | 0 | 0 | 0 | 0 | 0 | 4 | 74 | 11 | 1 | 0 | 0 | 0 | 0 | Y | 6957 | Chain B, Cobrotoxin |
| 110 | 1804 | 1COE | 155.73 | 63 | 61 | 0 | 0 | 0 | 0 | 0 | 61 | 63 | 61 | 42 | 0 | 0 | 0 | 0 | 2.6655E8 |  |  |  |  |  | 2.8883E7 | 2.1676E6 | 4.5532E7 | 3.2829E5 |  |  |  |  | 4 | 2 | 6 | 0 | 0 | 0 | 0 | 0 | 4 | 74 | 11 | 1 | 0 | 0 | 0 | 0 | Y | 6957 | Chain A, COBROTOXIN |
| 110 | 1805 | AAB25735.1 | 155.73 | 63 | 61 | 0 | 0 | 0 | 0 | 0 | 61 | 63 | 61 | 42 | 0 | 0 | 0 | 0 | 2.6655E8 |  |  |  |  |  | 2.8883E7 | 2.1676E6 | 4.5532E7 | 3.2829E5 |  |  |  |  | 4 | 2 | 6 | 0 | 0 | 0 | 0 | 0 | 4 | 74 | 11 | 1 | 0 | 0 | 0 | 0 | Y | 6957 | neurotoxin, NTX [Naja naja=Formosan cobra, ssp. atra, venom, Peptide, 62 aa] |
| 97 | 897 | P0DSM9.1 | 153.62 | 77 | 0 | 0 | 0 | 0 | 0 | 0 | 0 | 0 | 77 | 0 | 0 | 17 | 0 | 0 |  |  |  |  |  |  |  |  | 3.9843E7 |  |  |  |  |  | 6 | 1 | 0 | 0 | 0 | 0 | 0 | 0 | 0 | 0 | 126 | 0 | 0 | 4 | 0 | 0 | Y | 6139 | RecName: Full=Weak toxin NWT |
| 124 | 460 | AFJ50410.1 | 151.22 | 9 | 0 | 2 | 0 | 2 | 5 | 2 | 7 | 0 | 0 | 5 | 2 | 2 | 2 | 2 |  |  |  |  | 7.3573E5 |  |  |  |  |  |  |  |  |  | 5 | 1 | 0 | 1 | 0 | 1 | 4 | 3 | 36 | 0 | 0 | 4 | 1 | 1 | 1 | 2 | N | 53645 | Keratin, type II cytoskeletal cochleal-like [Crotalus adamanteus] |
| 124 | 461 | JAG44289.1 | 151.22 | 9 | 0 | 2 | 0 | 2 | 5 | 2 | 7 | 0 | 0 | 5 | 2 | 2 | 2 | 2 |  |  |  |  | 7.3573E5 |  |  |  |  |  |  |  |  |  | 5 | 1 | 0 | 1 | 0 | 1 | 4 | 3 | 36 | 0 | 0 | 4 | 1 | 1 | 1 | 2 | N | 53768 | Keratin, type II cytoskeletal cochleal-like [Crotalus horridus] |
| 105 | 921 | P25668.1 | 149.71 | 48 | 0 | 32 | 32 | 32 | 34 | 32 | 0 | 14 | 46 | 32 | 32 | 48 | 32 | 32 |  |  |  |  |  |  |  |  | 1.4714E7 |  |  | 9.4959E5 |  |  | 4 | 1 | 0 | 8 | 3 | 5 | 8 | 12 | 0 | 1 | 52 | 10 | 5 | 7 | 7 | 7 | Y | 7847 | RecName: Full=Long neurotoxin 1; AltName: Full=Toxin A |
| 121 | 515 | B8K1W0.1 | 149.15 | 10 | 0 | 0 | 2 | 7 | 4 | 3 | 0 | 0 | 0 | 0 | 0 | 0 | 0 | 2 |  |  |  | 4.5543E6 | 4.7866E5 | 1.0412E6 |  |  |  |  |  |  |  |  | 6 | 4 | 0 | 0 | 1 | 6 | 18 | 35 | 0 | 0 | 0 | 0 | 0 | 0 | 0 | 1 | Y | 69555 | RecName: Full=Zinc metalloproteinase-disintegrin-like daborhagin-K; AltName: Full=Haemorrhagic metalloproteinase russelysin; AltName: Full=Snake venom metalloproteinase; Short=SVMP; Flags: Precursor |
| 121 | 516 | AAZ39880.1 | 149.15 | 10 | 0 | 0 | 2 | 7 | 4 | 3 | 0 | 0 | 0 | 0 | 0 | 0 | 0 | 2 |  |  |  | 4.5543E6 | 4.7866E5 | 1.0412E6 |  |  |  |  |  |  |  |  | 6 | 4 | 0 | 0 | 1 | 6 | 18 | 35 | 0 | 0 | 0 | 0 | 0 | 0 | 0 | 1 | Y | 69555 | hemorrhagic metalloproteinase russelysin [Daboia russelii] |
| 72 | 2424 | Q9YGI4.1 | 149.05 | 23 | 0 | 15 | 15 | 15 | 0 | 0 | 0 | 0 | 15 | 21 | 21 | 23 | 15 | 15 |  |  |  |  |  |  |  |  |  |  |  | 2.5637E6 |  |  | 4 | 1 | 0 | 1 | 2 | 1 | 0 | 0 | 0 | 0 | 5 | 27 | 124 | 38 | 5 | 2 | Y | 9899 | RecName: Full=Probable weak neurotoxin NNAM2; AltName: Full=TA-N9; Flags: Precursor |
| 72 | 2425 | CAA04578.1 | 149.05 | 23 | 0 | 15 | 15 | 15 | 0 | 0 | 0 | 0 | 15 | 21 | 21 | 23 | 15 | 15 |  |  |  |  |  |  |  |  |  |  |  | 2.5637E6 |  |  | 4 | 1 | 0 | 1 | 2 | 1 | 0 | 0 | 0 | 0 | 5 | 27 | 124 | 38 | 5 | 2 | Y | 9885 | unnamed protein product (plasmid) [Naja atra] |
| 72 | 2426 | CAA10530.1 | 149.05 | 23 | 0 | 15 | 15 | 15 | 0 | 0 | 0 | 0 | 15 | 21 | 21 | 23 | 15 | 15 |  |  |  |  |  |  |  |  |  |  |  | 2.5637E6 |  |  | 4 | 1 | 0 | 1 | 2 | 1 | 0 | 0 | 0 | 0 | 5 | 27 | 124 | 38 | 5 | 2 | Y | 9899 | neurotoxin homolog [Naja atra] |
| 72 | 2809 | P60814.1 | 149.05 | 23 | 0 | 15 | 15 | 15 | 0 | 0 | 0 | 0 | 15 | 21 | 21 | 23 | 15 | 15 |  |  |  |  |  |  |  |  |  |  |  | 2.5637E6 |  |  | 4 | 1 | 0 | 1 | 2 | 1 | 0 | 0 | 0 | 0 | 5 | 27 | 124 | 38 | 5 | 2 | Y | 9811 | RecName: Full=Probable weak neurotoxin NNAM2I; Flags: Precursor |
| 72 | 2810 | CAA06888.1 | 149.05 | 23 | 0 | 15 | 15 | 15 | 0 | 0 | 0 | 0 | 15 | 21 | 21 | 23 | 15 | 15 |  |  |  |  |  |  |  |  |  |  |  | 2.5637E6 |  |  | 4 | 1 | 0 | 1 | 2 | 1 | 0 | 0 | 0 | 0 | 5 | 27 | 124 | 38 | 5 | 2 | Y | 9811 | neurotoxin [Naja atra] |
| 72 | 3034 | P82935.2 | 149.05 | 23 | 0 | 15 | 15 | 15 | 0 | 0 | 0 | 0 | 15 | 21 | 21 | 23 | 15 | 15 |  |  |  |  |  |  |  |  |  |  |  | 2.5637E6 |  |  | 4 | 1 | 0 | 1 | 2 | 1 | 0 | 0 | 0 | 0 | 5 | 27 | 124 | 38 | 5 | 2 | Y | 9915 | RecName: Full=Tryptophan-containing weak neurotoxin; Short=WTX; Flags: Precursor |
| 125 | 467 | Q98956.1 | 148.75 | 44 | 0 | 17 | 33 | 10 | 0 | 0 | 0 | 0 | 10 | 0 | 0 | 19 | 10 | 33 |  |  |  |  |  |  |  |  |  |  |  |  |  | 2.0971E6 | 7 | 1 | 0 | 1 | 30 | 1 | 0 | 0 | 0 | 0 | 1 | 0 | 0 | 2 | 1 | 8 | Y | 8894 | RecName: Full=Cytotoxin 1b; AltName: Full=Cardiotoxin-1b; Flags: Precursor |
| 125 | 468 | AAB18378.1 | 148.75 | 44 | 0 | 17 | 33 | 10 | 0 | 0 | 0 | 0 | 10 | 0 | 0 | 19 | 10 | 33 |  |  |  |  |  |  |  |  |  |  |  |  |  | 2.0971E6 | 7 | 1 | 0 | 1 | 30 | 1 | 0 | 0 | 0 | 0 | 1 | 0 | 0 | 2 | 1 | 8 | Y | 8894 | cardiotoxin 1b [Naja atra] |
| 178 | 1474 | JAS04550.1 | 146.72 | 14 | 0 | 0 | 9 | 14 | 0 | 0 | 0 | 0 | 0 | 0 | 0 | 0 | 0 | 0 |  |  |  | 2.2026E6 |  |  |  |  |  |  |  |  |  |  | 4 | 1 | 0 | 0 | 8 | 13 | 0 | 0 | 0 | 0 | 0 | 0 | 0 | 0 | 0 | 0 | Y | 26681 | Cysteine-rich secretory protein 1 [Agkistrodon piscivorus conanti] |
| 70 | 2650 | P25679.2 | 146.47 | 28 | 0 | 20 | 20 | 20 | 0 | 0 | 0 | 0 | 20 | 28 | 28 | 28 | 20 | 20 |  |  |  |  |  |  |  |  |  |  | 4.8427E7 |  |  |  | 4 | 1 | 0 | 1 | 2 | 1 | 0 | 0 | 0 | 0 | 5 | 27 | 125 | 36 | 5 | 2 | Y | 7438 | RecName: Full=Weak toxin CM-9a |
| 70 | 3033 | 2MJ0 | 146.47 | 27 | 0 | 20 | 20 | 20 | 0 | 0 | 0 | 0 | 20 | 27 | 27 | 27 | 20 | 20 |  |  |  |  |  |  |  |  |  |  | 4.8427E7 |  |  |  | 4 | 1 | 0 | 1 | 2 | 1 | 0 | 0 | 0 | 0 | 5 | 27 | 125 | 36 | 5 | 2 | Y | 7729 | Chain A, Weak tryptophan-containing neurotoxin |
| 70 | 4735 | C0HJW9.2 | 146.47 | 55 | 0 | 39 | 39 | 39 | 0 | 0 | 0 | 0 | 39 | 55 | 55 | 55 | 39 | 39 |  |  |  |  |  |  |  |  |  |  | 4.8427E7 |  |  |  | 4 | 1 | 0 | 1 | 2 | 1 | 0 | 0 | 0 | 0 | 5 | 27 | 125 | 36 | 5 | 2 | Y | 3943 | RecName: Full=Neurotoxin Nk-3FTx |
| 191 | 795 | XP\_015680068.1 | 145.97 | 10 | 0 | 2 | 0 | 0 | 0 | 0 | 0 | 0 | 0 | 0 | 0 | 0 | 9 | 10 |  | 2.7926E6 |  |  |  |  |  |  |  |  |  |  | 1.5102E7 | 1.3827E7 | 4 | 4 | 0 | 1 | 0 | 0 | 0 | 0 | 0 | 0 | 0 | 0 | 0 | 0 | 4 | 4 | Y | 47362 | transforming growth factor beta-1 proprotein [Protobothrops mucrosquamatus] |
| 191 | 830 | XP\_026544123.1 | 145.97 | 10 | 0 | 2 | 0 | 0 | 0 | 0 | 0 | 0 | 0 | 0 | 0 | 0 | 9 | 10 |  | 2.7926E6 |  |  |  |  |  |  |  |  |  |  | 1.5102E7 | 1.3827E7 | 4 | 4 | 0 | 1 | 0 | 0 | 0 | 0 | 0 | 0 | 0 | 0 | 0 | 0 | 4 | 4 | Y | 47310 | transforming growth factor beta-1 [Notechis scutatus] |
| 194 | 547 | G8XQX1.1 | 143.15 | 12 | 0 | 0 | 0 | 0 | 0 | 12 | 0 | 0 | 0 | 0 | 0 | 0 | 0 | 0 |  |  |  |  |  | 1.4191E6 |  |  |  |  |  |  |  |  | 6 | 2 | 0 | 0 | 0 | 0 | 0 | 14 | 0 | 0 | 0 | 0 | 0 | 0 | 0 | 0 | Y | 56888 | RecName: Full=L-amino-acid oxidase; Short=DrLAO; Short=LAAO; Flags: Precursor |
| 194 | 548 | ACF70483.1 | 143.15 | 12 | 0 | 0 | 0 | 0 | 0 | 12 | 0 | 0 | 0 | 0 | 0 | 0 | 0 | 0 |  |  |  |  |  | 1.4191E6 |  |  |  |  |  |  |  |  | 6 | 2 | 0 | 0 | 0 | 0 | 0 | 14 | 0 | 0 | 0 | 0 | 0 | 0 | 0 | 0 | Y | 56888 | secreted L-amino acid oxidase precursor [Daboia russelii] |
| 94 | 1549 | P60623.1 | 142.80 | 10 | 0 | 0 | 0 | 10 | 4 | 4 | 0 | 0 | 0 | 0 | 0 | 0 | 0 | 0 |  |  |  | 4.9771E6 |  |  |  |  |  |  |  |  |  |  | 4 | 1 | 0 | 0 | 0 | 127 | 10 | 1 | 0 | 0 | 0 | 0 | 0 | 0 | 0 | 0 | N | 26294 | RecName: Full=Cysteine-rich venom protein; Short=CRVP; AltName: Full=Cysteine-rich secretory protein; AltName: Full=Stecrisp; Flags: Precursor |
| 94 | 1550 | AAQ98964.1 | 142.80 | 10 | 0 | 0 | 0 | 10 | 4 | 4 | 0 | 0 | 0 | 0 | 0 | 0 | 0 | 0 |  |  |  | 4.9771E6 |  |  |  |  |  |  |  |  |  |  | 4 | 1 | 0 | 0 | 0 | 127 | 10 | 1 | 0 | 0 | 0 | 0 | 0 | 0 | 0 | 0 | N | 26294 | cysteine-rich secretory protein, partial [Trimeresurus stejnegeri] |
| 94 | 1551 | ACE73574.1 | 142.80 | 10 | 0 | 0 | 0 | 10 | 4 | 4 | 0 | 0 | 0 | 0 | 0 | 0 | 0 | 0 |  |  |  | 4.9771E6 |  |  |  |  |  |  |  |  |  |  | 4 | 1 | 0 | 0 | 0 | 127 | 10 | 1 | 0 | 0 | 0 | 0 | 0 | 0 | 0 | 0 | N | 27082 | cysteine-rich seceretory protein Ts-CRPM [Trimeresurus stejnegeri] |
| 94 | 1555 | ACE73572.1 | 142.80 | 10 | 0 | 0 | 0 | 10 | 4 | 4 | 0 | 0 | 0 | 0 | 0 | 0 | 0 | 0 |  |  |  | 4.9771E6 |  |  |  |  |  |  |  |  |  |  | 4 | 1 | 0 | 0 | 0 | 127 | 10 | 1 | 0 | 0 | 0 | 0 | 0 | 0 | 0 | 0 | N | 27054 | cysteine-rich seceretory protein Ts-CRPYa [Trimeresurus stejnegeri] |
| 94 | 1557 | 1RC9 | 142.80 | 10 | 0 | 0 | 0 | 10 | 5 | 5 | 0 | 0 | 0 | 0 | 0 | 0 | 0 | 0 |  |  |  | 4.9771E6 |  |  |  |  |  |  |  |  |  |  | 4 | 1 | 0 | 0 | 0 | 127 | 10 | 1 | 0 | 0 | 0 | 0 | 0 | 0 | 0 | 0 | N | 25129 | Chain A, Cysteine-rich Secretory Protein |
| 94 | 1560 | ACE73573.1 | 142.80 | 10 | 0 | 0 | 0 | 10 | 4 | 4 | 0 | 0 | 0 | 0 | 0 | 0 | 0 | 0 |  |  |  | 4.9771E6 |  |  |  |  |  |  |  |  |  |  | 4 | 1 | 0 | 0 | 0 | 127 | 10 | 1 | 0 | 0 | 0 | 0 | 0 | 0 | 0 | 0 | N | 27052 | cysteine-rich seceretory protein Ts-CRPYb [Trimeresurus stejnegeri] |
| 184 | 1172 | P82464.1 | 140.81 | 66 | 0 | 0 | 0 | 0 | 0 | 0 | 0 | 0 | 15 | 20 | 15 | 66 | 0 | 20 |  |  |  |  |  |  |  |  |  |  |  | 4.9409E6 |  |  | 5 | 1 | 0 | 0 | 0 | 0 | 0 | 0 | 0 | 0 | 4 | 1 | 1 | 6 | 0 | 1 | Y | 7624 | RecName: Full=Muscarinic toxin-like protein 3; Short=MTLP-3 |
| 176 | 646 | ETE73213.1 | 139.64 | 13 | 0 | 4 | 3 | 0 | 0 | 3 | 0 | 0 | 4 | 0 | 0 | 0 | 0 | 6 |  | 2.9535E5 | 1.213E6 |  |  | 8.8827E5 |  |  | 2.8969E7 |  |  |  |  | 3.9168E6 | 6 | 6 | 0 | 2 | 1 | 0 | 0 | 1 | 0 | 0 | 12 | 0 | 0 | 0 | 0 | 2 | N | 48455 | Calreticulin [Ophiophagus hannah] |
| 176 | 647 | XP\_026572586.1 | 139.64 | 13 | 0 | 4 | 3 | 0 | 0 | 3 | 0 | 0 | 4 | 0 | 0 | 0 | 0 | 6 |  | 2.9535E5 | 1.213E6 |  |  | 8.8827E5 |  |  | 2.8969E7 |  |  |  |  | 3.9168E6 | 6 | 6 | 0 | 2 | 1 | 0 | 0 | 1 | 0 | 0 | 12 | 0 | 0 | 0 | 0 | 2 | N | 48544 | calreticulin [Pseudonaja textilis] |
| 176 | 649 | JAC96061.1 | 139.64 | 13 | 0 | 4 | 3 | 0 | 0 | 3 | 0 | 0 | 4 | 0 | 0 | 0 | 0 | 6 |  | 2.9535E5 | 1.213E6 |  |  | 8.8827E5 |  |  | 2.8969E7 |  |  |  |  | 3.9168E6 | 6 | 6 | 0 | 2 | 1 | 0 | 0 | 1 | 0 | 0 | 12 | 0 | 0 | 0 | 0 | 2 | N | 48526 | Calreticulin [Hypsiglena sp. JMG-2014] |
| 26 | 1282 | 1CDT | 138.81 | 30 | 0 | 0 | 0 | 0 | 0 | 12 | 0 | 0 | 0 | 0 | 0 | 30 | 30 | 22 |  |  |  |  |  |  |  |  |  |  |  | 1.6406E6 | 1.808E6 | 1.7198E7 | 4 | 1 | 0 | 0 | 0 | 0 | 0 | 1 | 0 | 0 | 0 | 0 | 0 | 100 | 29 | 5 | Y | 6715 | Chain B, CARDIOTOXIN VII4 |
| 26 | 1283 | P01452.1 | 138.81 | 30 | 0 | 0 | 0 | 0 | 0 | 12 | 0 | 0 | 0 | 0 | 0 | 30 | 30 | 22 |  |  |  |  |  |  |  |  |  |  |  | 1.6406E6 | 1.808E6 | 1.7198E7 | 4 | 1 | 0 | 0 | 0 | 0 | 0 | 1 | 0 | 0 | 0 | 0 | 0 | 100 | 29 | 5 | Y | 6715 | RecName: Full=Cytotoxin 4; AltName: Full=CTX M3; AltName: Full=Cardiotoxin V(II)4 |
| 198 | 431 | ETE73206.1 | 138.48 | 19 | 0 | 0 | 0 | 0 | 19 | 3 | 0 | 0 | 0 | 0 | 0 | 0 | 0 | 0 |  |  |  |  | 1.3009E7 |  |  |  |  |  |  |  |  |  | 5 | 2 | 0 | 0 | 0 | 0 | 11 | 2 | 0 | 0 | 0 | 0 | 0 | 0 | 0 | 0 | N | 45572 | Deoxyribonuclease-2-alpha, partial [Ophiophagus hannah] |
| 198 | 438 | XP\_026572614.1 | 138.48 | 21 | 0 | 0 | 0 | 0 | 21 | 3 | 0 | 0 | 0 | 0 | 0 | 0 | 0 | 0 |  |  |  |  | 1.3009E7 |  |  |  |  |  |  |  |  |  | 5 | 2 | 0 | 0 | 0 | 0 | 11 | 2 | 0 | 0 | 0 | 0 | 0 | 0 | 0 | 0 | N | 40365 | deoxyribonuclease-2-alpha isoform X2 [Pseudonaja textilis] |
| 198 | 439 | XP\_026572616.1 | 138.48 | 21 | 0 | 0 | 0 | 0 | 21 | 3 | 0 | 0 | 0 | 0 | 0 | 0 | 0 | 0 |  |  |  |  | 1.3009E7 |  |  |  |  |  |  |  |  |  | 5 | 2 | 0 | 0 | 0 | 0 | 11 | 2 | 0 | 0 | 0 | 0 | 0 | 0 | 0 | 0 | N | 40365 | deoxyribonuclease-2-alpha isoform X2 [Pseudonaja textilis] |
| 198 | 440 | XP\_026572618.1 | 138.48 | 21 | 0 | 0 | 0 | 0 | 21 | 3 | 0 | 0 | 0 | 0 | 0 | 0 | 0 | 0 |  |  |  |  | 1.3009E7 |  |  |  |  |  |  |  |  |  | 5 | 2 | 0 | 0 | 0 | 0 | 11 | 2 | 0 | 0 | 0 | 0 | 0 | 0 | 0 | 0 | N | 40365 | deoxyribonuclease-2-alpha isoform X2 [Pseudonaja textilis] |
| 198 | 441 | XP\_026572617.1 | 138.48 | 21 | 0 | 0 | 0 | 0 | 21 | 3 | 0 | 0 | 0 | 0 | 0 | 0 | 0 | 0 |  |  |  |  | 1.3009E7 |  |  |  |  |  |  |  |  |  | 5 | 2 | 0 | 0 | 0 | 0 | 11 | 2 | 0 | 0 | 0 | 0 | 0 | 0 | 0 | 0 | N | 40365 | deoxyribonuclease-2-alpha isoform X2 [Pseudonaja textilis] |
| 198 | 442 | XP\_026572615.1 | 138.48 | 21 | 0 | 0 | 0 | 0 | 21 | 3 | 0 | 0 | 0 | 0 | 0 | 0 | 0 | 0 |  |  |  |  | 1.3009E7 |  |  |  |  |  |  |  |  |  | 5 | 2 | 0 | 0 | 0 | 0 | 11 | 2 | 0 | 0 | 0 | 0 | 0 | 0 | 0 | 0 | N | 40365 | deoxyribonuclease-2-alpha isoform X2 [Pseudonaja textilis] |
| 198 | 443 | XP\_026572612.1 | 138.48 | 17 | 0 | 0 | 0 | 0 | 17 | 3 | 0 | 0 | 0 | 0 | 0 | 0 | 0 | 0 |  |  |  |  | 1.3009E7 |  |  |  |  |  |  |  |  |  | 5 | 2 | 0 | 0 | 0 | 0 | 11 | 2 | 0 | 0 | 0 | 0 | 0 | 0 | 0 | 0 | N | 48788 | deoxyribonuclease-2-alpha isoform X1 [Pseudonaja textilis] |
| 198 | 446 | XP\_026535344.1 | 138.48 | 20 | 0 | 0 | 0 | 0 | 20 | 3 | 0 | 0 | 0 | 0 | 0 | 0 | 0 | 0 |  |  |  |  | 1.3009E7 |  |  |  |  |  |  |  |  |  | 5 | 2 | 0 | 0 | 0 | 0 | 11 | 2 | 0 | 0 | 0 | 0 | 0 | 0 | 0 | 0 | N | 41795 | deoxyribonuclease-2-alpha isoform X2 [Notechis scutatus] |
| 198 | 447 | XP\_026535343.1 | 138.48 | 20 | 0 | 0 | 0 | 0 | 20 | 3 | 0 | 0 | 0 | 0 | 0 | 0 | 0 | 0 |  |  |  |  | 1.3009E7 |  |  |  |  |  |  |  |  |  | 5 | 2 | 0 | 0 | 0 | 0 | 11 | 2 | 0 | 0 | 0 | 0 | 0 | 0 | 0 | 0 | N | 42582 | deoxyribonuclease-2-alpha isoform X1 [Notechis scutatus] |
| 198 | 448 | XP\_026535346.1 | 138.48 | 21 | 0 | 0 | 0 | 0 | 21 | 3 | 0 | 0 | 0 | 0 | 0 | 0 | 0 | 0 |  |  |  |  | 1.3009E7 |  |  |  |  |  |  |  |  |  | 5 | 2 | 0 | 0 | 0 | 0 | 11 | 2 | 0 | 0 | 0 | 0 | 0 | 0 | 0 | 0 | N | 40403 | deoxyribonuclease-2-alpha isoform X3 [Notechis scutatus] |
| 198 | 449 | XP\_026535348.1 | 138.48 | 21 | 0 | 0 | 0 | 0 | 21 | 3 | 0 | 0 | 0 | 0 | 0 | 0 | 0 | 0 |  |  |  |  | 1.3009E7 |  |  |  |  |  |  |  |  |  | 5 | 2 | 0 | 0 | 0 | 0 | 11 | 2 | 0 | 0 | 0 | 0 | 0 | 0 | 0 | 0 | N | 40403 | deoxyribonuclease-2-alpha isoform X3 [Notechis scutatus] |
| 198 | 450 | XP\_026535349.1 | 138.48 | 21 | 0 | 0 | 0 | 0 | 21 | 3 | 0 | 0 | 0 | 0 | 0 | 0 | 0 | 0 |  |  |  |  | 1.3009E7 |  |  |  |  |  |  |  |  |  | 5 | 2 | 0 | 0 | 0 | 0 | 11 | 2 | 0 | 0 | 0 | 0 | 0 | 0 | 0 | 0 | N | 40403 | deoxyribonuclease-2-alpha isoform X3 [Notechis scutatus] |
| 198 | 451 | XP\_026535350.1 | 138.48 | 21 | 0 | 0 | 0 | 0 | 21 | 3 | 0 | 0 | 0 | 0 | 0 | 0 | 0 | 0 |  |  |  |  | 1.3009E7 |  |  |  |  |  |  |  |  |  | 5 | 2 | 0 | 0 | 0 | 0 | 11 | 2 | 0 | 0 | 0 | 0 | 0 | 0 | 0 | 0 | N | 40403 | deoxyribonuclease-2-alpha isoform X3 [Notechis scutatus] |
| 198 | 452 | XP\_026535345.1 | 138.48 | 21 | 0 | 0 | 0 | 0 | 21 | 3 | 0 | 0 | 0 | 0 | 0 | 0 | 0 | 0 |  |  |  |  | 1.3009E7 |  |  |  |  |  |  |  |  |  | 5 | 2 | 0 | 0 | 0 | 0 | 11 | 2 | 0 | 0 | 0 | 0 | 0 | 0 | 0 | 0 | N | 40403 | deoxyribonuclease-2-alpha isoform X3 [Notechis scutatus] |
| 245 | 706 | XP\_026555457.1 | 137.20 | 11 | 0 | 0 | 0 | 11 | 0 | 0 | 0 | 0 | 0 | 0 | 0 | 0 | 0 | 0 |  |  |  | 4.5823E6 |  |  |  |  |  |  |  |  |  |  | 3 | 1 | 0 | 0 | 0 | 6 | 0 | 0 | 0 | 0 | 0 | 0 | 0 | 0 | 0 | 0 | Y | 37785 | N(4)-(beta-N-acetylglucosaminyl)-L-asparaginase [Pseudonaja textilis] |
| 245 | 719 | XP\_026522978.1 | 137.20 | 11 | 0 | 0 | 0 | 11 | 0 | 0 | 0 | 0 | 0 | 0 | 0 | 0 | 0 | 0 |  |  |  | 4.5823E6 |  |  |  |  |  |  |  |  |  |  | 3 | 1 | 0 | 0 | 0 | 6 | 0 | 0 | 0 | 0 | 0 | 0 | 0 | 0 | 0 | 0 | Y | 37717 | N(4)-(beta-N-acetylglucosaminyl)-L-asparaginase [Notechis scutatus] |
| 216 | 462 | XP\_026575334.1 | 136.86 | 6 | 0 | 0 | 2 | 3 | 4 | 0 | 0 | 0 | 0 | 0 | 0 | 0 | 0 | 0 |  |  | 3.5691E5 | 3.9029E6 | 1.998E6 |  |  |  |  |  |  |  |  |  | 5 | 5 | 0 | 0 | 1 | 7 | 3 | 0 | 0 | 0 | 0 | 0 | 0 | 0 | 0 | 0 | N | 109505 | peptidyl-glycine alpha-amidating monooxygenase isoform X2 [Pseudonaja textilis] |
| 216 | 463 | XP\_026575314.1 | 136.86 | 6 | 0 | 0 | 2 | 3 | 4 | 0 | 0 | 0 | 0 | 0 | 0 | 0 | 0 | 0 |  |  | 3.5691E5 | 3.9029E6 | 1.998E6 |  |  |  |  |  |  |  |  |  | 5 | 5 | 0 | 0 | 1 | 7 | 3 | 0 | 0 | 0 | 0 | 0 | 0 | 0 | 0 | 0 | N | 109576 | peptidyl-glycine alpha-amidating monooxygenase isoform X1 [Pseudonaja textilis] |
| 216 | 464 | XP\_026575319.1 | 136.86 | 6 | 0 | 0 | 2 | 3 | 4 | 0 | 0 | 0 | 0 | 0 | 0 | 0 | 0 | 0 |  |  | 3.5691E5 | 3.9029E6 | 1.998E6 |  |  |  |  |  |  |  |  |  | 5 | 5 | 0 | 0 | 1 | 7 | 3 | 0 | 0 | 0 | 0 | 0 | 0 | 0 | 0 | 0 | N | 109576 | peptidyl-glycine alpha-amidating monooxygenase isoform X1 [Pseudonaja textilis] |
| 216 | 465 | XP\_026575325.1 | 136.86 | 6 | 0 | 0 | 2 | 3 | 4 | 0 | 0 | 0 | 0 | 0 | 0 | 0 | 0 | 0 |  |  | 3.5691E5 | 3.9029E6 | 1.998E6 |  |  |  |  |  |  |  |  |  | 5 | 5 | 0 | 0 | 1 | 7 | 3 | 0 | 0 | 0 | 0 | 0 | 0 | 0 | 0 | 0 | N | 109576 | peptidyl-glycine alpha-amidating monooxygenase isoform X1 [Pseudonaja textilis] |
| 216 | 479 | XP\_026575343.1 | 136.86 | 7 | 0 | 0 | 2 | 3 | 5 | 0 | 0 | 0 | 0 | 0 | 0 | 0 | 0 | 0 |  |  | 3.5691E5 | 3.9029E6 | 1.998E6 |  |  |  |  |  |  |  |  |  | 5 | 5 | 0 | 0 | 1 | 7 | 3 | 0 | 0 | 0 | 0 | 0 | 0 | 0 | 0 | 0 | N | 97316 | peptidyl-glycine alpha-amidating monooxygenase isoform X3 [Pseudonaja textilis] |
| 246 | 943 | XP\_034273677.1 | 135.54 | 8 | 0 | 0 | 0 | 6 | 0 | 2 | 0 | 0 | 0 | 0 | 0 | 0 | 0 | 0 |  |  |  | 1.3337E7 |  | 2.0504E5 |  |  |  |  |  |  |  |  | 4 | 4 | 0 | 0 | 0 | 5 | 0 | 1 | 0 | 0 | 0 | 0 | 0 | 0 | 0 | 0 | N | 61767 | EH domain-containing protein 4 [Pantherophis guttatus] |
| 246 | 949 | LAA22316.1 | 135.54 | 8 | 0 | 0 | 0 | 6 | 0 | 2 | 0 | 0 | 0 | 0 | 0 | 0 | 0 | 0 |  |  |  | 1.3337E7 |  | 2.0504E5 |  |  |  |  |  |  |  |  | 4 | 4 | 0 | 0 | 0 | 5 | 0 | 1 | 0 | 0 | 0 | 0 | 0 | 0 | 0 | 0 | N | 61673 | hypothetical protein [Micrurus lemniscatus carvalhoi] |
| 246 | 950 | LAA22315.1 | 135.54 | 8 | 0 | 0 | 0 | 6 | 0 | 2 | 0 | 0 | 0 | 0 | 0 | 0 | 0 | 0 |  |  |  | 1.3337E7 |  | 2.0504E5 |  |  |  |  |  |  |  |  | 4 | 4 | 0 | 0 | 0 | 5 | 0 | 1 | 0 | 0 | 0 | 0 | 0 | 0 | 0 | 0 | N | 61673 | hypothetical protein [Micrurus lemniscatus carvalhoi] |
| 246 | 955 | JAC95688.1 | 135.54 | 8 | 0 | 0 | 0 | 6 | 0 | 2 | 0 | 0 | 0 | 0 | 0 | 0 | 0 | 0 |  |  |  | 1.3337E7 |  | 2.0504E5 |  |  |  |  |  |  |  |  | 4 | 4 | 0 | 0 | 0 | 5 | 0 | 1 | 0 | 0 | 0 | 0 | 0 | 0 | 0 | 0 | N | 61637 | EH domain-containing protein 4 [Hypsiglena sp. JMG-2014] |
| 246 | 956 | LAB64401.1 | 135.54 | 8 | 0 | 0 | 0 | 6 | 0 | 2 | 0 | 0 | 0 | 0 | 0 | 0 | 0 | 0 |  |  |  | 1.3337E7 |  | 2.0504E5 |  |  |  |  |  |  |  |  | 4 | 4 | 0 | 0 | 0 | 5 | 0 | 1 | 0 | 0 | 0 | 0 | 0 | 0 | 0 | 0 | N | 61672 | hypothetical protein [Micrurus surinamensis] |
| 246 | 960 | LAA61330.1 | 135.54 | 8 | 0 | 0 | 0 | 6 | 0 | 2 | 0 | 0 | 0 | 0 | 0 | 0 | 0 | 0 |  |  |  | 1.3337E7 |  | 2.0504E5 |  |  |  |  |  |  |  |  | 4 | 4 | 0 | 0 | 0 | 5 | 0 | 1 | 0 | 0 | 0 | 0 | 0 | 0 | 0 | 0 | N | 61709 | hypothetical protein [Micrurus corallinus] |
| 206 | 455 | XP\_026528360.1 | 135.22 | 5 | 0 | 0 | 0 | 0 | 3 | 5 | 0 | 0 | 0 | 0 | 0 | 0 | 0 | 0 |  |  |  |  | 1.2008E6 | 4.6406E6 |  |  |  |  |  |  |  |  | 4 | 4 | 0 | 0 | 0 | 0 | 3 | 7 | 0 | 0 | 0 | 0 | 0 | 0 | 0 | 0 | N | 83931 | neuroendocrine convertase 1 [Notechis scutatus] |
| 206 | 456 | XP\_034296604.1 | 135.22 | 5 | 0 | 0 | 0 | 0 | 3 | 5 | 0 | 0 | 0 | 0 | 0 | 0 | 0 | 0 |  |  |  |  | 1.2008E6 | 4.6406E6 |  |  |  |  |  |  |  |  | 4 | 4 | 0 | 0 | 0 | 0 | 3 | 7 | 0 | 0 | 0 | 0 | 0 | 0 | 0 | 0 | N | 83807 | neuroendocrine convertase 1 [Pantherophis guttatus] |
| 206 | 457 | XP\_026575034.1 | 135.22 | 5 | 0 | 0 | 0 | 0 | 3 | 5 | 0 | 0 | 0 | 0 | 0 | 0 | 0 | 0 |  |  |  |  | 1.2008E6 | 4.6406E6 |  |  |  |  |  |  |  |  | 4 | 4 | 0 | 0 | 0 | 0 | 3 | 7 | 0 | 0 | 0 | 0 | 0 | 0 | 0 | 0 | N | 83897 | neuroendocrine convertase 1 [Pseudonaja textilis] |
| 206 | 458 | ETE68499.1 | 135.22 | 5 | 0 | 0 | 0 | 0 | 3 | 5 | 0 | 0 | 0 | 0 | 0 | 0 | 0 | 0 |  |  |  |  | 1.2008E6 | 4.6406E6 |  |  |  |  |  |  |  |  | 4 | 4 | 0 | 0 | 0 | 0 | 3 | 7 | 0 | 0 | 0 | 0 | 0 | 0 | 0 | 0 | N | 83039 | Neuroendocrine convertase 1, partial [Ophiophagus hannah] |
| 206 | 474 | XP\_032069925.1 | 135.22 | 5 | 0 | 0 | 0 | 0 | 3 | 5 | 0 | 0 | 0 | 0 | 0 | 0 | 0 | 0 |  |  |  |  | 1.2008E6 | 4.6406E6 |  |  |  |  |  |  |  |  | 4 | 4 | 0 | 0 | 0 | 0 | 3 | 7 | 0 | 0 | 0 | 0 | 0 | 0 | 0 | 0 | N | 83866 | neuroendocrine convertase 1 [Thamnophis elegans] |
| 206 | 475 | XP\_013913235.1 | 135.22 | 5 | 0 | 0 | 0 | 0 | 3 | 5 | 0 | 0 | 0 | 0 | 0 | 0 | 0 | 0 |  |  |  |  | 1.2008E6 | 4.6406E6 |  |  |  |  |  |  |  |  | 4 | 4 | 0 | 0 | 0 | 0 | 3 | 7 | 0 | 0 | 0 | 0 | 0 | 0 | 0 | 0 | N | 83866 | PREDICTED: neuroendocrine convertase 1 [Thamnophis sirtalis] |
| 206 | 486 | XP\_015687426.1 | 135.22 | 5 | 0 | 0 | 0 | 0 | 3 | 5 | 0 | 0 | 0 | 0 | 0 | 0 | 0 | 0 |  |  |  |  | 1.2008E6 | 4.6406E6 |  |  |  |  |  |  |  |  | 4 | 4 | 0 | 0 | 0 | 0 | 3 | 7 | 0 | 0 | 0 | 0 | 0 | 0 | 0 | 0 | N | 83688 | neuroendocrine convertase 1 [Protobothrops mucrosquamatus] |
| 206 | 487 | XP\_007439683.1 | 135.22 | 5 | 0 | 0 | 0 | 0 | 3 | 5 | 0 | 0 | 0 | 0 | 0 | 0 | 0 | 0 |  |  |  |  | 1.2008E6 | 4.6406E6 |  |  |  |  |  |  |  |  | 4 | 4 | 0 | 0 | 0 | 0 | 3 | 7 | 0 | 0 | 0 | 0 | 0 | 0 | 0 | 0 | N | 83953 | neuroendocrine convertase 1 [Python bivittatus] |
| 135 | 1806 | P14613.1 | 132.01 | 61 | 61 | 0 | 0 | 0 | 0 | 0 | 61 | 19 | 19 | 0 | 0 | 0 | 0 | 0 | 6.0506E7 |  |  |  |  |  | 4.4306E6 |  |  |  |  |  |  |  | 3 | 1 | 5 | 0 | 0 | 0 | 0 | 0 | 7 | 42 | 3 | 0 | 0 | 0 | 0 | 0 | Y | 6983 | RecName: Full=Short neurotoxin 1; AltName: Full=Toxin C-6 |
| 174 | 549 | XP\_026571379.1 | 130.79 | 6 | 0 | 3 | 4 | 1 | 0 | 0 | 0 | 0 | 0 | 0 | 0 | 0 | 0 | 1 |  | 4.9489E6 | 1.8559E8 | 3.3547E6 |  |  |  |  |  |  |  |  |  |  | 4 | 2 | 0 | 4 | 14 | 3 | 0 | 0 | 0 | 0 | 0 | 0 | 0 | 0 | 0 | 3 | Y | 80026 | C4b-binding protein alpha chain-like isoform X1 [Pseudonaja textilis] |
| 181 | 1110 | XP\_026579406.1 | 130.66 | 21 | 0 | 0 | 0 | 0 | 0 | 0 | 0 | 0 | 0 | 20 | 15 | 7 | 7 | 0 |  |  |  |  |  |  |  |  |  | 3.5198E7 | 3.3284E7 |  |  |  | 4 | 3 | 0 | 0 | 0 | 0 | 0 | 0 | 0 | 0 | 0 | 9 | 5 | 3 | 1 | 0 | Y | 22428 | BPTI/Kunitz domain-containing protein-like isoform X3 [Pseudonaja textilis] |
| 181 | 1111 | XP\_026579408.1 | 130.66 | 21 | 0 | 0 | 0 | 0 | 0 | 0 | 0 | 0 | 0 | 20 | 15 | 7 | 7 | 0 |  |  |  |  |  |  |  |  |  | 3.5198E7 | 3.3284E7 |  |  |  | 4 | 3 | 0 | 0 | 0 | 0 | 0 | 0 | 0 | 0 | 0 | 9 | 5 | 3 | 1 | 0 | Y | 22376 | BPTI/Kunitz domain-containing protein-like isoform X1 [Pseudonaja textilis] |
| 181 | 1112 | XP\_026579405.1 | 130.66 | 21 | 0 | 0 | 0 | 0 | 0 | 0 | 0 | 0 | 0 | 20 | 15 | 7 | 7 | 0 |  |  |  |  |  |  |  |  |  | 3.5198E7 | 3.3284E7 |  |  |  | 4 | 3 | 0 | 0 | 0 | 0 | 0 | 0 | 0 | 0 | 0 | 9 | 5 | 3 | 1 | 0 | Y | 22372 | BPTI/Kunitz domain-containing protein-like isoform X2 [Pseudonaja textilis] |
| 181 | 1113 | XP\_026579409.1 | 130.66 | 21 | 0 | 0 | 0 | 0 | 0 | 0 | 0 | 0 | 0 | 20 | 15 | 7 | 7 | 0 |  |  |  |  |  |  |  |  |  | 3.5198E7 | 3.3284E7 |  |  |  | 4 | 3 | 0 | 0 | 0 | 0 | 0 | 0 | 0 | 0 | 0 | 9 | 5 | 3 | 1 | 0 | Y | 22372 | BPTI/Kunitz domain-containing protein-like isoform X2 [Pseudonaja textilis] |
| 181 | 1114 | XP\_026579404.1 | 130.66 | 21 | 0 | 0 | 0 | 0 | 0 | 0 | 0 | 0 | 0 | 20 | 15 | 7 | 7 | 0 |  |  |  |  |  |  |  |  |  | 3.5198E7 | 3.3284E7 |  |  |  | 4 | 3 | 0 | 0 | 0 | 0 | 0 | 0 | 0 | 0 | 0 | 9 | 5 | 3 | 1 | 0 | Y | 22376 | BPTI/Kunitz domain-containing protein-like isoform X1 [Pseudonaja textilis] |
| 162 | 688 | XP\_026544110.1 | 129.07 | 12 | 0 | 0 | 8 | 7 | 0 | 0 | 0 | 2 | 2 | 0 | 0 | 2 | 2 | 2 |  |  | 3.4722E7 | 3.9539E7 |  |  |  |  |  |  |  |  |  |  | 4 | 1 | 0 | 0 | 6 | 7 | 0 | 0 | 0 | 3 | 3 | 0 | 0 | 1 | 6 | 3 | Y | 50196 | hepatocyte growth factor activator [Notechis scutatus] |
| 120 | 1174 | AJB84503.1 | 128.08 | 6 | 0 | 0 | 2 | 2 | 3 | 2 | 0 | 0 | 3 | 0 | 0 | 2 | 2 | 2 |  |  |  |  |  |  |  |  | 6.1915E5 |  |  |  |  |  | 4 | 1 | 0 | 0 | 1 | 3 | 20 | 33 | 0 | 0 | 1 | 0 | 0 | 2 | 2 | 1 | Y | 68466 | venom metalloprotease precursor [Philodryas chamissonis] |
| 109 | 708 | Q90WI8.1 | 125.65 | 11 | 0 | 0 | 0 | 11 | 0 | 5 | 0 | 0 | 0 | 0 | 0 | 0 | 0 | 0 |  |  |  | 1.2445E9 |  | 0 |  |  |  |  |  |  |  |  | 3 | 2 | 0 | 0 | 0 | 85 | 0 | 2 | 0 | 0 | 0 | 0 | 0 | 0 | 0 | 0 | Y | 18638 | RecName: Full=C-type lectin BfL-1; Short=CTL; Flags: Precursor |
| 109 | 709 | AAK43584.1 | 125.65 | 11 | 0 | 0 | 0 | 11 | 0 | 5 | 0 | 0 | 0 | 0 | 0 | 0 | 0 | 0 |  |  |  | 1.2445E9 |  | 0 |  |  |  |  |  |  |  |  | 3 | 2 | 0 | 0 | 0 | 85 | 0 | 2 | 0 | 0 | 0 | 0 | 0 | 0 | 0 | 0 | Y | 18638 | C-type lectin-like protein 1 [Bungarus fasciatus] |
| 237 | 792 | XP\_026575982.1 | 125.13 | 10 | 0 | 0 | 0 | 10 | 0 | 0 | 0 | 0 | 0 | 0 | 0 | 0 | 0 | 0 |  |  |  | 2.0751E7 |  |  |  |  |  |  |  |  |  |  | 3 | 1 | 0 | 0 | 0 | 7 | 0 | 0 | 0 | 0 | 0 | 0 | 0 | 0 | 0 | 0 | Y | 37973 | cathepsin S [Pseudonaja textilis] |
| 237 | 793 | XP\_026575983.1 | 125.13 | 10 | 0 | 0 | 0 | 10 | 0 | 0 | 0 | 0 | 0 | 0 | 0 | 0 | 0 | 0 |  |  |  | 2.0751E7 |  |  |  |  |  |  |  |  |  |  | 3 | 1 | 0 | 0 | 0 | 7 | 0 | 0 | 0 | 0 | 0 | 0 | 0 | 0 | 0 | 0 | Y | 37973 | cathepsin S [Pseudonaja textilis] |
| 253 | 641 | XP\_034295297.1 | 121.93 | 10 | 0 | 0 | 0 | 0 | 0 | 10 | 0 | 0 | 0 | 0 | 0 | 0 | 0 | 0 |  |  |  |  |  | 3.4196E6 |  |  |  |  |  |  |  |  | 3 | 3 | 0 | 0 | 0 | 0 | 0 | 5 | 0 | 0 | 0 | 0 | 0 | 0 | 0 | 0 | N | 44043 | glia-derived nexin [Pantherophis guttatus] |
| 253 | 644 | JAG67228.1 | 121.93 | 10 | 0 | 0 | 0 | 0 | 0 | 10 | 0 | 0 | 0 | 0 | 0 | 0 | 0 | 0 |  |  |  |  |  | 3.4196E6 |  |  |  |  |  |  |  |  | 3 | 3 | 0 | 0 | 0 | 0 | 0 | 5 | 0 | 0 | 0 | 0 | 0 | 0 | 0 | 0 | N | 44109 | Glia-derived nexin-like protein [Boiga irregularis] |
| 212 | 571 | XP\_026575692.1 | 119.16 | 8 | 0 | 0 | 0 | 0 | 2 | 8 | 0 | 0 | 0 | 0 | 0 | 0 | 0 | 0 |  |  |  |  | 2.3107E6 | 1.3876E7 |  |  |  |  |  |  |  |  | 4 | 4 | 0 | 0 | 0 | 0 | 3 | 10 | 0 | 0 | 0 | 0 | 0 | 0 | 0 | 0 | N | 53443 | multiple inositol polyphosphate phosphatase 1 isoform X2 [Pseudonaja textilis] |
| 212 | 572 | XP\_026575691.1 | 119.16 | 8 | 0 | 0 | 0 | 0 | 2 | 8 | 0 | 0 | 0 | 0 | 0 | 0 | 0 | 0 |  |  |  |  | 2.3107E6 | 1.3876E7 |  |  |  |  |  |  |  |  | 4 | 4 | 0 | 0 | 0 | 0 | 3 | 10 | 0 | 0 | 0 | 0 | 0 | 0 | 0 | 0 | N | 53858 | multiple inositol polyphosphate phosphatase 1 isoform X1 [Pseudonaja textilis] |
| 212 | 630 | XP\_026542555.1 | 119.16 | 8 | 0 | 0 | 0 | 0 | 2 | 8 | 0 | 0 | 0 | 0 | 0 | 0 | 0 | 0 |  |  |  |  | 2.3107E6 | 1.3876E7 |  |  |  |  |  |  |  |  | 4 | 4 | 0 | 0 | 0 | 0 | 3 | 10 | 0 | 0 | 0 | 0 | 0 | 0 | 0 | 0 | N | 53135 | multiple inositol polyphosphate phosphatase 1 isoform X2 [Notechis scutatus] |
| 212 | 631 | XP\_026542553.1 | 119.16 | 8 | 0 | 0 | 0 | 0 | 2 | 8 | 0 | 0 | 0 | 0 | 0 | 0 | 0 | 0 |  |  |  |  | 2.3107E6 | 1.3876E7 |  |  |  |  |  |  |  |  | 4 | 4 | 0 | 0 | 0 | 0 | 3 | 10 | 0 | 0 | 0 | 0 | 0 | 0 | 0 | 0 | N | 53549 | multiple inositol polyphosphate phosphatase 1 isoform X1 [Notechis scutatus] |
| 212 | 653 | XP\_029139434.1 | 119.16 | 8 | 0 | 0 | 0 | 0 | 2 | 8 | 0 | 0 | 0 | 0 | 0 | 0 | 0 | 0 |  |  |  |  | 2.3107E6 | 1.3876E7 |  |  |  |  |  |  |  |  | 4 | 4 | 0 | 0 | 0 | 0 | 3 | 10 | 0 | 0 | 0 | 0 | 0 | 0 | 0 | 0 | N | 53980 | multiple inositol polyphosphate phosphatase 1 [Protobothrops mucrosquamatus] |
| 139 | 853 | XP\_013915209.1 | 118.11 | 5 | 0 | 2 | 0 | 2 | 0 | 0 | 4 | 0 | 0 | 2 | 0 | 0 | 0 | 0 |  |  |  | 1.4425E7 |  |  |  |  |  |  |  |  |  |  | 4 | 1 | 0 | 1 | 0 | 1 | 0 | 0 | 32 | 0 | 0 | 2 | 0 | 0 | 0 | 0 | N | 59764 | PREDICTED: keratin, type II cytoskeletal 6A-like [Thamnophis sirtalis] |
| 189 | 856 | XP\_026565129.1 | 117.92 | 7 | 0 | 0 | 0 | 0 | 7 | 5 | 0 | 0 | 0 | 0 | 0 | 0 | 0 | 0 |  |  |  |  | 4.8154E6 | 3.0574E6 |  |  |  |  |  |  |  |  | 3 | 1 | 0 | 0 | 0 | 0 | 8 | 7 | 0 | 0 | 0 | 0 | 0 | 0 | 0 | 0 | N | 53650 | lactadherin isoform X1 [Pseudonaja textilis] |
| 189 | 926 | XP\_026565130.1 | 117.92 | 7 | 0 | 0 | 0 | 0 | 7 | 5 | 0 | 0 | 0 | 0 | 0 | 0 | 0 | 0 |  |  |  |  | 4.8154E6 | 3.0574E6 |  |  |  |  |  |  |  |  | 3 | 1 | 0 | 0 | 0 | 0 | 8 | 7 | 0 | 0 | 0 | 0 | 0 | 0 | 0 | 0 | N | 52954 | lactadherin isoform X2 [Pseudonaja textilis] |
| 276 | 1658 | AFJ49577.1 | 117.13 | 18 | 0 | 0 | 0 | 0 | 0 | 18 | 0 | 0 | 0 | 0 | 0 | 0 | 0 | 0 |  |  |  |  |  | 2.5422E6 |  |  |  |  |  |  |  |  | 3 | 3 | 0 | 0 | 0 | 0 | 0 | 5 | 0 | 0 | 0 | 0 | 0 | 0 | 0 | 0 | N | 16838 | Calmodulin [Crotalus adamanteus] |
| 276 | 1659 | JAC96065.1 | 117.13 | 18 | 0 | 0 | 0 | 0 | 0 | 18 | 0 | 0 | 0 | 0 | 0 | 0 | 0 | 0 |  |  |  |  |  | 2.5422E6 |  |  |  |  |  |  |  |  | 3 | 3 | 0 | 0 | 0 | 0 | 0 | 5 | 0 | 0 | 0 | 0 | 0 | 0 | 0 | 0 | N | 16838 | Calmodulin [Hypsiglena sp. JMG-2014] |
| 276 | 1660 | JAB54566.1 | 117.13 | 18 | 0 | 0 | 0 | 0 | 0 | 18 | 0 | 0 | 0 | 0 | 0 | 0 | 0 | 0 |  |  |  |  |  | 2.5422E6 |  |  |  |  |  |  |  |  | 3 | 3 | 0 | 0 | 0 | 0 | 0 | 5 | 0 | 0 | 0 | 0 | 0 | 0 | 0 | 0 | N | 16838 | Calmodulin [Micrurus fulvius] |
| 276 | 1661 | JAA97540.1 | 117.13 | 18 | 0 | 0 | 0 | 0 | 0 | 18 | 0 | 0 | 0 | 0 | 0 | 0 | 0 | 0 |  |  |  |  |  | 2.5422E6 |  |  |  |  |  |  |  |  | 3 | 3 | 0 | 0 | 0 | 0 | 0 | 5 | 0 | 0 | 0 | 0 | 0 | 0 | 0 | 0 | N | 16838 | Calmodulin [Crotalus horridus] |
| 276 | 1662 | JAA97541.1 | 117.13 | 18 | 0 | 0 | 0 | 0 | 0 | 18 | 0 | 0 | 0 | 0 | 0 | 0 | 0 | 0 |  |  |  |  |  | 2.5422E6 |  |  |  |  |  |  |  |  | 3 | 3 | 0 | 0 | 0 | 0 | 0 | 5 | 0 | 0 | 0 | 0 | 0 | 0 | 0 | 0 | N | 16838 | Calmodulin [Crotalus horridus] |
| 276 | 1663 | JAA97542.1 | 117.13 | 18 | 0 | 0 | 0 | 0 | 0 | 18 | 0 | 0 | 0 | 0 | 0 | 0 | 0 | 0 |  |  |  |  |  | 2.5422E6 |  |  |  |  |  |  |  |  | 3 | 3 | 0 | 0 | 0 | 0 | 0 | 5 | 0 | 0 | 0 | 0 | 0 | 0 | 0 | 0 | N | 16838 | Calmodulin [Crotalus horridus] |
| 276 | 1664 | JAG47255.1 | 117.13 | 18 | 0 | 0 | 0 | 0 | 0 | 18 | 0 | 0 | 0 | 0 | 0 | 0 | 0 | 0 |  |  |  |  |  | 2.5422E6 |  |  |  |  |  |  |  |  | 3 | 3 | 0 | 0 | 0 | 0 | 0 | 5 | 0 | 0 | 0 | 0 | 0 | 0 | 0 | 0 | N | 16838 | Calmodulin [Crotalus horridus] |
| 276 | 1665 | JAC96069.1 | 117.13 | 18 | 0 | 0 | 0 | 0 | 0 | 18 | 0 | 0 | 0 | 0 | 0 | 0 | 0 | 0 |  |  |  |  |  | 2.5422E6 |  |  |  |  |  |  |  |  | 3 | 3 | 0 | 0 | 0 | 0 | 0 | 5 | 0 | 0 | 0 | 0 | 0 | 0 | 0 | 0 | N | 16838 | Calmodulin [Hypsiglena sp. JMG-2014] |
| 276 | 1666 | JAG68499.1 | 117.13 | 18 | 0 | 0 | 0 | 0 | 0 | 18 | 0 | 0 | 0 | 0 | 0 | 0 | 0 | 0 |  |  |  |  |  | 2.5422E6 |  |  |  |  |  |  |  |  | 3 | 3 | 0 | 0 | 0 | 0 | 0 | 5 | 0 | 0 | 0 | 0 | 0 | 0 | 0 | 0 | N | 16838 | Calmodulin [Boiga irregularis] |
| 276 | 1667 | JAG47256.1 | 117.13 | 18 | 0 | 0 | 0 | 0 | 0 | 18 | 0 | 0 | 0 | 0 | 0 | 0 | 0 | 0 |  |  |  |  |  | 2.5422E6 |  |  |  |  |  |  |  |  | 3 | 3 | 0 | 0 | 0 | 0 | 0 | 5 | 0 | 0 | 0 | 0 | 0 | 0 | 0 | 0 | N | 16838 | Calmodulin [Crotalus horridus] |
| 276 | 1668 | JAC96068.1 | 117.13 | 18 | 0 | 0 | 0 | 0 | 0 | 18 | 0 | 0 | 0 | 0 | 0 | 0 | 0 | 0 |  |  |  |  |  | 2.5422E6 |  |  |  |  |  |  |  |  | 3 | 3 | 0 | 0 | 0 | 0 | 0 | 5 | 0 | 0 | 0 | 0 | 0 | 0 | 0 | 0 | N | 16838 | calmodulin 2 [Hypsiglena sp. JMG-2014] |
| 276 | 1669 | AFJ49580.1 | 117.13 | 18 | 0 | 0 | 0 | 0 | 0 | 18 | 0 | 0 | 0 | 0 | 0 | 0 | 0 | 0 |  |  |  |  |  | 2.5422E6 |  |  |  |  |  |  |  |  | 3 | 3 | 0 | 0 | 0 | 0 | 0 | 5 | 0 | 0 | 0 | 0 | 0 | 0 | 0 | 0 | N | 16838 | Calmodulin-4 [Crotalus adamanteus] |
| 276 | 1670 | JAG47254.1 | 117.13 | 18 | 0 | 0 | 0 | 0 | 0 | 18 | 0 | 0 | 0 | 0 | 0 | 0 | 0 | 0 |  |  |  |  |  | 2.5422E6 |  |  |  |  |  |  |  |  | 3 | 3 | 0 | 0 | 0 | 0 | 0 | 5 | 0 | 0 | 0 | 0 | 0 | 0 | 0 | 0 | N | 16838 | Calmodulin [Crotalus horridus] |
| 276 | 1671 | JAG68498.1 | 117.13 | 18 | 0 | 0 | 0 | 0 | 0 | 18 | 0 | 0 | 0 | 0 | 0 | 0 | 0 | 0 |  |  |  |  |  | 2.5422E6 |  |  |  |  |  |  |  |  | 3 | 3 | 0 | 0 | 0 | 0 | 0 | 5 | 0 | 0 | 0 | 0 | 0 | 0 | 0 | 0 | N | 16838 | Calmodulin [Boiga irregularis] |
| 276 | 1672 | JAG45514.1 | 117.13 | 18 | 0 | 0 | 0 | 0 | 0 | 18 | 0 | 0 | 0 | 0 | 0 | 0 | 0 | 0 |  |  |  |  |  | 2.5422E6 |  |  |  |  |  |  |  |  | 3 | 3 | 0 | 0 | 0 | 0 | 0 | 5 | 0 | 0 | 0 | 0 | 0 | 0 | 0 | 0 | N | 16838 | Calmodulin [Crotalus horridus] |
| 276 | 1673 | JAG45513.1 | 117.13 | 18 | 0 | 0 | 0 | 0 | 0 | 18 | 0 | 0 | 0 | 0 | 0 | 0 | 0 | 0 |  |  |  |  |  | 2.5422E6 |  |  |  |  |  |  |  |  | 3 | 3 | 0 | 0 | 0 | 0 | 0 | 5 | 0 | 0 | 0 | 0 | 0 | 0 | 0 | 0 | N | 16838 | Calmodulin [Crotalus horridus] |
| 276 | 1674 | JAG45515.1 | 117.13 | 18 | 0 | 0 | 0 | 0 | 0 | 18 | 0 | 0 | 0 | 0 | 0 | 0 | 0 | 0 |  |  |  |  |  | 2.5422E6 |  |  |  |  |  |  |  |  | 3 | 3 | 0 | 0 | 0 | 0 | 0 | 5 | 0 | 0 | 0 | 0 | 0 | 0 | 0 | 0 | N | 16838 | Calmodulin [Crotalus horridus] |
| 276 | 1675 | JAC96066.1 | 117.13 | 18 | 0 | 0 | 0 | 0 | 0 | 18 | 0 | 0 | 0 | 0 | 0 | 0 | 0 | 0 |  |  |  |  |  | 2.5422E6 |  |  |  |  |  |  |  |  | 3 | 3 | 0 | 0 | 0 | 0 | 0 | 5 | 0 | 0 | 0 | 0 | 0 | 0 | 0 | 0 | N | 16838 | Calmodulin [Hypsiglena sp. JMG-2014] |
| 276 | 1676 | AFJ49579.1 | 117.13 | 18 | 0 | 0 | 0 | 0 | 0 | 18 | 0 | 0 | 0 | 0 | 0 | 0 | 0 | 0 |  |  |  |  |  | 2.5422E6 |  |  |  |  |  |  |  |  | 3 | 3 | 0 | 0 | 0 | 0 | 0 | 5 | 0 | 0 | 0 | 0 | 0 | 0 | 0 | 0 | N | 16838 | Calmodulin [Crotalus adamanteus] |
| 276 | 1677 | JAC96064.1 | 117.13 | 18 | 0 | 0 | 0 | 0 | 0 | 18 | 0 | 0 | 0 | 0 | 0 | 0 | 0 | 0 |  |  |  |  |  | 2.5422E6 |  |  |  |  |  |  |  |  | 3 | 3 | 0 | 0 | 0 | 0 | 0 | 5 | 0 | 0 | 0 | 0 | 0 | 0 | 0 | 0 | N | 16838 | calmodulin [Hypsiglena sp. JMG-2014] |
| 276 | 1678 | JAC96067.1 | 117.13 | 18 | 0 | 0 | 0 | 0 | 0 | 18 | 0 | 0 | 0 | 0 | 0 | 0 | 0 | 0 |  |  |  |  |  | 2.5422E6 |  |  |  |  |  |  |  |  | 3 | 3 | 0 | 0 | 0 | 0 | 0 | 5 | 0 | 0 | 0 | 0 | 0 | 0 | 0 | 0 | N | 16838 | Calmodulin [Hypsiglena sp. JMG-2014] |
| 276 | 1679 | JAG68500.1 | 117.13 | 18 | 0 | 0 | 0 | 0 | 0 | 18 | 0 | 0 | 0 | 0 | 0 | 0 | 0 | 0 |  |  |  |  |  | 2.5422E6 |  |  |  |  |  |  |  |  | 3 | 3 | 0 | 0 | 0 | 0 | 0 | 5 | 0 | 0 | 0 | 0 | 0 | 0 | 0 | 0 | N | 16838 | Calmodulin [Boiga irregularis] |
| 276 | 1680 | JAB54567.1 | 117.13 | 18 | 0 | 0 | 0 | 0 | 0 | 18 | 0 | 0 | 0 | 0 | 0 | 0 | 0 | 0 |  |  |  |  |  | 2.5422E6 |  |  |  |  |  |  |  |  | 3 | 3 | 0 | 0 | 0 | 0 | 0 | 5 | 0 | 0 | 0 | 0 | 0 | 0 | 0 | 0 | N | 16838 | Calmodulin [Micrurus fulvius] |
| 276 | 1681 | JAB54564.1 | 117.13 | 18 | 0 | 0 | 0 | 0 | 0 | 18 | 0 | 0 | 0 | 0 | 0 | 0 | 0 | 0 |  |  |  |  |  | 2.5422E6 |  |  |  |  |  |  |  |  | 3 | 3 | 0 | 0 | 0 | 0 | 0 | 5 | 0 | 0 | 0 | 0 | 0 | 0 | 0 | 0 | N | 16838 | Calmodulin-4 [Micrurus fulvius] |
| 276 | 1682 | ETE70432.1 | 117.13 | 15 | 0 | 0 | 0 | 0 | 0 | 15 | 0 | 0 | 0 | 0 | 0 | 0 | 0 | 0 |  |  |  |  |  | 2.5422E6 |  |  |  |  |  |  |  |  | 3 | 3 | 0 | 0 | 0 | 0 | 0 | 5 | 0 | 0 | 0 | 0 | 0 | 0 | 0 | 0 | N | 19920 | Calmodulin, partial [Ophiophagus hannah] |
| 276 | 1683 | ETE66325.1 | 117.13 | 15 | 0 | 0 | 0 | 0 | 0 | 15 | 0 | 0 | 0 | 0 | 0 | 0 | 0 | 0 |  |  |  |  |  | 2.5422E6 |  |  |  |  |  |  |  |  | 3 | 3 | 0 | 0 | 0 | 0 | 0 | 5 | 0 | 0 | 0 | 0 | 0 | 0 | 0 | 0 | N | 20320 | Calmodulin, partial [Ophiophagus hannah] |
| 276 | 1684 | JAG45516.1 | 117.13 | 18 | 0 | 0 | 0 | 0 | 0 | 18 | 0 | 0 | 0 | 0 | 0 | 0 | 0 | 0 |  |  |  |  |  | 2.5422E6 |  |  |  |  |  |  |  |  | 3 | 3 | 0 | 0 | 0 | 0 | 0 | 5 | 0 | 0 | 0 | 0 | 0 | 0 | 0 | 0 | N | 16840 | Calmodulin [Crotalus horridus] |
| 276 | 1685 | JAA97543.1 | 117.13 | 18 | 0 | 0 | 0 | 0 | 0 | 18 | 0 | 0 | 0 | 0 | 0 | 0 | 0 | 0 |  |  |  |  |  | 2.5422E6 |  |  |  |  |  |  |  |  | 3 | 3 | 0 | 0 | 0 | 0 | 0 | 5 | 0 | 0 | 0 | 0 | 0 | 0 | 0 | 0 | N | 16840 | Calmodulin [Crotalus horridus] |
| 276 | 1686 | JAG47257.1 | 117.13 | 18 | 0 | 0 | 0 | 0 | 0 | 18 | 0 | 0 | 0 | 0 | 0 | 0 | 0 | 0 |  |  |  |  |  | 2.5422E6 |  |  |  |  |  |  |  |  | 3 | 3 | 0 | 0 | 0 | 0 | 0 | 5 | 0 | 0 | 0 | 0 | 0 | 0 | 0 | 0 | N | 16840 | Calmodulin [Crotalus horridus] |
| 276 | 1687 | AFJ49578.1 | 117.13 | 18 | 0 | 0 | 0 | 0 | 0 | 18 | 0 | 0 | 0 | 0 | 0 | 0 | 0 | 0 |  |  |  |  |  | 2.5422E6 |  |  |  |  |  |  |  |  | 3 | 3 | 0 | 0 | 0 | 0 | 0 | 5 | 0 | 0 | 0 | 0 | 0 | 0 | 0 | 0 | N | 16840 | Calmodulin [Crotalus adamanteus] |
| 247 | 1023 | S29299 | 114.93 | 33 | 0 | 0 | 0 | 33 | 0 | 0 | 0 | 0 | 0 | 0 | 0 | 0 | 0 | 0 |  |  |  | 6.2289E6 |  |  |  |  |  |  |  |  |  |  | 3 | 3 | 0 | 0 | 0 | 7 | 0 | 0 | 0 | 0 | 0 | 0 | 0 | 0 | 0 | 0 | Y | 15421 | phospholipase A2 (EC 3.1.1.4) - Russell's viper |
| 247 | 1024 | ACD43466.1 | 114.93 | 33 | 0 | 0 | 0 | 33 | 0 | 0 | 0 | 0 | 0 | 0 | 0 | 0 | 0 | 0 |  |  |  | 6.2289E6 |  |  |  |  |  |  |  |  |  |  | 3 | 3 | 0 | 0 | 0 | 7 | 0 | 0 | 0 | 0 | 0 | 0 | 0 | 0 | 0 | 0 | Y | 15421 | acidic phospholipase A2 [Daboia siamensis] |
| 247 | 1025 | AAZ53184.1 | 114.93 | 33 | 0 | 0 | 0 | 33 | 0 | 0 | 0 | 0 | 0 | 0 | 0 | 0 | 0 | 0 |  |  |  | 6.2289E6 |  |  |  |  |  |  |  |  |  |  | 3 | 3 | 0 | 0 | 0 | 7 | 0 | 0 | 0 | 0 | 0 | 0 | 0 | 0 | 0 | 0 | Y | 15421 | acidic phospholipase A2 [Daboia russelii limitis] |
| 247 | 1026 | ACD43468.1 | 114.93 | 33 | 0 | 0 | 0 | 33 | 0 | 0 | 0 | 0 | 0 | 0 | 0 | 0 | 0 | 0 |  |  |  | 6.2289E6 |  |  |  |  |  |  |  |  |  |  | 3 | 3 | 0 | 0 | 0 | 7 | 0 | 0 | 0 | 0 | 0 | 0 | 0 | 0 | 0 | 0 | Y | 15421 | acidic phospholipase A2 [Daboia siamensis] |
| 247 | 1027 | P31100.1 | 114.93 | 33 | 0 | 0 | 0 | 33 | 0 | 0 | 0 | 0 | 0 | 0 | 0 | 0 | 0 | 0 |  |  |  | 6.2289E6 |  |  |  |  |  |  |  |  |  |  | 3 | 3 | 0 | 0 | 0 | 7 | 0 | 0 | 0 | 0 | 0 | 0 | 0 | 0 | 0 | 0 | Y | 15421 | RecName: Full=Acidic phospholipase A2 RV-7; Short=svPLA2; AltName: Full=F7; AltName: Full=Phosphatidylcholine 2-acylhydrolase; AltName: Full=Phospholipase A2 inhibitor; AltName: Full=S4; AltName: Full=Viperotoxin F; AltName: Full=... |
| 247 | 1028 | CAA48457.1 | 114.93 | 33 | 0 | 0 | 0 | 33 | 0 | 0 | 0 | 0 | 0 | 0 | 0 | 0 | 0 | 0 |  |  |  | 6.2289E6 |  |  |  |  |  |  |  |  |  |  | 3 | 3 | 0 | 0 | 0 | 7 | 0 | 0 | 0 | 0 | 0 | 0 | 0 | 0 | 0 | 0 | Y | 15421 | phospholipase a2 [Daboia russelii] |
| 239 | 827 | XP\_026532545.1 | 113.78 | 7 | 0 | 1 | 5 | 0 | 0 | 0 | 0 | 0 | 0 | 0 | 0 | 0 | 0 | 1 |  |  | 4.5399E6 |  |  |  |  |  |  |  |  |  |  |  | 3 | 1 | 0 | 1 | 3 | 0 | 0 | 0 | 0 | 0 | 0 | 0 | 0 | 0 | 0 | 3 | Y | 80178 | C4b-binding protein alpha chain-like [Notechis scutatus] |
| 185 | 1020 | XP\_026560089.1 | 112.99 | 20 | 0 | 0 | 0 | 0 | 0 | 0 | 0 | 0 | 0 | 0 | 0 | 20 | 20 | 0 |  |  |  |  |  |  |  |  |  |  |  | 9.6271E6 | 3.4875E7 |  | 3 | 3 | 0 | 0 | 0 | 0 | 0 | 0 | 0 | 0 | 0 | 0 | 0 | 9 | 10 | 0 | Y | 22535 | platelet-derived growth factor subunit A isoform X3 [Pseudonaja textilis] |
| 185 | 1021 | LAB42085.1 | 112.99 | 17 | 0 | 0 | 0 | 0 | 0 | 0 | 0 | 0 | 0 | 0 | 0 | 17 | 17 | 0 |  |  |  |  |  |  |  |  |  |  |  | 9.6271E6 | 3.4875E7 |  | 3 | 3 | 0 | 0 | 0 | 0 | 0 | 0 | 0 | 0 | 0 | 0 | 0 | 9 | 10 | 0 | Y | 25772 | hypothetical protein, partial [Micrurus spixii] |
| 185 | 1031 | XP\_026560087.1 | 112.99 | 18 | 0 | 0 | 0 | 0 | 0 | 0 | 0 | 0 | 0 | 0 | 0 | 18 | 18 | 0 |  |  |  |  |  |  |  |  |  |  |  | 9.6271E6 | 3.4875E7 |  | 3 | 3 | 0 | 0 | 0 | 0 | 0 | 0 | 0 | 0 | 0 | 0 | 0 | 9 | 10 | 0 | Y | 24924 | platelet-derived growth factor subunit A isoform X1 [Pseudonaja textilis] |
| 185 | 1032 | XP\_026560088.1 | 112.99 | 18 | 0 | 0 | 0 | 0 | 0 | 0 | 0 | 0 | 0 | 0 | 0 | 18 | 18 | 0 |  |  |  |  |  |  |  |  |  |  |  | 9.6271E6 | 3.4875E7 |  | 3 | 3 | 0 | 0 | 0 | 0 | 0 | 0 | 0 | 0 | 0 | 0 | 0 | 9 | 10 | 0 | Y | 24698 | platelet-derived growth factor subunit A isoform X2 [Pseudonaja textilis] |
| 185 | 1035 | LAB42082.1 | 112.99 | 14 | 0 | 0 | 0 | 0 | 0 | 0 | 0 | 0 | 0 | 0 | 0 | 14 | 14 | 0 |  |  |  |  |  |  |  |  |  |  |  | 9.6271E6 | 3.4875E7 |  | 3 | 3 | 0 | 0 | 0 | 0 | 0 | 0 | 0 | 0 | 0 | 0 | 0 | 9 | 10 | 0 | Y | 31036 | hypothetical protein, partial [Micrurus spixii] |
| 185 | 1036 | LAA51478.1 | 112.99 | 33 | 0 | 0 | 0 | 0 | 0 | 0 | 0 | 0 | 0 | 0 | 0 | 33 | 33 | 0 |  |  |  |  |  |  |  |  |  |  |  | 9.6271E6 | 3.4875E7 |  | 3 | 3 | 0 | 0 | 0 | 0 | 0 | 0 | 0 | 0 | 0 | 0 | 0 | 9 | 10 | 0 | Y | 13713 | hypothetical protein, partial [Micrurus corallinus] |
| 185 | 1038 | XP\_034286468.1 | 112.99 | 25 | 0 | 0 | 0 | 0 | 0 | 0 | 0 | 0 | 0 | 0 | 0 | 25 | 25 | 0 |  |  |  |  |  |  |  |  |  |  |  | 9.6271E6 | 3.4875E7 |  | 3 | 3 | 0 | 0 | 0 | 0 | 0 | 0 | 0 | 0 | 0 | 0 | 0 | 9 | 10 | 0 | Y | 18298 | platelet-derived growth factor subunit A, partial [Pantherophis guttatus] |
| 185 | 1047 | JAB53674.1 | 112.99 | 16 | 0 | 0 | 0 | 0 | 0 | 0 | 0 | 0 | 0 | 0 | 0 | 16 | 16 | 0 |  |  |  |  |  |  |  |  |  |  |  | 9.6271E6 | 3.4875E7 |  | 3 | 3 | 0 | 0 | 0 | 0 | 0 | 0 | 0 | 0 | 0 | 0 | 0 | 9 | 10 | 0 | Y | 27667 | platelet-derived growth factor A chain long form [Micrurus fulvius] |
| 185 | 1048 | XP\_026540879.1 | 112.99 | 18 | 0 | 0 | 0 | 0 | 0 | 0 | 0 | 0 | 0 | 0 | 0 | 18 | 18 | 0 |  |  |  |  |  |  |  |  |  |  |  | 9.6271E6 | 3.4875E7 |  | 3 | 3 | 0 | 0 | 0 | 0 | 0 | 0 | 0 | 0 | 0 | 0 | 0 | 9 | 10 | 0 | Y | 25552 | platelet-derived growth factor subunit A [Notechis scutatus] |
| 185 | 1055 | LAA51474.1 | 112.99 | 33 | 0 | 0 | 0 | 0 | 0 | 0 | 0 | 0 | 0 | 0 | 0 | 33 | 33 | 0 |  |  |  |  |  |  |  |  |  |  |  | 9.6271E6 | 3.4875E7 |  | 3 | 3 | 0 | 0 | 0 | 0 | 0 | 0 | 0 | 0 | 0 | 0 | 0 | 9 | 10 | 0 | Y | 13798 | hypothetical protein, partial [Micrurus corallinus] |
| 185 | 1115 | AFJ50955.1 | 112.99 | 20 | 0 | 0 | 0 | 0 | 0 | 0 | 0 | 0 | 0 | 0 | 0 | 20 | 20 | 0 |  |  |  |  |  |  |  |  |  |  |  | 9.6271E6 | 3.4875E7 |  | 3 | 3 | 0 | 0 | 0 | 0 | 0 | 0 | 0 | 0 | 0 | 0 | 0 | 9 | 10 | 0 | Y | 22576 | Platelet-derived growth factor subunit A preproprotein [Crotalus adamanteus] |
| 185 | 1116 | JAI12034.1 | 112.99 | 20 | 0 | 0 | 0 | 0 | 0 | 0 | 0 | 0 | 0 | 0 | 0 | 20 | 20 | 0 |  |  |  |  |  |  |  |  |  |  |  | 9.6271E6 | 3.4875E7 |  | 3 | 3 | 0 | 0 | 0 | 0 | 0 | 0 | 0 | 0 | 0 | 0 | 0 | 9 | 10 | 0 | Y | 22576 | platelet-derived growth factor subunit A [Crotalus adamanteus] |
| 185 | 1117 | XP\_015676418.1 | 112.99 | 20 | 0 | 0 | 0 | 0 | 0 | 0 | 0 | 0 | 0 | 0 | 0 | 20 | 20 | 0 |  |  |  |  |  |  |  |  |  |  |  | 9.6271E6 | 3.4875E7 |  | 3 | 3 | 0 | 0 | 0 | 0 | 0 | 0 | 0 | 0 | 0 | 0 | 0 | 9 | 10 | 0 | Y | 22562 | platelet-derived growth factor subunit A [Protobothrops mucrosquamatus] |
| 185 | 1122 | XP\_007426296.1 | 112.99 | 20 | 0 | 0 | 0 | 0 | 0 | 0 | 0 | 0 | 0 | 0 | 0 | 20 | 20 | 0 |  |  |  |  |  |  |  |  |  |  |  | 9.6271E6 | 3.4875E7 |  | 3 | 3 | 0 | 0 | 0 | 0 | 0 | 0 | 0 | 0 | 0 | 0 | 0 | 9 | 10 | 0 | Y | 22561 | platelet-derived growth factor subunit A isoform X3 [Python bivittatus] |
| 185 | 1123 | XP\_025021193.1 | 112.99 | 19 | 0 | 0 | 0 | 0 | 0 | 0 | 0 | 0 | 0 | 0 | 0 | 19 | 19 | 0 |  |  |  |  |  |  |  |  |  |  |  | 9.6271E6 | 3.4875E7 |  | 3 | 3 | 0 | 0 | 0 | 0 | 0 | 0 | 0 | 0 | 0 | 0 | 0 | 9 | 10 | 0 | Y | 23303 | platelet-derived growth factor subunit A isoform X2 [Python bivittatus] |
| 185 | 1124 | XP\_015743230.1 | 112.99 | 18 | 0 | 0 | 0 | 0 | 0 | 0 | 0 | 0 | 0 | 0 | 0 | 18 | 18 | 0 |  |  |  |  |  |  |  |  |  |  |  | 9.6271E6 | 3.4875E7 |  | 3 | 3 | 0 | 0 | 0 | 0 | 0 | 0 | 0 | 0 | 0 | 0 | 0 | 9 | 10 | 0 | Y | 24934 | platelet-derived growth factor subunit A isoform X1 [Python bivittatus] |
| 301 | 1088 | ETE62885.1 | 111.51 | 5 | 0 | 0 | 0 | 0 | 0 | 0 | 0 | 0 | 0 | 0 | 0 | 0 | 0 | 5 |  |  |  |  |  |  |  |  |  |  |  |  |  | 6.8985E6 | 1 | 1 | 0 | 0 | 0 | 0 | 0 | 0 | 0 | 0 | 0 | 0 | 0 | 0 | 0 | 1 | N | 38690 | Cathepsin L1 [Ophiophagus hannah] |
| 301 | 1089 | XP\_026530368.1 | 111.51 | 5 | 0 | 0 | 0 | 0 | 0 | 0 | 0 | 0 | 0 | 0 | 0 | 0 | 0 | 5 |  |  |  |  |  |  |  |  |  |  |  |  |  | 6.8985E6 | 1 | 1 | 0 | 0 | 0 | 0 | 0 | 0 | 0 | 0 | 0 | 0 | 0 | 0 | 0 | 1 | N | 38731 | cathepsin L1 [Notechis scutatus] |
| 301 | 1090 | XP\_026530376.1 | 111.51 | 5 | 0 | 0 | 0 | 0 | 0 | 0 | 0 | 0 | 0 | 0 | 0 | 0 | 0 | 5 |  |  |  |  |  |  |  |  |  |  |  |  |  | 6.8985E6 | 1 | 1 | 0 | 0 | 0 | 0 | 0 | 0 | 0 | 0 | 0 | 0 | 0 | 0 | 0 | 1 | N | 38731 | cathepsin L1 [Notechis scutatus] |
| 214 | 758 | XP\_032067737.1 | 110.09 | 13 | 0 | 0 | 0 | 0 | 13 | 3 | 0 | 0 | 0 | 0 | 0 | 0 | 0 | 0 |  |  |  |  | 1.2651E6 |  |  |  |  |  |  |  |  |  | 4 | 1 | 0 | 0 | 0 | 0 | 8 | 2 | 0 | 0 | 0 | 0 | 0 | 0 | 0 | 0 | N | 40510 | deoxyribonuclease-2-alpha [Thamnophis elegans] |
| 156 | 1744 | P00601.1 | 110.09 | 18 | 0 | 0 | 8 | 8 | 8 | 8 | 0 | 0 | 0 | 8 | 8 | 8 | 18 | 8 |  |  | 0 | 2.0387E6 | 1.6797E6 | 5.2055E6 |  |  |  | 6.4055E5 | 6.5361E6 | 4.8112E7 | 9.7922E7 | 6.6543E7 | 2 | 1 | 0 | 0 | 1 | 2 | 1 | 2 | 0 | 0 | 0 | 2 | 3 | 4 | 11 | 4 | Y | 13360 | RecName: Full=Acidic phospholipase A2 DE-III; Short=svPLA2; AltName: Full=Phosphatidylcholine 2-acylhydrolase |
| 156 | 1760 | P00600.1 | 110.09 | 18 | 0 | 0 | 8 | 8 | 8 | 8 | 0 | 0 | 0 | 8 | 8 | 8 | 18 | 8 |  |  | 0 | 2.0387E6 | 1.6797E6 | 5.2055E6 |  |  |  | 6.4055E5 | 6.5361E6 | 4.8112E7 | 9.7922E7 | 6.6543E7 | 2 | 1 | 0 | 0 | 1 | 2 | 1 | 2 | 0 | 0 | 0 | 2 | 3 | 4 | 11 | 4 | Y | 13427 | RecName: Full=Acidic phospholipase A2 DE-II; Short=svPLA2; AltName: Full=Phosphatidylcholine 2-acylhydrolase |
| 201 | 1029 | LAB32499.1 | 109.82 | 13 | 0 | 0 | 0 | 0 | 13 | 5 | 0 | 0 | 0 | 0 | 0 | 0 | 0 | 0 |  |  |  |  | 1.4306E6 |  |  |  |  |  |  |  |  |  | 3 | 1 | 0 | 0 | 0 | 0 | 8 | 5 | 0 | 0 | 0 | 0 | 0 | 0 | 0 | 0 | N | 27404 | hypothetical protein, partial [Micrurus spixii] |
| 221 | 965 | XP\_013928902.1 | 107.91 | 5 | 0 | 0 | 0 | 0 | 0 | 0 | 0 | 0 | 2 | 1 | 3 | 3 | 0 | 0 |  |  |  |  |  |  |  |  | 2.1671E6 |  |  |  |  |  | 3 | 1 | 0 | 0 | 0 | 0 | 0 | 0 | 0 | 0 | 1 | 1 | 4 | 2 | 0 | 0 | Y | 118178 | PREDICTED: inactive serine/threonine-protein kinase TEX14-like, partial [Thamnophis sirtalis] |
| 236 | 745 | JAG46496.1 | 106.92 | 11 | 0 | 0 | 0 | 11 | 0 | 0 | 0 | 0 | 0 | 0 | 0 | 0 | 0 | 0 |  |  |  | 1.9676E6 |  |  |  |  |  |  |  |  |  |  | 4 | 1 | 0 | 0 | 0 | 6 | 0 | 0 | 0 | 0 | 0 | 0 | 0 | 0 | 0 | 0 | Y | 49179 | legumain preproprotein [Crotalus horridus] |
| 207 | 1735 | ETE61477.1 | 104.08 | 35 | 0 | 0 | 35 | 35 | 0 | 0 | 0 | 0 | 0 | 0 | 0 | 0 | 0 | 0 |  |  | 2.9612E7 | 8.4681E6 |  |  |  |  |  |  |  |  |  |  | 2 | 1 | 0 | 0 | 6 | 8 | 0 | 0 | 0 | 0 | 0 | 0 | 0 | 0 | 0 | 0 | Y | 10789 | Hepatocyte growth factor activator, partial [Ophiophagus hannah] |
| 227 | 1184 | Q7LZI1.1 | 103.52 | 14 | 0 | 0 | 8 | 14 | 8 | 0 | 0 | 0 | 0 | 0 | 0 | 0 | 0 | 0 |  |  | 2.4516E6 | 2.4281E6 | 5.4922E5 |  |  |  |  |  |  |  |  |  | 2 | 2 | 0 | 0 | 2 | 3 | 1 | 0 | 0 | 0 | 0 | 0 | 0 | 0 | 0 | 0 | N | 20452 | RecName: Full=Phospholipase A2 inhibitor 31 kDa subunit; AltName: Full=gamma-PLI |
| 227 | 1185 | AAB32582.1 | 103.52 | 14 | 0 | 0 | 8 | 14 | 8 | 0 | 0 | 0 | 0 | 0 | 0 | 0 | 0 | 0 |  |  | 2.4516E6 | 2.4281E6 | 5.4922E5 |  |  |  |  |  |  |  |  |  | 2 | 2 | 0 | 0 | 2 | 3 | 1 | 0 | 0 | 0 | 0 | 0 | 0 | 0 | 0 | 0 | N | 20452 | phospholipase A2 31 kda subunit, PLA2 31 kda subunit=urokinase-type plasminogen activator receptor homolog [Naja naja kaouthia=Thailand cobras, blood, Peptide, 188 aa] |
| 255 | 1459 | ETE59007.1 | 100.89 | 33 | 0 | 0 | 0 | 33 | 0 | 0 | 0 | 0 | 0 | 0 | 0 | 0 | 0 | 0 |  |  |  | 3.8866E7 |  |  |  |  |  |  |  |  |  |  | 2 | 2 | 0 | 0 | 0 | 5 | 0 | 0 | 0 | 0 | 0 | 0 | 0 | 0 | 0 | 0 | N | 9348 | hypothetical protein L345\_15265, partial [Ophiophagus hannah] |
| 230 | 1360 | XP\_026572592.1 | 99.11 | 2 | 0 | 0 | 0 | 0 | 0 | 0 | 0 | 0 | 2 | 2 | 0 | 0 | 0 | 0 |  |  |  |  |  |  |  |  | 5.5253E6 | 5.9121E5 |  |  |  |  | 1 | 1 | 0 | 0 | 0 | 0 | 0 | 0 | 0 | 0 | 4 | 1 | 0 | 0 | 0 | 0 | Y | 100735 | low-density lipoprotein receptor [Pseudonaja textilis] |
| 230 | 1386 | XP\_015671074.1 | 99.11 | 2 | 0 | 0 | 0 | 0 | 0 | 0 | 0 | 0 | 2 | 2 | 0 | 0 | 0 | 0 |  |  |  |  |  |  |  |  | 5.5253E6 | 5.9121E5 |  |  |  |  | 1 | 1 | 0 | 0 | 0 | 0 | 0 | 0 | 0 | 0 | 4 | 1 | 0 | 0 | 0 | 0 | Y | 96598 | low-density lipoprotein receptor [Protobothrops mucrosquamatus] |
| 230 | 1387 | JAA96697.1 | 99.11 | 2 | 0 | 0 | 0 | 0 | 0 | 0 | 0 | 0 | 2 | 2 | 0 | 0 | 0 | 0 |  |  |  |  |  |  |  |  | 5.5253E6 | 5.9121E5 |  |  |  |  | 1 | 1 | 0 | 0 | 0 | 0 | 0 | 0 | 0 | 0 | 4 | 1 | 0 | 0 | 0 | 0 | Y | 97001 | Low-density lipoprotein receptor 1-like protein [Crotalus horridus] |
| 230 | 1388 | JAG44208.1 | 99.11 | 2 | 0 | 0 | 0 | 0 | 0 | 0 | 0 | 0 | 2 | 2 | 0 | 0 | 0 | 0 |  |  |  |  |  |  |  |  | 5.5253E6 | 5.9121E5 |  |  |  |  | 1 | 1 | 0 | 0 | 0 | 0 | 0 | 0 | 0 | 0 | 4 | 1 | 0 | 0 | 0 | 0 | Y | 97001 | Low-density lipoprotein receptor 1-like protein [Crotalus horridus] |
| 230 | 1389 | JAG46497.1 | 99.11 | 2 | 0 | 0 | 0 | 0 | 0 | 0 | 0 | 0 | 2 | 2 | 0 | 0 | 0 | 0 |  |  |  |  |  |  |  |  | 5.5253E6 | 5.9121E5 |  |  |  |  | 1 | 1 | 0 | 0 | 0 | 0 | 0 | 0 | 0 | 0 | 4 | 1 | 0 | 0 | 0 | 0 | Y | 97001 | Low-density lipoprotein receptor 1-like protein [Crotalus horridus] |
| 230 | 1746 | XP\_026548351.1 | 99.11 | 9 | 0 | 0 | 0 | 0 | 0 | 0 | 0 | 0 | 9 | 9 | 0 | 0 | 0 | 0 |  |  |  |  |  |  |  |  | 5.5253E6 | 5.9121E5 |  |  |  |  | 1 | 1 | 0 | 0 | 0 | 0 | 0 | 0 | 0 | 0 | 4 | 1 | 0 | 0 | 0 | 0 | Y | 24980 | low-density lipoprotein receptor 1-like, partial [Notechis scutatus] |
| 243 | 2309 | JAA75025.1 | 98.85 | 13 | 0 | 0 | 0 | 0 | 0 | 0 | 0 | 0 | 0 | 0 | 0 | 13 | 8 | 0 |  |  |  |  |  |  |  |  |  |  |  | 1.8335E6 |  |  | 2 | 1 | 0 | 0 | 0 | 0 | 0 | 0 | 0 | 0 | 0 | 0 | 0 | 1 | 6 | 0 | Y | 16595 | PLA2-Sut-8 [Suta fasciata] |
| 243 | 4866 | P10117.1 | 98.85 | 17 | 0 | 0 | 0 | 0 | 0 | 0 | 0 | 0 | 0 | 0 | 0 | 17 | 10 | 0 |  |  |  |  |  |  |  |  |  |  |  | 1.8335E6 |  |  | 2 | 1 | 0 | 0 | 0 | 0 | 0 | 0 | 0 | 0 | 0 | 0 | 0 | 1 | 6 | 0 | Y | 13024 | RecName: Full=Basic phospholipase A2 homolog 1; Short=svPLA2 homolog; AltName: Full=Phospholipase A2 homolog I; Short=PLH-I |
| 200 | 1092 | AAZ75607.1 | 98.55 | 17 | 0 | 0 | 0 | 17 | 7 | 7 | 0 | 0 | 0 | 0 | 0 | 0 | 0 | 0 |  |  |  | 2.1128E6 |  |  |  |  |  |  |  |  |  |  | 3 | 1 | 0 | 0 | 0 | 10 | 3 | 1 | 0 | 0 | 0 | 0 | 0 | 0 | 0 | 0 | Y | 26648 | CRISP-TRI1, partial [Trimorphodon biscutatus] |
| 200 | 1093 | Q2XXP4.1 | 98.55 | 17 | 0 | 0 | 0 | 17 | 7 | 7 | 0 | 0 | 0 | 0 | 0 | 0 | 0 | 0 |  |  |  | 2.1128E6 |  |  |  |  |  |  |  |  |  |  | 3 | 1 | 0 | 0 | 0 | 10 | 3 | 1 | 0 | 0 | 0 | 0 | 0 | 0 | 0 | 0 | Y | 26648 | RecName: Full=Cysteine-rich venom protein TRI1; Short=CRVP; AltName: Full=Cysteine-rich secretory protein TRI1; Short=CRISP-TRI1; Flags: Precursor |
| 229 | 5330 | P01415.1 | 96.84 | 41 | 0 | 0 | 0 | 0 | 0 | 0 | 0 | 0 | 0 | 30 | 0 | 41 | 0 | 0 |  |  |  |  |  |  |  |  |  | 6.7771E7 |  | 1.9791E6 |  |  | 3 | 3 | 0 | 0 | 0 | 0 | 0 | 0 | 0 | 0 | 0 | 1 | 0 | 2 | 0 | 0 | Y | 7033 | RecName: Full=Weak toxin CM-2 |
| 261 | 846 | ETE59734.1 | 95.88 | 10 | 0 | 0 | 0 | 0 | 0 | 10 | 0 | 0 | 0 | 0 | 0 | 0 | 0 | 0 |  |  |  |  |  | 1.8063E6 |  |  |  |  |  |  |  |  | 3 | 3 | 0 | 0 | 0 | 0 | 0 | 4 | 0 | 0 | 0 | 0 | 0 | 0 | 0 | 0 | Y | 46615 | Endoplasmic reticulum resident protein 44 [Ophiophagus hannah] |
| 261 | 847 | JAI10032.1 | 95.88 | 10 | 0 | 0 | 0 | 0 | 0 | 10 | 0 | 0 | 0 | 0 | 0 | 0 | 0 | 0 |  |  |  |  |  | 1.8063E6 |  |  |  |  |  |  |  |  | 3 | 3 | 0 | 0 | 0 | 0 | 0 | 4 | 0 | 0 | 0 | 0 | 0 | 0 | 0 | 0 | Y | 46721 | Endoplasmic reticulum resident protein 44 [Micrurus fulvius] |
| 261 | 848 | JAB54269.1 | 95.88 | 10 | 0 | 0 | 0 | 0 | 0 | 10 | 0 | 0 | 0 | 0 | 0 | 0 | 0 | 0 |  |  |  |  |  | 1.8063E6 |  |  |  |  |  |  |  |  | 3 | 3 | 0 | 0 | 0 | 0 | 0 | 4 | 0 | 0 | 0 | 0 | 0 | 0 | 0 | 0 | Y | 46721 | Endoplasmic reticulum resident protein 44 [Micrurus fulvius] |
| 261 | 867 | JAG68039.1 | 95.88 | 10 | 0 | 0 | 0 | 0 | 0 | 10 | 0 | 0 | 0 | 0 | 0 | 0 | 0 | 0 |  |  |  |  |  | 1.8063E6 |  |  |  |  |  |  |  |  | 3 | 3 | 0 | 0 | 0 | 0 | 0 | 4 | 0 | 0 | 0 | 0 | 0 | 0 | 0 | 0 | Y | 46584 | Endoplasmic reticulum resident protein 44 [Boiga irregularis] |
| 261 | 871 | JAG68042.1 | 95.88 | 10 | 0 | 0 | 0 | 0 | 0 | 10 | 0 | 0 | 0 | 0 | 0 | 0 | 0 | 0 |  |  |  |  |  | 1.8063E6 |  |  |  |  |  |  |  |  | 3 | 3 | 0 | 0 | 0 | 0 | 0 | 4 | 0 | 0 | 0 | 0 | 0 | 0 | 0 | 0 | Y | 47634 | Endoplasmic reticulum resident protein 44 [Boiga irregularis] |
| 261 | 877 | XP\_034272274.1 | 95.88 | 10 | 0 | 0 | 0 | 0 | 0 | 10 | 0 | 0 | 0 | 0 | 0 | 0 | 0 | 0 |  |  |  |  |  | 1.8063E6 |  |  |  |  |  |  |  |  | 3 | 3 | 0 | 0 | 0 | 0 | 0 | 4 | 0 | 0 | 0 | 0 | 0 | 0 | 0 | 0 | Y | 46557 | endoplasmic reticulum resident protein 44 isoform X1 [Pantherophis guttatus] |
| 261 | 878 | XP\_032091607.1 | 95.88 | 10 | 0 | 0 | 0 | 0 | 0 | 10 | 0 | 0 | 0 | 0 | 0 | 0 | 0 | 0 |  |  |  |  |  | 1.8063E6 |  |  |  |  |  |  |  |  | 3 | 3 | 0 | 0 | 0 | 0 | 0 | 4 | 0 | 0 | 0 | 0 | 0 | 0 | 0 | 0 | Y | 46539 | endoplasmic reticulum resident protein 44 isoform X2 [Thamnophis elegans] |
| 261 | 879 | XP\_013907890.1 | 95.88 | 10 | 0 | 0 | 0 | 0 | 0 | 10 | 0 | 0 | 0 | 0 | 0 | 0 | 0 | 0 |  |  |  |  |  | 1.8063E6 |  |  |  |  |  |  |  |  | 3 | 3 | 0 | 0 | 0 | 0 | 0 | 4 | 0 | 0 | 0 | 0 | 0 | 0 | 0 | 0 | Y | 46539 | PREDICTED: endoplasmic reticulum resident protein 44 [Thamnophis sirtalis] |
| 261 | 884 | JAC95662.1 | 95.88 | 10 | 0 | 0 | 0 | 0 | 0 | 10 | 0 | 0 | 0 | 0 | 0 | 0 | 0 | 0 |  |  |  |  |  | 1.8063E6 |  |  |  |  |  |  |  |  | 3 | 3 | 0 | 0 | 0 | 0 | 0 | 4 | 0 | 0 | 0 | 0 | 0 | 0 | 0 | 0 | Y | 46496 | Endoplasmic reticulum resident protein 44 [Hypsiglena sp. JMG-2014] |
| 266 | 1604 | JAB53957.1 | 95.73 | 16 | 0 | 0 | 0 | 16 | 0 | 0 | 0 | 0 | 0 | 0 | 0 | 0 | 0 | 0 |  |  |  | 6.7615E6 |  |  |  |  |  |  |  |  |  |  | 2 | 2 | 0 | 0 | 0 | 3 | 0 | 0 | 0 | 0 | 0 | 0 | 0 | 0 | 0 | 0 | N | 24793 | hypothetical protein LOC100566677 [Micrurus fulvius] |
| 266 | 1860 | ETE68407.1 | 95.73 | 17 | 0 | 0 | 0 | 17 | 0 | 0 | 0 | 0 | 0 | 0 | 0 | 0 | 0 | 0 |  |  |  | 6.7615E6 |  |  |  |  |  |  |  |  |  |  | 2 | 2 | 0 | 0 | 0 | 3 | 0 | 0 | 0 | 0 | 0 | 0 | 0 | 0 | 0 | 0 | N | 23297 | Artemin, partial [Ophiophagus hannah] |
| 248 | 876 | P18964.1 | 95.59 | 19 | 0 | 0 | 6 | 19 | 0 | 0 | 0 | 0 | 0 | 0 | 0 | 0 | 0 | 0 |  |  | 5.5226E5 | 4.2569E6 |  |  |  |  |  |  |  |  |  |  | 3 | 3 | 0 | 0 | 1 | 5 | 0 | 0 | 0 | 0 | 0 | 0 | 0 | 0 | 0 | 0 | Y | 26182 | RecName: Full=Factor V activator RVV-V alpha; AltName: Full=Russel's viper venom FV activator alpha; Short=RVV-V alpha; AltName: Full=Snake venom serine protease; Short=SVSP |
| 209 | 721 | AAB22477.1 | 95.56 | 5 | 0 | 0 | 0 | 5 | 3 | 3 | 0 | 0 | 0 | 0 | 0 | 0 | 0 | 0 |  |  |  | 2.532E6 | 1.6707E5 | 2.6478E5 |  |  |  |  |  |  |  |  | 2 | 2 | 0 | 0 | 0 | 4 | 2 | 1 | 0 | 0 | 0 | 0 | 0 | 0 | 0 | 0 | Y | 47975 | coagulation factor X activating enzyme heavy chain, RVV-X-heavy chain=metalloproteinase with disintegrin (platelet aggregation inhibitor)-like and C-type lectin-like domains [Vipera russelli=Russell's viper, Siamensis, venom, Peptide, 429 aa] |
| 209 | 725 | AAZ39881.1 | 95.56 | 4 | 0 | 0 | 0 | 4 | 2 | 2 | 0 | 0 | 0 | 0 | 0 | 0 | 0 | 0 |  |  |  | 2.532E6 | 1.6707E5 | 2.6478E5 |  |  |  |  |  |  |  |  | 2 | 2 | 0 | 0 | 0 | 4 | 2 | 1 | 0 | 0 | 0 | 0 | 0 | 0 | 0 | 0 | Y | 69648 | RVV-X-heavy chain [Daboia russelii] |
| 209 | 726 | Q7LZ61.2 | 95.56 | 4 | 0 | 0 | 0 | 4 | 2 | 2 | 0 | 0 | 0 | 0 | 0 | 0 | 0 | 0 |  |  |  | 2.532E6 | 1.6707E5 | 2.6478E5 |  |  |  |  |  |  |  |  | 2 | 2 | 0 | 0 | 0 | 4 | 2 | 1 | 0 | 0 | 0 | 0 | 0 | 0 | 0 | 0 | Y | 69648 | RecName: Full=Coagulation factor X-activating enzyme heavy chain; AltName: Full=Coagulation factor X-activating enzyme chain alpha; AltName: Full=RVV-X heavy chain; AltName: Full=Russellysin; AltName: Full=Snake venom metalloprote... |
| 263 | 1142 | LAA21878.1 | 95.10 | 2 | 0 | 0 | 0 | 2 | 0 | 0 | 0 | 0 | 0 | 0 | 0 | 0 | 0 | 0 |  |  |  | 7.586E5 |  |  |  |  |  |  |  |  |  |  | 1 | 1 | 0 | 0 | 0 | 1 | 0 | 0 | 0 | 0 | 0 | 0 | 0 | 0 | 0 | 0 | N | 76625 | hypothetical protein [Micrurus lemniscatus carvalhoi] |
| 263 | 1148 | XP\_026557455.1 | 95.10 | 2 | 0 | 0 | 0 | 2 | 0 | 0 | 0 | 0 | 0 | 0 | 0 | 0 | 0 | 0 |  |  |  | 7.586E5 |  |  |  |  |  |  |  |  |  |  | 1 | 1 | 0 | 0 | 0 | 1 | 0 | 0 | 0 | 0 | 0 | 0 | 0 | 0 | 0 | 0 | N | 77840 | serotransferrin [Pseudonaja textilis] |
| 263 | 1164 | XP\_026547769.1 | 95.10 | 2 | 0 | 0 | 0 | 2 | 0 | 0 | 0 | 0 | 0 | 0 | 0 | 0 | 0 | 0 |  |  |  | 7.586E5 |  |  |  |  |  |  |  |  |  |  | 1 | 1 | 0 | 0 | 0 | 1 | 0 | 0 | 0 | 0 | 0 | 0 | 0 | 0 | 0 | 0 | N | 78354 | serotransferrin [Notechis scutatus] |
| 292 | 1532 | S68801 | 94.99 | 29 | 0 | 0 | 0 | 0 | 0 | 29 | 0 | 0 | 0 | 0 | 0 | 0 | 0 | 0 |  |  |  |  |  | 1.0592E7 |  |  |  |  |  |  |  |  | 2 | 1 | 0 | 0 | 0 | 0 | 0 | 4 | 0 | 0 | 0 | 0 | 0 | 0 | 0 | 0 | N | 9793 | acetylcholinesterase (EC 3.1.1.7) - banded krait (fragments) |
| 240 | 923 | Q90WI7.1 | 92.83 | 11 | 0 | 0 | 0 | 11 | 0 | 0 | 0 | 0 | 0 | 0 | 0 | 0 | 0 | 6 |  |  |  | 9.7754E5 |  |  |  |  |  |  |  |  |  | 1.6621E6 | 2 | 1 | 0 | 0 | 0 | 3 | 0 | 0 | 0 | 0 | 0 | 0 | 0 | 0 | 0 | 2 | Y | 18254 | RecName: Full=C-type lectin BfL-2; Short=CTL; Flags: Precursor |
| 240 | 924 | AAK43585.1 | 92.83 | 11 | 0 | 0 | 0 | 11 | 0 | 0 | 0 | 0 | 0 | 0 | 0 | 0 | 0 | 6 |  |  |  | 9.7754E5 |  |  |  |  |  |  |  |  |  | 1.6621E6 | 2 | 1 | 0 | 0 | 0 | 3 | 0 | 0 | 0 | 0 | 0 | 0 | 0 | 0 | 0 | 2 | Y | 18254 | C-type lectin-like protein 2 [Bungarus fasciatus] |
| 148 | 1095 | ETE67452.1 | 92.08 | 8 | 0 | 0 | 0 | 2 | 0 | 0 | 0 | 0 | 0 | 3 | 6 | 6 | 0 | 0 |  |  |  | 8.8163E5 |  |  |  |  |  | 0 | 1.6244E7 | 1.1239E7 |  |  | 3 | 3 | 0 | 0 | 0 | 2 | 0 | 0 | 0 | 0 | 0 | 1 | 9 | 19 | 0 | 0 | Y | 39348 | Tumor necrosis factor receptor superfamily member 11B [Ophiophagus hannah] |
| 195 | 2500 | LAB42774.1 | 90.02 | 6 | 0 | 6 | 6 | 0 | 0 | 0 | 0 | 0 | 0 | 0 | 0 | 0 | 0 | 6 |  | 1.5685E7 | 3.324E7 |  |  |  |  |  |  |  |  |  |  | 3.3282E6 | 1 | 1 | 0 | 8 | 6 | 0 | 0 | 0 | 0 | 0 | 0 | 0 | 0 | 0 | 0 | 3 | Y | 40247 | hypothetical protein [Micrurus spixii] |
| 195 | 2504 | XP\_026558006.1 | 90.02 | 6 | 0 | 6 | 6 | 0 | 0 | 0 | 0 | 0 | 0 | 0 | 0 | 0 | 0 | 6 |  | 1.5685E7 | 3.324E7 |  |  |  |  |  |  |  |  |  |  | 3.3282E6 | 1 | 1 | 0 | 8 | 6 | 0 | 0 | 0 | 0 | 0 | 0 | 0 | 0 | 0 | 0 | 3 | Y | 40576 | MANSC domain-containing protein 1 [Pseudonaja textilis] |
| 151 | 2800 | P0DSN0.1 | 89.79 | 18 | 0 | 0 | 0 | 0 | 0 | 0 | 0 | 0 | 0 | 18 | 16 | 16 | 16 | 16 |  |  |  |  |  |  |  |  |  | 3.9201E7 | 3.0418E8 | 1.3918E7 | 3.0136E6 | 1.1066E6 | 3 | 3 | 0 | 0 | 0 | 0 | 0 | 0 | 0 | 0 | 0 | 4 | 9 | 2 | 1 | 1 | Y | 6854 | RecName: Full=Nakoroxin; AltName: Full=Naja kaouthia orphan toxin; AltName: Full=Three-finger toxin; Short=3FTx |
| 294 | 2909 | ETE70042.1 | 89.77 | 14 | 0 | 0 | 0 | 0 | 0 | 14 | 0 | 0 | 0 | 0 | 0 | 0 | 0 | 0 |  |  |  |  |  | 5.849E6 |  |  |  |  |  |  |  |  | 2 | 2 | 0 | 0 | 0 | 0 | 0 | 2 | 0 | 0 | 0 | 0 | 0 | 0 | 0 | 0 | N | 13780 | Sulfhydryl oxidase 1, partial [Ophiophagus hannah] |
| 259 | 1363 | JAS05177.1 | 87.92 | 9 | 0 | 0 | 0 | 0 | 0 | 0 | 0 | 0 | 0 | 4 | 0 | 0 | 4 | 9 |  |  |  |  |  |  |  |  |  | 2.7265E6 |  |  | 6.3332E5 | 1.2329E6 | 2 | 2 | 0 | 0 | 0 | 0 | 0 | 0 | 0 | 0 | 0 | 1 | 0 | 0 | 1 | 2 | Y | 48122 | coagulation factor VII [Micrurus tener] |
| 259 | 1364 | JAI09074.1 | 87.92 | 9 | 0 | 0 | 0 | 0 | 0 | 0 | 0 | 0 | 0 | 4 | 0 | 0 | 4 | 9 |  |  |  |  |  |  |  |  |  | 2.7265E6 |  |  | 6.3332E5 | 1.2329E6 | 2 | 2 | 0 | 0 | 0 | 0 | 0 | 0 | 0 | 0 | 0 | 1 | 0 | 0 | 1 | 2 | Y | 48122 | coagulation factor VII [Micrurus fulvius] |
| 259 | 1365 | JAS05063.1 | 87.92 | 9 | 0 | 0 | 0 | 0 | 0 | 0 | 0 | 0 | 0 | 4 | 0 | 0 | 4 | 9 |  |  |  |  |  |  |  |  |  | 2.7265E6 |  |  | 6.3332E5 | 1.2329E6 | 2 | 2 | 0 | 0 | 0 | 0 | 0 | 0 | 0 | 0 | 0 | 1 | 0 | 0 | 1 | 2 | Y | 48122 | coagulation factor VII [Micrurus fulvius] |
| 259 | 1366 | JAB54462.1 | 87.92 | 9 | 0 | 0 | 0 | 0 | 0 | 0 | 0 | 0 | 0 | 4 | 0 | 0 | 4 | 9 |  |  |  |  |  |  |  |  |  | 2.7265E6 |  |  | 6.3332E5 | 1.2329E6 | 2 | 2 | 0 | 0 | 0 | 0 | 0 | 0 | 0 | 0 | 0 | 1 | 0 | 0 | 1 | 2 | Y | 48122 | coagulation factor 7 [Micrurus fulvius] |
| 259 | 1391 | ETE73401.1 | 87.92 | 3 | 0 | 0 | 0 | 0 | 0 | 0 | 0 | 0 | 0 | 1 | 0 | 0 | 1 | 3 |  |  |  |  |  |  |  |  |  | 2.7265E6 |  |  | 6.3332E5 | 1.2329E6 | 2 | 2 | 0 | 0 | 0 | 0 | 0 | 0 | 0 | 0 | 0 | 1 | 0 | 0 | 1 | 2 | Y | 138992 | Coagulation factor X isoform 1, partial [Ophiophagus hannah] |
| 259 | 1536 | XP\_026548111.1 | 87.92 | 10 | 0 | 0 | 0 | 0 | 0 | 0 | 0 | 0 | 0 | 5 | 0 | 0 | 5 | 10 |  |  |  |  |  |  |  |  |  | 2.7265E6 |  |  | 6.3332E5 | 1.2329E6 | 2 | 2 | 0 | 0 | 0 | 0 | 0 | 0 | 0 | 0 | 0 | 1 | 0 | 0 | 1 | 2 | Y | 44790 | coagulation factor VII, partial [Notechis scutatus] |
| 254 | 1201 | LAB57775.1 | 87.57 | 8 | 0 | 0 | 0 | 0 | 0 | 0 | 0 | 0 | 4 | 0 | 4 | 4 | 0 | 0 |  |  |  |  |  |  |  |  | 6.7584E5 |  | 1.1776E6 | 6.5957E6 |  |  | 2 | 2 | 0 | 0 | 0 | 0 | 0 | 0 | 0 | 0 | 2 | 0 | 1 | 1 | 0 | 0 | N | 30373 | hypothetical protein, partial [Micrurus surinamensis] |
| 254 | 1205 | LAA86163.1 | 87.57 | 4 | 0 | 0 | 0 | 0 | 0 | 0 | 0 | 0 | 2 | 0 | 2 | 2 | 0 | 0 |  |  |  |  |  |  |  |  | 6.7584E5 |  | 1.1776E6 | 6.5957E6 |  |  | 2 | 2 | 0 | 0 | 0 | 0 | 0 | 0 | 0 | 0 | 2 | 0 | 1 | 1 | 0 | 0 | N | 67294 | hypothetical protein [Micrurus lemniscatus lemniscatus] |
| 254 | 1211 | LAA20302.1 | 87.57 | 6 | 0 | 0 | 0 | 0 | 0 | 0 | 0 | 0 | 3 | 0 | 3 | 3 | 0 | 0 |  |  |  |  |  |  |  |  | 6.7584E5 |  | 1.1776E6 | 6.5957E6 |  |  | 2 | 2 | 0 | 0 | 0 | 0 | 0 | 0 | 0 | 0 | 2 | 0 | 1 | 1 | 0 | 0 | N | 40689 | hypothetical protein, partial [Micrurus lemniscatus carvalhoi] |
| 280 | 1322 | XP\_034268532.1 | 87.20 | 10 | 0 | 0 | 4 | 10 | 0 | 0 | 0 | 0 | 0 | 0 | 0 | 0 | 0 | 0 |  |  |  | 9.8993E5 |  |  |  |  |  |  |  |  |  |  | 2 | 1 | 0 | 0 | 2 | 3 | 0 | 0 | 0 | 0 | 0 | 0 | 0 | 0 | 0 | 0 | N | 24760 | mammalian ependymin-related protein 1 isoform X1 [Pantherophis guttatus] |
| 280 | 1327 | XP\_026530630.1 | 87.20 | 10 | 0 | 0 | 4 | 10 | 0 | 0 | 0 | 0 | 0 | 0 | 0 | 0 | 0 | 0 |  |  |  | 9.8993E5 |  |  |  |  |  |  |  |  |  |  | 2 | 1 | 0 | 0 | 2 | 3 | 0 | 0 | 0 | 0 | 0 | 0 | 0 | 0 | 0 | 0 | N | 25176 | mammalian ependymin-related protein 1 [Notechis scutatus] |
| 280 | 1330 | XP\_026558033.1 | 87.20 | 10 | 0 | 0 | 4 | 10 | 0 | 0 | 0 | 0 | 0 | 0 | 0 | 0 | 0 | 0 |  |  |  | 9.8993E5 |  |  |  |  |  |  |  |  |  |  | 2 | 1 | 0 | 0 | 2 | 3 | 0 | 0 | 0 | 0 | 0 | 0 | 0 | 0 | 0 | 0 | N | 25146 | mammalian ependymin-related protein 1 [Pseudonaja textilis] |
| 280 | 1567 | XP\_032093281.1 | 87.20 | 10 | 0 | 0 | 4 | 10 | 0 | 0 | 0 | 0 | 0 | 0 | 0 | 0 | 0 | 0 |  |  |  | 9.8993E5 |  |  |  |  |  |  |  |  |  |  | 2 | 1 | 0 | 0 | 2 | 3 | 0 | 0 | 0 | 0 | 0 | 0 | 0 | 0 | 0 | 0 | N | 25155 | mammalian ependymin-related protein 1 [Thamnophis elegans] |
| 280 | 1617 | XP\_034268617.1 | 87.20 | 12 | 0 | 0 | 5 | 12 | 0 | 0 | 0 | 0 | 0 | 0 | 0 | 0 | 0 | 0 |  |  |  | 9.8993E5 |  |  |  |  |  |  |  |  |  |  | 2 | 1 | 0 | 0 | 2 | 3 | 0 | 0 | 0 | 0 | 0 | 0 | 0 | 0 | 0 | 0 | N | 21131 | mammalian ependymin-related protein 1 isoform X2 [Pantherophis guttatus] |
| 280 | 1816 | XP\_013924998.1 | 87.20 | 10 | 0 | 0 | 4 | 10 | 0 | 0 | 0 | 0 | 0 | 0 | 0 | 0 | 0 | 0 |  |  |  | 9.8993E5 |  |  |  |  |  |  |  |  |  |  | 2 | 1 | 0 | 0 | 2 | 3 | 0 | 0 | 0 | 0 | 0 | 0 | 0 | 0 | 0 | 0 | N | 25129 | PREDICTED: mammalian ependymin-related protein 1 [Thamnophis sirtalis] |
| 289 | 831 | XP\_026527210.1 | 86.28 | 10 | 0 | 0 | 0 | 0 | 0 | 10 | 0 | 0 | 0 | 0 | 0 | 0 | 0 | 0 |  |  |  |  |  | 2.4443E6 |  |  |  |  |  |  |  |  | 3 | 2 | 0 | 0 | 0 | 0 | 0 | 3 | 0 | 0 | 0 | 0 | 0 | 0 | 0 | 0 | N | 58703 | sialate O-acetylesterase [Notechis scutatus] |
| 289 | 832 | XP\_026573812.1 | 86.28 | 10 | 0 | 0 | 0 | 0 | 0 | 10 | 0 | 0 | 0 | 0 | 0 | 0 | 0 | 0 |  |  |  |  |  | 2.4443E6 |  |  |  |  |  |  |  |  | 3 | 2 | 0 | 0 | 0 | 0 | 0 | 3 | 0 | 0 | 0 | 0 | 0 | 0 | 0 | 0 | N | 58672 | sialate O-acetylesterase [Pseudonaja textilis] |
| 331 | 1268 | JAC95044.1 | 86.21 | 2 | 0 | 0 | 0 | 0 | 0 | 2 | 0 | 0 | 0 | 0 | 0 | 0 | 0 | 0 |  |  |  |  |  | 1.2413E6 |  |  |  |  |  |  |  |  | 1 | 1 | 0 | 0 | 0 | 0 | 0 | 1 | 0 | 0 | 0 | 0 | 0 | 0 | 0 | 0 | N | 86011 | Dipeptidylpeptidase 4 [Pantherophis guttatus] |
| 331 | 1430 | JAC94983.1 | 86.21 | 2 | 0 | 0 | 0 | 0 | 0 | 2 | 0 | 0 | 0 | 0 | 0 | 0 | 0 | 0 |  |  |  |  |  | 1.2413E6 |  |  |  |  |  |  |  |  | 1 | 1 | 0 | 0 | 0 | 0 | 0 | 1 | 0 | 0 | 0 | 0 | 0 | 0 | 0 | 0 | N | 86071 | Dipeptidylpeptidase 4 [Opheodrys aestivus] |
| 331 | 1434 | XP\_007421588.1 | 86.21 | 2 | 0 | 0 | 0 | 0 | 0 | 2 | 0 | 0 | 0 | 0 | 0 | 0 | 0 | 0 |  |  |  |  |  | 1.2413E6 |  |  |  |  |  |  |  |  | 1 | 1 | 0 | 0 | 0 | 0 | 0 | 1 | 0 | 0 | 0 | 0 | 0 | 0 | 0 | 0 | N | 85981 | dipeptidyl peptidase 4 isoform X2 [Python bivittatus] |
| 331 | 1435 | XP\_015745082.1 | 86.21 | 2 | 0 | 0 | 0 | 0 | 0 | 2 | 0 | 0 | 0 | 0 | 0 | 0 | 0 | 0 |  |  |  |  |  | 1.2413E6 |  |  |  |  |  |  |  |  | 1 | 1 | 0 | 0 | 0 | 0 | 0 | 1 | 0 | 0 | 0 | 0 | 0 | 0 | 0 | 0 | N | 86052 | dipeptidyl peptidase 4 isoform X1 [Python bivittatus] |
| 331 | 1436 | JAC94938.1 | 86.21 | 2 | 0 | 0 | 0 | 0 | 0 | 2 | 0 | 0 | 0 | 0 | 0 | 0 | 0 | 0 |  |  |  |  |  | 1.2413E6 |  |  |  |  |  |  |  |  | 1 | 1 | 0 | 0 | 0 | 0 | 0 | 1 | 0 | 0 | 0 | 0 | 0 | 0 | 0 | 0 | N | 86105 | Dipeptidylpeptidase 4 [Python regius] |
| 331 | 1438 | ABQ63106.1 | 86.21 | 2 | 0 | 0 | 0 | 0 | 0 | 2 | 0 | 0 | 0 | 0 | 0 | 0 | 0 | 0 |  |  |  |  |  | 1.2413E6 |  |  |  |  |  |  |  |  | 1 | 1 | 0 | 0 | 0 | 0 | 0 | 1 | 0 | 0 | 0 | 0 | 0 | 0 | 0 | 0 | N | 86405 | venom dipeptidylpeptidase IV [Hoplocephalus stephensii] |
| 331 | 1439 | ABQ63102.1 | 86.21 | 2 | 0 | 0 | 0 | 0 | 0 | 2 | 0 | 0 | 0 | 0 | 0 | 0 | 0 | 0 |  |  |  |  |  | 1.2413E6 |  |  |  |  |  |  |  |  | 1 | 1 | 0 | 0 | 0 | 0 | 0 | 1 | 0 | 0 | 0 | 0 | 0 | 0 | 0 | 0 | N | 86617 | venom dipeptidylpeptidase IV [Cryptophis nigrescens] |
| 331 | 1440 | ABQ63103.1 | 86.21 | 2 | 0 | 0 | 0 | 0 | 0 | 2 | 0 | 0 | 0 | 0 | 0 | 0 | 0 | 0 |  |  |  |  |  | 1.2413E6 |  |  |  |  |  |  |  |  | 1 | 1 | 0 | 0 | 0 | 0 | 0 | 1 | 0 | 0 | 0 | 0 | 0 | 0 | 0 | 0 | N | 86495 | venom dipeptidylpeptidase IV [Pseudechis australis] |
| 331 | 1441 | ABQ63107.1 | 86.21 | 2 | 0 | 0 | 0 | 0 | 0 | 2 | 0 | 0 | 0 | 0 | 0 | 0 | 0 | 0 |  |  |  |  |  | 1.2413E6 |  |  |  |  |  |  |  |  | 1 | 1 | 0 | 0 | 0 | 0 | 0 | 1 | 0 | 0 | 0 | 0 | 0 | 0 | 0 | 0 | N | 86457 | venom dipeptidylpeptidase IV [Tropidechis carinatus] |
| 331 | 1444 | ABQ63100.1 | 86.21 | 2 | 0 | 0 | 0 | 0 | 0 | 2 | 0 | 0 | 0 | 0 | 0 | 0 | 0 | 0 |  |  |  |  |  | 1.2413E6 |  |  |  |  |  |  |  |  | 1 | 1 | 0 | 0 | 0 | 0 | 0 | 1 | 0 | 0 | 0 | 0 | 0 | 0 | 0 | 0 | N | 86559 | venom dipeptidylpeptidase IV [Pseudonaja textilis] |
| 331 | 1445 | ETE70527.1 | 86.21 | 2 | 0 | 0 | 0 | 0 | 0 | 2 | 0 | 0 | 0 | 0 | 0 | 0 | 0 | 0 |  |  |  |  |  | 1.2413E6 |  |  |  |  |  |  |  |  | 1 | 1 | 0 | 0 | 0 | 0 | 0 | 1 | 0 | 0 | 0 | 0 | 0 | 0 | 0 | 0 | N | 86366 | Dipeptidyl peptidase 4 [Ophiophagus hannah] |
| 331 | 1558 | ABQ63104.1 | 86.21 | 2 | 0 | 0 | 0 | 0 | 0 | 2 | 0 | 0 | 0 | 0 | 0 | 0 | 0 | 0 |  |  |  |  |  | 1.2413E6 |  |  |  |  |  |  |  |  | 1 | 1 | 0 | 0 | 0 | 0 | 0 | 1 | 0 | 0 | 0 | 0 | 0 | 0 | 0 | 0 | N | 86607 | venom dipeptidylpeptidase IV [Pseudechis porphyriacus] |
| 331 | 1605 | ABQ63098.1 | 86.21 | 2 | 0 | 0 | 0 | 0 | 0 | 2 | 0 | 0 | 0 | 0 | 0 | 0 | 0 | 0 |  |  |  |  |  | 1.2413E6 |  |  |  |  |  |  |  |  | 1 | 1 | 0 | 0 | 0 | 0 | 0 | 1 | 0 | 0 | 0 | 0 | 0 | 0 | 0 | 0 | N | 86600 | venom dipeptidylpeptidase IV [Oxyuranus scutellatus] |
| 331 | 1606 | ABQ63099.1 | 86.21 | 2 | 0 | 0 | 0 | 0 | 0 | 2 | 0 | 0 | 0 | 0 | 0 | 0 | 0 | 0 |  |  |  |  |  | 1.2413E6 |  |  |  |  |  |  |  |  | 1 | 1 | 0 | 0 | 0 | 0 | 0 | 1 | 0 | 0 | 0 | 0 | 0 | 0 | 0 | 0 | N | 86590 | venom dipeptidylpeptidase IV [Oxyuranus microlepidotus] |
| 331 | 1611 | ABQ63101.1 | 86.21 | 2 | 0 | 0 | 0 | 0 | 0 | 2 | 0 | 0 | 0 | 0 | 0 | 0 | 0 | 0 |  |  |  |  |  | 1.2413E6 |  |  |  |  |  |  |  |  | 1 | 1 | 0 | 0 | 0 | 0 | 0 | 1 | 0 | 0 | 0 | 0 | 0 | 0 | 0 | 0 | N | 85960 | venom dipeptidylpeptidase IV [Demansia vestigiata] |
| 331 | 1620 | JAI10061.1 | 86.21 | 2 | 0 | 0 | 0 | 0 | 0 | 2 | 0 | 0 | 0 | 0 | 0 | 0 | 0 | 0 |  |  |  |  |  | 1.2413E6 |  |  |  |  |  |  |  |  | 1 | 1 | 0 | 0 | 0 | 0 | 0 | 1 | 0 | 0 | 0 | 0 | 0 | 0 | 0 | 0 | N | 86366 | dipeptidylpeptidase [Micrurus fulvius] |
| 331 | 1623 | BAN89364.1 | 86.21 | 2 | 0 | 0 | 0 | 0 | 0 | 2 | 0 | 0 | 0 | 0 | 0 | 0 | 0 | 0 |  |  |  |  |  | 1.2413E6 |  |  |  |  |  |  |  |  | 1 | 1 | 0 | 0 | 0 | 0 | 0 | 1 | 0 | 0 | 0 | 0 | 0 | 0 | 0 | 0 | N | 79450 | dipeptidyl peptidase iv [Protobothrops flavoviridis] |
| 331 | 1625 | BAP39988.1 | 86.21 | 2 | 0 | 0 | 0 | 0 | 0 | 2 | 0 | 0 | 0 | 0 | 0 | 0 | 0 | 0 |  |  |  |  |  | 1.2413E6 |  |  |  |  |  |  |  |  | 1 | 1 | 0 | 0 | 0 | 0 | 0 | 1 | 0 | 0 | 0 | 0 | 0 | 0 | 0 | 0 | N | 86179 | venom dipeptidylpeptidase IV [Protobothrops flavoviridis] |
| 331 | 1636 | JAC96553.1 | 86.21 | 2 | 0 | 0 | 0 | 0 | 0 | 2 | 0 | 0 | 0 | 0 | 0 | 0 | 0 | 0 |  |  |  |  |  | 1.2413E6 |  |  |  |  |  |  |  |  | 1 | 1 | 0 | 0 | 0 | 0 | 0 | 1 | 0 | 0 | 0 | 0 | 0 | 0 | 0 | 0 | N | 86518 | Dipeptidylpeptidase 4 [Echis coloratus] |
| 262 | 1143 | XP\_034262198.1 | 83.95 | 2 | 0 | 0 | 0 | 0 | 0 | 2 | 0 | 0 | 0 | 0 | 0 | 0 | 0 | 0 |  |  |  |  |  | 9.5255E5 |  |  |  |  |  |  |  |  | 2 | 2 | 0 | 0 | 0 | 0 | 0 | 3 | 0 | 0 | 0 | 0 | 0 | 0 | 0 | 0 | N | 140811 | carboxypeptidase D [Pantherophis guttatus] |
| 262 | 1149 | XP\_026545885.1 | 83.95 | 2 | 0 | 0 | 0 | 0 | 0 | 2 | 0 | 0 | 0 | 0 | 0 | 0 | 0 | 0 |  |  |  |  |  | 9.5255E5 |  |  |  |  |  |  |  |  | 2 | 2 | 0 | 0 | 0 | 0 | 0 | 3 | 0 | 0 | 0 | 0 | 0 | 0 | 0 | 0 | N | 143676 | carboxypeptidase D [Notechis scutatus] |
| 262 | 1150 | XP\_026578833.1 | 83.95 | 2 | 0 | 0 | 0 | 0 | 0 | 2 | 0 | 0 | 0 | 0 | 0 | 0 | 0 | 0 |  |  |  |  |  | 9.5255E5 |  |  |  |  |  |  |  |  | 2 | 2 | 0 | 0 | 0 | 0 | 0 | 3 | 0 | 0 | 0 | 0 | 0 | 0 | 0 | 0 | N | 151337 | carboxypeptidase D [Pseudonaja textilis] |
| 233 | 1271 | QGC85377.1 | 83.76 | 6 | 0 | 0 | 0 | 3 | 6 | 6 | 0 | 0 | 3 | 0 | 0 | 0 | 0 | 0 |  |  |  |  | 7.8532E6 | 1.2522E6 |  |  |  |  |  |  |  |  | 2 | 1 | 0 | 0 | 0 | 1 | 4 | 2 | 0 | 0 | 2 | 0 | 0 | 0 | 0 | 0 | Y | 55494 | metalloproteinase, partial [Dispholidus typus] |
| 282 | 824 | XP\_007422201.1 | 83.06 | 1 | 0 | 0 | 0 | 0 | 1 | 0 | 0 | 0 | 0 | 0 | 0 | 0 | 0 | 0 |  |  |  |  | 3.1218E5 |  |  |  |  |  |  |  |  |  | 1 | 1 | 0 | 0 | 0 | 0 | 1 | 0 | 0 | 0 | 0 | 0 | 0 | 0 | 0 | 0 | N | 329911 | desmoplakin isoform X1 [Python bivittatus] |
| 282 | 825 | XP\_015666325.1 | 83.06 | 1 | 0 | 0 | 0 | 0 | 1 | 0 | 0 | 0 | 0 | 0 | 0 | 0 | 0 | 0 |  |  |  |  | 3.1218E5 |  |  |  |  |  |  |  |  |  | 1 | 1 | 0 | 0 | 0 | 0 | 1 | 0 | 0 | 0 | 0 | 0 | 0 | 0 | 0 | 0 | N | 329546 | desmoplakin [Protobothrops mucrosquamatus] |
| 225 | 1442 | XP\_026578659.1 | 81.30 | 3 | 0 | 0 | 0 | 1 | 0 | 3 | 0 | 0 | 0 | 0 | 0 | 0 | 0 | 0 |  |  |  | 2.0877E6 |  | 6.9801E6 |  |  |  |  |  |  |  |  | 2 | 1 | 0 | 0 | 0 | 3 | 0 | 5 | 0 | 0 | 0 | 0 | 0 | 0 | 0 | 0 | N | 69487 | serum albumin-like [Pseudonaja textilis] |
| 225 | 1443 | XP\_026544429.1 | 81.30 | 4 | 0 | 0 | 0 | 2 | 0 | 4 | 0 | 0 | 0 | 0 | 0 | 0 | 0 | 0 |  |  |  | 2.0877E6 |  | 6.9801E6 |  |  |  |  |  |  |  |  | 2 | 1 | 0 | 0 | 0 | 3 | 0 | 5 | 0 | 0 | 0 | 0 | 0 | 0 | 0 | 0 | N | 54749 | serum albumin-like [Notechis scutatus] |
| 225 | 1446 | XP\_034279009.1 | 81.30 | 3 | 0 | 0 | 0 | 1 | 0 | 3 | 0 | 0 | 0 | 0 | 0 | 0 | 0 | 0 |  |  |  | 2.0877E6 |  | 6.9801E6 |  |  |  |  |  |  |  |  | 2 | 1 | 0 | 0 | 0 | 3 | 0 | 5 | 0 | 0 | 0 | 0 | 0 | 0 | 0 | 0 | N | 69338 | serum albumin-like [Pantherophis guttatus] |
| 225 | 1448 | ETE59846.1 | 81.30 | 3 | 0 | 0 | 0 | 1 | 0 | 3 | 0 | 0 | 0 | 0 | 0 | 0 | 0 | 0 |  |  |  | 2.0877E6 |  | 6.9801E6 |  |  |  |  |  |  |  |  | 2 | 1 | 0 | 0 | 0 | 3 | 0 | 5 | 0 | 0 | 0 | 0 | 0 | 0 | 0 | 0 | N | 72383 | Alpha-fetoprotein, partial [Ophiophagus hannah] |
| 225 | 1520 | XP\_015665591.1 | 81.30 | 3 | 0 | 0 | 0 | 1 | 0 | 3 | 0 | 0 | 0 | 0 | 0 | 0 | 0 | 0 |  |  |  | 2.0877E6 |  | 6.9801E6 |  |  |  |  |  |  |  |  | 2 | 1 | 0 | 0 | 0 | 3 | 0 | 5 | 0 | 0 | 0 | 0 | 0 | 0 | 0 | 0 | N | 69629 | serum albumin-like [Protobothrops mucrosquamatus] |
| 249 | 1769 | D9IX97.1 | 80.66 | 11 | 0 | 0 | 0 | 0 | 0 | 0 | 0 | 5 | 11 | 0 | 0 | 0 | 0 | 0 |  |  |  |  |  |  |  | 6.4809E5 | 4.4457E6 |  |  |  |  |  | 2 | 2 | 0 | 0 | 0 | 0 | 0 | 0 | 0 | 1 | 5 | 0 | 0 | 0 | 0 | 0 | Y | 17345 | RecName: Full=Natriuretic peptide Na-NP; Flags: Precursor |
| 249 | 1770 | ADK12001.1 | 80.66 | 11 | 0 | 0 | 0 | 0 | 0 | 0 | 0 | 5 | 11 | 0 | 0 | 0 | 0 | 0 |  |  |  |  |  |  |  | 6.4809E5 | 4.4457E6 |  |  |  |  |  | 2 | 2 | 0 | 0 | 0 | 0 | 0 | 0 | 0 | 1 | 5 | 0 | 0 | 0 | 0 | 0 | Y | 17345 | natriuretic peptide [Naja atra] |
| 297 | 1407 | JAB52844.1 | 79.88 | 4 | 0 | 0 | 0 | 4 | 0 | 0 | 0 | 0 | 0 | 0 | 0 | 0 | 0 | 0 |  |  |  | 1.6493E6 |  |  |  |  |  |  |  |  |  |  | 1 | 1 | 0 | 0 | 0 | 2 | 0 | 0 | 0 | 0 | 0 | 0 | 0 | 0 | 0 | 0 | Y | 39958 | cysteine-rich with EGF-like domain 1 [Micrurus fulvius] |
| 297 | 1408 | JAI10117.1 | 79.88 | 4 | 0 | 0 | 0 | 4 | 0 | 0 | 0 | 0 | 0 | 0 | 0 | 0 | 0 | 0 |  |  |  | 1.6493E6 |  |  |  |  |  |  |  |  |  |  | 1 | 1 | 0 | 0 | 0 | 2 | 0 | 0 | 0 | 0 | 0 | 0 | 0 | 0 | 0 | 0 | Y | 40613 | cysteine-rich with EGF-like domain protein [Micrurus fulvius] |
| 297 | 1411 | XP\_026524808.1 | 79.88 | 4 | 0 | 0 | 0 | 4 | 0 | 0 | 0 | 0 | 0 | 0 | 0 | 0 | 0 | 0 |  |  |  | 1.6493E6 |  |  |  |  |  |  |  |  |  |  | 1 | 1 | 0 | 0 | 0 | 2 | 0 | 0 | 0 | 0 | 0 | 0 | 0 | 0 | 0 | 0 | Y | 39756 | cysteine-rich with EGF-like domain protein 2 isoform X2 [Notechis scutatus] |
| 297 | 1416 | XP\_026569840.1 | 79.88 | 4 | 0 | 0 | 0 | 4 | 0 | 0 | 0 | 0 | 0 | 0 | 0 | 0 | 0 | 0 |  |  |  | 1.6493E6 |  |  |  |  |  |  |  |  |  |  | 1 | 1 | 0 | 0 | 0 | 2 | 0 | 0 | 0 | 0 | 0 | 0 | 0 | 0 | 0 | 0 | Y | 39728 | cysteine-rich with EGF-like domain protein 2 isoform X2 [Pseudonaja textilis] |
| 297 | 1417 | XP\_026569839.1 | 79.88 | 4 | 0 | 0 | 0 | 4 | 0 | 0 | 0 | 0 | 0 | 0 | 0 | 0 | 0 | 0 |  |  |  | 1.6493E6 |  |  |  |  |  |  |  |  |  |  | 1 | 1 | 0 | 0 | 0 | 2 | 0 | 0 | 0 | 0 | 0 | 0 | 0 | 0 | 0 | 0 | Y | 39857 | cysteine-rich with EGF-like domain protein 2 isoform X1 [Pseudonaja textilis] |
| 297 | 1418 | XP\_026524807.1 | 79.88 | 4 | 0 | 0 | 0 | 4 | 0 | 0 | 0 | 0 | 0 | 0 | 0 | 0 | 0 | 0 |  |  |  | 1.6493E6 |  |  |  |  |  |  |  |  |  |  | 1 | 1 | 0 | 0 | 0 | 2 | 0 | 0 | 0 | 0 | 0 | 0 | 0 | 0 | 0 | 0 | Y | 39885 | cysteine-rich with EGF-like domain protein 2 isoform X1 [Notechis scutatus] |
| 284 | 1570 | ABN72547.1 | 79.37 | 3 | 0 | 0 | 0 | 0 | 0 | 0 | 0 | 0 | 0 | 0 | 0 | 3 | 0 | 0 |  |  |  |  |  |  |  |  |  |  |  | 2.3064E6 |  |  | 1 | 1 | 0 | 0 | 0 | 0 | 0 | 0 | 0 | 0 | 0 | 0 | 0 | 2 | 0 | 0 | N | 69403 | metalloproteinase [Naja atra] |
| 284 | 1571 | A8QL59.1 | 79.37 | 3 | 0 | 0 | 0 | 0 | 0 | 0 | 0 | 0 | 0 | 0 | 0 | 3 | 0 | 0 |  |  |  |  |  |  |  |  |  |  |  | 2.3064E6 |  |  | 1 | 1 | 0 | 0 | 0 | 0 | 0 | 0 | 0 | 0 | 0 | 0 | 0 | 2 | 0 | 0 | N | 69403 | RecName: Full=Zinc metalloproteinase-disintegrin-like NaMP; AltName: Full=Snake venom metalloproteinase; Short=SVMP; Flags: Precursor |
| 308 | 1152 | ETE70348.1 | 78.03 | 2 | 0 | 0 | 0 | 0 | 0 | 2 | 0 | 0 | 0 | 0 | 0 | 0 | 0 | 0 |  |  |  |  |  | 2.8074E5 |  |  |  |  |  |  |  |  | 1 | 1 | 0 | 0 | 0 | 0 | 0 | 1 | 0 | 0 | 0 | 0 | 0 | 0 | 0 | 0 | N | 58260 | Protein disulfide-isomerase [Ophiophagus hannah] |
| 308 | 1157 | JAI09686.1 | 78.03 | 2 | 0 | 0 | 0 | 0 | 0 | 2 | 0 | 0 | 0 | 0 | 0 | 0 | 0 | 0 |  |  |  |  |  | 2.8074E5 |  |  |  |  |  |  |  |  | 1 | 1 | 0 | 0 | 0 | 0 | 0 | 1 | 0 | 0 | 0 | 0 | 0 | 0 | 0 | 0 | N | 58075 | PDI [Micrurus fulvius] |
| 308 | 1158 | JAB53739.1 | 78.03 | 2 | 0 | 0 | 0 | 0 | 0 | 2 | 0 | 0 | 0 | 0 | 0 | 0 | 0 | 0 |  |  |  |  |  | 2.8074E5 |  |  |  |  |  |  |  |  | 1 | 1 | 0 | 0 | 0 | 0 | 0 | 1 | 0 | 0 | 0 | 0 | 0 | 0 | 0 | 0 | N | 58075 | disulfide-isomerase-like protein [Micrurus fulvius] |
| 308 | 1163 | JAB53738.1 | 78.03 | 2 | 0 | 0 | 0 | 0 | 0 | 2 | 0 | 0 | 0 | 0 | 0 | 0 | 0 | 0 |  |  |  |  |  | 2.8074E5 |  |  |  |  |  |  |  |  | 1 | 1 | 0 | 0 | 0 | 0 | 0 | 1 | 0 | 0 | 0 | 0 | 0 | 0 | 0 | 0 | N | 52421 | disulfide-isomerase-like protein [Micrurus fulvius] |
| 308 | 1216 | JAG67097.1 | 78.03 | 2 | 0 | 0 | 0 | 0 | 0 | 2 | 0 | 0 | 0 | 0 | 0 | 0 | 0 | 0 |  |  |  |  |  | 2.8074E5 |  |  |  |  |  |  |  |  | 1 | 1 | 0 | 0 | 0 | 0 | 0 | 1 | 0 | 0 | 0 | 0 | 0 | 0 | 0 | 0 | N | 57896 | PDI [Boiga irregularis] |
| 308 | 1231 | XP\_015680645.1 | 78.03 | 2 | 0 | 0 | 0 | 0 | 0 | 2 | 0 | 0 | 0 | 0 | 0 | 0 | 0 | 0 |  |  |  |  |  | 2.8074E5 |  |  |  |  |  |  |  |  | 1 | 1 | 0 | 0 | 0 | 0 | 0 | 1 | 0 | 0 | 0 | 0 | 0 | 0 | 0 | 0 | N | 58330 | protein disulfide-isomerase [Protobothrops mucrosquamatus] |
| 308 | 1232 | JAV49704.1 | 78.03 | 2 | 0 | 0 | 0 | 0 | 0 | 2 | 0 | 0 | 0 | 0 | 0 | 0 | 0 | 0 |  |  |  |  |  | 2.8074E5 |  |  |  |  |  |  |  |  | 1 | 1 | 0 | 0 | 0 | 0 | 0 | 1 | 0 | 0 | 0 | 0 | 0 | 0 | 0 | 0 | N | 58415 | Pdi [Agkistrodon contortrix contortrix] |
| 308 | 1236 | XP\_032068234.1 | 78.03 | 2 | 0 | 0 | 0 | 0 | 0 | 2 | 0 | 0 | 0 | 0 | 0 | 0 | 0 | 0 |  |  |  |  |  | 2.8074E5 |  |  |  |  |  |  |  |  | 1 | 1 | 0 | 0 | 0 | 0 | 0 | 1 | 0 | 0 | 0 | 0 | 0 | 0 | 0 | 0 | N | 57670 | LOW QUALITY PROTEIN: protein disulfide-isomerase [Thamnophis elegans] |
| 308 | 1237 | XP\_013916852.1 | 78.03 | 2 | 0 | 0 | 0 | 0 | 0 | 2 | 0 | 0 | 0 | 0 | 0 | 0 | 0 | 0 |  |  |  |  |  | 2.8074E5 |  |  |  |  |  |  |  |  | 1 | 1 | 0 | 0 | 0 | 0 | 0 | 1 | 0 | 0 | 0 | 0 | 0 | 0 | 0 | 0 | N | 57930 | PREDICTED: protein disulfide-isomerase [Thamnophis sirtalis] |
| 308 | 1238 | XP\_034268776.1 | 78.03 | 2 | 0 | 0 | 0 | 0 | 0 | 2 | 0 | 0 | 0 | 0 | 0 | 0 | 0 | 0 |  |  |  |  |  | 2.8074E5 |  |  |  |  |  |  |  |  | 1 | 1 | 0 | 0 | 0 | 0 | 0 | 1 | 0 | 0 | 0 | 0 | 0 | 0 | 0 | 0 | N | 57950 | protein disulfide-isomerase [Pantherophis guttatus] |
| 308 | 1239 | AFJ50884.1 | 78.03 | 2 | 0 | 0 | 0 | 0 | 0 | 2 | 0 | 0 | 0 | 0 | 0 | 0 | 0 | 0 |  |  |  |  |  | 2.8074E5 |  |  |  |  |  |  |  |  | 1 | 1 | 0 | 0 | 0 | 0 | 0 | 1 | 0 | 0 | 0 | 0 | 0 | 0 | 0 | 0 | N | 58617 | PDI [Crotalus adamanteus] |
| 308 | 1240 | JAI12151.1 | 78.03 | 2 | 0 | 0 | 0 | 0 | 0 | 2 | 0 | 0 | 0 | 0 | 0 | 0 | 0 | 0 |  |  |  |  |  | 2.8074E5 |  |  |  |  |  |  |  |  | 1 | 1 | 0 | 0 | 0 | 0 | 0 | 1 | 0 | 0 | 0 | 0 | 0 | 0 | 0 | 0 | N | 58617 | PDI [Crotalus adamanteus] |
| 308 | 1243 | XP\_007436274.2 | 78.03 | 2 | 0 | 0 | 0 | 0 | 0 | 2 | 0 | 0 | 0 | 0 | 0 | 0 | 0 | 0 |  |  |  |  |  | 2.8074E5 |  |  |  |  |  |  |  |  | 1 | 1 | 0 | 0 | 0 | 0 | 0 | 1 | 0 | 0 | 0 | 0 | 0 | 0 | 0 | 0 | N | 58225 | protein disulfide-isomerase [Python bivittatus] |
| 308 | 1244 | JAA96208.1 | 78.03 | 2 | 0 | 0 | 0 | 0 | 0 | 2 | 0 | 0 | 0 | 0 | 0 | 0 | 0 | 0 |  |  |  |  |  | 2.8074E5 |  |  |  |  |  |  |  |  | 1 | 1 | 0 | 0 | 0 | 0 | 0 | 1 | 0 | 0 | 0 | 0 | 0 | 0 | 0 | 0 | N | 58430 | Protein disulfide-isomerase [Crotalus horridus] |
| 251 | 2897 | XP\_025026995.1 | 77.09 | 13 | 0 | 0 | 0 | 0 | 0 | 0 | 0 | 0 | 0 | 0 | 0 | 0 | 0 | 13 |  |  |  |  |  |  |  |  |  |  |  |  |  | 3.9964E7 | 1 | 1 | 0 | 0 | 0 | 0 | 0 | 0 | 0 | 0 | 0 | 0 | 0 | 0 | 0 | 7 | Y | 15909 | insulin-like growth factor I isoform X2 [Python bivittatus] |
| 251 | 2898 | XP\_007420001.1 | 77.09 | 11 | 0 | 0 | 0 | 0 | 0 | 0 | 0 | 0 | 0 | 0 | 0 | 0 | 0 | 11 |  |  |  |  |  |  |  |  |  |  |  |  |  | 3.9964E7 | 1 | 1 | 0 | 0 | 0 | 0 | 0 | 0 | 0 | 0 | 0 | 0 | 0 | 0 | 0 | 7 | Y | 18531 | insulin-like growth factor I isoform X1 [Python bivittatus] |
| 251 | 2998 | XP\_025027023.1 | 77.09 | 14 | 0 | 0 | 0 | 0 | 0 | 0 | 0 | 0 | 0 | 0 | 0 | 0 | 0 | 14 |  |  |  |  |  |  |  |  |  |  |  |  |  | 3.9964E7 | 1 | 1 | 0 | 0 | 0 | 0 | 0 | 0 | 0 | 0 | 0 | 0 | 0 | 0 | 0 | 7 | Y | 14515 | insulin-like growth factor I isoform X3 [Python bivittatus] |
| 287 | 1187 | JAG44810.1 | 75.99 | 3 | 0 | 0 | 0 | 0 | 0 | 3 | 0 | 0 | 0 | 0 | 0 | 0 | 0 | 0 |  |  |  |  |  | 1.1085E6 |  |  |  |  |  |  |  |  | 2 | 2 | 0 | 0 | 0 | 0 | 0 | 4 | 0 | 0 | 0 | 0 | 0 | 0 | 0 | 0 | N | 69323 | Ezrin-like protein [Crotalus horridus] |
| 287 | 1188 | JAA97085.1 | 75.99 | 3 | 0 | 0 | 0 | 0 | 0 | 3 | 0 | 0 | 0 | 0 | 0 | 0 | 0 | 0 |  |  |  |  |  | 1.1085E6 |  |  |  |  |  |  |  |  | 2 | 2 | 0 | 0 | 0 | 0 | 0 | 4 | 0 | 0 | 0 | 0 | 0 | 0 | 0 | 0 | N | 69323 | Ezrin-like protein [Crotalus horridus] |
| 287 | 1189 | JAG46842.1 | 75.99 | 3 | 0 | 0 | 0 | 0 | 0 | 3 | 0 | 0 | 0 | 0 | 0 | 0 | 0 | 0 |  |  |  |  |  | 1.1085E6 |  |  |  |  |  |  |  |  | 2 | 2 | 0 | 0 | 0 | 0 | 0 | 4 | 0 | 0 | 0 | 0 | 0 | 0 | 0 | 0 | N | 69323 | Ezrin-like protein [Crotalus horridus] |
| 287 | 1191 | XP\_015672332.1 | 75.99 | 3 | 0 | 0 | 0 | 0 | 0 | 3 | 0 | 0 | 0 | 0 | 0 | 0 | 0 | 0 |  |  |  |  |  | 1.1085E6 |  |  |  |  |  |  |  |  | 2 | 2 | 0 | 0 | 0 | 0 | 0 | 4 | 0 | 0 | 0 | 0 | 0 | 0 | 0 | 0 | N | 69373 | ezrin [Protobothrops mucrosquamatus] |
| 287 | 1192 | XP\_015672333.1 | 75.99 | 3 | 0 | 0 | 0 | 0 | 0 | 3 | 0 | 0 | 0 | 0 | 0 | 0 | 0 | 0 |  |  |  |  |  | 1.1085E6 |  |  |  |  |  |  |  |  | 2 | 2 | 0 | 0 | 0 | 0 | 0 | 4 | 0 | 0 | 0 | 0 | 0 | 0 | 0 | 0 | N | 69373 | ezrin [Protobothrops mucrosquamatus] |
| 287 | 1193 | JAI13359.1 | 75.99 | 3 | 0 | 0 | 0 | 0 | 0 | 3 | 0 | 0 | 0 | 0 | 0 | 0 | 0 | 0 |  |  |  |  |  | 1.1085E6 |  |  |  |  |  |  |  |  | 2 | 2 | 0 | 0 | 0 | 0 | 0 | 4 | 0 | 0 | 0 | 0 | 0 | 0 | 0 | 0 | N | 69296 | Ezrin-like protein [Crotalus adamanteus] |
| 287 | 1194 | AFJ50045.1 | 75.99 | 3 | 0 | 0 | 0 | 0 | 0 | 3 | 0 | 0 | 0 | 0 | 0 | 0 | 0 | 0 |  |  |  |  |  | 1.1085E6 |  |  |  |  |  |  |  |  | 2 | 2 | 0 | 0 | 0 | 0 | 0 | 4 | 0 | 0 | 0 | 0 | 0 | 0 | 0 | 0 | N | 69296 | Ezrin-like [Crotalus adamanteus] |
| 287 | 1195 | JAC95627.1 | 75.99 | 3 | 0 | 0 | 0 | 0 | 0 | 3 | 0 | 0 | 0 | 0 | 0 | 0 | 0 | 0 |  |  |  |  |  | 1.1085E6 |  |  |  |  |  |  |  |  | 2 | 2 | 0 | 0 | 0 | 0 | 0 | 4 | 0 | 0 | 0 | 0 | 0 | 0 | 0 | 0 | N | 69440 | Ezrin-like protein [Hypsiglena sp. JMG-2014] |
| 287 | 1196 | JAV50585.1 | 75.99 | 3 | 0 | 0 | 0 | 0 | 0 | 3 | 0 | 0 | 0 | 0 | 0 | 0 | 0 | 0 |  |  |  |  |  | 1.1085E6 |  |  |  |  |  |  |  |  | 2 | 2 | 0 | 0 | 0 | 0 | 0 | 4 | 0 | 0 | 0 | 0 | 0 | 0 | 0 | 0 | N | 69353 | Ezrin-like protein [Agkistrodon contortrix contortrix] |
| 287 | 1197 | XP\_026560859.1 | 75.99 | 3 | 0 | 0 | 0 | 0 | 0 | 3 | 0 | 0 | 0 | 0 | 0 | 0 | 0 | 0 |  |  |  |  |  | 1.1085E6 |  |  |  |  |  |  |  |  | 2 | 2 | 0 | 0 | 0 | 0 | 0 | 4 | 0 | 0 | 0 | 0 | 0 | 0 | 0 | 0 | N | 69465 | ezrin isoform X1 [Pseudonaja textilis] |
| 287 | 1198 | XP\_026531043.1 | 75.99 | 3 | 0 | 0 | 0 | 0 | 0 | 3 | 0 | 0 | 0 | 0 | 0 | 0 | 0 | 0 |  |  |  |  |  | 1.1085E6 |  |  |  |  |  |  |  |  | 2 | 2 | 0 | 0 | 0 | 0 | 0 | 4 | 0 | 0 | 0 | 0 | 0 | 0 | 0 | 0 | N | 69439 | ezrin [Notechis scutatus] |
| 287 | 1199 | XP\_026531042.1 | 75.99 | 3 | 0 | 0 | 0 | 0 | 0 | 3 | 0 | 0 | 0 | 0 | 0 | 0 | 0 | 0 |  |  |  |  |  | 1.1085E6 |  |  |  |  |  |  |  |  | 2 | 2 | 0 | 0 | 0 | 0 | 0 | 4 | 0 | 0 | 0 | 0 | 0 | 0 | 0 | 0 | N | 69439 | ezrin [Notechis scutatus] |
| 287 | 1200 | XP\_026560858.1 | 75.99 | 3 | 0 | 0 | 0 | 0 | 0 | 3 | 0 | 0 | 0 | 0 | 0 | 0 | 0 | 0 |  |  |  |  |  | 1.1085E6 |  |  |  |  |  |  |  |  | 2 | 2 | 0 | 0 | 0 | 0 | 0 | 4 | 0 | 0 | 0 | 0 | 0 | 0 | 0 | 0 | N | 69465 | ezrin isoform X1 [Pseudonaja textilis] |
| 287 | 1202 | JAI10013.1 | 75.99 | 3 | 0 | 0 | 0 | 0 | 0 | 3 | 0 | 0 | 0 | 0 | 0 | 0 | 0 | 0 |  |  |  |  |  | 1.1085E6 |  |  |  |  |  |  |  |  | 2 | 2 | 0 | 0 | 0 | 0 | 0 | 4 | 0 | 0 | 0 | 0 | 0 | 0 | 0 | 0 | N | 69529 | Ezrin-like protein [Micrurus fulvius] |
| 287 | 1203 | JAB54254.1 | 75.99 | 3 | 0 | 0 | 0 | 0 | 0 | 3 | 0 | 0 | 0 | 0 | 0 | 0 | 0 | 0 |  |  |  |  |  | 1.1085E6 |  |  |  |  |  |  |  |  | 2 | 2 | 0 | 0 | 0 | 0 | 0 | 4 | 0 | 0 | 0 | 0 | 0 | 0 | 0 | 0 | N | 69529 | Ezrin-like protein [Micrurus fulvius] |
| 287 | 1204 | XP\_032071606.1 | 75.99 | 3 | 0 | 0 | 0 | 0 | 0 | 3 | 0 | 0 | 0 | 0 | 0 | 0 | 0 | 0 |  |  |  |  |  | 1.1085E6 |  |  |  |  |  |  |  |  | 2 | 2 | 0 | 0 | 0 | 0 | 0 | 4 | 0 | 0 | 0 | 0 | 0 | 0 | 0 | 0 | N | 69381 | ezrin [Thamnophis elegans] |
| 287 | 1206 | XP\_013913154.1 | 75.99 | 3 | 0 | 0 | 0 | 0 | 0 | 3 | 0 | 0 | 0 | 0 | 0 | 0 | 0 | 0 |  |  |  |  |  | 1.1085E6 |  |  |  |  |  |  |  |  | 2 | 2 | 0 | 0 | 0 | 0 | 0 | 4 | 0 | 0 | 0 | 0 | 0 | 0 | 0 | 0 | N | 69377 | PREDICTED: ezrin [Thamnophis sirtalis] |
| 287 | 1207 | JAG67998.1 | 75.99 | 3 | 0 | 0 | 0 | 0 | 0 | 3 | 0 | 0 | 0 | 0 | 0 | 0 | 0 | 0 |  |  |  |  |  | 1.1085E6 |  |  |  |  |  |  |  |  | 2 | 2 | 0 | 0 | 0 | 0 | 0 | 4 | 0 | 0 | 0 | 0 | 0 | 0 | 0 | 0 | N | 69357 | Ezrin-like protein [Boiga irregularis] |
| 287 | 1208 | XP\_026560860.1 | 75.99 | 3 | 0 | 0 | 0 | 0 | 0 | 3 | 0 | 0 | 0 | 0 | 0 | 0 | 0 | 0 |  |  |  |  |  | 1.1085E6 |  |  |  |  |  |  |  |  | 2 | 2 | 0 | 0 | 0 | 0 | 0 | 4 | 0 | 0 | 0 | 0 | 0 | 0 | 0 | 0 | N | 69497 | ezrin isoform X2 [Pseudonaja textilis] |
| 287 | 1209 | XP\_034284439.1 | 75.99 | 3 | 0 | 0 | 0 | 0 | 0 | 3 | 0 | 0 | 0 | 0 | 0 | 0 | 0 | 0 |  |  |  |  |  | 1.1085E6 |  |  |  |  |  |  |  |  | 2 | 2 | 0 | 0 | 0 | 0 | 0 | 4 | 0 | 0 | 0 | 0 | 0 | 0 | 0 | 0 | N | 69251 | ezrin [Pantherophis guttatus] |
| 287 | 1210 | XP\_034284440.1 | 75.99 | 3 | 0 | 0 | 0 | 0 | 0 | 3 | 0 | 0 | 0 | 0 | 0 | 0 | 0 | 0 |  |  |  |  |  | 1.1085E6 |  |  |  |  |  |  |  |  | 2 | 2 | 0 | 0 | 0 | 0 | 0 | 4 | 0 | 0 | 0 | 0 | 0 | 0 | 0 | 0 | N | 69251 | ezrin [Pantherophis guttatus] |
| 287 | 1410 | LAA22923.1 | 75.99 | 4 | 0 | 0 | 0 | 0 | 0 | 4 | 0 | 0 | 0 | 0 | 0 | 0 | 0 | 0 |  |  |  |  |  | 1.1085E6 |  |  |  |  |  |  |  |  | 2 | 2 | 0 | 0 | 0 | 0 | 0 | 4 | 0 | 0 | 0 | 0 | 0 | 0 | 0 | 0 | N | 51369 | hypothetical protein [Micrurus lemniscatus carvalhoi] |
| 287 | 1421 | LAA22919.1 | 75.99 | 5 | 0 | 0 | 0 | 0 | 0 | 5 | 0 | 0 | 0 | 0 | 0 | 0 | 0 | 0 |  |  |  |  |  | 1.1085E6 |  |  |  |  |  |  |  |  | 2 | 2 | 0 | 0 | 0 | 0 | 0 | 4 | 0 | 0 | 0 | 0 | 0 | 0 | 0 | 0 | N | 45129 | hypothetical protein, partial [Micrurus lemniscatus carvalhoi] |
| 241 | 3169 | JAG66612.1 | 75.62 | 17 | 0 | 0 | 0 | 0 | 17 | 17 | 0 | 0 | 0 | 0 | 0 | 0 | 0 | 0 |  |  |  |  | 7.8745E5 | 9.2096E6 |  |  |  |  |  |  |  |  | 1 | 1 | 0 | 0 | 0 | 0 | 1 | 6 | 0 | 0 | 0 | 0 | 0 | 0 | 0 | 0 | N | 10163 | protein S100-A6 [Boiga irregularis] |
| 215 | 1633 | LAA17506.1 | 74.18 | 3 | 0 | 0 | 0 | 0 | 0 | 0 | 0 | 0 | 0 | 0 | 0 | 3 | 3 | 3 |  |  |  |  |  |  |  |  |  |  |  | 1.2537E6 | 3.2381E6 | 2.867E7 | 1 | 1 | 0 | 0 | 0 | 0 | 0 | 0 | 0 | 0 | 0 | 0 | 0 | 3 | 5 | 5 | N | 53369 | hypothetical protein, partial [Micrurus lemniscatus carvalhoi] |
| 215 | 1634 | LAB17921.1 | 74.18 | 3 | 0 | 0 | 0 | 0 | 0 | 0 | 0 | 0 | 0 | 0 | 0 | 3 | 3 | 3 |  |  |  |  |  |  |  |  |  |  |  | 1.2537E6 | 3.2381E6 | 2.867E7 | 1 | 1 | 0 | 0 | 0 | 0 | 0 | 0 | 0 | 0 | 0 | 0 | 0 | 3 | 5 | 5 | N | 53456 | hypothetical protein, partial [Micrurus spixii] |
| 215 | 1635 | XP\_013912093.1 | 74.18 | 3 | 0 | 0 | 0 | 0 | 0 | 0 | 0 | 0 | 0 | 0 | 0 | 3 | 3 | 3 |  |  |  |  |  |  |  |  |  |  |  | 1.2537E6 | 3.2381E6 | 2.867E7 | 1 | 1 | 0 | 0 | 0 | 0 | 0 | 0 | 0 | 0 | 0 | 0 | 0 | 3 | 5 | 5 | N | 47484 | PREDICTED: vascular endothelial growth factor C [Thamnophis sirtalis] |
| 215 | 1637 | XP\_034298588.1 | 74.18 | 3 | 0 | 0 | 0 | 0 | 0 | 0 | 0 | 0 | 0 | 0 | 0 | 3 | 3 | 3 |  |  |  |  |  |  |  |  |  |  |  | 1.2537E6 | 3.2381E6 | 2.867E7 | 1 | 1 | 0 | 0 | 0 | 0 | 0 | 0 | 0 | 0 | 0 | 0 | 0 | 3 | 5 | 5 | N | 47507 | vascular endothelial growth factor C isoform X1 [Pantherophis guttatus] |
| 215 | 1638 | JAC95037.1 | 74.18 | 3 | 0 | 0 | 0 | 0 | 0 | 0 | 0 | 0 | 0 | 0 | 0 | 3 | 3 | 3 |  |  |  |  |  |  |  |  |  |  |  | 1.2537E6 | 3.2381E6 | 2.867E7 | 1 | 1 | 0 | 0 | 0 | 0 | 0 | 0 | 0 | 0 | 0 | 0 | 0 | 3 | 5 | 5 | N | 47507 | Vascular endothelial growth factor C [Pantherophis guttatus] |
| 215 | 1641 | JAI08988.1 | 74.18 | 3 | 0 | 0 | 0 | 0 | 0 | 0 | 0 | 0 | 0 | 0 | 0 | 3 | 3 | 3 |  |  |  |  |  |  |  |  |  |  |  | 1.2537E6 | 3.2381E6 | 2.867E7 | 1 | 1 | 0 | 0 | 0 | 0 | 0 | 0 | 0 | 0 | 0 | 0 | 0 | 3 | 5 | 5 | N | 47564 | Vascular endothelial growth factor 2 [Micrurus fulvius] |
| 215 | 1642 | JAI09156.1 | 74.18 | 3 | 0 | 0 | 0 | 0 | 0 | 0 | 0 | 0 | 0 | 0 | 0 | 3 | 3 | 3 |  |  |  |  |  |  |  |  |  |  |  | 1.2537E6 | 3.2381E6 | 2.867E7 | 1 | 1 | 0 | 0 | 0 | 0 | 0 | 0 | 0 | 0 | 0 | 0 | 0 | 3 | 5 | 5 | N | 47564 | Vascular endothelial growth factor C-like protein [Micrurus fulvius] |
| 215 | 1643 | JAB52939.1 | 74.18 | 3 | 0 | 0 | 0 | 0 | 0 | 0 | 0 | 0 | 0 | 0 | 0 | 3 | 3 | 3 |  |  |  |  |  |  |  |  |  |  |  | 1.2537E6 | 3.2381E6 | 2.867E7 | 1 | 1 | 0 | 0 | 0 | 0 | 0 | 0 | 0 | 0 | 0 | 0 | 0 | 3 | 5 | 5 | N | 47564 | vascular endothelial growth factor C [Micrurus fulvius] |
| 215 | 1644 | JAS04977.1 | 74.18 | 3 | 0 | 0 | 0 | 0 | 0 | 0 | 0 | 0 | 0 | 0 | 0 | 3 | 3 | 3 |  |  |  |  |  |  |  |  |  |  |  | 1.2537E6 | 3.2381E6 | 2.867E7 | 1 | 1 | 0 | 0 | 0 | 0 | 0 | 0 | 0 | 0 | 0 | 0 | 0 | 3 | 5 | 5 | N | 47564 | Vascular endothelial growth factor 2 [Micrurus fulvius] |
| 215 | 1645 | XP\_026555459.1 | 74.18 | 3 | 0 | 0 | 0 | 0 | 0 | 0 | 0 | 0 | 0 | 0 | 0 | 3 | 3 | 3 |  |  |  |  |  |  |  |  |  |  |  | 1.2537E6 | 3.2381E6 | 2.867E7 | 1 | 1 | 0 | 0 | 0 | 0 | 0 | 0 | 0 | 0 | 0 | 0 | 0 | 3 | 5 | 5 | N | 47615 | vascular endothelial growth factor C [Pseudonaja textilis] |
| 215 | 1646 | XP\_026522979.1 | 74.18 | 3 | 0 | 0 | 0 | 0 | 0 | 0 | 0 | 0 | 0 | 0 | 0 | 3 | 3 | 3 |  |  |  |  |  |  |  |  |  |  |  | 1.2537E6 | 3.2381E6 | 2.867E7 | 1 | 1 | 0 | 0 | 0 | 0 | 0 | 0 | 0 | 0 | 0 | 0 | 0 | 3 | 5 | 5 | N | 47528 | vascular endothelial growth factor C [Notechis scutatus] |
| 215 | 1647 | ETE60014.1 | 74.18 | 3 | 0 | 0 | 0 | 0 | 0 | 0 | 0 | 0 | 0 | 0 | 0 | 3 | 3 | 3 |  |  |  |  |  |  |  |  |  |  |  | 1.2537E6 | 3.2381E6 | 2.867E7 | 1 | 1 | 0 | 0 | 0 | 0 | 0 | 0 | 0 | 0 | 0 | 0 | 0 | 3 | 5 | 5 | N | 47557 | Vascular endothelial growth factor C [Ophiophagus hannah] |
| 215 | 1649 | XP\_032080314.1 | 74.18 | 3 | 0 | 0 | 0 | 0 | 0 | 0 | 0 | 0 | 0 | 0 | 0 | 3 | 3 | 3 |  |  |  |  |  |  |  |  |  |  |  | 1.2537E6 | 3.2381E6 | 2.867E7 | 1 | 1 | 0 | 0 | 0 | 0 | 0 | 0 | 0 | 0 | 0 | 0 | 0 | 3 | 5 | 5 | N | 49464 | LOW QUALITY PROTEIN: vascular endothelial growth factor C [Thamnophis elegans] |
| 215 | 1772 | XP\_034298589.1 | 74.18 | 4 | 0 | 0 | 0 | 0 | 0 | 0 | 0 | 0 | 0 | 0 | 0 | 4 | 4 | 4 |  |  |  |  |  |  |  |  |  |  |  | 1.2537E6 | 3.2381E6 | 2.867E7 | 1 | 1 | 0 | 0 | 0 | 0 | 0 | 0 | 0 | 0 | 0 | 0 | 0 | 3 | 5 | 5 | N | 42840 | vascular endothelial growth factor C isoform X2 [Pantherophis guttatus] |
| 215 | 1780 | XP\_029139565.1 | 74.18 | 3 | 0 | 0 | 0 | 0 | 0 | 0 | 0 | 0 | 0 | 0 | 0 | 3 | 3 | 3 |  |  |  |  |  |  |  |  |  |  |  | 1.2537E6 | 3.2381E6 | 2.867E7 | 1 | 1 | 0 | 0 | 0 | 0 | 0 | 0 | 0 | 0 | 0 | 0 | 0 | 3 | 5 | 5 | N | 47709 | vascular endothelial growth factor C [Protobothrops mucrosquamatus] |
| 215 | 1809 | JAC96561.1 | 74.18 | 3 | 0 | 0 | 0 | 0 | 0 | 0 | 0 | 0 | 0 | 0 | 0 | 3 | 3 | 3 |  |  |  |  |  |  |  |  |  |  |  | 1.2537E6 | 3.2381E6 | 2.867E7 | 1 | 1 | 0 | 0 | 0 | 0 | 0 | 0 | 0 | 0 | 0 | 0 | 0 | 3 | 5 | 5 | N | 47649 | Vascular endothelial growth factor C [Echis coloratus] |
| 215 | 1812 | JAI10614.1 | 74.18 | 3 | 0 | 0 | 0 | 0 | 0 | 0 | 0 | 0 | 0 | 0 | 0 | 3 | 3 | 3 |  |  |  |  |  |  |  |  |  |  |  | 1.2537E6 | 3.2381E6 | 2.867E7 | 1 | 1 | 0 | 0 | 0 | 0 | 0 | 0 | 0 | 0 | 0 | 0 | 0 | 3 | 5 | 5 | N | 47697 | Vascular endothelial growth factor C-like protein [Crotalus adamanteus] |
| 215 | 1813 | SMD28268.1 | 74.18 | 3 | 0 | 0 | 0 | 0 | 0 | 0 | 0 | 0 | 0 | 0 | 0 | 3 | 3 | 3 |  |  |  |  |  |  |  |  |  |  |  | 1.2537E6 | 3.2381E6 | 2.867E7 | 1 | 1 | 0 | 0 | 0 | 0 | 0 | 0 | 0 | 0 | 0 | 0 | 0 | 3 | 5 | 5 | N | 47611 | vascular endothelial growth factor CdtVEGF3 [Crotalus durissus terrificus] |
| 215 | 1814 | JAA95033.1 | 74.18 | 3 | 0 | 0 | 0 | 0 | 0 | 0 | 0 | 0 | 0 | 0 | 0 | 3 | 3 | 3 |  |  |  |  |  |  |  |  |  |  |  | 1.2537E6 | 3.2381E6 | 2.867E7 | 1 | 1 | 0 | 0 | 0 | 0 | 0 | 0 | 0 | 0 | 0 | 0 | 0 | 3 | 5 | 5 | N | 47639 | Vascular endothelial growth factor C-like protein [Crotalus horridus] |
| 215 | 1815 | ASU45066.1 | 74.18 | 3 | 0 | 0 | 0 | 0 | 0 | 0 | 0 | 0 | 0 | 0 | 0 | 3 | 3 | 3 |  |  |  |  |  |  |  |  |  |  |  | 1.2537E6 | 3.2381E6 | 2.867E7 | 1 | 1 | 0 | 0 | 0 | 0 | 0 | 0 | 0 | 0 | 0 | 0 | 0 | 3 | 5 | 5 | N | 47699 | vascular endothelial growth factor C [Daboia russelii] |
| 215 | 1820 | JAV48556.1 | 74.18 | 3 | 0 | 0 | 0 | 0 | 0 | 0 | 0 | 0 | 0 | 0 | 0 | 3 | 3 | 3 |  |  |  |  |  |  |  |  |  |  |  | 1.2537E6 | 3.2381E6 | 2.867E7 | 1 | 1 | 0 | 0 | 0 | 0 | 0 | 0 | 0 | 0 | 0 | 0 | 0 | 3 | 5 | 5 | N | 47653 | Vascular endothelial growth factor C-like protein [Agkistrodon contortrix contortrix] |
| 215 | 1821 | XP\_034298590.1 | 74.18 | 4 | 0 | 0 | 0 | 0 | 0 | 0 | 0 | 0 | 0 | 0 | 0 | 4 | 4 | 4 |  |  |  |  |  |  |  |  |  |  |  | 1.2537E6 | 3.2381E6 | 2.867E7 | 1 | 1 | 0 | 0 | 0 | 0 | 0 | 0 | 0 | 0 | 0 | 0 | 0 | 3 | 5 | 5 | N | 40062 | vascular endothelial growth factor C isoform X3 [Pantherophis guttatus] |
| 215 | 1826 | JAC94973.1 | 74.18 | 4 | 0 | 0 | 0 | 0 | 0 | 0 | 0 | 0 | 0 | 0 | 0 | 4 | 4 | 4 |  |  |  |  |  |  |  |  |  |  |  | 1.2537E6 | 3.2381E6 | 2.867E7 | 1 | 1 | 0 | 0 | 0 | 0 | 0 | 0 | 0 | 0 | 0 | 0 | 0 | 3 | 5 | 5 | N | 40057 | Vascular endothelial growth factor C [Opheodrys aestivus] |
| 215 | 2227 | LAB17920.1 | 74.18 | 5 | 0 | 0 | 0 | 0 | 0 | 0 | 0 | 0 | 0 | 0 | 0 | 5 | 5 | 5 |  |  |  |  |  |  |  |  |  |  |  | 1.2537E6 | 3.2381E6 | 2.867E7 | 1 | 1 | 0 | 0 | 0 | 0 | 0 | 0 | 0 | 0 | 0 | 0 | 0 | 3 | 5 | 5 | N | 32584 | hypothetical protein [Micrurus spixii] |
| 215 | 2318 | JAC94939.1 | 74.18 | 3 | 0 | 0 | 0 | 0 | 0 | 0 | 0 | 0 | 0 | 0 | 0 | 3 | 3 | 3 |  |  |  |  |  |  |  |  |  |  |  | 1.2537E6 | 3.2381E6 | 2.867E7 | 1 | 1 | 0 | 0 | 0 | 0 | 0 | 0 | 0 | 0 | 0 | 0 | 0 | 3 | 5 | 5 | N | 47538 | Vascular endothelial growth factor C [Python regius] |
| 215 | 2361 | XP\_007425787.1 | 74.18 | 4 | 0 | 0 | 0 | 0 | 0 | 0 | 0 | 0 | 0 | 0 | 0 | 4 | 4 | 4 |  |  |  |  |  |  |  |  |  |  |  | 1.2537E6 | 3.2381E6 | 2.867E7 | 1 | 1 | 0 | 0 | 0 | 0 | 0 | 0 | 0 | 0 | 0 | 0 | 0 | 3 | 5 | 5 | N | 42635 | vascular endothelial growth factor C, partial [Python bivittatus] |
| 293 | 1872 | XP\_015666657.1 | 73.35 | 9 | 0 | 0 | 4 | 9 | 0 | 0 | 0 | 0 | 0 | 0 | 0 | 0 | 0 | 0 |  |  |  | 2.9914E5 |  |  |  |  |  |  |  |  |  |  | 2 | 1 | 0 | 0 | 2 | 2 | 0 | 0 | 0 | 0 | 0 | 0 | 0 | 0 | 0 | 0 | N | 25001 | mammalian ependymin-related protein 1 [Protobothrops mucrosquamatus] |
| 293 | 1902 | ETE58174.1 | 73.35 | 10 | 0 | 0 | 4 | 10 | 0 | 0 | 0 | 0 | 0 | 0 | 0 | 0 | 0 | 0 |  |  |  | 2.9914E5 |  |  |  |  |  |  |  |  |  |  | 2 | 1 | 0 | 0 | 2 | 2 | 0 | 0 | 0 | 0 | 0 | 0 | 0 | 0 | 0 | 0 | N | 24063 | Mammalian ependymin-related protein 1, partial [Ophiophagus hannah] |
| 187 | 1427 | XP\_032084681.1 | 73.27 | 2 | 0 | 0 | 0 | 0 | 2 | 2 | 0 | 0 | 0 | 0 | 0 | 0 | 0 | 0 |  |  |  |  | 1.4893E8 | 1.9296E8 |  |  |  |  |  |  |  |  | 1 | 1 | 0 | 0 | 0 | 0 | 2 | 13 | 0 | 0 | 0 | 0 | 0 | 0 | 0 | 0 | Y | 69288 | zinc metalloproteinase-disintegrin-like MTP9 [Thamnophis elegans] |
| 264 | 1455 | XP\_034280930.1 | 72.75 | 4 | 0 | 4 | 4 | 4 | 0 | 0 | 0 | 0 | 0 | 0 | 0 | 0 | 0 | 0 |  | 0 | 1.5627E6 | 1.9529E6 |  |  |  |  |  |  |  |  |  |  | 1 | 1 | 0 | 1 | 2 | 3 | 0 | 0 | 0 | 0 | 0 | 0 | 0 | 0 | 0 | 0 | N | 39235 | extracellular matrix protein 1 [Pantherophis guttatus] |
| 264 | 1879 | ETE63009.1 | 72.75 | 5 | 0 | 5 | 5 | 5 | 0 | 0 | 0 | 0 | 0 | 0 | 0 | 0 | 0 | 0 |  | 0 | 1.5627E6 | 1.9529E6 |  |  |  |  |  |  |  |  |  |  | 1 | 1 | 0 | 1 | 2 | 3 | 0 | 0 | 0 | 0 | 0 | 0 | 0 | 0 | 0 | 0 | N | 34271 | Extracellular matrix protein 1, partial [Ophiophagus hannah] |
| 305 | 1139 | XP\_015676332.1 | 72.07 | 4 | 0 | 0 | 0 | 0 | 2 | 2 | 0 | 0 | 0 | 0 | 0 | 0 | 0 | 0 |  |  |  |  | 2.0603E5 | 5.0967E5 |  |  |  |  |  |  |  |  | 2 | 2 | 0 | 0 | 0 | 0 | 1 | 1 | 0 | 0 | 0 | 0 | 0 | 0 | 0 | 0 | N | 61048 | N-acetylglucosamine-6-sulfatase isoform X1 [Protobothrops mucrosquamatus] |
| 305 | 1154 | LAA85609.1 | 72.07 | 4 | 0 | 0 | 0 | 0 | 2 | 2 | 0 | 0 | 0 | 0 | 0 | 0 | 0 | 0 |  |  |  |  | 2.0603E5 | 5.0967E5 |  |  |  |  |  |  |  |  | 2 | 2 | 0 | 0 | 0 | 0 | 1 | 1 | 0 | 0 | 0 | 0 | 0 | 0 | 0 | 0 | N | 61053 | hypothetical protein [Micrurus lemniscatus lemniscatus] |
| 305 | 1155 | LAA85607.1 | 72.07 | 4 | 0 | 0 | 0 | 0 | 2 | 2 | 0 | 0 | 0 | 0 | 0 | 0 | 0 | 0 |  |  |  |  | 2.0603E5 | 5.0967E5 |  |  |  |  |  |  |  |  | 2 | 2 | 0 | 0 | 0 | 0 | 1 | 1 | 0 | 0 | 0 | 0 | 0 | 0 | 0 | 0 | N | 61053 | hypothetical protein [Micrurus lemniscatus lemniscatus] |
| 305 | 1181 | XP\_013929985.1 | 72.07 | 4 | 0 | 0 | 0 | 0 | 2 | 2 | 0 | 0 | 0 | 0 | 0 | 0 | 0 | 0 |  |  |  |  | 2.0603E5 | 5.0967E5 |  |  |  |  |  |  |  |  | 2 | 2 | 0 | 0 | 0 | 0 | 1 | 1 | 0 | 0 | 0 | 0 | 0 | 0 | 0 | 0 | N | 61166 | PREDICTED: N-acetylglucosamine-6-sulfatase [Thamnophis sirtalis] |
| 305 | 1190 | XP\_032076778.1 | 72.07 | 4 | 0 | 0 | 0 | 0 | 2 | 2 | 0 | 0 | 0 | 0 | 0 | 0 | 0 | 0 |  |  |  |  | 2.0603E5 | 5.0967E5 |  |  |  |  |  |  |  |  | 2 | 2 | 0 | 0 | 0 | 0 | 1 | 1 | 0 | 0 | 0 | 0 | 0 | 0 | 0 | 0 | N | 61159 | N-acetylglucosamine-6-sulfatase [Thamnophis elegans] |
| 279 | 1252 | LAA30756.1 | 71.36 | 22 | 0 | 0 | 0 | 0 | 0 | 22 | 0 | 0 | 0 | 0 | 0 | 0 | 0 | 0 |  |  |  |  |  | 8.7627E6 |  |  |  |  |  |  |  |  | 2 | 1 | 0 | 0 | 0 | 0 | 0 | 2 | 0 | 0 | 0 | 0 | 0 | 0 | 0 | 0 | N | 12797 | hypothetical protein, partial [Micrurus lemniscatus carvalhoi] |
| 304 | 1125 | XP\_026545273.1 | 70.77 | 6 | 0 | 0 | 0 | 6 | 0 | 0 | 0 | 0 | 0 | 0 | 0 | 0 | 0 | 0 |  |  |  | 1.2281E6 |  |  |  |  |  |  |  |  |  |  | 2 | 2 | 0 | 0 | 0 | 3 | 0 | 0 | 0 | 0 | 0 | 0 | 0 | 0 | 0 | 0 | Y | 56767 | intestinal-type alkaline phosphatase [Notechis scutatus] |
| 304 | 1140 | XP\_026576786.1 | 70.77 | 6 | 0 | 0 | 0 | 6 | 0 | 0 | 0 | 0 | 0 | 0 | 0 | 0 | 0 | 0 |  |  |  | 1.2281E6 |  |  |  |  |  |  |  |  |  |  | 2 | 2 | 0 | 0 | 0 | 3 | 0 | 0 | 0 | 0 | 0 | 0 | 0 | 0 | 0 | 0 | Y | 56737 | intestinal-type alkaline phosphatase [Pseudonaja textilis] |
| 304 | 1576 | ETE58211.1 | 70.77 | 12 | 0 | 0 | 0 | 12 | 0 | 0 | 0 | 0 | 0 | 0 | 0 | 0 | 0 | 0 |  |  |  | 1.2281E6 |  |  |  |  |  |  |  |  |  |  | 2 | 2 | 0 | 0 | 0 | 3 | 0 | 0 | 0 | 0 | 0 | 0 | 0 | 0 | 0 | 0 | Y | 28374 | Intestinal-type alkaline phosphatase, partial [Ophiophagus hannah] |
| 274 | 2108 | P01433.1 | 68.39 | 38 | 13 | 0 | 0 | 0 | 0 | 0 | 25 | 0 | 0 | 0 | 0 | 0 | 0 | 0 |  |  |  |  |  |  | 0 |  |  |  |  |  |  |  | 2 | 1 | 1 | 0 | 0 | 0 | 0 | 0 | 1 | 0 | 0 | 0 | 0 | 0 | 0 | 0 | Y | 6832 | RecName: Full=Short neurotoxin 2; AltName: Full=Toxin IV |
| 290 | 1392 | ETE68149.1 | 68.11 | 2 | 0 | 0 | 0 | 0 | 2 | 2 | 0 | 0 | 0 | 0 | 0 | 0 | 0 | 0 |  |  |  |  | 5.8342E5 | 1.9512E6 |  |  |  |  |  |  |  |  | 1 | 1 | 0 | 0 | 0 | 0 | 1 | 2 | 0 | 0 | 0 | 0 | 0 | 0 | 0 | 0 | N | 45887 | Neuroserpin, partial [Ophiophagus hannah] |
| 290 | 1704 | XP\_034294930.1 | 68.11 | 2 | 0 | 0 | 0 | 0 | 2 | 2 | 0 | 0 | 0 | 0 | 0 | 0 | 0 | 0 |  |  |  |  | 5.8342E5 | 1.9512E6 |  |  |  |  |  |  |  |  | 1 | 1 | 0 | 0 | 0 | 0 | 1 | 2 | 0 | 0 | 0 | 0 | 0 | 0 | 0 | 0 | N | 46278 | neuroserpin [Pantherophis guttatus] |
| 290 | 1705 | LAA57072.1 | 68.11 | 2 | 0 | 0 | 0 | 0 | 2 | 2 | 0 | 0 | 0 | 0 | 0 | 0 | 0 | 0 |  |  |  |  | 5.8342E5 | 1.9512E6 |  |  |  |  |  |  |  |  | 1 | 1 | 0 | 0 | 0 | 0 | 1 | 2 | 0 | 0 | 0 | 0 | 0 | 0 | 0 | 0 | N | 46381 | hypothetical protein [Micrurus corallinus] |
| 290 | 1707 | LAA70840.1 | 68.11 | 2 | 0 | 0 | 0 | 0 | 2 | 2 | 0 | 0 | 0 | 0 | 0 | 0 | 0 | 0 |  |  |  |  | 5.8342E5 | 1.9512E6 |  |  |  |  |  |  |  |  | 1 | 1 | 0 | 0 | 0 | 0 | 1 | 2 | 0 | 0 | 0 | 0 | 0 | 0 | 0 | 0 | N | 46354 | hypothetical protein [Micrurus lemniscatus lemniscatus] |
| 309 | 1738 | ETE67626.1 | 67.78 | 8 | 0 | 0 | 8 | 0 | 0 | 0 | 0 | 0 | 0 | 0 | 0 | 0 | 0 | 0 |  |  | 1.0164E6 |  |  |  |  |  |  |  |  |  |  |  | 1 | 1 | 0 | 0 | 1 | 0 | 0 | 0 | 0 | 0 | 0 | 0 | 0 | 0 | 0 | 0 | N | 24156 | hypothetical protein L345\_06588, partial [Ophiophagus hannah] |
| 309 | 1773 | XP\_032090398.1 | 67.78 | 17 | 0 | 0 | 17 | 0 | 0 | 0 | 0 | 0 | 0 | 0 | 0 | 0 | 0 | 0 |  |  | 1.0164E6 |  |  |  |  |  |  |  |  |  |  |  | 1 | 1 | 0 | 0 | 1 | 0 | 0 | 0 | 0 | 0 | 0 | 0 | 0 | 0 | 0 | 0 | N | 11629 | cytochrome c [Thamnophis elegans] |
| 309 | 1774 | XP\_013909813.1 | 67.78 | 17 | 0 | 0 | 17 | 0 | 0 | 0 | 0 | 0 | 0 | 0 | 0 | 0 | 0 | 0 |  |  | 1.0164E6 |  |  |  |  |  |  |  |  |  |  |  | 1 | 1 | 0 | 0 | 1 | 0 | 0 | 0 | 0 | 0 | 0 | 0 | 0 | 0 | 0 | 0 | N | 11629 | PREDICTED: cytochrome c [Thamnophis sirtalis] |
| 309 | 1775 | XP\_013909814.1 | 67.78 | 17 | 0 | 0 | 17 | 0 | 0 | 0 | 0 | 0 | 0 | 0 | 0 | 0 | 0 | 0 |  |  | 1.0164E6 |  |  |  |  |  |  |  |  |  |  |  | 1 | 1 | 0 | 0 | 1 | 0 | 0 | 0 | 0 | 0 | 0 | 0 | 0 | 0 | 0 | 0 | N | 11629 | PREDICTED: cytochrome c [Thamnophis sirtalis] |
| 309 | 1776 | XP\_026529406.1 | 67.78 | 17 | 0 | 0 | 17 | 0 | 0 | 0 | 0 | 0 | 0 | 0 | 0 | 0 | 0 | 0 |  |  | 1.0164E6 |  |  |  |  |  |  |  |  |  |  |  | 1 | 1 | 0 | 0 | 1 | 0 | 0 | 0 | 0 | 0 | 0 | 0 | 0 | 0 | 0 | 0 | N | 11629 | cytochrome c [Notechis scutatus] |
| 309 | 1777 | XP\_026562211.1 | 67.78 | 17 | 0 | 0 | 17 | 0 | 0 | 0 | 0 | 0 | 0 | 0 | 0 | 0 | 0 | 0 |  |  | 1.0164E6 |  |  |  |  |  |  |  |  |  |  |  | 1 | 1 | 0 | 0 | 1 | 0 | 0 | 0 | 0 | 0 | 0 | 0 | 0 | 0 | 0 | 0 | N | 11629 | cytochrome c [Pseudonaja textilis] |
| 309 | 1778 | XP\_032090399.1 | 67.78 | 17 | 0 | 0 | 17 | 0 | 0 | 0 | 0 | 0 | 0 | 0 | 0 | 0 | 0 | 0 |  |  | 1.0164E6 |  |  |  |  |  |  |  |  |  |  |  | 1 | 1 | 0 | 0 | 1 | 0 | 0 | 0 | 0 | 0 | 0 | 0 | 0 | 0 | 0 | 0 | N | 11629 | cytochrome c [Thamnophis elegans] |
| 309 | 1839 | XP\_034257829.1 | 67.78 | 17 | 0 | 0 | 17 | 0 | 0 | 0 | 0 | 0 | 0 | 0 | 0 | 0 | 0 | 0 |  |  | 1.0164E6 |  |  |  |  |  |  |  |  |  |  |  | 1 | 1 | 0 | 0 | 1 | 0 | 0 | 0 | 0 | 0 | 0 | 0 | 0 | 0 | 0 | 0 | N | 11643 | cytochrome c [Pantherophis guttatus] |
| 309 | 1840 | JAG68241.1 | 67.78 | 17 | 0 | 0 | 17 | 0 | 0 | 0 | 0 | 0 | 0 | 0 | 0 | 0 | 0 | 0 |  |  | 1.0164E6 |  |  |  |  |  |  |  |  |  |  |  | 1 | 1 | 0 | 0 | 1 | 0 | 0 | 0 | 0 | 0 | 0 | 0 | 0 | 0 | 0 | 0 | N | 11643 | Cytochrome c [Boiga irregularis] |
| 309 | 1841 | JAG68226.1 | 67.78 | 17 | 0 | 0 | 17 | 0 | 0 | 0 | 0 | 0 | 0 | 0 | 0 | 0 | 0 | 0 |  |  | 1.0164E6 |  |  |  |  |  |  |  |  |  |  |  | 1 | 1 | 0 | 0 | 1 | 0 | 0 | 0 | 0 | 0 | 0 | 0 | 0 | 0 | 0 | 0 | N | 11643 | cytochrome c [Boiga irregularis] |
| 309 | 1842 | XP\_034257830.1 | 67.78 | 17 | 0 | 0 | 17 | 0 | 0 | 0 | 0 | 0 | 0 | 0 | 0 | 0 | 0 | 0 |  |  | 1.0164E6 |  |  |  |  |  |  |  |  |  |  |  | 1 | 1 | 0 | 0 | 1 | 0 | 0 | 0 | 0 | 0 | 0 | 0 | 0 | 0 | 0 | 0 | N | 11643 | cytochrome c [Pantherophis guttatus] |
| 232 | 1424 | JAS05411.1 | 67.25 | 4 | 0 | 0 | 0 | 0 | 0 | 3 | 0 | 0 | 0 | 0 | 0 | 1 | 0 | 0 |  |  |  |  |  | 2.4757E8 |  |  |  |  |  |  |  |  | 2 | 1 | 0 | 0 | 0 | 0 | 0 | 2 | 0 | 0 | 0 | 0 | 0 | 1 | 0 | 0 | Y | 68316 | Metalloproteinase type III 7b [Sistrurus miliarius barbouri] |
| 232 | 1425 | JAS05412.1 | 67.25 | 4 | 0 | 0 | 0 | 0 | 0 | 3 | 0 | 0 | 0 | 0 | 0 | 1 | 0 | 0 |  |  |  |  |  | 2.4757E8 |  |  |  |  |  |  |  |  | 2 | 1 | 0 | 0 | 0 | 0 | 0 | 2 | 0 | 0 | 0 | 0 | 0 | 1 | 0 | 0 | Y | 68472 | Metalloproteinase type III 7a [Sistrurus miliarius barbouri] |
| 232 | 1431 | QIV64956.1 | 67.25 | 4 | 0 | 0 | 0 | 0 | 0 | 3 | 0 | 0 | 0 | 0 | 0 | 1 | 0 | 0 |  |  |  |  |  | 2.4757E8 |  |  |  |  |  |  |  |  | 2 | 1 | 0 | 0 | 0 | 0 | 0 | 2 | 0 | 0 | 0 | 0 | 0 | 1 | 0 | 0 | Y | 68366 | MDC-8b [Crotalus atrox] |
| 203 | 2191 | XP\_032073626.1 | 66.84 | 3 | 0 | 3 | 3 | 3 | 0 | 3 | 0 | 0 | 0 | 0 | 0 | 0 | 0 | 3 |  | 3.7045E7 | 7.7031E8 | 5.0143E6 |  | 5.0515E5 |  |  |  |  |  |  |  | 1.8628E7 | 1 | 1 | 0 | 3 | 7 | 2 | 0 | 1 | 0 | 0 | 0 | 0 | 0 | 0 | 0 | 2 | Y | 38075 | zona pellucida sperm-binding protein 3 receptor-like isoform X3 [Thamnophis elegans] |
| 203 | 2192 | XP\_032073624.1 | 66.84 | 3 | 0 | 3 | 3 | 3 | 0 | 3 | 0 | 0 | 0 | 0 | 0 | 0 | 0 | 3 |  | 3.7045E7 | 7.7031E8 | 5.0143E6 |  | 5.0515E5 |  |  |  |  |  |  |  | 1.8628E7 | 1 | 1 | 0 | 3 | 7 | 2 | 0 | 1 | 0 | 0 | 0 | 0 | 0 | 0 | 0 | 2 | Y | 41736 | membrane cofactor protein-like isoform X1 [Thamnophis elegans] |
| 203 | 2307 | XP\_032073625.1 | 66.84 | 3 | 0 | 3 | 3 | 3 | 0 | 3 | 0 | 0 | 0 | 0 | 0 | 0 | 0 | 3 |  | 3.7045E7 | 7.7031E8 | 5.0143E6 |  | 5.0515E5 |  |  |  |  |  |  |  | 1.8628E7 | 1 | 1 | 0 | 3 | 7 | 2 | 0 | 1 | 0 | 0 | 0 | 0 | 0 | 0 | 0 | 2 | Y | 39347 | membrane cofactor protein-like isoform X2 [Thamnophis elegans] |
| 355 | 3392 | JAB53384.1 | 65.74 | 17 | 0 | 0 | 0 | 0 | 0 | 17 | 0 | 0 | 0 | 0 | 0 | 0 | 0 | 0 |  |  |  |  |  | 6.9347E6 |  |  |  |  |  |  |  |  | 1 | 1 | 0 | 0 | 0 | 0 | 0 | 1 | 0 | 0 | 0 | 0 | 0 | 0 | 0 | 0 | N | 10120 | S100-A6 [Micrurus fulvius] |
| 285 | 1478 | AAB28452.1 | 65.36 | 41 | 0 | 0 | 0 | 0 | 0 | 0 | 0 | 0 | 0 | 28 | 0 | 12 | 12 | 0 |  |  |  |  |  |  |  |  |  | 9.165E6 |  |  |  |  | 2 | 1 | 0 | 0 | 0 | 0 | 0 | 0 | 0 | 0 | 0 | 1 | 0 | 1 | 2 | 0 | Y | 7361 | m1-toxin=m1 muscarinic receptor specific antagonist [Dendroaspis angusticeps=Eastern green mambas, Africa, venom, Peptide, 64 aa] |
| 285 | 1923 | 3NEQ | 65.36 | 39 | 0 | 0 | 0 | 0 | 0 | 0 | 0 | 0 | 0 | 27 | 0 | 12 | 12 | 0 |  |  |  |  |  |  |  |  |  | 9.165E6 |  |  |  |  | 2 | 1 | 0 | 0 | 0 | 0 | 0 | 0 | 0 | 0 | 0 | 1 | 0 | 1 | 2 | 0 | Y | 7587 | Chain B, Three-finger Muscarinic Toxin 7 |
| 285 | 1958 | 6WJC | 65.36 | 38 | 0 | 0 | 0 | 0 | 0 | 0 | 0 | 0 | 0 | 26 | 0 | 12 | 12 | 0 |  |  |  |  |  |  |  |  |  | 9.165E6 |  |  |  |  | 2 | 1 | 0 | 0 | 0 | 0 | 0 | 0 | 0 | 0 | 0 | 1 | 0 | 1 | 2 | 0 | Y | 7779 | Chain C, Muscarinic toxin 7 |
| 285 | 1962 | AAM00185.1 | 65.36 | 30 | 0 | 0 | 0 | 0 | 0 | 0 | 0 | 0 | 0 | 21 | 0 | 9 | 9 | 0 |  |  |  |  |  |  |  |  |  | 9.165E6 |  |  |  |  | 2 | 1 | 0 | 0 | 0 | 0 | 0 | 0 | 0 | 0 | 0 | 1 | 0 | 1 | 2 | 0 | Y | 9771 | m1-toxin1 [Dendroaspis angusticeps] |
| 285 | 1963 | Q8QGR0.1 | 65.36 | 30 | 0 | 0 | 0 | 0 | 0 | 0 | 0 | 0 | 0 | 21 | 0 | 9 | 9 | 0 |  |  |  |  |  |  |  |  |  | 9.165E6 |  |  |  |  | 2 | 1 | 0 | 0 | 0 | 0 | 0 | 0 | 0 | 0 | 0 | 1 | 0 | 1 | 2 | 0 | Y | 9771 | RecName: Full=Muscarinic toxin 7; Short=MT-7; Short=MT7; AltName: Full=Muscarinic toxin 1; Short=m1-toxin; Flags: Precursor |
| 285 | 1970 | 2VLW | 65.36 | 40 | 0 | 0 | 0 | 0 | 0 | 0 | 0 | 0 | 0 | 28 | 0 | 12 | 12 | 0 |  |  |  |  |  |  |  |  |  | 9.165E6 |  |  |  |  | 2 | 1 | 0 | 0 | 0 | 0 | 0 | 0 | 0 | 0 | 0 | 1 | 0 | 1 | 2 | 0 | Y | 7436 | Chain B, Muscarinic M1-toxin1 |
| 580 | 2107 | XP\_007427942.1 | 65.01 | 4 | 0 | 0 | 0 | 4 | 0 | 0 | 0 | 0 | 0 | 0 | 0 | 0 | 0 | 0 |  |  |  | 4.0563E5 |  |  |  |  |  |  |  |  |  |  | 1 | 1 | 0 | 0 | 0 | 1 | 0 | 0 | 0 | 0 | 0 | 0 | 0 | 0 | 0 | 0 | N | 39050 | beta-1,4-galactosyltransferase 1 [Python bivittatus] |
| 580 | 2276 | XP\_032068558.1 | 65.01 | 4 | 0 | 0 | 0 | 4 | 0 | 0 | 0 | 0 | 0 | 0 | 0 | 0 | 0 | 0 |  |  |  | 4.0563E5 |  |  |  |  |  |  |  |  |  |  | 1 | 1 | 0 | 0 | 0 | 1 | 0 | 0 | 0 | 0 | 0 | 0 | 0 | 0 | 0 | 0 | N | 39108 | beta-1,4-galactosyltransferase 1 [Thamnophis elegans] |
| 580 | 2277 | XP\_013931643.1 | 65.01 | 4 | 0 | 0 | 0 | 4 | 0 | 0 | 0 | 0 | 0 | 0 | 0 | 0 | 0 | 0 |  |  |  | 4.0563E5 |  |  |  |  |  |  |  |  |  |  | 1 | 1 | 0 | 0 | 0 | 1 | 0 | 0 | 0 | 0 | 0 | 0 | 0 | 0 | 0 | 0 | N | 39108 | PREDICTED: beta-1,4-galactosyltransferase 1 [Thamnophis sirtalis] |
| 580 | 2281 | XP\_026528681.1 | 65.01 | 4 | 0 | 0 | 0 | 4 | 0 | 0 | 0 | 0 | 0 | 0 | 0 | 0 | 0 | 0 |  |  |  | 4.0563E5 |  |  |  |  |  |  |  |  |  |  | 1 | 1 | 0 | 0 | 0 | 1 | 0 | 0 | 0 | 0 | 0 | 0 | 0 | 0 | 0 | 0 | N | 39214 | beta-1,4-galactosyltransferase 1 [Notechis scutatus] |
| 580 | 2289 | JAA97000.1 | 65.01 | 4 | 0 | 0 | 0 | 4 | 0 | 0 | 0 | 0 | 0 | 0 | 0 | 0 | 0 | 0 |  |  |  | 4.0563E5 |  |  |  |  |  |  |  |  |  |  | 1 | 1 | 0 | 0 | 0 | 1 | 0 | 0 | 0 | 0 | 0 | 0 | 0 | 0 | 0 | 0 | N | 39194 | beta-1,4-galactosyltransferase 1-like protein [Crotalus horridus] |
| 580 | 2290 | JAG44675.1 | 65.01 | 4 | 0 | 0 | 0 | 4 | 0 | 0 | 0 | 0 | 0 | 0 | 0 | 0 | 0 | 0 |  |  |  | 4.0563E5 |  |  |  |  |  |  |  |  |  |  | 1 | 1 | 0 | 0 | 0 | 1 | 0 | 0 | 0 | 0 | 0 | 0 | 0 | 0 | 0 | 0 | N | 39194 | beta-1,4-galactosyltransferase 1-like protein [Crotalus horridus] |
| 580 | 2320 | XP\_015669310.1 | 65.01 | 4 | 0 | 0 | 0 | 4 | 0 | 0 | 0 | 0 | 0 | 0 | 0 | 0 | 0 | 0 |  |  |  | 4.0563E5 |  |  |  |  |  |  |  |  |  |  | 1 | 1 | 0 | 0 | 0 | 1 | 0 | 0 | 0 | 0 | 0 | 0 | 0 | 0 | 0 | 0 | N | 39171 | beta-1,4-galactosyltransferase 1 [Protobothrops mucrosquamatus] |
| 580 | 2335 | XP\_026568521.1 | 65.01 | 4 | 0 | 0 | 0 | 4 | 0 | 0 | 0 | 0 | 0 | 0 | 0 | 0 | 0 | 0 |  |  |  | 4.0563E5 |  |  |  |  |  |  |  |  |  |  | 1 | 1 | 0 | 0 | 0 | 1 | 0 | 0 | 0 | 0 | 0 | 0 | 0 | 0 | 0 | 0 | N | 39071 | beta-1,4-galactosyltransferase 1 [Pseudonaja textilis] |
| 580 | 2336 | JAB54194.1 | 65.01 | 4 | 0 | 0 | 0 | 4 | 0 | 0 | 0 | 0 | 0 | 0 | 0 | 0 | 0 | 0 |  |  |  | 4.0563E5 |  |  |  |  |  |  |  |  |  |  | 1 | 1 | 0 | 0 | 0 | 1 | 0 | 0 | 0 | 0 | 0 | 0 | 0 | 0 | 0 | 0 | N | 38969 | beta-1,4-galactosyltransferase 1 [Micrurus fulvius] |
| 580 | 2337 | XP\_034266717.1 | 65.01 | 5 | 0 | 0 | 0 | 5 | 0 | 0 | 0 | 0 | 0 | 0 | 0 | 0 | 0 | 0 |  |  |  | 4.0563E5 |  |  |  |  |  |  |  |  |  |  | 1 | 1 | 0 | 0 | 0 | 1 | 0 | 0 | 0 | 0 | 0 | 0 | 0 | 0 | 0 | 0 | N | 31995 | beta-1,4-galactosyltransferase 1 isoform X2 [Pantherophis guttatus] |
| 580 | 2338 | XP\_034266716.1 | 65.01 | 4 | 0 | 0 | 0 | 4 | 0 | 0 | 0 | 0 | 0 | 0 | 0 | 0 | 0 | 0 |  |  |  | 4.0563E5 |  |  |  |  |  |  |  |  |  |  | 1 | 1 | 0 | 0 | 0 | 1 | 0 | 0 | 0 | 0 | 0 | 0 | 0 | 0 | 0 | 0 | N | 39237 | beta-1,4-galactosyltransferase 1 isoform X1 [Pantherophis guttatus] |
| 580 | 2394 | ETE59419.1 | 65.01 | 4 | 0 | 0 | 0 | 4 | 0 | 0 | 0 | 0 | 0 | 0 | 0 | 0 | 0 | 0 |  |  |  | 4.0563E5 |  |  |  |  |  |  |  |  |  |  | 1 | 1 | 0 | 0 | 0 | 1 | 0 | 0 | 0 | 0 | 0 | 0 | 0 | 0 | 0 | 0 | N | 39003 | Beta-1,4-galactosyltransferase 1 [Ophiophagus hannah] |
| 580 | 2514 | LAB58625.1 | 65.01 | 6 | 0 | 0 | 0 | 6 | 0 | 0 | 0 | 0 | 0 | 0 | 0 | 0 | 0 | 0 |  |  |  | 4.0563E5 |  |  |  |  |  |  |  |  |  |  | 1 | 1 | 0 | 0 | 0 | 1 | 0 | 0 | 0 | 0 | 0 | 0 | 0 | 0 | 0 | 0 | N | 27774 | hypothetical protein, partial [Micrurus surinamensis] |
| 307 | 1822 | XP\_026551779.1 | 62.74 | 5 | 0 | 0 | 0 | 0 | 0 | 0 | 0 | 0 | 5 | 0 | 0 | 0 | 0 | 0 |  |  |  |  |  |  |  |  | 2.2158E5 |  |  |  |  |  | 1 | 1 | 0 | 0 | 0 | 0 | 0 | 0 | 0 | 0 | 1 | 0 | 0 | 0 | 0 | 0 | Y | 34151 | epithelial cell adhesion molecule [Pseudonaja textilis] |
| 307 | 1942 | XP\_026540478.1 | 62.74 | 5 | 0 | 0 | 0 | 0 | 0 | 0 | 0 | 0 | 5 | 0 | 0 | 0 | 0 | 0 |  |  |  |  |  |  |  |  | 2.2158E5 |  |  |  |  |  | 1 | 1 | 0 | 0 | 0 | 0 | 0 | 0 | 0 | 0 | 1 | 0 | 0 | 0 | 0 | 0 | Y | 33966 | epithelial cell adhesion molecule [Notechis scutatus] |
| 352 | 2010 | XP\_026577946.1 | 60.51 | 2 | 0 | 0 | 0 | 0 | 0 | 2 | 0 | 0 | 0 | 0 | 0 | 0 | 0 | 0 |  |  |  |  |  | 1.2578E5 |  |  |  |  |  |  |  |  | 1 | 1 | 0 | 0 | 0 | 0 | 0 | 1 | 0 | 0 | 0 | 0 | 0 | 0 | 0 | 0 | N | 61656 | cholinesterase-like [Pseudonaja textilis] |
| 354 | 3018 | LAA84028.1 | 60.42 | 7 | 0 | 0 | 0 | 0 | 7 | 7 | 0 | 0 | 0 | 0 | 0 | 0 | 0 | 0 |  |  |  |  | 3.6125E5 | 8.5646E5 |  |  |  |  |  |  |  |  | 1 | 1 | 0 | 0 | 0 | 0 | 1 | 1 | 0 | 0 | 0 | 0 | 0 | 0 | 0 | 0 | N | 25754 | hypothetical protein [Micrurus lemniscatus lemniscatus] |
| 267 | 4082 | XP\_007444677.1 | 60.11 | 6 | 0 | 0 | 0 | 0 | 0 | 6 | 0 | 0 | 0 | 0 | 0 | 0 | 0 | 0 |  |  |  |  |  | 2.6897E7 |  |  |  |  |  |  |  |  | 1 | 1 | 0 | 0 | 0 | 0 | 0 | 5 | 0 | 0 | 0 | 0 | 0 | 0 | 0 | 0 | N | 20584 | L-amino-acid oxidase-like, partial [Python bivittatus] |
| 283 | 1056 | XP\_013919280.1 | 59.97 | 1 | 0 | 0 | 0 | 0 | 1 | 1 | 0 | 0 | 0 | 0 | 0 | 0 | 0 | 0 |  |  |  |  | 7.9386E5 | 1.5067E5 |  |  |  |  |  |  |  |  | 1 | 1 | 0 | 0 | 0 | 0 | 1 | 1 | 0 | 0 | 0 | 0 | 0 | 0 | 0 | 0 | N | 81965 | PREDICTED: junction plakoglobin [Thamnophis sirtalis] |
| 283 | 1057 | XP\_032091066.1 | 59.97 | 1 | 0 | 0 | 0 | 0 | 1 | 1 | 0 | 0 | 0 | 0 | 0 | 0 | 0 | 0 |  |  |  |  | 7.9386E5 | 1.5067E5 |  |  |  |  |  |  |  |  | 1 | 1 | 0 | 0 | 0 | 0 | 1 | 1 | 0 | 0 | 0 | 0 | 0 | 0 | 0 | 0 | N | 86365 | junction plakoglobin [Thamnophis elegans] |
| 283 | 1058 | XP\_032091065.1 | 59.97 | 1 | 0 | 0 | 0 | 0 | 1 | 1 | 0 | 0 | 0 | 0 | 0 | 0 | 0 | 0 |  |  |  |  | 7.9386E5 | 1.5067E5 |  |  |  |  |  |  |  |  | 1 | 1 | 0 | 0 | 0 | 0 | 1 | 1 | 0 | 0 | 0 | 0 | 0 | 0 | 0 | 0 | N | 86365 | junction plakoglobin [Thamnophis elegans] |
| 283 | 1059 | JAG67632.1 | 59.97 | 1 | 0 | 0 | 0 | 0 | 1 | 1 | 0 | 0 | 0 | 0 | 0 | 0 | 0 | 0 |  |  |  |  | 7.9386E5 | 1.5067E5 |  |  |  |  |  |  |  |  | 1 | 1 | 0 | 0 | 0 | 0 | 1 | 1 | 0 | 0 | 0 | 0 | 0 | 0 | 0 | 0 | N | 81861 | junction plakoglobin [Boiga irregularis] |
| 283 | 1060 | XP\_034271617.1 | 59.97 | 1 | 0 | 0 | 0 | 0 | 1 | 1 | 0 | 0 | 0 | 0 | 0 | 0 | 0 | 0 |  |  |  |  | 7.9386E5 | 1.5067E5 |  |  |  |  |  |  |  |  | 1 | 1 | 0 | 0 | 0 | 0 | 1 | 1 | 0 | 0 | 0 | 0 | 0 | 0 | 0 | 0 | N | 81870 | junction plakoglobin isoform X2 [Pantherophis guttatus] |
| 283 | 1061 | XP\_034271615.1 | 59.97 | 1 | 0 | 0 | 0 | 0 | 1 | 1 | 0 | 0 | 0 | 0 | 0 | 0 | 0 | 0 |  |  |  |  | 7.9386E5 | 1.5067E5 |  |  |  |  |  |  |  |  | 1 | 1 | 0 | 0 | 0 | 0 | 1 | 1 | 0 | 0 | 0 | 0 | 0 | 0 | 0 | 0 | N | 81941 | junction plakoglobin isoform X1 [Pantherophis guttatus] |
| 283 | 1062 | XP\_034271618.1 | 59.97 | 1 | 0 | 0 | 0 | 0 | 1 | 1 | 0 | 0 | 0 | 0 | 0 | 0 | 0 | 0 |  |  |  |  | 7.9386E5 | 1.5067E5 |  |  |  |  |  |  |  |  | 1 | 1 | 0 | 0 | 0 | 0 | 1 | 1 | 0 | 0 | 0 | 0 | 0 | 0 | 0 | 0 | N | 81941 | junction plakoglobin isoform X1 [Pantherophis guttatus] |
| 283 | 1063 | XP\_034271616.1 | 59.97 | 1 | 0 | 0 | 0 | 0 | 1 | 1 | 0 | 0 | 0 | 0 | 0 | 0 | 0 | 0 |  |  |  |  | 7.9386E5 | 1.5067E5 |  |  |  |  |  |  |  |  | 1 | 1 | 0 | 0 | 0 | 0 | 1 | 1 | 0 | 0 | 0 | 0 | 0 | 0 | 0 | 0 | N | 81941 | junction plakoglobin isoform X1 [Pantherophis guttatus] |
| 283 | 1064 | XP\_015687775.1 | 59.97 | 1 | 0 | 0 | 0 | 0 | 1 | 1 | 0 | 0 | 0 | 0 | 0 | 0 | 0 | 0 |  |  |  |  | 7.9386E5 | 1.5067E5 |  |  |  |  |  |  |  |  | 1 | 1 | 0 | 0 | 0 | 0 | 1 | 1 | 0 | 0 | 0 | 0 | 0 | 0 | 0 | 0 | N | 81935 | junction plakoglobin [Protobothrops mucrosquamatus] |
| 283 | 1065 | XP\_015687778.1 | 59.97 | 1 | 0 | 0 | 0 | 0 | 1 | 1 | 0 | 0 | 0 | 0 | 0 | 0 | 0 | 0 |  |  |  |  | 7.9386E5 | 1.5067E5 |  |  |  |  |  |  |  |  | 1 | 1 | 0 | 0 | 0 | 0 | 1 | 1 | 0 | 0 | 0 | 0 | 0 | 0 | 0 | 0 | N | 81935 | junction plakoglobin [Protobothrops mucrosquamatus] |
| 283 | 1066 | XP\_015687777.1 | 59.97 | 1 | 0 | 0 | 0 | 0 | 1 | 1 | 0 | 0 | 0 | 0 | 0 | 0 | 0 | 0 |  |  |  |  | 7.9386E5 | 1.5067E5 |  |  |  |  |  |  |  |  | 1 | 1 | 0 | 0 | 0 | 0 | 1 | 1 | 0 | 0 | 0 | 0 | 0 | 0 | 0 | 0 | N | 81935 | junction plakoglobin [Protobothrops mucrosquamatus] |
| 283 | 1067 | XP\_015687776.1 | 59.97 | 1 | 0 | 0 | 0 | 0 | 1 | 1 | 0 | 0 | 0 | 0 | 0 | 0 | 0 | 0 |  |  |  |  | 7.9386E5 | 1.5067E5 |  |  |  |  |  |  |  |  | 1 | 1 | 0 | 0 | 0 | 0 | 1 | 1 | 0 | 0 | 0 | 0 | 0 | 0 | 0 | 0 | N | 81935 | junction plakoglobin [Protobothrops mucrosquamatus] |
| 283 | 1068 | XP\_026576301.1 | 59.97 | 1 | 0 | 0 | 0 | 0 | 1 | 1 | 0 | 0 | 0 | 0 | 0 | 0 | 0 | 0 |  |  |  |  | 7.9386E5 | 1.5067E5 |  |  |  |  |  |  |  |  | 1 | 1 | 0 | 0 | 0 | 0 | 1 | 1 | 0 | 0 | 0 | 0 | 0 | 0 | 0 | 0 | N | 81304 | junction plakoglobin isoform X2 [Pseudonaja textilis] |
| 283 | 1069 | XP\_026525656.1 | 59.97 | 1 | 0 | 0 | 0 | 0 | 1 | 1 | 0 | 0 | 0 | 0 | 0 | 0 | 0 | 0 |  |  |  |  | 7.9386E5 | 1.5067E5 |  |  |  |  |  |  |  |  | 1 | 1 | 0 | 0 | 0 | 0 | 1 | 1 | 0 | 0 | 0 | 0 | 0 | 0 | 0 | 0 | N | 81304 | junction plakoglobin isoform X2 [Notechis scutatus] |
| 283 | 1070 | XP\_026576299.1 | 59.97 | 1 | 0 | 0 | 0 | 0 | 1 | 1 | 0 | 0 | 0 | 0 | 0 | 0 | 0 | 0 |  |  |  |  | 7.9386E5 | 1.5067E5 |  |  |  |  |  |  |  |  | 1 | 1 | 0 | 0 | 0 | 0 | 1 | 1 | 0 | 0 | 0 | 0 | 0 | 0 | 0 | 0 | N | 81375 | junction plakoglobin isoform X1 [Pseudonaja textilis] |
| 283 | 1071 | XP\_026525655.1 | 59.97 | 1 | 0 | 0 | 0 | 0 | 1 | 1 | 0 | 0 | 0 | 0 | 0 | 0 | 0 | 0 |  |  |  |  | 7.9386E5 | 1.5067E5 |  |  |  |  |  |  |  |  | 1 | 1 | 0 | 0 | 0 | 0 | 1 | 1 | 0 | 0 | 0 | 0 | 0 | 0 | 0 | 0 | N | 81375 | junction plakoglobin isoform X1 [Notechis scutatus] |
| 283 | 1072 | XP\_026525654.1 | 59.97 | 1 | 0 | 0 | 0 | 0 | 1 | 1 | 0 | 0 | 0 | 0 | 0 | 0 | 0 | 0 |  |  |  |  | 7.9386E5 | 1.5067E5 |  |  |  |  |  |  |  |  | 1 | 1 | 0 | 0 | 0 | 0 | 1 | 1 | 0 | 0 | 0 | 0 | 0 | 0 | 0 | 0 | N | 81375 | junction plakoglobin isoform X1 [Notechis scutatus] |
| 283 | 1073 | XP\_026576298.1 | 59.97 | 1 | 0 | 0 | 0 | 0 | 1 | 1 | 0 | 0 | 0 | 0 | 0 | 0 | 0 | 0 |  |  |  |  | 7.9386E5 | 1.5067E5 |  |  |  |  |  |  |  |  | 1 | 1 | 0 | 0 | 0 | 0 | 1 | 1 | 0 | 0 | 0 | 0 | 0 | 0 | 0 | 0 | N | 81375 | junction plakoglobin isoform X1 [Pseudonaja textilis] |
| 283 | 1074 | JAG44306.1 | 59.97 | 1 | 0 | 0 | 0 | 0 | 1 | 1 | 0 | 0 | 0 | 0 | 0 | 0 | 0 | 0 |  |  |  |  | 7.9386E5 | 1.5067E5 |  |  |  |  |  |  |  |  | 1 | 1 | 0 | 0 | 0 | 0 | 1 | 1 | 0 | 0 | 0 | 0 | 0 | 0 | 0 | 0 | N | 81903 | junction plakoglobin [Crotalus horridus] |
| 283 | 1075 | JAA96758.1 | 59.97 | 1 | 0 | 0 | 0 | 0 | 1 | 1 | 0 | 0 | 0 | 0 | 0 | 0 | 0 | 0 |  |  |  |  | 7.9386E5 | 1.5067E5 |  |  |  |  |  |  |  |  | 1 | 1 | 0 | 0 | 0 | 0 | 1 | 1 | 0 | 0 | 0 | 0 | 0 | 0 | 0 | 0 | N | 81903 | junction plakoglobin [Crotalus horridus] |
| 283 | 1076 | JAG46538.1 | 59.97 | 1 | 0 | 0 | 0 | 0 | 1 | 1 | 0 | 0 | 0 | 0 | 0 | 0 | 0 | 0 |  |  |  |  | 7.9386E5 | 1.5067E5 |  |  |  |  |  |  |  |  | 1 | 1 | 0 | 0 | 0 | 0 | 1 | 1 | 0 | 0 | 0 | 0 | 0 | 0 | 0 | 0 | N | 81903 | junction plakoglobin [Crotalus horridus] |
| 283 | 1077 | JAI12884.1 | 59.97 | 1 | 0 | 0 | 0 | 0 | 1 | 1 | 0 | 0 | 0 | 0 | 0 | 0 | 0 | 0 |  |  |  |  | 7.9386E5 | 1.5067E5 |  |  |  |  |  |  |  |  | 1 | 1 | 0 | 0 | 0 | 0 | 1 | 1 | 0 | 0 | 0 | 0 | 0 | 0 | 0 | 0 | N | 81917 | junction plakoglobin [Crotalus adamanteus] |
| 283 | 1078 | JAV50234.1 | 59.97 | 1 | 0 | 0 | 0 | 0 | 1 | 1 | 0 | 0 | 0 | 0 | 0 | 0 | 0 | 0 |  |  |  |  | 7.9386E5 | 1.5067E5 |  |  |  |  |  |  |  |  | 1 | 1 | 0 | 0 | 0 | 0 | 1 | 1 | 0 | 0 | 0 | 0 | 0 | 0 | 0 | 0 | N | 81358 | JUP protein [Agkistrodon contortrix contortrix] |
| 283 | 1079 | LAB43566.1 | 59.97 | 1 | 0 | 0 | 0 | 0 | 1 | 1 | 0 | 0 | 0 | 0 | 0 | 0 | 0 | 0 |  |  |  |  | 7.9386E5 | 1.5067E5 |  |  |  |  |  |  |  |  | 1 | 1 | 0 | 0 | 0 | 0 | 1 | 1 | 0 | 0 | 0 | 0 | 0 | 0 | 0 | 0 | N | 83463 | hypothetical protein, partial [Micrurus spixii] |
| 283 | 1080 | ETE68218.1 | 59.97 | 1 | 0 | 0 | 0 | 0 | 1 | 1 | 0 | 0 | 0 | 0 | 0 | 0 | 0 | 0 |  |  |  |  | 7.9386E5 | 1.5067E5 |  |  |  |  |  |  |  |  | 1 | 1 | 0 | 0 | 0 | 0 | 1 | 1 | 0 | 0 | 0 | 0 | 0 | 0 | 0 | 0 | N | 83009 | Junction plakoglobin [Ophiophagus hannah] |
| 283 | 1084 | XP\_025022318.1 | 59.97 | 1 | 0 | 0 | 0 | 0 | 1 | 1 | 0 | 0 | 0 | 0 | 0 | 0 | 0 | 0 |  |  |  |  | 7.9386E5 | 1.5067E5 |  |  |  |  |  |  |  |  | 1 | 1 | 0 | 0 | 0 | 0 | 1 | 1 | 0 | 0 | 0 | 0 | 0 | 0 | 0 | 0 | N | 81985 | junction plakoglobin isoform X2 [Python bivittatus] |
| 283 | 1085 | XP\_025022317.1 | 59.97 | 1 | 0 | 0 | 0 | 0 | 1 | 1 | 0 | 0 | 0 | 0 | 0 | 0 | 0 | 0 |  |  |  |  | 7.9386E5 | 1.5067E5 |  |  |  |  |  |  |  |  | 1 | 1 | 0 | 0 | 0 | 0 | 1 | 1 | 0 | 0 | 0 | 0 | 0 | 0 | 0 | 0 | N | 82056 | junction plakoglobin isoform X1 [Python bivittatus] |
| 283 | 1086 | XP\_007428111.1 | 59.97 | 1 | 0 | 0 | 0 | 0 | 1 | 1 | 0 | 0 | 0 | 0 | 0 | 0 | 0 | 0 |  |  |  |  | 7.9386E5 | 1.5067E5 |  |  |  |  |  |  |  |  | 1 | 1 | 0 | 0 | 0 | 0 | 1 | 1 | 0 | 0 | 0 | 0 | 0 | 0 | 0 | 0 | N | 82056 | junction plakoglobin isoform X1 [Python bivittatus] |
| 383 | 1272 | ADJ51055.1 | 58.56 | 4 | 0 | 0 | 4 | 0 | 0 | 0 | 0 | 0 | 0 | 0 | 0 | 0 | 0 | 0 |  |  | 9.9291E6 |  |  |  |  |  |  |  |  |  |  |  | 1 | 1 | 0 | 0 | 1 | 0 | 0 | 0 | 0 | 0 | 0 | 0 | 0 | 0 | 0 | 0 | Y | 68956 | venom metalloproteinase [Cerberus rynchops] |
| 383 | 1273 | D8VNS0.1 | 58.56 | 4 | 0 | 0 | 4 | 0 | 0 | 0 | 0 | 0 | 0 | 0 | 0 | 0 | 0 | 0 |  |  | 9.9291E6 |  |  |  |  |  |  |  |  |  |  |  | 1 | 1 | 0 | 0 | 1 | 0 | 0 | 0 | 0 | 0 | 0 | 0 | 0 | 0 | 0 | 0 | Y | 68956 | RecName: Full=Zinc metalloproteinase-disintegrin-like; AltName: Full=Snake venom metalloproteinase; Short=SVMP; Flags: Precursor |
| 325 | 1540 | XP\_032070784.1 | 58.31 | 1 | 0 | 0 | 0 | 0 | 0 | 0 | 0 | 0 | 0 | 0 | 0 | 0 | 0 | 1 |  |  |  |  |  |  |  |  |  |  |  |  |  | 2.8873E5 | 1 | 1 | 0 | 0 | 0 | 0 | 0 | 0 | 0 | 0 | 0 | 0 | 0 | 0 | 0 | 1 | Y | 277331 | LOW QUALITY PROTEIN: cation-independent mannose-6-phosphate receptor [Thamnophis elegans] |
| 325 | 1691 | XP\_026560868.1 | 58.31 | 1 | 0 | 0 | 0 | 0 | 0 | 0 | 0 | 0 | 0 | 0 | 0 | 0 | 0 | 1 |  |  |  |  |  |  |  |  |  |  |  |  |  | 2.8873E5 | 1 | 1 | 0 | 0 | 0 | 0 | 0 | 0 | 0 | 0 | 0 | 0 | 0 | 0 | 0 | 1 | Y | 277539 | cation-independent mannose-6-phosphate receptor [Pseudonaja textilis] |
| 310 | 6651 | ADN67592.1 | 58.07 | 30 | 0 | 0 | 0 | 0 | 0 | 0 | 0 | 0 | 0 | 0 | 0 | 30 | 0 | 0 |  |  |  |  |  |  |  |  |  |  |  | 1.0921E7 |  |  | 2 | 2 | 0 | 0 | 0 | 0 | 0 | 0 | 0 | 0 | 0 | 0 | 0 | 2 | 0 | 0 | Y | 3364 | three-finger toxin precursor, partial [Bungarus multicinctus] |
| 310 | 6653 | 2H8U | 58.07 | 14 | 0 | 0 | 0 | 0 | 0 | 0 | 0 | 0 | 0 | 0 | 0 | 14 | 0 | 0 |  |  |  |  |  |  |  |  |  |  |  | 1.0921E7 |  |  | 2 | 2 | 0 | 0 | 0 | 0 | 0 | 0 | 0 | 0 | 0 | 0 | 0 | 2 | 0 | 0 | Y | 7216 | Chain B, Bucain |
| 310 | 6654 | P83346.1 | 58.07 | 14 | 0 | 0 | 0 | 0 | 0 | 0 | 0 | 0 | 0 | 0 | 0 | 14 | 0 | 0 |  |  |  |  |  |  |  |  |  |  |  | 1.0921E7 |  |  | 2 | 2 | 0 | 0 | 0 | 0 | 0 | 0 | 0 | 0 | 0 | 0 | 0 | 2 | 0 | 0 | Y | 7216 | RecName: Full=Bucain |
| 310 | 6655 | 1VYC | 58.07 | 14 | 0 | 0 | 0 | 0 | 0 | 0 | 0 | 0 | 0 | 0 | 0 | 14 | 0 | 0 |  |  |  |  |  |  |  |  |  |  |  | 1.0921E7 |  |  | 2 | 2 | 0 | 0 | 0 | 0 | 0 | 0 | 0 | 0 | 0 | 0 | 0 | 2 | 0 | 0 | Y | 7216 | Chain A, BUCAIN |
| 310 | 6656 | CAA06887.1 | 58.07 | 10 | 0 | 0 | 0 | 0 | 0 | 0 | 0 | 0 | 0 | 0 | 0 | 10 | 0 | 0 |  |  |  |  |  |  |  |  |  |  |  | 1.0921E7 |  |  | 2 | 2 | 0 | 0 | 0 | 0 | 0 | 0 | 0 | 0 | 0 | 0 | 0 | 2 | 0 | 0 | Y | 9519 | neurotoxin-like protein [Bungarus multicinctus multicinctus] |
| 310 | 6657 | Q9YGI8.1 | 58.07 | 10 | 0 | 0 | 0 | 0 | 0 | 0 | 0 | 0 | 0 | 0 | 0 | 10 | 0 | 0 |  |  |  |  |  |  |  |  |  |  |  | 1.0921E7 |  |  | 2 | 2 | 0 | 0 | 0 | 0 | 0 | 0 | 0 | 0 | 0 | 0 | 0 | 2 | 0 | 0 | Y | 9519 | RecName: Full=Short neurotoxin homolog NTL4; Flags: Precursor |
| 213 | 2271 | JAS03127.1 | 57.44 | 8 | 0 | 0 | 0 | 0 | 0 | 0 | 0 | 0 | 0 | 8 | 8 | 8 | 0 | 0 |  |  |  |  |  |  |  |  |  | 8.1733E6 | 2.6155E7 | 1.3093E6 |  |  | 1 | 1 | 0 | 0 | 0 | 0 | 0 | 0 | 0 | 0 | 0 | 4 | 6 | 2 | 0 | 0 | Y | 20534 | Papilin, partial [Phalotris mertensi] |
| 330 | 2121 | XP\_015669101.1 | 57.26 | 3 | 0 | 0 | 0 | 0 | 0 | 3 | 0 | 0 | 0 | 0 | 0 | 0 | 0 | 0 |  |  |  |  |  | 1.4289E5 |  |  |  |  |  |  |  |  | 1 | 1 | 0 | 0 | 0 | 0 | 0 | 2 | 0 | 0 | 0 | 0 | 0 | 0 | 0 | 0 | N | 57981 | prosaposin [Protobothrops mucrosquamatus] |
| 330 | 2127 | XP\_032088228.1 | 57.26 | 2 | 0 | 0 | 0 | 0 | 0 | 2 | 0 | 0 | 0 | 0 | 0 | 0 | 0 | 0 |  |  |  |  |  | 1.4289E5 |  |  |  |  |  |  |  |  | 1 | 1 | 0 | 0 | 0 | 0 | 0 | 2 | 0 | 0 | 0 | 0 | 0 | 0 | 0 | 0 | N | 58435 | prosaposin-like isoform X1 [Thamnophis elegans] |
| 330 | 2128 | XP\_013923763.1 | 57.26 | 3 | 0 | 0 | 0 | 0 | 0 | 3 | 0 | 0 | 0 | 0 | 0 | 0 | 0 | 0 |  |  |  |  |  | 1.4289E5 |  |  |  |  |  |  |  |  | 1 | 1 | 0 | 0 | 0 | 0 | 0 | 2 | 0 | 0 | 0 | 0 | 0 | 0 | 0 | 0 | N | 58050 | PREDICTED: prosaposin isoform X2 [Thamnophis sirtalis] |
| 330 | 2129 | XP\_013923762.1 | 57.26 | 2 | 0 | 0 | 0 | 0 | 0 | 2 | 0 | 0 | 0 | 0 | 0 | 0 | 0 | 0 |  |  |  |  |  | 1.4289E5 |  |  |  |  |  |  |  |  | 1 | 1 | 0 | 0 | 0 | 0 | 0 | 2 | 0 | 0 | 0 | 0 | 0 | 0 | 0 | 0 | N | 58435 | PREDICTED: prosaposin isoform X1 [Thamnophis sirtalis] |
| 330 | 2130 | XP\_032088229.1 | 57.26 | 3 | 0 | 0 | 0 | 0 | 0 | 3 | 0 | 0 | 0 | 0 | 0 | 0 | 0 | 0 |  |  |  |  |  | 1.4289E5 |  |  |  |  |  |  |  |  | 1 | 1 | 0 | 0 | 0 | 0 | 0 | 2 | 0 | 0 | 0 | 0 | 0 | 0 | 0 | 0 | N | 58050 | prosaposin-like isoform X2 [Thamnophis elegans] |
| 330 | 2164 | BAN82088.1 | 57.26 | 2 | 0 | 0 | 0 | 0 | 0 | 2 | 0 | 0 | 0 | 0 | 0 | 0 | 0 | 0 |  |  |  |  |  | 1.4289E5 |  |  |  |  |  |  |  |  | 1 | 1 | 0 | 0 | 0 | 0 | 0 | 2 | 0 | 0 | 0 | 0 | 0 | 0 | 0 | 0 | N | 58564 | hypothetical protein [Protobothrops flavoviridis] |
| 330 | 2166 | XP\_026569353.1 | 57.26 | 2 | 0 | 0 | 0 | 0 | 0 | 2 | 0 | 0 | 0 | 0 | 0 | 0 | 0 | 0 |  |  |  |  |  | 1.4289E5 |  |  |  |  |  |  |  |  | 1 | 1 | 0 | 0 | 0 | 0 | 0 | 2 | 0 | 0 | 0 | 0 | 0 | 0 | 0 | 0 | N | 58674 | prosaposin isoform X1 [Pseudonaja textilis] |
| 330 | 2168 | XP\_026569354.1 | 57.26 | 3 | 0 | 0 | 0 | 0 | 0 | 3 | 0 | 0 | 0 | 0 | 0 | 0 | 0 | 0 |  |  |  |  |  | 1.4289E5 |  |  |  |  |  |  |  |  | 1 | 1 | 0 | 0 | 0 | 0 | 0 | 2 | 0 | 0 | 0 | 0 | 0 | 0 | 0 | 0 | N | 58289 | prosaposin isoform X2 [Pseudonaja textilis] |
| 330 | 2177 | XP\_026528619.1 | 57.26 | 2 | 0 | 0 | 0 | 0 | 0 | 2 | 0 | 0 | 0 | 0 | 0 | 0 | 0 | 0 |  |  |  |  |  | 1.4289E5 |  |  |  |  |  |  |  |  | 1 | 1 | 0 | 0 | 0 | 0 | 0 | 2 | 0 | 0 | 0 | 0 | 0 | 0 | 0 | 0 | N | 58759 | prosaposin-like isoform X1 [Notechis scutatus] |
| 330 | 2186 | XP\_026528620.1 | 57.26 | 3 | 0 | 0 | 0 | 0 | 0 | 3 | 0 | 0 | 0 | 0 | 0 | 0 | 0 | 0 |  |  |  |  |  | 1.4289E5 |  |  |  |  |  |  |  |  | 1 | 1 | 0 | 0 | 0 | 0 | 0 | 2 | 0 | 0 | 0 | 0 | 0 | 0 | 0 | 0 | N | 58373 | prosaposin-like isoform X2 [Notechis scutatus] |
| 330 | 2196 | ETE61271.1 | 57.26 | 3 | 0 | 0 | 0 | 0 | 0 | 3 | 0 | 0 | 0 | 0 | 0 | 0 | 0 | 0 |  |  |  |  |  | 1.4289E5 |  |  |  |  |  |  |  |  | 1 | 1 | 0 | 0 | 0 | 0 | 0 | 2 | 0 | 0 | 0 | 0 | 0 | 0 | 0 | 0 | N | 54055 | Proactivator polypeptide [Ophiophagus hannah] |
| 330 | 2238 | AFJ51008.1 | 57.26 | 3 | 0 | 0 | 0 | 0 | 0 | 3 | 0 | 0 | 0 | 0 | 0 | 0 | 0 | 0 |  |  |  |  |  | 1.4289E5 |  |  |  |  |  |  |  |  | 1 | 1 | 0 | 0 | 0 | 0 | 0 | 2 | 0 | 0 | 0 | 0 | 0 | 0 | 0 | 0 | N | 58013 | Proactivator polypeptide-like [Crotalus adamanteus] |
| 330 | 2411 | XP\_007441356.1 | 57.26 | 3 | 0 | 0 | 0 | 0 | 0 | 3 | 0 | 0 | 0 | 0 | 0 | 0 | 0 | 0 |  |  |  |  |  | 1.4289E5 |  |  |  |  |  |  |  |  | 1 | 1 | 0 | 0 | 0 | 0 | 0 | 2 | 0 | 0 | 0 | 0 | 0 | 0 | 0 | 0 | N | 42934 | prosaposin-like [Python bivittatus] |
| 330 | 2701 | XP\_034291658.1 | 57.26 | 2 | 0 | 0 | 0 | 0 | 0 | 2 | 0 | 0 | 0 | 0 | 0 | 0 | 0 | 0 |  |  |  |  |  | 1.4289E5 |  |  |  |  |  |  |  |  | 1 | 1 | 0 | 0 | 0 | 0 | 0 | 2 | 0 | 0 | 0 | 0 | 0 | 0 | 0 | 0 | N | 58438 | prosaposin-like isoform X4 [Pantherophis guttatus] |
| 330 | 2702 | XP\_034291656.1 | 57.26 | 2 | 0 | 0 | 0 | 0 | 0 | 2 | 0 | 0 | 0 | 0 | 0 | 0 | 0 | 0 |  |  |  |  |  | 1.4289E5 |  |  |  |  |  |  |  |  | 1 | 1 | 0 | 0 | 0 | 0 | 0 | 2 | 0 | 0 | 0 | 0 | 0 | 0 | 0 | 0 | N | 58696 | prosaposin-like isoform X2 [Pantherophis guttatus] |
| 330 | 2703 | XP\_034291657.1 | 57.26 | 2 | 0 | 0 | 0 | 0 | 0 | 2 | 0 | 0 | 0 | 0 | 0 | 0 | 0 | 0 |  |  |  |  |  | 1.4289E5 |  |  |  |  |  |  |  |  | 1 | 1 | 0 | 0 | 0 | 0 | 0 | 2 | 0 | 0 | 0 | 0 | 0 | 0 | 0 | 0 | N | 58695 | prosaposin-like isoform X3 [Pantherophis guttatus] |
| 330 | 2704 | XP\_034291655.1 | 57.26 | 2 | 0 | 0 | 0 | 0 | 0 | 2 | 0 | 0 | 0 | 0 | 0 | 0 | 0 | 0 |  |  |  |  |  | 1.4289E5 |  |  |  |  |  |  |  |  | 1 | 1 | 0 | 0 | 0 | 0 | 0 | 2 | 0 | 0 | 0 | 0 | 0 | 0 | 0 | 0 | N | 58824 | prosaposin-like isoform X1 [Pantherophis guttatus] |
| 402 | 1808 | XP\_029139042.1 | 56.27 | 1 | 0 | 0 | 1 | 0 | 0 | 0 | 0 | 0 | 0 | 0 | 0 | 0 | 0 | 0 |  |  | 3.829E5 |  |  |  |  |  |  |  |  |  |  |  | 1 | 1 | 0 | 0 | 1 | 0 | 0 | 0 | 0 | 0 | 0 | 0 | 0 | 0 | 0 | 0 | N | 212259 | receptor-type tyrosine-protein phosphatase F [Protobothrops mucrosquamatus] |
| 402 | 1830 | XP\_032073554.1 | 56.27 | 1 | 0 | 0 | 1 | 0 | 0 | 0 | 0 | 0 | 0 | 0 | 0 | 0 | 0 | 0 |  |  | 3.829E5 |  |  |  |  |  |  |  |  |  |  |  | 1 | 1 | 0 | 0 | 1 | 0 | 0 | 0 | 0 | 0 | 0 | 0 | 0 | 0 | 0 | 0 | N | 211357 | receptor-type tyrosine-protein phosphatase F isoform X2 [Thamnophis elegans] |
| 402 | 1831 | XP\_032073552.1 | 56.27 | 1 | 0 | 0 | 1 | 0 | 0 | 0 | 0 | 0 | 0 | 0 | 0 | 0 | 0 | 0 |  |  | 3.829E5 |  |  |  |  |  |  |  |  |  |  |  | 1 | 1 | 0 | 0 | 1 | 0 | 0 | 0 | 0 | 0 | 0 | 0 | 0 | 0 | 0 | 0 | N | 212394 | receptor-type tyrosine-protein phosphatase F isoform X1 [Thamnophis elegans] |
| 402 | 1832 | XP\_032073553.1 | 56.27 | 1 | 0 | 0 | 1 | 0 | 0 | 0 | 0 | 0 | 0 | 0 | 0 | 0 | 0 | 0 |  |  | 3.829E5 |  |  |  |  |  |  |  |  |  |  |  | 1 | 1 | 0 | 0 | 1 | 0 | 0 | 0 | 0 | 0 | 0 | 0 | 0 | 0 | 0 | 0 | N | 212394 | receptor-type tyrosine-protein phosphatase F isoform X1 [Thamnophis elegans] |
| 402 | 1833 | XP\_032073551.1 | 56.27 | 1 | 0 | 0 | 1 | 0 | 0 | 0 | 0 | 0 | 0 | 0 | 0 | 0 | 0 | 0 |  |  | 3.829E5 |  |  |  |  |  |  |  |  |  |  |  | 1 | 1 | 0 | 0 | 1 | 0 | 0 | 0 | 0 | 0 | 0 | 0 | 0 | 0 | 0 | 0 | N | 212394 | receptor-type tyrosine-protein phosphatase F isoform X1 [Thamnophis elegans] |
| 402 | 1898 | XP\_026562780.1 | 56.27 | 1 | 0 | 0 | 1 | 0 | 0 | 0 | 0 | 0 | 0 | 0 | 0 | 0 | 0 | 0 |  |  | 3.829E5 |  |  |  |  |  |  |  |  |  |  |  | 1 | 1 | 0 | 0 | 1 | 0 | 0 | 0 | 0 | 0 | 0 | 0 | 0 | 0 | 0 | 0 | N | 212684 | receptor-type tyrosine-protein phosphatase F isoform X2 [Pseudonaja textilis] |
| 402 | 1899 | XP\_026562779.1 | 56.27 | 1 | 0 | 0 | 1 | 0 | 0 | 0 | 0 | 0 | 0 | 0 | 0 | 0 | 0 | 0 |  |  | 3.829E5 |  |  |  |  |  |  |  |  |  |  |  | 1 | 1 | 0 | 0 | 1 | 0 | 0 | 0 | 0 | 0 | 0 | 0 | 0 | 0 | 0 | 0 | N | 213720 | receptor-type tyrosine-protein phosphatase F isoform X1 [Pseudonaja textilis] |
| 402 | 1900 | XP\_026521506.1 | 56.27 | 1 | 0 | 0 | 1 | 0 | 0 | 0 | 0 | 0 | 0 | 0 | 0 | 0 | 0 | 0 |  |  | 3.829E5 |  |  |  |  |  |  |  |  |  |  |  | 1 | 1 | 0 | 0 | 1 | 0 | 0 | 0 | 0 | 0 | 0 | 0 | 0 | 0 | 0 | 0 | N | 211479 | receptor-type tyrosine-protein phosphatase F isoform X2 [Notechis scutatus] |
| 402 | 1901 | XP\_026521503.1 | 56.27 | 1 | 0 | 0 | 1 | 0 | 0 | 0 | 0 | 0 | 0 | 0 | 0 | 0 | 0 | 0 |  |  | 3.829E5 |  |  |  |  |  |  |  |  |  |  |  | 1 | 1 | 0 | 0 | 1 | 0 | 0 | 0 | 0 | 0 | 0 | 0 | 0 | 0 | 0 | 0 | N | 212515 | receptor-type tyrosine-protein phosphatase F isoform X1 [Notechis scutatus] |
| 402 | 1904 | XP\_013917183.1 | 56.27 | 1 | 0 | 0 | 1 | 0 | 0 | 0 | 0 | 0 | 0 | 0 | 0 | 0 | 0 | 0 |  |  | 3.829E5 |  |  |  |  |  |  |  |  |  |  |  | 1 | 1 | 0 | 0 | 1 | 0 | 0 | 0 | 0 | 0 | 0 | 0 | 0 | 0 | 0 | 0 | N | 213358 | PREDICTED: receptor-type tyrosine-protein phosphatase F [Thamnophis sirtalis] |
| 402 | 1905 | XP\_034270756.1 | 56.27 | 1 | 0 | 0 | 1 | 0 | 0 | 0 | 0 | 0 | 0 | 0 | 0 | 0 | 0 | 0 |  |  | 3.829E5 |  |  |  |  |  |  |  |  |  |  |  | 1 | 1 | 0 | 0 | 1 | 0 | 0 | 0 | 0 | 0 | 0 | 0 | 0 | 0 | 0 | 0 | N | 203456 | receptor-type tyrosine-protein phosphatase F isoform X12 [Pantherophis guttatus] |
| 402 | 1906 | XP\_034270755.1 | 56.27 | 1 | 0 | 0 | 1 | 0 | 0 | 0 | 0 | 0 | 0 | 0 | 0 | 0 | 0 | 0 |  |  | 3.829E5 |  |  |  |  |  |  |  |  |  |  |  | 1 | 1 | 0 | 0 | 1 | 0 | 0 | 0 | 0 | 0 | 0 | 0 | 0 | 0 | 0 | 0 | N | 211480 | receptor-type tyrosine-protein phosphatase F isoform X11 [Pantherophis guttatus] |
| 402 | 1907 | XP\_034270754.1 | 56.27 | 1 | 0 | 0 | 1 | 0 | 0 | 0 | 0 | 0 | 0 | 0 | 0 | 0 | 0 | 0 |  |  | 3.829E5 |  |  |  |  |  |  |  |  |  |  |  | 1 | 1 | 0 | 0 | 1 | 0 | 0 | 0 | 0 | 0 | 0 | 0 | 0 | 0 | 0 | 0 | N | 212061 | receptor-type tyrosine-protein phosphatase F isoform X10 [Pantherophis guttatus] |
| 402 | 1908 | XP\_034270753.1 | 56.27 | 1 | 0 | 0 | 1 | 0 | 0 | 0 | 0 | 0 | 0 | 0 | 0 | 0 | 0 | 0 |  |  | 3.829E5 |  |  |  |  |  |  |  |  |  |  |  | 1 | 1 | 0 | 0 | 1 | 0 | 0 | 0 | 0 | 0 | 0 | 0 | 0 | 0 | 0 | 0 | N | 212516 | receptor-type tyrosine-protein phosphatase F isoform X9 [Pantherophis guttatus] |
| 402 | 1909 | XP\_034270752.1 | 56.27 | 1 | 0 | 0 | 1 | 0 | 0 | 0 | 0 | 0 | 0 | 0 | 0 | 0 | 0 | 0 |  |  | 3.829E5 |  |  |  |  |  |  |  |  |  |  |  | 1 | 1 | 0 | 0 | 1 | 0 | 0 | 0 | 0 | 0 | 0 | 0 | 0 | 0 | 0 | 0 | N | 212988 | receptor-type tyrosine-protein phosphatase F isoform X8 [Pantherophis guttatus] |
| 402 | 1910 | XP\_034270751.1 | 56.27 | 1 | 0 | 0 | 1 | 0 | 0 | 0 | 0 | 0 | 0 | 0 | 0 | 0 | 0 | 0 |  |  | 3.829E5 |  |  |  |  |  |  |  |  |  |  |  | 1 | 1 | 0 | 0 | 1 | 0 | 0 | 0 | 0 | 0 | 0 | 0 | 0 | 0 | 0 | 0 | N | 212983 | receptor-type tyrosine-protein phosphatase F isoform X7 [Pantherophis guttatus] |
| 402 | 1911 | XP\_034270750.1 | 56.27 | 1 | 0 | 0 | 1 | 0 | 0 | 0 | 0 | 0 | 0 | 0 | 0 | 0 | 0 | 0 |  |  | 3.829E5 |  |  |  |  |  |  |  |  |  |  |  | 1 | 1 | 0 | 0 | 1 | 0 | 0 | 0 | 0 | 0 | 0 | 0 | 0 | 0 | 0 | 0 | N | 213097 | receptor-type tyrosine-protein phosphatase F isoform X6 [Pantherophis guttatus] |
| 402 | 1912 | XP\_034270749.1 | 56.27 | 1 | 0 | 0 | 1 | 0 | 0 | 0 | 0 | 0 | 0 | 0 | 0 | 0 | 0 | 0 |  |  | 3.829E5 |  |  |  |  |  |  |  |  |  |  |  | 1 | 1 | 0 | 0 | 1 | 0 | 0 | 0 | 0 | 0 | 0 | 0 | 0 | 0 | 0 | 0 | N | 213570 | receptor-type tyrosine-protein phosphatase F isoform X5 [Pantherophis guttatus] |
| 402 | 1913 | XP\_034270748.1 | 56.27 | 1 | 0 | 0 | 1 | 0 | 0 | 0 | 0 | 0 | 0 | 0 | 0 | 0 | 0 | 0 |  |  | 3.829E5 |  |  |  |  |  |  |  |  |  |  |  | 1 | 1 | 0 | 0 | 1 | 0 | 0 | 0 | 0 | 0 | 0 | 0 | 0 | 0 | 0 | 0 | N | 213565 | receptor-type tyrosine-protein phosphatase F isoform X4 [Pantherophis guttatus] |
| 402 | 1914 | XP\_034270747.1 | 56.27 | 1 | 0 | 0 | 1 | 0 | 0 | 0 | 0 | 0 | 0 | 0 | 0 | 0 | 0 | 0 |  |  | 3.829E5 |  |  |  |  |  |  |  |  |  |  |  | 1 | 1 | 0 | 0 | 1 | 0 | 0 | 0 | 0 | 0 | 0 | 0 | 0 | 0 | 0 | 0 | N | 214019 | receptor-type tyrosine-protein phosphatase F isoform X3 [Pantherophis guttatus] |
| 402 | 1915 | XP\_034270745.1 | 56.27 | 1 | 0 | 0 | 1 | 0 | 0 | 0 | 0 | 0 | 0 | 0 | 0 | 0 | 0 | 0 |  |  | 3.829E5 |  |  |  |  |  |  |  |  |  |  |  | 1 | 1 | 0 | 0 | 1 | 0 | 0 | 0 | 0 | 0 | 0 | 0 | 0 | 0 | 0 | 0 | N | 214128 | receptor-type tyrosine-protein phosphatase F isoform X2 [Pantherophis guttatus] |
| 402 | 1916 | XP\_034270743.1 | 56.27 | 1 | 0 | 0 | 1 | 0 | 0 | 0 | 0 | 0 | 0 | 0 | 0 | 0 | 0 | 0 |  |  | 3.829E5 |  |  |  |  |  |  |  |  |  |  |  | 1 | 1 | 0 | 0 | 1 | 0 | 0 | 0 | 0 | 0 | 0 | 0 | 0 | 0 | 0 | 0 | N | 214601 | receptor-type tyrosine-protein phosphatase F isoform X1 [Pantherophis guttatus] |
| 402 | 1917 | XP\_034270741.1 | 56.27 | 1 | 0 | 0 | 1 | 0 | 0 | 0 | 0 | 0 | 0 | 0 | 0 | 0 | 0 | 0 |  |  | 3.829E5 |  |  |  |  |  |  |  |  |  |  |  | 1 | 1 | 0 | 0 | 1 | 0 | 0 | 0 | 0 | 0 | 0 | 0 | 0 | 0 | 0 | 0 | N | 214601 | receptor-type tyrosine-protein phosphatase F isoform X1 [Pantherophis guttatus] |
| 402 | 1918 | XP\_034270742.1 | 56.27 | 1 | 0 | 0 | 1 | 0 | 0 | 0 | 0 | 0 | 0 | 0 | 0 | 0 | 0 | 0 |  |  | 3.829E5 |  |  |  |  |  |  |  |  |  |  |  | 1 | 1 | 0 | 0 | 1 | 0 | 0 | 0 | 0 | 0 | 0 | 0 | 0 | 0 | 0 | 0 | N | 214601 | receptor-type tyrosine-protein phosphatase F isoform X1 [Pantherophis guttatus] |
| 402 | 1919 | XP\_034270744.1 | 56.27 | 1 | 0 | 0 | 1 | 0 | 0 | 0 | 0 | 0 | 0 | 0 | 0 | 0 | 0 | 0 |  |  | 3.829E5 |  |  |  |  |  |  |  |  |  |  |  | 1 | 1 | 0 | 0 | 1 | 0 | 0 | 0 | 0 | 0 | 0 | 0 | 0 | 0 | 0 | 0 | N | 214601 | receptor-type tyrosine-protein phosphatase F isoform X1 [Pantherophis guttatus] |
| 402 | 2041 | XP\_025029121.1 | 56.27 | 1 | 0 | 0 | 1 | 0 | 0 | 0 | 0 | 0 | 0 | 0 | 0 | 0 | 0 | 0 |  |  | 3.829E5 |  |  |  |  |  |  |  |  |  |  |  | 1 | 1 | 0 | 0 | 1 | 0 | 0 | 0 | 0 | 0 | 0 | 0 | 0 | 0 | 0 | 0 | N | 211713 | receptor-type tyrosine-protein phosphatase F isoform X2 [Python bivittatus] |
| 402 | 2042 | XP\_025029079.1 | 56.27 | 1 | 0 | 0 | 1 | 0 | 0 | 0 | 0 | 0 | 0 | 0 | 0 | 0 | 0 | 0 |  |  | 3.829E5 |  |  |  |  |  |  |  |  |  |  |  | 1 | 1 | 0 | 0 | 1 | 0 | 0 | 0 | 0 | 0 | 0 | 0 | 0 | 0 | 0 | 0 | N | 212749 | receptor-type tyrosine-protein phosphatase F isoform X1 [Python bivittatus] |
| 402 | 2188 | JAI11788.1 | 56.27 | 1 | 0 | 0 | 1 | 0 | 0 | 0 | 0 | 0 | 0 | 0 | 0 | 0 | 0 | 0 |  |  | 3.829E5 |  |  |  |  |  |  |  |  |  |  |  | 1 | 1 | 0 | 0 | 1 | 0 | 0 | 0 | 0 | 0 | 0 | 0 | 0 | 0 | 0 | 0 | N | 202589 | receptor-type tyrosine-protein phosphatase F [Crotalus adamanteus] |
| 402 | 2241 | XP\_015683998.1 | 56.27 | 1 | 0 | 0 | 1 | 0 | 0 | 0 | 0 | 0 | 0 | 0 | 0 | 0 | 0 | 0 |  |  | 3.829E5 |  |  |  |  |  |  |  |  |  |  |  | 1 | 1 | 0 | 0 | 1 | 0 | 0 | 0 | 0 | 0 | 0 | 0 | 0 | 0 | 0 | 0 | N | 212719 | receptor-type tyrosine-protein phosphatase delta [Protobothrops mucrosquamatus] |
| 402 | 2242 | XP\_032070216.1 | 56.27 | 1 | 0 | 0 | 1 | 0 | 0 | 0 | 0 | 0 | 0 | 0 | 0 | 0 | 0 | 0 |  |  | 3.829E5 |  |  |  |  |  |  |  |  |  |  |  | 1 | 1 | 0 | 0 | 1 | 0 | 0 | 0 | 0 | 0 | 0 | 0 | 0 | 0 | 0 | 0 | N | 215121 | LOW QUALITY PROTEIN: receptor-type tyrosine-protein phosphatase delta [Thamnophis elegans] |
| 402 | 2243 | XP\_034266284.1 | 56.27 | 1 | 0 | 0 | 1 | 0 | 0 | 0 | 0 | 0 | 0 | 0 | 0 | 0 | 0 | 0 |  |  | 3.829E5 |  |  |  |  |  |  |  |  |  |  |  | 1 | 1 | 0 | 0 | 1 | 0 | 0 | 0 | 0 | 0 | 0 | 0 | 0 | 0 | 0 | 0 | N | 219291 | receptor-type tyrosine-protein phosphatase delta isoform X17 [Pantherophis guttatus] |
| 402 | 2244 | XP\_034266280.1 | 56.27 | 1 | 0 | 0 | 1 | 0 | 0 | 0 | 0 | 0 | 0 | 0 | 0 | 0 | 0 | 0 |  |  | 3.829E5 |  |  |  |  |  |  |  |  |  |  |  | 1 | 1 | 0 | 0 | 1 | 0 | 0 | 0 | 0 | 0 | 0 | 0 | 0 | 0 | 0 | 0 | N | 219345 | receptor-type tyrosine-protein phosphatase delta isoform X14 [Pantherophis guttatus] |
| 402 | 2245 | XP\_034266279.1 | 56.27 | 1 | 0 | 0 | 1 | 0 | 0 | 0 | 0 | 0 | 0 | 0 | 0 | 0 | 0 | 0 |  |  | 3.829E5 |  |  |  |  |  |  |  |  |  |  |  | 1 | 1 | 0 | 0 | 1 | 0 | 0 | 0 | 0 | 0 | 0 | 0 | 0 | 0 | 0 | 0 | N | 219620 | receptor-type tyrosine-protein phosphatase delta isoform X13 [Pantherophis guttatus] |
| 402 | 2246 | XP\_034266271.1 | 56.27 | 1 | 0 | 0 | 1 | 0 | 0 | 0 | 0 | 0 | 0 | 0 | 0 | 0 | 0 | 0 |  |  | 3.829E5 |  |  |  |  |  |  |  |  |  |  |  | 1 | 1 | 0 | 0 | 1 | 0 | 0 | 0 | 0 | 0 | 0 | 0 | 0 | 0 | 0 | 0 | N | 220115 | receptor-type tyrosine-protein phosphatase delta isoform X6 [Pantherophis guttatus] |
| 402 | 2247 | XP\_034266274.1 | 56.27 | 1 | 0 | 0 | 1 | 0 | 0 | 0 | 0 | 0 | 0 | 0 | 0 | 0 | 0 | 0 |  |  | 3.829E5 |  |  |  |  |  |  |  |  |  |  |  | 1 | 1 | 0 | 0 | 1 | 0 | 0 | 0 | 0 | 0 | 0 | 0 | 0 | 0 | 0 | 0 | N | 219872 | receptor-type tyrosine-protein phosphatase delta isoform X9 [Pantherophis guttatus] |
| 402 | 2248 | XP\_034266267.1 | 56.27 | 1 | 0 | 0 | 1 | 0 | 0 | 0 | 0 | 0 | 0 | 0 | 0 | 0 | 0 | 0 |  |  | 3.829E5 |  |  |  |  |  |  |  |  |  |  |  | 1 | 1 | 0 | 0 | 1 | 0 | 0 | 0 | 0 | 0 | 0 | 0 | 0 | 0 | 0 | 0 | N | 220202 | receptor-type tyrosine-protein phosphatase delta isoform X2 [Pantherophis guttatus] |
| 402 | 2262 | LAB52886.1 | 56.27 | 3 | 0 | 0 | 3 | 0 | 0 | 0 | 0 | 0 | 0 | 0 | 0 | 0 | 0 | 0 |  |  | 3.829E5 |  |  |  |  |  |  |  |  |  |  |  | 1 | 1 | 0 | 0 | 1 | 0 | 0 | 0 | 0 | 0 | 0 | 0 | 0 | 0 | 0 | 0 | N | 59480 | hypothetical protein, partial [Micrurus surinamensis] |
| 402 | 2265 | XP\_026527075.1 | 56.27 | 1 | 0 | 0 | 1 | 0 | 0 | 0 | 0 | 0 | 0 | 0 | 0 | 0 | 0 | 0 |  |  | 3.829E5 |  |  |  |  |  |  |  |  |  |  |  | 1 | 1 | 0 | 0 | 1 | 0 | 0 | 0 | 0 | 0 | 0 | 0 | 0 | 0 | 0 | 0 | N | 204516 | receptor-type tyrosine-protein phosphatase delta [Notechis scutatus] |
| 402 | 3757 | LAA68618.1 | 56.27 | 10 | 0 | 0 | 10 | 0 | 0 | 0 | 0 | 0 | 0 | 0 | 0 | 0 | 0 | 0 |  |  | 3.829E5 |  |  |  |  |  |  |  |  |  |  |  | 1 | 1 | 0 | 0 | 1 | 0 | 0 | 0 | 0 | 0 | 0 | 0 | 0 | 0 | 0 | 0 | N | 17102 | hypothetical protein, partial [Micrurus lemniscatus lemniscatus] |
| 402 | 4397 | ETE69571.1 | 56.27 | 16 | 0 | 0 | 16 | 0 | 0 | 0 | 0 | 0 | 0 | 0 | 0 | 0 | 0 | 0 |  |  | 3.829E5 |  |  |  |  |  |  |  |  |  |  |  | 1 | 1 | 0 | 0 | 1 | 0 | 0 | 0 | 0 | 0 | 0 | 0 | 0 | 0 | 0 | 0 | N | 9948 | Receptor-type tyrosine-protein phosphatase F, partial [Ophiophagus hannah] |
| 402 | 4401 | LAB52887.1 | 56.27 | 12 | 0 | 0 | 12 | 0 | 0 | 0 | 0 | 0 | 0 | 0 | 0 | 0 | 0 | 0 |  |  | 3.829E5 |  |  |  |  |  |  |  |  |  |  |  | 1 | 1 | 0 | 0 | 1 | 0 | 0 | 0 | 0 | 0 | 0 | 0 | 0 | 0 | 0 | 0 | N | 13606 | hypothetical protein [Micrurus surinamensis] |
| 402 | 4402 | LAA17479.1 | 56.27 | 12 | 0 | 0 | 12 | 0 | 0 | 0 | 0 | 0 | 0 | 0 | 0 | 0 | 0 | 0 |  |  | 3.829E5 |  |  |  |  |  |  |  |  |  |  |  | 1 | 1 | 0 | 0 | 1 | 0 | 0 | 0 | 0 | 0 | 0 | 0 | 0 | 0 | 0 | 0 | N | 13606 | hypothetical protein [Micrurus lemniscatus carvalhoi] |
| 402 | 4403 | LAA17480.1 | 56.27 | 12 | 0 | 0 | 12 | 0 | 0 | 0 | 0 | 0 | 0 | 0 | 0 | 0 | 0 | 0 |  |  | 3.829E5 |  |  |  |  |  |  |  |  |  |  |  | 1 | 1 | 0 | 0 | 1 | 0 | 0 | 0 | 0 | 0 | 0 | 0 | 0 | 0 | 0 | 0 | N | 13606 | hypothetical protein [Micrurus lemniscatus carvalhoi] |
| 402 | 4480 | XP\_007424914.1 | 56.27 | 12 | 0 | 0 | 12 | 0 | 0 | 0 | 0 | 0 | 0 | 0 | 0 | 0 | 0 | 0 |  |  | 3.829E5 |  |  |  |  |  |  |  |  |  |  |  | 1 | 1 | 0 | 0 | 1 | 0 | 0 | 0 | 0 | 0 | 0 | 0 | 0 | 0 | 0 | 0 | N | 13522 | receptor-type tyrosine-protein phosphatase delta-like, partial [Python bivittatus] |
| 402 | 4627 | ETE63588.1 | 56.27 | 13 | 0 | 0 | 13 | 0 | 0 | 0 | 0 | 0 | 0 | 0 | 0 | 0 | 0 | 0 |  |  | 3.829E5 |  |  |  |  |  |  |  |  |  |  |  | 1 | 1 | 0 | 0 | 1 | 0 | 0 | 0 | 0 | 0 | 0 | 0 | 0 | 0 | 0 | 0 | N | 13051 | Receptor-type tyrosine-protein phosphatase delta, partial [Ophiophagus hannah] |
| 335 | 1619 | XP\_015680353.1 | 53.40 | 3 | 0 | 0 | 0 | 3 | 0 | 0 | 0 | 0 | 0 | 0 | 0 | 0 | 0 | 0 |  |  |  | 1.368E6 |  |  |  |  |  |  |  |  |  |  | 1 | 1 | 0 | 0 | 0 | 1 | 0 | 0 | 0 | 0 | 0 | 0 | 0 | 0 | 0 | 0 | N | 33502 | cathepsin Z [Protobothrops mucrosquamatus] |
| 335 | 1640 | XP\_034289001.1 | 53.40 | 3 | 0 | 0 | 0 | 3 | 0 | 0 | 0 | 0 | 0 | 0 | 0 | 0 | 0 | 0 |  |  |  | 1.368E6 |  |  |  |  |  |  |  |  |  |  | 1 | 1 | 0 | 0 | 0 | 1 | 0 | 0 | 0 | 0 | 0 | 0 | 0 | 0 | 0 | 0 | N | 33408 | cathepsin Z-like [Pantherophis guttatus] |
| 335 | 1648 | ETE63943.1 | 53.40 | 2 | 0 | 0 | 0 | 2 | 0 | 0 | 0 | 0 | 0 | 0 | 0 | 0 | 0 | 0 |  |  |  | 1.368E6 |  |  |  |  |  |  |  |  |  |  | 1 | 1 | 0 | 0 | 0 | 1 | 0 | 0 | 0 | 0 | 0 | 0 | 0 | 0 | 0 | 0 | N | 48700 | Cathepsin Z, partial [Ophiophagus hannah] |
| 335 | 1656 | XP\_026520698.1 | 53.40 | 3 | 0 | 0 | 0 | 3 | 0 | 0 | 0 | 0 | 0 | 0 | 0 | 0 | 0 | 0 |  |  |  | 1.368E6 |  |  |  |  |  |  |  |  |  |  | 1 | 1 | 0 | 0 | 0 | 1 | 0 | 0 | 0 | 0 | 0 | 0 | 0 | 0 | 0 | 0 | N | 33585 | cathepsin Z [Notechis scutatus] |
| 358 | 4481 | ADP88560.1 | 53.13 | 6 | 0 | 0 | 0 | 6 | 0 | 0 | 0 | 0 | 0 | 0 | 0 | 0 | 0 | 0 |  |  |  | 1.3548E6 |  |  |  |  |  |  |  |  |  |  | 1 | 1 | 0 | 0 | 0 | 2 | 0 | 0 | 0 | 0 | 0 | 0 | 0 | 0 | 0 | 0 | N | 28035 | serine beta-fibrinogenase-like protein precursor [Daboia siamensis] |
| 358 | 4482 | E5L0E4.1 | 53.13 | 6 | 0 | 0 | 0 | 6 | 0 | 0 | 0 | 0 | 0 | 0 | 0 | 0 | 0 | 0 |  |  |  | 1.3548E6 |  |  |  |  |  |  |  |  |  |  | 1 | 1 | 0 | 0 | 0 | 2 | 0 | 0 | 0 | 0 | 0 | 0 | 0 | 0 | 0 | 0 | N | 28035 | RecName: Full=Beta-fibrinogenase-like; Short=RVBF; AltName: Full=Snake venom serine protease; Short=SVSP; Flags: Precursor |
| 349 | 2205 | ETE61970.1 | 52.07 | 2 | 0 | 0 | 0 | 0 | 0 | 2 | 0 | 0 | 0 | 0 | 0 | 0 | 0 | 0 |  |  |  |  |  | 7.9169E5 |  |  |  |  |  |  |  |  | 1 | 1 | 0 | 0 | 0 | 0 | 0 | 1 | 0 | 0 | 0 | 0 | 0 | 0 | 0 | 0 | N | 54724 | Glutathione reductase, mitochondrial, partial [Ophiophagus hannah] |
| 349 | 2528 | XP\_013910191.1 | 52.07 | 3 | 0 | 0 | 0 | 0 | 0 | 3 | 0 | 0 | 0 | 0 | 0 | 0 | 0 | 0 |  |  |  |  |  | 7.9169E5 |  |  |  |  |  |  |  |  | 1 | 1 | 0 | 0 | 0 | 0 | 0 | 1 | 0 | 0 | 0 | 0 | 0 | 0 | 0 | 0 | N | 45486 | PREDICTED: glutathione reductase, mitochondrial isoform X2 [Thamnophis sirtalis] |
| 349 | 2529 | XP\_013910190.1 | 52.07 | 2 | 0 | 0 | 0 | 0 | 0 | 2 | 0 | 0 | 0 | 0 | 0 | 0 | 0 | 0 |  |  |  |  |  | 7.9169E5 |  |  |  |  |  |  |  |  | 1 | 1 | 0 | 0 | 0 | 0 | 0 | 1 | 0 | 0 | 0 | 0 | 0 | 0 | 0 | 0 | N | 54671 | PREDICTED: glutathione reductase, mitochondrial isoform X1 [Thamnophis sirtalis] |
| 349 | 2700 | XP\_007427939.2 | 52.07 | 2 | 0 | 0 | 0 | 0 | 0 | 2 | 0 | 0 | 0 | 0 | 0 | 0 | 0 | 0 |  |  |  |  |  | 7.9169E5 |  |  |  |  |  |  |  |  | 1 | 1 | 0 | 0 | 0 | 0 | 0 | 1 | 0 | 0 | 0 | 0 | 0 | 0 | 0 | 0 | N | 53748 | glutathione reductase, mitochondrial isoform X1 [Python bivittatus] |
| 349 | 2712 | XP\_015669311.1 | 52.07 | 2 | 0 | 0 | 0 | 0 | 0 | 2 | 0 | 0 | 0 | 0 | 0 | 0 | 0 | 0 |  |  |  |  |  | 7.9169E5 |  |  |  |  |  |  |  |  | 1 | 1 | 0 | 0 | 0 | 0 | 0 | 1 | 0 | 0 | 0 | 0 | 0 | 0 | 0 | 0 | N | 54483 | glutathione reductase, mitochondrial [Protobothrops mucrosquamatus] |
| 349 | 2716 | XP\_026568582.1 | 52.07 | 2 | 0 | 0 | 0 | 0 | 0 | 2 | 0 | 0 | 0 | 0 | 0 | 0 | 0 | 0 |  |  |  |  |  | 7.9169E5 |  |  |  |  |  |  |  |  | 1 | 1 | 0 | 0 | 0 | 0 | 0 | 1 | 0 | 0 | 0 | 0 | 0 | 0 | 0 | 0 | N | 54031 | glutathione reductase, mitochondrial [Pseudonaja textilis] |
| 349 | 2717 | XP\_026528673.1 | 52.07 | 2 | 0 | 0 | 0 | 0 | 0 | 2 | 0 | 0 | 0 | 0 | 0 | 0 | 0 | 0 |  |  |  |  |  | 7.9169E5 |  |  |  |  |  |  |  |  | 1 | 1 | 0 | 0 | 0 | 0 | 0 | 1 | 0 | 0 | 0 | 0 | 0 | 0 | 0 | 0 | N | 54065 | glutathione reductase, mitochondrial [Notechis scutatus] |
| 349 | 2751 | XP\_025022218.1 | 52.07 | 3 | 0 | 0 | 0 | 0 | 0 | 3 | 0 | 0 | 0 | 0 | 0 | 0 | 0 | 0 |  |  |  |  |  | 7.9169E5 |  |  |  |  |  |  |  |  | 1 | 1 | 0 | 0 | 0 | 0 | 0 | 1 | 0 | 0 | 0 | 0 | 0 | 0 | 0 | 0 | N | 45265 | glutathione reductase, mitochondrial isoform X2 [Python bivittatus] |
| 349 | 2752 | XP\_007427940.1 | 52.07 | 3 | 0 | 0 | 0 | 0 | 0 | 3 | 0 | 0 | 0 | 0 | 0 | 0 | 0 | 0 |  |  |  |  |  | 7.9169E5 |  |  |  |  |  |  |  |  | 1 | 1 | 0 | 0 | 0 | 0 | 0 | 1 | 0 | 0 | 0 | 0 | 0 | 0 | 0 | 0 | N | 45265 | glutathione reductase, mitochondrial isoform X2 [Python bivittatus] |
| 349 | 2856 | XP\_032068554.1 | 52.07 | 2 | 0 | 0 | 0 | 0 | 0 | 2 | 0 | 0 | 0 | 0 | 0 | 0 | 0 | 0 |  |  |  |  |  | 7.9169E5 |  |  |  |  |  |  |  |  | 1 | 1 | 0 | 0 | 0 | 0 | 0 | 1 | 0 | 0 | 0 | 0 | 0 | 0 | 0 | 0 | N | 54697 | glutathione reductase, mitochondrial isoform X1 [Thamnophis elegans] |
| 349 | 2873 | XP\_034266719.1 | 52.07 | 2 | 0 | 0 | 0 | 0 | 0 | 2 | 0 | 0 | 0 | 0 | 0 | 0 | 0 | 0 |  |  |  |  |  | 7.9169E5 |  |  |  |  |  |  |  |  | 1 | 1 | 0 | 0 | 0 | 0 | 0 | 1 | 0 | 0 | 0 | 0 | 0 | 0 | 0 | 0 | N | 54204 | glutathione reductase, mitochondrial [Pantherophis guttatus] |
| 349 | 2991 | XP\_032068557.1 | 52.07 | 3 | 0 | 0 | 0 | 0 | 0 | 3 | 0 | 0 | 0 | 0 | 0 | 0 | 0 | 0 |  |  |  |  |  | 7.9169E5 |  |  |  |  |  |  |  |  | 1 | 1 | 0 | 0 | 0 | 0 | 0 | 1 | 0 | 0 | 0 | 0 | 0 | 0 | 0 | 0 | N | 45484 | glutathione reductase, mitochondrial isoform X2 [Thamnophis elegans] |
| 349 | 2992 | XP\_032068555.1 | 52.07 | 3 | 0 | 0 | 0 | 0 | 0 | 3 | 0 | 0 | 0 | 0 | 0 | 0 | 0 | 0 |  |  |  |  |  | 7.9169E5 |  |  |  |  |  |  |  |  | 1 | 1 | 0 | 0 | 0 | 0 | 0 | 1 | 0 | 0 | 0 | 0 | 0 | 0 | 0 | 0 | N | 45484 | glutathione reductase, mitochondrial isoform X2 [Thamnophis elegans] |
| 349 | 2993 | XP\_032068556.1 | 52.07 | 3 | 0 | 0 | 0 | 0 | 0 | 3 | 0 | 0 | 0 | 0 | 0 | 0 | 0 | 0 |  |  |  |  |  | 7.9169E5 |  |  |  |  |  |  |  |  | 1 | 1 | 0 | 0 | 0 | 0 | 0 | 1 | 0 | 0 | 0 | 0 | 0 | 0 | 0 | 0 | N | 45484 | glutathione reductase, mitochondrial isoform X2 [Thamnophis elegans] |
| 291 | 2358 | XP\_026520482.1 | 50.94 | 4 | 0 | 0 | 0 | 0 | 0 | 0 | 0 | 0 | 0 | 0 | 0 | 4 | 0 | 0 |  |  |  |  |  |  |  |  |  |  |  | 5.1341E7 |  |  | 1 | 1 | 0 | 0 | 0 | 0 | 0 | 0 | 0 | 0 | 0 | 0 | 0 | 4 | 0 | 0 | Y | 27411 | methylthioribulose-1-phosphate dehydratase isoform X2 [Notechis scutatus] |
| 291 | 2359 | LAB44896.1 | 50.94 | 4 | 0 | 0 | 0 | 0 | 0 | 0 | 0 | 0 | 0 | 0 | 0 | 4 | 0 | 0 |  |  |  |  |  |  |  |  |  |  |  | 5.1341E7 |  |  | 1 | 1 | 0 | 0 | 0 | 0 | 0 | 0 | 0 | 0 | 0 | 0 | 0 | 4 | 0 | 0 | Y | 27923 | hypothetical protein [Micrurus spixii] |
| 291 | 2369 | XP\_026520481.1 | 50.94 | 4 | 0 | 0 | 0 | 0 | 0 | 0 | 0 | 0 | 0 | 0 | 0 | 4 | 0 | 0 |  |  |  |  |  |  |  |  |  |  |  | 5.1341E7 |  |  | 1 | 1 | 0 | 0 | 0 | 0 | 0 | 0 | 0 | 0 | 0 | 0 | 0 | 4 | 0 | 0 | Y | 28275 | methylthioribulose-1-phosphate dehydratase isoform X1 [Notechis scutatus] |
| 291 | 2382 | XP\_007426488.2 | 50.94 | 3 | 0 | 0 | 0 | 0 | 0 | 0 | 0 | 0 | 0 | 0 | 0 | 3 | 0 | 0 |  |  |  |  |  |  |  |  |  |  |  | 5.1341E7 |  |  | 1 | 1 | 0 | 0 | 0 | 0 | 0 | 0 | 0 | 0 | 0 | 0 | 0 | 4 | 0 | 0 | Y | 39905 | methylthioribulose-1-phosphate dehydratase [Python bivittatus] |
| 291 | 2384 | XP\_032084080.1 | 50.94 | 4 | 0 | 0 | 0 | 0 | 0 | 0 | 0 | 0 | 0 | 0 | 0 | 4 | 0 | 0 |  |  |  |  |  |  |  |  |  |  |  | 5.1341E7 |  |  | 1 | 1 | 0 | 0 | 0 | 0 | 0 | 0 | 0 | 0 | 0 | 0 | 0 | 4 | 0 | 0 | Y | 27406 | methylthioribulose-1-phosphate dehydratase [Thamnophis elegans] |
| 291 | 2435 | LAA66582.1 | 50.94 | 4 | 0 | 0 | 0 | 0 | 0 | 0 | 0 | 0 | 0 | 0 | 0 | 4 | 0 | 0 |  |  |  |  |  |  |  |  |  |  |  | 5.1341E7 |  |  | 1 | 1 | 0 | 0 | 0 | 0 | 0 | 0 | 0 | 0 | 0 | 0 | 0 | 4 | 0 | 0 | Y | 27484 | hypothetical protein [Micrurus corallinus] |
| 291 | 2479 | JAG46376.1 | 50.94 | 4 | 0 | 0 | 0 | 0 | 0 | 0 | 0 | 0 | 0 | 0 | 0 | 4 | 0 | 0 |  |  |  |  |  |  |  |  |  |  |  | 5.1341E7 |  |  | 1 | 1 | 0 | 0 | 0 | 0 | 0 | 0 | 0 | 0 | 0 | 0 | 0 | 4 | 0 | 0 | Y | 27348 | Putative methylthioribulose-1-phosphate dehydratase -like protein [Crotalus horridus] |
| 291 | 2555 | XP\_026562924.1 | 50.94 | 4 | 0 | 0 | 0 | 0 | 0 | 0 | 0 | 0 | 0 | 0 | 0 | 4 | 0 | 0 |  |  |  |  |  |  |  |  |  |  |  | 5.1341E7 |  |  | 1 | 1 | 0 | 0 | 0 | 0 | 0 | 0 | 0 | 0 | 0 | 0 | 0 | 4 | 0 | 0 | Y | 27455 | methylthioribulose-1-phosphate dehydratase isoform X2 [Pseudonaja textilis] |
| 291 | 2572 | XP\_026562916.1 | 50.94 | 4 | 0 | 0 | 0 | 0 | 0 | 0 | 0 | 0 | 0 | 0 | 0 | 4 | 0 | 0 |  |  |  |  |  |  |  |  |  |  |  | 5.1341E7 |  |  | 1 | 1 | 0 | 0 | 0 | 0 | 0 | 0 | 0 | 0 | 0 | 0 | 0 | 4 | 0 | 0 | Y | 28320 | methylthioribulose-1-phosphate dehydratase isoform X1 [Pseudonaja textilis] |
| 291 | 2644 | JAG67440.1 | 50.94 | 4 | 0 | 0 | 0 | 0 | 0 | 0 | 0 | 0 | 0 | 0 | 0 | 4 | 0 | 0 |  |  |  |  |  |  |  |  |  |  |  | 5.1341E7 |  |  | 1 | 1 | 0 | 0 | 0 | 0 | 0 | 0 | 0 | 0 | 0 | 0 | 0 | 4 | 0 | 0 | Y | 27448 | putative methylthioribulose-1-phosphate dehydratase -like protein [Boiga irregularis] |
| 291 | 2730 | JAC95140.1 | 50.94 | 4 | 0 | 0 | 0 | 0 | 0 | 0 | 0 | 0 | 0 | 0 | 0 | 4 | 0 | 0 |  |  |  |  |  |  |  |  |  |  |  | 5.1341E7 |  |  | 1 | 1 | 0 | 0 | 0 | 0 | 0 | 0 | 0 | 0 | 0 | 0 | 0 | 4 | 0 | 0 | Y | 27408 | putative methylthioribulose-1-phosphate dehydratase -like protein [Hypsiglena sp. JMG-2014] |
| 291 | 2734 | ETE68137.1 | 50.94 | 5 | 0 | 0 | 0 | 0 | 0 | 0 | 0 | 0 | 0 | 0 | 0 | 5 | 0 | 0 |  |  |  |  |  |  |  |  |  |  |  | 5.1341E7 |  |  | 1 | 1 | 0 | 0 | 0 | 0 | 0 | 0 | 0 | 0 | 0 | 0 | 0 | 4 | 0 | 0 | Y | 21764 | putative methylthioribulose-1-phosphate dehydratase [Ophiophagus hannah] |
| 291 | 2951 | JAI12592.1 | 50.94 | 4 | 0 | 0 | 0 | 0 | 0 | 0 | 0 | 0 | 0 | 0 | 0 | 4 | 0 | 0 |  |  |  |  |  |  |  |  |  |  |  | 5.1341E7 |  |  | 1 | 1 | 0 | 0 | 0 | 0 | 0 | 0 | 0 | 0 | 0 | 0 | 0 | 4 | 0 | 0 | Y | 27330 | putative methylthioribulose-1-phosphate dehydratase -like protein [Crotalus adamanteus] |
| 291 | 2952 | JAG43994.1 | 50.94 | 4 | 0 | 0 | 0 | 0 | 0 | 0 | 0 | 0 | 0 | 0 | 0 | 4 | 0 | 0 |  |  |  |  |  |  |  |  |  |  |  | 5.1341E7 |  |  | 1 | 1 | 0 | 0 | 0 | 0 | 0 | 0 | 0 | 0 | 0 | 0 | 0 | 4 | 0 | 0 | Y | 27316 | Putative methylthioribulose-1-phosphate dehydratase -like protein [Crotalus horridus] |
| 291 | 2953 | JAA96544.1 | 50.94 | 4 | 0 | 0 | 0 | 0 | 0 | 0 | 0 | 0 | 0 | 0 | 0 | 4 | 0 | 0 |  |  |  |  |  |  |  |  |  |  |  | 5.1341E7 |  |  | 1 | 1 | 0 | 0 | 0 | 0 | 0 | 0 | 0 | 0 | 0 | 0 | 0 | 4 | 0 | 0 | Y | 27316 | Putative methylthioribulose-1-phosphate dehydratase-like protein [Crotalus horridus] |
| 291 | 3171 | XP\_034273253.1 | 50.94 | 4 | 0 | 0 | 0 | 0 | 0 | 0 | 0 | 0 | 0 | 0 | 0 | 4 | 0 | 0 |  |  |  |  |  |  |  |  |  |  |  | 5.1341E7 |  |  | 1 | 1 | 0 | 0 | 0 | 0 | 0 | 0 | 0 | 0 | 0 | 0 | 0 | 4 | 0 | 0 | Y | 27344 | methylthioribulose-1-phosphate dehydratase isoform X2 [Pantherophis guttatus] |
| 291 | 3172 | XP\_034273252.1 | 50.94 | 4 | 0 | 0 | 0 | 0 | 0 | 0 | 0 | 0 | 0 | 0 | 0 | 4 | 0 | 0 |  |  |  |  |  |  |  |  |  |  |  | 5.1341E7 |  |  | 1 | 1 | 0 | 0 | 0 | 0 | 0 | 0 | 0 | 0 | 0 | 0 | 0 | 4 | 0 | 0 | Y | 27431 | methylthioribulose-1-phosphate dehydratase isoform X1 [Pantherophis guttatus] |
| 291 | 3289 | XP\_013930126.1 | 50.94 | 5 | 0 | 0 | 0 | 0 | 0 | 0 | 0 | 0 | 0 | 0 | 0 | 5 | 0 | 0 |  |  |  |  |  |  |  |  |  |  |  | 5.1341E7 |  |  | 1 | 1 | 0 | 0 | 0 | 0 | 0 | 0 | 0 | 0 | 0 | 0 | 0 | 4 | 0 | 0 | Y | 21365 | PREDICTED: methylthioribulose-1-phosphate dehydratase, partial [Thamnophis sirtalis] |
| 291 | 3330 | XP\_015673888.1 | 50.94 | 4 | 0 | 0 | 0 | 0 | 0 | 0 | 0 | 0 | 0 | 0 | 0 | 4 | 0 | 0 |  |  |  |  |  |  |  |  |  |  |  | 5.1341E7 |  |  | 1 | 1 | 0 | 0 | 0 | 0 | 0 | 0 | 0 | 0 | 0 | 0 | 0 | 4 | 0 | 0 | Y | 27333 | methylthioribulose-1-phosphate dehydratase [Protobothrops mucrosquamatus] |
| 291 | 3341 | LAA66581.1 | 50.94 | 5 | 0 | 0 | 0 | 0 | 0 | 0 | 0 | 0 | 0 | 0 | 0 | 5 | 0 | 0 |  |  |  |  |  |  |  |  |  |  |  | 5.1341E7 |  |  | 1 | 1 | 0 | 0 | 0 | 0 | 0 | 0 | 0 | 0 | 0 | 0 | 0 | 4 | 0 | 0 | Y | 22262 | hypothetical protein [Micrurus corallinus] |
| 291 | 3343 | LAB44894.1 | 50.94 | 5 | 0 | 0 | 0 | 0 | 0 | 0 | 0 | 0 | 0 | 0 | 0 | 5 | 0 | 0 |  |  |  |  |  |  |  |  |  |  |  | 5.1341E7 |  |  | 1 | 1 | 0 | 0 | 0 | 0 | 0 | 0 | 0 | 0 | 0 | 0 | 0 | 4 | 0 | 0 | Y | 19318 | hypothetical protein [Micrurus spixii] |
| 291 | 3344 | LAA66585.1 | 50.94 | 5 | 0 | 0 | 0 | 0 | 0 | 0 | 0 | 0 | 0 | 0 | 0 | 5 | 0 | 0 |  |  |  |  |  |  |  |  |  |  |  | 5.1341E7 |  |  | 1 | 1 | 0 | 0 | 0 | 0 | 0 | 0 | 0 | 0 | 0 | 0 | 0 | 4 | 0 | 0 | Y | 19318 | hypothetical protein [Micrurus corallinus] |
| 250 | 1960 | XP\_026576072.1 | 48.91 | 3 | 0 | 3 | 3 | 3 | 0 | 0 | 0 | 0 | 0 | 0 | 0 | 0 | 0 | 0 |  | 2.0681E6 | 1.4041E4 | 1.5701E6 |  |  |  |  |  |  |  |  |  |  | 1 | 1 | 0 | 2 | 1 | 3 | 0 | 0 | 0 | 0 | 0 | 0 | 0 | 0 | 0 | 0 | N | 39960 | extracellular matrix protein 1 isoform X2 [Pseudonaja textilis] |
| 250 | 1961 | XP\_026576070.1 | 48.91 | 3 | 0 | 3 | 3 | 3 | 0 | 0 | 0 | 0 | 0 | 0 | 0 | 0 | 0 | 0 |  | 2.0681E6 | 1.4041E4 | 1.5701E6 |  |  |  |  |  |  |  |  |  |  | 1 | 1 | 0 | 2 | 1 | 3 | 0 | 0 | 0 | 0 | 0 | 0 | 0 | 0 | 0 | 0 | N | 40088 | extracellular matrix protein 1 isoform X1 [Pseudonaja textilis] |
| 348 | 2071 | JAB53705.1 | 46.16 | 6 | 0 | 0 | 0 | 6 | 0 | 0 | 0 | 0 | 0 | 0 | 0 | 0 | 0 | 0 |  |  |  | 4.6853E5 |  |  |  |  |  |  |  |  |  |  | 1 | 1 | 0 | 0 | 0 | 2 | 0 | 0 | 0 | 0 | 0 | 0 | 0 | 0 | 0 | 0 | N | 23247 | phosphatidylethanolamine-binding protein 4 [Micrurus fulvius] |
| 348 | 2387 | XP\_026570986.1 | 46.16 | 6 | 0 | 0 | 0 | 6 | 0 | 0 | 0 | 0 | 0 | 0 | 0 | 0 | 0 | 0 |  |  |  | 4.6853E5 |  |  |  |  |  |  |  |  |  |  | 1 | 1 | 0 | 0 | 0 | 2 | 0 | 0 | 0 | 0 | 0 | 0 | 0 | 0 | 0 | 0 | N | 23371 | phosphatidylethanolamine-binding protein 4 [Pseudonaja textilis] |
| 348 | 2575 | XP\_026539773.1 | 46.16 | 6 | 0 | 0 | 0 | 6 | 0 | 0 | 0 | 0 | 0 | 0 | 0 | 0 | 0 | 0 |  |  |  | 4.6853E5 |  |  |  |  |  |  |  |  |  |  | 1 | 1 | 0 | 0 | 0 | 2 | 0 | 0 | 0 | 0 | 0 | 0 | 0 | 0 | 0 | 0 | N | 23342 | phosphatidylethanolamine-binding protein 4 [Notechis scutatus] |
| 348 | 2576 | XP\_026539772.1 | 46.16 | 6 | 0 | 0 | 0 | 6 | 0 | 0 | 0 | 0 | 0 | 0 | 0 | 0 | 0 | 0 |  |  |  | 4.6853E5 |  |  |  |  |  |  |  |  |  |  | 1 | 1 | 0 | 0 | 0 | 2 | 0 | 0 | 0 | 0 | 0 | 0 | 0 | 0 | 0 | 0 | N | 23342 | phosphatidylethanolamine-binding protein 4 [Notechis scutatus] |
| 460 | 2085 | XP\_013925432.1 | 45.20 | 3 | 0 | 0 | 0 | 3 | 0 | 0 | 0 | 0 | 0 | 0 | 0 | 0 | 0 | 0 |  |  |  | 4.7985E5 |  |  |  |  |  |  |  |  |  |  | 1 | 1 | 0 | 0 | 0 | 1 | 0 | 0 | 0 | 0 | 0 | 0 | 0 | 0 | 0 | 0 | N | 55065 | PREDICTED: nucleobindin-2-like [Thamnophis sirtalis] |
| 460 | 2086 | XP\_032078463.1 | 45.20 | 3 | 0 | 0 | 0 | 3 | 0 | 0 | 0 | 0 | 0 | 0 | 0 | 0 | 0 | 0 |  |  |  | 4.7985E5 |  |  |  |  |  |  |  |  |  |  | 1 | 1 | 0 | 0 | 0 | 1 | 0 | 0 | 0 | 0 | 0 | 0 | 0 | 0 | 0 | 0 | N | 55065 | nucleobindin-2 [Thamnophis elegans] |
| 460 | 2090 | XP\_034272855.1 | 45.20 | 3 | 0 | 0 | 0 | 3 | 0 | 0 | 0 | 0 | 0 | 0 | 0 | 0 | 0 | 0 |  |  |  | 4.7985E5 |  |  |  |  |  |  |  |  |  |  | 1 | 1 | 0 | 0 | 0 | 1 | 0 | 0 | 0 | 0 | 0 | 0 | 0 | 0 | 0 | 0 | N | 54912 | nucleobindin-2 [Pantherophis guttatus] |
| 460 | 2092 | JAG67177.1 | 45.20 | 3 | 0 | 0 | 0 | 3 | 0 | 0 | 0 | 0 | 0 | 0 | 0 | 0 | 0 | 0 |  |  |  | 4.7985E5 |  |  |  |  |  |  |  |  |  |  | 1 | 1 | 0 | 0 | 0 | 1 | 0 | 0 | 0 | 0 | 0 | 0 | 0 | 0 | 0 | 0 | N | 55230 | Nucleobindin-2-like protein [Boiga irregularis] |
| 460 | 2151 | JAB53784.1 | 45.20 | 3 | 0 | 0 | 0 | 3 | 0 | 0 | 0 | 0 | 0 | 0 | 0 | 0 | 0 | 0 |  |  |  | 4.7985E5 |  |  |  |  |  |  |  |  |  |  | 1 | 1 | 0 | 0 | 0 | 1 | 0 | 0 | 0 | 0 | 0 | 0 | 0 | 0 | 0 | 0 | N | 54970 | Nucleobindin-2 [Micrurus fulvius] |
| 460 | 2174 | XP\_026562300.1 | 45.20 | 4 | 0 | 0 | 0 | 4 | 0 | 0 | 0 | 0 | 0 | 0 | 0 | 0 | 0 | 0 |  |  |  | 4.7985E5 |  |  |  |  |  |  |  |  |  |  | 1 | 1 | 0 | 0 | 0 | 1 | 0 | 0 | 0 | 0 | 0 | 0 | 0 | 0 | 0 | 0 | N | 51084 | nucleobindin-2 isoform X2 [Pseudonaja textilis] |
| 460 | 2175 | XP\_026562299.1 | 45.20 | 3 | 0 | 0 | 0 | 3 | 0 | 0 | 0 | 0 | 0 | 0 | 0 | 0 | 0 | 0 |  |  |  | 4.7985E5 |  |  |  |  |  |  |  |  |  |  | 1 | 1 | 0 | 0 | 0 | 1 | 0 | 0 | 0 | 0 | 0 | 0 | 0 | 0 | 0 | 0 | N | 55120 | nucleobindin-2 isoform X1 [Pseudonaja textilis] |
| 460 | 2176 | XP\_026524106.1 | 45.20 | 3 | 0 | 0 | 0 | 3 | 0 | 0 | 0 | 0 | 0 | 0 | 0 | 0 | 0 | 0 |  |  |  | 4.7985E5 |  |  |  |  |  |  |  |  |  |  | 1 | 1 | 0 | 0 | 0 | 1 | 0 | 0 | 0 | 0 | 0 | 0 | 0 | 0 | 0 | 0 | N | 55065 | nucleobindin-2 [Notechis scutatus] |
| 334 | 2215 | XP\_032084634.1 | 45.09 | 1 | 0 | 0 | 0 | 0 | 0 | 1 | 0 | 0 | 0 | 0 | 0 | 0 | 0 | 0 |  |  |  |  |  | 0 |  |  |  |  |  |  |  |  | 1 | 1 | 0 | 0 | 0 | 0 | 0 | 1 | 0 | 0 | 0 | 0 | 0 | 0 | 0 | 0 | Y | 73524 | LOW QUALITY PROTEIN: zinc metalloproteinase-disintegrin-like NaMP [Thamnophis elegans] |
| 334 | 2565 | JAC96591.1 | 45.09 | 3 | 0 | 0 | 0 | 0 | 0 | 3 | 0 | 0 | 0 | 0 | 0 | 0 | 0 | 0 |  |  |  |  |  | 0 |  |  |  |  |  |  |  |  | 1 | 1 | 0 | 0 | 0 | 0 | 0 | 1 | 0 | 0 | 0 | 0 | 0 | 0 | 0 | 0 | Y | 29512 | Snake venom metalloproteinase T, partial [Echis coloratus] |
| 473 | 2170 | XP\_013929526.1 | 44.98 | 1 | 0 | 0 | 0 | 0 | 0 | 0 | 0 | 0 | 0 | 0 | 0 | 0 | 0 | 1 |  |  |  |  |  |  |  |  |  |  |  |  |  | 6.8208E7 | 1 | 1 | 0 | 0 | 0 | 0 | 0 | 0 | 0 | 0 | 0 | 0 | 0 | 0 | 0 | 1 | Y | 110673 | PREDICTED: SWI/SNF-related matrix-associated actin-dependent regulator of chromatin subfamily A-like protein 1 [Thamnophis sirtalis] |
| 473 | 2200 | XP\_032074628.1 | 44.98 | 1 | 0 | 0 | 0 | 0 | 0 | 0 | 0 | 0 | 0 | 0 | 0 | 0 | 0 | 1 |  |  |  |  |  |  |  |  |  |  |  |  |  | 6.8208E7 | 1 | 1 | 0 | 0 | 0 | 0 | 0 | 0 | 0 | 0 | 0 | 0 | 0 | 0 | 0 | 1 | Y | 104987 | SWI/SNF-related matrix-associated actin-dependent regulator of chromatin subfamily A-like protein 1 isoform X2 [Thamnophis elegans] |
| 473 | 2201 | XP\_032074600.1 | 44.98 | 1 | 0 | 0 | 0 | 0 | 0 | 0 | 0 | 0 | 0 | 0 | 0 | 0 | 0 | 1 |  |  |  |  |  |  |  |  |  |  |  |  |  | 6.8208E7 | 1 | 1 | 0 | 0 | 0 | 0 | 0 | 0 | 0 | 0 | 0 | 0 | 0 | 0 | 0 | 1 | Y | 110970 | SWI/SNF-related matrix-associated actin-dependent regulator of chromatin subfamily A-like protein 1 isoform X1 [Thamnophis elegans] |
| 473 | 2202 | XP\_032074609.1 | 44.98 | 1 | 0 | 0 | 0 | 0 | 0 | 0 | 0 | 0 | 0 | 0 | 0 | 0 | 0 | 1 |  |  |  |  |  |  |  |  |  |  |  |  |  | 6.8208E7 | 1 | 1 | 0 | 0 | 0 | 0 | 0 | 0 | 0 | 0 | 0 | 0 | 0 | 0 | 0 | 1 | Y | 110970 | SWI/SNF-related matrix-associated actin-dependent regulator of chromatin subfamily A-like protein 1 isoform X1 [Thamnophis elegans] |
| 473 | 2203 | XP\_032074620.1 | 44.98 | 1 | 0 | 0 | 0 | 0 | 0 | 0 | 0 | 0 | 0 | 0 | 0 | 0 | 0 | 1 |  |  |  |  |  |  |  |  |  |  |  |  |  | 6.8208E7 | 1 | 1 | 0 | 0 | 0 | 0 | 0 | 0 | 0 | 0 | 0 | 0 | 0 | 0 | 0 | 1 | Y | 110970 | SWI/SNF-related matrix-associated actin-dependent regulator of chromatin subfamily A-like protein 1 isoform X1 [Thamnophis elegans] |
| 473 | 2204 | XP\_032074590.1 | 44.98 | 1 | 0 | 0 | 0 | 0 | 0 | 0 | 0 | 0 | 0 | 0 | 0 | 0 | 0 | 1 |  |  |  |  |  |  |  |  |  |  |  |  |  | 6.8208E7 | 1 | 1 | 0 | 0 | 0 | 0 | 0 | 0 | 0 | 0 | 0 | 0 | 0 | 0 | 0 | 1 | Y | 110970 | SWI/SNF-related matrix-associated actin-dependent regulator of chromatin subfamily A-like protein 1 isoform X1 [Thamnophis elegans] |
| 356 | 5252 | E5AJX2.1 | 44.90 | 4 | 0 | 0 | 0 | 4 | 0 | 0 | 0 | 0 | 0 | 0 | 0 | 0 | 0 | 0 |  |  |  | 1.0314E6 |  |  |  |  |  |  |  |  |  |  | 1 | 1 | 0 | 0 | 0 | 2 | 0 | 0 | 0 | 0 | 0 | 0 | 0 | 0 | 0 | 0 | N | 28216 | RecName: Full=Snake venom serine protease nikobin; Short=SVSP; Flags: Precursor |
| 356 | 5253 | CBW30778.1 | 44.90 | 4 | 0 | 0 | 0 | 4 | 0 | 0 | 0 | 0 | 0 | 0 | 0 | 0 | 0 | 0 |  |  |  | 1.0314E6 |  |  |  |  |  |  |  |  |  |  | 1 | 1 | 0 | 0 | 0 | 2 | 0 | 0 | 0 | 0 | 0 | 0 | 0 | 0 | 0 | 0 | N | 28216 | putative serine protease nikobin [Vipera berus nikolskii] |
| 627 | 1932 | LAA92466.1 | 44.62 | 6 | 0 | 0 | 0 | 0 | 0 | 0 | 0 | 0 | 0 | 0 | 0 | 6 | 0 | 0 |  |  |  |  |  |  |  |  |  |  |  | 3.8764E5 |  |  | 1 | 1 | 0 | 0 | 0 | 0 | 0 | 0 | 0 | 0 | 0 | 0 | 0 | 1 | 0 | 0 | Y | 20351 | hypothetical protein [Micrurus lemniscatus lemniscatus] |
| 627 | 1933 | LAA30014.1 | 44.62 | 6 | 0 | 0 | 0 | 0 | 0 | 0 | 0 | 0 | 0 | 0 | 0 | 6 | 0 | 0 |  |  |  |  |  |  |  |  |  |  |  | 3.8764E5 |  |  | 1 | 1 | 0 | 0 | 0 | 0 | 0 | 0 | 0 | 0 | 0 | 0 | 0 | 1 | 0 | 0 | Y | 20351 | hypothetical protein [Micrurus lemniscatus carvalhoi] |
| 627 | 2510 | XP\_026572403.1 | 44.62 | 6 | 0 | 0 | 0 | 0 | 0 | 0 | 0 | 0 | 0 | 0 | 0 | 6 | 0 | 0 |  |  |  |  |  |  |  |  |  |  |  | 3.8764E5 |  |  | 1 | 1 | 0 | 0 | 0 | 0 | 0 | 0 | 0 | 0 | 0 | 0 | 0 | 1 | 0 | 0 | Y | 20319 | endothelial cell-specific molecule 1 [Pseudonaja textilis] |
| 627 | 2511 | XP\_026543179.1 | 44.62 | 6 | 0 | 0 | 0 | 0 | 0 | 0 | 0 | 0 | 0 | 0 | 0 | 6 | 0 | 0 |  |  |  |  |  |  |  |  |  |  |  | 3.8764E5 |  |  | 1 | 1 | 0 | 0 | 0 | 0 | 0 | 0 | 0 | 0 | 0 | 0 | 0 | 1 | 0 | 0 | Y | 20291 | endothelial cell-specific molecule 1 [Notechis scutatus] |
| 627 | 2512 | XP\_034296763.1 | 44.62 | 6 | 0 | 0 | 0 | 0 | 0 | 0 | 0 | 0 | 0 | 0 | 0 | 6 | 0 | 0 |  |  |  |  |  |  |  |  |  |  |  | 3.8764E5 |  |  | 1 | 1 | 0 | 0 | 0 | 0 | 0 | 0 | 0 | 0 | 0 | 0 | 0 | 1 | 0 | 0 | Y | 20292 | endothelial cell-specific molecule 1 [Pantherophis guttatus] |
| 627 | 2646 | XP\_013918417.1 | 44.62 | 6 | 0 | 0 | 0 | 0 | 0 | 0 | 0 | 0 | 0 | 0 | 0 | 6 | 0 | 0 |  |  |  |  |  |  |  |  |  |  |  | 3.8764E5 |  |  | 1 | 1 | 0 | 0 | 0 | 0 | 0 | 0 | 0 | 0 | 0 | 0 | 0 | 1 | 0 | 0 | Y | 20307 | PREDICTED: endothelial cell-specific molecule 1 [Thamnophis sirtalis] |
| 627 | 2697 | XP\_015667264.1 | 44.62 | 6 | 0 | 0 | 0 | 0 | 0 | 0 | 0 | 0 | 0 | 0 | 0 | 6 | 0 | 0 |  |  |  |  |  |  |  |  |  |  |  | 3.8764E5 |  |  | 1 | 1 | 0 | 0 | 0 | 0 | 0 | 0 | 0 | 0 | 0 | 0 | 0 | 1 | 0 | 0 | Y | 20280 | endothelial cell-specific molecule 1 [Protobothrops mucrosquamatus] |
| 627 | 2698 | XP\_007421398.1 | 44.62 | 6 | 0 | 0 | 0 | 0 | 0 | 0 | 0 | 0 | 0 | 0 | 0 | 6 | 0 | 0 |  |  |  |  |  |  |  |  |  |  |  | 3.8764E5 |  |  | 1 | 1 | 0 | 0 | 0 | 0 | 0 | 0 | 0 | 0 | 0 | 0 | 0 | 1 | 0 | 0 | Y | 20392 | endothelial cell-specific molecule 1 [Python bivittatus] |
| 627 | 3382 | ETE72814.1 | 44.62 | 7 | 0 | 0 | 0 | 0 | 0 | 0 | 0 | 0 | 0 | 0 | 0 | 7 | 0 | 0 |  |  |  |  |  |  |  |  |  |  |  | 3.8764E5 |  |  | 1 | 1 | 0 | 0 | 0 | 0 | 0 | 0 | 0 | 0 | 0 | 0 | 0 | 1 | 0 | 0 | Y | 19894 | Endothelial cell-specific molecule 1, partial [Ophiophagus hannah] |
| 315 | 3359 | XP\_026552061.1 | 44.05 | 3 | 0 | 0 | 0 | 0 | 0 | 0 | 0 | 0 | 0 | 0 | 0 | 0 | 0 | 3 |  |  |  |  |  |  |  |  |  |  |  |  |  | 1.678E7 | 1 | 1 | 0 | 0 | 0 | 0 | 0 | 0 | 0 | 0 | 0 | 0 | 0 | 0 | 0 | 1 | N | 38038 | meiotic recombination protein DMC1/LIM15 homolog isoform X1 [Pseudonaja textilis] |
| 315 | 3416 | XP\_034259615.1 | 44.05 | 3 | 0 | 0 | 0 | 0 | 0 | 0 | 0 | 0 | 0 | 0 | 0 | 0 | 0 | 3 |  |  |  |  |  |  |  |  |  |  |  |  |  | 1.678E7 | 1 | 1 | 0 | 0 | 0 | 0 | 0 | 0 | 0 | 0 | 0 | 0 | 0 | 0 | 0 | 1 | N | 32798 | meiotic recombination protein DMC1/LIM15 homolog isoform X3 [Pantherophis guttatus] |
| 679 | 5883 | XP\_034291756.1 | 43.64 | 10 | 0 | 0 | 0 | 0 | 0 | 10 | 0 | 0 | 0 | 0 | 0 | 0 | 0 | 0 |  |  |  |  |  | 5.5479E5 |  |  |  |  |  |  |  |  | 1 | 1 | 0 | 0 | 0 | 0 | 0 | 1 | 0 | 0 | 0 | 0 | 0 | 0 | 0 | 0 | Y | 15604 | interleukin-18-binding protein [Pantherophis guttatus] |
| 497 | 2292 | ETE60133.1 | 42.38 | 2 | 0 | 0 | 2 | 0 | 0 | 0 | 0 | 0 | 0 | 0 | 0 | 0 | 0 | 0 |  |  | 0 |  |  |  |  |  |  |  |  |  |  |  | 1 | 1 | 0 | 0 | 1 | 0 | 0 | 0 | 0 | 0 | 0 | 0 | 0 | 0 | 0 | 0 | Y | 210699 | Hemicentin-1, partial [Ophiophagus hannah] |
| 319 | 1921 | AFJ49242.1 | 41.02 | 3 | 0 | 0 | 0 | 0 | 0 | 3 | 0 | 0 | 0 | 0 | 0 | 0 | 0 | 0 |  |  |  |  |  | 2.4757E8 |  |  |  |  |  |  |  |  | 1 | 1 | 0 | 0 | 0 | 0 | 0 | 2 | 0 | 0 | 0 | 0 | 0 | 0 | 0 | 0 | Y | 67993 | Snake venom metalloproteinase (type III) 4b [Crotalus adamanteus] |
| 319 | 1922 | JAS05277.1 | 41.02 | 3 | 0 | 0 | 0 | 0 | 0 | 3 | 0 | 0 | 0 | 0 | 0 | 0 | 0 | 0 |  |  |  |  |  | 2.4757E8 |  |  |  |  |  |  |  |  | 1 | 1 | 0 | 0 | 0 | 0 | 0 | 2 | 0 | 0 | 0 | 0 | 0 | 0 | 0 | 0 | Y | 67957 | Metalloproteinase type III 5b [Sistrurus tergeminus] |
| 319 | 1943 | JAS04444.1 | 41.02 | 3 | 0 | 0 | 0 | 0 | 0 | 3 | 0 | 0 | 0 | 0 | 0 | 0 | 0 | 0 |  |  |  |  |  | 2.4757E8 |  |  |  |  |  |  |  |  | 1 | 1 | 0 | 0 | 0 | 0 | 0 | 2 | 0 | 0 | 0 | 0 | 0 | 0 | 0 | 0 | Y | 68214 | Metalloproteinase type III 9b [Agkistrodon piscivorus conanti] |
| 319 | 1944 | JAS04445.1 | 41.02 | 3 | 0 | 0 | 0 | 0 | 0 | 3 | 0 | 0 | 0 | 0 | 0 | 0 | 0 | 0 |  |  |  |  |  | 2.4757E8 |  |  |  |  |  |  |  |  | 1 | 1 | 0 | 0 | 0 | 0 | 0 | 2 | 0 | 0 | 0 | 0 | 0 | 0 | 0 | 0 | Y | 68139 | Metalloproteinase type III 9a [Agkistrodon piscivorus conanti] |
| 319 | 1945 | JAS04456.1 | 41.02 | 3 | 0 | 0 | 0 | 0 | 0 | 3 | 0 | 0 | 0 | 0 | 0 | 0 | 0 | 0 |  |  |  |  |  | 2.4757E8 |  |  |  |  |  |  |  |  | 1 | 1 | 0 | 0 | 0 | 0 | 0 | 2 | 0 | 0 | 0 | 0 | 0 | 0 | 0 | 0 | Y | 68245 | Metalloproteinase type III 13 [Agkistrodon piscivorus conanti] |
| 319 | 1988 | JAG43634.1 | 41.02 | 3 | 0 | 0 | 0 | 0 | 0 | 3 | 0 | 0 | 0 | 0 | 0 | 0 | 0 | 0 |  |  |  |  |  | 2.4757E8 |  |  |  |  |  |  |  |  | 1 | 1 | 0 | 0 | 0 | 0 | 0 | 2 | 0 | 0 | 0 | 0 | 0 | 0 | 0 | 0 | Y | 67953 | Metalloproteinase (type III) 1 [Crotalus horridus] |
| 319 | 1989 | JAA94872.1 | 41.02 | 3 | 0 | 0 | 0 | 0 | 0 | 3 | 0 | 0 | 0 | 0 | 0 | 0 | 0 | 0 |  |  |  |  |  | 2.4757E8 |  |  |  |  |  |  |  |  | 1 | 1 | 0 | 0 | 0 | 0 | 0 | 2 | 0 | 0 | 0 | 0 | 0 | 0 | 0 | 0 | Y | 67953 | Snake venom metalloproteinase (type III) 1 [Crotalus horridus] |
| 319 | 1990 | JAS04764.1 | 41.02 | 3 | 0 | 0 | 0 | 0 | 0 | 3 | 0 | 0 | 0 | 0 | 0 | 0 | 0 | 0 |  |  |  |  |  | 2.4757E8 |  |  |  |  |  |  |  |  | 1 | 1 | 0 | 0 | 0 | 0 | 0 | 2 | 0 | 0 | 0 | 0 | 0 | 0 | 0 | 0 | Y | 67953 | Metalloproteinase type III 1 [Crotalus horridus] |
| 319 | 1994 | JAS04838.1 | 41.02 | 3 | 0 | 0 | 0 | 0 | 0 | 3 | 0 | 0 | 0 | 0 | 0 | 0 | 0 | 0 |  |  |  |  |  | 2.4757E8 |  |  |  |  |  |  |  |  | 1 | 1 | 0 | 0 | 0 | 0 | 0 | 2 | 0 | 0 | 0 | 0 | 0 | 0 | 0 | 0 | Y | 67842 | Metalloproteinase type III 5a [Crotalus horridus] |
| 319 | 1995 | JAG46104.1 | 41.02 | 3 | 0 | 0 | 0 | 0 | 0 | 3 | 0 | 0 | 0 | 0 | 0 | 0 | 0 | 0 |  |  |  |  |  | 2.4757E8 |  |  |  |  |  |  |  |  | 1 | 1 | 0 | 0 | 0 | 0 | 0 | 2 | 0 | 0 | 0 | 0 | 0 | 0 | 0 | 0 | Y | 67842 | Metalloproteinase (type III) 5a [Crotalus horridus] |
| 319 | 1996 | AEJ31988.1 | 41.02 | 3 | 0 | 0 | 0 | 0 | 0 | 3 | 0 | 0 | 0 | 0 | 0 | 0 | 0 | 0 |  |  |  |  |  | 2.4757E8 |  |  |  |  |  |  |  |  | 1 | 1 | 0 | 0 | 0 | 0 | 0 | 2 | 0 | 0 | 0 | 0 | 0 | 0 | 0 | 0 | Y | 68025 | metalloproteinase 5 [Crotalus adamanteus] |
| 319 | 1997 | QIP75708.1 | 41.02 | 3 | 0 | 0 | 0 | 0 | 0 | 3 | 0 | 0 | 0 | 0 | 0 | 0 | 0 | 0 |  |  |  |  |  | 2.4757E8 |  |  |  |  |  |  |  |  | 1 | 1 | 0 | 0 | 0 | 0 | 0 | 2 | 0 | 0 | 0 | 0 | 0 | 0 | 0 | 0 | Y | 67899 | MDC-2 [Crotalus horridus] |
| 319 | 1998 | QIV64743.1 | 41.02 | 3 | 0 | 0 | 0 | 0 | 0 | 3 | 0 | 0 | 0 | 0 | 0 | 0 | 0 | 0 |  |  |  |  |  | 2.4757E8 |  |  |  |  |  |  |  |  | 1 | 1 | 0 | 0 | 0 | 0 | 0 | 2 | 0 | 0 | 0 | 0 | 0 | 0 | 0 | 0 | Y | 67944 | MDC-2 [Crotalus horridus] |
| 319 | 1999 | JAS04837.1 | 41.02 | 3 | 0 | 0 | 0 | 0 | 0 | 3 | 0 | 0 | 0 | 0 | 0 | 0 | 0 | 0 |  |  |  |  |  | 2.4757E8 |  |  |  |  |  |  |  |  | 1 | 1 | 0 | 0 | 0 | 0 | 0 | 2 | 0 | 0 | 0 | 0 | 0 | 0 | 0 | 0 | Y | 67914 | Metalloproteinase type III 5b [Crotalus horridus] |
| 319 | 2000 | JAG46103.1 | 41.02 | 3 | 0 | 0 | 0 | 0 | 0 | 3 | 0 | 0 | 0 | 0 | 0 | 0 | 0 | 0 |  |  |  |  |  | 2.4757E8 |  |  |  |  |  |  |  |  | 1 | 1 | 0 | 0 | 0 | 0 | 0 | 2 | 0 | 0 | 0 | 0 | 0 | 0 | 0 | 0 | Y | 67914 | Metalloproteinase (type III) 5b [Crotalus horridus] |
| 319 | 2001 | JAV48404.1 | 41.02 | 3 | 0 | 0 | 0 | 0 | 0 | 3 | 0 | 0 | 0 | 0 | 0 | 0 | 0 | 0 |  |  |  |  |  | 2.4757E8 |  |  |  |  |  |  |  |  | 1 | 1 | 0 | 0 | 0 | 0 | 0 | 2 | 0 | 0 | 0 | 0 | 0 | 0 | 0 | 0 | Y | 67910 | Cadam10\_SVMPIII-10 [Crotalus adamanteus] |
| 319 | 2002 | AEJ31990.1 | 41.02 | 3 | 0 | 0 | 0 | 0 | 0 | 3 | 0 | 0 | 0 | 0 | 0 | 0 | 0 | 0 |  |  |  |  |  | 2.4757E8 |  |  |  |  |  |  |  |  | 1 | 1 | 0 | 0 | 0 | 0 | 0 | 2 | 0 | 0 | 0 | 0 | 0 | 0 | 0 | 0 | Y | 67910 | metalloproteinase 7 [Crotalus adamanteus] |
| 319 | 2003 | JAI10385.1 | 41.02 | 3 | 0 | 0 | 0 | 0 | 0 | 3 | 0 | 0 | 0 | 0 | 0 | 0 | 0 | 0 |  |  |  |  |  | 2.4757E8 |  |  |  |  |  |  |  |  | 1 | 1 | 0 | 0 | 0 | 0 | 0 | 2 | 0 | 0 | 0 | 0 | 0 | 0 | 0 | 0 | Y | 67910 | Metalloproteinase (type III) 3 [Crotalus adamanteus] |
| 319 | 2004 | F8S108.1 | 41.02 | 3 | 0 | 0 | 0 | 0 | 0 | 3 | 0 | 0 | 0 | 0 | 0 | 0 | 0 | 0 |  |  |  |  |  | 2.4757E8 |  |  |  |  |  |  |  |  | 1 | 1 | 0 | 0 | 0 | 0 | 0 | 2 | 0 | 0 | 0 | 0 | 0 | 0 | 0 | 0 | Y | 67910 | RecName: Full=Zinc metalloproteinase-disintegrin-like 4a; AltName: Full=Snake venom metalloproteinase 7; Short=SVMP; Flags: Precursor |
| 319 | 2005 | JAS04681.1 | 41.02 | 3 | 0 | 0 | 0 | 0 | 0 | 3 | 0 | 0 | 0 | 0 | 0 | 0 | 0 | 0 |  |  |  |  |  | 2.4757E8 |  |  |  |  |  |  |  |  | 1 | 1 | 0 | 0 | 0 | 0 | 0 | 2 | 0 | 0 | 0 | 0 | 0 | 0 | 0 | 0 | Y | 67910 | Metalloproteinase type III 3 [Crotalus adamanteus] |
| 319 | 2006 | AFJ49241.1 | 41.02 | 3 | 0 | 0 | 0 | 0 | 0 | 3 | 0 | 0 | 0 | 0 | 0 | 0 | 0 | 0 |  |  |  |  |  | 2.4757E8 |  |  |  |  |  |  |  |  | 1 | 1 | 0 | 0 | 0 | 0 | 0 | 2 | 0 | 0 | 0 | 0 | 0 | 0 | 0 | 0 | Y | 67910 | Snake venom metalloproteinase (type III) 4a [Crotalus adamanteus] |
| 319 | 2007 | Q9DGB9.1 | 41.02 | 3 | 0 | 0 | 0 | 0 | 0 | 3 | 0 | 0 | 0 | 0 | 0 | 0 | 0 | 0 |  |  |  |  |  | 2.4757E8 |  |  |  |  |  |  |  |  | 1 | 1 | 0 | 0 | 0 | 0 | 0 | 2 | 0 | 0 | 0 | 0 | 0 | 0 | 0 | 0 | Y | 67960 | RecName: Full=Zinc metalloproteinase-disintegrin-like VAP1; AltName: Full=Snake venom metalloproteinase; Short=SVMP; AltName: Full=Vascular apoptosis-inducing protein 1; Short=Vap-1; Flags: Precursor |
| 319 | 2008 | BAB18307.1 | 41.02 | 3 | 0 | 0 | 0 | 0 | 0 | 3 | 0 | 0 | 0 | 0 | 0 | 0 | 0 | 0 |  |  |  |  |  | 2.4757E8 |  |  |  |  |  |  |  |  | 1 | 1 | 0 | 0 | 0 | 0 | 0 | 2 | 0 | 0 | 0 | 0 | 0 | 0 | 0 | 0 | Y | 67960 | vascular apoptosis-inducing protein 1 [Crotalus atrox] |
| 319 | 2009 | QIV64929.1 | 41.02 | 3 | 0 | 0 | 0 | 0 | 0 | 3 | 0 | 0 | 0 | 0 | 0 | 0 | 0 | 0 |  |  |  |  |  | 2.4757E8 |  |  |  |  |  |  |  |  | 1 | 1 | 0 | 0 | 0 | 0 | 0 | 2 | 0 | 0 | 0 | 0 | 0 | 0 | 0 | 0 | Y | 67994 | MDC-2 [Crotalus atrox] |
| 319 | 2017 | JAS05278.1 | 41.02 | 3 | 0 | 0 | 0 | 0 | 0 | 3 | 0 | 0 | 0 | 0 | 0 | 0 | 0 | 0 |  |  |  |  |  | 2.4757E8 |  |  |  |  |  |  |  |  | 1 | 1 | 0 | 0 | 0 | 0 | 0 | 2 | 0 | 0 | 0 | 0 | 0 | 0 | 0 | 0 | Y | 67949 | Metalloproteinase type III 5a [Sistrurus tergeminus] |
| 319 | 2019 | JAS05416.1 | 41.02 | 3 | 0 | 0 | 0 | 0 | 0 | 3 | 0 | 0 | 0 | 0 | 0 | 0 | 0 | 0 |  |  |  |  |  | 2.4757E8 |  |  |  |  |  |  |  |  | 1 | 1 | 0 | 0 | 0 | 0 | 0 | 2 | 0 | 0 | 0 | 0 | 0 | 0 | 0 | 0 | Y | 68078 | Metalloproteinase type III 5a [Sistrurus miliarius barbouri] |
| 319 | 2020 | JAS05415.1 | 41.02 | 3 | 0 | 0 | 0 | 0 | 0 | 3 | 0 | 0 | 0 | 0 | 0 | 0 | 0 | 0 |  |  |  |  |  | 2.4757E8 |  |  |  |  |  |  |  |  | 1 | 1 | 0 | 0 | 0 | 0 | 0 | 2 | 0 | 0 | 0 | 0 | 0 | 0 | 0 | 0 | Y | 67951 | Metalloproteinase type III 5b [Sistrurus miliarius barbouri] |
| 319 | 2043 | QIV64767.1 | 41.02 | 3 | 0 | 0 | 0 | 0 | 0 | 3 | 0 | 0 | 0 | 0 | 0 | 0 | 0 | 0 |  |  |  |  |  | 2.4757E8 |  |  |  |  |  |  |  |  | 1 | 1 | 0 | 0 | 0 | 0 | 0 | 2 | 0 | 0 | 0 | 0 | 0 | 0 | 0 | 0 | Y | 67984 | MDC-2 [Crotalus scutulatus] |
| 319 | 2138 | JAS04457.1 | 41.02 | 3 | 0 | 0 | 0 | 0 | 0 | 3 | 0 | 0 | 0 | 0 | 0 | 0 | 0 | 0 |  |  |  |  |  | 2.4757E8 |  |  |  |  |  |  |  |  | 1 | 1 | 0 | 0 | 0 | 0 | 0 | 2 | 0 | 0 | 0 | 0 | 0 | 0 | 0 | 0 | Y | 67952 | Metalloproteinase type III 12b [Agkistrodon piscivorus conanti] |
| 319 | 2139 | JAS04458.1 | 41.02 | 3 | 0 | 0 | 0 | 0 | 0 | 3 | 0 | 0 | 0 | 0 | 0 | 0 | 0 | 0 |  |  |  |  |  | 2.4757E8 |  |  |  |  |  |  |  |  | 1 | 1 | 0 | 0 | 0 | 0 | 0 | 2 | 0 | 0 | 0 | 0 | 0 | 0 | 0 | 0 | Y | 67978 | Metalloproteinase type III 12a [Agkistrodon piscivorus conanti] |
| 319 | 2158 | 2ERQ | 41.02 | 4 | 0 | 0 | 0 | 0 | 0 | 4 | 0 | 0 | 0 | 0 | 0 | 0 | 0 | 0 |  |  |  |  |  | 2.4757E8 |  |  |  |  |  |  |  |  | 1 | 1 | 0 | 0 | 0 | 0 | 0 | 2 | 0 | 0 | 0 | 0 | 0 | 0 | 0 | 0 | Y | 47320 | Chain B, Vascular Apoptosis-inducing Protein 1 |
| 319 | 2159 | 2ERO | 41.02 | 4 | 0 | 0 | 0 | 0 | 0 | 4 | 0 | 0 | 0 | 0 | 0 | 0 | 0 | 0 |  |  |  |  |  | 2.4757E8 |  |  |  |  |  |  |  |  | 1 | 1 | 0 | 0 | 0 | 0 | 0 | 2 | 0 | 0 | 0 | 0 | 0 | 0 | 0 | 0 | Y | 47320 | Chain B, Vascular Apoptosis-inducing Protein 1 |
| 319 | 2160 | 2ERP | 41.02 | 4 | 0 | 0 | 0 | 0 | 0 | 4 | 0 | 0 | 0 | 0 | 0 | 0 | 0 | 0 |  |  |  |  |  | 2.4757E8 |  |  |  |  |  |  |  |  | 1 | 1 | 0 | 0 | 0 | 0 | 0 | 2 | 0 | 0 | 0 | 0 | 0 | 0 | 0 | 0 | Y | 47320 | Chain B, Vascular Apoptosis-inducing Protein 1 |
| 319 | 2194 | ACI02286.1 | 41.02 | 4 | 0 | 0 | 0 | 0 | 0 | 4 | 0 | 0 | 0 | 0 | 0 | 0 | 0 | 0 |  |  |  |  |  | 2.4757E8 |  |  |  |  |  |  |  |  | 1 | 1 | 0 | 0 | 0 | 0 | 0 | 2 | 0 | 0 | 0 | 0 | 0 | 0 | 0 | 0 | Y | 46165 | crotastatin-1, partial [Crotalus durissus cascavella] |
| 618 | 2383 | XP\_015676173.1 | 40.42 | 2 | 0 | 0 | 0 | 0 | 0 | 2 | 0 | 0 | 0 | 0 | 0 | 0 | 0 | 0 |  |  |  |  |  | 1.1981E5 |  |  |  |  |  |  |  |  | 1 | 1 | 0 | 0 | 0 | 0 | 0 | 1 | 0 | 0 | 0 | 0 | 0 | 0 | 0 | 0 | N | 62725 | iduronate 2-sulfatase [Protobothrops mucrosquamatus] |
| 573 | 5540 | XP\_026568545.1 | 40.35 | 1 | 1 | 0 | 0 | 0 | 0 | 0 | 0 | 0 | 0 | 0 | 0 | 0 | 0 | 0 | 5.5742E5 |  |  |  |  |  |  |  |  |  |  |  |  |  | 1 | 1 | 1 | 0 | 0 | 0 | 0 | 0 | 0 | 0 | 0 | 0 | 0 | 0 | 0 | 0 | N | 140699 | cyclin-G-associated kinase isoform X2 [Pseudonaja textilis] |
| 357 | 4894 | ETE69783.1 | 40.17 | 9 | 0 | 0 | 0 | 0 | 0 | 9 | 0 | 0 | 0 | 0 | 0 | 0 | 0 | 0 |  |  |  |  |  | 1.6326E6 |  |  |  |  |  |  |  |  | 1 | 1 | 0 | 0 | 0 | 0 | 0 | 1 | 0 | 0 | 0 | 0 | 0 | 0 | 0 | 0 | Y | 16760 | Epididymal secretory protein E1 [Ophiophagus hannah] |
| 357 | 4895 | XP\_032087352.1 | 40.17 | 9 | 0 | 0 | 0 | 0 | 0 | 9 | 0 | 0 | 0 | 0 | 0 | 0 | 0 | 0 |  |  |  |  |  | 1.6326E6 |  |  |  |  |  |  |  |  | 1 | 1 | 0 | 0 | 0 | 0 | 0 | 1 | 0 | 0 | 0 | 0 | 0 | 0 | 0 | 0 | Y | 16615 | NPC intracellular cholesterol transporter 2 [Thamnophis elegans] |
| 357 | 6038 | XP\_026520220.1 | 40.17 | 9 | 0 | 0 | 0 | 0 | 0 | 9 | 0 | 0 | 0 | 0 | 0 | 0 | 0 | 0 |  |  |  |  |  | 1.6326E6 |  |  |  |  |  |  |  |  | 1 | 1 | 0 | 0 | 0 | 0 | 0 | 1 | 0 | 0 | 0 | 0 | 0 | 0 | 0 | 0 | Y | 16760 | NPC intracellular cholesterol transporter 2 [Notechis scutatus] |
| 357 | 6039 | XP\_026567489.1 | 40.17 | 9 | 0 | 0 | 0 | 0 | 0 | 9 | 0 | 0 | 0 | 0 | 0 | 0 | 0 | 0 |  |  |  |  |  | 1.6326E6 |  |  |  |  |  |  |  |  | 1 | 1 | 0 | 0 | 0 | 0 | 0 | 1 | 0 | 0 | 0 | 0 | 0 | 0 | 0 | 0 | Y | 16769 | NPC intracellular cholesterol transporter 2 [Pseudonaja textilis] |
| 669 | 10281 | XP\_034279939.1 | 40.11 | 1 | 1 | 0 | 0 | 0 | 0 | 0 | 0 | 0 | 0 | 0 | 0 | 0 | 0 | 0 | 3.7938E5 |  |  |  |  |  |  |  |  |  |  |  |  |  | 1 | 1 | 1 | 0 | 0 | 0 | 0 | 0 | 0 | 0 | 0 | 0 | 0 | 0 | 0 | 0 | N | 62617 | H/ACA ribonucleoprotein complex subunit DKC1 isoform X1 [Pantherophis guttatus] |
| 641 | 2885 | LAA93895.1 | 40.00 | 5 | 0 | 0 | 0 | 5 | 0 | 0 | 0 | 0 | 0 | 0 | 0 | 0 | 0 | 0 |  |  |  | 4.833E5 |  |  |  |  |  |  |  |  |  |  | 1 | 1 | 0 | 0 | 0 | 1 | 0 | 0 | 0 | 0 | 0 | 0 | 0 | 0 | 0 | 0 | N | 19716 | hypothetical protein, partial [Micrurus lemniscatus lemniscatus] |
| 630 | 2886 | XP\_034263619.1 | 39.95 | 18 | 0 | 0 | 0 | 0 | 0 | 0 | 0 | 0 | 0 | 0 | 18 | 0 | 0 | 0 |  |  |  |  |  |  |  |  |  |  | 5.2493E5 |  |  |  | 1 | 1 | 0 | 0 | 0 | 0 | 0 | 0 | 0 | 0 | 0 | 0 | 1 | 0 | 0 | 0 | Y | 16240 | fibrillin-2-like, partial [Pantherophis guttatus] |
| 630 | 2887 | XP\_034261218.1 | 39.95 | 18 | 0 | 0 | 0 | 0 | 0 | 0 | 0 | 0 | 0 | 0 | 18 | 0 | 0 | 0 |  |  |  |  |  |  |  |  |  |  | 5.2493E5 |  |  |  | 1 | 1 | 0 | 0 | 0 | 0 | 0 | 0 | 0 | 0 | 0 | 0 | 1 | 0 | 0 | 0 | Y | 16164 | fibrillin-2-like, partial [Pantherophis guttatus] |
| 581 | 2327 | XP\_026544192.1 | 39.80 | 4 | 0 | 0 | 0 | 4 | 0 | 0 | 0 | 0 | 0 | 0 | 0 | 0 | 0 | 0 |  |  |  | 3.9684E5 |  |  |  |  |  |  |  |  |  |  | 1 | 1 | 0 | 0 | 0 | 1 | 0 | 0 | 0 | 0 | 0 | 0 | 0 | 0 | 0 | 0 | Y | 54034 | hemopexin [Notechis scutatus] |
| 611 | 3065 | XP\_034280025.1 | 39.24 | 3 | 0 | 0 | 0 | 0 | 0 | 0 | 0 | 0 | 0 | 3 | 0 | 0 | 0 | 0 |  |  |  |  |  |  |  |  |  | 0 |  |  |  |  | 1 | 1 | 0 | 0 | 0 | 0 | 0 | 0 | 0 | 0 | 0 | 1 | 0 | 0 | 0 | 0 | Y | 83201 | mitogen-activated protein kinase kinase kinase 19 isoform X3 [Pantherophis guttatus] |
| 472 | 1835 | XP\_032083351.1 | 38.30 | 1 | 0 | 0 | 1 | 0 | 0 | 0 | 0 | 0 | 0 | 0 | 0 | 0 | 0 | 0 |  |  | 0 |  |  |  |  |  |  |  |  |  |  |  | 1 | 1 | 0 | 0 | 1 | 0 | 0 | 0 | 0 | 0 | 0 | 0 | 0 | 0 | 0 | 0 | N | 161004 | collagen alpha-5(IV) chain isoform X4 [Thamnophis elegans] |
| 472 | 1836 | XP\_032083350.1 | 38.30 | 1 | 0 | 0 | 1 | 0 | 0 | 0 | 0 | 0 | 0 | 0 | 0 | 0 | 0 | 0 |  |  | 0 |  |  |  |  |  |  |  |  |  |  |  | 1 | 1 | 0 | 0 | 1 | 0 | 0 | 0 | 0 | 0 | 0 | 0 | 0 | 0 | 0 | 0 | N | 161276 | collagen alpha-5(IV) chain isoform X3 [Thamnophis elegans] |
| 472 | 2217 | XP\_034290930.1 | 38.30 | 1 | 0 | 0 | 1 | 0 | 0 | 0 | 0 | 0 | 0 | 0 | 0 | 0 | 0 | 0 |  |  | 0 |  |  |  |  |  |  |  |  |  |  |  | 1 | 1 | 0 | 0 | 1 | 0 | 0 | 0 | 0 | 0 | 0 | 0 | 0 | 0 | 0 | 0 | N | 161346 | collagen alpha-5(IV) chain isoform X3 [Pantherophis guttatus] |
| 472 | 2364 | XP\_026558860.1 | 38.30 | 1 | 0 | 0 | 1 | 0 | 0 | 0 | 0 | 0 | 0 | 0 | 0 | 0 | 0 | 0 |  |  | 0 |  |  |  |  |  |  |  |  |  |  |  | 1 | 1 | 0 | 0 | 1 | 0 | 0 | 0 | 0 | 0 | 0 | 0 | 0 | 0 | 0 | 0 | N | 147096 | collagen alpha-5(IV) chain [Pseudonaja textilis] |
| 502 | 4410 | ETE62997.1 | 38.02 | 1 | 0 | 0 | 0 | 0 | 0 | 0 | 0 | 0 | 0 | 1 | 0 | 0 | 0 | 0 |  |  |  |  |  |  |  |  |  | 4.1385E4 |  |  |  |  | 1 | 1 | 0 | 0 | 0 | 0 | 0 | 0 | 0 | 0 | 0 | 1 | 0 | 0 | 0 | 0 | N | 70661 | Vomeronasal type-2 receptor 1, partial [Ophiophagus hannah] |
| total 802 proteins |  |  |  |  |  |  |  |  |  |  |  |  |  |  |  |  |  |  |  |  |  |  |  |  |  |  |  |  |  |  |  |  |  |  |  |  |  |  |  |  |  |  |  |  |  |  |  |  |  |  |  |
| --- | --- | --- | --- | --- | --- | --- | --- | --- | --- | --- | --- | --- | --- | --- | --- | --- | --- | --- | --- | --- | --- | --- | --- | --- | --- | --- | --- | --- | --- | --- | --- | --- | --- | --- | --- | --- | --- | --- | --- | --- | --- | --- | --- | --- | --- | --- | --- | --- | --- | --- | --- |

  

5Z2G

back to list

  

| Protein Coverage
| Supporting Peptides
|

Protein Coverage:

Supporting Peptides:

| Peptide | Uniq | -10lgP | Mass | Length | ppm | m/z | z | RT | Fraction | Scan | Source File | Area F1 | Area F10 | Area F11 | Area F12 | Area F13 | Area F14 | Area F2 | Area F3 | Area F4 | Area F5 | Area F6 | Area F7 | Area F8 | Area F9 | #Feature | #Feature F1 | #Feature F10 | #Feature F11 | #Feature F12 | #Feature F13 | #Feature F14 | #Feature F2 | #Feature F3 | #Feature F4 | #Feature F5 | #Feature F6 | #Feature F7 | #Feature F8 | #Feature F9 | Start | End | PTM | AScore | Found By |
| --- | --- | --- | --- | --- | --- | --- | --- | --- | --- | --- | --- | --- | --- | --- | --- | --- | --- | --- | --- | --- | --- | --- | --- | --- | --- | --- | --- | --- | --- | --- | --- | --- | --- | --- | --- | --- | --- | --- | --- | --- | --- | --- | --- | --- | --- |
| K.HVVVVGAGMAGLSAAYVLAGAGHK.V | N | 107.42 | 2234.1992 | 24 | 1.2 | 745.7412 | 3 | 65.01 | 6 | F6:42957 | NaSa01\_F14.raw |  |  |  | 6.8911E6 | 4.2938E7 | 6.5431E8 |  |  |  |  |  |  |  |  | 25 | 0 | 0 | 0 | 2 | 2 | 21 | 0 | 0 | 0 | 0 | 0 | 0 | 0 | 0 | 54 | 77 |  |  | PEAKS DB |
| K.TC(+57.02)ADIVINDLSLIHDLPK.R | Y | 96.41 | 2036.0612 | 18 | 1.5 | 1019.0394 | 2 | 82.06 | 6 | F6:56577 | NaSa01\_F14.raw |  |  |  | 4.4983E6 | 7.6898E7 | 5.3211E8 |  |  |  |  |  |  |  |  | 13 | 0 | 0 | 0 | 1 | 4 | 8 | 0 | 0 | 0 | 0 | 0 | 0 | 0 | 0 | 405 | 422 | Carbamidomethylation | C2:Carbamidomethylation:1000.00 | PEAKS DB |
| K.HVVVVGAGM(+15.99)AGLSAAYVLAGAGHK.V | N | 95.06 | 2250.1943 | 24 | 0.9 | 751.0728 | 3 | 53.09 | 6 | F6:32085 | NaSa01\_F14.raw |  |  |  |  | 1.6389E7 | 1.5623E8 |  |  |  |  |  |  |  |  | 5 | 0 | 0 | 0 | 0 | 2 | 3 | 0 | 0 | 0 | 0 | 0 | 0 | 0 | 0 | 54 | 77 | Oxidation (M) | M9:Oxidation (M):1000.00 | PEAKS DB |
| F.TPYQFQDYFESAAAPVGR.I | Y | 94.96 | 2045.9482 | 18 | -0.1 | 1023.9813 | 2 | 72.54 | 6 | F6:48623 | NaSa01\_F14.raw |  |  |  |  | 1.3361E7 | 2.9427E8 |  |  |  |  |  |  |  |  | 3 | 0 | 0 | 0 | 0 | 1 | 2 | 0 | 0 | 0 | 0 | 0 | 0 | 0 | 0 | 450 | 467 |  |  | PEAKS DB |
| K.TFVTADYVIVC(+57.02)STSR.A | Y | 93.18 | 1717.8345 | 15 | 2.2 | 859.9264 | 2 | 60.90 | 6 | F6:39859 | NaSa01\_F14.raw |  |  |  |  | 5.5865E7 | 4.4428E8 |  |  |  |  |  |  | 5.5981E5 |  | 20 | 0 | 0 | 0 | 0 | 3 | 16 | 0 | 0 | 0 | 0 | 0 | 0 | 1 | 0 | 300 | 314 | Carbamidomethylation | C11:Carbamidomethylation:1000.00 | PEAKS DB |
| K.LNEFFQENENAWYYINNIR.K | Y | 90.36 | 2476.1448 | 19 | 0.7 | 1239.0806 | 2 | 91.17 | 6 | F6:64025 | NaSa01\_F14.raw |  |  |  |  | 6.6266E5 | 7.9615E7 |  |  |  |  |  |  |  |  | 3 | 0 | 0 | 0 | 0 | 1 | 2 | 0 | 0 | 0 | 0 | 0 | 0 | 0 | 0 | 128 | 146 |  |  | PEAKS DB |
| K.YTMGSITSFTPYQFQDYFESAAAPVGR.I | Y | 82.61 | 3033.3855 | 27 | 0.8 | 1012.1366 | 3 | 100.62 | 6 | F6:71855 | NaSa01\_F14.raw |  |  |  |  | 1.0072E7 | 2.7658E8 |  |  |  |  |  |  |  |  | 5 | 0 | 0 | 0 | 0 | 2 | 3 | 0 | 0 | 0 | 0 | 0 | 0 | 0 | 0 | 441 | 467 |  |  | PEAKS DB |
| R.FHGWIDSTIM(+15.99)TGLR.A | Y | 82.52 | 1648.8031 | 14 | 0.3 | 825.4091 | 2 | 56.10 | 6 | F6:34934 | NaSa01\_F14.raw |  |  |  |  | 1.0724E7 | 1.9786E8 |  |  |  |  |  |  |  |  | 15 | 0 | 0 | 0 | 0 | 2 | 13 | 0 | 0 | 0 | 0 | 0 | 0 | 0 | 0 | 478 | 491 | Oxidation (M) | M10:Oxidation (M):1000.00 | PEAKS DB |
| Y.VLADDSDFFQALDTK.T | Y | 82.09 | 1683.7991 | 15 | 0.1 | 842.9069 | 2 | 78.47 | 6 | F6:53277 | NaSa01\_F14.raw |  |  |  | 2.5191E5 | 2.6921E8 | 3.2203E9 |  |  |  |  |  |  |  |  | 9 | 0 | 0 | 0 | 1 | 2 | 6 | 0 | 0 | 0 | 0 | 0 | 0 | 0 | 0 | 390 | 404 |  |  | PEAKS DB |
| R.FHGWIDSTIMTGLR.A | Y | 81.76 | 1632.8082 | 14 | 0.3 | 817.4116 | 2 | 68.30 | 6 | F6:44954 | NaSa01\_F14.raw |  | 2.7707E6 |  | 6.8303E6 | 2.7763E7 | 7.2536E8 |  |  |  |  |  |  |  |  | 28 | 0 | 1 | 0 | 3 | 2 | 22 | 0 | 0 | 0 | 0 | 0 | 0 | 0 | 0 | 478 | 491 |  |  | PEAKS DB |
| M.AGLSAAYVLAGAGHK.V | N | 79.84 | 1384.7462 | 15 | 0.5 | 693.3807 | 2 | 36.23 | 6 | F6:17690 | NaSa01\_F14.raw |  |  |  |  |  | 4.4211E7 |  |  |  |  |  |  |  |  | 2 | 0 | 0 | 0 | 0 | 0 | 2 | 0 | 0 | 0 | 0 | 0 | 0 | 0 | 0 | 63 | 77 |  |  | PEAKS DB |
| M.AYVLADDSDFFQALDTK.T | Y | 79.60 | 1917.8995 | 17 | -0.3 | 959.9567 | 2 | 85.82 | 6 | F6:59496 | NaSa01\_F14.raw |  |  |  |  | 3.4939E6 | 3.9274E7 |  |  |  |  |  |  |  |  | 2 | 0 | 0 | 0 | 0 | 1 | 1 | 0 | 0 | 0 | 0 | 0 | 0 | 0 | 0 | 388 | 404 |  |  | PEAKS DB |
| K.REIQALC(+57.02)YPSIK.K | N | 77.78 | 1476.7759 | 12 | 0.2 | 493.2660 | 3 | 32.82 | 6 | F6:14068 | NaSa01\_F14.raw |  |  |  |  | 3.5291E7 | 3.7818E8 |  |  |  |  |  |  |  |  | 8 | 0 | 0 | 0 | 0 | 2 | 6 | 0 | 0 | 0 | 0 | 0 | 0 | 0 | 0 | 423 | 434 | Carbamidomethylation | C7:Carbamidomethylation:1000.00 | PEAKS DB |
| S.ITSFTPYQFQDYFESAAAPVGR.I | Y | 77.75 | 2494.1804 | 22 | 0.4 | 1248.0980 | 2 | 89.99 | 6 | F6:63003 | NaSa01\_F14.raw |  |  |  |  |  | 8.4867E6 |  |  |  |  |  |  |  |  | 1 | 0 | 0 | 0 | 0 | 0 | 1 | 0 | 0 | 0 | 0 | 0 | 0 | 0 | 0 | 446 | 467 |  |  | PEAKS DB |
| R.EIQALC(+57.02)YPSIK.K | N | 77.61 | 1320.6747 | 11 | 0.3 | 661.3448 | 2 | 47.88 | 6 | F6:27511 | NaSa01\_F14.raw |  |  |  | 3.0826E6 | 1.353E8 | 1.8533E9 |  |  |  |  |  |  |  |  | 18 | 0 | 0 | 0 | 1 | 1 | 16 | 0 | 0 | 0 | 0 | 0 | 0 | 0 | 0 | 424 | 434 | Carbamidomethylation | C6:Carbamidomethylation:1000.00 | PEAKS DB |
| A.GMAGLSAAYVLAGAGHK.V | N | 77.31 | 1572.8082 | 17 | 0.2 | 525.2768 | 3 | 50.77 | 6 | F6:29981 | NaSa01\_F14.raw |  |  |  |  | 5.5361E5 | 1.0582E7 |  |  |  |  |  |  |  |  | 3 | 0 | 0 | 0 | 0 | 1 | 2 | 0 | 0 | 0 | 0 | 0 | 0 | 0 | 0 | 61 | 77 |  |  | PEAKS DB |
| Y.QFQDYFESAAAPVGR.I | Y | 76.00 | 1684.7844 | 15 | 0.6 | 843.4000 | 2 | 59.29 | 6 | F6:37194 | NaSa01\_F14.raw |  |  |  | 8.1084E5 | 8.279E7 | 1.2226E9 |  |  |  |  |  |  |  |  | 13 | 0 | 0 | 0 | 1 | 3 | 9 | 0 | 0 | 0 | 0 | 0 | 0 | 0 | 0 | 453 | 467 |  |  | PEAKS DB |
| R.IYFEPPLPPKK.A | N | 74.78 | 1327.7539 | 11 | 0.3 | 664.8844 | 2 | 39.91 | 6 | F6:21060 | NaSa01\_F14.raw |  |  |  |  | 5.0279E7 | 4.7146E8 |  |  |  |  |  |  |  |  | 4 | 0 | 0 | 0 | 0 | 2 | 2 | 0 | 0 | 0 | 0 | 0 | 0 | 0 | 0 | 319 | 329 |  |  | PEAKS DB |
| R.EGWYVNMGPM(+15.99)R.L | N | 73.07 | 1354.5798 | 11 | 0.4 | 678.2975 | 2 | 56.26 | 6 | F6:34728 | NaSa01\_F14.raw |  |  |  |  | 3.9735E7 | 2.4523E8 |  |  |  |  |  |  |  |  | 7 | 0 | 0 | 0 | 0 | 4 | 3 | 0 | 0 | 0 | 0 | 0 | 0 | 0 | 0 | 99 | 109 | Oxidation (M) | M10:Oxidation (M):51.69 | PEAKS DB |
| R.IYFEPPLPPK.K | N | 72.12 | 1199.6589 | 10 | -0.1 | 600.8367 | 2 | 57.48 | 6 | F6:38014 | NaSa01\_F14.raw |  |  |  | 1.5815E6 | 1.7195E8 | 2.5686E9 |  |  | 1.0411E6 |  |  |  |  |  | 22 | 0 | 0 | 0 | 1 | 2 | 18 | 0 | 0 | 1 | 0 | 0 | 0 | 0 | 0 | 319 | 328 |  |  | PEAKS DB |
| R.EGWYVNMGPMR.L | N | 71.70 | 1338.5850 | 11 | 0.0 | 670.2997 | 2 | 59.12 | 6 | F6:37086 | NaSa01\_F14.raw |  |  |  |  | 1.3719E8 | 2.0017E9 |  |  |  |  |  |  |  |  | 6 | 0 | 0 | 0 | 0 | 3 | 3 | 0 | 0 | 0 | 0 | 0 | 0 | 0 | 0 | 99 | 109 |  |  | PEAKS DB |
| A.YVLADDSDFFQALDTK.T | Y | 71.22 | 1846.8624 | 16 | 0.6 | 924.4391 | 2 | 84.46 | 6 | F6:58413 | NaSa01\_F14.raw |  |  |  |  |  | 2.5549E6 |  |  |  |  |  |  |  |  | 1 | 0 | 0 | 0 | 0 | 0 | 1 | 0 | 0 | 0 | 0 | 0 | 0 | 0 | 0 | 389 | 404 |  |  | PEAKS DB |
| M.YQAIAEMVHLNAR.V | N | 70.47 | 1514.7664 | 13 | 0.6 | 505.9297 | 3 | 42.75 | 6 | F6:23121 | NaSa01\_F14.raw |  |  |  |  |  | 6.4909E6 |  |  |  |  |  |  |  |  | 2 | 0 | 0 | 0 | 0 | 0 | 2 | 0 | 0 | 0 | 0 | 0 | 0 | 0 | 0 | 267 | 279 |  |  | PEAKS DB |
| A.DIVINDLSLIHDLPK.R | Y | 70.43 | 1703.9458 | 15 | 0.0 | 568.9892 | 3 | 80.66 | 6 | F6:55094 | NaSa01\_F14.raw |  |  |  | 4.3515E6 | 5.5307E6 | 1.0082E7 |  |  |  |  |  | 7.9457E5 |  |  | 7 | 0 | 0 | 0 | 2 | 2 | 2 | 0 | 0 | 0 | 0 | 0 | 1 | 0 | 0 | 408 | 422 |  |  | PEAKS DB |
| K.YTM(+15.99)GSITSFTPYQFQDYFESAAAPVGR.I | Y | 68.73 | 3049.3804 | 27 | 1.3 | 1017.4688 | 3 | 94.25 | 6 | F6:66450 | NaSa01\_F14.raw |  |  |  | 7.1363E5 | 6.5743E6 | 9.733E7 |  |  |  |  |  |  |  |  | 4 | 0 | 0 | 0 | 1 | 1 | 2 | 0 | 0 | 0 | 0 | 0 | 0 | 0 | 0 | 441 | 467 | Oxidation (M) | M3:Oxidation (M):1000.00 | PEAKS DB |
| R.EGWYVNM(+15.99)GPMR.L | N | 67.93 | 1354.5798 | 11 | 0.8 | 678.2977 | 2 | 41.16 | 6 | F6:22021 | NaSa01\_F14.raw |  |  |  | 5.0564E5 | 1.7067E7 | 2.1752E8 |  |  |  |  |  |  |  |  | 5 | 0 | 0 | 0 | 1 | 2 | 2 | 0 | 0 | 0 | 0 | 0 | 0 | 0 | 0 | 99 | 109 | Oxidation (M) | M7:Oxidation (M):39.30 | PEAKS DB |
| K.SASQLYQESLR.K | Y | 67.67 | 1280.6360 | 11 | 0.1 | 641.3253 | 2 | 24.71 | 6 | F6:8085 | NaSa01\_F14.raw |  |  |  |  | 1.1171E8 | 7.0099E6 |  |  |  |  |  |  |  |  | 2 | 0 | 0 | 0 | 0 | 1 | 1 | 0 | 0 | 0 | 0 | 0 | 0 | 0 | 0 | 171 | 181 |  |  | PEAKS DB |
| K.YDSYSTKEYLIK.E | N | 67.50 | 1508.7399 | 12 | 0.6 | 503.9209 | 3 | 26.71 | 6 | F6:10035 | NaSa01\_F14.raw |  |  |  |  |  | 7.9504E6 |  |  |  |  |  |  |  |  | 2 | 0 | 0 | 0 | 0 | 0 | 2 | 0 | 0 | 0 | 0 | 0 | 0 | 0 | 0 | 199 | 210 |  |  | PEAKS DB |
| K.HVVVVGAGMAGLSAAY.V | N | 67.41 | 1500.7759 | 16 | 0.2 | 751.3954 | 2 | 68.19 | 6 | F6:44843 | NaSa01\_F14.raw |  |  |  |  | 2.067E7 | 2.0687E8 |  |  |  |  |  |  |  |  | 10 | 0 | 0 | 0 | 0 | 1 | 9 | 0 | 0 | 0 | 0 | 0 | 0 | 0 | 0 | 54 | 69 |  |  | PEAKS DB |
| Y.QAIAEMVHLNAR.V | N | 66.25 | 1351.7030 | 12 | -0.4 | 676.8585 | 2 | 31.55 | 6 | F6:13654 | NaSa01\_F14.raw |  |  |  |  |  | 7.8157E7 |  |  |  |  |  |  |  |  | 2 | 0 | 0 | 0 | 0 | 0 | 2 | 0 | 0 | 0 | 0 | 0 | 0 | 0 | 0 | 268 | 279 |  |  | PEAKS DB |
| R.IHFAGEYTGR.F | Y | 65.92 | 1149.5566 | 10 | -0.7 | 384.1926 | 3 | 11.57 | 6 | F6:1949 | NaSa01\_F14.raw |  |  |  |  | 1.5757E6 | 2.2033E7 |  |  |  |  |  |  |  |  | 9 | 0 | 0 | 0 | 0 | 3 | 6 | 0 | 0 | 0 | 0 | 0 | 0 | 0 | 0 | 468 | 477 |  |  | PEAKS DB |
| T.SFTPYQFQDYFESAAAPVGR.I | Y | 65.14 | 2280.0486 | 20 | 0.8 | 1141.0325 | 2 | 85.41 | 6 | F6:59216 | NaSa01\_F14.raw |  |  |  |  |  | 1.7558E6 |  |  |  |  |  |  |  |  | 1 | 0 | 0 | 0 | 0 | 0 | 1 | 0 | 0 | 0 | 0 | 0 | 0 | 0 | 0 | 448 | 467 |  |  | PEAKS DB |
| K.FWEADGIHGGKSTTDLPSR.F | N | 64.97 | 2072.9915 | 19 | 0.7 | 692.0049 | 3 | 29.66 | 6 | F6:12168 | NaSa01\_F14.raw |  |  |  |  |  | 2.4928E6 |  |  |  |  |  |  |  |  | 1 | 0 | 0 | 0 | 0 | 0 | 1 | 0 | 0 | 0 | 0 | 0 | 0 | 0 | 0 | 352 | 370 |  |  | PEAKS DB |
| R.RIYFEPPLPPKK.A | N | 64.76 | 1483.8550 | 12 | -0.5 | 495.6253 | 3 | 28.82 | 6 | F6:11398 | NaSa01\_F14.raw |  |  |  |  | 2.2402E7 | 1.4186E8 |  |  |  |  |  |  |  |  | 5 | 0 | 0 | 0 | 0 | 2 | 3 | 0 | 0 | 0 | 0 | 0 | 0 | 0 | 0 | 318 | 329 |  |  | PEAKS DB |
| L.SAAYVLAGAGHK.V | N | 63.01 | 1143.6036 | 12 | -1.1 | 572.8085 | 2 | 11.49 | 6 | F6:1894 | NaSa01\_F14.raw |  |  |  |  |  | 4.4228E6 |  |  |  |  |  |  |  |  | 2 | 0 | 0 | 0 | 0 | 0 | 2 | 0 | 0 | 0 | 0 | 0 | 0 | 0 | 0 | 66 | 77 |  |  | PEAKS DB |
| K.SDALFSYEK.R | N | 62.97 | 1058.4919 | 9 | 0.4 | 530.2535 | 2 | 34.28 | 6 | F6:16290 | NaSa01\_F14.raw |  |  |  |  | 5.08E7 | 1.3053E7 |  |  |  |  |  |  |  |  | 4 | 0 | 0 | 0 | 0 | 1 | 3 | 0 | 0 | 0 | 0 | 0 | 0 | 0 | 0 | 242 | 250 |  |  | PEAKS DB |
| F.QDYFESAAAPVGR.I | Y | 62.52 | 1409.6575 | 13 | 0.1 | 705.8361 | 2 | 39.67 | 6 | F6:20451 | NaSa01\_F14.raw |  |  |  |  |  | 3.65E6 |  |  |  |  |  |  |  |  | 1 | 0 | 0 | 0 | 0 | 0 | 1 | 0 | 0 | 0 | 0 | 0 | 0 | 0 | 0 | 455 | 467 |  |  | PEAKS DB |
| K.YPVKPSEEGK.S | N | 62.09 | 1132.5764 | 10 | 1.6 | 567.2964 | 2 | 11.44 | 6 | F6:1813 | NaSa01\_F14.raw |  |  |  |  |  | 1.8552E6 |  |  |  |  |  |  |  |  | 2 | 0 | 0 | 0 | 0 | 0 | 2 | 0 | 0 | 0 | 0 | 0 | 0 | 0 | 0 | 161 | 170 |  |  | PEAKS DB |
| D.SDFFQALDTK.T | Y | 61.64 | 1170.5557 | 10 | 0.6 | 586.2855 | 2 | 57.84 | 6 | F6:36028 | NaSa01\_F14.raw |  |  |  |  |  | 4.304E6 |  |  |  |  |  |  |  |  | 1 | 0 | 0 | 0 | 0 | 0 | 1 | 0 | 0 | 0 | 0 | 0 | 0 | 0 | 0 | 395 | 404 |  |  | PEAKS DB |
| R.RIYFEPPLPPK.K | N | 60.85 | 1355.7601 | 11 | 0.4 | 452.9275 | 3 | 43.97 | 6 | F6:24986 | NaSa01\_F14.raw |  |  |  |  | 9.9689E7 | 1.1129E9 |  |  |  |  |  |  |  |  | 4 | 0 | 0 | 0 | 0 | 2 | 2 | 0 | 0 | 0 | 0 | 0 | 0 | 0 | 0 | 318 | 328 |  |  | PEAKS DB |
| K.RFDEIVGGFDQLPISMYQA.I | N | 60.53 | 2185.0513 | 19 | -0.2 | 1093.5327 | 2 | 97.41 | 6 | F6:69051 | NaSa01\_F14.raw |  |  |  |  |  | 1.6822E7 |  |  |  |  |  |  |  |  | 1 | 0 | 0 | 0 | 0 | 0 | 1 | 0 | 0 | 0 | 0 | 0 | 0 | 0 | 0 | 251 | 269 |  |  | PEAKS DB |
| F.FQENENAWYYINNIR.K | Y | 60.04 | 1972.9067 | 15 | -0.4 | 987.4603 | 2 | 71.69 | 6 | F6:47797 | NaSa01\_F14.raw |  |  |  |  |  | 2.7056E6 |  |  |  |  |  |  |  |  | 1 | 0 | 0 | 0 | 0 | 0 | 1 | 0 | 0 | 0 | 0 | 0 | 0 | 0 | 0 | 132 | 146 |  |  | PEAKS DB |
| S.FTPYQFQDYFESAAAPVGR.I | Y | 59.98 | 2193.0166 | 19 | -0.2 | 1097.5154 | 2 | 83.99 | 6 | F6:57987 | NaSa01\_F14.raw |  |  |  |  |  | 1.8906E6 |  |  |  |  |  |  |  |  | 1 | 0 | 0 | 0 | 0 | 0 | 1 | 0 | 0 | 0 | 0 | 0 | 0 | 0 | 0 | 449 | 467 |  |  | PEAKS DB |
| D.DSDFFQALDTK.T | Y | 59.42 | 1285.5826 | 11 | -0.4 | 643.7983 | 2 | 67.26 | 6 | F6:44023 | NaSa01\_F14.raw |  |  |  |  |  | 2.633E6 |  |  |  |  |  |  |  |  | 1 | 0 | 0 | 0 | 0 | 0 | 1 | 0 | 0 | 0 | 0 | 0 | 0 | 0 | 0 | 394 | 404 |  |  | PEAKS DB |
| K.FWEADGIHGGK.S | N | 59.16 | 1215.5673 | 11 | 0.2 | 608.7910 | 2 | 11.57 | 6 | F6:1961 | NaSa01\_F14.raw |  |  |  |  | 4.0485E5 | 1.8815E7 |  |  |  |  |  |  |  |  | 9 | 0 | 0 | 0 | 0 | 1 | 8 | 0 | 0 | 0 | 0 | 0 | 0 | 0 | 0 | 352 | 362 |  |  | PEAKS DB |
| Y.HLSFMESLK.S | Y | 58.90 | 1090.5481 | 9 | 0.5 | 546.2816 | 2 | 34.90 | 6 | F6:16342 | NaSa01\_F14.raw |  |  |  |  |  | 1.1344E7 |  |  |  |  |  |  |  |  | 1 | 0 | 0 | 0 | 0 | 0 | 1 | 0 | 0 | 0 | 0 | 0 | 0 | 0 | 0 | 233 | 241 |  |  | PEAKS DB |
| A.GLSAAYVLAGAGHK.V | N | 58.41 | 1313.7091 | 14 | 0.3 | 438.9104 | 3 | 32.92 | 6 | F6:14801 | NaSa01\_F14.raw |  |  |  |  |  | 1.2419E6 |  |  |  |  |  |  |  |  | 2 | 0 | 0 | 0 | 0 | 0 | 2 | 0 | 0 | 0 | 0 | 0 | 0 | 0 | 0 | 64 | 77 |  |  | PEAKS DB |
| K.HVVVVGAGMAGL.S | N | 58.15 | 1108.6063 | 12 | 0.5 | 555.3107 | 2 | 51.19 | 6 | F6:30972 | NaSa01\_F14.raw |  |  |  |  | 1.7665E7 | 2.672E8 |  |  |  |  |  |  |  |  | 3 | 0 | 0 | 0 | 0 | 1 | 2 | 0 | 0 | 0 | 0 | 0 | 0 | 0 | 0 | 54 | 65 |  |  | PEAKS DB |
| L.SAAYVLAGAGHKVTLLEASER.V | N | 57.68 | 2142.1433 | 21 | 0.0 | 715.0551 | 3 | 41.78 | 6 | F6:22392 | NaSa01\_F14.raw |  |  |  |  |  | 9.3986E5 |  |  |  |  |  |  |  |  | 1 | 0 | 0 | 0 | 0 | 0 | 1 | 0 | 0 | 0 | 0 | 0 | 0 | 0 | 0 | 66 | 86 |  |  | PEAKS DB |
| K.TC(+57.02)ADIVINDLSLIHDLPKR.E | Y | 56.94 | 2192.1624 | 19 | 0.0 | 549.0479 | 4 | 70.00 | 6 | F6:46365 | NaSa01\_F14.raw |  |  |  |  |  | 6.8137E5 |  |  |  |  |  |  |  |  | 1 | 0 | 0 | 0 | 0 | 0 | 1 | 0 | 0 | 0 | 0 | 0 | 0 | 0 | 0 | 405 | 423 | Carbamidomethylation | C2:Carbamidomethylation:1000.00 | PEAKS DB |
| R.GAVDMIGDLLNEDSSYHLS.F | Y | 56.35 | 2034.9204 | 19 | 1.2 | 1018.4687 | 2 | 95.85 | 6 | F6:67896 | NaSa01\_F14.raw |  |  |  |  |  | 1.2201E7 |  |  |  |  |  |  |  |  | 1 | 0 | 0 | 0 | 0 | 0 | 1 | 0 | 0 | 0 | 0 | 0 | 0 | 0 | 0 | 217 | 235 |  |  | PEAKS DB |
| K.RFDEIVGGFDQLPISMY.Q | N | 56.32 | 1985.9557 | 17 | 1.0 | 993.9861 | 2 | 99.98 | 6 | F6:71504 | NaSa01\_F14.raw |  |  |  |  | 8.8677E6 | 1.2924E8 |  |  |  |  |  |  |  |  | 4 | 0 | 0 | 0 | 0 | 1 | 3 | 0 | 0 | 0 | 0 | 0 | 0 | 0 | 0 | 251 | 267 |  |  | PEAKS DB |
| K.RFDEIVGGFDQLPISM(+15.99)Y.Q | N | 56.17 | 2001.9506 | 17 | 0.5 | 1001.9830 | 2 | 91.18 | 6 | F6:63904 | NaSa01\_F14.raw |  |  |  |  | 1.3345E6 | 1.0983E7 |  |  |  |  |  |  |  |  | 3 | 0 | 0 | 0 | 0 | 1 | 2 | 0 | 0 | 0 | 0 | 0 | 0 | 0 | 0 | 251 | 267 | Oxidation (M) | M16:Oxidation (M):1000.00 | PEAKS DB |
| Q.FQDYFESAAAPVGR.I | Y | 55.52 | 1556.7258 | 14 | 0.1 | 779.3702 | 2 | 53.21 | 6 | F6:32139 | NaSa01\_F14.raw |  |  |  |  |  | 8.9133E5 |  |  |  |  |  |  |  |  | 1 | 0 | 0 | 0 | 0 | 0 | 1 | 0 | 0 | 0 | 0 | 0 | 0 | 0 | 0 | 454 | 467 |  |  | PEAKS DB |
| R.EGWYVNM(+15.99)GPM(+15.99)R.L | N | 54.52 | 1370.5747 | 11 | -0.6 | 686.2942 | 2 | 35.77 | 6 | F6:19242 | NaSa01\_F14.raw |  |  |  |  | 3.3829E6 | 5.1173E7 |  |  |  |  |  |  |  |  | 8 | 0 | 0 | 0 | 0 | 1 | 7 | 0 | 0 | 0 | 0 | 0 | 0 | 0 | 0 | 99 | 109 | Oxidation (M) | M7:Oxidation (M):1000.00;M10:Oxidation (M):1000.00 | PEAKS DB |
| R.FDEIVGGFDQLPISM(+15.99)YQAIAEMVHLNAR.V | N | 54.25 | 3179.5420 | 28 | 1.5 | 1060.8562 | 3 | 104.78 | 6 | F6:75217 | NaSa01\_F14.raw |  |  |  |  |  | 1.2736E6 |  |  |  |  |  |  |  |  | 1 | 0 | 0 | 0 | 0 | 0 | 1 | 0 | 0 | 0 | 0 | 0 | 0 | 0 | 0 | 252 | 279 | Oxidation (M) | M15:Oxidation (M):67.12 | PEAKS DB |
| L.ADDSDFFQALDTK.T | Y | 53.91 | 1471.6466 | 13 | 0.4 | 736.8309 | 2 | 66.91 | 6 | F6:43666 | NaSa01\_F14.raw |  |  |  |  |  | 8.4089E5 |  |  |  |  |  |  |  |  | 1 | 0 | 0 | 0 | 0 | 0 | 1 | 0 | 0 | 0 | 0 | 0 | 0 | 0 | 0 | 392 | 404 |  |  | PEAKS DB |
| K.TC(+57.02)ADIVINDLSLIHD.L | Y | 53.82 | 1697.8295 | 15 | 1.9 | 849.9236 | 2 | 84.53 | 6 | F6:58437 | NaSa01\_F14.raw |  |  |  |  |  | 3.1631E6 |  |  |  |  |  |  |  |  | 1 | 0 | 0 | 0 | 0 | 0 | 1 | 0 | 0 | 0 | 0 | 0 | 0 | 0 | 0 | 405 | 419 | Carbamidomethylation | C2:Carbamidomethylation:1000.00 | PEAKS DB |
| N.DLSLIHDLPK.R | N | 53.54 | 1149.6393 | 10 | 0.2 | 575.8270 | 2 | 52.53 | 6 | F6:31365 | NaSa01\_F14.raw |  |  |  | 3.056E5 |  | 2.4605E6 |  |  |  |  |  |  |  |  | 2 | 0 | 0 | 0 | 1 | 0 | 1 | 0 | 0 | 0 | 0 | 0 | 0 | 0 | 0 | 413 | 422 |  |  | PEAKS DB |
| W.IDSTIMTGLR.A | Y | 53.08 | 1105.5801 | 10 | 0.9 | 553.7978 | 2 | 43.38 | 6 | F6:23683 | NaSa01\_F14.raw |  |  |  |  |  | 8.1441E6 |  |  |  |  |  |  |  |  | 1 | 0 | 0 | 0 | 0 | 0 | 1 | 0 | 0 | 0 | 0 | 0 | 0 | 0 | 0 | 482 | 491 |  |  | PEAKS DB |
| K.RFDEIVGGFDQLPISM.Y | N | 52.02 | 1822.8923 | 16 | 1.0 | 912.4543 | 2 | 96.41 | 6 | F6:68325 | NaSa01\_F14.raw |  |  |  |  | 8.0404E5 | 1.1182E7 |  |  |  |  |  |  |  |  | 2 | 0 | 0 | 0 | 0 | 1 | 1 | 0 | 0 | 0 | 0 | 0 | 0 | 0 | 0 | 251 | 266 |  |  | PEAKS DB |
| K.KFWEADGIHGGK.S | N | 51.66 | 1343.6622 | 12 | -0.1 | 448.8947 | 3 | 11.66 | 5 | F5:2144 | NaSa01\_F13.raw |  |  |  |  | 1.996E6 | 1.1435E7 |  |  |  |  |  |  |  |  | 3 | 0 | 0 | 0 | 0 | 1 | 2 | 0 | 0 | 0 | 0 | 0 | 0 | 0 | 0 | 351 | 362 |  |  | PEAKS DB |
| M.GSITSFTPYQFQDYFESAAAPVGR.I | Y | 51.25 | 2638.2339 | 24 | 3.8 | 880.4219 | 3 | 92.69 | 6 | F6:65150 | NaSa01\_F14.raw |  |  |  |  |  | 7.6022E6 |  |  |  |  |  |  |  |  | 2 | 0 | 0 | 0 | 0 | 0 | 2 | 0 | 0 | 0 | 0 | 0 | 0 | 0 | 0 | 444 | 467 |  |  | PEAKS DB |
| K.VTLLEASER.V | N | 50.90 | 1016.5502 | 9 | 0.2 | 509.2825 | 2 | 25.50 | 6 | F6:8654 | NaSa01\_F14.raw |  |  |  |  |  | 9.4866E6 |  |  |  |  |  |  |  |  | 2 | 0 | 0 | 0 | 0 | 0 | 2 | 0 | 0 | 0 | 0 | 0 | 0 | 0 | 0 | 78 | 86 |  |  | PEAKS DB |
| R.VTYQTPAK.T | N | 50.89 | 906.4811 | 8 | 0.0 | 454.2478 | 2 | 11.43 | 6 | F6:1806 | NaSa01\_F14.raw |  |  |  |  |  | 3.9897E6 |  |  |  |  |  |  |  |  | 1 | 0 | 0 | 0 | 0 | 0 | 1 | 0 | 0 | 0 | 0 | 0 | 0 | 0 | 0 | 292 | 299 |  |  | PEAKS DB |
| Q.DYFESAAAPVGR.I | Y | 50.87 | 1281.5989 | 12 | 2.6 | 641.8083 | 2 | 42.78 | 6 | F6:23130 | NaSa01\_F14.raw |  |  |  |  |  | 0 |  |  |  |  |  |  |  |  | 0 | 0 | 0 | 0 | 0 | 0 | 0 | 0 | 0 | 0 | 0 | 0 | 0 | 0 | 0 | 456 | 467 |  |  | PEAKS DB |
| K.HVVVVGAGMAGLS.A | N | 50.60 | 1195.6383 | 13 | -0.2 | 598.8263 | 2 | 46.44 | 6 | F6:26217 | NaSa01\_F14.raw |  |  |  |  |  | 5.0901E6 |  |  |  |  |  |  |  |  | 1 | 0 | 0 | 0 | 0 | 0 | 1 | 0 | 0 | 0 | 0 | 0 | 0 | 0 | 0 | 54 | 66 |  |  | PEAKS DB |
| K.HVVVVGAGM(+15.99)AGLSAAY.V | N | 50.48 | 1516.7708 | 16 | 0.9 | 759.3934 | 2 | 46.65 | 6 | F6:26724 | NaSa01\_F14.raw |  |  |  |  |  | 2.0076E7 |  |  |  |  |  |  |  |  | 3 | 0 | 0 | 0 | 0 | 0 | 3 | 0 | 0 | 0 | 0 | 0 | 0 | 0 | 0 | 54 | 69 | Oxidation (M) | M9:Oxidation (M):1000.00 | PEAKS DB |
| K.HVVVVGAGMAGLSAA.Y | N | 50.47 | 1337.7125 | 15 | 0.6 | 669.8640 | 2 | 50.72 | 6 | F6:29947 | NaSa01\_F14.raw |  |  |  |  |  | 7.7421E6 |  |  |  |  |  |  |  |  | 1 | 0 | 0 | 0 | 0 | 0 | 1 | 0 | 0 | 0 | 0 | 0 | 0 | 0 | 0 | 54 | 68 |  |  | PEAKS DB |
| R.EIQALC(+57.02)YPSIKK.W | N | 49.90 | 1448.7697 | 12 | 0.7 | 483.9308 | 3 | 30.77 | 6 | F6:13252 | NaSa01\_F14.raw |  |  |  |  |  | 6.9213E6 |  |  |  |  |  |  |  |  | 2 | 0 | 0 | 0 | 0 | 0 | 2 | 0 | 0 | 0 | 0 | 0 | 0 | 0 | 0 | 424 | 435 | Carbamidomethylation | C6:Carbamidomethylation:1000.00 | PEAKS DB |
| K.RFDEIVGGFDQLPISMYQ.A | N | 49.87 | 2114.0142 | 18 | 1.4 | 1058.0159 | 2 | 96.41 | 6 | F6:68423 | NaSa01\_F14.raw |  |  |  |  |  | 4.0398E6 |  |  |  |  |  |  |  |  | 1 | 0 | 0 | 0 | 0 | 0 | 1 | 0 | 0 | 0 | 0 | 0 | 0 | 0 | 0 | 251 | 268 |  |  | PEAKS DB |
| H.GWIDSTIMTGLR.A | Y | 49.75 | 1348.6809 | 12 | 0.1 | 675.3478 | 2 | 78.70 | 6 | F6:53478 | NaSa01\_F14.raw |  |  |  |  |  | 2.7681E6 |  |  |  |  |  |  |  |  | 2 | 0 | 0 | 0 | 0 | 0 | 2 | 0 | 0 | 0 | 0 | 0 | 0 | 0 | 0 | 480 | 491 |  |  | PEAKS DB |
| I.YFEPPLPPK.K | N | 49.42 | 1086.5750 | 9 | 6.0 | 544.2952 | 2 | 42.50 | 8 | F8:25506 | NaSa01\_F3.raw |  |  |  |  |  |  |  | 6.3004E5 |  |  |  |  |  |  | 1 | 0 | 0 | 0 | 0 | 0 | 0 | 0 | 1 | 0 | 0 | 0 | 0 | 0 | 0 | 320 | 328 |  |  | PEAKS DB |
| K.HVVVVGAGMAGLSA.A | N | 48.82 | 1266.6754 | 14 | 0.3 | 634.3452 | 2 | 49.72 | 6 | F6:29252 | NaSa01\_F14.raw |  |  |  |  |  | 3.9558E6 |  |  |  |  |  |  |  |  | 1 | 0 | 0 | 0 | 0 | 0 | 1 | 0 | 0 | 0 | 0 | 0 | 0 | 0 | 0 | 54 | 67 |  |  | PEAKS DB |
| K.STTDLPSR.F | N | 48.78 | 875.4348 | 8 | 0.1 | 438.7247 | 2 | 11.44 | 6 | F6:1817 | NaSa01\_F14.raw |  |  |  |  |  | 1.0568E6 |  |  |  |  |  |  |  |  | 1 | 0 | 0 | 0 | 0 | 0 | 1 | 0 | 0 | 0 | 0 | 0 | 0 | 0 | 0 | 363 | 370 |  |  | PEAKS DB |
| R.GAVDMIGDLLNEDSSY.H | N | 48.29 | 1697.7454 | 16 | 1.7 | 849.8814 | 2 | 107.90 | 6 | F6:77609 | NaSa01\_F14.raw |  |  |  |  |  | 1.6853E7 |  |  |  |  |  |  |  |  | 1 | 0 | 0 | 0 | 0 | 0 | 1 | 0 | 0 | 0 | 0 | 0 | 0 | 0 | 0 | 217 | 232 |  |  | PEAKS DB |
| R.FDEIVGGFDQLPISM.Y | N | 48.24 | 1666.7913 | 15 | 1.2 | 834.4039 | 2 | 112.47 | 6 | F6:80862 | NaSa01\_F14.raw |  |  |  |  |  | 8.4104E6 |  |  |  |  |  |  |  |  | 1 | 0 | 0 | 0 | 0 | 0 | 1 | 0 | 0 | 0 | 0 | 0 | 0 | 0 | 0 | 252 | 266 |  |  | PEAKS DB |
| Y.QAIAEM(+15.99)VHLNAR.V | N | 47.05 | 1367.6980 | 12 | 0.8 | 456.9070 | 3 | 31.64 | 6 | F6:13767 | NaSa01\_F14.raw |  |  |  |  |  | 2.4624E6 |  |  |  |  |  |  |  |  | 3 | 0 | 0 | 0 | 0 | 0 | 3 | 0 | 0 | 0 | 0 | 0 | 0 | 0 | 0 | 268 | 279 | Oxidation (M) | M6:Oxidation (M):1000.00 | PEAKS DB |
| R.FHGWIDSTIM.T | Y | 46.94 | 1205.5540 | 10 | 0.7 | 603.7847 | 2 | 69.86 | 6 | F6:46616 | NaSa01\_F14.raw |  |  |  |  |  | 2.4473E7 |  |  |  |  |  |  |  |  | 1 | 0 | 0 | 0 | 0 | 0 | 1 | 0 | 0 | 0 | 0 | 0 | 0 | 0 | 0 | 478 | 487 |  |  | PEAKS DB |
| Y.TMGSITSFTPYQFQDYFESAAAPVGR.I | Y | 46.62 | 2870.3220 | 26 | 0.3 | 957.7816 | 3 | 97.15 | 6 | F6:68946 | NaSa01\_F14.raw |  |  |  |  |  | 1.4838E7 |  |  |  |  |  |  |  |  | 2 | 0 | 0 | 0 | 0 | 0 | 2 | 0 | 0 | 0 | 0 | 0 | 0 | 0 | 0 | 442 | 467 |  |  | PEAKS DB |
| K.TFVTADYVIVC(+57.02).S | Y | 46.30 | 1286.6217 | 11 | 0.6 | 644.3185 | 2 | 81.27 | 6 | F6:56092 | NaSa01\_F14.raw |  |  |  |  | 2.639E6 | 3.2724E7 |  |  |  |  |  |  |  |  | 2 | 0 | 0 | 0 | 0 | 1 | 1 | 0 | 0 | 0 | 0 | 0 | 0 | 0 | 0 | 300 | 310 | Carbamidomethylation | C11:Carbamidomethylation:1000.00 | PEAKS DB |
| K.KDPSLLKYPVKPSEEGK.S | Y | 46.30 | 1914.0461 | 17 | -0.5 | 479.5186 | 4 | 11.65 | 5 | F5:2119 | NaSa01\_F13.raw |  |  |  |  | 1.573E5 |  |  |  |  |  |  |  |  |  | 1 | 0 | 0 | 0 | 0 | 1 | 0 | 0 | 0 | 0 | 0 | 0 | 0 | 0 | 0 | 154 | 170 |  |  | PEAKS DB |
| A.DDSDFFQALDTK.T | Y | 44.68 | 1400.6095 | 12 | 1.0 | 701.3127 | 2 | 73.11 | 4 | F4:50624 | NaSa01\_F12.raw |  |  |  | 6.4171E5 |  |  |  |  |  |  |  |  |  |  | 1 | 0 | 0 | 0 | 1 | 0 | 0 | 0 | 0 | 0 | 0 | 0 | 0 | 0 | 0 | 393 | 404 |  |  | PEAKS DB |
| R.FDEIVGGFDQLPISMY.Q | N | 44.41 | 1829.8545 | 16 | 0.2 | 915.9347 | 2 | 116.62 | 6 | F6:83518 | NaSa01\_F14.raw |  |  |  |  |  | 6.1039E7 |  |  |  |  |  |  |  |  | 1 | 0 | 0 | 0 | 0 | 0 | 1 | 0 | 0 | 0 | 0 | 0 | 0 | 0 | 0 | 252 | 267 |  |  | PEAKS DB |
| A.GM(+15.99)AGLSAAYVLAGAGHK.V | N | 43.41 | 1588.8031 | 17 | 0.5 | 530.6086 | 3 | 39.38 | 6 | F6:20374 | NaSa01\_F14.raw |  |  |  |  |  | 2.4697E5 |  |  |  |  |  |  |  |  | 1 | 0 | 0 | 0 | 0 | 0 | 1 | 0 | 0 | 0 | 0 | 0 | 0 | 0 | 0 | 61 | 77 | Oxidation (M) | M2:Oxidation (M):1000.00 | PEAKS DB |
| G.SITSFTPYQFQDYFESAAAPVGR.I | Y | 43.36 | 2581.2124 | 23 | 0.0 | 1291.6135 | 2 | 92.73 | 6 | F6:65352 | NaSa01\_F14.raw |  |  |  |  |  | 4.3026E5 |  |  |  |  |  |  |  |  | 1 | 0 | 0 | 0 | 0 | 0 | 1 | 0 | 0 | 0 | 0 | 0 | 0 | 0 | 0 | 445 | 467 |  |  | PEAKS DB |
| R.FDEIVGGFDQLPISMYQ.A | N | 43.29 | 1957.9131 | 17 | 0.8 | 979.9646 | 2 | 111.22 | 6 | F6:80041 | NaSa01\_F14.raw |  |  |  |  |  | 3.0744E6 |  |  |  |  |  |  |  |  | 1 | 0 | 0 | 0 | 0 | 0 | 1 | 0 | 0 | 0 | 0 | 0 | 0 | 0 | 0 | 252 | 268 |  |  | PEAKS DB |
| K.HVVVVGAGM(+15.99)AGL.S | N | 43.09 | 1124.6012 | 12 | 0.5 | 563.3082 | 2 | 50.23 | 6 | F6:30439 | NaSa01\_F14.raw |  |  |  |  | 3.0223E6 | 1.1076E7 |  |  |  |  |  |  |  |  | 3 | 0 | 0 | 0 | 0 | 1 | 2 | 0 | 0 | 0 | 0 | 0 | 0 | 0 | 0 | 54 | 65 | Oxidation (M) | M9:Oxidation (M):1000.00 | PEAKS DB |
| E.IQALC(+57.02)YPSIK.K | N | 42.76 | 1191.6322 | 10 | -0.9 | 596.8228 | 2 | 42.19 | 6 | F6:22642 | NaSa01\_F14.raw |  |  |  |  |  | 1.1323E6 |  |  |  |  |  |  |  |  | 1 | 0 | 0 | 0 | 0 | 0 | 1 | 0 | 0 | 0 | 0 | 0 | 0 | 0 | 0 | 425 | 434 | Carbamidomethylation | C5:Carbamidomethylation:1000.00 | PEAKS DB |
| Y.TM(+15.99)GSITSFTPYQFQDYFESAAAPVGR.I | Y | 42.42 | 2886.3169 | 26 | 0.9 | 963.1138 | 3 | 92.52 | 6 | F6:65107 | NaSa01\_F14.raw |  |  |  |  |  | 1.1735E6 |  |  |  |  |  |  |  |  | 1 | 0 | 0 | 0 | 0 | 0 | 1 | 0 | 0 | 0 | 0 | 0 | 0 | 0 | 0 | 442 | 467 | Oxidation (M) | M2:Oxidation (M):1000.00 | PEAKS DB |
| V.GAGMAGLSAAYVLAGAGHK.V | N | 41.36 | 1700.8668 | 19 | -0.1 | 567.9628 | 3 | 53.80 | 6 | F6:32769 | NaSa01\_F14.raw |  |  |  |  |  | 3.5832E5 |  |  |  |  |  |  |  |  | 1 | 0 | 0 | 0 | 0 | 0 | 1 | 0 | 0 | 0 | 0 | 0 | 0 | 0 | 0 | 59 | 77 |  |  | PEAKS DB |
| R.FHGWIDST.I | Y | 40.89 | 961.4294 | 8 | -0.2 | 481.7219 | 2 | 38.55 | 6 | F6:19670 | NaSa01\_F14.raw |  |  |  |  |  | 1.042E6 |  |  |  |  |  |  |  |  | 1 | 0 | 0 | 0 | 0 | 0 | 1 | 0 | 0 | 0 | 0 | 0 | 0 | 0 | 0 | 478 | 485 |  |  | PEAKS DB |
| F.HGWIDSTIMTGLR.A | Y | 40.51 | 1485.7399 | 13 | -0.5 | 496.2537 | 3 | 60.76 | 6 | F6:38590 | NaSa01\_F14.raw |  |  |  |  |  | 1.5775E5 |  |  |  |  |  |  |  |  | 1 | 0 | 0 | 0 | 0 | 0 | 1 | 0 | 0 | 0 | 0 | 0 | 0 | 0 | 0 | 479 | 491 |  |  | PEAKS DB |
| G.MAGLSAAYVLAGAGHK.V | N | 39.83 | 1515.7867 | 16 | 0.3 | 506.2697 | 3 | 47.39 | 6 | F6:27145 | NaSa01\_F14.raw |  |  |  |  |  | 5.9167E5 |  |  |  |  |  |  |  |  | 1 | 0 | 0 | 0 | 0 | 0 | 1 | 0 | 0 | 0 | 0 | 0 | 0 | 0 | 0 | 62 | 77 |  |  | PEAKS DB |
| E.GWYVNMGPMR.L | N | 38.80 | 1209.5424 | 10 | 0.9 | 605.7790 | 2 | 55.07 | 6 | F6:33759 | NaSa01\_F14.raw |  |  |  |  |  | 8.4887E5 |  |  |  |  |  |  |  |  | 1 | 0 | 0 | 0 | 0 | 0 | 1 | 0 | 0 | 0 | 0 | 0 | 0 | 0 | 0 | 100 | 109 |  |  | PEAKS DB |
| R.FDEIVGGFDQLPISMYQA.I | N | 38.57 | 2028.9502 | 18 | 1.1 | 1015.4835 | 2 | 112.01 | 6 | F6:80759 | NaSa01\_F14.raw |  |  |  |  |  | 1.2262E7 |  |  |  |  |  |  |  |  | 1 | 0 | 0 | 0 | 0 | 0 | 1 | 0 | 0 | 0 | 0 | 0 | 0 | 0 | 0 | 252 | 269 |  |  | PEAKS DB |
| R.FDEIVGGFDQLPISM(+15.99)Y.Q | N | 38.39 | 1845.8495 | 16 | 0.4 | 923.9324 | 2 | 105.35 | 6 | F6:75636 | NaSa01\_F14.raw |  |  |  |  |  | 4.6489E6 |  |  |  |  |  |  |  |  | 1 | 0 | 0 | 0 | 0 | 0 | 1 | 0 | 0 | 0 | 0 | 0 | 0 | 0 | 0 | 252 | 267 | Oxidation (M) | M15:Oxidation (M):1000.00 | PEAKS DB |
| F.VTADYVIVC(+57.02)STSR.A | N | 38.16 | 1469.7184 | 13 | 0.8 | 490.9138 | 3 | 38.41 | 6 | F6:19506 | NaSa01\_F14.raw |  |  |  |  |  | 3.3852E5 |  |  |  |  |  |  |  |  | 1 | 0 | 0 | 0 | 0 | 0 | 1 | 0 | 0 | 0 | 0 | 0 | 0 | 0 | 0 | 302 | 314 | Carbamidomethylation | C9:Carbamidomethylation:1000.00 | PEAKS DB |
| total 98 peptides |  |  |  |  |  |  |  |  |  |  |  |  |  |  |  |  |  |  |  |  |  |  |  |  |  |  |  |  |  |  |  |  |  |  |  |  |  |  |  |  |  |  |  |  |  |
| --- | --- | --- | --- | --- | --- | --- | --- | --- | --- | --- | --- | --- | --- | --- | --- | --- | --- | --- | --- | --- | --- | --- | --- | --- | --- | --- | --- | --- | --- | --- | --- | --- | --- | --- | --- | --- | --- | --- | --- | --- | --- | --- | --- | --- | --- |

A8QL58.2

back to list

  

| Protein Coverage
| Supporting Peptides
|

Protein Coverage:

Supporting Peptides:

| Peptide | Uniq | -10lgP | Mass | Length | ppm | m/z | z | RT | Fraction | Scan | Source File | Area F1 | Area F10 | Area F11 | Area F12 | Area F13 | Area F14 | Area F2 | Area F3 | Area F4 | Area F5 | Area F6 | Area F7 | Area F8 | Area F9 | #Feature | #Feature F1 | #Feature F10 | #Feature F11 | #Feature F12 | #Feature F13 | #Feature F14 | #Feature F2 | #Feature F3 | #Feature F4 | #Feature F5 | #Feature F6 | #Feature F7 | #Feature F8 | #Feature F9 | Start | End | PTM | AScore | Found By |
| --- | --- | --- | --- | --- | --- | --- | --- | --- | --- | --- | --- | --- | --- | --- | --- | --- | --- | --- | --- | --- | --- | --- | --- | --- | --- | --- | --- | --- | --- | --- | --- | --- | --- | --- | --- | --- | --- | --- | --- | --- | --- | --- | --- | --- | --- |
| K.HVVVVGAGMAGLSAAYVLAGAGHK.V | N | 107.42 | 2234.1992 | 24 | 1.2 | 745.7412 | 3 | 65.01 | 6 | F6:42957 | NaSa01\_F14.raw |  |  |  | 6.8911E6 | 4.2938E7 | 6.5431E8 |  |  |  |  |  |  |  |  | 25 | 0 | 0 | 0 | 2 | 2 | 21 | 0 | 0 | 0 | 0 | 0 | 0 | 0 | 0 | 54 | 77 |  |  | PEAKS DB |
| K.TC(+57.02)ADIVINDLSLIHDLPK.R | Y | 96.41 | 2036.0612 | 18 | 1.5 | 1019.0394 | 2 | 82.06 | 6 | F6:56577 | NaSa01\_F14.raw |  |  |  | 4.4983E6 | 7.6898E7 | 5.3211E8 |  |  |  |  |  |  |  |  | 13 | 0 | 0 | 0 | 1 | 4 | 8 | 0 | 0 | 0 | 0 | 0 | 0 | 0 | 0 | 405 | 422 | Carbamidomethylation | C2:Carbamidomethylation:1000.00 | PEAKS DB |
| K.HVVVVGAGM(+15.99)AGLSAAYVLAGAGHK.V | N | 95.06 | 2250.1943 | 24 | 0.9 | 751.0728 | 3 | 53.09 | 6 | F6:32085 | NaSa01\_F14.raw |  |  |  |  | 1.6389E7 | 1.5623E8 |  |  |  |  |  |  |  |  | 5 | 0 | 0 | 0 | 0 | 2 | 3 | 0 | 0 | 0 | 0 | 0 | 0 | 0 | 0 | 54 | 77 | Oxidation (M) | M9:Oxidation (M):1000.00 | PEAKS DB |
| F.TPYQFQDYFESAAAPVGR.I | Y | 94.96 | 2045.9482 | 18 | -0.1 | 1023.9813 | 2 | 72.54 | 6 | F6:48623 | NaSa01\_F14.raw |  |  |  |  | 1.3361E7 | 2.9427E8 |  |  |  |  |  |  |  |  | 3 | 0 | 0 | 0 | 0 | 1 | 2 | 0 | 0 | 0 | 0 | 0 | 0 | 0 | 0 | 450 | 467 |  |  | PEAKS DB |
| K.TFVTADYVIVC(+57.02)STSR.A | Y | 93.18 | 1717.8345 | 15 | 2.2 | 859.9264 | 2 | 60.90 | 6 | F6:39859 | NaSa01\_F14.raw |  |  |  |  | 5.5865E7 | 4.4428E8 |  |  |  |  |  |  | 5.5981E5 |  | 20 | 0 | 0 | 0 | 0 | 3 | 16 | 0 | 0 | 0 | 0 | 0 | 0 | 1 | 0 | 300 | 314 | Carbamidomethylation | C11:Carbamidomethylation:1000.00 | PEAKS DB |
| K.LNEFFQENENAWYYINNIR.K | Y | 90.36 | 2476.1448 | 19 | 0.7 | 1239.0806 | 2 | 91.17 | 6 | F6:64025 | NaSa01\_F14.raw |  |  |  |  | 6.6266E5 | 7.9615E7 |  |  |  |  |  |  |  |  | 3 | 0 | 0 | 0 | 0 | 1 | 2 | 0 | 0 | 0 | 0 | 0 | 0 | 0 | 0 | 128 | 146 |  |  | PEAKS DB |
| K.YTMGSITSFTPYQFQDYFESAAAPVGR.I | Y | 82.61 | 3033.3855 | 27 | 0.8 | 1012.1366 | 3 | 100.62 | 6 | F6:71855 | NaSa01\_F14.raw |  |  |  |  | 1.0072E7 | 2.7658E8 |  |  |  |  |  |  |  |  | 5 | 0 | 0 | 0 | 0 | 2 | 3 | 0 | 0 | 0 | 0 | 0 | 0 | 0 | 0 | 441 | 467 |  |  | PEAKS DB |
| R.FHGWIDSTIM(+15.99)TGLR.A | Y | 82.52 | 1648.8031 | 14 | 0.3 | 825.4091 | 2 | 56.10 | 6 | F6:34934 | NaSa01\_F14.raw |  |  |  |  | 1.0724E7 | 1.9786E8 |  |  |  |  |  |  |  |  | 15 | 0 | 0 | 0 | 0 | 2 | 13 | 0 | 0 | 0 | 0 | 0 | 0 | 0 | 0 | 478 | 491 | Oxidation (M) | M10:Oxidation (M):1000.00 | PEAKS DB |
| Y.VLADDSDFFQALDTK.T | Y | 82.09 | 1683.7991 | 15 | 0.1 | 842.9069 | 2 | 78.47 | 6 | F6:53277 | NaSa01\_F14.raw |  |  |  | 2.5191E5 | 2.6921E8 | 3.2203E9 |  |  |  |  |  |  |  |  | 9 | 0 | 0 | 0 | 1 | 2 | 6 | 0 | 0 | 0 | 0 | 0 | 0 | 0 | 0 | 390 | 404 |  |  | PEAKS DB |
| R.FHGWIDSTIMTGLR.A | Y | 81.76 | 1632.8082 | 14 | 0.3 | 817.4116 | 2 | 68.30 | 6 | F6:44954 | NaSa01\_F14.raw |  | 2.7707E6 |  | 6.8303E6 | 2.7763E7 | 7.2536E8 |  |  |  |  |  |  |  |  | 28 | 0 | 1 | 0 | 3 | 2 | 22 | 0 | 0 | 0 | 0 | 0 | 0 | 0 | 0 | 478 | 491 |  |  | PEAKS DB |
| M.AGLSAAYVLAGAGHK.V | N | 79.84 | 1384.7462 | 15 | 0.5 | 693.3807 | 2 | 36.23 | 6 | F6:17690 | NaSa01\_F14.raw |  |  |  |  |  | 4.4211E7 |  |  |  |  |  |  |  |  | 2 | 0 | 0 | 0 | 0 | 0 | 2 | 0 | 0 | 0 | 0 | 0 | 0 | 0 | 0 | 63 | 77 |  |  | PEAKS DB |
| M.AYVLADDSDFFQALDTK.T | Y | 79.60 | 1917.8995 | 17 | -0.3 | 959.9567 | 2 | 85.82 | 6 | F6:59496 | NaSa01\_F14.raw |  |  |  |  | 3.4939E6 | 3.9274E7 |  |  |  |  |  |  |  |  | 2 | 0 | 0 | 0 | 0 | 1 | 1 | 0 | 0 | 0 | 0 | 0 | 0 | 0 | 0 | 388 | 404 |  |  | PEAKS DB |
| K.REIQALC(+57.02)YPSIK.K | N | 77.78 | 1476.7759 | 12 | 0.2 | 493.2660 | 3 | 32.82 | 6 | F6:14068 | NaSa01\_F14.raw |  |  |  |  | 3.5291E7 | 3.7818E8 |  |  |  |  |  |  |  |  | 8 | 0 | 0 | 0 | 0 | 2 | 6 | 0 | 0 | 0 | 0 | 0 | 0 | 0 | 0 | 423 | 434 | Carbamidomethylation | C7:Carbamidomethylation:1000.00 | PEAKS DB |
| S.ITSFTPYQFQDYFESAAAPVGR.I | Y | 77.75 | 2494.1804 | 22 | 0.4 | 1248.0980 | 2 | 89.99 | 6 | F6:63003 | NaSa01\_F14.raw |  |  |  |  |  | 8.4867E6 |  |  |  |  |  |  |  |  | 1 | 0 | 0 | 0 | 0 | 0 | 1 | 0 | 0 | 0 | 0 | 0 | 0 | 0 | 0 | 446 | 467 |  |  | PEAKS DB |
| R.EIQALC(+57.02)YPSIK.K | N | 77.61 | 1320.6747 | 11 | 0.3 | 661.3448 | 2 | 47.88 | 6 | F6:27511 | NaSa01\_F14.raw |  |  |  | 3.0826E6 | 1.353E8 | 1.8533E9 |  |  |  |  |  |  |  |  | 18 | 0 | 0 | 0 | 1 | 1 | 16 | 0 | 0 | 0 | 0 | 0 | 0 | 0 | 0 | 424 | 434 | Carbamidomethylation | C6:Carbamidomethylation:1000.00 | PEAKS DB |
| A.GMAGLSAAYVLAGAGHK.V | N | 77.31 | 1572.8082 | 17 | 0.2 | 525.2768 | 3 | 50.77 | 6 | F6:29981 | NaSa01\_F14.raw |  |  |  |  | 5.5361E5 | 1.0582E7 |  |  |  |  |  |  |  |  | 3 | 0 | 0 | 0 | 0 | 1 | 2 | 0 | 0 | 0 | 0 | 0 | 0 | 0 | 0 | 61 | 77 |  |  | PEAKS DB |
| Y.QFQDYFESAAAPVGR.I | Y | 76.00 | 1684.7844 | 15 | 0.6 | 843.4000 | 2 | 59.29 | 6 | F6:37194 | NaSa01\_F14.raw |  |  |  | 8.1084E5 | 8.279E7 | 1.2226E9 |  |  |  |  |  |  |  |  | 13 | 0 | 0 | 0 | 1 | 3 | 9 | 0 | 0 | 0 | 0 | 0 | 0 | 0 | 0 | 453 | 467 |  |  | PEAKS DB |
| R.IYFEPPLPPKK.A | N | 74.78 | 1327.7539 | 11 | 0.3 | 664.8844 | 2 | 39.91 | 6 | F6:21060 | NaSa01\_F14.raw |  |  |  |  | 5.0279E7 | 4.7146E8 |  |  |  |  |  |  |  |  | 4 | 0 | 0 | 0 | 0 | 2 | 2 | 0 | 0 | 0 | 0 | 0 | 0 | 0 | 0 | 319 | 329 |  |  | PEAKS DB |
| R.EGWYVNMGPM(+15.99)R.L | N | 73.07 | 1354.5798 | 11 | 0.4 | 678.2975 | 2 | 56.26 | 6 | F6:34728 | NaSa01\_F14.raw |  |  |  |  | 3.9735E7 | 2.4523E8 |  |  |  |  |  |  |  |  | 7 | 0 | 0 | 0 | 0 | 4 | 3 | 0 | 0 | 0 | 0 | 0 | 0 | 0 | 0 | 99 | 109 | Oxidation (M) | M10:Oxidation (M):51.69 | PEAKS DB |
| R.IYFEPPLPPK.K | N | 72.12 | 1199.6589 | 10 | -0.1 | 600.8367 | 2 | 57.48 | 6 | F6:38014 | NaSa01\_F14.raw |  |  |  | 1.5815E6 | 1.7195E8 | 2.5686E9 |  |  | 1.0411E6 |  |  |  |  |  | 22 | 0 | 0 | 0 | 1 | 2 | 18 | 0 | 0 | 1 | 0 | 0 | 0 | 0 | 0 | 319 | 328 |  |  | PEAKS DB |
| R.EGWYVNMGPMR.L | N | 71.70 | 1338.5850 | 11 | 0.0 | 670.2997 | 2 | 59.12 | 6 | F6:37086 | NaSa01\_F14.raw |  |  |  |  | 1.3719E8 | 2.0017E9 |  |  |  |  |  |  |  |  | 6 | 0 | 0 | 0 | 0 | 3 | 3 | 0 | 0 | 0 | 0 | 0 | 0 | 0 | 0 | 99 | 109 |  |  | PEAKS DB |
| A.YVLADDSDFFQALDTK.T | Y | 71.22 | 1846.8624 | 16 | 0.6 | 924.4391 | 2 | 84.46 | 6 | F6:58413 | NaSa01\_F14.raw |  |  |  |  |  | 2.5549E6 |  |  |  |  |  |  |  |  | 1 | 0 | 0 | 0 | 0 | 0 | 1 | 0 | 0 | 0 | 0 | 0 | 0 | 0 | 0 | 389 | 404 |  |  | PEAKS DB |
| M.YQAIAEMVHLNAR.V | N | 70.47 | 1514.7664 | 13 | 0.6 | 505.9297 | 3 | 42.75 | 6 | F6:23121 | NaSa01\_F14.raw |  |  |  |  |  | 6.4909E6 |  |  |  |  |  |  |  |  | 2 | 0 | 0 | 0 | 0 | 0 | 2 | 0 | 0 | 0 | 0 | 0 | 0 | 0 | 0 | 267 | 279 |  |  | PEAKS DB |
| A.DIVINDLSLIHDLPK.R | Y | 70.43 | 1703.9458 | 15 | 0.0 | 568.9892 | 3 | 80.66 | 6 | F6:55094 | NaSa01\_F14.raw |  |  |  | 4.3515E6 | 5.5307E6 | 1.0082E7 |  |  |  |  |  | 7.9457E5 |  |  | 7 | 0 | 0 | 0 | 2 | 2 | 2 | 0 | 0 | 0 | 0 | 0 | 1 | 0 | 0 | 408 | 422 |  |  | PEAKS DB |
| K.YTM(+15.99)GSITSFTPYQFQDYFESAAAPVGR.I | Y | 68.73 | 3049.3804 | 27 | 1.3 | 1017.4688 | 3 | 94.25 | 6 | F6:66450 | NaSa01\_F14.raw |  |  |  | 7.1363E5 | 6.5743E6 | 9.733E7 |  |  |  |  |  |  |  |  | 4 | 0 | 0 | 0 | 1 | 1 | 2 | 0 | 0 | 0 | 0 | 0 | 0 | 0 | 0 | 441 | 467 | Oxidation (M) | M3:Oxidation (M):1000.00 | PEAKS DB |
| R.EGWYVNM(+15.99)GPMR.L | N | 67.93 | 1354.5798 | 11 | 0.8 | 678.2977 | 2 | 41.16 | 6 | F6:22021 | NaSa01\_F14.raw |  |  |  | 5.0564E5 | 1.7067E7 | 2.1752E8 |  |  |  |  |  |  |  |  | 5 | 0 | 0 | 0 | 1 | 2 | 2 | 0 | 0 | 0 | 0 | 0 | 0 | 0 | 0 | 99 | 109 | Oxidation (M) | M7:Oxidation (M):39.30 | PEAKS DB |
| K.SASQLYQESLR.K | Y | 67.67 | 1280.6360 | 11 | 0.1 | 641.3253 | 2 | 24.71 | 6 | F6:8085 | NaSa01\_F14.raw |  |  |  |  | 1.1171E8 | 7.0099E6 |  |  |  |  |  |  |  |  | 2 | 0 | 0 | 0 | 0 | 1 | 1 | 0 | 0 | 0 | 0 | 0 | 0 | 0 | 0 | 171 | 181 |  |  | PEAKS DB |
| K.YDSYSTKEYLIK.E | N | 67.50 | 1508.7399 | 12 | 0.6 | 503.9209 | 3 | 26.71 | 6 | F6:10035 | NaSa01\_F14.raw |  |  |  |  |  | 7.9504E6 |  |  |  |  |  |  |  |  | 2 | 0 | 0 | 0 | 0 | 0 | 2 | 0 | 0 | 0 | 0 | 0 | 0 | 0 | 0 | 199 | 210 |  |  | PEAKS DB |
| K.HVVVVGAGMAGLSAAY.V | N | 67.41 | 1500.7759 | 16 | 0.2 | 751.3954 | 2 | 68.19 | 6 | F6:44843 | NaSa01\_F14.raw |  |  |  |  | 2.067E7 | 2.0687E8 |  |  |  |  |  |  |  |  | 10 | 0 | 0 | 0 | 0 | 1 | 9 | 0 | 0 | 0 | 0 | 0 | 0 | 0 | 0 | 54 | 69 |  |  | PEAKS DB |
| Y.QAIAEMVHLNAR.V | N | 66.25 | 1351.7030 | 12 | -0.4 | 676.8585 | 2 | 31.55 | 6 | F6:13654 | NaSa01\_F14.raw |  |  |  |  |  | 7.8157E7 |  |  |  |  |  |  |  |  | 2 | 0 | 0 | 0 | 0 | 0 | 2 | 0 | 0 | 0 | 0 | 0 | 0 | 0 | 0 | 268 | 279 |  |  | PEAKS DB |
| R.IHFAGEYTGR.F | Y | 65.92 | 1149.5566 | 10 | -0.7 | 384.1926 | 3 | 11.57 | 6 | F6:1949 | NaSa01\_F14.raw |  |  |  |  | 1.5757E6 | 2.2033E7 |  |  |  |  |  |  |  |  | 9 | 0 | 0 | 0 | 0 | 3 | 6 | 0 | 0 | 0 | 0 | 0 | 0 | 0 | 0 | 468 | 477 |  |  | PEAKS DB |
| T.SFTPYQFQDYFESAAAPVGR.I | Y | 65.14 | 2280.0486 | 20 | 0.8 | 1141.0325 | 2 | 85.41 | 6 | F6:59216 | NaSa01\_F14.raw |  |  |  |  |  | 1.7558E6 |  |  |  |  |  |  |  |  | 1 | 0 | 0 | 0 | 0 | 0 | 1 | 0 | 0 | 0 | 0 | 0 | 0 | 0 | 0 | 448 | 467 |  |  | PEAKS DB |
| K.FWEADGIHGGKSTTDLPSR.F | N | 64.97 | 2072.9915 | 19 | 0.7 | 692.0049 | 3 | 29.66 | 6 | F6:12168 | NaSa01\_F14.raw |  |  |  |  |  | 2.4928E6 |  |  |  |  |  |  |  |  | 1 | 0 | 0 | 0 | 0 | 0 | 1 | 0 | 0 | 0 | 0 | 0 | 0 | 0 | 0 | 352 | 370 |  |  | PEAKS DB |
| R.RIYFEPPLPPKK.A | N | 64.76 | 1483.8550 | 12 | -0.5 | 495.6253 | 3 | 28.82 | 6 | F6:11398 | NaSa01\_F14.raw |  |  |  |  | 2.2402E7 | 1.4186E8 |  |  |  |  |  |  |  |  | 5 | 0 | 0 | 0 | 0 | 2 | 3 | 0 | 0 | 0 | 0 | 0 | 0 | 0 | 0 | 318 | 329 |  |  | PEAKS DB |
| L.SAAYVLAGAGHK.V | N | 63.01 | 1143.6036 | 12 | -1.1 | 572.8085 | 2 | 11.49 | 6 | F6:1894 | NaSa01\_F14.raw |  |  |  |  |  | 4.4228E6 |  |  |  |  |  |  |  |  | 2 | 0 | 0 | 0 | 0 | 0 | 2 | 0 | 0 | 0 | 0 | 0 | 0 | 0 | 0 | 66 | 77 |  |  | PEAKS DB |
| K.SDALFSYEK.R | N | 62.97 | 1058.4919 | 9 | 0.4 | 530.2535 | 2 | 34.28 | 6 | F6:16290 | NaSa01\_F14.raw |  |  |  |  | 5.08E7 | 1.3053E7 |  |  |  |  |  |  |  |  | 4 | 0 | 0 | 0 | 0 | 1 | 3 | 0 | 0 | 0 | 0 | 0 | 0 | 0 | 0 | 242 | 250 |  |  | PEAKS DB |
| F.QDYFESAAAPVGR.I | Y | 62.52 | 1409.6575 | 13 | 0.1 | 705.8361 | 2 | 39.67 | 6 | F6:20451 | NaSa01\_F14.raw |  |  |  |  |  | 3.65E6 |  |  |  |  |  |  |  |  | 1 | 0 | 0 | 0 | 0 | 0 | 1 | 0 | 0 | 0 | 0 | 0 | 0 | 0 | 0 | 455 | 467 |  |  | PEAKS DB |
| K.YPVKPSEEGK.S | N | 62.09 | 1132.5764 | 10 | 1.6 | 567.2964 | 2 | 11.44 | 6 | F6:1813 | NaSa01\_F14.raw |  |  |  |  |  | 1.8552E6 |  |  |  |  |  |  |  |  | 2 | 0 | 0 | 0 | 0 | 0 | 2 | 0 | 0 | 0 | 0 | 0 | 0 | 0 | 0 | 161 | 170 |  |  | PEAKS DB |
| D.SDFFQALDTK.T | Y | 61.64 | 1170.5557 | 10 | 0.6 | 586.2855 | 2 | 57.84 | 6 | F6:36028 | NaSa01\_F14.raw |  |  |  |  |  | 4.304E6 |  |  |  |  |  |  |  |  | 1 | 0 | 0 | 0 | 0 | 0 | 1 | 0 | 0 | 0 | 0 | 0 | 0 | 0 | 0 | 395 | 404 |  |  | PEAKS DB |
| R.RIYFEPPLPPK.K | N | 60.85 | 1355.7601 | 11 | 0.4 | 452.9275 | 3 | 43.97 | 6 | F6:24986 | NaSa01\_F14.raw |  |  |  |  | 9.9689E7 | 1.1129E9 |  |  |  |  |  |  |  |  | 4 | 0 | 0 | 0 | 0 | 2 | 2 | 0 | 0 | 0 | 0 | 0 | 0 | 0 | 0 | 318 | 328 |  |  | PEAKS DB |
| K.RFDEIVGGFDQLPISMYQA.I | N | 60.53 | 2185.0513 | 19 | -0.2 | 1093.5327 | 2 | 97.41 | 6 | F6:69051 | NaSa01\_F14.raw |  |  |  |  |  | 1.6822E7 |  |  |  |  |  |  |  |  | 1 | 0 | 0 | 0 | 0 | 0 | 1 | 0 | 0 | 0 | 0 | 0 | 0 | 0 | 0 | 251 | 269 |  |  | PEAKS DB |
| F.FQENENAWYYINNIR.K | Y | 60.04 | 1972.9067 | 15 | -0.4 | 987.4603 | 2 | 71.69 | 6 | F6:47797 | NaSa01\_F14.raw |  |  |  |  |  | 2.7056E6 |  |  |  |  |  |  |  |  | 1 | 0 | 0 | 0 | 0 | 0 | 1 | 0 | 0 | 0 | 0 | 0 | 0 | 0 | 0 | 132 | 146 |  |  | PEAKS DB |
| S.FTPYQFQDYFESAAAPVGR.I | Y | 59.98 | 2193.0166 | 19 | -0.2 | 1097.5154 | 2 | 83.99 | 6 | F6:57987 | NaSa01\_F14.raw |  |  |  |  |  | 1.8906E6 |  |  |  |  |  |  |  |  | 1 | 0 | 0 | 0 | 0 | 0 | 1 | 0 | 0 | 0 | 0 | 0 | 0 | 0 | 0 | 449 | 467 |  |  | PEAKS DB |
| D.DSDFFQALDTK.T | Y | 59.42 | 1285.5826 | 11 | -0.4 | 643.7983 | 2 | 67.26 | 6 | F6:44023 | NaSa01\_F14.raw |  |  |  |  |  | 2.633E6 |  |  |  |  |  |  |  |  | 1 | 0 | 0 | 0 | 0 | 0 | 1 | 0 | 0 | 0 | 0 | 0 | 0 | 0 | 0 | 394 | 404 |  |  | PEAKS DB |
| K.FWEADGIHGGK.S | N | 59.16 | 1215.5673 | 11 | 0.2 | 608.7910 | 2 | 11.57 | 6 | F6:1961 | NaSa01\_F14.raw |  |  |  |  | 4.0485E5 | 1.8815E7 |  |  |  |  |  |  |  |  | 9 | 0 | 0 | 0 | 0 | 1 | 8 | 0 | 0 | 0 | 0 | 0 | 0 | 0 | 0 | 352 | 362 |  |  | PEAKS DB |
| Y.HLSFMESLK.S | Y | 58.90 | 1090.5481 | 9 | 0.5 | 546.2816 | 2 | 34.90 | 6 | F6:16342 | NaSa01\_F14.raw |  |  |  |  |  | 1.1344E7 |  |  |  |  |  |  |  |  | 1 | 0 | 0 | 0 | 0 | 0 | 1 | 0 | 0 | 0 | 0 | 0 | 0 | 0 | 0 | 233 | 241 |  |  | PEAKS DB |
| A.GLSAAYVLAGAGHK.V | N | 58.41 | 1313.7091 | 14 | 0.3 | 438.9104 | 3 | 32.92 | 6 | F6:14801 | NaSa01\_F14.raw |  |  |  |  |  | 1.2419E6 |  |  |  |  |  |  |  |  | 2 | 0 | 0 | 0 | 0 | 0 | 2 | 0 | 0 | 0 | 0 | 0 | 0 | 0 | 0 | 64 | 77 |  |  | PEAKS DB |
| K.HVVVVGAGMAGL.S | N | 58.15 | 1108.6063 | 12 | 0.5 | 555.3107 | 2 | 51.19 | 6 | F6:30972 | NaSa01\_F14.raw |  |  |  |  | 1.7665E7 | 2.672E8 |  |  |  |  |  |  |  |  | 3 | 0 | 0 | 0 | 0 | 1 | 2 | 0 | 0 | 0 | 0 | 0 | 0 | 0 | 0 | 54 | 65 |  |  | PEAKS DB |
| L.SAAYVLAGAGHKVTLLEASER.V | N | 57.68 | 2142.1433 | 21 | 0.0 | 715.0551 | 3 | 41.78 | 6 | F6:22392 | NaSa01\_F14.raw |  |  |  |  |  | 9.3986E5 |  |  |  |  |  |  |  |  | 1 | 0 | 0 | 0 | 0 | 0 | 1 | 0 | 0 | 0 | 0 | 0 | 0 | 0 | 0 | 66 | 86 |  |  | PEAKS DB |
| K.TC(+57.02)ADIVINDLSLIHDLPKR.E | Y | 56.94 | 2192.1624 | 19 | 0.0 | 549.0479 | 4 | 70.00 | 6 | F6:46365 | NaSa01\_F14.raw |  |  |  |  |  | 6.8137E5 |  |  |  |  |  |  |  |  | 1 | 0 | 0 | 0 | 0 | 0 | 1 | 0 | 0 | 0 | 0 | 0 | 0 | 0 | 0 | 405 | 423 | Carbamidomethylation | C2:Carbamidomethylation:1000.00 | PEAKS DB |
| R.GAVDMIGDLLNEDSSYHLS.F | Y | 56.35 | 2034.9204 | 19 | 1.2 | 1018.4687 | 2 | 95.85 | 6 | F6:67896 | NaSa01\_F14.raw |  |  |  |  |  | 1.2201E7 |  |  |  |  |  |  |  |  | 1 | 0 | 0 | 0 | 0 | 0 | 1 | 0 | 0 | 0 | 0 | 0 | 0 | 0 | 0 | 217 | 235 |  |  | PEAKS DB |
| K.RFDEIVGGFDQLPISMY.Q | N | 56.32 | 1985.9557 | 17 | 1.0 | 993.9861 | 2 | 99.98 | 6 | F6:71504 | NaSa01\_F14.raw |  |  |  |  | 8.8677E6 | 1.2924E8 |  |  |  |  |  |  |  |  | 4 | 0 | 0 | 0 | 0 | 1 | 3 | 0 | 0 | 0 | 0 | 0 | 0 | 0 | 0 | 251 | 267 |  |  | PEAKS DB |
| K.RFDEIVGGFDQLPISM(+15.99)Y.Q | N | 56.17 | 2001.9506 | 17 | 0.5 | 1001.9830 | 2 | 91.18 | 6 | F6:63904 | NaSa01\_F14.raw |  |  |  |  | 1.3345E6 | 1.0983E7 |  |  |  |  |  |  |  |  | 3 | 0 | 0 | 0 | 0 | 1 | 2 | 0 | 0 | 0 | 0 | 0 | 0 | 0 | 0 | 251 | 267 | Oxidation (M) | M16:Oxidation (M):1000.00 | PEAKS DB |
| Q.FQDYFESAAAPVGR.I | Y | 55.52 | 1556.7258 | 14 | 0.1 | 779.3702 | 2 | 53.21 | 6 | F6:32139 | NaSa01\_F14.raw |  |  |  |  |  | 8.9133E5 |  |  |  |  |  |  |  |  | 1 | 0 | 0 | 0 | 0 | 0 | 1 | 0 | 0 | 0 | 0 | 0 | 0 | 0 | 0 | 454 | 467 |  |  | PEAKS DB |
| R.EGWYVNM(+15.99)GPM(+15.99)R.L | N | 54.52 | 1370.5747 | 11 | -0.6 | 686.2942 | 2 | 35.77 | 6 | F6:19242 | NaSa01\_F14.raw |  |  |  |  | 3.3829E6 | 5.1173E7 |  |  |  |  |  |  |  |  | 8 | 0 | 0 | 0 | 0 | 1 | 7 | 0 | 0 | 0 | 0 | 0 | 0 | 0 | 0 | 99 | 109 | Oxidation (M) | M7:Oxidation (M):1000.00;M10:Oxidation (M):1000.00 | PEAKS DB |
| R.FDEIVGGFDQLPISM(+15.99)YQAIAEMVHLNAR.V | N | 54.25 | 3179.5420 | 28 | 1.5 | 1060.8562 | 3 | 104.78 | 6 | F6:75217 | NaSa01\_F14.raw |  |  |  |  |  | 1.2736E6 |  |  |  |  |  |  |  |  | 1 | 0 | 0 | 0 | 0 | 0 | 1 | 0 | 0 | 0 | 0 | 0 | 0 | 0 | 0 | 252 | 279 | Oxidation (M) | M15:Oxidation (M):67.12 | PEAKS DB |
| L.ADDSDFFQALDTK.T | Y | 53.91 | 1471.6466 | 13 | 0.4 | 736.8309 | 2 | 66.91 | 6 | F6:43666 | NaSa01\_F14.raw |  |  |  |  |  | 8.4089E5 |  |  |  |  |  |  |  |  | 1 | 0 | 0 | 0 | 0 | 0 | 1 | 0 | 0 | 0 | 0 | 0 | 0 | 0 | 0 | 392 | 404 |  |  | PEAKS DB |
| K.TC(+57.02)ADIVINDLSLIHD.L | Y | 53.82 | 1697.8295 | 15 | 1.9 | 849.9236 | 2 | 84.53 | 6 | F6:58437 | NaSa01\_F14.raw |  |  |  |  |  | 3.1631E6 |  |  |  |  |  |  |  |  | 1 | 0 | 0 | 0 | 0 | 0 | 1 | 0 | 0 | 0 | 0 | 0 | 0 | 0 | 0 | 405 | 419 | Carbamidomethylation | C2:Carbamidomethylation:1000.00 | PEAKS DB |
| N.DLSLIHDLPK.R | N | 53.54 | 1149.6393 | 10 | 0.2 | 575.8270 | 2 | 52.53 | 6 | F6:31365 | NaSa01\_F14.raw |  |  |  | 3.056E5 |  | 2.4605E6 |  |  |  |  |  |  |  |  | 2 | 0 | 0 | 0 | 1 | 0 | 1 | 0 | 0 | 0 | 0 | 0 | 0 | 0 | 0 | 413 | 422 |  |  | PEAKS DB |
| W.IDSTIMTGLR.A | Y | 53.08 | 1105.5801 | 10 | 0.9 | 553.7978 | 2 | 43.38 | 6 | F6:23683 | NaSa01\_F14.raw |  |  |  |  |  | 8.1441E6 |  |  |  |  |  |  |  |  | 1 | 0 | 0 | 0 | 0 | 0 | 1 | 0 | 0 | 0 | 0 | 0 | 0 | 0 | 0 | 482 | 491 |  |  | PEAKS DB |
| K.RFDEIVGGFDQLPISM.Y | N | 52.02 | 1822.8923 | 16 | 1.0 | 912.4543 | 2 | 96.41 | 6 | F6:68325 | NaSa01\_F14.raw |  |  |  |  | 8.0404E5 | 1.1182E7 |  |  |  |  |  |  |  |  | 2 | 0 | 0 | 0 | 0 | 1 | 1 | 0 | 0 | 0 | 0 | 0 | 0 | 0 | 0 | 251 | 266 |  |  | PEAKS DB |
| K.KFWEADGIHGGK.S | N | 51.66 | 1343.6622 | 12 | -0.1 | 448.8947 | 3 | 11.66 | 5 | F5:2144 | NaSa01\_F13.raw |  |  |  |  | 1.996E6 | 1.1435E7 |  |  |  |  |  |  |  |  | 3 | 0 | 0 | 0 | 0 | 1 | 2 | 0 | 0 | 0 | 0 | 0 | 0 | 0 | 0 | 351 | 362 |  |  | PEAKS DB |
| M.GSITSFTPYQFQDYFESAAAPVGR.I | Y | 51.25 | 2638.2339 | 24 | 3.8 | 880.4219 | 3 | 92.69 | 6 | F6:65150 | NaSa01\_F14.raw |  |  |  |  |  | 7.6022E6 |  |  |  |  |  |  |  |  | 2 | 0 | 0 | 0 | 0 | 0 | 2 | 0 | 0 | 0 | 0 | 0 | 0 | 0 | 0 | 444 | 467 |  |  | PEAKS DB |
| K.VTLLEASER.V | N | 50.90 | 1016.5502 | 9 | 0.2 | 509.2825 | 2 | 25.50 | 6 | F6:8654 | NaSa01\_F14.raw |  |  |  |  |  | 9.4866E6 |  |  |  |  |  |  |  |  | 2 | 0 | 0 | 0 | 0 | 0 | 2 | 0 | 0 | 0 | 0 | 0 | 0 | 0 | 0 | 78 | 86 |  |  | PEAKS DB |
| R.VTYQTPAK.T | N | 50.89 | 906.4811 | 8 | 0.0 | 454.2478 | 2 | 11.43 | 6 | F6:1806 | NaSa01\_F14.raw |  |  |  |  |  | 3.9897E6 |  |  |  |  |  |  |  |  | 1 | 0 | 0 | 0 | 0 | 0 | 1 | 0 | 0 | 0 | 0 | 0 | 0 | 0 | 0 | 292 | 299 |  |  | PEAKS DB |
| Q.DYFESAAAPVGR.I | Y | 50.87 | 1281.5989 | 12 | 2.6 | 641.8083 | 2 | 42.78 | 6 | F6:23130 | NaSa01\_F14.raw |  |  |  |  |  | 0 |  |  |  |  |  |  |  |  | 0 | 0 | 0 | 0 | 0 | 0 | 0 | 0 | 0 | 0 | 0 | 0 | 0 | 0 | 0 | 456 | 467 |  |  | PEAKS DB |
| K.HVVVVGAGMAGLS.A | N | 50.60 | 1195.6383 | 13 | -0.2 | 598.8263 | 2 | 46.44 | 6 | F6:26217 | NaSa01\_F14.raw |  |  |  |  |  | 5.0901E6 |  |  |  |  |  |  |  |  | 1 | 0 | 0 | 0 | 0 | 0 | 1 | 0 | 0 | 0 | 0 | 0 | 0 | 0 | 0 | 54 | 66 |  |  | PEAKS DB |
| K.HVVVVGAGM(+15.99)AGLSAAY.V | N | 50.48 | 1516.7708 | 16 | 0.9 | 759.3934 | 2 | 46.65 | 6 | F6:26724 | NaSa01\_F14.raw |  |  |  |  |  | 2.0076E7 |  |  |  |  |  |  |  |  | 3 | 0 | 0 | 0 | 0 | 0 | 3 | 0 | 0 | 0 | 0 | 0 | 0 | 0 | 0 | 54 | 69 | Oxidation (M) | M9:Oxidation (M):1000.00 | PEAKS DB |
| K.HVVVVGAGMAGLSAA.Y | N | 50.47 | 1337.7125 | 15 | 0.6 | 669.8640 | 2 | 50.72 | 6 | F6:29947 | NaSa01\_F14.raw |  |  |  |  |  | 7.7421E6 |  |  |  |  |  |  |  |  | 1 | 0 | 0 | 0 | 0 | 0 | 1 | 0 | 0 | 0 | 0 | 0 | 0 | 0 | 0 | 54 | 68 |  |  | PEAKS DB |
| R.EIQALC(+57.02)YPSIKK.W | N | 49.90 | 1448.7697 | 12 | 0.7 | 483.9308 | 3 | 30.77 | 6 | F6:13252 | NaSa01\_F14.raw |  |  |  |  |  | 6.9213E6 |  |  |  |  |  |  |  |  | 2 | 0 | 0 | 0 | 0 | 0 | 2 | 0 | 0 | 0 | 0 | 0 | 0 | 0 | 0 | 424 | 435 | Carbamidomethylation | C6:Carbamidomethylation:1000.00 | PEAKS DB |
| K.RFDEIVGGFDQLPISMYQ.A | N | 49.87 | 2114.0142 | 18 | 1.4 | 1058.0159 | 2 | 96.41 | 6 | F6:68423 | NaSa01\_F14.raw |  |  |  |  |  | 4.0398E6 |  |  |  |  |  |  |  |  | 1 | 0 | 0 | 0 | 0 | 0 | 1 | 0 | 0 | 0 | 0 | 0 | 0 | 0 | 0 | 251 | 268 |  |  | PEAKS DB |
| H.GWIDSTIMTGLR.A | Y | 49.75 | 1348.6809 | 12 | 0.1 | 675.3478 | 2 | 78.70 | 6 | F6:53478 | NaSa01\_F14.raw |  |  |  |  |  | 2.7681E6 |  |  |  |  |  |  |  |  | 2 | 0 | 0 | 0 | 0 | 0 | 2 | 0 | 0 | 0 | 0 | 0 | 0 | 0 | 0 | 480 | 491 |  |  | PEAKS DB |
| I.YFEPPLPPK.K | N | 49.42 | 1086.5750 | 9 | 6.0 | 544.2952 | 2 | 42.50 | 8 | F8:25506 | NaSa01\_F3.raw |  |  |  |  |  |  |  | 6.3004E5 |  |  |  |  |  |  | 1 | 0 | 0 | 0 | 0 | 0 | 0 | 0 | 1 | 0 | 0 | 0 | 0 | 0 | 0 | 320 | 328 |  |  | PEAKS DB |
| K.HVVVVGAGMAGLSA.A | N | 48.82 | 1266.6754 | 14 | 0.3 | 634.3452 | 2 | 49.72 | 6 | F6:29252 | NaSa01\_F14.raw |  |  |  |  |  | 3.9558E6 |  |  |  |  |  |  |  |  | 1 | 0 | 0 | 0 | 0 | 0 | 1 | 0 | 0 | 0 | 0 | 0 | 0 | 0 | 0 | 54 | 67 |  |  | PEAKS DB |
| K.STTDLPSR.F | N | 48.78 | 875.4348 | 8 | 0.1 | 438.7247 | 2 | 11.44 | 6 | F6:1817 | NaSa01\_F14.raw |  |  |  |  |  | 1.0568E6 |  |  |  |  |  |  |  |  | 1 | 0 | 0 | 0 | 0 | 0 | 1 | 0 | 0 | 0 | 0 | 0 | 0 | 0 | 0 | 363 | 370 |  |  | PEAKS DB |
| R.GAVDMIGDLLNEDSSY.H | N | 48.29 | 1697.7454 | 16 | 1.7 | 849.8814 | 2 | 107.90 | 6 | F6:77609 | NaSa01\_F14.raw |  |  |  |  |  | 1.6853E7 |  |  |  |  |  |  |  |  | 1 | 0 | 0 | 0 | 0 | 0 | 1 | 0 | 0 | 0 | 0 | 0 | 0 | 0 | 0 | 217 | 232 |  |  | PEAKS DB |
| R.FDEIVGGFDQLPISM.Y | N | 48.24 | 1666.7913 | 15 | 1.2 | 834.4039 | 2 | 112.47 | 6 | F6:80862 | NaSa01\_F14.raw |  |  |  |  |  | 8.4104E6 |  |  |  |  |  |  |  |  | 1 | 0 | 0 | 0 | 0 | 0 | 1 | 0 | 0 | 0 | 0 | 0 | 0 | 0 | 0 | 252 | 266 |  |  | PEAKS DB |
| Y.QAIAEM(+15.99)VHLNAR.V | N | 47.05 | 1367.6980 | 12 | 0.8 | 456.9070 | 3 | 31.64 | 6 | F6:13767 | NaSa01\_F14.raw |  |  |  |  |  | 2.4624E6 |  |  |  |  |  |  |  |  | 3 | 0 | 0 | 0 | 0 | 0 | 3 | 0 | 0 | 0 | 0 | 0 | 0 | 0 | 0 | 268 | 279 | Oxidation (M) | M6:Oxidation (M):1000.00 | PEAKS DB |
| R.FHGWIDSTIM.T | Y | 46.94 | 1205.5540 | 10 | 0.7 | 603.7847 | 2 | 69.86 | 6 | F6:46616 | NaSa01\_F14.raw |  |  |  |  |  | 2.4473E7 |  |  |  |  |  |  |  |  | 1 | 0 | 0 | 0 | 0 | 0 | 1 | 0 | 0 | 0 | 0 | 0 | 0 | 0 | 0 | 478 | 487 |  |  | PEAKS DB |
| Y.TMGSITSFTPYQFQDYFESAAAPVGR.I | Y | 46.62 | 2870.3220 | 26 | 0.3 | 957.7816 | 3 | 97.15 | 6 | F6:68946 | NaSa01\_F14.raw |  |  |  |  |  | 1.4838E7 |  |  |  |  |  |  |  |  | 2 | 0 | 0 | 0 | 0 | 0 | 2 | 0 | 0 | 0 | 0 | 0 | 0 | 0 | 0 | 442 | 467 |  |  | PEAKS DB |
| K.TFVTADYVIVC(+57.02).S | Y | 46.30 | 1286.6217 | 11 | 0.6 | 644.3185 | 2 | 81.27 | 6 | F6:56092 | NaSa01\_F14.raw |  |  |  |  | 2.639E6 | 3.2724E7 |  |  |  |  |  |  |  |  | 2 | 0 | 0 | 0 | 0 | 1 | 1 | 0 | 0 | 0 | 0 | 0 | 0 | 0 | 0 | 300 | 310 | Carbamidomethylation | C11:Carbamidomethylation:1000.00 | PEAKS DB |
| K.KDPSLLKYPVKPSEEGK.S | Y | 46.30 | 1914.0461 | 17 | -0.5 | 479.5186 | 4 | 11.65 | 5 | F5:2119 | NaSa01\_F13.raw |  |  |  |  | 1.573E5 |  |  |  |  |  |  |  |  |  | 1 | 0 | 0 | 0 | 0 | 1 | 0 | 0 | 0 | 0 | 0 | 0 | 0 | 0 | 0 | 154 | 170 |  |  | PEAKS DB |
| A.DDSDFFQALDTK.T | Y | 44.68 | 1400.6095 | 12 | 1.0 | 701.3127 | 2 | 73.11 | 4 | F4:50624 | NaSa01\_F12.raw |  |  |  | 6.4171E5 |  |  |  |  |  |  |  |  |  |  | 1 | 0 | 0 | 0 | 1 | 0 | 0 | 0 | 0 | 0 | 0 | 0 | 0 | 0 | 0 | 393 | 404 |  |  | PEAKS DB |
| R.FDEIVGGFDQLPISMY.Q | N | 44.41 | 1829.8545 | 16 | 0.2 | 915.9347 | 2 | 116.62 | 6 | F6:83518 | NaSa01\_F14.raw |  |  |  |  |  | 6.1039E7 |  |  |  |  |  |  |  |  | 1 | 0 | 0 | 0 | 0 | 0 | 1 | 0 | 0 | 0 | 0 | 0 | 0 | 0 | 0 | 252 | 267 |  |  | PEAKS DB |
| A.GM(+15.99)AGLSAAYVLAGAGHK.V | N | 43.41 | 1588.8031 | 17 | 0.5 | 530.6086 | 3 | 39.38 | 6 | F6:20374 | NaSa01\_F14.raw |  |  |  |  |  | 2.4697E5 |  |  |  |  |  |  |  |  | 1 | 0 | 0 | 0 | 0 | 0 | 1 | 0 | 0 | 0 | 0 | 0 | 0 | 0 | 0 | 61 | 77 | Oxidation (M) | M2:Oxidation (M):1000.00 | PEAKS DB |
| G.SITSFTPYQFQDYFESAAAPVGR.I | Y | 43.36 | 2581.2124 | 23 | 0.0 | 1291.6135 | 2 | 92.73 | 6 | F6:65352 | NaSa01\_F14.raw |  |  |  |  |  | 4.3026E5 |  |  |  |  |  |  |  |  | 1 | 0 | 0 | 0 | 0 | 0 | 1 | 0 | 0 | 0 | 0 | 0 | 0 | 0 | 0 | 445 | 467 |  |  | PEAKS DB |
| R.FDEIVGGFDQLPISMYQ.A | N | 43.29 | 1957.9131 | 17 | 0.8 | 979.9646 | 2 | 111.22 | 6 | F6:80041 | NaSa01\_F14.raw |  |  |  |  |  | 3.0744E6 |  |  |  |  |  |  |  |  | 1 | 0 | 0 | 0 | 0 | 0 | 1 | 0 | 0 | 0 | 0 | 0 | 0 | 0 | 0 | 252 | 268 |  |  | PEAKS DB |
| K.HVVVVGAGM(+15.99)AGL.S | N | 43.09 | 1124.6012 | 12 | 0.5 | 563.3082 | 2 | 50.23 | 6 | F6:30439 | NaSa01\_F14.raw |  |  |  |  | 3.0223E6 | 1.1076E7 |  |  |  |  |  |  |  |  | 3 | 0 | 0 | 0 | 0 | 1 | 2 | 0 | 0 | 0 | 0 | 0 | 0 | 0 | 0 | 54 | 65 | Oxidation (M) | M9:Oxidation (M):1000.00 | PEAKS DB |
| E.IQALC(+57.02)YPSIK.K | N | 42.76 | 1191.6322 | 10 | -0.9 | 596.8228 | 2 | 42.19 | 6 | F6:22642 | NaSa01\_F14.raw |  |  |  |  |  | 1.1323E6 |  |  |  |  |  |  |  |  | 1 | 0 | 0 | 0 | 0 | 0 | 1 | 0 | 0 | 0 | 0 | 0 | 0 | 0 | 0 | 425 | 434 | Carbamidomethylation | C5:Carbamidomethylation:1000.00 | PEAKS DB |
| Y.TM(+15.99)GSITSFTPYQFQDYFESAAAPVGR.I | Y | 42.42 | 2886.3169 | 26 | 0.9 | 963.1138 | 3 | 92.52 | 6 | F6:65107 | NaSa01\_F14.raw |  |  |  |  |  | 1.1735E6 |  |  |  |  |  |  |  |  | 1 | 0 | 0 | 0 | 0 | 0 | 1 | 0 | 0 | 0 | 0 | 0 | 0 | 0 | 0 | 442 | 467 | Oxidation (M) | M2:Oxidation (M):1000.00 | PEAKS DB |
| V.GAGMAGLSAAYVLAGAGHK.V | N | 41.36 | 1700.8668 | 19 | -0.1 | 567.9628 | 3 | 53.80 | 6 | F6:32769 | NaSa01\_F14.raw |  |  |  |  |  | 3.5832E5 |  |  |  |  |  |  |  |  | 1 | 0 | 0 | 0 | 0 | 0 | 1 | 0 | 0 | 0 | 0 | 0 | 0 | 0 | 0 | 59 | 77 |  |  | PEAKS DB |
| R.FHGWIDST.I | Y | 40.89 | 961.4294 | 8 | -0.2 | 481.7219 | 2 | 38.55 | 6 | F6:19670 | NaSa01\_F14.raw |  |  |  |  |  | 1.042E6 |  |  |  |  |  |  |  |  | 1 | 0 | 0 | 0 | 0 | 0 | 1 | 0 | 0 | 0 | 0 | 0 | 0 | 0 | 0 | 478 | 485 |  |  | PEAKS DB |
| F.HGWIDSTIMTGLR.A | Y | 40.51 | 1485.7399 | 13 | -0.5 | 496.2537 | 3 | 60.76 | 6 | F6:38590 | NaSa01\_F14.raw |  |  |  |  |  | 1.5775E5 |  |  |  |  |  |  |  |  | 1 | 0 | 0 | 0 | 0 | 0 | 1 | 0 | 0 | 0 | 0 | 0 | 0 | 0 | 0 | 479 | 491 |  |  | PEAKS DB |
| G.MAGLSAAYVLAGAGHK.V | N | 39.83 | 1515.7867 | 16 | 0.3 | 506.2697 | 3 | 47.39 | 6 | F6:27145 | NaSa01\_F14.raw |  |  |  |  |  | 5.9167E5 |  |  |  |  |  |  |  |  | 1 | 0 | 0 | 0 | 0 | 0 | 1 | 0 | 0 | 0 | 0 | 0 | 0 | 0 | 0 | 62 | 77 |  |  | PEAKS DB |
| E.GWYVNMGPMR.L | N | 38.80 | 1209.5424 | 10 | 0.9 | 605.7790 | 2 | 55.07 | 6 | F6:33759 | NaSa01\_F14.raw |  |  |  |  |  | 8.4887E5 |  |  |  |  |  |  |  |  | 1 | 0 | 0 | 0 | 0 | 0 | 1 | 0 | 0 | 0 | 0 | 0 | 0 | 0 | 0 | 100 | 109 |  |  | PEAKS DB |
| R.FDEIVGGFDQLPISMYQA.I | N | 38.57 | 2028.9502 | 18 | 1.1 | 1015.4835 | 2 | 112.01 | 6 | F6:80759 | NaSa01\_F14.raw |  |  |  |  |  | 1.2262E7 |  |  |  |  |  |  |  |  | 1 | 0 | 0 | 0 | 0 | 0 | 1 | 0 | 0 | 0 | 0 | 0 | 0 | 0 | 0 | 252 | 269 |  |  | PEAKS DB |
| R.FDEIVGGFDQLPISM(+15.99)Y.Q | N | 38.39 | 1845.8495 | 16 | 0.4 | 923.9324 | 2 | 105.35 | 6 | F6:75636 | NaSa01\_F14.raw |  |  |  |  |  | 4.6489E6 |  |  |  |  |  |  |  |  | 1 | 0 | 0 | 0 | 0 | 0 | 1 | 0 | 0 | 0 | 0 | 0 | 0 | 0 | 0 | 252 | 267 | Oxidation (M) | M15:Oxidation (M):1000.00 | PEAKS DB |
| F.VTADYVIVC(+57.02)STSR.A | N | 38.16 | 1469.7184 | 13 | 0.8 | 490.9138 | 3 | 38.41 | 6 | F6:19506 | NaSa01\_F14.raw |  |  |  |  |  | 3.3852E5 |  |  |  |  |  |  |  |  | 1 | 0 | 0 | 0 | 0 | 0 | 1 | 0 | 0 | 0 | 0 | 0 | 0 | 0 | 0 | 302 | 314 | Carbamidomethylation | C9:Carbamidomethylation:1000.00 | PEAKS DB |
| total 98 peptides |  |  |  |  |  |  |  |  |  |  |  |  |  |  |  |  |  |  |  |  |  |  |  |  |  |  |  |  |  |  |  |  |  |  |  |  |  |  |  |  |  |  |  |  |  |
| --- | --- | --- | --- | --- | --- | --- | --- | --- | --- | --- | --- | --- | --- | --- | --- | --- | --- | --- | --- | --- | --- | --- | --- | --- | --- | --- | --- | --- | --- | --- | --- | --- | --- | --- | --- | --- | --- | --- | --- | --- | --- | --- | --- | --- | --- |

AVX27607.1

back to list

  

| Protein Coverage
| Supporting Peptides
|

Protein Coverage:

Supporting Peptides:

| Peptide | Uniq | -10lgP | Mass | Length | ppm | m/z | z | RT | Fraction | Scan | Source File | Area F1 | Area F10 | Area F11 | Area F12 | Area F13 | Area F14 | Area F2 | Area F3 | Area F4 | Area F5 | Area F6 | Area F7 | Area F8 | Area F9 | #Feature | #Feature F1 | #Feature F10 | #Feature F11 | #Feature F12 | #Feature F13 | #Feature F14 | #Feature F2 | #Feature F3 | #Feature F4 | #Feature F5 | #Feature F6 | #Feature F7 | #Feature F8 | #Feature F9 | Start | End | PTM | AScore | Found By |
| --- | --- | --- | --- | --- | --- | --- | --- | --- | --- | --- | --- | --- | --- | --- | --- | --- | --- | --- | --- | --- | --- | --- | --- | --- | --- | --- | --- | --- | --- | --- | --- | --- | --- | --- | --- | --- | --- | --- | --- | --- | --- | --- | --- | --- | --- |
| K.HVVVVGAGMAGLSAAYVLAGAGHK.V | N | 107.42 | 2234.1992 | 24 | 1.2 | 745.7412 | 3 | 65.01 | 6 | F6:42957 | NaSa01\_F14.raw |  |  |  | 6.8911E6 | 4.2938E7 | 6.5431E8 |  |  |  |  |  |  |  |  | 25 | 0 | 0 | 0 | 2 | 2 | 21 | 0 | 0 | 0 | 0 | 0 | 0 | 0 | 0 | 54 | 77 |  |  | PEAKS DB |
| K.TC(+57.02)ADIVINDLSLIHDLPK.R | Y | 96.41 | 2036.0612 | 18 | 1.5 | 1019.0394 | 2 | 82.06 | 6 | F6:56577 | NaSa01\_F14.raw |  |  |  | 4.4983E6 | 7.6898E7 | 5.3211E8 |  |  |  |  |  |  |  |  | 13 | 0 | 0 | 0 | 1 | 4 | 8 | 0 | 0 | 0 | 0 | 0 | 0 | 0 | 0 | 405 | 422 | Carbamidomethylation | C2:Carbamidomethylation:1000.00 | PEAKS DB |
| K.HVVVVGAGM(+15.99)AGLSAAYVLAGAGHK.V | N | 95.06 | 2250.1943 | 24 | 0.9 | 751.0728 | 3 | 53.09 | 6 | F6:32085 | NaSa01\_F14.raw |  |  |  |  | 1.6389E7 | 1.5623E8 |  |  |  |  |  |  |  |  | 5 | 0 | 0 | 0 | 0 | 2 | 3 | 0 | 0 | 0 | 0 | 0 | 0 | 0 | 0 | 54 | 77 | Oxidation (M) | M9:Oxidation (M):1000.00 | PEAKS DB |
| F.TPYQFQDYFESAAAPVGR.I | Y | 94.96 | 2045.9482 | 18 | -0.1 | 1023.9813 | 2 | 72.54 | 6 | F6:48623 | NaSa01\_F14.raw |  |  |  |  | 1.3361E7 | 2.9427E8 |  |  |  |  |  |  |  |  | 3 | 0 | 0 | 0 | 0 | 1 | 2 | 0 | 0 | 0 | 0 | 0 | 0 | 0 | 0 | 450 | 467 |  |  | PEAKS DB |
| K.TFVTADYVIVC(+57.02)STSR.A | Y | 93.18 | 1717.8345 | 15 | 2.2 | 859.9264 | 2 | 60.90 | 6 | F6:39859 | NaSa01\_F14.raw |  |  |  |  | 5.5865E7 | 4.4428E8 |  |  |  |  |  |  | 5.5981E5 |  | 20 | 0 | 0 | 0 | 0 | 3 | 16 | 0 | 0 | 0 | 0 | 0 | 0 | 1 | 0 | 300 | 314 | Carbamidomethylation | C11:Carbamidomethylation:1000.00 | PEAKS DB |
| K.LNEFFQENENAWYYINNIR.K | Y | 90.36 | 2476.1448 | 19 | 0.7 | 1239.0806 | 2 | 91.17 | 6 | F6:64025 | NaSa01\_F14.raw |  |  |  |  | 6.6266E5 | 7.9615E7 |  |  |  |  |  |  |  |  | 3 | 0 | 0 | 0 | 0 | 1 | 2 | 0 | 0 | 0 | 0 | 0 | 0 | 0 | 0 | 128 | 146 |  |  | PEAKS DB |
| K.YTMGSITSFTPYQFQDYFESAAAPVGR.I | Y | 82.61 | 3033.3855 | 27 | 0.8 | 1012.1366 | 3 | 100.62 | 6 | F6:71855 | NaSa01\_F14.raw |  |  |  |  | 1.0072E7 | 2.7658E8 |  |  |  |  |  |  |  |  | 5 | 0 | 0 | 0 | 0 | 2 | 3 | 0 | 0 | 0 | 0 | 0 | 0 | 0 | 0 | 441 | 467 |  |  | PEAKS DB |
| R.FHGWIDSTIM(+15.99)TGLR.A | Y | 82.52 | 1648.8031 | 14 | 0.3 | 825.4091 | 2 | 56.10 | 6 | F6:34934 | NaSa01\_F14.raw |  |  |  |  | 1.0724E7 | 1.9786E8 |  |  |  |  |  |  |  |  | 15 | 0 | 0 | 0 | 0 | 2 | 13 | 0 | 0 | 0 | 0 | 0 | 0 | 0 | 0 | 478 | 491 | Oxidation (M) | M10:Oxidation (M):1000.00 | PEAKS DB |
| Y.VLADDSDFFQALDTK.T | Y | 82.09 | 1683.7991 | 15 | 0.1 | 842.9069 | 2 | 78.47 | 6 | F6:53277 | NaSa01\_F14.raw |  |  |  | 2.5191E5 | 2.6921E8 | 3.2203E9 |  |  |  |  |  |  |  |  | 9 | 0 | 0 | 0 | 1 | 2 | 6 | 0 | 0 | 0 | 0 | 0 | 0 | 0 | 0 | 390 | 404 |  |  | PEAKS DB |
| R.FHGWIDSTIMTGLR.A | Y | 81.76 | 1632.8082 | 14 | 0.3 | 817.4116 | 2 | 68.30 | 6 | F6:44954 | NaSa01\_F14.raw |  | 2.7707E6 |  | 6.8303E6 | 2.7763E7 | 7.2536E8 |  |  |  |  |  |  |  |  | 28 | 0 | 1 | 0 | 3 | 2 | 22 | 0 | 0 | 0 | 0 | 0 | 0 | 0 | 0 | 478 | 491 |  |  | PEAKS DB |
| M.AGLSAAYVLAGAGHK.V | N | 79.84 | 1384.7462 | 15 | 0.5 | 693.3807 | 2 | 36.23 | 6 | F6:17690 | NaSa01\_F14.raw |  |  |  |  |  | 4.4211E7 |  |  |  |  |  |  |  |  | 2 | 0 | 0 | 0 | 0 | 0 | 2 | 0 | 0 | 0 | 0 | 0 | 0 | 0 | 0 | 63 | 77 |  |  | PEAKS DB |
| M.AYVLADDSDFFQALDTK.T | Y | 79.60 | 1917.8995 | 17 | -0.3 | 959.9567 | 2 | 85.82 | 6 | F6:59496 | NaSa01\_F14.raw |  |  |  |  | 3.4939E6 | 3.9274E7 |  |  |  |  |  |  |  |  | 2 | 0 | 0 | 0 | 0 | 1 | 1 | 0 | 0 | 0 | 0 | 0 | 0 | 0 | 0 | 388 | 404 |  |  | PEAKS DB |
| K.REIQALC(+57.02)YPSIK.K | N | 77.78 | 1476.7759 | 12 | 0.2 | 493.2660 | 3 | 32.82 | 6 | F6:14068 | NaSa01\_F14.raw |  |  |  |  | 3.5291E7 | 3.7818E8 |  |  |  |  |  |  |  |  | 8 | 0 | 0 | 0 | 0 | 2 | 6 | 0 | 0 | 0 | 0 | 0 | 0 | 0 | 0 | 423 | 434 | Carbamidomethylation | C7:Carbamidomethylation:1000.00 | PEAKS DB |
| S.ITSFTPYQFQDYFESAAAPVGR.I | Y | 77.75 | 2494.1804 | 22 | 0.4 | 1248.0980 | 2 | 89.99 | 6 | F6:63003 | NaSa01\_F14.raw |  |  |  |  |  | 8.4867E6 |  |  |  |  |  |  |  |  | 1 | 0 | 0 | 0 | 0 | 0 | 1 | 0 | 0 | 0 | 0 | 0 | 0 | 0 | 0 | 446 | 467 |  |  | PEAKS DB |
| R.EIQALC(+57.02)YPSIK.K | N | 77.61 | 1320.6747 | 11 | 0.3 | 661.3448 | 2 | 47.88 | 6 | F6:27511 | NaSa01\_F14.raw |  |  |  | 3.0826E6 | 1.353E8 | 1.8533E9 |  |  |  |  |  |  |  |  | 18 | 0 | 0 | 0 | 1 | 1 | 16 | 0 | 0 | 0 | 0 | 0 | 0 | 0 | 0 | 424 | 434 | Carbamidomethylation | C6:Carbamidomethylation:1000.00 | PEAKS DB |
| A.GMAGLSAAYVLAGAGHK.V | N | 77.31 | 1572.8082 | 17 | 0.2 | 525.2768 | 3 | 50.77 | 6 | F6:29981 | NaSa01\_F14.raw |  |  |  |  | 5.5361E5 | 1.0582E7 |  |  |  |  |  |  |  |  | 3 | 0 | 0 | 0 | 0 | 1 | 2 | 0 | 0 | 0 | 0 | 0 | 0 | 0 | 0 | 61 | 77 |  |  | PEAKS DB |
| Y.QFQDYFESAAAPVGR.I | Y | 76.00 | 1684.7844 | 15 | 0.6 | 843.4000 | 2 | 59.29 | 6 | F6:37194 | NaSa01\_F14.raw |  |  |  | 8.1084E5 | 8.279E7 | 1.2226E9 |  |  |  |  |  |  |  |  | 13 | 0 | 0 | 0 | 1 | 3 | 9 | 0 | 0 | 0 | 0 | 0 | 0 | 0 | 0 | 453 | 467 |  |  | PEAKS DB |
| R.IYFEPPLPPKK.A | N | 74.78 | 1327.7539 | 11 | 0.3 | 664.8844 | 2 | 39.91 | 6 | F6:21060 | NaSa01\_F14.raw |  |  |  |  | 5.0279E7 | 4.7146E8 |  |  |  |  |  |  |  |  | 4 | 0 | 0 | 0 | 0 | 2 | 2 | 0 | 0 | 0 | 0 | 0 | 0 | 0 | 0 | 319 | 329 |  |  | PEAKS DB |
| R.EGWYVNMGPM(+15.99)R.L | N | 73.07 | 1354.5798 | 11 | 0.4 | 678.2975 | 2 | 56.26 | 6 | F6:34728 | NaSa01\_F14.raw |  |  |  |  | 3.9735E7 | 2.4523E8 |  |  |  |  |  |  |  |  | 7 | 0 | 0 | 0 | 0 | 4 | 3 | 0 | 0 | 0 | 0 | 0 | 0 | 0 | 0 | 99 | 109 | Oxidation (M) | M10:Oxidation (M):51.69 | PEAKS DB |
| R.IYFEPPLPPK.K | N | 72.12 | 1199.6589 | 10 | -0.1 | 600.8367 | 2 | 57.48 | 6 | F6:38014 | NaSa01\_F14.raw |  |  |  | 1.5815E6 | 1.7195E8 | 2.5686E9 |  |  | 1.0411E6 |  |  |  |  |  | 22 | 0 | 0 | 0 | 1 | 2 | 18 | 0 | 0 | 1 | 0 | 0 | 0 | 0 | 0 | 319 | 328 |  |  | PEAKS DB |
| R.EGWYVNMGPMR.L | N | 71.70 | 1338.5850 | 11 | 0.0 | 670.2997 | 2 | 59.12 | 6 | F6:37086 | NaSa01\_F14.raw |  |  |  |  | 1.3719E8 | 2.0017E9 |  |  |  |  |  |  |  |  | 6 | 0 | 0 | 0 | 0 | 3 | 3 | 0 | 0 | 0 | 0 | 0 | 0 | 0 | 0 | 99 | 109 |  |  | PEAKS DB |
| A.YVLADDSDFFQALDTK.T | Y | 71.22 | 1846.8624 | 16 | 0.6 | 924.4391 | 2 | 84.46 | 6 | F6:58413 | NaSa01\_F14.raw |  |  |  |  |  | 2.5549E6 |  |  |  |  |  |  |  |  | 1 | 0 | 0 | 0 | 0 | 0 | 1 | 0 | 0 | 0 | 0 | 0 | 0 | 0 | 0 | 389 | 404 |  |  | PEAKS DB |
| M.YQAIAEMVHLNAR.V | N | 70.47 | 1514.7664 | 13 | 0.6 | 505.9297 | 3 | 42.75 | 6 | F6:23121 | NaSa01\_F14.raw |  |  |  |  |  | 6.4909E6 |  |  |  |  |  |  |  |  | 2 | 0 | 0 | 0 | 0 | 0 | 2 | 0 | 0 | 0 | 0 | 0 | 0 | 0 | 0 | 267 | 279 |  |  | PEAKS DB |
| A.DIVINDLSLIHDLPK.R | Y | 70.43 | 1703.9458 | 15 | 0.0 | 568.9892 | 3 | 80.66 | 6 | F6:55094 | NaSa01\_F14.raw |  |  |  | 4.3515E6 | 5.5307E6 | 1.0082E7 |  |  |  |  |  | 7.9457E5 |  |  | 7 | 0 | 0 | 0 | 2 | 2 | 2 | 0 | 0 | 0 | 0 | 0 | 1 | 0 | 0 | 408 | 422 |  |  | PEAKS DB |
| K.YTM(+15.99)GSITSFTPYQFQDYFESAAAPVGR.I | Y | 68.73 | 3049.3804 | 27 | 1.3 | 1017.4688 | 3 | 94.25 | 6 | F6:66450 | NaSa01\_F14.raw |  |  |  | 7.1363E5 | 6.5743E6 | 9.733E7 |  |  |  |  |  |  |  |  | 4 | 0 | 0 | 0 | 1 | 1 | 2 | 0 | 0 | 0 | 0 | 0 | 0 | 0 | 0 | 441 | 467 | Oxidation (M) | M3:Oxidation (M):1000.00 | PEAKS DB |
| R.EGWYVNM(+15.99)GPMR.L | N | 67.93 | 1354.5798 | 11 | 0.8 | 678.2977 | 2 | 41.16 | 6 | F6:22021 | NaSa01\_F14.raw |  |  |  | 5.0564E5 | 1.7067E7 | 2.1752E8 |  |  |  |  |  |  |  |  | 5 | 0 | 0 | 0 | 1 | 2 | 2 | 0 | 0 | 0 | 0 | 0 | 0 | 0 | 0 | 99 | 109 | Oxidation (M) | M7:Oxidation (M):39.30 | PEAKS DB |
| K.SASQLYQESLR.K | Y | 67.67 | 1280.6360 | 11 | 0.1 | 641.3253 | 2 | 24.71 | 6 | F6:8085 | NaSa01\_F14.raw |  |  |  |  | 1.1171E8 | 7.0099E6 |  |  |  |  |  |  |  |  | 2 | 0 | 0 | 0 | 0 | 1 | 1 | 0 | 0 | 0 | 0 | 0 | 0 | 0 | 0 | 171 | 181 |  |  | PEAKS DB |
| K.YDSYSTKEYLIK.E | N | 67.50 | 1508.7399 | 12 | 0.6 | 503.9209 | 3 | 26.71 | 6 | F6:10035 | NaSa01\_F14.raw |  |  |  |  |  | 7.9504E6 |  |  |  |  |  |  |  |  | 2 | 0 | 0 | 0 | 0 | 0 | 2 | 0 | 0 | 0 | 0 | 0 | 0 | 0 | 0 | 199 | 210 |  |  | PEAKS DB |
| K.HVVVVGAGMAGLSAAY.V | N | 67.41 | 1500.7759 | 16 | 0.2 | 751.3954 | 2 | 68.19 | 6 | F6:44843 | NaSa01\_F14.raw |  |  |  |  | 2.067E7 | 2.0687E8 |  |  |  |  |  |  |  |  | 10 | 0 | 0 | 0 | 0 | 1 | 9 | 0 | 0 | 0 | 0 | 0 | 0 | 0 | 0 | 54 | 69 |  |  | PEAKS DB |
| Y.QAIAEMVHLNAR.V | N | 66.25 | 1351.7030 | 12 | -0.4 | 676.8585 | 2 | 31.55 | 6 | F6:13654 | NaSa01\_F14.raw |  |  |  |  |  | 7.8157E7 |  |  |  |  |  |  |  |  | 2 | 0 | 0 | 0 | 0 | 0 | 2 | 0 | 0 | 0 | 0 | 0 | 0 | 0 | 0 | 268 | 279 |  |  | PEAKS DB |
| R.IHFAGEYTGR.F | Y | 65.92 | 1149.5566 | 10 | -0.7 | 384.1926 | 3 | 11.57 | 6 | F6:1949 | NaSa01\_F14.raw |  |  |  |  | 1.5757E6 | 2.2033E7 |  |  |  |  |  |  |  |  | 9 | 0 | 0 | 0 | 0 | 3 | 6 | 0 | 0 | 0 | 0 | 0 | 0 | 0 | 0 | 468 | 477 |  |  | PEAKS DB |
| T.SFTPYQFQDYFESAAAPVGR.I | Y | 65.14 | 2280.0486 | 20 | 0.8 | 1141.0325 | 2 | 85.41 | 6 | F6:59216 | NaSa01\_F14.raw |  |  |  |  |  | 1.7558E6 |  |  |  |  |  |  |  |  | 1 | 0 | 0 | 0 | 0 | 0 | 1 | 0 | 0 | 0 | 0 | 0 | 0 | 0 | 0 | 448 | 467 |  |  | PEAKS DB |
| K.FWEADGIHGGKSTTDLPSR.F | N | 64.97 | 2072.9915 | 19 | 0.7 | 692.0049 | 3 | 29.66 | 6 | F6:12168 | NaSa01\_F14.raw |  |  |  |  |  | 2.4928E6 |  |  |  |  |  |  |  |  | 1 | 0 | 0 | 0 | 0 | 0 | 1 | 0 | 0 | 0 | 0 | 0 | 0 | 0 | 0 | 352 | 370 |  |  | PEAKS DB |
| R.RIYFEPPLPPKK.A | N | 64.76 | 1483.8550 | 12 | -0.5 | 495.6253 | 3 | 28.82 | 6 | F6:11398 | NaSa01\_F14.raw |  |  |  |  | 2.2402E7 | 1.4186E8 |  |  |  |  |  |  |  |  | 5 | 0 | 0 | 0 | 0 | 2 | 3 | 0 | 0 | 0 | 0 | 0 | 0 | 0 | 0 | 318 | 329 |  |  | PEAKS DB |
| L.SAAYVLAGAGHK.V | N | 63.01 | 1143.6036 | 12 | -1.1 | 572.8085 | 2 | 11.49 | 6 | F6:1894 | NaSa01\_F14.raw |  |  |  |  |  | 4.4228E6 |  |  |  |  |  |  |  |  | 2 | 0 | 0 | 0 | 0 | 0 | 2 | 0 | 0 | 0 | 0 | 0 | 0 | 0 | 0 | 66 | 77 |  |  | PEAKS DB |
| K.SDALFSYEK.R | N | 62.97 | 1058.4919 | 9 | 0.4 | 530.2535 | 2 | 34.28 | 6 | F6:16290 | NaSa01\_F14.raw |  |  |  |  | 5.08E7 | 1.3053E7 |  |  |  |  |  |  |  |  | 4 | 0 | 0 | 0 | 0 | 1 | 3 | 0 | 0 | 0 | 0 | 0 | 0 | 0 | 0 | 242 | 250 |  |  | PEAKS DB |
| F.QDYFESAAAPVGR.I | Y | 62.52 | 1409.6575 | 13 | 0.1 | 705.8361 | 2 | 39.67 | 6 | F6:20451 | NaSa01\_F14.raw |  |  |  |  |  | 3.65E6 |  |  |  |  |  |  |  |  | 1 | 0 | 0 | 0 | 0 | 0 | 1 | 0 | 0 | 0 | 0 | 0 | 0 | 0 | 0 | 455 | 467 |  |  | PEAKS DB |
| K.YPVKPSEEGK.S | N | 62.09 | 1132.5764 | 10 | 1.6 | 567.2964 | 2 | 11.44 | 6 | F6:1813 | NaSa01\_F14.raw |  |  |  |  |  | 1.8552E6 |  |  |  |  |  |  |  |  | 2 | 0 | 0 | 0 | 0 | 0 | 2 | 0 | 0 | 0 | 0 | 0 | 0 | 0 | 0 | 161 | 170 |  |  | PEAKS DB |
| D.SDFFQALDTK.T | Y | 61.64 | 1170.5557 | 10 | 0.6 | 586.2855 | 2 | 57.84 | 6 | F6:36028 | NaSa01\_F14.raw |  |  |  |  |  | 4.304E6 |  |  |  |  |  |  |  |  | 1 | 0 | 0 | 0 | 0 | 0 | 1 | 0 | 0 | 0 | 0 | 0 | 0 | 0 | 0 | 395 | 404 |  |  | PEAKS DB |
| R.RIYFEPPLPPK.K | N | 60.85 | 1355.7601 | 11 | 0.4 | 452.9275 | 3 | 43.97 | 6 | F6:24986 | NaSa01\_F14.raw |  |  |  |  | 9.9689E7 | 1.1129E9 |  |  |  |  |  |  |  |  | 4 | 0 | 0 | 0 | 0 | 2 | 2 | 0 | 0 | 0 | 0 | 0 | 0 | 0 | 0 | 318 | 328 |  |  | PEAKS DB |
| K.RFDEIVGGFDQLPISMYQA.I | N | 60.53 | 2185.0513 | 19 | -0.2 | 1093.5327 | 2 | 97.41 | 6 | F6:69051 | NaSa01\_F14.raw |  |  |  |  |  | 1.6822E7 |  |  |  |  |  |  |  |  | 1 | 0 | 0 | 0 | 0 | 0 | 1 | 0 | 0 | 0 | 0 | 0 | 0 | 0 | 0 | 251 | 269 |  |  | PEAKS DB |
| F.FQENENAWYYINNIR.K | Y | 60.04 | 1972.9067 | 15 | -0.4 | 987.4603 | 2 | 71.69 | 6 | F6:47797 | NaSa01\_F14.raw |  |  |  |  |  | 2.7056E6 |  |  |  |  |  |  |  |  | 1 | 0 | 0 | 0 | 0 | 0 | 1 | 0 | 0 | 0 | 0 | 0 | 0 | 0 | 0 | 132 | 146 |  |  | PEAKS DB |
| S.FTPYQFQDYFESAAAPVGR.I | Y | 59.98 | 2193.0166 | 19 | -0.2 | 1097.5154 | 2 | 83.99 | 6 | F6:57987 | NaSa01\_F14.raw |  |  |  |  |  | 1.8906E6 |  |  |  |  |  |  |  |  | 1 | 0 | 0 | 0 | 0 | 0 | 1 | 0 | 0 | 0 | 0 | 0 | 0 | 0 | 0 | 449 | 467 |  |  | PEAKS DB |
| D.DSDFFQALDTK.T | Y | 59.42 | 1285.5826 | 11 | -0.4 | 643.7983 | 2 | 67.26 | 6 | F6:44023 | NaSa01\_F14.raw |  |  |  |  |  | 2.633E6 |  |  |  |  |  |  |  |  | 1 | 0 | 0 | 0 | 0 | 0 | 1 | 0 | 0 | 0 | 0 | 0 | 0 | 0 | 0 | 394 | 404 |  |  | PEAKS DB |
| K.FWEADGIHGGK.S | N | 59.16 | 1215.5673 | 11 | 0.2 | 608.7910 | 2 | 11.57 | 6 | F6:1961 | NaSa01\_F14.raw |  |  |  |  | 4.0485E5 | 1.8815E7 |  |  |  |  |  |  |  |  | 9 | 0 | 0 | 0 | 0 | 1 | 8 | 0 | 0 | 0 | 0 | 0 | 0 | 0 | 0 | 352 | 362 |  |  | PEAKS DB |
| Y.HLSFMESLK.S | Y | 58.90 | 1090.5481 | 9 | 0.5 | 546.2816 | 2 | 34.90 | 6 | F6:16342 | NaSa01\_F14.raw |  |  |  |  |  | 1.1344E7 |  |  |  |  |  |  |  |  | 1 | 0 | 0 | 0 | 0 | 0 | 1 | 0 | 0 | 0 | 0 | 0 | 0 | 0 | 0 | 233 | 241 |  |  | PEAKS DB |
| A.GLSAAYVLAGAGHK.V | N | 58.41 | 1313.7091 | 14 | 0.3 | 438.9104 | 3 | 32.92 | 6 | F6:14801 | NaSa01\_F14.raw |  |  |  |  |  | 1.2419E6 |  |  |  |  |  |  |  |  | 2 | 0 | 0 | 0 | 0 | 0 | 2 | 0 | 0 | 0 | 0 | 0 | 0 | 0 | 0 | 64 | 77 |  |  | PEAKS DB |
| K.HVVVVGAGMAGL.S | N | 58.15 | 1108.6063 | 12 | 0.5 | 555.3107 | 2 | 51.19 | 6 | F6:30972 | NaSa01\_F14.raw |  |  |  |  | 1.7665E7 | 2.672E8 |  |  |  |  |  |  |  |  | 3 | 0 | 0 | 0 | 0 | 1 | 2 | 0 | 0 | 0 | 0 | 0 | 0 | 0 | 0 | 54 | 65 |  |  | PEAKS DB |
| L.SAAYVLAGAGHKVTLLEASER.V | N | 57.68 | 2142.1433 | 21 | 0.0 | 715.0551 | 3 | 41.78 | 6 | F6:22392 | NaSa01\_F14.raw |  |  |  |  |  | 9.3986E5 |  |  |  |  |  |  |  |  | 1 | 0 | 0 | 0 | 0 | 0 | 1 | 0 | 0 | 0 | 0 | 0 | 0 | 0 | 0 | 66 | 86 |  |  | PEAKS DB |
| K.TC(+57.02)ADIVINDLSLIHDLPKR.E | Y | 56.94 | 2192.1624 | 19 | 0.0 | 549.0479 | 4 | 70.00 | 6 | F6:46365 | NaSa01\_F14.raw |  |  |  |  |  | 6.8137E5 |  |  |  |  |  |  |  |  | 1 | 0 | 0 | 0 | 0 | 0 | 1 | 0 | 0 | 0 | 0 | 0 | 0 | 0 | 0 | 405 | 423 | Carbamidomethylation | C2:Carbamidomethylation:1000.00 | PEAKS DB |
| R.GAVDMIGDLLNEDSSYHLS.F | Y | 56.35 | 2034.9204 | 19 | 1.2 | 1018.4687 | 2 | 95.85 | 6 | F6:67896 | NaSa01\_F14.raw |  |  |  |  |  | 1.2201E7 |  |  |  |  |  |  |  |  | 1 | 0 | 0 | 0 | 0 | 0 | 1 | 0 | 0 | 0 | 0 | 0 | 0 | 0 | 0 | 217 | 235 |  |  | PEAKS DB |
| K.RFDEIVGGFDQLPISMY.Q | N | 56.32 | 1985.9557 | 17 | 1.0 | 993.9861 | 2 | 99.98 | 6 | F6:71504 | NaSa01\_F14.raw |  |  |  |  | 8.8677E6 | 1.2924E8 |  |  |  |  |  |  |  |  | 4 | 0 | 0 | 0 | 0 | 1 | 3 | 0 | 0 | 0 | 0 | 0 | 0 | 0 | 0 | 251 | 267 |  |  | PEAKS DB |
| K.RFDEIVGGFDQLPISM(+15.99)Y.Q | N | 56.17 | 2001.9506 | 17 | 0.5 | 1001.9830 | 2 | 91.18 | 6 | F6:63904 | NaSa01\_F14.raw |  |  |  |  | 1.3345E6 | 1.0983E7 |  |  |  |  |  |  |  |  | 3 | 0 | 0 | 0 | 0 | 1 | 2 | 0 | 0 | 0 | 0 | 0 | 0 | 0 | 0 | 251 | 267 | Oxidation (M) | M16:Oxidation (M):1000.00 | PEAKS DB |
| Q.FQDYFESAAAPVGR.I | Y | 55.52 | 1556.7258 | 14 | 0.1 | 779.3702 | 2 | 53.21 | 6 | F6:32139 | NaSa01\_F14.raw |  |  |  |  |  | 8.9133E5 |  |  |  |  |  |  |  |  | 1 | 0 | 0 | 0 | 0 | 0 | 1 | 0 | 0 | 0 | 0 | 0 | 0 | 0 | 0 | 454 | 467 |  |  | PEAKS DB |
| R.EGWYVNM(+15.99)GPM(+15.99)R.L | N | 54.52 | 1370.5747 | 11 | -0.6 | 686.2942 | 2 | 35.77 | 6 | F6:19242 | NaSa01\_F14.raw |  |  |  |  | 3.3829E6 | 5.1173E7 |  |  |  |  |  |  |  |  | 8 | 0 | 0 | 0 | 0 | 1 | 7 | 0 | 0 | 0 | 0 | 0 | 0 | 0 | 0 | 99 | 109 | Oxidation (M) | M7:Oxidation (M):1000.00;M10:Oxidation (M):1000.00 | PEAKS DB |
| R.FDEIVGGFDQLPISM(+15.99)YQAIAEMVHLNAR.V | N | 54.25 | 3179.5420 | 28 | 1.5 | 1060.8562 | 3 | 104.78 | 6 | F6:75217 | NaSa01\_F14.raw |  |  |  |  |  | 1.2736E6 |  |  |  |  |  |  |  |  | 1 | 0 | 0 | 0 | 0 | 0 | 1 | 0 | 0 | 0 | 0 | 0 | 0 | 0 | 0 | 252 | 279 | Oxidation (M) | M15:Oxidation (M):67.12 | PEAKS DB |
| L.ADDSDFFQALDTK.T | Y | 53.91 | 1471.6466 | 13 | 0.4 | 736.8309 | 2 | 66.91 | 6 | F6:43666 | NaSa01\_F14.raw |  |  |  |  |  | 8.4089E5 |  |  |  |  |  |  |  |  | 1 | 0 | 0 | 0 | 0 | 0 | 1 | 0 | 0 | 0 | 0 | 0 | 0 | 0 | 0 | 392 | 404 |  |  | PEAKS DB |
| K.TC(+57.02)ADIVINDLSLIHD.L | Y | 53.82 | 1697.8295 | 15 | 1.9 | 849.9236 | 2 | 84.53 | 6 | F6:58437 | NaSa01\_F14.raw |  |  |  |  |  | 3.1631E6 |  |  |  |  |  |  |  |  | 1 | 0 | 0 | 0 | 0 | 0 | 1 | 0 | 0 | 0 | 0 | 0 | 0 | 0 | 0 | 405 | 419 | Carbamidomethylation | C2:Carbamidomethylation:1000.00 | PEAKS DB |
| N.DLSLIHDLPK.R | N | 53.54 | 1149.6393 | 10 | 0.2 | 575.8270 | 2 | 52.53 | 6 | F6:31365 | NaSa01\_F14.raw |  |  |  | 3.056E5 |  | 2.4605E6 |  |  |  |  |  |  |  |  | 2 | 0 | 0 | 0 | 1 | 0 | 1 | 0 | 0 | 0 | 0 | 0 | 0 | 0 | 0 | 413 | 422 |  |  | PEAKS DB |
| W.IDSTIMTGLR.A | Y | 53.08 | 1105.5801 | 10 | 0.9 | 553.7978 | 2 | 43.38 | 6 | F6:23683 | NaSa01\_F14.raw |  |  |  |  |  | 8.1441E6 |  |  |  |  |  |  |  |  | 1 | 0 | 0 | 0 | 0 | 0 | 1 | 0 | 0 | 0 | 0 | 0 | 0 | 0 | 0 | 482 | 491 |  |  | PEAKS DB |
| K.RFDEIVGGFDQLPISM.Y | N | 52.02 | 1822.8923 | 16 | 1.0 | 912.4543 | 2 | 96.41 | 6 | F6:68325 | NaSa01\_F14.raw |  |  |  |  | 8.0404E5 | 1.1182E7 |  |  |  |  |  |  |  |  | 2 | 0 | 0 | 0 | 0 | 1 | 1 | 0 | 0 | 0 | 0 | 0 | 0 | 0 | 0 | 251 | 266 |  |  | PEAKS DB |
| K.KFWEADGIHGGK.S | N | 51.66 | 1343.6622 | 12 | -0.1 | 448.8947 | 3 | 11.66 | 5 | F5:2144 | NaSa01\_F13.raw |  |  |  |  | 1.996E6 | 1.1435E7 |  |  |  |  |  |  |  |  | 3 | 0 | 0 | 0 | 0 | 1 | 2 | 0 | 0 | 0 | 0 | 0 | 0 | 0 | 0 | 351 | 362 |  |  | PEAKS DB |
| M.GSITSFTPYQFQDYFESAAAPVGR.I | Y | 51.25 | 2638.2339 | 24 | 3.8 | 880.4219 | 3 | 92.69 | 6 | F6:65150 | NaSa01\_F14.raw |  |  |  |  |  | 7.6022E6 |  |  |  |  |  |  |  |  | 2 | 0 | 0 | 0 | 0 | 0 | 2 | 0 | 0 | 0 | 0 | 0 | 0 | 0 | 0 | 444 | 467 |  |  | PEAKS DB |
| K.VTLLEASER.V | N | 50.90 | 1016.5502 | 9 | 0.2 | 509.2825 | 2 | 25.50 | 6 | F6:8654 | NaSa01\_F14.raw |  |  |  |  |  | 9.4866E6 |  |  |  |  |  |  |  |  | 2 | 0 | 0 | 0 | 0 | 0 | 2 | 0 | 0 | 0 | 0 | 0 | 0 | 0 | 0 | 78 | 86 |  |  | PEAKS DB |
| R.VTYQTPAK.T | N | 50.89 | 906.4811 | 8 | 0.0 | 454.2478 | 2 | 11.43 | 6 | F6:1806 | NaSa01\_F14.raw |  |  |  |  |  | 3.9897E6 |  |  |  |  |  |  |  |  | 1 | 0 | 0 | 0 | 0 | 0 | 1 | 0 | 0 | 0 | 0 | 0 | 0 | 0 | 0 | 292 | 299 |  |  | PEAKS DB |
| Q.DYFESAAAPVGR.I | Y | 50.87 | 1281.5989 | 12 | 2.6 | 641.8083 | 2 | 42.78 | 6 | F6:23130 | NaSa01\_F14.raw |  |  |  |  |  | 0 |  |  |  |  |  |  |  |  | 0 | 0 | 0 | 0 | 0 | 0 | 0 | 0 | 0 | 0 | 0 | 0 | 0 | 0 | 0 | 456 | 467 |  |  | PEAKS DB |
| K.HVVVVGAGMAGLS.A | N | 50.60 | 1195.6383 | 13 | -0.2 | 598.8263 | 2 | 46.44 | 6 | F6:26217 | NaSa01\_F14.raw |  |  |  |  |  | 5.0901E6 |  |  |  |  |  |  |  |  | 1 | 0 | 0 | 0 | 0 | 0 | 1 | 0 | 0 | 0 | 0 | 0 | 0 | 0 | 0 | 54 | 66 |  |  | PEAKS DB |
| K.HVVVVGAGM(+15.99)AGLSAAY.V | N | 50.48 | 1516.7708 | 16 | 0.9 | 759.3934 | 2 | 46.65 | 6 | F6:26724 | NaSa01\_F14.raw |  |  |  |  |  | 2.0076E7 |  |  |  |  |  |  |  |  | 3 | 0 | 0 | 0 | 0 | 0 | 3 | 0 | 0 | 0 | 0 | 0 | 0 | 0 | 0 | 54 | 69 | Oxidation (M) | M9:Oxidation (M):1000.00 | PEAKS DB |
| K.HVVVVGAGMAGLSAA.Y | N | 50.47 | 1337.7125 | 15 | 0.6 | 669.8640 | 2 | 50.72 | 6 | F6:29947 | NaSa01\_F14.raw |  |  |  |  |  | 7.7421E6 |  |  |  |  |  |  |  |  | 1 | 0 | 0 | 0 | 0 | 0 | 1 | 0 | 0 | 0 | 0 | 0 | 0 | 0 | 0 | 54 | 68 |  |  | PEAKS DB |
| R.EIQALC(+57.02)YPSIKK.W | N | 49.90 | 1448.7697 | 12 | 0.7 | 483.9308 | 3 | 30.77 | 6 | F6:13252 | NaSa01\_F14.raw |  |  |  |  |  | 6.9213E6 |  |  |  |  |  |  |  |  | 2 | 0 | 0 | 0 | 0 | 0 | 2 | 0 | 0 | 0 | 0 | 0 | 0 | 0 | 0 | 424 | 435 | Carbamidomethylation | C6:Carbamidomethylation:1000.00 | PEAKS DB |
| K.RFDEIVGGFDQLPISMYQ.A | N | 49.87 | 2114.0142 | 18 | 1.4 | 1058.0159 | 2 | 96.41 | 6 | F6:68423 | NaSa01\_F14.raw |  |  |  |  |  | 4.0398E6 |  |  |  |  |  |  |  |  | 1 | 0 | 0 | 0 | 0 | 0 | 1 | 0 | 0 | 0 | 0 | 0 | 0 | 0 | 0 | 251 | 268 |  |  | PEAKS DB |
| H.GWIDSTIMTGLR.A | Y | 49.75 | 1348.6809 | 12 | 0.1 | 675.3478 | 2 | 78.70 | 6 | F6:53478 | NaSa01\_F14.raw |  |  |  |  |  | 2.7681E6 |  |  |  |  |  |  |  |  | 2 | 0 | 0 | 0 | 0 | 0 | 2 | 0 | 0 | 0 | 0 | 0 | 0 | 0 | 0 | 480 | 491 |  |  | PEAKS DB |
| I.YFEPPLPPK.K | N | 49.42 | 1086.5750 | 9 | 6.0 | 544.2952 | 2 | 42.50 | 8 | F8:25506 | NaSa01\_F3.raw |  |  |  |  |  |  |  | 6.3004E5 |  |  |  |  |  |  | 1 | 0 | 0 | 0 | 0 | 0 | 0 | 0 | 1 | 0 | 0 | 0 | 0 | 0 | 0 | 320 | 328 |  |  | PEAKS DB |
| K.HVVVVGAGMAGLSA.A | N | 48.82 | 1266.6754 | 14 | 0.3 | 634.3452 | 2 | 49.72 | 6 | F6:29252 | NaSa01\_F14.raw |  |  |  |  |  | 3.9558E6 |  |  |  |  |  |  |  |  | 1 | 0 | 0 | 0 | 0 | 0 | 1 | 0 | 0 | 0 | 0 | 0 | 0 | 0 | 0 | 54 | 67 |  |  | PEAKS DB |
| K.STTDLPSR.F | N | 48.78 | 875.4348 | 8 | 0.1 | 438.7247 | 2 | 11.44 | 6 | F6:1817 | NaSa01\_F14.raw |  |  |  |  |  | 1.0568E6 |  |  |  |  |  |  |  |  | 1 | 0 | 0 | 0 | 0 | 0 | 1 | 0 | 0 | 0 | 0 | 0 | 0 | 0 | 0 | 363 | 370 |  |  | PEAKS DB |
| R.GAVDMIGDLLNEDSSY.H | N | 48.29 | 1697.7454 | 16 | 1.7 | 849.8814 | 2 | 107.90 | 6 | F6:77609 | NaSa01\_F14.raw |  |  |  |  |  | 1.6853E7 |  |  |  |  |  |  |  |  | 1 | 0 | 0 | 0 | 0 | 0 | 1 | 0 | 0 | 0 | 0 | 0 | 0 | 0 | 0 | 217 | 232 |  |  | PEAKS DB |
| R.FDEIVGGFDQLPISM.Y | N | 48.24 | 1666.7913 | 15 | 1.2 | 834.4039 | 2 | 112.47 | 6 | F6:80862 | NaSa01\_F14.raw |  |  |  |  |  | 8.4104E6 |  |  |  |  |  |  |  |  | 1 | 0 | 0 | 0 | 0 | 0 | 1 | 0 | 0 | 0 | 0 | 0 | 0 | 0 | 0 | 252 | 266 |  |  | PEAKS DB |
| Y.QAIAEM(+15.99)VHLNAR.V | N | 47.05 | 1367.6980 | 12 | 0.8 | 456.9070 | 3 | 31.64 | 6 | F6:13767 | NaSa01\_F14.raw |  |  |  |  |  | 2.4624E6 |  |  |  |  |  |  |  |  | 3 | 0 | 0 | 0 | 0 | 0 | 3 | 0 | 0 | 0 | 0 | 0 | 0 | 0 | 0 | 268 | 279 | Oxidation (M) | M6:Oxidation (M):1000.00 | PEAKS DB |
| R.FHGWIDSTIM.T | Y | 46.94 | 1205.5540 | 10 | 0.7 | 603.7847 | 2 | 69.86 | 6 | F6:46616 | NaSa01\_F14.raw |  |  |  |  |  | 2.4473E7 |  |  |  |  |  |  |  |  | 1 | 0 | 0 | 0 | 0 | 0 | 1 | 0 | 0 | 0 | 0 | 0 | 0 | 0 | 0 | 478 | 487 |  |  | PEAKS DB |
| Y.TMGSITSFTPYQFQDYFESAAAPVGR.I | Y | 46.62 | 2870.3220 | 26 | 0.3 | 957.7816 | 3 | 97.15 | 6 | F6:68946 | NaSa01\_F14.raw |  |  |  |  |  | 1.4838E7 |  |  |  |  |  |  |  |  | 2 | 0 | 0 | 0 | 0 | 0 | 2 | 0 | 0 | 0 | 0 | 0 | 0 | 0 | 0 | 442 | 467 |  |  | PEAKS DB |
| K.TFVTADYVIVC(+57.02).S | Y | 46.30 | 1286.6217 | 11 | 0.6 | 644.3185 | 2 | 81.27 | 6 | F6:56092 | NaSa01\_F14.raw |  |  |  |  | 2.639E6 | 3.2724E7 |  |  |  |  |  |  |  |  | 2 | 0 | 0 | 0 | 0 | 1 | 1 | 0 | 0 | 0 | 0 | 0 | 0 | 0 | 0 | 300 | 310 | Carbamidomethylation | C11:Carbamidomethylation:1000.00 | PEAKS DB |
| K.KDPSLLKYPVKPSEEGK.S | Y | 46.30 | 1914.0461 | 17 | -0.5 | 479.5186 | 4 | 11.65 | 5 | F5:2119 | NaSa01\_F13.raw |  |  |  |  | 1.573E5 |  |  |  |  |  |  |  |  |  | 1 | 0 | 0 | 0 | 0 | 1 | 0 | 0 | 0 | 0 | 0 | 0 | 0 | 0 | 0 | 154 | 170 |  |  | PEAKS DB |
| A.DDSDFFQALDTK.T | Y | 44.68 | 1400.6095 | 12 | 1.0 | 701.3127 | 2 | 73.11 | 4 | F4:50624 | NaSa01\_F12.raw |  |  |  | 6.4171E5 |  |  |  |  |  |  |  |  |  |  | 1 | 0 | 0 | 0 | 1 | 0 | 0 | 0 | 0 | 0 | 0 | 0 | 0 | 0 | 0 | 393 | 404 |  |  | PEAKS DB |
| R.FDEIVGGFDQLPISMY.Q | N | 44.41 | 1829.8545 | 16 | 0.2 | 915.9347 | 2 | 116.62 | 6 | F6:83518 | NaSa01\_F14.raw |  |  |  |  |  | 6.1039E7 |  |  |  |  |  |  |  |  | 1 | 0 | 0 | 0 | 0 | 0 | 1 | 0 | 0 | 0 | 0 | 0 | 0 | 0 | 0 | 252 | 267 |  |  | PEAKS DB |
| A.GM(+15.99)AGLSAAYVLAGAGHK.V | N | 43.41 | 1588.8031 | 17 | 0.5 | 530.6086 | 3 | 39.38 | 6 | F6:20374 | NaSa01\_F14.raw |  |  |  |  |  | 2.4697E5 |  |  |  |  |  |  |  |  | 1 | 0 | 0 | 0 | 0 | 0 | 1 | 0 | 0 | 0 | 0 | 0 | 0 | 0 | 0 | 61 | 77 | Oxidation (M) | M2:Oxidation (M):1000.00 | PEAKS DB |
| G.SITSFTPYQFQDYFESAAAPVGR.I | Y | 43.36 | 2581.2124 | 23 | 0.0 | 1291.6135 | 2 | 92.73 | 6 | F6:65352 | NaSa01\_F14.raw |  |  |  |  |  | 4.3026E5 |  |  |  |  |  |  |  |  | 1 | 0 | 0 | 0 | 0 | 0 | 1 | 0 | 0 | 0 | 0 | 0 | 0 | 0 | 0 | 445 | 467 |  |  | PEAKS DB |
| R.FDEIVGGFDQLPISMYQ.A | N | 43.29 | 1957.9131 | 17 | 0.8 | 979.9646 | 2 | 111.22 | 6 | F6:80041 | NaSa01\_F14.raw |  |  |  |  |  | 3.0744E6 |  |  |  |  |  |  |  |  | 1 | 0 | 0 | 0 | 0 | 0 | 1 | 0 | 0 | 0 | 0 | 0 | 0 | 0 | 0 | 252 | 268 |  |  | PEAKS DB |
| K.HVVVVGAGM(+15.99)AGL.S | N | 43.09 | 1124.6012 | 12 | 0.5 | 563.3082 | 2 | 50.23 | 6 | F6:30439 | NaSa01\_F14.raw |  |  |  |  | 3.0223E6 | 1.1076E7 |  |  |  |  |  |  |  |  | 3 | 0 | 0 | 0 | 0 | 1 | 2 | 0 | 0 | 0 | 0 | 0 | 0 | 0 | 0 | 54 | 65 | Oxidation (M) | M9:Oxidation (M):1000.00 | PEAKS DB |
| E.IQALC(+57.02)YPSIK.K | N | 42.76 | 1191.6322 | 10 | -0.9 | 596.8228 | 2 | 42.19 | 6 | F6:22642 | NaSa01\_F14.raw |  |  |  |  |  | 1.1323E6 |  |  |  |  |  |  |  |  | 1 | 0 | 0 | 0 | 0 | 0 | 1 | 0 | 0 | 0 | 0 | 0 | 0 | 0 | 0 | 425 | 434 | Carbamidomethylation | C5:Carbamidomethylation:1000.00 | PEAKS DB |
| Y.TM(+15.99)GSITSFTPYQFQDYFESAAAPVGR.I | Y | 42.42 | 2886.3169 | 26 | 0.9 | 963.1138 | 3 | 92.52 | 6 | F6:65107 | NaSa01\_F14.raw |  |  |  |  |  | 1.1735E6 |  |  |  |  |  |  |  |  | 1 | 0 | 0 | 0 | 0 | 0 | 1 | 0 | 0 | 0 | 0 | 0 | 0 | 0 | 0 | 442 | 467 | Oxidation (M) | M2:Oxidation (M):1000.00 | PEAKS DB |
| V.GAGMAGLSAAYVLAGAGHK.V | N | 41.36 | 1700.8668 | 19 | -0.1 | 567.9628 | 3 | 53.80 | 6 | F6:32769 | NaSa01\_F14.raw |  |  |  |  |  | 3.5832E5 |  |  |  |  |  |  |  |  | 1 | 0 | 0 | 0 | 0 | 0 | 1 | 0 | 0 | 0 | 0 | 0 | 0 | 0 | 0 | 59 | 77 |  |  | PEAKS DB |
| R.FHGWIDST.I | Y | 40.89 | 961.4294 | 8 | -0.2 | 481.7219 | 2 | 38.55 | 6 | F6:19670 | NaSa01\_F14.raw |  |  |  |  |  | 1.042E6 |  |  |  |  |  |  |  |  | 1 | 0 | 0 | 0 | 0 | 0 | 1 | 0 | 0 | 0 | 0 | 0 | 0 | 0 | 0 | 478 | 485 |  |  | PEAKS DB |
| F.HGWIDSTIMTGLR.A | Y | 40.51 | 1485.7399 | 13 | -0.5 | 496.2537 | 3 | 60.76 | 6 | F6:38590 | NaSa01\_F14.raw |  |  |  |  |  | 1.5775E5 |  |  |  |  |  |  |  |  | 1 | 0 | 0 | 0 | 0 | 0 | 1 | 0 | 0 | 0 | 0 | 0 | 0 | 0 | 0 | 479 | 491 |  |  | PEAKS DB |
| G.MAGLSAAYVLAGAGHK.V | N | 39.83 | 1515.7867 | 16 | 0.3 | 506.2697 | 3 | 47.39 | 6 | F6:27145 | NaSa01\_F14.raw |  |  |  |  |  | 5.9167E5 |  |  |  |  |  |  |  |  | 1 | 0 | 0 | 0 | 0 | 0 | 1 | 0 | 0 | 0 | 0 | 0 | 0 | 0 | 0 | 62 | 77 |  |  | PEAKS DB |
| E.GWYVNMGPMR.L | N | 38.80 | 1209.5424 | 10 | 0.9 | 605.7790 | 2 | 55.07 | 6 | F6:33759 | NaSa01\_F14.raw |  |  |  |  |  | 8.4887E5 |  |  |  |  |  |  |  |  | 1 | 0 | 0 | 0 | 0 | 0 | 1 | 0 | 0 | 0 | 0 | 0 | 0 | 0 | 0 | 100 | 109 |  |  | PEAKS DB |
| R.FDEIVGGFDQLPISMYQA.I | N | 38.57 | 2028.9502 | 18 | 1.1 | 1015.4835 | 2 | 112.01 | 6 | F6:80759 | NaSa01\_F14.raw |  |  |  |  |  | 1.2262E7 |  |  |  |  |  |  |  |  | 1 | 0 | 0 | 0 | 0 | 0 | 1 | 0 | 0 | 0 | 0 | 0 | 0 | 0 | 0 | 252 | 269 |  |  | PEAKS DB |
| R.FDEIVGGFDQLPISM(+15.99)Y.Q | N | 38.39 | 1845.8495 | 16 | 0.4 | 923.9324 | 2 | 105.35 | 6 | F6:75636 | NaSa01\_F14.raw |  |  |  |  |  | 4.6489E6 |  |  |  |  |  |  |  |  | 1 | 0 | 0 | 0 | 0 | 0 | 1 | 0 | 0 | 0 | 0 | 0 | 0 | 0 | 0 | 252 | 267 | Oxidation (M) | M15:Oxidation (M):1000.00 | PEAKS DB |
| F.VTADYVIVC(+57.02)STSR.A | N | 38.16 | 1469.7184 | 13 | 0.8 | 490.9138 | 3 | 38.41 | 6 | F6:19506 | NaSa01\_F14.raw |  |  |  |  |  | 3.3852E5 |  |  |  |  |  |  |  |  | 1 | 0 | 0 | 0 | 0 | 0 | 1 | 0 | 0 | 0 | 0 | 0 | 0 | 0 | 0 | 302 | 314 | Carbamidomethylation | C9:Carbamidomethylation:1000.00 | PEAKS DB |
| total 98 peptides |  |  |  |  |  |  |  |  |  |  |  |  |  |  |  |  |  |  |  |  |  |  |  |  |  |  |  |  |  |  |  |  |  |  |  |  |  |  |  |  |  |  |  |  |  |
| --- | --- | --- | --- | --- | --- | --- | --- | --- | --- | --- | --- | --- | --- | --- | --- | --- | --- | --- | --- | --- | --- | --- | --- | --- | --- | --- | --- | --- | --- | --- | --- | --- | --- | --- | --- | --- | --- | --- | --- | --- | --- | --- | --- | --- | --- |

A0A2I4HXH5.1

back to list

  

| Protein Coverage
| Supporting Peptides
|

Protein Coverage:

Supporting Peptides:

| Peptide | Uniq | -10lgP | Mass | Length | ppm | m/z | z | RT | Fraction | Scan | Source File | Area F1 | Area F10 | Area F11 | Area F12 | Area F13 | Area F14 | Area F2 | Area F3 | Area F4 | Area F5 | Area F6 | Area F7 | Area F8 | Area F9 | #Feature | #Feature F1 | #Feature F10 | #Feature F11 | #Feature F12 | #Feature F13 | #Feature F14 | #Feature F2 | #Feature F3 | #Feature F4 | #Feature F5 | #Feature F6 | #Feature F7 | #Feature F8 | #Feature F9 | Start | End | PTM | AScore | Found By |
| --- | --- | --- | --- | --- | --- | --- | --- | --- | --- | --- | --- | --- | --- | --- | --- | --- | --- | --- | --- | --- | --- | --- | --- | --- | --- | --- | --- | --- | --- | --- | --- | --- | --- | --- | --- | --- | --- | --- | --- | --- | --- | --- | --- | --- | --- |
| R.HPDDNEWNHVSMC(+57.02)IVNGGGIR.S | N | 99.67 | 2406.0593 | 21 | -0.4 | 803.0267 | 3 | 49.93 | 6 | F6:29280 | NaSa01\_F14.raw |  |  |  |  | 6.5991E7 | 1.6043E8 |  |  |  |  |  |  |  | 7.6096E6 | 9 | 0 | 0 | 0 | 0 | 3 | 4 | 0 | 0 | 0 | 0 | 0 | 0 | 0 | 2 | 350 | 370 | Carbamidomethylation | C13:Carbamidomethylation:1000.00 | PEAKS DB |
| R.HGQGTGELLQVSGIK.V | N | 97.61 | 1522.8103 | 15 | 0.4 | 762.4127 | 2 | 31.05 | 6 | F6:13489 | NaSa01\_F14.raw |  |  |  | 4.9661E5 | 6.4612E8 | 1.2796E9 |  |  |  |  |  |  |  |  | 8 | 0 | 0 | 0 | 1 | 3 | 4 | 0 | 0 | 0 | 0 | 0 | 0 | 0 | 0 | 418 | 432 |  |  | PEAKS DB |
| K.IIALGHSGFMEDC(+57.02)R.I | Y | 94.07 | 1604.7439 | 14 | 0.2 | 803.3794 | 2 | 40.20 | 6 | F6:20779 | NaSa01\_F14.raw |  |  |  |  | 3.3701E8 | 6.7418E8 |  |  |  |  |  |  |  |  | 4 | 0 | 0 | 0 | 0 | 2 | 2 | 0 | 0 | 0 | 0 | 0 | 0 | 0 | 0 | 190 | 203 | Carbamidomethylation | C13:Carbamidomethylation:1000.00 | PEAKS DB |
| R.YDAM(+15.99)ALGNHEFDNGLNGLLDPLLK.N | N | 89.01 | 2645.2795 | 24 | 0.3 | 882.7674 | 3 | 88.88 | 6 | F6:61975 | NaSa01\_F14.raw |  |  |  |  | 1.9977E7 | 6.8848E7 |  |  |  |  |  |  |  |  | 3 | 0 | 0 | 0 | 0 | 1 | 2 | 0 | 0 | 0 | 0 | 0 | 0 | 0 | 0 | 85 | 108 | Oxidation (M) | M4:Oxidation (M):1000.00 | PEAKS DB |
| R.HPDDNEWNHVSM(+15.99)C(+57.02)IVNGGGIR.S | N | 86.62 | 2422.0542 | 21 | -0.3 | 808.3584 | 3 | 44.12 | 6 | F6:24148 | NaSa01\_F14.raw |  |  |  |  | 3.2766E7 | 9.9954E7 |  |  |  |  |  |  |  | 8.8723E6 | 8 | 0 | 0 | 0 | 0 | 2 | 4 | 0 | 0 | 0 | 0 | 0 | 0 | 0 | 2 | 350 | 370 | Oxidation (M); Carbamidomethylation | M12:Oxidation (M):1000.00;C13:Carbamidomethylation:1000.00 | PEAKS DB |
| K.VLLPSFLAAGGDGYYMLK.G | N | 84.56 | 1913.9961 | 18 | 1.4 | 639.0068 | 3 | 94.26 | 6 | F6:66829 | NaSa01\_F14.raw |  |  |  | 4.47E5 | 9.4519E7 | 6.4928E8 |  |  |  |  |  |  |  |  | 12 | 0 | 0 | 0 | 1 | 4 | 7 | 0 | 0 | 0 | 0 | 0 | 0 | 0 | 0 | 471 | 488 |  |  | PEAKS DB |
| K.ASGNPILLNKSIQEDPAVK.A | N | 83.17 | 1993.0844 | 19 | 0.6 | 665.3691 | 3 | 35.69 | 6 | F6:17128 | NaSa01\_F14.raw |  |  |  |  | 1.5436E6 | 2.8654E6 |  |  |  |  |  |  |  |  | 2 | 0 | 0 | 0 | 0 | 1 | 1 | 0 | 0 | 0 | 0 | 0 | 0 | 0 | 0 | 278 | 296 |  |  | PEAKS DB |
| K.VLLPSFLAAGGDGYYM(+15.99)LK.G | N | 82.89 | 1929.9910 | 18 | 0.7 | 966.0034 | 2 | 87.45 | 6 | F6:60748 | NaSa01\_F14.raw |  |  |  |  | 2.8899E7 | 1.2442E8 |  |  |  |  |  |  |  |  | 10 | 0 | 0 | 0 | 0 | 3 | 7 | 0 | 0 | 0 | 0 | 0 | 0 | 0 | 0 | 471 | 488 | Oxidation (M) | M16:Oxidation (M):1000.00 | PEAKS DB |
| R.FHEC(+57.02)NLGNLIC(+57.02)DAVVYNNLR.H | N | 82.50 | 2420.1365 | 20 | -0.2 | 807.7192 | 3 | 77.73 | 6 | F6:52912 | NaSa01\_F14.raw |  |  |  |  | 2.6877E7 | 9.4785E7 |  |  |  |  |  |  | 7.773E6 | 1.2217E8 | 9 | 0 | 0 | 0 | 0 | 1 | 3 | 0 | 0 | 0 | 0 | 0 | 0 | 2 | 3 | 330 | 349 | Carbamidomethylation | C4:Carbamidomethylation:1000.00;C11:Carbamidomethylation:1000.00 | PEAKS DB |
| K.ETPVLSNPGPYLEFR.D | N | 81.49 | 1717.8674 | 15 | 0.8 | 859.9417 | 2 | 69.77 | 6 | F6:46166 | NaSa01\_F14.raw |  |  |  | 7.2199E5 | 2.4678E8 | 7.6435E8 |  |  |  | 2.2582E6 |  |  |  |  | 7 | 0 | 0 | 0 | 1 | 3 | 2 | 0 | 0 | 0 | 1 | 0 | 0 | 0 | 0 | 155 | 169 |  |  | PEAKS DB |
| R.QVPVVQAYAFGK.Y | N | 80.72 | 1305.7081 | 12 | -0.2 | 653.8612 | 2 | 81.01 | 6 | F6:55346 | NaSa01\_F14.raw |  |  |  | 4.7562E5 | 3.326E8 | 6.8066E8 |  |  |  |  |  |  |  |  | 5 | 0 | 0 | 0 | 1 | 1 | 3 | 0 | 0 | 0 | 0 | 0 | 0 | 0 | 0 | 249 | 260 |  |  | PEAKS DB |
| R.EVVHFMNSLR.Y | N | 80.17 | 1230.6179 | 10 | 0.3 | 616.3164 | 2 | 32.92 | 6 | F6:14493 | NaSa01\_F14.raw |  |  |  |  | 2.3691E8 | 4.1556E8 |  |  |  |  |  |  |  |  | 11 | 0 | 0 | 0 | 0 | 3 | 8 | 0 | 0 | 0 | 0 | 0 | 0 | 0 | 0 | 75 | 84 |  |  | PEAKS DB |
| K.IIALGHSGFM(+15.99)EDC(+57.02)R.I | Y | 79.95 | 1620.7388 | 14 | -0.1 | 541.2535 | 3 | 27.89 | 6 | F6:10406 | NaSa01\_F14.raw |  |  |  |  | 5.8744E7 | 4.18E7 |  |  |  |  |  |  |  |  | 14 | 0 | 0 | 0 | 0 | 9 | 5 | 0 | 0 | 0 | 0 | 0 | 0 | 0 | 0 | 190 | 203 | Oxidation (M); Carbamidomethylation | M10:Oxidation (M):1000.00;C13:Carbamidomethylation:1000.00 | PEAKS DB |
| R.VVSLNVLC(+57.02)TEC(+57.02)R.V | N | 79.36 | 1448.7115 | 12 | 0.2 | 725.3632 | 2 | 49.71 | 6 | F6:29319 | NaSa01\_F14.raw |  |  |  | 0 | 2.7472E8 | 5.8042E8 |  |  |  |  |  | 8.8246E5 |  |  | 7 | 0 | 0 | 0 | 0 | 2 | 4 | 0 | 0 | 0 | 0 | 0 | 1 | 0 | 0 | 445 | 456 | Carbamidomethylation | C8:Carbamidomethylation:1000.00;C11:Carbamidomethylation:1000.00 | PEAKS DB |
| R.VPTYVPLEMEK.T | N | 79.12 | 1304.6686 | 11 | 0.1 | 653.3416 | 2 | 49.94 | 6 | F6:29351 | NaSa01\_F14.raw |  |  |  | 2.0159E6 | 5.6178E8 | 1.2743E9 |  |  |  |  |  |  |  |  | 9 | 0 | 0 | 0 | 1 | 1 | 7 | 0 | 0 | 0 | 0 | 0 | 0 | 0 | 0 | 457 | 467 |  |  | PEAKS DB |
| K.VQLQNYSSQEIGR.T | Y | 78.37 | 1520.7583 | 13 | 0.7 | 761.3870 | 2 | 27.88 | 6 | F6:10399 | NaSa01\_F14.raw |  |  |  |  | 5.4938E7 | 1.4351E8 |  |  |  |  |  |  |  |  | 2 | 0 | 0 | 0 | 0 | 1 | 1 | 0 | 0 | 0 | 0 | 0 | 0 | 0 | 0 | 304 | 316 |  |  | PEAKS DB |
| R.YDAMALGNHEFDNGLNGLLDPLLK.N | N | 78.14 | 2629.2847 | 24 | 0.8 | 877.4362 | 3 | 94.34 | 6 | F6:67114 | NaSa01\_F14.raw |  |  |  |  | 1.6292E7 | 1.1665E8 |  |  |  |  |  |  |  |  | 4 | 0 | 0 | 0 | 0 | 2 | 2 | 0 | 0 | 0 | 0 | 0 | 0 | 0 | 0 | 85 | 108 |  |  | PEAKS DB |
| E.LTILHTNDVHAR.L | N | 75.00 | 1388.7524 | 12 | 0.9 | 463.9252 | 3 | 11.51 | 6 | F6:1905 | NaSa01\_F14.raw |  |  |  |  | 1.6389E6 | 3.6507E6 |  |  |  |  |  |  |  |  | 4 | 0 | 0 | 0 | 0 | 2 | 2 | 0 | 0 | 0 | 0 | 0 | 0 | 0 | 0 | 5 | 16 |  |  | PEAKS DB |
| K.ETPVLSNPGPYLEFRDEVEELQK.H | N | 74.67 | 2688.3281 | 23 | 0.7 | 897.1173 | 3 | 77.38 | 6 | F6:52291 | NaSa01\_F14.raw |  |  |  |  | 3.5808E8 | 6.9841E8 |  |  |  |  |  |  |  |  | 4 | 0 | 0 | 0 | 0 | 2 | 2 | 0 | 0 | 0 | 0 | 0 | 0 | 0 | 0 | 155 | 177 |  |  | PEAKS DB |
| F.LYTGSPPSNEVAAGNYPFMQLSDDGR.Q | N | 71.64 | 2785.2654 | 26 | 0.3 | 1393.6404 | 2 | 73.27 | 6 | F6:49121 | NaSa01\_F14.raw |  |  |  |  | 3.0881E7 | 6.4244E7 |  |  |  |  |  |  |  |  | 4 | 0 | 0 | 0 | 0 | 2 | 2 | 0 | 0 | 0 | 0 | 0 | 0 | 0 | 0 | 223 | 248 |  |  | PEAKS DB |
| K.ASGNPILLNK.S | N | 69.82 | 1025.5869 | 10 | -0.4 | 513.8005 | 2 | 11.59 | 6 | F6:1963 | NaSa01\_F14.raw |  |  |  |  | 2.4579E6 | 4.7925E6 |  |  |  |  |  |  |  |  | 3 | 0 | 0 | 0 | 0 | 1 | 2 | 0 | 0 | 0 | 0 | 0 | 0 | 0 | 0 | 278 | 287 |  |  | PEAKS DB |
| K.VVYDLSQKPGK.R | Y | 69.45 | 1232.6764 | 11 | 0.4 | 617.3457 | 2 | 11.55 | 6 | F6:1927 | NaSa01\_F14.raw |  |  |  |  | 6.1516E6 | 7.9536E6 |  |  |  |  |  |  |  |  | 4 | 0 | 0 | 0 | 0 | 2 | 2 | 0 | 0 | 0 | 0 | 0 | 0 | 0 | 0 | 433 | 443 |  |  | PEAKS DB |
| Y.TGSPPSNEVAAGNYPFMQLSDDGR.Q | N | 69.41 | 2509.1179 | 24 | 0.8 | 837.3806 | 3 | 65.65 | 6 | F6:42541 | NaSa01\_F14.raw |  |  |  |  | 1.8833E7 | 4.2184E7 |  |  |  |  |  |  |  |  | 4 | 0 | 0 | 0 | 0 | 2 | 2 | 0 | 0 | 0 | 0 | 0 | 0 | 0 | 0 | 225 | 248 |  |  | PEAKS DB |
| S.FLAAGGDGYYMLK.G | N | 67.12 | 1404.6747 | 13 | 0.2 | 703.3447 | 2 | 58.91 | 6 | F6:37338 | NaSa01\_F14.raw |  |  |  |  | 1.8909E7 | 8.0208E7 |  |  |  |  |  |  |  |  | 3 | 0 | 0 | 0 | 0 | 1 | 2 | 0 | 0 | 0 | 0 | 0 | 0 | 0 | 0 | 476 | 488 |  |  | PEAKS DB |
| F.LAAGGDGYYMLK.G | N | 66.41 | 1257.6063 | 12 | 0.2 | 629.8105 | 2 | 41.73 | 6 | F6:22051 | NaSa01\_F14.raw |  |  |  |  |  | 5.7329E6 |  |  |  |  |  |  |  |  | 1 | 0 | 0 | 0 | 0 | 0 | 1 | 0 | 0 | 0 | 0 | 0 | 0 | 0 | 0 | 477 | 488 |  |  | PEAKS DB |
| R.EVVHFM(+15.99)NSLR.Y | N | 65.78 | 1246.6128 | 10 | 0.3 | 624.3138 | 2 | 31.71 | 6 | F6:14277 | NaSa01\_F14.raw |  |  |  |  | 3.2821E6 | 5.0137E7 |  |  |  |  |  |  |  |  | 13 | 0 | 0 | 0 | 0 | 2 | 11 | 0 | 0 | 0 | 0 | 0 | 0 | 0 | 0 | 75 | 84 | Oxidation (M) | M6:Oxidation (M):1000.00 | PEAKS DB |
| R.VPTYVPLEM(+15.99)EK.T | N | 64.86 | 1320.6635 | 11 | 0.4 | 661.3392 | 2 | 41.94 | 6 | F6:25976 | NaSa01\_F14.raw |  |  |  |  | 7.6238E7 | 9.8061E7 |  |  |  |  |  |  |  |  | 15 | 0 | 0 | 0 | 0 | 7 | 8 | 0 | 0 | 0 | 0 | 0 | 0 | 0 | 0 | 457 | 467 | Oxidation (M) | M9:Oxidation (M):1000.00 | PEAKS DB |
| K.GDSSNHSSGDLDISIVGDYIK.R | N | 64.63 | 2178.0076 | 21 | 1.0 | 727.0105 | 3 | 59.88 | 6 | F6:37927 | NaSa01\_F14.raw |  |  |  |  | 1.4469E6 | 1.4153E8 |  |  |  |  |  |  |  |  | 3 | 0 | 0 | 0 | 0 | 1 | 2 | 0 | 0 | 0 | 0 | 0 | 0 | 0 | 0 | 489 | 509 |  |  | PEAKS DB |
| R.VPTYVPLEMEKTYK.V | N | 64.21 | 1696.8745 | 14 | 0.2 | 566.6322 | 3 | 43.05 | 6 | F6:23317 | NaSa01\_F14.raw |  |  |  |  | 9.4948E5 | 1.8657E6 |  |  |  |  |  |  |  |  | 2 | 0 | 0 | 0 | 0 | 1 | 1 | 0 | 0 | 0 | 0 | 0 | 0 | 0 | 0 | 457 | 470 |  |  | PEAKS DB |
| K.FPILSANIRPK.G | N | 63.57 | 1254.7448 | 11 | 1.8 | 419.2563 | 3 | 38.83 | 6 | F6:20180 | NaSa01\_F14.raw |  |  |  | 3.6184E5 | 9.4886E7 | 1.2559E8 |  |  |  |  | 2.1098E6 |  |  |  | 10 | 0 | 0 | 0 | 1 | 2 | 5 | 0 | 0 | 0 | 0 | 2 | 0 | 0 | 0 | 112 | 122 |  |  | PEAKS DB |
| K.RVVSLNVLC(+57.02)TEC(+57.02)R.V | N | 62.55 | 1604.8126 | 13 | 0.2 | 535.9449 | 3 | 35.03 | 6 | F6:16539 | NaSa01\_F14.raw |  |  |  |  | 4.4155E6 | 6.0208E6 |  |  |  |  |  |  |  |  | 3 | 0 | 0 | 0 | 0 | 1 | 2 | 0 | 0 | 0 | 0 | 0 | 0 | 0 | 0 | 444 | 456 | Carbamidomethylation | C9:Carbamidomethylation:1000.00;C12:Carbamidomethylation:1000.00 | PEAKS DB |
| K.GVDVVVGGHTNTF.L | N | 61.79 | 1300.6411 | 13 | 1.1 | 651.3286 | 2 | 41.80 | 6 | F6:22128 | NaSa01\_F14.raw |  |  |  |  | 2.249E7 | 5.2209E7 |  |  |  |  |  |  |  |  | 2 | 0 | 0 | 0 | 0 | 1 | 1 | 0 | 0 | 0 | 0 | 0 | 0 | 0 | 0 | 210 | 222 |  |  | PEAKS DB |
| L.TAVLPFGGTFDLLQIK.G | N | 60.64 | 1718.9607 | 16 | -0.1 | 860.4875 | 2 | 99.74 | 6 | F6:71142 | NaSa01\_F14.raw |  |  | 2.8271E6 | 1.2518E6 | 2.1013E7 | 4.0691E6 |  |  |  |  |  |  |  |  | 4 | 0 | 0 | 1 | 1 | 1 | 1 | 0 | 0 | 0 | 0 | 0 | 0 | 0 | 0 | 388 | 403 |  |  | PEAKS DB |
| K.IINVGSEKVGIIGYTTK.E | N | 60.09 | 1791.0142 | 17 | -0.7 | 598.0116 | 3 | 45.32 | 6 | F6:25348 | NaSa01\_F14.raw |  |  |  |  | 9.4386E5 | 1.7014E6 |  |  |  |  |  |  |  |  | 2 | 0 | 0 | 0 | 0 | 1 | 1 | 0 | 0 | 0 | 0 | 0 | 0 | 0 | 0 | 138 | 154 |  |  | PEAKS DB |
| K.VFPAMEGR.M | N | 56.33 | 905.4429 | 8 | 0.2 | 453.7288 | 2 | 22.33 | 6 | F6:7722 | NaSa01\_F14.raw |  |  |  |  |  | 3.6244E6 |  |  |  |  |  |  |  |  | 2 | 0 | 0 | 0 | 0 | 0 | 2 | 0 | 0 | 0 | 0 | 0 | 0 | 0 | 0 | 514 | 521 |  |  | PEAKS DB |
| N.ISGYILPYK.I | N | 56.17 | 1052.5906 | 9 | 0.7 | 527.3029 | 2 | 45.82 | 6 | F6:26096 | NaSa01\_F14.raw |  |  |  | 2.2592E5 | 4.6763E8 | 8.5839E8 |  |  |  |  |  |  |  |  | 5 | 0 | 0 | 0 | 1 | 1 | 3 | 0 | 0 | 0 | 0 | 0 | 0 | 0 | 0 | 129 | 137 |  |  | PEAKS DB |
| S.FLAAGGDGYYM(+15.99)LK.G | N | 55.62 | 1420.6697 | 13 | -0.1 | 711.3420 | 2 | 48.69 | 6 | F6:28173 | NaSa01\_F14.raw |  |  |  |  |  | 5.7308E6 |  |  |  |  |  |  |  |  | 1 | 0 | 0 | 0 | 0 | 0 | 1 | 0 | 0 | 0 | 0 | 0 | 0 | 0 | 0 | 476 | 488 | Oxidation (M) | M11:Oxidation (M):1000.00 | PEAKS DB |
| H.GQGTGELLQVSGIK.V | N | 55.06 | 1385.7515 | 14 | 0.3 | 693.8832 | 2 | 45.20 | 6 | F6:25108 | NaSa01\_F14.raw |  |  |  |  |  | 1.2594E6 |  |  |  |  |  |  |  |  | 1 | 0 | 0 | 0 | 0 | 0 | 1 | 0 | 0 | 0 | 0 | 0 | 0 | 0 | 0 | 419 | 432 |  |  | PEAKS DB |
| K.GVDVVVGGHTNTFLY.T | N | 54.75 | 1576.7886 | 15 | 0.1 | 789.4016 | 2 | 63.80 | 6 | F6:41327 | NaSa01\_F14.raw |  |  |  |  | 1.2739E7 | 2.8201E7 |  |  |  |  |  |  |  |  | 2 | 0 | 0 | 0 | 0 | 1 | 1 | 0 | 0 | 0 | 0 | 0 | 0 | 0 | 0 | 210 | 224 |  |  | PEAKS DB |
| R.QVPVVQAYAF.G | N | 53.91 | 1120.5917 | 10 | 0.0 | 561.3031 | 2 | 73.70 | 6 | F6:49221 | NaSa01\_F14.raw |  |  |  |  | 7.1015E6 | 1.9997E7 |  |  |  |  |  |  |  |  | 2 | 0 | 0 | 0 | 0 | 1 | 1 | 0 | 0 | 0 | 0 | 0 | 0 | 0 | 0 | 249 | 258 |  |  | PEAKS DB |
| M.ALGNHEFDNGLNGLLDPLLK.N | N | 53.45 | 2149.1167 | 20 | 0.0 | 717.3795 | 3 | 83.56 | 6 | F6:57342 | NaSa01\_F14.raw |  |  |  |  | 5.3244E6 | 2.0787E7 |  |  |  |  |  |  |  |  | 3 | 0 | 0 | 0 | 0 | 1 | 2 | 0 | 0 | 0 | 0 | 0 | 0 | 0 | 0 | 89 | 108 |  |  | PEAKS DB |
| K.IIALGHSGFMED.C | Y | 52.82 | 1288.6122 | 12 | 1.2 | 645.3141 | 2 | 55.54 | 6 | F6:34199 | NaSa01\_F14.raw |  |  |  |  |  | 1.6459E6 |  |  |  |  |  |  |  |  | 1 | 0 | 0 | 0 | 0 | 0 | 1 | 0 | 0 | 0 | 0 | 0 | 0 | 0 | 0 | 190 | 201 |  |  | PEAKS DB |
| K.LTTLGVNK.I | N | 52.68 | 844.5018 | 8 | -1.0 | 423.2578 | 2 | 11.58 | 6 | F6:2148 | NaSa01\_F14.raw |  |  |  |  |  | 3.1326E6 |  |  |  |  |  |  |  |  | 1 | 0 | 0 | 0 | 0 | 0 | 1 | 0 | 0 | 0 | 0 | 0 | 0 | 0 | 0 | 182 | 189 |  |  | PEAKS DB |
| H.TNTFLYTGSPPSNEVAAGNYPFMQLSDDGR.Q | N | 52.38 | 3248.4719 | 30 | 1.3 | 1083.8326 | 3 | 85.33 | 6 | F6:59051 | NaSa01\_F14.raw |  |  |  |  |  | 8.2809E6 |  |  |  |  |  |  |  |  | 1 | 0 | 0 | 0 | 0 | 0 | 1 | 0 | 0 | 0 | 0 | 0 | 0 | 0 | 0 | 219 | 248 |  |  | PEAKS DB |
| K.GVDVVVGGHTNTFLYTGSPPSNEVAAGNYPFMQLSDDGR.Q | N | 51.80 | 4067.8960 | 39 | 0.4 | 1356.9731 | 3 | 83.86 | 6 | F6:58119 | NaSa01\_F14.raw |  |  |  |  |  | 2.3146E7 |  |  |  |  |  |  |  |  | 2 | 0 | 0 | 0 | 0 | 0 | 2 | 0 | 0 | 0 | 0 | 0 | 0 | 0 | 0 | 210 | 248 |  |  | PEAKS DB |
| K.VLLPSFLAAGGDGY.Y | N | 51.68 | 1378.7133 | 14 | 0.7 | 690.3644 | 2 | 100.88 | 6 | F6:72024 | NaSa01\_F14.raw |  |  |  |  | 1.3644E6 | 2.8748E6 |  |  |  |  |  |  |  |  | 2 | 0 | 0 | 0 | 0 | 1 | 1 | 0 | 0 | 0 | 0 | 0 | 0 | 0 | 0 | 471 | 484 |  |  | PEAKS DB |
| F.GGTFDLLQIK.G | N | 50.44 | 1090.6022 | 10 | -0.6 | 546.3080 | 2 | 64.67 | 6 | F6:41967 | NaSa01\_F14.raw |  |  |  |  |  | 9.7795E7 |  |  |  |  |  |  |  |  | 2 | 0 | 0 | 0 | 0 | 0 | 2 | 0 | 0 | 0 | 0 | 0 | 0 | 0 | 0 | 394 | 403 |  |  | PEAKS DB |
| K.VGIIGYTTK.E | N | 50.04 | 950.5436 | 9 | 0.3 | 476.2792 | 2 | 28.48 | 6 | F6:11194 | NaSa01\_F14.raw |  |  |  |  |  | 5.1319E6 |  |  |  |  |  |  |  |  | 2 | 0 | 0 | 0 | 0 | 0 | 2 | 0 | 0 | 0 | 0 | 0 | 0 | 0 | 0 | 146 | 154 |  |  | PEAKS DB |
| L.AAGGDGYYMLK.G | N | 49.71 | 1144.5222 | 11 | 0.7 | 573.2688 | 2 | 30.46 | 5 | F5:13468 | NaSa01\_F13.raw |  |  |  |  | 6.4456E6 |  |  |  |  |  |  |  |  |  | 1 | 0 | 0 | 0 | 0 | 1 | 0 | 0 | 0 | 0 | 0 | 0 | 0 | 0 | 0 | 478 | 488 |  |  | PEAKS DB |
| K.GVDVVVGGHTNTFLYTGSPPSNEVAAGNYPFM(+15.99)QLSDDGR.Q | N | 48.61 | 4083.8909 | 39 | 0.5 | 1362.3049 | 3 | 77.12 | 6 | F6:52373 | NaSa01\_F14.raw |  |  |  |  | 2.9373E6 | 7.6942E6 |  |  |  |  |  |  |  |  | 3 | 0 | 0 | 0 | 0 | 1 | 2 | 0 | 0 | 0 | 0 | 0 | 0 | 0 | 0 | 210 | 248 | Oxidation (M) | M32:Oxidation (M):1000.00 | PEAKS DB |
| R.FHEC(+57.02)NLGNLIC(+57.02)DAVVY.N | N | 48.35 | 1922.8655 | 16 | 1.4 | 962.4414 | 2 | 82.45 | 5 | F5:56634 | NaSa01\_F13.raw |  |  |  |  | 8.0322E7 | 1.7562E8 |  |  |  |  |  |  |  | 2.4692E7 | 3 | 0 | 0 | 0 | 0 | 1 | 1 | 0 | 0 | 0 | 0 | 0 | 0 | 0 | 1 | 330 | 345 | Carbamidomethylation | C4:Carbamidomethylation:1000.00;C11:Carbamidomethylation:1000.00 | PEAKS DB |
| R.HGQGTGELLQVSGIKVVYDLSQKPGK.R | Y | 48.22 | 2737.4763 | 26 | 0.2 | 685.3765 | 4 | 57.14 | 5 | F5:36175 | NaSa01\_F13.raw |  |  |  |  | 6.6345E5 |  |  |  |  |  |  |  |  |  | 1 | 0 | 0 | 0 | 0 | 1 | 0 | 0 | 0 | 0 | 0 | 0 | 0 | 0 | 0 | 418 | 443 |  |  | PEAKS DB |
| R.HPDDNEWNHVSMC(+57.02).I | N | 48.19 | 1639.6144 | 13 | 0.5 | 820.8149 | 2 | 26.12 | 5 | F5:10348 | NaSa01\_F13.raw |  |  |  |  | 8.8799E6 |  |  |  |  |  |  |  |  |  | 2 | 0 | 0 | 0 | 0 | 2 | 0 | 0 | 0 | 0 | 0 | 0 | 0 | 0 | 0 | 350 | 362 | Carbamidomethylation | C13:Carbamidomethylation:1000.00 | PEAKS DB |
| K.VLLPSFLAAGGD.G | N | 47.35 | 1158.6284 | 12 | -0.1 | 580.3214 | 2 | 96.95 | 6 | F6:68512 | NaSa01\_F14.raw |  |  |  |  |  | 1.1427E6 |  |  |  |  |  |  |  |  | 1 | 0 | 0 | 0 | 0 | 0 | 1 | 0 | 0 | 0 | 0 | 0 | 0 | 0 | 0 | 471 | 482 |  |  | PEAKS DB |
| R.HPDDNEWNHVSM.C | N | 47.22 | 1479.5837 | 12 | 1.1 | 740.8000 | 2 | 25.93 | 5 | F5:10070 | NaSa01\_F13.raw |  |  |  |  | 2.3098E6 |  |  |  |  |  |  |  |  |  | 1 | 0 | 0 | 0 | 0 | 1 | 0 | 0 | 0 | 0 | 0 | 0 | 0 | 0 | 0 | 350 | 361 |  |  | PEAKS DB |
| R.QVPVVQAY.A | N | 45.65 | 902.4861 | 8 | -0.5 | 452.2501 | 2 | 43.38 | 5 | F5:24541 | NaSa01\_F13.raw |  |  |  |  | 1.2052E7 |  |  |  |  |  |  |  |  |  | 1 | 0 | 0 | 0 | 0 | 1 | 0 | 0 | 0 | 0 | 0 | 0 | 0 | 0 | 0 | 249 | 256 |  |  | PEAKS DB |
| N.PGPYLEFR.D | N | 45.44 | 977.4970 | 8 | 0.7 | 489.7561 | 2 | 39.90 | 6 | F6:20609 | NaSa01\_F14.raw |  |  |  |  |  | 4.6995E5 |  |  |  |  |  |  |  |  | 1 | 0 | 0 | 0 | 0 | 0 | 1 | 0 | 0 | 0 | 0 | 0 | 0 | 0 | 0 | 162 | 169 |  |  | PEAKS DB |
| N.LGNLIC(+57.02)DAVVYNNLR.H | N | 45.20 | 1732.8931 | 15 | 1.0 | 867.4547 | 2 | 69.10 | 6 | F6:45743 | NaSa01\_F14.raw |  |  |  |  |  | 1.1316E6 |  |  |  |  |  |  |  |  | 1 | 0 | 0 | 0 | 0 | 0 | 1 | 0 | 0 | 0 | 0 | 0 | 0 | 0 | 0 | 335 | 349 | Carbamidomethylation | C6:Carbamidomethylation:1000.00 | PEAKS DB |
| L.YTGSPPSNEVAAGNYPFMQLSDDGR.Q | N | 45.19 | 2672.1812 | 25 | -0.8 | 1337.0968 | 2 | 68.20 | 6 | F6:44852 | NaSa01\_F14.raw |  |  |  |  |  | 0 |  |  |  |  |  |  |  |  | 0 | 0 | 0 | 0 | 0 | 0 | 0 | 0 | 0 | 0 | 0 | 0 | 0 | 0 | 0 | 224 | 248 |  |  | PEAKS DB |
| K.IINVGSEK.V | N | 44.70 | 858.4811 | 8 | -0.5 | 430.2476 | 2 | 11.48 | 6 | F6:1861 | NaSa01\_F14.raw |  |  |  |  |  | 1.3857E6 |  |  |  |  |  |  |  |  | 1 | 0 | 0 | 0 | 0 | 0 | 1 | 0 | 0 | 0 | 0 | 0 | 0 | 0 | 0 | 138 | 145 |  |  | PEAKS DB |
| K.VLLPSFLAAGGDGYY.M | N | 43.81 | 1541.7765 | 15 | 0.7 | 771.8961 | 2 | 105.28 | 6 | F6:75496 | NaSa01\_F14.raw |  |  |  |  | 1.4762E7 | 3.1893E7 |  |  |  |  |  |  |  |  | 2 | 0 | 0 | 0 | 0 | 1 | 1 | 0 | 0 | 0 | 0 | 0 | 0 | 0 | 0 | 471 | 485 |  |  | PEAKS DB |
| K.FPILSANIR.P | N | 43.38 | 1029.5970 | 9 | -0.4 | 515.8056 | 2 | 52.72 | 6 | F6:31843 | NaSa01\_F14.raw |  |  |  |  |  | 1.5657E8 |  |  |  |  |  |  |  |  | 1 | 0 | 0 | 0 | 0 | 0 | 1 | 0 | 0 | 0 | 0 | 0 | 0 | 0 | 0 | 112 | 120 |  |  | PEAKS DB |
| K.VLLPSFLAAGGDGYYM.L | N | 42.72 | 1672.8170 | 16 | 0.9 | 837.4166 | 2 | 111.51 | 6 | F6:80284 | NaSa01\_F14.raw |  |  |  |  |  | 1.1099E6 |  |  |  |  |  |  |  |  | 1 | 0 | 0 | 0 | 0 | 0 | 1 | 0 | 0 | 0 | 0 | 0 | 0 | 0 | 0 | 471 | 486 |  |  | PEAKS DB |
| Q.GTGELLQVSGIK.V | N | 42.14 | 1200.6714 | 12 | 0.9 | 601.3435 | 2 | 44.43 | 6 | F6:24568 | NaSa01\_F14.raw |  |  |  |  |  | 7.0353E5 |  |  |  |  |  |  |  |  | 1 | 0 | 0 | 0 | 0 | 0 | 1 | 0 | 0 | 0 | 0 | 0 | 0 | 0 | 0 | 421 | 432 |  |  | PEAKS DB |
| K.LTTLGVNKIIALGH.S | N | 41.05 | 1448.8715 | 14 | 0.1 | 483.9645 | 3 | 54.77 | 6 | F6:33352 | NaSa01\_F14.raw |  |  |  |  |  | 5.5856E5 |  |  |  |  |  |  |  |  | 1 | 0 | 0 | 0 | 0 | 0 | 1 | 0 | 0 | 0 | 0 | 0 | 0 | 0 | 0 | 182 | 195 |  |  | PEAKS DB |
| N.HEFDNGLNGLLDPLLK.N | N | 40.62 | 1793.9312 | 16 | 0.2 | 598.9844 | 3 | 81.57 | 6 | F6:55994 | NaSa01\_F14.raw |  |  |  |  |  | 5.7086E5 |  |  |  |  |  |  |  |  | 1 | 0 | 0 | 0 | 0 | 0 | 1 | 0 | 0 | 0 | 0 | 0 | 0 | 0 | 0 | 93 | 108 |  |  | PEAKS DB |
| N.YPFMQLSDDGR.Q | N | 40.57 | 1327.5867 | 11 | 0.4 | 664.8009 | 2 | 53.65 | 6 | F6:32287 | NaSa01\_F14.raw |  |  |  |  |  | 5.3395E5 |  |  |  |  |  |  |  |  | 1 | 0 | 0 | 0 | 0 | 0 | 1 | 0 | 0 | 0 | 0 | 0 | 0 | 0 | 0 | 238 | 248 |  |  | PEAKS DB |
| A.AGNYPFMQLSDDGR.Q | N | 40.07 | 1569.6881 | 14 | 0.1 | 785.8514 | 2 | 56.81 | 6 | F6:35126 | NaSa01\_F14.raw |  |  |  |  |  | 2.5054E5 |  |  |  |  |  |  |  |  | 1 | 0 | 0 | 0 | 0 | 0 | 1 | 0 | 0 | 0 | 0 | 0 | 0 | 0 | 0 | 235 | 248 |  |  | PEAKS DB |
| K.NVKFPILSAN.I | N | 39.82 | 1101.6182 | 10 | 0.5 | 551.8167 | 2 | 54.61 | 6 | F6:33186 | NaSa01\_F14.raw |  |  |  |  |  | 1.3385E6 |  |  |  |  |  |  |  |  | 1 | 0 | 0 | 0 | 0 | 0 | 1 | 0 | 0 | 0 | 0 | 0 | 0 | 0 | 0 | 109 | 118 |  |  | PEAKS DB |
| K.IIALGHSGF.M | N | 37.88 | 913.5021 | 9 | -1.2 | 457.7578 | 2 | 43.37 | 6 | F6:23538 | NaSa01\_F14.raw |  |  |  |  |  | 5.7946E6 |  |  |  |  |  |  |  |  | 1 | 0 | 0 | 0 | 0 | 0 | 1 | 0 | 0 | 0 | 0 | 0 | 0 | 0 | 0 | 190 | 198 |  |  | PEAKS DB |
| total 70 peptides |  |  |  |  |  |  |  |  |  |  |  |  |  |  |  |  |  |  |  |  |  |  |  |  |  |  |  |  |  |  |  |  |  |  |  |  |  |  |  |  |  |  |  |  |  |
| --- | --- | --- | --- | --- | --- | --- | --- | --- | --- | --- | --- | --- | --- | --- | --- | --- | --- | --- | --- | --- | --- | --- | --- | --- | --- | --- | --- | --- | --- | --- | --- | --- | --- | --- | --- | --- | --- | --- | --- | --- | --- | --- | --- | --- | --- |

5H7W

back to list

  

| Protein Coverage
| Supporting Peptides
|

Protein Coverage:

Supporting Peptides:

| Peptide | Uniq | -10lgP | Mass | Length | ppm | m/z | z | RT | Fraction | Scan | Source File | Area F1 | Area F10 | Area F11 | Area F12 | Area F13 | Area F14 | Area F2 | Area F3 | Area F4 | Area F5 | Area F6 | Area F7 | Area F8 | Area F9 | #Feature | #Feature F1 | #Feature F10 | #Feature F11 | #Feature F12 | #Feature F13 | #Feature F14 | #Feature F2 | #Feature F3 | #Feature F4 | #Feature F5 | #Feature F6 | #Feature F7 | #Feature F8 | #Feature F9 | Start | End | PTM | AScore | Found By |
| --- | --- | --- | --- | --- | --- | --- | --- | --- | --- | --- | --- | --- | --- | --- | --- | --- | --- | --- | --- | --- | --- | --- | --- | --- | --- | --- | --- | --- | --- | --- | --- | --- | --- | --- | --- | --- | --- | --- | --- | --- | --- | --- | --- | --- | --- |
| R.HPDDNEWNHVSMC(+57.02)IVNGGGIR.S | N | 99.67 | 2406.0593 | 21 | -0.4 | 803.0267 | 3 | 49.93 | 6 | F6:29280 | NaSa01\_F14.raw |  |  |  |  | 6.5991E7 | 1.6043E8 |  |  |  |  |  |  |  | 7.6096E6 | 9 | 0 | 0 | 0 | 0 | 3 | 4 | 0 | 0 | 0 | 0 | 0 | 0 | 0 | 2 | 350 | 370 | Carbamidomethylation | C13:Carbamidomethylation:1000.00 | PEAKS DB |
| R.HGQGTGELLQVSGIK.V | N | 97.61 | 1522.8103 | 15 | 0.4 | 762.4127 | 2 | 31.05 | 6 | F6:13489 | NaSa01\_F14.raw |  |  |  | 4.9661E5 | 6.4612E8 | 1.2796E9 |  |  |  |  |  |  |  |  | 8 | 0 | 0 | 0 | 1 | 3 | 4 | 0 | 0 | 0 | 0 | 0 | 0 | 0 | 0 | 418 | 432 |  |  | PEAKS DB |
| K.IIALGHSGFMEDC(+57.02)R.I | Y | 94.07 | 1604.7439 | 14 | 0.2 | 803.3794 | 2 | 40.20 | 6 | F6:20779 | NaSa01\_F14.raw |  |  |  |  | 3.3701E8 | 6.7418E8 |  |  |  |  |  |  |  |  | 4 | 0 | 0 | 0 | 0 | 2 | 2 | 0 | 0 | 0 | 0 | 0 | 0 | 0 | 0 | 190 | 203 | Carbamidomethylation | C13:Carbamidomethylation:1000.00 | PEAKS DB |
| R.YDAM(+15.99)ALGNHEFDNGLNGLLDPLLK.N | N | 89.01 | 2645.2795 | 24 | 0.3 | 882.7674 | 3 | 88.88 | 6 | F6:61975 | NaSa01\_F14.raw |  |  |  |  | 1.9977E7 | 6.8848E7 |  |  |  |  |  |  |  |  | 3 | 0 | 0 | 0 | 0 | 1 | 2 | 0 | 0 | 0 | 0 | 0 | 0 | 0 | 0 | 85 | 108 | Oxidation (M) | M4:Oxidation (M):1000.00 | PEAKS DB |
| R.HPDDNEWNHVSM(+15.99)C(+57.02)IVNGGGIR.S | N | 86.62 | 2422.0542 | 21 | -0.3 | 808.3584 | 3 | 44.12 | 6 | F6:24148 | NaSa01\_F14.raw |  |  |  |  | 3.2766E7 | 9.9954E7 |  |  |  |  |  |  |  | 8.8723E6 | 8 | 0 | 0 | 0 | 0 | 2 | 4 | 0 | 0 | 0 | 0 | 0 | 0 | 0 | 2 | 350 | 370 | Oxidation (M); Carbamidomethylation | M12:Oxidation (M):1000.00;C13:Carbamidomethylation:1000.00 | PEAKS DB |
| K.VLLPSFLAAGGDGYYMLK.G | N | 84.56 | 1913.9961 | 18 | 1.4 | 639.0068 | 3 | 94.26 | 6 | F6:66829 | NaSa01\_F14.raw |  |  |  | 4.47E5 | 9.4519E7 | 6.4928E8 |  |  |  |  |  |  |  |  | 12 | 0 | 0 | 0 | 1 | 4 | 7 | 0 | 0 | 0 | 0 | 0 | 0 | 0 | 0 | 471 | 488 |  |  | PEAKS DB |
| K.ASGNPILLNKSIQEDPAVK.A | N | 83.17 | 1993.0844 | 19 | 0.6 | 665.3691 | 3 | 35.69 | 6 | F6:17128 | NaSa01\_F14.raw |  |  |  |  | 1.5436E6 | 2.8654E6 |  |  |  |  |  |  |  |  | 2 | 0 | 0 | 0 | 0 | 1 | 1 | 0 | 0 | 0 | 0 | 0 | 0 | 0 | 0 | 278 | 296 |  |  | PEAKS DB |
| K.VLLPSFLAAGGDGYYM(+15.99)LK.G | N | 82.89 | 1929.9910 | 18 | 0.7 | 966.0034 | 2 | 87.45 | 6 | F6:60748 | NaSa01\_F14.raw |  |  |  |  | 2.8899E7 | 1.2442E8 |  |  |  |  |  |  |  |  | 10 | 0 | 0 | 0 | 0 | 3 | 7 | 0 | 0 | 0 | 0 | 0 | 0 | 0 | 0 | 471 | 488 | Oxidation (M) | M16:Oxidation (M):1000.00 | PEAKS DB |
| R.FHEC(+57.02)NLGNLIC(+57.02)DAVVYNNLR.H | N | 82.50 | 2420.1365 | 20 | -0.2 | 807.7192 | 3 | 77.73 | 6 | F6:52912 | NaSa01\_F14.raw |  |  |  |  | 2.6877E7 | 9.4785E7 |  |  |  |  |  |  | 7.773E6 | 1.2217E8 | 9 | 0 | 0 | 0 | 0 | 1 | 3 | 0 | 0 | 0 | 0 | 0 | 0 | 2 | 3 | 330 | 349 | Carbamidomethylation | C4:Carbamidomethylation:1000.00;C11:Carbamidomethylation:1000.00 | PEAKS DB |
| K.ETPVLSNPGPYLEFR.D | N | 81.49 | 1717.8674 | 15 | 0.8 | 859.9417 | 2 | 69.77 | 6 | F6:46166 | NaSa01\_F14.raw |  |  |  | 7.2199E5 | 2.4678E8 | 7.6435E8 |  |  |  | 2.2582E6 |  |  |  |  | 7 | 0 | 0 | 0 | 1 | 3 | 2 | 0 | 0 | 0 | 1 | 0 | 0 | 0 | 0 | 155 | 169 |  |  | PEAKS DB |
| R.QVPVVQAYAFGK.Y | N | 80.72 | 1305.7081 | 12 | -0.2 | 653.8612 | 2 | 81.01 | 6 | F6:55346 | NaSa01\_F14.raw |  |  |  | 4.7562E5 | 3.326E8 | 6.8066E8 |  |  |  |  |  |  |  |  | 5 | 0 | 0 | 0 | 1 | 1 | 3 | 0 | 0 | 0 | 0 | 0 | 0 | 0 | 0 | 249 | 260 |  |  | PEAKS DB |
| R.EVVHFMNSLR.Y | N | 80.17 | 1230.6179 | 10 | 0.3 | 616.3164 | 2 | 32.92 | 6 | F6:14493 | NaSa01\_F14.raw |  |  |  |  | 2.3691E8 | 4.1556E8 |  |  |  |  |  |  |  |  | 11 | 0 | 0 | 0 | 0 | 3 | 8 | 0 | 0 | 0 | 0 | 0 | 0 | 0 | 0 | 75 | 84 |  |  | PEAKS DB |
| K.IIALGHSGFM(+15.99)EDC(+57.02)R.I | Y | 79.95 | 1620.7388 | 14 | -0.1 | 541.2535 | 3 | 27.89 | 6 | F6:10406 | NaSa01\_F14.raw |  |  |  |  | 5.8744E7 | 4.18E7 |  |  |  |  |  |  |  |  | 14 | 0 | 0 | 0 | 0 | 9 | 5 | 0 | 0 | 0 | 0 | 0 | 0 | 0 | 0 | 190 | 203 | Oxidation (M); Carbamidomethylation | M10:Oxidation (M):1000.00;C13:Carbamidomethylation:1000.00 | PEAKS DB |
| R.VVSLNVLC(+57.02)TEC(+57.02)R.V | N | 79.36 | 1448.7115 | 12 | 0.2 | 725.3632 | 2 | 49.71 | 6 | F6:29319 | NaSa01\_F14.raw |  |  |  | 0 | 2.7472E8 | 5.8042E8 |  |  |  |  |  | 8.8246E5 |  |  | 7 | 0 | 0 | 0 | 0 | 2 | 4 | 0 | 0 | 0 | 0 | 0 | 1 | 0 | 0 | 445 | 456 | Carbamidomethylation | C8:Carbamidomethylation:1000.00;C11:Carbamidomethylation:1000.00 | PEAKS DB |
| R.VPTYVPLEMEK.T | N | 79.12 | 1304.6686 | 11 | 0.1 | 653.3416 | 2 | 49.94 | 6 | F6:29351 | NaSa01\_F14.raw |  |  |  | 2.0159E6 | 5.6178E8 | 1.2743E9 |  |  |  |  |  |  |  |  | 9 | 0 | 0 | 0 | 1 | 1 | 7 | 0 | 0 | 0 | 0 | 0 | 0 | 0 | 0 | 457 | 467 |  |  | PEAKS DB |
| K.VQLQNYSSQEIGR.T | Y | 78.37 | 1520.7583 | 13 | 0.7 | 761.3870 | 2 | 27.88 | 6 | F6:10399 | NaSa01\_F14.raw |  |  |  |  | 5.4938E7 | 1.4351E8 |  |  |  |  |  |  |  |  | 2 | 0 | 0 | 0 | 0 | 1 | 1 | 0 | 0 | 0 | 0 | 0 | 0 | 0 | 0 | 304 | 316 |  |  | PEAKS DB |
| R.YDAMALGNHEFDNGLNGLLDPLLK.N | N | 78.14 | 2629.2847 | 24 | 0.8 | 877.4362 | 3 | 94.34 | 6 | F6:67114 | NaSa01\_F14.raw |  |  |  |  | 1.6292E7 | 1.1665E8 |  |  |  |  |  |  |  |  | 4 | 0 | 0 | 0 | 0 | 2 | 2 | 0 | 0 | 0 | 0 | 0 | 0 | 0 | 0 | 85 | 108 |  |  | PEAKS DB |
| E.LTILHTNDVHAR.L | N | 75.00 | 1388.7524 | 12 | 0.9 | 463.9252 | 3 | 11.51 | 6 | F6:1905 | NaSa01\_F14.raw |  |  |  |  | 1.6389E6 | 3.6507E6 |  |  |  |  |  |  |  |  | 4 | 0 | 0 | 0 | 0 | 2 | 2 | 0 | 0 | 0 | 0 | 0 | 0 | 0 | 0 | 5 | 16 |  |  | PEAKS DB |
| K.ETPVLSNPGPYLEFRDEVEELQK.H | N | 74.67 | 2688.3281 | 23 | 0.7 | 897.1173 | 3 | 77.38 | 6 | F6:52291 | NaSa01\_F14.raw |  |  |  |  | 3.5808E8 | 6.9841E8 |  |  |  |  |  |  |  |  | 4 | 0 | 0 | 0 | 0 | 2 | 2 | 0 | 0 | 0 | 0 | 0 | 0 | 0 | 0 | 155 | 177 |  |  | PEAKS DB |
| F.LYTGSPPSNEVAAGNYPFMQLSDDGR.Q | N | 71.64 | 2785.2654 | 26 | 0.3 | 1393.6404 | 2 | 73.27 | 6 | F6:49121 | NaSa01\_F14.raw |  |  |  |  | 3.0881E7 | 6.4244E7 |  |  |  |  |  |  |  |  | 4 | 0 | 0 | 0 | 0 | 2 | 2 | 0 | 0 | 0 | 0 | 0 | 0 | 0 | 0 | 223 | 248 |  |  | PEAKS DB |
| K.ASGNPILLNK.S | N | 69.82 | 1025.5869 | 10 | -0.4 | 513.8005 | 2 | 11.59 | 6 | F6:1963 | NaSa01\_F14.raw |  |  |  |  | 2.4579E6 | 4.7925E6 |  |  |  |  |  |  |  |  | 3 | 0 | 0 | 0 | 0 | 1 | 2 | 0 | 0 | 0 | 0 | 0 | 0 | 0 | 0 | 278 | 287 |  |  | PEAKS DB |
| K.VVYDLSQKPGK.R | Y | 69.45 | 1232.6764 | 11 | 0.4 | 617.3457 | 2 | 11.55 | 6 | F6:1927 | NaSa01\_F14.raw |  |  |  |  | 6.1516E6 | 7.9536E6 |  |  |  |  |  |  |  |  | 4 | 0 | 0 | 0 | 0 | 2 | 2 | 0 | 0 | 0 | 0 | 0 | 0 | 0 | 0 | 433 | 443 |  |  | PEAKS DB |
| Y.TGSPPSNEVAAGNYPFMQLSDDGR.Q | N | 69.41 | 2509.1179 | 24 | 0.8 | 837.3806 | 3 | 65.65 | 6 | F6:42541 | NaSa01\_F14.raw |  |  |  |  | 1.8833E7 | 4.2184E7 |  |  |  |  |  |  |  |  | 4 | 0 | 0 | 0 | 0 | 2 | 2 | 0 | 0 | 0 | 0 | 0 | 0 | 0 | 0 | 225 | 248 |  |  | PEAKS DB |
| S.FLAAGGDGYYMLK.G | N | 67.12 | 1404.6747 | 13 | 0.2 | 703.3447 | 2 | 58.91 | 6 | F6:37338 | NaSa01\_F14.raw |  |  |  |  | 1.8909E7 | 8.0208E7 |  |  |  |  |  |  |  |  | 3 | 0 | 0 | 0 | 0 | 1 | 2 | 0 | 0 | 0 | 0 | 0 | 0 | 0 | 0 | 476 | 488 |  |  | PEAKS DB |
| F.LAAGGDGYYMLK.G | N | 66.41 | 1257.6063 | 12 | 0.2 | 629.8105 | 2 | 41.73 | 6 | F6:22051 | NaSa01\_F14.raw |  |  |  |  |  | 5.7329E6 |  |  |  |  |  |  |  |  | 1 | 0 | 0 | 0 | 0 | 0 | 1 | 0 | 0 | 0 | 0 | 0 | 0 | 0 | 0 | 477 | 488 |  |  | PEAKS DB |
| R.EVVHFM(+15.99)NSLR.Y | N | 65.78 | 1246.6128 | 10 | 0.3 | 624.3138 | 2 | 31.71 | 6 | F6:14277 | NaSa01\_F14.raw |  |  |  |  | 3.2821E6 | 5.0137E7 |  |  |  |  |  |  |  |  | 13 | 0 | 0 | 0 | 0 | 2 | 11 | 0 | 0 | 0 | 0 | 0 | 0 | 0 | 0 | 75 | 84 | Oxidation (M) | M6:Oxidation (M):1000.00 | PEAKS DB |
| R.VPTYVPLEM(+15.99)EK.T | N | 64.86 | 1320.6635 | 11 | 0.4 | 661.3392 | 2 | 41.94 | 6 | F6:25976 | NaSa01\_F14.raw |  |  |  |  | 7.6238E7 | 9.8061E7 |  |  |  |  |  |  |  |  | 15 | 0 | 0 | 0 | 0 | 7 | 8 | 0 | 0 | 0 | 0 | 0 | 0 | 0 | 0 | 457 | 467 | Oxidation (M) | M9:Oxidation (M):1000.00 | PEAKS DB |
| K.GDSSNHSSGDLDISIVGDYIK.R | N | 64.63 | 2178.0076 | 21 | 1.0 | 727.0105 | 3 | 59.88 | 6 | F6:37927 | NaSa01\_F14.raw |  |  |  |  | 1.4469E6 | 1.4153E8 |  |  |  |  |  |  |  |  | 3 | 0 | 0 | 0 | 0 | 1 | 2 | 0 | 0 | 0 | 0 | 0 | 0 | 0 | 0 | 489 | 509 |  |  | PEAKS DB |
| R.VPTYVPLEMEKTYK.V | N | 64.21 | 1696.8745 | 14 | 0.2 | 566.6322 | 3 | 43.05 | 6 | F6:23317 | NaSa01\_F14.raw |  |  |  |  | 9.4948E5 | 1.8657E6 |  |  |  |  |  |  |  |  | 2 | 0 | 0 | 0 | 0 | 1 | 1 | 0 | 0 | 0 | 0 | 0 | 0 | 0 | 0 | 457 | 470 |  |  | PEAKS DB |
| K.FPILSANIRPK.G | N | 63.57 | 1254.7448 | 11 | 1.8 | 419.2563 | 3 | 38.83 | 6 | F6:20180 | NaSa01\_F14.raw |  |  |  | 3.6184E5 | 9.4886E7 | 1.2559E8 |  |  |  |  | 2.1098E6 |  |  |  | 10 | 0 | 0 | 0 | 1 | 2 | 5 | 0 | 0 | 0 | 0 | 2 | 0 | 0 | 0 | 112 | 122 |  |  | PEAKS DB |
| K.RVVSLNVLC(+57.02)TEC(+57.02)R.V | N | 62.55 | 1604.8126 | 13 | 0.2 | 535.9449 | 3 | 35.03 | 6 | F6:16539 | NaSa01\_F14.raw |  |  |  |  | 4.4155E6 | 6.0208E6 |  |  |  |  |  |  |  |  | 3 | 0 | 0 | 0 | 0 | 1 | 2 | 0 | 0 | 0 | 0 | 0 | 0 | 0 | 0 | 444 | 456 | Carbamidomethylation | C9:Carbamidomethylation:1000.00;C12:Carbamidomethylation:1000.00 | PEAKS DB |
| K.GVDVVVGGHTNTF.L | N | 61.79 | 1300.6411 | 13 | 1.1 | 651.3286 | 2 | 41.80 | 6 | F6:22128 | NaSa01\_F14.raw |  |  |  |  | 2.249E7 | 5.2209E7 |  |  |  |  |  |  |  |  | 2 | 0 | 0 | 0 | 0 | 1 | 1 | 0 | 0 | 0 | 0 | 0 | 0 | 0 | 0 | 210 | 222 |  |  | PEAKS DB |
| L.TAVLPFGGTFDLLQIK.G | N | 60.64 | 1718.9607 | 16 | -0.1 | 860.4875 | 2 | 99.74 | 6 | F6:71142 | NaSa01\_F14.raw |  |  | 2.8271E6 | 1.2518E6 | 2.1013E7 | 4.0691E6 |  |  |  |  |  |  |  |  | 4 | 0 | 0 | 1 | 1 | 1 | 1 | 0 | 0 | 0 | 0 | 0 | 0 | 0 | 0 | 388 | 403 |  |  | PEAKS DB |
| K.IINVGSEKVGIIGYTTK.E | N | 60.09 | 1791.0142 | 17 | -0.7 | 598.0116 | 3 | 45.32 | 6 | F6:25348 | NaSa01\_F14.raw |  |  |  |  | 9.4386E5 | 1.7014E6 |  |  |  |  |  |  |  |  | 2 | 0 | 0 | 0 | 0 | 1 | 1 | 0 | 0 | 0 | 0 | 0 | 0 | 0 | 0 | 138 | 154 |  |  | PEAKS DB |
| K.VFPAMEGR.M | N | 56.33 | 905.4429 | 8 | 0.2 | 453.7288 | 2 | 22.33 | 6 | F6:7722 | NaSa01\_F14.raw |  |  |  |  |  | 3.6244E6 |  |  |  |  |  |  |  |  | 2 | 0 | 0 | 0 | 0 | 0 | 2 | 0 | 0 | 0 | 0 | 0 | 0 | 0 | 0 | 514 | 521 |  |  | PEAKS DB |
| N.ISGYILPYK.I | N | 56.17 | 1052.5906 | 9 | 0.7 | 527.3029 | 2 | 45.82 | 6 | F6:26096 | NaSa01\_F14.raw |  |  |  | 2.2592E5 | 4.6763E8 | 8.5839E8 |  |  |  |  |  |  |  |  | 5 | 0 | 0 | 0 | 1 | 1 | 3 | 0 | 0 | 0 | 0 | 0 | 0 | 0 | 0 | 129 | 137 |  |  | PEAKS DB |
| S.FLAAGGDGYYM(+15.99)LK.G | N | 55.62 | 1420.6697 | 13 | -0.1 | 711.3420 | 2 | 48.69 | 6 | F6:28173 | NaSa01\_F14.raw |  |  |  |  |  | 5.7308E6 |  |  |  |  |  |  |  |  | 1 | 0 | 0 | 0 | 0 | 0 | 1 | 0 | 0 | 0 | 0 | 0 | 0 | 0 | 0 | 476 | 488 | Oxidation (M) | M11:Oxidation (M):1000.00 | PEAKS DB |
| H.GQGTGELLQVSGIK.V | N | 55.06 | 1385.7515 | 14 | 0.3 | 693.8832 | 2 | 45.20 | 6 | F6:25108 | NaSa01\_F14.raw |  |  |  |  |  | 1.2594E6 |  |  |  |  |  |  |  |  | 1 | 0 | 0 | 0 | 0 | 0 | 1 | 0 | 0 | 0 | 0 | 0 | 0 | 0 | 0 | 419 | 432 |  |  | PEAKS DB |
| K.GVDVVVGGHTNTFLY.T | N | 54.75 | 1576.7886 | 15 | 0.1 | 789.4016 | 2 | 63.80 | 6 | F6:41327 | NaSa01\_F14.raw |  |  |  |  | 1.2739E7 | 2.8201E7 |  |  |  |  |  |  |  |  | 2 | 0 | 0 | 0 | 0 | 1 | 1 | 0 | 0 | 0 | 0 | 0 | 0 | 0 | 0 | 210 | 224 |  |  | PEAKS DB |
| R.QVPVVQAYAF.G | N | 53.91 | 1120.5917 | 10 | 0.0 | 561.3031 | 2 | 73.70 | 6 | F6:49221 | NaSa01\_F14.raw |  |  |  |  | 7.1015E6 | 1.9997E7 |  |  |  |  |  |  |  |  | 2 | 0 | 0 | 0 | 0 | 1 | 1 | 0 | 0 | 0 | 0 | 0 | 0 | 0 | 0 | 249 | 258 |  |  | PEAKS DB |
| M.ALGNHEFDNGLNGLLDPLLK.N | N | 53.45 | 2149.1167 | 20 | 0.0 | 717.3795 | 3 | 83.56 | 6 | F6:57342 | NaSa01\_F14.raw |  |  |  |  | 5.3244E6 | 2.0787E7 |  |  |  |  |  |  |  |  | 3 | 0 | 0 | 0 | 0 | 1 | 2 | 0 | 0 | 0 | 0 | 0 | 0 | 0 | 0 | 89 | 108 |  |  | PEAKS DB |
| K.IIALGHSGFMED.C | Y | 52.82 | 1288.6122 | 12 | 1.2 | 645.3141 | 2 | 55.54 | 6 | F6:34199 | NaSa01\_F14.raw |  |  |  |  |  | 1.6459E6 |  |  |  |  |  |  |  |  | 1 | 0 | 0 | 0 | 0 | 0 | 1 | 0 | 0 | 0 | 0 | 0 | 0 | 0 | 0 | 190 | 201 |  |  | PEAKS DB |
| K.LTTLGVNK.I | N | 52.68 | 844.5018 | 8 | -1.0 | 423.2578 | 2 | 11.58 | 6 | F6:2148 | NaSa01\_F14.raw |  |  |  |  |  | 3.1326E6 |  |  |  |  |  |  |  |  | 1 | 0 | 0 | 0 | 0 | 0 | 1 | 0 | 0 | 0 | 0 | 0 | 0 | 0 | 0 | 182 | 189 |  |  | PEAKS DB |
| H.TNTFLYTGSPPSNEVAAGNYPFMQLSDDGR.Q | N | 52.38 | 3248.4719 | 30 | 1.3 | 1083.8326 | 3 | 85.33 | 6 | F6:59051 | NaSa01\_F14.raw |  |  |  |  |  | 8.2809E6 |  |  |  |  |  |  |  |  | 1 | 0 | 0 | 0 | 0 | 0 | 1 | 0 | 0 | 0 | 0 | 0 | 0 | 0 | 0 | 219 | 248 |  |  | PEAKS DB |
| K.GVDVVVGGHTNTFLYTGSPPSNEVAAGNYPFMQLSDDGR.Q | N | 51.80 | 4067.8960 | 39 | 0.4 | 1356.9731 | 3 | 83.86 | 6 | F6:58119 | NaSa01\_F14.raw |  |  |  |  |  | 2.3146E7 |  |  |  |  |  |  |  |  | 2 | 0 | 0 | 0 | 0 | 0 | 2 | 0 | 0 | 0 | 0 | 0 | 0 | 0 | 0 | 210 | 248 |  |  | PEAKS DB |
| K.VLLPSFLAAGGDGY.Y | N | 51.68 | 1378.7133 | 14 | 0.7 | 690.3644 | 2 | 100.88 | 6 | F6:72024 | NaSa01\_F14.raw |  |  |  |  | 1.3644E6 | 2.8748E6 |  |  |  |  |  |  |  |  | 2 | 0 | 0 | 0 | 0 | 1 | 1 | 0 | 0 | 0 | 0 | 0 | 0 | 0 | 0 | 471 | 484 |  |  | PEAKS DB |
| F.GGTFDLLQIK.G | N | 50.44 | 1090.6022 | 10 | -0.6 | 546.3080 | 2 | 64.67 | 6 | F6:41967 | NaSa01\_F14.raw |  |  |  |  |  | 9.7795E7 |  |  |  |  |  |  |  |  | 2 | 0 | 0 | 0 | 0 | 0 | 2 | 0 | 0 | 0 | 0 | 0 | 0 | 0 | 0 | 394 | 403 |  |  | PEAKS DB |
| K.VGIIGYTTK.E | N | 50.04 | 950.5436 | 9 | 0.3 | 476.2792 | 2 | 28.48 | 6 | F6:11194 | NaSa01\_F14.raw |  |  |  |  |  | 5.1319E6 |  |  |  |  |  |  |  |  | 2 | 0 | 0 | 0 | 0 | 0 | 2 | 0 | 0 | 0 | 0 | 0 | 0 | 0 | 0 | 146 | 154 |  |  | PEAKS DB |
| L.AAGGDGYYMLK.G | N | 49.71 | 1144.5222 | 11 | 0.7 | 573.2688 | 2 | 30.46 | 5 | F5:13468 | NaSa01\_F13.raw |  |  |  |  | 6.4456E6 |  |  |  |  |  |  |  |  |  | 1 | 0 | 0 | 0 | 0 | 1 | 0 | 0 | 0 | 0 | 0 | 0 | 0 | 0 | 0 | 478 | 488 |  |  | PEAKS DB |
| K.GVDVVVGGHTNTFLYTGSPPSNEVAAGNYPFM(+15.99)QLSDDGR.Q | N | 48.61 | 4083.8909 | 39 | 0.5 | 1362.3049 | 3 | 77.12 | 6 | F6:52373 | NaSa01\_F14.raw |  |  |  |  | 2.9373E6 | 7.6942E6 |  |  |  |  |  |  |  |  | 3 | 0 | 0 | 0 | 0 | 1 | 2 | 0 | 0 | 0 | 0 | 0 | 0 | 0 | 0 | 210 | 248 | Oxidation (M) | M32:Oxidation (M):1000.00 | PEAKS DB |
| R.FHEC(+57.02)NLGNLIC(+57.02)DAVVY.N | N | 48.35 | 1922.8655 | 16 | 1.4 | 962.4414 | 2 | 82.45 | 5 | F5:56634 | NaSa01\_F13.raw |  |  |  |  | 8.0322E7 | 1.7562E8 |  |  |  |  |  |  |  | 2.4692E7 | 3 | 0 | 0 | 0 | 0 | 1 | 1 | 0 | 0 | 0 | 0 | 0 | 0 | 0 | 1 | 330 | 345 | Carbamidomethylation | C4:Carbamidomethylation:1000.00;C11:Carbamidomethylation:1000.00 | PEAKS DB |
| R.HGQGTGELLQVSGIKVVYDLSQKPGK.R | Y | 48.22 | 2737.4763 | 26 | 0.2 | 685.3765 | 4 | 57.14 | 5 | F5:36175 | NaSa01\_F13.raw |  |  |  |  | 6.6345E5 |  |  |  |  |  |  |  |  |  | 1 | 0 | 0 | 0 | 0 | 1 | 0 | 0 | 0 | 0 | 0 | 0 | 0 | 0 | 0 | 418 | 443 |  |  | PEAKS DB |
| R.HPDDNEWNHVSMC(+57.02).I | N | 48.19 | 1639.6144 | 13 | 0.5 | 820.8149 | 2 | 26.12 | 5 | F5:10348 | NaSa01\_F13.raw |  |  |  |  | 8.8799E6 |  |  |  |  |  |  |  |  |  | 2 | 0 | 0 | 0 | 0 | 2 | 0 | 0 | 0 | 0 | 0 | 0 | 0 | 0 | 0 | 350 | 362 | Carbamidomethylation | C13:Carbamidomethylation:1000.00 | PEAKS DB |
| K.VLLPSFLAAGGD.G | N | 47.35 | 1158.6284 | 12 | -0.1 | 580.3214 | 2 | 96.95 | 6 | F6:68512 | NaSa01\_F14.raw |  |  |  |  |  | 1.1427E6 |  |  |  |  |  |  |  |  | 1 | 0 | 0 | 0 | 0 | 0 | 1 | 0 | 0 | 0 | 0 | 0 | 0 | 0 | 0 | 471 | 482 |  |  | PEAKS DB |
| R.HPDDNEWNHVSM.C | N | 47.22 | 1479.5837 | 12 | 1.1 | 740.8000 | 2 | 25.93 | 5 | F5:10070 | NaSa01\_F13.raw |  |  |  |  | 2.3098E6 |  |  |  |  |  |  |  |  |  | 1 | 0 | 0 | 0 | 0 | 1 | 0 | 0 | 0 | 0 | 0 | 0 | 0 | 0 | 0 | 350 | 361 |  |  | PEAKS DB |
| R.QVPVVQAY.A | N | 45.65 | 902.4861 | 8 | -0.5 | 452.2501 | 2 | 43.38 | 5 | F5:24541 | NaSa01\_F13.raw |  |  |  |  | 1.2052E7 |  |  |  |  |  |  |  |  |  | 1 | 0 | 0 | 0 | 0 | 1 | 0 | 0 | 0 | 0 | 0 | 0 | 0 | 0 | 0 | 249 | 256 |  |  | PEAKS DB |
| N.PGPYLEFR.D | N | 45.44 | 977.4970 | 8 | 0.7 | 489.7561 | 2 | 39.90 | 6 | F6:20609 | NaSa01\_F14.raw |  |  |  |  |  | 4.6995E5 |  |  |  |  |  |  |  |  | 1 | 0 | 0 | 0 | 0 | 0 | 1 | 0 | 0 | 0 | 0 | 0 | 0 | 0 | 0 | 162 | 169 |  |  | PEAKS DB |
| N.LGNLIC(+57.02)DAVVYNNLR.H | N | 45.20 | 1732.8931 | 15 | 1.0 | 867.4547 | 2 | 69.10 | 6 | F6:45743 | NaSa01\_F14.raw |  |  |  |  |  | 1.1316E6 |  |  |  |  |  |  |  |  | 1 | 0 | 0 | 0 | 0 | 0 | 1 | 0 | 0 | 0 | 0 | 0 | 0 | 0 | 0 | 335 | 349 | Carbamidomethylation | C6:Carbamidomethylation:1000.00 | PEAKS DB |
| L.YTGSPPSNEVAAGNYPFMQLSDDGR.Q | N | 45.19 | 2672.1812 | 25 | -0.8 | 1337.0968 | 2 | 68.20 | 6 | F6:44852 | NaSa01\_F14.raw |  |  |  |  |  | 0 |  |  |  |  |  |  |  |  | 0 | 0 | 0 | 0 | 0 | 0 | 0 | 0 | 0 | 0 | 0 | 0 | 0 | 0 | 0 | 224 | 248 |  |  | PEAKS DB |
| K.IINVGSEK.V | N | 44.70 | 858.4811 | 8 | -0.5 | 430.2476 | 2 | 11.48 | 6 | F6:1861 | NaSa01\_F14.raw |  |  |  |  |  | 1.3857E6 |  |  |  |  |  |  |  |  | 1 | 0 | 0 | 0 | 0 | 0 | 1 | 0 | 0 | 0 | 0 | 0 | 0 | 0 | 0 | 138 | 145 |  |  | PEAKS DB |
| K.VLLPSFLAAGGDGYY.M | N | 43.81 | 1541.7765 | 15 | 0.7 | 771.8961 | 2 | 105.28 | 6 | F6:75496 | NaSa01\_F14.raw |  |  |  |  | 1.4762E7 | 3.1893E7 |  |  |  |  |  |  |  |  | 2 | 0 | 0 | 0 | 0 | 1 | 1 | 0 | 0 | 0 | 0 | 0 | 0 | 0 | 0 | 471 | 485 |  |  | PEAKS DB |
| K.FPILSANIR.P | N | 43.38 | 1029.5970 | 9 | -0.4 | 515.8056 | 2 | 52.72 | 6 | F6:31843 | NaSa01\_F14.raw |  |  |  |  |  | 1.5657E8 |  |  |  |  |  |  |  |  | 1 | 0 | 0 | 0 | 0 | 0 | 1 | 0 | 0 | 0 | 0 | 0 | 0 | 0 | 0 | 112 | 120 |  |  | PEAKS DB |
| K.VLLPSFLAAGGDGYYM.L | N | 42.72 | 1672.8170 | 16 | 0.9 | 837.4166 | 2 | 111.51 | 6 | F6:80284 | NaSa01\_F14.raw |  |  |  |  |  | 1.1099E6 |  |  |  |  |  |  |  |  | 1 | 0 | 0 | 0 | 0 | 0 | 1 | 0 | 0 | 0 | 0 | 0 | 0 | 0 | 0 | 471 | 486 |  |  | PEAKS DB |
| Q.GTGELLQVSGIK.V | N | 42.14 | 1200.6714 | 12 | 0.9 | 601.3435 | 2 | 44.43 | 6 | F6:24568 | NaSa01\_F14.raw |  |  |  |  |  | 7.0353E5 |  |  |  |  |  |  |  |  | 1 | 0 | 0 | 0 | 0 | 0 | 1 | 0 | 0 | 0 | 0 | 0 | 0 | 0 | 0 | 421 | 432 |  |  | PEAKS DB |
| K.LTTLGVNKIIALGH.S | N | 41.05 | 1448.8715 | 14 | 0.1 | 483.9645 | 3 | 54.77 | 6 | F6:33352 | NaSa01\_F14.raw |  |  |  |  |  | 5.5856E5 |  |  |  |  |  |  |  |  | 1 | 0 | 0 | 0 | 0 | 0 | 1 | 0 | 0 | 0 | 0 | 0 | 0 | 0 | 0 | 182 | 195 |  |  | PEAKS DB |
| N.HEFDNGLNGLLDPLLK.N | N | 40.62 | 1793.9312 | 16 | 0.2 | 598.9844 | 3 | 81.57 | 6 | F6:55994 | NaSa01\_F14.raw |  |  |  |  |  | 5.7086E5 |  |  |  |  |  |  |  |  | 1 | 0 | 0 | 0 | 0 | 0 | 1 | 0 | 0 | 0 | 0 | 0 | 0 | 0 | 0 | 93 | 108 |  |  | PEAKS DB |
| N.YPFMQLSDDGR.Q | N | 40.57 | 1327.5867 | 11 | 0.4 | 664.8009 | 2 | 53.65 | 6 | F6:32287 | NaSa01\_F14.raw |  |  |  |  |  | 5.3395E5 |  |  |  |  |  |  |  |  | 1 | 0 | 0 | 0 | 0 | 0 | 1 | 0 | 0 | 0 | 0 | 0 | 0 | 0 | 0 | 238 | 248 |  |  | PEAKS DB |
| A.AGNYPFMQLSDDGR.Q | N | 40.07 | 1569.6881 | 14 | 0.1 | 785.8514 | 2 | 56.81 | 6 | F6:35126 | NaSa01\_F14.raw |  |  |  |  |  | 2.5054E5 |  |  |  |  |  |  |  |  | 1 | 0 | 0 | 0 | 0 | 0 | 1 | 0 | 0 | 0 | 0 | 0 | 0 | 0 | 0 | 235 | 248 |  |  | PEAKS DB |
| K.NVKFPILSAN.I | N | 39.82 | 1101.6182 | 10 | 0.5 | 551.8167 | 2 | 54.61 | 6 | F6:33186 | NaSa01\_F14.raw |  |  |  |  |  | 1.3385E6 |  |  |  |  |  |  |  |  | 1 | 0 | 0 | 0 | 0 | 0 | 1 | 0 | 0 | 0 | 0 | 0 | 0 | 0 | 0 | 109 | 118 |  |  | PEAKS DB |
| K.IIALGHSGF.M | N | 37.88 | 913.5021 | 9 | -1.2 | 457.7578 | 2 | 43.37 | 6 | F6:23538 | NaSa01\_F14.raw |  |  |  |  |  | 5.7946E6 |  |  |  |  |  |  |  |  | 1 | 0 | 0 | 0 | 0 | 0 | 1 | 0 | 0 | 0 | 0 | 0 | 0 | 0 | 0 | 190 | 198 |  |  | PEAKS DB |
| total 70 peptides |  |  |  |  |  |  |  |  |  |  |  |  |  |  |  |  |  |  |  |  |  |  |  |  |  |  |  |  |  |  |  |  |  |  |  |  |  |  |  |  |  |  |  |  |  |
| --- | --- | --- | --- | --- | --- | --- | --- | --- | --- | --- | --- | --- | --- | --- | --- | --- | --- | --- | --- | --- | --- | --- | --- | --- | --- | --- | --- | --- | --- | --- | --- | --- | --- | --- | --- | --- | --- | --- | --- | --- | --- | --- | --- | --- | --- |

3PRX

back to list

  

| Protein Coverage
| Supporting Peptides
|

Protein Coverage:

Supporting Peptides:

| Peptide | Uniq | -10lgP | Mass | Length | ppm | m/z | z | RT | Fraction | Scan | Source File | Area F1 | Area F10 | Area F11 | Area F12 | Area F13 | Area F14 | Area F2 | Area F3 | Area F4 | Area F5 | Area F6 | Area F7 | Area F8 | Area F9 | #Feature | #Feature F1 | #Feature F10 | #Feature F11 | #Feature F12 | #Feature F13 | #Feature F14 | #Feature F2 | #Feature F3 | #Feature F4 | #Feature F5 | #Feature F6 | #Feature F7 | #Feature F8 | #Feature F9 | Start | End | PTM | AScore | Found By |
| --- | --- | --- | --- | --- | --- | --- | --- | --- | --- | --- | --- | --- | --- | --- | --- | --- | --- | --- | --- | --- | --- | --- | --- | --- | --- | --- | --- | --- | --- | --- | --- | --- | --- | --- | --- | --- | --- | --- | --- | --- | --- | --- | --- | --- | --- |
| R.AILHNYVNEDIYVR.V | Y | 89.83 | 1717.8788 | 14 | 0.3 | 859.9469 | 2 | 43.05 | 6 | F6:23378 | NaSa01\_F14.raw |  |  |  |  | 1.5001E7 | 1.9759E8 |  |  |  |  |  |  |  |  | 8 | 0 | 0 | 0 | 0 | 3 | 5 | 0 | 0 | 0 | 0 | 0 | 0 | 0 | 0 | 834 | 847 |  |  | PEAKS DB |
| R.AVPFVIVPLEQGLHDVEIK.A | Y | 87.23 | 2102.1775 | 19 | 0.5 | 701.7335 | 3 | 85.72 | 6 | F6:59617 | NaSa01\_F14.raw |  |  |  | 7.0958E5 | 1.6836E7 | 1.2181E8 |  |  |  |  |  |  |  |  | 7 | 0 | 0 | 0 | 1 | 2 | 4 | 0 | 0 | 0 | 0 | 0 | 0 | 0 | 0 | 879 | 897 |  |  | PEAKS DB |
| K.HFEVGFIQPGSVK.V | N | 85.09 | 1443.7510 | 13 | -0.2 | 722.8826 | 2 | 44.13 | 6 | F6:24159 | NaSa01\_F14.raw |  |  |  |  | 2.4365E7 | 2.5116E8 |  |  |  |  |  |  |  |  | 8 | 0 | 0 | 0 | 0 | 2 | 6 | 0 | 0 | 0 | 0 | 0 | 0 | 0 | 0 | 1445 | 1457 |  |  | PEAKS DB |
| R.RDGQNLVTM(+15.99)NLHITPDLIPSFR.F | N | 85.06 | 2552.3169 | 22 | 0.3 | 639.0867 | 4 | 69.77 | 6 | F6:46133 | NaSa01\_F14.raw |  |  |  |  |  | 2.5484E6 |  |  |  |  |  |  |  |  | 2 | 0 | 0 | 0 | 0 | 0 | 2 | 0 | 0 | 0 | 0 | 0 | 0 | 0 | 0 | 498 | 519 | Oxidation (M) | M9:Oxidation (M):1000.00 | PEAKS DB |
| R.QNQYVVVQVTGPQVR.L | N | 83.17 | 1713.9163 | 15 | 0.2 | 857.9656 | 2 | 43.83 | 6 | F6:24244 | NaSa01\_F14.raw |  |  |  |  | 1.4255E7 | 1.5135E8 |  |  |  |  |  |  |  |  | 5 | 0 | 0 | 0 | 0 | 2 | 3 | 0 | 0 | 0 | 0 | 0 | 0 | 0 | 0 | 100 | 114 |  |  | PEAKS DB |
| K.VFFIDLQMPYSVVK.N | Y | 82.52 | 1684.8898 | 14 | 0.3 | 843.4524 | 2 | 95.37 | 6 | F6:67466 | NaSa01\_F14.raw |  |  |  |  | 5.5288E5 | 4.4172E6 |  |  |  |  |  |  |  |  | 2 | 0 | 0 | 0 | 0 | 1 | 1 | 0 | 0 | 0 | 0 | 0 | 0 | 0 | 0 | 813 | 826 |  |  | PEAKS DB |
| K.VNDDYLIWGSR.S | Y | 81.98 | 1336.6411 | 11 | 1.0 | 669.3285 | 2 | 59.77 | 6 | F6:37640 | NaSa01\_F14.raw |  |  |  | 4.0113E7 | 1.558E7 | 1.2947E8 |  |  |  |  |  |  |  |  | 4 | 0 | 0 | 0 | 1 | 2 | 1 | 0 | 0 | 0 | 0 | 0 | 0 | 0 | 0 | 1577 | 1587 |  |  | PEAKS DB |
| R.IEEQDGNDIYVMDVLEVIK.Q | Y | 80.07 | 2221.0823 | 19 | 0.7 | 1111.5492 | 2 | 101.01 | 4 | F4:73173 | NaSa01\_F12.raw |  |  |  | 1.608E7 | 4.109E6 | 2.6808E7 |  |  |  |  |  |  |  |  | 11 | 0 | 0 | 0 | 4 | 3 | 4 | 0 | 0 | 0 | 0 | 0 | 0 | 0 | 0 | 1531 | 1549 |  |  | PEAKS DB |
| K.ATMTILTFYNAQLQEK.A | Y | 79.74 | 1870.9498 | 16 | -0.2 | 936.4820 | 2 | 77.02 | 6 | F6:52034 | NaSa01\_F14.raw |  |  | 5.3707E5 | 1.2422E6 | 3.5134E6 | 4.7877E7 |  |  |  |  |  |  |  |  | 6 | 0 | 0 | 1 | 1 | 1 | 3 | 0 | 0 | 0 | 0 | 0 | 0 | 0 | 0 | 1321 | 1336 |  |  | PEAKS DB |
| K.QLDIFVHDFPR.K | N | 79.15 | 1385.7091 | 11 | 0.0 | 693.8618 | 2 | 58.44 | 6 | F6:36683 | NaSa01\_F14.raw |  |  |  |  | 9.0136E6 | 8.647E7 |  |  |  |  |  |  |  |  | 4 | 0 | 0 | 0 | 0 | 2 | 2 | 0 | 0 | 0 | 0 | 0 | 0 | 0 | 0 | 53 | 63 |  |  | PEAKS DB |
| K.GDNLIQMPGAAMK.I | Y | 78.60 | 1344.6530 | 13 | 0.2 | 673.3339 | 2 | 49.26 | 6 | F6:28453 | NaSa01\_F14.raw |  |  |  |  |  | 1.0626E8 |  |  |  |  |  |  |  |  | 2 | 0 | 0 | 0 | 0 | 0 | 2 | 0 | 0 | 0 | 0 | 0 | 0 | 0 | 0 | 552 | 564 |  |  | PEAKS DB |
| F.VIVPLEQGLHDVEIK.A | Y | 76.27 | 1687.9508 | 15 | 0.0 | 844.9827 | 2 | 58.08 | 6 | F6:36376 | NaSa01\_F14.raw |  |  |  |  | 5.5586E6 | 9.3853E7 |  |  |  |  |  |  |  |  | 3 | 0 | 0 | 0 | 0 | 1 | 2 | 0 | 0 | 0 | 0 | 0 | 0 | 0 | 0 | 883 | 897 |  |  | PEAKS DB |
| R.DDNEDGFIADSDIISR.S | Y | 75.58 | 1780.7751 | 16 | 0.3 | 891.3951 | 2 | 68.40 | 6 | F6:44898 | NaSa01\_F14.raw |  |  |  |  | 9.0776E5 | 1.2244E7 |  |  |  |  |  |  |  |  | 4 | 0 | 0 | 0 | 0 | 1 | 3 | 0 | 0 | 0 | 0 | 0 | 0 | 0 | 0 | 733 | 748 |  |  | PEAKS DB |
| K.C(+57.02)C(+57.02)EDVMHENPMGYTC(+57.02)EK.R | Y | 74.09 | 2158.7886 | 17 | 7.4 | 720.6050 | 3 | 31.06 | 8 | F8:16109 | NaSa01\_F3.raw |  |  |  |  |  |  |  | 1.0485E7 | 5.6652E7 | 1.4617E7 |  |  |  |  | 6 | 0 | 0 | 0 | 0 | 0 | 0 | 0 | 1 | 3 | 2 | 0 | 0 | 0 | 0 | 677 | 693 | Carbamidomethylation | C1:Carbamidomethylation:1000.00;C2:Carbamidomethylation:1000.00;C15:Carbamidomethylation:1000.00 | PEAKS DB |
| R.INYENALLAR.T | Y | 74.07 | 1175.6299 | 10 | 0.1 | 588.8223 | 2 | 45.12 | 6 | F6:25480 | NaSa01\_F14.raw |  |  | 3.3741E5 | 7.6601E6 | 1.5972E7 | 1.5972E8 |  |  |  |  |  |  |  |  | 7 | 0 | 0 | 1 | 3 | 1 | 2 | 0 | 0 | 0 | 0 | 0 | 0 | 0 | 0 | 1292 | 1301 |  |  | PEAKS DB |
| Y.YQVGNNEIVADSVWVDVK.D | N | 73.99 | 2034.0057 | 18 | 0.2 | 1018.0104 | 2 | 70.65 | 6 | F6:46860 | NaSa01\_F14.raw |  |  |  |  |  | 6.5239E6 |  |  |  |  |  |  |  |  | 1 | 0 | 0 | 0 | 0 | 0 | 1 | 0 | 0 | 0 | 0 | 0 | 0 | 0 | 0 | 524 | 541 |  |  | PEAKS DB |
| K.ASVQEALWSDGVR.K | Y | 72.98 | 1416.6997 | 13 | 0.1 | 709.3572 | 2 | 49.71 | 6 | F6:29208 | NaSa01\_F14.raw |  |  |  |  | 1.3438E7 | 1.2825E8 |  |  |  |  |  |  |  |  | 7 | 0 | 0 | 0 | 0 | 1 | 6 | 0 | 0 | 0 | 0 | 0 | 0 | 0 | 0 | 898 | 910 |  |  | PEAKS DB |
| R.VELLYNPAFC(+57.02)SASTK.G | Y | 72.57 | 1698.8286 | 15 | -0.7 | 850.4210 | 2 | 59.22 | 6 | F6:37110 | NaSa01\_F14.raw |  |  |  |  | 1.6821E7 | 2.15E8 |  |  |  |  |  |  |  |  | 2 | 0 | 0 | 0 | 0 | 1 | 1 | 0 | 0 | 0 | 0 | 0 | 0 | 0 | 0 | 848 | 862 | Carbamidomethylation | C10:Carbamidomethylation:1000.00 | PEAKS DB |
| K.LNQDITVTASGDGK.A | Y | 72.09 | 1417.7048 | 14 | 0.8 | 709.8602 | 2 | 20.83 | 6 | F6:5302 | NaSa01\_F14.raw |  |  |  |  | 4.3744E5 | 4.5631E6 |  |  |  |  |  |  |  |  | 3 | 0 | 0 | 0 | 0 | 1 | 2 | 0 | 0 | 0 | 0 | 0 | 0 | 0 | 0 | 1307 | 1320 |  |  | PEAKS DB |
| K.LILNIPLNAQSLPITVR.T | Y | 71.60 | 1874.1353 | 17 | 0.5 | 625.7194 | 3 | 90.36 | 6 | F6:63322 | NaSa01\_F14.raw |  |  |  |  | 1.4111E7 | 4.901E7 |  |  |  |  |  |  |  |  | 4 | 0 | 0 | 0 | 0 | 2 | 2 | 0 | 0 | 0 | 0 | 0 | 0 | 0 | 0 | 398 | 414 |  |  | PEAKS DB |
| K.YFTYLILNK.G | N | 70.49 | 1173.6433 | 9 | 0.1 | 587.8290 | 2 | 64.67 | 6 | F6:41809 | NaSa01\_F14.raw |  |  |  |  | 1.0728E7 | 7.7707E7 |  |  |  |  |  |  |  |  | 2 | 0 | 0 | 0 | 0 | 1 | 1 | 0 | 0 | 0 | 0 | 0 | 0 | 0 | 0 | 478 | 486 |  |  | PEAKS DB |
| R.WPHEDEC(+57.02)QEEEFQK.L | Y | 69.62 | 1889.7526 | 14 | 0.9 | 945.8844 | 2 | 23.39 | 4 | F4:9200 | NaSa01\_F12.raw |  |  |  | 2.8919E7 |  | 3.166E6 |  |  |  |  |  |  |  |  | 3 | 0 | 0 | 0 | 2 | 0 | 1 | 0 | 0 | 0 | 0 | 0 | 0 | 0 | 0 | 1610 | 1623 | Carbamidomethylation | C7:Carbamidomethylation:1000.00 | PEAKS DB |
| K.SDFGC(+57.02)TAGSGQNNLGVFEDAGLALTTSTNLNTK.Q | N | 66.95 | 3359.5576 | 33 | 0.8 | 1120.8607 | 3 | 89.37 | 6 | F6:62425 | NaSa01\_F14.raw |  |  |  |  |  | 3.8512E7 |  |  |  |  |  |  |  |  | 1 | 0 | 0 | 0 | 0 | 0 | 1 | 0 | 0 | 0 | 0 | 0 | 0 | 0 | 0 | 605 | 637 | Carbamidomethylation | C5:Carbamidomethylation:1000.00 | PEAKS DB |
| R.DGQNLVTM(+15.99)NLHITPDLIPSFR.F | N | 66.34 | 2396.2158 | 21 | 0.4 | 799.7462 | 3 | 83.99 | 6 | F6:57985 | NaSa01\_F14.raw |  |  |  |  |  | 5.9574E6 |  |  |  |  |  |  |  |  | 1 | 0 | 0 | 0 | 0 | 0 | 1 | 0 | 0 | 0 | 0 | 0 | 0 | 0 | 0 | 499 | 519 | Oxidation (M) | M8:Oxidation (M):1000.00 | PEAKS DB |
| L.ARDDNEDGFIADSDIISR.S | Y | 66.31 | 2007.9133 | 18 | 1.0 | 670.3124 | 3 | 48.95 | 6 | F6:28381 | NaSa01\_F14.raw |  |  |  |  |  | 6.6726E6 |  |  |  |  |  |  |  |  | 2 | 0 | 0 | 0 | 0 | 0 | 2 | 0 | 0 | 0 | 0 | 0 | 0 | 0 | 0 | 731 | 748 |  |  | PEAKS DB |
| Y.VTNPDGSPAAHVPVVSEAFHSMGTTLSDGTAK.L | Y | 66.31 | 3179.5193 | 32 | 0.8 | 795.8877 | 4 | 59.12 | 6 | F6:37224 | NaSa01\_F14.raw |  |  |  |  |  | 2.3066E7 |  |  |  |  |  |  |  |  | 1 | 0 | 0 | 0 | 0 | 0 | 1 | 0 | 0 | 0 | 0 | 0 | 0 | 0 | 0 | 366 | 397 |  |  | PEAKS DB |
| K.C(+57.02)C(+57.02)EDVMHENPM(+15.99)GYTC(+57.02)EK.R | Y | 65.55 | 2174.7837 | 17 | 1.4 | 725.9362 | 3 | 11.70 | 9 | F9:2004 | NaSa01\_F4.raw |  |  |  |  |  |  |  |  | 1.0934E7 |  |  |  |  |  | 2 | 0 | 0 | 0 | 0 | 0 | 0 | 0 | 0 | 2 | 0 | 0 | 0 | 0 | 0 | 677 | 693 | Carbamidomethylation; Oxidation (M) | C1:Carbamidomethylation:1000.00;C2:Carbamidomethylation:1000.00;M11:Oxidation (M):43.99;C15:Carbamidomethylation:1000.00 | PEAKS DB |
| Y.NLPDLVSLGTWR.I | Y | 65.12 | 1369.7354 | 12 | 0.1 | 685.8750 | 2 | 81.18 | 6 | F6:55452 | NaSa01\_F14.raw |  |  |  |  | 6.3971E6 | 8.6753E7 |  |  |  |  |  |  |  |  | 2 | 0 | 0 | 0 | 0 | 1 | 1 | 0 | 0 | 0 | 0 | 0 | 0 | 0 | 0 | 187 | 198 |  |  | PEAKS DB |
| K.GDNLIQMPGAAM(+15.99)K.I | Y | 64.58 | 1360.6479 | 13 | 0.8 | 681.3318 | 2 | 38.53 | 6 | F6:19473 | NaSa01\_F14.raw |  |  |  |  | 9.6097E5 | 9.5549E6 |  |  |  |  |  |  |  |  | 3 | 0 | 0 | 0 | 0 | 1 | 2 | 0 | 0 | 0 | 0 | 0 | 0 | 0 | 0 | 552 | 564 | Oxidation (M) | M12:Oxidation (M):38.33 | PEAKS DB |
| E.VEGVAFVLFGVK.I | N | 62.96 | 1263.7227 | 12 | 0.2 | 632.8687 | 2 | 85.00 | 6 | F6:58754 | NaSa01\_F14.raw |  |  |  |  |  | 2.0835E6 |  |  |  |  |  |  |  |  | 1 | 0 | 0 | 0 | 0 | 0 | 1 | 0 | 0 | 0 | 0 | 0 | 0 | 0 | 0 | 257 | 268 |  |  | PEAKS DB |
| Y.QSSFLFIQTDK.G | Y | 62.43 | 1312.6663 | 11 | -0.4 | 657.3401 | 2 | 57.57 | 6 | F6:35846 | NaSa01\_F14.raw |  |  |  |  |  | 1.1659E7 |  |  |  |  |  |  |  |  | 1 | 0 | 0 | 0 | 0 | 0 | 1 | 0 | 0 | 0 | 0 | 0 | 0 | 0 | 0 | 124 | 134 |  |  | PEAKS DB |
| K.GVGGTQLEVIK.A | Y | 62.08 | 1099.6237 | 11 | 0.2 | 550.8192 | 2 | 30.32 | 12 | F12:15497 | NaSa01\_F7.raw |  |  |  |  | 1.4118E7 | 1.0253E6 |  |  |  |  |  | 1.7364E6 |  |  | 3 | 0 | 0 | 0 | 0 | 1 | 1 | 0 | 0 | 0 | 0 | 0 | 1 | 0 | 0 | 936 | 946 |  |  | PEAKS DB |
| K.ATM(+15.99)TILTFYNAQLQEK.A | Y | 62.07 | 1886.9448 | 16 | 0.4 | 944.4801 | 2 | 64.84 | 6 | F6:42088 | NaSa01\_F14.raw |  |  |  |  |  | 1.0319E7 |  |  |  |  |  |  |  |  | 2 | 0 | 0 | 0 | 0 | 0 | 2 | 0 | 0 | 0 | 0 | 0 | 0 | 0 | 0 | 1321 | 1336 | Oxidation (M) | M3:Oxidation (M):1000.00 | PEAKS DB |
| R.FPNLNELVGH.T | N | 62.05 | 1138.5770 | 10 | 0.8 | 570.2963 | 2 | 67.53 | 6 | F6:44045 | NaSa01\_F14.raw |  |  |  |  | 1.7762E6 | 2.8185E7 |  |  |  |  |  |  |  |  | 2 | 0 | 0 | 0 | 0 | 1 | 1 | 0 | 0 | 0 | 0 | 0 | 0 | 0 | 0 | 303 | 312 |  |  | PEAKS DB |
| K.VYSYYNLDEK.C | N | 61.93 | 1292.5924 | 10 | 1.2 | 647.3043 | 2 | 33.77 | 6 | F6:15693 | NaSa01\_F14.raw |  |  |  |  | 1.5304E7 | 1.0252E8 |  |  |  |  |  |  |  |  | 3 | 0 | 0 | 0 | 0 | 1 | 2 | 0 | 0 | 0 | 0 | 0 | 0 | 0 | 0 | 1458 | 1467 |  |  | PEAKS DB |
| M.TILTFYNAQLQEK.A | N | 61.57 | 1567.8246 | 13 | 0.3 | 784.9198 | 2 | 60.05 | 6 | F6:38047 | NaSa01\_F14.raw |  |  |  |  |  | 5.1346E6 |  |  |  |  |  |  |  |  | 1 | 0 | 0 | 0 | 0 | 0 | 1 | 0 | 0 | 0 | 0 | 0 | 0 | 0 | 0 | 1324 | 1336 |  |  | PEAKS DB |
| H.ITPDLIPSFR.F | N | 61.36 | 1157.6444 | 10 | 0.0 | 579.8295 | 2 | 65.01 | 6 | F6:42335 | NaSa01\_F14.raw |  |  |  |  | 3.0601E6 | 3.8923E7 |  |  |  |  |  |  |  |  | 3 | 0 | 0 | 0 | 0 | 1 | 2 | 0 | 0 | 0 | 0 | 0 | 0 | 0 | 0 | 510 | 519 |  |  | PEAKS DB |
| K.PGDNLPVNFNVK.G | Y | 60.28 | 1312.6775 | 12 | 0.4 | 657.3463 | 2 | 47.72 | 6 | F6:27452 | NaSa01\_F14.raw |  |  |  |  |  | 1.0823E6 |  |  |  |  |  |  |  |  | 1 | 0 | 0 | 0 | 0 | 0 | 1 | 0 | 0 | 0 | 0 | 0 | 0 | 0 | 0 | 456 | 467 |  |  | PEAKS DB |
| W.VVLAVSFTPTK.G | Y | 59.98 | 1160.6804 | 11 | 0.4 | 581.3477 | 2 | 51.95 | 6 | F6:30874 | NaSa01\_F14.raw |  |  |  |  |  | 3.7062E6 |  |  |  |  |  |  |  |  | 1 | 0 | 0 | 0 | 0 | 0 | 1 | 0 | 0 | 0 | 0 | 0 | 0 | 0 | 0 | 788 | 798 |  |  | PEAKS DB |
| K.YEHSPENYTAYFDVR.K | N | 59.87 | 1889.8220 | 15 | -0.9 | 630.9474 | 3 | 47.19 | 6 | F6:27480 | NaSa01\_F14.raw |  |  |  |  |  | 2.5484E6 |  |  |  |  |  |  |  |  | 2 | 0 | 0 | 0 | 0 | 0 | 2 | 0 | 0 | 0 | 0 | 0 | 0 | 0 | 0 | 203 | 217 |  |  | PEAKS DB |
| R.IDVPLQIEK.A | Y | 59.34 | 1053.6069 | 9 | 0.1 | 527.8108 | 2 | 47.86 | 6 | F6:27228 | NaSa01\_F14.raw |  |  |  | 3.0425E7 | 1.9664E7 | 1.6873E8 |  |  |  |  |  |  |  |  | 4 | 0 | 0 | 0 | 1 | 1 | 2 | 0 | 0 | 0 | 0 | 0 | 0 | 0 | 0 | 1506 | 1514 |  |  | PEAKS DB |
| K.IIIQGDPVAQIIENSIDGSK.L | Y | 59.31 | 2109.1316 | 20 | 0.6 | 1055.5737 | 2 | 96.62 | 6 | F6:68548 | NaSa01\_F14.raw |  |  | 1.4465E5 |  | 1.6001E5 | 2.5079E6 |  |  |  |  |  |  |  |  | 3 | 0 | 0 | 1 | 0 | 1 | 1 | 0 | 0 | 0 | 0 | 0 | 0 | 0 | 0 | 963 | 982 |  |  | PEAKS DB |
| K.GIC(+57.02)VAEPYEIR.V | Y | 59.06 | 1305.6387 | 11 | 0.4 | 653.8268 | 2 | 44.31 | 6 | F6:24643 | NaSa01\_F14.raw |  |  |  |  | 1.0302E7 | 1.0808E8 |  |  |  |  |  |  |  |  | 3 | 0 | 0 | 0 | 0 | 1 | 2 | 0 | 0 | 0 | 0 | 0 | 0 | 0 | 0 | 799 | 809 | Carbamidomethylation | C3:Carbamidomethylation:1000.00 | PEAKS DB |
| E.DGFIADSDIISR.S | Y | 58.62 | 1307.6357 | 12 | -0.1 | 654.8251 | 2 | 60.77 | 6 | F6:38594 | NaSa01\_F14.raw |  |  |  |  |  | 4.7593E6 |  |  |  |  |  |  |  |  | 1 | 0 | 0 | 0 | 0 | 0 | 1 | 0 | 0 | 0 | 0 | 0 | 0 | 0 | 0 | 737 | 748 |  |  | PEAKS DB |
| R.FPNLNELVGHTLY.A | N | 58.47 | 1515.7721 | 13 | 0.3 | 758.8936 | 2 | 85.62 | 6 | F6:59205 | NaSa01\_F14.raw |  |  |  |  |  | 1.9527E7 |  |  |  |  |  |  |  |  | 1 | 0 | 0 | 0 | 0 | 0 | 1 | 0 | 0 | 0 | 0 | 0 | 0 | 0 | 0 | 303 | 315 |  |  | PEAKS DB |
| S.MLTGFLPDAEDLTR.L | N | 58.03 | 1577.7759 | 14 | 0.5 | 789.8956 | 2 | 78.56 | 6 | F6:53520 | NaSa01\_F14.raw |  |  |  |  |  | 2.8623E6 |  |  |  |  |  |  |  |  | 1 | 0 | 0 | 0 | 0 | 0 | 1 | 0 | 0 | 0 | 0 | 0 | 0 | 0 | 0 | 1386 | 1399 |  |  | PEAKS DB |
| R.KYVLPSFEVR.L | Y | 56.74 | 1236.6866 | 10 | 0.3 | 413.2363 | 3 | 42.11 | 6 | F6:22829 | NaSa01\_F14.raw |  |  |  |  | 5.509E6 | 4.9733E7 |  |  |  |  |  |  |  |  | 6 | 0 | 0 | 0 | 0 | 2 | 4 | 0 | 0 | 0 | 0 | 0 | 0 | 0 | 0 | 218 | 227 |  |  | PEAKS DB |
| R.DGQNLVTMNLHITPDLIPSFR.F | N | 56.46 | 2380.2209 | 21 | 0.5 | 794.4147 | 3 | 91.58 | 6 | F6:64345 | NaSa01\_F14.raw |  |  |  |  |  | 2.1315E6 |  |  |  |  |  |  |  |  | 1 | 0 | 0 | 0 | 0 | 0 | 1 | 0 | 0 | 0 | 0 | 0 | 0 | 0 | 0 | 499 | 519 |  |  | PEAKS DB |
| K.VAVIIYLNK.V | Y | 56.21 | 1031.6379 | 9 | 0.8 | 516.8267 | 2 | 50.20 | 6 | F6:30133 | NaSa01\_F14.raw |  |  |  |  |  | 1.67E7 |  |  |  |  |  |  |  |  | 2 | 0 | 0 | 0 | 0 | 0 | 2 | 0 | 0 | 0 | 0 | 0 | 0 | 0 | 0 | 1421 | 1429 |  |  | PEAKS DB |
| G.ALYTLITPAVLR.T | N | 55.82 | 1329.8020 | 12 | 0.4 | 665.9086 | 2 | 75.95 | 6 | F6:51300 | NaSa01\_F14.raw |  |  |  | 1.7564E6 | 1.5853E7 | 2.019E8 |  |  |  |  |  |  |  |  | 6 | 0 | 0 | 0 | 1 | 1 | 4 | 0 | 0 | 0 | 0 | 0 | 0 | 0 | 0 | 23 | 34 |  |  | PEAKS DB |
| N.LHITPDLIPSFR.F | N | 52.36 | 1407.7874 | 12 | 0.4 | 470.2699 | 3 | 64.03 | 6 | F6:41508 | NaSa01\_F14.raw |  |  |  |  |  | 2.1751E7 |  |  |  |  |  |  |  |  | 2 | 0 | 0 | 0 | 0 | 0 | 2 | 0 | 0 | 0 | 0 | 0 | 0 | 0 | 0 | 508 | 519 |  |  | PEAKS DB |
| K.YVLPSFEVR.L | N | 51.77 | 1108.5917 | 9 | 0.6 | 555.3035 | 2 | 58.44 | 6 | F6:36495 | NaSa01\_F14.raw |  |  |  | 4.1589E5 | 1.253E7 | 1.3997E8 |  |  |  |  |  |  |  |  | 4 | 0 | 0 | 0 | 1 | 1 | 2 | 0 | 0 | 0 | 0 | 0 | 0 | 0 | 0 | 219 | 227 |  |  | PEAKS DB |
| K.TMSFYLR.D | Y | 51.69 | 916.4476 | 7 | 0.3 | 459.2312 | 2 | 41.67 | 6 | F6:22840 | NaSa01\_F14.raw |  |  |  |  |  | 5.6816E7 |  |  |  |  |  |  |  |  | 1 | 0 | 0 | 0 | 0 | 0 | 1 | 0 | 0 | 0 | 0 | 0 | 0 | 0 | 0 | 775 | 781 |  |  | PEAKS DB |
| R.DGQNLVTMNLH.I | N | 51.62 | 1240.5870 | 11 | 0.0 | 621.3008 | 2 | 49.19 | 6 | F6:28557 | NaSa01\_F14.raw |  |  |  |  |  | 4.925E6 |  |  |  |  |  |  |  |  | 1 | 0 | 0 | 0 | 0 | 0 | 1 | 0 | 0 | 0 | 0 | 0 | 0 | 0 | 0 | 499 | 509 |  |  | PEAKS DB |
| L.NIPLNAQSLPITVR.T | Y | 50.97 | 1534.8831 | 14 | -1.4 | 768.4477 | 2 | 61.34 | 6 | F6:39039 | NaSa01\_F14.raw |  |  |  |  |  | 1.6927E5 |  |  |  |  |  |  |  |  | 1 | 0 | 0 | 0 | 0 | 0 | 1 | 0 | 0 | 0 | 0 | 0 | 0 | 0 | 0 | 401 | 414 |  |  | PEAKS DB |
| K.C(+57.02)C(+57.02)EDVM(+15.99)HENPM(+15.99)GYTC(+57.02)EK.R | Y | 50.02 | 2190.7786 | 17 | 0.7 | 731.2673 | 3 | 11.49 | 9 | F9:1837 | NaSa01\_F4.raw |  |  |  |  |  |  |  |  | 4.1662E5 |  |  |  |  |  | 1 | 0 | 0 | 0 | 0 | 0 | 0 | 0 | 0 | 1 | 0 | 0 | 0 | 0 | 0 | 677 | 693 | Carbamidomethylation; Oxidation (M) | C1:Carbamidomethylation:1000.00;C2:Carbamidomethylation:1000.00;M6:Oxidation (M):1000.00;M11:Oxidation (M):1000.00;C15:Carbamidomethylation:1000.00 | PEAKS DB |
| K.GIYTPGSPVLYR.V | N | 49.98 | 1321.7030 | 12 | -8.2 | 661.8533 | 2 | 47.56 | 6 | F6:27271 | NaSa01\_F14.raw |  |  |  |  |  | 4.6433E6 |  |  |  |  |  |  |  |  | 1 | 0 | 0 | 0 | 0 | 0 | 1 | 0 | 0 | 0 | 0 | 0 | 0 | 0 | 0 | 135 | 146 |  |  | PEAKS DB |
| K.AC(+57.02)ETNVDYVYK.T | Y | 49.20 | 1360.5969 | 11 | 0.3 | 681.3060 | 2 | 22.05 | 6 | F6:6230 | NaSa01\_F14.raw |  |  |  | 2.4601E5 |  | 5.6943E5 |  |  |  |  |  |  |  |  | 2 | 0 | 0 | 0 | 1 | 0 | 1 | 0 | 0 | 0 | 0 | 0 | 0 | 0 | 0 | 1515 | 1525 | Carbamidomethylation | C2:Carbamidomethylation:1000.00 | PEAKS DB |
| M.LTGFLPDAEDLTR.L | N | 47.16 | 1446.7354 | 13 | 1.2 | 724.3758 | 2 | 69.30 | 6 | F6:45774 | NaSa01\_F14.raw |  |  |  |  |  | 7.2495E5 |  |  |  |  |  |  |  |  | 1 | 0 | 0 | 0 | 0 | 0 | 1 | 0 | 0 | 0 | 0 | 0 | 0 | 0 | 0 | 1387 | 1399 |  |  | PEAKS DB |
| R.VGLVAVDKAVYVLNDK.Y | N | 46.90 | 1701.9664 | 16 | 0.8 | 568.3299 | 3 | 57.39 | 6 | F6:35573 | NaSa01\_F14.raw |  |  |  |  |  | 1.8722E5 |  |  |  |  |  |  |  |  | 1 | 0 | 0 | 0 | 0 | 0 | 1 | 0 | 0 | 0 | 0 | 0 | 0 | 0 | 0 | 575 | 590 |  |  | PEAKS DB |
| K.THQYISQR.K | Y | 46.29 | 1031.5149 | 8 | 0.4 | 516.7650 | 2 | 11.44 | 6 | F6:1825 | NaSa01\_F14.raw |  |  |  |  |  | 1.3503E5 |  |  |  |  |  |  |  |  | 1 | 0 | 0 | 0 | 0 | 0 | 1 | 0 | 0 | 0 | 0 | 0 | 0 | 0 | 0 | 1560 | 1567 |  |  | PEAKS DB |
| D.DNEDGFIADSDIISR.S | Y | 46.28 | 1665.7482 | 15 | 0.7 | 833.8820 | 2 | 65.65 | 6 | F6:42702 | NaSa01\_F14.raw |  |  |  |  |  | 2.6489E5 |  |  |  |  |  |  |  |  | 1 | 0 | 0 | 0 | 0 | 0 | 1 | 0 | 0 | 0 | 0 | 0 | 0 | 0 | 0 | 734 | 748 |  |  | PEAKS DB |
| H.VAITSTEIKPGDNLPVNFNVK.G | Y | 46.15 | 2255.2161 | 21 | 0.2 | 752.7461 | 3 | 57.73 | 6 | F6:36049 | NaSa01\_F14.raw |  |  |  |  |  | 3.3349E6 |  |  |  |  |  |  |  |  | 1 | 0 | 0 | 0 | 0 | 0 | 1 | 0 | 0 | 0 | 0 | 0 | 0 | 0 | 0 | 447 | 467 |  |  | PEAKS DB |
| K.IIIQGDPVAQIIEN.S | Y | 45.71 | 1521.8402 | 14 | 0.0 | 761.9274 | 2 | 85.63 | 6 | F6:59337 | NaSa01\_F14.raw |  |  |  |  |  | 1.4516E7 |  |  |  |  |  |  |  |  | 1 | 0 | 0 | 0 | 0 | 0 | 1 | 0 | 0 | 0 | 0 | 0 | 0 | 0 | 0 | 963 | 976 |  |  | PEAKS DB |
| K.TM(+15.99)SFYLR.D | Y | 45.45 | 932.4426 | 7 | 0.2 | 467.2286 | 2 | 32.98 | 6 | F6:14848 | NaSa01\_F14.raw |  |  |  |  |  | 1.994E6 |  |  |  |  |  |  |  |  | 2 | 0 | 0 | 0 | 0 | 0 | 2 | 0 | 0 | 0 | 0 | 0 | 0 | 0 | 0 | 775 | 781 | Oxidation (M) | M2:Oxidation (M):1000.00 | PEAKS DB |
| N.DIYVMDVLEVIK.Q | N | 45.17 | 1435.7632 | 12 | 0.7 | 718.8894 | 2 | 106.25 | 4 | F4:76997 | NaSa01\_F12.raw |  |  |  | 2.8255E5 |  |  |  |  |  |  |  |  |  |  | 1 | 0 | 0 | 0 | 1 | 0 | 0 | 0 | 0 | 0 | 0 | 0 | 0 | 0 | 0 | 1538 | 1549 |  |  | PEAKS DB |
| K.YFKPGMPYELTVY.V | N | 44.78 | 1606.7742 | 13 | 0.4 | 804.3947 | 2 | 71.51 | 6 | F6:47852 | NaSa01\_F14.raw |  |  |  |  |  | 1.8453E7 |  |  |  |  |  |  |  |  | 1 | 0 | 0 | 0 | 0 | 0 | 1 | 0 | 0 | 0 | 0 | 0 | 0 | 0 | 0 | 353 | 365 |  |  | PEAKS DB |
| R.SRFPNLNELVGH.T | N | 44.24 | 1381.7102 | 12 | 0.6 | 461.5776 | 3 | 50.79 | 6 | F6:30122 | NaSa01\_F14.raw |  |  |  |  |  | 1.9009E7 |  |  |  |  |  |  |  |  | 3 | 0 | 0 | 0 | 0 | 0 | 3 | 0 | 0 | 0 | 0 | 0 | 0 | 0 | 0 | 301 | 312 |  |  | PEAKS DB |
| K.TVIVEFQTPEG.I | N | 43.94 | 1218.6132 | 11 | 3.8 | 610.3144 | 2 | 63.73 | 7 | F7:45532 | NaSa01\_F2.raw |  |  |  |  |  |  | 6.7633E6 | 2.6291E6 |  |  |  |  |  |  | 2 | 0 | 0 | 0 | 0 | 0 | 0 | 1 | 1 | 0 | 0 | 0 | 0 | 0 | 0 | 160 | 170 |  |  | PEAKS DB |
| M.NLHITPDLIPSFR.F | N | 43.77 | 1521.8303 | 13 | 0.6 | 508.2843 | 3 | 65.19 | 6 | F6:42284 | NaSa01\_F14.raw |  |  |  |  |  | 1.1088E7 |  |  |  |  |  |  |  |  | 2 | 0 | 0 | 0 | 0 | 0 | 2 | 0 | 0 | 0 | 0 | 0 | 0 | 0 | 0 | 507 | 519 |  |  | PEAKS DB |
| K.AAFLEC(+57.02)C(+57.02)R.Y | N | 43.14 | 1025.4423 | 8 | 0.7 | 513.7288 | 2 | 11.72 | 9 | F9:2016 | NaSa01\_F4.raw |  |  |  |  |  |  |  |  | 5.2365E5 |  |  |  |  |  | 1 | 0 | 0 | 0 | 0 | 0 | 0 | 0 | 0 | 1 | 0 | 0 | 0 | 0 | 0 | 706 | 713 | Carbamidomethylation | C6:Carbamidomethylation:1000.00;C7:Carbamidomethylation:1000.00 | PEAKS DB |
| L.SYQSSFLFIQTDK.G | Y | 42.37 | 1562.7616 | 13 | 1.2 | 782.3890 | 2 | 64.49 | 6 | F6:41739 | NaSa01\_F14.raw |  |  |  |  |  | 3.4858E5 |  |  |  |  |  |  |  |  | 1 | 0 | 0 | 0 | 0 | 0 | 1 | 0 | 0 | 0 | 0 | 0 | 0 | 0 | 0 | 122 | 134 |  |  | PEAKS DB |
| R.KC(+57.02)C(+57.02)EDVMHENPMGYTC(+57.02)EK.R | Y | 42.21 | 2286.8835 | 18 | 1.4 | 763.3029 | 3 | 20.88 | 10 | F10:7153 | NaSa01\_F5.raw |  |  |  |  |  |  |  |  | 7.1178E5 | 3.5368E6 |  |  |  |  | 3 | 0 | 0 | 0 | 0 | 0 | 0 | 0 | 0 | 1 | 2 | 0 | 0 | 0 | 0 | 676 | 693 | Carbamidomethylation | C2:Carbamidomethylation:1000.00;C3:Carbamidomethylation:1000.00;C16:Carbamidomethylation:1000.00 | PEAKS DB |
| R.IPIIDGDGK.A | Y | 42.15 | 926.5073 | 9 | 0.6 | 464.2612 | 2 | 32.92 | 6 | F6:14878 | NaSa01\_F14.raw |  |  |  |  |  | 3.4884E5 |  |  |  |  |  |  |  |  | 1 | 0 | 0 | 0 | 0 | 0 | 1 | 0 | 0 | 0 | 0 | 0 | 0 | 0 | 0 | 283 | 291 |  |  | PEAKS DB |
| Y.VLPSFEVR.L | N | 41.89 | 945.5283 | 8 | -0.2 | 473.7714 | 2 | 40.35 | 6 | F6:20803 | NaSa01\_F14.raw |  |  |  |  |  | 5.7172E5 |  |  |  |  |  |  |  |  | 1 | 0 | 0 | 0 | 0 | 0 | 1 | 0 | 0 | 0 | 0 | 0 | 0 | 0 | 0 | 220 | 227 |  |  | PEAKS DB |
| K.IC(+57.02)IGNVC(+57.02)R.C | Y | 41.79 | 990.4739 | 8 | -1.3 | 496.2436 | 2 | 11.58 | 6 | F6:1976 | NaSa01\_F14.raw |  |  |  |  |  | 2.2107E5 |  |  |  |  |  |  |  |  | 1 | 0 | 0 | 0 | 0 | 0 | 1 | 0 | 0 | 0 | 0 | 0 | 0 | 0 | 0 | 1484 | 1491 | Carbamidomethylation | C2:Carbamidomethylation:1000.00;C7:Carbamidomethylation:1000.00 | PEAKS DB |
| R.SRFPNLNELVGHTLY.A | N | 40.90 | 1758.9053 | 15 | 0.7 | 587.3094 | 3 | 70.95 | 6 | F6:46924 | NaSa01\_F14.raw |  |  |  |  |  | 5.6962E6 |  |  |  |  |  |  |  |  | 1 | 0 | 0 | 0 | 0 | 0 | 1 | 0 | 0 | 0 | 0 | 0 | 0 | 0 | 0 | 301 | 315 |  |  | PEAKS DB |
| Y.TLITPAVLR.T | N | 40.29 | 982.6175 | 9 | 0.4 | 492.3162 | 2 | 45.78 | 6 | F6:25572 | NaSa01\_F14.raw |  |  |  |  |  | 5.8349E7 |  |  |  |  |  |  |  |  | 1 | 0 | 0 | 0 | 0 | 0 | 1 | 0 | 0 | 0 | 0 | 0 | 0 | 0 | 0 | 26 | 34 |  |  | PEAKS DB |
| K.LILNIPLNAQ.S | Y | 40.28 | 1107.6652 | 10 | -0.2 | 554.8397 | 2 | 84.63 | 6 | F6:58512 | NaSa01\_F14.raw |  |  |  |  |  | 3.2621E6 |  |  |  |  |  |  |  |  | 1 | 0 | 0 | 0 | 0 | 0 | 1 | 0 | 0 | 0 | 0 | 0 | 0 | 0 | 0 | 398 | 407 |  |  | PEAKS DB |
| K.IIIQGDPVAQI.I | Y | 39.99 | 1165.6707 | 11 | 0.7 | 583.8430 | 2 | 72.27 | 6 | F6:48414 | NaSa01\_F14.raw |  |  |  |  |  | 1.2067E6 |  |  |  |  |  |  |  |  | 1 | 0 | 0 | 0 | 0 | 0 | 1 | 0 | 0 | 0 | 0 | 0 | 0 | 0 | 0 | 963 | 973 |  |  | PEAKS DB |
| Y.QVGNNEIVADSVWVDVK.D | N | 39.98 | 1870.9425 | 17 | 0.5 | 936.4790 | 2 | 64.84 | 6 | F6:41917 | NaSa01\_F14.raw |  |  |  |  |  | 1.8564E6 |  |  |  |  |  |  |  |  | 1 | 0 | 0 | 0 | 0 | 0 | 1 | 0 | 0 | 0 | 0 | 0 | 0 | 0 | 0 | 525 | 541 |  |  | PEAKS DB |
| N.IPLNAQSLPITVR.T | Y | 39.95 | 1420.8402 | 13 | 0.7 | 711.4279 | 2 | 58.51 | 6 | F6:36546 | NaSa01\_F14.raw |  |  |  |  |  | 5.2985E5 |  |  |  |  |  |  |  |  | 1 | 0 | 0 | 0 | 0 | 0 | 1 | 0 | 0 | 0 | 0 | 0 | 0 | 0 | 0 | 402 | 414 |  |  | PEAKS DB |
| K.SWLWLTK.D | Y | 39.91 | 932.5120 | 7 | 0.3 | 467.2634 | 2 | 68.87 | 6 | F6:45829 | NaSa01\_F14.raw |  |  |  |  |  | 5.8771E7 |  |  |  |  |  |  |  |  | 1 | 0 | 0 | 0 | 0 | 0 | 1 | 0 | 0 | 0 | 0 | 0 | 0 | 0 | 0 | 754 | 760 |  |  | PEAKS DB |
| K.IIIQGDPVAQ.I | Y | 39.88 | 1052.5865 | 10 | 0.0 | 527.3005 | 2 | 48.47 | 6 | F6:28020 | NaSa01\_F14.raw |  |  |  |  |  | 3.3932E7 |  |  |  |  |  |  |  |  | 1 | 0 | 0 | 0 | 0 | 0 | 1 | 0 | 0 | 0 | 0 | 0 | 0 | 0 | 0 | 963 | 972 |  |  | PEAKS DB |
| R.IEEQDGNDIYVM(+15.99)DVLEVIK.Q | Y | 39.47 | 2237.0774 | 19 | 1.1 | 1119.5472 | 2 | 90.84 | 4 | F4:65053 | NaSa01\_F12.raw |  |  |  | 8.9297E5 |  |  |  |  |  |  |  |  |  |  | 1 | 0 | 0 | 0 | 1 | 0 | 0 | 0 | 0 | 0 | 0 | 0 | 0 | 0 | 0 | 1531 | 1549 | Oxidation (M) | M12:Oxidation (M):1000.00 | PEAKS DB |
| K.VVLLSYQSSF.L | Y | 38.34 | 1141.6019 | 10 | 0.4 | 571.8085 | 2 | 79.64 | 6 | F6:54198 | NaSa01\_F14.raw |  |  |  |  |  | 2.3493E6 |  |  |  |  |  |  |  |  | 1 | 0 | 0 | 0 | 0 | 0 | 1 | 0 | 0 | 0 | 0 | 0 | 0 | 0 | 0 | 118 | 127 |  |  | PEAKS DB |
| K.ISYIITK.N | N | 38.08 | 836.5007 | 7 | -0.7 | 419.2574 | 2 | 26.29 | 6 | F6:9476 | NaSa01\_F14.raw |  |  |  |  |  | 6.6012E5 |  |  |  |  |  |  |  |  | 1 | 0 | 0 | 0 | 0 | 0 | 1 | 0 | 0 | 0 | 0 | 0 | 0 | 0 | 0 | 1597 | 1603 |  |  | PEAKS DB |
| total 87 peptides |  |  |  |  |  |  |  |  |  |  |  |  |  |  |  |  |  |  |  |  |  |  |  |  |  |  |  |  |  |  |  |  |  |  |  |  |  |  |  |  |  |  |  |  |  |
| --- | --- | --- | --- | --- | --- | --- | --- | --- | --- | --- | --- | --- | --- | --- | --- | --- | --- | --- | --- | --- | --- | --- | --- | --- | --- | --- | --- | --- | --- | --- | --- | --- | --- | --- | --- | --- | --- | --- | --- | --- | --- | --- | --- | --- | --- |

3PVM

back to list

  

| Protein Coverage
| Supporting Peptides
|

Protein Coverage:

Supporting Peptides:

| Peptide | Uniq | -10lgP | Mass | Length | ppm | m/z | z | RT | Fraction | Scan | Source File | Area F1 | Area F10 | Area F11 | Area F12 | Area F13 | Area F14 | Area F2 | Area F3 | Area F4 | Area F5 | Area F6 | Area F7 | Area F8 | Area F9 | #Feature | #Feature F1 | #Feature F10 | #Feature F11 | #Feature F12 | #Feature F13 | #Feature F14 | #Feature F2 | #Feature F3 | #Feature F4 | #Feature F5 | #Feature F6 | #Feature F7 | #Feature F8 | #Feature F9 | Start | End | PTM | AScore | Found By |
| --- | --- | --- | --- | --- | --- | --- | --- | --- | --- | --- | --- | --- | --- | --- | --- | --- | --- | --- | --- | --- | --- | --- | --- | --- | --- | --- | --- | --- | --- | --- | --- | --- | --- | --- | --- | --- | --- | --- | --- | --- | --- | --- | --- | --- | --- |
| R.AILHNYVNEDIYVR.V | Y | 89.83 | 1717.8788 | 14 | 0.3 | 859.9469 | 2 | 43.05 | 6 | F6:23378 | NaSa01\_F14.raw |  |  |  |  | 1.5001E7 | 1.9759E8 |  |  |  |  |  |  |  |  | 8 | 0 | 0 | 0 | 0 | 3 | 5 | 0 | 0 | 0 | 0 | 0 | 0 | 0 | 0 | 834 | 847 |  |  | PEAKS DB |
| R.AVPFVIVPLEQGLHDVEIK.A | Y | 87.23 | 2102.1775 | 19 | 0.5 | 701.7335 | 3 | 85.72 | 6 | F6:59617 | NaSa01\_F14.raw |  |  |  | 7.0958E5 | 1.6836E7 | 1.2181E8 |  |  |  |  |  |  |  |  | 7 | 0 | 0 | 0 | 1 | 2 | 4 | 0 | 0 | 0 | 0 | 0 | 0 | 0 | 0 | 879 | 897 |  |  | PEAKS DB |
| K.HFEVGFIQPGSVK.V | N | 85.09 | 1443.7510 | 13 | -0.2 | 722.8826 | 2 | 44.13 | 6 | F6:24159 | NaSa01\_F14.raw |  |  |  |  | 2.4365E7 | 2.5116E8 |  |  |  |  |  |  |  |  | 8 | 0 | 0 | 0 | 0 | 2 | 6 | 0 | 0 | 0 | 0 | 0 | 0 | 0 | 0 | 1445 | 1457 |  |  | PEAKS DB |
| R.RDGQNLVTM(+15.99)NLHITPDLIPSFR.F | N | 85.06 | 2552.3169 | 22 | 0.3 | 639.0867 | 4 | 69.77 | 6 | F6:46133 | NaSa01\_F14.raw |  |  |  |  |  | 2.5484E6 |  |  |  |  |  |  |  |  | 2 | 0 | 0 | 0 | 0 | 0 | 2 | 0 | 0 | 0 | 0 | 0 | 0 | 0 | 0 | 498 | 519 | Oxidation (M) | M9:Oxidation (M):1000.00 | PEAKS DB |
| R.QNQYVVVQVTGPQVR.L | N | 83.17 | 1713.9163 | 15 | 0.2 | 857.9656 | 2 | 43.83 | 6 | F6:24244 | NaSa01\_F14.raw |  |  |  |  | 1.4255E7 | 1.5135E8 |  |  |  |  |  |  |  |  | 5 | 0 | 0 | 0 | 0 | 2 | 3 | 0 | 0 | 0 | 0 | 0 | 0 | 0 | 0 | 100 | 114 |  |  | PEAKS DB |
| K.VFFIDLQMPYSVVK.N | Y | 82.52 | 1684.8898 | 14 | 0.3 | 843.4524 | 2 | 95.37 | 6 | F6:67466 | NaSa01\_F14.raw |  |  |  |  | 5.5288E5 | 4.4172E6 |  |  |  |  |  |  |  |  | 2 | 0 | 0 | 0 | 0 | 1 | 1 | 0 | 0 | 0 | 0 | 0 | 0 | 0 | 0 | 813 | 826 |  |  | PEAKS DB |
| K.VNDDYLIWGSR.S | Y | 81.98 | 1336.6411 | 11 | 1.0 | 669.3285 | 2 | 59.77 | 6 | F6:37640 | NaSa01\_F14.raw |  |  |  | 4.0113E7 | 1.558E7 | 1.2947E8 |  |  |  |  |  |  |  |  | 4 | 0 | 0 | 0 | 1 | 2 | 1 | 0 | 0 | 0 | 0 | 0 | 0 | 0 | 0 | 1577 | 1587 |  |  | PEAKS DB |
| R.IEEQDGNDIYVMDVLEVIK.Q | Y | 80.07 | 2221.0823 | 19 | 0.7 | 1111.5492 | 2 | 101.01 | 4 | F4:73173 | NaSa01\_F12.raw |  |  |  | 1.608E7 | 4.109E6 | 2.6808E7 |  |  |  |  |  |  |  |  | 11 | 0 | 0 | 0 | 4 | 3 | 4 | 0 | 0 | 0 | 0 | 0 | 0 | 0 | 0 | 1531 | 1549 |  |  | PEAKS DB |
| K.ATMTILTFYNAQLQEK.A | Y | 79.74 | 1870.9498 | 16 | -0.2 | 936.4820 | 2 | 77.02 | 6 | F6:52034 | NaSa01\_F14.raw |  |  | 5.3707E5 | 1.2422E6 | 3.5134E6 | 4.7877E7 |  |  |  |  |  |  |  |  | 6 | 0 | 0 | 1 | 1 | 1 | 3 | 0 | 0 | 0 | 0 | 0 | 0 | 0 | 0 | 1321 | 1336 |  |  | PEAKS DB |
| K.QLDIFVHDFPR.K | N | 79.15 | 1385.7091 | 11 | 0.0 | 693.8618 | 2 | 58.44 | 6 | F6:36683 | NaSa01\_F14.raw |  |  |  |  | 9.0136E6 | 8.647E7 |  |  |  |  |  |  |  |  | 4 | 0 | 0 | 0 | 0 | 2 | 2 | 0 | 0 | 0 | 0 | 0 | 0 | 0 | 0 | 53 | 63 |  |  | PEAKS DB |
| K.GDNLIQMPGAAMK.I | Y | 78.60 | 1344.6530 | 13 | 0.2 | 673.3339 | 2 | 49.26 | 6 | F6:28453 | NaSa01\_F14.raw |  |  |  |  |  | 1.0626E8 |  |  |  |  |  |  |  |  | 2 | 0 | 0 | 0 | 0 | 0 | 2 | 0 | 0 | 0 | 0 | 0 | 0 | 0 | 0 | 552 | 564 |  |  | PEAKS DB |
| F.VIVPLEQGLHDVEIK.A | Y | 76.27 | 1687.9508 | 15 | 0.0 | 844.9827 | 2 | 58.08 | 6 | F6:36376 | NaSa01\_F14.raw |  |  |  |  | 5.5586E6 | 9.3853E7 |  |  |  |  |  |  |  |  | 3 | 0 | 0 | 0 | 0 | 1 | 2 | 0 | 0 | 0 | 0 | 0 | 0 | 0 | 0 | 883 | 897 |  |  | PEAKS DB |
| R.DDNEDGFIADSDIISR.S | Y | 75.58 | 1780.7751 | 16 | 0.3 | 891.3951 | 2 | 68.40 | 6 | F6:44898 | NaSa01\_F14.raw |  |  |  |  | 9.0776E5 | 1.2244E7 |  |  |  |  |  |  |  |  | 4 | 0 | 0 | 0 | 0 | 1 | 3 | 0 | 0 | 0 | 0 | 0 | 0 | 0 | 0 | 733 | 748 |  |  | PEAKS DB |
| K.C(+57.02)C(+57.02)EDVMHENPMGYTC(+57.02)EK.R | Y | 74.09 | 2158.7886 | 17 | 7.4 | 720.6050 | 3 | 31.06 | 8 | F8:16109 | NaSa01\_F3.raw |  |  |  |  |  |  |  | 1.0485E7 | 5.6652E7 | 1.4617E7 |  |  |  |  | 6 | 0 | 0 | 0 | 0 | 0 | 0 | 0 | 1 | 3 | 2 | 0 | 0 | 0 | 0 | 677 | 693 | Carbamidomethylation | C1:Carbamidomethylation:1000.00;C2:Carbamidomethylation:1000.00;C15:Carbamidomethylation:1000.00 | PEAKS DB |
| R.INYENALLAR.T | Y | 74.07 | 1175.6299 | 10 | 0.1 | 588.8223 | 2 | 45.12 | 6 | F6:25480 | NaSa01\_F14.raw |  |  | 3.3741E5 | 7.6601E6 | 1.5972E7 | 1.5972E8 |  |  |  |  |  |  |  |  | 7 | 0 | 0 | 1 | 3 | 1 | 2 | 0 | 0 | 0 | 0 | 0 | 0 | 0 | 0 | 1292 | 1301 |  |  | PEAKS DB |
| Y.YQVGNNEIVADSVWVDVK.D | N | 73.99 | 2034.0057 | 18 | 0.2 | 1018.0104 | 2 | 70.65 | 6 | F6:46860 | NaSa01\_F14.raw |  |  |  |  |  | 6.5239E6 |  |  |  |  |  |  |  |  | 1 | 0 | 0 | 0 | 0 | 0 | 1 | 0 | 0 | 0 | 0 | 0 | 0 | 0 | 0 | 524 | 541 |  |  | PEAKS DB |
| K.ASVQEALWSDGVR.K | Y | 72.98 | 1416.6997 | 13 | 0.1 | 709.3572 | 2 | 49.71 | 6 | F6:29208 | NaSa01\_F14.raw |  |  |  |  | 1.3438E7 | 1.2825E8 |  |  |  |  |  |  |  |  | 7 | 0 | 0 | 0 | 0 | 1 | 6 | 0 | 0 | 0 | 0 | 0 | 0 | 0 | 0 | 898 | 910 |  |  | PEAKS DB |
| R.VELLYNPAFC(+57.02)SASTK.G | Y | 72.57 | 1698.8286 | 15 | -0.7 | 850.4210 | 2 | 59.22 | 6 | F6:37110 | NaSa01\_F14.raw |  |  |  |  | 1.6821E7 | 2.15E8 |  |  |  |  |  |  |  |  | 2 | 0 | 0 | 0 | 0 | 1 | 1 | 0 | 0 | 0 | 0 | 0 | 0 | 0 | 0 | 848 | 862 | Carbamidomethylation | C10:Carbamidomethylation:1000.00 | PEAKS DB |
| K.LNQDITVTASGDGK.A | Y | 72.09 | 1417.7048 | 14 | 0.8 | 709.8602 | 2 | 20.83 | 6 | F6:5302 | NaSa01\_F14.raw |  |  |  |  | 4.3744E5 | 4.5631E6 |  |  |  |  |  |  |  |  | 3 | 0 | 0 | 0 | 0 | 1 | 2 | 0 | 0 | 0 | 0 | 0 | 0 | 0 | 0 | 1307 | 1320 |  |  | PEAKS DB |
| K.LILNIPLNAQSLPITVR.T | Y | 71.60 | 1874.1353 | 17 | 0.5 | 625.7194 | 3 | 90.36 | 6 | F6:63322 | NaSa01\_F14.raw |  |  |  |  | 1.4111E7 | 4.901E7 |  |  |  |  |  |  |  |  | 4 | 0 | 0 | 0 | 0 | 2 | 2 | 0 | 0 | 0 | 0 | 0 | 0 | 0 | 0 | 398 | 414 |  |  | PEAKS DB |
| K.YFTYLILNK.G | N | 70.49 | 1173.6433 | 9 | 0.1 | 587.8290 | 2 | 64.67 | 6 | F6:41809 | NaSa01\_F14.raw |  |  |  |  | 1.0728E7 | 7.7707E7 |  |  |  |  |  |  |  |  | 2 | 0 | 0 | 0 | 0 | 1 | 1 | 0 | 0 | 0 | 0 | 0 | 0 | 0 | 0 | 478 | 486 |  |  | PEAKS DB |
| R.WPHEDEC(+57.02)QEEEFQK.L | Y | 69.62 | 1889.7526 | 14 | 0.9 | 945.8844 | 2 | 23.39 | 4 | F4:9200 | NaSa01\_F12.raw |  |  |  | 2.8919E7 |  | 3.166E6 |  |  |  |  |  |  |  |  | 3 | 0 | 0 | 0 | 2 | 0 | 1 | 0 | 0 | 0 | 0 | 0 | 0 | 0 | 0 | 1610 | 1623 | Carbamidomethylation | C7:Carbamidomethylation:1000.00 | PEAKS DB |
| K.SDFGC(+57.02)TAGSGQNNLGVFEDAGLALTTSTNLNTK.Q | N | 66.95 | 3359.5576 | 33 | 0.8 | 1120.8607 | 3 | 89.37 | 6 | F6:62425 | NaSa01\_F14.raw |  |  |  |  |  | 3.8512E7 |  |  |  |  |  |  |  |  | 1 | 0 | 0 | 0 | 0 | 0 | 1 | 0 | 0 | 0 | 0 | 0 | 0 | 0 | 0 | 605 | 637 | Carbamidomethylation | C5:Carbamidomethylation:1000.00 | PEAKS DB |
| R.DGQNLVTM(+15.99)NLHITPDLIPSFR.F | N | 66.34 | 2396.2158 | 21 | 0.4 | 799.7462 | 3 | 83.99 | 6 | F6:57985 | NaSa01\_F14.raw |  |  |  |  |  | 5.9574E6 |  |  |  |  |  |  |  |  | 1 | 0 | 0 | 0 | 0 | 0 | 1 | 0 | 0 | 0 | 0 | 0 | 0 | 0 | 0 | 499 | 519 | Oxidation (M) | M8:Oxidation (M):1000.00 | PEAKS DB |
| L.ARDDNEDGFIADSDIISR.S | Y | 66.31 | 2007.9133 | 18 | 1.0 | 670.3124 | 3 | 48.95 | 6 | F6:28381 | NaSa01\_F14.raw |  |  |  |  |  | 6.6726E6 |  |  |  |  |  |  |  |  | 2 | 0 | 0 | 0 | 0 | 0 | 2 | 0 | 0 | 0 | 0 | 0 | 0 | 0 | 0 | 731 | 748 |  |  | PEAKS DB |
| Y.VTNPDGSPAAHVPVVSEAFHSMGTTLSDGTAK.L | Y | 66.31 | 3179.5193 | 32 | 0.8 | 795.8877 | 4 | 59.12 | 6 | F6:37224 | NaSa01\_F14.raw |  |  |  |  |  | 2.3066E7 |  |  |  |  |  |  |  |  | 1 | 0 | 0 | 0 | 0 | 0 | 1 | 0 | 0 | 0 | 0 | 0 | 0 | 0 | 0 | 366 | 397 |  |  | PEAKS DB |
| K.C(+57.02)C(+57.02)EDVMHENPM(+15.99)GYTC(+57.02)EK.R | Y | 65.55 | 2174.7837 | 17 | 1.4 | 725.9362 | 3 | 11.70 | 9 | F9:2004 | NaSa01\_F4.raw |  |  |  |  |  |  |  |  | 1.0934E7 |  |  |  |  |  | 2 | 0 | 0 | 0 | 0 | 0 | 0 | 0 | 0 | 2 | 0 | 0 | 0 | 0 | 0 | 677 | 693 | Carbamidomethylation; Oxidation (M) | C1:Carbamidomethylation:1000.00;C2:Carbamidomethylation:1000.00;M11:Oxidation (M):43.99;C15:Carbamidomethylation:1000.00 | PEAKS DB |
| Y.NLPDLVSLGTWR.I | Y | 65.12 | 1369.7354 | 12 | 0.1 | 685.8750 | 2 | 81.18 | 6 | F6:55452 | NaSa01\_F14.raw |  |  |  |  | 6.3971E6 | 8.6753E7 |  |  |  |  |  |  |  |  | 2 | 0 | 0 | 0 | 0 | 1 | 1 | 0 | 0 | 0 | 0 | 0 | 0 | 0 | 0 | 187 | 198 |  |  | PEAKS DB |
| K.GDNLIQMPGAAM(+15.99)K.I | Y | 64.58 | 1360.6479 | 13 | 0.8 | 681.3318 | 2 | 38.53 | 6 | F6:19473 | NaSa01\_F14.raw |  |  |  |  | 9.6097E5 | 9.5549E6 |  |  |  |  |  |  |  |  | 3 | 0 | 0 | 0 | 0 | 1 | 2 | 0 | 0 | 0 | 0 | 0 | 0 | 0 | 0 | 552 | 564 | Oxidation (M) | M12:Oxidation (M):38.33 | PEAKS DB |
| E.VEGVAFVLFGVK.I | N | 62.96 | 1263.7227 | 12 | 0.2 | 632.8687 | 2 | 85.00 | 6 | F6:58754 | NaSa01\_F14.raw |  |  |  |  |  | 2.0835E6 |  |  |  |  |  |  |  |  | 1 | 0 | 0 | 0 | 0 | 0 | 1 | 0 | 0 | 0 | 0 | 0 | 0 | 0 | 0 | 257 | 268 |  |  | PEAKS DB |
| Y.QSSFLFIQTDK.G | Y | 62.43 | 1312.6663 | 11 | -0.4 | 657.3401 | 2 | 57.57 | 6 | F6:35846 | NaSa01\_F14.raw |  |  |  |  |  | 1.1659E7 |  |  |  |  |  |  |  |  | 1 | 0 | 0 | 0 | 0 | 0 | 1 | 0 | 0 | 0 | 0 | 0 | 0 | 0 | 0 | 124 | 134 |  |  | PEAKS DB |
| K.GVGGTQLEVIK.A | Y | 62.08 | 1099.6237 | 11 | 0.2 | 550.8192 | 2 | 30.32 | 12 | F12:15497 | NaSa01\_F7.raw |  |  |  |  | 1.4118E7 | 1.0253E6 |  |  |  |  |  | 1.7364E6 |  |  | 3 | 0 | 0 | 0 | 0 | 1 | 1 | 0 | 0 | 0 | 0 | 0 | 1 | 0 | 0 | 936 | 946 |  |  | PEAKS DB |
| K.ATM(+15.99)TILTFYNAQLQEK.A | Y | 62.07 | 1886.9448 | 16 | 0.4 | 944.4801 | 2 | 64.84 | 6 | F6:42088 | NaSa01\_F14.raw |  |  |  |  |  | 1.0319E7 |  |  |  |  |  |  |  |  | 2 | 0 | 0 | 0 | 0 | 0 | 2 | 0 | 0 | 0 | 0 | 0 | 0 | 0 | 0 | 1321 | 1336 | Oxidation (M) | M3:Oxidation (M):1000.00 | PEAKS DB |
| R.FPNLNELVGH.T | N | 62.05 | 1138.5770 | 10 | 0.8 | 570.2963 | 2 | 67.53 | 6 | F6:44045 | NaSa01\_F14.raw |  |  |  |  | 1.7762E6 | 2.8185E7 |  |  |  |  |  |  |  |  | 2 | 0 | 0 | 0 | 0 | 1 | 1 | 0 | 0 | 0 | 0 | 0 | 0 | 0 | 0 | 303 | 312 |  |  | PEAKS DB |
| K.VYSYYNLDEK.C | N | 61.93 | 1292.5924 | 10 | 1.2 | 647.3043 | 2 | 33.77 | 6 | F6:15693 | NaSa01\_F14.raw |  |  |  |  | 1.5304E7 | 1.0252E8 |  |  |  |  |  |  |  |  | 3 | 0 | 0 | 0 | 0 | 1 | 2 | 0 | 0 | 0 | 0 | 0 | 0 | 0 | 0 | 1458 | 1467 |  |  | PEAKS DB |
| M.TILTFYNAQLQEK.A | N | 61.57 | 1567.8246 | 13 | 0.3 | 784.9198 | 2 | 60.05 | 6 | F6:38047 | NaSa01\_F14.raw |  |  |  |  |  | 5.1346E6 |  |  |  |  |  |  |  |  | 1 | 0 | 0 | 0 | 0 | 0 | 1 | 0 | 0 | 0 | 0 | 0 | 0 | 0 | 0 | 1324 | 1336 |  |  | PEAKS DB |
| H.ITPDLIPSFR.F | N | 61.36 | 1157.6444 | 10 | 0.0 | 579.8295 | 2 | 65.01 | 6 | F6:42335 | NaSa01\_F14.raw |  |  |  |  | 3.0601E6 | 3.8923E7 |  |  |  |  |  |  |  |  | 3 | 0 | 0 | 0 | 0 | 1 | 2 | 0 | 0 | 0 | 0 | 0 | 0 | 0 | 0 | 510 | 519 |  |  | PEAKS DB |
| K.PGDNLPVNFNVK.G | Y | 60.28 | 1312.6775 | 12 | 0.4 | 657.3463 | 2 | 47.72 | 6 | F6:27452 | NaSa01\_F14.raw |  |  |  |  |  | 1.0823E6 |  |  |  |  |  |  |  |  | 1 | 0 | 0 | 0 | 0 | 0 | 1 | 0 | 0 | 0 | 0 | 0 | 0 | 0 | 0 | 456 | 467 |  |  | PEAKS DB |
| W.VVLAVSFTPTK.G | Y | 59.98 | 1160.6804 | 11 | 0.4 | 581.3477 | 2 | 51.95 | 6 | F6:30874 | NaSa01\_F14.raw |  |  |  |  |  | 3.7062E6 |  |  |  |  |  |  |  |  | 1 | 0 | 0 | 0 | 0 | 0 | 1 | 0 | 0 | 0 | 0 | 0 | 0 | 0 | 0 | 788 | 798 |  |  | PEAKS DB |
| K.YEHSPENYTAYFDVR.K | N | 59.87 | 1889.8220 | 15 | -0.9 | 630.9474 | 3 | 47.19 | 6 | F6:27480 | NaSa01\_F14.raw |  |  |  |  |  | 2.5484E6 |  |  |  |  |  |  |  |  | 2 | 0 | 0 | 0 | 0 | 0 | 2 | 0 | 0 | 0 | 0 | 0 | 0 | 0 | 0 | 203 | 217 |  |  | PEAKS DB |
| R.IDVPLQIEK.A | Y | 59.34 | 1053.6069 | 9 | 0.1 | 527.8108 | 2 | 47.86 | 6 | F6:27228 | NaSa01\_F14.raw |  |  |  | 3.0425E7 | 1.9664E7 | 1.6873E8 |  |  |  |  |  |  |  |  | 4 | 0 | 0 | 0 | 1 | 1 | 2 | 0 | 0 | 0 | 0 | 0 | 0 | 0 | 0 | 1506 | 1514 |  |  | PEAKS DB |
| K.IIIQGDPVAQIIENSIDGSK.L | Y | 59.31 | 2109.1316 | 20 | 0.6 | 1055.5737 | 2 | 96.62 | 6 | F6:68548 | NaSa01\_F14.raw |  |  | 1.4465E5 |  | 1.6001E5 | 2.5079E6 |  |  |  |  |  |  |  |  | 3 | 0 | 0 | 1 | 0 | 1 | 1 | 0 | 0 | 0 | 0 | 0 | 0 | 0 | 0 | 963 | 982 |  |  | PEAKS DB |
| K.GIC(+57.02)VAEPYEIR.V | Y | 59.06 | 1305.6387 | 11 | 0.4 | 653.8268 | 2 | 44.31 | 6 | F6:24643 | NaSa01\_F14.raw |  |  |  |  | 1.0302E7 | 1.0808E8 |  |  |  |  |  |  |  |  | 3 | 0 | 0 | 0 | 0 | 1 | 2 | 0 | 0 | 0 | 0 | 0 | 0 | 0 | 0 | 799 | 809 | Carbamidomethylation | C3:Carbamidomethylation:1000.00 | PEAKS DB |
| E.DGFIADSDIISR.S | Y | 58.62 | 1307.6357 | 12 | -0.1 | 654.8251 | 2 | 60.77 | 6 | F6:38594 | NaSa01\_F14.raw |  |  |  |  |  | 4.7593E6 |  |  |  |  |  |  |  |  | 1 | 0 | 0 | 0 | 0 | 0 | 1 | 0 | 0 | 0 | 0 | 0 | 0 | 0 | 0 | 737 | 748 |  |  | PEAKS DB |
| R.FPNLNELVGHTLY.A | N | 58.47 | 1515.7721 | 13 | 0.3 | 758.8936 | 2 | 85.62 | 6 | F6:59205 | NaSa01\_F14.raw |  |  |  |  |  | 1.9527E7 |  |  |  |  |  |  |  |  | 1 | 0 | 0 | 0 | 0 | 0 | 1 | 0 | 0 | 0 | 0 | 0 | 0 | 0 | 0 | 303 | 315 |  |  | PEAKS DB |
| S.MLTGFLPDAEDLTR.L | N | 58.03 | 1577.7759 | 14 | 0.5 | 789.8956 | 2 | 78.56 | 6 | F6:53520 | NaSa01\_F14.raw |  |  |  |  |  | 2.8623E6 |  |  |  |  |  |  |  |  | 1 | 0 | 0 | 0 | 0 | 0 | 1 | 0 | 0 | 0 | 0 | 0 | 0 | 0 | 0 | 1386 | 1399 |  |  | PEAKS DB |
| R.KYVLPSFEVR.L | Y | 56.74 | 1236.6866 | 10 | 0.3 | 413.2363 | 3 | 42.11 | 6 | F6:22829 | NaSa01\_F14.raw |  |  |  |  | 5.509E6 | 4.9733E7 |  |  |  |  |  |  |  |  | 6 | 0 | 0 | 0 | 0 | 2 | 4 | 0 | 0 | 0 | 0 | 0 | 0 | 0 | 0 | 218 | 227 |  |  | PEAKS DB |
| R.DGQNLVTMNLHITPDLIPSFR.F | N | 56.46 | 2380.2209 | 21 | 0.5 | 794.4147 | 3 | 91.58 | 6 | F6:64345 | NaSa01\_F14.raw |  |  |  |  |  | 2.1315E6 |  |  |  |  |  |  |  |  | 1 | 0 | 0 | 0 | 0 | 0 | 1 | 0 | 0 | 0 | 0 | 0 | 0 | 0 | 0 | 499 | 519 |  |  | PEAKS DB |
| K.VAVIIYLNK.V | Y | 56.21 | 1031.6379 | 9 | 0.8 | 516.8267 | 2 | 50.20 | 6 | F6:30133 | NaSa01\_F14.raw |  |  |  |  |  | 1.67E7 |  |  |  |  |  |  |  |  | 2 | 0 | 0 | 0 | 0 | 0 | 2 | 0 | 0 | 0 | 0 | 0 | 0 | 0 | 0 | 1421 | 1429 |  |  | PEAKS DB |
| G.ALYTLITPAVLR.T | N | 55.82 | 1329.8020 | 12 | 0.4 | 665.9086 | 2 | 75.95 | 6 | F6:51300 | NaSa01\_F14.raw |  |  |  | 1.7564E6 | 1.5853E7 | 2.019E8 |  |  |  |  |  |  |  |  | 6 | 0 | 0 | 0 | 1 | 1 | 4 | 0 | 0 | 0 | 0 | 0 | 0 | 0 | 0 | 23 | 34 |  |  | PEAKS DB |
| N.LHITPDLIPSFR.F | N | 52.36 | 1407.7874 | 12 | 0.4 | 470.2699 | 3 | 64.03 | 6 | F6:41508 | NaSa01\_F14.raw |  |  |  |  |  | 2.1751E7 |  |  |  |  |  |  |  |  | 2 | 0 | 0 | 0 | 0 | 0 | 2 | 0 | 0 | 0 | 0 | 0 | 0 | 0 | 0 | 508 | 519 |  |  | PEAKS DB |
| K.YVLPSFEVR.L | N | 51.77 | 1108.5917 | 9 | 0.6 | 555.3035 | 2 | 58.44 | 6 | F6:36495 | NaSa01\_F14.raw |  |  |  | 4.1589E5 | 1.253E7 | 1.3997E8 |  |  |  |  |  |  |  |  | 4 | 0 | 0 | 0 | 1 | 1 | 2 | 0 | 0 | 0 | 0 | 0 | 0 | 0 | 0 | 219 | 227 |  |  | PEAKS DB |
| K.TMSFYLR.D | Y | 51.69 | 916.4476 | 7 | 0.3 | 459.2312 | 2 | 41.67 | 6 | F6:22840 | NaSa01\_F14.raw |  |  |  |  |  | 5.6816E7 |  |  |  |  |  |  |  |  | 1 | 0 | 0 | 0 | 0 | 0 | 1 | 0 | 0 | 0 | 0 | 0 | 0 | 0 | 0 | 775 | 781 |  |  | PEAKS DB |
| R.DGQNLVTMNLH.I | N | 51.62 | 1240.5870 | 11 | 0.0 | 621.3008 | 2 | 49.19 | 6 | F6:28557 | NaSa01\_F14.raw |  |  |  |  |  | 4.925E6 |  |  |  |  |  |  |  |  | 1 | 0 | 0 | 0 | 0 | 0 | 1 | 0 | 0 | 0 | 0 | 0 | 0 | 0 | 0 | 499 | 509 |  |  | PEAKS DB |
| L.NIPLNAQSLPITVR.T | Y | 50.97 | 1534.8831 | 14 | -1.4 | 768.4477 | 2 | 61.34 | 6 | F6:39039 | NaSa01\_F14.raw |  |  |  |  |  | 1.6927E5 |  |  |  |  |  |  |  |  | 1 | 0 | 0 | 0 | 0 | 0 | 1 | 0 | 0 | 0 | 0 | 0 | 0 | 0 | 0 | 401 | 414 |  |  | PEAKS DB |
| K.C(+57.02)C(+57.02)EDVM(+15.99)HENPM(+15.99)GYTC(+57.02)EK.R | Y | 50.02 | 2190.7786 | 17 | 0.7 | 731.2673 | 3 | 11.49 | 9 | F9:1837 | NaSa01\_F4.raw |  |  |  |  |  |  |  |  | 4.1662E5 |  |  |  |  |  | 1 | 0 | 0 | 0 | 0 | 0 | 0 | 0 | 0 | 1 | 0 | 0 | 0 | 0 | 0 | 677 | 693 | Carbamidomethylation; Oxidation (M) | C1:Carbamidomethylation:1000.00;C2:Carbamidomethylation:1000.00;M6:Oxidation (M):1000.00;M11:Oxidation (M):1000.00;C15:Carbamidomethylation:1000.00 | PEAKS DB |
| K.GIYTPGSPVLYR.V | N | 49.98 | 1321.7030 | 12 | -8.2 | 661.8533 | 2 | 47.56 | 6 | F6:27271 | NaSa01\_F14.raw |  |  |  |  |  | 4.6433E6 |  |  |  |  |  |  |  |  | 1 | 0 | 0 | 0 | 0 | 0 | 1 | 0 | 0 | 0 | 0 | 0 | 0 | 0 | 0 | 135 | 146 |  |  | PEAKS DB |
| K.AC(+57.02)ETNVDYVYK.T | Y | 49.20 | 1360.5969 | 11 | 0.3 | 681.3060 | 2 | 22.05 | 6 | F6:6230 | NaSa01\_F14.raw |  |  |  | 2.4601E5 |  | 5.6943E5 |  |  |  |  |  |  |  |  | 2 | 0 | 0 | 0 | 1 | 0 | 1 | 0 | 0 | 0 | 0 | 0 | 0 | 0 | 0 | 1515 | 1525 | Carbamidomethylation | C2:Carbamidomethylation:1000.00 | PEAKS DB |
| M.LTGFLPDAEDLTR.L | N | 47.16 | 1446.7354 | 13 | 1.2 | 724.3758 | 2 | 69.30 | 6 | F6:45774 | NaSa01\_F14.raw |  |  |  |  |  | 7.2495E5 |  |  |  |  |  |  |  |  | 1 | 0 | 0 | 0 | 0 | 0 | 1 | 0 | 0 | 0 | 0 | 0 | 0 | 0 | 0 | 1387 | 1399 |  |  | PEAKS DB |
| R.VGLVAVDKAVYVLNDK.Y | N | 46.90 | 1701.9664 | 16 | 0.8 | 568.3299 | 3 | 57.39 | 6 | F6:35573 | NaSa01\_F14.raw |  |  |  |  |  | 1.8722E5 |  |  |  |  |  |  |  |  | 1 | 0 | 0 | 0 | 0 | 0 | 1 | 0 | 0 | 0 | 0 | 0 | 0 | 0 | 0 | 575 | 590 |  |  | PEAKS DB |
| K.THQYISQR.K | Y | 46.29 | 1031.5149 | 8 | 0.4 | 516.7650 | 2 | 11.44 | 6 | F6:1825 | NaSa01\_F14.raw |  |  |  |  |  | 1.3503E5 |  |  |  |  |  |  |  |  | 1 | 0 | 0 | 0 | 0 | 0 | 1 | 0 | 0 | 0 | 0 | 0 | 0 | 0 | 0 | 1560 | 1567 |  |  | PEAKS DB |
| D.DNEDGFIADSDIISR.S | Y | 46.28 | 1665.7482 | 15 | 0.7 | 833.8820 | 2 | 65.65 | 6 | F6:42702 | NaSa01\_F14.raw |  |  |  |  |  | 2.6489E5 |  |  |  |  |  |  |  |  | 1 | 0 | 0 | 0 | 0 | 0 | 1 | 0 | 0 | 0 | 0 | 0 | 0 | 0 | 0 | 734 | 748 |  |  | PEAKS DB |
| H.VAITSTEIKPGDNLPVNFNVK.G | Y | 46.15 | 2255.2161 | 21 | 0.2 | 752.7461 | 3 | 57.73 | 6 | F6:36049 | NaSa01\_F14.raw |  |  |  |  |  | 3.3349E6 |  |  |  |  |  |  |  |  | 1 | 0 | 0 | 0 | 0 | 0 | 1 | 0 | 0 | 0 | 0 | 0 | 0 | 0 | 0 | 447 | 467 |  |  | PEAKS DB |
| K.IIIQGDPVAQIIEN.S | Y | 45.71 | 1521.8402 | 14 | 0.0 | 761.9274 | 2 | 85.63 | 6 | F6:59337 | NaSa01\_F14.raw |  |  |  |  |  | 1.4516E7 |  |  |  |  |  |  |  |  | 1 | 0 | 0 | 0 | 0 | 0 | 1 | 0 | 0 | 0 | 0 | 0 | 0 | 0 | 0 | 963 | 976 |  |  | PEAKS DB |
| K.TM(+15.99)SFYLR.D | Y | 45.45 | 932.4426 | 7 | 0.2 | 467.2286 | 2 | 32.98 | 6 | F6:14848 | NaSa01\_F14.raw |  |  |  |  |  | 1.994E6 |  |  |  |  |  |  |  |  | 2 | 0 | 0 | 0 | 0 | 0 | 2 | 0 | 0 | 0 | 0 | 0 | 0 | 0 | 0 | 775 | 781 | Oxidation (M) | M2:Oxidation (M):1000.00 | PEAKS DB |
| N.DIYVMDVLEVIK.Q | N | 45.17 | 1435.7632 | 12 | 0.7 | 718.8894 | 2 | 106.25 | 4 | F4:76997 | NaSa01\_F12.raw |  |  |  | 2.8255E5 |  |  |  |  |  |  |  |  |  |  | 1 | 0 | 0 | 0 | 1 | 0 | 0 | 0 | 0 | 0 | 0 | 0 | 0 | 0 | 0 | 1538 | 1549 |  |  | PEAKS DB |
| K.YFKPGMPYELTVY.V | N | 44.78 | 1606.7742 | 13 | 0.4 | 804.3947 | 2 | 71.51 | 6 | F6:47852 | NaSa01\_F14.raw |  |  |  |  |  | 1.8453E7 |  |  |  |  |  |  |  |  | 1 | 0 | 0 | 0 | 0 | 0 | 1 | 0 | 0 | 0 | 0 | 0 | 0 | 0 | 0 | 353 | 365 |  |  | PEAKS DB |
| R.SRFPNLNELVGH.T | N | 44.24 | 1381.7102 | 12 | 0.6 | 461.5776 | 3 | 50.79 | 6 | F6:30122 | NaSa01\_F14.raw |  |  |  |  |  | 1.9009E7 |  |  |  |  |  |  |  |  | 3 | 0 | 0 | 0 | 0 | 0 | 3 | 0 | 0 | 0 | 0 | 0 | 0 | 0 | 0 | 301 | 312 |  |  | PEAKS DB |
| K.TVIVEFQTPEG.I | N | 43.94 | 1218.6132 | 11 | 3.8 | 610.3144 | 2 | 63.73 | 7 | F7:45532 | NaSa01\_F2.raw |  |  |  |  |  |  | 6.7633E6 | 2.6291E6 |  |  |  |  |  |  | 2 | 0 | 0 | 0 | 0 | 0 | 0 | 1 | 1 | 0 | 0 | 0 | 0 | 0 | 0 | 160 | 170 |  |  | PEAKS DB |
| M.NLHITPDLIPSFR.F | N | 43.77 | 1521.8303 | 13 | 0.6 | 508.2843 | 3 | 65.19 | 6 | F6:42284 | NaSa01\_F14.raw |  |  |  |  |  | 1.1088E7 |  |  |  |  |  |  |  |  | 2 | 0 | 0 | 0 | 0 | 0 | 2 | 0 | 0 | 0 | 0 | 0 | 0 | 0 | 0 | 507 | 519 |  |  | PEAKS DB |
| K.AAFLEC(+57.02)C(+57.02)R.Y | N | 43.14 | 1025.4423 | 8 | 0.7 | 513.7288 | 2 | 11.72 | 9 | F9:2016 | NaSa01\_F4.raw |  |  |  |  |  |  |  |  | 5.2365E5 |  |  |  |  |  | 1 | 0 | 0 | 0 | 0 | 0 | 0 | 0 | 0 | 1 | 0 | 0 | 0 | 0 | 0 | 706 | 713 | Carbamidomethylation | C6:Carbamidomethylation:1000.00;C7:Carbamidomethylation:1000.00 | PEAKS DB |
| L.SYQSSFLFIQTDK.G | Y | 42.37 | 1562.7616 | 13 | 1.2 | 782.3890 | 2 | 64.49 | 6 | F6:41739 | NaSa01\_F14.raw |  |  |  |  |  | 3.4858E5 |  |  |  |  |  |  |  |  | 1 | 0 | 0 | 0 | 0 | 0 | 1 | 0 | 0 | 0 | 0 | 0 | 0 | 0 | 0 | 122 | 134 |  |  | PEAKS DB |
| R.KC(+57.02)C(+57.02)EDVMHENPMGYTC(+57.02)EK.R | Y | 42.21 | 2286.8835 | 18 | 1.4 | 763.3029 | 3 | 20.88 | 10 | F10:7153 | NaSa01\_F5.raw |  |  |  |  |  |  |  |  | 7.1178E5 | 3.5368E6 |  |  |  |  | 3 | 0 | 0 | 0 | 0 | 0 | 0 | 0 | 0 | 1 | 2 | 0 | 0 | 0 | 0 | 676 | 693 | Carbamidomethylation | C2:Carbamidomethylation:1000.00;C3:Carbamidomethylation:1000.00;C16:Carbamidomethylation:1000.00 | PEAKS DB |
| R.IPIIDGDGK.A | Y | 42.15 | 926.5073 | 9 | 0.6 | 464.2612 | 2 | 32.92 | 6 | F6:14878 | NaSa01\_F14.raw |  |  |  |  |  | 3.4884E5 |  |  |  |  |  |  |  |  | 1 | 0 | 0 | 0 | 0 | 0 | 1 | 0 | 0 | 0 | 0 | 0 | 0 | 0 | 0 | 283 | 291 |  |  | PEAKS DB |
| Y.VLPSFEVR.L | N | 41.89 | 945.5283 | 8 | -0.2 | 473.7714 | 2 | 40.35 | 6 | F6:20803 | NaSa01\_F14.raw |  |  |  |  |  | 5.7172E5 |  |  |  |  |  |  |  |  | 1 | 0 | 0 | 0 | 0 | 0 | 1 | 0 | 0 | 0 | 0 | 0 | 0 | 0 | 0 | 220 | 227 |  |  | PEAKS DB |
| K.IC(+57.02)IGNVC(+57.02)R.C | Y | 41.79 | 990.4739 | 8 | -1.3 | 496.2436 | 2 | 11.58 | 6 | F6:1976 | NaSa01\_F14.raw |  |  |  |  |  | 2.2107E5 |  |  |  |  |  |  |  |  | 1 | 0 | 0 | 0 | 0 | 0 | 1 | 0 | 0 | 0 | 0 | 0 | 0 | 0 | 0 | 1484 | 1491 | Carbamidomethylation | C2:Carbamidomethylation:1000.00;C7:Carbamidomethylation:1000.00 | PEAKS DB |
| R.SRFPNLNELVGHTLY.A | N | 40.90 | 1758.9053 | 15 | 0.7 | 587.3094 | 3 | 70.95 | 6 | F6:46924 | NaSa01\_F14.raw |  |  |  |  |  | 5.6962E6 |  |  |  |  |  |  |  |  | 1 | 0 | 0 | 0 | 0 | 0 | 1 | 0 | 0 | 0 | 0 | 0 | 0 | 0 | 0 | 301 | 315 |  |  | PEAKS DB |
| Y.TLITPAVLR.T | N | 40.29 | 982.6175 | 9 | 0.4 | 492.3162 | 2 | 45.78 | 6 | F6:25572 | NaSa01\_F14.raw |  |  |  |  |  | 5.8349E7 |  |  |  |  |  |  |  |  | 1 | 0 | 0 | 0 | 0 | 0 | 1 | 0 | 0 | 0 | 0 | 0 | 0 | 0 | 0 | 26 | 34 |  |  | PEAKS DB |
| K.LILNIPLNAQ.S | Y | 40.28 | 1107.6652 | 10 | -0.2 | 554.8397 | 2 | 84.63 | 6 | F6:58512 | NaSa01\_F14.raw |  |  |  |  |  | 3.2621E6 |  |  |  |  |  |  |  |  | 1 | 0 | 0 | 0 | 0 | 0 | 1 | 0 | 0 | 0 | 0 | 0 | 0 | 0 | 0 | 398 | 407 |  |  | PEAKS DB |
| K.IIIQGDPVAQI.I | Y | 39.99 | 1165.6707 | 11 | 0.7 | 583.8430 | 2 | 72.27 | 6 | F6:48414 | NaSa01\_F14.raw |  |  |  |  |  | 1.2067E6 |  |  |  |  |  |  |  |  | 1 | 0 | 0 | 0 | 0 | 0 | 1 | 0 | 0 | 0 | 0 | 0 | 0 | 0 | 0 | 963 | 973 |  |  | PEAKS DB |
| Y.QVGNNEIVADSVWVDVK.D | N | 39.98 | 1870.9425 | 17 | 0.5 | 936.4790 | 2 | 64.84 | 6 | F6:41917 | NaSa01\_F14.raw |  |  |  |  |  | 1.8564E6 |  |  |  |  |  |  |  |  | 1 | 0 | 0 | 0 | 0 | 0 | 1 | 0 | 0 | 0 | 0 | 0 | 0 | 0 | 0 | 525 | 541 |  |  | PEAKS DB |
| N.IPLNAQSLPITVR.T | Y | 39.95 | 1420.8402 | 13 | 0.7 | 711.4279 | 2 | 58.51 | 6 | F6:36546 | NaSa01\_F14.raw |  |  |  |  |  | 5.2985E5 |  |  |  |  |  |  |  |  | 1 | 0 | 0 | 0 | 0 | 0 | 1 | 0 | 0 | 0 | 0 | 0 | 0 | 0 | 0 | 402 | 414 |  |  | PEAKS DB |
| K.SWLWLTK.D | Y | 39.91 | 932.5120 | 7 | 0.3 | 467.2634 | 2 | 68.87 | 6 | F6:45829 | NaSa01\_F14.raw |  |  |  |  |  | 5.8771E7 |  |  |  |  |  |  |  |  | 1 | 0 | 0 | 0 | 0 | 0 | 1 | 0 | 0 | 0 | 0 | 0 | 0 | 0 | 0 | 754 | 760 |  |  | PEAKS DB |
| K.IIIQGDPVAQ.I | Y | 39.88 | 1052.5865 | 10 | 0.0 | 527.3005 | 2 | 48.47 | 6 | F6:28020 | NaSa01\_F14.raw |  |  |  |  |  | 3.3932E7 |  |  |  |  |  |  |  |  | 1 | 0 | 0 | 0 | 0 | 0 | 1 | 0 | 0 | 0 | 0 | 0 | 0 | 0 | 0 | 963 | 972 |  |  | PEAKS DB |
| R.IEEQDGNDIYVM(+15.99)DVLEVIK.Q | Y | 39.47 | 2237.0774 | 19 | 1.1 | 1119.5472 | 2 | 90.84 | 4 | F4:65053 | NaSa01\_F12.raw |  |  |  | 8.9297E5 |  |  |  |  |  |  |  |  |  |  | 1 | 0 | 0 | 0 | 1 | 0 | 0 | 0 | 0 | 0 | 0 | 0 | 0 | 0 | 0 | 1531 | 1549 | Oxidation (M) | M12:Oxidation (M):1000.00 | PEAKS DB |
| K.VVLLSYQSSF.L | Y | 38.34 | 1141.6019 | 10 | 0.4 | 571.8085 | 2 | 79.64 | 6 | F6:54198 | NaSa01\_F14.raw |  |  |  |  |  | 2.3493E6 |  |  |  |  |  |  |  |  | 1 | 0 | 0 | 0 | 0 | 0 | 1 | 0 | 0 | 0 | 0 | 0 | 0 | 0 | 0 | 118 | 127 |  |  | PEAKS DB |
| K.ISYIITK.N | N | 38.08 | 836.5007 | 7 | -0.7 | 419.2574 | 2 | 26.29 | 6 | F6:9476 | NaSa01\_F14.raw |  |  |  |  |  | 6.6012E5 |  |  |  |  |  |  |  |  | 1 | 0 | 0 | 0 | 0 | 0 | 1 | 0 | 0 | 0 | 0 | 0 | 0 | 0 | 0 | 1597 | 1603 |  |  | PEAKS DB |
| total 87 peptides |  |  |  |  |  |  |  |  |  |  |  |  |  |  |  |  |  |  |  |  |  |  |  |  |  |  |  |  |  |  |  |  |  |  |  |  |  |  |  |  |  |  |  |  |  |
| --- | --- | --- | --- | --- | --- | --- | --- | --- | --- | --- | --- | --- | --- | --- | --- | --- | --- | --- | --- | --- | --- | --- | --- | --- | --- | --- | --- | --- | --- | --- | --- | --- | --- | --- | --- | --- | --- | --- | --- | --- | --- | --- | --- | --- | --- |

AAA68989.1

back to list

  

| Protein Coverage
| Supporting Peptides
|

Protein Coverage:

Supporting Peptides:

| Peptide | Uniq | -10lgP | Mass | Length | ppm | m/z | z | RT | Fraction | Scan | Source File | Area F1 | Area F10 | Area F11 | Area F12 | Area F13 | Area F14 | Area F2 | Area F3 | Area F4 | Area F5 | Area F6 | Area F7 | Area F8 | Area F9 | #Feature | #Feature F1 | #Feature F10 | #Feature F11 | #Feature F12 | #Feature F13 | #Feature F14 | #Feature F2 | #Feature F3 | #Feature F4 | #Feature F5 | #Feature F6 | #Feature F7 | #Feature F8 | #Feature F9 | Start | End | PTM | AScore | Found By |
| --- | --- | --- | --- | --- | --- | --- | --- | --- | --- | --- | --- | --- | --- | --- | --- | --- | --- | --- | --- | --- | --- | --- | --- | --- | --- | --- | --- | --- | --- | --- | --- | --- | --- | --- | --- | --- | --- | --- | --- | --- | --- | --- | --- | --- | --- |
| R.AILHNYVNEDIYVR.V | Y | 89.83 | 1717.8788 | 14 | 0.3 | 859.9469 | 2 | 43.05 | 6 | F6:23378 | NaSa01\_F14.raw |  |  |  |  | 1.5001E7 | 1.9759E8 |  |  |  |  |  |  |  |  | 8 | 0 | 0 | 0 | 0 | 3 | 5 | 0 | 0 | 0 | 0 | 0 | 0 | 0 | 0 | 834 | 847 |  |  | PEAKS DB |
| R.AVPFVIVPLEQGLHDVEIK.A | Y | 87.23 | 2102.1775 | 19 | 0.5 | 701.7335 | 3 | 85.72 | 6 | F6:59617 | NaSa01\_F14.raw |  |  |  | 7.0958E5 | 1.6836E7 | 1.2181E8 |  |  |  |  |  |  |  |  | 7 | 0 | 0 | 0 | 1 | 2 | 4 | 0 | 0 | 0 | 0 | 0 | 0 | 0 | 0 | 879 | 897 |  |  | PEAKS DB |
| K.HFEVGFIQPGSVK.V | N | 85.09 | 1443.7510 | 13 | -0.2 | 722.8826 | 2 | 44.13 | 6 | F6:24159 | NaSa01\_F14.raw |  |  |  |  | 2.4365E7 | 2.5116E8 |  |  |  |  |  |  |  |  | 8 | 0 | 0 | 0 | 0 | 2 | 6 | 0 | 0 | 0 | 0 | 0 | 0 | 0 | 0 | 1445 | 1457 |  |  | PEAKS DB |
| R.RDGQNLVTM(+15.99)NLHITPDLIPSFR.F | N | 85.06 | 2552.3169 | 22 | 0.3 | 639.0867 | 4 | 69.77 | 6 | F6:46133 | NaSa01\_F14.raw |  |  |  |  |  | 2.5484E6 |  |  |  |  |  |  |  |  | 2 | 0 | 0 | 0 | 0 | 0 | 2 | 0 | 0 | 0 | 0 | 0 | 0 | 0 | 0 | 498 | 519 | Oxidation (M) | M9:Oxidation (M):1000.00 | PEAKS DB |
| R.QNQYVVVQVTGPQVR.L | N | 83.17 | 1713.9163 | 15 | 0.2 | 857.9656 | 2 | 43.83 | 6 | F6:24244 | NaSa01\_F14.raw |  |  |  |  | 1.4255E7 | 1.5135E8 |  |  |  |  |  |  |  |  | 5 | 0 | 0 | 0 | 0 | 2 | 3 | 0 | 0 | 0 | 0 | 0 | 0 | 0 | 0 | 100 | 114 |  |  | PEAKS DB |
| K.VFFIDLQMPYSVVK.N | Y | 82.52 | 1684.8898 | 14 | 0.3 | 843.4524 | 2 | 95.37 | 6 | F6:67466 | NaSa01\_F14.raw |  |  |  |  | 5.5288E5 | 4.4172E6 |  |  |  |  |  |  |  |  | 2 | 0 | 0 | 0 | 0 | 1 | 1 | 0 | 0 | 0 | 0 | 0 | 0 | 0 | 0 | 813 | 826 |  |  | PEAKS DB |
| K.VNDDYLIWGSR.S | Y | 81.98 | 1336.6411 | 11 | 1.0 | 669.3285 | 2 | 59.77 | 6 | F6:37640 | NaSa01\_F14.raw |  |  |  | 4.0113E7 | 1.558E7 | 1.2947E8 |  |  |  |  |  |  |  |  | 4 | 0 | 0 | 0 | 1 | 2 | 1 | 0 | 0 | 0 | 0 | 0 | 0 | 0 | 0 | 1577 | 1587 |  |  | PEAKS DB |
| R.IEEQDGNDIYVMDVLEVIK.Q | Y | 80.07 | 2221.0823 | 19 | 0.7 | 1111.5492 | 2 | 101.01 | 4 | F4:73173 | NaSa01\_F12.raw |  |  |  | 1.608E7 | 4.109E6 | 2.6808E7 |  |  |  |  |  |  |  |  | 11 | 0 | 0 | 0 | 4 | 3 | 4 | 0 | 0 | 0 | 0 | 0 | 0 | 0 | 0 | 1531 | 1549 |  |  | PEAKS DB |
| K.ATMTILTFYNAQLQEK.A | Y | 79.74 | 1870.9498 | 16 | -0.2 | 936.4820 | 2 | 77.02 | 6 | F6:52034 | NaSa01\_F14.raw |  |  | 5.3707E5 | 1.2422E6 | 3.5134E6 | 4.7877E7 |  |  |  |  |  |  |  |  | 6 | 0 | 0 | 1 | 1 | 1 | 3 | 0 | 0 | 0 | 0 | 0 | 0 | 0 | 0 | 1321 | 1336 |  |  | PEAKS DB |
| K.QLDIFVHDFPR.K | N | 79.15 | 1385.7091 | 11 | 0.0 | 693.8618 | 2 | 58.44 | 6 | F6:36683 | NaSa01\_F14.raw |  |  |  |  | 9.0136E6 | 8.647E7 |  |  |  |  |  |  |  |  | 4 | 0 | 0 | 0 | 0 | 2 | 2 | 0 | 0 | 0 | 0 | 0 | 0 | 0 | 0 | 53 | 63 |  |  | PEAKS DB |
| K.GDNLIQMPGAAMK.I | Y | 78.60 | 1344.6530 | 13 | 0.2 | 673.3339 | 2 | 49.26 | 6 | F6:28453 | NaSa01\_F14.raw |  |  |  |  |  | 1.0626E8 |  |  |  |  |  |  |  |  | 2 | 0 | 0 | 0 | 0 | 0 | 2 | 0 | 0 | 0 | 0 | 0 | 0 | 0 | 0 | 552 | 564 |  |  | PEAKS DB |
| F.VIVPLEQGLHDVEIK.A | Y | 76.27 | 1687.9508 | 15 | 0.0 | 844.9827 | 2 | 58.08 | 6 | F6:36376 | NaSa01\_F14.raw |  |  |  |  | 5.5586E6 | 9.3853E7 |  |  |  |  |  |  |  |  | 3 | 0 | 0 | 0 | 0 | 1 | 2 | 0 | 0 | 0 | 0 | 0 | 0 | 0 | 0 | 883 | 897 |  |  | PEAKS DB |
| R.DDNEDGFIADSDIISR.S | Y | 75.58 | 1780.7751 | 16 | 0.3 | 891.3951 | 2 | 68.40 | 6 | F6:44898 | NaSa01\_F14.raw |  |  |  |  | 9.0776E5 | 1.2244E7 |  |  |  |  |  |  |  |  | 4 | 0 | 0 | 0 | 0 | 1 | 3 | 0 | 0 | 0 | 0 | 0 | 0 | 0 | 0 | 733 | 748 |  |  | PEAKS DB |
| K.C(+57.02)C(+57.02)EDVMHENPMGYTC(+57.02)EK.R | Y | 74.09 | 2158.7886 | 17 | 7.4 | 720.6050 | 3 | 31.06 | 8 | F8:16109 | NaSa01\_F3.raw |  |  |  |  |  |  |  | 1.0485E7 | 5.6652E7 | 1.4617E7 |  |  |  |  | 6 | 0 | 0 | 0 | 0 | 0 | 0 | 0 | 1 | 3 | 2 | 0 | 0 | 0 | 0 | 677 | 693 | Carbamidomethylation | C1:Carbamidomethylation:1000.00;C2:Carbamidomethylation:1000.00;C15:Carbamidomethylation:1000.00 | PEAKS DB |
| R.INYENALLAR.T | Y | 74.07 | 1175.6299 | 10 | 0.1 | 588.8223 | 2 | 45.12 | 6 | F6:25480 | NaSa01\_F14.raw |  |  | 3.3741E5 | 7.6601E6 | 1.5972E7 | 1.5972E8 |  |  |  |  |  |  |  |  | 7 | 0 | 0 | 1 | 3 | 1 | 2 | 0 | 0 | 0 | 0 | 0 | 0 | 0 | 0 | 1292 | 1301 |  |  | PEAKS DB |
| Y.YQVGNNEIVADSVWVDVK.D | N | 73.99 | 2034.0057 | 18 | 0.2 | 1018.0104 | 2 | 70.65 | 6 | F6:46860 | NaSa01\_F14.raw |  |  |  |  |  | 6.5239E6 |  |  |  |  |  |  |  |  | 1 | 0 | 0 | 0 | 0 | 0 | 1 | 0 | 0 | 0 | 0 | 0 | 0 | 0 | 0 | 524 | 541 |  |  | PEAKS DB |
| K.ASVQEALWSDGVR.K | Y | 72.98 | 1416.6997 | 13 | 0.1 | 709.3572 | 2 | 49.71 | 6 | F6:29208 | NaSa01\_F14.raw |  |  |  |  | 1.3438E7 | 1.2825E8 |  |  |  |  |  |  |  |  | 7 | 0 | 0 | 0 | 0 | 1 | 6 | 0 | 0 | 0 | 0 | 0 | 0 | 0 | 0 | 898 | 910 |  |  | PEAKS DB |
| R.VELLYNPAFC(+57.02)SASTK.G | Y | 72.57 | 1698.8286 | 15 | -0.7 | 850.4210 | 2 | 59.22 | 6 | F6:37110 | NaSa01\_F14.raw |  |  |  |  | 1.6821E7 | 2.15E8 |  |  |  |  |  |  |  |  | 2 | 0 | 0 | 0 | 0 | 1 | 1 | 0 | 0 | 0 | 0 | 0 | 0 | 0 | 0 | 848 | 862 | Carbamidomethylation | C10:Carbamidomethylation:1000.00 | PEAKS DB |
| K.LNQDITVTASGDGK.A | Y | 72.09 | 1417.7048 | 14 | 0.8 | 709.8602 | 2 | 20.83 | 6 | F6:5302 | NaSa01\_F14.raw |  |  |  |  | 4.3744E5 | 4.5631E6 |  |  |  |  |  |  |  |  | 3 | 0 | 0 | 0 | 0 | 1 | 2 | 0 | 0 | 0 | 0 | 0 | 0 | 0 | 0 | 1307 | 1320 |  |  | PEAKS DB |
| K.LILNIPLNAQSLPITVR.T | Y | 71.60 | 1874.1353 | 17 | 0.5 | 625.7194 | 3 | 90.36 | 6 | F6:63322 | NaSa01\_F14.raw |  |  |  |  | 1.4111E7 | 4.901E7 |  |  |  |  |  |  |  |  | 4 | 0 | 0 | 0 | 0 | 2 | 2 | 0 | 0 | 0 | 0 | 0 | 0 | 0 | 0 | 398 | 414 |  |  | PEAKS DB |
| K.YFTYLILNK.G | N | 70.49 | 1173.6433 | 9 | 0.1 | 587.8290 | 2 | 64.67 | 6 | F6:41809 | NaSa01\_F14.raw |  |  |  |  | 1.0728E7 | 7.7707E7 |  |  |  |  |  |  |  |  | 2 | 0 | 0 | 0 | 0 | 1 | 1 | 0 | 0 | 0 | 0 | 0 | 0 | 0 | 0 | 478 | 486 |  |  | PEAKS DB |
| R.WPHEDEC(+57.02)QEEEFQK.L | Y | 69.62 | 1889.7526 | 14 | 0.9 | 945.8844 | 2 | 23.39 | 4 | F4:9200 | NaSa01\_F12.raw |  |  |  | 2.8919E7 |  | 3.166E6 |  |  |  |  |  |  |  |  | 3 | 0 | 0 | 0 | 2 | 0 | 1 | 0 | 0 | 0 | 0 | 0 | 0 | 0 | 0 | 1610 | 1623 | Carbamidomethylation | C7:Carbamidomethylation:1000.00 | PEAKS DB |
| K.SDFGC(+57.02)TAGSGQNNLGVFEDAGLALTTSTNLNTK.Q | N | 66.95 | 3359.5576 | 33 | 0.8 | 1120.8607 | 3 | 89.37 | 6 | F6:62425 | NaSa01\_F14.raw |  |  |  |  |  | 3.8512E7 |  |  |  |  |  |  |  |  | 1 | 0 | 0 | 0 | 0 | 0 | 1 | 0 | 0 | 0 | 0 | 0 | 0 | 0 | 0 | 605 | 637 | Carbamidomethylation | C5:Carbamidomethylation:1000.00 | PEAKS DB |
| R.DGQNLVTM(+15.99)NLHITPDLIPSFR.F | N | 66.34 | 2396.2158 | 21 | 0.4 | 799.7462 | 3 | 83.99 | 6 | F6:57985 | NaSa01\_F14.raw |  |  |  |  |  | 5.9574E6 |  |  |  |  |  |  |  |  | 1 | 0 | 0 | 0 | 0 | 0 | 1 | 0 | 0 | 0 | 0 | 0 | 0 | 0 | 0 | 499 | 519 | Oxidation (M) | M8:Oxidation (M):1000.00 | PEAKS DB |
| L.ARDDNEDGFIADSDIISR.S | Y | 66.31 | 2007.9133 | 18 | 1.0 | 670.3124 | 3 | 48.95 | 6 | F6:28381 | NaSa01\_F14.raw |  |  |  |  |  | 6.6726E6 |  |  |  |  |  |  |  |  | 2 | 0 | 0 | 0 | 0 | 0 | 2 | 0 | 0 | 0 | 0 | 0 | 0 | 0 | 0 | 731 | 748 |  |  | PEAKS DB |
| Y.VTNPDGSPAAHVPVVSEAFHSMGTTLSDGTAK.L | Y | 66.31 | 3179.5193 | 32 | 0.8 | 795.8877 | 4 | 59.12 | 6 | F6:37224 | NaSa01\_F14.raw |  |  |  |  |  | 2.3066E7 |  |  |  |  |  |  |  |  | 1 | 0 | 0 | 0 | 0 | 0 | 1 | 0 | 0 | 0 | 0 | 0 | 0 | 0 | 0 | 366 | 397 |  |  | PEAKS DB |
| K.C(+57.02)C(+57.02)EDVMHENPM(+15.99)GYTC(+57.02)EK.R | Y | 65.55 | 2174.7837 | 17 | 1.4 | 725.9362 | 3 | 11.70 | 9 | F9:2004 | NaSa01\_F4.raw |  |  |  |  |  |  |  |  | 1.0934E7 |  |  |  |  |  | 2 | 0 | 0 | 0 | 0 | 0 | 0 | 0 | 0 | 2 | 0 | 0 | 0 | 0 | 0 | 677 | 693 | Carbamidomethylation; Oxidation (M) | C1:Carbamidomethylation:1000.00;C2:Carbamidomethylation:1000.00;M11:Oxidation (M):43.99;C15:Carbamidomethylation:1000.00 | PEAKS DB |
| Y.NLPDLVSLGTWR.I | Y | 65.12 | 1369.7354 | 12 | 0.1 | 685.8750 | 2 | 81.18 | 6 | F6:55452 | NaSa01\_F14.raw |  |  |  |  | 6.3971E6 | 8.6753E7 |  |  |  |  |  |  |  |  | 2 | 0 | 0 | 0 | 0 | 1 | 1 | 0 | 0 | 0 | 0 | 0 | 0 | 0 | 0 | 187 | 198 |  |  | PEAKS DB |
| K.GDNLIQMPGAAM(+15.99)K.I | Y | 64.58 | 1360.6479 | 13 | 0.8 | 681.3318 | 2 | 38.53 | 6 | F6:19473 | NaSa01\_F14.raw |  |  |  |  | 9.6097E5 | 9.5549E6 |  |  |  |  |  |  |  |  | 3 | 0 | 0 | 0 | 0 | 1 | 2 | 0 | 0 | 0 | 0 | 0 | 0 | 0 | 0 | 552 | 564 | Oxidation (M) | M12:Oxidation (M):38.33 | PEAKS DB |
| E.VEGVAFVLFGVK.I | N | 62.96 | 1263.7227 | 12 | 0.2 | 632.8687 | 2 | 85.00 | 6 | F6:58754 | NaSa01\_F14.raw |  |  |  |  |  | 2.0835E6 |  |  |  |  |  |  |  |  | 1 | 0 | 0 | 0 | 0 | 0 | 1 | 0 | 0 | 0 | 0 | 0 | 0 | 0 | 0 | 257 | 268 |  |  | PEAKS DB |
| Y.QSSFLFIQTDK.G | Y | 62.43 | 1312.6663 | 11 | -0.4 | 657.3401 | 2 | 57.57 | 6 | F6:35846 | NaSa01\_F14.raw |  |  |  |  |  | 1.1659E7 |  |  |  |  |  |  |  |  | 1 | 0 | 0 | 0 | 0 | 0 | 1 | 0 | 0 | 0 | 0 | 0 | 0 | 0 | 0 | 124 | 134 |  |  | PEAKS DB |
| K.GVGGTQLEVIK.A | Y | 62.08 | 1099.6237 | 11 | 0.2 | 550.8192 | 2 | 30.32 | 12 | F12:15497 | NaSa01\_F7.raw |  |  |  |  | 1.4118E7 | 1.0253E6 |  |  |  |  |  | 1.7364E6 |  |  | 3 | 0 | 0 | 0 | 0 | 1 | 1 | 0 | 0 | 0 | 0 | 0 | 1 | 0 | 0 | 936 | 946 |  |  | PEAKS DB |
| K.ATM(+15.99)TILTFYNAQLQEK.A | Y | 62.07 | 1886.9448 | 16 | 0.4 | 944.4801 | 2 | 64.84 | 6 | F6:42088 | NaSa01\_F14.raw |  |  |  |  |  | 1.0319E7 |  |  |  |  |  |  |  |  | 2 | 0 | 0 | 0 | 0 | 0 | 2 | 0 | 0 | 0 | 0 | 0 | 0 | 0 | 0 | 1321 | 1336 | Oxidation (M) | M3:Oxidation (M):1000.00 | PEAKS DB |
| R.FPNLNELVGH.T | N | 62.05 | 1138.5770 | 10 | 0.8 | 570.2963 | 2 | 67.53 | 6 | F6:44045 | NaSa01\_F14.raw |  |  |  |  | 1.7762E6 | 2.8185E7 |  |  |  |  |  |  |  |  | 2 | 0 | 0 | 0 | 0 | 1 | 1 | 0 | 0 | 0 | 0 | 0 | 0 | 0 | 0 | 303 | 312 |  |  | PEAKS DB |
| K.VYSYYNLDEK.C | N | 61.93 | 1292.5924 | 10 | 1.2 | 647.3043 | 2 | 33.77 | 6 | F6:15693 | NaSa01\_F14.raw |  |  |  |  | 1.5304E7 | 1.0252E8 |  |  |  |  |  |  |  |  | 3 | 0 | 0 | 0 | 0 | 1 | 2 | 0 | 0 | 0 | 0 | 0 | 0 | 0 | 0 | 1458 | 1467 |  |  | PEAKS DB |
| M.TILTFYNAQLQEK.A | N | 61.57 | 1567.8246 | 13 | 0.3 | 784.9198 | 2 | 60.05 | 6 | F6:38047 | NaSa01\_F14.raw |  |  |  |  |  | 5.1346E6 |  |  |  |  |  |  |  |  | 1 | 0 | 0 | 0 | 0 | 0 | 1 | 0 | 0 | 0 | 0 | 0 | 0 | 0 | 0 | 1324 | 1336 |  |  | PEAKS DB |
| H.ITPDLIPSFR.F | N | 61.36 | 1157.6444 | 10 | 0.0 | 579.8295 | 2 | 65.01 | 6 | F6:42335 | NaSa01\_F14.raw |  |  |  |  | 3.0601E6 | 3.8923E7 |  |  |  |  |  |  |  |  | 3 | 0 | 0 | 0 | 0 | 1 | 2 | 0 | 0 | 0 | 0 | 0 | 0 | 0 | 0 | 510 | 519 |  |  | PEAKS DB |
| K.PGDNLPVNFNVK.G | Y | 60.28 | 1312.6775 | 12 | 0.4 | 657.3463 | 2 | 47.72 | 6 | F6:27452 | NaSa01\_F14.raw |  |  |  |  |  | 1.0823E6 |  |  |  |  |  |  |  |  | 1 | 0 | 0 | 0 | 0 | 0 | 1 | 0 | 0 | 0 | 0 | 0 | 0 | 0 | 0 | 456 | 467 |  |  | PEAKS DB |
| W.VVLAVSFTPTK.G | Y | 59.98 | 1160.6804 | 11 | 0.4 | 581.3477 | 2 | 51.95 | 6 | F6:30874 | NaSa01\_F14.raw |  |  |  |  |  | 3.7062E6 |  |  |  |  |  |  |  |  | 1 | 0 | 0 | 0 | 0 | 0 | 1 | 0 | 0 | 0 | 0 | 0 | 0 | 0 | 0 | 788 | 798 |  |  | PEAKS DB |
| K.YEHSPENYTAYFDVR.K | N | 59.87 | 1889.8220 | 15 | -0.9 | 630.9474 | 3 | 47.19 | 6 | F6:27480 | NaSa01\_F14.raw |  |  |  |  |  | 2.5484E6 |  |  |  |  |  |  |  |  | 2 | 0 | 0 | 0 | 0 | 0 | 2 | 0 | 0 | 0 | 0 | 0 | 0 | 0 | 0 | 203 | 217 |  |  | PEAKS DB |
| R.IDVPLQIEK.A | Y | 59.34 | 1053.6069 | 9 | 0.1 | 527.8108 | 2 | 47.86 | 6 | F6:27228 | NaSa01\_F14.raw |  |  |  | 3.0425E7 | 1.9664E7 | 1.6873E8 |  |  |  |  |  |  |  |  | 4 | 0 | 0 | 0 | 1 | 1 | 2 | 0 | 0 | 0 | 0 | 0 | 0 | 0 | 0 | 1506 | 1514 |  |  | PEAKS DB |
| K.IIIQGDPVAQIIENSIDGSK.L | Y | 59.31 | 2109.1316 | 20 | 0.6 | 1055.5737 | 2 | 96.62 | 6 | F6:68548 | NaSa01\_F14.raw |  |  | 1.4465E5 |  | 1.6001E5 | 2.5079E6 |  |  |  |  |  |  |  |  | 3 | 0 | 0 | 1 | 0 | 1 | 1 | 0 | 0 | 0 | 0 | 0 | 0 | 0 | 0 | 963 | 982 |  |  | PEAKS DB |
| K.GIC(+57.02)VAEPYEIR.V | Y | 59.06 | 1305.6387 | 11 | 0.4 | 653.8268 | 2 | 44.31 | 6 | F6:24643 | NaSa01\_F14.raw |  |  |  |  | 1.0302E7 | 1.0808E8 |  |  |  |  |  |  |  |  | 3 | 0 | 0 | 0 | 0 | 1 | 2 | 0 | 0 | 0 | 0 | 0 | 0 | 0 | 0 | 799 | 809 | Carbamidomethylation | C3:Carbamidomethylation:1000.00 | PEAKS DB |
| E.DGFIADSDIISR.S | Y | 58.62 | 1307.6357 | 12 | -0.1 | 654.8251 | 2 | 60.77 | 6 | F6:38594 | NaSa01\_F14.raw |  |  |  |  |  | 4.7593E6 |  |  |  |  |  |  |  |  | 1 | 0 | 0 | 0 | 0 | 0 | 1 | 0 | 0 | 0 | 0 | 0 | 0 | 0 | 0 | 737 | 748 |  |  | PEAKS DB |
| R.FPNLNELVGHTLY.A | N | 58.47 | 1515.7721 | 13 | 0.3 | 758.8936 | 2 | 85.62 | 6 | F6:59205 | NaSa01\_F14.raw |  |  |  |  |  | 1.9527E7 |  |  |  |  |  |  |  |  | 1 | 0 | 0 | 0 | 0 | 0 | 1 | 0 | 0 | 0 | 0 | 0 | 0 | 0 | 0 | 303 | 315 |  |  | PEAKS DB |
| S.MLTGFLPDAEDLTR.L | N | 58.03 | 1577.7759 | 14 | 0.5 | 789.8956 | 2 | 78.56 | 6 | F6:53520 | NaSa01\_F14.raw |  |  |  |  |  | 2.8623E6 |  |  |  |  |  |  |  |  | 1 | 0 | 0 | 0 | 0 | 0 | 1 | 0 | 0 | 0 | 0 | 0 | 0 | 0 | 0 | 1386 | 1399 |  |  | PEAKS DB |
| R.KYVLPSFEVR.L | Y | 56.74 | 1236.6866 | 10 | 0.3 | 413.2363 | 3 | 42.11 | 6 | F6:22829 | NaSa01\_F14.raw |  |  |  |  | 5.509E6 | 4.9733E7 |  |  |  |  |  |  |  |  | 6 | 0 | 0 | 0 | 0 | 2 | 4 | 0 | 0 | 0 | 0 | 0 | 0 | 0 | 0 | 218 | 227 |  |  | PEAKS DB |
| R.DGQNLVTMNLHITPDLIPSFR.F | N | 56.46 | 2380.2209 | 21 | 0.5 | 794.4147 | 3 | 91.58 | 6 | F6:64345 | NaSa01\_F14.raw |  |  |  |  |  | 2.1315E6 |  |  |  |  |  |  |  |  | 1 | 0 | 0 | 0 | 0 | 0 | 1 | 0 | 0 | 0 | 0 | 0 | 0 | 0 | 0 | 499 | 519 |  |  | PEAKS DB |
| K.VAVIIYLNK.V | Y | 56.21 | 1031.6379 | 9 | 0.8 | 516.8267 | 2 | 50.20 | 6 | F6:30133 | NaSa01\_F14.raw |  |  |  |  |  | 1.67E7 |  |  |  |  |  |  |  |  | 2 | 0 | 0 | 0 | 0 | 0 | 2 | 0 | 0 | 0 | 0 | 0 | 0 | 0 | 0 | 1421 | 1429 |  |  | PEAKS DB |
| G.ALYTLITPAVLR.T | N | 55.82 | 1329.8020 | 12 | 0.4 | 665.9086 | 2 | 75.95 | 6 | F6:51300 | NaSa01\_F14.raw |  |  |  | 1.7564E6 | 1.5853E7 | 2.019E8 |  |  |  |  |  |  |  |  | 6 | 0 | 0 | 0 | 1 | 1 | 4 | 0 | 0 | 0 | 0 | 0 | 0 | 0 | 0 | 23 | 34 |  |  | PEAKS DB |
| N.LHITPDLIPSFR.F | N | 52.36 | 1407.7874 | 12 | 0.4 | 470.2699 | 3 | 64.03 | 6 | F6:41508 | NaSa01\_F14.raw |  |  |  |  |  | 2.1751E7 |  |  |  |  |  |  |  |  | 2 | 0 | 0 | 0 | 0 | 0 | 2 | 0 | 0 | 0 | 0 | 0 | 0 | 0 | 0 | 508 | 519 |  |  | PEAKS DB |
| K.YVLPSFEVR.L | N | 51.77 | 1108.5917 | 9 | 0.6 | 555.3035 | 2 | 58.44 | 6 | F6:36495 | NaSa01\_F14.raw |  |  |  | 4.1589E5 | 1.253E7 | 1.3997E8 |  |  |  |  |  |  |  |  | 4 | 0 | 0 | 0 | 1 | 1 | 2 | 0 | 0 | 0 | 0 | 0 | 0 | 0 | 0 | 219 | 227 |  |  | PEAKS DB |
| K.TMSFYLR.D | Y | 51.69 | 916.4476 | 7 | 0.3 | 459.2312 | 2 | 41.67 | 6 | F6:22840 | NaSa01\_F14.raw |  |  |  |  |  | 5.6816E7 |  |  |  |  |  |  |  |  | 1 | 0 | 0 | 0 | 0 | 0 | 1 | 0 | 0 | 0 | 0 | 0 | 0 | 0 | 0 | 775 | 781 |  |  | PEAKS DB |
| R.DGQNLVTMNLH.I | N | 51.62 | 1240.5870 | 11 | 0.0 | 621.3008 | 2 | 49.19 | 6 | F6:28557 | NaSa01\_F14.raw |  |  |  |  |  | 4.925E6 |  |  |  |  |  |  |  |  | 1 | 0 | 0 | 0 | 0 | 0 | 1 | 0 | 0 | 0 | 0 | 0 | 0 | 0 | 0 | 499 | 509 |  |  | PEAKS DB |
| L.NIPLNAQSLPITVR.T | Y | 50.97 | 1534.8831 | 14 | -1.4 | 768.4477 | 2 | 61.34 | 6 | F6:39039 | NaSa01\_F14.raw |  |  |  |  |  | 1.6927E5 |  |  |  |  |  |  |  |  | 1 | 0 | 0 | 0 | 0 | 0 | 1 | 0 | 0 | 0 | 0 | 0 | 0 | 0 | 0 | 401 | 414 |  |  | PEAKS DB |
| K.C(+57.02)C(+57.02)EDVM(+15.99)HENPM(+15.99)GYTC(+57.02)EK.R | Y | 50.02 | 2190.7786 | 17 | 0.7 | 731.2673 | 3 | 11.49 | 9 | F9:1837 | NaSa01\_F4.raw |  |  |  |  |  |  |  |  | 4.1662E5 |  |  |  |  |  | 1 | 0 | 0 | 0 | 0 | 0 | 0 | 0 | 0 | 1 | 0 | 0 | 0 | 0 | 0 | 677 | 693 | Carbamidomethylation; Oxidation (M) | C1:Carbamidomethylation:1000.00;C2:Carbamidomethylation:1000.00;M6:Oxidation (M):1000.00;M11:Oxidation (M):1000.00;C15:Carbamidomethylation:1000.00 | PEAKS DB |
| K.GIYTPGSPVLYR.V | N | 49.98 | 1321.7030 | 12 | -8.2 | 661.8533 | 2 | 47.56 | 6 | F6:27271 | NaSa01\_F14.raw |  |  |  |  |  | 4.6433E6 |  |  |  |  |  |  |  |  | 1 | 0 | 0 | 0 | 0 | 0 | 1 | 0 | 0 | 0 | 0 | 0 | 0 | 0 | 0 | 135 | 146 |  |  | PEAKS DB |
| K.AC(+57.02)ETNVDYVYK.T | Y | 49.20 | 1360.5969 | 11 | 0.3 | 681.3060 | 2 | 22.05 | 6 | F6:6230 | NaSa01\_F14.raw |  |  |  | 2.4601E5 |  | 5.6943E5 |  |  |  |  |  |  |  |  | 2 | 0 | 0 | 0 | 1 | 0 | 1 | 0 | 0 | 0 | 0 | 0 | 0 | 0 | 0 | 1515 | 1525 | Carbamidomethylation | C2:Carbamidomethylation:1000.00 | PEAKS DB |
| M.LTGFLPDAEDLTR.L | N | 47.16 | 1446.7354 | 13 | 1.2 | 724.3758 | 2 | 69.30 | 6 | F6:45774 | NaSa01\_F14.raw |  |  |  |  |  | 7.2495E5 |  |  |  |  |  |  |  |  | 1 | 0 | 0 | 0 | 0 | 0 | 1 | 0 | 0 | 0 | 0 | 0 | 0 | 0 | 0 | 1387 | 1399 |  |  | PEAKS DB |
| R.VGLVAVDKAVYVLNDK.Y | N | 46.90 | 1701.9664 | 16 | 0.8 | 568.3299 | 3 | 57.39 | 6 | F6:35573 | NaSa01\_F14.raw |  |  |  |  |  | 1.8722E5 |  |  |  |  |  |  |  |  | 1 | 0 | 0 | 0 | 0 | 0 | 1 | 0 | 0 | 0 | 0 | 0 | 0 | 0 | 0 | 575 | 590 |  |  | PEAKS DB |
| K.THQYISQR.K | Y | 46.29 | 1031.5149 | 8 | 0.4 | 516.7650 | 2 | 11.44 | 6 | F6:1825 | NaSa01\_F14.raw |  |  |  |  |  | 1.3503E5 |  |  |  |  |  |  |  |  | 1 | 0 | 0 | 0 | 0 | 0 | 1 | 0 | 0 | 0 | 0 | 0 | 0 | 0 | 0 | 1560 | 1567 |  |  | PEAKS DB |
| D.DNEDGFIADSDIISR.S | Y | 46.28 | 1665.7482 | 15 | 0.7 | 833.8820 | 2 | 65.65 | 6 | F6:42702 | NaSa01\_F14.raw |  |  |  |  |  | 2.6489E5 |  |  |  |  |  |  |  |  | 1 | 0 | 0 | 0 | 0 | 0 | 1 | 0 | 0 | 0 | 0 | 0 | 0 | 0 | 0 | 734 | 748 |  |  | PEAKS DB |
| H.VAITSTEIKPGDNLPVNFNVK.G | Y | 46.15 | 2255.2161 | 21 | 0.2 | 752.7461 | 3 | 57.73 | 6 | F6:36049 | NaSa01\_F14.raw |  |  |  |  |  | 3.3349E6 |  |  |  |  |  |  |  |  | 1 | 0 | 0 | 0 | 0 | 0 | 1 | 0 | 0 | 0 | 0 | 0 | 0 | 0 | 0 | 447 | 467 |  |  | PEAKS DB |
| K.IIIQGDPVAQIIEN.S | Y | 45.71 | 1521.8402 | 14 | 0.0 | 761.9274 | 2 | 85.63 | 6 | F6:59337 | NaSa01\_F14.raw |  |  |  |  |  | 1.4516E7 |  |  |  |  |  |  |  |  | 1 | 0 | 0 | 0 | 0 | 0 | 1 | 0 | 0 | 0 | 0 | 0 | 0 | 0 | 0 | 963 | 976 |  |  | PEAKS DB |
| K.TM(+15.99)SFYLR.D | Y | 45.45 | 932.4426 | 7 | 0.2 | 467.2286 | 2 | 32.98 | 6 | F6:14848 | NaSa01\_F14.raw |  |  |  |  |  | 1.994E6 |  |  |  |  |  |  |  |  | 2 | 0 | 0 | 0 | 0 | 0 | 2 | 0 | 0 | 0 | 0 | 0 | 0 | 0 | 0 | 775 | 781 | Oxidation (M) | M2:Oxidation (M):1000.00 | PEAKS DB |
| N.DIYVMDVLEVIK.Q | N | 45.17 | 1435.7632 | 12 | 0.7 | 718.8894 | 2 | 106.25 | 4 | F4:76997 | NaSa01\_F12.raw |  |  |  | 2.8255E5 |  |  |  |  |  |  |  |  |  |  | 1 | 0 | 0 | 0 | 1 | 0 | 0 | 0 | 0 | 0 | 0 | 0 | 0 | 0 | 0 | 1538 | 1549 |  |  | PEAKS DB |
| K.YFKPGMPYELTVY.V | N | 44.78 | 1606.7742 | 13 | 0.4 | 804.3947 | 2 | 71.51 | 6 | F6:47852 | NaSa01\_F14.raw |  |  |  |  |  | 1.8453E7 |  |  |  |  |  |  |  |  | 1 | 0 | 0 | 0 | 0 | 0 | 1 | 0 | 0 | 0 | 0 | 0 | 0 | 0 | 0 | 353 | 365 |  |  | PEAKS DB |
| R.SRFPNLNELVGH.T | N | 44.24 | 1381.7102 | 12 | 0.6 | 461.5776 | 3 | 50.79 | 6 | F6:30122 | NaSa01\_F14.raw |  |  |  |  |  | 1.9009E7 |  |  |  |  |  |  |  |  | 3 | 0 | 0 | 0 | 0 | 0 | 3 | 0 | 0 | 0 | 0 | 0 | 0 | 0 | 0 | 301 | 312 |  |  | PEAKS DB |
| K.TVIVEFQTPEG.I | N | 43.94 | 1218.6132 | 11 | 3.8 | 610.3144 | 2 | 63.73 | 7 | F7:45532 | NaSa01\_F2.raw |  |  |  |  |  |  | 6.7633E6 | 2.6291E6 |  |  |  |  |  |  | 2 | 0 | 0 | 0 | 0 | 0 | 0 | 1 | 1 | 0 | 0 | 0 | 0 | 0 | 0 | 160 | 170 |  |  | PEAKS DB |
| M.NLHITPDLIPSFR.F | N | 43.77 | 1521.8303 | 13 | 0.6 | 508.2843 | 3 | 65.19 | 6 | F6:42284 | NaSa01\_F14.raw |  |  |  |  |  | 1.1088E7 |  |  |  |  |  |  |  |  | 2 | 0 | 0 | 0 | 0 | 0 | 2 | 0 | 0 | 0 | 0 | 0 | 0 | 0 | 0 | 507 | 519 |  |  | PEAKS DB |
| K.AAFLEC(+57.02)C(+57.02)R.Y | N | 43.14 | 1025.4423 | 8 | 0.7 | 513.7288 | 2 | 11.72 | 9 | F9:2016 | NaSa01\_F4.raw |  |  |  |  |  |  |  |  | 5.2365E5 |  |  |  |  |  | 1 | 0 | 0 | 0 | 0 | 0 | 0 | 0 | 0 | 1 | 0 | 0 | 0 | 0 | 0 | 706 | 713 | Carbamidomethylation | C6:Carbamidomethylation:1000.00;C7:Carbamidomethylation:1000.00 | PEAKS DB |
| L.SYQSSFLFIQTDK.G | Y | 42.37 | 1562.7616 | 13 | 1.2 | 782.3890 | 2 | 64.49 | 6 | F6:41739 | NaSa01\_F14.raw |  |  |  |  |  | 3.4858E5 |  |  |  |  |  |  |  |  | 1 | 0 | 0 | 0 | 0 | 0 | 1 | 0 | 0 | 0 | 0 | 0 | 0 | 0 | 0 | 122 | 134 |  |  | PEAKS DB |
| R.KC(+57.02)C(+57.02)EDVMHENPMGYTC(+57.02)EK.R | Y | 42.21 | 2286.8835 | 18 | 1.4 | 763.3029 | 3 | 20.88 | 10 | F10:7153 | NaSa01\_F5.raw |  |  |  |  |  |  |  |  | 7.1178E5 | 3.5368E6 |  |  |  |  | 3 | 0 | 0 | 0 | 0 | 0 | 0 | 0 | 0 | 1 | 2 | 0 | 0 | 0 | 0 | 676 | 693 | Carbamidomethylation | C2:Carbamidomethylation:1000.00;C3:Carbamidomethylation:1000.00;C16:Carbamidomethylation:1000.00 | PEAKS DB |
| R.IPIIDGDGK.A | Y | 42.15 | 926.5073 | 9 | 0.6 | 464.2612 | 2 | 32.92 | 6 | F6:14878 | NaSa01\_F14.raw |  |  |  |  |  | 3.4884E5 |  |  |  |  |  |  |  |  | 1 | 0 | 0 | 0 | 0 | 0 | 1 | 0 | 0 | 0 | 0 | 0 | 0 | 0 | 0 | 283 | 291 |  |  | PEAKS DB |
| Y.VLPSFEVR.L | N | 41.89 | 945.5283 | 8 | -0.2 | 473.7714 | 2 | 40.35 | 6 | F6:20803 | NaSa01\_F14.raw |  |  |  |  |  | 5.7172E5 |  |  |  |  |  |  |  |  | 1 | 0 | 0 | 0 | 0 | 0 | 1 | 0 | 0 | 0 | 0 | 0 | 0 | 0 | 0 | 220 | 227 |  |  | PEAKS DB |
| K.IC(+57.02)IGNVC(+57.02)R.C | Y | 41.79 | 990.4739 | 8 | -1.3 | 496.2436 | 2 | 11.58 | 6 | F6:1976 | NaSa01\_F14.raw |  |  |  |  |  | 2.2107E5 |  |  |  |  |  |  |  |  | 1 | 0 | 0 | 0 | 0 | 0 | 1 | 0 | 0 | 0 | 0 | 0 | 0 | 0 | 0 | 1484 | 1491 | Carbamidomethylation | C2:Carbamidomethylation:1000.00;C7:Carbamidomethylation:1000.00 | PEAKS DB |
| R.SRFPNLNELVGHTLY.A | N | 40.90 | 1758.9053 | 15 | 0.7 | 587.3094 | 3 | 70.95 | 6 | F6:46924 | NaSa01\_F14.raw |  |  |  |  |  | 5.6962E6 |  |  |  |  |  |  |  |  | 1 | 0 | 0 | 0 | 0 | 0 | 1 | 0 | 0 | 0 | 0 | 0 | 0 | 0 | 0 | 301 | 315 |  |  | PEAKS DB |
| Y.TLITPAVLR.T | N | 40.29 | 982.6175 | 9 | 0.4 | 492.3162 | 2 | 45.78 | 6 | F6:25572 | NaSa01\_F14.raw |  |  |  |  |  | 5.8349E7 |  |  |  |  |  |  |  |  | 1 | 0 | 0 | 0 | 0 | 0 | 1 | 0 | 0 | 0 | 0 | 0 | 0 | 0 | 0 | 26 | 34 |  |  | PEAKS DB |
| K.LILNIPLNAQ.S | Y | 40.28 | 1107.6652 | 10 | -0.2 | 554.8397 | 2 | 84.63 | 6 | F6:58512 | NaSa01\_F14.raw |  |  |  |  |  | 3.2621E6 |  |  |  |  |  |  |  |  | 1 | 0 | 0 | 0 | 0 | 0 | 1 | 0 | 0 | 0 | 0 | 0 | 0 | 0 | 0 | 398 | 407 |  |  | PEAKS DB |
| K.IIIQGDPVAQI.I | Y | 39.99 | 1165.6707 | 11 | 0.7 | 583.8430 | 2 | 72.27 | 6 | F6:48414 | NaSa01\_F14.raw |  |  |  |  |  | 1.2067E6 |  |  |  |  |  |  |  |  | 1 | 0 | 0 | 0 | 0 | 0 | 1 | 0 | 0 | 0 | 0 | 0 | 0 | 0 | 0 | 963 | 973 |  |  | PEAKS DB |
| Y.QVGNNEIVADSVWVDVK.D | N | 39.98 | 1870.9425 | 17 | 0.5 | 936.4790 | 2 | 64.84 | 6 | F6:41917 | NaSa01\_F14.raw |  |  |  |  |  | 1.8564E6 |  |  |  |  |  |  |  |  | 1 | 0 | 0 | 0 | 0 | 0 | 1 | 0 | 0 | 0 | 0 | 0 | 0 | 0 | 0 | 525 | 541 |  |  | PEAKS DB |
| N.IPLNAQSLPITVR.T | Y | 39.95 | 1420.8402 | 13 | 0.7 | 711.4279 | 2 | 58.51 | 6 | F6:36546 | NaSa01\_F14.raw |  |  |  |  |  | 5.2985E5 |  |  |  |  |  |  |  |  | 1 | 0 | 0 | 0 | 0 | 0 | 1 | 0 | 0 | 0 | 0 | 0 | 0 | 0 | 0 | 402 | 414 |  |  | PEAKS DB |
| K.SWLWLTK.D | Y | 39.91 | 932.5120 | 7 | 0.3 | 467.2634 | 2 | 68.87 | 6 | F6:45829 | NaSa01\_F14.raw |  |  |  |  |  | 5.8771E7 |  |  |  |  |  |  |  |  | 1 | 0 | 0 | 0 | 0 | 0 | 1 | 0 | 0 | 0 | 0 | 0 | 0 | 0 | 0 | 754 | 760 |  |  | PEAKS DB |
| K.IIIQGDPVAQ.I | Y | 39.88 | 1052.5865 | 10 | 0.0 | 527.3005 | 2 | 48.47 | 6 | F6:28020 | NaSa01\_F14.raw |  |  |  |  |  | 3.3932E7 |  |  |  |  |  |  |  |  | 1 | 0 | 0 | 0 | 0 | 0 | 1 | 0 | 0 | 0 | 0 | 0 | 0 | 0 | 0 | 963 | 972 |  |  | PEAKS DB |
| R.IEEQDGNDIYVM(+15.99)DVLEVIK.Q | Y | 39.47 | 2237.0774 | 19 | 1.1 | 1119.5472 | 2 | 90.84 | 4 | F4:65053 | NaSa01\_F12.raw |  |  |  | 8.9297E5 |  |  |  |  |  |  |  |  |  |  | 1 | 0 | 0 | 0 | 1 | 0 | 0 | 0 | 0 | 0 | 0 | 0 | 0 | 0 | 0 | 1531 | 1549 | Oxidation (M) | M12:Oxidation (M):1000.00 | PEAKS DB |
| K.VVLLSYQSSF.L | Y | 38.34 | 1141.6019 | 10 | 0.4 | 571.8085 | 2 | 79.64 | 6 | F6:54198 | NaSa01\_F14.raw |  |  |  |  |  | 2.3493E6 |  |  |  |  |  |  |  |  | 1 | 0 | 0 | 0 | 0 | 0 | 1 | 0 | 0 | 0 | 0 | 0 | 0 | 0 | 0 | 118 | 127 |  |  | PEAKS DB |
| K.ISYIITK.N | N | 38.08 | 836.5007 | 7 | -0.7 | 419.2574 | 2 | 26.29 | 6 | F6:9476 | NaSa01\_F14.raw |  |  |  |  |  | 6.6012E5 |  |  |  |  |  |  |  |  | 1 | 0 | 0 | 0 | 0 | 0 | 1 | 0 | 0 | 0 | 0 | 0 | 0 | 0 | 0 | 1597 | 1603 |  |  | PEAKS DB |
| total 87 peptides |  |  |  |  |  |  |  |  |  |  |  |  |  |  |  |  |  |  |  |  |  |  |  |  |  |  |  |  |  |  |  |  |  |  |  |  |  |  |  |  |  |  |  |  |  |
| --- | --- | --- | --- | --- | --- | --- | --- | --- | --- | --- | --- | --- | --- | --- | --- | --- | --- | --- | --- | --- | --- | --- | --- | --- | --- | --- | --- | --- | --- | --- | --- | --- | --- | --- | --- | --- | --- | --- | --- | --- | --- | --- | --- | --- | --- |

Q91132.1

back to list

  

| Protein Coverage
| Supporting Peptides
|

Protein Coverage:

Supporting Peptides:

| Peptide | Uniq | -10lgP | Mass | Length | ppm | m/z | z | RT | Fraction | Scan | Source File | Area F1 | Area F10 | Area F11 | Area F12 | Area F13 | Area F14 | Area F2 | Area F3 | Area F4 | Area F5 | Area F6 | Area F7 | Area F8 | Area F9 | #Feature | #Feature F1 | #Feature F10 | #Feature F11 | #Feature F12 | #Feature F13 | #Feature F14 | #Feature F2 | #Feature F3 | #Feature F4 | #Feature F5 | #Feature F6 | #Feature F7 | #Feature F8 | #Feature F9 | Start | End | PTM | AScore | Found By |
| --- | --- | --- | --- | --- | --- | --- | --- | --- | --- | --- | --- | --- | --- | --- | --- | --- | --- | --- | --- | --- | --- | --- | --- | --- | --- | --- | --- | --- | --- | --- | --- | --- | --- | --- | --- | --- | --- | --- | --- | --- | --- | --- | --- | --- | --- |
| R.AILHNYVNEDIYVR.V | Y | 89.83 | 1717.8788 | 14 | 0.3 | 859.9469 | 2 | 43.05 | 6 | F6:23378 | NaSa01\_F14.raw |  |  |  |  | 1.5001E7 | 1.9759E8 |  |  |  |  |  |  |  |  | 8 | 0 | 0 | 0 | 0 | 3 | 5 | 0 | 0 | 0 | 0 | 0 | 0 | 0 | 0 | 834 | 847 |  |  | PEAKS DB |
| R.AVPFVIVPLEQGLHDVEIK.A | Y | 87.23 | 2102.1775 | 19 | 0.5 | 701.7335 | 3 | 85.72 | 6 | F6:59617 | NaSa01\_F14.raw |  |  |  | 7.0958E5 | 1.6836E7 | 1.2181E8 |  |  |  |  |  |  |  |  | 7 | 0 | 0 | 0 | 1 | 2 | 4 | 0 | 0 | 0 | 0 | 0 | 0 | 0 | 0 | 879 | 897 |  |  | PEAKS DB |
| K.HFEVGFIQPGSVK.V | N | 85.09 | 1443.7510 | 13 | -0.2 | 722.8826 | 2 | 44.13 | 6 | F6:24159 | NaSa01\_F14.raw |  |  |  |  | 2.4365E7 | 2.5116E8 |  |  |  |  |  |  |  |  | 8 | 0 | 0 | 0 | 0 | 2 | 6 | 0 | 0 | 0 | 0 | 0 | 0 | 0 | 0 | 1445 | 1457 |  |  | PEAKS DB |
| R.RDGQNLVTM(+15.99)NLHITPDLIPSFR.F | N | 85.06 | 2552.3169 | 22 | 0.3 | 639.0867 | 4 | 69.77 | 6 | F6:46133 | NaSa01\_F14.raw |  |  |  |  |  | 2.5484E6 |  |  |  |  |  |  |  |  | 2 | 0 | 0 | 0 | 0 | 0 | 2 | 0 | 0 | 0 | 0 | 0 | 0 | 0 | 0 | 498 | 519 | Oxidation (M) | M9:Oxidation (M):1000.00 | PEAKS DB |
| R.QNQYVVVQVTGPQVR.L | N | 83.17 | 1713.9163 | 15 | 0.2 | 857.9656 | 2 | 43.83 | 6 | F6:24244 | NaSa01\_F14.raw |  |  |  |  | 1.4255E7 | 1.5135E8 |  |  |  |  |  |  |  |  | 5 | 0 | 0 | 0 | 0 | 2 | 3 | 0 | 0 | 0 | 0 | 0 | 0 | 0 | 0 | 100 | 114 |  |  | PEAKS DB |
| K.VFFIDLQMPYSVVK.N | Y | 82.52 | 1684.8898 | 14 | 0.3 | 843.4524 | 2 | 95.37 | 6 | F6:67466 | NaSa01\_F14.raw |  |  |  |  | 5.5288E5 | 4.4172E6 |  |  |  |  |  |  |  |  | 2 | 0 | 0 | 0 | 0 | 1 | 1 | 0 | 0 | 0 | 0 | 0 | 0 | 0 | 0 | 813 | 826 |  |  | PEAKS DB |
| K.VNDDYLIWGSR.S | Y | 81.98 | 1336.6411 | 11 | 1.0 | 669.3285 | 2 | 59.77 | 6 | F6:37640 | NaSa01\_F14.raw |  |  |  | 4.0113E7 | 1.558E7 | 1.2947E8 |  |  |  |  |  |  |  |  | 4 | 0 | 0 | 0 | 1 | 2 | 1 | 0 | 0 | 0 | 0 | 0 | 0 | 0 | 0 | 1577 | 1587 |  |  | PEAKS DB |
| R.IEEQDGNDIYVMDVLEVIK.Q | Y | 80.07 | 2221.0823 | 19 | 0.7 | 1111.5492 | 2 | 101.01 | 4 | F4:73173 | NaSa01\_F12.raw |  |  |  | 1.608E7 | 4.109E6 | 2.6808E7 |  |  |  |  |  |  |  |  | 11 | 0 | 0 | 0 | 4 | 3 | 4 | 0 | 0 | 0 | 0 | 0 | 0 | 0 | 0 | 1531 | 1549 |  |  | PEAKS DB |
| K.ATMTILTFYNAQLQEK.A | Y | 79.74 | 1870.9498 | 16 | -0.2 | 936.4820 | 2 | 77.02 | 6 | F6:52034 | NaSa01\_F14.raw |  |  | 5.3707E5 | 1.2422E6 | 3.5134E6 | 4.7877E7 |  |  |  |  |  |  |  |  | 6 | 0 | 0 | 1 | 1 | 1 | 3 | 0 | 0 | 0 | 0 | 0 | 0 | 0 | 0 | 1321 | 1336 |  |  | PEAKS DB |
| K.QLDIFVHDFPR.K | N | 79.15 | 1385.7091 | 11 | 0.0 | 693.8618 | 2 | 58.44 | 6 | F6:36683 | NaSa01\_F14.raw |  |  |  |  | 9.0136E6 | 8.647E7 |  |  |  |  |  |  |  |  | 4 | 0 | 0 | 0 | 0 | 2 | 2 | 0 | 0 | 0 | 0 | 0 | 0 | 0 | 0 | 53 | 63 |  |  | PEAKS DB |
| K.GDNLIQMPGAAMK.I | Y | 78.60 | 1344.6530 | 13 | 0.2 | 673.3339 | 2 | 49.26 | 6 | F6:28453 | NaSa01\_F14.raw |  |  |  |  |  | 1.0626E8 |  |  |  |  |  |  |  |  | 2 | 0 | 0 | 0 | 0 | 0 | 2 | 0 | 0 | 0 | 0 | 0 | 0 | 0 | 0 | 552 | 564 |  |  | PEAKS DB |
| F.VIVPLEQGLHDVEIK.A | Y | 76.27 | 1687.9508 | 15 | 0.0 | 844.9827 | 2 | 58.08 | 6 | F6:36376 | NaSa01\_F14.raw |  |  |  |  | 5.5586E6 | 9.3853E7 |  |  |  |  |  |  |  |  | 3 | 0 | 0 | 0 | 0 | 1 | 2 | 0 | 0 | 0 | 0 | 0 | 0 | 0 | 0 | 883 | 897 |  |  | PEAKS DB |
| R.DDNEDGFIADSDIISR.S | Y | 75.58 | 1780.7751 | 16 | 0.3 | 891.3951 | 2 | 68.40 | 6 | F6:44898 | NaSa01\_F14.raw |  |  |  |  | 9.0776E5 | 1.2244E7 |  |  |  |  |  |  |  |  | 4 | 0 | 0 | 0 | 0 | 1 | 3 | 0 | 0 | 0 | 0 | 0 | 0 | 0 | 0 | 733 | 748 |  |  | PEAKS DB |
| K.C(+57.02)C(+57.02)EDVMHENPMGYTC(+57.02)EK.R | Y | 74.09 | 2158.7886 | 17 | 7.4 | 720.6050 | 3 | 31.06 | 8 | F8:16109 | NaSa01\_F3.raw |  |  |  |  |  |  |  | 1.0485E7 | 5.6652E7 | 1.4617E7 |  |  |  |  | 6 | 0 | 0 | 0 | 0 | 0 | 0 | 0 | 1 | 3 | 2 | 0 | 0 | 0 | 0 | 677 | 693 | Carbamidomethylation | C1:Carbamidomethylation:1000.00;C2:Carbamidomethylation:1000.00;C15:Carbamidomethylation:1000.00 | PEAKS DB |
| R.INYENALLAR.T | Y | 74.07 | 1175.6299 | 10 | 0.1 | 588.8223 | 2 | 45.12 | 6 | F6:25480 | NaSa01\_F14.raw |  |  | 3.3741E5 | 7.6601E6 | 1.5972E7 | 1.5972E8 |  |  |  |  |  |  |  |  | 7 | 0 | 0 | 1 | 3 | 1 | 2 | 0 | 0 | 0 | 0 | 0 | 0 | 0 | 0 | 1292 | 1301 |  |  | PEAKS DB |
| Y.YQVGNNEIVADSVWVDVK.D | N | 73.99 | 2034.0057 | 18 | 0.2 | 1018.0104 | 2 | 70.65 | 6 | F6:46860 | NaSa01\_F14.raw |  |  |  |  |  | 6.5239E6 |  |  |  |  |  |  |  |  | 1 | 0 | 0 | 0 | 0 | 0 | 1 | 0 | 0 | 0 | 0 | 0 | 0 | 0 | 0 | 524 | 541 |  |  | PEAKS DB |
| K.ASVQEALWSDGVR.K | Y | 72.98 | 1416.6997 | 13 | 0.1 | 709.3572 | 2 | 49.71 | 6 | F6:29208 | NaSa01\_F14.raw |  |  |  |  | 1.3438E7 | 1.2825E8 |  |  |  |  |  |  |  |  | 7 | 0 | 0 | 0 | 0 | 1 | 6 | 0 | 0 | 0 | 0 | 0 | 0 | 0 | 0 | 898 | 910 |  |  | PEAKS DB |
| R.VELLYNPAFC(+57.02)SASTK.G | Y | 72.57 | 1698.8286 | 15 | -0.7 | 850.4210 | 2 | 59.22 | 6 | F6:37110 | NaSa01\_F14.raw |  |  |  |  | 1.6821E7 | 2.15E8 |  |  |  |  |  |  |  |  | 2 | 0 | 0 | 0 | 0 | 1 | 1 | 0 | 0 | 0 | 0 | 0 | 0 | 0 | 0 | 848 | 862 | Carbamidomethylation | C10:Carbamidomethylation:1000.00 | PEAKS DB |
| K.LNQDITVTASGDGK.A | Y | 72.09 | 1417.7048 | 14 | 0.8 | 709.8602 | 2 | 20.83 | 6 | F6:5302 | NaSa01\_F14.raw |  |  |  |  | 4.3744E5 | 4.5631E6 |  |  |  |  |  |  |  |  | 3 | 0 | 0 | 0 | 0 | 1 | 2 | 0 | 0 | 0 | 0 | 0 | 0 | 0 | 0 | 1307 | 1320 |  |  | PEAKS DB |
| K.LILNIPLNAQSLPITVR.T | Y | 71.60 | 1874.1353 | 17 | 0.5 | 625.7194 | 3 | 90.36 | 6 | F6:63322 | NaSa01\_F14.raw |  |  |  |  | 1.4111E7 | 4.901E7 |  |  |  |  |  |  |  |  | 4 | 0 | 0 | 0 | 0 | 2 | 2 | 0 | 0 | 0 | 0 | 0 | 0 | 0 | 0 | 398 | 414 |  |  | PEAKS DB |
| K.YFTYLILNK.G | N | 70.49 | 1173.6433 | 9 | 0.1 | 587.8290 | 2 | 64.67 | 6 | F6:41809 | NaSa01\_F14.raw |  |  |  |  | 1.0728E7 | 7.7707E7 |  |  |  |  |  |  |  |  | 2 | 0 | 0 | 0 | 0 | 1 | 1 | 0 | 0 | 0 | 0 | 0 | 0 | 0 | 0 | 478 | 486 |  |  | PEAKS DB |
| R.WPHEDEC(+57.02)QEEEFQK.L | Y | 69.62 | 1889.7526 | 14 | 0.9 | 945.8844 | 2 | 23.39 | 4 | F4:9200 | NaSa01\_F12.raw |  |  |  | 2.8919E7 |  | 3.166E6 |  |  |  |  |  |  |  |  | 3 | 0 | 0 | 0 | 2 | 0 | 1 | 0 | 0 | 0 | 0 | 0 | 0 | 0 | 0 | 1610 | 1623 | Carbamidomethylation | C7:Carbamidomethylation:1000.00 | PEAKS DB |
| K.SDFGC(+57.02)TAGSGQNNLGVFEDAGLALTTSTNLNTK.Q | N | 66.95 | 3359.5576 | 33 | 0.8 | 1120.8607 | 3 | 89.37 | 6 | F6:62425 | NaSa01\_F14.raw |  |  |  |  |  | 3.8512E7 |  |  |  |  |  |  |  |  | 1 | 0 | 0 | 0 | 0 | 0 | 1 | 0 | 0 | 0 | 0 | 0 | 0 | 0 | 0 | 605 | 637 | Carbamidomethylation | C5:Carbamidomethylation:1000.00 | PEAKS DB |
| R.DGQNLVTM(+15.99)NLHITPDLIPSFR.F | N | 66.34 | 2396.2158 | 21 | 0.4 | 799.7462 | 3 | 83.99 | 6 | F6:57985 | NaSa01\_F14.raw |  |  |  |  |  | 5.9574E6 |  |  |  |  |  |  |  |  | 1 | 0 | 0 | 0 | 0 | 0 | 1 | 0 | 0 | 0 | 0 | 0 | 0 | 0 | 0 | 499 | 519 | Oxidation (M) | M8:Oxidation (M):1000.00 | PEAKS DB |
| L.ARDDNEDGFIADSDIISR.S | Y | 66.31 | 2007.9133 | 18 | 1.0 | 670.3124 | 3 | 48.95 | 6 | F6:28381 | NaSa01\_F14.raw |  |  |  |  |  | 6.6726E6 |  |  |  |  |  |  |  |  | 2 | 0 | 0 | 0 | 0 | 0 | 2 | 0 | 0 | 0 | 0 | 0 | 0 | 0 | 0 | 731 | 748 |  |  | PEAKS DB |
| Y.VTNPDGSPAAHVPVVSEAFHSMGTTLSDGTAK.L | Y | 66.31 | 3179.5193 | 32 | 0.8 | 795.8877 | 4 | 59.12 | 6 | F6:37224 | NaSa01\_F14.raw |  |  |  |  |  | 2.3066E7 |  |  |  |  |  |  |  |  | 1 | 0 | 0 | 0 | 0 | 0 | 1 | 0 | 0 | 0 | 0 | 0 | 0 | 0 | 0 | 366 | 397 |  |  | PEAKS DB |
| K.C(+57.02)C(+57.02)EDVMHENPM(+15.99)GYTC(+57.02)EK.R | Y | 65.55 | 2174.7837 | 17 | 1.4 | 725.9362 | 3 | 11.70 | 9 | F9:2004 | NaSa01\_F4.raw |  |  |  |  |  |  |  |  | 1.0934E7 |  |  |  |  |  | 2 | 0 | 0 | 0 | 0 | 0 | 0 | 0 | 0 | 2 | 0 | 0 | 0 | 0 | 0 | 677 | 693 | Carbamidomethylation; Oxidation (M) | C1:Carbamidomethylation:1000.00;C2:Carbamidomethylation:1000.00;M11:Oxidation (M):43.99;C15:Carbamidomethylation:1000.00 | PEAKS DB |
| Y.NLPDLVSLGTWR.I | Y | 65.12 | 1369.7354 | 12 | 0.1 | 685.8750 | 2 | 81.18 | 6 | F6:55452 | NaSa01\_F14.raw |  |  |  |  | 6.3971E6 | 8.6753E7 |  |  |  |  |  |  |  |  | 2 | 0 | 0 | 0 | 0 | 1 | 1 | 0 | 0 | 0 | 0 | 0 | 0 | 0 | 0 | 187 | 198 |  |  | PEAKS DB |
| K.GDNLIQMPGAAM(+15.99)K.I | Y | 64.58 | 1360.6479 | 13 | 0.8 | 681.3318 | 2 | 38.53 | 6 | F6:19473 | NaSa01\_F14.raw |  |  |  |  | 9.6097E5 | 9.5549E6 |  |  |  |  |  |  |  |  | 3 | 0 | 0 | 0 | 0 | 1 | 2 | 0 | 0 | 0 | 0 | 0 | 0 | 0 | 0 | 552 | 564 | Oxidation (M) | M12:Oxidation (M):38.33 | PEAKS DB |
| E.VEGVAFVLFGVK.I | N | 62.96 | 1263.7227 | 12 | 0.2 | 632.8687 | 2 | 85.00 | 6 | F6:58754 | NaSa01\_F14.raw |  |  |  |  |  | 2.0835E6 |  |  |  |  |  |  |  |  | 1 | 0 | 0 | 0 | 0 | 0 | 1 | 0 | 0 | 0 | 0 | 0 | 0 | 0 | 0 | 257 | 268 |  |  | PEAKS DB |
| Y.QSSFLFIQTDK.G | Y | 62.43 | 1312.6663 | 11 | -0.4 | 657.3401 | 2 | 57.57 | 6 | F6:35846 | NaSa01\_F14.raw |  |  |  |  |  | 1.1659E7 |  |  |  |  |  |  |  |  | 1 | 0 | 0 | 0 | 0 | 0 | 1 | 0 | 0 | 0 | 0 | 0 | 0 | 0 | 0 | 124 | 134 |  |  | PEAKS DB |
| K.GVGGTQLEVIK.A | Y | 62.08 | 1099.6237 | 11 | 0.2 | 550.8192 | 2 | 30.32 | 12 | F12:15497 | NaSa01\_F7.raw |  |  |  |  | 1.4118E7 | 1.0253E6 |  |  |  |  |  | 1.7364E6 |  |  | 3 | 0 | 0 | 0 | 0 | 1 | 1 | 0 | 0 | 0 | 0 | 0 | 1 | 0 | 0 | 936 | 946 |  |  | PEAKS DB |
| K.ATM(+15.99)TILTFYNAQLQEK.A | Y | 62.07 | 1886.9448 | 16 | 0.4 | 944.4801 | 2 | 64.84 | 6 | F6:42088 | NaSa01\_F14.raw |  |  |  |  |  | 1.0319E7 |  |  |  |  |  |  |  |  | 2 | 0 | 0 | 0 | 0 | 0 | 2 | 0 | 0 | 0 | 0 | 0 | 0 | 0 | 0 | 1321 | 1336 | Oxidation (M) | M3:Oxidation (M):1000.00 | PEAKS DB |
| R.FPNLNELVGH.T | N | 62.05 | 1138.5770 | 10 | 0.8 | 570.2963 | 2 | 67.53 | 6 | F6:44045 | NaSa01\_F14.raw |  |  |  |  | 1.7762E6 | 2.8185E7 |  |  |  |  |  |  |  |  | 2 | 0 | 0 | 0 | 0 | 1 | 1 | 0 | 0 | 0 | 0 | 0 | 0 | 0 | 0 | 303 | 312 |  |  | PEAKS DB |
| K.VYSYYNLDEK.C | N | 61.93 | 1292.5924 | 10 | 1.2 | 647.3043 | 2 | 33.77 | 6 | F6:15693 | NaSa01\_F14.raw |  |  |  |  | 1.5304E7 | 1.0252E8 |  |  |  |  |  |  |  |  | 3 | 0 | 0 | 0 | 0 | 1 | 2 | 0 | 0 | 0 | 0 | 0 | 0 | 0 | 0 | 1458 | 1467 |  |  | PEAKS DB |
| M.TILTFYNAQLQEK.A | N | 61.57 | 1567.8246 | 13 | 0.3 | 784.9198 | 2 | 60.05 | 6 | F6:38047 | NaSa01\_F14.raw |  |  |  |  |  | 5.1346E6 |  |  |  |  |  |  |  |  | 1 | 0 | 0 | 0 | 0 | 0 | 1 | 0 | 0 | 0 | 0 | 0 | 0 | 0 | 0 | 1324 | 1336 |  |  | PEAKS DB |
| H.ITPDLIPSFR.F | N | 61.36 | 1157.6444 | 10 | 0.0 | 579.8295 | 2 | 65.01 | 6 | F6:42335 | NaSa01\_F14.raw |  |  |  |  | 3.0601E6 | 3.8923E7 |  |  |  |  |  |  |  |  | 3 | 0 | 0 | 0 | 0 | 1 | 2 | 0 | 0 | 0 | 0 | 0 | 0 | 0 | 0 | 510 | 519 |  |  | PEAKS DB |
| K.PGDNLPVNFNVK.G | Y | 60.28 | 1312.6775 | 12 | 0.4 | 657.3463 | 2 | 47.72 | 6 | F6:27452 | NaSa01\_F14.raw |  |  |  |  |  | 1.0823E6 |  |  |  |  |  |  |  |  | 1 | 0 | 0 | 0 | 0 | 0 | 1 | 0 | 0 | 0 | 0 | 0 | 0 | 0 | 0 | 456 | 467 |  |  | PEAKS DB |
| W.VVLAVSFTPTK.G | Y | 59.98 | 1160.6804 | 11 | 0.4 | 581.3477 | 2 | 51.95 | 6 | F6:30874 | NaSa01\_F14.raw |  |  |  |  |  | 3.7062E6 |  |  |  |  |  |  |  |  | 1 | 0 | 0 | 0 | 0 | 0 | 1 | 0 | 0 | 0 | 0 | 0 | 0 | 0 | 0 | 788 | 798 |  |  | PEAKS DB |
| K.YEHSPENYTAYFDVR.K | N | 59.87 | 1889.8220 | 15 | -0.9 | 630.9474 | 3 | 47.19 | 6 | F6:27480 | NaSa01\_F14.raw |  |  |  |  |  | 2.5484E6 |  |  |  |  |  |  |  |  | 2 | 0 | 0 | 0 | 0 | 0 | 2 | 0 | 0 | 0 | 0 | 0 | 0 | 0 | 0 | 203 | 217 |  |  | PEAKS DB |
| R.IDVPLQIEK.A | Y | 59.34 | 1053.6069 | 9 | 0.1 | 527.8108 | 2 | 47.86 | 6 | F6:27228 | NaSa01\_F14.raw |  |  |  | 3.0425E7 | 1.9664E7 | 1.6873E8 |  |  |  |  |  |  |  |  | 4 | 0 | 0 | 0 | 1 | 1 | 2 | 0 | 0 | 0 | 0 | 0 | 0 | 0 | 0 | 1506 | 1514 |  |  | PEAKS DB |
| K.IIIQGDPVAQIIENSIDGSK.L | Y | 59.31 | 2109.1316 | 20 | 0.6 | 1055.5737 | 2 | 96.62 | 6 | F6:68548 | NaSa01\_F14.raw |  |  | 1.4465E5 |  | 1.6001E5 | 2.5079E6 |  |  |  |  |  |  |  |  | 3 | 0 | 0 | 1 | 0 | 1 | 1 | 0 | 0 | 0 | 0 | 0 | 0 | 0 | 0 | 963 | 982 |  |  | PEAKS DB |
| K.GIC(+57.02)VAEPYEIR.V | Y | 59.06 | 1305.6387 | 11 | 0.4 | 653.8268 | 2 | 44.31 | 6 | F6:24643 | NaSa01\_F14.raw |  |  |  |  | 1.0302E7 | 1.0808E8 |  |  |  |  |  |  |  |  | 3 | 0 | 0 | 0 | 0 | 1 | 2 | 0 | 0 | 0 | 0 | 0 | 0 | 0 | 0 | 799 | 809 | Carbamidomethylation | C3:Carbamidomethylation:1000.00 | PEAKS DB |
| E.DGFIADSDIISR.S | Y | 58.62 | 1307.6357 | 12 | -0.1 | 654.8251 | 2 | 60.77 | 6 | F6:38594 | NaSa01\_F14.raw |  |  |  |  |  | 4.7593E6 |  |  |  |  |  |  |  |  | 1 | 0 | 0 | 0 | 0 | 0 | 1 | 0 | 0 | 0 | 0 | 0 | 0 | 0 | 0 | 737 | 748 |  |  | PEAKS DB |
| R.FPNLNELVGHTLY.A | N | 58.47 | 1515.7721 | 13 | 0.3 | 758.8936 | 2 | 85.62 | 6 | F6:59205 | NaSa01\_F14.raw |  |  |  |  |  | 1.9527E7 |  |  |  |  |  |  |  |  | 1 | 0 | 0 | 0 | 0 | 0 | 1 | 0 | 0 | 0 | 0 | 0 | 0 | 0 | 0 | 303 | 315 |  |  | PEAKS DB |
| S.MLTGFLPDAEDLTR.L | N | 58.03 | 1577.7759 | 14 | 0.5 | 789.8956 | 2 | 78.56 | 6 | F6:53520 | NaSa01\_F14.raw |  |  |  |  |  | 2.8623E6 |  |  |  |  |  |  |  |  | 1 | 0 | 0 | 0 | 0 | 0 | 1 | 0 | 0 | 0 | 0 | 0 | 0 | 0 | 0 | 1386 | 1399 |  |  | PEAKS DB |
| R.KYVLPSFEVR.L | Y | 56.74 | 1236.6866 | 10 | 0.3 | 413.2363 | 3 | 42.11 | 6 | F6:22829 | NaSa01\_F14.raw |  |  |  |  | 5.509E6 | 4.9733E7 |  |  |  |  |  |  |  |  | 6 | 0 | 0 | 0 | 0 | 2 | 4 | 0 | 0 | 0 | 0 | 0 | 0 | 0 | 0 | 218 | 227 |  |  | PEAKS DB |
| R.DGQNLVTMNLHITPDLIPSFR.F | N | 56.46 | 2380.2209 | 21 | 0.5 | 794.4147 | 3 | 91.58 | 6 | F6:64345 | NaSa01\_F14.raw |  |  |  |  |  | 2.1315E6 |  |  |  |  |  |  |  |  | 1 | 0 | 0 | 0 | 0 | 0 | 1 | 0 | 0 | 0 | 0 | 0 | 0 | 0 | 0 | 499 | 519 |  |  | PEAKS DB |
| K.VAVIIYLNK.V | Y | 56.21 | 1031.6379 | 9 | 0.8 | 516.8267 | 2 | 50.20 | 6 | F6:30133 | NaSa01\_F14.raw |  |  |  |  |  | 1.67E7 |  |  |  |  |  |  |  |  | 2 | 0 | 0 | 0 | 0 | 0 | 2 | 0 | 0 | 0 | 0 | 0 | 0 | 0 | 0 | 1421 | 1429 |  |  | PEAKS DB |
| G.ALYTLITPAVLR.T | N | 55.82 | 1329.8020 | 12 | 0.4 | 665.9086 | 2 | 75.95 | 6 | F6:51300 | NaSa01\_F14.raw |  |  |  | 1.7564E6 | 1.5853E7 | 2.019E8 |  |  |  |  |  |  |  |  | 6 | 0 | 0 | 0 | 1 | 1 | 4 | 0 | 0 | 0 | 0 | 0 | 0 | 0 | 0 | 23 | 34 |  |  | PEAKS DB |
| N.LHITPDLIPSFR.F | N | 52.36 | 1407.7874 | 12 | 0.4 | 470.2699 | 3 | 64.03 | 6 | F6:41508 | NaSa01\_F14.raw |  |  |  |  |  | 2.1751E7 |  |  |  |  |  |  |  |  | 2 | 0 | 0 | 0 | 0 | 0 | 2 | 0 | 0 | 0 | 0 | 0 | 0 | 0 | 0 | 508 | 519 |  |  | PEAKS DB |
| K.YVLPSFEVR.L | N | 51.77 | 1108.5917 | 9 | 0.6 | 555.3035 | 2 | 58.44 | 6 | F6:36495 | NaSa01\_F14.raw |  |  |  | 4.1589E5 | 1.253E7 | 1.3997E8 |  |  |  |  |  |  |  |  | 4 | 0 | 0 | 0 | 1 | 1 | 2 | 0 | 0 | 0 | 0 | 0 | 0 | 0 | 0 | 219 | 227 |  |  | PEAKS DB |
| K.TMSFYLR.D | Y | 51.69 | 916.4476 | 7 | 0.3 | 459.2312 | 2 | 41.67 | 6 | F6:22840 | NaSa01\_F14.raw |  |  |  |  |  | 5.6816E7 |  |  |  |  |  |  |  |  | 1 | 0 | 0 | 0 | 0 | 0 | 1 | 0 | 0 | 0 | 0 | 0 | 0 | 0 | 0 | 775 | 781 |  |  | PEAKS DB |
| R.DGQNLVTMNLH.I | N | 51.62 | 1240.5870 | 11 | 0.0 | 621.3008 | 2 | 49.19 | 6 | F6:28557 | NaSa01\_F14.raw |  |  |  |  |  | 4.925E6 |  |  |  |  |  |  |  |  | 1 | 0 | 0 | 0 | 0 | 0 | 1 | 0 | 0 | 0 | 0 | 0 | 0 | 0 | 0 | 499 | 509 |  |  | PEAKS DB |
| L.NIPLNAQSLPITVR.T | Y | 50.97 | 1534.8831 | 14 | -1.4 | 768.4477 | 2 | 61.34 | 6 | F6:39039 | NaSa01\_F14.raw |  |  |  |  |  | 1.6927E5 |  |  |  |  |  |  |  |  | 1 | 0 | 0 | 0 | 0 | 0 | 1 | 0 | 0 | 0 | 0 | 0 | 0 | 0 | 0 | 401 | 414 |  |  | PEAKS DB |
| K.C(+57.02)C(+57.02)EDVM(+15.99)HENPM(+15.99)GYTC(+57.02)EK.R | Y | 50.02 | 2190.7786 | 17 | 0.7 | 731.2673 | 3 | 11.49 | 9 | F9:1837 | NaSa01\_F4.raw |  |  |  |  |  |  |  |  | 4.1662E5 |  |  |  |  |  | 1 | 0 | 0 | 0 | 0 | 0 | 0 | 0 | 0 | 1 | 0 | 0 | 0 | 0 | 0 | 677 | 693 | Carbamidomethylation; Oxidation (M) | C1:Carbamidomethylation:1000.00;C2:Carbamidomethylation:1000.00;M6:Oxidation (M):1000.00;M11:Oxidation (M):1000.00;C15:Carbamidomethylation:1000.00 | PEAKS DB |
| K.GIYTPGSPVLYR.V | N | 49.98 | 1321.7030 | 12 | -8.2 | 661.8533 | 2 | 47.56 | 6 | F6:27271 | NaSa01\_F14.raw |  |  |  |  |  | 4.6433E6 |  |  |  |  |  |  |  |  | 1 | 0 | 0 | 0 | 0 | 0 | 1 | 0 | 0 | 0 | 0 | 0 | 0 | 0 | 0 | 135 | 146 |  |  | PEAKS DB |
| K.AC(+57.02)ETNVDYVYK.T | Y | 49.20 | 1360.5969 | 11 | 0.3 | 681.3060 | 2 | 22.05 | 6 | F6:6230 | NaSa01\_F14.raw |  |  |  | 2.4601E5 |  | 5.6943E5 |  |  |  |  |  |  |  |  | 2 | 0 | 0 | 0 | 1 | 0 | 1 | 0 | 0 | 0 | 0 | 0 | 0 | 0 | 0 | 1515 | 1525 | Carbamidomethylation | C2:Carbamidomethylation:1000.00 | PEAKS DB |
| M.LTGFLPDAEDLTR.L | N | 47.16 | 1446.7354 | 13 | 1.2 | 724.3758 | 2 | 69.30 | 6 | F6:45774 | NaSa01\_F14.raw |  |  |  |  |  | 7.2495E5 |  |  |  |  |  |  |  |  | 1 | 0 | 0 | 0 | 0 | 0 | 1 | 0 | 0 | 0 | 0 | 0 | 0 | 0 | 0 | 1387 | 1399 |  |  | PEAKS DB |
| R.VGLVAVDKAVYVLNDK.Y | N | 46.90 | 1701.9664 | 16 | 0.8 | 568.3299 | 3 | 57.39 | 6 | F6:35573 | NaSa01\_F14.raw |  |  |  |  |  | 1.8722E5 |  |  |  |  |  |  |  |  | 1 | 0 | 0 | 0 | 0 | 0 | 1 | 0 | 0 | 0 | 0 | 0 | 0 | 0 | 0 | 575 | 590 |  |  | PEAKS DB |
| K.THQYISQR.K | Y | 46.29 | 1031.5149 | 8 | 0.4 | 516.7650 | 2 | 11.44 | 6 | F6:1825 | NaSa01\_F14.raw |  |  |  |  |  | 1.3503E5 |  |  |  |  |  |  |  |  | 1 | 0 | 0 | 0 | 0 | 0 | 1 | 0 | 0 | 0 | 0 | 0 | 0 | 0 | 0 | 1560 | 1567 |  |  | PEAKS DB |
| D.DNEDGFIADSDIISR.S | Y | 46.28 | 1665.7482 | 15 | 0.7 | 833.8820 | 2 | 65.65 | 6 | F6:42702 | NaSa01\_F14.raw |  |  |  |  |  | 2.6489E5 |  |  |  |  |  |  |  |  | 1 | 0 | 0 | 0 | 0 | 0 | 1 | 0 | 0 | 0 | 0 | 0 | 0 | 0 | 0 | 734 | 748 |  |  | PEAKS DB |
| H.VAITSTEIKPGDNLPVNFNVK.G | Y | 46.15 | 2255.2161 | 21 | 0.2 | 752.7461 | 3 | 57.73 | 6 | F6:36049 | NaSa01\_F14.raw |  |  |  |  |  | 3.3349E6 |  |  |  |  |  |  |  |  | 1 | 0 | 0 | 0 | 0 | 0 | 1 | 0 | 0 | 0 | 0 | 0 | 0 | 0 | 0 | 447 | 467 |  |  | PEAKS DB |
| K.IIIQGDPVAQIIEN.S | Y | 45.71 | 1521.8402 | 14 | 0.0 | 761.9274 | 2 | 85.63 | 6 | F6:59337 | NaSa01\_F14.raw |  |  |  |  |  | 1.4516E7 |  |  |  |  |  |  |  |  | 1 | 0 | 0 | 0 | 0 | 0 | 1 | 0 | 0 | 0 | 0 | 0 | 0 | 0 | 0 | 963 | 976 |  |  | PEAKS DB |
| K.TM(+15.99)SFYLR.D | Y | 45.45 | 932.4426 | 7 | 0.2 | 467.2286 | 2 | 32.98 | 6 | F6:14848 | NaSa01\_F14.raw |  |  |  |  |  | 1.994E6 |  |  |  |  |  |  |  |  | 2 | 0 | 0 | 0 | 0 | 0 | 2 | 0 | 0 | 0 | 0 | 0 | 0 | 0 | 0 | 775 | 781 | Oxidation (M) | M2:Oxidation (M):1000.00 | PEAKS DB |
| N.DIYVMDVLEVIK.Q | N | 45.17 | 1435.7632 | 12 | 0.7 | 718.8894 | 2 | 106.25 | 4 | F4:76997 | NaSa01\_F12.raw |  |  |  | 2.8255E5 |  |  |  |  |  |  |  |  |  |  | 1 | 0 | 0 | 0 | 1 | 0 | 0 | 0 | 0 | 0 | 0 | 0 | 0 | 0 | 0 | 1538 | 1549 |  |  | PEAKS DB |
| K.YFKPGMPYELTVY.V | N | 44.78 | 1606.7742 | 13 | 0.4 | 804.3947 | 2 | 71.51 | 6 | F6:47852 | NaSa01\_F14.raw |  |  |  |  |  | 1.8453E7 |  |  |  |  |  |  |  |  | 1 | 0 | 0 | 0 | 0 | 0 | 1 | 0 | 0 | 0 | 0 | 0 | 0 | 0 | 0 | 353 | 365 |  |  | PEAKS DB |
| R.SRFPNLNELVGH.T | N | 44.24 | 1381.7102 | 12 | 0.6 | 461.5776 | 3 | 50.79 | 6 | F6:30122 | NaSa01\_F14.raw |  |  |  |  |  | 1.9009E7 |  |  |  |  |  |  |  |  | 3 | 0 | 0 | 0 | 0 | 0 | 3 | 0 | 0 | 0 | 0 | 0 | 0 | 0 | 0 | 301 | 312 |  |  | PEAKS DB |
| K.TVIVEFQTPEG.I | N | 43.94 | 1218.6132 | 11 | 3.8 | 610.3144 | 2 | 63.73 | 7 | F7:45532 | NaSa01\_F2.raw |  |  |  |  |  |  | 6.7633E6 | 2.6291E6 |  |  |  |  |  |  | 2 | 0 | 0 | 0 | 0 | 0 | 0 | 1 | 1 | 0 | 0 | 0 | 0 | 0 | 0 | 160 | 170 |  |  | PEAKS DB |
| M.NLHITPDLIPSFR.F | N | 43.77 | 1521.8303 | 13 | 0.6 | 508.2843 | 3 | 65.19 | 6 | F6:42284 | NaSa01\_F14.raw |  |  |  |  |  | 1.1088E7 |  |  |  |  |  |  |  |  | 2 | 0 | 0 | 0 | 0 | 0 | 2 | 0 | 0 | 0 | 0 | 0 | 0 | 0 | 0 | 507 | 519 |  |  | PEAKS DB |
| K.AAFLEC(+57.02)C(+57.02)R.Y | N | 43.14 | 1025.4423 | 8 | 0.7 | 513.7288 | 2 | 11.72 | 9 | F9:2016 | NaSa01\_F4.raw |  |  |  |  |  |  |  |  | 5.2365E5 |  |  |  |  |  | 1 | 0 | 0 | 0 | 0 | 0 | 0 | 0 | 0 | 1 | 0 | 0 | 0 | 0 | 0 | 706 | 713 | Carbamidomethylation | C6:Carbamidomethylation:1000.00;C7:Carbamidomethylation:1000.00 | PEAKS DB |
| L.SYQSSFLFIQTDK.G | Y | 42.37 | 1562.7616 | 13 | 1.2 | 782.3890 | 2 | 64.49 | 6 | F6:41739 | NaSa01\_F14.raw |  |  |  |  |  | 3.4858E5 |  |  |  |  |  |  |  |  | 1 | 0 | 0 | 0 | 0 | 0 | 1 | 0 | 0 | 0 | 0 | 0 | 0 | 0 | 0 | 122 | 134 |  |  | PEAKS DB |
| R.KC(+57.02)C(+57.02)EDVMHENPMGYTC(+57.02)EK.R | Y | 42.21 | 2286.8835 | 18 | 1.4 | 763.3029 | 3 | 20.88 | 10 | F10:7153 | NaSa01\_F5.raw |  |  |  |  |  |  |  |  | 7.1178E5 | 3.5368E6 |  |  |  |  | 3 | 0 | 0 | 0 | 0 | 0 | 0 | 0 | 0 | 1 | 2 | 0 | 0 | 0 | 0 | 676 | 693 | Carbamidomethylation | C2:Carbamidomethylation:1000.00;C3:Carbamidomethylation:1000.00;C16:Carbamidomethylation:1000.00 | PEAKS DB |
| R.IPIIDGDGK.A | Y | 42.15 | 926.5073 | 9 | 0.6 | 464.2612 | 2 | 32.92 | 6 | F6:14878 | NaSa01\_F14.raw |  |  |  |  |  | 3.4884E5 |  |  |  |  |  |  |  |  | 1 | 0 | 0 | 0 | 0 | 0 | 1 | 0 | 0 | 0 | 0 | 0 | 0 | 0 | 0 | 283 | 291 |  |  | PEAKS DB |
| Y.VLPSFEVR.L | N | 41.89 | 945.5283 | 8 | -0.2 | 473.7714 | 2 | 40.35 | 6 | F6:20803 | NaSa01\_F14.raw |  |  |  |  |  | 5.7172E5 |  |  |  |  |  |  |  |  | 1 | 0 | 0 | 0 | 0 | 0 | 1 | 0 | 0 | 0 | 0 | 0 | 0 | 0 | 0 | 220 | 227 |  |  | PEAKS DB |
| K.IC(+57.02)IGNVC(+57.02)R.C | Y | 41.79 | 990.4739 | 8 | -1.3 | 496.2436 | 2 | 11.58 | 6 | F6:1976 | NaSa01\_F14.raw |  |  |  |  |  | 2.2107E5 |  |  |  |  |  |  |  |  | 1 | 0 | 0 | 0 | 0 | 0 | 1 | 0 | 0 | 0 | 0 | 0 | 0 | 0 | 0 | 1484 | 1491 | Carbamidomethylation | C2:Carbamidomethylation:1000.00;C7:Carbamidomethylation:1000.00 | PEAKS DB |
| R.SRFPNLNELVGHTLY.A | N | 40.90 | 1758.9053 | 15 | 0.7 | 587.3094 | 3 | 70.95 | 6 | F6:46924 | NaSa01\_F14.raw |  |  |  |  |  | 5.6962E6 |  |  |  |  |  |  |  |  | 1 | 0 | 0 | 0 | 0 | 0 | 1 | 0 | 0 | 0 | 0 | 0 | 0 | 0 | 0 | 301 | 315 |  |  | PEAKS DB |
| Y.TLITPAVLR.T | N | 40.29 | 982.6175 | 9 | 0.4 | 492.3162 | 2 | 45.78 | 6 | F6:25572 | NaSa01\_F14.raw |  |  |  |  |  | 5.8349E7 |  |  |  |  |  |  |  |  | 1 | 0 | 0 | 0 | 0 | 0 | 1 | 0 | 0 | 0 | 0 | 0 | 0 | 0 | 0 | 26 | 34 |  |  | PEAKS DB |
| K.LILNIPLNAQ.S | Y | 40.28 | 1107.6652 | 10 | -0.2 | 554.8397 | 2 | 84.63 | 6 | F6:58512 | NaSa01\_F14.raw |  |  |  |  |  | 3.2621E6 |  |  |  |  |  |  |  |  | 1 | 0 | 0 | 0 | 0 | 0 | 1 | 0 | 0 | 0 | 0 | 0 | 0 | 0 | 0 | 398 | 407 |  |  | PEAKS DB |
| K.IIIQGDPVAQI.I | Y | 39.99 | 1165.6707 | 11 | 0.7 | 583.8430 | 2 | 72.27 | 6 | F6:48414 | NaSa01\_F14.raw |  |  |  |  |  | 1.2067E6 |  |  |  |  |  |  |  |  | 1 | 0 | 0 | 0 | 0 | 0 | 1 | 0 | 0 | 0 | 0 | 0 | 0 | 0 | 0 | 963 | 973 |  |  | PEAKS DB |
| Y.QVGNNEIVADSVWVDVK.D | N | 39.98 | 1870.9425 | 17 | 0.5 | 936.4790 | 2 | 64.84 | 6 | F6:41917 | NaSa01\_F14.raw |  |  |  |  |  | 1.8564E6 |  |  |  |  |  |  |  |  | 1 | 0 | 0 | 0 | 0 | 0 | 1 | 0 | 0 | 0 | 0 | 0 | 0 | 0 | 0 | 525 | 541 |  |  | PEAKS DB |
| N.IPLNAQSLPITVR.T | Y | 39.95 | 1420.8402 | 13 | 0.7 | 711.4279 | 2 | 58.51 | 6 | F6:36546 | NaSa01\_F14.raw |  |  |  |  |  | 5.2985E5 |  |  |  |  |  |  |  |  | 1 | 0 | 0 | 0 | 0 | 0 | 1 | 0 | 0 | 0 | 0 | 0 | 0 | 0 | 0 | 402 | 414 |  |  | PEAKS DB |
| K.SWLWLTK.D | Y | 39.91 | 932.5120 | 7 | 0.3 | 467.2634 | 2 | 68.87 | 6 | F6:45829 | NaSa01\_F14.raw |  |  |  |  |  | 5.8771E7 |  |  |  |  |  |  |  |  | 1 | 0 | 0 | 0 | 0 | 0 | 1 | 0 | 0 | 0 | 0 | 0 | 0 | 0 | 0 | 754 | 760 |  |  | PEAKS DB |
| K.IIIQGDPVAQ.I | Y | 39.88 | 1052.5865 | 10 | 0.0 | 527.3005 | 2 | 48.47 | 6 | F6:28020 | NaSa01\_F14.raw |  |  |  |  |  | 3.3932E7 |  |  |  |  |  |  |  |  | 1 | 0 | 0 | 0 | 0 | 0 | 1 | 0 | 0 | 0 | 0 | 0 | 0 | 0 | 0 | 963 | 972 |  |  | PEAKS DB |
| R.IEEQDGNDIYVM(+15.99)DVLEVIK.Q | Y | 39.47 | 2237.0774 | 19 | 1.1 | 1119.5472 | 2 | 90.84 | 4 | F4:65053 | NaSa01\_F12.raw |  |  |  | 8.9297E5 |  |  |  |  |  |  |  |  |  |  | 1 | 0 | 0 | 0 | 1 | 0 | 0 | 0 | 0 | 0 | 0 | 0 | 0 | 0 | 0 | 1531 | 1549 | Oxidation (M) | M12:Oxidation (M):1000.00 | PEAKS DB |
| K.VVLLSYQSSF.L | Y | 38.34 | 1141.6019 | 10 | 0.4 | 571.8085 | 2 | 79.64 | 6 | F6:54198 | NaSa01\_F14.raw |  |  |  |  |  | 2.3493E6 |  |  |  |  |  |  |  |  | 1 | 0 | 0 | 0 | 0 | 0 | 1 | 0 | 0 | 0 | 0 | 0 | 0 | 0 | 0 | 118 | 127 |  |  | PEAKS DB |
| K.ISYIITK.N | N | 38.08 | 836.5007 | 7 | -0.7 | 419.2574 | 2 | 26.29 | 6 | F6:9476 | NaSa01\_F14.raw |  |  |  |  |  | 6.6012E5 |  |  |  |  |  |  |  |  | 1 | 0 | 0 | 0 | 0 | 0 | 1 | 0 | 0 | 0 | 0 | 0 | 0 | 0 | 0 | 1597 | 1603 |  |  | PEAKS DB |
| total 87 peptides |  |  |  |  |  |  |  |  |  |  |  |  |  |  |  |  |  |  |  |  |  |  |  |  |  |  |  |  |  |  |  |  |  |  |  |  |  |  |  |  |  |  |  |  |  |
| --- | --- | --- | --- | --- | --- | --- | --- | --- | --- | --- | --- | --- | --- | --- | --- | --- | --- | --- | --- | --- | --- | --- | --- | --- | --- | --- | --- | --- | --- | --- | --- | --- | --- | --- | --- | --- | --- | --- | --- | --- | --- | --- | --- | --- | --- |

CAI46845.1

back to list

  

| Protein Coverage
| Supporting Peptides
|

Protein Coverage:

Supporting Peptides:

| Peptide | Uniq | -10lgP | Mass | Length | ppm | m/z | z | RT | Fraction | Scan | Source File | Area F1 | Area F10 | Area F11 | Area F12 | Area F13 | Area F14 | Area F2 | Area F3 | Area F4 | Area F5 | Area F6 | Area F7 | Area F8 | Area F9 | #Feature | #Feature F1 | #Feature F10 | #Feature F11 | #Feature F12 | #Feature F13 | #Feature F14 | #Feature F2 | #Feature F3 | #Feature F4 | #Feature F5 | #Feature F6 | #Feature F7 | #Feature F8 | #Feature F9 | Start | End | PTM | AScore | Found By |
| --- | --- | --- | --- | --- | --- | --- | --- | --- | --- | --- | --- | --- | --- | --- | --- | --- | --- | --- | --- | --- | --- | --- | --- | --- | --- | --- | --- | --- | --- | --- | --- | --- | --- | --- | --- | --- | --- | --- | --- | --- | --- | --- | --- | --- | --- |
| R.AILHNYVNEDIYVR.V | Y | 89.83 | 1717.8788 | 14 | 0.3 | 859.9469 | 2 | 43.05 | 6 | F6:23378 | NaSa01\_F14.raw |  |  |  |  | 1.5001E7 | 1.9759E8 |  |  |  |  |  |  |  |  | 8 | 0 | 0 | 0 | 0 | 3 | 5 | 0 | 0 | 0 | 0 | 0 | 0 | 0 | 0 | 834 | 847 |  |  | PEAKS DB |
| R.AVPFVIVPLEQGLHDVEIK.A | Y | 87.23 | 2102.1775 | 19 | 0.5 | 701.7335 | 3 | 85.72 | 6 | F6:59617 | NaSa01\_F14.raw |  |  |  | 7.0958E5 | 1.6836E7 | 1.2181E8 |  |  |  |  |  |  |  |  | 7 | 0 | 0 | 0 | 1 | 2 | 4 | 0 | 0 | 0 | 0 | 0 | 0 | 0 | 0 | 879 | 897 |  |  | PEAKS DB |
| K.HFEVGFIQPGSVK.V | N | 85.09 | 1443.7510 | 13 | -0.2 | 722.8826 | 2 | 44.13 | 6 | F6:24159 | NaSa01\_F14.raw |  |  |  |  | 2.4365E7 | 2.5116E8 |  |  |  |  |  |  |  |  | 8 | 0 | 0 | 0 | 0 | 2 | 6 | 0 | 0 | 0 | 0 | 0 | 0 | 0 | 0 | 1445 | 1457 |  |  | PEAKS DB |
| R.RDGQNLVTM(+15.99)NLHITPDLIPSFR.F | N | 85.06 | 2552.3169 | 22 | 0.3 | 639.0867 | 4 | 69.77 | 6 | F6:46133 | NaSa01\_F14.raw |  |  |  |  |  | 2.5484E6 |  |  |  |  |  |  |  |  | 2 | 0 | 0 | 0 | 0 | 0 | 2 | 0 | 0 | 0 | 0 | 0 | 0 | 0 | 0 | 498 | 519 | Oxidation (M) | M9:Oxidation (M):1000.00 | PEAKS DB |
| R.QNQYVVVQVTGPQVR.L | N | 83.17 | 1713.9163 | 15 | 0.2 | 857.9656 | 2 | 43.83 | 6 | F6:24244 | NaSa01\_F14.raw |  |  |  |  | 1.4255E7 | 1.5135E8 |  |  |  |  |  |  |  |  | 5 | 0 | 0 | 0 | 0 | 2 | 3 | 0 | 0 | 0 | 0 | 0 | 0 | 0 | 0 | 100 | 114 |  |  | PEAKS DB |
| K.VFFIDLQMPYSVVK.N | Y | 82.52 | 1684.8898 | 14 | 0.3 | 843.4524 | 2 | 95.37 | 6 | F6:67466 | NaSa01\_F14.raw |  |  |  |  | 5.5288E5 | 4.4172E6 |  |  |  |  |  |  |  |  | 2 | 0 | 0 | 0 | 0 | 1 | 1 | 0 | 0 | 0 | 0 | 0 | 0 | 0 | 0 | 813 | 826 |  |  | PEAKS DB |
| K.VNDDYLIWGSR.S | Y | 81.98 | 1336.6411 | 11 | 1.0 | 669.3285 | 2 | 59.77 | 6 | F6:37640 | NaSa01\_F14.raw |  |  |  | 4.0113E7 | 1.558E7 | 1.2947E8 |  |  |  |  |  |  |  |  | 4 | 0 | 0 | 0 | 1 | 2 | 1 | 0 | 0 | 0 | 0 | 0 | 0 | 0 | 0 | 1577 | 1587 |  |  | PEAKS DB |
| R.IEEQDGNDIYVMDVLEVIK.Q | Y | 80.07 | 2221.0823 | 19 | 0.7 | 1111.5492 | 2 | 101.01 | 4 | F4:73173 | NaSa01\_F12.raw |  |  |  | 1.608E7 | 4.109E6 | 2.6808E7 |  |  |  |  |  |  |  |  | 11 | 0 | 0 | 0 | 4 | 3 | 4 | 0 | 0 | 0 | 0 | 0 | 0 | 0 | 0 | 1531 | 1549 |  |  | PEAKS DB |
| K.ATMTILTFYNAQLQEK.A | Y | 79.74 | 1870.9498 | 16 | -0.2 | 936.4820 | 2 | 77.02 | 6 | F6:52034 | NaSa01\_F14.raw |  |  | 5.3707E5 | 1.2422E6 | 3.5134E6 | 4.7877E7 |  |  |  |  |  |  |  |  | 6 | 0 | 0 | 1 | 1 | 1 | 3 | 0 | 0 | 0 | 0 | 0 | 0 | 0 | 0 | 1321 | 1336 |  |  | PEAKS DB |
| K.QLDIFVHDFPR.K | N | 79.15 | 1385.7091 | 11 | 0.0 | 693.8618 | 2 | 58.44 | 6 | F6:36683 | NaSa01\_F14.raw |  |  |  |  | 9.0136E6 | 8.647E7 |  |  |  |  |  |  |  |  | 4 | 0 | 0 | 0 | 0 | 2 | 2 | 0 | 0 | 0 | 0 | 0 | 0 | 0 | 0 | 53 | 63 |  |  | PEAKS DB |
| K.GDNLIQMPGAAMK.I | Y | 78.60 | 1344.6530 | 13 | 0.2 | 673.3339 | 2 | 49.26 | 6 | F6:28453 | NaSa01\_F14.raw |  |  |  |  |  | 1.0626E8 |  |  |  |  |  |  |  |  | 2 | 0 | 0 | 0 | 0 | 0 | 2 | 0 | 0 | 0 | 0 | 0 | 0 | 0 | 0 | 552 | 564 |  |  | PEAKS DB |
| F.VIVPLEQGLHDVEIK.A | Y | 76.27 | 1687.9508 | 15 | 0.0 | 844.9827 | 2 | 58.08 | 6 | F6:36376 | NaSa01\_F14.raw |  |  |  |  | 5.5586E6 | 9.3853E7 |  |  |  |  |  |  |  |  | 3 | 0 | 0 | 0 | 0 | 1 | 2 | 0 | 0 | 0 | 0 | 0 | 0 | 0 | 0 | 883 | 897 |  |  | PEAKS DB |
| R.DDNEDGFIADSDIISR.S | Y | 75.58 | 1780.7751 | 16 | 0.3 | 891.3951 | 2 | 68.40 | 6 | F6:44898 | NaSa01\_F14.raw |  |  |  |  | 9.0776E5 | 1.2244E7 |  |  |  |  |  |  |  |  | 4 | 0 | 0 | 0 | 0 | 1 | 3 | 0 | 0 | 0 | 0 | 0 | 0 | 0 | 0 | 733 | 748 |  |  | PEAKS DB |
| K.C(+57.02)C(+57.02)EDVMHENPMGYTC(+57.02)EK.R | Y | 74.09 | 2158.7886 | 17 | 7.4 | 720.6050 | 3 | 31.06 | 8 | F8:16109 | NaSa01\_F3.raw |  |  |  |  |  |  |  | 1.0485E7 | 5.6652E7 | 1.4617E7 |  |  |  |  | 6 | 0 | 0 | 0 | 0 | 0 | 0 | 0 | 1 | 3 | 2 | 0 | 0 | 0 | 0 | 677 | 693 | Carbamidomethylation | C1:Carbamidomethylation:1000.00;C2:Carbamidomethylation:1000.00;C15:Carbamidomethylation:1000.00 | PEAKS DB |
| R.INYENALLAR.T | Y | 74.07 | 1175.6299 | 10 | 0.1 | 588.8223 | 2 | 45.12 | 6 | F6:25480 | NaSa01\_F14.raw |  |  | 3.3741E5 | 7.6601E6 | 1.5972E7 | 1.5972E8 |  |  |  |  |  |  |  |  | 7 | 0 | 0 | 1 | 3 | 1 | 2 | 0 | 0 | 0 | 0 | 0 | 0 | 0 | 0 | 1292 | 1301 |  |  | PEAKS DB |
| Y.YQVGNNEIVADSVWVDVK.D | N | 73.99 | 2034.0057 | 18 | 0.2 | 1018.0104 | 2 | 70.65 | 6 | F6:46860 | NaSa01\_F14.raw |  |  |  |  |  | 6.5239E6 |  |  |  |  |  |  |  |  | 1 | 0 | 0 | 0 | 0 | 0 | 1 | 0 | 0 | 0 | 0 | 0 | 0 | 0 | 0 | 524 | 541 |  |  | PEAKS DB |
| K.ASVQEALWSDGVR.K | Y | 72.98 | 1416.6997 | 13 | 0.1 | 709.3572 | 2 | 49.71 | 6 | F6:29208 | NaSa01\_F14.raw |  |  |  |  | 1.3438E7 | 1.2825E8 |  |  |  |  |  |  |  |  | 7 | 0 | 0 | 0 | 0 | 1 | 6 | 0 | 0 | 0 | 0 | 0 | 0 | 0 | 0 | 898 | 910 |  |  | PEAKS DB |
| R.VELLYNPAFC(+57.02)SASTK.G | Y | 72.57 | 1698.8286 | 15 | -0.7 | 850.4210 | 2 | 59.22 | 6 | F6:37110 | NaSa01\_F14.raw |  |  |  |  | 1.6821E7 | 2.15E8 |  |  |  |  |  |  |  |  | 2 | 0 | 0 | 0 | 0 | 1 | 1 | 0 | 0 | 0 | 0 | 0 | 0 | 0 | 0 | 848 | 862 | Carbamidomethylation | C10:Carbamidomethylation:1000.00 | PEAKS DB |
| K.LNQDITVTASGDGK.A | Y | 72.09 | 1417.7048 | 14 | 0.8 | 709.8602 | 2 | 20.83 | 6 | F6:5302 | NaSa01\_F14.raw |  |  |  |  | 4.3744E5 | 4.5631E6 |  |  |  |  |  |  |  |  | 3 | 0 | 0 | 0 | 0 | 1 | 2 | 0 | 0 | 0 | 0 | 0 | 0 | 0 | 0 | 1307 | 1320 |  |  | PEAKS DB |
| K.LILNIPLNAQSLPITVR.T | Y | 71.60 | 1874.1353 | 17 | 0.5 | 625.7194 | 3 | 90.36 | 6 | F6:63322 | NaSa01\_F14.raw |  |  |  |  | 1.4111E7 | 4.901E7 |  |  |  |  |  |  |  |  | 4 | 0 | 0 | 0 | 0 | 2 | 2 | 0 | 0 | 0 | 0 | 0 | 0 | 0 | 0 | 398 | 414 |  |  | PEAKS DB |
| K.YFTYLILNK.G | N | 70.49 | 1173.6433 | 9 | 0.1 | 587.8290 | 2 | 64.67 | 6 | F6:41809 | NaSa01\_F14.raw |  |  |  |  | 1.0728E7 | 7.7707E7 |  |  |  |  |  |  |  |  | 2 | 0 | 0 | 0 | 0 | 1 | 1 | 0 | 0 | 0 | 0 | 0 | 0 | 0 | 0 | 478 | 486 |  |  | PEAKS DB |
| R.WPHEDEC(+57.02)QEEEFQK.L | Y | 69.62 | 1889.7526 | 14 | 0.9 | 945.8844 | 2 | 23.39 | 4 | F4:9200 | NaSa01\_F12.raw |  |  |  | 2.8919E7 |  | 3.166E6 |  |  |  |  |  |  |  |  | 3 | 0 | 0 | 0 | 2 | 0 | 1 | 0 | 0 | 0 | 0 | 0 | 0 | 0 | 0 | 1610 | 1623 | Carbamidomethylation | C7:Carbamidomethylation:1000.00 | PEAKS DB |
| K.SDFGC(+57.02)TAGSGQNNLGVFEDAGLALTTSTNLNTK.Q | N | 66.95 | 3359.5576 | 33 | 0.8 | 1120.8607 | 3 | 89.37 | 6 | F6:62425 | NaSa01\_F14.raw |  |  |  |  |  | 3.8512E7 |  |  |  |  |  |  |  |  | 1 | 0 | 0 | 0 | 0 | 0 | 1 | 0 | 0 | 0 | 0 | 0 | 0 | 0 | 0 | 605 | 637 | Carbamidomethylation | C5:Carbamidomethylation:1000.00 | PEAKS DB |
| R.DGQNLVTM(+15.99)NLHITPDLIPSFR.F | N | 66.34 | 2396.2158 | 21 | 0.4 | 799.7462 | 3 | 83.99 | 6 | F6:57985 | NaSa01\_F14.raw |  |  |  |  |  | 5.9574E6 |  |  |  |  |  |  |  |  | 1 | 0 | 0 | 0 | 0 | 0 | 1 | 0 | 0 | 0 | 0 | 0 | 0 | 0 | 0 | 499 | 519 | Oxidation (M) | M8:Oxidation (M):1000.00 | PEAKS DB |
| L.ARDDNEDGFIADSDIISR.S | Y | 66.31 | 2007.9133 | 18 | 1.0 | 670.3124 | 3 | 48.95 | 6 | F6:28381 | NaSa01\_F14.raw |  |  |  |  |  | 6.6726E6 |  |  |  |  |  |  |  |  | 2 | 0 | 0 | 0 | 0 | 0 | 2 | 0 | 0 | 0 | 0 | 0 | 0 | 0 | 0 | 731 | 748 |  |  | PEAKS DB |
| Y.VTNPDGSPAAHVPVVSEAFHSMGTTLSDGTAK.L | Y | 66.31 | 3179.5193 | 32 | 0.8 | 795.8877 | 4 | 59.12 | 6 | F6:37224 | NaSa01\_F14.raw |  |  |  |  |  | 2.3066E7 |  |  |  |  |  |  |  |  | 1 | 0 | 0 | 0 | 0 | 0 | 1 | 0 | 0 | 0 | 0 | 0 | 0 | 0 | 0 | 366 | 397 |  |  | PEAKS DB |
| K.C(+57.02)C(+57.02)EDVMHENPM(+15.99)GYTC(+57.02)EK.R | Y | 65.55 | 2174.7837 | 17 | 1.4 | 725.9362 | 3 | 11.70 | 9 | F9:2004 | NaSa01\_F4.raw |  |  |  |  |  |  |  |  | 1.0934E7 |  |  |  |  |  | 2 | 0 | 0 | 0 | 0 | 0 | 0 | 0 | 0 | 2 | 0 | 0 | 0 | 0 | 0 | 677 | 693 | Carbamidomethylation; Oxidation (M) | C1:Carbamidomethylation:1000.00;C2:Carbamidomethylation:1000.00;M11:Oxidation (M):43.99;C15:Carbamidomethylation:1000.00 | PEAKS DB |
| Y.NLPDLVSLGTWR.I | Y | 65.12 | 1369.7354 | 12 | 0.1 | 685.8750 | 2 | 81.18 | 6 | F6:55452 | NaSa01\_F14.raw |  |  |  |  | 6.3971E6 | 8.6753E7 |  |  |  |  |  |  |  |  | 2 | 0 | 0 | 0 | 0 | 1 | 1 | 0 | 0 | 0 | 0 | 0 | 0 | 0 | 0 | 187 | 198 |  |  | PEAKS DB |
| K.GDNLIQMPGAAM(+15.99)K.I | Y | 64.58 | 1360.6479 | 13 | 0.8 | 681.3318 | 2 | 38.53 | 6 | F6:19473 | NaSa01\_F14.raw |  |  |  |  | 9.6097E5 | 9.5549E6 |  |  |  |  |  |  |  |  | 3 | 0 | 0 | 0 | 0 | 1 | 2 | 0 | 0 | 0 | 0 | 0 | 0 | 0 | 0 | 552 | 564 | Oxidation (M) | M12:Oxidation (M):38.33 | PEAKS DB |
| E.VEGVAFVLFGVK.I | N | 62.96 | 1263.7227 | 12 | 0.2 | 632.8687 | 2 | 85.00 | 6 | F6:58754 | NaSa01\_F14.raw |  |  |  |  |  | 2.0835E6 |  |  |  |  |  |  |  |  | 1 | 0 | 0 | 0 | 0 | 0 | 1 | 0 | 0 | 0 | 0 | 0 | 0 | 0 | 0 | 257 | 268 |  |  | PEAKS DB |
| Y.QSSFLFIQTDK.G | Y | 62.43 | 1312.6663 | 11 | -0.4 | 657.3401 | 2 | 57.57 | 6 | F6:35846 | NaSa01\_F14.raw |  |  |  |  |  | 1.1659E7 |  |  |  |  |  |  |  |  | 1 | 0 | 0 | 0 | 0 | 0 | 1 | 0 | 0 | 0 | 0 | 0 | 0 | 0 | 0 | 124 | 134 |  |  | PEAKS DB |
| K.GVGGTQLEVIK.A | Y | 62.08 | 1099.6237 | 11 | 0.2 | 550.8192 | 2 | 30.32 | 12 | F12:15497 | NaSa01\_F7.raw |  |  |  |  | 1.4118E7 | 1.0253E6 |  |  |  |  |  | 1.7364E6 |  |  | 3 | 0 | 0 | 0 | 0 | 1 | 1 | 0 | 0 | 0 | 0 | 0 | 1 | 0 | 0 | 936 | 946 |  |  | PEAKS DB |
| K.ATM(+15.99)TILTFYNAQLQEK.A | Y | 62.07 | 1886.9448 | 16 | 0.4 | 944.4801 | 2 | 64.84 | 6 | F6:42088 | NaSa01\_F14.raw |  |  |  |  |  | 1.0319E7 |  |  |  |  |  |  |  |  | 2 | 0 | 0 | 0 | 0 | 0 | 2 | 0 | 0 | 0 | 0 | 0 | 0 | 0 | 0 | 1321 | 1336 | Oxidation (M) | M3:Oxidation (M):1000.00 | PEAKS DB |
| R.FPNLNELVGH.T | N | 62.05 | 1138.5770 | 10 | 0.8 | 570.2963 | 2 | 67.53 | 6 | F6:44045 | NaSa01\_F14.raw |  |  |  |  | 1.7762E6 | 2.8185E7 |  |  |  |  |  |  |  |  | 2 | 0 | 0 | 0 | 0 | 1 | 1 | 0 | 0 | 0 | 0 | 0 | 0 | 0 | 0 | 303 | 312 |  |  | PEAKS DB |
| K.VYSYYNLDEK.C | N | 61.93 | 1292.5924 | 10 | 1.2 | 647.3043 | 2 | 33.77 | 6 | F6:15693 | NaSa01\_F14.raw |  |  |  |  | 1.5304E7 | 1.0252E8 |  |  |  |  |  |  |  |  | 3 | 0 | 0 | 0 | 0 | 1 | 2 | 0 | 0 | 0 | 0 | 0 | 0 | 0 | 0 | 1458 | 1467 |  |  | PEAKS DB |
| M.TILTFYNAQLQEK.A | N | 61.57 | 1567.8246 | 13 | 0.3 | 784.9198 | 2 | 60.05 | 6 | F6:38047 | NaSa01\_F14.raw |  |  |  |  |  | 5.1346E6 |  |  |  |  |  |  |  |  | 1 | 0 | 0 | 0 | 0 | 0 | 1 | 0 | 0 | 0 | 0 | 0 | 0 | 0 | 0 | 1324 | 1336 |  |  | PEAKS DB |
| H.ITPDLIPSFR.F | N | 61.36 | 1157.6444 | 10 | 0.0 | 579.8295 | 2 | 65.01 | 6 | F6:42335 | NaSa01\_F14.raw |  |  |  |  | 3.0601E6 | 3.8923E7 |  |  |  |  |  |  |  |  | 3 | 0 | 0 | 0 | 0 | 1 | 2 | 0 | 0 | 0 | 0 | 0 | 0 | 0 | 0 | 510 | 519 |  |  | PEAKS DB |
| K.PGDNLPVNFNVK.G | Y | 60.28 | 1312.6775 | 12 | 0.4 | 657.3463 | 2 | 47.72 | 6 | F6:27452 | NaSa01\_F14.raw |  |  |  |  |  | 1.0823E6 |  |  |  |  |  |  |  |  | 1 | 0 | 0 | 0 | 0 | 0 | 1 | 0 | 0 | 0 | 0 | 0 | 0 | 0 | 0 | 456 | 467 |  |  | PEAKS DB |
| W.VVLAVSFTPTK.G | Y | 59.98 | 1160.6804 | 11 | 0.4 | 581.3477 | 2 | 51.95 | 6 | F6:30874 | NaSa01\_F14.raw |  |  |  |  |  | 3.7062E6 |  |  |  |  |  |  |  |  | 1 | 0 | 0 | 0 | 0 | 0 | 1 | 0 | 0 | 0 | 0 | 0 | 0 | 0 | 0 | 788 | 798 |  |  | PEAKS DB |
| K.YEHSPENYTAYFDVR.K | N | 59.87 | 1889.8220 | 15 | -0.9 | 630.9474 | 3 | 47.19 | 6 | F6:27480 | NaSa01\_F14.raw |  |  |  |  |  | 2.5484E6 |  |  |  |  |  |  |  |  | 2 | 0 | 0 | 0 | 0 | 0 | 2 | 0 | 0 | 0 | 0 | 0 | 0 | 0 | 0 | 203 | 217 |  |  | PEAKS DB |
| R.IDVPLQIEK.A | Y | 59.34 | 1053.6069 | 9 | 0.1 | 527.8108 | 2 | 47.86 | 6 | F6:27228 | NaSa01\_F14.raw |  |  |  | 3.0425E7 | 1.9664E7 | 1.6873E8 |  |  |  |  |  |  |  |  | 4 | 0 | 0 | 0 | 1 | 1 | 2 | 0 | 0 | 0 | 0 | 0 | 0 | 0 | 0 | 1506 | 1514 |  |  | PEAKS DB |
| K.IIIQGDPVAQIIENSIDGSK.L | Y | 59.31 | 2109.1316 | 20 | 0.6 | 1055.5737 | 2 | 96.62 | 6 | F6:68548 | NaSa01\_F14.raw |  |  | 1.4465E5 |  | 1.6001E5 | 2.5079E6 |  |  |  |  |  |  |  |  | 3 | 0 | 0 | 1 | 0 | 1 | 1 | 0 | 0 | 0 | 0 | 0 | 0 | 0 | 0 | 963 | 982 |  |  | PEAKS DB |
| K.GIC(+57.02)VAEPYEIR.V | Y | 59.06 | 1305.6387 | 11 | 0.4 | 653.8268 | 2 | 44.31 | 6 | F6:24643 | NaSa01\_F14.raw |  |  |  |  | 1.0302E7 | 1.0808E8 |  |  |  |  |  |  |  |  | 3 | 0 | 0 | 0 | 0 | 1 | 2 | 0 | 0 | 0 | 0 | 0 | 0 | 0 | 0 | 799 | 809 | Carbamidomethylation | C3:Carbamidomethylation:1000.00 | PEAKS DB |
| E.DGFIADSDIISR.S | Y | 58.62 | 1307.6357 | 12 | -0.1 | 654.8251 | 2 | 60.77 | 6 | F6:38594 | NaSa01\_F14.raw |  |  |  |  |  | 4.7593E6 |  |  |  |  |  |  |  |  | 1 | 0 | 0 | 0 | 0 | 0 | 1 | 0 | 0 | 0 | 0 | 0 | 0 | 0 | 0 | 737 | 748 |  |  | PEAKS DB |
| R.FPNLNELVGHTLY.A | N | 58.47 | 1515.7721 | 13 | 0.3 | 758.8936 | 2 | 85.62 | 6 | F6:59205 | NaSa01\_F14.raw |  |  |  |  |  | 1.9527E7 |  |  |  |  |  |  |  |  | 1 | 0 | 0 | 0 | 0 | 0 | 1 | 0 | 0 | 0 | 0 | 0 | 0 | 0 | 0 | 303 | 315 |  |  | PEAKS DB |
| S.MLTGFLPDAEDLTR.L | N | 58.03 | 1577.7759 | 14 | 0.5 | 789.8956 | 2 | 78.56 | 6 | F6:53520 | NaSa01\_F14.raw |  |  |  |  |  | 2.8623E6 |  |  |  |  |  |  |  |  | 1 | 0 | 0 | 0 | 0 | 0 | 1 | 0 | 0 | 0 | 0 | 0 | 0 | 0 | 0 | 1386 | 1399 |  |  | PEAKS DB |
| R.KYVLPSFEVR.L | Y | 56.74 | 1236.6866 | 10 | 0.3 | 413.2363 | 3 | 42.11 | 6 | F6:22829 | NaSa01\_F14.raw |  |  |  |  | 5.509E6 | 4.9733E7 |  |  |  |  |  |  |  |  | 6 | 0 | 0 | 0 | 0 | 2 | 4 | 0 | 0 | 0 | 0 | 0 | 0 | 0 | 0 | 218 | 227 |  |  | PEAKS DB |
| R.DGQNLVTMNLHITPDLIPSFR.F | N | 56.46 | 2380.2209 | 21 | 0.5 | 794.4147 | 3 | 91.58 | 6 | F6:64345 | NaSa01\_F14.raw |  |  |  |  |  | 2.1315E6 |  |  |  |  |  |  |  |  | 1 | 0 | 0 | 0 | 0 | 0 | 1 | 0 | 0 | 0 | 0 | 0 | 0 | 0 | 0 | 499 | 519 |  |  | PEAKS DB |
| K.VAVIIYLNK.V | Y | 56.21 | 1031.6379 | 9 | 0.8 | 516.8267 | 2 | 50.20 | 6 | F6:30133 | NaSa01\_F14.raw |  |  |  |  |  | 1.67E7 |  |  |  |  |  |  |  |  | 2 | 0 | 0 | 0 | 0 | 0 | 2 | 0 | 0 | 0 | 0 | 0 | 0 | 0 | 0 | 1421 | 1429 |  |  | PEAKS DB |
| G.ALYTLITPAVLR.T | N | 55.82 | 1329.8020 | 12 | 0.4 | 665.9086 | 2 | 75.95 | 6 | F6:51300 | NaSa01\_F14.raw |  |  |  | 1.7564E6 | 1.5853E7 | 2.019E8 |  |  |  |  |  |  |  |  | 6 | 0 | 0 | 0 | 1 | 1 | 4 | 0 | 0 | 0 | 0 | 0 | 0 | 0 | 0 | 23 | 34 |  |  | PEAKS DB |
| N.LHITPDLIPSFR.F | N | 52.36 | 1407.7874 | 12 | 0.4 | 470.2699 | 3 | 64.03 | 6 | F6:41508 | NaSa01\_F14.raw |  |  |  |  |  | 2.1751E7 |  |  |  |  |  |  |  |  | 2 | 0 | 0 | 0 | 0 | 0 | 2 | 0 | 0 | 0 | 0 | 0 | 0 | 0 | 0 | 508 | 519 |  |  | PEAKS DB |
| K.YVLPSFEVR.L | N | 51.77 | 1108.5917 | 9 | 0.6 | 555.3035 | 2 | 58.44 | 6 | F6:36495 | NaSa01\_F14.raw |  |  |  | 4.1589E5 | 1.253E7 | 1.3997E8 |  |  |  |  |  |  |  |  | 4 | 0 | 0 | 0 | 1 | 1 | 2 | 0 | 0 | 0 | 0 | 0 | 0 | 0 | 0 | 219 | 227 |  |  | PEAKS DB |
| K.TMSFYLR.D | Y | 51.69 | 916.4476 | 7 | 0.3 | 459.2312 | 2 | 41.67 | 6 | F6:22840 | NaSa01\_F14.raw |  |  |  |  |  | 5.6816E7 |  |  |  |  |  |  |  |  | 1 | 0 | 0 | 0 | 0 | 0 | 1 | 0 | 0 | 0 | 0 | 0 | 0 | 0 | 0 | 775 | 781 |  |  | PEAKS DB |
| R.DGQNLVTMNLH.I | N | 51.62 | 1240.5870 | 11 | 0.0 | 621.3008 | 2 | 49.19 | 6 | F6:28557 | NaSa01\_F14.raw |  |  |  |  |  | 4.925E6 |  |  |  |  |  |  |  |  | 1 | 0 | 0 | 0 | 0 | 0 | 1 | 0 | 0 | 0 | 0 | 0 | 0 | 0 | 0 | 499 | 509 |  |  | PEAKS DB |
| L.NIPLNAQSLPITVR.T | Y | 50.97 | 1534.8831 | 14 | -1.4 | 768.4477 | 2 | 61.34 | 6 | F6:39039 | NaSa01\_F14.raw |  |  |  |  |  | 1.6927E5 |  |  |  |  |  |  |  |  | 1 | 0 | 0 | 0 | 0 | 0 | 1 | 0 | 0 | 0 | 0 | 0 | 0 | 0 | 0 | 401 | 414 |  |  | PEAKS DB |
| K.C(+57.02)C(+57.02)EDVM(+15.99)HENPM(+15.99)GYTC(+57.02)EK.R | Y | 50.02 | 2190.7786 | 17 | 0.7 | 731.2673 | 3 | 11.49 | 9 | F9:1837 | NaSa01\_F4.raw |  |  |  |  |  |  |  |  | 4.1662E5 |  |  |  |  |  | 1 | 0 | 0 | 0 | 0 | 0 | 0 | 0 | 0 | 1 | 0 | 0 | 0 | 0 | 0 | 677 | 693 | Carbamidomethylation; Oxidation (M) | C1:Carbamidomethylation:1000.00;C2:Carbamidomethylation:1000.00;M6:Oxidation (M):1000.00;M11:Oxidation (M):1000.00;C15:Carbamidomethylation:1000.00 | PEAKS DB |
| K.GIYTPGSPVLYR.V | N | 49.98 | 1321.7030 | 12 | -8.2 | 661.8533 | 2 | 47.56 | 6 | F6:27271 | NaSa01\_F14.raw |  |  |  |  |  | 4.6433E6 |  |  |  |  |  |  |  |  | 1 | 0 | 0 | 0 | 0 | 0 | 1 | 0 | 0 | 0 | 0 | 0 | 0 | 0 | 0 | 135 | 146 |  |  | PEAKS DB |
| K.AC(+57.02)ETNVDYVYK.T | Y | 49.20 | 1360.5969 | 11 | 0.3 | 681.3060 | 2 | 22.05 | 6 | F6:6230 | NaSa01\_F14.raw |  |  |  | 2.4601E5 |  | 5.6943E5 |  |  |  |  |  |  |  |  | 2 | 0 | 0 | 0 | 1 | 0 | 1 | 0 | 0 | 0 | 0 | 0 | 0 | 0 | 0 | 1515 | 1525 | Carbamidomethylation | C2:Carbamidomethylation:1000.00 | PEAKS DB |
| M.LTGFLPDAEDLTR.L | N | 47.16 | 1446.7354 | 13 | 1.2 | 724.3758 | 2 | 69.30 | 6 | F6:45774 | NaSa01\_F14.raw |  |  |  |  |  | 7.2495E5 |  |  |  |  |  |  |  |  | 1 | 0 | 0 | 0 | 0 | 0 | 1 | 0 | 0 | 0 | 0 | 0 | 0 | 0 | 0 | 1387 | 1399 |  |  | PEAKS DB |
| R.VGLVAVDKAVYVLNDK.Y | N | 46.90 | 1701.9664 | 16 | 0.8 | 568.3299 | 3 | 57.39 | 6 | F6:35573 | NaSa01\_F14.raw |  |  |  |  |  | 1.8722E5 |  |  |  |  |  |  |  |  | 1 | 0 | 0 | 0 | 0 | 0 | 1 | 0 | 0 | 0 | 0 | 0 | 0 | 0 | 0 | 575 | 590 |  |  | PEAKS DB |
| K.THQYISQR.K | Y | 46.29 | 1031.5149 | 8 | 0.4 | 516.7650 | 2 | 11.44 | 6 | F6:1825 | NaSa01\_F14.raw |  |  |  |  |  | 1.3503E5 |  |  |  |  |  |  |  |  | 1 | 0 | 0 | 0 | 0 | 0 | 1 | 0 | 0 | 0 | 0 | 0 | 0 | 0 | 0 | 1560 | 1567 |  |  | PEAKS DB |
| D.DNEDGFIADSDIISR.S | Y | 46.28 | 1665.7482 | 15 | 0.7 | 833.8820 | 2 | 65.65 | 6 | F6:42702 | NaSa01\_F14.raw |  |  |  |  |  | 2.6489E5 |  |  |  |  |  |  |  |  | 1 | 0 | 0 | 0 | 0 | 0 | 1 | 0 | 0 | 0 | 0 | 0 | 0 | 0 | 0 | 734 | 748 |  |  | PEAKS DB |
| H.VAITSTEIKPGDNLPVNFNVK.G | Y | 46.15 | 2255.2161 | 21 | 0.2 | 752.7461 | 3 | 57.73 | 6 | F6:36049 | NaSa01\_F14.raw |  |  |  |  |  | 3.3349E6 |  |  |  |  |  |  |  |  | 1 | 0 | 0 | 0 | 0 | 0 | 1 | 0 | 0 | 0 | 0 | 0 | 0 | 0 | 0 | 447 | 467 |  |  | PEAKS DB |
| K.IIIQGDPVAQIIEN.S | Y | 45.71 | 1521.8402 | 14 | 0.0 | 761.9274 | 2 | 85.63 | 6 | F6:59337 | NaSa01\_F14.raw |  |  |  |  |  | 1.4516E7 |  |  |  |  |  |  |  |  | 1 | 0 | 0 | 0 | 0 | 0 | 1 | 0 | 0 | 0 | 0 | 0 | 0 | 0 | 0 | 963 | 976 |  |  | PEAKS DB |
| K.TM(+15.99)SFYLR.D | Y | 45.45 | 932.4426 | 7 | 0.2 | 467.2286 | 2 | 32.98 | 6 | F6:14848 | NaSa01\_F14.raw |  |  |  |  |  | 1.994E6 |  |  |  |  |  |  |  |  | 2 | 0 | 0 | 0 | 0 | 0 | 2 | 0 | 0 | 0 | 0 | 0 | 0 | 0 | 0 | 775 | 781 | Oxidation (M) | M2:Oxidation (M):1000.00 | PEAKS DB |
| N.DIYVMDVLEVIK.Q | N | 45.17 | 1435.7632 | 12 | 0.7 | 718.8894 | 2 | 106.25 | 4 | F4:76997 | NaSa01\_F12.raw |  |  |  | 2.8255E5 |  |  |  |  |  |  |  |  |  |  | 1 | 0 | 0 | 0 | 1 | 0 | 0 | 0 | 0 | 0 | 0 | 0 | 0 | 0 | 0 | 1538 | 1549 |  |  | PEAKS DB |
| K.YFKPGMPYELTVY.V | N | 44.78 | 1606.7742 | 13 | 0.4 | 804.3947 | 2 | 71.51 | 6 | F6:47852 | NaSa01\_F14.raw |  |  |  |  |  | 1.8453E7 |  |  |  |  |  |  |  |  | 1 | 0 | 0 | 0 | 0 | 0 | 1 | 0 | 0 | 0 | 0 | 0 | 0 | 0 | 0 | 353 | 365 |  |  | PEAKS DB |
| R.SRFPNLNELVGH.T | N | 44.24 | 1381.7102 | 12 | 0.6 | 461.5776 | 3 | 50.79 | 6 | F6:30122 | NaSa01\_F14.raw |  |  |  |  |  | 1.9009E7 |  |  |  |  |  |  |  |  | 3 | 0 | 0 | 0 | 0 | 0 | 3 | 0 | 0 | 0 | 0 | 0 | 0 | 0 | 0 | 301 | 312 |  |  | PEAKS DB |
| K.TVIVEFQTPEG.I | N | 43.94 | 1218.6132 | 11 | 3.8 | 610.3144 | 2 | 63.73 | 7 | F7:45532 | NaSa01\_F2.raw |  |  |  |  |  |  | 6.7633E6 | 2.6291E6 |  |  |  |  |  |  | 2 | 0 | 0 | 0 | 0 | 0 | 0 | 1 | 1 | 0 | 0 | 0 | 0 | 0 | 0 | 160 | 170 |  |  | PEAKS DB |
| M.NLHITPDLIPSFR.F | N | 43.77 | 1521.8303 | 13 | 0.6 | 508.2843 | 3 | 65.19 | 6 | F6:42284 | NaSa01\_F14.raw |  |  |  |  |  | 1.1088E7 |  |  |  |  |  |  |  |  | 2 | 0 | 0 | 0 | 0 | 0 | 2 | 0 | 0 | 0 | 0 | 0 | 0 | 0 | 0 | 507 | 519 |  |  | PEAKS DB |
| K.AAFLEC(+57.02)C(+57.02)R.Y | N | 43.14 | 1025.4423 | 8 | 0.7 | 513.7288 | 2 | 11.72 | 9 | F9:2016 | NaSa01\_F4.raw |  |  |  |  |  |  |  |  | 5.2365E5 |  |  |  |  |  | 1 | 0 | 0 | 0 | 0 | 0 | 0 | 0 | 0 | 1 | 0 | 0 | 0 | 0 | 0 | 706 | 713 | Carbamidomethylation | C6:Carbamidomethylation:1000.00;C7:Carbamidomethylation:1000.00 | PEAKS DB |
| L.SYQSSFLFIQTDK.G | Y | 42.37 | 1562.7616 | 13 | 1.2 | 782.3890 | 2 | 64.49 | 6 | F6:41739 | NaSa01\_F14.raw |  |  |  |  |  | 3.4858E5 |  |  |  |  |  |  |  |  | 1 | 0 | 0 | 0 | 0 | 0 | 1 | 0 | 0 | 0 | 0 | 0 | 0 | 0 | 0 | 122 | 134 |  |  | PEAKS DB |
| R.KC(+57.02)C(+57.02)EDVMHENPMGYTC(+57.02)EK.R | Y | 42.21 | 2286.8835 | 18 | 1.4 | 763.3029 | 3 | 20.88 | 10 | F10:7153 | NaSa01\_F5.raw |  |  |  |  |  |  |  |  | 7.1178E5 | 3.5368E6 |  |  |  |  | 3 | 0 | 0 | 0 | 0 | 0 | 0 | 0 | 0 | 1 | 2 | 0 | 0 | 0 | 0 | 676 | 693 | Carbamidomethylation | C2:Carbamidomethylation:1000.00;C3:Carbamidomethylation:1000.00;C16:Carbamidomethylation:1000.00 | PEAKS DB |
| R.IPIIDGDGK.A | Y | 42.15 | 926.5073 | 9 | 0.6 | 464.2612 | 2 | 32.92 | 6 | F6:14878 | NaSa01\_F14.raw |  |  |  |  |  | 3.4884E5 |  |  |  |  |  |  |  |  | 1 | 0 | 0 | 0 | 0 | 0 | 1 | 0 | 0 | 0 | 0 | 0 | 0 | 0 | 0 | 283 | 291 |  |  | PEAKS DB |
| Y.VLPSFEVR.L | N | 41.89 | 945.5283 | 8 | -0.2 | 473.7714 | 2 | 40.35 | 6 | F6:20803 | NaSa01\_F14.raw |  |  |  |  |  | 5.7172E5 |  |  |  |  |  |  |  |  | 1 | 0 | 0 | 0 | 0 | 0 | 1 | 0 | 0 | 0 | 0 | 0 | 0 | 0 | 0 | 220 | 227 |  |  | PEAKS DB |
| K.IC(+57.02)IGNVC(+57.02)R.C | Y | 41.79 | 990.4739 | 8 | -1.3 | 496.2436 | 2 | 11.58 | 6 | F6:1976 | NaSa01\_F14.raw |  |  |  |  |  | 2.2107E5 |  |  |  |  |  |  |  |  | 1 | 0 | 0 | 0 | 0 | 0 | 1 | 0 | 0 | 0 | 0 | 0 | 0 | 0 | 0 | 1484 | 1491 | Carbamidomethylation | C2:Carbamidomethylation:1000.00;C7:Carbamidomethylation:1000.00 | PEAKS DB |
| R.SRFPNLNELVGHTLY.A | N | 40.90 | 1758.9053 | 15 | 0.7 | 587.3094 | 3 | 70.95 | 6 | F6:46924 | NaSa01\_F14.raw |  |  |  |  |  | 5.6962E6 |  |  |  |  |  |  |  |  | 1 | 0 | 0 | 0 | 0 | 0 | 1 | 0 | 0 | 0 | 0 | 0 | 0 | 0 | 0 | 301 | 315 |  |  | PEAKS DB |
| Y.TLITPAVLR.T | N | 40.29 | 982.6175 | 9 | 0.4 | 492.3162 | 2 | 45.78 | 6 | F6:25572 | NaSa01\_F14.raw |  |  |  |  |  | 5.8349E7 |  |  |  |  |  |  |  |  | 1 | 0 | 0 | 0 | 0 | 0 | 1 | 0 | 0 | 0 | 0 | 0 | 0 | 0 | 0 | 26 | 34 |  |  | PEAKS DB |
| K.LILNIPLNAQ.S | Y | 40.28 | 1107.6652 | 10 | -0.2 | 554.8397 | 2 | 84.63 | 6 | F6:58512 | NaSa01\_F14.raw |  |  |  |  |  | 3.2621E6 |  |  |  |  |  |  |  |  | 1 | 0 | 0 | 0 | 0 | 0 | 1 | 0 | 0 | 0 | 0 | 0 | 0 | 0 | 0 | 398 | 407 |  |  | PEAKS DB |
| K.IIIQGDPVAQI.I | Y | 39.99 | 1165.6707 | 11 | 0.7 | 583.8430 | 2 | 72.27 | 6 | F6:48414 | NaSa01\_F14.raw |  |  |  |  |  | 1.2067E6 |  |  |  |  |  |  |  |  | 1 | 0 | 0 | 0 | 0 | 0 | 1 | 0 | 0 | 0 | 0 | 0 | 0 | 0 | 0 | 963 | 973 |  |  | PEAKS DB |
| Y.QVGNNEIVADSVWVDVK.D | N | 39.98 | 1870.9425 | 17 | 0.5 | 936.4790 | 2 | 64.84 | 6 | F6:41917 | NaSa01\_F14.raw |  |  |  |  |  | 1.8564E6 |  |  |  |  |  |  |  |  | 1 | 0 | 0 | 0 | 0 | 0 | 1 | 0 | 0 | 0 | 0 | 0 | 0 | 0 | 0 | 525 | 541 |  |  | PEAKS DB |
| N.IPLNAQSLPITVR.T | Y | 39.95 | 1420.8402 | 13 | 0.7 | 711.4279 | 2 | 58.51 | 6 | F6:36546 | NaSa01\_F14.raw |  |  |  |  |  | 5.2985E5 |  |  |  |  |  |  |  |  | 1 | 0 | 0 | 0 | 0 | 0 | 1 | 0 | 0 | 0 | 0 | 0 | 0 | 0 | 0 | 402 | 414 |  |  | PEAKS DB |
| K.SWLWLTK.D | Y | 39.91 | 932.5120 | 7 | 0.3 | 467.2634 | 2 | 68.87 | 6 | F6:45829 | NaSa01\_F14.raw |  |  |  |  |  | 5.8771E7 |  |  |  |  |  |  |  |  | 1 | 0 | 0 | 0 | 0 | 0 | 1 | 0 | 0 | 0 | 0 | 0 | 0 | 0 | 0 | 754 | 760 |  |  | PEAKS DB |
| K.IIIQGDPVAQ.I | Y | 39.88 | 1052.5865 | 10 | 0.0 | 527.3005 | 2 | 48.47 | 6 | F6:28020 | NaSa01\_F14.raw |  |  |  |  |  | 3.3932E7 |  |  |  |  |  |  |  |  | 1 | 0 | 0 | 0 | 0 | 0 | 1 | 0 | 0 | 0 | 0 | 0 | 0 | 0 | 0 | 963 | 972 |  |  | PEAKS DB |
| R.IEEQDGNDIYVM(+15.99)DVLEVIK.Q | Y | 39.47 | 2237.0774 | 19 | 1.1 | 1119.5472 | 2 | 90.84 | 4 | F4:65053 | NaSa01\_F12.raw |  |  |  | 8.9297E5 |  |  |  |  |  |  |  |  |  |  | 1 | 0 | 0 | 0 | 1 | 0 | 0 | 0 | 0 | 0 | 0 | 0 | 0 | 0 | 0 | 1531 | 1549 | Oxidation (M) | M12:Oxidation (M):1000.00 | PEAKS DB |
| K.VVLLSYQSSF.L | Y | 38.34 | 1141.6019 | 10 | 0.4 | 571.8085 | 2 | 79.64 | 6 | F6:54198 | NaSa01\_F14.raw |  |  |  |  |  | 2.3493E6 |  |  |  |  |  |  |  |  | 1 | 0 | 0 | 0 | 0 | 0 | 1 | 0 | 0 | 0 | 0 | 0 | 0 | 0 | 0 | 118 | 127 |  |  | PEAKS DB |
| K.ISYIITK.N | N | 38.08 | 836.5007 | 7 | -0.7 | 419.2574 | 2 | 26.29 | 6 | F6:9476 | NaSa01\_F14.raw |  |  |  |  |  | 6.6012E5 |  |  |  |  |  |  |  |  | 1 | 0 | 0 | 0 | 0 | 0 | 1 | 0 | 0 | 0 | 0 | 0 | 0 | 0 | 0 | 1597 | 1603 |  |  | PEAKS DB |
| total 87 peptides |  |  |  |  |  |  |  |  |  |  |  |  |  |  |  |  |  |  |  |  |  |  |  |  |  |  |  |  |  |  |  |  |  |  |  |  |  |  |  |  |  |  |  |  |  |
| --- | --- | --- | --- | --- | --- | --- | --- | --- | --- | --- | --- | --- | --- | --- | --- | --- | --- | --- | --- | --- | --- | --- | --- | --- | --- | --- | --- | --- | --- | --- | --- | --- | --- | --- | --- | --- | --- | --- | --- | --- | --- | --- | --- | --- | --- |

6I2X

back to list

  

| Protein Coverage
| Supporting Peptides
|

Protein Coverage:

Supporting Peptides:

| Peptide | Uniq | -10lgP | Mass | Length | ppm | m/z | z | RT | Fraction | Scan | Source File | Area F1 | Area F10 | Area F11 | Area F12 | Area F13 | Area F14 | Area F2 | Area F3 | Area F4 | Area F5 | Area F6 | Area F7 | Area F8 | Area F9 | #Feature | #Feature F1 | #Feature F10 | #Feature F11 | #Feature F12 | #Feature F13 | #Feature F14 | #Feature F2 | #Feature F3 | #Feature F4 | #Feature F5 | #Feature F6 | #Feature F7 | #Feature F8 | #Feature F9 | Start | End | PTM | AScore | Found By |
| --- | --- | --- | --- | --- | --- | --- | --- | --- | --- | --- | --- | --- | --- | --- | --- | --- | --- | --- | --- | --- | --- | --- | --- | --- | --- | --- | --- | --- | --- | --- | --- | --- | --- | --- | --- | --- | --- | --- | --- | --- | --- | --- | --- | --- | --- |
| R.AILHNYVNEDIYVR.V | Y | 89.83 | 1717.8788 | 14 | 0.3 | 859.9469 | 2 | 43.05 | 6 | F6:23378 | NaSa01\_F14.raw |  |  |  |  | 1.5001E7 | 1.9759E8 |  |  |  |  |  |  |  |  | 8 | 0 | 0 | 0 | 0 | 3 | 5 | 0 | 0 | 0 | 0 | 0 | 0 | 0 | 0 | 834 | 847 |  |  | PEAKS DB |
| R.AVPFVIVPLEQGLHDVEIK.A | Y | 87.23 | 2102.1775 | 19 | 0.5 | 701.7335 | 3 | 85.72 | 6 | F6:59617 | NaSa01\_F14.raw |  |  |  | 7.0958E5 | 1.6836E7 | 1.2181E8 |  |  |  |  |  |  |  |  | 7 | 0 | 0 | 0 | 1 | 2 | 4 | 0 | 0 | 0 | 0 | 0 | 0 | 0 | 0 | 879 | 897 |  |  | PEAKS DB |
| K.HFEVGFIQPGSVK.V | N | 85.09 | 1443.7510 | 13 | -0.2 | 722.8826 | 2 | 44.13 | 6 | F6:24159 | NaSa01\_F14.raw |  |  |  |  | 2.4365E7 | 2.5116E8 |  |  |  |  |  |  |  |  | 8 | 0 | 0 | 0 | 0 | 2 | 6 | 0 | 0 | 0 | 0 | 0 | 0 | 0 | 0 | 1445 | 1457 |  |  | PEAKS DB |
| R.RDGQNLVTM(+15.99)NLHITPDLIPSFR.F | N | 85.06 | 2552.3169 | 22 | 0.3 | 639.0867 | 4 | 69.77 | 6 | F6:46133 | NaSa01\_F14.raw |  |  |  |  |  | 2.5484E6 |  |  |  |  |  |  |  |  | 2 | 0 | 0 | 0 | 0 | 0 | 2 | 0 | 0 | 0 | 0 | 0 | 0 | 0 | 0 | 498 | 519 | Oxidation (M) | M9:Oxidation (M):1000.00 | PEAKS DB |
| R.QNQYVVVQVTGPQVR.L | N | 83.17 | 1713.9163 | 15 | 0.2 | 857.9656 | 2 | 43.83 | 6 | F6:24244 | NaSa01\_F14.raw |  |  |  |  | 1.4255E7 | 1.5135E8 |  |  |  |  |  |  |  |  | 5 | 0 | 0 | 0 | 0 | 2 | 3 | 0 | 0 | 0 | 0 | 0 | 0 | 0 | 0 | 100 | 114 |  |  | PEAKS DB |
| K.VFFIDLQMPYSVVK.N | Y | 82.52 | 1684.8898 | 14 | 0.3 | 843.4524 | 2 | 95.37 | 6 | F6:67466 | NaSa01\_F14.raw |  |  |  |  | 5.5288E5 | 4.4172E6 |  |  |  |  |  |  |  |  | 2 | 0 | 0 | 0 | 0 | 1 | 1 | 0 | 0 | 0 | 0 | 0 | 0 | 0 | 0 | 813 | 826 |  |  | PEAKS DB |
| K.VNDDYLIWGSR.S | Y | 81.98 | 1336.6411 | 11 | 1.0 | 669.3285 | 2 | 59.77 | 6 | F6:37640 | NaSa01\_F14.raw |  |  |  | 4.0113E7 | 1.558E7 | 1.2947E8 |  |  |  |  |  |  |  |  | 4 | 0 | 0 | 0 | 1 | 2 | 1 | 0 | 0 | 0 | 0 | 0 | 0 | 0 | 0 | 1577 | 1587 |  |  | PEAKS DB |
| R.IEEQDGNDIYVMDVLEVIK.Q | Y | 80.07 | 2221.0823 | 19 | 0.7 | 1111.5492 | 2 | 101.01 | 4 | F4:73173 | NaSa01\_F12.raw |  |  |  | 1.608E7 | 4.109E6 | 2.6808E7 |  |  |  |  |  |  |  |  | 11 | 0 | 0 | 0 | 4 | 3 | 4 | 0 | 0 | 0 | 0 | 0 | 0 | 0 | 0 | 1531 | 1549 |  |  | PEAKS DB |
| K.ATMTILTFYNAQLQEK.A | Y | 79.74 | 1870.9498 | 16 | -0.2 | 936.4820 | 2 | 77.02 | 6 | F6:52034 | NaSa01\_F14.raw |  |  | 5.3707E5 | 1.2422E6 | 3.5134E6 | 4.7877E7 |  |  |  |  |  |  |  |  | 6 | 0 | 0 | 1 | 1 | 1 | 3 | 0 | 0 | 0 | 0 | 0 | 0 | 0 | 0 | 1321 | 1336 |  |  | PEAKS DB |
| K.QLDIFVHDFPR.K | N | 79.15 | 1385.7091 | 11 | 0.0 | 693.8618 | 2 | 58.44 | 6 | F6:36683 | NaSa01\_F14.raw |  |  |  |  | 9.0136E6 | 8.647E7 |  |  |  |  |  |  |  |  | 4 | 0 | 0 | 0 | 0 | 2 | 2 | 0 | 0 | 0 | 0 | 0 | 0 | 0 | 0 | 53 | 63 |  |  | PEAKS DB |
| K.GDNLIQMPGAAMK.I | Y | 78.60 | 1344.6530 | 13 | 0.2 | 673.3339 | 2 | 49.26 | 6 | F6:28453 | NaSa01\_F14.raw |  |  |  |  |  | 1.0626E8 |  |  |  |  |  |  |  |  | 2 | 0 | 0 | 0 | 0 | 0 | 2 | 0 | 0 | 0 | 0 | 0 | 0 | 0 | 0 | 552 | 564 |  |  | PEAKS DB |
| F.VIVPLEQGLHDVEIK.A | Y | 76.27 | 1687.9508 | 15 | 0.0 | 844.9827 | 2 | 58.08 | 6 | F6:36376 | NaSa01\_F14.raw |  |  |  |  | 5.5586E6 | 9.3853E7 |  |  |  |  |  |  |  |  | 3 | 0 | 0 | 0 | 0 | 1 | 2 | 0 | 0 | 0 | 0 | 0 | 0 | 0 | 0 | 883 | 897 |  |  | PEAKS DB |
| R.DDNEDGFIADSDIISR.S | Y | 75.58 | 1780.7751 | 16 | 0.3 | 891.3951 | 2 | 68.40 | 6 | F6:44898 | NaSa01\_F14.raw |  |  |  |  | 9.0776E5 | 1.2244E7 |  |  |  |  |  |  |  |  | 4 | 0 | 0 | 0 | 0 | 1 | 3 | 0 | 0 | 0 | 0 | 0 | 0 | 0 | 0 | 733 | 748 |  |  | PEAKS DB |
| K.C(+57.02)C(+57.02)EDVMHENPMGYTC(+57.02)EK.R | Y | 74.09 | 2158.7886 | 17 | 7.4 | 720.6050 | 3 | 31.06 | 8 | F8:16109 | NaSa01\_F3.raw |  |  |  |  |  |  |  | 1.0485E7 | 5.6652E7 | 1.4617E7 |  |  |  |  | 6 | 0 | 0 | 0 | 0 | 0 | 0 | 0 | 1 | 3 | 2 | 0 | 0 | 0 | 0 | 677 | 693 | Carbamidomethylation | C1:Carbamidomethylation:1000.00;C2:Carbamidomethylation:1000.00;C15:Carbamidomethylation:1000.00 | PEAKS DB |
| R.INYENALLAR.T | Y | 74.07 | 1175.6299 | 10 | 0.1 | 588.8223 | 2 | 45.12 | 6 | F6:25480 | NaSa01\_F14.raw |  |  | 3.3741E5 | 7.6601E6 | 1.5972E7 | 1.5972E8 |  |  |  |  |  |  |  |  | 7 | 0 | 0 | 1 | 3 | 1 | 2 | 0 | 0 | 0 | 0 | 0 | 0 | 0 | 0 | 1292 | 1301 |  |  | PEAKS DB |
| Y.YQVGNNEIVADSVWVDVK.D | N | 73.99 | 2034.0057 | 18 | 0.2 | 1018.0104 | 2 | 70.65 | 6 | F6:46860 | NaSa01\_F14.raw |  |  |  |  |  | 6.5239E6 |  |  |  |  |  |  |  |  | 1 | 0 | 0 | 0 | 0 | 0 | 1 | 0 | 0 | 0 | 0 | 0 | 0 | 0 | 0 | 524 | 541 |  |  | PEAKS DB |
| K.ASVQEALWSDGVR.K | Y | 72.98 | 1416.6997 | 13 | 0.1 | 709.3572 | 2 | 49.71 | 6 | F6:29208 | NaSa01\_F14.raw |  |  |  |  | 1.3438E7 | 1.2825E8 |  |  |  |  |  |  |  |  | 7 | 0 | 0 | 0 | 0 | 1 | 6 | 0 | 0 | 0 | 0 | 0 | 0 | 0 | 0 | 898 | 910 |  |  | PEAKS DB |
| R.VELLYNPAFC(+57.02)SASTK.G | Y | 72.57 | 1698.8286 | 15 | -0.7 | 850.4210 | 2 | 59.22 | 6 | F6:37110 | NaSa01\_F14.raw |  |  |  |  | 1.6821E7 | 2.15E8 |  |  |  |  |  |  |  |  | 2 | 0 | 0 | 0 | 0 | 1 | 1 | 0 | 0 | 0 | 0 | 0 | 0 | 0 | 0 | 848 | 862 | Carbamidomethylation | C10:Carbamidomethylation:1000.00 | PEAKS DB |
| K.LNQDITVTASGDGK.A | Y | 72.09 | 1417.7048 | 14 | 0.8 | 709.8602 | 2 | 20.83 | 6 | F6:5302 | NaSa01\_F14.raw |  |  |  |  | 4.3744E5 | 4.5631E6 |  |  |  |  |  |  |  |  | 3 | 0 | 0 | 0 | 0 | 1 | 2 | 0 | 0 | 0 | 0 | 0 | 0 | 0 | 0 | 1307 | 1320 |  |  | PEAKS DB |
| K.LILNIPLNAQSLPITVR.T | Y | 71.60 | 1874.1353 | 17 | 0.5 | 625.7194 | 3 | 90.36 | 6 | F6:63322 | NaSa01\_F14.raw |  |  |  |  | 1.4111E7 | 4.901E7 |  |  |  |  |  |  |  |  | 4 | 0 | 0 | 0 | 0 | 2 | 2 | 0 | 0 | 0 | 0 | 0 | 0 | 0 | 0 | 398 | 414 |  |  | PEAKS DB |
| K.YFTYLILNK.G | N | 70.49 | 1173.6433 | 9 | 0.1 | 587.8290 | 2 | 64.67 | 6 | F6:41809 | NaSa01\_F14.raw |  |  |  |  | 1.0728E7 | 7.7707E7 |  |  |  |  |  |  |  |  | 2 | 0 | 0 | 0 | 0 | 1 | 1 | 0 | 0 | 0 | 0 | 0 | 0 | 0 | 0 | 478 | 486 |  |  | PEAKS DB |
| R.WPHEDEC(+57.02)QEEEFQK.L | Y | 69.62 | 1889.7526 | 14 | 0.9 | 945.8844 | 2 | 23.39 | 4 | F4:9200 | NaSa01\_F12.raw |  |  |  | 2.8919E7 |  | 3.166E6 |  |  |  |  |  |  |  |  | 3 | 0 | 0 | 0 | 2 | 0 | 1 | 0 | 0 | 0 | 0 | 0 | 0 | 0 | 0 | 1610 | 1623 | Carbamidomethylation | C7:Carbamidomethylation:1000.00 | PEAKS DB |
| K.SDFGC(+57.02)TAGSGQNNLGVFEDAGLALTTSTNLNTK.Q | N | 66.95 | 3359.5576 | 33 | 0.8 | 1120.8607 | 3 | 89.37 | 6 | F6:62425 | NaSa01\_F14.raw |  |  |  |  |  | 3.8512E7 |  |  |  |  |  |  |  |  | 1 | 0 | 0 | 0 | 0 | 0 | 1 | 0 | 0 | 0 | 0 | 0 | 0 | 0 | 0 | 605 | 637 | Carbamidomethylation | C5:Carbamidomethylation:1000.00 | PEAKS DB |
| R.DGQNLVTM(+15.99)NLHITPDLIPSFR.F | N | 66.34 | 2396.2158 | 21 | 0.4 | 799.7462 | 3 | 83.99 | 6 | F6:57985 | NaSa01\_F14.raw |  |  |  |  |  | 5.9574E6 |  |  |  |  |  |  |  |  | 1 | 0 | 0 | 0 | 0 | 0 | 1 | 0 | 0 | 0 | 0 | 0 | 0 | 0 | 0 | 499 | 519 | Oxidation (M) | M8:Oxidation (M):1000.00 | PEAKS DB |
| L.ARDDNEDGFIADSDIISR.S | Y | 66.31 | 2007.9133 | 18 | 1.0 | 670.3124 | 3 | 48.95 | 6 | F6:28381 | NaSa01\_F14.raw |  |  |  |  |  | 6.6726E6 |  |  |  |  |  |  |  |  | 2 | 0 | 0 | 0 | 0 | 0 | 2 | 0 | 0 | 0 | 0 | 0 | 0 | 0 | 0 | 731 | 748 |  |  | PEAKS DB |
| Y.VTNPDGSPAAHVPVVSEAFHSMGTTLSDGTAK.L | Y | 66.31 | 3179.5193 | 32 | 0.8 | 795.8877 | 4 | 59.12 | 6 | F6:37224 | NaSa01\_F14.raw |  |  |  |  |  | 2.3066E7 |  |  |  |  |  |  |  |  | 1 | 0 | 0 | 0 | 0 | 0 | 1 | 0 | 0 | 0 | 0 | 0 | 0 | 0 | 0 | 366 | 397 |  |  | PEAKS DB |
| K.C(+57.02)C(+57.02)EDVMHENPM(+15.99)GYTC(+57.02)EK.R | Y | 65.55 | 2174.7837 | 17 | 1.4 | 725.9362 | 3 | 11.70 | 9 | F9:2004 | NaSa01\_F4.raw |  |  |  |  |  |  |  |  | 1.0934E7 |  |  |  |  |  | 2 | 0 | 0 | 0 | 0 | 0 | 0 | 0 | 0 | 2 | 0 | 0 | 0 | 0 | 0 | 677 | 693 | Carbamidomethylation; Oxidation (M) | C1:Carbamidomethylation:1000.00;C2:Carbamidomethylation:1000.00;M11:Oxidation (M):43.99;C15:Carbamidomethylation:1000.00 | PEAKS DB |
| Y.NLPDLVSLGTWR.I | Y | 65.12 | 1369.7354 | 12 | 0.1 | 685.8750 | 2 | 81.18 | 6 | F6:55452 | NaSa01\_F14.raw |  |  |  |  | 6.3971E6 | 8.6753E7 |  |  |  |  |  |  |  |  | 2 | 0 | 0 | 0 | 0 | 1 | 1 | 0 | 0 | 0 | 0 | 0 | 0 | 0 | 0 | 187 | 198 |  |  | PEAKS DB |
| K.GDNLIQMPGAAM(+15.99)K.I | Y | 64.58 | 1360.6479 | 13 | 0.8 | 681.3318 | 2 | 38.53 | 6 | F6:19473 | NaSa01\_F14.raw |  |  |  |  | 9.6097E5 | 9.5549E6 |  |  |  |  |  |  |  |  | 3 | 0 | 0 | 0 | 0 | 1 | 2 | 0 | 0 | 0 | 0 | 0 | 0 | 0 | 0 | 552 | 564 | Oxidation (M) | M12:Oxidation (M):38.33 | PEAKS DB |
| E.VEGVAFVLFGVK.I | N | 62.96 | 1263.7227 | 12 | 0.2 | 632.8687 | 2 | 85.00 | 6 | F6:58754 | NaSa01\_F14.raw |  |  |  |  |  | 2.0835E6 |  |  |  |  |  |  |  |  | 1 | 0 | 0 | 0 | 0 | 0 | 1 | 0 | 0 | 0 | 0 | 0 | 0 | 0 | 0 | 257 | 268 |  |  | PEAKS DB |
| Y.QSSFLFIQTDK.G | Y | 62.43 | 1312.6663 | 11 | -0.4 | 657.3401 | 2 | 57.57 | 6 | F6:35846 | NaSa01\_F14.raw |  |  |  |  |  | 1.1659E7 |  |  |  |  |  |  |  |  | 1 | 0 | 0 | 0 | 0 | 0 | 1 | 0 | 0 | 0 | 0 | 0 | 0 | 0 | 0 | 124 | 134 |  |  | PEAKS DB |
| K.GVGGTQLEVIK.A | Y | 62.08 | 1099.6237 | 11 | 0.2 | 550.8192 | 2 | 30.32 | 12 | F12:15497 | NaSa01\_F7.raw |  |  |  |  | 1.4118E7 | 1.0253E6 |  |  |  |  |  | 1.7364E6 |  |  | 3 | 0 | 0 | 0 | 0 | 1 | 1 | 0 | 0 | 0 | 0 | 0 | 1 | 0 | 0 | 936 | 946 |  |  | PEAKS DB |
| K.ATM(+15.99)TILTFYNAQLQEK.A | Y | 62.07 | 1886.9448 | 16 | 0.4 | 944.4801 | 2 | 64.84 | 6 | F6:42088 | NaSa01\_F14.raw |  |  |  |  |  | 1.0319E7 |  |  |  |  |  |  |  |  | 2 | 0 | 0 | 0 | 0 | 0 | 2 | 0 | 0 | 0 | 0 | 0 | 0 | 0 | 0 | 1321 | 1336 | Oxidation (M) | M3:Oxidation (M):1000.00 | PEAKS DB |
| R.FPNLNELVGH.T | N | 62.05 | 1138.5770 | 10 | 0.8 | 570.2963 | 2 | 67.53 | 6 | F6:44045 | NaSa01\_F14.raw |  |  |  |  | 1.7762E6 | 2.8185E7 |  |  |  |  |  |  |  |  | 2 | 0 | 0 | 0 | 0 | 1 | 1 | 0 | 0 | 0 | 0 | 0 | 0 | 0 | 0 | 303 | 312 |  |  | PEAKS DB |
| K.VYSYYNLDEK.C | N | 61.93 | 1292.5924 | 10 | 1.2 | 647.3043 | 2 | 33.77 | 6 | F6:15693 | NaSa01\_F14.raw |  |  |  |  | 1.5304E7 | 1.0252E8 |  |  |  |  |  |  |  |  | 3 | 0 | 0 | 0 | 0 | 1 | 2 | 0 | 0 | 0 | 0 | 0 | 0 | 0 | 0 | 1458 | 1467 |  |  | PEAKS DB |
| M.TILTFYNAQLQEK.A | N | 61.57 | 1567.8246 | 13 | 0.3 | 784.9198 | 2 | 60.05 | 6 | F6:38047 | NaSa01\_F14.raw |  |  |  |  |  | 5.1346E6 |  |  |  |  |  |  |  |  | 1 | 0 | 0 | 0 | 0 | 0 | 1 | 0 | 0 | 0 | 0 | 0 | 0 | 0 | 0 | 1324 | 1336 |  |  | PEAKS DB |
| H.ITPDLIPSFR.F | N | 61.36 | 1157.6444 | 10 | 0.0 | 579.8295 | 2 | 65.01 | 6 | F6:42335 | NaSa01\_F14.raw |  |  |  |  | 3.0601E6 | 3.8923E7 |  |  |  |  |  |  |  |  | 3 | 0 | 0 | 0 | 0 | 1 | 2 | 0 | 0 | 0 | 0 | 0 | 0 | 0 | 0 | 510 | 519 |  |  | PEAKS DB |
| K.PGDNLPVNFNVK.G | Y | 60.28 | 1312.6775 | 12 | 0.4 | 657.3463 | 2 | 47.72 | 6 | F6:27452 | NaSa01\_F14.raw |  |  |  |  |  | 1.0823E6 |  |  |  |  |  |  |  |  | 1 | 0 | 0 | 0 | 0 | 0 | 1 | 0 | 0 | 0 | 0 | 0 | 0 | 0 | 0 | 456 | 467 |  |  | PEAKS DB |
| W.VVLAVSFTPTK.G | Y | 59.98 | 1160.6804 | 11 | 0.4 | 581.3477 | 2 | 51.95 | 6 | F6:30874 | NaSa01\_F14.raw |  |  |  |  |  | 3.7062E6 |  |  |  |  |  |  |  |  | 1 | 0 | 0 | 0 | 0 | 0 | 1 | 0 | 0 | 0 | 0 | 0 | 0 | 0 | 0 | 788 | 798 |  |  | PEAKS DB |
| K.YEHSPENYTAYFDVR.K | N | 59.87 | 1889.8220 | 15 | -0.9 | 630.9474 | 3 | 47.19 | 6 | F6:27480 | NaSa01\_F14.raw |  |  |  |  |  | 2.5484E6 |  |  |  |  |  |  |  |  | 2 | 0 | 0 | 0 | 0 | 0 | 2 | 0 | 0 | 0 | 0 | 0 | 0 | 0 | 0 | 203 | 217 |  |  | PEAKS DB |
| R.IDVPLQIEK.A | Y | 59.34 | 1053.6069 | 9 | 0.1 | 527.8108 | 2 | 47.86 | 6 | F6:27228 | NaSa01\_F14.raw |  |  |  | 3.0425E7 | 1.9664E7 | 1.6873E8 |  |  |  |  |  |  |  |  | 4 | 0 | 0 | 0 | 1 | 1 | 2 | 0 | 0 | 0 | 0 | 0 | 0 | 0 | 0 | 1506 | 1514 |  |  | PEAKS DB |
| K.IIIQGDPVAQIIENSIDGSK.L | Y | 59.31 | 2109.1316 | 20 | 0.6 | 1055.5737 | 2 | 96.62 | 6 | F6:68548 | NaSa01\_F14.raw |  |  | 1.4465E5 |  | 1.6001E5 | 2.5079E6 |  |  |  |  |  |  |  |  | 3 | 0 | 0 | 1 | 0 | 1 | 1 | 0 | 0 | 0 | 0 | 0 | 0 | 0 | 0 | 963 | 982 |  |  | PEAKS DB |
| K.GIC(+57.02)VAEPYEIR.V | Y | 59.06 | 1305.6387 | 11 | 0.4 | 653.8268 | 2 | 44.31 | 6 | F6:24643 | NaSa01\_F14.raw |  |  |  |  | 1.0302E7 | 1.0808E8 |  |  |  |  |  |  |  |  | 3 | 0 | 0 | 0 | 0 | 1 | 2 | 0 | 0 | 0 | 0 | 0 | 0 | 0 | 0 | 799 | 809 | Carbamidomethylation | C3:Carbamidomethylation:1000.00 | PEAKS DB |
| E.DGFIADSDIISR.S | Y | 58.62 | 1307.6357 | 12 | -0.1 | 654.8251 | 2 | 60.77 | 6 | F6:38594 | NaSa01\_F14.raw |  |  |  |  |  | 4.7593E6 |  |  |  |  |  |  |  |  | 1 | 0 | 0 | 0 | 0 | 0 | 1 | 0 | 0 | 0 | 0 | 0 | 0 | 0 | 0 | 737 | 748 |  |  | PEAKS DB |
| R.FPNLNELVGHTLY.A | N | 58.47 | 1515.7721 | 13 | 0.3 | 758.8936 | 2 | 85.62 | 6 | F6:59205 | NaSa01\_F14.raw |  |  |  |  |  | 1.9527E7 |  |  |  |  |  |  |  |  | 1 | 0 | 0 | 0 | 0 | 0 | 1 | 0 | 0 | 0 | 0 | 0 | 0 | 0 | 0 | 303 | 315 |  |  | PEAKS DB |
| S.MLTGFLPDAEDLTR.L | N | 58.03 | 1577.7759 | 14 | 0.5 | 789.8956 | 2 | 78.56 | 6 | F6:53520 | NaSa01\_F14.raw |  |  |  |  |  | 2.8623E6 |  |  |  |  |  |  |  |  | 1 | 0 | 0 | 0 | 0 | 0 | 1 | 0 | 0 | 0 | 0 | 0 | 0 | 0 | 0 | 1386 | 1399 |  |  | PEAKS DB |
| R.KYVLPSFEVR.L | Y | 56.74 | 1236.6866 | 10 | 0.3 | 413.2363 | 3 | 42.11 | 6 | F6:22829 | NaSa01\_F14.raw |  |  |  |  | 5.509E6 | 4.9733E7 |  |  |  |  |  |  |  |  | 6 | 0 | 0 | 0 | 0 | 2 | 4 | 0 | 0 | 0 | 0 | 0 | 0 | 0 | 0 | 218 | 227 |  |  | PEAKS DB |
| R.DGQNLVTMNLHITPDLIPSFR.F | N | 56.46 | 2380.2209 | 21 | 0.5 | 794.4147 | 3 | 91.58 | 6 | F6:64345 | NaSa01\_F14.raw |  |  |  |  |  | 2.1315E6 |  |  |  |  |  |  |  |  | 1 | 0 | 0 | 0 | 0 | 0 | 1 | 0 | 0 | 0 | 0 | 0 | 0 | 0 | 0 | 499 | 519 |  |  | PEAKS DB |
| K.VAVIIYLNK.V | Y | 56.21 | 1031.6379 | 9 | 0.8 | 516.8267 | 2 | 50.20 | 6 | F6:30133 | NaSa01\_F14.raw |  |  |  |  |  | 1.67E7 |  |  |  |  |  |  |  |  | 2 | 0 | 0 | 0 | 0 | 0 | 2 | 0 | 0 | 0 | 0 | 0 | 0 | 0 | 0 | 1421 | 1429 |  |  | PEAKS DB |
| G.ALYTLITPAVLR.T | N | 55.82 | 1329.8020 | 12 | 0.4 | 665.9086 | 2 | 75.95 | 6 | F6:51300 | NaSa01\_F14.raw |  |  |  | 1.7564E6 | 1.5853E7 | 2.019E8 |  |  |  |  |  |  |  |  | 6 | 0 | 0 | 0 | 1 | 1 | 4 | 0 | 0 | 0 | 0 | 0 | 0 | 0 | 0 | 23 | 34 |  |  | PEAKS DB |
| N.LHITPDLIPSFR.F | N | 52.36 | 1407.7874 | 12 | 0.4 | 470.2699 | 3 | 64.03 | 6 | F6:41508 | NaSa01\_F14.raw |  |  |  |  |  | 2.1751E7 |  |  |  |  |  |  |  |  | 2 | 0 | 0 | 0 | 0 | 0 | 2 | 0 | 0 | 0 | 0 | 0 | 0 | 0 | 0 | 508 | 519 |  |  | PEAKS DB |
| K.YVLPSFEVR.L | N | 51.77 | 1108.5917 | 9 | 0.6 | 555.3035 | 2 | 58.44 | 6 | F6:36495 | NaSa01\_F14.raw |  |  |  | 4.1589E5 | 1.253E7 | 1.3997E8 |  |  |  |  |  |  |  |  | 4 | 0 | 0 | 0 | 1 | 1 | 2 | 0 | 0 | 0 | 0 | 0 | 0 | 0 | 0 | 219 | 227 |  |  | PEAKS DB |
| K.TMSFYLR.D | Y | 51.69 | 916.4476 | 7 | 0.3 | 459.2312 | 2 | 41.67 | 6 | F6:22840 | NaSa01\_F14.raw |  |  |  |  |  | 5.6816E7 |  |  |  |  |  |  |  |  | 1 | 0 | 0 | 0 | 0 | 0 | 1 | 0 | 0 | 0 | 0 | 0 | 0 | 0 | 0 | 775 | 781 |  |  | PEAKS DB |
| R.DGQNLVTMNLH.I | N | 51.62 | 1240.5870 | 11 | 0.0 | 621.3008 | 2 | 49.19 | 6 | F6:28557 | NaSa01\_F14.raw |  |  |  |  |  | 4.925E6 |  |  |  |  |  |  |  |  | 1 | 0 | 0 | 0 | 0 | 0 | 1 | 0 | 0 | 0 | 0 | 0 | 0 | 0 | 0 | 499 | 509 |  |  | PEAKS DB |
| L.NIPLNAQSLPITVR.T | Y | 50.97 | 1534.8831 | 14 | -1.4 | 768.4477 | 2 | 61.34 | 6 | F6:39039 | NaSa01\_F14.raw |  |  |  |  |  | 1.6927E5 |  |  |  |  |  |  |  |  | 1 | 0 | 0 | 0 | 0 | 0 | 1 | 0 | 0 | 0 | 0 | 0 | 0 | 0 | 0 | 401 | 414 |  |  | PEAKS DB |
| K.C(+57.02)C(+57.02)EDVM(+15.99)HENPM(+15.99)GYTC(+57.02)EK.R | Y | 50.02 | 2190.7786 | 17 | 0.7 | 731.2673 | 3 | 11.49 | 9 | F9:1837 | NaSa01\_F4.raw |  |  |  |  |  |  |  |  | 4.1662E5 |  |  |  |  |  | 1 | 0 | 0 | 0 | 0 | 0 | 0 | 0 | 0 | 1 | 0 | 0 | 0 | 0 | 0 | 677 | 693 | Carbamidomethylation; Oxidation (M) | C1:Carbamidomethylation:1000.00;C2:Carbamidomethylation:1000.00;M6:Oxidation (M):1000.00;M11:Oxidation (M):1000.00;C15:Carbamidomethylation:1000.00 | PEAKS DB |
| K.GIYTPGSPVLYR.V | N | 49.98 | 1321.7030 | 12 | -8.2 | 661.8533 | 2 | 47.56 | 6 | F6:27271 | NaSa01\_F14.raw |  |  |  |  |  | 4.6433E6 |  |  |  |  |  |  |  |  | 1 | 0 | 0 | 0 | 0 | 0 | 1 | 0 | 0 | 0 | 0 | 0 | 0 | 0 | 0 | 135 | 146 |  |  | PEAKS DB |
| K.AC(+57.02)ETNVDYVYK.T | Y | 49.20 | 1360.5969 | 11 | 0.3 | 681.3060 | 2 | 22.05 | 6 | F6:6230 | NaSa01\_F14.raw |  |  |  | 2.4601E5 |  | 5.6943E5 |  |  |  |  |  |  |  |  | 2 | 0 | 0 | 0 | 1 | 0 | 1 | 0 | 0 | 0 | 0 | 0 | 0 | 0 | 0 | 1515 | 1525 | Carbamidomethylation | C2:Carbamidomethylation:1000.00 | PEAKS DB |
| M.LTGFLPDAEDLTR.L | N | 47.16 | 1446.7354 | 13 | 1.2 | 724.3758 | 2 | 69.30 | 6 | F6:45774 | NaSa01\_F14.raw |  |  |  |  |  | 7.2495E5 |  |  |  |  |  |  |  |  | 1 | 0 | 0 | 0 | 0 | 0 | 1 | 0 | 0 | 0 | 0 | 0 | 0 | 0 | 0 | 1387 | 1399 |  |  | PEAKS DB |
| R.VGLVAVDKAVYVLNDK.Y | N | 46.90 | 1701.9664 | 16 | 0.8 | 568.3299 | 3 | 57.39 | 6 | F6:35573 | NaSa01\_F14.raw |  |  |  |  |  | 1.8722E5 |  |  |  |  |  |  |  |  | 1 | 0 | 0 | 0 | 0 | 0 | 1 | 0 | 0 | 0 | 0 | 0 | 0 | 0 | 0 | 575 | 590 |  |  | PEAKS DB |
| K.THQYISQR.K | Y | 46.29 | 1031.5149 | 8 | 0.4 | 516.7650 | 2 | 11.44 | 6 | F6:1825 | NaSa01\_F14.raw |  |  |  |  |  | 1.3503E5 |  |  |  |  |  |  |  |  | 1 | 0 | 0 | 0 | 0 | 0 | 1 | 0 | 0 | 0 | 0 | 0 | 0 | 0 | 0 | 1560 | 1567 |  |  | PEAKS DB |
| D.DNEDGFIADSDIISR.S | Y | 46.28 | 1665.7482 | 15 | 0.7 | 833.8820 | 2 | 65.65 | 6 | F6:42702 | NaSa01\_F14.raw |  |  |  |  |  | 2.6489E5 |  |  |  |  |  |  |  |  | 1 | 0 | 0 | 0 | 0 | 0 | 1 | 0 | 0 | 0 | 0 | 0 | 0 | 0 | 0 | 734 | 748 |  |  | PEAKS DB |
| H.VAITSTEIKPGDNLPVNFNVK.G | Y | 46.15 | 2255.2161 | 21 | 0.2 | 752.7461 | 3 | 57.73 | 6 | F6:36049 | NaSa01\_F14.raw |  |  |  |  |  | 3.3349E6 |  |  |  |  |  |  |  |  | 1 | 0 | 0 | 0 | 0 | 0 | 1 | 0 | 0 | 0 | 0 | 0 | 0 | 0 | 0 | 447 | 467 |  |  | PEAKS DB |
| K.IIIQGDPVAQIIEN.S | Y | 45.71 | 1521.8402 | 14 | 0.0 | 761.9274 | 2 | 85.63 | 6 | F6:59337 | NaSa01\_F14.raw |  |  |  |  |  | 1.4516E7 |  |  |  |  |  |  |  |  | 1 | 0 | 0 | 0 | 0 | 0 | 1 | 0 | 0 | 0 | 0 | 0 | 0 | 0 | 0 | 963 | 976 |  |  | PEAKS DB |
| K.TM(+15.99)SFYLR.D | Y | 45.45 | 932.4426 | 7 | 0.2 | 467.2286 | 2 | 32.98 | 6 | F6:14848 | NaSa01\_F14.raw |  |  |  |  |  | 1.994E6 |  |  |  |  |  |  |  |  | 2 | 0 | 0 | 0 | 0 | 0 | 2 | 0 | 0 | 0 | 0 | 0 | 0 | 0 | 0 | 775 | 781 | Oxidation (M) | M2:Oxidation (M):1000.00 | PEAKS DB |
| N.DIYVMDVLEVIK.Q | N | 45.17 | 1435.7632 | 12 | 0.7 | 718.8894 | 2 | 106.25 | 4 | F4:76997 | NaSa01\_F12.raw |  |  |  | 2.8255E5 |  |  |  |  |  |  |  |  |  |  | 1 | 0 | 0 | 0 | 1 | 0 | 0 | 0 | 0 | 0 | 0 | 0 | 0 | 0 | 0 | 1538 | 1549 |  |  | PEAKS DB |
| K.YFKPGMPYELTVY.V | N | 44.78 | 1606.7742 | 13 | 0.4 | 804.3947 | 2 | 71.51 | 6 | F6:47852 | NaSa01\_F14.raw |  |  |  |  |  | 1.8453E7 |  |  |  |  |  |  |  |  | 1 | 0 | 0 | 0 | 0 | 0 | 1 | 0 | 0 | 0 | 0 | 0 | 0 | 0 | 0 | 353 | 365 |  |  | PEAKS DB |
| R.SRFPNLNELVGH.T | N | 44.24 | 1381.7102 | 12 | 0.6 | 461.5776 | 3 | 50.79 | 6 | F6:30122 | NaSa01\_F14.raw |  |  |  |  |  | 1.9009E7 |  |  |  |  |  |  |  |  | 3 | 0 | 0 | 0 | 0 | 0 | 3 | 0 | 0 | 0 | 0 | 0 | 0 | 0 | 0 | 301 | 312 |  |  | PEAKS DB |
| K.TVIVEFQTPEG.I | N | 43.94 | 1218.6132 | 11 | 3.8 | 610.3144 | 2 | 63.73 | 7 | F7:45532 | NaSa01\_F2.raw |  |  |  |  |  |  | 6.7633E6 | 2.6291E6 |  |  |  |  |  |  | 2 | 0 | 0 | 0 | 0 | 0 | 0 | 1 | 1 | 0 | 0 | 0 | 0 | 0 | 0 | 160 | 170 |  |  | PEAKS DB |
| M.NLHITPDLIPSFR.F | N | 43.77 | 1521.8303 | 13 | 0.6 | 508.2843 | 3 | 65.19 | 6 | F6:42284 | NaSa01\_F14.raw |  |  |  |  |  | 1.1088E7 |  |  |  |  |  |  |  |  | 2 | 0 | 0 | 0 | 0 | 0 | 2 | 0 | 0 | 0 | 0 | 0 | 0 | 0 | 0 | 507 | 519 |  |  | PEAKS DB |
| K.AAFLEC(+57.02)C(+57.02)R.Y | N | 43.14 | 1025.4423 | 8 | 0.7 | 513.7288 | 2 | 11.72 | 9 | F9:2016 | NaSa01\_F4.raw |  |  |  |  |  |  |  |  | 5.2365E5 |  |  |  |  |  | 1 | 0 | 0 | 0 | 0 | 0 | 0 | 0 | 0 | 1 | 0 | 0 | 0 | 0 | 0 | 706 | 713 | Carbamidomethylation | C6:Carbamidomethylation:1000.00;C7:Carbamidomethylation:1000.00 | PEAKS DB |
| L.SYQSSFLFIQTDK.G | Y | 42.37 | 1562.7616 | 13 | 1.2 | 782.3890 | 2 | 64.49 | 6 | F6:41739 | NaSa01\_F14.raw |  |  |  |  |  | 3.4858E5 |  |  |  |  |  |  |  |  | 1 | 0 | 0 | 0 | 0 | 0 | 1 | 0 | 0 | 0 | 0 | 0 | 0 | 0 | 0 | 122 | 134 |  |  | PEAKS DB |
| R.KC(+57.02)C(+57.02)EDVMHENPMGYTC(+57.02)EK.R | Y | 42.21 | 2286.8835 | 18 | 1.4 | 763.3029 | 3 | 20.88 | 10 | F10:7153 | NaSa01\_F5.raw |  |  |  |  |  |  |  |  | 7.1178E5 | 3.5368E6 |  |  |  |  | 3 | 0 | 0 | 0 | 0 | 0 | 0 | 0 | 0 | 1 | 2 | 0 | 0 | 0 | 0 | 676 | 693 | Carbamidomethylation | C2:Carbamidomethylation:1000.00;C3:Carbamidomethylation:1000.00;C16:Carbamidomethylation:1000.00 | PEAKS DB |
| R.IPIIDGDGK.A | Y | 42.15 | 926.5073 | 9 | 0.6 | 464.2612 | 2 | 32.92 | 6 | F6:14878 | NaSa01\_F14.raw |  |  |  |  |  | 3.4884E5 |  |  |  |  |  |  |  |  | 1 | 0 | 0 | 0 | 0 | 0 | 1 | 0 | 0 | 0 | 0 | 0 | 0 | 0 | 0 | 283 | 291 |  |  | PEAKS DB |
| Y.VLPSFEVR.L | N | 41.89 | 945.5283 | 8 | -0.2 | 473.7714 | 2 | 40.35 | 6 | F6:20803 | NaSa01\_F14.raw |  |  |  |  |  | 5.7172E5 |  |  |  |  |  |  |  |  | 1 | 0 | 0 | 0 | 0 | 0 | 1 | 0 | 0 | 0 | 0 | 0 | 0 | 0 | 0 | 220 | 227 |  |  | PEAKS DB |
| K.IC(+57.02)IGNVC(+57.02)R.C | Y | 41.79 | 990.4739 | 8 | -1.3 | 496.2436 | 2 | 11.58 | 6 | F6:1976 | NaSa01\_F14.raw |  |  |  |  |  | 2.2107E5 |  |  |  |  |  |  |  |  | 1 | 0 | 0 | 0 | 0 | 0 | 1 | 0 | 0 | 0 | 0 | 0 | 0 | 0 | 0 | 1484 | 1491 | Carbamidomethylation | C2:Carbamidomethylation:1000.00;C7:Carbamidomethylation:1000.00 | PEAKS DB |
| R.SRFPNLNELVGHTLY.A | N | 40.90 | 1758.9053 | 15 | 0.7 | 587.3094 | 3 | 70.95 | 6 | F6:46924 | NaSa01\_F14.raw |  |  |  |  |  | 5.6962E6 |  |  |  |  |  |  |  |  | 1 | 0 | 0 | 0 | 0 | 0 | 1 | 0 | 0 | 0 | 0 | 0 | 0 | 0 | 0 | 301 | 315 |  |  | PEAKS DB |
| Y.TLITPAVLR.T | N | 40.29 | 982.6175 | 9 | 0.4 | 492.3162 | 2 | 45.78 | 6 | F6:25572 | NaSa01\_F14.raw |  |  |  |  |  | 5.8349E7 |  |  |  |  |  |  |  |  | 1 | 0 | 0 | 0 | 0 | 0 | 1 | 0 | 0 | 0 | 0 | 0 | 0 | 0 | 0 | 26 | 34 |  |  | PEAKS DB |
| K.LILNIPLNAQ.S | Y | 40.28 | 1107.6652 | 10 | -0.2 | 554.8397 | 2 | 84.63 | 6 | F6:58512 | NaSa01\_F14.raw |  |  |  |  |  | 3.2621E6 |  |  |  |  |  |  |  |  | 1 | 0 | 0 | 0 | 0 | 0 | 1 | 0 | 0 | 0 | 0 | 0 | 0 | 0 | 0 | 398 | 407 |  |  | PEAKS DB |
| K.IIIQGDPVAQI.I | Y | 39.99 | 1165.6707 | 11 | 0.7 | 583.8430 | 2 | 72.27 | 6 | F6:48414 | NaSa01\_F14.raw |  |  |  |  |  | 1.2067E6 |  |  |  |  |  |  |  |  | 1 | 0 | 0 | 0 | 0 | 0 | 1 | 0 | 0 | 0 | 0 | 0 | 0 | 0 | 0 | 963 | 973 |  |  | PEAKS DB |
| Y.QVGNNEIVADSVWVDVK.D | N | 39.98 | 1870.9425 | 17 | 0.5 | 936.4790 | 2 | 64.84 | 6 | F6:41917 | NaSa01\_F14.raw |  |  |  |  |  | 1.8564E6 |  |  |  |  |  |  |  |  | 1 | 0 | 0 | 0 | 0 | 0 | 1 | 0 | 0 | 0 | 0 | 0 | 0 | 0 | 0 | 525 | 541 |  |  | PEAKS DB |
| N.IPLNAQSLPITVR.T | Y | 39.95 | 1420.8402 | 13 | 0.7 | 711.4279 | 2 | 58.51 | 6 | F6:36546 | NaSa01\_F14.raw |  |  |  |  |  | 5.2985E5 |  |  |  |  |  |  |  |  | 1 | 0 | 0 | 0 | 0 | 0 | 1 | 0 | 0 | 0 | 0 | 0 | 0 | 0 | 0 | 402 | 414 |  |  | PEAKS DB |
| K.SWLWLTK.D | Y | 39.91 | 932.5120 | 7 | 0.3 | 467.2634 | 2 | 68.87 | 6 | F6:45829 | NaSa01\_F14.raw |  |  |  |  |  | 5.8771E7 |  |  |  |  |  |  |  |  | 1 | 0 | 0 | 0 | 0 | 0 | 1 | 0 | 0 | 0 | 0 | 0 | 0 | 0 | 0 | 754 | 760 |  |  | PEAKS DB |
| K.IIIQGDPVAQ.I | Y | 39.88 | 1052.5865 | 10 | 0.0 | 527.3005 | 2 | 48.47 | 6 | F6:28020 | NaSa01\_F14.raw |  |  |  |  |  | 3.3932E7 |  |  |  |  |  |  |  |  | 1 | 0 | 0 | 0 | 0 | 0 | 1 | 0 | 0 | 0 | 0 | 0 | 0 | 0 | 0 | 963 | 972 |  |  | PEAKS DB |
| R.IEEQDGNDIYVM(+15.99)DVLEVIK.Q | Y | 39.47 | 2237.0774 | 19 | 1.1 | 1119.5472 | 2 | 90.84 | 4 | F4:65053 | NaSa01\_F12.raw |  |  |  | 8.9297E5 |  |  |  |  |  |  |  |  |  |  | 1 | 0 | 0 | 0 | 1 | 0 | 0 | 0 | 0 | 0 | 0 | 0 | 0 | 0 | 0 | 1531 | 1549 | Oxidation (M) | M12:Oxidation (M):1000.00 | PEAKS DB |
| K.VVLLSYQSSF.L | Y | 38.34 | 1141.6019 | 10 | 0.4 | 571.8085 | 2 | 79.64 | 6 | F6:54198 | NaSa01\_F14.raw |  |  |  |  |  | 2.3493E6 |  |  |  |  |  |  |  |  | 1 | 0 | 0 | 0 | 0 | 0 | 1 | 0 | 0 | 0 | 0 | 0 | 0 | 0 | 0 | 118 | 127 |  |  | PEAKS DB |
| K.ISYIITK.N | N | 38.08 | 836.5007 | 7 | -0.7 | 419.2574 | 2 | 26.29 | 6 | F6:9476 | NaSa01\_F14.raw |  |  |  |  |  | 6.6012E5 |  |  |  |  |  |  |  |  | 1 | 0 | 0 | 0 | 0 | 0 | 1 | 0 | 0 | 0 | 0 | 0 | 0 | 0 | 0 | 1597 | 1603 |  |  | PEAKS DB |
| total 87 peptides |  |  |  |  |  |  |  |  |  |  |  |  |  |  |  |  |  |  |  |  |  |  |  |  |  |  |  |  |  |  |  |  |  |  |  |  |  |  |  |  |  |  |  |  |  |
| --- | --- | --- | --- | --- | --- | --- | --- | --- | --- | --- | --- | --- | --- | --- | --- | --- | --- | --- | --- | --- | --- | --- | --- | --- | --- | --- | --- | --- | --- | --- | --- | --- | --- | --- | --- | --- | --- | --- | --- | --- | --- | --- | --- | --- | --- |

I51018

back to list

  

| Protein Coverage
| Supporting Peptides
|

Protein Coverage:

Supporting Peptides:

| Peptide | Uniq | -10lgP | Mass | Length | ppm | m/z | z | RT | Fraction | Scan | Source File | Area F1 | Area F10 | Area F11 | Area F12 | Area F13 | Area F14 | Area F2 | Area F3 | Area F4 | Area F5 | Area F6 | Area F7 | Area F8 | Area F9 | #Feature | #Feature F1 | #Feature F10 | #Feature F11 | #Feature F12 | #Feature F13 | #Feature F14 | #Feature F2 | #Feature F3 | #Feature F4 | #Feature F5 | #Feature F6 | #Feature F7 | #Feature F8 | #Feature F9 | Start | End | PTM | AScore | Found By |
| --- | --- | --- | --- | --- | --- | --- | --- | --- | --- | --- | --- | --- | --- | --- | --- | --- | --- | --- | --- | --- | --- | --- | --- | --- | --- | --- | --- | --- | --- | --- | --- | --- | --- | --- | --- | --- | --- | --- | --- | --- | --- | --- | --- | --- | --- |
| R.AILHNYVNEDIYVR.V | Y | 89.83 | 1717.8788 | 14 | 0.3 | 859.9469 | 2 | 43.05 | 6 | F6:23378 | NaSa01\_F14.raw |  |  |  |  | 1.5001E7 | 1.9759E8 |  |  |  |  |  |  |  |  | 8 | 0 | 0 | 0 | 0 | 3 | 5 | 0 | 0 | 0 | 0 | 0 | 0 | 0 | 0 | 834 | 847 |  |  | PEAKS DB |
| R.AVPFVIVPLEQGLHDVEIK.A | Y | 87.23 | 2102.1775 | 19 | 0.5 | 701.7335 | 3 | 85.72 | 6 | F6:59617 | NaSa01\_F14.raw |  |  |  | 7.0958E5 | 1.6836E7 | 1.2181E8 |  |  |  |  |  |  |  |  | 7 | 0 | 0 | 0 | 1 | 2 | 4 | 0 | 0 | 0 | 0 | 0 | 0 | 0 | 0 | 879 | 897 |  |  | PEAKS DB |
| K.HFEVGFIQPGSVK.V | N | 85.09 | 1443.7510 | 13 | -0.2 | 722.8826 | 2 | 44.13 | 6 | F6:24159 | NaSa01\_F14.raw |  |  |  |  | 2.4365E7 | 2.5116E8 |  |  |  |  |  |  |  |  | 8 | 0 | 0 | 0 | 0 | 2 | 6 | 0 | 0 | 0 | 0 | 0 | 0 | 0 | 0 | 1445 | 1457 |  |  | PEAKS DB |
| R.RDGQNLVTM(+15.99)NLHITPDLIPSFR.F | N | 85.06 | 2552.3169 | 22 | 0.3 | 639.0867 | 4 | 69.77 | 6 | F6:46133 | NaSa01\_F14.raw |  |  |  |  |  | 2.5484E6 |  |  |  |  |  |  |  |  | 2 | 0 | 0 | 0 | 0 | 0 | 2 | 0 | 0 | 0 | 0 | 0 | 0 | 0 | 0 | 498 | 519 | Oxidation (M) | M9:Oxidation (M):1000.00 | PEAKS DB |
| R.QNQYVVVQVTGPQVR.L | N | 83.17 | 1713.9163 | 15 | 0.2 | 857.9656 | 2 | 43.83 | 6 | F6:24244 | NaSa01\_F14.raw |  |  |  |  | 1.4255E7 | 1.5135E8 |  |  |  |  |  |  |  |  | 5 | 0 | 0 | 0 | 0 | 2 | 3 | 0 | 0 | 0 | 0 | 0 | 0 | 0 | 0 | 100 | 114 |  |  | PEAKS DB |
| K.VFFIDLQMPYSVVK.N | Y | 82.52 | 1684.8898 | 14 | 0.3 | 843.4524 | 2 | 95.37 | 6 | F6:67466 | NaSa01\_F14.raw |  |  |  |  | 5.5288E5 | 4.4172E6 |  |  |  |  |  |  |  |  | 2 | 0 | 0 | 0 | 0 | 1 | 1 | 0 | 0 | 0 | 0 | 0 | 0 | 0 | 0 | 813 | 826 |  |  | PEAKS DB |
| K.VNDDYLIWGSR.S | Y | 81.98 | 1336.6411 | 11 | 1.0 | 669.3285 | 2 | 59.77 | 6 | F6:37640 | NaSa01\_F14.raw |  |  |  | 4.0113E7 | 1.558E7 | 1.2947E8 |  |  |  |  |  |  |  |  | 4 | 0 | 0 | 0 | 1 | 2 | 1 | 0 | 0 | 0 | 0 | 0 | 0 | 0 | 0 | 1577 | 1587 |  |  | PEAKS DB |
| R.IEEQDGNDIYVMDVLEVIK.Q | Y | 80.07 | 2221.0823 | 19 | 0.7 | 1111.5492 | 2 | 101.01 | 4 | F4:73173 | NaSa01\_F12.raw |  |  |  | 1.608E7 | 4.109E6 | 2.6808E7 |  |  |  |  |  |  |  |  | 11 | 0 | 0 | 0 | 4 | 3 | 4 | 0 | 0 | 0 | 0 | 0 | 0 | 0 | 0 | 1531 | 1549 |  |  | PEAKS DB |
| K.ATMTILTFYNAQLQEK.A | Y | 79.74 | 1870.9498 | 16 | -0.2 | 936.4820 | 2 | 77.02 | 6 | F6:52034 | NaSa01\_F14.raw |  |  | 5.3707E5 | 1.2422E6 | 3.5134E6 | 4.7877E7 |  |  |  |  |  |  |  |  | 6 | 0 | 0 | 1 | 1 | 1 | 3 | 0 | 0 | 0 | 0 | 0 | 0 | 0 | 0 | 1321 | 1336 |  |  | PEAKS DB |
| K.QLDIFVHDFPR.K | N | 79.15 | 1385.7091 | 11 | 0.0 | 693.8618 | 2 | 58.44 | 6 | F6:36683 | NaSa01\_F14.raw |  |  |  |  | 9.0136E6 | 8.647E7 |  |  |  |  |  |  |  |  | 4 | 0 | 0 | 0 | 0 | 2 | 2 | 0 | 0 | 0 | 0 | 0 | 0 | 0 | 0 | 53 | 63 |  |  | PEAKS DB |
| K.GDNLIQMPGAAMK.I | Y | 78.60 | 1344.6530 | 13 | 0.2 | 673.3339 | 2 | 49.26 | 6 | F6:28453 | NaSa01\_F14.raw |  |  |  |  |  | 1.0626E8 |  |  |  |  |  |  |  |  | 2 | 0 | 0 | 0 | 0 | 0 | 2 | 0 | 0 | 0 | 0 | 0 | 0 | 0 | 0 | 552 | 564 |  |  | PEAKS DB |
| F.VIVPLEQGLHDVEIK.A | Y | 76.27 | 1687.9508 | 15 | 0.0 | 844.9827 | 2 | 58.08 | 6 | F6:36376 | NaSa01\_F14.raw |  |  |  |  | 5.5586E6 | 9.3853E7 |  |  |  |  |  |  |  |  | 3 | 0 | 0 | 0 | 0 | 1 | 2 | 0 | 0 | 0 | 0 | 0 | 0 | 0 | 0 | 883 | 897 |  |  | PEAKS DB |
| R.DDNEDGFIADSDIISR.S | Y | 75.58 | 1780.7751 | 16 | 0.3 | 891.3951 | 2 | 68.40 | 6 | F6:44898 | NaSa01\_F14.raw |  |  |  |  | 9.0776E5 | 1.2244E7 |  |  |  |  |  |  |  |  | 4 | 0 | 0 | 0 | 0 | 1 | 3 | 0 | 0 | 0 | 0 | 0 | 0 | 0 | 0 | 733 | 748 |  |  | PEAKS DB |
| K.C(+57.02)C(+57.02)EDVMHENPMGYTC(+57.02)EK.R | Y | 74.09 | 2158.7886 | 17 | 7.4 | 720.6050 | 3 | 31.06 | 8 | F8:16109 | NaSa01\_F3.raw |  |  |  |  |  |  |  | 1.0485E7 | 5.6652E7 | 1.4617E7 |  |  |  |  | 6 | 0 | 0 | 0 | 0 | 0 | 0 | 0 | 1 | 3 | 2 | 0 | 0 | 0 | 0 | 677 | 693 | Carbamidomethylation | C1:Carbamidomethylation:1000.00;C2:Carbamidomethylation:1000.00;C15:Carbamidomethylation:1000.00 | PEAKS DB |
| R.INYENALLAR.T | Y | 74.07 | 1175.6299 | 10 | 0.1 | 588.8223 | 2 | 45.12 | 6 | F6:25480 | NaSa01\_F14.raw |  |  | 3.3741E5 | 7.6601E6 | 1.5972E7 | 1.5972E8 |  |  |  |  |  |  |  |  | 7 | 0 | 0 | 1 | 3 | 1 | 2 | 0 | 0 | 0 | 0 | 0 | 0 | 0 | 0 | 1292 | 1301 |  |  | PEAKS DB |
| Y.YQVGNNEIVADSVWVDVK.D | N | 73.99 | 2034.0057 | 18 | 0.2 | 1018.0104 | 2 | 70.65 | 6 | F6:46860 | NaSa01\_F14.raw |  |  |  |  |  | 6.5239E6 |  |  |  |  |  |  |  |  | 1 | 0 | 0 | 0 | 0 | 0 | 1 | 0 | 0 | 0 | 0 | 0 | 0 | 0 | 0 | 524 | 541 |  |  | PEAKS DB |
| K.ASVQEALWSDGVR.K | Y | 72.98 | 1416.6997 | 13 | 0.1 | 709.3572 | 2 | 49.71 | 6 | F6:29208 | NaSa01\_F14.raw |  |  |  |  | 1.3438E7 | 1.2825E8 |  |  |  |  |  |  |  |  | 7 | 0 | 0 | 0 | 0 | 1 | 6 | 0 | 0 | 0 | 0 | 0 | 0 | 0 | 0 | 898 | 910 |  |  | PEAKS DB |
| R.VELLYNPAFC(+57.02)SASTK.G | Y | 72.57 | 1698.8286 | 15 | -0.7 | 850.4210 | 2 | 59.22 | 6 | F6:37110 | NaSa01\_F14.raw |  |  |  |  | 1.6821E7 | 2.15E8 |  |  |  |  |  |  |  |  | 2 | 0 | 0 | 0 | 0 | 1 | 1 | 0 | 0 | 0 | 0 | 0 | 0 | 0 | 0 | 848 | 862 | Carbamidomethylation | C10:Carbamidomethylation:1000.00 | PEAKS DB |
| K.LNQDITVTASGDGK.A | Y | 72.09 | 1417.7048 | 14 | 0.8 | 709.8602 | 2 | 20.83 | 6 | F6:5302 | NaSa01\_F14.raw |  |  |  |  | 4.3744E5 | 4.5631E6 |  |  |  |  |  |  |  |  | 3 | 0 | 0 | 0 | 0 | 1 | 2 | 0 | 0 | 0 | 0 | 0 | 0 | 0 | 0 | 1307 | 1320 |  |  | PEAKS DB |
| K.LILNIPLNAQSLPITVR.T | Y | 71.60 | 1874.1353 | 17 | 0.5 | 625.7194 | 3 | 90.36 | 6 | F6:63322 | NaSa01\_F14.raw |  |  |  |  | 1.4111E7 | 4.901E7 |  |  |  |  |  |  |  |  | 4 | 0 | 0 | 0 | 0 | 2 | 2 | 0 | 0 | 0 | 0 | 0 | 0 | 0 | 0 | 398 | 414 |  |  | PEAKS DB |
| K.YFTYLILNK.G | N | 70.49 | 1173.6433 | 9 | 0.1 | 587.8290 | 2 | 64.67 | 6 | F6:41809 | NaSa01\_F14.raw |  |  |  |  | 1.0728E7 | 7.7707E7 |  |  |  |  |  |  |  |  | 2 | 0 | 0 | 0 | 0 | 1 | 1 | 0 | 0 | 0 | 0 | 0 | 0 | 0 | 0 | 478 | 486 |  |  | PEAKS DB |
| R.WPHEDEC(+57.02)QEEEFQK.L | Y | 69.62 | 1889.7526 | 14 | 0.9 | 945.8844 | 2 | 23.39 | 4 | F4:9200 | NaSa01\_F12.raw |  |  |  | 2.8919E7 |  | 3.166E6 |  |  |  |  |  |  |  |  | 3 | 0 | 0 | 0 | 2 | 0 | 1 | 0 | 0 | 0 | 0 | 0 | 0 | 0 | 0 | 1610 | 1623 | Carbamidomethylation | C7:Carbamidomethylation:1000.00 | PEAKS DB |
| K.SDFGC(+57.02)TAGSGQNNLGVFEDAGLALTTSTNLNTK.Q | N | 66.95 | 3359.5576 | 33 | 0.8 | 1120.8607 | 3 | 89.37 | 6 | F6:62425 | NaSa01\_F14.raw |  |  |  |  |  | 3.8512E7 |  |  |  |  |  |  |  |  | 1 | 0 | 0 | 0 | 0 | 0 | 1 | 0 | 0 | 0 | 0 | 0 | 0 | 0 | 0 | 605 | 637 | Carbamidomethylation | C5:Carbamidomethylation:1000.00 | PEAKS DB |
| R.DGQNLVTM(+15.99)NLHITPDLIPSFR.F | N | 66.34 | 2396.2158 | 21 | 0.4 | 799.7462 | 3 | 83.99 | 6 | F6:57985 | NaSa01\_F14.raw |  |  |  |  |  | 5.9574E6 |  |  |  |  |  |  |  |  | 1 | 0 | 0 | 0 | 0 | 0 | 1 | 0 | 0 | 0 | 0 | 0 | 0 | 0 | 0 | 499 | 519 | Oxidation (M) | M8:Oxidation (M):1000.00 | PEAKS DB |
| L.ARDDNEDGFIADSDIISR.S | Y | 66.31 | 2007.9133 | 18 | 1.0 | 670.3124 | 3 | 48.95 | 6 | F6:28381 | NaSa01\_F14.raw |  |  |  |  |  | 6.6726E6 |  |  |  |  |  |  |  |  | 2 | 0 | 0 | 0 | 0 | 0 | 2 | 0 | 0 | 0 | 0 | 0 | 0 | 0 | 0 | 731 | 748 |  |  | PEAKS DB |
| Y.VTNPDGSPAAHVPVVSEAFHSMGTTLSDGTAK.L | Y | 66.31 | 3179.5193 | 32 | 0.8 | 795.8877 | 4 | 59.12 | 6 | F6:37224 | NaSa01\_F14.raw |  |  |  |  |  | 2.3066E7 |  |  |  |  |  |  |  |  | 1 | 0 | 0 | 0 | 0 | 0 | 1 | 0 | 0 | 0 | 0 | 0 | 0 | 0 | 0 | 366 | 397 |  |  | PEAKS DB |
| K.C(+57.02)C(+57.02)EDVMHENPM(+15.99)GYTC(+57.02)EK.R | Y | 65.55 | 2174.7837 | 17 | 1.4 | 725.9362 | 3 | 11.70 | 9 | F9:2004 | NaSa01\_F4.raw |  |  |  |  |  |  |  |  | 1.0934E7 |  |  |  |  |  | 2 | 0 | 0 | 0 | 0 | 0 | 0 | 0 | 0 | 2 | 0 | 0 | 0 | 0 | 0 | 677 | 693 | Carbamidomethylation; Oxidation (M) | C1:Carbamidomethylation:1000.00;C2:Carbamidomethylation:1000.00;M11:Oxidation (M):43.99;C15:Carbamidomethylation:1000.00 | PEAKS DB |
| Y.NLPDLVSLGTWR.I | Y | 65.12 | 1369.7354 | 12 | 0.1 | 685.8750 | 2 | 81.18 | 6 | F6:55452 | NaSa01\_F14.raw |  |  |  |  | 6.3971E6 | 8.6753E7 |  |  |  |  |  |  |  |  | 2 | 0 | 0 | 0 | 0 | 1 | 1 | 0 | 0 | 0 | 0 | 0 | 0 | 0 | 0 | 187 | 198 |  |  | PEAKS DB |
| K.GDNLIQMPGAAM(+15.99)K.I | Y | 64.58 | 1360.6479 | 13 | 0.8 | 681.3318 | 2 | 38.53 | 6 | F6:19473 | NaSa01\_F14.raw |  |  |  |  | 9.6097E5 | 9.5549E6 |  |  |  |  |  |  |  |  | 3 | 0 | 0 | 0 | 0 | 1 | 2 | 0 | 0 | 0 | 0 | 0 | 0 | 0 | 0 | 552 | 564 | Oxidation (M) | M12:Oxidation (M):38.33 | PEAKS DB |
| E.VEGVAFVLFGVK.I | N | 62.96 | 1263.7227 | 12 | 0.2 | 632.8687 | 2 | 85.00 | 6 | F6:58754 | NaSa01\_F14.raw |  |  |  |  |  | 2.0835E6 |  |  |  |  |  |  |  |  | 1 | 0 | 0 | 0 | 0 | 0 | 1 | 0 | 0 | 0 | 0 | 0 | 0 | 0 | 0 | 257 | 268 |  |  | PEAKS DB |
| Y.QSSFLFIQTDK.G | Y | 62.43 | 1312.6663 | 11 | -0.4 | 657.3401 | 2 | 57.57 | 6 | F6:35846 | NaSa01\_F14.raw |  |  |  |  |  | 1.1659E7 |  |  |  |  |  |  |  |  | 1 | 0 | 0 | 0 | 0 | 0 | 1 | 0 | 0 | 0 | 0 | 0 | 0 | 0 | 0 | 124 | 134 |  |  | PEAKS DB |
| K.GVGGTQLEVIK.A | Y | 62.08 | 1099.6237 | 11 | 0.2 | 550.8192 | 2 | 30.32 | 12 | F12:15497 | NaSa01\_F7.raw |  |  |  |  | 1.4118E7 | 1.0253E6 |  |  |  |  |  | 1.7364E6 |  |  | 3 | 0 | 0 | 0 | 0 | 1 | 1 | 0 | 0 | 0 | 0 | 0 | 1 | 0 | 0 | 936 | 946 |  |  | PEAKS DB |
| K.ATM(+15.99)TILTFYNAQLQEK.A | Y | 62.07 | 1886.9448 | 16 | 0.4 | 944.4801 | 2 | 64.84 | 6 | F6:42088 | NaSa01\_F14.raw |  |  |  |  |  | 1.0319E7 |  |  |  |  |  |  |  |  | 2 | 0 | 0 | 0 | 0 | 0 | 2 | 0 | 0 | 0 | 0 | 0 | 0 | 0 | 0 | 1321 | 1336 | Oxidation (M) | M3:Oxidation (M):1000.00 | PEAKS DB |
| R.FPNLNELVGH.T | N | 62.05 | 1138.5770 | 10 | 0.8 | 570.2963 | 2 | 67.53 | 6 | F6:44045 | NaSa01\_F14.raw |  |  |  |  | 1.7762E6 | 2.8185E7 |  |  |  |  |  |  |  |  | 2 | 0 | 0 | 0 | 0 | 1 | 1 | 0 | 0 | 0 | 0 | 0 | 0 | 0 | 0 | 303 | 312 |  |  | PEAKS DB |
| K.VYSYYNLDEK.C | N | 61.93 | 1292.5924 | 10 | 1.2 | 647.3043 | 2 | 33.77 | 6 | F6:15693 | NaSa01\_F14.raw |  |  |  |  | 1.5304E7 | 1.0252E8 |  |  |  |  |  |  |  |  | 3 | 0 | 0 | 0 | 0 | 1 | 2 | 0 | 0 | 0 | 0 | 0 | 0 | 0 | 0 | 1458 | 1467 |  |  | PEAKS DB |
| M.TILTFYNAQLQEK.A | N | 61.57 | 1567.8246 | 13 | 0.3 | 784.9198 | 2 | 60.05 | 6 | F6:38047 | NaSa01\_F14.raw |  |  |  |  |  | 5.1346E6 |  |  |  |  |  |  |  |  | 1 | 0 | 0 | 0 | 0 | 0 | 1 | 0 | 0 | 0 | 0 | 0 | 0 | 0 | 0 | 1324 | 1336 |  |  | PEAKS DB |
| H.ITPDLIPSFR.F | N | 61.36 | 1157.6444 | 10 | 0.0 | 579.8295 | 2 | 65.01 | 6 | F6:42335 | NaSa01\_F14.raw |  |  |  |  | 3.0601E6 | 3.8923E7 |  |  |  |  |  |  |  |  | 3 | 0 | 0 | 0 | 0 | 1 | 2 | 0 | 0 | 0 | 0 | 0 | 0 | 0 | 0 | 510 | 519 |  |  | PEAKS DB |
| K.PGDNLPVNFNVK.G | Y | 60.28 | 1312.6775 | 12 | 0.4 | 657.3463 | 2 | 47.72 | 6 | F6:27452 | NaSa01\_F14.raw |  |  |  |  |  | 1.0823E6 |  |  |  |  |  |  |  |  | 1 | 0 | 0 | 0 | 0 | 0 | 1 | 0 | 0 | 0 | 0 | 0 | 0 | 0 | 0 | 456 | 467 |  |  | PEAKS DB |
| W.VVLAVSFTPTK.G | Y | 59.98 | 1160.6804 | 11 | 0.4 | 581.3477 | 2 | 51.95 | 6 | F6:30874 | NaSa01\_F14.raw |  |  |  |  |  | 3.7062E6 |  |  |  |  |  |  |  |  | 1 | 0 | 0 | 0 | 0 | 0 | 1 | 0 | 0 | 0 | 0 | 0 | 0 | 0 | 0 | 788 | 798 |  |  | PEAKS DB |
| K.YEHSPENYTAYFDVR.K | N | 59.87 | 1889.8220 | 15 | -0.9 | 630.9474 | 3 | 47.19 | 6 | F6:27480 | NaSa01\_F14.raw |  |  |  |  |  | 2.5484E6 |  |  |  |  |  |  |  |  | 2 | 0 | 0 | 0 | 0 | 0 | 2 | 0 | 0 | 0 | 0 | 0 | 0 | 0 | 0 | 203 | 217 |  |  | PEAKS DB |
| R.IDVPLQIEK.A | Y | 59.34 | 1053.6069 | 9 | 0.1 | 527.8108 | 2 | 47.86 | 6 | F6:27228 | NaSa01\_F14.raw |  |  |  | 3.0425E7 | 1.9664E7 | 1.6873E8 |  |  |  |  |  |  |  |  | 4 | 0 | 0 | 0 | 1 | 1 | 2 | 0 | 0 | 0 | 0 | 0 | 0 | 0 | 0 | 1506 | 1514 |  |  | PEAKS DB |
| K.IIIQGDPVAQIIENSIDGSK.L | Y | 59.31 | 2109.1316 | 20 | 0.6 | 1055.5737 | 2 | 96.62 | 6 | F6:68548 | NaSa01\_F14.raw |  |  | 1.4465E5 |  | 1.6001E5 | 2.5079E6 |  |  |  |  |  |  |  |  | 3 | 0 | 0 | 1 | 0 | 1 | 1 | 0 | 0 | 0 | 0 | 0 | 0 | 0 | 0 | 963 | 982 |  |  | PEAKS DB |
| K.GIC(+57.02)VAEPYEIR.V | Y | 59.06 | 1305.6387 | 11 | 0.4 | 653.8268 | 2 | 44.31 | 6 | F6:24643 | NaSa01\_F14.raw |  |  |  |  | 1.0302E7 | 1.0808E8 |  |  |  |  |  |  |  |  | 3 | 0 | 0 | 0 | 0 | 1 | 2 | 0 | 0 | 0 | 0 | 0 | 0 | 0 | 0 | 799 | 809 | Carbamidomethylation | C3:Carbamidomethylation:1000.00 | PEAKS DB |
| E.DGFIADSDIISR.S | Y | 58.62 | 1307.6357 | 12 | -0.1 | 654.8251 | 2 | 60.77 | 6 | F6:38594 | NaSa01\_F14.raw |  |  |  |  |  | 4.7593E6 |  |  |  |  |  |  |  |  | 1 | 0 | 0 | 0 | 0 | 0 | 1 | 0 | 0 | 0 | 0 | 0 | 0 | 0 | 0 | 737 | 748 |  |  | PEAKS DB |
| R.FPNLNELVGHTLY.A | N | 58.47 | 1515.7721 | 13 | 0.3 | 758.8936 | 2 | 85.62 | 6 | F6:59205 | NaSa01\_F14.raw |  |  |  |  |  | 1.9527E7 |  |  |  |  |  |  |  |  | 1 | 0 | 0 | 0 | 0 | 0 | 1 | 0 | 0 | 0 | 0 | 0 | 0 | 0 | 0 | 303 | 315 |  |  | PEAKS DB |
| S.MLTGFLPDAEDLTR.L | N | 58.03 | 1577.7759 | 14 | 0.5 | 789.8956 | 2 | 78.56 | 6 | F6:53520 | NaSa01\_F14.raw |  |  |  |  |  | 2.8623E6 |  |  |  |  |  |  |  |  | 1 | 0 | 0 | 0 | 0 | 0 | 1 | 0 | 0 | 0 | 0 | 0 | 0 | 0 | 0 | 1386 | 1399 |  |  | PEAKS DB |
| R.KYVLPSFEVR.L | Y | 56.74 | 1236.6866 | 10 | 0.3 | 413.2363 | 3 | 42.11 | 6 | F6:22829 | NaSa01\_F14.raw |  |  |  |  | 5.509E6 | 4.9733E7 |  |  |  |  |  |  |  |  | 6 | 0 | 0 | 0 | 0 | 2 | 4 | 0 | 0 | 0 | 0 | 0 | 0 | 0 | 0 | 218 | 227 |  |  | PEAKS DB |
| R.DGQNLVTMNLHITPDLIPSFR.F | N | 56.46 | 2380.2209 | 21 | 0.5 | 794.4147 | 3 | 91.58 | 6 | F6:64345 | NaSa01\_F14.raw |  |  |  |  |  | 2.1315E6 |  |  |  |  |  |  |  |  | 1 | 0 | 0 | 0 | 0 | 0 | 1 | 0 | 0 | 0 | 0 | 0 | 0 | 0 | 0 | 499 | 519 |  |  | PEAKS DB |
| K.VAVIIYLNK.V | Y | 56.21 | 1031.6379 | 9 | 0.8 | 516.8267 | 2 | 50.20 | 6 | F6:30133 | NaSa01\_F14.raw |  |  |  |  |  | 1.67E7 |  |  |  |  |  |  |  |  | 2 | 0 | 0 | 0 | 0 | 0 | 2 | 0 | 0 | 0 | 0 | 0 | 0 | 0 | 0 | 1421 | 1429 |  |  | PEAKS DB |
| G.ALYTLITPAVLR.T | N | 55.82 | 1329.8020 | 12 | 0.4 | 665.9086 | 2 | 75.95 | 6 | F6:51300 | NaSa01\_F14.raw |  |  |  | 1.7564E6 | 1.5853E7 | 2.019E8 |  |  |  |  |  |  |  |  | 6 | 0 | 0 | 0 | 1 | 1 | 4 | 0 | 0 | 0 | 0 | 0 | 0 | 0 | 0 | 23 | 34 |  |  | PEAKS DB |
| N.LHITPDLIPSFR.F | N | 52.36 | 1407.7874 | 12 | 0.4 | 470.2699 | 3 | 64.03 | 6 | F6:41508 | NaSa01\_F14.raw |  |  |  |  |  | 2.1751E7 |  |  |  |  |  |  |  |  | 2 | 0 | 0 | 0 | 0 | 0 | 2 | 0 | 0 | 0 | 0 | 0 | 0 | 0 | 0 | 508 | 519 |  |  | PEAKS DB |
| K.YVLPSFEVR.L | N | 51.77 | 1108.5917 | 9 | 0.6 | 555.3035 | 2 | 58.44 | 6 | F6:36495 | NaSa01\_F14.raw |  |  |  | 4.1589E5 | 1.253E7 | 1.3997E8 |  |  |  |  |  |  |  |  | 4 | 0 | 0 | 0 | 1 | 1 | 2 | 0 | 0 | 0 | 0 | 0 | 0 | 0 | 0 | 219 | 227 |  |  | PEAKS DB |
| K.TMSFYLR.D | Y | 51.69 | 916.4476 | 7 | 0.3 | 459.2312 | 2 | 41.67 | 6 | F6:22840 | NaSa01\_F14.raw |  |  |  |  |  | 5.6816E7 |  |  |  |  |  |  |  |  | 1 | 0 | 0 | 0 | 0 | 0 | 1 | 0 | 0 | 0 | 0 | 0 | 0 | 0 | 0 | 775 | 781 |  |  | PEAKS DB |
| R.DGQNLVTMNLH.I | N | 51.62 | 1240.5870 | 11 | 0.0 | 621.3008 | 2 | 49.19 | 6 | F6:28557 | NaSa01\_F14.raw |  |  |  |  |  | 4.925E6 |  |  |  |  |  |  |  |  | 1 | 0 | 0 | 0 | 0 | 0 | 1 | 0 | 0 | 0 | 0 | 0 | 0 | 0 | 0 | 499 | 509 |  |  | PEAKS DB |
| L.NIPLNAQSLPITVR.T | Y | 50.97 | 1534.8831 | 14 | -1.4 | 768.4477 | 2 | 61.34 | 6 | F6:39039 | NaSa01\_F14.raw |  |  |  |  |  | 1.6927E5 |  |  |  |  |  |  |  |  | 1 | 0 | 0 | 0 | 0 | 0 | 1 | 0 | 0 | 0 | 0 | 0 | 0 | 0 | 0 | 401 | 414 |  |  | PEAKS DB |
| K.C(+57.02)C(+57.02)EDVM(+15.99)HENPM(+15.99)GYTC(+57.02)EK.R | Y | 50.02 | 2190.7786 | 17 | 0.7 | 731.2673 | 3 | 11.49 | 9 | F9:1837 | NaSa01\_F4.raw |  |  |  |  |  |  |  |  | 4.1662E5 |  |  |  |  |  | 1 | 0 | 0 | 0 | 0 | 0 | 0 | 0 | 0 | 1 | 0 | 0 | 0 | 0 | 0 | 677 | 693 | Carbamidomethylation; Oxidation (M) | C1:Carbamidomethylation:1000.00;C2:Carbamidomethylation:1000.00;M6:Oxidation (M):1000.00;M11:Oxidation (M):1000.00;C15:Carbamidomethylation:1000.00 | PEAKS DB |
| K.GIYTPGSPVLYR.V | N | 49.98 | 1321.7030 | 12 | -8.2 | 661.8533 | 2 | 47.56 | 6 | F6:27271 | NaSa01\_F14.raw |  |  |  |  |  | 4.6433E6 |  |  |  |  |  |  |  |  | 1 | 0 | 0 | 0 | 0 | 0 | 1 | 0 | 0 | 0 | 0 | 0 | 0 | 0 | 0 | 135 | 146 |  |  | PEAKS DB |
| K.AC(+57.02)ETNVDYVYK.T | Y | 49.20 | 1360.5969 | 11 | 0.3 | 681.3060 | 2 | 22.05 | 6 | F6:6230 | NaSa01\_F14.raw |  |  |  | 2.4601E5 |  | 5.6943E5 |  |  |  |  |  |  |  |  | 2 | 0 | 0 | 0 | 1 | 0 | 1 | 0 | 0 | 0 | 0 | 0 | 0 | 0 | 0 | 1515 | 1525 | Carbamidomethylation | C2:Carbamidomethylation:1000.00 | PEAKS DB |
| M.LTGFLPDAEDLTR.L | N | 47.16 | 1446.7354 | 13 | 1.2 | 724.3758 | 2 | 69.30 | 6 | F6:45774 | NaSa01\_F14.raw |  |  |  |  |  | 7.2495E5 |  |  |  |  |  |  |  |  | 1 | 0 | 0 | 0 | 0 | 0 | 1 | 0 | 0 | 0 | 0 | 0 | 0 | 0 | 0 | 1387 | 1399 |  |  | PEAKS DB |
| R.VGLVAVDKAVYVLNDK.Y | N | 46.90 | 1701.9664 | 16 | 0.8 | 568.3299 | 3 | 57.39 | 6 | F6:35573 | NaSa01\_F14.raw |  |  |  |  |  | 1.8722E5 |  |  |  |  |  |  |  |  | 1 | 0 | 0 | 0 | 0 | 0 | 1 | 0 | 0 | 0 | 0 | 0 | 0 | 0 | 0 | 575 | 590 |  |  | PEAKS DB |
| K.THQYISQR.K | Y | 46.29 | 1031.5149 | 8 | 0.4 | 516.7650 | 2 | 11.44 | 6 | F6:1825 | NaSa01\_F14.raw |  |  |  |  |  | 1.3503E5 |  |  |  |  |  |  |  |  | 1 | 0 | 0 | 0 | 0 | 0 | 1 | 0 | 0 | 0 | 0 | 0 | 0 | 0 | 0 | 1560 | 1567 |  |  | PEAKS DB |
| D.DNEDGFIADSDIISR.S | Y | 46.28 | 1665.7482 | 15 | 0.7 | 833.8820 | 2 | 65.65 | 6 | F6:42702 | NaSa01\_F14.raw |  |  |  |  |  | 2.6489E5 |  |  |  |  |  |  |  |  | 1 | 0 | 0 | 0 | 0 | 0 | 1 | 0 | 0 | 0 | 0 | 0 | 0 | 0 | 0 | 734 | 748 |  |  | PEAKS DB |
| H.VAITSTEIKPGDNLPVNFNVK.G | Y | 46.15 | 2255.2161 | 21 | 0.2 | 752.7461 | 3 | 57.73 | 6 | F6:36049 | NaSa01\_F14.raw |  |  |  |  |  | 3.3349E6 |  |  |  |  |  |  |  |  | 1 | 0 | 0 | 0 | 0 | 0 | 1 | 0 | 0 | 0 | 0 | 0 | 0 | 0 | 0 | 447 | 467 |  |  | PEAKS DB |
| K.IIIQGDPVAQIIEN.S | Y | 45.71 | 1521.8402 | 14 | 0.0 | 761.9274 | 2 | 85.63 | 6 | F6:59337 | NaSa01\_F14.raw |  |  |  |  |  | 1.4516E7 |  |  |  |  |  |  |  |  | 1 | 0 | 0 | 0 | 0 | 0 | 1 | 0 | 0 | 0 | 0 | 0 | 0 | 0 | 0 | 963 | 976 |  |  | PEAKS DB |
| K.TM(+15.99)SFYLR.D | Y | 45.45 | 932.4426 | 7 | 0.2 | 467.2286 | 2 | 32.98 | 6 | F6:14848 | NaSa01\_F14.raw |  |  |  |  |  | 1.994E6 |  |  |  |  |  |  |  |  | 2 | 0 | 0 | 0 | 0 | 0 | 2 | 0 | 0 | 0 | 0 | 0 | 0 | 0 | 0 | 775 | 781 | Oxidation (M) | M2:Oxidation (M):1000.00 | PEAKS DB |
| N.DIYVMDVLEVIK.Q | N | 45.17 | 1435.7632 | 12 | 0.7 | 718.8894 | 2 | 106.25 | 4 | F4:76997 | NaSa01\_F12.raw |  |  |  | 2.8255E5 |  |  |  |  |  |  |  |  |  |  | 1 | 0 | 0 | 0 | 1 | 0 | 0 | 0 | 0 | 0 | 0 | 0 | 0 | 0 | 0 | 1538 | 1549 |  |  | PEAKS DB |
| K.YFKPGMPYELTVY.V | N | 44.78 | 1606.7742 | 13 | 0.4 | 804.3947 | 2 | 71.51 | 6 | F6:47852 | NaSa01\_F14.raw |  |  |  |  |  | 1.8453E7 |  |  |  |  |  |  |  |  | 1 | 0 | 0 | 0 | 0 | 0 | 1 | 0 | 0 | 0 | 0 | 0 | 0 | 0 | 0 | 353 | 365 |  |  | PEAKS DB |
| R.SRFPNLNELVGH.T | N | 44.24 | 1381.7102 | 12 | 0.6 | 461.5776 | 3 | 50.79 | 6 | F6:30122 | NaSa01\_F14.raw |  |  |  |  |  | 1.9009E7 |  |  |  |  |  |  |  |  | 3 | 0 | 0 | 0 | 0 | 0 | 3 | 0 | 0 | 0 | 0 | 0 | 0 | 0 | 0 | 301 | 312 |  |  | PEAKS DB |
| K.TVIVEFQTPEG.I | N | 43.94 | 1218.6132 | 11 | 3.8 | 610.3144 | 2 | 63.73 | 7 | F7:45532 | NaSa01\_F2.raw |  |  |  |  |  |  | 6.7633E6 | 2.6291E6 |  |  |  |  |  |  | 2 | 0 | 0 | 0 | 0 | 0 | 0 | 1 | 1 | 0 | 0 | 0 | 0 | 0 | 0 | 160 | 170 |  |  | PEAKS DB |
| M.NLHITPDLIPSFR.F | N | 43.77 | 1521.8303 | 13 | 0.6 | 508.2843 | 3 | 65.19 | 6 | F6:42284 | NaSa01\_F14.raw |  |  |  |  |  | 1.1088E7 |  |  |  |  |  |  |  |  | 2 | 0 | 0 | 0 | 0 | 0 | 2 | 0 | 0 | 0 | 0 | 0 | 0 | 0 | 0 | 507 | 519 |  |  | PEAKS DB |
| K.AAFLEC(+57.02)C(+57.02)R.Y | N | 43.14 | 1025.4423 | 8 | 0.7 | 513.7288 | 2 | 11.72 | 9 | F9:2016 | NaSa01\_F4.raw |  |  |  |  |  |  |  |  | 5.2365E5 |  |  |  |  |  | 1 | 0 | 0 | 0 | 0 | 0 | 0 | 0 | 0 | 1 | 0 | 0 | 0 | 0 | 0 | 706 | 713 | Carbamidomethylation | C6:Carbamidomethylation:1000.00;C7:Carbamidomethylation:1000.00 | PEAKS DB |
| L.SYQSSFLFIQTDK.G | Y | 42.37 | 1562.7616 | 13 | 1.2 | 782.3890 | 2 | 64.49 | 6 | F6:41739 | NaSa01\_F14.raw |  |  |  |  |  | 3.4858E5 |  |  |  |  |  |  |  |  | 1 | 0 | 0 | 0 | 0 | 0 | 1 | 0 | 0 | 0 | 0 | 0 | 0 | 0 | 0 | 122 | 134 |  |  | PEAKS DB |
| R.KC(+57.02)C(+57.02)EDVMHENPMGYTC(+57.02)EK.R | Y | 42.21 | 2286.8835 | 18 | 1.4 | 763.3029 | 3 | 20.88 | 10 | F10:7153 | NaSa01\_F5.raw |  |  |  |  |  |  |  |  | 7.1178E5 | 3.5368E6 |  |  |  |  | 3 | 0 | 0 | 0 | 0 | 0 | 0 | 0 | 0 | 1 | 2 | 0 | 0 | 0 | 0 | 676 | 693 | Carbamidomethylation | C2:Carbamidomethylation:1000.00;C3:Carbamidomethylation:1000.00;C16:Carbamidomethylation:1000.00 | PEAKS DB |
| R.IPIIDGDGK.A | Y | 42.15 | 926.5073 | 9 | 0.6 | 464.2612 | 2 | 32.92 | 6 | F6:14878 | NaSa01\_F14.raw |  |  |  |  |  | 3.4884E5 |  |  |  |  |  |  |  |  | 1 | 0 | 0 | 0 | 0 | 0 | 1 | 0 | 0 | 0 | 0 | 0 | 0 | 0 | 0 | 283 | 291 |  |  | PEAKS DB |
| Y.VLPSFEVR.L | N | 41.89 | 945.5283 | 8 | -0.2 | 473.7714 | 2 | 40.35 | 6 | F6:20803 | NaSa01\_F14.raw |  |  |  |  |  | 5.7172E5 |  |  |  |  |  |  |  |  | 1 | 0 | 0 | 0 | 0 | 0 | 1 | 0 | 0 | 0 | 0 | 0 | 0 | 0 | 0 | 220 | 227 |  |  | PEAKS DB |
| K.IC(+57.02)IGNVC(+57.02)R.C | Y | 41.79 | 990.4739 | 8 | -1.3 | 496.2436 | 2 | 11.58 | 6 | F6:1976 | NaSa01\_F14.raw |  |  |  |  |  | 2.2107E5 |  |  |  |  |  |  |  |  | 1 | 0 | 0 | 0 | 0 | 0 | 1 | 0 | 0 | 0 | 0 | 0 | 0 | 0 | 0 | 1484 | 1491 | Carbamidomethylation | C2:Carbamidomethylation:1000.00;C7:Carbamidomethylation:1000.00 | PEAKS DB |
| R.SRFPNLNELVGHTLY.A | N | 40.90 | 1758.9053 | 15 | 0.7 | 587.3094 | 3 | 70.95 | 6 | F6:46924 | NaSa01\_F14.raw |  |  |  |  |  | 5.6962E6 |  |  |  |  |  |  |  |  | 1 | 0 | 0 | 0 | 0 | 0 | 1 | 0 | 0 | 0 | 0 | 0 | 0 | 0 | 0 | 301 | 315 |  |  | PEAKS DB |
| Y.TLITPAVLR.T | N | 40.29 | 982.6175 | 9 | 0.4 | 492.3162 | 2 | 45.78 | 6 | F6:25572 | NaSa01\_F14.raw |  |  |  |  |  | 5.8349E7 |  |  |  |  |  |  |  |  | 1 | 0 | 0 | 0 | 0 | 0 | 1 | 0 | 0 | 0 | 0 | 0 | 0 | 0 | 0 | 26 | 34 |  |  | PEAKS DB |
| K.LILNIPLNAQ.S | Y | 40.28 | 1107.6652 | 10 | -0.2 | 554.8397 | 2 | 84.63 | 6 | F6:58512 | NaSa01\_F14.raw |  |  |  |  |  | 3.2621E6 |  |  |  |  |  |  |  |  | 1 | 0 | 0 | 0 | 0 | 0 | 1 | 0 | 0 | 0 | 0 | 0 | 0 | 0 | 0 | 398 | 407 |  |  | PEAKS DB |
| K.IIIQGDPVAQI.I | Y | 39.99 | 1165.6707 | 11 | 0.7 | 583.8430 | 2 | 72.27 | 6 | F6:48414 | NaSa01\_F14.raw |  |  |  |  |  | 1.2067E6 |  |  |  |  |  |  |  |  | 1 | 0 | 0 | 0 | 0 | 0 | 1 | 0 | 0 | 0 | 0 | 0 | 0 | 0 | 0 | 963 | 973 |  |  | PEAKS DB |
| Y.QVGNNEIVADSVWVDVK.D | N | 39.98 | 1870.9425 | 17 | 0.5 | 936.4790 | 2 | 64.84 | 6 | F6:41917 | NaSa01\_F14.raw |  |  |  |  |  | 1.8564E6 |  |  |  |  |  |  |  |  | 1 | 0 | 0 | 0 | 0 | 0 | 1 | 0 | 0 | 0 | 0 | 0 | 0 | 0 | 0 | 525 | 541 |  |  | PEAKS DB |
| N.IPLNAQSLPITVR.T | Y | 39.95 | 1420.8402 | 13 | 0.7 | 711.4279 | 2 | 58.51 | 6 | F6:36546 | NaSa01\_F14.raw |  |  |  |  |  | 5.2985E5 |  |  |  |  |  |  |  |  | 1 | 0 | 0 | 0 | 0 | 0 | 1 | 0 | 0 | 0 | 0 | 0 | 0 | 0 | 0 | 402 | 414 |  |  | PEAKS DB |
| K.SWLWLTK.D | Y | 39.91 | 932.5120 | 7 | 0.3 | 467.2634 | 2 | 68.87 | 6 | F6:45829 | NaSa01\_F14.raw |  |  |  |  |  | 5.8771E7 |  |  |  |  |  |  |  |  | 1 | 0 | 0 | 0 | 0 | 0 | 1 | 0 | 0 | 0 | 0 | 0 | 0 | 0 | 0 | 754 | 760 |  |  | PEAKS DB |
| K.IIIQGDPVAQ.I | Y | 39.88 | 1052.5865 | 10 | 0.0 | 527.3005 | 2 | 48.47 | 6 | F6:28020 | NaSa01\_F14.raw |  |  |  |  |  | 3.3932E7 |  |  |  |  |  |  |  |  | 1 | 0 | 0 | 0 | 0 | 0 | 1 | 0 | 0 | 0 | 0 | 0 | 0 | 0 | 0 | 963 | 972 |  |  | PEAKS DB |
| R.IEEQDGNDIYVM(+15.99)DVLEVIK.Q | Y | 39.47 | 2237.0774 | 19 | 1.1 | 1119.5472 | 2 | 90.84 | 4 | F4:65053 | NaSa01\_F12.raw |  |  |  | 8.9297E5 |  |  |  |  |  |  |  |  |  |  | 1 | 0 | 0 | 0 | 1 | 0 | 0 | 0 | 0 | 0 | 0 | 0 | 0 | 0 | 0 | 1531 | 1549 | Oxidation (M) | M12:Oxidation (M):1000.00 | PEAKS DB |
| K.VVLLSYQSSF.L | Y | 38.34 | 1141.6019 | 10 | 0.4 | 571.8085 | 2 | 79.64 | 6 | F6:54198 | NaSa01\_F14.raw |  |  |  |  |  | 2.3493E6 |  |  |  |  |  |  |  |  | 1 | 0 | 0 | 0 | 0 | 0 | 1 | 0 | 0 | 0 | 0 | 0 | 0 | 0 | 0 | 118 | 127 |  |  | PEAKS DB |
| K.ISYIITK.N | N | 38.08 | 836.5007 | 7 | -0.7 | 419.2574 | 2 | 26.29 | 6 | F6:9476 | NaSa01\_F14.raw |  |  |  |  |  | 6.6012E5 |  |  |  |  |  |  |  |  | 1 | 0 | 0 | 0 | 0 | 0 | 1 | 0 | 0 | 0 | 0 | 0 | 0 | 0 | 0 | 1597 | 1603 |  |  | PEAKS DB |
| total 87 peptides |  |  |  |  |  |  |  |  |  |  |  |  |  |  |  |  |  |  |  |  |  |  |  |  |  |  |  |  |  |  |  |  |  |  |  |  |  |  |  |  |  |  |  |  |  |
| --- | --- | --- | --- | --- | --- | --- | --- | --- | --- | --- | --- | --- | --- | --- | --- | --- | --- | --- | --- | --- | --- | --- | --- | --- | --- | --- | --- | --- | --- | --- | --- | --- | --- | --- | --- | --- | --- | --- | --- | --- | --- | --- | --- | --- | --- |

2GIZ

back to list

  

| Protein Coverage
| Supporting Peptides
|

Protein Coverage:

Supporting Peptides:

| Peptide | Uniq | -10lgP | Mass | Length | ppm | m/z | z | RT | Fraction | Scan | Source File | Area F1 | Area F10 | Area F11 | Area F12 | Area F13 | Area F14 | Area F2 | Area F3 | Area F4 | Area F5 | Area F6 | Area F7 | Area F8 | Area F9 | #Feature | #Feature F1 | #Feature F10 | #Feature F11 | #Feature F12 | #Feature F13 | #Feature F14 | #Feature F2 | #Feature F3 | #Feature F4 | #Feature F5 | #Feature F6 | #Feature F7 | #Feature F8 | #Feature F9 | Start | End | PTM | AScore | Found By |
| --- | --- | --- | --- | --- | --- | --- | --- | --- | --- | --- | --- | --- | --- | --- | --- | --- | --- | --- | --- | --- | --- | --- | --- | --- | --- | --- | --- | --- | --- | --- | --- | --- | --- | --- | --- | --- | --- | --- | --- | --- | --- | --- | --- | --- | --- |
| R.TWTEIIHLWHDEYK.N | Y | 86.98 | 1869.9049 | 14 | -0.4 | 935.9594 | 2 | 69.11 | 4 | F4:46528 | NaSa01\_F12.raw |  |  | 1.6758E6 | 5.726E9 | 7.3513E6 |  |  |  |  |  |  |  |  |  | 9 | 0 | 0 | 1 | 6 | 2 | 0 | 0 | 0 | 0 | 0 | 0 | 0 | 0 | 0 | 85 | 98 |  |  | PEAKS DB |
| R.VLEGIQC(+57.02)GESIYMSSNAR.T | Y | 86.82 | 2012.9296 | 18 | -0.1 | 1007.4720 | 2 | 58.59 | 4 | F4:37662 | NaSa01\_F12.raw |  | 6.539E5 | 1.5229E7 | 1.9162E10 | 6.8803E7 | 1.1875E7 |  |  |  |  |  |  |  |  | 13 | 0 | 1 | 2 | 6 | 2 | 2 | 0 | 0 | 0 | 0 | 0 | 0 | 0 | 0 | 67 | 84 | Carbamidomethylation | C7:Carbamidomethylation:1000.00 | PEAKS DB |
| R.VLEGIQC(+57.02)GESIYM(+15.99)SSNAR.T | Y | 83.82 | 2028.9244 | 18 | -1.0 | 1015.4684 | 2 | 49.35 | 4 | F4:32620 | NaSa01\_F12.raw |  |  |  | 2.596E9 | 2.4998E7 | 3.3484E6 |  |  |  |  |  |  |  |  | 24 | 0 | 0 | 0 | 20 | 2 | 2 | 0 | 0 | 0 | 0 | 0 | 0 | 0 | 0 | 67 | 84 | Carbamidomethylation; Oxidation (M) | C7:Carbamidomethylation:1000.00;M13:Oxidation (M):1000.00 | PEAKS DB |
| R.WANTC(+57.02)SLNHSPDNLR.V | Y | 83.41 | 1783.8060 | 15 | -0.3 | 892.9100 | 2 | 20.24 | 4 | F4:7228 | NaSa01\_F12.raw |  |  |  | 4.9332E10 | 1.1987E8 | 6.2009E4 |  |  |  |  |  |  |  |  | 25 | 0 | 0 | 0 | 20 | 4 | 1 | 0 | 0 | 0 | 0 | 0 | 0 | 0 | 0 | 52 | 66 | Carbamidomethylation | C5:Carbamidomethylation:1000.00 | PEAKS DB |
| K.QSSC(+57.02)QDDWIKSNC(+57.02)PASC(+57.02)FC(+57.02)R.N | Y | 79.80 | 2504.9929 | 20 | 0.6 | 836.0054 | 3 | 39.02 | 4 | F4:22824 | NaSa01\_F12.raw |  |  |  | 7.3335E7 |  |  |  |  |  |  |  |  |  |  | 3 | 0 | 0 | 0 | 3 | 0 | 0 | 0 | 0 | 0 | 0 | 0 | 0 | 0 | 0 | 198 | 217 | Carbamidomethylation | C4:Carbamidomethylation:1000.00;C13:Carbamidomethylation:1000.00;C17:Carbamidomethylation:1000.00;C19:Carbamidomethylation:1000.00 | PEAKS DB |
| K.LTNC(+57.02)DSLLKQSSC(+57.02)QDDWIK.S | Y | 79.46 | 2310.0620 | 19 | -1.1 | 771.0271 | 3 | 45.31 | 4 | F4:28181 | NaSa01\_F12.raw |  |  |  | 1.962E8 |  |  |  |  |  |  |  |  |  |  | 4 | 0 | 0 | 0 | 4 | 0 | 0 | 0 | 0 | 0 | 0 | 0 | 0 | 0 | 0 | 189 | 207 | Carbamidomethylation | C4:Carbamidomethylation:1000.00;C13:Carbamidomethylation:1000.00 | PEAKS DB |
| D.C(+57.02)PSAC(+57.02)DNGLC(+57.02)TNPC(+57.02)TIYNK.L | Y | 78.07 | 2243.9067 | 19 | -0.3 | 1122.9603 | 2 | 35.53 | 4 | F4:19614 | NaSa01\_F12.raw |  |  |  | 1.255E8 |  |  |  |  |  |  |  |  |  |  | 3 | 0 | 0 | 0 | 3 | 0 | 0 | 0 | 0 | 0 | 0 | 0 | 0 | 0 | 0 | 170 | 188 | Carbamidomethylation | C1:Carbamidomethylation:1000.00;C5:Carbamidomethylation:1000.00;C10:Carbamidomethylation:1000.00;C14:Carbamidomethylation:1000.00 | PEAKS DB |
| K.NFVYGVGANPPGSVTGHYTQIVWYQTYR.A | Y | 77.64 | 3173.5359 | 28 | 0.0 | 1058.8525 | 3 | 74.13 | 4 | F4:51117 | NaSa01\_F12.raw |  |  |  | 1.5642E9 |  |  |  |  |  |  |  |  |  |  | 6 | 0 | 0 | 0 | 6 | 0 | 0 | 0 | 0 | 0 | 0 | 0 | 0 | 0 | 0 | 99 | 126 |  |  | PEAKS DB |
| Y.GVGANPPGSVTGHYTQIVWYQTYR.A | Y | 77.12 | 2650.2927 | 24 | 0.9 | 884.4390 | 3 | 57.12 | 4 | F4:37409 | NaSa01\_F12.raw |  |  | 5.2237E5 | 6.6355E7 |  |  |  |  |  |  |  |  |  |  | 2 | 0 | 0 | 1 | 1 | 0 | 0 | 0 | 0 | 0 | 0 | 0 | 0 | 0 | 0 | 103 | 126 |  |  | PEAKS DB |
| R.VSPTASNMLKMEWYPEAASNAER.W | N | 76.94 | 2581.1941 | 23 | 0.7 | 861.4059 | 3 | 60.25 | 4 | F4:39967 | NaSa01\_F12.raw |  |  |  | 1.7324E8 |  |  |  |  |  |  |  |  |  |  | 2 | 0 | 0 | 0 | 2 | 0 | 0 | 0 | 0 | 0 | 0 | 0 | 0 | 0 | 0 | 29 | 51 |  |  | PEAKS DB |
| K.MEWYPEAASNAER.W | N | 75.93 | 1552.6616 | 13 | -4.7 | 518.5587 | 3 | 42.47 | 4 | F4:25783 | NaSa01\_F12.raw |  |  | 8.9734E6 | 5.588E10 | 1.0906E8 | 2.4001E7 |  |  |  |  |  |  |  |  | 18 | 0 | 0 | 1 | 10 | 5 | 2 | 0 | 0 | 0 | 0 | 0 | 0 | 0 | 0 | 39 | 51 |  |  | PEAKS DB |
| W.SYFYVC(+57.02)QYC(+57.02)PSGNFQGK.T | Y | 74.81 | 2103.8818 | 17 | 0.9 | 1052.9491 | 2 | 56.78 | 4 | F4:37002 | NaSa01\_F12.raw |  |  |  | 7.9496E7 | 3.5056E6 |  |  |  |  |  |  |  |  |  | 3 | 0 | 0 | 0 | 2 | 1 | 0 | 0 | 0 | 0 | 0 | 0 | 0 | 0 | 0 | 140 | 156 | Carbamidomethylation | C6:Carbamidomethylation:1000.00;C9:Carbamidomethylation:1000.00 | PEAKS DB |
| K.QKEIVDLHNSLR.R | N | 73.79 | 1450.7892 | 12 | 0.4 | 484.6039 | 3 | 11.92 | 4 | F4:3036 | NaSa01\_F12.raw |  |  |  | 1.138E8 | 7.1992E5 |  |  |  |  |  |  |  |  |  | 16 | 0 | 0 | 0 | 15 | 1 | 0 | 0 | 0 | 0 | 0 | 0 | 0 | 0 | 0 | 15 | 26 |  |  | PEAKS DB |
| L.RVLEGIQC(+57.02)GESIYMSSNAR.T | Y | 73.68 | 2169.0308 | 19 | -0.1 | 724.0175 | 3 | 41.70 | 4 | F4:24692 | NaSa01\_F12.raw |  |  |  | 1.2641E7 |  |  |  |  |  |  |  |  |  |  | 1 | 0 | 0 | 0 | 1 | 0 | 0 | 0 | 0 | 0 | 0 | 0 | 0 | 0 | 0 | 66 | 84 | Carbamidomethylation | C8:Carbamidomethylation:1000.00 | PEAKS DB |
| C.PSAC(+57.02)DNGLC(+57.02)TNPC(+57.02)TIYNK.L | Y | 73.51 | 2083.8762 | 18 | 0.2 | 1042.9456 | 2 | 34.80 | 4 | F4:18795 | NaSa01\_F12.raw |  |  |  | 1.0016E7 |  |  |  |  |  |  |  |  |  |  | 1 | 0 | 0 | 0 | 1 | 0 | 0 | 0 | 0 | 0 | 0 | 0 | 0 | 0 | 0 | 171 | 188 | Carbamidomethylation | C4:Carbamidomethylation:1000.00;C9:Carbamidomethylation:1000.00;C13:Carbamidomethylation:1000.00 | PEAKS DB |
| S.SAWSYFYVC(+57.02)QYC(+57.02)PSGNFQGK.T | Y | 73.42 | 2448.0303 | 20 | -0.2 | 1225.0222 | 2 | 75.77 | 4 | F4:52609 | NaSa01\_F12.raw |  |  |  | 7.5549E7 |  |  |  |  |  |  |  |  |  |  | 2 | 0 | 0 | 0 | 2 | 0 | 0 | 0 | 0 | 0 | 0 | 0 | 0 | 0 | 0 | 137 | 156 | Carbamidomethylation | C9:Carbamidomethylation:1000.00;C12:Carbamidomethylation:1000.00 | PEAKS DB |
| K.M(+15.99)EWYPEAASNAER.W | N | 73.15 | 1568.6565 | 13 | 0.9 | 523.8932 | 3 | 33.16 | 4 | F4:17227 | NaSa01\_F12.raw |  |  |  | 1.0876E10 | 1.0638E7 | 4.7019E6 |  |  |  |  |  |  |  |  | 17 | 0 | 0 | 0 | 15 | 1 | 1 | 0 | 0 | 0 | 0 | 0 | 0 | 0 | 0 | 39 | 51 | Oxidation (M) | M1:Oxidation (M):1000.00 | PEAKS DB |
| K.EIVDLHNSLR.R | N | 71.95 | 1194.6356 | 10 | 0.4 | 598.3253 | 2 | 28.76 | 4 | F4:13650 | NaSa01\_F12.raw |  |  |  | 7.7068E9 | 7.5889E7 | 5.7777E4 |  |  |  |  |  |  |  |  | 27 | 0 | 0 | 0 | 23 | 3 | 1 | 0 | 0 | 0 | 0 | 0 | 0 | 0 | 0 | 17 | 26 |  |  | PEAKS DB |
| Y.C(+57.02)PSSAWSYFYVC(+57.02)QYC(+57.02)PSGNFQGK.T | Y | 71.68 | 2792.1458 | 23 | 1.2 | 931.7236 | 3 | 77.58 | 4 | F4:54131 | NaSa01\_F12.raw |  |  |  | 2.7393E7 |  |  |  |  |  |  |  |  |  |  | 2 | 0 | 0 | 0 | 2 | 0 | 0 | 0 | 0 | 0 | 0 | 0 | 0 | 0 | 0 | 134 | 156 | Carbamidomethylation | C1:Carbamidomethylation:1000.00;C12:Carbamidomethylation:1000.00;C15:Carbamidomethylation:1000.00 | PEAKS DB |
| Y.FYVC(+57.02)QYC(+57.02)PSGNFQGK.T | Y | 71.29 | 1853.7865 | 15 | 0.2 | 927.9008 | 2 | 45.22 | 4 | F4:27254 | NaSa01\_F12.raw |  |  |  | 6.7966E7 |  |  |  |  |  |  |  |  |  |  | 1 | 0 | 0 | 0 | 1 | 0 | 0 | 0 | 0 | 0 | 0 | 0 | 0 | 0 | 0 | 142 | 156 | Carbamidomethylation | C4:Carbamidomethylation:1000.00;C7:Carbamidomethylation:1000.00 | PEAKS DB |
| F.YVC(+57.02)QYC(+57.02)PSGNFQGK.T | Y | 70.89 | 1706.7181 | 14 | -0.4 | 854.3660 | 2 | 26.36 | 4 | F4:11724 | NaSa01\_F12.raw |  |  |  | 1.5606E8 | 1.5285E7 |  |  |  |  |  |  |  |  |  | 2 | 0 | 0 | 0 | 1 | 1 | 0 | 0 | 0 | 0 | 0 | 0 | 0 | 0 | 0 | 143 | 156 | Carbamidomethylation | C3:Carbamidomethylation:1000.00;C6:Carbamidomethylation:1000.00 | PEAKS DB |
| D.NGLC(+57.02)TNPC(+57.02)TIYNK.L | Y | 69.78 | 1553.6967 | 13 | 0.5 | 777.8560 | 2 | 28.19 | 4 | F4:13013 | NaSa01\_F12.raw |  |  |  | 3.1653E7 |  |  |  |  |  |  |  |  |  |  | 1 | 0 | 0 | 0 | 1 | 0 | 0 | 0 | 0 | 0 | 0 | 0 | 0 | 0 | 0 | 176 | 188 | Carbamidomethylation | C4:Carbamidomethylation:1000.00;C8:Carbamidomethylation:1000.00 | PEAKS DB |
| K.SNC(+57.02)PASC(+57.02)FC(+57.02)R.N | N | 69.18 | 1257.4689 | 10 | 0.2 | 629.7418 | 2 | 11.79 | 4 | F4:2692 | NaSa01\_F12.raw |  |  |  | 1.0262E8 |  |  |  |  |  |  |  |  |  |  | 1 | 0 | 0 | 0 | 1 | 0 | 0 | 0 | 0 | 0 | 0 | 0 | 0 | 0 | 0 | 208 | 217 | Carbamidomethylation | C3:Carbamidomethylation:1000.00;C7:Carbamidomethylation:1000.00;C9:Carbamidomethylation:1000.00 | PEAKS DB |
| K.QSSC(+57.02)QDDWIK.S | Y | 68.96 | 1265.5347 | 10 | -0.7 | 633.7742 | 2 | 11.92 | 4 | F4:3391 | NaSa01\_F12.raw |  |  |  | 3.0416E8 |  |  |  |  |  |  |  |  |  |  | 8 | 0 | 0 | 0 | 8 | 0 | 0 | 0 | 0 | 0 | 0 | 0 | 0 | 0 | 0 | 198 | 207 | Carbamidomethylation | C4:Carbamidomethylation:1000.00 | PEAKS DB |
| K.MEWYPEAASNAERWANTC(+57.02)SLNHSPDNLR.V | Y | 66.81 | 3318.4570 | 28 | 0.1 | 830.6216 | 4 | 60.99 | 4 | F4:40505 | NaSa01\_F12.raw |  |  |  | 1.1514E7 |  |  |  |  |  |  |  |  |  |  | 3 | 0 | 0 | 0 | 3 | 0 | 0 | 0 | 0 | 0 | 0 | 0 | 0 | 0 | 0 | 39 | 66 | Carbamidomethylation | C18:Carbamidomethylation:1000.00 | PEAKS DB |
| N.GLC(+57.02)TNPC(+57.02)TIYNK.L | Y | 66.27 | 1439.6537 | 12 | -0.2 | 720.8340 | 2 | 27.00 | 4 | F4:12069 | NaSa01\_F12.raw |  |  |  | 3.5701E7 |  |  |  |  |  |  |  |  |  |  | 1 | 0 | 0 | 0 | 1 | 0 | 0 | 0 | 0 | 0 | 0 | 0 | 0 | 0 | 0 | 177 | 188 | Carbamidomethylation | C3:Carbamidomethylation:1000.00;C7:Carbamidomethylation:1000.00 | PEAKS DB |
| E.GIQC(+57.02)GESIYMSSNAR.T | Y | 64.94 | 1671.7345 | 15 | 0.6 | 836.8750 | 2 | 29.68 | 4 | F4:14460 | NaSa01\_F12.raw |  |  |  | 1.9465E6 |  |  |  |  |  |  |  |  |  |  | 1 | 0 | 0 | 0 | 1 | 0 | 0 | 0 | 0 | 0 | 0 | 0 | 0 | 0 | 0 | 70 | 84 | Carbamidomethylation | C4:Carbamidomethylation:1000.00 | PEAKS DB |
| N.PPGSVTGHYTQIVWYQTYR.A | Y | 64.76 | 2252.1013 | 19 | 0.1 | 751.7078 | 3 | 53.16 | 4 | F4:33903 | NaSa01\_F12.raw |  |  |  | 4.1382E6 |  |  |  |  |  |  |  |  |  |  | 1 | 0 | 0 | 0 | 1 | 0 | 0 | 0 | 0 | 0 | 0 | 0 | 0 | 0 | 0 | 108 | 126 |  |  | PEAKS DB |
| K.NFVYGVGANPPGSVTGH.Y | N | 64.66 | 1671.8005 | 17 | 0.7 | 836.9081 | 2 | 48.12 | 4 | F4:29754 | NaSa01\_F12.raw |  |  |  | 2.7791E8 | 1.1488E7 | 2.2522E6 |  |  |  |  |  |  |  |  | 6 | 0 | 0 | 0 | 4 | 1 | 1 | 0 | 0 | 0 | 0 | 0 | 0 | 0 | 0 | 99 | 115 |  |  | PEAKS DB |
| NVDFNSESTR.R | N | 64.34 | 1167.5156 | 10 | 0.3 | 584.7653 | 2 | 12.14 | 4 | F4:3045 | NaSa01\_F12.raw |  |  |  | 1.4013E8 |  |  |  |  |  |  |  |  |  |  | 6 | 0 | 0 | 0 | 6 | 0 | 0 | 0 | 0 | 0 | 0 | 0 | 0 | 0 | 0 | 1 | 10 |  |  | PEAKS DB |
| K.LTNC(+57.02)DSLLK.Q | Y | 64.00 | 1062.5380 | 9 | -1.6 | 532.2754 | 2 | 21.02 | 4 | F4:7364 | NaSa01\_F12.raw |  |  |  | 2.9662E8 |  |  |  |  |  |  |  |  |  |  | 6 | 0 | 0 | 0 | 6 | 0 | 0 | 0 | 0 | 0 | 0 | 0 | 0 | 0 | 0 | 189 | 197 | Carbamidomethylation | C4:Carbamidomethylation:1000.00 | PEAKS DB |
| H.YTQIVWYQTYR.A | Y | 63.87 | 1519.7460 | 11 | 1.3 | 760.8812 | 2 | 55.25 | 4 | F4:35871 | NaSa01\_F12.raw |  |  |  | 1.2665E8 | 2.5476E6 |  |  |  |  |  |  |  |  |  | 3 | 0 | 0 | 0 | 2 | 1 | 0 | 0 | 0 | 0 | 0 | 0 | 0 | 0 | 0 | 116 | 126 |  |  | PEAKS DB |
| N.VDFNSESTR.R | N | 63.60 | 1053.4727 | 9 | 0.3 | 527.7438 | 2 | 11.74 | 4 | F4:2649 | NaSa01\_F12.raw |  |  |  | 4.6861E6 |  |  |  |  |  |  |  |  |  |  | 1 | 0 | 0 | 0 | 1 | 0 | 0 | 0 | 0 | 0 | 0 | 0 | 0 | 0 | 0 | 2 | 10 |  |  | PEAKS DB |
| C.DNGLC(+57.02)TNPC(+57.02)TIYNK.L | Y | 63.45 | 1668.7236 | 14 | 0.8 | 835.3698 | 2 | 36.30 | 4 | F4:20118 | NaSa01\_F12.raw |  |  |  | 3.0623E7 |  |  |  |  |  |  |  |  |  |  | 1 | 0 | 0 | 0 | 1 | 0 | 0 | 0 | 0 | 0 | 0 | 0 | 0 | 0 | 0 | 175 | 188 | Carbamidomethylation | C5:Carbamidomethylation:1000.00;C9:Carbamidomethylation:1000.00 | PEAKS DB |
| L.EGIQC(+57.02)GESIYMSSNAR.T | Y | 62.23 | 1800.7771 | 16 | 0.5 | 901.3963 | 2 | 35.36 | 4 | F4:19124 | NaSa01\_F12.raw |  |  |  | 1.9136E6 |  |  |  |  |  |  |  |  |  |  | 1 | 0 | 0 | 0 | 1 | 0 | 0 | 0 | 0 | 0 | 0 | 0 | 0 | 0 | 0 | 69 | 84 | Carbamidomethylation | C5:Carbamidomethylation:1000.00 | PEAKS DB |
| R.VSPTASNM(+15.99)LKMEWYPEAASNAER.W | N | 61.53 | 2597.1890 | 23 | 0.7 | 866.7375 | 3 | 47.88 | 4 | F4:29657 | NaSa01\_F12.raw |  |  |  | 1.0394E7 |  |  |  |  |  |  |  |  |  |  | 2 | 0 | 0 | 0 | 2 | 0 | 0 | 0 | 0 | 0 | 0 | 0 | 0 | 0 | 0 | 29 | 51 | Oxidation (M) | M8:Oxidation (M):24.24 | PEAKS DB |
| K.LGPPC(+57.02)GDC(+57.02)PSAC(+57.02)DNGLC(+57.02)TNPC(+57.02)TIYNK.L | Y | 61.29 | 2940.1970 | 26 | -0.2 | 981.0728 | 3 | 53.63 | 4 | F4:34247 | NaSa01\_F12.raw |  |  |  | 1.1603E10 |  | 3.4496E6 |  |  |  |  |  | 6.1046E6 |  |  | 9 | 0 | 0 | 0 | 7 | 0 | 1 | 0 | 0 | 0 | 0 | 0 | 1 | 0 | 0 | 163 | 188 | Carbamidomethylation | C5:Carbamidomethylation:1000.00;C8:Carbamidomethylation:1000.00;C12:Carbamidomethylation:1000.00;C17:Carbamidomethylation:1000.00;C21:Carbamidomethylation:1000.00 | PEAKS DB |
| K.NFVYGVGANPPGSVTGHY.T | N | 60.74 | 1834.8638 | 18 | 1.5 | 918.4406 | 2 | 53.13 | 4 | F4:33956 | NaSa01\_F12.raw |  |  |  | 5.187E6 |  |  |  |  |  |  |  |  |  |  | 1 | 0 | 0 | 0 | 1 | 0 | 0 | 0 | 0 | 0 | 0 | 0 | 0 | 0 | 0 | 99 | 116 |  |  | PEAKS DB |
| R.TWTEIIHLWH.D | Y | 59.61 | 1334.6771 | 10 | 0.6 | 668.3463 | 2 | 74.13 | 4 | F4:51227 | NaSa01\_F12.raw |  |  |  | 5.1108E6 |  |  |  |  |  |  |  |  |  |  | 1 | 0 | 0 | 0 | 1 | 0 | 0 | 0 | 0 | 0 | 0 | 0 | 0 | 0 | 0 | 85 | 94 |  |  | PEAKS DB |
| K.LGPPC(+57.02)GDC(+57.02)PSAC(+57.02)DNGLC(+57.02)TNPC(+57.02)TIYNKLTNC(+57.02)DSLLK.Q | Y | 59.18 | 3984.7244 | 35 | -0.2 | 1329.2484 | 3 | 74.37 | 4 | F4:51337 | NaSa01\_F12.raw |  |  |  | 2.2817E8 |  |  |  |  |  |  |  |  |  |  | 3 | 0 | 0 | 0 | 3 | 0 | 0 | 0 | 0 | 0 | 0 | 0 | 0 | 0 | 0 | 163 | 197 | Carbamidomethylation | C5:Carbamidomethylation:1000.00;C8:Carbamidomethylation:1000.00;C12:Carbamidomethylation:1000.00;C17:Carbamidomethylation:1000.00;C21:Carbamidomethylation:1000.00;C30:Carbamidomethylation:1000.00 | PEAKS DB |
| K.TATPYKLGPPC(+57.02)GDC(+57.02)PSAC(+57.02)DNGLC(+57.02)TNPC(+57.02)TIYNK.L | Y | 58.88 | 3601.5405 | 32 | 0.0 | 1201.5208 | 3 | 49.06 | 4 | F4:30641 | NaSa01\_F12.raw |  |  |  | 5.9598E7 |  |  |  |  |  |  |  |  |  |  | 1 | 0 | 0 | 0 | 1 | 0 | 0 | 0 | 0 | 0 | 0 | 0 | 0 | 0 | 0 | 157 | 188 | Carbamidomethylation | C11:Carbamidomethylation:1000.00;C14:Carbamidomethylation:1000.00;C18:Carbamidomethylation:1000.00;C23:Carbamidomethylation:1000.00;C27:Carbamidomethylation:1000.00 | PEAKS DB |
| G.LC(+57.02)TNPC(+57.02)TIYNK.L | Y | 58.71 | 1382.6323 | 11 | 0.6 | 692.3239 | 2 | 23.47 | 4 | F4:9229 | NaSa01\_F12.raw |  |  |  | 2.5631E6 |  |  |  |  |  |  |  | 1.5272E6 |  |  | 2 | 0 | 0 | 0 | 1 | 0 | 0 | 0 | 0 | 0 | 0 | 0 | 1 | 0 | 0 | 178 | 188 | Carbamidomethylation | C2:Carbamidomethylation:1000.00;C6:Carbamidomethylation:1000.00 | PEAKS DB |
| R.WANTC(+57.02)SLNHSPDNLRVLEGIQC(+57.02)GESIYMSSNAR.T | Y | 58.28 | 3778.7251 | 33 | -0.2 | 945.6884 | 4 | 66.79 | 4 | F4:45302 | NaSa01\_F12.raw |  |  |  | 2.4518E7 |  |  |  |  |  |  |  |  |  |  | 2 | 0 | 0 | 0 | 2 | 0 | 0 | 0 | 0 | 0 | 0 | 0 | 0 | 0 | 0 | 52 | 84 | Carbamidomethylation | C5:Carbamidomethylation:1000.00;C22:Carbamidomethylation:1000.00 | PEAKS DB |
| S.AC(+57.02)DNGLC(+57.02)TNPC(+57.02)TIYNK.L | Y | 58.09 | 1899.7914 | 16 | -0.8 | 950.9022 | 2 | 32.96 | 4 | F4:17191 | NaSa01\_F12.raw |  |  |  | 5.221E6 |  |  |  |  |  |  |  |  |  |  | 1 | 0 | 0 | 0 | 1 | 0 | 0 | 0 | 0 | 0 | 0 | 0 | 0 | 0 | 0 | 173 | 188 | Carbamidomethylation | C2:Carbamidomethylation:1000.00;C7:Carbamidomethylation:1000.00;C11:Carbamidomethylation:1000.00 | PEAKS DB |
| E.IVDLHNSLR.R | N | 58.01 | 1065.5930 | 9 | -1.6 | 533.8029 | 2 | 26.50 | 4 | F4:11729 | NaSa01\_F12.raw |  |  |  | 1.3096E7 |  |  |  |  |  |  |  |  |  |  | 1 | 0 | 0 | 0 | 1 | 0 | 0 | 0 | 0 | 0 | 0 | 0 | 0 | 0 | 0 | 18 | 26 |  |  | PEAKS DB |
| R.AGC(+57.02)AVSYC(+57.02)PSSAWSYFYVC(+57.02)QYC(+57.02)PSGNFQGK.T | Y | 57.96 | 3500.4358 | 30 | 0.3 | 1167.8196 | 3 | 84.20 | 4 | F4:60175 | NaSa01\_F12.raw |  |  |  | 8.8824E8 |  |  |  |  |  |  |  |  |  |  | 3 | 0 | 0 | 0 | 3 | 0 | 0 | 0 | 0 | 0 | 0 | 0 | 0 | 0 | 0 | 127 | 156 | Carbamidomethylation | C3:Carbamidomethylation:1000.00;C8:Carbamidomethylation:1000.00;C19:Carbamidomethylation:1000.00;C22:Carbamidomethylation:1000.00 | PEAKS DB |
| N.TC(+57.02)SLNHSPDNLR.V | Y | 57.81 | 1412.6466 | 12 | 0.4 | 471.8897 | 3 | 11.55 | 4 | F4:2523 | NaSa01\_F12.raw |  |  |  | 6.8334E6 |  |  |  |  |  |  |  |  |  |  | 2 | 0 | 0 | 0 | 2 | 0 | 0 | 0 | 0 | 0 | 0 | 0 | 0 | 0 | 0 | 55 | 66 | Carbamidomethylation | C2:Carbamidomethylation:1000.00 | PEAKS DB |
| R.VSPTASNMLK.M | N | 57.39 | 1046.5430 | 10 | 0.1 | 524.2788 | 2 | 11.91 | 4 | F4:3054 | NaSa01\_F12.raw |  |  |  | 1.9382E8 |  |  |  |  |  |  |  |  |  |  | 5 | 0 | 0 | 0 | 5 | 0 | 0 | 0 | 0 | 0 | 0 | 0 | 0 | 0 | 0 | 29 | 38 |  |  | PEAKS DB |
| L.KMEWYPEAASNAER.W | N | 57.28 | 1680.7566 | 14 | 0.7 | 561.2599 | 3 | 28.56 | 4 | F4:13207 | NaSa01\_F12.raw |  |  |  | 1.222E8 |  |  |  |  |  |  |  |  |  |  | 2 | 0 | 0 | 0 | 2 | 0 | 0 | 0 | 0 | 0 | 0 | 0 | 0 | 0 | 0 | 38 | 51 |  |  | PEAKS DB |
| R.VSPTASNMLKM(+15.99)EWYPEAASNAER.W | N | 57.05 | 2597.1890 | 23 | 0.7 | 866.7375 | 3 | 49.69 | 4 | F4:31613 | NaSa01\_F12.raw |  |  |  | 1.4277E7 |  |  |  |  |  |  |  |  |  |  | 1 | 0 | 0 | 0 | 1 | 0 | 0 | 0 | 0 | 0 | 0 | 0 | 0 | 0 | 0 | 29 | 51 | Oxidation (M) | M11:Oxidation (M):32.97 | PEAKS DB |
| W.TEIIHLWHDEYK.N | Y | 56.98 | 1582.7780 | 12 | 0.1 | 528.6000 | 3 | 36.11 | 4 | F4:19977 | NaSa01\_F12.raw |  |  |  | 9.0049E6 |  |  |  |  |  |  |  |  |  |  | 4 | 0 | 0 | 0 | 4 | 0 | 0 | 0 | 0 | 0 | 0 | 0 | 0 | 0 | 0 | 87 | 98 |  |  | PEAKS DB |
| R.VLEGIQC(+57.02)GESIYMSSN.A | Y | 56.56 | 1785.7914 | 16 | 0.3 | 893.9033 | 2 | 68.26 | 4 | F4:46624 | NaSa01\_F12.raw |  |  |  | 2.5137E8 |  |  |  |  |  |  |  |  |  |  | 1 | 0 | 0 | 0 | 1 | 0 | 0 | 0 | 0 | 0 | 0 | 0 | 0 | 0 | 0 | 67 | 82 | Carbamidomethylation | C7:Carbamidomethylation:1000.00 | PEAKS DB |
| E.IIHLWHDEYK.N | N | 55.71 | 1352.6877 | 10 | -0.7 | 451.9029 | 3 | 26.09 | 4 | F4:11533 | NaSa01\_F12.raw |  |  |  | 6.5239E5 |  |  |  |  |  |  |  |  |  |  | 1 | 0 | 0 | 0 | 1 | 0 | 0 | 0 | 0 | 0 | 0 | 0 | 0 | 0 | 0 | 89 | 98 |  |  | PEAKS DB |
| C.AVSYC(+57.02)PSSAWSYFYVC(+57.02)QYC(+57.02)PSGNFQGK.T | Y | 55.49 | 3212.3467 | 27 | 0.6 | 1071.7902 | 3 | 83.95 | 4 | F4:59535 | NaSa01\_F12.raw |  |  |  | 1.1276E8 |  |  |  |  |  |  |  |  |  |  | 1 | 0 | 0 | 0 | 1 | 0 | 0 | 0 | 0 | 0 | 0 | 0 | 0 | 0 | 0 | 130 | 156 | Carbamidomethylation | C5:Carbamidomethylation:1000.00;C16:Carbamidomethylation:1000.00;C19:Carbamidomethylation:1000.00 | PEAKS DB |
| M.EWYPEAASNAER.W | N | 54.75 | 1421.6211 | 12 | 0.7 | 711.8183 | 2 | 30.32 | 4 | F4:14915 | NaSa01\_F12.raw |  |  |  | 1.4758E7 |  |  |  |  |  |  |  |  |  |  | 1 | 0 | 0 | 0 | 1 | 0 | 0 | 0 | 0 | 0 | 0 | 0 | 0 | 0 | 0 | 40 | 51 |  |  | PEAKS DB |
| NVDFNSESTRR.K | N | 53.63 | 1323.6167 | 11 | 0.6 | 662.8160 | 2 | 11.55 | 4 | F4:2518 | NaSa01\_F12.raw |  |  |  | 1.6815E7 |  |  |  |  |  |  |  |  |  |  | 1 | 0 | 0 | 0 | 1 | 0 | 0 | 0 | 0 | 0 | 0 | 0 | 0 | 0 | 0 | 1 | 11 |  |  | PEAKS DB |
| C.SLNHSPDNLR.V | Y | 52.12 | 1151.5684 | 10 | 0.9 | 576.7920 | 2 | 11.55 | 4 | F4:2531 | NaSa01\_F12.raw |  |  |  | 3.5847E6 |  |  |  |  |  |  |  |  |  |  | 3 | 0 | 0 | 0 | 3 | 0 | 0 | 0 | 0 | 0 | 0 | 0 | 0 | 0 | 0 | 57 | 66 |  |  | PEAKS DB |
| C.GDC(+57.02)PSAC(+57.02)DNGLC(+57.02)TNPC(+57.02)TIYNK.L | Y | 51.17 | 2415.9553 | 21 | 0.4 | 1208.9855 | 2 | 38.69 | 4 | F4:22109 | NaSa01\_F12.raw |  |  |  | 3.1743E6 |  |  |  |  |  |  |  |  |  |  | 1 | 0 | 0 | 0 | 1 | 0 | 0 | 0 | 0 | 0 | 0 | 0 | 0 | 0 | 0 | 168 | 188 | Carbamidomethylation | C3:Carbamidomethylation:1000.00;C7:Carbamidomethylation:1000.00;C12:Carbamidomethylation:1000.00;C16:Carbamidomethylation:1000.00 | PEAKS DB |
| R.VLEGIQC(+57.02)GESIYM(+15.99)SSN.A | Y | 51.15 | 1801.7863 | 16 | -0.1 | 901.9003 | 2 | 59.33 | 4 | F4:39244 | NaSa01\_F12.raw |  |  |  | 2.7515E7 |  |  |  |  |  |  |  |  |  |  | 1 | 0 | 0 | 0 | 1 | 0 | 0 | 0 | 0 | 0 | 0 | 0 | 0 | 0 | 0 | 67 | 82 | Carbamidomethylation; Oxidation (M) | C7:Carbamidomethylation:1000.00;M13:Oxidation (M):1000.00 | PEAKS DB |
| N.FVYGVGANPPGSVTGHYTQIVWYQTYR.A | Y | 50.64 | 3059.4929 | 27 | -4.3 | 1020.8339 | 3 | 70.65 | 4 | F4:48404 | NaSa01\_F12.raw |  |  |  | 2.9259E6 |  |  |  |  |  |  |  |  |  |  | 1 | 0 | 0 | 0 | 1 | 0 | 0 | 0 | 0 | 0 | 0 | 0 | 0 | 0 | 0 | 100 | 126 |  |  | PEAKS DB |
| K.MEWYPEAASN.A | N | 49.85 | 1196.4808 | 10 | 0.2 | 599.2478 | 2 | 59.73 | 4 | F4:39536 | NaSa01\_F12.raw |  |  |  | 6.0961E6 |  |  |  |  |  |  |  |  |  |  | 1 | 0 | 0 | 0 | 1 | 0 | 0 | 0 | 0 | 0 | 0 | 0 | 0 | 0 | 0 | 39 | 48 |  |  | PEAKS DB |
| R.RVSPTASNMLK.M | Y | 49.81 | 1202.6442 | 11 | -0.6 | 602.3290 | 2 | 11.56 | 4 | F4:2521 | NaSa01\_F12.raw |  |  |  | 1.3713E7 |  |  |  |  |  |  |  |  |  |  | 1 | 0 | 0 | 0 | 1 | 0 | 0 | 0 | 0 | 0 | 0 | 0 | 0 | 0 | 0 | 28 | 38 |  |  | PEAKS DB |
| R.VSPTASNM(+15.99)LK.M | N | 49.40 | 1062.5380 | 10 | -0.6 | 532.2759 | 2 | 11.55 | 4 | F4:2520 | NaSa01\_F12.raw |  |  |  | 1.5436E7 |  |  |  |  |  |  |  |  |  |  | 1 | 0 | 0 | 0 | 1 | 0 | 0 | 0 | 0 | 0 | 0 | 0 | 0 | 0 | 0 | 29 | 38 | Oxidation (M) | M8:Oxidation (M):1000.00 | PEAKS DB |
| Q.IVWYQTYR.A | Y | 48.98 | 1127.5764 | 8 | -4.0 | 564.7932 | 2 | 42.65 | 4 | F4:25261 | NaSa01\_F12.raw |  |  |  | 4.5531E6 |  |  |  |  |  |  |  |  |  |  | 1 | 0 | 0 | 0 | 1 | 0 | 0 | 0 | 0 | 0 | 0 | 0 | 0 | 0 | 0 | 119 | 126 |  |  | PEAKS DB |
| N.MLKMEWYPEAASNAER.W | N | 48.97 | 1924.8811 | 16 | 0.5 | 642.6346 | 3 | 47.89 | 4 | F4:29582 | NaSa01\_F12.raw |  |  |  | 1.1253E6 |  |  |  |  |  |  |  |  |  |  | 1 | 0 | 0 | 0 | 1 | 0 | 0 | 0 | 0 | 0 | 0 | 0 | 0 | 0 | 0 | 36 | 51 |  |  | PEAKS DB |
| E.RWANTC(+57.02)SLNHSPDNLR.V | Y | 48.62 | 1939.9071 | 16 | -0.2 | 647.6428 | 3 | 11.82 | 4 | F4:2745 | NaSa01\_F12.raw |  |  |  | 1.9776E6 |  |  |  |  |  |  |  |  |  |  | 1 | 0 | 0 | 0 | 1 | 0 | 0 | 0 | 0 | 0 | 0 | 0 | 0 | 0 | 0 | 51 | 66 | Carbamidomethylation | C6:Carbamidomethylation:1000.00 | PEAKS DB |
| Y.TQIVWYQTYR.A | Y | 47.87 | 1356.6826 | 10 | -1.4 | 679.3477 | 2 | 49.12 | 4 | F4:30645 | NaSa01\_F12.raw |  |  |  | 3.7309E6 |  |  |  |  |  |  |  |  |  |  | 1 | 0 | 0 | 0 | 1 | 0 | 0 | 0 | 0 | 0 | 0 | 0 | 0 | 0 | 0 | 117 | 126 |  |  | PEAKS DB |
| C.QYC(+57.02)PSGNFQGK.T | Y | 47.85 | 1284.5557 | 11 | -0.3 | 643.2849 | 2 | 11.76 | 4 | F4:2671 | NaSa01\_F12.raw |  |  |  | 2.6916E5 |  |  |  |  |  |  |  |  |  |  | 1 | 0 | 0 | 0 | 1 | 0 | 0 | 0 | 0 | 0 | 0 | 0 | 0 | 0 | 0 | 146 | 156 | Carbamidomethylation | C3:Carbamidomethylation:1000.00 | PEAKS DB |
| K.LGPPC(+57.02)GDC(+57.02)PSAC(+57.02)DNGL.C | Y | 47.57 | 1688.6593 | 16 | 0.9 | 845.3376 | 2 | 47.39 | 4 | F4:29273 | NaSa01\_F12.raw |  |  |  | 1.4372E7 |  |  |  |  |  |  |  |  |  |  | 1 | 0 | 0 | 0 | 1 | 0 | 0 | 0 | 0 | 0 | 0 | 0 | 0 | 0 | 0 | 163 | 178 | Carbamidomethylation | C5:Carbamidomethylation:1000.00;C8:Carbamidomethylation:1000.00;C12:Carbamidomethylation:1000.00 | PEAKS DB |
| R.VSPTASNM(+15.99)LKM(+15.99)EWYPEAASNAER.W | N | 47.23 | 2613.1838 | 23 | 0.8 | 872.0693 | 3 | 38.90 | 4 | F4:22253 | NaSa01\_F12.raw |  |  |  | 7.9992E6 |  |  |  |  |  |  |  |  |  |  | 1 | 0 | 0 | 0 | 1 | 0 | 0 | 0 | 0 | 0 | 0 | 0 | 0 | 0 | 0 | 29 | 51 | Oxidation (M) | M8:Oxidation (M):1000.00;M11:Oxidation (M):1000.00 | PEAKS DB |
| T.QIVWYQTYR.A | Y | 47.15 | 1255.6349 | 9 | -0.2 | 628.8246 | 2 | 46.43 | 4 | F4:28539 | NaSa01\_F12.raw |  |  |  | 1.0049E6 |  |  |  |  |  |  |  |  |  |  | 1 | 0 | 0 | 0 | 1 | 0 | 0 | 0 | 0 | 0 | 0 | 0 | 0 | 0 | 0 | 118 | 126 |  |  | PEAKS DB |
| L.RVLEGIQC(+57.02)GESIYM(+15.99)SSNAR.T | Y | 45.38 | 2185.0256 | 19 | 0.3 | 729.3494 | 3 | 36.12 | 4 | F4:19992 | NaSa01\_F12.raw |  |  |  | 1.1935E6 |  |  |  |  |  |  |  |  |  |  | 1 | 0 | 0 | 0 | 1 | 0 | 0 | 0 | 0 | 0 | 0 | 0 | 0 | 0 | 0 | 66 | 84 | Carbamidomethylation; Oxidation (M) | C8:Carbamidomethylation:1000.00;M14:Oxidation (M):1000.00 | PEAKS DB |
| R.TWTEIIHLWHD.E | Y | 44.31 | 1449.7041 | 11 | -0.1 | 484.2419 | 3 | 76.43 | 4 | F4:53592 | NaSa01\_F12.raw |  |  |  | 6.3002E6 |  |  |  |  |  |  |  |  |  |  | 2 | 0 | 0 | 0 | 2 | 0 | 0 | 0 | 0 | 0 | 0 | 0 | 0 | 0 | 0 | 85 | 95 |  |  | PEAKS DB |
| K.MEWYPEAASNAE.R | N | 44.04 | 1396.5605 | 12 | 0.5 | 699.2879 | 2 | 61.61 | 4 | F4:41017 | NaSa01\_F12.raw |  |  |  | 2.7222E6 |  |  |  |  |  |  |  |  |  |  | 1 | 0 | 0 | 0 | 1 | 0 | 0 | 0 | 0 | 0 | 0 | 0 | 0 | 0 | 0 | 39 | 50 |  |  | PEAKS DB |
| I.VDLHNSLR.R | N | 42.85 | 952.5090 | 8 | -1.0 | 477.2613 | 2 | 26.51 | 4 | F4:11862 | NaSa01\_F12.raw |  |  |  | 2.8116E8 |  |  |  |  |  |  |  |  |  |  | 1 | 0 | 0 | 0 | 1 | 0 | 0 | 0 | 0 | 0 | 0 | 0 | 0 | 0 | 0 | 19 | 26 |  |  | PEAKS DB |
| R.RVSPTASNMLKMEWYPEAASNAER.W | Y | 42.34 | 2737.2952 | 24 | 2.2 | 913.4410 | 3 | 51.07 | 4 | F4:32424 | NaSa01\_F12.raw |  |  |  | 1.8869E6 |  |  |  |  |  |  |  |  |  |  | 1 | 0 | 0 | 0 | 1 | 0 | 0 | 0 | 0 | 0 | 0 | 0 | 0 | 0 | 0 | 28 | 51 |  |  | PEAKS DB |
| R.TWTEIIHL.W | Y | 41.70 | 1011.5389 | 8 | 0.0 | 506.7768 | 2 | 72.54 | 4 | F4:50028 | NaSa01\_F12.raw |  |  |  | 7.7511E6 |  |  |  |  |  |  |  |  |  |  | 1 | 0 | 0 | 0 | 1 | 0 | 0 | 0 | 0 | 0 | 0 | 0 | 0 | 0 | 0 | 85 | 92 |  |  | PEAKS DB |
| A.C(+57.02)DNGLC(+57.02)TNPC(+57.02)TIYNK.L | Y | 41.26 | 1828.7543 | 15 | -0.4 | 915.3840 | 2 | 33.09 | 4 | F4:17353 | NaSa01\_F12.raw |  |  |  | 2.4548E6 |  |  |  |  |  |  |  |  |  |  | 1 | 0 | 0 | 0 | 1 | 0 | 0 | 0 | 0 | 0 | 0 | 0 | 0 | 0 | 0 | 174 | 188 | Carbamidomethylation | C1:Carbamidomethylation:1000.00;C6:Carbamidomethylation:1000.00;C10:Carbamidomethylation:1000.00 | PEAKS DB |
| G.IQC(+57.02)GESIYMSSNAR.T | Y | 41.22 | 1614.7130 | 14 | -0.2 | 808.3636 | 2 | 24.59 | 4 | F4:10632 | NaSa01\_F12.raw |  |  |  | 1.3785E6 |  |  |  |  |  |  |  |  |  |  | 1 | 0 | 0 | 0 | 1 | 0 | 0 | 0 | 0 | 0 | 0 | 0 | 0 | 0 | 0 | 71 | 84 | Carbamidomethylation | C3:Carbamidomethylation:1000.00 | PEAKS DB |
| A.RTWTEIIHLWHDEYK.N | Y | 40.99 | 2026.0061 | 15 | 0.5 | 507.5091 | 4 | 61.62 | 4 | F4:41097 | NaSa01\_F12.raw |  |  |  | 3.775E6 |  |  |  |  |  |  |  |  |  |  | 1 | 0 | 0 | 0 | 1 | 0 | 0 | 0 | 0 | 0 | 0 | 0 | 0 | 0 | 0 | 84 | 98 |  |  | PEAKS DB |
| A.WSYFYVC(+57.02)QYC(+57.02)PSGNFQGK.T | Y | 40.64 | 2289.9612 | 18 | -2.7 | 1145.9847 | 2 | 70.96 | 4 | F4:48864 | NaSa01\_F12.raw |  |  |  | 1.2488E6 |  |  |  |  |  |  |  |  |  |  | 1 | 0 | 0 | 0 | 1 | 0 | 0 | 0 | 0 | 0 | 0 | 0 | 0 | 0 | 0 | 139 | 156 | Carbamidomethylation | C7:Carbamidomethylation:1000.00;C10:Carbamidomethylation:1000.00 | PEAKS DB |
| R.VLEGIQC(+57.02)GESIY.M | Y | 40.52 | 1366.6438 | 12 | 0.7 | 684.3297 | 2 | 63.89 | 4 | F4:43118 | NaSa01\_F12.raw |  |  |  | 1.0552E8 |  |  |  |  |  |  |  |  |  |  | 1 | 0 | 0 | 0 | 1 | 0 | 0 | 0 | 0 | 0 | 0 | 0 | 0 | 0 | 0 | 67 | 78 | Carbamidomethylation | C7:Carbamidomethylation:1000.00 | PEAKS DB |
| R.VLEGIQC(+57.02)GESIYM.S | Y | 40.40 | 1497.6843 | 13 | 1.7 | 749.8507 | 2 | 74.39 | 4 | F4:51502 | NaSa01\_F12.raw |  |  |  | 5.5836E7 |  |  |  |  |  |  |  |  |  |  | 1 | 0 | 0 | 0 | 1 | 0 | 0 | 0 | 0 | 0 | 0 | 0 | 0 | 0 | 0 | 67 | 79 | Carbamidomethylation | C7:Carbamidomethylation:1000.00 | PEAKS DB |
| R.AGC(+57.02)AVSYC(+57.02)PSSAW.S | Y | 39.73 | 1414.5646 | 13 | 0.5 | 708.2899 | 2 | 56.74 | 4 | F4:36991 | NaSa01\_F12.raw |  |  |  | 1.3783E7 |  |  |  |  |  |  |  |  |  |  | 1 | 0 | 0 | 0 | 1 | 0 | 0 | 0 | 0 | 0 | 0 | 0 | 0 | 0 | 0 | 127 | 139 | Carbamidomethylation | C3:Carbamidomethylation:1000.00;C8:Carbamidomethylation:1000.00 | PEAKS DB |
| P.SAC(+57.02)DNGLC(+57.02)TNPC(+57.02)TIYNK.L | Y | 39.15 | 1986.8234 | 17 | -0.6 | 994.4184 | 2 | 33.70 | 4 | F4:17779 | NaSa01\_F12.raw |  |  |  | 1.5428E6 |  |  |  |  |  |  |  |  |  |  | 1 | 0 | 0 | 0 | 1 | 0 | 0 | 0 | 0 | 0 | 0 | 0 | 0 | 0 | 0 | 172 | 188 | Carbamidomethylation | C3:Carbamidomethylation:1000.00;C8:Carbamidomethylation:1000.00;C12:Carbamidomethylation:1000.00 | PEAKS DB |
| T.EIIHLWHDEYK.N | Y | 38.91 | 1481.7302 | 11 | -0.1 | 494.9173 | 3 | 34.09 | 4 | F4:18145 | NaSa01\_F12.raw |  |  |  | 5.1609E5 |  |  |  |  |  |  |  |  |  |  | 1 | 0 | 0 | 0 | 1 | 0 | 0 | 0 | 0 | 0 | 0 | 0 | 0 | 0 | 0 | 88 | 98 |  |  | PEAKS DB |
| K.QKEIVDLHN.S | N | 38.54 | 1094.5720 | 9 | 1.2 | 548.2939 | 2 | 11.68 | 4 | F4:2630 | NaSa01\_F12.raw |  |  |  | 6.029E4 |  |  |  |  |  |  |  |  |  |  | 1 | 0 | 0 | 0 | 1 | 0 | 0 | 0 | 0 | 0 | 0 | 0 | 0 | 0 | 0 | 15 | 23 |  |  | PEAKS DB |
| Y.KLGPPC(+57.02)GDC(+57.02)PSAC(+57.02)DNGLC(+57.02)TNPC(+57.02)TIYNK.L | Y | 37.77 | 3068.2917 | 27 | 1.8 | 1023.7731 | 3 | 39.94 | 4 | F4:22895 | NaSa01\_F12.raw |  |  |  | 2.227E8 |  |  |  |  |  |  |  |  |  |  | 1 | 0 | 0 | 0 | 1 | 0 | 0 | 0 | 0 | 0 | 0 | 0 | 0 | 0 | 0 | 162 | 188 | Carbamidomethylation | C6:Carbamidomethylation:1000.00;C9:Carbamidomethylation:1000.00;C13:Carbamidomethylation:1000.00;C18:Carbamidomethylation:1000.00;C22:Carbamidomethylation:1000.00 | PEAKS DB |
| total 88 peptides |  |  |  |  |  |  |  |  |  |  |  |  |  |  |  |  |  |  |  |  |  |  |  |  |  |  |  |  |  |  |  |  |  |  |  |  |  |  |  |  |  |  |  |  |  |
| --- | --- | --- | --- | --- | --- | --- | --- | --- | --- | --- | --- | --- | --- | --- | --- | --- | --- | --- | --- | --- | --- | --- | --- | --- | --- | --- | --- | --- | --- | --- | --- | --- | --- | --- | --- | --- | --- | --- | --- | --- | --- | --- | --- | --- | --- |

1XX5

back to list

  

| Protein Coverage
| Supporting Peptides
|

Protein Coverage:

Supporting Peptides:

| Peptide | Uniq | -10lgP | Mass | Length | ppm | m/z | z | RT | Fraction | Scan | Source File | Area F1 | Area F10 | Area F11 | Area F12 | Area F13 | Area F14 | Area F2 | Area F3 | Area F4 | Area F5 | Area F6 | Area F7 | Area F8 | Area F9 | #Feature | #Feature F1 | #Feature F10 | #Feature F11 | #Feature F12 | #Feature F13 | #Feature F14 | #Feature F2 | #Feature F3 | #Feature F4 | #Feature F5 | #Feature F6 | #Feature F7 | #Feature F8 | #Feature F9 | Start | End | PTM | AScore | Found By |
| --- | --- | --- | --- | --- | --- | --- | --- | --- | --- | --- | --- | --- | --- | --- | --- | --- | --- | --- | --- | --- | --- | --- | --- | --- | --- | --- | --- | --- | --- | --- | --- | --- | --- | --- | --- | --- | --- | --- | --- | --- | --- | --- | --- | --- | --- |
| R.TWTEIIHLWHDEYK.N | Y | 86.98 | 1869.9049 | 14 | -0.4 | 935.9594 | 2 | 69.11 | 4 | F4:46528 | NaSa01\_F12.raw |  |  | 1.6758E6 | 5.726E9 | 7.3513E6 |  |  |  |  |  |  |  |  |  | 9 | 0 | 0 | 1 | 6 | 2 | 0 | 0 | 0 | 0 | 0 | 0 | 0 | 0 | 0 | 85 | 98 |  |  | PEAKS DB |
| R.VLEGIQC(+57.02)GESIYMSSNAR.T | Y | 86.82 | 2012.9296 | 18 | -0.1 | 1007.4720 | 2 | 58.59 | 4 | F4:37662 | NaSa01\_F12.raw |  | 6.539E5 | 1.5229E7 | 1.9162E10 | 6.8803E7 | 1.1875E7 |  |  |  |  |  |  |  |  | 13 | 0 | 1 | 2 | 6 | 2 | 2 | 0 | 0 | 0 | 0 | 0 | 0 | 0 | 0 | 67 | 84 | Carbamidomethylation | C7:Carbamidomethylation:1000.00 | PEAKS DB |
| R.VLEGIQC(+57.02)GESIYM(+15.99)SSNAR.T | Y | 83.82 | 2028.9244 | 18 | -1.0 | 1015.4684 | 2 | 49.35 | 4 | F4:32620 | NaSa01\_F12.raw |  |  |  | 2.596E9 | 2.4998E7 | 3.3484E6 |  |  |  |  |  |  |  |  | 24 | 0 | 0 | 0 | 20 | 2 | 2 | 0 | 0 | 0 | 0 | 0 | 0 | 0 | 0 | 67 | 84 | Carbamidomethylation; Oxidation (M) | C7:Carbamidomethylation:1000.00;M13:Oxidation (M):1000.00 | PEAKS DB |
| R.WANTC(+57.02)SLNHSPDNLR.V | Y | 83.41 | 1783.8060 | 15 | -0.3 | 892.9100 | 2 | 20.24 | 4 | F4:7228 | NaSa01\_F12.raw |  |  |  | 4.9332E10 | 1.1987E8 | 6.2009E4 |  |  |  |  |  |  |  |  | 25 | 0 | 0 | 0 | 20 | 4 | 1 | 0 | 0 | 0 | 0 | 0 | 0 | 0 | 0 | 52 | 66 | Carbamidomethylation | C5:Carbamidomethylation:1000.00 | PEAKS DB |
| K.QSSC(+57.02)QDDWIKSNC(+57.02)PASC(+57.02)FC(+57.02)R.N | Y | 79.80 | 2504.9929 | 20 | 0.6 | 836.0054 | 3 | 39.02 | 4 | F4:22824 | NaSa01\_F12.raw |  |  |  | 7.3335E7 |  |  |  |  |  |  |  |  |  |  | 3 | 0 | 0 | 0 | 3 | 0 | 0 | 0 | 0 | 0 | 0 | 0 | 0 | 0 | 0 | 198 | 217 | Carbamidomethylation | C4:Carbamidomethylation:1000.00;C13:Carbamidomethylation:1000.00;C17:Carbamidomethylation:1000.00;C19:Carbamidomethylation:1000.00 | PEAKS DB |
| K.LTNC(+57.02)DSLLKQSSC(+57.02)QDDWIK.S | Y | 79.46 | 2310.0620 | 19 | -1.1 | 771.0271 | 3 | 45.31 | 4 | F4:28181 | NaSa01\_F12.raw |  |  |  | 1.962E8 |  |  |  |  |  |  |  |  |  |  | 4 | 0 | 0 | 0 | 4 | 0 | 0 | 0 | 0 | 0 | 0 | 0 | 0 | 0 | 0 | 189 | 207 | Carbamidomethylation | C4:Carbamidomethylation:1000.00;C13:Carbamidomethylation:1000.00 | PEAKS DB |
| D.C(+57.02)PSAC(+57.02)DNGLC(+57.02)TNPC(+57.02)TIYNK.L | Y | 78.07 | 2243.9067 | 19 | -0.3 | 1122.9603 | 2 | 35.53 | 4 | F4:19614 | NaSa01\_F12.raw |  |  |  | 1.255E8 |  |  |  |  |  |  |  |  |  |  | 3 | 0 | 0 | 0 | 3 | 0 | 0 | 0 | 0 | 0 | 0 | 0 | 0 | 0 | 0 | 170 | 188 | Carbamidomethylation | C1:Carbamidomethylation:1000.00;C5:Carbamidomethylation:1000.00;C10:Carbamidomethylation:1000.00;C14:Carbamidomethylation:1000.00 | PEAKS DB |
| K.NFVYGVGANPPGSVTGHYTQIVWYQTYR.A | Y | 77.64 | 3173.5359 | 28 | 0.0 | 1058.8525 | 3 | 74.13 | 4 | F4:51117 | NaSa01\_F12.raw |  |  |  | 1.5642E9 |  |  |  |  |  |  |  |  |  |  | 6 | 0 | 0 | 0 | 6 | 0 | 0 | 0 | 0 | 0 | 0 | 0 | 0 | 0 | 0 | 99 | 126 |  |  | PEAKS DB |
| Y.GVGANPPGSVTGHYTQIVWYQTYR.A | Y | 77.12 | 2650.2927 | 24 | 0.9 | 884.4390 | 3 | 57.12 | 4 | F4:37409 | NaSa01\_F12.raw |  |  | 5.2237E5 | 6.6355E7 |  |  |  |  |  |  |  |  |  |  | 2 | 0 | 0 | 1 | 1 | 0 | 0 | 0 | 0 | 0 | 0 | 0 | 0 | 0 | 0 | 103 | 126 |  |  | PEAKS DB |
| R.VSPTASNMLKMEWYPEAASNAER.W | N | 76.94 | 2581.1941 | 23 | 0.7 | 861.4059 | 3 | 60.25 | 4 | F4:39967 | NaSa01\_F12.raw |  |  |  | 1.7324E8 |  |  |  |  |  |  |  |  |  |  | 2 | 0 | 0 | 0 | 2 | 0 | 0 | 0 | 0 | 0 | 0 | 0 | 0 | 0 | 0 | 29 | 51 |  |  | PEAKS DB |
| K.MEWYPEAASNAER.W | N | 75.93 | 1552.6616 | 13 | -4.7 | 518.5587 | 3 | 42.47 | 4 | F4:25783 | NaSa01\_F12.raw |  |  | 8.9734E6 | 5.588E10 | 1.0906E8 | 2.4001E7 |  |  |  |  |  |  |  |  | 18 | 0 | 0 | 1 | 10 | 5 | 2 | 0 | 0 | 0 | 0 | 0 | 0 | 0 | 0 | 39 | 51 |  |  | PEAKS DB |
| W.SYFYVC(+57.02)QYC(+57.02)PSGNFQGK.T | Y | 74.81 | 2103.8818 | 17 | 0.9 | 1052.9491 | 2 | 56.78 | 4 | F4:37002 | NaSa01\_F12.raw |  |  |  | 7.9496E7 | 3.5056E6 |  |  |  |  |  |  |  |  |  | 3 | 0 | 0 | 0 | 2 | 1 | 0 | 0 | 0 | 0 | 0 | 0 | 0 | 0 | 0 | 140 | 156 | Carbamidomethylation | C6:Carbamidomethylation:1000.00;C9:Carbamidomethylation:1000.00 | PEAKS DB |
| K.QKEIVDLHNSLR.R | N | 73.79 | 1450.7892 | 12 | 0.4 | 484.6039 | 3 | 11.92 | 4 | F4:3036 | NaSa01\_F12.raw |  |  |  | 1.138E8 | 7.1992E5 |  |  |  |  |  |  |  |  |  | 16 | 0 | 0 | 0 | 15 | 1 | 0 | 0 | 0 | 0 | 0 | 0 | 0 | 0 | 0 | 15 | 26 |  |  | PEAKS DB |
| L.RVLEGIQC(+57.02)GESIYMSSNAR.T | Y | 73.68 | 2169.0308 | 19 | -0.1 | 724.0175 | 3 | 41.70 | 4 | F4:24692 | NaSa01\_F12.raw |  |  |  | 1.2641E7 |  |  |  |  |  |  |  |  |  |  | 1 | 0 | 0 | 0 | 1 | 0 | 0 | 0 | 0 | 0 | 0 | 0 | 0 | 0 | 0 | 66 | 84 | Carbamidomethylation | C8:Carbamidomethylation:1000.00 | PEAKS DB |
| C.PSAC(+57.02)DNGLC(+57.02)TNPC(+57.02)TIYNK.L | Y | 73.51 | 2083.8762 | 18 | 0.2 | 1042.9456 | 2 | 34.80 | 4 | F4:18795 | NaSa01\_F12.raw |  |  |  | 1.0016E7 |  |  |  |  |  |  |  |  |  |  | 1 | 0 | 0 | 0 | 1 | 0 | 0 | 0 | 0 | 0 | 0 | 0 | 0 | 0 | 0 | 171 | 188 | Carbamidomethylation | C4:Carbamidomethylation:1000.00;C9:Carbamidomethylation:1000.00;C13:Carbamidomethylation:1000.00 | PEAKS DB |
| S.SAWSYFYVC(+57.02)QYC(+57.02)PSGNFQGK.T | Y | 73.42 | 2448.0303 | 20 | -0.2 | 1225.0222 | 2 | 75.77 | 4 | F4:52609 | NaSa01\_F12.raw |  |  |  | 7.5549E7 |  |  |  |  |  |  |  |  |  |  | 2 | 0 | 0 | 0 | 2 | 0 | 0 | 0 | 0 | 0 | 0 | 0 | 0 | 0 | 0 | 137 | 156 | Carbamidomethylation | C9:Carbamidomethylation:1000.00;C12:Carbamidomethylation:1000.00 | PEAKS DB |
| K.M(+15.99)EWYPEAASNAER.W | N | 73.15 | 1568.6565 | 13 | 0.9 | 523.8932 | 3 | 33.16 | 4 | F4:17227 | NaSa01\_F12.raw |  |  |  | 1.0876E10 | 1.0638E7 | 4.7019E6 |  |  |  |  |  |  |  |  | 17 | 0 | 0 | 0 | 15 | 1 | 1 | 0 | 0 | 0 | 0 | 0 | 0 | 0 | 0 | 39 | 51 | Oxidation (M) | M1:Oxidation (M):1000.00 | PEAKS DB |
| K.EIVDLHNSLR.R | N | 71.95 | 1194.6356 | 10 | 0.4 | 598.3253 | 2 | 28.76 | 4 | F4:13650 | NaSa01\_F12.raw |  |  |  | 7.7068E9 | 7.5889E7 | 5.7777E4 |  |  |  |  |  |  |  |  | 27 | 0 | 0 | 0 | 23 | 3 | 1 | 0 | 0 | 0 | 0 | 0 | 0 | 0 | 0 | 17 | 26 |  |  | PEAKS DB |
| Y.C(+57.02)PSSAWSYFYVC(+57.02)QYC(+57.02)PSGNFQGK.T | Y | 71.68 | 2792.1458 | 23 | 1.2 | 931.7236 | 3 | 77.58 | 4 | F4:54131 | NaSa01\_F12.raw |  |  |  | 2.7393E7 |  |  |  |  |  |  |  |  |  |  | 2 | 0 | 0 | 0 | 2 | 0 | 0 | 0 | 0 | 0 | 0 | 0 | 0 | 0 | 0 | 134 | 156 | Carbamidomethylation | C1:Carbamidomethylation:1000.00;C12:Carbamidomethylation:1000.00;C15:Carbamidomethylation:1000.00 | PEAKS DB |
| Y.FYVC(+57.02)QYC(+57.02)PSGNFQGK.T | Y | 71.29 | 1853.7865 | 15 | 0.2 | 927.9008 | 2 | 45.22 | 4 | F4:27254 | NaSa01\_F12.raw |  |  |  | 6.7966E7 |  |  |  |  |  |  |  |  |  |  | 1 | 0 | 0 | 0 | 1 | 0 | 0 | 0 | 0 | 0 | 0 | 0 | 0 | 0 | 0 | 142 | 156 | Carbamidomethylation | C4:Carbamidomethylation:1000.00;C7:Carbamidomethylation:1000.00 | PEAKS DB |
| F.YVC(+57.02)QYC(+57.02)PSGNFQGK.T | Y | 70.89 | 1706.7181 | 14 | -0.4 | 854.3660 | 2 | 26.36 | 4 | F4:11724 | NaSa01\_F12.raw |  |  |  | 1.5606E8 | 1.5285E7 |  |  |  |  |  |  |  |  |  | 2 | 0 | 0 | 0 | 1 | 1 | 0 | 0 | 0 | 0 | 0 | 0 | 0 | 0 | 0 | 143 | 156 | Carbamidomethylation | C3:Carbamidomethylation:1000.00;C6:Carbamidomethylation:1000.00 | PEAKS DB |
| D.NGLC(+57.02)TNPC(+57.02)TIYNK.L | Y | 69.78 | 1553.6967 | 13 | 0.5 | 777.8560 | 2 | 28.19 | 4 | F4:13013 | NaSa01\_F12.raw |  |  |  | 3.1653E7 |  |  |  |  |  |  |  |  |  |  | 1 | 0 | 0 | 0 | 1 | 0 | 0 | 0 | 0 | 0 | 0 | 0 | 0 | 0 | 0 | 176 | 188 | Carbamidomethylation | C4:Carbamidomethylation:1000.00;C8:Carbamidomethylation:1000.00 | PEAKS DB |
| K.SNC(+57.02)PASC(+57.02)FC(+57.02)R.N | N | 69.18 | 1257.4689 | 10 | 0.2 | 629.7418 | 2 | 11.79 | 4 | F4:2692 | NaSa01\_F12.raw |  |  |  | 1.0262E8 |  |  |  |  |  |  |  |  |  |  | 1 | 0 | 0 | 0 | 1 | 0 | 0 | 0 | 0 | 0 | 0 | 0 | 0 | 0 | 0 | 208 | 217 | Carbamidomethylation | C3:Carbamidomethylation:1000.00;C7:Carbamidomethylation:1000.00;C9:Carbamidomethylation:1000.00 | PEAKS DB |
| K.QSSC(+57.02)QDDWIK.S | Y | 68.96 | 1265.5347 | 10 | -0.7 | 633.7742 | 2 | 11.92 | 4 | F4:3391 | NaSa01\_F12.raw |  |  |  | 3.0416E8 |  |  |  |  |  |  |  |  |  |  | 8 | 0 | 0 | 0 | 8 | 0 | 0 | 0 | 0 | 0 | 0 | 0 | 0 | 0 | 0 | 198 | 207 | Carbamidomethylation | C4:Carbamidomethylation:1000.00 | PEAKS DB |
| K.MEWYPEAASNAERWANTC(+57.02)SLNHSPDNLR.V | Y | 66.81 | 3318.4570 | 28 | 0.1 | 830.6216 | 4 | 60.99 | 4 | F4:40505 | NaSa01\_F12.raw |  |  |  | 1.1514E7 |  |  |  |  |  |  |  |  |  |  | 3 | 0 | 0 | 0 | 3 | 0 | 0 | 0 | 0 | 0 | 0 | 0 | 0 | 0 | 0 | 39 | 66 | Carbamidomethylation | C18:Carbamidomethylation:1000.00 | PEAKS DB |
| N.GLC(+57.02)TNPC(+57.02)TIYNK.L | Y | 66.27 | 1439.6537 | 12 | -0.2 | 720.8340 | 2 | 27.00 | 4 | F4:12069 | NaSa01\_F12.raw |  |  |  | 3.5701E7 |  |  |  |  |  |  |  |  |  |  | 1 | 0 | 0 | 0 | 1 | 0 | 0 | 0 | 0 | 0 | 0 | 0 | 0 | 0 | 0 | 177 | 188 | Carbamidomethylation | C3:Carbamidomethylation:1000.00;C7:Carbamidomethylation:1000.00 | PEAKS DB |
| E.GIQC(+57.02)GESIYMSSNAR.T | Y | 64.94 | 1671.7345 | 15 | 0.6 | 836.8750 | 2 | 29.68 | 4 | F4:14460 | NaSa01\_F12.raw |  |  |  | 1.9465E6 |  |  |  |  |  |  |  |  |  |  | 1 | 0 | 0 | 0 | 1 | 0 | 0 | 0 | 0 | 0 | 0 | 0 | 0 | 0 | 0 | 70 | 84 | Carbamidomethylation | C4:Carbamidomethylation:1000.00 | PEAKS DB |
| N.PPGSVTGHYTQIVWYQTYR.A | Y | 64.76 | 2252.1013 | 19 | 0.1 | 751.7078 | 3 | 53.16 | 4 | F4:33903 | NaSa01\_F12.raw |  |  |  | 4.1382E6 |  |  |  |  |  |  |  |  |  |  | 1 | 0 | 0 | 0 | 1 | 0 | 0 | 0 | 0 | 0 | 0 | 0 | 0 | 0 | 0 | 108 | 126 |  |  | PEAKS DB |
| K.NFVYGVGANPPGSVTGH.Y | N | 64.66 | 1671.8005 | 17 | 0.7 | 836.9081 | 2 | 48.12 | 4 | F4:29754 | NaSa01\_F12.raw |  |  |  | 2.7791E8 | 1.1488E7 | 2.2522E6 |  |  |  |  |  |  |  |  | 6 | 0 | 0 | 0 | 4 | 1 | 1 | 0 | 0 | 0 | 0 | 0 | 0 | 0 | 0 | 99 | 115 |  |  | PEAKS DB |
| NVDFNSESTR.R | N | 64.34 | 1167.5156 | 10 | 0.3 | 584.7653 | 2 | 12.14 | 4 | F4:3045 | NaSa01\_F12.raw |  |  |  | 1.4013E8 |  |  |  |  |  |  |  |  |  |  | 6 | 0 | 0 | 0 | 6 | 0 | 0 | 0 | 0 | 0 | 0 | 0 | 0 | 0 | 0 | 1 | 10 |  |  | PEAKS DB |
| K.LTNC(+57.02)DSLLK.Q | Y | 64.00 | 1062.5380 | 9 | -1.6 | 532.2754 | 2 | 21.02 | 4 | F4:7364 | NaSa01\_F12.raw |  |  |  | 2.9662E8 |  |  |  |  |  |  |  |  |  |  | 6 | 0 | 0 | 0 | 6 | 0 | 0 | 0 | 0 | 0 | 0 | 0 | 0 | 0 | 0 | 189 | 197 | Carbamidomethylation | C4:Carbamidomethylation:1000.00 | PEAKS DB |
| H.YTQIVWYQTYR.A | Y | 63.87 | 1519.7460 | 11 | 1.3 | 760.8812 | 2 | 55.25 | 4 | F4:35871 | NaSa01\_F12.raw |  |  |  | 1.2665E8 | 2.5476E6 |  |  |  |  |  |  |  |  |  | 3 | 0 | 0 | 0 | 2 | 1 | 0 | 0 | 0 | 0 | 0 | 0 | 0 | 0 | 0 | 116 | 126 |  |  | PEAKS DB |
| N.VDFNSESTR.R | N | 63.60 | 1053.4727 | 9 | 0.3 | 527.7438 | 2 | 11.74 | 4 | F4:2649 | NaSa01\_F12.raw |  |  |  | 4.6861E6 |  |  |  |  |  |  |  |  |  |  | 1 | 0 | 0 | 0 | 1 | 0 | 0 | 0 | 0 | 0 | 0 | 0 | 0 | 0 | 0 | 2 | 10 |  |  | PEAKS DB |
| C.DNGLC(+57.02)TNPC(+57.02)TIYNK.L | Y | 63.45 | 1668.7236 | 14 | 0.8 | 835.3698 | 2 | 36.30 | 4 | F4:20118 | NaSa01\_F12.raw |  |  |  | 3.0623E7 |  |  |  |  |  |  |  |  |  |  | 1 | 0 | 0 | 0 | 1 | 0 | 0 | 0 | 0 | 0 | 0 | 0 | 0 | 0 | 0 | 175 | 188 | Carbamidomethylation | C5:Carbamidomethylation:1000.00;C9:Carbamidomethylation:1000.00 | PEAKS DB |
| L.EGIQC(+57.02)GESIYMSSNAR.T | Y | 62.23 | 1800.7771 | 16 | 0.5 | 901.3963 | 2 | 35.36 | 4 | F4:19124 | NaSa01\_F12.raw |  |  |  | 1.9136E6 |  |  |  |  |  |  |  |  |  |  | 1 | 0 | 0 | 0 | 1 | 0 | 0 | 0 | 0 | 0 | 0 | 0 | 0 | 0 | 0 | 69 | 84 | Carbamidomethylation | C5:Carbamidomethylation:1000.00 | PEAKS DB |
| R.VSPTASNM(+15.99)LKMEWYPEAASNAER.W | N | 61.53 | 2597.1890 | 23 | 0.7 | 866.7375 | 3 | 47.88 | 4 | F4:29657 | NaSa01\_F12.raw |  |  |  | 1.0394E7 |  |  |  |  |  |  |  |  |  |  | 2 | 0 | 0 | 0 | 2 | 0 | 0 | 0 | 0 | 0 | 0 | 0 | 0 | 0 | 0 | 29 | 51 | Oxidation (M) | M8:Oxidation (M):24.24 | PEAKS DB |
| K.LGPPC(+57.02)GDC(+57.02)PSAC(+57.02)DNGLC(+57.02)TNPC(+57.02)TIYNK.L | Y | 61.29 | 2940.1970 | 26 | -0.2 | 981.0728 | 3 | 53.63 | 4 | F4:34247 | NaSa01\_F12.raw |  |  |  | 1.1603E10 |  | 3.4496E6 |  |  |  |  |  | 6.1046E6 |  |  | 9 | 0 | 0 | 0 | 7 | 0 | 1 | 0 | 0 | 0 | 0 | 0 | 1 | 0 | 0 | 163 | 188 | Carbamidomethylation | C5:Carbamidomethylation:1000.00;C8:Carbamidomethylation:1000.00;C12:Carbamidomethylation:1000.00;C17:Carbamidomethylation:1000.00;C21:Carbamidomethylation:1000.00 | PEAKS DB |
| K.NFVYGVGANPPGSVTGHY.T | N | 60.74 | 1834.8638 | 18 | 1.5 | 918.4406 | 2 | 53.13 | 4 | F4:33956 | NaSa01\_F12.raw |  |  |  | 5.187E6 |  |  |  |  |  |  |  |  |  |  | 1 | 0 | 0 | 0 | 1 | 0 | 0 | 0 | 0 | 0 | 0 | 0 | 0 | 0 | 0 | 99 | 116 |  |  | PEAKS DB |
| R.TWTEIIHLWH.D | Y | 59.61 | 1334.6771 | 10 | 0.6 | 668.3463 | 2 | 74.13 | 4 | F4:51227 | NaSa01\_F12.raw |  |  |  | 5.1108E6 |  |  |  |  |  |  |  |  |  |  | 1 | 0 | 0 | 0 | 1 | 0 | 0 | 0 | 0 | 0 | 0 | 0 | 0 | 0 | 0 | 85 | 94 |  |  | PEAKS DB |
| K.LGPPC(+57.02)GDC(+57.02)PSAC(+57.02)DNGLC(+57.02)TNPC(+57.02)TIYNKLTNC(+57.02)DSLLK.Q | Y | 59.18 | 3984.7244 | 35 | -0.2 | 1329.2484 | 3 | 74.37 | 4 | F4:51337 | NaSa01\_F12.raw |  |  |  | 2.2817E8 |  |  |  |  |  |  |  |  |  |  | 3 | 0 | 0 | 0 | 3 | 0 | 0 | 0 | 0 | 0 | 0 | 0 | 0 | 0 | 0 | 163 | 197 | Carbamidomethylation | C5:Carbamidomethylation:1000.00;C8:Carbamidomethylation:1000.00;C12:Carbamidomethylation:1000.00;C17:Carbamidomethylation:1000.00;C21:Carbamidomethylation:1000.00;C30:Carbamidomethylation:1000.00 | PEAKS DB |
| K.TATPYKLGPPC(+57.02)GDC(+57.02)PSAC(+57.02)DNGLC(+57.02)TNPC(+57.02)TIYNK.L | Y | 58.88 | 3601.5405 | 32 | 0.0 | 1201.5208 | 3 | 49.06 | 4 | F4:30641 | NaSa01\_F12.raw |  |  |  | 5.9598E7 |  |  |  |  |  |  |  |  |  |  | 1 | 0 | 0 | 0 | 1 | 0 | 0 | 0 | 0 | 0 | 0 | 0 | 0 | 0 | 0 | 157 | 188 | Carbamidomethylation | C11:Carbamidomethylation:1000.00;C14:Carbamidomethylation:1000.00;C18:Carbamidomethylation:1000.00;C23:Carbamidomethylation:1000.00;C27:Carbamidomethylation:1000.00 | PEAKS DB |
| G.LC(+57.02)TNPC(+57.02)TIYNK.L | Y | 58.71 | 1382.6323 | 11 | 0.6 | 692.3239 | 2 | 23.47 | 4 | F4:9229 | NaSa01\_F12.raw |  |  |  | 2.5631E6 |  |  |  |  |  |  |  | 1.5272E6 |  |  | 2 | 0 | 0 | 0 | 1 | 0 | 0 | 0 | 0 | 0 | 0 | 0 | 1 | 0 | 0 | 178 | 188 | Carbamidomethylation | C2:Carbamidomethylation:1000.00;C6:Carbamidomethylation:1000.00 | PEAKS DB |
| R.WANTC(+57.02)SLNHSPDNLRVLEGIQC(+57.02)GESIYMSSNAR.T | Y | 58.28 | 3778.7251 | 33 | -0.2 | 945.6884 | 4 | 66.79 | 4 | F4:45302 | NaSa01\_F12.raw |  |  |  | 2.4518E7 |  |  |  |  |  |  |  |  |  |  | 2 | 0 | 0 | 0 | 2 | 0 | 0 | 0 | 0 | 0 | 0 | 0 | 0 | 0 | 0 | 52 | 84 | Carbamidomethylation | C5:Carbamidomethylation:1000.00;C22:Carbamidomethylation:1000.00 | PEAKS DB |
| S.AC(+57.02)DNGLC(+57.02)TNPC(+57.02)TIYNK.L | Y | 58.09 | 1899.7914 | 16 | -0.8 | 950.9022 | 2 | 32.96 | 4 | F4:17191 | NaSa01\_F12.raw |  |  |  | 5.221E6 |  |  |  |  |  |  |  |  |  |  | 1 | 0 | 0 | 0 | 1 | 0 | 0 | 0 | 0 | 0 | 0 | 0 | 0 | 0 | 0 | 173 | 188 | Carbamidomethylation | C2:Carbamidomethylation:1000.00;C7:Carbamidomethylation:1000.00;C11:Carbamidomethylation:1000.00 | PEAKS DB |
| E.IVDLHNSLR.R | N | 58.01 | 1065.5930 | 9 | -1.6 | 533.8029 | 2 | 26.50 | 4 | F4:11729 | NaSa01\_F12.raw |  |  |  | 1.3096E7 |  |  |  |  |  |  |  |  |  |  | 1 | 0 | 0 | 0 | 1 | 0 | 0 | 0 | 0 | 0 | 0 | 0 | 0 | 0 | 0 | 18 | 26 |  |  | PEAKS DB |
| R.AGC(+57.02)AVSYC(+57.02)PSSAWSYFYVC(+57.02)QYC(+57.02)PSGNFQGK.T | Y | 57.96 | 3500.4358 | 30 | 0.3 | 1167.8196 | 3 | 84.20 | 4 | F4:60175 | NaSa01\_F12.raw |  |  |  | 8.8824E8 |  |  |  |  |  |  |  |  |  |  | 3 | 0 | 0 | 0 | 3 | 0 | 0 | 0 | 0 | 0 | 0 | 0 | 0 | 0 | 0 | 127 | 156 | Carbamidomethylation | C3:Carbamidomethylation:1000.00;C8:Carbamidomethylation:1000.00;C19:Carbamidomethylation:1000.00;C22:Carbamidomethylation:1000.00 | PEAKS DB |
| N.TC(+57.02)SLNHSPDNLR.V | Y | 57.81 | 1412.6466 | 12 | 0.4 | 471.8897 | 3 | 11.55 | 4 | F4:2523 | NaSa01\_F12.raw |  |  |  | 6.8334E6 |  |  |  |  |  |  |  |  |  |  | 2 | 0 | 0 | 0 | 2 | 0 | 0 | 0 | 0 | 0 | 0 | 0 | 0 | 0 | 0 | 55 | 66 | Carbamidomethylation | C2:Carbamidomethylation:1000.00 | PEAKS DB |
| R.VSPTASNMLK.M | N | 57.39 | 1046.5430 | 10 | 0.1 | 524.2788 | 2 | 11.91 | 4 | F4:3054 | NaSa01\_F12.raw |  |  |  | 1.9382E8 |  |  |  |  |  |  |  |  |  |  | 5 | 0 | 0 | 0 | 5 | 0 | 0 | 0 | 0 | 0 | 0 | 0 | 0 | 0 | 0 | 29 | 38 |  |  | PEAKS DB |
| L.KMEWYPEAASNAER.W | N | 57.28 | 1680.7566 | 14 | 0.7 | 561.2599 | 3 | 28.56 | 4 | F4:13207 | NaSa01\_F12.raw |  |  |  | 1.222E8 |  |  |  |  |  |  |  |  |  |  | 2 | 0 | 0 | 0 | 2 | 0 | 0 | 0 | 0 | 0 | 0 | 0 | 0 | 0 | 0 | 38 | 51 |  |  | PEAKS DB |
| R.VSPTASNMLKM(+15.99)EWYPEAASNAER.W | N | 57.05 | 2597.1890 | 23 | 0.7 | 866.7375 | 3 | 49.69 | 4 | F4:31613 | NaSa01\_F12.raw |  |  |  | 1.4277E7 |  |  |  |  |  |  |  |  |  |  | 1 | 0 | 0 | 0 | 1 | 0 | 0 | 0 | 0 | 0 | 0 | 0 | 0 | 0 | 0 | 29 | 51 | Oxidation (M) | M11:Oxidation (M):32.97 | PEAKS DB |
| W.TEIIHLWHDEYK.N | Y | 56.98 | 1582.7780 | 12 | 0.1 | 528.6000 | 3 | 36.11 | 4 | F4:19977 | NaSa01\_F12.raw |  |  |  | 9.0049E6 |  |  |  |  |  |  |  |  |  |  | 4 | 0 | 0 | 0 | 4 | 0 | 0 | 0 | 0 | 0 | 0 | 0 | 0 | 0 | 0 | 87 | 98 |  |  | PEAKS DB |
| R.VLEGIQC(+57.02)GESIYMSSN.A | Y | 56.56 | 1785.7914 | 16 | 0.3 | 893.9033 | 2 | 68.26 | 4 | F4:46624 | NaSa01\_F12.raw |  |  |  | 2.5137E8 |  |  |  |  |  |  |  |  |  |  | 1 | 0 | 0 | 0 | 1 | 0 | 0 | 0 | 0 | 0 | 0 | 0 | 0 | 0 | 0 | 67 | 82 | Carbamidomethylation | C7:Carbamidomethylation:1000.00 | PEAKS DB |
| E.IIHLWHDEYK.N | N | 55.71 | 1352.6877 | 10 | -0.7 | 451.9029 | 3 | 26.09 | 4 | F4:11533 | NaSa01\_F12.raw |  |  |  | 6.5239E5 |  |  |  |  |  |  |  |  |  |  | 1 | 0 | 0 | 0 | 1 | 0 | 0 | 0 | 0 | 0 | 0 | 0 | 0 | 0 | 0 | 89 | 98 |  |  | PEAKS DB |
| C.AVSYC(+57.02)PSSAWSYFYVC(+57.02)QYC(+57.02)PSGNFQGK.T | Y | 55.49 | 3212.3467 | 27 | 0.6 | 1071.7902 | 3 | 83.95 | 4 | F4:59535 | NaSa01\_F12.raw |  |  |  | 1.1276E8 |  |  |  |  |  |  |  |  |  |  | 1 | 0 | 0 | 0 | 1 | 0 | 0 | 0 | 0 | 0 | 0 | 0 | 0 | 0 | 0 | 130 | 156 | Carbamidomethylation | C5:Carbamidomethylation:1000.00;C16:Carbamidomethylation:1000.00;C19:Carbamidomethylation:1000.00 | PEAKS DB |
| M.EWYPEAASNAER.W | N | 54.75 | 1421.6211 | 12 | 0.7 | 711.8183 | 2 | 30.32 | 4 | F4:14915 | NaSa01\_F12.raw |  |  |  | 1.4758E7 |  |  |  |  |  |  |  |  |  |  | 1 | 0 | 0 | 0 | 1 | 0 | 0 | 0 | 0 | 0 | 0 | 0 | 0 | 0 | 0 | 40 | 51 |  |  | PEAKS DB |
| NVDFNSESTRR.K | N | 53.63 | 1323.6167 | 11 | 0.6 | 662.8160 | 2 | 11.55 | 4 | F4:2518 | NaSa01\_F12.raw |  |  |  | 1.6815E7 |  |  |  |  |  |  |  |  |  |  | 1 | 0 | 0 | 0 | 1 | 0 | 0 | 0 | 0 | 0 | 0 | 0 | 0 | 0 | 0 | 1 | 11 |  |  | PEAKS DB |
| C.SLNHSPDNLR.V | Y | 52.12 | 1151.5684 | 10 | 0.9 | 576.7920 | 2 | 11.55 | 4 | F4:2531 | NaSa01\_F12.raw |  |  |  | 3.5847E6 |  |  |  |  |  |  |  |  |  |  | 3 | 0 | 0 | 0 | 3 | 0 | 0 | 0 | 0 | 0 | 0 | 0 | 0 | 0 | 0 | 57 | 66 |  |  | PEAKS DB |
| C.GDC(+57.02)PSAC(+57.02)DNGLC(+57.02)TNPC(+57.02)TIYNK.L | Y | 51.17 | 2415.9553 | 21 | 0.4 | 1208.9855 | 2 | 38.69 | 4 | F4:22109 | NaSa01\_F12.raw |  |  |  | 3.1743E6 |  |  |  |  |  |  |  |  |  |  | 1 | 0 | 0 | 0 | 1 | 0 | 0 | 0 | 0 | 0 | 0 | 0 | 0 | 0 | 0 | 168 | 188 | Carbamidomethylation | C3:Carbamidomethylation:1000.00;C7:Carbamidomethylation:1000.00;C12:Carbamidomethylation:1000.00;C16:Carbamidomethylation:1000.00 | PEAKS DB |
| R.VLEGIQC(+57.02)GESIYM(+15.99)SSN.A | Y | 51.15 | 1801.7863 | 16 | -0.1 | 901.9003 | 2 | 59.33 | 4 | F4:39244 | NaSa01\_F12.raw |  |  |  | 2.7515E7 |  |  |  |  |  |  |  |  |  |  | 1 | 0 | 0 | 0 | 1 | 0 | 0 | 0 | 0 | 0 | 0 | 0 | 0 | 0 | 0 | 67 | 82 | Carbamidomethylation; Oxidation (M) | C7:Carbamidomethylation:1000.00;M13:Oxidation (M):1000.00 | PEAKS DB |
| N.FVYGVGANPPGSVTGHYTQIVWYQTYR.A | Y | 50.64 | 3059.4929 | 27 | -4.3 | 1020.8339 | 3 | 70.65 | 4 | F4:48404 | NaSa01\_F12.raw |  |  |  | 2.9259E6 |  |  |  |  |  |  |  |  |  |  | 1 | 0 | 0 | 0 | 1 | 0 | 0 | 0 | 0 | 0 | 0 | 0 | 0 | 0 | 0 | 100 | 126 |  |  | PEAKS DB |
| K.MEWYPEAASN.A | N | 49.85 | 1196.4808 | 10 | 0.2 | 599.2478 | 2 | 59.73 | 4 | F4:39536 | NaSa01\_F12.raw |  |  |  | 6.0961E6 |  |  |  |  |  |  |  |  |  |  | 1 | 0 | 0 | 0 | 1 | 0 | 0 | 0 | 0 | 0 | 0 | 0 | 0 | 0 | 0 | 39 | 48 |  |  | PEAKS DB |
| R.RVSPTASNMLK.M | Y | 49.81 | 1202.6442 | 11 | -0.6 | 602.3290 | 2 | 11.56 | 4 | F4:2521 | NaSa01\_F12.raw |  |  |  | 1.3713E7 |  |  |  |  |  |  |  |  |  |  | 1 | 0 | 0 | 0 | 1 | 0 | 0 | 0 | 0 | 0 | 0 | 0 | 0 | 0 | 0 | 28 | 38 |  |  | PEAKS DB |
| R.VSPTASNM(+15.99)LK.M | N | 49.40 | 1062.5380 | 10 | -0.6 | 532.2759 | 2 | 11.55 | 4 | F4:2520 | NaSa01\_F12.raw |  |  |  | 1.5436E7 |  |  |  |  |  |  |  |  |  |  | 1 | 0 | 0 | 0 | 1 | 0 | 0 | 0 | 0 | 0 | 0 | 0 | 0 | 0 | 0 | 29 | 38 | Oxidation (M) | M8:Oxidation (M):1000.00 | PEAKS DB |
| Q.IVWYQTYR.A | Y | 48.98 | 1127.5764 | 8 | -4.0 | 564.7932 | 2 | 42.65 | 4 | F4:25261 | NaSa01\_F12.raw |  |  |  | 4.5531E6 |  |  |  |  |  |  |  |  |  |  | 1 | 0 | 0 | 0 | 1 | 0 | 0 | 0 | 0 | 0 | 0 | 0 | 0 | 0 | 0 | 119 | 126 |  |  | PEAKS DB |
| N.MLKMEWYPEAASNAER.W | N | 48.97 | 1924.8811 | 16 | 0.5 | 642.6346 | 3 | 47.89 | 4 | F4:29582 | NaSa01\_F12.raw |  |  |  | 1.1253E6 |  |  |  |  |  |  |  |  |  |  | 1 | 0 | 0 | 0 | 1 | 0 | 0 | 0 | 0 | 0 | 0 | 0 | 0 | 0 | 0 | 36 | 51 |  |  | PEAKS DB |
| E.RWANTC(+57.02)SLNHSPDNLR.V | Y | 48.62 | 1939.9071 | 16 | -0.2 | 647.6428 | 3 | 11.82 | 4 | F4:2745 | NaSa01\_F12.raw |  |  |  | 1.9776E6 |  |  |  |  |  |  |  |  |  |  | 1 | 0 | 0 | 0 | 1 | 0 | 0 | 0 | 0 | 0 | 0 | 0 | 0 | 0 | 0 | 51 | 66 | Carbamidomethylation | C6:Carbamidomethylation:1000.00 | PEAKS DB |
| Y.TQIVWYQTYR.A | Y | 47.87 | 1356.6826 | 10 | -1.4 | 679.3477 | 2 | 49.12 | 4 | F4:30645 | NaSa01\_F12.raw |  |  |  | 3.7309E6 |  |  |  |  |  |  |  |  |  |  | 1 | 0 | 0 | 0 | 1 | 0 | 0 | 0 | 0 | 0 | 0 | 0 | 0 | 0 | 0 | 117 | 126 |  |  | PEAKS DB |
| C.QYC(+57.02)PSGNFQGK.T | Y | 47.85 | 1284.5557 | 11 | -0.3 | 643.2849 | 2 | 11.76 | 4 | F4:2671 | NaSa01\_F12.raw |  |  |  | 2.6916E5 |  |  |  |  |  |  |  |  |  |  | 1 | 0 | 0 | 0 | 1 | 0 | 0 | 0 | 0 | 0 | 0 | 0 | 0 | 0 | 0 | 146 | 156 | Carbamidomethylation | C3:Carbamidomethylation:1000.00 | PEAKS DB |
| K.LGPPC(+57.02)GDC(+57.02)PSAC(+57.02)DNGL.C | Y | 47.57 | 1688.6593 | 16 | 0.9 | 845.3376 | 2 | 47.39 | 4 | F4:29273 | NaSa01\_F12.raw |  |  |  | 1.4372E7 |  |  |  |  |  |  |  |  |  |  | 1 | 0 | 0 | 0 | 1 | 0 | 0 | 0 | 0 | 0 | 0 | 0 | 0 | 0 | 0 | 163 | 178 | Carbamidomethylation | C5:Carbamidomethylation:1000.00;C8:Carbamidomethylation:1000.00;C12:Carbamidomethylation:1000.00 | PEAKS DB |
| R.VSPTASNM(+15.99)LKM(+15.99)EWYPEAASNAER.W | N | 47.23 | 2613.1838 | 23 | 0.8 | 872.0693 | 3 | 38.90 | 4 | F4:22253 | NaSa01\_F12.raw |  |  |  | 7.9992E6 |  |  |  |  |  |  |  |  |  |  | 1 | 0 | 0 | 0 | 1 | 0 | 0 | 0 | 0 | 0 | 0 | 0 | 0 | 0 | 0 | 29 | 51 | Oxidation (M) | M8:Oxidation (M):1000.00;M11:Oxidation (M):1000.00 | PEAKS DB |
| T.QIVWYQTYR.A | Y | 47.15 | 1255.6349 | 9 | -0.2 | 628.8246 | 2 | 46.43 | 4 | F4:28539 | NaSa01\_F12.raw |  |  |  | 1.0049E6 |  |  |  |  |  |  |  |  |  |  | 1 | 0 | 0 | 0 | 1 | 0 | 0 | 0 | 0 | 0 | 0 | 0 | 0 | 0 | 0 | 118 | 126 |  |  | PEAKS DB |
| L.RVLEGIQC(+57.02)GESIYM(+15.99)SSNAR.T | Y | 45.38 | 2185.0256 | 19 | 0.3 | 729.3494 | 3 | 36.12 | 4 | F4:19992 | NaSa01\_F12.raw |  |  |  | 1.1935E6 |  |  |  |  |  |  |  |  |  |  | 1 | 0 | 0 | 0 | 1 | 0 | 0 | 0 | 0 | 0 | 0 | 0 | 0 | 0 | 0 | 66 | 84 | Carbamidomethylation; Oxidation (M) | C8:Carbamidomethylation:1000.00;M14:Oxidation (M):1000.00 | PEAKS DB |
| R.TWTEIIHLWHD.E | Y | 44.31 | 1449.7041 | 11 | -0.1 | 484.2419 | 3 | 76.43 | 4 | F4:53592 | NaSa01\_F12.raw |  |  |  | 6.3002E6 |  |  |  |  |  |  |  |  |  |  | 2 | 0 | 0 | 0 | 2 | 0 | 0 | 0 | 0 | 0 | 0 | 0 | 0 | 0 | 0 | 85 | 95 |  |  | PEAKS DB |
| K.MEWYPEAASNAE.R | N | 44.04 | 1396.5605 | 12 | 0.5 | 699.2879 | 2 | 61.61 | 4 | F4:41017 | NaSa01\_F12.raw |  |  |  | 2.7222E6 |  |  |  |  |  |  |  |  |  |  | 1 | 0 | 0 | 0 | 1 | 0 | 0 | 0 | 0 | 0 | 0 | 0 | 0 | 0 | 0 | 39 | 50 |  |  | PEAKS DB |
| I.VDLHNSLR.R | N | 42.85 | 952.5090 | 8 | -1.0 | 477.2613 | 2 | 26.51 | 4 | F4:11862 | NaSa01\_F12.raw |  |  |  | 2.8116E8 |  |  |  |  |  |  |  |  |  |  | 1 | 0 | 0 | 0 | 1 | 0 | 0 | 0 | 0 | 0 | 0 | 0 | 0 | 0 | 0 | 19 | 26 |  |  | PEAKS DB |
| R.RVSPTASNMLKMEWYPEAASNAER.W | Y | 42.34 | 2737.2952 | 24 | 2.2 | 913.4410 | 3 | 51.07 | 4 | F4:32424 | NaSa01\_F12.raw |  |  |  | 1.8869E6 |  |  |  |  |  |  |  |  |  |  | 1 | 0 | 0 | 0 | 1 | 0 | 0 | 0 | 0 | 0 | 0 | 0 | 0 | 0 | 0 | 28 | 51 |  |  | PEAKS DB |
| R.TWTEIIHL.W | Y | 41.70 | 1011.5389 | 8 | 0.0 | 506.7768 | 2 | 72.54 | 4 | F4:50028 | NaSa01\_F12.raw |  |  |  | 7.7511E6 |  |  |  |  |  |  |  |  |  |  | 1 | 0 | 0 | 0 | 1 | 0 | 0 | 0 | 0 | 0 | 0 | 0 | 0 | 0 | 0 | 85 | 92 |  |  | PEAKS DB |
| A.C(+57.02)DNGLC(+57.02)TNPC(+57.02)TIYNK.L | Y | 41.26 | 1828.7543 | 15 | -0.4 | 915.3840 | 2 | 33.09 | 4 | F4:17353 | NaSa01\_F12.raw |  |  |  | 2.4548E6 |  |  |  |  |  |  |  |  |  |  | 1 | 0 | 0 | 0 | 1 | 0 | 0 | 0 | 0 | 0 | 0 | 0 | 0 | 0 | 0 | 174 | 188 | Carbamidomethylation | C1:Carbamidomethylation:1000.00;C6:Carbamidomethylation:1000.00;C10:Carbamidomethylation:1000.00 | PEAKS DB |
| G.IQC(+57.02)GESIYMSSNAR.T | Y | 41.22 | 1614.7130 | 14 | -0.2 | 808.3636 | 2 | 24.59 | 4 | F4:10632 | NaSa01\_F12.raw |  |  |  | 1.3785E6 |  |  |  |  |  |  |  |  |  |  | 1 | 0 | 0 | 0 | 1 | 0 | 0 | 0 | 0 | 0 | 0 | 0 | 0 | 0 | 0 | 71 | 84 | Carbamidomethylation | C3:Carbamidomethylation:1000.00 | PEAKS DB |
| A.RTWTEIIHLWHDEYK.N | Y | 40.99 | 2026.0061 | 15 | 0.5 | 507.5091 | 4 | 61.62 | 4 | F4:41097 | NaSa01\_F12.raw |  |  |  | 3.775E6 |  |  |  |  |  |  |  |  |  |  | 1 | 0 | 0 | 0 | 1 | 0 | 0 | 0 | 0 | 0 | 0 | 0 | 0 | 0 | 0 | 84 | 98 |  |  | PEAKS DB |
| A.WSYFYVC(+57.02)QYC(+57.02)PSGNFQGK.T | Y | 40.64 | 2289.9612 | 18 | -2.7 | 1145.9847 | 2 | 70.96 | 4 | F4:48864 | NaSa01\_F12.raw |  |  |  | 1.2488E6 |  |  |  |  |  |  |  |  |  |  | 1 | 0 | 0 | 0 | 1 | 0 | 0 | 0 | 0 | 0 | 0 | 0 | 0 | 0 | 0 | 139 | 156 | Carbamidomethylation | C7:Carbamidomethylation:1000.00;C10:Carbamidomethylation:1000.00 | PEAKS DB |
| R.VLEGIQC(+57.02)GESIY.M | Y | 40.52 | 1366.6438 | 12 | 0.7 | 684.3297 | 2 | 63.89 | 4 | F4:43118 | NaSa01\_F12.raw |  |  |  | 1.0552E8 |  |  |  |  |  |  |  |  |  |  | 1 | 0 | 0 | 0 | 1 | 0 | 0 | 0 | 0 | 0 | 0 | 0 | 0 | 0 | 0 | 67 | 78 | Carbamidomethylation | C7:Carbamidomethylation:1000.00 | PEAKS DB |
| R.VLEGIQC(+57.02)GESIYM.S | Y | 40.40 | 1497.6843 | 13 | 1.7 | 749.8507 | 2 | 74.39 | 4 | F4:51502 | NaSa01\_F12.raw |  |  |  | 5.5836E7 |  |  |  |  |  |  |  |  |  |  | 1 | 0 | 0 | 0 | 1 | 0 | 0 | 0 | 0 | 0 | 0 | 0 | 0 | 0 | 0 | 67 | 79 | Carbamidomethylation | C7:Carbamidomethylation:1000.00 | PEAKS DB |
| R.AGC(+57.02)AVSYC(+57.02)PSSAW.S | Y | 39.73 | 1414.5646 | 13 | 0.5 | 708.2899 | 2 | 56.74 | 4 | F4:36991 | NaSa01\_F12.raw |  |  |  | 1.3783E7 |  |  |  |  |  |  |  |  |  |  | 1 | 0 | 0 | 0 | 1 | 0 | 0 | 0 | 0 | 0 | 0 | 0 | 0 | 0 | 0 | 127 | 139 | Carbamidomethylation | C3:Carbamidomethylation:1000.00;C8:Carbamidomethylation:1000.00 | PEAKS DB |
| P.SAC(+57.02)DNGLC(+57.02)TNPC(+57.02)TIYNK.L | Y | 39.15 | 1986.8234 | 17 | -0.6 | 994.4184 | 2 | 33.70 | 4 | F4:17779 | NaSa01\_F12.raw |  |  |  | 1.5428E6 |  |  |  |  |  |  |  |  |  |  | 1 | 0 | 0 | 0 | 1 | 0 | 0 | 0 | 0 | 0 | 0 | 0 | 0 | 0 | 0 | 172 | 188 | Carbamidomethylation | C3:Carbamidomethylation:1000.00;C8:Carbamidomethylation:1000.00;C12:Carbamidomethylation:1000.00 | PEAKS DB |
| T.EIIHLWHDEYK.N | Y | 38.91 | 1481.7302 | 11 | -0.1 | 494.9173 | 3 | 34.09 | 4 | F4:18145 | NaSa01\_F12.raw |  |  |  | 5.1609E5 |  |  |  |  |  |  |  |  |  |  | 1 | 0 | 0 | 0 | 1 | 0 | 0 | 0 | 0 | 0 | 0 | 0 | 0 | 0 | 0 | 88 | 98 |  |  | PEAKS DB |
| K.QKEIVDLHN.S | N | 38.54 | 1094.5720 | 9 | 1.2 | 548.2939 | 2 | 11.68 | 4 | F4:2630 | NaSa01\_F12.raw |  |  |  | 6.029E4 |  |  |  |  |  |  |  |  |  |  | 1 | 0 | 0 | 0 | 1 | 0 | 0 | 0 | 0 | 0 | 0 | 0 | 0 | 0 | 0 | 15 | 23 |  |  | PEAKS DB |
| Y.KLGPPC(+57.02)GDC(+57.02)PSAC(+57.02)DNGLC(+57.02)TNPC(+57.02)TIYNK.L | Y | 37.77 | 3068.2917 | 27 | 1.8 | 1023.7731 | 3 | 39.94 | 4 | F4:22895 | NaSa01\_F12.raw |  |  |  | 2.227E8 |  |  |  |  |  |  |  |  |  |  | 1 | 0 | 0 | 0 | 1 | 0 | 0 | 0 | 0 | 0 | 0 | 0 | 0 | 0 | 0 | 162 | 188 | Carbamidomethylation | C6:Carbamidomethylation:1000.00;C9:Carbamidomethylation:1000.00;C13:Carbamidomethylation:1000.00;C18:Carbamidomethylation:1000.00;C22:Carbamidomethylation:1000.00 | PEAKS DB |
| total 88 peptides |  |  |  |  |  |  |  |  |  |  |  |  |  |  |  |  |  |  |  |  |  |  |  |  |  |  |  |  |  |  |  |  |  |  |  |  |  |  |  |  |  |  |  |  |  |
| --- | --- | --- | --- | --- | --- | --- | --- | --- | --- | --- | --- | --- | --- | --- | --- | --- | --- | --- | --- | --- | --- | --- | --- | --- | --- | --- | --- | --- | --- | --- | --- | --- | --- | --- | --- | --- | --- | --- | --- | --- | --- | --- | --- | --- | --- |

3MZ8

back to list

  

| Protein Coverage
| Supporting Peptides
|

Protein Coverage:

Supporting Peptides:

| Peptide | Uniq | -10lgP | Mass | Length | ppm | m/z | z | RT | Fraction | Scan | Source File | Area F1 | Area F10 | Area F11 | Area F12 | Area F13 | Area F14 | Area F2 | Area F3 | Area F4 | Area F5 | Area F6 | Area F7 | Area F8 | Area F9 | #Feature | #Feature F1 | #Feature F10 | #Feature F11 | #Feature F12 | #Feature F13 | #Feature F14 | #Feature F2 | #Feature F3 | #Feature F4 | #Feature F5 | #Feature F6 | #Feature F7 | #Feature F8 | #Feature F9 | Start | End | PTM | AScore | Found By |
| --- | --- | --- | --- | --- | --- | --- | --- | --- | --- | --- | --- | --- | --- | --- | --- | --- | --- | --- | --- | --- | --- | --- | --- | --- | --- | --- | --- | --- | --- | --- | --- | --- | --- | --- | --- | --- | --- | --- | --- | --- | --- | --- | --- | --- | --- |
| R.TWTEIIHLWHDEYK.N | Y | 86.98 | 1869.9049 | 14 | -0.4 | 935.9594 | 2 | 69.11 | 4 | F4:46528 | NaSa01\_F12.raw |  |  | 1.6758E6 | 5.726E9 | 7.3513E6 |  |  |  |  |  |  |  |  |  | 9 | 0 | 0 | 1 | 6 | 2 | 0 | 0 | 0 | 0 | 0 | 0 | 0 | 0 | 0 | 85 | 98 |  |  | PEAKS DB |
| R.VLEGIQC(+57.02)GESIYMSSNAR.T | Y | 86.82 | 2012.9296 | 18 | -0.1 | 1007.4720 | 2 | 58.59 | 4 | F4:37662 | NaSa01\_F12.raw |  | 6.539E5 | 1.5229E7 | 1.9162E10 | 6.8803E7 | 1.1875E7 |  |  |  |  |  |  |  |  | 13 | 0 | 1 | 2 | 6 | 2 | 2 | 0 | 0 | 0 | 0 | 0 | 0 | 0 | 0 | 67 | 84 | Carbamidomethylation | C7:Carbamidomethylation:1000.00 | PEAKS DB |
| R.VLEGIQC(+57.02)GESIYM(+15.99)SSNAR.T | Y | 83.82 | 2028.9244 | 18 | -1.0 | 1015.4684 | 2 | 49.35 | 4 | F4:32620 | NaSa01\_F12.raw |  |  |  | 2.596E9 | 2.4998E7 | 3.3484E6 |  |  |  |  |  |  |  |  | 24 | 0 | 0 | 0 | 20 | 2 | 2 | 0 | 0 | 0 | 0 | 0 | 0 | 0 | 0 | 67 | 84 | Carbamidomethylation; Oxidation (M) | C7:Carbamidomethylation:1000.00;M13:Oxidation (M):1000.00 | PEAKS DB |
| R.WANTC(+57.02)SLNHSPDNLR.V | Y | 83.41 | 1783.8060 | 15 | -0.3 | 892.9100 | 2 | 20.24 | 4 | F4:7228 | NaSa01\_F12.raw |  |  |  | 4.9332E10 | 1.1987E8 | 6.2009E4 |  |  |  |  |  |  |  |  | 25 | 0 | 0 | 0 | 20 | 4 | 1 | 0 | 0 | 0 | 0 | 0 | 0 | 0 | 0 | 52 | 66 | Carbamidomethylation | C5:Carbamidomethylation:1000.00 | PEAKS DB |
| K.QSSC(+57.02)QDDWIKSNC(+57.02)PASC(+57.02)FC(+57.02)R.N | Y | 79.80 | 2504.9929 | 20 | 0.6 | 836.0054 | 3 | 39.02 | 4 | F4:22824 | NaSa01\_F12.raw |  |  |  | 7.3335E7 |  |  |  |  |  |  |  |  |  |  | 3 | 0 | 0 | 0 | 3 | 0 | 0 | 0 | 0 | 0 | 0 | 0 | 0 | 0 | 0 | 198 | 217 | Carbamidomethylation | C4:Carbamidomethylation:1000.00;C13:Carbamidomethylation:1000.00;C17:Carbamidomethylation:1000.00;C19:Carbamidomethylation:1000.00 | PEAKS DB |
| K.LTNC(+57.02)DSLLKQSSC(+57.02)QDDWIK.S | Y | 79.46 | 2310.0620 | 19 | -1.1 | 771.0271 | 3 | 45.31 | 4 | F4:28181 | NaSa01\_F12.raw |  |  |  | 1.962E8 |  |  |  |  |  |  |  |  |  |  | 4 | 0 | 0 | 0 | 4 | 0 | 0 | 0 | 0 | 0 | 0 | 0 | 0 | 0 | 0 | 189 | 207 | Carbamidomethylation | C4:Carbamidomethylation:1000.00;C13:Carbamidomethylation:1000.00 | PEAKS DB |
| D.C(+57.02)PSAC(+57.02)DNGLC(+57.02)TNPC(+57.02)TIYNK.L | Y | 78.07 | 2243.9067 | 19 | -0.3 | 1122.9603 | 2 | 35.53 | 4 | F4:19614 | NaSa01\_F12.raw |  |  |  | 1.255E8 |  |  |  |  |  |  |  |  |  |  | 3 | 0 | 0 | 0 | 3 | 0 | 0 | 0 | 0 | 0 | 0 | 0 | 0 | 0 | 0 | 170 | 188 | Carbamidomethylation | C1:Carbamidomethylation:1000.00;C5:Carbamidomethylation:1000.00;C10:Carbamidomethylation:1000.00;C14:Carbamidomethylation:1000.00 | PEAKS DB |
| K.NFVYGVGANPPGSVTGHYTQIVWYQTYR.A | Y | 77.64 | 3173.5359 | 28 | 0.0 | 1058.8525 | 3 | 74.13 | 4 | F4:51117 | NaSa01\_F12.raw |  |  |  | 1.5642E9 |  |  |  |  |  |  |  |  |  |  | 6 | 0 | 0 | 0 | 6 | 0 | 0 | 0 | 0 | 0 | 0 | 0 | 0 | 0 | 0 | 99 | 126 |  |  | PEAKS DB |
| Y.GVGANPPGSVTGHYTQIVWYQTYR.A | Y | 77.12 | 2650.2927 | 24 | 0.9 | 884.4390 | 3 | 57.12 | 4 | F4:37409 | NaSa01\_F12.raw |  |  | 5.2237E5 | 6.6355E7 |  |  |  |  |  |  |  |  |  |  | 2 | 0 | 0 | 1 | 1 | 0 | 0 | 0 | 0 | 0 | 0 | 0 | 0 | 0 | 0 | 103 | 126 |  |  | PEAKS DB |
| R.VSPTASNMLKMEWYPEAASNAER.W | N | 76.94 | 2581.1941 | 23 | 0.7 | 861.4059 | 3 | 60.25 | 4 | F4:39967 | NaSa01\_F12.raw |  |  |  | 1.7324E8 |  |  |  |  |  |  |  |  |  |  | 2 | 0 | 0 | 0 | 2 | 0 | 0 | 0 | 0 | 0 | 0 | 0 | 0 | 0 | 0 | 29 | 51 |  |  | PEAKS DB |
| K.MEWYPEAASNAER.W | N | 75.93 | 1552.6616 | 13 | -4.7 | 518.5587 | 3 | 42.47 | 4 | F4:25783 | NaSa01\_F12.raw |  |  | 8.9734E6 | 5.588E10 | 1.0906E8 | 2.4001E7 |  |  |  |  |  |  |  |  | 18 | 0 | 0 | 1 | 10 | 5 | 2 | 0 | 0 | 0 | 0 | 0 | 0 | 0 | 0 | 39 | 51 |  |  | PEAKS DB |
| W.SYFYVC(+57.02)QYC(+57.02)PSGNFQGK.T | Y | 74.81 | 2103.8818 | 17 | 0.9 | 1052.9491 | 2 | 56.78 | 4 | F4:37002 | NaSa01\_F12.raw |  |  |  | 7.9496E7 | 3.5056E6 |  |  |  |  |  |  |  |  |  | 3 | 0 | 0 | 0 | 2 | 1 | 0 | 0 | 0 | 0 | 0 | 0 | 0 | 0 | 0 | 140 | 156 | Carbamidomethylation | C6:Carbamidomethylation:1000.00;C9:Carbamidomethylation:1000.00 | PEAKS DB |
| K.QKEIVDLHNSLR.R | N | 73.79 | 1450.7892 | 12 | 0.4 | 484.6039 | 3 | 11.92 | 4 | F4:3036 | NaSa01\_F12.raw |  |  |  | 1.138E8 | 7.1992E5 |  |  |  |  |  |  |  |  |  | 16 | 0 | 0 | 0 | 15 | 1 | 0 | 0 | 0 | 0 | 0 | 0 | 0 | 0 | 0 | 15 | 26 |  |  | PEAKS DB |
| L.RVLEGIQC(+57.02)GESIYMSSNAR.T | Y | 73.68 | 2169.0308 | 19 | -0.1 | 724.0175 | 3 | 41.70 | 4 | F4:24692 | NaSa01\_F12.raw |  |  |  | 1.2641E7 |  |  |  |  |  |  |  |  |  |  | 1 | 0 | 0 | 0 | 1 | 0 | 0 | 0 | 0 | 0 | 0 | 0 | 0 | 0 | 0 | 66 | 84 | Carbamidomethylation | C8:Carbamidomethylation:1000.00 | PEAKS DB |
| C.PSAC(+57.02)DNGLC(+57.02)TNPC(+57.02)TIYNK.L | Y | 73.51 | 2083.8762 | 18 | 0.2 | 1042.9456 | 2 | 34.80 | 4 | F4:18795 | NaSa01\_F12.raw |  |  |  | 1.0016E7 |  |  |  |  |  |  |  |  |  |  | 1 | 0 | 0 | 0 | 1 | 0 | 0 | 0 | 0 | 0 | 0 | 0 | 0 | 0 | 0 | 171 | 188 | Carbamidomethylation | C4:Carbamidomethylation:1000.00;C9:Carbamidomethylation:1000.00;C13:Carbamidomethylation:1000.00 | PEAKS DB |
| S.SAWSYFYVC(+57.02)QYC(+57.02)PSGNFQGK.T | Y | 73.42 | 2448.0303 | 20 | -0.2 | 1225.0222 | 2 | 75.77 | 4 | F4:52609 | NaSa01\_F12.raw |  |  |  | 7.5549E7 |  |  |  |  |  |  |  |  |  |  | 2 | 0 | 0 | 0 | 2 | 0 | 0 | 0 | 0 | 0 | 0 | 0 | 0 | 0 | 0 | 137 | 156 | Carbamidomethylation | C9:Carbamidomethylation:1000.00;C12:Carbamidomethylation:1000.00 | PEAKS DB |
| K.M(+15.99)EWYPEAASNAER.W | N | 73.15 | 1568.6565 | 13 | 0.9 | 523.8932 | 3 | 33.16 | 4 | F4:17227 | NaSa01\_F12.raw |  |  |  | 1.0876E10 | 1.0638E7 | 4.7019E6 |  |  |  |  |  |  |  |  | 17 | 0 | 0 | 0 | 15 | 1 | 1 | 0 | 0 | 0 | 0 | 0 | 0 | 0 | 0 | 39 | 51 | Oxidation (M) | M1:Oxidation (M):1000.00 | PEAKS DB |
| K.EIVDLHNSLR.R | N | 71.95 | 1194.6356 | 10 | 0.4 | 598.3253 | 2 | 28.76 | 4 | F4:13650 | NaSa01\_F12.raw |  |  |  | 7.7068E9 | 7.5889E7 | 5.7777E4 |  |  |  |  |  |  |  |  | 27 | 0 | 0 | 0 | 23 | 3 | 1 | 0 | 0 | 0 | 0 | 0 | 0 | 0 | 0 | 17 | 26 |  |  | PEAKS DB |
| Y.C(+57.02)PSSAWSYFYVC(+57.02)QYC(+57.02)PSGNFQGK.T | Y | 71.68 | 2792.1458 | 23 | 1.2 | 931.7236 | 3 | 77.58 | 4 | F4:54131 | NaSa01\_F12.raw |  |  |  | 2.7393E7 |  |  |  |  |  |  |  |  |  |  | 2 | 0 | 0 | 0 | 2 | 0 | 0 | 0 | 0 | 0 | 0 | 0 | 0 | 0 | 0 | 134 | 156 | Carbamidomethylation | C1:Carbamidomethylation:1000.00;C12:Carbamidomethylation:1000.00;C15:Carbamidomethylation:1000.00 | PEAKS DB |
| Y.FYVC(+57.02)QYC(+57.02)PSGNFQGK.T | Y | 71.29 | 1853.7865 | 15 | 0.2 | 927.9008 | 2 | 45.22 | 4 | F4:27254 | NaSa01\_F12.raw |  |  |  | 6.7966E7 |  |  |  |  |  |  |  |  |  |  | 1 | 0 | 0 | 0 | 1 | 0 | 0 | 0 | 0 | 0 | 0 | 0 | 0 | 0 | 0 | 142 | 156 | Carbamidomethylation | C4:Carbamidomethylation:1000.00;C7:Carbamidomethylation:1000.00 | PEAKS DB |
| F.YVC(+57.02)QYC(+57.02)PSGNFQGK.T | Y | 70.89 | 1706.7181 | 14 | -0.4 | 854.3660 | 2 | 26.36 | 4 | F4:11724 | NaSa01\_F12.raw |  |  |  | 1.5606E8 | 1.5285E7 |  |  |  |  |  |  |  |  |  | 2 | 0 | 0 | 0 | 1 | 1 | 0 | 0 | 0 | 0 | 0 | 0 | 0 | 0 | 0 | 143 | 156 | Carbamidomethylation | C3:Carbamidomethylation:1000.00;C6:Carbamidomethylation:1000.00 | PEAKS DB |
| D.NGLC(+57.02)TNPC(+57.02)TIYNK.L | Y | 69.78 | 1553.6967 | 13 | 0.5 | 777.8560 | 2 | 28.19 | 4 | F4:13013 | NaSa01\_F12.raw |  |  |  | 3.1653E7 |  |  |  |  |  |  |  |  |  |  | 1 | 0 | 0 | 0 | 1 | 0 | 0 | 0 | 0 | 0 | 0 | 0 | 0 | 0 | 0 | 176 | 188 | Carbamidomethylation | C4:Carbamidomethylation:1000.00;C8:Carbamidomethylation:1000.00 | PEAKS DB |
| K.SNC(+57.02)PASC(+57.02)FC(+57.02)R.N | N | 69.18 | 1257.4689 | 10 | 0.2 | 629.7418 | 2 | 11.79 | 4 | F4:2692 | NaSa01\_F12.raw |  |  |  | 1.0262E8 |  |  |  |  |  |  |  |  |  |  | 1 | 0 | 0 | 0 | 1 | 0 | 0 | 0 | 0 | 0 | 0 | 0 | 0 | 0 | 0 | 208 | 217 | Carbamidomethylation | C3:Carbamidomethylation:1000.00;C7:Carbamidomethylation:1000.00;C9:Carbamidomethylation:1000.00 | PEAKS DB |
| K.QSSC(+57.02)QDDWIK.S | Y | 68.96 | 1265.5347 | 10 | -0.7 | 633.7742 | 2 | 11.92 | 4 | F4:3391 | NaSa01\_F12.raw |  |  |  | 3.0416E8 |  |  |  |  |  |  |  |  |  |  | 8 | 0 | 0 | 0 | 8 | 0 | 0 | 0 | 0 | 0 | 0 | 0 | 0 | 0 | 0 | 198 | 207 | Carbamidomethylation | C4:Carbamidomethylation:1000.00 | PEAKS DB |
| K.MEWYPEAASNAERWANTC(+57.02)SLNHSPDNLR.V | Y | 66.81 | 3318.4570 | 28 | 0.1 | 830.6216 | 4 | 60.99 | 4 | F4:40505 | NaSa01\_F12.raw |  |  |  | 1.1514E7 |  |  |  |  |  |  |  |  |  |  | 3 | 0 | 0 | 0 | 3 | 0 | 0 | 0 | 0 | 0 | 0 | 0 | 0 | 0 | 0 | 39 | 66 | Carbamidomethylation | C18:Carbamidomethylation:1000.00 | PEAKS DB |
| N.GLC(+57.02)TNPC(+57.02)TIYNK.L | Y | 66.27 | 1439.6537 | 12 | -0.2 | 720.8340 | 2 | 27.00 | 4 | F4:12069 | NaSa01\_F12.raw |  |  |  | 3.5701E7 |  |  |  |  |  |  |  |  |  |  | 1 | 0 | 0 | 0 | 1 | 0 | 0 | 0 | 0 | 0 | 0 | 0 | 0 | 0 | 0 | 177 | 188 | Carbamidomethylation | C3:Carbamidomethylation:1000.00;C7:Carbamidomethylation:1000.00 | PEAKS DB |
| E.GIQC(+57.02)GESIYMSSNAR.T | Y | 64.94 | 1671.7345 | 15 | 0.6 | 836.8750 | 2 | 29.68 | 4 | F4:14460 | NaSa01\_F12.raw |  |  |  | 1.9465E6 |  |  |  |  |  |  |  |  |  |  | 1 | 0 | 0 | 0 | 1 | 0 | 0 | 0 | 0 | 0 | 0 | 0 | 0 | 0 | 0 | 70 | 84 | Carbamidomethylation | C4:Carbamidomethylation:1000.00 | PEAKS DB |
| N.PPGSVTGHYTQIVWYQTYR.A | Y | 64.76 | 2252.1013 | 19 | 0.1 | 751.7078 | 3 | 53.16 | 4 | F4:33903 | NaSa01\_F12.raw |  |  |  | 4.1382E6 |  |  |  |  |  |  |  |  |  |  | 1 | 0 | 0 | 0 | 1 | 0 | 0 | 0 | 0 | 0 | 0 | 0 | 0 | 0 | 0 | 108 | 126 |  |  | PEAKS DB |
| K.NFVYGVGANPPGSVTGH.Y | N | 64.66 | 1671.8005 | 17 | 0.7 | 836.9081 | 2 | 48.12 | 4 | F4:29754 | NaSa01\_F12.raw |  |  |  | 2.7791E8 | 1.1488E7 | 2.2522E6 |  |  |  |  |  |  |  |  | 6 | 0 | 0 | 0 | 4 | 1 | 1 | 0 | 0 | 0 | 0 | 0 | 0 | 0 | 0 | 99 | 115 |  |  | PEAKS DB |
| NVDFNSESTR.R | N | 64.34 | 1167.5156 | 10 | 0.3 | 584.7653 | 2 | 12.14 | 4 | F4:3045 | NaSa01\_F12.raw |  |  |  | 1.4013E8 |  |  |  |  |  |  |  |  |  |  | 6 | 0 | 0 | 0 | 6 | 0 | 0 | 0 | 0 | 0 | 0 | 0 | 0 | 0 | 0 | 1 | 10 |  |  | PEAKS DB |
| K.LTNC(+57.02)DSLLK.Q | Y | 64.00 | 1062.5380 | 9 | -1.6 | 532.2754 | 2 | 21.02 | 4 | F4:7364 | NaSa01\_F12.raw |  |  |  | 2.9662E8 |  |  |  |  |  |  |  |  |  |  | 6 | 0 | 0 | 0 | 6 | 0 | 0 | 0 | 0 | 0 | 0 | 0 | 0 | 0 | 0 | 189 | 197 | Carbamidomethylation | C4:Carbamidomethylation:1000.00 | PEAKS DB |
| H.YTQIVWYQTYR.A | Y | 63.87 | 1519.7460 | 11 | 1.3 | 760.8812 | 2 | 55.25 | 4 | F4:35871 | NaSa01\_F12.raw |  |  |  | 1.2665E8 | 2.5476E6 |  |  |  |  |  |  |  |  |  | 3 | 0 | 0 | 0 | 2 | 1 | 0 | 0 | 0 | 0 | 0 | 0 | 0 | 0 | 0 | 116 | 126 |  |  | PEAKS DB |
| N.VDFNSESTR.R | N | 63.60 | 1053.4727 | 9 | 0.3 | 527.7438 | 2 | 11.74 | 4 | F4:2649 | NaSa01\_F12.raw |  |  |  | 4.6861E6 |  |  |  |  |  |  |  |  |  |  | 1 | 0 | 0 | 0 | 1 | 0 | 0 | 0 | 0 | 0 | 0 | 0 | 0 | 0 | 0 | 2 | 10 |  |  | PEAKS DB |
| C.DNGLC(+57.02)TNPC(+57.02)TIYNK.L | Y | 63.45 | 1668.7236 | 14 | 0.8 | 835.3698 | 2 | 36.30 | 4 | F4:20118 | NaSa01\_F12.raw |  |  |  | 3.0623E7 |  |  |  |  |  |  |  |  |  |  | 1 | 0 | 0 | 0 | 1 | 0 | 0 | 0 | 0 | 0 | 0 | 0 | 0 | 0 | 0 | 175 | 188 | Carbamidomethylation | C5:Carbamidomethylation:1000.00;C9:Carbamidomethylation:1000.00 | PEAKS DB |
| L.EGIQC(+57.02)GESIYMSSNAR.T | Y | 62.23 | 1800.7771 | 16 | 0.5 | 901.3963 | 2 | 35.36 | 4 | F4:19124 | NaSa01\_F12.raw |  |  |  | 1.9136E6 |  |  |  |  |  |  |  |  |  |  | 1 | 0 | 0 | 0 | 1 | 0 | 0 | 0 | 0 | 0 | 0 | 0 | 0 | 0 | 0 | 69 | 84 | Carbamidomethylation | C5:Carbamidomethylation:1000.00 | PEAKS DB |
| R.VSPTASNM(+15.99)LKMEWYPEAASNAER.W | N | 61.53 | 2597.1890 | 23 | 0.7 | 866.7375 | 3 | 47.88 | 4 | F4:29657 | NaSa01\_F12.raw |  |  |  | 1.0394E7 |  |  |  |  |  |  |  |  |  |  | 2 | 0 | 0 | 0 | 2 | 0 | 0 | 0 | 0 | 0 | 0 | 0 | 0 | 0 | 0 | 29 | 51 | Oxidation (M) | M8:Oxidation (M):24.24 | PEAKS DB |
| K.LGPPC(+57.02)GDC(+57.02)PSAC(+57.02)DNGLC(+57.02)TNPC(+57.02)TIYNK.L | Y | 61.29 | 2940.1970 | 26 | -0.2 | 981.0728 | 3 | 53.63 | 4 | F4:34247 | NaSa01\_F12.raw |  |  |  | 1.1603E10 |  | 3.4496E6 |  |  |  |  |  | 6.1046E6 |  |  | 9 | 0 | 0 | 0 | 7 | 0 | 1 | 0 | 0 | 0 | 0 | 0 | 1 | 0 | 0 | 163 | 188 | Carbamidomethylation | C5:Carbamidomethylation:1000.00;C8:Carbamidomethylation:1000.00;C12:Carbamidomethylation:1000.00;C17:Carbamidomethylation:1000.00;C21:Carbamidomethylation:1000.00 | PEAKS DB |
| K.NFVYGVGANPPGSVTGHY.T | N | 60.74 | 1834.8638 | 18 | 1.5 | 918.4406 | 2 | 53.13 | 4 | F4:33956 | NaSa01\_F12.raw |  |  |  | 5.187E6 |  |  |  |  |  |  |  |  |  |  | 1 | 0 | 0 | 0 | 1 | 0 | 0 | 0 | 0 | 0 | 0 | 0 | 0 | 0 | 0 | 99 | 116 |  |  | PEAKS DB |
| R.TWTEIIHLWH.D | Y | 59.61 | 1334.6771 | 10 | 0.6 | 668.3463 | 2 | 74.13 | 4 | F4:51227 | NaSa01\_F12.raw |  |  |  | 5.1108E6 |  |  |  |  |  |  |  |  |  |  | 1 | 0 | 0 | 0 | 1 | 0 | 0 | 0 | 0 | 0 | 0 | 0 | 0 | 0 | 0 | 85 | 94 |  |  | PEAKS DB |
| K.LGPPC(+57.02)GDC(+57.02)PSAC(+57.02)DNGLC(+57.02)TNPC(+57.02)TIYNKLTNC(+57.02)DSLLK.Q | Y | 59.18 | 3984.7244 | 35 | -0.2 | 1329.2484 | 3 | 74.37 | 4 | F4:51337 | NaSa01\_F12.raw |  |  |  | 2.2817E8 |  |  |  |  |  |  |  |  |  |  | 3 | 0 | 0 | 0 | 3 | 0 | 0 | 0 | 0 | 0 | 0 | 0 | 0 | 0 | 0 | 163 | 197 | Carbamidomethylation | C5:Carbamidomethylation:1000.00;C8:Carbamidomethylation:1000.00;C12:Carbamidomethylation:1000.00;C17:Carbamidomethylation:1000.00;C21:Carbamidomethylation:1000.00;C30:Carbamidomethylation:1000.00 | PEAKS DB |
| K.TATPYKLGPPC(+57.02)GDC(+57.02)PSAC(+57.02)DNGLC(+57.02)TNPC(+57.02)TIYNK.L | Y | 58.88 | 3601.5405 | 32 | 0.0 | 1201.5208 | 3 | 49.06 | 4 | F4:30641 | NaSa01\_F12.raw |  |  |  | 5.9598E7 |  |  |  |  |  |  |  |  |  |  | 1 | 0 | 0 | 0 | 1 | 0 | 0 | 0 | 0 | 0 | 0 | 0 | 0 | 0 | 0 | 157 | 188 | Carbamidomethylation | C11:Carbamidomethylation:1000.00;C14:Carbamidomethylation:1000.00;C18:Carbamidomethylation:1000.00;C23:Carbamidomethylation:1000.00;C27:Carbamidomethylation:1000.00 | PEAKS DB |
| G.LC(+57.02)TNPC(+57.02)TIYNK.L | Y | 58.71 | 1382.6323 | 11 | 0.6 | 692.3239 | 2 | 23.47 | 4 | F4:9229 | NaSa01\_F12.raw |  |  |  | 2.5631E6 |  |  |  |  |  |  |  | 1.5272E6 |  |  | 2 | 0 | 0 | 0 | 1 | 0 | 0 | 0 | 0 | 0 | 0 | 0 | 1 | 0 | 0 | 178 | 188 | Carbamidomethylation | C2:Carbamidomethylation:1000.00;C6:Carbamidomethylation:1000.00 | PEAKS DB |
| R.WANTC(+57.02)SLNHSPDNLRVLEGIQC(+57.02)GESIYMSSNAR.T | Y | 58.28 | 3778.7251 | 33 | -0.2 | 945.6884 | 4 | 66.79 | 4 | F4:45302 | NaSa01\_F12.raw |  |  |  | 2.4518E7 |  |  |  |  |  |  |  |  |  |  | 2 | 0 | 0 | 0 | 2 | 0 | 0 | 0 | 0 | 0 | 0 | 0 | 0 | 0 | 0 | 52 | 84 | Carbamidomethylation | C5:Carbamidomethylation:1000.00;C22:Carbamidomethylation:1000.00 | PEAKS DB |
| S.AC(+57.02)DNGLC(+57.02)TNPC(+57.02)TIYNK.L | Y | 58.09 | 1899.7914 | 16 | -0.8 | 950.9022 | 2 | 32.96 | 4 | F4:17191 | NaSa01\_F12.raw |  |  |  | 5.221E6 |  |  |  |  |  |  |  |  |  |  | 1 | 0 | 0 | 0 | 1 | 0 | 0 | 0 | 0 | 0 | 0 | 0 | 0 | 0 | 0 | 173 | 188 | Carbamidomethylation | C2:Carbamidomethylation:1000.00;C7:Carbamidomethylation:1000.00;C11:Carbamidomethylation:1000.00 | PEAKS DB |
| E.IVDLHNSLR.R | N | 58.01 | 1065.5930 | 9 | -1.6 | 533.8029 | 2 | 26.50 | 4 | F4:11729 | NaSa01\_F12.raw |  |  |  | 1.3096E7 |  |  |  |  |  |  |  |  |  |  | 1 | 0 | 0 | 0 | 1 | 0 | 0 | 0 | 0 | 0 | 0 | 0 | 0 | 0 | 0 | 18 | 26 |  |  | PEAKS DB |
| R.AGC(+57.02)AVSYC(+57.02)PSSAWSYFYVC(+57.02)QYC(+57.02)PSGNFQGK.T | Y | 57.96 | 3500.4358 | 30 | 0.3 | 1167.8196 | 3 | 84.20 | 4 | F4:60175 | NaSa01\_F12.raw |  |  |  | 8.8824E8 |  |  |  |  |  |  |  |  |  |  | 3 | 0 | 0 | 0 | 3 | 0 | 0 | 0 | 0 | 0 | 0 | 0 | 0 | 0 | 0 | 127 | 156 | Carbamidomethylation | C3:Carbamidomethylation:1000.00;C8:Carbamidomethylation:1000.00;C19:Carbamidomethylation:1000.00;C22:Carbamidomethylation:1000.00 | PEAKS DB |
| N.TC(+57.02)SLNHSPDNLR.V | Y | 57.81 | 1412.6466 | 12 | 0.4 | 471.8897 | 3 | 11.55 | 4 | F4:2523 | NaSa01\_F12.raw |  |  |  | 6.8334E6 |  |  |  |  |  |  |  |  |  |  | 2 | 0 | 0 | 0 | 2 | 0 | 0 | 0 | 0 | 0 | 0 | 0 | 0 | 0 | 0 | 55 | 66 | Carbamidomethylation | C2:Carbamidomethylation:1000.00 | PEAKS DB |
| R.VSPTASNMLK.M | N | 57.39 | 1046.5430 | 10 | 0.1 | 524.2788 | 2 | 11.91 | 4 | F4:3054 | NaSa01\_F12.raw |  |  |  | 1.9382E8 |  |  |  |  |  |  |  |  |  |  | 5 | 0 | 0 | 0 | 5 | 0 | 0 | 0 | 0 | 0 | 0 | 0 | 0 | 0 | 0 | 29 | 38 |  |  | PEAKS DB |
| L.KMEWYPEAASNAER.W | N | 57.28 | 1680.7566 | 14 | 0.7 | 561.2599 | 3 | 28.56 | 4 | F4:13207 | NaSa01\_F12.raw |  |  |  | 1.222E8 |  |  |  |  |  |  |  |  |  |  | 2 | 0 | 0 | 0 | 2 | 0 | 0 | 0 | 0 | 0 | 0 | 0 | 0 | 0 | 0 | 38 | 51 |  |  | PEAKS DB |
| R.VSPTASNMLKM(+15.99)EWYPEAASNAER.W | N | 57.05 | 2597.1890 | 23 | 0.7 | 866.7375 | 3 | 49.69 | 4 | F4:31613 | NaSa01\_F12.raw |  |  |  | 1.4277E7 |  |  |  |  |  |  |  |  |  |  | 1 | 0 | 0 | 0 | 1 | 0 | 0 | 0 | 0 | 0 | 0 | 0 | 0 | 0 | 0 | 29 | 51 | Oxidation (M) | M11:Oxidation (M):32.97 | PEAKS DB |
| W.TEIIHLWHDEYK.N | Y | 56.98 | 1582.7780 | 12 | 0.1 | 528.6000 | 3 | 36.11 | 4 | F4:19977 | NaSa01\_F12.raw |  |  |  | 9.0049E6 |  |  |  |  |  |  |  |  |  |  | 4 | 0 | 0 | 0 | 4 | 0 | 0 | 0 | 0 | 0 | 0 | 0 | 0 | 0 | 0 | 87 | 98 |  |  | PEAKS DB |
| R.VLEGIQC(+57.02)GESIYMSSN.A | Y | 56.56 | 1785.7914 | 16 | 0.3 | 893.9033 | 2 | 68.26 | 4 | F4:46624 | NaSa01\_F12.raw |  |  |  | 2.5137E8 |  |  |  |  |  |  |  |  |  |  | 1 | 0 | 0 | 0 | 1 | 0 | 0 | 0 | 0 | 0 | 0 | 0 | 0 | 0 | 0 | 67 | 82 | Carbamidomethylation | C7:Carbamidomethylation:1000.00 | PEAKS DB |
| E.IIHLWHDEYK.N | N | 55.71 | 1352.6877 | 10 | -0.7 | 451.9029 | 3 | 26.09 | 4 | F4:11533 | NaSa01\_F12.raw |  |  |  | 6.5239E5 |  |  |  |  |  |  |  |  |  |  | 1 | 0 | 0 | 0 | 1 | 0 | 0 | 0 | 0 | 0 | 0 | 0 | 0 | 0 | 0 | 89 | 98 |  |  | PEAKS DB |
| C.AVSYC(+57.02)PSSAWSYFYVC(+57.02)QYC(+57.02)PSGNFQGK.T | Y | 55.49 | 3212.3467 | 27 | 0.6 | 1071.7902 | 3 | 83.95 | 4 | F4:59535 | NaSa01\_F12.raw |  |  |  | 1.1276E8 |  |  |  |  |  |  |  |  |  |  | 1 | 0 | 0 | 0 | 1 | 0 | 0 | 0 | 0 | 0 | 0 | 0 | 0 | 0 | 0 | 130 | 156 | Carbamidomethylation | C5:Carbamidomethylation:1000.00;C16:Carbamidomethylation:1000.00;C19:Carbamidomethylation:1000.00 | PEAKS DB |
| M.EWYPEAASNAER.W | N | 54.75 | 1421.6211 | 12 | 0.7 | 711.8183 | 2 | 30.32 | 4 | F4:14915 | NaSa01\_F12.raw |  |  |  | 1.4758E7 |  |  |  |  |  |  |  |  |  |  | 1 | 0 | 0 | 0 | 1 | 0 | 0 | 0 | 0 | 0 | 0 | 0 | 0 | 0 | 0 | 40 | 51 |  |  | PEAKS DB |
| NVDFNSESTRR.K | N | 53.63 | 1323.6167 | 11 | 0.6 | 662.8160 | 2 | 11.55 | 4 | F4:2518 | NaSa01\_F12.raw |  |  |  | 1.6815E7 |  |  |  |  |  |  |  |  |  |  | 1 | 0 | 0 | 0 | 1 | 0 | 0 | 0 | 0 | 0 | 0 | 0 | 0 | 0 | 0 | 1 | 11 |  |  | PEAKS DB |
| C.SLNHSPDNLR.V | Y | 52.12 | 1151.5684 | 10 | 0.9 | 576.7920 | 2 | 11.55 | 4 | F4:2531 | NaSa01\_F12.raw |  |  |  | 3.5847E6 |  |  |  |  |  |  |  |  |  |  | 3 | 0 | 0 | 0 | 3 | 0 | 0 | 0 | 0 | 0 | 0 | 0 | 0 | 0 | 0 | 57 | 66 |  |  | PEAKS DB |
| C.GDC(+57.02)PSAC(+57.02)DNGLC(+57.02)TNPC(+57.02)TIYNK.L | Y | 51.17 | 2415.9553 | 21 | 0.4 | 1208.9855 | 2 | 38.69 | 4 | F4:22109 | NaSa01\_F12.raw |  |  |  | 3.1743E6 |  |  |  |  |  |  |  |  |  |  | 1 | 0 | 0 | 0 | 1 | 0 | 0 | 0 | 0 | 0 | 0 | 0 | 0 | 0 | 0 | 168 | 188 | Carbamidomethylation | C3:Carbamidomethylation:1000.00;C7:Carbamidomethylation:1000.00;C12:Carbamidomethylation:1000.00;C16:Carbamidomethylation:1000.00 | PEAKS DB |
| R.VLEGIQC(+57.02)GESIYM(+15.99)SSN.A | Y | 51.15 | 1801.7863 | 16 | -0.1 | 901.9003 | 2 | 59.33 | 4 | F4:39244 | NaSa01\_F12.raw |  |  |  | 2.7515E7 |  |  |  |  |  |  |  |  |  |  | 1 | 0 | 0 | 0 | 1 | 0 | 0 | 0 | 0 | 0 | 0 | 0 | 0 | 0 | 0 | 67 | 82 | Carbamidomethylation; Oxidation (M) | C7:Carbamidomethylation:1000.00;M13:Oxidation (M):1000.00 | PEAKS DB |
| N.FVYGVGANPPGSVTGHYTQIVWYQTYR.A | Y | 50.64 | 3059.4929 | 27 | -4.3 | 1020.8339 | 3 | 70.65 | 4 | F4:48404 | NaSa01\_F12.raw |  |  |  | 2.9259E6 |  |  |  |  |  |  |  |  |  |  | 1 | 0 | 0 | 0 | 1 | 0 | 0 | 0 | 0 | 0 | 0 | 0 | 0 | 0 | 0 | 100 | 126 |  |  | PEAKS DB |
| K.MEWYPEAASN.A | N | 49.85 | 1196.4808 | 10 | 0.2 | 599.2478 | 2 | 59.73 | 4 | F4:39536 | NaSa01\_F12.raw |  |  |  | 6.0961E6 |  |  |  |  |  |  |  |  |  |  | 1 | 0 | 0 | 0 | 1 | 0 | 0 | 0 | 0 | 0 | 0 | 0 | 0 | 0 | 0 | 39 | 48 |  |  | PEAKS DB |
| R.RVSPTASNMLK.M | Y | 49.81 | 1202.6442 | 11 | -0.6 | 602.3290 | 2 | 11.56 | 4 | F4:2521 | NaSa01\_F12.raw |  |  |  | 1.3713E7 |  |  |  |  |  |  |  |  |  |  | 1 | 0 | 0 | 0 | 1 | 0 | 0 | 0 | 0 | 0 | 0 | 0 | 0 | 0 | 0 | 28 | 38 |  |  | PEAKS DB |
| R.VSPTASNM(+15.99)LK.M | N | 49.40 | 1062.5380 | 10 | -0.6 | 532.2759 | 2 | 11.55 | 4 | F4:2520 | NaSa01\_F12.raw |  |  |  | 1.5436E7 |  |  |  |  |  |  |  |  |  |  | 1 | 0 | 0 | 0 | 1 | 0 | 0 | 0 | 0 | 0 | 0 | 0 | 0 | 0 | 0 | 29 | 38 | Oxidation (M) | M8:Oxidation (M):1000.00 | PEAKS DB |
| Q.IVWYQTYR.A | Y | 48.98 | 1127.5764 | 8 | -4.0 | 564.7932 | 2 | 42.65 | 4 | F4:25261 | NaSa01\_F12.raw |  |  |  | 4.5531E6 |  |  |  |  |  |  |  |  |  |  | 1 | 0 | 0 | 0 | 1 | 0 | 0 | 0 | 0 | 0 | 0 | 0 | 0 | 0 | 0 | 119 | 126 |  |  | PEAKS DB |
| N.MLKMEWYPEAASNAER.W | N | 48.97 | 1924.8811 | 16 | 0.5 | 642.6346 | 3 | 47.89 | 4 | F4:29582 | NaSa01\_F12.raw |  |  |  | 1.1253E6 |  |  |  |  |  |  |  |  |  |  | 1 | 0 | 0 | 0 | 1 | 0 | 0 | 0 | 0 | 0 | 0 | 0 | 0 | 0 | 0 | 36 | 51 |  |  | PEAKS DB |
| E.RWANTC(+57.02)SLNHSPDNLR.V | Y | 48.62 | 1939.9071 | 16 | -0.2 | 647.6428 | 3 | 11.82 | 4 | F4:2745 | NaSa01\_F12.raw |  |  |  | 1.9776E6 |  |  |  |  |  |  |  |  |  |  | 1 | 0 | 0 | 0 | 1 | 0 | 0 | 0 | 0 | 0 | 0 | 0 | 0 | 0 | 0 | 51 | 66 | Carbamidomethylation | C6:Carbamidomethylation:1000.00 | PEAKS DB |
| Y.TQIVWYQTYR.A | Y | 47.87 | 1356.6826 | 10 | -1.4 | 679.3477 | 2 | 49.12 | 4 | F4:30645 | NaSa01\_F12.raw |  |  |  | 3.7309E6 |  |  |  |  |  |  |  |  |  |  | 1 | 0 | 0 | 0 | 1 | 0 | 0 | 0 | 0 | 0 | 0 | 0 | 0 | 0 | 0 | 117 | 126 |  |  | PEAKS DB |
| C.QYC(+57.02)PSGNFQGK.T | Y | 47.85 | 1284.5557 | 11 | -0.3 | 643.2849 | 2 | 11.76 | 4 | F4:2671 | NaSa01\_F12.raw |  |  |  | 2.6916E5 |  |  |  |  |  |  |  |  |  |  | 1 | 0 | 0 | 0 | 1 | 0 | 0 | 0 | 0 | 0 | 0 | 0 | 0 | 0 | 0 | 146 | 156 | Carbamidomethylation | C3:Carbamidomethylation:1000.00 | PEAKS DB |
| K.LGPPC(+57.02)GDC(+57.02)PSAC(+57.02)DNGL.C | Y | 47.57 | 1688.6593 | 16 | 0.9 | 845.3376 | 2 | 47.39 | 4 | F4:29273 | NaSa01\_F12.raw |  |  |  | 1.4372E7 |  |  |  |  |  |  |  |  |  |  | 1 | 0 | 0 | 0 | 1 | 0 | 0 | 0 | 0 | 0 | 0 | 0 | 0 | 0 | 0 | 163 | 178 | Carbamidomethylation | C5:Carbamidomethylation:1000.00;C8:Carbamidomethylation:1000.00;C12:Carbamidomethylation:1000.00 | PEAKS DB |
| R.VSPTASNM(+15.99)LKM(+15.99)EWYPEAASNAER.W | N | 47.23 | 2613.1838 | 23 | 0.8 | 872.0693 | 3 | 38.90 | 4 | F4:22253 | NaSa01\_F12.raw |  |  |  | 7.9992E6 |  |  |  |  |  |  |  |  |  |  | 1 | 0 | 0 | 0 | 1 | 0 | 0 | 0 | 0 | 0 | 0 | 0 | 0 | 0 | 0 | 29 | 51 | Oxidation (M) | M8:Oxidation (M):1000.00;M11:Oxidation (M):1000.00 | PEAKS DB |
| T.QIVWYQTYR.A | Y | 47.15 | 1255.6349 | 9 | -0.2 | 628.8246 | 2 | 46.43 | 4 | F4:28539 | NaSa01\_F12.raw |  |  |  | 1.0049E6 |  |  |  |  |  |  |  |  |  |  | 1 | 0 | 0 | 0 | 1 | 0 | 0 | 0 | 0 | 0 | 0 | 0 | 0 | 0 | 0 | 118 | 126 |  |  | PEAKS DB |
| L.RVLEGIQC(+57.02)GESIYM(+15.99)SSNAR.T | Y | 45.38 | 2185.0256 | 19 | 0.3 | 729.3494 | 3 | 36.12 | 4 | F4:19992 | NaSa01\_F12.raw |  |  |  | 1.1935E6 |  |  |  |  |  |  |  |  |  |  | 1 | 0 | 0 | 0 | 1 | 0 | 0 | 0 | 0 | 0 | 0 | 0 | 0 | 0 | 0 | 66 | 84 | Carbamidomethylation; Oxidation (M) | C8:Carbamidomethylation:1000.00;M14:Oxidation (M):1000.00 | PEAKS DB |
| R.TWTEIIHLWHD.E | Y | 44.31 | 1449.7041 | 11 | -0.1 | 484.2419 | 3 | 76.43 | 4 | F4:53592 | NaSa01\_F12.raw |  |  |  | 6.3002E6 |  |  |  |  |  |  |  |  |  |  | 2 | 0 | 0 | 0 | 2 | 0 | 0 | 0 | 0 | 0 | 0 | 0 | 0 | 0 | 0 | 85 | 95 |  |  | PEAKS DB |
| K.MEWYPEAASNAE.R | N | 44.04 | 1396.5605 | 12 | 0.5 | 699.2879 | 2 | 61.61 | 4 | F4:41017 | NaSa01\_F12.raw |  |  |  | 2.7222E6 |  |  |  |  |  |  |  |  |  |  | 1 | 0 | 0 | 0 | 1 | 0 | 0 | 0 | 0 | 0 | 0 | 0 | 0 | 0 | 0 | 39 | 50 |  |  | PEAKS DB |
| I.VDLHNSLR.R | N | 42.85 | 952.5090 | 8 | -1.0 | 477.2613 | 2 | 26.51 | 4 | F4:11862 | NaSa01\_F12.raw |  |  |  | 2.8116E8 |  |  |  |  |  |  |  |  |  |  | 1 | 0 | 0 | 0 | 1 | 0 | 0 | 0 | 0 | 0 | 0 | 0 | 0 | 0 | 0 | 19 | 26 |  |  | PEAKS DB |
| R.RVSPTASNMLKMEWYPEAASNAER.W | Y | 42.34 | 2737.2952 | 24 | 2.2 | 913.4410 | 3 | 51.07 | 4 | F4:32424 | NaSa01\_F12.raw |  |  |  | 1.8869E6 |  |  |  |  |  |  |  |  |  |  | 1 | 0 | 0 | 0 | 1 | 0 | 0 | 0 | 0 | 0 | 0 | 0 | 0 | 0 | 0 | 28 | 51 |  |  | PEAKS DB |
| R.TWTEIIHL.W | Y | 41.70 | 1011.5389 | 8 | 0.0 | 506.7768 | 2 | 72.54 | 4 | F4:50028 | NaSa01\_F12.raw |  |  |  | 7.7511E6 |  |  |  |  |  |  |  |  |  |  | 1 | 0 | 0 | 0 | 1 | 0 | 0 | 0 | 0 | 0 | 0 | 0 | 0 | 0 | 0 | 85 | 92 |  |  | PEAKS DB |
| A.C(+57.02)DNGLC(+57.02)TNPC(+57.02)TIYNK.L | Y | 41.26 | 1828.7543 | 15 | -0.4 | 915.3840 | 2 | 33.09 | 4 | F4:17353 | NaSa01\_F12.raw |  |  |  | 2.4548E6 |  |  |  |  |  |  |  |  |  |  | 1 | 0 | 0 | 0 | 1 | 0 | 0 | 0 | 0 | 0 | 0 | 0 | 0 | 0 | 0 | 174 | 188 | Carbamidomethylation | C1:Carbamidomethylation:1000.00;C6:Carbamidomethylation:1000.00;C10:Carbamidomethylation:1000.00 | PEAKS DB |
| G.IQC(+57.02)GESIYMSSNAR.T | Y | 41.22 | 1614.7130 | 14 | -0.2 | 808.3636 | 2 | 24.59 | 4 | F4:10632 | NaSa01\_F12.raw |  |  |  | 1.3785E6 |  |  |  |  |  |  |  |  |  |  | 1 | 0 | 0 | 0 | 1 | 0 | 0 | 0 | 0 | 0 | 0 | 0 | 0 | 0 | 0 | 71 | 84 | Carbamidomethylation | C3:Carbamidomethylation:1000.00 | PEAKS DB |
| A.RTWTEIIHLWHDEYK.N | Y | 40.99 | 2026.0061 | 15 | 0.5 | 507.5091 | 4 | 61.62 | 4 | F4:41097 | NaSa01\_F12.raw |  |  |  | 3.775E6 |  |  |  |  |  |  |  |  |  |  | 1 | 0 | 0 | 0 | 1 | 0 | 0 | 0 | 0 | 0 | 0 | 0 | 0 | 0 | 0 | 84 | 98 |  |  | PEAKS DB |
| A.WSYFYVC(+57.02)QYC(+57.02)PSGNFQGK.T | Y | 40.64 | 2289.9612 | 18 | -2.7 | 1145.9847 | 2 | 70.96 | 4 | F4:48864 | NaSa01\_F12.raw |  |  |  | 1.2488E6 |  |  |  |  |  |  |  |  |  |  | 1 | 0 | 0 | 0 | 1 | 0 | 0 | 0 | 0 | 0 | 0 | 0 | 0 | 0 | 0 | 139 | 156 | Carbamidomethylation | C7:Carbamidomethylation:1000.00;C10:Carbamidomethylation:1000.00 | PEAKS DB |
| R.VLEGIQC(+57.02)GESIY.M | Y | 40.52 | 1366.6438 | 12 | 0.7 | 684.3297 | 2 | 63.89 | 4 | F4:43118 | NaSa01\_F12.raw |  |  |  | 1.0552E8 |  |  |  |  |  |  |  |  |  |  | 1 | 0 | 0 | 0 | 1 | 0 | 0 | 0 | 0 | 0 | 0 | 0 | 0 | 0 | 0 | 67 | 78 | Carbamidomethylation | C7:Carbamidomethylation:1000.00 | PEAKS DB |
| R.VLEGIQC(+57.02)GESIYM.S | Y | 40.40 | 1497.6843 | 13 | 1.7 | 749.8507 | 2 | 74.39 | 4 | F4:51502 | NaSa01\_F12.raw |  |  |  | 5.5836E7 |  |  |  |  |  |  |  |  |  |  | 1 | 0 | 0 | 0 | 1 | 0 | 0 | 0 | 0 | 0 | 0 | 0 | 0 | 0 | 0 | 67 | 79 | Carbamidomethylation | C7:Carbamidomethylation:1000.00 | PEAKS DB |
| R.AGC(+57.02)AVSYC(+57.02)PSSAW.S | Y | 39.73 | 1414.5646 | 13 | 0.5 | 708.2899 | 2 | 56.74 | 4 | F4:36991 | NaSa01\_F12.raw |  |  |  | 1.3783E7 |  |  |  |  |  |  |  |  |  |  | 1 | 0 | 0 | 0 | 1 | 0 | 0 | 0 | 0 | 0 | 0 | 0 | 0 | 0 | 0 | 127 | 139 | Carbamidomethylation | C3:Carbamidomethylation:1000.00;C8:Carbamidomethylation:1000.00 | PEAKS DB |
| P.SAC(+57.02)DNGLC(+57.02)TNPC(+57.02)TIYNK.L | Y | 39.15 | 1986.8234 | 17 | -0.6 | 994.4184 | 2 | 33.70 | 4 | F4:17779 | NaSa01\_F12.raw |  |  |  | 1.5428E6 |  |  |  |  |  |  |  |  |  |  | 1 | 0 | 0 | 0 | 1 | 0 | 0 | 0 | 0 | 0 | 0 | 0 | 0 | 0 | 0 | 172 | 188 | Carbamidomethylation | C3:Carbamidomethylation:1000.00;C8:Carbamidomethylation:1000.00;C12:Carbamidomethylation:1000.00 | PEAKS DB |
| T.EIIHLWHDEYK.N | Y | 38.91 | 1481.7302 | 11 | -0.1 | 494.9173 | 3 | 34.09 | 4 | F4:18145 | NaSa01\_F12.raw |  |  |  | 5.1609E5 |  |  |  |  |  |  |  |  |  |  | 1 | 0 | 0 | 0 | 1 | 0 | 0 | 0 | 0 | 0 | 0 | 0 | 0 | 0 | 0 | 88 | 98 |  |  | PEAKS DB |
| K.QKEIVDLHN.S | N | 38.54 | 1094.5720 | 9 | 1.2 | 548.2939 | 2 | 11.68 | 4 | F4:2630 | NaSa01\_F12.raw |  |  |  | 6.029E4 |  |  |  |  |  |  |  |  |  |  | 1 | 0 | 0 | 0 | 1 | 0 | 0 | 0 | 0 | 0 | 0 | 0 | 0 | 0 | 0 | 15 | 23 |  |  | PEAKS DB |
| Y.KLGPPC(+57.02)GDC(+57.02)PSAC(+57.02)DNGLC(+57.02)TNPC(+57.02)TIYNK.L | Y | 37.77 | 3068.2917 | 27 | 1.8 | 1023.7731 | 3 | 39.94 | 4 | F4:22895 | NaSa01\_F12.raw |  |  |  | 2.227E8 |  |  |  |  |  |  |  |  |  |  | 1 | 0 | 0 | 0 | 1 | 0 | 0 | 0 | 0 | 0 | 0 | 0 | 0 | 0 | 0 | 162 | 188 | Carbamidomethylation | C6:Carbamidomethylation:1000.00;C9:Carbamidomethylation:1000.00;C13:Carbamidomethylation:1000.00;C18:Carbamidomethylation:1000.00;C22:Carbamidomethylation:1000.00 | PEAKS DB |
| total 88 peptides |  |  |  |  |  |  |  |  |  |  |  |  |  |  |  |  |  |  |  |  |  |  |  |  |  |  |  |  |  |  |  |  |  |  |  |  |  |  |  |  |  |  |  |  |  |
| --- | --- | --- | --- | --- | --- | --- | --- | --- | --- | --- | --- | --- | --- | --- | --- | --- | --- | --- | --- | --- | --- | --- | --- | --- | --- | --- | --- | --- | --- | --- | --- | --- | --- | --- | --- | --- | --- | --- | --- | --- | --- | --- | --- | --- | --- |

Q7T1K6.1

back to list

  

| Protein Coverage
| Supporting Peptides
|

Protein Coverage:

Supporting Peptides:

| Peptide | Uniq | -10lgP | Mass | Length | ppm | m/z | z | RT | Fraction | Scan | Source File | Area F1 | Area F10 | Area F11 | Area F12 | Area F13 | Area F14 | Area F2 | Area F3 | Area F4 | Area F5 | Area F6 | Area F7 | Area F8 | Area F9 | #Feature | #Feature F1 | #Feature F10 | #Feature F11 | #Feature F12 | #Feature F13 | #Feature F14 | #Feature F2 | #Feature F3 | #Feature F4 | #Feature F5 | #Feature F6 | #Feature F7 | #Feature F8 | #Feature F9 | Start | End | PTM | AScore | Found By |
| --- | --- | --- | --- | --- | --- | --- | --- | --- | --- | --- | --- | --- | --- | --- | --- | --- | --- | --- | --- | --- | --- | --- | --- | --- | --- | --- | --- | --- | --- | --- | --- | --- | --- | --- | --- | --- | --- | --- | --- | --- | --- | --- | --- | --- | --- |
| R.TWTEIIHLWHDEYK.N | Y | 86.98 | 1869.9049 | 14 | -0.4 | 935.9594 | 2 | 69.11 | 4 | F4:46528 | NaSa01\_F12.raw |  |  | 1.6758E6 | 5.726E9 | 7.3513E6 |  |  |  |  |  |  |  |  |  | 9 | 0 | 0 | 1 | 6 | 2 | 0 | 0 | 0 | 0 | 0 | 0 | 0 | 0 | 0 | 103 | 116 |  |  | PEAKS DB |
| R.VLEGIQC(+57.02)GESIYMSSNAR.T | Y | 86.82 | 2012.9296 | 18 | -0.1 | 1007.4720 | 2 | 58.59 | 4 | F4:37662 | NaSa01\_F12.raw |  | 6.539E5 | 1.5229E7 | 1.9162E10 | 6.8803E7 | 1.1875E7 |  |  |  |  |  |  |  |  | 13 | 0 | 1 | 2 | 6 | 2 | 2 | 0 | 0 | 0 | 0 | 0 | 0 | 0 | 0 | 85 | 102 | Carbamidomethylation | C7:Carbamidomethylation:1000.00 | PEAKS DB |
| R.VLEGIQC(+57.02)GESIYM(+15.99)SSNAR.T | Y | 83.82 | 2028.9244 | 18 | -1.0 | 1015.4684 | 2 | 49.35 | 4 | F4:32620 | NaSa01\_F12.raw |  |  |  | 2.596E9 | 2.4998E7 | 3.3484E6 |  |  |  |  |  |  |  |  | 24 | 0 | 0 | 0 | 20 | 2 | 2 | 0 | 0 | 0 | 0 | 0 | 0 | 0 | 0 | 85 | 102 | Carbamidomethylation; Oxidation (M) | C7:Carbamidomethylation:1000.00;M13:Oxidation (M):1000.00 | PEAKS DB |
| R.WANTC(+57.02)SLNHSPDNLR.V | Y | 83.41 | 1783.8060 | 15 | -0.3 | 892.9100 | 2 | 20.24 | 4 | F4:7228 | NaSa01\_F12.raw |  |  |  | 4.9332E10 | 1.1987E8 | 6.2009E4 |  |  |  |  |  |  |  |  | 25 | 0 | 0 | 0 | 20 | 4 | 1 | 0 | 0 | 0 | 0 | 0 | 0 | 0 | 0 | 70 | 84 | Carbamidomethylation | C5:Carbamidomethylation:1000.00 | PEAKS DB |
| K.QSSC(+57.02)QDDWIKSNC(+57.02)PASC(+57.02)FC(+57.02)R.N | Y | 79.80 | 2504.9929 | 20 | 0.6 | 836.0054 | 3 | 39.02 | 4 | F4:22824 | NaSa01\_F12.raw |  |  |  | 7.3335E7 |  |  |  |  |  |  |  |  |  |  | 3 | 0 | 0 | 0 | 3 | 0 | 0 | 0 | 0 | 0 | 0 | 0 | 0 | 0 | 0 | 216 | 235 | Carbamidomethylation | C4:Carbamidomethylation:1000.00;C13:Carbamidomethylation:1000.00;C17:Carbamidomethylation:1000.00;C19:Carbamidomethylation:1000.00 | PEAKS DB |
| K.LTNC(+57.02)DSLLKQSSC(+57.02)QDDWIK.S | Y | 79.46 | 2310.0620 | 19 | -1.1 | 771.0271 | 3 | 45.31 | 4 | F4:28181 | NaSa01\_F12.raw |  |  |  | 1.962E8 |  |  |  |  |  |  |  |  |  |  | 4 | 0 | 0 | 0 | 4 | 0 | 0 | 0 | 0 | 0 | 0 | 0 | 0 | 0 | 0 | 207 | 225 | Carbamidomethylation | C4:Carbamidomethylation:1000.00;C13:Carbamidomethylation:1000.00 | PEAKS DB |
| D.C(+57.02)PSAC(+57.02)DNGLC(+57.02)TNPC(+57.02)TIYNK.L | Y | 78.07 | 2243.9067 | 19 | -0.3 | 1122.9603 | 2 | 35.53 | 4 | F4:19614 | NaSa01\_F12.raw |  |  |  | 1.255E8 |  |  |  |  |  |  |  |  |  |  | 3 | 0 | 0 | 0 | 3 | 0 | 0 | 0 | 0 | 0 | 0 | 0 | 0 | 0 | 0 | 188 | 206 | Carbamidomethylation | C1:Carbamidomethylation:1000.00;C5:Carbamidomethylation:1000.00;C10:Carbamidomethylation:1000.00;C14:Carbamidomethylation:1000.00 | PEAKS DB |
| K.NFVYGVGANPPGSVTGHYTQIVWYQTYR.A | Y | 77.64 | 3173.5359 | 28 | 0.0 | 1058.8525 | 3 | 74.13 | 4 | F4:51117 | NaSa01\_F12.raw |  |  |  | 1.5642E9 |  |  |  |  |  |  |  |  |  |  | 6 | 0 | 0 | 0 | 6 | 0 | 0 | 0 | 0 | 0 | 0 | 0 | 0 | 0 | 0 | 117 | 144 |  |  | PEAKS DB |
| Y.GVGANPPGSVTGHYTQIVWYQTYR.A | Y | 77.12 | 2650.2927 | 24 | 0.9 | 884.4390 | 3 | 57.12 | 4 | F4:37409 | NaSa01\_F12.raw |  |  | 5.2237E5 | 6.6355E7 |  |  |  |  |  |  |  |  |  |  | 2 | 0 | 0 | 1 | 1 | 0 | 0 | 0 | 0 | 0 | 0 | 0 | 0 | 0 | 0 | 121 | 144 |  |  | PEAKS DB |
| R.VSPTASNMLKMEWYPEAASNAER.W | N | 76.94 | 2581.1941 | 23 | 0.7 | 861.4059 | 3 | 60.25 | 4 | F4:39967 | NaSa01\_F12.raw |  |  |  | 1.7324E8 |  |  |  |  |  |  |  |  |  |  | 2 | 0 | 0 | 0 | 2 | 0 | 0 | 0 | 0 | 0 | 0 | 0 | 0 | 0 | 0 | 47 | 69 |  |  | PEAKS DB |
| K.MEWYPEAASNAER.W | N | 75.93 | 1552.6616 | 13 | -4.7 | 518.5587 | 3 | 42.47 | 4 | F4:25783 | NaSa01\_F12.raw |  |  | 8.9734E6 | 5.588E10 | 1.0906E8 | 2.4001E7 |  |  |  |  |  |  |  |  | 18 | 0 | 0 | 1 | 10 | 5 | 2 | 0 | 0 | 0 | 0 | 0 | 0 | 0 | 0 | 57 | 69 |  |  | PEAKS DB |
| W.SYFYVC(+57.02)QYC(+57.02)PSGNFQGK.T | Y | 74.81 | 2103.8818 | 17 | 0.9 | 1052.9491 | 2 | 56.78 | 4 | F4:37002 | NaSa01\_F12.raw |  |  |  | 7.9496E7 | 3.5056E6 |  |  |  |  |  |  |  |  |  | 3 | 0 | 0 | 0 | 2 | 1 | 0 | 0 | 0 | 0 | 0 | 0 | 0 | 0 | 0 | 158 | 174 | Carbamidomethylation | C6:Carbamidomethylation:1000.00;C9:Carbamidomethylation:1000.00 | PEAKS DB |
| K.QKEIVDLHNSLR.R | N | 73.79 | 1450.7892 | 12 | 0.4 | 484.6039 | 3 | 11.92 | 4 | F4:3036 | NaSa01\_F12.raw |  |  |  | 1.138E8 | 7.1992E5 |  |  |  |  |  |  |  |  |  | 16 | 0 | 0 | 0 | 15 | 1 | 0 | 0 | 0 | 0 | 0 | 0 | 0 | 0 | 0 | 33 | 44 |  |  | PEAKS DB |
| L.RVLEGIQC(+57.02)GESIYMSSNAR.T | Y | 73.68 | 2169.0308 | 19 | -0.1 | 724.0175 | 3 | 41.70 | 4 | F4:24692 | NaSa01\_F12.raw |  |  |  | 1.2641E7 |  |  |  |  |  |  |  |  |  |  | 1 | 0 | 0 | 0 | 1 | 0 | 0 | 0 | 0 | 0 | 0 | 0 | 0 | 0 | 0 | 84 | 102 | Carbamidomethylation | C8:Carbamidomethylation:1000.00 | PEAKS DB |
| C.PSAC(+57.02)DNGLC(+57.02)TNPC(+57.02)TIYNK.L | Y | 73.51 | 2083.8762 | 18 | 0.2 | 1042.9456 | 2 | 34.80 | 4 | F4:18795 | NaSa01\_F12.raw |  |  |  | 1.0016E7 |  |  |  |  |  |  |  |  |  |  | 1 | 0 | 0 | 0 | 1 | 0 | 0 | 0 | 0 | 0 | 0 | 0 | 0 | 0 | 0 | 189 | 206 | Carbamidomethylation | C4:Carbamidomethylation:1000.00;C9:Carbamidomethylation:1000.00;C13:Carbamidomethylation:1000.00 | PEAKS DB |
| S.SAWSYFYVC(+57.02)QYC(+57.02)PSGNFQGK.T | Y | 73.42 | 2448.0303 | 20 | -0.2 | 1225.0222 | 2 | 75.77 | 4 | F4:52609 | NaSa01\_F12.raw |  |  |  | 7.5549E7 |  |  |  |  |  |  |  |  |  |  | 2 | 0 | 0 | 0 | 2 | 0 | 0 | 0 | 0 | 0 | 0 | 0 | 0 | 0 | 0 | 155 | 174 | Carbamidomethylation | C9:Carbamidomethylation:1000.00;C12:Carbamidomethylation:1000.00 | PEAKS DB |
| K.M(+15.99)EWYPEAASNAER.W | N | 73.15 | 1568.6565 | 13 | 0.9 | 523.8932 | 3 | 33.16 | 4 | F4:17227 | NaSa01\_F12.raw |  |  |  | 1.0876E10 | 1.0638E7 | 4.7019E6 |  |  |  |  |  |  |  |  | 17 | 0 | 0 | 0 | 15 | 1 | 1 | 0 | 0 | 0 | 0 | 0 | 0 | 0 | 0 | 57 | 69 | Oxidation (M) | M1:Oxidation (M):1000.00 | PEAKS DB |
| K.EIVDLHNSLR.R | N | 71.95 | 1194.6356 | 10 | 0.4 | 598.3253 | 2 | 28.76 | 4 | F4:13650 | NaSa01\_F12.raw |  |  |  | 7.7068E9 | 7.5889E7 | 5.7777E4 |  |  |  |  |  |  |  |  | 27 | 0 | 0 | 0 | 23 | 3 | 1 | 0 | 0 | 0 | 0 | 0 | 0 | 0 | 0 | 35 | 44 |  |  | PEAKS DB |
| Y.C(+57.02)PSSAWSYFYVC(+57.02)QYC(+57.02)PSGNFQGK.T | Y | 71.68 | 2792.1458 | 23 | 1.2 | 931.7236 | 3 | 77.58 | 4 | F4:54131 | NaSa01\_F12.raw |  |  |  | 2.7393E7 |  |  |  |  |  |  |  |  |  |  | 2 | 0 | 0 | 0 | 2 | 0 | 0 | 0 | 0 | 0 | 0 | 0 | 0 | 0 | 0 | 152 | 174 | Carbamidomethylation | C1:Carbamidomethylation:1000.00;C12:Carbamidomethylation:1000.00;C15:Carbamidomethylation:1000.00 | PEAKS DB |
| Y.FYVC(+57.02)QYC(+57.02)PSGNFQGK.T | Y | 71.29 | 1853.7865 | 15 | 0.2 | 927.9008 | 2 | 45.22 | 4 | F4:27254 | NaSa01\_F12.raw |  |  |  | 6.7966E7 |  |  |  |  |  |  |  |  |  |  | 1 | 0 | 0 | 0 | 1 | 0 | 0 | 0 | 0 | 0 | 0 | 0 | 0 | 0 | 0 | 160 | 174 | Carbamidomethylation | C4:Carbamidomethylation:1000.00;C7:Carbamidomethylation:1000.00 | PEAKS DB |
| F.YVC(+57.02)QYC(+57.02)PSGNFQGK.T | Y | 70.89 | 1706.7181 | 14 | -0.4 | 854.3660 | 2 | 26.36 | 4 | F4:11724 | NaSa01\_F12.raw |  |  |  | 1.5606E8 | 1.5285E7 |  |  |  |  |  |  |  |  |  | 2 | 0 | 0 | 0 | 1 | 1 | 0 | 0 | 0 | 0 | 0 | 0 | 0 | 0 | 0 | 161 | 174 | Carbamidomethylation | C3:Carbamidomethylation:1000.00;C6:Carbamidomethylation:1000.00 | PEAKS DB |
| D.NGLC(+57.02)TNPC(+57.02)TIYNK.L | Y | 69.78 | 1553.6967 | 13 | 0.5 | 777.8560 | 2 | 28.19 | 4 | F4:13013 | NaSa01\_F12.raw |  |  |  | 3.1653E7 |  |  |  |  |  |  |  |  |  |  | 1 | 0 | 0 | 0 | 1 | 0 | 0 | 0 | 0 | 0 | 0 | 0 | 0 | 0 | 0 | 194 | 206 | Carbamidomethylation | C4:Carbamidomethylation:1000.00;C8:Carbamidomethylation:1000.00 | PEAKS DB |
| K.SNC(+57.02)PASC(+57.02)FC(+57.02)R.N | N | 69.18 | 1257.4689 | 10 | 0.2 | 629.7418 | 2 | 11.79 | 4 | F4:2692 | NaSa01\_F12.raw |  |  |  | 1.0262E8 |  |  |  |  |  |  |  |  |  |  | 1 | 0 | 0 | 0 | 1 | 0 | 0 | 0 | 0 | 0 | 0 | 0 | 0 | 0 | 0 | 226 | 235 | Carbamidomethylation | C3:Carbamidomethylation:1000.00;C7:Carbamidomethylation:1000.00;C9:Carbamidomethylation:1000.00 | PEAKS DB |
| K.QSSC(+57.02)QDDWIK.S | Y | 68.96 | 1265.5347 | 10 | -0.7 | 633.7742 | 2 | 11.92 | 4 | F4:3391 | NaSa01\_F12.raw |  |  |  | 3.0416E8 |  |  |  |  |  |  |  |  |  |  | 8 | 0 | 0 | 0 | 8 | 0 | 0 | 0 | 0 | 0 | 0 | 0 | 0 | 0 | 0 | 216 | 225 | Carbamidomethylation | C4:Carbamidomethylation:1000.00 | PEAKS DB |
| K.MEWYPEAASNAERWANTC(+57.02)SLNHSPDNLR.V | Y | 66.81 | 3318.4570 | 28 | 0.1 | 830.6216 | 4 | 60.99 | 4 | F4:40505 | NaSa01\_F12.raw |  |  |  | 1.1514E7 |  |  |  |  |  |  |  |  |  |  | 3 | 0 | 0 | 0 | 3 | 0 | 0 | 0 | 0 | 0 | 0 | 0 | 0 | 0 | 0 | 57 | 84 | Carbamidomethylation | C18:Carbamidomethylation:1000.00 | PEAKS DB |
| N.GLC(+57.02)TNPC(+57.02)TIYNK.L | Y | 66.27 | 1439.6537 | 12 | -0.2 | 720.8340 | 2 | 27.00 | 4 | F4:12069 | NaSa01\_F12.raw |  |  |  | 3.5701E7 |  |  |  |  |  |  |  |  |  |  | 1 | 0 | 0 | 0 | 1 | 0 | 0 | 0 | 0 | 0 | 0 | 0 | 0 | 0 | 0 | 195 | 206 | Carbamidomethylation | C3:Carbamidomethylation:1000.00;C7:Carbamidomethylation:1000.00 | PEAKS DB |
| E.GIQC(+57.02)GESIYMSSNAR.T | Y | 64.94 | 1671.7345 | 15 | 0.6 | 836.8750 | 2 | 29.68 | 4 | F4:14460 | NaSa01\_F12.raw |  |  |  | 1.9465E6 |  |  |  |  |  |  |  |  |  |  | 1 | 0 | 0 | 0 | 1 | 0 | 0 | 0 | 0 | 0 | 0 | 0 | 0 | 0 | 0 | 88 | 102 | Carbamidomethylation | C4:Carbamidomethylation:1000.00 | PEAKS DB |
| N.PPGSVTGHYTQIVWYQTYR.A | Y | 64.76 | 2252.1013 | 19 | 0.1 | 751.7078 | 3 | 53.16 | 4 | F4:33903 | NaSa01\_F12.raw |  |  |  | 4.1382E6 |  |  |  |  |  |  |  |  |  |  | 1 | 0 | 0 | 0 | 1 | 0 | 0 | 0 | 0 | 0 | 0 | 0 | 0 | 0 | 0 | 126 | 144 |  |  | PEAKS DB |
| K.NFVYGVGANPPGSVTGH.Y | N | 64.66 | 1671.8005 | 17 | 0.7 | 836.9081 | 2 | 48.12 | 4 | F4:29754 | NaSa01\_F12.raw |  |  |  | 2.7791E8 | 1.1488E7 | 2.2522E6 |  |  |  |  |  |  |  |  | 6 | 0 | 0 | 0 | 4 | 1 | 1 | 0 | 0 | 0 | 0 | 0 | 0 | 0 | 0 | 117 | 133 |  |  | PEAKS DB |
| G.NVDFNSESTR.R | N | 64.34 | 1167.5156 | 10 | 0.3 | 584.7653 | 2 | 12.14 | 4 | F4:3045 | NaSa01\_F12.raw |  |  |  | 1.4013E8 |  |  |  |  |  |  |  |  |  |  | 6 | 0 | 0 | 0 | 6 | 0 | 0 | 0 | 0 | 0 | 0 | 0 | 0 | 0 | 0 | 19 | 28 |  |  | PEAKS DB |
| K.LTNC(+57.02)DSLLK.Q | Y | 64.00 | 1062.5380 | 9 | -1.6 | 532.2754 | 2 | 21.02 | 4 | F4:7364 | NaSa01\_F12.raw |  |  |  | 2.9662E8 |  |  |  |  |  |  |  |  |  |  | 6 | 0 | 0 | 0 | 6 | 0 | 0 | 0 | 0 | 0 | 0 | 0 | 0 | 0 | 0 | 207 | 215 | Carbamidomethylation | C4:Carbamidomethylation:1000.00 | PEAKS DB |
| H.YTQIVWYQTYR.A | Y | 63.87 | 1519.7460 | 11 | 1.3 | 760.8812 | 2 | 55.25 | 4 | F4:35871 | NaSa01\_F12.raw |  |  |  | 1.2665E8 | 2.5476E6 |  |  |  |  |  |  |  |  |  | 3 | 0 | 0 | 0 | 2 | 1 | 0 | 0 | 0 | 0 | 0 | 0 | 0 | 0 | 0 | 134 | 144 |  |  | PEAKS DB |
| N.VDFNSESTR.R | N | 63.60 | 1053.4727 | 9 | 0.3 | 527.7438 | 2 | 11.74 | 4 | F4:2649 | NaSa01\_F12.raw |  |  |  | 4.6861E6 |  |  |  |  |  |  |  |  |  |  | 1 | 0 | 0 | 0 | 1 | 0 | 0 | 0 | 0 | 0 | 0 | 0 | 0 | 0 | 0 | 20 | 28 |  |  | PEAKS DB |
| C.DNGLC(+57.02)TNPC(+57.02)TIYNK.L | Y | 63.45 | 1668.7236 | 14 | 0.8 | 835.3698 | 2 | 36.30 | 4 | F4:20118 | NaSa01\_F12.raw |  |  |  | 3.0623E7 |  |  |  |  |  |  |  |  |  |  | 1 | 0 | 0 | 0 | 1 | 0 | 0 | 0 | 0 | 0 | 0 | 0 | 0 | 0 | 0 | 193 | 206 | Carbamidomethylation | C5:Carbamidomethylation:1000.00;C9:Carbamidomethylation:1000.00 | PEAKS DB |
| L.EGIQC(+57.02)GESIYMSSNAR.T | Y | 62.23 | 1800.7771 | 16 | 0.5 | 901.3963 | 2 | 35.36 | 4 | F4:19124 | NaSa01\_F12.raw |  |  |  | 1.9136E6 |  |  |  |  |  |  |  |  |  |  | 1 | 0 | 0 | 0 | 1 | 0 | 0 | 0 | 0 | 0 | 0 | 0 | 0 | 0 | 0 | 87 | 102 | Carbamidomethylation | C5:Carbamidomethylation:1000.00 | PEAKS DB |
| R.VSPTASNM(+15.99)LKMEWYPEAASNAER.W | N | 61.53 | 2597.1890 | 23 | 0.7 | 866.7375 | 3 | 47.88 | 4 | F4:29657 | NaSa01\_F12.raw |  |  |  | 1.0394E7 |  |  |  |  |  |  |  |  |  |  | 2 | 0 | 0 | 0 | 2 | 0 | 0 | 0 | 0 | 0 | 0 | 0 | 0 | 0 | 0 | 47 | 69 | Oxidation (M) | M8:Oxidation (M):24.24 | PEAKS DB |
| K.LGPPC(+57.02)GDC(+57.02)PSAC(+57.02)DNGLC(+57.02)TNPC(+57.02)TIYNK.L | Y | 61.29 | 2940.1970 | 26 | -0.2 | 981.0728 | 3 | 53.63 | 4 | F4:34247 | NaSa01\_F12.raw |  |  |  | 1.1603E10 |  | 3.4496E6 |  |  |  |  |  | 6.1046E6 |  |  | 9 | 0 | 0 | 0 | 7 | 0 | 1 | 0 | 0 | 0 | 0 | 0 | 1 | 0 | 0 | 181 | 206 | Carbamidomethylation | C5:Carbamidomethylation:1000.00;C8:Carbamidomethylation:1000.00;C12:Carbamidomethylation:1000.00;C17:Carbamidomethylation:1000.00;C21:Carbamidomethylation:1000.00 | PEAKS DB |
| K.NFVYGVGANPPGSVTGHY.T | N | 60.74 | 1834.8638 | 18 | 1.5 | 918.4406 | 2 | 53.13 | 4 | F4:33956 | NaSa01\_F12.raw |  |  |  | 5.187E6 |  |  |  |  |  |  |  |  |  |  | 1 | 0 | 0 | 0 | 1 | 0 | 0 | 0 | 0 | 0 | 0 | 0 | 0 | 0 | 0 | 117 | 134 |  |  | PEAKS DB |
| R.TWTEIIHLWH.D | Y | 59.61 | 1334.6771 | 10 | 0.6 | 668.3463 | 2 | 74.13 | 4 | F4:51227 | NaSa01\_F12.raw |  |  |  | 5.1108E6 |  |  |  |  |  |  |  |  |  |  | 1 | 0 | 0 | 0 | 1 | 0 | 0 | 0 | 0 | 0 | 0 | 0 | 0 | 0 | 0 | 103 | 112 |  |  | PEAKS DB |
| K.LGPPC(+57.02)GDC(+57.02)PSAC(+57.02)DNGLC(+57.02)TNPC(+57.02)TIYNKLTNC(+57.02)DSLLK.Q | Y | 59.18 | 3984.7244 | 35 | -0.2 | 1329.2484 | 3 | 74.37 | 4 | F4:51337 | NaSa01\_F12.raw |  |  |  | 2.2817E8 |  |  |  |  |  |  |  |  |  |  | 3 | 0 | 0 | 0 | 3 | 0 | 0 | 0 | 0 | 0 | 0 | 0 | 0 | 0 | 0 | 181 | 215 | Carbamidomethylation | C5:Carbamidomethylation:1000.00;C8:Carbamidomethylation:1000.00;C12:Carbamidomethylation:1000.00;C17:Carbamidomethylation:1000.00;C21:Carbamidomethylation:1000.00;C30:Carbamidomethylation:1000.00 | PEAKS DB |
| K.TATPYKLGPPC(+57.02)GDC(+57.02)PSAC(+57.02)DNGLC(+57.02)TNPC(+57.02)TIYNK.L | Y | 58.88 | 3601.5405 | 32 | 0.0 | 1201.5208 | 3 | 49.06 | 4 | F4:30641 | NaSa01\_F12.raw |  |  |  | 5.9598E7 |  |  |  |  |  |  |  |  |  |  | 1 | 0 | 0 | 0 | 1 | 0 | 0 | 0 | 0 | 0 | 0 | 0 | 0 | 0 | 0 | 175 | 206 | Carbamidomethylation | C11:Carbamidomethylation:1000.00;C14:Carbamidomethylation:1000.00;C18:Carbamidomethylation:1000.00;C23:Carbamidomethylation:1000.00;C27:Carbamidomethylation:1000.00 | PEAKS DB |
| G.LC(+57.02)TNPC(+57.02)TIYNK.L | Y | 58.71 | 1382.6323 | 11 | 0.6 | 692.3239 | 2 | 23.47 | 4 | F4:9229 | NaSa01\_F12.raw |  |  |  | 2.5631E6 |  |  |  |  |  |  |  | 1.5272E6 |  |  | 2 | 0 | 0 | 0 | 1 | 0 | 0 | 0 | 0 | 0 | 0 | 0 | 1 | 0 | 0 | 196 | 206 | Carbamidomethylation | C2:Carbamidomethylation:1000.00;C6:Carbamidomethylation:1000.00 | PEAKS DB |
| R.WANTC(+57.02)SLNHSPDNLRVLEGIQC(+57.02)GESIYMSSNAR.T | Y | 58.28 | 3778.7251 | 33 | -0.2 | 945.6884 | 4 | 66.79 | 4 | F4:45302 | NaSa01\_F12.raw |  |  |  | 2.4518E7 |  |  |  |  |  |  |  |  |  |  | 2 | 0 | 0 | 0 | 2 | 0 | 0 | 0 | 0 | 0 | 0 | 0 | 0 | 0 | 0 | 70 | 102 | Carbamidomethylation | C5:Carbamidomethylation:1000.00;C22:Carbamidomethylation:1000.00 | PEAKS DB |
| S.AC(+57.02)DNGLC(+57.02)TNPC(+57.02)TIYNK.L | Y | 58.09 | 1899.7914 | 16 | -0.8 | 950.9022 | 2 | 32.96 | 4 | F4:17191 | NaSa01\_F12.raw |  |  |  | 5.221E6 |  |  |  |  |  |  |  |  |  |  | 1 | 0 | 0 | 0 | 1 | 0 | 0 | 0 | 0 | 0 | 0 | 0 | 0 | 0 | 0 | 191 | 206 | Carbamidomethylation | C2:Carbamidomethylation:1000.00;C7:Carbamidomethylation:1000.00;C11:Carbamidomethylation:1000.00 | PEAKS DB |
| E.IVDLHNSLR.R | N | 58.01 | 1065.5930 | 9 | -1.6 | 533.8029 | 2 | 26.50 | 4 | F4:11729 | NaSa01\_F12.raw |  |  |  | 1.3096E7 |  |  |  |  |  |  |  |  |  |  | 1 | 0 | 0 | 0 | 1 | 0 | 0 | 0 | 0 | 0 | 0 | 0 | 0 | 0 | 0 | 36 | 44 |  |  | PEAKS DB |
| R.AGC(+57.02)AVSYC(+57.02)PSSAWSYFYVC(+57.02)QYC(+57.02)PSGNFQGK.T | Y | 57.96 | 3500.4358 | 30 | 0.3 | 1167.8196 | 3 | 84.20 | 4 | F4:60175 | NaSa01\_F12.raw |  |  |  | 8.8824E8 |  |  |  |  |  |  |  |  |  |  | 3 | 0 | 0 | 0 | 3 | 0 | 0 | 0 | 0 | 0 | 0 | 0 | 0 | 0 | 0 | 145 | 174 | Carbamidomethylation | C3:Carbamidomethylation:1000.00;C8:Carbamidomethylation:1000.00;C19:Carbamidomethylation:1000.00;C22:Carbamidomethylation:1000.00 | PEAKS DB |
| N.TC(+57.02)SLNHSPDNLR.V | Y | 57.81 | 1412.6466 | 12 | 0.4 | 471.8897 | 3 | 11.55 | 4 | F4:2523 | NaSa01\_F12.raw |  |  |  | 6.8334E6 |  |  |  |  |  |  |  |  |  |  | 2 | 0 | 0 | 0 | 2 | 0 | 0 | 0 | 0 | 0 | 0 | 0 | 0 | 0 | 0 | 73 | 84 | Carbamidomethylation | C2:Carbamidomethylation:1000.00 | PEAKS DB |
| R.VSPTASNMLK.M | N | 57.39 | 1046.5430 | 10 | 0.1 | 524.2788 | 2 | 11.91 | 4 | F4:3054 | NaSa01\_F12.raw |  |  |  | 1.9382E8 |  |  |  |  |  |  |  |  |  |  | 5 | 0 | 0 | 0 | 5 | 0 | 0 | 0 | 0 | 0 | 0 | 0 | 0 | 0 | 0 | 47 | 56 |  |  | PEAKS DB |
| L.KMEWYPEAASNAER.W | N | 57.28 | 1680.7566 | 14 | 0.7 | 561.2599 | 3 | 28.56 | 4 | F4:13207 | NaSa01\_F12.raw |  |  |  | 1.222E8 |  |  |  |  |  |  |  |  |  |  | 2 | 0 | 0 | 0 | 2 | 0 | 0 | 0 | 0 | 0 | 0 | 0 | 0 | 0 | 0 | 56 | 69 |  |  | PEAKS DB |
| R.VSPTASNMLKM(+15.99)EWYPEAASNAER.W | N | 57.05 | 2597.1890 | 23 | 0.7 | 866.7375 | 3 | 49.69 | 4 | F4:31613 | NaSa01\_F12.raw |  |  |  | 1.4277E7 |  |  |  |  |  |  |  |  |  |  | 1 | 0 | 0 | 0 | 1 | 0 | 0 | 0 | 0 | 0 | 0 | 0 | 0 | 0 | 0 | 47 | 69 | Oxidation (M) | M11:Oxidation (M):32.97 | PEAKS DB |
| W.TEIIHLWHDEYK.N | Y | 56.98 | 1582.7780 | 12 | 0.1 | 528.6000 | 3 | 36.11 | 4 | F4:19977 | NaSa01\_F12.raw |  |  |  | 9.0049E6 |  |  |  |  |  |  |  |  |  |  | 4 | 0 | 0 | 0 | 4 | 0 | 0 | 0 | 0 | 0 | 0 | 0 | 0 | 0 | 0 | 105 | 116 |  |  | PEAKS DB |
| R.VLEGIQC(+57.02)GESIYMSSN.A | Y | 56.56 | 1785.7914 | 16 | 0.3 | 893.9033 | 2 | 68.26 | 4 | F4:46624 | NaSa01\_F12.raw |  |  |  | 2.5137E8 |  |  |  |  |  |  |  |  |  |  | 1 | 0 | 0 | 0 | 1 | 0 | 0 | 0 | 0 | 0 | 0 | 0 | 0 | 0 | 0 | 85 | 100 | Carbamidomethylation | C7:Carbamidomethylation:1000.00 | PEAKS DB |
| E.IIHLWHDEYK.N | N | 55.71 | 1352.6877 | 10 | -0.7 | 451.9029 | 3 | 26.09 | 4 | F4:11533 | NaSa01\_F12.raw |  |  |  | 6.5239E5 |  |  |  |  |  |  |  |  |  |  | 1 | 0 | 0 | 0 | 1 | 0 | 0 | 0 | 0 | 0 | 0 | 0 | 0 | 0 | 0 | 107 | 116 |  |  | PEAKS DB |
| C.AVSYC(+57.02)PSSAWSYFYVC(+57.02)QYC(+57.02)PSGNFQGK.T | Y | 55.49 | 3212.3467 | 27 | 0.6 | 1071.7902 | 3 | 83.95 | 4 | F4:59535 | NaSa01\_F12.raw |  |  |  | 1.1276E8 |  |  |  |  |  |  |  |  |  |  | 1 | 0 | 0 | 0 | 1 | 0 | 0 | 0 | 0 | 0 | 0 | 0 | 0 | 0 | 0 | 148 | 174 | Carbamidomethylation | C5:Carbamidomethylation:1000.00;C16:Carbamidomethylation:1000.00;C19:Carbamidomethylation:1000.00 | PEAKS DB |
| M.EWYPEAASNAER.W | N | 54.75 | 1421.6211 | 12 | 0.7 | 711.8183 | 2 | 30.32 | 4 | F4:14915 | NaSa01\_F12.raw |  |  |  | 1.4758E7 |  |  |  |  |  |  |  |  |  |  | 1 | 0 | 0 | 0 | 1 | 0 | 0 | 0 | 0 | 0 | 0 | 0 | 0 | 0 | 0 | 58 | 69 |  |  | PEAKS DB |
| G.NVDFNSESTRR.K | N | 53.63 | 1323.6167 | 11 | 0.6 | 662.8160 | 2 | 11.55 | 4 | F4:2518 | NaSa01\_F12.raw |  |  |  | 1.6815E7 |  |  |  |  |  |  |  |  |  |  | 1 | 0 | 0 | 0 | 1 | 0 | 0 | 0 | 0 | 0 | 0 | 0 | 0 | 0 | 0 | 19 | 29 |  |  | PEAKS DB |
| C.SLNHSPDNLR.V | Y | 52.12 | 1151.5684 | 10 | 0.9 | 576.7920 | 2 | 11.55 | 4 | F4:2531 | NaSa01\_F12.raw |  |  |  | 3.5847E6 |  |  |  |  |  |  |  |  |  |  | 3 | 0 | 0 | 0 | 3 | 0 | 0 | 0 | 0 | 0 | 0 | 0 | 0 | 0 | 0 | 75 | 84 |  |  | PEAKS DB |
| C.GDC(+57.02)PSAC(+57.02)DNGLC(+57.02)TNPC(+57.02)TIYNK.L | Y | 51.17 | 2415.9553 | 21 | 0.4 | 1208.9855 | 2 | 38.69 | 4 | F4:22109 | NaSa01\_F12.raw |  |  |  | 3.1743E6 |  |  |  |  |  |  |  |  |  |  | 1 | 0 | 0 | 0 | 1 | 0 | 0 | 0 | 0 | 0 | 0 | 0 | 0 | 0 | 0 | 186 | 206 | Carbamidomethylation | C3:Carbamidomethylation:1000.00;C7:Carbamidomethylation:1000.00;C12:Carbamidomethylation:1000.00;C16:Carbamidomethylation:1000.00 | PEAKS DB |
| R.VLEGIQC(+57.02)GESIYM(+15.99)SSN.A | Y | 51.15 | 1801.7863 | 16 | -0.1 | 901.9003 | 2 | 59.33 | 4 | F4:39244 | NaSa01\_F12.raw |  |  |  | 2.7515E7 |  |  |  |  |  |  |  |  |  |  | 1 | 0 | 0 | 0 | 1 | 0 | 0 | 0 | 0 | 0 | 0 | 0 | 0 | 0 | 0 | 85 | 100 | Carbamidomethylation; Oxidation (M) | C7:Carbamidomethylation:1000.00;M13:Oxidation (M):1000.00 | PEAKS DB |
| N.FVYGVGANPPGSVTGHYTQIVWYQTYR.A | Y | 50.64 | 3059.4929 | 27 | -4.3 | 1020.8339 | 3 | 70.65 | 4 | F4:48404 | NaSa01\_F12.raw |  |  |  | 2.9259E6 |  |  |  |  |  |  |  |  |  |  | 1 | 0 | 0 | 0 | 1 | 0 | 0 | 0 | 0 | 0 | 0 | 0 | 0 | 0 | 0 | 118 | 144 |  |  | PEAKS DB |
| K.MEWYPEAASN.A | N | 49.85 | 1196.4808 | 10 | 0.2 | 599.2478 | 2 | 59.73 | 4 | F4:39536 | NaSa01\_F12.raw |  |  |  | 6.0961E6 |  |  |  |  |  |  |  |  |  |  | 1 | 0 | 0 | 0 | 1 | 0 | 0 | 0 | 0 | 0 | 0 | 0 | 0 | 0 | 0 | 57 | 66 |  |  | PEAKS DB |
| R.RVSPTASNMLK.M | Y | 49.81 | 1202.6442 | 11 | -0.6 | 602.3290 | 2 | 11.56 | 4 | F4:2521 | NaSa01\_F12.raw |  |  |  | 1.3713E7 |  |  |  |  |  |  |  |  |  |  | 1 | 0 | 0 | 0 | 1 | 0 | 0 | 0 | 0 | 0 | 0 | 0 | 0 | 0 | 0 | 46 | 56 |  |  | PEAKS DB |
| R.VSPTASNM(+15.99)LK.M | N | 49.40 | 1062.5380 | 10 | -0.6 | 532.2759 | 2 | 11.55 | 4 | F4:2520 | NaSa01\_F12.raw |  |  |  | 1.5436E7 |  |  |  |  |  |  |  |  |  |  | 1 | 0 | 0 | 0 | 1 | 0 | 0 | 0 | 0 | 0 | 0 | 0 | 0 | 0 | 0 | 47 | 56 | Oxidation (M) | M8:Oxidation (M):1000.00 | PEAKS DB |
| Q.IVWYQTYR.A | Y | 48.98 | 1127.5764 | 8 | -4.0 | 564.7932 | 2 | 42.65 | 4 | F4:25261 | NaSa01\_F12.raw |  |  |  | 4.5531E6 |  |  |  |  |  |  |  |  |  |  | 1 | 0 | 0 | 0 | 1 | 0 | 0 | 0 | 0 | 0 | 0 | 0 | 0 | 0 | 0 | 137 | 144 |  |  | PEAKS DB |
| N.MLKMEWYPEAASNAER.W | N | 48.97 | 1924.8811 | 16 | 0.5 | 642.6346 | 3 | 47.89 | 4 | F4:29582 | NaSa01\_F12.raw |  |  |  | 1.1253E6 |  |  |  |  |  |  |  |  |  |  | 1 | 0 | 0 | 0 | 1 | 0 | 0 | 0 | 0 | 0 | 0 | 0 | 0 | 0 | 0 | 54 | 69 |  |  | PEAKS DB |
| E.RWANTC(+57.02)SLNHSPDNLR.V | Y | 48.62 | 1939.9071 | 16 | -0.2 | 647.6428 | 3 | 11.82 | 4 | F4:2745 | NaSa01\_F12.raw |  |  |  | 1.9776E6 |  |  |  |  |  |  |  |  |  |  | 1 | 0 | 0 | 0 | 1 | 0 | 0 | 0 | 0 | 0 | 0 | 0 | 0 | 0 | 0 | 69 | 84 | Carbamidomethylation | C6:Carbamidomethylation:1000.00 | PEAKS DB |
| Y.TQIVWYQTYR.A | Y | 47.87 | 1356.6826 | 10 | -1.4 | 679.3477 | 2 | 49.12 | 4 | F4:30645 | NaSa01\_F12.raw |  |  |  | 3.7309E6 |  |  |  |  |  |  |  |  |  |  | 1 | 0 | 0 | 0 | 1 | 0 | 0 | 0 | 0 | 0 | 0 | 0 | 0 | 0 | 0 | 135 | 144 |  |  | PEAKS DB |
| C.QYC(+57.02)PSGNFQGK.T | Y | 47.85 | 1284.5557 | 11 | -0.3 | 643.2849 | 2 | 11.76 | 4 | F4:2671 | NaSa01\_F12.raw |  |  |  | 2.6916E5 |  |  |  |  |  |  |  |  |  |  | 1 | 0 | 0 | 0 | 1 | 0 | 0 | 0 | 0 | 0 | 0 | 0 | 0 | 0 | 0 | 164 | 174 | Carbamidomethylation | C3:Carbamidomethylation:1000.00 | PEAKS DB |
| K.LGPPC(+57.02)GDC(+57.02)PSAC(+57.02)DNGL.C | Y | 47.57 | 1688.6593 | 16 | 0.9 | 845.3376 | 2 | 47.39 | 4 | F4:29273 | NaSa01\_F12.raw |  |  |  | 1.4372E7 |  |  |  |  |  |  |  |  |  |  | 1 | 0 | 0 | 0 | 1 | 0 | 0 | 0 | 0 | 0 | 0 | 0 | 0 | 0 | 0 | 181 | 196 | Carbamidomethylation | C5:Carbamidomethylation:1000.00;C8:Carbamidomethylation:1000.00;C12:Carbamidomethylation:1000.00 | PEAKS DB |
| R.VSPTASNM(+15.99)LKM(+15.99)EWYPEAASNAER.W | N | 47.23 | 2613.1838 | 23 | 0.8 | 872.0693 | 3 | 38.90 | 4 | F4:22253 | NaSa01\_F12.raw |  |  |  | 7.9992E6 |  |  |  |  |  |  |  |  |  |  | 1 | 0 | 0 | 0 | 1 | 0 | 0 | 0 | 0 | 0 | 0 | 0 | 0 | 0 | 0 | 47 | 69 | Oxidation (M) | M8:Oxidation (M):1000.00;M11:Oxidation (M):1000.00 | PEAKS DB |
| T.QIVWYQTYR.A | Y | 47.15 | 1255.6349 | 9 | -0.2 | 628.8246 | 2 | 46.43 | 4 | F4:28539 | NaSa01\_F12.raw |  |  |  | 1.0049E6 |  |  |  |  |  |  |  |  |  |  | 1 | 0 | 0 | 0 | 1 | 0 | 0 | 0 | 0 | 0 | 0 | 0 | 0 | 0 | 0 | 136 | 144 |  |  | PEAKS DB |
| L.RVLEGIQC(+57.02)GESIYM(+15.99)SSNAR.T | Y | 45.38 | 2185.0256 | 19 | 0.3 | 729.3494 | 3 | 36.12 | 4 | F4:19992 | NaSa01\_F12.raw |  |  |  | 1.1935E6 |  |  |  |  |  |  |  |  |  |  | 1 | 0 | 0 | 0 | 1 | 0 | 0 | 0 | 0 | 0 | 0 | 0 | 0 | 0 | 0 | 84 | 102 | Carbamidomethylation; Oxidation (M) | C8:Carbamidomethylation:1000.00;M14:Oxidation (M):1000.00 | PEAKS DB |
| R.TWTEIIHLWHD.E | Y | 44.31 | 1449.7041 | 11 | -0.1 | 484.2419 | 3 | 76.43 | 4 | F4:53592 | NaSa01\_F12.raw |  |  |  | 6.3002E6 |  |  |  |  |  |  |  |  |  |  | 2 | 0 | 0 | 0 | 2 | 0 | 0 | 0 | 0 | 0 | 0 | 0 | 0 | 0 | 0 | 103 | 113 |  |  | PEAKS DB |
| K.MEWYPEAASNAE.R | N | 44.04 | 1396.5605 | 12 | 0.5 | 699.2879 | 2 | 61.61 | 4 | F4:41017 | NaSa01\_F12.raw |  |  |  | 2.7222E6 |  |  |  |  |  |  |  |  |  |  | 1 | 0 | 0 | 0 | 1 | 0 | 0 | 0 | 0 | 0 | 0 | 0 | 0 | 0 | 0 | 57 | 68 |  |  | PEAKS DB |
| I.VDLHNSLR.R | N | 42.85 | 952.5090 | 8 | -1.0 | 477.2613 | 2 | 26.51 | 4 | F4:11862 | NaSa01\_F12.raw |  |  |  | 2.8116E8 |  |  |  |  |  |  |  |  |  |  | 1 | 0 | 0 | 0 | 1 | 0 | 0 | 0 | 0 | 0 | 0 | 0 | 0 | 0 | 0 | 37 | 44 |  |  | PEAKS DB |
| R.RVSPTASNMLKMEWYPEAASNAER.W | Y | 42.34 | 2737.2952 | 24 | 2.2 | 913.4410 | 3 | 51.07 | 4 | F4:32424 | NaSa01\_F12.raw |  |  |  | 1.8869E6 |  |  |  |  |  |  |  |  |  |  | 1 | 0 | 0 | 0 | 1 | 0 | 0 | 0 | 0 | 0 | 0 | 0 | 0 | 0 | 0 | 46 | 69 |  |  | PEAKS DB |
| R.TWTEIIHL.W | Y | 41.70 | 1011.5389 | 8 | 0.0 | 506.7768 | 2 | 72.54 | 4 | F4:50028 | NaSa01\_F12.raw |  |  |  | 7.7511E6 |  |  |  |  |  |  |  |  |  |  | 1 | 0 | 0 | 0 | 1 | 0 | 0 | 0 | 0 | 0 | 0 | 0 | 0 | 0 | 0 | 103 | 110 |  |  | PEAKS DB |
| A.C(+57.02)DNGLC(+57.02)TNPC(+57.02)TIYNK.L | Y | 41.26 | 1828.7543 | 15 | -0.4 | 915.3840 | 2 | 33.09 | 4 | F4:17353 | NaSa01\_F12.raw |  |  |  | 2.4548E6 |  |  |  |  |  |  |  |  |  |  | 1 | 0 | 0 | 0 | 1 | 0 | 0 | 0 | 0 | 0 | 0 | 0 | 0 | 0 | 0 | 192 | 206 | Carbamidomethylation | C1:Carbamidomethylation:1000.00;C6:Carbamidomethylation:1000.00;C10:Carbamidomethylation:1000.00 | PEAKS DB |
| G.IQC(+57.02)GESIYMSSNAR.T | Y | 41.22 | 1614.7130 | 14 | -0.2 | 808.3636 | 2 | 24.59 | 4 | F4:10632 | NaSa01\_F12.raw |  |  |  | 1.3785E6 |  |  |  |  |  |  |  |  |  |  | 1 | 0 | 0 | 0 | 1 | 0 | 0 | 0 | 0 | 0 | 0 | 0 | 0 | 0 | 0 | 89 | 102 | Carbamidomethylation | C3:Carbamidomethylation:1000.00 | PEAKS DB |
| A.RTWTEIIHLWHDEYK.N | Y | 40.99 | 2026.0061 | 15 | 0.5 | 507.5091 | 4 | 61.62 | 4 | F4:41097 | NaSa01\_F12.raw |  |  |  | 3.775E6 |  |  |  |  |  |  |  |  |  |  | 1 | 0 | 0 | 0 | 1 | 0 | 0 | 0 | 0 | 0 | 0 | 0 | 0 | 0 | 0 | 102 | 116 |  |  | PEAKS DB |
| A.WSYFYVC(+57.02)QYC(+57.02)PSGNFQGK.T | Y | 40.64 | 2289.9612 | 18 | -2.7 | 1145.9847 | 2 | 70.96 | 4 | F4:48864 | NaSa01\_F12.raw |  |  |  | 1.2488E6 |  |  |  |  |  |  |  |  |  |  | 1 | 0 | 0 | 0 | 1 | 0 | 0 | 0 | 0 | 0 | 0 | 0 | 0 | 0 | 0 | 157 | 174 | Carbamidomethylation | C7:Carbamidomethylation:1000.00;C10:Carbamidomethylation:1000.00 | PEAKS DB |
| R.VLEGIQC(+57.02)GESIY.M | Y | 40.52 | 1366.6438 | 12 | 0.7 | 684.3297 | 2 | 63.89 | 4 | F4:43118 | NaSa01\_F12.raw |  |  |  | 1.0552E8 |  |  |  |  |  |  |  |  |  |  | 1 | 0 | 0 | 0 | 1 | 0 | 0 | 0 | 0 | 0 | 0 | 0 | 0 | 0 | 0 | 85 | 96 | Carbamidomethylation | C7:Carbamidomethylation:1000.00 | PEAKS DB |
| R.VLEGIQC(+57.02)GESIYM.S | Y | 40.40 | 1497.6843 | 13 | 1.7 | 749.8507 | 2 | 74.39 | 4 | F4:51502 | NaSa01\_F12.raw |  |  |  | 5.5836E7 |  |  |  |  |  |  |  |  |  |  | 1 | 0 | 0 | 0 | 1 | 0 | 0 | 0 | 0 | 0 | 0 | 0 | 0 | 0 | 0 | 85 | 97 | Carbamidomethylation | C7:Carbamidomethylation:1000.00 | PEAKS DB |
| R.AGC(+57.02)AVSYC(+57.02)PSSAW.S | Y | 39.73 | 1414.5646 | 13 | 0.5 | 708.2899 | 2 | 56.74 | 4 | F4:36991 | NaSa01\_F12.raw |  |  |  | 1.3783E7 |  |  |  |  |  |  |  |  |  |  | 1 | 0 | 0 | 0 | 1 | 0 | 0 | 0 | 0 | 0 | 0 | 0 | 0 | 0 | 0 | 145 | 157 | Carbamidomethylation | C3:Carbamidomethylation:1000.00;C8:Carbamidomethylation:1000.00 | PEAKS DB |
| P.SAC(+57.02)DNGLC(+57.02)TNPC(+57.02)TIYNK.L | Y | 39.15 | 1986.8234 | 17 | -0.6 | 994.4184 | 2 | 33.70 | 4 | F4:17779 | NaSa01\_F12.raw |  |  |  | 1.5428E6 |  |  |  |  |  |  |  |  |  |  | 1 | 0 | 0 | 0 | 1 | 0 | 0 | 0 | 0 | 0 | 0 | 0 | 0 | 0 | 0 | 190 | 206 | Carbamidomethylation | C3:Carbamidomethylation:1000.00;C8:Carbamidomethylation:1000.00;C12:Carbamidomethylation:1000.00 | PEAKS DB |
| T.EIIHLWHDEYK.N | Y | 38.91 | 1481.7302 | 11 | -0.1 | 494.9173 | 3 | 34.09 | 4 | F4:18145 | NaSa01\_F12.raw |  |  |  | 5.1609E5 |  |  |  |  |  |  |  |  |  |  | 1 | 0 | 0 | 0 | 1 | 0 | 0 | 0 | 0 | 0 | 0 | 0 | 0 | 0 | 0 | 106 | 116 |  |  | PEAKS DB |
| K.QKEIVDLHN.S | N | 38.54 | 1094.5720 | 9 | 1.2 | 548.2939 | 2 | 11.68 | 4 | F4:2630 | NaSa01\_F12.raw |  |  |  | 6.029E4 |  |  |  |  |  |  |  |  |  |  | 1 | 0 | 0 | 0 | 1 | 0 | 0 | 0 | 0 | 0 | 0 | 0 | 0 | 0 | 0 | 33 | 41 |  |  | PEAKS DB |
| Y.KLGPPC(+57.02)GDC(+57.02)PSAC(+57.02)DNGLC(+57.02)TNPC(+57.02)TIYNK.L | Y | 37.77 | 3068.2917 | 27 | 1.8 | 1023.7731 | 3 | 39.94 | 4 | F4:22895 | NaSa01\_F12.raw |  |  |  | 2.227E8 |  |  |  |  |  |  |  |  |  |  | 1 | 0 | 0 | 0 | 1 | 0 | 0 | 0 | 0 | 0 | 0 | 0 | 0 | 0 | 0 | 180 | 206 | Carbamidomethylation | C6:Carbamidomethylation:1000.00;C9:Carbamidomethylation:1000.00;C13:Carbamidomethylation:1000.00;C18:Carbamidomethylation:1000.00;C22:Carbamidomethylation:1000.00 | PEAKS DB |
| total 88 peptides |  |  |  |  |  |  |  |  |  |  |  |  |  |  |  |  |  |  |  |  |  |  |  |  |  |  |  |  |  |  |  |  |  |  |  |  |  |  |  |  |  |  |  |  |  |
| --- | --- | --- | --- | --- | --- | --- | --- | --- | --- | --- | --- | --- | --- | --- | --- | --- | --- | --- | --- | --- | --- | --- | --- | --- | --- | --- | --- | --- | --- | --- | --- | --- | --- | --- | --- | --- | --- | --- | --- | --- | --- | --- | --- | --- | --- |

AAP85301.1

back to list

  

| Protein Coverage
| Supporting Peptides
|

Protein Coverage:

Supporting Peptides:

| Peptide | Uniq | -10lgP | Mass | Length | ppm | m/z | z | RT | Fraction | Scan | Source File | Area F1 | Area F10 | Area F11 | Area F12 | Area F13 | Area F14 | Area F2 | Area F3 | Area F4 | Area F5 | Area F6 | Area F7 | Area F8 | Area F9 | #Feature | #Feature F1 | #Feature F10 | #Feature F11 | #Feature F12 | #Feature F13 | #Feature F14 | #Feature F2 | #Feature F3 | #Feature F4 | #Feature F5 | #Feature F6 | #Feature F7 | #Feature F8 | #Feature F9 | Start | End | PTM | AScore | Found By |
| --- | --- | --- | --- | --- | --- | --- | --- | --- | --- | --- | --- | --- | --- | --- | --- | --- | --- | --- | --- | --- | --- | --- | --- | --- | --- | --- | --- | --- | --- | --- | --- | --- | --- | --- | --- | --- | --- | --- | --- | --- | --- | --- | --- | --- | --- |
| R.TWTEIIHLWHDEYK.N | Y | 86.98 | 1869.9049 | 14 | -0.4 | 935.9594 | 2 | 69.11 | 4 | F4:46528 | NaSa01\_F12.raw |  |  | 1.6758E6 | 5.726E9 | 7.3513E6 |  |  |  |  |  |  |  |  |  | 9 | 0 | 0 | 1 | 6 | 2 | 0 | 0 | 0 | 0 | 0 | 0 | 0 | 0 | 0 | 103 | 116 |  |  | PEAKS DB |
| R.VLEGIQC(+57.02)GESIYMSSNAR.T | Y | 86.82 | 2012.9296 | 18 | -0.1 | 1007.4720 | 2 | 58.59 | 4 | F4:37662 | NaSa01\_F12.raw |  | 6.539E5 | 1.5229E7 | 1.9162E10 | 6.8803E7 | 1.1875E7 |  |  |  |  |  |  |  |  | 13 | 0 | 1 | 2 | 6 | 2 | 2 | 0 | 0 | 0 | 0 | 0 | 0 | 0 | 0 | 85 | 102 | Carbamidomethylation | C7:Carbamidomethylation:1000.00 | PEAKS DB |
| R.VLEGIQC(+57.02)GESIYM(+15.99)SSNAR.T | Y | 83.82 | 2028.9244 | 18 | -1.0 | 1015.4684 | 2 | 49.35 | 4 | F4:32620 | NaSa01\_F12.raw |  |  |  | 2.596E9 | 2.4998E7 | 3.3484E6 |  |  |  |  |  |  |  |  | 24 | 0 | 0 | 0 | 20 | 2 | 2 | 0 | 0 | 0 | 0 | 0 | 0 | 0 | 0 | 85 | 102 | Carbamidomethylation; Oxidation (M) | C7:Carbamidomethylation:1000.00;M13:Oxidation (M):1000.00 | PEAKS DB |
| R.WANTC(+57.02)SLNHSPDNLR.V | Y | 83.41 | 1783.8060 | 15 | -0.3 | 892.9100 | 2 | 20.24 | 4 | F4:7228 | NaSa01\_F12.raw |  |  |  | 4.9332E10 | 1.1987E8 | 6.2009E4 |  |  |  |  |  |  |  |  | 25 | 0 | 0 | 0 | 20 | 4 | 1 | 0 | 0 | 0 | 0 | 0 | 0 | 0 | 0 | 70 | 84 | Carbamidomethylation | C5:Carbamidomethylation:1000.00 | PEAKS DB |
| K.QSSC(+57.02)QDDWIKSNC(+57.02)PASC(+57.02)FC(+57.02)R.N | Y | 79.80 | 2504.9929 | 20 | 0.6 | 836.0054 | 3 | 39.02 | 4 | F4:22824 | NaSa01\_F12.raw |  |  |  | 7.3335E7 |  |  |  |  |  |  |  |  |  |  | 3 | 0 | 0 | 0 | 3 | 0 | 0 | 0 | 0 | 0 | 0 | 0 | 0 | 0 | 0 | 216 | 235 | Carbamidomethylation | C4:Carbamidomethylation:1000.00;C13:Carbamidomethylation:1000.00;C17:Carbamidomethylation:1000.00;C19:Carbamidomethylation:1000.00 | PEAKS DB |
| K.LTNC(+57.02)DSLLKQSSC(+57.02)QDDWIK.S | Y | 79.46 | 2310.0620 | 19 | -1.1 | 771.0271 | 3 | 45.31 | 4 | F4:28181 | NaSa01\_F12.raw |  |  |  | 1.962E8 |  |  |  |  |  |  |  |  |  |  | 4 | 0 | 0 | 0 | 4 | 0 | 0 | 0 | 0 | 0 | 0 | 0 | 0 | 0 | 0 | 207 | 225 | Carbamidomethylation | C4:Carbamidomethylation:1000.00;C13:Carbamidomethylation:1000.00 | PEAKS DB |
| D.C(+57.02)PSAC(+57.02)DNGLC(+57.02)TNPC(+57.02)TIYNK.L | Y | 78.07 | 2243.9067 | 19 | -0.3 | 1122.9603 | 2 | 35.53 | 4 | F4:19614 | NaSa01\_F12.raw |  |  |  | 1.255E8 |  |  |  |  |  |  |  |  |  |  | 3 | 0 | 0 | 0 | 3 | 0 | 0 | 0 | 0 | 0 | 0 | 0 | 0 | 0 | 0 | 188 | 206 | Carbamidomethylation | C1:Carbamidomethylation:1000.00;C5:Carbamidomethylation:1000.00;C10:Carbamidomethylation:1000.00;C14:Carbamidomethylation:1000.00 | PEAKS DB |
| K.NFVYGVGANPPGSVTGHYTQIVWYQTYR.A | Y | 77.64 | 3173.5359 | 28 | 0.0 | 1058.8525 | 3 | 74.13 | 4 | F4:51117 | NaSa01\_F12.raw |  |  |  | 1.5642E9 |  |  |  |  |  |  |  |  |  |  | 6 | 0 | 0 | 0 | 6 | 0 | 0 | 0 | 0 | 0 | 0 | 0 | 0 | 0 | 0 | 117 | 144 |  |  | PEAKS DB |
| Y.GVGANPPGSVTGHYTQIVWYQTYR.A | Y | 77.12 | 2650.2927 | 24 | 0.9 | 884.4390 | 3 | 57.12 | 4 | F4:37409 | NaSa01\_F12.raw |  |  | 5.2237E5 | 6.6355E7 |  |  |  |  |  |  |  |  |  |  | 2 | 0 | 0 | 1 | 1 | 0 | 0 | 0 | 0 | 0 | 0 | 0 | 0 | 0 | 0 | 121 | 144 |  |  | PEAKS DB |
| R.VSPTASNMLKMEWYPEAASNAER.W | N | 76.94 | 2581.1941 | 23 | 0.7 | 861.4059 | 3 | 60.25 | 4 | F4:39967 | NaSa01\_F12.raw |  |  |  | 1.7324E8 |  |  |  |  |  |  |  |  |  |  | 2 | 0 | 0 | 0 | 2 | 0 | 0 | 0 | 0 | 0 | 0 | 0 | 0 | 0 | 0 | 47 | 69 |  |  | PEAKS DB |
| K.MEWYPEAASNAER.W | N | 75.93 | 1552.6616 | 13 | -4.7 | 518.5587 | 3 | 42.47 | 4 | F4:25783 | NaSa01\_F12.raw |  |  | 8.9734E6 | 5.588E10 | 1.0906E8 | 2.4001E7 |  |  |  |  |  |  |  |  | 18 | 0 | 0 | 1 | 10 | 5 | 2 | 0 | 0 | 0 | 0 | 0 | 0 | 0 | 0 | 57 | 69 |  |  | PEAKS DB |
| W.SYFYVC(+57.02)QYC(+57.02)PSGNFQGK.T | Y | 74.81 | 2103.8818 | 17 | 0.9 | 1052.9491 | 2 | 56.78 | 4 | F4:37002 | NaSa01\_F12.raw |  |  |  | 7.9496E7 | 3.5056E6 |  |  |  |  |  |  |  |  |  | 3 | 0 | 0 | 0 | 2 | 1 | 0 | 0 | 0 | 0 | 0 | 0 | 0 | 0 | 0 | 158 | 174 | Carbamidomethylation | C6:Carbamidomethylation:1000.00;C9:Carbamidomethylation:1000.00 | PEAKS DB |
| K.QKEIVDLHNSLR.R | N | 73.79 | 1450.7892 | 12 | 0.4 | 484.6039 | 3 | 11.92 | 4 | F4:3036 | NaSa01\_F12.raw |  |  |  | 1.138E8 | 7.1992E5 |  |  |  |  |  |  |  |  |  | 16 | 0 | 0 | 0 | 15 | 1 | 0 | 0 | 0 | 0 | 0 | 0 | 0 | 0 | 0 | 33 | 44 |  |  | PEAKS DB |
| L.RVLEGIQC(+57.02)GESIYMSSNAR.T | Y | 73.68 | 2169.0308 | 19 | -0.1 | 724.0175 | 3 | 41.70 | 4 | F4:24692 | NaSa01\_F12.raw |  |  |  | 1.2641E7 |  |  |  |  |  |  |  |  |  |  | 1 | 0 | 0 | 0 | 1 | 0 | 0 | 0 | 0 | 0 | 0 | 0 | 0 | 0 | 0 | 84 | 102 | Carbamidomethylation | C8:Carbamidomethylation:1000.00 | PEAKS DB |
| C.PSAC(+57.02)DNGLC(+57.02)TNPC(+57.02)TIYNK.L | Y | 73.51 | 2083.8762 | 18 | 0.2 | 1042.9456 | 2 | 34.80 | 4 | F4:18795 | NaSa01\_F12.raw |  |  |  | 1.0016E7 |  |  |  |  |  |  |  |  |  |  | 1 | 0 | 0 | 0 | 1 | 0 | 0 | 0 | 0 | 0 | 0 | 0 | 0 | 0 | 0 | 189 | 206 | Carbamidomethylation | C4:Carbamidomethylation:1000.00;C9:Carbamidomethylation:1000.00;C13:Carbamidomethylation:1000.00 | PEAKS DB |
| S.SAWSYFYVC(+57.02)QYC(+57.02)PSGNFQGK.T | Y | 73.42 | 2448.0303 | 20 | -0.2 | 1225.0222 | 2 | 75.77 | 4 | F4:52609 | NaSa01\_F12.raw |  |  |  | 7.5549E7 |  |  |  |  |  |  |  |  |  |  | 2 | 0 | 0 | 0 | 2 | 0 | 0 | 0 | 0 | 0 | 0 | 0 | 0 | 0 | 0 | 155 | 174 | Carbamidomethylation | C9:Carbamidomethylation:1000.00;C12:Carbamidomethylation:1000.00 | PEAKS DB |
| K.M(+15.99)EWYPEAASNAER.W | N | 73.15 | 1568.6565 | 13 | 0.9 | 523.8932 | 3 | 33.16 | 4 | F4:17227 | NaSa01\_F12.raw |  |  |  | 1.0876E10 | 1.0638E7 | 4.7019E6 |  |  |  |  |  |  |  |  | 17 | 0 | 0 | 0 | 15 | 1 | 1 | 0 | 0 | 0 | 0 | 0 | 0 | 0 | 0 | 57 | 69 | Oxidation (M) | M1:Oxidation (M):1000.00 | PEAKS DB |
| K.EIVDLHNSLR.R | N | 71.95 | 1194.6356 | 10 | 0.4 | 598.3253 | 2 | 28.76 | 4 | F4:13650 | NaSa01\_F12.raw |  |  |  | 7.7068E9 | 7.5889E7 | 5.7777E4 |  |  |  |  |  |  |  |  | 27 | 0 | 0 | 0 | 23 | 3 | 1 | 0 | 0 | 0 | 0 | 0 | 0 | 0 | 0 | 35 | 44 |  |  | PEAKS DB |
| Y.C(+57.02)PSSAWSYFYVC(+57.02)QYC(+57.02)PSGNFQGK.T | Y | 71.68 | 2792.1458 | 23 | 1.2 | 931.7236 | 3 | 77.58 | 4 | F4:54131 | NaSa01\_F12.raw |  |  |  | 2.7393E7 |  |  |  |  |  |  |  |  |  |  | 2 | 0 | 0 | 0 | 2 | 0 | 0 | 0 | 0 | 0 | 0 | 0 | 0 | 0 | 0 | 152 | 174 | Carbamidomethylation | C1:Carbamidomethylation:1000.00;C12:Carbamidomethylation:1000.00;C15:Carbamidomethylation:1000.00 | PEAKS DB |
| Y.FYVC(+57.02)QYC(+57.02)PSGNFQGK.T | Y | 71.29 | 1853.7865 | 15 | 0.2 | 927.9008 | 2 | 45.22 | 4 | F4:27254 | NaSa01\_F12.raw |  |  |  | 6.7966E7 |  |  |  |  |  |  |  |  |  |  | 1 | 0 | 0 | 0 | 1 | 0 | 0 | 0 | 0 | 0 | 0 | 0 | 0 | 0 | 0 | 160 | 174 | Carbamidomethylation | C4:Carbamidomethylation:1000.00;C7:Carbamidomethylation:1000.00 | PEAKS DB |
| F.YVC(+57.02)QYC(+57.02)PSGNFQGK.T | Y | 70.89 | 1706.7181 | 14 | -0.4 | 854.3660 | 2 | 26.36 | 4 | F4:11724 | NaSa01\_F12.raw |  |  |  | 1.5606E8 | 1.5285E7 |  |  |  |  |  |  |  |  |  | 2 | 0 | 0 | 0 | 1 | 1 | 0 | 0 | 0 | 0 | 0 | 0 | 0 | 0 | 0 | 161 | 174 | Carbamidomethylation | C3:Carbamidomethylation:1000.00;C6:Carbamidomethylation:1000.00 | PEAKS DB |
| D.NGLC(+57.02)TNPC(+57.02)TIYNK.L | Y | 69.78 | 1553.6967 | 13 | 0.5 | 777.8560 | 2 | 28.19 | 4 | F4:13013 | NaSa01\_F12.raw |  |  |  | 3.1653E7 |  |  |  |  |  |  |  |  |  |  | 1 | 0 | 0 | 0 | 1 | 0 | 0 | 0 | 0 | 0 | 0 | 0 | 0 | 0 | 0 | 194 | 206 | Carbamidomethylation | C4:Carbamidomethylation:1000.00;C8:Carbamidomethylation:1000.00 | PEAKS DB |
| K.SNC(+57.02)PASC(+57.02)FC(+57.02)R.N | N | 69.18 | 1257.4689 | 10 | 0.2 | 629.7418 | 2 | 11.79 | 4 | F4:2692 | NaSa01\_F12.raw |  |  |  | 1.0262E8 |  |  |  |  |  |  |  |  |  |  | 1 | 0 | 0 | 0 | 1 | 0 | 0 | 0 | 0 | 0 | 0 | 0 | 0 | 0 | 0 | 226 | 235 | Carbamidomethylation | C3:Carbamidomethylation:1000.00;C7:Carbamidomethylation:1000.00;C9:Carbamidomethylation:1000.00 | PEAKS DB |
| K.QSSC(+57.02)QDDWIK.S | Y | 68.96 | 1265.5347 | 10 | -0.7 | 633.7742 | 2 | 11.92 | 4 | F4:3391 | NaSa01\_F12.raw |  |  |  | 3.0416E8 |  |  |  |  |  |  |  |  |  |  | 8 | 0 | 0 | 0 | 8 | 0 | 0 | 0 | 0 | 0 | 0 | 0 | 0 | 0 | 0 | 216 | 225 | Carbamidomethylation | C4:Carbamidomethylation:1000.00 | PEAKS DB |
| K.MEWYPEAASNAERWANTC(+57.02)SLNHSPDNLR.V | Y | 66.81 | 3318.4570 | 28 | 0.1 | 830.6216 | 4 | 60.99 | 4 | F4:40505 | NaSa01\_F12.raw |  |  |  | 1.1514E7 |  |  |  |  |  |  |  |  |  |  | 3 | 0 | 0 | 0 | 3 | 0 | 0 | 0 | 0 | 0 | 0 | 0 | 0 | 0 | 0 | 57 | 84 | Carbamidomethylation | C18:Carbamidomethylation:1000.00 | PEAKS DB |
| N.GLC(+57.02)TNPC(+57.02)TIYNK.L | Y | 66.27 | 1439.6537 | 12 | -0.2 | 720.8340 | 2 | 27.00 | 4 | F4:12069 | NaSa01\_F12.raw |  |  |  | 3.5701E7 |  |  |  |  |  |  |  |  |  |  | 1 | 0 | 0 | 0 | 1 | 0 | 0 | 0 | 0 | 0 | 0 | 0 | 0 | 0 | 0 | 195 | 206 | Carbamidomethylation | C3:Carbamidomethylation:1000.00;C7:Carbamidomethylation:1000.00 | PEAKS DB |
| E.GIQC(+57.02)GESIYMSSNAR.T | Y | 64.94 | 1671.7345 | 15 | 0.6 | 836.8750 | 2 | 29.68 | 4 | F4:14460 | NaSa01\_F12.raw |  |  |  | 1.9465E6 |  |  |  |  |  |  |  |  |  |  | 1 | 0 | 0 | 0 | 1 | 0 | 0 | 0 | 0 | 0 | 0 | 0 | 0 | 0 | 0 | 88 | 102 | Carbamidomethylation | C4:Carbamidomethylation:1000.00 | PEAKS DB |
| N.PPGSVTGHYTQIVWYQTYR.A | Y | 64.76 | 2252.1013 | 19 | 0.1 | 751.7078 | 3 | 53.16 | 4 | F4:33903 | NaSa01\_F12.raw |  |  |  | 4.1382E6 |  |  |  |  |  |  |  |  |  |  | 1 | 0 | 0 | 0 | 1 | 0 | 0 | 0 | 0 | 0 | 0 | 0 | 0 | 0 | 0 | 126 | 144 |  |  | PEAKS DB |
| K.NFVYGVGANPPGSVTGH.Y | N | 64.66 | 1671.8005 | 17 | 0.7 | 836.9081 | 2 | 48.12 | 4 | F4:29754 | NaSa01\_F12.raw |  |  |  | 2.7791E8 | 1.1488E7 | 2.2522E6 |  |  |  |  |  |  |  |  | 6 | 0 | 0 | 0 | 4 | 1 | 1 | 0 | 0 | 0 | 0 | 0 | 0 | 0 | 0 | 117 | 133 |  |  | PEAKS DB |
| G.NVDFNSESTR.R | N | 64.34 | 1167.5156 | 10 | 0.3 | 584.7653 | 2 | 12.14 | 4 | F4:3045 | NaSa01\_F12.raw |  |  |  | 1.4013E8 |  |  |  |  |  |  |  |  |  |  | 6 | 0 | 0 | 0 | 6 | 0 | 0 | 0 | 0 | 0 | 0 | 0 | 0 | 0 | 0 | 19 | 28 |  |  | PEAKS DB |
| K.LTNC(+57.02)DSLLK.Q | Y | 64.00 | 1062.5380 | 9 | -1.6 | 532.2754 | 2 | 21.02 | 4 | F4:7364 | NaSa01\_F12.raw |  |  |  | 2.9662E8 |  |  |  |  |  |  |  |  |  |  | 6 | 0 | 0 | 0 | 6 | 0 | 0 | 0 | 0 | 0 | 0 | 0 | 0 | 0 | 0 | 207 | 215 | Carbamidomethylation | C4:Carbamidomethylation:1000.00 | PEAKS DB |
| H.YTQIVWYQTYR.A | Y | 63.87 | 1519.7460 | 11 | 1.3 | 760.8812 | 2 | 55.25 | 4 | F4:35871 | NaSa01\_F12.raw |  |  |  | 1.2665E8 | 2.5476E6 |  |  |  |  |  |  |  |  |  | 3 | 0 | 0 | 0 | 2 | 1 | 0 | 0 | 0 | 0 | 0 | 0 | 0 | 0 | 0 | 134 | 144 |  |  | PEAKS DB |
| N.VDFNSESTR.R | N | 63.60 | 1053.4727 | 9 | 0.3 | 527.7438 | 2 | 11.74 | 4 | F4:2649 | NaSa01\_F12.raw |  |  |  | 4.6861E6 |  |  |  |  |  |  |  |  |  |  | 1 | 0 | 0 | 0 | 1 | 0 | 0 | 0 | 0 | 0 | 0 | 0 | 0 | 0 | 0 | 20 | 28 |  |  | PEAKS DB |
| C.DNGLC(+57.02)TNPC(+57.02)TIYNK.L | Y | 63.45 | 1668.7236 | 14 | 0.8 | 835.3698 | 2 | 36.30 | 4 | F4:20118 | NaSa01\_F12.raw |  |  |  | 3.0623E7 |  |  |  |  |  |  |  |  |  |  | 1 | 0 | 0 | 0 | 1 | 0 | 0 | 0 | 0 | 0 | 0 | 0 | 0 | 0 | 0 | 193 | 206 | Carbamidomethylation | C5:Carbamidomethylation:1000.00;C9:Carbamidomethylation:1000.00 | PEAKS DB |
| L.EGIQC(+57.02)GESIYMSSNAR.T | Y | 62.23 | 1800.7771 | 16 | 0.5 | 901.3963 | 2 | 35.36 | 4 | F4:19124 | NaSa01\_F12.raw |  |  |  | 1.9136E6 |  |  |  |  |  |  |  |  |  |  | 1 | 0 | 0 | 0 | 1 | 0 | 0 | 0 | 0 | 0 | 0 | 0 | 0 | 0 | 0 | 87 | 102 | Carbamidomethylation | C5:Carbamidomethylation:1000.00 | PEAKS DB |
| R.VSPTASNM(+15.99)LKMEWYPEAASNAER.W | N | 61.53 | 2597.1890 | 23 | 0.7 | 866.7375 | 3 | 47.88 | 4 | F4:29657 | NaSa01\_F12.raw |  |  |  | 1.0394E7 |  |  |  |  |  |  |  |  |  |  | 2 | 0 | 0 | 0 | 2 | 0 | 0 | 0 | 0 | 0 | 0 | 0 | 0 | 0 | 0 | 47 | 69 | Oxidation (M) | M8:Oxidation (M):24.24 | PEAKS DB |
| K.LGPPC(+57.02)GDC(+57.02)PSAC(+57.02)DNGLC(+57.02)TNPC(+57.02)TIYNK.L | Y | 61.29 | 2940.1970 | 26 | -0.2 | 981.0728 | 3 | 53.63 | 4 | F4:34247 | NaSa01\_F12.raw |  |  |  | 1.1603E10 |  | 3.4496E6 |  |  |  |  |  | 6.1046E6 |  |  | 9 | 0 | 0 | 0 | 7 | 0 | 1 | 0 | 0 | 0 | 0 | 0 | 1 | 0 | 0 | 181 | 206 | Carbamidomethylation | C5:Carbamidomethylation:1000.00;C8:Carbamidomethylation:1000.00;C12:Carbamidomethylation:1000.00;C17:Carbamidomethylation:1000.00;C21:Carbamidomethylation:1000.00 | PEAKS DB |
| K.NFVYGVGANPPGSVTGHY.T | N | 60.74 | 1834.8638 | 18 | 1.5 | 918.4406 | 2 | 53.13 | 4 | F4:33956 | NaSa01\_F12.raw |  |  |  | 5.187E6 |  |  |  |  |  |  |  |  |  |  | 1 | 0 | 0 | 0 | 1 | 0 | 0 | 0 | 0 | 0 | 0 | 0 | 0 | 0 | 0 | 117 | 134 |  |  | PEAKS DB |
| R.TWTEIIHLWH.D | Y | 59.61 | 1334.6771 | 10 | 0.6 | 668.3463 | 2 | 74.13 | 4 | F4:51227 | NaSa01\_F12.raw |  |  |  | 5.1108E6 |  |  |  |  |  |  |  |  |  |  | 1 | 0 | 0 | 0 | 1 | 0 | 0 | 0 | 0 | 0 | 0 | 0 | 0 | 0 | 0 | 103 | 112 |  |  | PEAKS DB |
| K.LGPPC(+57.02)GDC(+57.02)PSAC(+57.02)DNGLC(+57.02)TNPC(+57.02)TIYNKLTNC(+57.02)DSLLK.Q | Y | 59.18 | 3984.7244 | 35 | -0.2 | 1329.2484 | 3 | 74.37 | 4 | F4:51337 | NaSa01\_F12.raw |  |  |  | 2.2817E8 |  |  |  |  |  |  |  |  |  |  | 3 | 0 | 0 | 0 | 3 | 0 | 0 | 0 | 0 | 0 | 0 | 0 | 0 | 0 | 0 | 181 | 215 | Carbamidomethylation | C5:Carbamidomethylation:1000.00;C8:Carbamidomethylation:1000.00;C12:Carbamidomethylation:1000.00;C17:Carbamidomethylation:1000.00;C21:Carbamidomethylation:1000.00;C30:Carbamidomethylation:1000.00 | PEAKS DB |
| K.TATPYKLGPPC(+57.02)GDC(+57.02)PSAC(+57.02)DNGLC(+57.02)TNPC(+57.02)TIYNK.L | Y | 58.88 | 3601.5405 | 32 | 0.0 | 1201.5208 | 3 | 49.06 | 4 | F4:30641 | NaSa01\_F12.raw |  |  |  | 5.9598E7 |  |  |  |  |  |  |  |  |  |  | 1 | 0 | 0 | 0 | 1 | 0 | 0 | 0 | 0 | 0 | 0 | 0 | 0 | 0 | 0 | 175 | 206 | Carbamidomethylation | C11:Carbamidomethylation:1000.00;C14:Carbamidomethylation:1000.00;C18:Carbamidomethylation:1000.00;C23:Carbamidomethylation:1000.00;C27:Carbamidomethylation:1000.00 | PEAKS DB |
| G.LC(+57.02)TNPC(+57.02)TIYNK.L | Y | 58.71 | 1382.6323 | 11 | 0.6 | 692.3239 | 2 | 23.47 | 4 | F4:9229 | NaSa01\_F12.raw |  |  |  | 2.5631E6 |  |  |  |  |  |  |  | 1.5272E6 |  |  | 2 | 0 | 0 | 0 | 1 | 0 | 0 | 0 | 0 | 0 | 0 | 0 | 1 | 0 | 0 | 196 | 206 | Carbamidomethylation | C2:Carbamidomethylation:1000.00;C6:Carbamidomethylation:1000.00 | PEAKS DB |
| R.WANTC(+57.02)SLNHSPDNLRVLEGIQC(+57.02)GESIYMSSNAR.T | Y | 58.28 | 3778.7251 | 33 | -0.2 | 945.6884 | 4 | 66.79 | 4 | F4:45302 | NaSa01\_F12.raw |  |  |  | 2.4518E7 |  |  |  |  |  |  |  |  |  |  | 2 | 0 | 0 | 0 | 2 | 0 | 0 | 0 | 0 | 0 | 0 | 0 | 0 | 0 | 0 | 70 | 102 | Carbamidomethylation | C5:Carbamidomethylation:1000.00;C22:Carbamidomethylation:1000.00 | PEAKS DB |
| S.AC(+57.02)DNGLC(+57.02)TNPC(+57.02)TIYNK.L | Y | 58.09 | 1899.7914 | 16 | -0.8 | 950.9022 | 2 | 32.96 | 4 | F4:17191 | NaSa01\_F12.raw |  |  |  | 5.221E6 |  |  |  |  |  |  |  |  |  |  | 1 | 0 | 0 | 0 | 1 | 0 | 0 | 0 | 0 | 0 | 0 | 0 | 0 | 0 | 0 | 191 | 206 | Carbamidomethylation | C2:Carbamidomethylation:1000.00;C7:Carbamidomethylation:1000.00;C11:Carbamidomethylation:1000.00 | PEAKS DB |
| E.IVDLHNSLR.R | N | 58.01 | 1065.5930 | 9 | -1.6 | 533.8029 | 2 | 26.50 | 4 | F4:11729 | NaSa01\_F12.raw |  |  |  | 1.3096E7 |  |  |  |  |  |  |  |  |  |  | 1 | 0 | 0 | 0 | 1 | 0 | 0 | 0 | 0 | 0 | 0 | 0 | 0 | 0 | 0 | 36 | 44 |  |  | PEAKS DB |
| R.AGC(+57.02)AVSYC(+57.02)PSSAWSYFYVC(+57.02)QYC(+57.02)PSGNFQGK.T | Y | 57.96 | 3500.4358 | 30 | 0.3 | 1167.8196 | 3 | 84.20 | 4 | F4:60175 | NaSa01\_F12.raw |  |  |  | 8.8824E8 |  |  |  |  |  |  |  |  |  |  | 3 | 0 | 0 | 0 | 3 | 0 | 0 | 0 | 0 | 0 | 0 | 0 | 0 | 0 | 0 | 145 | 174 | Carbamidomethylation | C3:Carbamidomethylation:1000.00;C8:Carbamidomethylation:1000.00;C19:Carbamidomethylation:1000.00;C22:Carbamidomethylation:1000.00 | PEAKS DB |
| N.TC(+57.02)SLNHSPDNLR.V | Y | 57.81 | 1412.6466 | 12 | 0.4 | 471.8897 | 3 | 11.55 | 4 | F4:2523 | NaSa01\_F12.raw |  |  |  | 6.8334E6 |  |  |  |  |  |  |  |  |  |  | 2 | 0 | 0 | 0 | 2 | 0 | 0 | 0 | 0 | 0 | 0 | 0 | 0 | 0 | 0 | 73 | 84 | Carbamidomethylation | C2:Carbamidomethylation:1000.00 | PEAKS DB |
| R.VSPTASNMLK.M | N | 57.39 | 1046.5430 | 10 | 0.1 | 524.2788 | 2 | 11.91 | 4 | F4:3054 | NaSa01\_F12.raw |  |  |  | 1.9382E8 |  |  |  |  |  |  |  |  |  |  | 5 | 0 | 0 | 0 | 5 | 0 | 0 | 0 | 0 | 0 | 0 | 0 | 0 | 0 | 0 | 47 | 56 |  |  | PEAKS DB |
| L.KMEWYPEAASNAER.W | N | 57.28 | 1680.7566 | 14 | 0.7 | 561.2599 | 3 | 28.56 | 4 | F4:13207 | NaSa01\_F12.raw |  |  |  | 1.222E8 |  |  |  |  |  |  |  |  |  |  | 2 | 0 | 0 | 0 | 2 | 0 | 0 | 0 | 0 | 0 | 0 | 0 | 0 | 0 | 0 | 56 | 69 |  |  | PEAKS DB |
| R.VSPTASNMLKM(+15.99)EWYPEAASNAER.W | N | 57.05 | 2597.1890 | 23 | 0.7 | 866.7375 | 3 | 49.69 | 4 | F4:31613 | NaSa01\_F12.raw |  |  |  | 1.4277E7 |  |  |  |  |  |  |  |  |  |  | 1 | 0 | 0 | 0 | 1 | 0 | 0 | 0 | 0 | 0 | 0 | 0 | 0 | 0 | 0 | 47 | 69 | Oxidation (M) | M11:Oxidation (M):32.97 | PEAKS DB |
| W.TEIIHLWHDEYK.N | Y | 56.98 | 1582.7780 | 12 | 0.1 | 528.6000 | 3 | 36.11 | 4 | F4:19977 | NaSa01\_F12.raw |  |  |  | 9.0049E6 |  |  |  |  |  |  |  |  |  |  | 4 | 0 | 0 | 0 | 4 | 0 | 0 | 0 | 0 | 0 | 0 | 0 | 0 | 0 | 0 | 105 | 116 |  |  | PEAKS DB |
| R.VLEGIQC(+57.02)GESIYMSSN.A | Y | 56.56 | 1785.7914 | 16 | 0.3 | 893.9033 | 2 | 68.26 | 4 | F4:46624 | NaSa01\_F12.raw |  |  |  | 2.5137E8 |  |  |  |  |  |  |  |  |  |  | 1 | 0 | 0 | 0 | 1 | 0 | 0 | 0 | 0 | 0 | 0 | 0 | 0 | 0 | 0 | 85 | 100 | Carbamidomethylation | C7:Carbamidomethylation:1000.00 | PEAKS DB |
| E.IIHLWHDEYK.N | N | 55.71 | 1352.6877 | 10 | -0.7 | 451.9029 | 3 | 26.09 | 4 | F4:11533 | NaSa01\_F12.raw |  |  |  | 6.5239E5 |  |  |  |  |  |  |  |  |  |  | 1 | 0 | 0 | 0 | 1 | 0 | 0 | 0 | 0 | 0 | 0 | 0 | 0 | 0 | 0 | 107 | 116 |  |  | PEAKS DB |
| C.AVSYC(+57.02)PSSAWSYFYVC(+57.02)QYC(+57.02)PSGNFQGK.T | Y | 55.49 | 3212.3467 | 27 | 0.6 | 1071.7902 | 3 | 83.95 | 4 | F4:59535 | NaSa01\_F12.raw |  |  |  | 1.1276E8 |  |  |  |  |  |  |  |  |  |  | 1 | 0 | 0 | 0 | 1 | 0 | 0 | 0 | 0 | 0 | 0 | 0 | 0 | 0 | 0 | 148 | 174 | Carbamidomethylation | C5:Carbamidomethylation:1000.00;C16:Carbamidomethylation:1000.00;C19:Carbamidomethylation:1000.00 | PEAKS DB |
| M.EWYPEAASNAER.W | N | 54.75 | 1421.6211 | 12 | 0.7 | 711.8183 | 2 | 30.32 | 4 | F4:14915 | NaSa01\_F12.raw |  |  |  | 1.4758E7 |  |  |  |  |  |  |  |  |  |  | 1 | 0 | 0 | 0 | 1 | 0 | 0 | 0 | 0 | 0 | 0 | 0 | 0 | 0 | 0 | 58 | 69 |  |  | PEAKS DB |
| G.NVDFNSESTRR.K | N | 53.63 | 1323.6167 | 11 | 0.6 | 662.8160 | 2 | 11.55 | 4 | F4:2518 | NaSa01\_F12.raw |  |  |  | 1.6815E7 |  |  |  |  |  |  |  |  |  |  | 1 | 0 | 0 | 0 | 1 | 0 | 0 | 0 | 0 | 0 | 0 | 0 | 0 | 0 | 0 | 19 | 29 |  |  | PEAKS DB |
| C.SLNHSPDNLR.V | Y | 52.12 | 1151.5684 | 10 | 0.9 | 576.7920 | 2 | 11.55 | 4 | F4:2531 | NaSa01\_F12.raw |  |  |  | 3.5847E6 |  |  |  |  |  |  |  |  |  |  | 3 | 0 | 0 | 0 | 3 | 0 | 0 | 0 | 0 | 0 | 0 | 0 | 0 | 0 | 0 | 75 | 84 |  |  | PEAKS DB |
| C.GDC(+57.02)PSAC(+57.02)DNGLC(+57.02)TNPC(+57.02)TIYNK.L | Y | 51.17 | 2415.9553 | 21 | 0.4 | 1208.9855 | 2 | 38.69 | 4 | F4:22109 | NaSa01\_F12.raw |  |  |  | 3.1743E6 |  |  |  |  |  |  |  |  |  |  | 1 | 0 | 0 | 0 | 1 | 0 | 0 | 0 | 0 | 0 | 0 | 0 | 0 | 0 | 0 | 186 | 206 | Carbamidomethylation | C3:Carbamidomethylation:1000.00;C7:Carbamidomethylation:1000.00;C12:Carbamidomethylation:1000.00;C16:Carbamidomethylation:1000.00 | PEAKS DB |
| R.VLEGIQC(+57.02)GESIYM(+15.99)SSN.A | Y | 51.15 | 1801.7863 | 16 | -0.1 | 901.9003 | 2 | 59.33 | 4 | F4:39244 | NaSa01\_F12.raw |  |  |  | 2.7515E7 |  |  |  |  |  |  |  |  |  |  | 1 | 0 | 0 | 0 | 1 | 0 | 0 | 0 | 0 | 0 | 0 | 0 | 0 | 0 | 0 | 85 | 100 | Carbamidomethylation; Oxidation (M) | C7:Carbamidomethylation:1000.00;M13:Oxidation (M):1000.00 | PEAKS DB |
| N.FVYGVGANPPGSVTGHYTQIVWYQTYR.A | Y | 50.64 | 3059.4929 | 27 | -4.3 | 1020.8339 | 3 | 70.65 | 4 | F4:48404 | NaSa01\_F12.raw |  |  |  | 2.9259E6 |  |  |  |  |  |  |  |  |  |  | 1 | 0 | 0 | 0 | 1 | 0 | 0 | 0 | 0 | 0 | 0 | 0 | 0 | 0 | 0 | 118 | 144 |  |  | PEAKS DB |
| K.MEWYPEAASN.A | N | 49.85 | 1196.4808 | 10 | 0.2 | 599.2478 | 2 | 59.73 | 4 | F4:39536 | NaSa01\_F12.raw |  |  |  | 6.0961E6 |  |  |  |  |  |  |  |  |  |  | 1 | 0 | 0 | 0 | 1 | 0 | 0 | 0 | 0 | 0 | 0 | 0 | 0 | 0 | 0 | 57 | 66 |  |  | PEAKS DB |
| R.RVSPTASNMLK.M | Y | 49.81 | 1202.6442 | 11 | -0.6 | 602.3290 | 2 | 11.56 | 4 | F4:2521 | NaSa01\_F12.raw |  |  |  | 1.3713E7 |  |  |  |  |  |  |  |  |  |  | 1 | 0 | 0 | 0 | 1 | 0 | 0 | 0 | 0 | 0 | 0 | 0 | 0 | 0 | 0 | 46 | 56 |  |  | PEAKS DB |
| R.VSPTASNM(+15.99)LK.M | N | 49.40 | 1062.5380 | 10 | -0.6 | 532.2759 | 2 | 11.55 | 4 | F4:2520 | NaSa01\_F12.raw |  |  |  | 1.5436E7 |  |  |  |  |  |  |  |  |  |  | 1 | 0 | 0 | 0 | 1 | 0 | 0 | 0 | 0 | 0 | 0 | 0 | 0 | 0 | 0 | 47 | 56 | Oxidation (M) | M8:Oxidation (M):1000.00 | PEAKS DB |
| Q.IVWYQTYR.A | Y | 48.98 | 1127.5764 | 8 | -4.0 | 564.7932 | 2 | 42.65 | 4 | F4:25261 | NaSa01\_F12.raw |  |  |  | 4.5531E6 |  |  |  |  |  |  |  |  |  |  | 1 | 0 | 0 | 0 | 1 | 0 | 0 | 0 | 0 | 0 | 0 | 0 | 0 | 0 | 0 | 137 | 144 |  |  | PEAKS DB |
| N.MLKMEWYPEAASNAER.W | N | 48.97 | 1924.8811 | 16 | 0.5 | 642.6346 | 3 | 47.89 | 4 | F4:29582 | NaSa01\_F12.raw |  |  |  | 1.1253E6 |  |  |  |  |  |  |  |  |  |  | 1 | 0 | 0 | 0 | 1 | 0 | 0 | 0 | 0 | 0 | 0 | 0 | 0 | 0 | 0 | 54 | 69 |  |  | PEAKS DB |
| E.RWANTC(+57.02)SLNHSPDNLR.V | Y | 48.62 | 1939.9071 | 16 | -0.2 | 647.6428 | 3 | 11.82 | 4 | F4:2745 | NaSa01\_F12.raw |  |  |  | 1.9776E6 |  |  |  |  |  |  |  |  |  |  | 1 | 0 | 0 | 0 | 1 | 0 | 0 | 0 | 0 | 0 | 0 | 0 | 0 | 0 | 0 | 69 | 84 | Carbamidomethylation | C6:Carbamidomethylation:1000.00 | PEAKS DB |
| Y.TQIVWYQTYR.A | Y | 47.87 | 1356.6826 | 10 | -1.4 | 679.3477 | 2 | 49.12 | 4 | F4:30645 | NaSa01\_F12.raw |  |  |  | 3.7309E6 |  |  |  |  |  |  |  |  |  |  | 1 | 0 | 0 | 0 | 1 | 0 | 0 | 0 | 0 | 0 | 0 | 0 | 0 | 0 | 0 | 135 | 144 |  |  | PEAKS DB |
| C.QYC(+57.02)PSGNFQGK.T | Y | 47.85 | 1284.5557 | 11 | -0.3 | 643.2849 | 2 | 11.76 | 4 | F4:2671 | NaSa01\_F12.raw |  |  |  | 2.6916E5 |  |  |  |  |  |  |  |  |  |  | 1 | 0 | 0 | 0 | 1 | 0 | 0 | 0 | 0 | 0 | 0 | 0 | 0 | 0 | 0 | 164 | 174 | Carbamidomethylation | C3:Carbamidomethylation:1000.00 | PEAKS DB |
| K.LGPPC(+57.02)GDC(+57.02)PSAC(+57.02)DNGL.C | Y | 47.57 | 1688.6593 | 16 | 0.9 | 845.3376 | 2 | 47.39 | 4 | F4:29273 | NaSa01\_F12.raw |  |  |  | 1.4372E7 |  |  |  |  |  |  |  |  |  |  | 1 | 0 | 0 | 0 | 1 | 0 | 0 | 0 | 0 | 0 | 0 | 0 | 0 | 0 | 0 | 181 | 196 | Carbamidomethylation | C5:Carbamidomethylation:1000.00;C8:Carbamidomethylation:1000.00;C12:Carbamidomethylation:1000.00 | PEAKS DB |
| R.VSPTASNM(+15.99)LKM(+15.99)EWYPEAASNAER.W | N | 47.23 | 2613.1838 | 23 | 0.8 | 872.0693 | 3 | 38.90 | 4 | F4:22253 | NaSa01\_F12.raw |  |  |  | 7.9992E6 |  |  |  |  |  |  |  |  |  |  | 1 | 0 | 0 | 0 | 1 | 0 | 0 | 0 | 0 | 0 | 0 | 0 | 0 | 0 | 0 | 47 | 69 | Oxidation (M) | M8:Oxidation (M):1000.00;M11:Oxidation (M):1000.00 | PEAKS DB |
| T.QIVWYQTYR.A | Y | 47.15 | 1255.6349 | 9 | -0.2 | 628.8246 | 2 | 46.43 | 4 | F4:28539 | NaSa01\_F12.raw |  |  |  | 1.0049E6 |  |  |  |  |  |  |  |  |  |  | 1 | 0 | 0 | 0 | 1 | 0 | 0 | 0 | 0 | 0 | 0 | 0 | 0 | 0 | 0 | 136 | 144 |  |  | PEAKS DB |
| L.RVLEGIQC(+57.02)GESIYM(+15.99)SSNAR.T | Y | 45.38 | 2185.0256 | 19 | 0.3 | 729.3494 | 3 | 36.12 | 4 | F4:19992 | NaSa01\_F12.raw |  |  |  | 1.1935E6 |  |  |  |  |  |  |  |  |  |  | 1 | 0 | 0 | 0 | 1 | 0 | 0 | 0 | 0 | 0 | 0 | 0 | 0 | 0 | 0 | 84 | 102 | Carbamidomethylation; Oxidation (M) | C8:Carbamidomethylation:1000.00;M14:Oxidation (M):1000.00 | PEAKS DB |
| R.TWTEIIHLWHD.E | Y | 44.31 | 1449.7041 | 11 | -0.1 | 484.2419 | 3 | 76.43 | 4 | F4:53592 | NaSa01\_F12.raw |  |  |  | 6.3002E6 |  |  |  |  |  |  |  |  |  |  | 2 | 0 | 0 | 0 | 2 | 0 | 0 | 0 | 0 | 0 | 0 | 0 | 0 | 0 | 0 | 103 | 113 |  |  | PEAKS DB |
| K.MEWYPEAASNAE.R | N | 44.04 | 1396.5605 | 12 | 0.5 | 699.2879 | 2 | 61.61 | 4 | F4:41017 | NaSa01\_F12.raw |  |  |  | 2.7222E6 |  |  |  |  |  |  |  |  |  |  | 1 | 0 | 0 | 0 | 1 | 0 | 0 | 0 | 0 | 0 | 0 | 0 | 0 | 0 | 0 | 57 | 68 |  |  | PEAKS DB |
| I.VDLHNSLR.R | N | 42.85 | 952.5090 | 8 | -1.0 | 477.2613 | 2 | 26.51 | 4 | F4:11862 | NaSa01\_F12.raw |  |  |  | 2.8116E8 |  |  |  |  |  |  |  |  |  |  | 1 | 0 | 0 | 0 | 1 | 0 | 0 | 0 | 0 | 0 | 0 | 0 | 0 | 0 | 0 | 37 | 44 |  |  | PEAKS DB |
| R.RVSPTASNMLKMEWYPEAASNAER.W | Y | 42.34 | 2737.2952 | 24 | 2.2 | 913.4410 | 3 | 51.07 | 4 | F4:32424 | NaSa01\_F12.raw |  |  |  | 1.8869E6 |  |  |  |  |  |  |  |  |  |  | 1 | 0 | 0 | 0 | 1 | 0 | 0 | 0 | 0 | 0 | 0 | 0 | 0 | 0 | 0 | 46 | 69 |  |  | PEAKS DB |
| R.TWTEIIHL.W | Y | 41.70 | 1011.5389 | 8 | 0.0 | 506.7768 | 2 | 72.54 | 4 | F4:50028 | NaSa01\_F12.raw |  |  |  | 7.7511E6 |  |  |  |  |  |  |  |  |  |  | 1 | 0 | 0 | 0 | 1 | 0 | 0 | 0 | 0 | 0 | 0 | 0 | 0 | 0 | 0 | 103 | 110 |  |  | PEAKS DB |
| A.C(+57.02)DNGLC(+57.02)TNPC(+57.02)TIYNK.L | Y | 41.26 | 1828.7543 | 15 | -0.4 | 915.3840 | 2 | 33.09 | 4 | F4:17353 | NaSa01\_F12.raw |  |  |  | 2.4548E6 |  |  |  |  |  |  |  |  |  |  | 1 | 0 | 0 | 0 | 1 | 0 | 0 | 0 | 0 | 0 | 0 | 0 | 0 | 0 | 0 | 192 | 206 | Carbamidomethylation | C1:Carbamidomethylation:1000.00;C6:Carbamidomethylation:1000.00;C10:Carbamidomethylation:1000.00 | PEAKS DB |
| G.IQC(+57.02)GESIYMSSNAR.T | Y | 41.22 | 1614.7130 | 14 | -0.2 | 808.3636 | 2 | 24.59 | 4 | F4:10632 | NaSa01\_F12.raw |  |  |  | 1.3785E6 |  |  |  |  |  |  |  |  |  |  | 1 | 0 | 0 | 0 | 1 | 0 | 0 | 0 | 0 | 0 | 0 | 0 | 0 | 0 | 0 | 89 | 102 | Carbamidomethylation | C3:Carbamidomethylation:1000.00 | PEAKS DB |
| A.RTWTEIIHLWHDEYK.N | Y | 40.99 | 2026.0061 | 15 | 0.5 | 507.5091 | 4 | 61.62 | 4 | F4:41097 | NaSa01\_F12.raw |  |  |  | 3.775E6 |  |  |  |  |  |  |  |  |  |  | 1 | 0 | 0 | 0 | 1 | 0 | 0 | 0 | 0 | 0 | 0 | 0 | 0 | 0 | 0 | 102 | 116 |  |  | PEAKS DB |
| A.WSYFYVC(+57.02)QYC(+57.02)PSGNFQGK.T | Y | 40.64 | 2289.9612 | 18 | -2.7 | 1145.9847 | 2 | 70.96 | 4 | F4:48864 | NaSa01\_F12.raw |  |  |  | 1.2488E6 |  |  |  |  |  |  |  |  |  |  | 1 | 0 | 0 | 0 | 1 | 0 | 0 | 0 | 0 | 0 | 0 | 0 | 0 | 0 | 0 | 157 | 174 | Carbamidomethylation | C7:Carbamidomethylation:1000.00;C10:Carbamidomethylation:1000.00 | PEAKS DB |
| R.VLEGIQC(+57.02)GESIY.M | Y | 40.52 | 1366.6438 | 12 | 0.7 | 684.3297 | 2 | 63.89 | 4 | F4:43118 | NaSa01\_F12.raw |  |  |  | 1.0552E8 |  |  |  |  |  |  |  |  |  |  | 1 | 0 | 0 | 0 | 1 | 0 | 0 | 0 | 0 | 0 | 0 | 0 | 0 | 0 | 0 | 85 | 96 | Carbamidomethylation | C7:Carbamidomethylation:1000.00 | PEAKS DB |
| R.VLEGIQC(+57.02)GESIYM.S | Y | 40.40 | 1497.6843 | 13 | 1.7 | 749.8507 | 2 | 74.39 | 4 | F4:51502 | NaSa01\_F12.raw |  |  |  | 5.5836E7 |  |  |  |  |  |  |  |  |  |  | 1 | 0 | 0 | 0 | 1 | 0 | 0 | 0 | 0 | 0 | 0 | 0 | 0 | 0 | 0 | 85 | 97 | Carbamidomethylation | C7:Carbamidomethylation:1000.00 | PEAKS DB |
| R.AGC(+57.02)AVSYC(+57.02)PSSAW.S | Y | 39.73 | 1414.5646 | 13 | 0.5 | 708.2899 | 2 | 56.74 | 4 | F4:36991 | NaSa01\_F12.raw |  |  |  | 1.3783E7 |  |  |  |  |  |  |  |  |  |  | 1 | 0 | 0 | 0 | 1 | 0 | 0 | 0 | 0 | 0 | 0 | 0 | 0 | 0 | 0 | 145 | 157 | Carbamidomethylation | C3:Carbamidomethylation:1000.00;C8:Carbamidomethylation:1000.00 | PEAKS DB |
| P.SAC(+57.02)DNGLC(+57.02)TNPC(+57.02)TIYNK.L | Y | 39.15 | 1986.8234 | 17 | -0.6 | 994.4184 | 2 | 33.70 | 4 | F4:17779 | NaSa01\_F12.raw |  |  |  | 1.5428E6 |  |  |  |  |  |  |  |  |  |  | 1 | 0 | 0 | 0 | 1 | 0 | 0 | 0 | 0 | 0 | 0 | 0 | 0 | 0 | 0 | 190 | 206 | Carbamidomethylation | C3:Carbamidomethylation:1000.00;C8:Carbamidomethylation:1000.00;C12:Carbamidomethylation:1000.00 | PEAKS DB |
| T.EIIHLWHDEYK.N | Y | 38.91 | 1481.7302 | 11 | -0.1 | 494.9173 | 3 | 34.09 | 4 | F4:18145 | NaSa01\_F12.raw |  |  |  | 5.1609E5 |  |  |  |  |  |  |  |  |  |  | 1 | 0 | 0 | 0 | 1 | 0 | 0 | 0 | 0 | 0 | 0 | 0 | 0 | 0 | 0 | 106 | 116 |  |  | PEAKS DB |
| K.QKEIVDLHN.S | N | 38.54 | 1094.5720 | 9 | 1.2 | 548.2939 | 2 | 11.68 | 4 | F4:2630 | NaSa01\_F12.raw |  |  |  | 6.029E4 |  |  |  |  |  |  |  |  |  |  | 1 | 0 | 0 | 0 | 1 | 0 | 0 | 0 | 0 | 0 | 0 | 0 | 0 | 0 | 0 | 33 | 41 |  |  | PEAKS DB |
| Y.KLGPPC(+57.02)GDC(+57.02)PSAC(+57.02)DNGLC(+57.02)TNPC(+57.02)TIYNK.L | Y | 37.77 | 3068.2917 | 27 | 1.8 | 1023.7731 | 3 | 39.94 | 4 | F4:22895 | NaSa01\_F12.raw |  |  |  | 2.227E8 |  |  |  |  |  |  |  |  |  |  | 1 | 0 | 0 | 0 | 1 | 0 | 0 | 0 | 0 | 0 | 0 | 0 | 0 | 0 | 0 | 180 | 206 | Carbamidomethylation | C6:Carbamidomethylation:1000.00;C9:Carbamidomethylation:1000.00;C13:Carbamidomethylation:1000.00;C18:Carbamidomethylation:1000.00;C22:Carbamidomethylation:1000.00 | PEAKS DB |
| total 88 peptides |  |  |  |  |  |  |  |  |  |  |  |  |  |  |  |  |  |  |  |  |  |  |  |  |  |  |  |  |  |  |  |  |  |  |  |  |  |  |  |  |  |  |  |  |  |
| --- | --- | --- | --- | --- | --- | --- | --- | --- | --- | --- | --- | --- | --- | --- | --- | --- | --- | --- | --- | --- | --- | --- | --- | --- | --- | --- | --- | --- | --- | --- | --- | --- | --- | --- | --- | --- | --- | --- | --- | --- | --- | --- | --- | --- | --- |

JAS05143.1

back to list

  

| Protein Coverage
| Supporting Peptides
|

Protein Coverage:

Supporting Peptides:

| Peptide | Uniq | -10lgP | Mass | Length | ppm | m/z | z | RT | Fraction | Scan | Source File | Area F1 | Area F10 | Area F11 | Area F12 | Area F13 | Area F14 | Area F2 | Area F3 | Area F4 | Area F5 | Area F6 | Area F7 | Area F8 | Area F9 | #Feature | #Feature F1 | #Feature F10 | #Feature F11 | #Feature F12 | #Feature F13 | #Feature F14 | #Feature F2 | #Feature F3 | #Feature F4 | #Feature F5 | #Feature F6 | #Feature F7 | #Feature F8 | #Feature F9 | Start | End | PTM | AScore | Found By |
| --- | --- | --- | --- | --- | --- | --- | --- | --- | --- | --- | --- | --- | --- | --- | --- | --- | --- | --- | --- | --- | --- | --- | --- | --- | --- | --- | --- | --- | --- | --- | --- | --- | --- | --- | --- | --- | --- | --- | --- | --- | --- | --- | --- | --- | --- |
| R.HPDDNEWNHVSMC(+57.02)IVNGGGIR.S | N | 99.67 | 2406.0593 | 21 | -0.4 | 803.0267 | 3 | 49.93 | 6 | F6:29280 | NaSa01\_F14.raw |  |  |  |  | 6.5991E7 | 1.6043E8 |  |  |  |  |  |  |  | 7.6096E6 | 9 | 0 | 0 | 0 | 0 | 3 | 4 | 0 | 0 | 0 | 0 | 0 | 0 | 0 | 2 | 389 | 409 | Carbamidomethylation | C13:Carbamidomethylation:1000.00 | PEAKS DB |
| R.HGQGTGELLQVSGIK.V | N | 97.61 | 1522.8103 | 15 | 0.4 | 762.4127 | 2 | 31.05 | 6 | F6:13489 | NaSa01\_F14.raw |  |  |  | 4.9661E5 | 6.4612E8 | 1.2796E9 |  |  |  |  |  |  |  |  | 8 | 0 | 0 | 0 | 1 | 3 | 4 | 0 | 0 | 0 | 0 | 0 | 0 | 0 | 0 | 457 | 471 |  |  | PEAKS DB |
| R.YDAM(+15.99)ALGNHEFDNGLNGLLDPLLK.N | N | 89.01 | 2645.2795 | 24 | 0.3 | 882.7674 | 3 | 88.88 | 6 | F6:61975 | NaSa01\_F14.raw |  |  |  |  | 1.9977E7 | 6.8848E7 |  |  |  |  |  |  |  |  | 3 | 0 | 0 | 0 | 0 | 1 | 2 | 0 | 0 | 0 | 0 | 0 | 0 | 0 | 0 | 124 | 147 | Oxidation (M) | M4:Oxidation (M):1000.00 | PEAKS DB |
| R.HPDDNEWNHVSM(+15.99)C(+57.02)IVNGGGIR.S | N | 86.62 | 2422.0542 | 21 | -0.3 | 808.3584 | 3 | 44.12 | 6 | F6:24148 | NaSa01\_F14.raw |  |  |  |  | 3.2766E7 | 9.9954E7 |  |  |  |  |  |  |  | 8.8723E6 | 8 | 0 | 0 | 0 | 0 | 2 | 4 | 0 | 0 | 0 | 0 | 0 | 0 | 0 | 2 | 389 | 409 | Oxidation (M); Carbamidomethylation | M12:Oxidation (M):1000.00;C13:Carbamidomethylation:1000.00 | PEAKS DB |
| K.VLLPSFLAAGGDGYYMLK.G | N | 84.56 | 1913.9961 | 18 | 1.4 | 639.0068 | 3 | 94.26 | 6 | F6:66829 | NaSa01\_F14.raw |  |  |  | 4.47E5 | 9.4519E7 | 6.4928E8 |  |  |  |  |  |  |  |  | 12 | 0 | 0 | 0 | 1 | 4 | 7 | 0 | 0 | 0 | 0 | 0 | 0 | 0 | 0 | 510 | 527 |  |  | PEAKS DB |
| K.ASGNPILLNKSIQEDPAVK.A | N | 83.17 | 1993.0844 | 19 | 0.6 | 665.3691 | 3 | 35.69 | 6 | F6:17128 | NaSa01\_F14.raw |  |  |  |  | 1.5436E6 | 2.8654E6 |  |  |  |  |  |  |  |  | 2 | 0 | 0 | 0 | 0 | 1 | 1 | 0 | 0 | 0 | 0 | 0 | 0 | 0 | 0 | 317 | 335 |  |  | PEAKS DB |
| K.VLLPSFLAAGGDGYYM(+15.99)LK.G | N | 82.89 | 1929.9910 | 18 | 0.7 | 966.0034 | 2 | 87.45 | 6 | F6:60748 | NaSa01\_F14.raw |  |  |  |  | 2.8899E7 | 1.2442E8 |  |  |  |  |  |  |  |  | 10 | 0 | 0 | 0 | 0 | 3 | 7 | 0 | 0 | 0 | 0 | 0 | 0 | 0 | 0 | 510 | 527 | Oxidation (M) | M16:Oxidation (M):1000.00 | PEAKS DB |
| R.FHEC(+57.02)NLGNLIC(+57.02)DAVVYNNLR.H | N | 82.50 | 2420.1365 | 20 | -0.2 | 807.7192 | 3 | 77.73 | 6 | F6:52912 | NaSa01\_F14.raw |  |  |  |  | 2.6877E7 | 9.4785E7 |  |  |  |  |  |  | 7.773E6 | 1.2217E8 | 9 | 0 | 0 | 0 | 0 | 1 | 3 | 0 | 0 | 0 | 0 | 0 | 0 | 2 | 3 | 369 | 388 | Carbamidomethylation | C4:Carbamidomethylation:1000.00;C11:Carbamidomethylation:1000.00 | PEAKS DB |
| K.ETPVLSNPGPYLEFR.D | N | 81.49 | 1717.8674 | 15 | 0.8 | 859.9417 | 2 | 69.77 | 6 | F6:46166 | NaSa01\_F14.raw |  |  |  | 7.2199E5 | 2.4678E8 | 7.6435E8 |  |  |  | 2.2582E6 |  |  |  |  | 7 | 0 | 0 | 0 | 1 | 3 | 2 | 0 | 0 | 0 | 1 | 0 | 0 | 0 | 0 | 194 | 208 |  |  | PEAKS DB |
| R.QVPVVQAYAFGK.Y | N | 80.72 | 1305.7081 | 12 | -0.2 | 653.8612 | 2 | 81.01 | 6 | F6:55346 | NaSa01\_F14.raw |  |  |  | 4.7562E5 | 3.326E8 | 6.8066E8 |  |  |  |  |  |  |  |  | 5 | 0 | 0 | 0 | 1 | 1 | 3 | 0 | 0 | 0 | 0 | 0 | 0 | 0 | 0 | 288 | 299 |  |  | PEAKS DB |
| R.EVVHFMNSLR.Y | N | 80.17 | 1230.6179 | 10 | 0.3 | 616.3164 | 2 | 32.92 | 6 | F6:14493 | NaSa01\_F14.raw |  |  |  |  | 2.3691E8 | 4.1556E8 |  |  |  |  |  |  |  |  | 11 | 0 | 0 | 0 | 0 | 3 | 8 | 0 | 0 | 0 | 0 | 0 | 0 | 0 | 0 | 114 | 123 |  |  | PEAKS DB |
| R.VVSLNVLC(+57.02)TEC(+57.02)R.V | N | 79.36 | 1448.7115 | 12 | 0.2 | 725.3632 | 2 | 49.71 | 6 | F6:29319 | NaSa01\_F14.raw |  |  |  | 0 | 2.7472E8 | 5.8042E8 |  |  |  |  |  | 8.8246E5 |  |  | 7 | 0 | 0 | 0 | 0 | 2 | 4 | 0 | 0 | 0 | 0 | 0 | 1 | 0 | 0 | 484 | 495 | Carbamidomethylation | C8:Carbamidomethylation:1000.00;C11:Carbamidomethylation:1000.00 | PEAKS DB |
| R.YDAMALGNHEFDNGLNGLLDPLLK.N | N | 78.14 | 2629.2847 | 24 | 0.8 | 877.4362 | 3 | 94.34 | 6 | F6:67114 | NaSa01\_F14.raw |  |  |  |  | 1.6292E7 | 1.1665E8 |  |  |  |  |  |  |  |  | 4 | 0 | 0 | 0 | 0 | 2 | 2 | 0 | 0 | 0 | 0 | 0 | 0 | 0 | 0 | 124 | 147 |  |  | PEAKS DB |
| K.YLGYLNVIFDDK.G | N | 76.79 | 1458.7394 | 12 | 0.2 | 730.3771 | 2 | 85.89 | 6 | F6:59730 | NaSa01\_F14.raw |  |  |  |  | 6.7598E7 | 9.6452E7 |  |  |  |  |  |  |  |  | 5 | 0 | 0 | 0 | 0 | 4 | 1 | 0 | 0 | 0 | 0 | 0 | 0 | 0 | 0 | 300 | 311 |  |  | PEAKS DB |
| K.LTILHTNDVHAR.V | N | 75.00 | 1388.7524 | 12 | 0.9 | 463.9252 | 3 | 11.51 | 6 | F6:1905 | NaSa01\_F14.raw |  |  |  |  | 1.6389E6 | 3.6507E6 |  |  |  |  |  |  |  |  | 4 | 0 | 0 | 0 | 0 | 2 | 2 | 0 | 0 | 0 | 0 | 0 | 0 | 0 | 0 | 44 | 55 |  |  | PEAKS DB |
| K.ETPVLSNPGPYLEFRDEVEELQK.Q | N | 74.67 | 2688.3281 | 23 | 0.7 | 897.1173 | 3 | 77.38 | 6 | F6:52291 | NaSa01\_F14.raw |  |  |  |  | 3.5808E8 | 6.9841E8 |  |  |  |  |  |  |  |  | 4 | 0 | 0 | 0 | 0 | 2 | 2 | 0 | 0 | 0 | 0 | 0 | 0 | 0 | 0 | 194 | 216 |  |  | PEAKS DB |
| K.ASGNPILLNK.S | N | 69.82 | 1025.5869 | 10 | -0.4 | 513.8005 | 2 | 11.59 | 6 | F6:1963 | NaSa01\_F14.raw |  |  |  |  | 2.4579E6 | 4.7925E6 |  |  |  |  |  |  |  |  | 3 | 0 | 0 | 0 | 0 | 1 | 2 | 0 | 0 | 0 | 0 | 0 | 0 | 0 | 0 | 317 | 326 |  |  | PEAKS DB |
| S.FLAAGGDGYYMLK.G | N | 67.12 | 1404.6747 | 13 | 0.2 | 703.3447 | 2 | 58.91 | 6 | F6:37338 | NaSa01\_F14.raw |  |  |  |  | 1.8909E7 | 8.0208E7 |  |  |  |  |  |  |  |  | 3 | 0 | 0 | 0 | 0 | 1 | 2 | 0 | 0 | 0 | 0 | 0 | 0 | 0 | 0 | 515 | 527 |  |  | PEAKS DB |
| F.LAAGGDGYYMLK.G | N | 66.41 | 1257.6063 | 12 | 0.2 | 629.8105 | 2 | 41.73 | 6 | F6:22051 | NaSa01\_F14.raw |  |  |  |  |  | 5.7329E6 |  |  |  |  |  |  |  |  | 1 | 0 | 0 | 0 | 0 | 0 | 1 | 0 | 0 | 0 | 0 | 0 | 0 | 0 | 0 | 516 | 527 |  |  | PEAKS DB |
| R.EVVHFM(+15.99)NSLR.Y | N | 65.78 | 1246.6128 | 10 | 0.3 | 624.3138 | 2 | 31.71 | 6 | F6:14277 | NaSa01\_F14.raw |  |  |  |  | 3.2821E6 | 5.0137E7 |  |  |  |  |  |  |  |  | 13 | 0 | 0 | 0 | 0 | 2 | 11 | 0 | 0 | 0 | 0 | 0 | 0 | 0 | 0 | 114 | 123 | Oxidation (M) | M6:Oxidation (M):1000.00 | PEAKS DB |
| K.FPILSANIRPK.G | N | 63.57 | 1254.7448 | 11 | 1.8 | 419.2563 | 3 | 38.83 | 6 | F6:20180 | NaSa01\_F14.raw |  |  |  | 3.6184E5 | 9.4886E7 | 1.2559E8 |  |  |  |  | 2.1098E6 |  |  |  | 10 | 0 | 0 | 0 | 1 | 2 | 5 | 0 | 0 | 0 | 0 | 2 | 0 | 0 | 0 | 151 | 161 |  |  | PEAKS DB |
| K.RVVSLNVLC(+57.02)TEC(+57.02)R.V | N | 62.55 | 1604.8126 | 13 | 0.2 | 535.9449 | 3 | 35.03 | 6 | F6:16539 | NaSa01\_F14.raw |  |  |  |  | 4.4155E6 | 6.0208E6 |  |  |  |  |  |  |  |  | 3 | 0 | 0 | 0 | 0 | 1 | 2 | 0 | 0 | 0 | 0 | 0 | 0 | 0 | 0 | 483 | 495 | Carbamidomethylation | C9:Carbamidomethylation:1000.00;C12:Carbamidomethylation:1000.00 | PEAKS DB |
| K.GVDVVVGGHTNTF.L | N | 61.79 | 1300.6411 | 13 | 1.1 | 651.3286 | 2 | 41.80 | 6 | F6:22128 | NaSa01\_F14.raw |  |  |  |  | 2.249E7 | 5.2209E7 |  |  |  |  |  |  |  |  | 2 | 0 | 0 | 0 | 0 | 1 | 1 | 0 | 0 | 0 | 0 | 0 | 0 | 0 | 0 | 249 | 261 |  |  | PEAKS DB |
| L.TAVLPFGGTFDLLQIK.G | N | 60.64 | 1718.9607 | 16 | -0.1 | 860.4875 | 2 | 99.74 | 6 | F6:71142 | NaSa01\_F14.raw |  |  | 2.8271E6 | 1.2518E6 | 2.1013E7 | 4.0691E6 |  |  |  |  |  |  |  |  | 4 | 0 | 0 | 1 | 1 | 1 | 1 | 0 | 0 | 0 | 0 | 0 | 0 | 0 | 0 | 427 | 442 |  |  | PEAKS DB |
| K.IINVGSEKVGIIGYTTK.E | N | 60.09 | 1791.0142 | 17 | -0.7 | 598.0116 | 3 | 45.32 | 6 | F6:25348 | NaSa01\_F14.raw |  |  |  |  | 9.4386E5 | 1.7014E6 |  |  |  |  |  |  |  |  | 2 | 0 | 0 | 0 | 0 | 1 | 1 | 0 | 0 | 0 | 0 | 0 | 0 | 0 | 0 | 177 | 193 |  |  | PEAKS DB |
| K.YLGYLNVIFDDKGK.V | Y | 58.45 | 1643.8558 | 14 | -0.3 | 548.9590 | 3 | 70.19 | 5 | F5:46798 | NaSa01\_F13.raw |  |  |  |  | 1.8927E6 |  |  |  |  |  |  |  |  |  | 2 | 0 | 0 | 0 | 0 | 2 | 0 | 0 | 0 | 0 | 0 | 0 | 0 | 0 | 0 | 300 | 313 |  |  | PEAKS DB |
| K.VFPAMEGR.V | N | 56.33 | 905.4429 | 8 | 0.2 | 453.7288 | 2 | 22.33 | 6 | F6:7722 | NaSa01\_F14.raw |  |  |  |  |  | 3.6244E6 |  |  |  |  |  |  |  |  | 2 | 0 | 0 | 0 | 0 | 0 | 2 | 0 | 0 | 0 | 0 | 0 | 0 | 0 | 0 | 553 | 560 |  |  | PEAKS DB |
| N.ISGYILPYK.I | N | 56.17 | 1052.5906 | 9 | 0.7 | 527.3029 | 2 | 45.82 | 6 | F6:26096 | NaSa01\_F14.raw |  |  |  | 2.2592E5 | 4.6763E8 | 8.5839E8 |  |  |  |  |  |  |  |  | 5 | 0 | 0 | 0 | 1 | 1 | 3 | 0 | 0 | 0 | 0 | 0 | 0 | 0 | 0 | 168 | 176 |  |  | PEAKS DB |
| S.FLAAGGDGYYM(+15.99)LK.G | N | 55.62 | 1420.6697 | 13 | -0.1 | 711.3420 | 2 | 48.69 | 6 | F6:28173 | NaSa01\_F14.raw |  |  |  |  |  | 5.7308E6 |  |  |  |  |  |  |  |  | 1 | 0 | 0 | 0 | 0 | 0 | 1 | 0 | 0 | 0 | 0 | 0 | 0 | 0 | 0 | 515 | 527 | Oxidation (M) | M11:Oxidation (M):1000.00 | PEAKS DB |
| H.GQGTGELLQVSGIK.V | N | 55.06 | 1385.7515 | 14 | 0.3 | 693.8832 | 2 | 45.20 | 6 | F6:25108 | NaSa01\_F14.raw |  |  |  |  |  | 1.2594E6 |  |  |  |  |  |  |  |  | 1 | 0 | 0 | 0 | 0 | 0 | 1 | 0 | 0 | 0 | 0 | 0 | 0 | 0 | 0 | 458 | 471 |  |  | PEAKS DB |
| K.GVDVVVGGHTNTFLY.T | N | 54.75 | 1576.7886 | 15 | 0.1 | 789.4016 | 2 | 63.80 | 6 | F6:41327 | NaSa01\_F14.raw |  |  |  |  | 1.2739E7 | 2.8201E7 |  |  |  |  |  |  |  |  | 2 | 0 | 0 | 0 | 0 | 1 | 1 | 0 | 0 | 0 | 0 | 0 | 0 | 0 | 0 | 249 | 263 |  |  | PEAKS DB |
| R.QVPVVQAYAF.G | N | 53.91 | 1120.5917 | 10 | 0.0 | 561.3031 | 2 | 73.70 | 6 | F6:49221 | NaSa01\_F14.raw |  |  |  |  | 7.1015E6 | 1.9997E7 |  |  |  |  |  |  |  |  | 2 | 0 | 0 | 0 | 0 | 1 | 1 | 0 | 0 | 0 | 0 | 0 | 0 | 0 | 0 | 288 | 297 |  |  | PEAKS DB |
| M.ALGNHEFDNGLNGLLDPLLK.N | N | 53.45 | 2149.1167 | 20 | 0.0 | 717.3795 | 3 | 83.56 | 6 | F6:57342 | NaSa01\_F14.raw |  |  |  |  | 5.3244E6 | 2.0787E7 |  |  |  |  |  |  |  |  | 3 | 0 | 0 | 0 | 0 | 1 | 2 | 0 | 0 | 0 | 0 | 0 | 0 | 0 | 0 | 128 | 147 |  |  | PEAKS DB |
| K.LTTLGVNK.I | N | 52.68 | 844.5018 | 8 | -1.0 | 423.2578 | 2 | 11.58 | 6 | F6:2148 | NaSa01\_F14.raw |  |  |  |  |  | 3.1326E6 |  |  |  |  |  |  |  |  | 1 | 0 | 0 | 0 | 0 | 0 | 1 | 0 | 0 | 0 | 0 | 0 | 0 | 0 | 0 | 221 | 228 |  |  | PEAKS DB |
| K.VLLPSFLAAGGDGY.Y | N | 51.68 | 1378.7133 | 14 | 0.7 | 690.3644 | 2 | 100.88 | 6 | F6:72024 | NaSa01\_F14.raw |  |  |  |  | 1.3644E6 | 2.8748E6 |  |  |  |  |  |  |  |  | 2 | 0 | 0 | 0 | 0 | 1 | 1 | 0 | 0 | 0 | 0 | 0 | 0 | 0 | 0 | 510 | 523 |  |  | PEAKS DB |
| F.GGTFDLLQIK.G | N | 50.44 | 1090.6022 | 10 | -0.6 | 546.3080 | 2 | 64.67 | 6 | F6:41967 | NaSa01\_F14.raw |  |  |  |  |  | 9.7795E7 |  |  |  |  |  |  |  |  | 2 | 0 | 0 | 0 | 0 | 0 | 2 | 0 | 0 | 0 | 0 | 0 | 0 | 0 | 0 | 433 | 442 |  |  | PEAKS DB |
| Y.LNVIFDDK.G | N | 50.15 | 962.5073 | 8 | -0.5 | 482.2607 | 2 | 40.33 | 6 | F6:21131 | NaSa01\_F14.raw |  |  |  |  |  | 1.4605E6 |  |  |  |  |  |  |  |  | 1 | 0 | 0 | 0 | 0 | 0 | 1 | 0 | 0 | 0 | 0 | 0 | 0 | 0 | 0 | 304 | 311 |  |  | PEAKS DB |
| K.VGIIGYTTK.E | N | 50.04 | 950.5436 | 9 | 0.3 | 476.2792 | 2 | 28.48 | 6 | F6:11194 | NaSa01\_F14.raw |  |  |  |  |  | 5.1319E6 |  |  |  |  |  |  |  |  | 2 | 0 | 0 | 0 | 0 | 0 | 2 | 0 | 0 | 0 | 0 | 0 | 0 | 0 | 0 | 185 | 193 |  |  | PEAKS DB |
| L.AAGGDGYYMLK.G | N | 49.71 | 1144.5222 | 11 | 0.7 | 573.2688 | 2 | 30.46 | 5 | F5:13468 | NaSa01\_F13.raw |  |  |  |  | 6.4456E6 |  |  |  |  |  |  |  |  |  | 1 | 0 | 0 | 0 | 0 | 1 | 0 | 0 | 0 | 0 | 0 | 0 | 0 | 0 | 0 | 517 | 527 |  |  | PEAKS DB |
| R.FHEC(+57.02)NLGNLIC(+57.02)DAVVY.N | N | 48.35 | 1922.8655 | 16 | 1.4 | 962.4414 | 2 | 82.45 | 5 | F5:56634 | NaSa01\_F13.raw |  |  |  |  | 8.0322E7 | 1.7562E8 |  |  |  |  |  |  |  | 2.4692E7 | 3 | 0 | 0 | 0 | 0 | 1 | 1 | 0 | 0 | 0 | 0 | 0 | 0 | 0 | 1 | 369 | 384 | Carbamidomethylation | C4:Carbamidomethylation:1000.00;C11:Carbamidomethylation:1000.00 | PEAKS DB |
| R.HPDDNEWNHVSMC(+57.02).I | N | 48.19 | 1639.6144 | 13 | 0.5 | 820.8149 | 2 | 26.12 | 5 | F5:10348 | NaSa01\_F13.raw |  |  |  |  | 8.8799E6 |  |  |  |  |  |  |  |  |  | 2 | 0 | 0 | 0 | 0 | 2 | 0 | 0 | 0 | 0 | 0 | 0 | 0 | 0 | 0 | 389 | 401 | Carbamidomethylation | C13:Carbamidomethylation:1000.00 | PEAKS DB |
| K.VLLPSFLAAGGD.G | N | 47.35 | 1158.6284 | 12 | -0.1 | 580.3214 | 2 | 96.95 | 6 | F6:68512 | NaSa01\_F14.raw |  |  |  |  |  | 1.1427E6 |  |  |  |  |  |  |  |  | 1 | 0 | 0 | 0 | 0 | 0 | 1 | 0 | 0 | 0 | 0 | 0 | 0 | 0 | 0 | 510 | 521 |  |  | PEAKS DB |
| R.HPDDNEWNHVSM.C | N | 47.22 | 1479.5837 | 12 | 1.1 | 740.8000 | 2 | 25.93 | 5 | F5:10070 | NaSa01\_F13.raw |  |  |  |  | 2.3098E6 |  |  |  |  |  |  |  |  |  | 1 | 0 | 0 | 0 | 0 | 1 | 0 | 0 | 0 | 0 | 0 | 0 | 0 | 0 | 0 | 389 | 400 |  |  | PEAKS DB |
| R.QVPVVQAY.A | N | 45.65 | 902.4861 | 8 | -0.5 | 452.2501 | 2 | 43.38 | 5 | F5:24541 | NaSa01\_F13.raw |  |  |  |  | 1.2052E7 |  |  |  |  |  |  |  |  |  | 1 | 0 | 0 | 0 | 0 | 1 | 0 | 0 | 0 | 0 | 0 | 0 | 0 | 0 | 0 | 288 | 295 |  |  | PEAKS DB |
| N.PGPYLEFR.D | N | 45.44 | 977.4970 | 8 | 0.7 | 489.7561 | 2 | 39.90 | 6 | F6:20609 | NaSa01\_F14.raw |  |  |  |  |  | 4.6995E5 |  |  |  |  |  |  |  |  | 1 | 0 | 0 | 0 | 0 | 0 | 1 | 0 | 0 | 0 | 0 | 0 | 0 | 0 | 0 | 201 | 208 |  |  | PEAKS DB |
| N.LGNLIC(+57.02)DAVVYNNLR.H | N | 45.20 | 1732.8931 | 15 | 1.0 | 867.4547 | 2 | 69.10 | 6 | F6:45743 | NaSa01\_F14.raw |  |  |  |  |  | 1.1316E6 |  |  |  |  |  |  |  |  | 1 | 0 | 0 | 0 | 0 | 0 | 1 | 0 | 0 | 0 | 0 | 0 | 0 | 0 | 0 | 374 | 388 | Carbamidomethylation | C6:Carbamidomethylation:1000.00 | PEAKS DB |
| K.IINVGSEK.V | N | 44.70 | 858.4811 | 8 | -0.5 | 430.2476 | 2 | 11.48 | 6 | F6:1861 | NaSa01\_F14.raw |  |  |  |  |  | 1.3857E6 |  |  |  |  |  |  |  |  | 1 | 0 | 0 | 0 | 0 | 0 | 1 | 0 | 0 | 0 | 0 | 0 | 0 | 0 | 0 | 177 | 184 |  |  | PEAKS DB |
| K.VLLPSFLAAGGDGYY.M | N | 43.81 | 1541.7765 | 15 | 0.7 | 771.8961 | 2 | 105.28 | 6 | F6:75496 | NaSa01\_F14.raw |  |  |  |  | 1.4762E7 | 3.1893E7 |  |  |  |  |  |  |  |  | 2 | 0 | 0 | 0 | 0 | 1 | 1 | 0 | 0 | 0 | 0 | 0 | 0 | 0 | 0 | 510 | 524 |  |  | PEAKS DB |
| K.FPILSANIR.P | N | 43.38 | 1029.5970 | 9 | -0.4 | 515.8056 | 2 | 52.72 | 6 | F6:31843 | NaSa01\_F14.raw |  |  |  |  |  | 1.5657E8 |  |  |  |  |  |  |  |  | 1 | 0 | 0 | 0 | 0 | 0 | 1 | 0 | 0 | 0 | 0 | 0 | 0 | 0 | 0 | 151 | 159 |  |  | PEAKS DB |
| K.VLLPSFLAAGGDGYYM.L | N | 42.72 | 1672.8170 | 16 | 0.9 | 837.4166 | 2 | 111.51 | 6 | F6:80284 | NaSa01\_F14.raw |  |  |  |  |  | 1.1099E6 |  |  |  |  |  |  |  |  | 1 | 0 | 0 | 0 | 0 | 0 | 1 | 0 | 0 | 0 | 0 | 0 | 0 | 0 | 0 | 510 | 525 |  |  | PEAKS DB |
| Q.GTGELLQVSGIK.V | N | 42.14 | 1200.6714 | 12 | 0.9 | 601.3435 | 2 | 44.43 | 6 | F6:24568 | NaSa01\_F14.raw |  |  |  |  |  | 7.0353E5 |  |  |  |  |  |  |  |  | 1 | 0 | 0 | 0 | 0 | 0 | 1 | 0 | 0 | 0 | 0 | 0 | 0 | 0 | 0 | 460 | 471 |  |  | PEAKS DB |
| Y.LGYLNVIFDDK.G | N | 41.11 | 1295.6761 | 11 | 0.9 | 648.8459 | 2 | 76.55 | 6 | F6:51743 | NaSa01\_F14.raw |  |  |  |  |  | 0 |  |  |  |  |  |  |  |  | 0 | 0 | 0 | 0 | 0 | 0 | 0 | 0 | 0 | 0 | 0 | 0 | 0 | 0 | 0 | 301 | 311 |  |  | PEAKS DB |
| K.LTTLGVNKIIALGH.S | N | 41.05 | 1448.8715 | 14 | 0.1 | 483.9645 | 3 | 54.77 | 6 | F6:33352 | NaSa01\_F14.raw |  |  |  |  |  | 5.5856E5 |  |  |  |  |  |  |  |  | 1 | 0 | 0 | 0 | 0 | 0 | 1 | 0 | 0 | 0 | 0 | 0 | 0 | 0 | 0 | 221 | 234 |  |  | PEAKS DB |
| N.HEFDNGLNGLLDPLLK.N | N | 40.62 | 1793.9312 | 16 | 0.2 | 598.9844 | 3 | 81.57 | 6 | F6:55994 | NaSa01\_F14.raw |  |  |  |  |  | 5.7086E5 |  |  |  |  |  |  |  |  | 1 | 0 | 0 | 0 | 0 | 0 | 1 | 0 | 0 | 0 | 0 | 0 | 0 | 0 | 0 | 132 | 147 |  |  | PEAKS DB |
| N.YPFMQLSDDGR.Q | N | 40.57 | 1327.5867 | 11 | 0.4 | 664.8009 | 2 | 53.65 | 6 | F6:32287 | NaSa01\_F14.raw |  |  |  |  |  | 5.3395E5 |  |  |  |  |  |  |  |  | 1 | 0 | 0 | 0 | 0 | 0 | 1 | 0 | 0 | 0 | 0 | 0 | 0 | 0 | 0 | 277 | 287 |  |  | PEAKS DB |
| P.AGNYPFMQLSDDGR.Q | N | 40.07 | 1569.6881 | 14 | 0.1 | 785.8514 | 2 | 56.81 | 6 | F6:35126 | NaSa01\_F14.raw |  |  |  |  |  | 2.5054E5 |  |  |  |  |  |  |  |  | 1 | 0 | 0 | 0 | 0 | 0 | 1 | 0 | 0 | 0 | 0 | 0 | 0 | 0 | 0 | 274 | 287 |  |  | PEAKS DB |
| K.NVKFPILSAN.I | N | 39.82 | 1101.6182 | 10 | 0.5 | 551.8167 | 2 | 54.61 | 6 | F6:33186 | NaSa01\_F14.raw |  |  |  |  |  | 1.3385E6 |  |  |  |  |  |  |  |  | 1 | 0 | 0 | 0 | 0 | 0 | 1 | 0 | 0 | 0 | 0 | 0 | 0 | 0 | 0 | 148 | 157 |  |  | PEAKS DB |
| K.IIALGHSGF.K | N | 37.88 | 913.5021 | 9 | -1.2 | 457.7578 | 2 | 43.37 | 6 | F6:23538 | NaSa01\_F14.raw |  |  |  |  |  | 5.7946E6 |  |  |  |  |  |  |  |  | 1 | 0 | 0 | 0 | 0 | 0 | 1 | 0 | 0 | 0 | 0 | 0 | 0 | 0 | 0 | 229 | 237 |  |  | PEAKS DB |
| total 58 peptides |  |  |  |  |  |  |  |  |  |  |  |  |  |  |  |  |  |  |  |  |  |  |  |  |  |  |  |  |  |  |  |  |  |  |  |  |  |  |  |  |  |  |  |  |  |
| --- | --- | --- | --- | --- | --- | --- | --- | --- | --- | --- | --- | --- | --- | --- | --- | --- | --- | --- | --- | --- | --- | --- | --- | --- | --- | --- | --- | --- | --- | --- | --- | --- | --- | --- | --- | --- | --- | --- | --- | --- | --- | --- | --- | --- | --- |

JAS05036.1

back to list

  

| Protein Coverage
| Supporting Peptides
|

Protein Coverage:

Supporting Peptides:

| Peptide | Uniq | -10lgP | Mass | Length | ppm | m/z | z | RT | Fraction | Scan | Source File | Area F1 | Area F10 | Area F11 | Area F12 | Area F13 | Area F14 | Area F2 | Area F3 | Area F4 | Area F5 | Area F6 | Area F7 | Area F8 | Area F9 | #Feature | #Feature F1 | #Feature F10 | #Feature F11 | #Feature F12 | #Feature F13 | #Feature F14 | #Feature F2 | #Feature F3 | #Feature F4 | #Feature F5 | #Feature F6 | #Feature F7 | #Feature F8 | #Feature F9 | Start | End | PTM | AScore | Found By |
| --- | --- | --- | --- | --- | --- | --- | --- | --- | --- | --- | --- | --- | --- | --- | --- | --- | --- | --- | --- | --- | --- | --- | --- | --- | --- | --- | --- | --- | --- | --- | --- | --- | --- | --- | --- | --- | --- | --- | --- | --- | --- | --- | --- | --- | --- |
| R.HPDDNEWNHVSMC(+57.02)IVNGGGIR.S | N | 99.67 | 2406.0593 | 21 | -0.4 | 803.0267 | 3 | 49.93 | 6 | F6:29280 | NaSa01\_F14.raw |  |  |  |  | 6.5991E7 | 1.6043E8 |  |  |  |  |  |  |  | 7.6096E6 | 9 | 0 | 0 | 0 | 0 | 3 | 4 | 0 | 0 | 0 | 0 | 0 | 0 | 0 | 2 | 389 | 409 | Carbamidomethylation | C13:Carbamidomethylation:1000.00 | PEAKS DB |
| R.HGQGTGELLQVSGIK.V | N | 97.61 | 1522.8103 | 15 | 0.4 | 762.4127 | 2 | 31.05 | 6 | F6:13489 | NaSa01\_F14.raw |  |  |  | 4.9661E5 | 6.4612E8 | 1.2796E9 |  |  |  |  |  |  |  |  | 8 | 0 | 0 | 0 | 1 | 3 | 4 | 0 | 0 | 0 | 0 | 0 | 0 | 0 | 0 | 457 | 471 |  |  | PEAKS DB |
| R.YDAM(+15.99)ALGNHEFDNGLNGLLDPLLK.N | N | 89.01 | 2645.2795 | 24 | 0.3 | 882.7674 | 3 | 88.88 | 6 | F6:61975 | NaSa01\_F14.raw |  |  |  |  | 1.9977E7 | 6.8848E7 |  |  |  |  |  |  |  |  | 3 | 0 | 0 | 0 | 0 | 1 | 2 | 0 | 0 | 0 | 0 | 0 | 0 | 0 | 0 | 124 | 147 | Oxidation (M) | M4:Oxidation (M):1000.00 | PEAKS DB |
| R.HPDDNEWNHVSM(+15.99)C(+57.02)IVNGGGIR.S | N | 86.62 | 2422.0542 | 21 | -0.3 | 808.3584 | 3 | 44.12 | 6 | F6:24148 | NaSa01\_F14.raw |  |  |  |  | 3.2766E7 | 9.9954E7 |  |  |  |  |  |  |  | 8.8723E6 | 8 | 0 | 0 | 0 | 0 | 2 | 4 | 0 | 0 | 0 | 0 | 0 | 0 | 0 | 2 | 389 | 409 | Oxidation (M); Carbamidomethylation | M12:Oxidation (M):1000.00;C13:Carbamidomethylation:1000.00 | PEAKS DB |
| K.VLLPSFLAAGGDGYYMLK.G | N | 84.56 | 1913.9961 | 18 | 1.4 | 639.0068 | 3 | 94.26 | 6 | F6:66829 | NaSa01\_F14.raw |  |  |  | 4.47E5 | 9.4519E7 | 6.4928E8 |  |  |  |  |  |  |  |  | 12 | 0 | 0 | 0 | 1 | 4 | 7 | 0 | 0 | 0 | 0 | 0 | 0 | 0 | 0 | 510 | 527 |  |  | PEAKS DB |
| K.ASGNPILLNKSIQEDPAVK.A | N | 83.17 | 1993.0844 | 19 | 0.6 | 665.3691 | 3 | 35.69 | 6 | F6:17128 | NaSa01\_F14.raw |  |  |  |  | 1.5436E6 | 2.8654E6 |  |  |  |  |  |  |  |  | 2 | 0 | 0 | 0 | 0 | 1 | 1 | 0 | 0 | 0 | 0 | 0 | 0 | 0 | 0 | 317 | 335 |  |  | PEAKS DB |
| K.VLLPSFLAAGGDGYYM(+15.99)LK.G | N | 82.89 | 1929.9910 | 18 | 0.7 | 966.0034 | 2 | 87.45 | 6 | F6:60748 | NaSa01\_F14.raw |  |  |  |  | 2.8899E7 | 1.2442E8 |  |  |  |  |  |  |  |  | 10 | 0 | 0 | 0 | 0 | 3 | 7 | 0 | 0 | 0 | 0 | 0 | 0 | 0 | 0 | 510 | 527 | Oxidation (M) | M16:Oxidation (M):1000.00 | PEAKS DB |
| R.FHEC(+57.02)NLGNLIC(+57.02)DAVVYNNLR.H | N | 82.50 | 2420.1365 | 20 | -0.2 | 807.7192 | 3 | 77.73 | 6 | F6:52912 | NaSa01\_F14.raw |  |  |  |  | 2.6877E7 | 9.4785E7 |  |  |  |  |  |  | 7.773E6 | 1.2217E8 | 9 | 0 | 0 | 0 | 0 | 1 | 3 | 0 | 0 | 0 | 0 | 0 | 0 | 2 | 3 | 369 | 388 | Carbamidomethylation | C4:Carbamidomethylation:1000.00;C11:Carbamidomethylation:1000.00 | PEAKS DB |
| K.ETPVLSNPGPYLEFR.D | N | 81.49 | 1717.8674 | 15 | 0.8 | 859.9417 | 2 | 69.77 | 6 | F6:46166 | NaSa01\_F14.raw |  |  |  | 7.2199E5 | 2.4678E8 | 7.6435E8 |  |  |  | 2.2582E6 |  |  |  |  | 7 | 0 | 0 | 0 | 1 | 3 | 2 | 0 | 0 | 0 | 1 | 0 | 0 | 0 | 0 | 194 | 208 |  |  | PEAKS DB |
| R.QVPVVQAYAFGK.Y | N | 80.72 | 1305.7081 | 12 | -0.2 | 653.8612 | 2 | 81.01 | 6 | F6:55346 | NaSa01\_F14.raw |  |  |  | 4.7562E5 | 3.326E8 | 6.8066E8 |  |  |  |  |  |  |  |  | 5 | 0 | 0 | 0 | 1 | 1 | 3 | 0 | 0 | 0 | 0 | 0 | 0 | 0 | 0 | 288 | 299 |  |  | PEAKS DB |
| R.EVVHFMNSLR.Y | N | 80.17 | 1230.6179 | 10 | 0.3 | 616.3164 | 2 | 32.92 | 6 | F6:14493 | NaSa01\_F14.raw |  |  |  |  | 2.3691E8 | 4.1556E8 |  |  |  |  |  |  |  |  | 11 | 0 | 0 | 0 | 0 | 3 | 8 | 0 | 0 | 0 | 0 | 0 | 0 | 0 | 0 | 114 | 123 |  |  | PEAKS DB |
| R.VVSLNVLC(+57.02)TEC(+57.02)R.V | N | 79.36 | 1448.7115 | 12 | 0.2 | 725.3632 | 2 | 49.71 | 6 | F6:29319 | NaSa01\_F14.raw |  |  |  | 0 | 2.7472E8 | 5.8042E8 |  |  |  |  |  | 8.8246E5 |  |  | 7 | 0 | 0 | 0 | 0 | 2 | 4 | 0 | 0 | 0 | 0 | 0 | 1 | 0 | 0 | 484 | 495 | Carbamidomethylation | C8:Carbamidomethylation:1000.00;C11:Carbamidomethylation:1000.00 | PEAKS DB |
| R.YDAMALGNHEFDNGLNGLLDPLLK.N | N | 78.14 | 2629.2847 | 24 | 0.8 | 877.4362 | 3 | 94.34 | 6 | F6:67114 | NaSa01\_F14.raw |  |  |  |  | 1.6292E7 | 1.1665E8 |  |  |  |  |  |  |  |  | 4 | 0 | 0 | 0 | 0 | 2 | 2 | 0 | 0 | 0 | 0 | 0 | 0 | 0 | 0 | 124 | 147 |  |  | PEAKS DB |
| K.YLGYLNVIFDDK.G | N | 76.79 | 1458.7394 | 12 | 0.2 | 730.3771 | 2 | 85.89 | 6 | F6:59730 | NaSa01\_F14.raw |  |  |  |  | 6.7598E7 | 9.6452E7 |  |  |  |  |  |  |  |  | 5 | 0 | 0 | 0 | 0 | 4 | 1 | 0 | 0 | 0 | 0 | 0 | 0 | 0 | 0 | 300 | 311 |  |  | PEAKS DB |
| K.LTILHTNDVHAR.V | N | 75.00 | 1388.7524 | 12 | 0.9 | 463.9252 | 3 | 11.51 | 6 | F6:1905 | NaSa01\_F14.raw |  |  |  |  | 1.6389E6 | 3.6507E6 |  |  |  |  |  |  |  |  | 4 | 0 | 0 | 0 | 0 | 2 | 2 | 0 | 0 | 0 | 0 | 0 | 0 | 0 | 0 | 44 | 55 |  |  | PEAKS DB |
| K.ETPVLSNPGPYLEFRDEVEELQK.Q | N | 74.67 | 2688.3281 | 23 | 0.7 | 897.1173 | 3 | 77.38 | 6 | F6:52291 | NaSa01\_F14.raw |  |  |  |  | 3.5808E8 | 6.9841E8 |  |  |  |  |  |  |  |  | 4 | 0 | 0 | 0 | 0 | 2 | 2 | 0 | 0 | 0 | 0 | 0 | 0 | 0 | 0 | 194 | 216 |  |  | PEAKS DB |
| K.ASGNPILLNK.S | N | 69.82 | 1025.5869 | 10 | -0.4 | 513.8005 | 2 | 11.59 | 6 | F6:1963 | NaSa01\_F14.raw |  |  |  |  | 2.4579E6 | 4.7925E6 |  |  |  |  |  |  |  |  | 3 | 0 | 0 | 0 | 0 | 1 | 2 | 0 | 0 | 0 | 0 | 0 | 0 | 0 | 0 | 317 | 326 |  |  | PEAKS DB |
| S.FLAAGGDGYYMLK.G | N | 67.12 | 1404.6747 | 13 | 0.2 | 703.3447 | 2 | 58.91 | 6 | F6:37338 | NaSa01\_F14.raw |  |  |  |  | 1.8909E7 | 8.0208E7 |  |  |  |  |  |  |  |  | 3 | 0 | 0 | 0 | 0 | 1 | 2 | 0 | 0 | 0 | 0 | 0 | 0 | 0 | 0 | 515 | 527 |  |  | PEAKS DB |
| F.LAAGGDGYYMLK.G | N | 66.41 | 1257.6063 | 12 | 0.2 | 629.8105 | 2 | 41.73 | 6 | F6:22051 | NaSa01\_F14.raw |  |  |  |  |  | 5.7329E6 |  |  |  |  |  |  |  |  | 1 | 0 | 0 | 0 | 0 | 0 | 1 | 0 | 0 | 0 | 0 | 0 | 0 | 0 | 0 | 516 | 527 |  |  | PEAKS DB |
| R.EVVHFM(+15.99)NSLR.Y | N | 65.78 | 1246.6128 | 10 | 0.3 | 624.3138 | 2 | 31.71 | 6 | F6:14277 | NaSa01\_F14.raw |  |  |  |  | 3.2821E6 | 5.0137E7 |  |  |  |  |  |  |  |  | 13 | 0 | 0 | 0 | 0 | 2 | 11 | 0 | 0 | 0 | 0 | 0 | 0 | 0 | 0 | 114 | 123 | Oxidation (M) | M6:Oxidation (M):1000.00 | PEAKS DB |
| K.FPILSANIRPK.G | N | 63.57 | 1254.7448 | 11 | 1.8 | 419.2563 | 3 | 38.83 | 6 | F6:20180 | NaSa01\_F14.raw |  |  |  | 3.6184E5 | 9.4886E7 | 1.2559E8 |  |  |  |  | 2.1098E6 |  |  |  | 10 | 0 | 0 | 0 | 1 | 2 | 5 | 0 | 0 | 0 | 0 | 2 | 0 | 0 | 0 | 151 | 161 |  |  | PEAKS DB |
| K.RVVSLNVLC(+57.02)TEC(+57.02)R.V | N | 62.55 | 1604.8126 | 13 | 0.2 | 535.9449 | 3 | 35.03 | 6 | F6:16539 | NaSa01\_F14.raw |  |  |  |  | 4.4155E6 | 6.0208E6 |  |  |  |  |  |  |  |  | 3 | 0 | 0 | 0 | 0 | 1 | 2 | 0 | 0 | 0 | 0 | 0 | 0 | 0 | 0 | 483 | 495 | Carbamidomethylation | C9:Carbamidomethylation:1000.00;C12:Carbamidomethylation:1000.00 | PEAKS DB |
| K.GVDVVVGGHTNTF.L | N | 61.79 | 1300.6411 | 13 | 1.1 | 651.3286 | 2 | 41.80 | 6 | F6:22128 | NaSa01\_F14.raw |  |  |  |  | 2.249E7 | 5.2209E7 |  |  |  |  |  |  |  |  | 2 | 0 | 0 | 0 | 0 | 1 | 1 | 0 | 0 | 0 | 0 | 0 | 0 | 0 | 0 | 249 | 261 |  |  | PEAKS DB |
| L.TAVLPFGGTFDLLQIK.G | N | 60.64 | 1718.9607 | 16 | -0.1 | 860.4875 | 2 | 99.74 | 6 | F6:71142 | NaSa01\_F14.raw |  |  | 2.8271E6 | 1.2518E6 | 2.1013E7 | 4.0691E6 |  |  |  |  |  |  |  |  | 4 | 0 | 0 | 1 | 1 | 1 | 1 | 0 | 0 | 0 | 0 | 0 | 0 | 0 | 0 | 427 | 442 |  |  | PEAKS DB |
| K.IINVGSEKVGIIGYTTK.E | N | 60.09 | 1791.0142 | 17 | -0.7 | 598.0116 | 3 | 45.32 | 6 | F6:25348 | NaSa01\_F14.raw |  |  |  |  | 9.4386E5 | 1.7014E6 |  |  |  |  |  |  |  |  | 2 | 0 | 0 | 0 | 0 | 1 | 1 | 0 | 0 | 0 | 0 | 0 | 0 | 0 | 0 | 177 | 193 |  |  | PEAKS DB |
| K.YLGYLNVIFDDKGK.V | Y | 58.45 | 1643.8558 | 14 | -0.3 | 548.9590 | 3 | 70.19 | 5 | F5:46798 | NaSa01\_F13.raw |  |  |  |  | 1.8927E6 |  |  |  |  |  |  |  |  |  | 2 | 0 | 0 | 0 | 0 | 2 | 0 | 0 | 0 | 0 | 0 | 0 | 0 | 0 | 0 | 300 | 313 |  |  | PEAKS DB |
| K.VFPAMEGR.V | N | 56.33 | 905.4429 | 8 | 0.2 | 453.7288 | 2 | 22.33 | 6 | F6:7722 | NaSa01\_F14.raw |  |  |  |  |  | 3.6244E6 |  |  |  |  |  |  |  |  | 2 | 0 | 0 | 0 | 0 | 0 | 2 | 0 | 0 | 0 | 0 | 0 | 0 | 0 | 0 | 553 | 560 |  |  | PEAKS DB |
| N.ISGYILPYK.I | N | 56.17 | 1052.5906 | 9 | 0.7 | 527.3029 | 2 | 45.82 | 6 | F6:26096 | NaSa01\_F14.raw |  |  |  | 2.2592E5 | 4.6763E8 | 8.5839E8 |  |  |  |  |  |  |  |  | 5 | 0 | 0 | 0 | 1 | 1 | 3 | 0 | 0 | 0 | 0 | 0 | 0 | 0 | 0 | 168 | 176 |  |  | PEAKS DB |
| S.FLAAGGDGYYM(+15.99)LK.G | N | 55.62 | 1420.6697 | 13 | -0.1 | 711.3420 | 2 | 48.69 | 6 | F6:28173 | NaSa01\_F14.raw |  |  |  |  |  | 5.7308E6 |  |  |  |  |  |  |  |  | 1 | 0 | 0 | 0 | 0 | 0 | 1 | 0 | 0 | 0 | 0 | 0 | 0 | 0 | 0 | 515 | 527 | Oxidation (M) | M11:Oxidation (M):1000.00 | PEAKS DB |
| H.GQGTGELLQVSGIK.V | N | 55.06 | 1385.7515 | 14 | 0.3 | 693.8832 | 2 | 45.20 | 6 | F6:25108 | NaSa01\_F14.raw |  |  |  |  |  | 1.2594E6 |  |  |  |  |  |  |  |  | 1 | 0 | 0 | 0 | 0 | 0 | 1 | 0 | 0 | 0 | 0 | 0 | 0 | 0 | 0 | 458 | 471 |  |  | PEAKS DB |
| K.GVDVVVGGHTNTFLY.T | N | 54.75 | 1576.7886 | 15 | 0.1 | 789.4016 | 2 | 63.80 | 6 | F6:41327 | NaSa01\_F14.raw |  |  |  |  | 1.2739E7 | 2.8201E7 |  |  |  |  |  |  |  |  | 2 | 0 | 0 | 0 | 0 | 1 | 1 | 0 | 0 | 0 | 0 | 0 | 0 | 0 | 0 | 249 | 263 |  |  | PEAKS DB |
| R.QVPVVQAYAF.G | N | 53.91 | 1120.5917 | 10 | 0.0 | 561.3031 | 2 | 73.70 | 6 | F6:49221 | NaSa01\_F14.raw |  |  |  |  | 7.1015E6 | 1.9997E7 |  |  |  |  |  |  |  |  | 2 | 0 | 0 | 0 | 0 | 1 | 1 | 0 | 0 | 0 | 0 | 0 | 0 | 0 | 0 | 288 | 297 |  |  | PEAKS DB |
| M.ALGNHEFDNGLNGLLDPLLK.N | N | 53.45 | 2149.1167 | 20 | 0.0 | 717.3795 | 3 | 83.56 | 6 | F6:57342 | NaSa01\_F14.raw |  |  |  |  | 5.3244E6 | 2.0787E7 |  |  |  |  |  |  |  |  | 3 | 0 | 0 | 0 | 0 | 1 | 2 | 0 | 0 | 0 | 0 | 0 | 0 | 0 | 0 | 128 | 147 |  |  | PEAKS DB |
| K.LTTLGVNK.I | N | 52.68 | 844.5018 | 8 | -1.0 | 423.2578 | 2 | 11.58 | 6 | F6:2148 | NaSa01\_F14.raw |  |  |  |  |  | 3.1326E6 |  |  |  |  |  |  |  |  | 1 | 0 | 0 | 0 | 0 | 0 | 1 | 0 | 0 | 0 | 0 | 0 | 0 | 0 | 0 | 221 | 228 |  |  | PEAKS DB |
| K.VLLPSFLAAGGDGY.Y | N | 51.68 | 1378.7133 | 14 | 0.7 | 690.3644 | 2 | 100.88 | 6 | F6:72024 | NaSa01\_F14.raw |  |  |  |  | 1.3644E6 | 2.8748E6 |  |  |  |  |  |  |  |  | 2 | 0 | 0 | 0 | 0 | 1 | 1 | 0 | 0 | 0 | 0 | 0 | 0 | 0 | 0 | 510 | 523 |  |  | PEAKS DB |
| F.GGTFDLLQIK.G | N | 50.44 | 1090.6022 | 10 | -0.6 | 546.3080 | 2 | 64.67 | 6 | F6:41967 | NaSa01\_F14.raw |  |  |  |  |  | 9.7795E7 |  |  |  |  |  |  |  |  | 2 | 0 | 0 | 0 | 0 | 0 | 2 | 0 | 0 | 0 | 0 | 0 | 0 | 0 | 0 | 433 | 442 |  |  | PEAKS DB |
| Y.LNVIFDDK.G | N | 50.15 | 962.5073 | 8 | -0.5 | 482.2607 | 2 | 40.33 | 6 | F6:21131 | NaSa01\_F14.raw |  |  |  |  |  | 1.4605E6 |  |  |  |  |  |  |  |  | 1 | 0 | 0 | 0 | 0 | 0 | 1 | 0 | 0 | 0 | 0 | 0 | 0 | 0 | 0 | 304 | 311 |  |  | PEAKS DB |
| K.VGIIGYTTK.E | N | 50.04 | 950.5436 | 9 | 0.3 | 476.2792 | 2 | 28.48 | 6 | F6:11194 | NaSa01\_F14.raw |  |  |  |  |  | 5.1319E6 |  |  |  |  |  |  |  |  | 2 | 0 | 0 | 0 | 0 | 0 | 2 | 0 | 0 | 0 | 0 | 0 | 0 | 0 | 0 | 185 | 193 |  |  | PEAKS DB |
| L.AAGGDGYYMLK.G | N | 49.71 | 1144.5222 | 11 | 0.7 | 573.2688 | 2 | 30.46 | 5 | F5:13468 | NaSa01\_F13.raw |  |  |  |  | 6.4456E6 |  |  |  |  |  |  |  |  |  | 1 | 0 | 0 | 0 | 0 | 1 | 0 | 0 | 0 | 0 | 0 | 0 | 0 | 0 | 0 | 517 | 527 |  |  | PEAKS DB |
| R.FHEC(+57.02)NLGNLIC(+57.02)DAVVY.N | N | 48.35 | 1922.8655 | 16 | 1.4 | 962.4414 | 2 | 82.45 | 5 | F5:56634 | NaSa01\_F13.raw |  |  |  |  | 8.0322E7 | 1.7562E8 |  |  |  |  |  |  |  | 2.4692E7 | 3 | 0 | 0 | 0 | 0 | 1 | 1 | 0 | 0 | 0 | 0 | 0 | 0 | 0 | 1 | 369 | 384 | Carbamidomethylation | C4:Carbamidomethylation:1000.00;C11:Carbamidomethylation:1000.00 | PEAKS DB |
| R.HPDDNEWNHVSMC(+57.02).I | N | 48.19 | 1639.6144 | 13 | 0.5 | 820.8149 | 2 | 26.12 | 5 | F5:10348 | NaSa01\_F13.raw |  |  |  |  | 8.8799E6 |  |  |  |  |  |  |  |  |  | 2 | 0 | 0 | 0 | 0 | 2 | 0 | 0 | 0 | 0 | 0 | 0 | 0 | 0 | 0 | 389 | 401 | Carbamidomethylation | C13:Carbamidomethylation:1000.00 | PEAKS DB |
| K.VLLPSFLAAGGD.G | N | 47.35 | 1158.6284 | 12 | -0.1 | 580.3214 | 2 | 96.95 | 6 | F6:68512 | NaSa01\_F14.raw |  |  |  |  |  | 1.1427E6 |  |  |  |  |  |  |  |  | 1 | 0 | 0 | 0 | 0 | 0 | 1 | 0 | 0 | 0 | 0 | 0 | 0 | 0 | 0 | 510 | 521 |  |  | PEAKS DB |
| R.HPDDNEWNHVSM.C | N | 47.22 | 1479.5837 | 12 | 1.1 | 740.8000 | 2 | 25.93 | 5 | F5:10070 | NaSa01\_F13.raw |  |  |  |  | 2.3098E6 |  |  |  |  |  |  |  |  |  | 1 | 0 | 0 | 0 | 0 | 1 | 0 | 0 | 0 | 0 | 0 | 0 | 0 | 0 | 0 | 389 | 400 |  |  | PEAKS DB |
| R.QVPVVQAY.A | N | 45.65 | 902.4861 | 8 | -0.5 | 452.2501 | 2 | 43.38 | 5 | F5:24541 | NaSa01\_F13.raw |  |  |  |  | 1.2052E7 |  |  |  |  |  |  |  |  |  | 1 | 0 | 0 | 0 | 0 | 1 | 0 | 0 | 0 | 0 | 0 | 0 | 0 | 0 | 0 | 288 | 295 |  |  | PEAKS DB |
| N.PGPYLEFR.D | N | 45.44 | 977.4970 | 8 | 0.7 | 489.7561 | 2 | 39.90 | 6 | F6:20609 | NaSa01\_F14.raw |  |  |  |  |  | 4.6995E5 |  |  |  |  |  |  |  |  | 1 | 0 | 0 | 0 | 0 | 0 | 1 | 0 | 0 | 0 | 0 | 0 | 0 | 0 | 0 | 201 | 208 |  |  | PEAKS DB |
| N.LGNLIC(+57.02)DAVVYNNLR.H | N | 45.20 | 1732.8931 | 15 | 1.0 | 867.4547 | 2 | 69.10 | 6 | F6:45743 | NaSa01\_F14.raw |  |  |  |  |  | 1.1316E6 |  |  |  |  |  |  |  |  | 1 | 0 | 0 | 0 | 0 | 0 | 1 | 0 | 0 | 0 | 0 | 0 | 0 | 0 | 0 | 374 | 388 | Carbamidomethylation | C6:Carbamidomethylation:1000.00 | PEAKS DB |
| K.IINVGSEK.V | N | 44.70 | 858.4811 | 8 | -0.5 | 430.2476 | 2 | 11.48 | 6 | F6:1861 | NaSa01\_F14.raw |  |  |  |  |  | 1.3857E6 |  |  |  |  |  |  |  |  | 1 | 0 | 0 | 0 | 0 | 0 | 1 | 0 | 0 | 0 | 0 | 0 | 0 | 0 | 0 | 177 | 184 |  |  | PEAKS DB |
| K.VLLPSFLAAGGDGYY.M | N | 43.81 | 1541.7765 | 15 | 0.7 | 771.8961 | 2 | 105.28 | 6 | F6:75496 | NaSa01\_F14.raw |  |  |  |  | 1.4762E7 | 3.1893E7 |  |  |  |  |  |  |  |  | 2 | 0 | 0 | 0 | 0 | 1 | 1 | 0 | 0 | 0 | 0 | 0 | 0 | 0 | 0 | 510 | 524 |  |  | PEAKS DB |
| K.FPILSANIR.P | N | 43.38 | 1029.5970 | 9 | -0.4 | 515.8056 | 2 | 52.72 | 6 | F6:31843 | NaSa01\_F14.raw |  |  |  |  |  | 1.5657E8 |  |  |  |  |  |  |  |  | 1 | 0 | 0 | 0 | 0 | 0 | 1 | 0 | 0 | 0 | 0 | 0 | 0 | 0 | 0 | 151 | 159 |  |  | PEAKS DB |
| K.VLLPSFLAAGGDGYYM.L | N | 42.72 | 1672.8170 | 16 | 0.9 | 837.4166 | 2 | 111.51 | 6 | F6:80284 | NaSa01\_F14.raw |  |  |  |  |  | 1.1099E6 |  |  |  |  |  |  |  |  | 1 | 0 | 0 | 0 | 0 | 0 | 1 | 0 | 0 | 0 | 0 | 0 | 0 | 0 | 0 | 510 | 525 |  |  | PEAKS DB |
| Q.GTGELLQVSGIK.V | N | 42.14 | 1200.6714 | 12 | 0.9 | 601.3435 | 2 | 44.43 | 6 | F6:24568 | NaSa01\_F14.raw |  |  |  |  |  | 7.0353E5 |  |  |  |  |  |  |  |  | 1 | 0 | 0 | 0 | 0 | 0 | 1 | 0 | 0 | 0 | 0 | 0 | 0 | 0 | 0 | 460 | 471 |  |  | PEAKS DB |
| Y.LGYLNVIFDDK.G | N | 41.11 | 1295.6761 | 11 | 0.9 | 648.8459 | 2 | 76.55 | 6 | F6:51743 | NaSa01\_F14.raw |  |  |  |  |  | 0 |  |  |  |  |  |  |  |  | 0 | 0 | 0 | 0 | 0 | 0 | 0 | 0 | 0 | 0 | 0 | 0 | 0 | 0 | 0 | 301 | 311 |  |  | PEAKS DB |
| K.LTTLGVNKIIALGH.S | N | 41.05 | 1448.8715 | 14 | 0.1 | 483.9645 | 3 | 54.77 | 6 | F6:33352 | NaSa01\_F14.raw |  |  |  |  |  | 5.5856E5 |  |  |  |  |  |  |  |  | 1 | 0 | 0 | 0 | 0 | 0 | 1 | 0 | 0 | 0 | 0 | 0 | 0 | 0 | 0 | 221 | 234 |  |  | PEAKS DB |
| N.HEFDNGLNGLLDPLLK.N | N | 40.62 | 1793.9312 | 16 | 0.2 | 598.9844 | 3 | 81.57 | 6 | F6:55994 | NaSa01\_F14.raw |  |  |  |  |  | 5.7086E5 |  |  |  |  |  |  |  |  | 1 | 0 | 0 | 0 | 0 | 0 | 1 | 0 | 0 | 0 | 0 | 0 | 0 | 0 | 0 | 132 | 147 |  |  | PEAKS DB |
| N.YPFMQLSDDGR.Q | N | 40.57 | 1327.5867 | 11 | 0.4 | 664.8009 | 2 | 53.65 | 6 | F6:32287 | NaSa01\_F14.raw |  |  |  |  |  | 5.3395E5 |  |  |  |  |  |  |  |  | 1 | 0 | 0 | 0 | 0 | 0 | 1 | 0 | 0 | 0 | 0 | 0 | 0 | 0 | 0 | 277 | 287 |  |  | PEAKS DB |
| P.AGNYPFMQLSDDGR.Q | N | 40.07 | 1569.6881 | 14 | 0.1 | 785.8514 | 2 | 56.81 | 6 | F6:35126 | NaSa01\_F14.raw |  |  |  |  |  | 2.5054E5 |  |  |  |  |  |  |  |  | 1 | 0 | 0 | 0 | 0 | 0 | 1 | 0 | 0 | 0 | 0 | 0 | 0 | 0 | 0 | 274 | 287 |  |  | PEAKS DB |
| K.NVKFPILSAN.I | N | 39.82 | 1101.6182 | 10 | 0.5 | 551.8167 | 2 | 54.61 | 6 | F6:33186 | NaSa01\_F14.raw |  |  |  |  |  | 1.3385E6 |  |  |  |  |  |  |  |  | 1 | 0 | 0 | 0 | 0 | 0 | 1 | 0 | 0 | 0 | 0 | 0 | 0 | 0 | 0 | 148 | 157 |  |  | PEAKS DB |
| K.IIALGHSGF.K | N | 37.88 | 913.5021 | 9 | -1.2 | 457.7578 | 2 | 43.37 | 6 | F6:23538 | NaSa01\_F14.raw |  |  |  |  |  | 5.7946E6 |  |  |  |  |  |  |  |  | 1 | 0 | 0 | 0 | 0 | 0 | 1 | 0 | 0 | 0 | 0 | 0 | 0 | 0 | 0 | 229 | 237 |  |  | PEAKS DB |
| total 58 peptides |  |  |  |  |  |  |  |  |  |  |  |  |  |  |  |  |  |  |  |  |  |  |  |  |  |  |  |  |  |  |  |  |  |  |  |  |  |  |  |  |  |  |  |  |  |
| --- | --- | --- | --- | --- | --- | --- | --- | --- | --- | --- | --- | --- | --- | --- | --- | --- | --- | --- | --- | --- | --- | --- | --- | --- | --- | --- | --- | --- | --- | --- | --- | --- | --- | --- | --- | --- | --- | --- | --- | --- | --- | --- | --- | --- | --- |

JAS05037.1

back to list

  

| Protein Coverage
| Supporting Peptides
|

Protein Coverage:

Supporting Peptides:

| Peptide | Uniq | -10lgP | Mass | Length | ppm | m/z | z | RT | Fraction | Scan | Source File | Area F1 | Area F10 | Area F11 | Area F12 | Area F13 | Area F14 | Area F2 | Area F3 | Area F4 | Area F5 | Area F6 | Area F7 | Area F8 | Area F9 | #Feature | #Feature F1 | #Feature F10 | #Feature F11 | #Feature F12 | #Feature F13 | #Feature F14 | #Feature F2 | #Feature F3 | #Feature F4 | #Feature F5 | #Feature F6 | #Feature F7 | #Feature F8 | #Feature F9 | Start | End | PTM | AScore | Found By |
| --- | --- | --- | --- | --- | --- | --- | --- | --- | --- | --- | --- | --- | --- | --- | --- | --- | --- | --- | --- | --- | --- | --- | --- | --- | --- | --- | --- | --- | --- | --- | --- | --- | --- | --- | --- | --- | --- | --- | --- | --- | --- | --- | --- | --- | --- |
| R.HPDDNEWNHVSMC(+57.02)IVNGGGIR.S | N | 99.67 | 2406.0593 | 21 | -0.4 | 803.0267 | 3 | 49.93 | 6 | F6:29280 | NaSa01\_F14.raw |  |  |  |  | 6.5991E7 | 1.6043E8 |  |  |  |  |  |  |  | 7.6096E6 | 9 | 0 | 0 | 0 | 0 | 3 | 4 | 0 | 0 | 0 | 0 | 0 | 0 | 0 | 2 | 389 | 409 | Carbamidomethylation | C13:Carbamidomethylation:1000.00 | PEAKS DB |
| R.HGQGTGELLQVSGIK.V | N | 97.61 | 1522.8103 | 15 | 0.4 | 762.4127 | 2 | 31.05 | 6 | F6:13489 | NaSa01\_F14.raw |  |  |  | 4.9661E5 | 6.4612E8 | 1.2796E9 |  |  |  |  |  |  |  |  | 8 | 0 | 0 | 0 | 1 | 3 | 4 | 0 | 0 | 0 | 0 | 0 | 0 | 0 | 0 | 457 | 471 |  |  | PEAKS DB |
| R.YDAM(+15.99)ALGNHEFDNGLNGLLDPLLK.N | N | 89.01 | 2645.2795 | 24 | 0.3 | 882.7674 | 3 | 88.88 | 6 | F6:61975 | NaSa01\_F14.raw |  |  |  |  | 1.9977E7 | 6.8848E7 |  |  |  |  |  |  |  |  | 3 | 0 | 0 | 0 | 0 | 1 | 2 | 0 | 0 | 0 | 0 | 0 | 0 | 0 | 0 | 124 | 147 | Oxidation (M) | M4:Oxidation (M):1000.00 | PEAKS DB |
| R.HPDDNEWNHVSM(+15.99)C(+57.02)IVNGGGIR.S | N | 86.62 | 2422.0542 | 21 | -0.3 | 808.3584 | 3 | 44.12 | 6 | F6:24148 | NaSa01\_F14.raw |  |  |  |  | 3.2766E7 | 9.9954E7 |  |  |  |  |  |  |  | 8.8723E6 | 8 | 0 | 0 | 0 | 0 | 2 | 4 | 0 | 0 | 0 | 0 | 0 | 0 | 0 | 2 | 389 | 409 | Oxidation (M); Carbamidomethylation | M12:Oxidation (M):1000.00;C13:Carbamidomethylation:1000.00 | PEAKS DB |
| K.VLLPSFLAAGGDGYYMLK.G | N | 84.56 | 1913.9961 | 18 | 1.4 | 639.0068 | 3 | 94.26 | 6 | F6:66829 | NaSa01\_F14.raw |  |  |  | 4.47E5 | 9.4519E7 | 6.4928E8 |  |  |  |  |  |  |  |  | 12 | 0 | 0 | 0 | 1 | 4 | 7 | 0 | 0 | 0 | 0 | 0 | 0 | 0 | 0 | 510 | 527 |  |  | PEAKS DB |
| K.ASGNPILLNKSIQEDPAVK.A | N | 83.17 | 1993.0844 | 19 | 0.6 | 665.3691 | 3 | 35.69 | 6 | F6:17128 | NaSa01\_F14.raw |  |  |  |  | 1.5436E6 | 2.8654E6 |  |  |  |  |  |  |  |  | 2 | 0 | 0 | 0 | 0 | 1 | 1 | 0 | 0 | 0 | 0 | 0 | 0 | 0 | 0 | 317 | 335 |  |  | PEAKS DB |
| K.VLLPSFLAAGGDGYYM(+15.99)LK.G | N | 82.89 | 1929.9910 | 18 | 0.7 | 966.0034 | 2 | 87.45 | 6 | F6:60748 | NaSa01\_F14.raw |  |  |  |  | 2.8899E7 | 1.2442E8 |  |  |  |  |  |  |  |  | 10 | 0 | 0 | 0 | 0 | 3 | 7 | 0 | 0 | 0 | 0 | 0 | 0 | 0 | 0 | 510 | 527 | Oxidation (M) | M16:Oxidation (M):1000.00 | PEAKS DB |
| R.FHEC(+57.02)NLGNLIC(+57.02)DAVVYNNLR.H | N | 82.50 | 2420.1365 | 20 | -0.2 | 807.7192 | 3 | 77.73 | 6 | F6:52912 | NaSa01\_F14.raw |  |  |  |  | 2.6877E7 | 9.4785E7 |  |  |  |  |  |  | 7.773E6 | 1.2217E8 | 9 | 0 | 0 | 0 | 0 | 1 | 3 | 0 | 0 | 0 | 0 | 0 | 0 | 2 | 3 | 369 | 388 | Carbamidomethylation | C4:Carbamidomethylation:1000.00;C11:Carbamidomethylation:1000.00 | PEAKS DB |
| K.ETPVLSNPGPYLEFR.D | N | 81.49 | 1717.8674 | 15 | 0.8 | 859.9417 | 2 | 69.77 | 6 | F6:46166 | NaSa01\_F14.raw |  |  |  | 7.2199E5 | 2.4678E8 | 7.6435E8 |  |  |  | 2.2582E6 |  |  |  |  | 7 | 0 | 0 | 0 | 1 | 3 | 2 | 0 | 0 | 0 | 1 | 0 | 0 | 0 | 0 | 194 | 208 |  |  | PEAKS DB |
| R.QVPVVQAYAFGK.Y | N | 80.72 | 1305.7081 | 12 | -0.2 | 653.8612 | 2 | 81.01 | 6 | F6:55346 | NaSa01\_F14.raw |  |  |  | 4.7562E5 | 3.326E8 | 6.8066E8 |  |  |  |  |  |  |  |  | 5 | 0 | 0 | 0 | 1 | 1 | 3 | 0 | 0 | 0 | 0 | 0 | 0 | 0 | 0 | 288 | 299 |  |  | PEAKS DB |
| R.EVVHFMNSLR.Y | N | 80.17 | 1230.6179 | 10 | 0.3 | 616.3164 | 2 | 32.92 | 6 | F6:14493 | NaSa01\_F14.raw |  |  |  |  | 2.3691E8 | 4.1556E8 |  |  |  |  |  |  |  |  | 11 | 0 | 0 | 0 | 0 | 3 | 8 | 0 | 0 | 0 | 0 | 0 | 0 | 0 | 0 | 114 | 123 |  |  | PEAKS DB |
| R.VVSLNVLC(+57.02)TEC(+57.02)R.V | N | 79.36 | 1448.7115 | 12 | 0.2 | 725.3632 | 2 | 49.71 | 6 | F6:29319 | NaSa01\_F14.raw |  |  |  | 0 | 2.7472E8 | 5.8042E8 |  |  |  |  |  | 8.8246E5 |  |  | 7 | 0 | 0 | 0 | 0 | 2 | 4 | 0 | 0 | 0 | 0 | 0 | 1 | 0 | 0 | 484 | 495 | Carbamidomethylation | C8:Carbamidomethylation:1000.00;C11:Carbamidomethylation:1000.00 | PEAKS DB |
| R.YDAMALGNHEFDNGLNGLLDPLLK.N | N | 78.14 | 2629.2847 | 24 | 0.8 | 877.4362 | 3 | 94.34 | 6 | F6:67114 | NaSa01\_F14.raw |  |  |  |  | 1.6292E7 | 1.1665E8 |  |  |  |  |  |  |  |  | 4 | 0 | 0 | 0 | 0 | 2 | 2 | 0 | 0 | 0 | 0 | 0 | 0 | 0 | 0 | 124 | 147 |  |  | PEAKS DB |
| K.YLGYLNVIFDDK.G | N | 76.79 | 1458.7394 | 12 | 0.2 | 730.3771 | 2 | 85.89 | 6 | F6:59730 | NaSa01\_F14.raw |  |  |  |  | 6.7598E7 | 9.6452E7 |  |  |  |  |  |  |  |  | 5 | 0 | 0 | 0 | 0 | 4 | 1 | 0 | 0 | 0 | 0 | 0 | 0 | 0 | 0 | 300 | 311 |  |  | PEAKS DB |
| K.LTILHTNDVHAR.V | N | 75.00 | 1388.7524 | 12 | 0.9 | 463.9252 | 3 | 11.51 | 6 | F6:1905 | NaSa01\_F14.raw |  |  |  |  | 1.6389E6 | 3.6507E6 |  |  |  |  |  |  |  |  | 4 | 0 | 0 | 0 | 0 | 2 | 2 | 0 | 0 | 0 | 0 | 0 | 0 | 0 | 0 | 44 | 55 |  |  | PEAKS DB |
| K.ETPVLSNPGPYLEFRDEVEELQK.Q | N | 74.67 | 2688.3281 | 23 | 0.7 | 897.1173 | 3 | 77.38 | 6 | F6:52291 | NaSa01\_F14.raw |  |  |  |  | 3.5808E8 | 6.9841E8 |  |  |  |  |  |  |  |  | 4 | 0 | 0 | 0 | 0 | 2 | 2 | 0 | 0 | 0 | 0 | 0 | 0 | 0 | 0 | 194 | 216 |  |  | PEAKS DB |
| K.ASGNPILLNK.S | N | 69.82 | 1025.5869 | 10 | -0.4 | 513.8005 | 2 | 11.59 | 6 | F6:1963 | NaSa01\_F14.raw |  |  |  |  | 2.4579E6 | 4.7925E6 |  |  |  |  |  |  |  |  | 3 | 0 | 0 | 0 | 0 | 1 | 2 | 0 | 0 | 0 | 0 | 0 | 0 | 0 | 0 | 317 | 326 |  |  | PEAKS DB |
| S.FLAAGGDGYYMLK.G | N | 67.12 | 1404.6747 | 13 | 0.2 | 703.3447 | 2 | 58.91 | 6 | F6:37338 | NaSa01\_F14.raw |  |  |  |  | 1.8909E7 | 8.0208E7 |  |  |  |  |  |  |  |  | 3 | 0 | 0 | 0 | 0 | 1 | 2 | 0 | 0 | 0 | 0 | 0 | 0 | 0 | 0 | 515 | 527 |  |  | PEAKS DB |
| F.LAAGGDGYYMLK.G | N | 66.41 | 1257.6063 | 12 | 0.2 | 629.8105 | 2 | 41.73 | 6 | F6:22051 | NaSa01\_F14.raw |  |  |  |  |  | 5.7329E6 |  |  |  |  |  |  |  |  | 1 | 0 | 0 | 0 | 0 | 0 | 1 | 0 | 0 | 0 | 0 | 0 | 0 | 0 | 0 | 516 | 527 |  |  | PEAKS DB |
| R.EVVHFM(+15.99)NSLR.Y | N | 65.78 | 1246.6128 | 10 | 0.3 | 624.3138 | 2 | 31.71 | 6 | F6:14277 | NaSa01\_F14.raw |  |  |  |  | 3.2821E6 | 5.0137E7 |  |  |  |  |  |  |  |  | 13 | 0 | 0 | 0 | 0 | 2 | 11 | 0 | 0 | 0 | 0 | 0 | 0 | 0 | 0 | 114 | 123 | Oxidation (M) | M6:Oxidation (M):1000.00 | PEAKS DB |
| K.FPILSANIRPK.G | N | 63.57 | 1254.7448 | 11 | 1.8 | 419.2563 | 3 | 38.83 | 6 | F6:20180 | NaSa01\_F14.raw |  |  |  | 3.6184E5 | 9.4886E7 | 1.2559E8 |  |  |  |  | 2.1098E6 |  |  |  | 10 | 0 | 0 | 0 | 1 | 2 | 5 | 0 | 0 | 0 | 0 | 2 | 0 | 0 | 0 | 151 | 161 |  |  | PEAKS DB |
| K.RVVSLNVLC(+57.02)TEC(+57.02)R.V | N | 62.55 | 1604.8126 | 13 | 0.2 | 535.9449 | 3 | 35.03 | 6 | F6:16539 | NaSa01\_F14.raw |  |  |  |  | 4.4155E6 | 6.0208E6 |  |  |  |  |  |  |  |  | 3 | 0 | 0 | 0 | 0 | 1 | 2 | 0 | 0 | 0 | 0 | 0 | 0 | 0 | 0 | 483 | 495 | Carbamidomethylation | C9:Carbamidomethylation:1000.00;C12:Carbamidomethylation:1000.00 | PEAKS DB |
| K.GVDVVVGGHTNTF.L | N | 61.79 | 1300.6411 | 13 | 1.1 | 651.3286 | 2 | 41.80 | 6 | F6:22128 | NaSa01\_F14.raw |  |  |  |  | 2.249E7 | 5.2209E7 |  |  |  |  |  |  |  |  | 2 | 0 | 0 | 0 | 0 | 1 | 1 | 0 | 0 | 0 | 0 | 0 | 0 | 0 | 0 | 249 | 261 |  |  | PEAKS DB |
| L.TAVLPFGGTFDLLQIK.G | N | 60.64 | 1718.9607 | 16 | -0.1 | 860.4875 | 2 | 99.74 | 6 | F6:71142 | NaSa01\_F14.raw |  |  | 2.8271E6 | 1.2518E6 | 2.1013E7 | 4.0691E6 |  |  |  |  |  |  |  |  | 4 | 0 | 0 | 1 | 1 | 1 | 1 | 0 | 0 | 0 | 0 | 0 | 0 | 0 | 0 | 427 | 442 |  |  | PEAKS DB |
| K.IINVGSEKVGIIGYTTK.E | N | 60.09 | 1791.0142 | 17 | -0.7 | 598.0116 | 3 | 45.32 | 6 | F6:25348 | NaSa01\_F14.raw |  |  |  |  | 9.4386E5 | 1.7014E6 |  |  |  |  |  |  |  |  | 2 | 0 | 0 | 0 | 0 | 1 | 1 | 0 | 0 | 0 | 0 | 0 | 0 | 0 | 0 | 177 | 193 |  |  | PEAKS DB |
| K.YLGYLNVIFDDKGK.V | Y | 58.45 | 1643.8558 | 14 | -0.3 | 548.9590 | 3 | 70.19 | 5 | F5:46798 | NaSa01\_F13.raw |  |  |  |  | 1.8927E6 |  |  |  |  |  |  |  |  |  | 2 | 0 | 0 | 0 | 0 | 2 | 0 | 0 | 0 | 0 | 0 | 0 | 0 | 0 | 0 | 300 | 313 |  |  | PEAKS DB |
| K.VFPAMEGR.V | N | 56.33 | 905.4429 | 8 | 0.2 | 453.7288 | 2 | 22.33 | 6 | F6:7722 | NaSa01\_F14.raw |  |  |  |  |  | 3.6244E6 |  |  |  |  |  |  |  |  | 2 | 0 | 0 | 0 | 0 | 0 | 2 | 0 | 0 | 0 | 0 | 0 | 0 | 0 | 0 | 553 | 560 |  |  | PEAKS DB |
| N.ISGYILPYK.I | N | 56.17 | 1052.5906 | 9 | 0.7 | 527.3029 | 2 | 45.82 | 6 | F6:26096 | NaSa01\_F14.raw |  |  |  | 2.2592E5 | 4.6763E8 | 8.5839E8 |  |  |  |  |  |  |  |  | 5 | 0 | 0 | 0 | 1 | 1 | 3 | 0 | 0 | 0 | 0 | 0 | 0 | 0 | 0 | 168 | 176 |  |  | PEAKS DB |
| S.FLAAGGDGYYM(+15.99)LK.G | N | 55.62 | 1420.6697 | 13 | -0.1 | 711.3420 | 2 | 48.69 | 6 | F6:28173 | NaSa01\_F14.raw |  |  |  |  |  | 5.7308E6 |  |  |  |  |  |  |  |  | 1 | 0 | 0 | 0 | 0 | 0 | 1 | 0 | 0 | 0 | 0 | 0 | 0 | 0 | 0 | 515 | 527 | Oxidation (M) | M11:Oxidation (M):1000.00 | PEAKS DB |
| H.GQGTGELLQVSGIK.V | N | 55.06 | 1385.7515 | 14 | 0.3 | 693.8832 | 2 | 45.20 | 6 | F6:25108 | NaSa01\_F14.raw |  |  |  |  |  | 1.2594E6 |  |  |  |  |  |  |  |  | 1 | 0 | 0 | 0 | 0 | 0 | 1 | 0 | 0 | 0 | 0 | 0 | 0 | 0 | 0 | 458 | 471 |  |  | PEAKS DB |
| K.GVDVVVGGHTNTFLY.T | N | 54.75 | 1576.7886 | 15 | 0.1 | 789.4016 | 2 | 63.80 | 6 | F6:41327 | NaSa01\_F14.raw |  |  |  |  | 1.2739E7 | 2.8201E7 |  |  |  |  |  |  |  |  | 2 | 0 | 0 | 0 | 0 | 1 | 1 | 0 | 0 | 0 | 0 | 0 | 0 | 0 | 0 | 249 | 263 |  |  | PEAKS DB |
| R.QVPVVQAYAF.G | N | 53.91 | 1120.5917 | 10 | 0.0 | 561.3031 | 2 | 73.70 | 6 | F6:49221 | NaSa01\_F14.raw |  |  |  |  | 7.1015E6 | 1.9997E7 |  |  |  |  |  |  |  |  | 2 | 0 | 0 | 0 | 0 | 1 | 1 | 0 | 0 | 0 | 0 | 0 | 0 | 0 | 0 | 288 | 297 |  |  | PEAKS DB |
| M.ALGNHEFDNGLNGLLDPLLK.N | N | 53.45 | 2149.1167 | 20 | 0.0 | 717.3795 | 3 | 83.56 | 6 | F6:57342 | NaSa01\_F14.raw |  |  |  |  | 5.3244E6 | 2.0787E7 |  |  |  |  |  |  |  |  | 3 | 0 | 0 | 0 | 0 | 1 | 2 | 0 | 0 | 0 | 0 | 0 | 0 | 0 | 0 | 128 | 147 |  |  | PEAKS DB |
| K.LTTLGVNK.I | N | 52.68 | 844.5018 | 8 | -1.0 | 423.2578 | 2 | 11.58 | 6 | F6:2148 | NaSa01\_F14.raw |  |  |  |  |  | 3.1326E6 |  |  |  |  |  |  |  |  | 1 | 0 | 0 | 0 | 0 | 0 | 1 | 0 | 0 | 0 | 0 | 0 | 0 | 0 | 0 | 221 | 228 |  |  | PEAKS DB |
| K.VLLPSFLAAGGDGY.Y | N | 51.68 | 1378.7133 | 14 | 0.7 | 690.3644 | 2 | 100.88 | 6 | F6:72024 | NaSa01\_F14.raw |  |  |  |  | 1.3644E6 | 2.8748E6 |  |  |  |  |  |  |  |  | 2 | 0 | 0 | 0 | 0 | 1 | 1 | 0 | 0 | 0 | 0 | 0 | 0 | 0 | 0 | 510 | 523 |  |  | PEAKS DB |
| F.GGTFDLLQIK.G | N | 50.44 | 1090.6022 | 10 | -0.6 | 546.3080 | 2 | 64.67 | 6 | F6:41967 | NaSa01\_F14.raw |  |  |  |  |  | 9.7795E7 |  |  |  |  |  |  |  |  | 2 | 0 | 0 | 0 | 0 | 0 | 2 | 0 | 0 | 0 | 0 | 0 | 0 | 0 | 0 | 433 | 442 |  |  | PEAKS DB |
| Y.LNVIFDDK.G | N | 50.15 | 962.5073 | 8 | -0.5 | 482.2607 | 2 | 40.33 | 6 | F6:21131 | NaSa01\_F14.raw |  |  |  |  |  | 1.4605E6 |  |  |  |  |  |  |  |  | 1 | 0 | 0 | 0 | 0 | 0 | 1 | 0 | 0 | 0 | 0 | 0 | 0 | 0 | 0 | 304 | 311 |  |  | PEAKS DB |
| K.VGIIGYTTK.E | N | 50.04 | 950.5436 | 9 | 0.3 | 476.2792 | 2 | 28.48 | 6 | F6:11194 | NaSa01\_F14.raw |  |  |  |  |  | 5.1319E6 |  |  |  |  |  |  |  |  | 2 | 0 | 0 | 0 | 0 | 0 | 2 | 0 | 0 | 0 | 0 | 0 | 0 | 0 | 0 | 185 | 193 |  |  | PEAKS DB |
| L.AAGGDGYYMLK.G | N | 49.71 | 1144.5222 | 11 | 0.7 | 573.2688 | 2 | 30.46 | 5 | F5:13468 | NaSa01\_F13.raw |  |  |  |  | 6.4456E6 |  |  |  |  |  |  |  |  |  | 1 | 0 | 0 | 0 | 0 | 1 | 0 | 0 | 0 | 0 | 0 | 0 | 0 | 0 | 0 | 517 | 527 |  |  | PEAKS DB |
| R.FHEC(+57.02)NLGNLIC(+57.02)DAVVY.N | N | 48.35 | 1922.8655 | 16 | 1.4 | 962.4414 | 2 | 82.45 | 5 | F5:56634 | NaSa01\_F13.raw |  |  |  |  | 8.0322E7 | 1.7562E8 |  |  |  |  |  |  |  | 2.4692E7 | 3 | 0 | 0 | 0 | 0 | 1 | 1 | 0 | 0 | 0 | 0 | 0 | 0 | 0 | 1 | 369 | 384 | Carbamidomethylation | C4:Carbamidomethylation:1000.00;C11:Carbamidomethylation:1000.00 | PEAKS DB |
| R.HPDDNEWNHVSMC(+57.02).I | N | 48.19 | 1639.6144 | 13 | 0.5 | 820.8149 | 2 | 26.12 | 5 | F5:10348 | NaSa01\_F13.raw |  |  |  |  | 8.8799E6 |  |  |  |  |  |  |  |  |  | 2 | 0 | 0 | 0 | 0 | 2 | 0 | 0 | 0 | 0 | 0 | 0 | 0 | 0 | 0 | 389 | 401 | Carbamidomethylation | C13:Carbamidomethylation:1000.00 | PEAKS DB |
| K.VLLPSFLAAGGD.G | N | 47.35 | 1158.6284 | 12 | -0.1 | 580.3214 | 2 | 96.95 | 6 | F6:68512 | NaSa01\_F14.raw |  |  |  |  |  | 1.1427E6 |  |  |  |  |  |  |  |  | 1 | 0 | 0 | 0 | 0 | 0 | 1 | 0 | 0 | 0 | 0 | 0 | 0 | 0 | 0 | 510 | 521 |  |  | PEAKS DB |
| R.HPDDNEWNHVSM.C | N | 47.22 | 1479.5837 | 12 | 1.1 | 740.8000 | 2 | 25.93 | 5 | F5:10070 | NaSa01\_F13.raw |  |  |  |  | 2.3098E6 |  |  |  |  |  |  |  |  |  | 1 | 0 | 0 | 0 | 0 | 1 | 0 | 0 | 0 | 0 | 0 | 0 | 0 | 0 | 0 | 389 | 400 |  |  | PEAKS DB |
| R.QVPVVQAY.A | N | 45.65 | 902.4861 | 8 | -0.5 | 452.2501 | 2 | 43.38 | 5 | F5:24541 | NaSa01\_F13.raw |  |  |  |  | 1.2052E7 |  |  |  |  |  |  |  |  |  | 1 | 0 | 0 | 0 | 0 | 1 | 0 | 0 | 0 | 0 | 0 | 0 | 0 | 0 | 0 | 288 | 295 |  |  | PEAKS DB |
| N.PGPYLEFR.D | N | 45.44 | 977.4970 | 8 | 0.7 | 489.7561 | 2 | 39.90 | 6 | F6:20609 | NaSa01\_F14.raw |  |  |  |  |  | 4.6995E5 |  |  |  |  |  |  |  |  | 1 | 0 | 0 | 0 | 0 | 0 | 1 | 0 | 0 | 0 | 0 | 0 | 0 | 0 | 0 | 201 | 208 |  |  | PEAKS DB |
| N.LGNLIC(+57.02)DAVVYNNLR.H | N | 45.20 | 1732.8931 | 15 | 1.0 | 867.4547 | 2 | 69.10 | 6 | F6:45743 | NaSa01\_F14.raw |  |  |  |  |  | 1.1316E6 |  |  |  |  |  |  |  |  | 1 | 0 | 0 | 0 | 0 | 0 | 1 | 0 | 0 | 0 | 0 | 0 | 0 | 0 | 0 | 374 | 388 | Carbamidomethylation | C6:Carbamidomethylation:1000.00 | PEAKS DB |
| K.IINVGSEK.V | N | 44.70 | 858.4811 | 8 | -0.5 | 430.2476 | 2 | 11.48 | 6 | F6:1861 | NaSa01\_F14.raw |  |  |  |  |  | 1.3857E6 |  |  |  |  |  |  |  |  | 1 | 0 | 0 | 0 | 0 | 0 | 1 | 0 | 0 | 0 | 0 | 0 | 0 | 0 | 0 | 177 | 184 |  |  | PEAKS DB |
| K.VLLPSFLAAGGDGYY.M | N | 43.81 | 1541.7765 | 15 | 0.7 | 771.8961 | 2 | 105.28 | 6 | F6:75496 | NaSa01\_F14.raw |  |  |  |  | 1.4762E7 | 3.1893E7 |  |  |  |  |  |  |  |  | 2 | 0 | 0 | 0 | 0 | 1 | 1 | 0 | 0 | 0 | 0 | 0 | 0 | 0 | 0 | 510 | 524 |  |  | PEAKS DB |
| K.FPILSANIR.P | N | 43.38 | 1029.5970 | 9 | -0.4 | 515.8056 | 2 | 52.72 | 6 | F6:31843 | NaSa01\_F14.raw |  |  |  |  |  | 1.5657E8 |  |  |  |  |  |  |  |  | 1 | 0 | 0 | 0 | 0 | 0 | 1 | 0 | 0 | 0 | 0 | 0 | 0 | 0 | 0 | 151 | 159 |  |  | PEAKS DB |
| K.VLLPSFLAAGGDGYYM.L | N | 42.72 | 1672.8170 | 16 | 0.9 | 837.4166 | 2 | 111.51 | 6 | F6:80284 | NaSa01\_F14.raw |  |  |  |  |  | 1.1099E6 |  |  |  |  |  |  |  |  | 1 | 0 | 0 | 0 | 0 | 0 | 1 | 0 | 0 | 0 | 0 | 0 | 0 | 0 | 0 | 510 | 525 |  |  | PEAKS DB |
| Q.GTGELLQVSGIK.V | N | 42.14 | 1200.6714 | 12 | 0.9 | 601.3435 | 2 | 44.43 | 6 | F6:24568 | NaSa01\_F14.raw |  |  |  |  |  | 7.0353E5 |  |  |  |  |  |  |  |  | 1 | 0 | 0 | 0 | 0 | 0 | 1 | 0 | 0 | 0 | 0 | 0 | 0 | 0 | 0 | 460 | 471 |  |  | PEAKS DB |
| Y.LGYLNVIFDDK.G | N | 41.11 | 1295.6761 | 11 | 0.9 | 648.8459 | 2 | 76.55 | 6 | F6:51743 | NaSa01\_F14.raw |  |  |  |  |  | 0 |  |  |  |  |  |  |  |  | 0 | 0 | 0 | 0 | 0 | 0 | 0 | 0 | 0 | 0 | 0 | 0 | 0 | 0 | 0 | 301 | 311 |  |  | PEAKS DB |
| K.LTTLGVNKIIALGH.S | N | 41.05 | 1448.8715 | 14 | 0.1 | 483.9645 | 3 | 54.77 | 6 | F6:33352 | NaSa01\_F14.raw |  |  |  |  |  | 5.5856E5 |  |  |  |  |  |  |  |  | 1 | 0 | 0 | 0 | 0 | 0 | 1 | 0 | 0 | 0 | 0 | 0 | 0 | 0 | 0 | 221 | 234 |  |  | PEAKS DB |
| N.HEFDNGLNGLLDPLLK.N | N | 40.62 | 1793.9312 | 16 | 0.2 | 598.9844 | 3 | 81.57 | 6 | F6:55994 | NaSa01\_F14.raw |  |  |  |  |  | 5.7086E5 |  |  |  |  |  |  |  |  | 1 | 0 | 0 | 0 | 0 | 0 | 1 | 0 | 0 | 0 | 0 | 0 | 0 | 0 | 0 | 132 | 147 |  |  | PEAKS DB |
| N.YPFMQLSDDGR.Q | N | 40.57 | 1327.5867 | 11 | 0.4 | 664.8009 | 2 | 53.65 | 6 | F6:32287 | NaSa01\_F14.raw |  |  |  |  |  | 5.3395E5 |  |  |  |  |  |  |  |  | 1 | 0 | 0 | 0 | 0 | 0 | 1 | 0 | 0 | 0 | 0 | 0 | 0 | 0 | 0 | 277 | 287 |  |  | PEAKS DB |
| P.AGNYPFMQLSDDGR.Q | N | 40.07 | 1569.6881 | 14 | 0.1 | 785.8514 | 2 | 56.81 | 6 | F6:35126 | NaSa01\_F14.raw |  |  |  |  |  | 2.5054E5 |  |  |  |  |  |  |  |  | 1 | 0 | 0 | 0 | 0 | 0 | 1 | 0 | 0 | 0 | 0 | 0 | 0 | 0 | 0 | 274 | 287 |  |  | PEAKS DB |
| K.NVKFPILSAN.I | N | 39.82 | 1101.6182 | 10 | 0.5 | 551.8167 | 2 | 54.61 | 6 | F6:33186 | NaSa01\_F14.raw |  |  |  |  |  | 1.3385E6 |  |  |  |  |  |  |  |  | 1 | 0 | 0 | 0 | 0 | 0 | 1 | 0 | 0 | 0 | 0 | 0 | 0 | 0 | 0 | 148 | 157 |  |  | PEAKS DB |
| K.IIALGHSGF.K | N | 37.88 | 913.5021 | 9 | -1.2 | 457.7578 | 2 | 43.37 | 6 | F6:23538 | NaSa01\_F14.raw |  |  |  |  |  | 5.7946E6 |  |  |  |  |  |  |  |  | 1 | 0 | 0 | 0 | 0 | 0 | 1 | 0 | 0 | 0 | 0 | 0 | 0 | 0 | 0 | 229 | 237 |  |  | PEAKS DB |
| total 58 peptides |  |  |  |  |  |  |  |  |  |  |  |  |  |  |  |  |  |  |  |  |  |  |  |  |  |  |  |  |  |  |  |  |  |  |  |  |  |  |  |  |  |  |  |  |  |
| --- | --- | --- | --- | --- | --- | --- | --- | --- | --- | --- | --- | --- | --- | --- | --- | --- | --- | --- | --- | --- | --- | --- | --- | --- | --- | --- | --- | --- | --- | --- | --- | --- | --- | --- | --- | --- | --- | --- | --- | --- | --- | --- | --- | --- | --- |

JAI09047.1

back to list

  

| Protein Coverage
| Supporting Peptides
|

Protein Coverage:

Supporting Peptides:

| Peptide | Uniq | -10lgP | Mass | Length | ppm | m/z | z | RT | Fraction | Scan | Source File | Area F1 | Area F10 | Area F11 | Area F12 | Area F13 | Area F14 | Area F2 | Area F3 | Area F4 | Area F5 | Area F6 | Area F7 | Area F8 | Area F9 | #Feature | #Feature F1 | #Feature F10 | #Feature F11 | #Feature F12 | #Feature F13 | #Feature F14 | #Feature F2 | #Feature F3 | #Feature F4 | #Feature F5 | #Feature F6 | #Feature F7 | #Feature F8 | #Feature F9 | Start | End | PTM | AScore | Found By |
| --- | --- | --- | --- | --- | --- | --- | --- | --- | --- | --- | --- | --- | --- | --- | --- | --- | --- | --- | --- | --- | --- | --- | --- | --- | --- | --- | --- | --- | --- | --- | --- | --- | --- | --- | --- | --- | --- | --- | --- | --- | --- | --- | --- | --- | --- |
| R.HPDDNEWNHVSMC(+57.02)IVNGGGIR.S | N | 99.67 | 2406.0593 | 21 | -0.4 | 803.0267 | 3 | 49.93 | 6 | F6:29280 | NaSa01\_F14.raw |  |  |  |  | 6.5991E7 | 1.6043E8 |  |  |  |  |  |  |  | 7.6096E6 | 9 | 0 | 0 | 0 | 0 | 3 | 4 | 0 | 0 | 0 | 0 | 0 | 0 | 0 | 2 | 389 | 409 | Carbamidomethylation | C13:Carbamidomethylation:1000.00 | PEAKS DB |
| R.HGQGTGELLQVSGIK.V | N | 97.61 | 1522.8103 | 15 | 0.4 | 762.4127 | 2 | 31.05 | 6 | F6:13489 | NaSa01\_F14.raw |  |  |  | 4.9661E5 | 6.4612E8 | 1.2796E9 |  |  |  |  |  |  |  |  | 8 | 0 | 0 | 0 | 1 | 3 | 4 | 0 | 0 | 0 | 0 | 0 | 0 | 0 | 0 | 457 | 471 |  |  | PEAKS DB |
| R.YDAM(+15.99)ALGNHEFDNGLNGLLDPLLK.N | N | 89.01 | 2645.2795 | 24 | 0.3 | 882.7674 | 3 | 88.88 | 6 | F6:61975 | NaSa01\_F14.raw |  |  |  |  | 1.9977E7 | 6.8848E7 |  |  |  |  |  |  |  |  | 3 | 0 | 0 | 0 | 0 | 1 | 2 | 0 | 0 | 0 | 0 | 0 | 0 | 0 | 0 | 124 | 147 | Oxidation (M) | M4:Oxidation (M):1000.00 | PEAKS DB |
| R.HPDDNEWNHVSM(+15.99)C(+57.02)IVNGGGIR.S | N | 86.62 | 2422.0542 | 21 | -0.3 | 808.3584 | 3 | 44.12 | 6 | F6:24148 | NaSa01\_F14.raw |  |  |  |  | 3.2766E7 | 9.9954E7 |  |  |  |  |  |  |  | 8.8723E6 | 8 | 0 | 0 | 0 | 0 | 2 | 4 | 0 | 0 | 0 | 0 | 0 | 0 | 0 | 2 | 389 | 409 | Oxidation (M); Carbamidomethylation | M12:Oxidation (M):1000.00;C13:Carbamidomethylation:1000.00 | PEAKS DB |
| K.VLLPSFLAAGGDGYYMLK.G | N | 84.56 | 1913.9961 | 18 | 1.4 | 639.0068 | 3 | 94.26 | 6 | F6:66829 | NaSa01\_F14.raw |  |  |  | 4.47E5 | 9.4519E7 | 6.4928E8 |  |  |  |  |  |  |  |  | 12 | 0 | 0 | 0 | 1 | 4 | 7 | 0 | 0 | 0 | 0 | 0 | 0 | 0 | 0 | 510 | 527 |  |  | PEAKS DB |
| K.ASGNPILLNKSIQEDPAVK.A | N | 83.17 | 1993.0844 | 19 | 0.6 | 665.3691 | 3 | 35.69 | 6 | F6:17128 | NaSa01\_F14.raw |  |  |  |  | 1.5436E6 | 2.8654E6 |  |  |  |  |  |  |  |  | 2 | 0 | 0 | 0 | 0 | 1 | 1 | 0 | 0 | 0 | 0 | 0 | 0 | 0 | 0 | 317 | 335 |  |  | PEAKS DB |
| K.VLLPSFLAAGGDGYYM(+15.99)LK.G | N | 82.89 | 1929.9910 | 18 | 0.7 | 966.0034 | 2 | 87.45 | 6 | F6:60748 | NaSa01\_F14.raw |  |  |  |  | 2.8899E7 | 1.2442E8 |  |  |  |  |  |  |  |  | 10 | 0 | 0 | 0 | 0 | 3 | 7 | 0 | 0 | 0 | 0 | 0 | 0 | 0 | 0 | 510 | 527 | Oxidation (M) | M16:Oxidation (M):1000.00 | PEAKS DB |
| R.FHEC(+57.02)NLGNLIC(+57.02)DAVVYNNLR.H | N | 82.50 | 2420.1365 | 20 | -0.2 | 807.7192 | 3 | 77.73 | 6 | F6:52912 | NaSa01\_F14.raw |  |  |  |  | 2.6877E7 | 9.4785E7 |  |  |  |  |  |  | 7.773E6 | 1.2217E8 | 9 | 0 | 0 | 0 | 0 | 1 | 3 | 0 | 0 | 0 | 0 | 0 | 0 | 2 | 3 | 369 | 388 | Carbamidomethylation | C4:Carbamidomethylation:1000.00;C11:Carbamidomethylation:1000.00 | PEAKS DB |
| K.ETPVLSNPGPYLEFR.D | N | 81.49 | 1717.8674 | 15 | 0.8 | 859.9417 | 2 | 69.77 | 6 | F6:46166 | NaSa01\_F14.raw |  |  |  | 7.2199E5 | 2.4678E8 | 7.6435E8 |  |  |  | 2.2582E6 |  |  |  |  | 7 | 0 | 0 | 0 | 1 | 3 | 2 | 0 | 0 | 0 | 1 | 0 | 0 | 0 | 0 | 194 | 208 |  |  | PEAKS DB |
| R.QVPVVQAYAFGK.Y | N | 80.72 | 1305.7081 | 12 | -0.2 | 653.8612 | 2 | 81.01 | 6 | F6:55346 | NaSa01\_F14.raw |  |  |  | 4.7562E5 | 3.326E8 | 6.8066E8 |  |  |  |  |  |  |  |  | 5 | 0 | 0 | 0 | 1 | 1 | 3 | 0 | 0 | 0 | 0 | 0 | 0 | 0 | 0 | 288 | 299 |  |  | PEAKS DB |
| R.EVVHFMNSLR.Y | N | 80.17 | 1230.6179 | 10 | 0.3 | 616.3164 | 2 | 32.92 | 6 | F6:14493 | NaSa01\_F14.raw |  |  |  |  | 2.3691E8 | 4.1556E8 |  |  |  |  |  |  |  |  | 11 | 0 | 0 | 0 | 0 | 3 | 8 | 0 | 0 | 0 | 0 | 0 | 0 | 0 | 0 | 114 | 123 |  |  | PEAKS DB |
| R.VVSLNVLC(+57.02)TEC(+57.02)R.V | N | 79.36 | 1448.7115 | 12 | 0.2 | 725.3632 | 2 | 49.71 | 6 | F6:29319 | NaSa01\_F14.raw |  |  |  | 0 | 2.7472E8 | 5.8042E8 |  |  |  |  |  | 8.8246E5 |  |  | 7 | 0 | 0 | 0 | 0 | 2 | 4 | 0 | 0 | 0 | 0 | 0 | 1 | 0 | 0 | 484 | 495 | Carbamidomethylation | C8:Carbamidomethylation:1000.00;C11:Carbamidomethylation:1000.00 | PEAKS DB |
| R.YDAMALGNHEFDNGLNGLLDPLLK.N | N | 78.14 | 2629.2847 | 24 | 0.8 | 877.4362 | 3 | 94.34 | 6 | F6:67114 | NaSa01\_F14.raw |  |  |  |  | 1.6292E7 | 1.1665E8 |  |  |  |  |  |  |  |  | 4 | 0 | 0 | 0 | 0 | 2 | 2 | 0 | 0 | 0 | 0 | 0 | 0 | 0 | 0 | 124 | 147 |  |  | PEAKS DB |
| K.YLGYLNVIFDDK.G | N | 76.79 | 1458.7394 | 12 | 0.2 | 730.3771 | 2 | 85.89 | 6 | F6:59730 | NaSa01\_F14.raw |  |  |  |  | 6.7598E7 | 9.6452E7 |  |  |  |  |  |  |  |  | 5 | 0 | 0 | 0 | 0 | 4 | 1 | 0 | 0 | 0 | 0 | 0 | 0 | 0 | 0 | 300 | 311 |  |  | PEAKS DB |
| K.LTILHTNDVHAR.V | N | 75.00 | 1388.7524 | 12 | 0.9 | 463.9252 | 3 | 11.51 | 6 | F6:1905 | NaSa01\_F14.raw |  |  |  |  | 1.6389E6 | 3.6507E6 |  |  |  |  |  |  |  |  | 4 | 0 | 0 | 0 | 0 | 2 | 2 | 0 | 0 | 0 | 0 | 0 | 0 | 0 | 0 | 44 | 55 |  |  | PEAKS DB |
| K.ETPVLSNPGPYLEFRDEVEELQK.Q | N | 74.67 | 2688.3281 | 23 | 0.7 | 897.1173 | 3 | 77.38 | 6 | F6:52291 | NaSa01\_F14.raw |  |  |  |  | 3.5808E8 | 6.9841E8 |  |  |  |  |  |  |  |  | 4 | 0 | 0 | 0 | 0 | 2 | 2 | 0 | 0 | 0 | 0 | 0 | 0 | 0 | 0 | 194 | 216 |  |  | PEAKS DB |
| K.ASGNPILLNK.S | N | 69.82 | 1025.5869 | 10 | -0.4 | 513.8005 | 2 | 11.59 | 6 | F6:1963 | NaSa01\_F14.raw |  |  |  |  | 2.4579E6 | 4.7925E6 |  |  |  |  |  |  |  |  | 3 | 0 | 0 | 0 | 0 | 1 | 2 | 0 | 0 | 0 | 0 | 0 | 0 | 0 | 0 | 317 | 326 |  |  | PEAKS DB |
| S.FLAAGGDGYYMLK.G | N | 67.12 | 1404.6747 | 13 | 0.2 | 703.3447 | 2 | 58.91 | 6 | F6:37338 | NaSa01\_F14.raw |  |  |  |  | 1.8909E7 | 8.0208E7 |  |  |  |  |  |  |  |  | 3 | 0 | 0 | 0 | 0 | 1 | 2 | 0 | 0 | 0 | 0 | 0 | 0 | 0 | 0 | 515 | 527 |  |  | PEAKS DB |
| F.LAAGGDGYYMLK.G | N | 66.41 | 1257.6063 | 12 | 0.2 | 629.8105 | 2 | 41.73 | 6 | F6:22051 | NaSa01\_F14.raw |  |  |  |  |  | 5.7329E6 |  |  |  |  |  |  |  |  | 1 | 0 | 0 | 0 | 0 | 0 | 1 | 0 | 0 | 0 | 0 | 0 | 0 | 0 | 0 | 516 | 527 |  |  | PEAKS DB |
| R.EVVHFM(+15.99)NSLR.Y | N | 65.78 | 1246.6128 | 10 | 0.3 | 624.3138 | 2 | 31.71 | 6 | F6:14277 | NaSa01\_F14.raw |  |  |  |  | 3.2821E6 | 5.0137E7 |  |  |  |  |  |  |  |  | 13 | 0 | 0 | 0 | 0 | 2 | 11 | 0 | 0 | 0 | 0 | 0 | 0 | 0 | 0 | 114 | 123 | Oxidation (M) | M6:Oxidation (M):1000.00 | PEAKS DB |
| K.FPILSANIRPK.G | N | 63.57 | 1254.7448 | 11 | 1.8 | 419.2563 | 3 | 38.83 | 6 | F6:20180 | NaSa01\_F14.raw |  |  |  | 3.6184E5 | 9.4886E7 | 1.2559E8 |  |  |  |  | 2.1098E6 |  |  |  | 10 | 0 | 0 | 0 | 1 | 2 | 5 | 0 | 0 | 0 | 0 | 2 | 0 | 0 | 0 | 151 | 161 |  |  | PEAKS DB |
| K.RVVSLNVLC(+57.02)TEC(+57.02)R.V | N | 62.55 | 1604.8126 | 13 | 0.2 | 535.9449 | 3 | 35.03 | 6 | F6:16539 | NaSa01\_F14.raw |  |  |  |  | 4.4155E6 | 6.0208E6 |  |  |  |  |  |  |  |  | 3 | 0 | 0 | 0 | 0 | 1 | 2 | 0 | 0 | 0 | 0 | 0 | 0 | 0 | 0 | 483 | 495 | Carbamidomethylation | C9:Carbamidomethylation:1000.00;C12:Carbamidomethylation:1000.00 | PEAKS DB |
| K.GVDVVVGGHTNTF.L | N | 61.79 | 1300.6411 | 13 | 1.1 | 651.3286 | 2 | 41.80 | 6 | F6:22128 | NaSa01\_F14.raw |  |  |  |  | 2.249E7 | 5.2209E7 |  |  |  |  |  |  |  |  | 2 | 0 | 0 | 0 | 0 | 1 | 1 | 0 | 0 | 0 | 0 | 0 | 0 | 0 | 0 | 249 | 261 |  |  | PEAKS DB |
| L.TAVLPFGGTFDLLQIK.G | N | 60.64 | 1718.9607 | 16 | -0.1 | 860.4875 | 2 | 99.74 | 6 | F6:71142 | NaSa01\_F14.raw |  |  | 2.8271E6 | 1.2518E6 | 2.1013E7 | 4.0691E6 |  |  |  |  |  |  |  |  | 4 | 0 | 0 | 1 | 1 | 1 | 1 | 0 | 0 | 0 | 0 | 0 | 0 | 0 | 0 | 427 | 442 |  |  | PEAKS DB |
| K.IINVGSEKVGIIGYTTK.E | N | 60.09 | 1791.0142 | 17 | -0.7 | 598.0116 | 3 | 45.32 | 6 | F6:25348 | NaSa01\_F14.raw |  |  |  |  | 9.4386E5 | 1.7014E6 |  |  |  |  |  |  |  |  | 2 | 0 | 0 | 0 | 0 | 1 | 1 | 0 | 0 | 0 | 0 | 0 | 0 | 0 | 0 | 177 | 193 |  |  | PEAKS DB |
| K.YLGYLNVIFDDKGK.V | Y | 58.45 | 1643.8558 | 14 | -0.3 | 548.9590 | 3 | 70.19 | 5 | F5:46798 | NaSa01\_F13.raw |  |  |  |  | 1.8927E6 |  |  |  |  |  |  |  |  |  | 2 | 0 | 0 | 0 | 0 | 2 | 0 | 0 | 0 | 0 | 0 | 0 | 0 | 0 | 0 | 300 | 313 |  |  | PEAKS DB |
| K.VFPAMEGR.V | N | 56.33 | 905.4429 | 8 | 0.2 | 453.7288 | 2 | 22.33 | 6 | F6:7722 | NaSa01\_F14.raw |  |  |  |  |  | 3.6244E6 |  |  |  |  |  |  |  |  | 2 | 0 | 0 | 0 | 0 | 0 | 2 | 0 | 0 | 0 | 0 | 0 | 0 | 0 | 0 | 553 | 560 |  |  | PEAKS DB |
| N.ISGYILPYK.I | N | 56.17 | 1052.5906 | 9 | 0.7 | 527.3029 | 2 | 45.82 | 6 | F6:26096 | NaSa01\_F14.raw |  |  |  | 2.2592E5 | 4.6763E8 | 8.5839E8 |  |  |  |  |  |  |  |  | 5 | 0 | 0 | 0 | 1 | 1 | 3 | 0 | 0 | 0 | 0 | 0 | 0 | 0 | 0 | 168 | 176 |  |  | PEAKS DB |
| S.FLAAGGDGYYM(+15.99)LK.G | N | 55.62 | 1420.6697 | 13 | -0.1 | 711.3420 | 2 | 48.69 | 6 | F6:28173 | NaSa01\_F14.raw |  |  |  |  |  | 5.7308E6 |  |  |  |  |  |  |  |  | 1 | 0 | 0 | 0 | 0 | 0 | 1 | 0 | 0 | 0 | 0 | 0 | 0 | 0 | 0 | 515 | 527 | Oxidation (M) | M11:Oxidation (M):1000.00 | PEAKS DB |
| H.GQGTGELLQVSGIK.V | N | 55.06 | 1385.7515 | 14 | 0.3 | 693.8832 | 2 | 45.20 | 6 | F6:25108 | NaSa01\_F14.raw |  |  |  |  |  | 1.2594E6 |  |  |  |  |  |  |  |  | 1 | 0 | 0 | 0 | 0 | 0 | 1 | 0 | 0 | 0 | 0 | 0 | 0 | 0 | 0 | 458 | 471 |  |  | PEAKS DB |
| K.GVDVVVGGHTNTFLY.T | N | 54.75 | 1576.7886 | 15 | 0.1 | 789.4016 | 2 | 63.80 | 6 | F6:41327 | NaSa01\_F14.raw |  |  |  |  | 1.2739E7 | 2.8201E7 |  |  |  |  |  |  |  |  | 2 | 0 | 0 | 0 | 0 | 1 | 1 | 0 | 0 | 0 | 0 | 0 | 0 | 0 | 0 | 249 | 263 |  |  | PEAKS DB |
| R.QVPVVQAYAF.G | N | 53.91 | 1120.5917 | 10 | 0.0 | 561.3031 | 2 | 73.70 | 6 | F6:49221 | NaSa01\_F14.raw |  |  |  |  | 7.1015E6 | 1.9997E7 |  |  |  |  |  |  |  |  | 2 | 0 | 0 | 0 | 0 | 1 | 1 | 0 | 0 | 0 | 0 | 0 | 0 | 0 | 0 | 288 | 297 |  |  | PEAKS DB |
| M.ALGNHEFDNGLNGLLDPLLK.N | N | 53.45 | 2149.1167 | 20 | 0.0 | 717.3795 | 3 | 83.56 | 6 | F6:57342 | NaSa01\_F14.raw |  |  |  |  | 5.3244E6 | 2.0787E7 |  |  |  |  |  |  |  |  | 3 | 0 | 0 | 0 | 0 | 1 | 2 | 0 | 0 | 0 | 0 | 0 | 0 | 0 | 0 | 128 | 147 |  |  | PEAKS DB |
| K.LTTLGVNK.I | N | 52.68 | 844.5018 | 8 | -1.0 | 423.2578 | 2 | 11.58 | 6 | F6:2148 | NaSa01\_F14.raw |  |  |  |  |  | 3.1326E6 |  |  |  |  |  |  |  |  | 1 | 0 | 0 | 0 | 0 | 0 | 1 | 0 | 0 | 0 | 0 | 0 | 0 | 0 | 0 | 221 | 228 |  |  | PEAKS DB |
| K.VLLPSFLAAGGDGY.Y | N | 51.68 | 1378.7133 | 14 | 0.7 | 690.3644 | 2 | 100.88 | 6 | F6:72024 | NaSa01\_F14.raw |  |  |  |  | 1.3644E6 | 2.8748E6 |  |  |  |  |  |  |  |  | 2 | 0 | 0 | 0 | 0 | 1 | 1 | 0 | 0 | 0 | 0 | 0 | 0 | 0 | 0 | 510 | 523 |  |  | PEAKS DB |
| F.GGTFDLLQIK.G | N | 50.44 | 1090.6022 | 10 | -0.6 | 546.3080 | 2 | 64.67 | 6 | F6:41967 | NaSa01\_F14.raw |  |  |  |  |  | 9.7795E7 |  |  |  |  |  |  |  |  | 2 | 0 | 0 | 0 | 0 | 0 | 2 | 0 | 0 | 0 | 0 | 0 | 0 | 0 | 0 | 433 | 442 |  |  | PEAKS DB |
| Y.LNVIFDDK.G | N | 50.15 | 962.5073 | 8 | -0.5 | 482.2607 | 2 | 40.33 | 6 | F6:21131 | NaSa01\_F14.raw |  |  |  |  |  | 1.4605E6 |  |  |  |  |  |  |  |  | 1 | 0 | 0 | 0 | 0 | 0 | 1 | 0 | 0 | 0 | 0 | 0 | 0 | 0 | 0 | 304 | 311 |  |  | PEAKS DB |
| K.VGIIGYTTK.E | N | 50.04 | 950.5436 | 9 | 0.3 | 476.2792 | 2 | 28.48 | 6 | F6:11194 | NaSa01\_F14.raw |  |  |  |  |  | 5.1319E6 |  |  |  |  |  |  |  |  | 2 | 0 | 0 | 0 | 0 | 0 | 2 | 0 | 0 | 0 | 0 | 0 | 0 | 0 | 0 | 185 | 193 |  |  | PEAKS DB |
| L.AAGGDGYYMLK.G | N | 49.71 | 1144.5222 | 11 | 0.7 | 573.2688 | 2 | 30.46 | 5 | F5:13468 | NaSa01\_F13.raw |  |  |  |  | 6.4456E6 |  |  |  |  |  |  |  |  |  | 1 | 0 | 0 | 0 | 0 | 1 | 0 | 0 | 0 | 0 | 0 | 0 | 0 | 0 | 0 | 517 | 527 |  |  | PEAKS DB |
| R.FHEC(+57.02)NLGNLIC(+57.02)DAVVY.N | N | 48.35 | 1922.8655 | 16 | 1.4 | 962.4414 | 2 | 82.45 | 5 | F5:56634 | NaSa01\_F13.raw |  |  |  |  | 8.0322E7 | 1.7562E8 |  |  |  |  |  |  |  | 2.4692E7 | 3 | 0 | 0 | 0 | 0 | 1 | 1 | 0 | 0 | 0 | 0 | 0 | 0 | 0 | 1 | 369 | 384 | Carbamidomethylation | C4:Carbamidomethylation:1000.00;C11:Carbamidomethylation:1000.00 | PEAKS DB |
| R.HPDDNEWNHVSMC(+57.02).I | N | 48.19 | 1639.6144 | 13 | 0.5 | 820.8149 | 2 | 26.12 | 5 | F5:10348 | NaSa01\_F13.raw |  |  |  |  | 8.8799E6 |  |  |  |  |  |  |  |  |  | 2 | 0 | 0 | 0 | 0 | 2 | 0 | 0 | 0 | 0 | 0 | 0 | 0 | 0 | 0 | 389 | 401 | Carbamidomethylation | C13:Carbamidomethylation:1000.00 | PEAKS DB |
| K.VLLPSFLAAGGD.G | N | 47.35 | 1158.6284 | 12 | -0.1 | 580.3214 | 2 | 96.95 | 6 | F6:68512 | NaSa01\_F14.raw |  |  |  |  |  | 1.1427E6 |  |  |  |  |  |  |  |  | 1 | 0 | 0 | 0 | 0 | 0 | 1 | 0 | 0 | 0 | 0 | 0 | 0 | 0 | 0 | 510 | 521 |  |  | PEAKS DB |
| R.HPDDNEWNHVSM.C | N | 47.22 | 1479.5837 | 12 | 1.1 | 740.8000 | 2 | 25.93 | 5 | F5:10070 | NaSa01\_F13.raw |  |  |  |  | 2.3098E6 |  |  |  |  |  |  |  |  |  | 1 | 0 | 0 | 0 | 0 | 1 | 0 | 0 | 0 | 0 | 0 | 0 | 0 | 0 | 0 | 389 | 400 |  |  | PEAKS DB |
| R.QVPVVQAY.A | N | 45.65 | 902.4861 | 8 | -0.5 | 452.2501 | 2 | 43.38 | 5 | F5:24541 | NaSa01\_F13.raw |  |  |  |  | 1.2052E7 |  |  |  |  |  |  |  |  |  | 1 | 0 | 0 | 0 | 0 | 1 | 0 | 0 | 0 | 0 | 0 | 0 | 0 | 0 | 0 | 288 | 295 |  |  | PEAKS DB |
| N.PGPYLEFR.D | N | 45.44 | 977.4970 | 8 | 0.7 | 489.7561 | 2 | 39.90 | 6 | F6:20609 | NaSa01\_F14.raw |  |  |  |  |  | 4.6995E5 |  |  |  |  |  |  |  |  | 1 | 0 | 0 | 0 | 0 | 0 | 1 | 0 | 0 | 0 | 0 | 0 | 0 | 0 | 0 | 201 | 208 |  |  | PEAKS DB |
| N.LGNLIC(+57.02)DAVVYNNLR.H | N | 45.20 | 1732.8931 | 15 | 1.0 | 867.4547 | 2 | 69.10 | 6 | F6:45743 | NaSa01\_F14.raw |  |  |  |  |  | 1.1316E6 |  |  |  |  |  |  |  |  | 1 | 0 | 0 | 0 | 0 | 0 | 1 | 0 | 0 | 0 | 0 | 0 | 0 | 0 | 0 | 374 | 388 | Carbamidomethylation | C6:Carbamidomethylation:1000.00 | PEAKS DB |
| K.IINVGSEK.V | N | 44.70 | 858.4811 | 8 | -0.5 | 430.2476 | 2 | 11.48 | 6 | F6:1861 | NaSa01\_F14.raw |  |  |  |  |  | 1.3857E6 |  |  |  |  |  |  |  |  | 1 | 0 | 0 | 0 | 0 | 0 | 1 | 0 | 0 | 0 | 0 | 0 | 0 | 0 | 0 | 177 | 184 |  |  | PEAKS DB |
| K.VLLPSFLAAGGDGYY.M | N | 43.81 | 1541.7765 | 15 | 0.7 | 771.8961 | 2 | 105.28 | 6 | F6:75496 | NaSa01\_F14.raw |  |  |  |  | 1.4762E7 | 3.1893E7 |  |  |  |  |  |  |  |  | 2 | 0 | 0 | 0 | 0 | 1 | 1 | 0 | 0 | 0 | 0 | 0 | 0 | 0 | 0 | 510 | 524 |  |  | PEAKS DB |
| K.FPILSANIR.P | N | 43.38 | 1029.5970 | 9 | -0.4 | 515.8056 | 2 | 52.72 | 6 | F6:31843 | NaSa01\_F14.raw |  |  |  |  |  | 1.5657E8 |  |  |  |  |  |  |  |  | 1 | 0 | 0 | 0 | 0 | 0 | 1 | 0 | 0 | 0 | 0 | 0 | 0 | 0 | 0 | 151 | 159 |  |  | PEAKS DB |
| K.VLLPSFLAAGGDGYYM.L | N | 42.72 | 1672.8170 | 16 | 0.9 | 837.4166 | 2 | 111.51 | 6 | F6:80284 | NaSa01\_F14.raw |  |  |  |  |  | 1.1099E6 |  |  |  |  |  |  |  |  | 1 | 0 | 0 | 0 | 0 | 0 | 1 | 0 | 0 | 0 | 0 | 0 | 0 | 0 | 0 | 510 | 525 |  |  | PEAKS DB |
| Q.GTGELLQVSGIK.V | N | 42.14 | 1200.6714 | 12 | 0.9 | 601.3435 | 2 | 44.43 | 6 | F6:24568 | NaSa01\_F14.raw |  |  |  |  |  | 7.0353E5 |  |  |  |  |  |  |  |  | 1 | 0 | 0 | 0 | 0 | 0 | 1 | 0 | 0 | 0 | 0 | 0 | 0 | 0 | 0 | 460 | 471 |  |  | PEAKS DB |
| Y.LGYLNVIFDDK.G | N | 41.11 | 1295.6761 | 11 | 0.9 | 648.8459 | 2 | 76.55 | 6 | F6:51743 | NaSa01\_F14.raw |  |  |  |  |  | 0 |  |  |  |  |  |  |  |  | 0 | 0 | 0 | 0 | 0 | 0 | 0 | 0 | 0 | 0 | 0 | 0 | 0 | 0 | 0 | 301 | 311 |  |  | PEAKS DB |
| K.LTTLGVNKIIALGH.S | N | 41.05 | 1448.8715 | 14 | 0.1 | 483.9645 | 3 | 54.77 | 6 | F6:33352 | NaSa01\_F14.raw |  |  |  |  |  | 5.5856E5 |  |  |  |  |  |  |  |  | 1 | 0 | 0 | 0 | 0 | 0 | 1 | 0 | 0 | 0 | 0 | 0 | 0 | 0 | 0 | 221 | 234 |  |  | PEAKS DB |
| N.HEFDNGLNGLLDPLLK.N | N | 40.62 | 1793.9312 | 16 | 0.2 | 598.9844 | 3 | 81.57 | 6 | F6:55994 | NaSa01\_F14.raw |  |  |  |  |  | 5.7086E5 |  |  |  |  |  |  |  |  | 1 | 0 | 0 | 0 | 0 | 0 | 1 | 0 | 0 | 0 | 0 | 0 | 0 | 0 | 0 | 132 | 147 |  |  | PEAKS DB |
| N.YPFMQLSDDGR.Q | N | 40.57 | 1327.5867 | 11 | 0.4 | 664.8009 | 2 | 53.65 | 6 | F6:32287 | NaSa01\_F14.raw |  |  |  |  |  | 5.3395E5 |  |  |  |  |  |  |  |  | 1 | 0 | 0 | 0 | 0 | 0 | 1 | 0 | 0 | 0 | 0 | 0 | 0 | 0 | 0 | 277 | 287 |  |  | PEAKS DB |
| P.AGNYPFMQLSDDGR.Q | N | 40.07 | 1569.6881 | 14 | 0.1 | 785.8514 | 2 | 56.81 | 6 | F6:35126 | NaSa01\_F14.raw |  |  |  |  |  | 2.5054E5 |  |  |  |  |  |  |  |  | 1 | 0 | 0 | 0 | 0 | 0 | 1 | 0 | 0 | 0 | 0 | 0 | 0 | 0 | 0 | 274 | 287 |  |  | PEAKS DB |
| K.NVKFPILSAN.I | N | 39.82 | 1101.6182 | 10 | 0.5 | 551.8167 | 2 | 54.61 | 6 | F6:33186 | NaSa01\_F14.raw |  |  |  |  |  | 1.3385E6 |  |  |  |  |  |  |  |  | 1 | 0 | 0 | 0 | 0 | 0 | 1 | 0 | 0 | 0 | 0 | 0 | 0 | 0 | 0 | 148 | 157 |  |  | PEAKS DB |
| K.IIALGHSGF.K | N | 37.88 | 913.5021 | 9 | -1.2 | 457.7578 | 2 | 43.37 | 6 | F6:23538 | NaSa01\_F14.raw |  |  |  |  |  | 5.7946E6 |  |  |  |  |  |  |  |  | 1 | 0 | 0 | 0 | 0 | 0 | 1 | 0 | 0 | 0 | 0 | 0 | 0 | 0 | 0 | 229 | 237 |  |  | PEAKS DB |
| total 58 peptides |  |  |  |  |  |  |  |  |  |  |  |  |  |  |  |  |  |  |  |  |  |  |  |  |  |  |  |  |  |  |  |  |  |  |  |  |  |  |  |  |  |  |  |  |  |
| --- | --- | --- | --- | --- | --- | --- | --- | --- | --- | --- | --- | --- | --- | --- | --- | --- | --- | --- | --- | --- | --- | --- | --- | --- | --- | --- | --- | --- | --- | --- | --- | --- | --- | --- | --- | --- | --- | --- | --- | --- | --- | --- | --- | --- | --- |

JAB52815.1

back to list

  

| Protein Coverage
| Supporting Peptides
|

Protein Coverage:

Supporting Peptides:

| Peptide | Uniq | -10lgP | Mass | Length | ppm | m/z | z | RT | Fraction | Scan | Source File | Area F1 | Area F10 | Area F11 | Area F12 | Area F13 | Area F14 | Area F2 | Area F3 | Area F4 | Area F5 | Area F6 | Area F7 | Area F8 | Area F9 | #Feature | #Feature F1 | #Feature F10 | #Feature F11 | #Feature F12 | #Feature F13 | #Feature F14 | #Feature F2 | #Feature F3 | #Feature F4 | #Feature F5 | #Feature F6 | #Feature F7 | #Feature F8 | #Feature F9 | Start | End | PTM | AScore | Found By |
| --- | --- | --- | --- | --- | --- | --- | --- | --- | --- | --- | --- | --- | --- | --- | --- | --- | --- | --- | --- | --- | --- | --- | --- | --- | --- | --- | --- | --- | --- | --- | --- | --- | --- | --- | --- | --- | --- | --- | --- | --- | --- | --- | --- | --- | --- |
| R.HPDDNEWNHVSMC(+57.02)IVNGGGIR.S | N | 99.67 | 2406.0593 | 21 | -0.4 | 803.0267 | 3 | 49.93 | 6 | F6:29280 | NaSa01\_F14.raw |  |  |  |  | 6.5991E7 | 1.6043E8 |  |  |  |  |  |  |  | 7.6096E6 | 9 | 0 | 0 | 0 | 0 | 3 | 4 | 0 | 0 | 0 | 0 | 0 | 0 | 0 | 2 | 389 | 409 | Carbamidomethylation | C13:Carbamidomethylation:1000.00 | PEAKS DB |
| R.HGQGTGELLQVSGIK.V | N | 97.61 | 1522.8103 | 15 | 0.4 | 762.4127 | 2 | 31.05 | 6 | F6:13489 | NaSa01\_F14.raw |  |  |  | 4.9661E5 | 6.4612E8 | 1.2796E9 |  |  |  |  |  |  |  |  | 8 | 0 | 0 | 0 | 1 | 3 | 4 | 0 | 0 | 0 | 0 | 0 | 0 | 0 | 0 | 457 | 471 |  |  | PEAKS DB |
| R.YDAM(+15.99)ALGNHEFDNGLNGLLDPLLK.N | N | 89.01 | 2645.2795 | 24 | 0.3 | 882.7674 | 3 | 88.88 | 6 | F6:61975 | NaSa01\_F14.raw |  |  |  |  | 1.9977E7 | 6.8848E7 |  |  |  |  |  |  |  |  | 3 | 0 | 0 | 0 | 0 | 1 | 2 | 0 | 0 | 0 | 0 | 0 | 0 | 0 | 0 | 124 | 147 | Oxidation (M) | M4:Oxidation (M):1000.00 | PEAKS DB |
| R.HPDDNEWNHVSM(+15.99)C(+57.02)IVNGGGIR.S | N | 86.62 | 2422.0542 | 21 | -0.3 | 808.3584 | 3 | 44.12 | 6 | F6:24148 | NaSa01\_F14.raw |  |  |  |  | 3.2766E7 | 9.9954E7 |  |  |  |  |  |  |  | 8.8723E6 | 8 | 0 | 0 | 0 | 0 | 2 | 4 | 0 | 0 | 0 | 0 | 0 | 0 | 0 | 2 | 389 | 409 | Oxidation (M); Carbamidomethylation | M12:Oxidation (M):1000.00;C13:Carbamidomethylation:1000.00 | PEAKS DB |
| K.VLLPSFLAAGGDGYYMLK.G | N | 84.56 | 1913.9961 | 18 | 1.4 | 639.0068 | 3 | 94.26 | 6 | F6:66829 | NaSa01\_F14.raw |  |  |  | 4.47E5 | 9.4519E7 | 6.4928E8 |  |  |  |  |  |  |  |  | 12 | 0 | 0 | 0 | 1 | 4 | 7 | 0 | 0 | 0 | 0 | 0 | 0 | 0 | 0 | 510 | 527 |  |  | PEAKS DB |
| K.ASGNPILLNKSIQEDPAVK.A | N | 83.17 | 1993.0844 | 19 | 0.6 | 665.3691 | 3 | 35.69 | 6 | F6:17128 | NaSa01\_F14.raw |  |  |  |  | 1.5436E6 | 2.8654E6 |  |  |  |  |  |  |  |  | 2 | 0 | 0 | 0 | 0 | 1 | 1 | 0 | 0 | 0 | 0 | 0 | 0 | 0 | 0 | 317 | 335 |  |  | PEAKS DB |
| K.VLLPSFLAAGGDGYYM(+15.99)LK.G | N | 82.89 | 1929.9910 | 18 | 0.7 | 966.0034 | 2 | 87.45 | 6 | F6:60748 | NaSa01\_F14.raw |  |  |  |  | 2.8899E7 | 1.2442E8 |  |  |  |  |  |  |  |  | 10 | 0 | 0 | 0 | 0 | 3 | 7 | 0 | 0 | 0 | 0 | 0 | 0 | 0 | 0 | 510 | 527 | Oxidation (M) | M16:Oxidation (M):1000.00 | PEAKS DB |
| R.FHEC(+57.02)NLGNLIC(+57.02)DAVVYNNLR.H | N | 82.50 | 2420.1365 | 20 | -0.2 | 807.7192 | 3 | 77.73 | 6 | F6:52912 | NaSa01\_F14.raw |  |  |  |  | 2.6877E7 | 9.4785E7 |  |  |  |  |  |  | 7.773E6 | 1.2217E8 | 9 | 0 | 0 | 0 | 0 | 1 | 3 | 0 | 0 | 0 | 0 | 0 | 0 | 2 | 3 | 369 | 388 | Carbamidomethylation | C4:Carbamidomethylation:1000.00;C11:Carbamidomethylation:1000.00 | PEAKS DB |
| K.ETPVLSNPGPYLEFR.D | N | 81.49 | 1717.8674 | 15 | 0.8 | 859.9417 | 2 | 69.77 | 6 | F6:46166 | NaSa01\_F14.raw |  |  |  | 7.2199E5 | 2.4678E8 | 7.6435E8 |  |  |  | 2.2582E6 |  |  |  |  | 7 | 0 | 0 | 0 | 1 | 3 | 2 | 0 | 0 | 0 | 1 | 0 | 0 | 0 | 0 | 194 | 208 |  |  | PEAKS DB |
| R.QVPVVQAYAFGK.Y | N | 80.72 | 1305.7081 | 12 | -0.2 | 653.8612 | 2 | 81.01 | 6 | F6:55346 | NaSa01\_F14.raw |  |  |  | 4.7562E5 | 3.326E8 | 6.8066E8 |  |  |  |  |  |  |  |  | 5 | 0 | 0 | 0 | 1 | 1 | 3 | 0 | 0 | 0 | 0 | 0 | 0 | 0 | 0 | 288 | 299 |  |  | PEAKS DB |
| R.EVVHFMNSLR.Y | N | 80.17 | 1230.6179 | 10 | 0.3 | 616.3164 | 2 | 32.92 | 6 | F6:14493 | NaSa01\_F14.raw |  |  |  |  | 2.3691E8 | 4.1556E8 |  |  |  |  |  |  |  |  | 11 | 0 | 0 | 0 | 0 | 3 | 8 | 0 | 0 | 0 | 0 | 0 | 0 | 0 | 0 | 114 | 123 |  |  | PEAKS DB |
| R.VVSLNVLC(+57.02)TEC(+57.02)R.V | N | 79.36 | 1448.7115 | 12 | 0.2 | 725.3632 | 2 | 49.71 | 6 | F6:29319 | NaSa01\_F14.raw |  |  |  | 0 | 2.7472E8 | 5.8042E8 |  |  |  |  |  | 8.8246E5 |  |  | 7 | 0 | 0 | 0 | 0 | 2 | 4 | 0 | 0 | 0 | 0 | 0 | 1 | 0 | 0 | 484 | 495 | Carbamidomethylation | C8:Carbamidomethylation:1000.00;C11:Carbamidomethylation:1000.00 | PEAKS DB |
| R.YDAMALGNHEFDNGLNGLLDPLLK.N | N | 78.14 | 2629.2847 | 24 | 0.8 | 877.4362 | 3 | 94.34 | 6 | F6:67114 | NaSa01\_F14.raw |  |  |  |  | 1.6292E7 | 1.1665E8 |  |  |  |  |  |  |  |  | 4 | 0 | 0 | 0 | 0 | 2 | 2 | 0 | 0 | 0 | 0 | 0 | 0 | 0 | 0 | 124 | 147 |  |  | PEAKS DB |
| K.YLGYLNVIFDDK.G | N | 76.79 | 1458.7394 | 12 | 0.2 | 730.3771 | 2 | 85.89 | 6 | F6:59730 | NaSa01\_F14.raw |  |  |  |  | 6.7598E7 | 9.6452E7 |  |  |  |  |  |  |  |  | 5 | 0 | 0 | 0 | 0 | 4 | 1 | 0 | 0 | 0 | 0 | 0 | 0 | 0 | 0 | 300 | 311 |  |  | PEAKS DB |
| K.LTILHTNDVHAR.V | N | 75.00 | 1388.7524 | 12 | 0.9 | 463.9252 | 3 | 11.51 | 6 | F6:1905 | NaSa01\_F14.raw |  |  |  |  | 1.6389E6 | 3.6507E6 |  |  |  |  |  |  |  |  | 4 | 0 | 0 | 0 | 0 | 2 | 2 | 0 | 0 | 0 | 0 | 0 | 0 | 0 | 0 | 44 | 55 |  |  | PEAKS DB |
| K.ETPVLSNPGPYLEFRDEVEELQK.Q | N | 74.67 | 2688.3281 | 23 | 0.7 | 897.1173 | 3 | 77.38 | 6 | F6:52291 | NaSa01\_F14.raw |  |  |  |  | 3.5808E8 | 6.9841E8 |  |  |  |  |  |  |  |  | 4 | 0 | 0 | 0 | 0 | 2 | 2 | 0 | 0 | 0 | 0 | 0 | 0 | 0 | 0 | 194 | 216 |  |  | PEAKS DB |
| K.ASGNPILLNK.S | N | 69.82 | 1025.5869 | 10 | -0.4 | 513.8005 | 2 | 11.59 | 6 | F6:1963 | NaSa01\_F14.raw |  |  |  |  | 2.4579E6 | 4.7925E6 |  |  |  |  |  |  |  |  | 3 | 0 | 0 | 0 | 0 | 1 | 2 | 0 | 0 | 0 | 0 | 0 | 0 | 0 | 0 | 317 | 326 |  |  | PEAKS DB |
| S.FLAAGGDGYYMLK.G | N | 67.12 | 1404.6747 | 13 | 0.2 | 703.3447 | 2 | 58.91 | 6 | F6:37338 | NaSa01\_F14.raw |  |  |  |  | 1.8909E7 | 8.0208E7 |  |  |  |  |  |  |  |  | 3 | 0 | 0 | 0 | 0 | 1 | 2 | 0 | 0 | 0 | 0 | 0 | 0 | 0 | 0 | 515 | 527 |  |  | PEAKS DB |
| F.LAAGGDGYYMLK.G | N | 66.41 | 1257.6063 | 12 | 0.2 | 629.8105 | 2 | 41.73 | 6 | F6:22051 | NaSa01\_F14.raw |  |  |  |  |  | 5.7329E6 |  |  |  |  |  |  |  |  | 1 | 0 | 0 | 0 | 0 | 0 | 1 | 0 | 0 | 0 | 0 | 0 | 0 | 0 | 0 | 516 | 527 |  |  | PEAKS DB |
| R.EVVHFM(+15.99)NSLR.Y | N | 65.78 | 1246.6128 | 10 | 0.3 | 624.3138 | 2 | 31.71 | 6 | F6:14277 | NaSa01\_F14.raw |  |  |  |  | 3.2821E6 | 5.0137E7 |  |  |  |  |  |  |  |  | 13 | 0 | 0 | 0 | 0 | 2 | 11 | 0 | 0 | 0 | 0 | 0 | 0 | 0 | 0 | 114 | 123 | Oxidation (M) | M6:Oxidation (M):1000.00 | PEAKS DB |
| K.FPILSANIRPK.G | N | 63.57 | 1254.7448 | 11 | 1.8 | 419.2563 | 3 | 38.83 | 6 | F6:20180 | NaSa01\_F14.raw |  |  |  | 3.6184E5 | 9.4886E7 | 1.2559E8 |  |  |  |  | 2.1098E6 |  |  |  | 10 | 0 | 0 | 0 | 1 | 2 | 5 | 0 | 0 | 0 | 0 | 2 | 0 | 0 | 0 | 151 | 161 |  |  | PEAKS DB |
| K.RVVSLNVLC(+57.02)TEC(+57.02)R.V | N | 62.55 | 1604.8126 | 13 | 0.2 | 535.9449 | 3 | 35.03 | 6 | F6:16539 | NaSa01\_F14.raw |  |  |  |  | 4.4155E6 | 6.0208E6 |  |  |  |  |  |  |  |  | 3 | 0 | 0 | 0 | 0 | 1 | 2 | 0 | 0 | 0 | 0 | 0 | 0 | 0 | 0 | 483 | 495 | Carbamidomethylation | C9:Carbamidomethylation:1000.00;C12:Carbamidomethylation:1000.00 | PEAKS DB |
| K.GVDVVVGGHTNTF.L | N | 61.79 | 1300.6411 | 13 | 1.1 | 651.3286 | 2 | 41.80 | 6 | F6:22128 | NaSa01\_F14.raw |  |  |  |  | 2.249E7 | 5.2209E7 |  |  |  |  |  |  |  |  | 2 | 0 | 0 | 0 | 0 | 1 | 1 | 0 | 0 | 0 | 0 | 0 | 0 | 0 | 0 | 249 | 261 |  |  | PEAKS DB |
| L.TAVLPFGGTFDLLQIK.G | N | 60.64 | 1718.9607 | 16 | -0.1 | 860.4875 | 2 | 99.74 | 6 | F6:71142 | NaSa01\_F14.raw |  |  | 2.8271E6 | 1.2518E6 | 2.1013E7 | 4.0691E6 |  |  |  |  |  |  |  |  | 4 | 0 | 0 | 1 | 1 | 1 | 1 | 0 | 0 | 0 | 0 | 0 | 0 | 0 | 0 | 427 | 442 |  |  | PEAKS DB |
| K.IINVGSEKVGIIGYTTK.E | N | 60.09 | 1791.0142 | 17 | -0.7 | 598.0116 | 3 | 45.32 | 6 | F6:25348 | NaSa01\_F14.raw |  |  |  |  | 9.4386E5 | 1.7014E6 |  |  |  |  |  |  |  |  | 2 | 0 | 0 | 0 | 0 | 1 | 1 | 0 | 0 | 0 | 0 | 0 | 0 | 0 | 0 | 177 | 193 |  |  | PEAKS DB |
| K.YLGYLNVIFDDKGK.V | Y | 58.45 | 1643.8558 | 14 | -0.3 | 548.9590 | 3 | 70.19 | 5 | F5:46798 | NaSa01\_F13.raw |  |  |  |  | 1.8927E6 |  |  |  |  |  |  |  |  |  | 2 | 0 | 0 | 0 | 0 | 2 | 0 | 0 | 0 | 0 | 0 | 0 | 0 | 0 | 0 | 300 | 313 |  |  | PEAKS DB |
| K.VFPAMEGR.V | N | 56.33 | 905.4429 | 8 | 0.2 | 453.7288 | 2 | 22.33 | 6 | F6:7722 | NaSa01\_F14.raw |  |  |  |  |  | 3.6244E6 |  |  |  |  |  |  |  |  | 2 | 0 | 0 | 0 | 0 | 0 | 2 | 0 | 0 | 0 | 0 | 0 | 0 | 0 | 0 | 553 | 560 |  |  | PEAKS DB |
| N.ISGYILPYK.I | N | 56.17 | 1052.5906 | 9 | 0.7 | 527.3029 | 2 | 45.82 | 6 | F6:26096 | NaSa01\_F14.raw |  |  |  | 2.2592E5 | 4.6763E8 | 8.5839E8 |  |  |  |  |  |  |  |  | 5 | 0 | 0 | 0 | 1 | 1 | 3 | 0 | 0 | 0 | 0 | 0 | 0 | 0 | 0 | 168 | 176 |  |  | PEAKS DB |
| S.FLAAGGDGYYM(+15.99)LK.G | N | 55.62 | 1420.6697 | 13 | -0.1 | 711.3420 | 2 | 48.69 | 6 | F6:28173 | NaSa01\_F14.raw |  |  |  |  |  | 5.7308E6 |  |  |  |  |  |  |  |  | 1 | 0 | 0 | 0 | 0 | 0 | 1 | 0 | 0 | 0 | 0 | 0 | 0 | 0 | 0 | 515 | 527 | Oxidation (M) | M11:Oxidation (M):1000.00 | PEAKS DB |
| H.GQGTGELLQVSGIK.V | N | 55.06 | 1385.7515 | 14 | 0.3 | 693.8832 | 2 | 45.20 | 6 | F6:25108 | NaSa01\_F14.raw |  |  |  |  |  | 1.2594E6 |  |  |  |  |  |  |  |  | 1 | 0 | 0 | 0 | 0 | 0 | 1 | 0 | 0 | 0 | 0 | 0 | 0 | 0 | 0 | 458 | 471 |  |  | PEAKS DB |
| K.GVDVVVGGHTNTFLY.T | N | 54.75 | 1576.7886 | 15 | 0.1 | 789.4016 | 2 | 63.80 | 6 | F6:41327 | NaSa01\_F14.raw |  |  |  |  | 1.2739E7 | 2.8201E7 |  |  |  |  |  |  |  |  | 2 | 0 | 0 | 0 | 0 | 1 | 1 | 0 | 0 | 0 | 0 | 0 | 0 | 0 | 0 | 249 | 263 |  |  | PEAKS DB |
| R.QVPVVQAYAF.G | N | 53.91 | 1120.5917 | 10 | 0.0 | 561.3031 | 2 | 73.70 | 6 | F6:49221 | NaSa01\_F14.raw |  |  |  |  | 7.1015E6 | 1.9997E7 |  |  |  |  |  |  |  |  | 2 | 0 | 0 | 0 | 0 | 1 | 1 | 0 | 0 | 0 | 0 | 0 | 0 | 0 | 0 | 288 | 297 |  |  | PEAKS DB |
| M.ALGNHEFDNGLNGLLDPLLK.N | N | 53.45 | 2149.1167 | 20 | 0.0 | 717.3795 | 3 | 83.56 | 6 | F6:57342 | NaSa01\_F14.raw |  |  |  |  | 5.3244E6 | 2.0787E7 |  |  |  |  |  |  |  |  | 3 | 0 | 0 | 0 | 0 | 1 | 2 | 0 | 0 | 0 | 0 | 0 | 0 | 0 | 0 | 128 | 147 |  |  | PEAKS DB |
| K.LTTLGVNK.I | N | 52.68 | 844.5018 | 8 | -1.0 | 423.2578 | 2 | 11.58 | 6 | F6:2148 | NaSa01\_F14.raw |  |  |  |  |  | 3.1326E6 |  |  |  |  |  |  |  |  | 1 | 0 | 0 | 0 | 0 | 0 | 1 | 0 | 0 | 0 | 0 | 0 | 0 | 0 | 0 | 221 | 228 |  |  | PEAKS DB |
| K.VLLPSFLAAGGDGY.Y | N | 51.68 | 1378.7133 | 14 | 0.7 | 690.3644 | 2 | 100.88 | 6 | F6:72024 | NaSa01\_F14.raw |  |  |  |  | 1.3644E6 | 2.8748E6 |  |  |  |  |  |  |  |  | 2 | 0 | 0 | 0 | 0 | 1 | 1 | 0 | 0 | 0 | 0 | 0 | 0 | 0 | 0 | 510 | 523 |  |  | PEAKS DB |
| F.GGTFDLLQIK.G | N | 50.44 | 1090.6022 | 10 | -0.6 | 546.3080 | 2 | 64.67 | 6 | F6:41967 | NaSa01\_F14.raw |  |  |  |  |  | 9.7795E7 |  |  |  |  |  |  |  |  | 2 | 0 | 0 | 0 | 0 | 0 | 2 | 0 | 0 | 0 | 0 | 0 | 0 | 0 | 0 | 433 | 442 |  |  | PEAKS DB |
| Y.LNVIFDDK.G | N | 50.15 | 962.5073 | 8 | -0.5 | 482.2607 | 2 | 40.33 | 6 | F6:21131 | NaSa01\_F14.raw |  |  |  |  |  | 1.4605E6 |  |  |  |  |  |  |  |  | 1 | 0 | 0 | 0 | 0 | 0 | 1 | 0 | 0 | 0 | 0 | 0 | 0 | 0 | 0 | 304 | 311 |  |  | PEAKS DB |
| K.VGIIGYTTK.E | N | 50.04 | 950.5436 | 9 | 0.3 | 476.2792 | 2 | 28.48 | 6 | F6:11194 | NaSa01\_F14.raw |  |  |  |  |  | 5.1319E6 |  |  |  |  |  |  |  |  | 2 | 0 | 0 | 0 | 0 | 0 | 2 | 0 | 0 | 0 | 0 | 0 | 0 | 0 | 0 | 185 | 193 |  |  | PEAKS DB |
| L.AAGGDGYYMLK.G | N | 49.71 | 1144.5222 | 11 | 0.7 | 573.2688 | 2 | 30.46 | 5 | F5:13468 | NaSa01\_F13.raw |  |  |  |  | 6.4456E6 |  |  |  |  |  |  |  |  |  | 1 | 0 | 0 | 0 | 0 | 1 | 0 | 0 | 0 | 0 | 0 | 0 | 0 | 0 | 0 | 517 | 527 |  |  | PEAKS DB |
| R.FHEC(+57.02)NLGNLIC(+57.02)DAVVY.N | N | 48.35 | 1922.8655 | 16 | 1.4 | 962.4414 | 2 | 82.45 | 5 | F5:56634 | NaSa01\_F13.raw |  |  |  |  | 8.0322E7 | 1.7562E8 |  |  |  |  |  |  |  | 2.4692E7 | 3 | 0 | 0 | 0 | 0 | 1 | 1 | 0 | 0 | 0 | 0 | 0 | 0 | 0 | 1 | 369 | 384 | Carbamidomethylation | C4:Carbamidomethylation:1000.00;C11:Carbamidomethylation:1000.00 | PEAKS DB |
| R.HPDDNEWNHVSMC(+57.02).I | N | 48.19 | 1639.6144 | 13 | 0.5 | 820.8149 | 2 | 26.12 | 5 | F5:10348 | NaSa01\_F13.raw |  |  |  |  | 8.8799E6 |  |  |  |  |  |  |  |  |  | 2 | 0 | 0 | 0 | 0 | 2 | 0 | 0 | 0 | 0 | 0 | 0 | 0 | 0 | 0 | 389 | 401 | Carbamidomethylation | C13:Carbamidomethylation:1000.00 | PEAKS DB |
| K.VLLPSFLAAGGD.G | N | 47.35 | 1158.6284 | 12 | -0.1 | 580.3214 | 2 | 96.95 | 6 | F6:68512 | NaSa01\_F14.raw |  |  |  |  |  | 1.1427E6 |  |  |  |  |  |  |  |  | 1 | 0 | 0 | 0 | 0 | 0 | 1 | 0 | 0 | 0 | 0 | 0 | 0 | 0 | 0 | 510 | 521 |  |  | PEAKS DB |
| R.HPDDNEWNHVSM.C | N | 47.22 | 1479.5837 | 12 | 1.1 | 740.8000 | 2 | 25.93 | 5 | F5:10070 | NaSa01\_F13.raw |  |  |  |  | 2.3098E6 |  |  |  |  |  |  |  |  |  | 1 | 0 | 0 | 0 | 0 | 1 | 0 | 0 | 0 | 0 | 0 | 0 | 0 | 0 | 0 | 389 | 400 |  |  | PEAKS DB |
| R.QVPVVQAY.A | N | 45.65 | 902.4861 | 8 | -0.5 | 452.2501 | 2 | 43.38 | 5 | F5:24541 | NaSa01\_F13.raw |  |  |  |  | 1.2052E7 |  |  |  |  |  |  |  |  |  | 1 | 0 | 0 | 0 | 0 | 1 | 0 | 0 | 0 | 0 | 0 | 0 | 0 | 0 | 0 | 288 | 295 |  |  | PEAKS DB |
| N.PGPYLEFR.D | N | 45.44 | 977.4970 | 8 | 0.7 | 489.7561 | 2 | 39.90 | 6 | F6:20609 | NaSa01\_F14.raw |  |  |  |  |  | 4.6995E5 |  |  |  |  |  |  |  |  | 1 | 0 | 0 | 0 | 0 | 0 | 1 | 0 | 0 | 0 | 0 | 0 | 0 | 0 | 0 | 201 | 208 |  |  | PEAKS DB |
| N.LGNLIC(+57.02)DAVVYNNLR.H | N | 45.20 | 1732.8931 | 15 | 1.0 | 867.4547 | 2 | 69.10 | 6 | F6:45743 | NaSa01\_F14.raw |  |  |  |  |  | 1.1316E6 |  |  |  |  |  |  |  |  | 1 | 0 | 0 | 0 | 0 | 0 | 1 | 0 | 0 | 0 | 0 | 0 | 0 | 0 | 0 | 374 | 388 | Carbamidomethylation | C6:Carbamidomethylation:1000.00 | PEAKS DB |
| K.IINVGSEK.V | N | 44.70 | 858.4811 | 8 | -0.5 | 430.2476 | 2 | 11.48 | 6 | F6:1861 | NaSa01\_F14.raw |  |  |  |  |  | 1.3857E6 |  |  |  |  |  |  |  |  | 1 | 0 | 0 | 0 | 0 | 0 | 1 | 0 | 0 | 0 | 0 | 0 | 0 | 0 | 0 | 177 | 184 |  |  | PEAKS DB |
| K.VLLPSFLAAGGDGYY.M | N | 43.81 | 1541.7765 | 15 | 0.7 | 771.8961 | 2 | 105.28 | 6 | F6:75496 | NaSa01\_F14.raw |  |  |  |  | 1.4762E7 | 3.1893E7 |  |  |  |  |  |  |  |  | 2 | 0 | 0 | 0 | 0 | 1 | 1 | 0 | 0 | 0 | 0 | 0 | 0 | 0 | 0 | 510 | 524 |  |  | PEAKS DB |
| K.FPILSANIR.P | N | 43.38 | 1029.5970 | 9 | -0.4 | 515.8056 | 2 | 52.72 | 6 | F6:31843 | NaSa01\_F14.raw |  |  |  |  |  | 1.5657E8 |  |  |  |  |  |  |  |  | 1 | 0 | 0 | 0 | 0 | 0 | 1 | 0 | 0 | 0 | 0 | 0 | 0 | 0 | 0 | 151 | 159 |  |  | PEAKS DB |
| K.VLLPSFLAAGGDGYYM.L | N | 42.72 | 1672.8170 | 16 | 0.9 | 837.4166 | 2 | 111.51 | 6 | F6:80284 | NaSa01\_F14.raw |  |  |  |  |  | 1.1099E6 |  |  |  |  |  |  |  |  | 1 | 0 | 0 | 0 | 0 | 0 | 1 | 0 | 0 | 0 | 0 | 0 | 0 | 0 | 0 | 510 | 525 |  |  | PEAKS DB |
| Q.GTGELLQVSGIK.V | N | 42.14 | 1200.6714 | 12 | 0.9 | 601.3435 | 2 | 44.43 | 6 | F6:24568 | NaSa01\_F14.raw |  |  |  |  |  | 7.0353E5 |  |  |  |  |  |  |  |  | 1 | 0 | 0 | 0 | 0 | 0 | 1 | 0 | 0 | 0 | 0 | 0 | 0 | 0 | 0 | 460 | 471 |  |  | PEAKS DB |
| Y.LGYLNVIFDDK.G | N | 41.11 | 1295.6761 | 11 | 0.9 | 648.8459 | 2 | 76.55 | 6 | F6:51743 | NaSa01\_F14.raw |  |  |  |  |  | 0 |  |  |  |  |  |  |  |  | 0 | 0 | 0 | 0 | 0 | 0 | 0 | 0 | 0 | 0 | 0 | 0 | 0 | 0 | 0 | 301 | 311 |  |  | PEAKS DB |
| K.LTTLGVNKIIALGH.S | N | 41.05 | 1448.8715 | 14 | 0.1 | 483.9645 | 3 | 54.77 | 6 | F6:33352 | NaSa01\_F14.raw |  |  |  |  |  | 5.5856E5 |  |  |  |  |  |  |  |  | 1 | 0 | 0 | 0 | 0 | 0 | 1 | 0 | 0 | 0 | 0 | 0 | 0 | 0 | 0 | 221 | 234 |  |  | PEAKS DB |
| N.HEFDNGLNGLLDPLLK.N | N | 40.62 | 1793.9312 | 16 | 0.2 | 598.9844 | 3 | 81.57 | 6 | F6:55994 | NaSa01\_F14.raw |  |  |  |  |  | 5.7086E5 |  |  |  |  |  |  |  |  | 1 | 0 | 0 | 0 | 0 | 0 | 1 | 0 | 0 | 0 | 0 | 0 | 0 | 0 | 0 | 132 | 147 |  |  | PEAKS DB |
| N.YPFMQLSDDGR.Q | N | 40.57 | 1327.5867 | 11 | 0.4 | 664.8009 | 2 | 53.65 | 6 | F6:32287 | NaSa01\_F14.raw |  |  |  |  |  | 5.3395E5 |  |  |  |  |  |  |  |  | 1 | 0 | 0 | 0 | 0 | 0 | 1 | 0 | 0 | 0 | 0 | 0 | 0 | 0 | 0 | 277 | 287 |  |  | PEAKS DB |
| P.AGNYPFMQLSDDGR.Q | N | 40.07 | 1569.6881 | 14 | 0.1 | 785.8514 | 2 | 56.81 | 6 | F6:35126 | NaSa01\_F14.raw |  |  |  |  |  | 2.5054E5 |  |  |  |  |  |  |  |  | 1 | 0 | 0 | 0 | 0 | 0 | 1 | 0 | 0 | 0 | 0 | 0 | 0 | 0 | 0 | 274 | 287 |  |  | PEAKS DB |
| K.NVKFPILSAN.I | N | 39.82 | 1101.6182 | 10 | 0.5 | 551.8167 | 2 | 54.61 | 6 | F6:33186 | NaSa01\_F14.raw |  |  |  |  |  | 1.3385E6 |  |  |  |  |  |  |  |  | 1 | 0 | 0 | 0 | 0 | 0 | 1 | 0 | 0 | 0 | 0 | 0 | 0 | 0 | 0 | 148 | 157 |  |  | PEAKS DB |
| K.IIALGHSGF.K | N | 37.88 | 913.5021 | 9 | -1.2 | 457.7578 | 2 | 43.37 | 6 | F6:23538 | NaSa01\_F14.raw |  |  |  |  |  | 5.7946E6 |  |  |  |  |  |  |  |  | 1 | 0 | 0 | 0 | 0 | 0 | 1 | 0 | 0 | 0 | 0 | 0 | 0 | 0 | 0 | 229 | 237 |  |  | PEAKS DB |
| total 58 peptides |  |  |  |  |  |  |  |  |  |  |  |  |  |  |  |  |  |  |  |  |  |  |  |  |  |  |  |  |  |  |  |  |  |  |  |  |  |  |  |  |  |  |  |  |  |
| --- | --- | --- | --- | --- | --- | --- | --- | --- | --- | --- | --- | --- | --- | --- | --- | --- | --- | --- | --- | --- | --- | --- | --- | --- | --- | --- | --- | --- | --- | --- | --- | --- | --- | --- | --- | --- | --- | --- | --- | --- | --- | --- | --- | --- | --- |

JAI09048.1

back to list

  

| Protein Coverage
| Supporting Peptides
|

Protein Coverage:

Supporting Peptides:

| Peptide | Uniq | -10lgP | Mass | Length | ppm | m/z | z | RT | Fraction | Scan | Source File | Area F1 | Area F10 | Area F11 | Area F12 | Area F13 | Area F14 | Area F2 | Area F3 | Area F4 | Area F5 | Area F6 | Area F7 | Area F8 | Area F9 | #Feature | #Feature F1 | #Feature F10 | #Feature F11 | #Feature F12 | #Feature F13 | #Feature F14 | #Feature F2 | #Feature F3 | #Feature F4 | #Feature F5 | #Feature F6 | #Feature F7 | #Feature F8 | #Feature F9 | Start | End | PTM | AScore | Found By |
| --- | --- | --- | --- | --- | --- | --- | --- | --- | --- | --- | --- | --- | --- | --- | --- | --- | --- | --- | --- | --- | --- | --- | --- | --- | --- | --- | --- | --- | --- | --- | --- | --- | --- | --- | --- | --- | --- | --- | --- | --- | --- | --- | --- | --- | --- |
| R.HPDDNEWNHVSMC(+57.02)IVNGGGIR.S | N | 99.67 | 2406.0593 | 21 | -0.4 | 803.0267 | 3 | 49.93 | 6 | F6:29280 | NaSa01\_F14.raw |  |  |  |  | 6.5991E7 | 1.6043E8 |  |  |  |  |  |  |  | 7.6096E6 | 9 | 0 | 0 | 0 | 0 | 3 | 4 | 0 | 0 | 0 | 0 | 0 | 0 | 0 | 2 | 389 | 409 | Carbamidomethylation | C13:Carbamidomethylation:1000.00 | PEAKS DB |
| R.HGQGTGELLQVSGIK.V | N | 97.61 | 1522.8103 | 15 | 0.4 | 762.4127 | 2 | 31.05 | 6 | F6:13489 | NaSa01\_F14.raw |  |  |  | 4.9661E5 | 6.4612E8 | 1.2796E9 |  |  |  |  |  |  |  |  | 8 | 0 | 0 | 0 | 1 | 3 | 4 | 0 | 0 | 0 | 0 | 0 | 0 | 0 | 0 | 457 | 471 |  |  | PEAKS DB |
| R.YDAM(+15.99)ALGNHEFDNGLNGLLDPLLK.N | N | 89.01 | 2645.2795 | 24 | 0.3 | 882.7674 | 3 | 88.88 | 6 | F6:61975 | NaSa01\_F14.raw |  |  |  |  | 1.9977E7 | 6.8848E7 |  |  |  |  |  |  |  |  | 3 | 0 | 0 | 0 | 0 | 1 | 2 | 0 | 0 | 0 | 0 | 0 | 0 | 0 | 0 | 124 | 147 | Oxidation (M) | M4:Oxidation (M):1000.00 | PEAKS DB |
| R.HPDDNEWNHVSM(+15.99)C(+57.02)IVNGGGIR.S | N | 86.62 | 2422.0542 | 21 | -0.3 | 808.3584 | 3 | 44.12 | 6 | F6:24148 | NaSa01\_F14.raw |  |  |  |  | 3.2766E7 | 9.9954E7 |  |  |  |  |  |  |  | 8.8723E6 | 8 | 0 | 0 | 0 | 0 | 2 | 4 | 0 | 0 | 0 | 0 | 0 | 0 | 0 | 2 | 389 | 409 | Oxidation (M); Carbamidomethylation | M12:Oxidation (M):1000.00;C13:Carbamidomethylation:1000.00 | PEAKS DB |
| K.VLLPSFLAAGGDGYYMLK.G | N | 84.56 | 1913.9961 | 18 | 1.4 | 639.0068 | 3 | 94.26 | 6 | F6:66829 | NaSa01\_F14.raw |  |  |  | 4.47E5 | 9.4519E7 | 6.4928E8 |  |  |  |  |  |  |  |  | 12 | 0 | 0 | 0 | 1 | 4 | 7 | 0 | 0 | 0 | 0 | 0 | 0 | 0 | 0 | 510 | 527 |  |  | PEAKS DB |
| K.ASGNPILLNKSIQEDPAVK.A | N | 83.17 | 1993.0844 | 19 | 0.6 | 665.3691 | 3 | 35.69 | 6 | F6:17128 | NaSa01\_F14.raw |  |  |  |  | 1.5436E6 | 2.8654E6 |  |  |  |  |  |  |  |  | 2 | 0 | 0 | 0 | 0 | 1 | 1 | 0 | 0 | 0 | 0 | 0 | 0 | 0 | 0 | 317 | 335 |  |  | PEAKS DB |
| K.VLLPSFLAAGGDGYYM(+15.99)LK.G | N | 82.89 | 1929.9910 | 18 | 0.7 | 966.0034 | 2 | 87.45 | 6 | F6:60748 | NaSa01\_F14.raw |  |  |  |  | 2.8899E7 | 1.2442E8 |  |  |  |  |  |  |  |  | 10 | 0 | 0 | 0 | 0 | 3 | 7 | 0 | 0 | 0 | 0 | 0 | 0 | 0 | 0 | 510 | 527 | Oxidation (M) | M16:Oxidation (M):1000.00 | PEAKS DB |
| R.FHEC(+57.02)NLGNLIC(+57.02)DAVVYNNLR.H | N | 82.50 | 2420.1365 | 20 | -0.2 | 807.7192 | 3 | 77.73 | 6 | F6:52912 | NaSa01\_F14.raw |  |  |  |  | 2.6877E7 | 9.4785E7 |  |  |  |  |  |  | 7.773E6 | 1.2217E8 | 9 | 0 | 0 | 0 | 0 | 1 | 3 | 0 | 0 | 0 | 0 | 0 | 0 | 2 | 3 | 369 | 388 | Carbamidomethylation | C4:Carbamidomethylation:1000.00;C11:Carbamidomethylation:1000.00 | PEAKS DB |
| K.ETPVLSNPGPYLEFR.D | N | 81.49 | 1717.8674 | 15 | 0.8 | 859.9417 | 2 | 69.77 | 6 | F6:46166 | NaSa01\_F14.raw |  |  |  | 7.2199E5 | 2.4678E8 | 7.6435E8 |  |  |  | 2.2582E6 |  |  |  |  | 7 | 0 | 0 | 0 | 1 | 3 | 2 | 0 | 0 | 0 | 1 | 0 | 0 | 0 | 0 | 194 | 208 |  |  | PEAKS DB |
| R.QVPVVQAYAFGK.Y | N | 80.72 | 1305.7081 | 12 | -0.2 | 653.8612 | 2 | 81.01 | 6 | F6:55346 | NaSa01\_F14.raw |  |  |  | 4.7562E5 | 3.326E8 | 6.8066E8 |  |  |  |  |  |  |  |  | 5 | 0 | 0 | 0 | 1 | 1 | 3 | 0 | 0 | 0 | 0 | 0 | 0 | 0 | 0 | 288 | 299 |  |  | PEAKS DB |
| R.EVVHFMNSLR.Y | N | 80.17 | 1230.6179 | 10 | 0.3 | 616.3164 | 2 | 32.92 | 6 | F6:14493 | NaSa01\_F14.raw |  |  |  |  | 2.3691E8 | 4.1556E8 |  |  |  |  |  |  |  |  | 11 | 0 | 0 | 0 | 0 | 3 | 8 | 0 | 0 | 0 | 0 | 0 | 0 | 0 | 0 | 114 | 123 |  |  | PEAKS DB |
| R.VVSLNVLC(+57.02)TEC(+57.02)R.V | N | 79.36 | 1448.7115 | 12 | 0.2 | 725.3632 | 2 | 49.71 | 6 | F6:29319 | NaSa01\_F14.raw |  |  |  | 0 | 2.7472E8 | 5.8042E8 |  |  |  |  |  | 8.8246E5 |  |  | 7 | 0 | 0 | 0 | 0 | 2 | 4 | 0 | 0 | 0 | 0 | 0 | 1 | 0 | 0 | 484 | 495 | Carbamidomethylation | C8:Carbamidomethylation:1000.00;C11:Carbamidomethylation:1000.00 | PEAKS DB |
| R.YDAMALGNHEFDNGLNGLLDPLLK.N | N | 78.14 | 2629.2847 | 24 | 0.8 | 877.4362 | 3 | 94.34 | 6 | F6:67114 | NaSa01\_F14.raw |  |  |  |  | 1.6292E7 | 1.1665E8 |  |  |  |  |  |  |  |  | 4 | 0 | 0 | 0 | 0 | 2 | 2 | 0 | 0 | 0 | 0 | 0 | 0 | 0 | 0 | 124 | 147 |  |  | PEAKS DB |
| K.YLGYLNVIFDDK.G | N | 76.79 | 1458.7394 | 12 | 0.2 | 730.3771 | 2 | 85.89 | 6 | F6:59730 | NaSa01\_F14.raw |  |  |  |  | 6.7598E7 | 9.6452E7 |  |  |  |  |  |  |  |  | 5 | 0 | 0 | 0 | 0 | 4 | 1 | 0 | 0 | 0 | 0 | 0 | 0 | 0 | 0 | 300 | 311 |  |  | PEAKS DB |
| K.LTILHTNDVHAR.V | N | 75.00 | 1388.7524 | 12 | 0.9 | 463.9252 | 3 | 11.51 | 6 | F6:1905 | NaSa01\_F14.raw |  |  |  |  | 1.6389E6 | 3.6507E6 |  |  |  |  |  |  |  |  | 4 | 0 | 0 | 0 | 0 | 2 | 2 | 0 | 0 | 0 | 0 | 0 | 0 | 0 | 0 | 44 | 55 |  |  | PEAKS DB |
| K.ETPVLSNPGPYLEFRDEVEELQK.Q | N | 74.67 | 2688.3281 | 23 | 0.7 | 897.1173 | 3 | 77.38 | 6 | F6:52291 | NaSa01\_F14.raw |  |  |  |  | 3.5808E8 | 6.9841E8 |  |  |  |  |  |  |  |  | 4 | 0 | 0 | 0 | 0 | 2 | 2 | 0 | 0 | 0 | 0 | 0 | 0 | 0 | 0 | 194 | 216 |  |  | PEAKS DB |
| K.ASGNPILLNK.S | N | 69.82 | 1025.5869 | 10 | -0.4 | 513.8005 | 2 | 11.59 | 6 | F6:1963 | NaSa01\_F14.raw |  |  |  |  | 2.4579E6 | 4.7925E6 |  |  |  |  |  |  |  |  | 3 | 0 | 0 | 0 | 0 | 1 | 2 | 0 | 0 | 0 | 0 | 0 | 0 | 0 | 0 | 317 | 326 |  |  | PEAKS DB |
| S.FLAAGGDGYYMLK.G | N | 67.12 | 1404.6747 | 13 | 0.2 | 703.3447 | 2 | 58.91 | 6 | F6:37338 | NaSa01\_F14.raw |  |  |  |  | 1.8909E7 | 8.0208E7 |  |  |  |  |  |  |  |  | 3 | 0 | 0 | 0 | 0 | 1 | 2 | 0 | 0 | 0 | 0 | 0 | 0 | 0 | 0 | 515 | 527 |  |  | PEAKS DB |
| F.LAAGGDGYYMLK.G | N | 66.41 | 1257.6063 | 12 | 0.2 | 629.8105 | 2 | 41.73 | 6 | F6:22051 | NaSa01\_F14.raw |  |  |  |  |  | 5.7329E6 |  |  |  |  |  |  |  |  | 1 | 0 | 0 | 0 | 0 | 0 | 1 | 0 | 0 | 0 | 0 | 0 | 0 | 0 | 0 | 516 | 527 |  |  | PEAKS DB |
| R.EVVHFM(+15.99)NSLR.Y | N | 65.78 | 1246.6128 | 10 | 0.3 | 624.3138 | 2 | 31.71 | 6 | F6:14277 | NaSa01\_F14.raw |  |  |  |  | 3.2821E6 | 5.0137E7 |  |  |  |  |  |  |  |  | 13 | 0 | 0 | 0 | 0 | 2 | 11 | 0 | 0 | 0 | 0 | 0 | 0 | 0 | 0 | 114 | 123 | Oxidation (M) | M6:Oxidation (M):1000.00 | PEAKS DB |
| K.FPILSANIRPK.G | N | 63.57 | 1254.7448 | 11 | 1.8 | 419.2563 | 3 | 38.83 | 6 | F6:20180 | NaSa01\_F14.raw |  |  |  | 3.6184E5 | 9.4886E7 | 1.2559E8 |  |  |  |  | 2.1098E6 |  |  |  | 10 | 0 | 0 | 0 | 1 | 2 | 5 | 0 | 0 | 0 | 0 | 2 | 0 | 0 | 0 | 151 | 161 |  |  | PEAKS DB |
| K.RVVSLNVLC(+57.02)TEC(+57.02)R.V | N | 62.55 | 1604.8126 | 13 | 0.2 | 535.9449 | 3 | 35.03 | 6 | F6:16539 | NaSa01\_F14.raw |  |  |  |  | 4.4155E6 | 6.0208E6 |  |  |  |  |  |  |  |  | 3 | 0 | 0 | 0 | 0 | 1 | 2 | 0 | 0 | 0 | 0 | 0 | 0 | 0 | 0 | 483 | 495 | Carbamidomethylation | C9:Carbamidomethylation:1000.00;C12:Carbamidomethylation:1000.00 | PEAKS DB |
| K.GVDVVVGGHTNTF.L | N | 61.79 | 1300.6411 | 13 | 1.1 | 651.3286 | 2 | 41.80 | 6 | F6:22128 | NaSa01\_F14.raw |  |  |  |  | 2.249E7 | 5.2209E7 |  |  |  |  |  |  |  |  | 2 | 0 | 0 | 0 | 0 | 1 | 1 | 0 | 0 | 0 | 0 | 0 | 0 | 0 | 0 | 249 | 261 |  |  | PEAKS DB |
| L.TAVLPFGGTFDLLQIK.G | N | 60.64 | 1718.9607 | 16 | -0.1 | 860.4875 | 2 | 99.74 | 6 | F6:71142 | NaSa01\_F14.raw |  |  | 2.8271E6 | 1.2518E6 | 2.1013E7 | 4.0691E6 |  |  |  |  |  |  |  |  | 4 | 0 | 0 | 1 | 1 | 1 | 1 | 0 | 0 | 0 | 0 | 0 | 0 | 0 | 0 | 427 | 442 |  |  | PEAKS DB |
| K.IINVGSEKVGIIGYTTK.E | N | 60.09 | 1791.0142 | 17 | -0.7 | 598.0116 | 3 | 45.32 | 6 | F6:25348 | NaSa01\_F14.raw |  |  |  |  | 9.4386E5 | 1.7014E6 |  |  |  |  |  |  |  |  | 2 | 0 | 0 | 0 | 0 | 1 | 1 | 0 | 0 | 0 | 0 | 0 | 0 | 0 | 0 | 177 | 193 |  |  | PEAKS DB |
| K.YLGYLNVIFDDKGK.V | Y | 58.45 | 1643.8558 | 14 | -0.3 | 548.9590 | 3 | 70.19 | 5 | F5:46798 | NaSa01\_F13.raw |  |  |  |  | 1.8927E6 |  |  |  |  |  |  |  |  |  | 2 | 0 | 0 | 0 | 0 | 2 | 0 | 0 | 0 | 0 | 0 | 0 | 0 | 0 | 0 | 300 | 313 |  |  | PEAKS DB |
| K.VFPAMEGR.V | N | 56.33 | 905.4429 | 8 | 0.2 | 453.7288 | 2 | 22.33 | 6 | F6:7722 | NaSa01\_F14.raw |  |  |  |  |  | 3.6244E6 |  |  |  |  |  |  |  |  | 2 | 0 | 0 | 0 | 0 | 0 | 2 | 0 | 0 | 0 | 0 | 0 | 0 | 0 | 0 | 553 | 560 |  |  | PEAKS DB |
| N.ISGYILPYK.I | N | 56.17 | 1052.5906 | 9 | 0.7 | 527.3029 | 2 | 45.82 | 6 | F6:26096 | NaSa01\_F14.raw |  |  |  | 2.2592E5 | 4.6763E8 | 8.5839E8 |  |  |  |  |  |  |  |  | 5 | 0 | 0 | 0 | 1 | 1 | 3 | 0 | 0 | 0 | 0 | 0 | 0 | 0 | 0 | 168 | 176 |  |  | PEAKS DB |
| S.FLAAGGDGYYM(+15.99)LK.G | N | 55.62 | 1420.6697 | 13 | -0.1 | 711.3420 | 2 | 48.69 | 6 | F6:28173 | NaSa01\_F14.raw |  |  |  |  |  | 5.7308E6 |  |  |  |  |  |  |  |  | 1 | 0 | 0 | 0 | 0 | 0 | 1 | 0 | 0 | 0 | 0 | 0 | 0 | 0 | 0 | 515 | 527 | Oxidation (M) | M11:Oxidation (M):1000.00 | PEAKS DB |
| H.GQGTGELLQVSGIK.V | N | 55.06 | 1385.7515 | 14 | 0.3 | 693.8832 | 2 | 45.20 | 6 | F6:25108 | NaSa01\_F14.raw |  |  |  |  |  | 1.2594E6 |  |  |  |  |  |  |  |  | 1 | 0 | 0 | 0 | 0 | 0 | 1 | 0 | 0 | 0 | 0 | 0 | 0 | 0 | 0 | 458 | 471 |  |  | PEAKS DB |
| K.GVDVVVGGHTNTFLY.T | N | 54.75 | 1576.7886 | 15 | 0.1 | 789.4016 | 2 | 63.80 | 6 | F6:41327 | NaSa01\_F14.raw |  |  |  |  | 1.2739E7 | 2.8201E7 |  |  |  |  |  |  |  |  | 2 | 0 | 0 | 0 | 0 | 1 | 1 | 0 | 0 | 0 | 0 | 0 | 0 | 0 | 0 | 249 | 263 |  |  | PEAKS DB |
| R.QVPVVQAYAF.G | N | 53.91 | 1120.5917 | 10 | 0.0 | 561.3031 | 2 | 73.70 | 6 | F6:49221 | NaSa01\_F14.raw |  |  |  |  | 7.1015E6 | 1.9997E7 |  |  |  |  |  |  |  |  | 2 | 0 | 0 | 0 | 0 | 1 | 1 | 0 | 0 | 0 | 0 | 0 | 0 | 0 | 0 | 288 | 297 |  |  | PEAKS DB |
| M.ALGNHEFDNGLNGLLDPLLK.N | N | 53.45 | 2149.1167 | 20 | 0.0 | 717.3795 | 3 | 83.56 | 6 | F6:57342 | NaSa01\_F14.raw |  |  |  |  | 5.3244E6 | 2.0787E7 |  |  |  |  |  |  |  |  | 3 | 0 | 0 | 0 | 0 | 1 | 2 | 0 | 0 | 0 | 0 | 0 | 0 | 0 | 0 | 128 | 147 |  |  | PEAKS DB |
| K.LTTLGVNK.I | N | 52.68 | 844.5018 | 8 | -1.0 | 423.2578 | 2 | 11.58 | 6 | F6:2148 | NaSa01\_F14.raw |  |  |  |  |  | 3.1326E6 |  |  |  |  |  |  |  |  | 1 | 0 | 0 | 0 | 0 | 0 | 1 | 0 | 0 | 0 | 0 | 0 | 0 | 0 | 0 | 221 | 228 |  |  | PEAKS DB |
| K.VLLPSFLAAGGDGY.Y | N | 51.68 | 1378.7133 | 14 | 0.7 | 690.3644 | 2 | 100.88 | 6 | F6:72024 | NaSa01\_F14.raw |  |  |  |  | 1.3644E6 | 2.8748E6 |  |  |  |  |  |  |  |  | 2 | 0 | 0 | 0 | 0 | 1 | 1 | 0 | 0 | 0 | 0 | 0 | 0 | 0 | 0 | 510 | 523 |  |  | PEAKS DB |
| F.GGTFDLLQIK.G | N | 50.44 | 1090.6022 | 10 | -0.6 | 546.3080 | 2 | 64.67 | 6 | F6:41967 | NaSa01\_F14.raw |  |  |  |  |  | 9.7795E7 |  |  |  |  |  |  |  |  | 2 | 0 | 0 | 0 | 0 | 0 | 2 | 0 | 0 | 0 | 0 | 0 | 0 | 0 | 0 | 433 | 442 |  |  | PEAKS DB |
| Y.LNVIFDDK.G | N | 50.15 | 962.5073 | 8 | -0.5 | 482.2607 | 2 | 40.33 | 6 | F6:21131 | NaSa01\_F14.raw |  |  |  |  |  | 1.4605E6 |  |  |  |  |  |  |  |  | 1 | 0 | 0 | 0 | 0 | 0 | 1 | 0 | 0 | 0 | 0 | 0 | 0 | 0 | 0 | 304 | 311 |  |  | PEAKS DB |
| K.VGIIGYTTK.E | N | 50.04 | 950.5436 | 9 | 0.3 | 476.2792 | 2 | 28.48 | 6 | F6:11194 | NaSa01\_F14.raw |  |  |  |  |  | 5.1319E6 |  |  |  |  |  |  |  |  | 2 | 0 | 0 | 0 | 0 | 0 | 2 | 0 | 0 | 0 | 0 | 0 | 0 | 0 | 0 | 185 | 193 |  |  | PEAKS DB |
| L.AAGGDGYYMLK.G | N | 49.71 | 1144.5222 | 11 | 0.7 | 573.2688 | 2 | 30.46 | 5 | F5:13468 | NaSa01\_F13.raw |  |  |  |  | 6.4456E6 |  |  |  |  |  |  |  |  |  | 1 | 0 | 0 | 0 | 0 | 1 | 0 | 0 | 0 | 0 | 0 | 0 | 0 | 0 | 0 | 517 | 527 |  |  | PEAKS DB |
| R.FHEC(+57.02)NLGNLIC(+57.02)DAVVY.N | N | 48.35 | 1922.8655 | 16 | 1.4 | 962.4414 | 2 | 82.45 | 5 | F5:56634 | NaSa01\_F13.raw |  |  |  |  | 8.0322E7 | 1.7562E8 |  |  |  |  |  |  |  | 2.4692E7 | 3 | 0 | 0 | 0 | 0 | 1 | 1 | 0 | 0 | 0 | 0 | 0 | 0 | 0 | 1 | 369 | 384 | Carbamidomethylation | C4:Carbamidomethylation:1000.00;C11:Carbamidomethylation:1000.00 | PEAKS DB |
| R.HPDDNEWNHVSMC(+57.02).I | N | 48.19 | 1639.6144 | 13 | 0.5 | 820.8149 | 2 | 26.12 | 5 | F5:10348 | NaSa01\_F13.raw |  |  |  |  | 8.8799E6 |  |  |  |  |  |  |  |  |  | 2 | 0 | 0 | 0 | 0 | 2 | 0 | 0 | 0 | 0 | 0 | 0 | 0 | 0 | 0 | 389 | 401 | Carbamidomethylation | C13:Carbamidomethylation:1000.00 | PEAKS DB |
| K.VLLPSFLAAGGD.G | N | 47.35 | 1158.6284 | 12 | -0.1 | 580.3214 | 2 | 96.95 | 6 | F6:68512 | NaSa01\_F14.raw |  |  |  |  |  | 1.1427E6 |  |  |  |  |  |  |  |  | 1 | 0 | 0 | 0 | 0 | 0 | 1 | 0 | 0 | 0 | 0 | 0 | 0 | 0 | 0 | 510 | 521 |  |  | PEAKS DB |
| R.HPDDNEWNHVSM.C | N | 47.22 | 1479.5837 | 12 | 1.1 | 740.8000 | 2 | 25.93 | 5 | F5:10070 | NaSa01\_F13.raw |  |  |  |  | 2.3098E6 |  |  |  |  |  |  |  |  |  | 1 | 0 | 0 | 0 | 0 | 1 | 0 | 0 | 0 | 0 | 0 | 0 | 0 | 0 | 0 | 389 | 400 |  |  | PEAKS DB |
| R.QVPVVQAY.A | N | 45.65 | 902.4861 | 8 | -0.5 | 452.2501 | 2 | 43.38 | 5 | F5:24541 | NaSa01\_F13.raw |  |  |  |  | 1.2052E7 |  |  |  |  |  |  |  |  |  | 1 | 0 | 0 | 0 | 0 | 1 | 0 | 0 | 0 | 0 | 0 | 0 | 0 | 0 | 0 | 288 | 295 |  |  | PEAKS DB |
| N.PGPYLEFR.D | N | 45.44 | 977.4970 | 8 | 0.7 | 489.7561 | 2 | 39.90 | 6 | F6:20609 | NaSa01\_F14.raw |  |  |  |  |  | 4.6995E5 |  |  |  |  |  |  |  |  | 1 | 0 | 0 | 0 | 0 | 0 | 1 | 0 | 0 | 0 | 0 | 0 | 0 | 0 | 0 | 201 | 208 |  |  | PEAKS DB |
| N.LGNLIC(+57.02)DAVVYNNLR.H | N | 45.20 | 1732.8931 | 15 | 1.0 | 867.4547 | 2 | 69.10 | 6 | F6:45743 | NaSa01\_F14.raw |  |  |  |  |  | 1.1316E6 |  |  |  |  |  |  |  |  | 1 | 0 | 0 | 0 | 0 | 0 | 1 | 0 | 0 | 0 | 0 | 0 | 0 | 0 | 0 | 374 | 388 | Carbamidomethylation | C6:Carbamidomethylation:1000.00 | PEAKS DB |
| K.IINVGSEK.V | N | 44.70 | 858.4811 | 8 | -0.5 | 430.2476 | 2 | 11.48 | 6 | F6:1861 | NaSa01\_F14.raw |  |  |  |  |  | 1.3857E6 |  |  |  |  |  |  |  |  | 1 | 0 | 0 | 0 | 0 | 0 | 1 | 0 | 0 | 0 | 0 | 0 | 0 | 0 | 0 | 177 | 184 |  |  | PEAKS DB |
| K.VLLPSFLAAGGDGYY.M | N | 43.81 | 1541.7765 | 15 | 0.7 | 771.8961 | 2 | 105.28 | 6 | F6:75496 | NaSa01\_F14.raw |  |  |  |  | 1.4762E7 | 3.1893E7 |  |  |  |  |  |  |  |  | 2 | 0 | 0 | 0 | 0 | 1 | 1 | 0 | 0 | 0 | 0 | 0 | 0 | 0 | 0 | 510 | 524 |  |  | PEAKS DB |
| K.FPILSANIR.P | N | 43.38 | 1029.5970 | 9 | -0.4 | 515.8056 | 2 | 52.72 | 6 | F6:31843 | NaSa01\_F14.raw |  |  |  |  |  | 1.5657E8 |  |  |  |  |  |  |  |  | 1 | 0 | 0 | 0 | 0 | 0 | 1 | 0 | 0 | 0 | 0 | 0 | 0 | 0 | 0 | 151 | 159 |  |  | PEAKS DB |
| K.VLLPSFLAAGGDGYYM.L | N | 42.72 | 1672.8170 | 16 | 0.9 | 837.4166 | 2 | 111.51 | 6 | F6:80284 | NaSa01\_F14.raw |  |  |  |  |  | 1.1099E6 |  |  |  |  |  |  |  |  | 1 | 0 | 0 | 0 | 0 | 0 | 1 | 0 | 0 | 0 | 0 | 0 | 0 | 0 | 0 | 510 | 525 |  |  | PEAKS DB |
| Q.GTGELLQVSGIK.V | N | 42.14 | 1200.6714 | 12 | 0.9 | 601.3435 | 2 | 44.43 | 6 | F6:24568 | NaSa01\_F14.raw |  |  |  |  |  | 7.0353E5 |  |  |  |  |  |  |  |  | 1 | 0 | 0 | 0 | 0 | 0 | 1 | 0 | 0 | 0 | 0 | 0 | 0 | 0 | 0 | 460 | 471 |  |  | PEAKS DB |
| Y.LGYLNVIFDDK.G | N | 41.11 | 1295.6761 | 11 | 0.9 | 648.8459 | 2 | 76.55 | 6 | F6:51743 | NaSa01\_F14.raw |  |  |  |  |  | 0 |  |  |  |  |  |  |  |  | 0 | 0 | 0 | 0 | 0 | 0 | 0 | 0 | 0 | 0 | 0 | 0 | 0 | 0 | 0 | 301 | 311 |  |  | PEAKS DB |
| K.LTTLGVNKIIALGH.S | N | 41.05 | 1448.8715 | 14 | 0.1 | 483.9645 | 3 | 54.77 | 6 | F6:33352 | NaSa01\_F14.raw |  |  |  |  |  | 5.5856E5 |  |  |  |  |  |  |  |  | 1 | 0 | 0 | 0 | 0 | 0 | 1 | 0 | 0 | 0 | 0 | 0 | 0 | 0 | 0 | 221 | 234 |  |  | PEAKS DB |
| N.HEFDNGLNGLLDPLLK.N | N | 40.62 | 1793.9312 | 16 | 0.2 | 598.9844 | 3 | 81.57 | 6 | F6:55994 | NaSa01\_F14.raw |  |  |  |  |  | 5.7086E5 |  |  |  |  |  |  |  |  | 1 | 0 | 0 | 0 | 0 | 0 | 1 | 0 | 0 | 0 | 0 | 0 | 0 | 0 | 0 | 132 | 147 |  |  | PEAKS DB |
| N.YPFMQLSDDGR.Q | N | 40.57 | 1327.5867 | 11 | 0.4 | 664.8009 | 2 | 53.65 | 6 | F6:32287 | NaSa01\_F14.raw |  |  |  |  |  | 5.3395E5 |  |  |  |  |  |  |  |  | 1 | 0 | 0 | 0 | 0 | 0 | 1 | 0 | 0 | 0 | 0 | 0 | 0 | 0 | 0 | 277 | 287 |  |  | PEAKS DB |
| P.AGNYPFMQLSDDGR.Q | N | 40.07 | 1569.6881 | 14 | 0.1 | 785.8514 | 2 | 56.81 | 6 | F6:35126 | NaSa01\_F14.raw |  |  |  |  |  | 2.5054E5 |  |  |  |  |  |  |  |  | 1 | 0 | 0 | 0 | 0 | 0 | 1 | 0 | 0 | 0 | 0 | 0 | 0 | 0 | 0 | 274 | 287 |  |  | PEAKS DB |
| K.NVKFPILSAN.I | N | 39.82 | 1101.6182 | 10 | 0.5 | 551.8167 | 2 | 54.61 | 6 | F6:33186 | NaSa01\_F14.raw |  |  |  |  |  | 1.3385E6 |  |  |  |  |  |  |  |  | 1 | 0 | 0 | 0 | 0 | 0 | 1 | 0 | 0 | 0 | 0 | 0 | 0 | 0 | 0 | 148 | 157 |  |  | PEAKS DB |
| K.IIALGHSGF.K | N | 37.88 | 913.5021 | 9 | -1.2 | 457.7578 | 2 | 43.37 | 6 | F6:23538 | NaSa01\_F14.raw |  |  |  |  |  | 5.7946E6 |  |  |  |  |  |  |  |  | 1 | 0 | 0 | 0 | 0 | 0 | 1 | 0 | 0 | 0 | 0 | 0 | 0 | 0 | 0 | 229 | 237 |  |  | PEAKS DB |
| total 58 peptides |  |  |  |  |  |  |  |  |  |  |  |  |  |  |  |  |  |  |  |  |  |  |  |  |  |  |  |  |  |  |  |  |  |  |  |  |  |  |  |  |  |  |  |  |  |
| --- | --- | --- | --- | --- | --- | --- | --- | --- | --- | --- | --- | --- | --- | --- | --- | --- | --- | --- | --- | --- | --- | --- | --- | --- | --- | --- | --- | --- | --- | --- | --- | --- | --- | --- | --- | --- | --- | --- | --- | --- | --- | --- | --- | --- | --- |

JAI09049.1

back to list

  

| Protein Coverage
| Supporting Peptides
|

Protein Coverage:

Supporting Peptides:

| Peptide | Uniq | -10lgP | Mass | Length | ppm | m/z | z | RT | Fraction | Scan | Source File | Area F1 | Area F10 | Area F11 | Area F12 | Area F13 | Area F14 | Area F2 | Area F3 | Area F4 | Area F5 | Area F6 | Area F7 | Area F8 | Area F9 | #Feature | #Feature F1 | #Feature F10 | #Feature F11 | #Feature F12 | #Feature F13 | #Feature F14 | #Feature F2 | #Feature F3 | #Feature F4 | #Feature F5 | #Feature F6 | #Feature F7 | #Feature F8 | #Feature F9 | Start | End | PTM | AScore | Found By |
| --- | --- | --- | --- | --- | --- | --- | --- | --- | --- | --- | --- | --- | --- | --- | --- | --- | --- | --- | --- | --- | --- | --- | --- | --- | --- | --- | --- | --- | --- | --- | --- | --- | --- | --- | --- | --- | --- | --- | --- | --- | --- | --- | --- | --- | --- |
| R.HPDDNEWNHVSMC(+57.02)IVNGGGIR.S | N | 99.67 | 2406.0593 | 21 | -0.4 | 803.0267 | 3 | 49.93 | 6 | F6:29280 | NaSa01\_F14.raw |  |  |  |  | 6.5991E7 | 1.6043E8 |  |  |  |  |  |  |  | 7.6096E6 | 9 | 0 | 0 | 0 | 0 | 3 | 4 | 0 | 0 | 0 | 0 | 0 | 0 | 0 | 2 | 389 | 409 | Carbamidomethylation | C13:Carbamidomethylation:1000.00 | PEAKS DB |
| R.HGQGTGELLQVSGIK.V | N | 97.61 | 1522.8103 | 15 | 0.4 | 762.4127 | 2 | 31.05 | 6 | F6:13489 | NaSa01\_F14.raw |  |  |  | 4.9661E5 | 6.4612E8 | 1.2796E9 |  |  |  |  |  |  |  |  | 8 | 0 | 0 | 0 | 1 | 3 | 4 | 0 | 0 | 0 | 0 | 0 | 0 | 0 | 0 | 457 | 471 |  |  | PEAKS DB |
| R.YDAM(+15.99)ALGNHEFDNGLNGLLDPLLK.N | N | 89.01 | 2645.2795 | 24 | 0.3 | 882.7674 | 3 | 88.88 | 6 | F6:61975 | NaSa01\_F14.raw |  |  |  |  | 1.9977E7 | 6.8848E7 |  |  |  |  |  |  |  |  | 3 | 0 | 0 | 0 | 0 | 1 | 2 | 0 | 0 | 0 | 0 | 0 | 0 | 0 | 0 | 124 | 147 | Oxidation (M) | M4:Oxidation (M):1000.00 | PEAKS DB |
| R.HPDDNEWNHVSM(+15.99)C(+57.02)IVNGGGIR.S | N | 86.62 | 2422.0542 | 21 | -0.3 | 808.3584 | 3 | 44.12 | 6 | F6:24148 | NaSa01\_F14.raw |  |  |  |  | 3.2766E7 | 9.9954E7 |  |  |  |  |  |  |  | 8.8723E6 | 8 | 0 | 0 | 0 | 0 | 2 | 4 | 0 | 0 | 0 | 0 | 0 | 0 | 0 | 2 | 389 | 409 | Oxidation (M); Carbamidomethylation | M12:Oxidation (M):1000.00;C13:Carbamidomethylation:1000.00 | PEAKS DB |
| K.VLLPSFLAAGGDGYYMLK.G | N | 84.56 | 1913.9961 | 18 | 1.4 | 639.0068 | 3 | 94.26 | 6 | F6:66829 | NaSa01\_F14.raw |  |  |  | 4.47E5 | 9.4519E7 | 6.4928E8 |  |  |  |  |  |  |  |  | 12 | 0 | 0 | 0 | 1 | 4 | 7 | 0 | 0 | 0 | 0 | 0 | 0 | 0 | 0 | 510 | 527 |  |  | PEAKS DB |
| K.ASGNPILLNKSIQEDPAVK.A | N | 83.17 | 1993.0844 | 19 | 0.6 | 665.3691 | 3 | 35.69 | 6 | F6:17128 | NaSa01\_F14.raw |  |  |  |  | 1.5436E6 | 2.8654E6 |  |  |  |  |  |  |  |  | 2 | 0 | 0 | 0 | 0 | 1 | 1 | 0 | 0 | 0 | 0 | 0 | 0 | 0 | 0 | 317 | 335 |  |  | PEAKS DB |
| K.VLLPSFLAAGGDGYYM(+15.99)LK.G | N | 82.89 | 1929.9910 | 18 | 0.7 | 966.0034 | 2 | 87.45 | 6 | F6:60748 | NaSa01\_F14.raw |  |  |  |  | 2.8899E7 | 1.2442E8 |  |  |  |  |  |  |  |  | 10 | 0 | 0 | 0 | 0 | 3 | 7 | 0 | 0 | 0 | 0 | 0 | 0 | 0 | 0 | 510 | 527 | Oxidation (M) | M16:Oxidation (M):1000.00 | PEAKS DB |
| R.FHEC(+57.02)NLGNLIC(+57.02)DAVVYNNLR.H | N | 82.50 | 2420.1365 | 20 | -0.2 | 807.7192 | 3 | 77.73 | 6 | F6:52912 | NaSa01\_F14.raw |  |  |  |  | 2.6877E7 | 9.4785E7 |  |  |  |  |  |  | 7.773E6 | 1.2217E8 | 9 | 0 | 0 | 0 | 0 | 1 | 3 | 0 | 0 | 0 | 0 | 0 | 0 | 2 | 3 | 369 | 388 | Carbamidomethylation | C4:Carbamidomethylation:1000.00;C11:Carbamidomethylation:1000.00 | PEAKS DB |
| K.ETPVLSNPGPYLEFR.D | N | 81.49 | 1717.8674 | 15 | 0.8 | 859.9417 | 2 | 69.77 | 6 | F6:46166 | NaSa01\_F14.raw |  |  |  | 7.2199E5 | 2.4678E8 | 7.6435E8 |  |  |  | 2.2582E6 |  |  |  |  | 7 | 0 | 0 | 0 | 1 | 3 | 2 | 0 | 0 | 0 | 1 | 0 | 0 | 0 | 0 | 194 | 208 |  |  | PEAKS DB |
| R.QVPVVQAYAFGK.Y | N | 80.72 | 1305.7081 | 12 | -0.2 | 653.8612 | 2 | 81.01 | 6 | F6:55346 | NaSa01\_F14.raw |  |  |  | 4.7562E5 | 3.326E8 | 6.8066E8 |  |  |  |  |  |  |  |  | 5 | 0 | 0 | 0 | 1 | 1 | 3 | 0 | 0 | 0 | 0 | 0 | 0 | 0 | 0 | 288 | 299 |  |  | PEAKS DB |
| R.EVVHFMNSLR.Y | N | 80.17 | 1230.6179 | 10 | 0.3 | 616.3164 | 2 | 32.92 | 6 | F6:14493 | NaSa01\_F14.raw |  |  |  |  | 2.3691E8 | 4.1556E8 |  |  |  |  |  |  |  |  | 11 | 0 | 0 | 0 | 0 | 3 | 8 | 0 | 0 | 0 | 0 | 0 | 0 | 0 | 0 | 114 | 123 |  |  | PEAKS DB |
| R.VVSLNVLC(+57.02)TEC(+57.02)R.V | N | 79.36 | 1448.7115 | 12 | 0.2 | 725.3632 | 2 | 49.71 | 6 | F6:29319 | NaSa01\_F14.raw |  |  |  | 0 | 2.7472E8 | 5.8042E8 |  |  |  |  |  | 8.8246E5 |  |  | 7 | 0 | 0 | 0 | 0 | 2 | 4 | 0 | 0 | 0 | 0 | 0 | 1 | 0 | 0 | 484 | 495 | Carbamidomethylation | C8:Carbamidomethylation:1000.00;C11:Carbamidomethylation:1000.00 | PEAKS DB |
| R.YDAMALGNHEFDNGLNGLLDPLLK.N | N | 78.14 | 2629.2847 | 24 | 0.8 | 877.4362 | 3 | 94.34 | 6 | F6:67114 | NaSa01\_F14.raw |  |  |  |  | 1.6292E7 | 1.1665E8 |  |  |  |  |  |  |  |  | 4 | 0 | 0 | 0 | 0 | 2 | 2 | 0 | 0 | 0 | 0 | 0 | 0 | 0 | 0 | 124 | 147 |  |  | PEAKS DB |
| K.YLGYLNVIFDDK.G | N | 76.79 | 1458.7394 | 12 | 0.2 | 730.3771 | 2 | 85.89 | 6 | F6:59730 | NaSa01\_F14.raw |  |  |  |  | 6.7598E7 | 9.6452E7 |  |  |  |  |  |  |  |  | 5 | 0 | 0 | 0 | 0 | 4 | 1 | 0 | 0 | 0 | 0 | 0 | 0 | 0 | 0 | 300 | 311 |  |  | PEAKS DB |
| K.LTILHTNDVHAR.V | N | 75.00 | 1388.7524 | 12 | 0.9 | 463.9252 | 3 | 11.51 | 6 | F6:1905 | NaSa01\_F14.raw |  |  |  |  | 1.6389E6 | 3.6507E6 |  |  |  |  |  |  |  |  | 4 | 0 | 0 | 0 | 0 | 2 | 2 | 0 | 0 | 0 | 0 | 0 | 0 | 0 | 0 | 44 | 55 |  |  | PEAKS DB |
| K.ETPVLSNPGPYLEFRDEVEELQK.Q | N | 74.67 | 2688.3281 | 23 | 0.7 | 897.1173 | 3 | 77.38 | 6 | F6:52291 | NaSa01\_F14.raw |  |  |  |  | 3.5808E8 | 6.9841E8 |  |  |  |  |  |  |  |  | 4 | 0 | 0 | 0 | 0 | 2 | 2 | 0 | 0 | 0 | 0 | 0 | 0 | 0 | 0 | 194 | 216 |  |  | PEAKS DB |
| K.ASGNPILLNK.S | N | 69.82 | 1025.5869 | 10 | -0.4 | 513.8005 | 2 | 11.59 | 6 | F6:1963 | NaSa01\_F14.raw |  |  |  |  | 2.4579E6 | 4.7925E6 |  |  |  |  |  |  |  |  | 3 | 0 | 0 | 0 | 0 | 1 | 2 | 0 | 0 | 0 | 0 | 0 | 0 | 0 | 0 | 317 | 326 |  |  | PEAKS DB |
| S.FLAAGGDGYYMLK.G | N | 67.12 | 1404.6747 | 13 | 0.2 | 703.3447 | 2 | 58.91 | 6 | F6:37338 | NaSa01\_F14.raw |  |  |  |  | 1.8909E7 | 8.0208E7 |  |  |  |  |  |  |  |  | 3 | 0 | 0 | 0 | 0 | 1 | 2 | 0 | 0 | 0 | 0 | 0 | 0 | 0 | 0 | 515 | 527 |  |  | PEAKS DB |
| F.LAAGGDGYYMLK.G | N | 66.41 | 1257.6063 | 12 | 0.2 | 629.8105 | 2 | 41.73 | 6 | F6:22051 | NaSa01\_F14.raw |  |  |  |  |  | 5.7329E6 |  |  |  |  |  |  |  |  | 1 | 0 | 0 | 0 | 0 | 0 | 1 | 0 | 0 | 0 | 0 | 0 | 0 | 0 | 0 | 516 | 527 |  |  | PEAKS DB |
| R.EVVHFM(+15.99)NSLR.Y | N | 65.78 | 1246.6128 | 10 | 0.3 | 624.3138 | 2 | 31.71 | 6 | F6:14277 | NaSa01\_F14.raw |  |  |  |  | 3.2821E6 | 5.0137E7 |  |  |  |  |  |  |  |  | 13 | 0 | 0 | 0 | 0 | 2 | 11 | 0 | 0 | 0 | 0 | 0 | 0 | 0 | 0 | 114 | 123 | Oxidation (M) | M6:Oxidation (M):1000.00 | PEAKS DB |
| K.FPILSANIRPK.G | N | 63.57 | 1254.7448 | 11 | 1.8 | 419.2563 | 3 | 38.83 | 6 | F6:20180 | NaSa01\_F14.raw |  |  |  | 3.6184E5 | 9.4886E7 | 1.2559E8 |  |  |  |  | 2.1098E6 |  |  |  | 10 | 0 | 0 | 0 | 1 | 2 | 5 | 0 | 0 | 0 | 0 | 2 | 0 | 0 | 0 | 151 | 161 |  |  | PEAKS DB |
| K.RVVSLNVLC(+57.02)TEC(+57.02)R.V | N | 62.55 | 1604.8126 | 13 | 0.2 | 535.9449 | 3 | 35.03 | 6 | F6:16539 | NaSa01\_F14.raw |  |  |  |  | 4.4155E6 | 6.0208E6 |  |  |  |  |  |  |  |  | 3 | 0 | 0 | 0 | 0 | 1 | 2 | 0 | 0 | 0 | 0 | 0 | 0 | 0 | 0 | 483 | 495 | Carbamidomethylation | C9:Carbamidomethylation:1000.00;C12:Carbamidomethylation:1000.00 | PEAKS DB |
| K.GVDVVVGGHTNTF.L | N | 61.79 | 1300.6411 | 13 | 1.1 | 651.3286 | 2 | 41.80 | 6 | F6:22128 | NaSa01\_F14.raw |  |  |  |  | 2.249E7 | 5.2209E7 |  |  |  |  |  |  |  |  | 2 | 0 | 0 | 0 | 0 | 1 | 1 | 0 | 0 | 0 | 0 | 0 | 0 | 0 | 0 | 249 | 261 |  |  | PEAKS DB |
| L.TAVLPFGGTFDLLQIK.G | N | 60.64 | 1718.9607 | 16 | -0.1 | 860.4875 | 2 | 99.74 | 6 | F6:71142 | NaSa01\_F14.raw |  |  | 2.8271E6 | 1.2518E6 | 2.1013E7 | 4.0691E6 |  |  |  |  |  |  |  |  | 4 | 0 | 0 | 1 | 1 | 1 | 1 | 0 | 0 | 0 | 0 | 0 | 0 | 0 | 0 | 427 | 442 |  |  | PEAKS DB |
| K.IINVGSEKVGIIGYTTK.E | N | 60.09 | 1791.0142 | 17 | -0.7 | 598.0116 | 3 | 45.32 | 6 | F6:25348 | NaSa01\_F14.raw |  |  |  |  | 9.4386E5 | 1.7014E6 |  |  |  |  |  |  |  |  | 2 | 0 | 0 | 0 | 0 | 1 | 1 | 0 | 0 | 0 | 0 | 0 | 0 | 0 | 0 | 177 | 193 |  |  | PEAKS DB |
| K.YLGYLNVIFDDKGK.V | Y | 58.45 | 1643.8558 | 14 | -0.3 | 548.9590 | 3 | 70.19 | 5 | F5:46798 | NaSa01\_F13.raw |  |  |  |  | 1.8927E6 |  |  |  |  |  |  |  |  |  | 2 | 0 | 0 | 0 | 0 | 2 | 0 | 0 | 0 | 0 | 0 | 0 | 0 | 0 | 0 | 300 | 313 |  |  | PEAKS DB |
| K.VFPAMEGR.V | N | 56.33 | 905.4429 | 8 | 0.2 | 453.7288 | 2 | 22.33 | 6 | F6:7722 | NaSa01\_F14.raw |  |  |  |  |  | 3.6244E6 |  |  |  |  |  |  |  |  | 2 | 0 | 0 | 0 | 0 | 0 | 2 | 0 | 0 | 0 | 0 | 0 | 0 | 0 | 0 | 553 | 560 |  |  | PEAKS DB |
| N.ISGYILPYK.I | N | 56.17 | 1052.5906 | 9 | 0.7 | 527.3029 | 2 | 45.82 | 6 | F6:26096 | NaSa01\_F14.raw |  |  |  | 2.2592E5 | 4.6763E8 | 8.5839E8 |  |  |  |  |  |  |  |  | 5 | 0 | 0 | 0 | 1 | 1 | 3 | 0 | 0 | 0 | 0 | 0 | 0 | 0 | 0 | 168 | 176 |  |  | PEAKS DB |
| S.FLAAGGDGYYM(+15.99)LK.G | N | 55.62 | 1420.6697 | 13 | -0.1 | 711.3420 | 2 | 48.69 | 6 | F6:28173 | NaSa01\_F14.raw |  |  |  |  |  | 5.7308E6 |  |  |  |  |  |  |  |  | 1 | 0 | 0 | 0 | 0 | 0 | 1 | 0 | 0 | 0 | 0 | 0 | 0 | 0 | 0 | 515 | 527 | Oxidation (M) | M11:Oxidation (M):1000.00 | PEAKS DB |
| H.GQGTGELLQVSGIK.V | N | 55.06 | 1385.7515 | 14 | 0.3 | 693.8832 | 2 | 45.20 | 6 | F6:25108 | NaSa01\_F14.raw |  |  |  |  |  | 1.2594E6 |  |  |  |  |  |  |  |  | 1 | 0 | 0 | 0 | 0 | 0 | 1 | 0 | 0 | 0 | 0 | 0 | 0 | 0 | 0 | 458 | 471 |  |  | PEAKS DB |
| K.GVDVVVGGHTNTFLY.T | N | 54.75 | 1576.7886 | 15 | 0.1 | 789.4016 | 2 | 63.80 | 6 | F6:41327 | NaSa01\_F14.raw |  |  |  |  | 1.2739E7 | 2.8201E7 |  |  |  |  |  |  |  |  | 2 | 0 | 0 | 0 | 0 | 1 | 1 | 0 | 0 | 0 | 0 | 0 | 0 | 0 | 0 | 249 | 263 |  |  | PEAKS DB |
| R.QVPVVQAYAF.G | N | 53.91 | 1120.5917 | 10 | 0.0 | 561.3031 | 2 | 73.70 | 6 | F6:49221 | NaSa01\_F14.raw |  |  |  |  | 7.1015E6 | 1.9997E7 |  |  |  |  |  |  |  |  | 2 | 0 | 0 | 0 | 0 | 1 | 1 | 0 | 0 | 0 | 0 | 0 | 0 | 0 | 0 | 288 | 297 |  |  | PEAKS DB |
| M.ALGNHEFDNGLNGLLDPLLK.N | N | 53.45 | 2149.1167 | 20 | 0.0 | 717.3795 | 3 | 83.56 | 6 | F6:57342 | NaSa01\_F14.raw |  |  |  |  | 5.3244E6 | 2.0787E7 |  |  |  |  |  |  |  |  | 3 | 0 | 0 | 0 | 0 | 1 | 2 | 0 | 0 | 0 | 0 | 0 | 0 | 0 | 0 | 128 | 147 |  |  | PEAKS DB |
| K.LTTLGVNK.I | N | 52.68 | 844.5018 | 8 | -1.0 | 423.2578 | 2 | 11.58 | 6 | F6:2148 | NaSa01\_F14.raw |  |  |  |  |  | 3.1326E6 |  |  |  |  |  |  |  |  | 1 | 0 | 0 | 0 | 0 | 0 | 1 | 0 | 0 | 0 | 0 | 0 | 0 | 0 | 0 | 221 | 228 |  |  | PEAKS DB |
| K.VLLPSFLAAGGDGY.Y | N | 51.68 | 1378.7133 | 14 | 0.7 | 690.3644 | 2 | 100.88 | 6 | F6:72024 | NaSa01\_F14.raw |  |  |  |  | 1.3644E6 | 2.8748E6 |  |  |  |  |  |  |  |  | 2 | 0 | 0 | 0 | 0 | 1 | 1 | 0 | 0 | 0 | 0 | 0 | 0 | 0 | 0 | 510 | 523 |  |  | PEAKS DB |
| F.GGTFDLLQIK.G | N | 50.44 | 1090.6022 | 10 | -0.6 | 546.3080 | 2 | 64.67 | 6 | F6:41967 | NaSa01\_F14.raw |  |  |  |  |  | 9.7795E7 |  |  |  |  |  |  |  |  | 2 | 0 | 0 | 0 | 0 | 0 | 2 | 0 | 0 | 0 | 0 | 0 | 0 | 0 | 0 | 433 | 442 |  |  | PEAKS DB |
| Y.LNVIFDDK.G | N | 50.15 | 962.5073 | 8 | -0.5 | 482.2607 | 2 | 40.33 | 6 | F6:21131 | NaSa01\_F14.raw |  |  |  |  |  | 1.4605E6 |  |  |  |  |  |  |  |  | 1 | 0 | 0 | 0 | 0 | 0 | 1 | 0 | 0 | 0 | 0 | 0 | 0 | 0 | 0 | 304 | 311 |  |  | PEAKS DB |
| K.VGIIGYTTK.E | N | 50.04 | 950.5436 | 9 | 0.3 | 476.2792 | 2 | 28.48 | 6 | F6:11194 | NaSa01\_F14.raw |  |  |  |  |  | 5.1319E6 |  |  |  |  |  |  |  |  | 2 | 0 | 0 | 0 | 0 | 0 | 2 | 0 | 0 | 0 | 0 | 0 | 0 | 0 | 0 | 185 | 193 |  |  | PEAKS DB |
| L.AAGGDGYYMLK.G | N | 49.71 | 1144.5222 | 11 | 0.7 | 573.2688 | 2 | 30.46 | 5 | F5:13468 | NaSa01\_F13.raw |  |  |  |  | 6.4456E6 |  |  |  |  |  |  |  |  |  | 1 | 0 | 0 | 0 | 0 | 1 | 0 | 0 | 0 | 0 | 0 | 0 | 0 | 0 | 0 | 517 | 527 |  |  | PEAKS DB |
| R.FHEC(+57.02)NLGNLIC(+57.02)DAVVY.N | N | 48.35 | 1922.8655 | 16 | 1.4 | 962.4414 | 2 | 82.45 | 5 | F5:56634 | NaSa01\_F13.raw |  |  |  |  | 8.0322E7 | 1.7562E8 |  |  |  |  |  |  |  | 2.4692E7 | 3 | 0 | 0 | 0 | 0 | 1 | 1 | 0 | 0 | 0 | 0 | 0 | 0 | 0 | 1 | 369 | 384 | Carbamidomethylation | C4:Carbamidomethylation:1000.00;C11:Carbamidomethylation:1000.00 | PEAKS DB |
| R.HPDDNEWNHVSMC(+57.02).I | N | 48.19 | 1639.6144 | 13 | 0.5 | 820.8149 | 2 | 26.12 | 5 | F5:10348 | NaSa01\_F13.raw |  |  |  |  | 8.8799E6 |  |  |  |  |  |  |  |  |  | 2 | 0 | 0 | 0 | 0 | 2 | 0 | 0 | 0 | 0 | 0 | 0 | 0 | 0 | 0 | 389 | 401 | Carbamidomethylation | C13:Carbamidomethylation:1000.00 | PEAKS DB |
| K.VLLPSFLAAGGD.G | N | 47.35 | 1158.6284 | 12 | -0.1 | 580.3214 | 2 | 96.95 | 6 | F6:68512 | NaSa01\_F14.raw |  |  |  |  |  | 1.1427E6 |  |  |  |  |  |  |  |  | 1 | 0 | 0 | 0 | 0 | 0 | 1 | 0 | 0 | 0 | 0 | 0 | 0 | 0 | 0 | 510 | 521 |  |  | PEAKS DB |
| R.HPDDNEWNHVSM.C | N | 47.22 | 1479.5837 | 12 | 1.1 | 740.8000 | 2 | 25.93 | 5 | F5:10070 | NaSa01\_F13.raw |  |  |  |  | 2.3098E6 |  |  |  |  |  |  |  |  |  | 1 | 0 | 0 | 0 | 0 | 1 | 0 | 0 | 0 | 0 | 0 | 0 | 0 | 0 | 0 | 389 | 400 |  |  | PEAKS DB |
| R.QVPVVQAY.A | N | 45.65 | 902.4861 | 8 | -0.5 | 452.2501 | 2 | 43.38 | 5 | F5:24541 | NaSa01\_F13.raw |  |  |  |  | 1.2052E7 |  |  |  |  |  |  |  |  |  | 1 | 0 | 0 | 0 | 0 | 1 | 0 | 0 | 0 | 0 | 0 | 0 | 0 | 0 | 0 | 288 | 295 |  |  | PEAKS DB |
| N.PGPYLEFR.D | N | 45.44 | 977.4970 | 8 | 0.7 | 489.7561 | 2 | 39.90 | 6 | F6:20609 | NaSa01\_F14.raw |  |  |  |  |  | 4.6995E5 |  |  |  |  |  |  |  |  | 1 | 0 | 0 | 0 | 0 | 0 | 1 | 0 | 0 | 0 | 0 | 0 | 0 | 0 | 0 | 201 | 208 |  |  | PEAKS DB |
| N.LGNLIC(+57.02)DAVVYNNLR.H | N | 45.20 | 1732.8931 | 15 | 1.0 | 867.4547 | 2 | 69.10 | 6 | F6:45743 | NaSa01\_F14.raw |  |  |  |  |  | 1.1316E6 |  |  |  |  |  |  |  |  | 1 | 0 | 0 | 0 | 0 | 0 | 1 | 0 | 0 | 0 | 0 | 0 | 0 | 0 | 0 | 374 | 388 | Carbamidomethylation | C6:Carbamidomethylation:1000.00 | PEAKS DB |
| K.IINVGSEK.V | N | 44.70 | 858.4811 | 8 | -0.5 | 430.2476 | 2 | 11.48 | 6 | F6:1861 | NaSa01\_F14.raw |  |  |  |  |  | 1.3857E6 |  |  |  |  |  |  |  |  | 1 | 0 | 0 | 0 | 0 | 0 | 1 | 0 | 0 | 0 | 0 | 0 | 0 | 0 | 0 | 177 | 184 |  |  | PEAKS DB |
| K.VLLPSFLAAGGDGYY.M | N | 43.81 | 1541.7765 | 15 | 0.7 | 771.8961 | 2 | 105.28 | 6 | F6:75496 | NaSa01\_F14.raw |  |  |  |  | 1.4762E7 | 3.1893E7 |  |  |  |  |  |  |  |  | 2 | 0 | 0 | 0 | 0 | 1 | 1 | 0 | 0 | 0 | 0 | 0 | 0 | 0 | 0 | 510 | 524 |  |  | PEAKS DB |
| K.FPILSANIR.P | N | 43.38 | 1029.5970 | 9 | -0.4 | 515.8056 | 2 | 52.72 | 6 | F6:31843 | NaSa01\_F14.raw |  |  |  |  |  | 1.5657E8 |  |  |  |  |  |  |  |  | 1 | 0 | 0 | 0 | 0 | 0 | 1 | 0 | 0 | 0 | 0 | 0 | 0 | 0 | 0 | 151 | 159 |  |  | PEAKS DB |
| K.VLLPSFLAAGGDGYYM.L | N | 42.72 | 1672.8170 | 16 | 0.9 | 837.4166 | 2 | 111.51 | 6 | F6:80284 | NaSa01\_F14.raw |  |  |  |  |  | 1.1099E6 |  |  |  |  |  |  |  |  | 1 | 0 | 0 | 0 | 0 | 0 | 1 | 0 | 0 | 0 | 0 | 0 | 0 | 0 | 0 | 510 | 525 |  |  | PEAKS DB |
| Q.GTGELLQVSGIK.V | N | 42.14 | 1200.6714 | 12 | 0.9 | 601.3435 | 2 | 44.43 | 6 | F6:24568 | NaSa01\_F14.raw |  |  |  |  |  | 7.0353E5 |  |  |  |  |  |  |  |  | 1 | 0 | 0 | 0 | 0 | 0 | 1 | 0 | 0 | 0 | 0 | 0 | 0 | 0 | 0 | 460 | 471 |  |  | PEAKS DB |
| Y.LGYLNVIFDDK.G | N | 41.11 | 1295.6761 | 11 | 0.9 | 648.8459 | 2 | 76.55 | 6 | F6:51743 | NaSa01\_F14.raw |  |  |  |  |  | 0 |  |  |  |  |  |  |  |  | 0 | 0 | 0 | 0 | 0 | 0 | 0 | 0 | 0 | 0 | 0 | 0 | 0 | 0 | 0 | 301 | 311 |  |  | PEAKS DB |
| K.LTTLGVNKIIALGH.S | N | 41.05 | 1448.8715 | 14 | 0.1 | 483.9645 | 3 | 54.77 | 6 | F6:33352 | NaSa01\_F14.raw |  |  |  |  |  | 5.5856E5 |  |  |  |  |  |  |  |  | 1 | 0 | 0 | 0 | 0 | 0 | 1 | 0 | 0 | 0 | 0 | 0 | 0 | 0 | 0 | 221 | 234 |  |  | PEAKS DB |
| N.HEFDNGLNGLLDPLLK.N | N | 40.62 | 1793.9312 | 16 | 0.2 | 598.9844 | 3 | 81.57 | 6 | F6:55994 | NaSa01\_F14.raw |  |  |  |  |  | 5.7086E5 |  |  |  |  |  |  |  |  | 1 | 0 | 0 | 0 | 0 | 0 | 1 | 0 | 0 | 0 | 0 | 0 | 0 | 0 | 0 | 132 | 147 |  |  | PEAKS DB |
| N.YPFMQLSDDGR.Q | N | 40.57 | 1327.5867 | 11 | 0.4 | 664.8009 | 2 | 53.65 | 6 | F6:32287 | NaSa01\_F14.raw |  |  |  |  |  | 5.3395E5 |  |  |  |  |  |  |  |  | 1 | 0 | 0 | 0 | 0 | 0 | 1 | 0 | 0 | 0 | 0 | 0 | 0 | 0 | 0 | 277 | 287 |  |  | PEAKS DB |
| P.AGNYPFMQLSDDGR.Q | N | 40.07 | 1569.6881 | 14 | 0.1 | 785.8514 | 2 | 56.81 | 6 | F6:35126 | NaSa01\_F14.raw |  |  |  |  |  | 2.5054E5 |  |  |  |  |  |  |  |  | 1 | 0 | 0 | 0 | 0 | 0 | 1 | 0 | 0 | 0 | 0 | 0 | 0 | 0 | 0 | 274 | 287 |  |  | PEAKS DB |
| K.NVKFPILSAN.I | N | 39.82 | 1101.6182 | 10 | 0.5 | 551.8167 | 2 | 54.61 | 6 | F6:33186 | NaSa01\_F14.raw |  |  |  |  |  | 1.3385E6 |  |  |  |  |  |  |  |  | 1 | 0 | 0 | 0 | 0 | 0 | 1 | 0 | 0 | 0 | 0 | 0 | 0 | 0 | 0 | 148 | 157 |  |  | PEAKS DB |
| K.IIALGHSGF.K | N | 37.88 | 913.5021 | 9 | -1.2 | 457.7578 | 2 | 43.37 | 6 | F6:23538 | NaSa01\_F14.raw |  |  |  |  |  | 5.7946E6 |  |  |  |  |  |  |  |  | 1 | 0 | 0 | 0 | 0 | 0 | 1 | 0 | 0 | 0 | 0 | 0 | 0 | 0 | 0 | 229 | 237 |  |  | PEAKS DB |
| total 58 peptides |  |  |  |  |  |  |  |  |  |  |  |  |  |  |  |  |  |  |  |  |  |  |  |  |  |  |  |  |  |  |  |  |  |  |  |  |  |  |  |  |  |  |  |  |  |
| --- | --- | --- | --- | --- | --- | --- | --- | --- | --- | --- | --- | --- | --- | --- | --- | --- | --- | --- | --- | --- | --- | --- | --- | --- | --- | --- | --- | --- | --- | --- | --- | --- | --- | --- | --- | --- | --- | --- | --- | --- | --- | --- | --- | --- | --- |

JAS05038.1

back to list

  

| Protein Coverage
| Supporting Peptides
|

Protein Coverage:

Supporting Peptides:

| Peptide | Uniq | -10lgP | Mass | Length | ppm | m/z | z | RT | Fraction | Scan | Source File | Area F1 | Area F10 | Area F11 | Area F12 | Area F13 | Area F14 | Area F2 | Area F3 | Area F4 | Area F5 | Area F6 | Area F7 | Area F8 | Area F9 | #Feature | #Feature F1 | #Feature F10 | #Feature F11 | #Feature F12 | #Feature F13 | #Feature F14 | #Feature F2 | #Feature F3 | #Feature F4 | #Feature F5 | #Feature F6 | #Feature F7 | #Feature F8 | #Feature F9 | Start | End | PTM | AScore | Found By |
| --- | --- | --- | --- | --- | --- | --- | --- | --- | --- | --- | --- | --- | --- | --- | --- | --- | --- | --- | --- | --- | --- | --- | --- | --- | --- | --- | --- | --- | --- | --- | --- | --- | --- | --- | --- | --- | --- | --- | --- | --- | --- | --- | --- | --- | --- |
| R.HPDDNEWNHVSMC(+57.02)IVNGGGIR.S | N | 99.67 | 2406.0593 | 21 | -0.4 | 803.0267 | 3 | 49.93 | 6 | F6:29280 | NaSa01\_F14.raw |  |  |  |  | 6.5991E7 | 1.6043E8 |  |  |  |  |  |  |  | 7.6096E6 | 9 | 0 | 0 | 0 | 0 | 3 | 4 | 0 | 0 | 0 | 0 | 0 | 0 | 0 | 2 | 389 | 409 | Carbamidomethylation | C13:Carbamidomethylation:1000.00 | PEAKS DB |
| R.HGQGTGELLQVSGIK.V | N | 97.61 | 1522.8103 | 15 | 0.4 | 762.4127 | 2 | 31.05 | 6 | F6:13489 | NaSa01\_F14.raw |  |  |  | 4.9661E5 | 6.4612E8 | 1.2796E9 |  |  |  |  |  |  |  |  | 8 | 0 | 0 | 0 | 1 | 3 | 4 | 0 | 0 | 0 | 0 | 0 | 0 | 0 | 0 | 457 | 471 |  |  | PEAKS DB |
| R.YDAM(+15.99)ALGNHEFDNGLNGLLDPLLK.N | N | 89.01 | 2645.2795 | 24 | 0.3 | 882.7674 | 3 | 88.88 | 6 | F6:61975 | NaSa01\_F14.raw |  |  |  |  | 1.9977E7 | 6.8848E7 |  |  |  |  |  |  |  |  | 3 | 0 | 0 | 0 | 0 | 1 | 2 | 0 | 0 | 0 | 0 | 0 | 0 | 0 | 0 | 124 | 147 | Oxidation (M) | M4:Oxidation (M):1000.00 | PEAKS DB |
| R.HPDDNEWNHVSM(+15.99)C(+57.02)IVNGGGIR.S | N | 86.62 | 2422.0542 | 21 | -0.3 | 808.3584 | 3 | 44.12 | 6 | F6:24148 | NaSa01\_F14.raw |  |  |  |  | 3.2766E7 | 9.9954E7 |  |  |  |  |  |  |  | 8.8723E6 | 8 | 0 | 0 | 0 | 0 | 2 | 4 | 0 | 0 | 0 | 0 | 0 | 0 | 0 | 2 | 389 | 409 | Oxidation (M); Carbamidomethylation | M12:Oxidation (M):1000.00;C13:Carbamidomethylation:1000.00 | PEAKS DB |
| K.VLLPSFLAAGGDGYYMLK.G | N | 84.56 | 1913.9961 | 18 | 1.4 | 639.0068 | 3 | 94.26 | 6 | F6:66829 | NaSa01\_F14.raw |  |  |  | 4.47E5 | 9.4519E7 | 6.4928E8 |  |  |  |  |  |  |  |  | 12 | 0 | 0 | 0 | 1 | 4 | 7 | 0 | 0 | 0 | 0 | 0 | 0 | 0 | 0 | 510 | 527 |  |  | PEAKS DB |
| K.ASGNPILLNKSIQEDPAVK.A | N | 83.17 | 1993.0844 | 19 | 0.6 | 665.3691 | 3 | 35.69 | 6 | F6:17128 | NaSa01\_F14.raw |  |  |  |  | 1.5436E6 | 2.8654E6 |  |  |  |  |  |  |  |  | 2 | 0 | 0 | 0 | 0 | 1 | 1 | 0 | 0 | 0 | 0 | 0 | 0 | 0 | 0 | 317 | 335 |  |  | PEAKS DB |
| K.VLLPSFLAAGGDGYYM(+15.99)LK.G | N | 82.89 | 1929.9910 | 18 | 0.7 | 966.0034 | 2 | 87.45 | 6 | F6:60748 | NaSa01\_F14.raw |  |  |  |  | 2.8899E7 | 1.2442E8 |  |  |  |  |  |  |  |  | 10 | 0 | 0 | 0 | 0 | 3 | 7 | 0 | 0 | 0 | 0 | 0 | 0 | 0 | 0 | 510 | 527 | Oxidation (M) | M16:Oxidation (M):1000.00 | PEAKS DB |
| R.FHEC(+57.02)NLGNLIC(+57.02)DAVVYNNLR.H | N | 82.50 | 2420.1365 | 20 | -0.2 | 807.7192 | 3 | 77.73 | 6 | F6:52912 | NaSa01\_F14.raw |  |  |  |  | 2.6877E7 | 9.4785E7 |  |  |  |  |  |  | 7.773E6 | 1.2217E8 | 9 | 0 | 0 | 0 | 0 | 1 | 3 | 0 | 0 | 0 | 0 | 0 | 0 | 2 | 3 | 369 | 388 | Carbamidomethylation | C4:Carbamidomethylation:1000.00;C11:Carbamidomethylation:1000.00 | PEAKS DB |
| K.ETPVLSNPGPYLEFR.D | N | 81.49 | 1717.8674 | 15 | 0.8 | 859.9417 | 2 | 69.77 | 6 | F6:46166 | NaSa01\_F14.raw |  |  |  | 7.2199E5 | 2.4678E8 | 7.6435E8 |  |  |  | 2.2582E6 |  |  |  |  | 7 | 0 | 0 | 0 | 1 | 3 | 2 | 0 | 0 | 0 | 1 | 0 | 0 | 0 | 0 | 194 | 208 |  |  | PEAKS DB |
| R.QVPVVQAYAFGK.Y | N | 80.72 | 1305.7081 | 12 | -0.2 | 653.8612 | 2 | 81.01 | 6 | F6:55346 | NaSa01\_F14.raw |  |  |  | 4.7562E5 | 3.326E8 | 6.8066E8 |  |  |  |  |  |  |  |  | 5 | 0 | 0 | 0 | 1 | 1 | 3 | 0 | 0 | 0 | 0 | 0 | 0 | 0 | 0 | 288 | 299 |  |  | PEAKS DB |
| R.EVVHFMNSLR.Y | N | 80.17 | 1230.6179 | 10 | 0.3 | 616.3164 | 2 | 32.92 | 6 | F6:14493 | NaSa01\_F14.raw |  |  |  |  | 2.3691E8 | 4.1556E8 |  |  |  |  |  |  |  |  | 11 | 0 | 0 | 0 | 0 | 3 | 8 | 0 | 0 | 0 | 0 | 0 | 0 | 0 | 0 | 114 | 123 |  |  | PEAKS DB |
| R.VVSLNVLC(+57.02)TEC(+57.02)R.V | N | 79.36 | 1448.7115 | 12 | 0.2 | 725.3632 | 2 | 49.71 | 6 | F6:29319 | NaSa01\_F14.raw |  |  |  | 0 | 2.7472E8 | 5.8042E8 |  |  |  |  |  | 8.8246E5 |  |  | 7 | 0 | 0 | 0 | 0 | 2 | 4 | 0 | 0 | 0 | 0 | 0 | 1 | 0 | 0 | 484 | 495 | Carbamidomethylation | C8:Carbamidomethylation:1000.00;C11:Carbamidomethylation:1000.00 | PEAKS DB |
| R.YDAMALGNHEFDNGLNGLLDPLLK.N | N | 78.14 | 2629.2847 | 24 | 0.8 | 877.4362 | 3 | 94.34 | 6 | F6:67114 | NaSa01\_F14.raw |  |  |  |  | 1.6292E7 | 1.1665E8 |  |  |  |  |  |  |  |  | 4 | 0 | 0 | 0 | 0 | 2 | 2 | 0 | 0 | 0 | 0 | 0 | 0 | 0 | 0 | 124 | 147 |  |  | PEAKS DB |
| K.YLGYLNVIFDDK.G | N | 76.79 | 1458.7394 | 12 | 0.2 | 730.3771 | 2 | 85.89 | 6 | F6:59730 | NaSa01\_F14.raw |  |  |  |  | 6.7598E7 | 9.6452E7 |  |  |  |  |  |  |  |  | 5 | 0 | 0 | 0 | 0 | 4 | 1 | 0 | 0 | 0 | 0 | 0 | 0 | 0 | 0 | 300 | 311 |  |  | PEAKS DB |
| K.LTILHTNDVHAR.V | N | 75.00 | 1388.7524 | 12 | 0.9 | 463.9252 | 3 | 11.51 | 6 | F6:1905 | NaSa01\_F14.raw |  |  |  |  | 1.6389E6 | 3.6507E6 |  |  |  |  |  |  |  |  | 4 | 0 | 0 | 0 | 0 | 2 | 2 | 0 | 0 | 0 | 0 | 0 | 0 | 0 | 0 | 44 | 55 |  |  | PEAKS DB |
| K.ETPVLSNPGPYLEFRDEVEELQK.Q | N | 74.67 | 2688.3281 | 23 | 0.7 | 897.1173 | 3 | 77.38 | 6 | F6:52291 | NaSa01\_F14.raw |  |  |  |  | 3.5808E8 | 6.9841E8 |  |  |  |  |  |  |  |  | 4 | 0 | 0 | 0 | 0 | 2 | 2 | 0 | 0 | 0 | 0 | 0 | 0 | 0 | 0 | 194 | 216 |  |  | PEAKS DB |
| K.ASGNPILLNK.S | N | 69.82 | 1025.5869 | 10 | -0.4 | 513.8005 | 2 | 11.59 | 6 | F6:1963 | NaSa01\_F14.raw |  |  |  |  | 2.4579E6 | 4.7925E6 |  |  |  |  |  |  |  |  | 3 | 0 | 0 | 0 | 0 | 1 | 2 | 0 | 0 | 0 | 0 | 0 | 0 | 0 | 0 | 317 | 326 |  |  | PEAKS DB |
| S.FLAAGGDGYYMLK.G | N | 67.12 | 1404.6747 | 13 | 0.2 | 703.3447 | 2 | 58.91 | 6 | F6:37338 | NaSa01\_F14.raw |  |  |  |  | 1.8909E7 | 8.0208E7 |  |  |  |  |  |  |  |  | 3 | 0 | 0 | 0 | 0 | 1 | 2 | 0 | 0 | 0 | 0 | 0 | 0 | 0 | 0 | 515 | 527 |  |  | PEAKS DB |
| F.LAAGGDGYYMLK.G | N | 66.41 | 1257.6063 | 12 | 0.2 | 629.8105 | 2 | 41.73 | 6 | F6:22051 | NaSa01\_F14.raw |  |  |  |  |  | 5.7329E6 |  |  |  |  |  |  |  |  | 1 | 0 | 0 | 0 | 0 | 0 | 1 | 0 | 0 | 0 | 0 | 0 | 0 | 0 | 0 | 516 | 527 |  |  | PEAKS DB |
| R.EVVHFM(+15.99)NSLR.Y | N | 65.78 | 1246.6128 | 10 | 0.3 | 624.3138 | 2 | 31.71 | 6 | F6:14277 | NaSa01\_F14.raw |  |  |  |  | 3.2821E6 | 5.0137E7 |  |  |  |  |  |  |  |  | 13 | 0 | 0 | 0 | 0 | 2 | 11 | 0 | 0 | 0 | 0 | 0 | 0 | 0 | 0 | 114 | 123 | Oxidation (M) | M6:Oxidation (M):1000.00 | PEAKS DB |
| K.FPILSANIRPK.G | N | 63.57 | 1254.7448 | 11 | 1.8 | 419.2563 | 3 | 38.83 | 6 | F6:20180 | NaSa01\_F14.raw |  |  |  | 3.6184E5 | 9.4886E7 | 1.2559E8 |  |  |  |  | 2.1098E6 |  |  |  | 10 | 0 | 0 | 0 | 1 | 2 | 5 | 0 | 0 | 0 | 0 | 2 | 0 | 0 | 0 | 151 | 161 |  |  | PEAKS DB |
| K.RVVSLNVLC(+57.02)TEC(+57.02)R.V | N | 62.55 | 1604.8126 | 13 | 0.2 | 535.9449 | 3 | 35.03 | 6 | F6:16539 | NaSa01\_F14.raw |  |  |  |  | 4.4155E6 | 6.0208E6 |  |  |  |  |  |  |  |  | 3 | 0 | 0 | 0 | 0 | 1 | 2 | 0 | 0 | 0 | 0 | 0 | 0 | 0 | 0 | 483 | 495 | Carbamidomethylation | C9:Carbamidomethylation:1000.00;C12:Carbamidomethylation:1000.00 | PEAKS DB |
| K.GVDVVVGGHTNTF.L | N | 61.79 | 1300.6411 | 13 | 1.1 | 651.3286 | 2 | 41.80 | 6 | F6:22128 | NaSa01\_F14.raw |  |  |  |  | 2.249E7 | 5.2209E7 |  |  |  |  |  |  |  |  | 2 | 0 | 0 | 0 | 0 | 1 | 1 | 0 | 0 | 0 | 0 | 0 | 0 | 0 | 0 | 249 | 261 |  |  | PEAKS DB |
| L.TAVLPFGGTFDLLQIK.G | N | 60.64 | 1718.9607 | 16 | -0.1 | 860.4875 | 2 | 99.74 | 6 | F6:71142 | NaSa01\_F14.raw |  |  | 2.8271E6 | 1.2518E6 | 2.1013E7 | 4.0691E6 |  |  |  |  |  |  |  |  | 4 | 0 | 0 | 1 | 1 | 1 | 1 | 0 | 0 | 0 | 0 | 0 | 0 | 0 | 0 | 427 | 442 |  |  | PEAKS DB |
| K.IINVGSEKVGIIGYTTK.E | N | 60.09 | 1791.0142 | 17 | -0.7 | 598.0116 | 3 | 45.32 | 6 | F6:25348 | NaSa01\_F14.raw |  |  |  |  | 9.4386E5 | 1.7014E6 |  |  |  |  |  |  |  |  | 2 | 0 | 0 | 0 | 0 | 1 | 1 | 0 | 0 | 0 | 0 | 0 | 0 | 0 | 0 | 177 | 193 |  |  | PEAKS DB |
| K.YLGYLNVIFDDKGK.V | Y | 58.45 | 1643.8558 | 14 | -0.3 | 548.9590 | 3 | 70.19 | 5 | F5:46798 | NaSa01\_F13.raw |  |  |  |  | 1.8927E6 |  |  |  |  |  |  |  |  |  | 2 | 0 | 0 | 0 | 0 | 2 | 0 | 0 | 0 | 0 | 0 | 0 | 0 | 0 | 0 | 300 | 313 |  |  | PEAKS DB |
| K.VFPAMEGR.V | N | 56.33 | 905.4429 | 8 | 0.2 | 453.7288 | 2 | 22.33 | 6 | F6:7722 | NaSa01\_F14.raw |  |  |  |  |  | 3.6244E6 |  |  |  |  |  |  |  |  | 2 | 0 | 0 | 0 | 0 | 0 | 2 | 0 | 0 | 0 | 0 | 0 | 0 | 0 | 0 | 553 | 560 |  |  | PEAKS DB |
| N.ISGYILPYK.I | N | 56.17 | 1052.5906 | 9 | 0.7 | 527.3029 | 2 | 45.82 | 6 | F6:26096 | NaSa01\_F14.raw |  |  |  | 2.2592E5 | 4.6763E8 | 8.5839E8 |  |  |  |  |  |  |  |  | 5 | 0 | 0 | 0 | 1 | 1 | 3 | 0 | 0 | 0 | 0 | 0 | 0 | 0 | 0 | 168 | 176 |  |  | PEAKS DB |
| S.FLAAGGDGYYM(+15.99)LK.G | N | 55.62 | 1420.6697 | 13 | -0.1 | 711.3420 | 2 | 48.69 | 6 | F6:28173 | NaSa01\_F14.raw |  |  |  |  |  | 5.7308E6 |  |  |  |  |  |  |  |  | 1 | 0 | 0 | 0 | 0 | 0 | 1 | 0 | 0 | 0 | 0 | 0 | 0 | 0 | 0 | 515 | 527 | Oxidation (M) | M11:Oxidation (M):1000.00 | PEAKS DB |
| H.GQGTGELLQVSGIK.V | N | 55.06 | 1385.7515 | 14 | 0.3 | 693.8832 | 2 | 45.20 | 6 | F6:25108 | NaSa01\_F14.raw |  |  |  |  |  | 1.2594E6 |  |  |  |  |  |  |  |  | 1 | 0 | 0 | 0 | 0 | 0 | 1 | 0 | 0 | 0 | 0 | 0 | 0 | 0 | 0 | 458 | 471 |  |  | PEAKS DB |
| K.GVDVVVGGHTNTFLY.T | N | 54.75 | 1576.7886 | 15 | 0.1 | 789.4016 | 2 | 63.80 | 6 | F6:41327 | NaSa01\_F14.raw |  |  |  |  | 1.2739E7 | 2.8201E7 |  |  |  |  |  |  |  |  | 2 | 0 | 0 | 0 | 0 | 1 | 1 | 0 | 0 | 0 | 0 | 0 | 0 | 0 | 0 | 249 | 263 |  |  | PEAKS DB |
| R.QVPVVQAYAF.G | N | 53.91 | 1120.5917 | 10 | 0.0 | 561.3031 | 2 | 73.70 | 6 | F6:49221 | NaSa01\_F14.raw |  |  |  |  | 7.1015E6 | 1.9997E7 |  |  |  |  |  |  |  |  | 2 | 0 | 0 | 0 | 0 | 1 | 1 | 0 | 0 | 0 | 0 | 0 | 0 | 0 | 0 | 288 | 297 |  |  | PEAKS DB |
| M.ALGNHEFDNGLNGLLDPLLK.N | N | 53.45 | 2149.1167 | 20 | 0.0 | 717.3795 | 3 | 83.56 | 6 | F6:57342 | NaSa01\_F14.raw |  |  |  |  | 5.3244E6 | 2.0787E7 |  |  |  |  |  |  |  |  | 3 | 0 | 0 | 0 | 0 | 1 | 2 | 0 | 0 | 0 | 0 | 0 | 0 | 0 | 0 | 128 | 147 |  |  | PEAKS DB |
| K.LTTLGVNK.I | N | 52.68 | 844.5018 | 8 | -1.0 | 423.2578 | 2 | 11.58 | 6 | F6:2148 | NaSa01\_F14.raw |  |  |  |  |  | 3.1326E6 |  |  |  |  |  |  |  |  | 1 | 0 | 0 | 0 | 0 | 0 | 1 | 0 | 0 | 0 | 0 | 0 | 0 | 0 | 0 | 221 | 228 |  |  | PEAKS DB |
| K.VLLPSFLAAGGDGY.Y | N | 51.68 | 1378.7133 | 14 | 0.7 | 690.3644 | 2 | 100.88 | 6 | F6:72024 | NaSa01\_F14.raw |  |  |  |  | 1.3644E6 | 2.8748E6 |  |  |  |  |  |  |  |  | 2 | 0 | 0 | 0 | 0 | 1 | 1 | 0 | 0 | 0 | 0 | 0 | 0 | 0 | 0 | 510 | 523 |  |  | PEAKS DB |
| F.GGTFDLLQIK.G | N | 50.44 | 1090.6022 | 10 | -0.6 | 546.3080 | 2 | 64.67 | 6 | F6:41967 | NaSa01\_F14.raw |  |  |  |  |  | 9.7795E7 |  |  |  |  |  |  |  |  | 2 | 0 | 0 | 0 | 0 | 0 | 2 | 0 | 0 | 0 | 0 | 0 | 0 | 0 | 0 | 433 | 442 |  |  | PEAKS DB |
| Y.LNVIFDDK.G | N | 50.15 | 962.5073 | 8 | -0.5 | 482.2607 | 2 | 40.33 | 6 | F6:21131 | NaSa01\_F14.raw |  |  |  |  |  | 1.4605E6 |  |  |  |  |  |  |  |  | 1 | 0 | 0 | 0 | 0 | 0 | 1 | 0 | 0 | 0 | 0 | 0 | 0 | 0 | 0 | 304 | 311 |  |  | PEAKS DB |
| K.VGIIGYTTK.E | N | 50.04 | 950.5436 | 9 | 0.3 | 476.2792 | 2 | 28.48 | 6 | F6:11194 | NaSa01\_F14.raw |  |  |  |  |  | 5.1319E6 |  |  |  |  |  |  |  |  | 2 | 0 | 0 | 0 | 0 | 0 | 2 | 0 | 0 | 0 | 0 | 0 | 0 | 0 | 0 | 185 | 193 |  |  | PEAKS DB |
| L.AAGGDGYYMLK.G | N | 49.71 | 1144.5222 | 11 | 0.7 | 573.2688 | 2 | 30.46 | 5 | F5:13468 | NaSa01\_F13.raw |  |  |  |  | 6.4456E6 |  |  |  |  |  |  |  |  |  | 1 | 0 | 0 | 0 | 0 | 1 | 0 | 0 | 0 | 0 | 0 | 0 | 0 | 0 | 0 | 517 | 527 |  |  | PEAKS DB |
| R.FHEC(+57.02)NLGNLIC(+57.02)DAVVY.N | N | 48.35 | 1922.8655 | 16 | 1.4 | 962.4414 | 2 | 82.45 | 5 | F5:56634 | NaSa01\_F13.raw |  |  |  |  | 8.0322E7 | 1.7562E8 |  |  |  |  |  |  |  | 2.4692E7 | 3 | 0 | 0 | 0 | 0 | 1 | 1 | 0 | 0 | 0 | 0 | 0 | 0 | 0 | 1 | 369 | 384 | Carbamidomethylation | C4:Carbamidomethylation:1000.00;C11:Carbamidomethylation:1000.00 | PEAKS DB |
| R.HPDDNEWNHVSMC(+57.02).I | N | 48.19 | 1639.6144 | 13 | 0.5 | 820.8149 | 2 | 26.12 | 5 | F5:10348 | NaSa01\_F13.raw |  |  |  |  | 8.8799E6 |  |  |  |  |  |  |  |  |  | 2 | 0 | 0 | 0 | 0 | 2 | 0 | 0 | 0 | 0 | 0 | 0 | 0 | 0 | 0 | 389 | 401 | Carbamidomethylation | C13:Carbamidomethylation:1000.00 | PEAKS DB |
| K.VLLPSFLAAGGD.G | N | 47.35 | 1158.6284 | 12 | -0.1 | 580.3214 | 2 | 96.95 | 6 | F6:68512 | NaSa01\_F14.raw |  |  |  |  |  | 1.1427E6 |  |  |  |  |  |  |  |  | 1 | 0 | 0 | 0 | 0 | 0 | 1 | 0 | 0 | 0 | 0 | 0 | 0 | 0 | 0 | 510 | 521 |  |  | PEAKS DB |
| R.HPDDNEWNHVSM.C | N | 47.22 | 1479.5837 | 12 | 1.1 | 740.8000 | 2 | 25.93 | 5 | F5:10070 | NaSa01\_F13.raw |  |  |  |  | 2.3098E6 |  |  |  |  |  |  |  |  |  | 1 | 0 | 0 | 0 | 0 | 1 | 0 | 0 | 0 | 0 | 0 | 0 | 0 | 0 | 0 | 389 | 400 |  |  | PEAKS DB |
| R.QVPVVQAY.A | N | 45.65 | 902.4861 | 8 | -0.5 | 452.2501 | 2 | 43.38 | 5 | F5:24541 | NaSa01\_F13.raw |  |  |  |  | 1.2052E7 |  |  |  |  |  |  |  |  |  | 1 | 0 | 0 | 0 | 0 | 1 | 0 | 0 | 0 | 0 | 0 | 0 | 0 | 0 | 0 | 288 | 295 |  |  | PEAKS DB |
| N.PGPYLEFR.D | N | 45.44 | 977.4970 | 8 | 0.7 | 489.7561 | 2 | 39.90 | 6 | F6:20609 | NaSa01\_F14.raw |  |  |  |  |  | 4.6995E5 |  |  |  |  |  |  |  |  | 1 | 0 | 0 | 0 | 0 | 0 | 1 | 0 | 0 | 0 | 0 | 0 | 0 | 0 | 0 | 201 | 208 |  |  | PEAKS DB |
| N.LGNLIC(+57.02)DAVVYNNLR.H | N | 45.20 | 1732.8931 | 15 | 1.0 | 867.4547 | 2 | 69.10 | 6 | F6:45743 | NaSa01\_F14.raw |  |  |  |  |  | 1.1316E6 |  |  |  |  |  |  |  |  | 1 | 0 | 0 | 0 | 0 | 0 | 1 | 0 | 0 | 0 | 0 | 0 | 0 | 0 | 0 | 374 | 388 | Carbamidomethylation | C6:Carbamidomethylation:1000.00 | PEAKS DB |
| K.IINVGSEK.V | N | 44.70 | 858.4811 | 8 | -0.5 | 430.2476 | 2 | 11.48 | 6 | F6:1861 | NaSa01\_F14.raw |  |  |  |  |  | 1.3857E6 |  |  |  |  |  |  |  |  | 1 | 0 | 0 | 0 | 0 | 0 | 1 | 0 | 0 | 0 | 0 | 0 | 0 | 0 | 0 | 177 | 184 |  |  | PEAKS DB |
| K.VLLPSFLAAGGDGYY.M | N | 43.81 | 1541.7765 | 15 | 0.7 | 771.8961 | 2 | 105.28 | 6 | F6:75496 | NaSa01\_F14.raw |  |  |  |  | 1.4762E7 | 3.1893E7 |  |  |  |  |  |  |  |  | 2 | 0 | 0 | 0 | 0 | 1 | 1 | 0 | 0 | 0 | 0 | 0 | 0 | 0 | 0 | 510 | 524 |  |  | PEAKS DB |
| K.FPILSANIR.P | N | 43.38 | 1029.5970 | 9 | -0.4 | 515.8056 | 2 | 52.72 | 6 | F6:31843 | NaSa01\_F14.raw |  |  |  |  |  | 1.5657E8 |  |  |  |  |  |  |  |  | 1 | 0 | 0 | 0 | 0 | 0 | 1 | 0 | 0 | 0 | 0 | 0 | 0 | 0 | 0 | 151 | 159 |  |  | PEAKS DB |
| K.VLLPSFLAAGGDGYYM.L | N | 42.72 | 1672.8170 | 16 | 0.9 | 837.4166 | 2 | 111.51 | 6 | F6:80284 | NaSa01\_F14.raw |  |  |  |  |  | 1.1099E6 |  |  |  |  |  |  |  |  | 1 | 0 | 0 | 0 | 0 | 0 | 1 | 0 | 0 | 0 | 0 | 0 | 0 | 0 | 0 | 510 | 525 |  |  | PEAKS DB |
| Q.GTGELLQVSGIK.V | N | 42.14 | 1200.6714 | 12 | 0.9 | 601.3435 | 2 | 44.43 | 6 | F6:24568 | NaSa01\_F14.raw |  |  |  |  |  | 7.0353E5 |  |  |  |  |  |  |  |  | 1 | 0 | 0 | 0 | 0 | 0 | 1 | 0 | 0 | 0 | 0 | 0 | 0 | 0 | 0 | 460 | 471 |  |  | PEAKS DB |
| Y.LGYLNVIFDDK.G | N | 41.11 | 1295.6761 | 11 | 0.9 | 648.8459 | 2 | 76.55 | 6 | F6:51743 | NaSa01\_F14.raw |  |  |  |  |  | 0 |  |  |  |  |  |  |  |  | 0 | 0 | 0 | 0 | 0 | 0 | 0 | 0 | 0 | 0 | 0 | 0 | 0 | 0 | 0 | 301 | 311 |  |  | PEAKS DB |
| K.LTTLGVNKIIALGH.S | N | 41.05 | 1448.8715 | 14 | 0.1 | 483.9645 | 3 | 54.77 | 6 | F6:33352 | NaSa01\_F14.raw |  |  |  |  |  | 5.5856E5 |  |  |  |  |  |  |  |  | 1 | 0 | 0 | 0 | 0 | 0 | 1 | 0 | 0 | 0 | 0 | 0 | 0 | 0 | 0 | 221 | 234 |  |  | PEAKS DB |
| N.HEFDNGLNGLLDPLLK.N | N | 40.62 | 1793.9312 | 16 | 0.2 | 598.9844 | 3 | 81.57 | 6 | F6:55994 | NaSa01\_F14.raw |  |  |  |  |  | 5.7086E5 |  |  |  |  |  |  |  |  | 1 | 0 | 0 | 0 | 0 | 0 | 1 | 0 | 0 | 0 | 0 | 0 | 0 | 0 | 0 | 132 | 147 |  |  | PEAKS DB |
| N.YPFMQLSDDGR.Q | N | 40.57 | 1327.5867 | 11 | 0.4 | 664.8009 | 2 | 53.65 | 6 | F6:32287 | NaSa01\_F14.raw |  |  |  |  |  | 5.3395E5 |  |  |  |  |  |  |  |  | 1 | 0 | 0 | 0 | 0 | 0 | 1 | 0 | 0 | 0 | 0 | 0 | 0 | 0 | 0 | 277 | 287 |  |  | PEAKS DB |
| P.AGNYPFMQLSDDGR.Q | N | 40.07 | 1569.6881 | 14 | 0.1 | 785.8514 | 2 | 56.81 | 6 | F6:35126 | NaSa01\_F14.raw |  |  |  |  |  | 2.5054E5 |  |  |  |  |  |  |  |  | 1 | 0 | 0 | 0 | 0 | 0 | 1 | 0 | 0 | 0 | 0 | 0 | 0 | 0 | 0 | 274 | 287 |  |  | PEAKS DB |
| K.NVKFPILSAN.I | N | 39.82 | 1101.6182 | 10 | 0.5 | 551.8167 | 2 | 54.61 | 6 | F6:33186 | NaSa01\_F14.raw |  |  |  |  |  | 1.3385E6 |  |  |  |  |  |  |  |  | 1 | 0 | 0 | 0 | 0 | 0 | 1 | 0 | 0 | 0 | 0 | 0 | 0 | 0 | 0 | 148 | 157 |  |  | PEAKS DB |
| K.IIALGHSGF.K | N | 37.88 | 913.5021 | 9 | -1.2 | 457.7578 | 2 | 43.37 | 6 | F6:23538 | NaSa01\_F14.raw |  |  |  |  |  | 5.7946E6 |  |  |  |  |  |  |  |  | 1 | 0 | 0 | 0 | 0 | 0 | 1 | 0 | 0 | 0 | 0 | 0 | 0 | 0 | 0 | 229 | 237 |  |  | PEAKS DB |
| total 58 peptides |  |  |  |  |  |  |  |  |  |  |  |  |  |  |  |  |  |  |  |  |  |  |  |  |  |  |  |  |  |  |  |  |  |  |  |  |  |  |  |  |  |  |  |  |  |
| --- | --- | --- | --- | --- | --- | --- | --- | --- | --- | --- | --- | --- | --- | --- | --- | --- | --- | --- | --- | --- | --- | --- | --- | --- | --- | --- | --- | --- | --- | --- | --- | --- | --- | --- | --- | --- | --- | --- | --- | --- | --- | --- | --- | --- | --- |

Q9PVK7.1

back to list

  

| Protein Coverage
| Supporting Peptides
|

Protein Coverage:

Supporting Peptides:

| Peptide | Uniq | -10lgP | Mass | Length | ppm | m/z | z | RT | Fraction | Scan | Source File | Area F1 | Area F10 | Area F11 | Area F12 | Area F13 | Area F14 | Area F2 | Area F3 | Area F4 | Area F5 | Area F6 | Area F7 | Area F8 | Area F9 | #Feature | #Feature F1 | #Feature F10 | #Feature F11 | #Feature F12 | #Feature F13 | #Feature F14 | #Feature F2 | #Feature F3 | #Feature F4 | #Feature F5 | #Feature F6 | #Feature F7 | #Feature F8 | #Feature F9 | Start | End | PTM | AScore | Found By |
| --- | --- | --- | --- | --- | --- | --- | --- | --- | --- | --- | --- | --- | --- | --- | --- | --- | --- | --- | --- | --- | --- | --- | --- | --- | --- | --- | --- | --- | --- | --- | --- | --- | --- | --- | --- | --- | --- | --- | --- | --- | --- | --- | --- | --- | --- |
| R.MVAITMAHEMGHNLGMNHDK.G | Y | 96.31 | 2236.0010 | 20 | -0.6 | 1119.0071 | 2 | 34.94 | 6 | F6:16576 | NaSa01\_F14.raw |  |  |  |  | 2.3112E6 | 4.5092E9 |  |  |  |  |  |  |  |  | 11 | 0 | 0 | 0 | 0 | 1 | 10 | 0 | 0 | 0 | 0 | 0 | 0 | 0 | 0 | 322 | 341 |  |  | PEAKS DB |
| K.DDC(+57.02)DLPELC(+57.02)TGQSAEC(+57.02)PTDVFQR.N | N | 95.78 | 2712.1101 | 23 | -0.8 | 1357.0613 | 2 | 72.67 | 6 | F6:48718 | NaSa01\_F14.raw |  |  |  |  |  | 6.3934E8 |  |  |  | 5.9579E5 |  |  |  |  | 10 | 0 | 0 | 0 | 0 | 0 | 9 | 0 | 0 | 0 | 1 | 0 | 0 | 0 | 0 | 459 | 481 | Carbamidomethylation | C3:Carbamidomethylation:1000.00;C9:Carbamidomethylation:1000.00;C16:Carbamidomethylation:1000.00 | PEAKS DB |
| K.RNDNAQLLTGIDFNGTPVGLAYIGSIC(+57.02)NPK.T | N | 88.48 | 3217.6189 | 30 | 0.1 | 1073.5470 | 3 | 89.06 | 6 | F6:62183 | NaSa01\_F14.raw |  |  |  |  |  | 1.5231E7 |  |  |  |  |  |  |  |  | 1 | 0 | 0 | 0 | 0 | 0 | 1 | 0 | 0 | 0 | 0 | 0 | 0 | 0 | 0 | 278 | 307 | Carbamidomethylation | C27:Carbamidomethylation:1000.00 | PEAKS DB |
| R.NDNAQLLTGIDFNGTPVGLAYIGSIC(+57.02)NPK.T | N | 82.31 | 3061.5178 | 29 | 1.9 | 1021.5151 | 3 | 99.98 | 6 | F6:71311 | NaSa01\_F14.raw |  |  | 2.6315E5 |  |  | 1.5398E8 |  |  |  |  |  |  |  |  | 4 | 0 | 0 | 1 | 0 | 0 | 3 | 0 | 0 | 0 | 0 | 0 | 0 | 0 | 0 | 279 | 307 | Carbamidomethylation | C26:Carbamidomethylation:1000.00 | PEAKS DB |
| N.GTPVGLAYIGSIC(+57.02)NPK.T | N | 82.13 | 1645.8497 | 16 | -0.6 | 823.9316 | 2 | 61.34 | 6 | F6:39114 | NaSa01\_F14.raw |  |  |  |  |  | 9.3648E6 |  |  |  |  |  |  |  |  | 1 | 0 | 0 | 0 | 0 | 0 | 1 | 0 | 0 | 0 | 0 | 0 | 0 | 0 | 0 | 292 | 307 | Carbamidomethylation | C13:Carbamidomethylation:1000.00 | PEAKS DB |
| R.MVAITM(+15.99)AHEMGHNLGMNHDK.G | Y | 82.10 | 2251.9958 | 20 | 0.6 | 751.6730 | 3 | 18.62 | 6 | F6:4489 | NaSa01\_F14.raw |  |  |  |  |  | 6.078E8 |  |  |  |  |  |  |  |  | 6 | 0 | 0 | 0 | 0 | 0 | 6 | 0 | 0 | 0 | 0 | 0 | 0 | 0 | 0 | 322 | 341 | Oxidation (M) | M6:Oxidation (M):39.35 | PEAKS DB |
| Y.IGSIC(+57.02)NPKTSAAVVQDYSK.S | Y | 81.96 | 2037.0200 | 19 | 0.6 | 680.0143 | 3 | 29.13 | 6 | F6:11490 | NaSa01\_F14.raw |  |  |  |  |  | 6.8292E6 |  |  |  |  |  |  |  |  | 1 | 0 | 0 | 0 | 0 | 0 | 1 | 0 | 0 | 0 | 0 | 0 | 0 | 0 | 0 | 300 | 318 | Carbamidomethylation | C5:Carbamidomethylation:1000.00 | PEAKS DB |
| R.M(+15.99)VAITMAHEMGHNLGMNHDK.G | Y | 80.21 | 2251.9958 | 20 | -0.3 | 751.6724 | 3 | 25.50 | 6 | F6:8718 | NaSa01\_F14.raw |  |  |  |  |  | 6.5118E8 |  |  |  |  |  |  |  |  | 2 | 0 | 0 | 0 | 0 | 0 | 2 | 0 | 0 | 0 | 0 | 0 | 0 | 0 | 0 | 322 | 341 | Oxidation (M) | M1:Oxidation (M):102.43 | PEAKS DB |
| R.MVAITMAHEMGHNLGM(+15.99)NHDK.G | Y | 79.63 | 2251.9958 | 20 | 1.0 | 564.0068 | 4 | 25.50 | 6 | F6:10496 | NaSa01\_F14.raw |  |  |  |  |  | 1.1394E9 |  |  |  |  |  |  |  |  | 7 | 0 | 0 | 0 | 0 | 0 | 7 | 0 | 0 | 0 | 0 | 0 | 0 | 0 | 0 | 322 | 341 | Oxidation (M) | M16:Oxidation (M):71.27 | PEAKS DB |
| R.AAKDDC(+57.02)DLPELC(+57.02)TGQSAEC(+57.02)PTDVFQR.N | N | 79.07 | 2982.2793 | 26 | 0.8 | 995.1012 | 3 | 55.91 | 6 | F6:34388 | NaSa01\_F14.raw |  |  |  |  |  | 1.5419E8 |  |  |  |  |  |  |  |  | 2 | 0 | 0 | 0 | 0 | 0 | 2 | 0 | 0 | 0 | 0 | 0 | 0 | 0 | 0 | 456 | 481 | Carbamidomethylation | C6:Carbamidomethylation:1000.00;C12:Carbamidomethylation:1000.00;C19:Carbamidomethylation:1000.00 | PEAKS DB |
| K.C(+57.02)PIMTNQC(+57.02)IALR.G | N | 77.84 | 1475.7047 | 12 | 0.0 | 738.8596 | 2 | 39.74 | 6 | F6:21215 | NaSa01\_F14.raw |  |  | 3.3287E5 | 1.3692E6 | 9.8113E6 | 2.4068E9 |  |  |  | 1.7213E7 | 6.4357E6 |  | 4.0715E6 | 1.3044E6 | 15 | 0 | 0 | 1 | 1 | 2 | 5 | 0 | 0 | 0 | 2 | 2 | 0 | 1 | 1 | 497 | 508 | Carbamidomethylation | C1:Carbamidomethylation:1000.00;C8:Carbamidomethylation:1000.00 | PEAKS DB |
| R.NSMIC(+57.02)NC(+57.02)SISPR.D | N | 77.03 | 1437.6163 | 12 | -0.1 | 719.8154 | 2 | 22.49 | 6 | F6:6540 | NaSa01\_F14.raw |  |  |  |  |  | 7.6177E6 |  |  |  |  |  |  |  |  | 2 | 0 | 0 | 0 | 0 | 0 | 2 | 0 | 0 | 0 | 0 | 0 | 0 | 0 | 0 | 558 | 569 | Carbamidomethylation | C5:Carbamidomethylation:1000.00;C7:Carbamidomethylation:1000.00 | PEAKS DB |
| R.DPSYGMVEPGTK.C | N | 75.11 | 1279.5754 | 12 | 0.3 | 640.7952 | 2 | 29.01 | 12 | F12:14288 | NaSa01\_F7.raw |  |  |  |  | 1.9762E6 |  |  |  |  | 6.0782E5 | 1.1257E7 | 6.592E6 | 1.3719E6 |  | 5 | 0 | 0 | 0 | 0 | 1 | 0 | 0 | 0 | 0 | 1 | 1 | 1 | 1 | 0 | 570 | 581 |  |  | PEAKS DB |
| K.VYEM(+15.99)INTMNMIYR.R | N | 74.23 | 1692.7673 | 13 | -0.2 | 847.3907 | 2 | 57.49 | 6 | F6:35287 | NaSa01\_F14.raw |  |  |  |  |  | 4.3266E8 |  |  |  |  |  |  |  |  | 9 | 0 | 0 | 0 | 0 | 0 | 9 | 0 | 0 | 0 | 0 | 0 | 0 | 0 | 0 | 220 | 232 | Oxidation (M) | M4:Oxidation (M):77.51 | PEAKS DB |
| K.VYEMINTMNMIYR.R | N | 73.76 | 1676.7725 | 13 | -0.3 | 839.3932 | 2 | 71.51 | 6 | F6:48424 | NaSa01\_F14.raw |  |  |  |  |  | 9.5037E8 |  |  |  |  |  |  |  |  | 12 | 0 | 0 | 0 | 0 | 0 | 12 | 0 | 0 | 0 | 0 | 0 | 0 | 0 | 0 | 220 | 232 |  |  | PEAKS DB |
| D.LPELC(+57.02)TGQSAEC(+57.02)PTDVFQR.N | N | 73.30 | 2206.9988 | 19 | 0.2 | 1104.5068 | 2 | 60.04 | 6 | F6:38037 | NaSa01\_F14.raw |  |  |  |  |  | 2.7029E6 |  |  |  |  |  |  |  |  | 1 | 0 | 0 | 0 | 0 | 0 | 1 | 0 | 0 | 0 | 0 | 0 | 0 | 0 | 0 | 463 | 481 | Carbamidomethylation | C5:Carbamidomethylation:1000.00;C12:Carbamidomethylation:1000.00 | PEAKS DB |
| K.C(+57.02)PIM(+15.99)TNQC(+57.02)IALR.G | N | 73.02 | 1491.6996 | 12 | 0.1 | 746.8572 | 2 | 30.97 | 6 | F6:13155 | NaSa01\_F14.raw |  |  |  |  | 1.4708E6 | 1.6503E8 |  |  |  |  |  |  |  |  | 5 | 0 | 0 | 0 | 0 | 1 | 4 | 0 | 0 | 0 | 0 | 0 | 0 | 0 | 0 | 497 | 508 | Carbamidomethylation; Oxidation (M) | C1:Carbamidomethylation:1000.00;M4:Oxidation (M):1000.00;C8:Carbamidomethylation:1000.00 | PEAKS DB |
| K.TSAAVVQDYSK.S | Y | 73.00 | 1167.5771 | 11 | -0.1 | 584.7958 | 2 | 11.56 | 6 | F6:2129 | NaSa01\_F14.raw |  |  |  |  |  | 1.6873E7 |  |  |  |  |  |  |  |  | 1 | 0 | 0 | 0 | 0 | 0 | 1 | 0 | 0 | 0 | 0 | 0 | 0 | 0 | 0 | 308 | 318 |  |  | PEAKS DB |
| R.KVYEMINTMNMIYR.R | N | 72.83 | 1804.8674 | 14 | 0.6 | 903.4415 | 2 | 58.08 | 6 | F6:36256 | NaSa01\_F14.raw |  |  |  |  |  | 3.0426E6 |  |  |  |  |  |  |  |  | 1 | 0 | 0 | 0 | 0 | 0 | 1 | 0 | 0 | 0 | 0 | 0 | 0 | 0 | 0 | 219 | 232 |  |  | PEAKS DB |
| R.TKPAYQFSSC(+57.02)SVR.E | N | 72.78 | 1529.7296 | 13 | 0.4 | 765.8724 | 2 | 11.56 | 6 | F6:1943 | NaSa01\_F14.raw |  |  |  |  |  | 1.656E7 |  |  |  |  |  |  |  |  | 3 | 0 | 0 | 0 | 0 | 0 | 3 | 0 | 0 | 0 | 0 | 0 | 0 | 0 | 0 | 358 | 370 | Carbamidomethylation | C10:Carbamidomethylation:1000.00 | PEAKS DB |
| K.VYEMINTM(+15.99)NMIYR.R | N | 72.14 | 1692.7673 | 13 | -0.2 | 847.3907 | 2 | 57.49 | 6 | F6:36159 | NaSa01\_F14.raw |  |  |  |  |  | 4.3022E8 |  |  |  |  |  |  |  |  | 8 | 0 | 0 | 0 | 0 | 0 | 8 | 0 | 0 | 0 | 0 | 0 | 0 | 0 | 0 | 220 | 232 | Oxidation (M) | M8:Oxidation (M):79.36 | PEAKS DB |
| K.VYEMINTMNM(+15.99)IYR.R | N | 71.71 | 1692.7673 | 13 | -2.0 | 565.2619 | 3 | 61.54 | 6 | F6:39303 | NaSa01\_F14.raw |  |  |  |  |  | 2.4734E8 |  |  |  |  |  |  |  |  | 6 | 0 | 0 | 0 | 0 | 0 | 6 | 0 | 0 | 0 | 0 | 0 | 0 | 0 | 0 | 220 | 232 | Oxidation (M) | M10:Oxidation (M):28.86 | PEAKS DB |
| R.MVAITMAHEMGH.N | N | 70.23 | 1326.5883 | 12 | 0.1 | 664.3015 | 2 | 30.77 | 6 | F6:12852 | NaSa01\_F14.raw |  |  |  |  | 5.8936E5 | 1.005E8 |  |  |  |  |  |  |  |  | 4 | 0 | 0 | 0 | 0 | 1 | 3 | 0 | 0 | 0 | 0 | 0 | 0 | 0 | 0 | 322 | 333 |  |  | PEAKS DB |
| L.EIWSNINEINVQSDVK.A | Y | 68.84 | 1886.9374 | 16 | 1.2 | 944.4771 | 2 | 64.03 | 6 | F6:41453 | NaSa01\_F14.raw |  |  |  |  |  | 5.0333E6 |  |  |  |  |  |  |  |  | 1 | 0 | 0 | 0 | 0 | 0 | 1 | 0 | 0 | 0 | 0 | 0 | 0 | 0 | 0 | 244 | 259 |  |  | PEAKS DB |
| R.MVAITMAHEM(+15.99)GHNLGMNHDK.G | Y | 68.79 | 2251.9958 | 20 | -0.1 | 751.6725 | 3 | 34.90 | 6 | F6:16250 | NaSa01\_F14.raw |  |  |  |  |  | 1.0488E9 |  |  |  |  |  |  |  |  | 7 | 0 | 0 | 0 | 0 | 0 | 7 | 0 | 0 | 0 | 0 | 0 | 0 | 0 | 0 | 322 | 341 | Oxidation (M) | M10:Oxidation (M):30.02 | PEAKS DB |
| R.KVYEMINTMNM(+15.99)IYR.R | N | 67.76 | 1820.8623 | 14 | 0.0 | 607.9614 | 3 | 49.96 | 6 | F6:29587 | NaSa01\_F14.raw |  |  |  |  |  | 7.8852E6 |  |  |  |  |  |  |  |  | 1 | 0 | 0 | 0 | 0 | 0 | 1 | 0 | 0 | 0 | 0 | 0 | 0 | 0 | 0 | 219 | 232 | Oxidation (M) | M11:Oxidation (M):36.05 | PEAKS DB |
| R.NSM(+15.99)IC(+57.02)NC(+57.02)SISPR.D | N | 66.06 | 1453.6112 | 12 | 0.3 | 727.8131 | 2 | 11.56 | 6 | F6:1942 | NaSa01\_F14.raw |  |  |  |  |  | 2.1578E6 |  |  |  |  |  |  |  |  | 4 | 0 | 0 | 0 | 0 | 0 | 4 | 0 | 0 | 0 | 0 | 0 | 0 | 0 | 0 | 558 | 569 | Oxidation (M); Carbamidomethylation | M3:Oxidation (M):1000.00;C5:Carbamidomethylation:1000.00;C7:Carbamidomethylation:1000.00 | PEAKS DB |
| K.YIEFYM(+15.99)VVDNIMYR.H | Y | 65.66 | 1870.8634 | 14 | 1.4 | 936.4403 | 2 | 94.34 | 6 | F6:66655 | NaSa01\_F14.raw |  |  |  |  |  | 7.0109E6 |  |  |  |  |  |  |  |  | 4 | 0 | 0 | 0 | 0 | 0 | 4 | 0 | 0 | 0 | 0 | 0 | 0 | 0 | 0 | 194 | 207 | Oxidation (M) | M6:Oxidation (M):56.47 | PEAKS DB |
| K.ATLDLFGEWR.E | N | 64.95 | 1206.6033 | 10 | -1.0 | 604.3083 | 2 | 78.54 | 6 | F6:53113 | NaSa01\_F14.raw |  |  | 1.7813E6 | 2.0969E6 | 9.7051E6 | 2.2371E9 |  |  |  |  |  |  |  | 1.3846E6 | 13 | 0 | 0 | 1 | 1 | 1 | 9 | 0 | 0 | 0 | 0 | 0 | 0 | 0 | 1 | 260 | 269 |  |  | PEAKS DB |
| R.DSC(+57.02)FTLNQR.T | N | 63.93 | 1139.5029 | 9 | -0.1 | 570.7587 | 2 | 25.28 | 6 | F6:8701 | NaSa01\_F14.raw |  |  |  |  |  | 8.7088E6 |  |  |  |  |  |  |  |  | 2 | 0 | 0 | 0 | 0 | 0 | 2 | 0 | 0 | 0 | 0 | 0 | 0 | 0 | 0 | 517 | 525 | Carbamidomethylation | C3:Carbamidomethylation:1000.00 | PEAKS DB |
| K.VYEM(+15.99)INTMNM(+15.99)IYR.R | N | 61.29 | 1708.7623 | 13 | -0.3 | 855.3882 | 2 | 45.10 | 6 | F6:25392 | NaSa01\_F14.raw |  |  |  |  |  | 4.4184E7 |  |  |  |  |  |  |  |  | 1 | 0 | 0 | 0 | 0 | 0 | 1 | 0 | 0 | 0 | 0 | 0 | 0 | 0 | 0 | 220 | 232 | Oxidation (M) | M4:Oxidation (M):79.36;M10:Oxidation (M):27.16 | PEAKS DB |
| R.MVAITMAHEMGHNLGMNHD.K | N | 59.40 | 2107.9060 | 19 | 0.0 | 703.6426 | 3 | 45.80 | 6 | F6:25307 | NaSa01\_F14.raw |  |  |  |  |  | 7.6575E6 |  |  |  |  |  |  |  |  | 1 | 0 | 0 | 0 | 0 | 0 | 1 | 0 | 0 | 0 | 0 | 0 | 0 | 0 | 0 | 322 | 340 |  |  | PEAKS DB |
| R.MVAITM(+15.99)AHEMGHNLGM(+15.99)NHDK.G | Y | 58.59 | 2267.9907 | 20 | -1.0 | 568.0044 | 4 | 25.51 | 6 | F6:9709 | NaSa01\_F14.raw |  |  |  |  |  | 6.2433E7 |  |  |  |  |  |  |  |  | 3 | 0 | 0 | 0 | 0 | 0 | 3 | 0 | 0 | 0 | 0 | 0 | 0 | 0 | 0 | 322 | 341 | Oxidation (M) | M6:Oxidation (M):29.93;M16:Oxidation (M):53.33 | PEAKS DB |
| R.DRPQC(+57.02)ILNKPLSTDIVSPPIC(+57.02)GN.Y | N | 58.34 | 2593.2993 | 23 | -0.3 | 1297.6565 | 2 | 63.62 | 6 | F6:40889 | NaSa01\_F14.raw |  |  |  |  |  | 2.087E9 |  |  |  |  |  |  |  |  | 4 | 0 | 0 | 0 | 0 | 0 | 4 | 0 | 0 | 0 | 0 | 0 | 0 | 0 | 0 | 379 | 401 | Carbamidomethylation | C5:Carbamidomethylation:1000.00;C21:Carbamidomethylation:1000.00 | PEAKS DB |
| K.VYEMINTM(+15.99)NM(+15.99)IYR.R | N | 57.62 | 1708.7623 | 13 | -0.3 | 855.3882 | 2 | 45.10 | 6 | F6:25162 | NaSa01\_F14.raw |  |  |  |  |  | 4.4184E7 |  |  |  |  |  |  |  |  | 1 | 0 | 0 | 0 | 0 | 0 | 1 | 0 | 0 | 0 | 0 | 0 | 0 | 0 | 0 | 220 | 232 | Oxidation (M) | M8:Oxidation (M):60.62;M10:Oxidation (M):65.06 | PEAKS DB |
| R.M(+15.99)VAITMAHEMGHNLGM(+15.99)NHDK.G | Y | 57.42 | 2267.9907 | 20 | -0.1 | 568.0049 | 4 | 19.11 | 6 | F6:4424 | NaSa01\_F14.raw |  |  |  |  |  | 6.6833E7 |  |  |  |  |  |  |  |  | 4 | 0 | 0 | 0 | 0 | 0 | 4 | 0 | 0 | 0 | 0 | 0 | 0 | 0 | 0 | 322 | 341 | Oxidation (M) | M1:Oxidation (M):76.17;M16:Oxidation (M):50.87 | PEAKS DB |
| Y.MVVDNIMYR.H | Y | 56.61 | 1139.5468 | 9 | 0.8 | 570.7811 | 2 | 45.30 | 6 | F6:25326 | NaSa01\_F14.raw |  |  |  |  |  | 1.66E9 |  |  |  |  |  |  |  |  | 5 | 0 | 0 | 0 | 0 | 0 | 5 | 0 | 0 | 0 | 0 | 0 | 0 | 0 | 0 | 199 | 207 |  |  | PEAKS DB |
| E.IWSNINEINVQSDVK.A | Y | 56.19 | 1757.8948 | 15 | 0.7 | 879.9553 | 2 | 55.02 | 6 | F6:33706 | NaSa01\_F14.raw |  |  |  |  |  | 1.6563E6 |  |  |  |  |  |  |  |  | 1 | 0 | 0 | 0 | 0 | 0 | 1 | 0 | 0 | 0 | 0 | 0 | 0 | 0 | 0 | 245 | 259 |  |  | PEAKS DB |
| Q.LLTGIDFNGTPVGLAYIGSIC(+57.02)NPK.T | N | 56.04 | 2519.3093 | 24 | 0.7 | 1260.6628 | 2 | 95.97 | 6 | F6:68185 | NaSa01\_F14.raw |  |  |  |  |  | 4.6093E6 |  |  |  |  |  |  |  |  | 2 | 0 | 0 | 0 | 0 | 0 | 2 | 0 | 0 | 0 | 0 | 0 | 0 | 0 | 0 | 284 | 307 | Carbamidomethylation | C21:Carbamidomethylation:1000.00 | PEAKS DB |
| F.YMVVDNIMYR.H | Y | 55.65 | 1302.6101 | 10 | 0.7 | 652.3128 | 2 | 55.69 | 6 | F6:34367 | NaSa01\_F14.raw |  |  |  |  |  | 4.341E7 |  |  |  |  |  |  |  |  | 1 | 0 | 0 | 0 | 0 | 0 | 1 | 0 | 0 | 0 | 0 | 0 | 0 | 0 | 0 | 198 | 207 |  |  | PEAKS DB |
| K.GFC(+57.02)TC(+57.02)GFNK.C | N | 54.46 | 1089.4371 | 9 | -0.5 | 545.7256 | 2 | 11.57 | 6 | F6:1962 | NaSa01\_F14.raw |  |  |  |  |  | 2.5576E6 |  |  |  |  |  |  |  |  | 1 | 0 | 0 | 0 | 0 | 0 | 1 | 0 | 0 | 0 | 0 | 0 | 0 | 0 | 0 | 342 | 350 | Carbamidomethylation | C3:Carbamidomethylation:1000.00;C5:Carbamidomethylation:1000.00 | PEAKS DB |
| Y.M(+15.99)VVDNIMYR.H | Y | 54.07 | 1155.5416 | 9 | 0.0 | 578.7781 | 2 | 31.32 | 6 | F6:15096 | NaSa01\_F14.raw |  |  |  |  |  | 1.7774E8 |  |  |  |  |  |  |  |  | 2 | 0 | 0 | 0 | 0 | 0 | 2 | 0 | 0 | 0 | 0 | 0 | 0 | 0 | 0 | 199 | 207 | Oxidation (M) | M1:Oxidation (M):115.44 | PEAKS DB |
| Y.EMINTMNMIYR.R | N | 53.03 | 1414.6407 | 11 | -0.2 | 708.3275 | 2 | 57.05 | 6 | F6:35453 | NaSa01\_F14.raw |  |  |  |  |  | 4.7389E6 |  |  |  |  |  |  |  |  | 1 | 0 | 0 | 0 | 0 | 0 | 1 | 0 | 0 | 0 | 0 | 0 | 0 | 0 | 0 | 222 | 232 |  |  | PEAKS DB |
| M.INTMNMIYR.R | N | 51.67 | 1154.5576 | 9 | 0.4 | 578.2863 | 2 | 38.32 | 6 | F6:19378 | NaSa01\_F14.raw |  |  |  |  |  | 0 |  |  |  |  |  |  |  |  | 0 | 0 | 0 | 0 | 0 | 0 | 0 | 0 | 0 | 0 | 0 | 0 | 0 | 0 | 0 | 224 | 232 |  |  | PEAKS DB |
| R.KVYEMINTMN.M | N | 51.42 | 1241.5785 | 10 | 0.3 | 621.7967 | 2 | 33.25 | 6 | F6:15075 | NaSa01\_F14.raw |  |  |  |  |  | 2.3194E7 |  |  |  |  |  |  |  |  | 1 | 0 | 0 | 0 | 0 | 0 | 1 | 0 | 0 | 0 | 0 | 0 | 0 | 0 | 0 | 219 | 228 |  |  | PEAKS DB |
| R.MVAITMAHEM.G | N | 51.15 | 1132.5079 | 10 | 1.1 | 567.2618 | 2 | 44.18 | 6 | F6:24291 | NaSa01\_F14.raw |  |  |  |  |  | 4.7043E6 |  |  |  |  |  |  |  |  | 1 | 0 | 0 | 0 | 0 | 0 | 1 | 0 | 0 | 0 | 0 | 0 | 0 | 0 | 0 | 322 | 331 |  |  | PEAKS DB |
| Y.MVVDNIM(+15.99)YR.H | Y | 51.01 | 1155.5416 | 9 | 0.0 | 578.7781 | 2 | 31.32 | 6 | F6:13340 | NaSa01\_F14.raw |  |  |  |  |  | 1.7774E8 |  |  |  |  |  |  |  |  | 2 | 0 | 0 | 0 | 0 | 0 | 2 | 0 | 0 | 0 | 0 | 0 | 0 | 0 | 0 | 199 | 207 | Oxidation (M) | M7:Oxidation (M):113.36 | PEAKS DB |
| F.YM(+15.99)VVDNIMYR.H | Y | 50.84 | 1318.6050 | 10 | 0.4 | 660.3101 | 2 | 45.71 | 6 | F6:25735 | NaSa01\_F14.raw |  |  |  |  |  | 5.0243E6 |  |  |  |  |  |  |  |  | 2 | 0 | 0 | 0 | 0 | 0 | 2 | 0 | 0 | 0 | 0 | 0 | 0 | 0 | 0 | 198 | 207 | Oxidation (M) | M2:Oxidation (M):111.20 | PEAKS DB |
| K.VYEM(+15.99)INTM(+15.99)NMIYR.R | N | 50.77 | 1708.7623 | 13 | -0.3 | 855.3882 | 2 | 45.10 | 6 | F6:25618 | NaSa01\_F14.raw |  |  |  |  |  | 5.1185E7 |  |  |  |  |  |  |  |  | 3 | 0 | 0 | 0 | 0 | 0 | 3 | 0 | 0 | 0 | 0 | 0 | 0 | 0 | 0 | 220 | 232 | Oxidation (M) | M4:Oxidation (M):83.65;M8:Oxidation (M):18.23 | PEAKS DB |
| R.TKPAYQFSSC(+57.02).S | N | 50.58 | 1187.5281 | 10 | 0.3 | 594.7715 | 2 | 11.58 | 6 | F6:1956 | NaSa01\_F14.raw |  |  |  |  |  | 3.6584E6 |  |  |  |  |  |  |  |  | 1 | 0 | 0 | 0 | 0 | 0 | 1 | 0 | 0 | 0 | 0 | 0 | 0 | 0 | 0 | 358 | 367 | Carbamidomethylation | C10:Carbamidomethylation:1000.00 | PEAKS DB |
| K.VYEMINTMN.M | N | 49.68 | 1113.4835 | 9 | 0.3 | 557.7492 | 2 | 52.12 | 6 | F6:31163 | NaSa01\_F14.raw |  |  |  |  |  | 1.6784E8 |  |  |  |  |  |  |  |  | 1 | 0 | 0 | 0 | 0 | 0 | 1 | 0 | 0 | 0 | 0 | 0 | 0 | 0 | 0 | 220 | 228 |  |  | PEAKS DB |
| R.MVAITMAHEM(+15.99)GHNLGM(+15.99)NHDK.G | Y | 49.53 | 2267.9907 | 20 | -1.0 | 568.0044 | 4 | 25.51 | 6 | F6:9498 | NaSa01\_F14.raw |  |  |  |  |  | 4.8512E7 |  |  |  |  |  |  |  |  | 1 | 0 | 0 | 0 | 0 | 0 | 1 | 0 | 0 | 0 | 0 | 0 | 0 | 0 | 0 | 322 | 341 | Oxidation (M) | M10:Oxidation (M):8.26;M16:Oxidation (M):21.51 | PEAKS DB |
| C.PIMTNQC(+57.02)IALR.G | N | 49.03 | 1315.6741 | 11 | 0.4 | 658.8446 | 2 | 37.06 | 6 | F6:18281 | NaSa01\_F14.raw |  |  |  |  |  | 1.277E6 |  |  |  |  |  |  |  |  | 1 | 0 | 0 | 0 | 0 | 0 | 1 | 0 | 0 | 0 | 0 | 0 | 0 | 0 | 0 | 498 | 508 | Carbamidomethylation | C7:Carbamidomethylation:1000.00 | PEAKS DB |
| K.RNDNAQLLTGIDFNGTPVGLAY.I | N | 47.41 | 2348.1760 | 22 | 0.6 | 1175.0959 | 2 | 90.55 | 6 | F6:63498 | NaSa01\_F14.raw |  |  |  |  |  | 4.2697E6 |  |  |  |  |  |  |  |  | 1 | 0 | 0 | 0 | 0 | 0 | 1 | 0 | 0 | 0 | 0 | 0 | 0 | 0 | 0 | 278 | 299 |  |  | PEAKS DB |
| K.VYEM(+15.99)INTM(+15.99)NM(+15.99)IYR.R | N | 46.27 | 1724.7572 | 13 | -0.7 | 863.3853 | 2 | 32.47 | 6 | F6:14255 | NaSa01\_F14.raw |  |  |  |  |  | 3.9176E6 |  |  |  |  |  |  |  |  | 1 | 0 | 0 | 0 | 0 | 0 | 1 | 0 | 0 | 0 | 0 | 0 | 0 | 0 | 0 | 220 | 232 | Oxidation (M) | M4:Oxidation (M):1000.00;M8:Oxidation (M):1000.00;M10:Oxidation (M):1000.00 | PEAKS DB |
| R.MVAITMAH.E | N | 45.87 | 872.4249 | 8 | -0.1 | 437.2197 | 2 | 30.29 | 6 | F6:13244 | NaSa01\_F14.raw |  |  |  |  | 1.6148E6 | 2.3115E6 |  |  |  |  |  |  |  |  | 4 | 0 | 0 | 0 | 0 | 2 | 2 | 0 | 0 | 0 | 0 | 0 | 0 | 0 | 0 | 322 | 329 |  |  | PEAKS DB |
| K.VYEMINTM(+15.99)N.M | N | 45.82 | 1129.4784 | 9 | 0.2 | 565.7466 | 2 | 42.89 | 6 | F6:23229 | NaSa01\_F14.raw |  |  |  |  |  | 0 |  |  |  |  |  |  |  |  | 0 | 0 | 0 | 0 | 0 | 0 | 0 | 0 | 0 | 0 | 0 | 0 | 0 | 0 | 0 | 220 | 228 | Oxidation (M) | M8:Oxidation (M):52.34 | PEAKS DB |
| R.MVAITMAHEMGHNLGM.N | N | 45.75 | 1741.7772 | 16 | 0.5 | 871.8964 | 2 | 58.51 | 6 | F6:36744 | NaSa01\_F14.raw |  |  |  |  |  | 2.3274E6 |  |  |  |  |  |  |  |  | 1 | 0 | 0 | 0 | 0 | 0 | 1 | 0 | 0 | 0 | 0 | 0 | 0 | 0 | 0 | 322 | 337 |  |  | PEAKS DB |
| R.EITTSPPVQDHC(+57.02)YYHGY.F | N | 44.97 | 2065.8840 | 17 | 6.3 | 689.6360 | 3 | 31.90 | 8 | F8:16761 | NaSa01\_F3.raw |  |  |  |  |  |  |  | 3.1849E6 |  |  |  |  |  |  | 1 | 0 | 0 | 0 | 0 | 0 | 0 | 0 | 1 | 0 | 0 | 0 | 0 | 0 | 0 | 85 | 101 | Carbamidomethylation | C12:Carbamidomethylation:1000.00 | PEAKS DB |
| R.NDNAQLLTGIDFNGTPVGL.A | N | 44.91 | 1957.9745 | 19 | 0.5 | 979.9950 | 2 | 104.18 | 6 | F6:74818 | NaSa01\_F14.raw |  |  |  |  |  | 2.0756E7 |  |  |  |  |  |  |  |  | 1 | 0 | 0 | 0 | 0 | 0 | 1 | 0 | 0 | 0 | 0 | 0 | 0 | 0 | 0 | 279 | 297 |  |  | PEAKS DB |
| L.TGIDFNGTPVGLAYIGSIC(+57.02)NPK.T | N | 44.70 | 2293.1411 | 22 | -0.2 | 1147.5776 | 2 | 85.72 | 6 | F6:59453 | NaSa01\_F14.raw |  |  |  |  |  | 3.0432E6 |  |  |  |  |  |  |  |  | 1 | 0 | 0 | 0 | 0 | 0 | 1 | 0 | 0 | 0 | 0 | 0 | 0 | 0 | 0 | 286 | 307 | Carbamidomethylation | C19:Carbamidomethylation:1000.00 | PEAKS DB |
| R.DRPQC(+57.02)ILNKPL.S | N | 44.62 | 1352.7234 | 11 | 1.1 | 451.9156 | 3 | 27.50 | 5 | F5:11578 | NaSa01\_F13.raw |  |  |  |  | 1.9485E7 |  |  |  |  |  |  |  |  |  | 1 | 0 | 0 | 0 | 0 | 1 | 0 | 0 | 0 | 0 | 0 | 0 | 0 | 0 | 0 | 379 | 389 | Carbamidomethylation | C5:Carbamidomethylation:1000.00 | PEAKS DB |
| L.TNTPEQDR.Y | N | 44.60 | 959.4308 | 8 | 0.1 | 480.7227 | 2 | 11.42 | 6 | F6:1814 | NaSa01\_F14.raw |  |  |  |  |  | 1.6427E5 |  |  |  |  |  |  |  |  | 1 | 0 | 0 | 0 | 0 | 0 | 1 | 0 | 0 | 0 | 0 | 0 | 0 | 0 | 0 | 180 | 187 |  |  | PEAKS DB |
| R.MVAITM(+15.99)AHEMGH.N | N | 44.44 | 1342.5833 | 12 | -0.2 | 672.2988 | 2 | 30.93 | 6 | F6:12991 | NaSa01\_F14.raw |  |  |  |  |  | 1.3599E7 |  |  |  |  |  |  |  |  | 2 | 0 | 0 | 0 | 0 | 0 | 2 | 0 | 0 | 0 | 0 | 0 | 0 | 0 | 0 | 322 | 333 | Oxidation (M) | M6:Oxidation (M):8.80 | PEAKS DB |
| F.YMVVDNIM(+15.99)YR.H | Y | 44.15 | 1318.6050 | 10 | 0.4 | 660.3101 | 2 | 44.13 | 6 | F6:24416 | NaSa01\_F14.raw |  |  |  |  |  | 3.8934E6 |  |  |  |  |  |  |  |  | 1 | 0 | 0 | 0 | 0 | 0 | 1 | 0 | 0 | 0 | 0 | 0 | 0 | 0 | 0 | 198 | 207 | Oxidation (M) | M8:Oxidation (M):87.26 | PEAKS DB |
| A.TLDLFGEWR.E | N | 43.65 | 1135.5662 | 9 | -0.8 | 568.7899 | 2 | 77.17 | 6 | F6:52295 | NaSa01\_F14.raw |  |  |  |  |  | 1.3701E6 |  |  |  |  |  |  |  |  | 1 | 0 | 0 | 0 | 0 | 0 | 1 | 0 | 0 | 0 | 0 | 0 | 0 | 0 | 0 | 261 | 269 |  |  | PEAKS DB |
| R.YLQAEKYIEFY.M | N | 43.34 | 1465.7129 | 11 | 0.9 | 733.8644 | 2 | 68.34 | 6 | F6:44984 | NaSa01\_F14.raw |  |  |  |  |  | 1.4083E6 |  |  |  |  |  |  |  |  | 1 | 0 | 0 | 0 | 0 | 0 | 1 | 0 | 0 | 0 | 0 | 0 | 0 | 0 | 0 | 188 | 198 |  |  | PEAKS DB |
| R.MVAITM(+15.99)AHEM(+15.99)GHNLGMNHDK.G | Y | 43.14 | 2267.9907 | 20 | -0.3 | 568.0048 | 4 | 11.55 | 6 | F6:1862 | NaSa01\_F14.raw |  |  |  |  |  | 1.3427E7 |  |  |  |  |  |  |  |  | 2 | 0 | 0 | 0 | 0 | 0 | 2 | 0 | 0 | 0 | 0 | 0 | 0 | 0 | 0 | 322 | 341 | Oxidation (M) | M6:Oxidation (M):51.63;M10:Oxidation (M):33.66 | PEAKS DB |
| G.TPVGLAYIGSIC(+57.02)NPK.T | N | 42.66 | 1588.8282 | 15 | 1.1 | 795.4222 | 2 | 58.70 | 12 | F12:38993 | NaSa01\_F7.raw |  |  |  |  |  |  |  |  |  |  |  | 1.3171E5 |  |  | 1 | 0 | 0 | 0 | 0 | 0 | 0 | 0 | 0 | 0 | 0 | 0 | 1 | 0 | 0 | 293 | 307 | Carbamidomethylation | C12:Carbamidomethylation:1000.00 | PEAKS DB |
| R.KVYEMINTM(+15.99)NM(+15.99)IYR.R | N | 41.47 | 1836.8573 | 14 | 0.6 | 613.2934 | 3 | 33.38 | 6 | F6:15316 | NaSa01\_F14.raw |  |  |  |  |  | 1.4433E6 |  |  |  |  |  |  |  |  | 1 | 0 | 0 | 0 | 0 | 0 | 1 | 0 | 0 | 0 | 0 | 0 | 0 | 0 | 0 | 219 | 232 | Oxidation (M) | M9:Oxidation (M):58.99;M11:Oxidation (M):78.19 | PEAKS DB |
| K.YIEFYM(+15.99)VVDNIM(+15.99)YR.H | Y | 41.29 | 1886.8583 | 14 | -0.3 | 944.4361 | 2 | 79.64 | 6 | F6:54338 | NaSa01\_F14.raw |  |  |  |  |  | 4.7293E6 |  |  |  |  |  |  |  |  | 2 | 0 | 0 | 0 | 0 | 0 | 2 | 0 | 0 | 0 | 0 | 0 | 0 | 0 | 0 | 194 | 207 | Oxidation (M) | M6:Oxidation (M):1000.00;M12:Oxidation (M):1000.00 | PEAKS DB |
| R.M(+15.99)VAITMAHEMGH.N | N | 40.25 | 1342.5833 | 12 | -0.3 | 448.5349 | 3 | 25.47 | 6 | F6:9076 | NaSa01\_F14.raw |  |  |  |  |  | 1.8423E7 |  |  |  |  |  |  |  |  | 2 | 0 | 0 | 0 | 0 | 0 | 2 | 0 | 0 | 0 | 0 | 0 | 0 | 0 | 0 | 322 | 333 | Oxidation (M) | M1:Oxidation (M):10.53 | PEAKS DB |
| G.LAYIGSIC(+57.02)NPK.T | N | 38.59 | 1234.6379 | 11 | 0.2 | 618.3264 | 2 | 37.01 | 12 | F12:20710 | NaSa01\_F7.raw |  |  |  |  |  |  |  |  |  |  |  | 3.5988E5 |  |  | 1 | 0 | 0 | 0 | 0 | 0 | 0 | 0 | 0 | 0 | 0 | 0 | 1 | 0 | 0 | 297 | 307 | Carbamidomethylation | C8:Carbamidomethylation:1000.00 | PEAKS DB |
| K.PAYQFSSC(+57.02)SVR.E | N | 38.45 | 1300.5870 | 11 | 0.0 | 651.3008 | 2 | 23.13 | 6 | F6:6974 | NaSa01\_F14.raw |  |  |  |  |  | 1.0807E6 |  |  |  |  |  |  |  |  | 1 | 0 | 0 | 0 | 0 | 0 | 1 | 0 | 0 | 0 | 0 | 0 | 0 | 0 | 0 | 360 | 370 | Carbamidomethylation | C8:Carbamidomethylation:1000.00 | PEAKS DB |
| F.NGTPVGLAYIGSIC(+57.02)NPK.T | N | 38.29 | 1759.8927 | 17 | -1.0 | 880.9528 | 2 | 61.44 | 6 | F6:39069 | NaSa01\_F14.raw |  |  |  |  |  | 6.4633E5 |  |  |  |  |  |  |  |  | 1 | 0 | 0 | 0 | 0 | 0 | 1 | 0 | 0 | 0 | 0 | 0 | 0 | 0 | 0 | 291 | 307 | Carbamidomethylation | C14:Carbamidomethylation:1000.00 | PEAKS DB |
| total 75 peptides |  |  |  |  |  |  |  |  |  |  |  |  |  |  |  |  |  |  |  |  |  |  |  |  |  |  |  |  |  |  |  |  |  |  |  |  |  |  |  |  |  |  |  |  |  |
| --- | --- | --- | --- | --- | --- | --- | --- | --- | --- | --- | --- | --- | --- | --- | --- | --- | --- | --- | --- | --- | --- | --- | --- | --- | --- | --- | --- | --- | --- | --- | --- | --- | --- | --- | --- | --- | --- | --- | --- | --- | --- | --- | --- | --- | --- |

AAF00693.1

back to list

  

| Protein Coverage
| Supporting Peptides
|

Protein Coverage:

Supporting Peptides:

| Peptide | Uniq | -10lgP | Mass | Length | ppm | m/z | z | RT | Fraction | Scan | Source File | Area F1 | Area F10 | Area F11 | Area F12 | Area F13 | Area F14 | Area F2 | Area F3 | Area F4 | Area F5 | Area F6 | Area F7 | Area F8 | Area F9 | #Feature | #Feature F1 | #Feature F10 | #Feature F11 | #Feature F12 | #Feature F13 | #Feature F14 | #Feature F2 | #Feature F3 | #Feature F4 | #Feature F5 | #Feature F6 | #Feature F7 | #Feature F8 | #Feature F9 | Start | End | PTM | AScore | Found By |
| --- | --- | --- | --- | --- | --- | --- | --- | --- | --- | --- | --- | --- | --- | --- | --- | --- | --- | --- | --- | --- | --- | --- | --- | --- | --- | --- | --- | --- | --- | --- | --- | --- | --- | --- | --- | --- | --- | --- | --- | --- | --- | --- | --- | --- | --- |
| R.MVAITMAHEMGHNLGMNHDK.G | Y | 96.31 | 2236.0010 | 20 | -0.6 | 1119.0071 | 2 | 34.94 | 6 | F6:16576 | NaSa01\_F14.raw |  |  |  |  | 2.3112E6 | 4.5092E9 |  |  |  |  |  |  |  |  | 11 | 0 | 0 | 0 | 0 | 1 | 10 | 0 | 0 | 0 | 0 | 0 | 0 | 0 | 0 | 322 | 341 |  |  | PEAKS DB |
| K.DDC(+57.02)DLPELC(+57.02)TGQSAEC(+57.02)PTDVFQR.N | N | 95.78 | 2712.1101 | 23 | -0.8 | 1357.0613 | 2 | 72.67 | 6 | F6:48718 | NaSa01\_F14.raw |  |  |  |  |  | 6.3934E8 |  |  |  | 5.9579E5 |  |  |  |  | 10 | 0 | 0 | 0 | 0 | 0 | 9 | 0 | 0 | 0 | 1 | 0 | 0 | 0 | 0 | 459 | 481 | Carbamidomethylation | C3:Carbamidomethylation:1000.00;C9:Carbamidomethylation:1000.00;C16:Carbamidomethylation:1000.00 | PEAKS DB |
| K.RNDNAQLLTGIDFNGTPVGLAYIGSIC(+57.02)NPK.T | N | 88.48 | 3217.6189 | 30 | 0.1 | 1073.5470 | 3 | 89.06 | 6 | F6:62183 | NaSa01\_F14.raw |  |  |  |  |  | 1.5231E7 |  |  |  |  |  |  |  |  | 1 | 0 | 0 | 0 | 0 | 0 | 1 | 0 | 0 | 0 | 0 | 0 | 0 | 0 | 0 | 278 | 307 | Carbamidomethylation | C27:Carbamidomethylation:1000.00 | PEAKS DB |
| R.NDNAQLLTGIDFNGTPVGLAYIGSIC(+57.02)NPK.T | N | 82.31 | 3061.5178 | 29 | 1.9 | 1021.5151 | 3 | 99.98 | 6 | F6:71311 | NaSa01\_F14.raw |  |  | 2.6315E5 |  |  | 1.5398E8 |  |  |  |  |  |  |  |  | 4 | 0 | 0 | 1 | 0 | 0 | 3 | 0 | 0 | 0 | 0 | 0 | 0 | 0 | 0 | 279 | 307 | Carbamidomethylation | C26:Carbamidomethylation:1000.00 | PEAKS DB |
| N.GTPVGLAYIGSIC(+57.02)NPK.T | N | 82.13 | 1645.8497 | 16 | -0.6 | 823.9316 | 2 | 61.34 | 6 | F6:39114 | NaSa01\_F14.raw |  |  |  |  |  | 9.3648E6 |  |  |  |  |  |  |  |  | 1 | 0 | 0 | 0 | 0 | 0 | 1 | 0 | 0 | 0 | 0 | 0 | 0 | 0 | 0 | 292 | 307 | Carbamidomethylation | C13:Carbamidomethylation:1000.00 | PEAKS DB |
| R.MVAITM(+15.99)AHEMGHNLGMNHDK.G | Y | 82.10 | 2251.9958 | 20 | 0.6 | 751.6730 | 3 | 18.62 | 6 | F6:4489 | NaSa01\_F14.raw |  |  |  |  |  | 6.078E8 |  |  |  |  |  |  |  |  | 6 | 0 | 0 | 0 | 0 | 0 | 6 | 0 | 0 | 0 | 0 | 0 | 0 | 0 | 0 | 322 | 341 | Oxidation (M) | M6:Oxidation (M):39.35 | PEAKS DB |
| Y.IGSIC(+57.02)NPKTSAAVVQDYSK.S | Y | 81.96 | 2037.0200 | 19 | 0.6 | 680.0143 | 3 | 29.13 | 6 | F6:11490 | NaSa01\_F14.raw |  |  |  |  |  | 6.8292E6 |  |  |  |  |  |  |  |  | 1 | 0 | 0 | 0 | 0 | 0 | 1 | 0 | 0 | 0 | 0 | 0 | 0 | 0 | 0 | 300 | 318 | Carbamidomethylation | C5:Carbamidomethylation:1000.00 | PEAKS DB |
| R.M(+15.99)VAITMAHEMGHNLGMNHDK.G | Y | 80.21 | 2251.9958 | 20 | -0.3 | 751.6724 | 3 | 25.50 | 6 | F6:8718 | NaSa01\_F14.raw |  |  |  |  |  | 6.5118E8 |  |  |  |  |  |  |  |  | 2 | 0 | 0 | 0 | 0 | 0 | 2 | 0 | 0 | 0 | 0 | 0 | 0 | 0 | 0 | 322 | 341 | Oxidation (M) | M1:Oxidation (M):102.43 | PEAKS DB |
| R.MVAITMAHEMGHNLGM(+15.99)NHDK.G | Y | 79.63 | 2251.9958 | 20 | 1.0 | 564.0068 | 4 | 25.50 | 6 | F6:10496 | NaSa01\_F14.raw |  |  |  |  |  | 1.1394E9 |  |  |  |  |  |  |  |  | 7 | 0 | 0 | 0 | 0 | 0 | 7 | 0 | 0 | 0 | 0 | 0 | 0 | 0 | 0 | 322 | 341 | Oxidation (M) | M16:Oxidation (M):71.27 | PEAKS DB |
| R.AAKDDC(+57.02)DLPELC(+57.02)TGQSAEC(+57.02)PTDVFQR.N | N | 79.07 | 2982.2793 | 26 | 0.8 | 995.1012 | 3 | 55.91 | 6 | F6:34388 | NaSa01\_F14.raw |  |  |  |  |  | 1.5419E8 |  |  |  |  |  |  |  |  | 2 | 0 | 0 | 0 | 0 | 0 | 2 | 0 | 0 | 0 | 0 | 0 | 0 | 0 | 0 | 456 | 481 | Carbamidomethylation | C6:Carbamidomethylation:1000.00;C12:Carbamidomethylation:1000.00;C19:Carbamidomethylation:1000.00 | PEAKS DB |
| K.C(+57.02)PIMTNQC(+57.02)IALR.G | N | 77.84 | 1475.7047 | 12 | 0.0 | 738.8596 | 2 | 39.74 | 6 | F6:21215 | NaSa01\_F14.raw |  |  | 3.3287E5 | 1.3692E6 | 9.8113E6 | 2.4068E9 |  |  |  | 1.7213E7 | 6.4357E6 |  | 4.0715E6 | 1.3044E6 | 15 | 0 | 0 | 1 | 1 | 2 | 5 | 0 | 0 | 0 | 2 | 2 | 0 | 1 | 1 | 497 | 508 | Carbamidomethylation | C1:Carbamidomethylation:1000.00;C8:Carbamidomethylation:1000.00 | PEAKS DB |
| R.NSMIC(+57.02)NC(+57.02)SISPR.D | N | 77.03 | 1437.6163 | 12 | -0.1 | 719.8154 | 2 | 22.49 | 6 | F6:6540 | NaSa01\_F14.raw |  |  |  |  |  | 7.6177E6 |  |  |  |  |  |  |  |  | 2 | 0 | 0 | 0 | 0 | 0 | 2 | 0 | 0 | 0 | 0 | 0 | 0 | 0 | 0 | 558 | 569 | Carbamidomethylation | C5:Carbamidomethylation:1000.00;C7:Carbamidomethylation:1000.00 | PEAKS DB |
| R.DPSYGMVEPGTK.C | N | 75.11 | 1279.5754 | 12 | 0.3 | 640.7952 | 2 | 29.01 | 12 | F12:14288 | NaSa01\_F7.raw |  |  |  |  | 1.9762E6 |  |  |  |  | 6.0782E5 | 1.1257E7 | 6.592E6 | 1.3719E6 |  | 5 | 0 | 0 | 0 | 0 | 1 | 0 | 0 | 0 | 0 | 1 | 1 | 1 | 1 | 0 | 570 | 581 |  |  | PEAKS DB |
| K.VYEM(+15.99)INTMNMIYR.R | N | 74.23 | 1692.7673 | 13 | -0.2 | 847.3907 | 2 | 57.49 | 6 | F6:35287 | NaSa01\_F14.raw |  |  |  |  |  | 4.3266E8 |  |  |  |  |  |  |  |  | 9 | 0 | 0 | 0 | 0 | 0 | 9 | 0 | 0 | 0 | 0 | 0 | 0 | 0 | 0 | 220 | 232 | Oxidation (M) | M4:Oxidation (M):77.51 | PEAKS DB |
| K.VYEMINTMNMIYR.R | N | 73.76 | 1676.7725 | 13 | -0.3 | 839.3932 | 2 | 71.51 | 6 | F6:48424 | NaSa01\_F14.raw |  |  |  |  |  | 9.5037E8 |  |  |  |  |  |  |  |  | 12 | 0 | 0 | 0 | 0 | 0 | 12 | 0 | 0 | 0 | 0 | 0 | 0 | 0 | 0 | 220 | 232 |  |  | PEAKS DB |
| D.LPELC(+57.02)TGQSAEC(+57.02)PTDVFQR.N | N | 73.30 | 2206.9988 | 19 | 0.2 | 1104.5068 | 2 | 60.04 | 6 | F6:38037 | NaSa01\_F14.raw |  |  |  |  |  | 2.7029E6 |  |  |  |  |  |  |  |  | 1 | 0 | 0 | 0 | 0 | 0 | 1 | 0 | 0 | 0 | 0 | 0 | 0 | 0 | 0 | 463 | 481 | Carbamidomethylation | C5:Carbamidomethylation:1000.00;C12:Carbamidomethylation:1000.00 | PEAKS DB |
| K.C(+57.02)PIM(+15.99)TNQC(+57.02)IALR.G | N | 73.02 | 1491.6996 | 12 | 0.1 | 746.8572 | 2 | 30.97 | 6 | F6:13155 | NaSa01\_F14.raw |  |  |  |  | 1.4708E6 | 1.6503E8 |  |  |  |  |  |  |  |  | 5 | 0 | 0 | 0 | 0 | 1 | 4 | 0 | 0 | 0 | 0 | 0 | 0 | 0 | 0 | 497 | 508 | Carbamidomethylation; Oxidation (M) | C1:Carbamidomethylation:1000.00;M4:Oxidation (M):1000.00;C8:Carbamidomethylation:1000.00 | PEAKS DB |
| K.TSAAVVQDYSK.S | Y | 73.00 | 1167.5771 | 11 | -0.1 | 584.7958 | 2 | 11.56 | 6 | F6:2129 | NaSa01\_F14.raw |  |  |  |  |  | 1.6873E7 |  |  |  |  |  |  |  |  | 1 | 0 | 0 | 0 | 0 | 0 | 1 | 0 | 0 | 0 | 0 | 0 | 0 | 0 | 0 | 308 | 318 |  |  | PEAKS DB |
| R.KVYEMINTMNMIYR.R | N | 72.83 | 1804.8674 | 14 | 0.6 | 903.4415 | 2 | 58.08 | 6 | F6:36256 | NaSa01\_F14.raw |  |  |  |  |  | 3.0426E6 |  |  |  |  |  |  |  |  | 1 | 0 | 0 | 0 | 0 | 0 | 1 | 0 | 0 | 0 | 0 | 0 | 0 | 0 | 0 | 219 | 232 |  |  | PEAKS DB |
| R.TKPAYQFSSC(+57.02)SVR.E | N | 72.78 | 1529.7296 | 13 | 0.4 | 765.8724 | 2 | 11.56 | 6 | F6:1943 | NaSa01\_F14.raw |  |  |  |  |  | 1.656E7 |  |  |  |  |  |  |  |  | 3 | 0 | 0 | 0 | 0 | 0 | 3 | 0 | 0 | 0 | 0 | 0 | 0 | 0 | 0 | 358 | 370 | Carbamidomethylation | C10:Carbamidomethylation:1000.00 | PEAKS DB |
| K.VYEMINTM(+15.99)NMIYR.R | N | 72.14 | 1692.7673 | 13 | -0.2 | 847.3907 | 2 | 57.49 | 6 | F6:36159 | NaSa01\_F14.raw |  |  |  |  |  | 4.3022E8 |  |  |  |  |  |  |  |  | 8 | 0 | 0 | 0 | 0 | 0 | 8 | 0 | 0 | 0 | 0 | 0 | 0 | 0 | 0 | 220 | 232 | Oxidation (M) | M8:Oxidation (M):79.36 | PEAKS DB |
| K.VYEMINTMNM(+15.99)IYR.R | N | 71.71 | 1692.7673 | 13 | -2.0 | 565.2619 | 3 | 61.54 | 6 | F6:39303 | NaSa01\_F14.raw |  |  |  |  |  | 2.4734E8 |  |  |  |  |  |  |  |  | 6 | 0 | 0 | 0 | 0 | 0 | 6 | 0 | 0 | 0 | 0 | 0 | 0 | 0 | 0 | 220 | 232 | Oxidation (M) | M10:Oxidation (M):28.86 | PEAKS DB |
| R.MVAITMAHEMGH.N | N | 70.23 | 1326.5883 | 12 | 0.1 | 664.3015 | 2 | 30.77 | 6 | F6:12852 | NaSa01\_F14.raw |  |  |  |  | 5.8936E5 | 1.005E8 |  |  |  |  |  |  |  |  | 4 | 0 | 0 | 0 | 0 | 1 | 3 | 0 | 0 | 0 | 0 | 0 | 0 | 0 | 0 | 322 | 333 |  |  | PEAKS DB |
| L.EIWSNINEINVQSDVK.A | Y | 68.84 | 1886.9374 | 16 | 1.2 | 944.4771 | 2 | 64.03 | 6 | F6:41453 | NaSa01\_F14.raw |  |  |  |  |  | 5.0333E6 |  |  |  |  |  |  |  |  | 1 | 0 | 0 | 0 | 0 | 0 | 1 | 0 | 0 | 0 | 0 | 0 | 0 | 0 | 0 | 244 | 259 |  |  | PEAKS DB |
| R.MVAITMAHEM(+15.99)GHNLGMNHDK.G | Y | 68.79 | 2251.9958 | 20 | -0.1 | 751.6725 | 3 | 34.90 | 6 | F6:16250 | NaSa01\_F14.raw |  |  |  |  |  | 1.0488E9 |  |  |  |  |  |  |  |  | 7 | 0 | 0 | 0 | 0 | 0 | 7 | 0 | 0 | 0 | 0 | 0 | 0 | 0 | 0 | 322 | 341 | Oxidation (M) | M10:Oxidation (M):30.02 | PEAKS DB |
| R.KVYEMINTMNM(+15.99)IYR.R | N | 67.76 | 1820.8623 | 14 | 0.0 | 607.9614 | 3 | 49.96 | 6 | F6:29587 | NaSa01\_F14.raw |  |  |  |  |  | 7.8852E6 |  |  |  |  |  |  |  |  | 1 | 0 | 0 | 0 | 0 | 0 | 1 | 0 | 0 | 0 | 0 | 0 | 0 | 0 | 0 | 219 | 232 | Oxidation (M) | M11:Oxidation (M):36.05 | PEAKS DB |
| R.NSM(+15.99)IC(+57.02)NC(+57.02)SISPR.D | N | 66.06 | 1453.6112 | 12 | 0.3 | 727.8131 | 2 | 11.56 | 6 | F6:1942 | NaSa01\_F14.raw |  |  |  |  |  | 2.1578E6 |  |  |  |  |  |  |  |  | 4 | 0 | 0 | 0 | 0 | 0 | 4 | 0 | 0 | 0 | 0 | 0 | 0 | 0 | 0 | 558 | 569 | Oxidation (M); Carbamidomethylation | M3:Oxidation (M):1000.00;C5:Carbamidomethylation:1000.00;C7:Carbamidomethylation:1000.00 | PEAKS DB |
| K.YIEFYM(+15.99)VVDNIMYR.H | Y | 65.66 | 1870.8634 | 14 | 1.4 | 936.4403 | 2 | 94.34 | 6 | F6:66655 | NaSa01\_F14.raw |  |  |  |  |  | 7.0109E6 |  |  |  |  |  |  |  |  | 4 | 0 | 0 | 0 | 0 | 0 | 4 | 0 | 0 | 0 | 0 | 0 | 0 | 0 | 0 | 194 | 207 | Oxidation (M) | M6:Oxidation (M):56.47 | PEAKS DB |
| K.ATLDLFGEWR.E | N | 64.95 | 1206.6033 | 10 | -1.0 | 604.3083 | 2 | 78.54 | 6 | F6:53113 | NaSa01\_F14.raw |  |  | 1.7813E6 | 2.0969E6 | 9.7051E6 | 2.2371E9 |  |  |  |  |  |  |  | 1.3846E6 | 13 | 0 | 0 | 1 | 1 | 1 | 9 | 0 | 0 | 0 | 0 | 0 | 0 | 0 | 1 | 260 | 269 |  |  | PEAKS DB |
| R.DSC(+57.02)FTLNQR.T | N | 63.93 | 1139.5029 | 9 | -0.1 | 570.7587 | 2 | 25.28 | 6 | F6:8701 | NaSa01\_F14.raw |  |  |  |  |  | 8.7088E6 |  |  |  |  |  |  |  |  | 2 | 0 | 0 | 0 | 0 | 0 | 2 | 0 | 0 | 0 | 0 | 0 | 0 | 0 | 0 | 517 | 525 | Carbamidomethylation | C3:Carbamidomethylation:1000.00 | PEAKS DB |
| K.VYEM(+15.99)INTMNM(+15.99)IYR.R | N | 61.29 | 1708.7623 | 13 | -0.3 | 855.3882 | 2 | 45.10 | 6 | F6:25392 | NaSa01\_F14.raw |  |  |  |  |  | 4.4184E7 |  |  |  |  |  |  |  |  | 1 | 0 | 0 | 0 | 0 | 0 | 1 | 0 | 0 | 0 | 0 | 0 | 0 | 0 | 0 | 220 | 232 | Oxidation (M) | M4:Oxidation (M):79.36;M10:Oxidation (M):27.16 | PEAKS DB |
| R.MVAITMAHEMGHNLGMNHD.K | N | 59.40 | 2107.9060 | 19 | 0.0 | 703.6426 | 3 | 45.80 | 6 | F6:25307 | NaSa01\_F14.raw |  |  |  |  |  | 7.6575E6 |  |  |  |  |  |  |  |  | 1 | 0 | 0 | 0 | 0 | 0 | 1 | 0 | 0 | 0 | 0 | 0 | 0 | 0 | 0 | 322 | 340 |  |  | PEAKS DB |
| R.MVAITM(+15.99)AHEMGHNLGM(+15.99)NHDK.G | Y | 58.59 | 2267.9907 | 20 | -1.0 | 568.0044 | 4 | 25.51 | 6 | F6:9709 | NaSa01\_F14.raw |  |  |  |  |  | 6.2433E7 |  |  |  |  |  |  |  |  | 3 | 0 | 0 | 0 | 0 | 0 | 3 | 0 | 0 | 0 | 0 | 0 | 0 | 0 | 0 | 322 | 341 | Oxidation (M) | M6:Oxidation (M):29.93;M16:Oxidation (M):53.33 | PEAKS DB |
| R.DRPQC(+57.02)ILNKPLSTDIVSPPIC(+57.02)GN.Y | N | 58.34 | 2593.2993 | 23 | -0.3 | 1297.6565 | 2 | 63.62 | 6 | F6:40889 | NaSa01\_F14.raw |  |  |  |  |  | 2.087E9 |  |  |  |  |  |  |  |  | 4 | 0 | 0 | 0 | 0 | 0 | 4 | 0 | 0 | 0 | 0 | 0 | 0 | 0 | 0 | 379 | 401 | Carbamidomethylation | C5:Carbamidomethylation:1000.00;C21:Carbamidomethylation:1000.00 | PEAKS DB |
| K.VYEMINTM(+15.99)NM(+15.99)IYR.R | N | 57.62 | 1708.7623 | 13 | -0.3 | 855.3882 | 2 | 45.10 | 6 | F6:25162 | NaSa01\_F14.raw |  |  |  |  |  | 4.4184E7 |  |  |  |  |  |  |  |  | 1 | 0 | 0 | 0 | 0 | 0 | 1 | 0 | 0 | 0 | 0 | 0 | 0 | 0 | 0 | 220 | 232 | Oxidation (M) | M8:Oxidation (M):60.62;M10:Oxidation (M):65.06 | PEAKS DB |
| R.M(+15.99)VAITMAHEMGHNLGM(+15.99)NHDK.G | Y | 57.42 | 2267.9907 | 20 | -0.1 | 568.0049 | 4 | 19.11 | 6 | F6:4424 | NaSa01\_F14.raw |  |  |  |  |  | 6.6833E7 |  |  |  |  |  |  |  |  | 4 | 0 | 0 | 0 | 0 | 0 | 4 | 0 | 0 | 0 | 0 | 0 | 0 | 0 | 0 | 322 | 341 | Oxidation (M) | M1:Oxidation (M):76.17;M16:Oxidation (M):50.87 | PEAKS DB |
| Y.MVVDNIMYR.H | Y | 56.61 | 1139.5468 | 9 | 0.8 | 570.7811 | 2 | 45.30 | 6 | F6:25326 | NaSa01\_F14.raw |  |  |  |  |  | 1.66E9 |  |  |  |  |  |  |  |  | 5 | 0 | 0 | 0 | 0 | 0 | 5 | 0 | 0 | 0 | 0 | 0 | 0 | 0 | 0 | 199 | 207 |  |  | PEAKS DB |
| E.IWSNINEINVQSDVK.A | Y | 56.19 | 1757.8948 | 15 | 0.7 | 879.9553 | 2 | 55.02 | 6 | F6:33706 | NaSa01\_F14.raw |  |  |  |  |  | 1.6563E6 |  |  |  |  |  |  |  |  | 1 | 0 | 0 | 0 | 0 | 0 | 1 | 0 | 0 | 0 | 0 | 0 | 0 | 0 | 0 | 245 | 259 |  |  | PEAKS DB |
| Q.LLTGIDFNGTPVGLAYIGSIC(+57.02)NPK.T | N | 56.04 | 2519.3093 | 24 | 0.7 | 1260.6628 | 2 | 95.97 | 6 | F6:68185 | NaSa01\_F14.raw |  |  |  |  |  | 4.6093E6 |  |  |  |  |  |  |  |  | 2 | 0 | 0 | 0 | 0 | 0 | 2 | 0 | 0 | 0 | 0 | 0 | 0 | 0 | 0 | 284 | 307 | Carbamidomethylation | C21:Carbamidomethylation:1000.00 | PEAKS DB |
| F.YMVVDNIMYR.H | Y | 55.65 | 1302.6101 | 10 | 0.7 | 652.3128 | 2 | 55.69 | 6 | F6:34367 | NaSa01\_F14.raw |  |  |  |  |  | 4.341E7 |  |  |  |  |  |  |  |  | 1 | 0 | 0 | 0 | 0 | 0 | 1 | 0 | 0 | 0 | 0 | 0 | 0 | 0 | 0 | 198 | 207 |  |  | PEAKS DB |
| K.GFC(+57.02)TC(+57.02)GFNK.C | N | 54.46 | 1089.4371 | 9 | -0.5 | 545.7256 | 2 | 11.57 | 6 | F6:1962 | NaSa01\_F14.raw |  |  |  |  |  | 2.5576E6 |  |  |  |  |  |  |  |  | 1 | 0 | 0 | 0 | 0 | 0 | 1 | 0 | 0 | 0 | 0 | 0 | 0 | 0 | 0 | 342 | 350 | Carbamidomethylation | C3:Carbamidomethylation:1000.00;C5:Carbamidomethylation:1000.00 | PEAKS DB |
| Y.M(+15.99)VVDNIMYR.H | Y | 54.07 | 1155.5416 | 9 | 0.0 | 578.7781 | 2 | 31.32 | 6 | F6:15096 | NaSa01\_F14.raw |  |  |  |  |  | 1.7774E8 |  |  |  |  |  |  |  |  | 2 | 0 | 0 | 0 | 0 | 0 | 2 | 0 | 0 | 0 | 0 | 0 | 0 | 0 | 0 | 199 | 207 | Oxidation (M) | M1:Oxidation (M):115.44 | PEAKS DB |
| Y.EMINTMNMIYR.R | N | 53.03 | 1414.6407 | 11 | -0.2 | 708.3275 | 2 | 57.05 | 6 | F6:35453 | NaSa01\_F14.raw |  |  |  |  |  | 4.7389E6 |  |  |  |  |  |  |  |  | 1 | 0 | 0 | 0 | 0 | 0 | 1 | 0 | 0 | 0 | 0 | 0 | 0 | 0 | 0 | 222 | 232 |  |  | PEAKS DB |
| M.INTMNMIYR.R | N | 51.67 | 1154.5576 | 9 | 0.4 | 578.2863 | 2 | 38.32 | 6 | F6:19378 | NaSa01\_F14.raw |  |  |  |  |  | 0 |  |  |  |  |  |  |  |  | 0 | 0 | 0 | 0 | 0 | 0 | 0 | 0 | 0 | 0 | 0 | 0 | 0 | 0 | 0 | 224 | 232 |  |  | PEAKS DB |
| R.KVYEMINTMN.M | N | 51.42 | 1241.5785 | 10 | 0.3 | 621.7967 | 2 | 33.25 | 6 | F6:15075 | NaSa01\_F14.raw |  |  |  |  |  | 2.3194E7 |  |  |  |  |  |  |  |  | 1 | 0 | 0 | 0 | 0 | 0 | 1 | 0 | 0 | 0 | 0 | 0 | 0 | 0 | 0 | 219 | 228 |  |  | PEAKS DB |
| R.MVAITMAHEM.G | N | 51.15 | 1132.5079 | 10 | 1.1 | 567.2618 | 2 | 44.18 | 6 | F6:24291 | NaSa01\_F14.raw |  |  |  |  |  | 4.7043E6 |  |  |  |  |  |  |  |  | 1 | 0 | 0 | 0 | 0 | 0 | 1 | 0 | 0 | 0 | 0 | 0 | 0 | 0 | 0 | 322 | 331 |  |  | PEAKS DB |
| Y.MVVDNIM(+15.99)YR.H | Y | 51.01 | 1155.5416 | 9 | 0.0 | 578.7781 | 2 | 31.32 | 6 | F6:13340 | NaSa01\_F14.raw |  |  |  |  |  | 1.7774E8 |  |  |  |  |  |  |  |  | 2 | 0 | 0 | 0 | 0 | 0 | 2 | 0 | 0 | 0 | 0 | 0 | 0 | 0 | 0 | 199 | 207 | Oxidation (M) | M7:Oxidation (M):113.36 | PEAKS DB |
| F.YM(+15.99)VVDNIMYR.H | Y | 50.84 | 1318.6050 | 10 | 0.4 | 660.3101 | 2 | 45.71 | 6 | F6:25735 | NaSa01\_F14.raw |  |  |  |  |  | 5.0243E6 |  |  |  |  |  |  |  |  | 2 | 0 | 0 | 0 | 0 | 0 | 2 | 0 | 0 | 0 | 0 | 0 | 0 | 0 | 0 | 198 | 207 | Oxidation (M) | M2:Oxidation (M):111.20 | PEAKS DB |
| K.VYEM(+15.99)INTM(+15.99)NMIYR.R | N | 50.77 | 1708.7623 | 13 | -0.3 | 855.3882 | 2 | 45.10 | 6 | F6:25618 | NaSa01\_F14.raw |  |  |  |  |  | 5.1185E7 |  |  |  |  |  |  |  |  | 3 | 0 | 0 | 0 | 0 | 0 | 3 | 0 | 0 | 0 | 0 | 0 | 0 | 0 | 0 | 220 | 232 | Oxidation (M) | M4:Oxidation (M):83.65;M8:Oxidation (M):18.23 | PEAKS DB |
| R.TKPAYQFSSC(+57.02).S | N | 50.58 | 1187.5281 | 10 | 0.3 | 594.7715 | 2 | 11.58 | 6 | F6:1956 | NaSa01\_F14.raw |  |  |  |  |  | 3.6584E6 |  |  |  |  |  |  |  |  | 1 | 0 | 0 | 0 | 0 | 0 | 1 | 0 | 0 | 0 | 0 | 0 | 0 | 0 | 0 | 358 | 367 | Carbamidomethylation | C10:Carbamidomethylation:1000.00 | PEAKS DB |
| K.VYEMINTMN.M | N | 49.68 | 1113.4835 | 9 | 0.3 | 557.7492 | 2 | 52.12 | 6 | F6:31163 | NaSa01\_F14.raw |  |  |  |  |  | 1.6784E8 |  |  |  |  |  |  |  |  | 1 | 0 | 0 | 0 | 0 | 0 | 1 | 0 | 0 | 0 | 0 | 0 | 0 | 0 | 0 | 220 | 228 |  |  | PEAKS DB |
| R.MVAITMAHEM(+15.99)GHNLGM(+15.99)NHDK.G | Y | 49.53 | 2267.9907 | 20 | -1.0 | 568.0044 | 4 | 25.51 | 6 | F6:9498 | NaSa01\_F14.raw |  |  |  |  |  | 4.8512E7 |  |  |  |  |  |  |  |  | 1 | 0 | 0 | 0 | 0 | 0 | 1 | 0 | 0 | 0 | 0 | 0 | 0 | 0 | 0 | 322 | 341 | Oxidation (M) | M10:Oxidation (M):8.26;M16:Oxidation (M):21.51 | PEAKS DB |
| C.PIMTNQC(+57.02)IALR.G | N | 49.03 | 1315.6741 | 11 | 0.4 | 658.8446 | 2 | 37.06 | 6 | F6:18281 | NaSa01\_F14.raw |  |  |  |  |  | 1.277E6 |  |  |  |  |  |  |  |  | 1 | 0 | 0 | 0 | 0 | 0 | 1 | 0 | 0 | 0 | 0 | 0 | 0 | 0 | 0 | 498 | 508 | Carbamidomethylation | C7:Carbamidomethylation:1000.00 | PEAKS DB |
| K.RNDNAQLLTGIDFNGTPVGLAY.I | N | 47.41 | 2348.1760 | 22 | 0.6 | 1175.0959 | 2 | 90.55 | 6 | F6:63498 | NaSa01\_F14.raw |  |  |  |  |  | 4.2697E6 |  |  |  |  |  |  |  |  | 1 | 0 | 0 | 0 | 0 | 0 | 1 | 0 | 0 | 0 | 0 | 0 | 0 | 0 | 0 | 278 | 299 |  |  | PEAKS DB |
| K.VYEM(+15.99)INTM(+15.99)NM(+15.99)IYR.R | N | 46.27 | 1724.7572 | 13 | -0.7 | 863.3853 | 2 | 32.47 | 6 | F6:14255 | NaSa01\_F14.raw |  |  |  |  |  | 3.9176E6 |  |  |  |  |  |  |  |  | 1 | 0 | 0 | 0 | 0 | 0 | 1 | 0 | 0 | 0 | 0 | 0 | 0 | 0 | 0 | 220 | 232 | Oxidation (M) | M4:Oxidation (M):1000.00;M8:Oxidation (M):1000.00;M10:Oxidation (M):1000.00 | PEAKS DB |
| R.MVAITMAH.E | N | 45.87 | 872.4249 | 8 | -0.1 | 437.2197 | 2 | 30.29 | 6 | F6:13244 | NaSa01\_F14.raw |  |  |  |  | 1.6148E6 | 2.3115E6 |  |  |  |  |  |  |  |  | 4 | 0 | 0 | 0 | 0 | 2 | 2 | 0 | 0 | 0 | 0 | 0 | 0 | 0 | 0 | 322 | 329 |  |  | PEAKS DB |
| K.VYEMINTM(+15.99)N.M | N | 45.82 | 1129.4784 | 9 | 0.2 | 565.7466 | 2 | 42.89 | 6 | F6:23229 | NaSa01\_F14.raw |  |  |  |  |  | 0 |  |  |  |  |  |  |  |  | 0 | 0 | 0 | 0 | 0 | 0 | 0 | 0 | 0 | 0 | 0 | 0 | 0 | 0 | 0 | 220 | 228 | Oxidation (M) | M8:Oxidation (M):52.34 | PEAKS DB |
| R.MVAITMAHEMGHNLGM.N | N | 45.75 | 1741.7772 | 16 | 0.5 | 871.8964 | 2 | 58.51 | 6 | F6:36744 | NaSa01\_F14.raw |  |  |  |  |  | 2.3274E6 |  |  |  |  |  |  |  |  | 1 | 0 | 0 | 0 | 0 | 0 | 1 | 0 | 0 | 0 | 0 | 0 | 0 | 0 | 0 | 322 | 337 |  |  | PEAKS DB |
| R.EITTSPPVQDHC(+57.02)YYHGY.F | N | 44.97 | 2065.8840 | 17 | 6.3 | 689.6360 | 3 | 31.90 | 8 | F8:16761 | NaSa01\_F3.raw |  |  |  |  |  |  |  | 3.1849E6 |  |  |  |  |  |  | 1 | 0 | 0 | 0 | 0 | 0 | 0 | 0 | 1 | 0 | 0 | 0 | 0 | 0 | 0 | 85 | 101 | Carbamidomethylation | C12:Carbamidomethylation:1000.00 | PEAKS DB |
| R.NDNAQLLTGIDFNGTPVGL.A | N | 44.91 | 1957.9745 | 19 | 0.5 | 979.9950 | 2 | 104.18 | 6 | F6:74818 | NaSa01\_F14.raw |  |  |  |  |  | 2.0756E7 |  |  |  |  |  |  |  |  | 1 | 0 | 0 | 0 | 0 | 0 | 1 | 0 | 0 | 0 | 0 | 0 | 0 | 0 | 0 | 279 | 297 |  |  | PEAKS DB |
| L.TGIDFNGTPVGLAYIGSIC(+57.02)NPK.T | N | 44.70 | 2293.1411 | 22 | -0.2 | 1147.5776 | 2 | 85.72 | 6 | F6:59453 | NaSa01\_F14.raw |  |  |  |  |  | 3.0432E6 |  |  |  |  |  |  |  |  | 1 | 0 | 0 | 0 | 0 | 0 | 1 | 0 | 0 | 0 | 0 | 0 | 0 | 0 | 0 | 286 | 307 | Carbamidomethylation | C19:Carbamidomethylation:1000.00 | PEAKS DB |
| R.DRPQC(+57.02)ILNKPL.S | N | 44.62 | 1352.7234 | 11 | 1.1 | 451.9156 | 3 | 27.50 | 5 | F5:11578 | NaSa01\_F13.raw |  |  |  |  | 1.9485E7 |  |  |  |  |  |  |  |  |  | 1 | 0 | 0 | 0 | 0 | 1 | 0 | 0 | 0 | 0 | 0 | 0 | 0 | 0 | 0 | 379 | 389 | Carbamidomethylation | C5:Carbamidomethylation:1000.00 | PEAKS DB |
| L.TNTPEQDR.Y | N | 44.60 | 959.4308 | 8 | 0.1 | 480.7227 | 2 | 11.42 | 6 | F6:1814 | NaSa01\_F14.raw |  |  |  |  |  | 1.6427E5 |  |  |  |  |  |  |  |  | 1 | 0 | 0 | 0 | 0 | 0 | 1 | 0 | 0 | 0 | 0 | 0 | 0 | 0 | 0 | 180 | 187 |  |  | PEAKS DB |
| R.MVAITM(+15.99)AHEMGH.N | N | 44.44 | 1342.5833 | 12 | -0.2 | 672.2988 | 2 | 30.93 | 6 | F6:12991 | NaSa01\_F14.raw |  |  |  |  |  | 1.3599E7 |  |  |  |  |  |  |  |  | 2 | 0 | 0 | 0 | 0 | 0 | 2 | 0 | 0 | 0 | 0 | 0 | 0 | 0 | 0 | 322 | 333 | Oxidation (M) | M6:Oxidation (M):8.80 | PEAKS DB |
| F.YMVVDNIM(+15.99)YR.H | Y | 44.15 | 1318.6050 | 10 | 0.4 | 660.3101 | 2 | 44.13 | 6 | F6:24416 | NaSa01\_F14.raw |  |  |  |  |  | 3.8934E6 |  |  |  |  |  |  |  |  | 1 | 0 | 0 | 0 | 0 | 0 | 1 | 0 | 0 | 0 | 0 | 0 | 0 | 0 | 0 | 198 | 207 | Oxidation (M) | M8:Oxidation (M):87.26 | PEAKS DB |
| A.TLDLFGEWR.E | N | 43.65 | 1135.5662 | 9 | -0.8 | 568.7899 | 2 | 77.17 | 6 | F6:52295 | NaSa01\_F14.raw |  |  |  |  |  | 1.3701E6 |  |  |  |  |  |  |  |  | 1 | 0 | 0 | 0 | 0 | 0 | 1 | 0 | 0 | 0 | 0 | 0 | 0 | 0 | 0 | 261 | 269 |  |  | PEAKS DB |
| R.YLQAEKYIEFY.M | N | 43.34 | 1465.7129 | 11 | 0.9 | 733.8644 | 2 | 68.34 | 6 | F6:44984 | NaSa01\_F14.raw |  |  |  |  |  | 1.4083E6 |  |  |  |  |  |  |  |  | 1 | 0 | 0 | 0 | 0 | 0 | 1 | 0 | 0 | 0 | 0 | 0 | 0 | 0 | 0 | 188 | 198 |  |  | PEAKS DB |
| R.MVAITM(+15.99)AHEM(+15.99)GHNLGMNHDK.G | Y | 43.14 | 2267.9907 | 20 | -0.3 | 568.0048 | 4 | 11.55 | 6 | F6:1862 | NaSa01\_F14.raw |  |  |  |  |  | 1.3427E7 |  |  |  |  |  |  |  |  | 2 | 0 | 0 | 0 | 0 | 0 | 2 | 0 | 0 | 0 | 0 | 0 | 0 | 0 | 0 | 322 | 341 | Oxidation (M) | M6:Oxidation (M):51.63;M10:Oxidation (M):33.66 | PEAKS DB |
| G.TPVGLAYIGSIC(+57.02)NPK.T | N | 42.66 | 1588.8282 | 15 | 1.1 | 795.4222 | 2 | 58.70 | 12 | F12:38993 | NaSa01\_F7.raw |  |  |  |  |  |  |  |  |  |  |  | 1.3171E5 |  |  | 1 | 0 | 0 | 0 | 0 | 0 | 0 | 0 | 0 | 0 | 0 | 0 | 1 | 0 | 0 | 293 | 307 | Carbamidomethylation | C12:Carbamidomethylation:1000.00 | PEAKS DB |
| R.KVYEMINTM(+15.99)NM(+15.99)IYR.R | N | 41.47 | 1836.8573 | 14 | 0.6 | 613.2934 | 3 | 33.38 | 6 | F6:15316 | NaSa01\_F14.raw |  |  |  |  |  | 1.4433E6 |  |  |  |  |  |  |  |  | 1 | 0 | 0 | 0 | 0 | 0 | 1 | 0 | 0 | 0 | 0 | 0 | 0 | 0 | 0 | 219 | 232 | Oxidation (M) | M9:Oxidation (M):58.99;M11:Oxidation (M):78.19 | PEAKS DB |
| K.YIEFYM(+15.99)VVDNIM(+15.99)YR.H | Y | 41.29 | 1886.8583 | 14 | -0.3 | 944.4361 | 2 | 79.64 | 6 | F6:54338 | NaSa01\_F14.raw |  |  |  |  |  | 4.7293E6 |  |  |  |  |  |  |  |  | 2 | 0 | 0 | 0 | 0 | 0 | 2 | 0 | 0 | 0 | 0 | 0 | 0 | 0 | 0 | 194 | 207 | Oxidation (M) | M6:Oxidation (M):1000.00;M12:Oxidation (M):1000.00 | PEAKS DB |
| R.M(+15.99)VAITMAHEMGH.N | N | 40.25 | 1342.5833 | 12 | -0.3 | 448.5349 | 3 | 25.47 | 6 | F6:9076 | NaSa01\_F14.raw |  |  |  |  |  | 1.8423E7 |  |  |  |  |  |  |  |  | 2 | 0 | 0 | 0 | 0 | 0 | 2 | 0 | 0 | 0 | 0 | 0 | 0 | 0 | 0 | 322 | 333 | Oxidation (M) | M1:Oxidation (M):10.53 | PEAKS DB |
| G.LAYIGSIC(+57.02)NPK.T | N | 38.59 | 1234.6379 | 11 | 0.2 | 618.3264 | 2 | 37.01 | 12 | F12:20710 | NaSa01\_F7.raw |  |  |  |  |  |  |  |  |  |  |  | 3.5988E5 |  |  | 1 | 0 | 0 | 0 | 0 | 0 | 0 | 0 | 0 | 0 | 0 | 0 | 1 | 0 | 0 | 297 | 307 | Carbamidomethylation | C8:Carbamidomethylation:1000.00 | PEAKS DB |
| K.PAYQFSSC(+57.02)SVR.E | N | 38.45 | 1300.5870 | 11 | 0.0 | 651.3008 | 2 | 23.13 | 6 | F6:6974 | NaSa01\_F14.raw |  |  |  |  |  | 1.0807E6 |  |  |  |  |  |  |  |  | 1 | 0 | 0 | 0 | 0 | 0 | 1 | 0 | 0 | 0 | 0 | 0 | 0 | 0 | 0 | 360 | 370 | Carbamidomethylation | C8:Carbamidomethylation:1000.00 | PEAKS DB |
| F.NGTPVGLAYIGSIC(+57.02)NPK.T | N | 38.29 | 1759.8927 | 17 | -1.0 | 880.9528 | 2 | 61.44 | 6 | F6:39069 | NaSa01\_F14.raw |  |  |  |  |  | 6.4633E5 |  |  |  |  |  |  |  |  | 1 | 0 | 0 | 0 | 0 | 0 | 1 | 0 | 0 | 0 | 0 | 0 | 0 | 0 | 0 | 291 | 307 | Carbamidomethylation | C14:Carbamidomethylation:1000.00 | PEAKS DB |
| total 75 peptides |  |  |  |  |  |  |  |  |  |  |  |  |  |  |  |  |  |  |  |  |  |  |  |  |  |  |  |  |  |  |  |  |  |  |  |  |  |  |  |  |  |  |  |  |  |
| --- | --- | --- | --- | --- | --- | --- | --- | --- | --- | --- | --- | --- | --- | --- | --- | --- | --- | --- | --- | --- | --- | --- | --- | --- | --- | --- | --- | --- | --- | --- | --- | --- | --- | --- | --- | --- | --- | --- | --- | --- | --- | --- | --- | --- | --- |

ACN50006.1

back to list

  

| Protein Coverage
| Supporting Peptides
|

Protein Coverage:

Supporting Peptides:

| Peptide | Uniq | -10lgP | Mass | Length | ppm | m/z | z | RT | Fraction | Scan | Source File | Area F1 | Area F10 | Area F11 | Area F12 | Area F13 | Area F14 | Area F2 | Area F3 | Area F4 | Area F5 | Area F6 | Area F7 | Area F8 | Area F9 | #Feature | #Feature F1 | #Feature F10 | #Feature F11 | #Feature F12 | #Feature F13 | #Feature F14 | #Feature F2 | #Feature F3 | #Feature F4 | #Feature F5 | #Feature F6 | #Feature F7 | #Feature F8 | #Feature F9 | Start | End | PTM | AScore | Found By |
| --- | --- | --- | --- | --- | --- | --- | --- | --- | --- | --- | --- | --- | --- | --- | --- | --- | --- | --- | --- | --- | --- | --- | --- | --- | --- | --- | --- | --- | --- | --- | --- | --- | --- | --- | --- | --- | --- | --- | --- | --- | --- | --- | --- | --- | --- |
| K.DDC(+57.02)DLPELC(+57.02)TGQSAEC(+57.02)PTDVFQR.N | N | 95.78 | 2712.1101 | 23 | -0.8 | 1357.0613 | 2 | 72.67 | 6 | F6:48718 | NaSa01\_F14.raw |  |  |  |  |  | 6.3934E8 |  |  |  | 5.9579E5 |  |  |  |  | 10 | 0 | 0 | 0 | 0 | 0 | 9 | 0 | 0 | 0 | 1 | 0 | 0 | 0 | 0 | 471 | 493 | Carbamidomethylation | C3:Carbamidomethylation:1000.00;C9:Carbamidomethylation:1000.00;C16:Carbamidomethylation:1000.00 | PEAKS DB |
| K.RNDNAQLLTGIDFNGTPVGLAYIGSIC(+57.02)NPK.T | N | 88.48 | 3217.6189 | 30 | 0.1 | 1073.5470 | 3 | 89.06 | 6 | F6:62183 | NaSa01\_F14.raw |  |  |  |  |  | 1.5231E7 |  |  |  |  |  |  |  |  | 1 | 0 | 0 | 0 | 0 | 0 | 1 | 0 | 0 | 0 | 0 | 0 | 0 | 0 | 0 | 290 | 319 | Carbamidomethylation | C27:Carbamidomethylation:1000.00 | PEAKS DB |
| R.DPNYGMVEPGTKC(+57.02)GDGMVC(+57.02)SNR.Q | Y | 85.41 | 2443.0024 | 22 | -4.2 | 815.3380 | 3 | 34.23 | 6 | F6:15877 | NaSa01\_F14.raw |  |  |  |  |  | 1.112E7 |  |  |  |  |  |  |  |  | 1 | 0 | 0 | 0 | 0 | 0 | 1 | 0 | 0 | 0 | 0 | 0 | 0 | 0 | 0 | 583 | 604 | Carbamidomethylation | C13:Carbamidomethylation:1000.00;C19:Carbamidomethylation:1000.00 | PEAKS DB |
| R.DPNYGMVEPGTK.C | Y | 82.42 | 1306.5863 | 12 | 0.8 | 654.3010 | 2 | 28.28 | 6 | F6:10918 | NaSa01\_F14.raw |  |  |  |  |  | 6.8446E6 |  |  |  | 1.1625E6 |  |  | 3.2642E6 |  | 3 | 0 | 0 | 0 | 0 | 0 | 1 | 0 | 0 | 0 | 1 | 0 | 0 | 1 | 0 | 583 | 594 |  |  | PEAKS DB |
| R.NDNAQLLTGIDFNGTPVGLAYIGSIC(+57.02)NPK.T | N | 82.31 | 3061.5178 | 29 | 1.9 | 1021.5151 | 3 | 99.98 | 6 | F6:71311 | NaSa01\_F14.raw |  |  | 2.6315E5 |  |  | 1.5398E8 |  |  |  |  |  |  |  |  | 4 | 0 | 0 | 1 | 0 | 0 | 3 | 0 | 0 | 0 | 0 | 0 | 0 | 0 | 0 | 291 | 319 | Carbamidomethylation | C26:Carbamidomethylation:1000.00 | PEAKS DB |
| N.GTPVGLAYIGSIC(+57.02)NPK.T | N | 82.13 | 1645.8497 | 16 | -0.6 | 823.9316 | 2 | 61.34 | 6 | F6:39114 | NaSa01\_F14.raw |  |  |  |  |  | 9.3648E6 |  |  |  |  |  |  |  |  | 1 | 0 | 0 | 0 | 0 | 0 | 1 | 0 | 0 | 0 | 0 | 0 | 0 | 0 | 0 | 304 | 319 | Carbamidomethylation | C13:Carbamidomethylation:1000.00 | PEAKS DB |
| R.AAKDDC(+57.02)DLPELC(+57.02)TGQSAEC(+57.02)PTDVFQR.N | N | 79.07 | 2982.2793 | 26 | 0.8 | 995.1012 | 3 | 55.91 | 6 | F6:34388 | NaSa01\_F14.raw |  |  |  |  |  | 1.5419E8 |  |  |  |  |  |  |  |  | 2 | 0 | 0 | 0 | 0 | 0 | 2 | 0 | 0 | 0 | 0 | 0 | 0 | 0 | 0 | 468 | 493 | Carbamidomethylation | C6:Carbamidomethylation:1000.00;C12:Carbamidomethylation:1000.00;C19:Carbamidomethylation:1000.00 | PEAKS DB |
| K.C(+57.02)PIMTNQC(+57.02)IALR.G | N | 77.84 | 1475.7047 | 12 | 0.0 | 738.8596 | 2 | 39.74 | 6 | F6:21215 | NaSa01\_F14.raw |  |  | 3.3287E5 | 1.3692E6 | 9.8113E6 | 2.4068E9 |  |  |  | 1.7213E7 | 6.4357E6 |  | 4.0715E6 | 1.3044E6 | 15 | 0 | 0 | 1 | 1 | 2 | 5 | 0 | 0 | 0 | 2 | 2 | 0 | 1 | 1 | 510 | 521 | Carbamidomethylation | C1:Carbamidomethylation:1000.00;C8:Carbamidomethylation:1000.00 | PEAKS DB |
| R.NSMIC(+57.02)NC(+57.02)SISPR.D | N | 77.03 | 1437.6163 | 12 | -0.1 | 719.8154 | 2 | 22.49 | 6 | F6:6540 | NaSa01\_F14.raw |  |  |  |  |  | 7.6177E6 |  |  |  |  |  |  |  |  | 2 | 0 | 0 | 0 | 0 | 0 | 2 | 0 | 0 | 0 | 0 | 0 | 0 | 0 | 0 | 571 | 582 | Carbamidomethylation | C5:Carbamidomethylation:1000.00;C7:Carbamidomethylation:1000.00 | PEAKS DB |
| K.VYEM(+15.99)INTMNMIYR.R | N | 74.23 | 1692.7673 | 13 | -0.2 | 847.3907 | 2 | 57.49 | 6 | F6:35287 | NaSa01\_F14.raw |  |  |  |  |  | 4.3266E8 |  |  |  |  |  |  |  |  | 9 | 0 | 0 | 0 | 0 | 0 | 9 | 0 | 0 | 0 | 0 | 0 | 0 | 0 | 0 | 232 | 244 | Oxidation (M) | M4:Oxidation (M):77.51 | PEAKS DB |
| K.VYEMINTMNMIYR.R | N | 73.76 | 1676.7725 | 13 | -0.3 | 839.3932 | 2 | 71.51 | 6 | F6:48424 | NaSa01\_F14.raw |  |  |  |  |  | 9.5037E8 |  |  |  |  |  |  |  |  | 12 | 0 | 0 | 0 | 0 | 0 | 12 | 0 | 0 | 0 | 0 | 0 | 0 | 0 | 0 | 232 | 244 |  |  | PEAKS DB |
| D.LPELC(+57.02)TGQSAEC(+57.02)PTDVFQR.N | N | 73.30 | 2206.9988 | 19 | 0.2 | 1104.5068 | 2 | 60.04 | 6 | F6:38037 | NaSa01\_F14.raw |  |  |  |  |  | 2.7029E6 |  |  |  |  |  |  |  |  | 1 | 0 | 0 | 0 | 0 | 0 | 1 | 0 | 0 | 0 | 0 | 0 | 0 | 0 | 0 | 475 | 493 | Carbamidomethylation | C5:Carbamidomethylation:1000.00;C12:Carbamidomethylation:1000.00 | PEAKS DB |
| K.C(+57.02)PIM(+15.99)TNQC(+57.02)IALR.G | N | 73.02 | 1491.6996 | 12 | 0.1 | 746.8572 | 2 | 30.97 | 6 | F6:13155 | NaSa01\_F14.raw |  |  |  |  | 1.4708E6 | 1.6503E8 |  |  |  |  |  |  |  |  | 5 | 0 | 0 | 0 | 0 | 1 | 4 | 0 | 0 | 0 | 0 | 0 | 0 | 0 | 0 | 510 | 521 | Carbamidomethylation; Oxidation (M) | C1:Carbamidomethylation:1000.00;M4:Oxidation (M):1000.00;C8:Carbamidomethylation:1000.00 | PEAKS DB |
| R.KVYEMINTMNMIYR.R | N | 72.83 | 1804.8674 | 14 | 0.6 | 903.4415 | 2 | 58.08 | 6 | F6:36256 | NaSa01\_F14.raw |  |  |  |  |  | 3.0426E6 |  |  |  |  |  |  |  |  | 1 | 0 | 0 | 0 | 0 | 0 | 1 | 0 | 0 | 0 | 0 | 0 | 0 | 0 | 0 | 231 | 244 |  |  | PEAKS DB |
| R.TKPAYQFSSC(+57.02)SVR.E | N | 72.78 | 1529.7296 | 13 | 0.4 | 765.8724 | 2 | 11.56 | 6 | F6:1943 | NaSa01\_F14.raw |  |  |  |  |  | 1.656E7 |  |  |  |  |  |  |  |  | 3 | 0 | 0 | 0 | 0 | 0 | 3 | 0 | 0 | 0 | 0 | 0 | 0 | 0 | 0 | 370 | 382 | Carbamidomethylation | C10:Carbamidomethylation:1000.00 | PEAKS DB |
| K.VYEMINTM(+15.99)NMIYR.R | N | 72.14 | 1692.7673 | 13 | -0.2 | 847.3907 | 2 | 57.49 | 6 | F6:36159 | NaSa01\_F14.raw |  |  |  |  |  | 4.3022E8 |  |  |  |  |  |  |  |  | 8 | 0 | 0 | 0 | 0 | 0 | 8 | 0 | 0 | 0 | 0 | 0 | 0 | 0 | 0 | 232 | 244 | Oxidation (M) | M8:Oxidation (M):79.36 | PEAKS DB |
| K.VYEMINTMNM(+15.99)IYR.R | N | 71.71 | 1692.7673 | 13 | -2.0 | 565.2619 | 3 | 61.54 | 6 | F6:39303 | NaSa01\_F14.raw |  |  |  |  |  | 2.4734E8 |  |  |  |  |  |  |  |  | 6 | 0 | 0 | 0 | 0 | 0 | 6 | 0 | 0 | 0 | 0 | 0 | 0 | 0 | 0 | 232 | 244 | Oxidation (M) | M10:Oxidation (M):28.86 | PEAKS DB |
| R.MVAITMAHEMGH.N | N | 70.23 | 1326.5883 | 12 | 0.1 | 664.3015 | 2 | 30.77 | 6 | F6:12852 | NaSa01\_F14.raw |  |  |  |  | 5.8936E5 | 1.005E8 |  |  |  |  |  |  |  |  | 4 | 0 | 0 | 0 | 0 | 1 | 3 | 0 | 0 | 0 | 0 | 0 | 0 | 0 | 0 | 334 | 345 |  |  | PEAKS DB |
| R.KVYEMINTMNM(+15.99)IYR.R | N | 67.76 | 1820.8623 | 14 | 0.0 | 607.9614 | 3 | 49.96 | 6 | F6:29587 | NaSa01\_F14.raw |  |  |  |  |  | 7.8852E6 |  |  |  |  |  |  |  |  | 1 | 0 | 0 | 0 | 0 | 0 | 1 | 0 | 0 | 0 | 0 | 0 | 0 | 0 | 0 | 231 | 244 | Oxidation (M) | M11:Oxidation (M):36.05 | PEAKS DB |
| R.NSM(+15.99)IC(+57.02)NC(+57.02)SISPR.D | N | 66.06 | 1453.6112 | 12 | 0.3 | 727.8131 | 2 | 11.56 | 6 | F6:1942 | NaSa01\_F14.raw |  |  |  |  |  | 2.1578E6 |  |  |  |  |  |  |  |  | 4 | 0 | 0 | 0 | 0 | 0 | 4 | 0 | 0 | 0 | 0 | 0 | 0 | 0 | 0 | 571 | 582 | Oxidation (M); Carbamidomethylation | M3:Oxidation (M):1000.00;C5:Carbamidomethylation:1000.00;C7:Carbamidomethylation:1000.00 | PEAKS DB |
| R.DSC(+57.02)FTLNQR.T | N | 63.93 | 1139.5029 | 9 | -0.1 | 570.7587 | 2 | 25.28 | 6 | F6:8701 | NaSa01\_F14.raw |  |  |  |  |  | 8.7088E6 |  |  |  |  |  |  |  |  | 2 | 0 | 0 | 0 | 0 | 0 | 2 | 0 | 0 | 0 | 0 | 0 | 0 | 0 | 0 | 530 | 538 | Carbamidomethylation | C3:Carbamidomethylation:1000.00 | PEAKS DB |
| K.VYEM(+15.99)INTMNM(+15.99)IYR.R | N | 61.29 | 1708.7623 | 13 | -0.3 | 855.3882 | 2 | 45.10 | 6 | F6:25392 | NaSa01\_F14.raw |  |  |  |  |  | 4.4184E7 |  |  |  |  |  |  |  |  | 1 | 0 | 0 | 0 | 0 | 0 | 1 | 0 | 0 | 0 | 0 | 0 | 0 | 0 | 0 | 232 | 244 | Oxidation (M) | M4:Oxidation (M):79.36;M10:Oxidation (M):27.16 | PEAKS DB |
| R.MVAITMAHEMGHNLGMNHD.R | N | 59.40 | 2107.9060 | 19 | 0.0 | 703.6426 | 3 | 45.80 | 6 | F6:25307 | NaSa01\_F14.raw |  |  |  |  |  | 7.6575E6 |  |  |  |  |  |  |  |  | 1 | 0 | 0 | 0 | 0 | 0 | 1 | 0 | 0 | 0 | 0 | 0 | 0 | 0 | 0 | 334 | 352 |  |  | PEAKS DB |
| R.DRPQC(+57.02)ILNKPLSTDIVSPPIC(+57.02)GN.Y | N | 58.34 | 2593.2993 | 23 | -0.3 | 1297.6565 | 2 | 63.62 | 6 | F6:40889 | NaSa01\_F14.raw |  |  |  |  |  | 2.087E9 |  |  |  |  |  |  |  |  | 4 | 0 | 0 | 0 | 0 | 0 | 4 | 0 | 0 | 0 | 0 | 0 | 0 | 0 | 0 | 391 | 413 | Carbamidomethylation | C5:Carbamidomethylation:1000.00;C21:Carbamidomethylation:1000.00 | PEAKS DB |
| K.VYEMINTM(+15.99)NM(+15.99)IYR.R | N | 57.62 | 1708.7623 | 13 | -0.3 | 855.3882 | 2 | 45.10 | 6 | F6:25162 | NaSa01\_F14.raw |  |  |  |  |  | 4.4184E7 |  |  |  |  |  |  |  |  | 1 | 0 | 0 | 0 | 0 | 0 | 1 | 0 | 0 | 0 | 0 | 0 | 0 | 0 | 0 | 232 | 244 | Oxidation (M) | M8:Oxidation (M):60.62;M10:Oxidation (M):65.06 | PEAKS DB |
| Q.LLTGIDFNGTPVGLAYIGSIC(+57.02)NPK.T | N | 56.04 | 2519.3093 | 24 | 0.7 | 1260.6628 | 2 | 95.97 | 6 | F6:68185 | NaSa01\_F14.raw |  |  |  |  |  | 4.6093E6 |  |  |  |  |  |  |  |  | 2 | 0 | 0 | 0 | 0 | 0 | 2 | 0 | 0 | 0 | 0 | 0 | 0 | 0 | 0 | 296 | 319 | Carbamidomethylation | C21:Carbamidomethylation:1000.00 | PEAKS DB |
| R.NGLPC(+57.02)QNNQGYC(+57.02)YNGK.C | N | 55.19 | 1885.7836 | 16 | -0.1 | 943.8989 | 2 | 11.56 | 6 | F6:1965 | NaSa01\_F14.raw |  |  |  |  |  | 1.6943E6 |  |  |  |  |  |  |  |  | 1 | 0 | 0 | 0 | 0 | 0 | 1 | 0 | 0 | 0 | 0 | 0 | 0 | 0 | 0 | 494 | 509 | Carbamidomethylation | C5:Carbamidomethylation:1000.00;C12:Carbamidomethylation:1000.00 | PEAKS DB |
| R.GFC(+57.02)TC(+57.02)GFNK.C | N | 54.46 | 1089.4371 | 9 | -0.5 | 545.7256 | 2 | 11.57 | 6 | F6:1962 | NaSa01\_F14.raw |  |  |  |  |  | 2.5576E6 |  |  |  |  |  |  |  |  | 1 | 0 | 0 | 0 | 0 | 0 | 1 | 0 | 0 | 0 | 0 | 0 | 0 | 0 | 0 | 354 | 362 | Carbamidomethylation | C3:Carbamidomethylation:1000.00;C5:Carbamidomethylation:1000.00 | PEAKS DB |
| Y.EMINTMNMIYR.R | N | 53.03 | 1414.6407 | 11 | -0.2 | 708.3275 | 2 | 57.05 | 6 | F6:35453 | NaSa01\_F14.raw |  |  |  |  |  | 4.7389E6 |  |  |  |  |  |  |  |  | 1 | 0 | 0 | 0 | 0 | 0 | 1 | 0 | 0 | 0 | 0 | 0 | 0 | 0 | 0 | 234 | 244 |  |  | PEAKS DB |
| M.INTMNMIYR.R | N | 51.67 | 1154.5576 | 9 | 0.4 | 578.2863 | 2 | 38.32 | 6 | F6:19378 | NaSa01\_F14.raw |  |  |  |  |  | 0 |  |  |  |  |  |  |  |  | 0 | 0 | 0 | 0 | 0 | 0 | 0 | 0 | 0 | 0 | 0 | 0 | 0 | 0 | 0 | 236 | 244 |  |  | PEAKS DB |
| R.KVYEMINTMN.M | N | 51.42 | 1241.5785 | 10 | 0.3 | 621.7967 | 2 | 33.25 | 6 | F6:15075 | NaSa01\_F14.raw |  |  |  |  |  | 2.3194E7 |  |  |  |  |  |  |  |  | 1 | 0 | 0 | 0 | 0 | 0 | 1 | 0 | 0 | 0 | 0 | 0 | 0 | 0 | 0 | 231 | 240 |  |  | PEAKS DB |
| R.MVAITMAHEM.G | N | 51.15 | 1132.5079 | 10 | 1.1 | 567.2618 | 2 | 44.18 | 6 | F6:24291 | NaSa01\_F14.raw |  |  |  |  |  | 4.7043E6 |  |  |  |  |  |  |  |  | 1 | 0 | 0 | 0 | 0 | 0 | 1 | 0 | 0 | 0 | 0 | 0 | 0 | 0 | 0 | 334 | 343 |  |  | PEAKS DB |
| K.VYEM(+15.99)INTM(+15.99)NMIYR.R | N | 50.77 | 1708.7623 | 13 | -0.3 | 855.3882 | 2 | 45.10 | 6 | F6:25618 | NaSa01\_F14.raw |  |  |  |  |  | 5.1185E7 |  |  |  |  |  |  |  |  | 3 | 0 | 0 | 0 | 0 | 0 | 3 | 0 | 0 | 0 | 0 | 0 | 0 | 0 | 0 | 232 | 244 | Oxidation (M) | M4:Oxidation (M):83.65;M8:Oxidation (M):18.23 | PEAKS DB |
| R.TKPAYQFSSC(+57.02).S | N | 50.58 | 1187.5281 | 10 | 0.3 | 594.7715 | 2 | 11.58 | 6 | F6:1956 | NaSa01\_F14.raw |  |  |  |  |  | 3.6584E6 |  |  |  |  |  |  |  |  | 1 | 0 | 0 | 0 | 0 | 0 | 1 | 0 | 0 | 0 | 0 | 0 | 0 | 0 | 0 | 370 | 379 | Carbamidomethylation | C10:Carbamidomethylation:1000.00 | PEAKS DB |
| K.VYEMINTMN.M | N | 49.68 | 1113.4835 | 9 | 0.3 | 557.7492 | 2 | 52.12 | 6 | F6:31163 | NaSa01\_F14.raw |  |  |  |  |  | 1.6784E8 |  |  |  |  |  |  |  |  | 1 | 0 | 0 | 0 | 0 | 0 | 1 | 0 | 0 | 0 | 0 | 0 | 0 | 0 | 0 | 232 | 240 |  |  | PEAKS DB |
| C.PIMTNQC(+57.02)IALR.G | N | 49.03 | 1315.6741 | 11 | 0.4 | 658.8446 | 2 | 37.06 | 6 | F6:18281 | NaSa01\_F14.raw |  |  |  |  |  | 1.277E6 |  |  |  |  |  |  |  |  | 1 | 0 | 0 | 0 | 0 | 0 | 1 | 0 | 0 | 0 | 0 | 0 | 0 | 0 | 0 | 511 | 521 | Carbamidomethylation | C7:Carbamidomethylation:1000.00 | PEAKS DB |
| R.DPNYGM(+15.99)VEPGTK.C | Y | 48.74 | 1322.5813 | 12 | -0.3 | 662.2977 | 2 | 11.56 | 6 | F6:1972 | NaSa01\_F14.raw |  |  |  |  |  | 4.7262E5 |  |  |  |  |  |  |  |  | 1 | 0 | 0 | 0 | 0 | 0 | 1 | 0 | 0 | 0 | 0 | 0 | 0 | 0 | 0 | 583 | 594 | Oxidation (M) | M6:Oxidation (M):1000.00 | PEAKS DB |
| K.RNDNAQLLTGIDFNGTPVGLAY.I | N | 47.41 | 2348.1760 | 22 | 0.6 | 1175.0959 | 2 | 90.55 | 6 | F6:63498 | NaSa01\_F14.raw |  |  |  |  |  | 4.2697E6 |  |  |  |  |  |  |  |  | 1 | 0 | 0 | 0 | 0 | 0 | 1 | 0 | 0 | 0 | 0 | 0 | 0 | 0 | 0 | 290 | 311 |  |  | PEAKS DB |
| K.VYEM(+15.99)INTM(+15.99)NM(+15.99)IYR.R | N | 46.27 | 1724.7572 | 13 | -0.7 | 863.3853 | 2 | 32.47 | 6 | F6:14255 | NaSa01\_F14.raw |  |  |  |  |  | 3.9176E6 |  |  |  |  |  |  |  |  | 1 | 0 | 0 | 0 | 0 | 0 | 1 | 0 | 0 | 0 | 0 | 0 | 0 | 0 | 0 | 232 | 244 | Oxidation (M) | M4:Oxidation (M):1000.00;M8:Oxidation (M):1000.00;M10:Oxidation (M):1000.00 | PEAKS DB |
| R.MVAITMAH.E | N | 45.87 | 872.4249 | 8 | -0.1 | 437.2197 | 2 | 30.29 | 6 | F6:13244 | NaSa01\_F14.raw |  |  |  |  | 1.6148E6 | 2.3115E6 |  |  |  |  |  |  |  |  | 4 | 0 | 0 | 0 | 0 | 2 | 2 | 0 | 0 | 0 | 0 | 0 | 0 | 0 | 0 | 334 | 341 |  |  | PEAKS DB |
| K.VYEMINTM(+15.99)N.M | N | 45.82 | 1129.4784 | 9 | 0.2 | 565.7466 | 2 | 42.89 | 6 | F6:23229 | NaSa01\_F14.raw |  |  |  |  |  | 0 |  |  |  |  |  |  |  |  | 0 | 0 | 0 | 0 | 0 | 0 | 0 | 0 | 0 | 0 | 0 | 0 | 0 | 0 | 0 | 232 | 240 | Oxidation (M) | M8:Oxidation (M):52.34 | PEAKS DB |
| R.MVAITMAHEMGHNLGM.N | N | 45.75 | 1741.7772 | 16 | 0.5 | 871.8964 | 2 | 58.51 | 6 | F6:36744 | NaSa01\_F14.raw |  |  |  |  |  | 2.3274E6 |  |  |  |  |  |  |  |  | 1 | 0 | 0 | 0 | 0 | 0 | 1 | 0 | 0 | 0 | 0 | 0 | 0 | 0 | 0 | 334 | 349 |  |  | PEAKS DB |
| R.EITTSPPVQDHC(+57.02)YYHGY.I | N | 44.97 | 2065.8840 | 17 | 6.3 | 689.6360 | 3 | 31.90 | 8 | F8:16761 | NaSa01\_F3.raw |  |  |  |  |  |  |  | 3.1849E6 |  |  |  |  |  |  | 1 | 0 | 0 | 0 | 0 | 0 | 0 | 0 | 1 | 0 | 0 | 0 | 0 | 0 | 0 | 97 | 113 | Carbamidomethylation | C12:Carbamidomethylation:1000.00 | PEAKS DB |
| R.NDNAQLLTGIDFNGTPVGL.A | N | 44.91 | 1957.9745 | 19 | 0.5 | 979.9950 | 2 | 104.18 | 6 | F6:74818 | NaSa01\_F14.raw |  |  |  |  |  | 2.0756E7 |  |  |  |  |  |  |  |  | 1 | 0 | 0 | 0 | 0 | 0 | 1 | 0 | 0 | 0 | 0 | 0 | 0 | 0 | 0 | 291 | 309 |  |  | PEAKS DB |
| L.TGIDFNGTPVGLAYIGSIC(+57.02)NPK.T | N | 44.70 | 2293.1411 | 22 | -0.2 | 1147.5776 | 2 | 85.72 | 6 | F6:59453 | NaSa01\_F14.raw |  |  |  |  |  | 3.0432E6 |  |  |  |  |  |  |  |  | 1 | 0 | 0 | 0 | 0 | 0 | 1 | 0 | 0 | 0 | 0 | 0 | 0 | 0 | 0 | 298 | 319 | Carbamidomethylation | C19:Carbamidomethylation:1000.00 | PEAKS DB |
| R.DRPQC(+57.02)ILNKPL.S | N | 44.62 | 1352.7234 | 11 | 1.1 | 451.9156 | 3 | 27.50 | 5 | F5:11578 | NaSa01\_F13.raw |  |  |  |  | 1.9485E7 |  |  |  |  |  |  |  |  |  | 1 | 0 | 0 | 0 | 0 | 1 | 0 | 0 | 0 | 0 | 0 | 0 | 0 | 0 | 0 | 391 | 401 | Carbamidomethylation | C5:Carbamidomethylation:1000.00 | PEAKS DB |
| L.TNTPEQDR.Y | N | 44.60 | 959.4308 | 8 | 0.1 | 480.7227 | 2 | 11.42 | 6 | F6:1814 | NaSa01\_F14.raw |  |  |  |  |  | 1.6427E5 |  |  |  |  |  |  |  |  | 1 | 0 | 0 | 0 | 0 | 0 | 1 | 0 | 0 | 0 | 0 | 0 | 0 | 0 | 0 | 192 | 199 |  |  | PEAKS DB |
| R.MVAITM(+15.99)AHEMGH.N | N | 44.44 | 1342.5833 | 12 | -0.2 | 672.2988 | 2 | 30.93 | 6 | F6:12991 | NaSa01\_F14.raw |  |  |  |  |  | 1.3599E7 |  |  |  |  |  |  |  |  | 2 | 0 | 0 | 0 | 0 | 0 | 2 | 0 | 0 | 0 | 0 | 0 | 0 | 0 | 0 | 334 | 345 | Oxidation (M) | M6:Oxidation (M):8.80 | PEAKS DB |
| G.TPVGLAYIGSIC(+57.02)NPK.T | N | 42.66 | 1588.8282 | 15 | 1.1 | 795.4222 | 2 | 58.70 | 12 | F12:38993 | NaSa01\_F7.raw |  |  |  |  |  |  |  |  |  |  |  | 1.3171E5 |  |  | 1 | 0 | 0 | 0 | 0 | 0 | 0 | 0 | 0 | 0 | 0 | 0 | 1 | 0 | 0 | 305 | 319 | Carbamidomethylation | C12:Carbamidomethylation:1000.00 | PEAKS DB |
| R.KVYEMINTM(+15.99)NM(+15.99)IYR.R | N | 41.47 | 1836.8573 | 14 | 0.6 | 613.2934 | 3 | 33.38 | 6 | F6:15316 | NaSa01\_F14.raw |  |  |  |  |  | 1.4433E6 |  |  |  |  |  |  |  |  | 1 | 0 | 0 | 0 | 0 | 0 | 1 | 0 | 0 | 0 | 0 | 0 | 0 | 0 | 0 | 231 | 244 | Oxidation (M) | M9:Oxidation (M):58.99;M11:Oxidation (M):78.19 | PEAKS DB |
| R.M(+15.99)VAITMAHEMGH.N | N | 40.25 | 1342.5833 | 12 | -0.3 | 448.5349 | 3 | 25.47 | 6 | F6:9076 | NaSa01\_F14.raw |  |  |  |  |  | 1.8423E7 |  |  |  |  |  |  |  |  | 2 | 0 | 0 | 0 | 0 | 0 | 2 | 0 | 0 | 0 | 0 | 0 | 0 | 0 | 0 | 334 | 345 | Oxidation (M) | M1:Oxidation (M):10.53 | PEAKS DB |
| G.LAYIGSIC(+57.02)NPK.T | N | 38.59 | 1234.6379 | 11 | 0.2 | 618.3264 | 2 | 37.01 | 12 | F12:20710 | NaSa01\_F7.raw |  |  |  |  |  |  |  |  |  |  |  | 3.5988E5 |  |  | 1 | 0 | 0 | 0 | 0 | 0 | 0 | 0 | 0 | 0 | 0 | 0 | 1 | 0 | 0 | 309 | 319 | Carbamidomethylation | C8:Carbamidomethylation:1000.00 | PEAKS DB |
| K.PAYQFSSC(+57.02)SVR.E | N | 38.45 | 1300.5870 | 11 | 0.0 | 651.3008 | 2 | 23.13 | 6 | F6:6974 | NaSa01\_F14.raw |  |  |  |  |  | 1.0807E6 |  |  |  |  |  |  |  |  | 1 | 0 | 0 | 0 | 0 | 0 | 1 | 0 | 0 | 0 | 0 | 0 | 0 | 0 | 0 | 372 | 382 | Carbamidomethylation | C8:Carbamidomethylation:1000.00 | PEAKS DB |
| F.NGTPVGLAYIGSIC(+57.02)NPK.T | N | 38.29 | 1759.8927 | 17 | -1.0 | 880.9528 | 2 | 61.44 | 6 | F6:39069 | NaSa01\_F14.raw |  |  |  |  |  | 6.4633E5 |  |  |  |  |  |  |  |  | 1 | 0 | 0 | 0 | 0 | 0 | 1 | 0 | 0 | 0 | 0 | 0 | 0 | 0 | 0 | 303 | 319 | Carbamidomethylation | C14:Carbamidomethylation:1000.00 | PEAKS DB |
| D.PNYGM(+15.99)VEPGTK.C | Y | 37.70 | 1207.5543 | 11 | 0.7 | 604.7849 | 2 | 11.49 | 6 | F6:1887 | NaSa01\_F14.raw |  |  |  |  |  | 6.0854E4 |  |  |  |  |  |  |  |  | 1 | 0 | 0 | 0 | 0 | 0 | 1 | 0 | 0 | 0 | 0 | 0 | 0 | 0 | 0 | 584 | 594 | Oxidation (M) | M5:Oxidation (M):1000.00 | PEAKS DB |
| total 55 peptides |  |  |  |  |  |  |  |  |  |  |  |  |  |  |  |  |  |  |  |  |  |  |  |  |  |  |  |  |  |  |  |  |  |  |  |  |  |  |  |  |  |  |  |  |  |
| --- | --- | --- | --- | --- | --- | --- | --- | --- | --- | --- | --- | --- | --- | --- | --- | --- | --- | --- | --- | --- | --- | --- | --- | --- | --- | --- | --- | --- | --- | --- | --- | --- | --- | --- | --- | --- | --- | --- | --- | --- | --- | --- | --- | --- | --- |

D3TTC2.1

back to list

  

| Protein Coverage
| Supporting Peptides
|

Protein Coverage:

Supporting Peptides:

| Peptide | Uniq | -10lgP | Mass | Length | ppm | m/z | z | RT | Fraction | Scan | Source File | Area F1 | Area F10 | Area F11 | Area F12 | Area F13 | Area F14 | Area F2 | Area F3 | Area F4 | Area F5 | Area F6 | Area F7 | Area F8 | Area F9 | #Feature | #Feature F1 | #Feature F10 | #Feature F11 | #Feature F12 | #Feature F13 | #Feature F14 | #Feature F2 | #Feature F3 | #Feature F4 | #Feature F5 | #Feature F6 | #Feature F7 | #Feature F8 | #Feature F9 | Start | End | PTM | AScore | Found By |
| --- | --- | --- | --- | --- | --- | --- | --- | --- | --- | --- | --- | --- | --- | --- | --- | --- | --- | --- | --- | --- | --- | --- | --- | --- | --- | --- | --- | --- | --- | --- | --- | --- | --- | --- | --- | --- | --- | --- | --- | --- | --- | --- | --- | --- | --- |
| K.DDC(+57.02)DLPELC(+57.02)TGQSAEC(+57.02)PTDVFQR.N | N | 95.78 | 2712.1101 | 23 | -0.8 | 1357.0613 | 2 | 72.67 | 6 | F6:48718 | NaSa01\_F14.raw |  |  |  |  |  | 6.3934E8 |  |  |  | 5.9579E5 |  |  |  |  | 10 | 0 | 0 | 0 | 0 | 0 | 9 | 0 | 0 | 0 | 1 | 0 | 0 | 0 | 0 | 471 | 493 | Carbamidomethylation | C3:Carbamidomethylation:1000.00;C9:Carbamidomethylation:1000.00;C16:Carbamidomethylation:1000.00 | PEAKS DB |
| K.RNDNAQLLTGIDFNGTPVGLAYIGSIC(+57.02)NPK.T | N | 88.48 | 3217.6189 | 30 | 0.1 | 1073.5470 | 3 | 89.06 | 6 | F6:62183 | NaSa01\_F14.raw |  |  |  |  |  | 1.5231E7 |  |  |  |  |  |  |  |  | 1 | 0 | 0 | 0 | 0 | 0 | 1 | 0 | 0 | 0 | 0 | 0 | 0 | 0 | 0 | 290 | 319 | Carbamidomethylation | C27:Carbamidomethylation:1000.00 | PEAKS DB |
| R.DPNYGMVEPGTKC(+57.02)GDGMVC(+57.02)SNR.Q | Y | 85.41 | 2443.0024 | 22 | -4.2 | 815.3380 | 3 | 34.23 | 6 | F6:15877 | NaSa01\_F14.raw |  |  |  |  |  | 1.112E7 |  |  |  |  |  |  |  |  | 1 | 0 | 0 | 0 | 0 | 0 | 1 | 0 | 0 | 0 | 0 | 0 | 0 | 0 | 0 | 583 | 604 | Carbamidomethylation | C13:Carbamidomethylation:1000.00;C19:Carbamidomethylation:1000.00 | PEAKS DB |
| R.DPNYGMVEPGTK.C | Y | 82.42 | 1306.5863 | 12 | 0.8 | 654.3010 | 2 | 28.28 | 6 | F6:10918 | NaSa01\_F14.raw |  |  |  |  |  | 6.8446E6 |  |  |  | 1.1625E6 |  |  | 3.2642E6 |  | 3 | 0 | 0 | 0 | 0 | 0 | 1 | 0 | 0 | 0 | 1 | 0 | 0 | 1 | 0 | 583 | 594 |  |  | PEAKS DB |
| R.NDNAQLLTGIDFNGTPVGLAYIGSIC(+57.02)NPK.T | N | 82.31 | 3061.5178 | 29 | 1.9 | 1021.5151 | 3 | 99.98 | 6 | F6:71311 | NaSa01\_F14.raw |  |  | 2.6315E5 |  |  | 1.5398E8 |  |  |  |  |  |  |  |  | 4 | 0 | 0 | 1 | 0 | 0 | 3 | 0 | 0 | 0 | 0 | 0 | 0 | 0 | 0 | 291 | 319 | Carbamidomethylation | C26:Carbamidomethylation:1000.00 | PEAKS DB |
| N.GTPVGLAYIGSIC(+57.02)NPK.T | N | 82.13 | 1645.8497 | 16 | -0.6 | 823.9316 | 2 | 61.34 | 6 | F6:39114 | NaSa01\_F14.raw |  |  |  |  |  | 9.3648E6 |  |  |  |  |  |  |  |  | 1 | 0 | 0 | 0 | 0 | 0 | 1 | 0 | 0 | 0 | 0 | 0 | 0 | 0 | 0 | 304 | 319 | Carbamidomethylation | C13:Carbamidomethylation:1000.00 | PEAKS DB |
| R.AAKDDC(+57.02)DLPELC(+57.02)TGQSAEC(+57.02)PTDVFQR.N | N | 79.07 | 2982.2793 | 26 | 0.8 | 995.1012 | 3 | 55.91 | 6 | F6:34388 | NaSa01\_F14.raw |  |  |  |  |  | 1.5419E8 |  |  |  |  |  |  |  |  | 2 | 0 | 0 | 0 | 0 | 0 | 2 | 0 | 0 | 0 | 0 | 0 | 0 | 0 | 0 | 468 | 493 | Carbamidomethylation | C6:Carbamidomethylation:1000.00;C12:Carbamidomethylation:1000.00;C19:Carbamidomethylation:1000.00 | PEAKS DB |
| K.C(+57.02)PIMTNQC(+57.02)IALR.G | N | 77.84 | 1475.7047 | 12 | 0.0 | 738.8596 | 2 | 39.74 | 6 | F6:21215 | NaSa01\_F14.raw |  |  | 3.3287E5 | 1.3692E6 | 9.8113E6 | 2.4068E9 |  |  |  | 1.7213E7 | 6.4357E6 |  | 4.0715E6 | 1.3044E6 | 15 | 0 | 0 | 1 | 1 | 2 | 5 | 0 | 0 | 0 | 2 | 2 | 0 | 1 | 1 | 510 | 521 | Carbamidomethylation | C1:Carbamidomethylation:1000.00;C8:Carbamidomethylation:1000.00 | PEAKS DB |
| R.NSMIC(+57.02)NC(+57.02)SISPR.D | N | 77.03 | 1437.6163 | 12 | -0.1 | 719.8154 | 2 | 22.49 | 6 | F6:6540 | NaSa01\_F14.raw |  |  |  |  |  | 7.6177E6 |  |  |  |  |  |  |  |  | 2 | 0 | 0 | 0 | 0 | 0 | 2 | 0 | 0 | 0 | 0 | 0 | 0 | 0 | 0 | 571 | 582 | Carbamidomethylation | C5:Carbamidomethylation:1000.00;C7:Carbamidomethylation:1000.00 | PEAKS DB |
| K.VYEM(+15.99)INTMNMIYR.R | N | 74.23 | 1692.7673 | 13 | -0.2 | 847.3907 | 2 | 57.49 | 6 | F6:35287 | NaSa01\_F14.raw |  |  |  |  |  | 4.3266E8 |  |  |  |  |  |  |  |  | 9 | 0 | 0 | 0 | 0 | 0 | 9 | 0 | 0 | 0 | 0 | 0 | 0 | 0 | 0 | 232 | 244 | Oxidation (M) | M4:Oxidation (M):77.51 | PEAKS DB |
| K.VYEMINTMNMIYR.R | N | 73.76 | 1676.7725 | 13 | -0.3 | 839.3932 | 2 | 71.51 | 6 | F6:48424 | NaSa01\_F14.raw |  |  |  |  |  | 9.5037E8 |  |  |  |  |  |  |  |  | 12 | 0 | 0 | 0 | 0 | 0 | 12 | 0 | 0 | 0 | 0 | 0 | 0 | 0 | 0 | 232 | 244 |  |  | PEAKS DB |
| D.LPELC(+57.02)TGQSAEC(+57.02)PTDVFQR.N | N | 73.30 | 2206.9988 | 19 | 0.2 | 1104.5068 | 2 | 60.04 | 6 | F6:38037 | NaSa01\_F14.raw |  |  |  |  |  | 2.7029E6 |  |  |  |  |  |  |  |  | 1 | 0 | 0 | 0 | 0 | 0 | 1 | 0 | 0 | 0 | 0 | 0 | 0 | 0 | 0 | 475 | 493 | Carbamidomethylation | C5:Carbamidomethylation:1000.00;C12:Carbamidomethylation:1000.00 | PEAKS DB |
| K.C(+57.02)PIM(+15.99)TNQC(+57.02)IALR.G | N | 73.02 | 1491.6996 | 12 | 0.1 | 746.8572 | 2 | 30.97 | 6 | F6:13155 | NaSa01\_F14.raw |  |  |  |  | 1.4708E6 | 1.6503E8 |  |  |  |  |  |  |  |  | 5 | 0 | 0 | 0 | 0 | 1 | 4 | 0 | 0 | 0 | 0 | 0 | 0 | 0 | 0 | 510 | 521 | Carbamidomethylation; Oxidation (M) | C1:Carbamidomethylation:1000.00;M4:Oxidation (M):1000.00;C8:Carbamidomethylation:1000.00 | PEAKS DB |
| R.KVYEMINTMNMIYR.R | N | 72.83 | 1804.8674 | 14 | 0.6 | 903.4415 | 2 | 58.08 | 6 | F6:36256 | NaSa01\_F14.raw |  |  |  |  |  | 3.0426E6 |  |  |  |  |  |  |  |  | 1 | 0 | 0 | 0 | 0 | 0 | 1 | 0 | 0 | 0 | 0 | 0 | 0 | 0 | 0 | 231 | 244 |  |  | PEAKS DB |
| R.TKPAYQFSSC(+57.02)SVR.E | N | 72.78 | 1529.7296 | 13 | 0.4 | 765.8724 | 2 | 11.56 | 6 | F6:1943 | NaSa01\_F14.raw |  |  |  |  |  | 1.656E7 |  |  |  |  |  |  |  |  | 3 | 0 | 0 | 0 | 0 | 0 | 3 | 0 | 0 | 0 | 0 | 0 | 0 | 0 | 0 | 370 | 382 | Carbamidomethylation | C10:Carbamidomethylation:1000.00 | PEAKS DB |
| K.VYEMINTM(+15.99)NMIYR.R | N | 72.14 | 1692.7673 | 13 | -0.2 | 847.3907 | 2 | 57.49 | 6 | F6:36159 | NaSa01\_F14.raw |  |  |  |  |  | 4.3022E8 |  |  |  |  |  |  |  |  | 8 | 0 | 0 | 0 | 0 | 0 | 8 | 0 | 0 | 0 | 0 | 0 | 0 | 0 | 0 | 232 | 244 | Oxidation (M) | M8:Oxidation (M):79.36 | PEAKS DB |
| K.VYEMINTMNM(+15.99)IYR.R | N | 71.71 | 1692.7673 | 13 | -2.0 | 565.2619 | 3 | 61.54 | 6 | F6:39303 | NaSa01\_F14.raw |  |  |  |  |  | 2.4734E8 |  |  |  |  |  |  |  |  | 6 | 0 | 0 | 0 | 0 | 0 | 6 | 0 | 0 | 0 | 0 | 0 | 0 | 0 | 0 | 232 | 244 | Oxidation (M) | M10:Oxidation (M):28.86 | PEAKS DB |
| R.MVAITMAHEMGH.N | N | 70.23 | 1326.5883 | 12 | 0.1 | 664.3015 | 2 | 30.77 | 6 | F6:12852 | NaSa01\_F14.raw |  |  |  |  | 5.8936E5 | 1.005E8 |  |  |  |  |  |  |  |  | 4 | 0 | 0 | 0 | 0 | 1 | 3 | 0 | 0 | 0 | 0 | 0 | 0 | 0 | 0 | 334 | 345 |  |  | PEAKS DB |
| R.KVYEMINTMNM(+15.99)IYR.R | N | 67.76 | 1820.8623 | 14 | 0.0 | 607.9614 | 3 | 49.96 | 6 | F6:29587 | NaSa01\_F14.raw |  |  |  |  |  | 7.8852E6 |  |  |  |  |  |  |  |  | 1 | 0 | 0 | 0 | 0 | 0 | 1 | 0 | 0 | 0 | 0 | 0 | 0 | 0 | 0 | 231 | 244 | Oxidation (M) | M11:Oxidation (M):36.05 | PEAKS DB |
| R.NSM(+15.99)IC(+57.02)NC(+57.02)SISPR.D | N | 66.06 | 1453.6112 | 12 | 0.3 | 727.8131 | 2 | 11.56 | 6 | F6:1942 | NaSa01\_F14.raw |  |  |  |  |  | 2.1578E6 |  |  |  |  |  |  |  |  | 4 | 0 | 0 | 0 | 0 | 0 | 4 | 0 | 0 | 0 | 0 | 0 | 0 | 0 | 0 | 571 | 582 | Oxidation (M); Carbamidomethylation | M3:Oxidation (M):1000.00;C5:Carbamidomethylation:1000.00;C7:Carbamidomethylation:1000.00 | PEAKS DB |
| R.DSC(+57.02)FTLNQR.T | N | 63.93 | 1139.5029 | 9 | -0.1 | 570.7587 | 2 | 25.28 | 6 | F6:8701 | NaSa01\_F14.raw |  |  |  |  |  | 8.7088E6 |  |  |  |  |  |  |  |  | 2 | 0 | 0 | 0 | 0 | 0 | 2 | 0 | 0 | 0 | 0 | 0 | 0 | 0 | 0 | 530 | 538 | Carbamidomethylation | C3:Carbamidomethylation:1000.00 | PEAKS DB |
| K.VYEM(+15.99)INTMNM(+15.99)IYR.R | N | 61.29 | 1708.7623 | 13 | -0.3 | 855.3882 | 2 | 45.10 | 6 | F6:25392 | NaSa01\_F14.raw |  |  |  |  |  | 4.4184E7 |  |  |  |  |  |  |  |  | 1 | 0 | 0 | 0 | 0 | 0 | 1 | 0 | 0 | 0 | 0 | 0 | 0 | 0 | 0 | 232 | 244 | Oxidation (M) | M4:Oxidation (M):79.36;M10:Oxidation (M):27.16 | PEAKS DB |
| R.MVAITMAHEMGHNLGMNHD.R | N | 59.40 | 2107.9060 | 19 | 0.0 | 703.6426 | 3 | 45.80 | 6 | F6:25307 | NaSa01\_F14.raw |  |  |  |  |  | 7.6575E6 |  |  |  |  |  |  |  |  | 1 | 0 | 0 | 0 | 0 | 0 | 1 | 0 | 0 | 0 | 0 | 0 | 0 | 0 | 0 | 334 | 352 |  |  | PEAKS DB |
| R.DRPQC(+57.02)ILNKPLSTDIVSPPIC(+57.02)GN.Y | N | 58.34 | 2593.2993 | 23 | -0.3 | 1297.6565 | 2 | 63.62 | 6 | F6:40889 | NaSa01\_F14.raw |  |  |  |  |  | 2.087E9 |  |  |  |  |  |  |  |  | 4 | 0 | 0 | 0 | 0 | 0 | 4 | 0 | 0 | 0 | 0 | 0 | 0 | 0 | 0 | 391 | 413 | Carbamidomethylation | C5:Carbamidomethylation:1000.00;C21:Carbamidomethylation:1000.00 | PEAKS DB |
| K.VYEMINTM(+15.99)NM(+15.99)IYR.R | N | 57.62 | 1708.7623 | 13 | -0.3 | 855.3882 | 2 | 45.10 | 6 | F6:25162 | NaSa01\_F14.raw |  |  |  |  |  | 4.4184E7 |  |  |  |  |  |  |  |  | 1 | 0 | 0 | 0 | 0 | 0 | 1 | 0 | 0 | 0 | 0 | 0 | 0 | 0 | 0 | 232 | 244 | Oxidation (M) | M8:Oxidation (M):60.62;M10:Oxidation (M):65.06 | PEAKS DB |
| Q.LLTGIDFNGTPVGLAYIGSIC(+57.02)NPK.T | N | 56.04 | 2519.3093 | 24 | 0.7 | 1260.6628 | 2 | 95.97 | 6 | F6:68185 | NaSa01\_F14.raw |  |  |  |  |  | 4.6093E6 |  |  |  |  |  |  |  |  | 2 | 0 | 0 | 0 | 0 | 0 | 2 | 0 | 0 | 0 | 0 | 0 | 0 | 0 | 0 | 296 | 319 | Carbamidomethylation | C21:Carbamidomethylation:1000.00 | PEAKS DB |
| R.NGLPC(+57.02)QNNQGYC(+57.02)YNGK.C | N | 55.19 | 1885.7836 | 16 | -0.1 | 943.8989 | 2 | 11.56 | 6 | F6:1965 | NaSa01\_F14.raw |  |  |  |  |  | 1.6943E6 |  |  |  |  |  |  |  |  | 1 | 0 | 0 | 0 | 0 | 0 | 1 | 0 | 0 | 0 | 0 | 0 | 0 | 0 | 0 | 494 | 509 | Carbamidomethylation | C5:Carbamidomethylation:1000.00;C12:Carbamidomethylation:1000.00 | PEAKS DB |
| R.GFC(+57.02)TC(+57.02)GFNK.C | N | 54.46 | 1089.4371 | 9 | -0.5 | 545.7256 | 2 | 11.57 | 6 | F6:1962 | NaSa01\_F14.raw |  |  |  |  |  | 2.5576E6 |  |  |  |  |  |  |  |  | 1 | 0 | 0 | 0 | 0 | 0 | 1 | 0 | 0 | 0 | 0 | 0 | 0 | 0 | 0 | 354 | 362 | Carbamidomethylation | C3:Carbamidomethylation:1000.00;C5:Carbamidomethylation:1000.00 | PEAKS DB |
| Y.EMINTMNMIYR.R | N | 53.03 | 1414.6407 | 11 | -0.2 | 708.3275 | 2 | 57.05 | 6 | F6:35453 | NaSa01\_F14.raw |  |  |  |  |  | 4.7389E6 |  |  |  |  |  |  |  |  | 1 | 0 | 0 | 0 | 0 | 0 | 1 | 0 | 0 | 0 | 0 | 0 | 0 | 0 | 0 | 234 | 244 |  |  | PEAKS DB |
| M.INTMNMIYR.R | N | 51.67 | 1154.5576 | 9 | 0.4 | 578.2863 | 2 | 38.32 | 6 | F6:19378 | NaSa01\_F14.raw |  |  |  |  |  | 0 |  |  |  |  |  |  |  |  | 0 | 0 | 0 | 0 | 0 | 0 | 0 | 0 | 0 | 0 | 0 | 0 | 0 | 0 | 0 | 236 | 244 |  |  | PEAKS DB |
| R.KVYEMINTMN.M | N | 51.42 | 1241.5785 | 10 | 0.3 | 621.7967 | 2 | 33.25 | 6 | F6:15075 | NaSa01\_F14.raw |  |  |  |  |  | 2.3194E7 |  |  |  |  |  |  |  |  | 1 | 0 | 0 | 0 | 0 | 0 | 1 | 0 | 0 | 0 | 0 | 0 | 0 | 0 | 0 | 231 | 240 |  |  | PEAKS DB |
| R.MVAITMAHEM.G | N | 51.15 | 1132.5079 | 10 | 1.1 | 567.2618 | 2 | 44.18 | 6 | F6:24291 | NaSa01\_F14.raw |  |  |  |  |  | 4.7043E6 |  |  |  |  |  |  |  |  | 1 | 0 | 0 | 0 | 0 | 0 | 1 | 0 | 0 | 0 | 0 | 0 | 0 | 0 | 0 | 334 | 343 |  |  | PEAKS DB |
| K.VYEM(+15.99)INTM(+15.99)NMIYR.R | N | 50.77 | 1708.7623 | 13 | -0.3 | 855.3882 | 2 | 45.10 | 6 | F6:25618 | NaSa01\_F14.raw |  |  |  |  |  | 5.1185E7 |  |  |  |  |  |  |  |  | 3 | 0 | 0 | 0 | 0 | 0 | 3 | 0 | 0 | 0 | 0 | 0 | 0 | 0 | 0 | 232 | 244 | Oxidation (M) | M4:Oxidation (M):83.65;M8:Oxidation (M):18.23 | PEAKS DB |
| R.TKPAYQFSSC(+57.02).S | N | 50.58 | 1187.5281 | 10 | 0.3 | 594.7715 | 2 | 11.58 | 6 | F6:1956 | NaSa01\_F14.raw |  |  |  |  |  | 3.6584E6 |  |  |  |  |  |  |  |  | 1 | 0 | 0 | 0 | 0 | 0 | 1 | 0 | 0 | 0 | 0 | 0 | 0 | 0 | 0 | 370 | 379 | Carbamidomethylation | C10:Carbamidomethylation:1000.00 | PEAKS DB |
| K.VYEMINTMN.M | N | 49.68 | 1113.4835 | 9 | 0.3 | 557.7492 | 2 | 52.12 | 6 | F6:31163 | NaSa01\_F14.raw |  |  |  |  |  | 1.6784E8 |  |  |  |  |  |  |  |  | 1 | 0 | 0 | 0 | 0 | 0 | 1 | 0 | 0 | 0 | 0 | 0 | 0 | 0 | 0 | 232 | 240 |  |  | PEAKS DB |
| C.PIMTNQC(+57.02)IALR.G | N | 49.03 | 1315.6741 | 11 | 0.4 | 658.8446 | 2 | 37.06 | 6 | F6:18281 | NaSa01\_F14.raw |  |  |  |  |  | 1.277E6 |  |  |  |  |  |  |  |  | 1 | 0 | 0 | 0 | 0 | 0 | 1 | 0 | 0 | 0 | 0 | 0 | 0 | 0 | 0 | 511 | 521 | Carbamidomethylation | C7:Carbamidomethylation:1000.00 | PEAKS DB |
| R.DPNYGM(+15.99)VEPGTK.C | Y | 48.74 | 1322.5813 | 12 | -0.3 | 662.2977 | 2 | 11.56 | 6 | F6:1972 | NaSa01\_F14.raw |  |  |  |  |  | 4.7262E5 |  |  |  |  |  |  |  |  | 1 | 0 | 0 | 0 | 0 | 0 | 1 | 0 | 0 | 0 | 0 | 0 | 0 | 0 | 0 | 583 | 594 | Oxidation (M) | M6:Oxidation (M):1000.00 | PEAKS DB |
| K.RNDNAQLLTGIDFNGTPVGLAY.I | N | 47.41 | 2348.1760 | 22 | 0.6 | 1175.0959 | 2 | 90.55 | 6 | F6:63498 | NaSa01\_F14.raw |  |  |  |  |  | 4.2697E6 |  |  |  |  |  |  |  |  | 1 | 0 | 0 | 0 | 0 | 0 | 1 | 0 | 0 | 0 | 0 | 0 | 0 | 0 | 0 | 290 | 311 |  |  | PEAKS DB |
| K.VYEM(+15.99)INTM(+15.99)NM(+15.99)IYR.R | N | 46.27 | 1724.7572 | 13 | -0.7 | 863.3853 | 2 | 32.47 | 6 | F6:14255 | NaSa01\_F14.raw |  |  |  |  |  | 3.9176E6 |  |  |  |  |  |  |  |  | 1 | 0 | 0 | 0 | 0 | 0 | 1 | 0 | 0 | 0 | 0 | 0 | 0 | 0 | 0 | 232 | 244 | Oxidation (M) | M4:Oxidation (M):1000.00;M8:Oxidation (M):1000.00;M10:Oxidation (M):1000.00 | PEAKS DB |
| R.MVAITMAH.E | N | 45.87 | 872.4249 | 8 | -0.1 | 437.2197 | 2 | 30.29 | 6 | F6:13244 | NaSa01\_F14.raw |  |  |  |  | 1.6148E6 | 2.3115E6 |  |  |  |  |  |  |  |  | 4 | 0 | 0 | 0 | 0 | 2 | 2 | 0 | 0 | 0 | 0 | 0 | 0 | 0 | 0 | 334 | 341 |  |  | PEAKS DB |
| K.VYEMINTM(+15.99)N.M | N | 45.82 | 1129.4784 | 9 | 0.2 | 565.7466 | 2 | 42.89 | 6 | F6:23229 | NaSa01\_F14.raw |  |  |  |  |  | 0 |  |  |  |  |  |  |  |  | 0 | 0 | 0 | 0 | 0 | 0 | 0 | 0 | 0 | 0 | 0 | 0 | 0 | 0 | 0 | 232 | 240 | Oxidation (M) | M8:Oxidation (M):52.34 | PEAKS DB |
| R.MVAITMAHEMGHNLGM.N | N | 45.75 | 1741.7772 | 16 | 0.5 | 871.8964 | 2 | 58.51 | 6 | F6:36744 | NaSa01\_F14.raw |  |  |  |  |  | 2.3274E6 |  |  |  |  |  |  |  |  | 1 | 0 | 0 | 0 | 0 | 0 | 1 | 0 | 0 | 0 | 0 | 0 | 0 | 0 | 0 | 334 | 349 |  |  | PEAKS DB |
| R.EITTSPPVQDHC(+57.02)YYHGY.I | N | 44.97 | 2065.8840 | 17 | 6.3 | 689.6360 | 3 | 31.90 | 8 | F8:16761 | NaSa01\_F3.raw |  |  |  |  |  |  |  | 3.1849E6 |  |  |  |  |  |  | 1 | 0 | 0 | 0 | 0 | 0 | 0 | 0 | 1 | 0 | 0 | 0 | 0 | 0 | 0 | 97 | 113 | Carbamidomethylation | C12:Carbamidomethylation:1000.00 | PEAKS DB |
| R.NDNAQLLTGIDFNGTPVGL.A | N | 44.91 | 1957.9745 | 19 | 0.5 | 979.9950 | 2 | 104.18 | 6 | F6:74818 | NaSa01\_F14.raw |  |  |  |  |  | 2.0756E7 |  |  |  |  |  |  |  |  | 1 | 0 | 0 | 0 | 0 | 0 | 1 | 0 | 0 | 0 | 0 | 0 | 0 | 0 | 0 | 291 | 309 |  |  | PEAKS DB |
| L.TGIDFNGTPVGLAYIGSIC(+57.02)NPK.T | N | 44.70 | 2293.1411 | 22 | -0.2 | 1147.5776 | 2 | 85.72 | 6 | F6:59453 | NaSa01\_F14.raw |  |  |  |  |  | 3.0432E6 |  |  |  |  |  |  |  |  | 1 | 0 | 0 | 0 | 0 | 0 | 1 | 0 | 0 | 0 | 0 | 0 | 0 | 0 | 0 | 298 | 319 | Carbamidomethylation | C19:Carbamidomethylation:1000.00 | PEAKS DB |
| R.DRPQC(+57.02)ILNKPL.S | N | 44.62 | 1352.7234 | 11 | 1.1 | 451.9156 | 3 | 27.50 | 5 | F5:11578 | NaSa01\_F13.raw |  |  |  |  | 1.9485E7 |  |  |  |  |  |  |  |  |  | 1 | 0 | 0 | 0 | 0 | 1 | 0 | 0 | 0 | 0 | 0 | 0 | 0 | 0 | 0 | 391 | 401 | Carbamidomethylation | C5:Carbamidomethylation:1000.00 | PEAKS DB |
| L.TNTPEQDR.Y | N | 44.60 | 959.4308 | 8 | 0.1 | 480.7227 | 2 | 11.42 | 6 | F6:1814 | NaSa01\_F14.raw |  |  |  |  |  | 1.6427E5 |  |  |  |  |  |  |  |  | 1 | 0 | 0 | 0 | 0 | 0 | 1 | 0 | 0 | 0 | 0 | 0 | 0 | 0 | 0 | 192 | 199 |  |  | PEAKS DB |
| R.MVAITM(+15.99)AHEMGH.N | N | 44.44 | 1342.5833 | 12 | -0.2 | 672.2988 | 2 | 30.93 | 6 | F6:12991 | NaSa01\_F14.raw |  |  |  |  |  | 1.3599E7 |  |  |  |  |  |  |  |  | 2 | 0 | 0 | 0 | 0 | 0 | 2 | 0 | 0 | 0 | 0 | 0 | 0 | 0 | 0 | 334 | 345 | Oxidation (M) | M6:Oxidation (M):8.80 | PEAKS DB |
| G.TPVGLAYIGSIC(+57.02)NPK.T | N | 42.66 | 1588.8282 | 15 | 1.1 | 795.4222 | 2 | 58.70 | 12 | F12:38993 | NaSa01\_F7.raw |  |  |  |  |  |  |  |  |  |  |  | 1.3171E5 |  |  | 1 | 0 | 0 | 0 | 0 | 0 | 0 | 0 | 0 | 0 | 0 | 0 | 1 | 0 | 0 | 305 | 319 | Carbamidomethylation | C12:Carbamidomethylation:1000.00 | PEAKS DB |
| R.KVYEMINTM(+15.99)NM(+15.99)IYR.R | N | 41.47 | 1836.8573 | 14 | 0.6 | 613.2934 | 3 | 33.38 | 6 | F6:15316 | NaSa01\_F14.raw |  |  |  |  |  | 1.4433E6 |  |  |  |  |  |  |  |  | 1 | 0 | 0 | 0 | 0 | 0 | 1 | 0 | 0 | 0 | 0 | 0 | 0 | 0 | 0 | 231 | 244 | Oxidation (M) | M9:Oxidation (M):58.99;M11:Oxidation (M):78.19 | PEAKS DB |
| R.M(+15.99)VAITMAHEMGH.N | N | 40.25 | 1342.5833 | 12 | -0.3 | 448.5349 | 3 | 25.47 | 6 | F6:9076 | NaSa01\_F14.raw |  |  |  |  |  | 1.8423E7 |  |  |  |  |  |  |  |  | 2 | 0 | 0 | 0 | 0 | 0 | 2 | 0 | 0 | 0 | 0 | 0 | 0 | 0 | 0 | 334 | 345 | Oxidation (M) | M1:Oxidation (M):10.53 | PEAKS DB |
| G.LAYIGSIC(+57.02)NPK.T | N | 38.59 | 1234.6379 | 11 | 0.2 | 618.3264 | 2 | 37.01 | 12 | F12:20710 | NaSa01\_F7.raw |  |  |  |  |  |  |  |  |  |  |  | 3.5988E5 |  |  | 1 | 0 | 0 | 0 | 0 | 0 | 0 | 0 | 0 | 0 | 0 | 0 | 1 | 0 | 0 | 309 | 319 | Carbamidomethylation | C8:Carbamidomethylation:1000.00 | PEAKS DB |
| K.PAYQFSSC(+57.02)SVR.E | N | 38.45 | 1300.5870 | 11 | 0.0 | 651.3008 | 2 | 23.13 | 6 | F6:6974 | NaSa01\_F14.raw |  |  |  |  |  | 1.0807E6 |  |  |  |  |  |  |  |  | 1 | 0 | 0 | 0 | 0 | 0 | 1 | 0 | 0 | 0 | 0 | 0 | 0 | 0 | 0 | 372 | 382 | Carbamidomethylation | C8:Carbamidomethylation:1000.00 | PEAKS DB |
| F.NGTPVGLAYIGSIC(+57.02)NPK.T | N | 38.29 | 1759.8927 | 17 | -1.0 | 880.9528 | 2 | 61.44 | 6 | F6:39069 | NaSa01\_F14.raw |  |  |  |  |  | 6.4633E5 |  |  |  |  |  |  |  |  | 1 | 0 | 0 | 0 | 0 | 0 | 1 | 0 | 0 | 0 | 0 | 0 | 0 | 0 | 0 | 303 | 319 | Carbamidomethylation | C14:Carbamidomethylation:1000.00 | PEAKS DB |
| D.PNYGM(+15.99)VEPGTK.C | Y | 37.70 | 1207.5543 | 11 | 0.7 | 604.7849 | 2 | 11.49 | 6 | F6:1887 | NaSa01\_F14.raw |  |  |  |  |  | 6.0854E4 |  |  |  |  |  |  |  |  | 1 | 0 | 0 | 0 | 0 | 0 | 1 | 0 | 0 | 0 | 0 | 0 | 0 | 0 | 0 | 584 | 594 | Oxidation (M) | M5:Oxidation (M):1000.00 | PEAKS DB |
| total 55 peptides |  |  |  |  |  |  |  |  |  |  |  |  |  |  |  |  |  |  |  |  |  |  |  |  |  |  |  |  |  |  |  |  |  |  |  |  |  |  |  |  |  |  |  |  |  |
| --- | --- | --- | --- | --- | --- | --- | --- | --- | --- | --- | --- | --- | --- | --- | --- | --- | --- | --- | --- | --- | --- | --- | --- | --- | --- | --- | --- | --- | --- | --- | --- | --- | --- | --- | --- | --- | --- | --- | --- | --- | --- | --- | --- | --- | --- |

D5LMJ3.1

back to list

  

| Protein Coverage
| Supporting Peptides
|

Protein Coverage:

Supporting Peptides:

| Peptide | Uniq | -10lgP | Mass | Length | ppm | m/z | z | RT | Fraction | Scan | Source File | Area F1 | Area F10 | Area F11 | Area F12 | Area F13 | Area F14 | Area F2 | Area F3 | Area F4 | Area F5 | Area F6 | Area F7 | Area F8 | Area F9 | #Feature | #Feature F1 | #Feature F10 | #Feature F11 | #Feature F12 | #Feature F13 | #Feature F14 | #Feature F2 | #Feature F3 | #Feature F4 | #Feature F5 | #Feature F6 | #Feature F7 | #Feature F8 | #Feature F9 | Start | End | PTM | AScore | Found By |
| --- | --- | --- | --- | --- | --- | --- | --- | --- | --- | --- | --- | --- | --- | --- | --- | --- | --- | --- | --- | --- | --- | --- | --- | --- | --- | --- | --- | --- | --- | --- | --- | --- | --- | --- | --- | --- | --- | --- | --- | --- | --- | --- | --- | --- | --- |
| R.RTILMASTMAHELGHNMGIHHDK.A | Y | 102.57 | 2599.2571 | 23 | 0.5 | 650.8219 | 4 | 25.79 | 3 | F3:9045 | NaSa01\_F11.raw |  | 9.1055E5 | 2.4907E8 | 3.4546E7 |  |  |  |  |  |  |  |  |  |  | 13 | 0 | 1 | 9 | 3 | 0 | 0 | 0 | 0 | 0 | 0 | 0 | 0 | 0 | 0 | 328 | 350 |  |  | PEAKS DB |
| R.TILMASTMAHELGHNMGIHHDK.A | Y | 92.32 | 2443.1560 | 22 | 1.4 | 611.7971 | 4 | 35.33 | 3 | F3:17779 | NaSa01\_F11.raw |  | 2.0692E7 | 4.6099E9 | 9.7683E8 | 2.583E7 | 2.3127E7 |  |  |  |  |  |  |  |  | 44 | 0 | 3 | 23 | 13 | 2 | 3 | 0 | 0 | 0 | 0 | 0 | 0 | 0 | 0 | 329 | 350 |  |  | PEAKS DB |
| R.TILM(+15.99)ASTMAHELGHNMGIHHDK.A | Y | 82.29 | 2459.1509 | 22 | 0.5 | 615.7953 | 4 | 25.21 | 3 | F3:11732 | NaSa01\_F11.raw |  |  | 5.9895E8 | 1.361E8 |  |  |  |  |  |  |  |  |  |  | 15 | 0 | 0 | 9 | 6 | 0 | 0 | 0 | 0 | 0 | 0 | 0 | 0 | 0 | 0 | 329 | 350 | Oxidation (M) | M4:Oxidation (M):97.69 | PEAKS DB |
| K.C(+57.02)PTMENQC(+57.02)ITLLGPNYTVGPAGC(+57.02)FK.N | Y | 81.11 | 2827.2803 | 25 | 1.5 | 943.4354 | 3 | 74.97 | 3 | F3:47101 | NaSa01\_F11.raw |  | 3.3769E6 | 9.888E8 | 4.7351E8 | 2.0131E7 | 1.4081E7 |  |  |  |  |  |  |  |  | 14 | 0 | 2 | 5 | 4 | 1 | 2 | 0 | 0 | 0 | 0 | 0 | 0 | 0 | 0 | 508 | 532 | Carbamidomethylation | C1:Carbamidomethylation:1000.00;C8:Carbamidomethylation:1000.00;C23:Carbamidomethylation:1000.00 | PEAKS DB |
| R.VYEMVNYLNTKYR.R | Y | 77.67 | 1691.8341 | 13 | -0.1 | 564.9519 | 3 | 45.38 | 4 | F4:27468 | NaSa01\_F12.raw |  |  | 1.1565E7 | 5.395E6 | 7.5997E5 |  |  |  |  |  |  |  |  |  | 4 | 0 | 0 | 2 | 1 | 1 | 0 | 0 | 0 | 0 | 0 | 0 | 0 | 0 | 0 | 231 | 243 |  |  | PEAKS DB |
| R.TRVYEMVNYLNTK.Y | Y | 76.18 | 1629.8185 | 13 | -4.5 | 815.9128 | 2 | 42.57 | 4 | F4:25251 | NaSa01\_F12.raw |  |  | 7.0755E6 | 0 |  |  |  |  |  |  |  |  |  |  | 2 | 0 | 0 | 2 | 0 | 0 | 0 | 0 | 0 | 0 | 0 | 0 | 0 | 0 | 0 | 229 | 241 |  |  | PEAKS DB |
| R.TILMASTM(+15.99)AHELGHNMGIHHDK.A | Y | 74.28 | 2459.1509 | 22 | 0.3 | 492.8376 | 5 | 20.73 | 4 | F4:7454 | NaSa01\_F12.raw |  |  | 5.4127E8 | 6.276E7 | 7.7545E6 |  |  |  |  |  |  |  |  |  | 20 | 0 | 0 | 12 | 6 | 2 | 0 | 0 | 0 | 0 | 0 | 0 | 0 | 0 | 0 | 329 | 350 | Oxidation (M) | M8:Oxidation (M):58.99 | PEAKS DB |
| R.TILMASTMAHELGHNM(+15.99)GIHHDK.A | Y | 74.07 | 2459.1509 | 22 | 0.8 | 492.8378 | 5 | 25.22 | 4 | F4:10852 | NaSa01\_F12.raw |  |  | 1.9866E8 | 7.6075E7 | 8.24E6 | 3.8636E6 |  |  |  |  |  |  |  |  | 17 | 0 | 0 | 9 | 4 | 3 | 1 | 0 | 0 | 0 | 0 | 0 | 0 | 0 | 0 | 329 | 350 | Oxidation (M) | M16:Oxidation (M):63.64 | PEAKS DB |
| K.LQPHAQC(+57.02)DSEEC(+57.02)C(+57.02)EK.C | Y | 72.87 | 1889.7343 | 15 | -0.1 | 630.9186 | 3 | 11.55 | 4 | F4:2524 | NaSa01\_F12.raw |  |  |  | 1.7758E6 |  |  |  |  |  |  |  |  |  |  | 2 | 0 | 0 | 0 | 2 | 0 | 0 | 0 | 0 | 0 | 0 | 0 | 0 | 0 | 0 | 440 | 454 | Carbamidomethylation | C7:Carbamidomethylation:1000.00;C12:Carbamidomethylation:1000.00;C13:Carbamidomethylation:1000.00 | PEAKS DB |
| K.C(+57.02)PTM(+15.99)ENQC(+57.02)ITLLGPNYTVGPAGC(+57.02)FK.N | Y | 72.54 | 2843.2751 | 25 | 1.3 | 948.7668 | 3 | 70.50 | 3 | F3:43399 | NaSa01\_F11.raw |  |  | 4.1576E7 | 4.2268E7 |  |  |  |  |  |  |  |  |  |  | 7 | 0 | 0 | 5 | 2 | 0 | 0 | 0 | 0 | 0 | 0 | 0 | 0 | 0 | 0 | 508 | 532 | Carbamidomethylation; Oxidation (M) | C1:Carbamidomethylation:1000.00;M4:Oxidation (M):1000.00;C8:Carbamidomethylation:1000.00;C23:Carbamidomethylation:1000.00 | PEAKS DB |
| R.VYEMVNYLNTK.Y | Y | 71.83 | 1372.6697 | 11 | 0.2 | 687.3423 | 2 | 49.73 | 6 | F6:29500 | NaSa01\_F14.raw |  | 3.5484E7 | 1.6202E9 | 1.498E9 | 2.2005E8 | 1.3641E8 |  |  |  |  |  |  |  |  | 23 | 0 | 2 | 10 | 8 | 1 | 2 | 0 | 0 | 0 | 0 | 0 | 0 | 0 | 0 | 231 | 241 |  |  | PEAKS DB |
| R.TILM(+15.99)ASTM(+15.99)AHELGHNMGIHHDK.A | Y | 70.98 | 2475.1458 | 22 | 0.7 | 619.7941 | 4 | 13.60 | 4 | F4:5130 | NaSa01\_F12.raw |  |  |  | 4.4221E7 |  |  |  |  |  |  |  |  |  |  | 8 | 0 | 0 | 0 | 8 | 0 | 0 | 0 | 0 | 0 | 0 | 0 | 0 | 0 | 0 | 329 | 350 | Oxidation (M) | M4:Oxidation (M):96.93;M8:Oxidation (M):53.45 | PEAKS DB |
| R.NGHPC(+57.02)QNNQGYC(+57.02)YNGK.C | N | 68.53 | 1909.7584 | 16 | -0.2 | 637.5933 | 3 | 11.55 | 4 | F4:2526 | NaSa01\_F12.raw |  |  |  | 1.1419E6 |  |  |  |  |  |  |  |  |  |  | 2 | 0 | 0 | 0 | 2 | 0 | 0 | 0 | 0 | 0 | 0 | 0 | 0 | 0 | 0 | 492 | 507 | Carbamidomethylation | C5:Carbamidomethylation:1000.00;C12:Carbamidomethylation:1000.00 | PEAKS DB |
| K.KYIEFYLVVDNK.M | Y | 67.91 | 1529.8130 | 12 | 0.6 | 765.9142 | 2 | 55.94 | 3 | F3:31344 | NaSa01\_F11.raw |  |  | 5.3689E7 | 1.4428E6 |  |  |  |  |  |  |  |  |  |  | 15 | 0 | 0 | 14 | 1 | 0 | 0 | 0 | 0 | 0 | 0 | 0 | 0 | 0 | 0 | 205 | 216 |  |  | PEAKS DB |
| L.GPNYTVGPAGC(+57.02)FK.N | Y | 67.57 | 1366.6339 | 13 | 0.7 | 684.3247 | 2 | 32.10 | 9 | F9:11219 | NaSa01\_F4.raw |  |  |  | 4.2089E6 |  |  |  |  | 1.5247E7 |  |  |  |  |  | 2 | 0 | 0 | 0 | 1 | 0 | 0 | 0 | 0 | 1 | 0 | 0 | 0 | 0 | 0 | 520 | 532 | Carbamidomethylation | C11:Carbamidomethylation:1000.00 | PEAKS DB |
| R.VYEM(+15.99)VNYLNTK.Y | Y | 67.21 | 1388.6646 | 11 | 0.1 | 695.3396 | 2 | 31.53 | 4 | F4:15763 | NaSa01\_F12.raw |  |  | 7.824E7 | 1.3006E8 | 1.9228E7 |  |  |  |  |  |  |  |  |  | 17 | 0 | 0 | 5 | 9 | 3 | 0 | 0 | 0 | 0 | 0 | 0 | 0 | 0 | 0 | 231 | 241 | Oxidation (M) | M4:Oxidation (M):1000.00 | PEAKS DB |
| R.TILMASTMAHELGH.N | Y | 66.37 | 1510.7272 | 14 | 0.5 | 756.3712 | 2 | 47.57 | 3 | F3:26108 | NaSa01\_F11.raw |  |  | 4.7059E7 | 1.2846E8 | 1.1854E7 | 3.9776E6 |  |  |  |  |  |  |  |  | 10 | 0 | 0 | 2 | 5 | 2 | 1 | 0 | 0 | 0 | 0 | 0 | 0 | 0 | 0 | 329 | 342 |  |  | PEAKS DB |
| R.RTILMASTMAHELGH.N | Y | 65.80 | 1666.8284 | 15 | -0.6 | 556.6164 | 3 | 32.97 | 4 | F4:17457 | NaSa01\_F12.raw |  |  | 1.2098E7 | 1.9622E7 |  |  |  |  |  |  |  |  |  |  | 4 | 0 | 0 | 2 | 2 | 0 | 0 | 0 | 0 | 0 | 0 | 0 | 0 | 0 | 0 | 328 | 342 |  |  | PEAKS DB |
| K.YIEFYLVVDNK.M | Y | 63.10 | 1401.7180 | 11 | 0.9 | 701.8669 | 2 | 74.33 | 4 | F4:51541 | NaSa01\_F12.raw |  |  | 3.2924E7 | 6.0017E5 |  |  |  |  |  |  |  |  |  |  | 17 | 0 | 0 | 15 | 2 | 0 | 0 | 0 | 0 | 0 | 0 | 0 | 0 | 0 | 0 | 206 | 216 |  |  | PEAKS DB |
| L.LGPNYTVGPAGC(+57.02)FK.N | Y | 62.86 | 1479.7180 | 14 | 0.9 | 740.8669 | 2 | 42.98 | 9 | F9:19580 | NaSa01\_F4.raw |  |  |  | 1.6597E6 |  |  |  |  | 2.6352E6 |  |  |  |  |  | 2 | 0 | 0 | 0 | 1 | 0 | 0 | 0 | 0 | 1 | 0 | 0 | 0 | 0 | 0 | 519 | 532 | Carbamidomethylation | C12:Carbamidomethylation:1000.00 | PEAKS DB |
| E.PFYEFSSC(+57.02)SVR.E | Y | 62.63 | 1377.6023 | 11 | 0.1 | 689.8085 | 2 | 45.23 | 4 | F4:27365 | NaSa01\_F12.raw |  |  | 1.0485E7 | 8.6274E6 | 1.867E6 |  |  |  |  |  |  |  |  |  | 3 | 0 | 0 | 1 | 1 | 1 | 0 | 0 | 0 | 0 | 0 | 0 | 0 | 0 | 0 | 370 | 380 | Carbamidomethylation | C8:Carbamidomethylation:1000.00 | PEAKS DB |
| W.GSVAVVQDYSR.R | Y | 60.81 | 1179.5884 | 11 | 0.5 | 590.8018 | 2 | 12.09 | 3 | F3:3668 | NaSa01\_F11.raw |  |  | 3.4234E6 | 1.6841E7 | 1.3633E6 | 7.0857E5 |  |  |  |  |  |  |  |  | 6 | 0 | 0 | 2 | 2 | 1 | 1 | 0 | 0 | 0 | 0 | 0 | 0 | 0 | 0 | 317 | 327 |  |  | PEAKS DB |
| R.TILMASTMAHELGHNMGIHHD.K | Y | 60.76 | 2315.0610 | 21 | 0.1 | 579.7726 | 4 | 43.04 | 3 | F3:23564 | NaSa01\_F11.raw |  |  | 1.7071E7 |  |  |  |  |  |  |  |  |  |  |  | 3 | 0 | 0 | 3 | 0 | 0 | 0 | 0 | 0 | 0 | 0 | 0 | 0 | 0 | 0 | 329 | 349 |  |  | PEAKS DB |
| R.ERPQC(+57.02)ILNKPSR.K | Y | 58.96 | 1496.7881 | 12 | -0.1 | 499.9366 | 3 | 11.55 | 4 | F4:2522 | NaSa01\_F12.raw |  |  | 1.2197E6 | 3.9803E6 | 7.6868E5 | 4.246E5 |  |  |  |  |  |  |  |  | 4 | 0 | 0 | 1 | 1 | 1 | 1 | 0 | 0 | 0 | 0 | 0 | 0 | 0 | 0 | 389 | 400 | Carbamidomethylation | C5:Carbamidomethylation:1000.00 | PEAKS DB |
| T.LLGPNYTVGPAGC(+57.02)FK.N | Y | 58.57 | 1592.8020 | 15 | 1.0 | 797.4091 | 2 | 52.28 | 9 | F9:26433 | NaSa01\_F4.raw |  |  |  |  |  |  |  |  | 2.6952E5 |  |  |  |  |  | 1 | 0 | 0 | 0 | 0 | 0 | 0 | 0 | 0 | 1 | 0 | 0 | 0 | 0 | 0 | 518 | 532 | Carbamidomethylation | C13:Carbamidomethylation:1000.00 | PEAKS DB |
| R.TILMASTM(+15.99)AHELGH.N | Y | 57.52 | 1526.7222 | 14 | -0.3 | 509.9145 | 3 | 27.52 | 4 | F4:12573 | NaSa01\_F12.raw |  |  |  | 6.3536E6 |  |  |  |  |  |  |  |  |  |  | 2 | 0 | 0 | 0 | 2 | 0 | 0 | 0 | 0 | 0 | 0 | 0 | 0 | 0 | 0 | 329 | 342 | Oxidation (M) | M8:Oxidation (M):52.86 | PEAKS DB |
| R.EHQEYLLR.E | Y | 57.26 | 1086.5458 | 8 | 0.1 | 544.2802 | 2 | 11.90 | 3 | F3:3535 | NaSa01\_F11.raw |  |  | 1.0425E6 | 4.1065E6 |  |  |  |  |  |  |  |  |  |  | 2 | 0 | 0 | 1 | 1 | 0 | 0 | 0 | 0 | 0 | 0 | 0 | 0 | 0 | 0 | 381 | 388 |  |  | PEAKS DB |
| T.RVYEMVNYLNTK.Y | Y | 56.14 | 1528.7708 | 12 | -0.5 | 510.5973 | 3 | 35.20 | 4 | F4:19240 | NaSa01\_F12.raw |  |  | 1.3359E7 | 3.6979E6 |  |  |  |  |  |  |  |  |  |  | 5 | 0 | 0 | 3 | 2 | 0 | 0 | 0 | 0 | 0 | 0 | 0 | 0 | 0 | 0 | 230 | 241 |  |  | PEAKS DB |
| R.TILM(+15.99)ASTMAHELGH.N | Y | 55.76 | 1526.7222 | 14 | 0.3 | 509.9148 | 3 | 38.08 | 4 | F4:21565 | NaSa01\_F12.raw |  |  | 1.8887E6 | 7.1713E6 |  |  |  |  |  |  |  |  |  |  | 2 | 0 | 0 | 1 | 1 | 0 | 0 | 0 | 0 | 0 | 0 | 0 | 0 | 0 | 0 | 329 | 342 | Oxidation (M) | M4:Oxidation (M):61.69 | PEAKS DB |
| L.MASTMAHELGHNMGIHHDK.A | Y | 54.98 | 2115.9402 | 19 | 0.2 | 529.9924 | 4 | 11.44 | 5 | F5:1956 | NaSa01\_F13.raw |  |  |  | 2.0596E5 | 1.5711E6 |  |  |  |  |  |  |  |  |  | 2 | 0 | 0 | 0 | 1 | 1 | 0 | 0 | 0 | 0 | 0 | 0 | 0 | 0 | 0 | 332 | 350 |  |  | PEAKS DB |
| Q.C(+57.02)ITLLGPNYTVGPAGC(+57.02)FK.N | Y | 54.02 | 1966.9645 | 18 | 1.1 | 984.4906 | 2 | 70.89 | 3 | F3:43873 | NaSa01\_F11.raw |  |  | 2.1114E6 |  |  |  |  |  |  |  |  |  |  |  | 1 | 0 | 0 | 1 | 0 | 0 | 0 | 0 | 0 | 0 | 0 | 0 | 0 | 0 | 0 | 515 | 532 | Carbamidomethylation | C1:Carbamidomethylation:1000.00;C16:Carbamidomethylation:1000.00 | PEAKS DB |
| R.RTILMASTM(+15.99)AHELGHNMGIHHDK.A | Y | 53.91 | 2615.2520 | 23 | 0.2 | 654.8204 | 4 | 30.70 | 3 | F3:12801 | NaSa01\_F11.raw |  |  | 4.0736E7 |  |  |  |  |  |  |  |  |  |  |  | 4 | 0 | 0 | 4 | 0 | 0 | 0 | 0 | 0 | 0 | 0 | 0 | 0 | 0 | 0 | 328 | 350 | Oxidation (M) | M9:Oxidation (M):25.70 | PEAKS DB |
| R.RTILM(+15.99)ASTMAHELGHNMGIHHDK.A | Y | 53.25 | 2615.2520 | 23 | -0.3 | 524.0575 | 5 | 24.40 | 3 | F3:8054 | NaSa01\_F11.raw |  |  | 4.2973E7 |  |  |  |  |  |  |  |  |  |  |  | 5 | 0 | 0 | 5 | 0 | 0 | 0 | 0 | 0 | 0 | 0 | 0 | 0 | 0 | 0 | 328 | 350 | Oxidation (M) | M5:Oxidation (M):64.16 | PEAKS DB |
| R.TILM(+15.99)ASTMAHELGHNM(+15.99)GIHHDK.A | Y | 52.59 | 2475.1458 | 22 | 0.3 | 496.0366 | 5 | 19.61 | 4 | F4:6768 | NaSa01\_F12.raw |  |  | 4.7701E6 | 6.6746E6 |  |  |  |  |  |  |  |  |  |  | 2 | 0 | 0 | 1 | 1 | 0 | 0 | 0 | 0 | 0 | 0 | 0 | 0 | 0 | 0 | 329 | 350 | Oxidation (M) | M4:Oxidation (M):42.68;M16:Oxidation (M):51.56 | PEAKS DB |
| P.TMENQC(+57.02)ITLLGPNYTVGPAGC(+57.02)FK.N | Y | 51.62 | 2570.1968 | 23 | 2.0 | 857.7412 | 3 | 73.82 | 3 | F3:46273 | NaSa01\_F11.raw |  |  | 3.2802E6 |  |  |  |  |  |  |  |  |  |  |  | 1 | 0 | 0 | 1 | 0 | 0 | 0 | 0 | 0 | 0 | 0 | 0 | 0 | 0 | 0 | 510 | 532 | Carbamidomethylation | C6:Carbamidomethylation:1000.00;C21:Carbamidomethylation:1000.00 | PEAKS DB |
| K.C(+57.02)PTMENQC(+57.02)ITLLGPNYTVGPAGC(+57.02)FKNNR.K | Y | 49.93 | 3211.4670 | 28 | 0.3 | 1071.4966 | 3 | 61.53 | 4 | F4:41028 | NaSa01\_F12.raw |  |  |  | 2.2392E6 |  |  |  |  |  |  |  |  |  |  | 1 | 0 | 0 | 0 | 1 | 0 | 0 | 0 | 0 | 0 | 0 | 0 | 0 | 0 | 0 | 508 | 535 | Carbamidomethylation | C1:Carbamidomethylation:1000.00;C8:Carbamidomethylation:1000.00;C23:Carbamidomethylation:1000.00 | PEAKS DB |
| R.TILMASTMAHELGHN.M | Y | 49.33 | 1624.7701 | 15 | 0.7 | 813.3929 | 2 | 44.27 | 3 | F3:23505 | NaSa01\_F11.raw |  |  | 5.7446E6 | 1.4069E6 |  |  |  |  |  |  |  |  |  |  | 4 | 0 | 0 | 2 | 2 | 0 | 0 | 0 | 0 | 0 | 0 | 0 | 0 | 0 | 0 | 329 | 343 |  |  | PEAKS DB |
| R.TRVYEM(+15.99)VNYLNTK.Y | Y | 45.97 | 1645.8134 | 13 | 0.1 | 549.6118 | 3 | 25.02 | 4 | F4:10544 | NaSa01\_F12.raw |  |  |  | 1.803E5 |  |  |  |  |  |  |  |  |  |  | 1 | 0 | 0 | 0 | 1 | 0 | 0 | 0 | 0 | 0 | 0 | 0 | 0 | 0 | 0 | 229 | 241 | Oxidation (M) | M6:Oxidation (M):1000.00 | PEAKS DB |
| R.EITTSPPVQDHC(+57.02)YYHGY.I | N | 44.97 | 2065.8840 | 17 | 6.3 | 689.6360 | 3 | 31.90 | 8 | F8:16761 | NaSa01\_F3.raw |  |  |  |  |  |  |  | 3.1849E6 |  |  |  |  |  |  | 1 | 0 | 0 | 0 | 0 | 0 | 0 | 0 | 1 | 0 | 0 | 0 | 0 | 0 | 0 | 97 | 113 | Carbamidomethylation | C12:Carbamidomethylation:1000.00 | PEAKS DB |
| L.TNTPEQDR.Y | N | 44.60 | 959.4308 | 8 | 0.1 | 480.7227 | 2 | 11.42 | 6 | F6:1814 | NaSa01\_F14.raw |  |  |  |  |  | 1.6427E5 |  |  |  |  |  |  |  |  | 1 | 0 | 0 | 0 | 0 | 0 | 1 | 0 | 0 | 0 | 0 | 0 | 0 | 0 | 0 | 192 | 199 |  |  | PEAKS DB |
| R.TILMASTMAH.E | Y | 42.60 | 1074.5203 | 10 | -0.6 | 538.2671 | 2 | 35.49 | 4 | F4:19537 | NaSa01\_F12.raw |  |  |  | 7.8179E6 |  |  |  |  |  |  |  |  |  |  | 1 | 0 | 0 | 0 | 1 | 0 | 0 | 0 | 0 | 0 | 0 | 0 | 0 | 0 | 0 | 329 | 338 |  |  | PEAKS DB |
| R.TILM(+15.99)ASTM(+15.99)AHELGHNM(+15.99)GIHHDK.A | Y | 42.49 | 2491.1406 | 22 | -1.2 | 623.7917 | 4 | 39.36 | 3 | F3:19544 | NaSa01\_F11.raw |  |  | 2.5863E6 | 3.4525E5 |  |  |  |  |  |  |  |  |  |  | 2 | 0 | 0 | 1 | 1 | 0 | 0 | 0 | 0 | 0 | 0 | 0 | 0 | 0 | 0 | 329 | 350 | Oxidation (M) | M4:Oxidation (M):1000.00;M8:Oxidation (M):1000.00;M16:Oxidation (M):1000.00 | PEAKS DB |
| R.RTILMASTMAHELGHNM(+15.99)GIHHDK.A | Y | 42.14 | 2615.2520 | 23 | 0.7 | 524.0580 | 5 | 16.24 | 3 | F3:5531 | NaSa01\_F11.raw |  |  | 1.6294E7 |  |  |  |  |  |  |  |  |  |  |  | 2 | 0 | 0 | 2 | 0 | 0 | 0 | 0 | 0 | 0 | 0 | 0 | 0 | 0 | 0 | 328 | 350 | Oxidation (M) | M17:Oxidation (M):19.61 | PEAKS DB |
| R.TILMASTMAHELGHNMGIHHDKANC(+57.02)R.C | Y | 41.64 | 2944.3677 | 26 | -0.1 | 589.8807 | 5 | 31.68 | 3 | F3:13190 | NaSa01\_F11.raw |  |  | 2.2096E6 |  |  |  |  |  |  |  |  |  |  |  | 1 | 0 | 0 | 1 | 0 | 0 | 0 | 0 | 0 | 0 | 0 | 0 | 0 | 0 | 0 | 329 | 354 | Carbamidomethylation | C25:Carbamidomethylation:1000.00 | PEAKS DB |
| R.TRVYEMVNYLNTKYR.R | Y | 41.54 | 1948.9829 | 15 | 0.3 | 650.6685 | 3 | 42.81 | 3 | F3:22275 | NaSa01\_F11.raw |  |  | 9.5602E4 |  |  |  |  |  |  |  |  |  |  |  | 1 | 0 | 0 | 1 | 0 | 0 | 0 | 0 | 0 | 0 | 0 | 0 | 0 | 0 | 0 | 229 | 243 |  |  | PEAKS DB |
| R.TILMASTM(+15.99)AHELGHNM(+15.99)GIHHDK.A | Y | 39.79 | 2475.1458 | 22 | 0.0 | 619.7937 | 4 | 11.50 | 5 | F5:2017 | NaSa01\_F13.raw |  |  |  | 7.4858E5 | 8.6635E5 |  |  |  |  |  |  |  |  |  | 2 | 0 | 0 | 0 | 1 | 1 | 0 | 0 | 0 | 0 | 0 | 0 | 0 | 0 | 0 | 329 | 350 | Oxidation (M) | M8:Oxidation (M):0.00;M16:Oxidation (M):47.66 | PEAKS DB |
| total 46 peptides |  |  |  |  |  |  |  |  |  |  |  |  |  |  |  |  |  |  |  |  |  |  |  |  |  |  |  |  |  |  |  |  |  |  |  |  |  |  |  |  |  |  |  |  |  |
| --- | --- | --- | --- | --- | --- | --- | --- | --- | --- | --- | --- | --- | --- | --- | --- | --- | --- | --- | --- | --- | --- | --- | --- | --- | --- | --- | --- | --- | --- | --- | --- | --- | --- | --- | --- | --- | --- | --- | --- | --- | --- | --- | --- | --- | --- |

ADF43026.1

back to list

  

| Protein Coverage
| Supporting Peptides
|

Protein Coverage:

Supporting Peptides:

| Peptide | Uniq | -10lgP | Mass | Length | ppm | m/z | z | RT | Fraction | Scan | Source File | Area F1 | Area F10 | Area F11 | Area F12 | Area F13 | Area F14 | Area F2 | Area F3 | Area F4 | Area F5 | Area F6 | Area F7 | Area F8 | Area F9 | #Feature | #Feature F1 | #Feature F10 | #Feature F11 | #Feature F12 | #Feature F13 | #Feature F14 | #Feature F2 | #Feature F3 | #Feature F4 | #Feature F5 | #Feature F6 | #Feature F7 | #Feature F8 | #Feature F9 | Start | End | PTM | AScore | Found By |
| --- | --- | --- | --- | --- | --- | --- | --- | --- | --- | --- | --- | --- | --- | --- | --- | --- | --- | --- | --- | --- | --- | --- | --- | --- | --- | --- | --- | --- | --- | --- | --- | --- | --- | --- | --- | --- | --- | --- | --- | --- | --- | --- | --- | --- | --- |
| R.RTILMASTMAHELGHNMGIHHDK.A | Y | 102.57 | 2599.2571 | 23 | 0.5 | 650.8219 | 4 | 25.79 | 3 | F3:9045 | NaSa01\_F11.raw |  | 9.1055E5 | 2.4907E8 | 3.4546E7 |  |  |  |  |  |  |  |  |  |  | 13 | 0 | 1 | 9 | 3 | 0 | 0 | 0 | 0 | 0 | 0 | 0 | 0 | 0 | 0 | 328 | 350 |  |  | PEAKS DB |
| R.TILMASTMAHELGHNMGIHHDK.A | Y | 92.32 | 2443.1560 | 22 | 1.4 | 611.7971 | 4 | 35.33 | 3 | F3:17779 | NaSa01\_F11.raw |  | 2.0692E7 | 4.6099E9 | 9.7683E8 | 2.583E7 | 2.3127E7 |  |  |  |  |  |  |  |  | 44 | 0 | 3 | 23 | 13 | 2 | 3 | 0 | 0 | 0 | 0 | 0 | 0 | 0 | 0 | 329 | 350 |  |  | PEAKS DB |
| R.TILM(+15.99)ASTMAHELGHNMGIHHDK.A | Y | 82.29 | 2459.1509 | 22 | 0.5 | 615.7953 | 4 | 25.21 | 3 | F3:11732 | NaSa01\_F11.raw |  |  | 5.9895E8 | 1.361E8 |  |  |  |  |  |  |  |  |  |  | 15 | 0 | 0 | 9 | 6 | 0 | 0 | 0 | 0 | 0 | 0 | 0 | 0 | 0 | 0 | 329 | 350 | Oxidation (M) | M4:Oxidation (M):97.69 | PEAKS DB |
| K.C(+57.02)PTMENQC(+57.02)ITLLGPNYTVGPAGC(+57.02)FK.N | Y | 81.11 | 2827.2803 | 25 | 1.5 | 943.4354 | 3 | 74.97 | 3 | F3:47101 | NaSa01\_F11.raw |  | 3.3769E6 | 9.888E8 | 4.7351E8 | 2.0131E7 | 1.4081E7 |  |  |  |  |  |  |  |  | 14 | 0 | 2 | 5 | 4 | 1 | 2 | 0 | 0 | 0 | 0 | 0 | 0 | 0 | 0 | 508 | 532 | Carbamidomethylation | C1:Carbamidomethylation:1000.00;C8:Carbamidomethylation:1000.00;C23:Carbamidomethylation:1000.00 | PEAKS DB |
| R.VYEMVNYLNTKYR.R | Y | 77.67 | 1691.8341 | 13 | -0.1 | 564.9519 | 3 | 45.38 | 4 | F4:27468 | NaSa01\_F12.raw |  |  | 1.1565E7 | 5.395E6 | 7.5997E5 |  |  |  |  |  |  |  |  |  | 4 | 0 | 0 | 2 | 1 | 1 | 0 | 0 | 0 | 0 | 0 | 0 | 0 | 0 | 0 | 231 | 243 |  |  | PEAKS DB |
| R.TRVYEMVNYLNTK.Y | Y | 76.18 | 1629.8185 | 13 | -4.5 | 815.9128 | 2 | 42.57 | 4 | F4:25251 | NaSa01\_F12.raw |  |  | 7.0755E6 | 0 |  |  |  |  |  |  |  |  |  |  | 2 | 0 | 0 | 2 | 0 | 0 | 0 | 0 | 0 | 0 | 0 | 0 | 0 | 0 | 0 | 229 | 241 |  |  | PEAKS DB |
| R.TILMASTM(+15.99)AHELGHNMGIHHDK.A | Y | 74.28 | 2459.1509 | 22 | 0.3 | 492.8376 | 5 | 20.73 | 4 | F4:7454 | NaSa01\_F12.raw |  |  | 5.4127E8 | 6.276E7 | 7.7545E6 |  |  |  |  |  |  |  |  |  | 20 | 0 | 0 | 12 | 6 | 2 | 0 | 0 | 0 | 0 | 0 | 0 | 0 | 0 | 0 | 329 | 350 | Oxidation (M) | M8:Oxidation (M):58.99 | PEAKS DB |
| R.TILMASTMAHELGHNM(+15.99)GIHHDK.A | Y | 74.07 | 2459.1509 | 22 | 0.8 | 492.8378 | 5 | 25.22 | 4 | F4:10852 | NaSa01\_F12.raw |  |  | 1.9866E8 | 7.6075E7 | 8.24E6 | 3.8636E6 |  |  |  |  |  |  |  |  | 17 | 0 | 0 | 9 | 4 | 3 | 1 | 0 | 0 | 0 | 0 | 0 | 0 | 0 | 0 | 329 | 350 | Oxidation (M) | M16:Oxidation (M):63.64 | PEAKS DB |
| K.LQPHAQC(+57.02)DSEEC(+57.02)C(+57.02)EK.C | Y | 72.87 | 1889.7343 | 15 | -0.1 | 630.9186 | 3 | 11.55 | 4 | F4:2524 | NaSa01\_F12.raw |  |  |  | 1.7758E6 |  |  |  |  |  |  |  |  |  |  | 2 | 0 | 0 | 0 | 2 | 0 | 0 | 0 | 0 | 0 | 0 | 0 | 0 | 0 | 0 | 440 | 454 | Carbamidomethylation | C7:Carbamidomethylation:1000.00;C12:Carbamidomethylation:1000.00;C13:Carbamidomethylation:1000.00 | PEAKS DB |
| K.C(+57.02)PTM(+15.99)ENQC(+57.02)ITLLGPNYTVGPAGC(+57.02)FK.N | Y | 72.54 | 2843.2751 | 25 | 1.3 | 948.7668 | 3 | 70.50 | 3 | F3:43399 | NaSa01\_F11.raw |  |  | 4.1576E7 | 4.2268E7 |  |  |  |  |  |  |  |  |  |  | 7 | 0 | 0 | 5 | 2 | 0 | 0 | 0 | 0 | 0 | 0 | 0 | 0 | 0 | 0 | 508 | 532 | Carbamidomethylation; Oxidation (M) | C1:Carbamidomethylation:1000.00;M4:Oxidation (M):1000.00;C8:Carbamidomethylation:1000.00;C23:Carbamidomethylation:1000.00 | PEAKS DB |
| R.VYEMVNYLNTK.Y | Y | 71.83 | 1372.6697 | 11 | 0.2 | 687.3423 | 2 | 49.73 | 6 | F6:29500 | NaSa01\_F14.raw |  | 3.5484E7 | 1.6202E9 | 1.498E9 | 2.2005E8 | 1.3641E8 |  |  |  |  |  |  |  |  | 23 | 0 | 2 | 10 | 8 | 1 | 2 | 0 | 0 | 0 | 0 | 0 | 0 | 0 | 0 | 231 | 241 |  |  | PEAKS DB |
| R.TILM(+15.99)ASTM(+15.99)AHELGHNMGIHHDK.A | Y | 70.98 | 2475.1458 | 22 | 0.7 | 619.7941 | 4 | 13.60 | 4 | F4:5130 | NaSa01\_F12.raw |  |  |  | 4.4221E7 |  |  |  |  |  |  |  |  |  |  | 8 | 0 | 0 | 0 | 8 | 0 | 0 | 0 | 0 | 0 | 0 | 0 | 0 | 0 | 0 | 329 | 350 | Oxidation (M) | M4:Oxidation (M):96.93;M8:Oxidation (M):53.45 | PEAKS DB |
| R.NGHPC(+57.02)QNNQGYC(+57.02)YNGK.C | N | 68.53 | 1909.7584 | 16 | -0.2 | 637.5933 | 3 | 11.55 | 4 | F4:2526 | NaSa01\_F12.raw |  |  |  | 1.1419E6 |  |  |  |  |  |  |  |  |  |  | 2 | 0 | 0 | 0 | 2 | 0 | 0 | 0 | 0 | 0 | 0 | 0 | 0 | 0 | 0 | 492 | 507 | Carbamidomethylation | C5:Carbamidomethylation:1000.00;C12:Carbamidomethylation:1000.00 | PEAKS DB |
| K.KYIEFYLVVDNK.M | Y | 67.91 | 1529.8130 | 12 | 0.6 | 765.9142 | 2 | 55.94 | 3 | F3:31344 | NaSa01\_F11.raw |  |  | 5.3689E7 | 1.4428E6 |  |  |  |  |  |  |  |  |  |  | 15 | 0 | 0 | 14 | 1 | 0 | 0 | 0 | 0 | 0 | 0 | 0 | 0 | 0 | 0 | 205 | 216 |  |  | PEAKS DB |
| L.GPNYTVGPAGC(+57.02)FK.N | Y | 67.57 | 1366.6339 | 13 | 0.7 | 684.3247 | 2 | 32.10 | 9 | F9:11219 | NaSa01\_F4.raw |  |  |  | 4.2089E6 |  |  |  |  | 1.5247E7 |  |  |  |  |  | 2 | 0 | 0 | 0 | 1 | 0 | 0 | 0 | 0 | 1 | 0 | 0 | 0 | 0 | 0 | 520 | 532 | Carbamidomethylation | C11:Carbamidomethylation:1000.00 | PEAKS DB |
| R.VYEM(+15.99)VNYLNTK.Y | Y | 67.21 | 1388.6646 | 11 | 0.1 | 695.3396 | 2 | 31.53 | 4 | F4:15763 | NaSa01\_F12.raw |  |  | 7.824E7 | 1.3006E8 | 1.9228E7 |  |  |  |  |  |  |  |  |  | 17 | 0 | 0 | 5 | 9 | 3 | 0 | 0 | 0 | 0 | 0 | 0 | 0 | 0 | 0 | 231 | 241 | Oxidation (M) | M4:Oxidation (M):1000.00 | PEAKS DB |
| R.TILMASTMAHELGH.N | Y | 66.37 | 1510.7272 | 14 | 0.5 | 756.3712 | 2 | 47.57 | 3 | F3:26108 | NaSa01\_F11.raw |  |  | 4.7059E7 | 1.2846E8 | 1.1854E7 | 3.9776E6 |  |  |  |  |  |  |  |  | 10 | 0 | 0 | 2 | 5 | 2 | 1 | 0 | 0 | 0 | 0 | 0 | 0 | 0 | 0 | 329 | 342 |  |  | PEAKS DB |
| R.RTILMASTMAHELGH.N | Y | 65.80 | 1666.8284 | 15 | -0.6 | 556.6164 | 3 | 32.97 | 4 | F4:17457 | NaSa01\_F12.raw |  |  | 1.2098E7 | 1.9622E7 |  |  |  |  |  |  |  |  |  |  | 4 | 0 | 0 | 2 | 2 | 0 | 0 | 0 | 0 | 0 | 0 | 0 | 0 | 0 | 0 | 328 | 342 |  |  | PEAKS DB |
| K.YIEFYLVVDNK.M | Y | 63.10 | 1401.7180 | 11 | 0.9 | 701.8669 | 2 | 74.33 | 4 | F4:51541 | NaSa01\_F12.raw |  |  | 3.2924E7 | 6.0017E5 |  |  |  |  |  |  |  |  |  |  | 17 | 0 | 0 | 15 | 2 | 0 | 0 | 0 | 0 | 0 | 0 | 0 | 0 | 0 | 0 | 206 | 216 |  |  | PEAKS DB |
| L.LGPNYTVGPAGC(+57.02)FK.N | Y | 62.86 | 1479.7180 | 14 | 0.9 | 740.8669 | 2 | 42.98 | 9 | F9:19580 | NaSa01\_F4.raw |  |  |  | 1.6597E6 |  |  |  |  | 2.6352E6 |  |  |  |  |  | 2 | 0 | 0 | 0 | 1 | 0 | 0 | 0 | 0 | 1 | 0 | 0 | 0 | 0 | 0 | 519 | 532 | Carbamidomethylation | C12:Carbamidomethylation:1000.00 | PEAKS DB |
| E.PFYEFSSC(+57.02)SVR.E | Y | 62.63 | 1377.6023 | 11 | 0.1 | 689.8085 | 2 | 45.23 | 4 | F4:27365 | NaSa01\_F12.raw |  |  | 1.0485E7 | 8.6274E6 | 1.867E6 |  |  |  |  |  |  |  |  |  | 3 | 0 | 0 | 1 | 1 | 1 | 0 | 0 | 0 | 0 | 0 | 0 | 0 | 0 | 0 | 370 | 380 | Carbamidomethylation | C8:Carbamidomethylation:1000.00 | PEAKS DB |
| W.GSVAVVQDYSR.R | Y | 60.81 | 1179.5884 | 11 | 0.5 | 590.8018 | 2 | 12.09 | 3 | F3:3668 | NaSa01\_F11.raw |  |  | 3.4234E6 | 1.6841E7 | 1.3633E6 | 7.0857E5 |  |  |  |  |  |  |  |  | 6 | 0 | 0 | 2 | 2 | 1 | 1 | 0 | 0 | 0 | 0 | 0 | 0 | 0 | 0 | 317 | 327 |  |  | PEAKS DB |
| R.TILMASTMAHELGHNMGIHHD.K | Y | 60.76 | 2315.0610 | 21 | 0.1 | 579.7726 | 4 | 43.04 | 3 | F3:23564 | NaSa01\_F11.raw |  |  | 1.7071E7 |  |  |  |  |  |  |  |  |  |  |  | 3 | 0 | 0 | 3 | 0 | 0 | 0 | 0 | 0 | 0 | 0 | 0 | 0 | 0 | 0 | 329 | 349 |  |  | PEAKS DB |
| R.ERPQC(+57.02)ILNKPSR.K | Y | 58.96 | 1496.7881 | 12 | -0.1 | 499.9366 | 3 | 11.55 | 4 | F4:2522 | NaSa01\_F12.raw |  |  | 1.2197E6 | 3.9803E6 | 7.6868E5 | 4.246E5 |  |  |  |  |  |  |  |  | 4 | 0 | 0 | 1 | 1 | 1 | 1 | 0 | 0 | 0 | 0 | 0 | 0 | 0 | 0 | 389 | 400 | Carbamidomethylation | C5:Carbamidomethylation:1000.00 | PEAKS DB |
| T.LLGPNYTVGPAGC(+57.02)FK.N | Y | 58.57 | 1592.8020 | 15 | 1.0 | 797.4091 | 2 | 52.28 | 9 | F9:26433 | NaSa01\_F4.raw |  |  |  |  |  |  |  |  | 2.6952E5 |  |  |  |  |  | 1 | 0 | 0 | 0 | 0 | 0 | 0 | 0 | 0 | 1 | 0 | 0 | 0 | 0 | 0 | 518 | 532 | Carbamidomethylation | C13:Carbamidomethylation:1000.00 | PEAKS DB |
| R.TILMASTM(+15.99)AHELGH.N | Y | 57.52 | 1526.7222 | 14 | -0.3 | 509.9145 | 3 | 27.52 | 4 | F4:12573 | NaSa01\_F12.raw |  |  |  | 6.3536E6 |  |  |  |  |  |  |  |  |  |  | 2 | 0 | 0 | 0 | 2 | 0 | 0 | 0 | 0 | 0 | 0 | 0 | 0 | 0 | 0 | 329 | 342 | Oxidation (M) | M8:Oxidation (M):52.86 | PEAKS DB |
| R.EHQEYLLR.E | Y | 57.26 | 1086.5458 | 8 | 0.1 | 544.2802 | 2 | 11.90 | 3 | F3:3535 | NaSa01\_F11.raw |  |  | 1.0425E6 | 4.1065E6 |  |  |  |  |  |  |  |  |  |  | 2 | 0 | 0 | 1 | 1 | 0 | 0 | 0 | 0 | 0 | 0 | 0 | 0 | 0 | 0 | 381 | 388 |  |  | PEAKS DB |
| T.RVYEMVNYLNTK.Y | Y | 56.14 | 1528.7708 | 12 | -0.5 | 510.5973 | 3 | 35.20 | 4 | F4:19240 | NaSa01\_F12.raw |  |  | 1.3359E7 | 3.6979E6 |  |  |  |  |  |  |  |  |  |  | 5 | 0 | 0 | 3 | 2 | 0 | 0 | 0 | 0 | 0 | 0 | 0 | 0 | 0 | 0 | 230 | 241 |  |  | PEAKS DB |
| R.TILM(+15.99)ASTMAHELGH.N | Y | 55.76 | 1526.7222 | 14 | 0.3 | 509.9148 | 3 | 38.08 | 4 | F4:21565 | NaSa01\_F12.raw |  |  | 1.8887E6 | 7.1713E6 |  |  |  |  |  |  |  |  |  |  | 2 | 0 | 0 | 1 | 1 | 0 | 0 | 0 | 0 | 0 | 0 | 0 | 0 | 0 | 0 | 329 | 342 | Oxidation (M) | M4:Oxidation (M):61.69 | PEAKS DB |
| L.MASTMAHELGHNMGIHHDK.A | Y | 54.98 | 2115.9402 | 19 | 0.2 | 529.9924 | 4 | 11.44 | 5 | F5:1956 | NaSa01\_F13.raw |  |  |  | 2.0596E5 | 1.5711E6 |  |  |  |  |  |  |  |  |  | 2 | 0 | 0 | 0 | 1 | 1 | 0 | 0 | 0 | 0 | 0 | 0 | 0 | 0 | 0 | 332 | 350 |  |  | PEAKS DB |
| Q.C(+57.02)ITLLGPNYTVGPAGC(+57.02)FK.N | Y | 54.02 | 1966.9645 | 18 | 1.1 | 984.4906 | 2 | 70.89 | 3 | F3:43873 | NaSa01\_F11.raw |  |  | 2.1114E6 |  |  |  |  |  |  |  |  |  |  |  | 1 | 0 | 0 | 1 | 0 | 0 | 0 | 0 | 0 | 0 | 0 | 0 | 0 | 0 | 0 | 515 | 532 | Carbamidomethylation | C1:Carbamidomethylation:1000.00;C16:Carbamidomethylation:1000.00 | PEAKS DB |
| R.RTILMASTM(+15.99)AHELGHNMGIHHDK.A | Y | 53.91 | 2615.2520 | 23 | 0.2 | 654.8204 | 4 | 30.70 | 3 | F3:12801 | NaSa01\_F11.raw |  |  | 4.0736E7 |  |  |  |  |  |  |  |  |  |  |  | 4 | 0 | 0 | 4 | 0 | 0 | 0 | 0 | 0 | 0 | 0 | 0 | 0 | 0 | 0 | 328 | 350 | Oxidation (M) | M9:Oxidation (M):25.70 | PEAKS DB |
| R.RTILM(+15.99)ASTMAHELGHNMGIHHDK.A | Y | 53.25 | 2615.2520 | 23 | -0.3 | 524.0575 | 5 | 24.40 | 3 | F3:8054 | NaSa01\_F11.raw |  |  | 4.2973E7 |  |  |  |  |  |  |  |  |  |  |  | 5 | 0 | 0 | 5 | 0 | 0 | 0 | 0 | 0 | 0 | 0 | 0 | 0 | 0 | 0 | 328 | 350 | Oxidation (M) | M5:Oxidation (M):64.16 | PEAKS DB |
| R.TILM(+15.99)ASTMAHELGHNM(+15.99)GIHHDK.A | Y | 52.59 | 2475.1458 | 22 | 0.3 | 496.0366 | 5 | 19.61 | 4 | F4:6768 | NaSa01\_F12.raw |  |  | 4.7701E6 | 6.6746E6 |  |  |  |  |  |  |  |  |  |  | 2 | 0 | 0 | 1 | 1 | 0 | 0 | 0 | 0 | 0 | 0 | 0 | 0 | 0 | 0 | 329 | 350 | Oxidation (M) | M4:Oxidation (M):42.68;M16:Oxidation (M):51.56 | PEAKS DB |
| P.TMENQC(+57.02)ITLLGPNYTVGPAGC(+57.02)FK.N | Y | 51.62 | 2570.1968 | 23 | 2.0 | 857.7412 | 3 | 73.82 | 3 | F3:46273 | NaSa01\_F11.raw |  |  | 3.2802E6 |  |  |  |  |  |  |  |  |  |  |  | 1 | 0 | 0 | 1 | 0 | 0 | 0 | 0 | 0 | 0 | 0 | 0 | 0 | 0 | 0 | 510 | 532 | Carbamidomethylation | C6:Carbamidomethylation:1000.00;C21:Carbamidomethylation:1000.00 | PEAKS DB |
| K.C(+57.02)PTMENQC(+57.02)ITLLGPNYTVGPAGC(+57.02)FKNNR.K | Y | 49.93 | 3211.4670 | 28 | 0.3 | 1071.4966 | 3 | 61.53 | 4 | F4:41028 | NaSa01\_F12.raw |  |  |  | 2.2392E6 |  |  |  |  |  |  |  |  |  |  | 1 | 0 | 0 | 0 | 1 | 0 | 0 | 0 | 0 | 0 | 0 | 0 | 0 | 0 | 0 | 508 | 535 | Carbamidomethylation | C1:Carbamidomethylation:1000.00;C8:Carbamidomethylation:1000.00;C23:Carbamidomethylation:1000.00 | PEAKS DB |
| R.TILMASTMAHELGHN.M | Y | 49.33 | 1624.7701 | 15 | 0.7 | 813.3929 | 2 | 44.27 | 3 | F3:23505 | NaSa01\_F11.raw |  |  | 5.7446E6 | 1.4069E6 |  |  |  |  |  |  |  |  |  |  | 4 | 0 | 0 | 2 | 2 | 0 | 0 | 0 | 0 | 0 | 0 | 0 | 0 | 0 | 0 | 329 | 343 |  |  | PEAKS DB |
| R.TRVYEM(+15.99)VNYLNTK.Y | Y | 45.97 | 1645.8134 | 13 | 0.1 | 549.6118 | 3 | 25.02 | 4 | F4:10544 | NaSa01\_F12.raw |  |  |  | 1.803E5 |  |  |  |  |  |  |  |  |  |  | 1 | 0 | 0 | 0 | 1 | 0 | 0 | 0 | 0 | 0 | 0 | 0 | 0 | 0 | 0 | 229 | 241 | Oxidation (M) | M6:Oxidation (M):1000.00 | PEAKS DB |
| R.EITTSPPVQDHC(+57.02)YYHGY.I | N | 44.97 | 2065.8840 | 17 | 6.3 | 689.6360 | 3 | 31.90 | 8 | F8:16761 | NaSa01\_F3.raw |  |  |  |  |  |  |  | 3.1849E6 |  |  |  |  |  |  | 1 | 0 | 0 | 0 | 0 | 0 | 0 | 0 | 1 | 0 | 0 | 0 | 0 | 0 | 0 | 97 | 113 | Carbamidomethylation | C12:Carbamidomethylation:1000.00 | PEAKS DB |
| L.TNTPEQDR.Y | N | 44.60 | 959.4308 | 8 | 0.1 | 480.7227 | 2 | 11.42 | 6 | F6:1814 | NaSa01\_F14.raw |  |  |  |  |  | 1.6427E5 |  |  |  |  |  |  |  |  | 1 | 0 | 0 | 0 | 0 | 0 | 1 | 0 | 0 | 0 | 0 | 0 | 0 | 0 | 0 | 192 | 199 |  |  | PEAKS DB |
| R.TILMASTMAH.E | Y | 42.60 | 1074.5203 | 10 | -0.6 | 538.2671 | 2 | 35.49 | 4 | F4:19537 | NaSa01\_F12.raw |  |  |  | 7.8179E6 |  |  |  |  |  |  |  |  |  |  | 1 | 0 | 0 | 0 | 1 | 0 | 0 | 0 | 0 | 0 | 0 | 0 | 0 | 0 | 0 | 329 | 338 |  |  | PEAKS DB |
| R.TILM(+15.99)ASTM(+15.99)AHELGHNM(+15.99)GIHHDK.A | Y | 42.49 | 2491.1406 | 22 | -1.2 | 623.7917 | 4 | 39.36 | 3 | F3:19544 | NaSa01\_F11.raw |  |  | 2.5863E6 | 3.4525E5 |  |  |  |  |  |  |  |  |  |  | 2 | 0 | 0 | 1 | 1 | 0 | 0 | 0 | 0 | 0 | 0 | 0 | 0 | 0 | 0 | 329 | 350 | Oxidation (M) | M4:Oxidation (M):1000.00;M8:Oxidation (M):1000.00;M16:Oxidation (M):1000.00 | PEAKS DB |
| R.RTILMASTMAHELGHNM(+15.99)GIHHDK.A | Y | 42.14 | 2615.2520 | 23 | 0.7 | 524.0580 | 5 | 16.24 | 3 | F3:5531 | NaSa01\_F11.raw |  |  | 1.6294E7 |  |  |  |  |  |  |  |  |  |  |  | 2 | 0 | 0 | 2 | 0 | 0 | 0 | 0 | 0 | 0 | 0 | 0 | 0 | 0 | 0 | 328 | 350 | Oxidation (M) | M17:Oxidation (M):19.61 | PEAKS DB |
| R.TILMASTMAHELGHNMGIHHDKANC(+57.02)R.C | Y | 41.64 | 2944.3677 | 26 | -0.1 | 589.8807 | 5 | 31.68 | 3 | F3:13190 | NaSa01\_F11.raw |  |  | 2.2096E6 |  |  |  |  |  |  |  |  |  |  |  | 1 | 0 | 0 | 1 | 0 | 0 | 0 | 0 | 0 | 0 | 0 | 0 | 0 | 0 | 0 | 329 | 354 | Carbamidomethylation | C25:Carbamidomethylation:1000.00 | PEAKS DB |
| R.TRVYEMVNYLNTKYR.R | Y | 41.54 | 1948.9829 | 15 | 0.3 | 650.6685 | 3 | 42.81 | 3 | F3:22275 | NaSa01\_F11.raw |  |  | 9.5602E4 |  |  |  |  |  |  |  |  |  |  |  | 1 | 0 | 0 | 1 | 0 | 0 | 0 | 0 | 0 | 0 | 0 | 0 | 0 | 0 | 0 | 229 | 243 |  |  | PEAKS DB |
| R.TILMASTM(+15.99)AHELGHNM(+15.99)GIHHDK.A | Y | 39.79 | 2475.1458 | 22 | 0.0 | 619.7937 | 4 | 11.50 | 5 | F5:2017 | NaSa01\_F13.raw |  |  |  | 7.4858E5 | 8.6635E5 |  |  |  |  |  |  |  |  |  | 2 | 0 | 0 | 0 | 1 | 1 | 0 | 0 | 0 | 0 | 0 | 0 | 0 | 0 | 0 | 329 | 350 | Oxidation (M) | M8:Oxidation (M):0.00;M16:Oxidation (M):47.66 | PEAKS DB |
| total 46 peptides |  |  |  |  |  |  |  |  |  |  |  |  |  |  |  |  |  |  |  |  |  |  |  |  |  |  |  |  |  |  |  |  |  |  |  |  |  |  |  |  |  |  |  |  |  |
| --- | --- | --- | --- | --- | --- | --- | --- | --- | --- | --- | --- | --- | --- | --- | --- | --- | --- | --- | --- | --- | --- | --- | --- | --- | --- | --- | --- | --- | --- | --- | --- | --- | --- | --- | --- | --- | --- | --- | --- | --- | --- | --- | --- | --- | --- |

ACH73168.1

back to list

  

| Protein Coverage
| Supporting Peptides
|

Protein Coverage:

Supporting Peptides:

| Peptide | Uniq | -10lgP | Mass | Length | ppm | m/z | z | RT | Fraction | Scan | Source File | Area F1 | Area F10 | Area F11 | Area F12 | Area F13 | Area F14 | Area F2 | Area F3 | Area F4 | Area F5 | Area F6 | Area F7 | Area F8 | Area F9 | #Feature | #Feature F1 | #Feature F10 | #Feature F11 | #Feature F12 | #Feature F13 | #Feature F14 | #Feature F2 | #Feature F3 | #Feature F4 | #Feature F5 | #Feature F6 | #Feature F7 | #Feature F8 | #Feature F9 | Start | End | PTM | AScore | Found By |
| --- | --- | --- | --- | --- | --- | --- | --- | --- | --- | --- | --- | --- | --- | --- | --- | --- | --- | --- | --- | --- | --- | --- | --- | --- | --- | --- | --- | --- | --- | --- | --- | --- | --- | --- | --- | --- | --- | --- | --- | --- | --- | --- | --- | --- | --- |
| K.YLYVC(+57.02)QYC(+57.02)PAGNIIGSIATPYK.S | Y | 89.41 | 2550.2288 | 22 | 1.7 | 1276.1238 | 2 | 83.59 | 4 | F4:59165 | NaSa01\_F12.raw |  | 2.1485E7 | 1.9793E9 | 8.4159E7 |  |  |  |  |  |  |  |  |  | 6.0302E6 | 9 | 0 | 2 | 3 | 2 | 0 | 0 | 0 | 0 | 0 | 0 | 0 | 0 | 0 | 2 | 158 | 179 | Carbamidomethylation | C5:Carbamidomethylation:1000.00;C8:Carbamidomethylation:1000.00 | PEAKS DB |
| K.SGPPC(+57.02)GDC(+57.02)PSAC(+57.02)VNGLC(+57.02)TNPC(+57.02)K.H | N | 84.62 | 2406.9482 | 22 | -0.2 | 1204.4812 | 2 | 33.07 | 3 | F3:14521 | NaSa01\_F11.raw |  |  | 1.2348E8 | 4.9674E7 |  |  |  |  |  |  |  |  |  |  | 4 | 0 | 0 | 2 | 2 | 0 | 0 | 0 | 0 | 0 | 0 | 0 | 0 | 0 | 0 | 180 | 201 | Carbamidomethylation | C5:Carbamidomethylation:1000.00;C8:Carbamidomethylation:1000.00;C12:Carbamidomethylation:1000.00;C17:Carbamidomethylation:1000.00;C21:Carbamidomethylation:1000.00 | PEAKS DB |
| K.KFVYGVGANPPGSVIGHYTQIVWYNSHLLGC(+57.02)GAAK.C | Y | 84.42 | 3759.8984 | 35 | 1.3 | 940.9831 | 4 | 73.63 | 3 | F3:46103 | NaSa01\_F11.raw |  |  | 2.4078E7 |  |  |  |  |  |  |  |  |  |  |  | 1 | 0 | 0 | 1 | 0 | 0 | 0 | 0 | 0 | 0 | 0 | 0 | 0 | 0 | 0 | 118 | 152 | Carbamidomethylation | C31:Carbamidomethylation:1000.00 | PEAKS DB |
| R.NMLQMEWNSNAAQNAK.R | Y | 81.87 | 1848.8247 | 16 | -2.7 | 925.4171 | 2 | 48.72 | 3 | F3:26583 | NaSa01\_F11.raw |  | 2.686E6 | 5.2892E8 | 1.5782E8 | 2.2797E6 | 1.3086E6 |  |  |  |  |  |  |  | 1.156E6 | 15 | 0 | 1 | 8 | 3 | 1 | 1 | 0 | 0 | 0 | 0 | 0 | 0 | 0 | 1 | 54 | 69 |  |  | PEAKS DB |
| K.IGC(+57.02)GENLFMSSQPYAWSR.V | Y | 81.73 | 2101.9351 | 18 | 0.5 | 1051.9753 | 2 | 71.46 | 3 | F3:44819 | NaSa01\_F11.raw |  | 2.2215E6 | 2.6316E9 | 1.5538E8 |  |  |  |  |  |  |  |  |  | 7.6663E5 | 19 | 0 | 1 | 13 | 4 | 0 | 0 | 0 | 0 | 0 | 0 | 0 | 0 | 0 | 1 | 90 | 107 | Carbamidomethylation | C3:Carbamidomethylation:1000.00 | PEAKS DB |
| K.FVYGVGANPPGSVIGHYTQIVWYNSHLLGC(+57.02)GAAK.C | Y | 80.80 | 3631.8035 | 34 | 2.6 | 908.9605 | 4 | 80.84 | 3 | F3:52182 | NaSa01\_F11.raw |  |  | 1.0245E10 | 1.892E7 |  |  |  |  |  |  |  |  |  |  | 4 | 0 | 0 | 3 | 1 | 0 | 0 | 0 | 0 | 0 | 0 | 0 | 0 | 0 | 0 | 119 | 152 | Carbamidomethylation | C30:Carbamidomethylation:1000.00 | PEAKS DB |
| K.IGC(+57.02)GENLFM(+15.99)SSQPYAWSR.V | Y | 80.66 | 2117.9299 | 18 | -0.1 | 1059.9722 | 2 | 64.23 | 3 | F3:38201 | NaSa01\_F11.raw |  | 3.1123E6 | 2.8772E8 | 1.7534E7 |  |  |  |  |  |  |  |  |  |  | 13 | 0 | 1 | 9 | 3 | 0 | 0 | 0 | 0 | 0 | 0 | 0 | 0 | 0 | 0 | 90 | 107 | Carbamidomethylation; Oxidation (M) | C3:Carbamidomethylation:1000.00;M9:Oxidation (M):1000.00 | PEAKS DB |
| Y.C(+57.02)PAGNIIGSIATPYK.S | Y | 77.71 | 1560.7970 | 15 | 1.4 | 781.4069 | 2 | 55.17 | 3 | F3:30489 | NaSa01\_F11.raw |  |  | 2.0089E7 |  |  |  |  |  |  |  |  |  |  |  | 2 | 0 | 0 | 2 | 0 | 0 | 0 | 0 | 0 | 0 | 0 | 0 | 0 | 0 | 0 | 165 | 179 | Carbamidomethylation | C1:Carbamidomethylation:1000.00 | PEAKS DB |
| R.NMLQM(+15.99)EWNSNAAQNAK.R | Y | 76.35 | 1864.8196 | 16 | -0.3 | 933.4167 | 2 | 36.59 | 3 | F3:17279 | NaSa01\_F11.raw |  |  | 8.5143E7 | 2.8164E7 |  |  |  |  |  |  |  |  |  |  | 5 | 0 | 0 | 4 | 1 | 0 | 0 | 0 | 0 | 0 | 0 | 0 | 0 | 0 | 0 | 54 | 69 | Oxidation (M) | M5:Oxidation (M):77.25 | PEAKS DB |
| R.NM(+15.99)LQMEWNSNAAQNAK.R | Y | 75.98 | 1864.8196 | 16 | -0.4 | 933.4167 | 2 | 35.10 | 3 | F3:16079 | NaSa01\_F11.raw |  |  | 1.071E8 | 2.8164E7 |  |  |  |  |  |  |  |  |  |  | 8 | 0 | 0 | 7 | 1 | 0 | 0 | 0 | 0 | 0 | 0 | 0 | 0 | 0 | 0 | 54 | 69 | Oxidation (M) | M2:Oxidation (M):101.94 | PEAKS DB |
| Y.VC(+57.02)QYC(+57.02)PAGNIIGSIATPYK.S | Y | 75.50 | 2111.0181 | 19 | 0.3 | 1056.5166 | 2 | 67.02 | 3 | F3:40488 | NaSa01\_F11.raw |  |  | 1.1991E7 |  |  |  |  |  |  |  |  |  |  |  | 1 | 0 | 0 | 1 | 0 | 0 | 0 | 0 | 0 | 0 | 0 | 0 | 0 | 0 | 0 | 161 | 179 | Carbamidomethylation | C2:Carbamidomethylation:1000.00;C5:Carbamidomethylation:1000.00 | PEAKS DB |
| H.YTQIVWYNSHLLGC(+57.02)GAAK.C | Y | 75.38 | 2080.0200 | 18 | 0.5 | 1041.0178 | 2 | 54.52 | 3 | F3:29919 | NaSa01\_F11.raw |  |  | 7.881E7 |  |  |  |  |  |  |  |  |  |  |  | 2 | 0 | 0 | 2 | 0 | 0 | 0 | 0 | 0 | 0 | 0 | 0 | 0 | 0 | 0 | 135 | 152 | Carbamidomethylation | C14:Carbamidomethylation:1000.00 | PEAKS DB |
| C.QYC(+57.02)PAGNIIGSIATPYK.S | Y | 71.89 | 1851.9188 | 17 | -0.2 | 926.9665 | 2 | 63.76 | 3 | F3:37806 | NaSa01\_F11.raw |  |  | 1.3036E7 | 9.2987E5 |  |  |  |  |  |  |  |  |  |  | 4 | 0 | 0 | 3 | 1 | 0 | 0 | 0 | 0 | 0 | 0 | 0 | 0 | 0 | 0 | 163 | 179 | Carbamidomethylation | C3:Carbamidomethylation:1000.00 | PEAKS DB |
| K.FVYGVGANPPGSVIGHY.T | Y | 67.79 | 1732.8573 | 17 | 1.7 | 867.4374 | 2 | 61.37 | 3 | F3:35819 | NaSa01\_F11.raw |  |  | 4.555E6 |  |  |  |  |  |  |  |  |  |  |  | 1 | 0 | 0 | 1 | 0 | 0 | 0 | 0 | 0 | 0 | 0 | 0 | 0 | 0 | 0 | 119 | 135 |  |  | PEAKS DB |
| R.TVGKIGC(+57.02)GENLFMSSQPYAWSR.V | Y | 65.61 | 2487.1675 | 22 | 1.0 | 830.0640 | 3 | 60.97 | 3 | F3:35553 | NaSa01\_F11.raw |  |  | 4.3623E6 |  |  |  |  |  |  |  |  |  |  |  | 1 | 0 | 0 | 1 | 0 | 0 | 0 | 0 | 0 | 0 | 0 | 0 | 0 | 0 | 0 | 86 | 107 | Carbamidomethylation | C7:Carbamidomethylation:1000.00 | PEAKS DB |
| K.FVYGVGANPPGSVIGH.Y | Y | 65.53 | 1569.7939 | 16 | 0.8 | 785.9049 | 2 | 53.98 | 3 | F3:29540 | NaSa01\_F11.raw |  |  | 0 | 2.2555E6 |  |  |  |  |  |  |  |  |  |  | 1 | 0 | 0 | 0 | 1 | 0 | 0 | 0 | 0 | 0 | 0 | 0 | 0 | 0 | 0 | 119 | 134 |  |  | PEAKS DB |
| Q.YC(+57.02)PAGNIIGSIATPYK.S | Y | 65.38 | 1723.8604 | 16 | 1.5 | 862.9387 | 2 | 62.96 | 3 | F3:37193 | NaSa01\_F11.raw |  |  | 4.9582E6 |  |  |  |  |  |  |  |  |  |  |  | 2 | 0 | 0 | 2 | 0 | 0 | 0 | 0 | 0 | 0 | 0 | 0 | 0 | 0 | 0 | 164 | 179 | Carbamidomethylation | C2:Carbamidomethylation:1000.00 | PEAKS DB |
| K.HHNVFSNC(+57.02)QSLAK.Q | Y | 64.16 | 1540.7205 | 13 | 0.6 | 514.5811 | 3 | 11.55 | 4 | F4:2538 | NaSa01\_F12.raw |  |  |  | 3.5364E5 |  |  |  |  |  |  |  |  |  |  | 1 | 0 | 0 | 0 | 1 | 0 | 0 | 0 | 0 | 0 | 0 | 0 | 0 | 0 | 0 | 202 | 214 | Carbamidomethylation | C8:Carbamidomethylation:1000.00 | PEAKS DB |
| R.NMLQMEWNSNAAQNAKR.W | Y | 63.89 | 2004.9258 | 17 | 0.7 | 669.3163 | 3 | 35.81 | 3 | F3:16595 | NaSa01\_F11.raw |  |  | 3.7952E6 |  |  |  |  |  |  |  |  |  |  |  | 1 | 0 | 0 | 1 | 0 | 0 | 0 | 0 | 0 | 0 | 0 | 0 | 0 | 0 | 0 | 54 | 70 |  |  | PEAKS DB |
| N.MLQMEWNSNAAQNAK.R | Y | 63.28 | 1734.7817 | 15 | 0.0 | 868.3981 | 2 | 41.92 | 3 | F3:21522 | NaSa01\_F11.raw |  |  | 3.684E6 |  |  |  |  |  |  |  |  |  |  |  | 1 | 0 | 0 | 1 | 0 | 0 | 0 | 0 | 0 | 0 | 0 | 0 | 0 | 0 | 0 | 55 | 69 |  |  | PEAKS DB |
| R.VIQSWYDENKK.F | Y | 63.03 | 1408.6986 | 11 | 0.5 | 705.3569 | 2 | 12.09 | 3 | F3:3655 | NaSa01\_F11.raw |  |  | 5.2553E6 |  |  |  |  |  |  |  |  |  |  |  | 2 | 0 | 0 | 2 | 0 | 0 | 0 | 0 | 0 | 0 | 0 | 0 | 0 | 0 | 0 | 108 | 118 |  |  | PEAKS DB |
| K.QNAC(+57.02)QTEWMK.S | Y | 62.52 | 1294.5435 | 10 | 0.5 | 648.2794 | 2 | 12.09 | 3 | F3:3722 | NaSa01\_F11.raw |  |  | 8.8238E5 |  |  |  |  |  |  |  |  |  |  |  | 1 | 0 | 0 | 1 | 0 | 0 | 0 | 0 | 0 | 0 | 0 | 0 | 0 | 0 | 0 | 215 | 224 | Carbamidomethylation | C4:Carbamidomethylation:1000.00 | PEAKS DB |
| R.VIQSWYDENK.K | Y | 61.22 | 1280.6036 | 10 | 0.1 | 641.3091 | 2 | 28.55 | 4 | F4:13454 | NaSa01\_F12.raw |  |  |  | 1.5337E6 |  |  |  |  |  |  |  |  |  |  | 1 | 0 | 0 | 0 | 1 | 0 | 0 | 0 | 0 | 0 | 0 | 0 | 0 | 0 | 0 | 108 | 117 |  |  | PEAKS DB |
| K.FVYGVGANPPGSVIGHYTQIVWYNSH.L | Y | 58.71 | 2861.3926 | 26 | 0.8 | 954.8056 | 3 | 79.87 | 3 | F3:51233 | NaSa01\_F11.raw |  |  | 2.3982E8 |  |  |  |  |  |  |  |  |  |  |  | 1 | 0 | 0 | 1 | 0 | 0 | 0 | 0 | 0 | 0 | 0 | 0 | 0 | 0 | 0 | 119 | 144 |  |  | PEAKS DB |
| Y.GVGANPPGSVIGHYTQIVWYNSHLLGC(+57.02)GAAK.C | Y | 56.40 | 3222.6033 | 31 | 1.3 | 806.6592 | 4 | 72.12 | 3 | F3:45003 | NaSa01\_F11.raw |  |  | 1.461E7 |  |  |  |  |  |  |  |  |  |  |  | 1 | 0 | 0 | 1 | 0 | 0 | 0 | 0 | 0 | 0 | 0 | 0 | 0 | 0 | 0 | 122 | 152 | Carbamidomethylation | C27:Carbamidomethylation:1000.00 | PEAKS DB |
| N.PPGSVIGHYTQIVWYNSHLLGC(+57.02)GAAK.C | Y | 53.07 | 2824.4119 | 26 | 0.9 | 707.1109 | 4 | 67.96 | 3 | F3:41292 | NaSa01\_F11.raw |  |  | 9.9415E6 |  |  |  |  |  |  |  |  |  |  |  | 1 | 0 | 0 | 1 | 0 | 0 | 0 | 0 | 0 | 0 | 0 | 0 | 0 | 0 | 0 | 127 | 152 | Carbamidomethylation | C22:Carbamidomethylation:1000.00 | PEAKS DB |
| R.C(+57.02)SFAHSPPHLR.T | Y | 52.73 | 1307.6193 | 11 | -0.3 | 436.8802 | 3 | 11.63 | 3 | F3:3321 | NaSa01\_F11.raw |  |  | 1.4433E6 | 3.0387E5 |  |  |  |  |  |  |  |  |  |  | 3 | 0 | 0 | 2 | 1 | 0 | 0 | 0 | 0 | 0 | 0 | 0 | 0 | 0 | 0 | 75 | 85 | Carbamidomethylation | C1:Carbamidomethylation:1000.00 | PEAKS DB |
| R.NM(+15.99)LQM(+15.99)EWNSNAAQNAK.R | Y | 52.09 | 1880.8146 | 16 | 0.0 | 941.4146 | 2 | 23.04 | 4 | F4:9277 | NaSa01\_F12.raw |  |  |  | 4.0019E6 |  |  |  |  |  |  |  |  |  |  | 1 | 0 | 0 | 0 | 1 | 0 | 0 | 0 | 0 | 0 | 0 | 0 | 0 | 0 | 0 | 54 | 69 | Oxidation (M) | M2:Oxidation (M):1000.00;M5:Oxidation (M):1000.00 | PEAKS DB |
| A.RNMLQMEWNSNAAQNAK.R | Y | 51.96 | 2004.9258 | 17 | 0.3 | 669.3161 | 3 | 34.50 | 3 | F3:15607 | NaSa01\_F11.raw |  |  | 1.5558E6 |  |  |  |  |  |  |  |  |  |  |  | 1 | 0 | 0 | 1 | 0 | 0 | 0 | 0 | 0 | 0 | 0 | 0 | 0 | 0 | 0 | 53 | 69 |  |  | PEAKS DB |
| Y.TQIVWYNSHLLGC(+57.02)GAAK.C | Y | 49.12 | 1916.9567 | 17 | 5.2 | 959.4906 | 2 | 49.71 | 3 | F3:26927 | NaSa01\_F11.raw |  |  | 0 |  |  |  |  |  |  |  |  |  |  |  | 0 | 0 | 0 | 0 | 0 | 0 | 0 | 0 | 0 | 0 | 0 | 0 | 0 | 0 | 0 | 136 | 152 | Carbamidomethylation | C13:Carbamidomethylation:1000.00 | PEAKS DB |
| K.FVYGVGANPPGSVIGHYTQ.I | Y | 47.10 | 1961.9635 | 19 | -1.9 | 981.9872 | 2 | 59.92 | 3 | F3:34550 | NaSa01\_F11.raw |  |  | 4.9922E6 |  |  |  |  |  |  |  |  |  |  |  | 1 | 0 | 0 | 1 | 0 | 0 | 0 | 0 | 0 | 0 | 0 | 0 | 0 | 0 | 0 | 119 | 137 |  |  | PEAKS DB |
| K.YLYVC(+57.02)QYC(+57.02)PAGN.I | Y | 46.56 | 1506.6272 | 12 | 0.5 | 754.3212 | 2 | 57.29 | 3 | F3:32393 | NaSa01\_F11.raw |  |  | 3.2068E7 |  |  |  |  |  |  |  |  |  |  |  | 1 | 0 | 0 | 1 | 0 | 0 | 0 | 0 | 0 | 0 | 0 | 0 | 0 | 0 | 0 | 158 | 169 | Carbamidomethylation | C5:Carbamidomethylation:1000.00;C8:Carbamidomethylation:1000.00 | PEAKS DB |
| K.C(+57.02)SSSKYLYVC(+57.02)QYC(+57.02)PAGNIIGSIATPYK.S | Y | 45.78 | 3099.4504 | 27 | 1.0 | 1034.1584 | 3 | 70.24 | 3 | F3:43311 | NaSa01\_F11.raw |  |  | 3.453E6 |  |  |  |  |  |  |  |  |  |  |  | 1 | 0 | 0 | 1 | 0 | 0 | 0 | 0 | 0 | 0 | 0 | 0 | 0 | 0 | 0 | 153 | 179 | Carbamidomethylation | C1:Carbamidomethylation:1000.00;C10:Carbamidomethylation:1000.00;C13:Carbamidomethylation:1000.00 | PEAKS DB |
| K.FVYGVGANPPGSVIGHYTQIVWYNSHLLGC(+57.02)GAAKC(+57.02)SSSK.Y | Y | 44.01 | 4181.0249 | 39 | 0.5 | 837.2126 | 5 | 73.79 | 3 | F3:46241 | NaSa01\_F11.raw |  |  | 5.4419E6 |  |  |  |  |  |  |  |  |  |  |  | 1 | 0 | 0 | 1 | 0 | 0 | 0 | 0 | 0 | 0 | 0 | 0 | 0 | 0 | 0 | 119 | 157 | Carbamidomethylation | C30:Carbamidomethylation:1000.00;C35:Carbamidomethylation:1000.00 | PEAKS DB |
| N.LFMSSQPYAWSR.V | Y | 43.90 | 1471.6918 | 12 | 0.4 | 736.8535 | 2 | 58.10 | 3 | F3:33122 | NaSa01\_F11.raw |  |  | 2.2543E6 |  |  |  |  |  |  |  |  |  |  |  | 1 | 0 | 0 | 1 | 0 | 0 | 0 | 0 | 0 | 0 | 0 | 0 | 0 | 0 | 0 | 96 | 107 |  |  | PEAKS DB |
| K.IGC(+57.02)GENLFMSSQPY.A | Y | 43.37 | 1601.6854 | 14 | 1.2 | 801.8510 | 2 | 75.97 | 3 | F3:47994 | NaSa01\_F11.raw |  |  | 3.1448E7 |  |  |  |  |  |  |  |  |  |  |  | 1 | 0 | 0 | 1 | 0 | 0 | 0 | 0 | 0 | 0 | 0 | 0 | 0 | 0 | 0 | 90 | 103 | Carbamidomethylation | C3:Carbamidomethylation:1000.00 | PEAKS DB |
| N.IIGSIATPYK.S | N | 41.07 | 1061.6121 | 10 | 0.6 | 531.8136 | 2 | 33.13 | 3 | F3:14657 | NaSa01\_F11.raw |  |  | 1.2631E6 | 7.4884E6 |  |  |  |  |  |  |  |  |  |  | 2 | 0 | 0 | 1 | 1 | 0 | 0 | 0 | 0 | 0 | 0 | 0 | 0 | 0 | 0 | 170 | 179 |  |  | PEAKS DB |
| S.VIGHYTQIVWYNSHLLGC(+57.02)GAAK.C | Y | 40.84 | 2486.2529 | 22 | 0.1 | 622.5706 | 4 | 53.86 | 3 | F3:29573 | NaSa01\_F11.raw |  |  | 4.7058E5 |  |  |  |  |  |  |  |  |  |  |  | 1 | 0 | 0 | 1 | 0 | 0 | 0 | 0 | 0 | 0 | 0 | 0 | 0 | 0 | 0 | 131 | 152 | Carbamidomethylation | C18:Carbamidomethylation:1000.00 | PEAKS DB |
| K.IGC(+57.02)GENLFMSSQPYAW.S | Y | 40.60 | 1858.8019 | 16 | 0.2 | 930.4084 | 2 | 96.00 | 3 | F3:64850 | NaSa01\_F11.raw |  |  | 4.4469E7 |  |  |  |  |  |  |  |  |  |  |  | 1 | 0 | 0 | 1 | 0 | 0 | 0 | 0 | 0 | 0 | 0 | 0 | 0 | 0 | 0 | 90 | 105 | Carbamidomethylation | C3:Carbamidomethylation:1000.00 | PEAKS DB |
| R.NMLQMEWNSN.A | N | 38.45 | 1265.5170 | 10 | 0.4 | 633.7660 | 2 | 70.12 | 3 | F3:43109 | NaSa01\_F11.raw |  |  | 6.3761E6 |  |  |  |  |  |  |  |  |  |  |  | 1 | 0 | 0 | 1 | 0 | 0 | 0 | 0 | 0 | 0 | 0 | 0 | 0 | 0 | 0 | 54 | 63 |  |  | PEAKS DB |
| total 40 peptides |  |  |  |  |  |  |  |  |  |  |  |  |  |  |  |  |  |  |  |  |  |  |  |  |  |  |  |  |  |  |  |  |  |  |  |  |  |  |  |  |  |  |  |  |  |
| --- | --- | --- | --- | --- | --- | --- | --- | --- | --- | --- | --- | --- | --- | --- | --- | --- | --- | --- | --- | --- | --- | --- | --- | --- | --- | --- | --- | --- | --- | --- | --- | --- | --- | --- | --- | --- | --- | --- | --- | --- | --- | --- | --- | --- | --- |

P84808.2

back to list

  

| Protein Coverage
| Supporting Peptides
|

Protein Coverage:

Supporting Peptides:

| Peptide | Uniq | -10lgP | Mass | Length | ppm | m/z | z | RT | Fraction | Scan | Source File | Area F1 | Area F10 | Area F11 | Area F12 | Area F13 | Area F14 | Area F2 | Area F3 | Area F4 | Area F5 | Area F6 | Area F7 | Area F8 | Area F9 | #Feature | #Feature F1 | #Feature F10 | #Feature F11 | #Feature F12 | #Feature F13 | #Feature F14 | #Feature F2 | #Feature F3 | #Feature F4 | #Feature F5 | #Feature F6 | #Feature F7 | #Feature F8 | #Feature F9 | Start | End | PTM | AScore | Found By |
| --- | --- | --- | --- | --- | --- | --- | --- | --- | --- | --- | --- | --- | --- | --- | --- | --- | --- | --- | --- | --- | --- | --- | --- | --- | --- | --- | --- | --- | --- | --- | --- | --- | --- | --- | --- | --- | --- | --- | --- | --- | --- | --- | --- | --- | --- |
| K.YLYVC(+57.02)QYC(+57.02)PAGNIIGSIATPYK.S | Y | 89.41 | 2550.2288 | 22 | 1.7 | 1276.1238 | 2 | 83.59 | 4 | F4:59165 | NaSa01\_F12.raw |  | 2.1485E7 | 1.9793E9 | 8.4159E7 |  |  |  |  |  |  |  |  |  | 6.0302E6 | 9 | 0 | 2 | 3 | 2 | 0 | 0 | 0 | 0 | 0 | 0 | 0 | 0 | 0 | 2 | 158 | 179 | Carbamidomethylation | C5:Carbamidomethylation:1000.00;C8:Carbamidomethylation:1000.00 | PEAKS DB |
| K.SGPPC(+57.02)GDC(+57.02)PSAC(+57.02)VNGLC(+57.02)TNPC(+57.02)K.H | N | 84.62 | 2406.9482 | 22 | -0.2 | 1204.4812 | 2 | 33.07 | 3 | F3:14521 | NaSa01\_F11.raw |  |  | 1.2348E8 | 4.9674E7 |  |  |  |  |  |  |  |  |  |  | 4 | 0 | 0 | 2 | 2 | 0 | 0 | 0 | 0 | 0 | 0 | 0 | 0 | 0 | 0 | 180 | 201 | Carbamidomethylation | C5:Carbamidomethylation:1000.00;C8:Carbamidomethylation:1000.00;C12:Carbamidomethylation:1000.00;C17:Carbamidomethylation:1000.00;C21:Carbamidomethylation:1000.00 | PEAKS DB |
| K.KFVYGVGANPPGSVIGHYTQIVWYNSHLLGC(+57.02)GAAK.C | Y | 84.42 | 3759.8984 | 35 | 1.3 | 940.9831 | 4 | 73.63 | 3 | F3:46103 | NaSa01\_F11.raw |  |  | 2.4078E7 |  |  |  |  |  |  |  |  |  |  |  | 1 | 0 | 0 | 1 | 0 | 0 | 0 | 0 | 0 | 0 | 0 | 0 | 0 | 0 | 0 | 118 | 152 | Carbamidomethylation | C31:Carbamidomethylation:1000.00 | PEAKS DB |
| R.NMLQMEWNSNAAQNAK.R | Y | 81.87 | 1848.8247 | 16 | -2.7 | 925.4171 | 2 | 48.72 | 3 | F3:26583 | NaSa01\_F11.raw |  | 2.686E6 | 5.2892E8 | 1.5782E8 | 2.2797E6 | 1.3086E6 |  |  |  |  |  |  |  | 1.156E6 | 15 | 0 | 1 | 8 | 3 | 1 | 1 | 0 | 0 | 0 | 0 | 0 | 0 | 0 | 1 | 54 | 69 |  |  | PEAKS DB |
| K.IGC(+57.02)GENLFMSSQPYAWSR.V | Y | 81.73 | 2101.9351 | 18 | 0.5 | 1051.9753 | 2 | 71.46 | 3 | F3:44819 | NaSa01\_F11.raw |  | 2.2215E6 | 2.6316E9 | 1.5538E8 |  |  |  |  |  |  |  |  |  | 7.6663E5 | 19 | 0 | 1 | 13 | 4 | 0 | 0 | 0 | 0 | 0 | 0 | 0 | 0 | 0 | 1 | 90 | 107 | Carbamidomethylation | C3:Carbamidomethylation:1000.00 | PEAKS DB |
| K.FVYGVGANPPGSVIGHYTQIVWYNSHLLGC(+57.02)GAAK.C | Y | 80.80 | 3631.8035 | 34 | 2.6 | 908.9605 | 4 | 80.84 | 3 | F3:52182 | NaSa01\_F11.raw |  |  | 1.0245E10 | 1.892E7 |  |  |  |  |  |  |  |  |  |  | 4 | 0 | 0 | 3 | 1 | 0 | 0 | 0 | 0 | 0 | 0 | 0 | 0 | 0 | 0 | 119 | 152 | Carbamidomethylation | C30:Carbamidomethylation:1000.00 | PEAKS DB |
| K.IGC(+57.02)GENLFM(+15.99)SSQPYAWSR.V | Y | 80.66 | 2117.9299 | 18 | -0.1 | 1059.9722 | 2 | 64.23 | 3 | F3:38201 | NaSa01\_F11.raw |  | 3.1123E6 | 2.8772E8 | 1.7534E7 |  |  |  |  |  |  |  |  |  |  | 13 | 0 | 1 | 9 | 3 | 0 | 0 | 0 | 0 | 0 | 0 | 0 | 0 | 0 | 0 | 90 | 107 | Carbamidomethylation; Oxidation (M) | C3:Carbamidomethylation:1000.00;M9:Oxidation (M):1000.00 | PEAKS DB |
| Y.C(+57.02)PAGNIIGSIATPYK.S | Y | 77.71 | 1560.7970 | 15 | 1.4 | 781.4069 | 2 | 55.17 | 3 | F3:30489 | NaSa01\_F11.raw |  |  | 2.0089E7 |  |  |  |  |  |  |  |  |  |  |  | 2 | 0 | 0 | 2 | 0 | 0 | 0 | 0 | 0 | 0 | 0 | 0 | 0 | 0 | 0 | 165 | 179 | Carbamidomethylation | C1:Carbamidomethylation:1000.00 | PEAKS DB |
| R.NMLQM(+15.99)EWNSNAAQNAK.R | Y | 76.35 | 1864.8196 | 16 | -0.3 | 933.4167 | 2 | 36.59 | 3 | F3:17279 | NaSa01\_F11.raw |  |  | 8.5143E7 | 2.8164E7 |  |  |  |  |  |  |  |  |  |  | 5 | 0 | 0 | 4 | 1 | 0 | 0 | 0 | 0 | 0 | 0 | 0 | 0 | 0 | 0 | 54 | 69 | Oxidation (M) | M5:Oxidation (M):77.25 | PEAKS DB |
| R.NM(+15.99)LQMEWNSNAAQNAK.R | Y | 75.98 | 1864.8196 | 16 | -0.4 | 933.4167 | 2 | 35.10 | 3 | F3:16079 | NaSa01\_F11.raw |  |  | 1.071E8 | 2.8164E7 |  |  |  |  |  |  |  |  |  |  | 8 | 0 | 0 | 7 | 1 | 0 | 0 | 0 | 0 | 0 | 0 | 0 | 0 | 0 | 0 | 54 | 69 | Oxidation (M) | M2:Oxidation (M):101.94 | PEAKS DB |
| Y.VC(+57.02)QYC(+57.02)PAGNIIGSIATPYK.S | Y | 75.50 | 2111.0181 | 19 | 0.3 | 1056.5166 | 2 | 67.02 | 3 | F3:40488 | NaSa01\_F11.raw |  |  | 1.1991E7 |  |  |  |  |  |  |  |  |  |  |  | 1 | 0 | 0 | 1 | 0 | 0 | 0 | 0 | 0 | 0 | 0 | 0 | 0 | 0 | 0 | 161 | 179 | Carbamidomethylation | C2:Carbamidomethylation:1000.00;C5:Carbamidomethylation:1000.00 | PEAKS DB |
| H.YTQIVWYNSHLLGC(+57.02)GAAK.C | Y | 75.38 | 2080.0200 | 18 | 0.5 | 1041.0178 | 2 | 54.52 | 3 | F3:29919 | NaSa01\_F11.raw |  |  | 7.881E7 |  |  |  |  |  |  |  |  |  |  |  | 2 | 0 | 0 | 2 | 0 | 0 | 0 | 0 | 0 | 0 | 0 | 0 | 0 | 0 | 0 | 135 | 152 | Carbamidomethylation | C14:Carbamidomethylation:1000.00 | PEAKS DB |
| C.QYC(+57.02)PAGNIIGSIATPYK.S | Y | 71.89 | 1851.9188 | 17 | -0.2 | 926.9665 | 2 | 63.76 | 3 | F3:37806 | NaSa01\_F11.raw |  |  | 1.3036E7 | 9.2987E5 |  |  |  |  |  |  |  |  |  |  | 4 | 0 | 0 | 3 | 1 | 0 | 0 | 0 | 0 | 0 | 0 | 0 | 0 | 0 | 0 | 163 | 179 | Carbamidomethylation | C3:Carbamidomethylation:1000.00 | PEAKS DB |
| K.FVYGVGANPPGSVIGHY.T | Y | 67.79 | 1732.8573 | 17 | 1.7 | 867.4374 | 2 | 61.37 | 3 | F3:35819 | NaSa01\_F11.raw |  |  | 4.555E6 |  |  |  |  |  |  |  |  |  |  |  | 1 | 0 | 0 | 1 | 0 | 0 | 0 | 0 | 0 | 0 | 0 | 0 | 0 | 0 | 0 | 119 | 135 |  |  | PEAKS DB |
| R.TVGKIGC(+57.02)GENLFMSSQPYAWSR.V | Y | 65.61 | 2487.1675 | 22 | 1.0 | 830.0640 | 3 | 60.97 | 3 | F3:35553 | NaSa01\_F11.raw |  |  | 4.3623E6 |  |  |  |  |  |  |  |  |  |  |  | 1 | 0 | 0 | 1 | 0 | 0 | 0 | 0 | 0 | 0 | 0 | 0 | 0 | 0 | 0 | 86 | 107 | Carbamidomethylation | C7:Carbamidomethylation:1000.00 | PEAKS DB |
| K.FVYGVGANPPGSVIGH.Y | Y | 65.53 | 1569.7939 | 16 | 0.8 | 785.9049 | 2 | 53.98 | 3 | F3:29540 | NaSa01\_F11.raw |  |  | 0 | 2.2555E6 |  |  |  |  |  |  |  |  |  |  | 1 | 0 | 0 | 0 | 1 | 0 | 0 | 0 | 0 | 0 | 0 | 0 | 0 | 0 | 0 | 119 | 134 |  |  | PEAKS DB |
| Q.YC(+57.02)PAGNIIGSIATPYK.S | Y | 65.38 | 1723.8604 | 16 | 1.5 | 862.9387 | 2 | 62.96 | 3 | F3:37193 | NaSa01\_F11.raw |  |  | 4.9582E6 |  |  |  |  |  |  |  |  |  |  |  | 2 | 0 | 0 | 2 | 0 | 0 | 0 | 0 | 0 | 0 | 0 | 0 | 0 | 0 | 0 | 164 | 179 | Carbamidomethylation | C2:Carbamidomethylation:1000.00 | PEAKS DB |
| K.HHNVFSNC(+57.02)QSLAK.Q | Y | 64.16 | 1540.7205 | 13 | 0.6 | 514.5811 | 3 | 11.55 | 4 | F4:2538 | NaSa01\_F12.raw |  |  |  | 3.5364E5 |  |  |  |  |  |  |  |  |  |  | 1 | 0 | 0 | 0 | 1 | 0 | 0 | 0 | 0 | 0 | 0 | 0 | 0 | 0 | 0 | 202 | 214 | Carbamidomethylation | C8:Carbamidomethylation:1000.00 | PEAKS DB |
| R.NMLQMEWNSNAAQNAKR.W | Y | 63.89 | 2004.9258 | 17 | 0.7 | 669.3163 | 3 | 35.81 | 3 | F3:16595 | NaSa01\_F11.raw |  |  | 3.7952E6 |  |  |  |  |  |  |  |  |  |  |  | 1 | 0 | 0 | 1 | 0 | 0 | 0 | 0 | 0 | 0 | 0 | 0 | 0 | 0 | 0 | 54 | 70 |  |  | PEAKS DB |
| N.MLQMEWNSNAAQNAK.R | Y | 63.28 | 1734.7817 | 15 | 0.0 | 868.3981 | 2 | 41.92 | 3 | F3:21522 | NaSa01\_F11.raw |  |  | 3.684E6 |  |  |  |  |  |  |  |  |  |  |  | 1 | 0 | 0 | 1 | 0 | 0 | 0 | 0 | 0 | 0 | 0 | 0 | 0 | 0 | 0 | 55 | 69 |  |  | PEAKS DB |
| R.VIQSWYDENKK.F | Y | 63.03 | 1408.6986 | 11 | 0.5 | 705.3569 | 2 | 12.09 | 3 | F3:3655 | NaSa01\_F11.raw |  |  | 5.2553E6 |  |  |  |  |  |  |  |  |  |  |  | 2 | 0 | 0 | 2 | 0 | 0 | 0 | 0 | 0 | 0 | 0 | 0 | 0 | 0 | 0 | 108 | 118 |  |  | PEAKS DB |
| K.QNAC(+57.02)QTEWMK.S | Y | 62.52 | 1294.5435 | 10 | 0.5 | 648.2794 | 2 | 12.09 | 3 | F3:3722 | NaSa01\_F11.raw |  |  | 8.8238E5 |  |  |  |  |  |  |  |  |  |  |  | 1 | 0 | 0 | 1 | 0 | 0 | 0 | 0 | 0 | 0 | 0 | 0 | 0 | 0 | 0 | 215 | 224 | Carbamidomethylation | C4:Carbamidomethylation:1000.00 | PEAKS DB |
| R.VIQSWYDENK.K | Y | 61.22 | 1280.6036 | 10 | 0.1 | 641.3091 | 2 | 28.55 | 4 | F4:13454 | NaSa01\_F12.raw |  |  |  | 1.5337E6 |  |  |  |  |  |  |  |  |  |  | 1 | 0 | 0 | 0 | 1 | 0 | 0 | 0 | 0 | 0 | 0 | 0 | 0 | 0 | 0 | 108 | 117 |  |  | PEAKS DB |
| K.FVYGVGANPPGSVIGHYTQIVWYNSH.L | Y | 58.71 | 2861.3926 | 26 | 0.8 | 954.8056 | 3 | 79.87 | 3 | F3:51233 | NaSa01\_F11.raw |  |  | 2.3982E8 |  |  |  |  |  |  |  |  |  |  |  | 1 | 0 | 0 | 1 | 0 | 0 | 0 | 0 | 0 | 0 | 0 | 0 | 0 | 0 | 0 | 119 | 144 |  |  | PEAKS DB |
| Y.GVGANPPGSVIGHYTQIVWYNSHLLGC(+57.02)GAAK.C | Y | 56.40 | 3222.6033 | 31 | 1.3 | 806.6592 | 4 | 72.12 | 3 | F3:45003 | NaSa01\_F11.raw |  |  | 1.461E7 |  |  |  |  |  |  |  |  |  |  |  | 1 | 0 | 0 | 1 | 0 | 0 | 0 | 0 | 0 | 0 | 0 | 0 | 0 | 0 | 0 | 122 | 152 | Carbamidomethylation | C27:Carbamidomethylation:1000.00 | PEAKS DB |
| N.PPGSVIGHYTQIVWYNSHLLGC(+57.02)GAAK.C | Y | 53.07 | 2824.4119 | 26 | 0.9 | 707.1109 | 4 | 67.96 | 3 | F3:41292 | NaSa01\_F11.raw |  |  | 9.9415E6 |  |  |  |  |  |  |  |  |  |  |  | 1 | 0 | 0 | 1 | 0 | 0 | 0 | 0 | 0 | 0 | 0 | 0 | 0 | 0 | 0 | 127 | 152 | Carbamidomethylation | C22:Carbamidomethylation:1000.00 | PEAKS DB |
| R.C(+57.02)SFAHSPPHLR.T | Y | 52.73 | 1307.6193 | 11 | -0.3 | 436.8802 | 3 | 11.63 | 3 | F3:3321 | NaSa01\_F11.raw |  |  | 1.4433E6 | 3.0387E5 |  |  |  |  |  |  |  |  |  |  | 3 | 0 | 0 | 2 | 1 | 0 | 0 | 0 | 0 | 0 | 0 | 0 | 0 | 0 | 0 | 75 | 85 | Carbamidomethylation | C1:Carbamidomethylation:1000.00 | PEAKS DB |
| R.NM(+15.99)LQM(+15.99)EWNSNAAQNAK.R | Y | 52.09 | 1880.8146 | 16 | 0.0 | 941.4146 | 2 | 23.04 | 4 | F4:9277 | NaSa01\_F12.raw |  |  |  | 4.0019E6 |  |  |  |  |  |  |  |  |  |  | 1 | 0 | 0 | 0 | 1 | 0 | 0 | 0 | 0 | 0 | 0 | 0 | 0 | 0 | 0 | 54 | 69 | Oxidation (M) | M2:Oxidation (M):1000.00;M5:Oxidation (M):1000.00 | PEAKS DB |
| A.RNMLQMEWNSNAAQNAK.R | Y | 51.96 | 2004.9258 | 17 | 0.3 | 669.3161 | 3 | 34.50 | 3 | F3:15607 | NaSa01\_F11.raw |  |  | 1.5558E6 |  |  |  |  |  |  |  |  |  |  |  | 1 | 0 | 0 | 1 | 0 | 0 | 0 | 0 | 0 | 0 | 0 | 0 | 0 | 0 | 0 | 53 | 69 |  |  | PEAKS DB |
| Y.TQIVWYNSHLLGC(+57.02)GAAK.C | Y | 49.12 | 1916.9567 | 17 | 5.2 | 959.4906 | 2 | 49.71 | 3 | F3:26927 | NaSa01\_F11.raw |  |  | 0 |  |  |  |  |  |  |  |  |  |  |  | 0 | 0 | 0 | 0 | 0 | 0 | 0 | 0 | 0 | 0 | 0 | 0 | 0 | 0 | 0 | 136 | 152 | Carbamidomethylation | C13:Carbamidomethylation:1000.00 | PEAKS DB |
| K.FVYGVGANPPGSVIGHYTQ.I | Y | 47.10 | 1961.9635 | 19 | -1.9 | 981.9872 | 2 | 59.92 | 3 | F3:34550 | NaSa01\_F11.raw |  |  | 4.9922E6 |  |  |  |  |  |  |  |  |  |  |  | 1 | 0 | 0 | 1 | 0 | 0 | 0 | 0 | 0 | 0 | 0 | 0 | 0 | 0 | 0 | 119 | 137 |  |  | PEAKS DB |
| K.YLYVC(+57.02)QYC(+57.02)PAGN.I | Y | 46.56 | 1506.6272 | 12 | 0.5 | 754.3212 | 2 | 57.29 | 3 | F3:32393 | NaSa01\_F11.raw |  |  | 3.2068E7 |  |  |  |  |  |  |  |  |  |  |  | 1 | 0 | 0 | 1 | 0 | 0 | 0 | 0 | 0 | 0 | 0 | 0 | 0 | 0 | 0 | 158 | 169 | Carbamidomethylation | C5:Carbamidomethylation:1000.00;C8:Carbamidomethylation:1000.00 | PEAKS DB |
| K.C(+57.02)SSSKYLYVC(+57.02)QYC(+57.02)PAGNIIGSIATPYK.S | Y | 45.78 | 3099.4504 | 27 | 1.0 | 1034.1584 | 3 | 70.24 | 3 | F3:43311 | NaSa01\_F11.raw |  |  | 3.453E6 |  |  |  |  |  |  |  |  |  |  |  | 1 | 0 | 0 | 1 | 0 | 0 | 0 | 0 | 0 | 0 | 0 | 0 | 0 | 0 | 0 | 153 | 179 | Carbamidomethylation | C1:Carbamidomethylation:1000.00;C10:Carbamidomethylation:1000.00;C13:Carbamidomethylation:1000.00 | PEAKS DB |
| K.FVYGVGANPPGSVIGHYTQIVWYNSHLLGC(+57.02)GAAKC(+57.02)SSSK.Y | Y | 44.01 | 4181.0249 | 39 | 0.5 | 837.2126 | 5 | 73.79 | 3 | F3:46241 | NaSa01\_F11.raw |  |  | 5.4419E6 |  |  |  |  |  |  |  |  |  |  |  | 1 | 0 | 0 | 1 | 0 | 0 | 0 | 0 | 0 | 0 | 0 | 0 | 0 | 0 | 0 | 119 | 157 | Carbamidomethylation | C30:Carbamidomethylation:1000.00;C35:Carbamidomethylation:1000.00 | PEAKS DB |
| N.LFMSSQPYAWSR.V | Y | 43.90 | 1471.6918 | 12 | 0.4 | 736.8535 | 2 | 58.10 | 3 | F3:33122 | NaSa01\_F11.raw |  |  | 2.2543E6 |  |  |  |  |  |  |  |  |  |  |  | 1 | 0 | 0 | 1 | 0 | 0 | 0 | 0 | 0 | 0 | 0 | 0 | 0 | 0 | 0 | 96 | 107 |  |  | PEAKS DB |
| K.IGC(+57.02)GENLFMSSQPY.A | Y | 43.37 | 1601.6854 | 14 | 1.2 | 801.8510 | 2 | 75.97 | 3 | F3:47994 | NaSa01\_F11.raw |  |  | 3.1448E7 |  |  |  |  |  |  |  |  |  |  |  | 1 | 0 | 0 | 1 | 0 | 0 | 0 | 0 | 0 | 0 | 0 | 0 | 0 | 0 | 0 | 90 | 103 | Carbamidomethylation | C3:Carbamidomethylation:1000.00 | PEAKS DB |
| N.IIGSIATPYK.S | N | 41.07 | 1061.6121 | 10 | 0.6 | 531.8136 | 2 | 33.13 | 3 | F3:14657 | NaSa01\_F11.raw |  |  | 1.2631E6 | 7.4884E6 |  |  |  |  |  |  |  |  |  |  | 2 | 0 | 0 | 1 | 1 | 0 | 0 | 0 | 0 | 0 | 0 | 0 | 0 | 0 | 0 | 170 | 179 |  |  | PEAKS DB |
| S.VIGHYTQIVWYNSHLLGC(+57.02)GAAK.C | Y | 40.84 | 2486.2529 | 22 | 0.1 | 622.5706 | 4 | 53.86 | 3 | F3:29573 | NaSa01\_F11.raw |  |  | 4.7058E5 |  |  |  |  |  |  |  |  |  |  |  | 1 | 0 | 0 | 1 | 0 | 0 | 0 | 0 | 0 | 0 | 0 | 0 | 0 | 0 | 0 | 131 | 152 | Carbamidomethylation | C18:Carbamidomethylation:1000.00 | PEAKS DB |
| K.IGC(+57.02)GENLFMSSQPYAW.S | Y | 40.60 | 1858.8019 | 16 | 0.2 | 930.4084 | 2 | 96.00 | 3 | F3:64850 | NaSa01\_F11.raw |  |  | 4.4469E7 |  |  |  |  |  |  |  |  |  |  |  | 1 | 0 | 0 | 1 | 0 | 0 | 0 | 0 | 0 | 0 | 0 | 0 | 0 | 0 | 0 | 90 | 105 | Carbamidomethylation | C3:Carbamidomethylation:1000.00 | PEAKS DB |
| R.NMLQMEWNSN.A | N | 38.45 | 1265.5170 | 10 | 0.4 | 633.7660 | 2 | 70.12 | 3 | F3:43109 | NaSa01\_F11.raw |  |  | 6.3761E6 |  |  |  |  |  |  |  |  |  |  |  | 1 | 0 | 0 | 1 | 0 | 0 | 0 | 0 | 0 | 0 | 0 | 0 | 0 | 0 | 0 | 54 | 63 |  |  | PEAKS DB |
| total 40 peptides |  |  |  |  |  |  |  |  |  |  |  |  |  |  |  |  |  |  |  |  |  |  |  |  |  |  |  |  |  |  |  |  |  |  |  |  |  |  |  |  |  |  |  |  |  |
| --- | --- | --- | --- | --- | --- | --- | --- | --- | --- | --- | --- | --- | --- | --- | --- | --- | --- | --- | --- | --- | --- | --- | --- | --- | --- | --- | --- | --- | --- | --- | --- | --- | --- | --- | --- | --- | --- | --- | --- | --- | --- | --- | --- | --- | --- |

AHZ08822.1

back to list

  

| Protein Coverage
| Supporting Peptides
|

Protein Coverage:

Supporting Peptides:

| Peptide | Uniq | -10lgP | Mass | Length | ppm | m/z | z | RT | Fraction | Scan | Source File | Area F1 | Area F10 | Area F11 | Area F12 | Area F13 | Area F14 | Area F2 | Area F3 | Area F4 | Area F5 | Area F6 | Area F7 | Area F8 | Area F9 | #Feature | #Feature F1 | #Feature F10 | #Feature F11 | #Feature F12 | #Feature F13 | #Feature F14 | #Feature F2 | #Feature F3 | #Feature F4 | #Feature F5 | #Feature F6 | #Feature F7 | #Feature F8 | #Feature F9 | Start | End | PTM | AScore | Found By |
| --- | --- | --- | --- | --- | --- | --- | --- | --- | --- | --- | --- | --- | --- | --- | --- | --- | --- | --- | --- | --- | --- | --- | --- | --- | --- | --- | --- | --- | --- | --- | --- | --- | --- | --- | --- | --- | --- | --- | --- | --- | --- | --- | --- | --- | --- |
| K.YLYVC(+57.02)QYC(+57.02)PAGNIIGSIATPYK.S | Y | 89.41 | 2550.2288 | 22 | 1.7 | 1276.1238 | 2 | 83.59 | 4 | F4:59165 | NaSa01\_F12.raw |  | 2.1485E7 | 1.9793E9 | 8.4159E7 |  |  |  |  |  |  |  |  |  | 6.0302E6 | 9 | 0 | 2 | 3 | 2 | 0 | 0 | 0 | 0 | 0 | 0 | 0 | 0 | 0 | 2 | 158 | 179 | Carbamidomethylation | C5:Carbamidomethylation:1000.00;C8:Carbamidomethylation:1000.00 | PEAKS DB |
| K.SGPPC(+57.02)GDC(+57.02)PSAC(+57.02)VNGLC(+57.02)TNPC(+57.02)K.H | N | 84.62 | 2406.9482 | 22 | -0.2 | 1204.4812 | 2 | 33.07 | 3 | F3:14521 | NaSa01\_F11.raw |  |  | 1.2348E8 | 4.9674E7 |  |  |  |  |  |  |  |  |  |  | 4 | 0 | 0 | 2 | 2 | 0 | 0 | 0 | 0 | 0 | 0 | 0 | 0 | 0 | 0 | 180 | 201 | Carbamidomethylation | C5:Carbamidomethylation:1000.00;C8:Carbamidomethylation:1000.00;C12:Carbamidomethylation:1000.00;C17:Carbamidomethylation:1000.00;C21:Carbamidomethylation:1000.00 | PEAKS DB |
| K.KFVYGVGANPPGSVIGHYTQIVWYNSHLLGC(+57.02)GAAK.C | Y | 84.42 | 3759.8984 | 35 | 1.3 | 940.9831 | 4 | 73.63 | 3 | F3:46103 | NaSa01\_F11.raw |  |  | 2.4078E7 |  |  |  |  |  |  |  |  |  |  |  | 1 | 0 | 0 | 1 | 0 | 0 | 0 | 0 | 0 | 0 | 0 | 0 | 0 | 0 | 0 | 118 | 152 | Carbamidomethylation | C31:Carbamidomethylation:1000.00 | PEAKS DB |
| R.NMLQMEWNSNAAQNAK.R | Y | 81.87 | 1848.8247 | 16 | -2.7 | 925.4171 | 2 | 48.72 | 3 | F3:26583 | NaSa01\_F11.raw |  | 2.686E6 | 5.2892E8 | 1.5782E8 | 2.2797E6 | 1.3086E6 |  |  |  |  |  |  |  | 1.156E6 | 15 | 0 | 1 | 8 | 3 | 1 | 1 | 0 | 0 | 0 | 0 | 0 | 0 | 0 | 1 | 54 | 69 |  |  | PEAKS DB |
| K.IGC(+57.02)GENLFMSSQPYAWSR.V | Y | 81.73 | 2101.9351 | 18 | 0.5 | 1051.9753 | 2 | 71.46 | 3 | F3:44819 | NaSa01\_F11.raw |  | 2.2215E6 | 2.6316E9 | 1.5538E8 |  |  |  |  |  |  |  |  |  | 7.6663E5 | 19 | 0 | 1 | 13 | 4 | 0 | 0 | 0 | 0 | 0 | 0 | 0 | 0 | 0 | 1 | 90 | 107 | Carbamidomethylation | C3:Carbamidomethylation:1000.00 | PEAKS DB |
| K.FVYGVGANPPGSVIGHYTQIVWYNSHLLGC(+57.02)GAAK.C | Y | 80.80 | 3631.8035 | 34 | 2.6 | 908.9605 | 4 | 80.84 | 3 | F3:52182 | NaSa01\_F11.raw |  |  | 1.0245E10 | 1.892E7 |  |  |  |  |  |  |  |  |  |  | 4 | 0 | 0 | 3 | 1 | 0 | 0 | 0 | 0 | 0 | 0 | 0 | 0 | 0 | 0 | 119 | 152 | Carbamidomethylation | C30:Carbamidomethylation:1000.00 | PEAKS DB |
| K.IGC(+57.02)GENLFM(+15.99)SSQPYAWSR.V | Y | 80.66 | 2117.9299 | 18 | -0.1 | 1059.9722 | 2 | 64.23 | 3 | F3:38201 | NaSa01\_F11.raw |  | 3.1123E6 | 2.8772E8 | 1.7534E7 |  |  |  |  |  |  |  |  |  |  | 13 | 0 | 1 | 9 | 3 | 0 | 0 | 0 | 0 | 0 | 0 | 0 | 0 | 0 | 0 | 90 | 107 | Carbamidomethylation; Oxidation (M) | C3:Carbamidomethylation:1000.00;M9:Oxidation (M):1000.00 | PEAKS DB |
| Y.C(+57.02)PAGNIIGSIATPYK.S | Y | 77.71 | 1560.7970 | 15 | 1.4 | 781.4069 | 2 | 55.17 | 3 | F3:30489 | NaSa01\_F11.raw |  |  | 2.0089E7 |  |  |  |  |  |  |  |  |  |  |  | 2 | 0 | 0 | 2 | 0 | 0 | 0 | 0 | 0 | 0 | 0 | 0 | 0 | 0 | 0 | 165 | 179 | Carbamidomethylation | C1:Carbamidomethylation:1000.00 | PEAKS DB |
| R.NMLQM(+15.99)EWNSNAAQNAK.R | Y | 76.35 | 1864.8196 | 16 | -0.3 | 933.4167 | 2 | 36.59 | 3 | F3:17279 | NaSa01\_F11.raw |  |  | 8.5143E7 | 2.8164E7 |  |  |  |  |  |  |  |  |  |  | 5 | 0 | 0 | 4 | 1 | 0 | 0 | 0 | 0 | 0 | 0 | 0 | 0 | 0 | 0 | 54 | 69 | Oxidation (M) | M5:Oxidation (M):77.25 | PEAKS DB |
| R.NM(+15.99)LQMEWNSNAAQNAK.R | Y | 75.98 | 1864.8196 | 16 | -0.4 | 933.4167 | 2 | 35.10 | 3 | F3:16079 | NaSa01\_F11.raw |  |  | 1.071E8 | 2.8164E7 |  |  |  |  |  |  |  |  |  |  | 8 | 0 | 0 | 7 | 1 | 0 | 0 | 0 | 0 | 0 | 0 | 0 | 0 | 0 | 0 | 54 | 69 | Oxidation (M) | M2:Oxidation (M):101.94 | PEAKS DB |
| Y.VC(+57.02)QYC(+57.02)PAGNIIGSIATPYK.S | Y | 75.50 | 2111.0181 | 19 | 0.3 | 1056.5166 | 2 | 67.02 | 3 | F3:40488 | NaSa01\_F11.raw |  |  | 1.1991E7 |  |  |  |  |  |  |  |  |  |  |  | 1 | 0 | 0 | 1 | 0 | 0 | 0 | 0 | 0 | 0 | 0 | 0 | 0 | 0 | 0 | 161 | 179 | Carbamidomethylation | C2:Carbamidomethylation:1000.00;C5:Carbamidomethylation:1000.00 | PEAKS DB |
| H.YTQIVWYNSHLLGC(+57.02)GAAK.C | Y | 75.38 | 2080.0200 | 18 | 0.5 | 1041.0178 | 2 | 54.52 | 3 | F3:29919 | NaSa01\_F11.raw |  |  | 7.881E7 |  |  |  |  |  |  |  |  |  |  |  | 2 | 0 | 0 | 2 | 0 | 0 | 0 | 0 | 0 | 0 | 0 | 0 | 0 | 0 | 0 | 135 | 152 | Carbamidomethylation | C14:Carbamidomethylation:1000.00 | PEAKS DB |
| C.QYC(+57.02)PAGNIIGSIATPYK.S | Y | 71.89 | 1851.9188 | 17 | -0.2 | 926.9665 | 2 | 63.76 | 3 | F3:37806 | NaSa01\_F11.raw |  |  | 1.3036E7 | 9.2987E5 |  |  |  |  |  |  |  |  |  |  | 4 | 0 | 0 | 3 | 1 | 0 | 0 | 0 | 0 | 0 | 0 | 0 | 0 | 0 | 0 | 163 | 179 | Carbamidomethylation | C3:Carbamidomethylation:1000.00 | PEAKS DB |
| K.FVYGVGANPPGSVIGHY.T | Y | 67.79 | 1732.8573 | 17 | 1.7 | 867.4374 | 2 | 61.37 | 3 | F3:35819 | NaSa01\_F11.raw |  |  | 4.555E6 |  |  |  |  |  |  |  |  |  |  |  | 1 | 0 | 0 | 1 | 0 | 0 | 0 | 0 | 0 | 0 | 0 | 0 | 0 | 0 | 0 | 119 | 135 |  |  | PEAKS DB |
| R.TVGKIGC(+57.02)GENLFMSSQPYAWSR.V | Y | 65.61 | 2487.1675 | 22 | 1.0 | 830.0640 | 3 | 60.97 | 3 | F3:35553 | NaSa01\_F11.raw |  |  | 4.3623E6 |  |  |  |  |  |  |  |  |  |  |  | 1 | 0 | 0 | 1 | 0 | 0 | 0 | 0 | 0 | 0 | 0 | 0 | 0 | 0 | 0 | 86 | 107 | Carbamidomethylation | C7:Carbamidomethylation:1000.00 | PEAKS DB |
| K.FVYGVGANPPGSVIGH.Y | Y | 65.53 | 1569.7939 | 16 | 0.8 | 785.9049 | 2 | 53.98 | 3 | F3:29540 | NaSa01\_F11.raw |  |  | 0 | 2.2555E6 |  |  |  |  |  |  |  |  |  |  | 1 | 0 | 0 | 0 | 1 | 0 | 0 | 0 | 0 | 0 | 0 | 0 | 0 | 0 | 0 | 119 | 134 |  |  | PEAKS DB |
| Q.YC(+57.02)PAGNIIGSIATPYK.S | Y | 65.38 | 1723.8604 | 16 | 1.5 | 862.9387 | 2 | 62.96 | 3 | F3:37193 | NaSa01\_F11.raw |  |  | 4.9582E6 |  |  |  |  |  |  |  |  |  |  |  | 2 | 0 | 0 | 2 | 0 | 0 | 0 | 0 | 0 | 0 | 0 | 0 | 0 | 0 | 0 | 164 | 179 | Carbamidomethylation | C2:Carbamidomethylation:1000.00 | PEAKS DB |
| K.HHNVFSNC(+57.02)QSLAK.Q | Y | 64.16 | 1540.7205 | 13 | 0.6 | 514.5811 | 3 | 11.55 | 4 | F4:2538 | NaSa01\_F12.raw |  |  |  | 3.5364E5 |  |  |  |  |  |  |  |  |  |  | 1 | 0 | 0 | 0 | 1 | 0 | 0 | 0 | 0 | 0 | 0 | 0 | 0 | 0 | 0 | 202 | 214 | Carbamidomethylation | C8:Carbamidomethylation:1000.00 | PEAKS DB |
| R.NMLQMEWNSNAAQNAKR.W | Y | 63.89 | 2004.9258 | 17 | 0.7 | 669.3163 | 3 | 35.81 | 3 | F3:16595 | NaSa01\_F11.raw |  |  | 3.7952E6 |  |  |  |  |  |  |  |  |  |  |  | 1 | 0 | 0 | 1 | 0 | 0 | 0 | 0 | 0 | 0 | 0 | 0 | 0 | 0 | 0 | 54 | 70 |  |  | PEAKS DB |
| N.MLQMEWNSNAAQNAK.R | Y | 63.28 | 1734.7817 | 15 | 0.0 | 868.3981 | 2 | 41.92 | 3 | F3:21522 | NaSa01\_F11.raw |  |  | 3.684E6 |  |  |  |  |  |  |  |  |  |  |  | 1 | 0 | 0 | 1 | 0 | 0 | 0 | 0 | 0 | 0 | 0 | 0 | 0 | 0 | 0 | 55 | 69 |  |  | PEAKS DB |
| R.VIQSWYDENKK.F | Y | 63.03 | 1408.6986 | 11 | 0.5 | 705.3569 | 2 | 12.09 | 3 | F3:3655 | NaSa01\_F11.raw |  |  | 5.2553E6 |  |  |  |  |  |  |  |  |  |  |  | 2 | 0 | 0 | 2 | 0 | 0 | 0 | 0 | 0 | 0 | 0 | 0 | 0 | 0 | 0 | 108 | 118 |  |  | PEAKS DB |
| K.QNAC(+57.02)QTEWMK.S | Y | 62.52 | 1294.5435 | 10 | 0.5 | 648.2794 | 2 | 12.09 | 3 | F3:3722 | NaSa01\_F11.raw |  |  | 8.8238E5 |  |  |  |  |  |  |  |  |  |  |  | 1 | 0 | 0 | 1 | 0 | 0 | 0 | 0 | 0 | 0 | 0 | 0 | 0 | 0 | 0 | 215 | 224 | Carbamidomethylation | C4:Carbamidomethylation:1000.00 | PEAKS DB |
| R.VIQSWYDENK.K | Y | 61.22 | 1280.6036 | 10 | 0.1 | 641.3091 | 2 | 28.55 | 4 | F4:13454 | NaSa01\_F12.raw |  |  |  | 1.5337E6 |  |  |  |  |  |  |  |  |  |  | 1 | 0 | 0 | 0 | 1 | 0 | 0 | 0 | 0 | 0 | 0 | 0 | 0 | 0 | 0 | 108 | 117 |  |  | PEAKS DB |
| K.FVYGVGANPPGSVIGHYTQIVWYNSH.L | Y | 58.71 | 2861.3926 | 26 | 0.8 | 954.8056 | 3 | 79.87 | 3 | F3:51233 | NaSa01\_F11.raw |  |  | 2.3982E8 |  |  |  |  |  |  |  |  |  |  |  | 1 | 0 | 0 | 1 | 0 | 0 | 0 | 0 | 0 | 0 | 0 | 0 | 0 | 0 | 0 | 119 | 144 |  |  | PEAKS DB |
| Y.GVGANPPGSVIGHYTQIVWYNSHLLGC(+57.02)GAAK.C | Y | 56.40 | 3222.6033 | 31 | 1.3 | 806.6592 | 4 | 72.12 | 3 | F3:45003 | NaSa01\_F11.raw |  |  | 1.461E7 |  |  |  |  |  |  |  |  |  |  |  | 1 | 0 | 0 | 1 | 0 | 0 | 0 | 0 | 0 | 0 | 0 | 0 | 0 | 0 | 0 | 122 | 152 | Carbamidomethylation | C27:Carbamidomethylation:1000.00 | PEAKS DB |
| N.PPGSVIGHYTQIVWYNSHLLGC(+57.02)GAAK.C | Y | 53.07 | 2824.4119 | 26 | 0.9 | 707.1109 | 4 | 67.96 | 3 | F3:41292 | NaSa01\_F11.raw |  |  | 9.9415E6 |  |  |  |  |  |  |  |  |  |  |  | 1 | 0 | 0 | 1 | 0 | 0 | 0 | 0 | 0 | 0 | 0 | 0 | 0 | 0 | 0 | 127 | 152 | Carbamidomethylation | C22:Carbamidomethylation:1000.00 | PEAKS DB |
| R.C(+57.02)SFAHSPPHLR.T | Y | 52.73 | 1307.6193 | 11 | -0.3 | 436.8802 | 3 | 11.63 | 3 | F3:3321 | NaSa01\_F11.raw |  |  | 1.4433E6 | 3.0387E5 |  |  |  |  |  |  |  |  |  |  | 3 | 0 | 0 | 2 | 1 | 0 | 0 | 0 | 0 | 0 | 0 | 0 | 0 | 0 | 0 | 75 | 85 | Carbamidomethylation | C1:Carbamidomethylation:1000.00 | PEAKS DB |
| R.NM(+15.99)LQM(+15.99)EWNSNAAQNAK.R | Y | 52.09 | 1880.8146 | 16 | 0.0 | 941.4146 | 2 | 23.04 | 4 | F4:9277 | NaSa01\_F12.raw |  |  |  | 4.0019E6 |  |  |  |  |  |  |  |  |  |  | 1 | 0 | 0 | 0 | 1 | 0 | 0 | 0 | 0 | 0 | 0 | 0 | 0 | 0 | 0 | 54 | 69 | Oxidation (M) | M2:Oxidation (M):1000.00;M5:Oxidation (M):1000.00 | PEAKS DB |
| A.RNMLQMEWNSNAAQNAK.R | Y | 51.96 | 2004.9258 | 17 | 0.3 | 669.3161 | 3 | 34.50 | 3 | F3:15607 | NaSa01\_F11.raw |  |  | 1.5558E6 |  |  |  |  |  |  |  |  |  |  |  | 1 | 0 | 0 | 1 | 0 | 0 | 0 | 0 | 0 | 0 | 0 | 0 | 0 | 0 | 0 | 53 | 69 |  |  | PEAKS DB |
| Y.TQIVWYNSHLLGC(+57.02)GAAK.C | Y | 49.12 | 1916.9567 | 17 | 5.2 | 959.4906 | 2 | 49.71 | 3 | F3:26927 | NaSa01\_F11.raw |  |  | 0 |  |  |  |  |  |  |  |  |  |  |  | 0 | 0 | 0 | 0 | 0 | 0 | 0 | 0 | 0 | 0 | 0 | 0 | 0 | 0 | 0 | 136 | 152 | Carbamidomethylation | C13:Carbamidomethylation:1000.00 | PEAKS DB |
| K.FVYGVGANPPGSVIGHYTQ.I | Y | 47.10 | 1961.9635 | 19 | -1.9 | 981.9872 | 2 | 59.92 | 3 | F3:34550 | NaSa01\_F11.raw |  |  | 4.9922E6 |  |  |  |  |  |  |  |  |  |  |  | 1 | 0 | 0 | 1 | 0 | 0 | 0 | 0 | 0 | 0 | 0 | 0 | 0 | 0 | 0 | 119 | 137 |  |  | PEAKS DB |
| K.YLYVC(+57.02)QYC(+57.02)PAGN.I | Y | 46.56 | 1506.6272 | 12 | 0.5 | 754.3212 | 2 | 57.29 | 3 | F3:32393 | NaSa01\_F11.raw |  |  | 3.2068E7 |  |  |  |  |  |  |  |  |  |  |  | 1 | 0 | 0 | 1 | 0 | 0 | 0 | 0 | 0 | 0 | 0 | 0 | 0 | 0 | 0 | 158 | 169 | Carbamidomethylation | C5:Carbamidomethylation:1000.00;C8:Carbamidomethylation:1000.00 | PEAKS DB |
| K.C(+57.02)SSSKYLYVC(+57.02)QYC(+57.02)PAGNIIGSIATPYK.S | Y | 45.78 | 3099.4504 | 27 | 1.0 | 1034.1584 | 3 | 70.24 | 3 | F3:43311 | NaSa01\_F11.raw |  |  | 3.453E6 |  |  |  |  |  |  |  |  |  |  |  | 1 | 0 | 0 | 1 | 0 | 0 | 0 | 0 | 0 | 0 | 0 | 0 | 0 | 0 | 0 | 153 | 179 | Carbamidomethylation | C1:Carbamidomethylation:1000.00;C10:Carbamidomethylation:1000.00;C13:Carbamidomethylation:1000.00 | PEAKS DB |
| K.FVYGVGANPPGSVIGHYTQIVWYNSHLLGC(+57.02)GAAKC(+57.02)SSSK.Y | Y | 44.01 | 4181.0249 | 39 | 0.5 | 837.2126 | 5 | 73.79 | 3 | F3:46241 | NaSa01\_F11.raw |  |  | 5.4419E6 |  |  |  |  |  |  |  |  |  |  |  | 1 | 0 | 0 | 1 | 0 | 0 | 0 | 0 | 0 | 0 | 0 | 0 | 0 | 0 | 0 | 119 | 157 | Carbamidomethylation | C30:Carbamidomethylation:1000.00;C35:Carbamidomethylation:1000.00 | PEAKS DB |
| N.LFMSSQPYAWSR.V | Y | 43.90 | 1471.6918 | 12 | 0.4 | 736.8535 | 2 | 58.10 | 3 | F3:33122 | NaSa01\_F11.raw |  |  | 2.2543E6 |  |  |  |  |  |  |  |  |  |  |  | 1 | 0 | 0 | 1 | 0 | 0 | 0 | 0 | 0 | 0 | 0 | 0 | 0 | 0 | 0 | 96 | 107 |  |  | PEAKS DB |
| K.IGC(+57.02)GENLFMSSQPY.A | Y | 43.37 | 1601.6854 | 14 | 1.2 | 801.8510 | 2 | 75.97 | 3 | F3:47994 | NaSa01\_F11.raw |  |  | 3.1448E7 |  |  |  |  |  |  |  |  |  |  |  | 1 | 0 | 0 | 1 | 0 | 0 | 0 | 0 | 0 | 0 | 0 | 0 | 0 | 0 | 0 | 90 | 103 | Carbamidomethylation | C3:Carbamidomethylation:1000.00 | PEAKS DB |
| N.IIGSIATPYK.S | N | 41.07 | 1061.6121 | 10 | 0.6 | 531.8136 | 2 | 33.13 | 3 | F3:14657 | NaSa01\_F11.raw |  |  | 1.2631E6 | 7.4884E6 |  |  |  |  |  |  |  |  |  |  | 2 | 0 | 0 | 1 | 1 | 0 | 0 | 0 | 0 | 0 | 0 | 0 | 0 | 0 | 0 | 170 | 179 |  |  | PEAKS DB |
| S.VIGHYTQIVWYNSHLLGC(+57.02)GAAK.C | Y | 40.84 | 2486.2529 | 22 | 0.1 | 622.5706 | 4 | 53.86 | 3 | F3:29573 | NaSa01\_F11.raw |  |  | 4.7058E5 |  |  |  |  |  |  |  |  |  |  |  | 1 | 0 | 0 | 1 | 0 | 0 | 0 | 0 | 0 | 0 | 0 | 0 | 0 | 0 | 0 | 131 | 152 | Carbamidomethylation | C18:Carbamidomethylation:1000.00 | PEAKS DB |
| K.IGC(+57.02)GENLFMSSQPYAW.S | Y | 40.60 | 1858.8019 | 16 | 0.2 | 930.4084 | 2 | 96.00 | 3 | F3:64850 | NaSa01\_F11.raw |  |  | 4.4469E7 |  |  |  |  |  |  |  |  |  |  |  | 1 | 0 | 0 | 1 | 0 | 0 | 0 | 0 | 0 | 0 | 0 | 0 | 0 | 0 | 0 | 90 | 105 | Carbamidomethylation | C3:Carbamidomethylation:1000.00 | PEAKS DB |
| R.NMLQMEWNSN.A | N | 38.45 | 1265.5170 | 10 | 0.4 | 633.7660 | 2 | 70.12 | 3 | F3:43109 | NaSa01\_F11.raw |  |  | 6.3761E6 |  |  |  |  |  |  |  |  |  |  |  | 1 | 0 | 0 | 1 | 0 | 0 | 0 | 0 | 0 | 0 | 0 | 0 | 0 | 0 | 0 | 54 | 63 |  |  | PEAKS DB |
| total 40 peptides |  |  |  |  |  |  |  |  |  |  |  |  |  |  |  |  |  |  |  |  |  |  |  |  |  |  |  |  |  |  |  |  |  |  |  |  |  |  |  |  |  |  |  |  |  |
| --- | --- | --- | --- | --- | --- | --- | --- | --- | --- | --- | --- | --- | --- | --- | --- | --- | --- | --- | --- | --- | --- | --- | --- | --- | --- | --- | --- | --- | --- | --- | --- | --- | --- | --- | --- | --- | --- | --- | --- | --- | --- | --- | --- | --- | --- |

P82942.1

back to list

  

| Protein Coverage
| Supporting Peptides
|

Protein Coverage:

Supporting Peptides:

| Peptide | Uniq | -10lgP | Mass | Length | ppm | m/z | z | RT | Fraction | Scan | Source File | Area F1 | Area F10 | Area F11 | Area F12 | Area F13 | Area F14 | Area F2 | Area F3 | Area F4 | Area F5 | Area F6 | Area F7 | Area F8 | Area F9 | #Feature | #Feature F1 | #Feature F10 | #Feature F11 | #Feature F12 | #Feature F13 | #Feature F14 | #Feature F2 | #Feature F3 | #Feature F4 | #Feature F5 | #Feature F6 | #Feature F7 | #Feature F8 | #Feature F9 | Start | End | PTM | AScore | Found By |
| --- | --- | --- | --- | --- | --- | --- | --- | --- | --- | --- | --- | --- | --- | --- | --- | --- | --- | --- | --- | --- | --- | --- | --- | --- | --- | --- | --- | --- | --- | --- | --- | --- | --- | --- | --- | --- | --- | --- | --- | --- | --- | --- | --- | --- | --- |
| K.HDC(+57.02)DLPELC(+57.02)TGQSAEC(+57.02)PTDSLQR.N | Y | 99.29 | 2688.1213 | 23 | -0.4 | 897.0474 | 3 | 43.83 | 6 | F6:24085 | NaSa01\_F14.raw |  |  | 3.1613E5 | 5.3539E7 | 3.9189E8 | 1.1241E8 |  |  |  |  |  | 1.7553E7 | 2.8805E7 |  | 14 | 0 | 0 | 1 | 2 | 4 | 3 | 0 | 0 | 0 | 0 | 0 | 2 | 2 | 0 | 262 | 284 | Carbamidomethylation | C3:Carbamidomethylation:1000.00;C9:Carbamidomethylation:1000.00;C16:Carbamidomethylation:1000.00 | PEAKS DB |
| K.C(+57.02)PTLTNQC(+57.02)IALLGPHFTVSPK.G | Y | 90.48 | 2353.1921 | 21 | 0.6 | 785.4051 | 3 | 63.42 | 6 | F6:40811 | NaSa01\_F14.raw |  |  |  | 5.7749E7 | 7.2143E8 | 1.7872E8 |  |  |  |  | 3.1018E6 | 5.6519E8 | 4.2468E7 |  | 15 | 0 | 0 | 0 | 1 | 4 | 4 | 0 | 0 | 0 | 0 | 1 | 3 | 2 | 0 | 301 | 321 | Carbamidomethylation | C1:Carbamidomethylation:1000.00;C8:Carbamidomethylation:1000.00 | PEAKS DB |
| R.TAPAFQFSSC(+57.02)SIR.D | N | 76.44 | 1470.6925 | 13 | -0.4 | 736.3532 | 2 | 46.46 | 6 | F6:26184 | NaSa01\_F14.raw |  |  |  | 2.702E7 | 6.1746E8 | 9.7374E7 |  |  |  |  |  | 2.3839E5 |  | 5.3658E6 | 12 | 0 | 0 | 0 | 1 | 4 | 5 | 0 | 0 | 0 | 0 | 0 | 1 | 0 | 1 | 178 | 190 | Carbamidomethylation | C10:Carbamidomethylation:1000.00 | PEAKS DB |
| K.NTMSC(+57.02)LIPPNPDGIMAEPGTK.C | N | 73.85 | 2242.0432 | 21 | -0.8 | 1122.0280 | 2 | 68.00 | 6 | F6:44885 | NaSa01\_F14.raw |  |  |  |  | 6.4544E8 | 1.0813E8 |  |  |  |  |  | 8.4111E7 | 2.0872E7 |  | 9 | 0 | 0 | 0 | 0 | 3 | 2 | 0 | 0 | 0 | 0 | 0 | 2 | 2 | 0 | 362 | 382 | Carbamidomethylation | C5:Carbamidomethylation:1000.00 | PEAKS DB |
| R.VYEMINAVNTK.F | N | 69.56 | 1280.6434 | 11 | 0.7 | 641.3295 | 2 | 33.85 | 6 | F6:15682 | NaSa01\_F14.raw |  |  |  | 2.984E7 | 1.408E9 | 8.928E6 |  |  |  |  |  |  |  |  | 6 | 0 | 0 | 0 | 2 | 2 | 2 | 0 | 0 | 0 | 0 | 0 | 0 | 0 | 0 | 41 | 51 |  |  | PEAKS DB |
| R.NGHPC(+57.02)QNNQGYC(+57.02)YNGK.C | N | 68.53 | 1909.7584 | 16 | -0.2 | 637.5933 | 3 | 11.55 | 4 | F4:2526 | NaSa01\_F12.raw |  |  |  | 1.1419E6 |  |  |  |  |  |  |  |  |  |  | 2 | 0 | 0 | 0 | 2 | 0 | 0 | 0 | 0 | 0 | 0 | 0 | 0 | 0 | 0 | 285 | 300 | Carbamidomethylation | C5:Carbamidomethylation:1000.00;C12:Carbamidomethylation:1000.00 | PEAKS DB |
| R.DRPQC(+57.02)ILNKPLSTDIVSPAIC(+57.02)GN.Y | Y | 68.07 | 2567.2834 | 23 | -0.1 | 856.7683 | 3 | 62.77 | 6 | F6:40514 | NaSa01\_F14.raw |  |  |  |  | 1.6702E8 | 6.4265E7 |  |  |  |  |  |  |  |  | 2 | 0 | 0 | 0 | 0 | 1 | 1 | 0 | 0 | 0 | 0 | 0 | 0 | 0 | 0 | 199 | 221 | Carbamidomethylation | C5:Carbamidomethylation:1000.00;C21:Carbamidomethylation:1000.00 | PEAKS DB |
| R.MSLVASTMTHELGH.N | Y | 67.39 | 1512.7065 | 14 | 0.5 | 757.3609 | 2 | 43.14 | 6 | F6:23499 | NaSa01\_F14.raw |  |  |  |  | 4.0367E7 | 1.5011E7 |  |  |  |  |  |  |  |  | 5 | 0 | 0 | 0 | 0 | 2 | 3 | 0 | 0 | 0 | 0 | 0 | 0 | 0 | 0 | 140 | 153 |  |  | PEAKS DB |
| K.C(+57.02)PTLTNQC(+57.02)IALLGPH.F | Y | 66.78 | 1693.8280 | 15 | 1.4 | 847.9224 | 2 | 59.86 | 12 | F12:40049 | NaSa01\_F7.raw |  |  |  |  | 7.7393E6 |  |  |  |  |  |  | 1.8331E6 |  |  | 2 | 0 | 0 | 0 | 0 | 1 | 0 | 0 | 0 | 0 | 0 | 0 | 1 | 0 | 0 | 301 | 315 | Carbamidomethylation | C1:Carbamidomethylation:1000.00;C8:Carbamidomethylation:1000.00 | PEAKS DB |
| D.LPELC(+57.02)TGQSAEC(+57.02)PTDSLQR.N | N | 65.21 | 2160.9780 | 19 | 0.2 | 1081.4965 | 2 | 45.39 | 6 | F6:25364 | NaSa01\_F14.raw |  |  | 1.3036E6 | 4.0582E6 | 1.0756E6 | 2.3359E5 |  |  |  |  |  |  |  |  | 4 | 0 | 0 | 1 | 1 | 1 | 1 | 0 | 0 | 0 | 0 | 0 | 0 | 0 | 0 | 266 | 284 | Carbamidomethylation | C5:Carbamidomethylation:1000.00;C12:Carbamidomethylation:1000.00 | PEAKS DB |
| K.FEVKPAASVTLK.S | N | 62.46 | 1288.7390 | 12 | -0.4 | 430.5868 | 3 | 26.91 | 4 | F4:12431 | NaSa01\_F12.raw |  |  |  | 1.5916E6 | 3.0771E7 | 9.3704E5 |  |  |  |  |  |  |  |  | 4 | 0 | 0 | 0 | 1 | 2 | 1 | 0 | 0 | 0 | 0 | 0 | 0 | 0 | 0 | 73 | 84 |  |  | PEAKS DB |
| R.DYQEYLLR.D | Y | 60.47 | 1098.5345 | 8 | 0.0 | 550.2745 | 2 | 52.07 | 6 | F6:31070 | NaSa01\_F14.raw |  |  | 7.7518E5 | 3.7751E7 | 8.7826E8 | 1.6689E8 |  |  |  |  |  |  |  |  | 7 | 0 | 0 | 1 | 1 | 2 | 3 | 0 | 0 | 0 | 0 | 0 | 0 | 0 | 0 | 191 | 198 |  |  | PEAKS DB |
| K.GQC(+57.02)VDVQTAY | N | 59.29 | 1139.4917 | 10 | 0.4 | 570.7534 | 2 | 32.72 | 4 | F4:17040 | NaSa01\_F12.raw |  |  |  | 1.3163E7 | 3.9751E7 |  |  |  |  |  |  | 4.4194E6 | 8.2572E6 |  | 4 | 0 | 0 | 0 | 1 | 1 | 0 | 0 | 0 | 0 | 0 | 0 | 1 | 1 | 0 | 392 | 401 | Carbamidomethylation | C3:Carbamidomethylation:1000.00 | PEAKS DB |
| K.RTAPAFQFSSC(+57.02)SIR.D | N | 58.20 | 1626.7936 | 14 | 1.0 | 543.2723 | 3 | 33.58 | 5 | F5:16454 | NaSa01\_F13.raw |  |  |  |  | 3.0352E6 |  |  |  |  |  |  |  |  |  | 1 | 0 | 0 | 0 | 0 | 1 | 0 | 0 | 0 | 0 | 0 | 0 | 0 | 0 | 0 | 177 | 190 | Carbamidomethylation | C11:Carbamidomethylation:1000.00 | PEAKS DB |
| E.DKFEVKPAASVTLK.S | N | 56.83 | 1531.8610 | 14 | -0.6 | 511.6273 | 3 | 23.42 | 5 | F5:8219 | NaSa01\_F13.raw |  |  |  | 1.0862E6 | 2.5201E7 |  |  |  |  |  |  |  |  |  | 5 | 0 | 0 | 0 | 2 | 3 | 0 | 0 | 0 | 0 | 0 | 0 | 0 | 0 | 0 | 71 | 84 |  |  | PEAKS DB |
| R.VYEM(+15.99)INAVNTK.F | N | 56.71 | 1296.6384 | 11 | 0.5 | 649.3268 | 2 | 30.38 | 5 | F5:16103 | NaSa01\_F13.raw |  |  |  |  | 1.2957E8 | 2.8787E5 |  |  |  |  |  |  |  |  | 7 | 0 | 0 | 0 | 0 | 6 | 1 | 0 | 0 | 0 | 0 | 0 | 0 | 0 | 0 | 41 | 51 | Oxidation (M) | M4:Oxidation (M):1000.00 | PEAKS DB |
| C.IALLGPHFTVSPK.G | Y | 56.41 | 1378.7972 | 13 | -0.1 | 460.6063 | 3 | 45.67 | 12 | F12:28040 | NaSa01\_F7.raw |  |  |  |  | 2.2751E7 | 5.7377E6 |  |  |  |  |  | 3.919E6 |  |  | 5 | 0 | 0 | 0 | 0 | 2 | 1 | 0 | 0 | 0 | 0 | 0 | 2 | 0 | 0 | 309 | 321 |  |  | PEAKS DB |
| Q.C(+57.02)IALLGPHFTVSPK.G | Y | 55.60 | 1538.8279 | 14 | 0.2 | 770.4214 | 2 | 51.19 | 6 | F6:30442 | NaSa01\_F14.raw |  |  |  |  | 9.4215E6 | 3.3026E6 |  |  |  |  |  | 2.1708E6 |  |  | 6 | 0 | 0 | 0 | 0 | 2 | 2 | 0 | 0 | 0 | 0 | 0 | 2 | 0 | 0 | 308 | 321 | Carbamidomethylation | C1:Carbamidomethylation:1000.00 | PEAKS DB |
| K.GC(+57.02)FDLNMR.G | N | 55.38 | 1011.4266 | 8 | -0.6 | 506.7203 | 2 | 36.41 | 6 | F6:17906 | NaSa01\_F14.raw |  |  |  | 2.4727E7 | 3.4283E8 | 2.0246E7 |  |  |  |  |  | 5.5964E7 | 1.5414E7 |  | 5 | 0 | 0 | 0 | 1 | 1 | 1 | 0 | 0 | 0 | 0 | 0 | 1 | 1 | 0 | 322 | 329 | Carbamidomethylation | C2:Carbamidomethylation:1000.00 | PEAKS DB |
| K.C(+57.02)PTLTNQC(+57.02)IALLGPHF.T | Y | 50.31 | 1840.8964 | 16 | 1.4 | 921.4568 | 2 | 105.67 | 5 | F5:74085 | NaSa01\_F13.raw |  |  |  |  | 2.5834E5 |  |  |  |  |  |  |  |  |  | 1 | 0 | 0 | 0 | 0 | 1 | 0 | 0 | 0 | 0 | 0 | 0 | 0 | 0 | 0 | 301 | 316 | Carbamidomethylation | C1:Carbamidomethylation:1000.00;C8:Carbamidomethylation:1000.00 | PEAKS DB |
| K.RTAPAFQFSSC(+57.02).S | N | 49.15 | 1270.5764 | 11 | 0.1 | 636.2955 | 2 | 35.53 | 5 | F5:18275 | NaSa01\_F13.raw |  |  |  |  | 1.061E7 |  |  |  |  |  |  |  |  |  | 1 | 0 | 0 | 0 | 0 | 1 | 0 | 0 | 0 | 0 | 0 | 0 | 0 | 0 | 0 | 177 | 187 | Carbamidomethylation | C11:Carbamidomethylation:1000.00 | PEAKS DB |
| K.NTM(+15.99)SC(+57.02)LIPPNPDGIMAEPGTK.C | N | 48.35 | 2258.0381 | 21 | 0.2 | 1130.0265 | 2 | 57.82 | 5 | F5:37637 | NaSa01\_F13.raw |  |  |  |  | 1.036E8 |  |  |  |  |  |  |  |  |  | 3 | 0 | 0 | 0 | 0 | 3 | 0 | 0 | 0 | 0 | 0 | 0 | 0 | 0 | 0 | 362 | 382 | Oxidation (M); Carbamidomethylation | M3:Oxidation (M):96.93;C5:Carbamidomethylation:1000.00 | PEAKS DB |
| N.QC(+57.02)IALLGPHFTVSPK.G | Y | 47.53 | 1666.8865 | 15 | 1.1 | 556.6367 | 3 | 48.86 | 12 | F12:30858 | NaSa01\_F7.raw |  |  |  |  | 5.9055E6 |  |  |  |  |  |  | 9.1446E5 |  |  | 3 | 0 | 0 | 0 | 0 | 2 | 0 | 0 | 0 | 0 | 0 | 0 | 1 | 0 | 0 | 307 | 321 | Carbamidomethylation | C2:Carbamidomethylation:1000.00 | PEAKS DB |
| H.DC(+57.02)DLPELC(+57.02)TGQSAEC(+57.02)PTDSLQR.N | N | 46.78 | 2551.0625 | 22 | 0.8 | 851.3621 | 3 | 56.81 | 9 | F9:28977 | NaSa01\_F4.raw |  |  |  |  |  |  |  |  | 9.1393E5 |  |  |  |  |  | 1 | 0 | 0 | 0 | 0 | 0 | 0 | 0 | 0 | 1 | 0 | 0 | 0 | 0 | 0 | 263 | 284 | Carbamidomethylation | C2:Carbamidomethylation:1000.00;C8:Carbamidomethylation:1000.00;C15:Carbamidomethylation:1000.00 | PEAKS DB |
| K.NTMSC(+57.02)LIPPNPDGIM(+15.99)AEPGTK.C | N | 46.28 | 2258.0381 | 21 | 0.2 | 1130.0265 | 2 | 57.82 | 5 | F5:36541 | NaSa01\_F13.raw |  |  |  |  | 7.128E7 |  |  |  |  |  |  |  |  |  | 2 | 0 | 0 | 0 | 0 | 2 | 0 | 0 | 0 | 0 | 0 | 0 | 0 | 0 | 0 | 362 | 382 | Carbamidomethylation; Oxidation (M) | C5:Carbamidomethylation:1000.00;M15:Oxidation (M):63.99 | PEAKS DB |
| P.TLTNQC(+57.02)IALLGPHFTVSPK.G | Y | 45.47 | 2096.1089 | 19 | 0.3 | 699.7104 | 3 | 59.27 | 5 | F5:37833 | NaSa01\_F13.raw |  |  |  |  | 4.3778E6 |  |  |  |  |  |  |  |  |  | 1 | 0 | 0 | 0 | 0 | 1 | 0 | 0 | 0 | 0 | 0 | 0 | 0 | 0 | 0 | 303 | 321 | Carbamidomethylation | C6:Carbamidomethylation:1000.00 | PEAKS DB |
| R.DRPQC(+57.02)ILNKPL.S | N | 44.62 | 1352.7234 | 11 | 1.1 | 451.9156 | 3 | 27.50 | 5 | F5:11578 | NaSa01\_F13.raw |  |  |  |  | 1.9485E7 |  |  |  |  |  |  |  |  |  | 1 | 0 | 0 | 0 | 0 | 1 | 0 | 0 | 0 | 0 | 0 | 0 | 0 | 0 | 0 | 199 | 209 | Carbamidomethylation | C5:Carbamidomethylation:1000.00 | PEAKS DB |
| TNTPEQDR.Y | N | 44.60 | 959.4308 | 8 | 0.1 | 480.7227 | 2 | 11.42 | 6 | F6:1814 | NaSa01\_F14.raw |  |  |  |  |  | 1.6427E5 |  |  |  |  |  |  |  |  | 1 | 0 | 0 | 0 | 0 | 0 | 1 | 0 | 0 | 0 | 0 | 0 | 0 | 0 | 0 | 1 | 8 |  |  | PEAKS DB |
| R.M(+15.99)SLVASTMTHELGH.N | Y | 44.40 | 1528.7014 | 14 | 0.6 | 510.5747 | 3 | 34.32 | 5 | F5:17163 | NaSa01\_F13.raw |  |  |  |  | 3.6152E6 |  |  |  |  |  |  |  |  |  | 2 | 0 | 0 | 0 | 0 | 2 | 0 | 0 | 0 | 0 | 0 | 0 | 0 | 0 | 0 | 140 | 153 | Oxidation (M) | M1:Oxidation (M):83.89 | PEAKS DB |
| R.YLQAEKYIEFY.V | N | 43.34 | 1465.7129 | 11 | 0.9 | 733.8644 | 2 | 68.34 | 6 | F6:44984 | NaSa01\_F14.raw |  |  |  |  |  | 1.4083E6 |  |  |  |  |  |  |  |  | 1 | 0 | 0 | 0 | 0 | 0 | 1 | 0 | 0 | 0 | 0 | 0 | 0 | 0 | 0 | 9 | 19 |  |  | PEAKS DB |
| H.NLGIHHDEASC(+57.02)IC(+57.02)IPGPC(+57.02)IMLK.K | Y | 43.30 | 2534.1902 | 22 | -0.2 | 634.5547 | 4 | 58.71 | 5 | F5:37330 | NaSa01\_F13.raw |  |  |  |  | 4.8646E6 |  |  |  |  |  |  |  |  |  | 1 | 0 | 0 | 0 | 0 | 1 | 0 | 0 | 0 | 0 | 0 | 0 | 0 | 0 | 0 | 154 | 175 | Carbamidomethylation | C11:Carbamidomethylation:1000.00;C13:Carbamidomethylation:1000.00;C18:Carbamidomethylation:1000.00 | PEAKS DB |
| C.LIPPNPDGIMAEPGTK.C | N | 43.18 | 1648.8494 | 16 | -0.1 | 825.4319 | 2 | 51.19 | 6 | F6:30226 | NaSa01\_F14.raw |  |  |  |  | 1.7141E7 | 3.7268E6 |  |  |  |  |  |  |  |  | 3 | 0 | 0 | 0 | 0 | 2 | 1 | 0 | 0 | 0 | 0 | 0 | 0 | 0 | 0 | 367 | 382 |  |  | PEAKS DB |
| R.MSLVASTMTHELGHN.L | Y | 43.11 | 1626.7494 | 15 | 0.9 | 543.2576 | 3 | 41.85 | 5 | F5:23327 | NaSa01\_F13.raw |  |  |  |  | 2.3934E6 |  |  |  |  |  |  |  |  |  | 1 | 0 | 0 | 0 | 0 | 1 | 0 | 0 | 0 | 0 | 0 | 0 | 0 | 0 | 0 | 140 | 154 |  |  | PEAKS DB |
| R.TAPAFQFSSC(+57.02).S | N | 41.82 | 1114.4753 | 10 | -0.1 | 558.2449 | 2 | 56.38 | 6 | F6:34716 | NaSa01\_F14.raw |  |  |  |  | 1.868E8 | 4.1539E7 |  |  |  |  |  |  |  |  | 2 | 0 | 0 | 0 | 0 | 1 | 1 | 0 | 0 | 0 | 0 | 0 | 0 | 0 | 0 | 178 | 187 | Carbamidomethylation | C10:Carbamidomethylation:1000.00 | PEAKS DB |
| R.QTVLLPR.K | Y | 41.51 | 825.5072 | 7 | 1.0 | 413.7613 | 2 | 22.03 | 6 | F6:6052 | NaSa01\_F14.raw |  |  |  |  |  | 1.0008E6 |  |  |  |  |  |  |  |  | 2 | 0 | 0 | 0 | 0 | 0 | 2 | 0 | 0 | 0 | 0 | 0 | 0 | 0 | 0 | 91 | 97 |  |  | PEAKS DB |
| R.VYEMINAVNTKFR.P | N | 40.30 | 1583.8130 | 13 | 0.5 | 528.9452 | 3 | 43.49 | 5 | F5:24661 | NaSa01\_F13.raw |  |  |  |  | 1.7264E6 |  |  |  |  |  |  |  |  |  | 1 | 0 | 0 | 0 | 0 | 1 | 0 | 0 | 0 | 0 | 0 | 0 | 0 | 0 | 0 | 41 | 53 |  |  | PEAKS DB |
| R.MSLVASTM(+15.99)THELGH.N | Y | 40.22 | 1528.7014 | 14 | 0.3 | 765.3582 | 2 | 25.73 | 5 | F5:10059 | NaSa01\_F13.raw |  |  |  |  | 3.6062E6 |  |  |  |  |  |  |  |  |  | 2 | 0 | 0 | 0 | 0 | 2 | 0 | 0 | 0 | 0 | 0 | 0 | 0 | 0 | 0 | 140 | 153 | Oxidation (M) | M8:Oxidation (M):75.93 | PEAKS DB |
| T.LTNQC(+57.02)IALLGPHFTVSPK.G | Y | 39.95 | 1995.0612 | 18 | 0.4 | 666.0280 | 3 | 56.27 | 5 | F5:35452 | NaSa01\_F13.raw |  |  |  |  | 1.5117E6 |  |  |  |  |  |  |  |  |  | 1 | 0 | 0 | 0 | 0 | 1 | 0 | 0 | 0 | 0 | 0 | 0 | 0 | 0 | 0 | 304 | 321 | Carbamidomethylation | C5:Carbamidomethylation:1000.00 | PEAKS DB |
| R.MSLVASTMTH.E | Y | 38.23 | 1076.4995 | 10 | -0.5 | 539.2568 | 2 | 33.08 | 5 | F5:16037 | NaSa01\_F13.raw |  |  |  |  | 2.1E6 |  |  |  |  |  |  |  |  |  | 1 | 0 | 0 | 0 | 0 | 1 | 0 | 0 | 0 | 0 | 0 | 0 | 0 | 0 | 0 | 140 | 149 |  |  | PEAKS DB |
| R.YYNYDKPAIK.I | Y | 37.95 | 1273.6343 | 10 | 0.5 | 425.5522 | 3 | 11.64 | 5 | F5:2139 | NaSa01\_F13.raw |  |  |  |  | 5.9822E5 |  |  |  |  |  |  |  |  |  | 1 | 0 | 0 | 0 | 0 | 1 | 0 | 0 | 0 | 0 | 0 | 0 | 0 | 0 | 0 | 29 | 38 |  |  | PEAKS DB |
| total 40 peptides |  |  |  |  |  |  |  |  |  |  |  |  |  |  |  |  |  |  |  |  |  |  |  |  |  |  |  |  |  |  |  |  |  |  |  |  |  |  |  |  |  |  |  |  |  |
| --- | --- | --- | --- | --- | --- | --- | --- | --- | --- | --- | --- | --- | --- | --- | --- | --- | --- | --- | --- | --- | --- | --- | --- | --- | --- | --- | --- | --- | --- | --- | --- | --- | --- | --- | --- | --- | --- | --- | --- | --- | --- | --- | --- | --- | --- |

JAG67188.1

back to list

  

| Protein Coverage
| Supporting Peptides
|

Protein Coverage:

Supporting Peptides:

| Peptide | Uniq | -10lgP | Mass | Length | ppm | m/z | z | RT | Fraction | Scan | Source File | Area F1 | Area F10 | Area F11 | Area F12 | Area F13 | Area F14 | Area F2 | Area F3 | Area F4 | Area F5 | Area F6 | Area F7 | Area F8 | Area F9 | #Feature | #Feature F1 | #Feature F10 | #Feature F11 | #Feature F12 | #Feature F13 | #Feature F14 | #Feature F2 | #Feature F3 | #Feature F4 | #Feature F5 | #Feature F6 | #Feature F7 | #Feature F8 | #Feature F9 | Start | End | PTM | AScore | Found By |
| --- | --- | --- | --- | --- | --- | --- | --- | --- | --- | --- | --- | --- | --- | --- | --- | --- | --- | --- | --- | --- | --- | --- | --- | --- | --- | --- | --- | --- | --- | --- | --- | --- | --- | --- | --- | --- | --- | --- | --- | --- | --- | --- | --- | --- | --- |
| R.HPDDNEWNHVSMC(+57.02)IVNGGGIR.S | N | 99.67 | 2406.0593 | 21 | -0.4 | 803.0267 | 3 | 49.93 | 6 | F6:29280 | NaSa01\_F14.raw |  |  |  |  | 6.5991E7 | 1.6043E8 |  |  |  |  |  |  |  | 7.6096E6 | 9 | 0 | 0 | 0 | 0 | 3 | 4 | 0 | 0 | 0 | 0 | 0 | 0 | 0 | 2 | 389 | 409 | Carbamidomethylation | C13:Carbamidomethylation:1000.00 | PEAKS DB |
| R.HGQGTGELLQVSGIK.V | N | 97.61 | 1522.8103 | 15 | 0.4 | 762.4127 | 2 | 31.05 | 6 | F6:13489 | NaSa01\_F14.raw |  |  |  | 4.9661E5 | 6.4612E8 | 1.2796E9 |  |  |  |  |  |  |  |  | 8 | 0 | 0 | 0 | 1 | 3 | 4 | 0 | 0 | 0 | 0 | 0 | 0 | 0 | 0 | 457 | 471 |  |  | PEAKS DB |
| R.HPDDNEWNHVSM(+15.99)C(+57.02)IVNGGGIR.S | N | 86.62 | 2422.0542 | 21 | -0.3 | 808.3584 | 3 | 44.12 | 6 | F6:24148 | NaSa01\_F14.raw |  |  |  |  | 3.2766E7 | 9.9954E7 |  |  |  |  |  |  |  | 8.8723E6 | 8 | 0 | 0 | 0 | 0 | 2 | 4 | 0 | 0 | 0 | 0 | 0 | 0 | 0 | 2 | 389 | 409 | Oxidation (M); Carbamidomethylation | M12:Oxidation (M):1000.00;C13:Carbamidomethylation:1000.00 | PEAKS DB |
| R.FHEC(+57.02)NLGNLIC(+57.02)DAVVYNNLR.H | N | 82.50 | 2420.1365 | 20 | -0.2 | 807.7192 | 3 | 77.73 | 6 | F6:52912 | NaSa01\_F14.raw |  |  |  |  | 2.6877E7 | 9.4785E7 |  |  |  |  |  |  | 7.773E6 | 1.2217E8 | 9 | 0 | 0 | 0 | 0 | 1 | 3 | 0 | 0 | 0 | 0 | 0 | 0 | 2 | 3 | 369 | 388 | Carbamidomethylation | C4:Carbamidomethylation:1000.00;C11:Carbamidomethylation:1000.00 | PEAKS DB |
| K.ETPVLSNPGPYLEFR.D | N | 81.49 | 1717.8674 | 15 | 0.8 | 859.9417 | 2 | 69.77 | 6 | F6:46166 | NaSa01\_F14.raw |  |  |  | 7.2199E5 | 2.4678E8 | 7.6435E8 |  |  |  | 2.2582E6 |  |  |  |  | 7 | 0 | 0 | 0 | 1 | 3 | 2 | 0 | 0 | 0 | 1 | 0 | 0 | 0 | 0 | 194 | 208 |  |  | PEAKS DB |
| R.QVPVVQAYAFGK.Y | N | 80.72 | 1305.7081 | 12 | -0.2 | 653.8612 | 2 | 81.01 | 6 | F6:55346 | NaSa01\_F14.raw |  |  |  | 4.7562E5 | 3.326E8 | 6.8066E8 |  |  |  |  |  |  |  |  | 5 | 0 | 0 | 0 | 1 | 1 | 3 | 0 | 0 | 0 | 0 | 0 | 0 | 0 | 0 | 288 | 299 |  |  | PEAKS DB |
| R.VPTYVPLEMEK.S | N | 79.12 | 1304.6686 | 11 | 0.1 | 653.3416 | 2 | 49.94 | 6 | F6:29351 | NaSa01\_F14.raw |  |  |  | 2.0159E6 | 5.6178E8 | 1.2743E9 |  |  |  |  |  |  |  |  | 9 | 0 | 0 | 0 | 1 | 1 | 7 | 0 | 0 | 0 | 0 | 0 | 0 | 0 | 0 | 496 | 506 |  |  | PEAKS DB |
| R.YDAMALGNHEFDNGLDGLLDPLLK.Y | N | 78.94 | 2630.2686 | 24 | 0.8 | 877.7642 | 3 | 91.53 | 6 | F6:64244 | NaSa01\_F14.raw |  |  |  |  | 4.8732E7 | 1.8229E8 |  |  |  |  |  |  |  |  | 8 | 0 | 0 | 0 | 0 | 2 | 6 | 0 | 0 | 0 | 0 | 0 | 0 | 0 | 0 | 124 | 147 |  |  | PEAKS DB |
| E.LTILHTNDVHAR.V | N | 75.00 | 1388.7524 | 12 | 0.9 | 463.9252 | 3 | 11.51 | 6 | F6:1905 | NaSa01\_F14.raw |  |  |  |  | 1.6389E6 | 3.6507E6 |  |  |  |  |  |  |  |  | 4 | 0 | 0 | 0 | 0 | 2 | 2 | 0 | 0 | 0 | 0 | 0 | 0 | 0 | 0 | 44 | 55 |  |  | PEAKS DB |
| K.ETPVLSNPGPYLEFRDEVEELQK.H | N | 74.67 | 2688.3281 | 23 | 0.7 | 897.1173 | 3 | 77.38 | 6 | F6:52291 | NaSa01\_F14.raw |  |  |  |  | 3.5808E8 | 6.9841E8 |  |  |  |  |  |  |  |  | 4 | 0 | 0 | 0 | 0 | 2 | 2 | 0 | 0 | 0 | 0 | 0 | 0 | 0 | 0 | 194 | 216 |  |  | PEAKS DB |
| R.YDAM(+15.99)ALGNHEFDNGLDGLLDPLLK.Y | N | 74.52 | 2646.2634 | 24 | 1.6 | 883.0965 | 3 | 92.75 | 6 | F6:65301 | NaSa01\_F14.raw |  |  |  |  | 1.8093E7 | 4.6859E7 |  |  |  |  |  |  |  |  | 5 | 0 | 0 | 0 | 0 | 2 | 3 | 0 | 0 | 0 | 0 | 0 | 0 | 0 | 0 | 124 | 147 | Oxidation (M) | M4:Oxidation (M):1000.00 | PEAKS DB |
| R.VPTYVPLEM(+15.99)EK.S | N | 64.86 | 1320.6635 | 11 | 0.4 | 661.3392 | 2 | 41.94 | 6 | F6:25976 | NaSa01\_F14.raw |  |  |  |  | 7.6238E7 | 9.8061E7 |  |  |  |  |  |  |  |  | 15 | 0 | 0 | 0 | 0 | 7 | 8 | 0 | 0 | 0 | 0 | 0 | 0 | 0 | 0 | 496 | 506 | Oxidation (M) | M9:Oxidation (M):1000.00 | PEAKS DB |
| R.EVVQFMNSLR.Y | N | 63.80 | 1221.6176 | 10 | 1.2 | 611.8168 | 2 | 53.88 | 6 | F6:32969 | NaSa01\_F14.raw |  |  |  |  | 1.6483E7 | 2.9301E7 |  |  |  |  |  |  |  |  | 3 | 0 | 0 | 0 | 0 | 1 | 2 | 0 | 0 | 0 | 0 | 0 | 0 | 0 | 0 | 114 | 123 |  |  | PEAKS DB |
| M.ALGNHEFDNGLDGLLDPLLK.Y | N | 59.79 | 2150.1008 | 20 | 0.1 | 717.7076 | 3 | 88.24 | 6 | F6:61193 | NaSa01\_F14.raw |  |  |  |  | 5.9279E6 | 1.5211E7 |  |  |  |  |  |  |  |  | 4 | 0 | 0 | 0 | 0 | 2 | 2 | 0 | 0 | 0 | 0 | 0 | 0 | 0 | 0 | 128 | 147 |  |  | PEAKS DB |
| N.ISGYILPYK.I | N | 56.17 | 1052.5906 | 9 | 0.7 | 527.3029 | 2 | 45.82 | 6 | F6:26096 | NaSa01\_F14.raw |  |  |  | 2.2592E5 | 4.6763E8 | 8.5839E8 |  |  |  |  |  |  |  |  | 5 | 0 | 0 | 0 | 1 | 1 | 3 | 0 | 0 | 0 | 0 | 0 | 0 | 0 | 0 | 168 | 176 |  |  | PEAKS DB |
| H.GQGTGELLQVSGIK.V | N | 55.06 | 1385.7515 | 14 | 0.3 | 693.8832 | 2 | 45.20 | 6 | F6:25108 | NaSa01\_F14.raw |  |  |  |  |  | 1.2594E6 |  |  |  |  |  |  |  |  | 1 | 0 | 0 | 0 | 0 | 0 | 1 | 0 | 0 | 0 | 0 | 0 | 0 | 0 | 0 | 458 | 471 |  |  | PEAKS DB |
| R.QVPVVQAYAF.G | N | 53.91 | 1120.5917 | 10 | 0.0 | 561.3031 | 2 | 73.70 | 6 | F6:49221 | NaSa01\_F14.raw |  |  |  |  | 7.1015E6 | 1.9997E7 |  |  |  |  |  |  |  |  | 2 | 0 | 0 | 0 | 0 | 1 | 1 | 0 | 0 | 0 | 0 | 0 | 0 | 0 | 0 | 288 | 297 |  |  | PEAKS DB |
| K.LTTLGVNK.I | N | 52.68 | 844.5018 | 8 | -1.0 | 423.2578 | 2 | 11.58 | 6 | F6:2148 | NaSa01\_F14.raw |  |  |  |  |  | 3.1326E6 |  |  |  |  |  |  |  |  | 1 | 0 | 0 | 0 | 0 | 0 | 1 | 0 | 0 | 0 | 0 | 0 | 0 | 0 | 0 | 221 | 228 |  |  | PEAKS DB |
| K.VGIIGYTTK.E | N | 50.04 | 950.5436 | 9 | 0.3 | 476.2792 | 2 | 28.48 | 6 | F6:11194 | NaSa01\_F14.raw |  |  |  |  |  | 5.1319E6 |  |  |  |  |  |  |  |  | 2 | 0 | 0 | 0 | 0 | 0 | 2 | 0 | 0 | 0 | 0 | 0 | 0 | 0 | 0 | 185 | 193 |  |  | PEAKS DB |
| R.FHEC(+57.02)NLGNLIC(+57.02)DAVVY.N | N | 48.35 | 1922.8655 | 16 | 1.4 | 962.4414 | 2 | 82.45 | 5 | F5:56634 | NaSa01\_F13.raw |  |  |  |  | 8.0322E7 | 1.7562E8 |  |  |  |  |  |  |  | 2.4692E7 | 3 | 0 | 0 | 0 | 0 | 1 | 1 | 0 | 0 | 0 | 0 | 0 | 0 | 0 | 1 | 369 | 384 | Carbamidomethylation | C4:Carbamidomethylation:1000.00;C11:Carbamidomethylation:1000.00 | PEAKS DB |
| R.HPDDNEWNHVSMC(+57.02).I | N | 48.19 | 1639.6144 | 13 | 0.5 | 820.8149 | 2 | 26.12 | 5 | F5:10348 | NaSa01\_F13.raw |  |  |  |  | 8.8799E6 |  |  |  |  |  |  |  |  |  | 2 | 0 | 0 | 0 | 0 | 2 | 0 | 0 | 0 | 0 | 0 | 0 | 0 | 0 | 0 | 389 | 401 | Carbamidomethylation | C13:Carbamidomethylation:1000.00 | PEAKS DB |
| R.HPDDNEWNHVSM.C | N | 47.22 | 1479.5837 | 12 | 1.1 | 740.8000 | 2 | 25.93 | 5 | F5:10070 | NaSa01\_F13.raw |  |  |  |  | 2.3098E6 |  |  |  |  |  |  |  |  |  | 1 | 0 | 0 | 0 | 0 | 1 | 0 | 0 | 0 | 0 | 0 | 0 | 0 | 0 | 0 | 389 | 400 |  |  | PEAKS DB |
| R.QVPVVQAY.A | N | 45.65 | 902.4861 | 8 | -0.5 | 452.2501 | 2 | 43.38 | 5 | F5:24541 | NaSa01\_F13.raw |  |  |  |  | 1.2052E7 |  |  |  |  |  |  |  |  |  | 1 | 0 | 0 | 0 | 0 | 1 | 0 | 0 | 0 | 0 | 0 | 0 | 0 | 0 | 0 | 288 | 295 |  |  | PEAKS DB |
| N.PGPYLEFR.D | N | 45.44 | 977.4970 | 8 | 0.7 | 489.7561 | 2 | 39.90 | 6 | F6:20609 | NaSa01\_F14.raw |  |  |  |  |  | 4.6995E5 |  |  |  |  |  |  |  |  | 1 | 0 | 0 | 0 | 0 | 0 | 1 | 0 | 0 | 0 | 0 | 0 | 0 | 0 | 0 | 201 | 208 |  |  | PEAKS DB |
| N.LGNLIC(+57.02)DAVVYNNLR.H | N | 45.20 | 1732.8931 | 15 | 1.0 | 867.4547 | 2 | 69.10 | 6 | F6:45743 | NaSa01\_F14.raw |  |  |  |  |  | 1.1316E6 |  |  |  |  |  |  |  |  | 1 | 0 | 0 | 0 | 0 | 0 | 1 | 0 | 0 | 0 | 0 | 0 | 0 | 0 | 0 | 374 | 388 | Carbamidomethylation | C6:Carbamidomethylation:1000.00 | PEAKS DB |
| K.VLLPSFLATGGDGYDMLK.G | Y | 44.51 | 1895.9702 | 18 | 9.1 | 949.0010 | 2 | 94.44 | 6 | F6:66763 | NaSa01\_F14.raw |  |  |  |  | 4.3E5 | 4.1793E6 |  |  |  |  |  |  |  |  | 2 | 0 | 0 | 0 | 0 | 1 | 1 | 0 | 0 | 0 | 0 | 0 | 0 | 0 | 0 | 510 | 527 |  |  | PEAKS DB |
| Q.GTGELLQVSGIK.V | N | 42.14 | 1200.6714 | 12 | 0.9 | 601.3435 | 2 | 44.43 | 6 | F6:24568 | NaSa01\_F14.raw |  |  |  |  |  | 7.0353E5 |  |  |  |  |  |  |  |  | 1 | 0 | 0 | 0 | 0 | 0 | 1 | 0 | 0 | 0 | 0 | 0 | 0 | 0 | 0 | 460 | 471 |  |  | PEAKS DB |
| K.LTTLGVNKIIALGH.S | N | 41.05 | 1448.8715 | 14 | 0.1 | 483.9645 | 3 | 54.77 | 6 | F6:33352 | NaSa01\_F14.raw |  |  |  |  |  | 5.5856E5 |  |  |  |  |  |  |  |  | 1 | 0 | 0 | 0 | 0 | 0 | 1 | 0 | 0 | 0 | 0 | 0 | 0 | 0 | 0 | 221 | 234 |  |  | PEAKS DB |
| R.EVVQFM(+15.99)NSLR.Y | N | 38.12 | 1237.6125 | 10 | 0.1 | 619.8136 | 2 | 33.80 | 5 | F5:16544 | NaSa01\_F13.raw |  |  |  |  | 2.1424E6 |  |  |  |  |  |  |  |  |  | 1 | 0 | 0 | 0 | 0 | 1 | 0 | 0 | 0 | 0 | 0 | 0 | 0 | 0 | 0 | 114 | 123 | Oxidation (M) | M6:Oxidation (M):1000.00 | PEAKS DB |
| K.IIALGHSGF.K | N | 37.88 | 913.5021 | 9 | -1.2 | 457.7578 | 2 | 43.37 | 6 | F6:23538 | NaSa01\_F14.raw |  |  |  |  |  | 5.7946E6 |  |  |  |  |  |  |  |  | 1 | 0 | 0 | 0 | 0 | 0 | 1 | 0 | 0 | 0 | 0 | 0 | 0 | 0 | 0 | 229 | 237 |  |  | PEAKS DB |
| total 30 peptides |  |  |  |  |  |  |  |  |  |  |  |  |  |  |  |  |  |  |  |  |  |  |  |  |  |  |  |  |  |  |  |  |  |  |  |  |  |  |  |  |  |  |  |  |  |
| --- | --- | --- | --- | --- | --- | --- | --- | --- | --- | --- | --- | --- | --- | --- | --- | --- | --- | --- | --- | --- | --- | --- | --- | --- | --- | --- | --- | --- | --- | --- | --- | --- | --- | --- | --- | --- | --- | --- | --- | --- | --- | --- | --- | --- | --- |

AXL95287.1

back to list

  

| Protein Coverage
| Supporting Peptides
|

Protein Coverage:

Supporting Peptides:

| Peptide | Uniq | -10lgP | Mass | Length | ppm | m/z | z | RT | Fraction | Scan | Source File | Area F1 | Area F10 | Area F11 | Area F12 | Area F13 | Area F14 | Area F2 | Area F3 | Area F4 | Area F5 | Area F6 | Area F7 | Area F8 | Area F9 | #Feature | #Feature F1 | #Feature F10 | #Feature F11 | #Feature F12 | #Feature F13 | #Feature F14 | #Feature F2 | #Feature F3 | #Feature F4 | #Feature F5 | #Feature F6 | #Feature F7 | #Feature F8 | #Feature F9 | Start | End | PTM | AScore | Found By |
| --- | --- | --- | --- | --- | --- | --- | --- | --- | --- | --- | --- | --- | --- | --- | --- | --- | --- | --- | --- | --- | --- | --- | --- | --- | --- | --- | --- | --- | --- | --- | --- | --- | --- | --- | --- | --- | --- | --- | --- | --- | --- | --- | --- | --- | --- |
| K.HVVVVGAGMAGLSAAYVLAGAGHK.V | N | 107.42 | 2234.1992 | 24 | 1.2 | 745.7412 | 3 | 65.01 | 6 | F6:42957 | NaSa01\_F14.raw |  |  |  | 6.8911E6 | 4.2938E7 | 6.5431E8 |  |  |  |  |  |  |  |  | 25 | 0 | 0 | 0 | 2 | 2 | 21 | 0 | 0 | 0 | 0 | 0 | 0 | 0 | 0 | 53 | 76 |  |  | PEAKS DB |
| K.HVVVVGAGM(+15.99)AGLSAAYVLAGAGHK.V | N | 95.06 | 2250.1943 | 24 | 0.9 | 751.0728 | 3 | 53.09 | 6 | F6:32085 | NaSa01\_F14.raw |  |  |  |  | 1.6389E7 | 1.5623E8 |  |  |  |  |  |  |  |  | 5 | 0 | 0 | 0 | 0 | 2 | 3 | 0 | 0 | 0 | 0 | 0 | 0 | 0 | 0 | 53 | 76 | Oxidation (M) | M9:Oxidation (M):1000.00 | PEAKS DB |
| M.AGLSAAYVLAGAGHK.V | N | 79.84 | 1384.7462 | 15 | 0.5 | 693.3807 | 2 | 36.23 | 6 | F6:17690 | NaSa01\_F14.raw |  |  |  |  |  | 4.4211E7 |  |  |  |  |  |  |  |  | 2 | 0 | 0 | 0 | 0 | 0 | 2 | 0 | 0 | 0 | 0 | 0 | 0 | 0 | 0 | 62 | 76 |  |  | PEAKS DB |
| A.GMAGLSAAYVLAGAGHK.V | N | 77.31 | 1572.8082 | 17 | 0.2 | 525.2768 | 3 | 50.77 | 6 | F6:29981 | NaSa01\_F14.raw |  |  |  |  | 5.5361E5 | 1.0582E7 |  |  |  |  |  |  |  |  | 3 | 0 | 0 | 0 | 0 | 1 | 2 | 0 | 0 | 0 | 0 | 0 | 0 | 0 | 0 | 60 | 76 |  |  | PEAKS DB |
| M.YQAIAEMVHLNAR.V | N | 70.47 | 1514.7664 | 13 | 0.6 | 505.9297 | 3 | 42.75 | 6 | F6:23121 | NaSa01\_F14.raw |  |  |  |  |  | 6.4909E6 |  |  |  |  |  |  |  |  | 2 | 0 | 0 | 0 | 0 | 0 | 2 | 0 | 0 | 0 | 0 | 0 | 0 | 0 | 0 | 266 | 278 |  |  | PEAKS DB |
| R.SPLEKC(+57.02)FQEDDYEEFLEIAR.N | Y | 68.07 | 2517.1370 | 20 | -7.0 | 1259.5669 | 2 | 88.94 | 6 | F6:62130 | NaSa01\_F14.raw |  |  |  |  |  | 1.5989E8 |  |  |  |  |  |  |  |  | 1 | 0 | 0 | 0 | 0 | 0 | 1 | 0 | 0 | 0 | 0 | 0 | 0 | 0 | 0 | 23 | 42 | Carbamidomethylation | C6:Carbamidomethylation:1000.00 | PEAKS DB |
| K.HVVVVGAGMAGLSAAY.V | N | 67.41 | 1500.7759 | 16 | 0.2 | 751.3954 | 2 | 68.19 | 6 | F6:44843 | NaSa01\_F14.raw |  |  |  |  | 2.067E7 | 2.0687E8 |  |  |  |  |  |  |  |  | 10 | 0 | 0 | 0 | 0 | 1 | 9 | 0 | 0 | 0 | 0 | 0 | 0 | 0 | 0 | 53 | 68 |  |  | PEAKS DB |
| Y.QAIAEMVHLNAR.V | N | 66.25 | 1351.7030 | 12 | -0.4 | 676.8585 | 2 | 31.55 | 6 | F6:13654 | NaSa01\_F14.raw |  |  |  |  |  | 7.8157E7 |  |  |  |  |  |  |  |  | 2 | 0 | 0 | 0 | 0 | 0 | 2 | 0 | 0 | 0 | 0 | 0 | 0 | 0 | 0 | 267 | 278 |  |  | PEAKS DB |
| K.FWEADGIHGGKSTTDLPSR.F | N | 64.97 | 2072.9915 | 19 | 0.7 | 692.0049 | 3 | 29.66 | 6 | F6:12168 | NaSa01\_F14.raw |  |  |  |  |  | 2.4928E6 |  |  |  |  |  |  |  |  | 1 | 0 | 0 | 0 | 0 | 0 | 1 | 0 | 0 | 0 | 0 | 0 | 0 | 0 | 0 | 353 | 371 |  |  | PEAKS DB |
| L.SAAYVLAGAGHK.V | N | 63.01 | 1143.6036 | 12 | -1.1 | 572.8085 | 2 | 11.49 | 6 | F6:1894 | NaSa01\_F14.raw |  |  |  |  |  | 4.4228E6 |  |  |  |  |  |  |  |  | 2 | 0 | 0 | 0 | 0 | 0 | 2 | 0 | 0 | 0 | 0 | 0 | 0 | 0 | 0 | 65 | 76 |  |  | PEAKS DB |
| K.YPVKPSEEGK.S | N | 62.09 | 1132.5764 | 10 | 1.6 | 567.2964 | 2 | 11.44 | 6 | F6:1813 | NaSa01\_F14.raw |  |  |  |  |  | 1.8552E6 |  |  |  |  |  |  |  |  | 2 | 0 | 0 | 0 | 0 | 0 | 2 | 0 | 0 | 0 | 0 | 0 | 0 | 0 | 0 | 160 | 169 |  |  | PEAKS DB |
| K.RFDEIVGGFDQLPISMYQA.I | N | 60.53 | 2185.0513 | 19 | -0.2 | 1093.5327 | 2 | 97.41 | 6 | F6:69051 | NaSa01\_F14.raw |  |  |  |  |  | 1.6822E7 |  |  |  |  |  |  |  |  | 1 | 0 | 0 | 0 | 0 | 0 | 1 | 0 | 0 | 0 | 0 | 0 | 0 | 0 | 0 | 250 | 268 |  |  | PEAKS DB |
| K.FWEADGIHGGK.S | N | 59.16 | 1215.5673 | 11 | 0.2 | 608.7910 | 2 | 11.57 | 6 | F6:1961 | NaSa01\_F14.raw |  |  |  |  | 4.0485E5 | 1.8815E7 |  |  |  |  |  |  |  |  | 9 | 0 | 0 | 0 | 0 | 1 | 8 | 0 | 0 | 0 | 0 | 0 | 0 | 0 | 0 | 353 | 363 |  |  | PEAKS DB |
| A.GLSAAYVLAGAGHK.V | N | 58.41 | 1313.7091 | 14 | 0.3 | 438.9104 | 3 | 32.92 | 6 | F6:14801 | NaSa01\_F14.raw |  |  |  |  |  | 1.2419E6 |  |  |  |  |  |  |  |  | 2 | 0 | 0 | 0 | 0 | 0 | 2 | 0 | 0 | 0 | 0 | 0 | 0 | 0 | 0 | 63 | 76 |  |  | PEAKS DB |
| K.HVVVVGAGMAGL.S | N | 58.15 | 1108.6063 | 12 | 0.5 | 555.3107 | 2 | 51.19 | 6 | F6:30972 | NaSa01\_F14.raw |  |  |  |  | 1.7665E7 | 2.672E8 |  |  |  |  |  |  |  |  | 3 | 0 | 0 | 0 | 0 | 1 | 2 | 0 | 0 | 0 | 0 | 0 | 0 | 0 | 0 | 53 | 64 |  |  | PEAKS DB |
| K.RFDEIVGGFDQLPISMY.Q | N | 56.32 | 1985.9557 | 17 | 1.0 | 993.9861 | 2 | 99.98 | 6 | F6:71504 | NaSa01\_F14.raw |  |  |  |  | 8.8677E6 | 1.2924E8 |  |  |  |  |  |  |  |  | 4 | 0 | 0 | 0 | 0 | 1 | 3 | 0 | 0 | 0 | 0 | 0 | 0 | 0 | 0 | 250 | 266 |  |  | PEAKS DB |
| K.RFDEIVGGFDQLPISM(+15.99)Y.Q | N | 56.17 | 2001.9506 | 17 | 0.5 | 1001.9830 | 2 | 91.18 | 6 | F6:63904 | NaSa01\_F14.raw |  |  |  |  | 1.3345E6 | 1.0983E7 |  |  |  |  |  |  |  |  | 3 | 0 | 0 | 0 | 0 | 1 | 2 | 0 | 0 | 0 | 0 | 0 | 0 | 0 | 0 | 250 | 266 | Oxidation (M) | M16:Oxidation (M):1000.00 | PEAKS DB |
| R.FDEIVGGFDQLPISM(+15.99)YQAIAEMVHLNAR.V | N | 54.25 | 3179.5420 | 28 | 1.5 | 1060.8562 | 3 | 104.78 | 6 | F6:75217 | NaSa01\_F14.raw |  |  |  |  |  | 1.2736E6 |  |  |  |  |  |  |  |  | 1 | 0 | 0 | 0 | 0 | 0 | 1 | 0 | 0 | 0 | 0 | 0 | 0 | 0 | 0 | 251 | 278 | Oxidation (M) | M15:Oxidation (M):67.12 | PEAKS DB |
| K.RFDEIVGGFDQLPISM.Y | N | 52.02 | 1822.8923 | 16 | 1.0 | 912.4543 | 2 | 96.41 | 6 | F6:68325 | NaSa01\_F14.raw |  |  |  |  | 8.0404E5 | 1.1182E7 |  |  |  |  |  |  |  |  | 2 | 0 | 0 | 0 | 0 | 1 | 1 | 0 | 0 | 0 | 0 | 0 | 0 | 0 | 0 | 250 | 265 |  |  | PEAKS DB |
| K.KFWEADGIHGGK.S | N | 51.66 | 1343.6622 | 12 | -0.1 | 448.8947 | 3 | 11.66 | 5 | F5:2144 | NaSa01\_F13.raw |  |  |  |  | 1.996E6 | 1.1435E7 |  |  |  |  |  |  |  |  | 3 | 0 | 0 | 0 | 0 | 1 | 2 | 0 | 0 | 0 | 0 | 0 | 0 | 0 | 0 | 352 | 363 |  |  | PEAKS DB |
| K.HVVVVGAGMAGLS.A | N | 50.60 | 1195.6383 | 13 | -0.2 | 598.8263 | 2 | 46.44 | 6 | F6:26217 | NaSa01\_F14.raw |  |  |  |  |  | 5.0901E6 |  |  |  |  |  |  |  |  | 1 | 0 | 0 | 0 | 0 | 0 | 1 | 0 | 0 | 0 | 0 | 0 | 0 | 0 | 0 | 53 | 65 |  |  | PEAKS DB |
| K.HVVVVGAGM(+15.99)AGLSAAY.V | N | 50.48 | 1516.7708 | 16 | 0.9 | 759.3934 | 2 | 46.65 | 6 | F6:26724 | NaSa01\_F14.raw |  |  |  |  |  | 2.0076E7 |  |  |  |  |  |  |  |  | 3 | 0 | 0 | 0 | 0 | 0 | 3 | 0 | 0 | 0 | 0 | 0 | 0 | 0 | 0 | 53 | 68 | Oxidation (M) | M9:Oxidation (M):1000.00 | PEAKS DB |
| K.HVVVVGAGMAGLSAA.Y | N | 50.47 | 1337.7125 | 15 | 0.6 | 669.8640 | 2 | 50.72 | 6 | F6:29947 | NaSa01\_F14.raw |  |  |  |  |  | 7.7421E6 |  |  |  |  |  |  |  |  | 1 | 0 | 0 | 0 | 0 | 0 | 1 | 0 | 0 | 0 | 0 | 0 | 0 | 0 | 0 | 53 | 67 |  |  | PEAKS DB |
| K.RFDEIVGGFDQLPISMYQ.A | N | 49.87 | 2114.0142 | 18 | 1.4 | 1058.0159 | 2 | 96.41 | 6 | F6:68423 | NaSa01\_F14.raw |  |  |  |  |  | 4.0398E6 |  |  |  |  |  |  |  |  | 1 | 0 | 0 | 0 | 0 | 0 | 1 | 0 | 0 | 0 | 0 | 0 | 0 | 0 | 0 | 250 | 267 |  |  | PEAKS DB |
| K.HVVVVGAGMAGLSA.A | N | 48.82 | 1266.6754 | 14 | 0.3 | 634.3452 | 2 | 49.72 | 6 | F6:29252 | NaSa01\_F14.raw |  |  |  |  |  | 3.9558E6 |  |  |  |  |  |  |  |  | 1 | 0 | 0 | 0 | 0 | 0 | 1 | 0 | 0 | 0 | 0 | 0 | 0 | 0 | 0 | 53 | 66 |  |  | PEAKS DB |
| K.STTDLPSR.F | N | 48.78 | 875.4348 | 8 | 0.1 | 438.7247 | 2 | 11.44 | 6 | F6:1817 | NaSa01\_F14.raw |  |  |  |  |  | 1.0568E6 |  |  |  |  |  |  |  |  | 1 | 0 | 0 | 0 | 0 | 0 | 1 | 0 | 0 | 0 | 0 | 0 | 0 | 0 | 0 | 364 | 371 |  |  | PEAKS DB |
| R.GAVDMIGDLLNEDSSY.Y | N | 48.29 | 1697.7454 | 16 | 1.7 | 849.8814 | 2 | 107.90 | 6 | F6:77609 | NaSa01\_F14.raw |  |  |  |  |  | 1.6853E7 |  |  |  |  |  |  |  |  | 1 | 0 | 0 | 0 | 0 | 0 | 1 | 0 | 0 | 0 | 0 | 0 | 0 | 0 | 0 | 216 | 231 |  |  | PEAKS DB |
| R.FDEIVGGFDQLPISM.Y | N | 48.24 | 1666.7913 | 15 | 1.2 | 834.4039 | 2 | 112.47 | 6 | F6:80862 | NaSa01\_F14.raw |  |  |  |  |  | 8.4104E6 |  |  |  |  |  |  |  |  | 1 | 0 | 0 | 0 | 0 | 0 | 1 | 0 | 0 | 0 | 0 | 0 | 0 | 0 | 0 | 251 | 265 |  |  | PEAKS DB |
| Y.QAIAEM(+15.99)VHLNAR.V | N | 47.05 | 1367.6980 | 12 | 0.8 | 456.9070 | 3 | 31.64 | 6 | F6:13767 | NaSa01\_F14.raw |  |  |  |  |  | 2.4624E6 |  |  |  |  |  |  |  |  | 3 | 0 | 0 | 0 | 0 | 0 | 3 | 0 | 0 | 0 | 0 | 0 | 0 | 0 | 0 | 267 | 278 | Oxidation (M) | M6:Oxidation (M):1000.00 | PEAKS DB |
| R.FDEIVGGFDQLPISMY.Q | N | 44.41 | 1829.8545 | 16 | 0.2 | 915.9347 | 2 | 116.62 | 6 | F6:83518 | NaSa01\_F14.raw |  |  |  |  |  | 6.1039E7 |  |  |  |  |  |  |  |  | 1 | 0 | 0 | 0 | 0 | 0 | 1 | 0 | 0 | 0 | 0 | 0 | 0 | 0 | 0 | 251 | 266 |  |  | PEAKS DB |
| D.YEEFLEIAR.N | N | 44.25 | 1168.5764 | 9 | -0.6 | 585.2951 | 2 | 55.17 | 6 | F6:33697 | NaSa01\_F14.raw |  |  |  |  |  | 4.5232E5 |  |  |  |  |  |  |  |  | 1 | 0 | 0 | 0 | 0 | 0 | 1 | 0 | 0 | 0 | 0 | 0 | 0 | 0 | 0 | 34 | 42 |  |  | PEAKS DB |
| A.GM(+15.99)AGLSAAYVLAGAGHK.V | N | 43.41 | 1588.8031 | 17 | 0.5 | 530.6086 | 3 | 39.38 | 6 | F6:20374 | NaSa01\_F14.raw |  |  |  |  |  | 2.4697E5 |  |  |  |  |  |  |  |  | 1 | 0 | 0 | 0 | 0 | 0 | 1 | 0 | 0 | 0 | 0 | 0 | 0 | 0 | 0 | 60 | 76 | Oxidation (M) | M2:Oxidation (M):1000.00 | PEAKS DB |
| R.FDEIVGGFDQLPISMYQ.A | N | 43.29 | 1957.9131 | 17 | 0.8 | 979.9646 | 2 | 111.22 | 6 | F6:80041 | NaSa01\_F14.raw |  |  |  |  |  | 3.0744E6 |  |  |  |  |  |  |  |  | 1 | 0 | 0 | 0 | 0 | 0 | 1 | 0 | 0 | 0 | 0 | 0 | 0 | 0 | 0 | 251 | 267 |  |  | PEAKS DB |
| K.HVVVVGAGM(+15.99)AGL.S | N | 43.09 | 1124.6012 | 12 | 0.5 | 563.3082 | 2 | 50.23 | 6 | F6:30439 | NaSa01\_F14.raw |  |  |  |  | 3.0223E6 | 1.1076E7 |  |  |  |  |  |  |  |  | 3 | 0 | 0 | 0 | 0 | 1 | 2 | 0 | 0 | 0 | 0 | 0 | 0 | 0 | 0 | 53 | 64 | Oxidation (M) | M9:Oxidation (M):1000.00 | PEAKS DB |
| V.GAGMAGLSAAYVLAGAGHK.V | N | 41.36 | 1700.8668 | 19 | -0.1 | 567.9628 | 3 | 53.80 | 6 | F6:32769 | NaSa01\_F14.raw |  |  |  |  |  | 3.5832E5 |  |  |  |  |  |  |  |  | 1 | 0 | 0 | 0 | 0 | 0 | 1 | 0 | 0 | 0 | 0 | 0 | 0 | 0 | 0 | 58 | 76 |  |  | PEAKS DB |
| D.DYEEFLEIAR.N | N | 39.92 | 1283.6033 | 10 | -6.5 | 642.8047 | 2 | 76.29 | 6 | F6:51560 | NaSa01\_F14.raw |  |  |  |  |  | 3.9682E6 |  |  |  |  |  |  |  |  | 1 | 0 | 0 | 0 | 0 | 0 | 1 | 0 | 0 | 0 | 0 | 0 | 0 | 0 | 0 | 33 | 42 |  |  | PEAKS DB |
| G.MAGLSAAYVLAGAGHK.V | N | 39.83 | 1515.7867 | 16 | 0.3 | 506.2697 | 3 | 47.39 | 6 | F6:27145 | NaSa01\_F14.raw |  |  |  |  |  | 5.9167E5 |  |  |  |  |  |  |  |  | 1 | 0 | 0 | 0 | 0 | 0 | 1 | 0 | 0 | 0 | 0 | 0 | 0 | 0 | 0 | 61 | 76 |  |  | PEAKS DB |
| R.FDEIVGGFDQLPISMYQA.I | N | 38.57 | 2028.9502 | 18 | 1.1 | 1015.4835 | 2 | 112.01 | 6 | F6:80759 | NaSa01\_F14.raw |  |  |  |  |  | 1.2262E7 |  |  |  |  |  |  |  |  | 1 | 0 | 0 | 0 | 0 | 0 | 1 | 0 | 0 | 0 | 0 | 0 | 0 | 0 | 0 | 251 | 268 |  |  | PEAKS DB |
| R.FDEIVGGFDQLPISM(+15.99)Y.Q | N | 38.39 | 1845.8495 | 16 | 0.4 | 923.9324 | 2 | 105.35 | 6 | F6:75636 | NaSa01\_F14.raw |  |  |  |  |  | 4.6489E6 |  |  |  |  |  |  |  |  | 1 | 0 | 0 | 0 | 0 | 0 | 1 | 0 | 0 | 0 | 0 | 0 | 0 | 0 | 0 | 251 | 266 | Oxidation (M) | M15:Oxidation (M):1000.00 | PEAKS DB |
| N.VTADYVIVC(+57.02)STSR.A | N | 38.16 | 1469.7184 | 13 | 0.8 | 490.9138 | 3 | 38.41 | 6 | F6:19506 | NaSa01\_F14.raw |  |  |  |  |  | 3.3852E5 |  |  |  |  |  |  |  |  | 1 | 0 | 0 | 0 | 0 | 0 | 1 | 0 | 0 | 0 | 0 | 0 | 0 | 0 | 0 | 303 | 315 | Carbamidomethylation | C9:Carbamidomethylation:1000.00 | PEAKS DB |
| total 40 peptides |  |  |  |  |  |  |  |  |  |  |  |  |  |  |  |  |  |  |  |  |  |  |  |  |  |  |  |  |  |  |  |  |  |  |  |  |  |  |  |  |  |  |  |  |  |
| --- | --- | --- | --- | --- | --- | --- | --- | --- | --- | --- | --- | --- | --- | --- | --- | --- | --- | --- | --- | --- | --- | --- | --- | --- | --- | --- | --- | --- | --- | --- | --- | --- | --- | --- | --- | --- | --- | --- | --- | --- | --- | --- | --- | --- | --- |

P01446.1

back to list

  

| Protein Coverage
| Supporting Peptides
|

Protein Coverage:

Supporting Peptides:

| Peptide | Uniq | -10lgP | Mass | Length | ppm | m/z | z | RT | Fraction | Scan | Source File | Area F1 | Area F10 | Area F11 | Area F12 | Area F13 | Area F14 | Area F2 | Area F3 | Area F4 | Area F5 | Area F6 | Area F7 | Area F8 | Area F9 | #Feature | #Feature F1 | #Feature F10 | #Feature F11 | #Feature F12 | #Feature F13 | #Feature F14 | #Feature F2 | #Feature F3 | #Feature F4 | #Feature F5 | #Feature F6 | #Feature F7 | #Feature F8 | #Feature F9 | Start | End | PTM | AScore | Found By |
| --- | --- | --- | --- | --- | --- | --- | --- | --- | --- | --- | --- | --- | --- | --- | --- | --- | --- | --- | --- | --- | --- | --- | --- | --- | --- | --- | --- | --- | --- | --- | --- | --- | --- | --- | --- | --- | --- | --- | --- | --- | --- | --- | --- | --- | --- |
| K.LIPLAYKTC(+57.02)PAGKNLC(+57.02)YK.M | N | 91.80 | 2109.1113 | 18 | 1.1 | 528.2857 | 4 | 32.09 | 13 | F13:20619 | NaSa01\_F8.raw |  |  |  |  |  |  |  |  |  |  |  |  | 1.789E8 |  | 3 | 0 | 0 | 0 | 0 | 0 | 0 | 0 | 0 | 0 | 0 | 0 | 0 | 3 | 0 | 6 | 23 | Carbamidomethylation | C9:Carbamidomethylation:1000.00;C16:Carbamidomethylation:1000.00 | PEAKS DB |
| K.NLC(+57.02)YKMFMVSNK.T | N | 83.26 | 1533.7142 | 12 | 0.0 | 767.8644 | 2 | 44.27 | 13 | F13:30550 | NaSa01\_F8.raw |  |  |  |  |  |  |  |  |  |  |  | 5.8556E7 | 8.639E7 |  | 8 | 0 | 0 | 0 | 0 | 0 | 0 | 0 | 0 | 0 | 0 | 0 | 4 | 4 | 0 | 19 | 30 | Carbamidomethylation | C3:Carbamidomethylation:1000.00 | PEAKS DB |
| R.GC(+57.02)IDAC(+57.02)PKNSLLVK.Y | Y | 81.46 | 1573.7957 | 14 | 0.1 | 787.9052 | 2 | 22.85 | 12 | F12:9149 | NaSa01\_F7.raw |  |  |  |  |  |  |  |  |  |  |  | 6.4589E7 | 1.4838E8 |  | 4 | 0 | 0 | 0 | 0 | 0 | 0 | 0 | 0 | 0 | 0 | 0 | 2 | 2 | 0 | 37 | 50 | Carbamidomethylation | C2:Carbamidomethylation:1000.00;C6:Carbamidomethylation:1000.00 | PEAKS DB |
| K.LIPLAYKTC(+57.02)PAGK.N | N | 76.62 | 1430.7955 | 13 | 0.4 | 477.9393 | 3 | 25.12 | 13 | F13:14589 | NaSa01\_F8.raw |  |  |  |  |  |  |  |  |  |  |  | 6.3345E7 | 1.8912E7 |  | 4 | 0 | 0 | 0 | 0 | 0 | 0 | 0 | 0 | 0 | 0 | 0 | 3 | 1 | 0 | 6 | 18 | Carbamidomethylation | C9:Carbamidomethylation:1000.00 | PEAKS DB |
| K.NSLLVKYVC(+57.02)C(+57.02)NTDR.C | N | 74.70 | 1740.8287 | 14 | 0.8 | 871.4223 | 2 | 32.27 | 14 | F14:13415 | NaSa01\_F9.raw |  | 5.2349E8 | 1.0561E6 | 0 |  |  |  |  |  |  |  | 5.0056E7 | 1.163E8 | 2.1488E7 | 22 | 0 | 6 | 1 | 0 | 0 | 0 | 0 | 0 | 0 | 0 | 0 | 5 | 8 | 2 | 45 | 58 | Carbamidomethylation | C9:Carbamidomethylation:1000.00;C10:Carbamidomethylation:1000.00 | PEAKS DB |
| K.MFMVSNKTVPVKR.G | N | 74.24 | 1535.8317 | 13 | -0.8 | 512.9507 | 3 | 12.10 | 13 | F13:4234 | NaSa01\_F8.raw |  |  |  |  |  |  |  |  |  |  |  | 4.272E7 | 1.085E7 |  | 12 | 0 | 0 | 0 | 0 | 0 | 0 | 0 | 0 | 0 | 0 | 0 | 7 | 5 | 0 | 24 | 36 |  |  | PEAKS DB |
| K.NSLLVKYVC(+57.02)C(+57.02)NTDRC(+57.02)N | N | 73.46 | 2014.9023 | 16 | -0.1 | 672.6413 | 3 | 34.16 | 13 | F13:22324 | NaSa01\_F8.raw |  | 1.6983E8 |  |  |  |  |  |  |  |  |  | 1.6282E7 | 8.7478E7 |  | 9 | 0 | 3 | 0 | 0 | 0 | 0 | 0 | 0 | 0 | 0 | 0 | 4 | 2 | 0 | 45 | 60 | Carbamidomethylation | C9:Carbamidomethylation:1000.00;C10:Carbamidomethylation:1000.00;C15:Carbamidomethylation:1000.00 | PEAKS DB |
| R.GC(+57.02)IDAC(+57.02)PKNSLLVKYVC(+57.02)C(+57.02)NTDR.C | Y | 73.44 | 2642.2073 | 22 | 0.9 | 661.5597 | 4 | 42.24 | 13 | F13:28750 | NaSa01\_F8.raw |  |  |  |  |  |  |  |  |  |  |  | 8.0537E6 | 1.3418E7 |  | 4 | 0 | 0 | 0 | 0 | 0 | 0 | 0 | 0 | 0 | 0 | 0 | 2 | 2 | 0 | 37 | 58 | Carbamidomethylation | C2:Carbamidomethylation:1000.00;C6:Carbamidomethylation:1000.00;C17:Carbamidomethylation:1000.00;C18:Carbamidomethylation:1000.00 | PEAKS DB |
| K.MFMVSNKTVPVK.R | N | 73.39 | 1379.7305 | 12 | -0.3 | 690.8723 | 2 | 26.75 | 13 | F13:17104 | NaSa01\_F8.raw |  |  |  |  |  |  |  |  |  |  |  | 1.14E8 | 1.4689E8 |  | 12 | 0 | 0 | 0 | 0 | 0 | 0 | 0 | 0 | 0 | 0 | 0 | 6 | 6 | 0 | 24 | 35 |  |  | PEAKS DB |
| K.NLC(+57.02)YKMFMVSNKTVPVK.R | N | 71.08 | 2058.0464 | 17 | 0.8 | 687.0233 | 3 | 40.94 | 12 | F12:24128 | NaSa01\_F7.raw |  |  |  |  |  |  |  |  |  |  |  | 1.0394E6 |  |  | 1 | 0 | 0 | 0 | 0 | 0 | 0 | 0 | 0 | 0 | 0 | 0 | 1 | 0 | 0 | 19 | 35 | Carbamidomethylation | C3:Carbamidomethylation:1000.00 | PEAKS DB |
| K.TC(+57.02)PAGKNLC(+57.02)YKMFMVSNK.T | N | 69.88 | 2147.9988 | 18 | 0.0 | 538.0070 | 4 | 34.25 | 13 | F13:22340 | NaSa01\_F8.raw |  |  |  |  |  |  |  |  |  |  |  | 1.6903E6 | 1.5976E7 |  | 3 | 0 | 0 | 0 | 0 | 0 | 0 | 0 | 0 | 0 | 0 | 0 | 1 | 2 | 0 | 13 | 30 | Carbamidomethylation | C2:Carbamidomethylation:1000.00;C9:Carbamidomethylation:1000.00 | PEAKS DB |
| LKC(+57.02)NKLIPLAYK.T | N | 67.34 | 1459.8584 | 12 | -0.2 | 487.6266 | 3 | 21.98 | 13 | F13:13390 | NaSa01\_F8.raw |  |  |  |  |  |  |  |  |  |  |  | 3.4641E6 | 6.8191E7 |  | 5 | 0 | 0 | 0 | 0 | 0 | 0 | 0 | 0 | 0 | 0 | 0 | 1 | 4 | 0 | 1 | 12 | Carbamidomethylation | C3:Carbamidomethylation:1000.00 | PEAKS DB |
| K.MFM(+15.99)VSNKTVPVKR.G | N | 61.14 | 1551.8265 | 13 | 1.0 | 518.2833 | 3 | 12.17 | 12 | F12:2509 | NaSa01\_F7.raw |  |  |  |  |  |  |  |  |  |  |  | 5.7886E6 |  |  | 1 | 0 | 0 | 0 | 0 | 0 | 0 | 0 | 0 | 0 | 0 | 0 | 1 | 0 | 0 | 24 | 36 | Oxidation (M) | M3:Oxidation (M):32.28 | PEAKS DB |
| K.MFM(+15.99)VSNKTVPVK.R | N | 58.76 | 1395.7255 | 12 | -0.3 | 466.2490 | 3 | 22.53 | 12 | F12:11691 | NaSa01\_F7.raw |  |  |  |  |  |  |  |  |  |  |  | 1.9991E7 | 4.7839E6 |  | 4 | 0 | 0 | 0 | 0 | 0 | 0 | 0 | 0 | 0 | 0 | 0 | 3 | 1 | 0 | 24 | 35 | Oxidation (M) | M3:Oxidation (M):38.40 | PEAKS DB |
| K.C(+57.02)NKLIPLAYK.T | N | 57.38 | 1218.6794 | 10 | 0.7 | 610.3474 | 2 | 26.64 | 12 | F12:12500 | NaSa01\_F7.raw |  |  |  |  |  |  |  |  |  |  |  | 2.6598E7 | 9.2193E6 |  | 6 | 0 | 0 | 0 | 0 | 0 | 0 | 0 | 0 | 0 | 0 | 0 | 2 | 4 | 0 | 3 | 12 | Carbamidomethylation | C1:Carbamidomethylation:1000.00 | PEAKS DB |
| K.YVC(+57.02)C(+57.02)NTDR.C | N | 50.96 | 1086.4222 | 8 | 0.1 | 544.2184 | 2 | 11.80 | 12 | F12:2224 | NaSa01\_F7.raw |  |  | 2.9228E7 | 4.0658E5 |  |  |  |  |  |  |  | 4.3073E6 | 5.5734E8 | 1.1711E7 | 6 | 0 | 0 | 1 | 1 | 0 | 0 | 0 | 0 | 0 | 0 | 0 | 1 | 2 | 1 | 51 | 58 | Carbamidomethylation | C3:Carbamidomethylation:1000.00;C4:Carbamidomethylation:1000.00 | PEAKS DB |
| K.MFMVSNK.T | N | 49.84 | 855.3983 | 7 | 1.2 | 428.7069 | 2 | 23.50 | 13 | F13:11144 | NaSa01\_F8.raw |  |  |  |  |  |  |  |  |  |  |  | 6.1293E7 | 2.6525E9 | 4.3192E6 | 12 | 0 | 0 | 0 | 0 | 0 | 0 | 0 | 0 | 0 | 0 | 0 | 5 | 6 | 1 | 24 | 30 |  |  | PEAKS DB |
| K.RGC(+57.02)IDAC(+57.02)PK.N | Y | 48.78 | 1075.4902 | 9 | -1.4 | 538.7516 | 2 | 11.62 | 13 | F13:3793 | NaSa01\_F8.raw |  |  |  |  |  |  |  |  |  |  |  |  | 6.6452E7 |  | 1 | 0 | 0 | 0 | 0 | 0 | 0 | 0 | 0 | 0 | 0 | 0 | 0 | 1 | 0 | 36 | 44 | Carbamidomethylation | C3:Carbamidomethylation:1000.00;C7:Carbamidomethylation:1000.00 | PEAKS DB |
| K.NLC(+57.02)YKMF.M | N | 46.68 | 974.4354 | 7 | -0.6 | 488.2247 | 2 | 44.70 | 12 | F12:27287 | NaSa01\_F7.raw |  |  | 1.8393E7 |  |  |  |  |  |  |  |  | 5.6016E6 |  |  | 2 | 0 | 0 | 1 | 0 | 0 | 0 | 0 | 0 | 0 | 0 | 0 | 1 | 0 | 0 | 19 | 25 | Carbamidomethylation | C3:Carbamidomethylation:1000.00 | PEAKS DB |
| R.GC(+57.02)IDAC(+57.02)PK.N | Y | 46.35 | 919.3892 | 8 | 0.1 | 460.7019 | 2 | 15.00 | 13 | F13:6375 | NaSa01\_F8.raw |  |  |  |  |  |  |  |  |  |  |  | 2.3124E6 | 9.8151E8 |  | 2 | 0 | 0 | 0 | 0 | 0 | 0 | 0 | 0 | 0 | 0 | 0 | 1 | 1 | 0 | 37 | 44 | Carbamidomethylation | C2:Carbamidomethylation:1000.00;C6:Carbamidomethylation:1000.00 | PEAKS DB |
| I.PLAYKTC(+57.02)PAGK.N | N | 46.34 | 1204.6274 | 11 | 0.6 | 603.3214 | 2 | 24.59 | 12 | F12:10693 | NaSa01\_F7.raw |  |  |  |  |  |  |  |  |  |  |  | 1.2674E6 |  |  | 1 | 0 | 0 | 0 | 0 | 0 | 0 | 0 | 0 | 0 | 0 | 0 | 1 | 0 | 0 | 8 | 18 | Carbamidomethylation | C7:Carbamidomethylation:1000.00 | PEAKS DB |
| K.YVC(+57.02)C(+57.02)NTDRC(+57.02)N | N | 41.38 | 1360.4958 | 10 | 0.0 | 681.2552 | 2 | 11.81 | 12 | F12:2256 | NaSa01\_F7.raw |  |  |  |  |  |  |  |  |  |  |  | 2.7635E5 |  |  | 1 | 0 | 0 | 0 | 0 | 0 | 0 | 0 | 0 | 0 | 0 | 0 | 1 | 0 | 0 | 51 | 60 | Carbamidomethylation | C3:Carbamidomethylation:1000.00;C4:Carbamidomethylation:1000.00;C9:Carbamidomethylation:1000.00 | PEAKS DB |
| K.M(+15.99)FMVSNK.T | N | 41.18 | 871.3932 | 7 | 0.5 | 436.7041 | 2 | 17.34 | 12 | F12:3406 | NaSa01\_F7.raw |  |  |  |  |  |  |  |  |  |  |  | 1.8251E7 |  |  | 1 | 0 | 0 | 0 | 0 | 0 | 0 | 0 | 0 | 0 | 0 | 0 | 1 | 0 | 0 | 24 | 30 | Oxidation (M) | M1:Oxidation (M):20.63 | PEAKS DB |
| K.LIPLAYK.T | N | 40.88 | 816.5109 | 7 | 0.0 | 409.2627 | 2 | 42.39 | 12 | F12:25348 | NaSa01\_F7.raw |  |  |  |  |  | 6.2114E5 |  |  |  |  |  | 1.9145E10 | 0 | 2.4516E6 | 8 | 0 | 0 | 0 | 0 | 0 | 1 | 0 | 0 | 0 | 0 | 0 | 6 | 0 | 1 | 6 | 12 |  |  | PEAKS DB |
| K.NLC(+57.02)YKMFM.V | N | 39.67 | 1105.4758 | 8 | 0.3 | 553.7454 | 2 | 57.88 | 3 | F3:32929 | NaSa01\_F11.raw |  |  | 1.6979E6 |  |  |  |  |  |  |  |  |  |  |  | 1 | 0 | 0 | 1 | 0 | 0 | 0 | 0 | 0 | 0 | 0 | 0 | 0 | 0 | 0 | 19 | 26 | Carbamidomethylation | C3:Carbamidomethylation:1000.00 | PEAKS DB |
| K.MFM(+15.99)VSNK.T | N | 38.79 | 871.3932 | 7 | 0.4 | 436.7040 | 2 | 76.10 | 13 | F13:57631 | NaSa01\_F8.raw |  |  |  |  |  |  |  |  |  |  |  | 1.8251E7 | 5.9537E6 |  | 3 | 0 | 0 | 0 | 0 | 0 | 0 | 0 | 0 | 0 | 0 | 0 | 1 | 2 | 0 | 24 | 30 | Oxidation (M) | M3:Oxidation (M):30.83 | PEAKS DB |
| total 26 peptides |  |  |  |  |  |  |  |  |  |  |  |  |  |  |  |  |  |  |  |  |  |  |  |  |  |  |  |  |  |  |  |  |  |  |  |  |  |  |  |  |  |  |  |  |  |
| --- | --- | --- | --- | --- | --- | --- | --- | --- | --- | --- | --- | --- | --- | --- | --- | --- | --- | --- | --- | --- | --- | --- | --- | --- | --- | --- | --- | --- | --- | --- | --- | --- | --- | --- | --- | --- | --- | --- | --- | --- | --- | --- | --- | --- | --- |

6RC7

back to list

  

| Protein Coverage
| Supporting Peptides
|

Protein Coverage:

Supporting Peptides:

| Peptide | Uniq | -10lgP | Mass | Length | ppm | m/z | z | RT | Fraction | Scan | Source File | Area F1 | Area F10 | Area F11 | Area F12 | Area F13 | Area F14 | Area F2 | Area F3 | Area F4 | Area F5 | Area F6 | Area F7 | Area F8 | Area F9 | #Feature | #Feature F1 | #Feature F10 | #Feature F11 | #Feature F12 | #Feature F13 | #Feature F14 | #Feature F2 | #Feature F3 | #Feature F4 | #Feature F5 | #Feature F6 | #Feature F7 | #Feature F8 | #Feature F9 | Start | End | PTM | AScore | Found By |
| --- | --- | --- | --- | --- | --- | --- | --- | --- | --- | --- | --- | --- | --- | --- | --- | --- | --- | --- | --- | --- | --- | --- | --- | --- | --- | --- | --- | --- | --- | --- | --- | --- | --- | --- | --- | --- | --- | --- | --- | --- | --- | --- | --- | --- | --- |
| K.LIPLAYKTC(+57.02)PAGKNLC(+57.02)YK.M | N | 91.80 | 2109.1113 | 18 | 1.1 | 528.2857 | 4 | 32.09 | 13 | F13:20619 | NaSa01\_F8.raw |  |  |  |  |  |  |  |  |  |  |  |  | 1.789E8 |  | 3 | 0 | 0 | 0 | 0 | 0 | 0 | 0 | 0 | 0 | 0 | 0 | 0 | 3 | 0 | 6 | 23 | Carbamidomethylation | C9:Carbamidomethylation:1000.00;C16:Carbamidomethylation:1000.00 | PEAKS DB |
| K.NLC(+57.02)YKMFMVSNK.T | N | 83.26 | 1533.7142 | 12 | 0.0 | 767.8644 | 2 | 44.27 | 13 | F13:30550 | NaSa01\_F8.raw |  |  |  |  |  |  |  |  |  |  |  | 5.8556E7 | 8.639E7 |  | 8 | 0 | 0 | 0 | 0 | 0 | 0 | 0 | 0 | 0 | 0 | 0 | 4 | 4 | 0 | 19 | 30 | Carbamidomethylation | C3:Carbamidomethylation:1000.00 | PEAKS DB |
| R.GC(+57.02)IDAC(+57.02)PKNSLLVK.Y | Y | 81.46 | 1573.7957 | 14 | 0.1 | 787.9052 | 2 | 22.85 | 12 | F12:9149 | NaSa01\_F7.raw |  |  |  |  |  |  |  |  |  |  |  | 6.4589E7 | 1.4838E8 |  | 4 | 0 | 0 | 0 | 0 | 0 | 0 | 0 | 0 | 0 | 0 | 0 | 2 | 2 | 0 | 37 | 50 | Carbamidomethylation | C2:Carbamidomethylation:1000.00;C6:Carbamidomethylation:1000.00 | PEAKS DB |
| K.LIPLAYKTC(+57.02)PAGK.N | N | 76.62 | 1430.7955 | 13 | 0.4 | 477.9393 | 3 | 25.12 | 13 | F13:14589 | NaSa01\_F8.raw |  |  |  |  |  |  |  |  |  |  |  | 6.3345E7 | 1.8912E7 |  | 4 | 0 | 0 | 0 | 0 | 0 | 0 | 0 | 0 | 0 | 0 | 0 | 3 | 1 | 0 | 6 | 18 | Carbamidomethylation | C9:Carbamidomethylation:1000.00 | PEAKS DB |
| K.NSLLVKYVC(+57.02)C(+57.02)NTDR.C | N | 74.70 | 1740.8287 | 14 | 0.8 | 871.4223 | 2 | 32.27 | 14 | F14:13415 | NaSa01\_F9.raw |  | 5.2349E8 | 1.0561E6 | 0 |  |  |  |  |  |  |  | 5.0056E7 | 1.163E8 | 2.1488E7 | 22 | 0 | 6 | 1 | 0 | 0 | 0 | 0 | 0 | 0 | 0 | 0 | 5 | 8 | 2 | 45 | 58 | Carbamidomethylation | C9:Carbamidomethylation:1000.00;C10:Carbamidomethylation:1000.00 | PEAKS DB |
| K.MFMVSNKTVPVKR.G | N | 74.24 | 1535.8317 | 13 | -0.8 | 512.9507 | 3 | 12.10 | 13 | F13:4234 | NaSa01\_F8.raw |  |  |  |  |  |  |  |  |  |  |  | 4.272E7 | 1.085E7 |  | 12 | 0 | 0 | 0 | 0 | 0 | 0 | 0 | 0 | 0 | 0 | 0 | 7 | 5 | 0 | 24 | 36 |  |  | PEAKS DB |
| K.NSLLVKYVC(+57.02)C(+57.02)NTDRC(+57.02)N | N | 73.46 | 2014.9023 | 16 | -0.1 | 672.6413 | 3 | 34.16 | 13 | F13:22324 | NaSa01\_F8.raw |  | 1.6983E8 |  |  |  |  |  |  |  |  |  | 1.6282E7 | 8.7478E7 |  | 9 | 0 | 3 | 0 | 0 | 0 | 0 | 0 | 0 | 0 | 0 | 0 | 4 | 2 | 0 | 45 | 60 | Carbamidomethylation | C9:Carbamidomethylation:1000.00;C10:Carbamidomethylation:1000.00;C15:Carbamidomethylation:1000.00 | PEAKS DB |
| R.GC(+57.02)IDAC(+57.02)PKNSLLVKYVC(+57.02)C(+57.02)NTDR.C | Y | 73.44 | 2642.2073 | 22 | 0.9 | 661.5597 | 4 | 42.24 | 13 | F13:28750 | NaSa01\_F8.raw |  |  |  |  |  |  |  |  |  |  |  | 8.0537E6 | 1.3418E7 |  | 4 | 0 | 0 | 0 | 0 | 0 | 0 | 0 | 0 | 0 | 0 | 0 | 2 | 2 | 0 | 37 | 58 | Carbamidomethylation | C2:Carbamidomethylation:1000.00;C6:Carbamidomethylation:1000.00;C17:Carbamidomethylation:1000.00;C18:Carbamidomethylation:1000.00 | PEAKS DB |
| K.MFMVSNKTVPVK.R | N | 73.39 | 1379.7305 | 12 | -0.3 | 690.8723 | 2 | 26.75 | 13 | F13:17104 | NaSa01\_F8.raw |  |  |  |  |  |  |  |  |  |  |  | 1.14E8 | 1.4689E8 |  | 12 | 0 | 0 | 0 | 0 | 0 | 0 | 0 | 0 | 0 | 0 | 0 | 6 | 6 | 0 | 24 | 35 |  |  | PEAKS DB |
| K.NLC(+57.02)YKMFMVSNKTVPVK.R | N | 71.08 | 2058.0464 | 17 | 0.8 | 687.0233 | 3 | 40.94 | 12 | F12:24128 | NaSa01\_F7.raw |  |  |  |  |  |  |  |  |  |  |  | 1.0394E6 |  |  | 1 | 0 | 0 | 0 | 0 | 0 | 0 | 0 | 0 | 0 | 0 | 0 | 1 | 0 | 0 | 19 | 35 | Carbamidomethylation | C3:Carbamidomethylation:1000.00 | PEAKS DB |
| K.TC(+57.02)PAGKNLC(+57.02)YKMFMVSNK.T | N | 69.88 | 2147.9988 | 18 | 0.0 | 538.0070 | 4 | 34.25 | 13 | F13:22340 | NaSa01\_F8.raw |  |  |  |  |  |  |  |  |  |  |  | 1.6903E6 | 1.5976E7 |  | 3 | 0 | 0 | 0 | 0 | 0 | 0 | 0 | 0 | 0 | 0 | 0 | 1 | 2 | 0 | 13 | 30 | Carbamidomethylation | C2:Carbamidomethylation:1000.00;C9:Carbamidomethylation:1000.00 | PEAKS DB |
| LKC(+57.02)NKLIPLAYK.T | N | 67.34 | 1459.8584 | 12 | -0.2 | 487.6266 | 3 | 21.98 | 13 | F13:13390 | NaSa01\_F8.raw |  |  |  |  |  |  |  |  |  |  |  | 3.4641E6 | 6.8191E7 |  | 5 | 0 | 0 | 0 | 0 | 0 | 0 | 0 | 0 | 0 | 0 | 0 | 1 | 4 | 0 | 1 | 12 | Carbamidomethylation | C3:Carbamidomethylation:1000.00 | PEAKS DB |
| K.MFM(+15.99)VSNKTVPVKR.G | N | 61.14 | 1551.8265 | 13 | 1.0 | 518.2833 | 3 | 12.17 | 12 | F12:2509 | NaSa01\_F7.raw |  |  |  |  |  |  |  |  |  |  |  | 5.7886E6 |  |  | 1 | 0 | 0 | 0 | 0 | 0 | 0 | 0 | 0 | 0 | 0 | 0 | 1 | 0 | 0 | 24 | 36 | Oxidation (M) | M3:Oxidation (M):32.28 | PEAKS DB |
| K.MFM(+15.99)VSNKTVPVK.R | N | 58.76 | 1395.7255 | 12 | -0.3 | 466.2490 | 3 | 22.53 | 12 | F12:11691 | NaSa01\_F7.raw |  |  |  |  |  |  |  |  |  |  |  | 1.9991E7 | 4.7839E6 |  | 4 | 0 | 0 | 0 | 0 | 0 | 0 | 0 | 0 | 0 | 0 | 0 | 3 | 1 | 0 | 24 | 35 | Oxidation (M) | M3:Oxidation (M):38.40 | PEAKS DB |
| K.C(+57.02)NKLIPLAYK.T | N | 57.38 | 1218.6794 | 10 | 0.7 | 610.3474 | 2 | 26.64 | 12 | F12:12500 | NaSa01\_F7.raw |  |  |  |  |  |  |  |  |  |  |  | 2.6598E7 | 9.2193E6 |  | 6 | 0 | 0 | 0 | 0 | 0 | 0 | 0 | 0 | 0 | 0 | 0 | 2 | 4 | 0 | 3 | 12 | Carbamidomethylation | C1:Carbamidomethylation:1000.00 | PEAKS DB |
| K.YVC(+57.02)C(+57.02)NTDR.C | N | 50.96 | 1086.4222 | 8 | 0.1 | 544.2184 | 2 | 11.80 | 12 | F12:2224 | NaSa01\_F7.raw |  |  | 2.9228E7 | 4.0658E5 |  |  |  |  |  |  |  | 4.3073E6 | 5.5734E8 | 1.1711E7 | 6 | 0 | 0 | 1 | 1 | 0 | 0 | 0 | 0 | 0 | 0 | 0 | 1 | 2 | 1 | 51 | 58 | Carbamidomethylation | C3:Carbamidomethylation:1000.00;C4:Carbamidomethylation:1000.00 | PEAKS DB |
| K.MFMVSNK.T | N | 49.84 | 855.3983 | 7 | 1.2 | 428.7069 | 2 | 23.50 | 13 | F13:11144 | NaSa01\_F8.raw |  |  |  |  |  |  |  |  |  |  |  | 6.1293E7 | 2.6525E9 | 4.3192E6 | 12 | 0 | 0 | 0 | 0 | 0 | 0 | 0 | 0 | 0 | 0 | 0 | 5 | 6 | 1 | 24 | 30 |  |  | PEAKS DB |
| K.RGC(+57.02)IDAC(+57.02)PK.N | Y | 48.78 | 1075.4902 | 9 | -1.4 | 538.7516 | 2 | 11.62 | 13 | F13:3793 | NaSa01\_F8.raw |  |  |  |  |  |  |  |  |  |  |  |  | 6.6452E7 |  | 1 | 0 | 0 | 0 | 0 | 0 | 0 | 0 | 0 | 0 | 0 | 0 | 0 | 1 | 0 | 36 | 44 | Carbamidomethylation | C3:Carbamidomethylation:1000.00;C7:Carbamidomethylation:1000.00 | PEAKS DB |
| K.NLC(+57.02)YKMF.M | N | 46.68 | 974.4354 | 7 | -0.6 | 488.2247 | 2 | 44.70 | 12 | F12:27287 | NaSa01\_F7.raw |  |  | 1.8393E7 |  |  |  |  |  |  |  |  | 5.6016E6 |  |  | 2 | 0 | 0 | 1 | 0 | 0 | 0 | 0 | 0 | 0 | 0 | 0 | 1 | 0 | 0 | 19 | 25 | Carbamidomethylation | C3:Carbamidomethylation:1000.00 | PEAKS DB |
| R.GC(+57.02)IDAC(+57.02)PK.N | Y | 46.35 | 919.3892 | 8 | 0.1 | 460.7019 | 2 | 15.00 | 13 | F13:6375 | NaSa01\_F8.raw |  |  |  |  |  |  |  |  |  |  |  | 2.3124E6 | 9.8151E8 |  | 2 | 0 | 0 | 0 | 0 | 0 | 0 | 0 | 0 | 0 | 0 | 0 | 1 | 1 | 0 | 37 | 44 | Carbamidomethylation | C2:Carbamidomethylation:1000.00;C6:Carbamidomethylation:1000.00 | PEAKS DB |
| I.PLAYKTC(+57.02)PAGK.N | N | 46.34 | 1204.6274 | 11 | 0.6 | 603.3214 | 2 | 24.59 | 12 | F12:10693 | NaSa01\_F7.raw |  |  |  |  |  |  |  |  |  |  |  | 1.2674E6 |  |  | 1 | 0 | 0 | 0 | 0 | 0 | 0 | 0 | 0 | 0 | 0 | 0 | 1 | 0 | 0 | 8 | 18 | Carbamidomethylation | C7:Carbamidomethylation:1000.00 | PEAKS DB |
| K.YVC(+57.02)C(+57.02)NTDRC(+57.02)N | N | 41.38 | 1360.4958 | 10 | 0.0 | 681.2552 | 2 | 11.81 | 12 | F12:2256 | NaSa01\_F7.raw |  |  |  |  |  |  |  |  |  |  |  | 2.7635E5 |  |  | 1 | 0 | 0 | 0 | 0 | 0 | 0 | 0 | 0 | 0 | 0 | 0 | 1 | 0 | 0 | 51 | 60 | Carbamidomethylation | C3:Carbamidomethylation:1000.00;C4:Carbamidomethylation:1000.00;C9:Carbamidomethylation:1000.00 | PEAKS DB |
| K.M(+15.99)FMVSNK.T | N | 41.18 | 871.3932 | 7 | 0.5 | 436.7041 | 2 | 17.34 | 12 | F12:3406 | NaSa01\_F7.raw |  |  |  |  |  |  |  |  |  |  |  | 1.8251E7 |  |  | 1 | 0 | 0 | 0 | 0 | 0 | 0 | 0 | 0 | 0 | 0 | 0 | 1 | 0 | 0 | 24 | 30 | Oxidation (M) | M1:Oxidation (M):20.63 | PEAKS DB |
| K.LIPLAYK.T | N | 40.88 | 816.5109 | 7 | 0.0 | 409.2627 | 2 | 42.39 | 12 | F12:25348 | NaSa01\_F7.raw |  |  |  |  |  | 6.2114E5 |  |  |  |  |  | 1.9145E10 | 0 | 2.4516E6 | 8 | 0 | 0 | 0 | 0 | 0 | 1 | 0 | 0 | 0 | 0 | 0 | 6 | 0 | 1 | 6 | 12 |  |  | PEAKS DB |
| K.NLC(+57.02)YKMFM.V | N | 39.67 | 1105.4758 | 8 | 0.3 | 553.7454 | 2 | 57.88 | 3 | F3:32929 | NaSa01\_F11.raw |  |  | 1.6979E6 |  |  |  |  |  |  |  |  |  |  |  | 1 | 0 | 0 | 1 | 0 | 0 | 0 | 0 | 0 | 0 | 0 | 0 | 0 | 0 | 0 | 19 | 26 | Carbamidomethylation | C3:Carbamidomethylation:1000.00 | PEAKS DB |
| K.MFM(+15.99)VSNK.T | N | 38.79 | 871.3932 | 7 | 0.4 | 436.7040 | 2 | 76.10 | 13 | F13:57631 | NaSa01\_F8.raw |  |  |  |  |  |  |  |  |  |  |  | 1.8251E7 | 5.9537E6 |  | 3 | 0 | 0 | 0 | 0 | 0 | 0 | 0 | 0 | 0 | 0 | 0 | 1 | 2 | 0 | 24 | 30 | Oxidation (M) | M3:Oxidation (M):30.83 | PEAKS DB |
| total 26 peptides |  |  |  |  |  |  |  |  |  |  |  |  |  |  |  |  |  |  |  |  |  |  |  |  |  |  |  |  |  |  |  |  |  |  |  |  |  |  |  |  |  |  |  |  |  |
| --- | --- | --- | --- | --- | --- | --- | --- | --- | --- | --- | --- | --- | --- | --- | --- | --- | --- | --- | --- | --- | --- | --- | --- | --- | --- | --- | --- | --- | --- | --- | --- | --- | --- | --- | --- | --- | --- | --- | --- | --- | --- | --- | --- | --- | --- |

JAC95028.1

back to list

  

| Protein Coverage
| Supporting Peptides
|

Protein Coverage:

Supporting Peptides:

| Peptide | Uniq | -10lgP | Mass | Length | ppm | m/z | z | RT | Fraction | Scan | Source File | Area F1 | Area F10 | Area F11 | Area F12 | Area F13 | Area F14 | Area F2 | Area F3 | Area F4 | Area F5 | Area F6 | Area F7 | Area F8 | Area F9 | #Feature | #Feature F1 | #Feature F10 | #Feature F11 | #Feature F12 | #Feature F13 | #Feature F14 | #Feature F2 | #Feature F3 | #Feature F4 | #Feature F5 | #Feature F6 | #Feature F7 | #Feature F8 | #Feature F9 | Start | End | PTM | AScore | Found By |
| --- | --- | --- | --- | --- | --- | --- | --- | --- | --- | --- | --- | --- | --- | --- | --- | --- | --- | --- | --- | --- | --- | --- | --- | --- | --- | --- | --- | --- | --- | --- | --- | --- | --- | --- | --- | --- | --- | --- | --- | --- | --- | --- | --- | --- | --- |
| K.HVVVVGAGMAGLSAAYVLAGAGHK.V | N | 107.42 | 2234.1992 | 24 | 1.2 | 745.7412 | 3 | 65.01 | 6 | F6:42957 | NaSa01\_F14.raw |  |  |  | 6.8911E6 | 4.2938E7 | 6.5431E8 |  |  |  |  |  |  |  |  | 25 | 0 | 0 | 0 | 2 | 2 | 21 | 0 | 0 | 0 | 0 | 0 | 0 | 0 | 0 | 53 | 76 |  |  | PEAKS DB |
| K.HVVVVGAGM(+15.99)AGLSAAYVLAGAGHK.V | N | 95.06 | 2250.1943 | 24 | 0.9 | 751.0728 | 3 | 53.09 | 6 | F6:32085 | NaSa01\_F14.raw |  |  |  |  | 1.6389E7 | 1.5623E8 |  |  |  |  |  |  |  |  | 5 | 0 | 0 | 0 | 0 | 2 | 3 | 0 | 0 | 0 | 0 | 0 | 0 | 0 | 0 | 53 | 76 | Oxidation (M) | M9:Oxidation (M):1000.00 | PEAKS DB |
| M.AGLSAAYVLAGAGHK.V | N | 79.84 | 1384.7462 | 15 | 0.5 | 693.3807 | 2 | 36.23 | 6 | F6:17690 | NaSa01\_F14.raw |  |  |  |  |  | 4.4211E7 |  |  |  |  |  |  |  |  | 2 | 0 | 0 | 0 | 0 | 0 | 2 | 0 | 0 | 0 | 0 | 0 | 0 | 0 | 0 | 62 | 76 |  |  | PEAKS DB |
| A.GMAGLSAAYVLAGAGHK.V | N | 77.31 | 1572.8082 | 17 | 0.2 | 525.2768 | 3 | 50.77 | 6 | F6:29981 | NaSa01\_F14.raw |  |  |  |  | 5.5361E5 | 1.0582E7 |  |  |  |  |  |  |  |  | 3 | 0 | 0 | 0 | 0 | 1 | 2 | 0 | 0 | 0 | 0 | 0 | 0 | 0 | 0 | 60 | 76 |  |  | PEAKS DB |
| R.SPLEEC(+57.02)FKEDDYEEFLEIAR.N | Y | 67.76 | 2518.1208 | 20 | -4.4 | 1260.0621 | 2 | 90.63 | 6 | F6:63444 | NaSa01\_F14.raw |  |  |  |  |  | 2.8702E6 |  |  |  |  |  |  |  |  | 1 | 0 | 0 | 0 | 0 | 0 | 1 | 0 | 0 | 0 | 0 | 0 | 0 | 0 | 0 | 23 | 42 | Carbamidomethylation | C6:Carbamidomethylation:1000.00 | PEAKS DB |
| K.HVVVVGAGMAGLSAAY.V | N | 67.41 | 1500.7759 | 16 | 0.2 | 751.3954 | 2 | 68.19 | 6 | F6:44843 | NaSa01\_F14.raw |  |  |  |  | 2.067E7 | 2.0687E8 |  |  |  |  |  |  |  |  | 10 | 0 | 0 | 0 | 0 | 1 | 9 | 0 | 0 | 0 | 0 | 0 | 0 | 0 | 0 | 53 | 68 |  |  | PEAKS DB |
| K.FWEADGIHGGKSTTDLPSR.F | N | 64.97 | 2072.9915 | 19 | 0.7 | 692.0049 | 3 | 29.66 | 6 | F6:12168 | NaSa01\_F14.raw |  |  |  |  |  | 2.4928E6 |  |  |  |  |  |  |  |  | 1 | 0 | 0 | 0 | 0 | 0 | 1 | 0 | 0 | 0 | 0 | 0 | 0 | 0 | 0 | 353 | 371 |  |  | PEAKS DB |
| L.SAAYVLAGAGHK.V | N | 63.01 | 1143.6036 | 12 | -1.1 | 572.8085 | 2 | 11.49 | 6 | F6:1894 | NaSa01\_F14.raw |  |  |  |  |  | 4.4228E6 |  |  |  |  |  |  |  |  | 2 | 0 | 0 | 0 | 0 | 0 | 2 | 0 | 0 | 0 | 0 | 0 | 0 | 0 | 0 | 65 | 76 |  |  | PEAKS DB |
| K.YPVKPSEEGK.S | N | 62.09 | 1132.5764 | 10 | 1.6 | 567.2964 | 2 | 11.44 | 6 | F6:1813 | NaSa01\_F14.raw |  |  |  |  |  | 1.8552E6 |  |  |  |  |  |  |  |  | 2 | 0 | 0 | 0 | 0 | 0 | 2 | 0 | 0 | 0 | 0 | 0 | 0 | 0 | 0 | 160 | 169 |  |  | PEAKS DB |
| K.FWEADGIHGGK.S | N | 59.16 | 1215.5673 | 11 | 0.2 | 608.7910 | 2 | 11.57 | 6 | F6:1961 | NaSa01\_F14.raw |  |  |  |  | 4.0485E5 | 1.8815E7 |  |  |  |  |  |  |  |  | 9 | 0 | 0 | 0 | 0 | 1 | 8 | 0 | 0 | 0 | 0 | 0 | 0 | 0 | 0 | 353 | 363 |  |  | PEAKS DB |
| A.GLSAAYVLAGAGHK.V | N | 58.41 | 1313.7091 | 14 | 0.3 | 438.9104 | 3 | 32.92 | 6 | F6:14801 | NaSa01\_F14.raw |  |  |  |  |  | 1.2419E6 |  |  |  |  |  |  |  |  | 2 | 0 | 0 | 0 | 0 | 0 | 2 | 0 | 0 | 0 | 0 | 0 | 0 | 0 | 0 | 63 | 76 |  |  | PEAKS DB |
| K.HVVVVGAGMAGL.S | N | 58.15 | 1108.6063 | 12 | 0.5 | 555.3107 | 2 | 51.19 | 6 | F6:30972 | NaSa01\_F14.raw |  |  |  |  | 1.7665E7 | 2.672E8 |  |  |  |  |  |  |  |  | 3 | 0 | 0 | 0 | 0 | 1 | 2 | 0 | 0 | 0 | 0 | 0 | 0 | 0 | 0 | 53 | 64 |  |  | PEAKS DB |
| K.KFWEADGIHGGK.S | N | 51.66 | 1343.6622 | 12 | -0.1 | 448.8947 | 3 | 11.66 | 5 | F5:2144 | NaSa01\_F13.raw |  |  |  |  | 1.996E6 | 1.1435E7 |  |  |  |  |  |  |  |  | 3 | 0 | 0 | 0 | 0 | 1 | 2 | 0 | 0 | 0 | 0 | 0 | 0 | 0 | 0 | 352 | 363 |  |  | PEAKS DB |
| K.HVVVVGAGMAGLS.A | N | 50.60 | 1195.6383 | 13 | -0.2 | 598.8263 | 2 | 46.44 | 6 | F6:26217 | NaSa01\_F14.raw |  |  |  |  |  | 5.0901E6 |  |  |  |  |  |  |  |  | 1 | 0 | 0 | 0 | 0 | 0 | 1 | 0 | 0 | 0 | 0 | 0 | 0 | 0 | 0 | 53 | 65 |  |  | PEAKS DB |
| K.HVVVVGAGM(+15.99)AGLSAAY.V | N | 50.48 | 1516.7708 | 16 | 0.9 | 759.3934 | 2 | 46.65 | 6 | F6:26724 | NaSa01\_F14.raw |  |  |  |  |  | 2.0076E7 |  |  |  |  |  |  |  |  | 3 | 0 | 0 | 0 | 0 | 0 | 3 | 0 | 0 | 0 | 0 | 0 | 0 | 0 | 0 | 53 | 68 | Oxidation (M) | M9:Oxidation (M):1000.00 | PEAKS DB |
| K.HVVVVGAGMAGLSAA.Y | N | 50.47 | 1337.7125 | 15 | 0.6 | 669.8640 | 2 | 50.72 | 6 | F6:29947 | NaSa01\_F14.raw |  |  |  |  |  | 7.7421E6 |  |  |  |  |  |  |  |  | 1 | 0 | 0 | 0 | 0 | 0 | 1 | 0 | 0 | 0 | 0 | 0 | 0 | 0 | 0 | 53 | 67 |  |  | PEAKS DB |
| K.HVVVVGAGMAGLSA.A | N | 48.82 | 1266.6754 | 14 | 0.3 | 634.3452 | 2 | 49.72 | 6 | F6:29252 | NaSa01\_F14.raw |  |  |  |  |  | 3.9558E6 |  |  |  |  |  |  |  |  | 1 | 0 | 0 | 0 | 0 | 0 | 1 | 0 | 0 | 0 | 0 | 0 | 0 | 0 | 0 | 53 | 66 |  |  | PEAKS DB |
| K.STTDLPSR.F | N | 48.78 | 875.4348 | 8 | 0.1 | 438.7247 | 2 | 11.44 | 6 | F6:1817 | NaSa01\_F14.raw |  |  |  |  |  | 1.0568E6 |  |  |  |  |  |  |  |  | 1 | 0 | 0 | 0 | 0 | 0 | 1 | 0 | 0 | 0 | 0 | 0 | 0 | 0 | 0 | 364 | 371 |  |  | PEAKS DB |
| D.YEEFLEIAR.N | N | 44.25 | 1168.5764 | 9 | -0.6 | 585.2951 | 2 | 55.17 | 6 | F6:33697 | NaSa01\_F14.raw |  |  |  |  |  | 4.5232E5 |  |  |  |  |  |  |  |  | 1 | 0 | 0 | 0 | 0 | 0 | 1 | 0 | 0 | 0 | 0 | 0 | 0 | 0 | 0 | 34 | 42 |  |  | PEAKS DB |
| A.GM(+15.99)AGLSAAYVLAGAGHK.V | N | 43.41 | 1588.8031 | 17 | 0.5 | 530.6086 | 3 | 39.38 | 6 | F6:20374 | NaSa01\_F14.raw |  |  |  |  |  | 2.4697E5 |  |  |  |  |  |  |  |  | 1 | 0 | 0 | 0 | 0 | 0 | 1 | 0 | 0 | 0 | 0 | 0 | 0 | 0 | 0 | 60 | 76 | Oxidation (M) | M2:Oxidation (M):1000.00 | PEAKS DB |
| K.HVVVVGAGM(+15.99)AGL.S | N | 43.09 | 1124.6012 | 12 | 0.5 | 563.3082 | 2 | 50.23 | 6 | F6:30439 | NaSa01\_F14.raw |  |  |  |  | 3.0223E6 | 1.1076E7 |  |  |  |  |  |  |  |  | 3 | 0 | 0 | 0 | 0 | 1 | 2 | 0 | 0 | 0 | 0 | 0 | 0 | 0 | 0 | 53 | 64 | Oxidation (M) | M9:Oxidation (M):1000.00 | PEAKS DB |
| V.GAGMAGLSAAYVLAGAGHK.V | N | 41.36 | 1700.8668 | 19 | -0.1 | 567.9628 | 3 | 53.80 | 6 | F6:32769 | NaSa01\_F14.raw |  |  |  |  |  | 3.5832E5 |  |  |  |  |  |  |  |  | 1 | 0 | 0 | 0 | 0 | 0 | 1 | 0 | 0 | 0 | 0 | 0 | 0 | 0 | 0 | 58 | 76 |  |  | PEAKS DB |
| D.DYEEFLEIAR.N | N | 39.92 | 1283.6033 | 10 | -6.5 | 642.8047 | 2 | 76.29 | 6 | F6:51560 | NaSa01\_F14.raw |  |  |  |  |  | 3.9682E6 |  |  |  |  |  |  |  |  | 1 | 0 | 0 | 0 | 0 | 0 | 1 | 0 | 0 | 0 | 0 | 0 | 0 | 0 | 0 | 33 | 42 |  |  | PEAKS DB |
| G.MAGLSAAYVLAGAGHK.V | N | 39.83 | 1515.7867 | 16 | 0.3 | 506.2697 | 3 | 47.39 | 6 | F6:27145 | NaSa01\_F14.raw |  |  |  |  |  | 5.9167E5 |  |  |  |  |  |  |  |  | 1 | 0 | 0 | 0 | 0 | 0 | 1 | 0 | 0 | 0 | 0 | 0 | 0 | 0 | 0 | 61 | 76 |  |  | PEAKS DB |
| N.VTADYVIVC(+57.02)STSR.A | N | 38.16 | 1469.7184 | 13 | 0.8 | 490.9138 | 3 | 38.41 | 6 | F6:19506 | NaSa01\_F14.raw |  |  |  |  |  | 3.3852E5 |  |  |  |  |  |  |  |  | 1 | 0 | 0 | 0 | 0 | 0 | 1 | 0 | 0 | 0 | 0 | 0 | 0 | 0 | 0 | 303 | 315 | Carbamidomethylation | C9:Carbamidomethylation:1000.00 | PEAKS DB |
| total 25 peptides |  |  |  |  |  |  |  |  |  |  |  |  |  |  |  |  |  |  |  |  |  |  |  |  |  |  |  |  |  |  |  |  |  |  |  |  |  |  |  |  |  |  |  |  |  |
| --- | --- | --- | --- | --- | --- | --- | --- | --- | --- | --- | --- | --- | --- | --- | --- | --- | --- | --- | --- | --- | --- | --- | --- | --- | --- | --- | --- | --- | --- | --- | --- | --- | --- | --- | --- | --- | --- | --- | --- | --- | --- | --- | --- | --- | --- |

P82885.1

back to list

  

| Protein Coverage
| Supporting Peptides
|

Protein Coverage:

Supporting Peptides:

| Peptide | Uniq | -10lgP | Mass | Length | ppm | m/z | z | RT | Fraction | Scan | Source File | Area F1 | Area F10 | Area F11 | Area F12 | Area F13 | Area F14 | Area F2 | Area F3 | Area F4 | Area F5 | Area F6 | Area F7 | Area F8 | Area F9 | #Feature | #Feature F1 | #Feature F10 | #Feature F11 | #Feature F12 | #Feature F13 | #Feature F14 | #Feature F2 | #Feature F3 | #Feature F4 | #Feature F5 | #Feature F6 | #Feature F7 | #Feature F8 | #Feature F9 | Start | End | PTM | AScore | Found By |
| --- | --- | --- | --- | --- | --- | --- | --- | --- | --- | --- | --- | --- | --- | --- | --- | --- | --- | --- | --- | --- | --- | --- | --- | --- | --- | --- | --- | --- | --- | --- | --- | --- | --- | --- | --- | --- | --- | --- | --- | --- | --- | --- | --- | --- | --- |
| K.ADVTFDSNTAFESLVVSPDKK.T | Y | 98.06 | 2269.1113 | 21 | -3.6 | 1135.5588 | 2 | 66.07 | 3 | F3:39729 | NaSa01\_F11.raw |  | 2.375E8 | 2.9114E9 | 1.0583E8 | 1.3543E7 | 2.178E7 |  |  |  |  | 7.2734E6 | 6.3185E7 | 1.604E6 | 7.1388E6 | 34 | 0 | 3 | 20 | 4 | 1 | 1 | 0 | 0 | 0 | 0 | 1 | 2 | 1 | 1 | 9 | 29 |  |  | PEAKS DB |
| K.ADVTFDSNTAFESLVVSPDK.K | Y | 86.68 | 2141.0164 | 20 | -0.1 | 1071.5154 | 2 | 79.42 | 3 | F3:51412 | NaSa01\_F11.raw |  |  | 3.2645E8 | 7.9909E6 | 9.5775E5 | 2.4125E6 |  |  |  |  |  |  |  |  | 8 | 0 | 0 | 5 | 1 | 1 | 1 | 0 | 0 | 0 | 0 | 0 | 0 | 0 | 0 | 9 | 28 |  |  | PEAKS DB |
| Q.KADVTFDSNTAFESLVVSPDK.K | Y | 80.41 | 2269.1113 | 21 | 0.5 | 757.3781 | 3 | 64.40 | 3 | F3:38445 | NaSa01\_F11.raw |  |  | 9.8894E7 |  |  |  |  |  |  |  |  |  |  |  | 2 | 0 | 0 | 2 | 0 | 0 | 0 | 0 | 0 | 0 | 0 | 0 | 0 | 0 | 0 | 8 | 28 |  |  | PEAKS DB |
| R.FDGSPC(+57.02)VLGSPGFR.S | Y | 78.37 | 1494.6925 | 14 | 0.2 | 748.3537 | 2 | 54.03 | 3 | F3:29585 | NaSa01\_F11.raw |  | 1.4026E8 | 4.56E9 | 4.1021E7 | 1.3342E7 | 1.3339E7 |  | 1.0417E5 |  |  |  |  |  |  | 18 | 0 | 4 | 10 | 1 | 1 | 1 | 0 | 1 | 0 | 0 | 0 | 0 | 0 | 0 | 47 | 60 | Carbamidomethylation | C6:Carbamidomethylation:1000.00 | PEAKS DB |
| SPPGNWQKADVTFDSNTAFESLVVSPDKK.T | Y | 77.59 | 3163.5461 | 29 | 1.2 | 791.8948 | 4 | 63.11 | 3 | F3:37248 | NaSa01\_F11.raw |  |  | 2.7851E7 |  |  |  |  |  |  |  |  |  |  |  | 2 | 0 | 0 | 2 | 0 | 0 | 0 | 0 | 0 | 0 | 0 | 0 | 0 | 0 | 0 | 1 | 29 |  |  | PEAKS DB |
| K.TVENVGVSQVAPDNPER.F | Y | 76.60 | 1809.8856 | 17 | 0.1 | 905.9502 | 2 | 28.91 | 4 | F4:13725 | NaSa01\_F12.raw |  | 2.023E7 | 8.243E8 | 8.5018E7 | 1.2569E7 | 3.6927E6 |  |  |  |  |  |  |  | 7.7507E5 | 18 | 0 | 2 | 9 | 2 | 2 | 2 | 0 | 0 | 0 | 0 | 0 | 0 | 0 | 1 | 30 | 46 |  |  | PEAKS DB |
| A.DVTFDSNTAFESLVVSPDKK.T | Y | 76.44 | 2198.0742 | 20 | 0.0 | 733.6987 | 3 | 70.55 | 3 | F3:43572 | NaSa01\_F11.raw |  |  | 1.7056E6 |  |  |  |  |  |  |  |  |  |  |  | 1 | 0 | 0 | 1 | 0 | 0 | 0 | 0 | 0 | 0 | 0 | 0 | 0 | 0 | 0 | 10 | 29 |  |  | PEAKS DB |
| D.VTFDSNTAFESLVVSPDKK.T | Y | 75.52 | 2083.0474 | 19 | 1.5 | 695.3574 | 3 | 61.71 | 3 | F3:36155 | NaSa01\_F11.raw |  |  | 1.8337E7 |  |  |  |  |  |  |  |  |  |  |  | 2 | 0 | 0 | 2 | 0 | 0 | 0 | 0 | 0 | 0 | 0 | 0 | 0 | 0 | 0 | 11 | 29 |  |  | PEAKS DB |
| K.TVENVGVSQVAPDNPERFDGSPC(+57.02)VLGSPGFR.S | Y | 69.73 | 3286.5676 | 31 | 2.0 | 1096.5320 | 3 | 60.97 | 3 | F3:35709 | NaSa01\_F11.raw |  | 1.4425E7 | 1.6535E8 |  |  |  |  |  |  |  |  |  |  |  | 3 | 0 | 1 | 2 | 0 | 0 | 0 | 0 | 0 | 0 | 0 | 0 | 0 | 0 | 0 | 30 | 60 | Carbamidomethylation | C23:Carbamidomethylation:1000.00 | PEAKS DB |
| D.SNTAFESLVVSPDKK.T | Y | 60.50 | 1620.8358 | 15 | -0.2 | 541.2858 | 3 | 35.15 | 3 | F3:16183 | NaSa01\_F11.raw |  |  | 5.5729E6 |  |  |  |  |  |  |  |  |  |  |  | 2 | 0 | 0 | 2 | 0 | 0 | 0 | 0 | 0 | 0 | 0 | 0 | 0 | 0 | 0 | 15 | 29 |  |  | PEAKS DB |
| R.EWAVGLAGK.S | N | 58.85 | 929.4970 | 9 | 0.0 | 465.7558 | 2 | 36.91 | 3 | F3:17707 | NaSa01\_F11.raw |  | 2.1239E6 | 6.0817E7 | 1.5418E7 |  |  |  |  |  |  |  |  |  |  | 3 | 0 | 1 | 1 | 1 | 0 | 0 | 0 | 0 | 0 | 0 | 0 | 0 | 0 | 0 | 75 | 83 |  |  | PEAKS DB |
| Q.KADVTFDSNTAFESLVVSPDKK.T | Y | 53.68 | 2397.2063 | 22 | -0.7 | 600.3084 | 4 | 53.54 | 3 | F3:29101 | NaSa01\_F11.raw |  |  | 9.6657E5 |  |  |  |  |  |  |  |  |  |  |  | 2 | 0 | 0 | 2 | 0 | 0 | 0 | 0 | 0 | 0 | 0 | 0 | 0 | 0 | 0 | 8 | 29 |  |  | PEAKS DB |
| K.TVENVGVSQVAP.D | Y | 47.81 | 1198.6194 | 12 | 0.1 | 600.3170 | 2 | 40.45 | 12 | F12:23737 | NaSa01\_F7.raw |  |  |  |  |  |  |  |  |  |  |  | 6.4408E6 |  |  | 1 | 0 | 0 | 0 | 0 | 0 | 0 | 0 | 0 | 0 | 0 | 0 | 1 | 0 | 0 | 30 | 41 |  |  | PEAKS DB |
| K.KTVENVGVSQVAPDNPERFDGSPC(+57.02)VLGSPGFR.S | Y | 47.31 | 3414.6626 | 32 | 1.7 | 854.6744 | 4 | 51.72 | 3 | F3:27806 | NaSa01\_F11.raw |  |  | 0 |  |  |  |  |  |  |  |  |  |  |  | 0 | 0 | 0 | 0 | 0 | 0 | 0 | 0 | 0 | 0 | 0 | 0 | 0 | 0 | 0 | 29 | 60 | Carbamidomethylation | C24:Carbamidomethylation:1000.00 | PEAKS DB |
| R.IWQKGLWWLG | Y | 46.61 | 1285.6971 | 10 | 0.4 | 643.8561 | 2 | 84.98 | 3 | F3:55694 | NaSa01\_F11.raw |  |  | 1.5708E7 |  |  |  |  |  |  |  |  |  |  |  | 1 | 0 | 0 | 1 | 0 | 0 | 0 | 0 | 0 | 0 | 0 | 0 | 0 | 0 | 0 | 99 | 108 |  |  | PEAKS DB |
| SPPGNWQKADVTFDSNTAFESLVVSPDK.K | Y | 43.54 | 3035.4512 | 28 | 0.1 | 1012.8245 | 3 | 72.51 | 3 | F3:45054 | NaSa01\_F11.raw |  |  | 2.6237E6 |  |  |  |  |  |  |  |  |  |  |  | 1 | 0 | 0 | 1 | 0 | 0 | 0 | 0 | 0 | 0 | 0 | 0 | 0 | 0 | 0 | 1 | 28 |  |  | PEAKS DB |
| K.ADVTFDSNTAFESLVVSPDKKTVENVGVSQVAPDNPER.F | Y | 43.03 | 4060.9863 | 38 | 0.8 | 1016.2546 | 4 | 68.18 | 3 | F3:41586 | NaSa01\_F11.raw |  |  | 1.0255E7 |  |  |  |  |  |  |  |  |  |  |  | 1 | 0 | 0 | 1 | 0 | 0 | 0 | 0 | 0 | 0 | 0 | 0 | 0 | 0 | 0 | 9 | 46 |  |  | PEAKS DB |
| SPPGNWQK.A | Y | 41.52 | 912.4453 | 8 | 0.4 | 457.2301 | 2 | 11.64 | 3 | F3:3323 | NaSa01\_F11.raw |  |  | 1.4817E6 |  |  |  |  |  |  |  |  |  |  |  | 1 | 0 | 0 | 1 | 0 | 0 | 0 | 0 | 0 | 0 | 0 | 0 | 0 | 0 | 0 | 1 | 8 |  |  | PEAKS DB |
| F.DGSPC(+57.02)VLGSPGFR.S | Y | 39.98 | 1347.6241 | 13 | 0.9 | 674.8199 | 2 | 44.02 | 9 | F9:20389 | NaSa01\_F4.raw |  |  |  |  |  |  |  | 6.8192E4 | 4.8282E5 |  |  |  |  |  | 2 | 0 | 0 | 0 | 0 | 0 | 0 | 0 | 1 | 1 | 0 | 0 | 0 | 0 | 0 | 48 | 60 | Carbamidomethylation | C5:Carbamidomethylation:1000.00 | PEAKS DB |
| total 19 peptides |  |  |  |  |  |  |  |  |  |  |  |  |  |  |  |  |  |  |  |  |  |  |  |  |  |  |  |  |  |  |  |  |  |  |  |  |  |  |  |  |  |  |  |  |  |
| --- | --- | --- | --- | --- | --- | --- | --- | --- | --- | --- | --- | --- | --- | --- | --- | --- | --- | --- | --- | --- | --- | --- | --- | --- | --- | --- | --- | --- | --- | --- | --- | --- | --- | --- | --- | --- | --- | --- | --- | --- | --- | --- | --- | --- | --- |

P82462.1

back to list

  

| Protein Coverage
| Supporting Peptides
|

Protein Coverage:

Supporting Peptides:

| Peptide | Uniq | -10lgP | Mass | Length | ppm | m/z | z | RT | Fraction | Scan | Source File | Area F1 | Area F10 | Area F11 | Area F12 | Area F13 | Area F14 | Area F2 | Area F3 | Area F4 | Area F5 | Area F6 | Area F7 | Area F8 | Area F9 | #Feature | #Feature F1 | #Feature F10 | #Feature F11 | #Feature F12 | #Feature F13 | #Feature F14 | #Feature F2 | #Feature F3 | #Feature F4 | #Feature F5 | #Feature F6 | #Feature F7 | #Feature F8 | #Feature F9 | Start | End | PTM | AScore | Found By |
| --- | --- | --- | --- | --- | --- | --- | --- | --- | --- | --- | --- | --- | --- | --- | --- | --- | --- | --- | --- | --- | --- | --- | --- | --- | --- | --- | --- | --- | --- | --- | --- | --- | --- | --- | --- | --- | --- | --- | --- | --- | --- | --- | --- | --- | --- |
| K.FLFSETTETC(+57.02)PDGQNVC(+57.02)FNQAHLIYPGK.Y | Y | 88.67 | 3272.4907 | 28 | -0.4 | 1091.8370 | 3 | 70.99 | 14 | F14:45388 | NaSa01\_F9.raw |  |  | 2.0449E7 | 1.1485E7 |  |  |  |  |  | 3.9373E7 | 6.4432E6 | 3.8477E10 | 5.6281E9 | 4.6676E8 | 24 | 0 | 0 | 1 | 1 | 0 | 0 | 0 | 0 | 0 | 1 | 1 | 9 | 8 | 3 | 8 | 35 | Carbamidomethylation | C10:Carbamidomethylation:1000.00;C17:Carbamidomethylation:1000.00 | PEAKS DB |
| K.EKFLFSETTETC(+57.02)PDGQNVC(+57.02)FNQAHLIYPGK.Y | Y | 85.22 | 3529.6282 | 30 | 1.0 | 883.4152 | 4 | 63.71 | 13 | F13:47313 | NaSa01\_F8.raw |  |  |  |  |  |  |  |  |  |  |  | 3.4996E9 | 2.6966E8 | 7.7848E6 | 9 | 0 | 0 | 0 | 0 | 0 | 0 | 0 | 0 | 0 | 0 | 0 | 5 | 3 | 1 | 6 | 35 | Carbamidomethylation | C12:Carbamidomethylation:1000.00;C19:Carbamidomethylation:1000.00 | PEAKS DB |
| Q.NVC(+57.02)FNQAHLIYPGK.Y | Y | 80.34 | 1659.8191 | 14 | 0.1 | 830.9169 | 2 | 38.36 | 12 | F12:21891 | NaSa01\_F7.raw |  |  |  |  |  |  |  |  |  |  |  | 4.3504E7 |  |  | 2 | 0 | 0 | 0 | 0 | 0 | 0 | 0 | 0 | 0 | 0 | 0 | 2 | 0 | 0 | 22 | 35 | Carbamidomethylation | C3:Carbamidomethylation:1000.00 | PEAKS DB |
| D.GQNVC(+57.02)FNQAHLIYPGK.Y | Y | 77.16 | 1844.8992 | 16 | 0.3 | 923.4572 | 2 | 37.52 | 12 | F12:21272 | NaSa01\_F7.raw |  |  |  |  |  |  |  |  |  |  |  | 5.8903E7 |  |  | 2 | 0 | 0 | 0 | 0 | 0 | 0 | 0 | 0 | 0 | 0 | 0 | 2 | 0 | 0 | 20 | 35 | Carbamidomethylation | C5:Carbamidomethylation:1000.00 | PEAKS DB |
| N.VC(+57.02)FNQAHLIYPGK.Y | Y | 76.05 | 1545.7762 | 13 | 0.5 | 773.8958 | 2 | 36.44 | 12 | F12:20305 | NaSa01\_F7.raw |  |  |  |  |  |  |  |  |  |  |  | 4.2791E7 |  |  | 2 | 0 | 0 | 0 | 0 | 0 | 0 | 0 | 0 | 0 | 0 | 0 | 2 | 0 | 0 | 23 | 35 | Carbamidomethylation | C2:Carbamidomethylation:1000.00 | PEAKS DB |
| R.DVIFC(+57.02)C(+57.02)STDKC(+57.02)NL | N | 72.84 | 1630.6790 | 13 | 0.4 | 816.3471 | 2 | 50.65 | 13 | F13:36195 | NaSa01\_F8.raw |  |  |  |  |  |  |  |  |  |  |  | 4.4239E8 | 1.6774E8 |  | 2 | 0 | 0 | 0 | 0 | 0 | 0 | 0 | 0 | 0 | 0 | 0 | 1 | 1 | 0 | 53 | 65 | Carbamidomethylation | C5:Carbamidomethylation:1000.00;C6:Carbamidomethylation:1000.00;C11:Carbamidomethylation:1000.00 | PEAKS DB |
| R.DVIFC(+57.02)C(+57.02)STDK.C | N | 69.60 | 1243.5212 | 10 | 0.3 | 622.7681 | 2 | 31.72 | 12 | F12:17503 | NaSa01\_F7.raw |  |  |  |  |  |  |  |  | 2.0789E5 |  |  | 1.5723E9 | 3.5662E7 |  | 9 | 0 | 0 | 0 | 0 | 0 | 0 | 0 | 0 | 1 | 0 | 0 | 6 | 2 | 0 | 53 | 62 | Carbamidomethylation | C5:Carbamidomethylation:1000.00;C6:Carbamidomethylation:1000.00 | PEAKS DB |
| F.SETTETC(+57.02)PDGQNVC(+57.02)FNQAHLIYPGK.Y | Y | 68.23 | 2865.2698 | 25 | 0.5 | 956.0977 | 3 | 45.50 | 12 | F12:28159 | NaSa01\_F7.raw |  |  |  |  |  |  |  |  |  |  |  | 3.8882E7 | 1.5041E7 |  | 2 | 0 | 0 | 0 | 0 | 0 | 0 | 0 | 0 | 0 | 0 | 0 | 1 | 1 | 0 | 11 | 35 | Carbamidomethylation | C7:Carbamidomethylation:1000.00;C14:Carbamidomethylation:1000.00 | PEAKS DB |
| C.FNQAHLIYPGK.Y | Y | 66.56 | 1286.6771 | 11 | 0.2 | 644.3459 | 2 | 22.48 | 12 | F12:8890 | NaSa01\_F7.raw |  |  |  |  |  |  |  |  |  |  |  | 7.9222E7 |  |  | 2 | 0 | 0 | 0 | 0 | 0 | 0 | 0 | 0 | 0 | 0 | 0 | 2 | 0 | 0 | 25 | 35 |  |  | PEAKS DB |
| K.FLFSETTETC(+57.02)PDGQNVC(+57.02)FNQAH.L | Y | 65.08 | 2601.0901 | 22 | -0.4 | 1301.5518 | 2 | 63.63 | 12 | F12:43136 | NaSa01\_F7.raw |  |  |  |  |  |  |  |  |  |  |  | 1.5424E9 | 2.1117E8 |  | 7 | 0 | 0 | 0 | 0 | 0 | 0 | 0 | 0 | 0 | 0 | 0 | 4 | 3 | 0 | 8 | 29 | Carbamidomethylation | C10:Carbamidomethylation:1000.00;C17:Carbamidomethylation:1000.00 | PEAKS DB |
| K.LQNRDVIFC(+57.02)C(+57.02)STDK.C | N | 63.01 | 1754.8080 | 14 | 1.1 | 585.9439 | 3 | 21.02 | 12 | F12:7452 | NaSa01\_F7.raw |  |  |  |  |  |  |  |  |  |  |  | 4.3734E6 |  |  | 3 | 0 | 0 | 0 | 0 | 0 | 0 | 0 | 0 | 0 | 0 | 0 | 3 | 0 | 0 | 49 | 62 | Carbamidomethylation | C9:Carbamidomethylation:1000.00;C10:Carbamidomethylation:1000.00 | PEAKS DB |
| K.LQNRDVIFC(+57.02)C(+57.02)STDKC(+57.02)NL | N | 62.61 | 2141.9656 | 17 | -1.3 | 714.9949 | 3 | 34.82 | 12 | F12:19053 | NaSa01\_F7.raw |  |  |  |  |  |  |  |  |  |  |  | 1.4792E7 | 8.7189E6 |  | 3 | 0 | 0 | 0 | 0 | 0 | 0 | 0 | 0 | 0 | 0 | 0 | 2 | 1 | 0 | 49 | 65 | Carbamidomethylation | C9:Carbamidomethylation:1000.00;C10:Carbamidomethylation:1000.00;C15:Carbamidomethylation:1000.00 | PEAKS DB |
| F.LFSETTETC(+57.02)PDGQNVC(+57.02)FNQAHLIYPGK.Y | Y | 56.80 | 3125.4221 | 27 | 0.2 | 1042.8148 | 3 | 57.51 | 12 | F12:38068 | NaSa01\_F7.raw |  |  |  |  |  |  |  |  |  |  |  | 6.4223E7 |  |  | 1 | 0 | 0 | 0 | 0 | 0 | 0 | 0 | 0 | 0 | 0 | 0 | 1 | 0 | 0 | 9 | 35 | Carbamidomethylation | C9:Carbamidomethylation:1000.00;C16:Carbamidomethylation:1000.00 | PEAKS DB |
| K.FLFSETTETC(+57.02)PDGQNVC(+57.02)FNQAHLIYPGKYK.R | Y | 54.64 | 3563.6489 | 30 | 0.7 | 891.9202 | 4 | 61.08 | 12 | F12:41112 | NaSa01\_F7.raw |  |  |  |  |  |  |  |  |  |  |  | 1.1525E7 |  |  | 1 | 0 | 0 | 0 | 0 | 0 | 0 | 0 | 0 | 0 | 0 | 0 | 1 | 0 | 0 | 8 | 37 | Carbamidomethylation | C10:Carbamidomethylation:1000.00;C17:Carbamidomethylation:1000.00 | PEAKS DB |
| K.EKFLFSETTETC(+57.02)PDGQNVC(+57.02)FNQAH.L | Y | 53.64 | 2858.2275 | 24 | 0.9 | 953.7506 | 3 | 54.11 | 13 | F13:39142 | NaSa01\_F8.raw |  |  |  |  |  |  |  |  |  |  |  | 9.7361E7 | 8.5776E6 |  | 3 | 0 | 0 | 0 | 0 | 0 | 0 | 0 | 0 | 0 | 0 | 0 | 2 | 1 | 0 | 6 | 29 | Carbamidomethylation | C12:Carbamidomethylation:1000.00;C19:Carbamidomethylation:1000.00 | PEAKS DB |
| S.ETTETC(+57.02)PDGQNVC(+57.02)FNQAHLIYPGK.Y | Y | 52.99 | 2778.2378 | 24 | 0.3 | 927.0868 | 3 | 45.97 | 12 | F12:28401 | NaSa01\_F7.raw |  |  |  |  |  |  |  |  |  |  |  | 5.0118E6 |  |  | 1 | 0 | 0 | 0 | 0 | 0 | 0 | 0 | 0 | 0 | 0 | 0 | 1 | 0 | 0 | 12 | 35 | Carbamidomethylation | C6:Carbamidomethylation:1000.00;C13:Carbamidomethylation:1000.00 | PEAKS DB |
| E.KFLFSETTETC(+57.02)PDGQNVC(+57.02)FNQAHLIYPGK.Y | Y | 49.86 | 3400.5857 | 29 | 1.0 | 851.1545 | 4 | 58.32 | 12 | F12:38937 | NaSa01\_F7.raw |  |  |  |  |  |  |  |  |  |  |  | 1.6495E7 |  |  | 1 | 0 | 0 | 0 | 0 | 0 | 0 | 0 | 0 | 0 | 0 | 0 | 1 | 0 | 0 | 7 | 35 | Carbamidomethylation | C11:Carbamidomethylation:1000.00;C18:Carbamidomethylation:1000.00 | PEAKS DB |
| K.FLFSETTETC(+57.02)PDGQNVC(+57.02)FNQAHL.I | Y | 46.26 | 2714.1741 | 23 | 0.1 | 905.7321 | 3 | 72.97 | 12 | F12:50499 | NaSa01\_F7.raw |  |  |  |  |  |  |  |  |  |  |  | 1.609E7 |  |  | 1 | 0 | 0 | 0 | 0 | 0 | 0 | 0 | 0 | 0 | 0 | 0 | 1 | 0 | 0 | 8 | 30 | Carbamidomethylation | C10:Carbamidomethylation:1000.00;C17:Carbamidomethylation:1000.00 | PEAKS DB |
| R.GC(+57.02)AATC(+57.02)PK.L | N | 45.47 | 863.3630 | 8 | -1.2 | 432.6882 | 2 | 11.63 | 13 | F13:3786 | NaSa01\_F8.raw |  |  |  |  |  |  |  |  |  |  |  | 1.055E6 | 1.1773E7 |  | 2 | 0 | 0 | 0 | 0 | 0 | 0 | 0 | 0 | 0 | 0 | 0 | 1 | 1 | 0 | 41 | 48 | Carbamidomethylation | C2:Carbamidomethylation:1000.00;C6:Carbamidomethylation:1000.00 | PEAKS DB |
| V.C(+57.02)FNQAHLIYPGK.Y | Y | 41.73 | 1446.7078 | 12 | -0.6 | 483.2429 | 3 | 27.45 | 12 | F12:12985 | NaSa01\_F7.raw |  |  |  |  |  |  |  |  |  |  |  | 8.4784E5 |  |  | 1 | 0 | 0 | 0 | 0 | 0 | 0 | 0 | 0 | 0 | 0 | 0 | 1 | 0 | 0 | 24 | 35 | Carbamidomethylation | C1:Carbamidomethylation:1000.00 | PEAKS DB |
| K.FLFSETTETC(+57.02)PDGQNVC(+57.02)FN.Q | Y | 40.62 | 2264.9353 | 19 | 2.5 | 1133.4778 | 2 | 80.92 | 12 | F12:57202 | NaSa01\_F7.raw |  |  |  |  |  |  |  |  |  |  |  | 5.4859E7 |  |  | 1 | 0 | 0 | 0 | 0 | 0 | 0 | 0 | 0 | 0 | 0 | 0 | 1 | 0 | 0 | 8 | 26 | Carbamidomethylation | C10:Carbamidomethylation:1000.00;C17:Carbamidomethylation:1000.00 | PEAKS DB |
| C.PDGQNVC(+57.02)FNQAHLIYPGK.Y | Y | 39.88 | 2056.9788 | 18 | 0.4 | 686.6671 | 3 | 42.07 | 12 | F12:25007 | NaSa01\_F7.raw |  |  |  |  |  |  |  |  |  |  |  | 2.6329E6 |  |  | 1 | 0 | 0 | 0 | 0 | 0 | 0 | 0 | 0 | 0 | 0 | 0 | 1 | 0 | 0 | 18 | 35 | Carbamidomethylation | C7:Carbamidomethylation:1000.00 | PEAKS DB |
| G.QNVC(+57.02)FNQAHLIYPGK.Y | Y | 38.70 | 1787.8777 | 15 | 0.4 | 596.9667 | 3 | 37.64 | 12 | F12:21469 | NaSa01\_F7.raw |  |  |  |  |  |  |  |  |  |  |  | 1.372E6 |  |  | 1 | 0 | 0 | 0 | 0 | 0 | 0 | 0 | 0 | 0 | 0 | 0 | 1 | 0 | 0 | 21 | 35 | Carbamidomethylation | C4:Carbamidomethylation:1000.00 | PEAKS DB |
| total 23 peptides |  |  |  |  |  |  |  |  |  |  |  |  |  |  |  |  |  |  |  |  |  |  |  |  |  |  |  |  |  |  |  |  |  |  |  |  |  |  |  |  |  |  |  |  |  |
| --- | --- | --- | --- | --- | --- | --- | --- | --- | --- | --- | --- | --- | --- | --- | --- | --- | --- | --- | --- | --- | --- | --- | --- | --- | --- | --- | --- | --- | --- | --- | --- | --- | --- | --- | --- | --- | --- | --- | --- | --- | --- | --- | --- | --- | --- |

5GZ4

back to list

  

| Protein Coverage
| Supporting Peptides
|

Protein Coverage:

Supporting Peptides:

| Peptide | Uniq | -10lgP | Mass | Length | ppm | m/z | z | RT | Fraction | Scan | Source File | Area F1 | Area F10 | Area F11 | Area F12 | Area F13 | Area F14 | Area F2 | Area F3 | Area F4 | Area F5 | Area F6 | Area F7 | Area F8 | Area F9 | #Feature | #Feature F1 | #Feature F10 | #Feature F11 | #Feature F12 | #Feature F13 | #Feature F14 | #Feature F2 | #Feature F3 | #Feature F4 | #Feature F5 | #Feature F6 | #Feature F7 | #Feature F8 | #Feature F9 | Start | End | PTM | AScore | Found By |
| --- | --- | --- | --- | --- | --- | --- | --- | --- | --- | --- | --- | --- | --- | --- | --- | --- | --- | --- | --- | --- | --- | --- | --- | --- | --- | --- | --- | --- | --- | --- | --- | --- | --- | --- | --- | --- | --- | --- | --- | --- | --- | --- | --- | --- | --- |
| R.AEYLETWDTLMPNINK.L | N | 88.53 | 1936.9241 | 16 | -0.1 | 969.4692 | 2 | 83.63 | 6 | F6:57629 | NaSa01\_F14.raw |  |  |  |  | 6.0648E6 | 1.774E7 |  |  |  |  |  |  |  |  | 2 | 0 | 0 | 0 | 0 | 1 | 1 | 0 | 0 | 0 | 0 | 0 | 0 | 0 | 0 | 128 | 143 |  |  | PEAKS DB |
| K.RPDFSTLYIEEPDTTGHK.F | N | 80.76 | 2105.0066 | 18 | 0.9 | 527.2594 | 4 | 40.34 | 6 | F6:20893 | NaSa01\_F14.raw |  |  |  |  | 1.5745E7 | 4.5771E7 |  |  |  |  |  |  |  |  | 4 | 0 | 0 | 0 | 0 | 2 | 2 | 0 | 0 | 0 | 0 | 0 | 0 | 0 | 0 | 270 | 287 |  |  | PEAKS DB |
| R.MANVLC(+57.02)SC(+57.02)SEDC(+57.02)LTK.K | Y | 78.04 | 1786.7358 | 15 | 0.6 | 894.3757 | 2 | 38.83 | 6 | F6:19925 | NaSa01\_F14.raw |  |  |  |  | 1.13E6 | 3.4903E6 |  |  | 2.0377E6 |  |  |  |  |  | 3 | 0 | 0 | 0 | 0 | 1 | 1 | 0 | 0 | 1 | 0 | 0 | 0 | 0 | 0 | 65 | 79 | Carbamidomethylation | C6:Carbamidomethylation:1000.00;C8:Carbamidomethylation:1000.00;C12:Carbamidomethylation:1000.00 | PEAKS DB |
| K.DFYTFDSEAIVK.K | Y | 77.32 | 1433.6714 | 12 | -0.1 | 717.8429 | 2 | 70.96 | 6 | F6:47358 | NaSa01\_F14.raw |  |  |  | 0 | 4.5792E6 | 1.3357E7 |  |  |  |  |  |  |  |  | 3 | 0 | 0 | 0 | 0 | 1 | 2 | 0 | 0 | 0 | 0 | 0 | 0 | 0 | 0 | 370 | 381 |  |  | PEAKS DB |
| K.SMEAIFLAHGPGFK.E | Y | 74.37 | 1503.7544 | 14 | 0.0 | 502.2587 | 3 | 57.26 | 6 | F6:35536 | NaSa01\_F14.raw |  |  |  |  | 2.8719E6 | 1.7218E7 |  |  |  |  |  |  |  |  | 3 | 0 | 0 | 0 | 0 | 1 | 2 | 0 | 0 | 0 | 0 | 0 | 0 | 0 | 0 | 447 | 460 |  |  | PEAKS DB |
| K.AATYFWPGSEVK.I | N | 70.49 | 1354.6558 | 12 | 0.3 | 678.3354 | 2 | 54.36 | 6 | F6:33101 | NaSa01\_F14.raw |  |  |  |  | 1.0228E7 | 2.5683E7 |  |  |  |  |  |  |  |  | 2 | 0 | 0 | 0 | 0 | 1 | 1 | 0 | 0 | 0 | 0 | 0 | 0 | 0 | 0 | 223 | 234 |  |  | PEAKS DB |
| R.AEYLETWDTLM(+15.99)PNINK.L | N | 67.48 | 1952.9189 | 16 | 0.7 | 977.4674 | 2 | 69.36 | 6 | F6:45873 | NaSa01\_F14.raw |  |  |  |  |  | 1.3972E6 |  |  |  |  |  |  |  |  | 1 | 0 | 0 | 0 | 0 | 0 | 1 | 0 | 0 | 0 | 0 | 0 | 0 | 0 | 0 | 128 | 143 | Oxidation (M) | M11:Oxidation (M):1000.00 | PEAKS DB |
| K.SM(+15.99)EAIFLAHGPGFK.E | Y | 66.90 | 1519.7493 | 14 | -0.3 | 507.5902 | 3 | 42.22 | 6 | F6:22676 | NaSa01\_F14.raw |  |  |  |  |  | 2.1827E6 |  |  |  |  |  |  |  |  | 2 | 0 | 0 | 0 | 0 | 0 | 2 | 0 | 0 | 0 | 0 | 0 | 0 | 0 | 0 | 447 | 460 | Oxidation (M) | M2:Oxidation (M):1000.00 | PEAKS DB |
| R.TLGMLMEGLK.Q | N | 64.68 | 1091.5719 | 10 | 0.2 | 546.7933 | 2 | 60.78 | 6 | F6:38703 | NaSa01\_F14.raw |  |  |  |  | 4.0846E6 | 1.61E7 |  |  |  |  |  |  |  |  | 4 | 0 | 0 | 0 | 0 | 1 | 3 | 0 | 0 | 0 | 0 | 0 | 0 | 0 | 0 | 305 | 314 |  |  | PEAKS DB |
| N.LILLADHGMEAISC(+57.02)NR.L | N | 63.44 | 1811.9022 | 16 | 0.9 | 604.9752 | 3 | 51.51 | 6 | F6:30752 | NaSa01\_F14.raw |  |  |  |  | 4.5419E5 | 1.5046E6 |  |  |  |  |  |  |  |  | 2 | 0 | 0 | 0 | 0 | 1 | 1 | 0 | 0 | 0 | 0 | 0 | 0 | 0 | 0 | 324 | 339 | Carbamidomethylation | C14:Carbamidomethylation:1000.00 | PEAKS DB |
| R.LWNYFHSTLLPK.Y | N | 62.93 | 1517.8031 | 12 | -0.7 | 506.9413 | 3 | 60.91 | 6 | F6:38724 | NaSa01\_F14.raw |  |  |  |  | 2.2688E6 | 6.808E6 |  |  |  |  |  |  |  |  | 2 | 0 | 0 | 0 | 0 | 1 | 1 | 0 | 0 | 0 | 0 | 0 | 0 | 0 | 0 | 677 | 688 |  |  | PEAKS DB |
| R.NLHNC(+57.02)VNLILLADHGMEAISC(+57.02)NR.L | N | 62.42 | 2663.2729 | 23 | 0.3 | 666.8257 | 4 | 72.12 | 6 | F6:48078 | NaSa01\_F14.raw |  |  |  |  |  | 4.0587E6 |  |  |  |  |  |  |  |  | 2 | 0 | 0 | 0 | 0 | 0 | 2 | 0 | 0 | 0 | 0 | 0 | 0 | 0 | 0 | 317 | 339 | Carbamidomethylation | C5:Carbamidomethylation:1000.00;C21:Carbamidomethylation:1000.00 | PEAKS DB |
| K.SPDNLWVEER.M | N | 62.04 | 1243.5833 | 10 | -0.2 | 622.7988 | 2 | 37.70 | 6 | F6:18933 | NaSa01\_F14.raw |  |  |  |  | 6.1024E5 | 2.9466E5 |  |  |  |  |  |  |  |  | 2 | 0 | 0 | 0 | 0 | 1 | 1 | 0 | 0 | 0 | 0 | 0 | 0 | 0 | 0 | 776 | 785 |  |  | PEAKS DB |
| K.C(+57.02)SSITDLEAVNQR.L | Y | 59.75 | 1491.6987 | 13 | -0.5 | 498.2400 | 3 | 34.80 | 6 | F6:16433 | NaSa01\_F14.raw |  |  |  |  | 2.3431E6 | 4.9159E5 |  |  |  |  |  |  |  |  | 2 | 0 | 0 | 0 | 0 | 1 | 1 | 0 | 0 | 0 | 0 | 0 | 0 | 0 | 0 | 533 | 545 | Carbamidomethylation | C1:Carbamidomethylation:1000.00 | PEAKS DB |
| R.NAAWWGGQPIWHTASYQGLK.A | Y | 59.41 | 2270.1021 | 20 | 2.9 | 757.7101 | 3 | 66.75 | 6 | F6:43636 | NaSa01\_F14.raw |  |  |  |  |  | 0 |  |  |  |  |  |  |  |  | 0 | 0 | 0 | 0 | 0 | 0 | 0 | 0 | 0 | 0 | 0 | 0 | 0 | 0 | 0 | 203 | 222 |  |  | PEAKS DB |
| K.TFLPIFINSVN | Y | 57.84 | 1263.6863 | 11 | 0.1 | 632.8505 | 2 | 104.39 | 6 | F6:74971 | NaSa01\_F14.raw |  |  |  |  | 4.9729E6 | 1.3757E7 |  |  |  |  |  |  |  |  | 2 | 0 | 0 | 0 | 0 | 1 | 1 | 0 | 0 | 0 | 0 | 0 | 0 | 0 | 0 | 820 | 830 |  |  | PEAKS DB |
| K.FGPVSGQVIK.S | Y | 54.50 | 1030.5811 | 10 | 0.9 | 516.2983 | 2 | 29.23 | 6 | F6:11699 | NaSa01\_F14.raw |  |  |  |  | 1.668E6 | 2.3367E5 |  |  |  |  |  |  |  |  | 2 | 0 | 0 | 0 | 0 | 1 | 1 | 0 | 0 | 0 | 0 | 0 | 0 | 0 | 0 | 288 | 297 |  |  | PEAKS DB |
| K.EQSPPLYC(+57.02)LFGPVPSPDVSGC(+57.02)K.C | Y | 50.09 | 2433.1345 | 22 | -0.3 | 1217.5742 | 2 | 85.35 | 5 | F5:58909 | NaSa01\_F13.raw |  |  |  |  | 6.9068E5 | 5.204E6 |  |  |  |  |  |  |  |  | 3 | 0 | 0 | 0 | 0 | 1 | 2 | 0 | 0 | 0 | 0 | 0 | 0 | 0 | 0 | 511 | 532 | Carbamidomethylation | C8:Carbamidomethylation:1000.00;C21:Carbamidomethylation:1000.00 | PEAKS DB |
| Y.SQDILMPLWNSYTISK.S | Y | 49.44 | 1894.9498 | 16 | 0.2 | 948.4824 | 2 | 86.89 | 6 | F6:60363 | NaSa01\_F14.raw |  |  |  |  |  | 1.4657E6 |  |  |  |  |  |  |  |  | 1 | 0 | 0 | 0 | 0 | 0 | 1 | 0 | 0 | 0 | 0 | 0 | 0 | 0 | 0 | 587 | 602 |  |  | PEAKS DB |
| R.TLGMLM(+15.99)EGLK.Q | N | 48.81 | 1107.5668 | 10 | 0.5 | 554.7910 | 2 | 43.05 | 6 | F6:23300 | NaSa01\_F14.raw |  |  |  |  |  | 7.4052E5 |  |  |  |  |  |  |  |  | 1 | 0 | 0 | 0 | 0 | 0 | 1 | 0 | 0 | 0 | 0 | 0 | 0 | 0 | 0 | 305 | 314 | Oxidation (M) | M6:Oxidation (M):24.24 | PEAKS DB |
| R.DVELLTGLDFYSALK.Q | Y | 47.44 | 1682.8767 | 15 | 0.0 | 842.4456 | 2 | 104.10 | 5 | F5:73135 | NaSa01\_F13.raw |  |  |  |  | 2.0127E5 | 5.6666E5 |  |  |  |  |  |  |  |  | 2 | 0 | 0 | 0 | 0 | 1 | 1 | 0 | 0 | 0 | 0 | 0 | 0 | 0 | 0 | 795 | 809 |  |  | PEAKS DB |
| R.M(+15.99)ANVLC(+57.02)SC(+57.02)SEDC(+57.02)LTK.K | Y | 45.84 | 1802.7307 | 15 | 1.3 | 902.3738 | 2 | 29.26 | 9 | F9:9349 | NaSa01\_F4.raw |  |  |  |  |  |  |  |  | 1.4442E6 |  |  |  |  |  | 1 | 0 | 0 | 0 | 0 | 0 | 0 | 0 | 0 | 1 | 0 | 0 | 0 | 0 | 0 | 65 | 79 | Oxidation (M); Carbamidomethylation | M1:Oxidation (M):1000.00;C6:Carbamidomethylation:1000.00;C8:Carbamidomethylation:1000.00;C12:Carbamidomethylation:1000.00 | PEAKS DB |
| R.TLGM(+15.99)LMEGLK.Q | N | 45.27 | 1107.5668 | 10 | 0.1 | 554.7907 | 2 | 53.78 | 6 | F6:32542 | NaSa01\_F14.raw |  |  |  |  |  | 3.4616E6 |  |  |  |  |  |  |  |  | 1 | 0 | 0 | 0 | 0 | 0 | 1 | 0 | 0 | 0 | 0 | 0 | 0 | 0 | 0 | 305 | 314 | Oxidation (M) | M4:Oxidation (M):30.83 | PEAKS DB |
| Y.TIVTGLYAETHGIIDNNMYDVK.L | Y | 44.89 | 2466.2100 | 22 | 0.9 | 823.0780 | 3 | 71.03 | 6 | F6:47206 | NaSa01\_F14.raw |  |  |  |  |  | 1.7277E6 |  |  |  |  |  |  |  |  | 1 | 0 | 0 | 0 | 0 | 0 | 1 | 0 | 0 | 0 | 0 | 0 | 0 | 0 | 0 | 168 | 189 |  |  | PEAKS DB |
| R.NAAWWGGQPIWH.T | Y | 43.83 | 1421.6628 | 12 | 0.3 | 711.8389 | 2 | 73.69 | 6 | F6:49226 | NaSa01\_F14.raw |  |  |  |  |  | 1.6228E6 |  |  |  |  |  |  |  |  | 1 | 0 | 0 | 0 | 0 | 0 | 1 | 0 | 0 | 0 | 0 | 0 | 0 | 0 | 0 | 203 | 214 |  |  | PEAKS DB |
| K.SMEAIFLAH.G | N | 41.99 | 1017.4954 | 9 | -0.5 | 509.7547 | 2 | 59.21 | 6 | F6:37583 | NaSa01\_F14.raw |  |  |  |  |  | 2.984E6 |  |  |  |  |  |  |  |  | 1 | 0 | 0 | 0 | 0 | 0 | 1 | 0 | 0 | 0 | 0 | 0 | 0 | 0 | 0 | 447 | 455 |  |  | PEAKS DB |
| K.YISAYSQDILMPLWNSYTISK.S | Y | 41.17 | 2492.2297 | 21 | 1.5 | 1247.1240 | 2 | 98.66 | 6 | F6:70259 | NaSa01\_F14.raw |  |  |  |  |  | 4.2372E5 |  |  |  |  |  |  |  |  | 1 | 0 | 0 | 0 | 0 | 0 | 1 | 0 | 0 | 0 | 0 | 0 | 0 | 0 | 0 | 582 | 602 |  |  | PEAKS DB |
| K.MQSEADNLPYGRPHVLQH.S | Y | 38.90 | 2090.9956 | 18 | 0.7 | 698.0063 | 3 | 25.78 | 6 | F6:8958 | NaSa01\_F14.raw |  |  |  |  |  | 1.8719E6 |  |  |  |  |  |  |  |  | 1 | 0 | 0 | 0 | 0 | 0 | 1 | 0 | 0 | 0 | 0 | 0 | 0 | 0 | 0 | 554 | 571 |  |  | PEAKS DB |
| total 28 peptides |  |  |  |  |  |  |  |  |  |  |  |  |  |  |  |  |  |  |  |  |  |  |  |  |  |  |  |  |  |  |  |  |  |  |  |  |  |  |  |  |  |  |  |  |  |
| --- | --- | --- | --- | --- | --- | --- | --- | --- | --- | --- | --- | --- | --- | --- | --- | --- | --- | --- | --- | --- | --- | --- | --- | --- | --- | --- | --- | --- | --- | --- | --- | --- | --- | --- | --- | --- | --- | --- | --- | --- | --- | --- | --- | --- | --- |

A0A2D0TC04.1

back to list

  

| Protein Coverage
| Supporting Peptides
|

Protein Coverage:

Supporting Peptides:

| Peptide | Uniq | -10lgP | Mass | Length | ppm | m/z | z | RT | Fraction | Scan | Source File | Area F1 | Area F10 | Area F11 | Area F12 | Area F13 | Area F14 | Area F2 | Area F3 | Area F4 | Area F5 | Area F6 | Area F7 | Area F8 | Area F9 | #Feature | #Feature F1 | #Feature F10 | #Feature F11 | #Feature F12 | #Feature F13 | #Feature F14 | #Feature F2 | #Feature F3 | #Feature F4 | #Feature F5 | #Feature F6 | #Feature F7 | #Feature F8 | #Feature F9 | Start | End | PTM | AScore | Found By |
| --- | --- | --- | --- | --- | --- | --- | --- | --- | --- | --- | --- | --- | --- | --- | --- | --- | --- | --- | --- | --- | --- | --- | --- | --- | --- | --- | --- | --- | --- | --- | --- | --- | --- | --- | --- | --- | --- | --- | --- | --- | --- | --- | --- | --- | --- |
| R.AEYLETWDTLMPNINK.L | N | 88.53 | 1936.9241 | 16 | -0.1 | 969.4692 | 2 | 83.63 | 6 | F6:57629 | NaSa01\_F14.raw |  |  |  |  | 6.0648E6 | 1.774E7 |  |  |  |  |  |  |  |  | 2 | 0 | 0 | 0 | 0 | 1 | 1 | 0 | 0 | 0 | 0 | 0 | 0 | 0 | 0 | 128 | 143 |  |  | PEAKS DB |
| K.RPDFSTLYIEEPDTTGHK.F | N | 80.76 | 2105.0066 | 18 | 0.9 | 527.2594 | 4 | 40.34 | 6 | F6:20893 | NaSa01\_F14.raw |  |  |  |  | 1.5745E7 | 4.5771E7 |  |  |  |  |  |  |  |  | 4 | 0 | 0 | 0 | 0 | 2 | 2 | 0 | 0 | 0 | 0 | 0 | 0 | 0 | 0 | 270 | 287 |  |  | PEAKS DB |
| R.MANVLC(+57.02)SC(+57.02)SEDC(+57.02)LTK.K | Y | 78.04 | 1786.7358 | 15 | 0.6 | 894.3757 | 2 | 38.83 | 6 | F6:19925 | NaSa01\_F14.raw |  |  |  |  | 1.13E6 | 3.4903E6 |  |  | 2.0377E6 |  |  |  |  |  | 3 | 0 | 0 | 0 | 0 | 1 | 1 | 0 | 0 | 1 | 0 | 0 | 0 | 0 | 0 | 65 | 79 | Carbamidomethylation | C6:Carbamidomethylation:1000.00;C8:Carbamidomethylation:1000.00;C12:Carbamidomethylation:1000.00 | PEAKS DB |
| K.DFYTFDSEAIVK.K | Y | 77.32 | 1433.6714 | 12 | -0.1 | 717.8429 | 2 | 70.96 | 6 | F6:47358 | NaSa01\_F14.raw |  |  |  | 0 | 4.5792E6 | 1.3357E7 |  |  |  |  |  |  |  |  | 3 | 0 | 0 | 0 | 0 | 1 | 2 | 0 | 0 | 0 | 0 | 0 | 0 | 0 | 0 | 370 | 381 |  |  | PEAKS DB |
| K.SMEAIFLAHGPGFK.E | Y | 74.37 | 1503.7544 | 14 | 0.0 | 502.2587 | 3 | 57.26 | 6 | F6:35536 | NaSa01\_F14.raw |  |  |  |  | 2.8719E6 | 1.7218E7 |  |  |  |  |  |  |  |  | 3 | 0 | 0 | 0 | 0 | 1 | 2 | 0 | 0 | 0 | 0 | 0 | 0 | 0 | 0 | 447 | 460 |  |  | PEAKS DB |
| K.AATYFWPGSEVK.I | N | 70.49 | 1354.6558 | 12 | 0.3 | 678.3354 | 2 | 54.36 | 6 | F6:33101 | NaSa01\_F14.raw |  |  |  |  | 1.0228E7 | 2.5683E7 |  |  |  |  |  |  |  |  | 2 | 0 | 0 | 0 | 0 | 1 | 1 | 0 | 0 | 0 | 0 | 0 | 0 | 0 | 0 | 223 | 234 |  |  | PEAKS DB |
| R.AEYLETWDTLM(+15.99)PNINK.L | N | 67.48 | 1952.9189 | 16 | 0.7 | 977.4674 | 2 | 69.36 | 6 | F6:45873 | NaSa01\_F14.raw |  |  |  |  |  | 1.3972E6 |  |  |  |  |  |  |  |  | 1 | 0 | 0 | 0 | 0 | 0 | 1 | 0 | 0 | 0 | 0 | 0 | 0 | 0 | 0 | 128 | 143 | Oxidation (M) | M11:Oxidation (M):1000.00 | PEAKS DB |
| K.SM(+15.99)EAIFLAHGPGFK.E | Y | 66.90 | 1519.7493 | 14 | -0.3 | 507.5902 | 3 | 42.22 | 6 | F6:22676 | NaSa01\_F14.raw |  |  |  |  |  | 2.1827E6 |  |  |  |  |  |  |  |  | 2 | 0 | 0 | 0 | 0 | 0 | 2 | 0 | 0 | 0 | 0 | 0 | 0 | 0 | 0 | 447 | 460 | Oxidation (M) | M2:Oxidation (M):1000.00 | PEAKS DB |
| R.TLGMLMEGLK.Q | N | 64.68 | 1091.5719 | 10 | 0.2 | 546.7933 | 2 | 60.78 | 6 | F6:38703 | NaSa01\_F14.raw |  |  |  |  | 4.0846E6 | 1.61E7 |  |  |  |  |  |  |  |  | 4 | 0 | 0 | 0 | 0 | 1 | 3 | 0 | 0 | 0 | 0 | 0 | 0 | 0 | 0 | 305 | 314 |  |  | PEAKS DB |
| N.LILLADHGMEAISC(+57.02)NR.L | N | 63.44 | 1811.9022 | 16 | 0.9 | 604.9752 | 3 | 51.51 | 6 | F6:30752 | NaSa01\_F14.raw |  |  |  |  | 4.5419E5 | 1.5046E6 |  |  |  |  |  |  |  |  | 2 | 0 | 0 | 0 | 0 | 1 | 1 | 0 | 0 | 0 | 0 | 0 | 0 | 0 | 0 | 324 | 339 | Carbamidomethylation | C14:Carbamidomethylation:1000.00 | PEAKS DB |
| R.LWNYFHSTLLPK.Y | N | 62.93 | 1517.8031 | 12 | -0.7 | 506.9413 | 3 | 60.91 | 6 | F6:38724 | NaSa01\_F14.raw |  |  |  |  | 2.2688E6 | 6.808E6 |  |  |  |  |  |  |  |  | 2 | 0 | 0 | 0 | 0 | 1 | 1 | 0 | 0 | 0 | 0 | 0 | 0 | 0 | 0 | 677 | 688 |  |  | PEAKS DB |
| R.NLHNC(+57.02)VNLILLADHGMEAISC(+57.02)NR.L | N | 62.42 | 2663.2729 | 23 | 0.3 | 666.8257 | 4 | 72.12 | 6 | F6:48078 | NaSa01\_F14.raw |  |  |  |  |  | 4.0587E6 |  |  |  |  |  |  |  |  | 2 | 0 | 0 | 0 | 0 | 0 | 2 | 0 | 0 | 0 | 0 | 0 | 0 | 0 | 0 | 317 | 339 | Carbamidomethylation | C5:Carbamidomethylation:1000.00;C21:Carbamidomethylation:1000.00 | PEAKS DB |
| K.SPDNLWVEER.M | N | 62.04 | 1243.5833 | 10 | -0.2 | 622.7988 | 2 | 37.70 | 6 | F6:18933 | NaSa01\_F14.raw |  |  |  |  | 6.1024E5 | 2.9466E5 |  |  |  |  |  |  |  |  | 2 | 0 | 0 | 0 | 0 | 1 | 1 | 0 | 0 | 0 | 0 | 0 | 0 | 0 | 0 | 776 | 785 |  |  | PEAKS DB |
| K.C(+57.02)SSITDLEAVNQR.L | Y | 59.75 | 1491.6987 | 13 | -0.5 | 498.2400 | 3 | 34.80 | 6 | F6:16433 | NaSa01\_F14.raw |  |  |  |  | 2.3431E6 | 4.9159E5 |  |  |  |  |  |  |  |  | 2 | 0 | 0 | 0 | 0 | 1 | 1 | 0 | 0 | 0 | 0 | 0 | 0 | 0 | 0 | 533 | 545 | Carbamidomethylation | C1:Carbamidomethylation:1000.00 | PEAKS DB |
| R.NAAWWGGQPIWHTASYQGLK.A | Y | 59.41 | 2270.1021 | 20 | 2.9 | 757.7101 | 3 | 66.75 | 6 | F6:43636 | NaSa01\_F14.raw |  |  |  |  |  | 0 |  |  |  |  |  |  |  |  | 0 | 0 | 0 | 0 | 0 | 0 | 0 | 0 | 0 | 0 | 0 | 0 | 0 | 0 | 0 | 203 | 222 |  |  | PEAKS DB |
| K.TFLPIFINSVN | Y | 57.84 | 1263.6863 | 11 | 0.1 | 632.8505 | 2 | 104.39 | 6 | F6:74971 | NaSa01\_F14.raw |  |  |  |  | 4.9729E6 | 1.3757E7 |  |  |  |  |  |  |  |  | 2 | 0 | 0 | 0 | 0 | 1 | 1 | 0 | 0 | 0 | 0 | 0 | 0 | 0 | 0 | 820 | 830 |  |  | PEAKS DB |
| K.FGPVSGQVIK.S | Y | 54.50 | 1030.5811 | 10 | 0.9 | 516.2983 | 2 | 29.23 | 6 | F6:11699 | NaSa01\_F14.raw |  |  |  |  | 1.668E6 | 2.3367E5 |  |  |  |  |  |  |  |  | 2 | 0 | 0 | 0 | 0 | 1 | 1 | 0 | 0 | 0 | 0 | 0 | 0 | 0 | 0 | 288 | 297 |  |  | PEAKS DB |
| K.EQSPPLYC(+57.02)LFGPVPSPDVSGC(+57.02)K.C | Y | 50.09 | 2433.1345 | 22 | -0.3 | 1217.5742 | 2 | 85.35 | 5 | F5:58909 | NaSa01\_F13.raw |  |  |  |  | 6.9068E5 | 5.204E6 |  |  |  |  |  |  |  |  | 3 | 0 | 0 | 0 | 0 | 1 | 2 | 0 | 0 | 0 | 0 | 0 | 0 | 0 | 0 | 511 | 532 | Carbamidomethylation | C8:Carbamidomethylation:1000.00;C21:Carbamidomethylation:1000.00 | PEAKS DB |
| Y.SQDILMPLWNSYTISK.S | Y | 49.44 | 1894.9498 | 16 | 0.2 | 948.4824 | 2 | 86.89 | 6 | F6:60363 | NaSa01\_F14.raw |  |  |  |  |  | 1.4657E6 |  |  |  |  |  |  |  |  | 1 | 0 | 0 | 0 | 0 | 0 | 1 | 0 | 0 | 0 | 0 | 0 | 0 | 0 | 0 | 587 | 602 |  |  | PEAKS DB |
| R.TLGMLM(+15.99)EGLK.Q | N | 48.81 | 1107.5668 | 10 | 0.5 | 554.7910 | 2 | 43.05 | 6 | F6:23300 | NaSa01\_F14.raw |  |  |  |  |  | 7.4052E5 |  |  |  |  |  |  |  |  | 1 | 0 | 0 | 0 | 0 | 0 | 1 | 0 | 0 | 0 | 0 | 0 | 0 | 0 | 0 | 305 | 314 | Oxidation (M) | M6:Oxidation (M):24.24 | PEAKS DB |
| R.DVELLTGLDFYSALK.Q | Y | 47.44 | 1682.8767 | 15 | 0.0 | 842.4456 | 2 | 104.10 | 5 | F5:73135 | NaSa01\_F13.raw |  |  |  |  | 2.0127E5 | 5.6666E5 |  |  |  |  |  |  |  |  | 2 | 0 | 0 | 0 | 0 | 1 | 1 | 0 | 0 | 0 | 0 | 0 | 0 | 0 | 0 | 795 | 809 |  |  | PEAKS DB |
| R.M(+15.99)ANVLC(+57.02)SC(+57.02)SEDC(+57.02)LTK.K | Y | 45.84 | 1802.7307 | 15 | 1.3 | 902.3738 | 2 | 29.26 | 9 | F9:9349 | NaSa01\_F4.raw |  |  |  |  |  |  |  |  | 1.4442E6 |  |  |  |  |  | 1 | 0 | 0 | 0 | 0 | 0 | 0 | 0 | 0 | 1 | 0 | 0 | 0 | 0 | 0 | 65 | 79 | Oxidation (M); Carbamidomethylation | M1:Oxidation (M):1000.00;C6:Carbamidomethylation:1000.00;C8:Carbamidomethylation:1000.00;C12:Carbamidomethylation:1000.00 | PEAKS DB |
| R.TLGM(+15.99)LMEGLK.Q | N | 45.27 | 1107.5668 | 10 | 0.1 | 554.7907 | 2 | 53.78 | 6 | F6:32542 | NaSa01\_F14.raw |  |  |  |  |  | 3.4616E6 |  |  |  |  |  |  |  |  | 1 | 0 | 0 | 0 | 0 | 0 | 1 | 0 | 0 | 0 | 0 | 0 | 0 | 0 | 0 | 305 | 314 | Oxidation (M) | M4:Oxidation (M):30.83 | PEAKS DB |
| Y.TIVTGLYAETHGIIDNNMYDVK.L | Y | 44.89 | 2466.2100 | 22 | 0.9 | 823.0780 | 3 | 71.03 | 6 | F6:47206 | NaSa01\_F14.raw |  |  |  |  |  | 1.7277E6 |  |  |  |  |  |  |  |  | 1 | 0 | 0 | 0 | 0 | 0 | 1 | 0 | 0 | 0 | 0 | 0 | 0 | 0 | 0 | 168 | 189 |  |  | PEAKS DB |
| R.NAAWWGGQPIWH.T | Y | 43.83 | 1421.6628 | 12 | 0.3 | 711.8389 | 2 | 73.69 | 6 | F6:49226 | NaSa01\_F14.raw |  |  |  |  |  | 1.6228E6 |  |  |  |  |  |  |  |  | 1 | 0 | 0 | 0 | 0 | 0 | 1 | 0 | 0 | 0 | 0 | 0 | 0 | 0 | 0 | 203 | 214 |  |  | PEAKS DB |
| K.SMEAIFLAH.G | N | 41.99 | 1017.4954 | 9 | -0.5 | 509.7547 | 2 | 59.21 | 6 | F6:37583 | NaSa01\_F14.raw |  |  |  |  |  | 2.984E6 |  |  |  |  |  |  |  |  | 1 | 0 | 0 | 0 | 0 | 0 | 1 | 0 | 0 | 0 | 0 | 0 | 0 | 0 | 0 | 447 | 455 |  |  | PEAKS DB |
| K.YISAYSQDILMPLWNSYTISK.S | Y | 41.17 | 2492.2297 | 21 | 1.5 | 1247.1240 | 2 | 98.66 | 6 | F6:70259 | NaSa01\_F14.raw |  |  |  |  |  | 4.2372E5 |  |  |  |  |  |  |  |  | 1 | 0 | 0 | 0 | 0 | 0 | 1 | 0 | 0 | 0 | 0 | 0 | 0 | 0 | 0 | 582 | 602 |  |  | PEAKS DB |
| K.MQSEADNLPYGRPHVLQH.S | Y | 38.90 | 2090.9956 | 18 | 0.7 | 698.0063 | 3 | 25.78 | 6 | F6:8958 | NaSa01\_F14.raw |  |  |  |  |  | 1.8719E6 |  |  |  |  |  |  |  |  | 1 | 0 | 0 | 0 | 0 | 0 | 1 | 0 | 0 | 0 | 0 | 0 | 0 | 0 | 0 | 554 | 571 |  |  | PEAKS DB |
| total 28 peptides |  |  |  |  |  |  |  |  |  |  |  |  |  |  |  |  |  |  |  |  |  |  |  |  |  |  |  |  |  |  |  |  |  |  |  |  |  |  |  |  |  |  |  |  |  |
| --- | --- | --- | --- | --- | --- | --- | --- | --- | --- | --- | --- | --- | --- | --- | --- | --- | --- | --- | --- | --- | --- | --- | --- | --- | --- | --- | --- | --- | --- | --- | --- | --- | --- | --- | --- | --- | --- | --- | --- | --- | --- | --- | --- | --- | --- |

5GZ5

back to list

  

| Protein Coverage
| Supporting Peptides
|

Protein Coverage:

Supporting Peptides:

| Peptide | Uniq | -10lgP | Mass | Length | ppm | m/z | z | RT | Fraction | Scan | Source File | Area F1 | Area F10 | Area F11 | Area F12 | Area F13 | Area F14 | Area F2 | Area F3 | Area F4 | Area F5 | Area F6 | Area F7 | Area F8 | Area F9 | #Feature | #Feature F1 | #Feature F10 | #Feature F11 | #Feature F12 | #Feature F13 | #Feature F14 | #Feature F2 | #Feature F3 | #Feature F4 | #Feature F5 | #Feature F6 | #Feature F7 | #Feature F8 | #Feature F9 | Start | End | PTM | AScore | Found By |
| --- | --- | --- | --- | --- | --- | --- | --- | --- | --- | --- | --- | --- | --- | --- | --- | --- | --- | --- | --- | --- | --- | --- | --- | --- | --- | --- | --- | --- | --- | --- | --- | --- | --- | --- | --- | --- | --- | --- | --- | --- | --- | --- | --- | --- | --- |
| R.AEYLETWDTLMPNINK.L | N | 88.53 | 1936.9241 | 16 | -0.1 | 969.4692 | 2 | 83.63 | 6 | F6:57629 | NaSa01\_F14.raw |  |  |  |  | 6.0648E6 | 1.774E7 |  |  |  |  |  |  |  |  | 2 | 0 | 0 | 0 | 0 | 1 | 1 | 0 | 0 | 0 | 0 | 0 | 0 | 0 | 0 | 128 | 143 |  |  | PEAKS DB |
| K.RPDFSTLYIEEPDTTGHK.F | N | 80.76 | 2105.0066 | 18 | 0.9 | 527.2594 | 4 | 40.34 | 6 | F6:20893 | NaSa01\_F14.raw |  |  |  |  | 1.5745E7 | 4.5771E7 |  |  |  |  |  |  |  |  | 4 | 0 | 0 | 0 | 0 | 2 | 2 | 0 | 0 | 0 | 0 | 0 | 0 | 0 | 0 | 270 | 287 |  |  | PEAKS DB |
| R.MANVLC(+57.02)SC(+57.02)SEDC(+57.02)LTK.K | Y | 78.04 | 1786.7358 | 15 | 0.6 | 894.3757 | 2 | 38.83 | 6 | F6:19925 | NaSa01\_F14.raw |  |  |  |  | 1.13E6 | 3.4903E6 |  |  | 2.0377E6 |  |  |  |  |  | 3 | 0 | 0 | 0 | 0 | 1 | 1 | 0 | 0 | 1 | 0 | 0 | 0 | 0 | 0 | 65 | 79 | Carbamidomethylation | C6:Carbamidomethylation:1000.00;C8:Carbamidomethylation:1000.00;C12:Carbamidomethylation:1000.00 | PEAKS DB |
| K.DFYTFDSEAIVK.K | Y | 77.32 | 1433.6714 | 12 | -0.1 | 717.8429 | 2 | 70.96 | 6 | F6:47358 | NaSa01\_F14.raw |  |  |  | 0 | 4.5792E6 | 1.3357E7 |  |  |  |  |  |  |  |  | 3 | 0 | 0 | 0 | 0 | 1 | 2 | 0 | 0 | 0 | 0 | 0 | 0 | 0 | 0 | 370 | 381 |  |  | PEAKS DB |
| K.SMEAIFLAHGPGFK.E | Y | 74.37 | 1503.7544 | 14 | 0.0 | 502.2587 | 3 | 57.26 | 6 | F6:35536 | NaSa01\_F14.raw |  |  |  |  | 2.8719E6 | 1.7218E7 |  |  |  |  |  |  |  |  | 3 | 0 | 0 | 0 | 0 | 1 | 2 | 0 | 0 | 0 | 0 | 0 | 0 | 0 | 0 | 447 | 460 |  |  | PEAKS DB |
| K.AATYFWPGSEVK.I | N | 70.49 | 1354.6558 | 12 | 0.3 | 678.3354 | 2 | 54.36 | 6 | F6:33101 | NaSa01\_F14.raw |  |  |  |  | 1.0228E7 | 2.5683E7 |  |  |  |  |  |  |  |  | 2 | 0 | 0 | 0 | 0 | 1 | 1 | 0 | 0 | 0 | 0 | 0 | 0 | 0 | 0 | 223 | 234 |  |  | PEAKS DB |
| R.AEYLETWDTLM(+15.99)PNINK.L | N | 67.48 | 1952.9189 | 16 | 0.7 | 977.4674 | 2 | 69.36 | 6 | F6:45873 | NaSa01\_F14.raw |  |  |  |  |  | 1.3972E6 |  |  |  |  |  |  |  |  | 1 | 0 | 0 | 0 | 0 | 0 | 1 | 0 | 0 | 0 | 0 | 0 | 0 | 0 | 0 | 128 | 143 | Oxidation (M) | M11:Oxidation (M):1000.00 | PEAKS DB |
| K.SM(+15.99)EAIFLAHGPGFK.E | Y | 66.90 | 1519.7493 | 14 | -0.3 | 507.5902 | 3 | 42.22 | 6 | F6:22676 | NaSa01\_F14.raw |  |  |  |  |  | 2.1827E6 |  |  |  |  |  |  |  |  | 2 | 0 | 0 | 0 | 0 | 0 | 2 | 0 | 0 | 0 | 0 | 0 | 0 | 0 | 0 | 447 | 460 | Oxidation (M) | M2:Oxidation (M):1000.00 | PEAKS DB |
| R.TLGMLMEGLK.Q | N | 64.68 | 1091.5719 | 10 | 0.2 | 546.7933 | 2 | 60.78 | 6 | F6:38703 | NaSa01\_F14.raw |  |  |  |  | 4.0846E6 | 1.61E7 |  |  |  |  |  |  |  |  | 4 | 0 | 0 | 0 | 0 | 1 | 3 | 0 | 0 | 0 | 0 | 0 | 0 | 0 | 0 | 305 | 314 |  |  | PEAKS DB |
| N.LILLADHGMEAISC(+57.02)NR.L | N | 63.44 | 1811.9022 | 16 | 0.9 | 604.9752 | 3 | 51.51 | 6 | F6:30752 | NaSa01\_F14.raw |  |  |  |  | 4.5419E5 | 1.5046E6 |  |  |  |  |  |  |  |  | 2 | 0 | 0 | 0 | 0 | 1 | 1 | 0 | 0 | 0 | 0 | 0 | 0 | 0 | 0 | 324 | 339 | Carbamidomethylation | C14:Carbamidomethylation:1000.00 | PEAKS DB |
| R.LWNYFHSTLLPK.Y | N | 62.93 | 1517.8031 | 12 | -0.7 | 506.9413 | 3 | 60.91 | 6 | F6:38724 | NaSa01\_F14.raw |  |  |  |  | 2.2688E6 | 6.808E6 |  |  |  |  |  |  |  |  | 2 | 0 | 0 | 0 | 0 | 1 | 1 | 0 | 0 | 0 | 0 | 0 | 0 | 0 | 0 | 677 | 688 |  |  | PEAKS DB |
| R.NLHNC(+57.02)VNLILLADHGMEAISC(+57.02)NR.L | N | 62.42 | 2663.2729 | 23 | 0.3 | 666.8257 | 4 | 72.12 | 6 | F6:48078 | NaSa01\_F14.raw |  |  |  |  |  | 4.0587E6 |  |  |  |  |  |  |  |  | 2 | 0 | 0 | 0 | 0 | 0 | 2 | 0 | 0 | 0 | 0 | 0 | 0 | 0 | 0 | 317 | 339 | Carbamidomethylation | C5:Carbamidomethylation:1000.00;C21:Carbamidomethylation:1000.00 | PEAKS DB |
| K.SPDNLWVEER.M | N | 62.04 | 1243.5833 | 10 | -0.2 | 622.7988 | 2 | 37.70 | 6 | F6:18933 | NaSa01\_F14.raw |  |  |  |  | 6.1024E5 | 2.9466E5 |  |  |  |  |  |  |  |  | 2 | 0 | 0 | 0 | 0 | 1 | 1 | 0 | 0 | 0 | 0 | 0 | 0 | 0 | 0 | 776 | 785 |  |  | PEAKS DB |
| K.C(+57.02)SSITDLEAVNQR.L | Y | 59.75 | 1491.6987 | 13 | -0.5 | 498.2400 | 3 | 34.80 | 6 | F6:16433 | NaSa01\_F14.raw |  |  |  |  | 2.3431E6 | 4.9159E5 |  |  |  |  |  |  |  |  | 2 | 0 | 0 | 0 | 0 | 1 | 1 | 0 | 0 | 0 | 0 | 0 | 0 | 0 | 0 | 533 | 545 | Carbamidomethylation | C1:Carbamidomethylation:1000.00 | PEAKS DB |
| R.NAAWWGGQPIWHTASYQGLK.A | Y | 59.41 | 2270.1021 | 20 | 2.9 | 757.7101 | 3 | 66.75 | 6 | F6:43636 | NaSa01\_F14.raw |  |  |  |  |  | 0 |  |  |  |  |  |  |  |  | 0 | 0 | 0 | 0 | 0 | 0 | 0 | 0 | 0 | 0 | 0 | 0 | 0 | 0 | 0 | 203 | 222 |  |  | PEAKS DB |
| K.TFLPIFINSVN | Y | 57.84 | 1263.6863 | 11 | 0.1 | 632.8505 | 2 | 104.39 | 6 | F6:74971 | NaSa01\_F14.raw |  |  |  |  | 4.9729E6 | 1.3757E7 |  |  |  |  |  |  |  |  | 2 | 0 | 0 | 0 | 0 | 1 | 1 | 0 | 0 | 0 | 0 | 0 | 0 | 0 | 0 | 820 | 830 |  |  | PEAKS DB |
| K.FGPVSGQVIK.S | Y | 54.50 | 1030.5811 | 10 | 0.9 | 516.2983 | 2 | 29.23 | 6 | F6:11699 | NaSa01\_F14.raw |  |  |  |  | 1.668E6 | 2.3367E5 |  |  |  |  |  |  |  |  | 2 | 0 | 0 | 0 | 0 | 1 | 1 | 0 | 0 | 0 | 0 | 0 | 0 | 0 | 0 | 288 | 297 |  |  | PEAKS DB |
| K.EQSPPLYC(+57.02)LFGPVPSPDVSGC(+57.02)K.C | Y | 50.09 | 2433.1345 | 22 | -0.3 | 1217.5742 | 2 | 85.35 | 5 | F5:58909 | NaSa01\_F13.raw |  |  |  |  | 6.9068E5 | 5.204E6 |  |  |  |  |  |  |  |  | 3 | 0 | 0 | 0 | 0 | 1 | 2 | 0 | 0 | 0 | 0 | 0 | 0 | 0 | 0 | 511 | 532 | Carbamidomethylation | C8:Carbamidomethylation:1000.00;C21:Carbamidomethylation:1000.00 | PEAKS DB |
| Y.SQDILMPLWNSYTISK.S | Y | 49.44 | 1894.9498 | 16 | 0.2 | 948.4824 | 2 | 86.89 | 6 | F6:60363 | NaSa01\_F14.raw |  |  |  |  |  | 1.4657E6 |  |  |  |  |  |  |  |  | 1 | 0 | 0 | 0 | 0 | 0 | 1 | 0 | 0 | 0 | 0 | 0 | 0 | 0 | 0 | 587 | 602 |  |  | PEAKS DB |
| R.TLGMLM(+15.99)EGLK.Q | N | 48.81 | 1107.5668 | 10 | 0.5 | 554.7910 | 2 | 43.05 | 6 | F6:23300 | NaSa01\_F14.raw |  |  |  |  |  | 7.4052E5 |  |  |  |  |  |  |  |  | 1 | 0 | 0 | 0 | 0 | 0 | 1 | 0 | 0 | 0 | 0 | 0 | 0 | 0 | 0 | 305 | 314 | Oxidation (M) | M6:Oxidation (M):24.24 | PEAKS DB |
| R.DVELLTGLDFYSALK.Q | Y | 47.44 | 1682.8767 | 15 | 0.0 | 842.4456 | 2 | 104.10 | 5 | F5:73135 | NaSa01\_F13.raw |  |  |  |  | 2.0127E5 | 5.6666E5 |  |  |  |  |  |  |  |  | 2 | 0 | 0 | 0 | 0 | 1 | 1 | 0 | 0 | 0 | 0 | 0 | 0 | 0 | 0 | 795 | 809 |  |  | PEAKS DB |
| R.M(+15.99)ANVLC(+57.02)SC(+57.02)SEDC(+57.02)LTK.K | Y | 45.84 | 1802.7307 | 15 | 1.3 | 902.3738 | 2 | 29.26 | 9 | F9:9349 | NaSa01\_F4.raw |  |  |  |  |  |  |  |  | 1.4442E6 |  |  |  |  |  | 1 | 0 | 0 | 0 | 0 | 0 | 0 | 0 | 0 | 1 | 0 | 0 | 0 | 0 | 0 | 65 | 79 | Oxidation (M); Carbamidomethylation | M1:Oxidation (M):1000.00;C6:Carbamidomethylation:1000.00;C8:Carbamidomethylation:1000.00;C12:Carbamidomethylation:1000.00 | PEAKS DB |
| R.TLGM(+15.99)LMEGLK.Q | N | 45.27 | 1107.5668 | 10 | 0.1 | 554.7907 | 2 | 53.78 | 6 | F6:32542 | NaSa01\_F14.raw |  |  |  |  |  | 3.4616E6 |  |  |  |  |  |  |  |  | 1 | 0 | 0 | 0 | 0 | 0 | 1 | 0 | 0 | 0 | 0 | 0 | 0 | 0 | 0 | 305 | 314 | Oxidation (M) | M4:Oxidation (M):30.83 | PEAKS DB |
| Y.TIVTGLYAETHGIIDNNMYDVK.L | Y | 44.89 | 2466.2100 | 22 | 0.9 | 823.0780 | 3 | 71.03 | 6 | F6:47206 | NaSa01\_F14.raw |  |  |  |  |  | 1.7277E6 |  |  |  |  |  |  |  |  | 1 | 0 | 0 | 0 | 0 | 0 | 1 | 0 | 0 | 0 | 0 | 0 | 0 | 0 | 0 | 168 | 189 |  |  | PEAKS DB |
| R.NAAWWGGQPIWH.T | Y | 43.83 | 1421.6628 | 12 | 0.3 | 711.8389 | 2 | 73.69 | 6 | F6:49226 | NaSa01\_F14.raw |  |  |  |  |  | 1.6228E6 |  |  |  |  |  |  |  |  | 1 | 0 | 0 | 0 | 0 | 0 | 1 | 0 | 0 | 0 | 0 | 0 | 0 | 0 | 0 | 203 | 214 |  |  | PEAKS DB |
| K.SMEAIFLAH.G | N | 41.99 | 1017.4954 | 9 | -0.5 | 509.7547 | 2 | 59.21 | 6 | F6:37583 | NaSa01\_F14.raw |  |  |  |  |  | 2.984E6 |  |  |  |  |  |  |  |  | 1 | 0 | 0 | 0 | 0 | 0 | 1 | 0 | 0 | 0 | 0 | 0 | 0 | 0 | 0 | 447 | 455 |  |  | PEAKS DB |
| K.YISAYSQDILMPLWNSYTISK.S | Y | 41.17 | 2492.2297 | 21 | 1.5 | 1247.1240 | 2 | 98.66 | 6 | F6:70259 | NaSa01\_F14.raw |  |  |  |  |  | 4.2372E5 |  |  |  |  |  |  |  |  | 1 | 0 | 0 | 0 | 0 | 0 | 1 | 0 | 0 | 0 | 0 | 0 | 0 | 0 | 0 | 582 | 602 |  |  | PEAKS DB |
| K.MQSEADNLPYGRPHVLQH.S | Y | 38.90 | 2090.9956 | 18 | 0.7 | 698.0063 | 3 | 25.78 | 6 | F6:8958 | NaSa01\_F14.raw |  |  |  |  |  | 1.8719E6 |  |  |  |  |  |  |  |  | 1 | 0 | 0 | 0 | 0 | 0 | 1 | 0 | 0 | 0 | 0 | 0 | 0 | 0 | 0 | 554 | 571 |  |  | PEAKS DB |
| total 28 peptides |  |  |  |  |  |  |  |  |  |  |  |  |  |  |  |  |  |  |  |  |  |  |  |  |  |  |  |  |  |  |  |  |  |  |  |  |  |  |  |  |  |  |  |  |  |
| --- | --- | --- | --- | --- | --- | --- | --- | --- | --- | --- | --- | --- | --- | --- | --- | --- | --- | --- | --- | --- | --- | --- | --- | --- | --- | --- | --- | --- | --- | --- | --- | --- | --- | --- | --- | --- | --- | --- | --- | --- | --- | --- | --- | --- | --- |

AAB25732.1

back to list

  

| Protein Coverage
| Supporting Peptides
|

Protein Coverage:

Supporting Peptides:

| Peptide | Uniq | -10lgP | Mass | Length | ppm | m/z | z | RT | Fraction | Scan | Source File | Area F1 | Area F10 | Area F11 | Area F12 | Area F13 | Area F14 | Area F2 | Area F3 | Area F4 | Area F5 | Area F6 | Area F7 | Area F8 | Area F9 | #Feature | #Feature F1 | #Feature F10 | #Feature F11 | #Feature F12 | #Feature F13 | #Feature F14 | #Feature F2 | #Feature F3 | #Feature F4 | #Feature F5 | #Feature F6 | #Feature F7 | #Feature F8 | #Feature F9 | Start | End | PTM | AScore | Found By |
| --- | --- | --- | --- | --- | --- | --- | --- | --- | --- | --- | --- | --- | --- | --- | --- | --- | --- | --- | --- | --- | --- | --- | --- | --- | --- | --- | --- | --- | --- | --- | --- | --- | --- | --- | --- | --- | --- | --- | --- | --- | --- | --- | --- | --- | --- |
| K.MFMMSDLTIPVKR.G | N | 85.65 | 1567.7925 | 13 | -1.5 | 784.9023 | 2 | 63.10 | 14 | F14:38560 | NaSa01\_F9.raw |  | 4.7371E11 | 1.047E10 | 8.8147E8 | 1.4813E8 | 9.2858E7 |  |  |  | 4.107E6 | 3.1034E8 | 8.3184E8 | 3.1024E9 | 7.3076E10 | 218 | 0 | 26 | 40 | 34 | 23 | 36 | 0 | 0 | 0 | 2 | 8 | 12 | 18 | 19 | 24 | 36 |  |  | PEAKS DB |
| K.MFMMSDLTIPVK.R | N | 82.34 | 1411.6914 | 12 | -4.0 | 706.8502 | 2 | 79.95 | 14 | F14:52999 | NaSa01\_F9.raw |  | 4.0982E11 | 4.2072E9 | 5.5477E7 | 1.2943E8 | 8.5453E7 |  |  |  | 1.5074E7 | 1.0924E8 | 6.1759E8 | 3.1595E9 | 1.014E11 | 74 | 0 | 16 | 12 | 12 | 7 | 12 | 0 | 0 | 0 | 1 | 3 | 1 | 8 | 2 | 24 | 35 |  |  | PEAKS DB |
| K.M(+15.99)FMMSDLTIPVKR.G | N | 80.41 | 1583.7874 | 13 | 0.7 | 792.9015 | 2 | 55.29 | 14 | F14:32906 | NaSa01\_F9.raw |  | 7.1578E10 | 8.8657E8 | 1.4796E8 | 4.3406E7 | 2.9474E7 |  |  |  |  | 3.8429E7 | 9.9949E7 | 1.1726E9 | 2.1199E10 | 109 | 0 | 13 | 32 | 17 | 5 | 7 | 0 | 0 | 0 | 0 | 3 | 6 | 7 | 19 | 24 | 36 | Oxidation (M) | M1:Oxidation (M):55.92 | PEAKS DB |
| K.MFM(+15.99)MSDLTIPVKR.G | N | 78.47 | 1583.7874 | 13 | 0.0 | 528.9364 | 3 | 75.60 | 14 | F14:50089 | NaSa01\_F9.raw |  | 2.9383E11 | 2.8198E9 | 3.4172E8 | 8.981E7 | 4.3835E7 |  |  |  |  | 8.0368E7 | 3.2862E8 | 2.9321E9 | 3.3649E10 | 222 | 0 | 32 | 42 | 37 | 13 | 17 | 0 | 0 | 0 | 0 | 12 | 12 | 17 | 40 | 24 | 36 | Oxidation (M) | M3:Oxidation (M):54.01 | PEAKS DB |
| K.MFM(+15.99)M(+15.99)SDLTIPVKR.G | N | 76.77 | 1599.7822 | 13 | -0.6 | 534.2677 | 3 | 41.17 | 14 | F14:20645 | NaSa01\_F9.raw |  | 1.219E10 | 1.8117E8 | 6.9219E6 |  |  |  |  |  |  |  | 2.2869E8 | 2.6415E8 | 4.2115E9 | 87 | 0 | 40 | 13 | 1 | 0 | 0 | 0 | 0 | 0 | 0 | 0 | 5 | 7 | 21 | 24 | 36 | Oxidation (M) | M3:Oxidation (M):45.01;M4:Oxidation (M):77.24 | PEAKS DB |
| K.MFM(+15.99)M(+15.99)SDLTIPVK.R | N | 76.55 | 1443.6812 | 12 | -0.4 | 722.8475 | 2 | 64.03 | 14 | F14:39885 | NaSa01\_F9.raw |  | 3.1787E9 | 8.6017E7 | 1.9756E7 |  | 7.1305E6 |  |  |  |  |  | 7.9747E7 | 3.9621E8 | 3.7162E9 | 56 | 0 | 14 | 6 | 3 | 0 | 2 | 0 | 0 | 0 | 0 | 0 | 6 | 5 | 20 | 24 | 35 | Oxidation (M) | M3:Oxidation (M):45.01;M4:Oxidation (M):77.24 | PEAKS DB |
| K.M(+15.99)FM(+15.99)MSDLTIPVKR.G | N | 76.18 | 1599.7822 | 13 | 0.1 | 800.8985 | 2 | 52.55 | 13 | F13:37541 | NaSa01\_F8.raw |  | 6.2475E9 | 1.2325E8 | 1.9501E7 |  |  |  |  |  |  |  | 1.322E7 | 3.1306E8 | 3.3439E9 | 85 | 0 | 32 | 13 | 3 | 0 | 0 | 0 | 0 | 0 | 0 | 0 | 2 | 14 | 21 | 24 | 36 | Oxidation (M) | M1:Oxidation (M):77.24;M3:Oxidation (M):40.00 | PEAKS DB |
| K.M(+15.99)FM(+15.99)MSDLTIPVK.R | N | 75.96 | 1443.6812 | 12 | 0.1 | 722.8479 | 2 | 66.96 | 14 | F14:41891 | NaSa01\_F9.raw |  | 1.7613E9 | 6.7882E7 | 1.8015E7 | 2.7301E6 | 2.7953E6 |  |  |  |  | 5.7125E5 | 9.4013E6 | 2.141E8 | 1.891E9 | 32 | 0 | 5 | 5 | 2 | 1 | 1 | 0 | 0 | 0 | 0 | 1 | 3 | 4 | 10 | 24 | 35 | Oxidation (M) | M1:Oxidation (M):77.24;M3:Oxidation (M):40.00 | PEAKS DB |
| K.M(+15.99)FMM(+15.99)SDLTIPVKR.G | N | 75.86 | 1599.7822 | 13 | -3.1 | 534.2664 | 3 | 53.53 | 14 | F14:31147 | NaSa01\_F9.raw |  | 9.5945E9 | 1.5873E8 | 8.0591E6 |  |  |  |  |  |  |  | 2.0718E8 | 3.8055E8 | 2.4029E9 | 60 | 0 | 20 | 10 | 2 | 0 | 0 | 0 | 0 | 0 | 0 | 0 | 4 | 9 | 15 | 24 | 36 | Oxidation (M) | M1:Oxidation (M):54.01;M4:Oxidation (M):33.98 | PEAKS DB |
| K.MFMM(+15.99)SDLTIPVKR.G | N | 75.76 | 1583.7874 | 13 | 0.1 | 792.9011 | 2 | 49.93 | 13 | F13:35686 | NaSa01\_F8.raw |  | 2.0778E11 | 3.8572E8 | 2.8286E8 | 1.9434E7 | 3.8596E7 |  |  |  |  | 4.7301E7 | 4.5689E8 | 1.602E9 | 1.958E10 | 122 | 0 | 10 | 29 | 25 | 3 | 9 | 0 | 0 | 0 | 0 | 4 | 10 | 12 | 20 | 24 | 36 | Oxidation (M) | M4:Oxidation (M):33.98 | PEAKS DB |
| K.M(+15.99)FMM(+15.99)SDLTIPVK.R | N | 75.60 | 1443.6812 | 12 | 0.5 | 722.8482 | 2 | 55.86 | 14 | F14:34026 | NaSa01\_F9.raw |  | 2.4381E9 | 1.3198E8 | 1.6318E7 | 7.3315E6 | 0 |  |  |  |  |  | 2.6576E7 | 4.0662E8 | 2.2655E9 | 41 | 0 | 7 | 8 | 3 | 2 | 0 | 0 | 0 | 0 | 0 | 0 | 4 | 7 | 10 | 24 | 35 | Oxidation (M) | M1:Oxidation (M):54.01;M4:Oxidation (M):40.00 | PEAKS DB |
| K.MFMM(+15.99)SDLTIPVK.R | N | 74.24 | 1427.6863 | 12 | -0.3 | 714.8502 | 2 | 65.10 | 14 | F14:41090 | NaSa01\_F9.raw |  | 5.4562E10 | 4.6107E8 | 4.5289E7 | 5.1599E6 | 1.8549E7 |  |  |  | 2.0841E6 |  | 3.2728E8 | 8.8298E8 | 1.1369E10 | 40 | 0 | 7 | 9 | 3 | 2 | 2 | 0 | 0 | 0 | 2 | 0 | 6 | 2 | 7 | 24 | 35 | Oxidation (M) | M4:Oxidation (M):40.00 | PEAKS DB |
| K.M(+15.99)FMMSDLTIPVK.R | N | 72.36 | 1427.6863 | 12 | -0.1 | 714.8503 | 2 | 68.89 | 14 | F14:46191 | NaSa01\_F9.raw |  | 4.9093E10 | 5.9003E8 | 1.2556E8 | 2.7662E7 | 4.0319E7 |  |  |  | 1.0406E6 | 1.9495E7 | 1.1389E8 | 6.1791E8 | 1.0114E10 | 36 | 0 | 4 | 6 | 5 | 1 | 6 | 0 | 0 | 0 | 1 | 4 | 2 | 5 | 2 | 24 | 35 | Oxidation (M) | M1:Oxidation (M):45.01 | PEAKS DB |
| Y.KMFMMSDLTIPVK.R | N | 71.87 | 1539.7863 | 13 | 0.0 | 770.9004 | 2 | 62.06 | 14 | F14:37991 | NaSa01\_F9.raw |  | 6.9914E6 |  |  |  |  |  |  |  |  |  |  |  | 1.7145E8 | 3 | 0 | 1 | 0 | 0 | 0 | 0 | 0 | 0 | 0 | 0 | 0 | 0 | 0 | 2 | 23 | 35 |  |  | PEAKS DB |
| K.MFM(+15.99)MSDLTIPVK.R | N | 71.67 | 1427.6863 | 12 | -0.1 | 714.8503 | 2 | 74.60 | 14 | F14:48885 | NaSa01\_F9.raw |  | 7.9492E10 | 1.095E9 | 1.2717E8 | 7.4402E7 | 6.7354E7 |  |  |  | 1.258E6 | 3.108E7 | 1.9949E8 | 1.5634E9 | 2.2848E10 | 82 | 0 | 11 | 17 | 6 | 10 | 10 | 0 | 0 | 0 | 1 | 8 | 2 | 9 | 8 | 24 | 35 | Oxidation (M) | M3:Oxidation (M):45.01 | PEAKS DB |
| K.M(+15.99)FM(+15.99)M(+15.99)SDLTIPVKR.G | N | 71.54 | 1615.7772 | 13 | -0.1 | 539.5996 | 3 | 34.45 | 14 | F14:15279 | NaSa01\_F9.raw |  | 9.5625E8 | 2.0938E7 | 5.6476E6 |  |  |  |  |  |  |  | 5.7431E6 | 5.031E6 | 2.3574E8 | 87 | 0 | 51 | 8 | 2 | 0 | 0 | 0 | 0 | 0 | 0 | 0 | 4 | 4 | 18 | 24 | 36 | Oxidation (M) | M1:Oxidation (M):1000.00;M3:Oxidation (M):1000.00;M4:Oxidation (M):1000.00 | PEAKS DB |
| F.MMSDLTIPVKR.G | N | 71.31 | 1289.6836 | 11 | -3.4 | 430.9003 | 3 | 33.03 | 14 | F14:14050 | NaSa01\_F9.raw |  | 1.075E10 | 9.8694E6 | 4.7242E6 |  | 6.142E5 |  |  |  |  |  | 8.4849E6 | 4.0319E7 | 3.4698E8 | 26 | 0 | 10 | 3 | 2 | 0 | 1 | 0 | 0 | 0 | 0 | 0 | 2 | 2 | 6 | 26 | 36 |  |  | PEAKS DB |
| F.MMSDLTIPVK.R | N | 69.57 | 1133.5824 | 10 | 0.0 | 567.7985 | 2 | 48.73 | 14 | F14:27397 | NaSa01\_F9.raw |  | 3.0593E9 | 1.0852E7 | 9.582E6 |  | 7.0856E6 |  |  |  |  | 1.4839E6 | 1.0886E7 | 5.4196E7 | 1.3756E9 | 15 | 0 | 6 | 1 | 1 | 0 | 1 | 0 | 0 | 0 | 0 | 1 | 1 | 1 | 3 | 26 | 35 |  |  | PEAKS DB |
| Y.KMFMMSDLTIPVKR.G | N | 69.46 | 1695.8875 | 14 | 0.0 | 566.3031 | 3 | 49.12 | 14 | F14:27997 | NaSa01\_F9.raw |  | 0 |  |  |  |  |  |  |  |  | 1.6069E6 |  |  | 1.3139E8 | 5 | 0 | 0 | 0 | 0 | 0 | 0 | 0 | 0 | 0 | 0 | 1 | 0 | 0 | 4 | 23 | 36 |  |  | PEAKS DB |
| M.FMMSDLTIPVK.R | N | 66.96 | 1280.6509 | 11 | 1.2 | 641.3335 | 2 | 70.13 | 14 | F14:44588 | NaSa01\_F9.raw |  | 1.632E8 | 3.5244E6 |  |  |  |  |  |  |  |  |  | 4.1395E6 | 4.8053E7 | 7 | 0 | 3 | 1 | 0 | 0 | 0 | 0 | 0 | 0 | 0 | 0 | 0 | 1 | 2 | 25 | 35 |  |  | PEAKS DB |
| K.M(+15.99)FM(+15.99)M(+15.99)SDLTIPVK.R | N | 66.85 | 1459.6761 | 12 | -0.1 | 730.8453 | 2 | 47.85 | 14 | F14:26577 | NaSa01\_F9.raw |  | 3.593E8 | 4.3429E6 | 1.9537E6 |  | 1.1632E6 |  |  |  |  |  |  | 1.4862E7 | 1.5886E8 | 44 | 0 | 18 | 3 | 1 | 0 | 1 | 0 | 0 | 0 | 0 | 0 | 0 | 6 | 15 | 24 | 35 | Oxidation (M) | M1:Oxidation (M):1000.00;M3:Oxidation (M):1000.00;M4:Oxidation (M):1000.00 | PEAKS DB |
| M.MSDLTIPVKR.G | N | 63.86 | 1158.6431 | 10 | -0.4 | 580.3286 | 2 | 23.12 | 12 | F12:9466 | NaSa01\_F7.raw |  | 9.0224E8 |  |  |  |  |  |  |  |  |  | 7.9413E6 |  | 3.9313E6 | 10 | 0 | 6 | 0 | 0 | 0 | 0 | 0 | 0 | 0 | 0 | 0 | 2 | 0 | 2 | 27 | 36 |  |  | PEAKS DB |
| F.MM(+15.99)SDLTIPVK.R | N | 63.21 | 1149.5774 | 10 | 0.5 | 575.7963 | 2 | 37.96 | 14 | F14:17920 | NaSa01\_F9.raw |  | 6.5672E8 |  |  |  |  |  |  |  |  |  | 4.229E5 | 1.7521E7 | 2.0128E8 | 10 | 0 | 3 | 0 | 0 | 0 | 0 | 0 | 0 | 0 | 0 | 0 | 1 | 4 | 2 | 26 | 35 | Oxidation (M) | M2:Oxidation (M):17.01 | PEAKS DB |
| M.MSDLTIPVK.R | N | 62.11 | 1002.5419 | 9 | -0.8 | 502.2778 | 2 | 37.80 | 14 | F14:17786 | NaSa01\_F9.raw |  | 2.9883E9 | 6.709E5 | 1.1163E7 | 4.8834E6 | 2.4036E6 |  |  |  |  | 1.1097E6 | 8.1712E6 | 7.7762E7 | 1.8451E7 | 9 | 0 | 1 | 1 | 1 | 1 | 1 | 0 | 0 | 0 | 0 | 1 | 1 | 1 | 1 | 27 | 35 |  |  | PEAKS DB |
| M.FMMSDLTIPVKR.G | N | 61.80 | 1436.7520 | 12 | -3.3 | 479.9230 | 3 | 53.99 | 14 | F14:31124 | NaSa01\_F9.raw |  | 3.3177E9 | 4.5283E6 |  |  |  |  |  |  |  |  |  | 3.6473E6 | 6.0815E7 | 12 | 0 | 6 | 2 | 0 | 0 | 0 | 0 | 0 | 0 | 0 | 0 | 0 | 1 | 3 | 25 | 36 |  |  | PEAKS DB |
| F.M(+15.99)MSDLTIPVK.R | N | 60.64 | 1149.5774 | 10 | -0.1 | 575.7959 | 2 | 41.56 | 14 | F14:23719 | NaSa01\_F9.raw |  | 4.309E8 |  |  |  |  |  |  |  |  |  | 1.2837E5 | 1.0805E7 | 1.559E8 | 5 | 0 | 1 | 0 | 0 | 0 | 0 | 0 | 0 | 0 | 0 | 0 | 1 | 2 | 1 | 26 | 35 | Oxidation (M) | M1:Oxidation (M):17.01 | PEAKS DB |
| Y.KMFM(+15.99)MSDLTIPVK.R | N | 60.11 | 1555.7812 | 13 | 0.4 | 519.6013 | 3 | 57.95 | 14 | F14:34531 | NaSa01\_F9.raw |  |  |  |  |  |  |  |  |  |  |  |  |  | 5.4637E7 | 2 | 0 | 0 | 0 | 0 | 0 | 0 | 0 | 0 | 0 | 0 | 0 | 0 | 0 | 2 | 23 | 35 | Oxidation (M) | M4:Oxidation (M):19.68 | PEAKS DB |
| Y.KMFM(+15.99)MSDLTIPVKR.G | N | 59.87 | 1711.8823 | 14 | -0.6 | 428.9776 | 4 | 45.90 | 14 | F14:24847 | NaSa01\_F9.raw |  | 9.6342E5 |  |  |  |  |  |  |  |  |  |  |  | 1.2363E7 | 4 | 0 | 1 | 0 | 0 | 0 | 0 | 0 | 0 | 0 | 0 | 0 | 0 | 0 | 3 | 23 | 36 | Oxidation (M) | M4:Oxidation (M):20.00 | PEAKS DB |
| M.FM(+15.99)MSDLTIPVKR.G | N | 58.43 | 1452.7468 | 12 | -1.1 | 485.2557 | 3 | 50.53 | 14 | F14:28589 | NaSa01\_F9.raw |  | 1.0041E7 |  |  |  |  |  |  |  |  |  |  |  | 1.7669E6 | 2 | 0 | 1 | 0 | 0 | 0 | 0 | 0 | 0 | 0 | 0 | 0 | 0 | 0 | 1 | 25 | 36 | Oxidation (M) | M2:Oxidation (M):40.00 | PEAKS DB |
| K.MFMMSDLTIPVKRGC(+57.02)IDVC(+57.02)PK.S | N | 57.63 | 2497.2024 | 21 | -0.2 | 625.3077 | 4 | 62.50 | 2 | F2:33823 | NaSa01\_F10.raw |  | 7.1436E7 |  |  |  |  |  |  |  |  |  |  |  |  | 4 | 0 | 4 | 0 | 0 | 0 | 0 | 0 | 0 | 0 | 0 | 0 | 0 | 0 | 0 | 24 | 44 | Carbamidomethylation | C15:Carbamidomethylation:1000.00;C19:Carbamidomethylation:1000.00 | PEAKS DB |
| M.FMM(+15.99)SDLTIPVKR.G | N | 57.50 | 1452.7468 | 12 | -0.4 | 485.2560 | 3 | 38.55 | 14 | F14:18443 | NaSa01\_F9.raw |  | 1.3989E7 |  |  |  |  |  |  |  |  |  |  |  | 3.9973E6 | 3 | 0 | 2 | 0 | 0 | 0 | 0 | 0 | 0 | 0 | 0 | 0 | 0 | 0 | 1 | 25 | 36 | Oxidation (M) | M3:Oxidation (M):33.98 | PEAKS DB |
| K.TC(+57.02)PAGKNLC(+57.02)YKMFMMSDLTIPVK.R | N | 53.75 | 2704.2920 | 23 | 4.1 | 677.0831 | 4 | 64.24 | 2 | F2:34760 | NaSa01\_F10.raw |  | 4.0898E7 |  |  |  |  |  |  |  |  |  |  |  |  | 3 | 0 | 3 | 0 | 0 | 0 | 0 | 0 | 0 | 0 | 0 | 0 | 0 | 0 | 0 | 13 | 35 | Carbamidomethylation | C2:Carbamidomethylation:1000.00;C9:Carbamidomethylation:1000.00 | PEAKS DB |
| Y.KM(+15.99)FMMSDLTIPVKR.G | N | 53.09 | 1711.8823 | 14 | 0.3 | 428.9780 | 4 | 41.16 | 14 | F14:20431 | NaSa01\_F9.raw |  |  |  |  |  |  |  |  |  |  |  |  |  | 3.2055E6 | 2 | 0 | 0 | 0 | 0 | 0 | 0 | 0 | 0 | 0 | 0 | 0 | 0 | 0 | 2 | 23 | 36 | Oxidation (M) | M2:Oxidation (M):30.83 | PEAKS DB |
| F.MM(+15.99)SDLTIPVKR.G | N | 52.73 | 1305.6785 | 11 | 0.1 | 436.2335 | 3 | 24.28 | 14 | F14:9504 | NaSa01\_F9.raw |  | 5.0593E8 |  |  |  |  |  |  |  |  |  |  |  | 5.9731E6 | 6 | 0 | 5 | 0 | 0 | 0 | 0 | 0 | 0 | 0 | 0 | 0 | 0 | 0 | 1 | 26 | 36 | Oxidation (M) | M2:Oxidation (M):17.01 | PEAKS DB |
| R.GC(+57.02)IDVC(+57.02)PK.S | N | 51.80 | 947.4205 | 8 | -0.1 | 474.7175 | 2 | 11.78 | 4 | F4:2753 | NaSa01\_F12.raw |  |  | 1.2016E8 | 2.8471E6 |  |  |  |  | 1.4583E5 |  |  | 9.3598E5 | 3.6007E7 | 2.198E7 | 6 | 0 | 0 | 1 | 1 | 0 | 0 | 0 | 0 | 1 | 0 | 0 | 1 | 1 | 1 | 37 | 44 | Carbamidomethylation | C2:Carbamidomethylation:1000.00;C6:Carbamidomethylation:1000.00 | PEAKS DB |
| K.NLC(+57.02)YKMFMMSDLTIPVKR.G | N | 51.76 | 2246.1084 | 18 | -0.5 | 749.7097 | 3 | 65.64 | 2 | F2:35544 | NaSa01\_F10.raw |  | 9.3276E8 | 1.0866E6 |  |  |  |  |  |  |  |  |  |  |  | 9 | 0 | 8 | 1 | 0 | 0 | 0 | 0 | 0 | 0 | 0 | 0 | 0 | 0 | 0 | 19 | 36 | Carbamidomethylation | C3:Carbamidomethylation:1000.00 | PEAKS DB |
| K.YVC(+57.02)C(+57.02)NTDR.C | N | 50.96 | 1086.4222 | 8 | 0.1 | 544.2184 | 2 | 11.80 | 12 | F12:2224 | NaSa01\_F7.raw |  |  | 2.9228E7 | 4.0658E5 |  |  |  |  |  |  |  | 4.3073E6 | 5.5734E8 | 1.1711E7 | 6 | 0 | 0 | 1 | 1 | 0 | 0 | 0 | 0 | 0 | 0 | 0 | 1 | 2 | 1 | 51 | 58 | Carbamidomethylation | C3:Carbamidomethylation:1000.00;C4:Carbamidomethylation:1000.00 | PEAKS DB |
| Y.KMFMM(+15.99)SDLTIPVKR.G | N | 50.68 | 1711.8823 | 14 | -0.2 | 428.9778 | 4 | 36.60 | 14 | F14:16557 | NaSa01\_F9.raw |  |  |  |  |  |  |  |  |  |  |  |  |  | 1.1337E6 | 1 | 0 | 0 | 0 | 0 | 0 | 0 | 0 | 0 | 0 | 0 | 0 | 0 | 0 | 1 | 23 | 36 | Oxidation (M) | M5:Oxidation (M):11.06 | PEAKS DB |
| F.M(+15.99)M(+15.99)SDLTIPVK.R | N | 50.22 | 1165.5723 | 10 | -0.4 | 583.7932 | 2 | 48.73 | 14 | F14:27374 | NaSa01\_F9.raw |  | 1.3942E7 |  |  |  |  |  |  |  |  |  |  |  | 3.1316E6 | 2 | 0 | 1 | 0 | 0 | 0 | 0 | 0 | 0 | 0 | 0 | 0 | 0 | 0 | 1 | 26 | 35 | Oxidation (M) | M1:Oxidation (M):1000.00;M2:Oxidation (M):1000.00 | PEAKS DB |
| F.M(+15.99)MSDLTIPVKR.G | N | 50.21 | 1305.6785 | 11 | 0.1 | 436.2335 | 3 | 24.28 | 14 | F14:8281 | NaSa01\_F9.raw |  | 4.535E8 |  |  |  |  |  |  |  |  |  |  |  | 5.9731E6 | 4 | 0 | 3 | 0 | 0 | 0 | 0 | 0 | 0 | 0 | 0 | 0 | 0 | 0 | 1 | 26 | 36 | Oxidation (M) | M1:Oxidation (M):17.01 | PEAKS DB |
| M.FM(+15.99)MSDLTIPVK.R | N | 49.89 | 1296.6458 | 11 | 0.2 | 649.3303 | 2 | 65.76 | 14 | F14:40737 | NaSa01\_F9.raw |  | 4.4142E6 |  |  |  |  |  |  |  |  |  |  |  | 4.3603E6 | 2 | 0 | 1 | 0 | 0 | 0 | 0 | 0 | 0 | 0 | 0 | 0 | 0 | 0 | 1 | 25 | 35 | Oxidation (M) | M2:Oxidation (M):30.46 | PEAKS DB |
| M.M(+15.99)SDLTIPVK.R | N | 48.61 | 1018.5369 | 9 | 0.5 | 510.2759 | 2 | 30.64 | 13 | F13:19291 | NaSa01\_F8.raw |  | 3.7301E8 |  |  |  |  |  |  |  |  |  | 2.7209E5 | 4.1866E6 |  | 7 | 0 | 4 | 0 | 0 | 0 | 0 | 0 | 0 | 0 | 0 | 0 | 1 | 2 | 0 | 27 | 35 | Oxidation (M) | M1:Oxidation (M):1000.00 | PEAKS DB |
| R.GC(+57.02)IDVC(+57.02)PKSNLLVK.Y | Y | 48.50 | 1601.8269 | 14 | 0.5 | 534.9498 | 3 | 32.27 | 14 | F14:13382 | NaSa01\_F9.raw |  |  |  |  |  |  |  |  |  |  |  |  |  | 6.6657E6 | 1 | 0 | 0 | 0 | 0 | 0 | 0 | 0 | 0 | 0 | 0 | 0 | 0 | 0 | 1 | 37 | 50 | Carbamidomethylation | C2:Carbamidomethylation:1000.00;C6:Carbamidomethylation:1000.00 | PEAKS DB |
| K.NLC(+57.02)YKMFMMSDLTIPVK.R | N | 48.07 | 2090.0073 | 17 | 1.1 | 697.6771 | 3 | 78.05 | 3 | F3:50007 | NaSa01\_F11.raw |  | 4.5969E6 | 6.7434E5 |  |  |  |  |  |  |  |  |  |  | 1.2654E7 | 5 | 0 | 1 | 1 | 0 | 0 | 0 | 0 | 0 | 0 | 0 | 0 | 0 | 0 | 3 | 19 | 35 | Carbamidomethylation | C3:Carbamidomethylation:1000.00 | PEAKS DB |
| K.MFMMSDLTIPVKRG.C | N | 47.35 | 1624.8140 | 14 | 0.2 | 813.4144 | 2 | 60.68 | 2 | F2:33100 | NaSa01\_F10.raw |  | 4.0607E7 |  |  |  |  |  |  |  |  |  |  |  |  | 2 | 0 | 2 | 0 | 0 | 0 | 0 | 0 | 0 | 0 | 0 | 0 | 0 | 0 | 0 | 24 | 37 |  |  | PEAKS DB |
| Y.KMFMM(+15.99)SDLTIPVK.R | N | 47.13 | 1555.7812 | 13 | 0.4 | 519.6013 | 3 | 49.04 | 14 | F14:27572 | NaSa01\_F9.raw |  |  |  |  |  |  |  |  |  |  |  |  |  | 3.076E7 | 4 | 0 | 0 | 0 | 0 | 0 | 0 | 0 | 0 | 0 | 0 | 0 | 0 | 0 | 4 | 23 | 35 | Oxidation (M) | M5:Oxidation (M):7.65 | PEAKS DB |
| K.NLC(+57.02)YKMF.M | N | 46.68 | 974.4354 | 7 | -0.6 | 488.2247 | 2 | 44.70 | 12 | F12:27287 | NaSa01\_F7.raw |  |  | 1.8393E7 |  |  |  |  |  |  |  |  | 5.6016E6 |  |  | 2 | 0 | 0 | 1 | 0 | 0 | 0 | 0 | 0 | 0 | 0 | 0 | 1 | 0 | 0 | 19 | 25 | Carbamidomethylation | C3:Carbamidomethylation:1000.00 | PEAKS DB |
| N.LC(+57.02)YKMFMMSDLTIPVKR.G | N | 46.21 | 2132.0654 | 17 | -1.3 | 711.6948 | 3 | 64.28 | 2 | F2:34648 | NaSa01\_F10.raw |  | 1.9189E6 |  |  |  |  |  |  |  |  |  |  |  |  | 1 | 0 | 1 | 0 | 0 | 0 | 0 | 0 | 0 | 0 | 0 | 0 | 0 | 0 | 0 | 20 | 36 | Carbamidomethylation | C2:Carbamidomethylation:1000.00 | PEAKS DB |
| R.GC(+57.02)IDVC(+57.02)PKSNLLVKYVC(+57.02)C(+57.02)NTDR.C | Y | 45.82 | 2670.2385 | 22 | -0.5 | 891.0863 | 3 | 46.18 | 2 | F2:23352 | NaSa01\_F10.raw |  | 7.2667E7 |  |  |  |  |  |  |  |  |  |  |  |  | 2 | 0 | 2 | 0 | 0 | 0 | 0 | 0 | 0 | 0 | 0 | 0 | 0 | 0 | 0 | 37 | 58 | Carbamidomethylation | C2:Carbamidomethylation:1000.00;C6:Carbamidomethylation:1000.00;C17:Carbamidomethylation:1000.00;C18:Carbamidomethylation:1000.00 | PEAKS DB |
| K.NLC(+57.02)YKMFMM(+15.99)SDLTIPVKR.G | N | 45.15 | 2262.1033 | 18 | -0.1 | 566.5330 | 4 | 56.99 | 2 | F2:31071 | NaSa01\_F10.raw |  | 0 |  |  |  |  |  |  |  |  |  |  |  |  | 0 | 0 | 0 | 0 | 0 | 0 | 0 | 0 | 0 | 0 | 0 | 0 | 0 | 0 | 0 | 19 | 36 | Carbamidomethylation; Oxidation (M) | C3:Carbamidomethylation:1000.00;M9:Oxidation (M):0.00 | PEAKS DB |
| K.LIPIASKTC(+57.02)PAGK.N | N | 44.16 | 1354.7642 | 13 | -0.7 | 452.5950 | 3 | 11.53 | 2 | F2:1937 | NaSa01\_F10.raw |  | 0 |  |  |  |  |  |  |  |  |  |  |  |  | 0 | 0 | 0 | 0 | 0 | 0 | 0 | 0 | 0 | 0 | 0 | 0 | 0 | 0 | 0 | 6 | 18 | Carbamidomethylation | C9:Carbamidomethylation:1000.00 | PEAKS DB |
| K.SNLLVKYVC(+57.02)C(+57.02)NTDRC(+57.02)N | Y | 42.89 | 2014.9023 | 16 | 0.4 | 1008.4589 | 2 | 32.29 | 12 | F12:17032 | NaSa01\_F7.raw |  |  |  |  |  |  |  |  |  |  |  | 1.4718E5 |  |  | 1 | 0 | 0 | 0 | 0 | 0 | 0 | 0 | 0 | 0 | 0 | 0 | 1 | 0 | 0 | 45 | 60 | Carbamidomethylation | C9:Carbamidomethylation:1000.00;C10:Carbamidomethylation:1000.00;C15:Carbamidomethylation:1000.00 | PEAKS DB |
| K.NLC(+57.02)YKM(+15.99)FMMSDLTIPVKR.G | N | 42.18 | 2262.1033 | 18 | -0.3 | 566.5329 | 4 | 58.44 | 2 | F2:31811 | NaSa01\_F10.raw |  | 0 |  |  |  |  |  |  |  |  |  |  |  | 1.9296E5 | 1 | 0 | 0 | 0 | 0 | 0 | 0 | 0 | 0 | 0 | 0 | 0 | 0 | 0 | 1 | 19 | 36 | Carbamidomethylation; Oxidation (M) | C3:Carbamidomethylation:1000.00;M6:Oxidation (M):36.05 | PEAKS DB |
| Y.KM(+15.99)FMMSDLTIPVK.R | N | 41.86 | 1555.7812 | 13 | 3.7 | 778.9008 | 2 | 53.74 | 14 | F14:31073 | NaSa01\_F9.raw |  |  |  |  |  |  |  |  |  |  |  |  |  | 0 | 0 | 0 | 0 | 0 | 0 | 0 | 0 | 0 | 0 | 0 | 0 | 0 | 0 | 0 | 0 | 23 | 35 | Oxidation (M) | M2:Oxidation (M):11.22 | PEAKS DB |
| K.YVC(+57.02)C(+57.02)NTDRC(+57.02)N | N | 41.38 | 1360.4958 | 10 | 0.0 | 681.2552 | 2 | 11.81 | 12 | F12:2256 | NaSa01\_F7.raw |  |  |  |  |  |  |  |  |  |  |  | 2.7635E5 |  |  | 1 | 0 | 0 | 0 | 0 | 0 | 0 | 0 | 0 | 0 | 0 | 0 | 1 | 0 | 0 | 51 | 60 | Carbamidomethylation | C3:Carbamidomethylation:1000.00;C4:Carbamidomethylation:1000.00;C9:Carbamidomethylation:1000.00 | PEAKS DB |
| K.RGC(+57.02)IDVC(+57.02)PK.S | N | 40.76 | 1103.5216 | 9 | 0.0 | 552.7681 | 2 | 11.31 | 14 | F14:1779 | NaSa01\_F9.raw |  |  | 5.5737E6 |  |  |  |  |  |  |  |  |  |  | 6.4252E5 | 2 | 0 | 0 | 1 | 0 | 0 | 0 | 0 | 0 | 0 | 0 | 0 | 0 | 0 | 1 | 36 | 44 | Carbamidomethylation | C3:Carbamidomethylation:1000.00;C7:Carbamidomethylation:1000.00 | PEAKS DB |
| Y.KM(+15.99)FM(+15.99)MSDLTIPVKR.G | N | 40.02 | 1727.8772 | 14 | 0.0 | 432.9766 | 4 | 37.36 | 2 | F2:16465 | NaSa01\_F10.raw |  | 9.6029E5 |  |  |  |  |  |  |  |  |  |  |  |  | 1 | 0 | 1 | 0 | 0 | 0 | 0 | 0 | 0 | 0 | 0 | 0 | 0 | 0 | 0 | 23 | 36 | Oxidation (M) | M2:Oxidation (M):19.27;M4:Oxidation (M):7.65 | PEAKS DB |
| K.NLC(+57.02)YKMFM.M | N | 39.67 | 1105.4758 | 8 | 0.3 | 553.7454 | 2 | 57.88 | 3 | F3:32929 | NaSa01\_F11.raw |  |  | 1.6979E6 |  |  |  |  |  |  |  |  |  |  |  | 1 | 0 | 0 | 1 | 0 | 0 | 0 | 0 | 0 | 0 | 0 | 0 | 0 | 0 | 0 | 19 | 26 | Carbamidomethylation | C3:Carbamidomethylation:1000.00 | PEAKS DB |
| K.SNLLVKYVC(+57.02)C(+57.02)NTDR.C | Y | 39.44 | 1740.8287 | 14 | 0.8 | 871.4223 | 2 | 32.27 | 14 | F14:12982 | NaSa01\_F9.raw |  |  |  |  |  |  |  |  |  |  |  |  |  | 1.0131E7 | 1 | 0 | 0 | 0 | 0 | 0 | 0 | 0 | 0 | 0 | 0 | 0 | 0 | 0 | 1 | 45 | 58 | Carbamidomethylation | C9:Carbamidomethylation:1000.00;C10:Carbamidomethylation:1000.00 | PEAKS DB |
| K.NLC(+57.02)YKMFM(+15.99)MSDLTIPVKR.G | N | 38.40 | 2262.1033 | 18 | 0.1 | 755.0418 | 3 | 64.43 | 2 | F2:34785 | NaSa01\_F10.raw |  | 0 |  |  |  |  |  |  |  |  |  |  |  |  | 0 | 0 | 0 | 0 | 0 | 0 | 0 | 0 | 0 | 0 | 0 | 0 | 0 | 0 | 0 | 19 | 36 | Carbamidomethylation; Oxidation (M) | C3:Carbamidomethylation:1000.00;M8:Oxidation (M):0.00 | PEAKS DB |
| total 60 peptides |  |  |  |  |  |  |  |  |  |  |  |  |  |  |  |  |  |  |  |  |  |  |  |  |  |  |  |  |  |  |  |  |  |  |  |  |  |  |  |  |  |  |  |  |  |
| --- | --- | --- | --- | --- | --- | --- | --- | --- | --- | --- | --- | --- | --- | --- | --- | --- | --- | --- | --- | --- | --- | --- | --- | --- | --- | --- | --- | --- | --- | --- | --- | --- | --- | --- | --- | --- | --- | --- | --- | --- | --- | --- | --- | --- | --- |

3K7N

back to list

  

| Protein Coverage
| Supporting Peptides
|

Protein Coverage:

Supporting Peptides:

| Peptide | Uniq | -10lgP | Mass | Length | ppm | m/z | z | RT | Fraction | Scan | Source File | Area F1 | Area F10 | Area F11 | Area F12 | Area F13 | Area F14 | Area F2 | Area F3 | Area F4 | Area F5 | Area F6 | Area F7 | Area F8 | Area F9 | #Feature | #Feature F1 | #Feature F10 | #Feature F11 | #Feature F12 | #Feature F13 | #Feature F14 | #Feature F2 | #Feature F3 | #Feature F4 | #Feature F5 | #Feature F6 | #Feature F7 | #Feature F8 | #Feature F9 | Start | End | PTM | AScore | Found By |
| --- | --- | --- | --- | --- | --- | --- | --- | --- | --- | --- | --- | --- | --- | --- | --- | --- | --- | --- | --- | --- | --- | --- | --- | --- | --- | --- | --- | --- | --- | --- | --- | --- | --- | --- | --- | --- | --- | --- | --- | --- | --- | --- | --- | --- | --- |
| K.DDC(+57.02)DLPELC(+57.02)TGQSAEC(+57.02)PTDSLQR.N | N | 83.48 | 2666.0894 | 23 | 0.0 | 1334.0520 | 2 | 59.11 | 3 | F3:33999 | NaSa01\_F11.raw |  | 1.2917E6 | 2.5708E8 | 4.7991E8 | 3.8851E7 | 2.8712E7 |  |  | 2.4126E8 |  |  |  | 5.6598E5 |  | 16 | 0 | 1 | 5 | 4 | 2 | 2 | 0 | 0 | 1 | 0 | 0 | 0 | 1 | 0 | 258 | 280 | Carbamidomethylation | C3:Carbamidomethylation:1000.00;C9:Carbamidomethylation:1000.00;C16:Carbamidomethylation:1000.00 | PEAKS DB |
| R.TAPAFQFSSC(+57.02)SIR.E | N | 76.44 | 1470.6925 | 13 | -0.4 | 736.3532 | 2 | 46.46 | 6 | F6:26184 | NaSa01\_F14.raw |  |  |  | 2.702E7 | 6.1746E8 | 9.7374E7 |  |  |  |  |  | 2.3839E5 |  | 5.3658E6 | 12 | 0 | 0 | 0 | 1 | 4 | 5 | 0 | 0 | 0 | 0 | 0 | 1 | 0 | 1 | 174 | 186 | Carbamidomethylation | C10:Carbamidomethylation:1000.00 | PEAKS DB |
| K.NTMSC(+57.02)LIPPNPDGIMAEPGTK.C | N | 73.85 | 2242.0432 | 21 | -0.8 | 1122.0280 | 2 | 68.00 | 6 | F6:44885 | NaSa01\_F14.raw |  |  |  |  | 6.4544E8 | 1.0813E8 |  |  |  |  |  | 8.4111E7 | 2.0872E7 |  | 9 | 0 | 0 | 0 | 0 | 3 | 2 | 0 | 0 | 0 | 0 | 0 | 2 | 2 | 0 | 358 | 378 | Carbamidomethylation | C5:Carbamidomethylation:1000.00 | PEAKS DB |
| K.VYEMINAVNTK.F | N | 69.56 | 1280.6434 | 11 | 0.7 | 641.3295 | 2 | 33.85 | 6 | F6:15682 | NaSa01\_F14.raw |  |  |  | 2.984E7 | 1.408E9 | 8.928E6 |  |  |  |  |  |  |  |  | 6 | 0 | 0 | 0 | 2 | 2 | 2 | 0 | 0 | 0 | 0 | 0 | 0 | 0 | 0 | 36 | 46 |  |  | PEAKS DB |
| D.LPELC(+57.02)TGQSAEC(+57.02)PTDSLQR.N | N | 65.21 | 2160.9780 | 19 | 0.2 | 1081.4965 | 2 | 45.39 | 6 | F6:25364 | NaSa01\_F14.raw |  |  | 1.3036E6 | 4.0582E6 | 1.0756E6 | 2.3359E5 |  |  |  |  |  |  |  |  | 4 | 0 | 0 | 1 | 1 | 1 | 1 | 0 | 0 | 0 | 0 | 0 | 0 | 0 | 0 | 262 | 280 | Carbamidomethylation | C5:Carbamidomethylation:1000.00;C12:Carbamidomethylation:1000.00 | PEAKS DB |
| K.FEVKPAASVTLK.S | N | 62.46 | 1288.7390 | 12 | -0.4 | 430.5868 | 3 | 26.91 | 4 | F4:12431 | NaSa01\_F12.raw |  |  |  | 1.5916E6 | 3.0771E7 | 9.3704E5 |  |  |  |  |  |  |  |  | 4 | 0 | 0 | 0 | 1 | 2 | 1 | 0 | 0 | 0 | 0 | 0 | 0 | 0 | 0 | 68 | 79 |  |  | PEAKS DB |
| R.YYNNDKPAIK.I | N | 61.61 | 1224.6139 | 10 | 0.6 | 613.3146 | 2 | 11.43 | 6 | F6:1822 | NaSa01\_F14.raw |  |  |  |  | 4.0982E6 | 2.3915E5 |  |  |  |  |  |  |  |  | 3 | 0 | 0 | 0 | 0 | 2 | 1 | 0 | 0 | 0 | 0 | 0 | 0 | 0 | 0 | 24 | 33 |  |  | PEAKS DB |
| I.HIALIGLEIWSNK.D | N | 61.43 | 1492.8402 | 13 | 1.3 | 747.4283 | 2 | 69.99 | 3 | F3:43121 | NaSa01\_F11.raw |  |  | 1.443E7 | 2.5435E6 |  |  |  |  |  |  |  |  |  |  | 4 | 0 | 0 | 2 | 2 | 0 | 0 | 0 | 0 | 0 | 0 | 0 | 0 | 0 | 0 | 53 | 65 |  |  | PEAKS DB |
| H.IALIGLEIWSNK.D | N | 60.96 | 1355.7812 | 12 | 0.4 | 678.8982 | 2 | 85.01 | 4 | F4:60240 | NaSa01\_F12.raw |  |  | 3.7546E7 | 2.0261E7 |  |  |  |  |  |  |  |  |  |  | 2 | 0 | 0 | 1 | 1 | 0 | 0 | 0 | 0 | 0 | 0 | 0 | 0 | 0 | 0 | 54 | 65 |  |  | PEAKS DB |
| K.GQC(+57.02)VDVQTAY | N | 59.29 | 1139.4917 | 10 | 0.4 | 570.7534 | 2 | 32.72 | 4 | F4:17040 | NaSa01\_F12.raw |  |  |  | 1.3163E7 | 3.9751E7 |  |  |  |  |  |  | 4.4194E6 | 8.2572E6 |  | 4 | 0 | 0 | 0 | 1 | 1 | 0 | 0 | 0 | 0 | 0 | 0 | 1 | 1 | 0 | 388 | 397 | Carbamidomethylation | C3:Carbamidomethylation:1000.00 | PEAKS DB |
| R.DRPQC(+57.02)ILNKPLSTDIVSPPIC(+57.02)GN.Y | N | 58.34 | 2593.2993 | 23 | -0.3 | 1297.6565 | 2 | 63.62 | 6 | F6:40889 | NaSa01\_F14.raw |  |  |  |  |  | 2.087E9 |  |  |  |  |  |  |  |  | 4 | 0 | 0 | 0 | 0 | 0 | 4 | 0 | 0 | 0 | 0 | 0 | 0 | 0 | 0 | 195 | 217 | Carbamidomethylation | C5:Carbamidomethylation:1000.00;C21:Carbamidomethylation:1000.00 | PEAKS DB |
| R.VAKDDC(+57.02)DLPELC(+57.02)TGQSAEC(+57.02)PTDSLQR.N | N | 58.33 | 2964.2898 | 26 | 0.4 | 989.1042 | 3 | 43.90 | 5 | F5:25010 | NaSa01\_F13.raw |  |  |  | 1.0528E7 | 1.1804E7 | 1.7291E6 |  |  |  |  |  |  |  |  | 4 | 0 | 0 | 0 | 2 | 1 | 1 | 0 | 0 | 0 | 0 | 0 | 0 | 0 | 0 | 255 | 280 | Carbamidomethylation | C6:Carbamidomethylation:1000.00;C12:Carbamidomethylation:1000.00;C19:Carbamidomethylation:1000.00 | PEAKS DB |
| K.RTAPAFQFSSC(+57.02)SIR.E | N | 58.20 | 1626.7936 | 14 | 1.0 | 543.2723 | 3 | 33.58 | 5 | F5:16454 | NaSa01\_F13.raw |  |  |  |  | 3.0352E6 |  |  |  |  |  |  |  |  |  | 1 | 0 | 0 | 0 | 0 | 1 | 0 | 0 | 0 | 0 | 0 | 0 | 0 | 0 | 0 | 173 | 186 | Carbamidomethylation | C11:Carbamidomethylation:1000.00 | PEAKS DB |
| K.DKFEVKPAASVTLK.S | N | 56.83 | 1531.8610 | 14 | -0.6 | 511.6273 | 3 | 23.42 | 5 | F5:8219 | NaSa01\_F13.raw |  |  |  | 1.0862E6 | 2.5201E7 |  |  |  |  |  |  |  |  |  | 5 | 0 | 0 | 0 | 2 | 3 | 0 | 0 | 0 | 0 | 0 | 0 | 0 | 0 | 0 | 66 | 79 |  |  | PEAKS DB |
| K.VYEM(+15.99)INAVNTK.F | N | 56.71 | 1296.6384 | 11 | 0.5 | 649.3268 | 2 | 30.38 | 5 | F5:16103 | NaSa01\_F13.raw |  |  |  |  | 1.2957E8 | 2.8787E5 |  |  |  |  |  |  |  |  | 7 | 0 | 0 | 0 | 0 | 6 | 1 | 0 | 0 | 0 | 0 | 0 | 0 | 0 | 0 | 36 | 46 | Oxidation (M) | M4:Oxidation (M):1000.00 | PEAKS DB |
| A.LIGLEIWSNK.D | N | 56.38 | 1171.6600 | 10 | 0.6 | 586.8376 | 2 | 69.41 | 4 | F4:47508 | NaSa01\_F12.raw |  |  | 2.9162E6 | 5.1163E6 |  |  |  |  |  |  |  |  |  |  | 2 | 0 | 0 | 1 | 1 | 0 | 0 | 0 | 0 | 0 | 0 | 0 | 0 | 0 | 0 | 56 | 65 |  |  | PEAKS DB |
| L.IGLEIWSNK.D | N | 54.47 | 1058.5760 | 9 | 0.3 | 530.2955 | 2 | 55.93 | 3 | F3:31172 | NaSa01\_F11.raw |  |  | 9.3204E6 | 1.0988E7 |  | 3.4788E6 |  |  |  |  |  | 6.1468E5 | 0 |  | 4 | 0 | 0 | 1 | 1 | 0 | 1 | 0 | 0 | 0 | 0 | 0 | 1 | 0 | 0 | 57 | 65 |  |  | PEAKS DB |
| H.IALIGLEIWSNKDK.F | N | 54.42 | 1598.9032 | 14 | 0.9 | 533.9755 | 3 | 69.06 | 3 | F3:42065 | NaSa01\_F11.raw |  |  | 3.9962E6 | 4.2508E6 |  |  |  |  |  |  |  |  |  |  | 4 | 0 | 0 | 2 | 2 | 0 | 0 | 0 | 0 | 0 | 0 | 0 | 0 | 0 | 0 | 54 | 67 |  |  | PEAKS DB |
| K.RTAPAFQFSSC(+57.02).S | N | 49.15 | 1270.5764 | 11 | 0.1 | 636.2955 | 2 | 35.53 | 5 | F5:18275 | NaSa01\_F13.raw |  |  |  |  | 1.061E7 |  |  |  |  |  |  |  |  |  | 1 | 0 | 0 | 0 | 0 | 1 | 0 | 0 | 0 | 0 | 0 | 0 | 0 | 0 | 0 | 173 | 183 | Carbamidomethylation | C11:Carbamidomethylation:1000.00 | PEAKS DB |
| K.NTM(+15.99)SC(+57.02)LIPPNPDGIMAEPGTK.C | N | 48.35 | 2258.0381 | 21 | 0.2 | 1130.0265 | 2 | 57.82 | 5 | F5:37637 | NaSa01\_F13.raw |  |  |  |  | 1.036E8 |  |  |  |  |  |  |  |  |  | 3 | 0 | 0 | 0 | 0 | 3 | 0 | 0 | 0 | 0 | 0 | 0 | 0 | 0 | 0 | 358 | 378 | Oxidation (M); Carbamidomethylation | M3:Oxidation (M):96.93;C5:Carbamidomethylation:1000.00 | PEAKS DB |
| D.DC(+57.02)DLPELC(+57.02)TGQSAEC(+57.02)PTDSLQR.N | N | 46.78 | 2551.0625 | 22 | 0.8 | 851.3621 | 3 | 56.81 | 9 | F9:28977 | NaSa01\_F4.raw |  |  |  |  |  |  |  |  | 9.1393E5 |  |  |  |  |  | 1 | 0 | 0 | 0 | 0 | 0 | 0 | 0 | 0 | 1 | 0 | 0 | 0 | 0 | 0 | 259 | 280 | Carbamidomethylation | C2:Carbamidomethylation:1000.00;C8:Carbamidomethylation:1000.00;C15:Carbamidomethylation:1000.00 | PEAKS DB |
| K.NTMSC(+57.02)LIPPNPDGIM(+15.99)AEPGTK.C | N | 46.28 | 2258.0381 | 21 | 0.2 | 1130.0265 | 2 | 57.82 | 5 | F5:36541 | NaSa01\_F13.raw |  |  |  |  | 7.128E7 |  |  |  |  |  |  |  |  |  | 2 | 0 | 0 | 0 | 0 | 2 | 0 | 0 | 0 | 0 | 0 | 0 | 0 | 0 | 0 | 358 | 378 | Carbamidomethylation; Oxidation (M) | C5:Carbamidomethylation:1000.00;M15:Oxidation (M):63.99 | PEAKS DB |
| I.HIALIGLEIWSNKDK.F | N | 46.03 | 1735.9620 | 15 | 1.1 | 579.6619 | 3 | 57.93 | 3 | F3:32893 | NaSa01\_F11.raw |  |  | 0 |  |  |  |  |  |  |  |  |  |  |  | 0 | 0 | 0 | 0 | 0 | 0 | 0 | 0 | 0 | 0 | 0 | 0 | 0 | 0 | 0 | 53 | 67 |  |  | PEAKS DB |
| R.DRPQC(+57.02)ILNKPL.S | N | 44.62 | 1352.7234 | 11 | 1.1 | 451.9156 | 3 | 27.50 | 5 | F5:11578 | NaSa01\_F13.raw |  |  |  |  | 1.9485E7 |  |  |  |  |  |  |  |  |  | 1 | 0 | 0 | 0 | 0 | 1 | 0 | 0 | 0 | 0 | 0 | 0 | 0 | 0 | 0 | 195 | 205 | Carbamidomethylation | C5:Carbamidomethylation:1000.00 | PEAKS DB |
| I.GLEIWSNK.D | N | 43.99 | 945.4919 | 8 | 0.7 | 473.7536 | 2 | 37.33 | 12 | F12:20994 | NaSa01\_F7.raw |  |  |  |  |  |  |  |  |  |  |  | 1.2994E6 |  |  | 1 | 0 | 0 | 0 | 0 | 0 | 0 | 0 | 0 | 0 | 0 | 0 | 1 | 0 | 0 | 58 | 65 |  |  | PEAKS DB |
| C.LIPPNPDGIMAEPGTK.C | N | 43.18 | 1648.8494 | 16 | -0.1 | 825.4319 | 2 | 51.19 | 6 | F6:30226 | NaSa01\_F14.raw |  |  |  |  | 1.7141E7 | 3.7268E6 |  |  |  |  |  |  |  |  | 3 | 0 | 0 | 0 | 0 | 2 | 1 | 0 | 0 | 0 | 0 | 0 | 0 | 0 | 0 | 363 | 378 |  |  | PEAKS DB |
| R.TAPAFQFSSC(+57.02).S | N | 41.82 | 1114.4753 | 10 | -0.1 | 558.2449 | 2 | 56.38 | 6 | F6:34716 | NaSa01\_F14.raw |  |  |  |  | 1.868E8 | 4.1539E7 |  |  |  |  |  |  |  |  | 2 | 0 | 0 | 0 | 0 | 1 | 1 | 0 | 0 | 0 | 0 | 0 | 0 | 0 | 0 | 174 | 183 | Carbamidomethylation | C10:Carbamidomethylation:1000.00 | PEAKS DB |
| I.KVYEMINAVNTK.F | Y | 41.04 | 1408.7384 | 12 | 0.3 | 705.3767 | 2 | 22.14 | 5 | F5:7087 | NaSa01\_F13.raw |  |  |  |  | 4.1409E5 |  |  |  |  |  |  |  |  |  | 1 | 0 | 0 | 0 | 0 | 1 | 0 | 0 | 0 | 0 | 0 | 0 | 0 | 0 | 0 | 35 | 46 |  |  | PEAKS DB |
| K.VYEMINAVNTKFR.P | N | 40.30 | 1583.8130 | 13 | 0.5 | 528.9452 | 3 | 43.49 | 5 | F5:24661 | NaSa01\_F13.raw |  |  |  |  | 1.7264E6 |  |  |  |  |  |  |  |  |  | 1 | 0 | 0 | 0 | 0 | 1 | 0 | 0 | 0 | 0 | 0 | 0 | 0 | 0 | 0 | 36 | 48 |  |  | PEAKS DB |
| R.ETVLLPR.K | N | 38.52 | 826.4912 | 7 | -0.8 | 414.2526 | 2 | 26.58 | 6 | F6:9693 | NaSa01\_F14.raw |  |  |  |  |  | 1.9593E5 |  |  |  |  |  |  |  |  | 1 | 0 | 0 | 0 | 0 | 0 | 1 | 0 | 0 | 0 | 0 | 0 | 0 | 0 | 0 | 86 | 92 |  |  | PEAKS DB |
| total 30 peptides |  |  |  |  |  |  |  |  |  |  |  |  |  |  |  |  |  |  |  |  |  |  |  |  |  |  |  |  |  |  |  |  |  |  |  |  |  |  |  |  |  |  |  |  |  |
| --- | --- | --- | --- | --- | --- | --- | --- | --- | --- | --- | --- | --- | --- | --- | --- | --- | --- | --- | --- | --- | --- | --- | --- | --- | --- | --- | --- | --- | --- | --- | --- | --- | --- | --- | --- | --- | --- | --- | --- | --- | --- | --- | --- | --- | --- |

P14541.1

back to list

  

| Protein Coverage
| Supporting Peptides
|

Protein Coverage:

Supporting Peptides:

| Peptide | Uniq | -10lgP | Mass | Length | ppm | m/z | z | RT | Fraction | Scan | Source File | Area F1 | Area F10 | Area F11 | Area F12 | Area F13 | Area F14 | Area F2 | Area F3 | Area F4 | Area F5 | Area F6 | Area F7 | Area F8 | Area F9 | #Feature | #Feature F1 | #Feature F10 | #Feature F11 | #Feature F12 | #Feature F13 | #Feature F14 | #Feature F2 | #Feature F3 | #Feature F4 | #Feature F5 | #Feature F6 | #Feature F7 | #Feature F8 | #Feature F9 | Start | End | PTM | AScore | Found By |
| --- | --- | --- | --- | --- | --- | --- | --- | --- | --- | --- | --- | --- | --- | --- | --- | --- | --- | --- | --- | --- | --- | --- | --- | --- | --- | --- | --- | --- | --- | --- | --- | --- | --- | --- | --- | --- | --- | --- | --- | --- | --- | --- | --- | --- | --- |
| K.NSALLKYVC(+57.02)C(+57.02)STDK.C | N | 100.92 | 1657.7804 | 14 | 0.7 | 829.8981 | 2 | 31.42 | 14 | F14:12724 | NaSa01\_F9.raw |  |  |  |  |  |  |  |  |  |  |  |  |  | 4.3202E7 | 4 | 0 | 0 | 0 | 0 | 0 | 0 | 0 | 0 | 0 | 0 | 0 | 0 | 0 | 4 | 47 | 60 | Carbamidomethylation | C9:Carbamidomethylation:1000.00;C10:Carbamidomethylation:1000.00 | PEAKS DB |
| K.C(+57.02)HNTQLPFIYK.T | N | 82.29 | 1419.6969 | 11 | 0.5 | 710.8561 | 2 | 34.28 | 13 | F13:22766 | NaSa01\_F8.raw |  | 1.3801E9 | 4.4376E5 | 1.8132E7 | 2.2584E6 | 1.2279E6 | 9.8764E6 | 5.1631E7 | 5.3159E7 | 7.773E7 | 1.016E8 | 1.4033E8 | 5.3138E9 | 3.141E10 | 79 | 0 | 5 | 1 | 2 | 2 | 1 | 2 | 2 | 2 | 3 | 2 | 2 | 7 | 48 | 3 | 13 | Carbamidomethylation | C1:Carbamidomethylation:1000.00 | PEAKS DB |
| K.NSALLKYVC(+57.02)C(+57.02)STDKC(+57.02)N | N | 79.80 | 1931.8540 | 16 | 0.3 | 966.9346 | 2 | 32.80 | 14 | F14:13832 | NaSa01\_F9.raw |  |  |  |  |  |  |  |  |  |  |  |  |  | 1.007E5 | 1 | 0 | 0 | 0 | 0 | 0 | 0 | 0 | 0 | 0 | 0 | 0 | 0 | 0 | 1 | 47 | 62 | Carbamidomethylation | C9:Carbamidomethylation:1000.00;C10:Carbamidomethylation:1000.00;C15:Carbamidomethylation:1000.00 | PEAKS DB |
| LKC(+57.02)HNTQLPFIYK.T | N | 77.22 | 1660.8759 | 13 | 0.7 | 554.6329 | 3 | 25.03 | 14 | F14:7795 | NaSa01\_F9.raw |  | 2.1934E6 |  |  |  |  |  |  |  |  |  |  | 6.6131E7 | 1.1355E7 | 9 | 0 | 1 | 0 | 0 | 0 | 0 | 0 | 0 | 0 | 0 | 0 | 0 | 6 | 2 | 1 | 13 | Carbamidomethylation | C3:Carbamidomethylation:1000.00 | PEAKS DB |
| C.HNTQLPFIYK.T | N | 67.07 | 1259.6663 | 10 | -1.3 | 630.8396 | 2 | 32.85 | 14 | F14:13757 | NaSa01\_F9.raw |  |  |  |  |  |  |  | 8.6663E5 |  |  |  | 2.7317E5 | 7.5843E6 | 8.409E6 | 6 | 0 | 0 | 0 | 0 | 0 | 0 | 0 | 1 | 0 | 0 | 0 | 1 | 1 | 3 | 4 | 13 |  |  | PEAKS DB |
| L.KC(+57.02)HNTQLPFIYK.T | N | 66.68 | 1547.7919 | 12 | 4.1 | 516.9385 | 3 | 21.07 | 7 | F7:8734 | NaSa01\_F2.raw |  |  |  |  |  |  | 3.6629E6 |  |  |  |  | 3.3702E6 |  |  | 5 | 0 | 0 | 0 | 0 | 0 | 0 | 2 | 0 | 0 | 0 | 0 | 3 | 0 | 0 | 2 | 13 | Carbamidomethylation | C2:Carbamidomethylation:1000.00 | PEAKS DB |
| K.YVC(+57.02)C(+57.02)STDK.C | N | 58.14 | 1031.4053 | 8 | -0.1 | 516.7098 | 2 | 11.31 | 14 | F14:1765 | NaSa01\_F9.raw |  |  |  |  |  |  |  |  |  |  |  |  | 6.3787E6 | 1.3063E7 | 2 | 0 | 0 | 0 | 0 | 0 | 0 | 0 | 0 | 0 | 0 | 0 | 0 | 1 | 1 | 53 | 60 | Carbamidomethylation | C3:Carbamidomethylation:1000.00;C4:Carbamidomethylation:1000.00 | PEAKS DB |
| K.C(+57.02)HNTQLPFIYKTC(+57.02)PEGK.N | N | 56.66 | 2091.9871 | 17 | 0.2 | 524.0042 | 4 | 26.01 | 14 | F14:8544 | NaSa01\_F9.raw |  |  |  |  |  |  |  |  |  |  |  |  |  | 1.7887E7 | 3 | 0 | 0 | 0 | 0 | 0 | 0 | 0 | 0 | 0 | 0 | 0 | 0 | 0 | 3 | 3 | 19 | Carbamidomethylation | C1:Carbamidomethylation:1000.00;C13:Carbamidomethylation:1000.00 | PEAKS DB |
| K.NSALLKYVC(+57.02)C(+57.02).S | N | 56.63 | 1226.5787 | 10 | 0.6 | 614.2970 | 2 | 44.68 | 14 | F14:23729 | NaSa01\_F9.raw |  |  |  |  |  |  |  |  |  |  |  |  |  | 3.31E6 | 1 | 0 | 0 | 0 | 0 | 0 | 0 | 0 | 0 | 0 | 0 | 0 | 0 | 0 | 1 | 47 | 56 | Carbamidomethylation | C9:Carbamidomethylation:1000.00;C10:Carbamidomethylation:1000.00 | PEAKS DB |
| K.YVC(+57.02)C(+57.02)STDKC(+57.02)N | N | 53.58 | 1305.4788 | 10 | 0.1 | 653.7467 | 2 | 11.31 | 14 | F14:1767 | NaSa01\_F9.raw |  |  |  |  |  |  |  |  |  |  |  |  |  | 4.2146E6 | 1 | 0 | 0 | 0 | 0 | 0 | 0 | 0 | 0 | 0 | 0 | 0 | 0 | 0 | 1 | 53 | 62 | Carbamidomethylation | C3:Carbamidomethylation:1000.00;C4:Carbamidomethylation:1000.00;C9:Carbamidomethylation:1000.00 | PEAKS DB |
| H.NTQLPFIYK.T | N | 48.33 | 1122.6073 | 9 | 4.9 | 562.3107 | 2 | 49.87 | 8 | F8:31269 | NaSa01\_F3.raw |  |  |  |  |  |  |  | 2.8317E6 | 2.5307E6 |  |  |  | 7.9211E6 | 2.426E8 | 4 | 0 | 0 | 0 | 0 | 0 | 0 | 0 | 1 | 1 | 0 | 0 | 0 | 1 | 1 | 5 | 13 |  |  | PEAKS DB |
| K.C(+57.02)HNTQLPFIY.K | N | 45.87 | 1291.6019 | 10 | -0.9 | 646.8077 | 2 | 58.35 | 14 | F14:34645 | NaSa01\_F9.raw |  |  | 1.2654E7 |  |  |  |  |  |  |  |  |  |  | 7.448E7 | 2 | 0 | 0 | 1 | 0 | 0 | 0 | 0 | 0 | 0 | 0 | 0 | 0 | 0 | 1 | 3 | 12 | Carbamidomethylation | C1:Carbamidomethylation:1000.00 | PEAKS DB |
| K.TC(+57.02)PEGKNLC(+57.02)FK.A | N | 45.83 | 1352.6217 | 11 | -0.4 | 451.8810 | 3 | 11.40 | 14 | F14:1892 | NaSa01\_F9.raw |  |  |  |  |  |  |  |  |  |  |  |  |  | 1.3943E5 | 1 | 0 | 0 | 0 | 0 | 0 | 0 | 0 | 0 | 0 | 0 | 0 | 0 | 0 | 1 | 14 | 24 | Carbamidomethylation | C2:Carbamidomethylation:1000.00;C9:Carbamidomethylation:1000.00 | PEAKS DB |
| R.GC(+57.02)ADNC(+57.02)PKNSALLKYVC(+57.02)C(+57.02)STDK.C | N | 45.25 | 2560.1179 | 22 | 0.5 | 854.3804 | 3 | 26.88 | 14 | F14:9131 | NaSa01\_F9.raw |  |  |  |  |  |  |  |  |  |  |  |  |  | 3.2866E6 | 1 | 0 | 0 | 0 | 0 | 0 | 0 | 0 | 0 | 0 | 0 | 0 | 0 | 0 | 1 | 39 | 60 | Carbamidomethylation | C2:Carbamidomethylation:1000.00;C6:Carbamidomethylation:1000.00;C17:Carbamidomethylation:1000.00;C18:Carbamidomethylation:1000.00 | PEAKS DB |
| K.NLC(+57.02)FKATLK.K | N | 44.60 | 1093.5953 | 9 | -0.2 | 547.8048 | 2 | 21.04 | 14 | F14:4634 | NaSa01\_F9.raw |  |  |  |  |  |  |  |  |  |  |  |  |  | 7.6054E5 | 1 | 0 | 0 | 0 | 0 | 0 | 0 | 0 | 0 | 0 | 0 | 0 | 0 | 0 | 1 | 20 | 28 | Carbamidomethylation | C3:Carbamidomethylation:1000.00 | PEAKS DB |
| K.FPLKIPIK.R | Y | 41.88 | 954.6266 | 8 | 0.9 | 478.3210 | 2 | 40.02 | 14 | F14:19530 | NaSa01\_F9.raw |  |  |  |  |  |  |  |  |  |  |  |  | 3.2872E7 | 1.0825E9 | 3 | 0 | 0 | 0 | 0 | 0 | 0 | 0 | 0 | 0 | 0 | 0 | 0 | 1 | 2 | 30 | 37 |  |  | PEAKS DB |
| K.C(+57.02)HNTQLPF.I | N | 40.89 | 1015.4545 | 8 | -2.2 | 508.7334 | 2 | 32.86 | 14 | F14:13929 | NaSa01\_F9.raw |  |  |  |  |  |  |  |  |  |  |  |  |  | 1.6001E8 | 2 | 0 | 0 | 0 | 0 | 0 | 0 | 0 | 0 | 0 | 0 | 0 | 0 | 0 | 2 | 3 | 10 | Carbamidomethylation | C1:Carbamidomethylation:1000.00 | PEAKS DB |
| L.LKYVC(+57.02)C(+57.02)STDK.C | N | 38.80 | 1272.5842 | 10 | 0.2 | 425.2021 | 3 | 11.31 | 14 | F14:1774 | NaSa01\_F9.raw |  |  |  |  |  |  |  |  |  |  |  |  |  | 8.1953E5 | 1 | 0 | 0 | 0 | 0 | 0 | 0 | 0 | 0 | 0 | 0 | 0 | 0 | 0 | 1 | 51 | 60 | Carbamidomethylation | C5:Carbamidomethylation:1000.00;C6:Carbamidomethylation:1000.00 | PEAKS DB |
| total 18 peptides |  |  |  |  |  |  |  |  |  |  |  |  |  |  |  |  |  |  |  |  |  |  |  |  |  |  |  |  |  |  |  |  |  |  |  |  |  |  |  |  |  |  |  |  |  |
| --- | --- | --- | --- | --- | --- | --- | --- | --- | --- | --- | --- | --- | --- | --- | --- | --- | --- | --- | --- | --- | --- | --- | --- | --- | --- | --- | --- | --- | --- | --- | --- | --- | --- | --- | --- | --- | --- | --- | --- | --- | --- | --- | --- | --- | --- |

C3NJ

back to list

  

| Protein Coverage
| Supporting Peptides
|

Protein Coverage:

Supporting Peptides:

| Peptide | Uniq | -10lgP | Mass | Length | ppm | m/z | z | RT | Fraction | Scan | Source File | Area F1 | Area F10 | Area F11 | Area F12 | Area F13 | Area F14 | Area F2 | Area F3 | Area F4 | Area F5 | Area F6 | Area F7 | Area F8 | Area F9 | #Feature | #Feature F1 | #Feature F10 | #Feature F11 | #Feature F12 | #Feature F13 | #Feature F14 | #Feature F2 | #Feature F3 | #Feature F4 | #Feature F5 | #Feature F6 | #Feature F7 | #Feature F8 | #Feature F9 | Start | End | PTM | AScore | Found By |
| --- | --- | --- | --- | --- | --- | --- | --- | --- | --- | --- | --- | --- | --- | --- | --- | --- | --- | --- | --- | --- | --- | --- | --- | --- | --- | --- | --- | --- | --- | --- | --- | --- | --- | --- | --- | --- | --- | --- | --- | --- | --- | --- | --- | --- | --- |
| K.HFEVGFIQPGSVK.V | N | 85.09 | 1443.7510 | 13 | -0.2 | 722.8826 | 2 | 44.13 | 6 | F6:24159 | NaSa01\_F14.raw |  |  |  |  | 2.4365E7 | 2.5116E8 |  |  |  |  |  |  |  |  | 8 | 0 | 0 | 0 | 0 | 2 | 6 | 0 | 0 | 0 | 0 | 0 | 0 | 0 | 0 | 1454 | 1466 |  |  | PEAKS DB |
| R.RDGQNLVTM(+15.99)NLHITPDLIPSFR.F | N | 85.06 | 2552.3169 | 22 | 0.3 | 639.0867 | 4 | 69.77 | 6 | F6:46133 | NaSa01\_F14.raw |  |  |  |  |  | 2.5484E6 |  |  |  |  |  |  |  |  | 2 | 0 | 0 | 0 | 0 | 0 | 2 | 0 | 0 | 0 | 0 | 0 | 0 | 0 | 0 | 500 | 521 | Oxidation (M) | M9:Oxidation (M):1000.00 | PEAKS DB |
| R.VELIYNPAFC(+57.02)SASTEGQR.Y | N | 79.25 | 2040.9574 | 18 | 0.2 | 1021.4862 | 2 | 60.91 | 6 | F6:38815 | NaSa01\_F14.raw |  |  |  |  | 6.4401E5 | 5.2886E6 |  |  |  |  |  |  |  |  | 2 | 0 | 0 | 0 | 0 | 1 | 1 | 0 | 0 | 0 | 0 | 0 | 0 | 0 | 0 | 854 | 871 | Carbamidomethylation | C10:Carbamidomethylation:1000.00 | PEAKS DB |
| Y.YQVGNNEIVADSVWVDVK.D | N | 73.99 | 2034.0057 | 18 | 0.2 | 1018.0104 | 2 | 70.65 | 6 | F6:46860 | NaSa01\_F14.raw |  |  |  |  |  | 6.5239E6 |  |  |  |  |  |  |  |  | 1 | 0 | 0 | 0 | 0 | 0 | 1 | 0 | 0 | 0 | 0 | 0 | 0 | 0 | 0 | 526 | 543 |  |  | PEAKS DB |
| K.YFTYLILNK.G | N | 70.49 | 1173.6433 | 9 | 0.1 | 587.8290 | 2 | 64.67 | 6 | F6:41809 | NaSa01\_F14.raw |  |  |  |  | 1.0728E7 | 7.7707E7 |  |  |  |  |  |  |  |  | 2 | 0 | 0 | 0 | 0 | 1 | 1 | 0 | 0 | 0 | 0 | 0 | 0 | 0 | 0 | 480 | 488 |  |  | PEAKS DB |
| K.SDFGC(+57.02)TAGSGQNNLGVFEDAGLALTTSTNLNTK.Q | N | 66.95 | 3359.5576 | 33 | 0.8 | 1120.8607 | 3 | 89.37 | 6 | F6:62425 | NaSa01\_F14.raw |  |  |  |  |  | 3.8512E7 |  |  |  |  |  |  |  |  | 1 | 0 | 0 | 0 | 0 | 0 | 1 | 0 | 0 | 0 | 0 | 0 | 0 | 0 | 0 | 611 | 643 | Carbamidomethylation | C5:Carbamidomethylation:1000.00 | PEAKS DB |
| R.DGQNLVTM(+15.99)NLHITPDLIPSFR.F | N | 66.34 | 2396.2158 | 21 | 0.4 | 799.7462 | 3 | 83.99 | 6 | F6:57985 | NaSa01\_F14.raw |  |  |  |  |  | 5.9574E6 |  |  |  |  |  |  |  |  | 1 | 0 | 0 | 0 | 0 | 0 | 1 | 0 | 0 | 0 | 0 | 0 | 0 | 0 | 0 | 501 | 521 | Oxidation (M) | M8:Oxidation (M):1000.00 | PEAKS DB |
| H.ITPDLIPSFR.F | N | 61.36 | 1157.6444 | 10 | 0.0 | 579.8295 | 2 | 65.01 | 6 | F6:42335 | NaSa01\_F14.raw |  |  |  |  | 3.0601E6 | 3.8923E7 |  |  |  |  |  |  |  |  | 3 | 0 | 0 | 0 | 0 | 1 | 2 | 0 | 0 | 0 | 0 | 0 | 0 | 0 | 0 | 512 | 521 |  |  | PEAKS DB |
| R.DGQNLVTMNLHITPDLIPSFR.F | N | 56.46 | 2380.2209 | 21 | 0.5 | 794.4147 | 3 | 91.58 | 6 | F6:64345 | NaSa01\_F14.raw |  |  |  |  |  | 2.1315E6 |  |  |  |  |  |  |  |  | 1 | 0 | 0 | 0 | 0 | 0 | 1 | 0 | 0 | 0 | 0 | 0 | 0 | 0 | 0 | 501 | 521 |  |  | PEAKS DB |
| G.ALYTLITPAVLR.T | N | 55.82 | 1329.8020 | 12 | 0.4 | 665.9086 | 2 | 75.95 | 6 | F6:51300 | NaSa01\_F14.raw |  |  |  | 1.7564E6 | 1.5853E7 | 2.019E8 |  |  |  |  |  |  |  |  | 6 | 0 | 0 | 0 | 1 | 1 | 4 | 0 | 0 | 0 | 0 | 0 | 0 | 0 | 0 | 23 | 34 |  |  | PEAKS DB |
| Y.VTNPDGSPAAHVPVVSEAIHSEGTTLSDGTAK.L | Y | 54.20 | 3143.5371 | 32 | 0.7 | 786.8921 | 4 | 45.04 | 6 | F6:25045 | NaSa01\_F14.raw |  |  |  |  |  | 6.2278E6 |  |  |  |  |  |  |  |  | 1 | 0 | 0 | 0 | 0 | 0 | 1 | 0 | 0 | 0 | 0 | 0 | 0 | 0 | 0 | 368 | 399 |  |  | PEAKS DB |
| N.LHITPDLIPSFR.F | N | 52.36 | 1407.7874 | 12 | 0.4 | 470.2699 | 3 | 64.03 | 6 | F6:41508 | NaSa01\_F14.raw |  |  |  |  |  | 2.1751E7 |  |  |  |  |  |  |  |  | 2 | 0 | 0 | 0 | 0 | 0 | 2 | 0 | 0 | 0 | 0 | 0 | 0 | 0 | 0 | 510 | 521 |  |  | PEAKS DB |
| E.YVLPSFEVR.L | N | 51.77 | 1108.5917 | 9 | 0.6 | 555.3035 | 2 | 58.44 | 6 | F6:36495 | NaSa01\_F14.raw |  |  |  | 4.1589E5 | 1.253E7 | 1.3997E8 |  |  |  |  |  |  |  |  | 4 | 0 | 0 | 0 | 1 | 1 | 2 | 0 | 0 | 0 | 0 | 0 | 0 | 0 | 0 | 221 | 229 |  |  | PEAKS DB |
| R.DGQNLVTMNLH.I | N | 51.62 | 1240.5870 | 11 | 0.0 | 621.3008 | 2 | 49.19 | 6 | F6:28557 | NaSa01\_F14.raw |  |  |  |  |  | 4.925E6 |  |  |  |  |  |  |  |  | 1 | 0 | 0 | 0 | 0 | 0 | 1 | 0 | 0 | 0 | 0 | 0 | 0 | 0 | 0 | 501 | 511 |  |  | PEAKS DB |
| R.VGLVAVDKAVYVLNDK.Y | N | 46.90 | 1701.9664 | 16 | 0.8 | 568.3299 | 3 | 57.39 | 6 | F6:35573 | NaSa01\_F14.raw |  |  |  |  |  | 1.8722E5 |  |  |  |  |  |  |  |  | 1 | 0 | 0 | 0 | 0 | 0 | 1 | 0 | 0 | 0 | 0 | 0 | 0 | 0 | 0 | 581 | 596 |  |  | PEAKS DB |
| K.YFKPGMPYELTVY.V | N | 44.78 | 1606.7742 | 13 | 0.4 | 804.3947 | 2 | 71.51 | 6 | F6:47852 | NaSa01\_F14.raw |  |  |  |  |  | 1.8453E7 |  |  |  |  |  |  |  |  | 1 | 0 | 0 | 0 | 0 | 0 | 1 | 0 | 0 | 0 | 0 | 0 | 0 | 0 | 0 | 355 | 367 |  |  | PEAKS DB |
| K.TVIVEFQTPEG.I | N | 43.94 | 1218.6132 | 11 | 3.8 | 610.3144 | 2 | 63.73 | 7 | F7:45532 | NaSa01\_F2.raw |  |  |  |  |  |  | 6.7633E6 | 2.6291E6 |  |  |  |  |  |  | 2 | 0 | 0 | 0 | 0 | 0 | 0 | 1 | 1 | 0 | 0 | 0 | 0 | 0 | 0 | 160 | 170 |  |  | PEAKS DB |
| M.NLHITPDLIPSFR.F | N | 43.77 | 1521.8303 | 13 | 0.6 | 508.2843 | 3 | 65.19 | 6 | F6:42284 | NaSa01\_F14.raw |  |  |  |  |  | 1.1088E7 |  |  |  |  |  |  |  |  | 2 | 0 | 0 | 0 | 0 | 0 | 2 | 0 | 0 | 0 | 0 | 0 | 0 | 0 | 0 | 509 | 521 |  |  | PEAKS DB |
| Y.VLPSFEVR.L | N | 41.89 | 945.5283 | 8 | -0.2 | 473.7714 | 2 | 40.35 | 6 | F6:20803 | NaSa01\_F14.raw |  |  |  |  |  | 5.7172E5 |  |  |  |  |  |  |  |  | 1 | 0 | 0 | 0 | 0 | 0 | 1 | 0 | 0 | 0 | 0 | 0 | 0 | 0 | 0 | 222 | 229 |  |  | PEAKS DB |
| Y.TLITPAVLR.T | N | 40.29 | 982.6175 | 9 | 0.4 | 492.3162 | 2 | 45.78 | 6 | F6:25572 | NaSa01\_F14.raw |  |  |  |  |  | 5.8349E7 |  |  |  |  |  |  |  |  | 1 | 0 | 0 | 0 | 0 | 0 | 1 | 0 | 0 | 0 | 0 | 0 | 0 | 0 | 0 | 26 | 34 |  |  | PEAKS DB |
| Y.QVGNNEIVADSVWVDVK.D | N | 39.98 | 1870.9425 | 17 | 0.5 | 936.4790 | 2 | 64.84 | 6 | F6:41917 | NaSa01\_F14.raw |  |  |  |  |  | 1.8564E6 |  |  |  |  |  |  |  |  | 1 | 0 | 0 | 0 | 0 | 0 | 1 | 0 | 0 | 0 | 0 | 0 | 0 | 0 | 0 | 527 | 543 |  |  | PEAKS DB |
| D.ISYLITK.N | N | 38.08 | 836.5007 | 7 | -0.7 | 419.2574 | 2 | 26.29 | 6 | F6:9476 | NaSa01\_F14.raw |  |  |  |  |  | 6.6012E5 |  |  |  |  |  |  |  |  | 1 | 0 | 0 | 0 | 0 | 0 | 1 | 0 | 0 | 0 | 0 | 0 | 0 | 0 | 0 | 1606 | 1612 |  |  | PEAKS DB |
| total 22 peptides |  |  |  |  |  |  |  |  |  |  |  |  |  |  |  |  |  |  |  |  |  |  |  |  |  |  |  |  |  |  |  |  |  |  |  |  |  |  |  |  |  |  |  |  |  |
| --- | --- | --- | --- | --- | --- | --- | --- | --- | --- | --- | --- | --- | --- | --- | --- | --- | --- | --- | --- | --- | --- | --- | --- | --- | --- | --- | --- | --- | --- | --- | --- | --- | --- | --- | --- | --- | --- | --- | --- | --- | --- | --- | --- | --- | --- |

Q01833.1

back to list

  

| Protein Coverage
| Supporting Peptides
|

Protein Coverage:

Supporting Peptides:

| Peptide | Uniq | -10lgP | Mass | Length | ppm | m/z | z | RT | Fraction | Scan | Source File | Area F1 | Area F10 | Area F11 | Area F12 | Area F13 | Area F14 | Area F2 | Area F3 | Area F4 | Area F5 | Area F6 | Area F7 | Area F8 | Area F9 | #Feature | #Feature F1 | #Feature F10 | #Feature F11 | #Feature F12 | #Feature F13 | #Feature F14 | #Feature F2 | #Feature F3 | #Feature F4 | #Feature F5 | #Feature F6 | #Feature F7 | #Feature F8 | #Feature F9 | Start | End | PTM | AScore | Found By |
| --- | --- | --- | --- | --- | --- | --- | --- | --- | --- | --- | --- | --- | --- | --- | --- | --- | --- | --- | --- | --- | --- | --- | --- | --- | --- | --- | --- | --- | --- | --- | --- | --- | --- | --- | --- | --- | --- | --- | --- | --- | --- | --- | --- | --- | --- |
| K.HFEVGFIQPGSVK.V | N | 85.09 | 1443.7510 | 13 | -0.2 | 722.8826 | 2 | 44.13 | 6 | F6:24159 | NaSa01\_F14.raw |  |  |  |  | 2.4365E7 | 2.5116E8 |  |  |  |  |  |  |  |  | 8 | 0 | 0 | 0 | 0 | 2 | 6 | 0 | 0 | 0 | 0 | 0 | 0 | 0 | 0 | 1454 | 1466 |  |  | PEAKS DB |
| R.RDGQNLVTM(+15.99)NLHITPDLIPSFR.F | N | 85.06 | 2552.3169 | 22 | 0.3 | 639.0867 | 4 | 69.77 | 6 | F6:46133 | NaSa01\_F14.raw |  |  |  |  |  | 2.5484E6 |  |  |  |  |  |  |  |  | 2 | 0 | 0 | 0 | 0 | 0 | 2 | 0 | 0 | 0 | 0 | 0 | 0 | 0 | 0 | 500 | 521 | Oxidation (M) | M9:Oxidation (M):1000.00 | PEAKS DB |
| R.VELIYNPAFC(+57.02)SASTEGQR.Y | N | 79.25 | 2040.9574 | 18 | 0.2 | 1021.4862 | 2 | 60.91 | 6 | F6:38815 | NaSa01\_F14.raw |  |  |  |  | 6.4401E5 | 5.2886E6 |  |  |  |  |  |  |  |  | 2 | 0 | 0 | 0 | 0 | 1 | 1 | 0 | 0 | 0 | 0 | 0 | 0 | 0 | 0 | 854 | 871 | Carbamidomethylation | C10:Carbamidomethylation:1000.00 | PEAKS DB |
| Y.YQVGNNEIVADSVWVDVK.D | N | 73.99 | 2034.0057 | 18 | 0.2 | 1018.0104 | 2 | 70.65 | 6 | F6:46860 | NaSa01\_F14.raw |  |  |  |  |  | 6.5239E6 |  |  |  |  |  |  |  |  | 1 | 0 | 0 | 0 | 0 | 0 | 1 | 0 | 0 | 0 | 0 | 0 | 0 | 0 | 0 | 526 | 543 |  |  | PEAKS DB |
| K.YFTYLILNK.G | N | 70.49 | 1173.6433 | 9 | 0.1 | 587.8290 | 2 | 64.67 | 6 | F6:41809 | NaSa01\_F14.raw |  |  |  |  | 1.0728E7 | 7.7707E7 |  |  |  |  |  |  |  |  | 2 | 0 | 0 | 0 | 0 | 1 | 1 | 0 | 0 | 0 | 0 | 0 | 0 | 0 | 0 | 480 | 488 |  |  | PEAKS DB |
| K.SDFGC(+57.02)TAGSGQNNLGVFEDAGLALTTSTNLNTK.Q | N | 66.95 | 3359.5576 | 33 | 0.8 | 1120.8607 | 3 | 89.37 | 6 | F6:62425 | NaSa01\_F14.raw |  |  |  |  |  | 3.8512E7 |  |  |  |  |  |  |  |  | 1 | 0 | 0 | 0 | 0 | 0 | 1 | 0 | 0 | 0 | 0 | 0 | 0 | 0 | 0 | 611 | 643 | Carbamidomethylation | C5:Carbamidomethylation:1000.00 | PEAKS DB |
| R.DGQNLVTM(+15.99)NLHITPDLIPSFR.F | N | 66.34 | 2396.2158 | 21 | 0.4 | 799.7462 | 3 | 83.99 | 6 | F6:57985 | NaSa01\_F14.raw |  |  |  |  |  | 5.9574E6 |  |  |  |  |  |  |  |  | 1 | 0 | 0 | 0 | 0 | 0 | 1 | 0 | 0 | 0 | 0 | 0 | 0 | 0 | 0 | 501 | 521 | Oxidation (M) | M8:Oxidation (M):1000.00 | PEAKS DB |
| H.ITPDLIPSFR.F | N | 61.36 | 1157.6444 | 10 | 0.0 | 579.8295 | 2 | 65.01 | 6 | F6:42335 | NaSa01\_F14.raw |  |  |  |  | 3.0601E6 | 3.8923E7 |  |  |  |  |  |  |  |  | 3 | 0 | 0 | 0 | 0 | 1 | 2 | 0 | 0 | 0 | 0 | 0 | 0 | 0 | 0 | 512 | 521 |  |  | PEAKS DB |
| R.DGQNLVTMNLHITPDLIPSFR.F | N | 56.46 | 2380.2209 | 21 | 0.5 | 794.4147 | 3 | 91.58 | 6 | F6:64345 | NaSa01\_F14.raw |  |  |  |  |  | 2.1315E6 |  |  |  |  |  |  |  |  | 1 | 0 | 0 | 0 | 0 | 0 | 1 | 0 | 0 | 0 | 0 | 0 | 0 | 0 | 0 | 501 | 521 |  |  | PEAKS DB |
| G.ALYTLITPAVLR.T | N | 55.82 | 1329.8020 | 12 | 0.4 | 665.9086 | 2 | 75.95 | 6 | F6:51300 | NaSa01\_F14.raw |  |  |  | 1.7564E6 | 1.5853E7 | 2.019E8 |  |  |  |  |  |  |  |  | 6 | 0 | 0 | 0 | 1 | 1 | 4 | 0 | 0 | 0 | 0 | 0 | 0 | 0 | 0 | 23 | 34 |  |  | PEAKS DB |
| Y.VTNPDGSPAAHVPVVSEAIHSEGTTLSDGTAK.L | Y | 54.20 | 3143.5371 | 32 | 0.7 | 786.8921 | 4 | 45.04 | 6 | F6:25045 | NaSa01\_F14.raw |  |  |  |  |  | 6.2278E6 |  |  |  |  |  |  |  |  | 1 | 0 | 0 | 0 | 0 | 0 | 1 | 0 | 0 | 0 | 0 | 0 | 0 | 0 | 0 | 368 | 399 |  |  | PEAKS DB |
| N.LHITPDLIPSFR.F | N | 52.36 | 1407.7874 | 12 | 0.4 | 470.2699 | 3 | 64.03 | 6 | F6:41508 | NaSa01\_F14.raw |  |  |  |  |  | 2.1751E7 |  |  |  |  |  |  |  |  | 2 | 0 | 0 | 0 | 0 | 0 | 2 | 0 | 0 | 0 | 0 | 0 | 0 | 0 | 0 | 510 | 521 |  |  | PEAKS DB |
| E.YVLPSFEVR.L | N | 51.77 | 1108.5917 | 9 | 0.6 | 555.3035 | 2 | 58.44 | 6 | F6:36495 | NaSa01\_F14.raw |  |  |  | 4.1589E5 | 1.253E7 | 1.3997E8 |  |  |  |  |  |  |  |  | 4 | 0 | 0 | 0 | 1 | 1 | 2 | 0 | 0 | 0 | 0 | 0 | 0 | 0 | 0 | 221 | 229 |  |  | PEAKS DB |
| R.DGQNLVTMNLH.I | N | 51.62 | 1240.5870 | 11 | 0.0 | 621.3008 | 2 | 49.19 | 6 | F6:28557 | NaSa01\_F14.raw |  |  |  |  |  | 4.925E6 |  |  |  |  |  |  |  |  | 1 | 0 | 0 | 0 | 0 | 0 | 1 | 0 | 0 | 0 | 0 | 0 | 0 | 0 | 0 | 501 | 511 |  |  | PEAKS DB |
| R.VGLVAVDKAVYVLNDK.Y | N | 46.90 | 1701.9664 | 16 | 0.8 | 568.3299 | 3 | 57.39 | 6 | F6:35573 | NaSa01\_F14.raw |  |  |  |  |  | 1.8722E5 |  |  |  |  |  |  |  |  | 1 | 0 | 0 | 0 | 0 | 0 | 1 | 0 | 0 | 0 | 0 | 0 | 0 | 0 | 0 | 581 | 596 |  |  | PEAKS DB |
| K.YFKPGMPYELTVY.V | N | 44.78 | 1606.7742 | 13 | 0.4 | 804.3947 | 2 | 71.51 | 6 | F6:47852 | NaSa01\_F14.raw |  |  |  |  |  | 1.8453E7 |  |  |  |  |  |  |  |  | 1 | 0 | 0 | 0 | 0 | 0 | 1 | 0 | 0 | 0 | 0 | 0 | 0 | 0 | 0 | 355 | 367 |  |  | PEAKS DB |
| K.TVIVEFQTPEG.I | N | 43.94 | 1218.6132 | 11 | 3.8 | 610.3144 | 2 | 63.73 | 7 | F7:45532 | NaSa01\_F2.raw |  |  |  |  |  |  | 6.7633E6 | 2.6291E6 |  |  |  |  |  |  | 2 | 0 | 0 | 0 | 0 | 0 | 0 | 1 | 1 | 0 | 0 | 0 | 0 | 0 | 0 | 160 | 170 |  |  | PEAKS DB |
| M.NLHITPDLIPSFR.F | N | 43.77 | 1521.8303 | 13 | 0.6 | 508.2843 | 3 | 65.19 | 6 | F6:42284 | NaSa01\_F14.raw |  |  |  |  |  | 1.1088E7 |  |  |  |  |  |  |  |  | 2 | 0 | 0 | 0 | 0 | 0 | 2 | 0 | 0 | 0 | 0 | 0 | 0 | 0 | 0 | 509 | 521 |  |  | PEAKS DB |
| Y.VLPSFEVR.L | N | 41.89 | 945.5283 | 8 | -0.2 | 473.7714 | 2 | 40.35 | 6 | F6:20803 | NaSa01\_F14.raw |  |  |  |  |  | 5.7172E5 |  |  |  |  |  |  |  |  | 1 | 0 | 0 | 0 | 0 | 0 | 1 | 0 | 0 | 0 | 0 | 0 | 0 | 0 | 0 | 222 | 229 |  |  | PEAKS DB |
| Y.TLITPAVLR.T | N | 40.29 | 982.6175 | 9 | 0.4 | 492.3162 | 2 | 45.78 | 6 | F6:25572 | NaSa01\_F14.raw |  |  |  |  |  | 5.8349E7 |  |  |  |  |  |  |  |  | 1 | 0 | 0 | 0 | 0 | 0 | 1 | 0 | 0 | 0 | 0 | 0 | 0 | 0 | 0 | 26 | 34 |  |  | PEAKS DB |
| Y.QVGNNEIVADSVWVDVK.D | N | 39.98 | 1870.9425 | 17 | 0.5 | 936.4790 | 2 | 64.84 | 6 | F6:41917 | NaSa01\_F14.raw |  |  |  |  |  | 1.8564E6 |  |  |  |  |  |  |  |  | 1 | 0 | 0 | 0 | 0 | 0 | 1 | 0 | 0 | 0 | 0 | 0 | 0 | 0 | 0 | 527 | 543 |  |  | PEAKS DB |
| D.ISYLITK.N | N | 38.08 | 836.5007 | 7 | -0.7 | 419.2574 | 2 | 26.29 | 6 | F6:9476 | NaSa01\_F14.raw |  |  |  |  |  | 6.6012E5 |  |  |  |  |  |  |  |  | 1 | 0 | 0 | 0 | 0 | 0 | 1 | 0 | 0 | 0 | 0 | 0 | 0 | 0 | 0 | 1606 | 1612 |  |  | PEAKS DB |
| total 22 peptides |  |  |  |  |  |  |  |  |  |  |  |  |  |  |  |  |  |  |  |  |  |  |  |  |  |  |  |  |  |  |  |  |  |  |  |  |  |  |  |  |  |  |  |  |  |
| --- | --- | --- | --- | --- | --- | --- | --- | --- | --- | --- | --- | --- | --- | --- | --- | --- | --- | --- | --- | --- | --- | --- | --- | --- | --- | --- | --- | --- | --- | --- | --- | --- | --- | --- | --- | --- | --- | --- | --- | --- | --- | --- | --- | --- | --- |

AAA49385.1

back to list

  

| Protein Coverage
| Supporting Peptides
|

Protein Coverage:

Supporting Peptides:

| Peptide | Uniq | -10lgP | Mass | Length | ppm | m/z | z | RT | Fraction | Scan | Source File | Area F1 | Area F10 | Area F11 | Area F12 | Area F13 | Area F14 | Area F2 | Area F3 | Area F4 | Area F5 | Area F6 | Area F7 | Area F8 | Area F9 | #Feature | #Feature F1 | #Feature F10 | #Feature F11 | #Feature F12 | #Feature F13 | #Feature F14 | #Feature F2 | #Feature F3 | #Feature F4 | #Feature F5 | #Feature F6 | #Feature F7 | #Feature F8 | #Feature F9 | Start | End | PTM | AScore | Found By |
| --- | --- | --- | --- | --- | --- | --- | --- | --- | --- | --- | --- | --- | --- | --- | --- | --- | --- | --- | --- | --- | --- | --- | --- | --- | --- | --- | --- | --- | --- | --- | --- | --- | --- | --- | --- | --- | --- | --- | --- | --- | --- | --- | --- | --- | --- |
| K.HFEVGFIQPGSVK.V | N | 85.09 | 1443.7510 | 13 | -0.2 | 722.8826 | 2 | 44.13 | 6 | F6:24159 | NaSa01\_F14.raw |  |  |  |  | 2.4365E7 | 2.5116E8 |  |  |  |  |  |  |  |  | 8 | 0 | 0 | 0 | 0 | 2 | 6 | 0 | 0 | 0 | 0 | 0 | 0 | 0 | 0 | 1454 | 1466 |  |  | PEAKS DB |
| R.RDGQNLVTM(+15.99)NLHITPDLIPSFR.F | N | 85.06 | 2552.3169 | 22 | 0.3 | 639.0867 | 4 | 69.77 | 6 | F6:46133 | NaSa01\_F14.raw |  |  |  |  |  | 2.5484E6 |  |  |  |  |  |  |  |  | 2 | 0 | 0 | 0 | 0 | 0 | 2 | 0 | 0 | 0 | 0 | 0 | 0 | 0 | 0 | 500 | 521 | Oxidation (M) | M9:Oxidation (M):1000.00 | PEAKS DB |
| R.VELIYNPAFC(+57.02)SASTEGQR.Y | N | 79.25 | 2040.9574 | 18 | 0.2 | 1021.4862 | 2 | 60.91 | 6 | F6:38815 | NaSa01\_F14.raw |  |  |  |  | 6.4401E5 | 5.2886E6 |  |  |  |  |  |  |  |  | 2 | 0 | 0 | 0 | 0 | 1 | 1 | 0 | 0 | 0 | 0 | 0 | 0 | 0 | 0 | 854 | 871 | Carbamidomethylation | C10:Carbamidomethylation:1000.00 | PEAKS DB |
| Y.YQVGNNEIVADSVWVDVK.D | N | 73.99 | 2034.0057 | 18 | 0.2 | 1018.0104 | 2 | 70.65 | 6 | F6:46860 | NaSa01\_F14.raw |  |  |  |  |  | 6.5239E6 |  |  |  |  |  |  |  |  | 1 | 0 | 0 | 0 | 0 | 0 | 1 | 0 | 0 | 0 | 0 | 0 | 0 | 0 | 0 | 526 | 543 |  |  | PEAKS DB |
| K.YFTYLILNK.G | N | 70.49 | 1173.6433 | 9 | 0.1 | 587.8290 | 2 | 64.67 | 6 | F6:41809 | NaSa01\_F14.raw |  |  |  |  | 1.0728E7 | 7.7707E7 |  |  |  |  |  |  |  |  | 2 | 0 | 0 | 0 | 0 | 1 | 1 | 0 | 0 | 0 | 0 | 0 | 0 | 0 | 0 | 480 | 488 |  |  | PEAKS DB |
| K.SDFGC(+57.02)TAGSGQNNLGVFEDAGLALTTSTNLNTK.Q | N | 66.95 | 3359.5576 | 33 | 0.8 | 1120.8607 | 3 | 89.37 | 6 | F6:62425 | NaSa01\_F14.raw |  |  |  |  |  | 3.8512E7 |  |  |  |  |  |  |  |  | 1 | 0 | 0 | 0 | 0 | 0 | 1 | 0 | 0 | 0 | 0 | 0 | 0 | 0 | 0 | 611 | 643 | Carbamidomethylation | C5:Carbamidomethylation:1000.00 | PEAKS DB |
| R.DGQNLVTM(+15.99)NLHITPDLIPSFR.F | N | 66.34 | 2396.2158 | 21 | 0.4 | 799.7462 | 3 | 83.99 | 6 | F6:57985 | NaSa01\_F14.raw |  |  |  |  |  | 5.9574E6 |  |  |  |  |  |  |  |  | 1 | 0 | 0 | 0 | 0 | 0 | 1 | 0 | 0 | 0 | 0 | 0 | 0 | 0 | 0 | 501 | 521 | Oxidation (M) | M8:Oxidation (M):1000.00 | PEAKS DB |
| H.ITPDLIPSFR.F | N | 61.36 | 1157.6444 | 10 | 0.0 | 579.8295 | 2 | 65.01 | 6 | F6:42335 | NaSa01\_F14.raw |  |  |  |  | 3.0601E6 | 3.8923E7 |  |  |  |  |  |  |  |  | 3 | 0 | 0 | 0 | 0 | 1 | 2 | 0 | 0 | 0 | 0 | 0 | 0 | 0 | 0 | 512 | 521 |  |  | PEAKS DB |
| R.DGQNLVTMNLHITPDLIPSFR.F | N | 56.46 | 2380.2209 | 21 | 0.5 | 794.4147 | 3 | 91.58 | 6 | F6:64345 | NaSa01\_F14.raw |  |  |  |  |  | 2.1315E6 |  |  |  |  |  |  |  |  | 1 | 0 | 0 | 0 | 0 | 0 | 1 | 0 | 0 | 0 | 0 | 0 | 0 | 0 | 0 | 501 | 521 |  |  | PEAKS DB |
| G.ALYTLITPAVLR.T | N | 55.82 | 1329.8020 | 12 | 0.4 | 665.9086 | 2 | 75.95 | 6 | F6:51300 | NaSa01\_F14.raw |  |  |  | 1.7564E6 | 1.5853E7 | 2.019E8 |  |  |  |  |  |  |  |  | 6 | 0 | 0 | 0 | 1 | 1 | 4 | 0 | 0 | 0 | 0 | 0 | 0 | 0 | 0 | 23 | 34 |  |  | PEAKS DB |
| Y.VTNPDGSPAAHVPVVSEAIHSEGTTLSDGTAK.L | Y | 54.20 | 3143.5371 | 32 | 0.7 | 786.8921 | 4 | 45.04 | 6 | F6:25045 | NaSa01\_F14.raw |  |  |  |  |  | 6.2278E6 |  |  |  |  |  |  |  |  | 1 | 0 | 0 | 0 | 0 | 0 | 1 | 0 | 0 | 0 | 0 | 0 | 0 | 0 | 0 | 368 | 399 |  |  | PEAKS DB |
| N.LHITPDLIPSFR.F | N | 52.36 | 1407.7874 | 12 | 0.4 | 470.2699 | 3 | 64.03 | 6 | F6:41508 | NaSa01\_F14.raw |  |  |  |  |  | 2.1751E7 |  |  |  |  |  |  |  |  | 2 | 0 | 0 | 0 | 0 | 0 | 2 | 0 | 0 | 0 | 0 | 0 | 0 | 0 | 0 | 510 | 521 |  |  | PEAKS DB |
| E.YVLPSFEVR.L | N | 51.77 | 1108.5917 | 9 | 0.6 | 555.3035 | 2 | 58.44 | 6 | F6:36495 | NaSa01\_F14.raw |  |  |  | 4.1589E5 | 1.253E7 | 1.3997E8 |  |  |  |  |  |  |  |  | 4 | 0 | 0 | 0 | 1 | 1 | 2 | 0 | 0 | 0 | 0 | 0 | 0 | 0 | 0 | 221 | 229 |  |  | PEAKS DB |
| R.DGQNLVTMNLH.I | N | 51.62 | 1240.5870 | 11 | 0.0 | 621.3008 | 2 | 49.19 | 6 | F6:28557 | NaSa01\_F14.raw |  |  |  |  |  | 4.925E6 |  |  |  |  |  |  |  |  | 1 | 0 | 0 | 0 | 0 | 0 | 1 | 0 | 0 | 0 | 0 | 0 | 0 | 0 | 0 | 501 | 511 |  |  | PEAKS DB |
| R.VGLVAVDKAVYVLNDK.Y | N | 46.90 | 1701.9664 | 16 | 0.8 | 568.3299 | 3 | 57.39 | 6 | F6:35573 | NaSa01\_F14.raw |  |  |  |  |  | 1.8722E5 |  |  |  |  |  |  |  |  | 1 | 0 | 0 | 0 | 0 | 0 | 1 | 0 | 0 | 0 | 0 | 0 | 0 | 0 | 0 | 581 | 596 |  |  | PEAKS DB |
| K.YFKPGMPYELTVY.V | N | 44.78 | 1606.7742 | 13 | 0.4 | 804.3947 | 2 | 71.51 | 6 | F6:47852 | NaSa01\_F14.raw |  |  |  |  |  | 1.8453E7 |  |  |  |  |  |  |  |  | 1 | 0 | 0 | 0 | 0 | 0 | 1 | 0 | 0 | 0 | 0 | 0 | 0 | 0 | 0 | 355 | 367 |  |  | PEAKS DB |
| K.TVIVEFQTPEG.I | N | 43.94 | 1218.6132 | 11 | 3.8 | 610.3144 | 2 | 63.73 | 7 | F7:45532 | NaSa01\_F2.raw |  |  |  |  |  |  | 6.7633E6 | 2.6291E6 |  |  |  |  |  |  | 2 | 0 | 0 | 0 | 0 | 0 | 0 | 1 | 1 | 0 | 0 | 0 | 0 | 0 | 0 | 160 | 170 |  |  | PEAKS DB |
| M.NLHITPDLIPSFR.F | N | 43.77 | 1521.8303 | 13 | 0.6 | 508.2843 | 3 | 65.19 | 6 | F6:42284 | NaSa01\_F14.raw |  |  |  |  |  | 1.1088E7 |  |  |  |  |  |  |  |  | 2 | 0 | 0 | 0 | 0 | 0 | 2 | 0 | 0 | 0 | 0 | 0 | 0 | 0 | 0 | 509 | 521 |  |  | PEAKS DB |
| Y.VLPSFEVR.L | N | 41.89 | 945.5283 | 8 | -0.2 | 473.7714 | 2 | 40.35 | 6 | F6:20803 | NaSa01\_F14.raw |  |  |  |  |  | 5.7172E5 |  |  |  |  |  |  |  |  | 1 | 0 | 0 | 0 | 0 | 0 | 1 | 0 | 0 | 0 | 0 | 0 | 0 | 0 | 0 | 222 | 229 |  |  | PEAKS DB |
| Y.TLITPAVLR.T | N | 40.29 | 982.6175 | 9 | 0.4 | 492.3162 | 2 | 45.78 | 6 | F6:25572 | NaSa01\_F14.raw |  |  |  |  |  | 5.8349E7 |  |  |  |  |  |  |  |  | 1 | 0 | 0 | 0 | 0 | 0 | 1 | 0 | 0 | 0 | 0 | 0 | 0 | 0 | 0 | 26 | 34 |  |  | PEAKS DB |
| Y.QVGNNEIVADSVWVDVK.D | N | 39.98 | 1870.9425 | 17 | 0.5 | 936.4790 | 2 | 64.84 | 6 | F6:41917 | NaSa01\_F14.raw |  |  |  |  |  | 1.8564E6 |  |  |  |  |  |  |  |  | 1 | 0 | 0 | 0 | 0 | 0 | 1 | 0 | 0 | 0 | 0 | 0 | 0 | 0 | 0 | 527 | 543 |  |  | PEAKS DB |
| D.ISYLITK.N | N | 38.08 | 836.5007 | 7 | -0.7 | 419.2574 | 2 | 26.29 | 6 | F6:9476 | NaSa01\_F14.raw |  |  |  |  |  | 6.6012E5 |  |  |  |  |  |  |  |  | 1 | 0 | 0 | 0 | 0 | 0 | 1 | 0 | 0 | 0 | 0 | 0 | 0 | 0 | 0 | 1606 | 1612 |  |  | PEAKS DB |
| total 22 peptides |  |  |  |  |  |  |  |  |  |  |  |  |  |  |  |  |  |  |  |  |  |  |  |  |  |  |  |  |  |  |  |  |  |  |  |  |  |  |  |  |  |  |  |  |  |
| --- | --- | --- | --- | --- | --- | --- | --- | --- | --- | --- | --- | --- | --- | --- | --- | --- | --- | --- | --- | --- | --- | --- | --- | --- | --- | --- | --- | --- | --- | --- | --- | --- | --- | --- | --- | --- | --- | --- | --- | --- | --- | --- | --- | --- | --- |

AAF82186.1

back to list

  

| Protein Coverage
| Supporting Peptides
|

Protein Coverage:

Supporting Peptides:

| Peptide | Uniq | -10lgP | Mass | Length | ppm | m/z | z | RT | Fraction | Scan | Source File | Area F1 | Area F10 | Area F11 | Area F12 | Area F13 | Area F14 | Area F2 | Area F3 | Area F4 | Area F5 | Area F6 | Area F7 | Area F8 | Area F9 | #Feature | #Feature F1 | #Feature F10 | #Feature F11 | #Feature F12 | #Feature F13 | #Feature F14 | #Feature F2 | #Feature F3 | #Feature F4 | #Feature F5 | #Feature F6 | #Feature F7 | #Feature F8 | #Feature F9 | Start | End | PTM | AScore | Found By |
| --- | --- | --- | --- | --- | --- | --- | --- | --- | --- | --- | --- | --- | --- | --- | --- | --- | --- | --- | --- | --- | --- | --- | --- | --- | --- | --- | --- | --- | --- | --- | --- | --- | --- | --- | --- | --- | --- | --- | --- | --- | --- | --- | --- | --- | --- |
| K.TYSYEC(+57.02)SQGTLTC(+57.02)K.G | N | 87.01 | 1696.7073 | 14 | -0.1 | 849.3608 | 2 | 21.82 | 13 | F13:11695 | NaSa01\_F8.raw |  | 2.9725E5 |  | 3.2683E6 | 3.3919E6 |  |  |  |  | 8.1372E5 | 5.9955E5 | 8.9065E7 | 1.107E10 | 1.3561E6 | 26 | 0 | 1 | 0 | 2 | 2 | 0 | 0 | 0 | 0 | 1 | 1 | 3 | 15 | 1 | 93 | 106 | Carbamidomethylation | C6:Carbamidomethylation:1000.00;C13:Carbamidomethylation:1000.00 | PEAKS DB |
| R.LAAIC(+57.02)FAGAPYNDNNYNIDLK.A | N | 75.95 | 2356.1157 | 21 | 0.2 | 1179.0654 | 2 | 73.89 | 14 | F14:48025 | NaSa01\_F9.raw |  | 2.4162E7 | 1.9876E7 | 1.2519E7 |  | 6.3392E6 |  |  |  |  |  | 2.2763E8 | 9.0788E9 | 8.5377E8 | 26 | 0 | 1 | 2 | 1 | 0 | 2 | 0 | 0 | 0 | 0 | 0 | 2 | 16 | 2 | 122 | 142 | Carbamidomethylation | C5:Carbamidomethylation:1000.00 | PEAKS DB |
| D.RLAAIC(+57.02)FAGAPYNDNNYNIDLK.A | N | 74.75 | 2512.2168 | 22 | 0.3 | 838.4131 | 3 | 63.00 | 13 | F13:46872 | NaSa01\_F8.raw |  |  |  |  |  |  |  |  |  |  |  | 2.3834E6 | 1.0646E8 | 4.9737E6 | 4 | 0 | 0 | 0 | 0 | 0 | 0 | 0 | 0 | 0 | 0 | 0 | 1 | 2 | 1 | 121 | 142 | Carbamidomethylation | C6:Carbamidomethylation:1000.00 | PEAKS DB |
| R.LAAIC(+57.02)FAGAPYNDNNYNIDLKAR.C | N | 74.35 | 2583.2539 | 23 | 0.6 | 862.0924 | 3 | 63.10 | 13 | F13:46916 | NaSa01\_F8.raw |  |  |  |  |  |  |  |  |  |  |  |  | 5.713E6 |  | 1 | 0 | 0 | 0 | 0 | 0 | 0 | 0 | 0 | 0 | 0 | 0 | 0 | 1 | 0 | 122 | 144 | Carbamidomethylation | C5:Carbamidomethylation:1000.00 | PEAKS DB |
| R.SWWDFADYGC(+57.02)YC(+57.02)GR.G | N | 73.88 | 1841.6926 | 14 | 1.1 | 921.8546 | 2 | 90.79 | 13 | F13:69941 | NaSa01\_F8.raw |  | 1.5441E7 | 2.3773E7 | 1.8946E6 |  |  |  |  |  |  | 1.0973E6 | 1.4857E8 | 3.3627E9 | 1.6201E8 | 15 | 0 | 1 | 1 | 1 | 0 | 0 | 0 | 0 | 0 | 0 | 1 | 1 | 7 | 3 | 44 | 57 | Carbamidomethylation | C10:Carbamidomethylation:1000.00;C12:Carbamidomethylation:1000.00 | PEAKS DB |
| F.AGAPYNDNNYNIDLK.A | N | 71.07 | 1680.7743 | 15 | 1.3 | 841.3955 | 2 | 40.11 | 13 | F13:26960 | NaSa01\_F8.raw |  |  |  |  |  | 3.6263E6 |  |  |  |  |  | 5.529E6 | 8.9066E8 | 9.7103E6 | 5 | 0 | 0 | 0 | 0 | 0 | 1 | 0 | 0 | 0 | 0 | 0 | 1 | 2 | 1 | 128 | 142 |  |  | PEAKS DB |
| G.APYNDNNYNIDLK.A | N | 68.37 | 1552.7157 | 13 | 0.6 | 777.3656 | 2 | 38.15 | 13 | F13:25157 | NaSa01\_F8.raw |  |  |  |  |  | 2.6345E6 |  |  |  |  |  | 2.4432E7 | 1.034E7 | 2.5874E6 | 4 | 0 | 0 | 0 | 0 | 0 | 1 | 0 | 0 | 0 | 0 | 0 | 1 | 1 | 1 | 130 | 142 |  |  | PEAKS DB |
| R.GGSGTPVDDLDR.C | N | 67.25 | 1187.5417 | 12 | -0.9 | 594.7776 | 2 | 12.12 | 13 | F13:4409 | NaSa01\_F8.raw |  |  |  |  |  |  |  |  |  |  |  |  | 9.3807E7 |  | 2 | 0 | 0 | 0 | 0 | 0 | 0 | 0 | 0 | 0 | 0 | 0 | 0 | 2 | 0 | 58 | 69 |  |  | PEAKS DB |
| N.RSWWDFADYGC(+57.02)YC(+57.02)GR.G | N | 66.86 | 1997.7937 | 15 | 0.9 | 666.9391 | 3 | 73.94 | 13 | F13:55816 | NaSa01\_F8.raw |  |  |  |  |  |  |  |  |  |  |  |  | 2.8562E6 |  | 3 | 0 | 0 | 0 | 0 | 0 | 0 | 0 | 0 | 0 | 0 | 0 | 0 | 3 | 0 | 43 | 57 | Carbamidomethylation | C11:Carbamidomethylation:1000.00;C13:Carbamidomethylation:1000.00 | PEAKS DB |
| C.FAGAPYNDNNYNIDLK.A | N | 65.61 | 1827.8428 | 16 | 0.0 | 914.9286 | 2 | 51.25 | 13 | F13:36712 | NaSa01\_F8.raw |  |  |  |  |  |  |  |  |  |  |  |  | 8.0677E7 |  | 1 | 0 | 0 | 0 | 0 | 0 | 0 | 0 | 0 | 0 | 0 | 0 | 0 | 1 | 0 | 127 | 142 |  |  | PEAKS DB |
| K.GGNDAC(+57.02)AAAVC(+57.02)DC(+57.02)DR.L | Y | 62.92 | 1610.5872 | 15 | 0.4 | 806.3011 | 2 | 11.75 | 13 | F13:4095 | NaSa01\_F8.raw |  |  |  |  |  |  |  |  |  |  |  |  | 3.3766E7 |  | 2 | 0 | 0 | 0 | 0 | 0 | 0 | 0 | 0 | 0 | 0 | 0 | 0 | 2 | 0 | 107 | 121 | Carbamidomethylation | C6:Carbamidomethylation:1000.00;C11:Carbamidomethylation:1000.00;C13:Carbamidomethylation:1000.00 | PEAKS DB |
| A.SSNRPMPLNLYQFK.N | N | 53.15 | 1693.8610 | 14 | 1.1 | 565.6282 | 3 | 45.07 | 14 | F14:24127 | NaSa01\_F9.raw |  |  |  |  |  |  |  |  |  |  |  |  |  | 7.2883E6 | 1 | 0 | 0 | 0 | 0 | 0 | 0 | 0 | 0 | 0 | 0 | 0 | 0 | 0 | 1 | 20 | 33 |  |  | PEAKS DB |
| N.DNNYNIDLK.A | N | 50.24 | 1107.5197 | 9 | -0.4 | 554.7669 | 2 | 31.93 | 13 | F13:20329 | NaSa01\_F8.raw |  |  |  |  |  |  |  |  |  |  |  |  | 1.0746E7 |  | 1 | 0 | 0 | 0 | 0 | 0 | 0 | 0 | 0 | 0 | 0 | 0 | 0 | 1 | 0 | 134 | 142 |  |  | PEAKS DB |
| W.WDFADYGC(+57.02)YC(+57.02)GR.G | N | 49.58 | 1568.5813 | 12 | 1.5 | 785.2991 | 2 | 61.99 | 13 | F13:45659 | NaSa01\_F8.raw |  |  |  |  |  |  |  |  |  |  |  |  | 1.1461E6 |  | 1 | 0 | 0 | 0 | 0 | 0 | 0 | 0 | 0 | 0 | 0 | 0 | 0 | 1 | 0 | 46 | 57 | Carbamidomethylation | C8:Carbamidomethylation:1000.00;C10:Carbamidomethylation:1000.00 | PEAKS DB |
| P.MPLNLYQFK.N | N | 47.16 | 1152.6001 | 9 | -0.2 | 577.3072 | 2 | 58.53 | 14 | F14:35134 | NaSa01\_F9.raw |  |  |  |  |  |  |  |  |  |  |  |  |  | 5.1142E6 | 1 | 0 | 0 | 0 | 0 | 0 | 0 | 0 | 0 | 0 | 0 | 0 | 0 | 0 | 1 | 25 | 33 |  |  | PEAKS DB |
| A.GAPYNDNNYNIDLK.A | N | 44.68 | 1609.7372 | 14 | 0.4 | 805.8762 | 2 | 38.90 | 13 | F13:26088 | NaSa01\_F8.raw |  |  |  |  |  |  |  |  |  |  |  |  | 7.5974E6 |  | 1 | 0 | 0 | 0 | 0 | 0 | 0 | 0 | 0 | 0 | 0 | 0 | 0 | 1 | 0 | 129 | 142 |  |  | PEAKS DB |
| R.LAAIC(+57.02)FAGAPYN.D | N | 44.68 | 1266.6067 | 12 | -0.1 | 634.3105 | 2 | 71.65 | 13 | F13:54114 | NaSa01\_F8.raw |  |  | 2.1689E6 | 3.0706E6 |  | 4.6852E6 |  |  |  |  | 4.8617E6 | 4.4367E7 | 2.0168E7 |  | 6 | 0 | 0 | 1 | 1 | 0 | 1 | 0 | 0 | 0 | 0 | 1 | 1 | 1 | 0 | 122 | 133 | Carbamidomethylation | C5:Carbamidomethylation:1000.00 | PEAKS DB |
| A.IC(+57.02)FAGAPYNDNNYNIDLK.A | N | 44.64 | 2100.9575 | 18 | 0.2 | 1051.4862 | 2 | 63.71 | 13 | F13:47153 | NaSa01\_F8.raw |  |  |  |  |  |  |  |  |  |  |  |  | 6.9015E6 |  | 1 | 0 | 0 | 0 | 0 | 0 | 0 | 0 | 0 | 0 | 0 | 0 | 0 | 1 | 0 | 125 | 142 | Carbamidomethylation | C2:Carbamidomethylation:1000.00 | PEAKS DB |
| Y.FKTYSYEC(+57.02)SQGTLTC(+57.02)K.G | N | 43.26 | 1971.8706 | 16 | 0.4 | 658.2977 | 3 | 23.27 | 13 | F13:13009 | NaSa01\_F8.raw |  |  |  |  |  |  |  |  |  |  |  |  | 9.3587E5 |  | 1 | 0 | 0 | 0 | 0 | 0 | 0 | 0 | 0 | 0 | 0 | 0 | 0 | 1 | 0 | 91 | 106 | Carbamidomethylation | C8:Carbamidomethylation:1000.00;C15:Carbamidomethylation:1000.00 | PEAKS DB |
| total 19 peptides |  |  |  |  |  |  |  |  |  |  |  |  |  |  |  |  |  |  |  |  |  |  |  |  |  |  |  |  |  |  |  |  |  |  |  |  |  |  |  |  |  |  |  |  |  |
| --- | --- | --- | --- | --- | --- | --- | --- | --- | --- | --- | --- | --- | --- | --- | --- | --- | --- | --- | --- | --- | --- | --- | --- | --- | --- | --- | --- | --- | --- | --- | --- | --- | --- | --- | --- | --- | --- | --- | --- | --- | --- | --- | --- | --- | --- |

Q9I900.1

back to list

  

| Protein Coverage
| Supporting Peptides
|

Protein Coverage:

Supporting Peptides:

| Peptide | Uniq | -10lgP | Mass | Length | ppm | m/z | z | RT | Fraction | Scan | Source File | Area F1 | Area F10 | Area F11 | Area F12 | Area F13 | Area F14 | Area F2 | Area F3 | Area F4 | Area F5 | Area F6 | Area F7 | Area F8 | Area F9 | #Feature | #Feature F1 | #Feature F10 | #Feature F11 | #Feature F12 | #Feature F13 | #Feature F14 | #Feature F2 | #Feature F3 | #Feature F4 | #Feature F5 | #Feature F6 | #Feature F7 | #Feature F8 | #Feature F9 | Start | End | PTM | AScore | Found By |
| --- | --- | --- | --- | --- | --- | --- | --- | --- | --- | --- | --- | --- | --- | --- | --- | --- | --- | --- | --- | --- | --- | --- | --- | --- | --- | --- | --- | --- | --- | --- | --- | --- | --- | --- | --- | --- | --- | --- | --- | --- | --- | --- | --- | --- | --- |
| K.TYSYEC(+57.02)SQGTLTC(+57.02)K.G | N | 87.01 | 1696.7073 | 14 | -0.1 | 849.3608 | 2 | 21.82 | 13 | F13:11695 | NaSa01\_F8.raw |  | 2.9725E5 |  | 3.2683E6 | 3.3919E6 |  |  |  |  | 8.1372E5 | 5.9955E5 | 8.9065E7 | 1.107E10 | 1.3561E6 | 26 | 0 | 1 | 0 | 2 | 2 | 0 | 0 | 0 | 0 | 1 | 1 | 3 | 15 | 1 | 93 | 106 | Carbamidomethylation | C6:Carbamidomethylation:1000.00;C13:Carbamidomethylation:1000.00 | PEAKS DB |
| R.LAAIC(+57.02)FAGAPYNDNNYNIDLK.A | N | 75.95 | 2356.1157 | 21 | 0.2 | 1179.0654 | 2 | 73.89 | 14 | F14:48025 | NaSa01\_F9.raw |  | 2.4162E7 | 1.9876E7 | 1.2519E7 |  | 6.3392E6 |  |  |  |  |  | 2.2763E8 | 9.0788E9 | 8.5377E8 | 26 | 0 | 1 | 2 | 1 | 0 | 2 | 0 | 0 | 0 | 0 | 0 | 2 | 16 | 2 | 122 | 142 | Carbamidomethylation | C5:Carbamidomethylation:1000.00 | PEAKS DB |
| D.RLAAIC(+57.02)FAGAPYNDNNYNIDLK.A | N | 74.75 | 2512.2168 | 22 | 0.3 | 838.4131 | 3 | 63.00 | 13 | F13:46872 | NaSa01\_F8.raw |  |  |  |  |  |  |  |  |  |  |  | 2.3834E6 | 1.0646E8 | 4.9737E6 | 4 | 0 | 0 | 0 | 0 | 0 | 0 | 0 | 0 | 0 | 0 | 0 | 1 | 2 | 1 | 121 | 142 | Carbamidomethylation | C6:Carbamidomethylation:1000.00 | PEAKS DB |
| R.LAAIC(+57.02)FAGAPYNDNNYNIDLKAR.C | N | 74.35 | 2583.2539 | 23 | 0.6 | 862.0924 | 3 | 63.10 | 13 | F13:46916 | NaSa01\_F8.raw |  |  |  |  |  |  |  |  |  |  |  |  | 5.713E6 |  | 1 | 0 | 0 | 0 | 0 | 0 | 0 | 0 | 0 | 0 | 0 | 0 | 0 | 1 | 0 | 122 | 144 | Carbamidomethylation | C5:Carbamidomethylation:1000.00 | PEAKS DB |
| R.SWWDFADYGC(+57.02)YC(+57.02)GR.G | N | 73.88 | 1841.6926 | 14 | 1.1 | 921.8546 | 2 | 90.79 | 13 | F13:69941 | NaSa01\_F8.raw |  | 1.5441E7 | 2.3773E7 | 1.8946E6 |  |  |  |  |  |  | 1.0973E6 | 1.4857E8 | 3.3627E9 | 1.6201E8 | 15 | 0 | 1 | 1 | 1 | 0 | 0 | 0 | 0 | 0 | 0 | 1 | 1 | 7 | 3 | 44 | 57 | Carbamidomethylation | C10:Carbamidomethylation:1000.00;C12:Carbamidomethylation:1000.00 | PEAKS DB |
| F.AGAPYNDNNYNIDLK.A | N | 71.07 | 1680.7743 | 15 | 1.3 | 841.3955 | 2 | 40.11 | 13 | F13:26960 | NaSa01\_F8.raw |  |  |  |  |  | 3.6263E6 |  |  |  |  |  | 5.529E6 | 8.9066E8 | 9.7103E6 | 5 | 0 | 0 | 0 | 0 | 0 | 1 | 0 | 0 | 0 | 0 | 0 | 1 | 2 | 1 | 128 | 142 |  |  | PEAKS DB |
| G.APYNDNNYNIDLK.A | N | 68.37 | 1552.7157 | 13 | 0.6 | 777.3656 | 2 | 38.15 | 13 | F13:25157 | NaSa01\_F8.raw |  |  |  |  |  | 2.6345E6 |  |  |  |  |  | 2.4432E7 | 1.034E7 | 2.5874E6 | 4 | 0 | 0 | 0 | 0 | 0 | 1 | 0 | 0 | 0 | 0 | 0 | 1 | 1 | 1 | 130 | 142 |  |  | PEAKS DB |
| R.GGSGTPVDDLDR.C | N | 67.25 | 1187.5417 | 12 | -0.9 | 594.7776 | 2 | 12.12 | 13 | F13:4409 | NaSa01\_F8.raw |  |  |  |  |  |  |  |  |  |  |  |  | 9.3807E7 |  | 2 | 0 | 0 | 0 | 0 | 0 | 0 | 0 | 0 | 0 | 0 | 0 | 0 | 2 | 0 | 58 | 69 |  |  | PEAKS DB |
| N.RSWWDFADYGC(+57.02)YC(+57.02)GR.G | N | 66.86 | 1997.7937 | 15 | 0.9 | 666.9391 | 3 | 73.94 | 13 | F13:55816 | NaSa01\_F8.raw |  |  |  |  |  |  |  |  |  |  |  |  | 2.8562E6 |  | 3 | 0 | 0 | 0 | 0 | 0 | 0 | 0 | 0 | 0 | 0 | 0 | 0 | 3 | 0 | 43 | 57 | Carbamidomethylation | C11:Carbamidomethylation:1000.00;C13:Carbamidomethylation:1000.00 | PEAKS DB |
| C.FAGAPYNDNNYNIDLK.A | N | 65.61 | 1827.8428 | 16 | 0.0 | 914.9286 | 2 | 51.25 | 13 | F13:36712 | NaSa01\_F8.raw |  |  |  |  |  |  |  |  |  |  |  |  | 8.0677E7 |  | 1 | 0 | 0 | 0 | 0 | 0 | 0 | 0 | 0 | 0 | 0 | 0 | 0 | 1 | 0 | 127 | 142 |  |  | PEAKS DB |
| K.GGNDAC(+57.02)AAAVC(+57.02)DC(+57.02)DR.L | Y | 62.92 | 1610.5872 | 15 | 0.4 | 806.3011 | 2 | 11.75 | 13 | F13:4095 | NaSa01\_F8.raw |  |  |  |  |  |  |  |  |  |  |  |  | 3.3766E7 |  | 2 | 0 | 0 | 0 | 0 | 0 | 0 | 0 | 0 | 0 | 0 | 0 | 0 | 2 | 0 | 107 | 121 | Carbamidomethylation | C6:Carbamidomethylation:1000.00;C11:Carbamidomethylation:1000.00;C13:Carbamidomethylation:1000.00 | PEAKS DB |
| A.SSNRPMPLNLYQFK.N | N | 53.15 | 1693.8610 | 14 | 1.1 | 565.6282 | 3 | 45.07 | 14 | F14:24127 | NaSa01\_F9.raw |  |  |  |  |  |  |  |  |  |  |  |  |  | 7.2883E6 | 1 | 0 | 0 | 0 | 0 | 0 | 0 | 0 | 0 | 0 | 0 | 0 | 0 | 0 | 1 | 20 | 33 |  |  | PEAKS DB |
| N.DNNYNIDLK.A | N | 50.24 | 1107.5197 | 9 | -0.4 | 554.7669 | 2 | 31.93 | 13 | F13:20329 | NaSa01\_F8.raw |  |  |  |  |  |  |  |  |  |  |  |  | 1.0746E7 |  | 1 | 0 | 0 | 0 | 0 | 0 | 0 | 0 | 0 | 0 | 0 | 0 | 0 | 1 | 0 | 134 | 142 |  |  | PEAKS DB |
| W.WDFADYGC(+57.02)YC(+57.02)GR.G | N | 49.58 | 1568.5813 | 12 | 1.5 | 785.2991 | 2 | 61.99 | 13 | F13:45659 | NaSa01\_F8.raw |  |  |  |  |  |  |  |  |  |  |  |  | 1.1461E6 |  | 1 | 0 | 0 | 0 | 0 | 0 | 0 | 0 | 0 | 0 | 0 | 0 | 0 | 1 | 0 | 46 | 57 | Carbamidomethylation | C8:Carbamidomethylation:1000.00;C10:Carbamidomethylation:1000.00 | PEAKS DB |
| P.MPLNLYQFK.N | N | 47.16 | 1152.6001 | 9 | -0.2 | 577.3072 | 2 | 58.53 | 14 | F14:35134 | NaSa01\_F9.raw |  |  |  |  |  |  |  |  |  |  |  |  |  | 5.1142E6 | 1 | 0 | 0 | 0 | 0 | 0 | 0 | 0 | 0 | 0 | 0 | 0 | 0 | 0 | 1 | 25 | 33 |  |  | PEAKS DB |
| A.GAPYNDNNYNIDLK.A | N | 44.68 | 1609.7372 | 14 | 0.4 | 805.8762 | 2 | 38.90 | 13 | F13:26088 | NaSa01\_F8.raw |  |  |  |  |  |  |  |  |  |  |  |  | 7.5974E6 |  | 1 | 0 | 0 | 0 | 0 | 0 | 0 | 0 | 0 | 0 | 0 | 0 | 0 | 1 | 0 | 129 | 142 |  |  | PEAKS DB |
| R.LAAIC(+57.02)FAGAPYN.D | N | 44.68 | 1266.6067 | 12 | -0.1 | 634.3105 | 2 | 71.65 | 13 | F13:54114 | NaSa01\_F8.raw |  |  | 2.1689E6 | 3.0706E6 |  | 4.6852E6 |  |  |  |  | 4.8617E6 | 4.4367E7 | 2.0168E7 |  | 6 | 0 | 0 | 1 | 1 | 0 | 1 | 0 | 0 | 0 | 0 | 1 | 1 | 1 | 0 | 122 | 133 | Carbamidomethylation | C5:Carbamidomethylation:1000.00 | PEAKS DB |
| A.IC(+57.02)FAGAPYNDNNYNIDLK.A | N | 44.64 | 2100.9575 | 18 | 0.2 | 1051.4862 | 2 | 63.71 | 13 | F13:47153 | NaSa01\_F8.raw |  |  |  |  |  |  |  |  |  |  |  |  | 6.9015E6 |  | 1 | 0 | 0 | 0 | 0 | 0 | 0 | 0 | 0 | 0 | 0 | 0 | 0 | 1 | 0 | 125 | 142 | Carbamidomethylation | C2:Carbamidomethylation:1000.00 | PEAKS DB |
| Y.FKTYSYEC(+57.02)SQGTLTC(+57.02)K.G | N | 43.26 | 1971.8706 | 16 | 0.4 | 658.2977 | 3 | 23.27 | 13 | F13:13009 | NaSa01\_F8.raw |  |  |  |  |  |  |  |  |  |  |  |  | 9.3587E5 |  | 1 | 0 | 0 | 0 | 0 | 0 | 0 | 0 | 0 | 0 | 0 | 0 | 0 | 1 | 0 | 91 | 106 | Carbamidomethylation | C8:Carbamidomethylation:1000.00;C15:Carbamidomethylation:1000.00 | PEAKS DB |
| total 19 peptides |  |  |  |  |  |  |  |  |  |  |  |  |  |  |  |  |  |  |  |  |  |  |  |  |  |  |  |  |  |  |  |  |  |  |  |  |  |  |  |  |  |  |  |  |  |
| --- | --- | --- | --- | --- | --- | --- | --- | --- | --- | --- | --- | --- | --- | --- | --- | --- | --- | --- | --- | --- | --- | --- | --- | --- | --- | --- | --- | --- | --- | --- | --- | --- | --- | --- | --- | --- | --- | --- | --- | --- | --- | --- | --- | --- | --- |

XP\_034258146.1

back to list

  

| Protein Coverage
| Supporting Peptides
|

Protein Coverage:

Supporting Peptides:

| Peptide | Uniq | -10lgP | Mass | Length | ppm | m/z | z | RT | Fraction | Scan | Source File | Area F1 | Area F10 | Area F11 | Area F12 | Area F13 | Area F14 | Area F2 | Area F3 | Area F4 | Area F5 | Area F6 | Area F7 | Area F8 | Area F9 | #Feature | #Feature F1 | #Feature F10 | #Feature F11 | #Feature F12 | #Feature F13 | #Feature F14 | #Feature F2 | #Feature F3 | #Feature F4 | #Feature F5 | #Feature F6 | #Feature F7 | #Feature F8 | #Feature F9 | Start | End | PTM | AScore | Found By |
| --- | --- | --- | --- | --- | --- | --- | --- | --- | --- | --- | --- | --- | --- | --- | --- | --- | --- | --- | --- | --- | --- | --- | --- | --- | --- | --- | --- | --- | --- | --- | --- | --- | --- | --- | --- | --- | --- | --- | --- | --- | --- | --- | --- | --- | --- |
| Q.GSAHDYGALSITGDYIAFR.N | Y | 98.22 | 2012.9591 | 19 | 0.2 | 1007.4871 | 2 | 65.79 | 6 | F6:42673 | NaSa01\_F14.raw |  |  |  |  | 2.8491E8 | 1.8035E9 |  |  |  |  |  |  |  |  | 8 | 0 | 0 | 0 | 0 | 3 | 5 | 0 | 0 | 0 | 0 | 0 | 0 | 0 | 0 | 69 | 87 |  |  | PEAKS DB |
| R.LVILGFPC(+57.02)NQFGK.Q | N | 80.93 | 1491.7908 | 13 | 0.3 | 746.9029 | 2 | 74.37 | 14 | F14:48186 | NaSa01\_F9.raw |  | 6.3482E6 | 4.5719E6 | 6.7588E6 | 7.3547E8 | 2.7574E9 |  |  |  |  |  | 1.9765E6 | 7.8579E6 | 5.5032E7 | 12 | 0 | 1 | 1 | 1 | 3 | 3 | 0 | 0 | 0 | 0 | 0 | 1 | 1 | 1 | 124 | 136 | Carbamidomethylation | C8:Carbamidomethylation:1000.00 | PEAKS DB |
| K.QEPGQNSEILQGIK.Y | N | 80.47 | 1539.7893 | 14 | 0.1 | 770.9020 | 2 | 34.92 | 6 | F6:17025 | NaSa01\_F14.raw |  |  | 7.9374E4 | 4.1222E6 | 3.3468E8 | 1.3102E9 |  |  |  |  |  |  | 0 |  | 8 | 0 | 0 | 1 | 1 | 2 | 4 | 0 | 0 | 0 | 0 | 0 | 0 | 0 | 0 | 137 | 150 |  |  | PEAKS DB |
| G.SAHDYGALSITGDYIAFR.N | Y | 73.82 | 1955.9377 | 18 | 0.1 | 652.9866 | 3 | 66.79 | 6 | F6:43601 | NaSa01\_F14.raw |  |  |  |  | 1.4153E6 | 8.9231E6 |  |  |  |  |  |  |  |  | 3 | 0 | 0 | 0 | 0 | 1 | 2 | 0 | 0 | 0 | 0 | 0 | 0 | 0 | 0 | 70 | 87 |  |  | PEAKS DB |
| H.DYGALSITGDYIAFR.N | Y | 72.24 | 1660.8096 | 15 | 0.9 | 831.4128 | 2 | 87.46 | 6 | F6:60801 | NaSa01\_F14.raw |  |  |  |  | 2.2648E6 | 8.0301E6 |  |  |  |  |  |  |  |  | 2 | 0 | 0 | 0 | 0 | 1 | 1 | 0 | 0 | 0 | 0 | 0 | 0 | 0 | 0 | 73 | 87 |  |  | PEAKS DB |
| A.LSITGDYIAFR.N | Y | 63.43 | 1254.6608 | 11 | 0.6 | 628.3380 | 2 | 66.28 | 6 | F6:43459 | NaSa01\_F14.raw |  |  |  |  |  | 1.4271E7 |  |  |  |  |  |  |  |  | 1 | 0 | 0 | 0 | 0 | 0 | 1 | 0 | 0 | 0 | 0 | 0 | 0 | 0 | 0 | 77 | 87 |  |  | PEAKS DB |
| L.VILGFPC(+57.02)NQFGK.Q | N | 58.48 | 1378.7067 | 12 | -0.1 | 690.3605 | 2 | 59.07 | 12 | F12:39406 | NaSa01\_F7.raw |  | 4.6143E6 | 1.4793E6 |  |  |  |  |  |  | 1.3101E7 | 1.8748E7 | 4.3606E7 | 7.4857E6 | 1.729E7 | 7 | 0 | 1 | 1 | 0 | 0 | 0 | 0 | 0 | 0 | 1 | 1 | 1 | 1 | 1 | 125 | 136 | Carbamidomethylation | C7:Carbamidomethylation:1000.00 | PEAKS DB |
| D.YGALSITGDYIAFR.N | Y | 57.29 | 1545.7827 | 14 | 0.5 | 773.8990 | 2 | 75.73 | 6 | F6:51089 | NaSa01\_F14.raw |  |  |  |  |  | 2.4249E6 |  |  |  |  |  |  |  |  | 1 | 0 | 0 | 0 | 0 | 0 | 1 | 0 | 0 | 0 | 0 | 0 | 0 | 0 | 0 | 74 | 87 |  |  | PEAKS DB |
| L.SITGDYIAFR.N | Y | 55.37 | 1141.5768 | 10 | 0.0 | 571.7957 | 2 | 51.71 | 6 | F6:31027 | NaSa01\_F14.raw |  |  |  |  |  | 6.5439E7 |  |  |  |  |  |  | 1.9563E6 |  | 3 | 0 | 0 | 0 | 0 | 0 | 2 | 0 | 0 | 0 | 0 | 0 | 0 | 1 | 0 | 78 | 87 |  |  | PEAKS DB |
| F.VPNFQLFQK.I | N | 54.38 | 1119.6077 | 9 | -0.6 | 560.8108 | 2 | 54.08 | 6 | F6:32772 | NaSa01\_F14.raw |  |  |  |  | 1.0977E6 | 4.3013E6 |  |  |  |  |  |  |  |  | 2 | 0 | 0 | 0 | 0 | 1 | 1 | 0 | 0 | 0 | 0 | 0 | 0 | 0 | 0 | 159 | 167 |  |  | PEAKS DB |
| R.LVILGFPC(+57.02)NQF.G | N | 49.68 | 1306.6743 | 11 | 0.4 | 654.3447 | 2 | 101.88 | 6 | F6:72830 | NaSa01\_F14.raw |  |  |  |  | 5.1302E6 | 2.3489E7 |  |  |  |  |  |  |  |  | 2 | 0 | 0 | 0 | 0 | 1 | 1 | 0 | 0 | 0 | 0 | 0 | 0 | 0 | 0 | 124 | 134 | Carbamidomethylation | C8:Carbamidomethylation:1000.00 | PEAKS DB |
| S.AHDYGALSITGDYIAFR.N | Y | 47.80 | 1868.9056 | 17 | 0.0 | 623.9758 | 3 | 66.37 | 6 | F6:43378 | NaSa01\_F14.raw |  |  |  |  |  | 9.0384E5 |  |  |  |  |  |  |  |  | 1 | 0 | 0 | 0 | 0 | 0 | 1 | 0 | 0 | 0 | 0 | 0 | 0 | 0 | 0 | 71 | 87 |  |  | PEAKS DB |
| G.ALSITGDYIAFR.N | Y | 47.03 | 1325.6979 | 12 | 0.4 | 663.8565 | 2 | 68.00 | 6 | F6:44721 | NaSa01\_F14.raw |  |  |  |  |  | 5.4223E5 |  |  |  |  |  |  |  |  | 1 | 0 | 0 | 0 | 0 | 0 | 1 | 0 | 0 | 0 | 0 | 0 | 0 | 0 | 0 | 76 | 87 |  |  | PEAKS DB |
| Y.GALSITGDYIAFR.N | Y | 45.69 | 1382.7194 | 13 | 0.5 | 692.3673 | 2 | 69.69 | 6 | F6:46167 | NaSa01\_F14.raw |  |  |  |  | 5.0563E5 | 2.2746E6 |  |  |  |  |  |  |  |  | 2 | 0 | 0 | 0 | 0 | 1 | 1 | 0 | 0 | 0 | 0 | 0 | 0 | 0 | 0 | 75 | 87 |  |  | PEAKS DB |
| S.ITGDYIAFR.N | Y | 45.15 | 1054.5447 | 9 | 0.0 | 528.2796 | 2 | 43.98 | 4 | F4:26138 | NaSa01\_F12.raw |  |  |  | 6.6915E5 |  | 7.114E5 |  |  |  |  |  |  |  |  | 2 | 0 | 0 | 0 | 1 | 0 | 1 | 0 | 0 | 0 | 0 | 0 | 0 | 0 | 0 | 79 | 87 |  |  | PEAKS DB |
| R.LVILGFPC(+57.02)NQFGKQEPGQNSEILQGIK.Y | N | 40.83 | 3013.5693 | 27 | 0.5 | 1005.5309 | 3 | 75.74 | 6 | F6:51193 | NaSa01\_F14.raw |  |  |  |  |  | 3.8526E6 |  |  |  |  |  |  |  |  | 1 | 0 | 0 | 0 | 0 | 0 | 1 | 0 | 0 | 0 | 0 | 0 | 0 | 0 | 0 | 124 | 150 | Carbamidomethylation | C8:Carbamidomethylation:1000.00 | PEAKS DB |
| total 16 peptides |  |  |  |  |  |  |  |  |  |  |  |  |  |  |  |  |  |  |  |  |  |  |  |  |  |  |  |  |  |  |  |  |  |  |  |  |  |  |  |  |  |  |  |  |  |
| --- | --- | --- | --- | --- | --- | --- | --- | --- | --- | --- | --- | --- | --- | --- | --- | --- | --- | --- | --- | --- | --- | --- | --- | --- | --- | --- | --- | --- | --- | --- | --- | --- | --- | --- | --- | --- | --- | --- | --- | --- | --- | --- | --- | --- | --- |

P0DI91.1

back to list

  

| Protein Coverage
| Supporting Peptides
|

Protein Coverage:

Supporting Peptides:

| Peptide | Uniq | -10lgP | Mass | Length | ppm | m/z | z | RT | Fraction | Scan | Source File | Area F1 | Area F10 | Area F11 | Area F12 | Area F13 | Area F14 | Area F2 | Area F3 | Area F4 | Area F5 | Area F6 | Area F7 | Area F8 | Area F9 | #Feature | #Feature F1 | #Feature F10 | #Feature F11 | #Feature F12 | #Feature F13 | #Feature F14 | #Feature F2 | #Feature F3 | #Feature F4 | #Feature F5 | #Feature F6 | #Feature F7 | #Feature F8 | #Feature F9 | Start | End | PTM | AScore | Found By |
| --- | --- | --- | --- | --- | --- | --- | --- | --- | --- | --- | --- | --- | --- | --- | --- | --- | --- | --- | --- | --- | --- | --- | --- | --- | --- | --- | --- | --- | --- | --- | --- | --- | --- | --- | --- | --- | --- | --- | --- | --- | --- | --- | --- | --- | --- |
| R.SPLEEC(+57.02)FQQNDYEEFLEIAR.N | Y | 97.57 | 2516.1165 | 20 | 1.6 | 1259.0675 | 2 | 89.00 | 6 | F6:62130 | NaSa01\_F14.raw |  |  | 1.4882E6 | 5.5854E6 | 1.7879E7 | 2.6263E8 |  |  |  |  |  |  |  |  | 8 | 0 | 0 | 2 | 2 | 2 | 2 | 0 | 0 | 0 | 0 | 0 | 0 | 0 | 0 | 5 | 24 | Carbamidomethylation | C6:Carbamidomethylation:1000.00 | PEAKS DB |
| R.RSPLEEC(+57.02)FQQNDYEEFLEIAR.N | Y | 88.25 | 2672.2175 | 21 | 0.4 | 891.7468 | 3 | 78.90 | 6 | F6:53813 | NaSa01\_F14.raw |  |  | 8.4096E4 | 4.9405E5 | 9.1254E5 | 1.1553E7 |  |  |  |  |  |  |  |  | 4 | 0 | 0 | 1 | 1 | 1 | 1 | 0 | 0 | 0 | 0 | 0 | 0 | 0 | 0 | 4 | 24 | Carbamidomethylation | C7:Carbamidomethylation:1000.00 | PEAKS DB |
| K.REIQALC(+57.02)YPSIK.K | N | 77.78 | 1476.7759 | 12 | 0.2 | 493.2660 | 3 | 32.82 | 6 | F6:14068 | NaSa01\_F14.raw |  |  |  |  | 3.5291E7 | 3.7818E8 |  |  |  |  |  |  |  |  | 8 | 0 | 0 | 0 | 0 | 2 | 6 | 0 | 0 | 0 | 0 | 0 | 0 | 0 | 0 | 83 | 94 | Carbamidomethylation | C7:Carbamidomethylation:1000.00 | PEAKS DB |
| R.EIQALC(+57.02)YPSIK.K | N | 77.61 | 1320.6747 | 11 | 0.3 | 661.3448 | 2 | 47.88 | 6 | F6:27511 | NaSa01\_F14.raw |  |  |  | 3.0826E6 | 1.353E8 | 1.8533E9 |  |  |  |  |  |  |  |  | 18 | 0 | 0 | 0 | 1 | 1 | 16 | 0 | 0 | 0 | 0 | 0 | 0 | 0 | 0 | 84 | 94 | Carbamidomethylation | C6:Carbamidomethylation:1000.00 | PEAKS DB |
| K.SDALFSYEK.K | N | 62.97 | 1058.4919 | 9 | 0.4 | 530.2535 | 2 | 34.28 | 6 | F6:16290 | NaSa01\_F14.raw |  |  |  |  | 5.08E7 | 1.3053E7 |  |  |  |  |  |  |  |  | 4 | 0 | 0 | 0 | 0 | 1 | 3 | 0 | 0 | 0 | 0 | 0 | 0 | 0 | 0 | 49 | 57 |  |  | PEAKS DB |
| K.FWEADGIHGGK.V | N | 59.16 | 1215.5673 | 11 | 0.2 | 608.7910 | 2 | 11.57 | 6 | F6:1961 | NaSa01\_F14.raw |  |  |  |  | 4.0485E5 | 1.8815E7 |  |  |  |  |  |  |  |  | 9 | 0 | 0 | 0 | 0 | 1 | 8 | 0 | 0 | 0 | 0 | 0 | 0 | 0 | 0 | 59 | 69 |  |  | PEAKS DB |
| F.QQNDYEEFLEIAR.N | Y | 58.88 | 1653.7634 | 13 | 0.9 | 827.8898 | 2 | 70.27 | 6 | F6:46520 | NaSa01\_F14.raw |  |  |  |  |  | 5.7188E5 |  |  |  |  |  |  |  |  | 1 | 0 | 0 | 0 | 0 | 0 | 1 | 0 | 0 | 0 | 0 | 0 | 0 | 0 | 0 | 12 | 24 |  |  | PEAKS DB |
| Y.HLSFIESLK.S | Y | 55.05 | 1072.5917 | 9 | 0.4 | 537.3033 | 2 | 38.32 | 6 | F6:19306 | NaSa01\_F14.raw |  |  |  |  | 6.3021E6 | 4.5683E7 |  |  |  |  |  |  |  |  | 2 | 0 | 0 | 0 | 0 | 1 | 1 | 0 | 0 | 0 | 0 | 0 | 0 | 0 | 0 | 40 | 48 |  |  | PEAKS DB |
| N.DLSLIHDLPK.R | N | 53.54 | 1149.6393 | 10 | 0.2 | 575.8270 | 2 | 52.53 | 6 | F6:31365 | NaSa01\_F14.raw |  |  |  | 3.056E5 |  | 2.4605E6 |  |  |  |  |  |  |  |  | 2 | 0 | 0 | 0 | 1 | 0 | 1 | 0 | 0 | 0 | 0 | 0 | 0 | 0 | 0 | 73 | 82 |  |  | PEAKS DB |
| C.FQQNDYEEFLEIAR.N | Y | 52.42 | 1800.8318 | 14 | -1.3 | 901.4220 | 2 | 76.48 | 6 | F6:51649 | NaSa01\_F14.raw |  |  |  |  |  | 1.8135E6 |  |  |  |  |  |  |  |  | 2 | 0 | 0 | 0 | 0 | 0 | 2 | 0 | 0 | 0 | 0 | 0 | 0 | 0 | 0 | 11 | 24 |  |  | PEAKS DB |
| K.KFWEADGIHGGK.V | N | 51.66 | 1343.6622 | 12 | -0.1 | 448.8947 | 3 | 11.66 | 5 | F5:2144 | NaSa01\_F13.raw |  |  |  |  | 1.996E6 | 1.1435E7 |  |  |  |  |  |  |  |  | 3 | 0 | 0 | 0 | 0 | 1 | 2 | 0 | 0 | 0 | 0 | 0 | 0 | 0 | 0 | 58 | 69 |  |  | PEAKS DB |
| R.EIQALC(+57.02)YPSIKK | N | 49.90 | 1448.7697 | 12 | 0.7 | 483.9308 | 3 | 30.77 | 6 | F6:13252 | NaSa01\_F14.raw |  |  |  |  |  | 6.9213E6 |  |  |  |  |  |  |  |  | 2 | 0 | 0 | 0 | 0 | 0 | 2 | 0 | 0 | 0 | 0 | 0 | 0 | 0 | 0 | 84 | 95 | Carbamidomethylation | C6:Carbamidomethylation:1000.00 | PEAKS DB |
| D.YEEFLEIAR.N | N | 44.25 | 1168.5764 | 9 | -0.6 | 585.2951 | 2 | 55.17 | 6 | F6:33697 | NaSa01\_F14.raw |  |  |  |  |  | 4.5232E5 |  |  |  |  |  |  |  |  | 1 | 0 | 0 | 0 | 0 | 0 | 1 | 0 | 0 | 0 | 0 | 0 | 0 | 0 | 0 | 16 | 24 |  |  | PEAKS DB |
| E.IQALC(+57.02)YPSIK.K | N | 42.76 | 1191.6322 | 10 | -0.9 | 596.8228 | 2 | 42.19 | 6 | F6:22642 | NaSa01\_F14.raw |  |  |  |  |  | 1.1323E6 |  |  |  |  |  |  |  |  | 1 | 0 | 0 | 0 | 0 | 0 | 1 | 0 | 0 | 0 | 0 | 0 | 0 | 0 | 0 | 85 | 94 | Carbamidomethylation | C5:Carbamidomethylation:1000.00 | PEAKS DB |
| N.DYEEFLEIAR.N | N | 39.92 | 1283.6033 | 10 | -6.5 | 642.8047 | 2 | 76.29 | 6 | F6:51560 | NaSa01\_F14.raw |  |  |  |  |  | 3.9682E6 |  |  |  |  |  |  |  |  | 1 | 0 | 0 | 0 | 0 | 0 | 1 | 0 | 0 | 0 | 0 | 0 | 0 | 0 | 0 | 15 | 24 |  |  | PEAKS DB |
| total 15 peptides |  |  |  |  |  |  |  |  |  |  |  |  |  |  |  |  |  |  |  |  |  |  |  |  |  |  |  |  |  |  |  |  |  |  |  |  |  |  |  |  |  |  |  |  |  |
| --- | --- | --- | --- | --- | --- | --- | --- | --- | --- | --- | --- | --- | --- | --- | --- | --- | --- | --- | --- | --- | --- | --- | --- | --- | --- | --- | --- | --- | --- | --- | --- | --- | --- | --- | --- | --- | --- | --- | --- | --- | --- | --- | --- | --- | --- |

XP\_026544671.1

back to list

  

| Protein Coverage
| Supporting Peptides
|

Protein Coverage:

Supporting Peptides:

| Peptide | Uniq | -10lgP | Mass | Length | ppm | m/z | z | RT | Fraction | Scan | Source File | Area F1 | Area F10 | Area F11 | Area F12 | Area F13 | Area F14 | Area F2 | Area F3 | Area F4 | Area F5 | Area F6 | Area F7 | Area F8 | Area F9 | #Feature | #Feature F1 | #Feature F10 | #Feature F11 | #Feature F12 | #Feature F13 | #Feature F14 | #Feature F2 | #Feature F3 | #Feature F4 | #Feature F5 | #Feature F6 | #Feature F7 | #Feature F8 | #Feature F9 | Start | End | PTM | AScore | Found By |
| --- | --- | --- | --- | --- | --- | --- | --- | --- | --- | --- | --- | --- | --- | --- | --- | --- | --- | --- | --- | --- | --- | --- | --- | --- | --- | --- | --- | --- | --- | --- | --- | --- | --- | --- | --- | --- | --- | --- | --- | --- | --- | --- | --- | --- | --- |
| K.GGAYSYIESHPWQAGIFVK.N | N | 86.81 | 2109.0320 | 19 | 0.9 | 1055.5242 | 2 | 65.14 | 4 | F4:44125 | NaSa01\_F12.raw |  | 3.9067E7 | 1.32E8 | 7.8017E7 | 7.0674E6 | 0 |  |  |  |  |  |  |  |  | 8 | 0 | 2 | 2 | 2 | 2 | 0 | 0 | 0 | 0 | 0 | 0 | 0 | 0 | 0 | 292 | 310 |  |  | PEAKS DB |
| Q.LPDWTEC(+57.02)EVSGYGK.H | N | 85.25 | 1639.7188 | 14 | 1.0 | 820.8674 | 2 | 52.78 | 3 | F3:28524 | NaSa01\_F11.raw |  | 0 | 2.6381E7 | 8.0628E7 | 2.9981E6 | 1.7302E6 |  |  |  |  |  |  | 3.668E5 |  | 5 | 0 | 0 | 1 | 1 | 1 | 1 | 0 | 0 | 0 | 0 | 0 | 0 | 1 | 0 | 416 | 429 | Carbamidomethylation | C7:Carbamidomethylation:1000.00 | PEAKS DB |
| K.ALYSPNLFIC(+57.02)FC(+57.02)PPGFSGK.F | N | 83.65 | 2174.0330 | 19 | -0.3 | 1088.0234 | 2 | 93.60 | 12 | F12:67510 | NaSa01\_F7.raw |  | 5.8401E7 | 6.3158E7 | 6.0423E6 |  |  |  |  |  |  | 2.7517E7 | 1.7226E8 | 4.6E6 | 6.0752E6 | 13 | 0 | 3 | 2 | 2 | 0 | 0 | 0 | 0 | 0 | 0 | 2 | 2 | 1 | 1 | 77 | 95 | Carbamidomethylation | C10:Carbamidomethylation:1000.00;C12:Carbamidomethylation:1000.00 | PEAKS DB |
| R.GTWSTTESGAEC(+57.02)VNWK.T | N | 81.48 | 1811.7784 | 16 | 0.3 | 906.8967 | 2 | 40.16 | 12 | F12:23328 | NaSa01\_F7.raw |  | 3.1844E6 | 5.7251E6 | 4.2946E6 |  | 2.742E5 |  |  |  |  |  | 6.7871E7 | 2.0844E6 |  | 8 | 0 | 1 | 1 | 1 | 0 | 1 | 0 | 0 | 0 | 0 | 0 | 3 | 1 | 0 | 115 | 130 | Carbamidomethylation | C12:Carbamidomethylation:1000.00 | PEAKS DB |
| R.TVTENMLC(+57.02)AGDTR.Q | N | 75.17 | 1466.6494 | 13 | 1.2 | 734.3329 | 2 | 28.26 | 4 | F4:13301 | NaSa01\_F12.raw |  |  |  | 3.07E7 | 7.9493E5 |  |  |  |  |  |  |  |  |  | 3 | 0 | 0 | 0 | 2 | 1 | 0 | 0 | 0 | 0 | 0 | 0 | 0 | 0 | 0 | 463 | 475 | Carbamidomethylation | C8:Carbamidomethylation:1000.00 | PEAKS DB |
| R.LYPDSFC(+57.02)TSAR.L | N | 68.01 | 1315.5867 | 11 | -0.3 | 658.8004 | 2 | 32.96 | 4 | F4:17051 | NaSa01\_F12.raw |  | 5.5415E6 | 3.7403E5 | 7.9385E7 | 3.5956E6 |  |  |  | 1.159E5 |  |  |  |  |  | 6 | 0 | 1 | 1 | 2 | 1 | 0 | 0 | 0 | 1 | 0 | 0 | 0 | 0 | 0 | 448 | 458 | Carbamidomethylation | C7:Carbamidomethylation:1000.00 | PEAKS DB |
| K.QAYNWKESWLR.R | Y | 66.78 | 1479.7258 | 11 | 0.0 | 494.2492 | 3 | 39.93 | 12 | F12:23307 | NaSa01\_F7.raw |  |  |  |  |  |  |  |  |  |  |  | 1.0505E7 |  |  | 2 | 0 | 0 | 0 | 0 | 0 | 0 | 0 | 0 | 0 | 0 | 0 | 2 | 0 | 0 | 27 | 37 |  |  | PEAKS DB |
| Y.SPNLFIC(+57.02)FC(+57.02)PPGFSGK.F | N | 66.41 | 1826.8484 | 16 | 0.7 | 914.4321 | 2 | 82.84 | 12 | F12:58627 | NaSa01\_F7.raw |  |  |  |  |  |  |  |  |  |  |  | 1.7423E6 |  |  | 1 | 0 | 0 | 0 | 0 | 0 | 0 | 0 | 0 | 0 | 0 | 0 | 1 | 0 | 0 | 80 | 95 | Carbamidomethylation | C7:Carbamidomethylation:1000.00;C9:Carbamidomethylation:1000.00 | PEAKS DB |
| Q.LGLGDHNYC(+57.02)R.N | N | 63.69 | 1203.5455 | 10 | 0.8 | 402.1895 | 3 | 12.19 | 12 | F12:2522 | NaSa01\_F7.raw |  |  |  |  |  |  |  |  |  |  |  | 8.9722E5 |  |  | 2 | 0 | 0 | 0 | 0 | 0 | 0 | 0 | 0 | 0 | 0 | 0 | 2 | 0 | 0 | 150 | 159 | Carbamidomethylation | C9:Carbamidomethylation:1000.00 | PEAKS DB |
| F.IC(+57.02)FC(+57.02)PPGFSGK.F | N | 63.52 | 1268.5681 | 11 | 0.2 | 635.2914 | 2 | 50.36 | 12 | F12:32061 | NaSa01\_F7.raw |  |  |  |  |  |  |  |  |  |  |  | 2.2109E7 |  |  | 1 | 0 | 0 | 0 | 0 | 0 | 0 | 0 | 0 | 0 | 0 | 0 | 1 | 0 | 0 | 85 | 95 | Carbamidomethylation | C2:Carbamidomethylation:1000.00;C4:Carbamidomethylation:1000.00 | PEAKS DB |
| N.LFIC(+57.02)FC(+57.02)PPGFSGK.F | N | 63.19 | 1528.7206 | 13 | -0.5 | 765.3672 | 2 | 78.45 | 12 | F12:54679 | NaSa01\_F7.raw |  |  | 4.4273E6 |  |  |  |  |  |  |  | 2.2158E6 | 5.365E7 |  |  | 3 | 0 | 0 | 1 | 0 | 0 | 0 | 0 | 0 | 0 | 0 | 1 | 1 | 0 | 0 | 83 | 95 | Carbamidomethylation | C4:Carbamidomethylation:1000.00;C6:Carbamidomethylation:1000.00 | PEAKS DB |
| R.TLDNDIALLK.L | N | 57.12 | 1114.6234 | 10 | 0.0 | 558.3190 | 2 | 52.92 | 4 | F4:34239 | NaSa01\_F12.raw |  | 4.5967E6 | 1.4483E7 | 6.6342E7 | 2.5476E6 |  |  |  |  |  |  |  |  |  | 6 | 0 | 1 | 1 | 3 | 1 | 0 | 0 | 0 | 0 | 0 | 0 | 0 | 0 | 0 | 380 | 389 |  |  | PEAKS DB |
| L.FIC(+57.02)FC(+57.02)PPGFSGK.F | N | 49.97 | 1415.6366 | 12 | -0.6 | 708.8251 | 2 | 63.43 | 12 | F12:43236 | NaSa01\_F7.raw |  |  |  |  |  |  |  |  |  |  |  | 3.3384E6 |  |  | 1 | 0 | 0 | 0 | 0 | 0 | 0 | 0 | 0 | 0 | 0 | 0 | 1 | 0 | 0 | 84 | 95 | Carbamidomethylation | C3:Carbamidomethylation:1000.00;C5:Carbamidomethylation:1000.00 | PEAKS DB |
| Y.SYIESHPWQAGIFVK.N | N | 43.01 | 1760.8885 | 15 | 0.2 | 587.9702 | 3 | 53.84 | 4 | F4:34590 | NaSa01\_F12.raw |  |  |  | 0 |  |  |  |  |  |  |  |  |  |  | 0 | 0 | 0 | 0 | 0 | 0 | 0 | 0 | 0 | 0 | 0 | 0 | 0 | 0 | 0 | 296 | 310 |  |  | PEAKS DB |
| total 14 peptides |  |  |  |  |  |  |  |  |  |  |  |  |  |  |  |  |  |  |  |  |  |  |  |  |  |  |  |  |  |  |  |  |  |  |  |  |  |  |  |  |  |  |  |  |  |
| --- | --- | --- | --- | --- | --- | --- | --- | --- | --- | --- | --- | --- | --- | --- | --- | --- | --- | --- | --- | --- | --- | --- | --- | --- | --- | --- | --- | --- | --- | --- | --- | --- | --- | --- | --- | --- | --- | --- | --- | --- | --- | --- | --- | --- | --- |

1POB

back to list

  

| Protein Coverage
| Supporting Peptides
|

Protein Coverage:

Supporting Peptides:

| Peptide | Uniq | -10lgP | Mass | Length | ppm | m/z | z | RT | Fraction | Scan | Source File | Area F1 | Area F10 | Area F11 | Area F12 | Area F13 | Area F14 | Area F2 | Area F3 | Area F4 | Area F5 | Area F6 | Area F7 | Area F8 | Area F9 | #Feature | #Feature F1 | #Feature F10 | #Feature F11 | #Feature F12 | #Feature F13 | #Feature F14 | #Feature F2 | #Feature F3 | #Feature F4 | #Feature F5 | #Feature F6 | #Feature F7 | #Feature F8 | #Feature F9 | Start | End | PTM | AScore | Found By |
| --- | --- | --- | --- | --- | --- | --- | --- | --- | --- | --- | --- | --- | --- | --- | --- | --- | --- | --- | --- | --- | --- | --- | --- | --- | --- | --- | --- | --- | --- | --- | --- | --- | --- | --- | --- | --- | --- | --- | --- | --- | --- | --- | --- | --- | --- |
| K.TYSYEC(+57.02)SQGTLTC(+57.02)K.G | N | 87.01 | 1696.7073 | 14 | -0.1 | 849.3608 | 2 | 21.82 | 13 | F13:11695 | NaSa01\_F8.raw |  | 2.9725E5 |  | 3.2683E6 | 3.3919E6 |  |  |  |  | 8.1372E5 | 5.9955E5 | 8.9065E7 | 1.107E10 | 1.3561E6 | 26 | 0 | 1 | 0 | 2 | 2 | 0 | 0 | 0 | 0 | 1 | 1 | 3 | 15 | 1 | 66 | 79 | Carbamidomethylation | C6:Carbamidomethylation:1000.00;C13:Carbamidomethylation:1000.00 | PEAKS DB |
| NLYQFKNMIQC(+57.02)TVPSR.S | N | 80.86 | 1997.9816 | 16 | 0.1 | 667.0012 | 3 | 54.61 | 13 | F13:39759 | NaSa01\_F8.raw |  |  |  |  |  |  |  |  |  |  |  |  | 1.2158E7 |  | 1 | 0 | 0 | 0 | 0 | 0 | 0 | 0 | 0 | 0 | 0 | 0 | 0 | 1 | 0 | 1 | 16 | Carbamidomethylation | C11:Carbamidomethylation:1000.00 | PEAKS DB |
| R.SWWDFADYGC(+57.02)YC(+57.02)GR.G | N | 73.88 | 1841.6926 | 14 | 1.1 | 921.8546 | 2 | 90.79 | 13 | F13:69941 | NaSa01\_F8.raw |  | 1.5441E7 | 2.3773E7 | 1.8946E6 |  |  |  |  |  |  | 1.0973E6 | 1.4857E8 | 3.3627E9 | 1.6201E8 | 15 | 0 | 1 | 1 | 1 | 0 | 0 | 0 | 0 | 0 | 0 | 1 | 1 | 7 | 3 | 17 | 30 | Carbamidomethylation | C10:Carbamidomethylation:1000.00;C12:Carbamidomethylation:1000.00 | PEAKS DB |
| R.C(+57.02)C(+57.02)QVHDNC(+57.02)YNEAEKISGC(+57.02)WPYFK.T | N | 73.59 | 2964.2087 | 23 | -0.1 | 989.0768 | 3 | 51.14 | 13 | F13:36581 | NaSa01\_F8.raw |  |  |  |  |  |  |  |  |  |  |  |  | 9.3007E7 |  | 2 | 0 | 0 | 0 | 0 | 0 | 0 | 0 | 0 | 0 | 0 | 0 | 0 | 2 | 0 | 43 | 65 | Carbamidomethylation | C1:Carbamidomethylation:1000.00;C2:Carbamidomethylation:1000.00;C8:Carbamidomethylation:1000.00;C18:Carbamidomethylation:1000.00 | PEAKS DB |
| K.ISGC(+57.02)WPYFK.T | N | 70.94 | 1156.5375 | 9 | -1.5 | 579.2751 | 2 | 55.58 | 13 | F13:41533 | NaSa01\_F8.raw |  | 2.8643E7 | 1.4353E7 | 8.5095E6 | 3.4707E6 | 1.0467E7 |  |  |  | 1.6642E6 | 3.0204E7 | 6.6628E8 | 1.5103E10 | 3.4384E8 | 20 | 0 | 1 | 1 | 1 | 1 | 1 | 0 | 0 | 0 | 1 | 1 | 3 | 8 | 2 | 57 | 65 | Carbamidomethylation | C4:Carbamidomethylation:1000.00 | PEAKS DB |
| R.C(+57.02)C(+57.02)QVHDNC(+57.02)YNEAEK.I | N | 70.07 | 1825.6818 | 14 | -1.6 | 913.8467 | 2 | 11.63 | 13 | F13:3808 | NaSa01\_F8.raw |  |  |  |  |  |  |  |  |  |  |  |  | 2.5276E7 |  | 2 | 0 | 0 | 0 | 0 | 0 | 0 | 0 | 0 | 0 | 0 | 0 | 0 | 2 | 0 | 43 | 56 | Carbamidomethylation | C1:Carbamidomethylation:1000.00;C2:Carbamidomethylation:1000.00;C8:Carbamidomethylation:1000.00 | PEAKS DB |
| R.GGSGTPVDDLDR.C | N | 67.25 | 1187.5417 | 12 | -0.9 | 594.7776 | 2 | 12.12 | 13 | F13:4409 | NaSa01\_F8.raw |  |  |  |  |  |  |  |  |  |  |  |  | 9.3807E7 |  | 2 | 0 | 0 | 0 | 0 | 0 | 0 | 0 | 0 | 0 | 0 | 0 | 0 | 2 | 0 | 31 | 42 |  |  | PEAKS DB |
| S.RSWWDFADYGC(+57.02)YC(+57.02)GR.G | N | 66.86 | 1997.7937 | 15 | 0.9 | 666.9391 | 3 | 73.94 | 13 | F13:55816 | NaSa01\_F8.raw |  |  |  |  |  |  |  |  |  |  |  |  | 2.8562E6 |  | 3 | 0 | 0 | 0 | 0 | 0 | 0 | 0 | 0 | 0 | 0 | 0 | 0 | 3 | 0 | 16 | 30 | Carbamidomethylation | C11:Carbamidomethylation:1000.00;C13:Carbamidomethylation:1000.00 | PEAKS DB |
| K.ISGC(+57.02)WPYFKTYSYEC(+57.02)SQGTLTC(+57.02)K.G | N | 64.07 | 2835.2341 | 23 | -0.7 | 946.0847 | 3 | 59.43 | 13 | F13:43496 | NaSa01\_F8.raw |  |  |  |  |  |  |  |  |  |  |  |  | 6.2715E6 |  | 1 | 0 | 0 | 0 | 0 | 0 | 0 | 0 | 0 | 0 | 0 | 0 | 0 | 1 | 0 | 57 | 79 | Carbamidomethylation | C4:Carbamidomethylation:1000.00;C15:Carbamidomethylation:1000.00;C22:Carbamidomethylation:1000.00 | PEAKS DB |
| R.GGSGTPVDDLDRC(+57.02)C(+57.02)QVHDNC(+57.02)YNEAEK.I | N | 55.65 | 2995.2131 | 26 | 0.6 | 749.8110 | 4 | 24.18 | 13 | F13:13902 | NaSa01\_F8.raw |  |  |  |  |  |  |  |  |  |  |  |  | 3.8626E7 |  | 2 | 0 | 0 | 0 | 0 | 0 | 0 | 0 | 0 | 0 | 0 | 0 | 0 | 2 | 0 | 31 | 56 | Carbamidomethylation | C13:Carbamidomethylation:1000.00;C14:Carbamidomethylation:1000.00;C20:Carbamidomethylation:1000.00 | PEAKS DB |
| K.NMIQC(+57.02)TVPSR.S | N | 51.98 | 1204.5692 | 10 | -0.2 | 603.2917 | 2 | 12.12 | 13 | F13:4212 | NaSa01\_F8.raw |  |  |  |  |  |  |  |  |  |  |  |  | 8.3493E7 |  | 2 | 0 | 0 | 0 | 0 | 0 | 0 | 0 | 0 | 0 | 0 | 0 | 0 | 2 | 0 | 7 | 16 | Carbamidomethylation | C5:Carbamidomethylation:1000.00 | PEAKS DB |
| K.NMIQC(+57.02)TVPSRSWWDFADYGC(+57.02)YC(+57.02)GR.G | N | 50.20 | 3028.2512 | 24 | -0.9 | 1010.4235 | 3 | 79.97 | 13 | F13:61019 | NaSa01\_F8.raw |  |  |  |  |  |  |  |  |  |  |  |  | 0 |  | 0 | 0 | 0 | 0 | 0 | 0 | 0 | 0 | 0 | 0 | 0 | 0 | 0 | 0 | 0 | 7 | 30 | Carbamidomethylation | C5:Carbamidomethylation:1000.00;C20:Carbamidomethylation:1000.00;C22:Carbamidomethylation:1000.00 | PEAKS DB |
| W.WDFADYGC(+57.02)YC(+57.02)GR.G | N | 49.58 | 1568.5813 | 12 | 1.5 | 785.2991 | 2 | 61.99 | 13 | F13:45659 | NaSa01\_F8.raw |  |  |  |  |  |  |  |  |  |  |  |  | 1.1461E6 |  | 1 | 0 | 0 | 0 | 0 | 0 | 0 | 0 | 0 | 0 | 0 | 0 | 0 | 1 | 0 | 19 | 30 | Carbamidomethylation | C8:Carbamidomethylation:1000.00;C10:Carbamidomethylation:1000.00 | PEAKS DB |
| R.LAAIC(+57.02)FAGAPYN.D | N | 44.68 | 1266.6067 | 12 | -0.1 | 634.3105 | 2 | 71.65 | 13 | F13:54114 | NaSa01\_F8.raw |  |  | 2.1689E6 | 3.0706E6 |  | 4.6852E6 |  |  |  |  | 4.8617E6 | 4.4367E7 | 2.0168E7 |  | 6 | 0 | 0 | 1 | 1 | 0 | 1 | 0 | 0 | 0 | 0 | 1 | 1 | 1 | 0 | 95 | 106 | Carbamidomethylation | C5:Carbamidomethylation:1000.00 | PEAKS DB |
| Y.FKTYSYEC(+57.02)SQGTLTC(+57.02)K.G | N | 43.26 | 1971.8706 | 16 | 0.4 | 658.2977 | 3 | 23.27 | 13 | F13:13009 | NaSa01\_F8.raw |  |  |  |  |  |  |  |  |  |  |  |  | 9.3587E5 |  | 1 | 0 | 0 | 0 | 0 | 0 | 0 | 0 | 0 | 0 | 0 | 0 | 0 | 1 | 0 | 64 | 79 | Carbamidomethylation | C8:Carbamidomethylation:1000.00;C15:Carbamidomethylation:1000.00 | PEAKS DB |
| R.LAAIC(+57.02)FAGAPYNDNDYNINLK.A | Y | 39.14 | 2356.1157 | 21 | 1.0 | 786.3800 | 3 | 73.65 | 4 | F4:51073 | NaSa01\_F12.raw |  |  |  | 5.4292E6 |  |  |  |  |  |  |  |  |  |  | 1 | 0 | 0 | 0 | 1 | 0 | 0 | 0 | 0 | 0 | 0 | 0 | 0 | 0 | 0 | 95 | 115 | Carbamidomethylation | C5:Carbamidomethylation:1000.00 | PEAKS DB |
| total 16 peptides |  |  |  |  |  |  |  |  |  |  |  |  |  |  |  |  |  |  |  |  |  |  |  |  |  |  |  |  |  |  |  |  |  |  |  |  |  |  |  |  |  |  |  |  |  |
| --- | --- | --- | --- | --- | --- | --- | --- | --- | --- | --- | --- | --- | --- | --- | --- | --- | --- | --- | --- | --- | --- | --- | --- | --- | --- | --- | --- | --- | --- | --- | --- | --- | --- | --- | --- | --- | --- | --- | --- | --- | --- | --- | --- | --- | --- |

1POA

back to list

  

| Protein Coverage
| Supporting Peptides
|

Protein Coverage:

Supporting Peptides:

| Peptide | Uniq | -10lgP | Mass | Length | ppm | m/z | z | RT | Fraction | Scan | Source File | Area F1 | Area F10 | Area F11 | Area F12 | Area F13 | Area F14 | Area F2 | Area F3 | Area F4 | Area F5 | Area F6 | Area F7 | Area F8 | Area F9 | #Feature | #Feature F1 | #Feature F10 | #Feature F11 | #Feature F12 | #Feature F13 | #Feature F14 | #Feature F2 | #Feature F3 | #Feature F4 | #Feature F5 | #Feature F6 | #Feature F7 | #Feature F8 | #Feature F9 | Start | End | PTM | AScore | Found By |
| --- | --- | --- | --- | --- | --- | --- | --- | --- | --- | --- | --- | --- | --- | --- | --- | --- | --- | --- | --- | --- | --- | --- | --- | --- | --- | --- | --- | --- | --- | --- | --- | --- | --- | --- | --- | --- | --- | --- | --- | --- | --- | --- | --- | --- | --- |
| K.TYSYEC(+57.02)SQGTLTC(+57.02)K.G | N | 87.01 | 1696.7073 | 14 | -0.1 | 849.3608 | 2 | 21.82 | 13 | F13:11695 | NaSa01\_F8.raw |  | 2.9725E5 |  | 3.2683E6 | 3.3919E6 |  |  |  |  | 8.1372E5 | 5.9955E5 | 8.9065E7 | 1.107E10 | 1.3561E6 | 26 | 0 | 1 | 0 | 2 | 2 | 0 | 0 | 0 | 0 | 1 | 1 | 3 | 15 | 1 | 66 | 79 | Carbamidomethylation | C6:Carbamidomethylation:1000.00;C13:Carbamidomethylation:1000.00 | PEAKS DB |
| NLYQFKNMIQC(+57.02)TVPSR.S | N | 80.86 | 1997.9816 | 16 | 0.1 | 667.0012 | 3 | 54.61 | 13 | F13:39759 | NaSa01\_F8.raw |  |  |  |  |  |  |  |  |  |  |  |  | 1.2158E7 |  | 1 | 0 | 0 | 0 | 0 | 0 | 0 | 0 | 0 | 0 | 0 | 0 | 0 | 1 | 0 | 1 | 16 | Carbamidomethylation | C11:Carbamidomethylation:1000.00 | PEAKS DB |
| R.SWWDFADYGC(+57.02)YC(+57.02)GR.G | N | 73.88 | 1841.6926 | 14 | 1.1 | 921.8546 | 2 | 90.79 | 13 | F13:69941 | NaSa01\_F8.raw |  | 1.5441E7 | 2.3773E7 | 1.8946E6 |  |  |  |  |  |  | 1.0973E6 | 1.4857E8 | 3.3627E9 | 1.6201E8 | 15 | 0 | 1 | 1 | 1 | 0 | 0 | 0 | 0 | 0 | 0 | 1 | 1 | 7 | 3 | 17 | 30 | Carbamidomethylation | C10:Carbamidomethylation:1000.00;C12:Carbamidomethylation:1000.00 | PEAKS DB |
| R.C(+57.02)C(+57.02)QVHDNC(+57.02)YNEAEKISGC(+57.02)WPYFK.T | N | 73.59 | 2964.2087 | 23 | -0.1 | 989.0768 | 3 | 51.14 | 13 | F13:36581 | NaSa01\_F8.raw |  |  |  |  |  |  |  |  |  |  |  |  | 9.3007E7 |  | 2 | 0 | 0 | 0 | 0 | 0 | 0 | 0 | 0 | 0 | 0 | 0 | 0 | 2 | 0 | 43 | 65 | Carbamidomethylation | C1:Carbamidomethylation:1000.00;C2:Carbamidomethylation:1000.00;C8:Carbamidomethylation:1000.00;C18:Carbamidomethylation:1000.00 | PEAKS DB |
| K.ISGC(+57.02)WPYFK.T | N | 70.94 | 1156.5375 | 9 | -1.5 | 579.2751 | 2 | 55.58 | 13 | F13:41533 | NaSa01\_F8.raw |  | 2.8643E7 | 1.4353E7 | 8.5095E6 | 3.4707E6 | 1.0467E7 |  |  |  | 1.6642E6 | 3.0204E7 | 6.6628E8 | 1.5103E10 | 3.4384E8 | 20 | 0 | 1 | 1 | 1 | 1 | 1 | 0 | 0 | 0 | 1 | 1 | 3 | 8 | 2 | 57 | 65 | Carbamidomethylation | C4:Carbamidomethylation:1000.00 | PEAKS DB |
| R.C(+57.02)C(+57.02)QVHDNC(+57.02)YNEAEK.I | N | 70.07 | 1825.6818 | 14 | -1.6 | 913.8467 | 2 | 11.63 | 13 | F13:3808 | NaSa01\_F8.raw |  |  |  |  |  |  |  |  |  |  |  |  | 2.5276E7 |  | 2 | 0 | 0 | 0 | 0 | 0 | 0 | 0 | 0 | 0 | 0 | 0 | 0 | 2 | 0 | 43 | 56 | Carbamidomethylation | C1:Carbamidomethylation:1000.00;C2:Carbamidomethylation:1000.00;C8:Carbamidomethylation:1000.00 | PEAKS DB |
| R.GGSGTPVDDLDR.C | N | 67.25 | 1187.5417 | 12 | -0.9 | 594.7776 | 2 | 12.12 | 13 | F13:4409 | NaSa01\_F8.raw |  |  |  |  |  |  |  |  |  |  |  |  | 9.3807E7 |  | 2 | 0 | 0 | 0 | 0 | 0 | 0 | 0 | 0 | 0 | 0 | 0 | 0 | 2 | 0 | 31 | 42 |  |  | PEAKS DB |
| S.RSWWDFADYGC(+57.02)YC(+57.02)GR.G | N | 66.86 | 1997.7937 | 15 | 0.9 | 666.9391 | 3 | 73.94 | 13 | F13:55816 | NaSa01\_F8.raw |  |  |  |  |  |  |  |  |  |  |  |  | 2.8562E6 |  | 3 | 0 | 0 | 0 | 0 | 0 | 0 | 0 | 0 | 0 | 0 | 0 | 0 | 3 | 0 | 16 | 30 | Carbamidomethylation | C11:Carbamidomethylation:1000.00;C13:Carbamidomethylation:1000.00 | PEAKS DB |
| K.ISGC(+57.02)WPYFKTYSYEC(+57.02)SQGTLTC(+57.02)K.G | N | 64.07 | 2835.2341 | 23 | -0.7 | 946.0847 | 3 | 59.43 | 13 | F13:43496 | NaSa01\_F8.raw |  |  |  |  |  |  |  |  |  |  |  |  | 6.2715E6 |  | 1 | 0 | 0 | 0 | 0 | 0 | 0 | 0 | 0 | 0 | 0 | 0 | 0 | 1 | 0 | 57 | 79 | Carbamidomethylation | C4:Carbamidomethylation:1000.00;C15:Carbamidomethylation:1000.00;C22:Carbamidomethylation:1000.00 | PEAKS DB |
| R.GGSGTPVDDLDRC(+57.02)C(+57.02)QVHDNC(+57.02)YNEAEK.I | N | 55.65 | 2995.2131 | 26 | 0.6 | 749.8110 | 4 | 24.18 | 13 | F13:13902 | NaSa01\_F8.raw |  |  |  |  |  |  |  |  |  |  |  |  | 3.8626E7 |  | 2 | 0 | 0 | 0 | 0 | 0 | 0 | 0 | 0 | 0 | 0 | 0 | 0 | 2 | 0 | 31 | 56 | Carbamidomethylation | C13:Carbamidomethylation:1000.00;C14:Carbamidomethylation:1000.00;C20:Carbamidomethylation:1000.00 | PEAKS DB |
| K.NMIQC(+57.02)TVPSR.S | N | 51.98 | 1204.5692 | 10 | -0.2 | 603.2917 | 2 | 12.12 | 13 | F13:4212 | NaSa01\_F8.raw |  |  |  |  |  |  |  |  |  |  |  |  | 8.3493E7 |  | 2 | 0 | 0 | 0 | 0 | 0 | 0 | 0 | 0 | 0 | 0 | 0 | 0 | 2 | 0 | 7 | 16 | Carbamidomethylation | C5:Carbamidomethylation:1000.00 | PEAKS DB |
| K.NMIQC(+57.02)TVPSRSWWDFADYGC(+57.02)YC(+57.02)GR.G | N | 50.20 | 3028.2512 | 24 | -0.9 | 1010.4235 | 3 | 79.97 | 13 | F13:61019 | NaSa01\_F8.raw |  |  |  |  |  |  |  |  |  |  |  |  | 0 |  | 0 | 0 | 0 | 0 | 0 | 0 | 0 | 0 | 0 | 0 | 0 | 0 | 0 | 0 | 0 | 7 | 30 | Carbamidomethylation | C5:Carbamidomethylation:1000.00;C20:Carbamidomethylation:1000.00;C22:Carbamidomethylation:1000.00 | PEAKS DB |
| W.WDFADYGC(+57.02)YC(+57.02)GR.G | N | 49.58 | 1568.5813 | 12 | 1.5 | 785.2991 | 2 | 61.99 | 13 | F13:45659 | NaSa01\_F8.raw |  |  |  |  |  |  |  |  |  |  |  |  | 1.1461E6 |  | 1 | 0 | 0 | 0 | 0 | 0 | 0 | 0 | 0 | 0 | 0 | 0 | 0 | 1 | 0 | 19 | 30 | Carbamidomethylation | C8:Carbamidomethylation:1000.00;C10:Carbamidomethylation:1000.00 | PEAKS DB |
| R.LAAIC(+57.02)FAGAPYN.D | N | 44.68 | 1266.6067 | 12 | -0.1 | 634.3105 | 2 | 71.65 | 13 | F13:54114 | NaSa01\_F8.raw |  |  | 2.1689E6 | 3.0706E6 |  | 4.6852E6 |  |  |  |  | 4.8617E6 | 4.4367E7 | 2.0168E7 |  | 6 | 0 | 0 | 1 | 1 | 0 | 1 | 0 | 0 | 0 | 0 | 1 | 1 | 1 | 0 | 95 | 106 | Carbamidomethylation | C5:Carbamidomethylation:1000.00 | PEAKS DB |
| Y.FKTYSYEC(+57.02)SQGTLTC(+57.02)K.G | N | 43.26 | 1971.8706 | 16 | 0.4 | 658.2977 | 3 | 23.27 | 13 | F13:13009 | NaSa01\_F8.raw |  |  |  |  |  |  |  |  |  |  |  |  | 9.3587E5 |  | 1 | 0 | 0 | 0 | 0 | 0 | 0 | 0 | 0 | 0 | 0 | 0 | 0 | 1 | 0 | 64 | 79 | Carbamidomethylation | C8:Carbamidomethylation:1000.00;C15:Carbamidomethylation:1000.00 | PEAKS DB |
| R.LAAIC(+57.02)FAGAPYNDNDYNINLK.A | Y | 39.14 | 2356.1157 | 21 | 1.0 | 786.3800 | 3 | 73.65 | 4 | F4:51073 | NaSa01\_F12.raw |  |  |  | 5.4292E6 |  |  |  |  |  |  |  |  |  |  | 1 | 0 | 0 | 0 | 1 | 0 | 0 | 0 | 0 | 0 | 0 | 0 | 0 | 0 | 0 | 95 | 115 | Carbamidomethylation | C5:Carbamidomethylation:1000.00 | PEAKS DB |
| total 16 peptides |  |  |  |  |  |  |  |  |  |  |  |  |  |  |  |  |  |  |  |  |  |  |  |  |  |  |  |  |  |  |  |  |  |  |  |  |  |  |  |  |  |  |  |  |  |
| --- | --- | --- | --- | --- | --- | --- | --- | --- | --- | --- | --- | --- | --- | --- | --- | --- | --- | --- | --- | --- | --- | --- | --- | --- | --- | --- | --- | --- | --- | --- | --- | --- | --- | --- | --- | --- | --- | --- | --- | --- | --- | --- | --- | --- | --- |

XP\_026546198.1

back to list

  

| Protein Coverage
| Supporting Peptides
|

Protein Coverage:

Supporting Peptides:

| Peptide | Uniq | -10lgP | Mass | Length | ppm | m/z | z | RT | Fraction | Scan | Source File | Area F1 | Area F10 | Area F11 | Area F12 | Area F13 | Area F14 | Area F2 | Area F3 | Area F4 | Area F5 | Area F6 | Area F7 | Area F8 | Area F9 | #Feature | #Feature F1 | #Feature F10 | #Feature F11 | #Feature F12 | #Feature F13 | #Feature F14 | #Feature F2 | #Feature F3 | #Feature F4 | #Feature F5 | #Feature F6 | #Feature F7 | #Feature F8 | #Feature F9 | Start | End | PTM | AScore | Found By |
| --- | --- | --- | --- | --- | --- | --- | --- | --- | --- | --- | --- | --- | --- | --- | --- | --- | --- | --- | --- | --- | --- | --- | --- | --- | --- | --- | --- | --- | --- | --- | --- | --- | --- | --- | --- | --- | --- | --- | --- | --- | --- | --- | --- | --- | --- |
| R.IPVYSAYVYNPGK.A | N | 84.05 | 1469.7554 | 13 | 0.9 | 735.8856 | 2 | 50.54 | 6 | F6:29743 | NaSa01\_F14.raw |  |  | 0 | 5.2943E6 | 9.5904E8 | 6.5068E6 |  |  |  | 1.4262E6 | 2.0524E6 | 5.8608E5 | 3.9528E5 | 3.6466E6 | 9 | 0 | 0 | 0 | 1 | 2 | 1 | 0 | 0 | 0 | 1 | 1 | 1 | 1 | 1 | 70 | 82 |  |  | PEAKS DB |
| K.LAQLYNVNHVSLFHSDC(+57.02)PR.Q | N | 83.18 | 2269.1062 | 19 | 0.1 | 757.3761 | 3 | 43.25 | 4 | F4:25798 | NaSa01\_F12.raw |  |  |  | 5.5169E7 | 7.3204E8 | 7.8894E6 |  |  |  |  |  |  |  |  | 11 | 0 | 0 | 0 | 4 | 5 | 2 | 0 | 0 | 0 | 0 | 0 | 0 | 0 | 0 | 255 | 273 | Carbamidomethylation | C17:Carbamidomethylation:1000.00 | PEAKS DB |
| G.EVVDSFQDHC(+57.02)PQFFLR.E | N | 71.48 | 2022.9258 | 16 | 0.5 | 675.3162 | 3 | 60.39 | 4 | F4:40242 | NaSa01\_F12.raw |  |  | 6.7523E6 | 9.5937E6 | 1.2968E9 | 1.0449E7 |  |  |  |  |  |  | 5.3462E6 |  | 8 | 0 | 0 | 1 | 1 | 4 | 1 | 0 | 0 | 0 | 0 | 0 | 0 | 1 | 0 | 20 | 35 | Carbamidomethylation | C10:Carbamidomethylation:1000.00 | PEAKS DB |
| K.SSTFTLTNIVPQFIK.L | N | 69.82 | 1694.9243 | 15 | 1.1 | 848.4704 | 2 | 89.01 | 4 | F4:63439 | NaSa01\_F12.raw |  |  |  | 6.2934E6 | 1.5029E9 | 8.7605E6 |  |  |  |  |  |  |  |  | 4 | 0 | 0 | 0 | 1 | 2 | 1 | 0 | 0 | 0 | 0 | 0 | 0 | 0 | 0 | 154 | 168 |  |  | PEAKS DB |
| R.GHLNPNGHQPDYSAK.S | N | 61.86 | 1633.7596 | 15 | 1.0 | 545.5944 | 3 | 11.43 | 5 | F5:1940 | NaSa01\_F13.raw |  |  |  |  | 1.4956E6 |  |  |  |  |  |  |  |  |  | 1 | 0 | 0 | 0 | 0 | 1 | 0 | 0 | 0 | 0 | 0 | 0 | 0 | 0 | 0 | 139 | 153 |  |  | PEAKS DB |
| R.FTLGQLEEK.L | Y | 60.48 | 1063.5549 | 9 | -0.4 | 532.7845 | 2 | 42.56 | 6 | F6:22936 | NaSa01\_F14.raw |  |  |  |  | 4.6886E8 | 5.0932E6 |  |  | 4.3246E5 |  |  |  |  |  | 3 | 0 | 0 | 0 | 0 | 1 | 1 | 0 | 0 | 1 | 0 | 0 | 0 | 0 | 0 | 246 | 254 |  |  | PEAKS DB |
| Y.NVNHVSLFHSDC(+57.02)PR.Q | N | 58.23 | 1680.7791 | 14 | 0.8 | 561.2674 | 3 | 11.62 | 5 | F5:2096 | NaSa01\_F13.raw |  |  |  |  | 2.3142E6 |  |  |  |  |  |  |  |  |  | 1 | 0 | 0 | 0 | 0 | 1 | 0 | 0 | 0 | 0 | 0 | 0 | 0 | 0 | 0 | 260 | 273 | Carbamidomethylation | C12:Carbamidomethylation:1000.00 | PEAKS DB |
| F.AVVGAVPGDTYIAR.G | N | 57.03 | 1387.7460 | 14 | 1.7 | 694.8815 | 2 | 40.72 | 6 | F6:21346 | NaSa01\_F14.raw |  |  |  | 4.6266E6 | 7.6974E8 | 7.3018E6 |  |  |  |  |  |  |  |  | 4 | 0 | 0 | 0 | 1 | 2 | 1 | 0 | 0 | 0 | 0 | 0 | 0 | 0 | 0 | 194 | 207 |  |  | PEAKS DB |
| Q.LYNVNHVSLFHSDC(+57.02)PR.Q | N | 53.73 | 1956.9264 | 16 | 1.2 | 490.2395 | 4 | 33.35 | 5 | F5:16269 | NaSa01\_F13.raw |  |  |  |  | 1.2213E6 |  |  |  |  |  |  |  |  |  | 1 | 0 | 0 | 0 | 0 | 1 | 0 | 0 | 0 | 0 | 0 | 0 | 0 | 0 | 0 | 258 | 273 | Carbamidomethylation | C14:Carbamidomethylation:1000.00 | PEAKS DB |
| R.VNRPSHLWSAAC(+57.02)C(+57.02).V | N | 51.05 | 1556.6976 | 13 | 0.0 | 779.3561 | 2 | 11.66 | 5 | F5:2173 | NaSa01\_F13.raw |  |  |  |  | 3.8373E6 |  |  |  |  |  |  |  |  |  | 2 | 0 | 0 | 0 | 0 | 2 | 0 | 0 | 0 | 0 | 0 | 0 | 0 | 0 | 0 | 210 | 222 | Carbamidomethylation | C12:Carbamidomethylation:1000.00;C13:Carbamidomethylation:1000.00 | PEAKS DB |
| K.LAQLYNVNHVSLF.H | N | 49.44 | 1516.8038 | 13 | -0.3 | 759.4089 | 2 | 71.90 | 5 | F5:48055 | NaSa01\_F13.raw |  |  |  |  | 1.3448E8 |  |  |  |  |  |  |  |  |  | 1 | 0 | 0 | 0 | 0 | 1 | 0 | 0 | 0 | 0 | 0 | 0 | 0 | 0 | 0 | 255 | 267 |  |  | PEAKS DB |
| P.VYSAYVYNPGK.A | N | 48.05 | 1259.6187 | 11 | 0.1 | 630.8167 | 2 | 28.78 | 5 | F5:12547 | NaSa01\_F13.raw |  |  |  |  | 1.6549E6 |  |  |  |  |  |  |  |  |  | 1 | 0 | 0 | 0 | 0 | 1 | 0 | 0 | 0 | 0 | 0 | 0 | 0 | 0 | 0 | 72 | 82 |  |  | PEAKS DB |
| R.VNRPSHLWSAAC(+57.02).C | N | 47.36 | 1396.6670 | 12 | 0.2 | 699.3409 | 2 | 11.69 | 5 | F5:2180 | NaSa01\_F13.raw |  |  |  |  | 6.1785E5 |  |  |  |  |  |  |  |  |  | 1 | 0 | 0 | 0 | 0 | 1 | 0 | 0 | 0 | 0 | 0 | 0 | 0 | 0 | 0 | 210 | 221 | Carbamidomethylation | C12:Carbamidomethylation:1000.00 | PEAKS DB |
| L.YNVNHVSLFHSDC(+57.02)PR.Q | N | 46.41 | 1843.8424 | 15 | 1.7 | 615.6224 | 3 | 25.29 | 5 | F5:9481 | NaSa01\_F13.raw |  |  |  |  | 7.5396E6 |  |  |  |  |  |  |  |  |  | 2 | 0 | 0 | 0 | 0 | 2 | 0 | 0 | 0 | 0 | 0 | 0 | 0 | 0 | 0 | 259 | 273 | Carbamidomethylation | C13:Carbamidomethylation:1000.00 | PEAKS DB |
| S.TFTLTNIVPQFIK.L | N | 45.77 | 1520.8602 | 13 | 0.0 | 761.4374 | 2 | 89.58 | 5 | F5:62408 | NaSa01\_F13.raw |  |  |  |  | 1.3379E6 |  |  |  |  |  |  |  |  |  | 1 | 0 | 0 | 0 | 0 | 1 | 0 | 0 | 0 | 0 | 0 | 0 | 0 | 0 | 0 | 156 | 168 |  |  | PEAKS DB |
| R.SWAILAR.N | N | 44.55 | 815.4653 | 7 | 0.1 | 408.7400 | 2 | 43.13 | 4 | F4:25699 | NaSa01\_F12.raw |  |  |  | 9.8044E6 |  | 9.814E6 |  |  |  |  |  |  |  |  | 3 | 0 | 0 | 0 | 1 | 0 | 2 | 0 | 0 | 0 | 0 | 0 | 0 | 0 | 0 | 231 | 237 |  |  | PEAKS DB |
| N.HVSLFHSDC(+57.02)PR.Q | N | 41.73 | 1353.6248 | 11 | -0.2 | 452.2154 | 3 | 11.44 | 5 | F5:2051 | NaSa01\_F13.raw |  |  |  |  | 5.6233E5 |  |  |  |  |  |  |  |  |  | 1 | 0 | 0 | 0 | 0 | 1 | 0 | 0 | 0 | 0 | 0 | 0 | 0 | 0 | 0 | 263 | 273 | Carbamidomethylation | C9:Carbamidomethylation:1000.00 | PEAKS DB |
| F.TLTNIVPQFIK.L | N | 40.01 | 1272.7441 | 11 | 0.2 | 637.3795 | 2 | 67.27 | 5 | F5:44583 | NaSa01\_F13.raw |  |  |  |  | 1.1734E8 |  |  |  |  |  |  |  |  |  | 1 | 0 | 0 | 0 | 0 | 1 | 0 | 0 | 0 | 0 | 0 | 0 | 0 | 0 | 0 | 158 | 168 |  |  | PEAKS DB |
| total 18 peptides |  |  |  |  |  |  |  |  |  |  |  |  |  |  |  |  |  |  |  |  |  |  |  |  |  |  |  |  |  |  |  |  |  |  |  |  |  |  |  |  |  |  |  |  |  |
| --- | --- | --- | --- | --- | --- | --- | --- | --- | --- | --- | --- | --- | --- | --- | --- | --- | --- | --- | --- | --- | --- | --- | --- | --- | --- | --- | --- | --- | --- | --- | --- | --- | --- | --- | --- | --- | --- | --- | --- | --- | --- | --- | --- | --- | --- |

XP\_026546199.1

back to list

  

| Protein Coverage
| Supporting Peptides
|

Protein Coverage:

Supporting Peptides:

| Peptide | Uniq | -10lgP | Mass | Length | ppm | m/z | z | RT | Fraction | Scan | Source File | Area F1 | Area F10 | Area F11 | Area F12 | Area F13 | Area F14 | Area F2 | Area F3 | Area F4 | Area F5 | Area F6 | Area F7 | Area F8 | Area F9 | #Feature | #Feature F1 | #Feature F10 | #Feature F11 | #Feature F12 | #Feature F13 | #Feature F14 | #Feature F2 | #Feature F3 | #Feature F4 | #Feature F5 | #Feature F6 | #Feature F7 | #Feature F8 | #Feature F9 | Start | End | PTM | AScore | Found By |
| --- | --- | --- | --- | --- | --- | --- | --- | --- | --- | --- | --- | --- | --- | --- | --- | --- | --- | --- | --- | --- | --- | --- | --- | --- | --- | --- | --- | --- | --- | --- | --- | --- | --- | --- | --- | --- | --- | --- | --- | --- | --- | --- | --- | --- | --- |
| R.IPVYSAYVYNPGK.A | N | 84.05 | 1469.7554 | 13 | 0.9 | 735.8856 | 2 | 50.54 | 6 | F6:29743 | NaSa01\_F14.raw |  |  | 0 | 5.2943E6 | 9.5904E8 | 6.5068E6 |  |  |  | 1.4262E6 | 2.0524E6 | 5.8608E5 | 3.9528E5 | 3.6466E6 | 9 | 0 | 0 | 0 | 1 | 2 | 1 | 0 | 0 | 0 | 1 | 1 | 1 | 1 | 1 | 70 | 82 |  |  | PEAKS DB |
| K.LAQLYNVNHVSLFHSDC(+57.02)PR.Q | N | 83.18 | 2269.1062 | 19 | 0.1 | 757.3761 | 3 | 43.25 | 4 | F4:25798 | NaSa01\_F12.raw |  |  |  | 5.5169E7 | 7.3204E8 | 7.8894E6 |  |  |  |  |  |  |  |  | 11 | 0 | 0 | 0 | 4 | 5 | 2 | 0 | 0 | 0 | 0 | 0 | 0 | 0 | 0 | 255 | 273 | Carbamidomethylation | C17:Carbamidomethylation:1000.00 | PEAKS DB |
| G.EVVDSFQDHC(+57.02)PQFFLR.E | N | 71.48 | 2022.9258 | 16 | 0.5 | 675.3162 | 3 | 60.39 | 4 | F4:40242 | NaSa01\_F12.raw |  |  | 6.7523E6 | 9.5937E6 | 1.2968E9 | 1.0449E7 |  |  |  |  |  |  | 5.3462E6 |  | 8 | 0 | 0 | 1 | 1 | 4 | 1 | 0 | 0 | 0 | 0 | 0 | 0 | 1 | 0 | 20 | 35 | Carbamidomethylation | C10:Carbamidomethylation:1000.00 | PEAKS DB |
| K.SSTFTLTNIVPQFIK.L | N | 69.82 | 1694.9243 | 15 | 1.1 | 848.4704 | 2 | 89.01 | 4 | F4:63439 | NaSa01\_F12.raw |  |  |  | 6.2934E6 | 1.5029E9 | 8.7605E6 |  |  |  |  |  |  |  |  | 4 | 0 | 0 | 0 | 1 | 2 | 1 | 0 | 0 | 0 | 0 | 0 | 0 | 0 | 0 | 154 | 168 |  |  | PEAKS DB |
| R.GHLNPNGHQPDYSAK.S | N | 61.86 | 1633.7596 | 15 | 1.0 | 545.5944 | 3 | 11.43 | 5 | F5:1940 | NaSa01\_F13.raw |  |  |  |  | 1.4956E6 |  |  |  |  |  |  |  |  |  | 1 | 0 | 0 | 0 | 0 | 1 | 0 | 0 | 0 | 0 | 0 | 0 | 0 | 0 | 0 | 139 | 153 |  |  | PEAKS DB |
| R.FTLGQLEEK.L | Y | 60.48 | 1063.5549 | 9 | -0.4 | 532.7845 | 2 | 42.56 | 6 | F6:22936 | NaSa01\_F14.raw |  |  |  |  | 4.6886E8 | 5.0932E6 |  |  | 4.3246E5 |  |  |  |  |  | 3 | 0 | 0 | 0 | 0 | 1 | 1 | 0 | 0 | 1 | 0 | 0 | 0 | 0 | 0 | 246 | 254 |  |  | PEAKS DB |
| Y.NVNHVSLFHSDC(+57.02)PR.Q | N | 58.23 | 1680.7791 | 14 | 0.8 | 561.2674 | 3 | 11.62 | 5 | F5:2096 | NaSa01\_F13.raw |  |  |  |  | 2.3142E6 |  |  |  |  |  |  |  |  |  | 1 | 0 | 0 | 0 | 0 | 1 | 0 | 0 | 0 | 0 | 0 | 0 | 0 | 0 | 0 | 260 | 273 | Carbamidomethylation | C12:Carbamidomethylation:1000.00 | PEAKS DB |
| F.AVVGAVPGDTYIAR.G | N | 57.03 | 1387.7460 | 14 | 1.7 | 694.8815 | 2 | 40.72 | 6 | F6:21346 | NaSa01\_F14.raw |  |  |  | 4.6266E6 | 7.6974E8 | 7.3018E6 |  |  |  |  |  |  |  |  | 4 | 0 | 0 | 0 | 1 | 2 | 1 | 0 | 0 | 0 | 0 | 0 | 0 | 0 | 0 | 194 | 207 |  |  | PEAKS DB |
| Q.LYNVNHVSLFHSDC(+57.02)PR.Q | N | 53.73 | 1956.9264 | 16 | 1.2 | 490.2395 | 4 | 33.35 | 5 | F5:16269 | NaSa01\_F13.raw |  |  |  |  | 1.2213E6 |  |  |  |  |  |  |  |  |  | 1 | 0 | 0 | 0 | 0 | 1 | 0 | 0 | 0 | 0 | 0 | 0 | 0 | 0 | 0 | 258 | 273 | Carbamidomethylation | C14:Carbamidomethylation:1000.00 | PEAKS DB |
| R.VNRPSHLWSAAC(+57.02)C(+57.02).V | N | 51.05 | 1556.6976 | 13 | 0.0 | 779.3561 | 2 | 11.66 | 5 | F5:2173 | NaSa01\_F13.raw |  |  |  |  | 3.8373E6 |  |  |  |  |  |  |  |  |  | 2 | 0 | 0 | 0 | 0 | 2 | 0 | 0 | 0 | 0 | 0 | 0 | 0 | 0 | 0 | 210 | 222 | Carbamidomethylation | C12:Carbamidomethylation:1000.00;C13:Carbamidomethylation:1000.00 | PEAKS DB |
| K.LAQLYNVNHVSLF.H | N | 49.44 | 1516.8038 | 13 | -0.3 | 759.4089 | 2 | 71.90 | 5 | F5:48055 | NaSa01\_F13.raw |  |  |  |  | 1.3448E8 |  |  |  |  |  |  |  |  |  | 1 | 0 | 0 | 0 | 0 | 1 | 0 | 0 | 0 | 0 | 0 | 0 | 0 | 0 | 0 | 255 | 267 |  |  | PEAKS DB |
| P.VYSAYVYNPGK.A | N | 48.05 | 1259.6187 | 11 | 0.1 | 630.8167 | 2 | 28.78 | 5 | F5:12547 | NaSa01\_F13.raw |  |  |  |  | 1.6549E6 |  |  |  |  |  |  |  |  |  | 1 | 0 | 0 | 0 | 0 | 1 | 0 | 0 | 0 | 0 | 0 | 0 | 0 | 0 | 0 | 72 | 82 |  |  | PEAKS DB |
| R.VNRPSHLWSAAC(+57.02).C | N | 47.36 | 1396.6670 | 12 | 0.2 | 699.3409 | 2 | 11.69 | 5 | F5:2180 | NaSa01\_F13.raw |  |  |  |  | 6.1785E5 |  |  |  |  |  |  |  |  |  | 1 | 0 | 0 | 0 | 0 | 1 | 0 | 0 | 0 | 0 | 0 | 0 | 0 | 0 | 0 | 210 | 221 | Carbamidomethylation | C12:Carbamidomethylation:1000.00 | PEAKS DB |
| L.YNVNHVSLFHSDC(+57.02)PR.Q | N | 46.41 | 1843.8424 | 15 | 1.7 | 615.6224 | 3 | 25.29 | 5 | F5:9481 | NaSa01\_F13.raw |  |  |  |  | 7.5396E6 |  |  |  |  |  |  |  |  |  | 2 | 0 | 0 | 0 | 0 | 2 | 0 | 0 | 0 | 0 | 0 | 0 | 0 | 0 | 0 | 259 | 273 | Carbamidomethylation | C13:Carbamidomethylation:1000.00 | PEAKS DB |
| S.TFTLTNIVPQFIK.L | N | 45.77 | 1520.8602 | 13 | 0.0 | 761.4374 | 2 | 89.58 | 5 | F5:62408 | NaSa01\_F13.raw |  |  |  |  | 1.3379E6 |  |  |  |  |  |  |  |  |  | 1 | 0 | 0 | 0 | 0 | 1 | 0 | 0 | 0 | 0 | 0 | 0 | 0 | 0 | 0 | 156 | 168 |  |  | PEAKS DB |
| R.SWAILAR.N | N | 44.55 | 815.4653 | 7 | 0.1 | 408.7400 | 2 | 43.13 | 4 | F4:25699 | NaSa01\_F12.raw |  |  |  | 9.8044E6 |  | 9.814E6 |  |  |  |  |  |  |  |  | 3 | 0 | 0 | 0 | 1 | 0 | 2 | 0 | 0 | 0 | 0 | 0 | 0 | 0 | 0 | 231 | 237 |  |  | PEAKS DB |
| N.HVSLFHSDC(+57.02)PR.Q | N | 41.73 | 1353.6248 | 11 | -0.2 | 452.2154 | 3 | 11.44 | 5 | F5:2051 | NaSa01\_F13.raw |  |  |  |  | 5.6233E5 |  |  |  |  |  |  |  |  |  | 1 | 0 | 0 | 0 | 0 | 1 | 0 | 0 | 0 | 0 | 0 | 0 | 0 | 0 | 0 | 263 | 273 | Carbamidomethylation | C9:Carbamidomethylation:1000.00 | PEAKS DB |
| F.TLTNIVPQFIK.L | N | 40.01 | 1272.7441 | 11 | 0.2 | 637.3795 | 2 | 67.27 | 5 | F5:44583 | NaSa01\_F13.raw |  |  |  |  | 1.1734E8 |  |  |  |  |  |  |  |  |  | 1 | 0 | 0 | 0 | 0 | 1 | 0 | 0 | 0 | 0 | 0 | 0 | 0 | 0 | 0 | 158 | 168 |  |  | PEAKS DB |
| total 18 peptides |  |  |  |  |  |  |  |  |  |  |  |  |  |  |  |  |  |  |  |  |  |  |  |  |  |  |  |  |  |  |  |  |  |  |  |  |  |  |  |  |  |  |  |  |  |
| --- | --- | --- | --- | --- | --- | --- | --- | --- | --- | --- | --- | --- | --- | --- | --- | --- | --- | --- | --- | --- | --- | --- | --- | --- | --- | --- | --- | --- | --- | --- | --- | --- | --- | --- | --- | --- | --- | --- | --- | --- | --- | --- | --- | --- | --- |

XP\_026546197.1

back to list

  

| Protein Coverage
| Supporting Peptides
|

Protein Coverage:

Supporting Peptides:

| Peptide | Uniq | -10lgP | Mass | Length | ppm | m/z | z | RT | Fraction | Scan | Source File | Area F1 | Area F10 | Area F11 | Area F12 | Area F13 | Area F14 | Area F2 | Area F3 | Area F4 | Area F5 | Area F6 | Area F7 | Area F8 | Area F9 | #Feature | #Feature F1 | #Feature F10 | #Feature F11 | #Feature F12 | #Feature F13 | #Feature F14 | #Feature F2 | #Feature F3 | #Feature F4 | #Feature F5 | #Feature F6 | #Feature F7 | #Feature F8 | #Feature F9 | Start | End | PTM | AScore | Found By |
| --- | --- | --- | --- | --- | --- | --- | --- | --- | --- | --- | --- | --- | --- | --- | --- | --- | --- | --- | --- | --- | --- | --- | --- | --- | --- | --- | --- | --- | --- | --- | --- | --- | --- | --- | --- | --- | --- | --- | --- | --- | --- | --- | --- | --- | --- |
| R.IPVYSAYVYNPGK.A | N | 84.05 | 1469.7554 | 13 | 0.9 | 735.8856 | 2 | 50.54 | 6 | F6:29743 | NaSa01\_F14.raw |  |  | 0 | 5.2943E6 | 9.5904E8 | 6.5068E6 |  |  |  | 1.4262E6 | 2.0524E6 | 5.8608E5 | 3.9528E5 | 3.6466E6 | 9 | 0 | 0 | 0 | 1 | 2 | 1 | 0 | 0 | 0 | 1 | 1 | 1 | 1 | 1 | 70 | 82 |  |  | PEAKS DB |
| K.LAQLYNVNHVSLFHSDC(+57.02)PR.Q | N | 83.18 | 2269.1062 | 19 | 0.1 | 757.3761 | 3 | 43.25 | 4 | F4:25798 | NaSa01\_F12.raw |  |  |  | 5.5169E7 | 7.3204E8 | 7.8894E6 |  |  |  |  |  |  |  |  | 11 | 0 | 0 | 0 | 4 | 5 | 2 | 0 | 0 | 0 | 0 | 0 | 0 | 0 | 0 | 255 | 273 | Carbamidomethylation | C17:Carbamidomethylation:1000.00 | PEAKS DB |
| G.EVVDSFQDHC(+57.02)PQFFLR.E | N | 71.48 | 2022.9258 | 16 | 0.5 | 675.3162 | 3 | 60.39 | 4 | F4:40242 | NaSa01\_F12.raw |  |  | 6.7523E6 | 9.5937E6 | 1.2968E9 | 1.0449E7 |  |  |  |  |  |  | 5.3462E6 |  | 8 | 0 | 0 | 1 | 1 | 4 | 1 | 0 | 0 | 0 | 0 | 0 | 0 | 1 | 0 | 20 | 35 | Carbamidomethylation | C10:Carbamidomethylation:1000.00 | PEAKS DB |
| K.SSTFTLTNIVPQFIK.L | N | 69.82 | 1694.9243 | 15 | 1.1 | 848.4704 | 2 | 89.01 | 4 | F4:63439 | NaSa01\_F12.raw |  |  |  | 6.2934E6 | 1.5029E9 | 8.7605E6 |  |  |  |  |  |  |  |  | 4 | 0 | 0 | 0 | 1 | 2 | 1 | 0 | 0 | 0 | 0 | 0 | 0 | 0 | 0 | 154 | 168 |  |  | PEAKS DB |
| R.GHLNPNGHQPDYSAK.S | N | 61.86 | 1633.7596 | 15 | 1.0 | 545.5944 | 3 | 11.43 | 5 | F5:1940 | NaSa01\_F13.raw |  |  |  |  | 1.4956E6 |  |  |  |  |  |  |  |  |  | 1 | 0 | 0 | 0 | 0 | 1 | 0 | 0 | 0 | 0 | 0 | 0 | 0 | 0 | 0 | 139 | 153 |  |  | PEAKS DB |
| R.FTLGQLEEK.L | Y | 60.48 | 1063.5549 | 9 | -0.4 | 532.7845 | 2 | 42.56 | 6 | F6:22936 | NaSa01\_F14.raw |  |  |  |  | 4.6886E8 | 5.0932E6 |  |  | 4.3246E5 |  |  |  |  |  | 3 | 0 | 0 | 0 | 0 | 1 | 1 | 0 | 0 | 1 | 0 | 0 | 0 | 0 | 0 | 246 | 254 |  |  | PEAKS DB |
| Y.NVNHVSLFHSDC(+57.02)PR.Q | N | 58.23 | 1680.7791 | 14 | 0.8 | 561.2674 | 3 | 11.62 | 5 | F5:2096 | NaSa01\_F13.raw |  |  |  |  | 2.3142E6 |  |  |  |  |  |  |  |  |  | 1 | 0 | 0 | 0 | 0 | 1 | 0 | 0 | 0 | 0 | 0 | 0 | 0 | 0 | 0 | 260 | 273 | Carbamidomethylation | C12:Carbamidomethylation:1000.00 | PEAKS DB |
| F.AVVGAVPGDTYIAR.G | N | 57.03 | 1387.7460 | 14 | 1.7 | 694.8815 | 2 | 40.72 | 6 | F6:21346 | NaSa01\_F14.raw |  |  |  | 4.6266E6 | 7.6974E8 | 7.3018E6 |  |  |  |  |  |  |  |  | 4 | 0 | 0 | 0 | 1 | 2 | 1 | 0 | 0 | 0 | 0 | 0 | 0 | 0 | 0 | 194 | 207 |  |  | PEAKS DB |
| Q.LYNVNHVSLFHSDC(+57.02)PR.Q | N | 53.73 | 1956.9264 | 16 | 1.2 | 490.2395 | 4 | 33.35 | 5 | F5:16269 | NaSa01\_F13.raw |  |  |  |  | 1.2213E6 |  |  |  |  |  |  |  |  |  | 1 | 0 | 0 | 0 | 0 | 1 | 0 | 0 | 0 | 0 | 0 | 0 | 0 | 0 | 0 | 258 | 273 | Carbamidomethylation | C14:Carbamidomethylation:1000.00 | PEAKS DB |
| R.VNRPSHLWSAAC(+57.02)C(+57.02).V | N | 51.05 | 1556.6976 | 13 | 0.0 | 779.3561 | 2 | 11.66 | 5 | F5:2173 | NaSa01\_F13.raw |  |  |  |  | 3.8373E6 |  |  |  |  |  |  |  |  |  | 2 | 0 | 0 | 0 | 0 | 2 | 0 | 0 | 0 | 0 | 0 | 0 | 0 | 0 | 0 | 210 | 222 | Carbamidomethylation | C12:Carbamidomethylation:1000.00;C13:Carbamidomethylation:1000.00 | PEAKS DB |
| K.LAQLYNVNHVSLF.H | N | 49.44 | 1516.8038 | 13 | -0.3 | 759.4089 | 2 | 71.90 | 5 | F5:48055 | NaSa01\_F13.raw |  |  |  |  | 1.3448E8 |  |  |  |  |  |  |  |  |  | 1 | 0 | 0 | 0 | 0 | 1 | 0 | 0 | 0 | 0 | 0 | 0 | 0 | 0 | 0 | 255 | 267 |  |  | PEAKS DB |
| P.VYSAYVYNPGK.A | N | 48.05 | 1259.6187 | 11 | 0.1 | 630.8167 | 2 | 28.78 | 5 | F5:12547 | NaSa01\_F13.raw |  |  |  |  | 1.6549E6 |  |  |  |  |  |  |  |  |  | 1 | 0 | 0 | 0 | 0 | 1 | 0 | 0 | 0 | 0 | 0 | 0 | 0 | 0 | 0 | 72 | 82 |  |  | PEAKS DB |
| R.VNRPSHLWSAAC(+57.02).C | N | 47.36 | 1396.6670 | 12 | 0.2 | 699.3409 | 2 | 11.69 | 5 | F5:2180 | NaSa01\_F13.raw |  |  |  |  | 6.1785E5 |  |  |  |  |  |  |  |  |  | 1 | 0 | 0 | 0 | 0 | 1 | 0 | 0 | 0 | 0 | 0 | 0 | 0 | 0 | 0 | 210 | 221 | Carbamidomethylation | C12:Carbamidomethylation:1000.00 | PEAKS DB |
| L.YNVNHVSLFHSDC(+57.02)PR.Q | N | 46.41 | 1843.8424 | 15 | 1.7 | 615.6224 | 3 | 25.29 | 5 | F5:9481 | NaSa01\_F13.raw |  |  |  |  | 7.5396E6 |  |  |  |  |  |  |  |  |  | 2 | 0 | 0 | 0 | 0 | 2 | 0 | 0 | 0 | 0 | 0 | 0 | 0 | 0 | 0 | 259 | 273 | Carbamidomethylation | C13:Carbamidomethylation:1000.00 | PEAKS DB |
| S.TFTLTNIVPQFIK.L | N | 45.77 | 1520.8602 | 13 | 0.0 | 761.4374 | 2 | 89.58 | 5 | F5:62408 | NaSa01\_F13.raw |  |  |  |  | 1.3379E6 |  |  |  |  |  |  |  |  |  | 1 | 0 | 0 | 0 | 0 | 1 | 0 | 0 | 0 | 0 | 0 | 0 | 0 | 0 | 0 | 156 | 168 |  |  | PEAKS DB |
| R.SWAILAR.N | N | 44.55 | 815.4653 | 7 | 0.1 | 408.7400 | 2 | 43.13 | 4 | F4:25699 | NaSa01\_F12.raw |  |  |  | 9.8044E6 |  | 9.814E6 |  |  |  |  |  |  |  |  | 3 | 0 | 0 | 0 | 1 | 0 | 2 | 0 | 0 | 0 | 0 | 0 | 0 | 0 | 0 | 231 | 237 |  |  | PEAKS DB |
| N.HVSLFHSDC(+57.02)PR.Q | N | 41.73 | 1353.6248 | 11 | -0.2 | 452.2154 | 3 | 11.44 | 5 | F5:2051 | NaSa01\_F13.raw |  |  |  |  | 5.6233E5 |  |  |  |  |  |  |  |  |  | 1 | 0 | 0 | 0 | 0 | 1 | 0 | 0 | 0 | 0 | 0 | 0 | 0 | 0 | 0 | 263 | 273 | Carbamidomethylation | C9:Carbamidomethylation:1000.00 | PEAKS DB |
| F.TLTNIVPQFIK.L | N | 40.01 | 1272.7441 | 11 | 0.2 | 637.3795 | 2 | 67.27 | 5 | F5:44583 | NaSa01\_F13.raw |  |  |  |  | 1.1734E8 |  |  |  |  |  |  |  |  |  | 1 | 0 | 0 | 0 | 0 | 1 | 0 | 0 | 0 | 0 | 0 | 0 | 0 | 0 | 0 | 158 | 168 |  |  | PEAKS DB |
| total 18 peptides |  |  |  |  |  |  |  |  |  |  |  |  |  |  |  |  |  |  |  |  |  |  |  |  |  |  |  |  |  |  |  |  |  |  |  |  |  |  |  |  |  |  |  |  |  |
| --- | --- | --- | --- | --- | --- | --- | --- | --- | --- | --- | --- | --- | --- | --- | --- | --- | --- | --- | --- | --- | --- | --- | --- | --- | --- | --- | --- | --- | --- | --- | --- | --- | --- | --- | --- | --- | --- | --- | --- | --- | --- | --- | --- | --- | --- |

XP\_026546196.1

back to list

  

| Protein Coverage
| Supporting Peptides
|

Protein Coverage:

Supporting Peptides:

| Peptide | Uniq | -10lgP | Mass | Length | ppm | m/z | z | RT | Fraction | Scan | Source File | Area F1 | Area F10 | Area F11 | Area F12 | Area F13 | Area F14 | Area F2 | Area F3 | Area F4 | Area F5 | Area F6 | Area F7 | Area F8 | Area F9 | #Feature | #Feature F1 | #Feature F10 | #Feature F11 | #Feature F12 | #Feature F13 | #Feature F14 | #Feature F2 | #Feature F3 | #Feature F4 | #Feature F5 | #Feature F6 | #Feature F7 | #Feature F8 | #Feature F9 | Start | End | PTM | AScore | Found By |
| --- | --- | --- | --- | --- | --- | --- | --- | --- | --- | --- | --- | --- | --- | --- | --- | --- | --- | --- | --- | --- | --- | --- | --- | --- | --- | --- | --- | --- | --- | --- | --- | --- | --- | --- | --- | --- | --- | --- | --- | --- | --- | --- | --- | --- | --- |
| R.IPVYSAYVYNPGK.A | N | 84.05 | 1469.7554 | 13 | 0.9 | 735.8856 | 2 | 50.54 | 6 | F6:29743 | NaSa01\_F14.raw |  |  | 0 | 5.2943E6 | 9.5904E8 | 6.5068E6 |  |  |  | 1.4262E6 | 2.0524E6 | 5.8608E5 | 3.9528E5 | 3.6466E6 | 9 | 0 | 0 | 0 | 1 | 2 | 1 | 0 | 0 | 0 | 1 | 1 | 1 | 1 | 1 | 84 | 96 |  |  | PEAKS DB |
| K.LAQLYNVNHVSLFHSDC(+57.02)PR.Q | N | 83.18 | 2269.1062 | 19 | 0.1 | 757.3761 | 3 | 43.25 | 4 | F4:25798 | NaSa01\_F12.raw |  |  |  | 5.5169E7 | 7.3204E8 | 7.8894E6 |  |  |  |  |  |  |  |  | 11 | 0 | 0 | 0 | 4 | 5 | 2 | 0 | 0 | 0 | 0 | 0 | 0 | 0 | 0 | 269 | 287 | Carbamidomethylation | C17:Carbamidomethylation:1000.00 | PEAKS DB |
| G.EVVDSFQDHC(+57.02)PQFFLR.E | N | 71.48 | 2022.9258 | 16 | 0.5 | 675.3162 | 3 | 60.39 | 4 | F4:40242 | NaSa01\_F12.raw |  |  | 6.7523E6 | 9.5937E6 | 1.2968E9 | 1.0449E7 |  |  |  |  |  |  | 5.3462E6 |  | 8 | 0 | 0 | 1 | 1 | 4 | 1 | 0 | 0 | 0 | 0 | 0 | 0 | 1 | 0 | 34 | 49 | Carbamidomethylation | C10:Carbamidomethylation:1000.00 | PEAKS DB |
| K.SSTFTLTNIVPQFIK.L | N | 69.82 | 1694.9243 | 15 | 1.1 | 848.4704 | 2 | 89.01 | 4 | F4:63439 | NaSa01\_F12.raw |  |  |  | 6.2934E6 | 1.5029E9 | 8.7605E6 |  |  |  |  |  |  |  |  | 4 | 0 | 0 | 0 | 1 | 2 | 1 | 0 | 0 | 0 | 0 | 0 | 0 | 0 | 0 | 168 | 182 |  |  | PEAKS DB |
| R.GHLNPNGHQPDYSAK.S | N | 61.86 | 1633.7596 | 15 | 1.0 | 545.5944 | 3 | 11.43 | 5 | F5:1940 | NaSa01\_F13.raw |  |  |  |  | 1.4956E6 |  |  |  |  |  |  |  |  |  | 1 | 0 | 0 | 0 | 0 | 1 | 0 | 0 | 0 | 0 | 0 | 0 | 0 | 0 | 0 | 153 | 167 |  |  | PEAKS DB |
| R.FTLGQLEEK.L | Y | 60.48 | 1063.5549 | 9 | -0.4 | 532.7845 | 2 | 42.56 | 6 | F6:22936 | NaSa01\_F14.raw |  |  |  |  | 4.6886E8 | 5.0932E6 |  |  | 4.3246E5 |  |  |  |  |  | 3 | 0 | 0 | 0 | 0 | 1 | 1 | 0 | 0 | 1 | 0 | 0 | 0 | 0 | 0 | 260 | 268 |  |  | PEAKS DB |
| Y.NVNHVSLFHSDC(+57.02)PR.Q | N | 58.23 | 1680.7791 | 14 | 0.8 | 561.2674 | 3 | 11.62 | 5 | F5:2096 | NaSa01\_F13.raw |  |  |  |  | 2.3142E6 |  |  |  |  |  |  |  |  |  | 1 | 0 | 0 | 0 | 0 | 1 | 0 | 0 | 0 | 0 | 0 | 0 | 0 | 0 | 0 | 274 | 287 | Carbamidomethylation | C12:Carbamidomethylation:1000.00 | PEAKS DB |
| F.AVVGAVPGDTYIAR.G | N | 57.03 | 1387.7460 | 14 | 1.7 | 694.8815 | 2 | 40.72 | 6 | F6:21346 | NaSa01\_F14.raw |  |  |  | 4.6266E6 | 7.6974E8 | 7.3018E6 |  |  |  |  |  |  |  |  | 4 | 0 | 0 | 0 | 1 | 2 | 1 | 0 | 0 | 0 | 0 | 0 | 0 | 0 | 0 | 208 | 221 |  |  | PEAKS DB |
| Q.LYNVNHVSLFHSDC(+57.02)PR.Q | N | 53.73 | 1956.9264 | 16 | 1.2 | 490.2395 | 4 | 33.35 | 5 | F5:16269 | NaSa01\_F13.raw |  |  |  |  | 1.2213E6 |  |  |  |  |  |  |  |  |  | 1 | 0 | 0 | 0 | 0 | 1 | 0 | 0 | 0 | 0 | 0 | 0 | 0 | 0 | 0 | 272 | 287 | Carbamidomethylation | C14:Carbamidomethylation:1000.00 | PEAKS DB |
| R.VNRPSHLWSAAC(+57.02)C(+57.02).V | N | 51.05 | 1556.6976 | 13 | 0.0 | 779.3561 | 2 | 11.66 | 5 | F5:2173 | NaSa01\_F13.raw |  |  |  |  | 3.8373E6 |  |  |  |  |  |  |  |  |  | 2 | 0 | 0 | 0 | 0 | 2 | 0 | 0 | 0 | 0 | 0 | 0 | 0 | 0 | 0 | 224 | 236 | Carbamidomethylation | C12:Carbamidomethylation:1000.00;C13:Carbamidomethylation:1000.00 | PEAKS DB |
| K.LAQLYNVNHVSLF.H | N | 49.44 | 1516.8038 | 13 | -0.3 | 759.4089 | 2 | 71.90 | 5 | F5:48055 | NaSa01\_F13.raw |  |  |  |  | 1.3448E8 |  |  |  |  |  |  |  |  |  | 1 | 0 | 0 | 0 | 0 | 1 | 0 | 0 | 0 | 0 | 0 | 0 | 0 | 0 | 0 | 269 | 281 |  |  | PEAKS DB |
| P.VYSAYVYNPGK.A | N | 48.05 | 1259.6187 | 11 | 0.1 | 630.8167 | 2 | 28.78 | 5 | F5:12547 | NaSa01\_F13.raw |  |  |  |  | 1.6549E6 |  |  |  |  |  |  |  |  |  | 1 | 0 | 0 | 0 | 0 | 1 | 0 | 0 | 0 | 0 | 0 | 0 | 0 | 0 | 0 | 86 | 96 |  |  | PEAKS DB |
| R.VNRPSHLWSAAC(+57.02).C | N | 47.36 | 1396.6670 | 12 | 0.2 | 699.3409 | 2 | 11.69 | 5 | F5:2180 | NaSa01\_F13.raw |  |  |  |  | 6.1785E5 |  |  |  |  |  |  |  |  |  | 1 | 0 | 0 | 0 | 0 | 1 | 0 | 0 | 0 | 0 | 0 | 0 | 0 | 0 | 0 | 224 | 235 | Carbamidomethylation | C12:Carbamidomethylation:1000.00 | PEAKS DB |
| L.YNVNHVSLFHSDC(+57.02)PR.Q | N | 46.41 | 1843.8424 | 15 | 1.7 | 615.6224 | 3 | 25.29 | 5 | F5:9481 | NaSa01\_F13.raw |  |  |  |  | 7.5396E6 |  |  |  |  |  |  |  |  |  | 2 | 0 | 0 | 0 | 0 | 2 | 0 | 0 | 0 | 0 | 0 | 0 | 0 | 0 | 0 | 273 | 287 | Carbamidomethylation | C13:Carbamidomethylation:1000.00 | PEAKS DB |
| S.TFTLTNIVPQFIK.L | N | 45.77 | 1520.8602 | 13 | 0.0 | 761.4374 | 2 | 89.58 | 5 | F5:62408 | NaSa01\_F13.raw |  |  |  |  | 1.3379E6 |  |  |  |  |  |  |  |  |  | 1 | 0 | 0 | 0 | 0 | 1 | 0 | 0 | 0 | 0 | 0 | 0 | 0 | 0 | 0 | 170 | 182 |  |  | PEAKS DB |
| R.SWAILAR.N | N | 44.55 | 815.4653 | 7 | 0.1 | 408.7400 | 2 | 43.13 | 4 | F4:25699 | NaSa01\_F12.raw |  |  |  | 9.8044E6 |  | 9.814E6 |  |  |  |  |  |  |  |  | 3 | 0 | 0 | 0 | 1 | 0 | 2 | 0 | 0 | 0 | 0 | 0 | 0 | 0 | 0 | 245 | 251 |  |  | PEAKS DB |
| N.HVSLFHSDC(+57.02)PR.Q | N | 41.73 | 1353.6248 | 11 | -0.2 | 452.2154 | 3 | 11.44 | 5 | F5:2051 | NaSa01\_F13.raw |  |  |  |  | 5.6233E5 |  |  |  |  |  |  |  |  |  | 1 | 0 | 0 | 0 | 0 | 1 | 0 | 0 | 0 | 0 | 0 | 0 | 0 | 0 | 0 | 277 | 287 | Carbamidomethylation | C9:Carbamidomethylation:1000.00 | PEAKS DB |
| F.TLTNIVPQFIK.L | N | 40.01 | 1272.7441 | 11 | 0.2 | 637.3795 | 2 | 67.27 | 5 | F5:44583 | NaSa01\_F13.raw |  |  |  |  | 1.1734E8 |  |  |  |  |  |  |  |  |  | 1 | 0 | 0 | 0 | 0 | 1 | 0 | 0 | 0 | 0 | 0 | 0 | 0 | 0 | 0 | 172 | 182 |  |  | PEAKS DB |
| total 18 peptides |  |  |  |  |  |  |  |  |  |  |  |  |  |  |  |  |  |  |  |  |  |  |  |  |  |  |  |  |  |  |  |  |  |  |  |  |  |  |  |  |  |  |  |  |  |
| --- | --- | --- | --- | --- | --- | --- | --- | --- | --- | --- | --- | --- | --- | --- | --- | --- | --- | --- | --- | --- | --- | --- | --- | --- | --- | --- | --- | --- | --- | --- | --- | --- | --- | --- | --- | --- | --- | --- | --- | --- | --- | --- | --- | --- | --- |

CAA55333.1

back to list

  

| Protein Coverage
| Supporting Peptides
|

Protein Coverage:

Supporting Peptides:

| Peptide | Uniq | -10lgP | Mass | Length | ppm | m/z | z | RT | Fraction | Scan | Source File | Area F1 | Area F10 | Area F11 | Area F12 | Area F13 | Area F14 | Area F2 | Area F3 | Area F4 | Area F5 | Area F6 | Area F7 | Area F8 | Area F9 | #Feature | #Feature F1 | #Feature F10 | #Feature F11 | #Feature F12 | #Feature F13 | #Feature F14 | #Feature F2 | #Feature F3 | #Feature F4 | #Feature F5 | #Feature F6 | #Feature F7 | #Feature F8 | #Feature F9 | Start | End | PTM | AScore | Found By |
| --- | --- | --- | --- | --- | --- | --- | --- | --- | --- | --- | --- | --- | --- | --- | --- | --- | --- | --- | --- | --- | --- | --- | --- | --- | --- | --- | --- | --- | --- | --- | --- | --- | --- | --- | --- | --- | --- | --- | --- | --- | --- | --- | --- | --- | --- |
| K.ELGDYFFTNEFLVK.Y | Y | 82.30 | 1720.8347 | 14 | 0.6 | 861.4252 | 2 | 94.44 | 6 | F6:66730 | NaSa01\_F14.raw |  |  |  |  |  | 5.9185E6 |  |  |  |  |  |  |  |  | 1 | 0 | 0 | 0 | 0 | 0 | 1 | 0 | 0 | 0 | 0 | 0 | 0 | 0 | 0 | 425 | 438 |  |  | PEAKS DB |
| R.DSVLAQYIFELSR.R | Y | 76.23 | 1539.7932 | 13 | 0.3 | 770.9041 | 2 | 100.59 | 6 | F6:71727 | NaSa01\_F14.raw |  |  |  |  | 2.9411E5 | 4.7114E6 |  |  |  |  |  |  |  |  | 2 | 0 | 0 | 0 | 0 | 1 | 1 | 0 | 0 | 0 | 0 | 0 | 0 | 0 | 0 | 160 | 172 |  |  | PEAKS DB |
| K.TSSTGTISPFVHPNAEEAC(+57.02)QAFQNDR.D | Y | 73.41 | 2863.2832 | 26 | 0.1 | 955.4351 | 3 | 50.78 | 6 | F6:29864 | NaSa01\_F14.raw |  |  |  |  |  | 6.679E6 |  |  |  |  |  |  |  |  | 1 | 0 | 0 | 0 | 0 | 0 | 1 | 0 | 0 | 0 | 0 | 0 | 0 | 0 | 0 | 134 | 159 | Carbamidomethylation | C19:Carbamidomethylation:1000.00 | PEAKS DB |
| K.PLVERPNC(+57.02)LATLANDAR.S | Y | 73.35 | 1908.9839 | 17 | 0.3 | 637.3354 | 3 | 42.83 | 6 | F6:23089 | NaSa01\_F14.raw |  |  |  |  |  | 3.3101E6 |  |  |  |  |  |  |  |  | 1 | 0 | 0 | 0 | 0 | 0 | 1 | 0 | 0 | 0 | 0 | 0 | 0 | 0 | 0 | 310 | 326 | Carbamidomethylation | C8:Carbamidomethylation:1000.00 | PEAKS DB |
| R.PNC(+57.02)LATLANDAR.S | Y | 69.07 | 1314.6350 | 12 | 0.3 | 658.3250 | 2 | 34.17 | 6 | F6:15985 | NaSa01\_F14.raw |  |  |  |  |  | 1.5892E6 |  |  |  |  |  |  |  |  | 1 | 0 | 0 | 0 | 0 | 0 | 1 | 0 | 0 | 0 | 0 | 0 | 0 | 0 | 0 | 315 | 326 | Carbamidomethylation | C3:Carbamidomethylation:1000.00 | PEAKS DB |
| K.LSAEIIELHK.K | Y | 66.19 | 1151.6550 | 10 | 0.0 | 576.8348 | 2 | 32.96 | 6 | F6:14725 | NaSa01\_F14.raw |  |  |  |  | 1.0805E6 | 1.0034E7 |  |  |  |  |  |  |  |  | 4 | 0 | 0 | 0 | 0 | 2 | 2 | 0 | 0 | 0 | 0 | 0 | 0 | 0 | 0 | 69 | 78 |  |  | PEAKS DB |
| R.YPTALSVVILESTK.T | Y | 66.10 | 1519.8497 | 14 | 0.6 | 760.9326 | 2 | 75.74 | 6 | F6:50955 | NaSa01\_F14.raw |  |  |  |  |  | 2.0303E6 |  |  |  |  |  |  |  |  | 2 | 0 | 0 | 0 | 0 | 0 | 2 | 0 | 0 | 0 | 0 | 0 | 0 | 0 | 0 | 174 | 187 |  |  | PEAKS DB |
| K.SKPNISEEELAATILTFR.E | Y | 65.45 | 2018.0684 | 18 | 0.2 | 673.6968 | 3 | 77.73 | 6 | F6:52814 | NaSa01\_F14.raw |  |  |  |  | 1.2042E6 | 6.9306E6 |  |  |  |  |  |  |  |  | 3 | 0 | 0 | 0 | 0 | 1 | 2 | 0 | 0 | 0 | 0 | 0 | 0 | 0 | 0 | 563 | 580 |  |  | PEAKS DB |
| R.RYPTALSVVILESTK.T | Y | 62.48 | 1675.9508 | 15 | 0.9 | 559.6580 | 3 | 59.32 | 6 | F6:37173 | NaSa01\_F14.raw |  |  |  |  |  | 5.6148E6 |  |  |  |  |  |  |  |  | 1 | 0 | 0 | 0 | 0 | 0 | 1 | 0 | 0 | 0 | 0 | 0 | 0 | 0 | 0 | 173 | 187 |  |  | PEAKS DB |
| R.MMPQAPTSFLIELTEK.V | Y | 60.34 | 1834.9209 | 16 | 0.4 | 612.6478 | 3 | 90.18 | 6 | F6:63179 | NaSa01\_F14.raw |  |  |  |  |  | 5.7731E6 |  |  |  |  |  |  |  |  | 2 | 0 | 0 | 0 | 0 | 0 | 2 | 0 | 0 | 0 | 0 | 0 | 0 | 0 | 0 | 442 | 457 |  |  | PEAKS DB |
| K.DAISSNVGHC(+57.02)C(+57.02)EKPLVERPNC(+57.02)LATLANDAR.S | Y | 60.30 | 3366.5867 | 30 | 0.8 | 842.6546 | 4 | 41.88 | 6 | F6:22328 | NaSa01\_F14.raw |  |  |  |  |  | 5.4202E6 |  |  |  |  |  |  |  |  | 2 | 0 | 0 | 0 | 0 | 0 | 2 | 0 | 0 | 0 | 0 | 0 | 0 | 0 | 0 | 297 | 326 | Carbamidomethylation | C10:Carbamidomethylation:1000.00;C11:Carbamidomethylation:1000.00;C21:Carbamidomethylation:1000.00 | PEAKS DB |
| R.EIMEEQEYTC(+57.02)YNLK.K | Y | 52.83 | 1848.7910 | 14 | 0.6 | 925.4033 | 2 | 46.58 | 6 | F6:26514 | NaSa01\_F14.raw |  |  |  |  |  | 2.0919E6 |  |  |  |  |  |  |  |  | 1 | 0 | 0 | 0 | 0 | 0 | 1 | 0 | 0 | 0 | 0 | 0 | 0 | 0 | 0 | 219 | 232 | Carbamidomethylation | C10:Carbamidomethylation:1000.00 | PEAKS DB |
| R.WEC(+57.02)ISNLGPDLSFVPPTFNPK.T | Y | 51.31 | 2417.1726 | 21 | 0.5 | 1209.5942 | 2 | 93.26 | 6 | F6:65690 | NaSa01\_F14.raw |  |  |  |  |  | 1.4003E6 |  |  |  |  |  |  |  |  | 1 | 0 | 0 | 0 | 0 | 0 | 1 | 0 | 0 | 0 | 0 | 0 | 0 | 0 | 0 | 513 | 533 | Carbamidomethylation | C3:Carbamidomethylation:1000.00 | PEAKS DB |
| K.ESFLFTLTR.N | N | 51.21 | 1112.5865 | 9 | 0.4 | 557.3008 | 2 | 70.68 | 6 | F6:46788 | NaSa01\_F14.raw |  |  |  |  |  | 3.5418E6 |  |  |  |  |  |  |  |  | 1 | 0 | 0 | 0 | 0 | 0 | 1 | 0 | 0 | 0 | 0 | 0 | 0 | 0 | 0 | 356 | 364 |  |  | PEAKS DB |
| R.SPDLPPPSEEILK.E | Y | 50.94 | 1420.7449 | 13 | 0.8 | 711.3802 | 2 | 49.25 | 6 | F6:28594 | NaSa01\_F14.raw |  |  |  |  |  | 1.1465E7 |  |  |  |  |  |  |  |  | 1 | 0 | 0 | 0 | 0 | 0 | 1 | 0 | 0 | 0 | 0 | 0 | 0 | 0 | 0 | 327 | 339 |  |  | PEAKS DB |
| K.KGLLSELVK.S | Y | 49.77 | 985.6171 | 9 | 0.4 | 493.8160 | 2 | 35.68 | 6 | F6:17294 | NaSa01\_F14.raw |  |  |  |  |  | 1.4145E6 |  |  |  |  |  |  |  |  | 1 | 0 | 0 | 0 | 0 | 0 | 1 | 0 | 0 | 0 | 0 | 0 | 0 | 0 | 0 | 554 | 562 |  |  | PEAKS DB |
| R.M(+15.99)MPQAPTSFLIELTEK.V | Y | 48.10 | 1850.9158 | 16 | 0.6 | 926.4658 | 2 | 86.33 | 6 | F6:60001 | NaSa01\_F14.raw |  |  |  |  |  | 9.0541E5 |  |  |  |  |  |  |  |  | 1 | 0 | 0 | 0 | 0 | 0 | 1 | 0 | 0 | 0 | 0 | 0 | 0 | 0 | 0 | 442 | 457 | Oxidation (M) | M1:Oxidation (M):0.00 | PEAKS DB |
| K.GLLSELVK.S | Y | 42.16 | 857.5222 | 8 | 0.8 | 429.7687 | 2 | 55.43 | 6 | F6:34022 | NaSa01\_F14.raw |  |  |  |  |  | 3.4826E6 |  |  |  |  |  |  |  |  | 1 | 0 | 0 | 0 | 0 | 0 | 1 | 0 | 0 | 0 | 0 | 0 | 0 | 0 | 0 | 555 | 562 |  |  | PEAKS DB |
| R.ENYKESFLFTLTR.N | Y | 39.72 | 1646.8303 | 13 | 1.9 | 549.9518 | 3 | 60.34 | 4 | F4:39973 | NaSa01\_F12.raw |  |  |  | 1.0464E5 |  |  |  |  |  |  |  |  |  |  | 1 | 0 | 0 | 0 | 1 | 0 | 0 | 0 | 0 | 0 | 0 | 0 | 0 | 0 | 0 | 352 | 364 |  |  | PEAKS DB |
| total 19 peptides |  |  |  |  |  |  |  |  |  |  |  |  |  |  |  |  |  |  |  |  |  |  |  |  |  |  |  |  |  |  |  |  |  |  |  |  |  |  |  |  |  |  |  |  |  |
| --- | --- | --- | --- | --- | --- | --- | --- | --- | --- | --- | --- | --- | --- | --- | --- | --- | --- | --- | --- | --- | --- | --- | --- | --- | --- | --- | --- | --- | --- | --- | --- | --- | --- | --- | --- | --- | --- | --- | --- | --- | --- | --- | --- | --- | --- |

S59517

back to list

  

| Protein Coverage
| Supporting Peptides
|

Protein Coverage:

Supporting Peptides:

| Peptide | Uniq | -10lgP | Mass | Length | ppm | m/z | z | RT | Fraction | Scan | Source File | Area F1 | Area F10 | Area F11 | Area F12 | Area F13 | Area F14 | Area F2 | Area F3 | Area F4 | Area F5 | Area F6 | Area F7 | Area F8 | Area F9 | #Feature | #Feature F1 | #Feature F10 | #Feature F11 | #Feature F12 | #Feature F13 | #Feature F14 | #Feature F2 | #Feature F3 | #Feature F4 | #Feature F5 | #Feature F6 | #Feature F7 | #Feature F8 | #Feature F9 | Start | End | PTM | AScore | Found By |
| --- | --- | --- | --- | --- | --- | --- | --- | --- | --- | --- | --- | --- | --- | --- | --- | --- | --- | --- | --- | --- | --- | --- | --- | --- | --- | --- | --- | --- | --- | --- | --- | --- | --- | --- | --- | --- | --- | --- | --- | --- | --- | --- | --- | --- | --- |
| K.ELGDYFFTNEFLVK.Y | Y | 82.30 | 1720.8347 | 14 | 0.6 | 861.4252 | 2 | 94.44 | 6 | F6:66730 | NaSa01\_F14.raw |  |  |  |  |  | 5.9185E6 |  |  |  |  |  |  |  |  | 1 | 0 | 0 | 0 | 0 | 0 | 1 | 0 | 0 | 0 | 0 | 0 | 0 | 0 | 0 | 425 | 438 |  |  | PEAKS DB |
| R.DSVLAQYIFELSR.R | Y | 76.23 | 1539.7932 | 13 | 0.3 | 770.9041 | 2 | 100.59 | 6 | F6:71727 | NaSa01\_F14.raw |  |  |  |  | 2.9411E5 | 4.7114E6 |  |  |  |  |  |  |  |  | 2 | 0 | 0 | 0 | 0 | 1 | 1 | 0 | 0 | 0 | 0 | 0 | 0 | 0 | 0 | 160 | 172 |  |  | PEAKS DB |
| K.TSSTGTISPFVHPNAEEAC(+57.02)QAFQNDR.D | Y | 73.41 | 2863.2832 | 26 | 0.1 | 955.4351 | 3 | 50.78 | 6 | F6:29864 | NaSa01\_F14.raw |  |  |  |  |  | 6.679E6 |  |  |  |  |  |  |  |  | 1 | 0 | 0 | 0 | 0 | 0 | 1 | 0 | 0 | 0 | 0 | 0 | 0 | 0 | 0 | 134 | 159 | Carbamidomethylation | C19:Carbamidomethylation:1000.00 | PEAKS DB |
| K.PLVERPNC(+57.02)LATLANDAR.S | Y | 73.35 | 1908.9839 | 17 | 0.3 | 637.3354 | 3 | 42.83 | 6 | F6:23089 | NaSa01\_F14.raw |  |  |  |  |  | 3.3101E6 |  |  |  |  |  |  |  |  | 1 | 0 | 0 | 0 | 0 | 0 | 1 | 0 | 0 | 0 | 0 | 0 | 0 | 0 | 0 | 310 | 326 | Carbamidomethylation | C8:Carbamidomethylation:1000.00 | PEAKS DB |
| R.PNC(+57.02)LATLANDAR.S | Y | 69.07 | 1314.6350 | 12 | 0.3 | 658.3250 | 2 | 34.17 | 6 | F6:15985 | NaSa01\_F14.raw |  |  |  |  |  | 1.5892E6 |  |  |  |  |  |  |  |  | 1 | 0 | 0 | 0 | 0 | 0 | 1 | 0 | 0 | 0 | 0 | 0 | 0 | 0 | 0 | 315 | 326 | Carbamidomethylation | C3:Carbamidomethylation:1000.00 | PEAKS DB |
| K.LSAEIIELHK.K | Y | 66.19 | 1151.6550 | 10 | 0.0 | 576.8348 | 2 | 32.96 | 6 | F6:14725 | NaSa01\_F14.raw |  |  |  |  | 1.0805E6 | 1.0034E7 |  |  |  |  |  |  |  |  | 4 | 0 | 0 | 0 | 0 | 2 | 2 | 0 | 0 | 0 | 0 | 0 | 0 | 0 | 0 | 69 | 78 |  |  | PEAKS DB |
| R.YPTALSVVILESTK.T | Y | 66.10 | 1519.8497 | 14 | 0.6 | 760.9326 | 2 | 75.74 | 6 | F6:50955 | NaSa01\_F14.raw |  |  |  |  |  | 2.0303E6 |  |  |  |  |  |  |  |  | 2 | 0 | 0 | 0 | 0 | 0 | 2 | 0 | 0 | 0 | 0 | 0 | 0 | 0 | 0 | 174 | 187 |  |  | PEAKS DB |
| K.SKPNISEEELAATILTFR.E | Y | 65.45 | 2018.0684 | 18 | 0.2 | 673.6968 | 3 | 77.73 | 6 | F6:52814 | NaSa01\_F14.raw |  |  |  |  | 1.2042E6 | 6.9306E6 |  |  |  |  |  |  |  |  | 3 | 0 | 0 | 0 | 0 | 1 | 2 | 0 | 0 | 0 | 0 | 0 | 0 | 0 | 0 | 563 | 580 |  |  | PEAKS DB |
| R.RYPTALSVVILESTK.T | Y | 62.48 | 1675.9508 | 15 | 0.9 | 559.6580 | 3 | 59.32 | 6 | F6:37173 | NaSa01\_F14.raw |  |  |  |  |  | 5.6148E6 |  |  |  |  |  |  |  |  | 1 | 0 | 0 | 0 | 0 | 0 | 1 | 0 | 0 | 0 | 0 | 0 | 0 | 0 | 0 | 173 | 187 |  |  | PEAKS DB |
| R.MMPQAPTSFLIELTEK.V | Y | 60.34 | 1834.9209 | 16 | 0.4 | 612.6478 | 3 | 90.18 | 6 | F6:63179 | NaSa01\_F14.raw |  |  |  |  |  | 5.7731E6 |  |  |  |  |  |  |  |  | 2 | 0 | 0 | 0 | 0 | 0 | 2 | 0 | 0 | 0 | 0 | 0 | 0 | 0 | 0 | 442 | 457 |  |  | PEAKS DB |
| K.DAISSNVGHC(+57.02)C(+57.02)EKPLVERPNC(+57.02)LATLANDAR.S | Y | 60.30 | 3366.5867 | 30 | 0.8 | 842.6546 | 4 | 41.88 | 6 | F6:22328 | NaSa01\_F14.raw |  |  |  |  |  | 5.4202E6 |  |  |  |  |  |  |  |  | 2 | 0 | 0 | 0 | 0 | 0 | 2 | 0 | 0 | 0 | 0 | 0 | 0 | 0 | 0 | 297 | 326 | Carbamidomethylation | C10:Carbamidomethylation:1000.00;C11:Carbamidomethylation:1000.00;C21:Carbamidomethylation:1000.00 | PEAKS DB |
| R.EIMEEQEYTC(+57.02)YNLK.K | Y | 52.83 | 1848.7910 | 14 | 0.6 | 925.4033 | 2 | 46.58 | 6 | F6:26514 | NaSa01\_F14.raw |  |  |  |  |  | 2.0919E6 |  |  |  |  |  |  |  |  | 1 | 0 | 0 | 0 | 0 | 0 | 1 | 0 | 0 | 0 | 0 | 0 | 0 | 0 | 0 | 219 | 232 | Carbamidomethylation | C10:Carbamidomethylation:1000.00 | PEAKS DB |
| R.WEC(+57.02)ISNLGPDLSFVPPTFNPK.T | Y | 51.31 | 2417.1726 | 21 | 0.5 | 1209.5942 | 2 | 93.26 | 6 | F6:65690 | NaSa01\_F14.raw |  |  |  |  |  | 1.4003E6 |  |  |  |  |  |  |  |  | 1 | 0 | 0 | 0 | 0 | 0 | 1 | 0 | 0 | 0 | 0 | 0 | 0 | 0 | 0 | 513 | 533 | Carbamidomethylation | C3:Carbamidomethylation:1000.00 | PEAKS DB |
| K.ESFLFTLTR.N | N | 51.21 | 1112.5865 | 9 | 0.4 | 557.3008 | 2 | 70.68 | 6 | F6:46788 | NaSa01\_F14.raw |  |  |  |  |  | 3.5418E6 |  |  |  |  |  |  |  |  | 1 | 0 | 0 | 0 | 0 | 0 | 1 | 0 | 0 | 0 | 0 | 0 | 0 | 0 | 0 | 356 | 364 |  |  | PEAKS DB |
| R.SPDLPPPSEEILK.E | Y | 50.94 | 1420.7449 | 13 | 0.8 | 711.3802 | 2 | 49.25 | 6 | F6:28594 | NaSa01\_F14.raw |  |  |  |  |  | 1.1465E7 |  |  |  |  |  |  |  |  | 1 | 0 | 0 | 0 | 0 | 0 | 1 | 0 | 0 | 0 | 0 | 0 | 0 | 0 | 0 | 327 | 339 |  |  | PEAKS DB |
| K.KGLLSELVK.S | Y | 49.77 | 985.6171 | 9 | 0.4 | 493.8160 | 2 | 35.68 | 6 | F6:17294 | NaSa01\_F14.raw |  |  |  |  |  | 1.4145E6 |  |  |  |  |  |  |  |  | 1 | 0 | 0 | 0 | 0 | 0 | 1 | 0 | 0 | 0 | 0 | 0 | 0 | 0 | 0 | 554 | 562 |  |  | PEAKS DB |
| R.M(+15.99)MPQAPTSFLIELTEK.V | Y | 48.10 | 1850.9158 | 16 | 0.6 | 926.4658 | 2 | 86.33 | 6 | F6:60001 | NaSa01\_F14.raw |  |  |  |  |  | 9.0541E5 |  |  |  |  |  |  |  |  | 1 | 0 | 0 | 0 | 0 | 0 | 1 | 0 | 0 | 0 | 0 | 0 | 0 | 0 | 0 | 442 | 457 | Oxidation (M) | M1:Oxidation (M):0.00 | PEAKS DB |
| K.GLLSELVK.S | Y | 42.16 | 857.5222 | 8 | 0.8 | 429.7687 | 2 | 55.43 | 6 | F6:34022 | NaSa01\_F14.raw |  |  |  |  |  | 3.4826E6 |  |  |  |  |  |  |  |  | 1 | 0 | 0 | 0 | 0 | 0 | 1 | 0 | 0 | 0 | 0 | 0 | 0 | 0 | 0 | 555 | 562 |  |  | PEAKS DB |
| R.ENYKESFLFTLTR.N | Y | 39.72 | 1646.8303 | 13 | 1.9 | 549.9518 | 3 | 60.34 | 4 | F4:39973 | NaSa01\_F12.raw |  |  |  | 1.0464E5 |  |  |  |  |  |  |  |  |  |  | 1 | 0 | 0 | 0 | 1 | 0 | 0 | 0 | 0 | 0 | 0 | 0 | 0 | 0 | 0 | 352 | 364 |  |  | PEAKS DB |
| total 19 peptides |  |  |  |  |  |  |  |  |  |  |  |  |  |  |  |  |  |  |  |  |  |  |  |  |  |  |  |  |  |  |  |  |  |  |  |  |  |  |  |  |  |  |  |  |  |
| --- | --- | --- | --- | --- | --- | --- | --- | --- | --- | --- | --- | --- | --- | --- | --- | --- | --- | --- | --- | --- | --- | --- | --- | --- | --- | --- | --- | --- | --- | --- | --- | --- | --- | --- | --- | --- | --- | --- | --- | --- | --- | --- | --- | --- | --- |

XP\_034291087.1

back to list

  

| Protein Coverage
| Supporting Peptides
|

Protein Coverage:

Supporting Peptides:

| Peptide | Uniq | -10lgP | Mass | Length | ppm | m/z | z | RT | Fraction | Scan | Source File | Area F1 | Area F10 | Area F11 | Area F12 | Area F13 | Area F14 | Area F2 | Area F3 | Area F4 | Area F5 | Area F6 | Area F7 | Area F8 | Area F9 | #Feature | #Feature F1 | #Feature F10 | #Feature F11 | #Feature F12 | #Feature F13 | #Feature F14 | #Feature F2 | #Feature F3 | #Feature F4 | #Feature F5 | #Feature F6 | #Feature F7 | #Feature F8 | #Feature F9 | Start | End | PTM | AScore | Found By |
| --- | --- | --- | --- | --- | --- | --- | --- | --- | --- | --- | --- | --- | --- | --- | --- | --- | --- | --- | --- | --- | --- | --- | --- | --- | --- | --- | --- | --- | --- | --- | --- | --- | --- | --- | --- | --- | --- | --- | --- | --- | --- | --- | --- | --- | --- |
| K.GGAYSYIESHPWQAGIFVK.N | N | 86.81 | 2109.0320 | 19 | 0.9 | 1055.5242 | 2 | 65.14 | 4 | F4:44125 | NaSa01\_F12.raw |  | 3.9067E7 | 1.32E8 | 7.8017E7 | 7.0674E6 | 0 |  |  |  |  |  |  |  |  | 8 | 0 | 2 | 2 | 2 | 2 | 0 | 0 | 0 | 0 | 0 | 0 | 0 | 0 | 0 | 315 | 333 |  |  | PEAKS DB |
| K.ALYSPNLFIC(+57.02)FC(+57.02)PPGFSGK.F | N | 83.65 | 2174.0330 | 19 | -0.3 | 1088.0234 | 2 | 93.60 | 12 | F12:67510 | NaSa01\_F7.raw |  | 5.8401E7 | 6.3158E7 | 6.0423E6 |  |  |  |  |  |  | 2.7517E7 | 1.7226E8 | 4.6E6 | 6.0752E6 | 13 | 0 | 3 | 2 | 2 | 0 | 0 | 0 | 0 | 0 | 0 | 2 | 2 | 1 | 1 | 101 | 119 | Carbamidomethylation | C10:Carbamidomethylation:1000.00;C12:Carbamidomethylation:1000.00 | PEAKS DB |
| R.TVTENMLC(+57.02)AGDTR.Q | N | 75.17 | 1466.6494 | 13 | 1.2 | 734.3329 | 2 | 28.26 | 4 | F4:13301 | NaSa01\_F12.raw |  |  |  | 3.07E7 | 7.9493E5 |  |  |  |  |  |  |  |  |  | 3 | 0 | 0 | 0 | 2 | 1 | 0 | 0 | 0 | 0 | 0 | 0 | 0 | 0 | 0 | 486 | 498 | Carbamidomethylation | C8:Carbamidomethylation:1000.00 | PEAKS DB |
| R.LYPDSFC(+57.02)TSAR.L | N | 68.01 | 1315.5867 | 11 | -0.3 | 658.8004 | 2 | 32.96 | 4 | F4:17051 | NaSa01\_F12.raw |  | 5.5415E6 | 3.7403E5 | 7.9385E7 | 3.5956E6 |  |  |  | 1.159E5 |  |  |  |  |  | 6 | 0 | 1 | 1 | 2 | 1 | 0 | 0 | 0 | 1 | 0 | 0 | 0 | 0 | 0 | 471 | 481 | Carbamidomethylation | C7:Carbamidomethylation:1000.00 | PEAKS DB |
| K.LIGIISWGVGC(+57.02)GK.K | Y | 67.64 | 1358.7380 | 13 | -0.1 | 680.3762 | 2 | 73.31 | 4 | F4:51304 | NaSa01\_F12.raw |  | 7.1754E7 | 9.3057E7 | 4.5149E7 | 9.3211E5 | 3.7275E5 |  |  |  |  |  |  | 1.646E5 | 1.1739E6 | 10 | 0 | 2 | 1 | 3 | 1 | 1 | 0 | 0 | 0 | 0 | 0 | 0 | 1 | 1 | 522 | 534 | Carbamidomethylation | C11:Carbamidomethylation:1000.00 | PEAKS DB |
| Y.SPNLFIC(+57.02)FC(+57.02)PPGFSGK.F | N | 66.41 | 1826.8484 | 16 | 0.7 | 914.4321 | 2 | 82.84 | 12 | F12:58627 | NaSa01\_F7.raw |  |  |  |  |  |  |  |  |  |  |  | 1.7423E6 |  |  | 1 | 0 | 0 | 0 | 0 | 0 | 0 | 0 | 0 | 0 | 0 | 0 | 1 | 0 | 0 | 104 | 119 | Carbamidomethylation | C7:Carbamidomethylation:1000.00;C9:Carbamidomethylation:1000.00 | PEAKS DB |
| Q.LGLGDHNYC(+57.02)R.N | N | 63.69 | 1203.5455 | 10 | 0.8 | 402.1895 | 3 | 12.19 | 12 | F12:2522 | NaSa01\_F7.raw |  |  |  |  |  |  |  |  |  |  |  | 8.9722E5 |  |  | 2 | 0 | 0 | 0 | 0 | 0 | 0 | 0 | 0 | 0 | 0 | 0 | 2 | 0 | 0 | 173 | 182 | Carbamidomethylation | C9:Carbamidomethylation:1000.00 | PEAKS DB |
| F.IC(+57.02)FC(+57.02)PPGFSGK.F | N | 63.52 | 1268.5681 | 11 | 0.2 | 635.2914 | 2 | 50.36 | 12 | F12:32061 | NaSa01\_F7.raw |  |  |  |  |  |  |  |  |  |  |  | 2.2109E7 |  |  | 1 | 0 | 0 | 0 | 0 | 0 | 0 | 0 | 0 | 0 | 0 | 0 | 1 | 0 | 0 | 109 | 119 | Carbamidomethylation | C2:Carbamidomethylation:1000.00;C4:Carbamidomethylation:1000.00 | PEAKS DB |
| K.SSVTEQIFQVER.Y | Y | 63.46 | 1421.7151 | 12 | 0.4 | 711.8651 | 2 | 49.57 | 4 | F4:31004 | NaSa01\_F12.raw |  | 8.5848E6 | 5.8299E6 | 5.0908E7 | 4.5635E6 |  |  |  |  |  |  |  |  |  | 5 | 0 | 2 | 1 | 1 | 1 | 0 | 0 | 0 | 0 | 0 | 0 | 0 | 0 | 0 | 381 | 392 |  |  | PEAKS DB |
| N.LFIC(+57.02)FC(+57.02)PPGFSGK.F | N | 63.19 | 1528.7206 | 13 | -0.5 | 765.3672 | 2 | 78.45 | 12 | F12:54679 | NaSa01\_F7.raw |  |  | 4.4273E6 |  |  |  |  |  |  |  | 2.2158E6 | 5.365E7 |  |  | 3 | 0 | 0 | 1 | 0 | 0 | 0 | 0 | 0 | 0 | 0 | 1 | 1 | 0 | 0 | 107 | 119 | Carbamidomethylation | C4:Carbamidomethylation:1000.00;C6:Carbamidomethylation:1000.00 | PEAKS DB |
| M.KLIGIISWGVGC(+57.02)GK.K | Y | 58.88 | 1486.8330 | 14 | 0.4 | 496.6185 | 3 | 57.05 | 3 | F3:32194 | NaSa01\_F11.raw |  | 1.1191E6 | 1.7845E6 |  |  |  |  |  |  |  |  |  |  |  | 3 | 0 | 2 | 1 | 0 | 0 | 0 | 0 | 0 | 0 | 0 | 0 | 0 | 0 | 0 | 521 | 534 | Carbamidomethylation | C12:Carbamidomethylation:1000.00 | PEAKS DB |
| R.TLDNDIALLK.L | N | 57.12 | 1114.6234 | 10 | 0.0 | 558.3190 | 2 | 52.92 | 4 | F4:34239 | NaSa01\_F12.raw |  | 4.5967E6 | 1.4483E7 | 6.6342E7 | 2.5476E6 |  |  |  |  |  |  |  |  |  | 6 | 0 | 1 | 1 | 3 | 1 | 0 | 0 | 0 | 0 | 0 | 0 | 0 | 0 | 0 | 403 | 412 |  |  | PEAKS DB |
| K.LIGIISWGVGC(+57.02)GKK.N | Y | 54.66 | 1486.8330 | 14 | 0.1 | 496.6183 | 3 | 54.77 | 4 | F4:35312 | NaSa01\_F12.raw |  | 2.0124E6 | 1.0274E6 | 3.3473E5 |  |  |  |  |  |  |  |  |  |  | 4 | 0 | 1 | 2 | 1 | 0 | 0 | 0 | 0 | 0 | 0 | 0 | 0 | 0 | 0 | 522 | 535 | Carbamidomethylation | C11:Carbamidomethylation:1000.00 | PEAKS DB |
| L.FIC(+57.02)FC(+57.02)PPGFSGK.F | N | 49.97 | 1415.6366 | 12 | -0.6 | 708.8251 | 2 | 63.43 | 12 | F12:43236 | NaSa01\_F7.raw |  |  |  |  |  |  |  |  |  |  |  | 3.3384E6 |  |  | 1 | 0 | 0 | 0 | 0 | 0 | 0 | 0 | 0 | 0 | 0 | 0 | 1 | 0 | 0 | 108 | 119 | Carbamidomethylation | C3:Carbamidomethylation:1000.00;C5:Carbamidomethylation:1000.00 | PEAKS DB |
| Y.SYIESHPWQAGIFVK.N | N | 43.01 | 1760.8885 | 15 | 0.2 | 587.9702 | 3 | 53.84 | 4 | F4:34590 | NaSa01\_F12.raw |  |  |  | 0 |  |  |  |  |  |  |  |  |  |  | 0 | 0 | 0 | 0 | 0 | 0 | 0 | 0 | 0 | 0 | 0 | 0 | 0 | 0 | 0 | 319 | 333 |  |  | PEAKS DB |
| total 15 peptides |  |  |  |  |  |  |  |  |  |  |  |  |  |  |  |  |  |  |  |  |  |  |  |  |  |  |  |  |  |  |  |  |  |  |  |  |  |  |  |  |  |  |  |  |  |
| --- | --- | --- | --- | --- | --- | --- | --- | --- | --- | --- | --- | --- | --- | --- | --- | --- | --- | --- | --- | --- | --- | --- | --- | --- | --- | --- | --- | --- | --- | --- | --- | --- | --- | --- | --- | --- | --- | --- | --- | --- | --- | --- | --- | --- | --- |

XP\_034291085.1

back to list

  

| Protein Coverage
| Supporting Peptides
|

Protein Coverage:

Supporting Peptides:

| Peptide | Uniq | -10lgP | Mass | Length | ppm | m/z | z | RT | Fraction | Scan | Source File | Area F1 | Area F10 | Area F11 | Area F12 | Area F13 | Area F14 | Area F2 | Area F3 | Area F4 | Area F5 | Area F6 | Area F7 | Area F8 | Area F9 | #Feature | #Feature F1 | #Feature F10 | #Feature F11 | #Feature F12 | #Feature F13 | #Feature F14 | #Feature F2 | #Feature F3 | #Feature F4 | #Feature F5 | #Feature F6 | #Feature F7 | #Feature F8 | #Feature F9 | Start | End | PTM | AScore | Found By |
| --- | --- | --- | --- | --- | --- | --- | --- | --- | --- | --- | --- | --- | --- | --- | --- | --- | --- | --- | --- | --- | --- | --- | --- | --- | --- | --- | --- | --- | --- | --- | --- | --- | --- | --- | --- | --- | --- | --- | --- | --- | --- | --- | --- | --- | --- |
| K.GGAYSYIESHPWQAGIFVK.N | N | 86.81 | 2109.0320 | 19 | 0.9 | 1055.5242 | 2 | 65.14 | 4 | F4:44125 | NaSa01\_F12.raw |  | 3.9067E7 | 1.32E8 | 7.8017E7 | 7.0674E6 | 0 |  |  |  |  |  |  |  |  | 8 | 0 | 2 | 2 | 2 | 2 | 0 | 0 | 0 | 0 | 0 | 0 | 0 | 0 | 0 | 315 | 333 |  |  | PEAKS DB |
| K.ALYSPNLFIC(+57.02)FC(+57.02)PPGFSGK.F | N | 83.65 | 2174.0330 | 19 | -0.3 | 1088.0234 | 2 | 93.60 | 12 | F12:67510 | NaSa01\_F7.raw |  | 5.8401E7 | 6.3158E7 | 6.0423E6 |  |  |  |  |  |  | 2.7517E7 | 1.7226E8 | 4.6E6 | 6.0752E6 | 13 | 0 | 3 | 2 | 2 | 0 | 0 | 0 | 0 | 0 | 0 | 2 | 2 | 1 | 1 | 101 | 119 | Carbamidomethylation | C10:Carbamidomethylation:1000.00;C12:Carbamidomethylation:1000.00 | PEAKS DB |
| R.TVTENMLC(+57.02)AGDTR.Q | N | 75.17 | 1466.6494 | 13 | 1.2 | 734.3329 | 2 | 28.26 | 4 | F4:13301 | NaSa01\_F12.raw |  |  |  | 3.07E7 | 7.9493E5 |  |  |  |  |  |  |  |  |  | 3 | 0 | 0 | 0 | 2 | 1 | 0 | 0 | 0 | 0 | 0 | 0 | 0 | 0 | 0 | 486 | 498 | Carbamidomethylation | C8:Carbamidomethylation:1000.00 | PEAKS DB |
| R.LYPDSFC(+57.02)TSAR.L | N | 68.01 | 1315.5867 | 11 | -0.3 | 658.8004 | 2 | 32.96 | 4 | F4:17051 | NaSa01\_F12.raw |  | 5.5415E6 | 3.7403E5 | 7.9385E7 | 3.5956E6 |  |  |  | 1.159E5 |  |  |  |  |  | 6 | 0 | 1 | 1 | 2 | 1 | 0 | 0 | 0 | 1 | 0 | 0 | 0 | 0 | 0 | 471 | 481 | Carbamidomethylation | C7:Carbamidomethylation:1000.00 | PEAKS DB |
| K.LIGIISWGVGC(+57.02)GK.K | Y | 67.64 | 1358.7380 | 13 | -0.1 | 680.3762 | 2 | 73.31 | 4 | F4:51304 | NaSa01\_F12.raw |  | 7.1754E7 | 9.3057E7 | 4.5149E7 | 9.3211E5 | 3.7275E5 |  |  |  |  |  |  | 1.646E5 | 1.1739E6 | 10 | 0 | 2 | 1 | 3 | 1 | 1 | 0 | 0 | 0 | 0 | 0 | 0 | 1 | 1 | 522 | 534 | Carbamidomethylation | C11:Carbamidomethylation:1000.00 | PEAKS DB |
| Y.SPNLFIC(+57.02)FC(+57.02)PPGFSGK.F | N | 66.41 | 1826.8484 | 16 | 0.7 | 914.4321 | 2 | 82.84 | 12 | F12:58627 | NaSa01\_F7.raw |  |  |  |  |  |  |  |  |  |  |  | 1.7423E6 |  |  | 1 | 0 | 0 | 0 | 0 | 0 | 0 | 0 | 0 | 0 | 0 | 0 | 1 | 0 | 0 | 104 | 119 | Carbamidomethylation | C7:Carbamidomethylation:1000.00;C9:Carbamidomethylation:1000.00 | PEAKS DB |
| Q.LGLGDHNYC(+57.02)R.N | N | 63.69 | 1203.5455 | 10 | 0.8 | 402.1895 | 3 | 12.19 | 12 | F12:2522 | NaSa01\_F7.raw |  |  |  |  |  |  |  |  |  |  |  | 8.9722E5 |  |  | 2 | 0 | 0 | 0 | 0 | 0 | 0 | 0 | 0 | 0 | 0 | 0 | 2 | 0 | 0 | 173 | 182 | Carbamidomethylation | C9:Carbamidomethylation:1000.00 | PEAKS DB |
| F.IC(+57.02)FC(+57.02)PPGFSGK.F | N | 63.52 | 1268.5681 | 11 | 0.2 | 635.2914 | 2 | 50.36 | 12 | F12:32061 | NaSa01\_F7.raw |  |  |  |  |  |  |  |  |  |  |  | 2.2109E7 |  |  | 1 | 0 | 0 | 0 | 0 | 0 | 0 | 0 | 0 | 0 | 0 | 0 | 1 | 0 | 0 | 109 | 119 | Carbamidomethylation | C2:Carbamidomethylation:1000.00;C4:Carbamidomethylation:1000.00 | PEAKS DB |
| K.SSVTEQIFQVER.Y | Y | 63.46 | 1421.7151 | 12 | 0.4 | 711.8651 | 2 | 49.57 | 4 | F4:31004 | NaSa01\_F12.raw |  | 8.5848E6 | 5.8299E6 | 5.0908E7 | 4.5635E6 |  |  |  |  |  |  |  |  |  | 5 | 0 | 2 | 1 | 1 | 1 | 0 | 0 | 0 | 0 | 0 | 0 | 0 | 0 | 0 | 381 | 392 |  |  | PEAKS DB |
| N.LFIC(+57.02)FC(+57.02)PPGFSGK.F | N | 63.19 | 1528.7206 | 13 | -0.5 | 765.3672 | 2 | 78.45 | 12 | F12:54679 | NaSa01\_F7.raw |  |  | 4.4273E6 |  |  |  |  |  |  |  | 2.2158E6 | 5.365E7 |  |  | 3 | 0 | 0 | 1 | 0 | 0 | 0 | 0 | 0 | 0 | 0 | 1 | 1 | 0 | 0 | 107 | 119 | Carbamidomethylation | C4:Carbamidomethylation:1000.00;C6:Carbamidomethylation:1000.00 | PEAKS DB |
| M.KLIGIISWGVGC(+57.02)GK.K | Y | 58.88 | 1486.8330 | 14 | 0.4 | 496.6185 | 3 | 57.05 | 3 | F3:32194 | NaSa01\_F11.raw |  | 1.1191E6 | 1.7845E6 |  |  |  |  |  |  |  |  |  |  |  | 3 | 0 | 2 | 1 | 0 | 0 | 0 | 0 | 0 | 0 | 0 | 0 | 0 | 0 | 0 | 521 | 534 | Carbamidomethylation | C12:Carbamidomethylation:1000.00 | PEAKS DB |
| R.TLDNDIALLK.L | N | 57.12 | 1114.6234 | 10 | 0.0 | 558.3190 | 2 | 52.92 | 4 | F4:34239 | NaSa01\_F12.raw |  | 4.5967E6 | 1.4483E7 | 6.6342E7 | 2.5476E6 |  |  |  |  |  |  |  |  |  | 6 | 0 | 1 | 1 | 3 | 1 | 0 | 0 | 0 | 0 | 0 | 0 | 0 | 0 | 0 | 403 | 412 |  |  | PEAKS DB |
| K.LIGIISWGVGC(+57.02)GKK.N | Y | 54.66 | 1486.8330 | 14 | 0.1 | 496.6183 | 3 | 54.77 | 4 | F4:35312 | NaSa01\_F12.raw |  | 2.0124E6 | 1.0274E6 | 3.3473E5 |  |  |  |  |  |  |  |  |  |  | 4 | 0 | 1 | 2 | 1 | 0 | 0 | 0 | 0 | 0 | 0 | 0 | 0 | 0 | 0 | 522 | 535 | Carbamidomethylation | C11:Carbamidomethylation:1000.00 | PEAKS DB |
| L.FIC(+57.02)FC(+57.02)PPGFSGK.F | N | 49.97 | 1415.6366 | 12 | -0.6 | 708.8251 | 2 | 63.43 | 12 | F12:43236 | NaSa01\_F7.raw |  |  |  |  |  |  |  |  |  |  |  | 3.3384E6 |  |  | 1 | 0 | 0 | 0 | 0 | 0 | 0 | 0 | 0 | 0 | 0 | 0 | 1 | 0 | 0 | 108 | 119 | Carbamidomethylation | C3:Carbamidomethylation:1000.00;C5:Carbamidomethylation:1000.00 | PEAKS DB |
| Y.SYIESHPWQAGIFVK.N | N | 43.01 | 1760.8885 | 15 | 0.2 | 587.9702 | 3 | 53.84 | 4 | F4:34590 | NaSa01\_F12.raw |  |  |  | 0 |  |  |  |  |  |  |  |  |  |  | 0 | 0 | 0 | 0 | 0 | 0 | 0 | 0 | 0 | 0 | 0 | 0 | 0 | 0 | 0 | 319 | 333 |  |  | PEAKS DB |
| total 15 peptides |  |  |  |  |  |  |  |  |  |  |  |  |  |  |  |  |  |  |  |  |  |  |  |  |  |  |  |  |  |  |  |  |  |  |  |  |  |  |  |  |  |  |  |  |  |
| --- | --- | --- | --- | --- | --- | --- | --- | --- | --- | --- | --- | --- | --- | --- | --- | --- | --- | --- | --- | --- | --- | --- | --- | --- | --- | --- | --- | --- | --- | --- | --- | --- | --- | --- | --- | --- | --- | --- | --- | --- | --- | --- | --- | --- | --- |

XP\_034291086.1

back to list

  

| Protein Coverage
| Supporting Peptides
|

Protein Coverage:

Supporting Peptides:

| Peptide | Uniq | -10lgP | Mass | Length | ppm | m/z | z | RT | Fraction | Scan | Source File | Area F1 | Area F10 | Area F11 | Area F12 | Area F13 | Area F14 | Area F2 | Area F3 | Area F4 | Area F5 | Area F6 | Area F7 | Area F8 | Area F9 | #Feature | #Feature F1 | #Feature F10 | #Feature F11 | #Feature F12 | #Feature F13 | #Feature F14 | #Feature F2 | #Feature F3 | #Feature F4 | #Feature F5 | #Feature F6 | #Feature F7 | #Feature F8 | #Feature F9 | Start | End | PTM | AScore | Found By |
| --- | --- | --- | --- | --- | --- | --- | --- | --- | --- | --- | --- | --- | --- | --- | --- | --- | --- | --- | --- | --- | --- | --- | --- | --- | --- | --- | --- | --- | --- | --- | --- | --- | --- | --- | --- | --- | --- | --- | --- | --- | --- | --- | --- | --- | --- |
| K.GGAYSYIESHPWQAGIFVK.N | N | 86.81 | 2109.0320 | 19 | 0.9 | 1055.5242 | 2 | 65.14 | 4 | F4:44125 | NaSa01\_F12.raw |  | 3.9067E7 | 1.32E8 | 7.8017E7 | 7.0674E6 | 0 |  |  |  |  |  |  |  |  | 8 | 0 | 2 | 2 | 2 | 2 | 0 | 0 | 0 | 0 | 0 | 0 | 0 | 0 | 0 | 315 | 333 |  |  | PEAKS DB |
| K.ALYSPNLFIC(+57.02)FC(+57.02)PPGFSGK.F | N | 83.65 | 2174.0330 | 19 | -0.3 | 1088.0234 | 2 | 93.60 | 12 | F12:67510 | NaSa01\_F7.raw |  | 5.8401E7 | 6.3158E7 | 6.0423E6 |  |  |  |  |  |  | 2.7517E7 | 1.7226E8 | 4.6E6 | 6.0752E6 | 13 | 0 | 3 | 2 | 2 | 0 | 0 | 0 | 0 | 0 | 0 | 2 | 2 | 1 | 1 | 101 | 119 | Carbamidomethylation | C10:Carbamidomethylation:1000.00;C12:Carbamidomethylation:1000.00 | PEAKS DB |
| R.TVTENMLC(+57.02)AGDTR.Q | N | 75.17 | 1466.6494 | 13 | 1.2 | 734.3329 | 2 | 28.26 | 4 | F4:13301 | NaSa01\_F12.raw |  |  |  | 3.07E7 | 7.9493E5 |  |  |  |  |  |  |  |  |  | 3 | 0 | 0 | 0 | 2 | 1 | 0 | 0 | 0 | 0 | 0 | 0 | 0 | 0 | 0 | 486 | 498 | Carbamidomethylation | C8:Carbamidomethylation:1000.00 | PEAKS DB |
| R.LYPDSFC(+57.02)TSAR.L | N | 68.01 | 1315.5867 | 11 | -0.3 | 658.8004 | 2 | 32.96 | 4 | F4:17051 | NaSa01\_F12.raw |  | 5.5415E6 | 3.7403E5 | 7.9385E7 | 3.5956E6 |  |  |  | 1.159E5 |  |  |  |  |  | 6 | 0 | 1 | 1 | 2 | 1 | 0 | 0 | 0 | 1 | 0 | 0 | 0 | 0 | 0 | 471 | 481 | Carbamidomethylation | C7:Carbamidomethylation:1000.00 | PEAKS DB |
| K.LIGIISWGVGC(+57.02)GK.K | Y | 67.64 | 1358.7380 | 13 | -0.1 | 680.3762 | 2 | 73.31 | 4 | F4:51304 | NaSa01\_F12.raw |  | 7.1754E7 | 9.3057E7 | 4.5149E7 | 9.3211E5 | 3.7275E5 |  |  |  |  |  |  | 1.646E5 | 1.1739E6 | 10 | 0 | 2 | 1 | 3 | 1 | 1 | 0 | 0 | 0 | 0 | 0 | 0 | 1 | 1 | 522 | 534 | Carbamidomethylation | C11:Carbamidomethylation:1000.00 | PEAKS DB |
| Y.SPNLFIC(+57.02)FC(+57.02)PPGFSGK.F | N | 66.41 | 1826.8484 | 16 | 0.7 | 914.4321 | 2 | 82.84 | 12 | F12:58627 | NaSa01\_F7.raw |  |  |  |  |  |  |  |  |  |  |  | 1.7423E6 |  |  | 1 | 0 | 0 | 0 | 0 | 0 | 0 | 0 | 0 | 0 | 0 | 0 | 1 | 0 | 0 | 104 | 119 | Carbamidomethylation | C7:Carbamidomethylation:1000.00;C9:Carbamidomethylation:1000.00 | PEAKS DB |
| Q.LGLGDHNYC(+57.02)R.N | N | 63.69 | 1203.5455 | 10 | 0.8 | 402.1895 | 3 | 12.19 | 12 | F12:2522 | NaSa01\_F7.raw |  |  |  |  |  |  |  |  |  |  |  | 8.9722E5 |  |  | 2 | 0 | 0 | 0 | 0 | 0 | 0 | 0 | 0 | 0 | 0 | 0 | 2 | 0 | 0 | 173 | 182 | Carbamidomethylation | C9:Carbamidomethylation:1000.00 | PEAKS DB |
| F.IC(+57.02)FC(+57.02)PPGFSGK.F | N | 63.52 | 1268.5681 | 11 | 0.2 | 635.2914 | 2 | 50.36 | 12 | F12:32061 | NaSa01\_F7.raw |  |  |  |  |  |  |  |  |  |  |  | 2.2109E7 |  |  | 1 | 0 | 0 | 0 | 0 | 0 | 0 | 0 | 0 | 0 | 0 | 0 | 1 | 0 | 0 | 109 | 119 | Carbamidomethylation | C2:Carbamidomethylation:1000.00;C4:Carbamidomethylation:1000.00 | PEAKS DB |
| K.SSVTEQIFQVER.Y | Y | 63.46 | 1421.7151 | 12 | 0.4 | 711.8651 | 2 | 49.57 | 4 | F4:31004 | NaSa01\_F12.raw |  | 8.5848E6 | 5.8299E6 | 5.0908E7 | 4.5635E6 |  |  |  |  |  |  |  |  |  | 5 | 0 | 2 | 1 | 1 | 1 | 0 | 0 | 0 | 0 | 0 | 0 | 0 | 0 | 0 | 381 | 392 |  |  | PEAKS DB |
| N.LFIC(+57.02)FC(+57.02)PPGFSGK.F | N | 63.19 | 1528.7206 | 13 | -0.5 | 765.3672 | 2 | 78.45 | 12 | F12:54679 | NaSa01\_F7.raw |  |  | 4.4273E6 |  |  |  |  |  |  |  | 2.2158E6 | 5.365E7 |  |  | 3 | 0 | 0 | 1 | 0 | 0 | 0 | 0 | 0 | 0 | 0 | 1 | 1 | 0 | 0 | 107 | 119 | Carbamidomethylation | C4:Carbamidomethylation:1000.00;C6:Carbamidomethylation:1000.00 | PEAKS DB |
| M.KLIGIISWGVGC(+57.02)GK.K | Y | 58.88 | 1486.8330 | 14 | 0.4 | 496.6185 | 3 | 57.05 | 3 | F3:32194 | NaSa01\_F11.raw |  | 1.1191E6 | 1.7845E6 |  |  |  |  |  |  |  |  |  |  |  | 3 | 0 | 2 | 1 | 0 | 0 | 0 | 0 | 0 | 0 | 0 | 0 | 0 | 0 | 0 | 521 | 534 | Carbamidomethylation | C12:Carbamidomethylation:1000.00 | PEAKS DB |
| R.TLDNDIALLK.L | N | 57.12 | 1114.6234 | 10 | 0.0 | 558.3190 | 2 | 52.92 | 4 | F4:34239 | NaSa01\_F12.raw |  | 4.5967E6 | 1.4483E7 | 6.6342E7 | 2.5476E6 |  |  |  |  |  |  |  |  |  | 6 | 0 | 1 | 1 | 3 | 1 | 0 | 0 | 0 | 0 | 0 | 0 | 0 | 0 | 0 | 403 | 412 |  |  | PEAKS DB |
| K.LIGIISWGVGC(+57.02)GKK.N | Y | 54.66 | 1486.8330 | 14 | 0.1 | 496.6183 | 3 | 54.77 | 4 | F4:35312 | NaSa01\_F12.raw |  | 2.0124E6 | 1.0274E6 | 3.3473E5 |  |  |  |  |  |  |  |  |  |  | 4 | 0 | 1 | 2 | 1 | 0 | 0 | 0 | 0 | 0 | 0 | 0 | 0 | 0 | 0 | 522 | 535 | Carbamidomethylation | C11:Carbamidomethylation:1000.00 | PEAKS DB |
| L.FIC(+57.02)FC(+57.02)PPGFSGK.F | N | 49.97 | 1415.6366 | 12 | -0.6 | 708.8251 | 2 | 63.43 | 12 | F12:43236 | NaSa01\_F7.raw |  |  |  |  |  |  |  |  |  |  |  | 3.3384E6 |  |  | 1 | 0 | 0 | 0 | 0 | 0 | 0 | 0 | 0 | 0 | 0 | 0 | 1 | 0 | 0 | 108 | 119 | Carbamidomethylation | C3:Carbamidomethylation:1000.00;C5:Carbamidomethylation:1000.00 | PEAKS DB |
| Y.SYIESHPWQAGIFVK.N | N | 43.01 | 1760.8885 | 15 | 0.2 | 587.9702 | 3 | 53.84 | 4 | F4:34590 | NaSa01\_F12.raw |  |  |  | 0 |  |  |  |  |  |  |  |  |  |  | 0 | 0 | 0 | 0 | 0 | 0 | 0 | 0 | 0 | 0 | 0 | 0 | 0 | 0 | 0 | 319 | 333 |  |  | PEAKS DB |
| total 15 peptides |  |  |  |  |  |  |  |  |  |  |  |  |  |  |  |  |  |  |  |  |  |  |  |  |  |  |  |  |  |  |  |  |  |  |  |  |  |  |  |  |  |  |  |  |  |
| --- | --- | --- | --- | --- | --- | --- | --- | --- | --- | --- | --- | --- | --- | --- | --- | --- | --- | --- | --- | --- | --- | --- | --- | --- | --- | --- | --- | --- | --- | --- | --- | --- | --- | --- | --- | --- | --- | --- | --- | --- | --- | --- | --- | --- | --- |

ETE56723.1

back to list

  

| Protein Coverage
| Supporting Peptides
|

Protein Coverage:

Supporting Peptides:

| Peptide | Uniq | -10lgP | Mass | Length | ppm | m/z | z | RT | Fraction | Scan | Source File | Area F1 | Area F10 | Area F11 | Area F12 | Area F13 | Area F14 | Area F2 | Area F3 | Area F4 | Area F5 | Area F6 | Area F7 | Area F8 | Area F9 | #Feature | #Feature F1 | #Feature F10 | #Feature F11 | #Feature F12 | #Feature F13 | #Feature F14 | #Feature F2 | #Feature F3 | #Feature F4 | #Feature F5 | #Feature F6 | #Feature F7 | #Feature F8 | #Feature F9 | Start | End | PTM | AScore | Found By |
| --- | --- | --- | --- | --- | --- | --- | --- | --- | --- | --- | --- | --- | --- | --- | --- | --- | --- | --- | --- | --- | --- | --- | --- | --- | --- | --- | --- | --- | --- | --- | --- | --- | --- | --- | --- | --- | --- | --- | --- | --- | --- | --- | --- | --- | --- |
| K.LAQLYNVNHVSLFHSDC(+57.02)PR.Q | N | 83.18 | 2269.1062 | 19 | 0.1 | 757.3761 | 3 | 43.25 | 4 | F4:25798 | NaSa01\_F12.raw |  |  |  | 5.5169E7 | 7.3204E8 | 7.8894E6 |  |  |  |  |  |  |  |  | 11 | 0 | 0 | 0 | 4 | 5 | 2 | 0 | 0 | 0 | 0 | 0 | 0 | 0 | 0 | 141 | 159 | Carbamidomethylation | C17:Carbamidomethylation:1000.00 | PEAKS DB |
| K.SSTFTLTNIVPQFIK.L | N | 69.82 | 1694.9243 | 15 | 1.1 | 848.4704 | 2 | 89.01 | 4 | F4:63439 | NaSa01\_F12.raw |  |  |  | 6.2934E6 | 1.5029E9 | 8.7605E6 |  |  |  |  |  |  |  |  | 4 | 0 | 0 | 0 | 1 | 2 | 1 | 0 | 0 | 0 | 0 | 0 | 0 | 0 | 0 | 51 | 65 |  |  | PEAKS DB |
| R.GC(+57.02)QQTFAVVGAVPGDTYIAR.G | Y | 67.92 | 2109.0312 | 20 | -0.4 | 1055.5225 | 2 | 62.95 | 6 | F6:40467 | NaSa01\_F14.raw |  |  |  | 2.7309E7 | 5.4108E8 | 2.9423E6 |  |  |  |  |  |  |  |  | 5 | 0 | 0 | 0 | 2 | 2 | 1 | 0 | 0 | 0 | 0 | 0 | 0 | 0 | 0 | 85 | 104 | Carbamidomethylation | C2:Carbamidomethylation:1000.00 | PEAKS DB |
| R.VNRPSHLWSAAC(+57.02)C(+57.02)LIDNNHLR.S | N | 67.46 | 2532.2227 | 21 | 0.1 | 634.0630 | 4 | 50.12 | 5 | F5:30674 | NaSa01\_F13.raw |  |  |  | 4.7154E6 | 5.3132E7 |  |  |  |  |  |  |  |  | 3.8075E6 | 6 | 0 | 0 | 0 | 2 | 3 | 0 | 0 | 0 | 0 | 0 | 0 | 0 | 0 | 1 | 107 | 127 | Carbamidomethylation | C12:Carbamidomethylation:1000.00;C13:Carbamidomethylation:1000.00 | PEAKS DB |
| R.GHLNPNGHQPDYSAK.S | N | 61.86 | 1633.7596 | 15 | 1.0 | 545.5944 | 3 | 11.43 | 5 | F5:1940 | NaSa01\_F13.raw |  |  |  |  | 1.4956E6 |  |  |  |  |  |  |  |  |  | 1 | 0 | 0 | 0 | 0 | 1 | 0 | 0 | 0 | 0 | 0 | 0 | 0 | 0 | 0 | 36 | 50 |  |  | PEAKS DB |
| W.SAAC(+57.02)C(+57.02)LIDNNHLR.S | N | 58.44 | 1542.7031 | 13 | 0.8 | 515.2421 | 3 | 20.79 | 5 | F5:6078 | NaSa01\_F13.raw |  |  |  |  | 3.0055E7 |  |  |  |  |  |  |  |  |  | 3 | 0 | 0 | 0 | 0 | 3 | 0 | 0 | 0 | 0 | 0 | 0 | 0 | 0 | 0 | 115 | 127 | Carbamidomethylation | C4:Carbamidomethylation:1000.00;C5:Carbamidomethylation:1000.00 | PEAKS DB |
| Y.NVNHVSLFHSDC(+57.02)PR.Q | N | 58.23 | 1680.7791 | 14 | 0.8 | 561.2674 | 3 | 11.62 | 5 | F5:2096 | NaSa01\_F13.raw |  |  |  |  | 2.3142E6 |  |  |  |  |  |  |  |  |  | 1 | 0 | 0 | 0 | 0 | 1 | 0 | 0 | 0 | 0 | 0 | 0 | 0 | 0 | 0 | 146 | 159 | Carbamidomethylation | C12:Carbamidomethylation:1000.00 | PEAKS DB |
| F.AVVGAVPGDTYIAR.G | N | 57.03 | 1387.7460 | 14 | 1.7 | 694.8815 | 2 | 40.72 | 6 | F6:21346 | NaSa01\_F14.raw |  |  |  | 4.6266E6 | 7.6974E8 | 7.3018E6 |  |  |  |  |  |  |  |  | 4 | 0 | 0 | 0 | 1 | 2 | 1 | 0 | 0 | 0 | 0 | 0 | 0 | 0 | 0 | 91 | 104 |  |  | PEAKS DB |
| Q.LYNVNHVSLFHSDC(+57.02)PR.Q | N | 53.73 | 1956.9264 | 16 | 1.2 | 490.2395 | 4 | 33.35 | 5 | F5:16269 | NaSa01\_F13.raw |  |  |  |  | 1.2213E6 |  |  |  |  |  |  |  |  |  | 1 | 0 | 0 | 0 | 0 | 1 | 0 | 0 | 0 | 0 | 0 | 0 | 0 | 0 | 0 | 144 | 159 | Carbamidomethylation | C14:Carbamidomethylation:1000.00 | PEAKS DB |
| R.PSHLWSAAC(+57.02)C(+57.02)LIDNNHLR.S | N | 52.90 | 2163.0103 | 18 | -0.4 | 541.7596 | 4 | 54.70 | 5 | F5:34354 | NaSa01\_F13.raw |  |  |  |  | 1.7436E7 |  |  |  |  |  |  |  |  |  | 2 | 0 | 0 | 0 | 0 | 2 | 0 | 0 | 0 | 0 | 0 | 0 | 0 | 0 | 0 | 110 | 127 | Carbamidomethylation | C9:Carbamidomethylation:1000.00;C10:Carbamidomethylation:1000.00 | PEAKS DB |
| R.VNRPSHLWSAAC(+57.02)C(+57.02).L | N | 51.05 | 1556.6976 | 13 | 0.0 | 779.3561 | 2 | 11.66 | 5 | F5:2173 | NaSa01\_F13.raw |  |  |  |  | 3.8373E6 |  |  |  |  |  |  |  |  |  | 2 | 0 | 0 | 0 | 0 | 2 | 0 | 0 | 0 | 0 | 0 | 0 | 0 | 0 | 0 | 107 | 119 | Carbamidomethylation | C12:Carbamidomethylation:1000.00;C13:Carbamidomethylation:1000.00 | PEAKS DB |
| K.LAQLYNVNHVSLF.H | N | 49.44 | 1516.8038 | 13 | -0.3 | 759.4089 | 2 | 71.90 | 5 | F5:48055 | NaSa01\_F13.raw |  |  |  |  | 1.3448E8 |  |  |  |  |  |  |  |  |  | 1 | 0 | 0 | 0 | 0 | 1 | 0 | 0 | 0 | 0 | 0 | 0 | 0 | 0 | 0 | 141 | 153 |  |  | PEAKS DB |
| R.VNRPSHLWSAAC(+57.02).C | N | 47.36 | 1396.6670 | 12 | 0.2 | 699.3409 | 2 | 11.69 | 5 | F5:2180 | NaSa01\_F13.raw |  |  |  |  | 6.1785E5 |  |  |  |  |  |  |  |  |  | 1 | 0 | 0 | 0 | 0 | 1 | 0 | 0 | 0 | 0 | 0 | 0 | 0 | 0 | 0 | 107 | 118 | Carbamidomethylation | C12:Carbamidomethylation:1000.00 | PEAKS DB |
| L.YNVNHVSLFHSDC(+57.02)PR.Q | N | 46.41 | 1843.8424 | 15 | 1.7 | 615.6224 | 3 | 25.29 | 5 | F5:9481 | NaSa01\_F13.raw |  |  |  |  | 7.5396E6 |  |  |  |  |  |  |  |  |  | 2 | 0 | 0 | 0 | 0 | 2 | 0 | 0 | 0 | 0 | 0 | 0 | 0 | 0 | 0 | 145 | 159 | Carbamidomethylation | C13:Carbamidomethylation:1000.00 | PEAKS DB |
| S.TFTLTNIVPQFIK.L | N | 45.77 | 1520.8602 | 13 | 0.0 | 761.4374 | 2 | 89.58 | 5 | F5:62408 | NaSa01\_F13.raw |  |  |  |  | 1.3379E6 |  |  |  |  |  |  |  |  |  | 1 | 0 | 0 | 0 | 0 | 1 | 0 | 0 | 0 | 0 | 0 | 0 | 0 | 0 | 0 | 53 | 65 |  |  | PEAKS DB |
| R.SWAILAR.N | N | 44.55 | 815.4653 | 7 | 0.1 | 408.7400 | 2 | 43.13 | 4 | F4:25699 | NaSa01\_F12.raw |  |  |  | 9.8044E6 |  | 9.814E6 |  |  |  |  |  |  |  |  | 3 | 0 | 0 | 0 | 1 | 0 | 2 | 0 | 0 | 0 | 0 | 0 | 0 | 0 | 0 | 128 | 134 |  |  | PEAKS DB |
| C.QQTFAVVGAVPGDTYIAR.G | Y | 41.91 | 1891.9791 | 18 | 0.8 | 946.9976 | 2 | 62.35 | 5 | F5:40357 | NaSa01\_F13.raw |  |  |  |  | 1.1476E6 |  |  |  |  |  |  |  |  |  | 1 | 0 | 0 | 0 | 0 | 1 | 0 | 0 | 0 | 0 | 0 | 0 | 0 | 0 | 0 | 87 | 104 |  |  | PEAKS DB |
| N.HVSLFHSDC(+57.02)PR.Q | N | 41.73 | 1353.6248 | 11 | -0.2 | 452.2154 | 3 | 11.44 | 5 | F5:2051 | NaSa01\_F13.raw |  |  |  |  | 5.6233E5 |  |  |  |  |  |  |  |  |  | 1 | 0 | 0 | 0 | 0 | 1 | 0 | 0 | 0 | 0 | 0 | 0 | 0 | 0 | 0 | 149 | 159 | Carbamidomethylation | C9:Carbamidomethylation:1000.00 | PEAKS DB |
| F.TLTNIVPQFIK.L | N | 40.01 | 1272.7441 | 11 | 0.2 | 637.3795 | 2 | 67.27 | 5 | F5:44583 | NaSa01\_F13.raw |  |  |  |  | 1.1734E8 |  |  |  |  |  |  |  |  |  | 1 | 0 | 0 | 0 | 0 | 1 | 0 | 0 | 0 | 0 | 0 | 0 | 0 | 0 | 0 | 55 | 65 |  |  | PEAKS DB |
| H.LWSAAC(+57.02)C(+57.02)LIDNNHLR.S | N | 39.30 | 1841.8665 | 15 | 1.1 | 614.9634 | 3 | 53.29 | 5 | F5:33016 | NaSa01\_F13.raw |  |  |  |  | 8.5064E5 |  |  |  |  |  |  |  |  |  | 1 | 0 | 0 | 0 | 0 | 1 | 0 | 0 | 0 | 0 | 0 | 0 | 0 | 0 | 0 | 113 | 127 | Carbamidomethylation | C6:Carbamidomethylation:1000.00;C7:Carbamidomethylation:1000.00 | PEAKS DB |
| R.GC(+57.02)QQTFAVVGAVPGDTY.I | Y | 38.71 | 1768.8090 | 17 | 0.7 | 885.4124 | 2 | 72.30 | 5 | F5:48511 | NaSa01\_F13.raw |  |  |  |  | 9.2887E6 |  |  |  |  |  |  |  |  |  | 1 | 0 | 0 | 0 | 0 | 1 | 0 | 0 | 0 | 0 | 0 | 0 | 0 | 0 | 0 | 85 | 101 | Carbamidomethylation | C2:Carbamidomethylation:1000.00 | PEAKS DB |
| total 21 peptides |  |  |  |  |  |  |  |  |  |  |  |  |  |  |  |  |  |  |  |  |  |  |  |  |  |  |  |  |  |  |  |  |  |  |  |  |  |  |  |  |  |  |  |  |  |
| --- | --- | --- | --- | --- | --- | --- | --- | --- | --- | --- | --- | --- | --- | --- | --- | --- | --- | --- | --- | --- | --- | --- | --- | --- | --- | --- | --- | --- | --- | --- | --- | --- | --- | --- | --- | --- | --- | --- | --- | --- | --- | --- | --- | --- | --- |

ETE68810.1

back to list

  

| Protein Coverage
| Supporting Peptides
|

Protein Coverage:

Supporting Peptides:

| Peptide | Uniq | -10lgP | Mass | Length | ppm | m/z | z | RT | Fraction | Scan | Source File | Area F1 | Area F10 | Area F11 | Area F12 | Area F13 | Area F14 | Area F2 | Area F3 | Area F4 | Area F5 | Area F6 | Area F7 | Area F8 | Area F9 | #Feature | #Feature F1 | #Feature F10 | #Feature F11 | #Feature F12 | #Feature F13 | #Feature F14 | #Feature F2 | #Feature F3 | #Feature F4 | #Feature F5 | #Feature F6 | #Feature F7 | #Feature F8 | #Feature F9 | Start | End | PTM | AScore | Found By |
| --- | --- | --- | --- | --- | --- | --- | --- | --- | --- | --- | --- | --- | --- | --- | --- | --- | --- | --- | --- | --- | --- | --- | --- | --- | --- | --- | --- | --- | --- | --- | --- | --- | --- | --- | --- | --- | --- | --- | --- | --- | --- | --- | --- | --- | --- |
| K.NSC(+57.02)PPVVETFGDTAK.L | Y | 84.63 | 1620.7454 | 15 | 0.5 | 811.3804 | 2 | 36.78 | 6 | F6:18221 | NaSa01\_F14.raw |  |  |  | 1.1558E6 | 8.6172E7 | 5.8583E7 |  |  |  |  |  |  |  |  | 4 | 0 | 0 | 0 | 1 | 2 | 1 | 0 | 0 | 0 | 0 | 0 | 0 | 0 | 0 | 197 | 211 | Carbamidomethylation | C3:Carbamidomethylation:1000.00 | PEAKS DB |
| R.LVILGFPC(+57.02)NQFGK.Q | N | 80.93 | 1491.7908 | 13 | 0.3 | 746.9029 | 2 | 74.37 | 14 | F14:48186 | NaSa01\_F9.raw |  | 6.3482E6 | 4.5719E6 | 6.7588E6 | 7.3547E8 | 2.7574E9 |  |  |  |  |  | 1.9765E6 | 7.8579E6 | 5.5032E7 | 12 | 0 | 1 | 1 | 1 | 3 | 3 | 0 | 0 | 0 | 0 | 0 | 1 | 1 | 1 | 137 | 149 | Carbamidomethylation | C8:Carbamidomethylation:1000.00 | PEAKS DB |
| K.QEPGQNSEILQGIK.H | N | 80.47 | 1539.7893 | 14 | 0.1 | 770.9020 | 2 | 34.92 | 6 | F6:17025 | NaSa01\_F14.raw |  |  | 7.9374E4 | 4.1222E6 | 3.3468E8 | 1.3102E9 |  |  |  |  |  |  | 0 |  | 8 | 0 | 0 | 1 | 1 | 2 | 4 | 0 | 0 | 0 | 0 | 0 | 0 | 0 | 0 | 150 | 163 |  |  | PEAKS DB |
| K.LFWSPLKIHDIK.W | N | 68.85 | 1495.8551 | 12 | 0.5 | 499.6259 | 3 | 54.14 | 6 | F6:32777 | NaSa01\_F14.raw |  |  |  |  |  | 4.8748E5 |  |  |  |  |  |  |  |  | 1 | 0 | 0 | 0 | 0 | 0 | 1 | 0 | 0 | 0 | 0 | 0 | 0 | 0 | 0 | 212 | 223 |  |  | PEAKS DB |
| K.WNFEKFLVNPQGK.P | N | 61.53 | 1605.8303 | 13 | 0.1 | 536.2841 | 3 | 64.13 | 6 | F6:41454 | NaSa01\_F14.raw |  |  |  |  |  | 3.4585E6 |  |  |  |  |  |  |  |  | 2 | 0 | 0 | 0 | 0 | 0 | 2 | 0 | 0 | 0 | 0 | 0 | 0 | 0 | 0 | 224 | 236 |  |  | PEAKS DB |
| L.VILGFPC(+57.02)NQFGK.Q | N | 58.48 | 1378.7067 | 12 | -0.1 | 690.3605 | 2 | 59.07 | 12 | F12:39406 | NaSa01\_F7.raw |  | 4.6143E6 | 1.4793E6 |  |  |  |  |  |  | 1.3101E7 | 1.8748E7 | 4.3606E7 | 7.4857E6 | 1.729E7 | 7 | 0 | 1 | 1 | 0 | 0 | 0 | 0 | 0 | 0 | 1 | 1 | 1 | 1 | 1 | 138 | 149 | Carbamidomethylation | C7:Carbamidomethylation:1000.00 | PEAKS DB |
| K.FLVNPQGKPVMR.W | N | 58.11 | 1384.7649 | 12 | -0.2 | 462.5955 | 3 | 25.87 | 5 | F5:9864 | NaSa01\_F13.raw |  |  |  |  | 9.037E6 |  |  |  |  |  |  |  |  |  | 2 | 0 | 0 | 0 | 0 | 2 | 0 | 0 | 0 | 0 | 0 | 0 | 0 | 0 | 0 | 229 | 240 |  |  | PEAKS DB |
| F.VPNFQLFQK.G | N | 54.38 | 1119.6077 | 9 | -0.6 | 560.8108 | 2 | 54.08 | 6 | F6:32772 | NaSa01\_F14.raw |  |  |  |  | 1.0977E6 | 4.3013E6 |  |  |  |  |  |  |  |  | 2 | 0 | 0 | 0 | 0 | 1 | 1 | 0 | 0 | 0 | 0 | 0 | 0 | 0 | 0 | 172 | 180 |  |  | PEAKS DB |
| K.GDVNGENEQKIYTFLK.N | Y | 53.46 | 1853.9159 | 16 | 0.6 | 618.9796 | 3 | 45.49 | 6 | F6:25522 | NaSa01\_F14.raw |  |  |  |  |  | 1.7469E6 |  |  |  |  |  |  |  |  | 1 | 0 | 0 | 0 | 0 | 0 | 1 | 0 | 0 | 0 | 0 | 0 | 0 | 0 | 0 | 181 | 196 |  |  | PEAKS DB |
| K.QEPGQNSEILQGIKHVR.P | Y | 51.51 | 1932.0177 | 17 | -0.2 | 484.0116 | 4 | 22.95 | 6 | F6:7040 | NaSa01\_F14.raw |  |  |  |  |  | 5.4601E6 |  |  |  |  |  |  |  |  | 2 | 0 | 0 | 0 | 0 | 0 | 2 | 0 | 0 | 0 | 0 | 0 | 0 | 0 | 0 | 150 | 166 |  |  | PEAKS DB |
| K.LFWSPLK.I | N | 50.63 | 889.5062 | 7 | -1.0 | 445.7599 | 2 | 61.33 | 6 | F6:38812 | NaSa01\_F14.raw |  |  |  |  |  | 1.0011E9 |  |  |  |  |  |  |  |  | 2 | 0 | 0 | 0 | 0 | 0 | 2 | 0 | 0 | 0 | 0 | 0 | 0 | 0 | 0 | 212 | 218 |  |  | PEAKS DB |
| R.LVILGFPC(+57.02)NQF.G | N | 49.68 | 1306.6743 | 11 | 0.4 | 654.3447 | 2 | 101.88 | 6 | F6:72830 | NaSa01\_F14.raw |  |  |  |  | 5.1302E6 | 2.3489E7 |  |  |  |  |  |  |  |  | 2 | 0 | 0 | 0 | 0 | 1 | 1 | 0 | 0 | 0 | 0 | 0 | 0 | 0 | 0 | 137 | 147 | Carbamidomethylation | C8:Carbamidomethylation:1000.00 | PEAKS DB |
| K.FLVNPQGKP.V | N | 41.99 | 998.5549 | 9 | -0.1 | 500.2847 | 2 | 25.59 | 12 | F12:11599 | NaSa01\_F7.raw |  |  |  |  |  |  |  |  |  |  |  | 8.4827E5 |  |  | 1 | 0 | 0 | 0 | 0 | 0 | 0 | 0 | 0 | 0 | 0 | 0 | 1 | 0 | 0 | 229 | 237 |  |  | PEAKS DB |
| K.FLVNPQGK.P | N | 41.78 | 901.5021 | 8 | 0.4 | 451.7585 | 2 | 11.58 | 6 | F6:1950 | NaSa01\_F14.raw |  |  |  |  |  | 1.0259E7 |  |  |  |  |  |  |  |  | 1 | 0 | 0 | 0 | 0 | 0 | 1 | 0 | 0 | 0 | 0 | 0 | 0 | 0 | 0 | 229 | 236 |  |  | PEAKS DB |
| R.LVILGFPC(+57.02)NQFGKQEPGQNSEILQGIK.H | N | 40.83 | 3013.5693 | 27 | 0.5 | 1005.5309 | 3 | 75.74 | 6 | F6:51193 | NaSa01\_F14.raw |  |  |  |  |  | 3.8526E6 |  |  |  |  |  |  |  |  | 1 | 0 | 0 | 0 | 0 | 0 | 1 | 0 | 0 | 0 | 0 | 0 | 0 | 0 | 0 | 137 | 163 | Carbamidomethylation | C8:Carbamidomethylation:1000.00 | PEAKS DB |
| K.VDC(+57.02)YDSVK.G | N | 38.28 | 984.4222 | 8 | 0.4 | 493.2186 | 2 | 11.46 | 6 | F6:1906 | NaSa01\_F14.raw |  |  |  |  |  | 8.6822E4 |  |  |  |  |  |  |  |  | 1 | 0 | 0 | 0 | 0 | 0 | 1 | 0 | 0 | 0 | 0 | 0 | 0 | 0 | 0 | 104 | 111 | Carbamidomethylation | C3:Carbamidomethylation:1000.00 | PEAKS DB |
| total 16 peptides |  |  |  |  |  |  |  |  |  |  |  |  |  |  |  |  |  |  |  |  |  |  |  |  |  |  |  |  |  |  |  |  |  |  |  |  |  |  |  |  |  |  |  |  |  |
| --- | --- | --- | --- | --- | --- | --- | --- | --- | --- | --- | --- | --- | --- | --- | --- | --- | --- | --- | --- | --- | --- | --- | --- | --- | --- | --- | --- | --- | --- | --- | --- | --- | --- | --- | --- | --- | --- | --- | --- | --- | --- | --- | --- | --- | --- |

P49122.1

back to list

  

| Protein Coverage
| Supporting Peptides
|

Protein Coverage:

Supporting Peptides:

| Peptide | Uniq | -10lgP | Mass | Length | ppm | m/z | z | RT | Fraction | Scan | Source File | Area F1 | Area F10 | Area F11 | Area F12 | Area F13 | Area F14 | Area F2 | Area F3 | Area F4 | Area F5 | Area F6 | Area F7 | Area F8 | Area F9 | #Feature | #Feature F1 | #Feature F10 | #Feature F11 | #Feature F12 | #Feature F13 | #Feature F14 | #Feature F2 | #Feature F3 | #Feature F4 | #Feature F5 | #Feature F6 | #Feature F7 | #Feature F8 | #Feature F9 | Start | End | PTM | AScore | Found By |
| --- | --- | --- | --- | --- | --- | --- | --- | --- | --- | --- | --- | --- | --- | --- | --- | --- | --- | --- | --- | --- | --- | --- | --- | --- | --- | --- | --- | --- | --- | --- | --- | --- | --- | --- | --- | --- | --- | --- | --- | --- | --- | --- | --- | --- | --- |
| K.C(+57.02)HNTQLPFIYNTC(+57.02)PEGK.N | Y | 97.57 | 2077.9351 | 17 | 0.3 | 1039.9751 | 2 | 41.17 | 14 | F14:20558 | NaSa01\_F9.raw |  | 3.0385E8 | 5.4446E9 | 1.8138E8 |  |  |  | 1.3973E6 | 1.8487E6 | 8.7169E6 | 6.8957E6 | 1.7246E7 | 1.87E7 | 8.7712E7 | 39 | 0 | 4 | 15 | 6 | 0 | 0 | 0 | 1 | 1 | 2 | 2 | 3 | 2 | 3 | 24 | 40 | Carbamidomethylation | C1:Carbamidomethylation:1000.00;C13:Carbamidomethylation:1000.00 | PEAKS DB |
| N.TQLPFIYNTC(+57.02)PEGK.N | Y | 64.68 | 1666.8025 | 14 | 0.8 | 834.4092 | 2 | 57.47 | 3 | F3:32437 | NaSa01\_F11.raw |  |  | 3.3356E7 | 3.534E6 |  |  |  | 2.8987E5 |  |  |  | 3.3921E5 |  |  | 4 | 0 | 0 | 1 | 1 | 0 | 0 | 0 | 1 | 0 | 0 | 0 | 1 | 0 | 0 | 27 | 40 | Carbamidomethylation | C10:Carbamidomethylation:1000.00 | PEAKS DB |
| H.NTQLPFIYNTC(+57.02)PEGK.N | Y | 63.52 | 1780.8453 | 15 | 1.6 | 891.4313 | 2 | 57.41 | 3 | F3:32438 | NaSa01\_F11.raw |  |  | 4.2403E6 |  |  |  |  |  |  |  |  |  |  |  | 1 | 0 | 0 | 1 | 0 | 0 | 0 | 0 | 0 | 0 | 0 | 0 | 0 | 0 | 0 | 26 | 40 | Carbamidomethylation | C11:Carbamidomethylation:1000.00 | PEAKS DB |
| T.QLPFIYNTC(+57.02)PEGK.N | Y | 63.16 | 1565.7548 | 13 | 5.6 | 783.8849 | 2 | 54.13 | 8 | F8:34354 | NaSa01\_F3.raw |  |  |  |  |  |  |  | 1.2778E6 | 3.044E5 |  |  |  |  |  | 2 | 0 | 0 | 0 | 0 | 0 | 0 | 0 | 1 | 1 | 0 | 0 | 0 | 0 | 0 | 28 | 40 | Carbamidomethylation | C9:Carbamidomethylation:1000.00 | PEAKS DB |
| Q.LPFIYNTC(+57.02)PEGK.N | Y | 60.06 | 1437.6962 | 12 | 0.7 | 719.8559 | 2 | 50.71 | 3 | F3:27298 | NaSa01\_F11.raw |  |  | 0 |  |  |  |  |  |  |  |  |  |  |  | 0 | 0 | 0 | 0 | 0 | 0 | 0 | 0 | 0 | 0 | 0 | 0 | 0 | 0 | 0 | 29 | 40 | Carbamidomethylation | C8:Carbamidomethylation:1000.00 | PEAKS DB |
| K.FPLKFPVK.R | N | 57.61 | 974.5953 | 8 | -0.6 | 488.3046 | 2 | 37.96 | 14 | F14:17689 | NaSa01\_F9.raw |  | 1.5411E8 |  | 1.0715E8 |  |  |  |  |  |  | 6.1047E6 | 4.2227E6 | 1.2449E7 | 2.4646E7 | 7 | 0 | 1 | 0 | 1 | 0 | 0 | 0 | 0 | 0 | 0 | 1 | 1 | 2 | 1 | 50 | 57 |  |  | PEAKS DB |
| K.C(+57.02)HNTQLPFIYN.T | Y | 55.76 | 1405.6449 | 11 | -0.2 | 703.8296 | 2 | 54.03 | 3 | F3:29508 | NaSa01\_F11.raw |  |  | 2.153E7 |  |  |  |  |  |  |  |  |  |  |  | 1 | 0 | 0 | 1 | 0 | 0 | 0 | 0 | 0 | 0 | 0 | 0 | 0 | 0 | 0 | 24 | 34 | Carbamidomethylation | C1:Carbamidomethylation:1000.00 | PEAKS DB |
| T.LKC(+57.02)HNTQLPFIYNTC(+57.02)PEGK.N | Y | 53.75 | 2319.1140 | 19 | 0.7 | 774.0458 | 3 | 33.41 | 3 | F3:14752 | NaSa01\_F11.raw |  |  | 5.1693E6 | 5.6635E6 |  |  |  |  |  |  |  |  |  |  | 4 | 0 | 0 | 2 | 2 | 0 | 0 | 0 | 0 | 0 | 0 | 0 | 0 | 0 | 0 | 22 | 40 | Carbamidomethylation | C3:Carbamidomethylation:1000.00;C15:Carbamidomethylation:1000.00 | PEAKS DB |
| K.ATLKFPLKFPVK.R | Y | 52.34 | 1387.8591 | 12 | -0.3 | 463.6268 | 3 | 43.64 | 3 | F3:23188 | NaSa01\_F11.raw |  |  | 5.6749E6 | 0 |  |  |  |  |  |  |  |  |  |  | 1 | 0 | 0 | 1 | 0 | 0 | 0 | 0 | 0 | 0 | 0 | 0 | 0 | 0 | 0 | 46 | 57 |  |  | PEAKS DB |
| K.ATLKFPLK.F | Y | 50.67 | 916.5745 | 8 | -0.6 | 459.2943 | 2 | 23.30 | 14 | F14:6238 | NaSa01\_F9.raw |  |  |  |  |  |  |  |  |  |  |  |  |  | 4.7482E5 | 1 | 0 | 0 | 0 | 0 | 0 | 0 | 0 | 0 | 0 | 0 | 0 | 0 | 0 | 1 | 46 | 53 |  |  | PEAKS DB |
| L.KC(+57.02)HNTQLPFIYNTC(+57.02)PEGK.N | Y | 49.65 | 2206.0300 | 18 | 1.0 | 552.5153 | 4 | 28.38 | 12 | F12:13814 | NaSa01\_F7.raw |  |  |  |  |  |  |  | 3.9773E5 |  |  |  | 2.8597E6 |  |  | 3 | 0 | 0 | 0 | 0 | 0 | 0 | 0 | 1 | 0 | 0 | 0 | 2 | 0 | 0 | 23 | 40 | Carbamidomethylation | C2:Carbamidomethylation:1000.00;C14:Carbamidomethylation:1000.00 | PEAKS DB |
| C.HNTQLPFIYNTC(+57.02)PEGK.N | Y | 49.15 | 1917.9043 | 16 | 0.5 | 959.9599 | 2 | 41.40 | 3 | F3:21100 | NaSa01\_F11.raw |  |  | 0 |  |  |  |  |  |  |  |  |  |  |  | 0 | 0 | 0 | 0 | 0 | 0 | 0 | 0 | 0 | 0 | 0 | 0 | 0 | 0 | 0 | 25 | 40 | Carbamidomethylation | C12:Carbamidomethylation:1000.00 | PEAKS DB |
| K.FPLKFPVKR.G | N | 47.73 | 1130.6964 | 9 | 0.8 | 377.9064 | 3 | 24.46 | 3 | F3:8053 | NaSa01\_F11.raw |  |  | 2.7756E6 | 5.2434E6 |  |  |  |  |  |  |  |  |  |  | 3 | 0 | 0 | 1 | 2 | 0 | 0 | 0 | 0 | 0 | 0 | 0 | 0 | 0 | 0 | 50 | 58 |  |  | PEAKS DB |
| K.C(+57.02)HNTQLPFIY.N | N | 45.87 | 1291.6019 | 10 | -0.9 | 646.8077 | 2 | 58.35 | 14 | F14:34645 | NaSa01\_F9.raw |  |  | 1.2654E7 |  |  |  |  |  |  |  |  |  |  | 7.448E7 | 2 | 0 | 0 | 1 | 0 | 0 | 0 | 0 | 0 | 0 | 0 | 0 | 0 | 0 | 1 | 24 | 33 | Carbamidomethylation | C1:Carbamidomethylation:1000.00 | PEAKS DB |
| N.TC(+57.02)PEGKNLC(+57.02)FK.A | N | 45.83 | 1352.6217 | 11 | -0.4 | 451.8810 | 3 | 11.40 | 14 | F14:1892 | NaSa01\_F9.raw |  |  |  |  |  |  |  |  |  |  |  |  |  | 1.3943E5 | 1 | 0 | 0 | 0 | 0 | 0 | 0 | 0 | 0 | 0 | 0 | 0 | 0 | 0 | 1 | 35 | 45 | Carbamidomethylation | C2:Carbamidomethylation:1000.00;C9:Carbamidomethylation:1000.00 | PEAKS DB |
| K.NLC(+57.02)FKATLK.F | N | 44.60 | 1093.5953 | 9 | -0.2 | 547.8048 | 2 | 21.04 | 14 | F14:4634 | NaSa01\_F9.raw |  |  |  |  |  |  |  |  |  |  |  |  |  | 7.6054E5 | 1 | 0 | 0 | 0 | 0 | 0 | 0 | 0 | 0 | 0 | 0 | 0 | 0 | 0 | 1 | 41 | 49 | Carbamidomethylation | C3:Carbamidomethylation:1000.00 | PEAKS DB |
| K.C(+57.02)HNTQLPF.I | N | 40.89 | 1015.4545 | 8 | -2.2 | 508.7334 | 2 | 32.86 | 14 | F14:13929 | NaSa01\_F9.raw |  |  |  |  |  |  |  |  |  |  |  |  |  | 1.6001E8 | 2 | 0 | 0 | 0 | 0 | 0 | 0 | 0 | 0 | 0 | 0 | 0 | 0 | 0 | 2 | 24 | 31 | Carbamidomethylation | C1:Carbamidomethylation:1000.00 | PEAKS DB |
| F.IYNTC(+57.02)PEGK.N | Y | 39.81 | 1080.4910 | 9 | 0.5 | 541.2531 | 2 | 11.63 | 3 | F3:3328 | NaSa01\_F11.raw |  |  | 7.0144E5 |  |  |  |  |  |  |  |  |  |  |  | 1 | 0 | 0 | 1 | 0 | 0 | 0 | 0 | 0 | 0 | 0 | 0 | 0 | 0 | 0 | 32 | 40 | Carbamidomethylation | C5:Carbamidomethylation:1000.00 | PEAKS DB |
| total 18 peptides |  |  |  |  |  |  |  |  |  |  |  |  |  |  |  |  |  |  |  |  |  |  |  |  |  |  |  |  |  |  |  |  |  |  |  |  |  |  |  |  |  |  |  |  |  |
| --- | --- | --- | --- | --- | --- | --- | --- | --- | --- | --- | --- | --- | --- | --- | --- | --- | --- | --- | --- | --- | --- | --- | --- | --- | --- | --- | --- | --- | --- | --- | --- | --- | --- | --- | --- | --- | --- | --- | --- | --- | --- | --- | --- | --- | --- |

CAA90967.1

back to list

  

| Protein Coverage
| Supporting Peptides
|

Protein Coverage:

Supporting Peptides:

| Peptide | Uniq | -10lgP | Mass | Length | ppm | m/z | z | RT | Fraction | Scan | Source File | Area F1 | Area F10 | Area F11 | Area F12 | Area F13 | Area F14 | Area F2 | Area F3 | Area F4 | Area F5 | Area F6 | Area F7 | Area F8 | Area F9 | #Feature | #Feature F1 | #Feature F10 | #Feature F11 | #Feature F12 | #Feature F13 | #Feature F14 | #Feature F2 | #Feature F3 | #Feature F4 | #Feature F5 | #Feature F6 | #Feature F7 | #Feature F8 | #Feature F9 | Start | End | PTM | AScore | Found By |
| --- | --- | --- | --- | --- | --- | --- | --- | --- | --- | --- | --- | --- | --- | --- | --- | --- | --- | --- | --- | --- | --- | --- | --- | --- | --- | --- | --- | --- | --- | --- | --- | --- | --- | --- | --- | --- | --- | --- | --- | --- | --- | --- | --- | --- | --- |
| K.C(+57.02)HNTQLPFIYNTC(+57.02)PEGK.N | Y | 97.57 | 2077.9351 | 17 | 0.3 | 1039.9751 | 2 | 41.17 | 14 | F14:20558 | NaSa01\_F9.raw |  | 3.0385E8 | 5.4446E9 | 1.8138E8 |  |  |  | 1.3973E6 | 1.8487E6 | 8.7169E6 | 6.8957E6 | 1.7246E7 | 1.87E7 | 8.7712E7 | 39 | 0 | 4 | 15 | 6 | 0 | 0 | 0 | 1 | 1 | 2 | 2 | 3 | 2 | 3 | 24 | 40 | Carbamidomethylation | C1:Carbamidomethylation:1000.00;C13:Carbamidomethylation:1000.00 | PEAKS DB |
| N.TQLPFIYNTC(+57.02)PEGK.N | Y | 64.68 | 1666.8025 | 14 | 0.8 | 834.4092 | 2 | 57.47 | 3 | F3:32437 | NaSa01\_F11.raw |  |  | 3.3356E7 | 3.534E6 |  |  |  | 2.8987E5 |  |  |  | 3.3921E5 |  |  | 4 | 0 | 0 | 1 | 1 | 0 | 0 | 0 | 1 | 0 | 0 | 0 | 1 | 0 | 0 | 27 | 40 | Carbamidomethylation | C10:Carbamidomethylation:1000.00 | PEAKS DB |
| H.NTQLPFIYNTC(+57.02)PEGK.N | Y | 63.52 | 1780.8453 | 15 | 1.6 | 891.4313 | 2 | 57.41 | 3 | F3:32438 | NaSa01\_F11.raw |  |  | 4.2403E6 |  |  |  |  |  |  |  |  |  |  |  | 1 | 0 | 0 | 1 | 0 | 0 | 0 | 0 | 0 | 0 | 0 | 0 | 0 | 0 | 0 | 26 | 40 | Carbamidomethylation | C11:Carbamidomethylation:1000.00 | PEAKS DB |
| T.QLPFIYNTC(+57.02)PEGK.N | Y | 63.16 | 1565.7548 | 13 | 5.6 | 783.8849 | 2 | 54.13 | 8 | F8:34354 | NaSa01\_F3.raw |  |  |  |  |  |  |  | 1.2778E6 | 3.044E5 |  |  |  |  |  | 2 | 0 | 0 | 0 | 0 | 0 | 0 | 0 | 1 | 1 | 0 | 0 | 0 | 0 | 0 | 28 | 40 | Carbamidomethylation | C9:Carbamidomethylation:1000.00 | PEAKS DB |
| Q.LPFIYNTC(+57.02)PEGK.N | Y | 60.06 | 1437.6962 | 12 | 0.7 | 719.8559 | 2 | 50.71 | 3 | F3:27298 | NaSa01\_F11.raw |  |  | 0 |  |  |  |  |  |  |  |  |  |  |  | 0 | 0 | 0 | 0 | 0 | 0 | 0 | 0 | 0 | 0 | 0 | 0 | 0 | 0 | 0 | 29 | 40 | Carbamidomethylation | C8:Carbamidomethylation:1000.00 | PEAKS DB |
| K.FPLKFPVK.R | N | 57.61 | 974.5953 | 8 | -0.6 | 488.3046 | 2 | 37.96 | 14 | F14:17689 | NaSa01\_F9.raw |  | 1.5411E8 |  | 1.0715E8 |  |  |  |  |  |  | 6.1047E6 | 4.2227E6 | 1.2449E7 | 2.4646E7 | 7 | 0 | 1 | 0 | 1 | 0 | 0 | 0 | 0 | 0 | 0 | 1 | 1 | 2 | 1 | 50 | 57 |  |  | PEAKS DB |
| K.C(+57.02)HNTQLPFIYN.T | Y | 55.76 | 1405.6449 | 11 | -0.2 | 703.8296 | 2 | 54.03 | 3 | F3:29508 | NaSa01\_F11.raw |  |  | 2.153E7 |  |  |  |  |  |  |  |  |  |  |  | 1 | 0 | 0 | 1 | 0 | 0 | 0 | 0 | 0 | 0 | 0 | 0 | 0 | 0 | 0 | 24 | 34 | Carbamidomethylation | C1:Carbamidomethylation:1000.00 | PEAKS DB |
| T.LKC(+57.02)HNTQLPFIYNTC(+57.02)PEGK.N | Y | 53.75 | 2319.1140 | 19 | 0.7 | 774.0458 | 3 | 33.41 | 3 | F3:14752 | NaSa01\_F11.raw |  |  | 5.1693E6 | 5.6635E6 |  |  |  |  |  |  |  |  |  |  | 4 | 0 | 0 | 2 | 2 | 0 | 0 | 0 | 0 | 0 | 0 | 0 | 0 | 0 | 0 | 22 | 40 | Carbamidomethylation | C3:Carbamidomethylation:1000.00;C15:Carbamidomethylation:1000.00 | PEAKS DB |
| K.ATLKFPLKFPVK.R | Y | 52.34 | 1387.8591 | 12 | -0.3 | 463.6268 | 3 | 43.64 | 3 | F3:23188 | NaSa01\_F11.raw |  |  | 5.6749E6 | 0 |  |  |  |  |  |  |  |  |  |  | 1 | 0 | 0 | 1 | 0 | 0 | 0 | 0 | 0 | 0 | 0 | 0 | 0 | 0 | 0 | 46 | 57 |  |  | PEAKS DB |
| K.ATLKFPLK.F | Y | 50.67 | 916.5745 | 8 | -0.6 | 459.2943 | 2 | 23.30 | 14 | F14:6238 | NaSa01\_F9.raw |  |  |  |  |  |  |  |  |  |  |  |  |  | 4.7482E5 | 1 | 0 | 0 | 0 | 0 | 0 | 0 | 0 | 0 | 0 | 0 | 0 | 0 | 0 | 1 | 46 | 53 |  |  | PEAKS DB |
| L.KC(+57.02)HNTQLPFIYNTC(+57.02)PEGK.N | Y | 49.65 | 2206.0300 | 18 | 1.0 | 552.5153 | 4 | 28.38 | 12 | F12:13814 | NaSa01\_F7.raw |  |  |  |  |  |  |  | 3.9773E5 |  |  |  | 2.8597E6 |  |  | 3 | 0 | 0 | 0 | 0 | 0 | 0 | 0 | 1 | 0 | 0 | 0 | 2 | 0 | 0 | 23 | 40 | Carbamidomethylation | C2:Carbamidomethylation:1000.00;C14:Carbamidomethylation:1000.00 | PEAKS DB |
| C.HNTQLPFIYNTC(+57.02)PEGK.N | Y | 49.15 | 1917.9043 | 16 | 0.5 | 959.9599 | 2 | 41.40 | 3 | F3:21100 | NaSa01\_F11.raw |  |  | 0 |  |  |  |  |  |  |  |  |  |  |  | 0 | 0 | 0 | 0 | 0 | 0 | 0 | 0 | 0 | 0 | 0 | 0 | 0 | 0 | 0 | 25 | 40 | Carbamidomethylation | C12:Carbamidomethylation:1000.00 | PEAKS DB |
| K.FPLKFPVKR.G | N | 47.73 | 1130.6964 | 9 | 0.8 | 377.9064 | 3 | 24.46 | 3 | F3:8053 | NaSa01\_F11.raw |  |  | 2.7756E6 | 5.2434E6 |  |  |  |  |  |  |  |  |  |  | 3 | 0 | 0 | 1 | 2 | 0 | 0 | 0 | 0 | 0 | 0 | 0 | 0 | 0 | 0 | 50 | 58 |  |  | PEAKS DB |
| K.C(+57.02)HNTQLPFIY.N | N | 45.87 | 1291.6019 | 10 | -0.9 | 646.8077 | 2 | 58.35 | 14 | F14:34645 | NaSa01\_F9.raw |  |  | 1.2654E7 |  |  |  |  |  |  |  |  |  |  | 7.448E7 | 2 | 0 | 0 | 1 | 0 | 0 | 0 | 0 | 0 | 0 | 0 | 0 | 0 | 0 | 1 | 24 | 33 | Carbamidomethylation | C1:Carbamidomethylation:1000.00 | PEAKS DB |
| N.TC(+57.02)PEGKNLC(+57.02)FK.A | N | 45.83 | 1352.6217 | 11 | -0.4 | 451.8810 | 3 | 11.40 | 14 | F14:1892 | NaSa01\_F9.raw |  |  |  |  |  |  |  |  |  |  |  |  |  | 1.3943E5 | 1 | 0 | 0 | 0 | 0 | 0 | 0 | 0 | 0 | 0 | 0 | 0 | 0 | 0 | 1 | 35 | 45 | Carbamidomethylation | C2:Carbamidomethylation:1000.00;C9:Carbamidomethylation:1000.00 | PEAKS DB |
| K.NLC(+57.02)FKATLK.F | N | 44.60 | 1093.5953 | 9 | -0.2 | 547.8048 | 2 | 21.04 | 14 | F14:4634 | NaSa01\_F9.raw |  |  |  |  |  |  |  |  |  |  |  |  |  | 7.6054E5 | 1 | 0 | 0 | 0 | 0 | 0 | 0 | 0 | 0 | 0 | 0 | 0 | 0 | 0 | 1 | 41 | 49 | Carbamidomethylation | C3:Carbamidomethylation:1000.00 | PEAKS DB |
| K.C(+57.02)HNTQLPF.I | N | 40.89 | 1015.4545 | 8 | -2.2 | 508.7334 | 2 | 32.86 | 14 | F14:13929 | NaSa01\_F9.raw |  |  |  |  |  |  |  |  |  |  |  |  |  | 1.6001E8 | 2 | 0 | 0 | 0 | 0 | 0 | 0 | 0 | 0 | 0 | 0 | 0 | 0 | 0 | 2 | 24 | 31 | Carbamidomethylation | C1:Carbamidomethylation:1000.00 | PEAKS DB |
| F.IYNTC(+57.02)PEGK.N | Y | 39.81 | 1080.4910 | 9 | 0.5 | 541.2531 | 2 | 11.63 | 3 | F3:3328 | NaSa01\_F11.raw |  |  | 7.0144E5 |  |  |  |  |  |  |  |  |  |  |  | 1 | 0 | 0 | 1 | 0 | 0 | 0 | 0 | 0 | 0 | 0 | 0 | 0 | 0 | 0 | 32 | 40 | Carbamidomethylation | C5:Carbamidomethylation:1000.00 | PEAKS DB |
| total 18 peptides |  |  |  |  |  |  |  |  |  |  |  |  |  |  |  |  |  |  |  |  |  |  |  |  |  |  |  |  |  |  |  |  |  |  |  |  |  |  |  |  |  |  |  |  |  |
| --- | --- | --- | --- | --- | --- | --- | --- | --- | --- | --- | --- | --- | --- | --- | --- | --- | --- | --- | --- | --- | --- | --- | --- | --- | --- | --- | --- | --- | --- | --- | --- | --- | --- | --- | --- | --- | --- | --- | --- | --- | --- | --- | --- | --- | --- |

CAA77017.1

back to list

  

| Protein Coverage
| Supporting Peptides
|

Protein Coverage:

Supporting Peptides:

| Peptide | Uniq | -10lgP | Mass | Length | ppm | m/z | z | RT | Fraction | Scan | Source File | Area F1 | Area F10 | Area F11 | Area F12 | Area F13 | Area F14 | Area F2 | Area F3 | Area F4 | Area F5 | Area F6 | Area F7 | Area F8 | Area F9 | #Feature | #Feature F1 | #Feature F10 | #Feature F11 | #Feature F12 | #Feature F13 | #Feature F14 | #Feature F2 | #Feature F3 | #Feature F4 | #Feature F5 | #Feature F6 | #Feature F7 | #Feature F8 | #Feature F9 | Start | End | PTM | AScore | Found By |
| --- | --- | --- | --- | --- | --- | --- | --- | --- | --- | --- | --- | --- | --- | --- | --- | --- | --- | --- | --- | --- | --- | --- | --- | --- | --- | --- | --- | --- | --- | --- | --- | --- | --- | --- | --- | --- | --- | --- | --- | --- | --- | --- | --- | --- | --- |
| K.C(+57.02)HNTQLPFIYNTC(+57.02)PEGK.N | Y | 97.57 | 2077.9351 | 17 | 0.3 | 1039.9751 | 2 | 41.17 | 14 | F14:20558 | NaSa01\_F9.raw |  | 3.0385E8 | 5.4446E9 | 1.8138E8 |  |  |  | 1.3973E6 | 1.8487E6 | 8.7169E6 | 6.8957E6 | 1.7246E7 | 1.87E7 | 8.7712E7 | 39 | 0 | 4 | 15 | 6 | 0 | 0 | 0 | 1 | 1 | 2 | 2 | 3 | 2 | 3 | 24 | 40 | Carbamidomethylation | C1:Carbamidomethylation:1000.00;C13:Carbamidomethylation:1000.00 | PEAKS DB |
| N.TQLPFIYNTC(+57.02)PEGK.N | Y | 64.68 | 1666.8025 | 14 | 0.8 | 834.4092 | 2 | 57.47 | 3 | F3:32437 | NaSa01\_F11.raw |  |  | 3.3356E7 | 3.534E6 |  |  |  | 2.8987E5 |  |  |  | 3.3921E5 |  |  | 4 | 0 | 0 | 1 | 1 | 0 | 0 | 0 | 1 | 0 | 0 | 0 | 1 | 0 | 0 | 27 | 40 | Carbamidomethylation | C10:Carbamidomethylation:1000.00 | PEAKS DB |
| H.NTQLPFIYNTC(+57.02)PEGK.N | Y | 63.52 | 1780.8453 | 15 | 1.6 | 891.4313 | 2 | 57.41 | 3 | F3:32438 | NaSa01\_F11.raw |  |  | 4.2403E6 |  |  |  |  |  |  |  |  |  |  |  | 1 | 0 | 0 | 1 | 0 | 0 | 0 | 0 | 0 | 0 | 0 | 0 | 0 | 0 | 0 | 26 | 40 | Carbamidomethylation | C11:Carbamidomethylation:1000.00 | PEAKS DB |
| T.QLPFIYNTC(+57.02)PEGK.N | Y | 63.16 | 1565.7548 | 13 | 5.6 | 783.8849 | 2 | 54.13 | 8 | F8:34354 | NaSa01\_F3.raw |  |  |  |  |  |  |  | 1.2778E6 | 3.044E5 |  |  |  |  |  | 2 | 0 | 0 | 0 | 0 | 0 | 0 | 0 | 1 | 1 | 0 | 0 | 0 | 0 | 0 | 28 | 40 | Carbamidomethylation | C9:Carbamidomethylation:1000.00 | PEAKS DB |
| Q.LPFIYNTC(+57.02)PEGK.N | Y | 60.06 | 1437.6962 | 12 | 0.7 | 719.8559 | 2 | 50.71 | 3 | F3:27298 | NaSa01\_F11.raw |  |  | 0 |  |  |  |  |  |  |  |  |  |  |  | 0 | 0 | 0 | 0 | 0 | 0 | 0 | 0 | 0 | 0 | 0 | 0 | 0 | 0 | 0 | 29 | 40 | Carbamidomethylation | C8:Carbamidomethylation:1000.00 | PEAKS DB |
| K.FPLKFPVK.R | N | 57.61 | 974.5953 | 8 | -0.6 | 488.3046 | 2 | 37.96 | 14 | F14:17689 | NaSa01\_F9.raw |  | 1.5411E8 |  | 1.0715E8 |  |  |  |  |  |  | 6.1047E6 | 4.2227E6 | 1.2449E7 | 2.4646E7 | 7 | 0 | 1 | 0 | 1 | 0 | 0 | 0 | 0 | 0 | 0 | 1 | 1 | 2 | 1 | 50 | 57 |  |  | PEAKS DB |
| K.C(+57.02)HNTQLPFIYN.T | Y | 55.76 | 1405.6449 | 11 | -0.2 | 703.8296 | 2 | 54.03 | 3 | F3:29508 | NaSa01\_F11.raw |  |  | 2.153E7 |  |  |  |  |  |  |  |  |  |  |  | 1 | 0 | 0 | 1 | 0 | 0 | 0 | 0 | 0 | 0 | 0 | 0 | 0 | 0 | 0 | 24 | 34 | Carbamidomethylation | C1:Carbamidomethylation:1000.00 | PEAKS DB |
| T.LKC(+57.02)HNTQLPFIYNTC(+57.02)PEGK.N | Y | 53.75 | 2319.1140 | 19 | 0.7 | 774.0458 | 3 | 33.41 | 3 | F3:14752 | NaSa01\_F11.raw |  |  | 5.1693E6 | 5.6635E6 |  |  |  |  |  |  |  |  |  |  | 4 | 0 | 0 | 2 | 2 | 0 | 0 | 0 | 0 | 0 | 0 | 0 | 0 | 0 | 0 | 22 | 40 | Carbamidomethylation | C3:Carbamidomethylation:1000.00;C15:Carbamidomethylation:1000.00 | PEAKS DB |
| K.ATLKFPLKFPVK.R | Y | 52.34 | 1387.8591 | 12 | -0.3 | 463.6268 | 3 | 43.64 | 3 | F3:23188 | NaSa01\_F11.raw |  |  | 5.6749E6 | 0 |  |  |  |  |  |  |  |  |  |  | 1 | 0 | 0 | 1 | 0 | 0 | 0 | 0 | 0 | 0 | 0 | 0 | 0 | 0 | 0 | 46 | 57 |  |  | PEAKS DB |
| K.ATLKFPLK.F | Y | 50.67 | 916.5745 | 8 | -0.6 | 459.2943 | 2 | 23.30 | 14 | F14:6238 | NaSa01\_F9.raw |  |  |  |  |  |  |  |  |  |  |  |  |  | 4.7482E5 | 1 | 0 | 0 | 0 | 0 | 0 | 0 | 0 | 0 | 0 | 0 | 0 | 0 | 0 | 1 | 46 | 53 |  |  | PEAKS DB |
| L.KC(+57.02)HNTQLPFIYNTC(+57.02)PEGK.N | Y | 49.65 | 2206.0300 | 18 | 1.0 | 552.5153 | 4 | 28.38 | 12 | F12:13814 | NaSa01\_F7.raw |  |  |  |  |  |  |  | 3.9773E5 |  |  |  | 2.8597E6 |  |  | 3 | 0 | 0 | 0 | 0 | 0 | 0 | 0 | 1 | 0 | 0 | 0 | 2 | 0 | 0 | 23 | 40 | Carbamidomethylation | C2:Carbamidomethylation:1000.00;C14:Carbamidomethylation:1000.00 | PEAKS DB |
| C.HNTQLPFIYNTC(+57.02)PEGK.N | Y | 49.15 | 1917.9043 | 16 | 0.5 | 959.9599 | 2 | 41.40 | 3 | F3:21100 | NaSa01\_F11.raw |  |  | 0 |  |  |  |  |  |  |  |  |  |  |  | 0 | 0 | 0 | 0 | 0 | 0 | 0 | 0 | 0 | 0 | 0 | 0 | 0 | 0 | 0 | 25 | 40 | Carbamidomethylation | C12:Carbamidomethylation:1000.00 | PEAKS DB |
| K.FPLKFPVKR.G | N | 47.73 | 1130.6964 | 9 | 0.8 | 377.9064 | 3 | 24.46 | 3 | F3:8053 | NaSa01\_F11.raw |  |  | 2.7756E6 | 5.2434E6 |  |  |  |  |  |  |  |  |  |  | 3 | 0 | 0 | 1 | 2 | 0 | 0 | 0 | 0 | 0 | 0 | 0 | 0 | 0 | 0 | 50 | 58 |  |  | PEAKS DB |
| K.C(+57.02)HNTQLPFIY.N | N | 45.87 | 1291.6019 | 10 | -0.9 | 646.8077 | 2 | 58.35 | 14 | F14:34645 | NaSa01\_F9.raw |  |  | 1.2654E7 |  |  |  |  |  |  |  |  |  |  | 7.448E7 | 2 | 0 | 0 | 1 | 0 | 0 | 0 | 0 | 0 | 0 | 0 | 0 | 0 | 0 | 1 | 24 | 33 | Carbamidomethylation | C1:Carbamidomethylation:1000.00 | PEAKS DB |
| N.TC(+57.02)PEGKNLC(+57.02)FK.A | N | 45.83 | 1352.6217 | 11 | -0.4 | 451.8810 | 3 | 11.40 | 14 | F14:1892 | NaSa01\_F9.raw |  |  |  |  |  |  |  |  |  |  |  |  |  | 1.3943E5 | 1 | 0 | 0 | 0 | 0 | 0 | 0 | 0 | 0 | 0 | 0 | 0 | 0 | 0 | 1 | 35 | 45 | Carbamidomethylation | C2:Carbamidomethylation:1000.00;C9:Carbamidomethylation:1000.00 | PEAKS DB |
| K.NLC(+57.02)FKATLK.F | N | 44.60 | 1093.5953 | 9 | -0.2 | 547.8048 | 2 | 21.04 | 14 | F14:4634 | NaSa01\_F9.raw |  |  |  |  |  |  |  |  |  |  |  |  |  | 7.6054E5 | 1 | 0 | 0 | 0 | 0 | 0 | 0 | 0 | 0 | 0 | 0 | 0 | 0 | 0 | 1 | 41 | 49 | Carbamidomethylation | C3:Carbamidomethylation:1000.00 | PEAKS DB |
| K.C(+57.02)HNTQLPF.I | N | 40.89 | 1015.4545 | 8 | -2.2 | 508.7334 | 2 | 32.86 | 14 | F14:13929 | NaSa01\_F9.raw |  |  |  |  |  |  |  |  |  |  |  |  |  | 1.6001E8 | 2 | 0 | 0 | 0 | 0 | 0 | 0 | 0 | 0 | 0 | 0 | 0 | 0 | 0 | 2 | 24 | 31 | Carbamidomethylation | C1:Carbamidomethylation:1000.00 | PEAKS DB |
| F.IYNTC(+57.02)PEGK.N | Y | 39.81 | 1080.4910 | 9 | 0.5 | 541.2531 | 2 | 11.63 | 3 | F3:3328 | NaSa01\_F11.raw |  |  | 7.0144E5 |  |  |  |  |  |  |  |  |  |  |  | 1 | 0 | 0 | 1 | 0 | 0 | 0 | 0 | 0 | 0 | 0 | 0 | 0 | 0 | 0 | 32 | 40 | Carbamidomethylation | C5:Carbamidomethylation:1000.00 | PEAKS DB |
| total 18 peptides |  |  |  |  |  |  |  |  |  |  |  |  |  |  |  |  |  |  |  |  |  |  |  |  |  |  |  |  |  |  |  |  |  |  |  |  |  |  |  |  |  |  |  |  |  |
| --- | --- | --- | --- | --- | --- | --- | --- | --- | --- | --- | --- | --- | --- | --- | --- | --- | --- | --- | --- | --- | --- | --- | --- | --- | --- | --- | --- | --- | --- | --- | --- | --- | --- | --- | --- | --- | --- | --- | --- | --- | --- | --- | --- | --- | --- |

XP\_026541908.1

back to list

  

| Protein Coverage
| Supporting Peptides
|

Protein Coverage:

Supporting Peptides:

| Peptide | Uniq | -10lgP | Mass | Length | ppm | m/z | z | RT | Fraction | Scan | Source File | Area F1 | Area F10 | Area F11 | Area F12 | Area F13 | Area F14 | Area F2 | Area F3 | Area F4 | Area F5 | Area F6 | Area F7 | Area F8 | Area F9 | #Feature | #Feature F1 | #Feature F10 | #Feature F11 | #Feature F12 | #Feature F13 | #Feature F14 | #Feature F2 | #Feature F3 | #Feature F4 | #Feature F5 | #Feature F6 | #Feature F7 | #Feature F8 | #Feature F9 | Start | End | PTM | AScore | Found By |
| --- | --- | --- | --- | --- | --- | --- | --- | --- | --- | --- | --- | --- | --- | --- | --- | --- | --- | --- | --- | --- | --- | --- | --- | --- | --- | --- | --- | --- | --- | --- | --- | --- | --- | --- | --- | --- | --- | --- | --- | --- | --- | --- | --- | --- | --- |
| R.LVILGFPC(+57.02)NQFGK.Q | N | 80.93 | 1491.7908 | 13 | 0.3 | 746.9029 | 2 | 74.37 | 14 | F14:48186 | NaSa01\_F9.raw |  | 6.3482E6 | 4.5719E6 | 6.7588E6 | 7.3547E8 | 2.7574E9 |  |  |  |  |  | 1.9765E6 | 7.8579E6 | 5.5032E7 | 12 | 0 | 1 | 1 | 1 | 3 | 3 | 0 | 0 | 0 | 0 | 0 | 1 | 1 | 1 | 124 | 136 | Carbamidomethylation | C8:Carbamidomethylation:1000.00 | PEAKS DB |
| K.QEPGQNSEILQGIK.Y | N | 80.47 | 1539.7893 | 14 | 0.1 | 770.9020 | 2 | 34.92 | 6 | F6:17025 | NaSa01\_F14.raw |  |  | 7.9374E4 | 4.1222E6 | 3.3468E8 | 1.3102E9 |  |  |  |  |  |  | 0 |  | 8 | 0 | 0 | 1 | 1 | 2 | 4 | 0 | 0 | 0 | 0 | 0 | 0 | 0 | 0 | 137 | 150 |  |  | PEAKS DB |
| R.PGGGYVPNFQLFQK.G | Y | 79.31 | 1550.7881 | 14 | 0.2 | 776.4015 | 2 | 68.00 | 6 | F6:44835 | NaSa01\_F14.raw |  |  |  | 1.3787E6 | 2.3027E8 | 1.1136E9 |  |  |  | 1.1813E7 |  | 5.021E7 |  |  | 14 | 0 | 0 | 0 | 1 | 3 | 7 | 0 | 0 | 0 | 1 | 0 | 2 | 0 | 0 | 154 | 167 |  |  | PEAKS DB |
| R.LFWSPLKIHDIK.W | N | 68.85 | 1495.8551 | 12 | 0.5 | 499.6259 | 3 | 54.14 | 6 | F6:32777 | NaSa01\_F14.raw |  |  |  |  |  | 4.8748E5 |  |  |  |  |  |  |  |  | 1 | 0 | 0 | 0 | 0 | 0 | 1 | 0 | 0 | 0 | 0 | 0 | 0 | 0 | 0 | 199 | 210 |  |  | PEAKS DB |
| K.WNFEKFLVNPQGK.P | N | 61.53 | 1605.8303 | 13 | 0.1 | 536.2841 | 3 | 64.13 | 6 | F6:41454 | NaSa01\_F14.raw |  |  |  |  |  | 3.4585E6 |  |  |  |  |  |  |  |  | 2 | 0 | 0 | 0 | 0 | 0 | 2 | 0 | 0 | 0 | 0 | 0 | 0 | 0 | 0 | 211 | 223 |  |  | PEAKS DB |
| L.VILGFPC(+57.02)NQFGK.Q | N | 58.48 | 1378.7067 | 12 | -0.1 | 690.3605 | 2 | 59.07 | 12 | F12:39406 | NaSa01\_F7.raw |  | 4.6143E6 | 1.4793E6 |  |  |  |  |  |  | 1.3101E7 | 1.8748E7 | 4.3606E7 | 7.4857E6 | 1.729E7 | 7 | 0 | 1 | 1 | 0 | 0 | 0 | 0 | 0 | 0 | 1 | 1 | 1 | 1 | 1 | 125 | 136 | Carbamidomethylation | C7:Carbamidomethylation:1000.00 | PEAKS DB |
| K.FLVNPQGKPVMR.W | N | 58.11 | 1384.7649 | 12 | -0.2 | 462.5955 | 3 | 25.87 | 5 | F5:9864 | NaSa01\_F13.raw |  |  |  |  | 9.037E6 |  |  |  |  |  |  |  |  |  | 2 | 0 | 0 | 0 | 0 | 2 | 0 | 0 | 0 | 0 | 0 | 0 | 0 | 0 | 0 | 216 | 227 |  |  | PEAKS DB |
| Y.VPNFQLFQK.G | N | 54.38 | 1119.6077 | 9 | -0.6 | 560.8108 | 2 | 54.08 | 6 | F6:32772 | NaSa01\_F14.raw |  |  |  |  | 1.0977E6 | 4.3013E6 |  |  |  |  |  |  |  |  | 2 | 0 | 0 | 0 | 0 | 1 | 1 | 0 | 0 | 0 | 0 | 0 | 0 | 0 | 0 | 159 | 167 |  |  | PEAKS DB |
| G.GGYVPNFQLFQK.G | Y | 52.92 | 1396.7139 | 12 | 0.1 | 699.3643 | 2 | 67.25 | 6 | F6:43947 | NaSa01\_F14.raw |  |  |  |  |  | 2.9962E6 |  |  |  |  |  |  |  |  | 1 | 0 | 0 | 0 | 0 | 0 | 1 | 0 | 0 | 0 | 0 | 0 | 0 | 0 | 0 | 156 | 167 |  |  | PEAKS DB |
| R.LFWSPLK.I | N | 50.63 | 889.5062 | 7 | -1.0 | 445.7599 | 2 | 61.33 | 6 | F6:38812 | NaSa01\_F14.raw |  |  |  |  |  | 1.0011E9 |  |  |  |  |  |  |  |  | 2 | 0 | 0 | 0 | 0 | 0 | 2 | 0 | 0 | 0 | 0 | 0 | 0 | 0 | 0 | 199 | 205 |  |  | PEAKS DB |
| R.LVILGFPC(+57.02)NQF.G | N | 49.68 | 1306.6743 | 11 | 0.4 | 654.3447 | 2 | 101.88 | 6 | F6:72830 | NaSa01\_F14.raw |  |  |  |  | 5.1302E6 | 2.3489E7 |  |  |  |  |  |  |  |  | 2 | 0 | 0 | 0 | 0 | 1 | 1 | 0 | 0 | 0 | 0 | 0 | 0 | 0 | 0 | 124 | 134 | Carbamidomethylation | C8:Carbamidomethylation:1000.00 | PEAKS DB |
| K.FLVNPQGKP.V | N | 41.99 | 998.5549 | 9 | -0.1 | 500.2847 | 2 | 25.59 | 12 | F12:11599 | NaSa01\_F7.raw |  |  |  |  |  |  |  |  |  |  |  | 8.4827E5 |  |  | 1 | 0 | 0 | 0 | 0 | 0 | 0 | 0 | 0 | 0 | 0 | 0 | 1 | 0 | 0 | 216 | 224 |  |  | PEAKS DB |
| K.FLVNPQGK.P | N | 41.78 | 901.5021 | 8 | 0.4 | 451.7585 | 2 | 11.58 | 6 | F6:1950 | NaSa01\_F14.raw |  |  |  |  |  | 1.0259E7 |  |  |  |  |  |  |  |  | 1 | 0 | 0 | 0 | 0 | 0 | 1 | 0 | 0 | 0 | 0 | 0 | 0 | 0 | 0 | 216 | 223 |  |  | PEAKS DB |
| R.LVILGFPC(+57.02)NQFGKQEPGQNSEILQGIK.Y | N | 40.83 | 3013.5693 | 27 | 0.5 | 1005.5309 | 3 | 75.74 | 6 | F6:51193 | NaSa01\_F14.raw |  |  |  |  |  | 3.8526E6 |  |  |  |  |  |  |  |  | 1 | 0 | 0 | 0 | 0 | 0 | 1 | 0 | 0 | 0 | 0 | 0 | 0 | 0 | 0 | 124 | 150 | Carbamidomethylation | C8:Carbamidomethylation:1000.00 | PEAKS DB |
| Y.VRPGGGYVPNFQLFQK.G | Y | 40.52 | 1805.9576 | 16 | 0.0 | 602.9932 | 3 | 56.10 | 6 | F6:34650 | NaSa01\_F14.raw |  |  |  |  |  | 4.2186E6 |  |  |  |  |  |  |  |  | 1 | 0 | 0 | 0 | 0 | 0 | 1 | 0 | 0 | 0 | 0 | 0 | 0 | 0 | 0 | 152 | 167 |  |  | PEAKS DB |
| K.VDC(+57.02)YDSVK.G | N | 38.28 | 984.4222 | 8 | 0.4 | 493.2186 | 2 | 11.46 | 6 | F6:1906 | NaSa01\_F14.raw |  |  |  |  |  | 8.6822E4 |  |  |  |  |  |  |  |  | 1 | 0 | 0 | 0 | 0 | 0 | 1 | 0 | 0 | 0 | 0 | 0 | 0 | 0 | 0 | 61 | 68 | Carbamidomethylation | C3:Carbamidomethylation:1000.00 | PEAKS DB |
| total 16 peptides |  |  |  |  |  |  |  |  |  |  |  |  |  |  |  |  |  |  |  |  |  |  |  |  |  |  |  |  |  |  |  |  |  |  |  |  |  |  |  |  |  |  |  |  |  |
| --- | --- | --- | --- | --- | --- | --- | --- | --- | --- | --- | --- | --- | --- | --- | --- | --- | --- | --- | --- | --- | --- | --- | --- | --- | --- | --- | --- | --- | --- | --- | --- | --- | --- | --- | --- | --- | --- | --- | --- | --- | --- | --- | --- | --- | --- |

XP\_026541916.1

back to list

  

| Protein Coverage
| Supporting Peptides
|

Protein Coverage:

Supporting Peptides:

| Peptide | Uniq | -10lgP | Mass | Length | ppm | m/z | z | RT | Fraction | Scan | Source File | Area F1 | Area F10 | Area F11 | Area F12 | Area F13 | Area F14 | Area F2 | Area F3 | Area F4 | Area F5 | Area F6 | Area F7 | Area F8 | Area F9 | #Feature | #Feature F1 | #Feature F10 | #Feature F11 | #Feature F12 | #Feature F13 | #Feature F14 | #Feature F2 | #Feature F3 | #Feature F4 | #Feature F5 | #Feature F6 | #Feature F7 | #Feature F8 | #Feature F9 | Start | End | PTM | AScore | Found By |
| --- | --- | --- | --- | --- | --- | --- | --- | --- | --- | --- | --- | --- | --- | --- | --- | --- | --- | --- | --- | --- | --- | --- | --- | --- | --- | --- | --- | --- | --- | --- | --- | --- | --- | --- | --- | --- | --- | --- | --- | --- | --- | --- | --- | --- | --- |
| R.LVILGFPC(+57.02)NQFGK.Q | N | 80.93 | 1491.7908 | 13 | 0.3 | 746.9029 | 2 | 74.37 | 14 | F14:48186 | NaSa01\_F9.raw |  | 6.3482E6 | 4.5719E6 | 6.7588E6 | 7.3547E8 | 2.7574E9 |  |  |  |  |  | 1.9765E6 | 7.8579E6 | 5.5032E7 | 12 | 0 | 1 | 1 | 1 | 3 | 3 | 0 | 0 | 0 | 0 | 0 | 1 | 1 | 1 | 93 | 105 | Carbamidomethylation | C8:Carbamidomethylation:1000.00 | PEAKS DB |
| K.QEPGQNSEILQGIK.Y | N | 80.47 | 1539.7893 | 14 | 0.1 | 770.9020 | 2 | 34.92 | 6 | F6:17025 | NaSa01\_F14.raw |  |  | 7.9374E4 | 4.1222E6 | 3.3468E8 | 1.3102E9 |  |  |  |  |  |  | 0 |  | 8 | 0 | 0 | 1 | 1 | 2 | 4 | 0 | 0 | 0 | 0 | 0 | 0 | 0 | 0 | 106 | 119 |  |  | PEAKS DB |
| R.PGGGYVPNFQLFQK.G | Y | 79.31 | 1550.7881 | 14 | 0.2 | 776.4015 | 2 | 68.00 | 6 | F6:44835 | NaSa01\_F14.raw |  |  |  | 1.3787E6 | 2.3027E8 | 1.1136E9 |  |  |  | 1.1813E7 |  | 5.021E7 |  |  | 14 | 0 | 0 | 0 | 1 | 3 | 7 | 0 | 0 | 0 | 1 | 0 | 2 | 0 | 0 | 123 | 136 |  |  | PEAKS DB |
| R.LFWSPLKIHDIK.W | N | 68.85 | 1495.8551 | 12 | 0.5 | 499.6259 | 3 | 54.14 | 6 | F6:32777 | NaSa01\_F14.raw |  |  |  |  |  | 4.8748E5 |  |  |  |  |  |  |  |  | 1 | 0 | 0 | 0 | 0 | 0 | 1 | 0 | 0 | 0 | 0 | 0 | 0 | 0 | 0 | 168 | 179 |  |  | PEAKS DB |
| K.WNFEKFLVNPQGK.P | N | 61.53 | 1605.8303 | 13 | 0.1 | 536.2841 | 3 | 64.13 | 6 | F6:41454 | NaSa01\_F14.raw |  |  |  |  |  | 3.4585E6 |  |  |  |  |  |  |  |  | 2 | 0 | 0 | 0 | 0 | 0 | 2 | 0 | 0 | 0 | 0 | 0 | 0 | 0 | 0 | 180 | 192 |  |  | PEAKS DB |
| L.VILGFPC(+57.02)NQFGK.Q | N | 58.48 | 1378.7067 | 12 | -0.1 | 690.3605 | 2 | 59.07 | 12 | F12:39406 | NaSa01\_F7.raw |  | 4.6143E6 | 1.4793E6 |  |  |  |  |  |  | 1.3101E7 | 1.8748E7 | 4.3606E7 | 7.4857E6 | 1.729E7 | 7 | 0 | 1 | 1 | 0 | 0 | 0 | 0 | 0 | 0 | 1 | 1 | 1 | 1 | 1 | 94 | 105 | Carbamidomethylation | C7:Carbamidomethylation:1000.00 | PEAKS DB |
| K.FLVNPQGKPVMR.W | N | 58.11 | 1384.7649 | 12 | -0.2 | 462.5955 | 3 | 25.87 | 5 | F5:9864 | NaSa01\_F13.raw |  |  |  |  | 9.037E6 |  |  |  |  |  |  |  |  |  | 2 | 0 | 0 | 0 | 0 | 2 | 0 | 0 | 0 | 0 | 0 | 0 | 0 | 0 | 0 | 185 | 196 |  |  | PEAKS DB |
| Y.VPNFQLFQK.G | N | 54.38 | 1119.6077 | 9 | -0.6 | 560.8108 | 2 | 54.08 | 6 | F6:32772 | NaSa01\_F14.raw |  |  |  |  | 1.0977E6 | 4.3013E6 |  |  |  |  |  |  |  |  | 2 | 0 | 0 | 0 | 0 | 1 | 1 | 0 | 0 | 0 | 0 | 0 | 0 | 0 | 0 | 128 | 136 |  |  | PEAKS DB |
| G.GGYVPNFQLFQK.G | Y | 52.92 | 1396.7139 | 12 | 0.1 | 699.3643 | 2 | 67.25 | 6 | F6:43947 | NaSa01\_F14.raw |  |  |  |  |  | 2.9962E6 |  |  |  |  |  |  |  |  | 1 | 0 | 0 | 0 | 0 | 0 | 1 | 0 | 0 | 0 | 0 | 0 | 0 | 0 | 0 | 125 | 136 |  |  | PEAKS DB |
| R.LFWSPLK.I | N | 50.63 | 889.5062 | 7 | -1.0 | 445.7599 | 2 | 61.33 | 6 | F6:38812 | NaSa01\_F14.raw |  |  |  |  |  | 1.0011E9 |  |  |  |  |  |  |  |  | 2 | 0 | 0 | 0 | 0 | 0 | 2 | 0 | 0 | 0 | 0 | 0 | 0 | 0 | 0 | 168 | 174 |  |  | PEAKS DB |
| R.LVILGFPC(+57.02)NQF.G | N | 49.68 | 1306.6743 | 11 | 0.4 | 654.3447 | 2 | 101.88 | 6 | F6:72830 | NaSa01\_F14.raw |  |  |  |  | 5.1302E6 | 2.3489E7 |  |  |  |  |  |  |  |  | 2 | 0 | 0 | 0 | 0 | 1 | 1 | 0 | 0 | 0 | 0 | 0 | 0 | 0 | 0 | 93 | 103 | Carbamidomethylation | C8:Carbamidomethylation:1000.00 | PEAKS DB |
| K.FLVNPQGKP.V | N | 41.99 | 998.5549 | 9 | -0.1 | 500.2847 | 2 | 25.59 | 12 | F12:11599 | NaSa01\_F7.raw |  |  |  |  |  |  |  |  |  |  |  | 8.4827E5 |  |  | 1 | 0 | 0 | 0 | 0 | 0 | 0 | 0 | 0 | 0 | 0 | 0 | 1 | 0 | 0 | 185 | 193 |  |  | PEAKS DB |
| K.FLVNPQGK.P | N | 41.78 | 901.5021 | 8 | 0.4 | 451.7585 | 2 | 11.58 | 6 | F6:1950 | NaSa01\_F14.raw |  |  |  |  |  | 1.0259E7 |  |  |  |  |  |  |  |  | 1 | 0 | 0 | 0 | 0 | 0 | 1 | 0 | 0 | 0 | 0 | 0 | 0 | 0 | 0 | 185 | 192 |  |  | PEAKS DB |
| R.LVILGFPC(+57.02)NQFGKQEPGQNSEILQGIK.Y | N | 40.83 | 3013.5693 | 27 | 0.5 | 1005.5309 | 3 | 75.74 | 6 | F6:51193 | NaSa01\_F14.raw |  |  |  |  |  | 3.8526E6 |  |  |  |  |  |  |  |  | 1 | 0 | 0 | 0 | 0 | 0 | 1 | 0 | 0 | 0 | 0 | 0 | 0 | 0 | 0 | 93 | 119 | Carbamidomethylation | C8:Carbamidomethylation:1000.00 | PEAKS DB |
| Y.VRPGGGYVPNFQLFQK.G | Y | 40.52 | 1805.9576 | 16 | 0.0 | 602.9932 | 3 | 56.10 | 6 | F6:34650 | NaSa01\_F14.raw |  |  |  |  |  | 4.2186E6 |  |  |  |  |  |  |  |  | 1 | 0 | 0 | 0 | 0 | 0 | 1 | 0 | 0 | 0 | 0 | 0 | 0 | 0 | 0 | 121 | 136 |  |  | PEAKS DB |
| E.VDC(+57.02)YDSVK.G | N | 38.28 | 984.4222 | 8 | 0.4 | 493.2186 | 2 | 11.46 | 6 | F6:1906 | NaSa01\_F14.raw |  |  |  |  |  | 8.6822E4 |  |  |  |  |  |  |  |  | 1 | 0 | 0 | 0 | 0 | 0 | 1 | 0 | 0 | 0 | 0 | 0 | 0 | 0 | 0 | 30 | 37 | Carbamidomethylation | C3:Carbamidomethylation:1000.00 | PEAKS DB |
| total 16 peptides |  |  |  |  |  |  |  |  |  |  |  |  |  |  |  |  |  |  |  |  |  |  |  |  |  |  |  |  |  |  |  |  |  |  |  |  |  |  |  |  |  |  |  |  |  |
| --- | --- | --- | --- | --- | --- | --- | --- | --- | --- | --- | --- | --- | --- | --- | --- | --- | --- | --- | --- | --- | --- | --- | --- | --- | --- | --- | --- | --- | --- | --- | --- | --- | --- | --- | --- | --- | --- | --- | --- | --- | --- | --- | --- | --- | --- |

XP\_026552407.1

back to list

  

| Protein Coverage
| Supporting Peptides
|

Protein Coverage:

Supporting Peptides:

| Peptide | Uniq | -10lgP | Mass | Length | ppm | m/z | z | RT | Fraction | Scan | Source File | Area F1 | Area F10 | Area F11 | Area F12 | Area F13 | Area F14 | Area F2 | Area F3 | Area F4 | Area F5 | Area F6 | Area F7 | Area F8 | Area F9 | #Feature | #Feature F1 | #Feature F10 | #Feature F11 | #Feature F12 | #Feature F13 | #Feature F14 | #Feature F2 | #Feature F3 | #Feature F4 | #Feature F5 | #Feature F6 | #Feature F7 | #Feature F8 | #Feature F9 | Start | End | PTM | AScore | Found By |
| --- | --- | --- | --- | --- | --- | --- | --- | --- | --- | --- | --- | --- | --- | --- | --- | --- | --- | --- | --- | --- | --- | --- | --- | --- | --- | --- | --- | --- | --- | --- | --- | --- | --- | --- | --- | --- | --- | --- | --- | --- | --- | --- | --- | --- | --- |
| R.LVILGFPC(+57.02)NQFGK.Q | N | 80.93 | 1491.7908 | 13 | 0.3 | 746.9029 | 2 | 74.37 | 14 | F14:48186 | NaSa01\_F9.raw |  | 6.3482E6 | 4.5719E6 | 6.7588E6 | 7.3547E8 | 2.7574E9 |  |  |  |  |  | 1.9765E6 | 7.8579E6 | 5.5032E7 | 12 | 0 | 1 | 1 | 1 | 3 | 3 | 0 | 0 | 0 | 0 | 0 | 1 | 1 | 1 | 93 | 105 | Carbamidomethylation | C8:Carbamidomethylation:1000.00 | PEAKS DB |
| K.QEPGQNSEILQGIK.Y | N | 80.47 | 1539.7893 | 14 | 0.1 | 770.9020 | 2 | 34.92 | 6 | F6:17025 | NaSa01\_F14.raw |  |  | 7.9374E4 | 4.1222E6 | 3.3468E8 | 1.3102E9 |  |  |  |  |  |  | 0 |  | 8 | 0 | 0 | 1 | 1 | 2 | 4 | 0 | 0 | 0 | 0 | 0 | 0 | 0 | 0 | 106 | 119 |  |  | PEAKS DB |
| R.PGGGYVPNFQLFQK.G | Y | 79.31 | 1550.7881 | 14 | 0.2 | 776.4015 | 2 | 68.00 | 6 | F6:44835 | NaSa01\_F14.raw |  |  |  | 1.3787E6 | 2.3027E8 | 1.1136E9 |  |  |  | 1.1813E7 |  | 5.021E7 |  |  | 14 | 0 | 0 | 0 | 1 | 3 | 7 | 0 | 0 | 0 | 1 | 0 | 2 | 0 | 0 | 123 | 136 |  |  | PEAKS DB |
| R.LFWSPLKIHDIK.W | N | 68.85 | 1495.8551 | 12 | 0.5 | 499.6259 | 3 | 54.14 | 6 | F6:32777 | NaSa01\_F14.raw |  |  |  |  |  | 4.8748E5 |  |  |  |  |  |  |  |  | 1 | 0 | 0 | 0 | 0 | 0 | 1 | 0 | 0 | 0 | 0 | 0 | 0 | 0 | 0 | 168 | 179 |  |  | PEAKS DB |
| K.WNFEKFLVNPQGK.P | N | 61.53 | 1605.8303 | 13 | 0.1 | 536.2841 | 3 | 64.13 | 6 | F6:41454 | NaSa01\_F14.raw |  |  |  |  |  | 3.4585E6 |  |  |  |  |  |  |  |  | 2 | 0 | 0 | 0 | 0 | 0 | 2 | 0 | 0 | 0 | 0 | 0 | 0 | 0 | 0 | 180 | 192 |  |  | PEAKS DB |
| L.VILGFPC(+57.02)NQFGK.Q | N | 58.48 | 1378.7067 | 12 | -0.1 | 690.3605 | 2 | 59.07 | 12 | F12:39406 | NaSa01\_F7.raw |  | 4.6143E6 | 1.4793E6 |  |  |  |  |  |  | 1.3101E7 | 1.8748E7 | 4.3606E7 | 7.4857E6 | 1.729E7 | 7 | 0 | 1 | 1 | 0 | 0 | 0 | 0 | 0 | 0 | 1 | 1 | 1 | 1 | 1 | 94 | 105 | Carbamidomethylation | C7:Carbamidomethylation:1000.00 | PEAKS DB |
| K.FLVNPQGKPVMR.W | N | 58.11 | 1384.7649 | 12 | -0.2 | 462.5955 | 3 | 25.87 | 5 | F5:9864 | NaSa01\_F13.raw |  |  |  |  | 9.037E6 |  |  |  |  |  |  |  |  |  | 2 | 0 | 0 | 0 | 0 | 2 | 0 | 0 | 0 | 0 | 0 | 0 | 0 | 0 | 0 | 185 | 196 |  |  | PEAKS DB |
| Y.VPNFQLFQK.G | N | 54.38 | 1119.6077 | 9 | -0.6 | 560.8108 | 2 | 54.08 | 6 | F6:32772 | NaSa01\_F14.raw |  |  |  |  | 1.0977E6 | 4.3013E6 |  |  |  |  |  |  |  |  | 2 | 0 | 0 | 0 | 0 | 1 | 1 | 0 | 0 | 0 | 0 | 0 | 0 | 0 | 0 | 128 | 136 |  |  | PEAKS DB |
| G.GGYVPNFQLFQK.G | Y | 52.92 | 1396.7139 | 12 | 0.1 | 699.3643 | 2 | 67.25 | 6 | F6:43947 | NaSa01\_F14.raw |  |  |  |  |  | 2.9962E6 |  |  |  |  |  |  |  |  | 1 | 0 | 0 | 0 | 0 | 0 | 1 | 0 | 0 | 0 | 0 | 0 | 0 | 0 | 0 | 125 | 136 |  |  | PEAKS DB |
| R.LFWSPLK.I | N | 50.63 | 889.5062 | 7 | -1.0 | 445.7599 | 2 | 61.33 | 6 | F6:38812 | NaSa01\_F14.raw |  |  |  |  |  | 1.0011E9 |  |  |  |  |  |  |  |  | 2 | 0 | 0 | 0 | 0 | 0 | 2 | 0 | 0 | 0 | 0 | 0 | 0 | 0 | 0 | 168 | 174 |  |  | PEAKS DB |
| R.LVILGFPC(+57.02)NQF.G | N | 49.68 | 1306.6743 | 11 | 0.4 | 654.3447 | 2 | 101.88 | 6 | F6:72830 | NaSa01\_F14.raw |  |  |  |  | 5.1302E6 | 2.3489E7 |  |  |  |  |  |  |  |  | 2 | 0 | 0 | 0 | 0 | 1 | 1 | 0 | 0 | 0 | 0 | 0 | 0 | 0 | 0 | 93 | 103 | Carbamidomethylation | C8:Carbamidomethylation:1000.00 | PEAKS DB |
| K.FLVNPQGKP.V | N | 41.99 | 998.5549 | 9 | -0.1 | 500.2847 | 2 | 25.59 | 12 | F12:11599 | NaSa01\_F7.raw |  |  |  |  |  |  |  |  |  |  |  | 8.4827E5 |  |  | 1 | 0 | 0 | 0 | 0 | 0 | 0 | 0 | 0 | 0 | 0 | 0 | 1 | 0 | 0 | 185 | 193 |  |  | PEAKS DB |
| K.FLVNPQGK.P | N | 41.78 | 901.5021 | 8 | 0.4 | 451.7585 | 2 | 11.58 | 6 | F6:1950 | NaSa01\_F14.raw |  |  |  |  |  | 1.0259E7 |  |  |  |  |  |  |  |  | 1 | 0 | 0 | 0 | 0 | 0 | 1 | 0 | 0 | 0 | 0 | 0 | 0 | 0 | 0 | 185 | 192 |  |  | PEAKS DB |
| R.LVILGFPC(+57.02)NQFGKQEPGQNSEILQGIK.Y | N | 40.83 | 3013.5693 | 27 | 0.5 | 1005.5309 | 3 | 75.74 | 6 | F6:51193 | NaSa01\_F14.raw |  |  |  |  |  | 3.8526E6 |  |  |  |  |  |  |  |  | 1 | 0 | 0 | 0 | 0 | 0 | 1 | 0 | 0 | 0 | 0 | 0 | 0 | 0 | 0 | 93 | 119 | Carbamidomethylation | C8:Carbamidomethylation:1000.00 | PEAKS DB |
| Y.VRPGGGYVPNFQLFQK.G | Y | 40.52 | 1805.9576 | 16 | 0.0 | 602.9932 | 3 | 56.10 | 6 | F6:34650 | NaSa01\_F14.raw |  |  |  |  |  | 4.2186E6 |  |  |  |  |  |  |  |  | 1 | 0 | 0 | 0 | 0 | 0 | 1 | 0 | 0 | 0 | 0 | 0 | 0 | 0 | 0 | 121 | 136 |  |  | PEAKS DB |
| E.VDC(+57.02)YDSVK.G | N | 38.28 | 984.4222 | 8 | 0.4 | 493.2186 | 2 | 11.46 | 6 | F6:1906 | NaSa01\_F14.raw |  |  |  |  |  | 8.6822E4 |  |  |  |  |  |  |  |  | 1 | 0 | 0 | 0 | 0 | 0 | 1 | 0 | 0 | 0 | 0 | 0 | 0 | 0 | 0 | 30 | 37 | Carbamidomethylation | C3:Carbamidomethylation:1000.00 | PEAKS DB |
| total 16 peptides |  |  |  |  |  |  |  |  |  |  |  |  |  |  |  |  |  |  |  |  |  |  |  |  |  |  |  |  |  |  |  |  |  |  |  |  |  |  |  |  |  |  |  |  |  |
| --- | --- | --- | --- | --- | --- | --- | --- | --- | --- | --- | --- | --- | --- | --- | --- | --- | --- | --- | --- | --- | --- | --- | --- | --- | --- | --- | --- | --- | --- | --- | --- | --- | --- | --- | --- | --- | --- | --- | --- | --- | --- | --- | --- | --- | --- |

XP\_026552406.1

back to list

  

| Protein Coverage
| Supporting Peptides
|

Protein Coverage:

Supporting Peptides:

| Peptide | Uniq | -10lgP | Mass | Length | ppm | m/z | z | RT | Fraction | Scan | Source File | Area F1 | Area F10 | Area F11 | Area F12 | Area F13 | Area F14 | Area F2 | Area F3 | Area F4 | Area F5 | Area F6 | Area F7 | Area F8 | Area F9 | #Feature | #Feature F1 | #Feature F10 | #Feature F11 | #Feature F12 | #Feature F13 | #Feature F14 | #Feature F2 | #Feature F3 | #Feature F4 | #Feature F5 | #Feature F6 | #Feature F7 | #Feature F8 | #Feature F9 | Start | End | PTM | AScore | Found By |
| --- | --- | --- | --- | --- | --- | --- | --- | --- | --- | --- | --- | --- | --- | --- | --- | --- | --- | --- | --- | --- | --- | --- | --- | --- | --- | --- | --- | --- | --- | --- | --- | --- | --- | --- | --- | --- | --- | --- | --- | --- | --- | --- | --- | --- | --- |
| R.LVILGFPC(+57.02)NQFGK.Q | N | 80.93 | 1491.7908 | 13 | 0.3 | 746.9029 | 2 | 74.37 | 14 | F14:48186 | NaSa01\_F9.raw |  | 6.3482E6 | 4.5719E6 | 6.7588E6 | 7.3547E8 | 2.7574E9 |  |  |  |  |  | 1.9765E6 | 7.8579E6 | 5.5032E7 | 12 | 0 | 1 | 1 | 1 | 3 | 3 | 0 | 0 | 0 | 0 | 0 | 1 | 1 | 1 | 124 | 136 | Carbamidomethylation | C8:Carbamidomethylation:1000.00 | PEAKS DB |
| K.QEPGQNSEILQGIK.Y | N | 80.47 | 1539.7893 | 14 | 0.1 | 770.9020 | 2 | 34.92 | 6 | F6:17025 | NaSa01\_F14.raw |  |  | 7.9374E4 | 4.1222E6 | 3.3468E8 | 1.3102E9 |  |  |  |  |  |  | 0 |  | 8 | 0 | 0 | 1 | 1 | 2 | 4 | 0 | 0 | 0 | 0 | 0 | 0 | 0 | 0 | 137 | 150 |  |  | PEAKS DB |
| R.PGGGYVPNFQLFQK.G | Y | 79.31 | 1550.7881 | 14 | 0.2 | 776.4015 | 2 | 68.00 | 6 | F6:44835 | NaSa01\_F14.raw |  |  |  | 1.3787E6 | 2.3027E8 | 1.1136E9 |  |  |  | 1.1813E7 |  | 5.021E7 |  |  | 14 | 0 | 0 | 0 | 1 | 3 | 7 | 0 | 0 | 0 | 1 | 0 | 2 | 0 | 0 | 154 | 167 |  |  | PEAKS DB |
| R.LFWSPLKIHDIK.W | N | 68.85 | 1495.8551 | 12 | 0.5 | 499.6259 | 3 | 54.14 | 6 | F6:32777 | NaSa01\_F14.raw |  |  |  |  |  | 4.8748E5 |  |  |  |  |  |  |  |  | 1 | 0 | 0 | 0 | 0 | 0 | 1 | 0 | 0 | 0 | 0 | 0 | 0 | 0 | 0 | 199 | 210 |  |  | PEAKS DB |
| K.WNFEKFLVNPQGK.P | N | 61.53 | 1605.8303 | 13 | 0.1 | 536.2841 | 3 | 64.13 | 6 | F6:41454 | NaSa01\_F14.raw |  |  |  |  |  | 3.4585E6 |  |  |  |  |  |  |  |  | 2 | 0 | 0 | 0 | 0 | 0 | 2 | 0 | 0 | 0 | 0 | 0 | 0 | 0 | 0 | 211 | 223 |  |  | PEAKS DB |
| L.VILGFPC(+57.02)NQFGK.Q | N | 58.48 | 1378.7067 | 12 | -0.1 | 690.3605 | 2 | 59.07 | 12 | F12:39406 | NaSa01\_F7.raw |  | 4.6143E6 | 1.4793E6 |  |  |  |  |  |  | 1.3101E7 | 1.8748E7 | 4.3606E7 | 7.4857E6 | 1.729E7 | 7 | 0 | 1 | 1 | 0 | 0 | 0 | 0 | 0 | 0 | 1 | 1 | 1 | 1 | 1 | 125 | 136 | Carbamidomethylation | C7:Carbamidomethylation:1000.00 | PEAKS DB |
| K.FLVNPQGKPVMR.W | N | 58.11 | 1384.7649 | 12 | -0.2 | 462.5955 | 3 | 25.87 | 5 | F5:9864 | NaSa01\_F13.raw |  |  |  |  | 9.037E6 |  |  |  |  |  |  |  |  |  | 2 | 0 | 0 | 0 | 0 | 2 | 0 | 0 | 0 | 0 | 0 | 0 | 0 | 0 | 0 | 216 | 227 |  |  | PEAKS DB |
| Y.VPNFQLFQK.G | N | 54.38 | 1119.6077 | 9 | -0.6 | 560.8108 | 2 | 54.08 | 6 | F6:32772 | NaSa01\_F14.raw |  |  |  |  | 1.0977E6 | 4.3013E6 |  |  |  |  |  |  |  |  | 2 | 0 | 0 | 0 | 0 | 1 | 1 | 0 | 0 | 0 | 0 | 0 | 0 | 0 | 0 | 159 | 167 |  |  | PEAKS DB |
| G.GGYVPNFQLFQK.G | Y | 52.92 | 1396.7139 | 12 | 0.1 | 699.3643 | 2 | 67.25 | 6 | F6:43947 | NaSa01\_F14.raw |  |  |  |  |  | 2.9962E6 |  |  |  |  |  |  |  |  | 1 | 0 | 0 | 0 | 0 | 0 | 1 | 0 | 0 | 0 | 0 | 0 | 0 | 0 | 0 | 156 | 167 |  |  | PEAKS DB |
| R.LFWSPLK.I | N | 50.63 | 889.5062 | 7 | -1.0 | 445.7599 | 2 | 61.33 | 6 | F6:38812 | NaSa01\_F14.raw |  |  |  |  |  | 1.0011E9 |  |  |  |  |  |  |  |  | 2 | 0 | 0 | 0 | 0 | 0 | 2 | 0 | 0 | 0 | 0 | 0 | 0 | 0 | 0 | 199 | 205 |  |  | PEAKS DB |
| R.LVILGFPC(+57.02)NQF.G | N | 49.68 | 1306.6743 | 11 | 0.4 | 654.3447 | 2 | 101.88 | 6 | F6:72830 | NaSa01\_F14.raw |  |  |  |  | 5.1302E6 | 2.3489E7 |  |  |  |  |  |  |  |  | 2 | 0 | 0 | 0 | 0 | 1 | 1 | 0 | 0 | 0 | 0 | 0 | 0 | 0 | 0 | 124 | 134 | Carbamidomethylation | C8:Carbamidomethylation:1000.00 | PEAKS DB |
| K.FLVNPQGKP.V | N | 41.99 | 998.5549 | 9 | -0.1 | 500.2847 | 2 | 25.59 | 12 | F12:11599 | NaSa01\_F7.raw |  |  |  |  |  |  |  |  |  |  |  | 8.4827E5 |  |  | 1 | 0 | 0 | 0 | 0 | 0 | 0 | 0 | 0 | 0 | 0 | 0 | 1 | 0 | 0 | 216 | 224 |  |  | PEAKS DB |
| K.FLVNPQGK.P | N | 41.78 | 901.5021 | 8 | 0.4 | 451.7585 | 2 | 11.58 | 6 | F6:1950 | NaSa01\_F14.raw |  |  |  |  |  | 1.0259E7 |  |  |  |  |  |  |  |  | 1 | 0 | 0 | 0 | 0 | 0 | 1 | 0 | 0 | 0 | 0 | 0 | 0 | 0 | 0 | 216 | 223 |  |  | PEAKS DB |
| R.LVILGFPC(+57.02)NQFGKQEPGQNSEILQGIK.Y | N | 40.83 | 3013.5693 | 27 | 0.5 | 1005.5309 | 3 | 75.74 | 6 | F6:51193 | NaSa01\_F14.raw |  |  |  |  |  | 3.8526E6 |  |  |  |  |  |  |  |  | 1 | 0 | 0 | 0 | 0 | 0 | 1 | 0 | 0 | 0 | 0 | 0 | 0 | 0 | 0 | 124 | 150 | Carbamidomethylation | C8:Carbamidomethylation:1000.00 | PEAKS DB |
| Y.VRPGGGYVPNFQLFQK.G | Y | 40.52 | 1805.9576 | 16 | 0.0 | 602.9932 | 3 | 56.10 | 6 | F6:34650 | NaSa01\_F14.raw |  |  |  |  |  | 4.2186E6 |  |  |  |  |  |  |  |  | 1 | 0 | 0 | 0 | 0 | 0 | 1 | 0 | 0 | 0 | 0 | 0 | 0 | 0 | 0 | 152 | 167 |  |  | PEAKS DB |
| K.VDC(+57.02)YDSVK.G | N | 38.28 | 984.4222 | 8 | 0.4 | 493.2186 | 2 | 11.46 | 6 | F6:1906 | NaSa01\_F14.raw |  |  |  |  |  | 8.6822E4 |  |  |  |  |  |  |  |  | 1 | 0 | 0 | 0 | 0 | 0 | 1 | 0 | 0 | 0 | 0 | 0 | 0 | 0 | 0 | 61 | 68 | Carbamidomethylation | C3:Carbamidomethylation:1000.00 | PEAKS DB |
| total 16 peptides |  |  |  |  |  |  |  |  |  |  |  |  |  |  |  |  |  |  |  |  |  |  |  |  |  |  |  |  |  |  |  |  |  |  |  |  |  |  |  |  |  |  |  |  |  |
| --- | --- | --- | --- | --- | --- | --- | --- | --- | --- | --- | --- | --- | --- | --- | --- | --- | --- | --- | --- | --- | --- | --- | --- | --- | --- | --- | --- | --- | --- | --- | --- | --- | --- | --- | --- | --- | --- | --- | --- | --- | --- | --- | --- | --- | --- |

XP\_029140080.1

back to list

  

| Protein Coverage
| Supporting Peptides
|

Protein Coverage:

Supporting Peptides:

| Peptide | Uniq | -10lgP | Mass | Length | ppm | m/z | z | RT | Fraction | Scan | Source File | Area F1 | Area F10 | Area F11 | Area F12 | Area F13 | Area F14 | Area F2 | Area F3 | Area F4 | Area F5 | Area F6 | Area F7 | Area F8 | Area F9 | #Feature | #Feature F1 | #Feature F10 | #Feature F11 | #Feature F12 | #Feature F13 | #Feature F14 | #Feature F2 | #Feature F3 | #Feature F4 | #Feature F5 | #Feature F6 | #Feature F7 | #Feature F8 | #Feature F9 | Start | End | PTM | AScore | Found By |
| --- | --- | --- | --- | --- | --- | --- | --- | --- | --- | --- | --- | --- | --- | --- | --- | --- | --- | --- | --- | --- | --- | --- | --- | --- | --- | --- | --- | --- | --- | --- | --- | --- | --- | --- | --- | --- | --- | --- | --- | --- | --- | --- | --- | --- | --- |
| Q.LPDWTEC(+57.02)EVSGYGK.H | N | 85.25 | 1639.7188 | 14 | 1.0 | 820.8674 | 2 | 52.78 | 3 | F3:28524 | NaSa01\_F11.raw |  | 0 | 2.6381E7 | 8.0628E7 | 2.9981E6 | 1.7302E6 |  |  |  |  |  |  | 3.668E5 |  | 5 | 0 | 0 | 1 | 1 | 1 | 1 | 0 | 0 | 0 | 0 | 0 | 0 | 1 | 0 | 436 | 449 | Carbamidomethylation | C7:Carbamidomethylation:1000.00 | PEAKS DB |
| Q.ALYSPNLFIC(+57.02)FC(+57.02)PPGFSGK.F | N | 83.65 | 2174.0330 | 19 | -0.3 | 1088.0234 | 2 | 93.60 | 12 | F12:67510 | NaSa01\_F7.raw |  | 5.8401E7 | 6.3158E7 | 6.0423E6 |  |  |  |  |  |  | 2.7517E7 | 1.7226E8 | 4.6E6 | 6.0752E6 | 13 | 0 | 3 | 2 | 2 | 0 | 0 | 0 | 0 | 0 | 0 | 2 | 2 | 1 | 1 | 99 | 117 | Carbamidomethylation | C10:Carbamidomethylation:1000.00;C12:Carbamidomethylation:1000.00 | PEAKS DB |
| R.GTWSTTESGAEC(+57.02)VNWK.T | N | 81.48 | 1811.7784 | 16 | 0.3 | 906.8967 | 2 | 40.16 | 12 | F12:23328 | NaSa01\_F7.raw |  | 3.1844E6 | 5.7251E6 | 4.2946E6 |  | 2.742E5 |  |  |  |  |  | 6.7871E7 | 2.0844E6 |  | 8 | 0 | 1 | 1 | 1 | 0 | 1 | 0 | 0 | 0 | 0 | 0 | 3 | 1 | 0 | 137 | 152 | Carbamidomethylation | C12:Carbamidomethylation:1000.00 | PEAKS DB |
| R.TVTENMLC(+57.02)AGDTR.H | N | 75.17 | 1466.6494 | 13 | 1.2 | 734.3329 | 2 | 28.26 | 4 | F4:13301 | NaSa01\_F12.raw |  |  |  | 3.07E7 | 7.9493E5 |  |  |  |  |  |  |  |  |  | 3 | 0 | 0 | 0 | 2 | 1 | 0 | 0 | 0 | 0 | 0 | 0 | 0 | 0 | 0 | 483 | 495 | Carbamidomethylation | C8:Carbamidomethylation:1000.00 | PEAKS DB |
| R.AVC(+57.02)YQDIGDTYR.G | Y | 73.93 | 1459.6401 | 12 | -0.7 | 730.8268 | 2 | 34.23 | 12 | F12:18680 | NaSa01\_F7.raw |  | 4.2652E6 | 1.0449E7 | 1.23E7 | 1.1923E6 | 6.1065E5 |  |  |  |  | 4.6972E6 | 8.5238E7 | 3.4801E5 | 1.4118E6 | 10 | 0 | 1 | 1 | 1 | 1 | 1 | 0 | 0 | 0 | 0 | 1 | 1 | 2 | 1 | 125 | 136 | Carbamidomethylation | C3:Carbamidomethylation:1000.00 | PEAKS DB |
| Y.SPNLFIC(+57.02)FC(+57.02)PPGFSGK.F | N | 66.41 | 1826.8484 | 16 | 0.7 | 914.4321 | 2 | 82.84 | 12 | F12:58627 | NaSa01\_F7.raw |  |  |  |  |  |  |  |  |  |  |  | 1.7423E6 |  |  | 1 | 0 | 0 | 0 | 0 | 0 | 0 | 0 | 0 | 0 | 0 | 0 | 1 | 0 | 0 | 102 | 117 | Carbamidomethylation | C7:Carbamidomethylation:1000.00;C9:Carbamidomethylation:1000.00 | PEAKS DB |
| L.LGLGDHNYC(+57.02)R.N | N | 63.69 | 1203.5455 | 10 | 0.8 | 402.1895 | 3 | 12.19 | 12 | F12:2522 | NaSa01\_F7.raw |  |  |  |  |  |  |  |  |  |  |  | 8.9722E5 |  |  | 2 | 0 | 0 | 0 | 0 | 0 | 0 | 0 | 0 | 0 | 0 | 0 | 2 | 0 | 0 | 171 | 180 | Carbamidomethylation | C9:Carbamidomethylation:1000.00 | PEAKS DB |
| F.IC(+57.02)FC(+57.02)PPGFSGK.F | N | 63.52 | 1268.5681 | 11 | 0.2 | 635.2914 | 2 | 50.36 | 12 | F12:32061 | NaSa01\_F7.raw |  |  |  |  |  |  |  |  |  |  |  | 2.2109E7 |  |  | 1 | 0 | 0 | 0 | 0 | 0 | 0 | 0 | 0 | 0 | 0 | 0 | 1 | 0 | 0 | 107 | 117 | Carbamidomethylation | C2:Carbamidomethylation:1000.00;C4:Carbamidomethylation:1000.00 | PEAKS DB |
| N.LFIC(+57.02)FC(+57.02)PPGFSGK.F | N | 63.19 | 1528.7206 | 13 | -0.5 | 765.3672 | 2 | 78.45 | 12 | F12:54679 | NaSa01\_F7.raw |  |  | 4.4273E6 |  |  |  |  |  |  |  | 2.2158E6 | 5.365E7 |  |  | 3 | 0 | 0 | 1 | 0 | 0 | 0 | 0 | 0 | 0 | 0 | 1 | 1 | 0 | 0 | 105 | 117 | Carbamidomethylation | C4:Carbamidomethylation:1000.00;C6:Carbamidomethylation:1000.00 | PEAKS DB |
| R.TLDNDIALLK.L | N | 57.12 | 1114.6234 | 10 | 0.0 | 558.3190 | 2 | 52.92 | 4 | F4:34239 | NaSa01\_F12.raw |  | 4.5967E6 | 1.4483E7 | 6.6342E7 | 2.5476E6 |  |  |  |  |  |  |  |  |  | 6 | 0 | 1 | 1 | 3 | 1 | 0 | 0 | 0 | 0 | 0 | 0 | 0 | 0 | 0 | 400 | 409 |  |  | PEAKS DB |
| L.FIC(+57.02)FC(+57.02)PPGFSGK.F | N | 49.97 | 1415.6366 | 12 | -0.6 | 708.8251 | 2 | 63.43 | 12 | F12:43236 | NaSa01\_F7.raw |  |  |  |  |  |  |  |  |  |  |  | 3.3384E6 |  |  | 1 | 0 | 0 | 0 | 0 | 0 | 0 | 0 | 0 | 0 | 0 | 0 | 1 | 0 | 0 | 106 | 117 | Carbamidomethylation | C3:Carbamidomethylation:1000.00;C5:Carbamidomethylation:1000.00 | PEAKS DB |
| total 11 peptides |  |  |  |  |  |  |  |  |  |  |  |  |  |  |  |  |  |  |  |  |  |  |  |  |  |  |  |  |  |  |  |  |  |  |  |  |  |  |  |  |  |  |  |  |  |
| --- | --- | --- | --- | --- | --- | --- | --- | --- | --- | --- | --- | --- | --- | --- | --- | --- | --- | --- | --- | --- | --- | --- | --- | --- | --- | --- | --- | --- | --- | --- | --- | --- | --- | --- | --- | --- | --- | --- | --- | --- | --- | --- | --- | --- | --- |

JAI11009.1

back to list

  

| Protein Coverage
| Supporting Peptides
|

Protein Coverage:

Supporting Peptides:

| Peptide | Uniq | -10lgP | Mass | Length | ppm | m/z | z | RT | Fraction | Scan | Source File | Area F1 | Area F10 | Area F11 | Area F12 | Area F13 | Area F14 | Area F2 | Area F3 | Area F4 | Area F5 | Area F6 | Area F7 | Area F8 | Area F9 | #Feature | #Feature F1 | #Feature F10 | #Feature F11 | #Feature F12 | #Feature F13 | #Feature F14 | #Feature F2 | #Feature F3 | #Feature F4 | #Feature F5 | #Feature F6 | #Feature F7 | #Feature F8 | #Feature F9 | Start | End | PTM | AScore | Found By |
| --- | --- | --- | --- | --- | --- | --- | --- | --- | --- | --- | --- | --- | --- | --- | --- | --- | --- | --- | --- | --- | --- | --- | --- | --- | --- | --- | --- | --- | --- | --- | --- | --- | --- | --- | --- | --- | --- | --- | --- | --- | --- | --- | --- | --- | --- |
| Q.LPDWTEC(+57.02)EVSGYGK.H | N | 85.25 | 1639.7188 | 14 | 1.0 | 820.8674 | 2 | 52.78 | 3 | F3:28524 | NaSa01\_F11.raw |  | 0 | 2.6381E7 | 8.0628E7 | 2.9981E6 | 1.7302E6 |  |  |  |  |  |  | 3.668E5 |  | 5 | 0 | 0 | 1 | 1 | 1 | 1 | 0 | 0 | 0 | 0 | 0 | 0 | 1 | 0 | 437 | 450 | Carbamidomethylation | C7:Carbamidomethylation:1000.00 | PEAKS DB |
| Q.ALYSPNLFIC(+57.02)FC(+57.02)PPGFSGK.F | N | 83.65 | 2174.0330 | 19 | -0.3 | 1088.0234 | 2 | 93.60 | 12 | F12:67510 | NaSa01\_F7.raw |  | 5.8401E7 | 6.3158E7 | 6.0423E6 |  |  |  |  |  |  | 2.7517E7 | 1.7226E8 | 4.6E6 | 6.0752E6 | 13 | 0 | 3 | 2 | 2 | 0 | 0 | 0 | 0 | 0 | 0 | 2 | 2 | 1 | 1 | 99 | 117 | Carbamidomethylation | C10:Carbamidomethylation:1000.00;C12:Carbamidomethylation:1000.00 | PEAKS DB |
| R.GTWSTTESGAEC(+57.02)VNWK.T | N | 81.48 | 1811.7784 | 16 | 0.3 | 906.8967 | 2 | 40.16 | 12 | F12:23328 | NaSa01\_F7.raw |  | 3.1844E6 | 5.7251E6 | 4.2946E6 |  | 2.742E5 |  |  |  |  |  | 6.7871E7 | 2.0844E6 |  | 8 | 0 | 1 | 1 | 1 | 0 | 1 | 0 | 0 | 0 | 0 | 0 | 3 | 1 | 0 | 137 | 152 | Carbamidomethylation | C12:Carbamidomethylation:1000.00 | PEAKS DB |
| R.TVTENMLC(+57.02)AGDTR.H | N | 75.17 | 1466.6494 | 13 | 1.2 | 734.3329 | 2 | 28.26 | 4 | F4:13301 | NaSa01\_F12.raw |  |  |  | 3.07E7 | 7.9493E5 |  |  |  |  |  |  |  |  |  | 3 | 0 | 0 | 0 | 2 | 1 | 0 | 0 | 0 | 0 | 0 | 0 | 0 | 0 | 0 | 484 | 496 | Carbamidomethylation | C8:Carbamidomethylation:1000.00 | PEAKS DB |
| R.AVC(+57.02)YQDIGDTYR.G | Y | 73.93 | 1459.6401 | 12 | -0.7 | 730.8268 | 2 | 34.23 | 12 | F12:18680 | NaSa01\_F7.raw |  | 4.2652E6 | 1.0449E7 | 1.23E7 | 1.1923E6 | 6.1065E5 |  |  |  |  | 4.6972E6 | 8.5238E7 | 3.4801E5 | 1.4118E6 | 10 | 0 | 1 | 1 | 1 | 1 | 1 | 0 | 0 | 0 | 0 | 1 | 1 | 2 | 1 | 125 | 136 | Carbamidomethylation | C3:Carbamidomethylation:1000.00 | PEAKS DB |
| Y.SPNLFIC(+57.02)FC(+57.02)PPGFSGK.F | N | 66.41 | 1826.8484 | 16 | 0.7 | 914.4321 | 2 | 82.84 | 12 | F12:58627 | NaSa01\_F7.raw |  |  |  |  |  |  |  |  |  |  |  | 1.7423E6 |  |  | 1 | 0 | 0 | 0 | 0 | 0 | 0 | 0 | 0 | 0 | 0 | 0 | 1 | 0 | 0 | 102 | 117 | Carbamidomethylation | C7:Carbamidomethylation:1000.00;C9:Carbamidomethylation:1000.00 | PEAKS DB |
| L.LGLGDHNYC(+57.02)R.N | N | 63.69 | 1203.5455 | 10 | 0.8 | 402.1895 | 3 | 12.19 | 12 | F12:2522 | NaSa01\_F7.raw |  |  |  |  |  |  |  |  |  |  |  | 8.9722E5 |  |  | 2 | 0 | 0 | 0 | 0 | 0 | 0 | 0 | 0 | 0 | 0 | 0 | 2 | 0 | 0 | 171 | 180 | Carbamidomethylation | C9:Carbamidomethylation:1000.00 | PEAKS DB |
| F.IC(+57.02)FC(+57.02)PPGFSGK.F | N | 63.52 | 1268.5681 | 11 | 0.2 | 635.2914 | 2 | 50.36 | 12 | F12:32061 | NaSa01\_F7.raw |  |  |  |  |  |  |  |  |  |  |  | 2.2109E7 |  |  | 1 | 0 | 0 | 0 | 0 | 0 | 0 | 0 | 0 | 0 | 0 | 0 | 1 | 0 | 0 | 107 | 117 | Carbamidomethylation | C2:Carbamidomethylation:1000.00;C4:Carbamidomethylation:1000.00 | PEAKS DB |
| N.LFIC(+57.02)FC(+57.02)PPGFSGK.F | N | 63.19 | 1528.7206 | 13 | -0.5 | 765.3672 | 2 | 78.45 | 12 | F12:54679 | NaSa01\_F7.raw |  |  | 4.4273E6 |  |  |  |  |  |  |  | 2.2158E6 | 5.365E7 |  |  | 3 | 0 | 0 | 1 | 0 | 0 | 0 | 0 | 0 | 0 | 0 | 1 | 1 | 0 | 0 | 105 | 117 | Carbamidomethylation | C4:Carbamidomethylation:1000.00;C6:Carbamidomethylation:1000.00 | PEAKS DB |
| R.TLDNDIALLK.L | N | 57.12 | 1114.6234 | 10 | 0.0 | 558.3190 | 2 | 52.92 | 4 | F4:34239 | NaSa01\_F12.raw |  | 4.5967E6 | 1.4483E7 | 6.6342E7 | 2.5476E6 |  |  |  |  |  |  |  |  |  | 6 | 0 | 1 | 1 | 3 | 1 | 0 | 0 | 0 | 0 | 0 | 0 | 0 | 0 | 0 | 401 | 410 |  |  | PEAKS DB |
| L.FIC(+57.02)FC(+57.02)PPGFSGK.F | N | 49.97 | 1415.6366 | 12 | -0.6 | 708.8251 | 2 | 63.43 | 12 | F12:43236 | NaSa01\_F7.raw |  |  |  |  |  |  |  |  |  |  |  | 3.3384E6 |  |  | 1 | 0 | 0 | 0 | 0 | 0 | 0 | 0 | 0 | 0 | 0 | 0 | 1 | 0 | 0 | 106 | 117 | Carbamidomethylation | C3:Carbamidomethylation:1000.00;C5:Carbamidomethylation:1000.00 | PEAKS DB |
| total 11 peptides |  |  |  |  |  |  |  |  |  |  |  |  |  |  |  |  |  |  |  |  |  |  |  |  |  |  |  |  |  |  |  |  |  |  |  |  |  |  |  |  |  |  |  |  |  |
| --- | --- | --- | --- | --- | --- | --- | --- | --- | --- | --- | --- | --- | --- | --- | --- | --- | --- | --- | --- | --- | --- | --- | --- | --- | --- | --- | --- | --- | --- | --- | --- | --- | --- | --- | --- | --- | --- | --- | --- | --- | --- | --- | --- | --- | --- |

XP\_026535930.1

back to list

  

| Protein Coverage
| Supporting Peptides
|

Protein Coverage:

Supporting Peptides:

| Peptide | Uniq | -10lgP | Mass | Length | ppm | m/z | z | RT | Fraction | Scan | Source File | Area F1 | Area F10 | Area F11 | Area F12 | Area F13 | Area F14 | Area F2 | Area F3 | Area F4 | Area F5 | Area F6 | Area F7 | Area F8 | Area F9 | #Feature | #Feature F1 | #Feature F10 | #Feature F11 | #Feature F12 | #Feature F13 | #Feature F14 | #Feature F2 | #Feature F3 | #Feature F4 | #Feature F5 | #Feature F6 | #Feature F7 | #Feature F8 | #Feature F9 | Start | End | PTM | AScore | Found By |
| --- | --- | --- | --- | --- | --- | --- | --- | --- | --- | --- | --- | --- | --- | --- | --- | --- | --- | --- | --- | --- | --- | --- | --- | --- | --- | --- | --- | --- | --- | --- | --- | --- | --- | --- | --- | --- | --- | --- | --- | --- | --- | --- | --- | --- | --- |
| K.TETGQELEC(+57.02)LQDHLDDLVTDC(+57.02)R.E | Y | 93.55 | 2646.1538 | 22 | 0.9 | 883.0593 | 3 | 77.87 | 14 | F14:50873 | NaSa01\_F9.raw |  |  | 1.4892E7 |  |  |  |  |  |  |  |  |  |  | 1.3075E7 | 2 | 0 | 0 | 1 | 0 | 0 | 0 | 0 | 0 | 0 | 0 | 0 | 0 | 0 | 1 | 633 | 654 | Carbamidomethylation | C9:Carbamidomethylation:1000.00;C21:Carbamidomethylation:1000.00 | PEAKS DB |
| R.QVSSEC(+57.02)QGEMLDYR.R | Y | 70.56 | 1700.7134 | 14 | -0.1 | 851.3639 | 2 | 30.02 | 4 | F4:14700 | NaSa01\_F12.raw |  |  |  | 1.5274E6 |  |  |  |  |  |  |  |  |  |  | 1 | 0 | 0 | 0 | 1 | 0 | 0 | 0 | 0 | 0 | 0 | 0 | 0 | 0 | 0 | 396 | 409 | Carbamidomethylation | C6:Carbamidomethylation:1000.00 | PEAKS DB |
| R.ALNEAC(+57.02)ESVIQTAC(+57.02)K.H | Y | 62.36 | 1692.7811 | 15 | -0.3 | 847.3976 | 2 | 32.95 | 4 | F4:17219 | NaSa01\_F12.raw |  |  |  | 5.582E5 |  |  |  |  |  |  |  |  |  |  | 1 | 0 | 0 | 0 | 1 | 0 | 0 | 0 | 0 | 0 | 0 | 0 | 0 | 0 | 0 | 484 | 498 | Carbamidomethylation | C6:Carbamidomethylation:1000.00;C14:Carbamidomethylation:1000.00 | PEAKS DB |
| A.DIFVDPVLHTAC(+57.02)ALDIK.H | Y | 61.91 | 1925.9921 | 17 | 0.6 | 643.0050 | 3 | 80.84 | 14 | F14:53034 | NaSa01\_F9.raw |  |  |  |  |  |  |  |  |  |  |  |  |  | 4.1632E6 | 2 | 0 | 0 | 0 | 0 | 0 | 0 | 0 | 0 | 0 | 0 | 0 | 0 | 0 | 2 | 1042 | 1058 | Carbamidomethylation | C12:Carbamidomethylation:1000.00 | PEAKS DB |
| R.LSYLLMC(+57.02)LESAVHR.G | Y | 61.57 | 1690.8535 | 14 | 0.0 | 564.6251 | 3 | 75.92 | 4 | F4:52849 | NaSa01\_F12.raw |  |  |  | 3.5748E5 |  |  |  |  |  |  |  |  |  |  | 2 | 0 | 0 | 0 | 2 | 0 | 0 | 0 | 0 | 0 | 0 | 0 | 0 | 0 | 0 | 380 | 393 | Carbamidomethylation | C7:Carbamidomethylation:1000.00 | PEAKS DB |
| K.MTAIIFSDYR.L | Y | 55.58 | 1215.5958 | 10 | 0.6 | 608.8055 | 2 | 57.64 | 4 | F4:37652 | NaSa01\_F12.raw |  |  | 1.4856E5 | 1.7582E6 |  |  |  |  |  |  |  |  |  |  | 2 | 0 | 0 | 1 | 1 | 0 | 0 | 0 | 0 | 0 | 0 | 0 | 0 | 0 | 0 | 205 | 214 |  |  | PEAKS DB |
| K.HSWANNLAVLEC(+57.02)LQDVR.E | Y | 55.40 | 2023.9897 | 17 | 0.1 | 675.6706 | 3 | 71.10 | 4 | F4:48795 | NaSa01\_F12.raw |  |  | 3.6424E5 | 1.7171E6 |  |  |  |  |  |  |  |  |  |  | 2 | 0 | 0 | 1 | 1 | 0 | 0 | 0 | 0 | 0 | 0 | 0 | 0 | 0 | 0 | 111 | 127 | Carbamidomethylation | C12:Carbamidomethylation:1000.00 | PEAKS DB |
| K.AKEESELEGQVISC(+57.02)LK.L | Y | 52.23 | 1818.9033 | 16 | 0.5 | 910.4594 | 2 | 43.69 | 13 | F13:30043 | NaSa01\_F8.raw |  |  |  |  |  |  |  |  |  |  |  |  | 4.3746E6 |  | 2 | 0 | 0 | 0 | 0 | 0 | 0 | 0 | 0 | 0 | 0 | 0 | 0 | 2 | 0 | 939 | 954 | Carbamidomethylation | C14:Carbamidomethylation:1000.00 | PEAKS DB |
| R.VAELSSDDFHLDR.H | Y | 51.43 | 1502.7001 | 13 | 0.2 | 501.9074 | 3 | 38.96 | 6 | F6:19788 | NaSa01\_F14.raw |  |  |  | 2.435E6 |  | 2.5942E5 |  |  |  |  |  |  |  |  | 3 | 0 | 0 | 0 | 2 | 0 | 1 | 0 | 0 | 0 | 0 | 0 | 0 | 0 | 0 | 280 | 292 |  |  | PEAKS DB |
| R.QMSC(+57.02)LMEALEDKR.V | Y | 48.60 | 1609.7262 | 13 | -0.3 | 537.5825 | 3 | 45.06 | 14 | F14:24054 | NaSa01\_F9.raw |  |  |  |  |  |  |  |  |  |  |  |  |  | 4.9085E5 | 1 | 0 | 0 | 0 | 0 | 0 | 0 | 0 | 0 | 0 | 0 | 0 | 0 | 0 | 1 | 1071 | 1083 | Carbamidomethylation | C4:Carbamidomethylation:1000.00 | PEAKS DB |
| R.MLMEDFSLSPEIILGC(+57.02)R.T | Y | 43.73 | 2009.9624 | 17 | 0.5 | 1005.9890 | 2 | 100.55 | 3 | F3:68781 | NaSa01\_F11.raw |  |  | 2.5783E5 |  |  |  |  |  |  |  |  |  |  |  | 1 | 0 | 0 | 1 | 0 | 0 | 0 | 0 | 0 | 0 | 0 | 0 | 0 | 0 | 0 | 411 | 427 | Carbamidomethylation | C16:Carbamidomethylation:1000.00 | PEAKS DB |
| T.EMMDPELDYTLMR.V | Y | 40.89 | 1642.7041 | 13 | 1.1 | 822.3602 | 2 | 70.47 | 11 | F11:49102 | NaSa01\_F6.raw |  |  |  |  |  |  |  |  |  |  | 5.4593E5 |  |  |  | 1 | 0 | 0 | 0 | 0 | 0 | 0 | 0 | 0 | 0 | 0 | 1 | 0 | 0 | 0 | 852 | 864 |  |  | PEAKS DB |
| R.LIC(+57.02)GFMDHC(+57.02)R.S | Y | 39.14 | 1307.5574 | 10 | 0.2 | 436.8598 | 3 | 25.71 | 4 | F4:11092 | NaSa01\_F12.raw |  |  |  | 1.668E5 |  |  |  |  |  |  |  |  |  |  | 1 | 0 | 0 | 0 | 1 | 0 | 0 | 0 | 0 | 0 | 0 | 0 | 0 | 0 | 0 | 215 | 224 | Carbamidomethylation | C3:Carbamidomethylation:1000.00;C9:Carbamidomethylation:1000.00 | PEAKS DB |
| R.LLELQYFISR.D | Y | 38.68 | 1280.7129 | 10 | 0.5 | 641.3640 | 2 | 79.02 | 4 | F4:55464 | NaSa01\_F12.raw |  |  |  | 4.8808E5 |  |  |  |  |  |  |  |  |  |  | 1 | 0 | 0 | 0 | 1 | 0 | 0 | 0 | 0 | 0 | 0 | 0 | 0 | 0 | 0 | 528 | 537 |  |  | PEAKS DB |
| total 14 peptides |  |  |  |  |  |  |  |  |  |  |  |  |  |  |  |  |  |  |  |  |  |  |  |  |  |  |  |  |  |  |  |  |  |  |  |  |  |  |  |  |  |  |  |  |  |
| --- | --- | --- | --- | --- | --- | --- | --- | --- | --- | --- | --- | --- | --- | --- | --- | --- | --- | --- | --- | --- | --- | --- | --- | --- | --- | --- | --- | --- | --- | --- | --- | --- | --- | --- | --- | --- | --- | --- | --- | --- | --- | --- | --- | --- | --- |

XP\_026570202.1

back to list

  

| Protein Coverage
| Supporting Peptides
|

Protein Coverage:

Supporting Peptides:

| Peptide | Uniq | -10lgP | Mass | Length | ppm | m/z | z | RT | Fraction | Scan | Source File | Area F1 | Area F10 | Area F11 | Area F12 | Area F13 | Area F14 | Area F2 | Area F3 | Area F4 | Area F5 | Area F6 | Area F7 | Area F8 | Area F9 | #Feature | #Feature F1 | #Feature F10 | #Feature F11 | #Feature F12 | #Feature F13 | #Feature F14 | #Feature F2 | #Feature F3 | #Feature F4 | #Feature F5 | #Feature F6 | #Feature F7 | #Feature F8 | #Feature F9 | Start | End | PTM | AScore | Found By |
| --- | --- | --- | --- | --- | --- | --- | --- | --- | --- | --- | --- | --- | --- | --- | --- | --- | --- | --- | --- | --- | --- | --- | --- | --- | --- | --- | --- | --- | --- | --- | --- | --- | --- | --- | --- | --- | --- | --- | --- | --- | --- | --- | --- | --- | --- |
| K.TETGQELEC(+57.02)LQDHLDDLVTDC(+57.02)R.E | Y | 93.55 | 2646.1538 | 22 | 0.9 | 883.0593 | 3 | 77.87 | 14 | F14:50873 | NaSa01\_F9.raw |  |  | 1.4892E7 |  |  |  |  |  |  |  |  |  |  | 1.3075E7 | 2 | 0 | 0 | 1 | 0 | 0 | 0 | 0 | 0 | 0 | 0 | 0 | 0 | 0 | 1 | 637 | 658 | Carbamidomethylation | C9:Carbamidomethylation:1000.00;C21:Carbamidomethylation:1000.00 | PEAKS DB |
| R.QVSSEC(+57.02)QGEMLDYR.R | Y | 70.56 | 1700.7134 | 14 | -0.1 | 851.3639 | 2 | 30.02 | 4 | F4:14700 | NaSa01\_F12.raw |  |  |  | 1.5274E6 |  |  |  |  |  |  |  |  |  |  | 1 | 0 | 0 | 0 | 1 | 0 | 0 | 0 | 0 | 0 | 0 | 0 | 0 | 0 | 0 | 400 | 413 | Carbamidomethylation | C6:Carbamidomethylation:1000.00 | PEAKS DB |
| R.ALNEAC(+57.02)ESVIQTAC(+57.02)K.H | Y | 62.36 | 1692.7811 | 15 | -0.3 | 847.3976 | 2 | 32.95 | 4 | F4:17219 | NaSa01\_F12.raw |  |  |  | 5.582E5 |  |  |  |  |  |  |  |  |  |  | 1 | 0 | 0 | 0 | 1 | 0 | 0 | 0 | 0 | 0 | 0 | 0 | 0 | 0 | 0 | 488 | 502 | Carbamidomethylation | C6:Carbamidomethylation:1000.00;C14:Carbamidomethylation:1000.00 | PEAKS DB |
| A.DIFVDPVLHTAC(+57.02)ALDIK.H | Y | 61.91 | 1925.9921 | 17 | 0.6 | 643.0050 | 3 | 80.84 | 14 | F14:53034 | NaSa01\_F9.raw |  |  |  |  |  |  |  |  |  |  |  |  |  | 4.1632E6 | 2 | 0 | 0 | 0 | 0 | 0 | 0 | 0 | 0 | 0 | 0 | 0 | 0 | 0 | 2 | 1046 | 1062 | Carbamidomethylation | C12:Carbamidomethylation:1000.00 | PEAKS DB |
| R.LSYLLMC(+57.02)LESAVHR.G | Y | 61.57 | 1690.8535 | 14 | 0.0 | 564.6251 | 3 | 75.92 | 4 | F4:52849 | NaSa01\_F12.raw |  |  |  | 3.5748E5 |  |  |  |  |  |  |  |  |  |  | 2 | 0 | 0 | 0 | 2 | 0 | 0 | 0 | 0 | 0 | 0 | 0 | 0 | 0 | 0 | 384 | 397 | Carbamidomethylation | C7:Carbamidomethylation:1000.00 | PEAKS DB |
| K.MTAIIFSDYR.L | Y | 55.58 | 1215.5958 | 10 | 0.6 | 608.8055 | 2 | 57.64 | 4 | F4:37652 | NaSa01\_F12.raw |  |  | 1.4856E5 | 1.7582E6 |  |  |  |  |  |  |  |  |  |  | 2 | 0 | 0 | 1 | 1 | 0 | 0 | 0 | 0 | 0 | 0 | 0 | 0 | 0 | 0 | 209 | 218 |  |  | PEAKS DB |
| K.HSWANNLAVLEC(+57.02)LQDVR.E | Y | 55.40 | 2023.9897 | 17 | 0.1 | 675.6706 | 3 | 71.10 | 4 | F4:48795 | NaSa01\_F12.raw |  |  | 3.6424E5 | 1.7171E6 |  |  |  |  |  |  |  |  |  |  | 2 | 0 | 0 | 1 | 1 | 0 | 0 | 0 | 0 | 0 | 0 | 0 | 0 | 0 | 0 | 115 | 131 | Carbamidomethylation | C12:Carbamidomethylation:1000.00 | PEAKS DB |
| K.AKEESELEGQVISC(+57.02)LK.L | Y | 52.23 | 1818.9033 | 16 | 0.5 | 910.4594 | 2 | 43.69 | 13 | F13:30043 | NaSa01\_F8.raw |  |  |  |  |  |  |  |  |  |  |  |  | 4.3746E6 |  | 2 | 0 | 0 | 0 | 0 | 0 | 0 | 0 | 0 | 0 | 0 | 0 | 0 | 2 | 0 | 943 | 958 | Carbamidomethylation | C14:Carbamidomethylation:1000.00 | PEAKS DB |
| R.VAELSSDDFHLDR.H | Y | 51.43 | 1502.7001 | 13 | 0.2 | 501.9074 | 3 | 38.96 | 6 | F6:19788 | NaSa01\_F14.raw |  |  |  | 2.435E6 |  | 2.5942E5 |  |  |  |  |  |  |  |  | 3 | 0 | 0 | 0 | 2 | 0 | 1 | 0 | 0 | 0 | 0 | 0 | 0 | 0 | 0 | 284 | 296 |  |  | PEAKS DB |
| R.QMSC(+57.02)LMEALEDKR.V | Y | 48.60 | 1609.7262 | 13 | -0.3 | 537.5825 | 3 | 45.06 | 14 | F14:24054 | NaSa01\_F9.raw |  |  |  |  |  |  |  |  |  |  |  |  |  | 4.9085E5 | 1 | 0 | 0 | 0 | 0 | 0 | 0 | 0 | 0 | 0 | 0 | 0 | 0 | 0 | 1 | 1075 | 1087 | Carbamidomethylation | C4:Carbamidomethylation:1000.00 | PEAKS DB |
| R.MLMEDFSLSPEIILGC(+57.02)R.T | Y | 43.73 | 2009.9624 | 17 | 0.5 | 1005.9890 | 2 | 100.55 | 3 | F3:68781 | NaSa01\_F11.raw |  |  | 2.5783E5 |  |  |  |  |  |  |  |  |  |  |  | 1 | 0 | 0 | 1 | 0 | 0 | 0 | 0 | 0 | 0 | 0 | 0 | 0 | 0 | 0 | 415 | 431 | Carbamidomethylation | C16:Carbamidomethylation:1000.00 | PEAKS DB |
| T.EMMDPELDYTLMR.V | Y | 40.89 | 1642.7041 | 13 | 1.1 | 822.3602 | 2 | 70.47 | 11 | F11:49102 | NaSa01\_F6.raw |  |  |  |  |  |  |  |  |  |  | 5.4593E5 |  |  |  | 1 | 0 | 0 | 0 | 0 | 0 | 0 | 0 | 0 | 0 | 0 | 1 | 0 | 0 | 0 | 856 | 868 |  |  | PEAKS DB |
| R.LIC(+57.02)GFMDHC(+57.02)R.S | Y | 39.14 | 1307.5574 | 10 | 0.2 | 436.8598 | 3 | 25.71 | 4 | F4:11092 | NaSa01\_F12.raw |  |  |  | 1.668E5 |  |  |  |  |  |  |  |  |  |  | 1 | 0 | 0 | 0 | 1 | 0 | 0 | 0 | 0 | 0 | 0 | 0 | 0 | 0 | 0 | 219 | 228 | Carbamidomethylation | C3:Carbamidomethylation:1000.00;C9:Carbamidomethylation:1000.00 | PEAKS DB |
| R.LLELQYFISR.D | Y | 38.68 | 1280.7129 | 10 | 0.5 | 641.3640 | 2 | 79.02 | 4 | F4:55464 | NaSa01\_F12.raw |  |  |  | 4.8808E5 |  |  |  |  |  |  |  |  |  |  | 1 | 0 | 0 | 0 | 1 | 0 | 0 | 0 | 0 | 0 | 0 | 0 | 0 | 0 | 0 | 532 | 541 |  |  | PEAKS DB |
| total 14 peptides |  |  |  |  |  |  |  |  |  |  |  |  |  |  |  |  |  |  |  |  |  |  |  |  |  |  |  |  |  |  |  |  |  |  |  |  |  |  |  |  |  |  |  |  |  |
| --- | --- | --- | --- | --- | --- | --- | --- | --- | --- | --- | --- | --- | --- | --- | --- | --- | --- | --- | --- | --- | --- | --- | --- | --- | --- | --- | --- | --- | --- | --- | --- | --- | --- | --- | --- | --- | --- | --- | --- | --- | --- | --- | --- | --- | --- |

ABN72539.1

back to list

  

| Protein Coverage
| Supporting Peptides
|

Protein Coverage:

Supporting Peptides:

| Peptide | Uniq | -10lgP | Mass | Length | ppm | m/z | z | RT | Fraction | Scan | Source File | Area F1 | Area F10 | Area F11 | Area F12 | Area F13 | Area F14 | Area F2 | Area F3 | Area F4 | Area F5 | Area F6 | Area F7 | Area F8 | Area F9 | #Feature | #Feature F1 | #Feature F10 | #Feature F11 | #Feature F12 | #Feature F13 | #Feature F14 | #Feature F2 | #Feature F3 | #Feature F4 | #Feature F5 | #Feature F6 | #Feature F7 | #Feature F8 | #Feature F9 | Start | End | PTM | AScore | Found By |
| --- | --- | --- | --- | --- | --- | --- | --- | --- | --- | --- | --- | --- | --- | --- | --- | --- | --- | --- | --- | --- | --- | --- | --- | --- | --- | --- | --- | --- | --- | --- | --- | --- | --- | --- | --- | --- | --- | --- | --- | --- | --- | --- | --- | --- | --- |
| K.EGWYVNMGPM(+15.99)R.L | N | 73.07 | 1354.5798 | 11 | 0.4 | 678.2975 | 2 | 56.26 | 6 | F6:34728 | NaSa01\_F14.raw |  |  |  |  | 3.9735E7 | 2.4523E8 |  |  |  |  |  |  |  |  | 7 | 0 | 0 | 0 | 0 | 4 | 3 | 0 | 0 | 0 | 0 | 0 | 0 | 0 | 0 | 99 | 109 | Oxidation (M) | M10:Oxidation (M):51.69 | PEAKS DB |
| K.EGWYVNMGPMR.L | N | 71.70 | 1338.5850 | 11 | 0.0 | 670.2997 | 2 | 59.12 | 6 | F6:37086 | NaSa01\_F14.raw |  |  |  |  | 1.3719E8 | 2.0017E9 |  |  |  |  |  |  |  |  | 6 | 0 | 0 | 0 | 0 | 3 | 3 | 0 | 0 | 0 | 0 | 0 | 0 | 0 | 0 | 99 | 109 |  |  | PEAKS DB |
| K.EGWYVNM(+15.99)GPMR.L | N | 67.93 | 1354.5798 | 11 | 0.8 | 678.2977 | 2 | 41.16 | 6 | F6:22021 | NaSa01\_F14.raw |  |  |  | 5.0564E5 | 1.7067E7 | 2.1752E8 |  |  |  |  |  |  |  |  | 5 | 0 | 0 | 0 | 1 | 2 | 2 | 0 | 0 | 0 | 0 | 0 | 0 | 0 | 0 | 99 | 109 | Oxidation (M) | M7:Oxidation (M):39.30 | PEAKS DB |
| K.YDSYSTKEYLIK.E | N | 67.50 | 1508.7399 | 12 | 0.6 | 503.9209 | 3 | 26.71 | 6 | F6:10035 | NaSa01\_F14.raw |  |  |  |  |  | 7.9504E6 |  |  |  |  |  |  |  |  | 2 | 0 | 0 | 0 | 0 | 0 | 2 | 0 | 0 | 0 | 0 | 0 | 0 | 0 | 0 | 199 | 210 |  |  | PEAKS DB |
| K.HVVVVGAGMAGLSAAY.V | N | 67.41 | 1500.7759 | 16 | 0.2 | 751.3954 | 2 | 68.19 | 6 | F6:44843 | NaSa01\_F14.raw |  |  |  |  | 2.067E7 | 2.0687E8 |  |  |  |  |  |  |  |  | 10 | 0 | 0 | 0 | 0 | 1 | 9 | 0 | 0 | 0 | 0 | 0 | 0 | 0 | 0 | 54 | 69 |  |  | PEAKS DB |
| K.FWEADGIHGGKSTTDLPSR.F | N | 64.97 | 2072.9915 | 19 | 0.7 | 692.0049 | 3 | 29.66 | 6 | F6:12168 | NaSa01\_F14.raw |  |  |  |  |  | 2.4928E6 |  |  |  |  |  |  |  |  | 1 | 0 | 0 | 0 | 0 | 0 | 1 | 0 | 0 | 0 | 0 | 0 | 0 | 0 | 0 | 354 | 372 |  |  | PEAKS DB |
| K.YPVKPSEEGK.S | N | 62.09 | 1132.5764 | 10 | 1.6 | 567.2964 | 2 | 11.44 | 6 | F6:1813 | NaSa01\_F14.raw |  |  |  |  |  | 1.8552E6 |  |  |  |  |  |  |  |  | 2 | 0 | 0 | 0 | 0 | 0 | 2 | 0 | 0 | 0 | 0 | 0 | 0 | 0 | 0 | 161 | 170 |  |  | PEAKS DB |
| K.FWEADGIHGGK.S | N | 59.16 | 1215.5673 | 11 | 0.2 | 608.7910 | 2 | 11.57 | 6 | F6:1961 | NaSa01\_F14.raw |  |  |  |  | 4.0485E5 | 1.8815E7 |  |  |  |  |  |  |  |  | 9 | 0 | 0 | 0 | 0 | 1 | 8 | 0 | 0 | 0 | 0 | 0 | 0 | 0 | 0 | 354 | 364 |  |  | PEAKS DB |
| K.HVVVVGAGMAGL.S | N | 58.15 | 1108.6063 | 12 | 0.5 | 555.3107 | 2 | 51.19 | 6 | F6:30972 | NaSa01\_F14.raw |  |  |  |  | 1.7665E7 | 2.672E8 |  |  |  |  |  |  |  |  | 3 | 0 | 0 | 0 | 0 | 1 | 2 | 0 | 0 | 0 | 0 | 0 | 0 | 0 | 0 | 54 | 65 |  |  | PEAKS DB |
| K.EGWYVNM(+15.99)GPM(+15.99)R.L | N | 54.52 | 1370.5747 | 11 | -0.6 | 686.2942 | 2 | 35.77 | 6 | F6:19242 | NaSa01\_F14.raw |  |  |  |  | 3.3829E6 | 5.1173E7 |  |  |  |  |  |  |  |  | 8 | 0 | 0 | 0 | 0 | 1 | 7 | 0 | 0 | 0 | 0 | 0 | 0 | 0 | 0 | 99 | 109 | Oxidation (M) | M7:Oxidation (M):1000.00;M10:Oxidation (M):1000.00 | PEAKS DB |
| Q.KFWEADGIHGGK.S | N | 51.66 | 1343.6622 | 12 | -0.1 | 448.8947 | 3 | 11.66 | 5 | F5:2144 | NaSa01\_F13.raw |  |  |  |  | 1.996E6 | 1.1435E7 |  |  |  |  |  |  |  |  | 3 | 0 | 0 | 0 | 0 | 1 | 2 | 0 | 0 | 0 | 0 | 0 | 0 | 0 | 0 | 353 | 364 |  |  | PEAKS DB |
| K.HVVVVGAGMAGLS.A | N | 50.60 | 1195.6383 | 13 | -0.2 | 598.8263 | 2 | 46.44 | 6 | F6:26217 | NaSa01\_F14.raw |  |  |  |  |  | 5.0901E6 |  |  |  |  |  |  |  |  | 1 | 0 | 0 | 0 | 0 | 0 | 1 | 0 | 0 | 0 | 0 | 0 | 0 | 0 | 0 | 54 | 66 |  |  | PEAKS DB |
| K.HVVVVGAGM(+15.99)AGLSAAY.V | N | 50.48 | 1516.7708 | 16 | 0.9 | 759.3934 | 2 | 46.65 | 6 | F6:26724 | NaSa01\_F14.raw |  |  |  |  |  | 2.0076E7 |  |  |  |  |  |  |  |  | 3 | 0 | 0 | 0 | 0 | 0 | 3 | 0 | 0 | 0 | 0 | 0 | 0 | 0 | 0 | 54 | 69 | Oxidation (M) | M9:Oxidation (M):1000.00 | PEAKS DB |
| K.HVVVVGAGMAGLSAA.Y | N | 50.47 | 1337.7125 | 15 | 0.6 | 669.8640 | 2 | 50.72 | 6 | F6:29947 | NaSa01\_F14.raw |  |  |  |  |  | 7.7421E6 |  |  |  |  |  |  |  |  | 1 | 0 | 0 | 0 | 0 | 0 | 1 | 0 | 0 | 0 | 0 | 0 | 0 | 0 | 0 | 54 | 68 |  |  | PEAKS DB |
| K.HVVVVGAGMAGLSA.A | N | 48.82 | 1266.6754 | 14 | 0.3 | 634.3452 | 2 | 49.72 | 6 | F6:29252 | NaSa01\_F14.raw |  |  |  |  |  | 3.9558E6 |  |  |  |  |  |  |  |  | 1 | 0 | 0 | 0 | 0 | 0 | 1 | 0 | 0 | 0 | 0 | 0 | 0 | 0 | 0 | 54 | 67 |  |  | PEAKS DB |
| K.STTDLPSR.F | N | 48.78 | 875.4348 | 8 | 0.1 | 438.7247 | 2 | 11.44 | 6 | F6:1817 | NaSa01\_F14.raw |  |  |  |  |  | 1.0568E6 |  |  |  |  |  |  |  |  | 1 | 0 | 0 | 0 | 0 | 0 | 1 | 0 | 0 | 0 | 0 | 0 | 0 | 0 | 0 | 365 | 372 |  |  | PEAKS DB |
| R.GAVDMIGDLLNEDSSY.Y | N | 48.29 | 1697.7454 | 16 | 1.7 | 849.8814 | 2 | 107.90 | 6 | F6:77609 | NaSa01\_F14.raw |  |  |  |  |  | 1.6853E7 |  |  |  |  |  |  |  |  | 1 | 0 | 0 | 0 | 0 | 0 | 1 | 0 | 0 | 0 | 0 | 0 | 0 | 0 | 0 | 217 | 232 |  |  | PEAKS DB |
| K.HVVVVGAGMAGLSAAYVLEKAGH.R | Y | 48.06 | 2235.1833 | 23 | 9.1 | 746.0752 | 3 | 66.13 | 6 | F6:43104 | NaSa01\_F14.raw |  |  |  |  |  | 0 |  |  |  |  |  |  |  |  | 0 | 0 | 0 | 0 | 0 | 0 | 0 | 0 | 0 | 0 | 0 | 0 | 0 | 0 | 0 | 54 | 76 |  |  | PEAKS DB |
| D.YEEFLEIAR.N | N | 44.25 | 1168.5764 | 9 | -0.6 | 585.2951 | 2 | 55.17 | 6 | F6:33697 | NaSa01\_F14.raw |  |  |  |  |  | 4.5232E5 |  |  |  |  |  |  |  |  | 1 | 0 | 0 | 0 | 0 | 0 | 1 | 0 | 0 | 0 | 0 | 0 | 0 | 0 | 0 | 35 | 43 |  |  | PEAKS DB |
| K.HVVVVGAGM(+15.99)AGL.S | N | 43.09 | 1124.6012 | 12 | 0.5 | 563.3082 | 2 | 50.23 | 6 | F6:30439 | NaSa01\_F14.raw |  |  |  |  | 3.0223E6 | 1.1076E7 |  |  |  |  |  |  |  |  | 3 | 0 | 0 | 0 | 0 | 1 | 2 | 0 | 0 | 0 | 0 | 0 | 0 | 0 | 0 | 54 | 65 | Oxidation (M) | M9:Oxidation (M):1000.00 | PEAKS DB |
| A.DYEEFLEIAR.N | N | 39.92 | 1283.6033 | 10 | -6.5 | 642.8047 | 2 | 76.29 | 6 | F6:51560 | NaSa01\_F14.raw |  |  |  |  |  | 3.9682E6 |  |  |  |  |  |  |  |  | 1 | 0 | 0 | 0 | 0 | 0 | 1 | 0 | 0 | 0 | 0 | 0 | 0 | 0 | 0 | 34 | 43 |  |  | PEAKS DB |
| E.GWYVNMGPMR.L | N | 38.80 | 1209.5424 | 10 | 0.9 | 605.7790 | 2 | 55.07 | 6 | F6:33759 | NaSa01\_F14.raw |  |  |  |  |  | 8.4887E5 |  |  |  |  |  |  |  |  | 1 | 0 | 0 | 0 | 0 | 0 | 1 | 0 | 0 | 0 | 0 | 0 | 0 | 0 | 0 | 100 | 109 |  |  | PEAKS DB |
| total 22 peptides |  |  |  |  |  |  |  |  |  |  |  |  |  |  |  |  |  |  |  |  |  |  |  |  |  |  |  |  |  |  |  |  |  |  |  |  |  |  |  |  |  |  |  |  |  |
| --- | --- | --- | --- | --- | --- | --- | --- | --- | --- | --- | --- | --- | --- | --- | --- | --- | --- | --- | --- | --- | --- | --- | --- | --- | --- | --- | --- | --- | --- | --- | --- | --- | --- | --- | --- | --- | --- | --- | --- | --- | --- | --- | --- | --- | --- |

A8QL51.1

back to list

  

| Protein Coverage
| Supporting Peptides
|

Protein Coverage:

Supporting Peptides:

| Peptide | Uniq | -10lgP | Mass | Length | ppm | m/z | z | RT | Fraction | Scan | Source File | Area F1 | Area F10 | Area F11 | Area F12 | Area F13 | Area F14 | Area F2 | Area F3 | Area F4 | Area F5 | Area F6 | Area F7 | Area F8 | Area F9 | #Feature | #Feature F1 | #Feature F10 | #Feature F11 | #Feature F12 | #Feature F13 | #Feature F14 | #Feature F2 | #Feature F3 | #Feature F4 | #Feature F5 | #Feature F6 | #Feature F7 | #Feature F8 | #Feature F9 | Start | End | PTM | AScore | Found By |
| --- | --- | --- | --- | --- | --- | --- | --- | --- | --- | --- | --- | --- | --- | --- | --- | --- | --- | --- | --- | --- | --- | --- | --- | --- | --- | --- | --- | --- | --- | --- | --- | --- | --- | --- | --- | --- | --- | --- | --- | --- | --- | --- | --- | --- | --- |
| K.EGWYVNMGPM(+15.99)R.L | N | 73.07 | 1354.5798 | 11 | 0.4 | 678.2975 | 2 | 56.26 | 6 | F6:34728 | NaSa01\_F14.raw |  |  |  |  | 3.9735E7 | 2.4523E8 |  |  |  |  |  |  |  |  | 7 | 0 | 0 | 0 | 0 | 4 | 3 | 0 | 0 | 0 | 0 | 0 | 0 | 0 | 0 | 99 | 109 | Oxidation (M) | M10:Oxidation (M):51.69 | PEAKS DB |
| K.EGWYVNMGPMR.L | N | 71.70 | 1338.5850 | 11 | 0.0 | 670.2997 | 2 | 59.12 | 6 | F6:37086 | NaSa01\_F14.raw |  |  |  |  | 1.3719E8 | 2.0017E9 |  |  |  |  |  |  |  |  | 6 | 0 | 0 | 0 | 0 | 3 | 3 | 0 | 0 | 0 | 0 | 0 | 0 | 0 | 0 | 99 | 109 |  |  | PEAKS DB |
| K.EGWYVNM(+15.99)GPMR.L | N | 67.93 | 1354.5798 | 11 | 0.8 | 678.2977 | 2 | 41.16 | 6 | F6:22021 | NaSa01\_F14.raw |  |  |  | 5.0564E5 | 1.7067E7 | 2.1752E8 |  |  |  |  |  |  |  |  | 5 | 0 | 0 | 0 | 1 | 2 | 2 | 0 | 0 | 0 | 0 | 0 | 0 | 0 | 0 | 99 | 109 | Oxidation (M) | M7:Oxidation (M):39.30 | PEAKS DB |
| K.YDSYSTKEYLIK.E | N | 67.50 | 1508.7399 | 12 | 0.6 | 503.9209 | 3 | 26.71 | 6 | F6:10035 | NaSa01\_F14.raw |  |  |  |  |  | 7.9504E6 |  |  |  |  |  |  |  |  | 2 | 0 | 0 | 0 | 0 | 0 | 2 | 0 | 0 | 0 | 0 | 0 | 0 | 0 | 0 | 199 | 210 |  |  | PEAKS DB |
| K.HVVVVGAGMAGLSAAY.V | N | 67.41 | 1500.7759 | 16 | 0.2 | 751.3954 | 2 | 68.19 | 6 | F6:44843 | NaSa01\_F14.raw |  |  |  |  | 2.067E7 | 2.0687E8 |  |  |  |  |  |  |  |  | 10 | 0 | 0 | 0 | 0 | 1 | 9 | 0 | 0 | 0 | 0 | 0 | 0 | 0 | 0 | 54 | 69 |  |  | PEAKS DB |
| K.FWEADGIHGGKSTTDLPSR.F | N | 64.97 | 2072.9915 | 19 | 0.7 | 692.0049 | 3 | 29.66 | 6 | F6:12168 | NaSa01\_F14.raw |  |  |  |  |  | 2.4928E6 |  |  |  |  |  |  |  |  | 1 | 0 | 0 | 0 | 0 | 0 | 1 | 0 | 0 | 0 | 0 | 0 | 0 | 0 | 0 | 354 | 372 |  |  | PEAKS DB |
| K.YPVKPSEEGK.S | N | 62.09 | 1132.5764 | 10 | 1.6 | 567.2964 | 2 | 11.44 | 6 | F6:1813 | NaSa01\_F14.raw |  |  |  |  |  | 1.8552E6 |  |  |  |  |  |  |  |  | 2 | 0 | 0 | 0 | 0 | 0 | 2 | 0 | 0 | 0 | 0 | 0 | 0 | 0 | 0 | 161 | 170 |  |  | PEAKS DB |
| K.FWEADGIHGGK.S | N | 59.16 | 1215.5673 | 11 | 0.2 | 608.7910 | 2 | 11.57 | 6 | F6:1961 | NaSa01\_F14.raw |  |  |  |  | 4.0485E5 | 1.8815E7 |  |  |  |  |  |  |  |  | 9 | 0 | 0 | 0 | 0 | 1 | 8 | 0 | 0 | 0 | 0 | 0 | 0 | 0 | 0 | 354 | 364 |  |  | PEAKS DB |
| K.HVVVVGAGMAGL.S | N | 58.15 | 1108.6063 | 12 | 0.5 | 555.3107 | 2 | 51.19 | 6 | F6:30972 | NaSa01\_F14.raw |  |  |  |  | 1.7665E7 | 2.672E8 |  |  |  |  |  |  |  |  | 3 | 0 | 0 | 0 | 0 | 1 | 2 | 0 | 0 | 0 | 0 | 0 | 0 | 0 | 0 | 54 | 65 |  |  | PEAKS DB |
| K.EGWYVNM(+15.99)GPM(+15.99)R.L | N | 54.52 | 1370.5747 | 11 | -0.6 | 686.2942 | 2 | 35.77 | 6 | F6:19242 | NaSa01\_F14.raw |  |  |  |  | 3.3829E6 | 5.1173E7 |  |  |  |  |  |  |  |  | 8 | 0 | 0 | 0 | 0 | 1 | 7 | 0 | 0 | 0 | 0 | 0 | 0 | 0 | 0 | 99 | 109 | Oxidation (M) | M7:Oxidation (M):1000.00;M10:Oxidation (M):1000.00 | PEAKS DB |
| Q.KFWEADGIHGGK.S | N | 51.66 | 1343.6622 | 12 | -0.1 | 448.8947 | 3 | 11.66 | 5 | F5:2144 | NaSa01\_F13.raw |  |  |  |  | 1.996E6 | 1.1435E7 |  |  |  |  |  |  |  |  | 3 | 0 | 0 | 0 | 0 | 1 | 2 | 0 | 0 | 0 | 0 | 0 | 0 | 0 | 0 | 353 | 364 |  |  | PEAKS DB |
| K.HVVVVGAGMAGLS.A | N | 50.60 | 1195.6383 | 13 | -0.2 | 598.8263 | 2 | 46.44 | 6 | F6:26217 | NaSa01\_F14.raw |  |  |  |  |  | 5.0901E6 |  |  |  |  |  |  |  |  | 1 | 0 | 0 | 0 | 0 | 0 | 1 | 0 | 0 | 0 | 0 | 0 | 0 | 0 | 0 | 54 | 66 |  |  | PEAKS DB |
| K.HVVVVGAGM(+15.99)AGLSAAY.V | N | 50.48 | 1516.7708 | 16 | 0.9 | 759.3934 | 2 | 46.65 | 6 | F6:26724 | NaSa01\_F14.raw |  |  |  |  |  | 2.0076E7 |  |  |  |  |  |  |  |  | 3 | 0 | 0 | 0 | 0 | 0 | 3 | 0 | 0 | 0 | 0 | 0 | 0 | 0 | 0 | 54 | 69 | Oxidation (M) | M9:Oxidation (M):1000.00 | PEAKS DB |
| K.HVVVVGAGMAGLSAA.Y | N | 50.47 | 1337.7125 | 15 | 0.6 | 669.8640 | 2 | 50.72 | 6 | F6:29947 | NaSa01\_F14.raw |  |  |  |  |  | 7.7421E6 |  |  |  |  |  |  |  |  | 1 | 0 | 0 | 0 | 0 | 0 | 1 | 0 | 0 | 0 | 0 | 0 | 0 | 0 | 0 | 54 | 68 |  |  | PEAKS DB |
| K.HVVVVGAGMAGLSA.A | N | 48.82 | 1266.6754 | 14 | 0.3 | 634.3452 | 2 | 49.72 | 6 | F6:29252 | NaSa01\_F14.raw |  |  |  |  |  | 3.9558E6 |  |  |  |  |  |  |  |  | 1 | 0 | 0 | 0 | 0 | 0 | 1 | 0 | 0 | 0 | 0 | 0 | 0 | 0 | 0 | 54 | 67 |  |  | PEAKS DB |
| K.STTDLPSR.F | N | 48.78 | 875.4348 | 8 | 0.1 | 438.7247 | 2 | 11.44 | 6 | F6:1817 | NaSa01\_F14.raw |  |  |  |  |  | 1.0568E6 |  |  |  |  |  |  |  |  | 1 | 0 | 0 | 0 | 0 | 0 | 1 | 0 | 0 | 0 | 0 | 0 | 0 | 0 | 0 | 365 | 372 |  |  | PEAKS DB |
| R.GAVDMIGDLLNEDSSY.Y | N | 48.29 | 1697.7454 | 16 | 1.7 | 849.8814 | 2 | 107.90 | 6 | F6:77609 | NaSa01\_F14.raw |  |  |  |  |  | 1.6853E7 |  |  |  |  |  |  |  |  | 1 | 0 | 0 | 0 | 0 | 0 | 1 | 0 | 0 | 0 | 0 | 0 | 0 | 0 | 0 | 217 | 232 |  |  | PEAKS DB |
| K.HVVVVGAGMAGLSAAYVLEKAGH.R | Y | 48.06 | 2235.1833 | 23 | 9.1 | 746.0752 | 3 | 66.13 | 6 | F6:43104 | NaSa01\_F14.raw |  |  |  |  |  | 0 |  |  |  |  |  |  |  |  | 0 | 0 | 0 | 0 | 0 | 0 | 0 | 0 | 0 | 0 | 0 | 0 | 0 | 0 | 0 | 54 | 76 |  |  | PEAKS DB |
| D.YEEFLEIAR.N | N | 44.25 | 1168.5764 | 9 | -0.6 | 585.2951 | 2 | 55.17 | 6 | F6:33697 | NaSa01\_F14.raw |  |  |  |  |  | 4.5232E5 |  |  |  |  |  |  |  |  | 1 | 0 | 0 | 0 | 0 | 0 | 1 | 0 | 0 | 0 | 0 | 0 | 0 | 0 | 0 | 35 | 43 |  |  | PEAKS DB |
| K.HVVVVGAGM(+15.99)AGL.S | N | 43.09 | 1124.6012 | 12 | 0.5 | 563.3082 | 2 | 50.23 | 6 | F6:30439 | NaSa01\_F14.raw |  |  |  |  | 3.0223E6 | 1.1076E7 |  |  |  |  |  |  |  |  | 3 | 0 | 0 | 0 | 0 | 1 | 2 | 0 | 0 | 0 | 0 | 0 | 0 | 0 | 0 | 54 | 65 | Oxidation (M) | M9:Oxidation (M):1000.00 | PEAKS DB |
| A.DYEEFLEIAR.N | N | 39.92 | 1283.6033 | 10 | -6.5 | 642.8047 | 2 | 76.29 | 6 | F6:51560 | NaSa01\_F14.raw |  |  |  |  |  | 3.9682E6 |  |  |  |  |  |  |  |  | 1 | 0 | 0 | 0 | 0 | 0 | 1 | 0 | 0 | 0 | 0 | 0 | 0 | 0 | 0 | 34 | 43 |  |  | PEAKS DB |
| E.GWYVNMGPMR.L | N | 38.80 | 1209.5424 | 10 | 0.9 | 605.7790 | 2 | 55.07 | 6 | F6:33759 | NaSa01\_F14.raw |  |  |  |  |  | 8.4887E5 |  |  |  |  |  |  |  |  | 1 | 0 | 0 | 0 | 0 | 0 | 1 | 0 | 0 | 0 | 0 | 0 | 0 | 0 | 0 | 100 | 109 |  |  | PEAKS DB |
| total 22 peptides |  |  |  |  |  |  |  |  |  |  |  |  |  |  |  |  |  |  |  |  |  |  |  |  |  |  |  |  |  |  |  |  |  |  |  |  |  |  |  |  |  |  |  |  |  |
| --- | --- | --- | --- | --- | --- | --- | --- | --- | --- | --- | --- | --- | --- | --- | --- | --- | --- | --- | --- | --- | --- | --- | --- | --- | --- | --- | --- | --- | --- | --- | --- | --- | --- | --- | --- | --- | --- | --- | --- | --- | --- | --- | --- | --- | --- |

P82463.1

back to list

  

| Protein Coverage
| Supporting Peptides
|

Protein Coverage:

Supporting Peptides:

| Peptide | Uniq | -10lgP | Mass | Length | ppm | m/z | z | RT | Fraction | Scan | Source File | Area F1 | Area F10 | Area F11 | Area F12 | Area F13 | Area F14 | Area F2 | Area F3 | Area F4 | Area F5 | Area F6 | Area F7 | Area F8 | Area F9 | #Feature | #Feature F1 | #Feature F10 | #Feature F11 | #Feature F12 | #Feature F13 | #Feature F14 | #Feature F2 | #Feature F3 | #Feature F4 | #Feature F5 | #Feature F6 | #Feature F7 | #Feature F8 | #Feature F9 | Start | End | PTM | AScore | Found By |
| --- | --- | --- | --- | --- | --- | --- | --- | --- | --- | --- | --- | --- | --- | --- | --- | --- | --- | --- | --- | --- | --- | --- | --- | --- | --- | --- | --- | --- | --- | --- | --- | --- | --- | --- | --- | --- | --- | --- | --- | --- | --- | --- | --- | --- | --- |
| K.SIFGVTTEDC(+57.02)PDGQNLC(+57.02)FK.R | Y | 88.73 | 2186.9612 | 19 | 0.6 | 1094.4885 | 2 | 66.81 | 14 | F14:41770 | NaSa01\_F9.raw |  | 2.7994E6 | 2.389E6 | 6.4607E6 |  |  |  |  |  | 3.8053E10 | 1.703E8 | 2.6228E7 | 1.411E7 | 8.1596E6 | 33 | 0 | 1 | 1 | 2 | 0 | 0 | 0 | 0 | 0 | 19 | 4 | 3 | 2 | 1 | 8 | 26 | Carbamidomethylation | C10:Carbamidomethylation:1000.00;C17:Carbamidomethylation:1000.00 | PEAKS DB |
| R.GC(+57.02)AATC(+57.02)PIAENRDVIEC(+57.02)C(+57.02)STDK.C | Y | 65.63 | 2526.0608 | 22 | 0.9 | 843.0283 | 3 | 26.17 | 10 | F10:11392 | NaSa01\_F5.raw |  |  |  |  |  |  |  |  |  | 1.4966E7 | 6.5965E6 |  |  |  | 3 | 0 | 0 | 0 | 0 | 0 | 0 | 0 | 0 | 0 | 2 | 1 | 0 | 0 | 0 | 41 | 62 | Carbamidomethylation | C2:Carbamidomethylation:1000.00;C6:Carbamidomethylation:1000.00;C17:Carbamidomethylation:1000.00;C18:Carbamidomethylation:1000.00 | PEAKS DB |
| K.SIFGVTTEDC(+57.02)PDGQNLC(+57.02)FKR.W | Y | 63.72 | 2343.0623 | 20 | 2.5 | 782.0300 | 3 | 51.62 | 10 | F10:32061 | NaSa01\_F5.raw |  |  |  |  |  |  |  |  |  | 1.0969E8 | 5.0693E6 |  |  |  | 4 | 0 | 0 | 0 | 0 | 0 | 0 | 0 | 0 | 0 | 2 | 2 | 0 | 0 | 0 | 8 | 27 | Carbamidomethylation | C10:Carbamidomethylation:1000.00;C17:Carbamidomethylation:1000.00 | PEAKS DB |
| R.WHMIVPGR.Y | N | 61.36 | 994.5171 | 8 | 0.0 | 498.2658 | 2 | 27.65 | 12 | F12:13546 | NaSa01\_F7.raw |  | 1.3757E6 |  | 8.2304E5 |  | 1.7633E5 |  |  |  | 1.1532E10 | 1.155E9 | 1.9845E7 | 3.841E6 |  | 29 | 0 | 1 | 0 | 1 | 0 | 1 | 0 | 0 | 0 | 22 | 2 | 1 | 1 | 0 | 28 | 35 |  |  | PEAKS DB |
| R.DVIEC(+57.02)C(+57.02)STDKC(+57.02)NL | Y | 59.29 | 1612.6531 | 13 | 0.4 | 807.3342 | 2 | 29.45 | 10 | F10:14056 | NaSa01\_F5.raw |  |  |  |  |  |  |  |  |  | 1.2405E7 |  |  |  |  | 1 | 0 | 0 | 0 | 0 | 0 | 0 | 0 | 0 | 0 | 1 | 0 | 0 | 0 | 0 | 53 | 65 | Carbamidomethylation | C5:Carbamidomethylation:1000.00;C6:Carbamidomethylation:1000.00;C11:Carbamidomethylation:1000.00 | PEAKS DB |
| K.EKSIFGVTTEDC(+57.02)PDGQNLC(+57.02)FK.R | Y | 58.19 | 2444.0989 | 21 | 1.0 | 815.7077 | 3 | 54.29 | 10 | F10:34536 | NaSa01\_F5.raw |  |  |  |  |  |  |  |  |  | 9.0188E6 |  |  |  |  | 1 | 0 | 0 | 0 | 0 | 0 | 0 | 0 | 0 | 0 | 1 | 0 | 0 | 0 | 0 | 6 | 26 | Carbamidomethylation | C12:Carbamidomethylation:1000.00;C19:Carbamidomethylation:1000.00 | PEAKS DB |
| F.GVTTEDC(+57.02)PDGQNLC(+57.02)FK.R | Y | 56.86 | 1839.7767 | 16 | 0.6 | 920.8962 | 2 | 34.21 | 10 | F10:17964 | NaSa01\_F5.raw |  |  |  |  |  |  |  |  |  | 1.6273E7 |  |  |  |  | 1 | 0 | 0 | 0 | 0 | 0 | 0 | 0 | 0 | 0 | 1 | 0 | 0 | 0 | 0 | 11 | 26 | Carbamidomethylation | C7:Carbamidomethylation:1000.00;C14:Carbamidomethylation:1000.00 | PEAKS DB |
| K.RWHMIVPGR.Y | N | 55.76 | 1150.6182 | 9 | -1.0 | 384.5463 | 3 | 12.23 | 10 | F10:2218 | NaSa01\_F5.raw |  |  |  |  |  |  |  |  |  | 4.0095E6 | 2.8217E5 |  |  |  | 3 | 0 | 0 | 0 | 0 | 0 | 0 | 0 | 0 | 0 | 2 | 1 | 0 | 0 | 0 | 27 | 35 |  |  | PEAKS DB |
| R.GC(+57.02)AATC(+57.02)PIAENR.D | Y | 54.24 | 1318.5758 | 12 | 1.1 | 660.2959 | 2 | 11.72 | 10 | F10:1790 | NaSa01\_F5.raw |  |  |  |  |  |  |  |  |  | 1.0191E7 |  |  |  |  | 2 | 0 | 0 | 0 | 0 | 0 | 0 | 0 | 0 | 0 | 2 | 0 | 0 | 0 | 0 | 41 | 52 | Carbamidomethylation | C2:Carbamidomethylation:1000.00;C6:Carbamidomethylation:1000.00 | PEAKS DB |
| R.WHM(+15.99)IVPGR.Y | N | 52.04 | 1010.5120 | 8 | 1.1 | 506.2639 | 2 | 17.03 | 10 | F10:6282 | NaSa01\_F5.raw |  |  |  |  |  |  |  |  |  | 4.9608E8 | 1.2332E8 |  |  |  | 14 | 0 | 0 | 0 | 0 | 0 | 0 | 0 | 0 | 0 | 8 | 6 | 0 | 0 | 0 | 28 | 35 | Oxidation (M) | M3:Oxidation (M):1000.00 | PEAKS DB |
| E.KSIFGVTTEDC(+57.02)PDGQNLC(+57.02)FK.R | Y | 51.57 | 2315.0562 | 20 | -0.5 | 772.6923 | 3 | 50.48 | 10 | F10:31632 | NaSa01\_F5.raw |  |  |  |  |  |  |  |  |  | 1.8804E7 |  |  |  |  | 2 | 0 | 0 | 0 | 0 | 0 | 0 | 0 | 0 | 0 | 2 | 0 | 0 | 0 | 0 | 7 | 26 | Carbamidomethylation | C11:Carbamidomethylation:1000.00;C18:Carbamidomethylation:1000.00 | PEAKS DB |
| LTC(+57.02)VKEKSIFGVTTEDC(+57.02)PDGQNLC(+57.02)FK.R | Y | 41.23 | 3045.4246 | 26 | 0.9 | 762.3641 | 4 | 47.17 | 10 | F10:28901 | NaSa01\_F5.raw |  |  |  |  |  |  |  |  |  | 4.4752E6 |  |  |  |  | 1 | 0 | 0 | 0 | 0 | 0 | 0 | 0 | 0 | 0 | 1 | 0 | 0 | 0 | 0 | 1 | 26 | Carbamidomethylation | C3:Carbamidomethylation:1000.00;C17:Carbamidomethylation:1000.00;C24:Carbamidomethylation:1000.00 | PEAKS DB |
| total 12 peptides |  |  |  |  |  |  |  |  |  |  |  |  |  |  |  |  |  |  |  |  |  |  |  |  |  |  |  |  |  |  |  |  |  |  |  |  |  |  |  |  |  |  |  |  |  |
| --- | --- | --- | --- | --- | --- | --- | --- | --- | --- | --- | --- | --- | --- | --- | --- | --- | --- | --- | --- | --- | --- | --- | --- | --- | --- | --- | --- | --- | --- | --- | --- | --- | --- | --- | --- | --- | --- | --- | --- | --- | --- | --- | --- | --- | --- |

AAM51550.1

back to list

  

| Protein Coverage
| Supporting Peptides
|

Protein Coverage:

Supporting Peptides:

| Peptide | Uniq | -10lgP | Mass | Length | ppm | m/z | z | RT | Fraction | Scan | Source File | Area F1 | Area F10 | Area F11 | Area F12 | Area F13 | Area F14 | Area F2 | Area F3 | Area F4 | Area F5 | Area F6 | Area F7 | Area F8 | Area F9 | #Feature | #Feature F1 | #Feature F10 | #Feature F11 | #Feature F12 | #Feature F13 | #Feature F14 | #Feature F2 | #Feature F3 | #Feature F4 | #Feature F5 | #Feature F6 | #Feature F7 | #Feature F8 | #Feature F9 | Start | End | PTM | AScore | Found By |
| --- | --- | --- | --- | --- | --- | --- | --- | --- | --- | --- | --- | --- | --- | --- | --- | --- | --- | --- | --- | --- | --- | --- | --- | --- | --- | --- | --- | --- | --- | --- | --- | --- | --- | --- | --- | --- | --- | --- | --- | --- | --- | --- | --- | --- | --- |
| R.VYEMVNALNTMYR.R | Y | 82.22 | 1602.7534 | 13 | -0.2 | 802.3838 | 2 | 64.72 | 6 | F6:42241 | NaSa01\_F14.raw |  |  |  |  | 1.2523E9 | 1.681E9 |  |  |  |  |  |  |  |  | 28 | 0 | 0 | 0 | 0 | 7 | 21 | 0 | 0 | 0 | 0 | 0 | 0 | 0 | 0 | 232 | 244 |  |  | PEAKS DB |
| R.VYEM(+15.99)VNALNTMYR.R | Y | 76.33 | 1618.7483 | 13 | -0.5 | 810.3810 | 2 | 46.45 | 6 | F6:26764 | NaSa01\_F14.raw |  |  |  |  | 1.4619E8 | 1.8182E8 |  |  |  |  |  |  |  |  | 15 | 0 | 0 | 0 | 0 | 8 | 7 | 0 | 0 | 0 | 0 | 0 | 0 | 0 | 0 | 232 | 244 | Oxidation (M) | M4:Oxidation (M):120.29 | PEAKS DB |
| R.VYEMVNALNTM(+15.99)YR.R | Y | 73.21 | 1618.7483 | 13 | 0.1 | 810.3815 | 2 | 51.71 | 6 | F6:30885 | NaSa01\_F14.raw |  |  |  |  | 1.6748E8 | 1.7646E8 |  |  |  |  |  |  |  |  | 11 | 0 | 0 | 0 | 0 | 7 | 4 | 0 | 0 | 0 | 0 | 0 | 0 | 0 | 0 | 232 | 244 | Oxidation (M) | M11:Oxidation (M):136.94 | PEAKS DB |
| R.VYEM(+15.99)VNALNTM(+15.99)YR.R | Y | 67.36 | 1634.7433 | 13 | 0.7 | 818.3795 | 2 | 35.13 | 6 | F6:16690 | NaSa01\_F14.raw |  |  |  |  | 1.6196E7 | 1.8076E7 |  |  |  |  |  |  |  |  | 5 | 0 | 0 | 0 | 0 | 2 | 3 | 0 | 0 | 0 | 0 | 0 | 0 | 0 | 0 | 232 | 244 | Oxidation (M) | M4:Oxidation (M):1000.00;M11:Oxidation (M):1000.00 | PEAKS DB |
| Q.ATLDLFGEWR.E | N | 64.95 | 1206.6033 | 10 | -1.0 | 604.3083 | 2 | 78.54 | 6 | F6:53113 | NaSa01\_F14.raw |  |  | 1.7813E6 | 2.0969E6 | 9.7051E6 | 2.2371E9 |  |  |  |  |  |  |  | 1.3846E6 | 13 | 0 | 0 | 1 | 1 | 1 | 9 | 0 | 0 | 0 | 0 | 0 | 0 | 0 | 1 | 272 | 281 |  |  | PEAKS DB |
| R.RVYEMVNALNTMYR.R | Y | 62.74 | 1758.8545 | 14 | 0.2 | 587.2922 | 3 | 52.94 | 5 | F5:32686 | NaSa01\_F13.raw |  |  |  |  | 3.4741E6 | 3.7312E5 |  |  |  |  |  |  |  |  | 2 | 0 | 0 | 0 | 0 | 1 | 1 | 0 | 0 | 0 | 0 | 0 | 0 | 0 | 0 | 231 | 244 |  |  | PEAKS DB |
| R.DRPQC(+57.02)ILNKPSR.K | Y | 62.57 | 1482.7725 | 12 | 0.1 | 495.2648 | 3 | 11.43 | 6 | F6:1820 | NaSa01\_F14.raw |  |  |  |  |  | 4.4523E5 |  |  |  |  |  |  |  |  | 1 | 0 | 0 | 0 | 0 | 0 | 1 | 0 | 0 | 0 | 0 | 0 | 0 | 0 | 0 | 391 | 402 | Carbamidomethylation | C5:Carbamidomethylation:1000.00 | PEAKS DB |
| Y.EMVNALNTMYR.R | Y | 57.94 | 1340.6217 | 11 | 0.0 | 671.3181 | 2 | 45.49 | 6 | F6:25491 | NaSa01\_F14.raw |  |  |  |  | 3.2449E6 | 3.9417E6 |  |  |  |  |  |  |  |  | 2 | 0 | 0 | 0 | 0 | 1 | 1 | 0 | 0 | 0 | 0 | 0 | 0 | 0 | 0 | 234 | 244 |  |  | PEAKS DB |
| K.SVAVVQDHSK.S | Y | 54.93 | 1068.5564 | 10 | 0.5 | 535.2858 | 2 | 11.43 | 6 | F6:1829 | NaSa01\_F14.raw |  |  |  |  |  | 5.6023E5 |  |  |  |  |  |  |  |  | 1 | 0 | 0 | 0 | 0 | 0 | 1 | 0 | 0 | 0 | 0 | 0 | 0 | 0 | 0 | 321 | 330 |  |  | PEAKS DB |
| L.TNTPEQDR.Y | N | 44.60 | 959.4308 | 8 | 0.1 | 480.7227 | 2 | 11.42 | 6 | F6:1814 | NaSa01\_F14.raw |  |  |  |  |  | 1.6427E5 |  |  |  |  |  |  |  |  | 1 | 0 | 0 | 0 | 0 | 0 | 1 | 0 | 0 | 0 | 0 | 0 | 0 | 0 | 0 | 192 | 199 |  |  | PEAKS DB |
| A.TLDLFGEWR.E | N | 43.65 | 1135.5662 | 9 | -0.8 | 568.7899 | 2 | 77.17 | 6 | F6:52295 | NaSa01\_F14.raw |  |  |  |  |  | 1.3701E6 |  |  |  |  |  |  |  |  | 1 | 0 | 0 | 0 | 0 | 0 | 1 | 0 | 0 | 0 | 0 | 0 | 0 | 0 | 0 | 273 | 281 |  |  | PEAKS DB |
| R.VYEMVNALN.T | Y | 43.56 | 1051.5009 | 9 | 0.8 | 526.7581 | 2 | 55.57 | 6 | F6:34337 | NaSa01\_F14.raw |  |  |  |  |  | 1.1362E7 |  |  |  |  |  |  |  |  | 1 | 0 | 0 | 0 | 0 | 0 | 1 | 0 | 0 | 0 | 0 | 0 | 0 | 0 | 0 | 232 | 240 |  |  | PEAKS DB |
| K.NDC(+57.02)DFPELC(+57.02)TGR.S | Y | 43.11 | 1482.5868 | 12 | -2.8 | 742.2986 | 2 | 57.74 | 6 | F6:35996 | NaSa01\_F14.raw |  |  |  |  |  | 0 |  |  |  |  |  |  |  |  | 0 | 0 | 0 | 0 | 0 | 0 | 0 | 0 | 0 | 0 | 0 | 0 | 0 | 0 | 0 | 471 | 482 | Carbamidomethylation | C3:Carbamidomethylation:1000.00;C9:Carbamidomethylation:1000.00 | PEAKS DB |
| R.RVYEMVNALNTM(+15.99)YR.R | Y | 42.50 | 1774.8495 | 14 | 0.4 | 592.6240 | 3 | 41.48 | 5 | F5:22847 | NaSa01\_F13.raw |  |  |  |  | 1.0527E6 |  |  |  |  |  |  |  |  |  | 1 | 0 | 0 | 0 | 0 | 1 | 0 | 0 | 0 | 0 | 0 | 0 | 0 | 0 | 0 | 231 | 244 | Oxidation (M) | M12:Oxidation (M):71.72 | PEAKS DB |
| K.MSPGLC(+57.02)FMLN.W | Y | 40.35 | 1168.5079 | 10 | 0.3 | 585.2614 | 2 | 87.24 | 6 | F6:60616 | NaSa01\_F14.raw |  |  |  |  |  | 8.3727E6 |  |  |  |  |  |  |  |  | 1 | 0 | 0 | 0 | 0 | 0 | 1 | 0 | 0 | 0 | 0 | 0 | 0 | 0 | 0 | 527 | 536 | Carbamidomethylation | C6:Carbamidomethylation:1000.00 | PEAKS DB |
| K.MSPGLC(+57.02)FM.L | Y | 39.01 | 941.3809 | 8 | 0.4 | 471.6979 | 2 | 76.54 | 6 | F6:51754 | NaSa01\_F14.raw |  |  |  |  |  | 6.3291E5 |  |  |  |  |  |  |  |  | 1 | 0 | 0 | 0 | 0 | 0 | 1 | 0 | 0 | 0 | 0 | 0 | 0 | 0 | 0 | 527 | 534 | Carbamidomethylation | C6:Carbamidomethylation:1000.00 | PEAKS DB |
| total 16 peptides |  |  |  |  |  |  |  |  |  |  |  |  |  |  |  |  |  |  |  |  |  |  |  |  |  |  |  |  |  |  |  |  |  |  |  |  |  |  |  |  |  |  |  |  |  |
| --- | --- | --- | --- | --- | --- | --- | --- | --- | --- | --- | --- | --- | --- | --- | --- | --- | --- | --- | --- | --- | --- | --- | --- | --- | --- | --- | --- | --- | --- | --- | --- | --- | --- | --- | --- | --- | --- | --- | --- | --- | --- | --- | --- | --- | --- |

Q10749.3

back to list

  

| Protein Coverage
| Supporting Peptides
|

Protein Coverage:

Supporting Peptides:

| Peptide | Uniq | -10lgP | Mass | Length | ppm | m/z | z | RT | Fraction | Scan | Source File | Area F1 | Area F10 | Area F11 | Area F12 | Area F13 | Area F14 | Area F2 | Area F3 | Area F4 | Area F5 | Area F6 | Area F7 | Area F8 | Area F9 | #Feature | #Feature F1 | #Feature F10 | #Feature F11 | #Feature F12 | #Feature F13 | #Feature F14 | #Feature F2 | #Feature F3 | #Feature F4 | #Feature F5 | #Feature F6 | #Feature F7 | #Feature F8 | #Feature F9 | Start | End | PTM | AScore | Found By |
| --- | --- | --- | --- | --- | --- | --- | --- | --- | --- | --- | --- | --- | --- | --- | --- | --- | --- | --- | --- | --- | --- | --- | --- | --- | --- | --- | --- | --- | --- | --- | --- | --- | --- | --- | --- | --- | --- | --- | --- | --- | --- | --- | --- | --- | --- |
| R.VYEMVNALNTMYR.R | Y | 82.22 | 1602.7534 | 13 | -0.2 | 802.3838 | 2 | 64.72 | 6 | F6:42241 | NaSa01\_F14.raw |  |  |  |  | 1.2523E9 | 1.681E9 |  |  |  |  |  |  |  |  | 28 | 0 | 0 | 0 | 0 | 7 | 21 | 0 | 0 | 0 | 0 | 0 | 0 | 0 | 0 | 232 | 244 |  |  | PEAKS DB |
| R.VYEM(+15.99)VNALNTMYR.R | Y | 76.33 | 1618.7483 | 13 | -0.5 | 810.3810 | 2 | 46.45 | 6 | F6:26764 | NaSa01\_F14.raw |  |  |  |  | 1.4619E8 | 1.8182E8 |  |  |  |  |  |  |  |  | 15 | 0 | 0 | 0 | 0 | 8 | 7 | 0 | 0 | 0 | 0 | 0 | 0 | 0 | 0 | 232 | 244 | Oxidation (M) | M4:Oxidation (M):120.29 | PEAKS DB |
| R.VYEMVNALNTM(+15.99)YR.R | Y | 73.21 | 1618.7483 | 13 | 0.1 | 810.3815 | 2 | 51.71 | 6 | F6:30885 | NaSa01\_F14.raw |  |  |  |  | 1.6748E8 | 1.7646E8 |  |  |  |  |  |  |  |  | 11 | 0 | 0 | 0 | 0 | 7 | 4 | 0 | 0 | 0 | 0 | 0 | 0 | 0 | 0 | 232 | 244 | Oxidation (M) | M11:Oxidation (M):136.94 | PEAKS DB |
| R.VYEM(+15.99)VNALNTM(+15.99)YR.R | Y | 67.36 | 1634.7433 | 13 | 0.7 | 818.3795 | 2 | 35.13 | 6 | F6:16690 | NaSa01\_F14.raw |  |  |  |  | 1.6196E7 | 1.8076E7 |  |  |  |  |  |  |  |  | 5 | 0 | 0 | 0 | 0 | 2 | 3 | 0 | 0 | 0 | 0 | 0 | 0 | 0 | 0 | 232 | 244 | Oxidation (M) | M4:Oxidation (M):1000.00;M11:Oxidation (M):1000.00 | PEAKS DB |
| Q.ATLDLFGEWR.E | N | 64.95 | 1206.6033 | 10 | -1.0 | 604.3083 | 2 | 78.54 | 6 | F6:53113 | NaSa01\_F14.raw |  |  | 1.7813E6 | 2.0969E6 | 9.7051E6 | 2.2371E9 |  |  |  |  |  |  |  | 1.3846E6 | 13 | 0 | 0 | 1 | 1 | 1 | 9 | 0 | 0 | 0 | 0 | 0 | 0 | 0 | 1 | 272 | 281 |  |  | PEAKS DB |
| R.RVYEMVNALNTMYR.R | Y | 62.74 | 1758.8545 | 14 | 0.2 | 587.2922 | 3 | 52.94 | 5 | F5:32686 | NaSa01\_F13.raw |  |  |  |  | 3.4741E6 | 3.7312E5 |  |  |  |  |  |  |  |  | 2 | 0 | 0 | 0 | 0 | 1 | 1 | 0 | 0 | 0 | 0 | 0 | 0 | 0 | 0 | 231 | 244 |  |  | PEAKS DB |
| R.DRPQC(+57.02)ILNKPSR.K | Y | 62.57 | 1482.7725 | 12 | 0.1 | 495.2648 | 3 | 11.43 | 6 | F6:1820 | NaSa01\_F14.raw |  |  |  |  |  | 4.4523E5 |  |  |  |  |  |  |  |  | 1 | 0 | 0 | 0 | 0 | 0 | 1 | 0 | 0 | 0 | 0 | 0 | 0 | 0 | 0 | 391 | 402 | Carbamidomethylation | C5:Carbamidomethylation:1000.00 | PEAKS DB |
| Y.EMVNALNTMYR.R | Y | 57.94 | 1340.6217 | 11 | 0.0 | 671.3181 | 2 | 45.49 | 6 | F6:25491 | NaSa01\_F14.raw |  |  |  |  | 3.2449E6 | 3.9417E6 |  |  |  |  |  |  |  |  | 2 | 0 | 0 | 0 | 0 | 1 | 1 | 0 | 0 | 0 | 0 | 0 | 0 | 0 | 0 | 234 | 244 |  |  | PEAKS DB |
| K.SVAVVQDHSK.S | Y | 54.93 | 1068.5564 | 10 | 0.5 | 535.2858 | 2 | 11.43 | 6 | F6:1829 | NaSa01\_F14.raw |  |  |  |  |  | 5.6023E5 |  |  |  |  |  |  |  |  | 1 | 0 | 0 | 0 | 0 | 0 | 1 | 0 | 0 | 0 | 0 | 0 | 0 | 0 | 0 | 321 | 330 |  |  | PEAKS DB |
| L.TNTPEQDR.Y | N | 44.60 | 959.4308 | 8 | 0.1 | 480.7227 | 2 | 11.42 | 6 | F6:1814 | NaSa01\_F14.raw |  |  |  |  |  | 1.6427E5 |  |  |  |  |  |  |  |  | 1 | 0 | 0 | 0 | 0 | 0 | 1 | 0 | 0 | 0 | 0 | 0 | 0 | 0 | 0 | 192 | 199 |  |  | PEAKS DB |
| A.TLDLFGEWR.E | N | 43.65 | 1135.5662 | 9 | -0.8 | 568.7899 | 2 | 77.17 | 6 | F6:52295 | NaSa01\_F14.raw |  |  |  |  |  | 1.3701E6 |  |  |  |  |  |  |  |  | 1 | 0 | 0 | 0 | 0 | 0 | 1 | 0 | 0 | 0 | 0 | 0 | 0 | 0 | 0 | 273 | 281 |  |  | PEAKS DB |
| R.VYEMVNALN.T | Y | 43.56 | 1051.5009 | 9 | 0.8 | 526.7581 | 2 | 55.57 | 6 | F6:34337 | NaSa01\_F14.raw |  |  |  |  |  | 1.1362E7 |  |  |  |  |  |  |  |  | 1 | 0 | 0 | 0 | 0 | 0 | 1 | 0 | 0 | 0 | 0 | 0 | 0 | 0 | 0 | 232 | 240 |  |  | PEAKS DB |
| K.NDC(+57.02)DFPELC(+57.02)TGR.S | Y | 43.11 | 1482.5868 | 12 | -2.8 | 742.2986 | 2 | 57.74 | 6 | F6:35996 | NaSa01\_F14.raw |  |  |  |  |  | 0 |  |  |  |  |  |  |  |  | 0 | 0 | 0 | 0 | 0 | 0 | 0 | 0 | 0 | 0 | 0 | 0 | 0 | 0 | 0 | 471 | 482 | Carbamidomethylation | C3:Carbamidomethylation:1000.00;C9:Carbamidomethylation:1000.00 | PEAKS DB |
| R.RVYEMVNALNTM(+15.99)YR.R | Y | 42.50 | 1774.8495 | 14 | 0.4 | 592.6240 | 3 | 41.48 | 5 | F5:22847 | NaSa01\_F13.raw |  |  |  |  | 1.0527E6 |  |  |  |  |  |  |  |  |  | 1 | 0 | 0 | 0 | 0 | 1 | 0 | 0 | 0 | 0 | 0 | 0 | 0 | 0 | 0 | 231 | 244 | Oxidation (M) | M12:Oxidation (M):71.72 | PEAKS DB |
| K.MSPGLC(+57.02)FMLN.W | Y | 40.35 | 1168.5079 | 10 | 0.3 | 585.2614 | 2 | 87.24 | 6 | F6:60616 | NaSa01\_F14.raw |  |  |  |  |  | 8.3727E6 |  |  |  |  |  |  |  |  | 1 | 0 | 0 | 0 | 0 | 0 | 1 | 0 | 0 | 0 | 0 | 0 | 0 | 0 | 0 | 527 | 536 | Carbamidomethylation | C6:Carbamidomethylation:1000.00 | PEAKS DB |
| K.MSPGLC(+57.02)FM.L | Y | 39.01 | 941.3809 | 8 | 0.4 | 471.6979 | 2 | 76.54 | 6 | F6:51754 | NaSa01\_F14.raw |  |  |  |  |  | 6.3291E5 |  |  |  |  |  |  |  |  | 1 | 0 | 0 | 0 | 0 | 0 | 1 | 0 | 0 | 0 | 0 | 0 | 0 | 0 | 0 | 527 | 534 | Carbamidomethylation | C6:Carbamidomethylation:1000.00 | PEAKS DB |
| total 16 peptides |  |  |  |  |  |  |  |  |  |  |  |  |  |  |  |  |  |  |  |  |  |  |  |  |  |  |  |  |  |  |  |  |  |  |  |  |  |  |  |  |  |  |  |  |  |
| --- | --- | --- | --- | --- | --- | --- | --- | --- | --- | --- | --- | --- | --- | --- | --- | --- | --- | --- | --- | --- | --- | --- | --- | --- | --- | --- | --- | --- | --- | --- | --- | --- | --- | --- | --- | --- | --- | --- | --- | --- | --- | --- | --- | --- | --- |

1JE9

back to list

  

| Protein Coverage
| Supporting Peptides
|

Protein Coverage:

Supporting Peptides:

| Peptide | Uniq | -10lgP | Mass | Length | ppm | m/z | z | RT | Fraction | Scan | Source File | Area F1 | Area F10 | Area F11 | Area F12 | Area F13 | Area F14 | Area F2 | Area F3 | Area F4 | Area F5 | Area F6 | Area F7 | Area F8 | Area F9 | #Feature | #Feature F1 | #Feature F10 | #Feature F11 | #Feature F12 | #Feature F13 | #Feature F14 | #Feature F2 | #Feature F3 | #Feature F4 | #Feature F5 | #Feature F6 | #Feature F7 | #Feature F8 | #Feature F9 | Start | End | PTM | AScore | Found By |
| --- | --- | --- | --- | --- | --- | --- | --- | --- | --- | --- | --- | --- | --- | --- | --- | --- | --- | --- | --- | --- | --- | --- | --- | --- | --- | --- | --- | --- | --- | --- | --- | --- | --- | --- | --- | --- | --- | --- | --- | --- | --- | --- | --- | --- | --- |
| LEC(+57.02)HNQQSSQAPTTK.T | Y | 94.12 | 1727.7897 | 15 | 2.6 | 864.9018 | 2 | 11.85 | 7 | F7:1793 | NaSa01\_F2.raw |  |  |  |  |  |  | 3.965E7 | 7.7122E5 |  |  |  |  |  |  | 4 | 0 | 0 | 0 | 0 | 0 | 0 | 3 | 1 | 0 | 0 | 0 | 0 | 0 | 0 | 1 | 15 | Carbamidomethylation | C3:Carbamidomethylation:1000.00 | PEAKS DB |
| K.VKPGVNLNC(+57.02)C(+57.02)R.T | N | 70.88 | 1315.6489 | 11 | 3.4 | 439.5571 | 3 | 15.40 | 7 | F7:4041 | NaSa01\_F2.raw | 6.4726E6 |  |  |  |  |  | 9.7026E8 | 5.2894E7 | 1.73E6 |  |  | 6.2028E5 |  |  | 29 | 1 | 0 | 0 | 0 | 0 | 0 | 21 | 5 | 1 | 0 | 0 | 1 | 0 | 0 | 45 | 55 | Carbamidomethylation | C9:Carbamidomethylation:1000.00;C10:Carbamidomethylation:1000.00 | PEAKS DB |
| K.PGVNLNC(+57.02)C(+57.02)R.T | N | 65.54 | 1088.4856 | 9 | 3.3 | 545.2502 | 2 | 11.84 | 7 | F7:1796 | NaSa01\_F2.raw |  |  |  |  |  |  | 1.1229E7 | 5.157E5 |  |  |  |  |  |  | 2 | 0 | 0 | 0 | 0 | 0 | 0 | 1 | 1 | 0 | 0 | 0 | 0 | 0 | 0 | 47 | 55 | Carbamidomethylation | C7:Carbamidomethylation:1000.00;C8:Carbamidomethylation:1000.00 | PEAKS DB |
| K.WWSDHRGTIIER.G | N | 65.49 | 1554.7692 | 12 | 2.5 | 519.2634 | 3 | 24.28 | 7 | F7:11177 | NaSa01\_F2.raw |  |  |  |  |  |  | 8.5193E7 |  |  |  |  |  |  |  | 6 | 0 | 0 | 0 | 0 | 0 | 0 | 6 | 0 | 0 | 0 | 0 | 0 | 0 | 0 | 27 | 38 |  |  | PEAKS DB |
| K.KWWSDHR.G | N | 64.93 | 1013.4832 | 7 | 3.6 | 507.7492 | 2 | 11.87 | 7 | F7:1799 | NaSa01\_F2.raw |  |  |  |  |  |  | 1.9831E7 | 2.8577E5 |  |  |  |  |  |  | 12 | 0 | 0 | 0 | 0 | 0 | 0 | 11 | 1 | 0 | 0 | 0 | 0 | 0 | 0 | 26 | 32 |  |  | PEAKS DB |
| K.TC(+57.02)SGETNC(+57.02)YKKWWSDHR.G | N | 62.15 | 2213.9370 | 17 | 3.9 | 554.4921 | 4 | 11.88 | 7 | F7:1862 | NaSa01\_F2.raw |  |  |  |  |  |  | 3.6341E6 |  |  |  |  |  |  |  | 1 | 0 | 0 | 0 | 0 | 0 | 0 | 1 | 0 | 0 | 0 | 0 | 0 | 0 | 0 | 16 | 32 | Carbamidomethylation | C2:Carbamidomethylation:1000.00;C8:Carbamidomethylation:1000.00 | PEAKS DB |
| K.TC(+57.02)SGETNC(+57.02)YK.K | N | 50.93 | 1218.4645 | 10 | 3.3 | 610.2397 | 2 | 11.85 | 7 | F7:1792 | NaSa01\_F2.raw |  |  |  |  |  |  | 5.231E6 |  |  |  |  |  |  |  | 1 | 0 | 0 | 0 | 0 | 0 | 0 | 1 | 0 | 0 | 0 | 0 | 0 | 0 | 0 | 16 | 25 | Carbamidomethylation | C2:Carbamidomethylation:1000.00;C8:Carbamidomethylation:1000.00 | PEAKS DB |
| K.VKPGVNLN.C | N | 43.70 | 839.4865 | 8 | 4.5 | 420.7512 | 2 | 12.01 | 7 | F7:1901 | NaSa01\_F2.raw |  |  |  |  |  |  | 4.946E7 |  |  |  |  |  |  |  | 1 | 0 | 0 | 0 | 0 | 0 | 0 | 1 | 0 | 0 | 0 | 0 | 0 | 0 | 0 | 45 | 52 |  |  | PEAKS DB |
| K.VKPGVNLNC(+57.02)C(+57.02).R | N | 41.90 | 1159.5479 | 10 | 4.0 | 580.7818 | 2 | 12.55 | 7 | F7:2420 | NaSa01\_F2.raw |  |  |  |  |  |  | 1.0026E5 |  |  |  |  |  |  |  | 1 | 0 | 0 | 0 | 0 | 0 | 0 | 1 | 0 | 0 | 0 | 0 | 0 | 0 | 0 | 45 | 54 | Carbamidomethylation | C9:Carbamidomethylation:1000.00;C10:Carbamidomethylation:1000.00 | PEAKS DB |
| total 9 peptides |  |  |  |  |  |  |  |  |  |  |  |  |  |  |  |  |  |  |  |  |  |  |  |  |  |  |  |  |  |  |  |  |  |  |  |  |  |  |  |  |  |  |  |  |  |
| --- | --- | --- | --- | --- | --- | --- | --- | --- | --- | --- | --- | --- | --- | --- | --- | --- | --- | --- | --- | --- | --- | --- | --- | --- | --- | --- | --- | --- | --- | --- | --- | --- | --- | --- | --- | --- | --- | --- | --- | --- | --- | --- | --- | --- | --- |

P59276.1

back to list

  

| Protein Coverage
| Supporting Peptides
|

Protein Coverage:

Supporting Peptides:

| Peptide | Uniq | -10lgP | Mass | Length | ppm | m/z | z | RT | Fraction | Scan | Source File | Area F1 | Area F10 | Area F11 | Area F12 | Area F13 | Area F14 | Area F2 | Area F3 | Area F4 | Area F5 | Area F6 | Area F7 | Area F8 | Area F9 | #Feature | #Feature F1 | #Feature F10 | #Feature F11 | #Feature F12 | #Feature F13 | #Feature F14 | #Feature F2 | #Feature F3 | #Feature F4 | #Feature F5 | #Feature F6 | #Feature F7 | #Feature F8 | #Feature F9 | Start | End | PTM | AScore | Found By |
| --- | --- | --- | --- | --- | --- | --- | --- | --- | --- | --- | --- | --- | --- | --- | --- | --- | --- | --- | --- | --- | --- | --- | --- | --- | --- | --- | --- | --- | --- | --- | --- | --- | --- | --- | --- | --- | --- | --- | --- | --- | --- | --- | --- | --- | --- |
| LEC(+57.02)HNQQSSQAPTTK.T | Y | 94.12 | 1727.7897 | 15 | 2.6 | 864.9018 | 2 | 11.85 | 7 | F7:1793 | NaSa01\_F2.raw |  |  |  |  |  |  | 3.965E7 | 7.7122E5 |  |  |  |  |  |  | 4 | 0 | 0 | 0 | 0 | 0 | 0 | 3 | 1 | 0 | 0 | 0 | 0 | 0 | 0 | 1 | 15 | Carbamidomethylation | C3:Carbamidomethylation:1000.00 | PEAKS DB |
| K.VKPGVNLNC(+57.02)C(+57.02)R.T | N | 70.88 | 1315.6489 | 11 | 3.4 | 439.5571 | 3 | 15.40 | 7 | F7:4041 | NaSa01\_F2.raw | 6.4726E6 |  |  |  |  |  | 9.7026E8 | 5.2894E7 | 1.73E6 |  |  | 6.2028E5 |  |  | 29 | 1 | 0 | 0 | 0 | 0 | 0 | 21 | 5 | 1 | 0 | 0 | 1 | 0 | 0 | 45 | 55 | Carbamidomethylation | C9:Carbamidomethylation:1000.00;C10:Carbamidomethylation:1000.00 | PEAKS DB |
| K.PGVNLNC(+57.02)C(+57.02)R.T | N | 65.54 | 1088.4856 | 9 | 3.3 | 545.2502 | 2 | 11.84 | 7 | F7:1796 | NaSa01\_F2.raw |  |  |  |  |  |  | 1.1229E7 | 5.157E5 |  |  |  |  |  |  | 2 | 0 | 0 | 0 | 0 | 0 | 0 | 1 | 1 | 0 | 0 | 0 | 0 | 0 | 0 | 47 | 55 | Carbamidomethylation | C7:Carbamidomethylation:1000.00;C8:Carbamidomethylation:1000.00 | PEAKS DB |
| K.WWSDHRGTIIER.G | N | 65.49 | 1554.7692 | 12 | 2.5 | 519.2634 | 3 | 24.28 | 7 | F7:11177 | NaSa01\_F2.raw |  |  |  |  |  |  | 8.5193E7 |  |  |  |  |  |  |  | 6 | 0 | 0 | 0 | 0 | 0 | 0 | 6 | 0 | 0 | 0 | 0 | 0 | 0 | 0 | 27 | 38 |  |  | PEAKS DB |
| K.KWWSDHR.G | N | 64.93 | 1013.4832 | 7 | 3.6 | 507.7492 | 2 | 11.87 | 7 | F7:1799 | NaSa01\_F2.raw |  |  |  |  |  |  | 1.9831E7 | 2.8577E5 |  |  |  |  |  |  | 12 | 0 | 0 | 0 | 0 | 0 | 0 | 11 | 1 | 0 | 0 | 0 | 0 | 0 | 0 | 26 | 32 |  |  | PEAKS DB |
| K.TC(+57.02)SGETNC(+57.02)YKKWWSDHR.G | N | 62.15 | 2213.9370 | 17 | 3.9 | 554.4921 | 4 | 11.88 | 7 | F7:1862 | NaSa01\_F2.raw |  |  |  |  |  |  | 3.6341E6 |  |  |  |  |  |  |  | 1 | 0 | 0 | 0 | 0 | 0 | 0 | 1 | 0 | 0 | 0 | 0 | 0 | 0 | 0 | 16 | 32 | Carbamidomethylation | C2:Carbamidomethylation:1000.00;C8:Carbamidomethylation:1000.00 | PEAKS DB |
| K.TC(+57.02)SGETNC(+57.02)YK.K | N | 50.93 | 1218.4645 | 10 | 3.3 | 610.2397 | 2 | 11.85 | 7 | F7:1792 | NaSa01\_F2.raw |  |  |  |  |  |  | 5.231E6 |  |  |  |  |  |  |  | 1 | 0 | 0 | 0 | 0 | 0 | 0 | 1 | 0 | 0 | 0 | 0 | 0 | 0 | 0 | 16 | 25 | Carbamidomethylation | C2:Carbamidomethylation:1000.00;C8:Carbamidomethylation:1000.00 | PEAKS DB |
| K.VKPGVNLN.C | N | 43.70 | 839.4865 | 8 | 4.5 | 420.7512 | 2 | 12.01 | 7 | F7:1901 | NaSa01\_F2.raw |  |  |  |  |  |  | 4.946E7 |  |  |  |  |  |  |  | 1 | 0 | 0 | 0 | 0 | 0 | 0 | 1 | 0 | 0 | 0 | 0 | 0 | 0 | 0 | 45 | 52 |  |  | PEAKS DB |
| K.VKPGVNLNC(+57.02)C(+57.02).R | N | 41.90 | 1159.5479 | 10 | 4.0 | 580.7818 | 2 | 12.55 | 7 | F7:2420 | NaSa01\_F2.raw |  |  |  |  |  |  | 1.0026E5 |  |  |  |  |  |  |  | 1 | 0 | 0 | 0 | 0 | 0 | 0 | 1 | 0 | 0 | 0 | 0 | 0 | 0 | 0 | 45 | 54 | Carbamidomethylation | C9:Carbamidomethylation:1000.00;C10:Carbamidomethylation:1000.00 | PEAKS DB |
| total 9 peptides |  |  |  |  |  |  |  |  |  |  |  |  |  |  |  |  |  |  |  |  |  |  |  |  |  |  |  |  |  |  |  |  |  |  |  |  |  |  |  |  |  |  |  |  |  |
| --- | --- | --- | --- | --- | --- | --- | --- | --- | --- | --- | --- | --- | --- | --- | --- | --- | --- | --- | --- | --- | --- | --- | --- | --- | --- | --- | --- | --- | --- | --- | --- | --- | --- | --- | --- | --- | --- | --- | --- | --- | --- | --- | --- | --- | --- |

Q9W727.1

back to list

  

| Protein Coverage
| Supporting Peptides
|

Protein Coverage:

Supporting Peptides:

| Peptide | Uniq | -10lgP | Mass | Length | ppm | m/z | z | RT | Fraction | Scan | Source File | Area F1 | Area F10 | Area F11 | Area F12 | Area F13 | Area F14 | Area F2 | Area F3 | Area F4 | Area F5 | Area F6 | Area F7 | Area F8 | Area F9 | #Feature | #Feature F1 | #Feature F10 | #Feature F11 | #Feature F12 | #Feature F13 | #Feature F14 | #Feature F2 | #Feature F3 | #Feature F4 | #Feature F5 | #Feature F6 | #Feature F7 | #Feature F8 | #Feature F9 | Start | End | PTM | AScore | Found By |
| --- | --- | --- | --- | --- | --- | --- | --- | --- | --- | --- | --- | --- | --- | --- | --- | --- | --- | --- | --- | --- | --- | --- | --- | --- | --- | --- | --- | --- | --- | --- | --- | --- | --- | --- | --- | --- | --- | --- | --- | --- | --- | --- | --- | --- | --- |
| R.TPETTEIC(+57.02)PDSWYFC(+57.02)YK.I | Y | 81.74 | 2195.9180 | 17 | -0.7 | 1098.9655 | 2 | 71.65 | 13 | F13:53840 | NaSa01\_F8.raw |  | 1.7723E6 | 2.8638E6 | 2.2356E6 |  |  |  |  |  | 5.0538E7 | 5.2497E8 | 4.9973E7 | 2.1128E7 | 1.097E7 | 12 | 0 | 1 | 2 | 1 | 0 | 0 | 0 | 0 | 0 | 1 | 3 | 2 | 1 | 1 | 31 | 47 | Carbamidomethylation | C8:Carbamidomethylation:1000.00;C15:Carbamidomethylation:1000.00 | PEAKS DB |
| R.GC(+57.02)TFTC(+57.02)PELRPTGK.Y | Y | 79.83 | 1622.7545 | 14 | 0.4 | 812.3848 | 2 | 22.47 | 12 | F12:8775 | NaSa01\_F7.raw |  |  |  | 9.3921E5 |  |  |  |  | 6.803E5 | 8.999E7 | 9.0442E9 | 1.5862E8 | 2.4179E7 |  | 12 | 0 | 0 | 0 | 1 | 0 | 0 | 0 | 0 | 1 | 2 | 4 | 2 | 2 | 0 | 61 | 74 | Carbamidomethylation | C2:Carbamidomethylation:1000.00;C6:Carbamidomethylation:1000.00 | PEAKS DB |
| T.TEIC(+57.02)PDSWYFC(+57.02)YK.I | N | 56.79 | 1767.7273 | 13 | 0.5 | 884.8713 | 2 | 66.72 | 12 | F12:45597 | NaSa01\_F7.raw |  |  |  |  |  |  |  |  |  | 1.292E6 |  | 1.6669E6 |  | 6.9702E5 | 3 | 0 | 0 | 0 | 0 | 0 | 0 | 0 | 0 | 0 | 1 | 0 | 1 | 0 | 1 | 35 | 47 | Carbamidomethylation | C4:Carbamidomethylation:1000.00;C11:Carbamidomethylation:1000.00 | PEAKS DB |
| K.RGC(+57.02)TFTC(+57.02)PELRPTGK.Y | Y | 55.40 | 1778.8556 | 15 | 0.2 | 445.7213 | 4 | 12.03 | 11 | F11:2167 | NaSa01\_F6.raw |  |  |  |  |  |  |  |  |  |  | 2.5597E6 | 5.36E5 |  |  | 2 | 0 | 0 | 0 | 0 | 0 | 0 | 0 | 0 | 0 | 0 | 1 | 1 | 0 | 0 | 60 | 74 | Carbamidomethylation | C3:Carbamidomethylation:1000.00;C7:Carbamidomethylation:1000.00 | PEAKS DB |
| G.YTTIC(+57.02)YNHLSR.T | Y | 54.48 | 1426.6664 | 11 | -0.3 | 476.5626 | 3 | 17.82 | 12 | F12:4946 | NaSa01\_F7.raw |  |  |  |  |  |  |  |  |  |  | 2.8348E5 | 3.8627E6 |  |  | 3 | 0 | 0 | 0 | 0 | 0 | 0 | 0 | 0 | 0 | 0 | 1 | 2 | 0 | 0 | 20 | 30 | Carbamidomethylation | C5:Carbamidomethylation:1000.00 | PEAKS DB |
| S.RTPETTEIC(+57.02)PDSWYFC(+57.02)YK.I | Y | 50.51 | 2352.0190 | 18 | 0.2 | 785.0138 | 3 | 56.62 | 11 | F11:38298 | NaSa01\_F6.raw |  |  |  |  |  |  |  |  |  |  | 1.4651E7 |  |  |  | 1 | 0 | 0 | 0 | 0 | 0 | 0 | 0 | 0 | 0 | 0 | 1 | 0 | 0 | 0 | 30 | 47 | Carbamidomethylation | C9:Carbamidomethylation:1000.00;C16:Carbamidomethylation:1000.00 | PEAKS DB |
| K.ISLADGNDVR.I | N | 50.19 | 1058.5356 | 10 | 0.1 | 530.2751 | 2 | 21.09 | 12 | F12:7712 | NaSa01\_F7.raw |  |  |  |  |  |  |  |  |  |  |  | 1.1584E5 |  |  | 1 | 0 | 0 | 0 | 0 | 0 | 0 | 0 | 0 | 0 | 0 | 0 | 1 | 0 | 0 | 48 | 57 |  |  | PEAKS DB |
| P.DSWYFC(+57.02)YK.I | N | 48.52 | 1167.4695 | 8 | 0.7 | 584.7424 | 2 | 58.18 | 12 | F12:38630 | NaSa01\_F7.raw |  |  |  |  |  |  |  |  |  |  |  | 1.6222E6 |  |  | 1 | 0 | 0 | 0 | 0 | 0 | 0 | 0 | 0 | 0 | 0 | 0 | 1 | 0 | 0 | 40 | 47 | Carbamidomethylation | C6:Carbamidomethylation:1000.00 | PEAKS DB |
| R.GC(+57.02)TFTC(+57.02)PELR.P | N | 47.93 | 1239.5376 | 10 | 0.1 | 620.7761 | 2 | 29.29 | 9 | F9:9432 | NaSa01\_F4.raw |  |  |  |  |  |  |  |  | 1.4766E6 |  | 2.7398E7 | 2.0728E6 |  |  | 3 | 0 | 0 | 0 | 0 | 0 | 0 | 0 | 0 | 1 | 0 | 1 | 1 | 0 | 0 | 61 | 70 | Carbamidomethylation | C2:Carbamidomethylation:1000.00;C6:Carbamidomethylation:1000.00 | PEAKS DB |
| R.TPETTEIC(+57.02)PDSWYF.C | Y | 38.78 | 1744.7290 | 14 | 2.3 | 873.3738 | 2 | 91.30 | 11 | F11:65616 | NaSa01\_F6.raw |  |  |  |  |  |  |  |  |  |  | 2.3407E6 |  |  |  | 1 | 0 | 0 | 0 | 0 | 0 | 0 | 0 | 0 | 0 | 0 | 1 | 0 | 0 | 0 | 31 | 44 | Carbamidomethylation | C8:Carbamidomethylation:1000.00 | PEAKS DB |
| total 10 peptides |  |  |  |  |  |  |  |  |  |  |  |  |  |  |  |  |  |  |  |  |  |  |  |  |  |  |  |  |  |  |  |  |  |  |  |  |  |  |  |  |  |  |  |  |  |
| --- | --- | --- | --- | --- | --- | --- | --- | --- | --- | --- | --- | --- | --- | --- | --- | --- | --- | --- | --- | --- | --- | --- | --- | --- | --- | --- | --- | --- | --- | --- | --- | --- | --- | --- | --- | --- | --- | --- | --- | --- | --- | --- | --- | --- | --- |

Q9DEQ3.1

back to list

  

| Protein Coverage
| Supporting Peptides
|

Protein Coverage:

Supporting Peptides:

| Peptide | Uniq | -10lgP | Mass | Length | ppm | m/z | z | RT | Fraction | Scan | Source File | Area F1 | Area F10 | Area F11 | Area F12 | Area F13 | Area F14 | Area F2 | Area F3 | Area F4 | Area F5 | Area F6 | Area F7 | Area F8 | Area F9 | #Feature | #Feature F1 | #Feature F10 | #Feature F11 | #Feature F12 | #Feature F13 | #Feature F14 | #Feature F2 | #Feature F3 | #Feature F4 | #Feature F5 | #Feature F6 | #Feature F7 | #Feature F8 | #Feature F9 | Start | End | PTM | AScore | Found By |
| --- | --- | --- | --- | --- | --- | --- | --- | --- | --- | --- | --- | --- | --- | --- | --- | --- | --- | --- | --- | --- | --- | --- | --- | --- | --- | --- | --- | --- | --- | --- | --- | --- | --- | --- | --- | --- | --- | --- | --- | --- | --- | --- | --- | --- | --- |
| R.TPETTEIC(+57.02)PDSWYFC(+57.02)YK.I | Y | 81.74 | 2195.9180 | 17 | -0.7 | 1098.9655 | 2 | 71.65 | 13 | F13:53840 | NaSa01\_F8.raw |  | 1.7723E6 | 2.8638E6 | 2.2356E6 |  |  |  |  |  | 5.0538E7 | 5.2497E8 | 4.9973E7 | 2.1128E7 | 1.097E7 | 12 | 0 | 1 | 2 | 1 | 0 | 0 | 0 | 0 | 0 | 1 | 3 | 2 | 1 | 1 | 31 | 47 | Carbamidomethylation | C8:Carbamidomethylation:1000.00;C15:Carbamidomethylation:1000.00 | PEAKS DB |
| R.GC(+57.02)TFTC(+57.02)PELRPTGK.Y | Y | 79.83 | 1622.7545 | 14 | 0.4 | 812.3848 | 2 | 22.47 | 12 | F12:8775 | NaSa01\_F7.raw |  |  |  | 9.3921E5 |  |  |  |  | 6.803E5 | 8.999E7 | 9.0442E9 | 1.5862E8 | 2.4179E7 |  | 12 | 0 | 0 | 0 | 1 | 0 | 0 | 0 | 0 | 1 | 2 | 4 | 2 | 2 | 0 | 61 | 74 | Carbamidomethylation | C2:Carbamidomethylation:1000.00;C6:Carbamidomethylation:1000.00 | PEAKS DB |
| T.TEIC(+57.02)PDSWYFC(+57.02)YK.I | N | 56.79 | 1767.7273 | 13 | 0.5 | 884.8713 | 2 | 66.72 | 12 | F12:45597 | NaSa01\_F7.raw |  |  |  |  |  |  |  |  |  | 1.292E6 |  | 1.6669E6 |  | 6.9702E5 | 3 | 0 | 0 | 0 | 0 | 0 | 0 | 0 | 0 | 0 | 1 | 0 | 1 | 0 | 1 | 35 | 47 | Carbamidomethylation | C4:Carbamidomethylation:1000.00;C11:Carbamidomethylation:1000.00 | PEAKS DB |
| K.RGC(+57.02)TFTC(+57.02)PELRPTGK.Y | Y | 55.40 | 1778.8556 | 15 | 0.2 | 445.7213 | 4 | 12.03 | 11 | F11:2167 | NaSa01\_F6.raw |  |  |  |  |  |  |  |  |  |  | 2.5597E6 | 5.36E5 |  |  | 2 | 0 | 0 | 0 | 0 | 0 | 0 | 0 | 0 | 0 | 0 | 1 | 1 | 0 | 0 | 60 | 74 | Carbamidomethylation | C3:Carbamidomethylation:1000.00;C7:Carbamidomethylation:1000.00 | PEAKS DB |
| G.YTTIC(+57.02)YNHLSR.T | Y | 54.48 | 1426.6664 | 11 | -0.3 | 476.5626 | 3 | 17.82 | 12 | F12:4946 | NaSa01\_F7.raw |  |  |  |  |  |  |  |  |  |  | 2.8348E5 | 3.8627E6 |  |  | 3 | 0 | 0 | 0 | 0 | 0 | 0 | 0 | 0 | 0 | 0 | 1 | 2 | 0 | 0 | 20 | 30 | Carbamidomethylation | C5:Carbamidomethylation:1000.00 | PEAKS DB |
| S.RTPETTEIC(+57.02)PDSWYFC(+57.02)YK.I | Y | 50.51 | 2352.0190 | 18 | 0.2 | 785.0138 | 3 | 56.62 | 11 | F11:38298 | NaSa01\_F6.raw |  |  |  |  |  |  |  |  |  |  | 1.4651E7 |  |  |  | 1 | 0 | 0 | 0 | 0 | 0 | 0 | 0 | 0 | 0 | 0 | 1 | 0 | 0 | 0 | 30 | 47 | Carbamidomethylation | C9:Carbamidomethylation:1000.00;C16:Carbamidomethylation:1000.00 | PEAKS DB |
| K.ISLADGNDVR.I | N | 50.19 | 1058.5356 | 10 | 0.1 | 530.2751 | 2 | 21.09 | 12 | F12:7712 | NaSa01\_F7.raw |  |  |  |  |  |  |  |  |  |  |  | 1.1584E5 |  |  | 1 | 0 | 0 | 0 | 0 | 0 | 0 | 0 | 0 | 0 | 0 | 0 | 1 | 0 | 0 | 48 | 57 |  |  | PEAKS DB |
| P.DSWYFC(+57.02)YK.I | N | 48.52 | 1167.4695 | 8 | 0.7 | 584.7424 | 2 | 58.18 | 12 | F12:38630 | NaSa01\_F7.raw |  |  |  |  |  |  |  |  |  |  |  | 1.6222E6 |  |  | 1 | 0 | 0 | 0 | 0 | 0 | 0 | 0 | 0 | 0 | 0 | 0 | 1 | 0 | 0 | 40 | 47 | Carbamidomethylation | C6:Carbamidomethylation:1000.00 | PEAKS DB |
| R.GC(+57.02)TFTC(+57.02)PELR.P | N | 47.93 | 1239.5376 | 10 | 0.1 | 620.7761 | 2 | 29.29 | 9 | F9:9432 | NaSa01\_F4.raw |  |  |  |  |  |  |  |  | 1.4766E6 |  | 2.7398E7 | 2.0728E6 |  |  | 3 | 0 | 0 | 0 | 0 | 0 | 0 | 0 | 0 | 1 | 0 | 1 | 1 | 0 | 0 | 61 | 70 | Carbamidomethylation | C2:Carbamidomethylation:1000.00;C6:Carbamidomethylation:1000.00 | PEAKS DB |
| R.TPETTEIC(+57.02)PDSWYF.C | Y | 38.78 | 1744.7290 | 14 | 2.3 | 873.3738 | 2 | 91.30 | 11 | F11:65616 | NaSa01\_F6.raw |  |  |  |  |  |  |  |  |  |  | 2.3407E6 |  |  |  | 1 | 0 | 0 | 0 | 0 | 0 | 0 | 0 | 0 | 0 | 0 | 1 | 0 | 0 | 0 | 31 | 44 | Carbamidomethylation | C8:Carbamidomethylation:1000.00 | PEAKS DB |
| total 10 peptides |  |  |  |  |  |  |  |  |  |  |  |  |  |  |  |  |  |  |  |  |  |  |  |  |  |  |  |  |  |  |  |  |  |  |  |  |  |  |  |  |  |  |  |  |  |
| --- | --- | --- | --- | --- | --- | --- | --- | --- | --- | --- | --- | --- | --- | --- | --- | --- | --- | --- | --- | --- | --- | --- | --- | --- | --- | --- | --- | --- | --- | --- | --- | --- | --- | --- | --- | --- | --- | --- | --- | --- | --- | --- | --- | --- | --- |

CAB50691.1

back to list

  

| Protein Coverage
| Supporting Peptides
|

Protein Coverage:

Supporting Peptides:

| Peptide | Uniq | -10lgP | Mass | Length | ppm | m/z | z | RT | Fraction | Scan | Source File | Area F1 | Area F10 | Area F11 | Area F12 | Area F13 | Area F14 | Area F2 | Area F3 | Area F4 | Area F5 | Area F6 | Area F7 | Area F8 | Area F9 | #Feature | #Feature F1 | #Feature F10 | #Feature F11 | #Feature F12 | #Feature F13 | #Feature F14 | #Feature F2 | #Feature F3 | #Feature F4 | #Feature F5 | #Feature F6 | #Feature F7 | #Feature F8 | #Feature F9 | Start | End | PTM | AScore | Found By |
| --- | --- | --- | --- | --- | --- | --- | --- | --- | --- | --- | --- | --- | --- | --- | --- | --- | --- | --- | --- | --- | --- | --- | --- | --- | --- | --- | --- | --- | --- | --- | --- | --- | --- | --- | --- | --- | --- | --- | --- | --- | --- | --- | --- | --- | --- |
| R.TPETTEIC(+57.02)PDSWYFC(+57.02)YK.I | Y | 81.74 | 2195.9180 | 17 | -0.7 | 1098.9655 | 2 | 71.65 | 13 | F13:53840 | NaSa01\_F8.raw |  | 1.7723E6 | 2.8638E6 | 2.2356E6 |  |  |  |  |  | 5.0538E7 | 5.2497E8 | 4.9973E7 | 2.1128E7 | 1.097E7 | 12 | 0 | 1 | 2 | 1 | 0 | 0 | 0 | 0 | 0 | 1 | 3 | 2 | 1 | 1 | 31 | 47 | Carbamidomethylation | C8:Carbamidomethylation:1000.00;C15:Carbamidomethylation:1000.00 | PEAKS DB |
| R.GC(+57.02)TFTC(+57.02)PELRPTGK.Y | Y | 79.83 | 1622.7545 | 14 | 0.4 | 812.3848 | 2 | 22.47 | 12 | F12:8775 | NaSa01\_F7.raw |  |  |  | 9.3921E5 |  |  |  |  | 6.803E5 | 8.999E7 | 9.0442E9 | 1.5862E8 | 2.4179E7 |  | 12 | 0 | 0 | 0 | 1 | 0 | 0 | 0 | 0 | 1 | 2 | 4 | 2 | 2 | 0 | 61 | 74 | Carbamidomethylation | C2:Carbamidomethylation:1000.00;C6:Carbamidomethylation:1000.00 | PEAKS DB |
| T.TEIC(+57.02)PDSWYFC(+57.02)YK.I | N | 56.79 | 1767.7273 | 13 | 0.5 | 884.8713 | 2 | 66.72 | 12 | F12:45597 | NaSa01\_F7.raw |  |  |  |  |  |  |  |  |  | 1.292E6 |  | 1.6669E6 |  | 6.9702E5 | 3 | 0 | 0 | 0 | 0 | 0 | 0 | 0 | 0 | 0 | 1 | 0 | 1 | 0 | 1 | 35 | 47 | Carbamidomethylation | C4:Carbamidomethylation:1000.00;C11:Carbamidomethylation:1000.00 | PEAKS DB |
| K.RGC(+57.02)TFTC(+57.02)PELRPTGK.Y | Y | 55.40 | 1778.8556 | 15 | 0.2 | 445.7213 | 4 | 12.03 | 11 | F11:2167 | NaSa01\_F6.raw |  |  |  |  |  |  |  |  |  |  | 2.5597E6 | 5.36E5 |  |  | 2 | 0 | 0 | 0 | 0 | 0 | 0 | 0 | 0 | 0 | 0 | 1 | 1 | 0 | 0 | 60 | 74 | Carbamidomethylation | C3:Carbamidomethylation:1000.00;C7:Carbamidomethylation:1000.00 | PEAKS DB |
| G.YTTIC(+57.02)YNHLSR.T | Y | 54.48 | 1426.6664 | 11 | -0.3 | 476.5626 | 3 | 17.82 | 12 | F12:4946 | NaSa01\_F7.raw |  |  |  |  |  |  |  |  |  |  | 2.8348E5 | 3.8627E6 |  |  | 3 | 0 | 0 | 0 | 0 | 0 | 0 | 0 | 0 | 0 | 0 | 1 | 2 | 0 | 0 | 20 | 30 | Carbamidomethylation | C5:Carbamidomethylation:1000.00 | PEAKS DB |
| S.RTPETTEIC(+57.02)PDSWYFC(+57.02)YK.I | Y | 50.51 | 2352.0190 | 18 | 0.2 | 785.0138 | 3 | 56.62 | 11 | F11:38298 | NaSa01\_F6.raw |  |  |  |  |  |  |  |  |  |  | 1.4651E7 |  |  |  | 1 | 0 | 0 | 0 | 0 | 0 | 0 | 0 | 0 | 0 | 0 | 1 | 0 | 0 | 0 | 30 | 47 | Carbamidomethylation | C9:Carbamidomethylation:1000.00;C16:Carbamidomethylation:1000.00 | PEAKS DB |
| K.ISLADGNDVR.I | N | 50.19 | 1058.5356 | 10 | 0.1 | 530.2751 | 2 | 21.09 | 12 | F12:7712 | NaSa01\_F7.raw |  |  |  |  |  |  |  |  |  |  |  | 1.1584E5 |  |  | 1 | 0 | 0 | 0 | 0 | 0 | 0 | 0 | 0 | 0 | 0 | 0 | 1 | 0 | 0 | 48 | 57 |  |  | PEAKS DB |
| P.DSWYFC(+57.02)YK.I | N | 48.52 | 1167.4695 | 8 | 0.7 | 584.7424 | 2 | 58.18 | 12 | F12:38630 | NaSa01\_F7.raw |  |  |  |  |  |  |  |  |  |  |  | 1.6222E6 |  |  | 1 | 0 | 0 | 0 | 0 | 0 | 0 | 0 | 0 | 0 | 0 | 0 | 1 | 0 | 0 | 40 | 47 | Carbamidomethylation | C6:Carbamidomethylation:1000.00 | PEAKS DB |
| R.GC(+57.02)TFTC(+57.02)PELR.P | N | 47.93 | 1239.5376 | 10 | 0.1 | 620.7761 | 2 | 29.29 | 9 | F9:9432 | NaSa01\_F4.raw |  |  |  |  |  |  |  |  | 1.4766E6 |  | 2.7398E7 | 2.0728E6 |  |  | 3 | 0 | 0 | 0 | 0 | 0 | 0 | 0 | 0 | 1 | 0 | 1 | 1 | 0 | 0 | 61 | 70 | Carbamidomethylation | C2:Carbamidomethylation:1000.00;C6:Carbamidomethylation:1000.00 | PEAKS DB |
| R.TPETTEIC(+57.02)PDSWYF.C | Y | 38.78 | 1744.7290 | 14 | 2.3 | 873.3738 | 2 | 91.30 | 11 | F11:65616 | NaSa01\_F6.raw |  |  |  |  |  |  |  |  |  |  | 2.3407E6 |  |  |  | 1 | 0 | 0 | 0 | 0 | 0 | 0 | 0 | 0 | 0 | 0 | 1 | 0 | 0 | 0 | 31 | 44 | Carbamidomethylation | C8:Carbamidomethylation:1000.00 | PEAKS DB |
| total 10 peptides |  |  |  |  |  |  |  |  |  |  |  |  |  |  |  |  |  |  |  |  |  |  |  |  |  |  |  |  |  |  |  |  |  |  |  |  |  |  |  |  |  |  |  |  |  |
| --- | --- | --- | --- | --- | --- | --- | --- | --- | --- | --- | --- | --- | --- | --- | --- | --- | --- | --- | --- | --- | --- | --- | --- | --- | --- | --- | --- | --- | --- | --- | --- | --- | --- | --- | --- | --- | --- | --- | --- | --- | --- | --- | --- | --- | --- |

CAC08183.1

back to list

  

| Protein Coverage
| Supporting Peptides
|

Protein Coverage:

Supporting Peptides:

| Peptide | Uniq | -10lgP | Mass | Length | ppm | m/z | z | RT | Fraction | Scan | Source File | Area F1 | Area F10 | Area F11 | Area F12 | Area F13 | Area F14 | Area F2 | Area F3 | Area F4 | Area F5 | Area F6 | Area F7 | Area F8 | Area F9 | #Feature | #Feature F1 | #Feature F10 | #Feature F11 | #Feature F12 | #Feature F13 | #Feature F14 | #Feature F2 | #Feature F3 | #Feature F4 | #Feature F5 | #Feature F6 | #Feature F7 | #Feature F8 | #Feature F9 | Start | End | PTM | AScore | Found By |
| --- | --- | --- | --- | --- | --- | --- | --- | --- | --- | --- | --- | --- | --- | --- | --- | --- | --- | --- | --- | --- | --- | --- | --- | --- | --- | --- | --- | --- | --- | --- | --- | --- | --- | --- | --- | --- | --- | --- | --- | --- | --- | --- | --- | --- | --- |
| R.TPETTEIC(+57.02)PDSWYFC(+57.02)YK.I | Y | 81.74 | 2195.9180 | 17 | -0.7 | 1098.9655 | 2 | 71.65 | 13 | F13:53840 | NaSa01\_F8.raw |  | 1.7723E6 | 2.8638E6 | 2.2356E6 |  |  |  |  |  | 5.0538E7 | 5.2497E8 | 4.9973E7 | 2.1128E7 | 1.097E7 | 12 | 0 | 1 | 2 | 1 | 0 | 0 | 0 | 0 | 0 | 1 | 3 | 2 | 1 | 1 | 31 | 47 | Carbamidomethylation | C8:Carbamidomethylation:1000.00;C15:Carbamidomethylation:1000.00 | PEAKS DB |
| R.GC(+57.02)TFTC(+57.02)PELRPTGK.Y | Y | 79.83 | 1622.7545 | 14 | 0.4 | 812.3848 | 2 | 22.47 | 12 | F12:8775 | NaSa01\_F7.raw |  |  |  | 9.3921E5 |  |  |  |  | 6.803E5 | 8.999E7 | 9.0442E9 | 1.5862E8 | 2.4179E7 |  | 12 | 0 | 0 | 0 | 1 | 0 | 0 | 0 | 0 | 1 | 2 | 4 | 2 | 2 | 0 | 61 | 74 | Carbamidomethylation | C2:Carbamidomethylation:1000.00;C6:Carbamidomethylation:1000.00 | PEAKS DB |
| T.TEIC(+57.02)PDSWYFC(+57.02)YK.I | N | 56.79 | 1767.7273 | 13 | 0.5 | 884.8713 | 2 | 66.72 | 12 | F12:45597 | NaSa01\_F7.raw |  |  |  |  |  |  |  |  |  | 1.292E6 |  | 1.6669E6 |  | 6.9702E5 | 3 | 0 | 0 | 0 | 0 | 0 | 0 | 0 | 0 | 0 | 1 | 0 | 1 | 0 | 1 | 35 | 47 | Carbamidomethylation | C4:Carbamidomethylation:1000.00;C11:Carbamidomethylation:1000.00 | PEAKS DB |
| K.RGC(+57.02)TFTC(+57.02)PELRPTGK.Y | Y | 55.40 | 1778.8556 | 15 | 0.2 | 445.7213 | 4 | 12.03 | 11 | F11:2167 | NaSa01\_F6.raw |  |  |  |  |  |  |  |  |  |  | 2.5597E6 | 5.36E5 |  |  | 2 | 0 | 0 | 0 | 0 | 0 | 0 | 0 | 0 | 0 | 0 | 1 | 1 | 0 | 0 | 60 | 74 | Carbamidomethylation | C3:Carbamidomethylation:1000.00;C7:Carbamidomethylation:1000.00 | PEAKS DB |
| G.YTTIC(+57.02)YNHLSR.T | Y | 54.48 | 1426.6664 | 11 | -0.3 | 476.5626 | 3 | 17.82 | 12 | F12:4946 | NaSa01\_F7.raw |  |  |  |  |  |  |  |  |  |  | 2.8348E5 | 3.8627E6 |  |  | 3 | 0 | 0 | 0 | 0 | 0 | 0 | 0 | 0 | 0 | 0 | 1 | 2 | 0 | 0 | 20 | 30 | Carbamidomethylation | C5:Carbamidomethylation:1000.00 | PEAKS DB |
| S.RTPETTEIC(+57.02)PDSWYFC(+57.02)YK.I | Y | 50.51 | 2352.0190 | 18 | 0.2 | 785.0138 | 3 | 56.62 | 11 | F11:38298 | NaSa01\_F6.raw |  |  |  |  |  |  |  |  |  |  | 1.4651E7 |  |  |  | 1 | 0 | 0 | 0 | 0 | 0 | 0 | 0 | 0 | 0 | 0 | 1 | 0 | 0 | 0 | 30 | 47 | Carbamidomethylation | C9:Carbamidomethylation:1000.00;C16:Carbamidomethylation:1000.00 | PEAKS DB |
| K.ISLADGNDVR.I | N | 50.19 | 1058.5356 | 10 | 0.1 | 530.2751 | 2 | 21.09 | 12 | F12:7712 | NaSa01\_F7.raw |  |  |  |  |  |  |  |  |  |  |  | 1.1584E5 |  |  | 1 | 0 | 0 | 0 | 0 | 0 | 0 | 0 | 0 | 0 | 0 | 0 | 1 | 0 | 0 | 48 | 57 |  |  | PEAKS DB |
| P.DSWYFC(+57.02)YK.I | N | 48.52 | 1167.4695 | 8 | 0.7 | 584.7424 | 2 | 58.18 | 12 | F12:38630 | NaSa01\_F7.raw |  |  |  |  |  |  |  |  |  |  |  | 1.6222E6 |  |  | 1 | 0 | 0 | 0 | 0 | 0 | 0 | 0 | 0 | 0 | 0 | 0 | 1 | 0 | 0 | 40 | 47 | Carbamidomethylation | C6:Carbamidomethylation:1000.00 | PEAKS DB |
| R.GC(+57.02)TFTC(+57.02)PELR.P | N | 47.93 | 1239.5376 | 10 | 0.1 | 620.7761 | 2 | 29.29 | 9 | F9:9432 | NaSa01\_F4.raw |  |  |  |  |  |  |  |  | 1.4766E6 |  | 2.7398E7 | 2.0728E6 |  |  | 3 | 0 | 0 | 0 | 0 | 0 | 0 | 0 | 0 | 1 | 0 | 1 | 1 | 0 | 0 | 61 | 70 | Carbamidomethylation | C2:Carbamidomethylation:1000.00;C6:Carbamidomethylation:1000.00 | PEAKS DB |
| R.TPETTEIC(+57.02)PDSWYF.C | Y | 38.78 | 1744.7290 | 14 | 2.3 | 873.3738 | 2 | 91.30 | 11 | F11:65616 | NaSa01\_F6.raw |  |  |  |  |  |  |  |  |  |  | 2.3407E6 |  |  |  | 1 | 0 | 0 | 0 | 0 | 0 | 0 | 0 | 0 | 0 | 0 | 1 | 0 | 0 | 0 | 31 | 44 | Carbamidomethylation | C8:Carbamidomethylation:1000.00 | PEAKS DB |
| total 10 peptides |  |  |  |  |  |  |  |  |  |  |  |  |  |  |  |  |  |  |  |  |  |  |  |  |  |  |  |  |  |  |  |  |  |  |  |  |  |  |  |  |  |  |  |  |  |
| --- | --- | --- | --- | --- | --- | --- | --- | --- | --- | --- | --- | --- | --- | --- | --- | --- | --- | --- | --- | --- | --- | --- | --- | --- | --- | --- | --- | --- | --- | --- | --- | --- | --- | --- | --- | --- | --- | --- | --- | --- | --- | --- | --- | --- | --- |

XP\_026522175.1

back to list

  

| Protein Coverage
| Supporting Peptides
|

Protein Coverage:

Supporting Peptides:

| Peptide | Uniq | -10lgP | Mass | Length | ppm | m/z | z | RT | Fraction | Scan | Source File | Area F1 | Area F10 | Area F11 | Area F12 | Area F13 | Area F14 | Area F2 | Area F3 | Area F4 | Area F5 | Area F6 | Area F7 | Area F8 | Area F9 | #Feature | #Feature F1 | #Feature F10 | #Feature F11 | #Feature F12 | #Feature F13 | #Feature F14 | #Feature F2 | #Feature F3 | #Feature F4 | #Feature F5 | #Feature F6 | #Feature F7 | #Feature F8 | #Feature F9 | Start | End | PTM | AScore | Found By |
| --- | --- | --- | --- | --- | --- | --- | --- | --- | --- | --- | --- | --- | --- | --- | --- | --- | --- | --- | --- | --- | --- | --- | --- | --- | --- | --- | --- | --- | --- | --- | --- | --- | --- | --- | --- | --- | --- | --- | --- | --- | --- | --- | --- | --- | --- |
| R.LGVHNVHVHYEDEQIR.V | N | 91.56 | 1943.9602 | 16 | 0.3 | 648.9942 | 3 | 16.71 | 4 | F4:5129 | NaSa01\_F12.raw |  |  |  | 1.0032E9 | 1.3103E7 | 3.1756E6 |  |  |  |  |  |  |  |  | 16 | 0 | 0 | 0 | 11 | 2 | 3 | 0 | 0 | 0 | 0 | 0 | 0 | 0 | 0 | 107 | 122 |  |  | PEAKS DB |
| R.LGVHNVHVHYEDEQIRVPK.E | N | 90.99 | 2268.1763 | 19 | 0.2 | 568.0515 | 4 | 21.69 | 4 | F4:8124 | NaSa01\_F12.raw |  |  |  | 2.4838E8 |  |  |  |  |  |  |  |  |  |  | 3 | 0 | 0 | 0 | 3 | 0 | 0 | 0 | 0 | 0 | 0 | 0 | 0 | 0 | 0 | 107 | 125 |  |  | PEAKS DB |
| R.FPC(+57.02)AQLLEPGVYTK.V | N | 85.07 | 1621.8174 | 14 | 0.0 | 811.9160 | 2 | 62.82 | 4 | F4:41970 | NaSa01\_F12.raw |  |  | 2.7296E6 | 1.441E9 | 1.3181E7 | 4.0365E6 |  |  |  |  |  |  |  |  | 25 | 0 | 0 | 1 | 22 | 1 | 1 | 0 | 0 | 0 | 0 | 0 | 0 | 0 | 0 | 254 | 267 | Carbamidomethylation | C3:Carbamidomethylation:1000.00 | PEAKS DB |
| H.NVHVHYEDEQIRVPK.E | N | 60.48 | 1861.9435 | 15 | -0.7 | 466.4928 | 4 | 11.83 | 4 | F4:2757 | NaSa01\_F12.raw |  |  |  | 5.6639E5 |  |  |  |  |  |  |  |  |  |  | 1 | 0 | 0 | 0 | 1 | 0 | 0 | 0 | 0 | 0 | 0 | 0 | 0 | 0 | 0 | 111 | 125 |  |  | PEAKS DB |
| C.AQLLEPGVYTK.V | N | 56.08 | 1217.6655 | 11 | 0.3 | 609.8402 | 2 | 35.74 | 4 | F4:19559 | NaSa01\_F12.raw |  |  |  | 7.9156E6 |  |  |  |  |  |  |  |  |  |  | 1 | 0 | 0 | 0 | 1 | 0 | 0 | 0 | 0 | 0 | 0 | 0 | 0 | 0 | 0 | 257 | 267 |  |  | PEAKS DB |
| G.QIQGIVSWGR.F | N | 53.28 | 1142.6196 | 10 | -0.2 | 572.3170 | 2 | 46.03 | 4 | F4:28225 | NaSa01\_F12.raw |  |  | 1.2578E6 | 4.548E7 |  |  |  |  |  |  |  |  |  |  | 3 | 0 | 0 | 1 | 2 | 0 | 0 | 0 | 0 | 0 | 0 | 0 | 0 | 0 | 0 | 244 | 253 |  |  | PEAKS DB |
| R.FPC(+57.02)AQLLEPGVY.T | Y | 44.08 | 1392.6747 | 12 | 0.0 | 697.3446 | 2 | 85.51 | 4 | F4:60830 | NaSa01\_F12.raw |  |  |  | 3.8557E6 |  |  |  |  |  |  |  |  |  |  | 1 | 0 | 0 | 0 | 1 | 0 | 0 | 0 | 0 | 0 | 0 | 0 | 0 | 0 | 0 | 254 | 265 | Carbamidomethylation | C3:Carbamidomethylation:1000.00 | PEAKS DB |
| total 7 peptides |  |  |  |  |  |  |  |  |  |  |  |  |  |  |  |  |  |  |  |  |  |  |  |  |  |  |  |  |  |  |  |  |  |  |  |  |  |  |  |  |  |  |  |  |  |
| --- | --- | --- | --- | --- | --- | --- | --- | --- | --- | --- | --- | --- | --- | --- | --- | --- | --- | --- | --- | --- | --- | --- | --- | --- | --- | --- | --- | --- | --- | --- | --- | --- | --- | --- | --- | --- | --- | --- | --- | --- | --- | --- | --- | --- | --- |

XP\_026522177.1

back to list

  

| Protein Coverage
| Supporting Peptides
|

Protein Coverage:

Supporting Peptides:

| Peptide | Uniq | -10lgP | Mass | Length | ppm | m/z | z | RT | Fraction | Scan | Source File | Area F1 | Area F10 | Area F11 | Area F12 | Area F13 | Area F14 | Area F2 | Area F3 | Area F4 | Area F5 | Area F6 | Area F7 | Area F8 | Area F9 | #Feature | #Feature F1 | #Feature F10 | #Feature F11 | #Feature F12 | #Feature F13 | #Feature F14 | #Feature F2 | #Feature F3 | #Feature F4 | #Feature F5 | #Feature F6 | #Feature F7 | #Feature F8 | #Feature F9 | Start | End | PTM | AScore | Found By |
| --- | --- | --- | --- | --- | --- | --- | --- | --- | --- | --- | --- | --- | --- | --- | --- | --- | --- | --- | --- | --- | --- | --- | --- | --- | --- | --- | --- | --- | --- | --- | --- | --- | --- | --- | --- | --- | --- | --- | --- | --- | --- | --- | --- | --- | --- |
| R.LGVHNVHVHYEDEQIR.V | N | 91.56 | 1943.9602 | 16 | 0.3 | 648.9942 | 3 | 16.71 | 4 | F4:5129 | NaSa01\_F12.raw |  |  |  | 1.0032E9 | 1.3103E7 | 3.1756E6 |  |  |  |  |  |  |  |  | 16 | 0 | 0 | 0 | 11 | 2 | 3 | 0 | 0 | 0 | 0 | 0 | 0 | 0 | 0 | 75 | 90 |  |  | PEAKS DB |
| R.LGVHNVHVHYEDEQIRVPK.E | N | 90.99 | 2268.1763 | 19 | 0.2 | 568.0515 | 4 | 21.69 | 4 | F4:8124 | NaSa01\_F12.raw |  |  |  | 2.4838E8 |  |  |  |  |  |  |  |  |  |  | 3 | 0 | 0 | 0 | 3 | 0 | 0 | 0 | 0 | 0 | 0 | 0 | 0 | 0 | 0 | 75 | 93 |  |  | PEAKS DB |
| R.FPC(+57.02)AQLLEPGVYTK.V | N | 85.07 | 1621.8174 | 14 | 0.0 | 811.9160 | 2 | 62.82 | 4 | F4:41970 | NaSa01\_F12.raw |  |  | 2.7296E6 | 1.441E9 | 1.3181E7 | 4.0365E6 |  |  |  |  |  |  |  |  | 25 | 0 | 0 | 1 | 22 | 1 | 1 | 0 | 0 | 0 | 0 | 0 | 0 | 0 | 0 | 222 | 235 | Carbamidomethylation | C3:Carbamidomethylation:1000.00 | PEAKS DB |
| H.NVHVHYEDEQIRVPK.E | N | 60.48 | 1861.9435 | 15 | -0.7 | 466.4928 | 4 | 11.83 | 4 | F4:2757 | NaSa01\_F12.raw |  |  |  | 5.6639E5 |  |  |  |  |  |  |  |  |  |  | 1 | 0 | 0 | 0 | 1 | 0 | 0 | 0 | 0 | 0 | 0 | 0 | 0 | 0 | 0 | 79 | 93 |  |  | PEAKS DB |
| C.AQLLEPGVYTK.V | N | 56.08 | 1217.6655 | 11 | 0.3 | 609.8402 | 2 | 35.74 | 4 | F4:19559 | NaSa01\_F12.raw |  |  |  | 7.9156E6 |  |  |  |  |  |  |  |  |  |  | 1 | 0 | 0 | 0 | 1 | 0 | 0 | 0 | 0 | 0 | 0 | 0 | 0 | 0 | 0 | 225 | 235 |  |  | PEAKS DB |
| G.QIQGIVSWGR.F | N | 53.28 | 1142.6196 | 10 | -0.2 | 572.3170 | 2 | 46.03 | 4 | F4:28225 | NaSa01\_F12.raw |  |  | 1.2578E6 | 4.548E7 |  |  |  |  |  |  |  |  |  |  | 3 | 0 | 0 | 1 | 2 | 0 | 0 | 0 | 0 | 0 | 0 | 0 | 0 | 0 | 0 | 212 | 221 |  |  | PEAKS DB |
| R.FPC(+57.02)AQLLEPGVY.T | Y | 44.08 | 1392.6747 | 12 | 0.0 | 697.3446 | 2 | 85.51 | 4 | F4:60830 | NaSa01\_F12.raw |  |  |  | 3.8557E6 |  |  |  |  |  |  |  |  |  |  | 1 | 0 | 0 | 0 | 1 | 0 | 0 | 0 | 0 | 0 | 0 | 0 | 0 | 0 | 0 | 222 | 233 | Carbamidomethylation | C3:Carbamidomethylation:1000.00 | PEAKS DB |
| total 7 peptides |  |  |  |  |  |  |  |  |  |  |  |  |  |  |  |  |  |  |  |  |  |  |  |  |  |  |  |  |  |  |  |  |  |  |  |  |  |  |  |  |  |  |  |  |  |
| --- | --- | --- | --- | --- | --- | --- | --- | --- | --- | --- | --- | --- | --- | --- | --- | --- | --- | --- | --- | --- | --- | --- | --- | --- | --- | --- | --- | --- | --- | --- | --- | --- | --- | --- | --- | --- | --- | --- | --- | --- | --- | --- | --- | --- | --- |

XP\_026522176.1

back to list

  

| Protein Coverage
| Supporting Peptides
|

Protein Coverage:

Supporting Peptides:

| Peptide | Uniq | -10lgP | Mass | Length | ppm | m/z | z | RT | Fraction | Scan | Source File | Area F1 | Area F10 | Area F11 | Area F12 | Area F13 | Area F14 | Area F2 | Area F3 | Area F4 | Area F5 | Area F6 | Area F7 | Area F8 | Area F9 | #Feature | #Feature F1 | #Feature F10 | #Feature F11 | #Feature F12 | #Feature F13 | #Feature F14 | #Feature F2 | #Feature F3 | #Feature F4 | #Feature F5 | #Feature F6 | #Feature F7 | #Feature F8 | #Feature F9 | Start | End | PTM | AScore | Found By |
| --- | --- | --- | --- | --- | --- | --- | --- | --- | --- | --- | --- | --- | --- | --- | --- | --- | --- | --- | --- | --- | --- | --- | --- | --- | --- | --- | --- | --- | --- | --- | --- | --- | --- | --- | --- | --- | --- | --- | --- | --- | --- | --- | --- | --- | --- |
| R.LGVHNVHVHYEDEQIR.V | N | 91.56 | 1943.9602 | 16 | 0.3 | 648.9942 | 3 | 16.71 | 4 | F4:5129 | NaSa01\_F12.raw |  |  |  | 1.0032E9 | 1.3103E7 | 3.1756E6 |  |  |  |  |  |  |  |  | 16 | 0 | 0 | 0 | 11 | 2 | 3 | 0 | 0 | 0 | 0 | 0 | 0 | 0 | 0 | 84 | 99 |  |  | PEAKS DB |
| R.LGVHNVHVHYEDEQIRVPK.E | N | 90.99 | 2268.1763 | 19 | 0.2 | 568.0515 | 4 | 21.69 | 4 | F4:8124 | NaSa01\_F12.raw |  |  |  | 2.4838E8 |  |  |  |  |  |  |  |  |  |  | 3 | 0 | 0 | 0 | 3 | 0 | 0 | 0 | 0 | 0 | 0 | 0 | 0 | 0 | 0 | 84 | 102 |  |  | PEAKS DB |
| R.FPC(+57.02)AQLLEPGVYTK.V | N | 85.07 | 1621.8174 | 14 | 0.0 | 811.9160 | 2 | 62.82 | 4 | F4:41970 | NaSa01\_F12.raw |  |  | 2.7296E6 | 1.441E9 | 1.3181E7 | 4.0365E6 |  |  |  |  |  |  |  |  | 25 | 0 | 0 | 1 | 22 | 1 | 1 | 0 | 0 | 0 | 0 | 0 | 0 | 0 | 0 | 231 | 244 | Carbamidomethylation | C3:Carbamidomethylation:1000.00 | PEAKS DB |
| H.NVHVHYEDEQIRVPK.E | N | 60.48 | 1861.9435 | 15 | -0.7 | 466.4928 | 4 | 11.83 | 4 | F4:2757 | NaSa01\_F12.raw |  |  |  | 5.6639E5 |  |  |  |  |  |  |  |  |  |  | 1 | 0 | 0 | 0 | 1 | 0 | 0 | 0 | 0 | 0 | 0 | 0 | 0 | 0 | 0 | 88 | 102 |  |  | PEAKS DB |
| C.AQLLEPGVYTK.V | N | 56.08 | 1217.6655 | 11 | 0.3 | 609.8402 | 2 | 35.74 | 4 | F4:19559 | NaSa01\_F12.raw |  |  |  | 7.9156E6 |  |  |  |  |  |  |  |  |  |  | 1 | 0 | 0 | 0 | 1 | 0 | 0 | 0 | 0 | 0 | 0 | 0 | 0 | 0 | 0 | 234 | 244 |  |  | PEAKS DB |
| G.QIQGIVSWGR.F | N | 53.28 | 1142.6196 | 10 | -0.2 | 572.3170 | 2 | 46.03 | 4 | F4:28225 | NaSa01\_F12.raw |  |  | 1.2578E6 | 4.548E7 |  |  |  |  |  |  |  |  |  |  | 3 | 0 | 0 | 1 | 2 | 0 | 0 | 0 | 0 | 0 | 0 | 0 | 0 | 0 | 0 | 221 | 230 |  |  | PEAKS DB |
| R.FPC(+57.02)AQLLEPGVY.T | Y | 44.08 | 1392.6747 | 12 | 0.0 | 697.3446 | 2 | 85.51 | 4 | F4:60830 | NaSa01\_F12.raw |  |  |  | 3.8557E6 |  |  |  |  |  |  |  |  |  |  | 1 | 0 | 0 | 0 | 1 | 0 | 0 | 0 | 0 | 0 | 0 | 0 | 0 | 0 | 0 | 231 | 242 | Carbamidomethylation | C3:Carbamidomethylation:1000.00 | PEAKS DB |
| total 7 peptides |  |  |  |  |  |  |  |  |  |  |  |  |  |  |  |  |  |  |  |  |  |  |  |  |  |  |  |  |  |  |  |  |  |  |  |  |  |  |  |  |  |  |  |  |  |
| --- | --- | --- | --- | --- | --- | --- | --- | --- | --- | --- | --- | --- | --- | --- | --- | --- | --- | --- | --- | --- | --- | --- | --- | --- | --- | --- | --- | --- | --- | --- | --- | --- | --- | --- | --- | --- | --- | --- | --- | --- | --- | --- | --- | --- | --- |

ABN72545.1

back to list

  

| Protein Coverage
| Supporting Peptides
|

Protein Coverage:

Supporting Peptides:

| Peptide | Uniq | -10lgP | Mass | Length | ppm | m/z | z | RT | Fraction | Scan | Source File | Area F1 | Area F10 | Area F11 | Area F12 | Area F13 | Area F14 | Area F2 | Area F3 | Area F4 | Area F5 | Area F6 | Area F7 | Area F8 | Area F9 | #Feature | #Feature F1 | #Feature F10 | #Feature F11 | #Feature F12 | #Feature F13 | #Feature F14 | #Feature F2 | #Feature F3 | #Feature F4 | #Feature F5 | #Feature F6 | #Feature F7 | #Feature F8 | #Feature F9 | Start | End | PTM | AScore | Found By |
| --- | --- | --- | --- | --- | --- | --- | --- | --- | --- | --- | --- | --- | --- | --- | --- | --- | --- | --- | --- | --- | --- | --- | --- | --- | --- | --- | --- | --- | --- | --- | --- | --- | --- | --- | --- | --- | --- | --- | --- | --- | --- | --- | --- | --- | --- |
| K.LGVHNVHVHYEDEQIR.V | N | 91.56 | 1943.9602 | 16 | 0.3 | 648.9942 | 3 | 16.71 | 4 | F4:5129 | NaSa01\_F12.raw |  |  |  | 1.0032E9 | 1.3103E7 | 3.1756E6 |  |  |  |  |  |  |  |  | 16 | 0 | 0 | 0 | 11 | 2 | 3 | 0 | 0 | 0 | 0 | 0 | 0 | 0 | 0 | 107 | 122 |  |  | PEAKS DB |
| K.LGVHNVHVHYEDEQIRVPK.E | N | 90.99 | 2268.1763 | 19 | 0.2 | 568.0515 | 4 | 21.69 | 4 | F4:8124 | NaSa01\_F12.raw |  |  |  | 2.4838E8 |  |  |  |  |  |  |  |  |  |  | 3 | 0 | 0 | 0 | 3 | 0 | 0 | 0 | 0 | 0 | 0 | 0 | 0 | 0 | 0 | 107 | 125 |  |  | PEAKS DB |
| R.FPC(+57.02)AQLLEPGVYTK.V | N | 85.07 | 1621.8174 | 14 | 0.0 | 811.9160 | 2 | 62.82 | 4 | F4:41970 | NaSa01\_F12.raw |  |  | 2.7296E6 | 1.441E9 | 1.3181E7 | 4.0365E6 |  |  |  |  |  |  |  |  | 25 | 0 | 0 | 1 | 22 | 1 | 1 | 0 | 0 | 0 | 0 | 0 | 0 | 0 | 0 | 254 | 267 | Carbamidomethylation | C3:Carbamidomethylation:1000.00 | PEAKS DB |
| H.NVHVHYEDEQIRVPK.E | N | 60.48 | 1861.9435 | 15 | -0.7 | 466.4928 | 4 | 11.83 | 4 | F4:2757 | NaSa01\_F12.raw |  |  |  | 5.6639E5 |  |  |  |  |  |  |  |  |  |  | 1 | 0 | 0 | 0 | 1 | 0 | 0 | 0 | 0 | 0 | 0 | 0 | 0 | 0 | 0 | 111 | 125 |  |  | PEAKS DB |
| C.AQLLEPGVYTK.V | N | 56.08 | 1217.6655 | 11 | 0.3 | 609.8402 | 2 | 35.74 | 4 | F4:19559 | NaSa01\_F12.raw |  |  |  | 7.9156E6 |  |  |  |  |  |  |  |  |  |  | 1 | 0 | 0 | 0 | 1 | 0 | 0 | 0 | 0 | 0 | 0 | 0 | 0 | 0 | 0 | 257 | 267 |  |  | PEAKS DB |
| G.QIQGIVSWGR.F | N | 53.28 | 1142.6196 | 10 | -0.2 | 572.3170 | 2 | 46.03 | 4 | F4:28225 | NaSa01\_F12.raw |  |  | 1.2578E6 | 4.548E7 |  |  |  |  |  |  |  |  |  |  | 3 | 0 | 0 | 1 | 2 | 0 | 0 | 0 | 0 | 0 | 0 | 0 | 0 | 0 | 0 | 244 | 253 |  |  | PEAKS DB |
| R.FPC(+57.02)AQLLEPGVY.T | Y | 44.08 | 1392.6747 | 12 | 0.0 | 697.3446 | 2 | 85.51 | 4 | F4:60830 | NaSa01\_F12.raw |  |  |  | 3.8557E6 |  |  |  |  |  |  |  |  |  |  | 1 | 0 | 0 | 0 | 1 | 0 | 0 | 0 | 0 | 0 | 0 | 0 | 0 | 0 | 0 | 254 | 265 | Carbamidomethylation | C3:Carbamidomethylation:1000.00 | PEAKS DB |
| total 7 peptides |  |  |  |  |  |  |  |  |  |  |  |  |  |  |  |  |  |  |  |  |  |  |  |  |  |  |  |  |  |  |  |  |  |  |  |  |  |  |  |  |  |  |  |  |  |
| --- | --- | --- | --- | --- | --- | --- | --- | --- | --- | --- | --- | --- | --- | --- | --- | --- | --- | --- | --- | --- | --- | --- | --- | --- | --- | --- | --- | --- | --- | --- | --- | --- | --- | --- | --- | --- | --- | --- | --- | --- | --- | --- | --- | --- | --- |

A8QL57.1

back to list

  

| Protein Coverage
| Supporting Peptides
|

Protein Coverage:

Supporting Peptides:

| Peptide | Uniq | -10lgP | Mass | Length | ppm | m/z | z | RT | Fraction | Scan | Source File | Area F1 | Area F10 | Area F11 | Area F12 | Area F13 | Area F14 | Area F2 | Area F3 | Area F4 | Area F5 | Area F6 | Area F7 | Area F8 | Area F9 | #Feature | #Feature F1 | #Feature F10 | #Feature F11 | #Feature F12 | #Feature F13 | #Feature F14 | #Feature F2 | #Feature F3 | #Feature F4 | #Feature F5 | #Feature F6 | #Feature F7 | #Feature F8 | #Feature F9 | Start | End | PTM | AScore | Found By |
| --- | --- | --- | --- | --- | --- | --- | --- | --- | --- | --- | --- | --- | --- | --- | --- | --- | --- | --- | --- | --- | --- | --- | --- | --- | --- | --- | --- | --- | --- | --- | --- | --- | --- | --- | --- | --- | --- | --- | --- | --- | --- | --- | --- | --- | --- |
| K.LGVHNVHVHYEDEQIR.V | N | 91.56 | 1943.9602 | 16 | 0.3 | 648.9942 | 3 | 16.71 | 4 | F4:5129 | NaSa01\_F12.raw |  |  |  | 1.0032E9 | 1.3103E7 | 3.1756E6 |  |  |  |  |  |  |  |  | 16 | 0 | 0 | 0 | 11 | 2 | 3 | 0 | 0 | 0 | 0 | 0 | 0 | 0 | 0 | 107 | 122 |  |  | PEAKS DB |
| K.LGVHNVHVHYEDEQIRVPK.E | N | 90.99 | 2268.1763 | 19 | 0.2 | 568.0515 | 4 | 21.69 | 4 | F4:8124 | NaSa01\_F12.raw |  |  |  | 2.4838E8 |  |  |  |  |  |  |  |  |  |  | 3 | 0 | 0 | 0 | 3 | 0 | 0 | 0 | 0 | 0 | 0 | 0 | 0 | 0 | 0 | 107 | 125 |  |  | PEAKS DB |
| R.FPC(+57.02)AQLLEPGVYTK.V | N | 85.07 | 1621.8174 | 14 | 0.0 | 811.9160 | 2 | 62.82 | 4 | F4:41970 | NaSa01\_F12.raw |  |  | 2.7296E6 | 1.441E9 | 1.3181E7 | 4.0365E6 |  |  |  |  |  |  |  |  | 25 | 0 | 0 | 1 | 22 | 1 | 1 | 0 | 0 | 0 | 0 | 0 | 0 | 0 | 0 | 254 | 267 | Carbamidomethylation | C3:Carbamidomethylation:1000.00 | PEAKS DB |
| H.NVHVHYEDEQIRVPK.E | N | 60.48 | 1861.9435 | 15 | -0.7 | 466.4928 | 4 | 11.83 | 4 | F4:2757 | NaSa01\_F12.raw |  |  |  | 5.6639E5 |  |  |  |  |  |  |  |  |  |  | 1 | 0 | 0 | 0 | 1 | 0 | 0 | 0 | 0 | 0 | 0 | 0 | 0 | 0 | 0 | 111 | 125 |  |  | PEAKS DB |
| C.AQLLEPGVYTK.V | N | 56.08 | 1217.6655 | 11 | 0.3 | 609.8402 | 2 | 35.74 | 4 | F4:19559 | NaSa01\_F12.raw |  |  |  | 7.9156E6 |  |  |  |  |  |  |  |  |  |  | 1 | 0 | 0 | 0 | 1 | 0 | 0 | 0 | 0 | 0 | 0 | 0 | 0 | 0 | 0 | 257 | 267 |  |  | PEAKS DB |
| G.QIQGIVSWGR.F | N | 53.28 | 1142.6196 | 10 | -0.2 | 572.3170 | 2 | 46.03 | 4 | F4:28225 | NaSa01\_F12.raw |  |  | 1.2578E6 | 4.548E7 |  |  |  |  |  |  |  |  |  |  | 3 | 0 | 0 | 1 | 2 | 0 | 0 | 0 | 0 | 0 | 0 | 0 | 0 | 0 | 0 | 244 | 253 |  |  | PEAKS DB |
| R.FPC(+57.02)AQLLEPGVY.T | Y | 44.08 | 1392.6747 | 12 | 0.0 | 697.3446 | 2 | 85.51 | 4 | F4:60830 | NaSa01\_F12.raw |  |  |  | 3.8557E6 |  |  |  |  |  |  |  |  |  |  | 1 | 0 | 0 | 0 | 1 | 0 | 0 | 0 | 0 | 0 | 0 | 0 | 0 | 0 | 0 | 254 | 265 | Carbamidomethylation | C3:Carbamidomethylation:1000.00 | PEAKS DB |
| total 7 peptides |  |  |  |  |  |  |  |  |  |  |  |  |  |  |  |  |  |  |  |  |  |  |  |  |  |  |  |  |  |  |  |  |  |  |  |  |  |  |  |  |  |  |  |  |  |
| --- | --- | --- | --- | --- | --- | --- | --- | --- | --- | --- | --- | --- | --- | --- | --- | --- | --- | --- | --- | --- | --- | --- | --- | --- | --- | --- | --- | --- | --- | --- | --- | --- | --- | --- | --- | --- | --- | --- | --- | --- | --- | --- | --- | --- | --- |

ABN72541.1

back to list

  

| Protein Coverage
| Supporting Peptides
|

Protein Coverage:

Supporting Peptides:

| Peptide | Uniq | -10lgP | Mass | Length | ppm | m/z | z | RT | Fraction | Scan | Source File | Area F1 | Area F10 | Area F11 | Area F12 | Area F13 | Area F14 | Area F2 | Area F3 | Area F4 | Area F5 | Area F6 | Area F7 | Area F8 | Area F9 | #Feature | #Feature F1 | #Feature F10 | #Feature F11 | #Feature F12 | #Feature F13 | #Feature F14 | #Feature F2 | #Feature F3 | #Feature F4 | #Feature F5 | #Feature F6 | #Feature F7 | #Feature F8 | #Feature F9 | Start | End | PTM | AScore | Found By |
| --- | --- | --- | --- | --- | --- | --- | --- | --- | --- | --- | --- | --- | --- | --- | --- | --- | --- | --- | --- | --- | --- | --- | --- | --- | --- | --- | --- | --- | --- | --- | --- | --- | --- | --- | --- | --- | --- | --- | --- | --- | --- | --- | --- | --- | --- |
| K.LGVHNVHVHYEDEQIR.V | N | 91.56 | 1943.9602 | 16 | 0.3 | 648.9942 | 3 | 16.71 | 4 | F4:5129 | NaSa01\_F12.raw |  |  |  | 1.0032E9 | 1.3103E7 | 3.1756E6 |  |  |  |  |  |  |  |  | 16 | 0 | 0 | 0 | 11 | 2 | 3 | 0 | 0 | 0 | 0 | 0 | 0 | 0 | 0 | 107 | 122 |  |  | PEAKS DB |
| K.LGVHNVHVHYEDEQIRVPK.E | N | 90.99 | 2268.1763 | 19 | 0.2 | 568.0515 | 4 | 21.69 | 4 | F4:8124 | NaSa01\_F12.raw |  |  |  | 2.4838E8 |  |  |  |  |  |  |  |  |  |  | 3 | 0 | 0 | 0 | 3 | 0 | 0 | 0 | 0 | 0 | 0 | 0 | 0 | 0 | 0 | 107 | 125 |  |  | PEAKS DB |
| R.FPC(+57.02)AQLLEPGVYTK.V | N | 85.07 | 1621.8174 | 14 | 0.0 | 811.9160 | 2 | 62.82 | 4 | F4:41970 | NaSa01\_F12.raw |  |  | 2.7296E6 | 1.441E9 | 1.3181E7 | 4.0365E6 |  |  |  |  |  |  |  |  | 25 | 0 | 0 | 1 | 22 | 1 | 1 | 0 | 0 | 0 | 0 | 0 | 0 | 0 | 0 | 254 | 267 | Carbamidomethylation | C3:Carbamidomethylation:1000.00 | PEAKS DB |
| H.NVHVHYEDEQIRVPK.E | N | 60.48 | 1861.9435 | 15 | -0.7 | 466.4928 | 4 | 11.83 | 4 | F4:2757 | NaSa01\_F12.raw |  |  |  | 5.6639E5 |  |  |  |  |  |  |  |  |  |  | 1 | 0 | 0 | 0 | 1 | 0 | 0 | 0 | 0 | 0 | 0 | 0 | 0 | 0 | 0 | 111 | 125 |  |  | PEAKS DB |
| C.AQLLEPGVYTK.V | N | 56.08 | 1217.6655 | 11 | 0.3 | 609.8402 | 2 | 35.74 | 4 | F4:19559 | NaSa01\_F12.raw |  |  |  | 7.9156E6 |  |  |  |  |  |  |  |  |  |  | 1 | 0 | 0 | 0 | 1 | 0 | 0 | 0 | 0 | 0 | 0 | 0 | 0 | 0 | 0 | 257 | 267 |  |  | PEAKS DB |
| G.QIQGIVSWGR.F | N | 53.28 | 1142.6196 | 10 | -0.2 | 572.3170 | 2 | 46.03 | 4 | F4:28225 | NaSa01\_F12.raw |  |  | 1.2578E6 | 4.548E7 |  |  |  |  |  |  |  |  |  |  | 3 | 0 | 0 | 1 | 2 | 0 | 0 | 0 | 0 | 0 | 0 | 0 | 0 | 0 | 0 | 244 | 253 |  |  | PEAKS DB |
| R.FPC(+57.02)AQLLEPGVY.T | Y | 44.08 | 1392.6747 | 12 | 0.0 | 697.3446 | 2 | 85.51 | 4 | F4:60830 | NaSa01\_F12.raw |  |  |  | 3.8557E6 |  |  |  |  |  |  |  |  |  |  | 1 | 0 | 0 | 0 | 1 | 0 | 0 | 0 | 0 | 0 | 0 | 0 | 0 | 0 | 0 | 254 | 265 | Carbamidomethylation | C3:Carbamidomethylation:1000.00 | PEAKS DB |
| total 7 peptides |  |  |  |  |  |  |  |  |  |  |  |  |  |  |  |  |  |  |  |  |  |  |  |  |  |  |  |  |  |  |  |  |  |  |  |  |  |  |  |  |  |  |  |  |  |
| --- | --- | --- | --- | --- | --- | --- | --- | --- | --- | --- | --- | --- | --- | --- | --- | --- | --- | --- | --- | --- | --- | --- | --- | --- | --- | --- | --- | --- | --- | --- | --- | --- | --- | --- | --- | --- | --- | --- | --- | --- | --- | --- | --- | --- | --- |

A8QL53.1

back to list

  

| Protein Coverage
| Supporting Peptides
|

Protein Coverage:

Supporting Peptides:

| Peptide | Uniq | -10lgP | Mass | Length | ppm | m/z | z | RT | Fraction | Scan | Source File | Area F1 | Area F10 | Area F11 | Area F12 | Area F13 | Area F14 | Area F2 | Area F3 | Area F4 | Area F5 | Area F6 | Area F7 | Area F8 | Area F9 | #Feature | #Feature F1 | #Feature F10 | #Feature F11 | #Feature F12 | #Feature F13 | #Feature F14 | #Feature F2 | #Feature F3 | #Feature F4 | #Feature F5 | #Feature F6 | #Feature F7 | #Feature F8 | #Feature F9 | Start | End | PTM | AScore | Found By |
| --- | --- | --- | --- | --- | --- | --- | --- | --- | --- | --- | --- | --- | --- | --- | --- | --- | --- | --- | --- | --- | --- | --- | --- | --- | --- | --- | --- | --- | --- | --- | --- | --- | --- | --- | --- | --- | --- | --- | --- | --- | --- | --- | --- | --- | --- |
| K.LGVHNVHVHYEDEQIR.V | N | 91.56 | 1943.9602 | 16 | 0.3 | 648.9942 | 3 | 16.71 | 4 | F4:5129 | NaSa01\_F12.raw |  |  |  | 1.0032E9 | 1.3103E7 | 3.1756E6 |  |  |  |  |  |  |  |  | 16 | 0 | 0 | 0 | 11 | 2 | 3 | 0 | 0 | 0 | 0 | 0 | 0 | 0 | 0 | 107 | 122 |  |  | PEAKS DB |
| K.LGVHNVHVHYEDEQIRVPK.E | N | 90.99 | 2268.1763 | 19 | 0.2 | 568.0515 | 4 | 21.69 | 4 | F4:8124 | NaSa01\_F12.raw |  |  |  | 2.4838E8 |  |  |  |  |  |  |  |  |  |  | 3 | 0 | 0 | 0 | 3 | 0 | 0 | 0 | 0 | 0 | 0 | 0 | 0 | 0 | 0 | 107 | 125 |  |  | PEAKS DB |
| R.FPC(+57.02)AQLLEPGVYTK.V | N | 85.07 | 1621.8174 | 14 | 0.0 | 811.9160 | 2 | 62.82 | 4 | F4:41970 | NaSa01\_F12.raw |  |  | 2.7296E6 | 1.441E9 | 1.3181E7 | 4.0365E6 |  |  |  |  |  |  |  |  | 25 | 0 | 0 | 1 | 22 | 1 | 1 | 0 | 0 | 0 | 0 | 0 | 0 | 0 | 0 | 254 | 267 | Carbamidomethylation | C3:Carbamidomethylation:1000.00 | PEAKS DB |
| H.NVHVHYEDEQIRVPK.E | N | 60.48 | 1861.9435 | 15 | -0.7 | 466.4928 | 4 | 11.83 | 4 | F4:2757 | NaSa01\_F12.raw |  |  |  | 5.6639E5 |  |  |  |  |  |  |  |  |  |  | 1 | 0 | 0 | 0 | 1 | 0 | 0 | 0 | 0 | 0 | 0 | 0 | 0 | 0 | 0 | 111 | 125 |  |  | PEAKS DB |
| C.AQLLEPGVYTK.V | N | 56.08 | 1217.6655 | 11 | 0.3 | 609.8402 | 2 | 35.74 | 4 | F4:19559 | NaSa01\_F12.raw |  |  |  | 7.9156E6 |  |  |  |  |  |  |  |  |  |  | 1 | 0 | 0 | 0 | 1 | 0 | 0 | 0 | 0 | 0 | 0 | 0 | 0 | 0 | 0 | 257 | 267 |  |  | PEAKS DB |
| G.QIQGIVSWGR.F | N | 53.28 | 1142.6196 | 10 | -0.2 | 572.3170 | 2 | 46.03 | 4 | F4:28225 | NaSa01\_F12.raw |  |  | 1.2578E6 | 4.548E7 |  |  |  |  |  |  |  |  |  |  | 3 | 0 | 0 | 1 | 2 | 0 | 0 | 0 | 0 | 0 | 0 | 0 | 0 | 0 | 0 | 244 | 253 |  |  | PEAKS DB |
| R.FPC(+57.02)AQLLEPGVY.T | Y | 44.08 | 1392.6747 | 12 | 0.0 | 697.3446 | 2 | 85.51 | 4 | F4:60830 | NaSa01\_F12.raw |  |  |  | 3.8557E6 |  |  |  |  |  |  |  |  |  |  | 1 | 0 | 0 | 0 | 1 | 0 | 0 | 0 | 0 | 0 | 0 | 0 | 0 | 0 | 0 | 254 | 265 | Carbamidomethylation | C3:Carbamidomethylation:1000.00 | PEAKS DB |
| total 7 peptides |  |  |  |  |  |  |  |  |  |  |  |  |  |  |  |  |  |  |  |  |  |  |  |  |  |  |  |  |  |  |  |  |  |  |  |  |  |  |  |  |  |  |  |  |  |
| --- | --- | --- | --- | --- | --- | --- | --- | --- | --- | --- | --- | --- | --- | --- | --- | --- | --- | --- | --- | --- | --- | --- | --- | --- | --- | --- | --- | --- | --- | --- | --- | --- | --- | --- | --- | --- | --- | --- | --- | --- | --- | --- | --- | --- | --- |

XP\_026574628.1

back to list

  

| Protein Coverage
| Supporting Peptides
|

Protein Coverage:

Supporting Peptides:

| Peptide | Uniq | -10lgP | Mass | Length | ppm | m/z | z | RT | Fraction | Scan | Source File | Area F1 | Area F10 | Area F11 | Area F12 | Area F13 | Area F14 | Area F2 | Area F3 | Area F4 | Area F5 | Area F6 | Area F7 | Area F8 | Area F9 | #Feature | #Feature F1 | #Feature F10 | #Feature F11 | #Feature F12 | #Feature F13 | #Feature F14 | #Feature F2 | #Feature F3 | #Feature F4 | #Feature F5 | #Feature F6 | #Feature F7 | #Feature F8 | #Feature F9 | Start | End | PTM | AScore | Found By |
| --- | --- | --- | --- | --- | --- | --- | --- | --- | --- | --- | --- | --- | --- | --- | --- | --- | --- | --- | --- | --- | --- | --- | --- | --- | --- | --- | --- | --- | --- | --- | --- | --- | --- | --- | --- | --- | --- | --- | --- | --- | --- | --- | --- | --- | --- |
| K.LGVHNVHVHYEDEQIR.V | N | 91.56 | 1943.9602 | 16 | 0.3 | 648.9942 | 3 | 16.71 | 4 | F4:5129 | NaSa01\_F12.raw |  |  |  | 1.0032E9 | 1.3103E7 | 3.1756E6 |  |  |  |  |  |  |  |  | 16 | 0 | 0 | 0 | 11 | 2 | 3 | 0 | 0 | 0 | 0 | 0 | 0 | 0 | 0 | 75 | 90 |  |  | PEAKS DB |
| K.LGVHNVHVHYEDEQIRVPK.E | N | 90.99 | 2268.1763 | 19 | 0.2 | 568.0515 | 4 | 21.69 | 4 | F4:8124 | NaSa01\_F12.raw |  |  |  | 2.4838E8 |  |  |  |  |  |  |  |  |  |  | 3 | 0 | 0 | 0 | 3 | 0 | 0 | 0 | 0 | 0 | 0 | 0 | 0 | 0 | 0 | 75 | 93 |  |  | PEAKS DB |
| R.FPC(+57.02)AQLLEPGVYTK.V | N | 85.07 | 1621.8174 | 14 | 0.0 | 811.9160 | 2 | 62.82 | 4 | F4:41970 | NaSa01\_F12.raw |  |  | 2.7296E6 | 1.441E9 | 1.3181E7 | 4.0365E6 |  |  |  |  |  |  |  |  | 25 | 0 | 0 | 1 | 22 | 1 | 1 | 0 | 0 | 0 | 0 | 0 | 0 | 0 | 0 | 222 | 235 | Carbamidomethylation | C3:Carbamidomethylation:1000.00 | PEAKS DB |
| H.NVHVHYEDEQIRVPK.E | N | 60.48 | 1861.9435 | 15 | -0.7 | 466.4928 | 4 | 11.83 | 4 | F4:2757 | NaSa01\_F12.raw |  |  |  | 5.6639E5 |  |  |  |  |  |  |  |  |  |  | 1 | 0 | 0 | 0 | 1 | 0 | 0 | 0 | 0 | 0 | 0 | 0 | 0 | 0 | 0 | 79 | 93 |  |  | PEAKS DB |
| C.AQLLEPGVYTK.V | N | 56.08 | 1217.6655 | 11 | 0.3 | 609.8402 | 2 | 35.74 | 4 | F4:19559 | NaSa01\_F12.raw |  |  |  | 7.9156E6 |  |  |  |  |  |  |  |  |  |  | 1 | 0 | 0 | 0 | 1 | 0 | 0 | 0 | 0 | 0 | 0 | 0 | 0 | 0 | 0 | 225 | 235 |  |  | PEAKS DB |
| G.QIQGIVSWGR.F | N | 53.28 | 1142.6196 | 10 | -0.2 | 572.3170 | 2 | 46.03 | 4 | F4:28225 | NaSa01\_F12.raw |  |  | 1.2578E6 | 4.548E7 |  |  |  |  |  |  |  |  |  |  | 3 | 0 | 0 | 1 | 2 | 0 | 0 | 0 | 0 | 0 | 0 | 0 | 0 | 0 | 0 | 212 | 221 |  |  | PEAKS DB |
| R.FPC(+57.02)AQLLEPGVY.T | Y | 44.08 | 1392.6747 | 12 | 0.0 | 697.3446 | 2 | 85.51 | 4 | F4:60830 | NaSa01\_F12.raw |  |  |  | 3.8557E6 |  |  |  |  |  |  |  |  |  |  | 1 | 0 | 0 | 0 | 1 | 0 | 0 | 0 | 0 | 0 | 0 | 0 | 0 | 0 | 0 | 222 | 233 | Carbamidomethylation | C3:Carbamidomethylation:1000.00 | PEAKS DB |
| total 7 peptides |  |  |  |  |  |  |  |  |  |  |  |  |  |  |  |  |  |  |  |  |  |  |  |  |  |  |  |  |  |  |  |  |  |  |  |  |  |  |  |  |  |  |  |  |  |
| --- | --- | --- | --- | --- | --- | --- | --- | --- | --- | --- | --- | --- | --- | --- | --- | --- | --- | --- | --- | --- | --- | --- | --- | --- | --- | --- | --- | --- | --- | --- | --- | --- | --- | --- | --- | --- | --- | --- | --- | --- | --- | --- | --- | --- | --- |

XP\_026574627.1

back to list

  

| Protein Coverage
| Supporting Peptides
|

Protein Coverage:

Supporting Peptides:

| Peptide | Uniq | -10lgP | Mass | Length | ppm | m/z | z | RT | Fraction | Scan | Source File | Area F1 | Area F10 | Area F11 | Area F12 | Area F13 | Area F14 | Area F2 | Area F3 | Area F4 | Area F5 | Area F6 | Area F7 | Area F8 | Area F9 | #Feature | #Feature F1 | #Feature F10 | #Feature F11 | #Feature F12 | #Feature F13 | #Feature F14 | #Feature F2 | #Feature F3 | #Feature F4 | #Feature F5 | #Feature F6 | #Feature F7 | #Feature F8 | #Feature F9 | Start | End | PTM | AScore | Found By |
| --- | --- | --- | --- | --- | --- | --- | --- | --- | --- | --- | --- | --- | --- | --- | --- | --- | --- | --- | --- | --- | --- | --- | --- | --- | --- | --- | --- | --- | --- | --- | --- | --- | --- | --- | --- | --- | --- | --- | --- | --- | --- | --- | --- | --- | --- |
| K.LGVHNVHVHYEDEQIR.V | N | 91.56 | 1943.9602 | 16 | 0.3 | 648.9942 | 3 | 16.71 | 4 | F4:5129 | NaSa01\_F12.raw |  |  |  | 1.0032E9 | 1.3103E7 | 3.1756E6 |  |  |  |  |  |  |  |  | 16 | 0 | 0 | 0 | 11 | 2 | 3 | 0 | 0 | 0 | 0 | 0 | 0 | 0 | 0 | 84 | 99 |  |  | PEAKS DB |
| K.LGVHNVHVHYEDEQIRVPK.E | N | 90.99 | 2268.1763 | 19 | 0.2 | 568.0515 | 4 | 21.69 | 4 | F4:8124 | NaSa01\_F12.raw |  |  |  | 2.4838E8 |  |  |  |  |  |  |  |  |  |  | 3 | 0 | 0 | 0 | 3 | 0 | 0 | 0 | 0 | 0 | 0 | 0 | 0 | 0 | 0 | 84 | 102 |  |  | PEAKS DB |
| R.FPC(+57.02)AQLLEPGVYTK.V | N | 85.07 | 1621.8174 | 14 | 0.0 | 811.9160 | 2 | 62.82 | 4 | F4:41970 | NaSa01\_F12.raw |  |  | 2.7296E6 | 1.441E9 | 1.3181E7 | 4.0365E6 |  |  |  |  |  |  |  |  | 25 | 0 | 0 | 1 | 22 | 1 | 1 | 0 | 0 | 0 | 0 | 0 | 0 | 0 | 0 | 231 | 244 | Carbamidomethylation | C3:Carbamidomethylation:1000.00 | PEAKS DB |
| H.NVHVHYEDEQIRVPK.E | N | 60.48 | 1861.9435 | 15 | -0.7 | 466.4928 | 4 | 11.83 | 4 | F4:2757 | NaSa01\_F12.raw |  |  |  | 5.6639E5 |  |  |  |  |  |  |  |  |  |  | 1 | 0 | 0 | 0 | 1 | 0 | 0 | 0 | 0 | 0 | 0 | 0 | 0 | 0 | 0 | 88 | 102 |  |  | PEAKS DB |
| C.AQLLEPGVYTK.V | N | 56.08 | 1217.6655 | 11 | 0.3 | 609.8402 | 2 | 35.74 | 4 | F4:19559 | NaSa01\_F12.raw |  |  |  | 7.9156E6 |  |  |  |  |  |  |  |  |  |  | 1 | 0 | 0 | 0 | 1 | 0 | 0 | 0 | 0 | 0 | 0 | 0 | 0 | 0 | 0 | 234 | 244 |  |  | PEAKS DB |
| G.QIQGIVSWGR.F | N | 53.28 | 1142.6196 | 10 | -0.2 | 572.3170 | 2 | 46.03 | 4 | F4:28225 | NaSa01\_F12.raw |  |  | 1.2578E6 | 4.548E7 |  |  |  |  |  |  |  |  |  |  | 3 | 0 | 0 | 1 | 2 | 0 | 0 | 0 | 0 | 0 | 0 | 0 | 0 | 0 | 0 | 221 | 230 |  |  | PEAKS DB |
| R.FPC(+57.02)AQLLEPGVY.T | Y | 44.08 | 1392.6747 | 12 | 0.0 | 697.3446 | 2 | 85.51 | 4 | F4:60830 | NaSa01\_F12.raw |  |  |  | 3.8557E6 |  |  |  |  |  |  |  |  |  |  | 1 | 0 | 0 | 0 | 1 | 0 | 0 | 0 | 0 | 0 | 0 | 0 | 0 | 0 | 0 | 231 | 242 | Carbamidomethylation | C3:Carbamidomethylation:1000.00 | PEAKS DB |
| total 7 peptides |  |  |  |  |  |  |  |  |  |  |  |  |  |  |  |  |  |  |  |  |  |  |  |  |  |  |  |  |  |  |  |  |  |  |  |  |  |  |  |  |  |  |  |  |  |
| --- | --- | --- | --- | --- | --- | --- | --- | --- | --- | --- | --- | --- | --- | --- | --- | --- | --- | --- | --- | --- | --- | --- | --- | --- | --- | --- | --- | --- | --- | --- | --- | --- | --- | --- | --- | --- | --- | --- | --- | --- | --- | --- | --- | --- | --- |

XP\_026524834.1

back to list

  

| Protein Coverage
| Supporting Peptides
|

Protein Coverage:

Supporting Peptides:

| Peptide | Uniq | -10lgP | Mass | Length | ppm | m/z | z | RT | Fraction | Scan | Source File | Area F1 | Area F10 | Area F11 | Area F12 | Area F13 | Area F14 | Area F2 | Area F3 | Area F4 | Area F5 | Area F6 | Area F7 | Area F8 | Area F9 | #Feature | #Feature F1 | #Feature F10 | #Feature F11 | #Feature F12 | #Feature F13 | #Feature F14 | #Feature F2 | #Feature F3 | #Feature F4 | #Feature F5 | #Feature F6 | #Feature F7 | #Feature F8 | #Feature F9 | Start | End | PTM | AScore | Found By |
| --- | --- | --- | --- | --- | --- | --- | --- | --- | --- | --- | --- | --- | --- | --- | --- | --- | --- | --- | --- | --- | --- | --- | --- | --- | --- | --- | --- | --- | --- | --- | --- | --- | --- | --- | --- | --- | --- | --- | --- | --- | --- | --- | --- | --- | --- |
| R.PFYAYTFEPLTK.E | Y | 75.08 | 1475.7336 | 12 | 0.2 | 738.8743 | 2 | 68.72 | 6 | F6:45340 | NaSa01\_F14.raw |  |  |  |  |  | 9.6668E6 |  |  |  |  |  |  |  |  | 1 | 0 | 0 | 0 | 0 | 0 | 1 | 0 | 0 | 0 | 0 | 0 | 0 | 0 | 0 | 314 | 325 |  |  | PEAKS DB |
| K.HSDSNAFLHLFPESFR.I | Y | 70.71 | 1902.9012 | 16 | 0.1 | 635.3077 | 3 | 63.63 | 6 | F6:41126 | NaSa01\_F14.raw |  |  |  |  |  | 1.477E7 |  |  |  |  |  |  |  |  | 3 | 0 | 0 | 0 | 0 | 0 | 3 | 0 | 0 | 0 | 0 | 0 | 0 | 0 | 0 | 398 | 413 |  |  | PEAKS DB |
| K.APMYPNEPFLVFWNAPTTQC(+57.02)QLR.Y | Y | 62.46 | 2779.3250 | 23 | 0.2 | 927.4491 | 3 | 97.44 | 6 | F6:69231 | NaSa01\_F14.raw |  |  |  |  |  | 3.3863E6 |  |  |  |  |  |  |  |  | 1 | 0 | 0 | 0 | 0 | 0 | 1 | 0 | 0 | 0 | 0 | 0 | 0 | 0 | 0 | 46 | 68 | Carbamidomethylation | C20:Carbamidomethylation:1000.00 | PEAKS DB |
| R.KDYALPVFVYAR.P | Y | 61.07 | 1440.7765 | 12 | 0.1 | 481.2662 | 3 | 57.57 | 6 | F6:35796 | NaSa01\_F14.raw |  |  |  |  |  | 1.2843E7 |  |  |  |  |  |  |  |  | 2 | 0 | 0 | 0 | 0 | 0 | 2 | 0 | 0 | 0 | 0 | 0 | 0 | 0 | 0 | 302 | 313 |  |  | PEAKS DB |
| R.NDQLLWLWR.D | Y | 57.24 | 1242.6509 | 9 | 1.1 | 622.3334 | 2 | 84.97 | 6 | F6:58743 | NaSa01\_F14.raw |  |  |  |  |  | 3.1787E6 |  |  |  |  |  |  |  |  | 1 | 0 | 0 | 0 | 0 | 0 | 1 | 0 | 0 | 0 | 0 | 0 | 0 | 0 | 0 | 253 | 261 |  |  | PEAKS DB |
| R.DTLLLAENMR.P | Y | 51.50 | 1174.6016 | 10 | -0.3 | 588.3079 | 2 | 54.08 | 6 | F6:32861 | NaSa01\_F14.raw |  |  |  |  |  | 6.4614E6 |  |  |  |  |  |  |  |  | 1 | 0 | 0 | 0 | 0 | 0 | 1 | 0 | 0 | 0 | 0 | 0 | 0 | 0 | 0 | 207 | 216 |  |  | PEAKS DB |
| K.NFMC(+57.02)QC(+57.02)YQGWK.G | Y | 49.22 | 1520.6000 | 11 | 0.1 | 761.3073 | 2 | 45.55 | 6 | F6:25449 | NaSa01\_F14.raw |  |  |  |  |  | 2.0824E6 |  |  |  |  |  |  |  |  | 1 | 0 | 0 | 0 | 0 | 0 | 1 | 0 | 0 | 0 | 0 | 0 | 0 | 0 | 0 | 442 | 452 | Carbamidomethylation | C4:Carbamidomethylation:1000.00;C6:Carbamidomethylation:1000.00 | PEAKS DB |
| K.DYALPVFVYAR.P | Y | 48.80 | 1312.6815 | 11 | 0.4 | 657.3483 | 2 | 83.06 | 6 | F6:57177 | NaSa01\_F14.raw |  |  |  |  |  | 3.8653E6 |  |  |  |  |  |  |  |  | 1 | 0 | 0 | 0 | 0 | 0 | 1 | 0 | 0 | 0 | 0 | 0 | 0 | 0 | 0 | 303 | 313 |  |  | PEAKS DB |
| R.KHSDSNAFLHLFPESFR.I | Y | 44.26 | 2030.9962 | 17 | -0.5 | 508.7561 | 4 | 56.62 | 6 | F6:35260 | NaSa01\_F14.raw |  |  |  |  |  | 3.9116E5 |  |  |  |  |  |  |  |  | 1 | 0 | 0 | 0 | 0 | 0 | 1 | 0 | 0 | 0 | 0 | 0 | 0 | 0 | 0 | 397 | 413 |  |  | PEAKS DB |
| K.TFHGLGVIDWENWR.P | Y | 43.98 | 1728.8372 | 14 | 0.5 | 577.2866 | 3 | 75.07 | 6 | F6:50512 | NaSa01\_F14.raw |  |  |  |  |  | 0 |  |  |  |  |  |  |  |  | 0 | 0 | 0 | 0 | 0 | 0 | 0 | 0 | 0 | 0 | 0 | 0 | 0 | 0 | 0 | 143 | 156 |  |  | PEAKS DB |
| Y.YLYPDC(+57.02)YNYNYK.K | Y | 39.32 | 1674.7024 | 12 | 0.3 | 838.3588 | 2 | 46.44 | 6 | F6:26475 | NaSa01\_F14.raw |  |  |  |  |  | 1.2437E6 |  |  |  |  |  |  |  |  | 1 | 0 | 0 | 0 | 0 | 0 | 1 | 0 | 0 | 0 | 0 | 0 | 0 | 0 | 0 | 224 | 235 | Carbamidomethylation | C6:Carbamidomethylation:1000.00 | PEAKS DB |
| total 11 peptides |  |  |  |  |  |  |  |  |  |  |  |  |  |  |  |  |  |  |  |  |  |  |  |  |  |  |  |  |  |  |  |  |  |  |  |  |  |  |  |  |  |  |  |  |  |
| --- | --- | --- | --- | --- | --- | --- | --- | --- | --- | --- | --- | --- | --- | --- | --- | --- | --- | --- | --- | --- | --- | --- | --- | --- | --- | --- | --- | --- | --- | --- | --- | --- | --- | --- | --- | --- | --- | --- | --- | --- | --- | --- | --- | --- | --- |

XP\_026524835.1

back to list

  

| Protein Coverage
| Supporting Peptides
|

Protein Coverage:

Supporting Peptides:

| Peptide | Uniq | -10lgP | Mass | Length | ppm | m/z | z | RT | Fraction | Scan | Source File | Area F1 | Area F10 | Area F11 | Area F12 | Area F13 | Area F14 | Area F2 | Area F3 | Area F4 | Area F5 | Area F6 | Area F7 | Area F8 | Area F9 | #Feature | #Feature F1 | #Feature F10 | #Feature F11 | #Feature F12 | #Feature F13 | #Feature F14 | #Feature F2 | #Feature F3 | #Feature F4 | #Feature F5 | #Feature F6 | #Feature F7 | #Feature F8 | #Feature F9 | Start | End | PTM | AScore | Found By |
| --- | --- | --- | --- | --- | --- | --- | --- | --- | --- | --- | --- | --- | --- | --- | --- | --- | --- | --- | --- | --- | --- | --- | --- | --- | --- | --- | --- | --- | --- | --- | --- | --- | --- | --- | --- | --- | --- | --- | --- | --- | --- | --- | --- | --- | --- |
| R.PFYAYTFEPLTK.E | Y | 75.08 | 1475.7336 | 12 | 0.2 | 738.8743 | 2 | 68.72 | 6 | F6:45340 | NaSa01\_F14.raw |  |  |  |  |  | 9.6668E6 |  |  |  |  |  |  |  |  | 1 | 0 | 0 | 0 | 0 | 0 | 1 | 0 | 0 | 0 | 0 | 0 | 0 | 0 | 0 | 314 | 325 |  |  | PEAKS DB |
| K.HSDSNAFLHLFPESFR.I | Y | 70.71 | 1902.9012 | 16 | 0.1 | 635.3077 | 3 | 63.63 | 6 | F6:41126 | NaSa01\_F14.raw |  |  |  |  |  | 1.477E7 |  |  |  |  |  |  |  |  | 3 | 0 | 0 | 0 | 0 | 0 | 3 | 0 | 0 | 0 | 0 | 0 | 0 | 0 | 0 | 398 | 413 |  |  | PEAKS DB |
| K.APMYPNEPFLVFWNAPTTQC(+57.02)QLR.Y | Y | 62.46 | 2779.3250 | 23 | 0.2 | 927.4491 | 3 | 97.44 | 6 | F6:69231 | NaSa01\_F14.raw |  |  |  |  |  | 3.3863E6 |  |  |  |  |  |  |  |  | 1 | 0 | 0 | 0 | 0 | 0 | 1 | 0 | 0 | 0 | 0 | 0 | 0 | 0 | 0 | 46 | 68 | Carbamidomethylation | C20:Carbamidomethylation:1000.00 | PEAKS DB |
| R.KDYALPVFVYAR.P | Y | 61.07 | 1440.7765 | 12 | 0.1 | 481.2662 | 3 | 57.57 | 6 | F6:35796 | NaSa01\_F14.raw |  |  |  |  |  | 1.2843E7 |  |  |  |  |  |  |  |  | 2 | 0 | 0 | 0 | 0 | 0 | 2 | 0 | 0 | 0 | 0 | 0 | 0 | 0 | 0 | 302 | 313 |  |  | PEAKS DB |
| R.NDQLLWLWR.D | Y | 57.24 | 1242.6509 | 9 | 1.1 | 622.3334 | 2 | 84.97 | 6 | F6:58743 | NaSa01\_F14.raw |  |  |  |  |  | 3.1787E6 |  |  |  |  |  |  |  |  | 1 | 0 | 0 | 0 | 0 | 0 | 1 | 0 | 0 | 0 | 0 | 0 | 0 | 0 | 0 | 253 | 261 |  |  | PEAKS DB |
| R.DTLLLAENMR.P | Y | 51.50 | 1174.6016 | 10 | -0.3 | 588.3079 | 2 | 54.08 | 6 | F6:32861 | NaSa01\_F14.raw |  |  |  |  |  | 6.4614E6 |  |  |  |  |  |  |  |  | 1 | 0 | 0 | 0 | 0 | 0 | 1 | 0 | 0 | 0 | 0 | 0 | 0 | 0 | 0 | 207 | 216 |  |  | PEAKS DB |
| K.NFMC(+57.02)QC(+57.02)YQGWK.G | Y | 49.22 | 1520.6000 | 11 | 0.1 | 761.3073 | 2 | 45.55 | 6 | F6:25449 | NaSa01\_F14.raw |  |  |  |  |  | 2.0824E6 |  |  |  |  |  |  |  |  | 1 | 0 | 0 | 0 | 0 | 0 | 1 | 0 | 0 | 0 | 0 | 0 | 0 | 0 | 0 | 442 | 452 | Carbamidomethylation | C4:Carbamidomethylation:1000.00;C6:Carbamidomethylation:1000.00 | PEAKS DB |
| K.DYALPVFVYAR.P | Y | 48.80 | 1312.6815 | 11 | 0.4 | 657.3483 | 2 | 83.06 | 6 | F6:57177 | NaSa01\_F14.raw |  |  |  |  |  | 3.8653E6 |  |  |  |  |  |  |  |  | 1 | 0 | 0 | 0 | 0 | 0 | 1 | 0 | 0 | 0 | 0 | 0 | 0 | 0 | 0 | 303 | 313 |  |  | PEAKS DB |
| R.KHSDSNAFLHLFPESFR.I | Y | 44.26 | 2030.9962 | 17 | -0.5 | 508.7561 | 4 | 56.62 | 6 | F6:35260 | NaSa01\_F14.raw |  |  |  |  |  | 3.9116E5 |  |  |  |  |  |  |  |  | 1 | 0 | 0 | 0 | 0 | 0 | 1 | 0 | 0 | 0 | 0 | 0 | 0 | 0 | 0 | 397 | 413 |  |  | PEAKS DB |
| K.TFHGLGVIDWENWR.P | Y | 43.98 | 1728.8372 | 14 | 0.5 | 577.2866 | 3 | 75.07 | 6 | F6:50512 | NaSa01\_F14.raw |  |  |  |  |  | 0 |  |  |  |  |  |  |  |  | 0 | 0 | 0 | 0 | 0 | 0 | 0 | 0 | 0 | 0 | 0 | 0 | 0 | 0 | 0 | 143 | 156 |  |  | PEAKS DB |
| Y.YLYPDC(+57.02)YNYNYK.K | Y | 39.32 | 1674.7024 | 12 | 0.3 | 838.3588 | 2 | 46.44 | 6 | F6:26475 | NaSa01\_F14.raw |  |  |  |  |  | 1.2437E6 |  |  |  |  |  |  |  |  | 1 | 0 | 0 | 0 | 0 | 0 | 1 | 0 | 0 | 0 | 0 | 0 | 0 | 0 | 0 | 224 | 235 | Carbamidomethylation | C6:Carbamidomethylation:1000.00 | PEAKS DB |
| total 11 peptides |  |  |  |  |  |  |  |  |  |  |  |  |  |  |  |  |  |  |  |  |  |  |  |  |  |  |  |  |  |  |  |  |  |  |  |  |  |  |  |  |  |  |  |  |  |
| --- | --- | --- | --- | --- | --- | --- | --- | --- | --- | --- | --- | --- | --- | --- | --- | --- | --- | --- | --- | --- | --- | --- | --- | --- | --- | --- | --- | --- | --- | --- | --- | --- | --- | --- | --- | --- | --- | --- | --- | --- | --- | --- | --- | --- | --- |

1T37

back to list

  

| Protein Coverage
| Supporting Peptides
|

Protein Coverage:

Supporting Peptides:

| Peptide | Uniq | -10lgP | Mass | Length | ppm | m/z | z | RT | Fraction | Scan | Source File | Area F1 | Area F10 | Area F11 | Area F12 | Area F13 | Area F14 | Area F2 | Area F3 | Area F4 | Area F5 | Area F6 | Area F7 | Area F8 | Area F9 | #Feature | #Feature F1 | #Feature F10 | #Feature F11 | #Feature F12 | #Feature F13 | #Feature F14 | #Feature F2 | #Feature F3 | #Feature F4 | #Feature F5 | #Feature F6 | #Feature F7 | #Feature F8 | #Feature F9 | Start | End | PTM | AScore | Found By |
| --- | --- | --- | --- | --- | --- | --- | --- | --- | --- | --- | --- | --- | --- | --- | --- | --- | --- | --- | --- | --- | --- | --- | --- | --- | --- | --- | --- | --- | --- | --- | --- | --- | --- | --- | --- | --- | --- | --- | --- | --- | --- | --- | --- | --- | --- |
| R.LAAIC(+57.02)FAGAPYNDDNYNIDLK.A | Y | 86.11 | 2357.0999 | 21 | 2.1 | 1179.5597 | 2 | 75.61 | 13 | F13:58613 | NaSa01\_F8.raw |  |  |  |  |  |  |  |  |  |  |  |  | 1.5729E9 | 2.4885E7 | 13 | 0 | 0 | 0 | 0 | 0 | 0 | 0 | 0 | 0 | 0 | 0 | 0 | 9 | 4 | 95 | 115 | Carbamidomethylation | C5:Carbamidomethylation:1000.00 | PEAKS DB |
| NLYQFKNMIQC(+57.02)TVPSR.S | N | 80.86 | 1997.9816 | 16 | 0.1 | 667.0012 | 3 | 54.61 | 13 | F13:39759 | NaSa01\_F8.raw |  |  |  |  |  |  |  |  |  |  |  |  | 1.2158E7 |  | 1 | 0 | 0 | 0 | 0 | 0 | 0 | 0 | 0 | 0 | 0 | 0 | 0 | 1 | 0 | 1 | 16 | Carbamidomethylation | C11:Carbamidomethylation:1000.00 | PEAKS DB |
| F.AGAPYNDDNYNIDLK.A | Y | 75.47 | 1681.7583 | 15 | 1.2 | 841.8875 | 2 | 42.16 | 13 | F13:28760 | NaSa01\_F8.raw |  |  |  |  |  |  |  |  |  |  |  |  | 5.1435E7 |  | 5 | 0 | 0 | 0 | 0 | 0 | 0 | 0 | 0 | 0 | 0 | 0 | 0 | 5 | 0 | 101 | 115 |  |  | PEAKS DB |
| K.GGSGTPVDDLDR.C | N | 67.25 | 1187.5417 | 12 | -0.9 | 594.7776 | 2 | 12.12 | 13 | F13:4409 | NaSa01\_F8.raw |  |  |  |  |  |  |  |  |  |  |  |  | 9.3807E7 |  | 2 | 0 | 0 | 0 | 0 | 0 | 0 | 0 | 0 | 0 | 0 | 0 | 0 | 2 | 0 | 31 | 42 |  |  | PEAKS DB |
| K.NMIQC(+57.02)TVPSR.S | N | 51.98 | 1204.5692 | 10 | -0.2 | 603.2917 | 2 | 12.12 | 13 | F13:4212 | NaSa01\_F8.raw |  |  |  |  |  |  |  |  |  |  |  |  | 8.3493E7 |  | 2 | 0 | 0 | 0 | 0 | 0 | 0 | 0 | 0 | 0 | 0 | 0 | 0 | 2 | 0 | 7 | 16 | Carbamidomethylation | C5:Carbamidomethylation:1000.00 | PEAKS DB |
| C.FAGAPYNDDNYNIDLK.A | Y | 50.79 | 1828.8268 | 16 | 0.7 | 915.4213 | 2 | 54.73 | 13 | F13:39782 | NaSa01\_F8.raw |  |  |  |  |  |  |  |  |  |  |  |  | 1.5272E6 |  | 1 | 0 | 0 | 0 | 0 | 0 | 0 | 0 | 0 | 0 | 0 | 0 | 0 | 1 | 0 | 100 | 115 |  |  | PEAKS DB |
| R.LAAIC(+57.02)FAGAPYN.D | N | 44.68 | 1266.6067 | 12 | -0.1 | 634.3105 | 2 | 71.65 | 13 | F13:54114 | NaSa01\_F8.raw |  |  | 2.1689E6 | 3.0706E6 |  | 4.6852E6 |  |  |  |  | 4.8617E6 | 4.4367E7 | 2.0168E7 |  | 6 | 0 | 0 | 1 | 1 | 0 | 1 | 0 | 0 | 0 | 0 | 1 | 1 | 1 | 0 | 95 | 106 | Carbamidomethylation | C5:Carbamidomethylation:1000.00 | PEAKS DB |
| D.RLAAIC(+57.02)FAGAPYNDDNYNIDLK.A | Y | 38.73 | 2513.2009 | 22 | 0.1 | 838.7410 | 3 | 66.87 | 13 | F13:49830 | NaSa01\_F8.raw |  |  |  |  |  |  |  |  |  |  |  |  | 3.144E6 |  | 1 | 0 | 0 | 0 | 0 | 0 | 0 | 0 | 0 | 0 | 0 | 0 | 0 | 1 | 0 | 94 | 115 | Carbamidomethylation | C6:Carbamidomethylation:1000.00 | PEAKS DB |
| total 8 peptides |  |  |  |  |  |  |  |  |  |  |  |  |  |  |  |  |  |  |  |  |  |  |  |  |  |  |  |  |  |  |  |  |  |  |  |  |  |  |  |  |  |  |  |  |  |
| --- | --- | --- | --- | --- | --- | --- | --- | --- | --- | --- | --- | --- | --- | --- | --- | --- | --- | --- | --- | --- | --- | --- | --- | --- | --- | --- | --- | --- | --- | --- | --- | --- | --- | --- | --- | --- | --- | --- | --- | --- | --- | --- | --- | --- | --- |

1ZM6

back to list

  

| Protein Coverage
| Supporting Peptides
|

Protein Coverage:

Supporting Peptides:

| Peptide | Uniq | -10lgP | Mass | Length | ppm | m/z | z | RT | Fraction | Scan | Source File | Area F1 | Area F10 | Area F11 | Area F12 | Area F13 | Area F14 | Area F2 | Area F3 | Area F4 | Area F5 | Area F6 | Area F7 | Area F8 | Area F9 | #Feature | #Feature F1 | #Feature F10 | #Feature F11 | #Feature F12 | #Feature F13 | #Feature F14 | #Feature F2 | #Feature F3 | #Feature F4 | #Feature F5 | #Feature F6 | #Feature F7 | #Feature F8 | #Feature F9 | Start | End | PTM | AScore | Found By |
| --- | --- | --- | --- | --- | --- | --- | --- | --- | --- | --- | --- | --- | --- | --- | --- | --- | --- | --- | --- | --- | --- | --- | --- | --- | --- | --- | --- | --- | --- | --- | --- | --- | --- | --- | --- | --- | --- | --- | --- | --- | --- | --- | --- | --- | --- |
| R.LAAIC(+57.02)FAGAPYNDDNYNIDLK.A | Y | 86.11 | 2357.0999 | 21 | 2.1 | 1179.5597 | 2 | 75.61 | 13 | F13:58613 | NaSa01\_F8.raw |  |  |  |  |  |  |  |  |  |  |  |  | 1.5729E9 | 2.4885E7 | 13 | 0 | 0 | 0 | 0 | 0 | 0 | 0 | 0 | 0 | 0 | 0 | 0 | 9 | 4 | 95 | 115 | Carbamidomethylation | C5:Carbamidomethylation:1000.00 | PEAKS DB |
| NLYQFKNMIQC(+57.02)TVPSR.S | N | 80.86 | 1997.9816 | 16 | 0.1 | 667.0012 | 3 | 54.61 | 13 | F13:39759 | NaSa01\_F8.raw |  |  |  |  |  |  |  |  |  |  |  |  | 1.2158E7 |  | 1 | 0 | 0 | 0 | 0 | 0 | 0 | 0 | 0 | 0 | 0 | 0 | 0 | 1 | 0 | 1 | 16 | Carbamidomethylation | C11:Carbamidomethylation:1000.00 | PEAKS DB |
| F.AGAPYNDDNYNIDLK.A | Y | 75.47 | 1681.7583 | 15 | 1.2 | 841.8875 | 2 | 42.16 | 13 | F13:28760 | NaSa01\_F8.raw |  |  |  |  |  |  |  |  |  |  |  |  | 5.1435E7 |  | 5 | 0 | 0 | 0 | 0 | 0 | 0 | 0 | 0 | 0 | 0 | 0 | 0 | 5 | 0 | 101 | 115 |  |  | PEAKS DB |
| K.GGSGTPVDDLDR.C | N | 67.25 | 1187.5417 | 12 | -0.9 | 594.7776 | 2 | 12.12 | 13 | F13:4409 | NaSa01\_F8.raw |  |  |  |  |  |  |  |  |  |  |  |  | 9.3807E7 |  | 2 | 0 | 0 | 0 | 0 | 0 | 0 | 0 | 0 | 0 | 0 | 0 | 0 | 2 | 0 | 31 | 42 |  |  | PEAKS DB |
| K.NMIQC(+57.02)TVPSR.S | N | 51.98 | 1204.5692 | 10 | -0.2 | 603.2917 | 2 | 12.12 | 13 | F13:4212 | NaSa01\_F8.raw |  |  |  |  |  |  |  |  |  |  |  |  | 8.3493E7 |  | 2 | 0 | 0 | 0 | 0 | 0 | 0 | 0 | 0 | 0 | 0 | 0 | 0 | 2 | 0 | 7 | 16 | Carbamidomethylation | C5:Carbamidomethylation:1000.00 | PEAKS DB |
| C.FAGAPYNDDNYNIDLK.A | Y | 50.79 | 1828.8268 | 16 | 0.7 | 915.4213 | 2 | 54.73 | 13 | F13:39782 | NaSa01\_F8.raw |  |  |  |  |  |  |  |  |  |  |  |  | 1.5272E6 |  | 1 | 0 | 0 | 0 | 0 | 0 | 0 | 0 | 0 | 0 | 0 | 0 | 0 | 1 | 0 | 100 | 115 |  |  | PEAKS DB |
| R.LAAIC(+57.02)FAGAPYN.D | N | 44.68 | 1266.6067 | 12 | -0.1 | 634.3105 | 2 | 71.65 | 13 | F13:54114 | NaSa01\_F8.raw |  |  | 2.1689E6 | 3.0706E6 |  | 4.6852E6 |  |  |  |  | 4.8617E6 | 4.4367E7 | 2.0168E7 |  | 6 | 0 | 0 | 1 | 1 | 0 | 1 | 0 | 0 | 0 | 0 | 1 | 1 | 1 | 0 | 95 | 106 | Carbamidomethylation | C5:Carbamidomethylation:1000.00 | PEAKS DB |
| D.RLAAIC(+57.02)FAGAPYNDDNYNIDLK.A | Y | 38.73 | 2513.2009 | 22 | 0.1 | 838.7410 | 3 | 66.87 | 13 | F13:49830 | NaSa01\_F8.raw |  |  |  |  |  |  |  |  |  |  |  |  | 3.144E6 |  | 1 | 0 | 0 | 0 | 0 | 0 | 0 | 0 | 0 | 0 | 0 | 0 | 0 | 1 | 0 | 94 | 115 | Carbamidomethylation | C6:Carbamidomethylation:1000.00 | PEAKS DB |
| total 8 peptides |  |  |  |  |  |  |  |  |  |  |  |  |  |  |  |  |  |  |  |  |  |  |  |  |  |  |  |  |  |  |  |  |  |  |  |  |  |  |  |  |  |  |  |  |  |
| --- | --- | --- | --- | --- | --- | --- | --- | --- | --- | --- | --- | --- | --- | --- | --- | --- | --- | --- | --- | --- | --- | --- | --- | --- | --- | --- | --- | --- | --- | --- | --- | --- | --- | --- | --- | --- | --- | --- | --- | --- | --- | --- | --- | --- | --- |

JAC94989.1

back to list

  

| Protein Coverage
| Supporting Peptides
|

Protein Coverage:

Supporting Peptides:

| Peptide | Uniq | -10lgP | Mass | Length | ppm | m/z | z | RT | Fraction | Scan | Source File | Area F1 | Area F10 | Area F11 | Area F12 | Area F13 | Area F14 | Area F2 | Area F3 | Area F4 | Area F5 | Area F6 | Area F7 | Area F8 | Area F9 | #Feature | #Feature F1 | #Feature F10 | #Feature F11 | #Feature F12 | #Feature F13 | #Feature F14 | #Feature F2 | #Feature F3 | #Feature F4 | #Feature F5 | #Feature F6 | #Feature F7 | #Feature F8 | #Feature F9 | Start | End | PTM | AScore | Found By |
| --- | --- | --- | --- | --- | --- | --- | --- | --- | --- | --- | --- | --- | --- | --- | --- | --- | --- | --- | --- | --- | --- | --- | --- | --- | --- | --- | --- | --- | --- | --- | --- | --- | --- | --- | --- | --- | --- | --- | --- | --- | --- | --- | --- | --- | --- |
| K.QNSGTYNNQYMILDTK.K | N | 84.72 | 1888.8625 | 16 | 0.2 | 945.4387 | 2 | 44.21 | 6 | F6:24307 | NaSa01\_F14.raw |  |  |  |  |  | 3.8863E7 |  |  |  |  |  |  |  |  | 3 | 0 | 0 | 0 | 0 | 0 | 3 | 0 | 0 | 0 | 0 | 0 | 0 | 0 | 0 | 351 | 366 |  |  | PEAKS DB |
| K.YGLDFSYEMAPR.A | N | 79.24 | 1447.6442 | 12 | -0.4 | 724.8291 | 2 | 65.86 | 6 | F6:42675 | NaSa01\_F14.raw |  |  |  |  | 1.2137E6 | 5.7636E7 |  |  |  |  |  |  |  |  | 2 | 0 | 0 | 0 | 0 | 1 | 1 | 0 | 0 | 0 | 0 | 0 | 0 | 0 | 0 | 427 | 438 |  |  | PEAKS DB |
| K.QVVPESLLAWER.V | Y | 71.30 | 1425.7616 | 12 | 0.8 | 713.8886 | 2 | 70.59 | 6 | F6:47248 | NaSa01\_F14.raw |  |  |  | 1.5221E6 | 1.9871E6 | 5.1446E7 |  |  |  |  |  |  |  |  | 5 | 0 | 0 | 0 | 1 | 1 | 3 | 0 | 0 | 0 | 0 | 0 | 0 | 0 | 0 | 319 | 330 |  |  | PEAKS DB |
| K.YGLDFSYEM(+15.99)APR.A | N | 68.49 | 1463.6390 | 12 | 0.2 | 732.8270 | 2 | 54.75 | 6 | F6:33400 | NaSa01\_F14.raw |  |  |  |  |  | 6.3581E6 |  |  |  |  |  |  |  |  | 1 | 0 | 0 | 0 | 0 | 0 | 1 | 0 | 0 | 0 | 0 | 0 | 0 | 0 | 0 | 427 | 438 | Oxidation (M) | M9:Oxidation (M):1000.00 | PEAKS DB |
| K.VLPGYENIYFAH.S | N | 52.82 | 1421.6979 | 12 | -0.2 | 711.8561 | 2 | 63.63 | 6 | F6:40911 | NaSa01\_F14.raw |  |  |  |  |  | 4.0012E7 |  |  |  |  |  |  |  |  | 1 | 0 | 0 | 0 | 0 | 0 | 1 | 0 | 0 | 0 | 0 | 0 | 0 | 0 | 0 | 239 | 250 |  |  | PEAKS DB |
| K.GYWPSYNIPFHN.I | Y | 50.10 | 1493.6727 | 12 | 0.9 | 747.8443 | 2 | 72.52 | 6 | F6:48403 | NaSa01\_F14.raw |  |  |  |  |  | 2.4618E6 |  |  |  |  |  |  |  |  | 1 | 0 | 0 | 0 | 0 | 0 | 1 | 0 | 0 | 0 | 0 | 0 | 0 | 0 | 0 | 401 | 412 |  |  | PEAKS DB |
| K.QNSGTYNNQYM(+15.99)ILDTK.K | N | 49.76 | 1904.8574 | 16 | -0.1 | 953.4359 | 2 | 35.20 | 6 | F6:16927 | NaSa01\_F14.raw |  |  |  |  |  | 2.3713E6 |  |  |  |  |  |  |  |  | 1 | 0 | 0 | 0 | 0 | 0 | 1 | 0 | 0 | 0 | 0 | 0 | 0 | 0 | 0 | 351 | 366 | Oxidation (M) | M11:Oxidation (M):1000.00 | PEAKS DB |
| H.SSWFTYAATLR.I | N | 49.21 | 1301.6404 | 11 | 0.0 | 651.8275 | 2 | 68.60 | 6 | F6:45310 | NaSa01\_F14.raw |  |  |  |  |  | 1.0363E7 |  |  |  |  |  |  |  |  | 1 | 0 | 0 | 0 | 0 | 0 | 1 | 0 | 0 | 0 | 0 | 0 | 0 | 0 | 0 | 251 | 261 |  |  | PEAKS DB |
| L.DFSYEMAPR.A | N | 43.68 | 1114.4753 | 9 | 0.5 | 558.2452 | 2 | 40.56 | 9 | F9:17621 | NaSa01\_F4.raw |  |  |  |  |  |  |  |  | 8.8862E5 |  |  |  |  |  | 1 | 0 | 0 | 0 | 0 | 0 | 0 | 0 | 0 | 1 | 0 | 0 | 0 | 0 | 0 | 430 | 438 |  |  | PEAKS DB |
| total 9 peptides |  |  |  |  |  |  |  |  |  |  |  |  |  |  |  |  |  |  |  |  |  |  |  |  |  |  |  |  |  |  |  |  |  |  |  |  |  |  |  |  |  |  |  |  |  |
| --- | --- | --- | --- | --- | --- | --- | --- | --- | --- | --- | --- | --- | --- | --- | --- | --- | --- | --- | --- | --- | --- | --- | --- | --- | --- | --- | --- | --- | --- | --- | --- | --- | --- | --- | --- | --- | --- | --- | --- | --- | --- | --- | --- | --- | --- |

AXL96651.1

back to list

  

| Protein Coverage
| Supporting Peptides
|

Protein Coverage:

Supporting Peptides:

| Peptide | Uniq | -10lgP | Mass | Length | ppm | m/z | z | RT | Fraction | Scan | Source File | Area F1 | Area F10 | Area F11 | Area F12 | Area F13 | Area F14 | Area F2 | Area F3 | Area F4 | Area F5 | Area F6 | Area F7 | Area F8 | Area F9 | #Feature | #Feature F1 | #Feature F10 | #Feature F11 | #Feature F12 | #Feature F13 | #Feature F14 | #Feature F2 | #Feature F3 | #Feature F4 | #Feature F5 | #Feature F6 | #Feature F7 | #Feature F8 | #Feature F9 | Start | End | PTM | AScore | Found By |
| --- | --- | --- | --- | --- | --- | --- | --- | --- | --- | --- | --- | --- | --- | --- | --- | --- | --- | --- | --- | --- | --- | --- | --- | --- | --- | --- | --- | --- | --- | --- | --- | --- | --- | --- | --- | --- | --- | --- | --- | --- | --- | --- | --- | --- | --- |
| K.YIELYM(+15.99)VVDNVMYR.K | Y | 73.66 | 1822.8634 | 14 | 1.9 | 912.4407 | 2 | 87.28 | 6 | F6:61115 | NaSa01\_F14.raw |  |  |  |  | 1.2196E7 | 1.6722E7 |  |  |  |  |  |  |  |  | 8 | 0 | 0 | 0 | 0 | 4 | 4 | 0 | 0 | 0 | 0 | 0 | 0 | 0 | 0 | 205 | 218 | Oxidation (M) | M6:Oxidation (M):129.15 | PEAKS DB |
| N.VTLDLFGEWR.E | N | 67.98 | 1234.6346 | 10 | 0.3 | 618.3248 | 2 | 83.54 | 6 | F6:57918 | NaSa01\_F14.raw |  |  | 1.1051E5 |  | 1.1401E8 | 1.4573E8 |  |  |  |  |  |  |  | 9.2511E5 | 13 | 0 | 0 | 1 | 0 | 2 | 9 | 0 | 0 | 0 | 0 | 0 | 0 | 0 | 1 | 271 | 280 |  |  | PEAKS DB |
| L.YMVVDNVMYR.K | Y | 62.88 | 1288.5944 | 10 | 0.3 | 645.3046 | 2 | 48.18 | 6 | F6:27670 | NaSa01\_F14.raw |  |  |  |  | 7.0602E6 | 1.025E7 |  |  |  |  |  |  |  |  | 2 | 0 | 0 | 0 | 0 | 1 | 1 | 0 | 0 | 0 | 0 | 0 | 0 | 0 | 0 | 209 | 218 |  |  | PEAKS DB |
| A.LIGLEIWSNK.D | N | 56.38 | 1171.6600 | 10 | 0.6 | 586.8376 | 2 | 69.41 | 4 | F4:47508 | NaSa01\_F12.raw |  |  | 2.9162E6 | 5.1163E6 |  |  |  |  |  |  |  |  |  |  | 2 | 0 | 0 | 1 | 1 | 0 | 0 | 0 | 0 | 0 | 0 | 0 | 0 | 0 | 0 | 251 | 260 |  |  | PEAKS DB |
| L.IGLEIWSNK.D | N | 54.47 | 1058.5760 | 9 | 0.3 | 530.2955 | 2 | 55.93 | 3 | F3:31172 | NaSa01\_F11.raw |  |  | 9.3204E6 | 1.0988E7 |  | 3.4788E6 |  |  |  |  |  | 6.1468E5 | 0 |  | 4 | 0 | 0 | 1 | 1 | 0 | 1 | 0 | 0 | 0 | 0 | 0 | 1 | 0 | 0 | 252 | 260 |  |  | PEAKS DB |
| K.YIELYM(+15.99)VVDNVM(+15.99)YR.K | Y | 48.86 | 1838.8583 | 14 | 1.7 | 920.4380 | 2 | 73.09 | 6 | F6:48946 | NaSa01\_F14.raw |  |  |  |  |  | 1.5205E6 |  |  |  |  |  |  |  |  | 1 | 0 | 0 | 0 | 0 | 0 | 1 | 0 | 0 | 0 | 0 | 0 | 0 | 0 | 0 | 205 | 218 | Oxidation (M) | M6:Oxidation (M):1000.00;M12:Oxidation (M):1000.00 | PEAKS DB |
| I.GLEIWSNK.D | N | 43.99 | 945.4919 | 8 | 0.7 | 473.7536 | 2 | 37.33 | 12 | F12:20994 | NaSa01\_F7.raw |  |  |  |  |  |  |  |  |  |  |  | 1.2994E6 |  |  | 1 | 0 | 0 | 0 | 0 | 0 | 0 | 0 | 0 | 0 | 0 | 0 | 1 | 0 | 0 | 253 | 260 |  |  | PEAKS DB |
| V.TLDLFGEWR.E | N | 43.65 | 1135.5662 | 9 | -0.8 | 568.7899 | 2 | 77.17 | 6 | F6:52295 | NaSa01\_F14.raw |  |  |  |  |  | 1.3701E6 |  |  |  |  |  |  |  |  | 1 | 0 | 0 | 0 | 0 | 0 | 1 | 0 | 0 | 0 | 0 | 0 | 0 | 0 | 0 | 272 | 280 |  |  | PEAKS DB |
| Y.MVVDNVMYR.K | Y | 42.43 | 1125.5311 | 9 | -0.5 | 563.7726 | 2 | 35.30 | 6 | F6:16991 | NaSa01\_F14.raw |  |  |  |  | 4.6299E8 | 1.6721E7 |  |  |  |  |  |  |  |  | 2 | 0 | 0 | 0 | 0 | 1 | 1 | 0 | 0 | 0 | 0 | 0 | 0 | 0 | 0 | 210 | 218 |  |  | PEAKS DB |
| total 9 peptides |  |  |  |  |  |  |  |  |  |  |  |  |  |  |  |  |  |  |  |  |  |  |  |  |  |  |  |  |  |  |  |  |  |  |  |  |  |  |  |  |  |  |  |  |  |
| --- | --- | --- | --- | --- | --- | --- | --- | --- | --- | --- | --- | --- | --- | --- | --- | --- | --- | --- | --- | --- | --- | --- | --- | --- | --- | --- | --- | --- | --- | --- | --- | --- | --- | --- | --- | --- | --- | --- | --- | --- | --- | --- | --- | --- | --- |

AXL96665.1

back to list

  

| Protein Coverage
| Supporting Peptides
|

Protein Coverage:

Supporting Peptides:

| Peptide | Uniq | -10lgP | Mass | Length | ppm | m/z | z | RT | Fraction | Scan | Source File | Area F1 | Area F10 | Area F11 | Area F12 | Area F13 | Area F14 | Area F2 | Area F3 | Area F4 | Area F5 | Area F6 | Area F7 | Area F8 | Area F9 | #Feature | #Feature F1 | #Feature F10 | #Feature F11 | #Feature F12 | #Feature F13 | #Feature F14 | #Feature F2 | #Feature F3 | #Feature F4 | #Feature F5 | #Feature F6 | #Feature F7 | #Feature F8 | #Feature F9 | Start | End | PTM | AScore | Found By |
| --- | --- | --- | --- | --- | --- | --- | --- | --- | --- | --- | --- | --- | --- | --- | --- | --- | --- | --- | --- | --- | --- | --- | --- | --- | --- | --- | --- | --- | --- | --- | --- | --- | --- | --- | --- | --- | --- | --- | --- | --- | --- | --- | --- | --- | --- |
| K.YIELYM(+15.99)VVDNVMYR.K | Y | 73.66 | 1822.8634 | 14 | 1.9 | 912.4407 | 2 | 87.28 | 6 | F6:61115 | NaSa01\_F14.raw |  |  |  |  | 1.2196E7 | 1.6722E7 |  |  |  |  |  |  |  |  | 8 | 0 | 0 | 0 | 0 | 4 | 4 | 0 | 0 | 0 | 0 | 0 | 0 | 0 | 0 | 205 | 218 | Oxidation (M) | M6:Oxidation (M):129.15 | PEAKS DB |
| N.VTLDLFGEWR.E | N | 67.98 | 1234.6346 | 10 | 0.3 | 618.3248 | 2 | 83.54 | 6 | F6:57918 | NaSa01\_F14.raw |  |  | 1.1051E5 |  | 1.1401E8 | 1.4573E8 |  |  |  |  |  |  |  | 9.2511E5 | 13 | 0 | 0 | 1 | 0 | 2 | 9 | 0 | 0 | 0 | 0 | 0 | 0 | 0 | 1 | 271 | 280 |  |  | PEAKS DB |
| L.YMVVDNVMYR.K | Y | 62.88 | 1288.5944 | 10 | 0.3 | 645.3046 | 2 | 48.18 | 6 | F6:27670 | NaSa01\_F14.raw |  |  |  |  | 7.0602E6 | 1.025E7 |  |  |  |  |  |  |  |  | 2 | 0 | 0 | 0 | 0 | 1 | 1 | 0 | 0 | 0 | 0 | 0 | 0 | 0 | 0 | 209 | 218 |  |  | PEAKS DB |
| A.LIGLEIWSNK.D | N | 56.38 | 1171.6600 | 10 | 0.6 | 586.8376 | 2 | 69.41 | 4 | F4:47508 | NaSa01\_F12.raw |  |  | 2.9162E6 | 5.1163E6 |  |  |  |  |  |  |  |  |  |  | 2 | 0 | 0 | 1 | 1 | 0 | 0 | 0 | 0 | 0 | 0 | 0 | 0 | 0 | 0 | 251 | 260 |  |  | PEAKS DB |
| L.IGLEIWSNK.D | N | 54.47 | 1058.5760 | 9 | 0.3 | 530.2955 | 2 | 55.93 | 3 | F3:31172 | NaSa01\_F11.raw |  |  | 9.3204E6 | 1.0988E7 |  | 3.4788E6 |  |  |  |  |  | 6.1468E5 | 0 |  | 4 | 0 | 0 | 1 | 1 | 0 | 1 | 0 | 0 | 0 | 0 | 0 | 1 | 0 | 0 | 252 | 260 |  |  | PEAKS DB |
| K.YIELYM(+15.99)VVDNVM(+15.99)YR.K | Y | 48.86 | 1838.8583 | 14 | 1.7 | 920.4380 | 2 | 73.09 | 6 | F6:48946 | NaSa01\_F14.raw |  |  |  |  |  | 1.5205E6 |  |  |  |  |  |  |  |  | 1 | 0 | 0 | 0 | 0 | 0 | 1 | 0 | 0 | 0 | 0 | 0 | 0 | 0 | 0 | 205 | 218 | Oxidation (M) | M6:Oxidation (M):1000.00;M12:Oxidation (M):1000.00 | PEAKS DB |
| I.GLEIWSNK.D | N | 43.99 | 945.4919 | 8 | 0.7 | 473.7536 | 2 | 37.33 | 12 | F12:20994 | NaSa01\_F7.raw |  |  |  |  |  |  |  |  |  |  |  | 1.2994E6 |  |  | 1 | 0 | 0 | 0 | 0 | 0 | 0 | 0 | 0 | 0 | 0 | 0 | 1 | 0 | 0 | 253 | 260 |  |  | PEAKS DB |
| V.TLDLFGEWR.E | N | 43.65 | 1135.5662 | 9 | -0.8 | 568.7899 | 2 | 77.17 | 6 | F6:52295 | NaSa01\_F14.raw |  |  |  |  |  | 1.3701E6 |  |  |  |  |  |  |  |  | 1 | 0 | 0 | 0 | 0 | 0 | 1 | 0 | 0 | 0 | 0 | 0 | 0 | 0 | 0 | 272 | 280 |  |  | PEAKS DB |
| Y.MVVDNVMYR.K | Y | 42.43 | 1125.5311 | 9 | -0.5 | 563.7726 | 2 | 35.30 | 6 | F6:16991 | NaSa01\_F14.raw |  |  |  |  | 4.6299E8 | 1.6721E7 |  |  |  |  |  |  |  |  | 2 | 0 | 0 | 0 | 0 | 1 | 1 | 0 | 0 | 0 | 0 | 0 | 0 | 0 | 0 | 210 | 218 |  |  | PEAKS DB |
| total 9 peptides |  |  |  |  |  |  |  |  |  |  |  |  |  |  |  |  |  |  |  |  |  |  |  |  |  |  |  |  |  |  |  |  |  |  |  |  |  |  |  |  |  |  |  |  |  |
| --- | --- | --- | --- | --- | --- | --- | --- | --- | --- | --- | --- | --- | --- | --- | --- | --- | --- | --- | --- | --- | --- | --- | --- | --- | --- | --- | --- | --- | --- | --- | --- | --- | --- | --- | --- | --- | --- | --- | --- | --- | --- | --- | --- | --- | --- |

AXL96660.1

back to list

  

| Protein Coverage
| Supporting Peptides
|

Protein Coverage:

Supporting Peptides:

| Peptide | Uniq | -10lgP | Mass | Length | ppm | m/z | z | RT | Fraction | Scan | Source File | Area F1 | Area F10 | Area F11 | Area F12 | Area F13 | Area F14 | Area F2 | Area F3 | Area F4 | Area F5 | Area F6 | Area F7 | Area F8 | Area F9 | #Feature | #Feature F1 | #Feature F10 | #Feature F11 | #Feature F12 | #Feature F13 | #Feature F14 | #Feature F2 | #Feature F3 | #Feature F4 | #Feature F5 | #Feature F6 | #Feature F7 | #Feature F8 | #Feature F9 | Start | End | PTM | AScore | Found By |
| --- | --- | --- | --- | --- | --- | --- | --- | --- | --- | --- | --- | --- | --- | --- | --- | --- | --- | --- | --- | --- | --- | --- | --- | --- | --- | --- | --- | --- | --- | --- | --- | --- | --- | --- | --- | --- | --- | --- | --- | --- | --- | --- | --- | --- | --- |
| K.YIELYM(+15.99)VVDNVMYR.K | Y | 73.66 | 1822.8634 | 14 | 1.9 | 912.4407 | 2 | 87.28 | 6 | F6:61115 | NaSa01\_F14.raw |  |  |  |  | 1.2196E7 | 1.6722E7 |  |  |  |  |  |  |  |  | 8 | 0 | 0 | 0 | 0 | 4 | 4 | 0 | 0 | 0 | 0 | 0 | 0 | 0 | 0 | 205 | 218 | Oxidation (M) | M6:Oxidation (M):129.15 | PEAKS DB |
| N.VTLDLFGEWR.E | N | 67.98 | 1234.6346 | 10 | 0.3 | 618.3248 | 2 | 83.54 | 6 | F6:57918 | NaSa01\_F14.raw |  |  | 1.1051E5 |  | 1.1401E8 | 1.4573E8 |  |  |  |  |  |  |  | 9.2511E5 | 13 | 0 | 0 | 1 | 0 | 2 | 9 | 0 | 0 | 0 | 0 | 0 | 0 | 0 | 1 | 271 | 280 |  |  | PEAKS DB |
| L.YMVVDNVMYR.K | Y | 62.88 | 1288.5944 | 10 | 0.3 | 645.3046 | 2 | 48.18 | 6 | F6:27670 | NaSa01\_F14.raw |  |  |  |  | 7.0602E6 | 1.025E7 |  |  |  |  |  |  |  |  | 2 | 0 | 0 | 0 | 0 | 1 | 1 | 0 | 0 | 0 | 0 | 0 | 0 | 0 | 0 | 209 | 218 |  |  | PEAKS DB |
| A.LIGLEIWSNK.D | N | 56.38 | 1171.6600 | 10 | 0.6 | 586.8376 | 2 | 69.41 | 4 | F4:47508 | NaSa01\_F12.raw |  |  | 2.9162E6 | 5.1163E6 |  |  |  |  |  |  |  |  |  |  | 2 | 0 | 0 | 1 | 1 | 0 | 0 | 0 | 0 | 0 | 0 | 0 | 0 | 0 | 0 | 251 | 260 |  |  | PEAKS DB |
| L.IGLEIWSNK.D | N | 54.47 | 1058.5760 | 9 | 0.3 | 530.2955 | 2 | 55.93 | 3 | F3:31172 | NaSa01\_F11.raw |  |  | 9.3204E6 | 1.0988E7 |  | 3.4788E6 |  |  |  |  |  | 6.1468E5 | 0 |  | 4 | 0 | 0 | 1 | 1 | 0 | 1 | 0 | 0 | 0 | 0 | 0 | 1 | 0 | 0 | 252 | 260 |  |  | PEAKS DB |
| K.YIELYM(+15.99)VVDNVM(+15.99)YR.K | Y | 48.86 | 1838.8583 | 14 | 1.7 | 920.4380 | 2 | 73.09 | 6 | F6:48946 | NaSa01\_F14.raw |  |  |  |  |  | 1.5205E6 |  |  |  |  |  |  |  |  | 1 | 0 | 0 | 0 | 0 | 0 | 1 | 0 | 0 | 0 | 0 | 0 | 0 | 0 | 0 | 205 | 218 | Oxidation (M) | M6:Oxidation (M):1000.00;M12:Oxidation (M):1000.00 | PEAKS DB |
| I.GLEIWSNK.D | N | 43.99 | 945.4919 | 8 | 0.7 | 473.7536 | 2 | 37.33 | 12 | F12:20994 | NaSa01\_F7.raw |  |  |  |  |  |  |  |  |  |  |  | 1.2994E6 |  |  | 1 | 0 | 0 | 0 | 0 | 0 | 0 | 0 | 0 | 0 | 0 | 0 | 1 | 0 | 0 | 253 | 260 |  |  | PEAKS DB |
| V.TLDLFGEWR.E | N | 43.65 | 1135.5662 | 9 | -0.8 | 568.7899 | 2 | 77.17 | 6 | F6:52295 | NaSa01\_F14.raw |  |  |  |  |  | 1.3701E6 |  |  |  |  |  |  |  |  | 1 | 0 | 0 | 0 | 0 | 0 | 1 | 0 | 0 | 0 | 0 | 0 | 0 | 0 | 0 | 272 | 280 |  |  | PEAKS DB |
| Y.MVVDNVMYR.K | Y | 42.43 | 1125.5311 | 9 | -0.5 | 563.7726 | 2 | 35.30 | 6 | F6:16991 | NaSa01\_F14.raw |  |  |  |  | 4.6299E8 | 1.6721E7 |  |  |  |  |  |  |  |  | 2 | 0 | 0 | 0 | 0 | 1 | 1 | 0 | 0 | 0 | 0 | 0 | 0 | 0 | 0 | 210 | 218 |  |  | PEAKS DB |
| total 9 peptides |  |  |  |  |  |  |  |  |  |  |  |  |  |  |  |  |  |  |  |  |  |  |  |  |  |  |  |  |  |  |  |  |  |  |  |  |  |  |  |  |  |  |  |  |  |
| --- | --- | --- | --- | --- | --- | --- | --- | --- | --- | --- | --- | --- | --- | --- | --- | --- | --- | --- | --- | --- | --- | --- | --- | --- | --- | --- | --- | --- | --- | --- | --- | --- | --- | --- | --- | --- | --- | --- | --- | --- | --- | --- | --- | --- | --- |

AXL96668.1

back to list

  

| Protein Coverage
| Supporting Peptides
|

Protein Coverage:

Supporting Peptides:

| Peptide | Uniq | -10lgP | Mass | Length | ppm | m/z | z | RT | Fraction | Scan | Source File | Area F1 | Area F10 | Area F11 | Area F12 | Area F13 | Area F14 | Area F2 | Area F3 | Area F4 | Area F5 | Area F6 | Area F7 | Area F8 | Area F9 | #Feature | #Feature F1 | #Feature F10 | #Feature F11 | #Feature F12 | #Feature F13 | #Feature F14 | #Feature F2 | #Feature F3 | #Feature F4 | #Feature F5 | #Feature F6 | #Feature F7 | #Feature F8 | #Feature F9 | Start | End | PTM | AScore | Found By |
| --- | --- | --- | --- | --- | --- | --- | --- | --- | --- | --- | --- | --- | --- | --- | --- | --- | --- | --- | --- | --- | --- | --- | --- | --- | --- | --- | --- | --- | --- | --- | --- | --- | --- | --- | --- | --- | --- | --- | --- | --- | --- | --- | --- | --- | --- |
| K.YIELYM(+15.99)VVDNVMYR.K | Y | 73.66 | 1822.8634 | 14 | 1.9 | 912.4407 | 2 | 87.28 | 6 | F6:61115 | NaSa01\_F14.raw |  |  |  |  | 1.2196E7 | 1.6722E7 |  |  |  |  |  |  |  |  | 8 | 0 | 0 | 0 | 0 | 4 | 4 | 0 | 0 | 0 | 0 | 0 | 0 | 0 | 0 | 205 | 218 | Oxidation (M) | M6:Oxidation (M):129.15 | PEAKS DB |
| N.VTLDLFGEWR.E | N | 67.98 | 1234.6346 | 10 | 0.3 | 618.3248 | 2 | 83.54 | 6 | F6:57918 | NaSa01\_F14.raw |  |  | 1.1051E5 |  | 1.1401E8 | 1.4573E8 |  |  |  |  |  |  |  | 9.2511E5 | 13 | 0 | 0 | 1 | 0 | 2 | 9 | 0 | 0 | 0 | 0 | 0 | 0 | 0 | 1 | 271 | 280 |  |  | PEAKS DB |
| L.YMVVDNVMYR.K | Y | 62.88 | 1288.5944 | 10 | 0.3 | 645.3046 | 2 | 48.18 | 6 | F6:27670 | NaSa01\_F14.raw |  |  |  |  | 7.0602E6 | 1.025E7 |  |  |  |  |  |  |  |  | 2 | 0 | 0 | 0 | 0 | 1 | 1 | 0 | 0 | 0 | 0 | 0 | 0 | 0 | 0 | 209 | 218 |  |  | PEAKS DB |
| A.LIGLEIWSNK.D | N | 56.38 | 1171.6600 | 10 | 0.6 | 586.8376 | 2 | 69.41 | 4 | F4:47508 | NaSa01\_F12.raw |  |  | 2.9162E6 | 5.1163E6 |  |  |  |  |  |  |  |  |  |  | 2 | 0 | 0 | 1 | 1 | 0 | 0 | 0 | 0 | 0 | 0 | 0 | 0 | 0 | 0 | 251 | 260 |  |  | PEAKS DB |
| L.IGLEIWSNK.D | N | 54.47 | 1058.5760 | 9 | 0.3 | 530.2955 | 2 | 55.93 | 3 | F3:31172 | NaSa01\_F11.raw |  |  | 9.3204E6 | 1.0988E7 |  | 3.4788E6 |  |  |  |  |  | 6.1468E5 | 0 |  | 4 | 0 | 0 | 1 | 1 | 0 | 1 | 0 | 0 | 0 | 0 | 0 | 1 | 0 | 0 | 252 | 260 |  |  | PEAKS DB |
| K.YIELYM(+15.99)VVDNVM(+15.99)YR.K | Y | 48.86 | 1838.8583 | 14 | 1.7 | 920.4380 | 2 | 73.09 | 6 | F6:48946 | NaSa01\_F14.raw |  |  |  |  |  | 1.5205E6 |  |  |  |  |  |  |  |  | 1 | 0 | 0 | 0 | 0 | 0 | 1 | 0 | 0 | 0 | 0 | 0 | 0 | 0 | 0 | 205 | 218 | Oxidation (M) | M6:Oxidation (M):1000.00;M12:Oxidation (M):1000.00 | PEAKS DB |
| I.GLEIWSNK.D | N | 43.99 | 945.4919 | 8 | 0.7 | 473.7536 | 2 | 37.33 | 12 | F12:20994 | NaSa01\_F7.raw |  |  |  |  |  |  |  |  |  |  |  | 1.2994E6 |  |  | 1 | 0 | 0 | 0 | 0 | 0 | 0 | 0 | 0 | 0 | 0 | 0 | 1 | 0 | 0 | 253 | 260 |  |  | PEAKS DB |
| V.TLDLFGEWR.E | N | 43.65 | 1135.5662 | 9 | -0.8 | 568.7899 | 2 | 77.17 | 6 | F6:52295 | NaSa01\_F14.raw |  |  |  |  |  | 1.3701E6 |  |  |  |  |  |  |  |  | 1 | 0 | 0 | 0 | 0 | 0 | 1 | 0 | 0 | 0 | 0 | 0 | 0 | 0 | 0 | 272 | 280 |  |  | PEAKS DB |
| Y.MVVDNVMYR.K | Y | 42.43 | 1125.5311 | 9 | -0.5 | 563.7726 | 2 | 35.30 | 6 | F6:16991 | NaSa01\_F14.raw |  |  |  |  | 4.6299E8 | 1.6721E7 |  |  |  |  |  |  |  |  | 2 | 0 | 0 | 0 | 0 | 1 | 1 | 0 | 0 | 0 | 0 | 0 | 0 | 0 | 0 | 210 | 218 |  |  | PEAKS DB |
| total 9 peptides |  |  |  |  |  |  |  |  |  |  |  |  |  |  |  |  |  |  |  |  |  |  |  |  |  |  |  |  |  |  |  |  |  |  |  |  |  |  |  |  |  |  |  |  |  |
| --- | --- | --- | --- | --- | --- | --- | --- | --- | --- | --- | --- | --- | --- | --- | --- | --- | --- | --- | --- | --- | --- | --- | --- | --- | --- | --- | --- | --- | --- | --- | --- | --- | --- | --- | --- | --- | --- | --- | --- | --- | --- | --- | --- | --- | --- |

AXL96656.1

back to list

  

| Protein Coverage
| Supporting Peptides
|

Protein Coverage:

Supporting Peptides:

| Peptide | Uniq | -10lgP | Mass | Length | ppm | m/z | z | RT | Fraction | Scan | Source File | Area F1 | Area F10 | Area F11 | Area F12 | Area F13 | Area F14 | Area F2 | Area F3 | Area F4 | Area F5 | Area F6 | Area F7 | Area F8 | Area F9 | #Feature | #Feature F1 | #Feature F10 | #Feature F11 | #Feature F12 | #Feature F13 | #Feature F14 | #Feature F2 | #Feature F3 | #Feature F4 | #Feature F5 | #Feature F6 | #Feature F7 | #Feature F8 | #Feature F9 | Start | End | PTM | AScore | Found By |
| --- | --- | --- | --- | --- | --- | --- | --- | --- | --- | --- | --- | --- | --- | --- | --- | --- | --- | --- | --- | --- | --- | --- | --- | --- | --- | --- | --- | --- | --- | --- | --- | --- | --- | --- | --- | --- | --- | --- | --- | --- | --- | --- | --- | --- | --- |
| K.YIELYM(+15.99)VVDNVMYR.K | Y | 73.66 | 1822.8634 | 14 | 1.9 | 912.4407 | 2 | 87.28 | 6 | F6:61115 | NaSa01\_F14.raw |  |  |  |  | 1.2196E7 | 1.6722E7 |  |  |  |  |  |  |  |  | 8 | 0 | 0 | 0 | 0 | 4 | 4 | 0 | 0 | 0 | 0 | 0 | 0 | 0 | 0 | 205 | 218 | Oxidation (M) | M6:Oxidation (M):129.15 | PEAKS DB |
| N.VTLDLFGEWR.E | N | 67.98 | 1234.6346 | 10 | 0.3 | 618.3248 | 2 | 83.54 | 6 | F6:57918 | NaSa01\_F14.raw |  |  | 1.1051E5 |  | 1.1401E8 | 1.4573E8 |  |  |  |  |  |  |  | 9.2511E5 | 13 | 0 | 0 | 1 | 0 | 2 | 9 | 0 | 0 | 0 | 0 | 0 | 0 | 0 | 1 | 271 | 280 |  |  | PEAKS DB |
| L.YMVVDNVMYR.K | Y | 62.88 | 1288.5944 | 10 | 0.3 | 645.3046 | 2 | 48.18 | 6 | F6:27670 | NaSa01\_F14.raw |  |  |  |  | 7.0602E6 | 1.025E7 |  |  |  |  |  |  |  |  | 2 | 0 | 0 | 0 | 0 | 1 | 1 | 0 | 0 | 0 | 0 | 0 | 0 | 0 | 0 | 209 | 218 |  |  | PEAKS DB |
| A.LIGLEIWSNK.D | N | 56.38 | 1171.6600 | 10 | 0.6 | 586.8376 | 2 | 69.41 | 4 | F4:47508 | NaSa01\_F12.raw |  |  | 2.9162E6 | 5.1163E6 |  |  |  |  |  |  |  |  |  |  | 2 | 0 | 0 | 1 | 1 | 0 | 0 | 0 | 0 | 0 | 0 | 0 | 0 | 0 | 0 | 251 | 260 |  |  | PEAKS DB |
| L.IGLEIWSNK.D | N | 54.47 | 1058.5760 | 9 | 0.3 | 530.2955 | 2 | 55.93 | 3 | F3:31172 | NaSa01\_F11.raw |  |  | 9.3204E6 | 1.0988E7 |  | 3.4788E6 |  |  |  |  |  | 6.1468E5 | 0 |  | 4 | 0 | 0 | 1 | 1 | 0 | 1 | 0 | 0 | 0 | 0 | 0 | 1 | 0 | 0 | 252 | 260 |  |  | PEAKS DB |
| K.YIELYM(+15.99)VVDNVM(+15.99)YR.K | Y | 48.86 | 1838.8583 | 14 | 1.7 | 920.4380 | 2 | 73.09 | 6 | F6:48946 | NaSa01\_F14.raw |  |  |  |  |  | 1.5205E6 |  |  |  |  |  |  |  |  | 1 | 0 | 0 | 0 | 0 | 0 | 1 | 0 | 0 | 0 | 0 | 0 | 0 | 0 | 0 | 205 | 218 | Oxidation (M) | M6:Oxidation (M):1000.00;M12:Oxidation (M):1000.00 | PEAKS DB |
| I.GLEIWSNK.D | N | 43.99 | 945.4919 | 8 | 0.7 | 473.7536 | 2 | 37.33 | 12 | F12:20994 | NaSa01\_F7.raw |  |  |  |  |  |  |  |  |  |  |  | 1.2994E6 |  |  | 1 | 0 | 0 | 0 | 0 | 0 | 0 | 0 | 0 | 0 | 0 | 0 | 1 | 0 | 0 | 253 | 260 |  |  | PEAKS DB |
| V.TLDLFGEWR.E | N | 43.65 | 1135.5662 | 9 | -0.8 | 568.7899 | 2 | 77.17 | 6 | F6:52295 | NaSa01\_F14.raw |  |  |  |  |  | 1.3701E6 |  |  |  |  |  |  |  |  | 1 | 0 | 0 | 0 | 0 | 0 | 1 | 0 | 0 | 0 | 0 | 0 | 0 | 0 | 0 | 272 | 280 |  |  | PEAKS DB |
| Y.MVVDNVMYR.K | Y | 42.43 | 1125.5311 | 9 | -0.5 | 563.7726 | 2 | 35.30 | 6 | F6:16991 | NaSa01\_F14.raw |  |  |  |  | 4.6299E8 | 1.6721E7 |  |  |  |  |  |  |  |  | 2 | 0 | 0 | 0 | 0 | 1 | 1 | 0 | 0 | 0 | 0 | 0 | 0 | 0 | 0 | 210 | 218 |  |  | PEAKS DB |
| total 9 peptides |  |  |  |  |  |  |  |  |  |  |  |  |  |  |  |  |  |  |  |  |  |  |  |  |  |  |  |  |  |  |  |  |  |  |  |  |  |  |  |  |  |  |  |  |  |
| --- | --- | --- | --- | --- | --- | --- | --- | --- | --- | --- | --- | --- | --- | --- | --- | --- | --- | --- | --- | --- | --- | --- | --- | --- | --- | --- | --- | --- | --- | --- | --- | --- | --- | --- | --- | --- | --- | --- | --- | --- | --- | --- | --- | --- | --- |

JAA74859.1

back to list

  

| Protein Coverage
| Supporting Peptides
|

Protein Coverage:

Supporting Peptides:

| Peptide | Uniq | -10lgP | Mass | Length | ppm | m/z | z | RT | Fraction | Scan | Source File | Area F1 | Area F10 | Area F11 | Area F12 | Area F13 | Area F14 | Area F2 | Area F3 | Area F4 | Area F5 | Area F6 | Area F7 | Area F8 | Area F9 | #Feature | #Feature F1 | #Feature F10 | #Feature F11 | #Feature F12 | #Feature F13 | #Feature F14 | #Feature F2 | #Feature F3 | #Feature F4 | #Feature F5 | #Feature F6 | #Feature F7 | #Feature F8 | #Feature F9 | Start | End | PTM | AScore | Found By |
| --- | --- | --- | --- | --- | --- | --- | --- | --- | --- | --- | --- | --- | --- | --- | --- | --- | --- | --- | --- | --- | --- | --- | --- | --- | --- | --- | --- | --- | --- | --- | --- | --- | --- | --- | --- | --- | --- | --- | --- | --- | --- | --- | --- | --- | --- |
| K.C(+57.02)PIMTNQC(+57.02)IALR.G | N | 77.84 | 1475.7047 | 12 | 0.0 | 738.8596 | 2 | 39.74 | 6 | F6:21215 | NaSa01\_F14.raw |  |  | 3.3287E5 | 1.3692E6 | 9.8113E6 | 2.4068E9 |  |  |  | 1.7213E7 | 6.4357E6 |  | 4.0715E6 | 1.3044E6 | 15 | 0 | 0 | 1 | 1 | 2 | 5 | 0 | 0 | 0 | 2 | 2 | 0 | 1 | 1 | 419 | 430 | Carbamidomethylation | C1:Carbamidomethylation:1000.00;C8:Carbamidomethylation:1000.00 | PEAKS DB |
| K.C(+57.02)PIM(+15.99)TNQC(+57.02)IALR.G | N | 73.02 | 1491.6996 | 12 | 0.1 | 746.8572 | 2 | 30.97 | 6 | F6:13155 | NaSa01\_F14.raw |  |  |  |  | 1.4708E6 | 1.6503E8 |  |  |  |  |  |  |  |  | 5 | 0 | 0 | 0 | 0 | 1 | 4 | 0 | 0 | 0 | 0 | 0 | 0 | 0 | 0 | 419 | 430 | Carbamidomethylation; Oxidation (M) | C1:Carbamidomethylation:1000.00;M4:Oxidation (M):1000.00;C8:Carbamidomethylation:1000.00 | PEAKS DB |
| R.NGHPC(+57.02)QNNQGYC(+57.02)YNGK.C | N | 68.53 | 1909.7584 | 16 | -0.2 | 637.5933 | 3 | 11.55 | 4 | F4:2526 | NaSa01\_F12.raw |  |  |  | 1.1419E6 |  |  |  |  |  |  |  |  |  |  | 2 | 0 | 0 | 0 | 2 | 0 | 0 | 0 | 0 | 0 | 0 | 0 | 0 | 0 | 0 | 403 | 418 | Carbamidomethylation | C5:Carbamidomethylation:1000.00;C12:Carbamidomethylation:1000.00 | PEAKS DB |
| K.YIEFYVVLDNK.M | Y | 50.73 | 1401.7180 | 11 | -0.1 | 701.8662 | 2 | 80.29 | 3 | F3:52363 | NaSa01\_F11.raw |  |  | 1.8228E6 |  |  |  |  |  |  |  |  |  |  |  | 6 | 0 | 0 | 6 | 0 | 0 | 0 | 0 | 0 | 0 | 0 | 0 | 0 | 0 | 0 | 116 | 126 |  |  | PEAKS DB |
| C.PIMTNQC(+57.02)IALR.G | N | 49.03 | 1315.6741 | 11 | 0.4 | 658.8446 | 2 | 37.06 | 6 | F6:18281 | NaSa01\_F14.raw |  |  |  |  |  | 1.277E6 |  |  |  |  |  |  |  |  | 1 | 0 | 0 | 0 | 0 | 0 | 1 | 0 | 0 | 0 | 0 | 0 | 0 | 0 | 0 | 420 | 430 | Carbamidomethylation | C7:Carbamidomethylation:1000.00 | PEAKS DB |
| R.VYEMINTLNM(+15.99)MYR.S | Y | 46.83 | 1692.7673 | 13 | 0.2 | 847.3911 | 2 | 66.28 | 6 | F6:47293 | NaSa01\_F14.raw |  |  |  |  |  | 3.1331E7 |  |  |  |  |  |  |  |  | 1 | 0 | 0 | 0 | 0 | 0 | 1 | 0 | 0 | 0 | 0 | 0 | 0 | 0 | 0 | 142 | 154 | Oxidation (M) | M10:Oxidation (M):0.00 | PEAKS DB |
| R.EITTSPPVQDHC(+57.02)YYHGY.I | N | 44.97 | 2065.8840 | 17 | 6.3 | 689.6360 | 3 | 31.90 | 8 | F8:16761 | NaSa01\_F3.raw |  |  |  |  |  |  |  | 3.1849E6 |  |  |  |  |  |  | 1 | 0 | 0 | 0 | 0 | 0 | 0 | 0 | 1 | 0 | 0 | 0 | 0 | 0 | 0 | 7 | 23 | Carbamidomethylation | C12:Carbamidomethylation:1000.00 | PEAKS DB |
| R.VYEMINTLNMMYR.S | Y | 44.76 | 1676.7725 | 13 | 0.3 | 839.3938 | 2 | 79.34 | 6 | F6:54680 | NaSa01\_F14.raw |  |  |  |  |  | 1.1827E7 |  |  |  |  |  |  |  |  | 5 | 0 | 0 | 0 | 0 | 0 | 5 | 0 | 0 | 0 | 0 | 0 | 0 | 0 | 0 | 142 | 154 |  |  | PEAKS DB |
| R.DRPQC(+57.02)ILNKPL.S | N | 44.62 | 1352.7234 | 11 | 1.1 | 451.9156 | 3 | 27.50 | 5 | F5:11578 | NaSa01\_F13.raw |  |  |  |  | 1.9485E7 |  |  |  |  |  |  |  |  |  | 1 | 0 | 0 | 0 | 0 | 1 | 0 | 0 | 0 | 0 | 0 | 0 | 0 | 0 | 0 | 302 | 312 | Carbamidomethylation | C5:Carbamidomethylation:1000.00 | PEAKS DB |
| R.ETVLLPR.K | N | 38.52 | 826.4912 | 7 | -0.8 | 414.2526 | 2 | 26.58 | 6 | F6:9693 | NaSa01\_F14.raw |  |  |  |  |  | 1.9593E5 |  |  |  |  |  |  |  |  | 1 | 0 | 0 | 0 | 0 | 0 | 1 | 0 | 0 | 0 | 0 | 0 | 0 | 0 | 0 | 192 | 198 |  |  | PEAKS DB |
| total 10 peptides |  |  |  |  |  |  |  |  |  |  |  |  |  |  |  |  |  |  |  |  |  |  |  |  |  |  |  |  |  |  |  |  |  |  |  |  |  |  |  |  |  |  |  |  |  |
| --- | --- | --- | --- | --- | --- | --- | --- | --- | --- | --- | --- | --- | --- | --- | --- | --- | --- | --- | --- | --- | --- | --- | --- | --- | --- | --- | --- | --- | --- | --- | --- | --- | --- | --- | --- | --- | --- | --- | --- | --- | --- | --- | --- | --- | --- |

XP\_026581281.1

back to list

  

| Protein Coverage
| Supporting Peptides
|

Protein Coverage:

Supporting Peptides:

| Peptide | Uniq | -10lgP | Mass | Length | ppm | m/z | z | RT | Fraction | Scan | Source File | Area F1 | Area F10 | Area F11 | Area F12 | Area F13 | Area F14 | Area F2 | Area F3 | Area F4 | Area F5 | Area F6 | Area F7 | Area F8 | Area F9 | #Feature | #Feature F1 | #Feature F10 | #Feature F11 | #Feature F12 | #Feature F13 | #Feature F14 | #Feature F2 | #Feature F3 | #Feature F4 | #Feature F5 | #Feature F6 | #Feature F7 | #Feature F8 | #Feature F9 | Start | End | PTM | AScore | Found By |
| --- | --- | --- | --- | --- | --- | --- | --- | --- | --- | --- | --- | --- | --- | --- | --- | --- | --- | --- | --- | --- | --- | --- | --- | --- | --- | --- | --- | --- | --- | --- | --- | --- | --- | --- | --- | --- | --- | --- | --- | --- | --- | --- | --- | --- | --- |
| S.AVTIFGESAGAASVGMHLLSTQSR.G | N | 74.72 | 2389.2061 | 24 | -2.4 | 797.4074 | 3 | 74.92 | 6 | F6:50334 | NaSa01\_F14.raw |  |  |  |  |  | 1.5428E7 |  |  |  |  |  |  |  |  | 1 | 0 | 0 | 0 | 0 | 0 | 1 | 0 | 0 | 0 | 0 | 0 | 0 | 0 | 0 | 224 | 247 |  |  | PEAKS DB |
| K.DEGSYFLIYGLPGFSK.D | N | 70.64 | 1791.8718 | 16 | 0.9 | 896.9440 | 2 | 103.46 | 6 | F6:74175 | NaSa01\_F14.raw |  |  |  |  |  | 2.75E6 |  |  |  |  |  |  |  |  | 1 | 0 | 0 | 0 | 0 | 0 | 1 | 0 | 0 | 0 | 0 | 0 | 0 | 0 | 0 | 357 | 372 |  |  | PEAKS DB |
| K.VYAYLFDHR.A | N | 60.29 | 1182.5822 | 9 | 0.6 | 592.2987 | 2 | 34.97 | 6 | F6:16603 | NaSa01\_F14.raw |  |  |  |  | 2.7878E6 | 1.0437E7 |  |  |  |  |  |  |  |  | 5 | 0 | 0 | 0 | 0 | 2 | 3 | 0 | 0 | 0 | 0 | 0 | 0 | 0 | 0 | 449 | 457 |  |  | PEAKS DB |
| R.AQIC(+57.02)AFWNHFLPK.L | N | 54.78 | 1630.8079 | 13 | -0.7 | 544.6095 | 3 | 69.19 | 4 | F4:47418 | NaSa01\_F12.raw |  |  |  | 1.5192E6 |  |  |  |  |  |  |  |  |  |  | 2 | 0 | 0 | 0 | 2 | 0 | 0 | 0 | 0 | 0 | 0 | 0 | 0 | 0 | 0 | 549 | 561 | Carbamidomethylation | C4:Carbamidomethylation:1000.00 | PEAKS DB |
| F.LGVPFAEPPVGR.M | Y | 54.69 | 1237.6819 | 12 | 0.1 | 619.8483 | 2 | 55.91 | 6 | F6:34315 | NaSa01\_F14.raw |  |  |  |  |  | 2.9886E6 |  |  |  |  |  |  |  |  | 1 | 0 | 0 | 0 | 0 | 0 | 1 | 0 | 0 | 0 | 0 | 0 | 0 | 0 | 0 | 62 | 73 |  |  | PEAKS DB |
| R.MGVPHANDIATEAVVLQYTDWQDQDNGEK.N | Y | 52.93 | 3243.4778 | 29 | -1.8 | 1082.1646 | 3 | 76.70 | 6 | F6:51869 | NaSa01\_F14.raw |  |  |  |  |  | 1.636E6 |  |  |  |  |  |  |  |  | 1 | 0 | 0 | 0 | 0 | 0 | 1 | 0 | 0 | 0 | 0 | 0 | 0 | 0 | 0 | 389 | 417 |  |  | PEAKS DB |
| R.YWVNFAR.T | N | 48.39 | 954.4712 | 7 | -0.1 | 478.2428 | 2 | 47.33 | 6 | F6:27012 | NaSa01\_F14.raw |  |  |  |  |  | 8.3404E6 |  |  |  |  |  |  |  |  | 1 | 0 | 0 | 0 | 0 | 0 | 1 | 0 | 0 | 0 | 0 | 0 | 0 | 0 | 0 | 503 | 509 |  |  | PEAKS DB |
| E.EWSVLPYK.S | N | 47.69 | 1020.5280 | 8 | 0.3 | 511.2715 | 2 | 53.29 | 6 | F6:31997 | NaSa01\_F14.raw |  |  |  |  |  | 6.877E6 |  |  |  |  |  |  |  |  | 1 | 0 | 0 | 0 | 0 | 0 | 1 | 0 | 0 | 0 | 0 | 0 | 0 | 0 | 0 | 309 | 316 |  |  | PEAKS DB |
| R.FLRPEPVKPWQH.V | N | 44.97 | 1532.8252 | 12 | 0.7 | 511.9493 | 3 | 25.51 | 6 | F6:8645 | NaSa01\_F14.raw |  |  |  |  |  | 2.5936E6 |  |  |  |  |  |  |  |  | 1 | 0 | 0 | 0 | 0 | 0 | 1 | 0 | 0 | 0 | 0 | 0 | 0 | 0 | 0 | 76 | 87 |  |  | PEAKS DB |
| total 9 peptides |  |  |  |  |  |  |  |  |  |  |  |  |  |  |  |  |  |  |  |  |  |  |  |  |  |  |  |  |  |  |  |  |  |  |  |  |  |  |  |  |  |  |  |  |  |
| --- | --- | --- | --- | --- | --- | --- | --- | --- | --- | --- | --- | --- | --- | --- | --- | --- | --- | --- | --- | --- | --- | --- | --- | --- | --- | --- | --- | --- | --- | --- | --- | --- | --- | --- | --- | --- | --- | --- | --- | --- | --- | --- | --- | --- | --- |

4GFY

back to list

  

| Protein Coverage
| Supporting Peptides
|

Protein Coverage:

Supporting Peptides:

| Peptide | Uniq | -10lgP | Mass | Length | ppm | m/z | z | RT | Fraction | Scan | Source File | Area F1 | Area F10 | Area F11 | Area F12 | Area F13 | Area F14 | Area F2 | Area F3 | Area F4 | Area F5 | Area F6 | Area F7 | Area F8 | Area F9 | #Feature | #Feature F1 | #Feature F10 | #Feature F11 | #Feature F12 | #Feature F13 | #Feature F14 | #Feature F2 | #Feature F3 | #Feature F4 | #Feature F5 | #Feature F6 | #Feature F7 | #Feature F8 | #Feature F9 | Start | End | PTM | AScore | Found By |
| --- | --- | --- | --- | --- | --- | --- | --- | --- | --- | --- | --- | --- | --- | --- | --- | --- | --- | --- | --- | --- | --- | --- | --- | --- | --- | --- | --- | --- | --- | --- | --- | --- | --- | --- | --- | --- | --- | --- | --- | --- | --- | --- | --- | --- | --- |
| K.LAIPSYSSYGC(+57.02)YC(+57.02)GWGGK.G | Y | 83.35 | 2024.8760 | 18 | 1.3 | 1013.4466 | 2 | 65.73 | 4 | F4:44422 | NaSa01\_F12.raw |  |  |  | 1.8132E7 |  |  |  |  |  |  |  |  |  |  | 1 | 0 | 0 | 0 | 1 | 0 | 0 | 0 | 0 | 0 | 0 | 0 | 0 | 0 | 0 | 16 | 33 | Carbamidomethylation | C11:Carbamidomethylation:1000.00;C13:Carbamidomethylation:1000.00 | PEAKS DB |
| R.C(+57.02)C(+57.02)FVHDC(+57.02)C(+57.02)YGNLPDC(+57.02)NPK.S | N | 73.65 | 2314.8687 | 18 | 0.7 | 772.6307 | 3 | 32.34 | 4 | F4:16838 | NaSa01\_F12.raw |  |  |  | 2.3387E7 |  | 7.5227E6 |  |  |  |  |  |  |  |  | 2 | 0 | 0 | 0 | 1 | 0 | 1 | 0 | 0 | 0 | 0 | 0 | 0 | 0 | 0 | 43 | 60 | Carbamidomethylation | C1:Carbamidomethylation:1000.00;C2:Carbamidomethylation:1000.00;C7:Carbamidomethylation:1000.00;C8:Carbamidomethylation:1000.00;C15:Carbamidomethylation:1000.00 | PEAKS DB |
| K.YMLYPDFLC(+57.02)K.G | N | 68.32 | 1348.6195 | 10 | 0.2 | 675.3171 | 2 | 75.10 | 4 | F4:52326 | NaSa01\_F12.raw |  |  |  | 2.1669E7 |  |  |  |  |  |  |  |  |  |  | 1 | 0 | 0 | 0 | 1 | 0 | 0 | 0 | 0 | 0 | 0 | 0 | 0 | 0 | 0 | 107 | 116 | Carbamidomethylation | C9:Carbamidomethylation:1000.00 | PEAKS DB |
| K.YM(+15.99)LYPDFLC(+57.02)K.G | N | 64.41 | 1364.6145 | 10 | 0.0 | 683.3145 | 2 | 68.02 | 4 | F4:46357 | NaSa01\_F12.raw |  |  |  | 2.115E6 |  |  |  |  |  |  |  |  |  |  | 1 | 0 | 0 | 0 | 1 | 0 | 0 | 0 | 0 | 0 | 0 | 0 | 0 | 0 | 0 | 107 | 116 | Oxidation (M); Carbamidomethylation | M2:Oxidation (M):1000.00;C9:Carbamidomethylation:1000.00 | PEAKS DB |
| K.KYMLYPDFLC(+57.02)K.G | Y | 60.31 | 1476.7145 | 11 | 0.3 | 493.2456 | 3 | 58.25 | 4 | F4:38516 | NaSa01\_F12.raw |  |  |  | 5.4533E6 |  |  |  |  |  |  |  |  |  |  | 2 | 0 | 0 | 0 | 2 | 0 | 0 | 0 | 0 | 0 | 0 | 0 | 0 | 0 | 0 | 106 | 116 | Carbamidomethylation | C10:Carbamidomethylation:1000.00 | PEAKS DB |
| S.YSSYGC(+57.02)YC(+57.02)GWGGK.G | N | 58.28 | 1543.5861 | 13 | 0.3 | 772.8005 | 2 | 35.31 | 6 | F6:16919 | NaSa01\_F14.raw |  |  |  |  |  | 5.8277E6 |  |  |  |  |  |  |  |  | 1 | 0 | 0 | 0 | 0 | 0 | 1 | 0 | 0 | 0 | 0 | 0 | 0 | 0 | 0 | 21 | 33 | Carbamidomethylation | C6:Carbamidomethylation:1000.00;C8:Carbamidomethylation:1000.00 | PEAKS DB |
| K.KYM(+15.99)LYPDFLC(+57.02)K.G | Y | 43.40 | 1492.7095 | 11 | -0.3 | 498.5770 | 3 | 51.59 | 4 | F4:32744 | NaSa01\_F12.raw |  |  |  | 7.4997E5 |  |  |  |  |  |  |  |  |  |  | 1 | 0 | 0 | 0 | 1 | 0 | 0 | 0 | 0 | 0 | 0 | 0 | 0 | 0 | 0 | 106 | 116 | Oxidation (M); Carbamidomethylation | M3:Oxidation (M):1000.00;C10:Carbamidomethylation:1000.00 | PEAKS DB |
| SLLEFGK.M | Y | 38.22 | 792.4381 | 7 | 0.1 | 397.2264 | 2 | 39.94 | 4 | F4:23423 | NaSa01\_F12.raw |  |  |  | 8.4704E6 |  |  |  |  |  |  |  |  |  |  | 1 | 0 | 0 | 0 | 1 | 0 | 0 | 0 | 0 | 0 | 0 | 0 | 0 | 0 | 0 | 1 | 7 |  |  | PEAKS DB |
| total 8 peptides |  |  |  |  |  |  |  |  |  |  |  |  |  |  |  |  |  |  |  |  |  |  |  |  |  |  |  |  |  |  |  |  |  |  |  |  |  |  |  |  |  |  |  |  |  |
| --- | --- | --- | --- | --- | --- | --- | --- | --- | --- | --- | --- | --- | --- | --- | --- | --- | --- | --- | --- | --- | --- | --- | --- | --- | --- | --- | --- | --- | --- | --- | --- | --- | --- | --- | --- | --- | --- | --- | --- | --- | --- | --- | --- | --- | --- |

2QU9

back to list

  

| Protein Coverage
| Supporting Peptides
|

Protein Coverage:

Supporting Peptides:

| Peptide | Uniq | -10lgP | Mass | Length | ppm | m/z | z | RT | Fraction | Scan | Source File | Area F1 | Area F10 | Area F11 | Area F12 | Area F13 | Area F14 | Area F2 | Area F3 | Area F4 | Area F5 | Area F6 | Area F7 | Area F8 | Area F9 | #Feature | #Feature F1 | #Feature F10 | #Feature F11 | #Feature F12 | #Feature F13 | #Feature F14 | #Feature F2 | #Feature F3 | #Feature F4 | #Feature F5 | #Feature F6 | #Feature F7 | #Feature F8 | #Feature F9 | Start | End | PTM | AScore | Found By |
| --- | --- | --- | --- | --- | --- | --- | --- | --- | --- | --- | --- | --- | --- | --- | --- | --- | --- | --- | --- | --- | --- | --- | --- | --- | --- | --- | --- | --- | --- | --- | --- | --- | --- | --- | --- | --- | --- | --- | --- | --- | --- | --- | --- | --- | --- |
| K.LAIPSYSSYGC(+57.02)YC(+57.02)GWGGK.G | Y | 83.35 | 2024.8760 | 18 | 1.3 | 1013.4466 | 2 | 65.73 | 4 | F4:44422 | NaSa01\_F12.raw |  |  |  | 1.8132E7 |  |  |  |  |  |  |  |  |  |  | 1 | 0 | 0 | 0 | 1 | 0 | 0 | 0 | 0 | 0 | 0 | 0 | 0 | 0 | 0 | 16 | 33 | Carbamidomethylation | C11:Carbamidomethylation:1000.00;C13:Carbamidomethylation:1000.00 | PEAKS DB |
| R.C(+57.02)C(+57.02)FVHDC(+57.02)C(+57.02)YGNLPDC(+57.02)NPK.S | N | 73.65 | 2314.8687 | 18 | 0.7 | 772.6307 | 3 | 32.34 | 4 | F4:16838 | NaSa01\_F12.raw |  |  |  | 2.3387E7 |  | 7.5227E6 |  |  |  |  |  |  |  |  | 2 | 0 | 0 | 0 | 1 | 0 | 1 | 0 | 0 | 0 | 0 | 0 | 0 | 0 | 0 | 43 | 60 | Carbamidomethylation | C1:Carbamidomethylation:1000.00;C2:Carbamidomethylation:1000.00;C7:Carbamidomethylation:1000.00;C8:Carbamidomethylation:1000.00;C15:Carbamidomethylation:1000.00 | PEAKS DB |
| K.YMLYPDFLC(+57.02)K.G | N | 68.32 | 1348.6195 | 10 | 0.2 | 675.3171 | 2 | 75.10 | 4 | F4:52326 | NaSa01\_F12.raw |  |  |  | 2.1669E7 |  |  |  |  |  |  |  |  |  |  | 1 | 0 | 0 | 0 | 1 | 0 | 0 | 0 | 0 | 0 | 0 | 0 | 0 | 0 | 0 | 107 | 116 | Carbamidomethylation | C9:Carbamidomethylation:1000.00 | PEAKS DB |
| K.YM(+15.99)LYPDFLC(+57.02)K.G | N | 64.41 | 1364.6145 | 10 | 0.0 | 683.3145 | 2 | 68.02 | 4 | F4:46357 | NaSa01\_F12.raw |  |  |  | 2.115E6 |  |  |  |  |  |  |  |  |  |  | 1 | 0 | 0 | 0 | 1 | 0 | 0 | 0 | 0 | 0 | 0 | 0 | 0 | 0 | 0 | 107 | 116 | Oxidation (M); Carbamidomethylation | M2:Oxidation (M):1000.00;C9:Carbamidomethylation:1000.00 | PEAKS DB |
| K.KYMLYPDFLC(+57.02)K.G | Y | 60.31 | 1476.7145 | 11 | 0.3 | 493.2456 | 3 | 58.25 | 4 | F4:38516 | NaSa01\_F12.raw |  |  |  | 5.4533E6 |  |  |  |  |  |  |  |  |  |  | 2 | 0 | 0 | 0 | 2 | 0 | 0 | 0 | 0 | 0 | 0 | 0 | 0 | 0 | 0 | 106 | 116 | Carbamidomethylation | C10:Carbamidomethylation:1000.00 | PEAKS DB |
| S.YSSYGC(+57.02)YC(+57.02)GWGGK.G | N | 58.28 | 1543.5861 | 13 | 0.3 | 772.8005 | 2 | 35.31 | 6 | F6:16919 | NaSa01\_F14.raw |  |  |  |  |  | 5.8277E6 |  |  |  |  |  |  |  |  | 1 | 0 | 0 | 0 | 0 | 0 | 1 | 0 | 0 | 0 | 0 | 0 | 0 | 0 | 0 | 21 | 33 | Carbamidomethylation | C6:Carbamidomethylation:1000.00;C8:Carbamidomethylation:1000.00 | PEAKS DB |
| K.KYM(+15.99)LYPDFLC(+57.02)K.G | Y | 43.40 | 1492.7095 | 11 | -0.3 | 498.5770 | 3 | 51.59 | 4 | F4:32744 | NaSa01\_F12.raw |  |  |  | 7.4997E5 |  |  |  |  |  |  |  |  |  |  | 1 | 0 | 0 | 0 | 1 | 0 | 0 | 0 | 0 | 0 | 0 | 0 | 0 | 0 | 0 | 106 | 116 | Oxidation (M); Carbamidomethylation | M3:Oxidation (M):1000.00;C10:Carbamidomethylation:1000.00 | PEAKS DB |
| SLLEFGK.M | Y | 38.22 | 792.4381 | 7 | 0.1 | 397.2264 | 2 | 39.94 | 4 | F4:23423 | NaSa01\_F12.raw |  |  |  | 8.4704E6 |  |  |  |  |  |  |  |  |  |  | 1 | 0 | 0 | 0 | 1 | 0 | 0 | 0 | 0 | 0 | 0 | 0 | 0 | 0 | 0 | 1 | 7 |  |  | PEAKS DB |
| total 8 peptides |  |  |  |  |  |  |  |  |  |  |  |  |  |  |  |  |  |  |  |  |  |  |  |  |  |  |  |  |  |  |  |  |  |  |  |  |  |  |  |  |  |  |  |  |  |
| --- | --- | --- | --- | --- | --- | --- | --- | --- | --- | --- | --- | --- | --- | --- | --- | --- | --- | --- | --- | --- | --- | --- | --- | --- | --- | --- | --- | --- | --- | --- | --- | --- | --- | --- | --- | --- | --- | --- | --- | --- | --- | --- | --- | --- | --- |

1SV9

back to list

  

| Protein Coverage
| Supporting Peptides
|

Protein Coverage:

Supporting Peptides:

| Peptide | Uniq | -10lgP | Mass | Length | ppm | m/z | z | RT | Fraction | Scan | Source File | Area F1 | Area F10 | Area F11 | Area F12 | Area F13 | Area F14 | Area F2 | Area F3 | Area F4 | Area F5 | Area F6 | Area F7 | Area F8 | Area F9 | #Feature | #Feature F1 | #Feature F10 | #Feature F11 | #Feature F12 | #Feature F13 | #Feature F14 | #Feature F2 | #Feature F3 | #Feature F4 | #Feature F5 | #Feature F6 | #Feature F7 | #Feature F8 | #Feature F9 | Start | End | PTM | AScore | Found By |
| --- | --- | --- | --- | --- | --- | --- | --- | --- | --- | --- | --- | --- | --- | --- | --- | --- | --- | --- | --- | --- | --- | --- | --- | --- | --- | --- | --- | --- | --- | --- | --- | --- | --- | --- | --- | --- | --- | --- | --- | --- | --- | --- | --- | --- | --- |
| K.LAIPSYSSYGC(+57.02)YC(+57.02)GWGGK.G | Y | 83.35 | 2024.8760 | 18 | 1.3 | 1013.4466 | 2 | 65.73 | 4 | F4:44422 | NaSa01\_F12.raw |  |  |  | 1.8132E7 |  |  |  |  |  |  |  |  |  |  | 1 | 0 | 0 | 0 | 1 | 0 | 0 | 0 | 0 | 0 | 0 | 0 | 0 | 0 | 0 | 16 | 33 | Carbamidomethylation | C11:Carbamidomethylation:1000.00;C13:Carbamidomethylation:1000.00 | PEAKS DB |
| R.C(+57.02)C(+57.02)FVHDC(+57.02)C(+57.02)YGNLPDC(+57.02)NPK.S | N | 73.65 | 2314.8687 | 18 | 0.7 | 772.6307 | 3 | 32.34 | 4 | F4:16838 | NaSa01\_F12.raw |  |  |  | 2.3387E7 |  | 7.5227E6 |  |  |  |  |  |  |  |  | 2 | 0 | 0 | 0 | 1 | 0 | 1 | 0 | 0 | 0 | 0 | 0 | 0 | 0 | 0 | 43 | 60 | Carbamidomethylation | C1:Carbamidomethylation:1000.00;C2:Carbamidomethylation:1000.00;C7:Carbamidomethylation:1000.00;C8:Carbamidomethylation:1000.00;C15:Carbamidomethylation:1000.00 | PEAKS DB |
| K.YMLYPDFLC(+57.02)K.G | N | 68.32 | 1348.6195 | 10 | 0.2 | 675.3171 | 2 | 75.10 | 4 | F4:52326 | NaSa01\_F12.raw |  |  |  | 2.1669E7 |  |  |  |  |  |  |  |  |  |  | 1 | 0 | 0 | 0 | 1 | 0 | 0 | 0 | 0 | 0 | 0 | 0 | 0 | 0 | 0 | 107 | 116 | Carbamidomethylation | C9:Carbamidomethylation:1000.00 | PEAKS DB |
| K.YM(+15.99)LYPDFLC(+57.02)K.G | N | 64.41 | 1364.6145 | 10 | 0.0 | 683.3145 | 2 | 68.02 | 4 | F4:46357 | NaSa01\_F12.raw |  |  |  | 2.115E6 |  |  |  |  |  |  |  |  |  |  | 1 | 0 | 0 | 0 | 1 | 0 | 0 | 0 | 0 | 0 | 0 | 0 | 0 | 0 | 0 | 107 | 116 | Oxidation (M); Carbamidomethylation | M2:Oxidation (M):1000.00;C9:Carbamidomethylation:1000.00 | PEAKS DB |
| K.KYMLYPDFLC(+57.02)K.G | Y | 60.31 | 1476.7145 | 11 | 0.3 | 493.2456 | 3 | 58.25 | 4 | F4:38516 | NaSa01\_F12.raw |  |  |  | 5.4533E6 |  |  |  |  |  |  |  |  |  |  | 2 | 0 | 0 | 0 | 2 | 0 | 0 | 0 | 0 | 0 | 0 | 0 | 0 | 0 | 0 | 106 | 116 | Carbamidomethylation | C10:Carbamidomethylation:1000.00 | PEAKS DB |
| S.YSSYGC(+57.02)YC(+57.02)GWGGK.G | N | 58.28 | 1543.5861 | 13 | 0.3 | 772.8005 | 2 | 35.31 | 6 | F6:16919 | NaSa01\_F14.raw |  |  |  |  |  | 5.8277E6 |  |  |  |  |  |  |  |  | 1 | 0 | 0 | 0 | 0 | 0 | 1 | 0 | 0 | 0 | 0 | 0 | 0 | 0 | 0 | 21 | 33 | Carbamidomethylation | C6:Carbamidomethylation:1000.00;C8:Carbamidomethylation:1000.00 | PEAKS DB |
| K.KYM(+15.99)LYPDFLC(+57.02)K.G | Y | 43.40 | 1492.7095 | 11 | -0.3 | 498.5770 | 3 | 51.59 | 4 | F4:32744 | NaSa01\_F12.raw |  |  |  | 7.4997E5 |  |  |  |  |  |  |  |  |  |  | 1 | 0 | 0 | 0 | 1 | 0 | 0 | 0 | 0 | 0 | 0 | 0 | 0 | 0 | 0 | 106 | 116 | Oxidation (M); Carbamidomethylation | M3:Oxidation (M):1000.00;C10:Carbamidomethylation:1000.00 | PEAKS DB |
| SLLEFGK.M | Y | 38.22 | 792.4381 | 7 | 0.1 | 397.2264 | 2 | 39.94 | 4 | F4:23423 | NaSa01\_F12.raw |  |  |  | 8.4704E6 |  |  |  |  |  |  |  |  |  |  | 1 | 0 | 0 | 0 | 1 | 0 | 0 | 0 | 0 | 0 | 0 | 0 | 0 | 0 | 0 | 1 | 7 |  |  | PEAKS DB |
| total 8 peptides |  |  |  |  |  |  |  |  |  |  |  |  |  |  |  |  |  |  |  |  |  |  |  |  |  |  |  |  |  |  |  |  |  |  |  |  |  |  |  |  |  |  |  |  |  |
| --- | --- | --- | --- | --- | --- | --- | --- | --- | --- | --- | --- | --- | --- | --- | --- | --- | --- | --- | --- | --- | --- | --- | --- | --- | --- | --- | --- | --- | --- | --- | --- | --- | --- | --- | --- | --- | --- | --- | --- | --- | --- | --- | --- | --- | --- |

3G8F

back to list

  

| Protein Coverage
| Supporting Peptides
|

Protein Coverage:

Supporting Peptides:

| Peptide | Uniq | -10lgP | Mass | Length | ppm | m/z | z | RT | Fraction | Scan | Source File | Area F1 | Area F10 | Area F11 | Area F12 | Area F13 | Area F14 | Area F2 | Area F3 | Area F4 | Area F5 | Area F6 | Area F7 | Area F8 | Area F9 | #Feature | #Feature F1 | #Feature F10 | #Feature F11 | #Feature F12 | #Feature F13 | #Feature F14 | #Feature F2 | #Feature F3 | #Feature F4 | #Feature F5 | #Feature F6 | #Feature F7 | #Feature F8 | #Feature F9 | Start | End | PTM | AScore | Found By |
| --- | --- | --- | --- | --- | --- | --- | --- | --- | --- | --- | --- | --- | --- | --- | --- | --- | --- | --- | --- | --- | --- | --- | --- | --- | --- | --- | --- | --- | --- | --- | --- | --- | --- | --- | --- | --- | --- | --- | --- | --- | --- | --- | --- | --- | --- |
| K.LAIPSYSSYGC(+57.02)YC(+57.02)GWGGK.G | Y | 83.35 | 2024.8760 | 18 | 1.3 | 1013.4466 | 2 | 65.73 | 4 | F4:44422 | NaSa01\_F12.raw |  |  |  | 1.8132E7 |  |  |  |  |  |  |  |  |  |  | 1 | 0 | 0 | 0 | 1 | 0 | 0 | 0 | 0 | 0 | 0 | 0 | 0 | 0 | 0 | 16 | 33 | Carbamidomethylation | C11:Carbamidomethylation:1000.00;C13:Carbamidomethylation:1000.00 | PEAKS DB |
| R.C(+57.02)C(+57.02)FVHDC(+57.02)C(+57.02)YGNLPDC(+57.02)NPK.S | N | 73.65 | 2314.8687 | 18 | 0.7 | 772.6307 | 3 | 32.34 | 4 | F4:16838 | NaSa01\_F12.raw |  |  |  | 2.3387E7 |  | 7.5227E6 |  |  |  |  |  |  |  |  | 2 | 0 | 0 | 0 | 1 | 0 | 1 | 0 | 0 | 0 | 0 | 0 | 0 | 0 | 0 | 43 | 60 | Carbamidomethylation | C1:Carbamidomethylation:1000.00;C2:Carbamidomethylation:1000.00;C7:Carbamidomethylation:1000.00;C8:Carbamidomethylation:1000.00;C15:Carbamidomethylation:1000.00 | PEAKS DB |
| K.YMLYPDFLC(+57.02)K.G | N | 68.32 | 1348.6195 | 10 | 0.2 | 675.3171 | 2 | 75.10 | 4 | F4:52326 | NaSa01\_F12.raw |  |  |  | 2.1669E7 |  |  |  |  |  |  |  |  |  |  | 1 | 0 | 0 | 0 | 1 | 0 | 0 | 0 | 0 | 0 | 0 | 0 | 0 | 0 | 0 | 107 | 116 | Carbamidomethylation | C9:Carbamidomethylation:1000.00 | PEAKS DB |
| K.YM(+15.99)LYPDFLC(+57.02)K.G | N | 64.41 | 1364.6145 | 10 | 0.0 | 683.3145 | 2 | 68.02 | 4 | F4:46357 | NaSa01\_F12.raw |  |  |  | 2.115E6 |  |  |  |  |  |  |  |  |  |  | 1 | 0 | 0 | 0 | 1 | 0 | 0 | 0 | 0 | 0 | 0 | 0 | 0 | 0 | 0 | 107 | 116 | Oxidation (M); Carbamidomethylation | M2:Oxidation (M):1000.00;C9:Carbamidomethylation:1000.00 | PEAKS DB |
| K.KYMLYPDFLC(+57.02)K.G | Y | 60.31 | 1476.7145 | 11 | 0.3 | 493.2456 | 3 | 58.25 | 4 | F4:38516 | NaSa01\_F12.raw |  |  |  | 5.4533E6 |  |  |  |  |  |  |  |  |  |  | 2 | 0 | 0 | 0 | 2 | 0 | 0 | 0 | 0 | 0 | 0 | 0 | 0 | 0 | 0 | 106 | 116 | Carbamidomethylation | C10:Carbamidomethylation:1000.00 | PEAKS DB |
| S.YSSYGC(+57.02)YC(+57.02)GWGGK.G | N | 58.28 | 1543.5861 | 13 | 0.3 | 772.8005 | 2 | 35.31 | 6 | F6:16919 | NaSa01\_F14.raw |  |  |  |  |  | 5.8277E6 |  |  |  |  |  |  |  |  | 1 | 0 | 0 | 0 | 0 | 0 | 1 | 0 | 0 | 0 | 0 | 0 | 0 | 0 | 0 | 21 | 33 | Carbamidomethylation | C6:Carbamidomethylation:1000.00;C8:Carbamidomethylation:1000.00 | PEAKS DB |
| K.KYM(+15.99)LYPDFLC(+57.02)K.G | Y | 43.40 | 1492.7095 | 11 | -0.3 | 498.5770 | 3 | 51.59 | 4 | F4:32744 | NaSa01\_F12.raw |  |  |  | 7.4997E5 |  |  |  |  |  |  |  |  |  |  | 1 | 0 | 0 | 0 | 1 | 0 | 0 | 0 | 0 | 0 | 0 | 0 | 0 | 0 | 0 | 106 | 116 | Oxidation (M); Carbamidomethylation | M3:Oxidation (M):1000.00;C10:Carbamidomethylation:1000.00 | PEAKS DB |
| SLLEFGK.M | Y | 38.22 | 792.4381 | 7 | 0.1 | 397.2264 | 2 | 39.94 | 4 | F4:23423 | NaSa01\_F12.raw |  |  |  | 8.4704E6 |  |  |  |  |  |  |  |  |  |  | 1 | 0 | 0 | 0 | 1 | 0 | 0 | 0 | 0 | 0 | 0 | 0 | 0 | 0 | 0 | 1 | 7 |  |  | PEAKS DB |
| total 8 peptides |  |  |  |  |  |  |  |  |  |  |  |  |  |  |  |  |  |  |  |  |  |  |  |  |  |  |  |  |  |  |  |  |  |  |  |  |  |  |  |  |  |  |  |  |  |
| --- | --- | --- | --- | --- | --- | --- | --- | --- | --- | --- | --- | --- | --- | --- | --- | --- | --- | --- | --- | --- | --- | --- | --- | --- | --- | --- | --- | --- | --- | --- | --- | --- | --- | --- | --- | --- | --- | --- | --- | --- | --- | --- | --- | --- | --- |

1Y38

back to list

  

| Protein Coverage
| Supporting Peptides
|

Protein Coverage:

Supporting Peptides:

| Peptide | Uniq | -10lgP | Mass | Length | ppm | m/z | z | RT | Fraction | Scan | Source File | Area F1 | Area F10 | Area F11 | Area F12 | Area F13 | Area F14 | Area F2 | Area F3 | Area F4 | Area F5 | Area F6 | Area F7 | Area F8 | Area F9 | #Feature | #Feature F1 | #Feature F10 | #Feature F11 | #Feature F12 | #Feature F13 | #Feature F14 | #Feature F2 | #Feature F3 | #Feature F4 | #Feature F5 | #Feature F6 | #Feature F7 | #Feature F8 | #Feature F9 | Start | End | PTM | AScore | Found By |
| --- | --- | --- | --- | --- | --- | --- | --- | --- | --- | --- | --- | --- | --- | --- | --- | --- | --- | --- | --- | --- | --- | --- | --- | --- | --- | --- | --- | --- | --- | --- | --- | --- | --- | --- | --- | --- | --- | --- | --- | --- | --- | --- | --- | --- | --- |
| K.LAIPSYSSYGC(+57.02)YC(+57.02)GWGGK.G | Y | 83.35 | 2024.8760 | 18 | 1.3 | 1013.4466 | 2 | 65.73 | 4 | F4:44422 | NaSa01\_F12.raw |  |  |  | 1.8132E7 |  |  |  |  |  |  |  |  |  |  | 1 | 0 | 0 | 0 | 1 | 0 | 0 | 0 | 0 | 0 | 0 | 0 | 0 | 0 | 0 | 16 | 33 | Carbamidomethylation | C11:Carbamidomethylation:1000.00;C13:Carbamidomethylation:1000.00 | PEAKS DB |
| R.C(+57.02)C(+57.02)FVHDC(+57.02)C(+57.02)YGNLPDC(+57.02)NPK.S | N | 73.65 | 2314.8687 | 18 | 0.7 | 772.6307 | 3 | 32.34 | 4 | F4:16838 | NaSa01\_F12.raw |  |  |  | 2.3387E7 |  | 7.5227E6 |  |  |  |  |  |  |  |  | 2 | 0 | 0 | 0 | 1 | 0 | 1 | 0 | 0 | 0 | 0 | 0 | 0 | 0 | 0 | 43 | 60 | Carbamidomethylation | C1:Carbamidomethylation:1000.00;C2:Carbamidomethylation:1000.00;C7:Carbamidomethylation:1000.00;C8:Carbamidomethylation:1000.00;C15:Carbamidomethylation:1000.00 | PEAKS DB |
| K.YMLYPDFLC(+57.02)K.G | N | 68.32 | 1348.6195 | 10 | 0.2 | 675.3171 | 2 | 75.10 | 4 | F4:52326 | NaSa01\_F12.raw |  |  |  | 2.1669E7 |  |  |  |  |  |  |  |  |  |  | 1 | 0 | 0 | 0 | 1 | 0 | 0 | 0 | 0 | 0 | 0 | 0 | 0 | 0 | 0 | 107 | 116 | Carbamidomethylation | C9:Carbamidomethylation:1000.00 | PEAKS DB |
| K.YM(+15.99)LYPDFLC(+57.02)K.G | N | 64.41 | 1364.6145 | 10 | 0.0 | 683.3145 | 2 | 68.02 | 4 | F4:46357 | NaSa01\_F12.raw |  |  |  | 2.115E6 |  |  |  |  |  |  |  |  |  |  | 1 | 0 | 0 | 0 | 1 | 0 | 0 | 0 | 0 | 0 | 0 | 0 | 0 | 0 | 0 | 107 | 116 | Oxidation (M); Carbamidomethylation | M2:Oxidation (M):1000.00;C9:Carbamidomethylation:1000.00 | PEAKS DB |
| K.KYMLYPDFLC(+57.02)K.G | Y | 60.31 | 1476.7145 | 11 | 0.3 | 493.2456 | 3 | 58.25 | 4 | F4:38516 | NaSa01\_F12.raw |  |  |  | 5.4533E6 |  |  |  |  |  |  |  |  |  |  | 2 | 0 | 0 | 0 | 2 | 0 | 0 | 0 | 0 | 0 | 0 | 0 | 0 | 0 | 0 | 106 | 116 | Carbamidomethylation | C10:Carbamidomethylation:1000.00 | PEAKS DB |
| S.YSSYGC(+57.02)YC(+57.02)GWGGK.G | N | 58.28 | 1543.5861 | 13 | 0.3 | 772.8005 | 2 | 35.31 | 6 | F6:16919 | NaSa01\_F14.raw |  |  |  |  |  | 5.8277E6 |  |  |  |  |  |  |  |  | 1 | 0 | 0 | 0 | 0 | 0 | 1 | 0 | 0 | 0 | 0 | 0 | 0 | 0 | 0 | 21 | 33 | Carbamidomethylation | C6:Carbamidomethylation:1000.00;C8:Carbamidomethylation:1000.00 | PEAKS DB |
| K.KYM(+15.99)LYPDFLC(+57.02)K.G | Y | 43.40 | 1492.7095 | 11 | -0.3 | 498.5770 | 3 | 51.59 | 4 | F4:32744 | NaSa01\_F12.raw |  |  |  | 7.4997E5 |  |  |  |  |  |  |  |  |  |  | 1 | 0 | 0 | 0 | 1 | 0 | 0 | 0 | 0 | 0 | 0 | 0 | 0 | 0 | 0 | 106 | 116 | Oxidation (M); Carbamidomethylation | M3:Oxidation (M):1000.00;C10:Carbamidomethylation:1000.00 | PEAKS DB |
| SLLEFGK.M | Y | 38.22 | 792.4381 | 7 | 0.1 | 397.2264 | 2 | 39.94 | 4 | F4:23423 | NaSa01\_F12.raw |  |  |  | 8.4704E6 |  |  |  |  |  |  |  |  |  |  | 1 | 0 | 0 | 0 | 1 | 0 | 0 | 0 | 0 | 0 | 0 | 0 | 0 | 0 | 0 | 1 | 7 |  |  | PEAKS DB |
| total 8 peptides |  |  |  |  |  |  |  |  |  |  |  |  |  |  |  |  |  |  |  |  |  |  |  |  |  |  |  |  |  |  |  |  |  |  |  |  |  |  |  |  |  |  |  |  |  |
| --- | --- | --- | --- | --- | --- | --- | --- | --- | --- | --- | --- | --- | --- | --- | --- | --- | --- | --- | --- | --- | --- | --- | --- | --- | --- | --- | --- | --- | --- | --- | --- | --- | --- | --- | --- | --- | --- | --- | --- | --- | --- | --- | --- | --- | --- |

1TK4

back to list

  

| Protein Coverage
| Supporting Peptides
|

Protein Coverage:

Supporting Peptides:

| Peptide | Uniq | -10lgP | Mass | Length | ppm | m/z | z | RT | Fraction | Scan | Source File | Area F1 | Area F10 | Area F11 | Area F12 | Area F13 | Area F14 | Area F2 | Area F3 | Area F4 | Area F5 | Area F6 | Area F7 | Area F8 | Area F9 | #Feature | #Feature F1 | #Feature F10 | #Feature F11 | #Feature F12 | #Feature F13 | #Feature F14 | #Feature F2 | #Feature F3 | #Feature F4 | #Feature F5 | #Feature F6 | #Feature F7 | #Feature F8 | #Feature F9 | Start | End | PTM | AScore | Found By |
| --- | --- | --- | --- | --- | --- | --- | --- | --- | --- | --- | --- | --- | --- | --- | --- | --- | --- | --- | --- | --- | --- | --- | --- | --- | --- | --- | --- | --- | --- | --- | --- | --- | --- | --- | --- | --- | --- | --- | --- | --- | --- | --- | --- | --- | --- |
| K.LAIPSYSSYGC(+57.02)YC(+57.02)GWGGK.G | Y | 83.35 | 2024.8760 | 18 | 1.3 | 1013.4466 | 2 | 65.73 | 4 | F4:44422 | NaSa01\_F12.raw |  |  |  | 1.8132E7 |  |  |  |  |  |  |  |  |  |  | 1 | 0 | 0 | 0 | 1 | 0 | 0 | 0 | 0 | 0 | 0 | 0 | 0 | 0 | 0 | 16 | 33 | Carbamidomethylation | C11:Carbamidomethylation:1000.00;C13:Carbamidomethylation:1000.00 | PEAKS DB |
| R.C(+57.02)C(+57.02)FVHDC(+57.02)C(+57.02)YGNLPDC(+57.02)NPK.S | N | 73.65 | 2314.8687 | 18 | 0.7 | 772.6307 | 3 | 32.34 | 4 | F4:16838 | NaSa01\_F12.raw |  |  |  | 2.3387E7 |  | 7.5227E6 |  |  |  |  |  |  |  |  | 2 | 0 | 0 | 0 | 1 | 0 | 1 | 0 | 0 | 0 | 0 | 0 | 0 | 0 | 0 | 43 | 60 | Carbamidomethylation | C1:Carbamidomethylation:1000.00;C2:Carbamidomethylation:1000.00;C7:Carbamidomethylation:1000.00;C8:Carbamidomethylation:1000.00;C15:Carbamidomethylation:1000.00 | PEAKS DB |
| K.YMLYPDFLC(+57.02)K.G | N | 68.32 | 1348.6195 | 10 | 0.2 | 675.3171 | 2 | 75.10 | 4 | F4:52326 | NaSa01\_F12.raw |  |  |  | 2.1669E7 |  |  |  |  |  |  |  |  |  |  | 1 | 0 | 0 | 0 | 1 | 0 | 0 | 0 | 0 | 0 | 0 | 0 | 0 | 0 | 0 | 107 | 116 | Carbamidomethylation | C9:Carbamidomethylation:1000.00 | PEAKS DB |
| K.YM(+15.99)LYPDFLC(+57.02)K.G | N | 64.41 | 1364.6145 | 10 | 0.0 | 683.3145 | 2 | 68.02 | 4 | F4:46357 | NaSa01\_F12.raw |  |  |  | 2.115E6 |  |  |  |  |  |  |  |  |  |  | 1 | 0 | 0 | 0 | 1 | 0 | 0 | 0 | 0 | 0 | 0 | 0 | 0 | 0 | 0 | 107 | 116 | Oxidation (M); Carbamidomethylation | M2:Oxidation (M):1000.00;C9:Carbamidomethylation:1000.00 | PEAKS DB |
| K.KYMLYPDFLC(+57.02)K.G | Y | 60.31 | 1476.7145 | 11 | 0.3 | 493.2456 | 3 | 58.25 | 4 | F4:38516 | NaSa01\_F12.raw |  |  |  | 5.4533E6 |  |  |  |  |  |  |  |  |  |  | 2 | 0 | 0 | 0 | 2 | 0 | 0 | 0 | 0 | 0 | 0 | 0 | 0 | 0 | 0 | 106 | 116 | Carbamidomethylation | C10:Carbamidomethylation:1000.00 | PEAKS DB |
| S.YSSYGC(+57.02)YC(+57.02)GWGGK.G | N | 58.28 | 1543.5861 | 13 | 0.3 | 772.8005 | 2 | 35.31 | 6 | F6:16919 | NaSa01\_F14.raw |  |  |  |  |  | 5.8277E6 |  |  |  |  |  |  |  |  | 1 | 0 | 0 | 0 | 0 | 0 | 1 | 0 | 0 | 0 | 0 | 0 | 0 | 0 | 0 | 21 | 33 | Carbamidomethylation | C6:Carbamidomethylation:1000.00;C8:Carbamidomethylation:1000.00 | PEAKS DB |
| K.KYM(+15.99)LYPDFLC(+57.02)K.G | Y | 43.40 | 1492.7095 | 11 | -0.3 | 498.5770 | 3 | 51.59 | 4 | F4:32744 | NaSa01\_F12.raw |  |  |  | 7.4997E5 |  |  |  |  |  |  |  |  |  |  | 1 | 0 | 0 | 0 | 1 | 0 | 0 | 0 | 0 | 0 | 0 | 0 | 0 | 0 | 0 | 106 | 116 | Oxidation (M); Carbamidomethylation | M3:Oxidation (M):1000.00;C10:Carbamidomethylation:1000.00 | PEAKS DB |
| SLLEFGK.M | Y | 38.22 | 792.4381 | 7 | 0.1 | 397.2264 | 2 | 39.94 | 4 | F4:23423 | NaSa01\_F12.raw |  |  |  | 8.4704E6 |  |  |  |  |  |  |  |  |  |  | 1 | 0 | 0 | 0 | 1 | 0 | 0 | 0 | 0 | 0 | 0 | 0 | 0 | 0 | 0 | 1 | 7 |  |  | PEAKS DB |
| total 8 peptides |  |  |  |  |  |  |  |  |  |  |  |  |  |  |  |  |  |  |  |  |  |  |  |  |  |  |  |  |  |  |  |  |  |  |  |  |  |  |  |  |  |  |  |  |  |
| --- | --- | --- | --- | --- | --- | --- | --- | --- | --- | --- | --- | --- | --- | --- | --- | --- | --- | --- | --- | --- | --- | --- | --- | --- | --- | --- | --- | --- | --- | --- | --- | --- | --- | --- | --- | --- | --- | --- | --- | --- | --- | --- | --- | --- | --- |

2QVD

back to list

  

| Protein Coverage
| Supporting Peptides
|

Protein Coverage:

Supporting Peptides:

| Peptide | Uniq | -10lgP | Mass | Length | ppm | m/z | z | RT | Fraction | Scan | Source File | Area F1 | Area F10 | Area F11 | Area F12 | Area F13 | Area F14 | Area F2 | Area F3 | Area F4 | Area F5 | Area F6 | Area F7 | Area F8 | Area F9 | #Feature | #Feature F1 | #Feature F10 | #Feature F11 | #Feature F12 | #Feature F13 | #Feature F14 | #Feature F2 | #Feature F3 | #Feature F4 | #Feature F5 | #Feature F6 | #Feature F7 | #Feature F8 | #Feature F9 | Start | End | PTM | AScore | Found By |
| --- | --- | --- | --- | --- | --- | --- | --- | --- | --- | --- | --- | --- | --- | --- | --- | --- | --- | --- | --- | --- | --- | --- | --- | --- | --- | --- | --- | --- | --- | --- | --- | --- | --- | --- | --- | --- | --- | --- | --- | --- | --- | --- | --- | --- | --- |
| K.LAIPSYSSYGC(+57.02)YC(+57.02)GWGGK.G | Y | 83.35 | 2024.8760 | 18 | 1.3 | 1013.4466 | 2 | 65.73 | 4 | F4:44422 | NaSa01\_F12.raw |  |  |  | 1.8132E7 |  |  |  |  |  |  |  |  |  |  | 1 | 0 | 0 | 0 | 1 | 0 | 0 | 0 | 0 | 0 | 0 | 0 | 0 | 0 | 0 | 16 | 33 | Carbamidomethylation | C11:Carbamidomethylation:1000.00;C13:Carbamidomethylation:1000.00 | PEAKS DB |
| R.C(+57.02)C(+57.02)FVHDC(+57.02)C(+57.02)YGNLPDC(+57.02)NPK.S | N | 73.65 | 2314.8687 | 18 | 0.7 | 772.6307 | 3 | 32.34 | 4 | F4:16838 | NaSa01\_F12.raw |  |  |  | 2.3387E7 |  | 7.5227E6 |  |  |  |  |  |  |  |  | 2 | 0 | 0 | 0 | 1 | 0 | 1 | 0 | 0 | 0 | 0 | 0 | 0 | 0 | 0 | 43 | 60 | Carbamidomethylation | C1:Carbamidomethylation:1000.00;C2:Carbamidomethylation:1000.00;C7:Carbamidomethylation:1000.00;C8:Carbamidomethylation:1000.00;C15:Carbamidomethylation:1000.00 | PEAKS DB |
| K.YMLYPDFLC(+57.02)K.G | N | 68.32 | 1348.6195 | 10 | 0.2 | 675.3171 | 2 | 75.10 | 4 | F4:52326 | NaSa01\_F12.raw |  |  |  | 2.1669E7 |  |  |  |  |  |  |  |  |  |  | 1 | 0 | 0 | 0 | 1 | 0 | 0 | 0 | 0 | 0 | 0 | 0 | 0 | 0 | 0 | 107 | 116 | Carbamidomethylation | C9:Carbamidomethylation:1000.00 | PEAKS DB |
| K.YM(+15.99)LYPDFLC(+57.02)K.G | N | 64.41 | 1364.6145 | 10 | 0.0 | 683.3145 | 2 | 68.02 | 4 | F4:46357 | NaSa01\_F12.raw |  |  |  | 2.115E6 |  |  |  |  |  |  |  |  |  |  | 1 | 0 | 0 | 0 | 1 | 0 | 0 | 0 | 0 | 0 | 0 | 0 | 0 | 0 | 0 | 107 | 116 | Oxidation (M); Carbamidomethylation | M2:Oxidation (M):1000.00;C9:Carbamidomethylation:1000.00 | PEAKS DB |
| K.KYMLYPDFLC(+57.02)K.G | Y | 60.31 | 1476.7145 | 11 | 0.3 | 493.2456 | 3 | 58.25 | 4 | F4:38516 | NaSa01\_F12.raw |  |  |  | 5.4533E6 |  |  |  |  |  |  |  |  |  |  | 2 | 0 | 0 | 0 | 2 | 0 | 0 | 0 | 0 | 0 | 0 | 0 | 0 | 0 | 0 | 106 | 116 | Carbamidomethylation | C10:Carbamidomethylation:1000.00 | PEAKS DB |
| S.YSSYGC(+57.02)YC(+57.02)GWGGK.G | N | 58.28 | 1543.5861 | 13 | 0.3 | 772.8005 | 2 | 35.31 | 6 | F6:16919 | NaSa01\_F14.raw |  |  |  |  |  | 5.8277E6 |  |  |  |  |  |  |  |  | 1 | 0 | 0 | 0 | 0 | 0 | 1 | 0 | 0 | 0 | 0 | 0 | 0 | 0 | 0 | 21 | 33 | Carbamidomethylation | C6:Carbamidomethylation:1000.00;C8:Carbamidomethylation:1000.00 | PEAKS DB |
| K.KYM(+15.99)LYPDFLC(+57.02)K.G | Y | 43.40 | 1492.7095 | 11 | -0.3 | 498.5770 | 3 | 51.59 | 4 | F4:32744 | NaSa01\_F12.raw |  |  |  | 7.4997E5 |  |  |  |  |  |  |  |  |  |  | 1 | 0 | 0 | 0 | 1 | 0 | 0 | 0 | 0 | 0 | 0 | 0 | 0 | 0 | 0 | 106 | 116 | Oxidation (M); Carbamidomethylation | M3:Oxidation (M):1000.00;C10:Carbamidomethylation:1000.00 | PEAKS DB |
| SLLEFGK.M | Y | 38.22 | 792.4381 | 7 | 0.1 | 397.2264 | 2 | 39.94 | 4 | F4:23423 | NaSa01\_F12.raw |  |  |  | 8.4704E6 |  |  |  |  |  |  |  |  |  |  | 1 | 0 | 0 | 0 | 1 | 0 | 0 | 0 | 0 | 0 | 0 | 0 | 0 | 0 | 0 | 1 | 7 |  |  | PEAKS DB |
| total 8 peptides |  |  |  |  |  |  |  |  |  |  |  |  |  |  |  |  |  |  |  |  |  |  |  |  |  |  |  |  |  |  |  |  |  |  |  |  |  |  |  |  |  |  |  |  |  |
| --- | --- | --- | --- | --- | --- | --- | --- | --- | --- | --- | --- | --- | --- | --- | --- | --- | --- | --- | --- | --- | --- | --- | --- | --- | --- | --- | --- | --- | --- | --- | --- | --- | --- | --- | --- | --- | --- | --- | --- | --- | --- | --- | --- | --- | --- |

4GLD

back to list

  

| Protein Coverage
| Supporting Peptides
|

Protein Coverage:

Supporting Peptides:

| Peptide | Uniq | -10lgP | Mass | Length | ppm | m/z | z | RT | Fraction | Scan | Source File | Area F1 | Area F10 | Area F11 | Area F12 | Area F13 | Area F14 | Area F2 | Area F3 | Area F4 | Area F5 | Area F6 | Area F7 | Area F8 | Area F9 | #Feature | #Feature F1 | #Feature F10 | #Feature F11 | #Feature F12 | #Feature F13 | #Feature F14 | #Feature F2 | #Feature F3 | #Feature F4 | #Feature F5 | #Feature F6 | #Feature F7 | #Feature F8 | #Feature F9 | Start | End | PTM | AScore | Found By |
| --- | --- | --- | --- | --- | --- | --- | --- | --- | --- | --- | --- | --- | --- | --- | --- | --- | --- | --- | --- | --- | --- | --- | --- | --- | --- | --- | --- | --- | --- | --- | --- | --- | --- | --- | --- | --- | --- | --- | --- | --- | --- | --- | --- | --- | --- |
| K.LAIPSYSSYGC(+57.02)YC(+57.02)GWGGK.G | Y | 83.35 | 2024.8760 | 18 | 1.3 | 1013.4466 | 2 | 65.73 | 4 | F4:44422 | NaSa01\_F12.raw |  |  |  | 1.8132E7 |  |  |  |  |  |  |  |  |  |  | 1 | 0 | 0 | 0 | 1 | 0 | 0 | 0 | 0 | 0 | 0 | 0 | 0 | 0 | 0 | 16 | 33 | Carbamidomethylation | C11:Carbamidomethylation:1000.00;C13:Carbamidomethylation:1000.00 | PEAKS DB |
| R.C(+57.02)C(+57.02)FVHDC(+57.02)C(+57.02)YGNLPDC(+57.02)NPK.S | N | 73.65 | 2314.8687 | 18 | 0.7 | 772.6307 | 3 | 32.34 | 4 | F4:16838 | NaSa01\_F12.raw |  |  |  | 2.3387E7 |  | 7.5227E6 |  |  |  |  |  |  |  |  | 2 | 0 | 0 | 0 | 1 | 0 | 1 | 0 | 0 | 0 | 0 | 0 | 0 | 0 | 0 | 43 | 60 | Carbamidomethylation | C1:Carbamidomethylation:1000.00;C2:Carbamidomethylation:1000.00;C7:Carbamidomethylation:1000.00;C8:Carbamidomethylation:1000.00;C15:Carbamidomethylation:1000.00 | PEAKS DB |
| K.YMLYPDFLC(+57.02)K.G | N | 68.32 | 1348.6195 | 10 | 0.2 | 675.3171 | 2 | 75.10 | 4 | F4:52326 | NaSa01\_F12.raw |  |  |  | 2.1669E7 |  |  |  |  |  |  |  |  |  |  | 1 | 0 | 0 | 0 | 1 | 0 | 0 | 0 | 0 | 0 | 0 | 0 | 0 | 0 | 0 | 107 | 116 | Carbamidomethylation | C9:Carbamidomethylation:1000.00 | PEAKS DB |
| K.YM(+15.99)LYPDFLC(+57.02)K.G | N | 64.41 | 1364.6145 | 10 | 0.0 | 683.3145 | 2 | 68.02 | 4 | F4:46357 | NaSa01\_F12.raw |  |  |  | 2.115E6 |  |  |  |  |  |  |  |  |  |  | 1 | 0 | 0 | 0 | 1 | 0 | 0 | 0 | 0 | 0 | 0 | 0 | 0 | 0 | 0 | 107 | 116 | Oxidation (M); Carbamidomethylation | M2:Oxidation (M):1000.00;C9:Carbamidomethylation:1000.00 | PEAKS DB |
| K.KYMLYPDFLC(+57.02)K.G | Y | 60.31 | 1476.7145 | 11 | 0.3 | 493.2456 | 3 | 58.25 | 4 | F4:38516 | NaSa01\_F12.raw |  |  |  | 5.4533E6 |  |  |  |  |  |  |  |  |  |  | 2 | 0 | 0 | 0 | 2 | 0 | 0 | 0 | 0 | 0 | 0 | 0 | 0 | 0 | 0 | 106 | 116 | Carbamidomethylation | C10:Carbamidomethylation:1000.00 | PEAKS DB |
| S.YSSYGC(+57.02)YC(+57.02)GWGGK.G | N | 58.28 | 1543.5861 | 13 | 0.3 | 772.8005 | 2 | 35.31 | 6 | F6:16919 | NaSa01\_F14.raw |  |  |  |  |  | 5.8277E6 |  |  |  |  |  |  |  |  | 1 | 0 | 0 | 0 | 0 | 0 | 1 | 0 | 0 | 0 | 0 | 0 | 0 | 0 | 0 | 21 | 33 | Carbamidomethylation | C6:Carbamidomethylation:1000.00;C8:Carbamidomethylation:1000.00 | PEAKS DB |
| K.KYM(+15.99)LYPDFLC(+57.02)K.G | Y | 43.40 | 1492.7095 | 11 | -0.3 | 498.5770 | 3 | 51.59 | 4 | F4:32744 | NaSa01\_F12.raw |  |  |  | 7.4997E5 |  |  |  |  |  |  |  |  |  |  | 1 | 0 | 0 | 0 | 1 | 0 | 0 | 0 | 0 | 0 | 0 | 0 | 0 | 0 | 0 | 106 | 116 | Oxidation (M); Carbamidomethylation | M3:Oxidation (M):1000.00;C10:Carbamidomethylation:1000.00 | PEAKS DB |
| SLLEFGK.M | Y | 38.22 | 792.4381 | 7 | 0.1 | 397.2264 | 2 | 39.94 | 4 | F4:23423 | NaSa01\_F12.raw |  |  |  | 8.4704E6 |  |  |  |  |  |  |  |  |  |  | 1 | 0 | 0 | 0 | 1 | 0 | 0 | 0 | 0 | 0 | 0 | 0 | 0 | 0 | 0 | 1 | 7 |  |  | PEAKS DB |
| total 8 peptides |  |  |  |  |  |  |  |  |  |  |  |  |  |  |  |  |  |  |  |  |  |  |  |  |  |  |  |  |  |  |  |  |  |  |  |  |  |  |  |  |  |  |  |  |  |
| --- | --- | --- | --- | --- | --- | --- | --- | --- | --- | --- | --- | --- | --- | --- | --- | --- | --- | --- | --- | --- | --- | --- | --- | --- | --- | --- | --- | --- | --- | --- | --- | --- | --- | --- | --- | --- | --- | --- | --- | --- | --- | --- | --- | --- | --- |

4EIX

back to list

  

| Protein Coverage
| Supporting Peptides
|

Protein Coverage:

Supporting Peptides:

| Peptide | Uniq | -10lgP | Mass | Length | ppm | m/z | z | RT | Fraction | Scan | Source File | Area F1 | Area F10 | Area F11 | Area F12 | Area F13 | Area F14 | Area F2 | Area F3 | Area F4 | Area F5 | Area F6 | Area F7 | Area F8 | Area F9 | #Feature | #Feature F1 | #Feature F10 | #Feature F11 | #Feature F12 | #Feature F13 | #Feature F14 | #Feature F2 | #Feature F3 | #Feature F4 | #Feature F5 | #Feature F6 | #Feature F7 | #Feature F8 | #Feature F9 | Start | End | PTM | AScore | Found By |
| --- | --- | --- | --- | --- | --- | --- | --- | --- | --- | --- | --- | --- | --- | --- | --- | --- | --- | --- | --- | --- | --- | --- | --- | --- | --- | --- | --- | --- | --- | --- | --- | --- | --- | --- | --- | --- | --- | --- | --- | --- | --- | --- | --- | --- | --- |
| K.LAIPSYSSYGC(+57.02)YC(+57.02)GWGGK.G | Y | 83.35 | 2024.8760 | 18 | 1.3 | 1013.4466 | 2 | 65.73 | 4 | F4:44422 | NaSa01\_F12.raw |  |  |  | 1.8132E7 |  |  |  |  |  |  |  |  |  |  | 1 | 0 | 0 | 0 | 1 | 0 | 0 | 0 | 0 | 0 | 0 | 0 | 0 | 0 | 0 | 16 | 33 | Carbamidomethylation | C11:Carbamidomethylation:1000.00;C13:Carbamidomethylation:1000.00 | PEAKS DB |
| R.C(+57.02)C(+57.02)FVHDC(+57.02)C(+57.02)YGNLPDC(+57.02)NPK.S | N | 73.65 | 2314.8687 | 18 | 0.7 | 772.6307 | 3 | 32.34 | 4 | F4:16838 | NaSa01\_F12.raw |  |  |  | 2.3387E7 |  | 7.5227E6 |  |  |  |  |  |  |  |  | 2 | 0 | 0 | 0 | 1 | 0 | 1 | 0 | 0 | 0 | 0 | 0 | 0 | 0 | 0 | 43 | 60 | Carbamidomethylation | C1:Carbamidomethylation:1000.00;C2:Carbamidomethylation:1000.00;C7:Carbamidomethylation:1000.00;C8:Carbamidomethylation:1000.00;C15:Carbamidomethylation:1000.00 | PEAKS DB |
| K.YMLYPDFLC(+57.02)K.G | N | 68.32 | 1348.6195 | 10 | 0.2 | 675.3171 | 2 | 75.10 | 4 | F4:52326 | NaSa01\_F12.raw |  |  |  | 2.1669E7 |  |  |  |  |  |  |  |  |  |  | 1 | 0 | 0 | 0 | 1 | 0 | 0 | 0 | 0 | 0 | 0 | 0 | 0 | 0 | 0 | 107 | 116 | Carbamidomethylation | C9:Carbamidomethylation:1000.00 | PEAKS DB |
| K.YM(+15.99)LYPDFLC(+57.02)K.G | N | 64.41 | 1364.6145 | 10 | 0.0 | 683.3145 | 2 | 68.02 | 4 | F4:46357 | NaSa01\_F12.raw |  |  |  | 2.115E6 |  |  |  |  |  |  |  |  |  |  | 1 | 0 | 0 | 0 | 1 | 0 | 0 | 0 | 0 | 0 | 0 | 0 | 0 | 0 | 0 | 107 | 116 | Oxidation (M); Carbamidomethylation | M2:Oxidation (M):1000.00;C9:Carbamidomethylation:1000.00 | PEAKS DB |
| K.KYMLYPDFLC(+57.02)K.G | Y | 60.31 | 1476.7145 | 11 | 0.3 | 493.2456 | 3 | 58.25 | 4 | F4:38516 | NaSa01\_F12.raw |  |  |  | 5.4533E6 |  |  |  |  |  |  |  |  |  |  | 2 | 0 | 0 | 0 | 2 | 0 | 0 | 0 | 0 | 0 | 0 | 0 | 0 | 0 | 0 | 106 | 116 | Carbamidomethylation | C10:Carbamidomethylation:1000.00 | PEAKS DB |
| S.YSSYGC(+57.02)YC(+57.02)GWGGK.G | N | 58.28 | 1543.5861 | 13 | 0.3 | 772.8005 | 2 | 35.31 | 6 | F6:16919 | NaSa01\_F14.raw |  |  |  |  |  | 5.8277E6 |  |  |  |  |  |  |  |  | 1 | 0 | 0 | 0 | 0 | 0 | 1 | 0 | 0 | 0 | 0 | 0 | 0 | 0 | 0 | 21 | 33 | Carbamidomethylation | C6:Carbamidomethylation:1000.00;C8:Carbamidomethylation:1000.00 | PEAKS DB |
| K.KYM(+15.99)LYPDFLC(+57.02)K.G | Y | 43.40 | 1492.7095 | 11 | -0.3 | 498.5770 | 3 | 51.59 | 4 | F4:32744 | NaSa01\_F12.raw |  |  |  | 7.4997E5 |  |  |  |  |  |  |  |  |  |  | 1 | 0 | 0 | 0 | 1 | 0 | 0 | 0 | 0 | 0 | 0 | 0 | 0 | 0 | 0 | 106 | 116 | Oxidation (M); Carbamidomethylation | M3:Oxidation (M):1000.00;C10:Carbamidomethylation:1000.00 | PEAKS DB |
| SLLEFGK.M | Y | 38.22 | 792.4381 | 7 | 0.1 | 397.2264 | 2 | 39.94 | 4 | F4:23423 | NaSa01\_F12.raw |  |  |  | 8.4704E6 |  |  |  |  |  |  |  |  |  |  | 1 | 0 | 0 | 0 | 1 | 0 | 0 | 0 | 0 | 0 | 0 | 0 | 0 | 0 | 0 | 1 | 7 |  |  | PEAKS DB |
| total 8 peptides |  |  |  |  |  |  |  |  |  |  |  |  |  |  |  |  |  |  |  |  |  |  |  |  |  |  |  |  |  |  |  |  |  |  |  |  |  |  |  |  |  |  |  |  |  |
| --- | --- | --- | --- | --- | --- | --- | --- | --- | --- | --- | --- | --- | --- | --- | --- | --- | --- | --- | --- | --- | --- | --- | --- | --- | --- | --- | --- | --- | --- | --- | --- | --- | --- | --- | --- | --- | --- | --- | --- | --- | --- | --- | --- | --- | --- |

3H1X

back to list

  

| Protein Coverage
| Supporting Peptides
|

Protein Coverage:

Supporting Peptides:

| Peptide | Uniq | -10lgP | Mass | Length | ppm | m/z | z | RT | Fraction | Scan | Source File | Area F1 | Area F10 | Area F11 | Area F12 | Area F13 | Area F14 | Area F2 | Area F3 | Area F4 | Area F5 | Area F6 | Area F7 | Area F8 | Area F9 | #Feature | #Feature F1 | #Feature F10 | #Feature F11 | #Feature F12 | #Feature F13 | #Feature F14 | #Feature F2 | #Feature F3 | #Feature F4 | #Feature F5 | #Feature F6 | #Feature F7 | #Feature F8 | #Feature F9 | Start | End | PTM | AScore | Found By |
| --- | --- | --- | --- | --- | --- | --- | --- | --- | --- | --- | --- | --- | --- | --- | --- | --- | --- | --- | --- | --- | --- | --- | --- | --- | --- | --- | --- | --- | --- | --- | --- | --- | --- | --- | --- | --- | --- | --- | --- | --- | --- | --- | --- | --- | --- |
| K.LAIPSYSSYGC(+57.02)YC(+57.02)GWGGK.G | Y | 83.35 | 2024.8760 | 18 | 1.3 | 1013.4466 | 2 | 65.73 | 4 | F4:44422 | NaSa01\_F12.raw |  |  |  | 1.8132E7 |  |  |  |  |  |  |  |  |  |  | 1 | 0 | 0 | 0 | 1 | 0 | 0 | 0 | 0 | 0 | 0 | 0 | 0 | 0 | 0 | 16 | 33 | Carbamidomethylation | C11:Carbamidomethylation:1000.00;C13:Carbamidomethylation:1000.00 | PEAKS DB |
| R.C(+57.02)C(+57.02)FVHDC(+57.02)C(+57.02)YGNLPDC(+57.02)NPK.S | N | 73.65 | 2314.8687 | 18 | 0.7 | 772.6307 | 3 | 32.34 | 4 | F4:16838 | NaSa01\_F12.raw |  |  |  | 2.3387E7 |  | 7.5227E6 |  |  |  |  |  |  |  |  | 2 | 0 | 0 | 0 | 1 | 0 | 1 | 0 | 0 | 0 | 0 | 0 | 0 | 0 | 0 | 43 | 60 | Carbamidomethylation | C1:Carbamidomethylation:1000.00;C2:Carbamidomethylation:1000.00;C7:Carbamidomethylation:1000.00;C8:Carbamidomethylation:1000.00;C15:Carbamidomethylation:1000.00 | PEAKS DB |
| K.YMLYPDFLC(+57.02)K.G | N | 68.32 | 1348.6195 | 10 | 0.2 | 675.3171 | 2 | 75.10 | 4 | F4:52326 | NaSa01\_F12.raw |  |  |  | 2.1669E7 |  |  |  |  |  |  |  |  |  |  | 1 | 0 | 0 | 0 | 1 | 0 | 0 | 0 | 0 | 0 | 0 | 0 | 0 | 0 | 0 | 107 | 116 | Carbamidomethylation | C9:Carbamidomethylation:1000.00 | PEAKS DB |
| K.YM(+15.99)LYPDFLC(+57.02)K.G | N | 64.41 | 1364.6145 | 10 | 0.0 | 683.3145 | 2 | 68.02 | 4 | F4:46357 | NaSa01\_F12.raw |  |  |  | 2.115E6 |  |  |  |  |  |  |  |  |  |  | 1 | 0 | 0 | 0 | 1 | 0 | 0 | 0 | 0 | 0 | 0 | 0 | 0 | 0 | 0 | 107 | 116 | Oxidation (M); Carbamidomethylation | M2:Oxidation (M):1000.00;C9:Carbamidomethylation:1000.00 | PEAKS DB |
| K.KYMLYPDFLC(+57.02)K.G | Y | 60.31 | 1476.7145 | 11 | 0.3 | 493.2456 | 3 | 58.25 | 4 | F4:38516 | NaSa01\_F12.raw |  |  |  | 5.4533E6 |  |  |  |  |  |  |  |  |  |  | 2 | 0 | 0 | 0 | 2 | 0 | 0 | 0 | 0 | 0 | 0 | 0 | 0 | 0 | 0 | 106 | 116 | Carbamidomethylation | C10:Carbamidomethylation:1000.00 | PEAKS DB |
| S.YSSYGC(+57.02)YC(+57.02)GWGGK.G | N | 58.28 | 1543.5861 | 13 | 0.3 | 772.8005 | 2 | 35.31 | 6 | F6:16919 | NaSa01\_F14.raw |  |  |  |  |  | 5.8277E6 |  |  |  |  |  |  |  |  | 1 | 0 | 0 | 0 | 0 | 0 | 1 | 0 | 0 | 0 | 0 | 0 | 0 | 0 | 0 | 21 | 33 | Carbamidomethylation | C6:Carbamidomethylation:1000.00;C8:Carbamidomethylation:1000.00 | PEAKS DB |
| K.KYM(+15.99)LYPDFLC(+57.02)K.G | Y | 43.40 | 1492.7095 | 11 | -0.3 | 498.5770 | 3 | 51.59 | 4 | F4:32744 | NaSa01\_F12.raw |  |  |  | 7.4997E5 |  |  |  |  |  |  |  |  |  |  | 1 | 0 | 0 | 0 | 1 | 0 | 0 | 0 | 0 | 0 | 0 | 0 | 0 | 0 | 0 | 106 | 116 | Oxidation (M); Carbamidomethylation | M3:Oxidation (M):1000.00;C10:Carbamidomethylation:1000.00 | PEAKS DB |
| SLLEFGK.M | Y | 38.22 | 792.4381 | 7 | 0.1 | 397.2264 | 2 | 39.94 | 4 | F4:23423 | NaSa01\_F12.raw |  |  |  | 8.4704E6 |  |  |  |  |  |  |  |  |  |  | 1 | 0 | 0 | 0 | 1 | 0 | 0 | 0 | 0 | 0 | 0 | 0 | 0 | 0 | 0 | 1 | 7 |  |  | PEAKS DB |
| total 8 peptides |  |  |  |  |  |  |  |  |  |  |  |  |  |  |  |  |  |  |  |  |  |  |  |  |  |  |  |  |  |  |  |  |  |  |  |  |  |  |  |  |  |  |  |  |  |
| --- | --- | --- | --- | --- | --- | --- | --- | --- | --- | --- | --- | --- | --- | --- | --- | --- | --- | --- | --- | --- | --- | --- | --- | --- | --- | --- | --- | --- | --- | --- | --- | --- | --- | --- | --- | --- | --- | --- | --- | --- | --- | --- | --- | --- | --- |

2B17

back to list

  

| Protein Coverage
| Supporting Peptides
|

Protein Coverage:

Supporting Peptides:

| Peptide | Uniq | -10lgP | Mass | Length | ppm | m/z | z | RT | Fraction | Scan | Source File | Area F1 | Area F10 | Area F11 | Area F12 | Area F13 | Area F14 | Area F2 | Area F3 | Area F4 | Area F5 | Area F6 | Area F7 | Area F8 | Area F9 | #Feature | #Feature F1 | #Feature F10 | #Feature F11 | #Feature F12 | #Feature F13 | #Feature F14 | #Feature F2 | #Feature F3 | #Feature F4 | #Feature F5 | #Feature F6 | #Feature F7 | #Feature F8 | #Feature F9 | Start | End | PTM | AScore | Found By |
| --- | --- | --- | --- | --- | --- | --- | --- | --- | --- | --- | --- | --- | --- | --- | --- | --- | --- | --- | --- | --- | --- | --- | --- | --- | --- | --- | --- | --- | --- | --- | --- | --- | --- | --- | --- | --- | --- | --- | --- | --- | --- | --- | --- | --- | --- |
| K.LAIPSYSSYGC(+57.02)YC(+57.02)GWGGK.G | Y | 83.35 | 2024.8760 | 18 | 1.3 | 1013.4466 | 2 | 65.73 | 4 | F4:44422 | NaSa01\_F12.raw |  |  |  | 1.8132E7 |  |  |  |  |  |  |  |  |  |  | 1 | 0 | 0 | 0 | 1 | 0 | 0 | 0 | 0 | 0 | 0 | 0 | 0 | 0 | 0 | 16 | 33 | Carbamidomethylation | C11:Carbamidomethylation:1000.00;C13:Carbamidomethylation:1000.00 | PEAKS DB |
| R.C(+57.02)C(+57.02)FVHDC(+57.02)C(+57.02)YGNLPDC(+57.02)NPK.S | N | 73.65 | 2314.8687 | 18 | 0.7 | 772.6307 | 3 | 32.34 | 4 | F4:16838 | NaSa01\_F12.raw |  |  |  | 2.3387E7 |  | 7.5227E6 |  |  |  |  |  |  |  |  | 2 | 0 | 0 | 0 | 1 | 0 | 1 | 0 | 0 | 0 | 0 | 0 | 0 | 0 | 0 | 43 | 60 | Carbamidomethylation | C1:Carbamidomethylation:1000.00;C2:Carbamidomethylation:1000.00;C7:Carbamidomethylation:1000.00;C8:Carbamidomethylation:1000.00;C15:Carbamidomethylation:1000.00 | PEAKS DB |
| K.YMLYPDFLC(+57.02)K.G | N | 68.32 | 1348.6195 | 10 | 0.2 | 675.3171 | 2 | 75.10 | 4 | F4:52326 | NaSa01\_F12.raw |  |  |  | 2.1669E7 |  |  |  |  |  |  |  |  |  |  | 1 | 0 | 0 | 0 | 1 | 0 | 0 | 0 | 0 | 0 | 0 | 0 | 0 | 0 | 0 | 107 | 116 | Carbamidomethylation | C9:Carbamidomethylation:1000.00 | PEAKS DB |
| K.YM(+15.99)LYPDFLC(+57.02)K.G | N | 64.41 | 1364.6145 | 10 | 0.0 | 683.3145 | 2 | 68.02 | 4 | F4:46357 | NaSa01\_F12.raw |  |  |  | 2.115E6 |  |  |  |  |  |  |  |  |  |  | 1 | 0 | 0 | 0 | 1 | 0 | 0 | 0 | 0 | 0 | 0 | 0 | 0 | 0 | 0 | 107 | 116 | Oxidation (M); Carbamidomethylation | M2:Oxidation (M):1000.00;C9:Carbamidomethylation:1000.00 | PEAKS DB |
| K.KYMLYPDFLC(+57.02)K.G | Y | 60.31 | 1476.7145 | 11 | 0.3 | 493.2456 | 3 | 58.25 | 4 | F4:38516 | NaSa01\_F12.raw |  |  |  | 5.4533E6 |  |  |  |  |  |  |  |  |  |  | 2 | 0 | 0 | 0 | 2 | 0 | 0 | 0 | 0 | 0 | 0 | 0 | 0 | 0 | 0 | 106 | 116 | Carbamidomethylation | C10:Carbamidomethylation:1000.00 | PEAKS DB |
| S.YSSYGC(+57.02)YC(+57.02)GWGGK.G | N | 58.28 | 1543.5861 | 13 | 0.3 | 772.8005 | 2 | 35.31 | 6 | F6:16919 | NaSa01\_F14.raw |  |  |  |  |  | 5.8277E6 |  |  |  |  |  |  |  |  | 1 | 0 | 0 | 0 | 0 | 0 | 1 | 0 | 0 | 0 | 0 | 0 | 0 | 0 | 0 | 21 | 33 | Carbamidomethylation | C6:Carbamidomethylation:1000.00;C8:Carbamidomethylation:1000.00 | PEAKS DB |
| K.KYM(+15.99)LYPDFLC(+57.02)K.G | Y | 43.40 | 1492.7095 | 11 | -0.3 | 498.5770 | 3 | 51.59 | 4 | F4:32744 | NaSa01\_F12.raw |  |  |  | 7.4997E5 |  |  |  |  |  |  |  |  |  |  | 1 | 0 | 0 | 0 | 1 | 0 | 0 | 0 | 0 | 0 | 0 | 0 | 0 | 0 | 0 | 106 | 116 | Oxidation (M); Carbamidomethylation | M3:Oxidation (M):1000.00;C10:Carbamidomethylation:1000.00 | PEAKS DB |
| SLLEFGK.M | Y | 38.22 | 792.4381 | 7 | 0.1 | 397.2264 | 2 | 39.94 | 4 | F4:23423 | NaSa01\_F12.raw |  |  |  | 8.4704E6 |  |  |  |  |  |  |  |  |  |  | 1 | 0 | 0 | 0 | 1 | 0 | 0 | 0 | 0 | 0 | 0 | 0 | 0 | 0 | 0 | 1 | 7 |  |  | PEAKS DB |
| total 8 peptides |  |  |  |  |  |  |  |  |  |  |  |  |  |  |  |  |  |  |  |  |  |  |  |  |  |  |  |  |  |  |  |  |  |  |  |  |  |  |  |  |  |  |  |  |  |
| --- | --- | --- | --- | --- | --- | --- | --- | --- | --- | --- | --- | --- | --- | --- | --- | --- | --- | --- | --- | --- | --- | --- | --- | --- | --- | --- | --- | --- | --- | --- | --- | --- | --- | --- | --- | --- | --- | --- | --- | --- | --- | --- | --- | --- | --- |

4FGA

back to list

  

| Protein Coverage
| Supporting Peptides
|

Protein Coverage:

Supporting Peptides:

| Peptide | Uniq | -10lgP | Mass | Length | ppm | m/z | z | RT | Fraction | Scan | Source File | Area F1 | Area F10 | Area F11 | Area F12 | Area F13 | Area F14 | Area F2 | Area F3 | Area F4 | Area F5 | Area F6 | Area F7 | Area F8 | Area F9 | #Feature | #Feature F1 | #Feature F10 | #Feature F11 | #Feature F12 | #Feature F13 | #Feature F14 | #Feature F2 | #Feature F3 | #Feature F4 | #Feature F5 | #Feature F6 | #Feature F7 | #Feature F8 | #Feature F9 | Start | End | PTM | AScore | Found By |
| --- | --- | --- | --- | --- | --- | --- | --- | --- | --- | --- | --- | --- | --- | --- | --- | --- | --- | --- | --- | --- | --- | --- | --- | --- | --- | --- | --- | --- | --- | --- | --- | --- | --- | --- | --- | --- | --- | --- | --- | --- | --- | --- | --- | --- | --- |
| K.LAIPSYSSYGC(+57.02)YC(+57.02)GWGGK.G | Y | 83.35 | 2024.8760 | 18 | 1.3 | 1013.4466 | 2 | 65.73 | 4 | F4:44422 | NaSa01\_F12.raw |  |  |  | 1.8132E7 |  |  |  |  |  |  |  |  |  |  | 1 | 0 | 0 | 0 | 1 | 0 | 0 | 0 | 0 | 0 | 0 | 0 | 0 | 0 | 0 | 16 | 33 | Carbamidomethylation | C11:Carbamidomethylation:1000.00;C13:Carbamidomethylation:1000.00 | PEAKS DB |
| R.C(+57.02)C(+57.02)FVHDC(+57.02)C(+57.02)YGNLPDC(+57.02)NPK.S | N | 73.65 | 2314.8687 | 18 | 0.7 | 772.6307 | 3 | 32.34 | 4 | F4:16838 | NaSa01\_F12.raw |  |  |  | 2.3387E7 |  | 7.5227E6 |  |  |  |  |  |  |  |  | 2 | 0 | 0 | 0 | 1 | 0 | 1 | 0 | 0 | 0 | 0 | 0 | 0 | 0 | 0 | 43 | 60 | Carbamidomethylation | C1:Carbamidomethylation:1000.00;C2:Carbamidomethylation:1000.00;C7:Carbamidomethylation:1000.00;C8:Carbamidomethylation:1000.00;C15:Carbamidomethylation:1000.00 | PEAKS DB |
| K.YMLYPDFLC(+57.02)K.G | N | 68.32 | 1348.6195 | 10 | 0.2 | 675.3171 | 2 | 75.10 | 4 | F4:52326 | NaSa01\_F12.raw |  |  |  | 2.1669E7 |  |  |  |  |  |  |  |  |  |  | 1 | 0 | 0 | 0 | 1 | 0 | 0 | 0 | 0 | 0 | 0 | 0 | 0 | 0 | 0 | 107 | 116 | Carbamidomethylation | C9:Carbamidomethylation:1000.00 | PEAKS DB |
| K.YM(+15.99)LYPDFLC(+57.02)K.G | N | 64.41 | 1364.6145 | 10 | 0.0 | 683.3145 | 2 | 68.02 | 4 | F4:46357 | NaSa01\_F12.raw |  |  |  | 2.115E6 |  |  |  |  |  |  |  |  |  |  | 1 | 0 | 0 | 0 | 1 | 0 | 0 | 0 | 0 | 0 | 0 | 0 | 0 | 0 | 0 | 107 | 116 | Oxidation (M); Carbamidomethylation | M2:Oxidation (M):1000.00;C9:Carbamidomethylation:1000.00 | PEAKS DB |
| K.KYMLYPDFLC(+57.02)K.G | Y | 60.31 | 1476.7145 | 11 | 0.3 | 493.2456 | 3 | 58.25 | 4 | F4:38516 | NaSa01\_F12.raw |  |  |  | 5.4533E6 |  |  |  |  |  |  |  |  |  |  | 2 | 0 | 0 | 0 | 2 | 0 | 0 | 0 | 0 | 0 | 0 | 0 | 0 | 0 | 0 | 106 | 116 | Carbamidomethylation | C10:Carbamidomethylation:1000.00 | PEAKS DB |
| S.YSSYGC(+57.02)YC(+57.02)GWGGK.G | N | 58.28 | 1543.5861 | 13 | 0.3 | 772.8005 | 2 | 35.31 | 6 | F6:16919 | NaSa01\_F14.raw |  |  |  |  |  | 5.8277E6 |  |  |  |  |  |  |  |  | 1 | 0 | 0 | 0 | 0 | 0 | 1 | 0 | 0 | 0 | 0 | 0 | 0 | 0 | 0 | 21 | 33 | Carbamidomethylation | C6:Carbamidomethylation:1000.00;C8:Carbamidomethylation:1000.00 | PEAKS DB |
| K.KYM(+15.99)LYPDFLC(+57.02)K.G | Y | 43.40 | 1492.7095 | 11 | -0.3 | 498.5770 | 3 | 51.59 | 4 | F4:32744 | NaSa01\_F12.raw |  |  |  | 7.4997E5 |  |  |  |  |  |  |  |  |  |  | 1 | 0 | 0 | 0 | 1 | 0 | 0 | 0 | 0 | 0 | 0 | 0 | 0 | 0 | 0 | 106 | 116 | Oxidation (M); Carbamidomethylation | M3:Oxidation (M):1000.00;C10:Carbamidomethylation:1000.00 | PEAKS DB |
| SLLEFGK.M | Y | 38.22 | 792.4381 | 7 | 0.1 | 397.2264 | 2 | 39.94 | 4 | F4:23423 | NaSa01\_F12.raw |  |  |  | 8.4704E6 |  |  |  |  |  |  |  |  |  |  | 1 | 0 | 0 | 0 | 1 | 0 | 0 | 0 | 0 | 0 | 0 | 0 | 0 | 0 | 0 | 1 | 7 |  |  | PEAKS DB |
| total 8 peptides |  |  |  |  |  |  |  |  |  |  |  |  |  |  |  |  |  |  |  |  |  |  |  |  |  |  |  |  |  |  |  |  |  |  |  |  |  |  |  |  |  |  |  |  |  |
| --- | --- | --- | --- | --- | --- | --- | --- | --- | --- | --- | --- | --- | --- | --- | --- | --- | --- | --- | --- | --- | --- | --- | --- | --- | --- | --- | --- | --- | --- | --- | --- | --- | --- | --- | --- | --- | --- | --- | --- | --- | --- | --- | --- | --- | --- |

2ARM

back to list

  

| Protein Coverage
| Supporting Peptides
|

Protein Coverage:

Supporting Peptides:

| Peptide | Uniq | -10lgP | Mass | Length | ppm | m/z | z | RT | Fraction | Scan | Source File | Area F1 | Area F10 | Area F11 | Area F12 | Area F13 | Area F14 | Area F2 | Area F3 | Area F4 | Area F5 | Area F6 | Area F7 | Area F8 | Area F9 | #Feature | #Feature F1 | #Feature F10 | #Feature F11 | #Feature F12 | #Feature F13 | #Feature F14 | #Feature F2 | #Feature F3 | #Feature F4 | #Feature F5 | #Feature F6 | #Feature F7 | #Feature F8 | #Feature F9 | Start | End | PTM | AScore | Found By |
| --- | --- | --- | --- | --- | --- | --- | --- | --- | --- | --- | --- | --- | --- | --- | --- | --- | --- | --- | --- | --- | --- | --- | --- | --- | --- | --- | --- | --- | --- | --- | --- | --- | --- | --- | --- | --- | --- | --- | --- | --- | --- | --- | --- | --- | --- |
| K.LAIPSYSSYGC(+57.02)YC(+57.02)GWGGK.G | Y | 83.35 | 2024.8760 | 18 | 1.3 | 1013.4466 | 2 | 65.73 | 4 | F4:44422 | NaSa01\_F12.raw |  |  |  | 1.8132E7 |  |  |  |  |  |  |  |  |  |  | 1 | 0 | 0 | 0 | 1 | 0 | 0 | 0 | 0 | 0 | 0 | 0 | 0 | 0 | 0 | 16 | 33 | Carbamidomethylation | C11:Carbamidomethylation:1000.00;C13:Carbamidomethylation:1000.00 | PEAKS DB |
| R.C(+57.02)C(+57.02)FVHDC(+57.02)C(+57.02)YGNLPDC(+57.02)NPK.S | N | 73.65 | 2314.8687 | 18 | 0.7 | 772.6307 | 3 | 32.34 | 4 | F4:16838 | NaSa01\_F12.raw |  |  |  | 2.3387E7 |  | 7.5227E6 |  |  |  |  |  |  |  |  | 2 | 0 | 0 | 0 | 1 | 0 | 1 | 0 | 0 | 0 | 0 | 0 | 0 | 0 | 0 | 43 | 60 | Carbamidomethylation | C1:Carbamidomethylation:1000.00;C2:Carbamidomethylation:1000.00;C7:Carbamidomethylation:1000.00;C8:Carbamidomethylation:1000.00;C15:Carbamidomethylation:1000.00 | PEAKS DB |
| K.YMLYPDFLC(+57.02)K.G | N | 68.32 | 1348.6195 | 10 | 0.2 | 675.3171 | 2 | 75.10 | 4 | F4:52326 | NaSa01\_F12.raw |  |  |  | 2.1669E7 |  |  |  |  |  |  |  |  |  |  | 1 | 0 | 0 | 0 | 1 | 0 | 0 | 0 | 0 | 0 | 0 | 0 | 0 | 0 | 0 | 107 | 116 | Carbamidomethylation | C9:Carbamidomethylation:1000.00 | PEAKS DB |
| K.YM(+15.99)LYPDFLC(+57.02)K.G | N | 64.41 | 1364.6145 | 10 | 0.0 | 683.3145 | 2 | 68.02 | 4 | F4:46357 | NaSa01\_F12.raw |  |  |  | 2.115E6 |  |  |  |  |  |  |  |  |  |  | 1 | 0 | 0 | 0 | 1 | 0 | 0 | 0 | 0 | 0 | 0 | 0 | 0 | 0 | 0 | 107 | 116 | Oxidation (M); Carbamidomethylation | M2:Oxidation (M):1000.00;C9:Carbamidomethylation:1000.00 | PEAKS DB |
| K.KYMLYPDFLC(+57.02)K.G | Y | 60.31 | 1476.7145 | 11 | 0.3 | 493.2456 | 3 | 58.25 | 4 | F4:38516 | NaSa01\_F12.raw |  |  |  | 5.4533E6 |  |  |  |  |  |  |  |  |  |  | 2 | 0 | 0 | 0 | 2 | 0 | 0 | 0 | 0 | 0 | 0 | 0 | 0 | 0 | 0 | 106 | 116 | Carbamidomethylation | C10:Carbamidomethylation:1000.00 | PEAKS DB |
| S.YSSYGC(+57.02)YC(+57.02)GWGGK.G | N | 58.28 | 1543.5861 | 13 | 0.3 | 772.8005 | 2 | 35.31 | 6 | F6:16919 | NaSa01\_F14.raw |  |  |  |  |  | 5.8277E6 |  |  |  |  |  |  |  |  | 1 | 0 | 0 | 0 | 0 | 0 | 1 | 0 | 0 | 0 | 0 | 0 | 0 | 0 | 0 | 21 | 33 | Carbamidomethylation | C6:Carbamidomethylation:1000.00;C8:Carbamidomethylation:1000.00 | PEAKS DB |
| K.KYM(+15.99)LYPDFLC(+57.02)K.G | Y | 43.40 | 1492.7095 | 11 | -0.3 | 498.5770 | 3 | 51.59 | 4 | F4:32744 | NaSa01\_F12.raw |  |  |  | 7.4997E5 |  |  |  |  |  |  |  |  |  |  | 1 | 0 | 0 | 0 | 1 | 0 | 0 | 0 | 0 | 0 | 0 | 0 | 0 | 0 | 0 | 106 | 116 | Oxidation (M); Carbamidomethylation | M3:Oxidation (M):1000.00;C10:Carbamidomethylation:1000.00 | PEAKS DB |
| SLLEFGK.M | Y | 38.22 | 792.4381 | 7 | 0.1 | 397.2264 | 2 | 39.94 | 4 | F4:23423 | NaSa01\_F12.raw |  |  |  | 8.4704E6 |  |  |  |  |  |  |  |  |  |  | 1 | 0 | 0 | 0 | 1 | 0 | 0 | 0 | 0 | 0 | 0 | 0 | 0 | 0 | 0 | 1 | 7 |  |  | PEAKS DB |
| total 8 peptides |  |  |  |  |  |  |  |  |  |  |  |  |  |  |  |  |  |  |  |  |  |  |  |  |  |  |  |  |  |  |  |  |  |  |  |  |  |  |  |  |  |  |  |  |  |
| --- | --- | --- | --- | --- | --- | --- | --- | --- | --- | --- | --- | --- | --- | --- | --- | --- | --- | --- | --- | --- | --- | --- | --- | --- | --- | --- | --- | --- | --- | --- | --- | --- | --- | --- | --- | --- | --- | --- | --- | --- | --- | --- | --- | --- | --- |

1ZR8

back to list

  

| Protein Coverage
| Supporting Peptides
|

Protein Coverage:

Supporting Peptides:

| Peptide | Uniq | -10lgP | Mass | Length | ppm | m/z | z | RT | Fraction | Scan | Source File | Area F1 | Area F10 | Area F11 | Area F12 | Area F13 | Area F14 | Area F2 | Area F3 | Area F4 | Area F5 | Area F6 | Area F7 | Area F8 | Area F9 | #Feature | #Feature F1 | #Feature F10 | #Feature F11 | #Feature F12 | #Feature F13 | #Feature F14 | #Feature F2 | #Feature F3 | #Feature F4 | #Feature F5 | #Feature F6 | #Feature F7 | #Feature F8 | #Feature F9 | Start | End | PTM | AScore | Found By |
| --- | --- | --- | --- | --- | --- | --- | --- | --- | --- | --- | --- | --- | --- | --- | --- | --- | --- | --- | --- | --- | --- | --- | --- | --- | --- | --- | --- | --- | --- | --- | --- | --- | --- | --- | --- | --- | --- | --- | --- | --- | --- | --- | --- | --- | --- |
| K.LAIPSYSSYGC(+57.02)YC(+57.02)GWGGK.G | Y | 83.35 | 2024.8760 | 18 | 1.3 | 1013.4466 | 2 | 65.73 | 4 | F4:44422 | NaSa01\_F12.raw |  |  |  | 1.8132E7 |  |  |  |  |  |  |  |  |  |  | 1 | 0 | 0 | 0 | 1 | 0 | 0 | 0 | 0 | 0 | 0 | 0 | 0 | 0 | 0 | 16 | 33 | Carbamidomethylation | C11:Carbamidomethylation:1000.00;C13:Carbamidomethylation:1000.00 | PEAKS DB |
| R.C(+57.02)C(+57.02)FVHDC(+57.02)C(+57.02)YGNLPDC(+57.02)NPK.S | N | 73.65 | 2314.8687 | 18 | 0.7 | 772.6307 | 3 | 32.34 | 4 | F4:16838 | NaSa01\_F12.raw |  |  |  | 2.3387E7 |  | 7.5227E6 |  |  |  |  |  |  |  |  | 2 | 0 | 0 | 0 | 1 | 0 | 1 | 0 | 0 | 0 | 0 | 0 | 0 | 0 | 0 | 43 | 60 | Carbamidomethylation | C1:Carbamidomethylation:1000.00;C2:Carbamidomethylation:1000.00;C7:Carbamidomethylation:1000.00;C8:Carbamidomethylation:1000.00;C15:Carbamidomethylation:1000.00 | PEAKS DB |
| K.YMLYPDFLC(+57.02)K.G | N | 68.32 | 1348.6195 | 10 | 0.2 | 675.3171 | 2 | 75.10 | 4 | F4:52326 | NaSa01\_F12.raw |  |  |  | 2.1669E7 |  |  |  |  |  |  |  |  |  |  | 1 | 0 | 0 | 0 | 1 | 0 | 0 | 0 | 0 | 0 | 0 | 0 | 0 | 0 | 0 | 107 | 116 | Carbamidomethylation | C9:Carbamidomethylation:1000.00 | PEAKS DB |
| K.YM(+15.99)LYPDFLC(+57.02)K.G | N | 64.41 | 1364.6145 | 10 | 0.0 | 683.3145 | 2 | 68.02 | 4 | F4:46357 | NaSa01\_F12.raw |  |  |  | 2.115E6 |  |  |  |  |  |  |  |  |  |  | 1 | 0 | 0 | 0 | 1 | 0 | 0 | 0 | 0 | 0 | 0 | 0 | 0 | 0 | 0 | 107 | 116 | Oxidation (M); Carbamidomethylation | M2:Oxidation (M):1000.00;C9:Carbamidomethylation:1000.00 | PEAKS DB |
| K.KYMLYPDFLC(+57.02)K.G | Y | 60.31 | 1476.7145 | 11 | 0.3 | 493.2456 | 3 | 58.25 | 4 | F4:38516 | NaSa01\_F12.raw |  |  |  | 5.4533E6 |  |  |  |  |  |  |  |  |  |  | 2 | 0 | 0 | 0 | 2 | 0 | 0 | 0 | 0 | 0 | 0 | 0 | 0 | 0 | 0 | 106 | 116 | Carbamidomethylation | C10:Carbamidomethylation:1000.00 | PEAKS DB |
| S.YSSYGC(+57.02)YC(+57.02)GWGGK.G | N | 58.28 | 1543.5861 | 13 | 0.3 | 772.8005 | 2 | 35.31 | 6 | F6:16919 | NaSa01\_F14.raw |  |  |  |  |  | 5.8277E6 |  |  |  |  |  |  |  |  | 1 | 0 | 0 | 0 | 0 | 0 | 1 | 0 | 0 | 0 | 0 | 0 | 0 | 0 | 0 | 21 | 33 | Carbamidomethylation | C6:Carbamidomethylation:1000.00;C8:Carbamidomethylation:1000.00 | PEAKS DB |
| K.KYM(+15.99)LYPDFLC(+57.02)K.G | Y | 43.40 | 1492.7095 | 11 | -0.3 | 498.5770 | 3 | 51.59 | 4 | F4:32744 | NaSa01\_F12.raw |  |  |  | 7.4997E5 |  |  |  |  |  |  |  |  |  |  | 1 | 0 | 0 | 0 | 1 | 0 | 0 | 0 | 0 | 0 | 0 | 0 | 0 | 0 | 0 | 106 | 116 | Oxidation (M); Carbamidomethylation | M3:Oxidation (M):1000.00;C10:Carbamidomethylation:1000.00 | PEAKS DB |
| SLLEFGK.M | Y | 38.22 | 792.4381 | 7 | 0.1 | 397.2264 | 2 | 39.94 | 4 | F4:23423 | NaSa01\_F12.raw |  |  |  | 8.4704E6 |  |  |  |  |  |  |  |  |  |  | 1 | 0 | 0 | 0 | 1 | 0 | 0 | 0 | 0 | 0 | 0 | 0 | 0 | 0 | 0 | 1 | 7 |  |  | PEAKS DB |
| total 8 peptides |  |  |  |  |  |  |  |  |  |  |  |  |  |  |  |  |  |  |  |  |  |  |  |  |  |  |  |  |  |  |  |  |  |  |  |  |  |  |  |  |  |  |  |  |  |
| --- | --- | --- | --- | --- | --- | --- | --- | --- | --- | --- | --- | --- | --- | --- | --- | --- | --- | --- | --- | --- | --- | --- | --- | --- | --- | --- | --- | --- | --- | --- | --- | --- | --- | --- | --- | --- | --- | --- | --- | --- | --- | --- | --- | --- | --- |

1TG4

back to list

  

| Protein Coverage
| Supporting Peptides
|

Protein Coverage:

Supporting Peptides:

| Peptide | Uniq | -10lgP | Mass | Length | ppm | m/z | z | RT | Fraction | Scan | Source File | Area F1 | Area F10 | Area F11 | Area F12 | Area F13 | Area F14 | Area F2 | Area F3 | Area F4 | Area F5 | Area F6 | Area F7 | Area F8 | Area F9 | #Feature | #Feature F1 | #Feature F10 | #Feature F11 | #Feature F12 | #Feature F13 | #Feature F14 | #Feature F2 | #Feature F3 | #Feature F4 | #Feature F5 | #Feature F6 | #Feature F7 | #Feature F8 | #Feature F9 | Start | End | PTM | AScore | Found By |
| --- | --- | --- | --- | --- | --- | --- | --- | --- | --- | --- | --- | --- | --- | --- | --- | --- | --- | --- | --- | --- | --- | --- | --- | --- | --- | --- | --- | --- | --- | --- | --- | --- | --- | --- | --- | --- | --- | --- | --- | --- | --- | --- | --- | --- | --- |
| K.LAIPSYSSYGC(+57.02)YC(+57.02)GWGGK.G | Y | 83.35 | 2024.8760 | 18 | 1.3 | 1013.4466 | 2 | 65.73 | 4 | F4:44422 | NaSa01\_F12.raw |  |  |  | 1.8132E7 |  |  |  |  |  |  |  |  |  |  | 1 | 0 | 0 | 0 | 1 | 0 | 0 | 0 | 0 | 0 | 0 | 0 | 0 | 0 | 0 | 16 | 33 | Carbamidomethylation | C11:Carbamidomethylation:1000.00;C13:Carbamidomethylation:1000.00 | PEAKS DB |
| R.C(+57.02)C(+57.02)FVHDC(+57.02)C(+57.02)YGNLPDC(+57.02)NPK.S | N | 73.65 | 2314.8687 | 18 | 0.7 | 772.6307 | 3 | 32.34 | 4 | F4:16838 | NaSa01\_F12.raw |  |  |  | 2.3387E7 |  | 7.5227E6 |  |  |  |  |  |  |  |  | 2 | 0 | 0 | 0 | 1 | 0 | 1 | 0 | 0 | 0 | 0 | 0 | 0 | 0 | 0 | 43 | 60 | Carbamidomethylation | C1:Carbamidomethylation:1000.00;C2:Carbamidomethylation:1000.00;C7:Carbamidomethylation:1000.00;C8:Carbamidomethylation:1000.00;C15:Carbamidomethylation:1000.00 | PEAKS DB |
| K.YMLYPDFLC(+57.02)K.G | N | 68.32 | 1348.6195 | 10 | 0.2 | 675.3171 | 2 | 75.10 | 4 | F4:52326 | NaSa01\_F12.raw |  |  |  | 2.1669E7 |  |  |  |  |  |  |  |  |  |  | 1 | 0 | 0 | 0 | 1 | 0 | 0 | 0 | 0 | 0 | 0 | 0 | 0 | 0 | 0 | 107 | 116 | Carbamidomethylation | C9:Carbamidomethylation:1000.00 | PEAKS DB |
| K.YM(+15.99)LYPDFLC(+57.02)K.G | N | 64.41 | 1364.6145 | 10 | 0.0 | 683.3145 | 2 | 68.02 | 4 | F4:46357 | NaSa01\_F12.raw |  |  |  | 2.115E6 |  |  |  |  |  |  |  |  |  |  | 1 | 0 | 0 | 0 | 1 | 0 | 0 | 0 | 0 | 0 | 0 | 0 | 0 | 0 | 0 | 107 | 116 | Oxidation (M); Carbamidomethylation | M2:Oxidation (M):1000.00;C9:Carbamidomethylation:1000.00 | PEAKS DB |
| K.KYMLYPDFLC(+57.02)K.G | Y | 60.31 | 1476.7145 | 11 | 0.3 | 493.2456 | 3 | 58.25 | 4 | F4:38516 | NaSa01\_F12.raw |  |  |  | 5.4533E6 |  |  |  |  |  |  |  |  |  |  | 2 | 0 | 0 | 0 | 2 | 0 | 0 | 0 | 0 | 0 | 0 | 0 | 0 | 0 | 0 | 106 | 116 | Carbamidomethylation | C10:Carbamidomethylation:1000.00 | PEAKS DB |
| S.YSSYGC(+57.02)YC(+57.02)GWGGK.G | N | 58.28 | 1543.5861 | 13 | 0.3 | 772.8005 | 2 | 35.31 | 6 | F6:16919 | NaSa01\_F14.raw |  |  |  |  |  | 5.8277E6 |  |  |  |  |  |  |  |  | 1 | 0 | 0 | 0 | 0 | 0 | 1 | 0 | 0 | 0 | 0 | 0 | 0 | 0 | 0 | 21 | 33 | Carbamidomethylation | C6:Carbamidomethylation:1000.00;C8:Carbamidomethylation:1000.00 | PEAKS DB |
| K.KYM(+15.99)LYPDFLC(+57.02)K.G | Y | 43.40 | 1492.7095 | 11 | -0.3 | 498.5770 | 3 | 51.59 | 4 | F4:32744 | NaSa01\_F12.raw |  |  |  | 7.4997E5 |  |  |  |  |  |  |  |  |  |  | 1 | 0 | 0 | 0 | 1 | 0 | 0 | 0 | 0 | 0 | 0 | 0 | 0 | 0 | 0 | 106 | 116 | Oxidation (M); Carbamidomethylation | M3:Oxidation (M):1000.00;C10:Carbamidomethylation:1000.00 | PEAKS DB |
| SLLEFGK.M | Y | 38.22 | 792.4381 | 7 | 0.1 | 397.2264 | 2 | 39.94 | 4 | F4:23423 | NaSa01\_F12.raw |  |  |  | 8.4704E6 |  |  |  |  |  |  |  |  |  |  | 1 | 0 | 0 | 0 | 1 | 0 | 0 | 0 | 0 | 0 | 0 | 0 | 0 | 0 | 0 | 1 | 7 |  |  | PEAKS DB |
| total 8 peptides |  |  |  |  |  |  |  |  |  |  |  |  |  |  |  |  |  |  |  |  |  |  |  |  |  |  |  |  |  |  |  |  |  |  |  |  |  |  |  |  |  |  |  |  |  |
| --- | --- | --- | --- | --- | --- | --- | --- | --- | --- | --- | --- | --- | --- | --- | --- | --- | --- | --- | --- | --- | --- | --- | --- | --- | --- | --- | --- | --- | --- | --- | --- | --- | --- | --- | --- | --- | --- | --- | --- | --- | --- | --- | --- | --- | --- |

1SQZ

back to list

  

| Protein Coverage
| Supporting Peptides
|

Protein Coverage:

Supporting Peptides:

| Peptide | Uniq | -10lgP | Mass | Length | ppm | m/z | z | RT | Fraction | Scan | Source File | Area F1 | Area F10 | Area F11 | Area F12 | Area F13 | Area F14 | Area F2 | Area F3 | Area F4 | Area F5 | Area F6 | Area F7 | Area F8 | Area F9 | #Feature | #Feature F1 | #Feature F10 | #Feature F11 | #Feature F12 | #Feature F13 | #Feature F14 | #Feature F2 | #Feature F3 | #Feature F4 | #Feature F5 | #Feature F6 | #Feature F7 | #Feature F8 | #Feature F9 | Start | End | PTM | AScore | Found By |
| --- | --- | --- | --- | --- | --- | --- | --- | --- | --- | --- | --- | --- | --- | --- | --- | --- | --- | --- | --- | --- | --- | --- | --- | --- | --- | --- | --- | --- | --- | --- | --- | --- | --- | --- | --- | --- | --- | --- | --- | --- | --- | --- | --- | --- | --- |
| K.LAIPSYSSYGC(+57.02)YC(+57.02)GWGGK.G | Y | 83.35 | 2024.8760 | 18 | 1.3 | 1013.4466 | 2 | 65.73 | 4 | F4:44422 | NaSa01\_F12.raw |  |  |  | 1.8132E7 |  |  |  |  |  |  |  |  |  |  | 1 | 0 | 0 | 0 | 1 | 0 | 0 | 0 | 0 | 0 | 0 | 0 | 0 | 0 | 0 | 16 | 33 | Carbamidomethylation | C11:Carbamidomethylation:1000.00;C13:Carbamidomethylation:1000.00 | PEAKS DB |
| R.C(+57.02)C(+57.02)FVHDC(+57.02)C(+57.02)YGNLPDC(+57.02)NPK.S | N | 73.65 | 2314.8687 | 18 | 0.7 | 772.6307 | 3 | 32.34 | 4 | F4:16838 | NaSa01\_F12.raw |  |  |  | 2.3387E7 |  | 7.5227E6 |  |  |  |  |  |  |  |  | 2 | 0 | 0 | 0 | 1 | 0 | 1 | 0 | 0 | 0 | 0 | 0 | 0 | 0 | 0 | 43 | 60 | Carbamidomethylation | C1:Carbamidomethylation:1000.00;C2:Carbamidomethylation:1000.00;C7:Carbamidomethylation:1000.00;C8:Carbamidomethylation:1000.00;C15:Carbamidomethylation:1000.00 | PEAKS DB |
| K.YMLYPDFLC(+57.02)K.G | N | 68.32 | 1348.6195 | 10 | 0.2 | 675.3171 | 2 | 75.10 | 4 | F4:52326 | NaSa01\_F12.raw |  |  |  | 2.1669E7 |  |  |  |  |  |  |  |  |  |  | 1 | 0 | 0 | 0 | 1 | 0 | 0 | 0 | 0 | 0 | 0 | 0 | 0 | 0 | 0 | 107 | 116 | Carbamidomethylation | C9:Carbamidomethylation:1000.00 | PEAKS DB |
| K.YM(+15.99)LYPDFLC(+57.02)K.G | N | 64.41 | 1364.6145 | 10 | 0.0 | 683.3145 | 2 | 68.02 | 4 | F4:46357 | NaSa01\_F12.raw |  |  |  | 2.115E6 |  |  |  |  |  |  |  |  |  |  | 1 | 0 | 0 | 0 | 1 | 0 | 0 | 0 | 0 | 0 | 0 | 0 | 0 | 0 | 0 | 107 | 116 | Oxidation (M); Carbamidomethylation | M2:Oxidation (M):1000.00;C9:Carbamidomethylation:1000.00 | PEAKS DB |
| K.KYMLYPDFLC(+57.02)K.G | Y | 60.31 | 1476.7145 | 11 | 0.3 | 493.2456 | 3 | 58.25 | 4 | F4:38516 | NaSa01\_F12.raw |  |  |  | 5.4533E6 |  |  |  |  |  |  |  |  |  |  | 2 | 0 | 0 | 0 | 2 | 0 | 0 | 0 | 0 | 0 | 0 | 0 | 0 | 0 | 0 | 106 | 116 | Carbamidomethylation | C10:Carbamidomethylation:1000.00 | PEAKS DB |
| S.YSSYGC(+57.02)YC(+57.02)GWGGK.G | N | 58.28 | 1543.5861 | 13 | 0.3 | 772.8005 | 2 | 35.31 | 6 | F6:16919 | NaSa01\_F14.raw |  |  |  |  |  | 5.8277E6 |  |  |  |  |  |  |  |  | 1 | 0 | 0 | 0 | 0 | 0 | 1 | 0 | 0 | 0 | 0 | 0 | 0 | 0 | 0 | 21 | 33 | Carbamidomethylation | C6:Carbamidomethylation:1000.00;C8:Carbamidomethylation:1000.00 | PEAKS DB |
| K.KYM(+15.99)LYPDFLC(+57.02)K.G | Y | 43.40 | 1492.7095 | 11 | -0.3 | 498.5770 | 3 | 51.59 | 4 | F4:32744 | NaSa01\_F12.raw |  |  |  | 7.4997E5 |  |  |  |  |  |  |  |  |  |  | 1 | 0 | 0 | 0 | 1 | 0 | 0 | 0 | 0 | 0 | 0 | 0 | 0 | 0 | 0 | 106 | 116 | Oxidation (M); Carbamidomethylation | M3:Oxidation (M):1000.00;C10:Carbamidomethylation:1000.00 | PEAKS DB |
| SLLEFGK.M | Y | 38.22 | 792.4381 | 7 | 0.1 | 397.2264 | 2 | 39.94 | 4 | F4:23423 | NaSa01\_F12.raw |  |  |  | 8.4704E6 |  |  |  |  |  |  |  |  |  |  | 1 | 0 | 0 | 0 | 1 | 0 | 0 | 0 | 0 | 0 | 0 | 0 | 0 | 0 | 0 | 1 | 7 |  |  | PEAKS DB |
| total 8 peptides |  |  |  |  |  |  |  |  |  |  |  |  |  |  |  |  |  |  |  |  |  |  |  |  |  |  |  |  |  |  |  |  |  |  |  |  |  |  |  |  |  |  |  |  |  |
| --- | --- | --- | --- | --- | --- | --- | --- | --- | --- | --- | --- | --- | --- | --- | --- | --- | --- | --- | --- | --- | --- | --- | --- | --- | --- | --- | --- | --- | --- | --- | --- | --- | --- | --- | --- | --- | --- | --- | --- | --- | --- | --- | --- | --- | --- |

3FG5

back to list

  

| Protein Coverage
| Supporting Peptides
|

Protein Coverage:

Supporting Peptides:

| Peptide | Uniq | -10lgP | Mass | Length | ppm | m/z | z | RT | Fraction | Scan | Source File | Area F1 | Area F10 | Area F11 | Area F12 | Area F13 | Area F14 | Area F2 | Area F3 | Area F4 | Area F5 | Area F6 | Area F7 | Area F8 | Area F9 | #Feature | #Feature F1 | #Feature F10 | #Feature F11 | #Feature F12 | #Feature F13 | #Feature F14 | #Feature F2 | #Feature F3 | #Feature F4 | #Feature F5 | #Feature F6 | #Feature F7 | #Feature F8 | #Feature F9 | Start | End | PTM | AScore | Found By |
| --- | --- | --- | --- | --- | --- | --- | --- | --- | --- | --- | --- | --- | --- | --- | --- | --- | --- | --- | --- | --- | --- | --- | --- | --- | --- | --- | --- | --- | --- | --- | --- | --- | --- | --- | --- | --- | --- | --- | --- | --- | --- | --- | --- | --- | --- |
| K.LAIPSYSSYGC(+57.02)YC(+57.02)GWGGK.G | Y | 83.35 | 2024.8760 | 18 | 1.3 | 1013.4466 | 2 | 65.73 | 4 | F4:44422 | NaSa01\_F12.raw |  |  |  | 1.8132E7 |  |  |  |  |  |  |  |  |  |  | 1 | 0 | 0 | 0 | 1 | 0 | 0 | 0 | 0 | 0 | 0 | 0 | 0 | 0 | 0 | 16 | 33 | Carbamidomethylation | C11:Carbamidomethylation:1000.00;C13:Carbamidomethylation:1000.00 | PEAKS DB |
| R.C(+57.02)C(+57.02)FVHDC(+57.02)C(+57.02)YGNLPDC(+57.02)NPK.S | N | 73.65 | 2314.8687 | 18 | 0.7 | 772.6307 | 3 | 32.34 | 4 | F4:16838 | NaSa01\_F12.raw |  |  |  | 2.3387E7 |  | 7.5227E6 |  |  |  |  |  |  |  |  | 2 | 0 | 0 | 0 | 1 | 0 | 1 | 0 | 0 | 0 | 0 | 0 | 0 | 0 | 0 | 43 | 60 | Carbamidomethylation | C1:Carbamidomethylation:1000.00;C2:Carbamidomethylation:1000.00;C7:Carbamidomethylation:1000.00;C8:Carbamidomethylation:1000.00;C15:Carbamidomethylation:1000.00 | PEAKS DB |
| K.YMLYPDFLC(+57.02)K.G | N | 68.32 | 1348.6195 | 10 | 0.2 | 675.3171 | 2 | 75.10 | 4 | F4:52326 | NaSa01\_F12.raw |  |  |  | 2.1669E7 |  |  |  |  |  |  |  |  |  |  | 1 | 0 | 0 | 0 | 1 | 0 | 0 | 0 | 0 | 0 | 0 | 0 | 0 | 0 | 0 | 107 | 116 | Carbamidomethylation | C9:Carbamidomethylation:1000.00 | PEAKS DB |
| K.YM(+15.99)LYPDFLC(+57.02)K.G | N | 64.41 | 1364.6145 | 10 | 0.0 | 683.3145 | 2 | 68.02 | 4 | F4:46357 | NaSa01\_F12.raw |  |  |  | 2.115E6 |  |  |  |  |  |  |  |  |  |  | 1 | 0 | 0 | 0 | 1 | 0 | 0 | 0 | 0 | 0 | 0 | 0 | 0 | 0 | 0 | 107 | 116 | Oxidation (M); Carbamidomethylation | M2:Oxidation (M):1000.00;C9:Carbamidomethylation:1000.00 | PEAKS DB |
| K.KYMLYPDFLC(+57.02)K.G | Y | 60.31 | 1476.7145 | 11 | 0.3 | 493.2456 | 3 | 58.25 | 4 | F4:38516 | NaSa01\_F12.raw |  |  |  | 5.4533E6 |  |  |  |  |  |  |  |  |  |  | 2 | 0 | 0 | 0 | 2 | 0 | 0 | 0 | 0 | 0 | 0 | 0 | 0 | 0 | 0 | 106 | 116 | Carbamidomethylation | C10:Carbamidomethylation:1000.00 | PEAKS DB |
| S.YSSYGC(+57.02)YC(+57.02)GWGGK.G | N | 58.28 | 1543.5861 | 13 | 0.3 | 772.8005 | 2 | 35.31 | 6 | F6:16919 | NaSa01\_F14.raw |  |  |  |  |  | 5.8277E6 |  |  |  |  |  |  |  |  | 1 | 0 | 0 | 0 | 0 | 0 | 1 | 0 | 0 | 0 | 0 | 0 | 0 | 0 | 0 | 21 | 33 | Carbamidomethylation | C6:Carbamidomethylation:1000.00;C8:Carbamidomethylation:1000.00 | PEAKS DB |
| K.KYM(+15.99)LYPDFLC(+57.02)K.G | Y | 43.40 | 1492.7095 | 11 | -0.3 | 498.5770 | 3 | 51.59 | 4 | F4:32744 | NaSa01\_F12.raw |  |  |  | 7.4997E5 |  |  |  |  |  |  |  |  |  |  | 1 | 0 | 0 | 0 | 1 | 0 | 0 | 0 | 0 | 0 | 0 | 0 | 0 | 0 | 0 | 106 | 116 | Oxidation (M); Carbamidomethylation | M3:Oxidation (M):1000.00;C10:Carbamidomethylation:1000.00 | PEAKS DB |
| SLLEFGK.M | Y | 38.22 | 792.4381 | 7 | 0.1 | 397.2264 | 2 | 39.94 | 4 | F4:23423 | NaSa01\_F12.raw |  |  |  | 8.4704E6 |  |  |  |  |  |  |  |  |  |  | 1 | 0 | 0 | 0 | 1 | 0 | 0 | 0 | 0 | 0 | 0 | 0 | 0 | 0 | 0 | 1 | 7 |  |  | PEAKS DB |
| total 8 peptides |  |  |  |  |  |  |  |  |  |  |  |  |  |  |  |  |  |  |  |  |  |  |  |  |  |  |  |  |  |  |  |  |  |  |  |  |  |  |  |  |  |  |  |  |  |
| --- | --- | --- | --- | --- | --- | --- | --- | --- | --- | --- | --- | --- | --- | --- | --- | --- | --- | --- | --- | --- | --- | --- | --- | --- | --- | --- | --- | --- | --- | --- | --- | --- | --- | --- | --- | --- | --- | --- | --- | --- | --- | --- | --- | --- | --- |

1TP2

back to list

  

| Protein Coverage
| Supporting Peptides
|

Protein Coverage:

Supporting Peptides:

| Peptide | Uniq | -10lgP | Mass | Length | ppm | m/z | z | RT | Fraction | Scan | Source File | Area F1 | Area F10 | Area F11 | Area F12 | Area F13 | Area F14 | Area F2 | Area F3 | Area F4 | Area F5 | Area F6 | Area F7 | Area F8 | Area F9 | #Feature | #Feature F1 | #Feature F10 | #Feature F11 | #Feature F12 | #Feature F13 | #Feature F14 | #Feature F2 | #Feature F3 | #Feature F4 | #Feature F5 | #Feature F6 | #Feature F7 | #Feature F8 | #Feature F9 | Start | End | PTM | AScore | Found By |
| --- | --- | --- | --- | --- | --- | --- | --- | --- | --- | --- | --- | --- | --- | --- | --- | --- | --- | --- | --- | --- | --- | --- | --- | --- | --- | --- | --- | --- | --- | --- | --- | --- | --- | --- | --- | --- | --- | --- | --- | --- | --- | --- | --- | --- | --- |
| K.LAIPSYSSYGC(+57.02)YC(+57.02)GWGGK.G | Y | 83.35 | 2024.8760 | 18 | 1.3 | 1013.4466 | 2 | 65.73 | 4 | F4:44422 | NaSa01\_F12.raw |  |  |  | 1.8132E7 |  |  |  |  |  |  |  |  |  |  | 1 | 0 | 0 | 0 | 1 | 0 | 0 | 0 | 0 | 0 | 0 | 0 | 0 | 0 | 0 | 16 | 33 | Carbamidomethylation | C11:Carbamidomethylation:1000.00;C13:Carbamidomethylation:1000.00 | PEAKS DB |
| R.C(+57.02)C(+57.02)FVHDC(+57.02)C(+57.02)YGNLPDC(+57.02)NPK.S | N | 73.65 | 2314.8687 | 18 | 0.7 | 772.6307 | 3 | 32.34 | 4 | F4:16838 | NaSa01\_F12.raw |  |  |  | 2.3387E7 |  | 7.5227E6 |  |  |  |  |  |  |  |  | 2 | 0 | 0 | 0 | 1 | 0 | 1 | 0 | 0 | 0 | 0 | 0 | 0 | 0 | 0 | 43 | 60 | Carbamidomethylation | C1:Carbamidomethylation:1000.00;C2:Carbamidomethylation:1000.00;C7:Carbamidomethylation:1000.00;C8:Carbamidomethylation:1000.00;C15:Carbamidomethylation:1000.00 | PEAKS DB |
| K.YMLYPDFLC(+57.02)K.G | N | 68.32 | 1348.6195 | 10 | 0.2 | 675.3171 | 2 | 75.10 | 4 | F4:52326 | NaSa01\_F12.raw |  |  |  | 2.1669E7 |  |  |  |  |  |  |  |  |  |  | 1 | 0 | 0 | 0 | 1 | 0 | 0 | 0 | 0 | 0 | 0 | 0 | 0 | 0 | 0 | 107 | 116 | Carbamidomethylation | C9:Carbamidomethylation:1000.00 | PEAKS DB |
| K.YM(+15.99)LYPDFLC(+57.02)K.G | N | 64.41 | 1364.6145 | 10 | 0.0 | 683.3145 | 2 | 68.02 | 4 | F4:46357 | NaSa01\_F12.raw |  |  |  | 2.115E6 |  |  |  |  |  |  |  |  |  |  | 1 | 0 | 0 | 0 | 1 | 0 | 0 | 0 | 0 | 0 | 0 | 0 | 0 | 0 | 0 | 107 | 116 | Oxidation (M); Carbamidomethylation | M2:Oxidation (M):1000.00;C9:Carbamidomethylation:1000.00 | PEAKS DB |
| K.KYMLYPDFLC(+57.02)K.G | Y | 60.31 | 1476.7145 | 11 | 0.3 | 493.2456 | 3 | 58.25 | 4 | F4:38516 | NaSa01\_F12.raw |  |  |  | 5.4533E6 |  |  |  |  |  |  |  |  |  |  | 2 | 0 | 0 | 0 | 2 | 0 | 0 | 0 | 0 | 0 | 0 | 0 | 0 | 0 | 0 | 106 | 116 | Carbamidomethylation | C10:Carbamidomethylation:1000.00 | PEAKS DB |
| S.YSSYGC(+57.02)YC(+57.02)GWGGK.G | N | 58.28 | 1543.5861 | 13 | 0.3 | 772.8005 | 2 | 35.31 | 6 | F6:16919 | NaSa01\_F14.raw |  |  |  |  |  | 5.8277E6 |  |  |  |  |  |  |  |  | 1 | 0 | 0 | 0 | 0 | 0 | 1 | 0 | 0 | 0 | 0 | 0 | 0 | 0 | 0 | 21 | 33 | Carbamidomethylation | C6:Carbamidomethylation:1000.00;C8:Carbamidomethylation:1000.00 | PEAKS DB |
| K.KYM(+15.99)LYPDFLC(+57.02)K.G | Y | 43.40 | 1492.7095 | 11 | -0.3 | 498.5770 | 3 | 51.59 | 4 | F4:32744 | NaSa01\_F12.raw |  |  |  | 7.4997E5 |  |  |  |  |  |  |  |  |  |  | 1 | 0 | 0 | 0 | 1 | 0 | 0 | 0 | 0 | 0 | 0 | 0 | 0 | 0 | 0 | 106 | 116 | Oxidation (M); Carbamidomethylation | M3:Oxidation (M):1000.00;C10:Carbamidomethylation:1000.00 | PEAKS DB |
| SLLEFGK.M | Y | 38.22 | 792.4381 | 7 | 0.1 | 397.2264 | 2 | 39.94 | 4 | F4:23423 | NaSa01\_F12.raw |  |  |  | 8.4704E6 |  |  |  |  |  |  |  |  |  |  | 1 | 0 | 0 | 0 | 1 | 0 | 0 | 0 | 0 | 0 | 0 | 0 | 0 | 0 | 0 | 1 | 7 |  |  | PEAKS DB |
| total 8 peptides |  |  |  |  |  |  |  |  |  |  |  |  |  |  |  |  |  |  |  |  |  |  |  |  |  |  |  |  |  |  |  |  |  |  |  |  |  |  |  |  |  |  |  |  |  |
| --- | --- | --- | --- | --- | --- | --- | --- | --- | --- | --- | --- | --- | --- | --- | --- | --- | --- | --- | --- | --- | --- | --- | --- | --- | --- | --- | --- | --- | --- | --- | --- | --- | --- | --- | --- | --- | --- | --- | --- | --- | --- | --- | --- | --- | --- |

1Q7A

back to list

  

| Protein Coverage
| Supporting Peptides
|

Protein Coverage:

Supporting Peptides:

| Peptide | Uniq | -10lgP | Mass | Length | ppm | m/z | z | RT | Fraction | Scan | Source File | Area F1 | Area F10 | Area F11 | Area F12 | Area F13 | Area F14 | Area F2 | Area F3 | Area F4 | Area F5 | Area F6 | Area F7 | Area F8 | Area F9 | #Feature | #Feature F1 | #Feature F10 | #Feature F11 | #Feature F12 | #Feature F13 | #Feature F14 | #Feature F2 | #Feature F3 | #Feature F4 | #Feature F5 | #Feature F6 | #Feature F7 | #Feature F8 | #Feature F9 | Start | End | PTM | AScore | Found By |
| --- | --- | --- | --- | --- | --- | --- | --- | --- | --- | --- | --- | --- | --- | --- | --- | --- | --- | --- | --- | --- | --- | --- | --- | --- | --- | --- | --- | --- | --- | --- | --- | --- | --- | --- | --- | --- | --- | --- | --- | --- | --- | --- | --- | --- | --- |
| K.LAIPSYSSYGC(+57.02)YC(+57.02)GWGGK.G | Y | 83.35 | 2024.8760 | 18 | 1.3 | 1013.4466 | 2 | 65.73 | 4 | F4:44422 | NaSa01\_F12.raw |  |  |  | 1.8132E7 |  |  |  |  |  |  |  |  |  |  | 1 | 0 | 0 | 0 | 1 | 0 | 0 | 0 | 0 | 0 | 0 | 0 | 0 | 0 | 0 | 16 | 33 | Carbamidomethylation | C11:Carbamidomethylation:1000.00;C13:Carbamidomethylation:1000.00 | PEAKS DB |
| R.C(+57.02)C(+57.02)FVHDC(+57.02)C(+57.02)YGNLPDC(+57.02)NPK.S | N | 73.65 | 2314.8687 | 18 | 0.7 | 772.6307 | 3 | 32.34 | 4 | F4:16838 | NaSa01\_F12.raw |  |  |  | 2.3387E7 |  | 7.5227E6 |  |  |  |  |  |  |  |  | 2 | 0 | 0 | 0 | 1 | 0 | 1 | 0 | 0 | 0 | 0 | 0 | 0 | 0 | 0 | 43 | 60 | Carbamidomethylation | C1:Carbamidomethylation:1000.00;C2:Carbamidomethylation:1000.00;C7:Carbamidomethylation:1000.00;C8:Carbamidomethylation:1000.00;C15:Carbamidomethylation:1000.00 | PEAKS DB |
| K.YMLYPDFLC(+57.02)K.G | N | 68.32 | 1348.6195 | 10 | 0.2 | 675.3171 | 2 | 75.10 | 4 | F4:52326 | NaSa01\_F12.raw |  |  |  | 2.1669E7 |  |  |  |  |  |  |  |  |  |  | 1 | 0 | 0 | 0 | 1 | 0 | 0 | 0 | 0 | 0 | 0 | 0 | 0 | 0 | 0 | 107 | 116 | Carbamidomethylation | C9:Carbamidomethylation:1000.00 | PEAKS DB |
| K.YM(+15.99)LYPDFLC(+57.02)K.G | N | 64.41 | 1364.6145 | 10 | 0.0 | 683.3145 | 2 | 68.02 | 4 | F4:46357 | NaSa01\_F12.raw |  |  |  | 2.115E6 |  |  |  |  |  |  |  |  |  |  | 1 | 0 | 0 | 0 | 1 | 0 | 0 | 0 | 0 | 0 | 0 | 0 | 0 | 0 | 0 | 107 | 116 | Oxidation (M); Carbamidomethylation | M2:Oxidation (M):1000.00;C9:Carbamidomethylation:1000.00 | PEAKS DB |
| K.KYMLYPDFLC(+57.02)K.G | Y | 60.31 | 1476.7145 | 11 | 0.3 | 493.2456 | 3 | 58.25 | 4 | F4:38516 | NaSa01\_F12.raw |  |  |  | 5.4533E6 |  |  |  |  |  |  |  |  |  |  | 2 | 0 | 0 | 0 | 2 | 0 | 0 | 0 | 0 | 0 | 0 | 0 | 0 | 0 | 0 | 106 | 116 | Carbamidomethylation | C10:Carbamidomethylation:1000.00 | PEAKS DB |
| S.YSSYGC(+57.02)YC(+57.02)GWGGK.G | N | 58.28 | 1543.5861 | 13 | 0.3 | 772.8005 | 2 | 35.31 | 6 | F6:16919 | NaSa01\_F14.raw |  |  |  |  |  | 5.8277E6 |  |  |  |  |  |  |  |  | 1 | 0 | 0 | 0 | 0 | 0 | 1 | 0 | 0 | 0 | 0 | 0 | 0 | 0 | 0 | 21 | 33 | Carbamidomethylation | C6:Carbamidomethylation:1000.00;C8:Carbamidomethylation:1000.00 | PEAKS DB |
| K.KYM(+15.99)LYPDFLC(+57.02)K.G | Y | 43.40 | 1492.7095 | 11 | -0.3 | 498.5770 | 3 | 51.59 | 4 | F4:32744 | NaSa01\_F12.raw |  |  |  | 7.4997E5 |  |  |  |  |  |  |  |  |  |  | 1 | 0 | 0 | 0 | 1 | 0 | 0 | 0 | 0 | 0 | 0 | 0 | 0 | 0 | 0 | 106 | 116 | Oxidation (M); Carbamidomethylation | M3:Oxidation (M):1000.00;C10:Carbamidomethylation:1000.00 | PEAKS DB |
| SLLEFGK.M | Y | 38.22 | 792.4381 | 7 | 0.1 | 397.2264 | 2 | 39.94 | 4 | F4:23423 | NaSa01\_F12.raw |  |  |  | 8.4704E6 |  |  |  |  |  |  |  |  |  |  | 1 | 0 | 0 | 0 | 1 | 0 | 0 | 0 | 0 | 0 | 0 | 0 | 0 | 0 | 0 | 1 | 7 |  |  | PEAKS DB |
| total 8 peptides |  |  |  |  |  |  |  |  |  |  |  |  |  |  |  |  |  |  |  |  |  |  |  |  |  |  |  |  |  |  |  |  |  |  |  |  |  |  |  |  |  |  |  |  |  |
| --- | --- | --- | --- | --- | --- | --- | --- | --- | --- | --- | --- | --- | --- | --- | --- | --- | --- | --- | --- | --- | --- | --- | --- | --- | --- | --- | --- | --- | --- | --- | --- | --- | --- | --- | --- | --- | --- | --- | --- | --- | --- | --- | --- | --- | --- |

1TH6

back to list

  

| Protein Coverage
| Supporting Peptides
|

Protein Coverage:

Supporting Peptides:

| Peptide | Uniq | -10lgP | Mass | Length | ppm | m/z | z | RT | Fraction | Scan | Source File | Area F1 | Area F10 | Area F11 | Area F12 | Area F13 | Area F14 | Area F2 | Area F3 | Area F4 | Area F5 | Area F6 | Area F7 | Area F8 | Area F9 | #Feature | #Feature F1 | #Feature F10 | #Feature F11 | #Feature F12 | #Feature F13 | #Feature F14 | #Feature F2 | #Feature F3 | #Feature F4 | #Feature F5 | #Feature F6 | #Feature F7 | #Feature F8 | #Feature F9 | Start | End | PTM | AScore | Found By |
| --- | --- | --- | --- | --- | --- | --- | --- | --- | --- | --- | --- | --- | --- | --- | --- | --- | --- | --- | --- | --- | --- | --- | --- | --- | --- | --- | --- | --- | --- | --- | --- | --- | --- | --- | --- | --- | --- | --- | --- | --- | --- | --- | --- | --- | --- |
| K.LAIPSYSSYGC(+57.02)YC(+57.02)GWGGK.G | Y | 83.35 | 2024.8760 | 18 | 1.3 | 1013.4466 | 2 | 65.73 | 4 | F4:44422 | NaSa01\_F12.raw |  |  |  | 1.8132E7 |  |  |  |  |  |  |  |  |  |  | 1 | 0 | 0 | 0 | 1 | 0 | 0 | 0 | 0 | 0 | 0 | 0 | 0 | 0 | 0 | 16 | 33 | Carbamidomethylation | C11:Carbamidomethylation:1000.00;C13:Carbamidomethylation:1000.00 | PEAKS DB |
| R.C(+57.02)C(+57.02)FVHDC(+57.02)C(+57.02)YGNLPDC(+57.02)NPK.S | N | 73.65 | 2314.8687 | 18 | 0.7 | 772.6307 | 3 | 32.34 | 4 | F4:16838 | NaSa01\_F12.raw |  |  |  | 2.3387E7 |  | 7.5227E6 |  |  |  |  |  |  |  |  | 2 | 0 | 0 | 0 | 1 | 0 | 1 | 0 | 0 | 0 | 0 | 0 | 0 | 0 | 0 | 43 | 60 | Carbamidomethylation | C1:Carbamidomethylation:1000.00;C2:Carbamidomethylation:1000.00;C7:Carbamidomethylation:1000.00;C8:Carbamidomethylation:1000.00;C15:Carbamidomethylation:1000.00 | PEAKS DB |
| K.YMLYPDFLC(+57.02)K.G | N | 68.32 | 1348.6195 | 10 | 0.2 | 675.3171 | 2 | 75.10 | 4 | F4:52326 | NaSa01\_F12.raw |  |  |  | 2.1669E7 |  |  |  |  |  |  |  |  |  |  | 1 | 0 | 0 | 0 | 1 | 0 | 0 | 0 | 0 | 0 | 0 | 0 | 0 | 0 | 0 | 107 | 116 | Carbamidomethylation | C9:Carbamidomethylation:1000.00 | PEAKS DB |
| K.YM(+15.99)LYPDFLC(+57.02)K.G | N | 64.41 | 1364.6145 | 10 | 0.0 | 683.3145 | 2 | 68.02 | 4 | F4:46357 | NaSa01\_F12.raw |  |  |  | 2.115E6 |  |  |  |  |  |  |  |  |  |  | 1 | 0 | 0 | 0 | 1 | 0 | 0 | 0 | 0 | 0 | 0 | 0 | 0 | 0 | 0 | 107 | 116 | Oxidation (M); Carbamidomethylation | M2:Oxidation (M):1000.00;C9:Carbamidomethylation:1000.00 | PEAKS DB |
| K.KYMLYPDFLC(+57.02)K.G | Y | 60.31 | 1476.7145 | 11 | 0.3 | 493.2456 | 3 | 58.25 | 4 | F4:38516 | NaSa01\_F12.raw |  |  |  | 5.4533E6 |  |  |  |  |  |  |  |  |  |  | 2 | 0 | 0 | 0 | 2 | 0 | 0 | 0 | 0 | 0 | 0 | 0 | 0 | 0 | 0 | 106 | 116 | Carbamidomethylation | C10:Carbamidomethylation:1000.00 | PEAKS DB |
| S.YSSYGC(+57.02)YC(+57.02)GWGGK.G | N | 58.28 | 1543.5861 | 13 | 0.3 | 772.8005 | 2 | 35.31 | 6 | F6:16919 | NaSa01\_F14.raw |  |  |  |  |  | 5.8277E6 |  |  |  |  |  |  |  |  | 1 | 0 | 0 | 0 | 0 | 0 | 1 | 0 | 0 | 0 | 0 | 0 | 0 | 0 | 0 | 21 | 33 | Carbamidomethylation | C6:Carbamidomethylation:1000.00;C8:Carbamidomethylation:1000.00 | PEAKS DB |
| K.KYM(+15.99)LYPDFLC(+57.02)K.G | Y | 43.40 | 1492.7095 | 11 | -0.3 | 498.5770 | 3 | 51.59 | 4 | F4:32744 | NaSa01\_F12.raw |  |  |  | 7.4997E5 |  |  |  |  |  |  |  |  |  |  | 1 | 0 | 0 | 0 | 1 | 0 | 0 | 0 | 0 | 0 | 0 | 0 | 0 | 0 | 0 | 106 | 116 | Oxidation (M); Carbamidomethylation | M3:Oxidation (M):1000.00;C10:Carbamidomethylation:1000.00 | PEAKS DB |
| SLLEFGK.M | Y | 38.22 | 792.4381 | 7 | 0.1 | 397.2264 | 2 | 39.94 | 4 | F4:23423 | NaSa01\_F12.raw |  |  |  | 8.4704E6 |  |  |  |  |  |  |  |  |  |  | 1 | 0 | 0 | 0 | 1 | 0 | 0 | 0 | 0 | 0 | 0 | 0 | 0 | 0 | 0 | 1 | 7 |  |  | PEAKS DB |
| total 8 peptides |  |  |  |  |  |  |  |  |  |  |  |  |  |  |  |  |  |  |  |  |  |  |  |  |  |  |  |  |  |  |  |  |  |  |  |  |  |  |  |  |  |  |  |  |  |
| --- | --- | --- | --- | --- | --- | --- | --- | --- | --- | --- | --- | --- | --- | --- | --- | --- | --- | --- | --- | --- | --- | --- | --- | --- | --- | --- | --- | --- | --- | --- | --- | --- | --- | --- | --- | --- | --- | --- | --- | --- | --- | --- | --- | --- | --- |

4HMB

back to list

  

| Protein Coverage
| Supporting Peptides
|

Protein Coverage:

Supporting Peptides:

| Peptide | Uniq | -10lgP | Mass | Length | ppm | m/z | z | RT | Fraction | Scan | Source File | Area F1 | Area F10 | Area F11 | Area F12 | Area F13 | Area F14 | Area F2 | Area F3 | Area F4 | Area F5 | Area F6 | Area F7 | Area F8 | Area F9 | #Feature | #Feature F1 | #Feature F10 | #Feature F11 | #Feature F12 | #Feature F13 | #Feature F14 | #Feature F2 | #Feature F3 | #Feature F4 | #Feature F5 | #Feature F6 | #Feature F7 | #Feature F8 | #Feature F9 | Start | End | PTM | AScore | Found By |
| --- | --- | --- | --- | --- | --- | --- | --- | --- | --- | --- | --- | --- | --- | --- | --- | --- | --- | --- | --- | --- | --- | --- | --- | --- | --- | --- | --- | --- | --- | --- | --- | --- | --- | --- | --- | --- | --- | --- | --- | --- | --- | --- | --- | --- | --- |
| K.LAIPSYSSYGC(+57.02)YC(+57.02)GWGGK.G | Y | 83.35 | 2024.8760 | 18 | 1.3 | 1013.4466 | 2 | 65.73 | 4 | F4:44422 | NaSa01\_F12.raw |  |  |  | 1.8132E7 |  |  |  |  |  |  |  |  |  |  | 1 | 0 | 0 | 0 | 1 | 0 | 0 | 0 | 0 | 0 | 0 | 0 | 0 | 0 | 0 | 16 | 33 | Carbamidomethylation | C11:Carbamidomethylation:1000.00;C13:Carbamidomethylation:1000.00 | PEAKS DB |
| R.C(+57.02)C(+57.02)FVHDC(+57.02)C(+57.02)YGNLPDC(+57.02)NPK.S | N | 73.65 | 2314.8687 | 18 | 0.7 | 772.6307 | 3 | 32.34 | 4 | F4:16838 | NaSa01\_F12.raw |  |  |  | 2.3387E7 |  | 7.5227E6 |  |  |  |  |  |  |  |  | 2 | 0 | 0 | 0 | 1 | 0 | 1 | 0 | 0 | 0 | 0 | 0 | 0 | 0 | 0 | 43 | 60 | Carbamidomethylation | C1:Carbamidomethylation:1000.00;C2:Carbamidomethylation:1000.00;C7:Carbamidomethylation:1000.00;C8:Carbamidomethylation:1000.00;C15:Carbamidomethylation:1000.00 | PEAKS DB |
| K.YMLYPDFLC(+57.02)K.G | N | 68.32 | 1348.6195 | 10 | 0.2 | 675.3171 | 2 | 75.10 | 4 | F4:52326 | NaSa01\_F12.raw |  |  |  | 2.1669E7 |  |  |  |  |  |  |  |  |  |  | 1 | 0 | 0 | 0 | 1 | 0 | 0 | 0 | 0 | 0 | 0 | 0 | 0 | 0 | 0 | 107 | 116 | Carbamidomethylation | C9:Carbamidomethylation:1000.00 | PEAKS DB |
| K.YM(+15.99)LYPDFLC(+57.02)K.G | N | 64.41 | 1364.6145 | 10 | 0.0 | 683.3145 | 2 | 68.02 | 4 | F4:46357 | NaSa01\_F12.raw |  |  |  | 2.115E6 |  |  |  |  |  |  |  |  |  |  | 1 | 0 | 0 | 0 | 1 | 0 | 0 | 0 | 0 | 0 | 0 | 0 | 0 | 0 | 0 | 107 | 116 | Oxidation (M); Carbamidomethylation | M2:Oxidation (M):1000.00;C9:Carbamidomethylation:1000.00 | PEAKS DB |
| K.KYMLYPDFLC(+57.02)K.G | Y | 60.31 | 1476.7145 | 11 | 0.3 | 493.2456 | 3 | 58.25 | 4 | F4:38516 | NaSa01\_F12.raw |  |  |  | 5.4533E6 |  |  |  |  |  |  |  |  |  |  | 2 | 0 | 0 | 0 | 2 | 0 | 0 | 0 | 0 | 0 | 0 | 0 | 0 | 0 | 0 | 106 | 116 | Carbamidomethylation | C10:Carbamidomethylation:1000.00 | PEAKS DB |
| S.YSSYGC(+57.02)YC(+57.02)GWGGK.G | N | 58.28 | 1543.5861 | 13 | 0.3 | 772.8005 | 2 | 35.31 | 6 | F6:16919 | NaSa01\_F14.raw |  |  |  |  |  | 5.8277E6 |  |  |  |  |  |  |  |  | 1 | 0 | 0 | 0 | 0 | 0 | 1 | 0 | 0 | 0 | 0 | 0 | 0 | 0 | 0 | 21 | 33 | Carbamidomethylation | C6:Carbamidomethylation:1000.00;C8:Carbamidomethylation:1000.00 | PEAKS DB |
| K.KYM(+15.99)LYPDFLC(+57.02)K.G | Y | 43.40 | 1492.7095 | 11 | -0.3 | 498.5770 | 3 | 51.59 | 4 | F4:32744 | NaSa01\_F12.raw |  |  |  | 7.4997E5 |  |  |  |  |  |  |  |  |  |  | 1 | 0 | 0 | 0 | 1 | 0 | 0 | 0 | 0 | 0 | 0 | 0 | 0 | 0 | 0 | 106 | 116 | Oxidation (M); Carbamidomethylation | M3:Oxidation (M):1000.00;C10:Carbamidomethylation:1000.00 | PEAKS DB |
| SLLEFGK.M | Y | 38.22 | 792.4381 | 7 | 0.1 | 397.2264 | 2 | 39.94 | 4 | F4:23423 | NaSa01\_F12.raw |  |  |  | 8.4704E6 |  |  |  |  |  |  |  |  |  |  | 1 | 0 | 0 | 0 | 1 | 0 | 0 | 0 | 0 | 0 | 0 | 0 | 0 | 0 | 0 | 1 | 7 |  |  | PEAKS DB |
| total 8 peptides |  |  |  |  |  |  |  |  |  |  |  |  |  |  |  |  |  |  |  |  |  |  |  |  |  |  |  |  |  |  |  |  |  |  |  |  |  |  |  |  |  |  |  |  |  |
| --- | --- | --- | --- | --- | --- | --- | --- | --- | --- | --- | --- | --- | --- | --- | --- | --- | --- | --- | --- | --- | --- | --- | --- | --- | --- | --- | --- | --- | --- | --- | --- | --- | --- | --- | --- | --- | --- | --- | --- | --- | --- | --- | --- | --- | --- |

3CBI

back to list

  

| Protein Coverage
| Supporting Peptides
|

Protein Coverage:

Supporting Peptides:

| Peptide | Uniq | -10lgP | Mass | Length | ppm | m/z | z | RT | Fraction | Scan | Source File | Area F1 | Area F10 | Area F11 | Area F12 | Area F13 | Area F14 | Area F2 | Area F3 | Area F4 | Area F5 | Area F6 | Area F7 | Area F8 | Area F9 | #Feature | #Feature F1 | #Feature F10 | #Feature F11 | #Feature F12 | #Feature F13 | #Feature F14 | #Feature F2 | #Feature F3 | #Feature F4 | #Feature F5 | #Feature F6 | #Feature F7 | #Feature F8 | #Feature F9 | Start | End | PTM | AScore | Found By |
| --- | --- | --- | --- | --- | --- | --- | --- | --- | --- | --- | --- | --- | --- | --- | --- | --- | --- | --- | --- | --- | --- | --- | --- | --- | --- | --- | --- | --- | --- | --- | --- | --- | --- | --- | --- | --- | --- | --- | --- | --- | --- | --- | --- | --- | --- |
| K.LAIPSYSSYGC(+57.02)YC(+57.02)GWGGK.G | Y | 83.35 | 2024.8760 | 18 | 1.3 | 1013.4466 | 2 | 65.73 | 4 | F4:44422 | NaSa01\_F12.raw |  |  |  | 1.8132E7 |  |  |  |  |  |  |  |  |  |  | 1 | 0 | 0 | 0 | 1 | 0 | 0 | 0 | 0 | 0 | 0 | 0 | 0 | 0 | 0 | 16 | 33 | Carbamidomethylation | C11:Carbamidomethylation:1000.00;C13:Carbamidomethylation:1000.00 | PEAKS DB |
| R.C(+57.02)C(+57.02)FVHDC(+57.02)C(+57.02)YGNLPDC(+57.02)NPK.S | N | 73.65 | 2314.8687 | 18 | 0.7 | 772.6307 | 3 | 32.34 | 4 | F4:16838 | NaSa01\_F12.raw |  |  |  | 2.3387E7 |  | 7.5227E6 |  |  |  |  |  |  |  |  | 2 | 0 | 0 | 0 | 1 | 0 | 1 | 0 | 0 | 0 | 0 | 0 | 0 | 0 | 0 | 43 | 60 | Carbamidomethylation | C1:Carbamidomethylation:1000.00;C2:Carbamidomethylation:1000.00;C7:Carbamidomethylation:1000.00;C8:Carbamidomethylation:1000.00;C15:Carbamidomethylation:1000.00 | PEAKS DB |
| K.YMLYPDFLC(+57.02)K.G | N | 68.32 | 1348.6195 | 10 | 0.2 | 675.3171 | 2 | 75.10 | 4 | F4:52326 | NaSa01\_F12.raw |  |  |  | 2.1669E7 |  |  |  |  |  |  |  |  |  |  | 1 | 0 | 0 | 0 | 1 | 0 | 0 | 0 | 0 | 0 | 0 | 0 | 0 | 0 | 0 | 107 | 116 | Carbamidomethylation | C9:Carbamidomethylation:1000.00 | PEAKS DB |
| K.YM(+15.99)LYPDFLC(+57.02)K.G | N | 64.41 | 1364.6145 | 10 | 0.0 | 683.3145 | 2 | 68.02 | 4 | F4:46357 | NaSa01\_F12.raw |  |  |  | 2.115E6 |  |  |  |  |  |  |  |  |  |  | 1 | 0 | 0 | 0 | 1 | 0 | 0 | 0 | 0 | 0 | 0 | 0 | 0 | 0 | 0 | 107 | 116 | Oxidation (M); Carbamidomethylation | M2:Oxidation (M):1000.00;C9:Carbamidomethylation:1000.00 | PEAKS DB |
| K.KYMLYPDFLC(+57.02)K.G | Y | 60.31 | 1476.7145 | 11 | 0.3 | 493.2456 | 3 | 58.25 | 4 | F4:38516 | NaSa01\_F12.raw |  |  |  | 5.4533E6 |  |  |  |  |  |  |  |  |  |  | 2 | 0 | 0 | 0 | 2 | 0 | 0 | 0 | 0 | 0 | 0 | 0 | 0 | 0 | 0 | 106 | 116 | Carbamidomethylation | C10:Carbamidomethylation:1000.00 | PEAKS DB |
| S.YSSYGC(+57.02)YC(+57.02)GWGGK.G | N | 58.28 | 1543.5861 | 13 | 0.3 | 772.8005 | 2 | 35.31 | 6 | F6:16919 | NaSa01\_F14.raw |  |  |  |  |  | 5.8277E6 |  |  |  |  |  |  |  |  | 1 | 0 | 0 | 0 | 0 | 0 | 1 | 0 | 0 | 0 | 0 | 0 | 0 | 0 | 0 | 21 | 33 | Carbamidomethylation | C6:Carbamidomethylation:1000.00;C8:Carbamidomethylation:1000.00 | PEAKS DB |
| K.KYM(+15.99)LYPDFLC(+57.02)K.G | Y | 43.40 | 1492.7095 | 11 | -0.3 | 498.5770 | 3 | 51.59 | 4 | F4:32744 | NaSa01\_F12.raw |  |  |  | 7.4997E5 |  |  |  |  |  |  |  |  |  |  | 1 | 0 | 0 | 0 | 1 | 0 | 0 | 0 | 0 | 0 | 0 | 0 | 0 | 0 | 0 | 106 | 116 | Oxidation (M); Carbamidomethylation | M3:Oxidation (M):1000.00;C10:Carbamidomethylation:1000.00 | PEAKS DB |
| SLLEFGK.M | Y | 38.22 | 792.4381 | 7 | 0.1 | 397.2264 | 2 | 39.94 | 4 | F4:23423 | NaSa01\_F12.raw |  |  |  | 8.4704E6 |  |  |  |  |  |  |  |  |  |  | 1 | 0 | 0 | 0 | 1 | 0 | 0 | 0 | 0 | 0 | 0 | 0 | 0 | 0 | 0 | 1 | 7 |  |  | PEAKS DB |
| total 8 peptides |  |  |  |  |  |  |  |  |  |  |  |  |  |  |  |  |  |  |  |  |  |  |  |  |  |  |  |  |  |  |  |  |  |  |  |  |  |  |  |  |  |  |  |  |  |
| --- | --- | --- | --- | --- | --- | --- | --- | --- | --- | --- | --- | --- | --- | --- | --- | --- | --- | --- | --- | --- | --- | --- | --- | --- | --- | --- | --- | --- | --- | --- | --- | --- | --- | --- | --- | --- | --- | --- | --- | --- | --- | --- | --- | --- | --- |

1OYF

back to list

  

| Protein Coverage
| Supporting Peptides
|

Protein Coverage:

Supporting Peptides:

| Peptide | Uniq | -10lgP | Mass | Length | ppm | m/z | z | RT | Fraction | Scan | Source File | Area F1 | Area F10 | Area F11 | Area F12 | Area F13 | Area F14 | Area F2 | Area F3 | Area F4 | Area F5 | Area F6 | Area F7 | Area F8 | Area F9 | #Feature | #Feature F1 | #Feature F10 | #Feature F11 | #Feature F12 | #Feature F13 | #Feature F14 | #Feature F2 | #Feature F3 | #Feature F4 | #Feature F5 | #Feature F6 | #Feature F7 | #Feature F8 | #Feature F9 | Start | End | PTM | AScore | Found By |
| --- | --- | --- | --- | --- | --- | --- | --- | --- | --- | --- | --- | --- | --- | --- | --- | --- | --- | --- | --- | --- | --- | --- | --- | --- | --- | --- | --- | --- | --- | --- | --- | --- | --- | --- | --- | --- | --- | --- | --- | --- | --- | --- | --- | --- | --- |
| R.LAIPSYSSYGC(+57.02)YC(+57.02)GWGGK.G | Y | 83.35 | 2024.8760 | 18 | 1.3 | 1013.4466 | 2 | 65.73 | 4 | F4:44422 | NaSa01\_F12.raw |  |  |  | 1.8132E7 |  |  |  |  |  |  |  |  |  |  | 1 | 0 | 0 | 0 | 1 | 0 | 0 | 0 | 0 | 0 | 0 | 0 | 0 | 0 | 0 | 16 | 33 | Carbamidomethylation | C11:Carbamidomethylation:1000.00;C13:Carbamidomethylation:1000.00 | PEAKS DB |
| R.C(+57.02)C(+57.02)FVHDC(+57.02)C(+57.02)YGNLPDC(+57.02)NPK.S | N | 73.65 | 2314.8687 | 18 | 0.7 | 772.6307 | 3 | 32.34 | 4 | F4:16838 | NaSa01\_F12.raw |  |  |  | 2.3387E7 |  | 7.5227E6 |  |  |  |  |  |  |  |  | 2 | 0 | 0 | 0 | 1 | 0 | 1 | 0 | 0 | 0 | 0 | 0 | 0 | 0 | 0 | 43 | 60 | Carbamidomethylation | C1:Carbamidomethylation:1000.00;C2:Carbamidomethylation:1000.00;C7:Carbamidomethylation:1000.00;C8:Carbamidomethylation:1000.00;C15:Carbamidomethylation:1000.00 | PEAKS DB |
| K.YMLYPDFLC(+57.02)K.G | N | 68.32 | 1348.6195 | 10 | 0.2 | 675.3171 | 2 | 75.10 | 4 | F4:52326 | NaSa01\_F12.raw |  |  |  | 2.1669E7 |  |  |  |  |  |  |  |  |  |  | 1 | 0 | 0 | 0 | 1 | 0 | 0 | 0 | 0 | 0 | 0 | 0 | 0 | 0 | 0 | 107 | 116 | Carbamidomethylation | C9:Carbamidomethylation:1000.00 | PEAKS DB |
| K.YM(+15.99)LYPDFLC(+57.02)K.G | N | 64.41 | 1364.6145 | 10 | 0.0 | 683.3145 | 2 | 68.02 | 4 | F4:46357 | NaSa01\_F12.raw |  |  |  | 2.115E6 |  |  |  |  |  |  |  |  |  |  | 1 | 0 | 0 | 0 | 1 | 0 | 0 | 0 | 0 | 0 | 0 | 0 | 0 | 0 | 0 | 107 | 116 | Oxidation (M); Carbamidomethylation | M2:Oxidation (M):1000.00;C9:Carbamidomethylation:1000.00 | PEAKS DB |
| K.KYMLYPDFLC(+57.02)K.G | Y | 60.31 | 1476.7145 | 11 | 0.3 | 493.2456 | 3 | 58.25 | 4 | F4:38516 | NaSa01\_F12.raw |  |  |  | 5.4533E6 |  |  |  |  |  |  |  |  |  |  | 2 | 0 | 0 | 0 | 2 | 0 | 0 | 0 | 0 | 0 | 0 | 0 | 0 | 0 | 0 | 106 | 116 | Carbamidomethylation | C10:Carbamidomethylation:1000.00 | PEAKS DB |
| S.YSSYGC(+57.02)YC(+57.02)GWGGK.G | N | 58.28 | 1543.5861 | 13 | 0.3 | 772.8005 | 2 | 35.31 | 6 | F6:16919 | NaSa01\_F14.raw |  |  |  |  |  | 5.8277E6 |  |  |  |  |  |  |  |  | 1 | 0 | 0 | 0 | 0 | 0 | 1 | 0 | 0 | 0 | 0 | 0 | 0 | 0 | 0 | 21 | 33 | Carbamidomethylation | C6:Carbamidomethylation:1000.00;C8:Carbamidomethylation:1000.00 | PEAKS DB |
| K.KYM(+15.99)LYPDFLC(+57.02)K.G | Y | 43.40 | 1492.7095 | 11 | -0.3 | 498.5770 | 3 | 51.59 | 4 | F4:32744 | NaSa01\_F12.raw |  |  |  | 7.4997E5 |  |  |  |  |  |  |  |  |  |  | 1 | 0 | 0 | 0 | 1 | 0 | 0 | 0 | 0 | 0 | 0 | 0 | 0 | 0 | 0 | 106 | 116 | Oxidation (M); Carbamidomethylation | M3:Oxidation (M):1000.00;C10:Carbamidomethylation:1000.00 | PEAKS DB |
| SLLEFGK.M | Y | 38.22 | 792.4381 | 7 | 0.1 | 397.2264 | 2 | 39.94 | 4 | F4:23423 | NaSa01\_F12.raw |  |  |  | 8.4704E6 |  |  |  |  |  |  |  |  |  |  | 1 | 0 | 0 | 0 | 1 | 0 | 0 | 0 | 0 | 0 | 0 | 0 | 0 | 0 | 0 | 1 | 7 |  |  | PEAKS DB |
| total 8 peptides |  |  |  |  |  |  |  |  |  |  |  |  |  |  |  |  |  |  |  |  |  |  |  |  |  |  |  |  |  |  |  |  |  |  |  |  |  |  |  |  |  |  |  |  |  |
| --- | --- | --- | --- | --- | --- | --- | --- | --- | --- | --- | --- | --- | --- | --- | --- | --- | --- | --- | --- | --- | --- | --- | --- | --- | --- | --- | --- | --- | --- | --- | --- | --- | --- | --- | --- | --- | --- | --- | --- | --- | --- | --- | --- | --- | --- |

2QUE

back to list

  

| Protein Coverage
| Supporting Peptides
|

Protein Coverage:

Supporting Peptides:

| Peptide | Uniq | -10lgP | Mass | Length | ppm | m/z | z | RT | Fraction | Scan | Source File | Area F1 | Area F10 | Area F11 | Area F12 | Area F13 | Area F14 | Area F2 | Area F3 | Area F4 | Area F5 | Area F6 | Area F7 | Area F8 | Area F9 | #Feature | #Feature F1 | #Feature F10 | #Feature F11 | #Feature F12 | #Feature F13 | #Feature F14 | #Feature F2 | #Feature F3 | #Feature F4 | #Feature F5 | #Feature F6 | #Feature F7 | #Feature F8 | #Feature F9 | Start | End | PTM | AScore | Found By |
| --- | --- | --- | --- | --- | --- | --- | --- | --- | --- | --- | --- | --- | --- | --- | --- | --- | --- | --- | --- | --- | --- | --- | --- | --- | --- | --- | --- | --- | --- | --- | --- | --- | --- | --- | --- | --- | --- | --- | --- | --- | --- | --- | --- | --- | --- |
| K.LAIPSYSSYGC(+57.02)YC(+57.02)GWGGK.G | Y | 83.35 | 2024.8760 | 18 | 1.3 | 1013.4466 | 2 | 65.73 | 4 | F4:44422 | NaSa01\_F12.raw |  |  |  | 1.8132E7 |  |  |  |  |  |  |  |  |  |  | 1 | 0 | 0 | 0 | 1 | 0 | 0 | 0 | 0 | 0 | 0 | 0 | 0 | 0 | 0 | 16 | 33 | Carbamidomethylation | C11:Carbamidomethylation:1000.00;C13:Carbamidomethylation:1000.00 | PEAKS DB |
| R.C(+57.02)C(+57.02)FVHDC(+57.02)C(+57.02)YGNLPDC(+57.02)NPK.S | N | 73.65 | 2314.8687 | 18 | 0.7 | 772.6307 | 3 | 32.34 | 4 | F4:16838 | NaSa01\_F12.raw |  |  |  | 2.3387E7 |  | 7.5227E6 |  |  |  |  |  |  |  |  | 2 | 0 | 0 | 0 | 1 | 0 | 1 | 0 | 0 | 0 | 0 | 0 | 0 | 0 | 0 | 43 | 60 | Carbamidomethylation | C1:Carbamidomethylation:1000.00;C2:Carbamidomethylation:1000.00;C7:Carbamidomethylation:1000.00;C8:Carbamidomethylation:1000.00;C15:Carbamidomethylation:1000.00 | PEAKS DB |
| K.YMLYPDFLC(+57.02)K.G | N | 68.32 | 1348.6195 | 10 | 0.2 | 675.3171 | 2 | 75.10 | 4 | F4:52326 | NaSa01\_F12.raw |  |  |  | 2.1669E7 |  |  |  |  |  |  |  |  |  |  | 1 | 0 | 0 | 0 | 1 | 0 | 0 | 0 | 0 | 0 | 0 | 0 | 0 | 0 | 0 | 107 | 116 | Carbamidomethylation | C9:Carbamidomethylation:1000.00 | PEAKS DB |
| K.YM(+15.99)LYPDFLC(+57.02)K.G | N | 64.41 | 1364.6145 | 10 | 0.0 | 683.3145 | 2 | 68.02 | 4 | F4:46357 | NaSa01\_F12.raw |  |  |  | 2.115E6 |  |  |  |  |  |  |  |  |  |  | 1 | 0 | 0 | 0 | 1 | 0 | 0 | 0 | 0 | 0 | 0 | 0 | 0 | 0 | 0 | 107 | 116 | Oxidation (M); Carbamidomethylation | M2:Oxidation (M):1000.00;C9:Carbamidomethylation:1000.00 | PEAKS DB |
| K.KYMLYPDFLC(+57.02)K.G | Y | 60.31 | 1476.7145 | 11 | 0.3 | 493.2456 | 3 | 58.25 | 4 | F4:38516 | NaSa01\_F12.raw |  |  |  | 5.4533E6 |  |  |  |  |  |  |  |  |  |  | 2 | 0 | 0 | 0 | 2 | 0 | 0 | 0 | 0 | 0 | 0 | 0 | 0 | 0 | 0 | 106 | 116 | Carbamidomethylation | C10:Carbamidomethylation:1000.00 | PEAKS DB |
| S.YSSYGC(+57.02)YC(+57.02)GWGGK.G | N | 58.28 | 1543.5861 | 13 | 0.3 | 772.8005 | 2 | 35.31 | 6 | F6:16919 | NaSa01\_F14.raw |  |  |  |  |  | 5.8277E6 |  |  |  |  |  |  |  |  | 1 | 0 | 0 | 0 | 0 | 0 | 1 | 0 | 0 | 0 | 0 | 0 | 0 | 0 | 0 | 21 | 33 | Carbamidomethylation | C6:Carbamidomethylation:1000.00;C8:Carbamidomethylation:1000.00 | PEAKS DB |
| K.KYM(+15.99)LYPDFLC(+57.02)K.G | Y | 43.40 | 1492.7095 | 11 | -0.3 | 498.5770 | 3 | 51.59 | 4 | F4:32744 | NaSa01\_F12.raw |  |  |  | 7.4997E5 |  |  |  |  |  |  |  |  |  |  | 1 | 0 | 0 | 0 | 1 | 0 | 0 | 0 | 0 | 0 | 0 | 0 | 0 | 0 | 0 | 106 | 116 | Oxidation (M); Carbamidomethylation | M3:Oxidation (M):1000.00;C10:Carbamidomethylation:1000.00 | PEAKS DB |
| SLLEFGK.M | Y | 38.22 | 792.4381 | 7 | 0.1 | 397.2264 | 2 | 39.94 | 4 | F4:23423 | NaSa01\_F12.raw |  |  |  | 8.4704E6 |  |  |  |  |  |  |  |  |  |  | 1 | 0 | 0 | 0 | 1 | 0 | 0 | 0 | 0 | 0 | 0 | 0 | 0 | 0 | 0 | 1 | 7 |  |  | PEAKS DB |
| total 8 peptides |  |  |  |  |  |  |  |  |  |  |  |  |  |  |  |  |  |  |  |  |  |  |  |  |  |  |  |  |  |  |  |  |  |  |  |  |  |  |  |  |  |  |  |  |  |
| --- | --- | --- | --- | --- | --- | --- | --- | --- | --- | --- | --- | --- | --- | --- | --- | --- | --- | --- | --- | --- | --- | --- | --- | --- | --- | --- | --- | --- | --- | --- | --- | --- | --- | --- | --- | --- | --- | --- | --- | --- | --- | --- | --- | --- | --- |

1ZWP

back to list

  

| Protein Coverage
| Supporting Peptides
|

Protein Coverage:

Supporting Peptides:

| Peptide | Uniq | -10lgP | Mass | Length | ppm | m/z | z | RT | Fraction | Scan | Source File | Area F1 | Area F10 | Area F11 | Area F12 | Area F13 | Area F14 | Area F2 | Area F3 | Area F4 | Area F5 | Area F6 | Area F7 | Area F8 | Area F9 | #Feature | #Feature F1 | #Feature F10 | #Feature F11 | #Feature F12 | #Feature F13 | #Feature F14 | #Feature F2 | #Feature F3 | #Feature F4 | #Feature F5 | #Feature F6 | #Feature F7 | #Feature F8 | #Feature F9 | Start | End | PTM | AScore | Found By |
| --- | --- | --- | --- | --- | --- | --- | --- | --- | --- | --- | --- | --- | --- | --- | --- | --- | --- | --- | --- | --- | --- | --- | --- | --- | --- | --- | --- | --- | --- | --- | --- | --- | --- | --- | --- | --- | --- | --- | --- | --- | --- | --- | --- | --- | --- |
| K.LAIPSYSSYGC(+57.02)YC(+57.02)GWGGK.G | Y | 83.35 | 2024.8760 | 18 | 1.3 | 1013.4466 | 2 | 65.73 | 4 | F4:44422 | NaSa01\_F12.raw |  |  |  | 1.8132E7 |  |  |  |  |  |  |  |  |  |  | 1 | 0 | 0 | 0 | 1 | 0 | 0 | 0 | 0 | 0 | 0 | 0 | 0 | 0 | 0 | 16 | 33 | Carbamidomethylation | C11:Carbamidomethylation:1000.00;C13:Carbamidomethylation:1000.00 | PEAKS DB |
| R.C(+57.02)C(+57.02)FVHDC(+57.02)C(+57.02)YGNLPDC(+57.02)NPK.S | N | 73.65 | 2314.8687 | 18 | 0.7 | 772.6307 | 3 | 32.34 | 4 | F4:16838 | NaSa01\_F12.raw |  |  |  | 2.3387E7 |  | 7.5227E6 |  |  |  |  |  |  |  |  | 2 | 0 | 0 | 0 | 1 | 0 | 1 | 0 | 0 | 0 | 0 | 0 | 0 | 0 | 0 | 43 | 60 | Carbamidomethylation | C1:Carbamidomethylation:1000.00;C2:Carbamidomethylation:1000.00;C7:Carbamidomethylation:1000.00;C8:Carbamidomethylation:1000.00;C15:Carbamidomethylation:1000.00 | PEAKS DB |
| K.YMLYPDFLC(+57.02)K.G | N | 68.32 | 1348.6195 | 10 | 0.2 | 675.3171 | 2 | 75.10 | 4 | F4:52326 | NaSa01\_F12.raw |  |  |  | 2.1669E7 |  |  |  |  |  |  |  |  |  |  | 1 | 0 | 0 | 0 | 1 | 0 | 0 | 0 | 0 | 0 | 0 | 0 | 0 | 0 | 0 | 107 | 116 | Carbamidomethylation | C9:Carbamidomethylation:1000.00 | PEAKS DB |
| K.YM(+15.99)LYPDFLC(+57.02)K.G | N | 64.41 | 1364.6145 | 10 | 0.0 | 683.3145 | 2 | 68.02 | 4 | F4:46357 | NaSa01\_F12.raw |  |  |  | 2.115E6 |  |  |  |  |  |  |  |  |  |  | 1 | 0 | 0 | 0 | 1 | 0 | 0 | 0 | 0 | 0 | 0 | 0 | 0 | 0 | 0 | 107 | 116 | Oxidation (M); Carbamidomethylation | M2:Oxidation (M):1000.00;C9:Carbamidomethylation:1000.00 | PEAKS DB |
| K.KYMLYPDFLC(+57.02)K.G | Y | 60.31 | 1476.7145 | 11 | 0.3 | 493.2456 | 3 | 58.25 | 4 | F4:38516 | NaSa01\_F12.raw |  |  |  | 5.4533E6 |  |  |  |  |  |  |  |  |  |  | 2 | 0 | 0 | 0 | 2 | 0 | 0 | 0 | 0 | 0 | 0 | 0 | 0 | 0 | 0 | 106 | 116 | Carbamidomethylation | C10:Carbamidomethylation:1000.00 | PEAKS DB |
| S.YSSYGC(+57.02)YC(+57.02)GWGGK.G | N | 58.28 | 1543.5861 | 13 | 0.3 | 772.8005 | 2 | 35.31 | 6 | F6:16919 | NaSa01\_F14.raw |  |  |  |  |  | 5.8277E6 |  |  |  |  |  |  |  |  | 1 | 0 | 0 | 0 | 0 | 0 | 1 | 0 | 0 | 0 | 0 | 0 | 0 | 0 | 0 | 21 | 33 | Carbamidomethylation | C6:Carbamidomethylation:1000.00;C8:Carbamidomethylation:1000.00 | PEAKS DB |
| K.KYM(+15.99)LYPDFLC(+57.02)K.G | Y | 43.40 | 1492.7095 | 11 | -0.3 | 498.5770 | 3 | 51.59 | 4 | F4:32744 | NaSa01\_F12.raw |  |  |  | 7.4997E5 |  |  |  |  |  |  |  |  |  |  | 1 | 0 | 0 | 0 | 1 | 0 | 0 | 0 | 0 | 0 | 0 | 0 | 0 | 0 | 0 | 106 | 116 | Oxidation (M); Carbamidomethylation | M3:Oxidation (M):1000.00;C10:Carbamidomethylation:1000.00 | PEAKS DB |
| SLLEFGK.M | Y | 38.22 | 792.4381 | 7 | 0.1 | 397.2264 | 2 | 39.94 | 4 | F4:23423 | NaSa01\_F12.raw |  |  |  | 8.4704E6 |  |  |  |  |  |  |  |  |  |  | 1 | 0 | 0 | 0 | 1 | 0 | 0 | 0 | 0 | 0 | 0 | 0 | 0 | 0 | 0 | 1 | 7 |  |  | PEAKS DB |
| total 8 peptides |  |  |  |  |  |  |  |  |  |  |  |  |  |  |  |  |  |  |  |  |  |  |  |  |  |  |  |  |  |  |  |  |  |  |  |  |  |  |  |  |  |  |  |  |  |
| --- | --- | --- | --- | --- | --- | --- | --- | --- | --- | --- | --- | --- | --- | --- | --- | --- | --- | --- | --- | --- | --- | --- | --- | --- | --- | --- | --- | --- | --- | --- | --- | --- | --- | --- | --- | --- | --- | --- | --- | --- | --- | --- | --- | --- | --- |

3FO7

back to list

  

| Protein Coverage
| Supporting Peptides
|

Protein Coverage:

Supporting Peptides:

| Peptide | Uniq | -10lgP | Mass | Length | ppm | m/z | z | RT | Fraction | Scan | Source File | Area F1 | Area F10 | Area F11 | Area F12 | Area F13 | Area F14 | Area F2 | Area F3 | Area F4 | Area F5 | Area F6 | Area F7 | Area F8 | Area F9 | #Feature | #Feature F1 | #Feature F10 | #Feature F11 | #Feature F12 | #Feature F13 | #Feature F14 | #Feature F2 | #Feature F3 | #Feature F4 | #Feature F5 | #Feature F6 | #Feature F7 | #Feature F8 | #Feature F9 | Start | End | PTM | AScore | Found By |
| --- | --- | --- | --- | --- | --- | --- | --- | --- | --- | --- | --- | --- | --- | --- | --- | --- | --- | --- | --- | --- | --- | --- | --- | --- | --- | --- | --- | --- | --- | --- | --- | --- | --- | --- | --- | --- | --- | --- | --- | --- | --- | --- | --- | --- | --- |
| K.LAIPSYSSYGC(+57.02)YC(+57.02)GWGGK.G | Y | 83.35 | 2024.8760 | 18 | 1.3 | 1013.4466 | 2 | 65.73 | 4 | F4:44422 | NaSa01\_F12.raw |  |  |  | 1.8132E7 |  |  |  |  |  |  |  |  |  |  | 1 | 0 | 0 | 0 | 1 | 0 | 0 | 0 | 0 | 0 | 0 | 0 | 0 | 0 | 0 | 16 | 33 | Carbamidomethylation | C11:Carbamidomethylation:1000.00;C13:Carbamidomethylation:1000.00 | PEAKS DB |
| R.C(+57.02)C(+57.02)FVHDC(+57.02)C(+57.02)YGNLPDC(+57.02)NPK.S | N | 73.65 | 2314.8687 | 18 | 0.7 | 772.6307 | 3 | 32.34 | 4 | F4:16838 | NaSa01\_F12.raw |  |  |  | 2.3387E7 |  | 7.5227E6 |  |  |  |  |  |  |  |  | 2 | 0 | 0 | 0 | 1 | 0 | 1 | 0 | 0 | 0 | 0 | 0 | 0 | 0 | 0 | 43 | 60 | Carbamidomethylation | C1:Carbamidomethylation:1000.00;C2:Carbamidomethylation:1000.00;C7:Carbamidomethylation:1000.00;C8:Carbamidomethylation:1000.00;C15:Carbamidomethylation:1000.00 | PEAKS DB |
| K.YMLYPDFLC(+57.02)K.G | N | 68.32 | 1348.6195 | 10 | 0.2 | 675.3171 | 2 | 75.10 | 4 | F4:52326 | NaSa01\_F12.raw |  |  |  | 2.1669E7 |  |  |  |  |  |  |  |  |  |  | 1 | 0 | 0 | 0 | 1 | 0 | 0 | 0 | 0 | 0 | 0 | 0 | 0 | 0 | 0 | 107 | 116 | Carbamidomethylation | C9:Carbamidomethylation:1000.00 | PEAKS DB |
| K.YM(+15.99)LYPDFLC(+57.02)K.G | N | 64.41 | 1364.6145 | 10 | 0.0 | 683.3145 | 2 | 68.02 | 4 | F4:46357 | NaSa01\_F12.raw |  |  |  | 2.115E6 |  |  |  |  |  |  |  |  |  |  | 1 | 0 | 0 | 0 | 1 | 0 | 0 | 0 | 0 | 0 | 0 | 0 | 0 | 0 | 0 | 107 | 116 | Oxidation (M); Carbamidomethylation | M2:Oxidation (M):1000.00;C9:Carbamidomethylation:1000.00 | PEAKS DB |
| K.KYMLYPDFLC(+57.02)K.G | Y | 60.31 | 1476.7145 | 11 | 0.3 | 493.2456 | 3 | 58.25 | 4 | F4:38516 | NaSa01\_F12.raw |  |  |  | 5.4533E6 |  |  |  |  |  |  |  |  |  |  | 2 | 0 | 0 | 0 | 2 | 0 | 0 | 0 | 0 | 0 | 0 | 0 | 0 | 0 | 0 | 106 | 116 | Carbamidomethylation | C10:Carbamidomethylation:1000.00 | PEAKS DB |
| S.YSSYGC(+57.02)YC(+57.02)GWGGK.G | N | 58.28 | 1543.5861 | 13 | 0.3 | 772.8005 | 2 | 35.31 | 6 | F6:16919 | NaSa01\_F14.raw |  |  |  |  |  | 5.8277E6 |  |  |  |  |  |  |  |  | 1 | 0 | 0 | 0 | 0 | 0 | 1 | 0 | 0 | 0 | 0 | 0 | 0 | 0 | 0 | 21 | 33 | Carbamidomethylation | C6:Carbamidomethylation:1000.00;C8:Carbamidomethylation:1000.00 | PEAKS DB |
| K.KYM(+15.99)LYPDFLC(+57.02)K.G | Y | 43.40 | 1492.7095 | 11 | -0.3 | 498.5770 | 3 | 51.59 | 4 | F4:32744 | NaSa01\_F12.raw |  |  |  | 7.4997E5 |  |  |  |  |  |  |  |  |  |  | 1 | 0 | 0 | 0 | 1 | 0 | 0 | 0 | 0 | 0 | 0 | 0 | 0 | 0 | 0 | 106 | 116 | Oxidation (M); Carbamidomethylation | M3:Oxidation (M):1000.00;C10:Carbamidomethylation:1000.00 | PEAKS DB |
| SLLEFGK.M | Y | 38.22 | 792.4381 | 7 | 0.1 | 397.2264 | 2 | 39.94 | 4 | F4:23423 | NaSa01\_F12.raw |  |  |  | 8.4704E6 |  |  |  |  |  |  |  |  |  |  | 1 | 0 | 0 | 0 | 1 | 0 | 0 | 0 | 0 | 0 | 0 | 0 | 0 | 0 | 0 | 1 | 7 |  |  | PEAKS DB |
| total 8 peptides |  |  |  |  |  |  |  |  |  |  |  |  |  |  |  |  |  |  |  |  |  |  |  |  |  |  |  |  |  |  |  |  |  |  |  |  |  |  |  |  |  |  |  |  |  |
| --- | --- | --- | --- | --- | --- | --- | --- | --- | --- | --- | --- | --- | --- | --- | --- | --- | --- | --- | --- | --- | --- | --- | --- | --- | --- | --- | --- | --- | --- | --- | --- | --- | --- | --- | --- | --- | --- | --- | --- | --- | --- | --- | --- | --- | --- |

AAZ53186.1

back to list

  

| Protein Coverage
| Supporting Peptides
|

Protein Coverage:

Supporting Peptides:

| Peptide | Uniq | -10lgP | Mass | Length | ppm | m/z | z | RT | Fraction | Scan | Source File | Area F1 | Area F10 | Area F11 | Area F12 | Area F13 | Area F14 | Area F2 | Area F3 | Area F4 | Area F5 | Area F6 | Area F7 | Area F8 | Area F9 | #Feature | #Feature F1 | #Feature F10 | #Feature F11 | #Feature F12 | #Feature F13 | #Feature F14 | #Feature F2 | #Feature F3 | #Feature F4 | #Feature F5 | #Feature F6 | #Feature F7 | #Feature F8 | #Feature F9 | Start | End | PTM | AScore | Found By |
| --- | --- | --- | --- | --- | --- | --- | --- | --- | --- | --- | --- | --- | --- | --- | --- | --- | --- | --- | --- | --- | --- | --- | --- | --- | --- | --- | --- | --- | --- | --- | --- | --- | --- | --- | --- | --- | --- | --- | --- | --- | --- | --- | --- | --- | --- |
| K.LAIPSYSSYGC(+57.02)YC(+57.02)GWGGK.G | Y | 83.35 | 2024.8760 | 18 | 1.3 | 1013.4466 | 2 | 65.73 | 4 | F4:44422 | NaSa01\_F12.raw |  |  |  | 1.8132E7 |  |  |  |  |  |  |  |  |  |  | 1 | 0 | 0 | 0 | 1 | 0 | 0 | 0 | 0 | 0 | 0 | 0 | 0 | 0 | 0 | 32 | 49 | Carbamidomethylation | C11:Carbamidomethylation:1000.00;C13:Carbamidomethylation:1000.00 | PEAKS DB |
| R.C(+57.02)C(+57.02)FVHDC(+57.02)C(+57.02)YGNLPDC(+57.02)NPK.S | N | 73.65 | 2314.8687 | 18 | 0.7 | 772.6307 | 3 | 32.34 | 4 | F4:16838 | NaSa01\_F12.raw |  |  |  | 2.3387E7 |  | 7.5227E6 |  |  |  |  |  |  |  |  | 2 | 0 | 0 | 0 | 1 | 0 | 1 | 0 | 0 | 0 | 0 | 0 | 0 | 0 | 0 | 59 | 76 | Carbamidomethylation | C1:Carbamidomethylation:1000.00;C2:Carbamidomethylation:1000.00;C7:Carbamidomethylation:1000.00;C8:Carbamidomethylation:1000.00;C15:Carbamidomethylation:1000.00 | PEAKS DB |
| K.YMLYPDFLC(+57.02)K.G | N | 68.32 | 1348.6195 | 10 | 0.2 | 675.3171 | 2 | 75.10 | 4 | F4:52326 | NaSa01\_F12.raw |  |  |  | 2.1669E7 |  |  |  |  |  |  |  |  |  |  | 1 | 0 | 0 | 0 | 1 | 0 | 0 | 0 | 0 | 0 | 0 | 0 | 0 | 0 | 0 | 123 | 132 | Carbamidomethylation | C9:Carbamidomethylation:1000.00 | PEAKS DB |
| K.YM(+15.99)LYPDFLC(+57.02)K.G | N | 64.41 | 1364.6145 | 10 | 0.0 | 683.3145 | 2 | 68.02 | 4 | F4:46357 | NaSa01\_F12.raw |  |  |  | 2.115E6 |  |  |  |  |  |  |  |  |  |  | 1 | 0 | 0 | 0 | 1 | 0 | 0 | 0 | 0 | 0 | 0 | 0 | 0 | 0 | 0 | 123 | 132 | Oxidation (M); Carbamidomethylation | M2:Oxidation (M):1000.00;C9:Carbamidomethylation:1000.00 | PEAKS DB |
| K.KYMLYPDFLC(+57.02)K.G | Y | 60.31 | 1476.7145 | 11 | 0.3 | 493.2456 | 3 | 58.25 | 4 | F4:38516 | NaSa01\_F12.raw |  |  |  | 5.4533E6 |  |  |  |  |  |  |  |  |  |  | 2 | 0 | 0 | 0 | 2 | 0 | 0 | 0 | 0 | 0 | 0 | 0 | 0 | 0 | 0 | 122 | 132 | Carbamidomethylation | C10:Carbamidomethylation:1000.00 | PEAKS DB |
| S.YSSYGC(+57.02)YC(+57.02)GWGGK.G | N | 58.28 | 1543.5861 | 13 | 0.3 | 772.8005 | 2 | 35.31 | 6 | F6:16919 | NaSa01\_F14.raw |  |  |  |  |  | 5.8277E6 |  |  |  |  |  |  |  |  | 1 | 0 | 0 | 0 | 0 | 0 | 1 | 0 | 0 | 0 | 0 | 0 | 0 | 0 | 0 | 37 | 49 | Carbamidomethylation | C6:Carbamidomethylation:1000.00;C8:Carbamidomethylation:1000.00 | PEAKS DB |
| K.KYM(+15.99)LYPDFLC(+57.02)K.G | Y | 43.40 | 1492.7095 | 11 | -0.3 | 498.5770 | 3 | 51.59 | 4 | F4:32744 | NaSa01\_F12.raw |  |  |  | 7.4997E5 |  |  |  |  |  |  |  |  |  |  | 1 | 0 | 0 | 0 | 1 | 0 | 0 | 0 | 0 | 0 | 0 | 0 | 0 | 0 | 0 | 122 | 132 | Oxidation (M); Carbamidomethylation | M3:Oxidation (M):1000.00;C10:Carbamidomethylation:1000.00 | PEAKS DB |
| G.SLLEFGK.M | Y | 38.22 | 792.4381 | 7 | 0.1 | 397.2264 | 2 | 39.94 | 4 | F4:23423 | NaSa01\_F12.raw |  |  |  | 8.4704E6 |  |  |  |  |  |  |  |  |  |  | 1 | 0 | 0 | 0 | 1 | 0 | 0 | 0 | 0 | 0 | 0 | 0 | 0 | 0 | 0 | 17 | 23 |  |  | PEAKS DB |
| total 8 peptides |  |  |  |  |  |  |  |  |  |  |  |  |  |  |  |  |  |  |  |  |  |  |  |  |  |  |  |  |  |  |  |  |  |  |  |  |  |  |  |  |  |  |  |  |  |
| --- | --- | --- | --- | --- | --- | --- | --- | --- | --- | --- | --- | --- | --- | --- | --- | --- | --- | --- | --- | --- | --- | --- | --- | --- | --- | --- | --- | --- | --- | --- | --- | --- | --- | --- | --- | --- | --- | --- | --- | --- | --- | --- | --- | --- | --- |

AAZ53179.1

back to list

  

| Protein Coverage
| Supporting Peptides
|

Protein Coverage:

Supporting Peptides:

| Peptide | Uniq | -10lgP | Mass | Length | ppm | m/z | z | RT | Fraction | Scan | Source File | Area F1 | Area F10 | Area F11 | Area F12 | Area F13 | Area F14 | Area F2 | Area F3 | Area F4 | Area F5 | Area F6 | Area F7 | Area F8 | Area F9 | #Feature | #Feature F1 | #Feature F10 | #Feature F11 | #Feature F12 | #Feature F13 | #Feature F14 | #Feature F2 | #Feature F3 | #Feature F4 | #Feature F5 | #Feature F6 | #Feature F7 | #Feature F8 | #Feature F9 | Start | End | PTM | AScore | Found By |
| --- | --- | --- | --- | --- | --- | --- | --- | --- | --- | --- | --- | --- | --- | --- | --- | --- | --- | --- | --- | --- | --- | --- | --- | --- | --- | --- | --- | --- | --- | --- | --- | --- | --- | --- | --- | --- | --- | --- | --- | --- | --- | --- | --- | --- | --- |
| K.LAIPSYSSYGC(+57.02)YC(+57.02)GWGGK.G | Y | 83.35 | 2024.8760 | 18 | 1.3 | 1013.4466 | 2 | 65.73 | 4 | F4:44422 | NaSa01\_F12.raw |  |  |  | 1.8132E7 |  |  |  |  |  |  |  |  |  |  | 1 | 0 | 0 | 0 | 1 | 0 | 0 | 0 | 0 | 0 | 0 | 0 | 0 | 0 | 0 | 32 | 49 | Carbamidomethylation | C11:Carbamidomethylation:1000.00;C13:Carbamidomethylation:1000.00 | PEAKS DB |
| R.C(+57.02)C(+57.02)FVHDC(+57.02)C(+57.02)YGNLPDC(+57.02)NPK.S | N | 73.65 | 2314.8687 | 18 | 0.7 | 772.6307 | 3 | 32.34 | 4 | F4:16838 | NaSa01\_F12.raw |  |  |  | 2.3387E7 |  | 7.5227E6 |  |  |  |  |  |  |  |  | 2 | 0 | 0 | 0 | 1 | 0 | 1 | 0 | 0 | 0 | 0 | 0 | 0 | 0 | 0 | 59 | 76 | Carbamidomethylation | C1:Carbamidomethylation:1000.00;C2:Carbamidomethylation:1000.00;C7:Carbamidomethylation:1000.00;C8:Carbamidomethylation:1000.00;C15:Carbamidomethylation:1000.00 | PEAKS DB |
| K.YMLYPDFLC(+57.02)K.G | N | 68.32 | 1348.6195 | 10 | 0.2 | 675.3171 | 2 | 75.10 | 4 | F4:52326 | NaSa01\_F12.raw |  |  |  | 2.1669E7 |  |  |  |  |  |  |  |  |  |  | 1 | 0 | 0 | 0 | 1 | 0 | 0 | 0 | 0 | 0 | 0 | 0 | 0 | 0 | 0 | 123 | 132 | Carbamidomethylation | C9:Carbamidomethylation:1000.00 | PEAKS DB |
| K.YM(+15.99)LYPDFLC(+57.02)K.G | N | 64.41 | 1364.6145 | 10 | 0.0 | 683.3145 | 2 | 68.02 | 4 | F4:46357 | NaSa01\_F12.raw |  |  |  | 2.115E6 |  |  |  |  |  |  |  |  |  |  | 1 | 0 | 0 | 0 | 1 | 0 | 0 | 0 | 0 | 0 | 0 | 0 | 0 | 0 | 0 | 123 | 132 | Oxidation (M); Carbamidomethylation | M2:Oxidation (M):1000.00;C9:Carbamidomethylation:1000.00 | PEAKS DB |
| K.KYMLYPDFLC(+57.02)K.G | Y | 60.31 | 1476.7145 | 11 | 0.3 | 493.2456 | 3 | 58.25 | 4 | F4:38516 | NaSa01\_F12.raw |  |  |  | 5.4533E6 |  |  |  |  |  |  |  |  |  |  | 2 | 0 | 0 | 0 | 2 | 0 | 0 | 0 | 0 | 0 | 0 | 0 | 0 | 0 | 0 | 122 | 132 | Carbamidomethylation | C10:Carbamidomethylation:1000.00 | PEAKS DB |
| S.YSSYGC(+57.02)YC(+57.02)GWGGK.G | N | 58.28 | 1543.5861 | 13 | 0.3 | 772.8005 | 2 | 35.31 | 6 | F6:16919 | NaSa01\_F14.raw |  |  |  |  |  | 5.8277E6 |  |  |  |  |  |  |  |  | 1 | 0 | 0 | 0 | 0 | 0 | 1 | 0 | 0 | 0 | 0 | 0 | 0 | 0 | 0 | 37 | 49 | Carbamidomethylation | C6:Carbamidomethylation:1000.00;C8:Carbamidomethylation:1000.00 | PEAKS DB |
| K.KYM(+15.99)LYPDFLC(+57.02)K.G | Y | 43.40 | 1492.7095 | 11 | -0.3 | 498.5770 | 3 | 51.59 | 4 | F4:32744 | NaSa01\_F12.raw |  |  |  | 7.4997E5 |  |  |  |  |  |  |  |  |  |  | 1 | 0 | 0 | 0 | 1 | 0 | 0 | 0 | 0 | 0 | 0 | 0 | 0 | 0 | 0 | 122 | 132 | Oxidation (M); Carbamidomethylation | M3:Oxidation (M):1000.00;C10:Carbamidomethylation:1000.00 | PEAKS DB |
| G.SLLEFGK.M | Y | 38.22 | 792.4381 | 7 | 0.1 | 397.2264 | 2 | 39.94 | 4 | F4:23423 | NaSa01\_F12.raw |  |  |  | 8.4704E6 |  |  |  |  |  |  |  |  |  |  | 1 | 0 | 0 | 0 | 1 | 0 | 0 | 0 | 0 | 0 | 0 | 0 | 0 | 0 | 0 | 17 | 23 |  |  | PEAKS DB |
| total 8 peptides |  |  |  |  |  |  |  |  |  |  |  |  |  |  |  |  |  |  |  |  |  |  |  |  |  |  |  |  |  |  |  |  |  |  |  |  |  |  |  |  |  |  |  |  |  |
| --- | --- | --- | --- | --- | --- | --- | --- | --- | --- | --- | --- | --- | --- | --- | --- | --- | --- | --- | --- | --- | --- | --- | --- | --- | --- | --- | --- | --- | --- | --- | --- | --- | --- | --- | --- | --- | --- | --- | --- | --- | --- | --- | --- | --- | --- |

LAA27322.1

back to list

  

| Protein Coverage
| Supporting Peptides
|

Protein Coverage:

Supporting Peptides:

| Peptide | Uniq | -10lgP | Mass | Length | ppm | m/z | z | RT | Fraction | Scan | Source File | Area F1 | Area F10 | Area F11 | Area F12 | Area F13 | Area F14 | Area F2 | Area F3 | Area F4 | Area F5 | Area F6 | Area F7 | Area F8 | Area F9 | #Feature | #Feature F1 | #Feature F10 | #Feature F11 | #Feature F12 | #Feature F13 | #Feature F14 | #Feature F2 | #Feature F3 | #Feature F4 | #Feature F5 | #Feature F6 | #Feature F7 | #Feature F8 | #Feature F9 | Start | End | PTM | AScore | Found By |
| --- | --- | --- | --- | --- | --- | --- | --- | --- | --- | --- | --- | --- | --- | --- | --- | --- | --- | --- | --- | --- | --- | --- | --- | --- | --- | --- | --- | --- | --- | --- | --- | --- | --- | --- | --- | --- | --- | --- | --- | --- | --- | --- | --- | --- | --- |
| K.QNSGTYNNQYMILDTK.K | N | 84.72 | 1888.8625 | 16 | 0.2 | 945.4387 | 2 | 44.21 | 6 | F6:24307 | NaSa01\_F14.raw |  |  |  |  |  | 3.8863E7 |  |  |  |  |  |  |  |  | 3 | 0 | 0 | 0 | 0 | 0 | 3 | 0 | 0 | 0 | 0 | 0 | 0 | 0 | 0 | 351 | 366 |  |  | PEAKS DB |
| K.TTHQGLPESYNFDFVTMK.P | N | 66.93 | 2113.9778 | 18 | 0.6 | 705.6670 | 3 | 60.44 | 6 | F6:38288 | NaSa01\_F14.raw |  |  |  |  |  | 1.2388E7 |  |  |  |  |  |  |  |  | 1 | 0 | 0 | 0 | 0 | 0 | 1 | 0 | 0 | 0 | 0 | 0 | 0 | 0 | 0 | 529 | 546 |  |  | PEAKS DB |
| K.TTHQGLPESYNFDFVTM(+15.99)KPVL | N | 61.25 | 2439.1780 | 21 | 0.3 | 814.0668 | 3 | 68.67 | 6 | F6:45195 | NaSa01\_F14.raw |  |  |  |  |  | 1.177E7 |  |  |  |  |  |  |  |  | 1 | 0 | 0 | 0 | 0 | 0 | 1 | 0 | 0 | 0 | 0 | 0 | 0 | 0 | 0 | 529 | 549 | Oxidation (M) | M17:Oxidation (M):1000.00 | PEAKS DB |
| K.FTAYVINGPPVEK.G | Y | 60.41 | 1433.7554 | 13 | 0.1 | 717.8850 | 2 | 51.71 | 6 | F6:30779 | NaSa01\_F14.raw |  |  |  |  |  | 2.3974E7 |  |  |  |  |  |  |  |  | 1 | 0 | 0 | 0 | 0 | 0 | 1 | 0 | 0 | 0 | 0 | 0 | 0 | 0 | 0 | 504 | 516 |  |  | PEAKS DB |
| K.TTHQGLPESYNFDFVTMKPVL | N | 51.11 | 2423.1831 | 21 | -2.3 | 808.7332 | 3 | 77.66 | 6 | F6:52507 | NaSa01\_F14.raw |  |  |  |  |  | 5.5103E7 |  |  |  |  |  |  |  |  | 1 | 0 | 0 | 0 | 0 | 0 | 1 | 0 | 0 | 0 | 0 | 0 | 0 | 0 | 0 | 529 | 549 |  |  | PEAKS DB |
| K.QNSGTYNNQYM(+15.99)ILDTK.K | N | 49.76 | 1904.8574 | 16 | -0.1 | 953.4359 | 2 | 35.20 | 6 | F6:16927 | NaSa01\_F14.raw |  |  |  |  |  | 2.3713E6 |  |  |  |  |  |  |  |  | 1 | 0 | 0 | 0 | 0 | 0 | 1 | 0 | 0 | 0 | 0 | 0 | 0 | 0 | 0 | 351 | 366 | Oxidation (M) | M11:Oxidation (M):1000.00 | PEAKS DB |
| H.SSWFTYAATLR.I | N | 49.21 | 1301.6404 | 11 | 0.0 | 651.8275 | 2 | 68.60 | 6 | F6:45310 | NaSa01\_F14.raw |  |  |  |  |  | 1.0363E7 |  |  |  |  |  |  |  |  | 1 | 0 | 0 | 0 | 0 | 0 | 1 | 0 | 0 | 0 | 0 | 0 | 0 | 0 | 0 | 251 | 261 |  |  | PEAKS DB |
| L.DFSYEMAPR.A | N | 43.68 | 1114.4753 | 9 | 0.5 | 558.2452 | 2 | 40.56 | 9 | F9:17621 | NaSa01\_F4.raw |  |  |  |  |  |  |  |  | 8.8862E5 |  |  |  |  |  | 1 | 0 | 0 | 0 | 0 | 0 | 0 | 0 | 0 | 1 | 0 | 0 | 0 | 0 | 0 | 426 | 434 |  |  | PEAKS DB |
| total 8 peptides |  |  |  |  |  |  |  |  |  |  |  |  |  |  |  |  |  |  |  |  |  |  |  |  |  |  |  |  |  |  |  |  |  |  |  |  |  |  |  |  |  |  |  |  |  |
| --- | --- | --- | --- | --- | --- | --- | --- | --- | --- | --- | --- | --- | --- | --- | --- | --- | --- | --- | --- | --- | --- | --- | --- | --- | --- | --- | --- | --- | --- | --- | --- | --- | --- | --- | --- | --- | --- | --- | --- | --- | --- | --- | --- | --- | --- |

LAA73439.1

back to list

  

| Protein Coverage
| Supporting Peptides
|

Protein Coverage:

Supporting Peptides:

| Peptide | Uniq | -10lgP | Mass | Length | ppm | m/z | z | RT | Fraction | Scan | Source File | Area F1 | Area F10 | Area F11 | Area F12 | Area F13 | Area F14 | Area F2 | Area F3 | Area F4 | Area F5 | Area F6 | Area F7 | Area F8 | Area F9 | #Feature | #Feature F1 | #Feature F10 | #Feature F11 | #Feature F12 | #Feature F13 | #Feature F14 | #Feature F2 | #Feature F3 | #Feature F4 | #Feature F5 | #Feature F6 | #Feature F7 | #Feature F8 | #Feature F9 | Start | End | PTM | AScore | Found By |
| --- | --- | --- | --- | --- | --- | --- | --- | --- | --- | --- | --- | --- | --- | --- | --- | --- | --- | --- | --- | --- | --- | --- | --- | --- | --- | --- | --- | --- | --- | --- | --- | --- | --- | --- | --- | --- | --- | --- | --- | --- | --- | --- | --- | --- | --- |
| K.QNSGTYNNQYMILDTK.K | N | 84.72 | 1888.8625 | 16 | 0.2 | 945.4387 | 2 | 44.21 | 6 | F6:24307 | NaSa01\_F14.raw |  |  |  |  |  | 3.8863E7 |  |  |  |  |  |  |  |  | 3 | 0 | 0 | 0 | 0 | 0 | 3 | 0 | 0 | 0 | 0 | 0 | 0 | 0 | 0 | 349 | 364 |  |  | PEAKS DB |
| K.TTHQGLPESYNFDFVTMK.P | N | 66.93 | 2113.9778 | 18 | 0.6 | 705.6670 | 3 | 60.44 | 6 | F6:38288 | NaSa01\_F14.raw |  |  |  |  |  | 1.2388E7 |  |  |  |  |  |  |  |  | 1 | 0 | 0 | 0 | 0 | 0 | 1 | 0 | 0 | 0 | 0 | 0 | 0 | 0 | 0 | 527 | 544 |  |  | PEAKS DB |
| K.TTHQGLPESYNFDFVTM(+15.99)KPVL | N | 61.25 | 2439.1780 | 21 | 0.3 | 814.0668 | 3 | 68.67 | 6 | F6:45195 | NaSa01\_F14.raw |  |  |  |  |  | 1.177E7 |  |  |  |  |  |  |  |  | 1 | 0 | 0 | 0 | 0 | 0 | 1 | 0 | 0 | 0 | 0 | 0 | 0 | 0 | 0 | 527 | 547 | Oxidation (M) | M17:Oxidation (M):1000.00 | PEAKS DB |
| K.FTAYVINGPPVEK.G | Y | 60.41 | 1433.7554 | 13 | 0.1 | 717.8850 | 2 | 51.71 | 6 | F6:30779 | NaSa01\_F14.raw |  |  |  |  |  | 2.3974E7 |  |  |  |  |  |  |  |  | 1 | 0 | 0 | 0 | 0 | 0 | 1 | 0 | 0 | 0 | 0 | 0 | 0 | 0 | 0 | 502 | 514 |  |  | PEAKS DB |
| K.TTHQGLPESYNFDFVTMKPVL | N | 51.11 | 2423.1831 | 21 | -2.3 | 808.7332 | 3 | 77.66 | 6 | F6:52507 | NaSa01\_F14.raw |  |  |  |  |  | 5.5103E7 |  |  |  |  |  |  |  |  | 1 | 0 | 0 | 0 | 0 | 0 | 1 | 0 | 0 | 0 | 0 | 0 | 0 | 0 | 0 | 527 | 547 |  |  | PEAKS DB |
| K.QNSGTYNNQYM(+15.99)ILDTK.K | N | 49.76 | 1904.8574 | 16 | -0.1 | 953.4359 | 2 | 35.20 | 6 | F6:16927 | NaSa01\_F14.raw |  |  |  |  |  | 2.3713E6 |  |  |  |  |  |  |  |  | 1 | 0 | 0 | 0 | 0 | 0 | 1 | 0 | 0 | 0 | 0 | 0 | 0 | 0 | 0 | 349 | 364 | Oxidation (M) | M11:Oxidation (M):1000.00 | PEAKS DB |
| H.SSWFTYAATLR.I | N | 49.21 | 1301.6404 | 11 | 0.0 | 651.8275 | 2 | 68.60 | 6 | F6:45310 | NaSa01\_F14.raw |  |  |  |  |  | 1.0363E7 |  |  |  |  |  |  |  |  | 1 | 0 | 0 | 0 | 0 | 0 | 1 | 0 | 0 | 0 | 0 | 0 | 0 | 0 | 0 | 249 | 259 |  |  | PEAKS DB |
| L.DFSYEMAPR.A | N | 43.68 | 1114.4753 | 9 | 0.5 | 558.2452 | 2 | 40.56 | 9 | F9:17621 | NaSa01\_F4.raw |  |  |  |  |  |  |  |  | 8.8862E5 |  |  |  |  |  | 1 | 0 | 0 | 0 | 0 | 0 | 0 | 0 | 0 | 1 | 0 | 0 | 0 | 0 | 0 | 424 | 432 |  |  | PEAKS DB |
| total 8 peptides |  |  |  |  |  |  |  |  |  |  |  |  |  |  |  |  |  |  |  |  |  |  |  |  |  |  |  |  |  |  |  |  |  |  |  |  |  |  |  |  |  |  |  |  |  |
| --- | --- | --- | --- | --- | --- | --- | --- | --- | --- | --- | --- | --- | --- | --- | --- | --- | --- | --- | --- | --- | --- | --- | --- | --- | --- | --- | --- | --- | --- | --- | --- | --- | --- | --- | --- | --- | --- | --- | --- | --- | --- | --- | --- | --- | --- |

P0DI84.1

back to list

  

| Protein Coverage
| Supporting Peptides
|

Protein Coverage:

Supporting Peptides:

| Peptide | Uniq | -10lgP | Mass | Length | ppm | m/z | z | RT | Fraction | Scan | Source File | Area F1 | Area F10 | Area F11 | Area F12 | Area F13 | Area F14 | Area F2 | Area F3 | Area F4 | Area F5 | Area F6 | Area F7 | Area F8 | Area F9 | #Feature | #Feature F1 | #Feature F10 | #Feature F11 | #Feature F12 | #Feature F13 | #Feature F14 | #Feature F2 | #Feature F3 | #Feature F4 | #Feature F5 | #Feature F6 | #Feature F7 | #Feature F8 | #Feature F9 | Start | End | PTM | AScore | Found By |
| --- | --- | --- | --- | --- | --- | --- | --- | --- | --- | --- | --- | --- | --- | --- | --- | --- | --- | --- | --- | --- | --- | --- | --- | --- | --- | --- | --- | --- | --- | --- | --- | --- | --- | --- | --- | --- | --- | --- | --- | --- | --- | --- | --- | --- | --- |
| K.HVVVVGAGMSGLSAAYVLAGAGHK.V | Y | 66.54 | 2250.1943 | 24 | 0.9 | 751.0728 | 3 | 53.09 | 6 | F6:33685 | NaSa01\_F14.raw |  |  |  |  |  | 1.5605E8 |  |  |  |  |  |  |  |  | 3 | 0 | 0 | 0 | 0 | 0 | 3 | 0 | 0 | 0 | 0 | 0 | 0 | 0 | 0 | 33 | 56 |  |  | PEAKS DB |
| L.SAAYVLAGAGHK.V | N | 63.01 | 1143.6036 | 12 | -1.1 | 572.8085 | 2 | 11.49 | 6 | F6:1894 | NaSa01\_F14.raw |  |  |  |  |  | 4.4228E6 |  |  |  |  |  |  |  |  | 2 | 0 | 0 | 0 | 0 | 0 | 2 | 0 | 0 | 0 | 0 | 0 | 0 | 0 | 0 | 45 | 56 |  |  | PEAKS DB |
| K.YPVKPSEEGK.S | N | 62.09 | 1132.5764 | 10 | 1.6 | 567.2964 | 2 | 11.44 | 6 | F6:1813 | NaSa01\_F14.raw |  |  |  |  |  | 1.8552E6 |  |  |  |  |  |  |  |  | 2 | 0 | 0 | 0 | 0 | 0 | 2 | 0 | 0 | 0 | 0 | 0 | 0 | 0 | 0 | 140 | 149 |  |  | PEAKS DB |
| S.GLSAAYVLAGAGHK.V | N | 58.41 | 1313.7091 | 14 | 0.3 | 438.9104 | 3 | 32.92 | 6 | F6:14801 | NaSa01\_F14.raw |  |  |  |  |  | 1.2419E6 |  |  |  |  |  |  |  |  | 2 | 0 | 0 | 0 | 0 | 0 | 2 | 0 | 0 | 0 | 0 | 0 | 0 | 0 | 0 | 43 | 56 |  |  | PEAKS DB |
| K.STTDLPSR.F | N | 48.78 | 875.4348 | 8 | 0.1 | 438.7247 | 2 | 11.44 | 6 | F6:1817 | NaSa01\_F14.raw |  |  |  |  |  | 1.0568E6 |  |  |  |  |  |  |  |  | 1 | 0 | 0 | 0 | 0 | 0 | 1 | 0 | 0 | 0 | 0 | 0 | 0 | 0 | 0 | 344 | 351 |  |  | PEAKS DB |
| R.FDEIVGGM(+15.99)DQLPTSMYR.A | N | 44.47 | 1973.8862 | 17 | 1.5 | 987.9519 | 2 | 63.33 | 6 | F6:40829 | NaSa01\_F14.raw |  |  |  |  |  | 4.221E4 |  |  |  |  |  |  |  |  | 1 | 0 | 0 | 0 | 0 | 0 | 1 | 0 | 0 | 0 | 0 | 0 | 0 | 0 | 0 | 231 | 247 | Oxidation (M) | M8:Oxidation (M):44.44 | PEAKS DB |
| D.YEEFLEIAR.N | N | 44.25 | 1168.5764 | 9 | -0.6 | 585.2951 | 2 | 55.17 | 6 | F6:33697 | NaSa01\_F14.raw |  |  |  |  |  | 4.5232E5 |  |  |  |  |  |  |  |  | 1 | 0 | 0 | 0 | 0 | 0 | 1 | 0 | 0 | 0 | 0 | 0 | 0 | 0 | 0 | 14 | 22 |  |  | PEAKS DB |
| K.HVVVVGAGMSGLSAAY.V | N | 43.59 | 1516.7708 | 16 | -0.3 | 759.3925 | 2 | 46.90 | 5 | F5:27565 | NaSa01\_F13.raw |  |  |  |  | 1.9176E6 |  |  |  |  |  |  |  |  |  | 1 | 0 | 0 | 0 | 0 | 1 | 0 | 0 | 0 | 0 | 0 | 0 | 0 | 0 | 0 | 33 | 48 |  |  | PEAKS DB |
| R.FDEIVGGMDQLPTSMYR.A | N | 42.71 | 1957.8914 | 17 | 1.2 | 979.9541 | 2 | 75.35 | 6 | F6:50859 | NaSa01\_F14.raw |  |  |  |  |  | 6.6822E5 |  |  |  |  |  |  |  |  | 1 | 0 | 0 | 0 | 0 | 0 | 1 | 0 | 0 | 0 | 0 | 0 | 0 | 0 | 0 | 231 | 247 |  |  | PEAKS DB |
| T.DYEEFLEIAR.N | N | 39.92 | 1283.6033 | 10 | -6.5 | 642.8047 | 2 | 76.29 | 6 | F6:51560 | NaSa01\_F14.raw |  |  |  |  |  | 3.9682E6 |  |  |  |  |  |  |  |  | 1 | 0 | 0 | 0 | 0 | 0 | 1 | 0 | 0 | 0 | 0 | 0 | 0 | 0 | 0 | 13 | 22 |  |  | PEAKS DB |
| total 10 peptides |  |  |  |  |  |  |  |  |  |  |  |  |  |  |  |  |  |  |  |  |  |  |  |  |  |  |  |  |  |  |  |  |  |  |  |  |  |  |  |  |  |  |  |  |  |
| --- | --- | --- | --- | --- | --- | --- | --- | --- | --- | --- | --- | --- | --- | --- | --- | --- | --- | --- | --- | --- | --- | --- | --- | --- | --- | --- | --- | --- | --- | --- | --- | --- | --- | --- | --- | --- | --- | --- | --- | --- | --- | --- | --- | --- | --- |

3KVE

back to list

  

| Protein Coverage
| Supporting Peptides
|

Protein Coverage:

Supporting Peptides:

| Peptide | Uniq | -10lgP | Mass | Length | ppm | m/z | z | RT | Fraction | Scan | Source File | Area F1 | Area F10 | Area F11 | Area F12 | Area F13 | Area F14 | Area F2 | Area F3 | Area F4 | Area F5 | Area F6 | Area F7 | Area F8 | Area F9 | #Feature | #Feature F1 | #Feature F10 | #Feature F11 | #Feature F12 | #Feature F13 | #Feature F14 | #Feature F2 | #Feature F3 | #Feature F4 | #Feature F5 | #Feature F6 | #Feature F7 | #Feature F8 | #Feature F9 | Start | End | PTM | AScore | Found By |
| --- | --- | --- | --- | --- | --- | --- | --- | --- | --- | --- | --- | --- | --- | --- | --- | --- | --- | --- | --- | --- | --- | --- | --- | --- | --- | --- | --- | --- | --- | --- | --- | --- | --- | --- | --- | --- | --- | --- | --- | --- | --- | --- | --- | --- | --- |
| K.HVVVVGAGMSGLSAAYVLAGAGHK.V | Y | 66.54 | 2250.1943 | 24 | 0.9 | 751.0728 | 3 | 53.09 | 6 | F6:33685 | NaSa01\_F14.raw |  |  |  |  |  | 1.5605E8 |  |  |  |  |  |  |  |  | 3 | 0 | 0 | 0 | 0 | 0 | 3 | 0 | 0 | 0 | 0 | 0 | 0 | 0 | 0 | 35 | 58 |  |  | PEAKS DB |
| L.SAAYVLAGAGHK.V | N | 63.01 | 1143.6036 | 12 | -1.1 | 572.8085 | 2 | 11.49 | 6 | F6:1894 | NaSa01\_F14.raw |  |  |  |  |  | 4.4228E6 |  |  |  |  |  |  |  |  | 2 | 0 | 0 | 0 | 0 | 0 | 2 | 0 | 0 | 0 | 0 | 0 | 0 | 0 | 0 | 47 | 58 |  |  | PEAKS DB |
| K.YPVKPSEEGK.S | N | 62.09 | 1132.5764 | 10 | 1.6 | 567.2964 | 2 | 11.44 | 6 | F6:1813 | NaSa01\_F14.raw |  |  |  |  |  | 1.8552E6 |  |  |  |  |  |  |  |  | 2 | 0 | 0 | 0 | 0 | 0 | 2 | 0 | 0 | 0 | 0 | 0 | 0 | 0 | 0 | 142 | 151 |  |  | PEAKS DB |
| S.GLSAAYVLAGAGHK.V | N | 58.41 | 1313.7091 | 14 | 0.3 | 438.9104 | 3 | 32.92 | 6 | F6:14801 | NaSa01\_F14.raw |  |  |  |  |  | 1.2419E6 |  |  |  |  |  |  |  |  | 2 | 0 | 0 | 0 | 0 | 0 | 2 | 0 | 0 | 0 | 0 | 0 | 0 | 0 | 0 | 45 | 58 |  |  | PEAKS DB |
| K.STTDLPSR.F | N | 48.78 | 875.4348 | 8 | 0.1 | 438.7247 | 2 | 11.44 | 6 | F6:1817 | NaSa01\_F14.raw |  |  |  |  |  | 1.0568E6 |  |  |  |  |  |  |  |  | 1 | 0 | 0 | 0 | 0 | 0 | 1 | 0 | 0 | 0 | 0 | 0 | 0 | 0 | 0 | 346 | 353 |  |  | PEAKS DB |
| R.FDEIVGGM(+15.99)DQLPTSMYR.A | N | 44.47 | 1973.8862 | 17 | 1.5 | 987.9519 | 2 | 63.33 | 6 | F6:40829 | NaSa01\_F14.raw |  |  |  |  |  | 4.221E4 |  |  |  |  |  |  |  |  | 1 | 0 | 0 | 0 | 0 | 0 | 1 | 0 | 0 | 0 | 0 | 0 | 0 | 0 | 0 | 233 | 249 | Oxidation (M) | M8:Oxidation (M):44.44 | PEAKS DB |
| D.YEEFLEIAR.N | N | 44.25 | 1168.5764 | 9 | -0.6 | 585.2951 | 2 | 55.17 | 6 | F6:33697 | NaSa01\_F14.raw |  |  |  |  |  | 4.5232E5 |  |  |  |  |  |  |  |  | 1 | 0 | 0 | 0 | 0 | 0 | 1 | 0 | 0 | 0 | 0 | 0 | 0 | 0 | 0 | 16 | 24 |  |  | PEAKS DB |
| K.HVVVVGAGMSGLSAAY.V | N | 43.59 | 1516.7708 | 16 | -0.3 | 759.3925 | 2 | 46.90 | 5 | F5:27565 | NaSa01\_F13.raw |  |  |  |  | 1.9176E6 |  |  |  |  |  |  |  |  |  | 1 | 0 | 0 | 0 | 0 | 1 | 0 | 0 | 0 | 0 | 0 | 0 | 0 | 0 | 0 | 35 | 50 |  |  | PEAKS DB |
| R.FDEIVGGMDQLPTSMYR.A | N | 42.71 | 1957.8914 | 17 | 1.2 | 979.9541 | 2 | 75.35 | 6 | F6:50859 | NaSa01\_F14.raw |  |  |  |  |  | 6.6822E5 |  |  |  |  |  |  |  |  | 1 | 0 | 0 | 0 | 0 | 0 | 1 | 0 | 0 | 0 | 0 | 0 | 0 | 0 | 0 | 233 | 249 |  |  | PEAKS DB |
| T.DYEEFLEIAR.N | N | 39.92 | 1283.6033 | 10 | -6.5 | 642.8047 | 2 | 76.29 | 6 | F6:51560 | NaSa01\_F14.raw |  |  |  |  |  | 3.9682E6 |  |  |  |  |  |  |  |  | 1 | 0 | 0 | 0 | 0 | 0 | 1 | 0 | 0 | 0 | 0 | 0 | 0 | 0 | 0 | 15 | 24 |  |  | PEAKS DB |
| total 10 peptides |  |  |  |  |  |  |  |  |  |  |  |  |  |  |  |  |  |  |  |  |  |  |  |  |  |  |  |  |  |  |  |  |  |  |  |  |  |  |  |  |  |  |  |  |  |
| --- | --- | --- | --- | --- | --- | --- | --- | --- | --- | --- | --- | --- | --- | --- | --- | --- | --- | --- | --- | --- | --- | --- | --- | --- | --- | --- | --- | --- | --- | --- | --- | --- | --- | --- | --- | --- | --- | --- | --- | --- | --- | --- | --- | --- | --- |

XP\_013911763.1

back to list

  

| Protein Coverage
| Supporting Peptides
|

Protein Coverage:

Supporting Peptides:

| Peptide | Uniq | -10lgP | Mass | Length | ppm | m/z | z | RT | Fraction | Scan | Source File | Area F1 | Area F10 | Area F11 | Area F12 | Area F13 | Area F14 | Area F2 | Area F3 | Area F4 | Area F5 | Area F6 | Area F7 | Area F8 | Area F9 | #Feature | #Feature F1 | #Feature F10 | #Feature F11 | #Feature F12 | #Feature F13 | #Feature F14 | #Feature F2 | #Feature F3 | #Feature F4 | #Feature F5 | #Feature F6 | #Feature F7 | #Feature F8 | #Feature F9 | Start | End | PTM | AScore | Found By |
| --- | --- | --- | --- | --- | --- | --- | --- | --- | --- | --- | --- | --- | --- | --- | --- | --- | --- | --- | --- | --- | --- | --- | --- | --- | --- | --- | --- | --- | --- | --- | --- | --- | --- | --- | --- | --- | --- | --- | --- | --- | --- | --- | --- | --- | --- |
| R.MEWYPEAASNAER.W | N | 75.93 | 1552.6616 | 13 | -4.7 | 518.5587 | 3 | 42.47 | 4 | F4:25783 | NaSa01\_F12.raw |  |  | 8.9734E6 | 5.588E10 | 1.0906E8 | 2.4001E7 |  |  |  |  |  |  |  |  | 18 | 0 | 0 | 1 | 10 | 5 | 2 | 0 | 0 | 0 | 0 | 0 | 0 | 0 | 0 | 57 | 69 |  |  | PEAKS DB |
| R.M(+15.99)EWYPEAASNAER.W | N | 73.15 | 1568.6565 | 13 | 0.9 | 523.8932 | 3 | 33.16 | 4 | F4:17227 | NaSa01\_F12.raw |  |  |  | 1.0876E10 | 1.0638E7 | 4.7019E6 |  |  |  |  |  |  |  |  | 17 | 0 | 0 | 0 | 15 | 1 | 1 | 0 | 0 | 0 | 0 | 0 | 0 | 0 | 0 | 57 | 69 | Oxidation (M) | M1:Oxidation (M):1000.00 | PEAKS DB |
| L.RMEWYPEAASNAER.W | Y | 61.57 | 1708.7627 | 14 | 1.5 | 570.5957 | 3 | 29.80 | 4 | F4:14589 | NaSa01\_F12.raw |  |  |  | 1.6053E8 |  |  |  |  |  |  |  |  |  |  | 2 | 0 | 0 | 0 | 2 | 0 | 0 | 0 | 0 | 0 | 0 | 0 | 0 | 0 | 0 | 56 | 69 |  |  | PEAKS DB |
| S.VSPTASNMLRMEWYPEAASNAER.W | Y | 61.31 | 2609.2002 | 23 | -3.9 | 870.7373 | 3 | 75.89 | 4 | F4:52732 | NaSa01\_F12.raw |  |  |  | 2.002E7 |  |  |  |  |  |  |  |  |  |  | 2 | 0 | 0 | 0 | 2 | 0 | 0 | 0 | 0 | 0 | 0 | 0 | 0 | 0 | 0 | 47 | 69 |  |  | PEAKS DB |
| M.EWYPEAASNAER.W | N | 54.75 | 1421.6211 | 12 | 0.7 | 711.8183 | 2 | 30.32 | 4 | F4:14915 | NaSa01\_F12.raw |  |  |  | 1.4758E7 |  |  |  |  |  |  |  |  |  |  | 1 | 0 | 0 | 0 | 1 | 0 | 0 | 0 | 0 | 0 | 0 | 0 | 0 | 0 | 0 | 58 | 69 |  |  | PEAKS DB |
| R.MEWYPEAASN.A | N | 49.85 | 1196.4808 | 10 | 0.2 | 599.2478 | 2 | 59.73 | 4 | F4:39536 | NaSa01\_F12.raw |  |  |  | 6.0961E6 |  |  |  |  |  |  |  |  |  |  | 1 | 0 | 0 | 0 | 1 | 0 | 0 | 0 | 0 | 0 | 0 | 0 | 0 | 0 | 0 | 57 | 66 |  |  | PEAKS DB |
| L.RM(+15.99)EWYPEAASNAER.W | Y | 45.97 | 1724.7576 | 14 | 0.7 | 575.9269 | 3 | 21.34 | 4 | F4:7743 | NaSa01\_F12.raw |  |  |  | 2.1175E6 |  |  |  |  |  |  |  |  |  |  | 1 | 0 | 0 | 0 | 1 | 0 | 0 | 0 | 0 | 0 | 0 | 0 | 0 | 0 | 0 | 56 | 69 | Oxidation (M) | M2:Oxidation (M):1000.00 | PEAKS DB |
| R.MEWYPEAASNAE.R | N | 44.04 | 1396.5605 | 12 | 0.5 | 699.2879 | 2 | 61.61 | 4 | F4:41017 | NaSa01\_F12.raw |  |  |  | 2.7222E6 |  |  |  |  |  |  |  |  |  |  | 1 | 0 | 0 | 0 | 1 | 0 | 0 | 0 | 0 | 0 | 0 | 0 | 0 | 0 | 0 | 57 | 68 |  |  | PEAKS DB |
| P.TASNMLRMEWYPEAASNAER.W | Y | 42.07 | 2326.0471 | 20 | 5.6 | 776.3607 | 3 | 61.06 | 4 | F4:40604 | NaSa01\_F12.raw |  |  |  | 4.6724E4 |  |  |  |  |  |  |  |  |  |  | 1 | 0 | 0 | 0 | 1 | 0 | 0 | 0 | 0 | 0 | 0 | 0 | 0 | 0 | 0 | 50 | 69 |  |  | PEAKS DB |
| total 9 peptides |  |  |  |  |  |  |  |  |  |  |  |  |  |  |  |  |  |  |  |  |  |  |  |  |  |  |  |  |  |  |  |  |  |  |  |  |  |  |  |  |  |  |  |  |  |
| --- | --- | --- | --- | --- | --- | --- | --- | --- | --- | --- | --- | --- | --- | --- | --- | --- | --- | --- | --- | --- | --- | --- | --- | --- | --- | --- | --- | --- | --- | --- | --- | --- | --- | --- | --- | --- | --- | --- | --- | --- | --- | --- | --- | --- | --- |

XP\_032069584.1

back to list

  

| Protein Coverage
| Supporting Peptides
|

Protein Coverage:

Supporting Peptides:

| Peptide | Uniq | -10lgP | Mass | Length | ppm | m/z | z | RT | Fraction | Scan | Source File | Area F1 | Area F10 | Area F11 | Area F12 | Area F13 | Area F14 | Area F2 | Area F3 | Area F4 | Area F5 | Area F6 | Area F7 | Area F8 | Area F9 | #Feature | #Feature F1 | #Feature F10 | #Feature F11 | #Feature F12 | #Feature F13 | #Feature F14 | #Feature F2 | #Feature F3 | #Feature F4 | #Feature F5 | #Feature F6 | #Feature F7 | #Feature F8 | #Feature F9 | Start | End | PTM | AScore | Found By |
| --- | --- | --- | --- | --- | --- | --- | --- | --- | --- | --- | --- | --- | --- | --- | --- | --- | --- | --- | --- | --- | --- | --- | --- | --- | --- | --- | --- | --- | --- | --- | --- | --- | --- | --- | --- | --- | --- | --- | --- | --- | --- | --- | --- | --- | --- |
| R.MEWYPEAASNAER.W | N | 75.93 | 1552.6616 | 13 | -4.7 | 518.5587 | 3 | 42.47 | 4 | F4:25783 | NaSa01\_F12.raw |  |  | 8.9734E6 | 5.588E10 | 1.0906E8 | 2.4001E7 |  |  |  |  |  |  |  |  | 18 | 0 | 0 | 1 | 10 | 5 | 2 | 0 | 0 | 0 | 0 | 0 | 0 | 0 | 0 | 57 | 69 |  |  | PEAKS DB |
| R.M(+15.99)EWYPEAASNAER.W | N | 73.15 | 1568.6565 | 13 | 0.9 | 523.8932 | 3 | 33.16 | 4 | F4:17227 | NaSa01\_F12.raw |  |  |  | 1.0876E10 | 1.0638E7 | 4.7019E6 |  |  |  |  |  |  |  |  | 17 | 0 | 0 | 0 | 15 | 1 | 1 | 0 | 0 | 0 | 0 | 0 | 0 | 0 | 0 | 57 | 69 | Oxidation (M) | M1:Oxidation (M):1000.00 | PEAKS DB |
| L.RMEWYPEAASNAER.W | Y | 61.57 | 1708.7627 | 14 | 1.5 | 570.5957 | 3 | 29.80 | 4 | F4:14589 | NaSa01\_F12.raw |  |  |  | 1.6053E8 |  |  |  |  |  |  |  |  |  |  | 2 | 0 | 0 | 0 | 2 | 0 | 0 | 0 | 0 | 0 | 0 | 0 | 0 | 0 | 0 | 56 | 69 |  |  | PEAKS DB |
| S.VSPTASNMLRMEWYPEAASNAER.W | Y | 61.31 | 2609.2002 | 23 | -3.9 | 870.7373 | 3 | 75.89 | 4 | F4:52732 | NaSa01\_F12.raw |  |  |  | 2.002E7 |  |  |  |  |  |  |  |  |  |  | 2 | 0 | 0 | 0 | 2 | 0 | 0 | 0 | 0 | 0 | 0 | 0 | 0 | 0 | 0 | 47 | 69 |  |  | PEAKS DB |
| M.EWYPEAASNAER.W | N | 54.75 | 1421.6211 | 12 | 0.7 | 711.8183 | 2 | 30.32 | 4 | F4:14915 | NaSa01\_F12.raw |  |  |  | 1.4758E7 |  |  |  |  |  |  |  |  |  |  | 1 | 0 | 0 | 0 | 1 | 0 | 0 | 0 | 0 | 0 | 0 | 0 | 0 | 0 | 0 | 58 | 69 |  |  | PEAKS DB |
| R.MEWYPEAASN.A | N | 49.85 | 1196.4808 | 10 | 0.2 | 599.2478 | 2 | 59.73 | 4 | F4:39536 | NaSa01\_F12.raw |  |  |  | 6.0961E6 |  |  |  |  |  |  |  |  |  |  | 1 | 0 | 0 | 0 | 1 | 0 | 0 | 0 | 0 | 0 | 0 | 0 | 0 | 0 | 0 | 57 | 66 |  |  | PEAKS DB |
| L.RM(+15.99)EWYPEAASNAER.W | Y | 45.97 | 1724.7576 | 14 | 0.7 | 575.9269 | 3 | 21.34 | 4 | F4:7743 | NaSa01\_F12.raw |  |  |  | 2.1175E6 |  |  |  |  |  |  |  |  |  |  | 1 | 0 | 0 | 0 | 1 | 0 | 0 | 0 | 0 | 0 | 0 | 0 | 0 | 0 | 0 | 56 | 69 | Oxidation (M) | M2:Oxidation (M):1000.00 | PEAKS DB |
| R.MEWYPEAASNAE.R | N | 44.04 | 1396.5605 | 12 | 0.5 | 699.2879 | 2 | 61.61 | 4 | F4:41017 | NaSa01\_F12.raw |  |  |  | 2.7222E6 |  |  |  |  |  |  |  |  |  |  | 1 | 0 | 0 | 0 | 1 | 0 | 0 | 0 | 0 | 0 | 0 | 0 | 0 | 0 | 0 | 57 | 68 |  |  | PEAKS DB |
| P.TASNMLRMEWYPEAASNAER.W | Y | 42.07 | 2326.0471 | 20 | 5.6 | 776.3607 | 3 | 61.06 | 4 | F4:40604 | NaSa01\_F12.raw |  |  |  | 4.6724E4 |  |  |  |  |  |  |  |  |  |  | 1 | 0 | 0 | 0 | 1 | 0 | 0 | 0 | 0 | 0 | 0 | 0 | 0 | 0 | 0 | 50 | 69 |  |  | PEAKS DB |
| total 9 peptides |  |  |  |  |  |  |  |  |  |  |  |  |  |  |  |  |  |  |  |  |  |  |  |  |  |  |  |  |  |  |  |  |  |  |  |  |  |  |  |  |  |  |  |  |  |
| --- | --- | --- | --- | --- | --- | --- | --- | --- | --- | --- | --- | --- | --- | --- | --- | --- | --- | --- | --- | --- | --- | --- | --- | --- | --- | --- | --- | --- | --- | --- | --- | --- | --- | --- | --- | --- | --- | --- | --- | --- | --- | --- | --- | --- | --- |

P86368.1

back to list

  

| Protein Coverage
| Supporting Peptides
|

Protein Coverage:

Supporting Peptides:

| Peptide | Uniq | -10lgP | Mass | Length | ppm | m/z | z | RT | Fraction | Scan | Source File | Area F1 | Area F10 | Area F11 | Area F12 | Area F13 | Area F14 | Area F2 | Area F3 | Area F4 | Area F5 | Area F6 | Area F7 | Area F8 | Area F9 | #Feature | #Feature F1 | #Feature F10 | #Feature F11 | #Feature F12 | #Feature F13 | #Feature F14 | #Feature F2 | #Feature F3 | #Feature F4 | #Feature F5 | #Feature F6 | #Feature F7 | #Feature F8 | #Feature F9 | Start | End | PTM | AScore | Found By |
| --- | --- | --- | --- | --- | --- | --- | --- | --- | --- | --- | --- | --- | --- | --- | --- | --- | --- | --- | --- | --- | --- | --- | --- | --- | --- | --- | --- | --- | --- | --- | --- | --- | --- | --- | --- | --- | --- | --- | --- | --- | --- | --- | --- | --- | --- |
| R.C(+57.02)C(+57.02)FVHDC(+57.02)C(+57.02)YGNLPDC(+57.02)NPK.S | N | 73.65 | 2314.8687 | 18 | 0.7 | 772.6307 | 3 | 32.34 | 4 | F4:16838 | NaSa01\_F12.raw |  |  |  | 2.3387E7 |  | 7.5227E6 |  |  |  |  |  |  |  |  | 2 | 0 | 0 | 0 | 1 | 0 | 1 | 0 | 0 | 0 | 0 | 0 | 0 | 0 | 0 | 43 | 60 | Carbamidomethylation | C1:Carbamidomethylation:1000.00;C2:Carbamidomethylation:1000.00;C7:Carbamidomethylation:1000.00;C8:Carbamidomethylation:1000.00;C15:Carbamidomethylation:1000.00 | PEAKS DB |
| K.LAVPFYSSYGC(+57.02)YC(+57.02)GWGGK.A | Y | 71.85 | 2070.8967 | 18 | -0.1 | 1036.4556 | 2 | 75.90 | 6 | F6:51213 | NaSa01\_F14.raw |  |  |  |  |  | 2.0527E6 |  |  |  |  |  |  |  |  | 1 | 0 | 0 | 0 | 0 | 0 | 1 | 0 | 0 | 0 | 0 | 0 | 0 | 0 | 0 | 16 | 33 | Carbamidomethylation | C11:Carbamidomethylation:1000.00;C13:Carbamidomethylation:1000.00 | PEAKS DB |
| K.IYMLYPDFLC(+57.02)K.G | Y | 70.76 | 1461.7036 | 11 | 0.1 | 731.8591 | 2 | 83.77 | 6 | F6:57698 | NaSa01\_F14.raw |  |  |  | 1.8441E5 | 1.2227E6 | 2.3995E7 |  |  |  |  |  |  |  |  | 5 | 0 | 0 | 0 | 1 | 1 | 3 | 0 | 0 | 0 | 0 | 0 | 0 | 0 | 0 | 106 | 116 | Carbamidomethylation | C10:Carbamidomethylation:1000.00 | PEAKS DB |
| I.YMLYPDFLC(+57.02)K.G | N | 68.32 | 1348.6195 | 10 | 0.2 | 675.3171 | 2 | 75.10 | 4 | F4:52326 | NaSa01\_F12.raw |  |  |  | 2.1669E7 |  |  |  |  |  |  |  |  |  |  | 1 | 0 | 0 | 0 | 1 | 0 | 0 | 0 | 0 | 0 | 0 | 0 | 0 | 0 | 0 | 107 | 116 | Carbamidomethylation | C9:Carbamidomethylation:1000.00 | PEAKS DB |
| I.YM(+15.99)LYPDFLC(+57.02)K.G | N | 64.41 | 1364.6145 | 10 | 0.0 | 683.3145 | 2 | 68.02 | 4 | F4:46357 | NaSa01\_F12.raw |  |  |  | 2.115E6 |  |  |  |  |  |  |  |  |  |  | 1 | 0 | 0 | 0 | 1 | 0 | 0 | 0 | 0 | 0 | 0 | 0 | 0 | 0 | 0 | 107 | 116 | Oxidation (M); Carbamidomethylation | M2:Oxidation (M):1000.00;C9:Carbamidomethylation:1000.00 | PEAKS DB |
| F.YSSYGC(+57.02)YC(+57.02)GWGGK.A | N | 58.28 | 1543.5861 | 13 | 0.3 | 772.8005 | 2 | 35.31 | 6 | F6:16919 | NaSa01\_F14.raw |  |  |  |  |  | 5.8277E6 |  |  |  |  |  |  |  |  | 1 | 0 | 0 | 0 | 0 | 0 | 1 | 0 | 0 | 0 | 0 | 0 | 0 | 0 | 0 | 21 | 33 | Carbamidomethylation | C6:Carbamidomethylation:1000.00;C8:Carbamidomethylation:1000.00 | PEAKS DB |
| K.IYM(+15.99)LYPDFLC(+57.02)K.G | Y | 42.94 | 1477.6985 | 11 | 0.7 | 739.8571 | 2 | 73.98 | 6 | F6:50010 | NaSa01\_F14.raw |  |  |  |  |  | 3.3884E6 |  |  |  |  |  |  |  |  | 1 | 0 | 0 | 0 | 0 | 0 | 1 | 0 | 0 | 0 | 0 | 0 | 0 | 0 | 0 | 106 | 116 | Oxidation (M); Carbamidomethylation | M3:Oxidation (M):1000.00;C10:Carbamidomethylation:1000.00 | PEAKS DB |
| total 7 peptides |  |  |  |  |  |  |  |  |  |  |  |  |  |  |  |  |  |  |  |  |  |  |  |  |  |  |  |  |  |  |  |  |  |  |  |  |  |  |  |  |  |  |  |  |  |
| --- | --- | --- | --- | --- | --- | --- | --- | --- | --- | --- | --- | --- | --- | --- | --- | --- | --- | --- | --- | --- | --- | --- | --- | --- | --- | --- | --- | --- | --- | --- | --- | --- | --- | --- | --- | --- | --- | --- | --- | --- | --- | --- | --- | --- | --- |

AAS94269.1

back to list

  

| Protein Coverage
| Supporting Peptides
|

Protein Coverage:

Supporting Peptides:

| Peptide | Uniq | -10lgP | Mass | Length | ppm | m/z | z | RT | Fraction | Scan | Source File | Area F1 | Area F10 | Area F11 | Area F12 | Area F13 | Area F14 | Area F2 | Area F3 | Area F4 | Area F5 | Area F6 | Area F7 | Area F8 | Area F9 | #Feature | #Feature F1 | #Feature F10 | #Feature F11 | #Feature F12 | #Feature F13 | #Feature F14 | #Feature F2 | #Feature F3 | #Feature F4 | #Feature F5 | #Feature F6 | #Feature F7 | #Feature F8 | #Feature F9 | Start | End | PTM | AScore | Found By |
| --- | --- | --- | --- | --- | --- | --- | --- | --- | --- | --- | --- | --- | --- | --- | --- | --- | --- | --- | --- | --- | --- | --- | --- | --- | --- | --- | --- | --- | --- | --- | --- | --- | --- | --- | --- | --- | --- | --- | --- | --- | --- | --- | --- | --- | --- |
| R.EDHPVHNLGEHSVC(+57.02)DSVSAWVTK.T | Y | 84.66 | 2602.1870 | 23 | 0.3 | 651.5542 | 4 | 37.09 | 14 | F14:17045 | NaSa01\_F9.raw |  |  |  |  |  |  |  |  |  |  |  |  |  | 2.8907E7 | 2 | 0 | 0 | 0 | 0 | 0 | 0 | 0 | 0 | 0 | 0 | 0 | 0 | 0 | 2 | 126 | 148 | Carbamidomethylation | C14:Carbamidomethylation:1000.00 | PEAKS DB |
| R.GIDSSHWNSYC(+57.02)TETDTFIK.A | Y | 74.18 | 2259.9744 | 19 | 0.8 | 754.3326 | 3 | 51.08 | 14 | F14:29210 | NaSa01\_F9.raw |  | 0 |  |  |  |  |  |  |  |  |  |  |  | 2.1049E7 | 2 | 0 | 0 | 0 | 0 | 0 | 0 | 0 | 0 | 0 | 0 | 0 | 0 | 0 | 2 | 193 | 211 | Carbamidomethylation | C11:Carbamidomethylation:1000.00 | PEAKS DB |
| K.GNTVTVMENVNLDNK.V | Y | 72.22 | 1646.7933 | 15 | 0.6 | 824.4044 | 2 | 40.82 | 14 | F14:20386 | NaSa01\_F9.raw |  |  |  | 2.9768E5 |  |  |  |  |  |  |  |  | 8.3395E5 | 6.5179E6 | 3 | 0 | 0 | 0 | 1 | 0 | 0 | 0 | 0 | 0 | 0 | 0 | 0 | 1 | 1 | 156 | 170 |  |  | PEAKS DB |
| K.ALTMEGNQASWR.F | Y | 55.04 | 1362.6350 | 12 | 0.1 | 682.3248 | 2 | 32.66 | 4 | F4:16876 | NaSa01\_F12.raw |  | 9.701E5 |  | 4.8138E5 |  |  |  |  |  |  |  |  | 1.0698E6 | 6.7641E5 | 4 | 0 | 1 | 0 | 1 | 0 | 0 | 0 | 0 | 0 | 0 | 0 | 0 | 1 | 1 | 212 | 223 |  |  | PEAKS DB |
| R.IDTAC(+57.02)VC(+57.02)VITK.K | Y | 39.67 | 1278.6312 | 11 | 0.2 | 640.3230 | 2 | 33.56 | 13 | F13:21699 | NaSa01\_F8.raw |  |  |  |  |  |  |  |  |  |  |  |  | 4.9169E5 |  | 1 | 0 | 0 | 0 | 0 | 0 | 0 | 0 | 0 | 0 | 0 | 0 | 0 | 1 | 0 | 227 | 237 | Carbamidomethylation | C5:Carbamidomethylation:1000.00;C7:Carbamidomethylation:1000.00 | PEAKS DB |
| total 5 peptides |  |  |  |  |  |  |  |  |  |  |  |  |  |  |  |  |  |  |  |  |  |  |  |  |  |  |  |  |  |  |  |  |  |  |  |  |  |  |  |  |  |  |  |  |  |
| --- | --- | --- | --- | --- | --- | --- | --- | --- | --- | --- | --- | --- | --- | --- | --- | --- | --- | --- | --- | --- | --- | --- | --- | --- | --- | --- | --- | --- | --- | --- | --- | --- | --- | --- | --- | --- | --- | --- | --- | --- | --- | --- | --- | --- | --- |

Q5YF89.1

back to list

  

| Protein Coverage
| Supporting Peptides
|

Protein Coverage:

Supporting Peptides:

| Peptide | Uniq | -10lgP | Mass | Length | ppm | m/z | z | RT | Fraction | Scan | Source File | Area F1 | Area F10 | Area F11 | Area F12 | Area F13 | Area F14 | Area F2 | Area F3 | Area F4 | Area F5 | Area F6 | Area F7 | Area F8 | Area F9 | #Feature | #Feature F1 | #Feature F10 | #Feature F11 | #Feature F12 | #Feature F13 | #Feature F14 | #Feature F2 | #Feature F3 | #Feature F4 | #Feature F5 | #Feature F6 | #Feature F7 | #Feature F8 | #Feature F9 | Start | End | PTM | AScore | Found By |
| --- | --- | --- | --- | --- | --- | --- | --- | --- | --- | --- | --- | --- | --- | --- | --- | --- | --- | --- | --- | --- | --- | --- | --- | --- | --- | --- | --- | --- | --- | --- | --- | --- | --- | --- | --- | --- | --- | --- | --- | --- | --- | --- | --- | --- | --- |
| R.EDHPVHNLGEHSVC(+57.02)DSVSAWVTK.T | Y | 84.66 | 2602.1870 | 23 | 0.3 | 651.5542 | 4 | 37.09 | 14 | F14:17045 | NaSa01\_F9.raw |  |  |  |  |  |  |  |  |  |  |  |  |  | 2.8907E7 | 2 | 0 | 0 | 0 | 0 | 0 | 0 | 0 | 0 | 0 | 0 | 0 | 0 | 0 | 2 | 126 | 148 | Carbamidomethylation | C14:Carbamidomethylation:1000.00 | PEAKS DB |
| R.GIDSSHWNSYC(+57.02)TETDTFIK.A | Y | 74.18 | 2259.9744 | 19 | 0.8 | 754.3326 | 3 | 51.08 | 14 | F14:29210 | NaSa01\_F9.raw |  | 0 |  |  |  |  |  |  |  |  |  |  |  | 2.1049E7 | 2 | 0 | 0 | 0 | 0 | 0 | 0 | 0 | 0 | 0 | 0 | 0 | 0 | 0 | 2 | 193 | 211 | Carbamidomethylation | C11:Carbamidomethylation:1000.00 | PEAKS DB |
| K.GNTVTVMENVNLDNK.V | Y | 72.22 | 1646.7933 | 15 | 0.6 | 824.4044 | 2 | 40.82 | 14 | F14:20386 | NaSa01\_F9.raw |  |  |  | 2.9768E5 |  |  |  |  |  |  |  |  | 8.3395E5 | 6.5179E6 | 3 | 0 | 0 | 0 | 1 | 0 | 0 | 0 | 0 | 0 | 0 | 0 | 0 | 1 | 1 | 156 | 170 |  |  | PEAKS DB |
| K.ALTMEGNQASWR.F | Y | 55.04 | 1362.6350 | 12 | 0.1 | 682.3248 | 2 | 32.66 | 4 | F4:16876 | NaSa01\_F12.raw |  | 9.701E5 |  | 4.8138E5 |  |  |  |  |  |  |  |  | 1.0698E6 | 6.7641E5 | 4 | 0 | 1 | 0 | 1 | 0 | 0 | 0 | 0 | 0 | 0 | 0 | 0 | 1 | 1 | 212 | 223 |  |  | PEAKS DB |
| R.IDTAC(+57.02)VC(+57.02)VITK.K | Y | 39.67 | 1278.6312 | 11 | 0.2 | 640.3230 | 2 | 33.56 | 13 | F13:21699 | NaSa01\_F8.raw |  |  |  |  |  |  |  |  |  |  |  |  | 4.9169E5 |  | 1 | 0 | 0 | 0 | 0 | 0 | 0 | 0 | 0 | 0 | 0 | 0 | 0 | 1 | 0 | 227 | 237 | Carbamidomethylation | C5:Carbamidomethylation:1000.00;C7:Carbamidomethylation:1000.00 | PEAKS DB |
| total 5 peptides |  |  |  |  |  |  |  |  |  |  |  |  |  |  |  |  |  |  |  |  |  |  |  |  |  |  |  |  |  |  |  |  |  |  |  |  |  |  |  |  |  |  |  |  |  |
| --- | --- | --- | --- | --- | --- | --- | --- | --- | --- | --- | --- | --- | --- | --- | --- | --- | --- | --- | --- | --- | --- | --- | --- | --- | --- | --- | --- | --- | --- | --- | --- | --- | --- | --- | --- | --- | --- | --- | --- | --- | --- | --- | --- | --- | --- |

P01140.2

back to list

  

| Protein Coverage
| Supporting Peptides
|

Protein Coverage:

Supporting Peptides:

| Peptide | Uniq | -10lgP | Mass | Length | ppm | m/z | z | RT | Fraction | Scan | Source File | Area F1 | Area F10 | Area F11 | Area F12 | Area F13 | Area F14 | Area F2 | Area F3 | Area F4 | Area F5 | Area F6 | Area F7 | Area F8 | Area F9 | #Feature | #Feature F1 | #Feature F10 | #Feature F11 | #Feature F12 | #Feature F13 | #Feature F14 | #Feature F2 | #Feature F3 | #Feature F4 | #Feature F5 | #Feature F6 | #Feature F7 | #Feature F8 | #Feature F9 | Start | End | PTM | AScore | Found By |
| --- | --- | --- | --- | --- | --- | --- | --- | --- | --- | --- | --- | --- | --- | --- | --- | --- | --- | --- | --- | --- | --- | --- | --- | --- | --- | --- | --- | --- | --- | --- | --- | --- | --- | --- | --- | --- | --- | --- | --- | --- | --- | --- | --- | --- | --- |
| EDHPVHNLGEHSVC(+57.02)DSVSAWVTK.T | Y | 84.66 | 2602.1870 | 23 | 0.3 | 651.5542 | 4 | 37.09 | 14 | F14:17045 | NaSa01\_F9.raw |  |  |  |  |  |  |  |  |  |  |  |  |  | 2.8907E7 | 2 | 0 | 0 | 0 | 0 | 0 | 0 | 0 | 0 | 0 | 0 | 0 | 0 | 0 | 2 | 1 | 23 | Carbamidomethylation | C14:Carbamidomethylation:1000.00 | PEAKS DB |
| R.GIDSSHWNSYC(+57.02)TETDTFIK.A | Y | 74.18 | 2259.9744 | 19 | 0.8 | 754.3326 | 3 | 51.08 | 14 | F14:29210 | NaSa01\_F9.raw |  | 0 |  |  |  |  |  |  |  |  |  |  |  | 2.1049E7 | 2 | 0 | 0 | 0 | 0 | 0 | 0 | 0 | 0 | 0 | 0 | 0 | 0 | 0 | 2 | 68 | 86 | Carbamidomethylation | C11:Carbamidomethylation:1000.00 | PEAKS DB |
| K.GNTVTVMENVNLDNK.V | Y | 72.22 | 1646.7933 | 15 | 0.6 | 824.4044 | 2 | 40.82 | 14 | F14:20386 | NaSa01\_F9.raw |  |  |  | 2.9768E5 |  |  |  |  |  |  |  |  | 8.3395E5 | 6.5179E6 | 3 | 0 | 0 | 0 | 1 | 0 | 0 | 0 | 0 | 0 | 0 | 0 | 0 | 1 | 1 | 31 | 45 |  |  | PEAKS DB |
| K.ALTMEGNQASWR.F | Y | 55.04 | 1362.6350 | 12 | 0.1 | 682.3248 | 2 | 32.66 | 4 | F4:16876 | NaSa01\_F12.raw |  | 9.701E5 |  | 4.8138E5 |  |  |  |  |  |  |  |  | 1.0698E6 | 6.7641E5 | 4 | 0 | 1 | 0 | 1 | 0 | 0 | 0 | 0 | 0 | 0 | 0 | 0 | 1 | 1 | 87 | 98 |  |  | PEAKS DB |
| R.IDTAC(+57.02)VC(+57.02)VITK.K | Y | 39.67 | 1278.6312 | 11 | 0.2 | 640.3230 | 2 | 33.56 | 13 | F13:21699 | NaSa01\_F8.raw |  |  |  |  |  |  |  |  |  |  |  |  | 4.9169E5 |  | 1 | 0 | 0 | 0 | 0 | 0 | 0 | 0 | 0 | 0 | 0 | 0 | 0 | 1 | 0 | 102 | 112 | Carbamidomethylation | C5:Carbamidomethylation:1000.00;C7:Carbamidomethylation:1000.00 | PEAKS DB |
| total 5 peptides |  |  |  |  |  |  |  |  |  |  |  |  |  |  |  |  |  |  |  |  |  |  |  |  |  |  |  |  |  |  |  |  |  |  |  |  |  |  |  |  |  |  |  |  |  |
| --- | --- | --- | --- | --- | --- | --- | --- | --- | --- | --- | --- | --- | --- | --- | --- | --- | --- | --- | --- | --- | --- | --- | --- | --- | --- | --- | --- | --- | --- | --- | --- | --- | --- | --- | --- | --- | --- | --- | --- | --- | --- | --- | --- | --- | --- |

ACR83850.1

back to list

  

| Protein Coverage
| Supporting Peptides
|

Protein Coverage:

Supporting Peptides:

| Peptide | Uniq | -10lgP | Mass | Length | ppm | m/z | z | RT | Fraction | Scan | Source File | Area F1 | Area F10 | Area F11 | Area F12 | Area F13 | Area F14 | Area F2 | Area F3 | Area F4 | Area F5 | Area F6 | Area F7 | Area F8 | Area F9 | #Feature | #Feature F1 | #Feature F10 | #Feature F11 | #Feature F12 | #Feature F13 | #Feature F14 | #Feature F2 | #Feature F3 | #Feature F4 | #Feature F5 | #Feature F6 | #Feature F7 | #Feature F8 | #Feature F9 | Start | End | PTM | AScore | Found By |
| --- | --- | --- | --- | --- | --- | --- | --- | --- | --- | --- | --- | --- | --- | --- | --- | --- | --- | --- | --- | --- | --- | --- | --- | --- | --- | --- | --- | --- | --- | --- | --- | --- | --- | --- | --- | --- | --- | --- | --- | --- | --- | --- | --- | --- | --- |
| K.LTC(+57.02)HFQVWSRPWLDK.T | Y | 74.63 | 1971.9778 | 15 | 0.2 | 494.0018 | 4 | 48.21 | 14 | F14:26843 | NaSa01\_F9.raw |  | 2.1776E7 |  |  |  |  |  |  |  |  |  |  |  | 7.6624E6 | 4 | 0 | 2 | 0 | 0 | 0 | 0 | 0 | 0 | 0 | 0 | 0 | 0 | 0 | 2 | 118 | 132 | Carbamidomethylation | C3:Carbamidomethylation:1000.00 | PEAKS DB |
| K.YYLMMELVK.T | Y | 68.59 | 1188.5923 | 9 | -2.4 | 595.3020 | 2 | 70.87 | 14 | F14:45465 | NaSa01\_F9.raw |  | 7.6239E7 |  |  |  |  |  |  |  |  |  |  |  | 1.7105E8 | 4 | 0 | 2 | 0 | 0 | 0 | 0 | 0 | 0 | 0 | 0 | 0 | 0 | 0 | 2 | 80 | 88 |  |  | PEAKS DB |
| K.YYLMM(+15.99)ELVK.T | Y | 66.27 | 1204.5872 | 9 | -0.7 | 603.3004 | 2 | 58.27 | 14 | F14:34889 | NaSa01\_F9.raw |  |  |  |  |  |  |  |  |  |  |  |  |  | 1.0294E7 | 1 | 0 | 0 | 0 | 0 | 0 | 0 | 0 | 0 | 0 | 0 | 0 | 0 | 0 | 1 | 80 | 88 | Oxidation (M) | M5:Oxidation (M):40.00 | PEAKS DB |
| R.VVEAQSQVVAGEK.Y | Y | 61.79 | 1342.7092 | 13 | 0.4 | 672.3622 | 2 | 11.40 | 14 | F14:1843 | NaSa01\_F9.raw |  |  |  |  |  |  |  |  |  |  |  |  |  | 2.3597E5 | 1 | 0 | 0 | 0 | 0 | 0 | 0 | 0 | 0 | 0 | 0 | 0 | 0 | 0 | 1 | 67 | 79 |  |  | PEAKS DB |
| K.AAAFAVQEYNAR.S | Y | 58.55 | 1309.6415 | 12 | 0.5 | 655.8284 | 2 | 28.37 | 14 | F14:10203 | NaSa01\_F9.raw |  |  |  |  |  |  |  |  |  |  |  |  |  | 6.1518E5 | 1 | 0 | 0 | 0 | 0 | 0 | 0 | 0 | 0 | 0 | 0 | 0 | 0 | 0 | 1 | 44 | 55 |  |  | PEAKS DB |
| K.LTC(+57.02)HFQVWSRPWLDKTELTK.M | Y | 55.93 | 2544.2947 | 20 | 0.2 | 509.8663 | 5 | 47.53 | 2 | F2:24527 | NaSa01\_F10.raw |  | 1.353E7 |  |  |  |  |  |  |  |  |  |  |  |  | 3 | 0 | 3 | 0 | 0 | 0 | 0 | 0 | 0 | 0 | 0 | 0 | 0 | 0 | 0 | 118 | 137 | Carbamidomethylation | C3:Carbamidomethylation:1000.00 | PEAKS DB |
| K.YYLM(+15.99)MELVK.T | Y | 49.86 | 1204.5872 | 9 | 0.0 | 603.3008 | 2 | 56.88 | 14 | F14:33376 | NaSa01\_F9.raw |  |  |  |  |  |  |  |  |  |  |  |  |  | 1.56E7 | 1 | 0 | 0 | 0 | 0 | 0 | 0 | 0 | 0 | 0 | 0 | 0 | 0 | 0 | 1 | 80 | 88 | Oxidation (M) | M4:Oxidation (M):26.02 | PEAKS DB |
| K.LTC(+57.02)HFQVWSR.P | Y | 39.89 | 1332.6398 | 10 | 0.2 | 667.3273 | 2 | 29.82 | 2 | F2:10696 | NaSa01\_F10.raw |  | 1.4862E6 |  |  |  |  |  |  |  |  |  |  |  |  | 1 | 0 | 1 | 0 | 0 | 0 | 0 | 0 | 0 | 0 | 0 | 0 | 0 | 0 | 0 | 118 | 127 | Carbamidomethylation | C3:Carbamidomethylation:1000.00 | PEAKS DB |
| total 8 peptides |  |  |  |  |  |  |  |  |  |  |  |  |  |  |  |  |  |  |  |  |  |  |  |  |  |  |  |  |  |  |  |  |  |  |  |  |  |  |  |  |  |  |  |  |  |
| --- | --- | --- | --- | --- | --- | --- | --- | --- | --- | --- | --- | --- | --- | --- | --- | --- | --- | --- | --- | --- | --- | --- | --- | --- | --- | --- | --- | --- | --- | --- | --- | --- | --- | --- | --- | --- | --- | --- | --- | --- | --- | --- | --- | --- | --- |

E3P6P4.1

back to list

  

| Protein Coverage
| Supporting Peptides
|

Protein Coverage:

Supporting Peptides:

| Peptide | Uniq | -10lgP | Mass | Length | ppm | m/z | z | RT | Fraction | Scan | Source File | Area F1 | Area F10 | Area F11 | Area F12 | Area F13 | Area F14 | Area F2 | Area F3 | Area F4 | Area F5 | Area F6 | Area F7 | Area F8 | Area F9 | #Feature | #Feature F1 | #Feature F10 | #Feature F11 | #Feature F12 | #Feature F13 | #Feature F14 | #Feature F2 | #Feature F3 | #Feature F4 | #Feature F5 | #Feature F6 | #Feature F7 | #Feature F8 | #Feature F9 | Start | End | PTM | AScore | Found By |
| --- | --- | --- | --- | --- | --- | --- | --- | --- | --- | --- | --- | --- | --- | --- | --- | --- | --- | --- | --- | --- | --- | --- | --- | --- | --- | --- | --- | --- | --- | --- | --- | --- | --- | --- | --- | --- | --- | --- | --- | --- | --- | --- | --- | --- | --- |
| K.LTC(+57.02)HFQVWSRPWLDK.T | Y | 74.63 | 1971.9778 | 15 | 0.2 | 494.0018 | 4 | 48.21 | 14 | F14:26843 | NaSa01\_F9.raw |  | 2.1776E7 |  |  |  |  |  |  |  |  |  |  |  | 7.6624E6 | 4 | 0 | 2 | 0 | 0 | 0 | 0 | 0 | 0 | 0 | 0 | 0 | 0 | 0 | 2 | 118 | 132 | Carbamidomethylation | C3:Carbamidomethylation:1000.00 | PEAKS DB |
| K.YYLMMELVK.T | Y | 68.59 | 1188.5923 | 9 | -2.4 | 595.3020 | 2 | 70.87 | 14 | F14:45465 | NaSa01\_F9.raw |  | 7.6239E7 |  |  |  |  |  |  |  |  |  |  |  | 1.7105E8 | 4 | 0 | 2 | 0 | 0 | 0 | 0 | 0 | 0 | 0 | 0 | 0 | 0 | 0 | 2 | 80 | 88 |  |  | PEAKS DB |
| K.YYLMM(+15.99)ELVK.T | Y | 66.27 | 1204.5872 | 9 | -0.7 | 603.3004 | 2 | 58.27 | 14 | F14:34889 | NaSa01\_F9.raw |  |  |  |  |  |  |  |  |  |  |  |  |  | 1.0294E7 | 1 | 0 | 0 | 0 | 0 | 0 | 0 | 0 | 0 | 0 | 0 | 0 | 0 | 0 | 1 | 80 | 88 | Oxidation (M) | M5:Oxidation (M):40.00 | PEAKS DB |
| R.VVEAQSQVVAGEK.Y | Y | 61.79 | 1342.7092 | 13 | 0.4 | 672.3622 | 2 | 11.40 | 14 | F14:1843 | NaSa01\_F9.raw |  |  |  |  |  |  |  |  |  |  |  |  |  | 2.3597E5 | 1 | 0 | 0 | 0 | 0 | 0 | 0 | 0 | 0 | 0 | 0 | 0 | 0 | 0 | 1 | 67 | 79 |  |  | PEAKS DB |
| K.AAAFAVQEYNAR.S | Y | 58.55 | 1309.6415 | 12 | 0.5 | 655.8284 | 2 | 28.37 | 14 | F14:10203 | NaSa01\_F9.raw |  |  |  |  |  |  |  |  |  |  |  |  |  | 6.1518E5 | 1 | 0 | 0 | 0 | 0 | 0 | 0 | 0 | 0 | 0 | 0 | 0 | 0 | 0 | 1 | 44 | 55 |  |  | PEAKS DB |
| K.LTC(+57.02)HFQVWSRPWLDKTELTK.M | Y | 55.93 | 2544.2947 | 20 | 0.2 | 509.8663 | 5 | 47.53 | 2 | F2:24527 | NaSa01\_F10.raw |  | 1.353E7 |  |  |  |  |  |  |  |  |  |  |  |  | 3 | 0 | 3 | 0 | 0 | 0 | 0 | 0 | 0 | 0 | 0 | 0 | 0 | 0 | 0 | 118 | 137 | Carbamidomethylation | C3:Carbamidomethylation:1000.00 | PEAKS DB |
| K.YYLM(+15.99)MELVK.T | Y | 49.86 | 1204.5872 | 9 | 0.0 | 603.3008 | 2 | 56.88 | 14 | F14:33376 | NaSa01\_F9.raw |  |  |  |  |  |  |  |  |  |  |  |  |  | 1.56E7 | 1 | 0 | 0 | 0 | 0 | 0 | 0 | 0 | 0 | 0 | 0 | 0 | 0 | 0 | 1 | 80 | 88 | Oxidation (M) | M4:Oxidation (M):26.02 | PEAKS DB |
| K.LTC(+57.02)HFQVWSR.P | Y | 39.89 | 1332.6398 | 10 | 0.2 | 667.3273 | 2 | 29.82 | 2 | F2:10696 | NaSa01\_F10.raw |  | 1.4862E6 |  |  |  |  |  |  |  |  |  |  |  |  | 1 | 0 | 1 | 0 | 0 | 0 | 0 | 0 | 0 | 0 | 0 | 0 | 0 | 0 | 0 | 118 | 127 | Carbamidomethylation | C3:Carbamidomethylation:1000.00 | PEAKS DB |
| total 8 peptides |  |  |  |  |  |  |  |  |  |  |  |  |  |  |  |  |  |  |  |  |  |  |  |  |  |  |  |  |  |  |  |  |  |  |  |  |  |  |  |  |  |  |  |  |  |
| --- | --- | --- | --- | --- | --- | --- | --- | --- | --- | --- | --- | --- | --- | --- | --- | --- | --- | --- | --- | --- | --- | --- | --- | --- | --- | --- | --- | --- | --- | --- | --- | --- | --- | --- | --- | --- | --- | --- | --- | --- | --- | --- | --- | --- | --- |

JAS05092.1

back to list

  

| Protein Coverage
| Supporting Peptides
|

Protein Coverage:

Supporting Peptides:

| Peptide | Uniq | -10lgP | Mass | Length | ppm | m/z | z | RT | Fraction | Scan | Source File | Area F1 | Area F10 | Area F11 | Area F12 | Area F13 | Area F14 | Area F2 | Area F3 | Area F4 | Area F5 | Area F6 | Area F7 | Area F8 | Area F9 | #Feature | #Feature F1 | #Feature F10 | #Feature F11 | #Feature F12 | #Feature F13 | #Feature F14 | #Feature F2 | #Feature F3 | #Feature F4 | #Feature F5 | #Feature F6 | #Feature F7 | #Feature F8 | #Feature F9 | Start | End | PTM | AScore | Found By |
| --- | --- | --- | --- | --- | --- | --- | --- | --- | --- | --- | --- | --- | --- | --- | --- | --- | --- | --- | --- | --- | --- | --- | --- | --- | --- | --- | --- | --- | --- | --- | --- | --- | --- | --- | --- | --- | --- | --- | --- | --- | --- | --- | --- | --- | --- |
| R.NGHPC(+57.02)QNNQGYC(+57.02)YNGK.C | N | 68.53 | 1909.7584 | 16 | -0.2 | 637.5933 | 3 | 11.55 | 4 | F4:2526 | NaSa01\_F12.raw |  |  |  | 1.1419E6 |  |  |  |  |  |  |  |  |  |  | 2 | 0 | 0 | 0 | 2 | 0 | 0 | 0 | 0 | 0 | 0 | 0 | 0 | 0 | 0 | 494 | 509 | Carbamidomethylation | C5:Carbamidomethylation:1000.00;C12:Carbamidomethylation:1000.00 | PEAKS DB |
| N.LVASAMAHEMGHNLGINHDR.A | Y | 65.70 | 2172.0317 | 20 | -0.1 | 544.0151 | 4 | 30.26 | 6 | F6:12452 | NaSa01\_F14.raw |  |  |  |  | 2.1472E7 | 7.564E6 |  |  |  |  |  |  |  |  | 6 | 0 | 0 | 0 | 0 | 2 | 4 | 0 | 0 | 0 | 0 | 0 | 0 | 0 | 0 | 334 | 353 |  |  | PEAKS DB |
| R.AAKDDC(+57.02)DLPEFC(+57.02)TGR.S | Y | 60.59 | 1753.7400 | 15 | 0.5 | 877.8777 | 2 | 28.75 | 6 | F6:11315 | NaSa01\_F14.raw |  |  |  |  | 2.6538E7 | 4.4121E6 |  |  |  |  |  |  |  |  | 2 | 0 | 0 | 0 | 0 | 1 | 1 | 0 | 0 | 0 | 0 | 0 | 0 | 0 | 0 | 468 | 482 | Carbamidomethylation | C6:Carbamidomethylation:1000.00;C12:Carbamidomethylation:1000.00 | PEAKS DB |
| E.C(+57.02)PTDSFQR.N | N | 57.13 | 1009.4287 | 8 | 0.6 | 505.7219 | 2 | 11.48 | 6 | F6:1863 | NaSa01\_F14.raw |  |  |  |  | 8.893E5 | 9.5733E5 |  |  |  |  |  |  |  |  | 2 | 0 | 0 | 0 | 0 | 1 | 1 | 0 | 0 | 0 | 0 | 0 | 0 | 0 | 0 | 486 | 493 | Carbamidomethylation | C1:Carbamidomethylation:1000.00 | PEAKS DB |
| N.LVASAMAHEM(+15.99)GHNLGINHDR.A | Y | 46.53 | 2188.0266 | 20 | 0.3 | 548.0141 | 4 | 11.60 | 5 | F5:2063 | NaSa01\_F13.raw |  |  |  |  | 4.3361E6 |  |  |  |  |  |  |  |  |  | 2 | 0 | 0 | 0 | 0 | 2 | 0 | 0 | 0 | 0 | 0 | 0 | 0 | 0 | 0 | 334 | 353 | Oxidation (M) | M10:Oxidation (M):19.86 | PEAKS DB |
| R.DRPQC(+57.02)ILNKPL.S | N | 44.62 | 1352.7234 | 11 | 1.1 | 451.9156 | 3 | 27.50 | 5 | F5:11578 | NaSa01\_F13.raw |  |  |  |  | 1.9485E7 |  |  |  |  |  |  |  |  |  | 1 | 0 | 0 | 0 | 0 | 1 | 0 | 0 | 0 | 0 | 0 | 0 | 0 | 0 | 0 | 392 | 402 | Carbamidomethylation | C5:Carbamidomethylation:1000.00 | PEAKS DB |
| L.TNTPEQDR.Y | N | 44.60 | 959.4308 | 8 | 0.1 | 480.7227 | 2 | 11.42 | 6 | F6:1814 | NaSa01\_F14.raw |  |  |  |  |  | 1.6427E5 |  |  |  |  |  |  |  |  | 1 | 0 | 0 | 0 | 0 | 0 | 1 | 0 | 0 | 0 | 0 | 0 | 0 | 0 | 0 | 192 | 199 |  |  | PEAKS DB |
| total 7 peptides |  |  |  |  |  |  |  |  |  |  |  |  |  |  |  |  |  |  |  |  |  |  |  |  |  |  |  |  |  |  |  |  |  |  |  |  |  |  |  |  |  |  |  |  |  |
| --- | --- | --- | --- | --- | --- | --- | --- | --- | --- | --- | --- | --- | --- | --- | --- | --- | --- | --- | --- | --- | --- | --- | --- | --- | --- | --- | --- | --- | --- | --- | --- | --- | --- | --- | --- | --- | --- | --- | --- | --- | --- | --- | --- | --- | --- |

2CTX

back to list

  

| Protein Coverage
| Supporting Peptides
|

Protein Coverage:

Supporting Peptides:

| Peptide | Uniq | -10lgP | Mass | Length | ppm | m/z | z | RT | Fraction | Scan | Source File | Area F1 | Area F10 | Area F11 | Area F12 | Area F13 | Area F14 | Area F2 | Area F3 | Area F4 | Area F5 | Area F6 | Area F7 | Area F8 | Area F9 | #Feature | #Feature F1 | #Feature F10 | #Feature F11 | #Feature F12 | #Feature F13 | #Feature F14 | #Feature F2 | #Feature F3 | #Feature F4 | #Feature F5 | #Feature F6 | #Feature F7 | #Feature F8 | #Feature F9 | Start | End | PTM | AScore | Found By |
| --- | --- | --- | --- | --- | --- | --- | --- | --- | --- | --- | --- | --- | --- | --- | --- | --- | --- | --- | --- | --- | --- | --- | --- | --- | --- | --- | --- | --- | --- | --- | --- | --- | --- | --- | --- | --- | --- | --- | --- | --- | --- | --- | --- | --- | --- |
| K.TGVDIQC(+57.02)C(+57.02)STDNC(+57.02)NPFPTR.K | Y | 81.18 | 2240.9248 | 19 | 0.2 | 1121.4698 | 2 | 44.50 | 13 | F13:30830 | NaSa01\_F8.raw |  | 3.1989E6 | 1.2538E6 | 1.652E7 | 2.9518E6 | 1.2399E7 |  |  | 1.1947E8 | 3.3128E7 |  | 4.2361E6 | 7.1424E6 | 1.221E7 | 16 | 0 | 2 | 1 | 2 | 1 | 2 | 0 | 0 | 2 | 2 | 0 | 1 | 1 | 2 | 50 | 68 | Carbamidomethylation | C7:Carbamidomethylation:1000.00;C8:Carbamidomethylation:1000.00;C13:Carbamidomethylation:1000.00 | PEAKS DB |
| R.VDLGC(+57.02)AATC(+57.02)PTVK.T | N | 78.55 | 1390.6584 | 13 | 0.2 | 696.3367 | 2 | 26.62 | 9 | F9:7117 | NaSa01\_F4.raw |  |  |  | 2.5017E6 | 1.5468E6 |  |  |  | 1.9328E7 | 1.5111E7 | 1.1416E6 | 1.9075E6 | 3.9599E6 |  | 7 | 0 | 0 | 0 | 1 | 1 | 0 | 0 | 0 | 1 | 1 | 1 | 1 | 1 | 0 | 37 | 49 | Carbamidomethylation | C5:Carbamidomethylation:1000.00;C9:Carbamidomethylation:1000.00 | PEAKS DB |
| R.C(+57.02)FITPDITSK.D | N | 68.13 | 1180.5798 | 10 | 0.4 | 591.2974 | 2 | 41.82 | 14 | F14:21115 | NaSa01\_F9.raw |  | 2.9364E6 | 3.7851E6 | 1.1007E6 | 7.2035E6 | 3.3336E7 |  | 2.9701E5 | 2.5797E8 | 4.8308E7 | 4.5629E6 | 9.9249E6 | 2.4521E7 | 9.2393E6 | 24 | 0 | 1 | 1 | 1 | 2 | 3 | 0 | 1 | 4 | 3 | 2 | 1 | 3 | 2 | 3 | 12 | Carbamidomethylation | C1:Carbamidomethylation:1000.00 | PEAKS DB |
| V.KTGVDIQC(+57.02)C(+57.02)STDNC(+57.02)NPFPTR.K | Y | 39.58 | 2369.0198 | 20 | 0.6 | 790.6810 | 3 | 30.76 | 9 | F9:9895 | NaSa01\_F4.raw |  |  |  |  |  |  |  |  | 1.0333E6 |  |  |  |  |  | 1 | 0 | 0 | 0 | 0 | 0 | 0 | 0 | 0 | 1 | 0 | 0 | 0 | 0 | 0 | 49 | 68 | Carbamidomethylation | C8:Carbamidomethylation:1000.00;C9:Carbamidomethylation:1000.00;C14:Carbamidomethylation:1000.00 | PEAKS DB |
| total 4 peptides |  |  |  |  |  |  |  |  |  |  |  |  |  |  |  |  |  |  |  |  |  |  |  |  |  |  |  |  |  |  |  |  |  |  |  |  |  |  |  |  |  |  |  |  |  |
| --- | --- | --- | --- | --- | --- | --- | --- | --- | --- | --- | --- | --- | --- | --- | --- | --- | --- | --- | --- | --- | --- | --- | --- | --- | --- | --- | --- | --- | --- | --- | --- | --- | --- | --- | --- | --- | --- | --- | --- | --- | --- | --- | --- | --- | --- |

P01391.1

back to list

  

| Protein Coverage
| Supporting Peptides
|

Protein Coverage:

Supporting Peptides:

| Peptide | Uniq | -10lgP | Mass | Length | ppm | m/z | z | RT | Fraction | Scan | Source File | Area F1 | Area F10 | Area F11 | Area F12 | Area F13 | Area F14 | Area F2 | Area F3 | Area F4 | Area F5 | Area F6 | Area F7 | Area F8 | Area F9 | #Feature | #Feature F1 | #Feature F10 | #Feature F11 | #Feature F12 | #Feature F13 | #Feature F14 | #Feature F2 | #Feature F3 | #Feature F4 | #Feature F5 | #Feature F6 | #Feature F7 | #Feature F8 | #Feature F9 | Start | End | PTM | AScore | Found By |
| --- | --- | --- | --- | --- | --- | --- | --- | --- | --- | --- | --- | --- | --- | --- | --- | --- | --- | --- | --- | --- | --- | --- | --- | --- | --- | --- | --- | --- | --- | --- | --- | --- | --- | --- | --- | --- | --- | --- | --- | --- | --- | --- | --- | --- | --- |
| K.TGVDIQC(+57.02)C(+57.02)STDNC(+57.02)NPFPTR.K | Y | 81.18 | 2240.9248 | 19 | 0.2 | 1121.4698 | 2 | 44.50 | 13 | F13:30830 | NaSa01\_F8.raw |  | 3.1989E6 | 1.2538E6 | 1.652E7 | 2.9518E6 | 1.2399E7 |  |  | 1.1947E8 | 3.3128E7 |  | 4.2361E6 | 7.1424E6 | 1.221E7 | 16 | 0 | 2 | 1 | 2 | 1 | 2 | 0 | 0 | 2 | 2 | 0 | 1 | 1 | 2 | 50 | 68 | Carbamidomethylation | C7:Carbamidomethylation:1000.00;C8:Carbamidomethylation:1000.00;C13:Carbamidomethylation:1000.00 | PEAKS DB |
| R.VDLGC(+57.02)AATC(+57.02)PTVK.T | N | 78.55 | 1390.6584 | 13 | 0.2 | 696.3367 | 2 | 26.62 | 9 | F9:7117 | NaSa01\_F4.raw |  |  |  | 2.5017E6 | 1.5468E6 |  |  |  | 1.9328E7 | 1.5111E7 | 1.1416E6 | 1.9075E6 | 3.9599E6 |  | 7 | 0 | 0 | 0 | 1 | 1 | 0 | 0 | 0 | 1 | 1 | 1 | 1 | 1 | 0 | 37 | 49 | Carbamidomethylation | C5:Carbamidomethylation:1000.00;C9:Carbamidomethylation:1000.00 | PEAKS DB |
| R.C(+57.02)FITPDITSK.D | N | 68.13 | 1180.5798 | 10 | 0.4 | 591.2974 | 2 | 41.82 | 14 | F14:21115 | NaSa01\_F9.raw |  | 2.9364E6 | 3.7851E6 | 1.1007E6 | 7.2035E6 | 3.3336E7 |  | 2.9701E5 | 2.5797E8 | 4.8308E7 | 4.5629E6 | 9.9249E6 | 2.4521E7 | 9.2393E6 | 24 | 0 | 1 | 1 | 1 | 2 | 3 | 0 | 1 | 4 | 3 | 2 | 1 | 3 | 2 | 3 | 12 | Carbamidomethylation | C1:Carbamidomethylation:1000.00 | PEAKS DB |
| V.KTGVDIQC(+57.02)C(+57.02)STDNC(+57.02)NPFPTR.K | Y | 39.58 | 2369.0198 | 20 | 0.6 | 790.6810 | 3 | 30.76 | 9 | F9:9895 | NaSa01\_F4.raw |  |  |  |  |  |  |  |  | 1.0333E6 |  |  |  |  |  | 1 | 0 | 0 | 0 | 0 | 0 | 0 | 0 | 0 | 1 | 0 | 0 | 0 | 0 | 0 | 49 | 68 | Carbamidomethylation | C8:Carbamidomethylation:1000.00;C9:Carbamidomethylation:1000.00;C14:Carbamidomethylation:1000.00 | PEAKS DB |
| total 4 peptides |  |  |  |  |  |  |  |  |  |  |  |  |  |  |  |  |  |  |  |  |  |  |  |  |  |  |  |  |  |  |  |  |  |  |  |  |  |  |  |  |  |  |  |  |  |
| --- | --- | --- | --- | --- | --- | --- | --- | --- | --- | --- | --- | --- | --- | --- | --- | --- | --- | --- | --- | --- | --- | --- | --- | --- | --- | --- | --- | --- | --- | --- | --- | --- | --- | --- | --- | --- | --- | --- | --- | --- | --- | --- | --- | --- | --- |

1YI5

back to list

  

| Protein Coverage
| Supporting Peptides
|

Protein Coverage:

Supporting Peptides:

| Peptide | Uniq | -10lgP | Mass | Length | ppm | m/z | z | RT | Fraction | Scan | Source File | Area F1 | Area F10 | Area F11 | Area F12 | Area F13 | Area F14 | Area F2 | Area F3 | Area F4 | Area F5 | Area F6 | Area F7 | Area F8 | Area F9 | #Feature | #Feature F1 | #Feature F10 | #Feature F11 | #Feature F12 | #Feature F13 | #Feature F14 | #Feature F2 | #Feature F3 | #Feature F4 | #Feature F5 | #Feature F6 | #Feature F7 | #Feature F8 | #Feature F9 | Start | End | PTM | AScore | Found By |
| --- | --- | --- | --- | --- | --- | --- | --- | --- | --- | --- | --- | --- | --- | --- | --- | --- | --- | --- | --- | --- | --- | --- | --- | --- | --- | --- | --- | --- | --- | --- | --- | --- | --- | --- | --- | --- | --- | --- | --- | --- | --- | --- | --- | --- | --- |
| K.TGVDIQC(+57.02)C(+57.02)STDNC(+57.02)NPFPTR.K | Y | 81.18 | 2240.9248 | 19 | 0.2 | 1121.4698 | 2 | 44.50 | 13 | F13:30830 | NaSa01\_F8.raw |  | 3.1989E6 | 1.2538E6 | 1.652E7 | 2.9518E6 | 1.2399E7 |  |  | 1.1947E8 | 3.3128E7 |  | 4.2361E6 | 7.1424E6 | 1.221E7 | 16 | 0 | 2 | 1 | 2 | 1 | 2 | 0 | 0 | 2 | 2 | 0 | 1 | 1 | 2 | 50 | 68 | Carbamidomethylation | C7:Carbamidomethylation:1000.00;C8:Carbamidomethylation:1000.00;C13:Carbamidomethylation:1000.00 | PEAKS DB |
| R.VDLGC(+57.02)AATC(+57.02)PTVK.T | N | 78.55 | 1390.6584 | 13 | 0.2 | 696.3367 | 2 | 26.62 | 9 | F9:7117 | NaSa01\_F4.raw |  |  |  | 2.5017E6 | 1.5468E6 |  |  |  | 1.9328E7 | 1.5111E7 | 1.1416E6 | 1.9075E6 | 3.9599E6 |  | 7 | 0 | 0 | 0 | 1 | 1 | 0 | 0 | 0 | 1 | 1 | 1 | 1 | 1 | 0 | 37 | 49 | Carbamidomethylation | C5:Carbamidomethylation:1000.00;C9:Carbamidomethylation:1000.00 | PEAKS DB |
| R.C(+57.02)FITPDITSK.D | N | 68.13 | 1180.5798 | 10 | 0.4 | 591.2974 | 2 | 41.82 | 14 | F14:21115 | NaSa01\_F9.raw |  | 2.9364E6 | 3.7851E6 | 1.1007E6 | 7.2035E6 | 3.3336E7 |  | 2.9701E5 | 2.5797E8 | 4.8308E7 | 4.5629E6 | 9.9249E6 | 2.4521E7 | 9.2393E6 | 24 | 0 | 1 | 1 | 1 | 2 | 3 | 0 | 1 | 4 | 3 | 2 | 1 | 3 | 2 | 3 | 12 | Carbamidomethylation | C1:Carbamidomethylation:1000.00 | PEAKS DB |
| V.KTGVDIQC(+57.02)C(+57.02)STDNC(+57.02)NPFPTR.K | Y | 39.58 | 2369.0198 | 20 | 0.6 | 790.6810 | 3 | 30.76 | 9 | F9:9895 | NaSa01\_F4.raw |  |  |  |  |  |  |  |  | 1.0333E6 |  |  |  |  |  | 1 | 0 | 0 | 0 | 0 | 0 | 0 | 0 | 0 | 1 | 0 | 0 | 0 | 0 | 0 | 49 | 68 | Carbamidomethylation | C8:Carbamidomethylation:1000.00;C9:Carbamidomethylation:1000.00;C14:Carbamidomethylation:1000.00 | PEAKS DB |
| total 4 peptides |  |  |  |  |  |  |  |  |  |  |  |  |  |  |  |  |  |  |  |  |  |  |  |  |  |  |  |  |  |  |  |  |  |  |  |  |  |  |  |  |  |  |  |  |  |
| --- | --- | --- | --- | --- | --- | --- | --- | --- | --- | --- | --- | --- | --- | --- | --- | --- | --- | --- | --- | --- | --- | --- | --- | --- | --- | --- | --- | --- | --- | --- | --- | --- | --- | --- | --- | --- | --- | --- | --- | --- | --- | --- | --- | --- | --- |

1LXH

back to list

  

| Protein Coverage
| Supporting Peptides
|

Protein Coverage:

Supporting Peptides:

| Peptide | Uniq | -10lgP | Mass | Length | ppm | m/z | z | RT | Fraction | Scan | Source File | Area F1 | Area F10 | Area F11 | Area F12 | Area F13 | Area F14 | Area F2 | Area F3 | Area F4 | Area F5 | Area F6 | Area F7 | Area F8 | Area F9 | #Feature | #Feature F1 | #Feature F10 | #Feature F11 | #Feature F12 | #Feature F13 | #Feature F14 | #Feature F2 | #Feature F3 | #Feature F4 | #Feature F5 | #Feature F6 | #Feature F7 | #Feature F8 | #Feature F9 | Start | End | PTM | AScore | Found By |
| --- | --- | --- | --- | --- | --- | --- | --- | --- | --- | --- | --- | --- | --- | --- | --- | --- | --- | --- | --- | --- | --- | --- | --- | --- | --- | --- | --- | --- | --- | --- | --- | --- | --- | --- | --- | --- | --- | --- | --- | --- | --- | --- | --- | --- | --- |
| K.TGVDIQC(+57.02)C(+57.02)STDNC(+57.02)NPFPTR.K | Y | 81.18 | 2240.9248 | 19 | 0.2 | 1121.4698 | 2 | 44.50 | 13 | F13:30830 | NaSa01\_F8.raw |  | 3.1989E6 | 1.2538E6 | 1.652E7 | 2.9518E6 | 1.2399E7 |  |  | 1.1947E8 | 3.3128E7 |  | 4.2361E6 | 7.1424E6 | 1.221E7 | 16 | 0 | 2 | 1 | 2 | 1 | 2 | 0 | 0 | 2 | 2 | 0 | 1 | 1 | 2 | 50 | 68 | Carbamidomethylation | C7:Carbamidomethylation:1000.00;C8:Carbamidomethylation:1000.00;C13:Carbamidomethylation:1000.00 | PEAKS DB |
| R.VDLGC(+57.02)AATC(+57.02)PTVK.T | N | 78.55 | 1390.6584 | 13 | 0.2 | 696.3367 | 2 | 26.62 | 9 | F9:7117 | NaSa01\_F4.raw |  |  |  | 2.5017E6 | 1.5468E6 |  |  |  | 1.9328E7 | 1.5111E7 | 1.1416E6 | 1.9075E6 | 3.9599E6 |  | 7 | 0 | 0 | 0 | 1 | 1 | 0 | 0 | 0 | 1 | 1 | 1 | 1 | 1 | 0 | 37 | 49 | Carbamidomethylation | C5:Carbamidomethylation:1000.00;C9:Carbamidomethylation:1000.00 | PEAKS DB |
| R.C(+57.02)FITPDITSK.D | N | 68.13 | 1180.5798 | 10 | 0.4 | 591.2974 | 2 | 41.82 | 14 | F14:21115 | NaSa01\_F9.raw |  | 2.9364E6 | 3.7851E6 | 1.1007E6 | 7.2035E6 | 3.3336E7 |  | 2.9701E5 | 2.5797E8 | 4.8308E7 | 4.5629E6 | 9.9249E6 | 2.4521E7 | 9.2393E6 | 24 | 0 | 1 | 1 | 1 | 2 | 3 | 0 | 1 | 4 | 3 | 2 | 1 | 3 | 2 | 3 | 12 | Carbamidomethylation | C1:Carbamidomethylation:1000.00 | PEAKS DB |
| V.KTGVDIQC(+57.02)C(+57.02)STDNC(+57.02)NPFPTR.K | Y | 39.58 | 2369.0198 | 20 | 0.6 | 790.6810 | 3 | 30.76 | 9 | F9:9895 | NaSa01\_F4.raw |  |  |  |  |  |  |  |  | 1.0333E6 |  |  |  |  |  | 1 | 0 | 0 | 0 | 0 | 0 | 0 | 0 | 0 | 1 | 0 | 0 | 0 | 0 | 0 | 49 | 68 | Carbamidomethylation | C8:Carbamidomethylation:1000.00;C9:Carbamidomethylation:1000.00;C14:Carbamidomethylation:1000.00 | PEAKS DB |
| total 4 peptides |  |  |  |  |  |  |  |  |  |  |  |  |  |  |  |  |  |  |  |  |  |  |  |  |  |  |  |  |  |  |  |  |  |  |  |  |  |  |  |  |  |  |  |  |  |
| --- | --- | --- | --- | --- | --- | --- | --- | --- | --- | --- | --- | --- | --- | --- | --- | --- | --- | --- | --- | --- | --- | --- | --- | --- | --- | --- | --- | --- | --- | --- | --- | --- | --- | --- | --- | --- | --- | --- | --- | --- | --- | --- | --- | --- | --- |

1LXG

back to list

  

| Protein Coverage
| Supporting Peptides
|

Protein Coverage:

Supporting Peptides:

| Peptide | Uniq | -10lgP | Mass | Length | ppm | m/z | z | RT | Fraction | Scan | Source File | Area F1 | Area F10 | Area F11 | Area F12 | Area F13 | Area F14 | Area F2 | Area F3 | Area F4 | Area F5 | Area F6 | Area F7 | Area F8 | Area F9 | #Feature | #Feature F1 | #Feature F10 | #Feature F11 | #Feature F12 | #Feature F13 | #Feature F14 | #Feature F2 | #Feature F3 | #Feature F4 | #Feature F5 | #Feature F6 | #Feature F7 | #Feature F8 | #Feature F9 | Start | End | PTM | AScore | Found By |
| --- | --- | --- | --- | --- | --- | --- | --- | --- | --- | --- | --- | --- | --- | --- | --- | --- | --- | --- | --- | --- | --- | --- | --- | --- | --- | --- | --- | --- | --- | --- | --- | --- | --- | --- | --- | --- | --- | --- | --- | --- | --- | --- | --- | --- | --- |
| K.TGVDIQC(+57.02)C(+57.02)STDNC(+57.02)NPFPTR.K | Y | 81.18 | 2240.9248 | 19 | 0.2 | 1121.4698 | 2 | 44.50 | 13 | F13:30830 | NaSa01\_F8.raw |  | 3.1989E6 | 1.2538E6 | 1.652E7 | 2.9518E6 | 1.2399E7 |  |  | 1.1947E8 | 3.3128E7 |  | 4.2361E6 | 7.1424E6 | 1.221E7 | 16 | 0 | 2 | 1 | 2 | 1 | 2 | 0 | 0 | 2 | 2 | 0 | 1 | 1 | 2 | 50 | 68 | Carbamidomethylation | C7:Carbamidomethylation:1000.00;C8:Carbamidomethylation:1000.00;C13:Carbamidomethylation:1000.00 | PEAKS DB |
| R.VDLGC(+57.02)AATC(+57.02)PTVK.T | N | 78.55 | 1390.6584 | 13 | 0.2 | 696.3367 | 2 | 26.62 | 9 | F9:7117 | NaSa01\_F4.raw |  |  |  | 2.5017E6 | 1.5468E6 |  |  |  | 1.9328E7 | 1.5111E7 | 1.1416E6 | 1.9075E6 | 3.9599E6 |  | 7 | 0 | 0 | 0 | 1 | 1 | 0 | 0 | 0 | 1 | 1 | 1 | 1 | 1 | 0 | 37 | 49 | Carbamidomethylation | C5:Carbamidomethylation:1000.00;C9:Carbamidomethylation:1000.00 | PEAKS DB |
| R.C(+57.02)FITPDITSK.D | N | 68.13 | 1180.5798 | 10 | 0.4 | 591.2974 | 2 | 41.82 | 14 | F14:21115 | NaSa01\_F9.raw |  | 2.9364E6 | 3.7851E6 | 1.1007E6 | 7.2035E6 | 3.3336E7 |  | 2.9701E5 | 2.5797E8 | 4.8308E7 | 4.5629E6 | 9.9249E6 | 2.4521E7 | 9.2393E6 | 24 | 0 | 1 | 1 | 1 | 2 | 3 | 0 | 1 | 4 | 3 | 2 | 1 | 3 | 2 | 3 | 12 | Carbamidomethylation | C1:Carbamidomethylation:1000.00 | PEAKS DB |
| V.KTGVDIQC(+57.02)C(+57.02)STDNC(+57.02)NPFPTR.K | Y | 39.58 | 2369.0198 | 20 | 0.6 | 790.6810 | 3 | 30.76 | 9 | F9:9895 | NaSa01\_F4.raw |  |  |  |  |  |  |  |  | 1.0333E6 |  |  |  |  |  | 1 | 0 | 0 | 0 | 0 | 0 | 0 | 0 | 0 | 1 | 0 | 0 | 0 | 0 | 0 | 49 | 68 | Carbamidomethylation | C8:Carbamidomethylation:1000.00;C9:Carbamidomethylation:1000.00;C14:Carbamidomethylation:1000.00 | PEAKS DB |
| total 4 peptides |  |  |  |  |  |  |  |  |  |  |  |  |  |  |  |  |  |  |  |  |  |  |  |  |  |  |  |  |  |  |  |  |  |  |  |  |  |  |  |  |  |  |  |  |  |
| --- | --- | --- | --- | --- | --- | --- | --- | --- | --- | --- | --- | --- | --- | --- | --- | --- | --- | --- | --- | --- | --- | --- | --- | --- | --- | --- | --- | --- | --- | --- | --- | --- | --- | --- | --- | --- | --- | --- | --- | --- | --- | --- | --- | --- | --- |

4AEA

back to list

  

| Protein Coverage
| Supporting Peptides
|

Protein Coverage:

Supporting Peptides:

| Peptide | Uniq | -10lgP | Mass | Length | ppm | m/z | z | RT | Fraction | Scan | Source File | Area F1 | Area F10 | Area F11 | Area F12 | Area F13 | Area F14 | Area F2 | Area F3 | Area F4 | Area F5 | Area F6 | Area F7 | Area F8 | Area F9 | #Feature | #Feature F1 | #Feature F10 | #Feature F11 | #Feature F12 | #Feature F13 | #Feature F14 | #Feature F2 | #Feature F3 | #Feature F4 | #Feature F5 | #Feature F6 | #Feature F7 | #Feature F8 | #Feature F9 | Start | End | PTM | AScore | Found By |
| --- | --- | --- | --- | --- | --- | --- | --- | --- | --- | --- | --- | --- | --- | --- | --- | --- | --- | --- | --- | --- | --- | --- | --- | --- | --- | --- | --- | --- | --- | --- | --- | --- | --- | --- | --- | --- | --- | --- | --- | --- | --- | --- | --- | --- | --- |
| K.TGVDIQC(+57.02)C(+57.02)STDNC(+57.02)NPFPTR.K | Y | 81.18 | 2240.9248 | 19 | 0.2 | 1121.4698 | 2 | 44.50 | 13 | F13:30830 | NaSa01\_F8.raw |  | 3.1989E6 | 1.2538E6 | 1.652E7 | 2.9518E6 | 1.2399E7 |  |  | 1.1947E8 | 3.3128E7 |  | 4.2361E6 | 7.1424E6 | 1.221E7 | 16 | 0 | 2 | 1 | 2 | 1 | 2 | 0 | 0 | 2 | 2 | 0 | 1 | 1 | 2 | 50 | 68 | Carbamidomethylation | C7:Carbamidomethylation:1000.00;C8:Carbamidomethylation:1000.00;C13:Carbamidomethylation:1000.00 | PEAKS DB |
| R.VDLGC(+57.02)AATC(+57.02)PTVK.T | N | 78.55 | 1390.6584 | 13 | 0.2 | 696.3367 | 2 | 26.62 | 9 | F9:7117 | NaSa01\_F4.raw |  |  |  | 2.5017E6 | 1.5468E6 |  |  |  | 1.9328E7 | 1.5111E7 | 1.1416E6 | 1.9075E6 | 3.9599E6 |  | 7 | 0 | 0 | 0 | 1 | 1 | 0 | 0 | 0 | 1 | 1 | 1 | 1 | 1 | 0 | 37 | 49 | Carbamidomethylation | C5:Carbamidomethylation:1000.00;C9:Carbamidomethylation:1000.00 | PEAKS DB |
| R.C(+57.02)FITPDITSK.D | N | 68.13 | 1180.5798 | 10 | 0.4 | 591.2974 | 2 | 41.82 | 14 | F14:21115 | NaSa01\_F9.raw |  | 2.9364E6 | 3.7851E6 | 1.1007E6 | 7.2035E6 | 3.3336E7 |  | 2.9701E5 | 2.5797E8 | 4.8308E7 | 4.5629E6 | 9.9249E6 | 2.4521E7 | 9.2393E6 | 24 | 0 | 1 | 1 | 1 | 2 | 3 | 0 | 1 | 4 | 3 | 2 | 1 | 3 | 2 | 3 | 12 | Carbamidomethylation | C1:Carbamidomethylation:1000.00 | PEAKS DB |
| V.KTGVDIQC(+57.02)C(+57.02)STDNC(+57.02)NPFPTR.K | Y | 39.58 | 2369.0198 | 20 | 0.6 | 790.6810 | 3 | 30.76 | 9 | F9:9895 | NaSa01\_F4.raw |  |  |  |  |  |  |  |  | 1.0333E6 |  |  |  |  |  | 1 | 0 | 0 | 0 | 0 | 0 | 0 | 0 | 0 | 1 | 0 | 0 | 0 | 0 | 0 | 49 | 68 | Carbamidomethylation | C8:Carbamidomethylation:1000.00;C9:Carbamidomethylation:1000.00;C14:Carbamidomethylation:1000.00 | PEAKS DB |
| total 4 peptides |  |  |  |  |  |  |  |  |  |  |  |  |  |  |  |  |  |  |  |  |  |  |  |  |  |  |  |  |  |  |  |  |  |  |  |  |  |  |  |  |  |  |  |  |  |
| --- | --- | --- | --- | --- | --- | --- | --- | --- | --- | --- | --- | --- | --- | --- | --- | --- | --- | --- | --- | --- | --- | --- | --- | --- | --- | --- | --- | --- | --- | --- | --- | --- | --- | --- | --- | --- | --- | --- | --- | --- | --- | --- | --- | --- | --- |

1CTX

back to list

  

| Protein Coverage
| Supporting Peptides
|

Protein Coverage:

Supporting Peptides:

| Peptide | Uniq | -10lgP | Mass | Length | ppm | m/z | z | RT | Fraction | Scan | Source File | Area F1 | Area F10 | Area F11 | Area F12 | Area F13 | Area F14 | Area F2 | Area F3 | Area F4 | Area F5 | Area F6 | Area F7 | Area F8 | Area F9 | #Feature | #Feature F1 | #Feature F10 | #Feature F11 | #Feature F12 | #Feature F13 | #Feature F14 | #Feature F2 | #Feature F3 | #Feature F4 | #Feature F5 | #Feature F6 | #Feature F7 | #Feature F8 | #Feature F9 | Start | End | PTM | AScore | Found By |
| --- | --- | --- | --- | --- | --- | --- | --- | --- | --- | --- | --- | --- | --- | --- | --- | --- | --- | --- | --- | --- | --- | --- | --- | --- | --- | --- | --- | --- | --- | --- | --- | --- | --- | --- | --- | --- | --- | --- | --- | --- | --- | --- | --- | --- | --- |
| K.TGVDIQC(+57.02)C(+57.02)STDNC(+57.02)NPFPTR.K | Y | 81.18 | 2240.9248 | 19 | 0.2 | 1121.4698 | 2 | 44.50 | 13 | F13:30830 | NaSa01\_F8.raw |  | 3.1989E6 | 1.2538E6 | 1.652E7 | 2.9518E6 | 1.2399E7 |  |  | 1.1947E8 | 3.3128E7 |  | 4.2361E6 | 7.1424E6 | 1.221E7 | 16 | 0 | 2 | 1 | 2 | 1 | 2 | 0 | 0 | 2 | 2 | 0 | 1 | 1 | 2 | 50 | 68 | Carbamidomethylation | C7:Carbamidomethylation:1000.00;C8:Carbamidomethylation:1000.00;C13:Carbamidomethylation:1000.00 | PEAKS DB |
| R.VDLGC(+57.02)AATC(+57.02)PTVK.T | N | 78.55 | 1390.6584 | 13 | 0.2 | 696.3367 | 2 | 26.62 | 9 | F9:7117 | NaSa01\_F4.raw |  |  |  | 2.5017E6 | 1.5468E6 |  |  |  | 1.9328E7 | 1.5111E7 | 1.1416E6 | 1.9075E6 | 3.9599E6 |  | 7 | 0 | 0 | 0 | 1 | 1 | 0 | 0 | 0 | 1 | 1 | 1 | 1 | 1 | 0 | 37 | 49 | Carbamidomethylation | C5:Carbamidomethylation:1000.00;C9:Carbamidomethylation:1000.00 | PEAKS DB |
| R.C(+57.02)FITPDITSK.D | N | 68.13 | 1180.5798 | 10 | 0.4 | 591.2974 | 2 | 41.82 | 14 | F14:21115 | NaSa01\_F9.raw |  | 2.9364E6 | 3.7851E6 | 1.1007E6 | 7.2035E6 | 3.3336E7 |  | 2.9701E5 | 2.5797E8 | 4.8308E7 | 4.5629E6 | 9.9249E6 | 2.4521E7 | 9.2393E6 | 24 | 0 | 1 | 1 | 1 | 2 | 3 | 0 | 1 | 4 | 3 | 2 | 1 | 3 | 2 | 3 | 12 | Carbamidomethylation | C1:Carbamidomethylation:1000.00 | PEAKS DB |
| V.KTGVDIQC(+57.02)C(+57.02)STDNC(+57.02)NPFPTR.K | Y | 39.58 | 2369.0198 | 20 | 0.6 | 790.6810 | 3 | 30.76 | 9 | F9:9895 | NaSa01\_F4.raw |  |  |  |  |  |  |  |  | 1.0333E6 |  |  |  |  |  | 1 | 0 | 0 | 0 | 0 | 0 | 0 | 0 | 0 | 1 | 0 | 0 | 0 | 0 | 0 | 49 | 68 | Carbamidomethylation | C8:Carbamidomethylation:1000.00;C9:Carbamidomethylation:1000.00;C14:Carbamidomethylation:1000.00 | PEAKS DB |
| total 4 peptides |  |  |  |  |  |  |  |  |  |  |  |  |  |  |  |  |  |  |  |  |  |  |  |  |  |  |  |  |  |  |  |  |  |  |  |  |  |  |  |  |  |  |  |  |  |
| --- | --- | --- | --- | --- | --- | --- | --- | --- | --- | --- | --- | --- | --- | --- | --- | --- | --- | --- | --- | --- | --- | --- | --- | --- | --- | --- | --- | --- | --- | --- | --- | --- | --- | --- | --- | --- | --- | --- | --- | --- | --- | --- | --- | --- | --- |

XP\_026568326.1

back to list

  

| Protein Coverage
| Supporting Peptides
|

Protein Coverage:

Supporting Peptides:

| Peptide | Uniq | -10lgP | Mass | Length | ppm | m/z | z | RT | Fraction | Scan | Source File | Area F1 | Area F10 | Area F11 | Area F12 | Area F13 | Area F14 | Area F2 | Area F3 | Area F4 | Area F5 | Area F6 | Area F7 | Area F8 | Area F9 | #Feature | #Feature F1 | #Feature F10 | #Feature F11 | #Feature F12 | #Feature F13 | #Feature F14 | #Feature F2 | #Feature F3 | #Feature F4 | #Feature F5 | #Feature F6 | #Feature F7 | #Feature F8 | #Feature F9 | Start | End | PTM | AScore | Found By |
| --- | --- | --- | --- | --- | --- | --- | --- | --- | --- | --- | --- | --- | --- | --- | --- | --- | --- | --- | --- | --- | --- | --- | --- | --- | --- | --- | --- | --- | --- | --- | --- | --- | --- | --- | --- | --- | --- | --- | --- | --- | --- | --- | --- | --- | --- |
| K.VTHAVVTVPAYFNDAQR.Q | Y | 61.81 | 1886.9639 | 17 | 0.2 | 629.9954 | 3 | 42.58 | 6 | F6:23016 | NaSa01\_F14.raw |  |  |  |  | 5.2872E5 | 1.0452E6 |  |  |  |  |  |  |  |  | 2 | 0 | 0 | 0 | 0 | 1 | 1 | 0 | 0 | 0 | 0 | 0 | 0 | 0 | 0 | 165 | 181 |  |  | PEAKS DB |
| R.ITPSYVAFTPEGER.L | Y | 58.58 | 1565.7725 | 14 | 0.5 | 783.8939 | 2 | 48.69 | 6 | F6:28273 | NaSa01\_F14.raw |  |  |  |  | 4.3411E5 | 9.2165E5 |  |  |  |  |  |  |  |  | 2 | 0 | 0 | 0 | 0 | 1 | 1 | 0 | 0 | 0 | 0 | 0 | 0 | 0 | 0 | 61 | 74 |  |  | PEAKS DB |
| K.SQIFSTASDNQPTVTIK.V | Y | 58.52 | 1835.9265 | 17 | -0.1 | 918.9705 | 2 | 44.59 | 6 | F6:24768 | NaSa01\_F14.raw |  |  |  |  | 1.4375E6 | 2.2847E6 |  |  |  |  |  |  |  |  | 2 | 0 | 0 | 0 | 0 | 1 | 1 | 0 | 0 | 0 | 0 | 0 | 0 | 0 | 0 | 448 | 464 |  |  | PEAKS DB |
| K.NQLTSNPENTVFDAK.R | Y | 54.59 | 1676.8005 | 15 | 0.2 | 839.4077 | 2 | 38.01 | 6 | F6:19036 | NaSa01\_F14.raw |  |  |  |  |  | 7.3924E5 |  |  |  |  |  |  |  |  | 1 | 0 | 0 | 0 | 0 | 0 | 1 | 0 | 0 | 0 | 0 | 0 | 0 | 0 | 0 | 82 | 96 |  |  | PEAKS DB |
| K.TFAPEEISAMVLTK.M | Y | 50.71 | 1535.7905 | 14 | 0.1 | 768.9026 | 2 | 75.48 | 6 | F6:50956 | NaSa01\_F14.raw |  |  |  |  |  | 5.2163E5 |  |  |  |  |  |  |  |  | 1 | 0 | 0 | 0 | 0 | 0 | 1 | 0 | 0 | 0 | 0 | 0 | 0 | 0 | 0 | 139 | 152 |  |  | PEAKS DB |
| K.DNHLLGTFDLTGIPPAPR.G | Y | 49.44 | 1933.0057 | 18 | 1.9 | 645.3438 | 3 | 75.60 | 6 | F6:51163 | NaSa01\_F14.raw |  |  |  |  | 9.7125E5 | 6.634E5 |  |  |  |  |  |  |  |  | 2 | 0 | 0 | 0 | 0 | 1 | 1 | 0 | 0 | 0 | 0 | 0 | 0 | 0 | 0 | 475 | 492 |  |  | PEAKS DB |
| K.DAGTIAGLNVMR.I | Y | 45.79 | 1216.6234 | 12 | 0.0 | 609.3190 | 2 | 54.07 | 6 | F6:32718 | NaSa01\_F14.raw |  |  |  |  |  | 6.7879E5 |  |  |  |  |  |  |  |  | 1 | 0 | 0 | 0 | 0 | 0 | 1 | 0 | 0 | 0 | 0 | 0 | 0 | 0 | 0 | 186 | 197 |  |  | PEAKS DB |
| total 7 peptides |  |  |  |  |  |  |  |  |  |  |  |  |  |  |  |  |  |  |  |  |  |  |  |  |  |  |  |  |  |  |  |  |  |  |  |  |  |  |  |  |  |  |  |  |  |
| --- | --- | --- | --- | --- | --- | --- | --- | --- | --- | --- | --- | --- | --- | --- | --- | --- | --- | --- | --- | --- | --- | --- | --- | --- | --- | --- | --- | --- | --- | --- | --- | --- | --- | --- | --- | --- | --- | --- | --- | --- | --- | --- | --- | --- | --- |

CAB45156.1

back to list

  

| Protein Coverage
| Supporting Peptides
|

Protein Coverage:

Supporting Peptides:

| Peptide | Uniq | -10lgP | Mass | Length | ppm | m/z | z | RT | Fraction | Scan | Source File | Area F1 | Area F10 | Area F11 | Area F12 | Area F13 | Area F14 | Area F2 | Area F3 | Area F4 | Area F5 | Area F6 | Area F7 | Area F8 | Area F9 | #Feature | #Feature F1 | #Feature F10 | #Feature F11 | #Feature F12 | #Feature F13 | #Feature F14 | #Feature F2 | #Feature F3 | #Feature F4 | #Feature F5 | #Feature F6 | #Feature F7 | #Feature F8 | #Feature F9 | Start | End | PTM | AScore | Found By |
| --- | --- | --- | --- | --- | --- | --- | --- | --- | --- | --- | --- | --- | --- | --- | --- | --- | --- | --- | --- | --- | --- | --- | --- | --- | --- | --- | --- | --- | --- | --- | --- | --- | --- | --- | --- | --- | --- | --- | --- | --- | --- | --- | --- | --- | --- |
| F.SETIEIC(+57.02)PDGHNFC(+57.02)FK.K | Y | 81.16 | 1952.8396 | 16 | 0.6 | 977.4277 | 2 | 49.30 | 14 | F14:27737 | NaSa01\_F9.raw |  |  |  |  |  |  |  |  |  |  |  |  | 9.4527E7 | 4.2686E7 | 4 | 0 | 0 | 0 | 0 | 0 | 0 | 0 | 0 | 0 | 0 | 0 | 0 | 2 | 2 | 32 | 47 | Carbamidomethylation | C7:Carbamidomethylation:1000.00;C14:Carbamidomethylation:1000.00 | PEAKS DB |
| K.GITRLPWVIR.G | Y | 57.91 | 1209.7346 | 10 | 0.9 | 404.2525 | 3 | 51.91 | 13 | F13:36710 | NaSa01\_F8.raw |  |  |  |  |  |  |  |  |  |  |  |  | 4.8481E7 | 3.1634E6 | 4 | 0 | 0 | 0 | 0 | 0 | 0 | 0 | 0 | 0 | 0 | 0 | 0 | 2 | 2 | 52 | 61 |  |  | PEAKS DB |
| R.VYVDC(+57.02)C(+57.02)AR.D | Y | 57.59 | 1041.4371 | 8 | -1.9 | 521.7249 | 2 | 11.64 | 13 | F13:3824 | NaSa01\_F8.raw |  |  |  |  |  |  |  |  |  |  |  |  | 1.2669E7 |  | 1 | 0 | 0 | 0 | 0 | 0 | 0 | 0 | 0 | 0 | 0 | 0 | 0 | 1 | 0 | 74 | 81 | Carbamidomethylation | C5:Carbamidomethylation:1000.00;C6:Carbamidomethylation:1000.00 | PEAKS DB |
| K.GITRLPWVIRGC(+57.02)AATC(+57.02)PK.A | Y | 51.16 | 2055.0869 | 18 | 0.1 | 514.7791 | 4 | 41.28 | 13 | F13:28062 | NaSa01\_F8.raw |  |  |  |  |  |  |  |  |  |  |  |  | 8.9241E5 |  | 1 | 0 | 0 | 0 | 0 | 0 | 0 | 0 | 0 | 0 | 0 | 0 | 0 | 1 | 0 | 52 | 69 | Carbamidomethylation | C12:Carbamidomethylation:1000.00;C16:Carbamidomethylation:1000.00 | PEAKS DB |
| R.GC(+57.02)AATC(+57.02)PK.A | N | 45.47 | 863.3630 | 8 | -1.2 | 432.6882 | 2 | 11.63 | 13 | F13:3786 | NaSa01\_F8.raw |  |  |  |  |  |  |  |  |  |  |  | 1.055E6 | 1.1773E7 |  | 2 | 0 | 0 | 0 | 0 | 0 | 0 | 0 | 0 | 0 | 0 | 0 | 1 | 1 | 0 | 62 | 69 | Carbamidomethylation | C2:Carbamidomethylation:1000.00;C6:Carbamidomethylation:1000.00 | PEAKS DB |
| Y.SIFSETIEIC(+57.02)PDGHNFC(+57.02)FK.K | Y | 41.89 | 2300.0242 | 19 | 1.0 | 767.6827 | 3 | 74.25 | 13 | F13:56279 | NaSa01\_F8.raw |  |  |  |  |  |  |  |  |  |  |  |  | 3.2313E6 |  | 1 | 0 | 0 | 0 | 0 | 0 | 0 | 0 | 0 | 0 | 0 | 0 | 0 | 1 | 0 | 29 | 47 | Carbamidomethylation | C10:Carbamidomethylation:1000.00;C17:Carbamidomethylation:1000.00 | PEAKS DB |
| I.EIC(+57.02)PDGHNFC(+57.02)FK.K | Y | 41.09 | 1522.6333 | 12 | -0.4 | 508.5515 | 3 | 29.70 | 9 | F9:9458 | NaSa01\_F4.raw |  |  |  |  |  |  |  |  | 3.2308E5 |  |  |  |  |  | 1 | 0 | 0 | 0 | 0 | 0 | 0 | 0 | 0 | 1 | 0 | 0 | 0 | 0 | 0 | 36 | 47 | Carbamidomethylation | C3:Carbamidomethylation:1000.00;C10:Carbamidomethylation:1000.00 | PEAKS DB |
| total 7 peptides |  |  |  |  |  |  |  |  |  |  |  |  |  |  |  |  |  |  |  |  |  |  |  |  |  |  |  |  |  |  |  |  |  |  |  |  |  |  |  |  |  |  |  |  |  |
| --- | --- | --- | --- | --- | --- | --- | --- | --- | --- | --- | --- | --- | --- | --- | --- | --- | --- | --- | --- | --- | --- | --- | --- | --- | --- | --- | --- | --- | --- | --- | --- | --- | --- | --- | --- | --- | --- | --- | --- | --- | --- | --- | --- | --- | --- |

Q9W717.1

back to list

  

| Protein Coverage
| Supporting Peptides
|

Protein Coverage:

Supporting Peptides:

| Peptide | Uniq | -10lgP | Mass | Length | ppm | m/z | z | RT | Fraction | Scan | Source File | Area F1 | Area F10 | Area F11 | Area F12 | Area F13 | Area F14 | Area F2 | Area F3 | Area F4 | Area F5 | Area F6 | Area F7 | Area F8 | Area F9 | #Feature | #Feature F1 | #Feature F10 | #Feature F11 | #Feature F12 | #Feature F13 | #Feature F14 | #Feature F2 | #Feature F3 | #Feature F4 | #Feature F5 | #Feature F6 | #Feature F7 | #Feature F8 | #Feature F9 | Start | End | PTM | AScore | Found By |
| --- | --- | --- | --- | --- | --- | --- | --- | --- | --- | --- | --- | --- | --- | --- | --- | --- | --- | --- | --- | --- | --- | --- | --- | --- | --- | --- | --- | --- | --- | --- | --- | --- | --- | --- | --- | --- | --- | --- | --- | --- | --- | --- | --- | --- | --- |
| F.SETIEIC(+57.02)PDGHNFC(+57.02)FK.K | Y | 81.16 | 1952.8396 | 16 | 0.6 | 977.4277 | 2 | 49.30 | 14 | F14:27737 | NaSa01\_F9.raw |  |  |  |  |  |  |  |  |  |  |  |  | 9.4527E7 | 4.2686E7 | 4 | 0 | 0 | 0 | 0 | 0 | 0 | 0 | 0 | 0 | 0 | 0 | 0 | 2 | 2 | 32 | 47 | Carbamidomethylation | C7:Carbamidomethylation:1000.00;C14:Carbamidomethylation:1000.00 | PEAKS DB |
| K.GITRLPWVIR.G | Y | 57.91 | 1209.7346 | 10 | 0.9 | 404.2525 | 3 | 51.91 | 13 | F13:36710 | NaSa01\_F8.raw |  |  |  |  |  |  |  |  |  |  |  |  | 4.8481E7 | 3.1634E6 | 4 | 0 | 0 | 0 | 0 | 0 | 0 | 0 | 0 | 0 | 0 | 0 | 0 | 2 | 2 | 52 | 61 |  |  | PEAKS DB |
| R.VYVDC(+57.02)C(+57.02)AR.D | Y | 57.59 | 1041.4371 | 8 | -1.9 | 521.7249 | 2 | 11.64 | 13 | F13:3824 | NaSa01\_F8.raw |  |  |  |  |  |  |  |  |  |  |  |  | 1.2669E7 |  | 1 | 0 | 0 | 0 | 0 | 0 | 0 | 0 | 0 | 0 | 0 | 0 | 0 | 1 | 0 | 74 | 81 | Carbamidomethylation | C5:Carbamidomethylation:1000.00;C6:Carbamidomethylation:1000.00 | PEAKS DB |
| K.GITRLPWVIRGC(+57.02)AATC(+57.02)PK.A | Y | 51.16 | 2055.0869 | 18 | 0.1 | 514.7791 | 4 | 41.28 | 13 | F13:28062 | NaSa01\_F8.raw |  |  |  |  |  |  |  |  |  |  |  |  | 8.9241E5 |  | 1 | 0 | 0 | 0 | 0 | 0 | 0 | 0 | 0 | 0 | 0 | 0 | 0 | 1 | 0 | 52 | 69 | Carbamidomethylation | C12:Carbamidomethylation:1000.00;C16:Carbamidomethylation:1000.00 | PEAKS DB |
| R.GC(+57.02)AATC(+57.02)PK.A | N | 45.47 | 863.3630 | 8 | -1.2 | 432.6882 | 2 | 11.63 | 13 | F13:3786 | NaSa01\_F8.raw |  |  |  |  |  |  |  |  |  |  |  | 1.055E6 | 1.1773E7 |  | 2 | 0 | 0 | 0 | 0 | 0 | 0 | 0 | 0 | 0 | 0 | 0 | 1 | 1 | 0 | 62 | 69 | Carbamidomethylation | C2:Carbamidomethylation:1000.00;C6:Carbamidomethylation:1000.00 | PEAKS DB |
| Y.SIFSETIEIC(+57.02)PDGHNFC(+57.02)FK.K | Y | 41.89 | 2300.0242 | 19 | 1.0 | 767.6827 | 3 | 74.25 | 13 | F13:56279 | NaSa01\_F8.raw |  |  |  |  |  |  |  |  |  |  |  |  | 3.2313E6 |  | 1 | 0 | 0 | 0 | 0 | 0 | 0 | 0 | 0 | 0 | 0 | 0 | 0 | 1 | 0 | 29 | 47 | Carbamidomethylation | C10:Carbamidomethylation:1000.00;C17:Carbamidomethylation:1000.00 | PEAKS DB |
| I.EIC(+57.02)PDGHNFC(+57.02)FK.K | Y | 41.09 | 1522.6333 | 12 | -0.4 | 508.5515 | 3 | 29.70 | 9 | F9:9458 | NaSa01\_F4.raw |  |  |  |  |  |  |  |  | 3.2308E5 |  |  |  |  |  | 1 | 0 | 0 | 0 | 0 | 0 | 0 | 0 | 0 | 1 | 0 | 0 | 0 | 0 | 0 | 36 | 47 | Carbamidomethylation | C3:Carbamidomethylation:1000.00;C10:Carbamidomethylation:1000.00 | PEAKS DB |
| total 7 peptides |  |  |  |  |  |  |  |  |  |  |  |  |  |  |  |  |  |  |  |  |  |  |  |  |  |  |  |  |  |  |  |  |  |  |  |  |  |  |  |  |  |  |  |  |  |
| --- | --- | --- | --- | --- | --- | --- | --- | --- | --- | --- | --- | --- | --- | --- | --- | --- | --- | --- | --- | --- | --- | --- | --- | --- | --- | --- | --- | --- | --- | --- | --- | --- | --- | --- | --- | --- | --- | --- | --- | --- | --- | --- | --- | --- | --- |

APB88857.1

back to list

  

| Protein Coverage
| Supporting Peptides
|

Protein Coverage:

Supporting Peptides:

| Peptide | Uniq | -10lgP | Mass | Length | ppm | m/z | z | RT | Fraction | Scan | Source File | Area F1 | Area F10 | Area F11 | Area F12 | Area F13 | Area F14 | Area F2 | Area F3 | Area F4 | Area F5 | Area F6 | Area F7 | Area F8 | Area F9 | #Feature | #Feature F1 | #Feature F10 | #Feature F11 | #Feature F12 | #Feature F13 | #Feature F14 | #Feature F2 | #Feature F3 | #Feature F4 | #Feature F5 | #Feature F6 | #Feature F7 | #Feature F8 | #Feature F9 | Start | End | PTM | AScore | Found By |
| --- | --- | --- | --- | --- | --- | --- | --- | --- | --- | --- | --- | --- | --- | --- | --- | --- | --- | --- | --- | --- | --- | --- | --- | --- | --- | --- | --- | --- | --- | --- | --- | --- | --- | --- | --- | --- | --- | --- | --- | --- | --- | --- | --- | --- | --- |
| F.SETIEIC(+57.02)PDGHNFC(+57.02)FK.K | Y | 81.16 | 1952.8396 | 16 | 0.6 | 977.4277 | 2 | 49.30 | 14 | F14:27737 | NaSa01\_F9.raw |  |  |  |  |  |  |  |  |  |  |  |  | 9.4527E7 | 4.2686E7 | 4 | 0 | 0 | 0 | 0 | 0 | 0 | 0 | 0 | 0 | 0 | 0 | 0 | 2 | 2 | 32 | 47 | Carbamidomethylation | C7:Carbamidomethylation:1000.00;C14:Carbamidomethylation:1000.00 | PEAKS DB |
| K.GITRLPWVIR.G | Y | 57.91 | 1209.7346 | 10 | 0.9 | 404.2525 | 3 | 51.91 | 13 | F13:36710 | NaSa01\_F8.raw |  |  |  |  |  |  |  |  |  |  |  |  | 4.8481E7 | 3.1634E6 | 4 | 0 | 0 | 0 | 0 | 0 | 0 | 0 | 0 | 0 | 0 | 0 | 0 | 2 | 2 | 52 | 61 |  |  | PEAKS DB |
| R.VYVDC(+57.02)C(+57.02)AR.D | Y | 57.59 | 1041.4371 | 8 | -1.9 | 521.7249 | 2 | 11.64 | 13 | F13:3824 | NaSa01\_F8.raw |  |  |  |  |  |  |  |  |  |  |  |  | 1.2669E7 |  | 1 | 0 | 0 | 0 | 0 | 0 | 0 | 0 | 0 | 0 | 0 | 0 | 0 | 1 | 0 | 74 | 81 | Carbamidomethylation | C5:Carbamidomethylation:1000.00;C6:Carbamidomethylation:1000.00 | PEAKS DB |
| K.GITRLPWVIRGC(+57.02)AATC(+57.02)PK.A | Y | 51.16 | 2055.0869 | 18 | 0.1 | 514.7791 | 4 | 41.28 | 13 | F13:28062 | NaSa01\_F8.raw |  |  |  |  |  |  |  |  |  |  |  |  | 8.9241E5 |  | 1 | 0 | 0 | 0 | 0 | 0 | 0 | 0 | 0 | 0 | 0 | 0 | 0 | 1 | 0 | 52 | 69 | Carbamidomethylation | C12:Carbamidomethylation:1000.00;C16:Carbamidomethylation:1000.00 | PEAKS DB |
| R.GC(+57.02)AATC(+57.02)PK.A | N | 45.47 | 863.3630 | 8 | -1.2 | 432.6882 | 2 | 11.63 | 13 | F13:3786 | NaSa01\_F8.raw |  |  |  |  |  |  |  |  |  |  |  | 1.055E6 | 1.1773E7 |  | 2 | 0 | 0 | 0 | 0 | 0 | 0 | 0 | 0 | 0 | 0 | 0 | 1 | 1 | 0 | 62 | 69 | Carbamidomethylation | C2:Carbamidomethylation:1000.00;C6:Carbamidomethylation:1000.00 | PEAKS DB |
| Y.SIFSETIEIC(+57.02)PDGHNFC(+57.02)FK.K | Y | 41.89 | 2300.0242 | 19 | 1.0 | 767.6827 | 3 | 74.25 | 13 | F13:56279 | NaSa01\_F8.raw |  |  |  |  |  |  |  |  |  |  |  |  | 3.2313E6 |  | 1 | 0 | 0 | 0 | 0 | 0 | 0 | 0 | 0 | 0 | 0 | 0 | 0 | 1 | 0 | 29 | 47 | Carbamidomethylation | C10:Carbamidomethylation:1000.00;C17:Carbamidomethylation:1000.00 | PEAKS DB |
| I.EIC(+57.02)PDGHNFC(+57.02)FK.K | Y | 41.09 | 1522.6333 | 12 | -0.4 | 508.5515 | 3 | 29.70 | 9 | F9:9458 | NaSa01\_F4.raw |  |  |  |  |  |  |  |  | 3.2308E5 |  |  |  |  |  | 1 | 0 | 0 | 0 | 0 | 0 | 0 | 0 | 0 | 1 | 0 | 0 | 0 | 0 | 0 | 36 | 47 | Carbamidomethylation | C3:Carbamidomethylation:1000.00;C10:Carbamidomethylation:1000.00 | PEAKS DB |
| total 7 peptides |  |  |  |  |  |  |  |  |  |  |  |  |  |  |  |  |  |  |  |  |  |  |  |  |  |  |  |  |  |  |  |  |  |  |  |  |  |  |  |  |  |  |  |  |  |
| --- | --- | --- | --- | --- | --- | --- | --- | --- | --- | --- | --- | --- | --- | --- | --- | --- | --- | --- | --- | --- | --- | --- | --- | --- | --- | --- | --- | --- | --- | --- | --- | --- | --- | --- | --- | --- | --- | --- | --- | --- | --- | --- | --- | --- | --- |

XP\_026530096.1

back to list

  

| Protein Coverage
| Supporting Peptides
|

Protein Coverage:

Supporting Peptides:

| Peptide | Uniq | -10lgP | Mass | Length | ppm | m/z | z | RT | Fraction | Scan | Source File | Area F1 | Area F10 | Area F11 | Area F12 | Area F13 | Area F14 | Area F2 | Area F3 | Area F4 | Area F5 | Area F6 | Area F7 | Area F8 | Area F9 | #Feature | #Feature F1 | #Feature F10 | #Feature F11 | #Feature F12 | #Feature F13 | #Feature F14 | #Feature F2 | #Feature F3 | #Feature F4 | #Feature F5 | #Feature F6 | #Feature F7 | #Feature F8 | #Feature F9 | Start | End | PTM | AScore | Found By |
| --- | --- | --- | --- | --- | --- | --- | --- | --- | --- | --- | --- | --- | --- | --- | --- | --- | --- | --- | --- | --- | --- | --- | --- | --- | --- | --- | --- | --- | --- | --- | --- | --- | --- | --- | --- | --- | --- | --- | --- | --- | --- | --- | --- | --- | --- |
| K.LGAVDATVHQMLASR.Y | Y | 78.42 | 1567.8141 | 15 | -0.2 | 784.9142 | 2 | 49.26 | 6 | F6:28796 | NaSa01\_F14.raw |  |  |  | 3.9315E5 |  | 4.9926E6 |  |  |  |  |  |  |  |  | 3 | 0 | 0 | 0 | 1 | 0 | 2 | 0 | 0 | 0 | 0 | 0 | 0 | 0 | 0 | 224 | 238 |  |  | PEAKS DB |
| K.GSFSDQGINEFLR.E | Y | 71.19 | 1468.6946 | 13 | 0.1 | 735.3546 | 2 | 62.58 | 6 | F6:40100 | NaSa01\_F14.raw |  |  |  |  |  | 5.7323E6 |  |  |  |  |  |  |  |  | 1 | 0 | 0 | 0 | 0 | 0 | 1 | 0 | 0 | 0 | 0 | 0 | 0 | 0 | 0 | 381 | 393 |  |  | PEAKS DB |
| K.NLEPEWASAATEVK.E | Y | 71.03 | 1543.7518 | 14 | 1.4 | 772.8843 | 2 | 51.41 | 6 | F6:30516 | NaSa01\_F14.raw |  |  |  |  |  | 2.9729E6 |  |  |  |  |  |  |  |  | 1 | 0 | 0 | 0 | 0 | 0 | 1 | 0 | 0 | 0 | 0 | 0 | 0 | 0 | 0 | 202 | 215 |  |  | PEAKS DB |
| K.DVIELTDDNFDK.T | Y | 58.42 | 1422.6514 | 12 | 0.4 | 712.3333 | 2 | 59.10 | 4 | F4:38909 | NaSa01\_F12.raw |  |  |  | 4.6725E5 |  |  |  |  |  |  |  |  |  |  | 1 | 0 | 0 | 0 | 1 | 0 | 0 | 0 | 0 | 0 | 0 | 0 | 0 | 0 | 0 | 168 | 179 |  |  | PEAKS DB |
| R.NAYLEVMLK.M | Y | 50.88 | 1079.5685 | 9 | 0.9 | 540.7920 | 2 | 53.78 | 6 | F6:32729 | NaSa01\_F14.raw |  |  |  |  |  | 1.1958E6 |  |  |  |  |  |  |  |  | 1 | 0 | 0 | 0 | 0 | 0 | 1 | 0 | 0 | 0 | 0 | 0 | 0 | 0 | 0 | 321 | 329 |  |  | PEAKS DB |
| total 5 peptides |  |  |  |  |  |  |  |  |  |  |  |  |  |  |  |  |  |  |  |  |  |  |  |  |  |  |  |  |  |  |  |  |  |  |  |  |  |  |  |  |  |  |  |  |  |
| --- | --- | --- | --- | --- | --- | --- | --- | --- | --- | --- | --- | --- | --- | --- | --- | --- | --- | --- | --- | --- | --- | --- | --- | --- | --- | --- | --- | --- | --- | --- | --- | --- | --- | --- | --- | --- | --- | --- | --- | --- | --- | --- | --- | --- | --- |

ETE67495.1

back to list

  

| Protein Coverage
| Supporting Peptides
|

Protein Coverage:

Supporting Peptides:

| Peptide | Uniq | -10lgP | Mass | Length | ppm | m/z | z | RT | Fraction | Scan | Source File | Area F1 | Area F10 | Area F11 | Area F12 | Area F13 | Area F14 | Area F2 | Area F3 | Area F4 | Area F5 | Area F6 | Area F7 | Area F8 | Area F9 | #Feature | #Feature F1 | #Feature F10 | #Feature F11 | #Feature F12 | #Feature F13 | #Feature F14 | #Feature F2 | #Feature F3 | #Feature F4 | #Feature F5 | #Feature F6 | #Feature F7 | #Feature F8 | #Feature F9 | Start | End | PTM | AScore | Found By |
| --- | --- | --- | --- | --- | --- | --- | --- | --- | --- | --- | --- | --- | --- | --- | --- | --- | --- | --- | --- | --- | --- | --- | --- | --- | --- | --- | --- | --- | --- | --- | --- | --- | --- | --- | --- | --- | --- | --- | --- | --- | --- | --- | --- | --- | --- |
| K.LGAVDATVHQMLASR.Y | Y | 78.42 | 1567.8141 | 15 | -0.2 | 784.9142 | 2 | 49.26 | 6 | F6:28796 | NaSa01\_F14.raw |  |  |  | 3.9315E5 |  | 4.9926E6 |  |  |  |  |  |  |  |  | 3 | 0 | 0 | 0 | 1 | 0 | 2 | 0 | 0 | 0 | 0 | 0 | 0 | 0 | 0 | 194 | 208 |  |  | PEAKS DB |
| K.GSFSDQGINEFLR.E | Y | 71.19 | 1468.6946 | 13 | 0.1 | 735.3546 | 2 | 62.58 | 6 | F6:40100 | NaSa01\_F14.raw |  |  |  |  |  | 5.7323E6 |  |  |  |  |  |  |  |  | 1 | 0 | 0 | 0 | 0 | 0 | 1 | 0 | 0 | 0 | 0 | 0 | 0 | 0 | 0 | 351 | 363 |  |  | PEAKS DB |
| K.NLEPEWASAATEVK.E | Y | 71.03 | 1543.7518 | 14 | 1.4 | 772.8843 | 2 | 51.41 | 6 | F6:30516 | NaSa01\_F14.raw |  |  |  |  |  | 2.9729E6 |  |  |  |  |  |  |  |  | 1 | 0 | 0 | 0 | 0 | 0 | 1 | 0 | 0 | 0 | 0 | 0 | 0 | 0 | 0 | 172 | 185 |  |  | PEAKS DB |
| K.DVIELTDDNFDK.T | Y | 58.42 | 1422.6514 | 12 | 0.4 | 712.3333 | 2 | 59.10 | 4 | F4:38909 | NaSa01\_F12.raw |  |  |  | 4.6725E5 |  |  |  |  |  |  |  |  |  |  | 1 | 0 | 0 | 0 | 1 | 0 | 0 | 0 | 0 | 0 | 0 | 0 | 0 | 0 | 0 | 138 | 149 |  |  | PEAKS DB |
| R.NAYLEVMLK.M | Y | 50.88 | 1079.5685 | 9 | 0.9 | 540.7920 | 2 | 53.78 | 6 | F6:32729 | NaSa01\_F14.raw |  |  |  |  |  | 1.1958E6 |  |  |  |  |  |  |  |  | 1 | 0 | 0 | 0 | 0 | 0 | 1 | 0 | 0 | 0 | 0 | 0 | 0 | 0 | 0 | 291 | 299 |  |  | PEAKS DB |
| total 5 peptides |  |  |  |  |  |  |  |  |  |  |  |  |  |  |  |  |  |  |  |  |  |  |  |  |  |  |  |  |  |  |  |  |  |  |  |  |  |  |  |  |  |  |  |  |  |
| --- | --- | --- | --- | --- | --- | --- | --- | --- | --- | --- | --- | --- | --- | --- | --- | --- | --- | --- | --- | --- | --- | --- | --- | --- | --- | --- | --- | --- | --- | --- | --- | --- | --- | --- | --- | --- | --- | --- | --- | --- | --- | --- | --- | --- | --- |

ABQ01132.1

back to list

  

| Protein Coverage
| Supporting Peptides
|

Protein Coverage:

Supporting Peptides:

| Peptide | Uniq | -10lgP | Mass | Length | ppm | m/z | z | RT | Fraction | Scan | Source File | Area F1 | Area F10 | Area F11 | Area F12 | Area F13 | Area F14 | Area F2 | Area F3 | Area F4 | Area F5 | Area F6 | Area F7 | Area F8 | Area F9 | #Feature | #Feature F1 | #Feature F10 | #Feature F11 | #Feature F12 | #Feature F13 | #Feature F14 | #Feature F2 | #Feature F3 | #Feature F4 | #Feature F5 | #Feature F6 | #Feature F7 | #Feature F8 | #Feature F9 | Start | End | PTM | AScore | Found By |
| --- | --- | --- | --- | --- | --- | --- | --- | --- | --- | --- | --- | --- | --- | --- | --- | --- | --- | --- | --- | --- | --- | --- | --- | --- | --- | --- | --- | --- | --- | --- | --- | --- | --- | --- | --- | --- | --- | --- | --- | --- | --- | --- | --- | --- | --- |
| R.NGHPC(+57.02)QNNQGYC(+57.02)YNGK.C | N | 68.53 | 1909.7584 | 16 | -0.2 | 637.5933 | 3 | 11.55 | 4 | F4:2526 | NaSa01\_F12.raw |  |  |  | 1.1419E6 |  |  |  |  |  |  |  |  |  |  | 2 | 0 | 0 | 0 | 2 | 0 | 0 | 0 | 0 | 0 | 0 | 0 | 0 | 0 | 0 | 490 | 505 | Carbamidomethylation | C5:Carbamidomethylation:1000.00;C12:Carbamidomethylation:1000.00 | PEAKS DB |
| R.DRPQC(+57.02)ILNKPLSTDIVTPPVC(+57.02)GN.Y | Y | 58.26 | 2593.2993 | 23 | 0.6 | 865.4409 | 3 | 60.97 | 6 | F6:39298 | NaSa01\_F14.raw |  |  |  |  | 5.0409E8 | 1.3552E9 |  |  |  |  |  |  |  |  | 5 | 0 | 0 | 0 | 0 | 3 | 2 | 0 | 0 | 0 | 0 | 0 | 0 | 0 | 0 | 387 | 409 | Carbamidomethylation | C5:Carbamidomethylation:1000.00;C21:Carbamidomethylation:1000.00 | PEAKS DB |
| E.C(+57.02)PTDSFQR.N | N | 57.13 | 1009.4287 | 8 | 0.6 | 505.7219 | 2 | 11.48 | 6 | F6:1863 | NaSa01\_F14.raw |  |  |  |  | 8.893E5 | 9.5733E5 |  |  |  |  |  |  |  |  | 2 | 0 | 0 | 0 | 0 | 1 | 1 | 0 | 0 | 0 | 0 | 0 | 0 | 0 | 0 | 482 | 489 | Carbamidomethylation | C1:Carbamidomethylation:1000.00 | PEAKS DB |
| R.EITTSPPVQDHC(+57.02)YYHGY.I | N | 44.97 | 2065.8840 | 17 | 6.3 | 689.6360 | 3 | 31.90 | 8 | F8:16761 | NaSa01\_F3.raw |  |  |  |  |  |  |  | 3.1849E6 |  |  |  |  |  |  | 1 | 0 | 0 | 0 | 0 | 0 | 0 | 0 | 1 | 0 | 0 | 0 | 0 | 0 | 0 | 92 | 108 | Carbamidomethylation | C12:Carbamidomethylation:1000.00 | PEAKS DB |
| R.DRPQC(+57.02)ILNKPL.S | N | 44.62 | 1352.7234 | 11 | 1.1 | 451.9156 | 3 | 27.50 | 5 | F5:11578 | NaSa01\_F13.raw |  |  |  |  | 1.9485E7 |  |  |  |  |  |  |  |  |  | 1 | 0 | 0 | 0 | 0 | 1 | 0 | 0 | 0 | 0 | 0 | 0 | 0 | 0 | 0 | 387 | 397 | Carbamidomethylation | C5:Carbamidomethylation:1000.00 | PEAKS DB |
| L.TNTPEQDR.Y | N | 44.60 | 959.4308 | 8 | 0.1 | 480.7227 | 2 | 11.42 | 6 | F6:1814 | NaSa01\_F14.raw |  |  |  |  |  | 1.6427E5 |  |  |  |  |  |  |  |  | 1 | 0 | 0 | 0 | 0 | 0 | 1 | 0 | 0 | 0 | 0 | 0 | 0 | 0 | 0 | 187 | 194 |  |  | PEAKS DB |
| R.ETVLLPR.K | N | 38.52 | 826.4912 | 7 | -0.8 | 414.2526 | 2 | 26.58 | 6 | F6:9693 | NaSa01\_F14.raw |  |  |  |  |  | 1.9593E5 |  |  |  |  |  |  |  |  | 1 | 0 | 0 | 0 | 0 | 0 | 1 | 0 | 0 | 0 | 0 | 0 | 0 | 0 | 0 | 277 | 283 |  |  | PEAKS DB |
| total 7 peptides |  |  |  |  |  |  |  |  |  |  |  |  |  |  |  |  |  |  |  |  |  |  |  |  |  |  |  |  |  |  |  |  |  |  |  |  |  |  |  |  |  |  |  |  |  |
| --- | --- | --- | --- | --- | --- | --- | --- | --- | --- | --- | --- | --- | --- | --- | --- | --- | --- | --- | --- | --- | --- | --- | --- | --- | --- | --- | --- | --- | --- | --- | --- | --- | --- | --- | --- | --- | --- | --- | --- | --- | --- | --- | --- | --- | --- |

JAI08992.1

back to list

  

| Protein Coverage
| Supporting Peptides
|

Protein Coverage:

Supporting Peptides:

| Peptide | Uniq | -10lgP | Mass | Length | ppm | m/z | z | RT | Fraction | Scan | Source File | Area F1 | Area F10 | Area F11 | Area F12 | Area F13 | Area F14 | Area F2 | Area F3 | Area F4 | Area F5 | Area F6 | Area F7 | Area F8 | Area F9 | #Feature | #Feature F1 | #Feature F10 | #Feature F11 | #Feature F12 | #Feature F13 | #Feature F14 | #Feature F2 | #Feature F3 | #Feature F4 | #Feature F5 | #Feature F6 | #Feature F7 | #Feature F8 | #Feature F9 | Start | End | PTM | AScore | Found By |
| --- | --- | --- | --- | --- | --- | --- | --- | --- | --- | --- | --- | --- | --- | --- | --- | --- | --- | --- | --- | --- | --- | --- | --- | --- | --- | --- | --- | --- | --- | --- | --- | --- | --- | --- | --- | --- | --- | --- | --- | --- | --- | --- | --- | --- | --- |
| R.NGHPC(+57.02)QNNQGYC(+57.02)YNGK.C | N | 68.53 | 1909.7584 | 16 | -0.2 | 637.5933 | 3 | 11.55 | 4 | F4:2526 | NaSa01\_F12.raw |  |  |  | 1.1419E6 |  |  |  |  |  |  |  |  |  |  | 2 | 0 | 0 | 0 | 2 | 0 | 0 | 0 | 0 | 0 | 0 | 0 | 0 | 0 | 0 | 494 | 509 | Carbamidomethylation | C5:Carbamidomethylation:1000.00;C12:Carbamidomethylation:1000.00 | PEAKS DB |
| K.C(+57.02)PTDSFQR.N | N | 57.13 | 1009.4287 | 8 | 0.6 | 505.7219 | 2 | 11.48 | 6 | F6:1863 | NaSa01\_F14.raw |  |  |  |  | 8.893E5 | 9.5733E5 |  |  |  |  |  |  |  |  | 2 | 0 | 0 | 0 | 0 | 1 | 1 | 0 | 0 | 0 | 0 | 0 | 0 | 0 | 0 | 486 | 493 | Carbamidomethylation | C1:Carbamidomethylation:1000.00 | PEAKS DB |
| R.TKPAYQFSSC(+57.02).S | N | 50.58 | 1187.5281 | 10 | 0.3 | 594.7715 | 2 | 11.58 | 6 | F6:1956 | NaSa01\_F14.raw |  |  |  |  |  | 3.6584E6 |  |  |  |  |  |  |  |  | 1 | 0 | 0 | 0 | 0 | 0 | 1 | 0 | 0 | 0 | 0 | 0 | 0 | 0 | 0 | 370 | 379 | Carbamidomethylation | C10:Carbamidomethylation:1000.00 | PEAKS DB |
| R.HLNFHIAL.T | Y | 44.76 | 963.5290 | 8 | 0.0 | 482.7718 | 2 | 48.94 | 6 | F6:28411 | NaSa01\_F14.raw |  |  |  |  |  | 1.1544E7 |  |  |  |  |  |  |  |  | 1 | 0 | 0 | 0 | 0 | 0 | 1 | 0 | 0 | 0 | 0 | 0 | 0 | 0 | 0 | 245 | 252 |  |  | PEAKS DB |
| R.DRPQC(+57.02)ILNKPL.S | N | 44.62 | 1352.7234 | 11 | 1.1 | 451.9156 | 3 | 27.50 | 5 | F5:11578 | NaSa01\_F13.raw |  |  |  |  | 1.9485E7 |  |  |  |  |  |  |  |  |  | 1 | 0 | 0 | 0 | 0 | 1 | 0 | 0 | 0 | 0 | 0 | 0 | 0 | 0 | 0 | 391 | 401 | Carbamidomethylation | C5:Carbamidomethylation:1000.00 | PEAKS DB |
| L.TNTPEQDR.Y | N | 44.60 | 959.4308 | 8 | 0.1 | 480.7227 | 2 | 11.42 | 6 | F6:1814 | NaSa01\_F14.raw |  |  |  |  |  | 1.6427E5 |  |  |  |  |  |  |  |  | 1 | 0 | 0 | 0 | 0 | 0 | 1 | 0 | 0 | 0 | 0 | 0 | 0 | 0 | 0 | 192 | 199 |  |  | PEAKS DB |
| total 6 peptides |  |  |  |  |  |  |  |  |  |  |  |  |  |  |  |  |  |  |  |  |  |  |  |  |  |  |  |  |  |  |  |  |  |  |  |  |  |  |  |  |  |  |  |  |  |
| --- | --- | --- | --- | --- | --- | --- | --- | --- | --- | --- | --- | --- | --- | --- | --- | --- | --- | --- | --- | --- | --- | --- | --- | --- | --- | --- | --- | --- | --- | --- | --- | --- | --- | --- | --- | --- | --- | --- | --- | --- | --- | --- | --- | --- | --- |

JAB52758.1

back to list

  

| Protein Coverage
| Supporting Peptides
|

Protein Coverage:

Supporting Peptides:

| Peptide | Uniq | -10lgP | Mass | Length | ppm | m/z | z | RT | Fraction | Scan | Source File | Area F1 | Area F10 | Area F11 | Area F12 | Area F13 | Area F14 | Area F2 | Area F3 | Area F4 | Area F5 | Area F6 | Area F7 | Area F8 | Area F9 | #Feature | #Feature F1 | #Feature F10 | #Feature F11 | #Feature F12 | #Feature F13 | #Feature F14 | #Feature F2 | #Feature F3 | #Feature F4 | #Feature F5 | #Feature F6 | #Feature F7 | #Feature F8 | #Feature F9 | Start | End | PTM | AScore | Found By |
| --- | --- | --- | --- | --- | --- | --- | --- | --- | --- | --- | --- | --- | --- | --- | --- | --- | --- | --- | --- | --- | --- | --- | --- | --- | --- | --- | --- | --- | --- | --- | --- | --- | --- | --- | --- | --- | --- | --- | --- | --- | --- | --- | --- | --- | --- |
| R.NGHPC(+57.02)QNNQGYC(+57.02)YNGK.C | N | 68.53 | 1909.7584 | 16 | -0.2 | 637.5933 | 3 | 11.55 | 4 | F4:2526 | NaSa01\_F12.raw |  |  |  | 1.1419E6 |  |  |  |  |  |  |  |  |  |  | 2 | 0 | 0 | 0 | 2 | 0 | 0 | 0 | 0 | 0 | 0 | 0 | 0 | 0 | 0 | 494 | 509 | Carbamidomethylation | C5:Carbamidomethylation:1000.00;C12:Carbamidomethylation:1000.00 | PEAKS DB |
| K.C(+57.02)PTDSFQR.N | N | 57.13 | 1009.4287 | 8 | 0.6 | 505.7219 | 2 | 11.48 | 6 | F6:1863 | NaSa01\_F14.raw |  |  |  |  | 8.893E5 | 9.5733E5 |  |  |  |  |  |  |  |  | 2 | 0 | 0 | 0 | 0 | 1 | 1 | 0 | 0 | 0 | 0 | 0 | 0 | 0 | 0 | 486 | 493 | Carbamidomethylation | C1:Carbamidomethylation:1000.00 | PEAKS DB |
| R.TKPAYQFSSC(+57.02).S | N | 50.58 | 1187.5281 | 10 | 0.3 | 594.7715 | 2 | 11.58 | 6 | F6:1956 | NaSa01\_F14.raw |  |  |  |  |  | 3.6584E6 |  |  |  |  |  |  |  |  | 1 | 0 | 0 | 0 | 0 | 0 | 1 | 0 | 0 | 0 | 0 | 0 | 0 | 0 | 0 | 370 | 379 | Carbamidomethylation | C10:Carbamidomethylation:1000.00 | PEAKS DB |
| R.HLNFHIAL.T | Y | 44.76 | 963.5290 | 8 | 0.0 | 482.7718 | 2 | 48.94 | 6 | F6:28411 | NaSa01\_F14.raw |  |  |  |  |  | 1.1544E7 |  |  |  |  |  |  |  |  | 1 | 0 | 0 | 0 | 0 | 0 | 1 | 0 | 0 | 0 | 0 | 0 | 0 | 0 | 0 | 245 | 252 |  |  | PEAKS DB |
| R.DRPQC(+57.02)ILNKPL.S | N | 44.62 | 1352.7234 | 11 | 1.1 | 451.9156 | 3 | 27.50 | 5 | F5:11578 | NaSa01\_F13.raw |  |  |  |  | 1.9485E7 |  |  |  |  |  |  |  |  |  | 1 | 0 | 0 | 0 | 0 | 1 | 0 | 0 | 0 | 0 | 0 | 0 | 0 | 0 | 0 | 391 | 401 | Carbamidomethylation | C5:Carbamidomethylation:1000.00 | PEAKS DB |
| L.TNTPEQDR.Y | N | 44.60 | 959.4308 | 8 | 0.1 | 480.7227 | 2 | 11.42 | 6 | F6:1814 | NaSa01\_F14.raw |  |  |  |  |  | 1.6427E5 |  |  |  |  |  |  |  |  | 1 | 0 | 0 | 0 | 0 | 0 | 1 | 0 | 0 | 0 | 0 | 0 | 0 | 0 | 0 | 192 | 199 |  |  | PEAKS DB |
| total 6 peptides |  |  |  |  |  |  |  |  |  |  |  |  |  |  |  |  |  |  |  |  |  |  |  |  |  |  |  |  |  |  |  |  |  |  |  |  |  |  |  |  |  |  |  |  |  |
| --- | --- | --- | --- | --- | --- | --- | --- | --- | --- | --- | --- | --- | --- | --- | --- | --- | --- | --- | --- | --- | --- | --- | --- | --- | --- | --- | --- | --- | --- | --- | --- | --- | --- | --- | --- | --- | --- | --- | --- | --- | --- | --- | --- | --- | --- |

JAS04981.1

back to list

  

| Protein Coverage
| Supporting Peptides
|

Protein Coverage:

Supporting Peptides:

| Peptide | Uniq | -10lgP | Mass | Length | ppm | m/z | z | RT | Fraction | Scan | Source File | Area F1 | Area F10 | Area F11 | Area F12 | Area F13 | Area F14 | Area F2 | Area F3 | Area F4 | Area F5 | Area F6 | Area F7 | Area F8 | Area F9 | #Feature | #Feature F1 | #Feature F10 | #Feature F11 | #Feature F12 | #Feature F13 | #Feature F14 | #Feature F2 | #Feature F3 | #Feature F4 | #Feature F5 | #Feature F6 | #Feature F7 | #Feature F8 | #Feature F9 | Start | End | PTM | AScore | Found By |
| --- | --- | --- | --- | --- | --- | --- | --- | --- | --- | --- | --- | --- | --- | --- | --- | --- | --- | --- | --- | --- | --- | --- | --- | --- | --- | --- | --- | --- | --- | --- | --- | --- | --- | --- | --- | --- | --- | --- | --- | --- | --- | --- | --- | --- | --- |
| R.NGHPC(+57.02)QNNQGYC(+57.02)YNGK.C | N | 68.53 | 1909.7584 | 16 | -0.2 | 637.5933 | 3 | 11.55 | 4 | F4:2526 | NaSa01\_F12.raw |  |  |  | 1.1419E6 |  |  |  |  |  |  |  |  |  |  | 2 | 0 | 0 | 0 | 2 | 0 | 0 | 0 | 0 | 0 | 0 | 0 | 0 | 0 | 0 | 494 | 509 | Carbamidomethylation | C5:Carbamidomethylation:1000.00;C12:Carbamidomethylation:1000.00 | PEAKS DB |
| K.C(+57.02)PTDSFQR.N | N | 57.13 | 1009.4287 | 8 | 0.6 | 505.7219 | 2 | 11.48 | 6 | F6:1863 | NaSa01\_F14.raw |  |  |  |  | 8.893E5 | 9.5733E5 |  |  |  |  |  |  |  |  | 2 | 0 | 0 | 0 | 0 | 1 | 1 | 0 | 0 | 0 | 0 | 0 | 0 | 0 | 0 | 486 | 493 | Carbamidomethylation | C1:Carbamidomethylation:1000.00 | PEAKS DB |
| R.TKPAYQFSSC(+57.02).S | N | 50.58 | 1187.5281 | 10 | 0.3 | 594.7715 | 2 | 11.58 | 6 | F6:1956 | NaSa01\_F14.raw |  |  |  |  |  | 3.6584E6 |  |  |  |  |  |  |  |  | 1 | 0 | 0 | 0 | 0 | 0 | 1 | 0 | 0 | 0 | 0 | 0 | 0 | 0 | 0 | 370 | 379 | Carbamidomethylation | C10:Carbamidomethylation:1000.00 | PEAKS DB |
| R.HLNFHIAL.T | Y | 44.76 | 963.5290 | 8 | 0.0 | 482.7718 | 2 | 48.94 | 6 | F6:28411 | NaSa01\_F14.raw |  |  |  |  |  | 1.1544E7 |  |  |  |  |  |  |  |  | 1 | 0 | 0 | 0 | 0 | 0 | 1 | 0 | 0 | 0 | 0 | 0 | 0 | 0 | 0 | 245 | 252 |  |  | PEAKS DB |
| R.DRPQC(+57.02)ILNKPL.S | N | 44.62 | 1352.7234 | 11 | 1.1 | 451.9156 | 3 | 27.50 | 5 | F5:11578 | NaSa01\_F13.raw |  |  |  |  | 1.9485E7 |  |  |  |  |  |  |  |  |  | 1 | 0 | 0 | 0 | 0 | 1 | 0 | 0 | 0 | 0 | 0 | 0 | 0 | 0 | 0 | 391 | 401 | Carbamidomethylation | C5:Carbamidomethylation:1000.00 | PEAKS DB |
| L.TNTPEQDR.Y | N | 44.60 | 959.4308 | 8 | 0.1 | 480.7227 | 2 | 11.42 | 6 | F6:1814 | NaSa01\_F14.raw |  |  |  |  |  | 1.6427E5 |  |  |  |  |  |  |  |  | 1 | 0 | 0 | 0 | 0 | 0 | 1 | 0 | 0 | 0 | 0 | 0 | 0 | 0 | 0 | 192 | 199 |  |  | PEAKS DB |
| total 6 peptides |  |  |  |  |  |  |  |  |  |  |  |  |  |  |  |  |  |  |  |  |  |  |  |  |  |  |  |  |  |  |  |  |  |  |  |  |  |  |  |  |  |  |  |  |  |
| --- | --- | --- | --- | --- | --- | --- | --- | --- | --- | --- | --- | --- | --- | --- | --- | --- | --- | --- | --- | --- | --- | --- | --- | --- | --- | --- | --- | --- | --- | --- | --- | --- | --- | --- | --- | --- | --- | --- | --- | --- | --- | --- | --- | --- | --- |

JAS05093.1

back to list

  

| Protein Coverage
| Supporting Peptides
|

Protein Coverage:

Supporting Peptides:

| Peptide | Uniq | -10lgP | Mass | Length | ppm | m/z | z | RT | Fraction | Scan | Source File | Area F1 | Area F10 | Area F11 | Area F12 | Area F13 | Area F14 | Area F2 | Area F3 | Area F4 | Area F5 | Area F6 | Area F7 | Area F8 | Area F9 | #Feature | #Feature F1 | #Feature F10 | #Feature F11 | #Feature F12 | #Feature F13 | #Feature F14 | #Feature F2 | #Feature F3 | #Feature F4 | #Feature F5 | #Feature F6 | #Feature F7 | #Feature F8 | #Feature F9 | Start | End | PTM | AScore | Found By |
| --- | --- | --- | --- | --- | --- | --- | --- | --- | --- | --- | --- | --- | --- | --- | --- | --- | --- | --- | --- | --- | --- | --- | --- | --- | --- | --- | --- | --- | --- | --- | --- | --- | --- | --- | --- | --- | --- | --- | --- | --- | --- | --- | --- | --- | --- |
| R.NGHPC(+57.02)QNNQGYC(+57.02)YNGK.C | N | 68.53 | 1909.7584 | 16 | -0.2 | 637.5933 | 3 | 11.55 | 4 | F4:2526 | NaSa01\_F12.raw |  |  |  | 1.1419E6 |  |  |  |  |  |  |  |  |  |  | 2 | 0 | 0 | 0 | 2 | 0 | 0 | 0 | 0 | 0 | 0 | 0 | 0 | 0 | 0 | 494 | 509 | Carbamidomethylation | C5:Carbamidomethylation:1000.00;C12:Carbamidomethylation:1000.00 | PEAKS DB |
| E.C(+57.02)PTDSFQR.N | N | 57.13 | 1009.4287 | 8 | 0.6 | 505.7219 | 2 | 11.48 | 6 | F6:1863 | NaSa01\_F14.raw |  |  |  |  | 8.893E5 | 9.5733E5 |  |  |  |  |  |  |  |  | 2 | 0 | 0 | 0 | 0 | 1 | 1 | 0 | 0 | 0 | 0 | 0 | 0 | 0 | 0 | 486 | 493 | Carbamidomethylation | C1:Carbamidomethylation:1000.00 | PEAKS DB |
| R.TKPAYQFSSC(+57.02).S | N | 50.58 | 1187.5281 | 10 | 0.3 | 594.7715 | 2 | 11.58 | 6 | F6:1956 | NaSa01\_F14.raw |  |  |  |  |  | 3.6584E6 |  |  |  |  |  |  |  |  | 1 | 0 | 0 | 0 | 0 | 0 | 1 | 0 | 0 | 0 | 0 | 0 | 0 | 0 | 0 | 370 | 379 | Carbamidomethylation | C10:Carbamidomethylation:1000.00 | PEAKS DB |
| R.HLNFHIAL.T | Y | 44.76 | 963.5290 | 8 | 0.0 | 482.7718 | 2 | 48.94 | 6 | F6:28411 | NaSa01\_F14.raw |  |  |  |  |  | 1.1544E7 |  |  |  |  |  |  |  |  | 1 | 0 | 0 | 0 | 0 | 0 | 1 | 0 | 0 | 0 | 0 | 0 | 0 | 0 | 0 | 245 | 252 |  |  | PEAKS DB |
| R.DRPQC(+57.02)ILNKPL.S | N | 44.62 | 1352.7234 | 11 | 1.1 | 451.9156 | 3 | 27.50 | 5 | F5:11578 | NaSa01\_F13.raw |  |  |  |  | 1.9485E7 |  |  |  |  |  |  |  |  |  | 1 | 0 | 0 | 0 | 0 | 1 | 0 | 0 | 0 | 0 | 0 | 0 | 0 | 0 | 0 | 391 | 401 | Carbamidomethylation | C5:Carbamidomethylation:1000.00 | PEAKS DB |
| L.TNTPEQDR.Y | N | 44.60 | 959.4308 | 8 | 0.1 | 480.7227 | 2 | 11.42 | 6 | F6:1814 | NaSa01\_F14.raw |  |  |  |  |  | 1.6427E5 |  |  |  |  |  |  |  |  | 1 | 0 | 0 | 0 | 0 | 0 | 1 | 0 | 0 | 0 | 0 | 0 | 0 | 0 | 0 | 192 | 199 |  |  | PEAKS DB |
| total 6 peptides |  |  |  |  |  |  |  |  |  |  |  |  |  |  |  |  |  |  |  |  |  |  |  |  |  |  |  |  |  |  |  |  |  |  |  |  |  |  |  |  |  |  |  |  |  |
| --- | --- | --- | --- | --- | --- | --- | --- | --- | --- | --- | --- | --- | --- | --- | --- | --- | --- | --- | --- | --- | --- | --- | --- | --- | --- | --- | --- | --- | --- | --- | --- | --- | --- | --- | --- | --- | --- | --- | --- | --- | --- | --- | --- | --- | --- |

ADN67584.1

back to list

  

| Protein Coverage
| Supporting Peptides
|

Protein Coverage:

Supporting Peptides:

| Peptide | Uniq | -10lgP | Mass | Length | ppm | m/z | z | RT | Fraction | Scan | Source File | Area F1 | Area F10 | Area F11 | Area F12 | Area F13 | Area F14 | Area F2 | Area F3 | Area F4 | Area F5 | Area F6 | Area F7 | Area F8 | Area F9 | #Feature | #Feature F1 | #Feature F10 | #Feature F11 | #Feature F12 | #Feature F13 | #Feature F14 | #Feature F2 | #Feature F3 | #Feature F4 | #Feature F5 | #Feature F6 | #Feature F7 | #Feature F8 | #Feature F9 | Start | End | PTM | AScore | Found By |
| --- | --- | --- | --- | --- | --- | --- | --- | --- | --- | --- | --- | --- | --- | --- | --- | --- | --- | --- | --- | --- | --- | --- | --- | --- | --- | --- | --- | --- | --- | --- | --- | --- | --- | --- | --- | --- | --- | --- | --- | --- | --- | --- | --- | --- | --- |
| T.LEC(+57.02)HNQQSSQTPTTTGC(+57.02)SGGETNC(+57.02)YK.K | Y | 85.04 | 2944.2021 | 26 | 2.7 | 982.4077 | 3 | 11.84 | 7 | F7:1790 | NaSa01\_F2.raw | 2.6655E8 |  |  |  |  |  | 2.8883E7 | 0 | 4.5532E7 | 3.2829E5 |  |  |  |  | 5 | 2 | 0 | 0 | 0 | 0 | 0 | 1 | 0 | 1 | 1 | 0 | 0 | 0 | 0 | 3 | 28 | Carbamidomethylation | C3:Carbamidomethylation:1000.00;C17:Carbamidomethylation:1000.00;C24:Carbamidomethylation:1000.00 | PEAKS DB |
| K.NGIEINC(+57.02)C(+57.02)TTDR.C | N | 76.35 | 1451.6133 | 12 | 3.3 | 726.8141 | 2 | 19.60 | 7 | F7:7461 | NaSa01\_F2.raw | 0 |  |  |  |  |  | 6.7801E6 | 5.7301E7 | 9.911E5 |  |  |  |  |  | 8 | 0 | 0 | 0 | 0 | 0 | 0 | 1 | 6 | 1 | 0 | 0 | 0 | 0 | 0 | 50 | 61 | Carbamidomethylation | C7:Carbamidomethylation:1000.00;C8:Carbamidomethylation:1000.00 | PEAKS DB |
| T.LEC(+57.02)HNQQSSQTPTTTGC(+57.02)SGGETNC(+57.02)YKK.R | Y | 59.86 | 3072.2971 | 27 | 5.5 | 769.0817 | 4 | 11.26 | 8 | F8:1677 | NaSa01\_F3.raw |  |  |  |  |  |  |  | 2.1676E6 |  |  |  |  |  |  | 3 | 0 | 0 | 0 | 0 | 0 | 0 | 0 | 3 | 0 | 0 | 0 | 0 | 0 | 0 | 3 | 29 | Carbamidomethylation | C3:Carbamidomethylation:1000.00;C17:Carbamidomethylation:1000.00;C24:Carbamidomethylation:1000.00 | PEAKS DB |
| N.GIEINC(+57.02)C(+57.02)TTDR.C | N | 50.21 | 1337.5704 | 11 | 6.3 | 669.7932 | 2 | 11.47 | 8 | F8:2008 | NaSa01\_F3.raw |  |  |  |  |  |  |  | 1.1583E6 |  |  |  |  |  |  | 1 | 0 | 0 | 0 | 0 | 0 | 0 | 0 | 1 | 0 | 0 | 0 | 0 | 0 | 0 | 51 | 61 | Carbamidomethylation | C6:Carbamidomethylation:1000.00;C7:Carbamidomethylation:1000.00 | PEAKS DB |
| total 4 peptides |  |  |  |  |  |  |  |  |  |  |  |  |  |  |  |  |  |  |  |  |  |  |  |  |  |  |  |  |  |  |  |  |  |  |  |  |  |  |  |  |  |  |  |  |  |
| --- | --- | --- | --- | --- | --- | --- | --- | --- | --- | --- | --- | --- | --- | --- | --- | --- | --- | --- | --- | --- | --- | --- | --- | --- | --- | --- | --- | --- | --- | --- | --- | --- | --- | --- | --- | --- | --- | --- | --- | --- | --- | --- | --- | --- | --- |

AAB36932.1

back to list

  

| Protein Coverage
| Supporting Peptides
|

Protein Coverage:

Supporting Peptides:

| Peptide | Uniq | -10lgP | Mass | Length | ppm | m/z | z | RT | Fraction | Scan | Source File | Area F1 | Area F10 | Area F11 | Area F12 | Area F13 | Area F14 | Area F2 | Area F3 | Area F4 | Area F5 | Area F6 | Area F7 | Area F8 | Area F9 | #Feature | #Feature F1 | #Feature F10 | #Feature F11 | #Feature F12 | #Feature F13 | #Feature F14 | #Feature F2 | #Feature F3 | #Feature F4 | #Feature F5 | #Feature F6 | #Feature F7 | #Feature F8 | #Feature F9 | Start | End | PTM | AScore | Found By |
| --- | --- | --- | --- | --- | --- | --- | --- | --- | --- | --- | --- | --- | --- | --- | --- | --- | --- | --- | --- | --- | --- | --- | --- | --- | --- | --- | --- | --- | --- | --- | --- | --- | --- | --- | --- | --- | --- | --- | --- | --- | --- | --- | --- | --- | --- |
| T.LEC(+57.02)HNQQSSQTPTTTGC(+57.02)SGGETNC(+57.02)YK.K | Y | 85.04 | 2944.2021 | 26 | 2.7 | 982.4077 | 3 | 11.84 | 7 | F7:1790 | NaSa01\_F2.raw | 2.6655E8 |  |  |  |  |  | 2.8883E7 | 0 | 4.5532E7 | 3.2829E5 |  |  |  |  | 5 | 2 | 0 | 0 | 0 | 0 | 0 | 1 | 0 | 1 | 1 | 0 | 0 | 0 | 0 | 22 | 47 | Carbamidomethylation | C3:Carbamidomethylation:1000.00;C17:Carbamidomethylation:1000.00;C24:Carbamidomethylation:1000.00 | PEAKS DB |
| K.NGIEINC(+57.02)C(+57.02)TTDR.C | N | 76.35 | 1451.6133 | 12 | 3.3 | 726.8141 | 2 | 19.60 | 7 | F7:7461 | NaSa01\_F2.raw | 0 |  |  |  |  |  | 6.7801E6 | 5.7301E7 | 9.911E5 |  |  |  |  |  | 8 | 0 | 0 | 0 | 0 | 0 | 0 | 1 | 6 | 1 | 0 | 0 | 0 | 0 | 0 | 69 | 80 | Carbamidomethylation | C7:Carbamidomethylation:1000.00;C8:Carbamidomethylation:1000.00 | PEAKS DB |
| T.LEC(+57.02)HNQQSSQTPTTTGC(+57.02)SGGETNC(+57.02)YKK.R | Y | 59.86 | 3072.2971 | 27 | 5.5 | 769.0817 | 4 | 11.26 | 8 | F8:1677 | NaSa01\_F3.raw |  |  |  |  |  |  |  | 2.1676E6 |  |  |  |  |  |  | 3 | 0 | 0 | 0 | 0 | 0 | 0 | 0 | 3 | 0 | 0 | 0 | 0 | 0 | 0 | 22 | 48 | Carbamidomethylation | C3:Carbamidomethylation:1000.00;C17:Carbamidomethylation:1000.00;C24:Carbamidomethylation:1000.00 | PEAKS DB |
| N.GIEINC(+57.02)C(+57.02)TTDR.C | N | 50.21 | 1337.5704 | 11 | 6.3 | 669.7932 | 2 | 11.47 | 8 | F8:2008 | NaSa01\_F3.raw |  |  |  |  |  |  |  | 1.1583E6 |  |  |  |  |  |  | 1 | 0 | 0 | 0 | 0 | 0 | 0 | 0 | 1 | 0 | 0 | 0 | 0 | 0 | 0 | 70 | 80 | Carbamidomethylation | C6:Carbamidomethylation:1000.00;C7:Carbamidomethylation:1000.00 | PEAKS DB |
| total 4 peptides |  |  |  |  |  |  |  |  |  |  |  |  |  |  |  |  |  |  |  |  |  |  |  |  |  |  |  |  |  |  |  |  |  |  |  |  |  |  |  |  |  |  |  |  |  |
| --- | --- | --- | --- | --- | --- | --- | --- | --- | --- | --- | --- | --- | --- | --- | --- | --- | --- | --- | --- | --- | --- | --- | --- | --- | --- | --- | --- | --- | --- | --- | --- | --- | --- | --- | --- | --- | --- | --- | --- | --- | --- | --- | --- | --- | --- |

AAB03221.1

back to list

  

| Protein Coverage
| Supporting Peptides
|

Protein Coverage:

Supporting Peptides:

| Peptide | Uniq | -10lgP | Mass | Length | ppm | m/z | z | RT | Fraction | Scan | Source File | Area F1 | Area F10 | Area F11 | Area F12 | Area F13 | Area F14 | Area F2 | Area F3 | Area F4 | Area F5 | Area F6 | Area F7 | Area F8 | Area F9 | #Feature | #Feature F1 | #Feature F10 | #Feature F11 | #Feature F12 | #Feature F13 | #Feature F14 | #Feature F2 | #Feature F3 | #Feature F4 | #Feature F5 | #Feature F6 | #Feature F7 | #Feature F8 | #Feature F9 | Start | End | PTM | AScore | Found By |
| --- | --- | --- | --- | --- | --- | --- | --- | --- | --- | --- | --- | --- | --- | --- | --- | --- | --- | --- | --- | --- | --- | --- | --- | --- | --- | --- | --- | --- | --- | --- | --- | --- | --- | --- | --- | --- | --- | --- | --- | --- | --- | --- | --- | --- | --- |
| T.LEC(+57.02)HNQQSSQTPTTTGC(+57.02)SGGETNC(+57.02)YK.K | Y | 85.04 | 2944.2021 | 26 | 2.7 | 982.4077 | 3 | 11.84 | 7 | F7:1790 | NaSa01\_F2.raw | 2.6655E8 |  |  |  |  |  | 2.8883E7 | 0 | 4.5532E7 | 3.2829E5 |  |  |  |  | 5 | 2 | 0 | 0 | 0 | 0 | 0 | 1 | 0 | 1 | 1 | 0 | 0 | 0 | 0 | 22 | 47 | Carbamidomethylation | C3:Carbamidomethylation:1000.00;C17:Carbamidomethylation:1000.00;C24:Carbamidomethylation:1000.00 | PEAKS DB |
| K.NGIEINC(+57.02)C(+57.02)TTDR.C | N | 76.35 | 1451.6133 | 12 | 3.3 | 726.8141 | 2 | 19.60 | 7 | F7:7461 | NaSa01\_F2.raw | 0 |  |  |  |  |  | 6.7801E6 | 5.7301E7 | 9.911E5 |  |  |  |  |  | 8 | 0 | 0 | 0 | 0 | 0 | 0 | 1 | 6 | 1 | 0 | 0 | 0 | 0 | 0 | 69 | 80 | Carbamidomethylation | C7:Carbamidomethylation:1000.00;C8:Carbamidomethylation:1000.00 | PEAKS DB |
| T.LEC(+57.02)HNQQSSQTPTTTGC(+57.02)SGGETNC(+57.02)YKK.R | Y | 59.86 | 3072.2971 | 27 | 5.5 | 769.0817 | 4 | 11.26 | 8 | F8:1677 | NaSa01\_F3.raw |  |  |  |  |  |  |  | 2.1676E6 |  |  |  |  |  |  | 3 | 0 | 0 | 0 | 0 | 0 | 0 | 0 | 3 | 0 | 0 | 0 | 0 | 0 | 0 | 22 | 48 | Carbamidomethylation | C3:Carbamidomethylation:1000.00;C17:Carbamidomethylation:1000.00;C24:Carbamidomethylation:1000.00 | PEAKS DB |
| N.GIEINC(+57.02)C(+57.02)TTDR.C | N | 50.21 | 1337.5704 | 11 | 6.3 | 669.7932 | 2 | 11.47 | 8 | F8:2008 | NaSa01\_F3.raw |  |  |  |  |  |  |  | 1.1583E6 |  |  |  |  |  |  | 1 | 0 | 0 | 0 | 0 | 0 | 0 | 0 | 1 | 0 | 0 | 0 | 0 | 0 | 0 | 70 | 80 | Carbamidomethylation | C6:Carbamidomethylation:1000.00;C7:Carbamidomethylation:1000.00 | PEAKS DB |
| total 4 peptides |  |  |  |  |  |  |  |  |  |  |  |  |  |  |  |  |  |  |  |  |  |  |  |  |  |  |  |  |  |  |  |  |  |  |  |  |  |  |  |  |  |  |  |  |  |
| --- | --- | --- | --- | --- | --- | --- | --- | --- | --- | --- | --- | --- | --- | --- | --- | --- | --- | --- | --- | --- | --- | --- | --- | --- | --- | --- | --- | --- | --- | --- | --- | --- | --- | --- | --- | --- | --- | --- | --- | --- | --- | --- | --- | --- | --- |

ADN67574.1

back to list

  

| Protein Coverage
| Supporting Peptides
|

Protein Coverage:

Supporting Peptides:

| Peptide | Uniq | -10lgP | Mass | Length | ppm | m/z | z | RT | Fraction | Scan | Source File | Area F1 | Area F10 | Area F11 | Area F12 | Area F13 | Area F14 | Area F2 | Area F3 | Area F4 | Area F5 | Area F6 | Area F7 | Area F8 | Area F9 | #Feature | #Feature F1 | #Feature F10 | #Feature F11 | #Feature F12 | #Feature F13 | #Feature F14 | #Feature F2 | #Feature F3 | #Feature F4 | #Feature F5 | #Feature F6 | #Feature F7 | #Feature F8 | #Feature F9 | Start | End | PTM | AScore | Found By |
| --- | --- | --- | --- | --- | --- | --- | --- | --- | --- | --- | --- | --- | --- | --- | --- | --- | --- | --- | --- | --- | --- | --- | --- | --- | --- | --- | --- | --- | --- | --- | --- | --- | --- | --- | --- | --- | --- | --- | --- | --- | --- | --- | --- | --- | --- |
| T.LEC(+57.02)HNQQSSQTPTTTGC(+57.02)SGGETNC(+57.02)YK.K | Y | 85.04 | 2944.2021 | 26 | 2.7 | 982.4077 | 3 | 11.84 | 7 | F7:1790 | NaSa01\_F2.raw | 2.6655E8 |  |  |  |  |  | 2.8883E7 | 0 | 4.5532E7 | 3.2829E5 |  |  |  |  | 5 | 2 | 0 | 0 | 0 | 0 | 0 | 1 | 0 | 1 | 1 | 0 | 0 | 0 | 0 | 22 | 47 | Carbamidomethylation | C3:Carbamidomethylation:1000.00;C17:Carbamidomethylation:1000.00;C24:Carbamidomethylation:1000.00 | PEAKS DB |
| K.NGIEINC(+57.02)C(+57.02)TTDR.C | N | 76.35 | 1451.6133 | 12 | 3.3 | 726.8141 | 2 | 19.60 | 7 | F7:7461 | NaSa01\_F2.raw | 0 |  |  |  |  |  | 6.7801E6 | 5.7301E7 | 9.911E5 |  |  |  |  |  | 8 | 0 | 0 | 0 | 0 | 0 | 0 | 1 | 6 | 1 | 0 | 0 | 0 | 0 | 0 | 69 | 80 | Carbamidomethylation | C7:Carbamidomethylation:1000.00;C8:Carbamidomethylation:1000.00 | PEAKS DB |
| T.LEC(+57.02)HNQQSSQTPTTTGC(+57.02)SGGETNC(+57.02)YKK.R | Y | 59.86 | 3072.2971 | 27 | 5.5 | 769.0817 | 4 | 11.26 | 8 | F8:1677 | NaSa01\_F3.raw |  |  |  |  |  |  |  | 2.1676E6 |  |  |  |  |  |  | 3 | 0 | 0 | 0 | 0 | 0 | 0 | 0 | 3 | 0 | 0 | 0 | 0 | 0 | 0 | 22 | 48 | Carbamidomethylation | C3:Carbamidomethylation:1000.00;C17:Carbamidomethylation:1000.00;C24:Carbamidomethylation:1000.00 | PEAKS DB |
| N.GIEINC(+57.02)C(+57.02)TTDR.C | N | 50.21 | 1337.5704 | 11 | 6.3 | 669.7932 | 2 | 11.47 | 8 | F8:2008 | NaSa01\_F3.raw |  |  |  |  |  |  |  | 1.1583E6 |  |  |  |  |  |  | 1 | 0 | 0 | 0 | 0 | 0 | 0 | 0 | 1 | 0 | 0 | 0 | 0 | 0 | 0 | 70 | 80 | Carbamidomethylation | C6:Carbamidomethylation:1000.00;C7:Carbamidomethylation:1000.00 | PEAKS DB |
| total 4 peptides |  |  |  |  |  |  |  |  |  |  |  |  |  |  |  |  |  |  |  |  |  |  |  |  |  |  |  |  |  |  |  |  |  |  |  |  |  |  |  |  |  |  |  |  |  |
| --- | --- | --- | --- | --- | --- | --- | --- | --- | --- | --- | --- | --- | --- | --- | --- | --- | --- | --- | --- | --- | --- | --- | --- | --- | --- | --- | --- | --- | --- | --- | --- | --- | --- | --- | --- | --- | --- | --- | --- | --- | --- | --- | --- | --- | --- |

AAB03223.1

back to list

  

| Protein Coverage
| Supporting Peptides
|

Protein Coverage:

Supporting Peptides:

| Peptide | Uniq | -10lgP | Mass | Length | ppm | m/z | z | RT | Fraction | Scan | Source File | Area F1 | Area F10 | Area F11 | Area F12 | Area F13 | Area F14 | Area F2 | Area F3 | Area F4 | Area F5 | Area F6 | Area F7 | Area F8 | Area F9 | #Feature | #Feature F1 | #Feature F10 | #Feature F11 | #Feature F12 | #Feature F13 | #Feature F14 | #Feature F2 | #Feature F3 | #Feature F4 | #Feature F5 | #Feature F6 | #Feature F7 | #Feature F8 | #Feature F9 | Start | End | PTM | AScore | Found By |
| --- | --- | --- | --- | --- | --- | --- | --- | --- | --- | --- | --- | --- | --- | --- | --- | --- | --- | --- | --- | --- | --- | --- | --- | --- | --- | --- | --- | --- | --- | --- | --- | --- | --- | --- | --- | --- | --- | --- | --- | --- | --- | --- | --- | --- | --- |
| T.LEC(+57.02)HNQQSSQTPTTTGC(+57.02)SGGETNC(+57.02)YK.K | Y | 85.04 | 2944.2021 | 26 | 2.7 | 982.4077 | 3 | 11.84 | 7 | F7:1790 | NaSa01\_F2.raw | 2.6655E8 |  |  |  |  |  | 2.8883E7 | 0 | 4.5532E7 | 3.2829E5 |  |  |  |  | 5 | 2 | 0 | 0 | 0 | 0 | 0 | 1 | 0 | 1 | 1 | 0 | 0 | 0 | 0 | 22 | 47 | Carbamidomethylation | C3:Carbamidomethylation:1000.00;C17:Carbamidomethylation:1000.00;C24:Carbamidomethylation:1000.00 | PEAKS DB |
| K.NGIEINC(+57.02)C(+57.02)TTDR.C | N | 76.35 | 1451.6133 | 12 | 3.3 | 726.8141 | 2 | 19.60 | 7 | F7:7461 | NaSa01\_F2.raw | 0 |  |  |  |  |  | 6.7801E6 | 5.7301E7 | 9.911E5 |  |  |  |  |  | 8 | 0 | 0 | 0 | 0 | 0 | 0 | 1 | 6 | 1 | 0 | 0 | 0 | 0 | 0 | 69 | 80 | Carbamidomethylation | C7:Carbamidomethylation:1000.00;C8:Carbamidomethylation:1000.00 | PEAKS DB |
| T.LEC(+57.02)HNQQSSQTPTTTGC(+57.02)SGGETNC(+57.02)YKK.R | Y | 59.86 | 3072.2971 | 27 | 5.5 | 769.0817 | 4 | 11.26 | 8 | F8:1677 | NaSa01\_F3.raw |  |  |  |  |  |  |  | 2.1676E6 |  |  |  |  |  |  | 3 | 0 | 0 | 0 | 0 | 0 | 0 | 0 | 3 | 0 | 0 | 0 | 0 | 0 | 0 | 22 | 48 | Carbamidomethylation | C3:Carbamidomethylation:1000.00;C17:Carbamidomethylation:1000.00;C24:Carbamidomethylation:1000.00 | PEAKS DB |
| N.GIEINC(+57.02)C(+57.02)TTDR.C | N | 50.21 | 1337.5704 | 11 | 6.3 | 669.7932 | 2 | 11.47 | 8 | F8:2008 | NaSa01\_F3.raw |  |  |  |  |  |  |  | 1.1583E6 |  |  |  |  |  |  | 1 | 0 | 0 | 0 | 0 | 0 | 0 | 0 | 1 | 0 | 0 | 0 | 0 | 0 | 0 | 70 | 80 | Carbamidomethylation | C6:Carbamidomethylation:1000.00;C7:Carbamidomethylation:1000.00 | PEAKS DB |
| total 4 peptides |  |  |  |  |  |  |  |  |  |  |  |  |  |  |  |  |  |  |  |  |  |  |  |  |  |  |  |  |  |  |  |  |  |  |  |  |  |  |  |  |  |  |  |  |  |
| --- | --- | --- | --- | --- | --- | --- | --- | --- | --- | --- | --- | --- | --- | --- | --- | --- | --- | --- | --- | --- | --- | --- | --- | --- | --- | --- | --- | --- | --- | --- | --- | --- | --- | --- | --- | --- | --- | --- | --- | --- | --- | --- | --- | --- | --- |

Q9PTT0.1

back to list

  

| Protein Coverage
| Supporting Peptides
|

Protein Coverage:

Supporting Peptides:

| Peptide | Uniq | -10lgP | Mass | Length | ppm | m/z | z | RT | Fraction | Scan | Source File | Area F1 | Area F10 | Area F11 | Area F12 | Area F13 | Area F14 | Area F2 | Area F3 | Area F4 | Area F5 | Area F6 | Area F7 | Area F8 | Area F9 | #Feature | #Feature F1 | #Feature F10 | #Feature F11 | #Feature F12 | #Feature F13 | #Feature F14 | #Feature F2 | #Feature F3 | #Feature F4 | #Feature F5 | #Feature F6 | #Feature F7 | #Feature F8 | #Feature F9 | Start | End | PTM | AScore | Found By |
| --- | --- | --- | --- | --- | --- | --- | --- | --- | --- | --- | --- | --- | --- | --- | --- | --- | --- | --- | --- | --- | --- | --- | --- | --- | --- | --- | --- | --- | --- | --- | --- | --- | --- | --- | --- | --- | --- | --- | --- | --- | --- | --- | --- | --- | --- |
| T.LEC(+57.02)HNQQSSQTPTTTGC(+57.02)SGGETNC(+57.02)YK.K | Y | 85.04 | 2944.2021 | 26 | 2.7 | 982.4077 | 3 | 11.84 | 7 | F7:1790 | NaSa01\_F2.raw | 2.6655E8 |  |  |  |  |  | 2.8883E7 | 0 | 4.5532E7 | 3.2829E5 |  |  |  |  | 5 | 2 | 0 | 0 | 0 | 0 | 0 | 1 | 0 | 1 | 1 | 0 | 0 | 0 | 0 | 22 | 47 | Carbamidomethylation | C3:Carbamidomethylation:1000.00;C17:Carbamidomethylation:1000.00;C24:Carbamidomethylation:1000.00 | PEAKS DB |
| K.NGIEINC(+57.02)C(+57.02)TTDR.C | N | 76.35 | 1451.6133 | 12 | 3.3 | 726.8141 | 2 | 19.60 | 7 | F7:7461 | NaSa01\_F2.raw | 0 |  |  |  |  |  | 6.7801E6 | 5.7301E7 | 9.911E5 |  |  |  |  |  | 8 | 0 | 0 | 0 | 0 | 0 | 0 | 1 | 6 | 1 | 0 | 0 | 0 | 0 | 0 | 69 | 80 | Carbamidomethylation | C7:Carbamidomethylation:1000.00;C8:Carbamidomethylation:1000.00 | PEAKS DB |
| T.LEC(+57.02)HNQQSSQTPTTTGC(+57.02)SGGETNC(+57.02)YKK.R | Y | 59.86 | 3072.2971 | 27 | 5.5 | 769.0817 | 4 | 11.26 | 8 | F8:1677 | NaSa01\_F3.raw |  |  |  |  |  |  |  | 2.1676E6 |  |  |  |  |  |  | 3 | 0 | 0 | 0 | 0 | 0 | 0 | 0 | 3 | 0 | 0 | 0 | 0 | 0 | 0 | 22 | 48 | Carbamidomethylation | C3:Carbamidomethylation:1000.00;C17:Carbamidomethylation:1000.00;C24:Carbamidomethylation:1000.00 | PEAKS DB |
| N.GIEINC(+57.02)C(+57.02)TTDR.C | N | 50.21 | 1337.5704 | 11 | 6.3 | 669.7932 | 2 | 11.47 | 8 | F8:2008 | NaSa01\_F3.raw |  |  |  |  |  |  |  | 1.1583E6 |  |  |  |  |  |  | 1 | 0 | 0 | 0 | 0 | 0 | 0 | 0 | 1 | 0 | 0 | 0 | 0 | 0 | 0 | 70 | 80 | Carbamidomethylation | C6:Carbamidomethylation:1000.00;C7:Carbamidomethylation:1000.00 | PEAKS DB |
| total 4 peptides |  |  |  |  |  |  |  |  |  |  |  |  |  |  |  |  |  |  |  |  |  |  |  |  |  |  |  |  |  |  |  |  |  |  |  |  |  |  |  |  |  |  |  |  |  |
| --- | --- | --- | --- | --- | --- | --- | --- | --- | --- | --- | --- | --- | --- | --- | --- | --- | --- | --- | --- | --- | --- | --- | --- | --- | --- | --- | --- | --- | --- | --- | --- | --- | --- | --- | --- | --- | --- | --- | --- | --- | --- | --- | --- | --- | --- |

P60770.1

back to list

  

| Protein Coverage
| Supporting Peptides
|

Protein Coverage:

Supporting Peptides:

| Peptide | Uniq | -10lgP | Mass | Length | ppm | m/z | z | RT | Fraction | Scan | Source File | Area F1 | Area F10 | Area F11 | Area F12 | Area F13 | Area F14 | Area F2 | Area F3 | Area F4 | Area F5 | Area F6 | Area F7 | Area F8 | Area F9 | #Feature | #Feature F1 | #Feature F10 | #Feature F11 | #Feature F12 | #Feature F13 | #Feature F14 | #Feature F2 | #Feature F3 | #Feature F4 | #Feature F5 | #Feature F6 | #Feature F7 | #Feature F8 | #Feature F9 | Start | End | PTM | AScore | Found By |
| --- | --- | --- | --- | --- | --- | --- | --- | --- | --- | --- | --- | --- | --- | --- | --- | --- | --- | --- | --- | --- | --- | --- | --- | --- | --- | --- | --- | --- | --- | --- | --- | --- | --- | --- | --- | --- | --- | --- | --- | --- | --- | --- | --- | --- | --- |
| T.LEC(+57.02)HNQQSSQTPTTTGC(+57.02)SGGETNC(+57.02)YK.K | Y | 85.04 | 2944.2021 | 26 | 2.7 | 982.4077 | 3 | 11.84 | 7 | F7:1790 | NaSa01\_F2.raw | 2.6655E8 |  |  |  |  |  | 2.8883E7 | 0 | 4.5532E7 | 3.2829E5 |  |  |  |  | 5 | 2 | 0 | 0 | 0 | 0 | 0 | 1 | 0 | 1 | 1 | 0 | 0 | 0 | 0 | 22 | 47 | Carbamidomethylation | C3:Carbamidomethylation:1000.00;C17:Carbamidomethylation:1000.00;C24:Carbamidomethylation:1000.00 | PEAKS DB |
| K.NGIEINC(+57.02)C(+57.02)TTDR.C | N | 76.35 | 1451.6133 | 12 | 3.3 | 726.8141 | 2 | 19.60 | 7 | F7:7461 | NaSa01\_F2.raw | 0 |  |  |  |  |  | 6.7801E6 | 5.7301E7 | 9.911E5 |  |  |  |  |  | 8 | 0 | 0 | 0 | 0 | 0 | 0 | 1 | 6 | 1 | 0 | 0 | 0 | 0 | 0 | 69 | 80 | Carbamidomethylation | C7:Carbamidomethylation:1000.00;C8:Carbamidomethylation:1000.00 | PEAKS DB |
| T.LEC(+57.02)HNQQSSQTPTTTGC(+57.02)SGGETNC(+57.02)YKK.R | Y | 59.86 | 3072.2971 | 27 | 5.5 | 769.0817 | 4 | 11.26 | 8 | F8:1677 | NaSa01\_F3.raw |  |  |  |  |  |  |  | 2.1676E6 |  |  |  |  |  |  | 3 | 0 | 0 | 0 | 0 | 0 | 0 | 0 | 3 | 0 | 0 | 0 | 0 | 0 | 0 | 22 | 48 | Carbamidomethylation | C3:Carbamidomethylation:1000.00;C17:Carbamidomethylation:1000.00;C24:Carbamidomethylation:1000.00 | PEAKS DB |
| N.GIEINC(+57.02)C(+57.02)TTDR.C | N | 50.21 | 1337.5704 | 11 | 6.3 | 669.7932 | 2 | 11.47 | 8 | F8:2008 | NaSa01\_F3.raw |  |  |  |  |  |  |  | 1.1583E6 |  |  |  |  |  |  | 1 | 0 | 0 | 0 | 0 | 0 | 0 | 0 | 1 | 0 | 0 | 0 | 0 | 0 | 0 | 70 | 80 | Carbamidomethylation | C6:Carbamidomethylation:1000.00;C7:Carbamidomethylation:1000.00 | PEAKS DB |
| total 4 peptides |  |  |  |  |  |  |  |  |  |  |  |  |  |  |  |  |  |  |  |  |  |  |  |  |  |  |  |  |  |  |  |  |  |  |  |  |  |  |  |  |  |  |  |  |  |
| --- | --- | --- | --- | --- | --- | --- | --- | --- | --- | --- | --- | --- | --- | --- | --- | --- | --- | --- | --- | --- | --- | --- | --- | --- | --- | --- | --- | --- | --- | --- | --- | --- | --- | --- | --- | --- | --- | --- | --- | --- | --- | --- | --- | --- | --- |

P60771.2

back to list

  

| Protein Coverage
| Supporting Peptides
|

Protein Coverage:

Supporting Peptides:

| Peptide | Uniq | -10lgP | Mass | Length | ppm | m/z | z | RT | Fraction | Scan | Source File | Area F1 | Area F10 | Area F11 | Area F12 | Area F13 | Area F14 | Area F2 | Area F3 | Area F4 | Area F5 | Area F6 | Area F7 | Area F8 | Area F9 | #Feature | #Feature F1 | #Feature F10 | #Feature F11 | #Feature F12 | #Feature F13 | #Feature F14 | #Feature F2 | #Feature F3 | #Feature F4 | #Feature F5 | #Feature F6 | #Feature F7 | #Feature F8 | #Feature F9 | Start | End | PTM | AScore | Found By |
| --- | --- | --- | --- | --- | --- | --- | --- | --- | --- | --- | --- | --- | --- | --- | --- | --- | --- | --- | --- | --- | --- | --- | --- | --- | --- | --- | --- | --- | --- | --- | --- | --- | --- | --- | --- | --- | --- | --- | --- | --- | --- | --- | --- | --- | --- |
| T.LEC(+57.02)HNQQSSQTPTTTGC(+57.02)SGGETNC(+57.02)YK.K | Y | 85.04 | 2944.2021 | 26 | 2.7 | 982.4077 | 3 | 11.84 | 7 | F7:1790 | NaSa01\_F2.raw | 2.6655E8 |  |  |  |  |  | 2.8883E7 | 0 | 4.5532E7 | 3.2829E5 |  |  |  |  | 5 | 2 | 0 | 0 | 0 | 0 | 0 | 1 | 0 | 1 | 1 | 0 | 0 | 0 | 0 | 22 | 47 | Carbamidomethylation | C3:Carbamidomethylation:1000.00;C17:Carbamidomethylation:1000.00;C24:Carbamidomethylation:1000.00 | PEAKS DB |
| K.NGIEINC(+57.02)C(+57.02)TTDR.C | N | 76.35 | 1451.6133 | 12 | 3.3 | 726.8141 | 2 | 19.60 | 7 | F7:7461 | NaSa01\_F2.raw | 0 |  |  |  |  |  | 6.7801E6 | 5.7301E7 | 9.911E5 |  |  |  |  |  | 8 | 0 | 0 | 0 | 0 | 0 | 0 | 1 | 6 | 1 | 0 | 0 | 0 | 0 | 0 | 69 | 80 | Carbamidomethylation | C7:Carbamidomethylation:1000.00;C8:Carbamidomethylation:1000.00 | PEAKS DB |
| T.LEC(+57.02)HNQQSSQTPTTTGC(+57.02)SGGETNC(+57.02)YKK.R | Y | 59.86 | 3072.2971 | 27 | 5.5 | 769.0817 | 4 | 11.26 | 8 | F8:1677 | NaSa01\_F3.raw |  |  |  |  |  |  |  | 2.1676E6 |  |  |  |  |  |  | 3 | 0 | 0 | 0 | 0 | 0 | 0 | 0 | 3 | 0 | 0 | 0 | 0 | 0 | 0 | 22 | 48 | Carbamidomethylation | C3:Carbamidomethylation:1000.00;C17:Carbamidomethylation:1000.00;C24:Carbamidomethylation:1000.00 | PEAKS DB |
| N.GIEINC(+57.02)C(+57.02)TTDR.C | N | 50.21 | 1337.5704 | 11 | 6.3 | 669.7932 | 2 | 11.47 | 8 | F8:2008 | NaSa01\_F3.raw |  |  |  |  |  |  |  | 1.1583E6 |  |  |  |  |  |  | 1 | 0 | 0 | 0 | 0 | 0 | 0 | 0 | 1 | 0 | 0 | 0 | 0 | 0 | 0 | 70 | 80 | Carbamidomethylation | C6:Carbamidomethylation:1000.00;C7:Carbamidomethylation:1000.00 | PEAKS DB |
| total 4 peptides |  |  |  |  |  |  |  |  |  |  |  |  |  |  |  |  |  |  |  |  |  |  |  |  |  |  |  |  |  |  |  |  |  |  |  |  |  |  |  |  |  |  |  |  |  |
| --- | --- | --- | --- | --- | --- | --- | --- | --- | --- | --- | --- | --- | --- | --- | --- | --- | --- | --- | --- | --- | --- | --- | --- | --- | --- | --- | --- | --- | --- | --- | --- | --- | --- | --- | --- | --- | --- | --- | --- | --- | --- | --- | --- | --- | --- |

AAB36931.1

back to list

  

| Protein Coverage
| Supporting Peptides
|

Protein Coverage:

Supporting Peptides:

| Peptide | Uniq | -10lgP | Mass | Length | ppm | m/z | z | RT | Fraction | Scan | Source File | Area F1 | Area F10 | Area F11 | Area F12 | Area F13 | Area F14 | Area F2 | Area F3 | Area F4 | Area F5 | Area F6 | Area F7 | Area F8 | Area F9 | #Feature | #Feature F1 | #Feature F10 | #Feature F11 | #Feature F12 | #Feature F13 | #Feature F14 | #Feature F2 | #Feature F3 | #Feature F4 | #Feature F5 | #Feature F6 | #Feature F7 | #Feature F8 | #Feature F9 | Start | End | PTM | AScore | Found By |
| --- | --- | --- | --- | --- | --- | --- | --- | --- | --- | --- | --- | --- | --- | --- | --- | --- | --- | --- | --- | --- | --- | --- | --- | --- | --- | --- | --- | --- | --- | --- | --- | --- | --- | --- | --- | --- | --- | --- | --- | --- | --- | --- | --- | --- | --- |
| T.LEC(+57.02)HNQQSSQTPTTTGC(+57.02)SGGETNC(+57.02)YK.K | Y | 85.04 | 2944.2021 | 26 | 2.7 | 982.4077 | 3 | 11.84 | 7 | F7:1790 | NaSa01\_F2.raw | 2.6655E8 |  |  |  |  |  | 2.8883E7 | 0 | 4.5532E7 | 3.2829E5 |  |  |  |  | 5 | 2 | 0 | 0 | 0 | 0 | 0 | 1 | 0 | 1 | 1 | 0 | 0 | 0 | 0 | 22 | 47 | Carbamidomethylation | C3:Carbamidomethylation:1000.00;C17:Carbamidomethylation:1000.00;C24:Carbamidomethylation:1000.00 | PEAKS DB |
| K.NGIEINC(+57.02)C(+57.02)TTDR.C | N | 76.35 | 1451.6133 | 12 | 3.3 | 726.8141 | 2 | 19.60 | 7 | F7:7461 | NaSa01\_F2.raw | 0 |  |  |  |  |  | 6.7801E6 | 5.7301E7 | 9.911E5 |  |  |  |  |  | 8 | 0 | 0 | 0 | 0 | 0 | 0 | 1 | 6 | 1 | 0 | 0 | 0 | 0 | 0 | 69 | 80 | Carbamidomethylation | C7:Carbamidomethylation:1000.00;C8:Carbamidomethylation:1000.00 | PEAKS DB |
| T.LEC(+57.02)HNQQSSQTPTTTGC(+57.02)SGGETNC(+57.02)YKK.R | Y | 59.86 | 3072.2971 | 27 | 5.5 | 769.0817 | 4 | 11.26 | 8 | F8:1677 | NaSa01\_F3.raw |  |  |  |  |  |  |  | 2.1676E6 |  |  |  |  |  |  | 3 | 0 | 0 | 0 | 0 | 0 | 0 | 0 | 3 | 0 | 0 | 0 | 0 | 0 | 0 | 22 | 48 | Carbamidomethylation | C3:Carbamidomethylation:1000.00;C17:Carbamidomethylation:1000.00;C24:Carbamidomethylation:1000.00 | PEAKS DB |
| N.GIEINC(+57.02)C(+57.02)TTDR.C | N | 50.21 | 1337.5704 | 11 | 6.3 | 669.7932 | 2 | 11.47 | 8 | F8:2008 | NaSa01\_F3.raw |  |  |  |  |  |  |  | 1.1583E6 |  |  |  |  |  |  | 1 | 0 | 0 | 0 | 0 | 0 | 0 | 0 | 1 | 0 | 0 | 0 | 0 | 0 | 0 | 70 | 80 | Carbamidomethylation | C6:Carbamidomethylation:1000.00;C7:Carbamidomethylation:1000.00 | PEAKS DB |
| total 4 peptides |  |  |  |  |  |  |  |  |  |  |  |  |  |  |  |  |  |  |  |  |  |  |  |  |  |  |  |  |  |  |  |  |  |  |  |  |  |  |  |  |  |  |  |  |  |
| --- | --- | --- | --- | --- | --- | --- | --- | --- | --- | --- | --- | --- | --- | --- | --- | --- | --- | --- | --- | --- | --- | --- | --- | --- | --- | --- | --- | --- | --- | --- | --- | --- | --- | --- | --- | --- | --- | --- | --- | --- | --- | --- | --- | --- | --- |

AAB01538.1

back to list

  

| Protein Coverage
| Supporting Peptides
|

Protein Coverage:

Supporting Peptides:

| Peptide | Uniq | -10lgP | Mass | Length | ppm | m/z | z | RT | Fraction | Scan | Source File | Area F1 | Area F10 | Area F11 | Area F12 | Area F13 | Area F14 | Area F2 | Area F3 | Area F4 | Area F5 | Area F6 | Area F7 | Area F8 | Area F9 | #Feature | #Feature F1 | #Feature F10 | #Feature F11 | #Feature F12 | #Feature F13 | #Feature F14 | #Feature F2 | #Feature F3 | #Feature F4 | #Feature F5 | #Feature F6 | #Feature F7 | #Feature F8 | #Feature F9 | Start | End | PTM | AScore | Found By |
| --- | --- | --- | --- | --- | --- | --- | --- | --- | --- | --- | --- | --- | --- | --- | --- | --- | --- | --- | --- | --- | --- | --- | --- | --- | --- | --- | --- | --- | --- | --- | --- | --- | --- | --- | --- | --- | --- | --- | --- | --- | --- | --- | --- | --- | --- |
| T.LEC(+57.02)HNQQSSQTPTTTGC(+57.02)SGGETNC(+57.02)YK.K | Y | 85.04 | 2944.2021 | 26 | 2.7 | 982.4077 | 3 | 11.84 | 7 | F7:1790 | NaSa01\_F2.raw | 2.6655E8 |  |  |  |  |  | 2.8883E7 | 0 | 4.5532E7 | 3.2829E5 |  |  |  |  | 5 | 2 | 0 | 0 | 0 | 0 | 0 | 1 | 0 | 1 | 1 | 0 | 0 | 0 | 0 | 22 | 47 | Carbamidomethylation | C3:Carbamidomethylation:1000.00;C17:Carbamidomethylation:1000.00;C24:Carbamidomethylation:1000.00 | PEAKS DB |
| K.NGIEINC(+57.02)C(+57.02)TTDR.C | N | 76.35 | 1451.6133 | 12 | 3.3 | 726.8141 | 2 | 19.60 | 7 | F7:7461 | NaSa01\_F2.raw | 0 |  |  |  |  |  | 6.7801E6 | 5.7301E7 | 9.911E5 |  |  |  |  |  | 8 | 0 | 0 | 0 | 0 | 0 | 0 | 1 | 6 | 1 | 0 | 0 | 0 | 0 | 0 | 69 | 80 | Carbamidomethylation | C7:Carbamidomethylation:1000.00;C8:Carbamidomethylation:1000.00 | PEAKS DB |
| T.LEC(+57.02)HNQQSSQTPTTTGC(+57.02)SGGETNC(+57.02)YKK.R | Y | 59.86 | 3072.2971 | 27 | 5.5 | 769.0817 | 4 | 11.26 | 8 | F8:1677 | NaSa01\_F3.raw |  |  |  |  |  |  |  | 2.1676E6 |  |  |  |  |  |  | 3 | 0 | 0 | 0 | 0 | 0 | 0 | 0 | 3 | 0 | 0 | 0 | 0 | 0 | 0 | 22 | 48 | Carbamidomethylation | C3:Carbamidomethylation:1000.00;C17:Carbamidomethylation:1000.00;C24:Carbamidomethylation:1000.00 | PEAKS DB |
| N.GIEINC(+57.02)C(+57.02)TTDR.C | N | 50.21 | 1337.5704 | 11 | 6.3 | 669.7932 | 2 | 11.47 | 8 | F8:2008 | NaSa01\_F3.raw |  |  |  |  |  |  |  | 1.1583E6 |  |  |  |  |  |  | 1 | 0 | 0 | 0 | 0 | 0 | 0 | 0 | 1 | 0 | 0 | 0 | 0 | 0 | 0 | 70 | 80 | Carbamidomethylation | C6:Carbamidomethylation:1000.00;C7:Carbamidomethylation:1000.00 | PEAKS DB |
| total 4 peptides |  |  |  |  |  |  |  |  |  |  |  |  |  |  |  |  |  |  |  |  |  |  |  |  |  |  |  |  |  |  |  |  |  |  |  |  |  |  |  |  |  |  |  |  |  |
| --- | --- | --- | --- | --- | --- | --- | --- | --- | --- | --- | --- | --- | --- | --- | --- | --- | --- | --- | --- | --- | --- | --- | --- | --- | --- | --- | --- | --- | --- | --- | --- | --- | --- | --- | --- | --- | --- | --- | --- | --- | --- | --- | --- | --- | --- |

AAY63884.1

back to list

  

| Protein Coverage
| Supporting Peptides
|

Protein Coverage:

Supporting Peptides:

| Peptide | Uniq | -10lgP | Mass | Length | ppm | m/z | z | RT | Fraction | Scan | Source File | Area F1 | Area F10 | Area F11 | Area F12 | Area F13 | Area F14 | Area F2 | Area F3 | Area F4 | Area F5 | Area F6 | Area F7 | Area F8 | Area F9 | #Feature | #Feature F1 | #Feature F10 | #Feature F11 | #Feature F12 | #Feature F13 | #Feature F14 | #Feature F2 | #Feature F3 | #Feature F4 | #Feature F5 | #Feature F6 | #Feature F7 | #Feature F8 | #Feature F9 | Start | End | PTM | AScore | Found By |
| --- | --- | --- | --- | --- | --- | --- | --- | --- | --- | --- | --- | --- | --- | --- | --- | --- | --- | --- | --- | --- | --- | --- | --- | --- | --- | --- | --- | --- | --- | --- | --- | --- | --- | --- | --- | --- | --- | --- | --- | --- | --- | --- | --- | --- | --- |
| T.LEC(+57.02)HNQQSSQTPTTTGC(+57.02)SGGETNC(+57.02)YK.K | Y | 85.04 | 2944.2021 | 26 | 2.7 | 982.4077 | 3 | 11.84 | 7 | F7:1790 | NaSa01\_F2.raw | 2.6655E8 |  |  |  |  |  | 2.8883E7 | 0 | 4.5532E7 | 3.2829E5 |  |  |  |  | 5 | 2 | 0 | 0 | 0 | 0 | 0 | 1 | 0 | 1 | 1 | 0 | 0 | 0 | 0 | 22 | 47 | Carbamidomethylation | C3:Carbamidomethylation:1000.00;C17:Carbamidomethylation:1000.00;C24:Carbamidomethylation:1000.00 | PEAKS DB |
| K.NGIEINC(+57.02)C(+57.02)TTDR.C | N | 76.35 | 1451.6133 | 12 | 3.3 | 726.8141 | 2 | 19.60 | 7 | F7:7461 | NaSa01\_F2.raw | 0 |  |  |  |  |  | 6.7801E6 | 5.7301E7 | 9.911E5 |  |  |  |  |  | 8 | 0 | 0 | 0 | 0 | 0 | 0 | 1 | 6 | 1 | 0 | 0 | 0 | 0 | 0 | 69 | 80 | Carbamidomethylation | C7:Carbamidomethylation:1000.00;C8:Carbamidomethylation:1000.00 | PEAKS DB |
| T.LEC(+57.02)HNQQSSQTPTTTGC(+57.02)SGGETNC(+57.02)YKK.R | Y | 59.86 | 3072.2971 | 27 | 5.5 | 769.0817 | 4 | 11.26 | 8 | F8:1677 | NaSa01\_F3.raw |  |  |  |  |  |  |  | 2.1676E6 |  |  |  |  |  |  | 3 | 0 | 0 | 0 | 0 | 0 | 0 | 0 | 3 | 0 | 0 | 0 | 0 | 0 | 0 | 22 | 48 | Carbamidomethylation | C3:Carbamidomethylation:1000.00;C17:Carbamidomethylation:1000.00;C24:Carbamidomethylation:1000.00 | PEAKS DB |
| N.GIEINC(+57.02)C(+57.02)TTDR.C | N | 50.21 | 1337.5704 | 11 | 6.3 | 669.7932 | 2 | 11.47 | 8 | F8:2008 | NaSa01\_F3.raw |  |  |  |  |  |  |  | 1.1583E6 |  |  |  |  |  |  | 1 | 0 | 0 | 0 | 0 | 0 | 0 | 0 | 1 | 0 | 0 | 0 | 0 | 0 | 0 | 70 | 80 | Carbamidomethylation | C6:Carbamidomethylation:1000.00;C7:Carbamidomethylation:1000.00 | PEAKS DB |
| total 4 peptides |  |  |  |  |  |  |  |  |  |  |  |  |  |  |  |  |  |  |  |  |  |  |  |  |  |  |  |  |  |  |  |  |  |  |  |  |  |  |  |  |  |  |  |  |  |
| --- | --- | --- | --- | --- | --- | --- | --- | --- | --- | --- | --- | --- | --- | --- | --- | --- | --- | --- | --- | --- | --- | --- | --- | --- | --- | --- | --- | --- | --- | --- | --- | --- | --- | --- | --- | --- | --- | --- | --- | --- | --- | --- | --- | --- | --- |

AAB36930.1

back to list

  

| Protein Coverage
| Supporting Peptides
|

Protein Coverage:

Supporting Peptides:

| Peptide | Uniq | -10lgP | Mass | Length | ppm | m/z | z | RT | Fraction | Scan | Source File | Area F1 | Area F10 | Area F11 | Area F12 | Area F13 | Area F14 | Area F2 | Area F3 | Area F4 | Area F5 | Area F6 | Area F7 | Area F8 | Area F9 | #Feature | #Feature F1 | #Feature F10 | #Feature F11 | #Feature F12 | #Feature F13 | #Feature F14 | #Feature F2 | #Feature F3 | #Feature F4 | #Feature F5 | #Feature F6 | #Feature F7 | #Feature F8 | #Feature F9 | Start | End | PTM | AScore | Found By |
| --- | --- | --- | --- | --- | --- | --- | --- | --- | --- | --- | --- | --- | --- | --- | --- | --- | --- | --- | --- | --- | --- | --- | --- | --- | --- | --- | --- | --- | --- | --- | --- | --- | --- | --- | --- | --- | --- | --- | --- | --- | --- | --- | --- | --- | --- |
| T.LEC(+57.02)HNQQSSQTPTTTGC(+57.02)SGGETNC(+57.02)YK.K | Y | 85.04 | 2944.2021 | 26 | 2.7 | 982.4077 | 3 | 11.84 | 7 | F7:1790 | NaSa01\_F2.raw | 2.6655E8 |  |  |  |  |  | 2.8883E7 | 0 | 4.5532E7 | 3.2829E5 |  |  |  |  | 5 | 2 | 0 | 0 | 0 | 0 | 0 | 1 | 0 | 1 | 1 | 0 | 0 | 0 | 0 | 22 | 47 | Carbamidomethylation | C3:Carbamidomethylation:1000.00;C17:Carbamidomethylation:1000.00;C24:Carbamidomethylation:1000.00 | PEAKS DB |
| K.NGIEINC(+57.02)C(+57.02)TTDR.C | N | 76.35 | 1451.6133 | 12 | 3.3 | 726.8141 | 2 | 19.60 | 7 | F7:7461 | NaSa01\_F2.raw | 0 |  |  |  |  |  | 6.7801E6 | 5.7301E7 | 9.911E5 |  |  |  |  |  | 8 | 0 | 0 | 0 | 0 | 0 | 0 | 1 | 6 | 1 | 0 | 0 | 0 | 0 | 0 | 69 | 80 | Carbamidomethylation | C7:Carbamidomethylation:1000.00;C8:Carbamidomethylation:1000.00 | PEAKS DB |
| T.LEC(+57.02)HNQQSSQTPTTTGC(+57.02)SGGETNC(+57.02)YKK.R | Y | 59.86 | 3072.2971 | 27 | 5.5 | 769.0817 | 4 | 11.26 | 8 | F8:1677 | NaSa01\_F3.raw |  |  |  |  |  |  |  | 2.1676E6 |  |  |  |  |  |  | 3 | 0 | 0 | 0 | 0 | 0 | 0 | 0 | 3 | 0 | 0 | 0 | 0 | 0 | 0 | 22 | 48 | Carbamidomethylation | C3:Carbamidomethylation:1000.00;C17:Carbamidomethylation:1000.00;C24:Carbamidomethylation:1000.00 | PEAKS DB |
| N.GIEINC(+57.02)C(+57.02)TTDR.C | N | 50.21 | 1337.5704 | 11 | 6.3 | 669.7932 | 2 | 11.47 | 8 | F8:2008 | NaSa01\_F3.raw |  |  |  |  |  |  |  | 1.1583E6 |  |  |  |  |  |  | 1 | 0 | 0 | 0 | 0 | 0 | 0 | 0 | 1 | 0 | 0 | 0 | 0 | 0 | 0 | 70 | 80 | Carbamidomethylation | C6:Carbamidomethylation:1000.00;C7:Carbamidomethylation:1000.00 | PEAKS DB |
| total 4 peptides |  |  |  |  |  |  |  |  |  |  |  |  |  |  |  |  |  |  |  |  |  |  |  |  |  |  |  |  |  |  |  |  |  |  |  |  |  |  |  |  |  |  |  |  |  |
| --- | --- | --- | --- | --- | --- | --- | --- | --- | --- | --- | --- | --- | --- | --- | --- | --- | --- | --- | --- | --- | --- | --- | --- | --- | --- | --- | --- | --- | --- | --- | --- | --- | --- | --- | --- | --- | --- | --- | --- | --- | --- | --- | --- | --- | --- |

AAB03222.1

back to list

  

| Protein Coverage
| Supporting Peptides
|

Protein Coverage:

Supporting Peptides:

| Peptide | Uniq | -10lgP | Mass | Length | ppm | m/z | z | RT | Fraction | Scan | Source File | Area F1 | Area F10 | Area F11 | Area F12 | Area F13 | Area F14 | Area F2 | Area F3 | Area F4 | Area F5 | Area F6 | Area F7 | Area F8 | Area F9 | #Feature | #Feature F1 | #Feature F10 | #Feature F11 | #Feature F12 | #Feature F13 | #Feature F14 | #Feature F2 | #Feature F3 | #Feature F4 | #Feature F5 | #Feature F6 | #Feature F7 | #Feature F8 | #Feature F9 | Start | End | PTM | AScore | Found By |
| --- | --- | --- | --- | --- | --- | --- | --- | --- | --- | --- | --- | --- | --- | --- | --- | --- | --- | --- | --- | --- | --- | --- | --- | --- | --- | --- | --- | --- | --- | --- | --- | --- | --- | --- | --- | --- | --- | --- | --- | --- | --- | --- | --- | --- | --- |
| T.LEC(+57.02)HNQQSSQTPTTTGC(+57.02)SGGETNC(+57.02)YK.K | Y | 85.04 | 2944.2021 | 26 | 2.7 | 982.4077 | 3 | 11.84 | 7 | F7:1790 | NaSa01\_F2.raw | 2.6655E8 |  |  |  |  |  | 2.8883E7 | 0 | 4.5532E7 | 3.2829E5 |  |  |  |  | 5 | 2 | 0 | 0 | 0 | 0 | 0 | 1 | 0 | 1 | 1 | 0 | 0 | 0 | 0 | 22 | 47 | Carbamidomethylation | C3:Carbamidomethylation:1000.00;C17:Carbamidomethylation:1000.00;C24:Carbamidomethylation:1000.00 | PEAKS DB |
| K.NGIEINC(+57.02)C(+57.02)TTDR.C | N | 76.35 | 1451.6133 | 12 | 3.3 | 726.8141 | 2 | 19.60 | 7 | F7:7461 | NaSa01\_F2.raw | 0 |  |  |  |  |  | 6.7801E6 | 5.7301E7 | 9.911E5 |  |  |  |  |  | 8 | 0 | 0 | 0 | 0 | 0 | 0 | 1 | 6 | 1 | 0 | 0 | 0 | 0 | 0 | 69 | 80 | Carbamidomethylation | C7:Carbamidomethylation:1000.00;C8:Carbamidomethylation:1000.00 | PEAKS DB |
| T.LEC(+57.02)HNQQSSQTPTTTGC(+57.02)SGGETNC(+57.02)YKK.R | Y | 59.86 | 3072.2971 | 27 | 5.5 | 769.0817 | 4 | 11.26 | 8 | F8:1677 | NaSa01\_F3.raw |  |  |  |  |  |  |  | 2.1676E6 |  |  |  |  |  |  | 3 | 0 | 0 | 0 | 0 | 0 | 0 | 0 | 3 | 0 | 0 | 0 | 0 | 0 | 0 | 22 | 48 | Carbamidomethylation | C3:Carbamidomethylation:1000.00;C17:Carbamidomethylation:1000.00;C24:Carbamidomethylation:1000.00 | PEAKS DB |
| N.GIEINC(+57.02)C(+57.02)TTDR.C | N | 50.21 | 1337.5704 | 11 | 6.3 | 669.7932 | 2 | 11.47 | 8 | F8:2008 | NaSa01\_F3.raw |  |  |  |  |  |  |  | 1.1583E6 |  |  |  |  |  |  | 1 | 0 | 0 | 0 | 0 | 0 | 0 | 0 | 1 | 0 | 0 | 0 | 0 | 0 | 0 | 70 | 80 | Carbamidomethylation | C6:Carbamidomethylation:1000.00;C7:Carbamidomethylation:1000.00 | PEAKS DB |
| total 4 peptides |  |  |  |  |  |  |  |  |  |  |  |  |  |  |  |  |  |  |  |  |  |  |  |  |  |  |  |  |  |  |  |  |  |  |  |  |  |  |  |  |  |  |  |  |  |
| --- | --- | --- | --- | --- | --- | --- | --- | --- | --- | --- | --- | --- | --- | --- | --- | --- | --- | --- | --- | --- | --- | --- | --- | --- | --- | --- | --- | --- | --- | --- | --- | --- | --- | --- | --- | --- | --- | --- | --- | --- | --- | --- | --- | --- | --- |

AAF21774.1

back to list

  

| Protein Coverage
| Supporting Peptides
|

Protein Coverage:

Supporting Peptides:

| Peptide | Uniq | -10lgP | Mass | Length | ppm | m/z | z | RT | Fraction | Scan | Source File | Area F1 | Area F10 | Area F11 | Area F12 | Area F13 | Area F14 | Area F2 | Area F3 | Area F4 | Area F5 | Area F6 | Area F7 | Area F8 | Area F9 | #Feature | #Feature F1 | #Feature F10 | #Feature F11 | #Feature F12 | #Feature F13 | #Feature F14 | #Feature F2 | #Feature F3 | #Feature F4 | #Feature F5 | #Feature F6 | #Feature F7 | #Feature F8 | #Feature F9 | Start | End | PTM | AScore | Found By |
| --- | --- | --- | --- | --- | --- | --- | --- | --- | --- | --- | --- | --- | --- | --- | --- | --- | --- | --- | --- | --- | --- | --- | --- | --- | --- | --- | --- | --- | --- | --- | --- | --- | --- | --- | --- | --- | --- | --- | --- | --- | --- | --- | --- | --- | --- |
| T.LEC(+57.02)HNQQSSQTPTTTGC(+57.02)SGGETNC(+57.02)YK.K | Y | 85.04 | 2944.2021 | 26 | 2.7 | 982.4077 | 3 | 11.84 | 7 | F7:1790 | NaSa01\_F2.raw | 2.6655E8 |  |  |  |  |  | 2.8883E7 | 0 | 4.5532E7 | 3.2829E5 |  |  |  |  | 5 | 2 | 0 | 0 | 0 | 0 | 0 | 1 | 0 | 1 | 1 | 0 | 0 | 0 | 0 | 22 | 47 | Carbamidomethylation | C3:Carbamidomethylation:1000.00;C17:Carbamidomethylation:1000.00;C24:Carbamidomethylation:1000.00 | PEAKS DB |
| K.NGIEINC(+57.02)C(+57.02)TTDR.C | N | 76.35 | 1451.6133 | 12 | 3.3 | 726.8141 | 2 | 19.60 | 7 | F7:7461 | NaSa01\_F2.raw | 0 |  |  |  |  |  | 6.7801E6 | 5.7301E7 | 9.911E5 |  |  |  |  |  | 8 | 0 | 0 | 0 | 0 | 0 | 0 | 1 | 6 | 1 | 0 | 0 | 0 | 0 | 0 | 69 | 80 | Carbamidomethylation | C7:Carbamidomethylation:1000.00;C8:Carbamidomethylation:1000.00 | PEAKS DB |
| T.LEC(+57.02)HNQQSSQTPTTTGC(+57.02)SGGETNC(+57.02)YKK.R | Y | 59.86 | 3072.2971 | 27 | 5.5 | 769.0817 | 4 | 11.26 | 8 | F8:1677 | NaSa01\_F3.raw |  |  |  |  |  |  |  | 2.1676E6 |  |  |  |  |  |  | 3 | 0 | 0 | 0 | 0 | 0 | 0 | 0 | 3 | 0 | 0 | 0 | 0 | 0 | 0 | 22 | 48 | Carbamidomethylation | C3:Carbamidomethylation:1000.00;C17:Carbamidomethylation:1000.00;C24:Carbamidomethylation:1000.00 | PEAKS DB |
| N.GIEINC(+57.02)C(+57.02)TTDR.C | N | 50.21 | 1337.5704 | 11 | 6.3 | 669.7932 | 2 | 11.47 | 8 | F8:2008 | NaSa01\_F3.raw |  |  |  |  |  |  |  | 1.1583E6 |  |  |  |  |  |  | 1 | 0 | 0 | 0 | 0 | 0 | 0 | 0 | 1 | 0 | 0 | 0 | 0 | 0 | 0 | 70 | 80 | Carbamidomethylation | C6:Carbamidomethylation:1000.00;C7:Carbamidomethylation:1000.00 | PEAKS DB |
| total 4 peptides |  |  |  |  |  |  |  |  |  |  |  |  |  |  |  |  |  |  |  |  |  |  |  |  |  |  |  |  |  |  |  |  |  |  |  |  |  |  |  |  |  |  |  |  |  |
| --- | --- | --- | --- | --- | --- | --- | --- | --- | --- | --- | --- | --- | --- | --- | --- | --- | --- | --- | --- | --- | --- | --- | --- | --- | --- | --- | --- | --- | --- | --- | --- | --- | --- | --- | --- | --- | --- | --- | --- | --- | --- | --- | --- | --- | --- |

AAR33035.1

back to list

  

| Protein Coverage
| Supporting Peptides
|

Protein Coverage:

Supporting Peptides:

| Peptide | Uniq | -10lgP | Mass | Length | ppm | m/z | z | RT | Fraction | Scan | Source File | Area F1 | Area F10 | Area F11 | Area F12 | Area F13 | Area F14 | Area F2 | Area F3 | Area F4 | Area F5 | Area F6 | Area F7 | Area F8 | Area F9 | #Feature | #Feature F1 | #Feature F10 | #Feature F11 | #Feature F12 | #Feature F13 | #Feature F14 | #Feature F2 | #Feature F3 | #Feature F4 | #Feature F5 | #Feature F6 | #Feature F7 | #Feature F8 | #Feature F9 | Start | End | PTM | AScore | Found By |
| --- | --- | --- | --- | --- | --- | --- | --- | --- | --- | --- | --- | --- | --- | --- | --- | --- | --- | --- | --- | --- | --- | --- | --- | --- | --- | --- | --- | --- | --- | --- | --- | --- | --- | --- | --- | --- | --- | --- | --- | --- | --- | --- | --- | --- | --- |
| T.LEC(+57.02)HNQQSSQTPTTTGC(+57.02)SGGETNC(+57.02)YK.K | Y | 85.04 | 2944.2021 | 26 | 2.7 | 982.4077 | 3 | 11.84 | 7 | F7:1790 | NaSa01\_F2.raw | 2.6655E8 |  |  |  |  |  | 2.8883E7 | 0 | 4.5532E7 | 3.2829E5 |  |  |  |  | 5 | 2 | 0 | 0 | 0 | 0 | 0 | 1 | 0 | 1 | 1 | 0 | 0 | 0 | 0 | 22 | 47 | Carbamidomethylation | C3:Carbamidomethylation:1000.00;C17:Carbamidomethylation:1000.00;C24:Carbamidomethylation:1000.00 | PEAKS DB |
| K.NGIEINC(+57.02)C(+57.02)TTDR.C | N | 76.35 | 1451.6133 | 12 | 3.3 | 726.8141 | 2 | 19.60 | 7 | F7:7461 | NaSa01\_F2.raw | 0 |  |  |  |  |  | 6.7801E6 | 5.7301E7 | 9.911E5 |  |  |  |  |  | 8 | 0 | 0 | 0 | 0 | 0 | 0 | 1 | 6 | 1 | 0 | 0 | 0 | 0 | 0 | 69 | 80 | Carbamidomethylation | C7:Carbamidomethylation:1000.00;C8:Carbamidomethylation:1000.00 | PEAKS DB |
| T.LEC(+57.02)HNQQSSQTPTTTGC(+57.02)SGGETNC(+57.02)YKK.R | Y | 59.86 | 3072.2971 | 27 | 5.5 | 769.0817 | 4 | 11.26 | 8 | F8:1677 | NaSa01\_F3.raw |  |  |  |  |  |  |  | 2.1676E6 |  |  |  |  |  |  | 3 | 0 | 0 | 0 | 0 | 0 | 0 | 0 | 3 | 0 | 0 | 0 | 0 | 0 | 0 | 22 | 48 | Carbamidomethylation | C3:Carbamidomethylation:1000.00;C17:Carbamidomethylation:1000.00;C24:Carbamidomethylation:1000.00 | PEAKS DB |
| N.GIEINC(+57.02)C(+57.02)TTDR.C | N | 50.21 | 1337.5704 | 11 | 6.3 | 669.7932 | 2 | 11.47 | 8 | F8:2008 | NaSa01\_F3.raw |  |  |  |  |  |  |  | 1.1583E6 |  |  |  |  |  |  | 1 | 0 | 0 | 0 | 0 | 0 | 0 | 0 | 1 | 0 | 0 | 0 | 0 | 0 | 0 | 70 | 80 | Carbamidomethylation | C6:Carbamidomethylation:1000.00;C7:Carbamidomethylation:1000.00 | PEAKS DB |
| total 4 peptides |  |  |  |  |  |  |  |  |  |  |  |  |  |  |  |  |  |  |  |  |  |  |  |  |  |  |  |  |  |  |  |  |  |  |  |  |  |  |  |  |  |  |  |  |  |
| --- | --- | --- | --- | --- | --- | --- | --- | --- | --- | --- | --- | --- | --- | --- | --- | --- | --- | --- | --- | --- | --- | --- | --- | --- | --- | --- | --- | --- | --- | --- | --- | --- | --- | --- | --- | --- | --- | --- | --- | --- | --- | --- | --- | --- | --- |

CAA73097.2

back to list

  

| Protein Coverage
| Supporting Peptides
|

Protein Coverage:

Supporting Peptides:

| Peptide | Uniq | -10lgP | Mass | Length | ppm | m/z | z | RT | Fraction | Scan | Source File | Area F1 | Area F10 | Area F11 | Area F12 | Area F13 | Area F14 | Area F2 | Area F3 | Area F4 | Area F5 | Area F6 | Area F7 | Area F8 | Area F9 | #Feature | #Feature F1 | #Feature F10 | #Feature F11 | #Feature F12 | #Feature F13 | #Feature F14 | #Feature F2 | #Feature F3 | #Feature F4 | #Feature F5 | #Feature F6 | #Feature F7 | #Feature F8 | #Feature F9 | Start | End | PTM | AScore | Found By |
| --- | --- | --- | --- | --- | --- | --- | --- | --- | --- | --- | --- | --- | --- | --- | --- | --- | --- | --- | --- | --- | --- | --- | --- | --- | --- | --- | --- | --- | --- | --- | --- | --- | --- | --- | --- | --- | --- | --- | --- | --- | --- | --- | --- | --- | --- |
| T.LEC(+57.02)HNQQSSQTPTTTGC(+57.02)SGGETNC(+57.02)YK.K | Y | 85.04 | 2944.2021 | 26 | 2.7 | 982.4077 | 3 | 11.84 | 7 | F7:1790 | NaSa01\_F2.raw | 2.6655E8 |  |  |  |  |  | 2.8883E7 | 0 | 4.5532E7 | 3.2829E5 |  |  |  |  | 5 | 2 | 0 | 0 | 0 | 0 | 0 | 1 | 0 | 1 | 1 | 0 | 0 | 0 | 0 | 22 | 47 | Carbamidomethylation | C3:Carbamidomethylation:1000.00;C17:Carbamidomethylation:1000.00;C24:Carbamidomethylation:1000.00 | PEAKS DB |
| K.NGIEINC(+57.02)C(+57.02)TTDR.C | N | 76.35 | 1451.6133 | 12 | 3.3 | 726.8141 | 2 | 19.60 | 7 | F7:7461 | NaSa01\_F2.raw | 0 |  |  |  |  |  | 6.7801E6 | 5.7301E7 | 9.911E5 |  |  |  |  |  | 8 | 0 | 0 | 0 | 0 | 0 | 0 | 1 | 6 | 1 | 0 | 0 | 0 | 0 | 0 | 69 | 80 | Carbamidomethylation | C7:Carbamidomethylation:1000.00;C8:Carbamidomethylation:1000.00 | PEAKS DB |
| T.LEC(+57.02)HNQQSSQTPTTTGC(+57.02)SGGETNC(+57.02)YKK.R | Y | 59.86 | 3072.2971 | 27 | 5.5 | 769.0817 | 4 | 11.26 | 8 | F8:1677 | NaSa01\_F3.raw |  |  |  |  |  |  |  | 2.1676E6 |  |  |  |  |  |  | 3 | 0 | 0 | 0 | 0 | 0 | 0 | 0 | 3 | 0 | 0 | 0 | 0 | 0 | 0 | 22 | 48 | Carbamidomethylation | C3:Carbamidomethylation:1000.00;C17:Carbamidomethylation:1000.00;C24:Carbamidomethylation:1000.00 | PEAKS DB |
| N.GIEINC(+57.02)C(+57.02)TTDR.C | N | 50.21 | 1337.5704 | 11 | 6.3 | 669.7932 | 2 | 11.47 | 8 | F8:2008 | NaSa01\_F3.raw |  |  |  |  |  |  |  | 1.1583E6 |  |  |  |  |  |  | 1 | 0 | 0 | 0 | 0 | 0 | 0 | 0 | 1 | 0 | 0 | 0 | 0 | 0 | 0 | 70 | 80 | Carbamidomethylation | C6:Carbamidomethylation:1000.00;C7:Carbamidomethylation:1000.00 | PEAKS DB |
| total 4 peptides |  |  |  |  |  |  |  |  |  |  |  |  |  |  |  |  |  |  |  |  |  |  |  |  |  |  |  |  |  |  |  |  |  |  |  |  |  |  |  |  |  |  |  |  |  |
| --- | --- | --- | --- | --- | --- | --- | --- | --- | --- | --- | --- | --- | --- | --- | --- | --- | --- | --- | --- | --- | --- | --- | --- | --- | --- | --- | --- | --- | --- | --- | --- | --- | --- | --- | --- | --- | --- | --- | --- | --- | --- | --- | --- | --- | --- |

1COD

back to list

  

| Protein Coverage
| Supporting Peptides
|

Protein Coverage:

Supporting Peptides:

| Peptide | Uniq | -10lgP | Mass | Length | ppm | m/z | z | RT | Fraction | Scan | Source File | Area F1 | Area F10 | Area F11 | Area F12 | Area F13 | Area F14 | Area F2 | Area F3 | Area F4 | Area F5 | Area F6 | Area F7 | Area F8 | Area F9 | #Feature | #Feature F1 | #Feature F10 | #Feature F11 | #Feature F12 | #Feature F13 | #Feature F14 | #Feature F2 | #Feature F3 | #Feature F4 | #Feature F5 | #Feature F6 | #Feature F7 | #Feature F8 | #Feature F9 | Start | End | PTM | AScore | Found By |
| --- | --- | --- | --- | --- | --- | --- | --- | --- | --- | --- | --- | --- | --- | --- | --- | --- | --- | --- | --- | --- | --- | --- | --- | --- | --- | --- | --- | --- | --- | --- | --- | --- | --- | --- | --- | --- | --- | --- | --- | --- | --- | --- | --- | --- | --- |
| LEC(+57.02)HNQQSSQTPTTTGC(+57.02)SGGETNC(+57.02)YK.K | Y | 85.04 | 2944.2021 | 26 | 2.7 | 982.4077 | 3 | 11.84 | 7 | F7:1790 | NaSa01\_F2.raw | 2.6655E8 |  |  |  |  |  | 2.8883E7 | 0 | 4.5532E7 | 3.2829E5 |  |  |  |  | 5 | 2 | 0 | 0 | 0 | 0 | 0 | 1 | 0 | 1 | 1 | 0 | 0 | 0 | 0 | 1 | 26 | Carbamidomethylation | C3:Carbamidomethylation:1000.00;C17:Carbamidomethylation:1000.00;C24:Carbamidomethylation:1000.00 | PEAKS DB |
| K.NGIEINC(+57.02)C(+57.02)TTDR.C | N | 76.35 | 1451.6133 | 12 | 3.3 | 726.8141 | 2 | 19.60 | 7 | F7:7461 | NaSa01\_F2.raw | 0 |  |  |  |  |  | 6.7801E6 | 5.7301E7 | 9.911E5 |  |  |  |  |  | 8 | 0 | 0 | 0 | 0 | 0 | 0 | 1 | 6 | 1 | 0 | 0 | 0 | 0 | 0 | 48 | 59 | Carbamidomethylation | C7:Carbamidomethylation:1000.00;C8:Carbamidomethylation:1000.00 | PEAKS DB |
| LEC(+57.02)HNQQSSQTPTTTGC(+57.02)SGGETNC(+57.02)YKK.R | Y | 59.86 | 3072.2971 | 27 | 5.5 | 769.0817 | 4 | 11.26 | 8 | F8:1677 | NaSa01\_F3.raw |  |  |  |  |  |  |  | 2.1676E6 |  |  |  |  |  |  | 3 | 0 | 0 | 0 | 0 | 0 | 0 | 0 | 3 | 0 | 0 | 0 | 0 | 0 | 0 | 1 | 27 | Carbamidomethylation | C3:Carbamidomethylation:1000.00;C17:Carbamidomethylation:1000.00;C24:Carbamidomethylation:1000.00 | PEAKS DB |
| N.GIEINC(+57.02)C(+57.02)TTDR.C | N | 50.21 | 1337.5704 | 11 | 6.3 | 669.7932 | 2 | 11.47 | 8 | F8:2008 | NaSa01\_F3.raw |  |  |  |  |  |  |  | 1.1583E6 |  |  |  |  |  |  | 1 | 0 | 0 | 0 | 0 | 0 | 0 | 0 | 1 | 0 | 0 | 0 | 0 | 0 | 0 | 49 | 59 | Carbamidomethylation | C6:Carbamidomethylation:1000.00;C7:Carbamidomethylation:1000.00 | PEAKS DB |
| total 4 peptides |  |  |  |  |  |  |  |  |  |  |  |  |  |  |  |  |  |  |  |  |  |  |  |  |  |  |  |  |  |  |  |  |  |  |  |  |  |  |  |  |  |  |  |  |  |
| --- | --- | --- | --- | --- | --- | --- | --- | --- | --- | --- | --- | --- | --- | --- | --- | --- | --- | --- | --- | --- | --- | --- | --- | --- | --- | --- | --- | --- | --- | --- | --- | --- | --- | --- | --- | --- | --- | --- | --- | --- | --- | --- | --- | --- | --- |

1V6P

back to list

  

| Protein Coverage
| Supporting Peptides
|

Protein Coverage:

Supporting Peptides:

| Peptide | Uniq | -10lgP | Mass | Length | ppm | m/z | z | RT | Fraction | Scan | Source File | Area F1 | Area F10 | Area F11 | Area F12 | Area F13 | Area F14 | Area F2 | Area F3 | Area F4 | Area F5 | Area F6 | Area F7 | Area F8 | Area F9 | #Feature | #Feature F1 | #Feature F10 | #Feature F11 | #Feature F12 | #Feature F13 | #Feature F14 | #Feature F2 | #Feature F3 | #Feature F4 | #Feature F5 | #Feature F6 | #Feature F7 | #Feature F8 | #Feature F9 | Start | End | PTM | AScore | Found By |
| --- | --- | --- | --- | --- | --- | --- | --- | --- | --- | --- | --- | --- | --- | --- | --- | --- | --- | --- | --- | --- | --- | --- | --- | --- | --- | --- | --- | --- | --- | --- | --- | --- | --- | --- | --- | --- | --- | --- | --- | --- | --- | --- | --- | --- | --- |
| LEC(+57.02)HNQQSSQTPTTTGC(+57.02)SGGETNC(+57.02)YK.K | Y | 85.04 | 2944.2021 | 26 | 2.7 | 982.4077 | 3 | 11.84 | 7 | F7:1790 | NaSa01\_F2.raw | 2.6655E8 |  |  |  |  |  | 2.8883E7 | 0 | 4.5532E7 | 3.2829E5 |  |  |  |  | 5 | 2 | 0 | 0 | 0 | 0 | 0 | 1 | 0 | 1 | 1 | 0 | 0 | 0 | 0 | 1 | 26 | Carbamidomethylation | C3:Carbamidomethylation:1000.00;C17:Carbamidomethylation:1000.00;C24:Carbamidomethylation:1000.00 | PEAKS DB |
| K.NGIEINC(+57.02)C(+57.02)TTDR.C | N | 76.35 | 1451.6133 | 12 | 3.3 | 726.8141 | 2 | 19.60 | 7 | F7:7461 | NaSa01\_F2.raw | 0 |  |  |  |  |  | 6.7801E6 | 5.7301E7 | 9.911E5 |  |  |  |  |  | 8 | 0 | 0 | 0 | 0 | 0 | 0 | 1 | 6 | 1 | 0 | 0 | 0 | 0 | 0 | 48 | 59 | Carbamidomethylation | C7:Carbamidomethylation:1000.00;C8:Carbamidomethylation:1000.00 | PEAKS DB |
| LEC(+57.02)HNQQSSQTPTTTGC(+57.02)SGGETNC(+57.02)YKK.R | Y | 59.86 | 3072.2971 | 27 | 5.5 | 769.0817 | 4 | 11.26 | 8 | F8:1677 | NaSa01\_F3.raw |  |  |  |  |  |  |  | 2.1676E6 |  |  |  |  |  |  | 3 | 0 | 0 | 0 | 0 | 0 | 0 | 0 | 3 | 0 | 0 | 0 | 0 | 0 | 0 | 1 | 27 | Carbamidomethylation | C3:Carbamidomethylation:1000.00;C17:Carbamidomethylation:1000.00;C24:Carbamidomethylation:1000.00 | PEAKS DB |
| N.GIEINC(+57.02)C(+57.02)TTDR.C | N | 50.21 | 1337.5704 | 11 | 6.3 | 669.7932 | 2 | 11.47 | 8 | F8:2008 | NaSa01\_F3.raw |  |  |  |  |  |  |  | 1.1583E6 |  |  |  |  |  |  | 1 | 0 | 0 | 0 | 0 | 0 | 0 | 0 | 1 | 0 | 0 | 0 | 0 | 0 | 0 | 49 | 59 | Carbamidomethylation | C6:Carbamidomethylation:1000.00;C7:Carbamidomethylation:1000.00 | PEAKS DB |
| total 4 peptides |  |  |  |  |  |  |  |  |  |  |  |  |  |  |  |  |  |  |  |  |  |  |  |  |  |  |  |  |  |  |  |  |  |  |  |  |  |  |  |  |  |  |  |  |  |
| --- | --- | --- | --- | --- | --- | --- | --- | --- | --- | --- | --- | --- | --- | --- | --- | --- | --- | --- | --- | --- | --- | --- | --- | --- | --- | --- | --- | --- | --- | --- | --- | --- | --- | --- | --- | --- | --- | --- | --- | --- | --- | --- | --- | --- | --- |

1COE

back to list

  

| Protein Coverage
| Supporting Peptides
|

Protein Coverage:

Supporting Peptides:

| Peptide | Uniq | -10lgP | Mass | Length | ppm | m/z | z | RT | Fraction | Scan | Source File | Area F1 | Area F10 | Area F11 | Area F12 | Area F13 | Area F14 | Area F2 | Area F3 | Area F4 | Area F5 | Area F6 | Area F7 | Area F8 | Area F9 | #Feature | #Feature F1 | #Feature F10 | #Feature F11 | #Feature F12 | #Feature F13 | #Feature F14 | #Feature F2 | #Feature F3 | #Feature F4 | #Feature F5 | #Feature F6 | #Feature F7 | #Feature F8 | #Feature F9 | Start | End | PTM | AScore | Found By |
| --- | --- | --- | --- | --- | --- | --- | --- | --- | --- | --- | --- | --- | --- | --- | --- | --- | --- | --- | --- | --- | --- | --- | --- | --- | --- | --- | --- | --- | --- | --- | --- | --- | --- | --- | --- | --- | --- | --- | --- | --- | --- | --- | --- | --- | --- |
| LEC(+57.02)HNQQSSQTPTTTGC(+57.02)SGGETNC(+57.02)YK.K | Y | 85.04 | 2944.2021 | 26 | 2.7 | 982.4077 | 3 | 11.84 | 7 | F7:1790 | NaSa01\_F2.raw | 2.6655E8 |  |  |  |  |  | 2.8883E7 | 0 | 4.5532E7 | 3.2829E5 |  |  |  |  | 5 | 2 | 0 | 0 | 0 | 0 | 0 | 1 | 0 | 1 | 1 | 0 | 0 | 0 | 0 | 1 | 26 | Carbamidomethylation | C3:Carbamidomethylation:1000.00;C17:Carbamidomethylation:1000.00;C24:Carbamidomethylation:1000.00 | PEAKS DB |
| K.NGIEINC(+57.02)C(+57.02)TTDR.C | N | 76.35 | 1451.6133 | 12 | 3.3 | 726.8141 | 2 | 19.60 | 7 | F7:7461 | NaSa01\_F2.raw | 0 |  |  |  |  |  | 6.7801E6 | 5.7301E7 | 9.911E5 |  |  |  |  |  | 8 | 0 | 0 | 0 | 0 | 0 | 0 | 1 | 6 | 1 | 0 | 0 | 0 | 0 | 0 | 48 | 59 | Carbamidomethylation | C7:Carbamidomethylation:1000.00;C8:Carbamidomethylation:1000.00 | PEAKS DB |
| LEC(+57.02)HNQQSSQTPTTTGC(+57.02)SGGETNC(+57.02)YKK.R | Y | 59.86 | 3072.2971 | 27 | 5.5 | 769.0817 | 4 | 11.26 | 8 | F8:1677 | NaSa01\_F3.raw |  |  |  |  |  |  |  | 2.1676E6 |  |  |  |  |  |  | 3 | 0 | 0 | 0 | 0 | 0 | 0 | 0 | 3 | 0 | 0 | 0 | 0 | 0 | 0 | 1 | 27 | Carbamidomethylation | C3:Carbamidomethylation:1000.00;C17:Carbamidomethylation:1000.00;C24:Carbamidomethylation:1000.00 | PEAKS DB |
| N.GIEINC(+57.02)C(+57.02)TTDR.C | N | 50.21 | 1337.5704 | 11 | 6.3 | 669.7932 | 2 | 11.47 | 8 | F8:2008 | NaSa01\_F3.raw |  |  |  |  |  |  |  | 1.1583E6 |  |  |  |  |  |  | 1 | 0 | 0 | 0 | 0 | 0 | 0 | 0 | 1 | 0 | 0 | 0 | 0 | 0 | 0 | 49 | 59 | Carbamidomethylation | C6:Carbamidomethylation:1000.00;C7:Carbamidomethylation:1000.00 | PEAKS DB |
| total 4 peptides |  |  |  |  |  |  |  |  |  |  |  |  |  |  |  |  |  |  |  |  |  |  |  |  |  |  |  |  |  |  |  |  |  |  |  |  |  |  |  |  |  |  |  |  |  |
| --- | --- | --- | --- | --- | --- | --- | --- | --- | --- | --- | --- | --- | --- | --- | --- | --- | --- | --- | --- | --- | --- | --- | --- | --- | --- | --- | --- | --- | --- | --- | --- | --- | --- | --- | --- | --- | --- | --- | --- | --- | --- | --- | --- | --- | --- |

AAB25735.1

back to list

  

| Protein Coverage
| Supporting Peptides
|

Protein Coverage:

Supporting Peptides:

| Peptide | Uniq | -10lgP | Mass | Length | ppm | m/z | z | RT | Fraction | Scan | Source File | Area F1 | Area F10 | Area F11 | Area F12 | Area F13 | Area F14 | Area F2 | Area F3 | Area F4 | Area F5 | Area F6 | Area F7 | Area F8 | Area F9 | #Feature | #Feature F1 | #Feature F10 | #Feature F11 | #Feature F12 | #Feature F13 | #Feature F14 | #Feature F2 | #Feature F3 | #Feature F4 | #Feature F5 | #Feature F6 | #Feature F7 | #Feature F8 | #Feature F9 | Start | End | PTM | AScore | Found By |
| --- | --- | --- | --- | --- | --- | --- | --- | --- | --- | --- | --- | --- | --- | --- | --- | --- | --- | --- | --- | --- | --- | --- | --- | --- | --- | --- | --- | --- | --- | --- | --- | --- | --- | --- | --- | --- | --- | --- | --- | --- | --- | --- | --- | --- | --- |
| LEC(+57.02)HNQQSSQTPTTTGC(+57.02)SGGETNC(+57.02)YK.K | Y | 85.04 | 2944.2021 | 26 | 2.7 | 982.4077 | 3 | 11.84 | 7 | F7:1790 | NaSa01\_F2.raw | 2.6655E8 |  |  |  |  |  | 2.8883E7 | 0 | 4.5532E7 | 3.2829E5 |  |  |  |  | 5 | 2 | 0 | 0 | 0 | 0 | 0 | 1 | 0 | 1 | 1 | 0 | 0 | 0 | 0 | 1 | 26 | Carbamidomethylation | C3:Carbamidomethylation:1000.00;C17:Carbamidomethylation:1000.00;C24:Carbamidomethylation:1000.00 | PEAKS DB |
| K.NGIEINC(+57.02)C(+57.02)TTDR.C | N | 76.35 | 1451.6133 | 12 | 3.3 | 726.8141 | 2 | 19.60 | 7 | F7:7461 | NaSa01\_F2.raw | 0 |  |  |  |  |  | 6.7801E6 | 5.7301E7 | 9.911E5 |  |  |  |  |  | 8 | 0 | 0 | 0 | 0 | 0 | 0 | 1 | 6 | 1 | 0 | 0 | 0 | 0 | 0 | 48 | 59 | Carbamidomethylation | C7:Carbamidomethylation:1000.00;C8:Carbamidomethylation:1000.00 | PEAKS DB |
| LEC(+57.02)HNQQSSQTPTTTGC(+57.02)SGGETNC(+57.02)YKK.R | Y | 59.86 | 3072.2971 | 27 | 5.5 | 769.0817 | 4 | 11.26 | 8 | F8:1677 | NaSa01\_F3.raw |  |  |  |  |  |  |  | 2.1676E6 |  |  |  |  |  |  | 3 | 0 | 0 | 0 | 0 | 0 | 0 | 0 | 3 | 0 | 0 | 0 | 0 | 0 | 0 | 1 | 27 | Carbamidomethylation | C3:Carbamidomethylation:1000.00;C17:Carbamidomethylation:1000.00;C24:Carbamidomethylation:1000.00 | PEAKS DB |
| N.GIEINC(+57.02)C(+57.02)TTDR.C | N | 50.21 | 1337.5704 | 11 | 6.3 | 669.7932 | 2 | 11.47 | 8 | F8:2008 | NaSa01\_F3.raw |  |  |  |  |  |  |  | 1.1583E6 |  |  |  |  |  |  | 1 | 0 | 0 | 0 | 0 | 0 | 0 | 0 | 1 | 0 | 0 | 0 | 0 | 0 | 0 | 49 | 59 | Carbamidomethylation | C6:Carbamidomethylation:1000.00;C7:Carbamidomethylation:1000.00 | PEAKS DB |
| total 4 peptides |  |  |  |  |  |  |  |  |  |  |  |  |  |  |  |  |  |  |  |  |  |  |  |  |  |  |  |  |  |  |  |  |  |  |  |  |  |  |  |  |  |  |  |  |  |
| --- | --- | --- | --- | --- | --- | --- | --- | --- | --- | --- | --- | --- | --- | --- | --- | --- | --- | --- | --- | --- | --- | --- | --- | --- | --- | --- | --- | --- | --- | --- | --- | --- | --- | --- | --- | --- | --- | --- | --- | --- | --- | --- | --- | --- | --- |

P0DSM9.1

back to list

  

| Protein Coverage
| Supporting Peptides
|

Protein Coverage:

Supporting Peptides:

| Peptide | Uniq | -10lgP | Mass | Length | ppm | m/z | z | RT | Fraction | Scan | Source File | Area F1 | Area F10 | Area F11 | Area F12 | Area F13 | Area F14 | Area F2 | Area F3 | Area F4 | Area F5 | Area F6 | Area F7 | Area F8 | Area F9 | #Feature | #Feature F1 | #Feature F10 | #Feature F11 | #Feature F12 | #Feature F13 | #Feature F14 | #Feature F2 | #Feature F3 | #Feature F4 | #Feature F5 | #Feature F6 | #Feature F7 | #Feature F8 | #Feature F9 | Start | End | PTM | AScore | Found By |
| --- | --- | --- | --- | --- | --- | --- | --- | --- | --- | --- | --- | --- | --- | --- | --- | --- | --- | --- | --- | --- | --- | --- | --- | --- | --- | --- | --- | --- | --- | --- | --- | --- | --- | --- | --- | --- | --- | --- | --- | --- | --- | --- | --- | --- | --- |
| LTC(+57.02)LIC(+57.02)PEKYC(+57.02)NK.V | N | 69.22 | 1697.7939 | 13 | 0.5 | 849.9047 | 2 | 27.21 | 9 | F9:7726 | NaSa01\_F4.raw |  |  |  |  |  |  |  |  | 3.9596E7 |  |  |  |  |  | 3 | 0 | 0 | 0 | 0 | 0 | 0 | 0 | 0 | 3 | 0 | 0 | 0 | 0 | 0 | 1 | 13 | Carbamidomethylation | C3:Carbamidomethylation:1000.00;C6:Carbamidomethylation:1000.00;C11:Carbamidomethylation:1000.00 | PEAKS DB |
| K.VHTC(+57.02)LNGEK.I | N | 64.61 | 1056.5022 | 9 | -0.1 | 529.2583 | 2 | 11.41 | 9 | F9:1761 | NaSa01\_F4.raw |  |  |  |  |  |  |  |  | 4.0964E6 |  |  |  |  |  | 1 | 0 | 0 | 0 | 0 | 0 | 0 | 0 | 0 | 1 | 0 | 0 | 0 | 0 | 0 | 14 | 22 | Carbamidomethylation | C4:Carbamidomethylation:1000.00 | PEAKS DB |
| LTC(+57.02)LIC(+57.02)PEK.Y | N | 56.98 | 1132.5620 | 9 | 1.2 | 567.2889 | 2 | 28.45 | 12 | F12:13908 | NaSa01\_F7.raw |  |  |  |  |  |  |  |  | 4.2053E8 |  |  | 1.641E7 |  |  | 3 | 0 | 0 | 0 | 0 | 0 | 0 | 0 | 0 | 2 | 0 | 0 | 1 | 0 | 0 | 1 | 9 | Carbamidomethylation | C3:Carbamidomethylation:1000.00;C6:Carbamidomethylation:1000.00 | PEAKS DB |
| R.EIVEC(+57.02)C(+57.02)STDK | N | 54.49 | 1239.5111 | 10 | -0.8 | 620.7623 | 2 | 11.41 | 9 | F9:1762 | NaSa01\_F4.raw |  |  |  |  |  |  |  |  | 4.202E6 |  |  |  |  |  | 1 | 0 | 0 | 0 | 0 | 0 | 0 | 0 | 0 | 1 | 0 | 0 | 0 | 0 | 0 | 44 | 53 | Carbamidomethylation | C5:Carbamidomethylation:1000.00;C6:Carbamidomethylation:1000.00 | PEAKS DB |
| LTC(+57.02)LIC(+57.02)PEKY.C | Y | 53.13 | 1295.6254 | 10 | 0.8 | 648.8205 | 2 | 43.31 | 9 | F9:19711 | NaSa01\_F4.raw |  |  |  |  |  |  |  |  | 3.9843E7 |  |  |  |  |  | 1 | 0 | 0 | 0 | 0 | 0 | 0 | 0 | 0 | 1 | 0 | 0 | 0 | 0 | 0 | 1 | 10 | Carbamidomethylation | C3:Carbamidomethylation:1000.00;C6:Carbamidomethylation:1000.00 | PEAKS DB |
| R.GC(+57.02)ADTC(+57.02)PVR.K | N | 53.07 | 1034.4274 | 9 | 0.3 | 518.2211 | 2 | 11.41 | 9 | F9:1771 | NaSa01\_F4.raw |  |  |  |  |  |  |  |  | 1.2529E7 |  |  |  |  |  | 1 | 0 | 0 | 0 | 0 | 0 | 0 | 0 | 0 | 1 | 0 | 0 | 0 | 0 | 0 | 32 | 40 | Carbamidomethylation | C2:Carbamidomethylation:1000.00;C6:Carbamidomethylation:1000.00 | PEAKS DB |
| total 6 peptides |  |  |  |  |  |  |  |  |  |  |  |  |  |  |  |  |  |  |  |  |  |  |  |  |  |  |  |  |  |  |  |  |  |  |  |  |  |  |  |  |  |  |  |  |  |
| --- | --- | --- | --- | --- | --- | --- | --- | --- | --- | --- | --- | --- | --- | --- | --- | --- | --- | --- | --- | --- | --- | --- | --- | --- | --- | --- | --- | --- | --- | --- | --- | --- | --- | --- | --- | --- | --- | --- | --- | --- | --- | --- | --- | --- | --- |

AFJ50410.1

back to list

  

| Protein Coverage
| Supporting Peptides
|

Protein Coverage:

Supporting Peptides:

| Peptide | Uniq | -10lgP | Mass | Length | ppm | m/z | z | RT | Fraction | Scan | Source File | Area F1 | Area F10 | Area F11 | Area F12 | Area F13 | Area F14 | Area F2 | Area F3 | Area F4 | Area F5 | Area F6 | Area F7 | Area F8 | Area F9 | #Feature | #Feature F1 | #Feature F10 | #Feature F11 | #Feature F12 | #Feature F13 | #Feature F14 | #Feature F2 | #Feature F3 | #Feature F4 | #Feature F5 | #Feature F6 | #Feature F7 | #Feature F8 | #Feature F9 | Start | End | PTM | AScore | Found By |
| --- | --- | --- | --- | --- | --- | --- | --- | --- | --- | --- | --- | --- | --- | --- | --- | --- | --- | --- | --- | --- | --- | --- | --- | --- | --- | --- | --- | --- | --- | --- | --- | --- | --- | --- | --- | --- | --- | --- | --- | --- | --- | --- | --- | --- | --- |
| R.NLDLDSIIAEVK.A | N | 73.65 | 1328.7188 | 12 | 3.0 | 665.3667 | 2 | 83.23 | 7 | F7:61182 | NaSa01\_F2.raw |  |  |  | 3.098E6 | 8.9275E6 | 6.3667E6 | 7.3552E6 |  |  | 7.5532E5 | 9.4354E4 | 8.1815E4 | 4.3244E5 | 1.7496E6 | 9 | 0 | 0 | 0 | 1 | 1 | 1 | 1 | 0 | 0 | 1 | 1 | 1 | 1 | 1 | 266 | 277 |  |  | PEAKS DB |
| K.LALDIEIATYR.K | N | 62.08 | 1276.7026 | 11 | 3.6 | 639.3590 | 2 | 67.89 | 7 | F7:49102 | NaSa01\_F2.raw |  | 1.1426E5 |  |  |  |  | 4.6527E6 |  |  | 7.8211E5 |  |  |  |  | 10 | 0 | 1 | 0 | 0 | 0 | 0 | 8 | 0 | 0 | 1 | 0 | 0 | 0 | 0 | 395 | 405 |  |  | PEAKS DB |
| K.FASFIDKVR.F | N | 57.41 | 1081.5920 | 9 | 2.8 | 541.8032 | 2 | 29.83 | 7 | F7:16135 | NaSa01\_F2.raw |  |  |  |  |  |  | 5.676E5 |  |  |  |  |  |  |  | 1 | 0 | 0 | 0 | 0 | 0 | 0 | 1 | 0 | 0 | 0 | 0 | 0 | 0 | 0 | 114 | 122 |  |  | PEAKS DB |
| K.FASFIDK.V | N | 53.06 | 826.4225 | 7 | 2.1 | 414.2182 | 2 | 30.61 | 7 | F7:16768 | NaSa01\_F2.raw |  |  |  |  |  |  | 6.2128E5 |  |  |  |  |  |  |  | 1 | 0 | 0 | 0 | 0 | 0 | 0 | 1 | 0 | 0 | 0 | 0 | 0 | 0 | 0 | 114 | 120 |  |  | PEAKS DB |
| K.NMQDLVEDFK.N | Y | 40.97 | 1237.5648 | 10 | -0.2 | 619.7896 | 2 | 61.05 | 5 | F5:39380 | NaSa01\_F13.raw |  |  |  |  | 7.3573E5 |  |  |  |  |  |  |  |  |  | 1 | 0 | 0 | 0 | 0 | 1 | 0 | 0 | 0 | 0 | 0 | 0 | 0 | 0 | 0 | 180 | 189 |  |  | PEAKS DB |
| total 5 peptides |  |  |  |  |  |  |  |  |  |  |  |  |  |  |  |  |  |  |  |  |  |  |  |  |  |  |  |  |  |  |  |  |  |  |  |  |  |  |  |  |  |  |  |  |  |
| --- | --- | --- | --- | --- | --- | --- | --- | --- | --- | --- | --- | --- | --- | --- | --- | --- | --- | --- | --- | --- | --- | --- | --- | --- | --- | --- | --- | --- | --- | --- | --- | --- | --- | --- | --- | --- | --- | --- | --- | --- | --- | --- | --- | --- | --- |

JAG44289.1

back to list

  

| Protein Coverage
| Supporting Peptides
|

Protein Coverage:

Supporting Peptides:

| Peptide | Uniq | -10lgP | Mass | Length | ppm | m/z | z | RT | Fraction | Scan | Source File | Area F1 | Area F10 | Area F11 | Area F12 | Area F13 | Area F14 | Area F2 | Area F3 | Area F4 | Area F5 | Area F6 | Area F7 | Area F8 | Area F9 | #Feature | #Feature F1 | #Feature F10 | #Feature F11 | #Feature F12 | #Feature F13 | #Feature F14 | #Feature F2 | #Feature F3 | #Feature F4 | #Feature F5 | #Feature F6 | #Feature F7 | #Feature F8 | #Feature F9 | Start | End | PTM | AScore | Found By |
| --- | --- | --- | --- | --- | --- | --- | --- | --- | --- | --- | --- | --- | --- | --- | --- | --- | --- | --- | --- | --- | --- | --- | --- | --- | --- | --- | --- | --- | --- | --- | --- | --- | --- | --- | --- | --- | --- | --- | --- | --- | --- | --- | --- | --- | --- |
| R.NLDLDSIIAEVK.A | N | 73.65 | 1328.7188 | 12 | 3.0 | 665.3667 | 2 | 83.23 | 7 | F7:61182 | NaSa01\_F2.raw |  |  |  | 3.098E6 | 8.9275E6 | 6.3667E6 | 7.3552E6 |  |  | 7.5532E5 | 9.4354E4 | 8.1815E4 | 4.3244E5 | 1.7496E6 | 9 | 0 | 0 | 0 | 1 | 1 | 1 | 1 | 0 | 0 | 1 | 1 | 1 | 1 | 1 | 266 | 277 |  |  | PEAKS DB |
| K.LALDIEIATYR.K | N | 62.08 | 1276.7026 | 11 | 3.6 | 639.3590 | 2 | 67.89 | 7 | F7:49102 | NaSa01\_F2.raw |  | 1.1426E5 |  |  |  |  | 4.6527E6 |  |  | 7.8211E5 |  |  |  |  | 10 | 0 | 1 | 0 | 0 | 0 | 0 | 8 | 0 | 0 | 1 | 0 | 0 | 0 | 0 | 395 | 405 |  |  | PEAKS DB |
| K.FASFIDKVR.F | N | 57.41 | 1081.5920 | 9 | 2.8 | 541.8032 | 2 | 29.83 | 7 | F7:16135 | NaSa01\_F2.raw |  |  |  |  |  |  | 5.676E5 |  |  |  |  |  |  |  | 1 | 0 | 0 | 0 | 0 | 0 | 0 | 1 | 0 | 0 | 0 | 0 | 0 | 0 | 0 | 114 | 122 |  |  | PEAKS DB |
| K.FASFIDK.V | N | 53.06 | 826.4225 | 7 | 2.1 | 414.2182 | 2 | 30.61 | 7 | F7:16768 | NaSa01\_F2.raw |  |  |  |  |  |  | 6.2128E5 |  |  |  |  |  |  |  | 1 | 0 | 0 | 0 | 0 | 0 | 0 | 1 | 0 | 0 | 0 | 0 | 0 | 0 | 0 | 114 | 120 |  |  | PEAKS DB |
| K.NMQDLVEDFK.N | Y | 40.97 | 1237.5648 | 10 | -0.2 | 619.7896 | 2 | 61.05 | 5 | F5:39380 | NaSa01\_F13.raw |  |  |  |  | 7.3573E5 |  |  |  |  |  |  |  |  |  | 1 | 0 | 0 | 0 | 0 | 1 | 0 | 0 | 0 | 0 | 0 | 0 | 0 | 0 | 0 | 180 | 189 |  |  | PEAKS DB |
| total 5 peptides |  |  |  |  |  |  |  |  |  |  |  |  |  |  |  |  |  |  |  |  |  |  |  |  |  |  |  |  |  |  |  |  |  |  |  |  |  |  |  |  |  |  |  |  |  |
| --- | --- | --- | --- | --- | --- | --- | --- | --- | --- | --- | --- | --- | --- | --- | --- | --- | --- | --- | --- | --- | --- | --- | --- | --- | --- | --- | --- | --- | --- | --- | --- | --- | --- | --- | --- | --- | --- | --- | --- | --- | --- | --- | --- | --- | --- |

P25668.1

back to list

  

| Protein Coverage
| Supporting Peptides
|

Protein Coverage:

Supporting Peptides:

| Peptide | Uniq | -10lgP | Mass | Length | ppm | m/z | z | RT | Fraction | Scan | Source File | Area F1 | Area F10 | Area F11 | Area F12 | Area F13 | Area F14 | Area F2 | Area F3 | Area F4 | Area F5 | Area F6 | Area F7 | Area F8 | Area F9 | #Feature | #Feature F1 | #Feature F10 | #Feature F11 | #Feature F12 | #Feature F13 | #Feature F14 | #Feature F2 | #Feature F3 | #Feature F4 | #Feature F5 | #Feature F6 | #Feature F7 | #Feature F8 | #Feature F9 | Start | End | PTM | AScore | Found By |
| --- | --- | --- | --- | --- | --- | --- | --- | --- | --- | --- | --- | --- | --- | --- | --- | --- | --- | --- | --- | --- | --- | --- | --- | --- | --- | --- | --- | --- | --- | --- | --- | --- | --- | --- | --- | --- | --- | --- | --- | --- | --- | --- | --- | --- | --- |
| R.VDLGC(+57.02)AATC(+57.02)PTVR.T | N | 78.45 | 1418.6646 | 13 | 1.4 | 710.3405 | 2 | 28.73 | 9 | F9:8677 | NaSa01\_F4.raw |  | 2.0713E6 | 6.8229E5 | 1.2855E7 | 6.5654E6 | 5.8169E5 |  |  | 3.1693E8 | 2.84E7 | 3.5942E6 | 7.844E6 | 1.3579E7 | 4.7725E5 | 16 | 0 | 3 | 1 | 1 | 2 | 1 | 0 | 0 | 2 | 1 | 1 | 1 | 1 | 2 | 37 | 49 | Carbamidomethylation | C5:Carbamidomethylation:1000.00;C9:Carbamidomethylation:1000.00 | PEAKS DB |
| R.C(+57.02)FITPDITSK.D | N | 68.13 | 1180.5798 | 10 | 0.4 | 591.2974 | 2 | 41.82 | 14 | F14:21115 | NaSa01\_F9.raw |  | 2.9364E6 | 3.7851E6 | 1.1007E6 | 7.2035E6 | 3.3336E7 |  | 2.9701E5 | 2.5797E8 | 4.8308E7 | 4.5629E6 | 9.9249E6 | 2.4521E7 | 9.2393E6 | 24 | 0 | 1 | 1 | 1 | 2 | 3 | 0 | 1 | 4 | 3 | 2 | 1 | 3 | 2 | 3 | 12 | Carbamidomethylation | C1:Carbamidomethylation:1000.00 | PEAKS DB |
| K.RVDLGC(+57.02)AATC(+57.02)PTVR.T | N | 56.49 | 1574.7657 | 14 | 0.7 | 525.9296 | 3 | 19.41 | 12 | F12:6390 | NaSa01\_F7.raw |  |  |  |  | 2.7551E5 |  |  |  |  |  |  | 1.0933E6 |  |  | 2 | 0 | 0 | 0 | 0 | 1 | 0 | 0 | 0 | 0 | 0 | 0 | 1 | 0 | 0 | 36 | 49 | Carbamidomethylation | C6:Carbamidomethylation:1000.00;C10:Carbamidomethylation:1000.00 | PEAKS DB |
| K.TWC(+57.02)DGFC(+57.02)SIR.G | Y | 47.78 | 1300.5328 | 10 | 0.5 | 651.2740 | 2 | 46.59 | 9 | F9:22282 | NaSa01\_F4.raw |  |  |  |  |  |  |  |  | 1.4714E7 |  |  | 9.4959E5 |  |  | 2 | 0 | 0 | 0 | 0 | 0 | 0 | 0 | 0 | 1 | 0 | 0 | 1 | 0 | 0 | 24 | 33 | Carbamidomethylation | C3:Carbamidomethylation:1000.00;C7:Carbamidomethylation:1000.00 | PEAKS DB |
| total 4 peptides |  |  |  |  |  |  |  |  |  |  |  |  |  |  |  |  |  |  |  |  |  |  |  |  |  |  |  |  |  |  |  |  |  |  |  |  |  |  |  |  |  |  |  |  |  |
| --- | --- | --- | --- | --- | --- | --- | --- | --- | --- | --- | --- | --- | --- | --- | --- | --- | --- | --- | --- | --- | --- | --- | --- | --- | --- | --- | --- | --- | --- | --- | --- | --- | --- | --- | --- | --- | --- | --- | --- | --- | --- | --- | --- | --- | --- |

B8K1W0.1

back to list

  

| Protein Coverage
| Supporting Peptides
|

Protein Coverage:

Supporting Peptides:

| Peptide | Uniq | -10lgP | Mass | Length | ppm | m/z | z | RT | Fraction | Scan | Source File | Area F1 | Area F10 | Area F11 | Area F12 | Area F13 | Area F14 | Area F2 | Area F3 | Area F4 | Area F5 | Area F6 | Area F7 | Area F8 | Area F9 | #Feature | #Feature F1 | #Feature F10 | #Feature F11 | #Feature F12 | #Feature F13 | #Feature F14 | #Feature F2 | #Feature F3 | #Feature F4 | #Feature F5 | #Feature F6 | #Feature F7 | #Feature F8 | #Feature F9 | Start | End | PTM | AScore | Found By |
| --- | --- | --- | --- | --- | --- | --- | --- | --- | --- | --- | --- | --- | --- | --- | --- | --- | --- | --- | --- | --- | --- | --- | --- | --- | --- | --- | --- | --- | --- | --- | --- | --- | --- | --- | --- | --- | --- | --- | --- | --- | --- | --- | --- | --- | --- |
| N.VTLDLFGEWR.K | N | 67.98 | 1234.6346 | 10 | 0.3 | 618.3248 | 2 | 83.54 | 6 | F6:57918 | NaSa01\_F14.raw |  |  | 1.1051E5 |  | 1.1401E8 | 1.4573E8 |  |  |  |  |  |  |  | 9.2511E5 | 13 | 0 | 0 | 1 | 0 | 2 | 9 | 0 | 0 | 0 | 0 | 0 | 0 | 0 | 1 | 270 | 279 |  |  | PEAKS DB |
| R.FLTEHNPEC(+57.02)IINPPLR.T | Y | 62.51 | 1948.9829 | 16 | 1.2 | 650.6690 | 3 | 49.67 | 4 | F4:30977 | NaSa01\_F12.raw |  |  |  | 1.8634E6 | 4.7866E5 |  |  |  |  |  |  |  |  |  | 2 | 0 | 0 | 0 | 1 | 1 | 0 | 0 | 0 | 0 | 0 | 0 | 0 | 0 | 0 | 386 | 401 | Carbamidomethylation | C9:Carbamidomethylation:1000.00 | PEAKS DB |
| R.TDIVSPPAC(+57.02)GNELLER.G | Y | 56.02 | 1769.8618 | 16 | 1.3 | 885.9393 | 2 | 51.49 | 4 | F4:32577 | NaSa01\_F12.raw |  |  |  | 2.5073E6 |  |  |  |  |  |  |  |  |  |  | 1 | 0 | 0 | 0 | 1 | 0 | 0 | 0 | 0 | 0 | 0 | 0 | 0 | 0 | 0 | 402 | 417 | Carbamidomethylation | C9:Carbamidomethylation:1000.00 | PEAKS DB |
| R.VPLVGLEIWK.N | Y | 46.80 | 1152.6907 | 10 | 0.1 | 577.3527 | 2 | 76.94 | 6 | F6:52016 | NaSa01\_F14.raw |  |  |  |  |  | 1.0412E6 |  |  |  |  |  |  |  |  | 2 | 0 | 0 | 0 | 0 | 0 | 2 | 0 | 0 | 0 | 0 | 0 | 0 | 0 | 0 | 248 | 257 |  |  | PEAKS DB |
| V.TLDLFGEWR.K | N | 43.65 | 1135.5662 | 9 | -0.8 | 568.7899 | 2 | 77.17 | 6 | F6:52295 | NaSa01\_F14.raw |  |  |  |  |  | 1.3701E6 |  |  |  |  |  |  |  |  | 1 | 0 | 0 | 0 | 0 | 0 | 1 | 0 | 0 | 0 | 0 | 0 | 0 | 0 | 0 | 271 | 279 |  |  | PEAKS DB |
| R.LGVYYAYC(+57.02)R.K | Y | 38.12 | 1163.5433 | 9 | 0.7 | 582.7794 | 2 | 36.11 | 4 | F4:19825 | NaSa01\_F12.raw |  |  |  | 1.8356E5 |  |  |  |  |  |  |  |  |  |  | 1 | 0 | 0 | 0 | 1 | 0 | 0 | 0 | 0 | 0 | 0 | 0 | 0 | 0 | 0 | 537 | 545 | Carbamidomethylation | C8:Carbamidomethylation:1000.00 | PEAKS DB |
| total 6 peptides |  |  |  |  |  |  |  |  |  |  |  |  |  |  |  |  |  |  |  |  |  |  |  |  |  |  |  |  |  |  |  |  |  |  |  |  |  |  |  |  |  |  |  |  |  |
| --- | --- | --- | --- | --- | --- | --- | --- | --- | --- | --- | --- | --- | --- | --- | --- | --- | --- | --- | --- | --- | --- | --- | --- | --- | --- | --- | --- | --- | --- | --- | --- | --- | --- | --- | --- | --- | --- | --- | --- | --- | --- | --- | --- | --- | --- |

AAZ39880.1

back to list

  

| Protein Coverage
| Supporting Peptides
|

Protein Coverage:

Supporting Peptides:

| Peptide | Uniq | -10lgP | Mass | Length | ppm | m/z | z | RT | Fraction | Scan | Source File | Area F1 | Area F10 | Area F11 | Area F12 | Area F13 | Area F14 | Area F2 | Area F3 | Area F4 | Area F5 | Area F6 | Area F7 | Area F8 | Area F9 | #Feature | #Feature F1 | #Feature F10 | #Feature F11 | #Feature F12 | #Feature F13 | #Feature F14 | #Feature F2 | #Feature F3 | #Feature F4 | #Feature F5 | #Feature F6 | #Feature F7 | #Feature F8 | #Feature F9 | Start | End | PTM | AScore | Found By |
| --- | --- | --- | --- | --- | --- | --- | --- | --- | --- | --- | --- | --- | --- | --- | --- | --- | --- | --- | --- | --- | --- | --- | --- | --- | --- | --- | --- | --- | --- | --- | --- | --- | --- | --- | --- | --- | --- | --- | --- | --- | --- | --- | --- | --- | --- |
| N.VTLDLFGEWR.K | N | 67.98 | 1234.6346 | 10 | 0.3 | 618.3248 | 2 | 83.54 | 6 | F6:57918 | NaSa01\_F14.raw |  |  | 1.1051E5 |  | 1.1401E8 | 1.4573E8 |  |  |  |  |  |  |  | 9.2511E5 | 13 | 0 | 0 | 1 | 0 | 2 | 9 | 0 | 0 | 0 | 0 | 0 | 0 | 0 | 1 | 270 | 279 |  |  | PEAKS DB |
| R.FLTEHNPEC(+57.02)IINPPLR.T | Y | 62.51 | 1948.9829 | 16 | 1.2 | 650.6690 | 3 | 49.67 | 4 | F4:30977 | NaSa01\_F12.raw |  |  |  | 1.8634E6 | 4.7866E5 |  |  |  |  |  |  |  |  |  | 2 | 0 | 0 | 0 | 1 | 1 | 0 | 0 | 0 | 0 | 0 | 0 | 0 | 0 | 0 | 386 | 401 | Carbamidomethylation | C9:Carbamidomethylation:1000.00 | PEAKS DB |
| R.TDIVSPPAC(+57.02)GNELLER.G | Y | 56.02 | 1769.8618 | 16 | 1.3 | 885.9393 | 2 | 51.49 | 4 | F4:32577 | NaSa01\_F12.raw |  |  |  | 2.5073E6 |  |  |  |  |  |  |  |  |  |  | 1 | 0 | 0 | 0 | 1 | 0 | 0 | 0 | 0 | 0 | 0 | 0 | 0 | 0 | 0 | 402 | 417 | Carbamidomethylation | C9:Carbamidomethylation:1000.00 | PEAKS DB |
| R.VPLVGLEIWK.N | Y | 46.80 | 1152.6907 | 10 | 0.1 | 577.3527 | 2 | 76.94 | 6 | F6:52016 | NaSa01\_F14.raw |  |  |  |  |  | 1.0412E6 |  |  |  |  |  |  |  |  | 2 | 0 | 0 | 0 | 0 | 0 | 2 | 0 | 0 | 0 | 0 | 0 | 0 | 0 | 0 | 248 | 257 |  |  | PEAKS DB |
| V.TLDLFGEWR.K | N | 43.65 | 1135.5662 | 9 | -0.8 | 568.7899 | 2 | 77.17 | 6 | F6:52295 | NaSa01\_F14.raw |  |  |  |  |  | 1.3701E6 |  |  |  |  |  |  |  |  | 1 | 0 | 0 | 0 | 0 | 0 | 1 | 0 | 0 | 0 | 0 | 0 | 0 | 0 | 0 | 271 | 279 |  |  | PEAKS DB |
| R.LGVYYAYC(+57.02)R.K | Y | 38.12 | 1163.5433 | 9 | 0.7 | 582.7794 | 2 | 36.11 | 4 | F4:19825 | NaSa01\_F12.raw |  |  |  | 1.8356E5 |  |  |  |  |  |  |  |  |  |  | 1 | 0 | 0 | 0 | 1 | 0 | 0 | 0 | 0 | 0 | 0 | 0 | 0 | 0 | 0 | 537 | 545 | Carbamidomethylation | C8:Carbamidomethylation:1000.00 | PEAKS DB |
| total 6 peptides |  |  |  |  |  |  |  |  |  |  |  |  |  |  |  |  |  |  |  |  |  |  |  |  |  |  |  |  |  |  |  |  |  |  |  |  |  |  |  |  |  |  |  |  |  |
| --- | --- | --- | --- | --- | --- | --- | --- | --- | --- | --- | --- | --- | --- | --- | --- | --- | --- | --- | --- | --- | --- | --- | --- | --- | --- | --- | --- | --- | --- | --- | --- | --- | --- | --- | --- | --- | --- | --- | --- | --- | --- | --- | --- | --- | --- |

Q9YGI4.1

back to list

  

| Protein Coverage
| Supporting Peptides
|

Protein Coverage:

Supporting Peptides:

| Peptide | Uniq | -10lgP | Mass | Length | ppm | m/z | z | RT | Fraction | Scan | Source File | Area F1 | Area F10 | Area F11 | Area F12 | Area F13 | Area F14 | Area F2 | Area F3 | Area F4 | Area F5 | Area F6 | Area F7 | Area F8 | Area F9 | #Feature | #Feature F1 | #Feature F10 | #Feature F11 | #Feature F12 | #Feature F13 | #Feature F14 | #Feature F2 | #Feature F3 | #Feature F4 | #Feature F5 | #Feature F6 | #Feature F7 | #Feature F8 | #Feature F9 | Start | End | PTM | AScore | Found By |
| --- | --- | --- | --- | --- | --- | --- | --- | --- | --- | --- | --- | --- | --- | --- | --- | --- | --- | --- | --- | --- | --- | --- | --- | --- | --- | --- | --- | --- | --- | --- | --- | --- | --- | --- | --- | --- | --- | --- | --- | --- | --- | --- | --- | --- | --- |
| T.LTC(+57.02)LNC(+57.02)PEMFC(+57.02)GK.F | N | 87.07 | 1628.6819 | 13 | 0.7 | 815.3488 | 2 | 45.64 | 14 | F14:24540 | NaSa01\_F9.raw |  | 3.1696E6 | 1.6649E6 | 1.8659E6 |  |  |  |  | 2.1823E6 | 6.7455E8 | 8.6607E9 | 5.3693E8 | 5.7295E7 | 1.4835E7 | 19 | 0 | 1 | 1 | 1 | 0 | 0 | 0 | 0 | 1 | 1 | 10 | 2 | 1 | 1 | 22 | 34 | Carbamidomethylation | C3:Carbamidomethylation:1000.00;C6:Carbamidomethylation:1000.00;C11:Carbamidomethylation:1000.00 | PEAKS DB |
| T.LTC(+57.02)LNC(+57.02)PEM(+15.99)FC(+57.02)GK.F | N | 67.45 | 1644.6769 | 13 | 0.7 | 823.3463 | 2 | 32.98 | 12 | F12:17393 | NaSa01\_F7.raw |  |  |  |  |  |  |  |  | 9.1936E5 | 1.0943E8 | 7.6473E8 | 5.7873E7 |  |  | 24 | 0 | 0 | 0 | 0 | 0 | 0 | 0 | 0 | 1 | 7 | 10 | 6 | 0 | 0 | 22 | 34 | Carbamidomethylation; Oxidation (M) | C3:Carbamidomethylation:1000.00;C6:Carbamidomethylation:1000.00;M9:Oxidation (M):1000.00;C11:Carbamidomethylation:1000.00 | PEAKS DB |
| T.LTC(+57.02)LNC(+57.02)PEMFC(+57.02)GKFQIC(+57.02)R.N | N | 62.75 | 2333.0247 | 18 | 0.9 | 778.6829 | 3 | 56.34 | 11 | F11:37844 | NaSa01\_F6.raw |  |  |  |  |  |  |  |  |  | 1.2723E6 | 1.8262E7 | 9.6113E5 |  |  | 3 | 0 | 0 | 0 | 0 | 0 | 0 | 0 | 0 | 0 | 1 | 1 | 1 | 0 | 0 | 22 | 39 | Carbamidomethylation | C3:Carbamidomethylation:1000.00;C6:Carbamidomethylation:1000.00;C11:Carbamidomethylation:1000.00;C17:Carbamidomethylation:1000.00 | PEAKS DB |
| L.TC(+57.02)LNC(+57.02)PEMFC(+57.02)GK.F | N | 53.17 | 1515.5979 | 12 | -0.4 | 758.8059 | 2 | 36.00 | 9 | F9:14208 | NaSa01\_F4.raw |  |  |  |  |  |  |  |  | 2.1699E6 |  | 1.4872E6 | 1.3767E6 | 1.0968E6 |  | 4 | 0 | 0 | 0 | 0 | 0 | 0 | 0 | 0 | 1 | 0 | 1 | 1 | 1 | 0 | 23 | 34 | Carbamidomethylation | C2:Carbamidomethylation:1000.00;C5:Carbamidomethylation:1000.00;C10:Carbamidomethylation:1000.00 | PEAKS DB |
| G.YTLTC(+57.02)LNC(+57.02)PEMFC(+57.02)GK.F | Y | 51.51 | 1892.7930 | 15 | 0.6 | 947.4044 | 2 | 60.30 | 12 | F12:40225 | NaSa01\_F7.raw |  |  |  |  |  |  |  |  |  |  |  | 2.5637E6 |  |  | 1 | 0 | 0 | 0 | 0 | 0 | 0 | 0 | 0 | 0 | 0 | 0 | 1 | 0 | 0 | 20 | 34 | Carbamidomethylation | C5:Carbamidomethylation:1000.00;C8:Carbamidomethylation:1000.00;C13:Carbamidomethylation:1000.00 | PEAKS DB |
| total 5 peptides |  |  |  |  |  |  |  |  |  |  |  |  |  |  |  |  |  |  |  |  |  |  |  |  |  |  |  |  |  |  |  |  |  |  |  |  |  |  |  |  |  |  |  |  |  |
| --- | --- | --- | --- | --- | --- | --- | --- | --- | --- | --- | --- | --- | --- | --- | --- | --- | --- | --- | --- | --- | --- | --- | --- | --- | --- | --- | --- | --- | --- | --- | --- | --- | --- | --- | --- | --- | --- | --- | --- | --- | --- | --- | --- | --- | --- |

CAA04578.1

back to list

  

| Protein Coverage
| Supporting Peptides
|

Protein Coverage:

Supporting Peptides:

| Peptide | Uniq | -10lgP | Mass | Length | ppm | m/z | z | RT | Fraction | Scan | Source File | Area F1 | Area F10 | Area F11 | Area F12 | Area F13 | Area F14 | Area F2 | Area F3 | Area F4 | Area F5 | Area F6 | Area F7 | Area F8 | Area F9 | #Feature | #Feature F1 | #Feature F10 | #Feature F11 | #Feature F12 | #Feature F13 | #Feature F14 | #Feature F2 | #Feature F3 | #Feature F4 | #Feature F5 | #Feature F6 | #Feature F7 | #Feature F8 | #Feature F9 | Start | End | PTM | AScore | Found By |
| --- | --- | --- | --- | --- | --- | --- | --- | --- | --- | --- | --- | --- | --- | --- | --- | --- | --- | --- | --- | --- | --- | --- | --- | --- | --- | --- | --- | --- | --- | --- | --- | --- | --- | --- | --- | --- | --- | --- | --- | --- | --- | --- | --- | --- | --- |
| T.LTC(+57.02)LNC(+57.02)PEMFC(+57.02)GK.F | N | 87.07 | 1628.6819 | 13 | 0.7 | 815.3488 | 2 | 45.64 | 14 | F14:24540 | NaSa01\_F9.raw |  | 3.1696E6 | 1.6649E6 | 1.8659E6 |  |  |  |  | 2.1823E6 | 6.7455E8 | 8.6607E9 | 5.3693E8 | 5.7295E7 | 1.4835E7 | 19 | 0 | 1 | 1 | 1 | 0 | 0 | 0 | 0 | 1 | 1 | 10 | 2 | 1 | 1 | 22 | 34 | Carbamidomethylation | C3:Carbamidomethylation:1000.00;C6:Carbamidomethylation:1000.00;C11:Carbamidomethylation:1000.00 | PEAKS DB |
| T.LTC(+57.02)LNC(+57.02)PEM(+15.99)FC(+57.02)GK.F | N | 67.45 | 1644.6769 | 13 | 0.7 | 823.3463 | 2 | 32.98 | 12 | F12:17393 | NaSa01\_F7.raw |  |  |  |  |  |  |  |  | 9.1936E5 | 1.0943E8 | 7.6473E8 | 5.7873E7 |  |  | 24 | 0 | 0 | 0 | 0 | 0 | 0 | 0 | 0 | 1 | 7 | 10 | 6 | 0 | 0 | 22 | 34 | Carbamidomethylation; Oxidation (M) | C3:Carbamidomethylation:1000.00;C6:Carbamidomethylation:1000.00;M9:Oxidation (M):1000.00;C11:Carbamidomethylation:1000.00 | PEAKS DB |
| T.LTC(+57.02)LNC(+57.02)PEMFC(+57.02)GKFQIC(+57.02)R.N | N | 62.75 | 2333.0247 | 18 | 0.9 | 778.6829 | 3 | 56.34 | 11 | F11:37844 | NaSa01\_F6.raw |  |  |  |  |  |  |  |  |  | 1.2723E6 | 1.8262E7 | 9.6113E5 |  |  | 3 | 0 | 0 | 0 | 0 | 0 | 0 | 0 | 0 | 0 | 1 | 1 | 1 | 0 | 0 | 22 | 39 | Carbamidomethylation | C3:Carbamidomethylation:1000.00;C6:Carbamidomethylation:1000.00;C11:Carbamidomethylation:1000.00;C17:Carbamidomethylation:1000.00 | PEAKS DB |
| L.TC(+57.02)LNC(+57.02)PEMFC(+57.02)GK.F | N | 53.17 | 1515.5979 | 12 | -0.4 | 758.8059 | 2 | 36.00 | 9 | F9:14208 | NaSa01\_F4.raw |  |  |  |  |  |  |  |  | 2.1699E6 |  | 1.4872E6 | 1.3767E6 | 1.0968E6 |  | 4 | 0 | 0 | 0 | 0 | 0 | 0 | 0 | 0 | 1 | 0 | 1 | 1 | 1 | 0 | 23 | 34 | Carbamidomethylation | C2:Carbamidomethylation:1000.00;C5:Carbamidomethylation:1000.00;C10:Carbamidomethylation:1000.00 | PEAKS DB |
| G.YTLTC(+57.02)LNC(+57.02)PEMFC(+57.02)GK.F | Y | 51.51 | 1892.7930 | 15 | 0.6 | 947.4044 | 2 | 60.30 | 12 | F12:40225 | NaSa01\_F7.raw |  |  |  |  |  |  |  |  |  |  |  | 2.5637E6 |  |  | 1 | 0 | 0 | 0 | 0 | 0 | 0 | 0 | 0 | 0 | 0 | 0 | 1 | 0 | 0 | 20 | 34 | Carbamidomethylation | C5:Carbamidomethylation:1000.00;C8:Carbamidomethylation:1000.00;C13:Carbamidomethylation:1000.00 | PEAKS DB |
| total 5 peptides |  |  |  |  |  |  |  |  |  |  |  |  |  |  |  |  |  |  |  |  |  |  |  |  |  |  |  |  |  |  |  |  |  |  |  |  |  |  |  |  |  |  |  |  |  |
| --- | --- | --- | --- | --- | --- | --- | --- | --- | --- | --- | --- | --- | --- | --- | --- | --- | --- | --- | --- | --- | --- | --- | --- | --- | --- | --- | --- | --- | --- | --- | --- | --- | --- | --- | --- | --- | --- | --- | --- | --- | --- | --- | --- | --- | --- |

CAA10530.1

back to list

  

| Protein Coverage
| Supporting Peptides
|

Protein Coverage:

Supporting Peptides:

| Peptide | Uniq | -10lgP | Mass | Length | ppm | m/z | z | RT | Fraction | Scan | Source File | Area F1 | Area F10 | Area F11 | Area F12 | Area F13 | Area F14 | Area F2 | Area F3 | Area F4 | Area F5 | Area F6 | Area F7 | Area F8 | Area F9 | #Feature | #Feature F1 | #Feature F10 | #Feature F11 | #Feature F12 | #Feature F13 | #Feature F14 | #Feature F2 | #Feature F3 | #Feature F4 | #Feature F5 | #Feature F6 | #Feature F7 | #Feature F8 | #Feature F9 | Start | End | PTM | AScore | Found By |
| --- | --- | --- | --- | --- | --- | --- | --- | --- | --- | --- | --- | --- | --- | --- | --- | --- | --- | --- | --- | --- | --- | --- | --- | --- | --- | --- | --- | --- | --- | --- | --- | --- | --- | --- | --- | --- | --- | --- | --- | --- | --- | --- | --- | --- | --- |
| T.LTC(+57.02)LNC(+57.02)PEMFC(+57.02)GK.F | N | 87.07 | 1628.6819 | 13 | 0.7 | 815.3488 | 2 | 45.64 | 14 | F14:24540 | NaSa01\_F9.raw |  | 3.1696E6 | 1.6649E6 | 1.8659E6 |  |  |  |  | 2.1823E6 | 6.7455E8 | 8.6607E9 | 5.3693E8 | 5.7295E7 | 1.4835E7 | 19 | 0 | 1 | 1 | 1 | 0 | 0 | 0 | 0 | 1 | 1 | 10 | 2 | 1 | 1 | 22 | 34 | Carbamidomethylation | C3:Carbamidomethylation:1000.00;C6:Carbamidomethylation:1000.00;C11:Carbamidomethylation:1000.00 | PEAKS DB |
| T.LTC(+57.02)LNC(+57.02)PEM(+15.99)FC(+57.02)GK.F | N | 67.45 | 1644.6769 | 13 | 0.7 | 823.3463 | 2 | 32.98 | 12 | F12:17393 | NaSa01\_F7.raw |  |  |  |  |  |  |  |  | 9.1936E5 | 1.0943E8 | 7.6473E8 | 5.7873E7 |  |  | 24 | 0 | 0 | 0 | 0 | 0 | 0 | 0 | 0 | 1 | 7 | 10 | 6 | 0 | 0 | 22 | 34 | Carbamidomethylation; Oxidation (M) | C3:Carbamidomethylation:1000.00;C6:Carbamidomethylation:1000.00;M9:Oxidation (M):1000.00;C11:Carbamidomethylation:1000.00 | PEAKS DB |
| T.LTC(+57.02)LNC(+57.02)PEMFC(+57.02)GKFQIC(+57.02)R.N | N | 62.75 | 2333.0247 | 18 | 0.9 | 778.6829 | 3 | 56.34 | 11 | F11:37844 | NaSa01\_F6.raw |  |  |  |  |  |  |  |  |  | 1.2723E6 | 1.8262E7 | 9.6113E5 |  |  | 3 | 0 | 0 | 0 | 0 | 0 | 0 | 0 | 0 | 0 | 1 | 1 | 1 | 0 | 0 | 22 | 39 | Carbamidomethylation | C3:Carbamidomethylation:1000.00;C6:Carbamidomethylation:1000.00;C11:Carbamidomethylation:1000.00;C17:Carbamidomethylation:1000.00 | PEAKS DB |
| L.TC(+57.02)LNC(+57.02)PEMFC(+57.02)GK.F | N | 53.17 | 1515.5979 | 12 | -0.4 | 758.8059 | 2 | 36.00 | 9 | F9:14208 | NaSa01\_F4.raw |  |  |  |  |  |  |  |  | 2.1699E6 |  | 1.4872E6 | 1.3767E6 | 1.0968E6 |  | 4 | 0 | 0 | 0 | 0 | 0 | 0 | 0 | 0 | 1 | 0 | 1 | 1 | 1 | 0 | 23 | 34 | Carbamidomethylation | C2:Carbamidomethylation:1000.00;C5:Carbamidomethylation:1000.00;C10:Carbamidomethylation:1000.00 | PEAKS DB |
| G.YTLTC(+57.02)LNC(+57.02)PEMFC(+57.02)GK.F | Y | 51.51 | 1892.7930 | 15 | 0.6 | 947.4044 | 2 | 60.30 | 12 | F12:40225 | NaSa01\_F7.raw |  |  |  |  |  |  |  |  |  |  |  | 2.5637E6 |  |  | 1 | 0 | 0 | 0 | 0 | 0 | 0 | 0 | 0 | 0 | 0 | 0 | 1 | 0 | 0 | 20 | 34 | Carbamidomethylation | C5:Carbamidomethylation:1000.00;C8:Carbamidomethylation:1000.00;C13:Carbamidomethylation:1000.00 | PEAKS DB |
| total 5 peptides |  |  |  |  |  |  |  |  |  |  |  |  |  |  |  |  |  |  |  |  |  |  |  |  |  |  |  |  |  |  |  |  |  |  |  |  |  |  |  |  |  |  |  |  |  |
| --- | --- | --- | --- | --- | --- | --- | --- | --- | --- | --- | --- | --- | --- | --- | --- | --- | --- | --- | --- | --- | --- | --- | --- | --- | --- | --- | --- | --- | --- | --- | --- | --- | --- | --- | --- | --- | --- | --- | --- | --- | --- | --- | --- | --- | --- |

P60814.1

back to list

  

| Protein Coverage
| Supporting Peptides
|

Protein Coverage:

Supporting Peptides:

| Peptide | Uniq | -10lgP | Mass | Length | ppm | m/z | z | RT | Fraction | Scan | Source File | Area F1 | Area F10 | Area F11 | Area F12 | Area F13 | Area F14 | Area F2 | Area F3 | Area F4 | Area F5 | Area F6 | Area F7 | Area F8 | Area F9 | #Feature | #Feature F1 | #Feature F10 | #Feature F11 | #Feature F12 | #Feature F13 | #Feature F14 | #Feature F2 | #Feature F3 | #Feature F4 | #Feature F5 | #Feature F6 | #Feature F7 | #Feature F8 | #Feature F9 | Start | End | PTM | AScore | Found By |
| --- | --- | --- | --- | --- | --- | --- | --- | --- | --- | --- | --- | --- | --- | --- | --- | --- | --- | --- | --- | --- | --- | --- | --- | --- | --- | --- | --- | --- | --- | --- | --- | --- | --- | --- | --- | --- | --- | --- | --- | --- | --- | --- | --- | --- | --- |
| T.LTC(+57.02)LNC(+57.02)PEMFC(+57.02)GK.F | N | 87.07 | 1628.6819 | 13 | 0.7 | 815.3488 | 2 | 45.64 | 14 | F14:24540 | NaSa01\_F9.raw |  | 3.1696E6 | 1.6649E6 | 1.8659E6 |  |  |  |  | 2.1823E6 | 6.7455E8 | 8.6607E9 | 5.3693E8 | 5.7295E7 | 1.4835E7 | 19 | 0 | 1 | 1 | 1 | 0 | 0 | 0 | 0 | 1 | 1 | 10 | 2 | 1 | 1 | 22 | 34 | Carbamidomethylation | C3:Carbamidomethylation:1000.00;C6:Carbamidomethylation:1000.00;C11:Carbamidomethylation:1000.00 | PEAKS DB |
| T.LTC(+57.02)LNC(+57.02)PEM(+15.99)FC(+57.02)GK.F | N | 67.45 | 1644.6769 | 13 | 0.7 | 823.3463 | 2 | 32.98 | 12 | F12:17393 | NaSa01\_F7.raw |  |  |  |  |  |  |  |  | 9.1936E5 | 1.0943E8 | 7.6473E8 | 5.7873E7 |  |  | 24 | 0 | 0 | 0 | 0 | 0 | 0 | 0 | 0 | 1 | 7 | 10 | 6 | 0 | 0 | 22 | 34 | Carbamidomethylation; Oxidation (M) | C3:Carbamidomethylation:1000.00;C6:Carbamidomethylation:1000.00;M9:Oxidation (M):1000.00;C11:Carbamidomethylation:1000.00 | PEAKS DB |
| T.LTC(+57.02)LNC(+57.02)PEMFC(+57.02)GKFQIC(+57.02)R.N | N | 62.75 | 2333.0247 | 18 | 0.9 | 778.6829 | 3 | 56.34 | 11 | F11:37844 | NaSa01\_F6.raw |  |  |  |  |  |  |  |  |  | 1.2723E6 | 1.8262E7 | 9.6113E5 |  |  | 3 | 0 | 0 | 0 | 0 | 0 | 0 | 0 | 0 | 0 | 1 | 1 | 1 | 0 | 0 | 22 | 39 | Carbamidomethylation | C3:Carbamidomethylation:1000.00;C6:Carbamidomethylation:1000.00;C11:Carbamidomethylation:1000.00;C17:Carbamidomethylation:1000.00 | PEAKS DB |
| L.TC(+57.02)LNC(+57.02)PEMFC(+57.02)GK.F | N | 53.17 | 1515.5979 | 12 | -0.4 | 758.8059 | 2 | 36.00 | 9 | F9:14208 | NaSa01\_F4.raw |  |  |  |  |  |  |  |  | 2.1699E6 |  | 1.4872E6 | 1.3767E6 | 1.0968E6 |  | 4 | 0 | 0 | 0 | 0 | 0 | 0 | 0 | 0 | 1 | 0 | 1 | 1 | 1 | 0 | 23 | 34 | Carbamidomethylation | C2:Carbamidomethylation:1000.00;C5:Carbamidomethylation:1000.00;C10:Carbamidomethylation:1000.00 | PEAKS DB |
| G.YTLTC(+57.02)LNC(+57.02)PEMFC(+57.02)GK.F | Y | 51.51 | 1892.7930 | 15 | 0.6 | 947.4044 | 2 | 60.30 | 12 | F12:40225 | NaSa01\_F7.raw |  |  |  |  |  |  |  |  |  |  |  | 2.5637E6 |  |  | 1 | 0 | 0 | 0 | 0 | 0 | 0 | 0 | 0 | 0 | 0 | 0 | 1 | 0 | 0 | 20 | 34 | Carbamidomethylation | C5:Carbamidomethylation:1000.00;C8:Carbamidomethylation:1000.00;C13:Carbamidomethylation:1000.00 | PEAKS DB |
| total 5 peptides |  |  |  |  |  |  |  |  |  |  |  |  |  |  |  |  |  |  |  |  |  |  |  |  |  |  |  |  |  |  |  |  |  |  |  |  |  |  |  |  |  |  |  |  |  |
| --- | --- | --- | --- | --- | --- | --- | --- | --- | --- | --- | --- | --- | --- | --- | --- | --- | --- | --- | --- | --- | --- | --- | --- | --- | --- | --- | --- | --- | --- | --- | --- | --- | --- | --- | --- | --- | --- | --- | --- | --- | --- | --- | --- | --- | --- |

CAA06888.1

back to list

  

| Protein Coverage
| Supporting Peptides
|

Protein Coverage:

Supporting Peptides:

| Peptide | Uniq | -10lgP | Mass | Length | ppm | m/z | z | RT | Fraction | Scan | Source File | Area F1 | Area F10 | Area F11 | Area F12 | Area F13 | Area F14 | Area F2 | Area F3 | Area F4 | Area F5 | Area F6 | Area F7 | Area F8 | Area F9 | #Feature | #Feature F1 | #Feature F10 | #Feature F11 | #Feature F12 | #Feature F13 | #Feature F14 | #Feature F2 | #Feature F3 | #Feature F4 | #Feature F5 | #Feature F6 | #Feature F7 | #Feature F8 | #Feature F9 | Start | End | PTM | AScore | Found By |
| --- | --- | --- | --- | --- | --- | --- | --- | --- | --- | --- | --- | --- | --- | --- | --- | --- | --- | --- | --- | --- | --- | --- | --- | --- | --- | --- | --- | --- | --- | --- | --- | --- | --- | --- | --- | --- | --- | --- | --- | --- | --- | --- | --- | --- | --- |
| T.LTC(+57.02)LNC(+57.02)PEMFC(+57.02)GK.F | N | 87.07 | 1628.6819 | 13 | 0.7 | 815.3488 | 2 | 45.64 | 14 | F14:24540 | NaSa01\_F9.raw |  | 3.1696E6 | 1.6649E6 | 1.8659E6 |  |  |  |  | 2.1823E6 | 6.7455E8 | 8.6607E9 | 5.3693E8 | 5.7295E7 | 1.4835E7 | 19 | 0 | 1 | 1 | 1 | 0 | 0 | 0 | 0 | 1 | 1 | 10 | 2 | 1 | 1 | 22 | 34 | Carbamidomethylation | C3:Carbamidomethylation:1000.00;C6:Carbamidomethylation:1000.00;C11:Carbamidomethylation:1000.00 | PEAKS DB |
| T.LTC(+57.02)LNC(+57.02)PEM(+15.99)FC(+57.02)GK.F | N | 67.45 | 1644.6769 | 13 | 0.7 | 823.3463 | 2 | 32.98 | 12 | F12:17393 | NaSa01\_F7.raw |  |  |  |  |  |  |  |  | 9.1936E5 | 1.0943E8 | 7.6473E8 | 5.7873E7 |  |  | 24 | 0 | 0 | 0 | 0 | 0 | 0 | 0 | 0 | 1 | 7 | 10 | 6 | 0 | 0 | 22 | 34 | Carbamidomethylation; Oxidation (M) | C3:Carbamidomethylation:1000.00;C6:Carbamidomethylation:1000.00;M9:Oxidation (M):1000.00;C11:Carbamidomethylation:1000.00 | PEAKS DB |
| T.LTC(+57.02)LNC(+57.02)PEMFC(+57.02)GKFQIC(+57.02)R.N | N | 62.75 | 2333.0247 | 18 | 0.9 | 778.6829 | 3 | 56.34 | 11 | F11:37844 | NaSa01\_F6.raw |  |  |  |  |  |  |  |  |  | 1.2723E6 | 1.8262E7 | 9.6113E5 |  |  | 3 | 0 | 0 | 0 | 0 | 0 | 0 | 0 | 0 | 0 | 1 | 1 | 1 | 0 | 0 | 22 | 39 | Carbamidomethylation | C3:Carbamidomethylation:1000.00;C6:Carbamidomethylation:1000.00;C11:Carbamidomethylation:1000.00;C17:Carbamidomethylation:1000.00 | PEAKS DB |
| L.TC(+57.02)LNC(+57.02)PEMFC(+57.02)GK.F | N | 53.17 | 1515.5979 | 12 | -0.4 | 758.8059 | 2 | 36.00 | 9 | F9:14208 | NaSa01\_F4.raw |  |  |  |  |  |  |  |  | 2.1699E6 |  | 1.4872E6 | 1.3767E6 | 1.0968E6 |  | 4 | 0 | 0 | 0 | 0 | 0 | 0 | 0 | 0 | 1 | 0 | 1 | 1 | 1 | 0 | 23 | 34 | Carbamidomethylation | C2:Carbamidomethylation:1000.00;C5:Carbamidomethylation:1000.00;C10:Carbamidomethylation:1000.00 | PEAKS DB |
| G.YTLTC(+57.02)LNC(+57.02)PEMFC(+57.02)GK.F | Y | 51.51 | 1892.7930 | 15 | 0.6 | 947.4044 | 2 | 60.30 | 12 | F12:40225 | NaSa01\_F7.raw |  |  |  |  |  |  |  |  |  |  |  | 2.5637E6 |  |  | 1 | 0 | 0 | 0 | 0 | 0 | 0 | 0 | 0 | 0 | 0 | 0 | 1 | 0 | 0 | 20 | 34 | Carbamidomethylation | C5:Carbamidomethylation:1000.00;C8:Carbamidomethylation:1000.00;C13:Carbamidomethylation:1000.00 | PEAKS DB |
| total 5 peptides |  |  |  |  |  |  |  |  |  |  |  |  |  |  |  |  |  |  |  |  |  |  |  |  |  |  |  |  |  |  |  |  |  |  |  |  |  |  |  |  |  |  |  |  |  |
| --- | --- | --- | --- | --- | --- | --- | --- | --- | --- | --- | --- | --- | --- | --- | --- | --- | --- | --- | --- | --- | --- | --- | --- | --- | --- | --- | --- | --- | --- | --- | --- | --- | --- | --- | --- | --- | --- | --- | --- | --- | --- | --- | --- | --- | --- |

P82935.2

back to list

  

| Protein Coverage
| Supporting Peptides
|

Protein Coverage:

Supporting Peptides:

| Peptide | Uniq | -10lgP | Mass | Length | ppm | m/z | z | RT | Fraction | Scan | Source File | Area F1 | Area F10 | Area F11 | Area F12 | Area F13 | Area F14 | Area F2 | Area F3 | Area F4 | Area F5 | Area F6 | Area F7 | Area F8 | Area F9 | #Feature | #Feature F1 | #Feature F10 | #Feature F11 | #Feature F12 | #Feature F13 | #Feature F14 | #Feature F2 | #Feature F3 | #Feature F4 | #Feature F5 | #Feature F6 | #Feature F7 | #Feature F8 | #Feature F9 | Start | End | PTM | AScore | Found By |
| --- | --- | --- | --- | --- | --- | --- | --- | --- | --- | --- | --- | --- | --- | --- | --- | --- | --- | --- | --- | --- | --- | --- | --- | --- | --- | --- | --- | --- | --- | --- | --- | --- | --- | --- | --- | --- | --- | --- | --- | --- | --- | --- | --- | --- | --- |
| T.LTC(+57.02)LNC(+57.02)PEMFC(+57.02)GK.F | N | 87.07 | 1628.6819 | 13 | 0.7 | 815.3488 | 2 | 45.64 | 14 | F14:24540 | NaSa01\_F9.raw |  | 3.1696E6 | 1.6649E6 | 1.8659E6 |  |  |  |  | 2.1823E6 | 6.7455E8 | 8.6607E9 | 5.3693E8 | 5.7295E7 | 1.4835E7 | 19 | 0 | 1 | 1 | 1 | 0 | 0 | 0 | 0 | 1 | 1 | 10 | 2 | 1 | 1 | 22 | 34 | Carbamidomethylation | C3:Carbamidomethylation:1000.00;C6:Carbamidomethylation:1000.00;C11:Carbamidomethylation:1000.00 | PEAKS DB |
| T.LTC(+57.02)LNC(+57.02)PEM(+15.99)FC(+57.02)GK.F | N | 67.45 | 1644.6769 | 13 | 0.7 | 823.3463 | 2 | 32.98 | 12 | F12:17393 | NaSa01\_F7.raw |  |  |  |  |  |  |  |  | 9.1936E5 | 1.0943E8 | 7.6473E8 | 5.7873E7 |  |  | 24 | 0 | 0 | 0 | 0 | 0 | 0 | 0 | 0 | 1 | 7 | 10 | 6 | 0 | 0 | 22 | 34 | Carbamidomethylation; Oxidation (M) | C3:Carbamidomethylation:1000.00;C6:Carbamidomethylation:1000.00;M9:Oxidation (M):1000.00;C11:Carbamidomethylation:1000.00 | PEAKS DB |
| T.LTC(+57.02)LNC(+57.02)PEMFC(+57.02)GKFQIC(+57.02)R.N | N | 62.75 | 2333.0247 | 18 | 0.9 | 778.6829 | 3 | 56.34 | 11 | F11:37844 | NaSa01\_F6.raw |  |  |  |  |  |  |  |  |  | 1.2723E6 | 1.8262E7 | 9.6113E5 |  |  | 3 | 0 | 0 | 0 | 0 | 0 | 0 | 0 | 0 | 0 | 1 | 1 | 1 | 0 | 0 | 22 | 39 | Carbamidomethylation | C3:Carbamidomethylation:1000.00;C6:Carbamidomethylation:1000.00;C11:Carbamidomethylation:1000.00;C17:Carbamidomethylation:1000.00 | PEAKS DB |
| L.TC(+57.02)LNC(+57.02)PEMFC(+57.02)GK.F | N | 53.17 | 1515.5979 | 12 | -0.4 | 758.8059 | 2 | 36.00 | 9 | F9:14208 | NaSa01\_F4.raw |  |  |  |  |  |  |  |  | 2.1699E6 |  | 1.4872E6 | 1.3767E6 | 1.0968E6 |  | 4 | 0 | 0 | 0 | 0 | 0 | 0 | 0 | 0 | 1 | 0 | 1 | 1 | 1 | 0 | 23 | 34 | Carbamidomethylation | C2:Carbamidomethylation:1000.00;C5:Carbamidomethylation:1000.00;C10:Carbamidomethylation:1000.00 | PEAKS DB |
| G.YTLTC(+57.02)LNC(+57.02)PEMFC(+57.02)GK.F | Y | 51.51 | 1892.7930 | 15 | 0.6 | 947.4044 | 2 | 60.30 | 12 | F12:40225 | NaSa01\_F7.raw |  |  |  |  |  |  |  |  |  |  |  | 2.5637E6 |  |  | 1 | 0 | 0 | 0 | 0 | 0 | 0 | 0 | 0 | 0 | 0 | 0 | 1 | 0 | 0 | 20 | 34 | Carbamidomethylation | C5:Carbamidomethylation:1000.00;C8:Carbamidomethylation:1000.00;C13:Carbamidomethylation:1000.00 | PEAKS DB |
| total 5 peptides |  |  |  |  |  |  |  |  |  |  |  |  |  |  |  |  |  |  |  |  |  |  |  |  |  |  |  |  |  |  |  |  |  |  |  |  |  |  |  |  |  |  |  |  |  |
| --- | --- | --- | --- | --- | --- | --- | --- | --- | --- | --- | --- | --- | --- | --- | --- | --- | --- | --- | --- | --- | --- | --- | --- | --- | --- | --- | --- | --- | --- | --- | --- | --- | --- | --- | --- | --- | --- | --- | --- | --- | --- | --- | --- | --- | --- |

Q98956.1

back to list

  

| Protein Coverage
| Supporting Peptides
|

Protein Coverage:

Supporting Peptides:

| Peptide | Uniq | -10lgP | Mass | Length | ppm | m/z | z | RT | Fraction | Scan | Source File | Area F1 | Area F10 | Area F11 | Area F12 | Area F13 | Area F14 | Area F2 | Area F3 | Area F4 | Area F5 | Area F6 | Area F7 | Area F8 | Area F9 | #Feature | #Feature F1 | #Feature F10 | #Feature F11 | #Feature F12 | #Feature F13 | #Feature F14 | #Feature F2 | #Feature F3 | #Feature F4 | #Feature F5 | #Feature F6 | #Feature F7 | #Feature F8 | #Feature F9 | Start | End | PTM | AScore | Found By |
| --- | --- | --- | --- | --- | --- | --- | --- | --- | --- | --- | --- | --- | --- | --- | --- | --- | --- | --- | --- | --- | --- | --- | --- | --- | --- | --- | --- | --- | --- | --- | --- | --- | --- | --- | --- | --- | --- | --- | --- | --- | --- | --- | --- | --- | --- |
| R.GC(+57.02)IDVC(+57.02)PKSSLLVK.Y | N | 59.56 | 1574.8160 | 14 | 0.4 | 788.4156 | 2 | 32.94 | 3 | F3:14389 | NaSa01\_F11.raw |  | 1.6206E6 | 1.1319E7 |  |  |  |  |  |  |  |  |  |  | 1.3923E6 | 3 | 0 | 1 | 1 | 0 | 0 | 0 | 0 | 0 | 0 | 0 | 0 | 0 | 0 | 1 | 58 | 71 | Carbamidomethylation | C2:Carbamidomethylation:1000.00;C6:Carbamidomethylation:1000.00 | PEAKS DB |
| K.MFMMSDLTVPVK.R | Y | 54.99 | 1397.6757 | 12 | -1.3 | 699.8442 | 2 | 82.97 | 14 | F14:55741 | NaSa01\_F9.raw |  |  |  |  |  |  |  |  |  |  |  |  |  | 2.0971E6 | 2 | 0 | 0 | 0 | 0 | 0 | 0 | 0 | 0 | 0 | 0 | 0 | 0 | 0 | 2 | 45 | 56 |  |  | PEAKS DB |
| R.GC(+57.02)IDVC(+57.02)PK.S | N | 51.80 | 947.4205 | 8 | -0.1 | 474.7175 | 2 | 11.78 | 4 | F4:2753 | NaSa01\_F12.raw |  |  | 1.2016E8 | 2.8471E6 |  |  |  |  | 1.4583E5 |  |  | 9.3598E5 | 3.6007E7 | 2.198E7 | 6 | 0 | 0 | 1 | 1 | 0 | 0 | 0 | 0 | 1 | 0 | 0 | 1 | 1 | 1 | 58 | 65 | Carbamidomethylation | C2:Carbamidomethylation:1000.00;C6:Carbamidomethylation:1000.00 | PEAKS DB |
| K.NLC(+57.02)YKMF.M | N | 46.68 | 974.4354 | 7 | -0.6 | 488.2247 | 2 | 44.70 | 12 | F12:27287 | NaSa01\_F7.raw |  |  | 1.8393E7 |  |  |  |  |  |  |  |  | 5.6016E6 |  |  | 2 | 0 | 0 | 1 | 0 | 0 | 0 | 0 | 0 | 0 | 0 | 0 | 1 | 0 | 0 | 40 | 46 | Carbamidomethylation | C3:Carbamidomethylation:1000.00 | PEAKS DB |
| K.RGC(+57.02)IDVC(+57.02)PK.S | N | 40.76 | 1103.5216 | 9 | 0.0 | 552.7681 | 2 | 11.31 | 14 | F14:1779 | NaSa01\_F9.raw |  |  | 5.5737E6 |  |  |  |  |  |  |  |  |  |  | 6.4252E5 | 2 | 0 | 0 | 1 | 0 | 0 | 0 | 0 | 0 | 0 | 0 | 0 | 0 | 0 | 1 | 57 | 65 | Carbamidomethylation | C3:Carbamidomethylation:1000.00;C7:Carbamidomethylation:1000.00 | PEAKS DB |
| K.SSLLVKYVC(+57.02)C(+57.02).N | N | 40.24 | 1227.5991 | 10 | -0.1 | 614.8068 | 2 | 46.30 | 3 | F3:25259 | NaSa01\_F11.raw |  |  | 2.1505E6 |  |  |  |  |  |  |  |  |  |  |  | 1 | 0 | 0 | 1 | 0 | 0 | 0 | 0 | 0 | 0 | 0 | 0 | 0 | 0 | 0 | 66 | 75 | Carbamidomethylation | C9:Carbamidomethylation:1000.00;C10:Carbamidomethylation:1000.00 | PEAKS DB |
| K.NLC(+57.02)YKMFM.M | N | 39.67 | 1105.4758 | 8 | 0.3 | 553.7454 | 2 | 57.88 | 3 | F3:32929 | NaSa01\_F11.raw |  |  | 1.6979E6 |  |  |  |  |  |  |  |  |  |  |  | 1 | 0 | 0 | 1 | 0 | 0 | 0 | 0 | 0 | 0 | 0 | 0 | 0 | 0 | 0 | 40 | 47 | Carbamidomethylation | C3:Carbamidomethylation:1000.00 | PEAKS DB |
| total 7 peptides |  |  |  |  |  |  |  |  |  |  |  |  |  |  |  |  |  |  |  |  |  |  |  |  |  |  |  |  |  |  |  |  |  |  |  |  |  |  |  |  |  |  |  |  |  |
| --- | --- | --- | --- | --- | --- | --- | --- | --- | --- | --- | --- | --- | --- | --- | --- | --- | --- | --- | --- | --- | --- | --- | --- | --- | --- | --- | --- | --- | --- | --- | --- | --- | --- | --- | --- | --- | --- | --- | --- | --- | --- | --- | --- | --- | --- |

AAB18378.1

back to list

  

| Protein Coverage
| Supporting Peptides
|

Protein Coverage:

Supporting Peptides:

| Peptide | Uniq | -10lgP | Mass | Length | ppm | m/z | z | RT | Fraction | Scan | Source File | Area F1 | Area F10 | Area F11 | Area F12 | Area F13 | Area F14 | Area F2 | Area F3 | Area F4 | Area F5 | Area F6 | Area F7 | Area F8 | Area F9 | #Feature | #Feature F1 | #Feature F10 | #Feature F11 | #Feature F12 | #Feature F13 | #Feature F14 | #Feature F2 | #Feature F3 | #Feature F4 | #Feature F5 | #Feature F6 | #Feature F7 | #Feature F8 | #Feature F9 | Start | End | PTM | AScore | Found By |
| --- | --- | --- | --- | --- | --- | --- | --- | --- | --- | --- | --- | --- | --- | --- | --- | --- | --- | --- | --- | --- | --- | --- | --- | --- | --- | --- | --- | --- | --- | --- | --- | --- | --- | --- | --- | --- | --- | --- | --- | --- | --- | --- | --- | --- | --- |
| R.GC(+57.02)IDVC(+57.02)PKSSLLVK.Y | N | 59.56 | 1574.8160 | 14 | 0.4 | 788.4156 | 2 | 32.94 | 3 | F3:14389 | NaSa01\_F11.raw |  | 1.6206E6 | 1.1319E7 |  |  |  |  |  |  |  |  |  |  | 1.3923E6 | 3 | 0 | 1 | 1 | 0 | 0 | 0 | 0 | 0 | 0 | 0 | 0 | 0 | 0 | 1 | 58 | 71 | Carbamidomethylation | C2:Carbamidomethylation:1000.00;C6:Carbamidomethylation:1000.00 | PEAKS DB |
| K.MFMMSDLTVPVK.R | Y | 54.99 | 1397.6757 | 12 | -1.3 | 699.8442 | 2 | 82.97 | 14 | F14:55741 | NaSa01\_F9.raw |  |  |  |  |  |  |  |  |  |  |  |  |  | 2.0971E6 | 2 | 0 | 0 | 0 | 0 | 0 | 0 | 0 | 0 | 0 | 0 | 0 | 0 | 0 | 2 | 45 | 56 |  |  | PEAKS DB |
| R.GC(+57.02)IDVC(+57.02)PK.S | N | 51.80 | 947.4205 | 8 | -0.1 | 474.7175 | 2 | 11.78 | 4 | F4:2753 | NaSa01\_F12.raw |  |  | 1.2016E8 | 2.8471E6 |  |  |  |  | 1.4583E5 |  |  | 9.3598E5 | 3.6007E7 | 2.198E7 | 6 | 0 | 0 | 1 | 1 | 0 | 0 | 0 | 0 | 1 | 0 | 0 | 1 | 1 | 1 | 58 | 65 | Carbamidomethylation | C2:Carbamidomethylation:1000.00;C6:Carbamidomethylation:1000.00 | PEAKS DB |
| K.NLC(+57.02)YKMF.M | N | 46.68 | 974.4354 | 7 | -0.6 | 488.2247 | 2 | 44.70 | 12 | F12:27287 | NaSa01\_F7.raw |  |  | 1.8393E7 |  |  |  |  |  |  |  |  | 5.6016E6 |  |  | 2 | 0 | 0 | 1 | 0 | 0 | 0 | 0 | 0 | 0 | 0 | 0 | 1 | 0 | 0 | 40 | 46 | Carbamidomethylation | C3:Carbamidomethylation:1000.00 | PEAKS DB |
| K.RGC(+57.02)IDVC(+57.02)PK.S | N | 40.76 | 1103.5216 | 9 | 0.0 | 552.7681 | 2 | 11.31 | 14 | F14:1779 | NaSa01\_F9.raw |  |  | 5.5737E6 |  |  |  |  |  |  |  |  |  |  | 6.4252E5 | 2 | 0 | 0 | 1 | 0 | 0 | 0 | 0 | 0 | 0 | 0 | 0 | 0 | 0 | 1 | 57 | 65 | Carbamidomethylation | C3:Carbamidomethylation:1000.00;C7:Carbamidomethylation:1000.00 | PEAKS DB |
| K.SSLLVKYVC(+57.02)C(+57.02).N | N | 40.24 | 1227.5991 | 10 | -0.1 | 614.8068 | 2 | 46.30 | 3 | F3:25259 | NaSa01\_F11.raw |  |  | 2.1505E6 |  |  |  |  |  |  |  |  |  |  |  | 1 | 0 | 0 | 1 | 0 | 0 | 0 | 0 | 0 | 0 | 0 | 0 | 0 | 0 | 0 | 66 | 75 | Carbamidomethylation | C9:Carbamidomethylation:1000.00;C10:Carbamidomethylation:1000.00 | PEAKS DB |
| K.NLC(+57.02)YKMFM.M | N | 39.67 | 1105.4758 | 8 | 0.3 | 553.7454 | 2 | 57.88 | 3 | F3:32929 | NaSa01\_F11.raw |  |  | 1.6979E6 |  |  |  |  |  |  |  |  |  |  |  | 1 | 0 | 0 | 1 | 0 | 0 | 0 | 0 | 0 | 0 | 0 | 0 | 0 | 0 | 0 | 40 | 47 | Carbamidomethylation | C3:Carbamidomethylation:1000.00 | PEAKS DB |
| total 7 peptides |  |  |  |  |  |  |  |  |  |  |  |  |  |  |  |  |  |  |  |  |  |  |  |  |  |  |  |  |  |  |  |  |  |  |  |  |  |  |  |  |  |  |  |  |  |
| --- | --- | --- | --- | --- | --- | --- | --- | --- | --- | --- | --- | --- | --- | --- | --- | --- | --- | --- | --- | --- | --- | --- | --- | --- | --- | --- | --- | --- | --- | --- | --- | --- | --- | --- | --- | --- | --- | --- | --- | --- | --- | --- | --- | --- | --- |

JAS04550.1

back to list

  

| Protein Coverage
| Supporting Peptides
|

Protein Coverage:

Supporting Peptides:

| Peptide | Uniq | -10lgP | Mass | Length | ppm | m/z | z | RT | Fraction | Scan | Source File | Area F1 | Area F10 | Area F11 | Area F12 | Area F13 | Area F14 | Area F2 | Area F3 | Area F4 | Area F5 | Area F6 | Area F7 | Area F8 | Area F9 | #Feature | #Feature F1 | #Feature F10 | #Feature F11 | #Feature F12 | #Feature F13 | #Feature F14 | #Feature F2 | #Feature F3 | #Feature F4 | #Feature F5 | #Feature F6 | #Feature F7 | #Feature F8 | #Feature F9 | Start | End | PTM | AScore | Found By |
| --- | --- | --- | --- | --- | --- | --- | --- | --- | --- | --- | --- | --- | --- | --- | --- | --- | --- | --- | --- | --- | --- | --- | --- | --- | --- | --- | --- | --- | --- | --- | --- | --- | --- | --- | --- | --- | --- | --- | --- | --- | --- | --- | --- | --- | --- |
| K.SGPPC(+57.02)GDC(+57.02)PSAC(+57.02)VNGLC(+57.02)TNPC(+57.02)K.H | N | 84.62 | 2406.9482 | 22 | -0.2 | 1204.4812 | 2 | 33.07 | 3 | F3:14521 | NaSa01\_F11.raw |  |  | 1.2348E8 | 4.9674E7 |  |  |  |  |  |  |  |  |  |  | 4 | 0 | 0 | 2 | 2 | 0 | 0 | 0 | 0 | 0 | 0 | 0 | 0 | 0 | 0 | 182 | 203 | Carbamidomethylation | C5:Carbamidomethylation:1000.00;C8:Carbamidomethylation:1000.00;C12:Carbamidomethylation:1000.00;C17:Carbamidomethylation:1000.00;C21:Carbamidomethylation:1000.00 | PEAKS DB |
| Q.IVDLHNSLR.R | N | 58.01 | 1065.5930 | 9 | -1.6 | 533.8029 | 2 | 26.50 | 4 | F4:11729 | NaSa01\_F12.raw |  |  |  | 1.3096E7 |  |  |  |  |  |  |  |  |  |  | 1 | 0 | 0 | 0 | 1 | 0 | 0 | 0 | 0 | 0 | 0 | 0 | 0 | 0 | 0 | 37 | 45 |  |  | PEAKS DB |
| I.QNQIVDLHNSLR.R | Y | 45.47 | 1435.7532 | 12 | -7.5 | 479.5881 | 3 | 30.87 | 4 | F4:15432 | NaSa01\_F12.raw |  |  |  | 2.2026E6 |  |  |  |  |  |  |  |  |  |  | 2 | 0 | 0 | 0 | 2 | 0 | 0 | 0 | 0 | 0 | 0 | 0 | 0 | 0 | 0 | 34 | 45 |  |  | PEAKS DB |
| I.VDLHNSLR.R | N | 42.85 | 952.5090 | 8 | -1.0 | 477.2613 | 2 | 26.51 | 4 | F4:11862 | NaSa01\_F12.raw |  |  |  | 2.8116E8 |  |  |  |  |  |  |  |  |  |  | 1 | 0 | 0 | 0 | 1 | 0 | 0 | 0 | 0 | 0 | 0 | 0 | 0 | 0 | 0 | 38 | 45 |  |  | PEAKS DB |
| total 4 peptides |  |  |  |  |  |  |  |  |  |  |  |  |  |  |  |  |  |  |  |  |  |  |  |  |  |  |  |  |  |  |  |  |  |  |  |  |  |  |  |  |  |  |  |  |  |
| --- | --- | --- | --- | --- | --- | --- | --- | --- | --- | --- | --- | --- | --- | --- | --- | --- | --- | --- | --- | --- | --- | --- | --- | --- | --- | --- | --- | --- | --- | --- | --- | --- | --- | --- | --- | --- | --- | --- | --- | --- | --- | --- | --- | --- | --- |

P25679.2

back to list

  

| Protein Coverage
| Supporting Peptides
|

Protein Coverage:

Supporting Peptides:

| Peptide | Uniq | -10lgP | Mass | Length | ppm | m/z | z | RT | Fraction | Scan | Source File | Area F1 | Area F10 | Area F11 | Area F12 | Area F13 | Area F14 | Area F2 | Area F3 | Area F4 | Area F5 | Area F6 | Area F7 | Area F8 | Area F9 | #Feature | #Feature F1 | #Feature F10 | #Feature F11 | #Feature F12 | #Feature F13 | #Feature F14 | #Feature F2 | #Feature F3 | #Feature F4 | #Feature F5 | #Feature F6 | #Feature F7 | #Feature F8 | #Feature F9 | Start | End | PTM | AScore | Found By |
| --- | --- | --- | --- | --- | --- | --- | --- | --- | --- | --- | --- | --- | --- | --- | --- | --- | --- | --- | --- | --- | --- | --- | --- | --- | --- | --- | --- | --- | --- | --- | --- | --- | --- | --- | --- | --- | --- | --- | --- | --- | --- | --- | --- | --- | --- |
| LTC(+57.02)LNC(+57.02)PEMFC(+57.02)GK.F | N | 87.07 | 1628.6819 | 13 | 0.7 | 815.3488 | 2 | 45.64 | 14 | F14:24540 | NaSa01\_F9.raw |  | 3.1696E6 | 1.6649E6 | 1.8659E6 |  |  |  |  | 2.1823E6 | 6.7455E8 | 8.6607E9 | 5.3693E8 | 5.7295E7 | 1.4835E7 | 19 | 0 | 1 | 1 | 1 | 0 | 0 | 0 | 0 | 1 | 1 | 10 | 2 | 1 | 1 | 1 | 13 | Carbamidomethylation | C3:Carbamidomethylation:1000.00;C6:Carbamidomethylation:1000.00;C11:Carbamidomethylation:1000.00 | PEAKS DB |
| LTC(+57.02)LNC(+57.02)PEM(+15.99)FC(+57.02)GK.F | N | 67.45 | 1644.6769 | 13 | 0.7 | 823.3463 | 2 | 32.98 | 12 | F12:17393 | NaSa01\_F7.raw |  |  |  |  |  |  |  |  | 9.1936E5 | 1.0943E8 | 7.6473E8 | 5.7873E7 |  |  | 24 | 0 | 0 | 0 | 0 | 0 | 0 | 0 | 0 | 1 | 7 | 10 | 6 | 0 | 0 | 1 | 13 | Carbamidomethylation; Oxidation (M) | C3:Carbamidomethylation:1000.00;C6:Carbamidomethylation:1000.00;M9:Oxidation (M):1000.00;C11:Carbamidomethylation:1000.00 | PEAKS DB |
| LTC(+57.02)LNC(+57.02)PEMFC(+57.02)GKFQIC(+57.02)R.N | N | 62.75 | 2333.0247 | 18 | 0.9 | 778.6829 | 3 | 56.34 | 11 | F11:37844 | NaSa01\_F6.raw |  |  |  |  |  |  |  |  |  | 1.2723E6 | 1.8262E7 | 9.6113E5 |  |  | 3 | 0 | 0 | 0 | 0 | 0 | 0 | 0 | 0 | 0 | 1 | 1 | 1 | 0 | 0 | 1 | 18 | Carbamidomethylation | C3:Carbamidomethylation:1000.00;C6:Carbamidomethylation:1000.00;C11:Carbamidomethylation:1000.00;C17:Carbamidomethylation:1000.00 | PEAKS DB |
| L.TC(+57.02)LNC(+57.02)PEMFC(+57.02)GK.F | N | 53.17 | 1515.5979 | 12 | -0.4 | 758.8059 | 2 | 36.00 | 9 | F9:14208 | NaSa01\_F4.raw |  |  |  |  |  |  |  |  | 2.1699E6 |  | 1.4872E6 | 1.3767E6 | 1.0968E6 |  | 4 | 0 | 0 | 0 | 0 | 0 | 0 | 0 | 0 | 1 | 0 | 1 | 1 | 1 | 0 | 2 | 13 | Carbamidomethylation | C2:Carbamidomethylation:1000.00;C5:Carbamidomethylation:1000.00;C10:Carbamidomethylation:1000.00 | PEAKS DB |
| LTC(+57.02)LNC(+57.02)PEMF.C | Y | 41.19 | 1283.5349 | 10 | 0.1 | 642.7748 | 2 | 69.02 | 11 | F11:47789 | NaSa01\_F6.raw |  |  |  |  |  |  |  |  |  |  | 4.8427E7 |  |  |  | 1 | 0 | 0 | 0 | 0 | 0 | 0 | 0 | 0 | 0 | 0 | 1 | 0 | 0 | 0 | 1 | 10 | Carbamidomethylation | C3:Carbamidomethylation:1000.00;C6:Carbamidomethylation:1000.00 | PEAKS DB |
| total 5 peptides |  |  |  |  |  |  |  |  |  |  |  |  |  |  |  |  |  |  |  |  |  |  |  |  |  |  |  |  |  |  |  |  |  |  |  |  |  |  |  |  |  |  |  |  |  |
| --- | --- | --- | --- | --- | --- | --- | --- | --- | --- | --- | --- | --- | --- | --- | --- | --- | --- | --- | --- | --- | --- | --- | --- | --- | --- | --- | --- | --- | --- | --- | --- | --- | --- | --- | --- | --- | --- | --- | --- | --- | --- | --- | --- | --- | --- |

2MJ0

back to list

  

| Protein Coverage
| Supporting Peptides
|

Protein Coverage:

Supporting Peptides:

| Peptide | Uniq | -10lgP | Mass | Length | ppm | m/z | z | RT | Fraction | Scan | Source File | Area F1 | Area F10 | Area F11 | Area F12 | Area F13 | Area F14 | Area F2 | Area F3 | Area F4 | Area F5 | Area F6 | Area F7 | Area F8 | Area F9 | #Feature | #Feature F1 | #Feature F10 | #Feature F11 | #Feature F12 | #Feature F13 | #Feature F14 | #Feature F2 | #Feature F3 | #Feature F4 | #Feature F5 | #Feature F6 | #Feature F7 | #Feature F8 | #Feature F9 | Start | End | PTM | AScore | Found By |
| --- | --- | --- | --- | --- | --- | --- | --- | --- | --- | --- | --- | --- | --- | --- | --- | --- | --- | --- | --- | --- | --- | --- | --- | --- | --- | --- | --- | --- | --- | --- | --- | --- | --- | --- | --- | --- | --- | --- | --- | --- | --- | --- | --- | --- | --- |
| M.LTC(+57.02)LNC(+57.02)PEMFC(+57.02)GK.F | N | 87.07 | 1628.6819 | 13 | 0.7 | 815.3488 | 2 | 45.64 | 14 | F14:24540 | NaSa01\_F9.raw |  | 3.1696E6 | 1.6649E6 | 1.8659E6 |  |  |  |  | 2.1823E6 | 6.7455E8 | 8.6607E9 | 5.3693E8 | 5.7295E7 | 1.4835E7 | 19 | 0 | 1 | 1 | 1 | 0 | 0 | 0 | 0 | 1 | 1 | 10 | 2 | 1 | 1 | 2 | 14 | Carbamidomethylation | C3:Carbamidomethylation:1000.00;C6:Carbamidomethylation:1000.00;C11:Carbamidomethylation:1000.00 | PEAKS DB |
| M.LTC(+57.02)LNC(+57.02)PEM(+15.99)FC(+57.02)GK.F | N | 67.45 | 1644.6769 | 13 | 0.7 | 823.3463 | 2 | 32.98 | 12 | F12:17393 | NaSa01\_F7.raw |  |  |  |  |  |  |  |  | 9.1936E5 | 1.0943E8 | 7.6473E8 | 5.7873E7 |  |  | 24 | 0 | 0 | 0 | 0 | 0 | 0 | 0 | 0 | 1 | 7 | 10 | 6 | 0 | 0 | 2 | 14 | Carbamidomethylation; Oxidation (M) | C3:Carbamidomethylation:1000.00;C6:Carbamidomethylation:1000.00;M9:Oxidation (M):1000.00;C11:Carbamidomethylation:1000.00 | PEAKS DB |
| M.LTC(+57.02)LNC(+57.02)PEMFC(+57.02)GKFQIC(+57.02)R.N | N | 62.75 | 2333.0247 | 18 | 0.9 | 778.6829 | 3 | 56.34 | 11 | F11:37844 | NaSa01\_F6.raw |  |  |  |  |  |  |  |  |  | 1.2723E6 | 1.8262E7 | 9.6113E5 |  |  | 3 | 0 | 0 | 0 | 0 | 0 | 0 | 0 | 0 | 0 | 1 | 1 | 1 | 0 | 0 | 2 | 19 | Carbamidomethylation | C3:Carbamidomethylation:1000.00;C6:Carbamidomethylation:1000.00;C11:Carbamidomethylation:1000.00;C17:Carbamidomethylation:1000.00 | PEAKS DB |
| L.TC(+57.02)LNC(+57.02)PEMFC(+57.02)GK.F | N | 53.17 | 1515.5979 | 12 | -0.4 | 758.8059 | 2 | 36.00 | 9 | F9:14208 | NaSa01\_F4.raw |  |  |  |  |  |  |  |  | 2.1699E6 |  | 1.4872E6 | 1.3767E6 | 1.0968E6 |  | 4 | 0 | 0 | 0 | 0 | 0 | 0 | 0 | 0 | 1 | 0 | 1 | 1 | 1 | 0 | 3 | 14 | Carbamidomethylation | C2:Carbamidomethylation:1000.00;C5:Carbamidomethylation:1000.00;C10:Carbamidomethylation:1000.00 | PEAKS DB |
| M.LTC(+57.02)LNC(+57.02)PEMF.C | Y | 41.19 | 1283.5349 | 10 | 0.1 | 642.7748 | 2 | 69.02 | 11 | F11:47789 | NaSa01\_F6.raw |  |  |  |  |  |  |  |  |  |  | 4.8427E7 |  |  |  | 1 | 0 | 0 | 0 | 0 | 0 | 0 | 0 | 0 | 0 | 0 | 1 | 0 | 0 | 0 | 2 | 11 | Carbamidomethylation | C3:Carbamidomethylation:1000.00;C6:Carbamidomethylation:1000.00 | PEAKS DB |
| total 5 peptides |  |  |  |  |  |  |  |  |  |  |  |  |  |  |  |  |  |  |  |  |  |  |  |  |  |  |  |  |  |  |  |  |  |  |  |  |  |  |  |  |  |  |  |  |  |
| --- | --- | --- | --- | --- | --- | --- | --- | --- | --- | --- | --- | --- | --- | --- | --- | --- | --- | --- | --- | --- | --- | --- | --- | --- | --- | --- | --- | --- | --- | --- | --- | --- | --- | --- | --- | --- | --- | --- | --- | --- | --- | --- | --- | --- | --- |

C0HJW9.2

back to list

  

| Protein Coverage
| Supporting Peptides
|

Protein Coverage:

Supporting Peptides:

| Peptide | Uniq | -10lgP | Mass | Length | ppm | m/z | z | RT | Fraction | Scan | Source File | Area F1 | Area F10 | Area F11 | Area F12 | Area F13 | Area F14 | Area F2 | Area F3 | Area F4 | Area F5 | Area F6 | Area F7 | Area F8 | Area F9 | #Feature | #Feature F1 | #Feature F10 | #Feature F11 | #Feature F12 | #Feature F13 | #Feature F14 | #Feature F2 | #Feature F3 | #Feature F4 | #Feature F5 | #Feature F6 | #Feature F7 | #Feature F8 | #Feature F9 | Start | End | PTM | AScore | Found By |
| --- | --- | --- | --- | --- | --- | --- | --- | --- | --- | --- | --- | --- | --- | --- | --- | --- | --- | --- | --- | --- | --- | --- | --- | --- | --- | --- | --- | --- | --- | --- | --- | --- | --- | --- | --- | --- | --- | --- | --- | --- | --- | --- | --- | --- | --- |
| LTC(+57.02)LNC(+57.02)PEMFC(+57.02)GK.F | N | 87.07 | 1628.6819 | 13 | 0.7 | 815.3488 | 2 | 45.64 | 14 | F14:24540 | NaSa01\_F9.raw |  | 3.1696E6 | 1.6649E6 | 1.8659E6 |  |  |  |  | 2.1823E6 | 6.7455E8 | 8.6607E9 | 5.3693E8 | 5.7295E7 | 1.4835E7 | 19 | 0 | 1 | 1 | 1 | 0 | 0 | 0 | 0 | 1 | 1 | 10 | 2 | 1 | 1 | 1 | 13 | Carbamidomethylation | C3:Carbamidomethylation:1000.00;C6:Carbamidomethylation:1000.00;C11:Carbamidomethylation:1000.00 | PEAKS DB |
| LTC(+57.02)LNC(+57.02)PEM(+15.99)FC(+57.02)GK.F | N | 67.45 | 1644.6769 | 13 | 0.7 | 823.3463 | 2 | 32.98 | 12 | F12:17393 | NaSa01\_F7.raw |  |  |  |  |  |  |  |  | 9.1936E5 | 1.0943E8 | 7.6473E8 | 5.7873E7 |  |  | 24 | 0 | 0 | 0 | 0 | 0 | 0 | 0 | 0 | 1 | 7 | 10 | 6 | 0 | 0 | 1 | 13 | Carbamidomethylation; Oxidation (M) | C3:Carbamidomethylation:1000.00;C6:Carbamidomethylation:1000.00;M9:Oxidation (M):1000.00;C11:Carbamidomethylation:1000.00 | PEAKS DB |
| LTC(+57.02)LNC(+57.02)PEMFC(+57.02)GKFQIC(+57.02)R.N | N | 62.75 | 2333.0247 | 18 | 0.9 | 778.6829 | 3 | 56.34 | 11 | F11:37844 | NaSa01\_F6.raw |  |  |  |  |  |  |  |  |  | 1.2723E6 | 1.8262E7 | 9.6113E5 |  |  | 3 | 0 | 0 | 0 | 0 | 0 | 0 | 0 | 0 | 0 | 1 | 1 | 1 | 0 | 0 | 1 | 18 | Carbamidomethylation | C3:Carbamidomethylation:1000.00;C6:Carbamidomethylation:1000.00;C11:Carbamidomethylation:1000.00;C17:Carbamidomethylation:1000.00 | PEAKS DB |
| L.TC(+57.02)LNC(+57.02)PEMFC(+57.02)GK.F | N | 53.17 | 1515.5979 | 12 | -0.4 | 758.8059 | 2 | 36.00 | 9 | F9:14208 | NaSa01\_F4.raw |  |  |  |  |  |  |  |  | 2.1699E6 |  | 1.4872E6 | 1.3767E6 | 1.0968E6 |  | 4 | 0 | 0 | 0 | 0 | 0 | 0 | 0 | 0 | 1 | 0 | 1 | 1 | 1 | 0 | 2 | 13 | Carbamidomethylation | C2:Carbamidomethylation:1000.00;C5:Carbamidomethylation:1000.00;C10:Carbamidomethylation:1000.00 | PEAKS DB |
| LTC(+57.02)LNC(+57.02)PEMF.C | Y | 41.19 | 1283.5349 | 10 | 0.1 | 642.7748 | 2 | 69.02 | 11 | F11:47789 | NaSa01\_F6.raw |  |  |  |  |  |  |  |  |  |  | 4.8427E7 |  |  |  | 1 | 0 | 0 | 0 | 0 | 0 | 0 | 0 | 0 | 0 | 0 | 1 | 0 | 0 | 0 | 1 | 10 | Carbamidomethylation | C3:Carbamidomethylation:1000.00;C6:Carbamidomethylation:1000.00 | PEAKS DB |
| total 5 peptides |  |  |  |  |  |  |  |  |  |  |  |  |  |  |  |  |  |  |  |  |  |  |  |  |  |  |  |  |  |  |  |  |  |  |  |  |  |  |  |  |  |  |  |  |  |
| --- | --- | --- | --- | --- | --- | --- | --- | --- | --- | --- | --- | --- | --- | --- | --- | --- | --- | --- | --- | --- | --- | --- | --- | --- | --- | --- | --- | --- | --- | --- | --- | --- | --- | --- | --- | --- | --- | --- | --- | --- | --- | --- | --- | --- | --- |

XP\_015680068.1

back to list

  

| Protein Coverage
| Supporting Peptides
|

Protein Coverage:

Supporting Peptides:

| Peptide | Uniq | -10lgP | Mass | Length | ppm | m/z | z | RT | Fraction | Scan | Source File | Area F1 | Area F10 | Area F11 | Area F12 | Area F13 | Area F14 | Area F2 | Area F3 | Area F4 | Area F5 | Area F6 | Area F7 | Area F8 | Area F9 | #Feature | #Feature F1 | #Feature F10 | #Feature F11 | #Feature F12 | #Feature F13 | #Feature F14 | #Feature F2 | #Feature F3 | #Feature F4 | #Feature F5 | #Feature F6 | #Feature F7 | #Feature F8 | #Feature F9 | Start | End | PTM | AScore | Found By |
| --- | --- | --- | --- | --- | --- | --- | --- | --- | --- | --- | --- | --- | --- | --- | --- | --- | --- | --- | --- | --- | --- | --- | --- | --- | --- | --- | --- | --- | --- | --- | --- | --- | --- | --- | --- | --- | --- | --- | --- | --- | --- | --- | --- | --- | --- |
| K.VEQLSNMIVK.S | Y | 66.68 | 1159.6271 | 10 | 0.6 | 580.8212 | 2 | 37.54 | 13 | F13:24751 | NaSa01\_F8.raw |  | 2.7926E6 |  |  |  |  |  |  |  |  |  |  | 1.2889E7 | 2.2176E6 | 3 | 0 | 1 | 0 | 0 | 0 | 0 | 0 | 0 | 0 | 0 | 0 | 0 | 1 | 1 | 399 | 408 |  |  | PEAKS DB |
| L.YNLHNPSASAAPC(+57.02)C(+57.02)VPDELEPLGIIYYVGR.Q | Y | 56.95 | 3374.6062 | 30 | 0.4 | 1125.8765 | 3 | 86.73 | 14 | F14:57917 | NaSa01\_F9.raw |  |  |  |  |  |  |  |  |  |  |  |  |  | 1.0113E7 | 1 | 0 | 0 | 0 | 0 | 0 | 0 | 0 | 0 | 0 | 0 | 0 | 0 | 0 | 1 | 366 | 395 | Carbamidomethylation | C13:Carbamidomethylation:1000.00;C14:Carbamidomethylation:1000.00 | PEAKS DB |
| C.C(+57.02)VPDELEPLGIIYYVGR.Q | Y | 48.76 | 1992.0026 | 17 | 1.5 | 997.0100 | 2 | 97.12 | 14 | F14:66923 | NaSa01\_F9.raw |  |  |  |  |  |  |  |  |  |  |  |  |  | 1.4968E6 | 2 | 0 | 0 | 0 | 0 | 0 | 0 | 0 | 0 | 0 | 0 | 0 | 0 | 0 | 2 | 379 | 395 | Carbamidomethylation | C1:Carbamidomethylation:1000.00 | PEAKS DB |
| L.HNPSASAAPC(+57.02)C(+57.02)VPDELEPLGIIYYVGR.Q | Y | 45.59 | 2984.4160 | 27 | -0.5 | 995.8121 | 3 | 84.11 | 13 | F13:64277 | NaSa01\_F8.raw |  |  |  |  |  |  |  |  |  |  |  |  | 2.213E6 |  | 1 | 0 | 0 | 0 | 0 | 0 | 0 | 0 | 0 | 0 | 0 | 0 | 0 | 1 | 0 | 369 | 395 | Carbamidomethylation | C10:Carbamidomethylation:1000.00;C11:Carbamidomethylation:1000.00 | PEAKS DB |
| total 4 peptides |  |  |  |  |  |  |  |  |  |  |  |  |  |  |  |  |  |  |  |  |  |  |  |  |  |  |  |  |  |  |  |  |  |  |  |  |  |  |  |  |  |  |  |  |  |
| --- | --- | --- | --- | --- | --- | --- | --- | --- | --- | --- | --- | --- | --- | --- | --- | --- | --- | --- | --- | --- | --- | --- | --- | --- | --- | --- | --- | --- | --- | --- | --- | --- | --- | --- | --- | --- | --- | --- | --- | --- | --- | --- | --- | --- | --- |

XP\_026544123.1

back to list

  

| Protein Coverage
| Supporting Peptides
|

Protein Coverage:

Supporting Peptides:

| Peptide | Uniq | -10lgP | Mass | Length | ppm | m/z | z | RT | Fraction | Scan | Source File | Area F1 | Area F10 | Area F11 | Area F12 | Area F13 | Area F14 | Area F2 | Area F3 | Area F4 | Area F5 | Area F6 | Area F7 | Area F8 | Area F9 | #Feature | #Feature F1 | #Feature F10 | #Feature F11 | #Feature F12 | #Feature F13 | #Feature F14 | #Feature F2 | #Feature F3 | #Feature F4 | #Feature F5 | #Feature F6 | #Feature F7 | #Feature F8 | #Feature F9 | Start | End | PTM | AScore | Found By |
| --- | --- | --- | --- | --- | --- | --- | --- | --- | --- | --- | --- | --- | --- | --- | --- | --- | --- | --- | --- | --- | --- | --- | --- | --- | --- | --- | --- | --- | --- | --- | --- | --- | --- | --- | --- | --- | --- | --- | --- | --- | --- | --- | --- | --- | --- |
| K.VEQLSNMIVK.S | Y | 66.68 | 1159.6271 | 10 | 0.6 | 580.8212 | 2 | 37.54 | 13 | F13:24751 | NaSa01\_F8.raw |  | 2.7926E6 |  |  |  |  |  |  |  |  |  |  | 1.2889E7 | 2.2176E6 | 3 | 0 | 1 | 0 | 0 | 0 | 0 | 0 | 0 | 0 | 0 | 0 | 0 | 1 | 1 | 401 | 410 |  |  | PEAKS DB |
| L.YNLHNPSASAAPC(+57.02)C(+57.02)VPDELEPLGIIYYVGR.Q | Y | 56.95 | 3374.6062 | 30 | 0.4 | 1125.8765 | 3 | 86.73 | 14 | F14:57917 | NaSa01\_F9.raw |  |  |  |  |  |  |  |  |  |  |  |  |  | 1.0113E7 | 1 | 0 | 0 | 0 | 0 | 0 | 0 | 0 | 0 | 0 | 0 | 0 | 0 | 0 | 1 | 368 | 397 | Carbamidomethylation | C13:Carbamidomethylation:1000.00;C14:Carbamidomethylation:1000.00 | PEAKS DB |
| C.C(+57.02)VPDELEPLGIIYYVGR.Q | Y | 48.76 | 1992.0026 | 17 | 1.5 | 997.0100 | 2 | 97.12 | 14 | F14:66923 | NaSa01\_F9.raw |  |  |  |  |  |  |  |  |  |  |  |  |  | 1.4968E6 | 2 | 0 | 0 | 0 | 0 | 0 | 0 | 0 | 0 | 0 | 0 | 0 | 0 | 0 | 2 | 381 | 397 | Carbamidomethylation | C1:Carbamidomethylation:1000.00 | PEAKS DB |
| L.HNPSASAAPC(+57.02)C(+57.02)VPDELEPLGIIYYVGR.Q | Y | 45.59 | 2984.4160 | 27 | -0.5 | 995.8121 | 3 | 84.11 | 13 | F13:64277 | NaSa01\_F8.raw |  |  |  |  |  |  |  |  |  |  |  |  | 2.213E6 |  | 1 | 0 | 0 | 0 | 0 | 0 | 0 | 0 | 0 | 0 | 0 | 0 | 0 | 1 | 0 | 371 | 397 | Carbamidomethylation | C10:Carbamidomethylation:1000.00;C11:Carbamidomethylation:1000.00 | PEAKS DB |
| total 4 peptides |  |  |  |  |  |  |  |  |  |  |  |  |  |  |  |  |  |  |  |  |  |  |  |  |  |  |  |  |  |  |  |  |  |  |  |  |  |  |  |  |  |  |  |  |  |
| --- | --- | --- | --- | --- | --- | --- | --- | --- | --- | --- | --- | --- | --- | --- | --- | --- | --- | --- | --- | --- | --- | --- | --- | --- | --- | --- | --- | --- | --- | --- | --- | --- | --- | --- | --- | --- | --- | --- | --- | --- | --- | --- | --- | --- | --- |

G8XQX1.1

back to list

  

| Protein Coverage
| Supporting Peptides
|

Protein Coverage:

Supporting Peptides:

| Peptide | Uniq | -10lgP | Mass | Length | ppm | m/z | z | RT | Fraction | Scan | Source File | Area F1 | Area F10 | Area F11 | Area F12 | Area F13 | Area F14 | Area F2 | Area F3 | Area F4 | Area F5 | Area F6 | Area F7 | Area F8 | Area F9 | #Feature | #Feature F1 | #Feature F10 | #Feature F11 | #Feature F12 | #Feature F13 | #Feature F14 | #Feature F2 | #Feature F3 | #Feature F4 | #Feature F5 | #Feature F6 | #Feature F7 | #Feature F8 | #Feature F9 | Start | End | PTM | AScore | Found By |
| --- | --- | --- | --- | --- | --- | --- | --- | --- | --- | --- | --- | --- | --- | --- | --- | --- | --- | --- | --- | --- | --- | --- | --- | --- | --- | --- | --- | --- | --- | --- | --- | --- | --- | --- | --- | --- | --- | --- | --- | --- | --- | --- | --- | --- | --- |
| L.SAAYVLAGAGHK.V | N | 63.01 | 1143.6036 | 12 | -1.1 | 572.8085 | 2 | 11.49 | 6 | F6:1894 | NaSa01\_F14.raw |  |  |  |  |  | 4.4228E6 |  |  |  |  |  |  |  |  | 2 | 0 | 0 | 0 | 0 | 0 | 2 | 0 | 0 | 0 | 0 | 0 | 0 | 0 | 0 | 65 | 76 |  |  | PEAKS DB |
| S.GLSAAYVLAGAGHK.V | N | 58.41 | 1313.7091 | 14 | 0.3 | 438.9104 | 3 | 32.92 | 6 | F6:14801 | NaSa01\_F14.raw |  |  |  |  |  | 1.2419E6 |  |  |  |  |  |  |  |  | 2 | 0 | 0 | 0 | 0 | 0 | 2 | 0 | 0 | 0 | 0 | 0 | 0 | 0 | 0 | 63 | 76 |  |  | PEAKS DB |
| R.ITFKPPLPPK.K | Y | 54.32 | 1136.6957 | 10 | 0.1 | 379.9059 | 3 | 33.54 | 6 | F6:15341 | NaSa01\_F14.raw |  |  |  |  |  | 1.2519E6 |  |  |  |  |  |  |  |  | 1 | 0 | 0 | 0 | 0 | 0 | 1 | 0 | 0 | 0 | 0 | 0 | 0 | 0 | 0 | 320 | 329 |  |  | PEAKS DB |
| K.STTDLPSR.F | N | 48.78 | 875.4348 | 8 | 0.1 | 438.7247 | 2 | 11.44 | 6 | F6:1817 | NaSa01\_F14.raw |  |  |  |  |  | 1.0568E6 |  |  |  |  |  |  |  |  | 1 | 0 | 0 | 0 | 0 | 0 | 1 | 0 | 0 | 0 | 0 | 0 | 0 | 0 | 0 | 364 | 371 |  |  | PEAKS DB |
| R.FDEIVGGM(+15.99)DQLPTSMYR.A | N | 44.47 | 1973.8862 | 17 | 1.5 | 987.9519 | 2 | 63.33 | 6 | F6:40829 | NaSa01\_F14.raw |  |  |  |  |  | 4.221E4 |  |  |  |  |  |  |  |  | 1 | 0 | 0 | 0 | 0 | 0 | 1 | 0 | 0 | 0 | 0 | 0 | 0 | 0 | 0 | 251 | 267 | Oxidation (M) | M8:Oxidation (M):44.44 | PEAKS DB |
| R.FDEIVGGMDQLPTSMYR.A | N | 42.71 | 1957.8914 | 17 | 1.2 | 979.9541 | 2 | 75.35 | 6 | F6:50859 | NaSa01\_F14.raw |  |  |  |  |  | 6.6822E5 |  |  |  |  |  |  |  |  | 1 | 0 | 0 | 0 | 0 | 0 | 1 | 0 | 0 | 0 | 0 | 0 | 0 | 0 | 0 | 251 | 267 |  |  | PEAKS DB |
| K.HIVIVGAGM(+15.99)SGLSAAYVLAGAGHK.V | Y | 42.54 | 2294.2205 | 24 | 0.6 | 574.5627 | 4 | 56.32 | 6 | F6:34802 | NaSa01\_F14.raw |  |  |  |  |  | 1.6712E5 |  |  |  |  |  |  |  |  | 1 | 0 | 0 | 0 | 0 | 0 | 1 | 0 | 0 | 0 | 0 | 0 | 0 | 0 | 0 | 53 | 76 | Oxidation (M) | M9:Oxidation (M):1000.00 | PEAKS DB |
| total 7 peptides |  |  |  |  |  |  |  |  |  |  |  |  |  |  |  |  |  |  |  |  |  |  |  |  |  |  |  |  |  |  |  |  |  |  |  |  |  |  |  |  |  |  |  |  |  |
| --- | --- | --- | --- | --- | --- | --- | --- | --- | --- | --- | --- | --- | --- | --- | --- | --- | --- | --- | --- | --- | --- | --- | --- | --- | --- | --- | --- | --- | --- | --- | --- | --- | --- | --- | --- | --- | --- | --- | --- | --- | --- | --- | --- | --- | --- |

ACF70483.1

back to list

  

| Protein Coverage
| Supporting Peptides
|

Protein Coverage:

Supporting Peptides:

| Peptide | Uniq | -10lgP | Mass | Length | ppm | m/z | z | RT | Fraction | Scan | Source File | Area F1 | Area F10 | Area F11 | Area F12 | Area F13 | Area F14 | Area F2 | Area F3 | Area F4 | Area F5 | Area F6 | Area F7 | Area F8 | Area F9 | #Feature | #Feature F1 | #Feature F10 | #Feature F11 | #Feature F12 | #Feature F13 | #Feature F14 | #Feature F2 | #Feature F3 | #Feature F4 | #Feature F5 | #Feature F6 | #Feature F7 | #Feature F8 | #Feature F9 | Start | End | PTM | AScore | Found By |
| --- | --- | --- | --- | --- | --- | --- | --- | --- | --- | --- | --- | --- | --- | --- | --- | --- | --- | --- | --- | --- | --- | --- | --- | --- | --- | --- | --- | --- | --- | --- | --- | --- | --- | --- | --- | --- | --- | --- | --- | --- | --- | --- | --- | --- | --- |
| L.SAAYVLAGAGHK.V | N | 63.01 | 1143.6036 | 12 | -1.1 | 572.8085 | 2 | 11.49 | 6 | F6:1894 | NaSa01\_F14.raw |  |  |  |  |  | 4.4228E6 |  |  |  |  |  |  |  |  | 2 | 0 | 0 | 0 | 0 | 0 | 2 | 0 | 0 | 0 | 0 | 0 | 0 | 0 | 0 | 65 | 76 |  |  | PEAKS DB |
| S.GLSAAYVLAGAGHK.V | N | 58.41 | 1313.7091 | 14 | 0.3 | 438.9104 | 3 | 32.92 | 6 | F6:14801 | NaSa01\_F14.raw |  |  |  |  |  | 1.2419E6 |  |  |  |  |  |  |  |  | 2 | 0 | 0 | 0 | 0 | 0 | 2 | 0 | 0 | 0 | 0 | 0 | 0 | 0 | 0 | 63 | 76 |  |  | PEAKS DB |
| R.ITFKPPLPPK.K | Y | 54.32 | 1136.6957 | 10 | 0.1 | 379.9059 | 3 | 33.54 | 6 | F6:15341 | NaSa01\_F14.raw |  |  |  |  |  | 1.2519E6 |  |  |  |  |  |  |  |  | 1 | 0 | 0 | 0 | 0 | 0 | 1 | 0 | 0 | 0 | 0 | 0 | 0 | 0 | 0 | 320 | 329 |  |  | PEAKS DB |
| K.STTDLPSR.F | N | 48.78 | 875.4348 | 8 | 0.1 | 438.7247 | 2 | 11.44 | 6 | F6:1817 | NaSa01\_F14.raw |  |  |  |  |  | 1.0568E6 |  |  |  |  |  |  |  |  | 1 | 0 | 0 | 0 | 0 | 0 | 1 | 0 | 0 | 0 | 0 | 0 | 0 | 0 | 0 | 364 | 371 |  |  | PEAKS DB |
| R.FDEIVGGM(+15.99)DQLPTSMYR.A | N | 44.47 | 1973.8862 | 17 | 1.5 | 987.9519 | 2 | 63.33 | 6 | F6:40829 | NaSa01\_F14.raw |  |  |  |  |  | 4.221E4 |  |  |  |  |  |  |  |  | 1 | 0 | 0 | 0 | 0 | 0 | 1 | 0 | 0 | 0 | 0 | 0 | 0 | 0 | 0 | 251 | 267 | Oxidation (M) | M8:Oxidation (M):44.44 | PEAKS DB |
| R.FDEIVGGMDQLPTSMYR.A | N | 42.71 | 1957.8914 | 17 | 1.2 | 979.9541 | 2 | 75.35 | 6 | F6:50859 | NaSa01\_F14.raw |  |  |  |  |  | 6.6822E5 |  |  |  |  |  |  |  |  | 1 | 0 | 0 | 0 | 0 | 0 | 1 | 0 | 0 | 0 | 0 | 0 | 0 | 0 | 0 | 251 | 267 |  |  | PEAKS DB |
| K.HIVIVGAGM(+15.99)SGLSAAYVLAGAGHK.V | Y | 42.54 | 2294.2205 | 24 | 0.6 | 574.5627 | 4 | 56.32 | 6 | F6:34802 | NaSa01\_F14.raw |  |  |  |  |  | 1.6712E5 |  |  |  |  |  |  |  |  | 1 | 0 | 0 | 0 | 0 | 0 | 1 | 0 | 0 | 0 | 0 | 0 | 0 | 0 | 0 | 53 | 76 | Oxidation (M) | M9:Oxidation (M):1000.00 | PEAKS DB |
| total 7 peptides |  |  |  |  |  |  |  |  |  |  |  |  |  |  |  |  |  |  |  |  |  |  |  |  |  |  |  |  |  |  |  |  |  |  |  |  |  |  |  |  |  |  |  |  |  |
| --- | --- | --- | --- | --- | --- | --- | --- | --- | --- | --- | --- | --- | --- | --- | --- | --- | --- | --- | --- | --- | --- | --- | --- | --- | --- | --- | --- | --- | --- | --- | --- | --- | --- | --- | --- | --- | --- | --- | --- | --- | --- | --- | --- | --- | --- |

P60623.1

back to list

  

| Protein Coverage
| Supporting Peptides
|

Protein Coverage:

Supporting Peptides:

| Peptide | Uniq | -10lgP | Mass | Length | ppm | m/z | z | RT | Fraction | Scan | Source File | Area F1 | Area F10 | Area F11 | Area F12 | Area F13 | Area F14 | Area F2 | Area F3 | Area F4 | Area F5 | Area F6 | Area F7 | Area F8 | Area F9 | #Feature | #Feature F1 | #Feature F10 | #Feature F11 | #Feature F12 | #Feature F13 | #Feature F14 | #Feature F2 | #Feature F3 | #Feature F4 | #Feature F5 | #Feature F6 | #Feature F7 | #Feature F8 | #Feature F9 | Start | End | PTM | AScore | Found By |
| --- | --- | --- | --- | --- | --- | --- | --- | --- | --- | --- | --- | --- | --- | --- | --- | --- | --- | --- | --- | --- | --- | --- | --- | --- | --- | --- | --- | --- | --- | --- | --- | --- | --- | --- | --- | --- | --- | --- | --- | --- | --- | --- | --- | --- | --- |
| N.EIVDLHNSLR.R | N | 71.95 | 1194.6356 | 10 | 0.4 | 598.3253 | 2 | 28.76 | 4 | F4:13650 | NaSa01\_F12.raw |  |  |  | 7.7068E9 | 7.5889E7 | 5.7777E4 |  |  |  |  |  |  |  |  | 27 | 0 | 0 | 0 | 23 | 3 | 1 | 0 | 0 | 0 | 0 | 0 | 0 | 0 | 0 | 29 | 38 |  |  | PEAKS DB |
| E.IVDLHNSLR.R | N | 58.01 | 1065.5930 | 9 | -1.6 | 533.8029 | 2 | 26.50 | 4 | F4:11729 | NaSa01\_F12.raw |  |  |  | 1.3096E7 |  |  |  |  |  |  |  |  |  |  | 1 | 0 | 0 | 0 | 1 | 0 | 0 | 0 | 0 | 0 | 0 | 0 | 0 | 0 | 0 | 30 | 38 |  |  | PEAKS DB |
| R.MEWYPEAADNAER.W | Y | 54.86 | 1580.6565 | 13 | 0.2 | 791.3357 | 2 | 46.81 | 4 | F4:28766 | NaSa01\_F12.raw |  |  |  | 4.9771E6 |  |  |  |  |  |  |  |  |  |  | 1 | 0 | 0 | 0 | 1 | 0 | 0 | 0 | 0 | 0 | 0 | 0 | 0 | 0 | 0 | 51 | 63 |  |  | PEAKS DB |
| I.VDLHNSLR.R | N | 42.85 | 952.5090 | 8 | -1.0 | 477.2613 | 2 | 26.51 | 4 | F4:11862 | NaSa01\_F12.raw |  |  |  | 2.8116E8 |  |  |  |  |  |  |  |  |  |  | 1 | 0 | 0 | 0 | 1 | 0 | 0 | 0 | 0 | 0 | 0 | 0 | 0 | 0 | 0 | 31 | 38 |  |  | PEAKS DB |
| total 4 peptides |  |  |  |  |  |  |  |  |  |  |  |  |  |  |  |  |  |  |  |  |  |  |  |  |  |  |  |  |  |  |  |  |  |  |  |  |  |  |  |  |  |  |  |  |  |
| --- | --- | --- | --- | --- | --- | --- | --- | --- | --- | --- | --- | --- | --- | --- | --- | --- | --- | --- | --- | --- | --- | --- | --- | --- | --- | --- | --- | --- | --- | --- | --- | --- | --- | --- | --- | --- | --- | --- | --- | --- | --- | --- | --- | --- | --- |

AAQ98964.1

back to list

  

| Protein Coverage
| Supporting Peptides
|

Protein Coverage:

Supporting Peptides:

| Peptide | Uniq | -10lgP | Mass | Length | ppm | m/z | z | RT | Fraction | Scan | Source File | Area F1 | Area F10 | Area F11 | Area F12 | Area F13 | Area F14 | Area F2 | Area F3 | Area F4 | Area F5 | Area F6 | Area F7 | Area F8 | Area F9 | #Feature | #Feature F1 | #Feature F10 | #Feature F11 | #Feature F12 | #Feature F13 | #Feature F14 | #Feature F2 | #Feature F3 | #Feature F4 | #Feature F5 | #Feature F6 | #Feature F7 | #Feature F8 | #Feature F9 | Start | End | PTM | AScore | Found By |
| --- | --- | --- | --- | --- | --- | --- | --- | --- | --- | --- | --- | --- | --- | --- | --- | --- | --- | --- | --- | --- | --- | --- | --- | --- | --- | --- | --- | --- | --- | --- | --- | --- | --- | --- | --- | --- | --- | --- | --- | --- | --- | --- | --- | --- | --- |
| N.EIVDLHNSLR.R | N | 71.95 | 1194.6356 | 10 | 0.4 | 598.3253 | 2 | 28.76 | 4 | F4:13650 | NaSa01\_F12.raw |  |  |  | 7.7068E9 | 7.5889E7 | 5.7777E4 |  |  |  |  |  |  |  |  | 27 | 0 | 0 | 0 | 23 | 3 | 1 | 0 | 0 | 0 | 0 | 0 | 0 | 0 | 0 | 29 | 38 |  |  | PEAKS DB |
| E.IVDLHNSLR.R | N | 58.01 | 1065.5930 | 9 | -1.6 | 533.8029 | 2 | 26.50 | 4 | F4:11729 | NaSa01\_F12.raw |  |  |  | 1.3096E7 |  |  |  |  |  |  |  |  |  |  | 1 | 0 | 0 | 0 | 1 | 0 | 0 | 0 | 0 | 0 | 0 | 0 | 0 | 0 | 0 | 30 | 38 |  |  | PEAKS DB |
| R.MEWYPEAADNAER.W | Y | 54.86 | 1580.6565 | 13 | 0.2 | 791.3357 | 2 | 46.81 | 4 | F4:28766 | NaSa01\_F12.raw |  |  |  | 4.9771E6 |  |  |  |  |  |  |  |  |  |  | 1 | 0 | 0 | 0 | 1 | 0 | 0 | 0 | 0 | 0 | 0 | 0 | 0 | 0 | 0 | 51 | 63 |  |  | PEAKS DB |
| I.VDLHNSLR.R | N | 42.85 | 952.5090 | 8 | -1.0 | 477.2613 | 2 | 26.51 | 4 | F4:11862 | NaSa01\_F12.raw |  |  |  | 2.8116E8 |  |  |  |  |  |  |  |  |  |  | 1 | 0 | 0 | 0 | 1 | 0 | 0 | 0 | 0 | 0 | 0 | 0 | 0 | 0 | 0 | 31 | 38 |  |  | PEAKS DB |
| total 4 peptides |  |  |  |  |  |  |  |  |  |  |  |  |  |  |  |  |  |  |  |  |  |  |  |  |  |  |  |  |  |  |  |  |  |  |  |  |  |  |  |  |  |  |  |  |  |
| --- | --- | --- | --- | --- | --- | --- | --- | --- | --- | --- | --- | --- | --- | --- | --- | --- | --- | --- | --- | --- | --- | --- | --- | --- | --- | --- | --- | --- | --- | --- | --- | --- | --- | --- | --- | --- | --- | --- | --- | --- | --- | --- | --- | --- | --- |

ACE73574.1

back to list

  

| Protein Coverage
| Supporting Peptides
|

Protein Coverage:

Supporting Peptides:

| Peptide | Uniq | -10lgP | Mass | Length | ppm | m/z | z | RT | Fraction | Scan | Source File | Area F1 | Area F10 | Area F11 | Area F12 | Area F13 | Area F14 | Area F2 | Area F3 | Area F4 | Area F5 | Area F6 | Area F7 | Area F8 | Area F9 | #Feature | #Feature F1 | #Feature F10 | #Feature F11 | #Feature F12 | #Feature F13 | #Feature F14 | #Feature F2 | #Feature F3 | #Feature F4 | #Feature F5 | #Feature F6 | #Feature F7 | #Feature F8 | #Feature F9 | Start | End | PTM | AScore | Found By |
| --- | --- | --- | --- | --- | --- | --- | --- | --- | --- | --- | --- | --- | --- | --- | --- | --- | --- | --- | --- | --- | --- | --- | --- | --- | --- | --- | --- | --- | --- | --- | --- | --- | --- | --- | --- | --- | --- | --- | --- | --- | --- | --- | --- | --- | --- |
| N.EIVDLHNSLR.R | N | 71.95 | 1194.6356 | 10 | 0.4 | 598.3253 | 2 | 28.76 | 4 | F4:13650 | NaSa01\_F12.raw |  |  |  | 7.7068E9 | 7.5889E7 | 5.7777E4 |  |  |  |  |  |  |  |  | 27 | 0 | 0 | 0 | 23 | 3 | 1 | 0 | 0 | 0 | 0 | 0 | 0 | 0 | 0 | 36 | 45 |  |  | PEAKS DB |
| E.IVDLHNSLR.R | N | 58.01 | 1065.5930 | 9 | -1.6 | 533.8029 | 2 | 26.50 | 4 | F4:11729 | NaSa01\_F12.raw |  |  |  | 1.3096E7 |  |  |  |  |  |  |  |  |  |  | 1 | 0 | 0 | 0 | 1 | 0 | 0 | 0 | 0 | 0 | 0 | 0 | 0 | 0 | 0 | 37 | 45 |  |  | PEAKS DB |
| R.MEWYPEAADNAER.W | Y | 54.86 | 1580.6565 | 13 | 0.2 | 791.3357 | 2 | 46.81 | 4 | F4:28766 | NaSa01\_F12.raw |  |  |  | 4.9771E6 |  |  |  |  |  |  |  |  |  |  | 1 | 0 | 0 | 0 | 1 | 0 | 0 | 0 | 0 | 0 | 0 | 0 | 0 | 0 | 0 | 58 | 70 |  |  | PEAKS DB |
| I.VDLHNSLR.R | N | 42.85 | 952.5090 | 8 | -1.0 | 477.2613 | 2 | 26.51 | 4 | F4:11862 | NaSa01\_F12.raw |  |  |  | 2.8116E8 |  |  |  |  |  |  |  |  |  |  | 1 | 0 | 0 | 0 | 1 | 0 | 0 | 0 | 0 | 0 | 0 | 0 | 0 | 0 | 0 | 38 | 45 |  |  | PEAKS DB |
| total 4 peptides |  |  |  |  |  |  |  |  |  |  |  |  |  |  |  |  |  |  |  |  |  |  |  |  |  |  |  |  |  |  |  |  |  |  |  |  |  |  |  |  |  |  |  |  |  |
| --- | --- | --- | --- | --- | --- | --- | --- | --- | --- | --- | --- | --- | --- | --- | --- | --- | --- | --- | --- | --- | --- | --- | --- | --- | --- | --- | --- | --- | --- | --- | --- | --- | --- | --- | --- | --- | --- | --- | --- | --- | --- | --- | --- | --- | --- |

ACE73572.1

back to list

  

| Protein Coverage
| Supporting Peptides
|

Protein Coverage:

Supporting Peptides:

| Peptide | Uniq | -10lgP | Mass | Length | ppm | m/z | z | RT | Fraction | Scan | Source File | Area F1 | Area F10 | Area F11 | Area F12 | Area F13 | Area F14 | Area F2 | Area F3 | Area F4 | Area F5 | Area F6 | Area F7 | Area F8 | Area F9 | #Feature | #Feature F1 | #Feature F10 | #Feature F11 | #Feature F12 | #Feature F13 | #Feature F14 | #Feature F2 | #Feature F3 | #Feature F4 | #Feature F5 | #Feature F6 | #Feature F7 | #Feature F8 | #Feature F9 | Start | End | PTM | AScore | Found By |
| --- | --- | --- | --- | --- | --- | --- | --- | --- | --- | --- | --- | --- | --- | --- | --- | --- | --- | --- | --- | --- | --- | --- | --- | --- | --- | --- | --- | --- | --- | --- | --- | --- | --- | --- | --- | --- | --- | --- | --- | --- | --- | --- | --- | --- | --- |
| N.EIVDLHNSLR.R | N | 71.95 | 1194.6356 | 10 | 0.4 | 598.3253 | 2 | 28.76 | 4 | F4:13650 | NaSa01\_F12.raw |  |  |  | 7.7068E9 | 7.5889E7 | 5.7777E4 |  |  |  |  |  |  |  |  | 27 | 0 | 0 | 0 | 23 | 3 | 1 | 0 | 0 | 0 | 0 | 0 | 0 | 0 | 0 | 36 | 45 |  |  | PEAKS DB |
| E.IVDLHNSLR.R | N | 58.01 | 1065.5930 | 9 | -1.6 | 533.8029 | 2 | 26.50 | 4 | F4:11729 | NaSa01\_F12.raw |  |  |  | 1.3096E7 |  |  |  |  |  |  |  |  |  |  | 1 | 0 | 0 | 0 | 1 | 0 | 0 | 0 | 0 | 0 | 0 | 0 | 0 | 0 | 0 | 37 | 45 |  |  | PEAKS DB |
| R.MEWYPEAADNAER.W | Y | 54.86 | 1580.6565 | 13 | 0.2 | 791.3357 | 2 | 46.81 | 4 | F4:28766 | NaSa01\_F12.raw |  |  |  | 4.9771E6 |  |  |  |  |  |  |  |  |  |  | 1 | 0 | 0 | 0 | 1 | 0 | 0 | 0 | 0 | 0 | 0 | 0 | 0 | 0 | 0 | 58 | 70 |  |  | PEAKS DB |
| I.VDLHNSLR.R | N | 42.85 | 952.5090 | 8 | -1.0 | 477.2613 | 2 | 26.51 | 4 | F4:11862 | NaSa01\_F12.raw |  |  |  | 2.8116E8 |  |  |  |  |  |  |  |  |  |  | 1 | 0 | 0 | 0 | 1 | 0 | 0 | 0 | 0 | 0 | 0 | 0 | 0 | 0 | 0 | 38 | 45 |  |  | PEAKS DB |
| total 4 peptides |  |  |  |  |  |  |  |  |  |  |  |  |  |  |  |  |  |  |  |  |  |  |  |  |  |  |  |  |  |  |  |  |  |  |  |  |  |  |  |  |  |  |  |  |  |
| --- | --- | --- | --- | --- | --- | --- | --- | --- | --- | --- | --- | --- | --- | --- | --- | --- | --- | --- | --- | --- | --- | --- | --- | --- | --- | --- | --- | --- | --- | --- | --- | --- | --- | --- | --- | --- | --- | --- | --- | --- | --- | --- | --- | --- | --- |

1RC9

back to list

  

| Protein Coverage
| Supporting Peptides
|

Protein Coverage:

Supporting Peptides:

| Peptide | Uniq | -10lgP | Mass | Length | ppm | m/z | z | RT | Fraction | Scan | Source File | Area F1 | Area F10 | Area F11 | Area F12 | Area F13 | Area F14 | Area F2 | Area F3 | Area F4 | Area F5 | Area F6 | Area F7 | Area F8 | Area F9 | #Feature | #Feature F1 | #Feature F10 | #Feature F11 | #Feature F12 | #Feature F13 | #Feature F14 | #Feature F2 | #Feature F3 | #Feature F4 | #Feature F5 | #Feature F6 | #Feature F7 | #Feature F8 | #Feature F9 | Start | End | PTM | AScore | Found By |
| --- | --- | --- | --- | --- | --- | --- | --- | --- | --- | --- | --- | --- | --- | --- | --- | --- | --- | --- | --- | --- | --- | --- | --- | --- | --- | --- | --- | --- | --- | --- | --- | --- | --- | --- | --- | --- | --- | --- | --- | --- | --- | --- | --- | --- | --- |
| N.EIVDLHNSLR.R | N | 71.95 | 1194.6356 | 10 | 0.4 | 598.3253 | 2 | 28.76 | 4 | F4:13650 | NaSa01\_F12.raw |  |  |  | 7.7068E9 | 7.5889E7 | 5.7777E4 |  |  |  |  |  |  |  |  | 27 | 0 | 0 | 0 | 23 | 3 | 1 | 0 | 0 | 0 | 0 | 0 | 0 | 0 | 0 | 17 | 26 |  |  | PEAKS DB |
| E.IVDLHNSLR.R | N | 58.01 | 1065.5930 | 9 | -1.6 | 533.8029 | 2 | 26.50 | 4 | F4:11729 | NaSa01\_F12.raw |  |  |  | 1.3096E7 |  |  |  |  |  |  |  |  |  |  | 1 | 0 | 0 | 0 | 1 | 0 | 0 | 0 | 0 | 0 | 0 | 0 | 0 | 0 | 0 | 18 | 26 |  |  | PEAKS DB |
| R.MEWYPEAADNAER.W | Y | 54.86 | 1580.6565 | 13 | 0.2 | 791.3357 | 2 | 46.81 | 4 | F4:28766 | NaSa01\_F12.raw |  |  |  | 4.9771E6 |  |  |  |  |  |  |  |  |  |  | 1 | 0 | 0 | 0 | 1 | 0 | 0 | 0 | 0 | 0 | 0 | 0 | 0 | 0 | 0 | 39 | 51 |  |  | PEAKS DB |
| I.VDLHNSLR.R | N | 42.85 | 952.5090 | 8 | -1.0 | 477.2613 | 2 | 26.51 | 4 | F4:11862 | NaSa01\_F12.raw |  |  |  | 2.8116E8 |  |  |  |  |  |  |  |  |  |  | 1 | 0 | 0 | 0 | 1 | 0 | 0 | 0 | 0 | 0 | 0 | 0 | 0 | 0 | 0 | 19 | 26 |  |  | PEAKS DB |
| total 4 peptides |  |  |  |  |  |  |  |  |  |  |  |  |  |  |  |  |  |  |  |  |  |  |  |  |  |  |  |  |  |  |  |  |  |  |  |  |  |  |  |  |  |  |  |  |  |
| --- | --- | --- | --- | --- | --- | --- | --- | --- | --- | --- | --- | --- | --- | --- | --- | --- | --- | --- | --- | --- | --- | --- | --- | --- | --- | --- | --- | --- | --- | --- | --- | --- | --- | --- | --- | --- | --- | --- | --- | --- | --- | --- | --- | --- | --- |

ACE73573.1

back to list

  

| Protein Coverage
| Supporting Peptides
|

Protein Coverage:

Supporting Peptides:

| Peptide | Uniq | -10lgP | Mass | Length | ppm | m/z | z | RT | Fraction | Scan | Source File | Area F1 | Area F10 | Area F11 | Area F12 | Area F13 | Area F14 | Area F2 | Area F3 | Area F4 | Area F5 | Area F6 | Area F7 | Area F8 | Area F9 | #Feature | #Feature F1 | #Feature F10 | #Feature F11 | #Feature F12 | #Feature F13 | #Feature F14 | #Feature F2 | #Feature F3 | #Feature F4 | #Feature F5 | #Feature F6 | #Feature F7 | #Feature F8 | #Feature F9 | Start | End | PTM | AScore | Found By |
| --- | --- | --- | --- | --- | --- | --- | --- | --- | --- | --- | --- | --- | --- | --- | --- | --- | --- | --- | --- | --- | --- | --- | --- | --- | --- | --- | --- | --- | --- | --- | --- | --- | --- | --- | --- | --- | --- | --- | --- | --- | --- | --- | --- | --- | --- |
| N.EIVDLHNSLR.R | N | 71.95 | 1194.6356 | 10 | 0.4 | 598.3253 | 2 | 28.76 | 4 | F4:13650 | NaSa01\_F12.raw |  |  |  | 7.7068E9 | 7.5889E7 | 5.7777E4 |  |  |  |  |  |  |  |  | 27 | 0 | 0 | 0 | 23 | 3 | 1 | 0 | 0 | 0 | 0 | 0 | 0 | 0 | 0 | 36 | 45 |  |  | PEAKS DB |
| E.IVDLHNSLR.R | N | 58.01 | 1065.5930 | 9 | -1.6 | 533.8029 | 2 | 26.50 | 4 | F4:11729 | NaSa01\_F12.raw |  |  |  | 1.3096E7 |  |  |  |  |  |  |  |  |  |  | 1 | 0 | 0 | 0 | 1 | 0 | 0 | 0 | 0 | 0 | 0 | 0 | 0 | 0 | 0 | 37 | 45 |  |  | PEAKS DB |
| R.MEWYPEAADNAER.W | Y | 54.86 | 1580.6565 | 13 | 0.2 | 791.3357 | 2 | 46.81 | 4 | F4:28766 | NaSa01\_F12.raw |  |  |  | 4.9771E6 |  |  |  |  |  |  |  |  |  |  | 1 | 0 | 0 | 0 | 1 | 0 | 0 | 0 | 0 | 0 | 0 | 0 | 0 | 0 | 0 | 58 | 70 |  |  | PEAKS DB |
| I.VDLHNSLR.R | N | 42.85 | 952.5090 | 8 | -1.0 | 477.2613 | 2 | 26.51 | 4 | F4:11862 | NaSa01\_F12.raw |  |  |  | 2.8116E8 |  |  |  |  |  |  |  |  |  |  | 1 | 0 | 0 | 0 | 1 | 0 | 0 | 0 | 0 | 0 | 0 | 0 | 0 | 0 | 0 | 38 | 45 |  |  | PEAKS DB |
| total 4 peptides |  |  |  |  |  |  |  |  |  |  |  |  |  |  |  |  |  |  |  |  |  |  |  |  |  |  |  |  |  |  |  |  |  |  |  |  |  |  |  |  |  |  |  |  |  |
| --- | --- | --- | --- | --- | --- | --- | --- | --- | --- | --- | --- | --- | --- | --- | --- | --- | --- | --- | --- | --- | --- | --- | --- | --- | --- | --- | --- | --- | --- | --- | --- | --- | --- | --- | --- | --- | --- | --- | --- | --- | --- | --- | --- | --- | --- |

P82464.1

back to list

  

| Protein Coverage
| Supporting Peptides
|

Protein Coverage:

Supporting Peptides:

| Peptide | Uniq | -10lgP | Mass | Length | ppm | m/z | z | RT | Fraction | Scan | Source File | Area F1 | Area F10 | Area F11 | Area F12 | Area F13 | Area F14 | Area F2 | Area F3 | Area F4 | Area F5 | Area F6 | Area F7 | Area F8 | Area F9 | #Feature | #Feature F1 | #Feature F10 | #Feature F11 | #Feature F12 | #Feature F13 | #Feature F14 | #Feature F2 | #Feature F3 | #Feature F4 | #Feature F5 | #Feature F6 | #Feature F7 | #Feature F8 | #Feature F9 | Start | End | PTM | AScore | Found By |
| --- | --- | --- | --- | --- | --- | --- | --- | --- | --- | --- | --- | --- | --- | --- | --- | --- | --- | --- | --- | --- | --- | --- | --- | --- | --- | --- | --- | --- | --- | --- | --- | --- | --- | --- | --- | --- | --- | --- | --- | --- | --- | --- | --- | --- | --- |
| R.GC(+57.02)TFTC(+57.02)PELRPTGIYVYC(+57.02)C(+57.02)R.R | Y | 58.67 | 2509.1011 | 20 | 0.0 | 837.3743 | 3 | 53.61 | 12 | F12:34923 | NaSa01\_F7.raw |  |  |  |  |  |  |  |  |  |  |  | 4.9409E6 |  |  | 1 | 0 | 0 | 0 | 0 | 0 | 0 | 0 | 0 | 0 | 0 | 0 | 1 | 0 | 0 | 40 | 59 | Carbamidomethylation | C2:Carbamidomethylation:1000.00;C6:Carbamidomethylation:1000.00;C18:Carbamidomethylation:1000.00;C19:Carbamidomethylation:1000.00 | PEAKS DB |
| T.TEIC(+57.02)PDSWYFC(+57.02)YK.I | N | 56.79 | 1767.7273 | 13 | 0.5 | 884.8713 | 2 | 66.72 | 12 | F12:45597 | NaSa01\_F7.raw |  |  |  |  |  |  |  |  |  | 1.292E6 |  | 1.6669E6 |  | 6.9702E5 | 3 | 0 | 0 | 0 | 0 | 0 | 0 | 0 | 0 | 0 | 1 | 0 | 1 | 0 | 1 | 14 | 26 | Carbamidomethylation | C4:Carbamidomethylation:1000.00;C11:Carbamidomethylation:1000.00 | PEAKS DB |
| K.ISLADGNDVR.I | N | 50.19 | 1058.5356 | 10 | 0.1 | 530.2751 | 2 | 21.09 | 12 | F12:7712 | NaSa01\_F7.raw |  |  |  |  |  |  |  |  |  |  |  | 1.1584E5 |  |  | 1 | 0 | 0 | 0 | 0 | 0 | 0 | 0 | 0 | 0 | 0 | 0 | 1 | 0 | 0 | 27 | 36 |  |  | PEAKS DB |
| P.DSWYFC(+57.02)YK.I | N | 48.52 | 1167.4695 | 8 | 0.7 | 584.7424 | 2 | 58.18 | 12 | F12:38630 | NaSa01\_F7.raw |  |  |  |  |  |  |  |  |  |  |  | 1.6222E6 |  |  | 1 | 0 | 0 | 0 | 0 | 0 | 0 | 0 | 0 | 0 | 0 | 0 | 1 | 0 | 0 | 19 | 26 | Carbamidomethylation | C6:Carbamidomethylation:1000.00 | PEAKS DB |
| R.GC(+57.02)TFTC(+57.02)PELR.P | N | 47.93 | 1239.5376 | 10 | 0.1 | 620.7761 | 2 | 29.29 | 9 | F9:9432 | NaSa01\_F4.raw |  |  |  |  |  |  |  |  | 1.4766E6 |  | 2.7398E7 | 2.0728E6 |  |  | 3 | 0 | 0 | 0 | 0 | 0 | 0 | 0 | 0 | 1 | 0 | 1 | 1 | 0 | 0 | 40 | 49 | Carbamidomethylation | C2:Carbamidomethylation:1000.00;C6:Carbamidomethylation:1000.00 | PEAKS DB |
| total 5 peptides |  |  |  |  |  |  |  |  |  |  |  |  |  |  |  |  |  |  |  |  |  |  |  |  |  |  |  |  |  |  |  |  |  |  |  |  |  |  |  |  |  |  |  |  |  |
| --- | --- | --- | --- | --- | --- | --- | --- | --- | --- | --- | --- | --- | --- | --- | --- | --- | --- | --- | --- | --- | --- | --- | --- | --- | --- | --- | --- | --- | --- | --- | --- | --- | --- | --- | --- | --- | --- | --- | --- | --- | --- | --- | --- | --- | --- |

ETE73213.1

back to list

  

| Protein Coverage
| Supporting Peptides
|

Protein Coverage:

Supporting Peptides:

| Peptide | Uniq | -10lgP | Mass | Length | ppm | m/z | z | RT | Fraction | Scan | Source File | Area F1 | Area F10 | Area F11 | Area F12 | Area F13 | Area F14 | Area F2 | Area F3 | Area F4 | Area F5 | Area F6 | Area F7 | Area F8 | Area F9 | #Feature | #Feature F1 | #Feature F10 | #Feature F11 | #Feature F12 | #Feature F13 | #Feature F14 | #Feature F2 | #Feature F3 | #Feature F4 | #Feature F5 | #Feature F6 | #Feature F7 | #Feature F8 | #Feature F9 | Start | End | PTM | AScore | Found By |
| --- | --- | --- | --- | --- | --- | --- | --- | --- | --- | --- | --- | --- | --- | --- | --- | --- | --- | --- | --- | --- | --- | --- | --- | --- | --- | --- | --- | --- | --- | --- | --- | --- | --- | --- | --- | --- | --- | --- | --- | --- | --- | --- | --- | --- | --- |
| P.EDWDKPEHIPDPDAK.K | Y | 67.36 | 1790.8110 | 15 | 1.6 | 597.9453 | 3 | 24.09 | 9 | F9:5835 | NaSa01\_F4.raw |  |  |  |  |  |  |  |  | 1.5562E7 |  |  |  |  |  | 3 | 0 | 0 | 0 | 0 | 0 | 0 | 0 | 0 | 3 | 0 | 0 | 0 | 0 | 0 | 233 | 247 |  |  | PEAKS DB |
| E.DWDKPEHIPDPDAK.K | Y | 57.62 | 1661.7684 | 14 | 1.6 | 554.9310 | 3 | 24.03 | 9 | F9:5784 | NaSa01\_F4.raw |  |  |  |  |  |  |  |  | 1.3407E7 |  |  |  |  |  | 3 | 0 | 0 | 0 | 0 | 0 | 0 | 0 | 0 | 3 | 0 | 0 | 0 | 0 | 0 | 234 | 247 |  |  | PEAKS DB |
| K.TLVVQFTVK.H | Y | 52.20 | 1033.6172 | 9 | -0.3 | 517.8157 | 2 | 48.10 | 14 | F14:26607 | NaSa01\_F9.raw |  |  |  |  |  |  |  |  |  |  |  |  |  | 2.022E6 | 1 | 0 | 0 | 0 | 0 | 0 | 0 | 0 | 0 | 0 | 0 | 0 | 0 | 0 | 1 | 89 | 97 |  |  | PEAKS DB |
| R.FYAISASFDSFSNK.D | Y | 46.34 | 1582.7303 | 14 | 0.3 | 792.3727 | 2 | 66.62 | 14 | F14:41575 | NaSa01\_F9.raw |  |  | 1.213E6 |  |  |  |  |  |  |  |  |  |  | 1.8947E6 | 2 | 0 | 0 | 1 | 0 | 0 | 0 | 0 | 0 | 0 | 0 | 0 | 0 | 0 | 1 | 73 | 86 |  |  | PEAKS DB |
| R.FYAISASFDSFSNKDK.T | Y | 41.05 | 1825.8522 | 16 | 1.4 | 913.9346 | 2 | 52.44 | 2 | F2:27907 | NaSa01\_F10.raw |  | 2.9535E5 |  |  |  |  |  |  |  |  |  |  |  |  | 2 | 0 | 2 | 0 | 0 | 0 | 0 | 0 | 0 | 0 | 0 | 0 | 0 | 0 | 0 | 73 | 88 |  |  | PEAKS DB |
| K.FAEEFGNETWGATK.D | Y | 37.61 | 1585.7048 | 14 | 0.0 | 793.8597 | 2 | 50.73 | 6 | F6:30017 | NaSa01\_F14.raw |  |  |  |  |  | 8.8827E5 |  |  |  |  |  |  |  |  | 1 | 0 | 0 | 0 | 0 | 0 | 1 | 0 | 0 | 0 | 0 | 0 | 0 | 0 | 0 | 337 | 350 |  |  | PEAKS DB |
| total 6 peptides |  |  |  |  |  |  |  |  |  |  |  |  |  |  |  |  |  |  |  |  |  |  |  |  |  |  |  |  |  |  |  |  |  |  |  |  |  |  |  |  |  |  |  |  |  |
| --- | --- | --- | --- | --- | --- | --- | --- | --- | --- | --- | --- | --- | --- | --- | --- | --- | --- | --- | --- | --- | --- | --- | --- | --- | --- | --- | --- | --- | --- | --- | --- | --- | --- | --- | --- | --- | --- | --- | --- | --- | --- | --- | --- | --- | --- |

XP\_026572586.1

back to list

  

| Protein Coverage
| Supporting Peptides
|

Protein Coverage:

Supporting Peptides:

| Peptide | Uniq | -10lgP | Mass | Length | ppm | m/z | z | RT | Fraction | Scan | Source File | Area F1 | Area F10 | Area F11 | Area F12 | Area F13 | Area F14 | Area F2 | Area F3 | Area F4 | Area F5 | Area F6 | Area F7 | Area F8 | Area F9 | #Feature | #Feature F1 | #Feature F10 | #Feature F11 | #Feature F12 | #Feature F13 | #Feature F14 | #Feature F2 | #Feature F3 | #Feature F4 | #Feature F5 | #Feature F6 | #Feature F7 | #Feature F8 | #Feature F9 | Start | End | PTM | AScore | Found By |
| --- | --- | --- | --- | --- | --- | --- | --- | --- | --- | --- | --- | --- | --- | --- | --- | --- | --- | --- | --- | --- | --- | --- | --- | --- | --- | --- | --- | --- | --- | --- | --- | --- | --- | --- | --- | --- | --- | --- | --- | --- | --- | --- | --- | --- | --- |
| P.EDWDKPEHIPDPDAK.K | Y | 67.36 | 1790.8110 | 15 | 1.6 | 597.9453 | 3 | 24.09 | 9 | F9:5835 | NaSa01\_F4.raw |  |  |  |  |  |  |  |  | 1.5562E7 |  |  |  |  |  | 3 | 0 | 0 | 0 | 0 | 0 | 0 | 0 | 0 | 3 | 0 | 0 | 0 | 0 | 0 | 233 | 247 |  |  | PEAKS DB |
| E.DWDKPEHIPDPDAK.K | Y | 57.62 | 1661.7684 | 14 | 1.6 | 554.9310 | 3 | 24.03 | 9 | F9:5784 | NaSa01\_F4.raw |  |  |  |  |  |  |  |  | 1.3407E7 |  |  |  |  |  | 3 | 0 | 0 | 0 | 0 | 0 | 0 | 0 | 0 | 3 | 0 | 0 | 0 | 0 | 0 | 234 | 247 |  |  | PEAKS DB |
| K.TLVVQFTVK.H | Y | 52.20 | 1033.6172 | 9 | -0.3 | 517.8157 | 2 | 48.10 | 14 | F14:26607 | NaSa01\_F9.raw |  |  |  |  |  |  |  |  |  |  |  |  |  | 2.022E6 | 1 | 0 | 0 | 0 | 0 | 0 | 0 | 0 | 0 | 0 | 0 | 0 | 0 | 0 | 1 | 89 | 97 |  |  | PEAKS DB |
| R.FYAISASFDSFSNK.D | Y | 46.34 | 1582.7303 | 14 | 0.3 | 792.3727 | 2 | 66.62 | 14 | F14:41575 | NaSa01\_F9.raw |  |  | 1.213E6 |  |  |  |  |  |  |  |  |  |  | 1.8947E6 | 2 | 0 | 0 | 1 | 0 | 0 | 0 | 0 | 0 | 0 | 0 | 0 | 0 | 0 | 1 | 73 | 86 |  |  | PEAKS DB |
| R.FYAISASFDSFSNKDK.T | Y | 41.05 | 1825.8522 | 16 | 1.4 | 913.9346 | 2 | 52.44 | 2 | F2:27907 | NaSa01\_F10.raw |  | 2.9535E5 |  |  |  |  |  |  |  |  |  |  |  |  | 2 | 0 | 2 | 0 | 0 | 0 | 0 | 0 | 0 | 0 | 0 | 0 | 0 | 0 | 0 | 73 | 88 |  |  | PEAKS DB |
| K.FAEEFGNETWGATK.D | Y | 37.61 | 1585.7048 | 14 | 0.0 | 793.8597 | 2 | 50.73 | 6 | F6:30017 | NaSa01\_F14.raw |  |  |  |  |  | 8.8827E5 |  |  |  |  |  |  |  |  | 1 | 0 | 0 | 0 | 0 | 0 | 1 | 0 | 0 | 0 | 0 | 0 | 0 | 0 | 0 | 337 | 350 |  |  | PEAKS DB |
| total 6 peptides |  |  |  |  |  |  |  |  |  |  |  |  |  |  |  |  |  |  |  |  |  |  |  |  |  |  |  |  |  |  |  |  |  |  |  |  |  |  |  |  |  |  |  |  |  |
| --- | --- | --- | --- | --- | --- | --- | --- | --- | --- | --- | --- | --- | --- | --- | --- | --- | --- | --- | --- | --- | --- | --- | --- | --- | --- | --- | --- | --- | --- | --- | --- | --- | --- | --- | --- | --- | --- | --- | --- | --- | --- | --- | --- | --- | --- |

JAC96061.1

back to list

  

| Protein Coverage
| Supporting Peptides
|

Protein Coverage:

Supporting Peptides:

| Peptide | Uniq | -10lgP | Mass | Length | ppm | m/z | z | RT | Fraction | Scan | Source File | Area F1 | Area F10 | Area F11 | Area F12 | Area F13 | Area F14 | Area F2 | Area F3 | Area F4 | Area F5 | Area F6 | Area F7 | Area F8 | Area F9 | #Feature | #Feature F1 | #Feature F10 | #Feature F11 | #Feature F12 | #Feature F13 | #Feature F14 | #Feature F2 | #Feature F3 | #Feature F4 | #Feature F5 | #Feature F6 | #Feature F7 | #Feature F8 | #Feature F9 | Start | End | PTM | AScore | Found By |
| --- | --- | --- | --- | --- | --- | --- | --- | --- | --- | --- | --- | --- | --- | --- | --- | --- | --- | --- | --- | --- | --- | --- | --- | --- | --- | --- | --- | --- | --- | --- | --- | --- | --- | --- | --- | --- | --- | --- | --- | --- | --- | --- | --- | --- | --- |
| P.EDWDKPEHIPDPDAK.K | Y | 67.36 | 1790.8110 | 15 | 1.6 | 597.9453 | 3 | 24.09 | 9 | F9:5835 | NaSa01\_F4.raw |  |  |  |  |  |  |  |  | 1.5562E7 |  |  |  |  |  | 3 | 0 | 0 | 0 | 0 | 0 | 0 | 0 | 0 | 3 | 0 | 0 | 0 | 0 | 0 | 233 | 247 |  |  | PEAKS DB |
| E.DWDKPEHIPDPDAK.K | Y | 57.62 | 1661.7684 | 14 | 1.6 | 554.9310 | 3 | 24.03 | 9 | F9:5784 | NaSa01\_F4.raw |  |  |  |  |  |  |  |  | 1.3407E7 |  |  |  |  |  | 3 | 0 | 0 | 0 | 0 | 0 | 0 | 0 | 0 | 3 | 0 | 0 | 0 | 0 | 0 | 234 | 247 |  |  | PEAKS DB |
| K.TLVVQFTVK.H | Y | 52.20 | 1033.6172 | 9 | -0.3 | 517.8157 | 2 | 48.10 | 14 | F14:26607 | NaSa01\_F9.raw |  |  |  |  |  |  |  |  |  |  |  |  |  | 2.022E6 | 1 | 0 | 0 | 0 | 0 | 0 | 0 | 0 | 0 | 0 | 0 | 0 | 0 | 0 | 1 | 89 | 97 |  |  | PEAKS DB |
| R.FYAISASFDSFSNK.D | Y | 46.34 | 1582.7303 | 14 | 0.3 | 792.3727 | 2 | 66.62 | 14 | F14:41575 | NaSa01\_F9.raw |  |  | 1.213E6 |  |  |  |  |  |  |  |  |  |  | 1.8947E6 | 2 | 0 | 0 | 1 | 0 | 0 | 0 | 0 | 0 | 0 | 0 | 0 | 0 | 0 | 1 | 73 | 86 |  |  | PEAKS DB |
| R.FYAISASFDSFSNKDK.T | Y | 41.05 | 1825.8522 | 16 | 1.4 | 913.9346 | 2 | 52.44 | 2 | F2:27907 | NaSa01\_F10.raw |  | 2.9535E5 |  |  |  |  |  |  |  |  |  |  |  |  | 2 | 0 | 2 | 0 | 0 | 0 | 0 | 0 | 0 | 0 | 0 | 0 | 0 | 0 | 0 | 73 | 88 |  |  | PEAKS DB |
| K.FAEEFGNETWGATK.D | Y | 37.61 | 1585.7048 | 14 | 0.0 | 793.8597 | 2 | 50.73 | 6 | F6:30017 | NaSa01\_F14.raw |  |  |  |  |  | 8.8827E5 |  |  |  |  |  |  |  |  | 1 | 0 | 0 | 0 | 0 | 0 | 1 | 0 | 0 | 0 | 0 | 0 | 0 | 0 | 0 | 337 | 350 |  |  | PEAKS DB |
| total 6 peptides |  |  |  |  |  |  |  |  |  |  |  |  |  |  |  |  |  |  |  |  |  |  |  |  |  |  |  |  |  |  |  |  |  |  |  |  |  |  |  |  |  |  |  |  |  |
| --- | --- | --- | --- | --- | --- | --- | --- | --- | --- | --- | --- | --- | --- | --- | --- | --- | --- | --- | --- | --- | --- | --- | --- | --- | --- | --- | --- | --- | --- | --- | --- | --- | --- | --- | --- | --- | --- | --- | --- | --- | --- | --- | --- | --- | --- |

1CDT

back to list

  

| Protein Coverage
| Supporting Peptides
|

Protein Coverage:

Supporting Peptides:

| Peptide | Uniq | -10lgP | Mass | Length | ppm | m/z | z | RT | Fraction | Scan | Source File | Area F1 | Area F10 | Area F11 | Area F12 | Area F13 | Area F14 | Area F2 | Area F3 | Area F4 | Area F5 | Area F6 | Area F7 | Area F8 | Area F9 | #Feature | #Feature F1 | #Feature F10 | #Feature F11 | #Feature F12 | #Feature F13 | #Feature F14 | #Feature F2 | #Feature F3 | #Feature F4 | #Feature F5 | #Feature F6 | #Feature F7 | #Feature F8 | #Feature F9 | Start | End | PTM | AScore | Found By |
| --- | --- | --- | --- | --- | --- | --- | --- | --- | --- | --- | --- | --- | --- | --- | --- | --- | --- | --- | --- | --- | --- | --- | --- | --- | --- | --- | --- | --- | --- | --- | --- | --- | --- | --- | --- | --- | --- | --- | --- | --- | --- | --- | --- | --- | --- |
| LKC(+57.02)NKLIPIAYK.T | N | 67.34 | 1459.8584 | 12 | -0.2 | 487.6266 | 3 | 21.98 | 13 | F13:13390 | NaSa01\_F8.raw |  |  |  |  |  |  |  |  |  |  |  | 3.4641E6 | 6.8191E7 |  | 5 | 0 | 0 | 0 | 0 | 0 | 0 | 0 | 0 | 0 | 0 | 0 | 1 | 4 | 0 | 1 | 12 | Carbamidomethylation | C3:Carbamidomethylation:1000.00 | PEAKS DB |
| K.C(+57.02)NKLIPIAYK.T | N | 57.38 | 1218.6794 | 10 | 0.7 | 610.3474 | 2 | 26.64 | 12 | F12:12500 | NaSa01\_F7.raw |  |  |  |  |  |  |  |  |  |  |  | 2.6598E7 | 9.2193E6 |  | 6 | 0 | 0 | 0 | 0 | 0 | 0 | 0 | 0 | 0 | 0 | 0 | 2 | 4 | 0 | 3 | 12 | Carbamidomethylation | C1:Carbamidomethylation:1000.00 | PEAKS DB |
| K.LIPIAYKTC(+57.02)PEGK.N | Y | 46.94 | 1488.8010 | 13 | 0.8 | 745.4084 | 2 | 38.02 | 13 | F13:25181 | NaSa01\_F8.raw |  |  |  |  |  |  |  |  |  |  |  | 1.6406E6 | 1.808E6 | 1.7198E7 | 4 | 0 | 0 | 0 | 0 | 0 | 0 | 0 | 0 | 0 | 0 | 0 | 1 | 1 | 2 | 6 | 18 | Carbamidomethylation | C9:Carbamidomethylation:1000.00 | PEAKS DB |
| K.LIPIAYK.T | N | 40.88 | 816.5109 | 7 | 0.0 | 409.2627 | 2 | 42.39 | 12 | F12:25348 | NaSa01\_F7.raw |  |  |  |  |  | 6.2114E5 |  |  |  |  |  | 1.9145E10 | 0 | 2.4516E6 | 8 | 0 | 0 | 0 | 0 | 0 | 1 | 0 | 0 | 0 | 0 | 0 | 6 | 0 | 1 | 6 | 12 |  |  | PEAKS DB |
| total 4 peptides |  |  |  |  |  |  |  |  |  |  |  |  |  |  |  |  |  |  |  |  |  |  |  |  |  |  |  |  |  |  |  |  |  |  |  |  |  |  |  |  |  |  |  |  |  |
| --- | --- | --- | --- | --- | --- | --- | --- | --- | --- | --- | --- | --- | --- | --- | --- | --- | --- | --- | --- | --- | --- | --- | --- | --- | --- | --- | --- | --- | --- | --- | --- | --- | --- | --- | --- | --- | --- | --- | --- | --- | --- | --- | --- | --- | --- |

P01452.1

back to list

  

| Protein Coverage
| Supporting Peptides
|

Protein Coverage:

Supporting Peptides:

| Peptide | Uniq | -10lgP | Mass | Length | ppm | m/z | z | RT | Fraction | Scan | Source File | Area F1 | Area F10 | Area F11 | Area F12 | Area F13 | Area F14 | Area F2 | Area F3 | Area F4 | Area F5 | Area F6 | Area F7 | Area F8 | Area F9 | #Feature | #Feature F1 | #Feature F10 | #Feature F11 | #Feature F12 | #Feature F13 | #Feature F14 | #Feature F2 | #Feature F3 | #Feature F4 | #Feature F5 | #Feature F6 | #Feature F7 | #Feature F8 | #Feature F9 | Start | End | PTM | AScore | Found By |
| --- | --- | --- | --- | --- | --- | --- | --- | --- | --- | --- | --- | --- | --- | --- | --- | --- | --- | --- | --- | --- | --- | --- | --- | --- | --- | --- | --- | --- | --- | --- | --- | --- | --- | --- | --- | --- | --- | --- | --- | --- | --- | --- | --- | --- | --- |
| LKC(+57.02)NKLIPIAYK.T | N | 67.34 | 1459.8584 | 12 | -0.2 | 487.6266 | 3 | 21.98 | 13 | F13:13390 | NaSa01\_F8.raw |  |  |  |  |  |  |  |  |  |  |  | 3.4641E6 | 6.8191E7 |  | 5 | 0 | 0 | 0 | 0 | 0 | 0 | 0 | 0 | 0 | 0 | 0 | 1 | 4 | 0 | 1 | 12 | Carbamidomethylation | C3:Carbamidomethylation:1000.00 | PEAKS DB |
| K.C(+57.02)NKLIPIAYK.T | N | 57.38 | 1218.6794 | 10 | 0.7 | 610.3474 | 2 | 26.64 | 12 | F12:12500 | NaSa01\_F7.raw |  |  |  |  |  |  |  |  |  |  |  | 2.6598E7 | 9.2193E6 |  | 6 | 0 | 0 | 0 | 0 | 0 | 0 | 0 | 0 | 0 | 0 | 0 | 2 | 4 | 0 | 3 | 12 | Carbamidomethylation | C1:Carbamidomethylation:1000.00 | PEAKS DB |
| K.LIPIAYKTC(+57.02)PEGK.N | Y | 46.94 | 1488.8010 | 13 | 0.8 | 745.4084 | 2 | 38.02 | 13 | F13:25181 | NaSa01\_F8.raw |  |  |  |  |  |  |  |  |  |  |  | 1.6406E6 | 1.808E6 | 1.7198E7 | 4 | 0 | 0 | 0 | 0 | 0 | 0 | 0 | 0 | 0 | 0 | 0 | 1 | 1 | 2 | 6 | 18 | Carbamidomethylation | C9:Carbamidomethylation:1000.00 | PEAKS DB |
| K.LIPIAYK.T | N | 40.88 | 816.5109 | 7 | 0.0 | 409.2627 | 2 | 42.39 | 12 | F12:25348 | NaSa01\_F7.raw |  |  |  |  |  | 6.2114E5 |  |  |  |  |  | 1.9145E10 | 0 | 2.4516E6 | 8 | 0 | 0 | 0 | 0 | 0 | 1 | 0 | 0 | 0 | 0 | 0 | 6 | 0 | 1 | 6 | 12 |  |  | PEAKS DB |
| total 4 peptides |  |  |  |  |  |  |  |  |  |  |  |  |  |  |  |  |  |  |  |  |  |  |  |  |  |  |  |  |  |  |  |  |  |  |  |  |  |  |  |  |  |  |  |  |  |
| --- | --- | --- | --- | --- | --- | --- | --- | --- | --- | --- | --- | --- | --- | --- | --- | --- | --- | --- | --- | --- | --- | --- | --- | --- | --- | --- | --- | --- | --- | --- | --- | --- | --- | --- | --- | --- | --- | --- | --- | --- | --- | --- | --- | --- | --- |

ETE73206.1

back to list

  

| Protein Coverage
| Supporting Peptides
|

Protein Coverage:

Supporting Peptides:

| Peptide | Uniq | -10lgP | Mass | Length | ppm | m/z | z | RT | Fraction | Scan | Source File | Area F1 | Area F10 | Area F11 | Area F12 | Area F13 | Area F14 | Area F2 | Area F3 | Area F4 | Area F5 | Area F6 | Area F7 | Area F8 | Area F9 | #Feature | #Feature F1 | #Feature F10 | #Feature F11 | #Feature F12 | #Feature F13 | #Feature F14 | #Feature F2 | #Feature F3 | #Feature F4 | #Feature F5 | #Feature F6 | #Feature F7 | #Feature F8 | #Feature F9 | Start | End | PTM | AScore | Found By |
| --- | --- | --- | --- | --- | --- | --- | --- | --- | --- | --- | --- | --- | --- | --- | --- | --- | --- | --- | --- | --- | --- | --- | --- | --- | --- | --- | --- | --- | --- | --- | --- | --- | --- | --- | --- | --- | --- | --- | --- | --- | --- | --- | --- | --- | --- |
| R.TQGFWLVHSTPR.F | N | 63.89 | 1427.7310 | 12 | 0.3 | 714.8730 | 2 | 38.99 | 5 | F5:20956 | NaSa01\_F13.raw |  |  |  |  | 5.2297E6 | 7.3135E5 |  |  |  |  |  |  |  |  | 3 | 0 | 0 | 0 | 0 | 2 | 1 | 0 | 0 | 0 | 0 | 0 | 0 | 0 | 0 | 172 | 183 |  |  | PEAKS DB |
| R.VYNVEQLAFQDPATSFFSTSDHSK.W | Y | 61.48 | 2717.2607 | 24 | 0.2 | 906.7610 | 3 | 80.41 | 5 | F5:54990 | NaSa01\_F13.raw |  |  |  |  | 6.6368E6 |  |  |  |  |  |  |  |  |  | 1 | 0 | 0 | 0 | 0 | 1 | 0 | 0 | 0 | 0 | 0 | 0 | 0 | 0 | 0 | 321 | 344 |  |  | PEAKS DB |
| E.HVQEEPWNSQVTLTSK.G | Y | 55.77 | 1881.9221 | 16 | 0.6 | 941.9689 | 2 | 33.62 | 5 | F5:16414 | NaSa01\_F13.raw |  |  |  |  | 6.3719E6 |  |  |  |  |  |  |  |  |  | 2 | 0 | 0 | 0 | 0 | 2 | 0 | 0 | 0 | 0 | 0 | 0 | 0 | 0 | 0 | 253 | 268 |  |  | PEAKS DB |
| H.VYDYQVQGAFAR.D | N | 44.07 | 1415.6833 | 12 | -0.1 | 708.8489 | 2 | 41.51 | 5 | F5:23007 | NaSa01\_F13.raw |  |  |  |  | 1.0731E6 |  |  |  |  |  |  |  |  |  | 1 | 0 | 0 | 0 | 0 | 1 | 0 | 0 | 0 | 0 | 0 | 0 | 0 | 0 | 0 | 229 | 240 |  |  | PEAKS DB |
| R.TLNQLYLEAANR.Q | N | 40.76 | 1404.7361 | 12 | 0.2 | 703.3754 | 2 | 46.99 | 5 | F5:27638 | NaSa01\_F13.raw |  |  |  |  | 6.988E6 |  |  |  |  |  |  |  |  |  | 1 | 0 | 0 | 0 | 0 | 1 | 0 | 0 | 0 | 0 | 0 | 0 | 0 | 0 | 0 | 125 | 136 |  |  | PEAKS DB |
| total 5 peptides |  |  |  |  |  |  |  |  |  |  |  |  |  |  |  |  |  |  |  |  |  |  |  |  |  |  |  |  |  |  |  |  |  |  |  |  |  |  |  |  |  |  |  |  |  |
| --- | --- | --- | --- | --- | --- | --- | --- | --- | --- | --- | --- | --- | --- | --- | --- | --- | --- | --- | --- | --- | --- | --- | --- | --- | --- | --- | --- | --- | --- | --- | --- | --- | --- | --- | --- | --- | --- | --- | --- | --- | --- | --- | --- | --- | --- |

XP\_026572614.1

back to list

  

| Protein Coverage
| Supporting Peptides
|

Protein Coverage:

Supporting Peptides:

| Peptide | Uniq | -10lgP | Mass | Length | ppm | m/z | z | RT | Fraction | Scan | Source File | Area F1 | Area F10 | Area F11 | Area F12 | Area F13 | Area F14 | Area F2 | Area F3 | Area F4 | Area F5 | Area F6 | Area F7 | Area F8 | Area F9 | #Feature | #Feature F1 | #Feature F10 | #Feature F11 | #Feature F12 | #Feature F13 | #Feature F14 | #Feature F2 | #Feature F3 | #Feature F4 | #Feature F5 | #Feature F6 | #Feature F7 | #Feature F8 | #Feature F9 | Start | End | PTM | AScore | Found By |
| --- | --- | --- | --- | --- | --- | --- | --- | --- | --- | --- | --- | --- | --- | --- | --- | --- | --- | --- | --- | --- | --- | --- | --- | --- | --- | --- | --- | --- | --- | --- | --- | --- | --- | --- | --- | --- | --- | --- | --- | --- | --- | --- | --- | --- | --- |
| R.TQGFWLVHSTPR.F | N | 63.89 | 1427.7310 | 12 | 0.3 | 714.8730 | 2 | 38.99 | 5 | F5:20956 | NaSa01\_F13.raw |  |  |  |  | 5.2297E6 | 7.3135E5 |  |  |  |  |  |  |  |  | 3 | 0 | 0 | 0 | 0 | 2 | 1 | 0 | 0 | 0 | 0 | 0 | 0 | 0 | 0 | 126 | 137 |  |  | PEAKS DB |
| R.VYNVEQLAFQDPATSFFSTSDHSK.W | Y | 61.48 | 2717.2607 | 24 | 0.2 | 906.7610 | 3 | 80.41 | 5 | F5:54990 | NaSa01\_F13.raw |  |  |  |  | 6.6368E6 |  |  |  |  |  |  |  |  |  | 1 | 0 | 0 | 0 | 0 | 1 | 0 | 0 | 0 | 0 | 0 | 0 | 0 | 0 | 0 | 276 | 299 |  |  | PEAKS DB |
| E.HVQEEPWNSQVTLTSK.G | Y | 55.77 | 1881.9221 | 16 | 0.6 | 941.9689 | 2 | 33.62 | 5 | F5:16414 | NaSa01\_F13.raw |  |  |  |  | 6.3719E6 |  |  |  |  |  |  |  |  |  | 2 | 0 | 0 | 0 | 0 | 2 | 0 | 0 | 0 | 0 | 0 | 0 | 0 | 0 | 0 | 208 | 223 |  |  | PEAKS DB |
| H.VYDYQVQGAFAR.D | N | 44.07 | 1415.6833 | 12 | -0.1 | 708.8489 | 2 | 41.51 | 5 | F5:23007 | NaSa01\_F13.raw |  |  |  |  | 1.0731E6 |  |  |  |  |  |  |  |  |  | 1 | 0 | 0 | 0 | 0 | 1 | 0 | 0 | 0 | 0 | 0 | 0 | 0 | 0 | 0 | 184 | 195 |  |  | PEAKS DB |
| R.TLNQLYLEAANR.Q | N | 40.76 | 1404.7361 | 12 | 0.2 | 703.3754 | 2 | 46.99 | 5 | F5:27638 | NaSa01\_F13.raw |  |  |  |  | 6.988E6 |  |  |  |  |  |  |  |  |  | 1 | 0 | 0 | 0 | 0 | 1 | 0 | 0 | 0 | 0 | 0 | 0 | 0 | 0 | 0 | 79 | 90 |  |  | PEAKS DB |
| total 5 peptides |  |  |  |  |  |  |  |  |  |  |  |  |  |  |  |  |  |  |  |  |  |  |  |  |  |  |  |  |  |  |  |  |  |  |  |  |  |  |  |  |  |  |  |  |  |
| --- | --- | --- | --- | --- | --- | --- | --- | --- | --- | --- | --- | --- | --- | --- | --- | --- | --- | --- | --- | --- | --- | --- | --- | --- | --- | --- | --- | --- | --- | --- | --- | --- | --- | --- | --- | --- | --- | --- | --- | --- | --- | --- | --- | --- | --- |

XP\_026572616.1

back to list

  

| Protein Coverage
| Supporting Peptides
|

Protein Coverage:

Supporting Peptides:

| Peptide | Uniq | -10lgP | Mass | Length | ppm | m/z | z | RT | Fraction | Scan | Source File | Area F1 | Area F10 | Area F11 | Area F12 | Area F13 | Area F14 | Area F2 | Area F3 | Area F4 | Area F5 | Area F6 | Area F7 | Area F8 | Area F9 | #Feature | #Feature F1 | #Feature F10 | #Feature F11 | #Feature F12 | #Feature F13 | #Feature F14 | #Feature F2 | #Feature F3 | #Feature F4 | #Feature F5 | #Feature F6 | #Feature F7 | #Feature F8 | #Feature F9 | Start | End | PTM | AScore | Found By |
| --- | --- | --- | --- | --- | --- | --- | --- | --- | --- | --- | --- | --- | --- | --- | --- | --- | --- | --- | --- | --- | --- | --- | --- | --- | --- | --- | --- | --- | --- | --- | --- | --- | --- | --- | --- | --- | --- | --- | --- | --- | --- | --- | --- | --- | --- |
| R.TQGFWLVHSTPR.F | N | 63.89 | 1427.7310 | 12 | 0.3 | 714.8730 | 2 | 38.99 | 5 | F5:20956 | NaSa01\_F13.raw |  |  |  |  | 5.2297E6 | 7.3135E5 |  |  |  |  |  |  |  |  | 3 | 0 | 0 | 0 | 0 | 2 | 1 | 0 | 0 | 0 | 0 | 0 | 0 | 0 | 0 | 126 | 137 |  |  | PEAKS DB |
| R.VYNVEQLAFQDPATSFFSTSDHSK.W | Y | 61.48 | 2717.2607 | 24 | 0.2 | 906.7610 | 3 | 80.41 | 5 | F5:54990 | NaSa01\_F13.raw |  |  |  |  | 6.6368E6 |  |  |  |  |  |  |  |  |  | 1 | 0 | 0 | 0 | 0 | 1 | 0 | 0 | 0 | 0 | 0 | 0 | 0 | 0 | 0 | 276 | 299 |  |  | PEAKS DB |
| E.HVQEEPWNSQVTLTSK.G | Y | 55.77 | 1881.9221 | 16 | 0.6 | 941.9689 | 2 | 33.62 | 5 | F5:16414 | NaSa01\_F13.raw |  |  |  |  | 6.3719E6 |  |  |  |  |  |  |  |  |  | 2 | 0 | 0 | 0 | 0 | 2 | 0 | 0 | 0 | 0 | 0 | 0 | 0 | 0 | 0 | 208 | 223 |  |  | PEAKS DB |
| H.VYDYQVQGAFAR.D | N | 44.07 | 1415.6833 | 12 | -0.1 | 708.8489 | 2 | 41.51 | 5 | F5:23007 | NaSa01\_F13.raw |  |  |  |  | 1.0731E6 |  |  |  |  |  |  |  |  |  | 1 | 0 | 0 | 0 | 0 | 1 | 0 | 0 | 0 | 0 | 0 | 0 | 0 | 0 | 0 | 184 | 195 |  |  | PEAKS DB |
| R.TLNQLYLEAANR.Q | N | 40.76 | 1404.7361 | 12 | 0.2 | 703.3754 | 2 | 46.99 | 5 | F5:27638 | NaSa01\_F13.raw |  |  |  |  | 6.988E6 |  |  |  |  |  |  |  |  |  | 1 | 0 | 0 | 0 | 0 | 1 | 0 | 0 | 0 | 0 | 0 | 0 | 0 | 0 | 0 | 79 | 90 |  |  | PEAKS DB |
| total 5 peptides |  |  |  |  |  |  |  |  |  |  |  |  |  |  |  |  |  |  |  |  |  |  |  |  |  |  |  |  |  |  |  |  |  |  |  |  |  |  |  |  |  |  |  |  |  |
| --- | --- | --- | --- | --- | --- | --- | --- | --- | --- | --- | --- | --- | --- | --- | --- | --- | --- | --- | --- | --- | --- | --- | --- | --- | --- | --- | --- | --- | --- | --- | --- | --- | --- | --- | --- | --- | --- | --- | --- | --- | --- | --- | --- | --- | --- |

XP\_026572618.1

back to list

  

| Protein Coverage
| Supporting Peptides
|

Protein Coverage:

Supporting Peptides:

| Peptide | Uniq | -10lgP | Mass | Length | ppm | m/z | z | RT | Fraction | Scan | Source File | Area F1 | Area F10 | Area F11 | Area F12 | Area F13 | Area F14 | Area F2 | Area F3 | Area F4 | Area F5 | Area F6 | Area F7 | Area F8 | Area F9 | #Feature | #Feature F1 | #Feature F10 | #Feature F11 | #Feature F12 | #Feature F13 | #Feature F14 | #Feature F2 | #Feature F3 | #Feature F4 | #Feature F5 | #Feature F6 | #Feature F7 | #Feature F8 | #Feature F9 | Start | End | PTM | AScore | Found By |
| --- | --- | --- | --- | --- | --- | --- | --- | --- | --- | --- | --- | --- | --- | --- | --- | --- | --- | --- | --- | --- | --- | --- | --- | --- | --- | --- | --- | --- | --- | --- | --- | --- | --- | --- | --- | --- | --- | --- | --- | --- | --- | --- | --- | --- | --- |
| R.TQGFWLVHSTPR.F | N | 63.89 | 1427.7310 | 12 | 0.3 | 714.8730 | 2 | 38.99 | 5 | F5:20956 | NaSa01\_F13.raw |  |  |  |  | 5.2297E6 | 7.3135E5 |  |  |  |  |  |  |  |  | 3 | 0 | 0 | 0 | 0 | 2 | 1 | 0 | 0 | 0 | 0 | 0 | 0 | 0 | 0 | 126 | 137 |  |  | PEAKS DB |
| R.VYNVEQLAFQDPATSFFSTSDHSK.W | Y | 61.48 | 2717.2607 | 24 | 0.2 | 906.7610 | 3 | 80.41 | 5 | F5:54990 | NaSa01\_F13.raw |  |  |  |  | 6.6368E6 |  |  |  |  |  |  |  |  |  | 1 | 0 | 0 | 0 | 0 | 1 | 0 | 0 | 0 | 0 | 0 | 0 | 0 | 0 | 0 | 276 | 299 |  |  | PEAKS DB |
| E.HVQEEPWNSQVTLTSK.G | Y | 55.77 | 1881.9221 | 16 | 0.6 | 941.9689 | 2 | 33.62 | 5 | F5:16414 | NaSa01\_F13.raw |  |  |  |  | 6.3719E6 |  |  |  |  |  |  |  |  |  | 2 | 0 | 0 | 0 | 0 | 2 | 0 | 0 | 0 | 0 | 0 | 0 | 0 | 0 | 0 | 208 | 223 |  |  | PEAKS DB |
| H.VYDYQVQGAFAR.D | N | 44.07 | 1415.6833 | 12 | -0.1 | 708.8489 | 2 | 41.51 | 5 | F5:23007 | NaSa01\_F13.raw |  |  |  |  | 1.0731E6 |  |  |  |  |  |  |  |  |  | 1 | 0 | 0 | 0 | 0 | 1 | 0 | 0 | 0 | 0 | 0 | 0 | 0 | 0 | 0 | 184 | 195 |  |  | PEAKS DB |
| R.TLNQLYLEAANR.Q | N | 40.76 | 1404.7361 | 12 | 0.2 | 703.3754 | 2 | 46.99 | 5 | F5:27638 | NaSa01\_F13.raw |  |  |  |  | 6.988E6 |  |  |  |  |  |  |  |  |  | 1 | 0 | 0 | 0 | 0 | 1 | 0 | 0 | 0 | 0 | 0 | 0 | 0 | 0 | 0 | 79 | 90 |  |  | PEAKS DB |
| total 5 peptides |  |  |  |  |  |  |  |  |  |  |  |  |  |  |  |  |  |  |  |  |  |  |  |  |  |  |  |  |  |  |  |  |  |  |  |  |  |  |  |  |  |  |  |  |  |
| --- | --- | --- | --- | --- | --- | --- | --- | --- | --- | --- | --- | --- | --- | --- | --- | --- | --- | --- | --- | --- | --- | --- | --- | --- | --- | --- | --- | --- | --- | --- | --- | --- | --- | --- | --- | --- | --- | --- | --- | --- | --- | --- | --- | --- | --- |

XP\_026572617.1

back to list

  

| Protein Coverage
| Supporting Peptides
|

Protein Coverage:

Supporting Peptides:

| Peptide | Uniq | -10lgP | Mass | Length | ppm | m/z | z | RT | Fraction | Scan | Source File | Area F1 | Area F10 | Area F11 | Area F12 | Area F13 | Area F14 | Area F2 | Area F3 | Area F4 | Area F5 | Area F6 | Area F7 | Area F8 | Area F9 | #Feature | #Feature F1 | #Feature F10 | #Feature F11 | #Feature F12 | #Feature F13 | #Feature F14 | #Feature F2 | #Feature F3 | #Feature F4 | #Feature F5 | #Feature F6 | #Feature F7 | #Feature F8 | #Feature F9 | Start | End | PTM | AScore | Found By |
| --- | --- | --- | --- | --- | --- | --- | --- | --- | --- | --- | --- | --- | --- | --- | --- | --- | --- | --- | --- | --- | --- | --- | --- | --- | --- | --- | --- | --- | --- | --- | --- | --- | --- | --- | --- | --- | --- | --- | --- | --- | --- | --- | --- | --- | --- |
| R.TQGFWLVHSTPR.F | N | 63.89 | 1427.7310 | 12 | 0.3 | 714.8730 | 2 | 38.99 | 5 | F5:20956 | NaSa01\_F13.raw |  |  |  |  | 5.2297E6 | 7.3135E5 |  |  |  |  |  |  |  |  | 3 | 0 | 0 | 0 | 0 | 2 | 1 | 0 | 0 | 0 | 0 | 0 | 0 | 0 | 0 | 126 | 137 |  |  | PEAKS DB |
| R.VYNVEQLAFQDPATSFFSTSDHSK.W | Y | 61.48 | 2717.2607 | 24 | 0.2 | 906.7610 | 3 | 80.41 | 5 | F5:54990 | NaSa01\_F13.raw |  |  |  |  | 6.6368E6 |  |  |  |  |  |  |  |  |  | 1 | 0 | 0 | 0 | 0 | 1 | 0 | 0 | 0 | 0 | 0 | 0 | 0 | 0 | 0 | 276 | 299 |  |  | PEAKS DB |
| E.HVQEEPWNSQVTLTSK.G | Y | 55.77 | 1881.9221 | 16 | 0.6 | 941.9689 | 2 | 33.62 | 5 | F5:16414 | NaSa01\_F13.raw |  |  |  |  | 6.3719E6 |  |  |  |  |  |  |  |  |  | 2 | 0 | 0 | 0 | 0 | 2 | 0 | 0 | 0 | 0 | 0 | 0 | 0 | 0 | 0 | 208 | 223 |  |  | PEAKS DB |
| H.VYDYQVQGAFAR.D | N | 44.07 | 1415.6833 | 12 | -0.1 | 708.8489 | 2 | 41.51 | 5 | F5:23007 | NaSa01\_F13.raw |  |  |  |  | 1.0731E6 |  |  |  |  |  |  |  |  |  | 1 | 0 | 0 | 0 | 0 | 1 | 0 | 0 | 0 | 0 | 0 | 0 | 0 | 0 | 0 | 184 | 195 |  |  | PEAKS DB |
| R.TLNQLYLEAANR.Q | N | 40.76 | 1404.7361 | 12 | 0.2 | 703.3754 | 2 | 46.99 | 5 | F5:27638 | NaSa01\_F13.raw |  |  |  |  | 6.988E6 |  |  |  |  |  |  |  |  |  | 1 | 0 | 0 | 0 | 0 | 1 | 0 | 0 | 0 | 0 | 0 | 0 | 0 | 0 | 0 | 79 | 90 |  |  | PEAKS DB |
| total 5 peptides |  |  |  |  |  |  |  |  |  |  |  |  |  |  |  |  |  |  |  |  |  |  |  |  |  |  |  |  |  |  |  |  |  |  |  |  |  |  |  |  |  |  |  |  |  |
| --- | --- | --- | --- | --- | --- | --- | --- | --- | --- | --- | --- | --- | --- | --- | --- | --- | --- | --- | --- | --- | --- | --- | --- | --- | --- | --- | --- | --- | --- | --- | --- | --- | --- | --- | --- | --- | --- | --- | --- | --- | --- | --- | --- | --- | --- |

XP\_026572615.1

back to list

  

| Protein Coverage
| Supporting Peptides
|

Protein Coverage:

Supporting Peptides:

| Peptide | Uniq | -10lgP | Mass | Length | ppm | m/z | z | RT | Fraction | Scan | Source File | Area F1 | Area F10 | Area F11 | Area F12 | Area F13 | Area F14 | Area F2 | Area F3 | Area F4 | Area F5 | Area F6 | Area F7 | Area F8 | Area F9 | #Feature | #Feature F1 | #Feature F10 | #Feature F11 | #Feature F12 | #Feature F13 | #Feature F14 | #Feature F2 | #Feature F3 | #Feature F4 | #Feature F5 | #Feature F6 | #Feature F7 | #Feature F8 | #Feature F9 | Start | End | PTM | AScore | Found By |
| --- | --- | --- | --- | --- | --- | --- | --- | --- | --- | --- | --- | --- | --- | --- | --- | --- | --- | --- | --- | --- | --- | --- | --- | --- | --- | --- | --- | --- | --- | --- | --- | --- | --- | --- | --- | --- | --- | --- | --- | --- | --- | --- | --- | --- | --- |
| R.TQGFWLVHSTPR.F | N | 63.89 | 1427.7310 | 12 | 0.3 | 714.8730 | 2 | 38.99 | 5 | F5:20956 | NaSa01\_F13.raw |  |  |  |  | 5.2297E6 | 7.3135E5 |  |  |  |  |  |  |  |  | 3 | 0 | 0 | 0 | 0 | 2 | 1 | 0 | 0 | 0 | 0 | 0 | 0 | 0 | 0 | 126 | 137 |  |  | PEAKS DB |
| R.VYNVEQLAFQDPATSFFSTSDHSK.W | Y | 61.48 | 2717.2607 | 24 | 0.2 | 906.7610 | 3 | 80.41 | 5 | F5:54990 | NaSa01\_F13.raw |  |  |  |  | 6.6368E6 |  |  |  |  |  |  |  |  |  | 1 | 0 | 0 | 0 | 0 | 1 | 0 | 0 | 0 | 0 | 0 | 0 | 0 | 0 | 0 | 276 | 299 |  |  | PEAKS DB |
| E.HVQEEPWNSQVTLTSK.G | Y | 55.77 | 1881.9221 | 16 | 0.6 | 941.9689 | 2 | 33.62 | 5 | F5:16414 | NaSa01\_F13.raw |  |  |  |  | 6.3719E6 |  |  |  |  |  |  |  |  |  | 2 | 0 | 0 | 0 | 0 | 2 | 0 | 0 | 0 | 0 | 0 | 0 | 0 | 0 | 0 | 208 | 223 |  |  | PEAKS DB |
| H.VYDYQVQGAFAR.D | N | 44.07 | 1415.6833 | 12 | -0.1 | 708.8489 | 2 | 41.51 | 5 | F5:23007 | NaSa01\_F13.raw |  |  |  |  | 1.0731E6 |  |  |  |  |  |  |  |  |  | 1 | 0 | 0 | 0 | 0 | 1 | 0 | 0 | 0 | 0 | 0 | 0 | 0 | 0 | 0 | 184 | 195 |  |  | PEAKS DB |
| R.TLNQLYLEAANR.Q | N | 40.76 | 1404.7361 | 12 | 0.2 | 703.3754 | 2 | 46.99 | 5 | F5:27638 | NaSa01\_F13.raw |  |  |  |  | 6.988E6 |  |  |  |  |  |  |  |  |  | 1 | 0 | 0 | 0 | 0 | 1 | 0 | 0 | 0 | 0 | 0 | 0 | 0 | 0 | 0 | 79 | 90 |  |  | PEAKS DB |
| total 5 peptides |  |  |  |  |  |  |  |  |  |  |  |  |  |  |  |  |  |  |  |  |  |  |  |  |  |  |  |  |  |  |  |  |  |  |  |  |  |  |  |  |  |  |  |  |  |
| --- | --- | --- | --- | --- | --- | --- | --- | --- | --- | --- | --- | --- | --- | --- | --- | --- | --- | --- | --- | --- | --- | --- | --- | --- | --- | --- | --- | --- | --- | --- | --- | --- | --- | --- | --- | --- | --- | --- | --- | --- | --- | --- | --- | --- | --- |

XP\_026572612.1

back to list

  

| Protein Coverage
| Supporting Peptides
|

Protein Coverage:

Supporting Peptides:

| Peptide | Uniq | -10lgP | Mass | Length | ppm | m/z | z | RT | Fraction | Scan | Source File | Area F1 | Area F10 | Area F11 | Area F12 | Area F13 | Area F14 | Area F2 | Area F3 | Area F4 | Area F5 | Area F6 | Area F7 | Area F8 | Area F9 | #Feature | #Feature F1 | #Feature F10 | #Feature F11 | #Feature F12 | #Feature F13 | #Feature F14 | #Feature F2 | #Feature F3 | #Feature F4 | #Feature F5 | #Feature F6 | #Feature F7 | #Feature F8 | #Feature F9 | Start | End | PTM | AScore | Found By |
| --- | --- | --- | --- | --- | --- | --- | --- | --- | --- | --- | --- | --- | --- | --- | --- | --- | --- | --- | --- | --- | --- | --- | --- | --- | --- | --- | --- | --- | --- | --- | --- | --- | --- | --- | --- | --- | --- | --- | --- | --- | --- | --- | --- | --- | --- |
| R.TQGFWLVHSTPR.F | N | 63.89 | 1427.7310 | 12 | 0.3 | 714.8730 | 2 | 38.99 | 5 | F5:20956 | NaSa01\_F13.raw |  |  |  |  | 5.2297E6 | 7.3135E5 |  |  |  |  |  |  |  |  | 3 | 0 | 0 | 0 | 0 | 2 | 1 | 0 | 0 | 0 | 0 | 0 | 0 | 0 | 0 | 205 | 216 |  |  | PEAKS DB |
| R.VYNVEQLAFQDPATSFFSTSDHSK.W | Y | 61.48 | 2717.2607 | 24 | 0.2 | 906.7610 | 3 | 80.41 | 5 | F5:54990 | NaSa01\_F13.raw |  |  |  |  | 6.6368E6 |  |  |  |  |  |  |  |  |  | 1 | 0 | 0 | 0 | 0 | 1 | 0 | 0 | 0 | 0 | 0 | 0 | 0 | 0 | 0 | 355 | 378 |  |  | PEAKS DB |
| E.HVQEEPWNSQVTLTSK.G | Y | 55.77 | 1881.9221 | 16 | 0.6 | 941.9689 | 2 | 33.62 | 5 | F5:16414 | NaSa01\_F13.raw |  |  |  |  | 6.3719E6 |  |  |  |  |  |  |  |  |  | 2 | 0 | 0 | 0 | 0 | 2 | 0 | 0 | 0 | 0 | 0 | 0 | 0 | 0 | 0 | 287 | 302 |  |  | PEAKS DB |
| H.VYDYQVQGAFAR.D | N | 44.07 | 1415.6833 | 12 | -0.1 | 708.8489 | 2 | 41.51 | 5 | F5:23007 | NaSa01\_F13.raw |  |  |  |  | 1.0731E6 |  |  |  |  |  |  |  |  |  | 1 | 0 | 0 | 0 | 0 | 1 | 0 | 0 | 0 | 0 | 0 | 0 | 0 | 0 | 0 | 263 | 274 |  |  | PEAKS DB |
| R.TLNQLYLEAANR.Q | N | 40.76 | 1404.7361 | 12 | 0.2 | 703.3754 | 2 | 46.99 | 5 | F5:27638 | NaSa01\_F13.raw |  |  |  |  | 6.988E6 |  |  |  |  |  |  |  |  |  | 1 | 0 | 0 | 0 | 0 | 1 | 0 | 0 | 0 | 0 | 0 | 0 | 0 | 0 | 0 | 158 | 169 |  |  | PEAKS DB |
| total 5 peptides |  |  |  |  |  |  |  |  |  |  |  |  |  |  |  |  |  |  |  |  |  |  |  |  |  |  |  |  |  |  |  |  |  |  |  |  |  |  |  |  |  |  |  |  |  |
| --- | --- | --- | --- | --- | --- | --- | --- | --- | --- | --- | --- | --- | --- | --- | --- | --- | --- | --- | --- | --- | --- | --- | --- | --- | --- | --- | --- | --- | --- | --- | --- | --- | --- | --- | --- | --- | --- | --- | --- | --- | --- | --- | --- | --- | --- |

XP\_026535344.1

back to list

  

| Protein Coverage
| Supporting Peptides
|

Protein Coverage:

Supporting Peptides:

| Peptide | Uniq | -10lgP | Mass | Length | ppm | m/z | z | RT | Fraction | Scan | Source File | Area F1 | Area F10 | Area F11 | Area F12 | Area F13 | Area F14 | Area F2 | Area F3 | Area F4 | Area F5 | Area F6 | Area F7 | Area F8 | Area F9 | #Feature | #Feature F1 | #Feature F10 | #Feature F11 | #Feature F12 | #Feature F13 | #Feature F14 | #Feature F2 | #Feature F3 | #Feature F4 | #Feature F5 | #Feature F6 | #Feature F7 | #Feature F8 | #Feature F9 | Start | End | PTM | AScore | Found By |
| --- | --- | --- | --- | --- | --- | --- | --- | --- | --- | --- | --- | --- | --- | --- | --- | --- | --- | --- | --- | --- | --- | --- | --- | --- | --- | --- | --- | --- | --- | --- | --- | --- | --- | --- | --- | --- | --- | --- | --- | --- | --- | --- | --- | --- | --- |
| R.TQGFWLVHSTPR.F | N | 63.89 | 1427.7310 | 12 | 0.3 | 714.8730 | 2 | 38.99 | 5 | F5:20956 | NaSa01\_F13.raw |  |  |  |  | 5.2297E6 | 7.3135E5 |  |  |  |  |  |  |  |  | 3 | 0 | 0 | 0 | 0 | 2 | 1 | 0 | 0 | 0 | 0 | 0 | 0 | 0 | 0 | 139 | 150 |  |  | PEAKS DB |
| R.VYNVEQLAFQDPATSFFSTSDHSK.W | Y | 61.48 | 2717.2607 | 24 | 0.2 | 906.7610 | 3 | 80.41 | 5 | F5:54990 | NaSa01\_F13.raw |  |  |  |  | 6.6368E6 |  |  |  |  |  |  |  |  |  | 1 | 0 | 0 | 0 | 0 | 1 | 0 | 0 | 0 | 0 | 0 | 0 | 0 | 0 | 0 | 289 | 312 |  |  | PEAKS DB |
| E.HVQEEPWNSQVTLTSK.G | Y | 55.77 | 1881.9221 | 16 | 0.6 | 941.9689 | 2 | 33.62 | 5 | F5:16414 | NaSa01\_F13.raw |  |  |  |  | 6.3719E6 |  |  |  |  |  |  |  |  |  | 2 | 0 | 0 | 0 | 0 | 2 | 0 | 0 | 0 | 0 | 0 | 0 | 0 | 0 | 0 | 221 | 236 |  |  | PEAKS DB |
| R.VYDYQVQGAFAR.D | N | 44.07 | 1415.6833 | 12 | -0.1 | 708.8489 | 2 | 41.51 | 5 | F5:23007 | NaSa01\_F13.raw |  |  |  |  | 1.0731E6 |  |  |  |  |  |  |  |  |  | 1 | 0 | 0 | 0 | 0 | 1 | 0 | 0 | 0 | 0 | 0 | 0 | 0 | 0 | 0 | 197 | 208 |  |  | PEAKS DB |
| R.TLNQLYLEAANR.Q | N | 40.76 | 1404.7361 | 12 | 0.2 | 703.3754 | 2 | 46.99 | 5 | F5:27638 | NaSa01\_F13.raw |  |  |  |  | 6.988E6 |  |  |  |  |  |  |  |  |  | 1 | 0 | 0 | 0 | 0 | 1 | 0 | 0 | 0 | 0 | 0 | 0 | 0 | 0 | 0 | 92 | 103 |  |  | PEAKS DB |
| total 5 peptides |  |  |  |  |  |  |  |  |  |  |  |  |  |  |  |  |  |  |  |  |  |  |  |  |  |  |  |  |  |  |  |  |  |  |  |  |  |  |  |  |  |  |  |  |  |
| --- | --- | --- | --- | --- | --- | --- | --- | --- | --- | --- | --- | --- | --- | --- | --- | --- | --- | --- | --- | --- | --- | --- | --- | --- | --- | --- | --- | --- | --- | --- | --- | --- | --- | --- | --- | --- | --- | --- | --- | --- | --- | --- | --- | --- | --- |

XP\_026535343.1

back to list

  

| Protein Coverage
| Supporting Peptides
|

Protein Coverage:

Supporting Peptides:

| Peptide | Uniq | -10lgP | Mass | Length | ppm | m/z | z | RT | Fraction | Scan | Source File | Area F1 | Area F10 | Area F11 | Area F12 | Area F13 | Area F14 | Area F2 | Area F3 | Area F4 | Area F5 | Area F6 | Area F7 | Area F8 | Area F9 | #Feature | #Feature F1 | #Feature F10 | #Feature F11 | #Feature F12 | #Feature F13 | #Feature F14 | #Feature F2 | #Feature F3 | #Feature F4 | #Feature F5 | #Feature F6 | #Feature F7 | #Feature F8 | #Feature F9 | Start | End | PTM | AScore | Found By |
| --- | --- | --- | --- | --- | --- | --- | --- | --- | --- | --- | --- | --- | --- | --- | --- | --- | --- | --- | --- | --- | --- | --- | --- | --- | --- | --- | --- | --- | --- | --- | --- | --- | --- | --- | --- | --- | --- | --- | --- | --- | --- | --- | --- | --- | --- |
| R.TQGFWLVHSTPR.F | N | 63.89 | 1427.7310 | 12 | 0.3 | 714.8730 | 2 | 38.99 | 5 | F5:20956 | NaSa01\_F13.raw |  |  |  |  | 5.2297E6 | 7.3135E5 |  |  |  |  |  |  |  |  | 3 | 0 | 0 | 0 | 0 | 2 | 1 | 0 | 0 | 0 | 0 | 0 | 0 | 0 | 0 | 145 | 156 |  |  | PEAKS DB |
| R.VYNVEQLAFQDPATSFFSTSDHSK.W | Y | 61.48 | 2717.2607 | 24 | 0.2 | 906.7610 | 3 | 80.41 | 5 | F5:54990 | NaSa01\_F13.raw |  |  |  |  | 6.6368E6 |  |  |  |  |  |  |  |  |  | 1 | 0 | 0 | 0 | 0 | 1 | 0 | 0 | 0 | 0 | 0 | 0 | 0 | 0 | 0 | 295 | 318 |  |  | PEAKS DB |
| E.HVQEEPWNSQVTLTSK.G | Y | 55.77 | 1881.9221 | 16 | 0.6 | 941.9689 | 2 | 33.62 | 5 | F5:16414 | NaSa01\_F13.raw |  |  |  |  | 6.3719E6 |  |  |  |  |  |  |  |  |  | 2 | 0 | 0 | 0 | 0 | 2 | 0 | 0 | 0 | 0 | 0 | 0 | 0 | 0 | 0 | 227 | 242 |  |  | PEAKS DB |
| R.VYDYQVQGAFAR.D | N | 44.07 | 1415.6833 | 12 | -0.1 | 708.8489 | 2 | 41.51 | 5 | F5:23007 | NaSa01\_F13.raw |  |  |  |  | 1.0731E6 |  |  |  |  |  |  |  |  |  | 1 | 0 | 0 | 0 | 0 | 1 | 0 | 0 | 0 | 0 | 0 | 0 | 0 | 0 | 0 | 203 | 214 |  |  | PEAKS DB |
| R.TLNQLYLEAANR.Q | N | 40.76 | 1404.7361 | 12 | 0.2 | 703.3754 | 2 | 46.99 | 5 | F5:27638 | NaSa01\_F13.raw |  |  |  |  | 6.988E6 |  |  |  |  |  |  |  |  |  | 1 | 0 | 0 | 0 | 0 | 1 | 0 | 0 | 0 | 0 | 0 | 0 | 0 | 0 | 0 | 98 | 109 |  |  | PEAKS DB |
| total 5 peptides |  |  |  |  |  |  |  |  |  |  |  |  |  |  |  |  |  |  |  |  |  |  |  |  |  |  |  |  |  |  |  |  |  |  |  |  |  |  |  |  |  |  |  |  |  |
| --- | --- | --- | --- | --- | --- | --- | --- | --- | --- | --- | --- | --- | --- | --- | --- | --- | --- | --- | --- | --- | --- | --- | --- | --- | --- | --- | --- | --- | --- | --- | --- | --- | --- | --- | --- | --- | --- | --- | --- | --- | --- | --- | --- | --- | --- |

XP\_026535346.1

back to list

  

| Protein Coverage
| Supporting Peptides
|

Protein Coverage:

Supporting Peptides:

| Peptide | Uniq | -10lgP | Mass | Length | ppm | m/z | z | RT | Fraction | Scan | Source File | Area F1 | Area F10 | Area F11 | Area F12 | Area F13 | Area F14 | Area F2 | Area F3 | Area F4 | Area F5 | Area F6 | Area F7 | Area F8 | Area F9 | #Feature | #Feature F1 | #Feature F10 | #Feature F11 | #Feature F12 | #Feature F13 | #Feature F14 | #Feature F2 | #Feature F3 | #Feature F4 | #Feature F5 | #Feature F6 | #Feature F7 | #Feature F8 | #Feature F9 | Start | End | PTM | AScore | Found By |
| --- | --- | --- | --- | --- | --- | --- | --- | --- | --- | --- | --- | --- | --- | --- | --- | --- | --- | --- | --- | --- | --- | --- | --- | --- | --- | --- | --- | --- | --- | --- | --- | --- | --- | --- | --- | --- | --- | --- | --- | --- | --- | --- | --- | --- | --- |
| R.TQGFWLVHSTPR.F | N | 63.89 | 1427.7310 | 12 | 0.3 | 714.8730 | 2 | 38.99 | 5 | F5:20956 | NaSa01\_F13.raw |  |  |  |  | 5.2297E6 | 7.3135E5 |  |  |  |  |  |  |  |  | 3 | 0 | 0 | 0 | 0 | 2 | 1 | 0 | 0 | 0 | 0 | 0 | 0 | 0 | 0 | 126 | 137 |  |  | PEAKS DB |
| R.VYNVEQLAFQDPATSFFSTSDHSK.W | Y | 61.48 | 2717.2607 | 24 | 0.2 | 906.7610 | 3 | 80.41 | 5 | F5:54990 | NaSa01\_F13.raw |  |  |  |  | 6.6368E6 |  |  |  |  |  |  |  |  |  | 1 | 0 | 0 | 0 | 0 | 1 | 0 | 0 | 0 | 0 | 0 | 0 | 0 | 0 | 0 | 276 | 299 |  |  | PEAKS DB |
| E.HVQEEPWNSQVTLTSK.G | Y | 55.77 | 1881.9221 | 16 | 0.6 | 941.9689 | 2 | 33.62 | 5 | F5:16414 | NaSa01\_F13.raw |  |  |  |  | 6.3719E6 |  |  |  |  |  |  |  |  |  | 2 | 0 | 0 | 0 | 0 | 2 | 0 | 0 | 0 | 0 | 0 | 0 | 0 | 0 | 0 | 208 | 223 |  |  | PEAKS DB |
| R.VYDYQVQGAFAR.D | N | 44.07 | 1415.6833 | 12 | -0.1 | 708.8489 | 2 | 41.51 | 5 | F5:23007 | NaSa01\_F13.raw |  |  |  |  | 1.0731E6 |  |  |  |  |  |  |  |  |  | 1 | 0 | 0 | 0 | 0 | 1 | 0 | 0 | 0 | 0 | 0 | 0 | 0 | 0 | 0 | 184 | 195 |  |  | PEAKS DB |
| R.TLNQLYLEAANR.Q | N | 40.76 | 1404.7361 | 12 | 0.2 | 703.3754 | 2 | 46.99 | 5 | F5:27638 | NaSa01\_F13.raw |  |  |  |  | 6.988E6 |  |  |  |  |  |  |  |  |  | 1 | 0 | 0 | 0 | 0 | 1 | 0 | 0 | 0 | 0 | 0 | 0 | 0 | 0 | 0 | 79 | 90 |  |  | PEAKS DB |
| total 5 peptides |  |  |  |  |  |  |  |  |  |  |  |  |  |  |  |  |  |  |  |  |  |  |  |  |  |  |  |  |  |  |  |  |  |  |  |  |  |  |  |  |  |  |  |  |  |
| --- | --- | --- | --- | --- | --- | --- | --- | --- | --- | --- | --- | --- | --- | --- | --- | --- | --- | --- | --- | --- | --- | --- | --- | --- | --- | --- | --- | --- | --- | --- | --- | --- | --- | --- | --- | --- | --- | --- | --- | --- | --- | --- | --- | --- | --- |

XP\_026535348.1

back to list

  

| Protein Coverage
| Supporting Peptides
|

Protein Coverage:

Supporting Peptides:

| Peptide | Uniq | -10lgP | Mass | Length | ppm | m/z | z | RT | Fraction | Scan | Source File | Area F1 | Area F10 | Area F11 | Area F12 | Area F13 | Area F14 | Area F2 | Area F3 | Area F4 | Area F5 | Area F6 | Area F7 | Area F8 | Area F9 | #Feature | #Feature F1 | #Feature F10 | #Feature F11 | #Feature F12 | #Feature F13 | #Feature F14 | #Feature F2 | #Feature F3 | #Feature F4 | #Feature F5 | #Feature F6 | #Feature F7 | #Feature F8 | #Feature F9 | Start | End | PTM | AScore | Found By |
| --- | --- | --- | --- | --- | --- | --- | --- | --- | --- | --- | --- | --- | --- | --- | --- | --- | --- | --- | --- | --- | --- | --- | --- | --- | --- | --- | --- | --- | --- | --- | --- | --- | --- | --- | --- | --- | --- | --- | --- | --- | --- | --- | --- | --- | --- |
| R.TQGFWLVHSTPR.F | N | 63.89 | 1427.7310 | 12 | 0.3 | 714.8730 | 2 | 38.99 | 5 | F5:20956 | NaSa01\_F13.raw |  |  |  |  | 5.2297E6 | 7.3135E5 |  |  |  |  |  |  |  |  | 3 | 0 | 0 | 0 | 0 | 2 | 1 | 0 | 0 | 0 | 0 | 0 | 0 | 0 | 0 | 126 | 137 |  |  | PEAKS DB |
| R.VYNVEQLAFQDPATSFFSTSDHSK.W | Y | 61.48 | 2717.2607 | 24 | 0.2 | 906.7610 | 3 | 80.41 | 5 | F5:54990 | NaSa01\_F13.raw |  |  |  |  | 6.6368E6 |  |  |  |  |  |  |  |  |  | 1 | 0 | 0 | 0 | 0 | 1 | 0 | 0 | 0 | 0 | 0 | 0 | 0 | 0 | 0 | 276 | 299 |  |  | PEAKS DB |
| E.HVQEEPWNSQVTLTSK.G | Y | 55.77 | 1881.9221 | 16 | 0.6 | 941.9689 | 2 | 33.62 | 5 | F5:16414 | NaSa01\_F13.raw |  |  |  |  | 6.3719E6 |  |  |  |  |  |  |  |  |  | 2 | 0 | 0 | 0 | 0 | 2 | 0 | 0 | 0 | 0 | 0 | 0 | 0 | 0 | 0 | 208 | 223 |  |  | PEAKS DB |
| R.VYDYQVQGAFAR.D | N | 44.07 | 1415.6833 | 12 | -0.1 | 708.8489 | 2 | 41.51 | 5 | F5:23007 | NaSa01\_F13.raw |  |  |  |  | 1.0731E6 |  |  |  |  |  |  |  |  |  | 1 | 0 | 0 | 0 | 0 | 1 | 0 | 0 | 0 | 0 | 0 | 0 | 0 | 0 | 0 | 184 | 195 |  |  | PEAKS DB |
| R.TLNQLYLEAANR.Q | N | 40.76 | 1404.7361 | 12 | 0.2 | 703.3754 | 2 | 46.99 | 5 | F5:27638 | NaSa01\_F13.raw |  |  |  |  | 6.988E6 |  |  |  |  |  |  |  |  |  | 1 | 0 | 0 | 0 | 0 | 1 | 0 | 0 | 0 | 0 | 0 | 0 | 0 | 0 | 0 | 79 | 90 |  |  | PEAKS DB |
| total 5 peptides |  |  |  |  |  |  |  |  |  |  |  |  |  |  |  |  |  |  |  |  |  |  |  |  |  |  |  |  |  |  |  |  |  |  |  |  |  |  |  |  |  |  |  |  |  |
| --- | --- | --- | --- | --- | --- | --- | --- | --- | --- | --- | --- | --- | --- | --- | --- | --- | --- | --- | --- | --- | --- | --- | --- | --- | --- | --- | --- | --- | --- | --- | --- | --- | --- | --- | --- | --- | --- | --- | --- | --- | --- | --- | --- | --- | --- |

XP\_026535349.1

back to list

  

| Protein Coverage
| Supporting Peptides
|

Protein Coverage:

Supporting Peptides:

| Peptide | Uniq | -10lgP | Mass | Length | ppm | m/z | z | RT | Fraction | Scan | Source File | Area F1 | Area F10 | Area F11 | Area F12 | Area F13 | Area F14 | Area F2 | Area F3 | Area F4 | Area F5 | Area F6 | Area F7 | Area F8 | Area F9 | #Feature | #Feature F1 | #Feature F10 | #Feature F11 | #Feature F12 | #Feature F13 | #Feature F14 | #Feature F2 | #Feature F3 | #Feature F4 | #Feature F5 | #Feature F6 | #Feature F7 | #Feature F8 | #Feature F9 | Start | End | PTM | AScore | Found By |
| --- | --- | --- | --- | --- | --- | --- | --- | --- | --- | --- | --- | --- | --- | --- | --- | --- | --- | --- | --- | --- | --- | --- | --- | --- | --- | --- | --- | --- | --- | --- | --- | --- | --- | --- | --- | --- | --- | --- | --- | --- | --- | --- | --- | --- | --- |
| R.TQGFWLVHSTPR.F | N | 63.89 | 1427.7310 | 12 | 0.3 | 714.8730 | 2 | 38.99 | 5 | F5:20956 | NaSa01\_F13.raw |  |  |  |  | 5.2297E6 | 7.3135E5 |  |  |  |  |  |  |  |  | 3 | 0 | 0 | 0 | 0 | 2 | 1 | 0 | 0 | 0 | 0 | 0 | 0 | 0 | 0 | 126 | 137 |  |  | PEAKS DB |
| R.VYNVEQLAFQDPATSFFSTSDHSK.W | Y | 61.48 | 2717.2607 | 24 | 0.2 | 906.7610 | 3 | 80.41 | 5 | F5:54990 | NaSa01\_F13.raw |  |  |  |  | 6.6368E6 |  |  |  |  |  |  |  |  |  | 1 | 0 | 0 | 0 | 0 | 1 | 0 | 0 | 0 | 0 | 0 | 0 | 0 | 0 | 0 | 276 | 299 |  |  | PEAKS DB |
| E.HVQEEPWNSQVTLTSK.G | Y | 55.77 | 1881.9221 | 16 | 0.6 | 941.9689 | 2 | 33.62 | 5 | F5:16414 | NaSa01\_F13.raw |  |  |  |  | 6.3719E6 |  |  |  |  |  |  |  |  |  | 2 | 0 | 0 | 0 | 0 | 2 | 0 | 0 | 0 | 0 | 0 | 0 | 0 | 0 | 0 | 208 | 223 |  |  | PEAKS DB |
| R.VYDYQVQGAFAR.D | N | 44.07 | 1415.6833 | 12 | -0.1 | 708.8489 | 2 | 41.51 | 5 | F5:23007 | NaSa01\_F13.raw |  |  |  |  | 1.0731E6 |  |  |  |  |  |  |  |  |  | 1 | 0 | 0 | 0 | 0 | 1 | 0 | 0 | 0 | 0 | 0 | 0 | 0 | 0 | 0 | 184 | 195 |  |  | PEAKS DB |
| R.TLNQLYLEAANR.Q | N | 40.76 | 1404.7361 | 12 | 0.2 | 703.3754 | 2 | 46.99 | 5 | F5:27638 | NaSa01\_F13.raw |  |  |  |  | 6.988E6 |  |  |  |  |  |  |  |  |  | 1 | 0 | 0 | 0 | 0 | 1 | 0 | 0 | 0 | 0 | 0 | 0 | 0 | 0 | 0 | 79 | 90 |  |  | PEAKS DB |
| total 5 peptides |  |  |  |  |  |  |  |  |  |  |  |  |  |  |  |  |  |  |  |  |  |  |  |  |  |  |  |  |  |  |  |  |  |  |  |  |  |  |  |  |  |  |  |  |  |
| --- | --- | --- | --- | --- | --- | --- | --- | --- | --- | --- | --- | --- | --- | --- | --- | --- | --- | --- | --- | --- | --- | --- | --- | --- | --- | --- | --- | --- | --- | --- | --- | --- | --- | --- | --- | --- | --- | --- | --- | --- | --- | --- | --- | --- | --- |

XP\_026535350.1

back to list

  

| Protein Coverage
| Supporting Peptides
|

Protein Coverage:

Supporting Peptides:

| Peptide | Uniq | -10lgP | Mass | Length | ppm | m/z | z | RT | Fraction | Scan | Source File | Area F1 | Area F10 | Area F11 | Area F12 | Area F13 | Area F14 | Area F2 | Area F3 | Area F4 | Area F5 | Area F6 | Area F7 | Area F8 | Area F9 | #Feature | #Feature F1 | #Feature F10 | #Feature F11 | #Feature F12 | #Feature F13 | #Feature F14 | #Feature F2 | #Feature F3 | #Feature F4 | #Feature F5 | #Feature F6 | #Feature F7 | #Feature F8 | #Feature F9 | Start | End | PTM | AScore | Found By |
| --- | --- | --- | --- | --- | --- | --- | --- | --- | --- | --- | --- | --- | --- | --- | --- | --- | --- | --- | --- | --- | --- | --- | --- | --- | --- | --- | --- | --- | --- | --- | --- | --- | --- | --- | --- | --- | --- | --- | --- | --- | --- | --- | --- | --- | --- |
| R.TQGFWLVHSTPR.F | N | 63.89 | 1427.7310 | 12 | 0.3 | 714.8730 | 2 | 38.99 | 5 | F5:20956 | NaSa01\_F13.raw |  |  |  |  | 5.2297E6 | 7.3135E5 |  |  |  |  |  |  |  |  | 3 | 0 | 0 | 0 | 0 | 2 | 1 | 0 | 0 | 0 | 0 | 0 | 0 | 0 | 0 | 126 | 137 |  |  | PEAKS DB |
| R.VYNVEQLAFQDPATSFFSTSDHSK.W | Y | 61.48 | 2717.2607 | 24 | 0.2 | 906.7610 | 3 | 80.41 | 5 | F5:54990 | NaSa01\_F13.raw |  |  |  |  | 6.6368E6 |  |  |  |  |  |  |  |  |  | 1 | 0 | 0 | 0 | 0 | 1 | 0 | 0 | 0 | 0 | 0 | 0 | 0 | 0 | 0 | 276 | 299 |  |  | PEAKS DB |
| E.HVQEEPWNSQVTLTSK.G | Y | 55.77 | 1881.9221 | 16 | 0.6 | 941.9689 | 2 | 33.62 | 5 | F5:16414 | NaSa01\_F13.raw |  |  |  |  | 6.3719E6 |  |  |  |  |  |  |  |  |  | 2 | 0 | 0 | 0 | 0 | 2 | 0 | 0 | 0 | 0 | 0 | 0 | 0 | 0 | 0 | 208 | 223 |  |  | PEAKS DB |
| R.VYDYQVQGAFAR.D | N | 44.07 | 1415.6833 | 12 | -0.1 | 708.8489 | 2 | 41.51 | 5 | F5:23007 | NaSa01\_F13.raw |  |  |  |  | 1.0731E6 |  |  |  |  |  |  |  |  |  | 1 | 0 | 0 | 0 | 0 | 1 | 0 | 0 | 0 | 0 | 0 | 0 | 0 | 0 | 0 | 184 | 195 |  |  | PEAKS DB |
| R.TLNQLYLEAANR.Q | N | 40.76 | 1404.7361 | 12 | 0.2 | 703.3754 | 2 | 46.99 | 5 | F5:27638 | NaSa01\_F13.raw |  |  |  |  | 6.988E6 |  |  |  |  |  |  |  |  |  | 1 | 0 | 0 | 0 | 0 | 1 | 0 | 0 | 0 | 0 | 0 | 0 | 0 | 0 | 0 | 79 | 90 |  |  | PEAKS DB |
| total 5 peptides |  |  |  |  |  |  |  |  |  |  |  |  |  |  |  |  |  |  |  |  |  |  |  |  |  |  |  |  |  |  |  |  |  |  |  |  |  |  |  |  |  |  |  |  |  |
| --- | --- | --- | --- | --- | --- | --- | --- | --- | --- | --- | --- | --- | --- | --- | --- | --- | --- | --- | --- | --- | --- | --- | --- | --- | --- | --- | --- | --- | --- | --- | --- | --- | --- | --- | --- | --- | --- | --- | --- | --- | --- | --- | --- | --- | --- |

XP\_026535345.1

back to list

  

| Protein Coverage
| Supporting Peptides
|

Protein Coverage:

Supporting Peptides:

| Peptide | Uniq | -10lgP | Mass | Length | ppm | m/z | z | RT | Fraction | Scan | Source File | Area F1 | Area F10 | Area F11 | Area F12 | Area F13 | Area F14 | Area F2 | Area F3 | Area F4 | Area F5 | Area F6 | Area F7 | Area F8 | Area F9 | #Feature | #Feature F1 | #Feature F10 | #Feature F11 | #Feature F12 | #Feature F13 | #Feature F14 | #Feature F2 | #Feature F3 | #Feature F4 | #Feature F5 | #Feature F6 | #Feature F7 | #Feature F8 | #Feature F9 | Start | End | PTM | AScore | Found By |
| --- | --- | --- | --- | --- | --- | --- | --- | --- | --- | --- | --- | --- | --- | --- | --- | --- | --- | --- | --- | --- | --- | --- | --- | --- | --- | --- | --- | --- | --- | --- | --- | --- | --- | --- | --- | --- | --- | --- | --- | --- | --- | --- | --- | --- | --- |
| R.TQGFWLVHSTPR.F | N | 63.89 | 1427.7310 | 12 | 0.3 | 714.8730 | 2 | 38.99 | 5 | F5:20956 | NaSa01\_F13.raw |  |  |  |  | 5.2297E6 | 7.3135E5 |  |  |  |  |  |  |  |  | 3 | 0 | 0 | 0 | 0 | 2 | 1 | 0 | 0 | 0 | 0 | 0 | 0 | 0 | 0 | 126 | 137 |  |  | PEAKS DB |
| R.VYNVEQLAFQDPATSFFSTSDHSK.W | Y | 61.48 | 2717.2607 | 24 | 0.2 | 906.7610 | 3 | 80.41 | 5 | F5:54990 | NaSa01\_F13.raw |  |  |  |  | 6.6368E6 |  |  |  |  |  |  |  |  |  | 1 | 0 | 0 | 0 | 0 | 1 | 0 | 0 | 0 | 0 | 0 | 0 | 0 | 0 | 0 | 276 | 299 |  |  | PEAKS DB |
| E.HVQEEPWNSQVTLTSK.G | Y | 55.77 | 1881.9221 | 16 | 0.6 | 941.9689 | 2 | 33.62 | 5 | F5:16414 | NaSa01\_F13.raw |  |  |  |  | 6.3719E6 |  |  |  |  |  |  |  |  |  | 2 | 0 | 0 | 0 | 0 | 2 | 0 | 0 | 0 | 0 | 0 | 0 | 0 | 0 | 0 | 208 | 223 |  |  | PEAKS DB |
| R.VYDYQVQGAFAR.D | N | 44.07 | 1415.6833 | 12 | -0.1 | 708.8489 | 2 | 41.51 | 5 | F5:23007 | NaSa01\_F13.raw |  |  |  |  | 1.0731E6 |  |  |  |  |  |  |  |  |  | 1 | 0 | 0 | 0 | 0 | 1 | 0 | 0 | 0 | 0 | 0 | 0 | 0 | 0 | 0 | 184 | 195 |  |  | PEAKS DB |
| R.TLNQLYLEAANR.Q | N | 40.76 | 1404.7361 | 12 | 0.2 | 703.3754 | 2 | 46.99 | 5 | F5:27638 | NaSa01\_F13.raw |  |  |  |  | 6.988E6 |  |  |  |  |  |  |  |  |  | 1 | 0 | 0 | 0 | 0 | 1 | 0 | 0 | 0 | 0 | 0 | 0 | 0 | 0 | 0 | 79 | 90 |  |  | PEAKS DB |
| total 5 peptides |  |  |  |  |  |  |  |  |  |  |  |  |  |  |  |  |  |  |  |  |  |  |  |  |  |  |  |  |  |  |  |  |  |  |  |  |  |  |  |  |  |  |  |  |  |
| --- | --- | --- | --- | --- | --- | --- | --- | --- | --- | --- | --- | --- | --- | --- | --- | --- | --- | --- | --- | --- | --- | --- | --- | --- | --- | --- | --- | --- | --- | --- | --- | --- | --- | --- | --- | --- | --- | --- | --- | --- | --- | --- | --- | --- | --- |

XP\_026555457.1

back to list

  

| Protein Coverage
| Supporting Peptides
|

Protein Coverage:

Supporting Peptides:

| Peptide | Uniq | -10lgP | Mass | Length | ppm | m/z | z | RT | Fraction | Scan | Source File | Area F1 | Area F10 | Area F11 | Area F12 | Area F13 | Area F14 | Area F2 | Area F3 | Area F4 | Area F5 | Area F6 | Area F7 | Area F8 | Area F9 | #Feature | #Feature F1 | #Feature F10 | #Feature F11 | #Feature F12 | #Feature F13 | #Feature F14 | #Feature F2 | #Feature F3 | #Feature F4 | #Feature F5 | #Feature F6 | #Feature F7 | #Feature F8 | #Feature F9 | Start | End | PTM | AScore | Found By |
| --- | --- | --- | --- | --- | --- | --- | --- | --- | --- | --- | --- | --- | --- | --- | --- | --- | --- | --- | --- | --- | --- | --- | --- | --- | --- | --- | --- | --- | --- | --- | --- | --- | --- | --- | --- | --- | --- | --- | --- | --- | --- | --- | --- | --- | --- |
| R.VPGFTQFHFMASNPILK.Q | Y | 75.72 | 1932.9919 | 17 | 0.1 | 645.3380 | 3 | 71.41 | 4 | F4:49019 | NaSa01\_F12.raw |  |  |  | 4.5823E6 |  |  |  |  |  |  |  |  |  |  | 1 | 0 | 0 | 0 | 1 | 0 | 0 | 0 | 0 | 0 | 0 | 0 | 0 | 0 | 0 | 327 | 343 |  |  | PEAKS DB |
| R.FLPSYQAVEYMR.L | N | 63.59 | 1502.7228 | 12 | 0.5 | 752.3690 | 2 | 59.73 | 4 | F4:39481 | NaSa01\_F12.raw |  |  |  | 4.6244E6 |  |  |  |  |  |  |  |  |  |  | 1 | 0 | 0 | 0 | 1 | 0 | 0 | 0 | 0 | 0 | 0 | 0 | 0 | 0 | 0 | 273 | 284 |  |  | PEAKS DB |
| K.QPSEQIVDC(+57.02)I | N | 41.14 | 1187.5492 | 10 | -1.7 | 594.7809 | 2 | 54.02 | 4 | F4:34712 | NaSa01\_F12.raw |  |  |  | 5.6474E5 |  |  |  |  |  |  |  |  |  |  | 1 | 0 | 0 | 0 | 1 | 0 | 0 | 0 | 0 | 0 | 0 | 0 | 0 | 0 | 0 | 344 | 353 | Carbamidomethylation | C9:Carbamidomethylation:1000.00 | PEAKS DB |
| total 3 peptides |  |  |  |  |  |  |  |  |  |  |  |  |  |  |  |  |  |  |  |  |  |  |  |  |  |  |  |  |  |  |  |  |  |  |  |  |  |  |  |  |  |  |  |  |  |
| --- | --- | --- | --- | --- | --- | --- | --- | --- | --- | --- | --- | --- | --- | --- | --- | --- | --- | --- | --- | --- | --- | --- | --- | --- | --- | --- | --- | --- | --- | --- | --- | --- | --- | --- | --- | --- | --- | --- | --- | --- | --- | --- | --- | --- | --- |

XP\_026522978.1

back to list

  

| Protein Coverage
| Supporting Peptides
|

Protein Coverage:

Supporting Peptides:

| Peptide | Uniq | -10lgP | Mass | Length | ppm | m/z | z | RT | Fraction | Scan | Source File | Area F1 | Area F10 | Area F11 | Area F12 | Area F13 | Area F14 | Area F2 | Area F3 | Area F4 | Area F5 | Area F6 | Area F7 | Area F8 | Area F9 | #Feature | #Feature F1 | #Feature F10 | #Feature F11 | #Feature F12 | #Feature F13 | #Feature F14 | #Feature F2 | #Feature F3 | #Feature F4 | #Feature F5 | #Feature F6 | #Feature F7 | #Feature F8 | #Feature F9 | Start | End | PTM | AScore | Found By |
| --- | --- | --- | --- | --- | --- | --- | --- | --- | --- | --- | --- | --- | --- | --- | --- | --- | --- | --- | --- | --- | --- | --- | --- | --- | --- | --- | --- | --- | --- | --- | --- | --- | --- | --- | --- | --- | --- | --- | --- | --- | --- | --- | --- | --- | --- |
| R.VPGFTQFHFMASNPILK.Q | Y | 75.72 | 1932.9919 | 17 | 0.1 | 645.3380 | 3 | 71.41 | 4 | F4:49019 | NaSa01\_F12.raw |  |  |  | 4.5823E6 |  |  |  |  |  |  |  |  |  |  | 1 | 0 | 0 | 0 | 1 | 0 | 0 | 0 | 0 | 0 | 0 | 0 | 0 | 0 | 0 | 327 | 343 |  |  | PEAKS DB |
| R.FLPSYQAVEYMR.L | N | 63.59 | 1502.7228 | 12 | 0.5 | 752.3690 | 2 | 59.73 | 4 | F4:39481 | NaSa01\_F12.raw |  |  |  | 4.6244E6 |  |  |  |  |  |  |  |  |  |  | 1 | 0 | 0 | 0 | 1 | 0 | 0 | 0 | 0 | 0 | 0 | 0 | 0 | 0 | 0 | 273 | 284 |  |  | PEAKS DB |
| K.QPSEQIVDC(+57.02)I | N | 41.14 | 1187.5492 | 10 | -1.7 | 594.7809 | 2 | 54.02 | 4 | F4:34712 | NaSa01\_F12.raw |  |  |  | 5.6474E5 |  |  |  |  |  |  |  |  |  |  | 1 | 0 | 0 | 0 | 1 | 0 | 0 | 0 | 0 | 0 | 0 | 0 | 0 | 0 | 0 | 344 | 353 | Carbamidomethylation | C9:Carbamidomethylation:1000.00 | PEAKS DB |
| total 3 peptides |  |  |  |  |  |  |  |  |  |  |  |  |  |  |  |  |  |  |  |  |  |  |  |  |  |  |  |  |  |  |  |  |  |  |  |  |  |  |  |  |  |  |  |  |  |
| --- | --- | --- | --- | --- | --- | --- | --- | --- | --- | --- | --- | --- | --- | --- | --- | --- | --- | --- | --- | --- | --- | --- | --- | --- | --- | --- | --- | --- | --- | --- | --- | --- | --- | --- | --- | --- | --- | --- | --- | --- | --- | --- | --- | --- | --- |

XP\_026575334.1

back to list

  

| Protein Coverage
| Supporting Peptides
|

Protein Coverage:

Supporting Peptides:

| Peptide | Uniq | -10lgP | Mass | Length | ppm | m/z | z | RT | Fraction | Scan | Source File | Area F1 | Area F10 | Area F11 | Area F12 | Area F13 | Area F14 | Area F2 | Area F3 | Area F4 | Area F5 | Area F6 | Area F7 | Area F8 | Area F9 | #Feature | #Feature F1 | #Feature F10 | #Feature F11 | #Feature F12 | #Feature F13 | #Feature F14 | #Feature F2 | #Feature F3 | #Feature F4 | #Feature F5 | #Feature F6 | #Feature F7 | #Feature F8 | #Feature F9 | Start | End | PTM | AScore | Found By |
| --- | --- | --- | --- | --- | --- | --- | --- | --- | --- | --- | --- | --- | --- | --- | --- | --- | --- | --- | --- | --- | --- | --- | --- | --- | --- | --- | --- | --- | --- | --- | --- | --- | --- | --- | --- | --- | --- | --- | --- | --- | --- | --- | --- | --- | --- |
| R.QLVPIGLSEFALDIR.M | Y | 61.03 | 1669.9402 | 15 | 1.0 | 835.9782 | 2 | 95.44 | 4 | F4:68666 | NaSa01\_F12.raw |  |  |  | 1.5973E6 | 3.2172E5 |  |  |  |  |  |  |  |  |  | 3 | 0 | 0 | 0 | 2 | 1 | 0 | 0 | 0 | 0 | 0 | 0 | 0 | 0 | 0 | 55 | 69 |  |  | PEAKS DB |
| P.IGLSEFALDIR.M | Y | 59.20 | 1232.6764 | 11 | 0.3 | 617.3456 | 2 | 76.06 | 4 | F4:53048 | NaSa01\_F12.raw |  |  |  | 1.0579E6 |  |  |  |  |  |  |  |  |  |  | 1 | 0 | 0 | 0 | 1 | 0 | 0 | 0 | 0 | 0 | 0 | 0 | 0 | 0 | 0 | 59 | 69 |  |  | PEAKS DB |
| R.LPVEEEAYVVDFKPH.A | Y | 52.76 | 1770.8828 | 15 | 0.4 | 591.3018 | 3 | 55.27 | 4 | F4:35730 | NaSa01\_F12.raw |  |  |  | 1.2477E6 | 1.5253E6 |  |  |  |  |  |  |  |  |  | 2 | 0 | 0 | 0 | 1 | 1 | 0 | 0 | 0 | 0 | 0 | 0 | 0 | 0 | 0 | 88 | 102 |  |  | PEAKS DB |
| K.YFVLQVHYGDVGVFK.D | Y | 42.63 | 1769.9141 | 15 | 0.6 | 590.9790 | 3 | 67.89 | 3 | F3:41163 | NaSa01\_F11.raw |  |  | 3.5691E5 |  |  |  |  |  |  |  |  |  |  |  | 1 | 0 | 0 | 1 | 0 | 0 | 0 | 0 | 0 | 0 | 0 | 0 | 0 | 0 | 0 | 168 | 182 |  |  | PEAKS DB |
| K.IEEVADWPGITLK.L | Y | 41.37 | 1469.7766 | 13 | 0.0 | 735.8956 | 2 | 70.61 | 5 | F5:47070 | NaSa01\_F13.raw |  |  |  |  | 1.5089E5 |  |  |  |  |  |  |  |  |  | 1 | 0 | 0 | 0 | 0 | 1 | 0 | 0 | 0 | 0 | 0 | 0 | 0 | 0 | 0 | 505 | 517 |  |  | PEAKS DB |
| total 5 peptides |  |  |  |  |  |  |  |  |  |  |  |  |  |  |  |  |  |  |  |  |  |  |  |  |  |  |  |  |  |  |  |  |  |  |  |  |  |  |  |  |  |  |  |  |  |
| --- | --- | --- | --- | --- | --- | --- | --- | --- | --- | --- | --- | --- | --- | --- | --- | --- | --- | --- | --- | --- | --- | --- | --- | --- | --- | --- | --- | --- | --- | --- | --- | --- | --- | --- | --- | --- | --- | --- | --- | --- | --- | --- | --- | --- | --- |

XP\_026575314.1

back to list

  

| Protein Coverage
| Supporting Peptides
|

Protein Coverage:

Supporting Peptides:

| Peptide | Uniq | -10lgP | Mass | Length | ppm | m/z | z | RT | Fraction | Scan | Source File | Area F1 | Area F10 | Area F11 | Area F12 | Area F13 | Area F14 | Area F2 | Area F3 | Area F4 | Area F5 | Area F6 | Area F7 | Area F8 | Area F9 | #Feature | #Feature F1 | #Feature F10 | #Feature F11 | #Feature F12 | #Feature F13 | #Feature F14 | #Feature F2 | #Feature F3 | #Feature F4 | #Feature F5 | #Feature F6 | #Feature F7 | #Feature F8 | #Feature F9 | Start | End | PTM | AScore | Found By |
| --- | --- | --- | --- | --- | --- | --- | --- | --- | --- | --- | --- | --- | --- | --- | --- | --- | --- | --- | --- | --- | --- | --- | --- | --- | --- | --- | --- | --- | --- | --- | --- | --- | --- | --- | --- | --- | --- | --- | --- | --- | --- | --- | --- | --- | --- |
| R.QLVPIGLSEFALDIR.M | Y | 61.03 | 1669.9402 | 15 | 1.0 | 835.9782 | 2 | 95.44 | 4 | F4:68666 | NaSa01\_F12.raw |  |  |  | 1.5973E6 | 3.2172E5 |  |  |  |  |  |  |  |  |  | 3 | 0 | 0 | 0 | 2 | 1 | 0 | 0 | 0 | 0 | 0 | 0 | 0 | 0 | 0 | 55 | 69 |  |  | PEAKS DB |
| P.IGLSEFALDIR.M | Y | 59.20 | 1232.6764 | 11 | 0.3 | 617.3456 | 2 | 76.06 | 4 | F4:53048 | NaSa01\_F12.raw |  |  |  | 1.0579E6 |  |  |  |  |  |  |  |  |  |  | 1 | 0 | 0 | 0 | 1 | 0 | 0 | 0 | 0 | 0 | 0 | 0 | 0 | 0 | 0 | 59 | 69 |  |  | PEAKS DB |
| R.LPVEEEAYVVDFKPH.A | Y | 52.76 | 1770.8828 | 15 | 0.4 | 591.3018 | 3 | 55.27 | 4 | F4:35730 | NaSa01\_F12.raw |  |  |  | 1.2477E6 | 1.5253E6 |  |  |  |  |  |  |  |  |  | 2 | 0 | 0 | 0 | 1 | 1 | 0 | 0 | 0 | 0 | 0 | 0 | 0 | 0 | 0 | 88 | 102 |  |  | PEAKS DB |
| K.YFVLQVHYGDVGVFK.D | Y | 42.63 | 1769.9141 | 15 | 0.6 | 590.9790 | 3 | 67.89 | 3 | F3:41163 | NaSa01\_F11.raw |  |  | 3.5691E5 |  |  |  |  |  |  |  |  |  |  |  | 1 | 0 | 0 | 1 | 0 | 0 | 0 | 0 | 0 | 0 | 0 | 0 | 0 | 0 | 0 | 168 | 182 |  |  | PEAKS DB |
| K.IEEVADWPGITLK.L | Y | 41.37 | 1469.7766 | 13 | 0.0 | 735.8956 | 2 | 70.61 | 5 | F5:47070 | NaSa01\_F13.raw |  |  |  |  | 1.5089E5 |  |  |  |  |  |  |  |  |  | 1 | 0 | 0 | 0 | 0 | 1 | 0 | 0 | 0 | 0 | 0 | 0 | 0 | 0 | 0 | 505 | 517 |  |  | PEAKS DB |
| total 5 peptides |  |  |  |  |  |  |  |  |  |  |  |  |  |  |  |  |  |  |  |  |  |  |  |  |  |  |  |  |  |  |  |  |  |  |  |  |  |  |  |  |  |  |  |  |  |
| --- | --- | --- | --- | --- | --- | --- | --- | --- | --- | --- | --- | --- | --- | --- | --- | --- | --- | --- | --- | --- | --- | --- | --- | --- | --- | --- | --- | --- | --- | --- | --- | --- | --- | --- | --- | --- | --- | --- | --- | --- | --- | --- | --- | --- | --- |

XP\_026575319.1

back to list

  

| Protein Coverage
| Supporting Peptides
|

Protein Coverage:

Supporting Peptides:

| Peptide | Uniq | -10lgP | Mass | Length | ppm | m/z | z | RT | Fraction | Scan | Source File | Area F1 | Area F10 | Area F11 | Area F12 | Area F13 | Area F14 | Area F2 | Area F3 | Area F4 | Area F5 | Area F6 | Area F7 | Area F8 | Area F9 | #Feature | #Feature F1 | #Feature F10 | #Feature F11 | #Feature F12 | #Feature F13 | #Feature F14 | #Feature F2 | #Feature F3 | #Feature F4 | #Feature F5 | #Feature F6 | #Feature F7 | #Feature F8 | #Feature F9 | Start | End | PTM | AScore | Found By |
| --- | --- | --- | --- | --- | --- | --- | --- | --- | --- | --- | --- | --- | --- | --- | --- | --- | --- | --- | --- | --- | --- | --- | --- | --- | --- | --- | --- | --- | --- | --- | --- | --- | --- | --- | --- | --- | --- | --- | --- | --- | --- | --- | --- | --- | --- |
| R.QLVPIGLSEFALDIR.M | Y | 61.03 | 1669.9402 | 15 | 1.0 | 835.9782 | 2 | 95.44 | 4 | F4:68666 | NaSa01\_F12.raw |  |  |  | 1.5973E6 | 3.2172E5 |  |  |  |  |  |  |  |  |  | 3 | 0 | 0 | 0 | 2 | 1 | 0 | 0 | 0 | 0 | 0 | 0 | 0 | 0 | 0 | 55 | 69 |  |  | PEAKS DB |
| P.IGLSEFALDIR.M | Y | 59.20 | 1232.6764 | 11 | 0.3 | 617.3456 | 2 | 76.06 | 4 | F4:53048 | NaSa01\_F12.raw |  |  |  | 1.0579E6 |  |  |  |  |  |  |  |  |  |  | 1 | 0 | 0 | 0 | 1 | 0 | 0 | 0 | 0 | 0 | 0 | 0 | 0 | 0 | 0 | 59 | 69 |  |  | PEAKS DB |
| R.LPVEEEAYVVDFKPH.A | Y | 52.76 | 1770.8828 | 15 | 0.4 | 591.3018 | 3 | 55.27 | 4 | F4:35730 | NaSa01\_F12.raw |  |  |  | 1.2477E6 | 1.5253E6 |  |  |  |  |  |  |  |  |  | 2 | 0 | 0 | 0 | 1 | 1 | 0 | 0 | 0 | 0 | 0 | 0 | 0 | 0 | 0 | 88 | 102 |  |  | PEAKS DB |
| K.YFVLQVHYGDVGVFK.D | Y | 42.63 | 1769.9141 | 15 | 0.6 | 590.9790 | 3 | 67.89 | 3 | F3:41163 | NaSa01\_F11.raw |  |  | 3.5691E5 |  |  |  |  |  |  |  |  |  |  |  | 1 | 0 | 0 | 1 | 0 | 0 | 0 | 0 | 0 | 0 | 0 | 0 | 0 | 0 | 0 | 168 | 182 |  |  | PEAKS DB |
| K.IEEVADWPGITLK.L | Y | 41.37 | 1469.7766 | 13 | 0.0 | 735.8956 | 2 | 70.61 | 5 | F5:47070 | NaSa01\_F13.raw |  |  |  |  | 1.5089E5 |  |  |  |  |  |  |  |  |  | 1 | 0 | 0 | 0 | 0 | 1 | 0 | 0 | 0 | 0 | 0 | 0 | 0 | 0 | 0 | 505 | 517 |  |  | PEAKS DB |
| total 5 peptides |  |  |  |  |  |  |  |  |  |  |  |  |  |  |  |  |  |  |  |  |  |  |  |  |  |  |  |  |  |  |  |  |  |  |  |  |  |  |  |  |  |  |  |  |  |
| --- | --- | --- | --- | --- | --- | --- | --- | --- | --- | --- | --- | --- | --- | --- | --- | --- | --- | --- | --- | --- | --- | --- | --- | --- | --- | --- | --- | --- | --- | --- | --- | --- | --- | --- | --- | --- | --- | --- | --- | --- | --- | --- | --- | --- | --- |

XP\_026575325.1

back to list

  

| Protein Coverage
| Supporting Peptides
|

Protein Coverage:

Supporting Peptides:

| Peptide | Uniq | -10lgP | Mass | Length | ppm | m/z | z | RT | Fraction | Scan | Source File | Area F1 | Area F10 | Area F11 | Area F12 | Area F13 | Area F14 | Area F2 | Area F3 | Area F4 | Area F5 | Area F6 | Area F7 | Area F8 | Area F9 | #Feature | #Feature F1 | #Feature F10 | #Feature F11 | #Feature F12 | #Feature F13 | #Feature F14 | #Feature F2 | #Feature F3 | #Feature F4 | #Feature F5 | #Feature F6 | #Feature F7 | #Feature F8 | #Feature F9 | Start | End | PTM | AScore | Found By |
| --- | --- | --- | --- | --- | --- | --- | --- | --- | --- | --- | --- | --- | --- | --- | --- | --- | --- | --- | --- | --- | --- | --- | --- | --- | --- | --- | --- | --- | --- | --- | --- | --- | --- | --- | --- | --- | --- | --- | --- | --- | --- | --- | --- | --- | --- |
| R.QLVPIGLSEFALDIR.M | Y | 61.03 | 1669.9402 | 15 | 1.0 | 835.9782 | 2 | 95.44 | 4 | F4:68666 | NaSa01\_F12.raw |  |  |  | 1.5973E6 | 3.2172E5 |  |  |  |  |  |  |  |  |  | 3 | 0 | 0 | 0 | 2 | 1 | 0 | 0 | 0 | 0 | 0 | 0 | 0 | 0 | 0 | 55 | 69 |  |  | PEAKS DB |
| P.IGLSEFALDIR.M | Y | 59.20 | 1232.6764 | 11 | 0.3 | 617.3456 | 2 | 76.06 | 4 | F4:53048 | NaSa01\_F12.raw |  |  |  | 1.0579E6 |  |  |  |  |  |  |  |  |  |  | 1 | 0 | 0 | 0 | 1 | 0 | 0 | 0 | 0 | 0 | 0 | 0 | 0 | 0 | 0 | 59 | 69 |  |  | PEAKS DB |
| R.LPVEEEAYVVDFKPH.A | Y | 52.76 | 1770.8828 | 15 | 0.4 | 591.3018 | 3 | 55.27 | 4 | F4:35730 | NaSa01\_F12.raw |  |  |  | 1.2477E6 | 1.5253E6 |  |  |  |  |  |  |  |  |  | 2 | 0 | 0 | 0 | 1 | 1 | 0 | 0 | 0 | 0 | 0 | 0 | 0 | 0 | 0 | 88 | 102 |  |  | PEAKS DB |
| K.YFVLQVHYGDVGVFK.D | Y | 42.63 | 1769.9141 | 15 | 0.6 | 590.9790 | 3 | 67.89 | 3 | F3:41163 | NaSa01\_F11.raw |  |  | 3.5691E5 |  |  |  |  |  |  |  |  |  |  |  | 1 | 0 | 0 | 1 | 0 | 0 | 0 | 0 | 0 | 0 | 0 | 0 | 0 | 0 | 0 | 168 | 182 |  |  | PEAKS DB |
| K.IEEVADWPGITLK.L | Y | 41.37 | 1469.7766 | 13 | 0.0 | 735.8956 | 2 | 70.61 | 5 | F5:47070 | NaSa01\_F13.raw |  |  |  |  | 1.5089E5 |  |  |  |  |  |  |  |  |  | 1 | 0 | 0 | 0 | 0 | 1 | 0 | 0 | 0 | 0 | 0 | 0 | 0 | 0 | 0 | 505 | 517 |  |  | PEAKS DB |
| total 5 peptides |  |  |  |  |  |  |  |  |  |  |  |  |  |  |  |  |  |  |  |  |  |  |  |  |  |  |  |  |  |  |  |  |  |  |  |  |  |  |  |  |  |  |  |  |  |
| --- | --- | --- | --- | --- | --- | --- | --- | --- | --- | --- | --- | --- | --- | --- | --- | --- | --- | --- | --- | --- | --- | --- | --- | --- | --- | --- | --- | --- | --- | --- | --- | --- | --- | --- | --- | --- | --- | --- | --- | --- | --- | --- | --- | --- | --- |

XP\_026575343.1

back to list

  

| Protein Coverage
| Supporting Peptides
|

Protein Coverage:

Supporting Peptides:

| Peptide | Uniq | -10lgP | Mass | Length | ppm | m/z | z | RT | Fraction | Scan | Source File | Area F1 | Area F10 | Area F11 | Area F12 | Area F13 | Area F14 | Area F2 | Area F3 | Area F4 | Area F5 | Area F6 | Area F7 | Area F8 | Area F9 | #Feature | #Feature F1 | #Feature F10 | #Feature F11 | #Feature F12 | #Feature F13 | #Feature F14 | #Feature F2 | #Feature F3 | #Feature F4 | #Feature F5 | #Feature F6 | #Feature F7 | #Feature F8 | #Feature F9 | Start | End | PTM | AScore | Found By |
| --- | --- | --- | --- | --- | --- | --- | --- | --- | --- | --- | --- | --- | --- | --- | --- | --- | --- | --- | --- | --- | --- | --- | --- | --- | --- | --- | --- | --- | --- | --- | --- | --- | --- | --- | --- | --- | --- | --- | --- | --- | --- | --- | --- | --- | --- |
| R.QLVPIGLSEFALDIR.M | Y | 61.03 | 1669.9402 | 15 | 1.0 | 835.9782 | 2 | 95.44 | 4 | F4:68666 | NaSa01\_F12.raw |  |  |  | 1.5973E6 | 3.2172E5 |  |  |  |  |  |  |  |  |  | 3 | 0 | 0 | 0 | 2 | 1 | 0 | 0 | 0 | 0 | 0 | 0 | 0 | 0 | 0 | 55 | 69 |  |  | PEAKS DB |
| P.IGLSEFALDIR.M | Y | 59.20 | 1232.6764 | 11 | 0.3 | 617.3456 | 2 | 76.06 | 4 | F4:53048 | NaSa01\_F12.raw |  |  |  | 1.0579E6 |  |  |  |  |  |  |  |  |  |  | 1 | 0 | 0 | 0 | 1 | 0 | 0 | 0 | 0 | 0 | 0 | 0 | 0 | 0 | 0 | 59 | 69 |  |  | PEAKS DB |
| R.LPVEEEAYVVDFKPH.A | Y | 52.76 | 1770.8828 | 15 | 0.4 | 591.3018 | 3 | 55.27 | 4 | F4:35730 | NaSa01\_F12.raw |  |  |  | 1.2477E6 | 1.5253E6 |  |  |  |  |  |  |  |  |  | 2 | 0 | 0 | 0 | 1 | 1 | 0 | 0 | 0 | 0 | 0 | 0 | 0 | 0 | 0 | 88 | 102 |  |  | PEAKS DB |
| K.YFVLQVHYGDVGVFK.D | Y | 42.63 | 1769.9141 | 15 | 0.6 | 590.9790 | 3 | 67.89 | 3 | F3:41163 | NaSa01\_F11.raw |  |  | 3.5691E5 |  |  |  |  |  |  |  |  |  |  |  | 1 | 0 | 0 | 1 | 0 | 0 | 0 | 0 | 0 | 0 | 0 | 0 | 0 | 0 | 0 | 168 | 182 |  |  | PEAKS DB |
| K.IEEVADWPGITLK.L | Y | 41.37 | 1469.7766 | 13 | 0.0 | 735.8956 | 2 | 70.61 | 5 | F5:47070 | NaSa01\_F13.raw |  |  |  |  | 1.5089E5 |  |  |  |  |  |  |  |  |  | 1 | 0 | 0 | 0 | 0 | 1 | 0 | 0 | 0 | 0 | 0 | 0 | 0 | 0 | 0 | 399 | 411 |  |  | PEAKS DB |
| total 5 peptides |  |  |  |  |  |  |  |  |  |  |  |  |  |  |  |  |  |  |  |  |  |  |  |  |  |  |  |  |  |  |  |  |  |  |  |  |  |  |  |  |  |  |  |  |  |
| --- | --- | --- | --- | --- | --- | --- | --- | --- | --- | --- | --- | --- | --- | --- | --- | --- | --- | --- | --- | --- | --- | --- | --- | --- | --- | --- | --- | --- | --- | --- | --- | --- | --- | --- | --- | --- | --- | --- | --- | --- | --- | --- | --- | --- | --- |

XP\_034273677.1

back to list

  

| Protein Coverage
| Supporting Peptides
|

Protein Coverage:

Supporting Peptides:

| Peptide | Uniq | -10lgP | Mass | Length | ppm | m/z | z | RT | Fraction | Scan | Source File | Area F1 | Area F10 | Area F11 | Area F12 | Area F13 | Area F14 | Area F2 | Area F3 | Area F4 | Area F5 | Area F6 | Area F7 | Area F8 | Area F9 | #Feature | #Feature F1 | #Feature F10 | #Feature F11 | #Feature F12 | #Feature F13 | #Feature F14 | #Feature F2 | #Feature F3 | #Feature F4 | #Feature F5 | #Feature F6 | #Feature F7 | #Feature F8 | #Feature F9 | Start | End | PTM | AScore | Found By |
| --- | --- | --- | --- | --- | --- | --- | --- | --- | --- | --- | --- | --- | --- | --- | --- | --- | --- | --- | --- | --- | --- | --- | --- | --- | --- | --- | --- | --- | --- | --- | --- | --- | --- | --- | --- | --- | --- | --- | --- | --- | --- | --- | --- | --- | --- |
| K.DKPTYDEIFYTLSPVNGR.I | Y | 69.24 | 2114.0320 | 18 | 0.3 | 705.6848 | 3 | 73.01 | 4 | F4:50499 | NaSa01\_F12.raw |  |  |  | 9.1545E6 |  |  |  |  |  |  |  |  |  |  | 1 | 0 | 0 | 0 | 1 | 0 | 0 | 0 | 0 | 0 | 0 | 0 | 0 | 0 | 0 | 446 | 463 |  |  | PEAKS DB |
| K.EGADEEEWIVAK.D | Y | 61.73 | 1374.6302 | 12 | 0.4 | 688.3227 | 2 | 44.40 | 4 | F4:26481 | NaSa01\_F12.raw |  |  |  | 2.3141E6 |  |  |  |  |  |  |  |  |  |  | 1 | 0 | 0 | 0 | 1 | 0 | 0 | 0 | 0 | 0 | 0 | 0 | 0 | 0 | 0 | 434 | 445 |  |  | PEAKS DB |
| A.DEEEWIVAK.D | Y | 52.41 | 1117.5291 | 9 | -0.2 | 559.7717 | 2 | 44.89 | 4 | F4:27080 | NaSa01\_F12.raw |  |  |  | 1.868E6 |  |  |  |  |  |  |  |  |  |  | 1 | 0 | 0 | 0 | 1 | 0 | 0 | 0 | 0 | 0 | 0 | 0 | 0 | 0 | 0 | 437 | 445 |  |  | PEAKS DB |
| K.LIEAVDNMLANK.V | Y | 41.75 | 1329.6962 | 12 | 0.2 | 665.8555 | 2 | 55.54 | 6 | F6:34135 | NaSa01\_F14.raw |  |  |  |  |  | 2.0504E5 |  |  |  |  |  |  |  |  | 1 | 0 | 0 | 0 | 0 | 0 | 1 | 0 | 0 | 0 | 0 | 0 | 0 | 0 | 0 | 380 | 391 |  |  | PEAKS DB |
| total 4 peptides |  |  |  |  |  |  |  |  |  |  |  |  |  |  |  |  |  |  |  |  |  |  |  |  |  |  |  |  |  |  |  |  |  |  |  |  |  |  |  |  |  |  |  |  |  |
| --- | --- | --- | --- | --- | --- | --- | --- | --- | --- | --- | --- | --- | --- | --- | --- | --- | --- | --- | --- | --- | --- | --- | --- | --- | --- | --- | --- | --- | --- | --- | --- | --- | --- | --- | --- | --- | --- | --- | --- | --- | --- | --- | --- | --- | --- |

LAA22316.1

back to list

  

| Protein Coverage
| Supporting Peptides
|

Protein Coverage:

Supporting Peptides:

| Peptide | Uniq | -10lgP | Mass | Length | ppm | m/z | z | RT | Fraction | Scan | Source File | Area F1 | Area F10 | Area F11 | Area F12 | Area F13 | Area F14 | Area F2 | Area F3 | Area F4 | Area F5 | Area F6 | Area F7 | Area F8 | Area F9 | #Feature | #Feature F1 | #Feature F10 | #Feature F11 | #Feature F12 | #Feature F13 | #Feature F14 | #Feature F2 | #Feature F3 | #Feature F4 | #Feature F5 | #Feature F6 | #Feature F7 | #Feature F8 | #Feature F9 | Start | End | PTM | AScore | Found By |
| --- | --- | --- | --- | --- | --- | --- | --- | --- | --- | --- | --- | --- | --- | --- | --- | --- | --- | --- | --- | --- | --- | --- | --- | --- | --- | --- | --- | --- | --- | --- | --- | --- | --- | --- | --- | --- | --- | --- | --- | --- | --- | --- | --- | --- | --- |
| K.DKPTYDEIFYTLSPVNGR.I | Y | 69.24 | 2114.0320 | 18 | 0.3 | 705.6848 | 3 | 73.01 | 4 | F4:50499 | NaSa01\_F12.raw |  |  |  | 9.1545E6 |  |  |  |  |  |  |  |  |  |  | 1 | 0 | 0 | 0 | 1 | 0 | 0 | 0 | 0 | 0 | 0 | 0 | 0 | 0 | 0 | 446 | 463 |  |  | PEAKS DB |
| K.EGADEEEWIVAK.D | Y | 61.73 | 1374.6302 | 12 | 0.4 | 688.3227 | 2 | 44.40 | 4 | F4:26481 | NaSa01\_F12.raw |  |  |  | 2.3141E6 |  |  |  |  |  |  |  |  |  |  | 1 | 0 | 0 | 0 | 1 | 0 | 0 | 0 | 0 | 0 | 0 | 0 | 0 | 0 | 0 | 434 | 445 |  |  | PEAKS DB |
| A.DEEEWIVAK.D | Y | 52.41 | 1117.5291 | 9 | -0.2 | 559.7717 | 2 | 44.89 | 4 | F4:27080 | NaSa01\_F12.raw |  |  |  | 1.868E6 |  |  |  |  |  |  |  |  |  |  | 1 | 0 | 0 | 0 | 1 | 0 | 0 | 0 | 0 | 0 | 0 | 0 | 0 | 0 | 0 | 437 | 445 |  |  | PEAKS DB |
| K.LIEAVDNMLANK.V | Y | 41.75 | 1329.6962 | 12 | 0.2 | 665.8555 | 2 | 55.54 | 6 | F6:34135 | NaSa01\_F14.raw |  |  |  |  |  | 2.0504E5 |  |  |  |  |  |  |  |  | 1 | 0 | 0 | 0 | 0 | 0 | 1 | 0 | 0 | 0 | 0 | 0 | 0 | 0 | 0 | 380 | 391 |  |  | PEAKS DB |
| total 4 peptides |  |  |  |  |  |  |  |  |  |  |  |  |  |  |  |  |  |  |  |  |  |  |  |  |  |  |  |  |  |  |  |  |  |  |  |  |  |  |  |  |  |  |  |  |  |
| --- | --- | --- | --- | --- | --- | --- | --- | --- | --- | --- | --- | --- | --- | --- | --- | --- | --- | --- | --- | --- | --- | --- | --- | --- | --- | --- | --- | --- | --- | --- | --- | --- | --- | --- | --- | --- | --- | --- | --- | --- | --- | --- | --- | --- | --- |

LAA22315.1

back to list

  

| Protein Coverage
| Supporting Peptides
|

Protein Coverage:

Supporting Peptides:

| Peptide | Uniq | -10lgP | Mass | Length | ppm | m/z | z | RT | Fraction | Scan | Source File | Area F1 | Area F10 | Area F11 | Area F12 | Area F13 | Area F14 | Area F2 | Area F3 | Area F4 | Area F5 | Area F6 | Area F7 | Area F8 | Area F9 | #Feature | #Feature F1 | #Feature F10 | #Feature F11 | #Feature F12 | #Feature F13 | #Feature F14 | #Feature F2 | #Feature F3 | #Feature F4 | #Feature F5 | #Feature F6 | #Feature F7 | #Feature F8 | #Feature F9 | Start | End | PTM | AScore | Found By |
| --- | --- | --- | --- | --- | --- | --- | --- | --- | --- | --- | --- | --- | --- | --- | --- | --- | --- | --- | --- | --- | --- | --- | --- | --- | --- | --- | --- | --- | --- | --- | --- | --- | --- | --- | --- | --- | --- | --- | --- | --- | --- | --- | --- | --- | --- |
| K.DKPTYDEIFYTLSPVNGR.I | Y | 69.24 | 2114.0320 | 18 | 0.3 | 705.6848 | 3 | 73.01 | 4 | F4:50499 | NaSa01\_F12.raw |  |  |  | 9.1545E6 |  |  |  |  |  |  |  |  |  |  | 1 | 0 | 0 | 0 | 1 | 0 | 0 | 0 | 0 | 0 | 0 | 0 | 0 | 0 | 0 | 446 | 463 |  |  | PEAKS DB |
| K.EGADEEEWIVAK.D | Y | 61.73 | 1374.6302 | 12 | 0.4 | 688.3227 | 2 | 44.40 | 4 | F4:26481 | NaSa01\_F12.raw |  |  |  | 2.3141E6 |  |  |  |  |  |  |  |  |  |  | 1 | 0 | 0 | 0 | 1 | 0 | 0 | 0 | 0 | 0 | 0 | 0 | 0 | 0 | 0 | 434 | 445 |  |  | PEAKS DB |
| A.DEEEWIVAK.D | Y | 52.41 | 1117.5291 | 9 | -0.2 | 559.7717 | 2 | 44.89 | 4 | F4:27080 | NaSa01\_F12.raw |  |  |  | 1.868E6 |  |  |  |  |  |  |  |  |  |  | 1 | 0 | 0 | 0 | 1 | 0 | 0 | 0 | 0 | 0 | 0 | 0 | 0 | 0 | 0 | 437 | 445 |  |  | PEAKS DB |
| K.LIEAVDNMLANK.V | Y | 41.75 | 1329.6962 | 12 | 0.2 | 665.8555 | 2 | 55.54 | 6 | F6:34135 | NaSa01\_F14.raw |  |  |  |  |  | 2.0504E5 |  |  |  |  |  |  |  |  | 1 | 0 | 0 | 0 | 0 | 0 | 1 | 0 | 0 | 0 | 0 | 0 | 0 | 0 | 0 | 380 | 391 |  |  | PEAKS DB |
| total 4 peptides |  |  |  |  |  |  |  |  |  |  |  |  |  |  |  |  |  |  |  |  |  |  |  |  |  |  |  |  |  |  |  |  |  |  |  |  |  |  |  |  |  |  |  |  |  |
| --- | --- | --- | --- | --- | --- | --- | --- | --- | --- | --- | --- | --- | --- | --- | --- | --- | --- | --- | --- | --- | --- | --- | --- | --- | --- | --- | --- | --- | --- | --- | --- | --- | --- | --- | --- | --- | --- | --- | --- | --- | --- | --- | --- | --- | --- |

JAC95688.1

back to list

  

| Protein Coverage
| Supporting Peptides
|

Protein Coverage:

Supporting Peptides:

| Peptide | Uniq | -10lgP | Mass | Length | ppm | m/z | z | RT | Fraction | Scan | Source File | Area F1 | Area F10 | Area F11 | Area F12 | Area F13 | Area F14 | Area F2 | Area F3 | Area F4 | Area F5 | Area F6 | Area F7 | Area F8 | Area F9 | #Feature | #Feature F1 | #Feature F10 | #Feature F11 | #Feature F12 | #Feature F13 | #Feature F14 | #Feature F2 | #Feature F3 | #Feature F4 | #Feature F5 | #Feature F6 | #Feature F7 | #Feature F8 | #Feature F9 | Start | End | PTM | AScore | Found By |
| --- | --- | --- | --- | --- | --- | --- | --- | --- | --- | --- | --- | --- | --- | --- | --- | --- | --- | --- | --- | --- | --- | --- | --- | --- | --- | --- | --- | --- | --- | --- | --- | --- | --- | --- | --- | --- | --- | --- | --- | --- | --- | --- | --- | --- | --- |
| K.DKPTYDEIFYTLSPVNGR.I | Y | 69.24 | 2114.0320 | 18 | 0.3 | 705.6848 | 3 | 73.01 | 4 | F4:50499 | NaSa01\_F12.raw |  |  |  | 9.1545E6 |  |  |  |  |  |  |  |  |  |  | 1 | 0 | 0 | 0 | 1 | 0 | 0 | 0 | 0 | 0 | 0 | 0 | 0 | 0 | 0 | 446 | 463 |  |  | PEAKS DB |
| K.EGADEEEWIVAK.D | Y | 61.73 | 1374.6302 | 12 | 0.4 | 688.3227 | 2 | 44.40 | 4 | F4:26481 | NaSa01\_F12.raw |  |  |  | 2.3141E6 |  |  |  |  |  |  |  |  |  |  | 1 | 0 | 0 | 0 | 1 | 0 | 0 | 0 | 0 | 0 | 0 | 0 | 0 | 0 | 0 | 434 | 445 |  |  | PEAKS DB |
| A.DEEEWIVAK.D | Y | 52.41 | 1117.5291 | 9 | -0.2 | 559.7717 | 2 | 44.89 | 4 | F4:27080 | NaSa01\_F12.raw |  |  |  | 1.868E6 |  |  |  |  |  |  |  |  |  |  | 1 | 0 | 0 | 0 | 1 | 0 | 0 | 0 | 0 | 0 | 0 | 0 | 0 | 0 | 0 | 437 | 445 |  |  | PEAKS DB |
| K.LIEAVDNMLANK.V | Y | 41.75 | 1329.6962 | 12 | 0.2 | 665.8555 | 2 | 55.54 | 6 | F6:34135 | NaSa01\_F14.raw |  |  |  |  |  | 2.0504E5 |  |  |  |  |  |  |  |  | 1 | 0 | 0 | 0 | 0 | 0 | 1 | 0 | 0 | 0 | 0 | 0 | 0 | 0 | 0 | 380 | 391 |  |  | PEAKS DB |
| total 4 peptides |  |  |  |  |  |  |  |  |  |  |  |  |  |  |  |  |  |  |  |  |  |  |  |  |  |  |  |  |  |  |  |  |  |  |  |  |  |  |  |  |  |  |  |  |  |
| --- | --- | --- | --- | --- | --- | --- | --- | --- | --- | --- | --- | --- | --- | --- | --- | --- | --- | --- | --- | --- | --- | --- | --- | --- | --- | --- | --- | --- | --- | --- | --- | --- | --- | --- | --- | --- | --- | --- | --- | --- | --- | --- | --- | --- | --- |

LAB64401.1

back to list

  

| Protein Coverage
| Supporting Peptides
|

Protein Coverage:

Supporting Peptides:

| Peptide | Uniq | -10lgP | Mass | Length | ppm | m/z | z | RT | Fraction | Scan | Source File | Area F1 | Area F10 | Area F11 | Area F12 | Area F13 | Area F14 | Area F2 | Area F3 | Area F4 | Area F5 | Area F6 | Area F7 | Area F8 | Area F9 | #Feature | #Feature F1 | #Feature F10 | #Feature F11 | #Feature F12 | #Feature F13 | #Feature F14 | #Feature F2 | #Feature F3 | #Feature F4 | #Feature F5 | #Feature F6 | #Feature F7 | #Feature F8 | #Feature F9 | Start | End | PTM | AScore | Found By |
| --- | --- | --- | --- | --- | --- | --- | --- | --- | --- | --- | --- | --- | --- | --- | --- | --- | --- | --- | --- | --- | --- | --- | --- | --- | --- | --- | --- | --- | --- | --- | --- | --- | --- | --- | --- | --- | --- | --- | --- | --- | --- | --- | --- | --- | --- |
| K.DKPTYDEIFYTLSPVNGR.I | Y | 69.24 | 2114.0320 | 18 | 0.3 | 705.6848 | 3 | 73.01 | 4 | F4:50499 | NaSa01\_F12.raw |  |  |  | 9.1545E6 |  |  |  |  |  |  |  |  |  |  | 1 | 0 | 0 | 0 | 1 | 0 | 0 | 0 | 0 | 0 | 0 | 0 | 0 | 0 | 0 | 446 | 463 |  |  | PEAKS DB |
| K.EGADEEEWIVAK.D | Y | 61.73 | 1374.6302 | 12 | 0.4 | 688.3227 | 2 | 44.40 | 4 | F4:26481 | NaSa01\_F12.raw |  |  |  | 2.3141E6 |  |  |  |  |  |  |  |  |  |  | 1 | 0 | 0 | 0 | 1 | 0 | 0 | 0 | 0 | 0 | 0 | 0 | 0 | 0 | 0 | 434 | 445 |  |  | PEAKS DB |
| A.DEEEWIVAK.D | Y | 52.41 | 1117.5291 | 9 | -0.2 | 559.7717 | 2 | 44.89 | 4 | F4:27080 | NaSa01\_F12.raw |  |  |  | 1.868E6 |  |  |  |  |  |  |  |  |  |  | 1 | 0 | 0 | 0 | 1 | 0 | 0 | 0 | 0 | 0 | 0 | 0 | 0 | 0 | 0 | 437 | 445 |  |  | PEAKS DB |
| K.LIEAVDNMLANK.V | Y | 41.75 | 1329.6962 | 12 | 0.2 | 665.8555 | 2 | 55.54 | 6 | F6:34135 | NaSa01\_F14.raw |  |  |  |  |  | 2.0504E5 |  |  |  |  |  |  |  |  | 1 | 0 | 0 | 0 | 0 | 0 | 1 | 0 | 0 | 0 | 0 | 0 | 0 | 0 | 0 | 380 | 391 |  |  | PEAKS DB |
| total 4 peptides |  |  |  |  |  |  |  |  |  |  |  |  |  |  |  |  |  |  |  |  |  |  |  |  |  |  |  |  |  |  |  |  |  |  |  |  |  |  |  |  |  |  |  |  |  |
| --- | --- | --- | --- | --- | --- | --- | --- | --- | --- | --- | --- | --- | --- | --- | --- | --- | --- | --- | --- | --- | --- | --- | --- | --- | --- | --- | --- | --- | --- | --- | --- | --- | --- | --- | --- | --- | --- | --- | --- | --- | --- | --- | --- | --- | --- |

LAA61330.1

back to list

  

| Protein Coverage
| Supporting Peptides
|

Protein Coverage:

Supporting Peptides:

| Peptide | Uniq | -10lgP | Mass | Length | ppm | m/z | z | RT | Fraction | Scan | Source File | Area F1 | Area F10 | Area F11 | Area F12 | Area F13 | Area F14 | Area F2 | Area F3 | Area F4 | Area F5 | Area F6 | Area F7 | Area F8 | Area F9 | #Feature | #Feature F1 | #Feature F10 | #Feature F11 | #Feature F12 | #Feature F13 | #Feature F14 | #Feature F2 | #Feature F3 | #Feature F4 | #Feature F5 | #Feature F6 | #Feature F7 | #Feature F8 | #Feature F9 | Start | End | PTM | AScore | Found By |
| --- | --- | --- | --- | --- | --- | --- | --- | --- | --- | --- | --- | --- | --- | --- | --- | --- | --- | --- | --- | --- | --- | --- | --- | --- | --- | --- | --- | --- | --- | --- | --- | --- | --- | --- | --- | --- | --- | --- | --- | --- | --- | --- | --- | --- | --- |
| K.DKPTYDEIFYTLSPVNGR.I | Y | 69.24 | 2114.0320 | 18 | 0.3 | 705.6848 | 3 | 73.01 | 4 | F4:50499 | NaSa01\_F12.raw |  |  |  | 9.1545E6 |  |  |  |  |  |  |  |  |  |  | 1 | 0 | 0 | 0 | 1 | 0 | 0 | 0 | 0 | 0 | 0 | 0 | 0 | 0 | 0 | 446 | 463 |  |  | PEAKS DB |
| K.EGADEEEWIVAK.D | Y | 61.73 | 1374.6302 | 12 | 0.4 | 688.3227 | 2 | 44.40 | 4 | F4:26481 | NaSa01\_F12.raw |  |  |  | 2.3141E6 |  |  |  |  |  |  |  |  |  |  | 1 | 0 | 0 | 0 | 1 | 0 | 0 | 0 | 0 | 0 | 0 | 0 | 0 | 0 | 0 | 434 | 445 |  |  | PEAKS DB |
| A.DEEEWIVAK.D | Y | 52.41 | 1117.5291 | 9 | -0.2 | 559.7717 | 2 | 44.89 | 4 | F4:27080 | NaSa01\_F12.raw |  |  |  | 1.868E6 |  |  |  |  |  |  |  |  |  |  | 1 | 0 | 0 | 0 | 1 | 0 | 0 | 0 | 0 | 0 | 0 | 0 | 0 | 0 | 0 | 437 | 445 |  |  | PEAKS DB |
| K.LIEAVDNMLANK.V | Y | 41.75 | 1329.6962 | 12 | 0.2 | 665.8555 | 2 | 55.54 | 6 | F6:34135 | NaSa01\_F14.raw |  |  |  |  |  | 2.0504E5 |  |  |  |  |  |  |  |  | 1 | 0 | 0 | 0 | 0 | 0 | 1 | 0 | 0 | 0 | 0 | 0 | 0 | 0 | 0 | 380 | 391 |  |  | PEAKS DB |
| total 4 peptides |  |  |  |  |  |  |  |  |  |  |  |  |  |  |  |  |  |  |  |  |  |  |  |  |  |  |  |  |  |  |  |  |  |  |  |  |  |  |  |  |  |  |  |  |  |
| --- | --- | --- | --- | --- | --- | --- | --- | --- | --- | --- | --- | --- | --- | --- | --- | --- | --- | --- | --- | --- | --- | --- | --- | --- | --- | --- | --- | --- | --- | --- | --- | --- | --- | --- | --- | --- | --- | --- | --- | --- | --- | --- | --- | --- | --- |

XP\_026528360.1

back to list

  

| Protein Coverage
| Supporting Peptides
|

Protein Coverage:

Supporting Peptides:

| Peptide | Uniq | -10lgP | Mass | Length | ppm | m/z | z | RT | Fraction | Scan | Source File | Area F1 | Area F10 | Area F11 | Area F12 | Area F13 | Area F14 | Area F2 | Area F3 | Area F4 | Area F5 | Area F6 | Area F7 | Area F8 | Area F9 | #Feature | #Feature F1 | #Feature F10 | #Feature F11 | #Feature F12 | #Feature F13 | #Feature F14 | #Feature F2 | #Feature F3 | #Feature F4 | #Feature F5 | #Feature F6 | #Feature F7 | #Feature F8 | #Feature F9 | Start | End | PTM | AScore | Found By |
| --- | --- | --- | --- | --- | --- | --- | --- | --- | --- | --- | --- | --- | --- | --- | --- | --- | --- | --- | --- | --- | --- | --- | --- | --- | --- | --- | --- | --- | --- | --- | --- | --- | --- | --- | --- | --- | --- | --- | --- | --- | --- | --- | --- | --- | --- |
| R.KLDLHVLPVWQK.G | Y | 65.49 | 1474.8660 | 12 | 1.1 | 492.6298 | 3 | 51.50 | 6 | F6:30729 | NaSa01\_F14.raw |  |  |  |  | 1.7504E5 | 1.6967E6 |  |  |  |  |  |  |  |  | 2 | 0 | 0 | 0 | 0 | 1 | 1 | 0 | 0 | 0 | 0 | 0 | 0 | 0 | 0 | 143 | 154 |  |  | PEAKS DB |
| R.FGFGLLNAK.A | Y | 55.32 | 965.5334 | 9 | -2.0 | 483.7730 | 2 | 61.54 | 6 | F6:39287 | NaSa01\_F14.raw |  |  |  |  | 1.0258E6 | 2.205E6 |  |  |  |  |  |  |  |  | 2 | 0 | 0 | 0 | 0 | 1 | 1 | 0 | 0 | 0 | 0 | 0 | 0 | 0 | 0 | 439 | 447 |  |  | PEAKS DB |
| L.ALEANPNLTWR.D | Y | 41.95 | 1283.6622 | 11 | -1.4 | 642.8375 | 2 | 43.42 | 6 | F6:23684 | NaSa01\_F14.raw |  |  |  |  |  | 0 |  |  |  |  |  |  |  |  | 0 | 0 | 0 | 0 | 0 | 0 | 0 | 0 | 0 | 0 | 0 | 0 | 0 | 0 | 0 | 395 | 405 |  |  | PEAKS DB |
| F.ALALEANPNLTWR.D | Y | 41.90 | 1467.7833 | 13 | 0.1 | 734.8990 | 2 | 60.67 | 6 | F6:38618 | NaSa01\_F14.raw |  |  |  |  |  | 7.3887E5 |  |  |  |  |  |  |  |  | 1 | 0 | 0 | 0 | 0 | 0 | 1 | 0 | 0 | 0 | 0 | 0 | 0 | 0 | 0 | 393 | 405 |  |  | PEAKS DB |
| total 4 peptides |  |  |  |  |  |  |  |  |  |  |  |  |  |  |  |  |  |  |  |  |  |  |  |  |  |  |  |  |  |  |  |  |  |  |  |  |  |  |  |  |  |  |  |  |  |
| --- | --- | --- | --- | --- | --- | --- | --- | --- | --- | --- | --- | --- | --- | --- | --- | --- | --- | --- | --- | --- | --- | --- | --- | --- | --- | --- | --- | --- | --- | --- | --- | --- | --- | --- | --- | --- | --- | --- | --- | --- | --- | --- | --- | --- | --- |

XP\_034296604.1

back to list

  

| Protein Coverage
| Supporting Peptides
|

Protein Coverage:

Supporting Peptides:

| Peptide | Uniq | -10lgP | Mass | Length | ppm | m/z | z | RT | Fraction | Scan | Source File | Area F1 | Area F10 | Area F11 | Area F12 | Area F13 | Area F14 | Area F2 | Area F3 | Area F4 | Area F5 | Area F6 | Area F7 | Area F8 | Area F9 | #Feature | #Feature F1 | #Feature F10 | #Feature F11 | #Feature F12 | #Feature F13 | #Feature F14 | #Feature F2 | #Feature F3 | #Feature F4 | #Feature F5 | #Feature F6 | #Feature F7 | #Feature F8 | #Feature F9 | Start | End | PTM | AScore | Found By |
| --- | --- | --- | --- | --- | --- | --- | --- | --- | --- | --- | --- | --- | --- | --- | --- | --- | --- | --- | --- | --- | --- | --- | --- | --- | --- | --- | --- | --- | --- | --- | --- | --- | --- | --- | --- | --- | --- | --- | --- | --- | --- | --- | --- | --- | --- |
| R.KLDLHVLPVWQK.G | Y | 65.49 | 1474.8660 | 12 | 1.1 | 492.6298 | 3 | 51.50 | 6 | F6:30729 | NaSa01\_F14.raw |  |  |  |  | 1.7504E5 | 1.6967E6 |  |  |  |  |  |  |  |  | 2 | 0 | 0 | 0 | 0 | 1 | 1 | 0 | 0 | 0 | 0 | 0 | 0 | 0 | 0 | 143 | 154 |  |  | PEAKS DB |
| R.FGFGLLNAK.A | Y | 55.32 | 965.5334 | 9 | -2.0 | 483.7730 | 2 | 61.54 | 6 | F6:39287 | NaSa01\_F14.raw |  |  |  |  | 1.0258E6 | 2.205E6 |  |  |  |  |  |  |  |  | 2 | 0 | 0 | 0 | 0 | 1 | 1 | 0 | 0 | 0 | 0 | 0 | 0 | 0 | 0 | 439 | 447 |  |  | PEAKS DB |
| L.ALEANPNLTWR.D | Y | 41.95 | 1283.6622 | 11 | -1.4 | 642.8375 | 2 | 43.42 | 6 | F6:23684 | NaSa01\_F14.raw |  |  |  |  |  | 0 |  |  |  |  |  |  |  |  | 0 | 0 | 0 | 0 | 0 | 0 | 0 | 0 | 0 | 0 | 0 | 0 | 0 | 0 | 0 | 395 | 405 |  |  | PEAKS DB |
| F.ALALEANPNLTWR.D | Y | 41.90 | 1467.7833 | 13 | 0.1 | 734.8990 | 2 | 60.67 | 6 | F6:38618 | NaSa01\_F14.raw |  |  |  |  |  | 7.3887E5 |  |  |  |  |  |  |  |  | 1 | 0 | 0 | 0 | 0 | 0 | 1 | 0 | 0 | 0 | 0 | 0 | 0 | 0 | 0 | 393 | 405 |  |  | PEAKS DB |
| total 4 peptides |  |  |  |  |  |  |  |  |  |  |  |  |  |  |  |  |  |  |  |  |  |  |  |  |  |  |  |  |  |  |  |  |  |  |  |  |  |  |  |  |  |  |  |  |  |
| --- | --- | --- | --- | --- | --- | --- | --- | --- | --- | --- | --- | --- | --- | --- | --- | --- | --- | --- | --- | --- | --- | --- | --- | --- | --- | --- | --- | --- | --- | --- | --- | --- | --- | --- | --- | --- | --- | --- | --- | --- | --- | --- | --- | --- | --- |

XP\_026575034.1

back to list

  

| Protein Coverage
| Supporting Peptides
|

Protein Coverage:

Supporting Peptides:

| Peptide | Uniq | -10lgP | Mass | Length | ppm | m/z | z | RT | Fraction | Scan | Source File | Area F1 | Area F10 | Area F11 | Area F12 | Area F13 | Area F14 | Area F2 | Area F3 | Area F4 | Area F5 | Area F6 | Area F7 | Area F8 | Area F9 | #Feature | #Feature F1 | #Feature F10 | #Feature F11 | #Feature F12 | #Feature F13 | #Feature F14 | #Feature F2 | #Feature F3 | #Feature F4 | #Feature F5 | #Feature F6 | #Feature F7 | #Feature F8 | #Feature F9 | Start | End | PTM | AScore | Found By |
| --- | --- | --- | --- | --- | --- | --- | --- | --- | --- | --- | --- | --- | --- | --- | --- | --- | --- | --- | --- | --- | --- | --- | --- | --- | --- | --- | --- | --- | --- | --- | --- | --- | --- | --- | --- | --- | --- | --- | --- | --- | --- | --- | --- | --- | --- |
| R.KLDLHVLPVWQK.G | Y | 65.49 | 1474.8660 | 12 | 1.1 | 492.6298 | 3 | 51.50 | 6 | F6:30729 | NaSa01\_F14.raw |  |  |  |  | 1.7504E5 | 1.6967E6 |  |  |  |  |  |  |  |  | 2 | 0 | 0 | 0 | 0 | 1 | 1 | 0 | 0 | 0 | 0 | 0 | 0 | 0 | 0 | 143 | 154 |  |  | PEAKS DB |
| R.FGFGLLNAK.A | Y | 55.32 | 965.5334 | 9 | -2.0 | 483.7730 | 2 | 61.54 | 6 | F6:39287 | NaSa01\_F14.raw |  |  |  |  | 1.0258E6 | 2.205E6 |  |  |  |  |  |  |  |  | 2 | 0 | 0 | 0 | 0 | 1 | 1 | 0 | 0 | 0 | 0 | 0 | 0 | 0 | 0 | 439 | 447 |  |  | PEAKS DB |
| L.ALEANPNLTWR.D | Y | 41.95 | 1283.6622 | 11 | -1.4 | 642.8375 | 2 | 43.42 | 6 | F6:23684 | NaSa01\_F14.raw |  |  |  |  |  | 0 |  |  |  |  |  |  |  |  | 0 | 0 | 0 | 0 | 0 | 0 | 0 | 0 | 0 | 0 | 0 | 0 | 0 | 0 | 0 | 395 | 405 |  |  | PEAKS DB |
| F.ALALEANPNLTWR.D | Y | 41.90 | 1467.7833 | 13 | 0.1 | 734.8990 | 2 | 60.67 | 6 | F6:38618 | NaSa01\_F14.raw |  |  |  |  |  | 7.3887E5 |  |  |  |  |  |  |  |  | 1 | 0 | 0 | 0 | 0 | 0 | 1 | 0 | 0 | 0 | 0 | 0 | 0 | 0 | 0 | 393 | 405 |  |  | PEAKS DB |
| total 4 peptides |  |  |  |  |  |  |  |  |  |  |  |  |  |  |  |  |  |  |  |  |  |  |  |  |  |  |  |  |  |  |  |  |  |  |  |  |  |  |  |  |  |  |  |  |  |
| --- | --- | --- | --- | --- | --- | --- | --- | --- | --- | --- | --- | --- | --- | --- | --- | --- | --- | --- | --- | --- | --- | --- | --- | --- | --- | --- | --- | --- | --- | --- | --- | --- | --- | --- | --- | --- | --- | --- | --- | --- | --- | --- | --- | --- | --- |

ETE68499.1

back to list

  

| Protein Coverage
| Supporting Peptides
|

Protein Coverage:

Supporting Peptides:

| Peptide | Uniq | -10lgP | Mass | Length | ppm | m/z | z | RT | Fraction | Scan | Source File | Area F1 | Area F10 | Area F11 | Area F12 | Area F13 | Area F14 | Area F2 | Area F3 | Area F4 | Area F5 | Area F6 | Area F7 | Area F8 | Area F9 | #Feature | #Feature F1 | #Feature F10 | #Feature F11 | #Feature F12 | #Feature F13 | #Feature F14 | #Feature F2 | #Feature F3 | #Feature F4 | #Feature F5 | #Feature F6 | #Feature F7 | #Feature F8 | #Feature F9 | Start | End | PTM | AScore | Found By |
| --- | --- | --- | --- | --- | --- | --- | --- | --- | --- | --- | --- | --- | --- | --- | --- | --- | --- | --- | --- | --- | --- | --- | --- | --- | --- | --- | --- | --- | --- | --- | --- | --- | --- | --- | --- | --- | --- | --- | --- | --- | --- | --- | --- | --- | --- |
| R.KLDLHVLPVWQK.G | Y | 65.49 | 1474.8660 | 12 | 1.1 | 492.6298 | 3 | 51.50 | 6 | F6:30729 | NaSa01\_F14.raw |  |  |  |  | 1.7504E5 | 1.6967E6 |  |  |  |  |  |  |  |  | 2 | 0 | 0 | 0 | 0 | 1 | 1 | 0 | 0 | 0 | 0 | 0 | 0 | 0 | 0 | 143 | 154 |  |  | PEAKS DB |
| R.FGFGLLNAK.A | Y | 55.32 | 965.5334 | 9 | -2.0 | 483.7730 | 2 | 61.54 | 6 | F6:39287 | NaSa01\_F14.raw |  |  |  |  | 1.0258E6 | 2.205E6 |  |  |  |  |  |  |  |  | 2 | 0 | 0 | 0 | 0 | 1 | 1 | 0 | 0 | 0 | 0 | 0 | 0 | 0 | 0 | 431 | 439 |  |  | PEAKS DB |
| L.ALEANPNLTWR.D | Y | 41.95 | 1283.6622 | 11 | -1.4 | 642.8375 | 2 | 43.42 | 6 | F6:23684 | NaSa01\_F14.raw |  |  |  |  |  | 0 |  |  |  |  |  |  |  |  | 0 | 0 | 0 | 0 | 0 | 0 | 0 | 0 | 0 | 0 | 0 | 0 | 0 | 0 | 0 | 387 | 397 |  |  | PEAKS DB |
| F.ALALEANPNLTWR.D | Y | 41.90 | 1467.7833 | 13 | 0.1 | 734.8990 | 2 | 60.67 | 6 | F6:38618 | NaSa01\_F14.raw |  |  |  |  |  | 7.3887E5 |  |  |  |  |  |  |  |  | 1 | 0 | 0 | 0 | 0 | 0 | 1 | 0 | 0 | 0 | 0 | 0 | 0 | 0 | 0 | 385 | 397 |  |  | PEAKS DB |
| total 4 peptides |  |  |  |  |  |  |  |  |  |  |  |  |  |  |  |  |  |  |  |  |  |  |  |  |  |  |  |  |  |  |  |  |  |  |  |  |  |  |  |  |  |  |  |  |  |
| --- | --- | --- | --- | --- | --- | --- | --- | --- | --- | --- | --- | --- | --- | --- | --- | --- | --- | --- | --- | --- | --- | --- | --- | --- | --- | --- | --- | --- | --- | --- | --- | --- | --- | --- | --- | --- | --- | --- | --- | --- | --- | --- | --- | --- | --- |

XP\_032069925.1

back to list

  

| Protein Coverage
| Supporting Peptides
|

Protein Coverage:

Supporting Peptides:

| Peptide | Uniq | -10lgP | Mass | Length | ppm | m/z | z | RT | Fraction | Scan | Source File | Area F1 | Area F10 | Area F11 | Area F12 | Area F13 | Area F14 | Area F2 | Area F3 | Area F4 | Area F5 | Area F6 | Area F7 | Area F8 | Area F9 | #Feature | #Feature F1 | #Feature F10 | #Feature F11 | #Feature F12 | #Feature F13 | #Feature F14 | #Feature F2 | #Feature F3 | #Feature F4 | #Feature F5 | #Feature F6 | #Feature F7 | #Feature F8 | #Feature F9 | Start | End | PTM | AScore | Found By |
| --- | --- | --- | --- | --- | --- | --- | --- | --- | --- | --- | --- | --- | --- | --- | --- | --- | --- | --- | --- | --- | --- | --- | --- | --- | --- | --- | --- | --- | --- | --- | --- | --- | --- | --- | --- | --- | --- | --- | --- | --- | --- | --- | --- | --- | --- |
| R.KLDLHVLPVWQK.G | Y | 65.49 | 1474.8660 | 12 | 1.1 | 492.6298 | 3 | 51.50 | 6 | F6:30729 | NaSa01\_F14.raw |  |  |  |  | 1.7504E5 | 1.6967E6 |  |  |  |  |  |  |  |  | 2 | 0 | 0 | 0 | 0 | 1 | 1 | 0 | 0 | 0 | 0 | 0 | 0 | 0 | 0 | 143 | 154 |  |  | PEAKS DB |
| R.FGFGLLNAK.A | Y | 55.32 | 965.5334 | 9 | -2.0 | 483.7730 | 2 | 61.54 | 6 | F6:39287 | NaSa01\_F14.raw |  |  |  |  | 1.0258E6 | 2.205E6 |  |  |  |  |  |  |  |  | 2 | 0 | 0 | 0 | 0 | 1 | 1 | 0 | 0 | 0 | 0 | 0 | 0 | 0 | 0 | 439 | 447 |  |  | PEAKS DB |
| L.ALEANPNLTWR.D | Y | 41.95 | 1283.6622 | 11 | -1.4 | 642.8375 | 2 | 43.42 | 6 | F6:23684 | NaSa01\_F14.raw |  |  |  |  |  | 0 |  |  |  |  |  |  |  |  | 0 | 0 | 0 | 0 | 0 | 0 | 0 | 0 | 0 | 0 | 0 | 0 | 0 | 0 | 0 | 395 | 405 |  |  | PEAKS DB |
| F.ALALEANPNLTWR.D | Y | 41.90 | 1467.7833 | 13 | 0.1 | 734.8990 | 2 | 60.67 | 6 | F6:38618 | NaSa01\_F14.raw |  |  |  |  |  | 7.3887E5 |  |  |  |  |  |  |  |  | 1 | 0 | 0 | 0 | 0 | 0 | 1 | 0 | 0 | 0 | 0 | 0 | 0 | 0 | 0 | 393 | 405 |  |  | PEAKS DB |
| total 4 peptides |  |  |  |  |  |  |  |  |  |  |  |  |  |  |  |  |  |  |  |  |  |  |  |  |  |  |  |  |  |  |  |  |  |  |  |  |  |  |  |  |  |  |  |  |  |
| --- | --- | --- | --- | --- | --- | --- | --- | --- | --- | --- | --- | --- | --- | --- | --- | --- | --- | --- | --- | --- | --- | --- | --- | --- | --- | --- | --- | --- | --- | --- | --- | --- | --- | --- | --- | --- | --- | --- | --- | --- | --- | --- | --- | --- | --- |

XP\_013913235.1

back to list

  

| Protein Coverage
| Supporting Peptides
|

Protein Coverage:

Supporting Peptides:

| Peptide | Uniq | -10lgP | Mass | Length | ppm | m/z | z | RT | Fraction | Scan | Source File | Area F1 | Area F10 | Area F11 | Area F12 | Area F13 | Area F14 | Area F2 | Area F3 | Area F4 | Area F5 | Area F6 | Area F7 | Area F8 | Area F9 | #Feature | #Feature F1 | #Feature F10 | #Feature F11 | #Feature F12 | #Feature F13 | #Feature F14 | #Feature F2 | #Feature F3 | #Feature F4 | #Feature F5 | #Feature F6 | #Feature F7 | #Feature F8 | #Feature F9 | Start | End | PTM | AScore | Found By |
| --- | --- | --- | --- | --- | --- | --- | --- | --- | --- | --- | --- | --- | --- | --- | --- | --- | --- | --- | --- | --- | --- | --- | --- | --- | --- | --- | --- | --- | --- | --- | --- | --- | --- | --- | --- | --- | --- | --- | --- | --- | --- | --- | --- | --- | --- |
| R.KLDLHVLPVWQK.G | Y | 65.49 | 1474.8660 | 12 | 1.1 | 492.6298 | 3 | 51.50 | 6 | F6:30729 | NaSa01\_F14.raw |  |  |  |  | 1.7504E5 | 1.6967E6 |  |  |  |  |  |  |  |  | 2 | 0 | 0 | 0 | 0 | 1 | 1 | 0 | 0 | 0 | 0 | 0 | 0 | 0 | 0 | 143 | 154 |  |  | PEAKS DB |
| R.FGFGLLNAK.A | Y | 55.32 | 965.5334 | 9 | -2.0 | 483.7730 | 2 | 61.54 | 6 | F6:39287 | NaSa01\_F14.raw |  |  |  |  | 1.0258E6 | 2.205E6 |  |  |  |  |  |  |  |  | 2 | 0 | 0 | 0 | 0 | 1 | 1 | 0 | 0 | 0 | 0 | 0 | 0 | 0 | 0 | 439 | 447 |  |  | PEAKS DB |
| L.ALEANPNLTWR.D | Y | 41.95 | 1283.6622 | 11 | -1.4 | 642.8375 | 2 | 43.42 | 6 | F6:23684 | NaSa01\_F14.raw |  |  |  |  |  | 0 |  |  |  |  |  |  |  |  | 0 | 0 | 0 | 0 | 0 | 0 | 0 | 0 | 0 | 0 | 0 | 0 | 0 | 0 | 0 | 395 | 405 |  |  | PEAKS DB |
| F.ALALEANPNLTWR.D | Y | 41.90 | 1467.7833 | 13 | 0.1 | 734.8990 | 2 | 60.67 | 6 | F6:38618 | NaSa01\_F14.raw |  |  |  |  |  | 7.3887E5 |  |  |  |  |  |  |  |  | 1 | 0 | 0 | 0 | 0 | 0 | 1 | 0 | 0 | 0 | 0 | 0 | 0 | 0 | 0 | 393 | 405 |  |  | PEAKS DB |
| total 4 peptides |  |  |  |  |  |  |  |  |  |  |  |  |  |  |  |  |  |  |  |  |  |  |  |  |  |  |  |  |  |  |  |  |  |  |  |  |  |  |  |  |  |  |  |  |  |
| --- | --- | --- | --- | --- | --- | --- | --- | --- | --- | --- | --- | --- | --- | --- | --- | --- | --- | --- | --- | --- | --- | --- | --- | --- | --- | --- | --- | --- | --- | --- | --- | --- | --- | --- | --- | --- | --- | --- | --- | --- | --- | --- | --- | --- | --- |

XP\_015687426.1

back to list

  

| Protein Coverage
| Supporting Peptides
|

Protein Coverage:

Supporting Peptides:

| Peptide | Uniq | -10lgP | Mass | Length | ppm | m/z | z | RT | Fraction | Scan | Source File | Area F1 | Area F10 | Area F11 | Area F12 | Area F13 | Area F14 | Area F2 | Area F3 | Area F4 | Area F5 | Area F6 | Area F7 | Area F8 | Area F9 | #Feature | #Feature F1 | #Feature F10 | #Feature F11 | #Feature F12 | #Feature F13 | #Feature F14 | #Feature F2 | #Feature F3 | #Feature F4 | #Feature F5 | #Feature F6 | #Feature F7 | #Feature F8 | #Feature F9 | Start | End | PTM | AScore | Found By |
| --- | --- | --- | --- | --- | --- | --- | --- | --- | --- | --- | --- | --- | --- | --- | --- | --- | --- | --- | --- | --- | --- | --- | --- | --- | --- | --- | --- | --- | --- | --- | --- | --- | --- | --- | --- | --- | --- | --- | --- | --- | --- | --- | --- | --- | --- |
| R.KLDLHVLPVWQK.G | Y | 65.49 | 1474.8660 | 12 | 1.1 | 492.6298 | 3 | 51.50 | 6 | F6:30729 | NaSa01\_F14.raw |  |  |  |  | 1.7504E5 | 1.6967E6 |  |  |  |  |  |  |  |  | 2 | 0 | 0 | 0 | 0 | 1 | 1 | 0 | 0 | 0 | 0 | 0 | 0 | 0 | 0 | 143 | 154 |  |  | PEAKS DB |
| R.FGFGLLNAK.A | Y | 55.32 | 965.5334 | 9 | -2.0 | 483.7730 | 2 | 61.54 | 6 | F6:39287 | NaSa01\_F14.raw |  |  |  |  | 1.0258E6 | 2.205E6 |  |  |  |  |  |  |  |  | 2 | 0 | 0 | 0 | 0 | 1 | 1 | 0 | 0 | 0 | 0 | 0 | 0 | 0 | 0 | 439 | 447 |  |  | PEAKS DB |
| L.ALEANPNLTWR.D | Y | 41.95 | 1283.6622 | 11 | -1.4 | 642.8375 | 2 | 43.42 | 6 | F6:23684 | NaSa01\_F14.raw |  |  |  |  |  | 0 |  |  |  |  |  |  |  |  | 0 | 0 | 0 | 0 | 0 | 0 | 0 | 0 | 0 | 0 | 0 | 0 | 0 | 0 | 0 | 395 | 405 |  |  | PEAKS DB |
| F.ALALEANPNLTWR.D | Y | 41.90 | 1467.7833 | 13 | 0.1 | 734.8990 | 2 | 60.67 | 6 | F6:38618 | NaSa01\_F14.raw |  |  |  |  |  | 7.3887E5 |  |  |  |  |  |  |  |  | 1 | 0 | 0 | 0 | 0 | 0 | 1 | 0 | 0 | 0 | 0 | 0 | 0 | 0 | 0 | 393 | 405 |  |  | PEAKS DB |
| total 4 peptides |  |  |  |  |  |  |  |  |  |  |  |  |  |  |  |  |  |  |  |  |  |  |  |  |  |  |  |  |  |  |  |  |  |  |  |  |  |  |  |  |  |  |  |  |  |
| --- | --- | --- | --- | --- | --- | --- | --- | --- | --- | --- | --- | --- | --- | --- | --- | --- | --- | --- | --- | --- | --- | --- | --- | --- | --- | --- | --- | --- | --- | --- | --- | --- | --- | --- | --- | --- | --- | --- | --- | --- | --- | --- | --- | --- | --- |

XP\_007439683.1

back to list

  

| Protein Coverage
| Supporting Peptides
|

Protein Coverage:

Supporting Peptides:

| Peptide | Uniq | -10lgP | Mass | Length | ppm | m/z | z | RT | Fraction | Scan | Source File | Area F1 | Area F10 | Area F11 | Area F12 | Area F13 | Area F14 | Area F2 | Area F3 | Area F4 | Area F5 | Area F6 | Area F7 | Area F8 | Area F9 | #Feature | #Feature F1 | #Feature F10 | #Feature F11 | #Feature F12 | #Feature F13 | #Feature F14 | #Feature F2 | #Feature F3 | #Feature F4 | #Feature F5 | #Feature F6 | #Feature F7 | #Feature F8 | #Feature F9 | Start | End | PTM | AScore | Found By |
| --- | --- | --- | --- | --- | --- | --- | --- | --- | --- | --- | --- | --- | --- | --- | --- | --- | --- | --- | --- | --- | --- | --- | --- | --- | --- | --- | --- | --- | --- | --- | --- | --- | --- | --- | --- | --- | --- | --- | --- | --- | --- | --- | --- | --- | --- |
| R.KLDLHVLPVWQK.G | Y | 65.49 | 1474.8660 | 12 | 1.1 | 492.6298 | 3 | 51.50 | 6 | F6:30729 | NaSa01\_F14.raw |  |  |  |  | 1.7504E5 | 1.6967E6 |  |  |  |  |  |  |  |  | 2 | 0 | 0 | 0 | 0 | 1 | 1 | 0 | 0 | 0 | 0 | 0 | 0 | 0 | 0 | 143 | 154 |  |  | PEAKS DB |
| R.FGFGLLNAK.A | Y | 55.32 | 965.5334 | 9 | -2.0 | 483.7730 | 2 | 61.54 | 6 | F6:39287 | NaSa01\_F14.raw |  |  |  |  | 1.0258E6 | 2.205E6 |  |  |  |  |  |  |  |  | 2 | 0 | 0 | 0 | 0 | 1 | 1 | 0 | 0 | 0 | 0 | 0 | 0 | 0 | 0 | 439 | 447 |  |  | PEAKS DB |
| L.ALEANPNLTWR.D | Y | 41.95 | 1283.6622 | 11 | -1.4 | 642.8375 | 2 | 43.42 | 6 | F6:23684 | NaSa01\_F14.raw |  |  |  |  |  | 0 |  |  |  |  |  |  |  |  | 0 | 0 | 0 | 0 | 0 | 0 | 0 | 0 | 0 | 0 | 0 | 0 | 0 | 0 | 0 | 395 | 405 |  |  | PEAKS DB |
| F.ALALEANPNLTWR.D | Y | 41.90 | 1467.7833 | 13 | 0.1 | 734.8990 | 2 | 60.67 | 6 | F6:38618 | NaSa01\_F14.raw |  |  |  |  |  | 7.3887E5 |  |  |  |  |  |  |  |  | 1 | 0 | 0 | 0 | 0 | 0 | 1 | 0 | 0 | 0 | 0 | 0 | 0 | 0 | 0 | 393 | 405 |  |  | PEAKS DB |
| total 4 peptides |  |  |  |  |  |  |  |  |  |  |  |  |  |  |  |  |  |  |  |  |  |  |  |  |  |  |  |  |  |  |  |  |  |  |  |  |  |  |  |  |  |  |  |  |  |
| --- | --- | --- | --- | --- | --- | --- | --- | --- | --- | --- | --- | --- | --- | --- | --- | --- | --- | --- | --- | --- | --- | --- | --- | --- | --- | --- | --- | --- | --- | --- | --- | --- | --- | --- | --- | --- | --- | --- | --- | --- | --- | --- | --- | --- | --- |

P14613.1

back to list

  

| Protein Coverage
| Supporting Peptides
|

Protein Coverage:

Supporting Peptides:

| Peptide | Uniq | -10lgP | Mass | Length | ppm | m/z | z | RT | Fraction | Scan | Source File | Area F1 | Area F10 | Area F11 | Area F12 | Area F13 | Area F14 | Area F2 | Area F3 | Area F4 | Area F5 | Area F6 | Area F7 | Area F8 | Area F9 | #Feature | #Feature F1 | #Feature F10 | #Feature F11 | #Feature F12 | #Feature F13 | #Feature F14 | #Feature F2 | #Feature F3 | #Feature F4 | #Feature F5 | #Feature F6 | #Feature F7 | #Feature F8 | #Feature F9 | Start | End | PTM | AScore | Found By |
| --- | --- | --- | --- | --- | --- | --- | --- | --- | --- | --- | --- | --- | --- | --- | --- | --- | --- | --- | --- | --- | --- | --- | --- | --- | --- | --- | --- | --- | --- | --- | --- | --- | --- | --- | --- | --- | --- | --- | --- | --- | --- | --- | --- | --- | --- |
| LEC(+57.02)HNQQSIQTPTTTGC(+57.02)SGGETNC(+57.02)YK.K | Y | 77.10 | 2970.2542 | 26 | 2.3 | 991.0834 | 3 | 12.23 | 1 | F1:2157 | NaSa01\_F1.raw | 6.0506E7 |  |  |  |  |  | 4.4306E6 |  |  |  |  |  |  |  | 4 | 2 | 0 | 0 | 0 | 0 | 0 | 2 | 0 | 0 | 0 | 0 | 0 | 0 | 0 | 1 | 26 | Carbamidomethylation | C3:Carbamidomethylation:1000.00;C17:Carbamidomethylation:1000.00;C24:Carbamidomethylation:1000.00 | PEAKS DB |
| K.NGIEINC(+57.02)C(+57.02)TTDR.C | N | 76.35 | 1451.6133 | 12 | 3.3 | 726.8141 | 2 | 19.60 | 7 | F7:7461 | NaSa01\_F2.raw | 0 |  |  |  |  |  | 6.7801E6 | 5.7301E7 | 9.911E5 |  |  |  |  |  | 8 | 0 | 0 | 0 | 0 | 0 | 0 | 1 | 6 | 1 | 0 | 0 | 0 | 0 | 0 | 48 | 59 | Carbamidomethylation | C7:Carbamidomethylation:1000.00;C8:Carbamidomethylation:1000.00 | PEAKS DB |
| N.GIEINC(+57.02)C(+57.02)TTDR.C | N | 50.21 | 1337.5704 | 11 | 6.3 | 669.7932 | 2 | 11.47 | 8 | F8:2008 | NaSa01\_F3.raw |  |  |  |  |  |  |  | 1.1583E6 |  |  |  |  |  |  | 1 | 0 | 0 | 0 | 0 | 0 | 0 | 0 | 1 | 0 | 0 | 0 | 0 | 0 | 0 | 49 | 59 | Carbamidomethylation | C6:Carbamidomethylation:1000.00;C7:Carbamidomethylation:1000.00 | PEAKS DB |
| total 3 peptides |  |  |  |  |  |  |  |  |  |  |  |  |  |  |  |  |  |  |  |  |  |  |  |  |  |  |  |  |  |  |  |  |  |  |  |  |  |  |  |  |  |  |  |  |  |
| --- | --- | --- | --- | --- | --- | --- | --- | --- | --- | --- | --- | --- | --- | --- | --- | --- | --- | --- | --- | --- | --- | --- | --- | --- | --- | --- | --- | --- | --- | --- | --- | --- | --- | --- | --- | --- | --- | --- | --- | --- | --- | --- | --- | --- | --- |

XP\_026571379.1

back to list

  

| Protein Coverage
| Supporting Peptides
|

Protein Coverage:

Supporting Peptides:

| Peptide | Uniq | -10lgP | Mass | Length | ppm | m/z | z | RT | Fraction | Scan | Source File | Area F1 | Area F10 | Area F11 | Area F12 | Area F13 | Area F14 | Area F2 | Area F3 | Area F4 | Area F5 | Area F6 | Area F7 | Area F8 | Area F9 | #Feature | #Feature F1 | #Feature F10 | #Feature F11 | #Feature F12 | #Feature F13 | #Feature F14 | #Feature F2 | #Feature F3 | #Feature F4 | #Feature F5 | #Feature F6 | #Feature F7 | #Feature F8 | #Feature F9 | Start | End | PTM | AScore | Found By |
| --- | --- | --- | --- | --- | --- | --- | --- | --- | --- | --- | --- | --- | --- | --- | --- | --- | --- | --- | --- | --- | --- | --- | --- | --- | --- | --- | --- | --- | --- | --- | --- | --- | --- | --- | --- | --- | --- | --- | --- | --- | --- | --- | --- | --- | --- |
| K.WSDLPTLC(+57.02)QGK.R | N | 64.57 | 1303.6230 | 11 | 0.5 | 652.8192 | 2 | 45.17 | 14 | F14:24063 | NaSa01\_F9.raw |  | 1.6687E6 |  |  |  |  |  |  |  |  |  |  |  | 1.3852E7 | 2 | 0 | 1 | 0 | 0 | 0 | 0 | 0 | 0 | 0 | 0 | 0 | 0 | 0 | 1 | 82 | 92 | Carbamidomethylation | C8:Carbamidomethylation:1000.00 | PEAKS DB |
| K.WNLPSPEC(+57.02)K.K | Y | 58.59 | 1129.5226 | 9 | 0.0 | 565.7686 | 2 | 38.23 | 3 | F3:18594 | NaSa01\_F11.raw |  | 4.9489E6 | 1.8498E8 | 3.3547E6 |  |  |  |  |  |  |  |  |  |  | 4 | 0 | 1 | 2 | 1 | 0 | 0 | 0 | 0 | 0 | 0 | 0 | 0 | 0 | 0 | 209 | 217 | Carbamidomethylation | C8:Carbamidomethylation:1000.00 | PEAKS DB |
| S.DC(+57.02)PRPVLPPHSSLR.G | N | 53.89 | 1629.8409 | 14 | 0.2 | 544.2877 | 3 | 12.08 | 3 | F3:3644 | NaSa01\_F11.raw |  |  | 1.6489E6 |  |  |  |  |  |  |  |  |  |  |  | 2 | 0 | 0 | 2 | 0 | 0 | 0 | 0 | 0 | 0 | 0 | 0 | 0 | 0 | 0 | 27 | 40 | Carbamidomethylation | C2:Carbamidomethylation:1000.00 | PEAKS DB |
| K.IVSSDDLR.L | Y | 46.00 | 903.4661 | 8 | -0.7 | 452.7400 | 2 | 11.89 | 3 | F3:3499 | NaSa01\_F11.raw |  |  | 6.1482E5 |  |  |  |  |  |  |  |  |  |  |  | 1 | 0 | 0 | 1 | 0 | 0 | 0 | 0 | 0 | 0 | 0 | 0 | 0 | 0 | 0 | 104 | 111 |  |  | PEAKS DB |
| total 4 peptides |  |  |  |  |  |  |  |  |  |  |  |  |  |  |  |  |  |  |  |  |  |  |  |  |  |  |  |  |  |  |  |  |  |  |  |  |  |  |  |  |  |  |  |  |  |
| --- | --- | --- | --- | --- | --- | --- | --- | --- | --- | --- | --- | --- | --- | --- | --- | --- | --- | --- | --- | --- | --- | --- | --- | --- | --- | --- | --- | --- | --- | --- | --- | --- | --- | --- | --- | --- | --- | --- | --- | --- | --- | --- | --- | --- | --- |

XP\_026579406.1

back to list

  

| Protein Coverage
| Supporting Peptides
|

Protein Coverage:

Supporting Peptides:

| Peptide | Uniq | -10lgP | Mass | Length | ppm | m/z | z | RT | Fraction | Scan | Source File | Area F1 | Area F10 | Area F11 | Area F12 | Area F13 | Area F14 | Area F2 | Area F3 | Area F4 | Area F5 | Area F6 | Area F7 | Area F8 | Area F9 | #Feature | #Feature F1 | #Feature F10 | #Feature F11 | #Feature F12 | #Feature F13 | #Feature F14 | #Feature F2 | #Feature F3 | #Feature F4 | #Feature F5 | #Feature F6 | #Feature F7 | #Feature F8 | #Feature F9 | Start | End | PTM | AScore | Found By |
| --- | --- | --- | --- | --- | --- | --- | --- | --- | --- | --- | --- | --- | --- | --- | --- | --- | --- | --- | --- | --- | --- | --- | --- | --- | --- | --- | --- | --- | --- | --- | --- | --- | --- | --- | --- | --- | --- | --- | --- | --- | --- | --- | --- | --- | --- |
| K.FTYGGC(+57.02)AGNANNFK.K | N | 74.51 | 1519.6514 | 14 | 0.5 | 760.8334 | 2 | 24.87 | 12 | F12:10904 | NaSa01\_F7.raw |  |  |  |  |  |  |  |  |  | 3.1463E8 | 1.1098E8 | 2.7258E6 | 8.8381E5 |  | 4 | 0 | 0 | 0 | 0 | 0 | 0 | 0 | 0 | 0 | 1 | 1 | 1 | 1 | 0 | 170 | 183 | Carbamidomethylation | C6:Carbamidomethylation:1000.00 | PEAKS DB |
| L.FEGSC(+57.02)SIFPNDSQR.C | Y | 57.60 | 1642.7046 | 14 | 0.4 | 822.3599 | 2 | 39.73 | 10 | F10:22558 | NaSa01\_F5.raw |  |  |  |  |  |  |  |  |  | 8.4936E6 |  |  |  |  | 1 | 0 | 0 | 0 | 0 | 0 | 0 | 0 | 0 | 0 | 1 | 0 | 0 | 0 | 0 | 34 | 47 | Carbamidomethylation | C5:Carbamidomethylation:1000.00 | PEAKS DB |
| G.LFEGSC(+57.02)SIFPNDSQR.C | Y | 48.78 | 1755.7886 | 15 | -0.2 | 878.9014 | 2 | 54.77 | 11 | F11:36750 | NaSa01\_F6.raw |  |  |  |  |  |  |  |  |  |  | 3.3284E7 |  |  |  | 1 | 0 | 0 | 0 | 0 | 0 | 0 | 0 | 0 | 0 | 0 | 1 | 0 | 0 | 0 | 33 | 47 | Carbamidomethylation | C6:Carbamidomethylation:1000.00 | PEAKS DB |
| K.C(+57.02)C(+57.02)SYGC(+57.02)ALYC(+57.02)MK.P | Y | 44.36 | 1571.5699 | 12 | -0.5 | 786.7919 | 2 | 36.34 | 10 | F10:19760 | NaSa01\_F5.raw |  |  |  |  |  |  |  |  |  | 2.6705E7 |  |  |  |  | 1 | 0 | 0 | 0 | 0 | 0 | 0 | 0 | 0 | 0 | 1 | 0 | 0 | 0 | 0 | 125 | 136 | Carbamidomethylation | C1:Carbamidomethylation:1000.00;C2:Carbamidomethylation:1000.00;C6:Carbamidomethylation:1000.00;C10:Carbamidomethylation:1000.00 | PEAKS DB |
| total 4 peptides |  |  |  |  |  |  |  |  |  |  |  |  |  |  |  |  |  |  |  |  |  |  |  |  |  |  |  |  |  |  |  |  |  |  |  |  |  |  |  |  |  |  |  |  |  |
| --- | --- | --- | --- | --- | --- | --- | --- | --- | --- | --- | --- | --- | --- | --- | --- | --- | --- | --- | --- | --- | --- | --- | --- | --- | --- | --- | --- | --- | --- | --- | --- | --- | --- | --- | --- | --- | --- | --- | --- | --- | --- | --- | --- | --- | --- |

XP\_026579408.1

back to list

  

| Protein Coverage
| Supporting Peptides
|

Protein Coverage:

Supporting Peptides:

| Peptide | Uniq | -10lgP | Mass | Length | ppm | m/z | z | RT | Fraction | Scan | Source File | Area F1 | Area F10 | Area F11 | Area F12 | Area F13 | Area F14 | Area F2 | Area F3 | Area F4 | Area F5 | Area F6 | Area F7 | Area F8 | Area F9 | #Feature | #Feature F1 | #Feature F10 | #Feature F11 | #Feature F12 | #Feature F13 | #Feature F14 | #Feature F2 | #Feature F3 | #Feature F4 | #Feature F5 | #Feature F6 | #Feature F7 | #Feature F8 | #Feature F9 | Start | End | PTM | AScore | Found By |
| --- | --- | --- | --- | --- | --- | --- | --- | --- | --- | --- | --- | --- | --- | --- | --- | --- | --- | --- | --- | --- | --- | --- | --- | --- | --- | --- | --- | --- | --- | --- | --- | --- | --- | --- | --- | --- | --- | --- | --- | --- | --- | --- | --- | --- | --- |
| K.FTYGGC(+57.02)AGNANNFK.K | N | 74.51 | 1519.6514 | 14 | 0.5 | 760.8334 | 2 | 24.87 | 12 | F12:10904 | NaSa01\_F7.raw |  |  |  |  |  |  |  |  |  | 3.1463E8 | 1.1098E8 | 2.7258E6 | 8.8381E5 |  | 4 | 0 | 0 | 0 | 0 | 0 | 0 | 0 | 0 | 0 | 1 | 1 | 1 | 1 | 0 | 170 | 183 | Carbamidomethylation | C6:Carbamidomethylation:1000.00 | PEAKS DB |
| L.FEGSC(+57.02)SIFPNDSQR.C | Y | 57.60 | 1642.7046 | 14 | 0.4 | 822.3599 | 2 | 39.73 | 10 | F10:22558 | NaSa01\_F5.raw |  |  |  |  |  |  |  |  |  | 8.4936E6 |  |  |  |  | 1 | 0 | 0 | 0 | 0 | 0 | 0 | 0 | 0 | 0 | 1 | 0 | 0 | 0 | 0 | 34 | 47 | Carbamidomethylation | C5:Carbamidomethylation:1000.00 | PEAKS DB |
| G.LFEGSC(+57.02)SIFPNDSQR.C | Y | 48.78 | 1755.7886 | 15 | -0.2 | 878.9014 | 2 | 54.77 | 11 | F11:36750 | NaSa01\_F6.raw |  |  |  |  |  |  |  |  |  |  | 3.3284E7 |  |  |  | 1 | 0 | 0 | 0 | 0 | 0 | 0 | 0 | 0 | 0 | 0 | 1 | 0 | 0 | 0 | 33 | 47 | Carbamidomethylation | C6:Carbamidomethylation:1000.00 | PEAKS DB |
| K.C(+57.02)C(+57.02)SYGC(+57.02)ALYC(+57.02)MK.P | Y | 44.36 | 1571.5699 | 12 | -0.5 | 786.7919 | 2 | 36.34 | 10 | F10:19760 | NaSa01\_F5.raw |  |  |  |  |  |  |  |  |  | 2.6705E7 |  |  |  |  | 1 | 0 | 0 | 0 | 0 | 0 | 0 | 0 | 0 | 0 | 1 | 0 | 0 | 0 | 0 | 125 | 136 | Carbamidomethylation | C1:Carbamidomethylation:1000.00;C2:Carbamidomethylation:1000.00;C6:Carbamidomethylation:1000.00;C10:Carbamidomethylation:1000.00 | PEAKS DB |
| total 4 peptides |  |  |  |  |  |  |  |  |  |  |  |  |  |  |  |  |  |  |  |  |  |  |  |  |  |  |  |  |  |  |  |  |  |  |  |  |  |  |  |  |  |  |  |  |  |
| --- | --- | --- | --- | --- | --- | --- | --- | --- | --- | --- | --- | --- | --- | --- | --- | --- | --- | --- | --- | --- | --- | --- | --- | --- | --- | --- | --- | --- | --- | --- | --- | --- | --- | --- | --- | --- | --- | --- | --- | --- | --- | --- | --- | --- | --- |

XP\_026579405.1

back to list

  

| Protein Coverage
| Supporting Peptides
|

Protein Coverage:

Supporting Peptides:

| Peptide | Uniq | -10lgP | Mass | Length | ppm | m/z | z | RT | Fraction | Scan | Source File | Area F1 | Area F10 | Area F11 | Area F12 | Area F13 | Area F14 | Area F2 | Area F3 | Area F4 | Area F5 | Area F6 | Area F7 | Area F8 | Area F9 | #Feature | #Feature F1 | #Feature F10 | #Feature F11 | #Feature F12 | #Feature F13 | #Feature F14 | #Feature F2 | #Feature F3 | #Feature F4 | #Feature F5 | #Feature F6 | #Feature F7 | #Feature F8 | #Feature F9 | Start | End | PTM | AScore | Found By |
| --- | --- | --- | --- | --- | --- | --- | --- | --- | --- | --- | --- | --- | --- | --- | --- | --- | --- | --- | --- | --- | --- | --- | --- | --- | --- | --- | --- | --- | --- | --- | --- | --- | --- | --- | --- | --- | --- | --- | --- | --- | --- | --- | --- | --- | --- |
| K.FTYGGC(+57.02)AGNANNFK.K | N | 74.51 | 1519.6514 | 14 | 0.5 | 760.8334 | 2 | 24.87 | 12 | F12:10904 | NaSa01\_F7.raw |  |  |  |  |  |  |  |  |  | 3.1463E8 | 1.1098E8 | 2.7258E6 | 8.8381E5 |  | 4 | 0 | 0 | 0 | 0 | 0 | 0 | 0 | 0 | 0 | 1 | 1 | 1 | 1 | 0 | 170 | 183 | Carbamidomethylation | C6:Carbamidomethylation:1000.00 | PEAKS DB |
| L.FEGSC(+57.02)SIFPNDSQR.C | Y | 57.60 | 1642.7046 | 14 | 0.4 | 822.3599 | 2 | 39.73 | 10 | F10:22558 | NaSa01\_F5.raw |  |  |  |  |  |  |  |  |  | 8.4936E6 |  |  |  |  | 1 | 0 | 0 | 0 | 0 | 0 | 0 | 0 | 0 | 0 | 1 | 0 | 0 | 0 | 0 | 34 | 47 | Carbamidomethylation | C5:Carbamidomethylation:1000.00 | PEAKS DB |
| G.LFEGSC(+57.02)SIFPNDSQR.C | Y | 48.78 | 1755.7886 | 15 | -0.2 | 878.9014 | 2 | 54.77 | 11 | F11:36750 | NaSa01\_F6.raw |  |  |  |  |  |  |  |  |  |  | 3.3284E7 |  |  |  | 1 | 0 | 0 | 0 | 0 | 0 | 0 | 0 | 0 | 0 | 0 | 1 | 0 | 0 | 0 | 33 | 47 | Carbamidomethylation | C6:Carbamidomethylation:1000.00 | PEAKS DB |
| K.C(+57.02)C(+57.02)SYGC(+57.02)ALYC(+57.02)MK.P | Y | 44.36 | 1571.5699 | 12 | -0.5 | 786.7919 | 2 | 36.34 | 10 | F10:19760 | NaSa01\_F5.raw |  |  |  |  |  |  |  |  |  | 2.6705E7 |  |  |  |  | 1 | 0 | 0 | 0 | 0 | 0 | 0 | 0 | 0 | 0 | 1 | 0 | 0 | 0 | 0 | 125 | 136 | Carbamidomethylation | C1:Carbamidomethylation:1000.00;C2:Carbamidomethylation:1000.00;C6:Carbamidomethylation:1000.00;C10:Carbamidomethylation:1000.00 | PEAKS DB |
| total 4 peptides |  |  |  |  |  |  |  |  |  |  |  |  |  |  |  |  |  |  |  |  |  |  |  |  |  |  |  |  |  |  |  |  |  |  |  |  |  |  |  |  |  |  |  |  |  |
| --- | --- | --- | --- | --- | --- | --- | --- | --- | --- | --- | --- | --- | --- | --- | --- | --- | --- | --- | --- | --- | --- | --- | --- | --- | --- | --- | --- | --- | --- | --- | --- | --- | --- | --- | --- | --- | --- | --- | --- | --- | --- | --- | --- | --- | --- |

XP\_026579409.1

back to list

  

| Protein Coverage
| Supporting Peptides
|

Protein Coverage:

Supporting Peptides:

| Peptide | Uniq | -10lgP | Mass | Length | ppm | m/z | z | RT | Fraction | Scan | Source File | Area F1 | Area F10 | Area F11 | Area F12 | Area F13 | Area F14 | Area F2 | Area F3 | Area F4 | Area F5 | Area F6 | Area F7 | Area F8 | Area F9 | #Feature | #Feature F1 | #Feature F10 | #Feature F11 | #Feature F12 | #Feature F13 | #Feature F14 | #Feature F2 | #Feature F3 | #Feature F4 | #Feature F5 | #Feature F6 | #Feature F7 | #Feature F8 | #Feature F9 | Start | End | PTM | AScore | Found By |
| --- | --- | --- | --- | --- | --- | --- | --- | --- | --- | --- | --- | --- | --- | --- | --- | --- | --- | --- | --- | --- | --- | --- | --- | --- | --- | --- | --- | --- | --- | --- | --- | --- | --- | --- | --- | --- | --- | --- | --- | --- | --- | --- | --- | --- | --- |
| K.FTYGGC(+57.02)AGNANNFK.K | N | 74.51 | 1519.6514 | 14 | 0.5 | 760.8334 | 2 | 24.87 | 12 | F12:10904 | NaSa01\_F7.raw |  |  |  |  |  |  |  |  |  | 3.1463E8 | 1.1098E8 | 2.7258E6 | 8.8381E5 |  | 4 | 0 | 0 | 0 | 0 | 0 | 0 | 0 | 0 | 0 | 1 | 1 | 1 | 1 | 0 | 170 | 183 | Carbamidomethylation | C6:Carbamidomethylation:1000.00 | PEAKS DB |
| L.FEGSC(+57.02)SIFPNDSQR.C | Y | 57.60 | 1642.7046 | 14 | 0.4 | 822.3599 | 2 | 39.73 | 10 | F10:22558 | NaSa01\_F5.raw |  |  |  |  |  |  |  |  |  | 8.4936E6 |  |  |  |  | 1 | 0 | 0 | 0 | 0 | 0 | 0 | 0 | 0 | 0 | 1 | 0 | 0 | 0 | 0 | 34 | 47 | Carbamidomethylation | C5:Carbamidomethylation:1000.00 | PEAKS DB |
| G.LFEGSC(+57.02)SIFPNDSQR.C | Y | 48.78 | 1755.7886 | 15 | -0.2 | 878.9014 | 2 | 54.77 | 11 | F11:36750 | NaSa01\_F6.raw |  |  |  |  |  |  |  |  |  |  | 3.3284E7 |  |  |  | 1 | 0 | 0 | 0 | 0 | 0 | 0 | 0 | 0 | 0 | 0 | 1 | 0 | 0 | 0 | 33 | 47 | Carbamidomethylation | C6:Carbamidomethylation:1000.00 | PEAKS DB |
| K.C(+57.02)C(+57.02)SYGC(+57.02)ALYC(+57.02)MK.P | Y | 44.36 | 1571.5699 | 12 | -0.5 | 786.7919 | 2 | 36.34 | 10 | F10:19760 | NaSa01\_F5.raw |  |  |  |  |  |  |  |  |  | 2.6705E7 |  |  |  |  | 1 | 0 | 0 | 0 | 0 | 0 | 0 | 0 | 0 | 0 | 1 | 0 | 0 | 0 | 0 | 125 | 136 | Carbamidomethylation | C1:Carbamidomethylation:1000.00;C2:Carbamidomethylation:1000.00;C6:Carbamidomethylation:1000.00;C10:Carbamidomethylation:1000.00 | PEAKS DB |
| total 4 peptides |  |  |  |  |  |  |  |  |  |  |  |  |  |  |  |  |  |  |  |  |  |  |  |  |  |  |  |  |  |  |  |  |  |  |  |  |  |  |  |  |  |  |  |  |  |
| --- | --- | --- | --- | --- | --- | --- | --- | --- | --- | --- | --- | --- | --- | --- | --- | --- | --- | --- | --- | --- | --- | --- | --- | --- | --- | --- | --- | --- | --- | --- | --- | --- | --- | --- | --- | --- | --- | --- | --- | --- | --- | --- | --- | --- | --- |

XP\_026579404.1

back to list

  

| Protein Coverage
| Supporting Peptides
|

Protein Coverage:

Supporting Peptides:

| Peptide | Uniq | -10lgP | Mass | Length | ppm | m/z | z | RT | Fraction | Scan | Source File | Area F1 | Area F10 | Area F11 | Area F12 | Area F13 | Area F14 | Area F2 | Area F3 | Area F4 | Area F5 | Area F6 | Area F7 | Area F8 | Area F9 | #Feature | #Feature F1 | #Feature F10 | #Feature F11 | #Feature F12 | #Feature F13 | #Feature F14 | #Feature F2 | #Feature F3 | #Feature F4 | #Feature F5 | #Feature F6 | #Feature F7 | #Feature F8 | #Feature F9 | Start | End | PTM | AScore | Found By |
| --- | --- | --- | --- | --- | --- | --- | --- | --- | --- | --- | --- | --- | --- | --- | --- | --- | --- | --- | --- | --- | --- | --- | --- | --- | --- | --- | --- | --- | --- | --- | --- | --- | --- | --- | --- | --- | --- | --- | --- | --- | --- | --- | --- | --- | --- |
| K.FTYGGC(+57.02)AGNANNFK.K | N | 74.51 | 1519.6514 | 14 | 0.5 | 760.8334 | 2 | 24.87 | 12 | F12:10904 | NaSa01\_F7.raw |  |  |  |  |  |  |  |  |  | 3.1463E8 | 1.1098E8 | 2.7258E6 | 8.8381E5 |  | 4 | 0 | 0 | 0 | 0 | 0 | 0 | 0 | 0 | 0 | 1 | 1 | 1 | 1 | 0 | 170 | 183 | Carbamidomethylation | C6:Carbamidomethylation:1000.00 | PEAKS DB |
| L.FEGSC(+57.02)SIFPNDSQR.C | Y | 57.60 | 1642.7046 | 14 | 0.4 | 822.3599 | 2 | 39.73 | 10 | F10:22558 | NaSa01\_F5.raw |  |  |  |  |  |  |  |  |  | 8.4936E6 |  |  |  |  | 1 | 0 | 0 | 0 | 0 | 0 | 0 | 0 | 0 | 0 | 1 | 0 | 0 | 0 | 0 | 34 | 47 | Carbamidomethylation | C5:Carbamidomethylation:1000.00 | PEAKS DB |
| G.LFEGSC(+57.02)SIFPNDSQR.C | Y | 48.78 | 1755.7886 | 15 | -0.2 | 878.9014 | 2 | 54.77 | 11 | F11:36750 | NaSa01\_F6.raw |  |  |  |  |  |  |  |  |  |  | 3.3284E7 |  |  |  | 1 | 0 | 0 | 0 | 0 | 0 | 0 | 0 | 0 | 0 | 0 | 1 | 0 | 0 | 0 | 33 | 47 | Carbamidomethylation | C6:Carbamidomethylation:1000.00 | PEAKS DB |
| K.C(+57.02)C(+57.02)SYGC(+57.02)ALYC(+57.02)MK.P | Y | 44.36 | 1571.5699 | 12 | -0.5 | 786.7919 | 2 | 36.34 | 10 | F10:19760 | NaSa01\_F5.raw |  |  |  |  |  |  |  |  |  | 2.6705E7 |  |  |  |  | 1 | 0 | 0 | 0 | 0 | 0 | 0 | 0 | 0 | 0 | 1 | 0 | 0 | 0 | 0 | 125 | 136 | Carbamidomethylation | C1:Carbamidomethylation:1000.00;C2:Carbamidomethylation:1000.00;C6:Carbamidomethylation:1000.00;C10:Carbamidomethylation:1000.00 | PEAKS DB |
| total 4 peptides |  |  |  |  |  |  |  |  |  |  |  |  |  |  |  |  |  |  |  |  |  |  |  |  |  |  |  |  |  |  |  |  |  |  |  |  |  |  |  |  |  |  |  |  |  |
| --- | --- | --- | --- | --- | --- | --- | --- | --- | --- | --- | --- | --- | --- | --- | --- | --- | --- | --- | --- | --- | --- | --- | --- | --- | --- | --- | --- | --- | --- | --- | --- | --- | --- | --- | --- | --- | --- | --- | --- | --- | --- | --- | --- | --- | --- |

XP\_026544110.1

back to list

  

| Protein Coverage
| Supporting Peptides
|

Protein Coverage:

Supporting Peptides:

| Peptide | Uniq | -10lgP | Mass | Length | ppm | m/z | z | RT | Fraction | Scan | Source File | Area F1 | Area F10 | Area F11 | Area F12 | Area F13 | Area F14 | Area F2 | Area F3 | Area F4 | Area F5 | Area F6 | Area F7 | Area F8 | Area F9 | #Feature | #Feature F1 | #Feature F10 | #Feature F11 | #Feature F12 | #Feature F13 | #Feature F14 | #Feature F2 | #Feature F3 | #Feature F4 | #Feature F5 | #Feature F6 | #Feature F7 | #Feature F8 | #Feature F9 | Start | End | PTM | AScore | Found By |
| --- | --- | --- | --- | --- | --- | --- | --- | --- | --- | --- | --- | --- | --- | --- | --- | --- | --- | --- | --- | --- | --- | --- | --- | --- | --- | --- | --- | --- | --- | --- | --- | --- | --- | --- | --- | --- | --- | --- | --- | --- | --- | --- | --- | --- | --- |
| K.YIMHPDYSVFNPTENDIVLIK.L | Y | 68.38 | 2507.2407 | 21 | 1.3 | 836.7553 | 3 | 77.40 | 4 | F4:54054 | NaSa01\_F12.raw |  |  | 3.3068E7 | 3.592E7 |  |  |  |  |  |  |  |  |  |  | 4 | 0 | 0 | 2 | 2 | 0 | 0 | 0 | 0 | 0 | 0 | 0 | 0 | 0 | 0 | 378 | 398 |  |  | PEAKS DB |
| R.C(+57.02)IC(+57.02)PAEFMGK.Q | N | 65.19 | 1211.5137 | 10 | 0.6 | 606.7645 | 2 | 43.69 | 13 | F13:29925 | NaSa01\_F8.raw |  |  |  |  |  |  |  | 4.6428E6 | 3.4963E6 |  |  | 2.5429E6 | 4.0807E7 | 5.061E6 | 6 | 0 | 0 | 0 | 0 | 0 | 0 | 0 | 1 | 1 | 0 | 0 | 1 | 2 | 1 | 82 | 91 | Carbamidomethylation | C1:Carbamidomethylation:1000.00;C3:Carbamidomethylation:1000.00 | PEAKS DB |
| K.YIM(+15.99)HPDYSVFNPTENDIVLIK.L | Y | 52.68 | 2523.2356 | 21 | 1.2 | 842.0868 | 3 | 70.99 | 3 | F3:43928 | NaSa01\_F11.raw |  |  | 1.6539E6 | 3.6188E6 |  |  |  |  |  |  |  |  |  |  | 2 | 0 | 0 | 1 | 1 | 0 | 0 | 0 | 0 | 0 | 0 | 0 | 0 | 0 | 0 | 378 | 398 | Oxidation (M) | M3:Oxidation (M):1000.00 | PEAKS DB |
| R.NFC(+57.02)GGSLIR.S | N | 49.30 | 1022.4967 | 9 | -0.5 | 512.2554 | 2 | 27.48 | 4 | F4:12517 | NaSa01\_F12.raw |  |  |  | 1.0049E5 |  |  |  |  |  |  |  |  |  |  | 1 | 0 | 0 | 0 | 1 | 0 | 0 | 0 | 0 | 0 | 0 | 0 | 0 | 0 | 0 | 326 | 334 | Carbamidomethylation | C3:Carbamidomethylation:1000.00 | PEAKS DB |
| R.SPDEDEQPWC(+57.02)YFIK.D | N | 46.63 | 1812.7665 | 14 | 0.0 | 907.3905 | 2 | 79.81 | 3 | F3:51099 | NaSa01\_F11.raw |  |  | 8.6299E5 |  |  |  |  |  |  |  |  |  |  |  | 1 | 0 | 0 | 1 | 0 | 0 | 0 | 0 | 0 | 0 | 0 | 0 | 0 | 0 | 0 | 235 | 248 | Carbamidomethylation | C10:Carbamidomethylation:1000.00 | PEAKS DB |
| total 5 peptides |  |  |  |  |  |  |  |  |  |  |  |  |  |  |  |  |  |  |  |  |  |  |  |  |  |  |  |  |  |  |  |  |  |  |  |  |  |  |  |  |  |  |  |  |  |
| --- | --- | --- | --- | --- | --- | --- | --- | --- | --- | --- | --- | --- | --- | --- | --- | --- | --- | --- | --- | --- | --- | --- | --- | --- | --- | --- | --- | --- | --- | --- | --- | --- | --- | --- | --- | --- | --- | --- | --- | --- | --- | --- | --- | --- | --- |

AJB84503.1

back to list

  

| Protein Coverage
| Supporting Peptides
|

Protein Coverage:

Supporting Peptides:

| Peptide | Uniq | -10lgP | Mass | Length | ppm | m/z | z | RT | Fraction | Scan | Source File | Area F1 | Area F10 | Area F11 | Area F12 | Area F13 | Area F14 | Area F2 | Area F3 | Area F4 | Area F5 | Area F6 | Area F7 | Area F8 | Area F9 | #Feature | #Feature F1 | #Feature F10 | #Feature F11 | #Feature F12 | #Feature F13 | #Feature F14 | #Feature F2 | #Feature F3 | #Feature F4 | #Feature F5 | #Feature F6 | #Feature F7 | #Feature F8 | #Feature F9 | Start | End | PTM | AScore | Found By |
| --- | --- | --- | --- | --- | --- | --- | --- | --- | --- | --- | --- | --- | --- | --- | --- | --- | --- | --- | --- | --- | --- | --- | --- | --- | --- | --- | --- | --- | --- | --- | --- | --- | --- | --- | --- | --- | --- | --- | --- | --- | --- | --- | --- | --- | --- |
| N.VTLDLFGEWR.K | N | 67.98 | 1234.6346 | 10 | 0.3 | 618.3248 | 2 | 83.54 | 6 | F6:57918 | NaSa01\_F14.raw |  |  | 1.1051E5 |  | 1.1401E8 | 1.4573E8 |  |  |  |  |  |  |  | 9.2511E5 | 13 | 0 | 0 | 1 | 0 | 2 | 9 | 0 | 0 | 0 | 0 | 0 | 0 | 0 | 1 | 273 | 282 |  |  | PEAKS DB |
| H.GQC(+57.02)VDVQTAY | N | 59.29 | 1139.4917 | 10 | 0.4 | 570.7534 | 2 | 32.72 | 4 | F4:17040 | NaSa01\_F12.raw |  |  |  | 1.3163E7 | 3.9751E7 |  |  |  |  |  |  | 4.4194E6 | 8.2572E6 |  | 4 | 0 | 0 | 0 | 1 | 1 | 0 | 0 | 0 | 0 | 0 | 0 | 1 | 1 | 0 | 606 | 615 | Carbamidomethylation | C3:Carbamidomethylation:1000.00 | PEAKS DB |
| V.TLDLFGEWR.K | N | 43.65 | 1135.5662 | 9 | -0.8 | 568.7899 | 2 | 77.17 | 6 | F6:52295 | NaSa01\_F14.raw |  |  |  |  |  | 1.3701E6 |  |  |  |  |  |  |  |  | 1 | 0 | 0 | 0 | 0 | 0 | 1 | 0 | 0 | 0 | 0 | 0 | 0 | 0 | 0 | 274 | 282 |  |  | PEAKS DB |
| R.DGHPC(+57.02)QNNQGYC(+57.02)YNGK.C | Y | 38.68 | 1910.7424 | 16 | 1.5 | 637.9224 | 3 | 11.41 | 9 | F9:1815 | NaSa01\_F4.raw |  |  |  |  |  |  |  |  | 6.1915E5 |  |  |  |  |  | 1 | 0 | 0 | 0 | 0 | 0 | 0 | 0 | 0 | 1 | 0 | 0 | 0 | 0 | 0 | 496 | 511 | Carbamidomethylation | C5:Carbamidomethylation:1000.00;C12:Carbamidomethylation:1000.00 | PEAKS DB |
| total 4 peptides |  |  |  |  |  |  |  |  |  |  |  |  |  |  |  |  |  |  |  |  |  |  |  |  |  |  |  |  |  |  |  |  |  |  |  |  |  |  |  |  |  |  |  |  |  |
| --- | --- | --- | --- | --- | --- | --- | --- | --- | --- | --- | --- | --- | --- | --- | --- | --- | --- | --- | --- | --- | --- | --- | --- | --- | --- | --- | --- | --- | --- | --- | --- | --- | --- | --- | --- | --- | --- | --- | --- | --- | --- | --- | --- | --- | --- |

Q90WI8.1

back to list

  

| Protein Coverage
| Supporting Peptides
|

Protein Coverage:

Supporting Peptides:

| Peptide | Uniq | -10lgP | Mass | Length | ppm | m/z | z | RT | Fraction | Scan | Source File | Area F1 | Area F10 | Area F11 | Area F12 | Area F13 | Area F14 | Area F2 | Area F3 | Area F4 | Area F5 | Area F6 | Area F7 | Area F8 | Area F9 | #Feature | #Feature F1 | #Feature F10 | #Feature F11 | #Feature F12 | #Feature F13 | #Feature F14 | #Feature F2 | #Feature F3 | #Feature F4 | #Feature F5 | #Feature F6 | #Feature F7 | #Feature F8 | #Feature F9 | Start | End | PTM | AScore | Found By |
| --- | --- | --- | --- | --- | --- | --- | --- | --- | --- | --- | --- | --- | --- | --- | --- | --- | --- | --- | --- | --- | --- | --- | --- | --- | --- | --- | --- | --- | --- | --- | --- | --- | --- | --- | --- | --- | --- | --- | --- | --- | --- | --- | --- | --- | --- |
| K.KYIWEWTDR.S | Y | 63.28 | 1295.6299 | 9 | 0.1 | 648.8223 | 2 | 51.37 | 4 | F4:32829 | NaSa01\_F12.raw |  |  |  | 1.6479E8 |  |  |  |  |  |  |  |  |  |  | 2 | 0 | 0 | 0 | 2 | 0 | 0 | 0 | 0 | 0 | 0 | 0 | 0 | 0 | 0 | 99 | 107 |  |  | PEAKS DB |
| K.YIWEWTDR.S | Y | 55.19 | 1167.5349 | 8 | 0.3 | 584.7749 | 2 | 65.79 | 4 | F4:44510 | NaSa01\_F12.raw |  |  |  | 1.0797E9 |  | 0 |  |  |  |  |  |  |  |  | 10 | 0 | 0 | 0 | 10 | 0 | 0 | 0 | 0 | 0 | 0 | 0 | 0 | 0 | 0 | 100 | 107 |  |  | PEAKS DB |
| T.C(+57.02)PIDWLPK.N | N | 38.20 | 1027.5161 | 8 | -0.6 | 514.7650 | 2 | 63.31 | 4 | F4:42545 | NaSa01\_F12.raw |  |  |  | 5.0664E5 |  |  |  |  |  |  |  |  |  |  | 1 | 0 | 0 | 0 | 1 | 0 | 0 | 0 | 0 | 0 | 0 | 0 | 0 | 0 | 0 | 26 | 33 | Carbamidomethylation | C1:Carbamidomethylation:1000.00 | PEAKS DB |
| total 3 peptides |  |  |  |  |  |  |  |  |  |  |  |  |  |  |  |  |  |  |  |  |  |  |  |  |  |  |  |  |  |  |  |  |  |  |  |  |  |  |  |  |  |  |  |  |  |
| --- | --- | --- | --- | --- | --- | --- | --- | --- | --- | --- | --- | --- | --- | --- | --- | --- | --- | --- | --- | --- | --- | --- | --- | --- | --- | --- | --- | --- | --- | --- | --- | --- | --- | --- | --- | --- | --- | --- | --- | --- | --- | --- | --- | --- | --- |

AAK43584.1

back to list

  

| Protein Coverage
| Supporting Peptides
|

Protein Coverage:

Supporting Peptides:

| Peptide | Uniq | -10lgP | Mass | Length | ppm | m/z | z | RT | Fraction | Scan | Source File | Area F1 | Area F10 | Area F11 | Area F12 | Area F13 | Area F14 | Area F2 | Area F3 | Area F4 | Area F5 | Area F6 | Area F7 | Area F8 | Area F9 | #Feature | #Feature F1 | #Feature F10 | #Feature F11 | #Feature F12 | #Feature F13 | #Feature F14 | #Feature F2 | #Feature F3 | #Feature F4 | #Feature F5 | #Feature F6 | #Feature F7 | #Feature F8 | #Feature F9 | Start | End | PTM | AScore | Found By |
| --- | --- | --- | --- | --- | --- | --- | --- | --- | --- | --- | --- | --- | --- | --- | --- | --- | --- | --- | --- | --- | --- | --- | --- | --- | --- | --- | --- | --- | --- | --- | --- | --- | --- | --- | --- | --- | --- | --- | --- | --- | --- | --- | --- | --- | --- |
| K.KYIWEWTDR.S | Y | 63.28 | 1295.6299 | 9 | 0.1 | 648.8223 | 2 | 51.37 | 4 | F4:32829 | NaSa01\_F12.raw |  |  |  | 1.6479E8 |  |  |  |  |  |  |  |  |  |  | 2 | 0 | 0 | 0 | 2 | 0 | 0 | 0 | 0 | 0 | 0 | 0 | 0 | 0 | 0 | 99 | 107 |  |  | PEAKS DB |
| K.YIWEWTDR.S | Y | 55.19 | 1167.5349 | 8 | 0.3 | 584.7749 | 2 | 65.79 | 4 | F4:44510 | NaSa01\_F12.raw |  |  |  | 1.0797E9 |  | 0 |  |  |  |  |  |  |  |  | 10 | 0 | 0 | 0 | 10 | 0 | 0 | 0 | 0 | 0 | 0 | 0 | 0 | 0 | 0 | 100 | 107 |  |  | PEAKS DB |
| T.C(+57.02)PIDWLPK.N | N | 38.20 | 1027.5161 | 8 | -0.6 | 514.7650 | 2 | 63.31 | 4 | F4:42545 | NaSa01\_F12.raw |  |  |  | 5.0664E5 |  |  |  |  |  |  |  |  |  |  | 1 | 0 | 0 | 0 | 1 | 0 | 0 | 0 | 0 | 0 | 0 | 0 | 0 | 0 | 0 | 26 | 33 | Carbamidomethylation | C1:Carbamidomethylation:1000.00 | PEAKS DB |
| total 3 peptides |  |  |  |  |  |  |  |  |  |  |  |  |  |  |  |  |  |  |  |  |  |  |  |  |  |  |  |  |  |  |  |  |  |  |  |  |  |  |  |  |  |  |  |  |  |
| --- | --- | --- | --- | --- | --- | --- | --- | --- | --- | --- | --- | --- | --- | --- | --- | --- | --- | --- | --- | --- | --- | --- | --- | --- | --- | --- | --- | --- | --- | --- | --- | --- | --- | --- | --- | --- | --- | --- | --- | --- | --- | --- | --- | --- | --- |

XP\_026575982.1

back to list

  

| Protein Coverage
| Supporting Peptides
|

Protein Coverage:

Supporting Peptides:

| Peptide | Uniq | -10lgP | Mass | Length | ppm | m/z | z | RT | Fraction | Scan | Source File | Area F1 | Area F10 | Area F11 | Area F12 | Area F13 | Area F14 | Area F2 | Area F3 | Area F4 | Area F5 | Area F6 | Area F7 | Area F8 | Area F9 | #Feature | #Feature F1 | #Feature F10 | #Feature F11 | #Feature F12 | #Feature F13 | #Feature F14 | #Feature F2 | #Feature F3 | #Feature F4 | #Feature F5 | #Feature F6 | #Feature F7 | #Feature F8 | #Feature F9 | Start | End | PTM | AScore | Found By |
| --- | --- | --- | --- | --- | --- | --- | --- | --- | --- | --- | --- | --- | --- | --- | --- | --- | --- | --- | --- | --- | --- | --- | --- | --- | --- | --- | --- | --- | --- | --- | --- | --- | --- | --- | --- | --- | --- | --- | --- | --- | --- | --- | --- | --- | --- |
| K.SNVGIGIPESIDWR.D | Y | 73.87 | 1541.7838 | 14 | 0.3 | 771.8994 | 2 | 69.76 | 4 | F4:47776 | NaSa01\_F12.raw |  |  |  | 2.0751E7 |  |  |  |  |  |  |  |  |  |  | 1 | 0 | 0 | 0 | 1 | 0 | 0 | 0 | 0 | 0 | 0 | 0 | 0 | 0 | 0 | 115 | 128 |  |  | PEAKS DB |
| F.SAVGALEAQLK.L | N | 44.82 | 1085.6080 | 11 | 0.9 | 543.8118 | 2 | 40.05 | 4 | F4:23158 | NaSa01\_F12.raw |  |  |  | 1.883E6 |  |  |  |  |  |  |  |  |  |  | 1 | 0 | 0 | 0 | 1 | 0 | 0 | 0 | 0 | 0 | 0 | 0 | 0 | 0 | 0 | 149 | 159 |  |  | PEAKS DB |
| K.ENGM(+15.99)DYWLVK.N | N | 38.81 | 1269.5699 | 10 | 0.4 | 635.7925 | 2 | 47.13 | 4 | F4:29053 | NaSa01\_F12.raw |  |  |  | 4.0851E5 |  |  |  |  |  |  |  |  |  |  | 1 | 0 | 0 | 0 | 1 | 0 | 0 | 0 | 0 | 0 | 0 | 0 | 0 | 0 | 0 | 294 | 303 | Oxidation (M) | M4:Oxidation (M):1000.00 | PEAKS DB |
| total 3 peptides |  |  |  |  |  |  |  |  |  |  |  |  |  |  |  |  |  |  |  |  |  |  |  |  |  |  |  |  |  |  |  |  |  |  |  |  |  |  |  |  |  |  |  |  |  |
| --- | --- | --- | --- | --- | --- | --- | --- | --- | --- | --- | --- | --- | --- | --- | --- | --- | --- | --- | --- | --- | --- | --- | --- | --- | --- | --- | --- | --- | --- | --- | --- | --- | --- | --- | --- | --- | --- | --- | --- | --- | --- | --- | --- | --- | --- |

XP\_026575983.1

back to list

  

| Protein Coverage
| Supporting Peptides
|

Protein Coverage:

Supporting Peptides:

| Peptide | Uniq | -10lgP | Mass | Length | ppm | m/z | z | RT | Fraction | Scan | Source File | Area F1 | Area F10 | Area F11 | Area F12 | Area F13 | Area F14 | Area F2 | Area F3 | Area F4 | Area F5 | Area F6 | Area F7 | Area F8 | Area F9 | #Feature | #Feature F1 | #Feature F10 | #Feature F11 | #Feature F12 | #Feature F13 | #Feature F14 | #Feature F2 | #Feature F3 | #Feature F4 | #Feature F5 | #Feature F6 | #Feature F7 | #Feature F8 | #Feature F9 | Start | End | PTM | AScore | Found By |
| --- | --- | --- | --- | --- | --- | --- | --- | --- | --- | --- | --- | --- | --- | --- | --- | --- | --- | --- | --- | --- | --- | --- | --- | --- | --- | --- | --- | --- | --- | --- | --- | --- | --- | --- | --- | --- | --- | --- | --- | --- | --- | --- | --- | --- | --- |
| K.SNVGIGIPESIDWR.D | Y | 73.87 | 1541.7838 | 14 | 0.3 | 771.8994 | 2 | 69.76 | 4 | F4:47776 | NaSa01\_F12.raw |  |  |  | 2.0751E7 |  |  |  |  |  |  |  |  |  |  | 1 | 0 | 0 | 0 | 1 | 0 | 0 | 0 | 0 | 0 | 0 | 0 | 0 | 0 | 0 | 115 | 128 |  |  | PEAKS DB |
| F.SAVGALEAQLK.L | N | 44.82 | 1085.6080 | 11 | 0.9 | 543.8118 | 2 | 40.05 | 4 | F4:23158 | NaSa01\_F12.raw |  |  |  | 1.883E6 |  |  |  |  |  |  |  |  |  |  | 1 | 0 | 0 | 0 | 1 | 0 | 0 | 0 | 0 | 0 | 0 | 0 | 0 | 0 | 0 | 149 | 159 |  |  | PEAKS DB |
| K.ENGM(+15.99)DYWLVK.N | N | 38.81 | 1269.5699 | 10 | 0.4 | 635.7925 | 2 | 47.13 | 4 | F4:29053 | NaSa01\_F12.raw |  |  |  | 4.0851E5 |  |  |  |  |  |  |  |  |  |  | 1 | 0 | 0 | 0 | 1 | 0 | 0 | 0 | 0 | 0 | 0 | 0 | 0 | 0 | 0 | 294 | 303 | Oxidation (M) | M4:Oxidation (M):1000.00 | PEAKS DB |
| total 3 peptides |  |  |  |  |  |  |  |  |  |  |  |  |  |  |  |  |  |  |  |  |  |  |  |  |  |  |  |  |  |  |  |  |  |  |  |  |  |  |  |  |  |  |  |  |  |
| --- | --- | --- | --- | --- | --- | --- | --- | --- | --- | --- | --- | --- | --- | --- | --- | --- | --- | --- | --- | --- | --- | --- | --- | --- | --- | --- | --- | --- | --- | --- | --- | --- | --- | --- | --- | --- | --- | --- | --- | --- | --- | --- | --- | --- | --- |

XP\_034295297.1

back to list

  

| Protein Coverage
| Supporting Peptides
|

Protein Coverage:

Supporting Peptides:

| Peptide | Uniq | -10lgP | Mass | Length | ppm | m/z | z | RT | Fraction | Scan | Source File | Area F1 | Area F10 | Area F11 | Area F12 | Area F13 | Area F14 | Area F2 | Area F3 | Area F4 | Area F5 | Area F6 | Area F7 | Area F8 | Area F9 | #Feature | #Feature F1 | #Feature F10 | #Feature F11 | #Feature F12 | #Feature F13 | #Feature F14 | #Feature F2 | #Feature F3 | #Feature F4 | #Feature F5 | #Feature F6 | #Feature F7 | #Feature F8 | #Feature F9 | Start | End | PTM | AScore | Found By |
| --- | --- | --- | --- | --- | --- | --- | --- | --- | --- | --- | --- | --- | --- | --- | --- | --- | --- | --- | --- | --- | --- | --- | --- | --- | --- | --- | --- | --- | --- | --- | --- | --- | --- | --- | --- | --- | --- | --- | --- | --- | --- | --- | --- | --- | --- |
| K.IANIVFANSGLK.M | Y | 67.09 | 1245.7081 | 12 | 0.4 | 623.8616 | 2 | 50.42 | 6 | F6:29701 | NaSa01\_F14.raw |  |  |  |  |  | 1.4021E6 |  |  |  |  |  |  |  |  | 1 | 0 | 0 | 0 | 0 | 0 | 1 | 0 | 0 | 0 | 0 | 0 | 0 | 0 | 0 | 109 | 120 |  |  | PEAKS DB |
| R.SENIYVSQLLQK.A | Y | 51.25 | 1420.7561 | 12 | 0.0 | 711.3853 | 2 | 55.02 | 6 | F6:33499 | NaSa01\_F14.raw |  |  |  |  |  | 1.449E6 |  |  |  |  |  |  |  |  | 1 | 0 | 0 | 0 | 0 | 0 | 1 | 0 | 0 | 0 | 0 | 0 | 0 | 0 | 0 | 329 | 340 |  |  | PEAKS DB |
| K.FTAEAETDLKEPLK.E | Y | 43.41 | 1590.8141 | 14 | -0.5 | 531.2784 | 3 | 29.23 | 6 | F6:11791 | NaSa01\_F14.raw |  |  |  |  |  | 5.6849E5 |  |  |  |  |  |  |  |  | 1 | 0 | 0 | 0 | 0 | 0 | 1 | 0 | 0 | 0 | 0 | 0 | 0 | 0 | 0 | 295 | 308 |  |  | PEAKS DB |
| total 3 peptides |  |  |  |  |  |  |  |  |  |  |  |  |  |  |  |  |  |  |  |  |  |  |  |  |  |  |  |  |  |  |  |  |  |  |  |  |  |  |  |  |  |  |  |  |  |
| --- | --- | --- | --- | --- | --- | --- | --- | --- | --- | --- | --- | --- | --- | --- | --- | --- | --- | --- | --- | --- | --- | --- | --- | --- | --- | --- | --- | --- | --- | --- | --- | --- | --- | --- | --- | --- | --- | --- | --- | --- | --- | --- | --- | --- | --- |

JAG67228.1

back to list

  

| Protein Coverage
| Supporting Peptides
|

Protein Coverage:

Supporting Peptides:

| Peptide | Uniq | -10lgP | Mass | Length | ppm | m/z | z | RT | Fraction | Scan | Source File | Area F1 | Area F10 | Area F11 | Area F12 | Area F13 | Area F14 | Area F2 | Area F3 | Area F4 | Area F5 | Area F6 | Area F7 | Area F8 | Area F9 | #Feature | #Feature F1 | #Feature F10 | #Feature F11 | #Feature F12 | #Feature F13 | #Feature F14 | #Feature F2 | #Feature F3 | #Feature F4 | #Feature F5 | #Feature F6 | #Feature F7 | #Feature F8 | #Feature F9 | Start | End | PTM | AScore | Found By |
| --- | --- | --- | --- | --- | --- | --- | --- | --- | --- | --- | --- | --- | --- | --- | --- | --- | --- | --- | --- | --- | --- | --- | --- | --- | --- | --- | --- | --- | --- | --- | --- | --- | --- | --- | --- | --- | --- | --- | --- | --- | --- | --- | --- | --- | --- |
| K.IANIVFANSGLK.M | Y | 67.09 | 1245.7081 | 12 | 0.4 | 623.8616 | 2 | 50.42 | 6 | F6:29701 | NaSa01\_F14.raw |  |  |  |  |  | 1.4021E6 |  |  |  |  |  |  |  |  | 1 | 0 | 0 | 0 | 0 | 0 | 1 | 0 | 0 | 0 | 0 | 0 | 0 | 0 | 0 | 109 | 120 |  |  | PEAKS DB |
| R.SENIYVSQLLQK.A | Y | 51.25 | 1420.7561 | 12 | 0.0 | 711.3853 | 2 | 55.02 | 6 | F6:33499 | NaSa01\_F14.raw |  |  |  |  |  | 1.449E6 |  |  |  |  |  |  |  |  | 1 | 0 | 0 | 0 | 0 | 0 | 1 | 0 | 0 | 0 | 0 | 0 | 0 | 0 | 0 | 329 | 340 |  |  | PEAKS DB |
| K.FTAEAETDLKEPLK.E | Y | 43.41 | 1590.8141 | 14 | -0.5 | 531.2784 | 3 | 29.23 | 6 | F6:11791 | NaSa01\_F14.raw |  |  |  |  |  | 5.6849E5 |  |  |  |  |  |  |  |  | 1 | 0 | 0 | 0 | 0 | 0 | 1 | 0 | 0 | 0 | 0 | 0 | 0 | 0 | 0 | 295 | 308 |  |  | PEAKS DB |
| total 3 peptides |  |  |  |  |  |  |  |  |  |  |  |  |  |  |  |  |  |  |  |  |  |  |  |  |  |  |  |  |  |  |  |  |  |  |  |  |  |  |  |  |  |  |  |  |  |
| --- | --- | --- | --- | --- | --- | --- | --- | --- | --- | --- | --- | --- | --- | --- | --- | --- | --- | --- | --- | --- | --- | --- | --- | --- | --- | --- | --- | --- | --- | --- | --- | --- | --- | --- | --- | --- | --- | --- | --- | --- | --- | --- | --- | --- | --- |

XP\_026575692.1

back to list

  

| Protein Coverage
| Supporting Peptides
|

Protein Coverage:

Supporting Peptides:

| Peptide | Uniq | -10lgP | Mass | Length | ppm | m/z | z | RT | Fraction | Scan | Source File | Area F1 | Area F10 | Area F11 | Area F12 | Area F13 | Area F14 | Area F2 | Area F3 | Area F4 | Area F5 | Area F6 | Area F7 | Area F8 | Area F9 | #Feature | #Feature F1 | #Feature F10 | #Feature F11 | #Feature F12 | #Feature F13 | #Feature F14 | #Feature F2 | #Feature F3 | #Feature F4 | #Feature F5 | #Feature F6 | #Feature F7 | #Feature F8 | #Feature F9 | Start | End | PTM | AScore | Found By |
| --- | --- | --- | --- | --- | --- | --- | --- | --- | --- | --- | --- | --- | --- | --- | --- | --- | --- | --- | --- | --- | --- | --- | --- | --- | --- | --- | --- | --- | --- | --- | --- | --- | --- | --- | --- | --- | --- | --- | --- | --- | --- | --- | --- | --- | --- |
| R.FPSLLAPGR.R | Y | 61.36 | 956.5443 | 9 | 0.2 | 479.2795 | 2 | 50.55 | 6 | F6:29572 | NaSa01\_F14.raw |  |  |  |  | 2.3107E6 | 7.499E6 |  |  |  |  |  |  |  |  | 3 | 0 | 0 | 0 | 0 | 1 | 2 | 0 | 0 | 0 | 0 | 0 | 0 | 0 | 0 | 155 | 163 |  |  | PEAKS DB |
| R.ATLLSPFFGTK.S | Y | 56.01 | 1180.6492 | 11 | 0.3 | 591.3320 | 2 | 72.54 | 6 | F6:48521 | NaSa01\_F14.raw |  |  |  |  |  | 2.2981E6 |  |  |  |  |  |  |  |  | 1 | 0 | 0 | 0 | 0 | 0 | 1 | 0 | 0 | 0 | 0 | 0 | 0 | 0 | 0 | 33 | 43 |  |  | PEAKS DB |
| K.VLEYLNDLK.Q | Y | 53.85 | 1105.6019 | 9 | 0.6 | 553.8086 | 2 | 53.29 | 6 | F6:32096 | NaSa01\_F14.raw |  |  |  |  |  | 3.8387E6 |  |  |  |  |  |  |  |  | 1 | 0 | 0 | 0 | 0 | 0 | 1 | 0 | 0 | 0 | 0 | 0 | 0 | 0 | 0 | 296 | 304 |  |  | PEAKS DB |
| K.LLPFTYSGK.T | Y | 47.33 | 1024.5593 | 9 | 0.1 | 513.2870 | 2 | 47.87 | 6 | F6:27515 | NaSa01\_F14.raw |  |  |  |  |  | 2.3988E5 |  |  |  |  |  |  |  |  | 1 | 0 | 0 | 0 | 0 | 0 | 1 | 0 | 0 | 0 | 0 | 0 | 0 | 0 | 0 | 426 | 434 |  |  | PEAKS DB |
| total 4 peptides |  |  |  |  |  |  |  |  |  |  |  |  |  |  |  |  |  |  |  |  |  |  |  |  |  |  |  |  |  |  |  |  |  |  |  |  |  |  |  |  |  |  |  |  |  |
| --- | --- | --- | --- | --- | --- | --- | --- | --- | --- | --- | --- | --- | --- | --- | --- | --- | --- | --- | --- | --- | --- | --- | --- | --- | --- | --- | --- | --- | --- | --- | --- | --- | --- | --- | --- | --- | --- | --- | --- | --- | --- | --- | --- | --- | --- |

XP\_026575691.1

back to list

  

| Protein Coverage
| Supporting Peptides
|

Protein Coverage:

Supporting Peptides:

| Peptide | Uniq | -10lgP | Mass | Length | ppm | m/z | z | RT | Fraction | Scan | Source File | Area F1 | Area F10 | Area F11 | Area F12 | Area F13 | Area F14 | Area F2 | Area F3 | Area F4 | Area F5 | Area F6 | Area F7 | Area F8 | Area F9 | #Feature | #Feature F1 | #Feature F10 | #Feature F11 | #Feature F12 | #Feature F13 | #Feature F14 | #Feature F2 | #Feature F3 | #Feature F4 | #Feature F5 | #Feature F6 | #Feature F7 | #Feature F8 | #Feature F9 | Start | End | PTM | AScore | Found By |
| --- | --- | --- | --- | --- | --- | --- | --- | --- | --- | --- | --- | --- | --- | --- | --- | --- | --- | --- | --- | --- | --- | --- | --- | --- | --- | --- | --- | --- | --- | --- | --- | --- | --- | --- | --- | --- | --- | --- | --- | --- | --- | --- | --- | --- | --- |
| R.FPSLLAPGR.R | Y | 61.36 | 956.5443 | 9 | 0.2 | 479.2795 | 2 | 50.55 | 6 | F6:29572 | NaSa01\_F14.raw |  |  |  |  | 2.3107E6 | 7.499E6 |  |  |  |  |  |  |  |  | 3 | 0 | 0 | 0 | 0 | 1 | 2 | 0 | 0 | 0 | 0 | 0 | 0 | 0 | 0 | 155 | 163 |  |  | PEAKS DB |
| R.ATLLSPFFGTK.S | Y | 56.01 | 1180.6492 | 11 | 0.3 | 591.3320 | 2 | 72.54 | 6 | F6:48521 | NaSa01\_F14.raw |  |  |  |  |  | 2.2981E6 |  |  |  |  |  |  |  |  | 1 | 0 | 0 | 0 | 0 | 0 | 1 | 0 | 0 | 0 | 0 | 0 | 0 | 0 | 0 | 33 | 43 |  |  | PEAKS DB |
| K.VLEYLNDLK.Q | Y | 53.85 | 1105.6019 | 9 | 0.6 | 553.8086 | 2 | 53.29 | 6 | F6:32096 | NaSa01\_F14.raw |  |  |  |  |  | 3.8387E6 |  |  |  |  |  |  |  |  | 1 | 0 | 0 | 0 | 0 | 0 | 1 | 0 | 0 | 0 | 0 | 0 | 0 | 0 | 0 | 300 | 308 |  |  | PEAKS DB |
| K.LLPFTYSGK.T | Y | 47.33 | 1024.5593 | 9 | 0.1 | 513.2870 | 2 | 47.87 | 6 | F6:27515 | NaSa01\_F14.raw |  |  |  |  |  | 2.3988E5 |  |  |  |  |  |  |  |  | 1 | 0 | 0 | 0 | 0 | 0 | 1 | 0 | 0 | 0 | 0 | 0 | 0 | 0 | 0 | 430 | 438 |  |  | PEAKS DB |
| total 4 peptides |  |  |  |  |  |  |  |  |  |  |  |  |  |  |  |  |  |  |  |  |  |  |  |  |  |  |  |  |  |  |  |  |  |  |  |  |  |  |  |  |  |  |  |  |  |
| --- | --- | --- | --- | --- | --- | --- | --- | --- | --- | --- | --- | --- | --- | --- | --- | --- | --- | --- | --- | --- | --- | --- | --- | --- | --- | --- | --- | --- | --- | --- | --- | --- | --- | --- | --- | --- | --- | --- | --- | --- | --- | --- | --- | --- | --- |

XP\_026542555.1

back to list

  

| Protein Coverage
| Supporting Peptides
|

Protein Coverage:

Supporting Peptides:

| Peptide | Uniq | -10lgP | Mass | Length | ppm | m/z | z | RT | Fraction | Scan | Source File | Area F1 | Area F10 | Area F11 | Area F12 | Area F13 | Area F14 | Area F2 | Area F3 | Area F4 | Area F5 | Area F6 | Area F7 | Area F8 | Area F9 | #Feature | #Feature F1 | #Feature F10 | #Feature F11 | #Feature F12 | #Feature F13 | #Feature F14 | #Feature F2 | #Feature F3 | #Feature F4 | #Feature F5 | #Feature F6 | #Feature F7 | #Feature F8 | #Feature F9 | Start | End | PTM | AScore | Found By |
| --- | --- | --- | --- | --- | --- | --- | --- | --- | --- | --- | --- | --- | --- | --- | --- | --- | --- | --- | --- | --- | --- | --- | --- | --- | --- | --- | --- | --- | --- | --- | --- | --- | --- | --- | --- | --- | --- | --- | --- | --- | --- | --- | --- | --- | --- |
| R.FPSLLAPGR.R | Y | 61.36 | 956.5443 | 9 | 0.2 | 479.2795 | 2 | 50.55 | 6 | F6:29572 | NaSa01\_F14.raw |  |  |  |  | 2.3107E6 | 7.499E6 |  |  |  |  |  |  |  |  | 3 | 0 | 0 | 0 | 0 | 1 | 2 | 0 | 0 | 0 | 0 | 0 | 0 | 0 | 0 | 152 | 160 |  |  | PEAKS DB |
| R.ATLLSPFFGTK.S | Y | 56.01 | 1180.6492 | 11 | 0.3 | 591.3320 | 2 | 72.54 | 6 | F6:48521 | NaSa01\_F14.raw |  |  |  |  |  | 2.2981E6 |  |  |  |  |  |  |  |  | 1 | 0 | 0 | 0 | 0 | 0 | 1 | 0 | 0 | 0 | 0 | 0 | 0 | 0 | 0 | 31 | 41 |  |  | PEAKS DB |
| K.VLEYLNDLK.Q | Y | 53.85 | 1105.6019 | 9 | 0.6 | 553.8086 | 2 | 53.29 | 6 | F6:32096 | NaSa01\_F14.raw |  |  |  |  |  | 3.8387E6 |  |  |  |  |  |  |  |  | 1 | 0 | 0 | 0 | 0 | 0 | 1 | 0 | 0 | 0 | 0 | 0 | 0 | 0 | 0 | 293 | 301 |  |  | PEAKS DB |
| K.LLPFTYSGK.T | Y | 47.33 | 1024.5593 | 9 | 0.1 | 513.2870 | 2 | 47.87 | 6 | F6:27515 | NaSa01\_F14.raw |  |  |  |  |  | 2.3988E5 |  |  |  |  |  |  |  |  | 1 | 0 | 0 | 0 | 0 | 0 | 1 | 0 | 0 | 0 | 0 | 0 | 0 | 0 | 0 | 423 | 431 |  |  | PEAKS DB |
| total 4 peptides |  |  |  |  |  |  |  |  |  |  |  |  |  |  |  |  |  |  |  |  |  |  |  |  |  |  |  |  |  |  |  |  |  |  |  |  |  |  |  |  |  |  |  |  |  |
| --- | --- | --- | --- | --- | --- | --- | --- | --- | --- | --- | --- | --- | --- | --- | --- | --- | --- | --- | --- | --- | --- | --- | --- | --- | --- | --- | --- | --- | --- | --- | --- | --- | --- | --- | --- | --- | --- | --- | --- | --- | --- | --- | --- | --- | --- |

XP\_026542553.1

back to list

  

| Protein Coverage
| Supporting Peptides
|

Protein Coverage:

Supporting Peptides:

| Peptide | Uniq | -10lgP | Mass | Length | ppm | m/z | z | RT | Fraction | Scan | Source File | Area F1 | Area F10 | Area F11 | Area F12 | Area F13 | Area F14 | Area F2 | Area F3 | Area F4 | Area F5 | Area F6 | Area F7 | Area F8 | Area F9 | #Feature | #Feature F1 | #Feature F10 | #Feature F11 | #Feature F12 | #Feature F13 | #Feature F14 | #Feature F2 | #Feature F3 | #Feature F4 | #Feature F5 | #Feature F6 | #Feature F7 | #Feature F8 | #Feature F9 | Start | End | PTM | AScore | Found By |
| --- | --- | --- | --- | --- | --- | --- | --- | --- | --- | --- | --- | --- | --- | --- | --- | --- | --- | --- | --- | --- | --- | --- | --- | --- | --- | --- | --- | --- | --- | --- | --- | --- | --- | --- | --- | --- | --- | --- | --- | --- | --- | --- | --- | --- | --- |
| R.FPSLLAPGR.R | Y | 61.36 | 956.5443 | 9 | 0.2 | 479.2795 | 2 | 50.55 | 6 | F6:29572 | NaSa01\_F14.raw |  |  |  |  | 2.3107E6 | 7.499E6 |  |  |  |  |  |  |  |  | 3 | 0 | 0 | 0 | 0 | 1 | 2 | 0 | 0 | 0 | 0 | 0 | 0 | 0 | 0 | 152 | 160 |  |  | PEAKS DB |
| R.ATLLSPFFGTK.S | Y | 56.01 | 1180.6492 | 11 | 0.3 | 591.3320 | 2 | 72.54 | 6 | F6:48521 | NaSa01\_F14.raw |  |  |  |  |  | 2.2981E6 |  |  |  |  |  |  |  |  | 1 | 0 | 0 | 0 | 0 | 0 | 1 | 0 | 0 | 0 | 0 | 0 | 0 | 0 | 0 | 31 | 41 |  |  | PEAKS DB |
| K.VLEYLNDLK.Q | Y | 53.85 | 1105.6019 | 9 | 0.6 | 553.8086 | 2 | 53.29 | 6 | F6:32096 | NaSa01\_F14.raw |  |  |  |  |  | 3.8387E6 |  |  |  |  |  |  |  |  | 1 | 0 | 0 | 0 | 0 | 0 | 1 | 0 | 0 | 0 | 0 | 0 | 0 | 0 | 0 | 297 | 305 |  |  | PEAKS DB |
| K.LLPFTYSGK.T | Y | 47.33 | 1024.5593 | 9 | 0.1 | 513.2870 | 2 | 47.87 | 6 | F6:27515 | NaSa01\_F14.raw |  |  |  |  |  | 2.3988E5 |  |  |  |  |  |  |  |  | 1 | 0 | 0 | 0 | 0 | 0 | 1 | 0 | 0 | 0 | 0 | 0 | 0 | 0 | 0 | 427 | 435 |  |  | PEAKS DB |
| total 4 peptides |  |  |  |  |  |  |  |  |  |  |  |  |  |  |  |  |  |  |  |  |  |  |  |  |  |  |  |  |  |  |  |  |  |  |  |  |  |  |  |  |  |  |  |  |  |
| --- | --- | --- | --- | --- | --- | --- | --- | --- | --- | --- | --- | --- | --- | --- | --- | --- | --- | --- | --- | --- | --- | --- | --- | --- | --- | --- | --- | --- | --- | --- | --- | --- | --- | --- | --- | --- | --- | --- | --- | --- | --- | --- | --- | --- | --- |

XP\_029139434.1

back to list

  

| Protein Coverage
| Supporting Peptides
|

Protein Coverage:

Supporting Peptides:

| Peptide | Uniq | -10lgP | Mass | Length | ppm | m/z | z | RT | Fraction | Scan | Source File | Area F1 | Area F10 | Area F11 | Area F12 | Area F13 | Area F14 | Area F2 | Area F3 | Area F4 | Area F5 | Area F6 | Area F7 | Area F8 | Area F9 | #Feature | #Feature F1 | #Feature F10 | #Feature F11 | #Feature F12 | #Feature F13 | #Feature F14 | #Feature F2 | #Feature F3 | #Feature F4 | #Feature F5 | #Feature F6 | #Feature F7 | #Feature F8 | #Feature F9 | Start | End | PTM | AScore | Found By |
| --- | --- | --- | --- | --- | --- | --- | --- | --- | --- | --- | --- | --- | --- | --- | --- | --- | --- | --- | --- | --- | --- | --- | --- | --- | --- | --- | --- | --- | --- | --- | --- | --- | --- | --- | --- | --- | --- | --- | --- | --- | --- | --- | --- | --- | --- |
| R.FPSLLAPGR.R | Y | 61.36 | 956.5443 | 9 | 0.2 | 479.2795 | 2 | 50.55 | 6 | F6:29572 | NaSa01\_F14.raw |  |  |  |  | 2.3107E6 | 7.499E6 |  |  |  |  |  |  |  |  | 3 | 0 | 0 | 0 | 0 | 1 | 2 | 0 | 0 | 0 | 0 | 0 | 0 | 0 | 0 | 154 | 162 |  |  | PEAKS DB |
| R.ATLLSPFFGTK.S | Y | 56.01 | 1180.6492 | 11 | 0.3 | 591.3320 | 2 | 72.54 | 6 | F6:48521 | NaSa01\_F14.raw |  |  |  |  |  | 2.2981E6 |  |  |  |  |  |  |  |  | 1 | 0 | 0 | 0 | 0 | 0 | 1 | 0 | 0 | 0 | 0 | 0 | 0 | 0 | 0 | 35 | 45 |  |  | PEAKS DB |
| K.VLEYLNDLK.Q | Y | 53.85 | 1105.6019 | 9 | 0.6 | 553.8086 | 2 | 53.29 | 6 | F6:32096 | NaSa01\_F14.raw |  |  |  |  |  | 3.8387E6 |  |  |  |  |  |  |  |  | 1 | 0 | 0 | 0 | 0 | 0 | 1 | 0 | 0 | 0 | 0 | 0 | 0 | 0 | 0 | 299 | 307 |  |  | PEAKS DB |
| K.LLPFTYSGK.T | Y | 47.33 | 1024.5593 | 9 | 0.1 | 513.2870 | 2 | 47.87 | 6 | F6:27515 | NaSa01\_F14.raw |  |  |  |  |  | 2.3988E5 |  |  |  |  |  |  |  |  | 1 | 0 | 0 | 0 | 0 | 0 | 1 | 0 | 0 | 0 | 0 | 0 | 0 | 0 | 0 | 429 | 437 |  |  | PEAKS DB |
| total 4 peptides |  |  |  |  |  |  |  |  |  |  |  |  |  |  |  |  |  |  |  |  |  |  |  |  |  |  |  |  |  |  |  |  |  |  |  |  |  |  |  |  |  |  |  |  |  |
| --- | --- | --- | --- | --- | --- | --- | --- | --- | --- | --- | --- | --- | --- | --- | --- | --- | --- | --- | --- | --- | --- | --- | --- | --- | --- | --- | --- | --- | --- | --- | --- | --- | --- | --- | --- | --- | --- | --- | --- | --- | --- | --- | --- | --- | --- |

XP\_013915209.1

back to list

  

| Protein Coverage
| Supporting Peptides
|

Protein Coverage:

Supporting Peptides:

| Peptide | Uniq | -10lgP | Mass | Length | ppm | m/z | z | RT | Fraction | Scan | Source File | Area F1 | Area F10 | Area F11 | Area F12 | Area F13 | Area F14 | Area F2 | Area F3 | Area F4 | Area F5 | Area F6 | Area F7 | Area F8 | Area F9 | #Feature | #Feature F1 | #Feature F10 | #Feature F11 | #Feature F12 | #Feature F13 | #Feature F14 | #Feature F2 | #Feature F3 | #Feature F4 | #Feature F5 | #Feature F6 | #Feature F7 | #Feature F8 | #Feature F9 | Start | End | PTM | AScore | Found By |
| --- | --- | --- | --- | --- | --- | --- | --- | --- | --- | --- | --- | --- | --- | --- | --- | --- | --- | --- | --- | --- | --- | --- | --- | --- | --- | --- | --- | --- | --- | --- | --- | --- | --- | --- | --- | --- | --- | --- | --- | --- | --- | --- | --- | --- | --- |
| K.LALDIEIATYR.K | N | 62.08 | 1276.7026 | 11 | 3.6 | 639.3590 | 2 | 67.89 | 7 | F7:49102 | NaSa01\_F2.raw |  | 1.1426E5 |  |  |  |  | 4.6527E6 |  |  | 7.8211E5 |  |  |  |  | 10 | 0 | 1 | 0 | 0 | 0 | 0 | 8 | 0 | 0 | 1 | 0 | 0 | 0 | 0 | 444 | 454 |  |  | PEAKS DB |
| K.FASFIDKVR.F | N | 57.41 | 1081.5920 | 9 | 2.8 | 541.8032 | 2 | 29.83 | 7 | F7:16135 | NaSa01\_F2.raw |  |  |  |  |  |  | 5.676E5 |  |  |  |  |  |  |  | 1 | 0 | 0 | 0 | 0 | 0 | 0 | 1 | 0 | 0 | 0 | 0 | 0 | 0 | 0 | 161 | 169 |  |  | PEAKS DB |
| K.FASFIDK.V | N | 53.06 | 826.4225 | 7 | 2.1 | 414.2182 | 2 | 30.61 | 7 | F7:16768 | NaSa01\_F2.raw |  |  |  |  |  |  | 6.2128E5 |  |  |  |  |  |  |  | 1 | 0 | 0 | 0 | 0 | 0 | 0 | 1 | 0 | 0 | 0 | 0 | 0 | 0 | 0 | 161 | 167 |  |  | PEAKS DB |
| K.YEELQVSVGR.H | Y | 38.53 | 1178.5931 | 10 | 0.5 | 590.3041 | 2 | 30.87 | 4 | F4:16507 | NaSa01\_F12.raw |  |  |  | 1.4425E7 |  |  |  |  |  |  |  |  |  |  | 1 | 0 | 0 | 0 | 1 | 0 | 0 | 0 | 0 | 0 | 0 | 0 | 0 | 0 | 0 | 348 | 357 |  |  | PEAKS DB |
| total 4 peptides |  |  |  |  |  |  |  |  |  |  |  |  |  |  |  |  |  |  |  |  |  |  |  |  |  |  |  |  |  |  |  |  |  |  |  |  |  |  |  |  |  |  |  |  |  |
| --- | --- | --- | --- | --- | --- | --- | --- | --- | --- | --- | --- | --- | --- | --- | --- | --- | --- | --- | --- | --- | --- | --- | --- | --- | --- | --- | --- | --- | --- | --- | --- | --- | --- | --- | --- | --- | --- | --- | --- | --- | --- | --- | --- | --- | --- |

XP\_026565129.1

back to list

  

| Protein Coverage
| Supporting Peptides
|

Protein Coverage:

Supporting Peptides:

| Peptide | Uniq | -10lgP | Mass | Length | ppm | m/z | z | RT | Fraction | Scan | Source File | Area F1 | Area F10 | Area F11 | Area F12 | Area F13 | Area F14 | Area F2 | Area F3 | Area F4 | Area F5 | Area F6 | Area F7 | Area F8 | Area F9 | #Feature | #Feature F1 | #Feature F10 | #Feature F11 | #Feature F12 | #Feature F13 | #Feature F14 | #Feature F2 | #Feature F3 | #Feature F4 | #Feature F5 | #Feature F6 | #Feature F7 | #Feature F8 | #Feature F9 | Start | End | PTM | AScore | Found By |
| --- | --- | --- | --- | --- | --- | --- | --- | --- | --- | --- | --- | --- | --- | --- | --- | --- | --- | --- | --- | --- | --- | --- | --- | --- | --- | --- | --- | --- | --- | --- | --- | --- | --- | --- | --- | --- | --- | --- | --- | --- | --- | --- | --- | --- | --- |
| R.DFGHIQYVAAYK.V | N | 74.98 | 1410.6931 | 12 | 0.1 | 706.3539 | 2 | 41.87 | 6 | F6:22401 | NaSa01\_F14.raw |  |  |  |  | 3.9336E6 | 2.3002E6 |  |  |  |  |  |  |  |  | 4 | 0 | 0 | 0 | 0 | 2 | 2 | 0 | 0 | 0 | 0 | 0 | 0 | 0 | 0 | 394 | 405 |  |  | PEAKS DB |
| K.NMFDVPIYAR.F | Y | 57.13 | 1224.5961 | 10 | 0.4 | 613.3055 | 2 | 64.99 | 6 | F6:42193 | NaSa01\_F14.raw |  |  |  |  | 4.8154E6 | 3.0574E6 |  |  |  |  |  |  |  |  | 2 | 0 | 0 | 0 | 0 | 1 | 1 | 0 | 0 | 0 | 0 | 0 | 0 | 0 | 0 | 441 | 450 |  |  | PEAKS DB |
| R.IFPEAWHNR.I | N | 43.13 | 1168.5778 | 9 | -0.1 | 390.5332 | 3 | 24.18 | 5 | F5:9021 | NaSa01\_F13.raw |  |  |  |  | 2.5333E6 |  |  |  |  |  |  |  |  |  | 1 | 0 | 0 | 0 | 0 | 1 | 0 | 0 | 0 | 0 | 0 | 0 | 0 | 0 | 0 | 454 | 462 |  |  | PEAKS DB |
| total 3 peptides |  |  |  |  |  |  |  |  |  |  |  |  |  |  |  |  |  |  |  |  |  |  |  |  |  |  |  |  |  |  |  |  |  |  |  |  |  |  |  |  |  |  |  |  |  |
| --- | --- | --- | --- | --- | --- | --- | --- | --- | --- | --- | --- | --- | --- | --- | --- | --- | --- | --- | --- | --- | --- | --- | --- | --- | --- | --- | --- | --- | --- | --- | --- | --- | --- | --- | --- | --- | --- | --- | --- | --- | --- | --- | --- | --- | --- |

XP\_026565130.1

back to list

  

| Protein Coverage
| Supporting Peptides
|

Protein Coverage:

Supporting Peptides:

| Peptide | Uniq | -10lgP | Mass | Length | ppm | m/z | z | RT | Fraction | Scan | Source File | Area F1 | Area F10 | Area F11 | Area F12 | Area F13 | Area F14 | Area F2 | Area F3 | Area F4 | Area F5 | Area F6 | Area F7 | Area F8 | Area F9 | #Feature | #Feature F1 | #Feature F10 | #Feature F11 | #Feature F12 | #Feature F13 | #Feature F14 | #Feature F2 | #Feature F3 | #Feature F4 | #Feature F5 | #Feature F6 | #Feature F7 | #Feature F8 | #Feature F9 | Start | End | PTM | AScore | Found By |
| --- | --- | --- | --- | --- | --- | --- | --- | --- | --- | --- | --- | --- | --- | --- | --- | --- | --- | --- | --- | --- | --- | --- | --- | --- | --- | --- | --- | --- | --- | --- | --- | --- | --- | --- | --- | --- | --- | --- | --- | --- | --- | --- | --- | --- | --- |
| R.DFGHIQYVAAYK.V | N | 74.98 | 1410.6931 | 12 | 0.1 | 706.3539 | 2 | 41.87 | 6 | F6:22401 | NaSa01\_F14.raw |  |  |  |  | 3.9336E6 | 2.3002E6 |  |  |  |  |  |  |  |  | 4 | 0 | 0 | 0 | 0 | 2 | 2 | 0 | 0 | 0 | 0 | 0 | 0 | 0 | 0 | 388 | 399 |  |  | PEAKS DB |
| K.NMFDVPIYAR.F | Y | 57.13 | 1224.5961 | 10 | 0.4 | 613.3055 | 2 | 64.99 | 6 | F6:42193 | NaSa01\_F14.raw |  |  |  |  | 4.8154E6 | 3.0574E6 |  |  |  |  |  |  |  |  | 2 | 0 | 0 | 0 | 0 | 1 | 1 | 0 | 0 | 0 | 0 | 0 | 0 | 0 | 0 | 435 | 444 |  |  | PEAKS DB |
| R.IFPEAWHNR.I | N | 43.13 | 1168.5778 | 9 | -0.1 | 390.5332 | 3 | 24.18 | 5 | F5:9021 | NaSa01\_F13.raw |  |  |  |  | 2.5333E6 |  |  |  |  |  |  |  |  |  | 1 | 0 | 0 | 0 | 0 | 1 | 0 | 0 | 0 | 0 | 0 | 0 | 0 | 0 | 0 | 448 | 456 |  |  | PEAKS DB |
| total 3 peptides |  |  |  |  |  |  |  |  |  |  |  |  |  |  |  |  |  |  |  |  |  |  |  |  |  |  |  |  |  |  |  |  |  |  |  |  |  |  |  |  |  |  |  |  |  |
| --- | --- | --- | --- | --- | --- | --- | --- | --- | --- | --- | --- | --- | --- | --- | --- | --- | --- | --- | --- | --- | --- | --- | --- | --- | --- | --- | --- | --- | --- | --- | --- | --- | --- | --- | --- | --- | --- | --- | --- | --- | --- | --- | --- | --- | --- |

AFJ49577.1

back to list

  

| Protein Coverage
| Supporting Peptides
|

Protein Coverage:

Supporting Peptides:

| Peptide | Uniq | -10lgP | Mass | Length | ppm | m/z | z | RT | Fraction | Scan | Source File | Area F1 | Area F10 | Area F11 | Area F12 | Area F13 | Area F14 | Area F2 | Area F3 | Area F4 | Area F5 | Area F6 | Area F7 | Area F8 | Area F9 | #Feature | #Feature F1 | #Feature F10 | #Feature F11 | #Feature F12 | #Feature F13 | #Feature F14 | #Feature F2 | #Feature F3 | #Feature F4 | #Feature F5 | #Feature F6 | #Feature F7 | #Feature F8 | #Feature F9 | Start | End | PTM | AScore | Found By |
| --- | --- | --- | --- | --- | --- | --- | --- | --- | --- | --- | --- | --- | --- | --- | --- | --- | --- | --- | --- | --- | --- | --- | --- | --- | --- | --- | --- | --- | --- | --- | --- | --- | --- | --- | --- | --- | --- | --- | --- | --- | --- | --- | --- | --- | --- |
| K.EAFSLFDKDGDGTITTK.E | Y | 72.19 | 1843.8839 | 17 | 0.4 | 615.6355 | 3 | 50.84 | 6 | F6:30148 | NaSa01\_F14.raw |  |  |  |  |  | 1.2973E6 |  |  |  |  |  |  |  |  | 1 | 0 | 0 | 0 | 0 | 0 | 1 | 0 | 0 | 0 | 0 | 0 | 0 | 0 | 0 | 15 | 31 |  |  | PEAKS DB |
| Q.LTEEQIAEFK.E | Y | 54.42 | 1206.6132 | 10 | 0.6 | 604.3142 | 2 | 38.33 | 6 | F6:19496 | NaSa01\_F14.raw |  |  |  |  |  | 7.101E5 |  |  |  |  |  |  |  |  | 1 | 0 | 0 | 0 | 0 | 0 | 1 | 0 | 0 | 0 | 0 | 0 | 0 | 0 | 0 | 5 | 14 |  |  | PEAKS DB |
| K.EAFSLFDK.D | Y | 53.17 | 955.4650 | 8 | 0.7 | 478.7401 | 2 | 53.29 | 6 | F6:32330 | NaSa01\_F14.raw |  |  |  |  |  | 5.3478E5 |  |  |  |  |  |  |  |  | 1 | 0 | 0 | 0 | 0 | 0 | 1 | 0 | 0 | 0 | 0 | 0 | 0 | 0 | 0 | 15 | 22 |  |  | PEAKS DB |
| total 3 peptides |  |  |  |  |  |  |  |  |  |  |  |  |  |  |  |  |  |  |  |  |  |  |  |  |  |  |  |  |  |  |  |  |  |  |  |  |  |  |  |  |  |  |  |  |  |
| --- | --- | --- | --- | --- | --- | --- | --- | --- | --- | --- | --- | --- | --- | --- | --- | --- | --- | --- | --- | --- | --- | --- | --- | --- | --- | --- | --- | --- | --- | --- | --- | --- | --- | --- | --- | --- | --- | --- | --- | --- | --- | --- | --- | --- | --- |

JAC96065.1

back to list

  

| Protein Coverage
| Supporting Peptides
|

Protein Coverage:

Supporting Peptides:

| Peptide | Uniq | -10lgP | Mass | Length | ppm | m/z | z | RT | Fraction | Scan | Source File | Area F1 | Area F10 | Area F11 | Area F12 | Area F13 | Area F14 | Area F2 | Area F3 | Area F4 | Area F5 | Area F6 | Area F7 | Area F8 | Area F9 | #Feature | #Feature F1 | #Feature F10 | #Feature F11 | #Feature F12 | #Feature F13 | #Feature F14 | #Feature F2 | #Feature F3 | #Feature F4 | #Feature F5 | #Feature F6 | #Feature F7 | #Feature F8 | #Feature F9 | Start | End | PTM | AScore | Found By |
| --- | --- | --- | --- | --- | --- | --- | --- | --- | --- | --- | --- | --- | --- | --- | --- | --- | --- | --- | --- | --- | --- | --- | --- | --- | --- | --- | --- | --- | --- | --- | --- | --- | --- | --- | --- | --- | --- | --- | --- | --- | --- | --- | --- | --- | --- |
| K.EAFSLFDKDGDGTITTK.E | Y | 72.19 | 1843.8839 | 17 | 0.4 | 615.6355 | 3 | 50.84 | 6 | F6:30148 | NaSa01\_F14.raw |  |  |  |  |  | 1.2973E6 |  |  |  |  |  |  |  |  | 1 | 0 | 0 | 0 | 0 | 0 | 1 | 0 | 0 | 0 | 0 | 0 | 0 | 0 | 0 | 15 | 31 |  |  | PEAKS DB |
| Q.LTEEQIAEFK.E | Y | 54.42 | 1206.6132 | 10 | 0.6 | 604.3142 | 2 | 38.33 | 6 | F6:19496 | NaSa01\_F14.raw |  |  |  |  |  | 7.101E5 |  |  |  |  |  |  |  |  | 1 | 0 | 0 | 0 | 0 | 0 | 1 | 0 | 0 | 0 | 0 | 0 | 0 | 0 | 0 | 5 | 14 |  |  | PEAKS DB |
| K.EAFSLFDK.D | Y | 53.17 | 955.4650 | 8 | 0.7 | 478.7401 | 2 | 53.29 | 6 | F6:32330 | NaSa01\_F14.raw |  |  |  |  |  | 5.3478E5 |  |  |  |  |  |  |  |  | 1 | 0 | 0 | 0 | 0 | 0 | 1 | 0 | 0 | 0 | 0 | 0 | 0 | 0 | 0 | 15 | 22 |  |  | PEAKS DB |
| total 3 peptides |  |  |  |  |  |  |  |  |  |  |  |  |  |  |  |  |  |  |  |  |  |  |  |  |  |  |  |  |  |  |  |  |  |  |  |  |  |  |  |  |  |  |  |  |  |
| --- | --- | --- | --- | --- | --- | --- | --- | --- | --- | --- | --- | --- | --- | --- | --- | --- | --- | --- | --- | --- | --- | --- | --- | --- | --- | --- | --- | --- | --- | --- | --- | --- | --- | --- | --- | --- | --- | --- | --- | --- | --- | --- | --- | --- | --- |

JAB54566.1

back to list

  

| Protein Coverage
| Supporting Peptides
|

Protein Coverage:

Supporting Peptides:

| Peptide | Uniq | -10lgP | Mass | Length | ppm | m/z | z | RT | Fraction | Scan | Source File | Area F1 | Area F10 | Area F11 | Area F12 | Area F13 | Area F14 | Area F2 | Area F3 | Area F4 | Area F5 | Area F6 | Area F7 | Area F8 | Area F9 | #Feature | #Feature F1 | #Feature F10 | #Feature F11 | #Feature F12 | #Feature F13 | #Feature F14 | #Feature F2 | #Feature F3 | #Feature F4 | #Feature F5 | #Feature F6 | #Feature F7 | #Feature F8 | #Feature F9 | Start | End | PTM | AScore | Found By |
| --- | --- | --- | --- | --- | --- | --- | --- | --- | --- | --- | --- | --- | --- | --- | --- | --- | --- | --- | --- | --- | --- | --- | --- | --- | --- | --- | --- | --- | --- | --- | --- | --- | --- | --- | --- | --- | --- | --- | --- | --- | --- | --- | --- | --- | --- |
| K.EAFSLFDKDGDGTITTK.E | Y | 72.19 | 1843.8839 | 17 | 0.4 | 615.6355 | 3 | 50.84 | 6 | F6:30148 | NaSa01\_F14.raw |  |  |  |  |  | 1.2973E6 |  |  |  |  |  |  |  |  | 1 | 0 | 0 | 0 | 0 | 0 | 1 | 0 | 0 | 0 | 0 | 0 | 0 | 0 | 0 | 15 | 31 |  |  | PEAKS DB |
| Q.LTEEQIAEFK.E | Y | 54.42 | 1206.6132 | 10 | 0.6 | 604.3142 | 2 | 38.33 | 6 | F6:19496 | NaSa01\_F14.raw |  |  |  |  |  | 7.101E5 |  |  |  |  |  |  |  |  | 1 | 0 | 0 | 0 | 0 | 0 | 1 | 0 | 0 | 0 | 0 | 0 | 0 | 0 | 0 | 5 | 14 |  |  | PEAKS DB |
| K.EAFSLFDK.D | Y | 53.17 | 955.4650 | 8 | 0.7 | 478.7401 | 2 | 53.29 | 6 | F6:32330 | NaSa01\_F14.raw |  |  |  |  |  | 5.3478E5 |  |  |  |  |  |  |  |  | 1 | 0 | 0 | 0 | 0 | 0 | 1 | 0 | 0 | 0 | 0 | 0 | 0 | 0 | 0 | 15 | 22 |  |  | PEAKS DB |
| total 3 peptides |  |  |  |  |  |  |  |  |  |  |  |  |  |  |  |  |  |  |  |  |  |  |  |  |  |  |  |  |  |  |  |  |  |  |  |  |  |  |  |  |  |  |  |  |  |
| --- | --- | --- | --- | --- | --- | --- | --- | --- | --- | --- | --- | --- | --- | --- | --- | --- | --- | --- | --- | --- | --- | --- | --- | --- | --- | --- | --- | --- | --- | --- | --- | --- | --- | --- | --- | --- | --- | --- | --- | --- | --- | --- | --- | --- | --- |

JAA97540.1

back to list

  

| Protein Coverage
| Supporting Peptides
|

Protein Coverage:

Supporting Peptides:

| Peptide | Uniq | -10lgP | Mass | Length | ppm | m/z | z | RT | Fraction | Scan | Source File | Area F1 | Area F10 | Area F11 | Area F12 | Area F13 | Area F14 | Area F2 | Area F3 | Area F4 | Area F5 | Area F6 | Area F7 | Area F8 | Area F9 | #Feature | #Feature F1 | #Feature F10 | #Feature F11 | #Feature F12 | #Feature F13 | #Feature F14 | #Feature F2 | #Feature F3 | #Feature F4 | #Feature F5 | #Feature F6 | #Feature F7 | #Feature F8 | #Feature F9 | Start | End | PTM | AScore | Found By |
| --- | --- | --- | --- | --- | --- | --- | --- | --- | --- | --- | --- | --- | --- | --- | --- | --- | --- | --- | --- | --- | --- | --- | --- | --- | --- | --- | --- | --- | --- | --- | --- | --- | --- | --- | --- | --- | --- | --- | --- | --- | --- | --- | --- | --- | --- |
| K.EAFSLFDKDGDGTITTK.E | Y | 72.19 | 1843.8839 | 17 | 0.4 | 615.6355 | 3 | 50.84 | 6 | F6:30148 | NaSa01\_F14.raw |  |  |  |  |  | 1.2973E6 |  |  |  |  |  |  |  |  | 1 | 0 | 0 | 0 | 0 | 0 | 1 | 0 | 0 | 0 | 0 | 0 | 0 | 0 | 0 | 15 | 31 |  |  | PEAKS DB |
| Q.LTEEQIAEFK.E | Y | 54.42 | 1206.6132 | 10 | 0.6 | 604.3142 | 2 | 38.33 | 6 | F6:19496 | NaSa01\_F14.raw |  |  |  |  |  | 7.101E5 |  |  |  |  |  |  |  |  | 1 | 0 | 0 | 0 | 0 | 0 | 1 | 0 | 0 | 0 | 0 | 0 | 0 | 0 | 0 | 5 | 14 |  |  | PEAKS DB |
| K.EAFSLFDK.D | Y | 53.17 | 955.4650 | 8 | 0.7 | 478.7401 | 2 | 53.29 | 6 | F6:32330 | NaSa01\_F14.raw |  |  |  |  |  | 5.3478E5 |  |  |  |  |  |  |  |  | 1 | 0 | 0 | 0 | 0 | 0 | 1 | 0 | 0 | 0 | 0 | 0 | 0 | 0 | 0 | 15 | 22 |  |  | PEAKS DB |
| total 3 peptides |  |  |  |  |  |  |  |  |  |  |  |  |  |  |  |  |  |  |  |  |  |  |  |  |  |  |  |  |  |  |  |  |  |  |  |  |  |  |  |  |  |  |  |  |  |
| --- | --- | --- | --- | --- | --- | --- | --- | --- | --- | --- | --- | --- | --- | --- | --- | --- | --- | --- | --- | --- | --- | --- | --- | --- | --- | --- | --- | --- | --- | --- | --- | --- | --- | --- | --- | --- | --- | --- | --- | --- | --- | --- | --- | --- | --- |

JAA97541.1

back to list

  

| Protein Coverage
| Supporting Peptides
|

Protein Coverage:

Supporting Peptides:

| Peptide | Uniq | -10lgP | Mass | Length | ppm | m/z | z | RT | Fraction | Scan | Source File | Area F1 | Area F10 | Area F11 | Area F12 | Area F13 | Area F14 | Area F2 | Area F3 | Area F4 | Area F5 | Area F6 | Area F7 | Area F8 | Area F9 | #Feature | #Feature F1 | #Feature F10 | #Feature F11 | #Feature F12 | #Feature F13 | #Feature F14 | #Feature F2 | #Feature F3 | #Feature F4 | #Feature F5 | #Feature F6 | #Feature F7 | #Feature F8 | #Feature F9 | Start | End | PTM | AScore | Found By |
| --- | --- | --- | --- | --- | --- | --- | --- | --- | --- | --- | --- | --- | --- | --- | --- | --- | --- | --- | --- | --- | --- | --- | --- | --- | --- | --- | --- | --- | --- | --- | --- | --- | --- | --- | --- | --- | --- | --- | --- | --- | --- | --- | --- | --- | --- |
| K.EAFSLFDKDGDGTITTK.E | Y | 72.19 | 1843.8839 | 17 | 0.4 | 615.6355 | 3 | 50.84 | 6 | F6:30148 | NaSa01\_F14.raw |  |  |  |  |  | 1.2973E6 |  |  |  |  |  |  |  |  | 1 | 0 | 0 | 0 | 0 | 0 | 1 | 0 | 0 | 0 | 0 | 0 | 0 | 0 | 0 | 15 | 31 |  |  | PEAKS DB |
| Q.LTEEQIAEFK.E | Y | 54.42 | 1206.6132 | 10 | 0.6 | 604.3142 | 2 | 38.33 | 6 | F6:19496 | NaSa01\_F14.raw |  |  |  |  |  | 7.101E5 |  |  |  |  |  |  |  |  | 1 | 0 | 0 | 0 | 0 | 0 | 1 | 0 | 0 | 0 | 0 | 0 | 0 | 0 | 0 | 5 | 14 |  |  | PEAKS DB |
| K.EAFSLFDK.D | Y | 53.17 | 955.4650 | 8 | 0.7 | 478.7401 | 2 | 53.29 | 6 | F6:32330 | NaSa01\_F14.raw |  |  |  |  |  | 5.3478E5 |  |  |  |  |  |  |  |  | 1 | 0 | 0 | 0 | 0 | 0 | 1 | 0 | 0 | 0 | 0 | 0 | 0 | 0 | 0 | 15 | 22 |  |  | PEAKS DB |
| total 3 peptides |  |  |  |  |  |  |  |  |  |  |  |  |  |  |  |  |  |  |  |  |  |  |  |  |  |  |  |  |  |  |  |  |  |  |  |  |  |  |  |  |  |  |  |  |  |
| --- | --- | --- | --- | --- | --- | --- | --- | --- | --- | --- | --- | --- | --- | --- | --- | --- | --- | --- | --- | --- | --- | --- | --- | --- | --- | --- | --- | --- | --- | --- | --- | --- | --- | --- | --- | --- | --- | --- | --- | --- | --- | --- | --- | --- | --- |

JAA97542.1

back to list

  

| Protein Coverage
| Supporting Peptides
|

Protein Coverage:

Supporting Peptides:

| Peptide | Uniq | -10lgP | Mass | Length | ppm | m/z | z | RT | Fraction | Scan | Source File | Area F1 | Area F10 | Area F11 | Area F12 | Area F13 | Area F14 | Area F2 | Area F3 | Area F4 | Area F5 | Area F6 | Area F7 | Area F8 | Area F9 | #Feature | #Feature F1 | #Feature F10 | #Feature F11 | #Feature F12 | #Feature F13 | #Feature F14 | #Feature F2 | #Feature F3 | #Feature F4 | #Feature F5 | #Feature F6 | #Feature F7 | #Feature F8 | #Feature F9 | Start | End | PTM | AScore | Found By |
| --- | --- | --- | --- | --- | --- | --- | --- | --- | --- | --- | --- | --- | --- | --- | --- | --- | --- | --- | --- | --- | --- | --- | --- | --- | --- | --- | --- | --- | --- | --- | --- | --- | --- | --- | --- | --- | --- | --- | --- | --- | --- | --- | --- | --- | --- |
| K.EAFSLFDKDGDGTITTK.E | Y | 72.19 | 1843.8839 | 17 | 0.4 | 615.6355 | 3 | 50.84 | 6 | F6:30148 | NaSa01\_F14.raw |  |  |  |  |  | 1.2973E6 |  |  |  |  |  |  |  |  | 1 | 0 | 0 | 0 | 0 | 0 | 1 | 0 | 0 | 0 | 0 | 0 | 0 | 0 | 0 | 15 | 31 |  |  | PEAKS DB |
| Q.LTEEQIAEFK.E | Y | 54.42 | 1206.6132 | 10 | 0.6 | 604.3142 | 2 | 38.33 | 6 | F6:19496 | NaSa01\_F14.raw |  |  |  |  |  | 7.101E5 |  |  |  |  |  |  |  |  | 1 | 0 | 0 | 0 | 0 | 0 | 1 | 0 | 0 | 0 | 0 | 0 | 0 | 0 | 0 | 5 | 14 |  |  | PEAKS DB |
| K.EAFSLFDK.D | Y | 53.17 | 955.4650 | 8 | 0.7 | 478.7401 | 2 | 53.29 | 6 | F6:32330 | NaSa01\_F14.raw |  |  |  |  |  | 5.3478E5 |  |  |  |  |  |  |  |  | 1 | 0 | 0 | 0 | 0 | 0 | 1 | 0 | 0 | 0 | 0 | 0 | 0 | 0 | 0 | 15 | 22 |  |  | PEAKS DB |
| total 3 peptides |  |  |  |  |  |  |  |  |  |  |  |  |  |  |  |  |  |  |  |  |  |  |  |  |  |  |  |  |  |  |  |  |  |  |  |  |  |  |  |  |  |  |  |  |  |
| --- | --- | --- | --- | --- | --- | --- | --- | --- | --- | --- | --- | --- | --- | --- | --- | --- | --- | --- | --- | --- | --- | --- | --- | --- | --- | --- | --- | --- | --- | --- | --- | --- | --- | --- | --- | --- | --- | --- | --- | --- | --- | --- | --- | --- | --- |

JAG47255.1

back to list

  

| Protein Coverage
| Supporting Peptides
|

Protein Coverage:

Supporting Peptides:

| Peptide | Uniq | -10lgP | Mass | Length | ppm | m/z | z | RT | Fraction | Scan | Source File | Area F1 | Area F10 | Area F11 | Area F12 | Area F13 | Area F14 | Area F2 | Area F3 | Area F4 | Area F5 | Area F6 | Area F7 | Area F8 | Area F9 | #Feature | #Feature F1 | #Feature F10 | #Feature F11 | #Feature F12 | #Feature F13 | #Feature F14 | #Feature F2 | #Feature F3 | #Feature F4 | #Feature F5 | #Feature F6 | #Feature F7 | #Feature F8 | #Feature F9 | Start | End | PTM | AScore | Found By |
[truncated: 888,672 more chars]
